# Supplementary material for: Intrinsic circadian organization of the spinal cord and dorsal root ganglia
Source: Pain Rep. 2026 Oct 8;11(6):e1505. doi: 10.1097/PR9.0000000000001505 (PMC13652437; doi:10.1097/PR9.0000000000001505)
Supplement: Supplementary file 1 [file painreports-11-e1505-s001.pdf]

Supplementary Figure legends

Supplementary Figure 1. Validation of circadian rhythmicity in dorsal root ganglia. (A) Permutation-based significance testing of circadian rhythmicity in dorsal root ganglia (DRGs). Histogram shows the distribution of the number of rhythmic genes identified after random permutation of circadian time labels (1,000 permutations). The observed number of rhythmic genes in the real dataset (n = 626; red line) lies far outside the null distribution, yielding an empirical P = 0.000999 and indicating that rhythmic transcription in DRGs exceeds chance expectation. (B) Number and percentage of expressed genes identified as rhythmic by each rhythm-detection algorithm at $q < 0.15$. (C) Heatmap showing rhythm-detection support for 10 canonical core clock genes across RAIN, JTK_CYCLE, ARSER, and CircaCompare. Tile color represents $-\log10(q)$, capped at 4; white circles indicate $q < 0.15$. (D) Time-course RNA-seq expression profiles (FPKM) of representative clock genes (*Per1*, *Per3*, *Cry1*, *Cry2*, *Clock*, *Nr1d1*, *Nr1d2*, and *Dbp*) in mouse DRGs sampled across a 52-h circadian time course under constant darkness. Points represent expression values at each time point, connected by lines for visualization. Vertical dashed lines denote successive circadian cycles. RAIN q-values are shown for each gene and reflect rhythmicity significance following multiple-testing correction. These profiles demonstrate heterogeneous rhythmic strength across individual clock components in DRGs.

Supplementary Figure 2. Phase-resolved transcriptional programs in dorsal root ganglia. (A, C, E) Z-score-normalized expression trajectories of rhythmic DRG genes binned by peak circadian phase, showing genes peaking at CT3-6 (A), CT6-9 (C), and CT12-15 (E). Each line represents an individual rhythmic gene identified by RAIN analysis ($q < 0.15$). Vertical dashed lines indicate circadian cycle boundaries (CT0, CT24, CT48). (B, D, F) Gene Ontology (GO) biological process enrichment networks corresponding to each phase bin shown at left. Nodes represent enriched GO terms (beige) and associated genes (colored), with node size proportional to the number of genes contributing to each term. Networks are shown for terms meeting an exploratory enrichment threshold (Benjamini-Hochberg-adjusted $p < 0.1$).

Supplementary Figure 3. Baseline circadian modulation of neuropathic pain-associated genes in dorsal root ganglia.
(A) Representative circadian expression profiles of genes overlapping between baseline circadian transcription and neuropathic pain-associated datasets (*Nr1d1*, *Dbp*, *Xdh* and *Lrrc17*) in DRGs under constant darkness. These genes exhibit defined circadian phase relationships under baseline conditions. RAIN q-values are shown for each gene. (B) Circadian expression profiles of potassium channel-related transcripts (*Kcnk2*, *Kcnk10*, and *Kcnk13*) implicated in neuropathic pain-associated signaling pathways. These genes display modest baseline circadian modulation in DRGs, consistent with temporal organization of excitability-related components under non-injured conditions.

Supplementary File 1: Time-series DRG expression matrix and circadian rhythmicity metrics (RAIN/ARS).
DRG RNA-seq time-course (CT0-52, 4-h resolution) with per-gene FPKM values and circadian rhythmicity metrics from RAIN and ARS, including phase/period estimates, amplitudes.

Supplementary File 2: Secondary rhythm-detection analysis of the expressed DRG transcriptome.

For each of the 17,394 expressed genes, this file reports q values and rhythmicity calls ($q < 0.15$) from RAIN, JTK_CYCLE, ARSER, and CircaCompare, together with the number of supporting methods, method-intersection category, and canonical core clock gene annotation. Overall, 724 genes were identified by at least one method (RAIN, 626; JTK_CYCLE, 3; ARSER, 42; CircaCompare, 389). Canonical core clock genes include *Arntl, Clock, Npas2, Per1, Per2, Per3, Cry1, Cry2, Nr1d1, and Nr1d2*.

Supplementary File 3: Phase-resolved Gene Ontology enrichment across circadian time in DRGs.

Gene Ontology (GO) enrichment analyses for circadian genes in dorsal root ganglia (DRGs), stratified into consecutive 3-hour circadian time (CT) bins. Enrichment results are provided for Biological Process (BP), Molecular Function (MF), and Cellular Component (CC) categories.

Supplementary Figure 1. Validation of circadian rhythmicity in dorsal root ganglia.

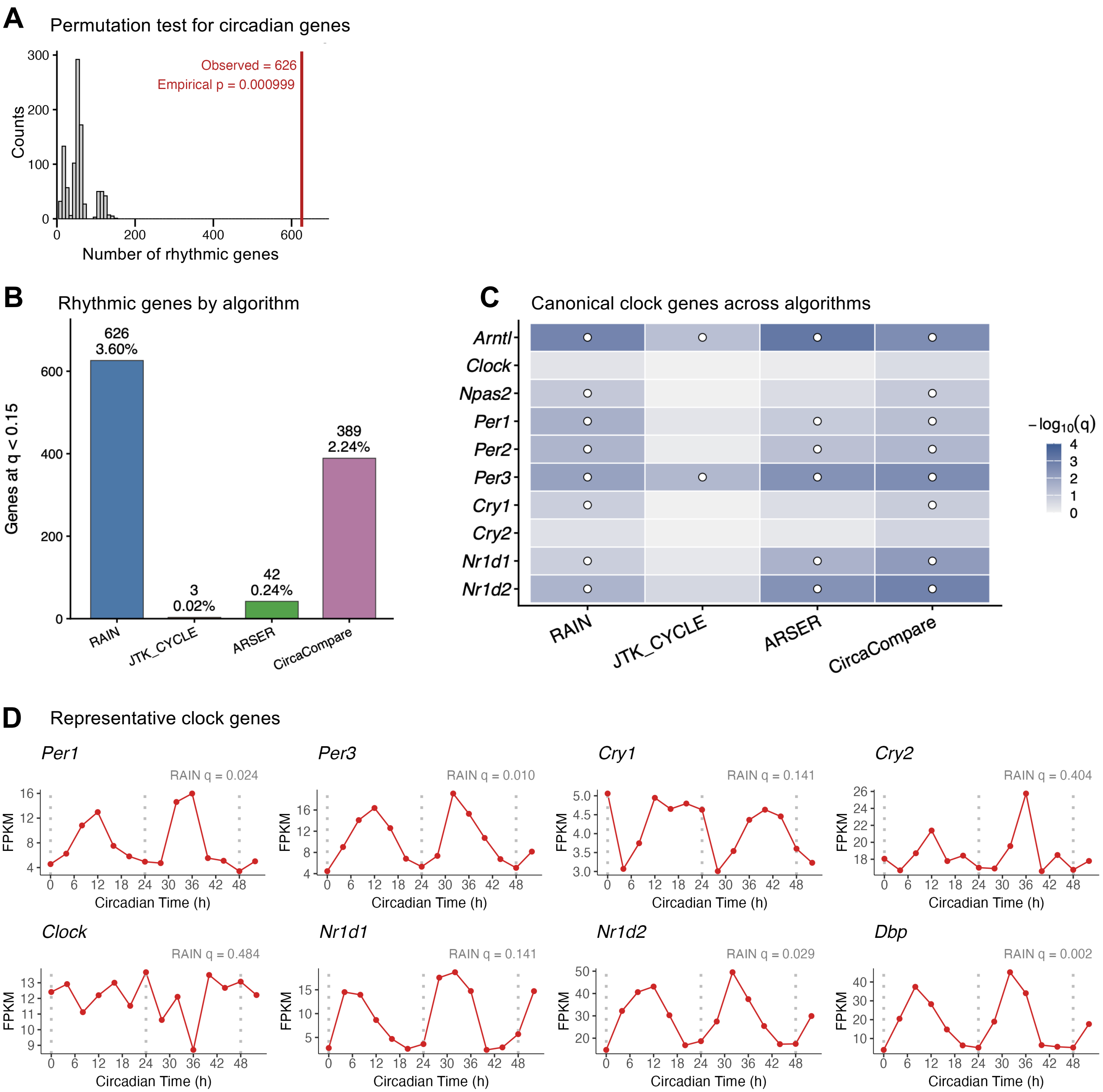

Supplementary Figure 2. Phase-resolved transcriptional programs in dorsal root ganglia.

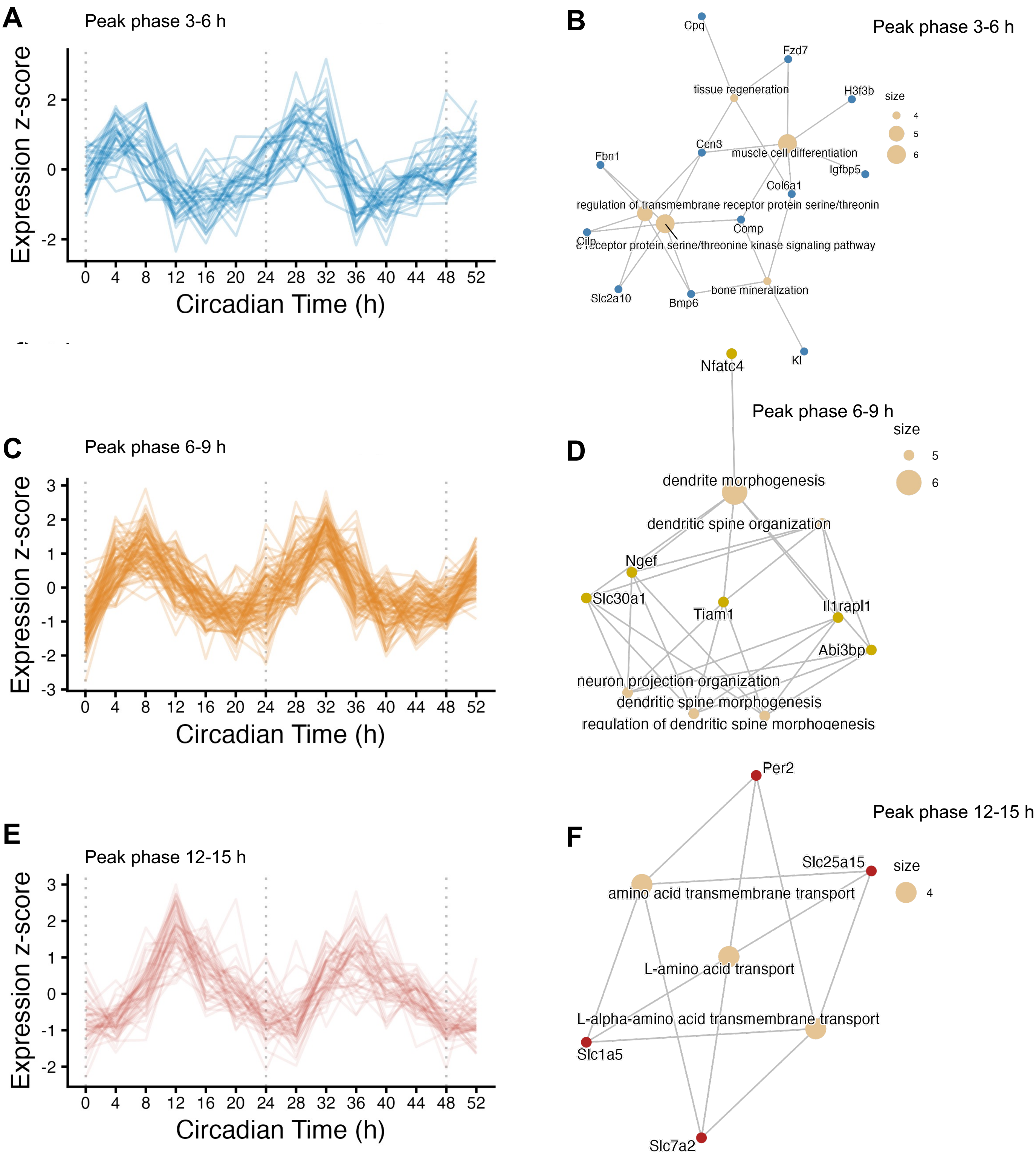

Supplementary Figure 3. Baseline circadian modulation of neuropathic pain–associated genes in dorsal root ganglia.

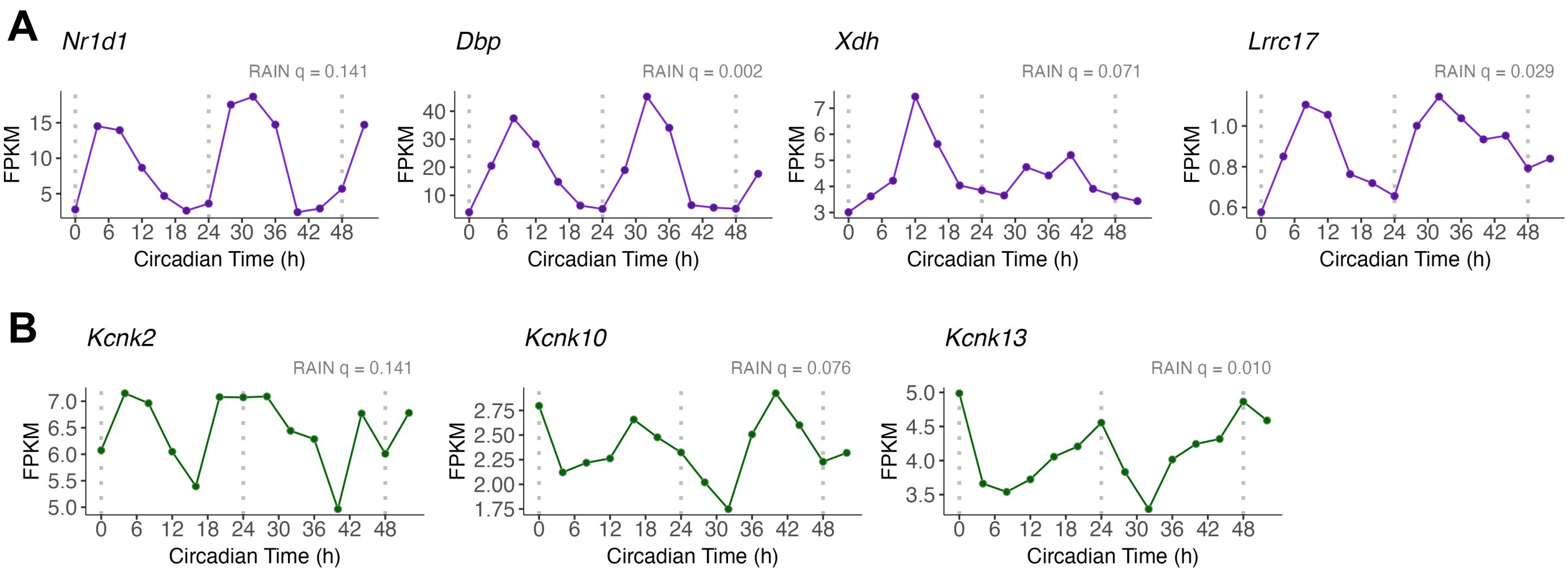

gene_id	gene_short_name	gene_description	T0_DRG_FPKM	T4_DRG_FPKM	T8_DRG_FPKM	T12_DRG_FPKM	T16_DRG_FPKM	T20_DRG_FPKM	T24_DRG_FPKM	T28_DRG_FPKM	T32_DRG_FPKM	T36_DRG_FPKM	T40_DRG_FPKM	T44_DRG_FPKM	T48_DRG_FPKM	T52_DRG_FPKM	rain_p	rain_q	rain_phase	rain_peak_shape	rain_period	ARS_pvalue	ARS_BH.Q	ARS_period	ARS_adjphase	ARS_amplitude
ENSMUSG00000000001	Gnai3	guanine nucleotide binding protein (G protein), alpha inhibiting 3 [Source:MGI Symbol;Acc:MGI:95773]	34.3867	35.3814	34.622	34.6402	36.1219	32.6901	33.1317	31.6351	34.4295	31.7052	37.9546	33.7293	35.2665	34.0828	0.849179819	0.966018836	20	4	24	0.615445216	0.867093272	24	15.98728172	0.027062573
ENSMUSG00000000028	Cdc45	cell division cycle 45 [Source:MGI Symbol;Acc:MGI:1338073]	1.83874	1.82264	2.48479	2.14479	1.90328	1.82672	1.8856	1.68267	1.8072	2.20594	1.90414	2.05584	2.3405	2.2223	0.891690964	0.966018836	16	16	24	0.566304823	0.843471947	24	10.00084743	0.046815246
ENSMUSG00000000031	H19	H19, imprinted maternally expressed transcript [Source:MGI Symbol;Acc:MGI:95891]	26.8837	32.2288	29.683	30.8894	36.1532	26.9469	30.7141	61.5561	30.0584	25.3921	26.8917	24.1697	25.7451	23.4977	0.919119843	0.966018836	8	16	24	0.634163501	0.875590992	24	5.977799444	0.126609638
ENSMUSG00000000037	Scml2	Scm polycomb group protein like 2 [Source:MGI Symbol;Acc:MGI:1340042]	0.69929	1.47822	1.45655	1.23775	0.851524	0.819561	0.772974	1.43412	0.862585	0.728952	0.730264	0.856659	0.935672	1.0275	0.076438025	0.453790923	8	12	24	0.050306025	0.644435324	24	6.470625735	0.171413962
ENSMUSG00000000049	Apoh	apolipoprotein H [Source:MGI Symbol;Acc:MGI:88058]	2.2402	1.91141	1.93764	2.13175	2.73341	2.26417	1.27397	1.52454	2.25247	1.48082	2.24789	2.04243	2.12569	1.88968	0.101443845	0.511659635	20	12	24	0.269292585	0.768040178	24	16.54822145	0.11727636
ENSMUSG00000000056	Narf	nuclear prelamin A recognition factor [Source:MGI Symbol;Acc:MGI:1914858]	11.2357	11.1187	10.6958	11.5196	10.376	11.6697	11.0696	12.2933	11.5655	12.1024	11.1705	11.4986	12.1983	10.8441	0.872380105	0.966018836	24	20	24	0.884053249	0.966895597	24	3.392569638	0.012427617
ENSMUSG00000000058	Cav2	caveolin 2 [Source:MGI Symbol;Acc:MGI:107571]	62.7755	73.8871	66.2151	68.9394	72.3849	55.6403	68.4299	62.5169	73.5859	56.3153	72.5341	55.5932	57.9513	58.4134	0.251986462	0.75129457	12	12	24	0.442365821	0.805393068	24	10.14269583	0.072046409
ENSMUSG00000000078	Klf6	Kruppel-like factor 6 [Source:MGI Symbol;Acc:MGI:1346318]	24.9835	23.1103	21.456	24.915	25.1355	22.7247	24.8548	21.9294	25.2477	23.736	26.2114	21.7792	24.695	23.1288	0.702630284	0.966018836	20	12	24	0.525808369	0.822781291	24	16.08332083	0.039639028
ENSMUSG00000000085	Scmh1	sex comb on midleg homolog 1 [Source:MGI Symbol;Acc:MGI:1352762]	12.3602	14.1451	12.3405	11.9144	15.3373	13.8749	13.4352	16.8306	13.8907	15.3185	13.753	14.9496	14.5683	13.9692	0.873350015	0.966018836	8	4	24	0.955764132	0.988639288	24	23.24827028	0.013876007
ENSMUSG00000000088	Cox5a	cytochrome c oxidase subunit 5A [Source:MGI Symbol;Acc:MGI:88474]	194.44	194.293	196.094	236.524	196.626	217.254	195.497	231.16	202.751	245.627	235.006	223.248	252.614	237.284	0.932671476	0.966018836	16	12	24	0.598102199	0.858819856	24	12.66935654	0.039184233
ENSMUSG00000000093	Tbx2	T-box 2 [Source:MGI Symbol;Acc:MGI:98494]	3.08473	3.24624	2.84793	2.86271	3.07435	2.78263	3.45089	3.54516	3.35713	4.96434	3.98812	3.99994	4.58622	4.22942	0.874672901	0.966018836	8	4	24	0.900391747	0.971794631	24	6.090638771	0.02349397
ENSMUSG00000000120	Ngfr	nerve growth factor receptor (TNFR superfamily, member 16) [Source:MGI Symbol;Acc:MGI:97323]	118.594	68.6531	76.1295	80.2059	86.3105	102.031	87.9107	103.33	54.7785	121.381	87.4332	92.3263	87.4981	90.1127	0.217827365	0.704989686	24	12	24	0.296796304	0.768040178	24	20.08193645	0.185747197
ENSMUSG00000000126	Wnt9a	wingless-type MMTV integration site family, member 9A [Source:MGI Symbol;Acc:MGI:2446084]	4.45239	3.43569	2.48464	3.17092	3.58216	3.59889	3.63466	3.38369	3.52182	3.68563	3.86593	3.92229	3.82009	3.87496	0.094000699	0.490854203	4	8	24	0.069280645	0.673520298	24	21.29764181	0.119922277
ENSMUSG00000000127	Fer	fer (fms/fps related) protein kinase [Source:MGI Symbol;Acc:MGI:105917]	9.82305	9.45199	8.91601	9.39798	10.1123	9.34131	9.55281	8.72649	9.61628	8.45763	10.5632	9.6973	10.781	9.58838	0.230338208	0.730447182	20	20	24	0.184521504	0.768040178	24	20.30383559	0.061223905
ENSMUSG00000000131	Xpo6	exportin 6 [Source:MGI Symbol;Acc:MGI:2429950]	21.4981	20.0591	22.0751	20.3217	22.9782	21.6543	19.2121	22.1824	21.0377	24.4965	20.7763	21.7401	20.8455	17.7976	0.399942636	0.932395417	16	16	24	0.17146574	0.768040178	24	14.59495129	0.073326125
ENSMUSG00000000134	Tfe3	transcription factor E3 [Source:MGI Symbol;Acc:MGI:98511]	13.6246	10.7228	11.4517	12.4407	12.3697	11.9094	13.4881	12.9501	11.2414	14.6966	10.8922	11.125	11.6961	11.5216	0.93717134	0.966018836	16	20	24	0.973186084	0.993305115	24	17.33218705	0.011959911
ENSMUSG00000000142	Axin2	axin 2 [Source:MGI Symbol;Acc:MGI:1270862]	2.87429	2.9902	2.74727	2.33838	2.66976	2.50895	2.51321	3.5013	2.78479	3.60741	2.66442	2.71899	2.43775	2.85047	0.226403091	0.722427815	8	16	24	0.338444942	0.773151665	24	7.269887757	0.079473176
ENSMUSG00000000148	Brat1	BRCA1-associated ATM activator 1 [Source:MGI Symbol;Acc:MGI:1891679]	5.93498	5.69847	5.35901	5.50549	6.12013	5.74738	5.44278	6.44809	6.35732	7.14799	5.74663	6.48836	6.16723	6.07003	0.761468226	0.966018836	24	4	24	0.809329345	0.939184036	24	11.28052901	0.02260615
ENSMUSG00000000149	Gna12	guanine nucleotide binding protein, alpha 12 [Source:MGI Symbol;Acc:MGI:95767]	33.2229	28.2546	28.3267	29.3067	31.9642	32.3318	34.7898	30.7971	27.4871	36.8527	27.4036	31.5674	31.3323	28.5485	0.114657038	0.534533508	24	12	24	0.327770315	0.768040178	24	20.04218087	0.076695589
ENSMUSG00000000154	Slc22a18	solute carrier family 22 (organic cation transporter), member 18 [Source:MGI Symbol;Acc:MGI:1336884]	5.76377	4.6656	5.34353	5.46709	4.89874	5.93023	5.60195	5.63895	4.97003	6.49652	4.67539	5.4382	5.42201	5.32274	0.652290823	0.966018836	24	20	24	0.946079053	0.986887534	24	21.2931521	0.015674898
ENSMUSG00000000157	Itgb2l	integrin beta 2-like [Source:MGI Symbol;Acc:MGI:1277979]	0.546395	1.21779	2.29192	1.24005	1.06986	1.18604	1.04735	0.803002	1.11196	0.609237	0.74046	1.2168	1.35835	0.886001	0.860993221	0.966018836	12	8	24	0.815647031	0.941221389	24	7.795687046	0.071846467
ENSMUSG00000000167	Pih1d2	PIH1 domain containing 2 [Source:MGI Symbol;Acc:MGI:1919864]	1.78546	2.01743	2.45682	2.2609	1.84688	2.08811	1.8524	2.31273	1.99893	1.33027	1.80674	1.6386	2.41305	2.28374	0.208071297	0.698704663	8	12	24	0.20770167	0.768040178	24	4.54227888	0.107819181
ENSMUSG00000000168	Dlat	dihydrolipoamide S-acetyltransferase (E2 component of pyruvate dehydrogenase complex) [Source:MGI Symbol;Acc:MGI:2385311]	43.7204	36.3023	39.9365	50.9733	33.8576	41.8342	40.3279	43.0078	34.6803	53.379	38.7031	39.912	48.4666	44.3998	0.909604019	0.966018836	16	4	24	0.947110765	0.987072308	24	9.239290615	0.02593515
ENSMUSG00000000171	Sdhd	succinate dehydrogenase complex, subunit D, integral membrane protein [Source:MGI Symbol;Acc:MGI:1914175]	113.297	128.963	128.822	151.365	129.309	118.705	118.095	133.34	140.314	121.504	146.8	125.531	144.889	136.019	0.365996436	0.893596186	20	4	24	0.184169283	0.768040178	24	10.27231429	0.083421138
ENSMUSG00000000184	Ccnd2	cyclin D2 [Source:MGI Symbol;Acc:MGI:88314]	3.58372	3.64094	3.81159	3.35035	3.96459	3.19831	4.05273	3.48206	4.18809	3.34954	3.49181	3.52676	3.44369	3.53941	0.44861185	0.966018836	12	4	24	0.658632891	0.884596426	24	6.331618081	0.032948707
ENSMUSG00000000194	Gpr107	G protein-coupled receptor 107 [Source:MGI Symbol;Acc:MGI:2139054]	10.6912	10.4209	10.9161	11.5583	11.1523	12.4185	11.86	12.29	9.54324	12.4272	9.99684	10.4352	10.5059	10.7293	0.877082091	0.966018836	16	20	24	0.892683346	0.969717376	24	18.15931457	0.021505677
ENSMUSG00000000197	Nalcn	sodium leak channel, non-selective [Source:MGI Symbol;Acc:MGI:2444306]	10.0558	10.0511	11.3032	10.343	8.63553	10.0077	9.77891	9.01227	9.04927	9.97611	9.30476	10.5891	9.9525	11.2371	0.85387321	0.966018836	12	8	24	0.647418804	0.880898698	24	4.445956734	0.038630439
ENSMUSG00000000202	Btbd17	BTB (POZ) domain containing 17 [Source:MGI Symbol;Acc:MGI:1919264]	1.46702	1.63471	2.49745	2.29721	1.70512	1.35138	2.12868	1.76253	3.22952	2.32696	1.6465	1.09976	0.927031	2.00333	7.55E-04	0.071344007	12	12	24	0.002396566	0.39630666	24	9.258361757	0.340285563
ENSMUSG00000000204	Slfn4	schlafen 4 [Source:MGI Symbol;Acc:MGI:1329010]	1.40533	1.84583	3.51855	2.05095	2.65691	2.45386	2.35128	2.01727	2.00952	1.0969	1.70916	1.20444	1.68768	1.28404	0.873350015	0.966018836	12	20	24	0.889043397	0.968820559	24	11.00222615	0.052704784
ENSMUSG00000000214	Th	tyrosine hydroxylase [Source:MGI Symbol;Acc:MGI:98735]	48.018	34.4076	33.3094	41.6228	34.5007	44.4489	42.43	41.907	28.9523	50.8308	39.5509	48.7322	41.6658	59.8706	0.245061943	0.741580974	24	12	24	0.472271314	0.805393068	24	22.16737976	0.121728126
ENSMUSG00000000223	Drp2	dystrophin related protein 2 [Source:MGI Symbol;Acc:MGI:107432]	136.215	134.933	113.414	135.448	160.166	124.175	160.18	126.691	148.853	138.274	132.561	141.732	143.018	118.611	0.800065124	0.966018836	4	4	24	0.450547216	0.805393068	24	18.64451812	0.074385549
ENSMUSG00000000244	Tspan32	tetraspanin 32 [Source:MGI Symbol;Acc:MGI:1350360]	0.843482	1.28221	1.86831	1.53614	1.36776	1.34967	1.32955	1.45589	1.45283	0.970082	1.09781	1.16773	1.5827	1.1006	0.939103211	0.966018836	12	12	24	0.554613162	0.837869569	24	8.049036315	0.074875658
ENSMUSG00000000253	Gmpr	guanosine monophosphate reductase [Source:MGI Symbol;Acc:MGI:1913605]	25.6261	24.3355	23.945	24.8479	22.7567	27.4241	25.0169	28.8336	22.9593	29.1812	22.7254	23.9851	25.9824	28.6527	0.305564675	0.811201458	8	12	24	0.438995868	0.805393068	24	3.604699635	0.059294324
ENSMUSG00000000263	Glra1	glycine receptor, alpha 1 subunit [Source:MGI Symbol;Acc:MGI:95747]	0.572731	2.20998	4.55307	1.20992	0.734713	2.11274	3.54199	0.735086	3.00293	1.08463	0.36836	1.84454	0.346784	2.36895	0.10951302	0.524035616	12	8	24	0.369704283	0.792235171	24	6.222806384	0.368930835
ENSMUSG00000000266	Mid2	midline 2 [Source:MGI Symbol;Acc:MGI:1344333]	11.9144	10.9725	11.2711	11.6332	11.2374	9.89085	10.5831	9.46144	11.2214	10.7533	10.7518	11.2316	10.5892	11.2516	0.868638024	0.966018836	12	12	24	0.727777133	0.91070186	24	12.05426126	0.024989107
ENSMUSG00000000275	Trim25	tripartite motif-containing 25 [Source:MGI Symbol;Acc:MGI:102749]	7.93877	6.58421	6.56826	6.31442	7.35913	7.67573	6.71148	7.69978	5.78148	7.30163	7.07076	6.88049	7.07348	7.12343	0.289496388	0.794869797	24	12	24	0.139903776	0.74956518	24	21.05921067	0.08309642
ENSMUSG00000000276	Dgke	diacylglycerol kinase, epsilon [Source:MGI Symbol;Acc:MGI:1889276]	9.12336	9.96361	10.3997	8.41275	9.0092	9.4075	8.44027	9.44379	9.25276	10.041	9.2567	9.92753	9.68958	10.6651	0.521072381	0.966018836	8	12	24	0.338876643	0.773455168	24	5.93860584	0.050624806
ENSMUSG00000000278	Scpep1	serine carboxypeptidase 1 [Source:MGI Symbol;Acc:MGI:1921867]	19.4759	17.5106	18.1194	17.0457	20.0488	19.7064	20.2491	21.3987	16.2601	17.894	18.6762	17.6877	18.6197	18.554	0.195388358	0.673387182	4	8	24	0.148084659	0.756225271	24	22.1913377	0.076477658
ENSMUSG00000000282	Mnt	max binding protein [Source:MGI Symbol;Acc:MGI:109150]	5.41428	4.82763	5.02837	5.21762	5.06665	5.18721	5.57295	5.72252	5.3496	7.61662	5.05355	6.17115	5.5591	5.58115	0.915082018	0.966018836	4	16	24	0.839832328	0.951411283	24	12.07445492	0.029822835
ENSMUSG00000000290	Itgb2	integrin beta 2 [Source:MGI Symbol;Acc:MGI:96611]	2.19302	3.30931	4.20498	3.7431	3.37692	3.59237	3.71146	3.23637	3.54355	2.30915	2.97142	2.98802	3.18267	2.42928	0.94086481	0.966018836	12	16	24	0.85874663	0.958405859	24	10.91178557	0.045028638
ENSMUSG00000000295	Hddc2	HD domain containing 2 [Source:MGI Symbol;Acc:MGI:1916942]	19.8404	24.4368	24.7527	23.6016	21.177	20.9682	19.5576	21.1537	25.7287	18.2997	23.8181	23.2209	24.1244	23.44	0.210566453	0.69936851	12	12	24	0.456573619	0.805393068	24	7.62764047	0.072236384
ENSMUSG00000000296	Tpd52l1	tumor protein D52-like 1 [Source:MGI Symbol;Acc:MGI:1298386]	4.34447	6.4394	7.92766	5.49472	6.02274	5.04737	5.16472	6.00213	6.34755	6.55617	7.02452	5.83932	4.62989	6.59115	0.009118941	0.192760771	12	16	24	0.018371047	0.559812298	24	9.981022643	0.202039304
ENSMUSG00000000301	Pemt	phosphatidylethanolamine N-methyltransferase [Source:MGI Symbol;Acc:MGI:104535]	6.96165	7.20706	8.84651	6.74083	7.57074	8.15844	6.03724	6.10913	6.73877	5.93851	7.19193	7.52997	7.86366	7.07176	0.895493752	0.966018836	24	16	24	0.750166929	0.918827661	24	19.18614474	0.046001882
ENSMUSG00000000303	Cdh1	cadherin 1 [Source:MGI Symbol;Acc:MGI:88354]	45.474	35.838	33.5225	37.6467	41.4947	40.988	39.7373	40.5224	31.0854	46.987	33.7527	40.4376	40.6428	36.9847	0.138202217	0.580158868	24	12	24	0.235316625	0.768040178	24	20.35094407	0.110775407
ENSMUSG00000000305	Cdh4	cadherin 4 [Source:MGI Symbol;Acc:MGI:99218]	1.46094	1.31353	1.79919	1.69632	1.26039	1.52265	1.37848	1.48834	1.28376	2.2062	1.41904	1.56604	1.54998	1.57944	0.945451136	0.966018836	16	4	24	0.35451973	0.781577622	24	11.0412763	0.074161377
ENSMUSG00000000308	Ckmt1	creatine kinase, mitochondrial 1, ubiquitous [Source:MGI Symbol;Acc:MGI:99441]	115.494	104.977	100.188	99.1608	89.357	110.789	105.859	102.014	85.4799	118.258	85.9961	102.911	98.9608	107.978	0.467283192	0.966018836	4	16	24	0.397957346	0.799546792	24	0.638821796	0.074279481
ENSMUSG00000000317	Bcl6b	B cell CLL/lymphoma 6, member B [Source:MGI Symbol;Acc:MGI:1278332]	0.358258	0.584995	0.626524	0.513441	0.391023	0.494323	0.745479	0.911055	0.812013	0.710189	0.666052	0.634802	1.01327	0.709773	0.714044782	0.966018836	4	20	24	0.038834824	0.611732283	24	5.30530174	0.111066057
ENSMUSG00000000318	Clec10a	C-type lectin domain family 10, member A [Source:MGI Symbol;Acc:MGI:96975]	1.1131	1.20123	1.41015	1.45237	1.64749	0.947325	2.00568	1.84379	1.59657	0.873714	1.22206	1.73003	0.872521	1.5359	0.823246279	0.966018836	8	8	24	0.827846263	0.946180429	24	5.135279799	0.056177291
ENSMUSG00000000320	Alox12	arachidonate 12-lipoxygenase [Source:MGI Symbol;Acc:MGI:87998]	0.604656	1.07183	1.42235	0.943528	0.777094	0.988701	0.780584	1.15609	1.14942	0.803258	0.817375	1.0349	1.09237	0.697708	0.424718357	0.966018836	12	8	24	0.390849571	0.799546792	24	7.557447509	0.090128082
ENSMUSG00000000326	Comt	catechol-O-methyltransferase [Source:MGI Symbol;Acc:MGI:88470]	46.4721	40.6085	40.4282	42.094	45.7225	44.1536	45.0081	46.6497	40.8312	45.006	48.3	44.5284	48.7993	42.5697	0.060394272	0.407012001	4	8	24	0.056254491	0.65534544	24	20.02609747	0.073740006
ENSMUSG00000000339	Rtca	RNA 3'-terminal phosphate cyclase [Source:MGI Symbol;Acc:MGI:1913618]	45.9817	44.919	43.5125	45.2647	43.6203	46.8271	41.4503	44.3044	42.1197	45.2885	48.3559	48.5803	50.7926	47.5214	0.14313801	0.585944911	24	12	24	0.280906057	0.768040178	24	20.30100771	0.044400875
ENSMUSG00000000340	Dbt	dihydrolipoamide branched chain transacylase E2 [Source:MGI Symbol;Acc:MGI:105386]	11.2423	10.7318	10.241	13.7009	9.85929	10.5395	10.0996	11.129	11.4844	12.1066	11.263	11.8872	11.9879	10.6721	0.865490576	0.966018836	16	16	24	0.51472639	0.816837918	24	11.98356643	0.051656952
ENSMUSG00000000346	Dazap2	DAZ associated protein 2 [Source:MGI Symbol;Acc:MGI:1344344]	73.8177	78.1398	80.0769	76.0311	71.6894	73.4145	74.9366	78.0111	76.1036	80.8016	71.0479	72.7203	74.8752	76.5987	4.29E-04	0.054040373	12	8	24	0.00317363	0.414576087	24	7.168558115	0.062579544
ENSMUSG00000000355	Mcts1	malignant T cell amplified sequence 1 [Source:MGI Symbol;Acc:MGI:1916245]	84.4003	88.0422	76.7085	80.3644	81.4742	77.4286	78.7798	79.7283	87.6797	72.3698	87.8085	80.2909	84.5691	83.0343	0.92721845	0.966018836	8	8	24	0.667320387	0.887459929	24	1.861146946	0.02889394
ENSMUSG00000000359	Rem1	rad and gem related GTP binding protein 1 [Source:MGI Symbol;Acc:MGI:1097696]	0.668349	0.815426	0.76594	0.669253	1.30266	0.659433	1.01235	0.948921	0.763406	0.636671	1.03331	1.1338	0.914351	0.783824	0.129903614	0.560263689	20	20	24	0.371216292	0.792948894	24	19.354503	0.0834989
ENSMUSG00000000374	Trappc10	trafficking protein particle complex 10 [Source:MGI Symbol;Acc:MGI:1336209]	18.6438	16.8356	17.7705	18.868	18.3093	19.2411	19.3692	19.1537	15.4309	21.4746	16.674	18.4479	16.5269	18.3107	0.914502381	0.966018836	24	12	24	0.73378183	0.9131851	24	17.27299683	0.036504823
ENSMUSG00000000378	Ccm2	cerebral cavernous malformation 2 [Source:MGI Symbol;Acc:MGI:2384924]	24.3832	19.5653	23.2909	23.343	22.4004	23.5128	22.7051	21.5954	18.9672	24.4524	22.0609	22.2839	21.8536	20.2967	0.128810631	0.560209134	16	16	24	0.130101246	0.740637625	24	17.55228118	0.078593989
ENSMUSG00000000384	Tbrg4	transforming growth factor beta regulated gene 4 [Source:MGI Symbol;Acc:MGI:1100868]	10.8199	9.56566	10.8319	11.4986	10.9671	11.1743	12.0782	11.9731	9.93221	13.1244	11.1199	10.7453	11.5206	11.0743	0.922029406	0.966018836	16	20	24	0.745637834	0.91691176	24	15.47692058	0.030458288
ENSMUSG00000000386	Mx1	MX dynamin-like GTPase 1 [Source:MGI Symbol;Acc:MGI:97243]	0.909488	0.943555	0.848336	0.902492	0.810096	0.777472	0.505403	1.2249	0.676492	0.736913	1.34572	0.562097	0.70535	0.761396	0.94086481	0.966018836	8	20	24	0.735953325	0.913953743	24	10.98036824	0.054402511
ENSMUSG00000000392	Fap	fibroblast activation protein [Source:MGI Symbol;Acc:MGI:109608]	3.76781	3.82776	3.56255	3.42683	3.95881	3.25754	3.33527	3.89793	4.75927	2.74818	3.96876	3.44648	3.69847	3.59187	0.658942124	0.966018836	12	12	24	0.68101079	0.893426659	24	5.469818308	0.050370846
ENSMUSG00000000399	Ndufa9	NADH:ubiquinone oxidoreductase subunit A9 [Source:MGI Symbol;Acc:MGI:1913358]	89.5213	96.3295	99.2135	109.046	96.9835	92.9535	86.4772	99.1943	96.9353	86.5833	100.581	88.1482	97.3558	94.9492	0.201875332	0.688108864	20	4	24	0.255682306	0.768040178	24	10.28790774	0.059544717
ENSMUSG00000000402	Egfl6	EGF-like-domain, multiple 6 [Source:MGI Symbol;Acc:MGI:1858599]	0.24925	0.650321	0.898636	0.485312	0.50452	0.676358	0.457085	0.630195	0.746673	0.462949	0.516568	0.434994	0.527374	0.8067	0.110888527	0.528581815	12	4	24	0.077357951	0.687324958	24	7.481556014	0.133535213
ENSMUSG00000000409	Lck	lymphocyte protein tyrosine kinase [Source:MGI Symbol;Acc:MGI:96756]	1.36542	0.713766	1.15435	1.68814	1.39307	1.63341	0.959471	1.16074	0.926664	0.84869	0.708698	1.014	1.09779	1.6404	0.761468226	0.966018836	24	20	24	0.91868954	0.978028197	24	20.34880272	0.03835504
ENSMUSG00000000416	Cttnbp2	cortactin binding protein 2 [Source:MGI Symbol;Acc:MGI:1353467]	7.94269	7.88824	7.63888	7.509	8.6599	7.26489	10.036	6.22796	8.96524	6.47325	7.86513	6.94454	6.97065	6.45782	0.92769718	0.966018836	20	12	24	0.810284888	0.939224719	24	19.07386319	0.043069396
ENSMUSG00000000420	Galnt1	polypeptide N-acetylgalactosaminyltransferase 1 [Source:MGI Symbol;Acc:MGI:894693]	19.3752	21.0288	23.4442	22.2903	21.72	20.7475	21.7159	19.9031	22.0588	20.9126	21.642	20.808	21.4611	21.4511	0.106193509	0.519440352	12	20	24	0.126716215	0.739868076	24	10.79386742	0.05006061
ENSMUSG00000000439	Mkrn2	makorin, ring finger protein, 2 [Source:MGI Symbol;Acc:MGI:1914277]	9.07413	9.06043	8.40989	8.01489	8.17004	10.0142	7.73289	9.14346	8.30079	8.61389	8.11889	9.86726	8.35347	9.13116	0.344645202	0.86981408	24	20	24	0.393523308	0.799546792	24	22.97168949	0.054431691
ENSMUSG00000000440	Pparg	peroxisome proliferator activated receptor gamma [Source:MGI Symbol;Acc:MGI:97747]	2.09171	1.69036	3.1041	6.63501	2.38854	2.14728	3.03539	3.38958	3.27077	4.09215	5.41637	3.67045	4.70191	4.34571	0.787022682	0.966018836	16	16	24	0.138172978	0.747972781	24	11.84626483	0.261158922
ENSMUSG00000000441	Raf1	v-raf-leukemia viral oncogene 1 [Source:MGI Symbol;Acc:MGI:97847]	36.1453	34.4992	33.8125	35.88	39.29	38.8245	35.5059	35.4775	32.8405	37.0757	33.4917	33.363	35.2059	37.6197	0.834434272	0.966018836	16	20	24	0.581853183	0.85096264	24	18.75892138	0.033508035
ENSMUSG00000000486	Septin1	septin 1 [Source:MGI Symbol;Acc:MGI:1858916]	3.91366	4.41896	5.72229	5.27973	5.66247	4.86215	5.68812	5.43925	5.13065	4.67699	5.75541	4.46739	5.58662	4.93357	0.965971228	0.969272194	20	4	24	0.577035287	0.848822643	24	11.77163933	0.057787507
ENSMUSG00000000489	Pdgfb	platelet derived growth factor, B polypeptide [Source:MGI Symbol;Acc:MGI:97528]	2.26587	2.86075	3.11248	2.54772	2.55486	2.54156	2.54445	4.06672	2.9249	3.84186	2.69529	2.70592	3.24672	3.01413	0.246839642	0.745922296	8	16	24	0.071088319	0.676901434	24	7.44781252	0.143339846
ENSMUSG00000000530	Acvrl1	activin A receptor, type II-like 1 [Source:MGI Symbol;Acc:MGI:1338946]	4.17689	4.09541	3.57653	4.17649	4.42849	3.47076	3.66572	4.9375	4.77705	4.39919	4.50445	3.9426	3.95559	3.40127	0.833406028	0.966018836	20	4	24	0.443873869	0.805393068	24	11.35228272	0.067904106
ENSMUSG00000000531	Tamalin	trafficking regulator and scaffold protein tamalin [Source:MGI Symbol;Acc:MGI:1860303]	1.4192	1.95126	1.59054	1.8312	1.40245	1.64395	1.0355	2.01711	1.66722	1.95854	1.38906	1.72201	2.1103	1.60086	0.942429894	0.966018836	8	12	24	0.44310057	0.805393068	24	7.627299808	0.085198918
ENSMUSG00000000532	Acvr1b	activin A receptor, type 1B [Source:MGI Symbol;Acc:MGI:1338944]	9.40757	8.34625	9.33022	8.97701	8.05419	9.34197	8.99312	9.37512	7.80758	12.7375	7.93811	9.73136	8.5901	9.48711	0.961261938	0.967043964	24	20	24	0.912549697	0.975319419	24	10.77291021	0.027811242
ENSMUSG00000000538	Tom1l2	target of myb1-like 2 (chicken) [Source:MGI Symbol;Acc:MGI:2443306]	64.4274	56.0403	57.7824	60.0373	59.5427	65.1993	61.9523	64.0593	50.5788	75.4393	53.1274	61.6333	58.1767	60.2651	0.390686537	0.915479136	24	12	24	0.793625972	0.933825946	24	19.3780338	0.039232033
ENSMUSG00000000552	Zfp385a	zinc finger protein 385A [Source:MGI Symbol;Acc:MGI:1352495]	6.74284	6.93487	7.4904	6.85838	5.52865	6.46246	7.83903	8.85343	9.25338	10.2111	7.01909	7.9112	6.95075	7.90518	0.409451058	0.94909271	12	8	24	0.064077908	0.663876076	24	7.473848745	0.163030249
ENSMUSG00000000555	Itga5	integrin alpha 5 (fibronectin receptor alpha) [Source:MGI Symbol;Acc:MGI:96604]	1.33131	1.86474	2.24433	1.89832	2.1504	1.72343	1.60529	1.85665	1.92061	1.47593	1.71635	1.59532	1.66026	1.65742	0.055920623	0.401074772	12	16	24	0.199393251	0.768040178	24	10.26281249	0.086590524
ENSMUSG00000000560	Gabra2	gamma-aminobutyric acid (GABA) A receptor, subunit alpha 2 [Source:MGI Symbol;Acc:MGI:95614]	11.7545	10.9436	12.0833	10.0647	10.4266	10.46	11.5617	9.17842	10.5416	9.29014	10.7536	10.388	10.1016	10.7382	0.942429894	0.966018836	4	12	24	0.525156674	0.822381894	24	0.142803607	0.041911354
ENSMUSG00000000561	Wdr77	WD repeat domain 77 [Source:MGI Symbol;Acc:MGI:1917715]	18.9521	18.515	19.1589	18.7876	19.9389	19.5695	19.387	18.2007	17.8968	21.3139	17.2031	19.475	18.1909	19.3299	0.622132989	0.966018836	16	20	24	0.727563148	0.910646611	24	15.66745245	0.022925258
ENSMUSG00000000563	Atp5pb	ATP synthase peripheral stalk-membrane subunit b [Source:MGI Symbol;Acc:MGI:1100495]	89.3132	94.752	93.6429	94.3754	91.0476	83.1068	91.9903	92.7776	99.4029	86.1537	97.2572	88.2492	96.1687	94.3758	0.148073804	0.590732968	12	12	24	0.15653254	0.762206981	24	7.143119157	0.05181609
ENSMUSG00000000567	Sox9	SRY (sex determining region Y)-box 9 [Source:MGI Symbol;Acc:MGI:98371]	0.946118	1.29327	1.89721	0.977726	1.00618	1.13342	1.59556	1.1378	1.33034	0.848368	1.23939	1.30726	1.05051	1.25785	0.129903614	0.560263689	12	4	24	0.487959052	0.805393068	24	4.660929313	0.081713171
ENSMUSG00000000568	Hnrnpd	heterogeneous nuclear ribonucleoprotein D [Source:MGI Symbol;Acc:MGI:101947]	32.648	38.8502	42.0934	41.3092	32.6416	35.7628	34.0124	37.6669	38.7873	41.7923	30.1188	37.4179	36.4291	40.5403	0.002094047	0.100255953	16	4	24	0.020079921	0.565108098	24	7.85558913	0.146379519
ENSMUSG00000000579	Dynlt1c	dynein light chain Tctex-type 1C [Source:MGI Symbol;Acc:MGI:3807476]	2.33643	1.98542	2.87254	2.13985	3.0029	1.57274	2.12145	3.30684	3.92476	2.95164	3.15269	1.78445	2.74711	3.32824	0.133266423	0.567728672	12	12	24	0.035262964	0.608551791	24	8.553942498	0.245826388
ENSMUSG00000000581	C1d	C1D nuclear receptor co-repressor [Source:MGI Symbol;Acc:MGI:1927354]	50.0894	57.0554	59.7558	57.3759	55.0512	47.5681	54.5742	53.5865	69.3164	45.4735	60.9309	55.1919	56.8085	59.5658	0.148073804	0.590732968	12	12	24	0.279247013	0.768040178	24	7.701014795	0.095215296
ENSMUSG00000000594	Gm2a	GM2 ganglioside activator protein [Source:MGI Symbol;Acc:MGI:95762]	14.1007	16.1299	17.0225	15.0182	15.537	16.0824	17.6232	16.7333	16.4407	16.0198	14.944	15.7568	15.1539	15.5228	0.581070934	0.966018836	12	8	24	0.453334042	0.805393068	24	6.106130271	0.040390143
ENSMUSG00000000600	Krit1	KRIT1, ankyrin repeat containing [Source:MGI Symbol;Acc:MGI:1930618]	24.5452	21.5145	20.7605	21.968	23.2172	20.8137	23.9936	19.0687	21.2718	16.7245	24.4857	19.9092	21.3092	19.4655	0.365996436	0.893596186	4	4	24	0.223308203	0.768040178	24	20.40090879	0.092785392
ENSMUSG00000000605	Clcn4	chloride channel, voltage-sensitive 4 [Source:MGI Symbol;Acc:MGI:104571]	42.6683	42.0032	40.8338	40.381	40.7339	42.5903	42.5137	39.3021	37.4731	43.9148	41.9135	41.9756	41.1832	43.4457	0.649212555	0.966018836	4	8	24	0.376701211	0.796713484	24	20.56063595	0.033193075
ENSMUSG00000000627	Sema4f	sema domain, immunoglobulin domain (Ig), TM domain, and short cytoplasmic domain [Source:MGI Symbol;Acc:MGI:1340055]	15.3879	11.7695	11.8333	13.3796	12.6016	14.6593	12.4152	14.6955	9.91363	17.3532	12.7033	13.3027	13.4712	13.3416	0.628286596	0.966018836	24	12	24	0.597240349	0.858318759	24	19.24588304	0.076054561
ENSMUSG00000000628	Hk2	hexokinase 2 [Source:MGI Symbol;Acc:MGI:1315197]	3.12465	5.02251	5.08096	13.7547	7.69446	5.93099	5.76028	9.16113	8.33891	12.157	10.7533	6.63541	7.89059	6.72772	0.032263213	0.324385159	16	12	24	0.005204627	0.4424469	24	12.28563638	0.466038715
ENSMUSG00000000631	Myo18a	myosin XVIIIA [Source:MGI Symbol;Acc:MGI:2667185]	33.6242	29.454	30.576	30.6356	29.1366	32.8961	30.0014	36.1458	24.5323	42.3718	27.8154	32.702	30.6133	31.4799	0.869721097	0.966018836	24	12	24	0.977657534	0.994290879	24	20.02326761	0.015982581
ENSMUSG00000000632	Sez6	seizure related gene 6 [Source:MGI Symbol;Acc:MGI:104745]	5.54933	4.75551	5.48846	4.36484	4.87567	5.03008	5.25546	4.67399	4.32514	6.17645	3.60732	4.62459	4.01395	5.24002	0.860560779	0.966018836	16	4	24	0.803557271	0.937199097	24	5.15321625	0.043094325
ENSMUSG00000000673	Haao	3-hydroxyanthranilate 3,4-dioxygenase [Source:MGI Symbol;Acc:MGI:1349444]	0.344492	0.764146	0.675792	0.836878	0.662906	0.720188	0.450563	0.910445	1.19915	0.532648	0.414349	0.682123	0.767633	0.521081	0.671726933	0.966018836	12	8	24	0.291351956	0.768040178	24	8.202210125	0.119009577
ENSMUSG00000000682	Cd52	CD52 antigen [Source:MGI Symbol;Acc:MGI:1346088]	13.3525	22.1301	28.8587	21.6067	19.2365	17.7838	16.2305	18.5542	26.0495	11.0621	18.0358	17.0346	19.4329	15.2758	0.122530972	0.550724475	12	16	24	0.415381268	0.800626647	24	8.22374284	0.178222089
ENSMUSG00000000686	Abhd15	abhydrolase domain containing 15 [Source:MGI Symbol;Acc:MGI:1914727]	0.545791	0.581722	0.681671	1.0252	0.610035	0.664633	0.512396	0.619339	0.789956	0.910028	0.856516	0.811108	0.794744	0.826126	0.017417473	0.255326033	16	12	24	0.010118137	0.503967859	24	12.07398326	0.109866603
ENSMUSG00000000690	Hoxb6	homeobox B6 [Source:MGI Symbol;Acc:MGI:96187]	5.58309	5.18829	4.98788	4.96167	4.62373	4.96785	6.81542	5.682	5.87528	5.13332	5.8745	5.66621	5.64682	6.24493	0.268178777	0.774280302	4	12	24	0.068071903	0.672292246	24	1.972209395	0.09286066
ENSMUSG00000000693	Loxl3	lysyl oxidase-like 3 [Source:MGI Symbol;Acc:MGI:1337004]	4.26242	5.57863	5.06582	4.6026	5.11932	5.12571	4.35373	5.12423	6.38615	4.58652	3.72049	3.92187	5.01849	4.38789	0.339853834	0.859092804	12	8	24	0.172143096	0.768040178	24	6.878670854	0.11993735
ENSMUSG00000000708	Kat2b	K(lysine) acetyltransferase 2B [Source:MGI Symbol;Acc:MGI:1343094]	14.6955	15.2947	15.8015	16.5814	14.8981	14.2358	15.0244	13.9969	17.4402	15.0709	14.1412	14.7448	14.3025	13.6001	0.146343412	0.590732968	12	20	24	0.079051421	0.690902773	24	10.26965254	0.071093044
ENSMUSG00000000711	Rab5b	RAB5B, member RAS oncogene family [Source:MGI Symbol;Acc:MGI:105938]	53.3961	46.6807	47.4662	49.0676	47.6276	51.3198	50.1688	51.4794	44.2559	54.6115	46.4162	51.4272	51.4786	50.1302	0.267533088	0.774280302	4	8	24	0.371820463	0.793398709	24	21.87424696	0.046662378
ENSMUSG00000000732	Icosl	icos ligand [Source:MGI Symbol;Acc:MGI:1354701]	1.46182	1.94375	2.01424	2.30245	2.42186	2.02138	1.97373	1.87692	2.32046	2.22052	2.10928	2.13661	1.78981	1.8594	0.002010614	0.100255953	20	8	24	0.003067443	0.414576087	24	12.98795079	0.130989822
ENSMUSG00000000738	Spg7	SPG7, paraplegin matrix AAA peptidase subunit [Source:MGI Symbol;Acc:MGI:2385906]	23.0438	20.7733	19.826	22.2579	20.3433	22.8784	20.9704	23.1222	21.298	23.6613	20.7948	20.6753	20.6147	21.3571	0.907068194	0.966018836	16	4	24	0.998442328	0.999730606	24	11.89334835	0.001746328
ENSMUSG00000000739	Sult5a1	sulfotransferase family 5A, member 1 [Source:MGI Symbol;Acc:MGI:1931463]	1.5905	2.39033	2.713	3.20148	3.15729	3.26038	2.12056	2.5417	2.57071	1.89589	3.45671	3.25097	2.50951	1.87004	0.006176201	0.16812024	24	4	24	0.06929976	0.673520298	24	15.75828619	0.205464948
ENSMUSG00000000740	Rpl13	ribosomal protein L13 [Source:MGI Symbol;Acc:MGI:105922]	20.1877	18.2477	18.1481	16.5204	17.495	17.2362	15.9609	17.7434	21.7723	14.7423	18.9974	18.9441	18.8864	16.0157	0.879206539	0.966018836	12	4	24	0.817323175	0.941863087	24	0.615176254	0.035208781
ENSMUSG00000000743	Chmp1a	charged multivesicular body protein 1A [Source:MGI Symbol;Acc:MGI:1920159]	44.3312	39.4711	41.272	41.7759	39.6263	43.4911	39.7425	44.3049	38.6001	43.3497	43.5079	42.3055	43.1368	41.7869	0.648082424	0.966018836	24	12	24	0.576913822	0.848735805	24	20.3396453	0.027849252
ENSMUSG00000000751	Rpa1	replication protein A1 [Source:MGI Symbol;Acc:MGI:1915525]	36.9869	31.457	31.2593	31.2818	28.5732	33.7781	34.0625	33.625	28.3061	37.6121	30.4983	33.8764	32.9953	33.5807	0.091160192	0.483721897	4	16	24	0.381388576	0.799546792	24	23.52096819	0.063312722
ENSMUSG00000000753	Serpinf1	serine (or cysteine) peptidase inhibitor, clade F, member 1 [Source:MGI Symbol;Acc:MGI:108080]	82.0498	70.5214	66.6775	69.8157	75.1673	67.6339	71.4562	78.6825	83.1679	58.3086	63.9015	62.1098	65.6201	63.8806	0.835401638	0.966018836	12	4	24	0.444365851	0.805393068	24	3.097010446	0.064418437
ENSMUSG00000000759	Tubgcp3	tubulin, gamma complex associated protein 3 [Source:MGI Symbol;Acc:MGI:2183752]	18.1423	18.196	17.5295	18.7176	18.4805	17.1368	17.5736	18.0547	19.7584	17.2073	18.8264	18.2901	17.9397	18.9455	0.697196105	0.966018836	12	16	24	0.513834063	0.816381038	24	9.014413995	0.025054646
ENSMUSG00000000766	Oprm1	opioid receptor, mu 1 [Source:MGI Symbol;Acc:MGI:97441]	2.25976	1.88108	2.32427	2.42622	2.83363	2.1609	1.93075	1.54739	2.34906	0.829506	2.99233	2.51317	2.94401	2.90504	0.702630284	0.966018836	20	12	24	0.626244436	0.872596805	24	20.45487671	0.119772937
ENSMUSG00000000776	Polr3d	polymerase (RNA) III (DNA directed) polypeptide D [Source:MGI Symbol;Acc:MGI:1914315]	8.71451	7.67796	6.96559	8.26875	8.10768	7.77147	7.32657	8.13397	8.18701	8.75342	7.54342	9.00073	8.40945	8.60595	0.880631923	0.966018836	24	12	24	0.909592099	0.974606327	24	19.90834506	0.016044271

ENSMUSG00000000782	Tcf7	transcription factor 7, T cell specific [Source:MGI Symbol;Acc:MGI:98507]	0.765872	1.21757	0.552016	0.929999	0.966091	0.660255	1.06681	1.14094	1.29648	1.09334	0.851022	0.849299	0.764866	0.753459	0.725061903	0.966018836	8	16	24	0.564378327	0.843106073	24	8.006900027	0.073178933
ENSMUSG00000000787	Ddx3x	DEAD box helicase 3, X-linked [Source:MGI Symbol;Acc:MGI:103064]	92.2869	102.514	101.264	99.7247	99.357	91.324	92.9339	91.8	101.628	92.2694	103.93	96.1724	98.7107	99.6857	0.387380622	0.915479136	12	12	24	0.30370157	0.768040178	24	10.10812824	0.0403483
ENSMUSG00000000792	Slc5a5	solute carrier family 5 (sodium iodide symporter), member 5 [Source:MGI Symbol;Acc:MGI:2149330]	4.68067	3.14826	2.8823	2.82485	2.82526	4.07326	3.65772	4.16983	2.94063	3.90398	3.09565	3.36046	3.20159	3.72603	0.080635668	0.459861248	4	16	24	0.14087432	0.751014756	24	23.84921605	0.13583717
ENSMUSG00000000794	Kcnn3	potassium intermediate/small conductance calcium-activated channel, subfamily N, member 3 [Source:MGI Symbol;Acc:MGI:2153183]	0.8162	1.11257	1.45973	0.847468	0.912883	1.02984	1.04331	0.873958	1.05745	1.01813	0.9274	1.25597	0.697861	1.54687	0.519468828	0.966018836	12	16	24	0.461989607	0.805393068	24	7.085294303	0.0826123
ENSMUSG00000000804	Usp32	ubiquitin specific peptidase 32 [Source:MGI Symbol;Acc:MGI:2144475]	37.7467	33.5037	33.076	33.2557	34.5382	33.772	33.982	29.0906	29.2923	32.657	32.9144	34.8245	35.2665	34.6143	0.008389425	0.192760771	4	8	24	0.065640136	0.667622064	24	20.79940809	0.082741119
ENSMUSG00000000805	Car4	carbonic anhydrase 4 [Source:MGI Symbol;Acc:MGI:1096574]	3.64656	4.253	4.17915	4.17018	2.61212	4.27648	4.47254	6.18945	4.76207	4.29809	4.84207	4.05344	4.88819	3.90145	0.832293447	0.966018836	8	16	24	0.270320318	0.768040178	24	4.945379214	0.130570844
ENSMUSG00000000811	Txnrd3	thioredoxin reductase 3 [Source:MGI Symbol;Acc:MGI:2386711]	4.85686	4.54585	5.20578	4.9896	4.82977	4.9409	4.10959	4.75266	4.6237	5.74011	4.36844	4.60411	4.62914	5.07069	0.428730128	0.966018836	16	12	24	0.171254205	0.768040178	24	10.67888117	0.069614877
ENSMUSG00000000823	Zfp512b	zinc finger protein 512B [Source:MGI Symbol;Acc:MGI:2685478]	11.8621	14.539	14.1094	12.1623	14.5202	13.0101	12.0074	14.3909	15.6344	14.3002	14.5031	16.2691	14.195	15.6696	0.949874888	0.966018836	8	20	24	0.434946936	0.805393068	24	8.892223564	0.056498495
ENSMUSG00000000826	Dnajc5	DnaJ heat shock protein family (Hsp40) member C5 [Source:MGI Symbol;Acc:MGI:892995]	52.0224	43.1728	48.7615	44.7741	43.6774	52.458	49.9056	48.2352	37.9975	57.5836	42.5121	49.8779	46.7385	50.922	0.777666236	0.966018836	24	20	24	0.630361482	0.873885049	24	21.96426695	0.059408837
ENSMUSG00000000827	Tpd52l2	tumor protein D52-like 2 [Source:MGI Symbol;Acc:MGI:1913564]	70.8455	68.1012	69.2777	70.2333	74.0648	70.0651	70.402	68.4536	70.8023	60.8186	76.5785	69.7534	71.859	70.5808	0.339604637	0.858712466	20	20	24	0.418628003	0.803070657	24	19.86307246	0.038999962
ENSMUSG00000000838	Fmr1	FMRP translational regulator 1 [Source:MGI Symbol;Acc:MGI:95564]	26.8961	29.3339	28.4788	26.207	28.4946	25.8029	27.3529	25.6248	25.1975	23.7256	27.7482	21.7618	20.7486	23.3888	0.914502381	0.966018836	20	4	24	0.615193352	0.866892532	24	11.85720634	0.036157263
ENSMUSG00000000861	Bcl11a	B cell CLL/lymphoma 11A (zinc finger protein) [Source:MGI Symbol;Acc:MGI:106190]	1.30421	1.4673	1.92389	1.04132	1.23689	1.55533	1.12774	1.12931	1.57432	1.29143	1.30704	1.50402	1.18561	1.31409	0.745118544	0.966018836	12	16	24	0.831856386	0.947968019	24	8.342940334	0.034804988
ENSMUSG00000000869	Il4	interleukin 4 [Source:MGI Symbol;Acc:MGI:96556]	7.49875	6.35625	6.78026	8.39917	7.21189	8.1257	6.82014	7.70764	7.03367	6.54674	7.3564	5.92054	5.95667	5.85187	0.848840613	0.966018836	20	12	24	0.288362045	0.768040178	24	15.07840211	0.073464149
ENSMUSG00000000876	Pxmp4	peroxisomal membrane protein 4 [Source:MGI Symbol;Acc:MGI:1891701]	6.83767	7.24484	7.4966	7.62117	6.65577	7.10836	8.02608	8.89324	7.6531	9.30653	7.33808	7.61428	7.73676	7.69881	0.598711142	0.966018836	16	4	24	0.212447113	0.768040178	24	7.672143866	0.07347679
ENSMUSG00000000881	Dlg3	discs large MAGUK scaffold protein 3 [Source:MGI Symbol;Acc:MGI:1888986]	5.7546	6.70637	6.51269	6.89022	7.1977	6.09867	6.89435	5.69243	7.45186	6.52121	5.60447	6.61357	5.55727	5.94567	0.649148181	0.966018836	16	12	24	0.293570469	0.768040178	24	11.57897196	0.070290049
ENSMUSG00000000884	Gnb1l	guanine nucleotide binding protein (G protein), beta polypeptide 1-like [Source:MGI Symbol;Acc:MGI:1338057]	2.13069	2.42065	1.9116	1.50909	1.96858	2.14651	2.39727	2.11839	1.83142	1.92511	1.7928	2.42731	2.51643	2.10552	0.004196064	0.140598674	4	12	24	8.11E-04	0.319044294	24	23.88588514	0.154856576
ENSMUSG00000000889	Dbh	dopamine beta hydroxylase [Source:MGI Symbol;Acc:MGI:94864]	0.0331425	0.0843588	1.17233	2.61903	0.598977	4.93126	6.6244	0.0297907	0.0340377	4.51976	4.41827	2.83985	0.011001	14.4083	0.95261238	0.966018836	24	4	24	0.650968485	0.882698308	24	16.02947154	0.4445185
ENSMUSG00000000901	Mmp11	matrix metallopeptidase 11 [Source:MGI Symbol;Acc:MGI:97008]	1.90999	2.56904	1.72609	1.69213	1.21602	1.63978	2.0978	2.41396	2.50316	2.00919	2.75283	3.06738	2.09424	3.34519	0.351315142	0.870418653	8	8	24	0.158950733	0.765350752	24	3.937440961	0.153410729
ENSMUSG00000000902	Smarcb1	SWI/SNF related, matrix associated, actin dependent regulator of chromatin, subfamily b, member 1 [Source:MGI Symbol;Acc:MGI:1328366]	20.1165	18.8592	17.8421	19.9475	18.9033	19.3086	18.7118	19.2218	18.6216	21.847	18.1014	21.0115	19.353	19.0061	0.245061943	0.741580974	24	12	24	0.716323144	0.90635549	24	17.38566081	0.025164787
ENSMUSG00000000903	Vpreb3	pre-B lymphocyte gene 3 [Source:MGI Symbol;Acc:MGI:98938]	1.20959	1.6131	1.59569	2.3627	1.28445	0.92527	1.26208	0.616134	1.88274	1.08509	1.41841	1.66154	2.28443	1.10872	0.88067614	0.966018836	12	20	24	0.76726011	0.924784773	24	11.96291399	0.085098167
ENSMUSG00000000915	Hip1r	huntingtin interacting protein 1 related [Source:MGI Symbol;Acc:MGI:1352504]	11.005	9.77288	11.1104	10.8673	10.458	9.7458	10.6742	12.0803	10.1425	11.0894	11.1543	12.0726	10.5137	10.3395	0.7866493	0.966018836	16	20	24	0.935461519	0.983819603	24	14.96601779	0.012492645
ENSMUSG00000000916	Nsun5	NOL1/NOP2/Sun domain family, member 5 [Source:MGI Symbol;Acc:MGI:2140844]	7.56714	7.7044	7.96954	7.98311	7.16969	7.54226	7.45307	7.9703	7.97337	6.86003	8.38341	7.12483	7.31305	7.90087	0.204218286	0.690546825	12	12	24	0.407561205	0.799546792	24	7.355933618	0.038683403
ENSMUSG00000000934	Top1mt	DNA topoisomerase 1, mitochondrial [Source:MGI Symbol;Acc:MGI:1920210]	5.123	5.07268	5.31519	3.72575	4.74916	4.6892	4.81356	5.48086	4.0045	5.08898	5.34475	5.00465	5.0817	4.78554	0.91241026	0.966018836	8	8	24	0.434827907	0.805393068	24	23.7709254	0.064566148
ENSMUSG00000000938	Hoxa10	homeobox A10 [Source:MGI Symbol;Acc:MGI:96171]	8.39474	9.60938	9.71253	8.36301	7.44461	9.24443	9.91675	8.33225	7.86333	8.22983	7.09685	8.45354	6.7925	8.4562	0.644063931	0.966018836	8	12	24	0.39509053	0.799546792	24	3.407282822	0.067038291
ENSMUSG00000000942	Hoxa4	homeobox A4 [Source:MGI Symbol;Acc:MGI:96176]	1.93911	1.38118	1.20565	1.30765	1.03793	1.40519	1.62084	1.61635	1.6458	1.55524	1.54584	1.67297	1.61408	1.52168	0.287843582	0.793714531	4	16	24	0.153631926	0.760223561	24	0.844570018	0.091102882
ENSMUSG00000000948	Gm38393	predicted gene, 38393 [Source:MGI Symbol;Acc:MGI:5613898]	6.51244	9.50355	7.5037	8.39627	10.2327	6.08557	8.26077	6.13869	7.34959	4.34644	9.4381	5.06559	4.82537	6.88533	0.503972924	0.966018836	20	4	24	0.782874758	0.930229965	24	12.66847006	0.082128521
ENSMUSG00000000957	Mmp14	matrix metallopeptidase 14 (membrane-inserted) [Source:MGI Symbol;Acc:MGI:101900]	11.8629	11.714	8.35036	9.3335	9.84032	10.4953	12.178	12.2244	8.90124	8.67021	9.25952	10.8533	12.2129	9.95641	3.15E-04	0.049770315	4	8	24	1.63E-04	0.140635355	24	23.75925709	0.218465993
ENSMUSG00000000958	Slc7a7	solute carrier family 7 (cationic amino acid transporter, y+ system), member 7 [Source:MGI Symbol;Acc:MGI:1337120]	35.3354	23.9223	24.4058	22.6959	27.4416	29.8749	25.607	28.3995	17.4485	27.5632	27.7433	26.2627	25.6217	25.3323	0.077301512	0.453790923	24	12	24	0.054497717	0.655263328	24	20.71047507	0.194009318
ENSMUSG00000000959	Oxa1l	oxidase assembly 1-like [Source:MGI Symbol;Acc:MGI:1916339]	15.4584	16.3095	16.8085	17.4351	16.8371	15.8293	17.8149	17.1395	16.7153	19.9422	15.3723	17.3006	16.3726	15.9197	0.718720526	0.966018836	16	4	24	0.337683408	0.772537663	24	11.04033591	0.052734058
ENSMUSG00000000976	Heatr6	HEAT repeat containing 6 [Source:MGI Symbol;Acc:MGI:1919790]	6.48344	5.3829	6.48798	5.56091	5.75226	6.53462	6.93121	6.61876	5.36469	6.8164	5.7843	6.64801	6.32207	6.04854	0.483881213	0.966018836	24	12	24	0.337687198	0.772537663	24	22.28418497	0.063354411
ENSMUSG00000001016	Ilf2	interleukin enhancer binding factor 2 [Source:MGI Symbol;Acc:MGI:1915031]	36.014	35.5566	36.9953	34.5427	34.5895	34.492	32.2912	32.2902	34.8519	32.8784	34.2604	34.7376	32.6252	35.2594	0.892044196	0.966018836	12	4	24	0.809443076	0.939184036	24	9.571769743	0.014015575
ENSMUSG00000001017	Chtop	chromatin target of PRMT1 [Source:MGI Symbol;Acc:MGI:1913761]	53.3321	48.4408	50.4403	48.5123	48.6037	48.2132	52.0007	48.8227	48.6167	51.3609	49.9814	52.277	51.9375	51.678	0.249881062	0.749643185	4	12	24	0.269473977	0.768040178	24	23.49919553	0.029778238
ENSMUSG00000001018	Snapin	SNAP-associated protein [Source:MGI Symbol;Acc:MGI:1333745]	58.5291	64.6722	62.0493	63.8503	58.8716	60.9118	55.2893	62.8458	65.6368	63.8381	63.4651	65.4173	65.3416	66.1365	0.760769894	0.966018836	20	24	24	0.150236617	0.758865539	24	8.626925973	0.048771362
ENSMUSG00000001020	S100a4	S100 calcium binding protein A4 [Source:MGI Symbol;Acc:MGI:1330282]	101.74	126.593	121.119	114.9	133.165	97.1897	104.822	99.6154	139.814	78.1821	113.284	96.9123	113.815	106.623	0.085890569	0.466404237	12	12	24	0.710498293	0.90429771	24	7.954338907	0.071964736
ENSMUSG00000001021	S100a3	S100 calcium binding protein A3 [Source:MGI Symbol;Acc:MGI:1338849]	1.13246	0.467367	0.961672	0.259381	0.573167	0.506697	0.767502	0.295886	0.408783	0.357627	0.463866	0.404662	0.616676	0.365629	0.036134798	0.331852526	4	12	24	0.227952248	0.768040178	24	23.68883021	0.129999499
ENSMUSG00000001025	S100a6	S100 calcium binding protein A6 (calcyclin) [Source:MGI Symbol;Acc:MGI:1339467]	1077.33	1200.74	1114.61	1219.31	1419.49	1300.37	1051.64	1142.04	1164.75	914.715	1176.91	990.903	1105.64	986.109	0.885025617	0.966018836	20	8	24	0.428517097	0.805393068	24	16.75704568	0.076020595
ENSMUSG00000001027	Scn4a	sodium channel, voltage-gated, type IV, alpha [Source:MGI Symbol;Acc:MGI:98250]	1.04456	1.61499	2.04715	2.33346	1.65638	2.10115	1.40556	4.43401	2.93421	5.3691	1.24882	1.61838	2.0374	1.75222	0.101443845	0.511659635	16	12	24	0.094041071	0.71175955	24	9.2972581	0.374984207
ENSMUSG00000001029	Icam2	intercellular adhesion molecule 2 [Source:MGI Symbol;Acc:MGI:96394]	3.35575	3.38473	3.00197	2.58488	2.40892	3.06132	3.55106	4.73916	3.62104	2.60513	2.42438	2.58591	3.09035	2.25287	0.05945106	0.406067671	8	12	24	0.060408634	0.65893609	24	3.113955375	0.18098365
ENSMUSG00000001034	Mapk7	mitogen-activated protein kinase 7 [Source:MGI Symbol;Acc:MGI:1346347]	9.9123	8.04452	6.79671	7.80211	8.62059	8.1194	8.73401	8.4954	8.35632	9.04135	8.73341	8.5198	8.47547	7.90401	0.339274762	0.858712466	20	16	24	0.229945054	0.768040178	24	20.5031267	0.074468152
ENSMUSG00000001036	Epn2	epsin 2 [Source:MGI Symbol;Acc:MGI:1333766]	22.089	19.9714	20.9612	20.9382	20.5691	22.0126	21.5014	20.6227	18.5239	24.8063	20.7423	21.9043	18.9061	20.3117	0.454881285	0.966018836	24	8	24	0.360130872	0.785733735	24	16.64249113	0.054796649
ENSMUSG00000001039	B9d1	B9 protein domain 1 [Source:MGI Symbol;Acc:MGI:1351471]	8.53062	9.75584	10.1448	9.12372	10.5526	9.55571	10.6432	9.79284	9.93686	8.68955	10.3016	9.52599	9.98804	9.75904	0.883051071	0.966018836	20	20	24	0.974356242	0.993545331	24	23.22525063	0.007891582
ENSMUSG00000001052	Sec24b	Sec24 related gene family, member B (S. cerevisiae) [Source:MGI Symbol;Acc:MGI:2139764]	17.185	17.9366	18.0623	15.9104	17.5901	17.6615	16.7165	18.0151	14.9273	18.2247	18.8405	18.0374	20.3479	18.1778	0.804027134	0.966018836	8	4	24	0.469849157	0.805393068	24	21.91790497	0.046440459
ENSMUSG00000001053	N4bp3	NEDD4 binding protein 3 [Source:MGI Symbol;Acc:MGI:2442218]	1.22045	1.11567	1.19269	0.974357	0.960263	1.09296	1.2737	1.52362	1.66036	1.59014	1.34962	1.46898	1.15703	1.3593	0.886838024	0.966018836	12	8	24	0.31327166	0.768040178	24	6.890302855	0.072474654
ENSMUSG00000001054	Rmnd5b	required for meiotic nuclear division 5 homolog B [Source:MGI Symbol;Acc:MGI:1913339]	15.9844	13.3439	12.2223	12.7443	12.92	15.7455	12.6026	13.5909	13.0055	13.4247	13.9327	14.5243	13.841	13.2865	0.020957534	0.262822894	24	12	24	0.037869482	0.61140397	24	21.01219829	0.099569605
ENSMUSG00000001056	Nhp2	NHP2 ribonucleoprotein [Source:MGI Symbol;Acc:MGI:1098547]	22.6044	22.5863	22.1911	21.9406	23.2529	20.6361	21.7857	24.2934	23.6786	19.6354	27.3149	24.1791	27.2104	24.8518	0.89563591	0.966018836	8	8	24	0.684258697	0.895093979	24	0.468569691	0.038323202
ENSMUSG00000001062	Vps9d1	VPS9 domain containing 1 [Source:MGI Symbol;Acc:MGI:1914143]	8.46095	8.28614	7.75547	8.5375	8.68726	8.24855	9.25288	8.96626	8.82374	11.8309	9.00148	8.57731	8.59618	9.43979	0.860709018	0.966018836	20	4	24	0.476460852	0.805393068	24	11.35051338	0.05501336
ENSMUSG00000001065	Zfp276	zinc finger protein (C2H2 type) 276 [Source:MGI Symbol;Acc:MGI:1888495]	14.2796	12.0641	14.12	19.1303	15.0526	11.8526	14.5917	11.9318	17.5006	16.1618	16.3387	12.3158	12.9715	11.747	0.008483961	0.192760771	16	16	24	0.015124025	0.537924552	24	12.53205186	0.207583305
ENSMUSG00000001076	C1ql4	complement component 1, q subcomponent-like 4 [Source:MGI Symbol;Acc:MGI:3579909]	2.9339	2.35411	2.50099	2.57579	2.63532	2.10215	2.43836	2.98572	2.27555	2.99675	3.02356	2.88523	3.41834	2.83103	0.945282252	0.966018836	4	8	24	0.921098981	0.979223229	24	22.37873495	0.020559865
ENSMUSG00000001082	Mfsd10	major facilitator superfamily domain containing 10 [Source:MGI Symbol;Acc:MGI:1915544]	13.1793	13.8983	12.1855	14.0468	14.7757	12.0753	13.9984	13.9779	13.9464	14.9074	15.672	14.2039	12.9131	12.9334	0.125880375	0.555618842	20	8	24	0.180214702	0.768040178	24	14.16702876	0.067902003
ENSMUSG00000001089	Luzp1	leucine zipper protein 1 [Source:MGI Symbol;Acc:MGI:107629]	19.9833	16.7696	18.0257	15.4442	17.0824	18.4463	17.8909	15.745	14.3966	17.2632	16.55	17.0333	17.1135	18.1712	0.402483494	0.934810776	4	12	24	0.094157305	0.71175955	24	22.10072473	0.08951328
ENSMUSG00000001098	Kctd10	potassium channel tetramerisation domain containing 10 [Source:MGI Symbol;Acc:MGI:2141207]	55.3292	40.0059	40.7217	45.2314	42.7624	48.5201	44.8607	46.7655	38.0176	53.8439	42.2438	47.7528	47.7104	46.2078	0.071575474	0.440262583	24	12	24	0.341916401	0.776129793	24	20.8600997	0.088803435
ENSMUSG00000001100	Poldip2	polymerase (DNA-directed), delta interacting protein 2 [Source:MGI Symbol;Acc:MGI:1915061]	27.4901	27.5133	29.2529	30.477	24.975	29.4503	29.4786	29.8951	29.0631	30.8717	26.608	28.2115	27.6602	29.4844	0.087978327	0.472021905	16	4	24	0.243743903	0.768040178	24	7.246654838	0.053556364
ENSMUSG00000001105	Ift20	intraflagellar transport 20 [Source:MGI Symbol;Acc:MGI:1915585]	47.8748	48.8822	47.7953	45.375	50.0437	43.6522	48.4402	46.2336	49.6424	40.1689	46.9751	43.5883	48.6062	45.4693	0.481811994	0.966018836	12	12	24	0.502467992	0.809624734	24	2.4931481	0.037425288
ENSMUSG00000001119	Col6a1	collagen, type VI, alpha 1 [Source:MGI Symbol;Acc:MGI:88459]	16.4181	20.0586	16.5706	14.6453	19.2033	16.1639	19.6488	21.494	23.301	12.4938	15.832	16.0436	16.5912	16.8324	0.004182163	0.140598674	8	8	24	0.204553427	0.768040178	24	3.506395227	0.147994127
ENSMUSG00000001120	Pcbp3	poly(rC) binding protein 3 [Source:MGI Symbol;Acc:MGI:1890470]	91.6324	68.4414	64.4821	75.8337	78.3473	83.3815	71.8957	80.7181	57.4323	97.4941	69.7729	79.7415	78.3394	82.2343	0.245061943	0.741580974	24	12	24	0.422042274	0.804917634	24	19.95104141	0.104974679
ENSMUSG00000001123	Lgals9	lectin, galactose binding, soluble 9 [Source:MGI Symbol;Acc:MGI:109496]	52.3855	38.3376	45.2202	37.476	45.1164	54.2489	43.0636	46.4241	33.6297	45.0231	43.821	39.4628	42.4752	41.4578	0.838532766	0.966018836	24	16	24	0.155173485	0.760294912	24	20.64590579	0.130512333
ENSMUSG00000001127	Araf	Araf proto-oncogene, serine/threonine kinase [Source:MGI Symbol;Acc:MGI:88065]	112.87	116.282	109.934	106.693	110.854	106.603	106.272	102.214	103.604	99.9608	109.351	101.228	103.654	111.661	0.920858634	0.966018836	8	16	24	0.695696227	0.899034004	24	1.581419832	0.018172333
ENSMUSG00000001128	Cfp	complement factor properdin [Source:MGI Symbol;Acc:MGI:97545]	7.56362	5.8231	7.08792	7.20224	7.64165	6.13338	6.43947	5.85756	6.86975	3.78816	7.36659	5.48356	5.25145	6.13507	0.581070934	0.966018836	20	12	24	0.882262311	0.966683041	24	18.14577425	0.04261569
ENSMUSG00000001131	Timp1	tissue inhibitor of metalloproteinase 1 [Source:MGI Symbol;Acc:MGI:98752]	4.82865	4.27659	5.19735	2.82957	6.41855	4.86027	4.22505	4.19562	3.6813	2.35029	5.35593	3.81574	3.51542	3.38536	0.129903614	0.560263689	20	20	24	0.292067777	0.768040178	24	20.17428616	0.17699607
ENSMUSG00000001134	Uxt	ubiquitously expressed prefoldin like chaperone [Source:MGI Symbol;Acc:MGI:1277988]	1.53287	2.50326	2.664	2.4659	1.25168	2.44162	1.99706	1.66873	2.42915	1.67135	2.3394	2.03141	1.74621	2.19575	0.43565473	0.966018836	12	16	24	0.507276602	0.812455891	24	8.196428505	0.102030275
ENSMUSG00000001138	Cnnm3	cyclin M3 [Source:MGI Symbol;Acc:MGI:2151055]	6.20591	5.20119	7.34279	7.69915	6.55669	6.19254	6.94904	6.84554	6.92241	6.47758	6.03825	6.63452	6.02626	6.40794	0.360033827	0.883787544	16	4	24	0.315820531	0.768040178	24	11.02131854	0.071635369
ENSMUSG00000001143	Lman2l	lectin, mannose-binding 2-like [Source:MGI Symbol;Acc:MGI:2443010]	16.0039	13.9317	13.4942	15.3415	14.107	16.9269	16.6494	15.5373	12.0598	16.1486	15.209	14.7946	15.2832	14.1763	0.079234726	0.458678242	24	12	24	0.084485363	0.702579245	24	20.32079711	0.104743464
ENSMUSG00000001150	Mcm3ap	minichromosome maintenance complex component 3 associated protein [Source:MGI Symbol;Acc:MGI:1930089]	12.4778	12.9816	13.5293	11.7578	13.1431	15.5093	11.3716	13.8047	12.0987	11.2165	14.2248	11.4169	12.5054	14.7469	0.888991156	0.966018836	8	8	24	0.903249117	0.973124041	24	0.290173235	0.024808158
ENSMUSG00000001151	Pcnt	pericentrin (kendrin) [Source:MGI Symbol;Acc:MGI:102722]	4.99298	5.1453	5.80635	4.88398	5.88832	6.3314	6.6623	6.2879	6.25334	6.50952	5.36236	6.19493	5.42884	5.17911	0.844511766	0.966018836	24	8	24	0.929270378	0.981515518	24	17.18380252	0.020016164
ENSMUSG00000001156	Mxd1	MAX dimerization protein 1 [Source:MGI Symbol;Acc:MGI:96908]	4.16261	4.09868	4.56431	5.2059	3.95568	3.93216	4.53754	4.17807	4.54287	3.71248	4.09639	3.92546	4.1033	3.67808	0.455507782	0.966018836	12	12	24	0.545354906	0.832868977	24	9.445777324	0.043903721
ENSMUSG00000001157	Gmcl1	germ cell-less, spermatogenesis associated 1 [Source:MGI Symbol;Acc:MGI:1345156]	17.2742	16.3336	15.9889	15.956	16.3799	16.3045	16.9183	14.9512	14.6205	16.3159	16.004	16.4707	15.9042	16.4155	0.16456443	0.628002127	4	8	24	0.08007702	0.69470896	24	20.63703495	0.048668336
ENSMUSG00000001158	Snrnp27	small nuclear ribonucleoprotein 27 (U4/U6.U5) [Source:MGI Symbol;Acc:MGI:1913868]	45.5681	52.8943	46.5575	49.2352	47.4124	43.3298	48.6997	45.6484	54.7732	41.5558	51.9681	48.901	53.0968	53.5918	0.909604019	0.966018836	12	4	24	0.456830316	0.805393068	24	4.847636321	0.056104499
ENSMUSG00000001166	Oas1c	2'-5' oligoadenylate synthetase 1C [Source:MGI Symbol;Acc:MGI:2149633]	4.98448	5.79161	5.80582	5.42025	4.4543	4.93727	4.18516	4.79861	4.97343	4.29242	5.49596	4.87236	5.1228	5.24762	0.702220766	0.966018836	12	12	24	0.539155806	0.829144433	24	7.115511995	0.054637643
ENSMUSG00000001173	Ocrl	OCRL, inositol polyphosphate-5-phosphatase [Source:MGI Symbol;Acc:MGI:109589]	22.7415	21.8386	20.9078	22.7861	22.0588	22.8064	18.4801	21.5954	20.8535	22.7828	19.9532	22.311	21.9313	22.5573	0.44861185	0.966018836	16	20	24	0.910825848	0.974769498	24	14.27344682	0.014461035
ENSMUSG00000001175	Calm1	calmodulin 1 [Source:MGI Symbol;Acc:MGI:88251]	626.912	554.81	547.085	546.548	515.268	576.706	552.7	550.333	492.303	615.238	575.789	607.275	605.168	624.817	0.235177833	0.73629392	4	8	24	0.310548755	0.768040178	24	22.48700678	0.060038642
ENSMUSG00000001211	Agpat3	1-acylglycerol-3-phosphate O-acyltransferase 3 [Source:MGI Symbol;Acc:MGI:1336186]	41.8412	34.9966	41.4762	49.0592	37.1815	43.4844	45.3702	44.391	37.5495	49.9452	39.8178	42.1675	43.2842	43.2014	0.93717134	0.966018836	16	20	24	0.886192489	0.967667196	24	13.45833737	0.02699471
ENSMUSG00000001227	Sema6b	sema domain, transmembrane domain (TM), and cytoplasmic domain, (semaphorin) 6B [Source:MGI Symbol;Acc:MGI:1202889]	0.974989	1.39889	2.13374	1.33088	1.02141	1.27888	1.69261	1.21101	1.66695	1.69421	1.10672	1.64921	1.08133	1.70044	0.181522804	0.659020016	12	8	24	0.156321648	0.761842768	24	7.88444217	0.149323141
ENSMUSG00000001228	Uhrf1	ubiquitin-like, containing PHD and RING finger domains, 1 [Source:MGI Symbol;Acc:MGI:1338889]	0.69458	0.589086	1.26993	0.771009	0.871608	0.726016	0.581513	1.10317	0.516414	0.697206	1.03614	0.739747	0.770046	0.474623	0.622293809	0.966018836	20	12	24	0.581777346	0.85096264	24	13.93898399	0.071832354
ENSMUSG00000001229	Dpp9	dipeptidylpeptidase 9 [Source:MGI Symbol;Acc:MGI:2443967]	14.0002	11.1155	11.1317	12.1278	13.0745	13.3832	14.7553	13.4595	10.2001	15.8331	11.8088	12.7911	12.8267	12.6195	0.112800839	0.530777648	4	8	24	0.337478258	0.772409896	24	20.27398451	0.093200604
ENSMUSG00000001240	Ramp2	receptor (calcitonin) activity modifying protein 2 [Source:MGI Symbol;Acc:MGI:1859650]	16.7758	16.4526	17.4879	23.2888	21.2802	19.5769	17.1445	18.6379	22.9148	19.5553	18.7941	15.7109	15.0945	14.2799	0.026323104	0.301028315	16	16	24	0.008911122	0.502965379	24	12.87221595	0.195645919
ENSMUSG00000001247	Lsr	lipolysis stimulated lipoprotein receptor [Source:MGI Symbol;Acc:MGI:1927471]	1.89084	1.97104	1.71491	1.45722	2.02079	1.89414	2.22757	2.30961	1.89024	1.45371	1.75674	2.18944	1.81433	1.63032	0.132411359	0.567728672	24	16	24	0.062664405	0.661961741	24	23.25811777	0.114252036
ENSMUSG00000001248	Gramd1a	GRAM domain containing 1A [Source:MGI Symbol;Acc:MGI:105490]	32.8852	28.6975	30.5222	37.5537	31.4418	31.3278	29.6926	29.2204	32.8793	33.7476	32.2814	30.6458	30.125	32.4927	0.01579111	0.253395174	16	16	24	0.059726196	0.658304792	24	12.83063674	0.082355553
ENSMUSG00000001249	Hpn	hepsin [Source:MGI Symbol;Acc:MGI:1196620]	1.74838	2.43065	1.49411	2.23939	1.70388	1.57182	2.54353	1.49419	2.0058	2.00293	2.35033	1.34852	2.1978	2.72463	0.773177465	0.966018836	8	16	24	0.641151262	0.878540546	24	5.838677836	0.080380298
ENSMUSG00000001260	Gabrg1	gamma-aminobutyric acid (GABA) A receptor, subunit gamma 1 [Source:MGI Symbol;Acc:MGI:103156]	6.8699	8.15455	8.52904	8.10748	7.61063	6.44037	7.59264	6.81962	7.79222	6.60698	7.37769	5.89487	6.57352	7.84093	0.004516345	0.140598674	12	12	24	0.03903108	0.613211607	24	8.294037554	0.116731946
ENSMUSG00000001270	Ckb	creatine kinase, brain [Source:MGI Symbol;Acc:MGI:88407]	181.799	164.56	185.837	140.133	119.638	177.861	190.418	180.573	149.159	195.478	146.009	175.112	157.933	182.555	0.522551072	0.966018836	8	12	24	0.174198166	0.768040178	24	2.720926461	0.140194754
ENSMUSG00000001280	Sp1	trans-acting transcription factor 1 [Source:MGI Symbol;Acc:MGI:98372]	7.90762	8.73488	8.80238	8.71768	9.02857	7.52539	8.38287	7.93185	9.50305	7.48635	8.38401	8.44617	8.20152	7.9679	0.44861185	0.966018836	12	16	24	0.466962171	0.805393068	24	9.931734171	0.043128356
ENSMUSG00000001281	Itgb7	integrin beta 7 [Source:MGI Symbol;Acc:MGI:96616]	2.90408	3.12637	2.4011	3.31115	2.18801	2.3122	1.87754	3.11989	4.0072	1.78948	2.3861	3.26925	2.60291	2.81611	0.607427393	0.966018836	12	8	24	0.428146839	0.805393068	24	5.761028133	0.125147904
ENSMUSG00000001285	Myg1	melanocyte proliferating gene 1 [Source:MGI Symbol;Acc:MGI:1929864]	34.1547	31.1895	34.3617	32.4016	31.8211	32.625	31.4574	31.4136	30.7188	26.8734	33.007	32.8961	34.8704	30.1907	0.32670903	0.845515883	4	4	24	0.223668504	0.768040178	24	21.87622505	0.060622411
ENSMUSG00000001288	Rarg	retinoic acid receptor, gamma [Source:MGI Symbol;Acc:MGI:97858]	15.902	13.8606	12.1781	15.1523	14.5985	14.369	17.9358	14.8106	17.0074	16.8914	16.6791	14.9036	16.6341	13.9363	0.887010723	0.966018836	4	4	24	0.710289526	0.90416311	24	19.07869351	0.04581446
ENSMUSG00000001289	Pfdn5	prefoldin 5 [Source:MGI Symbol;Acc:MGI:1928753]	148.154	158.129	158.377	144.383	181.217	149.153	149.774	152.028	162.766	108.957	145.854	135.635	138.774	138.798	0.300823756	0.809362476	12	4	24	0.91394669	0.975882466	24	0.323249324	0.024803623
ENSMUSG00000001300	Efnb2	ephrin B2 [Source:MGI Symbol;Acc:MGI:105097]	7.0816	6.58412	5.1611	6.07917	5.88829	6.09851	6.88524	6.6636	5.92051	7.13433	6.805	7.02425	6.32785	7.20102	0.46238066	0.966018836	8	4	24	0.349363567	0.779170188	24	22.95242685	0.062472158
ENSMUSG00000001305	Rrp15	ribosomal RNA processing 15 homolog [Source:MGI Symbol;Acc:MGI:1914473]	4.77694	5.13591	5.49694	5.86563	5.59265	5.32843	5.00229	5.65948	5.63341	4.99002	4.97203	5.68758	4.9919	5.40077	0.351315142	0.870418653	12	16	24	0.339838694	0.774150091	24	10.69499349	0.045631105
ENSMUSG00000001313	Rnd2	Rho family GTPase 2 [Source:MGI Symbol;Acc:MGI:1338755]	13.3023	19.6584	26.2202	14.8925	15.4693	19.5774	20.4856	15.826	19.416	14.5754	13.6204	17.2947	13.9663	18.8564	0.435739761	0.966018836	12	4	24	0.358545048	0.785198873	24	5.726096247	0.149600319
ENSMUSG00000001323	Srr	serine racemase [Source:MGI Symbol;Acc:MGI:1351636]	21.1042	18.6114	17.1465	19.5447	20.6474	20.4847	18.6091	18.137	18.9716	18.4	20.6524	17.4687	19.339	18.0858	0.212359178	0.701530532	20	12	24	0.088971197	0.708139275	24	18.38160756	0.072300113
ENSMUSG00000001333	Sync	syncoilin [Source:MGI Symbol;Acc:MGI:1916078]	8.34201	6.10312	5.91289	5.42439	6.61448	7.49931	7.12057	7.58989	5.0914	7.56071	6.28532	6.71647	7.37684	6.91094	0.033248781	0.324908923	4	8	24	0.050987533	0.645887282	24	22.36887418	0.155686683
ENSMUSG00000001334	Fndc5	fibronectin type III domain containing 5 [Source:MGI Symbol;Acc:MGI:1917614]	16.0309	15.2067	16.4707	15.5292	14.9874	16.7779	17.2353	16.5881	14.438	15.1813	13.6099	13.7959	13.834	14.8985	0.886952527	0.966018836	4	16	24	0.468649901	0.805393068	24	2.608415248	0.0400856
ENSMUSG00000001348	Acp5	acid phosphatase 5, tartrate resistant [Source:MGI Symbol;Acc:MGI:87883]	5.13447	5.92469	9.84386	11.7841	6.07302	9.86333	5.91241	10.8594	5.92265	7.7764	9.96862	7.41597	11.7531	6.10196	0.881355613	0.966018836	16	12	24	0.658601603	0.884596426	24	13.41873959	0.134755931
ENSMUSG00000001349	Cnn1	calponin 1 [Source:MGI Symbol;Acc:MGI:104979]	1.73785	0.970878	1.11798	1.61686	0.752644	1.29075	0.654744	2.57893	1.43688	0.982007	0.924794	1.01008	1.30218	0.864099	0.939486266	0.966018836	12	8	24	0.579954068	0.850085818	24	5.051921569	0.121590514
ENSMUSG00000001366	Fbxo9	f-box protein 9 [Source:MGI Symbol;Acc:MGI:1918788]	58.5947	56.5131	56.0974	58.0389	51.7586	59.0424	56.5848	51.4213	52.002	61.9605	50.1066	54.341	58.6569	56.3208	0.625825704	0.966018836	4	16	24	0.88788447	0.968476596	24	0.510329698	0.017220266
ENSMUSG00000001376	Vps50	VPS50 EARP/GARPII complex subunit [Source:MGI Symbol;Acc:MGI:1920538]	25.5555	30.1604	28.6147	29.1635	28.8781	26.4917	25.737	25.4467	28.3919	26.2269	29.2828	27.3963	25.8162	28.3612	0.05211941	0.391073722	20	8	24	0.154105522	0.760257003	24	11.40276051	0.0594817
ENSMUSG00000001380	Hars	histidyl-tRNA synthetase [Source:MGI Symbol;Acc:MGI:108087]	63.085	54.0828	57.7247	55.0715	57.8549	60.9391	54.0783	56.7171	46.0392	58.7663	56.6627	58.2463	54.7858	57.5281	0.515451644	0.966018836	24	8	24	0.174411051	0.768040178	24	19.80032382	0.074753053
ENSMUSG00000001383	Zmat2	zinc finger, matrin type 2 [Source:MGI Symbol;Acc:MGI:1913742]	52.8149	56.4981	56.6353	57.1625	57.3508	55.9125	50.2515	49.8751	57.8822	50.8682	54.933	55.3061	59.4682	58.7742	0.844511766	0.966018836	12	16	24	0.891120626	0.969291345	24	10.85725388	0.016060192
ENSMUSG00000001403	Ube2c	ubiquitin-conjugating enzyme E2C [Source:MGI Symbol;Acc:MGI:1915862]	1.26299	2.78928	4.09586	2.92171	2.11961	2.62888	2.65547	2.5366	2.67251	2.2966	2.95691	3.51508	4.01137	2.15174	0.844448841	0.966018836	12	20	24	0.857145233	0.957722653	24	10.26428097	0.065726493
ENSMUSG00000001415	Smg5	SMG5 nonsense mediated mRNA decay factor [Source:MGI Symbol;Acc:MGI:2447364]	28.1557	26.0427	27.9498	28.1973	30.9381	29.1467	27.7023	30.394	28.7576	30.2342	30.6429	29.1273	27.9092	29.3433	0.028180201	0.314814652	20	8	24	0.080365611	0.695795041	24	14.83002716	0.048646074
ENSMUSG00000001416	Cct3	chaperonin containing Tcp1, subunit 3 (gamma) [Source:MGI Symbol;Acc:MGI:104708]	127.047	114.6	116.196	117.318	136.244	125.619	108.161	112.427	100.987	108.3	119.325	115.216	121.035	117.199	0.229694208	0.729069536	20	16	24	0.060721158	0.660111271	24	19.17671297	0.09181999
ENSMUSG00000001418	Glmp	glycosylated lysosomal membrane protein [Source:MGI Symbol;Acc:MGI:1913318]	50.3312	44.2376	37.7943	44.917	49.3711	50.1634	49.3873	50.4319	41.5337	44.3615	39.9478	39.0229	46.0309	45.1219	0.170659409	0.643496587	4	8	24	0.216448883	0.768040178	24	22.83920757	0.091775385
ENSMUSG00000001419	Mef2d	myocyte enhancer factor 2D [Source:MGI Symbol;Acc:MGI:99533]	13.6364	12.6148	13.0085	13.9991	12.3359	13.5233	14.3603	15.253	14.9095	19.2452	13.1645	13.8804	14.5156	15.542	0.351315142	0.870418653	16	4	24	0.319603231	0.768040178	24	8.309084788	0.080118106
ENSMUSG00000001420	Tmem79	transmembrane protein 79 [Source:MGI Symbol;Acc:MGI:1919163]	30.828	21.0124	19.7921	23.6337	24.6032	29.1864	22.1831	26.8729	17.3132	31.9794	25.1435	26.021	25.8168	23.2188	0.0444686	0.365196805	24	12	24	0.14910686	0.757645213	24	19.31449845	0.178636329
ENSMUSG00000001424	Snd1	staphylococcal nuclease and tudor domain containing 1 [Source:MGI Symbol;Acc:MGI:1929266]	17.4017	17.2773	19.0749	20.5187	18.678	18.3177	17.9525	18.8853	16.9542	20.3636	14.5336	23.2943	20.6465	18.1741	0.93447422	0.966018836	16	16	24	0.963735635	0.99092303	24	18.94180484	0.017283367
ENSMUSG00000001435	Col18a1	collagen, type XVIII, alpha 1 [Source:MGI Symbol;Acc:MGI:88451]	6.46869	6.4659	6.62101	6.19377	7.56296	6.31309	6.99265	7.73655	6.43188	6.66053	7.40096	6.68161	6.11559	5.50771	0.883024237	0.966018836	20	12	24	0.486427081	0.805393068	24	16.47285826	0.053182564
ENSMUSG00000001436	Slc19a1	solute carrier family 19 (folate transporter), member 1 [Source:MGI Symbol;Acc:MGI:103182]	10.253	9.38375	11.0036	8.9489	10.1117	10.8698	7.67413	11.6957	8.15726	11.8396	10.2277	10.3393	9.64924	9.80568	0.890235289	0.966018836	24	4	24	0.795940535	0.934803902	24	14.54925976	0.043684902
ENSMUSG00000001440	Kpnb1	karyopherin (importin) beta 1 [Source:MGI Symbol;Acc:MGI:107532]	48.0787	40.8928	41.5419	41.1856	45.695	44.0933	43.5417	41.6364	36.2925	45.1465	41.348	44.5898	40.7903	43.9497	0.435739761	0.966018836	24	12	24	0.109923957	0.72082227	24	19.98772027	0.076785694
ENSMUSG00000001441	Npepps	aminopeptidase puromycin sensitive [Source:MGI Symbol;Acc:MGI:1101358]	58.4977	46.0102	47.1154	49.302	47.9056	53.9681	50.6463	50.0925	42.7924	59.8197	49.2537	54.8842	53.9273	55.5042	0.217827365	0.704989686	24	12	24	0.322867132	0.768040178	24	20.94133677	0.078482271

ENSMUSG00000001445	Mrpl10	mitochondrial ribosomal protein L10 [Source:MGI Symbol;Acc:MGI:1333801]	25.3	23.8941	24.5871	26.2019	24.108	26.4695	25.5116	25.0585	22.4947	28.062	25.2787	25.1609	25.5601	25.4436	0.300823756	0.809362476	16	20	24	0.515727261	0.817414863	24	17.41228267	0.032603899
ENSMUSG00000001467	Cyp51	cytochrome P450, family 51 [Source:MGI Symbol;Acc:MGI:106040]	54.6825	50.1731	48.0439	48.1124	54.0495	50.4476	53.0266	47.2053	51.0539	46.7254	57.055	50.3368	52.2943	49.7433	0.048507657	0.379129806	20	20	24	0.093697306	0.71175955	24	19.96902588	0.069342127
ENSMUSG00000001472	Tcf25	transcription factor 25 (basic helix-loop-helix) [Source:MGI Symbol;Acc:MGI:1914105]	187.489	155.311	166.408	170.545	156.662	189.675	170.332	176.08	146.546	200.246	159.19	172.393	162.141	177.213	0.954106009	0.966018836	24	12	24	0.771602719	0.926353417	24	20.3063131	0.037282888
ENSMUSG00000001473	Tubb6	tubulin, beta 6 class V [Source:MGI Symbol;Acc:MGI:1915201]	9.299	8.25535	8.36074	7.99028	9.75099	9.15814	8.74286	9.26356	7.61585	7.83399	10.2162	7.67651	7.45163	6.50501	0.328944792	0.847277612	20	16	24	0.08572115	0.703577714	24	18.02600103	0.115626029
ENSMUSG00000001482	Def8	differentially expressed in FDCP 8 [Source:MGI Symbol;Acc:MGI:1346331]	21.9496	19.3139	19.6734	20.5422	19.2594	22.3963	19.7167	21.497	16.76	23.2446	18.4477	20.7632	21.1724	19.9019	0.581070934	0.966018836	24	12	24	0.519043181	0.819011931	24	20.94889575	0.054866511
ENSMUSG00000001493	Meox1	mesenchyme homeobox 1 [Source:MGI Symbol;Acc:MGI:103220]	2.05196	2.15722	1.33573	1.1941	2.45378	1.66409	2.50809	2.05065	2.37607	1.20319	1.92002	1.656	2.26673	1.91717	0.068668966	0.432567661	4	12	24	0.134164358	0.744382556	24	0.185272873	0.171528039
ENSMUSG00000001497	Pax9	paired box 9 [Source:MGI Symbol;Acc:MGI:97493]	0.753252	0.605832	0.631746	0.525023	0.709141	0.519942	0.804074	0.619767	0.449868	0.471847	0.839829	0.453505	0.56886	0.513581	0.791413662	0.966018836	4	12	24	0.429392841	0.805393068	24	20.9013683	0.057247418
ENSMUSG00000001504	Irx2	Iroquois homeobox 2 [Source:MGI Symbol;Acc:MGI:1197526]	0.300414	0.728269	1.23931	0.234986	0.346918	0.645209	0.91007	0.38433	0.82902	0.449514	0.500425	0.58546	0.0793878	0.671983	0.369262716	0.893596186	12	4	24	0.522749902	0.820977872	24	6.828913455	0.128238343
ENSMUSG00000001506	Col1a1	collagen, type I, alpha 1 [Source:MGI Symbol;Acc:MGI:88467]	35.5965	38.7014	36.1762	35.0157	39.758	33.3409	42.5237	47.7019	50.2755	43.416	39.4896	35.599	44.0138	33.3809	0.937921962	0.966018836	12	12	24	0.471117794	0.805393068	24	7.239739276	0.090415834
ENSMUSG00000001507	Itga3	integrin alpha 3 [Source:MGI Symbol;Acc:MGI:96602]	13.9261	10.1415	11.4561	11.0137	12.4706	13.4141	10.9968	11.8465	10.3547	14.8319	11.8438	12.2114	11.5213	11.2377	0.269657278	0.777976231	24	12	24	0.203796784	0.768040178	24	17.71877348	0.100719363
ENSMUSG00000001508	Sgca	sarcoglycan, alpha (dystrophin-associated glycoprotein) [Source:MGI Symbol;Acc:MGI:894698]	0.85277	2.45686	2.38342	4.19904	1.90646	3.09642	1.97829	6.97321	4.52989	5.6221	2.18965	2.61892	3.64572	2.11621	0.510431574	0.966018836	16	4	24	0.153662704	0.760223561	24	9.35995956	0.391406965
ENSMUSG00000001517	Foxm1	forkhead box M1 [Source:MGI Symbol;Acc:MGI:1347487]	0.862355	0.848074	1.79927	0.979434	1.00111	0.986081	0.815279	1.38266	0.873991	1.13991	1.08304	0.701687	0.838114	0.958985	0.066875194	0.425513244	16	12	24	0.144419849	0.752803875	24	9.372514419	0.136159767
ENSMUSG00000001518	Itfg2	integrin alpha FG-GAP repeat containing 2 [Source:MGI Symbol;Acc:MGI:1915450]	9.50722	7.67395	7.64189	6.96692	7.3932	8.763	9.24997	9.74623	7.09305	8.93622	6.8918	7.98258	7.08847	7.52033	0.34864256	0.870418653	4	16	24	0.251576356	0.768040178	24	0.00911417	0.09727106
ENSMUSG00000001520	Nrip2	nuclear receptor interacting protein 2 [Source:MGI Symbol;Acc:MGI:1891884]	10.5138	9.65195	10.905	8.93328	9.33566	10.9585	11.273	9.44401	9.86503	11.5016	8.68292	10.1989	9.5851	10.8094	0.952127548	0.966018836	24	20	24	0.640000602	0.878006652	24	2.170150508	0.043280467
ENSMUSG00000001521	Tulp3	tubby-like protein 3 [Source:MGI Symbol;Acc:MGI:1329045]	9.48319	9.61659	9.4353	10.1356	10.4022	9.26374	9.79946	10.3228	10.5412	10.7426	10.099	9.90105	10.112	10.1038	0.287843582	0.793714531	16	8	24	0.05422996	0.654887447	24	10.8452711	0.044014938
ENSMUSG00000001524	Gtf2h4	general transcription factor II H, polypeptide 4 [Source:MGI Symbol;Acc:MGI:1338799]	7.69994	7.12538	7.58452	8.13208	7.18312	7.23935	6.58461	8.453	7.27583	8.16953	7.5902	7.19701	9.00534	7.40615	0.885059765	0.966018836	16	8	24	0.736649339	0.914180155	24	8.400800936	0.033196373
ENSMUSG00000001525	Tubb5	tubulin, beta 5 class I [Source:MGI Symbol;Acc:MGI:107812]	352.195	268.274	275.765	282.541	291.941	352.432	302.197	311.996	221.878	349.992	271.733	302.068	291.175	288.344	0.085890569	0.466404237	24	12	24	0.173093095	0.768040178	24	20.44809957	0.130950965
ENSMUSG00000001542	Ell2	elongation factor for RNA polymerase II 2 [Source:MGI Symbol;Acc:MGI:2183438]	9.40776	8.15558	8.4312	8.2772	8.58085	8.44153	9.25508	8.50758	6.49629	8.63982	9.65456	7.79858	7.74068	8.46118	0.912923872	0.966018836	20	16	24	0.359186545	0.785405943	24	19.57423902	0.070498295
ENSMUSG00000001552	Jup	junction plakoglobin [Source:MGI Symbol;Acc:MGI:96650]	30.1429	21.9348	22.2404	25.4743	27.2384	28.4188	26.0613	29.9936	20.5608	37.9183	26.1148	27.8551	27.4392	25.9161	0.077301512	0.453790923	24	12	24	0.42220975	0.804917634	24	18.31527055	0.110536709
ENSMUSG00000001555	Fkbp10	FK506 binding protein 10 [Source:MGI Symbol;Acc:MGI:104769]	5.29841	6.4113	6.93566	5.2858	5.69427	6.00156	4.96289	6.66447	7.30958	5.37899	5.30092	5.35119	5.65828	5.53152	0.128989168	0.560209134	12	4	24	0.07605475	0.686372559	24	7.096861154	0.123218218
ENSMUSG00000001569	Nom1	nucleolar protein with MIF4G domain 1 [Source:MGI Symbol;Acc:MGI:1861749]	11.48	10.7741	9.67217	12.7657	11.0818	10.8751	10.9896	14.6327	10.562	11.7095	10.3226	9.75474	10.9926	10.5289	0.92769718	0.966018836	16	20	24	0.82209801	0.943977421	24	6.463194587	0.036668176
ENSMUSG00000001576	Ergic1	endoplasmic reticulum-golgi intermediate compartment (ERGIC) 1 [Source:MGI Symbol;Acc:MGI:1914708]	22.7444	19.2295	19.7064	17.7208	20.3744	21.0687	20.3746	20.4558	17.2846	23.4009	17.3622	19.0917	18.6641	18.6683	0.920858634	0.966018836	4	8	24	0.63062942	0.874000625	24	21.53245959	0.047552494
ENSMUSG00000001588	Acap1	ArfGAP with coiled-coil, ankyrin repeat and PH domains 1 [Source:MGI Symbol;Acc:MGI:2388270]	1.11496	1.39332	1.90063	1.57175	1.52697	1.54077	1.77015	1.42755	1.42447	1.08328	0.992815	1.34578	1.6131	0.894767	0.860560779	0.966018836	4	4	24	0.964457213	0.991159965	24	0.358608967	0.017957957
ENSMUSG00000001604	Tcea3	transcription elongation factor A (SII), 3 [Source:MGI Symbol;Acc:MGI:1196908]	1.60957	3.87643	5.35619	5.74401	3.63929	4.69471	3.83633	8.42248	8.66171	8.84568	3.29496	3.85041	5.04666	4.78107	0.012443448	0.220221977	16	4	24	0.015189576	0.537924552	24	9.024486988	0.502530082
ENSMUSG00000001627	Ifrd1	interferon-related developmental regulator 1 [Source:MGI Symbol;Acc:MGI:1316717]	27.9253	32.3841	32.3246	36.374	36.2894	30.7362	31.1505	32.2248	35.6334	34.7784	34.2539	28.5176	38.5889	30.9474	0.084464639	0.466404237	16	8	24	0.14162595	0.751766185	24	11.36796675	0.096857096
ENSMUSG00000001630	Stk38l	serine/threonine kinase 38 like [Source:MGI Symbol;Acc:MGI:1922250]	9.22632	8.25216	8.51925	8.2592	8.58413	8.77993	7.91741	8.14399	7.48632	7.9362	8.2297	8.31384	8.4521	8.44552	0.472013662	0.966018836	24	12	24	0.098039194	0.71175955	24	21.09223951	0.049201938
ENSMUSG00000001632	Brpf1	bromodomain and PHD finger containing, 1 [Source:MGI Symbol;Acc:MGI:1926033]	4.73204	5.27131	6.08942	6.18434	6.05088	5.84257	5.26303	5.84148	6.7068	6.05856	5.62311	5.28842	5.90496	5.68643	0.004320961	0.140598674	12	16	24	0.008779692	0.502965379	24	10.68875908	0.109362941
ENSMUSG00000001642	Akr1b3	aldo-keto reductase family 1, member B3 (aldose reductase) [Source:MGI Symbol;Acc:MGI:1353494]	14.8631	13.8699	13.9196	15.8214	17.4891	14.6289	14.2973	16.5728	18.6435	11.6657	19.414	13.3771	16.1395	13.2151	0.953350544	0.966018836	20	12	24	0.774078842	0.927465562	24	15.18634393	0.054969833
ENSMUSG00000001656	Hoxc11	homeobox C11 [Source:MGI Symbol;Acc:MGI:96193]	0.277149	0.705261	1.09042	0.638911	0.513272	0.948933	1.30274	0.34474	0.977615	0.999691	0.294321	0.758273	0.495598	0.647675	0.830364743	0.966018836	12	16	24	0.764412784	0.92403577	24	8.757215437	0.082336881
ENSMUSG00000001657	Hoxc8	homeobox C8 [Source:MGI Symbol;Acc:MGI:96198]	2.85205	2.86079	2.91002	2.42738	2.69415	2.85709	3.23784	3.02548	2.76219	3.99154	3.21196	3.54499	3.288	3.55679	0.887010723	0.966018836	8	4	24	0.874370472	0.964836189	24	0.736037837	0.021382037
ENSMUSG00000001661	Hoxc6	homeobox C6 [Source:MGI Symbol;Acc:MGI:96197]	6.81955	5.56208	4.97767	6.69693	7.38552	6.01802	7.64996	6.58097	5.84309	5.36189	7.6921	6.59303	5.53124	6.6949	0.185796491	0.662239322	20	16	24	0.214976691	0.768040178	24	19.28205331	0.11771223
ENSMUSG00000001663	Gstt1	glutathione S-transferase, theta 1 [Source:MGI Symbol;Acc:MGI:107379]	13.7785	17.2904	16.3295	19.3302	19.6363	17.1995	13.5131	15.7635	22.7879	15.6711	19.7428	17.8286	15.8823	16.872	0.028180201	0.314814652	20	8	24	0.0572527	0.656248849	24	12.38562727	0.164362366
ENSMUSG00000001665	Gstt3	glutathione S-transferase, theta 3 [Source:MGI Symbol;Acc:MGI:2143526]	1.87052	2.46063	3.33672	2.78414	2.13543	2.27319	2.48728	2.74706	3.7624	2.52723	2.22578	1.95103	2.27619	2.6713	7.90E-04	0.071344007	12	12	24	8.64E-04	0.319044294	24	8.162841509	0.240495834
ENSMUSG00000001666	Ddt	D-dopachrome tautomerase [Source:MGI Symbol;Acc:MGI:1298381]	68.5072	60.8465	65.2008	64.658	55.179	70.4405	63.7215	70.0038	57.8997	68.6426	59.3898	61.2255	65.4608	67.3638	0.628286596	0.966018836	4	16	24	0.421967881	0.804917634	24	2.321198408	0.054000205
ENSMUSG00000001672	Marveld3	MARVEL (membrane-associating) domain containing 3 [Source:MGI Symbol;Acc:MGI:1920858]	1.04635	0.758052	0.606579	0.989286	0.88759	0.836031	0.90099	0.847495	0.781264	0.886535	0.999598	1.07932	0.991144	1.18936	0.277087842	0.789849983	4	8	24	0.268657629	0.768040178	24	20.67953953	0.063061788
ENSMUSG00000001674	Ddx18	DEAD box helicase 18 [Source:MGI Symbol;Acc:MGI:1914192]	4.11268	3.68773	4.62269	4.11253	3.75615	3.4742	2.80497	3.93943	3.5856	3.06477	3.51159	3.18387	3.48389	3.20202	0.787283372	0.966018836	12	12	24	0.415107236	0.800303366	24	9.054225024	0.06317231
ENSMUSG00000001687	Ubl3	ubiquitin-like 3 [Source:MGI Symbol;Acc:MGI:1344373]	61.6112	56.9088	61.712	55.7451	55.4247	58.8698	59.7639	50.6235	47.8287	72.8903	51.4435	58.9659	55.2739	56.0501	0.887862353	0.966018836	4	16	24	0.830681605	0.947596159	24	17.26629058	0.035750134
ENSMUSG00000001700	Gramd3	GRAM domain containing 3 [Source:MGI Symbol;Acc:MGI:1914815]	2.50547	3.56241	3.96703	2.61125	2.75295	3.09852	3.58065	3.45104	3.72882	2.64645	2.73497	2.74412	2.57027	2.95525	0.129903614	0.560263689	12	4	24	0.091425275	0.71175955	24	5.862665879	0.137397163
ENSMUSG00000001707	Eef1e1	eukaryotic translation elongation factor 1 epsilon 1 [Source:MGI Symbol;Acc:MGI:1913393]	15.5035	15.5879	15.5782	16.5631	18.9399	15.4812	12.9808	14.4971	17.4354	13.6531	17.0134	15.1228	16.4843	17.0997	0.718086778	0.966018836	20	4	24	0.556228062	0.838702296	24	13.22796768	0.057327899
ENSMUSG00000001729	Akt1	thymoma viral proto-oncogene 1 [Source:MGI Symbol;Acc:MGI:87986]	61.0523	47.2389	48.2384	51.3505	53.3288	56.6051	54.2579	53.4784	44.9655	61.5169	46.6327	54.4334	50.2041	50.8534	0.077301512	0.453790923	24	12	24	0.313274487	0.768040178	24	19.97720055	0.081853416
ENSMUSG00000001739	Cldn15	claudin 15 [Source:MGI Symbol;Acc:MGI:1913103]	1.12733	0.921829	0.819642	1.40967	0.918469	1.20427	1.03009	1.38759	1.00694	1.00826	1.18586	1.19944	1.18529	0.932183	0.245061943	0.741580974	24	12	24	0.587086753	0.853586751	24	19.27661541	0.05137245
ENSMUSG00000001741	Il16	interleukin 16 [Source:MGI Symbol;Acc:MGI:1270855]	14.2355	15.9461	15.0728	19.2978	19.4359	15.7013	17.5147	16.4623	18.8178	16.5633	15.8487	16.3208	16.4182	13.3949	0.478349072	0.966018836	16	16	24	0.190409879	0.768040178	24	13.77918679	0.098997171
ENSMUSG00000001750	Tcirg1	T cell, immune regulator 1, ATPase, H+ transporting, lysosomal V0 protein A3 [Source:MGI Symbol;Acc:MGI:1350931]	9.03889	11.696	11.0125	11.1084	15.7311	11.7333	15.6897	11.1784	13.205	10.6779	13.1131	11.8476	10.9427	10.5029	0.541384199	0.966018836	20	12	24	0.50065203	0.808986978	24	16.51725132	0.094668907
ENSMUSG00000001751	Naglu	alpha-N-acetylglucosaminidase (Sanfilippo disease IIIB) [Source:MGI Symbol;Acc:MGI:1351641]	4.12896	4.29679	4.45794	4.49657	4.94996	4.83936	4.00741	5.56227	5.44054	5.42084	4.00454	4.38177	4.28699	4.45071	0.058525295	0.405089131	12	16	24	0.201144627	0.768040178	24	9.804116054	0.093427795
ENSMUSG00000001755	Coasy	Coenzyme A synthase [Source:MGI Symbol;Acc:MGI:1918993]	14.9291	13.8131	15.8613	13.8823	13.6786	15.7788	14.7276	15.4041	12.4572	16.5916	14.4956	15.9263	15.0328	15.6659	0.879201926	0.966018836	24	20	24	0.754364546	0.920489464	24	21.85382922	0.031393832
ENSMUSG00000001761	Smo	smoothened, frizzled class receptor [Source:MGI Symbol;Acc:MGI:108075]	6.82233	7.51345	7.01064	7.22243	8.07025	6.48913	8.0529	7.66734	7.92414	6.77976	6.82084	7.14146	7.40442	7.5435	0.570349842	0.966018836	8	16	24	0.443997568	0.805393068	24	5.030462485	0.044348775
ENSMUSG00000001763	Tspan33	tetraspanin 33 [Source:MGI Symbol;Acc:MGI:1919012]	1.36597	2.05466	3.51104	2.02411	1.17777	2.13731	2.57322	1.83741	2.04302	1.36027	1.3422	1.88762	1.79067	2.0668	0.219464938	0.708495385	12	8	24	0.219680074	0.768040178	24	5.143559541	0.182477744
ENSMUSG00000001767	Crnkl1	crooked neck pre-mRNA splicing factor 1 [Source:MGI Symbol;Acc:MGI:1914127]	8.81556	7.68769	7.44838	7.6479	7.66175	7.34344	7.27616	7.30291	7.26886	7.39657	7.90299	7.21539	7.95885	7.05765	0.917867465	0.966018836	20	12	24	0.429245048	0.805393068	24	20.58230373	0.034401366
ENSMUSG00000001768	Rin2	Ras and Rab interactor 2 [Source:MGI Symbol;Acc:MGI:1921280]	8.47177	9.0048	8.85178	8.7279	8.80728	8.43276	8.3924	8.64813	9.24127	8.13876	8.00065	8.80589	8.40816	8.35561	0.625825704	0.966018836	12	16	24	0.402879671	0.799546792	24	6.78590877	0.025773353
ENSMUSG00000001773	Folh1	folate hydrolase 1 [Source:MGI Symbol;Acc:MGI:1858193]	7.48372	8.77839	8.88235	8.97226	9.17406	7.7801	8.33226	6.61197	9.25069	7.59707	9.97701	7.27321	7.51721	8.37596	0.038580592	0.335882166	20	4	24	0.159712358	0.76564405	24	12.35120635	0.104329415
ENSMUSG00000001774	Chordc1	cysteine and histidine-rich domain (CHORD)-containing, zinc-binding protein 1 [Source:MGI Symbol;Acc:MGI:1914167]	52.6875	41.865	32.9726	38.7076	44.3621	41.7237	46.0926	42.0733	37.6747	31.0543	53.989	42.2211	48.1317	42.0172	0.00455947	0.140598674	4	12	24	0.021815368	0.574400012	24	21.60794573	0.216187932
ENSMUSG00000001783	Rtcb	RNA 2',3'-cyclic phosphate and 5'-OH ligase [Source:MGI Symbol;Acc:MGI:106379]	62.7486	64.087	62.7601	62.6581	64.8965	64.4641	61.7614	59.3708	60.2823	59.4089	59.7869	62.3509	63.5716	66.1757	0.906657897	0.966018836	24	16	24	0.480489753	0.805393068	24	22.98386761	0.023375115
ENSMUSG00000001785	Pwp1	PWP1 homolog, endonuclein [Source:MGI Symbol;Acc:MGI:1914735]	14.0371	13.2458	14.6133	12.2471	12.6384	12.8126	11.3507	12.6139	12.6013	12.5832	13.0828	13.1757	13.1931	12.6927	0.725061903	0.966018836	4	12	24	0.943237716	0.986054554	24	4.763191148	0.010765522
ENSMUSG00000001786	Fbxo7	F-box protein 7 [Source:MGI Symbol;Acc:MGI:1917004]	21.8883	24.7393	23.6991	21.148	25.8023	22.4408	22.3242	23.6889	24.412	23.7824	22.2832	21.8134	24.3924	20.8223	0.891742979	0.966018836	12	12	24	0.827104818	0.946112955	24	9.569989291	0.022403675
ENSMUSG00000001794	Capns1	calpain, small subunit 1 [Source:MGI Symbol;Acc:MGI:88266]	437.499	345.49	351.085	352.74	356.189	408.936	359.828	363.118	293.248	381.109	342.427	348.444	360.352	348.395	0.43280837	0.966018836	4	8	24	0.08935441	0.708706636	24	21.01882438	0.102814328
ENSMUSG00000001802	Lrp3	low density lipoprotein receptor-related protein 3 [Source:MGI Symbol;Acc:MGI:3584516]	13.7845	13.2652	13.7711	13.5653	12.1061	14.2377	14.9644	16.2232	14.1704	18.424	13.148	15.8385	14.5432	15.7437	0.644063931	0.966018836	8	12	24	0.553742291	0.837374348	24	5.65978313	0.05436496
ENSMUSG00000001829	Clpb	ClpB caseinolytic peptidase B [Source:MGI Symbol;Acc:MGI:1100517]	11.2278	9.83893	10.179	10.8762	11.0493	11.3998	11.3822	11.253	9.23674	11.923	9.90354	11.164	10.8643	11.0333	0.245061943	0.741580974	24	12	24	0.339733305	0.774150091	24	20.29034022	0.055262488
ENSMUSG00000001833	Septin7	septin 7 [Source:MGI Symbol;Acc:MGI:1335094]	162.927	173.636	169.894	156.154	167.492	151.9	165.313	145.435	165.409	138.754	170.455	151.808	151.548	158.626	0.904387595	0.966018836	12	12	24	0.97179832	0.993121308	24	3.401660087	0.008302404
ENSMUSG00000001844	Zdhhc4	zinc finger, DHHC domain containing 4 [Source:MGI Symbol;Acc:MGI:1920131]	22.3845	20.2774	22.8889	24.3215	23.2083	21.767	21.7356	23.5301	20.0201	24.4987	21.8422	19.6972	20.2586	20.755	0.219464938	0.708495385	16	8	24	0.135196336	0.746923165	24	12.63457569	0.068986145
ENSMUSG00000001847	Rac1	Rac family small GTPase 1 [Source:MGI Symbol;Acc:MGI:97845]	153.269	133.651	144.695	145.896	150.944	153.6	146.32	148.052	129.623	169.194	134.669	145.61	141.551	141.751	0.649212555	0.966018836	16	20	24	0.509924211	0.813985618	24	17.21395569	0.044520741
ENSMUSG00000001855	Nup214	nucleoporin 214 [Source:MGI Symbol;Acc:MGI:1095411]	8.91406	7.06975	9.95738	7.58346	8.39861	10.4712	7.2906	8.82613	6.49306	9.62142	6.92316	7.11211	7.8741	8.01364	0.732668271	0.966018836	24	20	24	0.956859574	0.988639288	24	17.59570859	0.022511935
ENSMUSG00000001864	Aif1l	allograft inflammatory factor 1-like [Source:MGI Symbol;Acc:MGI:1919598]	27.281	29.1364	26.3542	22.8062	28.5546	21.2163	32.2457	27.1422	32.3152	23.9944	27.0533	26.5911	29.9111	25.5616	0.247964668	0.746745468	4	12	24	0.342731394	0.776697379	24	3.043339432	0.093966857
ENSMUSG00000001865	Cpa3	carboxypeptidase A3, mast cell [Source:MGI Symbol;Acc:MGI:88479]	1.6035	2.44702	1.34197	1.9161	1.87588	1.51058	1.43124	2.04762	1.89805	0.998048	1.95567	1.41205	2.05015	1.4892	0.954106009	0.966018836	8	16	24	0.755708123	0.921118175	24	2.624167853	0.05982923
ENSMUSG00000001870	Ltbp1	latent transforming growth factor beta binding protein 1 [Source:MGI Symbol;Acc:MGI:109151]	2.47162	3.14324	3.5887	3.33856	3.26279	2.90112	3.45717	3.21064	3.33276	2.59384	2.96571	2.9696	2.84163	2.65652	0.649212555	0.966018836	12	16	24	0.554419399	0.837783583	24	9.640653058	0.051821304
ENSMUSG00000001891	Ugp2	UDP-glucose pyrophosphorylase 2 [Source:MGI Symbol;Acc:MGI:2183447]	81.8202	81.0924	87.7543	88.2563	79.3574	82.5504	82.0035	96.7156	86.1499	88.0095	87.7126	84.816	91.79	87.0327	0.858269825	0.966018836	16	4	24	0.201525195	0.768040178	24	7.307061718	0.047900719
ENSMUSG00000001901	Kcnh6	potassium voltage-gated channel, subfamily H (eag-related), member 6 [Source:MGI Symbol;Acc:MGI:2684139]	10.4248	6.96877	6.66691	8.19364	8.01287	8.32286	7.61534	8.70989	6.44647	10.5505	7.86038	8.53685	9.14931	7.62286	0.210566453	0.69936851	24	12	24	0.365177956	0.789549788	24	19.62155199	0.108277477
ENSMUSG00000001909	Trmt1	tRNA methyltransferase 1 [Source:MGI Symbol;Acc:MGI:1289155]	26.793	25.623	25.4703	25.6894	24.9905	21.6248	25.4718	27.5509	27.7602	24.2977	27.9652	24.2313	26.6037	25.0846	0.591351901	0.966018836	12	12	24	0.359461701	0.785405943	24	6.5948093	0.053755929
ENSMUSG00000001910	Nacc1	nucleus accumbens associated 1, BEN and BTB (POZ) domain containing [Source:MGI Symbol;Acc:MGI:1914080]	15.8222	12.2211	12.766	14.0369	13.8113	14.9128	14.1395	15.0475	11.1188	17.9513	12.9968	13.7507	13.4896	14.3863	0.953141285	0.966018836	4	8	24	0.697417162	0.899338675	24	18.89535419	0.055190836
ENSMUSG00000001911	Nfix	nuclear factor I/X [Source:MGI Symbol;Acc:MGI:97311]	4.60849	5.65047	6.6572	5.75123	4.81205	4.51688	6.57371	6.17653	7.91487	8.47515	5.19914	5.71959	6.10673	7.55839	0.023460887	0.278499043	12	12	24	0.001319181	0.346832852	24	7.796008766	0.255356431
ENSMUSG00000001918	Slc1a5	solute carrier family 1 (neutral amino acid transporter), member 5 [Source:MGI Symbol;Acc:MGI:105305]	24.7035	20.8326	28.7292	46.2049	31.3038	26.5787	22.5272	26.6522	30.8657	37.1497	34.0496	24.6137	30.6465	25.521	0.004122392	0.140598674	16	16	24	0.002133935	0.395705644	24	12.74484528	0.320815919
ENSMUSG00000001924	Uba1	ubiquitin-like modifier activating enzyme 1 [Source:MGI Symbol;Acc:MGI:98890]	123.667	92.0375	100.307	103.639	92.4081	122.224	106.115	108.499	78.8463	126.725	94.2408	103.285	99.032	101.202	0.836635326	0.966018836	24	12	24	0.477096298	0.805393068	24	20.57157514	0.091334679
ENSMUSG00000001930	Vwf	Von Willebrand factor [Source:MGI Symbol;Acc:MGI:98941]	5.61531	8.79013	8.53173	8.71544	7.32859	7.68792	7.26148	10.5863	7.9685	9.10508	6.74538	6.54241	8.08711	5.65369	0.224668574	0.719154429	16	8	24	0.250782734	0.768040178	24	9.048260371	0.149508721
ENSMUSG00000001942	Siae	sialic acid acetylesterase [Source:MGI Symbol;Acc:MGI:104803]	10.5187	9.04067	9.33272	8.74798	8.59491	9.2178	9.92441	9.6344	8.16144	9.93262	8.98137	9.53309	8.71342	9.83534	0.570349842	0.966018836	4	16	24	0.320557167	0.768040178	24	0.171185385	0.052363563
ENSMUSG00000001943	Vsig2	V-set and immunoglobulin domain containing 2 [Source:MGI Symbol;Acc:MGI:1928009]	2.21356	3.02056	2.39872	1.60105	2.77134	2.67146	2.9812	2.45496	3.1653	2.80748	2.82229	2.21849	2.42733	2.27516	0.890648739	0.966018836	12	12	24	0.962042993	0.9903359	24	3.006354769	0.019818611
ENSMUSG00000001946	Esam	endothelial cell-specific adhesion molecule [Source:MGI Symbol;Acc:MGI:1916774]	11.294	10.9659	9.5898	10.1766	11.9089	11.3461	11.9383	12.1781	9.46092	10.8275	9.36085	10.1863	10.6393	9.18098	0.742579234	0.966018836	4	8	24	0.173049685	0.768040178	24	21.76868331	0.08498957
ENSMUSG00000001948	Spa17	sperm autoantigenic protein 17 [Source:MGI Symbol;Acc:MGI:1333778]	6.87215	7.97804	7.14288	7.77053	6.81332	6.08322	6.83273	8.2618	7.39235	6.07974	7.73303	7.82163	6.97652	8.75366	0.104170975	0.518002936	8	12	24	0.224759031	0.768040178	24	5.52045508	0.089432725
ENSMUSG00000001962	Fam50a	family with sequence similarity 50, member A [Source:MGI Symbol;Acc:MGI:1351626]	17.5941	16.8226	14.7562	15.1412	16.132	18.0084	16.8496	15.2639	14.9068	16.9747	16.1948	17.552	16.8801	15.4861	9.08E-05	0.029372629	24	12	24	0.002528264	0.396968951	24	20.66250631	0.103071691
ENSMUSG00000001964	Emd	emerin [Source:MGI Symbol;Acc:MGI:108117]	23.8434	20.6529	22.1844	19.3664	21.6279	21.8175	22.9548	22.4853	20.1247	21.8657	20.0337	20.7127	21.9958	21.0235	0.032045518	0.324385159	4	16	24	0.082740809	0.699403344	24	0.010146539	0.06259525
ENSMUSG00000001983	Taco1	translational activator of mitochondrially encoded cytochrome c oxidase I [Source:MGI Symbol;Acc:MGI:1917457]	11.2093	9.27247	9.82454	10.3223	9.63671	10.9321	11.1536	11.4962	10.0921	12.2982	12.2561	11.1194	11.3543	11.7078	0.853736882	0.966018836	8	4	24	0.694435677	0.898522833	24	19.9945776	0.031936147
ENSMUSG00000001985	Grik3	glutamate receptor, ionotropic, kainate 3 [Source:MGI Symbol;Acc:MGI:95816]	2.33333	2.03887	2.26189	2.26183	2.29276	2.42758	2.63499	2.12867	1.7436	2.72586	1.84419	2.0926	2.07664	2.08144	0.83638114	0.966018836	4	4	24	0.669978436	0.888925819	24	19.01545158	0.043605672
ENSMUSG00000001986	Gria3	glutamate receptor, ionotropic, AMPA3 (alpha 3) [Source:MGI Symbol;Acc:MGI:95810]	9.39486	11.2162	12.5242	11.1233	10.5316	10.7736	11.8316	10.083	12.3952	9.83268	10.9545	10.5162	9.94179	10.851	0.195343269	0.673387182	12	16	24	0.317796312	0.768040178	24	8.753202495	0.067745409
ENSMUSG00000001995	Sipa1l2	signal-induced proliferation-associated 1 like 2 [Source:MGI Symbol;Acc:MGI:2676970]	6.76571	5.62409	6.33422	6.92172	6.05989	6.25156	7.31461	5.90342	6.38407	8.26965	6.66492	6.81764	6.1625	5.66031	0.002034622	0.100255953	16	16	24	0.025555139	0.586541342	24	15.4948241	0.137583683
ENSMUSG00000001998	Ap4e1	adaptor-related protein complex AP-4, epsilon 1 [Source:MGI Symbol;Acc:MGI:1336993]	6.12158	5.94298	5.68104	6.34567	6.40341	5.9341	6.01859	5.99263	6.7626	5.45949	6.16231	6.71227	6.45166	6.70208	0.81544087	0.966018836	20	20	24	0.84627234	0.953507513	24	23.04477536	0.01691457
ENSMUSG00000001999	Blvra	biliverdin reductase A [Source:MGI Symbol;Acc:MGI:88170]	22.3155	24.8401	24.7474	23.727	24.1951	23.7141	21.7469	23.2959	26.7709	19.5655	26.413	23.636	25.2565	24.2285	0.913451828	0.966018836	12	16	24	0.922354229	0.979411448	24	7.334653309	0.01875751
ENSMUSG00000002006	Pdzd4	PDZ domain containing 4 [Source:MGI Symbol;Acc:MGI:2443483]	16.7406	13.7086	14.1547	15.4531	14.0407	16.9155	14.2529	14.6056	12.9446	16.2882	14.5811	17.0922	15.2825	16.0484	0.022634137	0.27189101	24	12	24	0.198054345	0.768040178	24	19.98125151	0.081823262
ENSMUSG00000002007	Srpk3	serine/arginine-rich protein specific kinase 3 [Source:MGI Symbol;Acc:MGI:1891338]	5.76679	7.12999	8.11409	5.25415	9.15088	6.89705	8.00467	5.06339	7.81151	7.87028	7.76234	5.21497	5.47249	5.88434	0.465197787	0.966018836	20	4	24	0.345526209	0.778420274	24	13.18247574	0.137502277
ENSMUSG00000002010	Idh3g	isocitrate dehydrogenase 3 (NAD+), gamma [Source:MGI Symbol;Acc:MGI:1099463]	74.034	79.3447	89.2009	102.043	84.773	80.9243	83.2549	89.283	87.1434	88.5255	90.8463	82.4828	91.6716	88.1089	0.447071126	0.966018836	16	8	24	0.035409484	0.608560667	24	10.88308021	0.095236822
ENSMUSG00000002012	Pnck	pregnancy upregulated non-ubiquitously expressed CaM kinase [Source:MGI Symbol;Acc:MGI:1347357]	10.3567	11.8068	15.3179	11.1386	10.0617	12.1189	11.3387	9.66319	13.7182	10.4037	8.76329	11.0164	9.61143	12.1661	0.064422003	0.414867207	12	8	24	0.191255348	0.768040178	24	7.05494246	0.135261268
ENSMUSG00000002014	Ssr4	signal sequence receptor, delta [Source:MGI Symbol;Acc:MGI:1099464]	55.5579	61.9045	60.1886	56.9024	61.8118	55.7686	56.5573	60.9973	69.5221	49.9105	64.6151	60.4413	61.6223	60.9667	0.652290823	0.966018836	12	8	24	0.710746917	0.904451957	24	6.209376445	0.037613197
ENSMUSG00000002015	Bcap31	B cell receptor associated protein 31 [Source:MGI Symbol;Acc:MGI:1350933]	126.015	128.751	122.155	126.516	130.351	119.579	119.348	121.478	123.414	103.899	127.993	120.99	127.896	124.663	0.890235289	0.966018836	20	4	24	0.671767933	0.889332808	24	23.20074004	0.028373012
ENSMUSG00000002017	Fam98a	family with sequence similarity 98, member A [Source:MGI Symbol;Acc:MGI:1919972]	35.2718	33.3271	32.7768	32.5182	32.3415	33.1278	31.5222	29.674	28.6719	33.4926	30.0931	31.8893	31.0716	31.2052	0.895137379	0.966018836	20	20	24	0.173058331	0.768040178	24	21.75901286	0.044248003
ENSMUSG00000002020	Ltbp2	latent transforming growth factor beta binding protein 2 [Source:MGI Symbol;Acc:MGI:99502]	0.673685	0.586265	0.976715	1.81325	0.68528	0.945878	1.08055	0.985103	0.930038	1.40495	1.59973	0.818466	1.09976	0.801743	0.350544145	0.870418653	16	16	24	0.086856029	0.70616511	24	12.99283971	0.186878738
ENSMUSG00000002028	Kmt2a	lysine (K)-specific methyltransferase 2A [Source:MGI Symbol;Acc:MGI:96995]	7.58979	7.56188	7.27397	8.07795	7.6969	6.84769	8.46283	6.98808	8.29827	8.04433	8.27741	7.64374	7.77428	8.07667	0.935593607	0.966018836	16	16	24	0.612527204	0.865571792	24	11.27413384	0.030584062
ENSMUSG00000002031	Ift46	intraflagellar transport 46 [Source:MGI Symbol;Acc:MGI:1923818]	49.4657	54.8625	51.6363	50.226	54.6071	55.763	49.097	50.0264	47.8336	50.315	48.3756	53.0147	52.1804	56.0544	0.939103211	0.966018836	8	4	24	0.682809067	0.894290982	24	22.32103926	0.027164342
ENSMUSG00000002032	Tmem25	transmembrane protein 25 [Source:MGI Symbol;Acc:MGI:1918937]	33.1586	24.2919	25.3523	26.9434	25.6609	31.4092	27.1523	29.8819	21.3867	34.6154	22.5396	27.4948	25.832	27.3587	0.601647512	0.966018836	24	12	24	0.637612345	0.876752586	24	21.30220324	0.074446735
ENSMUSG00000002043	Trappc6a	trafficking protein particle complex 6A [Source:MGI Symbol;Acc:MGI:1914341]	16.8582	14.6076	16.8349	16.1084	15.5539	15.8022	16.2343	14.9639	15.0417	16.6369	18.5593	14.7047	16.8479	17.1372	0.865514257	0.966018836	4	4	24	0.784374658	0.930811164	24	15.12518803	0.025219097
ENSMUSG00000002052	Supt6	SPT6, histone chaperone and transcription elongation factor [Source:MGI Symbol;Acc:MGI:107726]	20.8592	16.14	17.8355	17.732	17.2967	19.0375	17.8706	18.726	14.1268	21.7131	14.8844	17.7259	16.4436	17.0052	0.916556753	0.966018836	12	4	24	0.76849335	0.925385814	24	20.38381585	0.046324479
ENSMUSG00000002055	Spag5	sperm associated antigen 5 [Source:MGI Symbol;Acc:MGI:1927470]	1.10432	1.93809	2.12693	2.06656	1.1102	1.71189	2.33688	1.24761	1.63127	1.41635	1.03599	2.06887	1.22109	1.88963	0.966749204	0.969592092	12	8	24	0.64670414	0.880504888	24	5.580471178	0.09368569
ENSMUSG00000002058	Unc119	unc-119 lipid binding chaperone [Source:MGI Symbol;Acc:MGI:1328357]	28.1672	24.5392	23.3272	25.2283	24.1677	27.8381	24.569	23.3204	20.605	25.8229	23.0669	25.1572	23.0762	26.5026	0.891105762	0.966018836	24	12	24	0.22850775	0.768040178	24	20.81589248	0.076098235
ENSMUSG00000002059	Rab34	RAB34, member RAS oncogene family [Source:MGI Symbol;Acc:MGI:104606]	12.5554	18.5887	20.9016	18.1927	20.7053	17.1405	14.3515	17.7546	21.0329	16.543	16.4084	15.6726	15.3886	16.8283	1.18E-04	0.029372629	12	16	24	0.026119524	0.586541342	24	10.27792044	0.191489729
ENSMUSG00000002064	Sdf2	stromal cell derived factor 2 [Source:MGI Symbol;Acc:MGI:108019]	57.1001	58.8535	56.1166	55.9805	61.382	59.1739	52.1979	54.9283	59.6538	46.914	56.6805	56.1395	56.5658	55.3544	0.918497267	0.966018836	20	20	24	0.737902237	0.91451866	24	21.06579947	0.028904904
ENSMUSG00000002068	Ccne1	cyclin E1 [Source:MGI Symbol;Acc:MGI:88316]	4.71593	3.82952	3.5407	4.67727	4.17432	4.42024	4.16654	3.89977	3.39563	4.41689	3.79646	4.037	3.88508	3.89243	0.118446606	0.541945448	16	20	24	0.145890073	0.754579257	24	18.75750319	0.081383733
ENSMUSG00000002083	Bbc3	BCL2 binding component 3 [Source:MGI Symbol;Acc:MGI:2181667]	2.11694	2.37581	2.96687	2.08686	1.46916	1.60181	2.22675	2.13922	2.70999	3.02016	3.83342	2.78469	3.30665	3.15872	0.844488441	0.966018836	12	24	24	0.353919164	0.781118317	24	7.735260469	0.129607314
ENSMUSG00000002102	Psmc3	proteasome (prosome, macropain) 26S subunit, ATPase 3 [Source:MGI Symbol;Acc:MGI:1098754]	150.927	152.108	145.103	148.649	156.152	146.257	140.072	143.223	145.789	136.141	150.517	146.43	149.572	142.488	0.865233232	0.966018836	20	20	24	0.405519505	0.799546792	24	18.97521384	0.026001108
ENSMUSG00000002103	Acp2	acid phosphatase 2, lysosomal [Source:MGI Symbol;Acc:MGI:87882]	25.6964	25.2533	25.4705	25.3823	25.2059	25.3456	23.7456	25.1429	23.8998	24.9649	25.9437	26.0176	26.0043	25.5366	0.65208848	0.966018836	12	24	24	0.534811975	0.826740551	24	19.0452453	0.017595671
ENSMUSG00000002104	Rapsn	receptor-associated protein of the synapse [Source:MGI Symbol;Acc:MGI:99422]	3.89064	4.28828	4.43481	4.47247	5.4973	4.87994	4.80196	4.65425	4.65269	5.35459	4.14663	4.34197	4.43611	3.74482	0.688644847	0.966018836	16	16	24	0.209625528	0.768040178	24	14.56564227	0.083719539
ENSMUSG00000002105	Slc39a13	solute carrier family 39 (metal ion transporter), member 13 [Source:MGI Symbol;Acc:MGI:1915677]	20.9824	19.2613	18.4694	20.9489	21.9172	18.5445	21.0499	18.92	21.3081	20.8836	18.7271	20.1843	19.7038	17.9716	0.965521737	0.969272194	20	12	24	0.500431726	0.808986978	24	15.68920103	0.039654458
ENSMUSG00000002107	Celf2	CUGBP, Elav-like family member 2 [Source:MGI Symbol;Acc:MGI:1338822]	20.0995	24.3925	21.9912	24.6701	24.4016	21.4668	23.5067	24.077	25.5584	23.8393	20.8153	22.1911	21.1836	21.017	0.203788871	0.689899499	12	16	24	0.151131455	0.758865539	24	9.870395717	0.077977991

ENSMUSG00000002108	Nr1h3	nuclear receptor subfamily 1, group H, member 3 [Source:MGI Symbol;Acc:MGI:1352462]	6.55394	5.37053	5.68447	7.13277	8.95286	5.82542	7.69868	6.02673	6.31745	5.49596	8.09285	5.66661	7.14844	5.72213	0.295379186	0.805301811	20	12	24	0.206606709	0.768040178	24	17.41412687	0.138751523
ENSMUSG00000002109	Ddb2	damage specific DNA binding protein 2 [Source:MGI Symbol;Acc:MGI:1355314]	5.9076	7.37854	7.05288	6.60028	6.52575	6.67751	6.0683	6.04398	8.07778	6.39874	6.34636	6.20386	6.81853	6.75194	0.277087842	0.789849983	12	16	24	0.129290171	0.740162193	24	8.243357682	0.08382089
ENSMUSG00000002111	Spi1	spleen focus forming virus (SFFV) proviral integration oncogene [Source:MGI Symbol;Acc:MGI:98282]	2.59856	2.96719	2.82703	2.06829	2.91526	3.0771	3.61046	3.46396	3.7277	2.50266	2.96722	3.42432	2.12881	2.20448	0.945728192	0.966018836	24	16	24	0.870435128	0.963313607	24	22.75588228	0.04451562
ENSMUSG00000002129	Sf3a1	splicing factor 3a, subunit 1 [Source:MGI Symbol;Acc:MGI:1914715]	10.4501	8.19265	8.79324	8.98289	8.78906	9.22726	9.03523	8.7065	8.05715	11.0728	8.40416	9.30359	8.50102	9.05901	0.665691261	0.966018836	24	12	24	0.711144721	0.904572639	24	17.71114284	0.038473897
ENSMUSG00000002147	Stat6	signal transducer and activator of transcription 6 [Source:MGI Symbol;Acc:MGI:103034]	35.9978	29.6721	29.1335	32.2864	35.5463	33.7402	33.6496	33.3129	28.5627	33.4458	32.2059	31.9023	33.7149	29.3782	0.029412383	0.322165609	4	8	24	0.020295983	0.565108098	24	19.50100961	0.102469017
ENSMUSG00000002190	Clgn	calmegin [Source:MGI Symbol;Acc:MGI:107472]	55.3874	49.2819	46.4487	49.0513	47.7775	51.2569	44.6181	47.2271	42.4708	51.0913	49.4723	51.697	53.7658	52.9728	0.112800839	0.530777648	4	8	24	0.269487115	0.768040178	24	21.25893894	0.066275291
ENSMUSG00000002204	Napsa	napsin A aspartic peptidase [Source:MGI Symbol;Acc:MGI:109365]	2.15854	1.77115	2.92984	1.85515	1.91868	1.79799	2.63537	1.78372	1.95204	1.20078	1.57249	1.96829	1.74086	1.63622	0.846840613	0.966018836	12	4	24	0.492227098	0.805393068	24	1.381637194	0.083638378
ENSMUSG00000002205	Vrk3	vaccinia related kinase 3 [Source:MGI Symbol;Acc:MGI:2182465]	11.2552	10.1577	11.3164	10.4706	11.3864	11.7702	12.0076	12.9249	10.9615	14.207	10.596	11.4023	11.4263	9.67996	0.941885246	0.966018836	4	4	24	0.856606424	0.957505126	24	15.77969304	0.028934207
ENSMUSG00000002210	Smg9	SMG9 nonsense mediated mRNA decay factor [Source:MGI Symbol;Acc:MGI:1919247]	10.4335	10.6687	9.57886	10.6612	11.4081	10.0111	9.14898	11.573	10.1223	11.7257	8.88807	10.5252	10.5801	10.8366	0.570349842	0.966018836	8	16	24	0.757623693	0.921675291	24	8.306160707	0.033840554
ENSMUSG00000002221	Paxip1	PAX interacting (with transcription-activation domain) protein 1 [Source:MGI Symbol;Acc:MGI:1890430]	7.29174	8.14539	8.49571	8.94777	8.53842	7.82846	8.05239	8.78457	7.99435	8.95306	7.88596	8.27084	7.23248	7.99415	0.0043709	0.140598674	16	12	24	0.027676716	0.590220615	24	11.18667956	0.080375207
ENSMUSG00000002222	Rmnd5a	required for meiotic nuclear division 5 homolog A [Source:MGI Symbol;Acc:MGI:1915727]	30.3526	32.4629	30.9164	32.7548	30.9347	30.2096	32.2567	30.3071	32.223	31.8339	30.6383	30.9955	30.2891	31.7868	0.441501717	0.966018836	16	8	24	0.180140888	0.768040178	24	8.901010595	0.029252943
ENSMUSG00000002227	Mov10	Mov10 RISC complex RNA helicase [Source:MGI Symbol;Acc:MGI:97054]	1.48117	1.87383	1.56148	1.3993	1.84784	1.63425	2.09148	2.07872	1.76024	1.65174	1.73231	1.58465	1.52983	1.56475	0.88067614	0.966018836	8	20	24	0.724292403	0.90965493	24	2.309673393	0.035256463
ENSMUSG00000002228	Ppm1j	protein phosphatase 1J [Source:MGI Symbol;Acc:MGI:1919137]	62.0974	45.1721	43.5166	47.3881	51.5085	50.5271	45.6364	51.5853	42.642	53.0879	51.1369	47.8404	53.3303	47.3879	0.203305541	0.689605535	20	16	24	0.203346708	0.768040178	24	20.24491451	0.098254967
ENSMUSG00000002233	Rhoc	ras homolog family member C [Source:MGI Symbol;Acc:MGI:106028]	53.3931	44.6304	43.7525	36.366	50.7662	41.9768	48.182	47.9674	41.4728	33.6968	49.8975	42.459	46.3783	39.769	0.033553741	0.324908923	4	12	24	0.076070766	0.686372559	24	22.40062045	0.149315173
ENSMUSG00000002249	Tead3	TEA domain family member 3 [Source:MGI Symbol;Acc:MGI:109241]	3.95746	3.67125	4.17064	5.96394	5.12415	4.14611	4.26338	4.09669	5.40834	4.9493	4.50544	4.08359	3.98463	3.31492	8.58E-04	0.071344007	16	16	24	0.004739025	0.434006302	24	13.15006933	0.193406722
ENSMUSG00000002250	Ppard	peroxisome proliferator activator receptor delta [Source:MGI Symbol;Acc:MGI:101884]	4.67935	3.53252	3.97057	3.9951	4.16847	4.35552	4.48822	4.63657	4.33363	5.08391	4.4187	4.13304	3.93001	4.14311	0.832293447	0.966018836	4	4	24	0.777288385	0.928633759	24	15.71397707	0.029521005
ENSMUSG00000002257	Def6	differentially expressed in FDCP 6 [Source:MGI Symbol;Acc:MGI:1346328]	8.61201	5.80571	6.05708	6.34398	5.87761	6.83921	7.48004	7.667	5.35124	8.19333	6.94186	7.84477	7.74599	6.89315	0.105280783	0.518002936	4	8	24	0.174924123	0.768040178	24	21.94804138	0.127052161
ENSMUSG00000002265	Peg3	paternally expressed 3 [Source:MGI Symbol;Acc:MGI:104748]	46.0358	49.2003	51.1445	47.2742	42.7805	47.6148	46.048	42.4033	52.4779	47.3329	46.9484	53.0973	48.0508	55.3403	0.870491976	0.966018836	12	8	24	0.459033099	0.805393068	24	6.421363965	0.050922337
ENSMUSG00000002266	Zim1	zinc finger, imprinted 1 [Source:MGI Symbol;Acc:MGI:1341879]	1.58328	1.61656	1.49843	1.4397	1.61328	1.54557	1.60232	1.3738	1.79799	1.15101	1.93735	1.69012	1.48039	1.23618	0.510431574	0.966018836	20	20	24	0.403356982	0.799546792	24	18.86617646	0.064149993
ENSMUSG00000002274	Metrn	meteorin, glial cell differentiation regulator [Source:MGI Symbol;Acc:MGI:1917333]	29.5433	39.4757	42.3213	31.7509	40.3749	36.7608	42.5117	36.8864	42.2038	30.9829	37.8062	35.6336	37.9003	34.5969	0.908406451	0.966018836	12	4	24	0.854354122	0.956491441	24	4.92873658	0.037911267
ENSMUSG00000002279	Lmf1	lipase maturation factor 1 [Source:MGI Symbol;Acc:MGI:1923733]	11.2181	9.01966	9.53836	9.40435	10.1136	9.20198	10.0756	9.76541	8.5041	11.0742	9.63992	10.2216	10.1551	9.72453	0.267533088	0.774280302	4	8	24	0.435112093	0.805393068	24	20.07653108	0.015476701
ENSMUSG00000002280	Ciao3	cytosolic iron-sulfur assembly component 3 [Source:MGI Symbol;Acc:MGI:1914813]	9.17041	9.67972	9.62009	10.5506	10.3525	9.43289	11.4397	9.93574	10.3894	10.0767	9.21828	9.37022	10.2682	9.36148	0.945728192	0.966018836	16	8	24	0.885469957	0.967390811	24	8.85254368	0.017252826
ENSMUSG00000002289	Angptl4	angiopoietin-like 4 [Source:MGI Symbol;Acc:MGI:1888999]	11.2239	8.70466	7.44888	8.99856	8.59043	9.16447	13.7052	19.4779	7.88838	8.39869	8.61093	12.3152	17.1102	12.8335	0.0014632	0.099807435	4	8	24	0.009951661	0.503967859	24	0.788633534	0.368265043
ENSMUSG00000002297	Dbf4	DBF4 zinc finger [Source:MGI Symbol;Acc:MGI:1351328]	1.49116	1.09549	1.86468	1.71539	1.69077	1.56929	1.6194	1.51324	1.3232	1.24868	1.5637	1.2317	1.51494	1.22297	0.239653024	0.741580974	12	20	24	0.509937074	0.813985618	24	15.90828459	0.056303605
ENSMUSG00000002307	Daxx	Fas death domain-associated protein [Source:MGI Symbol;Acc:MGI:1197015]	8.25981	6.11103	6.63998	7.56678	7.46629	7.54816	7.1223	6.83882	8.37011	8.54037	6.34327	7.86964	7.36421	7.16277	0.5169953	0.966018836	16	16	24	0.742717154	0.916215545	24	15.49924879	0.039194081
ENSMUSG00000002308	Cd320	CD320 antigen [Source:MGI Symbol;Acc:MGI:1860083]	12.1023	13.1588	12.172	12.3232	14.1823	12.6375	13.0195	12.9044	14.4824	12.5906	16.0393	15.5123	16.3301	13.1246	0.826609618	0.966018836	20	20	24	0.351941775	0.780447911	24	18.77213099	0.061579932
ENSMUSG00000002319	Ipo4	importin 4 [Source:MGI Symbol;Acc:MGI:1923001]	27.0611	31.0321	25.3599	32.3107	25.2359	27.2281	28.1842	26.0915	24.674	28.7546	26.8781	27.175	30.0528	27.5218	0.723119329	0.966018836	4	8	24	0.964223308	0.991092813	24	23.83661166	0.012054777
ENSMUSG00000002320	Tm9sf1	transmembrane 9 superfamily member 1 [Source:MGI Symbol;Acc:MGI:1921390]	16.7074	15.3591	15.7317	16.9886	15.9494	16.6382	16.908	17.1187	15.4873	17.8464	14.8312	15.4189	15.3804	16.3762	0.890648739	0.966018836	16	4	24	0.928931297	0.981515518	24	8.174234885	0.011670104
ENSMUSG00000002324	Rec8	REC8 meiotic recombination protein [Source:MGI Symbol;Acc:MGI:1929645]	1.03911	1.33669	1.59555	0.998459	1.03048	0.859354	0.92028	0.821537	1.17101	0.84095	0.686575	1.2001	0.727827	1.06245	0.446653834	0.966018836	12	8	24	0.289763592	0.768040178	24	6.913784904	0.095895574
ENSMUSG00000002325	Irf9	interferon regulatory factor 9 [Source:MGI Symbol;Acc:MGI:107587]	13.105	12.178	11.778	13.1792	14.0543	12.8112	12.2046	13.0033	13.5111	10.867	14.2956	11.6557	11.0829	12.4548	0.89563591	0.966018836	20	8	24	0.573398027	0.847005958	24	15.44609191	0.044503861
ENSMUSG00000002326	Gmpr2	guanosine monophosphate reductase 2 [Source:MGI Symbol;Acc:MGI:1917903]	19.8328	21.8014	20.0947	21.6461	23.8329	20.1996	19.0633	18.4218	20.4284	16.5564	23.4818	20.2451	21.6691	19.8101	0.454605943	0.966018836	20	12	24	0.435378385	0.805393068	24	17.46793583	0.066775509
ENSMUSG00000002329	Mdp1	magnesium-dependent phosphatase 1 [Source:MGI Symbol;Acc:MGI:1915131]	27.9986	27.0307	25.9454	28.7027	26.8478	26.1018	27.0338	25.6678	26.5613	28.0862	25.8707	26.517	27.518	27.4566	0.745469888	0.966018836	16	4	24	0.953484811	0.988601783	24	10.73556944	0.006007183
ENSMUSG00000002332	Dhrs1	dehydrogenase/reductase (SDR family) member 1 [Source:MGI Symbol;Acc:MGI:1196314]	94.4678	89.709	90.7807	89.3384	98.8805	103.602	85.8939	94.08	81.5444	89.8534	98.2736	93.1968	87.9005	86.283	0.140905066	0.585944911	20	16	24	0.014751864	0.537860185	24	18.29272776	0.09014471
ENSMUSG00000002341	Ncan	neurocan [Source:MGI Symbol;Acc:MGI:104694]	1.35502	1.49494	1.94041	1.30628	1.12429	1.62837	1.76765	1.65985	1.39088	1.87857	1.27968	1.36825	1.22289	1.76277	0.235141005	0.73629392	12	8	24	0.228108415	0.768040178	24	6.100910111	0.10020188
ENSMUSG00000002342	Tmem161a	transmembrane protein 161A [Source:MGI Symbol;Acc:MGI:2384577]	14.0857	13.5418	14.4054	12.4014	14.2852	14.6415	16.344	18.7671	13.8986	15.5585	14.4873	15.2175	15.73	16.2968	0.465197787	0.966018836	8	4	24	0.146786404	0.755260456	24	1.939227552	0.084511858
ENSMUSG00000002343	Armc6	armadillo repeat containing 6 [Source:MGI Symbol;Acc:MGI:1924063]	18.6846	19.1038	21.8161	19.0799	21.4348	21.395	20.558	20.7715	16.1887	17.9976	18.7297	17.6933	19.8448	18.5854	0.773386789	0.966018836	20	20	24	0.602507782	0.861114219	24	21.13440509	0.045313359
ENSMUSG00000002345	Borcs8	BLOC-1 related complex subunit 8 [Source:MGI Symbol;Acc:MGI:1919618]	33.0301	32.6698	30.5645	26.7298	32.7586	30.5896	29.3382	29.6775	29.9016	28.9654	31.2466	31.8536	32.6621	30.3937	0.280810068	0.792139038	20	20	24	0.127776281	0.740162193	24	22.35163934	0.063940311
ENSMUSG00000002346	Slc25a42	solute carrier family 25, member 42 [Source:MGI Symbol;Acc:MGI:1920345]	5.54924	5.43087	6.46904	7.93244	6.64588	5.37365	6.86465	6.43285	6.41069	8.10907	5.89702	5.72746	6.74845	7.10821	0.185796491	0.662239322	16	8	24	0.076133085	0.686372559	24	10.23628632	0.132607762
ENSMUSG00000002365	Snx9	sorting nexin 9 [Source:MGI Symbol;Acc:MGI:1913866]	13.8446	11.9423	12.1581	12.3585	12.5779	13.4278	12.8531	13.67	11.8705	13.6108	12.4841	12.9899	13.8151	12.906	0.017495149	0.255326033	4	8	24	0.159630147	0.765591735	24	22.08054421	0.051634485
ENSMUSG00000002372	Ranbp3	RAN binding protein 3 [Source:MGI Symbol;Acc:MGI:1919060]	18.2882	17.9131	19.4953	17.1583	17.5655	18.8438	17.9574	19.0955	17.2839	18.6519	16.8782	18.1575	17.4377	18.3029	0.850398351	0.966018836	8	12	24	0.491830519	0.805393068	24	3.756584529	0.02667783
ENSMUSG00000002379	Ndufa11	NADH:ubiquinone oxidoreductase subunit A11 [Source:MGI Symbol;Acc:MGI:1917125]	183.179	192.869	190.406	180.292	188.876	178.615	177.733	186.947	207.702	172.143	205.577	183.736	200.529	192.349	0.697196105	0.966018836	12	12	24	0.651581461	0.882823265	24	6.70513649	0.029337434
ENSMUSG00000002393	Nr2f6	nuclear receptor subfamily 2, group F, member 6 [Source:MGI Symbol;Acc:MGI:1352453]	5.26565	4.71561	5.03897	7.68409	5.31961	5.13408	10.6566	4.89258	5.30228	6.49389	7.00166	6.32183	6.00845	6.48196	0.326899754	0.845515883	16	16	24	0.759052256	0.92214435	24	18.05214757	0.081816071
ENSMUSG00000002395	Use1	unconventional SNARE in the ER 1 homolog (S. cerevisiae) [Source:MGI Symbol;Acc:MGI:1914273]	72.9924	90.342	86.4651	82.8538	83.0452	77.031	73.4335	81.6509	100.363	72.6335	86.2123	84.8755	92.9858	82.5749	0.834434272	0.966018836	12	4	24	0.413958226	0.799546792	24	7.803976527	0.071159042
ENSMUSG00000002396	Ocel1	occludin/ELL domain containing 1 [Source:MGI Symbol;Acc:MGI:1924340]	6.95684	5.00277	4.64671	6.18466	5.97236	5.53	5.09842	5.38967	5.56999	5.30056	5.50171	5.5235	5.40719	5.20485	0.258307762	0.761738864	20	12	24	0.298432927	0.768040178	24	18.5262555	0.072125896
ENSMUSG00000002409	Dyrk1b	dual-specificity tyrosine-(Y)-phosphorylation regulated kinase 1b [Source:MGI Symbol;Acc:MGI:1330302]	4.97702	4.58808	5.77185	5.08646	4.33649	4.96099	4.41675	5.97679	6.5687	6.68094	5.56899	5.85473	4.9994	5.93333	0.448583581	0.966018836	8	20	24	0.09267539	0.71175955	24	9.29173373	0.112307174
ENSMUSG00000002413	Braf	Braf transforming gene [Source:MGI Symbol;Acc:MGI:88190]	9.28425	10.4962	10.4426	10.4008	9.90466	9.57197	10.0911	9.05518	10.5692	9.24987	9.62535	9.91742	9.39941	10.8084	0.569060064	0.966018836	12	16	24	0.31386212	0.768040178	24	7.912088263	0.04600977
ENSMUSG00000002416	Ndufb2	NADH:ubiquinone oxidoreductase subunit B2 [Source:MGI Symbol;Acc:MGI:1915448]	121.642	51.6905	125.556	132.718	138.816	127.887	115.123	131.48	157.456	118.025	206.455	178.395	120.646	85.0535	0.033553741	0.324908923	20	12	24	0.059864632	0.658304792	24	15.96078661	0.380557606
ENSMUSG00000002428	Hltf	helicase-like transcription factor [Source:MGI Symbol;Acc:MGI:1196437]	10.4088	11.4248	10.9626	10.3492	11.1905	10.1441	10.9151	9.63573	10.9334	8.54607	12.2511	9.8125	10.1343	10.336	0.953760335	0.966018836	20	4	24	0.954991009	0.988639288	24	19.27948222	0.01412821
ENSMUSG00000002455	Prpf6	pre-mRNA splicing factor 6 [Source:MGI Symbol;Acc:MGI:1922946]	20.3044	17.4736	20.0274	17.5993	18.6321	21.1612	17.9358	20.2185	17.2291	21.0464	16.5048	19.4037	19.5329	19.4305	0.95032543	0.966018836	24	20	24	0.725510068	0.90999235	24	23.57869572	0.035003314
ENSMUSG00000002458	Rgs19	regulator of G-protein signaling 19 [Source:MGI Symbol;Acc:MGI:1915153]	10.3726	8.89165	10.4612	11.1968	11.2837	10.3897	11.6356	10.4345	9.84586	7.38362	11.0399	10.2489	9.68508	7.25757	0.305564675	0.811201458	20	12	24	0.189091851	0.768040178	24	18.76894714	0.124616724
ENSMUSG00000002459	Rgs20	regulator of G-protein signaling 20 [Source:MGI Symbol;Acc:MGI:1929866]	1.64951	1.28606	2.32337	1.14789	1.66657	1.60302	1.62546	1.25593	0.99032	1.23131	1.85421	1.45621	0.990659	1.93857	0.865490576	0.966018836	20	20	24	0.894976629	0.970212381	24	19.02301722	0.042548528
ENSMUSG00000002475	Abhd3	abhydrolase domain containing 3 [Source:MGI Symbol;Acc:MGI:2147183]	14.4673	13.9546	15.552	13.0158	11.7612	14.8389	14.2894	12.1253	10.4587	11.1256	11.4427	10.5044	10.0885	12.2533	0.82502117	0.966018836	24	20	24	0.488304082	0.805393068	24	1.123115172	0.058839833
ENSMUSG00000002477	Snrpd1	small nuclear ribonucleoprotein D1 [Source:MGI Symbol;Acc:MGI:98344]	30.5272	33.655	32.3135	29.2063	34.1723	30.427	33.0433	30.5848	34.7936	25.9163	34.3534	29.4726	35.3409	36.6087	0.431765373	0.966018836	8	8	24	0.419704822	0.803639585	24	2.769201509	0.065533506
ENSMUSG00000002486	Tchp	trichoplein, keratin filament binding [Source:MGI Symbol;Acc:MGI:1925082]	4.71945	4.39779	4.7363	4.87833	5.04754	5.07042	5.87786	4.50615	4.70923	5.61109	5.35388	4.79266	4.41076	4.22139	0.004021228	0.140598674	20	12	24	0.095422569	0.71175955	24	16.26240792	0.091173533
ENSMUSG00000002489	Tiam1	T cell lymphoma invasion and metastasis 1 [Source:MGI Symbol;Acc:MGI:103306]	4.35549	4.60653	4.91053	4.64852	4.45975	4.21992	4.65685	4.89834	5.26326	5.27901	3.81807	4.17194	4.40732	4.44092	0.001971398	0.100255953	12	12	24	0.010791433	0.51149922	24	7.886816895	0.110422857
ENSMUSG00000002496	Tsc2	TSC complex subunit 2 [Source:MGI Symbol;Acc:MGI:102548]	26.4511	28.1245	28.2935	30.97	33.812	28.3134	30.3232	28.7226	28.1553	29.4896	27.8544	24.4796	27.806	24.8861	0.335107163	0.85758369	16	12	24	0.191474022	0.768040178	24	13.69659999	0.071612105
ENSMUSG00000002500	Rpl3l	ribosomal protein L3-like [Source:MGI Symbol;Acc:MGI:1913461]	2.42182	7.45041	8.43814	10.6054	6.41273	8.8	4.87613	19.1593	13.9231	14.948	7.00053	5.51715	8.69143	7.13522	0.082282215	0.462922111	16	12	24	0.032995581	0.606585237	24	9.370580539	0.592575308
ENSMUSG00000002504	Slc9a3r2	solute carrier family 9 (sodium/hydrogen exchanger), member 3 regulator 2 [Source:MGI Symbol;Acc:MGI:1890662]	52.0727	43.0103	41.0284	44.5842	47.0295	47.0262	48.1325	49.3892	43.4303	54.2262	42.0232	47.0657	46.6006	44.5572	0.43565473	0.966018836	4	8	24	0.569028395	0.844691814	24	21.00037212	0.049218903
ENSMUSG00000002524	Puf60	poly-U binding splicing factor 60 [Source:MGI Symbol;Acc:MGI:1915209]	67.838	63.6417	63.9578	60.4573	61.4069	62.5399	66.167	67.456	63.8326	69.7911	65.7433	67.3764	69.9322	64.2845	0.20729371	0.698501896	4	12	24	0.36182418	0.787031962	24	0.704663624	0.03178947
ENSMUSG00000002546	Golga2	golgi autoantigen, golgin subfamily a, 2 [Source:MGI Symbol;Acc:MGI:2139395]	27.8201	22.9344	24.855	23.7183	24.8678	25.6635	26.0725	26.6331	21.584	30.0921	21.301	25.8461	25.4076	25.4093	0.439800617	0.966018836	4	8	24	0.702153956	0.901574817	24	22.93111428	0.04402361
ENSMUSG00000002550	Uck1	uridine-cytidine kinase 1 [Source:MGI Symbol;Acc:MGI:98904]	20.2341	16.9708	19.2461	23.7828	18.6664	19.022	19.2791	19.1574	17.2812	18.6666	22.4728	18.4031	22.4797	20.2034	0.94086481	0.966018836	4	8	24	0.681743737	0.893856974	24	16.67724212	0.046793709
ENSMUSG00000002565	Scin	scinderin [Source:MGI Symbol;Acc:MGI:1306794]	5.42221	4.8169	3.83315	4.67744	4.33776	4.69572	4.80135	4.54866	4.04814	4.13445	4.4226	4.43334	5.63982	4.60873	1.18E-04	0.029372629	4	8	24	0.011466352	0.519508195	24	23.09033687	0.126206385
ENSMUSG00000002578	Ikzf4	IKAROS family zinc finger 4 [Source:MGI Symbol;Acc:MGI:1343139]	0.596863	0.595071	1.2808	0.649796	0.824895	0.557158	0.484921	0.603396	0.673541	0.511245	0.709497	1.01232	0.718987	0.675229	0.92769718	0.966018836	20	8	24	0.766797943	0.92458474	24	12.01725541	0.048848247
ENSMUSG00000002580	Mien1	migration and invasion enhancer 1 [Source:MGI Symbol;Acc:MGI:1913678]	114.583	102.526	100.298	95.2715	106.721	102.764	95.5003	101.203	92.5542	82.5274	105.798	98.6842	103.289	99.2053	0.082113105	0.462922111	20	20	24	0.064979241	0.663876076	24	22.08312205	0.089773029
ENSMUSG00000002588	Pon1	paraoxonase 1 [Source:MGI Symbol;Acc:MGI:103295]	0.323126	0.922783	1.05271	1.82542	0.462934	0.702828	0.523473	1.18615	0.923619	1.16349	0.890097	1.00856	1.81521	1.95213	0.311445561	0.821921422	16	4	24	0.101819096	0.713240751	24	7.883935154	0.245234183
ENSMUSG00000002602	Axl	AXL receptor tyrosine kinase [Source:MGI Symbol;Acc:MGI:1347244]	12.4444	14.2481	16.07	14.0188	15.4762	13.5024	12.4276	14.3153	17.0178	12.2512	13.7345	14.1411	12.4553	13.4892	0.019594567	0.258568924	12	16	24	0.176535719	0.768040178	24	9.902508176	0.096029461
ENSMUSG00000002603	Tgfb1	transforming growth factor, beta 1 [Source:MGI Symbol;Acc:MGI:98725]	1.42512	1.59147	1.63174	1.51801	1.28406	1.29712	1.76862	2.20838	1.96881	1.42136	2.39649	1.76821	2.00216	1.70642	0.557756884	0.966018836	8	8	24	0.516588985	0.817817397	24	5.070171236	0.068674866
ENSMUSG00000002608	Ccdc97	coiled-coil domain containing 97 [Source:MGI Symbol;Acc:MGI:1196455]	11.0655	9.03593	9.24793	11.02	9.69139	9.94018	11.9878	10.9313	10.7674	12.8895	11.073	11.1441	10.5624	9.74677	0.450844752	0.966018836	4	4	24	0.673968924	0.890473027	24	16.07745457	0.043924322
ENSMUSG00000002617	Zfp40	zinc finger protein 40 [Source:MGI Symbol;Acc:MGI:99185]	14.2668	17.3764	14.485	17.8625	16.2677	14.1444	14.4519	14.855	17.8622	12.8195	17.1406	15.1255	15.0328	15.9371	0.358866865	0.883787544	12	16	24	0.602758954	0.861114219	24	10.12140007	0.055959241
ENSMUSG00000002625	Akap8l	A kinase (PRKA) anchor protein 8-like [Source:MGI Symbol;Acc:MGI:1860606]	36.3553	39.028	36.6646	33.3518	40.7785	32.9319	37.9326	37.0564	41.2669	36.6652	36.3607	36.6799	39.2962	38.2756	0.636884864	0.966018836	8	16	24	0.451525054	0.805393068	24	5.286183176	0.044833085
ENSMUSG00000002633	Shh	sonic hedgehog [Source:MGI Symbol;Acc:MGI:98297]	4.80679	3.03184	2.85704	2.87542	2.99848	3.89194	3.42472	3.94995	2.45318	4.62516	3.36179	3.73327	3.76653	3.82667	0.109040811	0.524035616	4	8	24	0.249698226	0.768040178	24	22.1419844	0.138036116
ENSMUSG00000002635	Pdcd2l	programmed cell death 2-like [Source:MGI Symbol;Acc:MGI:1915329]	7.07238	9.07465	8.78175	10.5406	10.8115	10.5198	9.73327	10.0367	10.4099	8.89436	8.89218	8.61853	9.64842	9.80482	0.928780444	0.966018836	16	12	24	0.598062163	0.858819856	24	11.56582128	0.057953274
ENSMUSG00000002658	Gtf2f1	general transcription factor IIF, polypeptide 1 [Source:MGI Symbol;Acc:MGI:1923848]	32.6175	32.0125	31.5793	29.4908	33.436	32.0782	30.4426	29.2646	29.558	30.2515	31.967	31.5876	31.6376	29.7785	0.108253011	0.524035616	20	20	24	0.026304244	0.586541342	24	19.4054326	0.054483948
ENSMUSG00000002660	Clpp	caseinolytic mitochondrial matrix peptidase proteolytic subunit [Source:MGI Symbol;Acc:MGI:1858213]	26.4754	24.4852	25.249	26.2457	26.1211	26.248	25.0783	27.0797	25.6549	27.4462	26.6373	24.3318	26.5268	25.6014	0.928127947	0.966018836	20	4	24	0.583704423	0.85190598	24	13.23478616	0.020117816
ENSMUSG00000002661	Alkbh7	alkB homolog 7 [Source:MGI Symbol;Acc:MGI:1913650]	10.9989	10.7779	10.6913	10.4367	9.33657	11.0764	11.5451	13.8778	12.801	10.2381	15.1225	12.5541	13.803	12.2195	0.588682825	0.966018836	4	12	24	0.464510058	0.805393068	24	1.725435052	0.068348275
ENSMUSG00000002668	Dennd1c	DENN/MADD domain containing 1C [Source:MGI Symbol;Acc:MGI:1918035]	1.1987	1.26462	1.01147	1.87291	1.60422	1.3995	1.93823	1.46798	1.32732	0.927449	1.9969	1.18389	1.09752	1.24817	0.865490576	0.966018836	20	16	24	0.484746223	0.805393068	24	17.37893307	0.096884128
ENSMUSG00000002679	Med6	mediator complex subunit 6 [Source:MGI Symbol;Acc:MGI:1917042]	18.1182	23.5138	19.5432	19.4299	24.1591	20.4381	19.9804	19.7723	21.3835	16.8777	19.7535	20.3343	20.5599	21.3163	0.920620634	0.966018836	8	8	24	0.895160381	0.970250894	24	0.422390485	0.023943811
ENSMUSG00000002688	Prkd1	protein kinase D1 [Source:MGI Symbol;Acc:MGI:99879]	7.76546	7.05412	6.51555	7.25197	7.17785	7.56392	7.04243	7.06606	6.08769	8.24223	6.78614	7.55673	7.8903	6.29064	0.157813674	0.614233844	24	12	24	0.140982724	0.751014756	24	19.36694564	0.08434278
ENSMUSG00000002699	Lcp2	lymphocyte cytosolic protein 2 [Source:MGI Symbol;Acc:MGI:1321402]	1.43153	2.42442	2.00882	2.03303	1.49738	2.02619	1.48654	2.0737	2.33134	1.72383	1.27585	2.08929	2.33231	1.60985	0.602156008	0.966018836	8	12	24	0.348300906	0.779069631	24	5.688600856	0.110392221
ENSMUSG00000002718	Cse1l	chromosome segregation 1-like (S. cerevisiae) [Source:MGI Symbol;Acc:MGI:1339951]	28.322	31.4974	30.193	34.309	30.9416	30.5185	30.2479	29.1504	33.6349	24.7536	33.5864	29.9285	30.5415	29.4414	0.550682606	0.966018836	20	8	24	0.868417296	0.96247509	24	13.63115538	0.024106567
ENSMUSG00000002728	Naa20	N(alpha)-acetyltransferase 20, NatB catalytic subunit [Source:MGI Symbol;Acc:MGI:1915127]	33.7986	38.278	35.8798	39.3438	36.754	33.7007	38.472	34.0928	40.7659	28.7296	39.253	34.9384	39.9439	41.7182	0.867888967	0.966018836	12	12	24	0.635620624	0.876201318	24	4.435789332	0.052711109
ENSMUSG00000002731	Prkra	protein kinase, interferon inducible double stranded RNA dependent activator [Source:MGI Symbol;Acc:MGI:1344375]	20.7065	22.1368	21.0656	19.8238	20.8863	18.9505	20.1426	18.3851	21.0357	17.8581	21.7235	21.1608	20.7357	21.7741	0.945282252	0.966018836	8	8	24	0.822080703	0.943977421	24	1.597319139	0.022166722
ENSMUSG00000002732	Fkbp7	FK506 binding protein 7 [Source:MGI Symbol;Acc:MGI:1336879]	15.6617	20.8014	17.9177	17.5297	17.8228	15.0722	15.2933	19.2301	24.2651	13.8029	17.2467	17.1957	18.0197	16.9622	0.133266423	0.567728672	12	12	24	0.199513775	0.768040178	24	6.823999102	0.136360544
ENSMUSG00000002733	Plekha3	pleckstrin homology domain-containing, family A (phosphoinositide binding specific) member 3 [Source:MGI Symbol;Acc:MGI:1932515]	16.9224	13.5658	14.9893	15.0051	15.3326	15.2858	14.7016	14.1338	11.4528	15.6293	14.3906	13.1552	11.9001	13.7589	0.581070934	0.966018836	16	16	24	0.301961484	0.768040178	24	17.28120652	0.071830678
ENSMUSG00000002741	Ykt6	YKT6 v-SNARE homolog (S. cerevisiae) [Source:MGI Symbol;Acc:MGI:1927550]	39.4613	38.9061	37.3284	39.2983	39.1309	39.2989	37.5972	39.8226	36.1549	41.605	36.9314	39.8931	39.7167	40.0109	0.954237589	0.966018836	8	4	24	0.855020579	0.956831965	24	21.08569811	0.012263409
ENSMUSG00000002748	Baz1b	bromodomain adjacent to zinc finger domain, 1B [Source:MGI Symbol;Acc:MGI:1353499]	16.5781	15.6235	16.3897	17.7344	17.5027	16.085	16.9807	14.3681	16.4841	15.0576	17.1605	15.6718	16.0895	15.7057	0.117050591	0.538362324	20	12	24	0.17412533	0.768040178	24	16.16248354	0.0533835
ENSMUSG00000002763	Pex6	peroxisomal biogenesis factor 6 [Source:MGI Symbol;Acc:MGI:2385054]	9.47608	7.92208	7.12467	8.26519	8.01356	7.99494	8.51241	9.48539	8.04625	10.2259	9.18763	9.28872	9.55138	8.59195	0.887010723	0.966018836	4	8	24	0.649757631	0.882162033	24	20.87827233	0.043448099
ENSMUSG00000002767	Mrpl2	mitochondrial ribosomal protein L2 [Source:MGI Symbol;Acc:MGI:1351622]	18.2578	18.1932	18.5345	19.5529	16.8644	17.8718	17.5243	21.8015	19.0514	23.2991	16.8832	19.2304	19.5166	20.3348	0.085045716	0.466404237	16	4	24	0.134962771	0.74653384	24	7.50685352	0.092000275
ENSMUSG00000002768	Mea1	male enhanced antigen 1 [Source:MGI Symbol;Acc:MGI:96957]	86.1461	84.9145	78.8304	83.5987	89.4989	84.5197	77.8104	78.4823	81.7912	68.3593	92.3552	77.9847	84.1269	77.6007	0.401437339	0.933249185	20	12	24	0.317443415	0.768040178	24	19.19168014	0.062251025
ENSMUSG00000002769	Gnmt	glycine N-methyltransferase [Source:MGI Symbol;Acc:MGI:1202304]	0.911121	0.917405	0.652506	0.790843	0.477688	0.660302	1.03755	0.549253	0.923615	0.837285	0.594393	0.677509	0.358732	0.68286	0.885025617	0.966018836	12	8	24	0.687734467	0.896387271	24	6.033060094	0.054071582
ENSMUSG00000002771	Grin2d	glutamate receptor, ionotropic, NMDA2D (epsilon 4) [Source:MGI Symbol;Acc:MGI:95823]	0.602027	0.876831	1.31648	0.743044	0.607378	0.817393	0.933083	0.753597	1.13294	0.889863	0.604283	0.752573	0.672405	0.969136	0.010576629	0.197206032	12	8	24	0.032640743	0.606585237	24	7.246194281	0.151136796
ENSMUSG00000002778	Kdelr1	KDEL (Lys-Asp-Glu-Leu) endoplasmic reticulum protein retention receptor 1 [Source:MGI Symbol;Acc:MGI:1915387]	32.0947	31.6257	32.3784	30.3228	31.8146	32.3954	30.6535	33.107	32.0697	34.0431	29.2702	30.9154	32.0153	31.123	0.917370979	0.966018836	12	8	24	0.553973941	0.837374348	24	6.272948752	0.023465675
ENSMUSG00000002781	Tmem143	transmembrane protein 143 [Source:MGI Symbol;Acc:MGI:1917459]	11.1118	8.85799	10.1228	13.8431	9.45151	12.212	11.2579	12.9074	11.2054	13.6524	11.7487	13.5113	13.1574	12.8516	0.94791825	0.966018836	16	20	24	0.876692097	0.965723021	24	15.32943727	0.03031642
ENSMUSG00000002797	Ggct	gamma-glutamyl cyclotransferase [Source:MGI Symbol;Acc:MGI:95700]	21.1877	19.7214	21.7859	22.1273	21.225	18.5842	21.7416	19.7872	20.5784	19.2118	20.6248	19.4254	21.0065	18.4098	0.699015352	0.966018836	12	20	24	0.839749091	0.95137802	24	13.17826241	0.017300703
ENSMUSG00000002799	Jag2	jagged 2 [Source:MGI Symbol;Acc:MGI:1098270]	13.2058	10.0834	10.6962	9.63464	13.0977	10.4472	12.0416	11.9019	8.89602	14.3027	9.86533	10.9313	11.5809	11.1087	0.43565473	0.966018836	4	8	24	0.672884249	0.889786694	24	20.87021349	0.065747861
ENSMUSG00000002803	Btbd6	BTB (POZ) domain containing 6 [Source:MGI Symbol;Acc:MGI:3026623]	10.548	9.51969	10.9475	9.49124	10.3867	11.4904	11.2247	11.3418	10.1692	12.5037	8.80036	10.0545	9.54313	9.8156	0.776152743	0.966018836	24	20	24	0.984351576	0.996767829	24	6.461732953	0.009227257
ENSMUSG00000002804	Nudt14	nudix (nucleoside diphosphate linked moiety X)-type motif 14 [Source:MGI Symbol;Acc:MGI:1913424]	10.1689	11.5958	10.2579	12.0693	12.6115	10.1648	10.5006	10.3592	12.6417	10.2652	11.8958	9.65031	12.2406	12.9636	0.387380622	0.915479136	12	12	24	0.475770838	0.805393068	24	9.252547983	0.06533739
ENSMUSG00000002808	Epdr1	ependymin related protein 1 (zebrafish) [Source:MGI Symbol;Acc:MGI:2145369]	146.964	132.19	123.898	124.055	130.133	133.377	134.807	137.162	122.068	143.899	137.636	142.163	144.533	140.937	0.018894761	0.256345714	4	8	24	0.073001824	0.6801051	24	22.31889757	0.067741524
ENSMUSG00000002812	Flii	flightless I actin binding protein [Source:MGI Symbol;Acc:MGI:1342286]	32.0283	27.2214	28.3758	28.3475	29.7194	30.7345	29.0309	32.8894	26.1064	35.1821	26.037	29.4247	28.9131	27.354	0.835401638	0.966018836	24	12	24	0.853128121	0.956107729	24	19.88020186	0.028551589
ENSMUSG00000002814	Top3a	topoisomerase (DNA) III alpha [Source:MGI Symbol;Acc:MGI:1197527]	2.54388	1.9395	2.40293	1.71613	2.46285	2.10987	2.04605	2.31098	2.17228	2.06541	1.99787	2.35507	2.02213	2.00733	0.906657897	0.966018836	24	16	24	0.666257288	0.88724859	24	21.95365402	0.038549489
ENSMUSG00000002820	Atg4d	autophagy related 4D, cysteine peptidase [Source:MGI Symbol;Acc:MGI:2444308]	13.6675	12.9875	11.645	13.6591	12.8378	14.6321	13.8051	14.7213	12.3398	16.6981	14.0643	14.3177	14.9796	14.6663	0.46238066	0.966018836	24	12	24	0.629630422	0.873514169	24	19.97085311	0.040723016
ENSMUSG00000002825	Qtrt1	queuine tRNA-ribosyltransferase catalytic subunit 1 [Source:MGI Symbol;Acc:MGI:1931441]	5.68156	8.3149	7.2557	4.99032	7.75229	7.52479	6.0998	5.67087	5.89431	5.26255	5.81645	6.92646	6.21465	5.81692	0.581070934	0.966018836	24	16	24	0.532833278	0.825678486	24	21.53153741	0.08435729
ENSMUSG00000002831	Plin4	perilipin 4 [Source:MGI Symbol;Acc:MGI:1929709]	1.27288	1.47641	2.97106	6.49633	2.27179	2.77907	1.76451	4.91773	3.03975	6.47912	3.08954	2.62211	3.90611	3.53246	0.275008207	0.789849983	16	12	24	0.033287987	0.606585237	24	10.91025238	0.447435788
ENSMUSG00000002833	Hdgfl2	HDGF like 2 [Source:MGI Symbol;Acc:MGI:1194492]	21.1254	20.456	22.9768	23.9213	20.0995	19.8779	20.9197	20.9877	19.4216	24.4774	20.2691	18.4287	19.6339	19.1898	0.179603275	0.655480352	16	8	24	0.068341482	0.672656585	24	10.84930666	0.088186441
ENSMUSG00000002835	Chaf1a	chromatin assembly factor 1, subunit A (p150) [Source:MGI Symbol;Acc:MGI:1351331]	0.967937	1.07087	1.11131	1.47464	0.969626	1.16173	0.777397	1.05769	0.930676	0.859683	0.859216	0.991022	0.982896	1.08983	0.843639218	0.966018836	8	20	24	0.705735121	0.902918222	24	9.840352962	0.038105426
ENSMUSG00000002844	Adprh	ADP-ribosylarginine hydrolase [Source:MGI Symbol;Acc:MGI:1098234]	22.3372	19.4384	18.7957	18.0395	18.3708	20.1227	19.5251	20.9981	18.7291	22.6582	17.9709	20.8649	20.2696	20.6627	0.223774686	0.717349224	4	16	24	0.335706549	0.771468587	24	0.891217979	0.057263351
ENSMUSG00000002845	Tmem39a	transmembrane protein 39a [Source:MGI Symbol;Acc:MGI:1915096]	15.3397	14.3812	16.1011	18.8673	17.2434	17.067	21.0509	14.8341	17.4042	12.423	17.2139	13.984	14.3971	12.732	0.658895034	0.966018836	20	12	24	0.563133568	0.842499957	24	17.40484756	0.080786915
ENSMUSG00000002846	Timmdc1	translocase of inner mitochondrial membrane domain containing 1 [Source:MGI Symbol;Acc:MGI:1922139]	15.2299	14.0717	14.9506	14.5704	14.5441	16.3206	14.7952	15.7415	14.0296	13.9834	16.5562	15.6481	16.9764	15.6334	0.167216842	0.634643192	24	16	24	0.069263455	0.673520298	24	21.50248434	0.065931684

ENSMUSG00000002847	Pla1a	phospholipase A1 member A [Source:MGI Symbol;Acc:MGI:1934677]	1.0383	1.5585	1.47595	1.70007	1.65054	1.06089	0.775224	1.62117	2.02099	1.02571	1.38013	1.25046	1.11875	1.28063	0.033248781	0.324908923	12	16	24	0.064702111	0.663876076	24	9.771781686	0.180110161
ENSMUSG00000002870	Mcm2	minichromosome maintenance complex component 2 [Source:MGI Symbol;Acc:MGI:105380]	1.70167	2.19722	2.36248	2.27788	2.06221	2.09327	1.97582	2.38407	2.17076	1.99497	1.79744	2.26811	2.10316	2.16147	0.146381309	0.590732968	12	8	24	0.230155508	0.768040178	24	7.470798921	0.06375131
ENSMUSG00000002871	Tpra1	transmembrane protein, adipocyte asscociated 1 [Source:MGI Symbol;Acc:MGI:1345190]	21.6212	21.7939	22.4046	22.4075	20.4329	24.5678	21.144	21.7661	21.206	20.6302	21.9251	18.8078	22.1393	18.8603	0.782779623	0.966018836	12	20	24	0.909686006	0.974611343	24	18.17135065	0.015119652
ENSMUSG00000002881	Nab1	Ngfi-A binding protein 1 [Source:MGI Symbol;Acc:MGI:107564]	19.7337	17.2443	17.0354	18.7528	18.786	16.8213	19.5549	18.205	17.2378	17.6787	17.7582	17.1392	17.3667	17.1727	0.823657258	0.966018836	4	8	24	0.636236596	0.876420089	24	20.55452299	0.025990589
ENSMUSG00000002885	Adgre5	adhesion G protein-coupled receptor E5 [Source:MGI Symbol;Acc:MGI:1347095]	7.75343	8.09896	7.85541	8.7434	7.98811	7.3883	8.81705	9.11867	7.11613	6.77113	6.45113	6.70392	8.32027	6.59947	0.718086778	0.966018836	4	20	24	0.392069475	0.799546792	24	2.425274214	0.067164538
ENSMUSG00000002897	Il17ra	interleukin 17 receptor A [Source:MGI Symbol;Acc:MGI:107399]	1.17248	1.81652	2.1882	1.73508	1.59404	1.55754	1.54866	1.56993	1.79817	1.31473	1.37004	1.70123	1.40309	1.43434	0.112800839	0.530777648	12	16	24	0.290523736	0.768040178	24	8.74624967	0.084589519
ENSMUSG00000002900	Lamb1	laminin B1 [Source:MGI Symbol;Acc:MGI:96743]	35.1896	38.2856	37.0791	38.1119	44.9141	34.0476	42.3376	38.5185	40.3192	43.4383	34.3559	34.0532	40.0338	31.1331	0.959856266	0.967043964	16	8	24	0.630748222	0.874071894	24	11.97546688	0.055609172
ENSMUSG00000002908	Kcnn1	potassium intermediate/small conductance calcium-activated channel, subfamily N, member 1 [Source:MGI Symbol;Acc:MGI:1933993]	15.2269	11.1598	10.576	10.3948	12.9309	13.664	13.0374	12.8316	9.63963	12.6922	11.9226	13.6903	14.0991	12.422	1.08E-04	0.029372629	4	8	24	0.0018737	0.384740371	24	21.49469658	0.196514798
ENSMUSG00000002910	Arrdc2	arrestin domain containing 2 [Source:MGI Symbol;Acc:MGI:1918057]	7.758	6.80718	8.31582	11.8866	7.86149	7.63721	7.57022	8.18329	9.34501	12.4718	7.70713	8.6559	6.8969	6.51823	0.008483961	0.192760771	16	16	24	0.01382218	0.529470441	24	12.26408924	0.244466833
ENSMUSG00000002944	Cd36	CD36 molecule [Source:MGI Symbol;Acc:MGI:107899]	8.43764	12.6208	22.2242	40.1433	14.4241	14.5945	16.0152	31.6256	20.3859	27.7632	28.4714	20.3803	31.1746	26.9703	0.784278077	0.966018836	16	8	24	0.054875419	0.655263328	24	10.23260707	0.431307513
ENSMUSG00000002948	Map2k7	mitogen-activated protein kinase kinase 7 [Source:MGI Symbol;Acc:MGI:1346871]	20.2019	19.8052	20.0538	19.8549	20.1961	21.5239	19.8733	20.5452	20.5235	22.2017	19.5015	20.635	20.8909	20.1963	0.905762334	0.966018836	24	20	24	0.893931804	0.969958849	24	15.61035827	0.008786715
ENSMUSG00000002949	Timm44	translocase of inner mitochondrial membrane 44 [Source:MGI Symbol;Acc:MGI:1343262]	23.0325	28.0473	27.0443	28.0538	28.552	28.7199	27.4262	28.1423	29.5002	29.6292	26.7398	28.2862	27.7292	28.1657	0.417653118	0.963884303	16	12	24	0.189752675	0.768040178	24	10.96899621	0.053407976
ENSMUSG00000002957	Ap2a2	adaptor-related protein complex 2, alpha 2 subunit [Source:MGI Symbol;Acc:MGI:101920]	53.6763	40.3501	43.2538	44.0689	44.1264	52.4251	45.2334	48.2267	35.3285	56.8083	43.8077	46.4437	45.369	45.9717	0.245061943	0.741580974	24	12	24	0.379008008	0.79834227	24	19.80532123	0.095610577
ENSMUSG00000002963	Pnkp	polynucleotide kinase 3'- phosphatase [Source:MGI Symbol;Acc:MGI:1891698]	11.5435	11.0273	13.5751	10.7323	10.6376	12.9633	11.1084	12.6688	11.4882	13.1532	11.5108	12.6454	11.408	10.7457	0.896773941	0.966018836	12	20	24	0.942262622	0.985601401	24	12.09070004	0.015506991
ENSMUSG00000002968	Med25	mediator complex subunit 25 [Source:MGI Symbol;Acc:MGI:1922863]	13.002	11.3102	11.5427	12.0122	12.6742	12.0656	11.9588	14.5224	12.7012	16.2396	13.2091	13.045	12.3948	11.4037	0.622132989	0.966018836	20	12	24	0.489504174	0.805393068	24	13.66474773	0.059502464
ENSMUSG00000002980	Bcam	basal cell adhesion molecule [Source:MGI Symbol;Acc:MGI:1929940]	147.546	99.2271	105.42	103.962	107.491	129.391	117.411	133.111	88.7878	151.581	119.019	126.711	121.841	119.552	0.245061943	0.741580974	24	12	24	0.322544218	0.768040178	24	20.8663663	0.127981475
ENSMUSG00000002981	Clptm1	cleft lip and palate associated transmembrane protein 1 [Source:MGI Symbol;Acc:MGI:1927155]	51.2236	40.1749	43.6624	44.3104	42.1451	50.8937	48.762	46.6766	37.8022	50.867	39.0834	43.9416	45.2416	42.2438	0.216933149	0.704989686	4	8	24	0.325522377	0.768040178	24	21.54202229	0.082356115
ENSMUSG00000002983	Relb	avian reticuloendotheliosis viral (v-rel) oncogene related B [Source:MGI Symbol;Acc:MGI:103289]	2.77706	3.14484	2.90359	2.05662	3.06351	2.68556	3.51389	4.07898	3.39813	3.20059	3.25041	2.9338	3.83335	3.34936	0.348367381	0.870418653	4	20	24	0.092995257	0.71175955	24	3.075882907	0.123486825
ENSMUSG00000002984	Tomm40	translocase of outer mitochondrial membrane 40 [Source:MGI Symbol;Acc:MGI:1858259]	21.6183	17.603	19.2595	19.0929	20.1714	20.2649	19.5659	20.0612	19.2813	24.1026	20.0783	21.3599	20.9543	21.2659	0.522715778	0.966018836	24	12	24	0.623752491	0.87183699	24	17.27134229	0.035356435
ENSMUSG00000002985	Apoe	apolipoprotein E [Source:MGI Symbol;Acc:MGI:88057]	1561.75	1476.4	1497.88	1233.39	1329.72	1408.76	1492.7	1614.69	1421.64	1534.45	1709.09	1571.96	1601.46	1592.76	0.664568268	0.966018836	8	8	24	0.246386106	0.768040178	24	1.169499529	0.067373007
ENSMUSG00000002996	Hbp1	high mobility group box transcription factor 1 [Source:MGI Symbol;Acc:MGI:894659]	33.1375	37.7238	35.2381	36.2975	35.3289	33.5708	33.9713	36.2541	41.5323	32.408	38.7444	33.6063	36.3789	37.3787	0.46238066	0.966018836	12	12	24	0.191872443	0.768040178	24	7.838738279	0.067705887
ENSMUSG00000002997	Prkar2b	protein kinase, cAMP dependent regulatory, type II beta [Source:MGI Symbol;Acc:MGI:97760]	169.825	148.603	143.366	162.638	150.547	154.281	140.316	146.536	130.205	150.097	158.3	148.125	152.932	151.853	0.251986462	0.75129457	20	16	24	0.26457594	0.768040178	24	19.0324211	0.058568814
ENSMUSG00000003031	Cdkn1b	cyclin-dependent kinase inhibitor 1B [Source:MGI Symbol;Acc:MGI:104565]	53.4064	49.7159	49.2633	52.2049	47.0893	46.528	56.8908	48.9585	50.5739	55.3307	49.5775	48.1072	45.883	45.6923	0.916746631	0.966018836	16	8	24	0.866085245	0.961251055	24	10.71062605	0.019891102
ENSMUSG00000003032	Klf4	Kruppel-like factor 4 (gut) [Source:MGI Symbol;Acc:MGI:1342287]	3.23124	3.00909	2.40484	2.70944	2.13277	2.18588	3.17415	2.85575	3.69748	2.59892	2.89117	2.53525	2.46523	3.24685	0.261325393	0.768150062	8	16	24	0.096693859	0.71175955	24	5.070363033	0.136268516
ENSMUSG00000003033	Ap1m1	adaptor-related protein complex AP-1, mu subunit 1 [Source:MGI Symbol;Acc:MGI:102776]	61.3602	50.1735	49.0174	54.6775	57.184	59.3902	56.481	56.2422	45.6547	59.131	55.0538	54.1962	54.9418	52.6146	0.051610136	0.389629649	24	12	24	0.042216863	0.623594798	24	19.57728839	0.105785362
ENSMUSG00000003037	Rab8a	RAB8A, member RAS oncogene family [Source:MGI Symbol;Acc:MGI:96960]	18.4605	17.8792	16.4596	16.7717	16.9437	17.4119	19.1549	16.4793	16.4031	14.787	15.0377	17.3492	19.0732	19.2061	0.001845448	0.100255953	4	12	24	0.002198943	0.395705644	24	0.585535636	0.124217454
ENSMUSG00000003038	Hmgn2	high mobility group nucleosomal binding domain 2 [Source:MGI Symbol;Acc:MGI:96136]	17.1964	20.8865	24.688	21.6717	17.7403	18.0643	17.5913	18.6556	25.1936	18.9999	21.6034	19.8127	19.9602	19.8201	0.016795343	0.253395174	12	16	24	0.025368104	0.586541342	24	9.20214762	0.160326685
ENSMUSG00000003039	Fam32a	family with sequence similarity 32, member A [Source:MGI Symbol;Acc:MGI:1915172]	34.2478	32.3596	33.4007	33.5447	31.1092	32.2822	31.6062	31.431	32.6365	32.8178	32.2789	33.986	34.9388	35.773	0.944053021	0.966018836	4	16	24	0.526887402	0.823139162	24	3.393062907	0.023798983
ENSMUSG00000003062	Stard3nl	STARD3 N-terminal like [Source:MGI Symbol;Acc:MGI:1923455]	80.4701	74.8176	70.032	73.4996	74.7439	75.1282	70.5372	74.6062	71.7186	74.0514	78.9353	75.3739	78.5027	77.3161	0.061697418	0.409814092	4	8	24	0.250818871	0.768040178	24	21.13241691	0.037490285
ENSMUSG00000003068	Stk11	serine/threonine kinase 11 [Source:MGI Symbol;Acc:MGI:1341870]	31.0305	27.3758	29.5772	29.4633	29.7721	28.5408	29.5861	26.8402	28.9874	31.9178	27.8343	26.9206	28.3894	28.1622	0.350544145	0.870418653	16	16	24	0.479844338	0.805393068	24	13.06473414	0.03154648
ENSMUSG00000003070	Efna2	ephrin A2 [Source:MGI Symbol;Acc:MGI:102707]	0.945861	1.05502	0.810349	0.788855	0.681501	0.850024	1.03307	1.13915	0.826502	1.11645	0.97257	0.984043	0.94891	0.939132	0.45229395	0.966018836	8	12	24	0.265803939	0.768040178	24	2.530724515	0.058718845
ENSMUSG00000003072	Atp5d	ATP synthase, H+ transporting, mitochondrial F1 complex, delta subunit [Source:MGI Symbol;Acc:MGI:1913293]	169.016	138.285	145.219	158.438	149.504	156.646	148.703	171.302	149.276	170.027	188.589	172.41	195.256	170.804	0.814576414	0.966018836	24	12	24	0.451908726	0.805393068	24	19.90576876	0.055260929
ENSMUSG00000003099	Ppp5c	protein phosphatase 5, catalytic subunit [Source:MGI Symbol;Acc:MGI:102666]	29.8809	25.6625	26.0443	28.1832	25.009	28.02	30.1187	29.4512	25.8833	33.7936	24.9855	28.7721	27.743	28.9857	0.5169953	0.966018836	24	16	24	0.845646599	0.953396028	24	1.634328839	0.026975271
ENSMUSG00000003119	Cdk12	cyclin-dependent kinase 12 [Source:MGI Symbol;Acc:MGI:1098802]	6.24183	6.06164	6.54817	5.81173	6.76129	6.20868	5.9865	6.26275	5.9982	7.05547	5.9937	6.83932	6.72643	7.32056	0.761979	0.966018836	8	4	24	0.979506696	0.994983268	24	5.619267166	0.006436376
ENSMUSG00000003123	Lipe	lipase, hormone sensitive [Source:MGI Symbol;Acc:MGI:96790]	5.59165	4.20239	9.85061	13.9883	8.11758	7.08583	7.45696	11.8138	7.41618	14.0931	11.6974	7.92697	17.2073	10.9832	0.588208558	0.966018836	16	12	24	0.254308576	0.768040178	24	11.52831861	0.262343941
ENSMUSG00000003131	Pafah1b2	platelet-activating factor acetylhydrolase, isoform 1b, subunit 2 [Source:MGI Symbol;Acc:MGI:108415]	75.9238	64.9547	67.9141	68.3832	72.5312	74.9485	70.2487	64.2365	61.5189	67.0483	70.2066	70.5233	66.4787	66.1141	0.001920665	0.100255953	24	8	24	3.46E-04	0.227979988	24	19.24083055	0.097841914
ENSMUSG00000003134	Tbc1d8	TBC1 domain family, member 8 [Source:MGI Symbol;Acc:MGI:1927225]	5.79532	5.97317	4.68317	5.68781	5.85003	5.87978	5.39775	5.33309	5.04267	6.72543	5.63131	5.05012	5.53309	5.2803	0.265774856	0.773510818	16	20	24	0.455767774	0.805393068	24	16.59851648	0.053758023
ENSMUSG00000003135	Cnot11	CCR4-NOT transcription complex, subunit 11 [Source:MGI Symbol;Acc:MGI:106580]	8.25437	8.15214	9.55306	9.70236	8.22513	8.73167	7.9808	9.63499	8.38574	9.19283	7.45535	10.2329	8.36264	8.96829	0.581070934	0.966018836	16	12	24	0.671647945	0.889236096	24	9.038514876	0.043252407
ENSMUSG00000003153	Slc2a3	solute carrier family 2 (facilitated glucose transporter), member 3 [Source:MGI Symbol;Acc:MGI:95757]	21.3241	18.4548	20.8612	19.4941	20.0908	23.0529	20.69	20.0177	15.9046	23.2983	18.9678	20.7784	18.6769	22.1503	0.919571723	0.966018836	24	12	24	0.570723229	0.84547694	24	19.53581254	0.060001003
ENSMUSG00000003154	Foxj2	forkhead box J2 [Source:MGI Symbol;Acc:MGI:1926805]	5.78567	5.85226	5.42834	5.99664	6.11747	5.98442	6.42694	6.44304	6.11601	7.20802	6.17317	6.01588	5.94565	5.95763	0.575202741	0.966018836	16	20	24	0.594895412	0.857607093	24	13.33066668	0.029955097
ENSMUSG00000003161	Sri	sorcin [Source:MGI Symbol;Acc:MGI:98419]	117.681	109.025	108.851	107.322	113.88	123.58	109.3	108.029	99.6934	106.818	108.562	111.67	123.395	110.769	0.002261192	0.104113924	24	16	24	0.009091596	0.502965379	24	21.26339719	0.087738415
ENSMUSG00000003166	Dgcr2	DiGeorge syndrome critical region gene 2 [Source:MGI Symbol;Acc:MGI:892866]	19.8388	16.7195	19.0112	18.4356	17.7392	20.456	20.0256	18.962	16.0674	23.0414	16.0855	19.5238	17.571	18.6385	0.68887295	0.966018836	24	12	24	0.843981231	0.952749764	24	19.58922735	0.033329246
ENSMUSG00000003184	Irf3	interferon regulatory factor 3 [Source:MGI Symbol;Acc:MGI:1859179]	29.1038	29.45	26.4409	29.8333	31.276	26.3578	31.4827	30.0326	34.0446	26.1232	33.7049	28.8374	28.4941	26.262	0.88067614	0.966018836	20	4	24	0.865174599	0.960982718	24	15.35072235	0.026582323
ENSMUSG00000003190	Bcl2l12	BCL2-like 12 (proline rich) [Source:MGI Symbol;Acc:MGI:1922986]	3.2202	2.62787	2.95034	3.88109	4.16053	2.13401	3.64439	3.32493	3.39203	2.78977	3.03927	3.02498	3.0556	2.53131	0.93717134	0.966018836	20	4	24	0.665106838	0.886947643	24	13.43527067	0.066491732
ENSMUSG00000003198	Zfp959	zinc finger protein 959 [Source:MGI Symbol;Acc:MGI:2385058]	4.21586	4.27376	3.8919	4.18673	3.82298	3.73459	3.77182	4.07552	4.02905	3.27824	3.59956	4.21636	4.46742	3.8566	0.397360907	0.927993503	4	16	24	0.146434033	0.755124657	24	1.480719472	0.066341131
ENSMUSG00000003199	Mpnd	MPN domain containing [Source:MGI Symbol;Acc:MGI:1915297]	47.7278	43.4871	43.4906	45.1829	53.1259	52.5711	47.16	47.8133	38.8304	47.6633	44.8473	44.1575	48.452	42.3885	0.193690311	0.67239758	20	16	24	0.036438963	0.609176161	24	19.26760317	0.109697421
ENSMUSG00000003200	Sh3glb1	SH3-domain GRB2-like 1 [Source:MGI Symbol;Acc:MGI:700010]	13.6384	13.7585	12.8584	13.1466	14.2387	13.329	15.6508	14.8308	14.2608	14.9938	14.0117	14.1974	14.727	12.3421	0.856752753	0.966018836	4	8	24	0.736768472	0.914230051	24	20.68553754	0.028103449
ENSMUSG00000003206	Ebi3	Epstein-Barr virus induced gene 3 [Source:MGI Symbol;Acc:MGI:1354171]	0.592586	0.545482	1.3072	0.455266	0.696379	0.865582	0.978039	0.815926	0.913261	0.555735	0.794836	0.765327	0.875677	0.656508	0.722296281	0.966018836	12	4	24	0.814798974	0.940847489	24	2.778827214	0.0439625
ENSMUSG00000003208	Yju2	YJU2 splicing factor [Source:MGI Symbol;Acc:MGI:1920136]	4.38257	4.02927	4.18935	3.06444	3.90286	4.31998	4.25745	4.67517	4.24583	4.3434	4.66607	4.70416	4.71441	4.39299	0.296502765	0.807732044	24	16	24	0.128696531	0.740162193	24	23.84769737	0.081061636
ENSMUSG00000003226	Ranbp2	RAN binding protein 2 [Source:MGI Symbol;Acc:MGI:894323]	16.7956	18.6616	17.2199	17.4449	18.6636	15.8534	16.5338	16.2877	19.6374	13.9033	19.4122	18.0225	18.3259	18.4718	0.95032543	0.966018836	20	4	24	0.970010423	0.993003194	24	4.310290841	0.012596793
ENSMUSG00000003228	Grk5	G protein-coupled receptor kinase 5 [Source:MGI Symbol;Acc:MGI:109161]	5.92349	4.74221	5.15684	4.394	5.1219	5.0904	5.22343	4.66931	4.51218	5.37119	5.10406	5.45089	4.96445	0.035553741	0.324908923	4	12	24	0.013364806	0.529134128	24	21.29921555	0.118762779
ENSMUSG00000003233	Dvl3	dishevelled segment polarity protein 3 [Source:MGI Symbol;Acc:MGI:108100]	8.24302	8.78776	8.34598	9.27871	8.63374	9.16454	9.01829	8.71713	9.51216	11.7811	9.14132	9.1329	9.82537	9.62338	0.883870616	0.966018836	16	16	24	0.250837013	0.768040178	24	11.47710306	0.059938954
ENSMUSG00000003234	Abcf3	ATP-binding cassette, sub-family F (GCN20), member 3 [Source:MGI Symbol;Acc:MGI:1351656]	32.7907	33.2188	33.161	34.197	33.9445	34.3118	32.2561	32.2565	30.8768	30.1186	31.4746	32.1093	32.0907	30.9985	0.860993221	0.966018836	24	12	24	0.181929659	0.768040178	24	19.48872422	0.029837637
ENSMUSG00000003235	Eif2b5	eukaryotic translation initiation factor 2B, subunit 5 epsilon [Source:MGI Symbol;Acc:MGI:2446176]	27.9844	25.9662	26.5645	26.076	28.4137	29.086	27.326	27.6752	23.8992	25.9104	26.1128	26.0205	27.2686	25.9458	0.121789249	0.549378164	24	16	24	0.023309901	0.581965446	24	20.81866843	0.065350961
ENSMUSG00000003269	Cyth2	cytohesin 2 [Source:MGI Symbol;Acc:MGI:1334255]	32.1981	31.1157	32.5758	35.2102	35.2771	37.0967	32.1013	33.9086	31.9949	28.8782	26.9977	32.0513	33.879	33.0716	0.844448841	0.966018836	24	12	24	0.668048123	0.887831556	24	23.14379438	0.040106868
ENSMUSG00000003271	Sult2b1	sulfotransferase family, cytosolic, 2B, member 1 [Source:MGI Symbol;Acc:MGI:1926342]	0.555971	0.530405	0.722497	0.927968	0.546581	0.502885	0.406668	0.308699	0.636203	0.553431	0.659837	0.984776	0.59367	0.562812	0.449341601	0.966018836	12	20	24	0.236406169	0.768040178	24	14.18965911	0.100668324
ENSMUSG00000003273	Car11	carbonic anhydrase 11 [Source:MGI Symbol;Acc:MGI:1336193]	38.7361	35.8212	35.7716	34.7969	39.3927	36.4853	37.0335	37.2316	38.0845	39.649	41.7169	40.2344	38.9285	37.6027	0.287843582	0.793714531	20	16	24	0.130183198	0.740697538	24	17.78497668	0.04636679
ENSMUSG00000003279	Dlgap1	DLG associated protein 1 [Source:MGI Symbol;Acc:MGI:1346065]	8.82648	7.82573	9.27239	7.19848	6.97891	8.54022	8.94631	7.72906	6.99761	9.98097	6.38667	8.48289	8.0155	8.97029	0.714044782	0.966018836	4	16	24	0.504754156	0.811111334	24	2.519487408	0.07478945
ENSMUSG00000003282	Plag1	pleiomorphic adenoma gene 1 [Source:MGI Symbol;Acc:MGI:1891916]	0.967097	1.52287	1.28667	1.02437	1.39604	1.13346	1.21475	1.23376	1.66819	1.00467	1.65004	1.56174	1.15253	1.43997	0.745475226	0.966018836	12	16	24	0.890954471	0.969222457	24	9.142754729	0.028552186
ENSMUSG00000003283	Hck	hemopoietic cell kinase [Source:MGI Symbol;Acc:MGI:96052]	1.08918	1.297	2.01297	1.30303	1.59285	1.50137	1.48196	1.51407	1.57549	0.977181	1.40457	1.50094	1.48774	0.974316	0.723192329	0.966018836	12	20	24	0.959462544	0.989375125	24	15.5504612	0.018802537
ENSMUSG00000003299	Mrpl4	mitochondrial ribosomal protein L4 [Source:MGI Symbol;Acc:MGI:2137210]	18.1958	14.0527	17.6085	15.9159	13.5868	15.3462	18.6587	19.2575	16.3175	18.9375	17.8758	16.2903	16.8091	17.784	0.783267549	0.966018836	4	20	24	0.527882838	0.82357362	24	4.888851037	0.062838448
ENSMUSG00000003308	Keap1	kelch-like ECH-associated protein 1 [Source:MGI Symbol;Acc:MGI:1858732]	13.8036	13.0535	13.0737	13.0072	12.3371	14.149	13.6863	15.5336	13.5261	17.2394	13.2194	15.4022	15.2302	14.9706	0.767183862	0.966018836	24	20	24	0.741186974	0.915608309	24	3.316500879	0.030816895
ENSMUSG00000003316	Glg1	golgi apparatus protein 1 [Source:MGI Symbol;Acc:MGI:104967]	40.8216	32.5667	33.9953	36.1509	34.1736	38.6556	36.8596	36.5758	29.518	49.4313	31.9586	41.0778	36.3851	40.1533	0.245061943	0.741580974	24	12	24	0.778564913	0.928832041	24	19.91523302	0.052833293
ENSMUSG00000003344	Btbd2	BTB (POZ) domain containing 2 [Source:MGI Symbol;Acc:MGI:1933831]	26.9581	20.5436	22.3861	22.9805	20.8304	24.9234	23.2027	23.8225	19.5134	31.406	20.2355	22.7132	21.708	22.4976	0.823677655	0.966018836	24	12	24	0.937775013	0.984610794	24	18.19736721	0.025823733
ENSMUSG00000003345	Csnk1g2	casein kinase 1, gamma 2 [Source:MGI Symbol;Acc:MGI:1920014]	44.4965	35.9299	35.5389	39.3422	35.7962	35.0821	40.6125	43.3929	37.5774	43.791	37.6247	43.2648	41.7289	40.2115	0.515451644	0.966018836	4	8	24	0.579156839	0.849723241	24	0.92001808	0.046782737
ENSMUSG00000003346	Abhd17a	abhydrolase domain containing 17A [Source:MGI Symbol;Acc:MGI:106388]	32.1726	26.7607	23.3561	24.4998	22.5133	23.0621	29.8705	31.1236	28.4419	32.7431	27.395	28.6183	33.2355	30.2133	0.138719141	0.580158868	4	16	24	0.12942308	0.740162193	24	2.662898596	0.123340323
ENSMUSG00000003348	Mob3a	MOB kinase activator 3A [Source:MGI Symbol;Acc:MGI:3050117]	14.8819	10.9043	11.9169	12.8061	12.9738	13.182	12.9825	12.214	9.8529	12.0593	12.8029	12.1928	12.723	11.1846	0.021949812	0.267229408	4	8	24	0.0124985	0.525280095	24	19.75308262	0.131046945
ENSMUSG00000003352	Cacnb3	calcium channel, voltage-dependent, beta 3 subunit [Source:MGI Symbol;Acc:MGI:103307]	77.4406	52.3136	50.8525	56.3335	62.5085	63.3983	57.2846	61.9431	45.5614	72.9941	57.3604	61.4755	60.1306	57.6235	0.074438493	0.448301997	24	12	24	0.188884711	0.768040178	24	19.8163189	0.139127912
ENSMUSG00000003354	Ccdc65	coiled-coil domain containing 65 [Source:MGI Symbol;Acc:MGI:2146001]	1.05411	0.789285	0.943163	0.446903	0.686239	0.957295	1.0034	0.696534	0.813252	1.20234	0.98318	0.927357	0.469692	1.23998	0.745947816	0.966018836	8	4	24	0.968239781	0.992326888	24	1.593208377	0.019299635
ENSMUSG00000003355	Fkbp11	FK506 binding protein 11 [Source:MGI Symbol;Acc:MGI:1913370]	1.3629	1.72977	2.32961	1.11331	1.77075	1.25038	1.05756	1.29576	2.0772	1.24399	1.27846	1.44907	2.1169	1.99461	0.265774856	0.773510818	12	4	24	0.246239537	0.768040178	24	6.282046313	0.15075723
ENSMUSG00000003360	Ddx23	DEAD box helicase 23 [Source:MGI Symbol;Acc:MGI:1921601]	20.8025	19.282	19.1518	20.3853	19.7615	19.6747	20.1047	21.3365	17.9787	21.8772	19.4313	19.241	18.3299	20.1172	0.935732159	0.966018836	16	20	24	0.951629116	0.988339231	24	13.40785591	0.009224737
ENSMUSG00000003363	Pld3	phospholipase D family, member 3 [Source:MGI Symbol;Acc:MGI:1333782]	57.821	46.69	54.2517	51.2283	51.9695	60.9138	47.1658	54.1913	39.5018	59.7407	46.5056	52.0043	47.3447	49.3604	0.923635729	0.966018836	24	12	24	0.483389317	0.805393068	24	18.49733572	0.077864568
ENSMUSG00000003378	Grik5	glutamate receptor, ionotropic, kainate 5 (gamma 2) [Source:MGI Symbol;Acc:MGI:95818]	8.96836	9.37029	10.9869	9.48198	10.0259	10.9073	10.0935	10.1821	10.5319	13.1003	9.84544	11.3025	10.4411	10.8792	0.960269811	0.967043964	24	4	24	0.372674236	0.793998231	24	11.64826073	0.057869294
ENSMUSG00000003379	Cd79a	CD79A antigen (immunoglobulin-associated alpha) [Source:MGI Symbol;Acc:MGI:101774]	0.766129	1.35623	1.5337	1.41058	1.12779	1.39653	1.16036	1.62247	1.94918	0.74895	1.1408	1.34478	1.85402	1.16113	0.338172159	0.85758369	12	8	24	0.487522127	0.805393068	24	5.62564167	0.105130678
ENSMUSG00000003380	Rabac1	Rab acceptor 1 (prenylated) [Source:MGI Symbol;Acc:MGI:1201692]	250.361	237.93	231.953	245.22	239.659	231.573	229.067	240.217	264.67	213.792	273.82	231.084	251.283	228.906	0.859936714	0.966018836	20	12	24	0.96384886	0.990952901	24	15.96772409	0.010146941
ENSMUSG00000003382	Etv3	ets variant 3 [Source:MGI Symbol;Acc:MGI:1350926]	7.2786	6.12468	6.86382	7.1561	6.84815	6.62564	7.26582	6.64411	6.59287	7.51248	6.60675	7.20276	6.89392	6.78008	0.622132989	0.966018836	4	4	24	0.594258513	0.857257137	24	16.91156416	0.027658832
ENSMUSG00000003402	Prkcsh	protein kinase C substrate 80K-H [Source:MGI Symbol;Acc:MGI:107877]	26.1913	20.4272	22.0915	21.6477	23.278	25.6566	25.8233	23.1399	19.1366	25.9225	22.2031	23.1902	22.311	22.1807	0.061697418	0.409814092	4	8	24	0.104886332	0.717030134	24	20.18809823	0.105329851
ENSMUSG00000003410	Elavl3	ELAV like RNA binding protein 3 [Source:MGI Symbol;Acc:MGI:109157]	25.4926	21.4365	24.1772	21.3732	21.2124	24.4162	22.4661	21.9094	20.4617	27.7762	22.7798	24.9028	22.4611	24.5022	0.867888967	0.966018836	24	12	24	0.826521116	0.945827456	24	19.18357816	0.030553184
ENSMUSG00000003411	Rab3b	RAB3B, member RAS oncogene family [Source:MGI Symbol;Acc:MGI:1917158]	8.5181	9.29515	9.26109	10.2503	7.92367	12.2136	10.0643	8.1795	6.55811	11.8236	9.05287	9.91405	8.53261	14.9612	0.897570341	0.966018836	24	12	24	0.972282267	0.993238187	24	21.1517435	0.025935958
ENSMUSG00000003420	Fcgrt	Fc fragment of IgG receptor and transporter [Source:MGI Symbol;Acc:MGI:103017]	28.1875	35.1897	32.3276	36.2079	35.8719	30.562	34.1132	34.6872	42.6105	32.1328	33.7001	30.6166	33.8515	31.6702	0.236893212	0.7385769	12	12	24	0.135919266	0.747972781	24	9.400751298	0.106352482
ENSMUSG00000003421	Nosip	nitric oxide synthase interacting protein [Source:MGI Symbol;Acc:MGI:1913644]	31.5738	28.4612	29.296	27.8115	27.3388	29.6251	27.6707	30.1971	28.3335	31.2991	29.7408	31.7521	29.721	29.0836	0.924770496	0.966018836	24	20	24	0.707136754	0.903307866	24	21.72431436	0.02272289
ENSMUSG00000003423	Pih1d1	PIH1 domain containing 1 [Source:MGI Symbol;Acc:MGI:1916095]	29.4271	29.0857	30.4198	29.5547	31.7621	33.8639	29.5317	32.6475	29.3169	28.8018	32.6692	29.9437	31.0911	28.5337	0.192997592	0.670595308	20	12	24	0.217501978	0.768040178	24	18.60913494	0.051806537
ENSMUSG00000003429	Rps11	ribosomal protein S11 [Source:MGI Symbol;Acc:MGI:1351329]	207.66	198.324	193.353	189.984	195.871	201.843	193.91	188.731	211.168	176.769	191.201	200.755	206.979	195.272	0.101443845	0.511659635	4	12	24	0.172727707	0.768040178	24	23.76367152	0.046667418
ENSMUSG00000003435	Supt5	suppressor of Ty 5, DSIF elongation factor subunit [Source:MGI Symbol;Acc:MGI:1202400]	39.0605	32.5265	34.0685	33.3908	35.6499	39.1562	34.9993	36.8621	29.4559	41.3457	30.6116	35.2095	32.2208	33.9411	0.953350544	0.966018836	24	12	24	0.568173775	0.844316218	24	19.83405031	0.05770661
ENSMUSG00000003437	Paf1	Paf1, RNA polymerase II complex component [Source:MGI Symbol;Acc:MGI:1923988]	28.0316	26.0229	25.6121	28.1569	29.5413	26.8638	25.4399	22.8862	25.2094	27.2477	24.5151	21.7608	24.0615	24.6881	0.575387897	0.966018836	16	16	24	0.197694819	0.768040178	24	14.2385637	0.061184891
ENSMUSG00000003438	Timm50	translocase of inner mitochondrial membrane 50 [Source:MGI Symbol;Acc:MGI:1913775]	18.7456	17.6348	20.0881	21.4619	17.7547	19.5844	19.1399	19.9957	19.2807	20.1823	22.0723	18.801	20.0875	19.4121	0.347794523	0.870418653	16	12	24	0.282144526	0.768040178	24	12.31265162	0.049603491
ENSMUSG00000003444	Med29	mediator complex subunit 29 [Source:MGI Symbol;Acc:MGI:1914474]	2.91016	3.14047	3.39744	3.26433	2.90274	2.62569	2.77348	3.27833	3.78505	3.49306	4.66978	3.20723	3.72984	3.48517	0.360140629	0.883787544	12	12	24	0.14089327	0.751014756	24	10.29769682	0.105838123
ENSMUSG00000003452	Bicd1	BICD cargo adaptor 1 [Source:MGI Symbol;Acc:MGI:1101760]	14.4367	14.3883	13.4852	14.8474	15.5462	13.1395	14.2732	11.0672	14.1452	10.9957	15.2595	13.6256	12.5963	13.7877	0.836635326	0.966018836	20	12	24	0.506049279	0.811839584	24	17.60830257	0.063476159
ENSMUSG00000003458	Ncstn	nicastrin [Source:MGI Symbol;Acc:MGI:1891700]	24.7012	21.7508	22.7523	22.0859	22.8323	23.7828	23.283	23.5343	21.2781	25.9734	19.5285	23.9772	22.0417	23.7851	0.909604019	0.966018836	24	12	24	0.793583464	0.933825946	24	23.98239765	0.026559369
ENSMUSG00000003464	Pex19	peroxisomal biogenesis factor 19 [Source:MGI Symbol;Acc:MGI:1334458]	52.8796	51.3104	52.2911	57.9492	52.809	55.4124	51.3899	53.4249	50.366	59.4144	51.1189	50.6686	53.256	50.783	0.911685024	0.966018836	16	20	24	0.254911623	0.768040178	24	13.48913721	0.044140986
ENSMUSG00000003469	Phyhip	phytanoyl-CoA hydroxylase interacting protein [Source:MGI Symbol;Acc:MGI:1860417]	1.67779	2.58384	5.15356	2.52708	1.71646	3.53382	3.7447	2.00873	2.98897	3.18547	1.62544	2.75282	1.31601	3.41439	0.801995186	0.966018836	12	8	24	0.44676096	0.805393068	24	7.918202519	0.206113805
ENSMUSG00000003476	Crhr2	corticotropin releasing hormone receptor 2 [Source:MGI Symbol;Acc:MGI:894312]	0.56355	1.04573	1.21606	0.991616	1.37388	1.2825	1.0314	1.45507	1.26858	1.82852	0.963259	1.10422	1.20826	1.16123	0.881718085	0.966018836	16	12	24	0.193951205	0.768040178	24	10.72180004	0.124852286
ENSMUSG00000003477	Inmt	indolethylamine N-methyltransferase [Source:MGI Symbol;Acc:MGI:102963]	2.17259	4.61382	6.49466	10.3693	5.20533	3.27547	2.38644	3.71001	11.4686	5.37212	4.65143	4.54832	4.66539	4.34118	0.002004172	0.100255953	12	16	24	0.002220882	0.395705644	24	10.60021347	0.62555843
ENSMUSG00000003484	Cyp4f18	cytochrome P450, family 4, subfamily f, polypeptide 18 [Source:MGI Symbol;Acc:MGI:1919304]	0.640954	1.50162	1.83708	0.919434	1.05924	0.937356	1.06402	0.994242	1.1972	0.442222	1.08136	0.91423	0.814153	1.05038	0.287843582	0.793714531	12	12	24	0.428182285	0.805393068	24	6.456821479	0.119373767
ENSMUSG00000003500	Impdh1	inosine monophosphate dehydrogenase 1 [Source:MGI Symbol;Acc:MGI:96567]	42.6685	31.1064	30.6614	36.2721	34.8435	38.1029	34.2019	38.0093	25.6786	50.3833	32.2875	38.7858	37.0895	36.3583	0.077301512	0.453790923	24	12	24	0.520991074	0.820180873	24	19.24835835	0.104932434
ENSMUSG00000003518	Dusp3	dual specificity phosphatase 3 (vaccinia virus phosphatase VH1-related) [Source:MGI Symbol;Acc:MGI:1919599]	47.0742	38.3179	40.0659	39.3587	40.4386	45.0167	42.4602	42.7666	35.3873	48.0147	40.3418	42.8386	44.6805	41.4478	0.038115943	0.334069441	24	12	24	0.214172605	0.768040178	24	20.92355798	0.080164499
ENSMUSG00000003526	Prodh	proline dehydrogenase [Source:MGI Symbol;Acc:MGI:97770]	5.04646	5.43815	6.93296	9.36185	6.03481	7.3674	5.02954	5.032	7.26045	6.2034	5.07318	6.13721	4.62976	5.53436	0.004545736	0.140598674	16	12	24	0.044825427	0.628432994	24	12.24918868	0.208929459
ENSMUSG00000003527	Ess2	ess-2 splicing factor [Source:MGI Symbol;Acc:MGI:107854]	7.46901	7.2712	8.56163	7.79479	8.43767	7.72924	7.18851	7.56382	8.23003	8.67969	6.37379	7.742	8.17882	7.7708	0.137758149	0.580158868	16	12	24	0.398415483	0.799546792	24	9.665656868	0.057008352
ENSMUSG00000003528	Slc25a1	solute carrier family 25 (mitochondrial carrier, citrate transporter), member 1 [Source:MGI Symbol;Acc:MGI:1345283]	72.7546	60.4614	69.8846	74.5257	76.5636	72.5393	76.1862	72.7701	62.1678	81.199	70.4707	70.1738	73.302	73.0661	0.676344318	0.966018836	16	20	24	0.363410426	0.788241076	24	17.05274073	0.060616055
ENSMUSG00000003531	Dgcr6	DiGeorge syndrome critical region gene 6 [Source:MGI Symbol;Acc:MGI:1202877]	41.2779	42.4411	38.4916	44.4424	42.8126	42.8731	38.2581	42.6296	39.8557	38.3421	39.7267	42.3968	41.5159	41.8007	0.911685024	0.966018836	24	4	24	0.717047196	0.906751867	24	20.269781	0.022838824
ENSMUSG00000003534	Ddr1	discoidin domain receptor family, member 1 [Source:MGI Symbol;Acc:MGI:99216]	12.5173	12.9593	16.6313	13.4532	12.3088	12.8174	16.259	14.1334	14.8324	19.5519	11.5268	14.693	12.7633	15.0163	0.064951807	0.41673616	12	8	24	0.214046115	0.768040178	24	8.202010596	0.132728175
ENSMUSG00000003541	Ier3	immediate early response 3 [Source:MGI Symbol;Acc:MGI:104814]	16.6935	13.2	12.3131	12.8144	12.1446	14.4572	12.6669	15.9172	11.2117	14.9839	13.8348	13.5581	14.2496	14.7004	0.648082424	0.966018836	8	4	24	0.317474975	0.768040178	24	23.49494141	0.087116255
ENSMUSG00000003546	Klc4	kinesin light chain 4 [Source:MGI Symbol;Acc:MGI:1922014]	11.5833	10.3157	10.5092	10.872	10.3943	11.2956	11.5055	11.1548	10.2476	12.2854	10.2909	11.6314	11.2514	11.4814	0.200145651	0.684570416	24	12	24	0.52612892	0.822969183	24	22.51226632	0.032926093
ENSMUSG00000003549	Ercc1	excision repair cross-complementing rodent repair deficiency, complementation group 1 [Source:MGI Symbol;Acc:MGI:95412]	20.2459	17.8226	19.4822	19.8964	17.1805	17.8464	17.3368	19.3808	20.1444	18.0255	18.9013	17.912	18.3446	17.9996	0.480187506	0.966018836	12	12	24	0.307065014	0.768040178	24	8.060463762	0.045879502
ENSMUSG00000003559	As3mt	arsenite methyltransferase [Source:MGI Symbol;Acc:MGI:1929882]	9.91655	11.5072	12.3413	12.3513	11.2842	10.8146	11.4363	12.6403	12.0159	11.4806	10.5074	11.0395	11.375	10.5656	0.057005497	0.401309455	12	12	24	0.069717405	0.674183933	24	8.673489555	0.079278833
ENSMUSG00000003573	Homer3	homer scaffolding protein 3 [Source:MGI Symbol;Acc:MGI:1347359]	7.49897	7.02599	6.33792	6.63008	6.88841	5.55095	7.26561	5.94385	7.64149	7.19765	6.73974	5.9761	6.46103	5.26703	0.851656735	0.966018836	12	12	24	0.786328645	0.93134108	24	11.88675761	0.035931008
ENSMUSG00000003575	Crtc1	CREB regulated transcription coactivator 1 [Source:MGI Symbol;Acc:MGI:2142523]	7.2848	6.67708	7.49287	8.08423	7.14976	7.19899	6.73513	6.82529	7.9986	9.48946	6.69736	7.71628	7.57737	7.44632	0.238201401	0.741459408	16	4	24	0.108943393	0.72082227	24	11.40916587	0.092492746
ENSMUSG00000003581	Rnf215	ring finger protein 215 [Source:MGI Symbol;Acc:MGI:1918923]	17.2157	14.2989	15.4647	15.5936	15.2267	15.496	15.3103	14.7842	14.6294	14.8643	16.0958	14.0746	15.068	13.3557	0.17953325	0.655480352	20	12	24	0.196964244	0.768040178	24	18.14821012	0.048575429

ENSMUSG00000003585	Sec14l2	SEC14-like lipid binding 2 [Source:MGI Symbol;Acc:MGI:1915065]	5.41386	5.15082	5.93059	4.98061	4.43163	5.55288	5.64416	5.20784	4.52304	5.78012	4.34563	5.55461	4.98584	5.54464	0.929527443	0.966018836	24	20	24	0.446963898	0.805393068	24	2.457231416	0.059283987
ENSMUSG00000003604	Aven	apoptosis, caspase activation inhibitor [Source:MGI Symbol;Acc:MGI:1921518]	9.41471	7.36105	5.91434	9.11546	9.27728	8.85025	8.52565	7.60274	7.40995	8.33702	7.95345	8.3543	7.2898	8.00116	0.145592178	0.590732968	24	12	24	0.062409743	0.661961741	24	18.59121284	0.13828887
ENSMUSG00000003617	Cp	ceruloplasmin [Source:MGI Symbol;Acc:MGI:88476]	36.9817	45.7728	36.6236	36.3854	40.1954	30.0871	40.0348	37.1722	47.2907	25.7258	42.268	38.4279	38.7997	35.0825	0.91241026	0.966018836	20	20	24	0.690600074	0.897604733	24	3.158616708	0.074296428
ENSMUSG00000003623	Crot	carnitine O-octanoyltransferase [Source:MGI Symbol;Acc:MGI:1921364]	39.3666	41.3272	38.7664	40.2207	41.3615	37.8539	37.0118	37.0269	39.4299	35.1254	42.9722	37.8986	40.3295	38.7367	0.89563591	0.966018836	20	4	24	0.796581847	0.935045591	24	16.55590089	0.020251279
ENSMUSG00000003644	Rps6ka1	ribosomal protein S6 kinase polypeptide 1 [Source:MGI Symbol;Acc:MGI:104558]	4.26089	4.44077	4.69026	5.0778	4.27118	4.75108	5.70459	4.25187	4.26538	4.91526	3.785	4.24729	4.2054	4.12761	0.892555977	0.966018836	16	4	24	0.995316097	0.998759942	24	9.778607508	0.004733592
ENSMUSG00000003657	Calb2	calbindin 2 [Source:MGI Symbol;Acc:MGI:101914]	6.97452	11.3787	17.4899	8.16886	5.82479	12.0243	12.3768	8.68359	12.5643	10.1434	5.66996	8.05644	6.27995	13.456	0.010576629	0.197206032	12	8	24	0.087859145	0.707747429	24	6.228722523	0.373591118
ENSMUSG00000003660	Snrnp200	small nuclear ribonucleoprotein 200 (U5) [Source:MGI Symbol;Acc:MGI:2444401]	19.4386	18.0978	19.3455	19.5079	18.4607	20.5913	18.4832	18.88	16.8236	20.595	17.7402	20.4771	18.4575	19.9324	0.823657258	0.966018836	24	12	24	0.670053321	0.888925819	24	18.89332944	0.030579352
ENSMUSG00000003662	Ciao1	cytosolic iron-sulfur protein assembly 1 [Source:MGI Symbol;Acc:MGI:1346998]	34.0278	32.8165	30.0545	33.1028	33.6076	37.1032	31.8099	35.6402	29.3257	33.092	36.2654	30.0409	36.9487	33.6931	0.280810068	0.792139038	24	12	24	0.353913128	0.781118317	24	20.64498671	0.061585263
ENSMUSG00000003680	Taf6l	TATA-box binding protein associated factor 6 like [Source:MGI Symbol;Acc:MGI:2444957]	2.45132	3.02505	2.50607	4.12956	2.63584	2.86452	3.26974	3.44009	2.8235	3.54874	2.77707	2.79044	3.31848	3.03953	0.886952527	0.966018836	16	4	24	0.614960997	0.866797126	24	9.267282558	0.06332375
ENSMUSG00000003721	Insig2	insulin induced gene 2 [Source:MGI Symbol;Acc:MGI:1920249]	20.5435	22.1567	20.4707	20.623	22.013	23.7963	23.9196	21.5107	21.9206	21.9478	20.4816	20.0072	21.8062	20.8278	0.865514257	0.966018836	4	8	24	0.636519806	0.876483853	24	22.73148852	0.029036208
ENSMUSG00000003731	Kpna6	karyopherin (importin) alpha 6 [Source:MGI Symbol;Acc:MGI:1100836]	17.7064	16.0843	16.5538	17.0113	16.2948	17.801	17.8238	17.6158	15.1873	18.9061	16.7584	17.55	16.6383	17.7088	0.648082424	0.966018836	24	12	24	0.610823746	0.864861374	24	20.52966774	0.030689049
ENSMUSG00000003746	Man1a	mannosidase 1, alpha [Source:MGI Symbol;Acc:MGI:104677]	3.90743	5.16819	4.47498	4.79824	4.21176	4.0778	4.60249	4.30859	5.80157	4.1052	4.72219	4.72893	4.13494	4.39334	0.631736745	0.966018836	12	16	24	0.282332193	0.768040178	24	8.594232223	0.08045622
ENSMUSG00000003752	Itpkc	inositol 1,4,5-trisphosphate 3-kinase C [Source:MGI Symbol;Acc:MGI:2442554]	2.49545	2.40827	3.10418	2.50634	2.51271	2.68394	2.47378	2.83584	2.39614	2.46266	2.22607	2.28942	2.37923	2.25417	0.801273005	0.966018836	16	4	24	0.602728649	0.861114219	24	7.244364861	0.033139935
ENSMUSG00000003762	Coq8b	coenzyme Q8B [Source:MGI Symbol;Acc:MGI:1924139]	6.08108	6.00802	7.05468	6.47692	6.8479	6.57582	6.23103	6.61989	6.84383	6.0657	6.24888	5.77736	6.21908	5.99468	0.590017078	0.966018836	12	20	24	0.27833042	0.768040178	24	10.63855464	0.043712515
ENSMUSG00000003778	Brd8	bromodomain containing 8 [Source:MGI Symbol;Acc:MGI:1925906]	15.5558	17.0182	15.8199	15.8539	16.9014	16.0391	16.9666	15.4437	16.186	15.3015	15.3966	16.5007	16.7384	16.2105	0.512510541	0.966018836	4	12	24	0.365452863	0.789712692	24	23.37291792	0.028542441
ENSMUSG00000003779	Kif20a	kinesin family member 20A [Source:MGI Symbol;Acc:MGI:1201682]	0.953252	0.695899	1.09875	0.801262	0.645148	0.967203	0.857169	0.897554	1.12369	0.537133	0.862164	0.622535	0.839035	0.810081	0.676344318	0.966018836	12	4	24	0.427201455	0.805393068	24	4.516400496	0.068182488
ENSMUSG00000003808	Farsa	phenylalanyl-tRNA synthetase, alpha subunit [Source:MGI Symbol;Acc:MGI:1913840]	14.6505	13.9242	12.74	13.9723	12.7681	13.3555	13.5296	13.9913	13.2861	14.0478	12.7156	13.5891	13.483	13.8252	0.295379186	0.805301811	8	12	24	0.286524857	0.768040178	24	2.490100855	0.033300128
ENSMUSG00000003809	Gcdh	glutaryl-Coenzyme A dehydrogenase [Source:MGI Symbol;Acc:MGI:104541]	13.7134	13.3321	14.4776	14.4643	14.387	12.9423	12.6701	13.0959	14.6804	13.5139	13.9986	12.9402	13.7454	13.6	0.041292272	0.346472636	12	12	24	0.025975558	0.586541342	24	10.92145321	0.060027929
ENSMUSG00000003810	Mast2	microtubule associated serine/threonine kinase 2 [Source:MGI Symbol;Acc:MGI:894676]	30.4672	29.3093	25.3129	26.9474	32.1922	31.114	32.2055	31.0604	29.0846	33.1749	26.8759	29.9966	28.9678	28.2928	0.822170541	0.966018836	24	12	24	0.416796181	0.801546829	24	20.86523416	0.058558426
ENSMUSG00000003812	Dnase2a	deoxyribonuclease II alpha [Source:MGI Symbol;Acc:MGI:1329019]	2.7225	4.22077	3.59641	3.67476	3.65738	4.22758	3.39994	3.30074	4.62968	4.12458	3.36155	3.6094	4.51359	4.15066	0.880247609	0.966018836	12	16	24	0.465319872	0.805393068	24	8.511395848	0.075774947
ENSMUSG00000003813	Rad23a	RAD23 homolog A, nucleotide excision repair protein [Source:MGI Symbol;Acc:MGI:105126]	26.1821	26.2256	27.533	29.7914	28.0234	30.2157	30.1311	28.9704	26.2054	34.403	28.0383	27.4906	28.1203	26.8894	0.65208848	0.966018836	16	20	24	0.312173937	0.768040178	24	14.46534778	0.059779683
ENSMUSG00000003814	Calr	calreticulin [Source:MGI Symbol;Acc:MGI:88252]	190.802	132.917	128.358	119.799	154.048	158.934	170.425	154.716	101.497	120.453	142.234	142.466	141.118	130.42	0.001980441	0.100255953	4	12	24	5.90E-04	0.293325979	24	21.7216833	0.26640885
ENSMUSG00000003824	Syce2	synaptonemal complex central element protein 2 [Source:MGI Symbol;Acc:MGI:1919096]	1.75811	1.76122	1.50957	1.65106	1.77085	1.27978	1.18926	1.24802	1.80775	1.06135	1.06083	1.49472	1.24983	1.86147	0.608615915	0.966018836	8	20	24	0.536321046	0.827399772	24	5.698513118	0.073517681
ENSMUSG00000003847	Nfat5	nuclear factor of activated T cells 5 [Source:MGI Symbol;Acc:MGI:1859333]	14.1117	15.4058	14.061	14.3489	14.4992	13.3173	14.9134	14.1571	15.5061	13.3136	15.3879	14.8111	14.7886	15.6658	0.949874888	0.966018836	8	8	24	0.484742249	0.805393068	24	4.427273571	0.032071345
ENSMUSG00000003848	Nob1	NIN1/RPN12 binding protein 1 homolog [Source:MGI Symbol;Acc:MGI:1914869]	13.6157	11.7278	12.8246	14.8998	13.9652	13.022	14.1661	13.7698	13.9203	11.5678	13.9244	13.3681	13.9255	13.0825	0.892578749	0.966018836	4	4	24	0.717920772	0.907012192	24	18.89839329	0.031951514
ENSMUSG00000003849	Nqo1	NAD(P)H dehydrogenase, quinone 1 [Source:MGI Symbol;Acc:MGI:103187]	7.98602	9.31554	9.52699	8.47092	9.12903	8.30551	8.64072	7.5265	10.3597	7.4601	9.18484	8.26888	8.58788	8.44955	0.601647512	0.966018836	12	12	24	0.643480744	0.87892845	24	9.562344446	0.045006523
ENSMUSG00000003863	Ppfia3	protein tyrosine phosphatase, receptor type, f polypeptide (PTPRF), interacting protein (liprin), alpha 3 [Source:MGI Symbol;Acc:MGI:1924037]	11.4024	9.08759	9.70334	9.20148	9.25889	10.0369	9.79571	11.1716	9.15666	13.5752	9.97889	11.8992	10.8537	11.4858	0.85447369	0.966018836	24	12	24	0.903959211	0.97351114	24	23.13506556	0.025889444
ENSMUSG00000003865	Gys1	glycogen synthase 1, muscle [Source:MGI Symbol;Acc:MGI:101805]	12.8459	10.8127	12.894	15.4716	13.1476	14.8995	11.9442	18.2398	11.6674	20.4017	12.3686	13.006	14.023	13.6901	0.941955432	0.966018836	16	20	24	0.563603023	0.842665559	24	11.8860301	0.09697258
ENSMUSG00000003868	Ruvbl2	RuvB-like protein 2 [Source:MGI Symbol;Acc:MGI:1342299]	13.9583	10.2164	10.5923	13.0589	11.1773	11.8127	14.443	11.4205	9.0823	12.8908	11.2967	11.7056	11.3604	10.9915	0.121546923	0.548996929	4	8	24	0.236009273	0.768040178	24	20.12509301	0.10950696
ENSMUSG00000003872	Lin7b	lin-7 homolog B (C. elegans) [Source:MGI Symbol;Acc:MGI:1330858]	12.2298	13.694	14.206	15.807	10.7204	12.9618	13.0252	12.712	12.5755	14.4246	12.0034	13.4552	14.2297	14.155	0.129903614	0.560263689	16	4	24	0.334328356	0.770442964	24	7.372945765	0.076744019
ENSMUSG00000003873	Bax	BCL2-associated X protein [Source:MGI Symbol;Acc:MGI:99702]	34.5611	30.0134	32.1082	33.1296	34.6224	31.8525	33.0216	33.6961	31.3523	27.8865	34.7664	33.3433	33.4125	30.118	0.205280539	0.693330038	20	20	24	0.154464436	0.760257003	24	19.97190341	0.066874421
ENSMUSG00000003923	Tfam	transcription factor A, mitochondrial [Source:MGI Symbol;Acc:MGI:107810]	16.6465	17.4277	17.2859	17.1705	16.012	14.2673	17.8211	15.1407	14.5142	13.76	12.3275	14.3676	15.154	14.6895	0.236246476	0.7385769	4	20	24	0.137652399	0.747972781	24	3.263530391	0.077335544
ENSMUSG00000003929	Zfp81	zinc finger protein 81 [Source:MGI Symbol;Acc:MGI:1890752]	4.33276	5.84453	5.55452	4.7347	5.38127	4.69464	4.42488	4.85486	6.17473	3.63971	5.13719	5.62879	6.23144	5.73857	0.777867261	0.966018836	12	4	24	0.557298221	0.839072789	24	4.296670327	0.076594443
ENSMUSG00000003934	Efnb3	ephrin B3 [Source:MGI Symbol;Acc:MGI:109196]	2.48966	5.27736	9.3707	4.03859	3.11022	4.35875	5.75196	3.74704	6.02156	3.2363	3.0245	4.38917	2.27829	5.29869	0.10560184	0.518002936	12	16	24	0.175709633	0.768040178	24	7.151325507	0.323070985
ENSMUSG00000003948	Mmd	monocyte to macrophage differentiation-associated [Source:MGI Symbol;Acc:MGI:1914718]	8.71817	7.77949	9.96527	12.7755	8.59487	9.50501	9.79259	9.57819	7.87195	10.6638	10.0904	9.21495	9.32802	11.0403	0.814077113	0.966018836	16	12	24	0.426239243	0.805393068	24	12.38166491	0.085560471
ENSMUSG00000003949	Hlf	hepatic leukemia factor [Source:MGI Symbol;Acc:MGI:96108]	3.34893	6.13025	6.94871	5.57776	4.85454	3.8762	3.92862	3.83197	7.92706	5.52891	3.99853	3.98766	2.91978	4.93805	9.10E-06	0.009754434	12	16	24	0.001030758	0.336500758	24	9.480109434	0.391056688
ENSMUSG00000003955	Fam162a	family with sequence similarity 162, member A [Source:MGI Symbol;Acc:MGI:1917436]	63.3591	63.8303	59.9998	61.0212	57.7905	56.098	63.213	64.6769	69.5975	56.139	76.267	66.11	71.3683	68.743	0.916746631	0.966018836	8	8	24	0.542655472	0.831042618	24	2.927093407	0.047601055
ENSMUSG00000003970	Rpl8	ribosomal protein L8 [Source:MGI Symbol;Acc:MGI:1350927]	277.271	278.802	285.435	259.833	262.914	266.463	271.406	281.807	293.528	259.453	278.062	280.053	288.87	280.089	0.004644371	0.140598674	12	4	24	0.052796057	0.650526959	24	3.776922107	0.046592046
ENSMUSG00000003974	Grm3	glutamate receptor, metabotropic 3 [Source:MGI Symbol;Acc:MGI:1351340]	0.342821	0.809891	1.1493	0.633102	0.371943	0.885541	0.888635	0.530201	0.817009	0.543342	0.255844	0.643813	0.336727	0.851681	0.424718357	0.966018836	12	8	24	0.261840128	0.768040178	24	6.106261914	0.148949146
ENSMUSG00000003992	Ssbp2	single-stranded DNA binding protein 2 [Source:MGI Symbol;Acc:MGI:1914220]	42.8268	40.9736	40.7727	40.9814	40.21	41.1445	41.3865	39.9204	38.8351	42.0709	41.9374	45.403	44.8236	44.3736	0.098024777	0.501188411	4	8	24	0.128739648	0.740162193	24	22.02090469	0.042198211
ENSMUSG00000004018	Fancl	Fanconi anemia, complementation group L [Source:MGI Symbol;Acc:MGI:1914280]	2.2575	3.25591	2.43186	3.41317	2.80878	2.66495	2.5334	2.21624	3.77524	1.76759	3.25572	3.20955	3.09353	3.30243	0.882617464	0.966018836	12	16	24	0.97946409	0.994983268	24	9.396046918	0.018362111
ENSMUSG00000004031	Brinp2	bone morphogenetic protein/retinoic acid inducible neural-specific 2 [Source:MGI Symbol;Acc:MGI:2443333]	9.22956	7.13062	7.77966	7.58572	6.20967	7.98155	7.83837	8.5553	6.28427	9.75852	6.22422	7.63293	8.40019	8.78613	0.231491055	0.731037655	4	16	24	0.383055299	0.799546792	24	2.451578514	0.09707036
ENSMUSG00000004032	Gstm5	glutathione S-transferase, mu 5 [Source:MGI Symbol;Acc:MGI:1309466]	108.104	131.789	118.765	111.194	121.304	109.406	112.744	106.266	130.918	90.9373	119.571	111.298	119.951	119.057	0.916556571	0.966018836	12	4	24	0.671825098	0.889340174	24	4.235246635	0.047854238
ENSMUSG00000004035	Gstm7	glutathione S-transferase, mu 7 [Source:MGI Symbol;Acc:MGI:1915562]	4.31276	5.84025	6.5342	4.42651	3.4397	5.05131	5.61079	5.77536	5.4228	4.53092	3.35713	4.78413	5.00743	5.36384	0.002098032	0.100255953	12	8	24	0.003752095	0.426426863	24	4.562121419	0.246570335
ENSMUSG00000004040	Stat3	signal transducer and activator of transcription 3 [Source:MGI Symbol;Acc:MGI:103038]	66.8528	51.036	49.925	56.4614	56.2124	60.8202	58.463	60.2419	45.9261	70.1094	53.9244	59.75	58.6538	55.6418	0.074438493	0.448301997	24	12	24	0.276586139	0.768040178	24	20.01311906	0.101205642
ENSMUSG00000004043	Stat5a	signal transducer and activator of transcription 5A [Source:MGI Symbol;Acc:MGI:103036]	2.01165	2.14656	2.361	2.58456	2.54128	2.29515	2.76172	3.08171	2.82411	3.30819	2.68125	2.65122	3.37415	2.58545	0.794858476	0.966018836	16	8	24	0.483554771	0.805393068	24	9.940695395	0.054562096
ENSMUSG00000004044	Cavin1	caveolae associated 1 [Source:MGI Symbol;Acc:MGI:1277968]	37.9736	38.3493	33.7512	37.5295	37.0521	32.1712	37.9038	42.1881	45.7341	38.975	40.8368	42.2857	46.3154	40.217	0.890176313	0.966018836	8	8	24	0.45119942	0.805393068	24	4.912732148	0.056402949
ENSMUSG00000004054	Map3k11	mitogen-activated protein kinase kinase kinase 11 [Source:MGI Symbol;Acc:MGI:1346880]	11.8221	10.444	11.3682	11.532	11.3908	11.3627	11.8889	10.8207	10.3805	12.5254	10.6153	11.3027	11.6427	10.4264	0.347075276	0.870418653	4	4	24	0.395197778	0.799546792	24	17.9199527	0.040737185
ENSMUSG00000004056	Akt2	thymoma viral proto-oncogene 2 [Source:MGI Symbol;Acc:MGI:104874]	12.6298	11.4632	11.6959	14.1528	13.2194	12.3144	13.4591	14.4378	13.3715	14.8312	13.405	12.8213	14.9806	12.7982	0.897570341	0.966018836	16	20	24	0.719281359	0.907919194	24	13.01652568	0.030547295
ENSMUSG00000004069	Dnaja3	DnaJ heat shock protein family (Hsp40) member A3 [Source:MGI Symbol;Acc:MGI:1933786]	20.8984	17.8479	19.8339	23.3509	19.448	21.1653	19.6361	21.3019	17.7231	24.0011	19.4865	20.6698	20.7206	21.1956	0.934152439	0.966018836	16	4	24	0.642048966	0.878809617	24	14.57231605	0.043055468
ENSMUSG00000004070	Hmox2	heme oxygenase 2 [Source:MGI Symbol;Acc:MGI:109373]	73.7473	71.2997	68.9959	70.2346	71.4106	72.1024	67.5146	64.6005	69.8341	66.8957	72.3515	68.2113	71.8334	69.6511	0.443191651	0.966018836	20	12	24	0.323051269	0.768040178	24	19.30520837	0.029982355
ENSMUSG00000004071	Cdip1	cell death inducing Trp53 target 1 [Source:MGI Symbol;Acc:MGI:1913876]	40.922	38.3229	42.3709	39.043	37.8081	44.3825	42.976	41.6521	36.5362	47.6468	33.8283	40.1207	36.8468	41.4226	0.84100102	0.966018836	24	20	24	0.917905746	0.977818518	24	3.7612317	0.020731511
ENSMUSG00000004085	Map3k20	mitogen-activated protein kinase kinase kinase 20 [Source:MGI Symbol;Acc:MGI:2443258]	4.94445	5.34606	5.33437	5.26762	4.93092	5.47967	5.43303	8.17573	5.49935	8.11675	5.02432	4.97261	5.60801	5.76191	0.109247218	0.524035616	8	12	24	0.283833591	0.768040178	24	7.479198106	0.126494274
ENSMUSG00000004096	Cwc15	CWC15 spliceosome-associated protein [Source:MGI Symbol;Acc:MGI:1913320]	73.4003	75.1958	76.3888	72.0956	74.6099	71.9752	74.9733	70.5387	69.9856	68.148	71.7265	67.1304	71.8528	69.7837	0.890311217	0.966018836	4	20	24	0.706758717	0.903131193	24	1.311021748	0.012535644
ENSMUSG00000004098	Col5a3	collagen, type V, alpha 3 [Source:MGI Symbol;Acc:MGI:1858212]	31.2606	35.2927	33.5007	29.5161	33.3669	27.2668	35.5675	35.9141	38.7467	39.1507	36.3541	32.3872	35.6334	34.5589	0.718720526	0.966018836	12	12	24	0.12641309	0.739868076	24	7.579728988	0.100708762
ENSMUSG00000004099	Dnmt1	DNA methyltransferase (cytosine-5) 1 [Source:MGI Symbol;Acc:MGI:94912]	6.48632	5.69854	6.9481	6.59891	5.805	6.02405	6.52215	6.60884	5.59478	6.8142	5.57288	5.98923	5.56567	6.2134	0.676344318	0.966018836	16	4	24	0.600525422	0.860118739	24	8.02357657	0.039019605
ENSMUSG00000004100	Ppan	peter pan homolog [Source:MGI Symbol;Acc:MGI:2178445]	5.74043	6.1858	6.40098	5.50969	6.207	5.41086	5.84734	7.05766	5.83504	6.10789	5.86087	6.42952	5.8051	6.04348	0.77643975	0.966018836	8	20	24	0.456449363	0.805393068	24	5.949809797	0.042770874
ENSMUSG00000004105	Angptl2	angiopoietin-like 2 [Source:MGI Symbol;Acc:MGI:1347002]	7.59962	7.8912	10.0906	10.8014	12.0779	9.11155	8.32931	10.3921	11.7563	11.5948	9.84215	7.7951	7.42803	7.77588	0.004021228	0.140598674	16	12	24	0.001054958	0.336500758	24	11.97908986	0.262451931
ENSMUSG00000004110	Cacna1e	calcium channel, voltage-dependent, R type, alpha 1E subunit [Source:MGI Symbol;Acc:MGI:106217]	2.15504	2.95365	3.10165	2.71318	2.97434	2.81506	2.88942	2.39175	3.15716	2.37653	2.57501	3.11278	2.57365	3.26965	0.962348911	0.967745676	12	16	24	0.768097309	0.925197875	24	8.186966775	0.039279164
ENSMUSG00000004113	Cacna1b	calcium channel, voltage-dependent, N type, alpha 1B subunit [Source:MGI Symbol;Acc:MGI:88296]	20.7221	16.1185	16.3704	18.0902	17.9222	20.6818	15.0652	16.5411	15.2365	23.7939	16.3034	19.5211	16.5653	17.2767	0.433658121	0.966018836	16	20	24	0.291366527	0.768040178	24	16.7995479	0.110771953
ENSMUSG00000004127	Trmt10a	tRNA methyltransferase 10A [Source:MGI Symbol;Acc:MGI:1920421]	6.68296	7.27361	6.12337	6.42747	7.54165	6.28575	7.08489	7.19136	7.86676	7.08177	8.02764	8.07173	8.62652	8.31124	0.948454836	0.966018836	8	4	24	0.655795092	0.883738698	24	0.695956038	0.030893611
ENSMUSG00000004151	Etv1	ets variant 1 [Source:MGI Symbol;Acc:MGI:99254]	19.7182	18.3501	16.7635	18.9554	16.2536	18.2708	18.2983	18.7908	15.09	22.9069	17.786	16.5615	17.4402	18.6778	0.658895034	0.966018836	8	12	24	0.993756831	0.998755922	24	9.064856002	0.006430321
ENSMUSG00000004187	Kifc2	kinesin family member C2 [Source:MGI Symbol;Acc:MGI:109187]	18.8056	19.9175	20.9359	18.0072	18.1493	20.3352	19.3067	18.8176	20.0111	20.9284	18.2672	20.9719	19.5414	21.0327	0.836635326	0.966018836	12	8	24	0.480555909	0.805393068	24	5.105116977	0.035810601
ENSMUSG00000004207	Psap	prosaposin [Source:MGI Symbol;Acc:MGI:97783]	400.181	326.889	350.753	344.867	327.683	387.957	383.95	388.459	297.477	459.281	310.122	375.068	347.284	365.018	0.591351901	0.966018836	4	16	24	0.766612228	0.924541938	24	22.45222226	0.049248465
ENSMUSG00000004221	Ikbkg	inhibitor of kappaB kinase gamma [Source:MGI Symbol;Acc:MGI:1338074]	13.6599	13.3383	11.6212	13.7445	14.5294	13.5891	11.8732	12.5939	13.4128	12.7037	13.1129	13.7059	12.5145	12.826	0.272257525	0.783528688	24	4	24	0.154979642	0.760294912	24	17.01993485	0.059211145
ENSMUSG00000004233	Wars2	tryptophanyl tRNA synthetase 2 (mitochondrial) [Source:MGI Symbol;Acc:MGI:1917810]	2.83184	1.89729	1.92835	2.51301	2.02858	2.25287	1.98301	1.83499	1.83748	2.01846	2.02207	2.53569	2.31291	2.02924	0.041292272	0.346472636	24	12	24	0.115104123	0.727799063	24	19.93617266	0.10624222
ENSMUSG00000004263	Atn1	atrophin 1 [Source:MGI Symbol;Acc:MGI:104725]	9.74467	10.4491	9.67016	9.21295	10.2132	9.52911	9.66547	10.8042	11.4208	14.6773	10.6928	11.5867	10.7804	12.161	0.783267549	0.966018836	8	16	24	0.293567442	0.768040178	24	9.500357678	0.079375509
ENSMUSG00000004264	Phb2	prohibitin 2 [Source:MGI Symbol;Acc:MGI:102520]	29.5817	27.8283	31.8631	32.2466	29.8	30.3863	29.4172	31.9962	29.8787	37.3908	32.2312	32.9247	34.389	30.4047	0.7207001	0.966018836	16	16	24	0.230031735	0.768040178	24	13.08692745	0.058398845
ENSMUSG00000004266	Ptpn6	protein tyrosine phosphatase, non-receptor type 6 [Source:MGI Symbol;Acc:MGI:96055]	2.96584	3.94448	5.3982	3.66392	3.61674	4.42387	3.52893	3.19526	4.17364	2.95019	3.68349	3.85558	3.82054	3.27742	0.94086481	0.966018836	20	16	24	0.882671751	0.966732748	24	10.70334959	0.03728169
ENSMUSG00000004267	Eno2	enolase 2, gamma neuronal [Source:MGI Symbol;Acc:MGI:95394]	187.863	156.183	170.15	154.893	150.237	183.264	169.84	167.615	135.232	206.053	138.463	163.086	154.576	178.684	0.952127548	0.966018836	4	16	24	0.779417653	0.929103303	24	0.351775617	0.047108581
ENSMUSG00000004268	Emg1	EMG1 N1-specific pseudouridine methyltransferase [Source:MGI Symbol;Acc:MGI:1315195]	25.138	26.7734	26.3022	26.5381	28.1246	25.5285	28.3959	25.8072	28.1792	22.2772	28.2618	24.1934	27.7113	27.1301	0.90332508	0.966018836	20	4	24	0.866831384	0.961669572	24	2.346761422	0.020521948
ENSMUSG00000004270	Lpcat3	lysophosphatidylcholine acyltransferase 3 [Source:MGI Symbol;Acc:MGI:1315211]	20.9376	17.0097	17.8017	17.0178	17.2494	18.9718	19.7821	20.325	18.1762	17.6552	17.4751	17.9041	17.5992	16.9724	0.439800617	0.966018836	4	12	24	0.115446637	0.727799063	24	23.76193688	0.070113187
ENSMUSG00000004285	Atp6v1f	ATPase, H+ transporting, lysosomal V1 subunit F [Source:MGI Symbol;Acc:MGI:1913394]	154.259	159.42	143.556	141.991	159.074	149.567	142.308	147.297	157.623	119.583	166.172	147.686	152.886	144.358	0.369262716	0.893596186	20	20	24	0.535172565	0.82691856	24	21.82078073	0.051279026
ENSMUSG00000004317	Clcn5	chloride channel, voltage-sensitive 5 [Source:MGI Symbol;Acc:MGI:99486]	8.8031	7.04802	7.86803	6.79493	7.64439	7.47051	7.05697	6.76916	6.47303	7.17678	7.29016	7.47867	7.62	7.36348	0.22438807	0.718653303	4	4	24	0.166194488	0.767020009	24	21.13111842	0.068402393
ENSMUSG00000004319	Clcn3	chloride channel, voltage-sensitive 3 [Source:MGI Symbol;Acc:MGI:103555]	43.1912	44.1262	47.1446	43.373	42.9425	41.6501	41.0864	39.7052	43.8226	43.0971	43.0545	42.8723	43.7857	44.8014	0.020119233	0.259417302	12	12	24	0.322934203	0.768040178	24	9.020748644	0.034166566
ENSMUSG00000004328	Hif3a	hypoxia inducible factor 3, alpha subunit [Source:MGI Symbol;Acc:MGI:1859778]	0.354909	0.351856	1.07921	1.75776	0.530759	0.760017	0.279092	0.308	0.464667	1.29055	0.627801	0.514147	0.210143	0.219783	4.10E-04	0.054040373	16	12	24	0.001424776	0.354367925	24	12.84354289	0.38723681
ENSMUSG00000004347	Pde1c	phosphodiesterase 1C [Source:MGI Symbol;Acc:MGI:108413]	16.3121	14.3429	15.1077	15.6903	15.4294	15.1706	12.8362	13.5644	13.7609	14.0749	16.8686	16.4051	17.3084	16.5498	0.570592509	0.966018836	20	16	24	0.421648522	0.804769987	24	18.75072812	0.063222363
ENSMUSG00000004356	Utp20	UTP20 small subunit processome component [Source:MGI Symbol;Acc:MGI:1917933]	6.0039	5.04424	5.41707	6.19609	5.14451	4.99752	5.15262	4.97072	5.8455	4.20327	5.24383	4.9058	4.92075	4.8068	0.920992137	0.966018836	12	20	24	0.966640944	0.992039848	24	9.629487543	0.011177575
ENSMUSG00000004360	9330159F19Rik	RIKEN cDNA 9330159F19 gene [Source:MGI Symbol;Acc:MGI:3036239]	33.0308	20.905	22.7544	26.4735	27.9718	26.945	26.2456	20.2093	19.7568	21.6037	26.9389	21.4366	22.277	20.7502	0.030972684	0.324150946	20	12	24	0.009562522	0.502965379	24	18.81619205	0.197651254
ENSMUSG00000004364	Cul3	cullin 3 [Source:MGI Symbol;Acc:MGI:1347360]	67.9552	68.0776	65.3835	62.6972	65.4796	66.8872	63.0644	66.6773	66.651	72.1788	65.938	69.7713	66.5507	71.4855	0.849179819	0.966018836	8	12	24	0.833820032	0.94886119	24	5.043863946	0.012925756
ENSMUSG00000004366	Sst	somatostatin [Source:MGI Symbol;Acc:MGI:98326]	9.99728	13.4477	15.5994	10.0345	6.97275	10.8174	12.2677	11.6696	16.3092	11.9397	9.00648	15.4848	10.4889	14.1159	0.01142276	0.206107348	12	8	24	0.061388508	0.661066697	24	5.608512342	0.26931257
ENSMUSG00000004383	Large1	LARGE xylosyl- and glucuronyltransferase 1 [Source:MGI Symbol;Acc:MGI:1342270]	24.3301	20.6923	20.6462	19.8776	21.2863	24.3047	22.5002	22.8248	17.5454	26.8329	18.5912	23.0097	22.7594	23.3634	0.287703522	0.793714531	24	12	24	0.385466757	0.799546792	24	22.35986322	0.085871359
ENSMUSG00000004393	Ddx56	DEAD box helicase 56 [Source:MGI Symbol;Acc:MGI:1277172]	16.5656	15.2739	15.2046	15.4112	15.459	15.3643	15.0463	16.4619	14.0614	18.1613	13.9828	15.2977	14.5385	15.5476	0.877082091	0.966018836	16	20	24	0.950959177	0.988261248	24	8.854611695	0.012058681
ENSMUSG00000004394	Tmed4	transmembrane p24 trafficking protein 4 [Source:MGI Symbol;Acc:MGI:1915070]	36.1976	36.2247	37.116	35.8021	34.6226	37.3329	37.0775	36.6552	34.9819	35.0631	34.3797	35.5181	35.4517	35.5737	0.239653024	0.741580974	24	20	24	0.08659714	0.705877484	24	1.573119267	0.02643827
ENSMUSG00000004415	Col26a1	collagen, type XXVI, alpha 1 [Source:MGI Symbol;Acc:MGI:2155345]	5.24856	6.42511	6.65305	5.4987	7.65217	4.82687	8.37738	6.92277	7.25257	6.85378	6.32868	5.78164	6.47351	4.95936	0.907068194	0.966018836	12	12	24	0.792345989	0.933580505	24	9.370664911	0.057928888
ENSMUSG00000004446	Bid	BH3 interacting domain death agonist [Source:MGI Symbol;Acc:MGI:108093]	15.0715	13.5552	13.8089	13.5355	13.4207	14.998	12.7118	12.6191	12.4881	13.2425	14.1499	14.4929	13.6779	14.6193	0.308023687	0.814993005	24	12	24	0.190073019	0.768040178	24	20.23963778	0.059280691
ENSMUSG00000004451	Ralb	v-ral simian leukemia viral oncogene B [Source:MGI Symbol;Acc:MGI:1927244]	11.0142	10.5501	10.123	10.2838	9.88316	10.622	11.216	10.5663	11.0219	10.8191	10.8825	10.4011	11.3453	10.5848	0.163587268	0.626886304	4	12	24	0.220479369	0.768040178	24	0.963151574	0.031895254
ENSMUSG00000004455	Ppp1cc	protein phosphatase 1 catalytic subunit gamma [Source:MGI Symbol;Acc:MGI:104872]	6.82171	7.25777	6.70785	7.32285	6.89959	6.73446	7.81687	7.20044	7.45946	6.33405	7.16697	6.58183	6.54881	6.75721	0.923635729	0.966018836	8	16	24	0.764472927	0.92403577	24	4.825046688	0.021126096
ENSMUSG00000004460	Dnajb11	DnaJ heat shock protein family (Hsp40) member B11 [Source:MGI Symbol;Acc:MGI:1915088]	25.1864	18.9075	20.5114	18.5075	21.8693	21.6305	23.1392	20.4551	17.2144	21.2129	21.9315	21.5114	22.0308	21.1652	0.002004172	0.100255953	4	8	24	0.017696045	0.553087548	24	21.12607314	0.132763958
ENSMUSG00000004462	Tbccd1	TBCC domain containing 1 [Source:MGI Symbol;Acc:MGI:1917823]	11.3674	10.0988	10.9651	11.4869	10.6408	11.145	10.0846	11.1425	9.28668	12.1155	10.292	10.7747	10.5593	10.4324	0.718086778	0.966018836	16	20	24	0.567034098	0.843683291	24	15.66465258	0.036313253
ENSMUSG00000004473	Clec11a	C-type lectin domain family 11, member a [Source:MGI Symbol;Acc:MGI:1298219]	1.23722	1.18108	1.10181	1.02226	1.10158	1.10871	0.825117	1.46638	1.68488	0.752047	1.14633	1.15073	1.325	1.24755	0.029264055	0.322165609	8	8	24	0.317834977	0.768040178	24	4.345749764	0.091249062
ENSMUSG00000004500	Zfp324	zinc finger protein 324 [Source:MGI Symbol;Acc:MGI:2444641]	2.98161	2.51354	2.21048	2.50399	2.40078	2.82313	2.81907	2.46249	2.55504	2.70718	2.74557	3.08702	2.89236	3.04855	0.059229521	0.406067671	4	8	24	0.01688543	0.547196997	24	21.99926453	0.088830209
ENSMUSG00000004508	Gab2	growth factor receptor bound protein 2-associated protein 2 [Source:MGI Symbol;Acc:MGI:1333854]	7.19059	6.7598	6.69293	6.23456	6.6683	7.40892	6.53364	7.26771	5.72186	8.15437	6.16371	7.5816	6.54985	7.3615	0.942429894	0.966018836	24	12	24	0.726660289	0.91035631	24	21.88506541	0.039028713
ENSMUSG00000004530	Coro1c	coronin, actin binding protein 1C [Source:MGI Symbol;Acc:MGI:1345964]	23.1244	20.6353	21.878	18.694	21.9307	22.2612	18.3101	19.3936	18.2944	25.0928	20.1782	23.9393	20.0294	21.0432	0.658895034	0.966018836	24	12	24	0.4606187	0.805393068	24	17.60277446	0.068365558
ENSMUSG00000004535	Tax1bp1	Tax1 (human T cell leukemia virus type I) binding protein 1 [Source:MGI Symbol;Acc:MGI:1289308]	95.4349	93.5487	85.4706	88.3682	91.6716	88.3248	90.3882	86.3791	91.9865	85.5906	98.0882	94.9783	100.305	97.5292	0.310940564	0.820837787	4	12	24	0.199950713	0.768040178	24	22.55212434	0.047063478
ENSMUSG00000004552	Ctse	cathepsin E [Source:MGI Symbol;Acc:MGI:107361]	0.922636	2.04153	3.49315	3.11402	1.59709	2.56211	1.46024	1.84904	2.21845	1.45624	1.5618	2.19657	2.87985	1.36002	0.956794505	0.96629412	12	16	24	0.643084199	0.878809617	24	10.40824527	0.133076025
ENSMUSG00000004558	Ndrg2	N-myc downstream regulated gene 2 [Source:MGI Symbol;Acc:MGI:1352498]	109.382	141.993	183.72	158.629	130.739	168.73	159.168	179.829	160.927	171.45	127.237	139.441	123.176	144.008	0.158091814	0.614628746	12	16	24	0.155630091	0.761083296	24	8.912650801	0.166890792
ENSMUSG00000004561	Mettl17	methyltransferase like 17 [Source:MGI Symbol;Acc:MGI:1098577]	7.19213	8.192	8.04632	8.22875	8.19496	8.46636	8.89586	8.8088	9.8118	8.06143	6.74297	8.30748	8.97626	8.37621	0.588682825	0.966018836	4	16	24	0.282610848	0.768040178	24	5.073288767	0.071219691
ENSMUSG00000004562	Arhgef40	Rho guanine nucleotide exchange factor (GEF) 40 [Source:MGI Symbol;Acc:MGI:2685515]	29.5497	33.682	27.559	31.0189	36.4612	28.1742	38.7375	32.3315	39.2958	34.1014	32.965	29.5938	37.6845	29.0604	0.859936714	0.966018836	4	20	24	0.974252471	0.993545331	24	7.705182775	0.015949125
ENSMUSG00000004565	Pnpla6	patatin-like phospholipase domain containing 6 [Source:MGI Symbol;Acc:MGI:1354723]	33.975	28.8566	30.2967	30.5685	35.4954	32.1033	35.6649	31.2553	29.782	31.3161	31.8963	30.8689	30.7078	32.4334	0.152374472	0.60072565	4	8	24	0.161523857	0.76636332	24	19.96857326	0.065274447
ENSMUSG00000004567	Mcoln1	mucolipin 1 [Source:MGI Symbol;Acc:MGI:1890498]	33.8706	32.6983	32.3676	33.2792	32.5385	33.0728	30.1615	32.8536	33.3133	35.5581	30.3046	32.666	33.6665	33.8231	0.904831057	0.966018836	16	4	24	0.573608223	0.847005958	24	8.177399492	0.025888809
ENSMUSG00000004568	Arhgef18	rho/rac guanine nucleotide exchange factor (GEF) 18 [Source:MGI Symbol;Acc:MGI:2142567]	9.21072	7.39264	7.11978	8.0116	8.05819	8.2989	7.65132	9.16939	7.57435	9.93977	7.78792	8.15694	8.76258	7.68825	0.321662653	0.83607295	24	12	24	0.794414873	0.934055013	24	19.39993021	0.034560164
ENSMUSG00000004591	Pkn2	protein kinase N2 [Source:MGI Symbol;Acc:MGI:109211]	7.47382	8.14056	7.5547	8.91001	8.20452	7.37271	9.09695	7.77656	7.96722	7.48894	8.75037	7.63468	7.77003	8.0041	0.879201926	0.966018836	20	4	24	0.840255159	0.951645964	24	13.69105426	0.020573478
ENSMUSG00000004609	Cd33	CD33 antigen [Source:MGI Symbol;Acc:MGI:99440]	1.11617	2.53814	2.82625	1.98661	1.65246	1.79525	1.78758	1.62813	2.8192	1.29531	2.26457	1.70535	1.8311	1.63454	0.658895034	0.966018836	12	16	24	0.27901669	0.768040178	24	8.755911876	0.160725135
ENSMUSG00000004610	Etfb	electron transferring flavoprotein, beta polypeptide [Source:MGI Symbol;Acc:MGI:106098]	80.938	76.5188	81.3058	104.832	86.263	83.1718	79.8664	96.2211	85.2706	95.4277	89.9537	89.3732	102.509	90.2174	0.849179819	0.966018836	16	12	24	0.418654337	0.803070657	24	12.25574565	0.060296886
ENSMUSG00000004612	Nkg7	natural killer cell group 7 sequence [Source:MGI Symbol;Acc:MGI:1931250]	0.768117	0.731142	1.44595	0.50769	1.05651	1.09128	1.01332	0.743457	0.746836	0.731998	0.527205	0.897125	1.1803	0.580214	0.439800617	0.966018836	24	16	24	0.609154366	0.864393298	24	22.46429733	0.081622506
ENSMUSG00000004626	Stxbp2	syntaxin binding protein 2 [Source:MGI Symbol;Acc:MGI:107370]	11.2898	13.6743	12.7338	13.8861	12.2988	12.6155	13.0196	13.4891	14.1335	13.8994	13.1722	13.1368	13.3726	12.5499	0.151191877	0.598641364	16	8	24	0.095302013	0.71175955	24	9.607114688	0.062371334
ENSMUSG00000004631	Sgce	sarcoglycan, epsilon [Source:MGI Symbol;Acc:MGI:1329042]	25.5347	26.2794	23.5911	23.8591	26.6403	24.5416	25.2298	24.1761	26.2059	22.7504	23.8737	24.7885	24.0239	24.1001	0.598711142	0.966018836	4	12	24	0.500430231	0.808986978	24	23.03651975	0.027906636

ENSMUSG00000004633	Chn2	chimerin 2 [Source:MGI Symbol;Acc:MGI:1917243]	19.9686	13.315	14.638	14.4365	13.5955	15.8742	16.1739	14.5563	12.8109	15.594	12.5072	14.5695	13.2534	13.6293	0.777666236	0.966018836	24	20	24	0.230478907	0.768040178	24	22.26462047	0.096867063
ENSMUSG00000004637	Wwox	WW domain-containing oxidoreductase [Source:MGI Symbol;Acc:MGI:1931237]	8.07797	6.98945	7.53957	7.4498	6.48031	7.68615	7.67478	7.64955	7.63825	8.43114	6.39842	8.08941	6.73825	7.85111	0.676344318	0.966018836	24	20	24	0.668646096	0.888027111	24	4.819863547	0.038697297
ENSMUSG00000004642	Slbp	stem-loop binding protein [Source:MGI Symbol;Acc:MGI:108402]	16.4703	15.7537	18.9908	14.3113	15.2818	17.6867	18.0434	16.1971	16.5623	17.9069	17.3641	15.735	17.9077	16.4806	0.915082018	0.966018836	4	16	24	0.814463916	0.940820791	24	2.198193497	0.026312526
ENSMUSG00000004655	Aqp1	aquaporin 1 [Source:MGI Symbol;Acc:MGI:103201]	110.549	84.3238	93.5154	95.7182	98.8752	112.669	88.2467	105.426	68.9829	100.54	93.7449	94.1745	96.07	88.9129	0.428730128	0.966018836	24	12	24	0.144566252	0.753177589	24	19.64320846	0.13149861
ENSMUSG00000004661	Arid3b	AT rich interactive domain 3B (BRIGHT-like) [Source:MGI Symbol;Acc:MGI:1930768]	2.13285	1.85202	1.67347	1.76858	2.47745	1.81736	1.75481	2.14518	1.84611	2.36518	1.99254	1.97596	2.11672	2.2476	0.916963526	0.966018836	8	4	24	0.850185386	0.9550524	24	16.81564966	0.02535445
ENSMUSG00000004665	Cnn2	calponin 2 [Source:MGI Symbol;Acc:MGI:105093]	7.9368	8.9935	9.15458	7.575	9.00665	7.68786	8.80773	9.56489	10.5651	7.50412	7.75319	9.07844	9.32631	8.59576	0.058209189	0.40386543	12	4	24	0.150410465	0.758865539	24	4.631922997	0.096430694
ENSMUSG00000004667	Polr2e	polymerase (RNA) II (DNA directed) polypeptide E [Source:MGI Symbol;Acc:MGI:1913670]	30.6929	30.2482	32.2153	32.2726	32.0053	31.4177	31.2641	33.9068	31.1325	30.7745	33.0625	30.6811	30.2462	30.6579	0.244732441	0.741580974	20	8	24	0.450103273	0.805393068	24	12.46199063	0.02353766
ENSMUSG00000004677	Myo9b	myosin IXb [Source:MGI Symbol;Acc:MGI:106624]	17.3989	15.8634	16.8749	16.4316	19.9937	16.568	17.6218	16.7962	17.392	17.1326	17.5129	16.3427	16.7893	15.0142	0.192357053	0.669841558	20	12	24	0.134683892	0.746461599	24	16.37583179	0.063515135
ENSMUSG00000004698	Hdac9	histone deacetylase 9 [Source:MGI Symbol;Acc:MGI:1931221]	7.61318	7.12515	7.63774	7.54011	7.25722	7.61224	7.17037	6.69668	6.31875	8.25512	6.5703	7.4418	6.61213	7.55819	0.887862353	0.966018836	16	16	24	0.739975906	0.915232675	24	15.38230238	0.028237425
ENSMUSG00000004707	Ly9	lymphocyte antigen 9 [Source:MGI Symbol;Acc:MGI:96885]	1.56699	1.91422	1.96939	2.06727	2.12333	1.76292	2.06794	1.87184	2.12462	1.46436	1.65822	1.87485	1.52324	1.64948	0.851656735	0.966018836	12	16	24	0.73993364	0.915232675	24	10.5665604	0.034827422
ENSMUSG00000004730	Adgre1	adhesion G protein-coupled receptor E1 [Source:MGI Symbol;Acc:MGI:106912]	2.70243	3.04053	2.95557	2.34112	3.45494	2.11836	3.63488	2.76581	2.94188	1.56616	3.46845	2.95061	2.82965	2.29231	0.8635847	0.966018836	20	20	24	0.592791902	0.856634016	24	22.33407916	0.094778911
ENSMUSG00000004748	Mtfp1	mitochondrial fission process 1 [Source:MGI Symbol;Acc:MGI:1916686]	6.86818	7.02257	7.51725	7.56944	7.11216	7.9093	7.02849	9.41471	7.71988	9.40235	6.46319	7.00275	7.10378	8.58779	0.256685902	0.76168256	8	20	24	0.154708823	0.760257003	24	7.809374247	0.108779482
ENSMUSG00000004768	Rab23	RAB23, member RAS oncogene family [Source:MGI Symbol;Acc:MGI:99833]	9.17723	8.1693	10.2765	8.67149	8.5028	8.49874	8.98861	8.86247	9.25243	8.84002	9.47078	8.53339	8.46118	9.77393	0.390686537	0.915479136	12	12	24	0.289652491	0.768040178	24	7.999249757	0.052107522
ENSMUSG00000004771	Rab11a	RAB11A, member RAS oncogene family [Source:MGI Symbol;Acc:MGI:1858202]	187.447	178.259	174.201	188.545	190.233	192.271	156.085	170.389	167.845	155.076	174.644	159.009	185.524	156.54	0.70641939	0.966018836	20	12	24	0.357738702	0.784709986	24	18.48380324	0.055127427
ENSMUSG00000004788	Eif2b2	eukaryotic translation initiation factor 2B, subunit 2 beta [Source:MGI Symbol;Acc:MGI:2145118]	22.2304	19.2268	17.4282	21.0144	19.9882	19.6474	19.6885	22.8321	19.9899	22.2221	23.1833	18.8564	19.6707	22.1365	0.921442568	0.966018836	20	4	24	0.943059565	0.985996972	24	12.29106886	0.015384315
ENSMUSG00000004789	Dlst	dihydrolipoamide S-succinyltransferase (E2 component of 2-oxo-glutarate complex) [Source:MGI Symbol;Acc:MGI:1926170]	73.5399	67.8899	71.9102	87.8632	73.2357	75.5143	75.0668	77.039	66.1814	85.1067	76.0388	72.0451	81.1069	77.331	0.676344318	0.966018836	16	20	24	0.552763699	0.837013734	24	14.2349602	0.045881735
ENSMUSG00000004791	Pgf	placental growth factor [Source:MGI Symbol;Acc:MGI:105095]	4.54334	5.30648	4.63146	5.44819	5.35307	4.93935	4.81134	5.12846	4.42262	4.98966	4.88194	4.88598	4.45937	4.73948	0.251986462	0.75129457	16	12	24	0.333471171	0.769685083	24	14.50499747	0.043017114
ENSMUSG00000004798	Ulk2	unc-51 like kinase 2 [Source:MGI Symbol;Acc:MGI:1352758]	24.3502	22.2592	22.3625	23.8526	23.5559	24.9196	24.007	22.9357	21.4237	26.553	22.7596	23.5312	23.3515	22.1435	0.061697418	0.409814092	16	20	24	0.164160422	0.767020009	24	17.77522754	0.055687422
ENSMUSG00000004814	Ccl24	chemokine (C-C motif) ligand 24 [Source:MGI Symbol;Acc:MGI:1928953]	0.708617	0.481349	0.64818	0.429787	0.369064	0.449349	0.244059	0.792264	0.993585	0.304272	0.68935	0.698865	1.00516	0.529445	0.664568268	0.966018836	12	4	24	0.50992	0.813985618	24	3.985956181	0.09380123
ENSMUSG00000004815	Dgkq	diacylglycerol kinase, theta [Source:MGI Symbol;Acc:MGI:102918]	14.0316	12.8639	16.5492	16.051	16.4679	16.0797	15.7534	15.5784	15.821	15.3666	14.1376	14.5326	13.5376	15.1514	0.25851124	0.761738864	12	20	24	0.259702534	0.768040178	24	12.24779891	0.065332286
ENSMUSG00000004837	Grap	GRB2-related adaptor protein [Source:MGI Symbol;Acc:MGI:1918770]	2.43257	4.28615	3.42369	3.07539	4.03896	2.44196	2.61664	3.20475	3.61791	2.70692	3.5004	2.63852	2.75867	2.51143	0.160013844	0.619746337	12	16	24	0.23820297	0.768040178	24	10.3175875	0.130411396
ENSMUSG00000004843	Chmp2b	charged multivesicular body protein 2B [Source:MGI Symbol;Acc:MGI:1916192]	42.1716	41.517	39.1968	38.4002	40.401	40.6938	41.8979	38.1552	35.106	42.6618	36.1885	38.0326	35.715	37.8867	0.847312766	0.966018836	4	8	24	0.624454793	0.871960321	24	20.76467235	0.029603349
ENSMUSG00000004846	Plod3	procollagen-lysine, 2-oxoglutarate 5-dioxygenase 3 [Source:MGI Symbol;Acc:MGI:1347008]	21.9182	17.7162	20.3598	18.4424	24.0693	21.1673	18.7023	23.0938	16.4178	17.9045	20.3362	19.47	15.8579	16.7813	0.623812175	0.966018836	20	16	24	0.208623999	0.768040178	24	18.21383802	0.107934566
ENSMUSG00000004849	Ap1s1	adaptor protein complex AP-1, sigma 1 [Source:MGI Symbol;Acc:MGI:1098244]	85.5099	78.9821	85.2434	72.8155	77.5373	80.8419	79.9669	73.9186	67.3591	73.4308	80.2882	78.0378	80.1735	77.663	0.057224139	0.401309455	4	12	24	0.08255318	0.699403344	24	21.9394755	0.072798494
ENSMUSG00000004864	Mapk13	mitogen-activated protein kinase 13 [Source:MGI Symbol;Acc:MGI:1346864]	1.00841	0.976062	1.84391	1.31131	0.763777	0.972255	0.908458	1.0879	0.877403	0.686521	0.882333	1.2493	1.0096	0.836534	0.90332508	0.966018836	24	20	24	0.81260032	0.94007069	24	6.492229631	0.047895213
ENSMUSG00000004865	Srpk1	serine/arginine-rich protein specific kinase 1 [Source:MGI Symbol;Acc:MGI:106908]	31.493	24.7497	27.6924	25.9886	27.7876	27.7545	30.0728	26.4085	22.0424	26.6144	27.5618	27.7618	29.7236	26.7775	0.003383846	0.140598674	4	8	24	0.022494072	0.579292623	24	21.22554991	0.119426215
ENSMUSG00000004880	Lbr	lamin B receptor [Source:MGI Symbol;Acc:MGI:2138281]	9.2253	9.22542	11.7688	11.9592	10.4393	9.05846	9.30173	9.8143	11.4031	9.19639	10.3608	9.04405	9.62563	8.4506	0.074438493	0.448301997	12	12	24	0.027589045	0.590220615	24	11.21434107	0.126662863
ENSMUSG00000004885	Crabp2	cellular retinoic acid binding protein II [Source:MGI Symbol;Acc:MGI:88491]	13.0815	13.9598	8.94312	8.94066	11.3849	9.12482	9.58009	11.7983	13.7738	3.65941	10.5669	9.74052	8.50817	9.91098	0.5169953	0.966018836	8	8	24	0.299238117	0.768040178	24	1.296046897	0.232323599
ENSMUSG00000004891	Nes	nestin [Source:MGI Symbol;Acc:MGI:101784]	21.8729	24.8913	22.6819	25.822	34.3542	21.8755	27.7034	23.6266	29.0411	30.4688	24.1293	25.8651	26.4568	22.313	0.612141595	0.966018836	16	16	24	0.246794042	0.768040178	24	13.67912147	0.117308835
ENSMUSG00000004892	Bcan	brevican [Source:MGI Symbol;Acc:MGI:1096385]	22.9222	24.293	31.0086	22.2373	21.1918	25.8543	26.9851	23.3007	23.003	25.7739	20.185	21.1662	18.0387	25.2629	0.607427393	0.966018836	12	8	24	0.338060883	0.772742827	24	6.962926325	0.103230273
ENSMUSG00000004894	Hapln2	hyaluronan and proteoglycan link protein 2 [Source:MGI Symbol;Acc:MGI:2137300]	1.82996	3.2639	5.45107	2.30211	2.04425	2.4437	4.70283	1.88965	4.11888	1.81272	1.73769	2.9391	1.02387	2.73816	0.338172159	0.85758369	12	8	24	0.433633018	0.805393068	24	6.769804188	0.232157765
ENSMUSG00000004895	Prcc	papillary renal cell carcinoma (translocation-associated) [Source:MGI Symbol;Acc:MGI:2137738]	11.9066	9.85017	10.0856	10.6603	9.9602	10.7189	11.2767	11.4143	10.1941	13.7951	10.1547	12.0437	11.1276	11.9878	0.851656735	0.966018836	24	20	24	0.927841495	0.98127006	24	0.218965609	0.018219487
ENSMUSG00000004896	Rrnad1	ribosomal RNA adenine dimethylase domain containing 1 [Source:MGI Symbol;Acc:MGI:2387197]	10.3159	9.1051	9.35647	10.042	10.7359	9.51296	10.2771	9.70539	11.2941	10.4028	10.9121	9.9365	10.5512	9.89572	0.17953325	0.655480352	20	12	24	0.381038843	0.799546792	24	14.74395482	0.039703997
ENSMUSG00000004897	Hdgf	heparin binding growth factor [Source:MGI Symbol;Acc:MGI:1194494]	70.2945	61.4589	65.5109	69.3897	68.0486	72.8249	64.6154	70.1449	65.1846	81.3178	68.1544	69.9789	72.4032	69.8103	0.601647512	0.966018836	24	12	24	0.43597584	0.805393068	24	15.75381125	0.043868251
ENSMUSG00000004902	Slc25a18	solute carrier family 25 (mitochondrial carrier), member 18 [Source:MGI Symbol;Acc:MGI:1919053]	2.01713	4.61359	7.991	3.21249	2.86399	3.76267	4.45915	2.68718	4.52902	2.84408	2.31497	3.45545	1.50555	5.00643	0.256524739	0.76168256	12	8	24	0.2172658	0.768040178	24	7.523369637	0.318658365
ENSMUSG00000004929	Thop1	thimet oligopeptidase 1 [Source:MGI Symbol;Acc:MGI:1354165]	13.1961	9.59452	10.8578	9.24556	11.8206	12.6742	10.4647	11.5813	10.1711	13.1513	11.3525	11.6993	11.011	10.6929	0.314193491	0.825291692	24	12	24	0.250659942	0.768040178	24	19.20806285	0.095009304
ENSMUSG00000004931	Apba3	amyloid beta (A4) precursor protein-binding, family A, member 3 [Source:MGI Symbol;Acc:MGI:1888527]	14.0236	9.18868	9.80974	14.9323	13.1115	12.3971	13.2517	13.7843	12.4455	11.2334	13.5509	10.4751	10.8007	9.49224	0.894563866	0.966018836	20	12	24	0.331111041	0.768040178	24	16.83185546	0.119272204
ENSMUSG00000004933	Matk	megakaryocyte-associated tyrosine kinase [Source:MGI Symbol;Acc:MGI:99259]	2.03982	4.12855	6.6245	3.10757	2.24458	3.68051	4.03808	2.41164	5.43608	3.69242	2.32293	3.26022	2.32773	4.92532	0.010576629	0.197206032	12	8	24	0.087541554	0.707747429	24	7.495483915	0.34511347
ENSMUSG00000004934	Pias4	protein inhibitor of activated STAT 4 [Source:MGI Symbol;Acc:MGI:2136940]	8.87308	6.43708	6.92282	8.24736	8.04946	8.98487	7.60976	7.7434	7.15651	9.87905	7.88015	7.0685	7.6851	7.09007	0.108029159	0.524035616	16	20	24	0.190771434	0.768040178	24	16.52922875	0.103153329
ENSMUSG00000004936	Map2k1	mitogen-activated protein kinase kinase 1 [Source:MGI Symbol;Acc:MGI:1346866]	63.061	54.0391	56.5198	55.9922	55.1708	61.6342	56.7405	58.7287	48.7784	62.448	54.8279	55.23	54.1131	55.843	0.890176313	0.966018836	4	16	24	0.493580882	0.806117438	24	20.21414005	0.045063941
ENSMUSG00000004937	Sgta	small glutamine-rich tetratricopeptide repeat (TPR)-containing, alpha [Source:MGI Symbol;Acc:MGI:1098703]	28.9554	27.3457	30.8914	28.9582	28.5976	29.8547	32.457	30.9936	29.1478	34.5204	25.9137	30.655	29.7647	28.8641	0.903207257	0.966018836	24	20	24	0.906886274	0.974587616	24	6.383917749	0.018108844
ENSMUSG00000004939	Nmrk2	nicotinamide riboside kinase 2 [Source:MGI Symbol;Acc:MGI:1916814]	0.773226	3.55546	2.04492	2.30059	0.853973	1.71795	1.74297	9.56571	4.34491	2.12459	1.13818	1.37494	2.89747	2.21732	0.016024075	0.253395174	8	12	24	0.022348335	0.578664588	24	5.961457232	0.653396341
ENSMUSG00000004945	Tmem242	transmembrane protein 242 [Source:MGI Symbol;Acc:MGI:1917794]	78.1288	86.2641	79.4029	83.9369	78.9919	78.2391	80.6582	83.4971	91.1665	74.1463	87.4062	84.7455	91.843	90.2878	0.935593607	0.966018836	8	8	24	0.282857113	0.768040178	24	4.500485469	0.049690082
ENSMUSG00000004947	Dtx2	deltex 2, E3 ubiquitin ligase [Source:MGI Symbol;Acc:MGI:1921448]	5.11821	4.21818	4.07264	4.82363	3.87069	4.44222	4.25539	4.3865	3.92223	4.54332	4.52432	4.77182	5.17263	4.72254	0.454881285	0.966018836	4	8	24	0.344023578	0.777470047	24	22.39179635	0.059632773
ENSMUSG00000004951	Hspb1	heat shock protein 1 [Source:MGI Symbol;Acc:MGI:96240]	57.3926	36.1137	34.0104	39.1645	45.6164	46.7906	44.0208	49.9747	29.9985	44.2589	53.0543	44.0845	42.9617	37.0513	0.018923071	0.256345714	20	16	24	0.02878708	0.594683	24	19.87969305	0.242909242
ENSMUSG00000004952	Rasa4	RAS p21 protein activator 4 [Source:MGI Symbol;Acc:MGI:1858600]	12.0667	12.8312	12.4347	13.3986	13.9267	13.6372	11.7208	11.7298	12.8672	12.1271	15.2606	11.5933	12.9156	12.1956	0.138719141	0.580158868	20	8	24	0.075299554	0.685243407	24	15.17009718	0.085596227
ENSMUSG00000004961	Syt5	synaptotagmin V [Source:MGI Symbol;Acc:MGI:1926368]	46.2269	31.1953	36.8921	34.131	29.8327	33.9082	33.4017	39.2103	34.8875	46.0149	40.454	45.6815	42.2918	43.0708	0.849180783	0.966018836	4	16	24	0.856268	0.957469471	24	0.647688498	0.040842684
ENSMUSG00000004980	Hnrnpa2b1	heterogeneous nuclear ribonucleoprotein A2/B1 [Source:MGI Symbol;Acc:MGI:104819]	335.916	325	321.469	328.796	330.988	302.912	348.354	308.83	281.635	282.958	326.721	261.603	272.153	280.442	0.91241026	0.966018836	20	4	24	0.82775741	0.946180429	24	18.39857982	0.022342045
ENSMUSG00000004988	Fxyd4	FXYD domain-containing ion transport regulator 4 [Source:MGI Symbol;Acc:MGI:1889005]	0.586319	0.589626	0.814144	0.613873	0.874153	0.517217	0.382629	1.03833	0.934438	0.428242	1.03818	1.01083	0.750026	0.542088	0.916746631	0.966018836	20	8	24	0.790515993	0.932883394	24	13.07865892	0.051729092
ENSMUSG00000004994	Ccdc130	coiled-coil domain containing 130 [Source:MGI Symbol;Acc:MGI:1914986]	7.87704	8.71086	9.24839	10.8058	8.41304	8.47585	5.91099	11.4158	7.83912	8.84219	7.63026	8.79922	5.98889	7.72256	0.113148904	0.530777648	16	12	24	0.189050221	0.768040178	24	10.71197992	0.155758354
ENSMUSG00000004996	Mri1	methylthioribose-1-phosphate isomerase 1 [Source:MGI Symbol;Acc:MGI:1915123]	5.672	5.86946	6.67884	6.26977	5.72049	5.07312	5.39287	5.50405	6.07238	6.52218	6.04318	5.79809	6.33738	6.11762	0.061697418	0.409814092	16	8	24	0.024788339	0.586541342	24	9.64225759	0.090311554
ENSMUSG00000005034	Prkacb	protein kinase, cAMP dependent, catalytic, beta [Source:MGI Symbol;Acc:MGI:97594]	80.3826	70.6181	75.2166	70.3928	61.8314	76.3353	74.4244	69.3379	58.0641	78.2221	64.0883	68.6703	64.6625	74.1563	0.93447422	0.966018836	4	16	24	0.624548604	0.871960321	24	0.458465499	0.049252797
ENSMUSG00000005043	Sgsh	N-sulfoglucosamine sulfohydrolase (sulfamidase) [Source:MGI Symbol;Acc:MGI:1350341]	2.27871	2.01528	1.93552	2.09127	1.99404	2.16039	2.05175	1.9739	2.05797	2.38945	1.73336	2.3136	2.12267	1.95614	0.289496388	0.794869797	24	12	24	0.742176875	0.916130449	24	20.58335974	0.025962581
ENSMUSG00000005045	Chd5	chromodomain helicase DNA binding protein 5 [Source:MGI Symbol;Acc:MGI:3036258]	8.07991	7.34042	8.76233	7.79911	6.91285	8.70973	8.02547	7.3649	7.59888	11.1872	6.29737	8.61905	7.76589	9.76987	0.911685024	0.966018836	24	20	24	0.740654495	0.915293122	24	7.396876982	0.058894158
ENSMUSG00000005054	Cstb	cystatin B [Source:MGI Symbol;Acc:MGI:109514]	87.5889	93.201	87.948	82.7769	91.3457	87.8198	83.286	83.617	85.5768	65.0394	88.7312	82.788	92.2371	82.3193	0.922029406	0.966018836	20	20	24	0.299687878	0.768040178	24	23.12404265	0.072772642
ENSMUSG00000005057	Sh2b2	SH2B adaptor protein 2 [Source:MGI Symbol;Acc:MGI:1345171]	1.71697	0.887692	1.3687	2.00771	1.41464	1.02571	1.31054	1.35765	1.47315	2.24229	2.71752	1.56631	2.22512	2.23857	0.630352198	0.966018836	20	4	24	0.337017554	0.771960538	24	13.06428416	0.129695713
ENSMUSG00000005069	Pex5	peroxisomal biogenesis factor 5 [Source:MGI Symbol;Acc:MGI:1098808]	12.8198	10.9161	13.2996	12.6594	12.7135	13.669	12.9817	13.3385	12.7191	15.5837	13.9762	13.0753	13.303	13.3021	0.854443826	0.966018836	20	16	24	0.25751093	0.768040178	24	14.2867733	0.054965062
ENSMUSG00000005078	Jkamp	JNK1/MAPK8-associated membrane protein [Source:MGI Symbol;Acc:MGI:1915057]	49.2875	46.868	46.0696	45.3888	45.6295	44.1858	45.8214	44.5666	46.9474	40.0932	48.4304	45.1078	49.3515	47.5213	0.247964668	0.746745468	4	12	24	0.260470943	0.768040178	24	0.640963107	0.046714064
ENSMUSG00000005087	Cd44	CD44 antigen [Source:MGI Symbol;Acc:MGI:88338]	57.1588	46.6904	51.9648	47.5329	49.2853	57.8997	45.7159	48.6919	38.8664	58.3075	50.5268	51.4705	49.017	50.0601	0.476029854	0.966018836	24	8	24	0.297194413	0.768040178	24	18.82557589	0.093121505
ENSMUSG00000005089	Slc1a2	solute carrier family 1 (glial high affinity glutamate transporter), member 2 [Source:MGI Symbol;Acc:MGI:101931]	4.30124	15.4876	28.1154	9.48705	7.62005	14.3608	18.9871	8.09559	19.3666	10.035	5.44787	11.6302	4.14208	16.3061	0.10560184	0.518002936	12	8	24	0.270192508	0.768040178	24	7.392580359	0.48982537
ENSMUSG00000005102	Eif2ak4	eukaryotic translation initiation factor 2 alpha kinase 4 [Source:MGI Symbol;Acc:MGI:1353427]	4.17639	3.70284	3.96255	3.57849	3.4889	3.94372	3.82421	3.46233	3.91094	5.19812	3.40019	3.99483	4.06879	4.0726	0.840483986	0.966018836	4	16	24	0.904553758	0.973594617	24	7.917166841	0.023066872
ENSMUSG00000005103	Wdr1	WD repeat domain 1 [Source:MGI Symbol;Acc:MGI:1337100]	86.3795	70.9183	75.1036	70.1845	77.2236	84.7773	75.5821	78.4165	58.1499	82.5862	74.7763	75.3698	76.1513	76.678	0.595037318	0.966018836	4	8	24	0.146713738	0.755260456	24	20.64736371	0.103938654
ENSMUSG00000005107	Slc2a9	solute carrier family 2 (facilitated glucose transporter), member 9 [Source:MGI Symbol;Acc:MGI:2152844]	0.885446	1.18116	1.15588	1.43096	1.17132	1.17051	0.982422	0.965935	1.28078	1.37988	0.994288	1.19953	1.49664	1.3252	0.892555977	0.966018836	16	12	24	0.288448316	0.768040178	24	10.47877756	0.069935467
ENSMUSG00000005124	Ccn4	cellular communication network factor 4 [Source:MGI Symbol;Acc:MGI:1197008]	3.3199	3.10689	2.77171	2.4538	3.64537	2.55618	3.4412	3.23942	3.61724	2.85222	2.74944	2.51529	3.16221	2.82392	0.495929335	0.966018836	4	20	24	0.508951819	0.813786183	24	3.136634794	0.064662589
ENSMUSG00000005125	Ndrg1	N-myc downstream regulated gene 1 [Source:MGI Symbol;Acc:MGI:1341799]	300.531	272.378	241.441	256.079	320.239	265.875	335.791	283.451	271.904	287.441	262.85	302.064	299.635	247.048	0.058157448	0.40386543	4	8	24	0.102335682	0.713699364	24	20.96662758	0.113846514
ENSMUSG00000005131	4930550C14Rik	RIKEN cDNA 4930550C14 gene [Source:MGI Symbol;Acc:MGI:1922561]	0.860343	1.19266	0.518319	1.04667	0.883759	0.632697	0.578107	1.12052	1.02481	0.994174	1.28008	1.18084	1.02541	1.03084	0.895493752	0.966018836	8	20	24	0.84796867	0.954100589	24	11.0001386	0.039373519
ENSMUSG00000005142	Man2b1	mannosidase 2, alpha B1 [Source:MGI Symbol;Acc:MGI:107286]	8.81128	8.62293	9.02663	9.65589	8.43777	8.57784	9.11635	9.3823	9.61218	10.0269	8.05397	8.5631	8.54659	8.70983	0.001270265	0.087678517	16	4	24	0.062031425	0.661961741	24	8.682958268	0.070533245
ENSMUSG00000005148	Klf5	Kruppel-like factor 5 [Source:MGI Symbol;Acc:MGI:1338056]	9.43107	8.05832	6.77389	7.81166	7.50969	8.024	7.43101	7.57733	7.80565	9.01846	8.0828	8.65808	7.84939	8.18975	0.93447422	0.966018836	24	12	24	0.584207847	0.852087247	24	20.10057005	0.045692079
ENSMUSG00000005150	Wdr83	WD repeat domain containing 83 [Source:MGI Symbol;Acc:MGI:1915086]	14.8124	13.9462	13.8123	15.6387	15.9454	13.8145	13.7926	13.6083	14.2928	13.298	13.5871	14.3237	14.5393	14.303	0.866861748	0.966018836	4	8	24	0.621137725	0.870633098	24	16.74539995	0.026996648
ENSMUSG00000005161	Prdx2	peroxiredoxin 2 [Source:MGI Symbol;Acc:MGI:109486]	232.749	249.037	233.87	246.224	253.787	243.08	221.473	229.402	235.285	207.444	259.339	234.046	244.368	236.046	0.671726933	0.966018836	20	8	24	0.575927813	0.848285086	24	17.88604505	0.035020319
ENSMUSG00000005198	Polr2a	polymerase (RNA) II (DNA directed) polypeptide A [Source:MGI Symbol;Acc:MGI:98086]	9.04883	8.75753	9.1703	9.34291	9.65112	9.61108	9.39841	9.90747	8.54328	14.3891	7.83385	9.48153	8.99684	9.2812	0.954237589	0.966018836	16	20	24	0.599917006	0.860011178	24	11.93923285	0.071540984
ENSMUSG00000005204	Senp3	SUMO/sentrin specific peptidase 3 [Source:MGI Symbol;Acc:MGI:2158736]	42.8586	33.3378	33.1939	35.4763	40.5412	39.6104	41.9084	37.167	38.3793	35.5188	43.202	36.5505	31.7384	40.6407	0.745199643	0.966018836	16	20	24	0.396935481	0.799546792	24	19.27424859	0.077734378
ENSMUSG00000005225	Plekha8	pleckstrin homology domain containing, family A (phosphoinositide binding specific) member 8 [Source:MGI Symbol;Acc:MGI:2681164]	3.66771	3.25243	3.0831	3.32182	3.711	3.09019	3.77318	2.94381	3.18894	2.94195	3.63937	3.22758	3.04223	3.34475	0.791413662	0.966018836	20	20	24	0.293442484	0.768040178	24	19.49788437	0.058625417
ENSMUSG00000005233	Spc25	SPC25, NDC80 kinetochore complex component, homolog (S. cerevisiae) [Source:MGI Symbol;Acc:MGI:1913692]	1.18304	1.38795	1.85587	1.6884	1.44723	1.75257	1.22423	1.28875	1.2212	1.02658	1.35525	1.29188	1.69645	1.09736	0.910436409	0.966018836	20	20	24	0.783984633	0.930589812	24	17.01498856	0.041649028
ENSMUSG00000005251	Ripk4	receptor-interacting serine-threonine kinase 4 [Source:MGI Symbol;Acc:MGI:1919638]	0.600171	0.5642	0.628174	0.679666	0.626363	0.560884	0.67747	0.498276	0.608717	0.716443	0.534518	0.512433	0.513094	0.520242	0.080518813	0.4593055	16	16	24	0.131374373	0.741500219	24	12.25691808	0.064302339
ENSMUSG00000005262	Ufd1	ubiquitin recognition factor in ER-associated degradation 1 [Source:MGI Symbol;Acc:MGI:109353]	42.2129	46.4028	48.4754	45.8343	42.5432	46.3162	43.3509	45.4953	46.7002	46.723	45.9122	46.0241	48.1149	45.8024	0.590017078	0.966018836	12	8	24	0.221724874	0.768040178	24	8.420061621	0.037500597
ENSMUSG00000005267	Zfp287	zinc finger protein 287 [Source:MGI Symbol;Acc:MGI:2176561]	5.20902	5.0594	5.34102	4.65293	4.51804	5.07146	4.99929	4.77679	5.6397	5.46063	5.24267	5.26635	5.49816	5.17442	0.915475528	0.966018836	12	20	24	0.607747606	0.864036051	24	5.48615692	0.028545229
ENSMUSG00000005268	Prlr	prolactin receptor [Source:MGI Symbol;Acc:MGI:97763]	0.65426	0.939312	0.856188	0.70111	0.808422	0.650177	0.690623	0.599306	0.810185	0.608509	0.762346	0.740376	0.535967	0.791896	0.286642595	0.793714531	12	16	24	0.497945279	0.807927978	24	9.237611304	0.042762612
ENSMUSG00000005299	Letm1	leucine zipper-EF-hand containing transmembrane protein 1 [Source:MGI Symbol;Acc:MGI:1932557]	19.216	14.3484	15.0942	17.1252	15.4164	17.3698	16.9449	18.3269	14.3147	21.4221	17.6353	16.169	16.7986	17.9235	0.865490576	0.966018836	16	20	24	0.847712694	0.954054095	24	18.16961891	0.035690987
ENSMUSG00000005312	Ubqln1	ubiquilin 1 [Source:MGI Symbol;Acc:MGI:1860276]	58.9593	47.1888	48.5092	51.7755	48.0782	54.5395	53.0165	52.3357	39.0547	56.1193	45.002	48.9059	47.69	49.9307	0.725061903	0.966018836	4	8	24	0.394976474	0.799546792	24	21.40061697	0.076382428
ENSMUSG00000005338	Cadm3	cell adhesion molecule 3 [Source:MGI Symbol;Acc:MGI:2137858]	108.932	90.9184	87.4366	93.2917	104.627	101.565	105.528	99.5929	84.9048	125.763	87.5417	102.227	98.5329	97.9215	0.085329704	0.466404237	24	12	24	0.503704643	0.810360343	24	19.62128731	0.071612312
ENSMUSG00000005354	Txn2	thioredoxin 2 [Source:MGI Symbol;Acc:MGI:1929468]	64.2162	65.6692	66.8824	74.1359	66.9063	68.8606	61.8422	70.7447	64.3737	67.1646	69.5005	63.5099	69.7404	64.757	0.455507762	0.966018836	16	12	24	0.345207809	0.778298718	24	12.7500674	0.039167473
ENSMUSG00000005357	Slc1a6	solute carrier family 1 (high affinity aspartate/glutamate transporter), member 6 [Source:MGI Symbol;Acc:MGI:1096331]	2.37749	2.06349	2.49489	2.25108	1.94847	2.68925	2.30892	2.11678	2.13773	2.81794	1.87558	2.25153	2.22763	2.67416	0.914502391	0.966018836	24	20	24	0.854464743	0.95651302	24	5.369631963	0.028135655
ENSMUSG00000005360	Slc1a3	solute carrier family 1 (glial high affinity glutamate transporter), member 3 [Source:MGI Symbol;Acc:MGI:99917]	7.04808	8.68401	14.7796	8.45627	7.10701	8.57346	10.072	7.36297	9.12583	7.93687	7.39157	8.96763	5.84248	10.5709	0.3964038	0.927127564	12	8	24	0.307477092	0.768040178	24	7.656896935	0.17697347
ENSMUSG00000005362	Crbn	cereblon [Source:MGI Symbol;Acc:MGI:1913277]	97.7072	104.835	91.2226	99.3277	96.1995	90.3675	91.5803	89.9342	101.989	84.9464	105.516	97.8679	105.265	101.506	0.824031576	0.966018836	20	20	24	0.872677681	0.964187898	24	1.250958911	0.020064999
ENSMUSG00000005370	Msh6	mutS homolog 6 [Source:MGI Symbol;Acc:MGI:1343961]	5.29289	5.10996	5.54052	4.81705	5.09254	5.53959	5.62116	5.15325	4.7991	5.31619	5.27183	5.40548	5.51398	4.93094	0.149537283	0.594118663	4	12	24	0.105395432	0.717252017	24	21.33646063	0.051260712
ENSMUSG00000005371	Fbxo11	F-box protein 11 [Source:MGI Symbol;Acc:MGI:2147134]	35.2082	34.4203	34.3863	33.544	33.1492	32.0975	36.3224	30.5311	31.8133	33.3645	31.9805	34.1759	34.5015	35.5097	0.0521941	0.391073722	4	16	24	0.367493713	0.790832497	24	0.531186212	0.037038193
ENSMUSG00000005373	Mlxipl	MLX interacting protein-like [Source:MGI Symbol;Acc:MGI:1927999]	2.18667	1.69858	3.1242	6.69232	4.03681	3.0636	4.11368	6.05482	3.39611	5.43975	10.053	4.16651	7.37647	4.2344	0.823657258	0.966018836	16	20	24	0.187071759	0.768040178	24	14.16143138	0.299422464
ENSMUSG00000005374	Tbl2	transducin (beta)-like 2 [Source:MGI Symbol;Acc:MGI:1351652]	5.26646	4.06412	4.78999	3.78261	5.20635	5.23111	4.29587	5.01327	4.00383	5.03778	5.10302	4.51256	4.75191	4.5265	0.40165019	0.933249185	20	16	24	0.284048181	0.768040178	24	19.29369866	0.082306839
ENSMUSG00000005378	Bud23	BUD23, rRNA methyltransferase and ribosome maturation factor [Source:MGI Symbol;Acc:MGI:1913388]	17.0976	14.832	15.0782	18.6454	16.8257	15.6124	20.2972	16.358	15.7848	16.5794	18.9426	16.367	15.2019	14.433	0.059513104	0.406067671	20	12	24	0.241051205	0.768040178	24	17.12923451	0.088076531
ENSMUSG00000005397	Nid1	nidogen 1 [Source:MGI Symbol;Acc:MGI:97342]	27.817	27.9113	27.7205	28.1222	36.6691	28.6308	34.4154	31.6785	28.4083	31.0233	28.7173	28.4976	27.384	24.5043	0.392165785	0.918821614	20	12	24	0.378889577	0.798235801	24	17.02448543	0.07663397
ENSMUSG00000005410	Mcm5	minichromosome maintenance complex component 5 [Source:MGI Symbol;Acc:MGI:103197]	1.88125	2.35707	2.93998	2.42019	2.53092	2.4829	1.8238	2.02418	2.03321	1.96158	2.05004	2.1934	2.27208	1.7612	0.427762381	0.966018836	24	4	24	0.40832813	0.799546792	24	13.87910847	0.067064731
ENSMUSG00000005413	Hmox1	heme oxygenase 1 [Source:MGI Symbol;Acc:MGI:96163]	27.6315	19.9664	22.9599	24.3126	29.9177	26.9135	25.0868	23.9751	21.1408	22.6924	26.4769	22.1759	24.6378	20.1849	0.030972684	0.324150946	20	12	24	0.009695848	0.502965379	24	18.37133189	0.16766364
ENSMUSG00000005417	Mprip	myosin phosphatase Rho interacting protein [Source:MGI Symbol;Acc:MGI:1349438]	75.4541	63.9614	63.3203	70.5842	75.04	73.0877	71.8868	68.939	66.1913	75.983	69.6472	68.506	69.608	64.0345	0.048507657	0.379129806	16	20	24	0.037444853	0.609717533	24	17.98206243	0.081211555
ENSMUSG00000005442	Cic	capicua transcriptional repressor [Source:MGI Symbol;Acc:MGI:1918972]	27.6333	27.7519	31.8715	34.7482	36.454	29.5801	33.8766	25.5599	29.1061	36.4554	27.6645	27.0972	33.8708	28.7763	0.148627651	0.59172107	16	16	24	0.274174879	0.768040178	24	13.74274433	0.101692738
ENSMUSG00000005447	Pafah1b3	platelet-activating factor acetylhydrolase, isoform 1b, subunit 3 [Source:MGI Symbol;Acc:MGI:108414]	22.5218	22.9099	20.4034	24.0249	20.8739	24.5511	24.0269	21.6158	20.7613	18.1492	21.2474	22.4055	21.2009	21.7691	0.12833063	0.560209134	24	12	24	0.091548699	0.71175955	24	22.28632875	0.085374836
ENSMUSG00000005469	Prkaca	protein kinase, cAMP dependent, catalytic, alpha [Source:MGI Symbol;Acc:MGI:97592]	57.515	45.9596	48.8071	50.0733	48.6169	55.843	54.4501	53.3866	40.9631	72.899	44.9823	53.2458	51.0327	56.2416	0.455507762	0.966018836	4	8	24	0.878245833	0.965980735	24	20.33686456	0.041385993
ENSMUSG00000005470	Asf1b	anti-silencing function 1B histone chaperone [Source:MGI Symbol;Acc:MGI:1914179]	0.877856	0.905815	1.7456	1.48887	1.08181	1.02913	1.3231	0.613646	1.0144	0.724748	1.08854	1.25505	1.38785	0.813791	0.492524295	0.966018836	24	8	24	0.743987444	0.916218012	24	16.13688661	0.065627701
ENSMUSG00000005481	Ddx39a	DEAD box helicase 39a [Source:MGI Symbol;Acc:MGI:1915528]	5.00232	5.84458	7.25523	6.47164	6.34576	6.23231	6.36802	6.97891	6.289	5.64377	6.82318	5.83126	6.38205	5.51747	0.918497267	0.966018836	12	4	24	0.531376642	0.825282851	24	11.16653688	0.055386113
ENSMUSG00000005483	Dnajb1	DnaJ heat shock protein family (Hsp40) member B1 [Source:MGI Symbol;Acc:MGI:1931874]	24.5869	13.6658	14.4149	20.6625	19.0858	18.2824	21.2312	16.4297	12.5377	14.645	23.7954	15.7841	17.1592	15.1228	0.033622862	0.324980923	20	16	24	0.04201531	0.622498612	24	19.26841421	0.258879416
ENSMUSG00000005501	Usp40	ubiquitin specific peptidase 40 [Source:MGI Symbol;Acc:MGI:2443184]	8.32965	8.99141	9.05202	8.29304	8.99463	8.51392	8.85144	7.86683	9.19546	8.18368	8.8941	8.84162	8.2226	8.42321	0.708020493	0.966018836	20	16	24	0.787190564	0.931456435	24	14.0454422	0.016571513
ENSMUSG00000005505	Kbtbd4	kelch repeat and BTB (POZ) domain containing 4 [Source:MGI Symbol;Acc:MGI:1914386]	8.64017	7.42911	8.27684	7.45544	7.01276	8.21376	8.29087	7.20369	7.15089	7.88842	7.66458	7.68641	7.38516	8.00553	0.70641939	0.966018836	4	16	24	0.457070003	0.805393068	24	22.99322181	0.03979136
ENSMUSG00000005506	Celf1	CUGBP, Elav-like family member 1 [Source:MGI Symbol;Acc:MGI:1342295]	19.3449	20.0689	20.3486	21.0761	19.4218	18.7178	19.6819	17.7631	19.3852	20.8013	20.1018	18.9817	19.7603	19.0658	0.020499994	0.261611804	16	8	24	0.055361583	0.65534544	24	11.39031657	0.042864279
ENSMUSG00000005510	Ndufs3	NADH:ubiquinone oxidoreductase core subunit S3 [Source:MGI Symbol;Acc:MGI:1915599]	20.0182	16.9733	19.0712	20.5225	17.8622	16.5888	19.0979	20.4368	19.131	18.2978	19.112	18.1452	17.6776	19.1286	0.591351961	0.966018836	12	4	24	0.421918228	0.804917634	24	7.969563979	0.047375109
ENSMUSG00000005514	Por	cytochrome p450 oxidoreductase [Source:MGI Symbol;Acc:MGI:97744]	14.1672	14.6669	18.5378	17.4637	17.3687	13.1382	14.2889	17.9962	18.0538	20.0175	14.5604	13.7226	14.4573	16.6327	2.34E-05	0.019409398	12	12	24	2.71E-04	0.191526658	24	9.58395588	0.223959363
ENSMUSG00000005533	Igf1r	insulin-like growth factor I receptor [Source:MGI Symbol;Acc:MGI:96433]	7.70462	7.41669	6.53958	7.08073	6.79076	7.18524	7.30742	7.41669	7.04543	8.46262	6.6879	7.64908	7.9837	7.57529	0.124211791	0.552144108	4	16	24	0.509584614	0.813985618	24	1.15415612	0.038018705
ENSMUSG00000005534	Insr	insulin receptor [Source:MGI Symbol;Acc:MGI:96575]	6.43636	6.80082	7.54933	7.83147	6.49653	6.73587	6.1388	7.10285	8.08362	7.36999	6.34359	7.27081	7.32271	7.46798	0.020678668	0.262822894	16	8	24	0.017881903	0.555944581	24	8.68687552	0.103956081
ENSMUSG00000005566	Trim28	tripartite motif-containing 28 [Source:MGI Symbol;Acc:MGI:109274]	38.214	34.4852	38.081	39.0179	37.4366	40.5237	36.9156	38.2064	36.7627	36.5979	34.0786	33.1951	35.8606	37.1159	0.759203569	0.966018836	16	4	24	0.937893251	0.984659033	24	7.978306435	0.010546772

ENSMUSG00000005575	Ube2m	ubiquitin-conjugating enzyme E2M [Source:MGI Symbol;Acc:MGI:108278]	88.962	83.1471	80.4895	81.569	81.7193	88.9757	82.0719	82.1353	74.4562	96.2487	76.6927	88.4781	82.1933	85.4562	0.14313801	0.585944911	24	12	24	0.618589823	0.868995186	24	20.31211231	0.038144698
ENSMUSG00000005580	Adcy9	adenylate cyclase 9 [Source:MGI Symbol;Acc:MGI:108450]	5.82673	4.5537	5.47591	5.56828	4.68251	5.55518	5.85873	5.66225	4.43016	6.98299	4.5887	5.28245	5.03561	5.57817	0.833406028	0.966018836	4	16	24	0.994074235	0.998755922	24	4.668071892	0.006801982
ENSMUSG00000005583	Mef2c	myocyte enhancer factor 2C [Source:MGI Symbol;Acc:MGI:99458]	8.57823	10.9007	10.2813	11.2103	9.80356	11.8288	11.5004	13.4597	13.0418	15.4204	10.0981	11.2801	12.7642	12.1507	0.718720526	0.966018836	8	12	24	0.201100144	0.768040178	24	8.027404017	0.115815477
ENSMUSG00000005609	Ctr9	CTR9 homolog, Paf1/RNA polymerase II complex component [Source:MGI Symbol;Acc:MGI:109345]	19.7777	17.9007	17.7769	17.4267	16.6501	18.0083	17.436	18.2426	16.3023	17.8011	17.8406	18.7448	17.7609	17.998	0.186105764	0.662239322	8	4	24	0.136256232	0.747972781	24	23.02149918	0.047431028
ENSMUSG00000005610	Eif4g2	eukaryotic translation initiation factor 4, gamma 2 [Source:MGI Symbol;Acc:MGI:109207]	325.301	298.323	300.131	310.114	309.86	297.131	304.334	294.79	281.752	285.051	319.506	289.922	294.642	278.814	0.652290823	0.966018836	20	12	24	0.069463932	0.673533451	24	18.70582838	0.046085721
ENSMUSG00000005611	Irag1	inositol 1,4,5-triphosphate receptor associated 1 [Source:MGI Symbol;Acc:MGI:1338023]	1.29713	1.54798	1.57998	1.58645	1.24726	1.74958	1.82021	1.59401	1.91622	1.85276	1.74681	1.65577	1.63685	1.31652	0.935593607	0.966018836	12	20	24	0.742119543	0.916130449	24	11.57481767	0.035183217
ENSMUSG00000005615	Pcyt1a	phosphate cytidylyltransferase 1, choline, alpha isoform [Source:MGI Symbol;Acc:MGI:88557]	23.029	20.0812	20.8853	22.4387	21.5709	22.2216	20.7763	21.5096	19.0486	23.8949	20.2874	20.3687	20.3996	21.0719	0.874607729	0.966018836	16	20	24	0.572690466	0.846721006	24	16.14053812	0.03358134
ENSMUSG00000005621	Zfp592	zinc finger protein 592 [Source:MGI Symbol;Acc:MGI:2443541]	7.07455	4.75971	5.60493	6.27894	5.91228	6.26511	5.41067	6.12132	6.95245	8.69692	6.12737	6.94698	6.85128	6.32885	0.90703203	0.966018836	16	16	24	0.511472284	0.81494984	24	13.95650343	0.072777173
ENSMUSG00000005625	Psmd4	proteasome (prosome, macropain) 26S subunit, non-ATPase, 4 [Source:MGI Symbol;Acc:MGI:1201670]	89.6274	83.5751	88.4932	85.9757	84.9608	91.5972	81.982	87.4387	82.4332	91.218	85.1617	87.1396	90.5249	86.9632	0.890311217	0.966018836	24	12	24	0.782840259	0.930229965	24	19.42975721	0.015061598
ENSMUSG00000005628	Tmod4	tropomodulin 4 [Source:MGI Symbol;Acc:MGI:1355285]	3.51346	10.1373	9.52355	15.8297	7.89332	10.117	7.44039	19.7623	20.6175	14.3997	7.39044	7.95091	11.0868	8.23263	0.154574418	0.607059703	16	4	24	0.044336079	0.628432994	24	9.089893207	0.539275222
ENSMUSG00000005640	Insrr	insulin receptor-related receptor [Source:MGI Symbol;Acc:MGI:1346037]	0.591008	0.571055	0.563597	0.88413	0.774186	0.684492	0.939901	0.809528	0.647877	0.850813	0.996247	1.07138	0.743567	1.32808	0.628286596	0.966018836	20	16	24	0.809342742	0.939184036	24	17.02979853	0.030344813
ENSMUSG00000005656	Snx6	sorting nexin 6 [Source:MGI Symbol;Acc:MGI:1919433]	86.6758	82.8388	81.137	77.9898	86.5557	78.4655	75.9389	78.3176	78.4454	71.2811	90.6693	76.984	88.825	79.9693	0.911685024	0.966018836	20	20	24	0.56010377	0.840893245	24	20.95265246	0.042444861
ENSMUSG00000005667	Mthfd2	methylenetetrahydrofolate dehydrogenase (NAD+ dependent), methenyltetrahydrofolate cyclohydrolase [Source:MGI Symbol;Acc:MGI:1338850]	13.8729	10.773	12.7443	11.3779	10.3886	12.8997	11.6054	11.1541	8.37915	11.7347	10.8925	10.585	10.5536	10.2253	0.882263435	0.966018836	4	4	24	0.313620766	0.768040178	24	20.7622639	0.084410948
ENSMUSG00000005672	Kit	KIT proto-oncogene receptor tyrosine kinase [Source:MGI Symbol;Acc:MGI:96677]	18.3668	15.4908	14.9584	16.8154	15.7321	17.5935	16.9175	16.6384	12.7716	18.3458	15.2818	14.9286	14.3602	15.132	0.927004797	0.966018836	16	20	24	0.440044233	0.805393068	24	19.32447895	0.066007596
ENSMUSG00000005674	Tomm40l	translocase of outer mitochondrial membrane 40-like [Source:MGI Symbol;Acc:MGI:3589112]	26.8799	22.3776	22.3718	23.1952	24.2027	23.7139	22.2575	23.5484	20.9306	24.7423	25.1833	25.2263	24.9837	23.3454	0.046934151	0.375011045	24	12	24	0.072230645	0.679008169	24	19.44153789	0.077995301
ENSMUSG00000005681	Apoa2	apolipoprotein A-II [Source:MGI Symbol;Acc:MGI:88050]	0.914518	0.727158	0.85063	1.53696	1.05975	1.72437	1.18396	1.04283	0.661811	1.43948	1.38652	0.758336	1.90604	1.45733	0.777666236	0.966018836	16	20	24	0.568904394	0.84466645	24	18.05028957	0.103683484
ENSMUSG00000005682	Pan2	PAN2 poly(A) specific ribonuclease subunit [Source:MGI Symbol;Acc:MGI:1918984]	7.2282	7.67458	7.97678	7.51659	7.95358	7.70201	7.9375	7.57936	8.16294	8.0593	7.53259	7.51908	7.69496	7.74712	0.46238066	0.966018836	12	12	24	0.17526606	0.768040178	24	9.695124895	0.030549871
ENSMUSG00000005683	Cs	citrate synthase [Source:MGI Symbol;Acc:MGI:88529]	84.7835	70.9002	84.7689	97.1219	77.1243	91.3131	88.3737	99.9791	70.9288	121.574	80.4838	84.4715	93.3398	92.0725	0.911685024	0.966018836	16	4	24	0.874809106	0.964976615	24	12.02054042	0.040580757
ENSMUSG00000005686	Ampd3	adenosine monophosphate deaminase 3 [Source:MGI Symbol;Acc:MGI:1096344]	14.2128	12.6095	12.398	11.997	11.7185	15.0701	13.398	14.533	11.2607	14.8619	12.7347	13.3748	13.0397	14.9496	0.313239838	0.824470524	8	4	24	0.323785002	0.768040178	24	23.71961887	0.07253843
ENSMUSG00000005687	Bcas2	BCAS2 pre-mRNA processing factor [Source:MGI Symbol;Acc:MGI:1915433]	31.422	30.2884	31.3974	30.7279	29.2411	29.1363	27.3538	29.739	30.2588	32.994	32.2849	32.5015	30.5989	33.3492	0.880247609	0.966018836	16	12	24	0.427105827	0.805393068	24	10.55540028	0.036605585
ENSMUSG00000005696	Sh2d1a	SH2 domain containing 1A [Source:MGI Symbol;Acc:MGI:1328352]	0.654858	0.998629	1.64786	1.67942	0.741098	1.21166	0.818756	1.17356	1.49506	1.35953	1.09649	1.03141	1.21923	1.33995	0.024438424	0.287293295	12	16	24	0.010890892	0.51149922	24	9.406089418	0.217398112
ENSMUSG00000005698	Ctcf	CCCTC-binding factor [Source:MGI Symbol;Acc:MGI:109447]	14.9197	15.8675	16.3221	16.4832	15.8491	15.597	16.2332	15.1443	15.5269	16.6875	15.6872	16.4794	15.6529	15.7302	0.192357053	0.669841558	12	16	24	0.175663361	0.768040178	24	12.9356913	0.030694823
ENSMUSG00000005699	Pard6a	par-6 family cell polarity regulator alpha [Source:MGI Symbol;Acc:MGI:1927223]	10.2404	8.65406	9.58003	9.28511	8.99626	10.9717	9.91715	10.2654	8.15148	9.99006	10.5758	9.50632	9.89984	10.9248	0.834424969	0.966018836	8	4	24	0.356937556	0.783888514	24	21.48989389	0.061855381
ENSMUSG00000005705	Agrp	agouti related neuropeptide [Source:MGI Symbol;Acc:MGI:892013]	0.875555	0.523874	1.04435	1.22576	0.947944	1.20867	1.92375	1.69097	1.51842	0.575963	1.40923	0.863097	0.746956	0.895235	0.801251296	0.966018836	12	20	24	0.995629234	0.998763985	24	0.467267083	0.010622665
ENSMUSG00000005716	Pvalb	parvalbumin [Source:MGI Symbol;Acc:MGI:97821]	135.574	346.56	340.807	416.24	283.094	368.548	295.754	705.012	762.475	623.533	317.605	348.743	410.329	295.884	0.328944792	0.847277612	16	4	24	0.064132661	0.663876076	24	9.28783561	0.512069325
ENSMUSG00000005718	Tfap4	transcription factor AP4 [Source:MGI Symbol;Acc:MGI:103239]	1.52086	1.66359	1.65441	1.35852	1.85597	1.78713	2.06713	1.92951	2.15674	1.80711	1.70571	1.65289	1.9053	1.8854	0.881718085	0.966018836	8	8	24	0.457964001	0.805393068	24	3.764930301	0.0478988
ENSMUSG00000005732	Ranbp1	RAN binding protein 1 [Source:MGI Symbol;Acc:MGI:96269]	38.0151	39.0626	39.4202	39.9502	37.7127	35.3951	38.719	35.0318	39.904	36.4585	40.348	35.6164	37.1906	39.1479	0.09184219	0.484826421	12	12	24	0.306655507	0.768040178	24	9.355181518	0.040975966
ENSMUSG00000005774	Rfx5	regulatory factor X, 5 (influences HLA class II expression) [Source:MGI Symbol;Acc:MGI:1858421]	2.96235	3.45382	4.23915	2.91332	2.96854	3.26768	2.88058	3.51855	3.85643	3.23831	2.50049	3.58317	2.5288	3.32466	0.0528092	0.391647859	12	16	24	0.130996326	0.741427049	24	7.229984275	0.126226001
ENSMUSG00000005779	Psmb4	proteasome (prosome, macropain) subunit, beta type 4 [Source:MGI Symbol;Acc:MGI:1098257]	161.571	160.175	160.096	159.712	168.687	164.181	147.683	161.624	156.949	144.561	158.244	146.61	158.733	153.786	0.832742389	0.966018836	20	20	24	0.948043189	0.987280175	24	19.15923139	0.007657453
ENSMUSG00000005800	Mmp8	matrix metallopeptidase 8 [Source:MGI Symbol;Acc:MGI:1202395]	1.92539	5.35495	7.7153	6.19808	4.00625	5.26775	4.55749	3.4295	4.61359	3.07485	4.21896	4.2778	5.20624	3.20006	0.906657897	0.966018836	12	8	24	0.638295697	0.877195603	24	10.92975554	0.148326141
ENSMUSG00000005802	Slc30a4	solute carrier family 30 (zinc transporter), member 4 [Source:MGI Symbol;Acc:MGI:1345282]	5.90869	7.67224	7.5533	7.64611	6.98573	6.29449	6.36729	6.68024	7.95203	6.85998	7.61868	6.7055	6.99421	7.50525	0.018263889	0.256345714	12	16	24	0.009698155	0.502965379	24	9.545216145	0.117205985
ENSMUSG00000005803	Sqor	sulfide quinone oxidoreductase [Source:MGI Symbol;Acc:MGI:1929899]	6.95023	7.39375	8.30185	9.32559	8.29562	7.02692	8.24473	7.922	8.12392	7.41653	8.80405	8.45382	8.77369	7.99571	0.522551072	0.966018836	20	12	24	0.404596344	0.799546792	24	12.53496396	0.054140436
ENSMUSG00000005804	Bloc1s6	biogenesis of lysosomal organelles complex-1, subunit 6, pallidin [Source:MGI Symbol;Acc:MGI:1927580]	18.3529	16.4604	17.0921	16.582	15.3154	15.2771	14.5053	17.7453	15.4312	19.2011	17.5347	16.3806	18.1486	16.2183	0.886572916	0.966018836	16	8	24	0.815162212	0.940901154	24	8.81881766	0.029490221
ENSMUSG00000005813	Metap1	methionyl aminopeptidase 1 [Source:MGI Symbol;Acc:MGI:1922874]	24.9801	22.8101	22.6162	24.3483	23.1718	24.7406	24.5592	24.3924	21.3391	26.1039	25.0023	23.4904	23.0729	24.0117	0.181442559	0.659020016	16	20	24	0.405053851	0.799546792	24	18.2612577	0.039050631
ENSMUSG00000005823	Gpr108	G protein-coupled receptor 108 [Source:MGI Symbol;Acc:MGI:1925558]	21.585	19.9968	20.0924	20.2394	20.4691	22.4498	20.5214	21.0741	18.7271	20.1756	20.1663	20.2433	20.7626	19.4139	0.016795343	0.253395174	4	8	24	0.01479193	0.537924552	24	20.74976123	0.05846505
ENSMUSG00000005846	Rsl1d1	ribosomal L1 domain containing 1 [Source:MGI Symbol;Acc:MGI:1913659]	34.9486	35.9914	35.3481	33.2823	32.7074	31.341	31.7367	32.0173	35.8127	30.6975	35.4611	34.0209	35.5126	37.1312	0.202903295	0.689605535	8	8	24	0.327145319	0.768040178	24	4.964946045	0.049165584
ENSMUSG00000005871	Apc	APC, WNT signaling pathway regulator [Source:MGI Symbol;Acc:MGI:88039]	30.3218	30.027	29.5062	28.9728	30.0157	28.965	28.7526	27.3417	27.5469	28.7212	29.8642	30.8753	29.4158	31.8157	0.892578749	0.966018836	24	12	24	0.545769655	0.833198211	24	20.92840335	0.025700702
ENSMUSG00000005873	Reep5	receptor accessory protein 5 [Source:MGI Symbol;Acc:MGI:1270152]	641.337	535.515	497.854	556.437	522.555	584.243	544.934	569.087	444.65	617.285	552.371	563.28	575.421	570.169	0.143321297	0.585944911	24	12	24	0.234684652	0.768040178	24	21.06952161	0.087133891
ENSMUSG00000005881	Ergic3	ERGIC and golgi 3 [Source:MGI Symbol;Acc:MGI:1913616]	57.1334	59.57	62.4249	59.7301	59.2432	64.2509	66.117	67.8097	59.7202	59.3885	61.6693	56.4069	61.6317	57.8499	0.844448841	0.966018836	12	12	24	0.779046789	0.928962679	24	2.440563474	0.021381293
ENSMUSG00000005882	Uqcc1	ubiquinol-cytochrome c reductase complex assembly factor 1 [Source:MGI Symbol;Acc:MGI:1929472]	46.0914	42.4556	42.8879	42.1013	38.6401	43.4791	42.0637	43.1028	36.6788	46.1346	43.1032	43.9598	49.0079	47.866	0.590017078	0.966018836	4	8	24	0.31504314	0.768040178	24	0.214140928	0.059573764
ENSMUSG00000005886	Ncoa2	nuclear receptor coactivator 2 [Source:MGI Symbol;Acc:MGI:1276533]	13.2782	12.7483	12.9102	13.2465	12.1563	12.9635	13.2839	12.6018	11.7344	13.5492	12.2138	13.3326	12.3905	12.5922	0.772122688	0.966018836	24	12	24	0.72370446	0.909392141	24	20.53271193	0.01842749
ENSMUSG00000005893	Nr2c2	nuclear receptor subfamily 2, group C, member 2 [Source:MGI Symbol;Acc:MGI:1352466]	11.2314	11.6424	12.4613	10.7535	11.7596	11.2874	10.6532	10.3253	12.2291	11.5323	11.1694	12.4731	11.1084	11.9218	0.643325306	0.966018836	12	16	24	0.693706332	0.898364349	24	10.83046862	0.026633443
ENSMUSG00000005897	Nr2c1	nuclear receptor subfamily 2, group C, member 1 [Source:MGI Symbol;Acc:MGI:1352465]	3.65811	4.03598	3.77685	3.71219	3.64324	3.68883	4.09391	3.89852	3.59147	4.84172	3.53254	3.71762	3.86864	4.12782	0.033553741	0.324909923	8	12	24	0.018161145	0.518566719	24	7.003633106	0.043580966
ENSMUSG00000005899	Smpd4	sphingomyelin phosphodiesterase 4 [Source:MGI Symbol;Acc:MGI:1924876]	13.0727	11.1055	10.1547	12.3332	12.8726	12.0021	12.5199	11.0626	10.4995	11.0349	12.282	11.9706	11.5574	10.934	0.005162915	0.145313511	20	16	24	0.001715372	0.374946468	24	19.28990729	0.117417325
ENSMUSG00000005907	Pex1	peroxisomal biogenesis factor 1 [Source:MGI Symbol;Acc:MGI:1918632]	11.9301	14.8736	13.3128	12.7948	13.5884	12.8022	12.8174	11.9008	14.2243	12.5796	14.5446	12.6278	13.0748	11.0898	0.960338592	0.967043964	20	12	24	0.480616567	0.805393068	24	13.64982213	0.049456553
ENSMUSG00000005936	Kctd20	potassium channel tetramerisation domain containing 20 [Source:MGI Symbol;Acc:MGI:1914239]	29.8907	25.6273	26.7776	28.6311	28.3513	28.0243	28.8179	28.208	25.1671	29.1813	27.2188	27.9222	28.6519	27.0692	0.109040811	0.524035616	4	8	24	0.180217651	0.768040178	24	19.6044423	0.049198784
ENSMUSG00000005949	Ctns	cystinosis, nephropathic [Source:MGI Symbol;Acc:MGI:1932872]	18.8531	18.6406	17.9772	17.5977	21.2339	19.418	19.2062	20.0742	19.9396	19.3927	18.2342	18.2564	19.9768	17.146	0.890648739	0.966018836	4	8	24	0.755010926	0.920960002	24	19.47008056	0.02521665
ENSMUSG00000005950	P2rx5	purinergic receptor P2X, ligand-gated ion channel, 5 [Source:MGI Symbol;Acc:MGI:2137026]	4.13327	3.5849	3.72617	5.01276	4.03332	4.28495	3.75142	4.55972	3.53406	5.5062	4.58192	3.99773	4.39315	4.77986	0.369262716	0.893596186	16	20	24	0.36637241	0.790213221	24	13.28882274	0.077891601
ENSMUSG00000005951	Shpk	sedoheptulokinase [Source:MGI Symbol;Acc:MGI:1921887]	10.6099	13.219	12.6442	10.8231	12.619	11.9796	11.98	12.708	14.0896	11.9948	11.3888	13.062	12.9896	11.4362	0.843639218	0.966018836	12	4	24	0.627713161	0.872795898	24	6.013776313	0.041585306
ENSMUSG00000005952	Trpv1	transient receptor potential cation channel, subfamily V, member 1 [Source:MGI Symbol;Acc:MGI:1341787]	35.9901	28.7004	32.3479	34.6611	33.0965	40.469	30.3249	32.1899	23.8807	37.0694	30.1429	32.2843	29.0783	28.7576	0.125855811	0.555618842	16	16	24	0.112666491	0.726919094	24	17.90023047	0.137344169
ENSMUSG00000005958	Ephb3	Eph receptor B3 [Source:MGI Symbol;Acc:MGI:104770]	2.24113	1.90578	1.88398	2.00508	2.22503	2.01475	2.45208	2.24726	1.88298	2.32968	2.29198	1.95279	2.31266	1.93011	0.105280783	0.518002936	4	8	24	0.37445573	0.794636296	24	19.93498987	0.051727545
ENSMUSG00000005968	Tuft1	tuftelin 1 [Source:MGI Symbol;Acc:MGI:109572]	1.32407	1.21373	1.54285	1.57704	1.21354	1.35485	1.15326	1.60668	0.997181	1.2983	1.2044	1.53437	1.405	1.27039	0.858266942	0.966018836	24	20	24	0.990121871	0.998148205	24	20.57785984	0.006671823
ENSMUSG00000005973	Rcn1	reticulocalbin 1 [Source:MGI Symbol;Acc:MGI:104559]	31.6164	34.0318	29.2743	28.8085	34.973	29.052	33.6326	31.113	32.4602	28.013	28.3074	30.2417	32.2466	29.8731	0.097542159	0.501112642	4	12	24	0.251654049	0.768040178	24	0.796283104	0.06141042
ENSMUSG00000005980	Dnase1	deoxyribonuclease I [Source:MGI Symbol;Acc:MGI:103157]	4.38174	3.84475	3.17865	3.72502	3.87268	3.64664	3.39753	3.88694	2.81346	3.39722	3.62613	4.34567	3.56269	4.09987	0.188831826	0.664348865	8	4	24	0.132127589	0.742323615	24	21.54703625	0.104403846
ENSMUSG00000005981	Trap1	TNF receptor-associated protein 1 [Source:MGI Symbol;Acc:MGI:1915265]	35.3933	33.9479	34.745	37.026	34.8243	35.8299	34.0404	37.1357	30.8049	37.0929	34.7728	35.186	36.0187	34.743	0.06317972	0.967965568	16	20	24	0.686297657	0.895774045	24	17.94406457	0.023984233
ENSMUSG00000005982	Naa60	N(alpha)-acetyltransferase 60, NatF catalytic subunit [Source:MGI Symbol;Acc:MGI:1922013]	26.6604	21.9273	24.9122	23.8185	25.6995	26.3385	22.6923	22.6459	21.8652	31.2979	24.0584	25.5254	22.1402	23.0163	0.100757394	0.620479606	24	8	24	0.116162805	0.727799063	24	15.66366527	0.105501825
ENSMUSG00000005983	1700037C18Rik	RIKEN cDNA 1700037C18 gene [Source:MGI Symbol;Acc:MGI:1920511]	2.63863	2.32941	1.99167	1.91687	1.67285	2.83385	1.4523	2.03752	2.44794	1.99778	2.0872	2.39585	2.19365	1.64551	0.942429894	0.966018836	24	16	24	0.709668668	0.903951515	24	21.0603158	0.061560069
ENSMUSG00000005986	Ankrd13d	ankyrin repeat domain 13 family, member D [Source:MGI Symbol;Acc:MGI:1915673]	25.4801	21.3575	19.6852	21.6225	21.4656	24.7139	21.9545	22.2205	19.2888	25.8336	21.0967	22.7969	23.0578	21.6902	0.038115943	0.334069441	24	12	24	0.190679273	0.768040178	24	20.44073043	0.085713481
ENSMUSG00000005994	Tyrp1	tyrosinase-related protein 1 [Source:MGI Symbol;Acc:MGI:98881]	8.22422	5.33135	5.78997	5.92651	6.63171	6.396	6.44748	4.64442	4.31599	4.40706	8.10776	6.32427	6.00271	5.69423	0.01813461	0.256345714	20	16	24	0.01374542	0.52940102	24	19.78157653	0.245919767
ENSMUSG00000006005	Tpr	translocated promoter region, nuclear basket protein [Source:MGI Symbol;Acc:MGI:1922066]	47.123	48.9982	46.3789	54.1339	43.6552	46.3401	43.85	46.6796	44.5906	44.7882	43.5453	49.1691	50.0573	43.2278	0.924770496	0.966018836	4	16	24	0.98687826	0.997339197	24	22.95909522	0.00597051
ENSMUSG00000006010	Odr4	odr4 GPCR localization factor homolog [Source:MGI Symbol;Acc:MGI:2385108]	51.1045	49.5209	43.1239	51.6428	50.6189	47.9435	50.078	45.5836	49.5598	42.7064	52.7471	45.6547	50.446	49.9618	0.580049522	0.966018836	20	16	24	0.556107681	0.838702296	24	20.74592716	0.042153573
ENSMUSG00000006014	Prg4	proteoglycan 4 (megakaryocyte stimulating factor, articular superficial zone protein) [Source:MGI Symbol;Acc:MGI:1891344]	12.5374	15.0234	9.66174	9.41033	11.8117	9.55317	9.49359	12.9379	15.3327	8.67391	11.6794	14.6743	9.07145	11.6848	0.446653834	0.966018836	8	8	24	0.696375506	0.89922881	24	3.388720162	0.089148783
ENSMUSG00000006019	Dhx34	DEAH (Asp-Glu-Ala-His) box polypeptide 34 [Source:MGI Symbol;Acc:MGI:1918973]	2.56498	1.94328	1.72925	1.85275	1.71785	1.97077	2.57568	2.30154	2.45227	2.78068	2.65221	2.58288	2.07763	2.4565	0.868508291	0.966018836	24	12	24	0.854558511	0.956587667	24	23.74102415	0.032665685
ENSMUSG00000006021	Kptn	kaptin [Source:MGI Symbol;Acc:MGI:1890380]	10.3197	9.23191	10.1931	9.23798	8.68636	10.4731	8.78205	9.12383	8.6347	10.0082	9.17777	9.89556	8.68087	9.97961	0.858266942	0.966018836	24	20	24	0.930724888	0.982191975	24	20.3092424	0.014464077
ENSMUSG00000006024	Napa	N-ethylmaleimide sensitive fusion protein attachment protein alpha [Source:MGI Symbol;Acc:MGI:104563]	88.1969	74.9	74.214	76.7868	78.1937	91.1877	83.559	86.3536	66.7486	91.8354	75.533	80.8894	74.8762	76.6314	0.148073804	0.590732968	24	12	24	0.27591242	0.768040178	24	20.26117107	0.083126534
ENSMUSG00000006050	Sra1	steroid receptor RNA activator 1 [Source:MGI Symbol;Acc:MGI:1344414]	43.2163	43.2366	43.3406	40.2994	45.1658	43.6499	39.916	45.5434	41.6821	38.7467	45.305	39.295	42.7864	41.4864	0.890311217	0.966018836	20	20	24	0.919168385	0.978264112	24	23.59005659	0.012938746
ENSMUSG00000006056	Calcoco2	calcium binding and coiled-coil domain 2 [Source:MGI Symbol;Acc:MGI:1343177]	0.462178	0.982089	0.857866	1.12695	1.06247	0.89882	1.42821	0.87863	1.5437	0.843972	1.8001	1.08875	0.885344	0.847489	0.628895575	0.966018836	20	12	24	0.364427856	0.788837108	24	13.56538738	0.132173844
ENSMUSG00000006057	Atp5g1	ATP synthase, H+ transporting, mitochondrial F0 complex, subunit C1 (subunit 9) [Source:MGI Symbol;Acc:MGI:107653]	76.8003	80.0309	76.4037	75.1198	86.4379	86.6537	76.9	95.9179	90.9778	77.2129	82.9619	80.1257	84.8656	79.741	0.907068194	0.966018836	20	16	24	0.920055518	0.978750626	24	3.029162542	0.016507663
ENSMUSG00000006058	Snf8	SNF8, ESCRT-II complex subunit, homolog (S. cerevisiae) [Source:MGI Symbol;Acc:MGI:1343161]	21.0476	19.1195	18.9906	20.1429	19.6524	21.5457	18.7237	21.5355	17.9607	20.3427	19.4121	18.7075	21.4508	19.3601	0.868022237	0.966018836	4	8	24	0.513522087	0.816307374	24	21.55716076	0.038205484
ENSMUSG00000006095	Tbcb	tubulin folding cofactor B [Source:MGI Symbol;Acc:MGI:1913661]	95.7778	88.1931	86.5641	82.8012	91.174	93.1092	95.6035	81.6069	87.56	88.2697	89.2062	83.1287	85.4609	89.9873	0.496745696	0.966018836	4	8	24	0.283813329	0.768040178	24	21.33262934	0.043723127
ENSMUSG00000006127	Inpp5k	inositol polyphosphate 5-phosphatase K [Source:MGI Symbol;Acc:MGI:1194899]	32.2815	33.7374	32.2614	33.3162	31.8145	32.2507	29.3996	31.9537	33.4331	33.4058	31.2744	32.879	33.3294	33.5717	0.215777641	0.704989686	8	12	24	0.308967619	0.768040178	24	8.394675401	0.031792562
ENSMUSG00000006134	Crkl	v-crk avian sarcoma virus CT10 oncogene homolog-like [Source:MGI Symbol;Acc:MGI:104686]	15.3365	12.8019	12.9905	13.5517	13.0222	14.1301	14.0588	13.6011	11.9742	15.1816	13.1306	14.2556	14.1381	13.3442	0.026841899	0.305155548	24	12	24	0.207938736	0.768040178	24	20.75052703	0.06317724
ENSMUSG00000006154	Eps8l1	EPS8-like 1 [Source:MGI Symbol;Acc:MGI:1914675]	8.33315	5.91314	6.18858	7.59533	8.40955	8.10689	11.1504	6.67942	6.68351	6.4178	5.7605	7.43144	7.2233	9.91618	0.510031987	0.966018836	4	8	24	0.235632139	0.768040178	24	22.76506908	0.162189267
ENSMUSG00000006169	Clint1	clathrin interactor 1 [Source:MGI Symbol;Acc:MGI:2144243]	19.8591	16.7011	17.7533	17.0655	16.3617	18.0572	18.2009	17.5504	13.8434	18.6042	15.4498	16.0283	15.245	15.6493	0.858514388	0.966018836	4	16	24	0.548233122	0.834194881	24	21.4363264	0.047781287
ENSMUSG00000006191	Cdkal1	CDK5 regulatory subunit associated protein 1-like 1 [Source:MGI Symbol;Acc:MGI:1921765]	4.4975	4.9702	5.02375	5.68303	5.41631	5.17685	4.46585	4.85857	5.83133	4.26204	5.14197	5.10695	4.58186	5.58482	0.112953113	0.530777648	12	16	24	0.386957789	0.799546792	24	11.08856079	0.062637466
ENSMUSG00000006205	Htra1	HtrA serine peptidase 1 [Source:MGI Symbol;Acc:MGI:1929076]	53.3322	46.0886	47.9207	51.3778	48.1227	55.0055	51.2555	51.149	42.2539	49.0261	46.5906	46.098	45.1079	45.1046	0.622132989	0.966018836	16	20	24	0.202037768	0.768040178	24	19.93631048	0.063285667
ENSMUSG00000006215	Zbtb17	zinc finger and BTB domain containing 17 [Source:MGI Symbol;Acc:MGI:107410]	6.79596	5.23956	5.11074	4.88619	5.65284	5.91306	4.67552	5.18576	5.63973	5.90251	5.9581	5.56998	5.72586	4.83064	0.400729905	0.933249185	20	12	24	0.278309805	0.768040178	24	18.58184384	0.078498956
ENSMUSG00000006218	Fam131c	family with sequence similarity 131, member C [Source:MGI Symbol;Acc:MGI:2685539]	0.530849	0.698899	0.815358	0.682662	0.492454	0.640286	0.839072	0.784537	0.918287	1.0313	0.645746	0.814295	0.851223	0.85851	0.235187365	0.73629392	12	8	24	0.037474755	0.609919408	24	7.105198572	0.097037728
ENSMUSG00000006219	Fblim1	filamin binding LIM protein 1 [Source:MGI Symbol;Acc:MGI:1921452]	0.67497	0.995903	0.929017	0.747166	0.773992	0.953858	0.867541	1.11474	1.11307	0.971586	1.05452	0.849038	1.03116	1.15092	0.173541845	0.648600527	8	16	24	0.040669992	0.616921492	24	6.338514868	0.081345629
ENSMUSG00000006221	Hspb7	heat shock protein family, member 7 (cardiovascular) [Source:MGI Symbol;Acc:MGI:1352494]	1.30624	3.30188	6.18293	5.88893	3.61525	5.4149	3.54682	9.46295	4.77385	8.68653	3.01163	2.45786	6.22051	3.8087	0.328842658	0.847277612	16	4	24	0.141558262	0.751766185	24	9.190564409	0.443548362
ENSMUSG00000006235	Epor	erythropoietin receptor [Source:MGI Symbol;Acc:MGI:95408]	0.322375	0.942794	0.971424	1.16599	0.850015	0.862641	0.757502	0.779388	0.780515	0.633996	0.715753	0.559604	0.963639	0.830839	0.801995186	0.966018836	12	12	24	0.37832238	0.797706855	24	9.475161407	0.097165124
ENSMUSG00000006241	Ccdc159	coiled-coil domain containing 159 [Source:MGI Symbol;Acc:MGI:1914369]	8.6173	7.8347	8.13289	8.0468	8.79078	8.78598	9.40279	8.56328	7.47654	7.73867	8.95559	6.65832	8.71021	7.19407	0.210789085	0.699573621	20	16	24	0.424310665	0.805393068	24	20.37611871	0.062095364
ENSMUSG00000006262	Mob1b	MOB kinase activator 1B [Source:MGI Symbol;Acc:MGI:1915723]	5.47151	5.38299	5.75398	5.60622	5.37341	5.18377	5.24755	5.62625	6.16859	4.97957	5.97029	5.27532	4.65639	5.79367	0.068712455	0.432567661	12	12	24	0.212230604	0.768040178	24	9.135688106	0.061989398
ENSMUSG00000006273	Atp6v1b2	ATPase, H+ transporting, lysosomal V1 subunit B2 [Source:MGI Symbol;Acc:MGI:109618]	215.747	185.657	194.37	194.026	182.527	212.949	195.326	194.248	157.856	208.24	191.329	201.72	199.87	209.612	0.215777641	0.704989686	24	20	24	0.252692254	0.768040178	24	21.49543397	0.074630549
ENSMUSG00000006276	Eps15l1	epidermal growth factor receptor pathway substrate 15-like 1 [Source:MGI Symbol;Acc:MGI:104582]	24.5007	21.9352	23.2482	24.7915	24.8304	23.977	23.2033	23.1918	23.0278	25.5682	21.619	22.2408	22.7284	23.0176	0.5169953	0.966018836	16	16	24	0.391067445	0.799546792	24	13.60790403	0.034385241
ENSMUSG00000006281	Tep1	telomerase associated protein 1 [Source:MGI Symbol;Acc:MGI:109573]	6.47725	7.95826	5.39966	6.45503	7.86075	7.15685	9.69444	5.76079	9.36101	7.10615	8.78012	7.68972	7.32128	6.94153	0.840483986	0.966018836	20	20	24	0.633106288	0.875319941	24	18.95291856	0.081981514
ENSMUSG00000006288	Ttc5	tetratricopeptide repeat domain 5 [Source:MGI Symbol;Acc:MGI:2683584]	13.4217	15.5504	14.5593	16.2104	14.7924	14.4652	12.5301	15.8947	15.9946	13.6396	15.7802	14.2033	15.2815	15.0312	0.216404185	0.704989686	12	16	24	0.249519287	0.768040178	24	9.558655558	0.066654418
ENSMUSG00000006289	Osgep	O-sialoglycoprotein endopeptidase [Source:MGI Symbol;Acc:MGI:1913496]	10.9322	9.23381	10.7378	8.01582	11.1623	10.4687	9.69777	9.27349	11.2009	10.7966	10.1577	11.8626	11.7714	8.53567	0.406611082	0.943138173	24	8	24	0.401272936	0.799546792	24	19.18004528	0.08460336
ENSMUSG00000006299	Aamp	angio-associated migratory protein [Source:MGI Symbol;Acc:MGI:107809]	56.1659	54.197	56.0649	56.3419	56.38	55.9659	57.174	57.3014	54.2738	55.7568	54.1329	55.888	56.7379	55.2681	0.671726933	0.966018836	4	8	24	0.502061029	0.809301297	24	22.5438631	0.012443294
ENSMUSG00000006301	Tmbim1	transmembrane BAX inhibitor motif containing 1 [Source:MGI Symbol;Acc:MGI:1916910]	38.0695	36.6224	37.4643	37.208	42.8513	37.4742	44.3507	39.0404	38.5344	40.599	35.8135	37.9335	38.598	33.3567	0.897975397	0.966018836	4	4	24	0.620826421	0.870334916	24	18.4792392	0.03943444
ENSMUSG00000006304	Arpc2	actin related protein 2/3 complex, subunit 2 [Source:MGI Symbol;Acc:MGI:1923959]	112.865	103.936	106.507	103.876	108.901	110.258	95.9159	108.497	104.64	115.29	114.237	110.166	112.89	107.741	0.511348134	0.966018836	20	16	24	0.439610067	0.805393068	24	16.39535272	0.033586461
ENSMUSG00000006307	Kmt2b	lysine (K)-specific methyltransferase 2B [Source:MGI Symbol;Acc:MGI:109565]	7.67309	8.92449	9.46161	10.2279	9.59774	9.19098	8.78491	9.55882	10.3827	9.42099	7.40403	8.90043	9.19957	10.7022	0.108524038	0.524035616	12	16	24	0.084709458	0.70291653	24	8.131003158	0.111139331
ENSMUSG00000006310	Zbtb32	zinc finger and BTB domain containing 32 [Source:MGI Symbol;Acc:MGI:1891838]	0.368732	0.557823	0.745559	0.198226	0.514372	0.597253	0.510621	0.460199	0.665659	0.694736	0.373133	0.730335	0.568641	0.853856	0.932092277	0.966018836	12	16	24	0.472817847	0.805393068	24	5.055825821	0.073380937
ENSMUSG00000006315	Tmem147	transmembrane protein 147 [Source:MGI Symbol;Acc:MGI:1915011]	69.6636	68.6403	69.0788	69.2315	73.0706	71.2088	67.0497	71.7294	68.4819	64.1849	69.4563	68.2678	68.8706	67.1959	0.934152439	0.966018836	20	20	24	0.48859548	0.805393068	24	19.74286188	0.020683458
ENSMUSG00000006333	Rps9	ribosomal protein S9 [Source:MGI Symbol;Acc:MGI:1924096]	285.746	307.503	300.37	308.577	301.409	298.014	282.741	305.991	335.508	284.069	317.859	314.11	339.368	334.843	0.858266942	0.966018836	8	20	24	0.459356788	0.805393068	24	6.427819831	0.035220027
ENSMUSG00000006335	Tfpt	TCF3 (E2A) fusion partner [Source:MGI Symbol;Acc:MGI:1916964]	10.7898	10.2016	9.98959	8.03546	9.91314	11.0799	10.4931	9.9813	9.27561	12.9468	10.4642	12.5051	11.0764	12.0342	0.14313801	0.585944911	24	12	24	0.389481698	0.799546792	24	21.97524593	0.076025881
ENSMUSG00000006342	Susd2	sushi domain containing 2 [Source:MGI Symbol;Acc:MGI:1918983]	76.6175	51.8612	49.8306	57.3368	55.5651	74.4493	67.662	70.3978	41.0648	90.16	53.2076	61.9344	55.8494	60.0166	0.217827365	0.704989686	24	12	24	0.45795347	0.805393068	24	20.51917676	0.148451374
ENSMUSG00000006344	Ggt5	gamma-glutamyltransferase 5 [Source:MGI Symbol;Acc:MGI:1346063]	2.19881	1.95141	2.2766	1.77129	3.23849	1.80235	2.01876	2.06993	1.964	1.42434	2.6363	1.46367	1.85432	1.74181	0.928148816	0.966018836	20	4	24	0.692267258	0.898055706	24	16.61722431	0.068079223
ENSMUSG00000006345	Ggt1	gamma-glutamyltransferase 1 [Source:MGI Symbol;Acc:MGI:95706]	0.561827	0.716405	0.679964	0.671455	0.768853	0.542782	1.15635	0.857486	0.702122	0.647016	0.687239	0.819825	0.631641	0.749384	0.942877366	0.966018836	8	8	24	0.701119969	0.900900643	24	2.073302043	0.039298109
ENSMUSG00000006356	Crip2	cysteine rich protein 2 [Source:MGI Symbol;Acc:MGI:1915587]	171.896	126.965	113.313	124.184	130.195	141.17	148.211	149.508	121.99	169.021	137.467	146.61	144.142	140.144	0.009848456	0.194720062	4	8	24	0.234067282	0.768040178	24	21.75880763	0.111426328
ENSMUSG00000006360	Crip1	cysteine-rich protein 1 (intestinal) [Source:MGI Symbol;Acc:MGI:88501]	88.8908	107.122	88.6489	96.23	107.473	86.978	90.3208	101.647	125.443	74.0453	101.33	99.7538	104.967	100.539	0.935316621	0.966018836	8	16	24	0.731921981	0.912550805	24	5.199143848	0.057179049
ENSMUSG00000006362	Cbfa2t3	CBFA2/RUNX1 translocation partner 3 [Source:MGI Symbol;Acc:MGI:1338013]	1.31579	1.04748	0.785694	1.06466	0.92913	0.954715	1.17069	1.5676	0.910362	1.37841	1.05245	0.940808	1.03986	0.907586	0.949152198	0.966018836	4	8	24	0.811228765	0.939484534	24	1.579294036	0.036925183
ENSMUSG00000006369	Fbln1	fibulin 1 [Source:MGI Symbol;Acc:MGI:95487]	4.76873	5.49268	4.35334	3.87868	4.49985	4.51139	4.04288	6.11044	5.55971	2.23126	4.45118	5.01255	3.37657	4.10844	0.446653834	0.966018836	8	8	24	0.428935831	0.805393068	24	1.835100431	0.135681047
ENSMUSG00000006373	Pgrmc1	progesterone receptor membrane component 1 [Source:MGI Symbol;Acc:MGI:1858305]	110.629	114.833	113.285	106.234	109.182	103.918	100.147	105.83	110.836	109.476	106.056	108.846	106.854	114.414	0.128989168	0.560209134	12	12	24	0.166646149	0.767020009	24	7.403717009	0.040389449
ENSMUSG00000006378	Gcat	glycine C-acetyltransferase (2-amino-3-ketobutyrate-coenzyme A ligase) [Source:MGI Symbol;Acc:MGI:1349389]	9.35022	8.02841	7.32833	7.10898	10.1301	7.81392	7.92263	8.13886	8.73996	6.5093	8.11015	9.30794	8.87426	7.46957	0.136853693	0.578758359	20	20	24	0.142216169	0.751766185	24	21.17311147	0.118241451
ENSMUSG00000006386	Tek	TEK receptor tyrosine kinase [Source:MGI Symbol;Acc:MGI:98664]	2.4275	3.4536	2.97223	2.44679	2.561	2.68148	2.63375	4.32573	3.10189	2.83415	2.23566	2.73684	3.01832	2.68362	0.032263213	0.324385159	8	12	24	0.046752876	0.633507364	24	4.993951322	0.161922566
ENSMUSG00000006390	Elovl1	elongation of very long chain fatty acids (FEN1/Elo2, SUR4/Elo3, yeast)-like 1 [Source:MGI Symbol;Acc:MGI:1858959]	109.922	85.8262	92.1913	105.934	128.294	99.8268	110.569	87.0444	97.4355	97.1037	102.707	83.1863	92.3725	72.8802	0.036134798	0.331852526	20	12	24	0.03292546	0.606585237	24	16.76324975	0.164730208
ENSMUSG00000006392	Med8	mediator complex subunit 8 [Source:MGI Symbol;Acc:MGI:1915269]	33.687	30.1847	32.0856	28.8146	31.53	32.1703	33.8714	29.0483	28.5538	30.9347	30.9541	29.1469	28.2171	28.2822	0.742579234	0.966018836	4	4	24	0.170240116	0.767957732	24	19.86048348	0.05319096

ENSMUSG00000006395	Hyi	hydroxypyruvate isomerase (putative) [Source:MGI Symbol;Acc:MGI:1915430]	21.5823	20.5392	19.7848	21.2598	21.4862	21.7925	20.8037	21.1101	20.9543	19.2156	22.038	23.0032	23.6798	20.4229	0.019280932	0.257669847	24	12	24	0.022125666	0.576704253	24	20.67014475	0.071795861
ENSMUSG00000006398	Cdc20	cell division cycle 20 [Source:MGI Symbol;Acc:MGI:1859866]	0.896376	1.04684	1.41945	1.03594	0.877714	1.23857	0.999554	0.982801	1.21898	0.666598	0.83564	1.09975	1.36747	0.803132	0.933421033	0.966018836	12	4	24	0.667291894	0.887459929	24	2.039438135	0.054104199
ENSMUSG00000006403	Adamts4	a disintegrin-like and metallopeptidase (reprolysin type) with thrombospondin type 1 motif, 4 [Source:MGI Symbol;Acc:MGI:1339949]	1.20462	1.69369	2.50198	0.930339	0.882724	1.57515	2.33978	1.46691	1.64896	0.97639	0.673387	1.30913	0.807172	1.72913	0.046820236	0.375011045	12	8	24	0.074936897	0.68394125	24	4.124784896	0.260029854
ENSMUSG00000006411	Nectin4	nectin cell adhesion molecule 4 [Source:MGI Symbol;Acc:MGI:1918990]	6.48592	5.6348	5.26398	5.09593	5.74197	6.33713	5.53946	5.26473	3.99975	6.60594	6.14302	5.67068	5.75918	6.14754	0.287488775	0.793714531	24	12	24	0.175960851	0.768040178	24	19.96014345	0.113422321
ENSMUSG00000006412	Pfdn2	prefoldin 2 [Source:MGI Symbol;Acc:MGI:1276111]	51.6873	48.7224	48.6517	53.8615	52.8506	51.0939	52.2636	49.2142	51.4688	48.9693	43.9264	49.733	49.1526	46.9682	0.846840613	0.966018836	16	16	24	0.863130895	0.960158019	24	19.26124703	0.014864036
ENSMUSG00000006418	Rnf114	ring finger protein 114 [Source:MGI Symbol;Acc:MGI:1933159]	23.5957	21.1854	21.3768	21.5578	22.1895	23.4793	21.4458	23.6121	20.1076	22.7741	21.6572	20.2624	21.3919	20.9402	0.83638114	0.966018836	20	16	24	0.600251296	0.860027394	24	20.25461338	0.028298296
ENSMUSG00000006423	Steep1	STING1 ER exit protein 1 [Source:MGI Symbol;Acc:MGI:1924894]	15.4856	16.0153	14.939	15.5164	14.7745	15.0869	15.331	13.2878	14.4574	13.6648	14.9642	13.2364	14.4248	14.5812	0.788254138	0.966018836	4	8	24	0.896554964	0.970591958	24	23.0164502	0.011647585
ENSMUSG00000006435	Neurl1a	neuralized E3 ubiquitin protein ligase 1A [Source:MGI Symbol;Acc:MGI:1334263]	6.5743	6.04042	7.41755	6.75684	6.33574	7.05798	6.0779	8.17749	6.60791	10.6212	6.47259	7.60308	8.06572	8.16887	0.911135828	0.966018836	16	4	24	0.481255781	0.805393068	24	9.155917848	0.083739843
ENSMUSG00000006442	Srm	spermidine synthase [Source:MGI Symbol;Acc:MGI:102690]	19.1089	14.9717	17.3121	15.0655	16.2187	17.4607	15.4518	18.3783	14.6783	18.4189	17.2847	16.668	15.5132	15.972	0.911371589	0.966018836	24	12	24	0.738977379	0.914862699	24	19.05743078	0.037441647
ENSMUSG00000006445	Epha2	Eph receptor A2 [Source:MGI Symbol;Acc:MGI:95278]	1.90903	1.71515	1.47745	1.82748	2.06779	1.65312	1.62556	1.66738	1.80089	1.99153	1.64145	1.4713	1.81274	1.27211	0.560714538	0.966018836	16	16	24	0.313730953	0.768040178	24	15.29673597	0.063127342
ENSMUSG00000006456	Rbm14	RNA binding motif protein 14 [Source:MGI Symbol;Acc:MGI:1929092]	5.41183	4.11027	4.2926	4.86179	4.91542	4.66011	5.64575	4.58588	4.83573	5.10891	5.3443	4.83681	4.92774	4.49593	0.032547207	0.324908923	4	4	24	0.191350535	0.768040178	24	18.67631638	0.074358736
ENSMUSG00000006457	Actn3	actinin alpha 3 [Source:MGI Symbol;Acc:MGI:99678]	15.2165	41.3103	45.2945	55.5153	36.9083	56.2665	38.3493	104.472	92.3218	114.625	39.4855	52.8983	51.8022	34.0762	0.47028201	0.966018836	16	12	24	0.122586159	0.735333681	24	10.03922874	0.532330707
ENSMUSG00000006463	Zdhhc24	zinc finger, DHHC domain containing 24 [Source:MGI Symbol;Acc:MGI:1917855]	11.7418	10.0603	10.2652	11.8828	11.8102	11.3296	11.0254	12.051	9.7578	11.9917	11.4009	11.7668	10.7969	10.6712	0.022785983	0.272961011	16	20	24	0.105711848	0.717832234	24	17.57984516	0.073958084
ENSMUSG00000006464	Bbs1	Bardet-Biedl syndrome 1 (human) [Source:MGI Symbol;Acc:MGI:1277215]	3.45128	3.19864	2.99948	2.93705	2.78763	3.52205	3.22096	3.24712	3.10846	3.4029	2.52744	3.50368	2.94014	3.20625	0.022000259	0.267229408	24	20	24	0.296524486	0.768040178	24	0.782366141	0.060860618
ENSMUSG00000006471	Ndor1	NADPH dependent diflavin oxidoreductase 1 [Source:MGI Symbol;Acc:MGI:1926047]	11.7556	10.4995	10.7539	8.37191	10.3996	10.0018	11.8432	12.3832	13.6445	12.085	10.6216	12.575	11.8382	10.901	0.823657258	0.966018836	4	16	24	0.342886353	0.776697379	24	2.944839187	0.082504386
ENSMUSG00000006476	Nsmf	NMDA receptor synaptonuclear signaling and neuronal migration factor [Source:MGI Symbol;Acc:MGI:1861755]	56.8822	60.9286	59.7657	59.8194	65.1604	57.7083	64.9015	57.9924	63.2307	59.3372	54.7207	53.2353	55.9377	54.5977	0.834424969	0.966018836	12	12	24	0.602510826	0.861114219	24	9.933784186	0.032201195
ENSMUSG00000006494	Pdk1	pyruvate dehydrogenase kinase, isoenzyme 1 [Source:MGI Symbol;Acc:MGI:1926119]	10.759	11.7808	11.828	14.7747	10.7352	11.6299	10.2833	12.3204	11.9703	12.6691	11.7417	12.4604	13.1042	12.1354	0.510431574	0.966018836	16	4	24	0.243612374	0.768040178	24	10.37084415	0.076513938
ENSMUSG00000006498	Ptbp1	polypyrimidine tract binding protein 1 [Source:MGI Symbol;Acc:MGI:97791]	16.7473	20.064	21.7967	19.8432	20.6647	18.709	20.5682	19.6251	21.2607	18.5644	17.2437	18.6623	19.2814	18.3689	0.377663653	0.903463289	12	12	24	0.221897563	0.768040178	24	8.07971161	0.06963176
ENSMUSG00000006517	Mvd	mevalonate (diphospho) decarboxylase [Source:MGI Symbol;Acc:MGI:2179327]	32.7402	22.8055	24.6327	23.3827	27.3632	32.6465	27.7509	26.5755	21.6623	27.3725	26.1389	27.047	26.3417	23.6675	0.043240321	0.357642487	24	12	24	0.006426305	0.470633744	24	20.2960633	0.180788018
ENSMUSG00000006519	Cyba	cytochrome b-245, alpha polypeptide [Source:MGI Symbol;Acc:MGI:1316658]	10.8795	14.7149	17.2444	13.503	12.8125	10.6771	14.8886	13.7895	16.51	9.5272	14.8092	12.6768	14.2576	11.6508	0.148073804	0.590732968	12	12	24	0.441926646	0.805393068	24	6.74671477	0.118184234
ENSMUSG00000006522	Itih3	inter-alpha trypsin inhibitor, heavy chain 3 [Source:MGI Symbol;Acc:MGI:96620]	1.70641	4.86526	10.0537	3.62919	2.34785	5.87609	5.6953	3.32572	5.98584	3.23034	1.89708	4.35086	1.46745	6.1463	0.203305541	0.689605535	12	8	24	0.28901241	0.768040178	24	7.023047497	0.394817115
ENSMUSG00000006526	Stimate	STIM activating enhancer [Source:MGI Symbol;Acc:MGI:1921500]	7.73953	9.06527	8.73673	9.21557	7.71451	9.1878	8.1299	9.6198	7.40737	9.91425	7.4689	8.36765	7.67019	9.30178	0.481811994	0.966018836	8	12	24	0.509293341	0.813985618	24	7.737907645	0.062220004
ENSMUSG00000006527	Sfmbt1	Scm-like with four mbt domains 1 [Source:MGI Symbol;Acc:MGI:1859609]	4.0826	3.92057	4.31352	4.08742	4.05953	4.45121	4.08784	3.95294	3.89599	4.22189	4.11569	4.44993	4.06522	4.19932	0.43565473	0.966018836	24	8	24	0.234500753	0.768040178	24	18.14034129	0.032140402
ENSMUSG00000006542	Prkag3	protein kinase, AMP-activated, gamma 3 non-catalytic subunit [Source:MGI Symbol;Acc:MGI:1891343]	0.608852	1.35334	0.887763	1.11447	0.860159	1.49375	0.960946	2.65731	2.81743	3.10229	1.15167	1.32484	0.943658	0.614831	0.830364743	0.966018836	16	12	24	0.281363552	0.768040178	24	9.932581474	0.274917947
ENSMUSG00000006546	Cryba2	crystallin, beta A2 [Source:MGI Symbol;Acc:MGI:104336]	1.35923	1.19831	1.47724	1.14417	1.15659	1.03989	1.04594	1.43391	1.74884	1.82976	1.7653	1.45031	1.24186	1.09752	0.244732441	0.741580974	12	16	24	0.149656927	0.758377578	24	11.04990195	0.117646069
ENSMUSG00000006574	Slc4a1	solute carrier family 4 (anion exchanger), member 1 [Source:MGI Symbol;Acc:MGI:109393]	1.81113	4.86804	7.49725	6.74627	3.451	4.24915	4.12882	4.03603	4.52029	2.70604	3.4702	4.46394	6.52043	3.31255	0.888379597	0.966018836	12	8	24	0.639868807	0.87800173	24	8.437358463	0.168064411
ENSMUSG00000006575	Rundc3a	RUN domain containing 3A [Source:MGI Symbol;Acc:MGI:1858752]	90.2559	70.1523	72.2972	66.0456	70.2688	72.4158	71.1606	80.4042	66.847	84.5455	77.0517	81.3154	81.6705	80.1355	0.450844752	0.966018836	4	8	24	0.364078471	0.788727838	24	23.18536107	0.071528369
ENSMUSG00000006576	Slc4a3	solute carrier family 4 (anion exchanger), member 3 [Source:MGI Symbol;Acc:MGI:109350]	11.3253	14.9944	16.6155	12.5458	13.2279	13.2817	13.5379	14.4611	15.8583	15.6767	11.9675	15.3753	14.2941	14.6872	0.021288455	0.264116537	12	8	24	0.082645125	0.699403344	24	7.373529744	0.123852787
ENSMUSG00000006585	Cdt1	chromatin licensing and DNA replication factor 1 [Source:MGI Symbol;Acc:MGI:1914427]	0.527776	0.751424	1.26583	0.746274	1.01818	0.547705	0.743614	0.867432	0.816202	0.530205	0.845736	0.899538	1.01005	0.738314	0.892044196	0.966018836	12	16	24	0.773768071	0.92732018	24	8.741812462	0.048454396
ENSMUSG00000006586	Runx1t1	RUNX1 translocation partner 1 [Source:MGI Symbol;Acc:MGI:104793]	4.19293	3.84705	3.56347	4.05535	3.64776	3.98686	4.14601	3.65091	3.69964	3.84906	3.86931	4.03004	3.94311	3.77732	0.016795343	0.253395174	4	8	24	0.058766781	0.658304792	24	20.99990584	0.05117498
ENSMUSG00000006589	Aprt	adenine phosphoribosyl transferase [Source:MGI Symbol;Acc:MGI:88061]	39.8565	34.6673	37.8121	34.7898	38.6016	35.5562	39.6603	39.3965	36.0151	31.2696	39.5775	33.7049	39.019	35.3019	0.068668966	0.432567661	4	12	24	0.313032039	0.768040178	24	23.34637996	0.061557081
ENSMUSG00000006599	Gtf2h1	general transcription factor II H, polypeptide 1 [Source:MGI Symbol;Acc:MGI:1277216]	19.907	18.9413	16.7078	17.3156	18.2818	16.8917	17.3707	16.9761	17.9216	16.6706	19.2579	18.5102	19.3948	18.8736	0.117050591	0.538362324	4	12	24	0.287793438	0.768040178	24	22.76415445	0.05089686
ENSMUSG00000006611	Hfe	homeostatic iron regulator [Source:MGI Symbol;Acc:MGI:109191]	5.25069	7.21248	6.80199	6.73919	5.6362	5.69756	6.29507	6.07887	8.4472	5.33717	7.11402	5.01454	6.1198	5.45149	0.245061943	0.741580974	12	12	24	0.166001458	0.767020009	24	8.535423319	0.134632128
ENSMUSG00000006638	Abhd1	abhydrolase domain containing 1 [Source:MGI Symbol;Acc:MGI:1931013]	0.978319	0.983533	1.27486	1.49994	1.24484	1.13596	0.999926	1.1815	1.19552	1.00305	1.01231	0.949562	1.22006	1.09727	0.260026661	0.765037845	16	8	24	0.237527795	0.768040178	24	10.59977344	0.066773456
ENSMUSG00000006641	Slc5a6	solute carrier family 5 (sodium-dependent vitamin transporter), member 6 [Source:MGI Symbol;Acc:MGI:2660847]	4.54221	4.63387	4.92239	4.6155	4.95927	4.4924	5.59148	5.37343	5.77209	5.26677	4.83447	4.4576	3.96131	5.80526	0.085329704	0.466404237	12	12	24	0.128988484	0.740162193	24	7.798915948	0.103692072
ENSMUSG00000006651	Aplp1	amyloid beta (A4) precursor-like protein 1 [Source:MGI Symbol;Acc:MGI:88046]	61.2484	67.5378	92.6118	61.731	52.2554	73.7589	75.6268	66.4065	68.5828	70.6214	55.9182	69.1854	53.8045	72.3524	0.838532766	0.966018836	12	8	24	0.312822133	0.768040178	24	6.127464273	0.129078094
ENSMUSG00000006673	Qrich1	glutamine-rich 1 [Source:MGI Symbol;Acc:MGI:1916482]	30.8791	29.0479	28.466	26.4956	26.6196	30.2069	30.3846	30.8264	28.673	32.6327	26.2221	31.9234	31.4645	29.2596	0.038123942	0.334069441	4	16	24	0.157224079	0.763172075	24	0.832407243	0.067895514
ENSMUSG00000006675	P4htm	prolyl 4-hydroxylase, transmembrane (endoplasmic reticulum) [Source:MGI Symbol;Acc:MGI:1921693]	20.1887	17.372	18.1809	17.1353	15.7255	20.353	17.6808	19.2707	16.1428	20.9512	14.8089	19.6292	16.5559	18.8171	0.503972924	0.966018836	24	20	24	0.636039482	0.876344329	24	0.912141598	0.054129285
ENSMUSG00000006676	Usp19	ubiquitin specific peptidase 19 [Source:MGI Symbol;Acc:MGI:1918722]	46.2216	41.7295	41.6897	46.7008	45.3376	42.9677	46.6043	49.8234	43.1274	52.8423	44.9782	42.9483	44.1341	44.5188	0.941955432	0.966018836	16	20	24	0.728912278	0.91134678	24	12.01821526	0.029785814
ENSMUSG00000006678	Pola1	polymerase (DNA directed), alpha 1 [Source:MGI Symbol;Acc:MGI:99660]	2.22799	2.26557	2.05998	2.30381	2.15249	2.06557	1.98788	1.95675	2.34161	1.83396	2.53481	2.13302	2.43416	2.21897	0.81544087	0.966018836	20	12	24	0.958466134	0.989144435	24	18.67968004	0.010645146
ENSMUSG00000006699	Cdc42	cell division cycle 42 [Source:MGI Symbol;Acc:MGI:106211]	267.099	275.473	260.925	259.989	275.563	258.059	254.657	250.12	263.098	247.607	270.291	264.504	267.199	264.955	0.676344318	0.966018836	20	20	24	0.710427634	0.904241141	24	20.11146983	0.015816563
ENSMUSG00000006705	Pknox1	Pbx/knotted 1 homeobox [Source:MGI Symbol;Acc:MGI:1201409]	5.99399	5.35922	5.53847	6.05563	5.54361	5.12655	6.18313	5.32902	5.24505	5.23764	5.62379	5.04184	5.94913	5.03915	0.885025617	0.966018836	4	20	24	0.760058585	0.922505796	24	21.33048921	0.024604097
ENSMUSG00000006711	D130043K22Rik	RIKEN cDNA D130043K22 gene [Source:MGI Symbol;Acc:MGI:3036268]	21.8468	17.2137	16.2811	17.9353	17.7765	19.7657	18.9329	19.8807	14.1923	22.2432	18.4789	19.7333	20.0082	19.8188	0.062631097	0.410322149	4	8	24	0.217340569	0.768040178	24	21.14494883	0.108722299
ENSMUSG00000006715	Gmnn	geminin [Source:MGI Symbol;Acc:MGI:1927344]	6.16036	6.25888	7.07718	6.61793	7.31273	6.71095	6.97022	7.26595	6.59838	6.18105	7.03062	7.87416	8.25384	7.52288	0.945728192	0.966018836	24	16	24	0.444430703	0.805393068	24	22.44312919	0.043992628
ENSMUSG00000006717	Acot13	acyl-CoA thioesterase 13 [Source:MGI Symbol;Acc:MGI:1914084]	70.8379	79.4037	76.8456	81.9073	78.2013	68.3754	74.6068	81.9702	83.8451	62.0107	87.76	79.4859	88.3807	83.0503	0.896311169	0.966018836	8	16	24	0.738628806	0.914690529	24	5.019738001	0.041934239
ENSMUSG00000006728	Cdk4	cyclin-dependent kinase 4 [Source:MGI Symbol;Acc:MGI:88357]	39.165	35.2479	38.7025	36.0485	34.8017	32.9699	36.2574	34.1379	38.8732	31.3757	35.1024	34.6591	32.3805	29.3935	0.935732159	0.966018836	12	12	24	0.970469131	0.993003194	24	10.26123123	0.008880181
ENSMUSG00000006731	B4galnt1	beta-1,4-N-acetyl-galactosaminyl transferase 1 [Source:MGI Symbol;Acc:MGI:1342057]	10.9315	8.85078	9.91644	10.189	9.22824	10.3782	9.86729	9.96981	8.88755	11.0981	9.22367	9.94726	9.51549	10.0523	0.916556573	0.966018836	16	20	24	0.877529339	0.965881478	24	19.27144911	0.019420698
ENSMUSG00000006732	Mettl1	methyltransferase like 1 [Source:MGI Symbol;Acc:MGI:1339986]	8.30854	7.41363	8.28012	6.98589	7.63295	8.45935	7.57984	8.85755	8.00628	8.33414	7.73088	8.08986	9.37891	7.74757	0.91695334	0.966018836	24	20	24	0.390649707	0.799546792	24	0.723545118	0.049052488
ENSMUSG00000006736	Tspan31	tetraspanin 31 [Source:MGI Symbol;Acc:MGI:1914375]	54.4951	44.2793	45.7612	45.8098	50.252	51.3864	50.0077	49.2324	41.7358	46.7485	50.6002	46.5899	50.4779	47.6708	0.008754183	0.192760771	4	8	24	0.013593549	0.529134128	24	20.80133764	0.101656381
ENSMUSG00000006740	Kif5b	kinesin family member 5B [Source:MGI Symbol;Acc:MGI:1098268]	146.06	147.28	138.119	143.717	147.658	136.853	141.218	128.189	152.428	124.408	157.168	149.864	155.948	150.745	0.873171835	0.966018836	20	20	24	0.705287488	0.90280684	24	21.23948775	0.033484415
ENSMUSG00000006763	Saal1	serum amyloid A-like 1 [Source:MGI Symbol;Acc:MGI:1926185]	2.67532	2.64696	3.42582	2.58153	2.37637	2.7101	2.80743	2.97261	2.85771	2.27732	2.99152	2.90194	2.98063	2.50104	0.923635729	0.966018836	12	4	24	0.637488854	0.876711629	24	2.993208373	0.042033399
ENSMUSG00000006764	Tph2	tryptophan hydroxylase 2 [Source:MGI Symbol;Acc:MGI:2651811]	0.675042	0.638209	0.612261	0.741727	0.544688	0.615124	0.510872	0.404539	0.550239	0.520098	0.588434	0.642639	0.608151	0.66593	0.832742389	0.966018836	24	20	24	0.86600566	0.961251055	24	17.79490037	0.016954534
ENSMUSG00000006782	Cnp	2',3'-cyclic nucleotide 3' phosphodiesterase [Source:MGI Symbol;Acc:MGI:88437]	177.49	179.29	188.078	168.339	187.729	170.093	203.807	174.719	192.135	174.173	163.863	186.356	179.412	165.212	0.870491976	0.966018836	12	4	24	0.754915663	0.920907411	24	1.26653628	0.026099167
ENSMUSG00000006784	Odad4	outer dynein arm complex subunit 4 [Source:MGI Symbol;Acc:MGI:1921657]	0.730402	1.18031	0.97547	1.84026	1.17379	1.15959	1.43868	0.953403	1.21213	1.09891	2.20678	1.44463	1.29027	1.30961	0.796193867	0.966018836	20	12	24	0.169707493	0.767809411	24	15.0604212	0.143350301
ENSMUSG00000006800	Sulf2	sulfatase 2 [Source:MGI Symbol;Acc:MGI:1919293]	9.63431	10.4638	11.8956	10.0062	9.88277	10.7445	10.6788	11.443	10.9176	11.8408	8.78416	10.241	8.48204	10.8166	0.057264003	0.401309455	12	8	24	0.138934205	0.748341961	24	7.872224689	0.100056229
ENSMUSG00000006818	Sod2	superoxide dismutase 2, mitochondrial [Source:MGI Symbol;Acc:MGI:98352]	50.5507	47.0021	51.1682	59.1991	50.247	51.7158	52.257	56.0539	49.2956	59.5803	66.6242	59.2877	68.0845	63.531	0.921572192	0.966018836	16	20	24	0.67612416	0.891686075	24	15.94993982	0.037679717
ENSMUSG00000006850	Tmco6	transmembrane and coiled-coil domains 6 [Source:MGI Symbol;Acc:MGI:1919233]	6.54926	6.65306	6.2421	5.86174	7.19899	7.28519	7.9764	7.5013	6.92558	6.59218	6.77278	7.21861	6.82987	6.43327	0.173410058	0.648600527	24	16	24	0.11883831	0.729829751	24	22.0313386	0.077254224
ENSMUSG00000006906	Stambp	STAM binding protein [Source:MGI Symbol;Acc:MGI:1917777]	19.4792	18.1156	17.3103	19.5186	16.9722	18.476	19.4514	18.3161	16.3477	20.2156	17.4236	18.6151	17.5477	19.2582	0.628286596	0.966018836	24	16	24	0.849292363	0.954760051	24	22.69803906	0.02008897
ENSMUSG00000006920	Ezh1	enhancer of zeste 1 polycomb repressive complex 2 subunit [Source:MGI Symbol;Acc:MGI:1097695]	25.8459	24.8267	24.0411	25.0505	23.6536	24.7591	25.3722	26.5967	23.1623	25.2037	23.4257	25.9706	24.5876	25.2723	0.238201401	0.741459408	8	4	24	0.181383123	0.768040178	24	0.684347416	0.038834535
ENSMUSG00000006930	Hap1	huntingtin-associated protein 1 [Source:MGI Symbol;Acc:MGI:1261831]	5.12672	7.88606	13.3628	6.95847	5.12211	8.51459	9.06942	5.74482	10.4011	7.20417	4.95133	8.49915	3.63294	10.0834	0.064422003	0.414867207	12	8	24	0.288824393	0.768040178	24	7.625390741	0.281918659
ENSMUSG00000006931	P3h4	prolyl 3-hydroxylase family member 4 (non-enzymatic) [Source:MGI Symbol;Acc:MGI:1913430]	4.16669	5.04005	4.62014	6.31942	6.25528	5.02918	4.93595	6.10154	4.95088	5.17727	4.26096	4.41798	4.17659	4.61566	0.06180207	0.409814092	16	12	24	0.286201699	0.768040178	24	11.57568232	0.100812143
ENSMUSG00000006932	Ctnnb1	catenin (cadherin associated protein), beta 1 [Source:MGI Symbol;Acc:MGI:88276]	272.69	267.227	259.363	270.247	284.158	274.808	275.211	252.594	247.809	242.602	235.297	229.996	248.265	245.85	0.881996584	0.966018836	4	20	24	0.531666576	0.82547989	24	21.55981258	0.027990179
ENSMUSG00000006941	Eif1b	eukaryotic translation initiation factor 1B [Source:MGI Symbol;Acc:MGI:1916219]	113.435	94.7992	95.7205	98.6601	97.0715	102.161	104.014	94.7539	92.4909	101.396	105.803	104.175	104.167	104.957	0.055920623	0.401074772	4	8	24	0.050872307	0.645589244	24	20.9491814	0.07175218
ENSMUSG00000006958	Chrd	chordin [Source:MGI Symbol;Acc:MGI:1313268]	1.11769	1.07901	1.61763	0.945204	0.938853	1.09124	1.4306	1.18169	1.47733	0.699174	0.903842	0.983074	0.945097	0.965027	0.022000259	0.267229408	12	4	24	0.196736977	0.768040178	24	4.309527589	0.108507543
ENSMUSG00000006998	Psmd2	proteasome (prosome, macropain) 26S subunit, non-ATPase, 2 [Source:MGI Symbol;Acc:MGI:1096584]	75.508	68.2781	66.9377	73.9627	71.2325	75.7927	68.3569	73.4535	61.5953	69.8608	68.068	63.4809	71.3744	62.1433	0.446967971	0.966018836	12	12	24	0.250454414	0.768040178	24	19.50798247	0.05548571
ENSMUSG00000007021	Syngr3	synaptogyrin 3 [Source:MGI Symbol;Acc:MGI:1341881]	64.6753	47.589	49.8179	50.9574	49.3753	57.5863	52.0655	54.6608	39.3732	67.2664	51.1032	57.7543	54.612	59.1245	0.46238066	0.966018836	24	12	24	0.448322067	0.805393068	24	20.86854277	0.097763214
ENSMUSG00000007029	Vars	valyl-tRNA synthetase [Source:MGI Symbol;Acc:MGI:90675]	9.98853	9.51281	10.047	7.70725	9.36824	8.85512	8.66445	9.64538	9.43276	11.375	8.70854	8.71678	9.1245	9.94376	0.297756231	0.80820842	12	12	24	0.429211014	0.805393068	24	7.453669271	0.063800125
ENSMUSG00000007030	Vwa7	von Willebrand factor A domain containing 7 [Source:MGI Symbol;Acc:MGI:1306798]	12.3826	9.62004	8.12713	10.8194	10.1225	10.3866	10.0375	11.7555	9.4481	15.0535	10.8694	12.055	11.7848	11.1329	0.387380622	0.915479136	12	12	24	0.632395224	0.875020466	24	18.37193622	0.069832151
ENSMUSG00000007033	Hspa1l	heat shock protein 1-like [Source:MGI Symbol;Acc:MGI:96231]	1.09636	1.48475	1.53894	1.80168	1.14782	1.68867	1.63819	1.89228	1.90432	1.95825	1.58549	1.3979	1.94894	1.69556	0.676344318	0.966018836	16	4	24	0.118830344	0.729829751	24	7.967239412	0.10944847
ENSMUSG00000007035	Msh5	mutS homolog 5 [Source:MGI Symbol;Acc:MGI:1329021]	2.20382	2.48483	2.09032	2.41324	2.1068	2.06733	2.14963	2.03161	3.00118	2.8574	1.6421	2.06648	2.38413	2.43012	0.127415507	0.560209134	16	4	24	0.117790157	0.728323355	24	7.610490393	0.121826358
ENSMUSG00000007036	Abhd16a	abhydrolase domain containing 16A [Source:MGI Symbol;Acc:MGI:99476]	42.6595	37.7529	39.3522	38.9578	40.2638	38.9502	38.3535	40.9533	40.054	46.9479	42.5826	43.4917	44.7117	43.1964	0.858266942	0.966018836	16	20	24	0.850074357	0.955046946	24	15.66646549	0.016043312
ENSMUSG00000007038	Neu1	neuraminidase 1 [Source:MGI Symbol;Acc:MGI:97305]	16.6878	14.2731	14.2991	15.1351	15.4462	15.1186	15.7666	14.5537	14.2552	15.7804	15.2173	15.8606	16.3069	15.6794	0.009118941	0.192760771	4	8	24	0.062581857	0.661961741	24	20.66615022	0.058012937
ENSMUSG00000007039	Ddah2	dimethylarginine dimethylaminohydrolase 2 [Source:MGI Symbol;Acc:MGI:1859016]	21.4515	21.5809	20.9512	19.8959	22.7133	18.7284	23.0822	20.4493	23.5727	20.9442	20.0501	21.1026	23.8584	22.1306	0.247964668	0.746745468	4	12	24	0.363408004	0.788241076	24	3.711375851	0.051200479
ENSMUSG00000007041	Clic1	chloride intracellular channel 1 [Source:MGI Symbol;Acc:MGI:2148924]	73.7022	66.4312	65.9439	58.1069	71.1752	66.9635	59.6119	63.7798	47.6904	56.103	69.4526	62.098	67.8341	64.6725	0.175185919	0.651010555	20	20	24	0.091845051	0.71175955	24	21.16245106	0.129170815
ENSMUSG00000007050	Lsm2	LSM2 homolog, U6 small nuclear RNA and mRNA degradation associated [Source:MGI Symbol;Acc:MGI:90676]	5.91914	6.67928	7.23248	6.60024	7.65608	6.0566	5.63141	6.72283	6.85349	7.73211	6.64822	7.30477	6.85689	6.98268	0.321666243	0.83607295	16	12	24	0.053065282	0.650879532	24	11.43021859	0.095798068
ENSMUSG00000007080	Pole	polymerase (DNA directed), epsilon [Source:MGI Symbol;Acc:MGI:1196391]	0.718514	0.963129	1.48762	1.16659	1.30276	1.21899	1.02931	1.28584	1.43278	1.33683	1.01669	1.0757	1.02921	1.16811	0.004140921	0.140598674	12	16	24	0.023764464	0.584955728	24	10.36084757	0.135129663
ENSMUSG00000007097	Atp1a2	ATPase, Na+/K+ transporting, alpha 2 polypeptide [Source:MGI Symbol;Acc:MGI:88106]	101.953	126.057	138.284	116.214	107.291	121.637	127.345	135.457	137.941	112.582	98.3087	107.89	93.8045	125.192	0.010153564	0.194720062	12	8	24	0.032944187	0.606585237	24	6.737428158	0.168234664
ENSMUSG00000007122	Casq1	calsequestrin 1 [Source:MGI Symbol;Acc:MGI:1309468]	25.0071	53.5232	59.3029	69.5346	51.3626	66.012	46.1884	131.548	102.745	120.759	48.4918	54.1656	67.1502	45.413	0.387380622	0.915479136	16	12	24	0.100756433	0.712056576	24	9.594774631	0.48076191
ENSMUSG00000007207	Stx1a	syntaxin 1A (brain) [Source:MGI Symbol;Acc:MGI:109355]	20.8624	13.2244	15.5029	15.3033	16.0876	17.565	16.0233	16.4517	12.6408	17.6115	16.5004	15.7088	16.6812	15.4038	0.174295904	0.650395613	4	8	24	0.139929457	0.749574609	24	20.10611239	0.12609183
ENSMUSG00000007216	Zfp775	zinc finger protein 775 [Source:MGI Symbol;Acc:MGI:2683557]	1.50581	1.95107	1.53893	1.64235	1.63469	1.82513	2.13977	2.17701	1.44341	1.81262	1.88181	2.24699	2.02423	2.20486	0.076593464	0.453790923	8	4	24	0.101544643	0.713208839	24	23.91014088	0.089728587
ENSMUSG00000007279	Scube2	signal peptide, CUB domain, EGF-like 2 [Source:MGI Symbol;Acc:MGI:1928765]	0.376687	0.418216	0.474563	0.430003	0.578602	0.618621	0.487928	0.622841	0.53403	0.656659	0.478097	0.499152	0.347383	0.645308	0.658895034	0.966018836	16	12	24	0.516619939	0.817817397	24	11.2887693	0.042005095
ENSMUSG00000007338	Mrpl49	mitochondrial ribosomal protein L49 [Source:MGI Symbol;Acc:MGI:108180]	24.2287	23.7566	23.9218	24.2393	23.7398	27.0723	23.0322	25.4271	21.0849	25.1285	23.2056	24.5436	25.4063	26.5341	0.834434272	0.966018836	24	20	24	0.541720194	0.830485817	24	22.35957602	0.038156611
ENSMUSG00000007411	Mark3	MAP/microtubule affinity regulating kinase 3 [Source:MGI Symbol;Acc:MGI:1341865]	21.8173	21.4258	21.4629	23.3013	20.1998	22.2582	24.2529	20.5245	22.0909	20.2578	25.4249	21.9267	21.0111	19.4352	0.510431574	0.966018836	24	8	24	0.388858474	0.799546792	24	17.7256159	0.055326914
ENSMUSG00000007415	Gatad1	GATA zinc finger domain containing 1 [Source:MGI Symbol;Acc:MGI:1914460]	54.8271	51.4486	48.2688	48.9729	52.7485	52.3925	53.0514	50.8809	47.6575	49.6218	50.9041	52.5944	54.7721	51.3505	9.53E-06	0.009754434	4	8	24	6.52E-05	0.096004883	24	21.89619796	0.077332383
ENSMUSG00000007440	Pcdha10	protocadherin alpha 10 [Source:MGI Symbol;Acc:MGI:1298408]	1.57657	1.17117	1.31317	1.61799	1.33224	1.46061	1.6343	1.79816	1.10703	1.82441	1.12636	1.45604	1.31687	1.67984	0.951846915	0.966018836	8	4	24	0.895969735	0.970467191	24	2.399204329	0.026436731
ENSMUSG00000007458	M6pr	mannose-6-phosphate receptor, cation dependent [Source:MGI Symbol;Acc:MGI:96904]	45.5262	44.5058	45.1709	42.5495	45.2089	44.8289	44.245	42.9533	47.1297	42.3042	44.6683	45.3656	45.8192	43.958	0.47028201	0.966018836	4	12	24	0.59318026	0.856743157	24	22.89895743	0.017410618
ENSMUSG00000007476	Lrrc8a	leucine rich repeat containing 8A VRAC subunit A [Source:MGI Symbol;Acc:MGI:2652847]	10.8834	8.18711	9.30967	10.6466	9.12324	10.1288	10.3658	10.0067	7.55839	13.3747	8.78802	10.0125	9.55357	10.1061	0.608967886	0.966018836	16	20	24	0.791178273	0.933169725	24	17.35961485	0.050121697
ENSMUSG00000007564	Ppp2r1a	protein phosphatase 2, regulatory subunit A, alpha [Source:MGI Symbol;Acc:MGI:1926334]	124.737	106.364	111.792	115.862	111.946	135.962	121.213	123.805	95.5806	143.485	109.739	119.217	104.83	114.563	0.895493752	0.966018836	24	12	24	0.517203935	0.81821315	24	18.61535462	0.070705132
ENSMUSG00000007570	Fance	Fanconi anemia, complementation group E [Source:MGI Symbol;Acc:MGI:1920025]	9.01081	8.12302	11.0883	9.33466	8.81856	8.56649	8.24892	9.37472	9.70362	9.92676	8.3536	8.89216	8.60711	8.08145	0.10338739	0.517709628	12	20	24	0.063643214	0.663836692	24	10.13082453	0.09738747
ENSMUSG00000007589	Tinf2	Terf1 (TRF1)-interacting nuclear factor 2 [Source:MGI Symbol;Acc:MGI:107246]	8.49405	8.58642	8.27601	7.30208	8.81614	7.74131	8.90654	6.69382	9.07483	8.90655	9.25928	7.93739	8.16458	9.76917	0.868022237	0.966018836	20	4	24	0.937224488	0.984478515	24	10.43123315	0.018593774
ENSMUSG00000007594	Hapln4	hyaluronan and proteoglycan link protein 4 [Source:MGI Symbol;Acc:MGI:2679531]	8.11248	7.77404	9.48854	7.29505	5.74346	8.92944	9.89992	8.35162	6.86581	12.5453	6.46447	8.27215	7.36057	10.5285	0.714044782	0.966018836	8	12	24	0.516588102	0.817817397	24	5.115129386	0.121006601
ENSMUSG00000007603	Dus3l	dihydrouridine synthase 3-like (S. cerevisiae) [Source:MGI Symbol;Acc:MGI:2147092]	14.881	15.1774	15.937	15.5518	14.3675	14.9743	15.9446	14.4039	16.7401	15.8502	15.745	16.3952	16.1841	15.4182	0.891742979	0.966018836	12	20	24	0.711031735	0.904572112	24	9.223521664	0.019303247
ENSMUSG00000007610	Gtpbp3	GTP binding protein 3 [Source:MGI Symbol;Acc:MGI:1917609]	7.7811	7.24788	8.22946	7.29238	6.5724	8.14718	7.46015	9.19334	7.79199	9.27701	8.54859	7.54084	8.66088	8.63908	0.850906022	0.966018836	12	8	24	0.400582102	0.799546792	24	6.237892455	0.059311043
ENSMUSG00000007613	Tgfbr1	transforming growth factor, beta receptor I [Source:MGI Symbol;Acc:MGI:98728]	6.12177	7.21633	6.99665	7.13192	6.9871	5.95159	6.89095	6.75985	7.08131	5.97063	6.98893	6.24025	5.99908	6.50419	0.074438493	0.448301997	12	12	24	0.205813586	0.768040178	24	9.19525825	0.060729302
ENSMUSG00000007617	Homer1	homer scaffolding protein 1 [Source:MGI Symbol;Acc:MGI:1347345]	7.10552	7.19322	6.6513	7.87704	7.46795	6.7461	7.56189	7.34976	7.53003	6.95525	7.97596	6.50176	6.91522	6.92753	0.369262716	0.893596186	20	4	24	0.518334213	0.818644475	24	12.43714538	0.035212192
ENSMUSG00000007646	Rad51c	RAD51 paralog C [Source:MGI Symbol;Acc:MGI:2150020]	2.47304	1.75673	1.85186	1.95335	1.55228	2.15134	2.15458	1.60696	1.27863	1.41668	2.26351	2.30881	1.88873	1.71903	0.038115943	0.334069441	24	12	24	0.012523536	0.525280095	24	20.84658755	0.185696927
ENSMUSG00000007653	Gabrb2	gamma-aminobutyric acid (GABA) A receptor, subunit beta 2 [Source:MGI Symbol;Acc:MGI:95620]	2.43625	2.97171	2.73308	2.70622	2.59877	2.64874	3.07271	2.34858	2.98484	2.31299	2.62043	3.17746	2.70831	3.14167	0.575202741	0.966018836	24	20	24	0.59675005	0.858074191	24	2.04148935	0.042246845
ENSMUSG00000007655	Cav1	caveolin 1, caveolae protein [Source:MGI Symbol;Acc:MGI:102709]	82.8851	98.1028	96.0967	95.9069	109.329	79.4633	91.5306	95.3422	108.667	98.5142	104.346	85.7574	97.6202	85.2695	0.021214536	0.264116537	20	4	24	0.076810211	0.687041208	24	11.01977677	0.116618022
ENSMUSG00000007656	Arpp19	cAMP-regulated phosphoprotein 19 [Source:MGI Symbol;Acc:MGI:1891691]	76.1561	73.2382	72.1168	69.0177	70.3918	73.2007	76.6019	67.7962	69.9192	63.3962	71.3268	71.7752	75.446	70.2463	1.11E-04	0.029372629	4	12	24	0.004093581	0.427431763	24	23.01318583	0.075208719
ENSMUSG00000007659	Bcl2l1	BCL2-like 1 [Source:MGI Symbol;Acc:MGI:88139]	23.6301	19.6771	22.2543	28.0539	23.9733	24.6879	22.482	20.5147	21.3551	27.0222	20.1624	23.6986	19.8201	20.179	0.004349449	0.140598674	16	16	24	0.048133278	0.638366916	24	14.96378191	0.131617182
ENSMUSG00000007670	Khsrp	KH-type splicing regulatory protein [Source:MGI Symbol;Acc:MGI:1336214]	11.1238	10.5867	9.91819	12.0571	10.6928	10.1325	12.4688	10.9955	13.0224	13.3389	12.6708	11.4517	12.5578	10.9555	0.949152198	0.966018836	16	16	24	0.573487745	0.847005958	24	12.27921972	0.046442948
ENSMUSG00000007682	Dio2	deiodinase, iodothyronine, type II [Source:MGI Symbol;Acc:MGI:1338833]	2.28083	1.1795	1.37862	1.8919	1.18755	1.53333	1.51477	1.88882	1.67305	1.28895	1.80016	1.46345	1.57762	1.95171	0.915082018	0.966018836	4	16	24	0.653644417	0.883312134	24	2.04233252	0.063118017
ENSMUSG00000007721	Ccdc124	coiled-coil domain containing 124 [Source:MGI Symbol;Acc:MGI:1916403]	42.0204	34.8095	35.9019	30.6706	28.4667	31.6114	37.5091	39.988	36.4085	38.1941	41.062	36.9184	39.82	36.7	0.373702442	0.896700272	4	12	24	0.231311862	0.768040178	24	2.499367727	0.097268345
ENSMUSG00000007739	Cct4	chaperonin containing Tcp1, subunit 4 (delta) [Source:MGI Symbol;Acc:MGI:104689]	79.002	74.6747	71.1914	74.4076	79.0857	73.1934	74.4107	69.1396	74.3539	66.6749	85.9362	76.7269	84.2923	78.235	0.175185919	0.651010555	20	20	24	0.237225345	0.768040178	24	20.36747791	0.064792981
ENSMUSG00000007777	0610009B22Rik	RIKEN cDNA 0610009B22 gene [Source:MGI Symbol;Acc:MGI:1913300]	35.8354	39.2432	36.0487	36.79	36.6713	34.0514	34.4869	33.7218	34.7082	29.8266	35.893	35.7692	38.0439	40.3343	0.890235289	0.966018836	8	16	24	0.4336073	0.805393068	24	1.987688444	0.051398418
ENSMUSG00000007783	Cpt1c	carnitine palmitoyltransferase 1c [Source:MGI Symbol;Acc:MGI:2446526]	17.7461	15.3821	16.8595	16.7846	16.4502	16.2126	17.4647	16.3847	16.1971	18.6408	14.7423	17.1391	16.0953	16.8014	0.860560779	0.966018836	16	4	24	0.988972328	0.997679907	24	6.937510263	0.005005068
ENSMUSG00000007812	Zfp655	zinc finger protein 655 [Source:MGI Symbol;Acc:MGI:1919861]	14.6215	16.5763	16.1287	17.0271	15.5962	14.7237	14.1974	14.8025	16.6355	14.6088	16.3633	14.0996	15.6089	16.0085	0.036527779	0.332441566	12	12	24	0.067606626	0.67146536	24	9.506286962	0.074322433
ENSMUSG00000007815	Rhoa	ras homolog family member A [Source:MGI Symbol;Acc:MGI:1096342]	85.0286	79.5465	77.8593	77.5305	81.6776	77.8283	82.542	82.0162	77.9924	83.0013	84.6732	81.1714	81.4716	82.0581	0.722296281	0.966018836	4	8	24	0.420029591	0.803834751	24	21.36677244	0.022929297

ENSMUSG00000007817	Zmiz1	zinc finger, MIZ-type containing 1 [Source:MGI Symbol;Acc:MGI:3040693]	8.09015	7.62129	7.20638	8.28896	8.18947	7.964	8.87101	8.34444	7.59231	12.1758	7.89366	8.34704	8.13913	8.49225	0.834434272	0.966018836	16	20	24	0.639417043	0.877927579	24	13.41028463	0.056424079
ENSMUSG00000007827	Ankrd26	ankyrin repeat domain 26 [Source:MGI Symbol;Acc:MGI:1917887]	4.47465	4.27603	3.78479	3.61304	3.862	3.52134	4.70393	3.37081	4.63168	3.63627	4.66789	4.21274	4.27945	3.83635	0.305564675	0.811201458	4	12	24	0.604832298	0.862149054	24	22.7605257	0.052967945
ENSMUSG00000007833	Aldh16a1	aldehyde dehydrogenase 16 family, member A1 [Source:MGI Symbol;Acc:MGI:1916998]	6.03622	5.09035	5.70376	5.7503	6.53638	5.32709	4.76208	5.88192	7.61153	5.60088	5.1952	5.80494	5.38598	5.30363	0.922029406	0.966018836	12	20	24	0.373972817	0.794557071	24	10.75543929	0.079439061
ENSMUSG00000007836	Hnrnpa0	heterogeneous nuclear ribonucleoprotein A0 [Source:MGI Symbol;Acc:MGI:1924384]	19.5485	19.2468	21.3623	20.0488	17.2241	17.2724	19.8195	20.0634	19.1078	24.6761	19.4954	21.9455	19.9556	21.5301	0.960338592	0.967043964	16	4	24	0.295114377	0.768040178	24	8.304200431	0.074205844
ENSMUSG00000007837	Prrg2	proline-rich Gla (G-carboxyglutamic acid) polypeptide 2 [Source:MGI Symbol;Acc:MGI:1929596]	6.65271	7.2681	5.66826	7.55472	6.4611	6.21211	6.48732	9.10691	6.74148	7.50244	8.13717	7.48288	8.44678	7.12373	0.896753532	0.966018836	8	4	24	0.932278768	0.982622317	24	4.015912477	0.021473489
ENSMUSG00000007850	Hnrnph1	heterogeneous nuclear ribonucleoprotein H1 [Source:MGI Symbol;Acc:MGI:1891925]	167.311	167.293	162.858	171.238	175.561	149.509	173.767	149.068	154.832	132.491	179.362	132.957	138.083	138.448	0.920992137	0.966018836	20	12	24	0.65737344	0.884466305	24	15.87502153	0.043298702
ENSMUSG00000007867	Ift43	intraflagellar transport 43 [Source:MGI Symbol;Acc:MGI:1923661]	13.3044	12.3382	12.3573	10.6767	13.39	11.7642	13.9584	15.0223	13.2622	11.4687	13.1028	13.3442	11.5842	12.0726	0.849179819	0.966018836	4	12	24	0.461295332	0.805393068	24	0.370728171	0.058959012
ENSMUSG00000007872	Id3	inhibitor of DNA binding 3 [Source:MGI Symbol;Acc:MGI:96398]	39.3464	36.6875	34.4823	44.0832	31.6643	33.9332	32.337	36.1184	42.4649	42.9013	45.93	38.7591	40.6619	39.4556	0.671726933	0.966018836	16	8	24	0.293906626	0.768040178	24	11.03692362	0.093200989
ENSMUSG00000007877	Tcap	titin-cap [Source:MGI Symbol;Acc:MGI:1330233]	15.0893	51.6668	83.3631	119.722	66.3427	70.3124	32.8628	131.727	112.34	170.986	56.6694	48.6996	65.5113	57.0145	0.008244783	0.192760771	16	12	24	0.005896067	0.461723519	24	10.49901374	0.906455148
ENSMUSG00000007880	Arid1a	AT rich interactive domain 1A (SWI-like) [Source:MGI Symbol;Acc:MGI:1935147]	7.94038	7.54966	7.63443	8.64664	7.82515	7.95569	8.33293	8.24596	7.99796	10.2001	7.75851	8.12571	7.93408	7.93958	0.93717134	0.966018836	16	4	24	0.341685907	0.77606964	24	12.22008065	0.053474119
ENSMUSG00000007888	Crlf1	cytokine receptor-like factor 1 [Source:MGI Symbol;Acc:MGI:1340030]	1.01798	1.56364	1.31809	1.37102	1.42929	1.59117	0.475977	1.34336	1.97594	1.13391	2.04663	1.05485	0.980867	1.33813	0.050258003	0.385784509	20	8	24	0.176161009	0.768040178	24	11.54682259	0.186102818
ENSMUSG00000007891	Ctsd	cathepsin D [Source:MGI Symbol;Acc:MGI:88562]	385.641	335.219	348.15	347.131	366.101	393.244	343.877	392.759	299.006	360.972	362.664	350.247	361.035	348.806	0.1982019	0.680119125	24	12	24	0.160514693	0.765941695	24	20.36395997	0.075695139
ENSMUSG00000007892	Rplp1	ribosomal protein, large, P1 [Source:MGI Symbol;Acc:MGI:1927099]	397.172	408.698	380.644	309.232	334.618	281.563	380.125	434.972	521.218	345.976	489.551	420.154	459.682	416.474	0.767183862	0.966018836	12	4	24	0.240354867	0.768040178	24	4.757182251	0.145747386
ENSMUSG00000007908	Hmgcll1	3-hydroxymethyl-3-methylglutaryl-Coenzyme A lyase-like 1 [Source:MGI Symbol;Acc:MGI:2446108]	4.70622	4.48707	4.19987	3.83286	4.24689	4.68687	4.71092	4.13226	3.69464	4.37101	4.63994	4.60357	4.49589	4.36812	0.001837158	0.100255953	4	8	24	0.004485075	0.427431763	24	21.39787576	0.097884407
ENSMUSG00000007944	Ttc9b	tetratricopeptide repeat domain 9B [Source:MGI Symbol;Acc:MGI:1920282]	7.57771	6.2199	5.7068	6.27989	6.07744	7.30129	6.46664	6.54274	6.1036	8.38681	7.67766	7.46657	7.71486	7.73892	0.868638024	0.966018836	4	8	24	0.330031211	0.768040178	24	20.10829259	0.074483938
ENSMUSG00000007950	Abhd8	abhydrolase domain containing 8 [Source:MGI Symbol;Acc:MGI:1918946]	28.6249	22.3857	23.8528	21.3556	19.9416	23.8656	25.1541	26.5198	24.3965	31.3433	27.9233	31.3927	29.4016	31.3853	0.745469888	0.966018836	24	20	24	0.434294056	0.805393068	24	0.997809231	0.076432833
ENSMUSG00000007987	Ift22	intraflagellar transport 22 [Source:MGI Symbol;Acc:MGI:1914536]	24.436	22.1843	23.6016	22.8158	22.4153	24.2541	19.0947	24.8588	21.372	21.8558	23.3942	22.0211	21.4063	17.9639	0.846563228	0.966018836	24	4	24	0.462182123	0.805393068	24	16.41470948	0.053037587
ENSMUSG00000007989	Fzd3	frizzled class receptor 3 [Source:MGI Symbol;Acc:MGI:108476]	8.5302	9.1892	8.32201	8.69351	10.0025	7.50265	9.94806	8.0224	9.77722	7.77492	9.33458	8.25035	8.90042	8.27474	0.93717134	0.966018836	20	4	24	0.99051711	0.998233223	24	14.94386642	0.006553922
ENSMUSG00000008035	Mid1ip1	Mid1 interacting protein 1 (gastrulation specific G12-like (zebrafish)) [Source:MGI Symbol;Acc:MGI:1915291]	78.3249	62.2194	66.5706	66.0323	64.2653	69.6734	70.8148	70.5067	59.6481	83.7257	66.0776	75.0728	69.8465	69.6858	0.267533088	0.774280302	4	8	24	0.589669703	0.854432964	24	20.62780963	0.053538287
ENSMUSG00000008036	Ap2s1	adaptor-related protein complex 2, sigma 1 subunit [Source:MGI Symbol;Acc:MGI:2141861]	146.824	116.501	121.05	112.788	113.133	129.631	123.487	139.761	120.551	148.398	144.636	141.641	141.89	140.28	0.68887295	0.966018836	4	8	24	0.483076116	0.805393068	24	22.6869447	0.059326386
ENSMUSG00000008090	Fgfrl1	fibroblast growth factor receptor-like 1 [Source:MGI Symbol;Acc:MGI:2150920]	3.73263	4.14152	4.76314	4.26145	3.87081	4.27557	5.24739	5.04928	4.40905	5.047	3.72558	4.04074	4.3838	4.48383	0.217933169	0.704989686	12	8	24	0.170888101	0.768040178	24	6.167951711	0.093694538
ENSMUSG00000008129	Brme1	break repair meiotic recombinase recruitment factor 1 [Source:MGI Symbol;Acc:MGI:1921916]	0.69514	0.59569	0.743326	0.752874	0.548357	0.691882	0.871912	0.710276	0.669136	0.597421	0.722755	0.882382	0.630061	1.17877	0.82502117	0.966018836	24	20	24	0.440137498	0.805393068	24	2.807292416	0.056219549
ENSMUSG00000008136	Fhl2	four and a half LIM domains 2 [Source:MGI Symbol;Acc:MGI:1338762]	2.97329	4.74493	3.80184	4.56385	3.72345	3.29207	3.6322	4.59029	5.52051	2.58109	4.23744	3.85265	3.91487	3.6161	0.593560247	0.966018836	12	12	24	0.402593238	0.799546792	24	7.282818983	0.123354642
ENSMUSG00000008140	Emc10	ER membrane protein complex subunit 10 [Source:MGI Symbol;Acc:MGI:1916933]	58.7129	51.0542	50.6252	52.7449	52.7025	55.6411	54.4211	57.756	48.8303	62.9947	54.4181	57.8727	59.5274	53.2358	0.029412383	0.322165609	4	8	24	0.346862027	0.778844143	24	20.43756716	0.056513772
ENSMUSG00000008153	Clstn3	calsyntenin 3 [Source:MGI Symbol;Acc:MGI:2178323]	17.6804	17.1268	22.0868	20.297	15.1178	18.5838	20.1991	19.2165	16.3546	22.0749	16.4606	21.0637	18.8641	23.8506	0.956178643	0.96629412	16	4	24	0.479257889	0.805393068	24	5.956412978	0.084736256
ENSMUSG00000008167	Fbxw9	F-box and WD-40 domain protein 9 [Source:MGI Symbol;Acc:MGI:1915878]	7.18676	8.60885	7.83657	8.38501	8.46925	7.42013	8.74109	7.87546	9.71548	8.60071	8.95819	8.40807	9.71476	9.08055	0.879201926	0.966018836	12	12	24	0.366664291	0.790399468	24	8.481176217	0.050598218
ENSMUSG00000008193	Spib	Spi-B transcription factor (Spi-1/PU.1 related) [Source:MGI Symbol;Acc:MGI:892986]	0.437284	1.03063	1.18016	0.991791	0.738267	0.947648	1.08768	0.943375	1.20805	0.714659	0.883383	0.956863	1.3837	0.836496	0.745118544	0.966018836	12	8	24	0.552103259	0.836587563	24	6.150193959	0.077111269
ENSMUSG00000008200	Fnbp4	formin binding protein 4 [Source:MGI Symbol;Acc:MGI:1860513]	17.6322	24.7734	22.5793	20.1137	20.9401	19.702	17.8447	20.2228	24.2645	20.492	19.9649	19.4679	20.9883	21.77	0.015345591	0.253245931	12	12	24	0.03797216	0.61140397	24	8.102677441	0.127631183
ENSMUSG00000008206	Cers4	ceramide synthase 4 [Source:MGI Symbol;Acc:MGI:1914510]	32.4042	28.9639	31.0597	30.3135	35.0975	34.794	31.79	31.8466	27.2238	35.206	29.5724	30.4053	33.3618	29.6221	0.442512808	0.966018836	16	20	24	0.198466083	0.768040178	24	18.67923253	0.076141648
ENSMUSG00000008226	Scrn3	secernin 3 [Source:MGI Symbol;Acc:MGI:1921866]	27.8788	27.7576	25.4266	25.4585	27.487	26.8433	26.0469	25.885	25.347	25.8316	29.533	26.053	27.2815	27.2019	0.05282315	0.391647859	20	16	24	0.259040489	0.768040178	24	20.27279147	0.040877154
ENSMUSG00000008301	Phax	phosphorylated adaptor for RNA export [Source:MGI Symbol;Acc:MGI:1891839]	22.4276	25.1822	22.2689	22.598	23.998	25.9665	23.8488	22.3675	23.5994	21.4935	24.6056	22.6961	24.4196	24.1047	0.297756231	0.80820842	20	20	24	0.340747092	0.775204417	24	21.65413285	0.043938742
ENSMUSG00000008305	Tle1	transducin-like enhancer of split 1 [Source:MGI Symbol;Acc:MGI:104636]	25.5156	19.7795	19.2346	21.8374	21.5727	21.5722	21.9207	20.6114	18.656	23.8474	21.3588	22.1184	22.1708	20.122	0.03452758	0.328311403	4	8	24	0.096750801	0.71175955	24	19.49682589	0.093818474
ENSMUSG00000008307	1700109H08Rik	RIKEN cDNA 1700109H08 gene [Source:MGI Symbol;Acc:MGI:1924286]	1.33636	1.93789	2.56292	1.72272	1.42207	1.10589	1.13987	1.20247	2.4842	1.53682	1.48792	2.13934	1.66236	1.47834	0.101520755	0.511659635	12	16	24	0.159971031	0.76564405	24	9.071504612	0.181930322
ENSMUSG00000008318	Relt	RELT tumor necrosis factor receptor [Source:MGI Symbol;Acc:MGI:2443373]	3.8726	4.27302	4.31744	3.49699	3.20636	3.55041	3.78061	3.94512	3.6577	5.02595	3.70312	3.92774	4.48532	4.44118	0.254637229	0.757509828	8	12	24	0.16499042	0.767020009	24	5.653792082	0.09511204
ENSMUSG00000008333	Snrpb2	U2 small nuclear ribonucleoprotein B [Source:MGI Symbol;Acc:MGI:104805]	18.2586	23.0876	19.5611	19.4269	19.1038	18.8468	17.3261	20.0066	25.0751	20.0408	21.9995	21.2053	24.5259	23.1001	0.791413662	0.966018836	8	20	24	0.225804754	0.768040178	24	6.819562509	0.090055826
ENSMUSG00000008348	Ubc	ubiquitin C [Source:MGI Symbol;Acc:MGI:98889]	48.4878	43.5723	36.4922	42.665	43.4003	48.8622	45.8753	53.6827	41.3353	60.2172	43.6416	52.6855	53.9851	48.6401	0.138543921	0.580158868	24	12	24	0.463619421	0.805393068	24	21.98610742	0.080527404
ENSMUSG00000008373	Prpf31	pre-mRNA processing factor 31 [Source:MGI Symbol;Acc:MGI:1916238]	26.8682	23.5954	23.9701	21.6326	23.3336	26.7288	23.6909	24.7543	21.9796	21.2452	24.457	25.6086	24.5325	25.3283	0.004947794	0.141782417	24	16	24	0.004709988	0.434006302	24	22.61846968	0.108939136
ENSMUSG00000008384	Sertad1	SERTA domain containing 1 [Source:MGI Symbol;Acc:MGI:1913438]	14.168	15.6193	13.4753	16.7112	16.2204	14.0054	13.6304	14.9653	16.3528	15.9494	16.9108	14.5904	15.002	13.8025	0.115169104	0.536202194	20	8	24	0.038705494	0.61140397	24	12.9812564	0.095591092
ENSMUSG00000008393	Carhsp1	calcium regulated heat stable protein 1 [Source:MGI Symbol;Acc:MGI:1196368]	38.9484	31.7478	35.3113	33.2356	33.1344	37.3588	33.8111	33.8421	29.1951	35.6526	35.5553	37.3232	36.2327	35.8333	0.12833063	0.560209134	24	12	24	0.092955065	0.71175955	24	20.65416746	0.086357473
ENSMUSG00000008398	Elk3	ELK3, member of ETS oncogene family [Source:MGI Symbol;Acc:MGI:101762]	3.111	3.18123	3.01492	3.11231	2.89852	3.26581	2.75682	3.77904	2.99327	3.31316	2.84732	3.36743	3.28183	2.55381	0.853736882	0.966018836	24	20	24	0.959264585	0.98927861	24	22.96486222	0.012534239
ENSMUSG00000008429	Herpud2	HERPUD family member 2 [Source:MGI Symbol;Acc:MGI:1915393]	9.2501	9.5453	8.94088	10.0127	9.31912	8.67684	10.8936	9.60123	9.38884	11.607	9.01147	8.69475	8.14113	9.37271	0.173410058	0.648600527	16	8	24	0.408647972	0.799546792	24	10.01764561	0.063956636
ENSMUSG00000008435	Rdh13	retinol dehydrogenase 13 (all-trans and 9-cis) [Source:MGI Symbol;Acc:MGI:1918732]	10.7461	10.7578	12.7807	11.4031	11.5819	13.1443	10.8783	10.9638	12.2403	11.3148	11.88	10.5136	11.759	11.7737	0.569606064	0.966018836	12	16	24	0.536767951	0.827516282	24	11.88841651	0.039242843
ENSMUSG00000008450	Nutf2	nuclear transport factor 2 [Source:MGI Symbol;Acc:MGI:1915301]	2.90642	1.48218	1.71035	2.17176	1.35309	1.72066	1.47801	2.01432	1.85674	2.46002	2.17526	1.71967	1.94196	2.25115	0.353736882	0.966018836	16	4	24	0.895502713	0.970467191	24	8.134402819	0.040071442
ENSMUSG00000008475	Arpc5	actin related protein 2/3 complex, subunit 5 [Source:MGI Symbol;Acc:MGI:1915021]	127.104	121.022	115.731	120.821	118.328	115.403	118.257	118.256	125.571	127.514	133.89	127.647	134.134	126.356	0.906657897	0.966018836	20	16	24	0.918551515	0.978028197	24	18.6767561	0.009893076
ENSMUSG00000008489	Elavl2	ELAV like RNA binding protein 1 [Source:MGI Symbol;Acc:MGI:1100887]	92.4342	89.0262	80.8339	84.3415	83.6978	82.7705	83.3442	78.2408	86.5465	77.1945	94.451	87.9883	92.6308	91.3382	0.775247401	0.966018836	4	12	24	0.382881188	0.799546792	24	22.53475478	0.04913879
ENSMUSG00000008496	Pou2f2	POU domain, class 2, transcription factor 2 [Source:MGI Symbol;Acc:MGI:101897]	0.623666	0.874714	0.821874	0.630661	0.541559	0.71864	0.917421	0.699449	1.08044	0.78993	0.777676	0.788424	0.845825	0.939143	0.195388358	0.673387182	12	8	24	0.052876834	0.650698269	24	5.43261269	0.091600885
ENSMUSG00000008540	Mgst1	microsomal glutathione S-transferase 1 [Source:MGI Symbol;Acc:MGI:1913850]	26.3848	32.2872	49.3839	69.4688	37.5926	37.3802	38.3337	45.7351	42.7442	39.1397	57.129	42.0571	51.0053	46.8055	0.612141595	0.966018836	16	8	24	0.129220557	0.740162193	24	11.40903163	0.23637802
ENSMUSG00000008575	Nfib	nuclear factor I/B [Source:MGI Symbol;Acc:MGI:103188]	6.96159	8.21515	8.06335	7.90581	7.78665	6.77869	7.30097	7.35694	9.74831	8.35267	8.6488	7.88782	7.87699	8.36312	0.051446973	0.38924256	12	16	24	0.009236568	0.502965379	24	9.64069019	0.11667875
ENSMUSG00000008590	Htr3b	5-hydroxytryptamine (serotonin) receptor 3B [Source:MGI Symbol;Acc:MGI:1861899]	5.58327	5.15523	4.48553	5.01775	4.78701	5.42121	4.95191	5.04964	4.02739	5.92056	5.267	5.40027	4.75935	4.88887	0.148073804	0.590732968	24	12	24	0.221425113	0.768040178	24	19.12281728	0.079354962
ENSMUSG00000008604	Ubqln4	ubiquilin 4 [Source:MGI Symbol;Acc:MGI:2150152]	16.4312	15.9388	16.852	15.7781	16.3779	17.5219	17.403	17.6076	15.2852	18.1431	17.6457	16.4361	17.1445	15.6822	0.711168999	0.966018836	20	16	24	0.475195289	0.805393068	24	18.77529283	0.035177643
ENSMUSG00000008658	Rbfox1	RNA binding protein, fox-1 homolog (C. elegans) 1 [Source:MGI Symbol;Acc:MGI:1926224]	47.0113	37.6625	38.0717	39.6157	33.4757	40.1594	40.2592	41.2535	34.1625	52.6947	36.1377	40.1101	37.792	42.5531	0.622773557	0.966018836	4	16	24	0.91496597	0.976279382	24	2.720811819	0.028294111
ENSMUSG00000008668	Rps18	ribosomal protein S18 [Source:MGI Symbol;Acc:MGI:98146]	47.9254	58.8602	55.6725	63.1017	66.3041	59.7553	45.5534	54.9744	66.7573	39.3563	52.7869	42.3866	44.6329	41.7823	0.708020493	0.966018836	12	16	24	0.404132952	0.799546792	24	12.07822608	0.111927484
ENSMUSG00000008682	Rpl10	ribosomal protein L10 [Source:MGI Symbol;Acc:MGI:105943]	16.4602	14.6911	12.9988	13.4196	15.2397	14.669	14.2312	14.4418	16.4736	11.7038	15.2018	13.8855	16.4741	12.9636	0.702630284	0.966018836	4	12	24	0.257973556	0.768040178	24	22.56167838	0.087011949
ENSMUSG00000008683	Rps15a	ribosomal protein S15A [Source:MGI Symbol;Acc:MGI:2389091]	72.0292	112.216	100.13	81.2522	103.125	91.0846	83.6238	79.698	99.6573	56.4084	89.9193	75.2828	84.8109	78.6155	0.920858634	0.966018836	12	16	24	0.937499984	0.984526908	24	4.74384761	0.034523361
ENSMUSG00000008690	Ncaph2	non-SMC condensin II complex, subunit H2 [Source:MGI Symbol;Acc:MGI:1289164]	29.9707	28.1437	27.215	29.3432	30.3347	30.8234	29.4095	33.0141	28.581	31.1299	29.1916	28.8043	28.2324	29.4833	0.880631923	0.966018836	16	20	24	0.872107821	0.963835087	24	18.56665489	0.014934867
ENSMUSG00000008730	Hipk1	homeodomain interacting protein kinase 1 [Source:MGI Symbol;Acc:MGI:1314873]	17.16	17.1473	18.2559	19.1495	17.4936	16.479	17.9751	15.4795	16.6531	18.4643	16.9463	16.9803	15.9744	16.2447	0.08333408	0.462922111	16	16	24	0.054195994	0.654887447	24	13.08258932	0.062005641
ENSMUSG00000008734	Gprc5b	G protein-coupled receptor, family C, group 5, member B [Source:MGI Symbol;Acc:MGI:1927596]	49.4925	53.5385	54.5304	50.3265	52.499	48.3131	59.2922	50.9359	57.4669	61.4197	45.2204	59.6263	56.0345	55.063	0.897570341	0.966018836	16	4	24	0.555925356	0.838702296	24	5.995103591	0.052347982
ENSMUSG00000008763	Man1a2	mannosidase, alpha, class 1A, member 2 [Source:MGI Symbol;Acc:MGI:104676]	25.7571	23.2099	21.9189	23.564	23.0509	22.162	21.9704	20.8471	21.4482	22.2257	24.3615	23.5279	23.6747	23.0972	0.351840494	0.870418653	20	16	24	0.246584147	0.768040178	24	19.65431037	0.050635975
ENSMUSG00000008822	Acyp1	acylphosphatase 1, erythrocyte (common) type [Source:MGI Symbol;Acc:MGI:1913454]	38.1642	37.223	29.8915	32.2014	32.0431	31.4236	33.7079	31.2219	37.3799	30.3696	36.0977	35.4626	36.9816	37.2456	0.512510541	0.966018836	4	12	24	0.233386615	0.768040178	24	0.898321083	0.077685595
ENSMUSG00000008843	Cldn13	claudin 13 [Source:MGI Symbol;Acc:MGI:1913102]	0.483285	0.826343	2.48476	1.76534	0.724149	1.49782	0.857922	0.90534	1.0506	0.522453	0.755784	0.979798	1.78423	0.688049	0.906657897	0.966018836	12	8	24	0.855585651	0.957116652	24	9.21297632	0.084467745
ENSMUSG00000008845	Cd163	CD163 antigen [Source:MGI Symbol;Acc:MGI:2135946]	2.32442	2.35748	3.07737	4.73562	3.02065	2.41652	2.22255	2.45253	3.79164	2.55154	2.67457	2.84677	2.20887	1.83874	0.003172351	0.138295404	16	12	24	0.01540196	0.539649237	24	12.32756567	0.243924218
ENSMUSG00000008855	Hdac5	histone deacetylase 5 [Source:MGI Symbol;Acc:MGI:1333784]	18.3803	18.9897	19.7904	20.8838	19.2958	20.1783	23.276	20.4289	21.8556	21.9091	20.3803	20.7756	17.3117	22.4601	0.864782464	0.966018836	16	12	24	0.482699886	0.805393068	24	9.790683182	0.052026168
ENSMUSG00000008859	Rala	v-ral simian leukemia viral oncogene A (ras related) [Source:MGI Symbol;Acc:MGI:1927243]	41.7009	38.7719	39.6639	36.3936	39.2002	38.2135	39.5203	38.7419	35.7133	39.3934	41.4356	39.9855	40.8764	39.9427	0.235141005	0.73629392	4	8	24	0.158120939	0.764630328	24	21.77321387	0.04508581
ENSMUSG00000008874	Clec3a	C-type lectin domain family 3, member a [Source:MGI Symbol;Acc:MGI:2685642]	0.840045	0.473232	0.304152	0.743564	0.34646	0.735575	0.113572	0.997614	0.190581	2.52978	0.191358	0.299456	0.562139	0.352976	0.90332508	0.966018836	16	4	24	0.736631285	0.914180155	24	11.38890466	0.135882275
ENSMUSG00000008892	Vdac3	voltage-dependent anion channel 3 [Source:MGI Symbol;Acc:MGI:106922]	48.6351	52.8578	51.878	51.3484	48.4942	50.6333	48.2269	52.6008	52.516	50.6633	53.3009	48.7612	56.4666	52.4446	0.338172159	0.85758369	8	16	24	0.224918109	0.768040178	24	6.566038048	0.040189758
ENSMUSG00000008958	Vps72	vacuolar protein sorting 72 [Source:MGI Symbol;Acc:MGI:1202305]	13.2554	11.449	11.0552	12.7259	11.2361	11.4403	13.3705	13.4482	11.0813	11.9103	11.6027	12.5047	11.6616	12.7731	0.128989168	0.560209134	8	4	24	0.282606382	0.768040178	24	0.629675187	0.058211109
ENSMUSG00000008976	Gabpa	GA repeat binding protein, alpha [Source:MGI Symbol;Acc:MGI:95610]	11.837	10.8045	11.1166	11.8945	11.2423	9.91329	12.347	11.456	10.5782	9.44346	12.1156	10.4294	10.7107	9.93544	0.910436409	0.966018836	4	12	24	0.7980896	0.935424343	24	21.02094285	0.027755661
ENSMUSG00000008999	Bmp7	bone morphogenetic protein 7 [Source:MGI Symbol;Acc:MGI:103302]	7.26523	8.33541	6.70497	7.21684	8.70776	6.9728	8.42924	8.41601	9.28043	7.72543	6.63628	7.8205	8.57802	7.11857	0.90767227	0.966018836	4	20	24	0.653329139	0.883312134	24	3.601788552	0.04997025
ENSMUSG00000009013	Dynll1	dynein light chain LC8-type 1 [Source:MGI Symbol;Acc:MGI:1861457]	383.135	306.087	286.186	297.419	349.355	330.394	343.419	312.344	271.922	275.433	341.831	294.902	320.098	282.926	0.024438424	0.287293295	4	8	24	0.00307666	0.414576087	24	20.75125789	0.154276981
ENSMUSG00000009030	Pdcl	phosducin-like [Source:MGI Symbol;Acc:MGI:1914716]	23.0841	24.2759	22.7713	22.9868	23.3559	22.3525	20.8301	21.359	21.0737	20.6651	23.8599	20.223	21.1203	21.2373	0.891742979	0.966018836	20	4	24	0.616565153	0.867682235	24	15.03915216	0.023943617
ENSMUSG00000009035	Tmem184b	transmembrane protein 184b [Source:MGI Symbol;Acc:MGI:2445179]	20.6209	16.2751	16.2168	18.8753	17.2634	19.0699	20.041	21.1361	16.3755	24.4675	19.3349	21.8217	20.0091	19.7125	0.300823756	0.809362476	4	8	24	0.554298947	0.837681708	24	19.82028494	0.066159909
ENSMUSG00000009073	Nf2	neurofibromin 2 [Source:MGI Symbol;Acc:MGI:97307]	21.5515	17.9675	20.769	22.3292	21.1727	20.3747	21.3551	20.1838	17.9952	22.1642	19.6284	20.4948	19.4816	20.3662	0.231491055	0.731037655	16	16	24	0.365756533	0.789934347	24	16.27092074	0.050169833
ENSMUSG00000009075	Cabp7	calcium binding protein 7 [Source:MGI Symbol;Acc:MGI:2183437]	9.4008	9.51206	15.8105	9.08911	7.05881	11.3511	12.2453	9.4271	9.6326	11.8058	6.75273	10.4565	7.16529	11.7257	0.424718357	0.966018836	12	8	24	0.386036633	0.799546792	24	5.978367623	0.169255899
ENSMUSG00000009076	Zmat5	zinc finger, matrin type 5 [Source:MGI Symbol;Acc:MGI:1914428]	23.7724	23.6124	24.2947	25.187	25.3218	25.9327	22.2966	23.1604	24.0118	20.3914	26.156	23.1657	24.2427	21.9172	0.033553741	0.324908923	20	12	24	0.247250429	0.768040178	24	17.45232953	0.059071317
ENSMUSG00000009079	Ewsr1	Ewing sarcoma breakpoint region 1 [Source:MGI Symbol;Acc:MGI:99960]	79.2988	86.3704	80.2597	84.1049	91.548	78.6352	85.5195	76.8599	85.8432	79.1711	81.2395	76.398	78.1889	80.0772	0.44861185	0.966018836	20	4	24	0.562839681	0.842470381	24	12.34017147	0.029192707
ENSMUSG00000009090	Ap1b1	adaptor protein complex AP-1, beta 1 subunit [Source:MGI Symbol;Acc:MGI:1096368]	33.7854	27.0367	29.0589	28.6034	27.1822	34.9813	29.968	31.2026	25.7985	37.8368	23.8629	30.4542	28.2343	30.9442	0.969141379	0.970883022	24	20	24	0.819774307	0.942844249	24	22.89834905	0.044316661
ENSMUSG00000009092	Derl3	Der1-like domain family, member 3 [Source:MGI Symbol;Acc:MGI:1917627]	0.650097	0.436078	0.485032	0.548036	0.398989	0.454469	0.511949	0.696716	0.249127	0.802717	0.936275	0.455588	0.22071	0.420894	0.920872048	0.966018836	16	20	24	0.492576918	0.805558817	24	14.70412562	0.085151653
ENSMUSG00000009097	Tbx1	T-box 1 [Source:MGI Symbol;Acc:MGI:98493]	0.427218	0.382486	0.288206	0.391227	0.593263	0.468653	0.599512	0.655235	0.647549	0.540462	0.720689	0.530016	0.737909	0.424217	0.843639218	0.966018836	20	16	24	0.556598019	0.838702296	24	19.26156725	0.04731358
ENSMUSG00000009112	Bcl2l13	BCL2-like 13 (apoptosis facilitator) [Source:MGI Symbol;Acc:MGI:2136959]	13.8223	10.2253	12.6967	11.3572	10.2997	11.7169	11.6899	15.8966	10.3465	17.1795	13.1532	13.8982	13.346	13.2545	0.88067614	0.966018836	24	12	24	0.973418782	0.993305115	24	4.356804237	0.01839305
ENSMUSG00000009145	Dqx1	DEAQ RNA-dependent ATPase [Source:MGI Symbol;Acc:MGI:2136388]	1.53475	1.69736	1.29479	1.70787	1.66734	1.61589	1.47274	1.46266	1.53757	1.65461	1.82081	1.36304	1.68046	1.57573	0.46694635	0.966018836	16	20	24	0.44471386	0.805393068	24	15.42349412	0.040549259
ENSMUSG00000009185	Ccl8	chemokine (C-C motif) ligand 8 [Source:MGI Symbol;Acc:MGI:101878]	1.7261	1.3986	1.24922	0.913294	1.19129	1.03227	0.419523	1.63578	2.58039	0.30436	1.56817	0.814665	0.308703	0.875637	0.883024237	0.966018836	12	4	24	0.673355451	0.890057965	24	7.972759872	0.146208186
ENSMUSG00000009207	Lnpk	lunapark, ER junction formation factor [Source:MGI Symbol;Acc:MGI:1918115]	27.7218	25.9982	25.6625	25.1843	24.3526	26.2039	26.0549	24.7078	21.9641	24.7023	29.0694	25.139	26.0475	26.402	0.268052211	0.774280302	4	12	24	0.226743775	0.768040178	24	21.03611315	0.061147986
ENSMUSG00000009216	Fam163b	family with sequence similarity 163, member B [Source:MGI Symbol;Acc:MGI:1926106]	0.82099	1.26118	2.23402	1.29059	0.725151	1.22947	1.71338	0.827264	1.76419	1.4489	0.479251	1.34719	0.760963	1.68523	0.021288455	0.264116537	12	8	24	0.171360984	0.768040178	24	7.084063067	0.235555016
ENSMUSG00000009281	Rarres2	retinoic acid receptor responder (tazarotene induced) 2 [Source:MGI Symbol;Acc:MGI:1918910]	78.1651	86.7024	81.5641	68.8536	94.0665	69.3966	78.1931	79.3155	93.9369	57.2747	84.1096	75.424	82.1902	76.1421	0.649212555	0.966018836	12	4	24	0.759466902	0.922276859	24	2.504821931	0.053944562
ENSMUSG00000009291	Pttg1ip	pituitary tumor-transforming 1 interacting protein [Source:MGI Symbol;Acc:MGI:2652132]	74.3323	72.0355	71.3449	72.567	79.1046	72.1427	83.4548	77.3476	77.5997	77.2732	71.3778	73.7625	73.4016	66.7574	0.865514257	0.966018836	4	4	24	0.863425494	0.960304254	24	18.77666791	0.017726146
ENSMUSG00000009292	Trpm2	transient receptor potential cation channel, subfamily M, member 2 [Source:MGI Symbol;Acc:MGI:1351901]	7.65547	8.78482	7.57514	9.08337	8.92137	9.07563	7.79737	9.3104	8.00653	9.85383	7.27047	8.00741	7.00607	8.99008	0.192357053	0.669841558	16	12	24	0.484976969	0.805393068	24	10.68997939	0.064580161
ENSMUSG00000009293	Ube2g2	ubiquitin-conjugating enzyme E2G 2 [Source:MGI Symbol;Acc:MGI:1343188]	22.5208	19.724	19.2229	21.3092	20.0366	22.6772	19.2466	22.8508	18.3954	20.4252	21.3513	19.5493	20.1384	18.4139	0.496745696	0.966018836	24	12	24	0.308484857	0.768040178	24	19.32140353	0.058851289
ENSMUSG00000009350	Mpo	myeloperoxidase [Source:MGI Symbol;Acc:MGI:97137]	2.55062	5.03285	10.6089	5.11155	3.79504	6.41525	3.67274	4.1402	4.44932	2.45004	2.71663	3.69039	5.1291	2.73966	0.869755137	0.966018836	12	4	24	0.772837302	0.926921854	24	7.205755003	0.133302306
ENSMUSG00000009376	Met	met proto-oncogene [Source:MGI Symbol;Acc:MGI:96969]	2.18076	2.05458	1.97545	1.96605	2.73999	1.79439	2.41617	1.97578	2.04241	1.66195	2.17143	2.14913	2.2082	1.81269	0.06180207	0.409814092	4	12	24	0.284027253	0.768040178	24	20.07413807	0.077767523
ENSMUSG00000009378	Slc16a12	solute carrier family 16 (monocarboxylic acid transporters), member 12 [Source:MGI Symbol;Acc:MGI:2147716]	5.86055	7.38851	7.33208	6.43956	6.38223	6.53419	5.83582	6.88275	7.39487	6.41643	6.78097	7.07792	6.84452	8.07905	0.097975419	0.501188411	8	20	24	0.063691012	0.663876076	24	6.998796236	0.09315122
ENSMUSG00000009394	Syn2	synapsin II [Source:MGI Symbol;Acc:MGI:103020]	42.4829	35.8185	38.2158	36.5363	32.7463	44.0038	41.814	37.9574	30.9483	51.4544	33.5713	41.7292	35.309	46.2687	0.94693335	0.966018836	24	20	24	0.82825777	0.946496082	24	23.46143037	0.049847919
ENSMUSG00000009406	Elk1	ELK1, member of ETS oncogene family [Source:MGI Symbol;Acc:MGI:101833]	6.11743	4.91389	5.47787	5.64309	5.71816	5.19444	5.7213	4.31685	5.2756	5.17641	5.89862	5.21767	5.40616	4.94369	0.037425327	0.334069441	20	12	24	0.127926972	0.740162193	24	17.42961118	0.079636558
ENSMUSG00000009418	Nav1	neuron navigator 1 [Source:MGI Symbol;Acc:MGI:2183683]	23.3287	24.0769	24.8882	23.8129	24.2927	23.3776	20.8356	22.9973	26.2299	29.6951	21.3457	24.7507	26.2949	24.7581	0.476029854	0.966018836	12	16	24	0.229327184	0.768040178	24	9.687862803	0.080084059
ENSMUSG00000009470	Tnpo1	transportin 1 [Source:MGI Symbol;Acc:MGI:2681523]	13.5676	13.5864	13.9557	13.9013	14.3799	12.5691	13.7566	12.3939	13.1936	11.129	14.9057	11.8949	11.2555	12.5903	0.664568268	0.966018836	20	4	24	0.635092527	0.87594009	24	14.28769562	0.037651339
ENSMUSG00000009535	Rnmt	RNA (guanine-7-) methyltransferase [Source:MGI Symbol;Acc:MGI:1915147]	21.465	23.4954	22.3126	21.9877	21.05	22.1352	19.9532	20.9401	24.1403	21.6972	22.8472	23.8185	23.235	24.474	0.920858634	0.966018836	12	16	24	0.573731819	0.847005958	24	7.134969781	0.032207585
ENSMUSG00000009549	Srp14	signal recognition particle 14 [Source:MGI Symbol;Acc:MGI:107169]	186.449	194.546	185.324	175.629	188.957	172.124	185.155	178.386	207.131	148.657	197.84	180.577	196.166	185.083	0.951846915	0.966018836	4	12	24	0.57138827	0.845838471	24	2.075653452	0.047022747
ENSMUSG00000009551	6330409D20Rik	RIKEN cDNA 6330409D20 gene [Source:MGI Symbol;Acc:MGI:1917980]	2.04774	1.63575	1.43871	1.92297	1.84045	1.77399	1.7274	1.7932	0.996305	2.44707	1.37491	2.02976	1.70902	1.67709	0.187699199	0.662239322	16	20	24	0.449137531	0.805393068	24	19.12640621	0.096319046
ENSMUSG00000009555	Cdk9	cyclin-dependent kinase 9 (CDC2-related kinase) [Source:MGI Symbol;Acc:MGI:1328368]	26.2466	25.3543	25.8903	27.9449	27.1802	24.823	28.3661	27.7512	27.3206	28.9951	26.809	26.1698	25.973	26.1764	0.560714538	0.966018836	16	8	24	0.272577928	0.768040178	24	11.16900105	0.038469436
ENSMUSG00000009563	Tor2a	torsin family 2, member A [Source:MGI Symbol;Acc:MGI:1353596]	6.78912	6.43481	6.82099	6.94201	7.23502	6.45647	6.62248	6.57333	7.22073	6.05612	7.15402	6.33318	7.38079	7.00669	0.896799496	0.966018836	20	4	24	0.970327886	0.993003194	24	9.31784114	0.007502832
ENSMUSG00000009566	Fpgs	folylpolyglutamyl synthetase [Source:MGI Symbol;Acc:MGI:95576]	7.82073	6.67865	5.94436	7.32792	7.25109	6.68895	7.23043	6.97538	7.49428	7.43314	6.87179	6.44222	7.29476	7.29287	0.951945551	0.966018836	4	20	24	0.983754896	0.996593965	24	0.968056821	0.006533281
ENSMUSG00000009569	Mrtfb	myocardin related transcription factor B [Source:MGI Symbol;Acc:MGI:3050795]	5.91695	5.9186	6.24314	6.06692	5.28816	6.15606	5.99026	6.86817	5.81999	6.77999	5.08486	5.63255	5.5067	6.41763	0.109247218	0.524035616	8	12	24	0.113617308	0.727440321	24	6.223447031	0.084218285
ENSMUSG00000009575	Cbx5	chromobox 5 [Source:MGI Symbol;Acc:MGI:109372]	20.4342	22.0064	20.7009	21.5956	22.3293	20.0088	22.2912	19.8423	23.1298	18.3889	21.6391	21.9402	21.5344	21.5719	0.845386467	0.966018836	20	8	24	0.93274671	0.98297963	24	0.180360983	0.012461262
ENSMUSG00000009585	Apobec3	apolipoprotein B mRNA editing enzyme, catalytic polypeptide 3 [Source:MGI Symbol;Acc:MGI:1933111]	3.00713	3.82131	4.01706	3.60295	3.38117	3.45802	3.37699	4.18235	4.49681	3.33629	3.60367	3.22167	3.68623	3.14534	0.217827365	0.704989686	12	12	24	0.089933024	0.7097999	24	7.946819818	0.107493811
ENSMUSG00000009614	Sardh	sarcosine dehydrogenase [Source:MGI Symbol;Acc:MGI:2183102]	3.5747	4.09617	4.10879	4.70508	5.04666	3.72154	4.9771	4.24398	4.8007	4.63739	4.01508	3.8604	4.51039	3.24903	0.519468828	0.966018836	16	20	24	0.387904619	0.799546792	24	12.6347943	0.081063076
ENSMUSG00000009621	Vav2	vav 2 oncogene [Source:MGI Symbol;Acc:MGI:102718]	12.4672	10.7665	11.1389	13.2973	14.2006	13.2778	11.1034	12.241	9.92858	12.5335	11.9297	12.3196	11.2	11.7762	0.031295391	0.324385159	16	20	24	0.020933942	0.596174862	24	17.0732813	0.118585771
ENSMUSG00000009630	Ppp2cb	protein phosphatase 2 (formerly 2A), catalytic subunit, beta isoform [Source:MGI Symbol;Acc:MGI:1321161]	63.731	54.8618	52.4912	55.661	54.527	57.0786	60.8218	53.2403	49.1119	58.6212	52.874	57.0969	57.6091	52.6447	0.002254694	0.104113924	4	8	24	0.036138257	0.609176161	24	21.23826608	0.087511922
ENSMUSG00000009633	G0s2	G0/G1 switch gene 2 [Source:MGI Symbol;Acc:MGI:1316737]	13.5954	11.5584	16.6751	25.5488	18.561	14.7449	13.8394	12.1626	20.3617	23.2742	29.6374	16.3154	21.0088	16.2701	0.001790467	0.100255953	16	16	24	0.001698468	0.374946468	24	13.48248827	0.393076461
ENSMUSG00000009640	Dmap1	DNA methyltransferase 1-associated protein 1 [Source:MGI Symbol;Acc:MGI:1913483]	11.8821	10.8386	10.9566	8.70275	9.90222	11.3592	9.57539	12.133	10.0598	11.4542	10.2434	10.4199	10.6312	11.33	0.522551072	0.966018836	8	12	24	0.288798258	0.768040178	24	2.331102609	0.071299176

ENSMUSG00000009647	Mcu	mitochondrial calcium uniporter [Source:MGI Symbol;Acc:MGI:3026965]	14.6648	10.8638	11.3351	11.9648	10.8536	13.9316	12.1579	12.7465	10.3061	15.2148	10.6113	11.7263	12.183	11.91	0.371875382	0.894537464	4	8	24	0.675436583	0.891226827	24	21.64481874	0.058145456
ENSMUSG00000009681	Bcr	BCR activator of RhoGEF and GTPase [Source:MGI Symbol;Acc:MGI:88141]	13.2998	11.3551	10.6997	12.2449	11.743	13.8871	12.927	12.8825	10.8934	15.5951	11.5447	12.6896	11.6153	12.2552	0.074438493	0.448301997	24	12	24	0.558717106	0.839978322	24	18.99069426	0.0600556
ENSMUSG00000009687	Fxyd5	FXYD domain-containing ion transport regulator 5 [Source:MGI Symbol;Acc:MGI:1201785]	39.019	42.0416	38.5209	31.4683	42.4663	34.9304	34.68	39.8126	47.419	20.6519	38.3252	39.3079	35.6791	34.4075	0.938993891	0.966018836	12	4	24	0.535251055	0.82691856	24	1.031206627	0.116337248
ENSMUSG00000009731	Kcnd1	potassium voltage-gated channel, Shal-related family, member 1 [Source:MGI Symbol;Acc:MGI:96671]	41.0059	26.9419	27.7353	30.151	31.1395	36.8673	30.9641	35.2381	21.4215	38.9735	29.4482	31.6404	31.7531	29.8007	0.076187236	0.453790923	24	12	24	0.207136591	0.768040178	24	20.31540106	0.160986011
ENSMUSG00000009733	Tfcp2	transcription factor CP2 [Source:MGI Symbol;Acc:MGI:98509]	8.3395	7.58632	7.49906	7.91907	8.3234	8.10965	8.15543	7.89101	8.8191	9.19311	8.56721	8.67618	8.26556	8.56476	0.852772142	0.966018836	20	12	24	0.313427253	0.768040178	24	15.14437045	0.033339267
ENSMUSG00000009739	Pou6f1	POU domain, class 6, transcription factor 1 [Source:MGI Symbol;Acc:MGI:102935]	15.551	15.4537	17.7806	17.0026	16.4273	16.035	16.473	16.7887	15.6537	16.7776	14.0889	16.0806	15.063	17.1899	0.266953338	0.774280302	12	16	24	0.307791844	0.768040178	24	8.001269363	0.050079371
ENSMUSG00000009741	Ubp1	upstream binding protein 1 [Source:MGI Symbol;Acc:MGI:104889]	19.6904	23.5595	21.825	19.9595	22.5831	22.3689	20.4194	21.6679	24.139	21.5297	19.1354	21.5338	18.7691	19.3336	0.43565473	0.966018836	12	16	24	0.593987927	0.857184401	24	9.600610461	0.042612021
ENSMUSG00000009772	Nuak2	NUAK family, SNF1-like kinase, 2 [Source:MGI Symbol;Acc:MGI:1921387]	0.58568	0.650273	0.731587	0.498453	0.739459	0.60837	0.436261	0.769961	0.734537	0.537101	0.679919	0.659786	0.729832	0.570287	0.94086481	0.966018836	12	16	24	0.893503274	0.969902527	24	7.844072579	0.018325211
ENSMUSG00000009828	Cilk1	ciliogenesis associated kinase 1 [Source:MGI Symbol;Acc:MGI:1934157]	7.32441	6.74282	6.95595	6.04597	7.1672	6.47503	7.52225	6.41917	6.85251	7.11826	6.62852	7.08641	6.99932	6.81942	0.676344318	0.966018836	4	4	24	0.434207243	0.805393068	24	22.83030821	0.037265895
ENSMUSG00000009863	Sdhb	succinate dehydrogenase complex, subunit B, iron sulfur (Ip) [Source:MGI Symbol;Acc:MGI:1914930]	125.433	128.509	137.691	147.89	128.563	129.722	126.643	144.76	143.441	140.474	151.856	134.108	156.177	149.051	0.910436409	0.966018836	16	12	24	0.110802056	0.722405302	24	9.268859162	0.064463227
ENSMUSG00000009876	Cox4i2	cytochrome c oxidase subunit 4I2 [Source:MGI Symbol;Acc:MGI:2135755]	1.05965	1.4315	1.84267	2.01792	1.36925	1.87242	1.54406	1.76651	1.68576	2.24091	1.49824	1.35347	1.50607	1.11479	0.105345531	0.518002936	16	12	24	0.083192521	0.699403344	24	11.60710892	0.152171827
ENSMUSG00000009894	Snap47	synaptosomal-associated protein, 47 [Source:MGI Symbol;Acc:MGI:1915076]	100.223	101.799	102.237	96.4694	97.1641	104.133	97.3075	94.8964	94.0672	97.1714	98.0212	98.9453	96.2221	106.197	0.886952527	0.966018836	24	16	24	0.654457219	0.883569804	24	0.274692812	0.018557512
ENSMUSG00000009905	Kdsr	3-ketodihydrosphingosine reductase [Source:MGI Symbol;Acc:MGI:1918000]	9.48208	9.04564	9.26962	9.56764	9.22485	8.70009	8.32832	8.66543	8.8608	8.71031	9.08623	9.30609	9.27157	9.06535	0.904831057	0.966018836	16	16	24	0.767754159	0.924971564	24	14.73118516	0.014400279
ENSMUSG00000009907	Vps4b	vacuolar protein sorting 4B [Source:MGI Symbol;Acc:MGI:1100499]	44.0469	40.4881	45.1799	40.2388	43.0194	44.6054	40.9144	40.6638	37.5024	41.5171	41.8436	42.9577	40.8296	41.0948	0.244223134	0.741580974	24	8	24	0.158416123	0.764909967	24	19.33419955	0.049002493
ENSMUSG00000009927	Rps25	ribosomal protein S25 [Source:MGI Symbol;Acc:MGI:1922867]	67.7792	89.7999	88.1855	84.9065	89.8387	80.7223	75.7137	78.8668	88.9175	54.3262	79.7464	66.9287	71.3045	66.652	0.759899911	0.966018836	12	16	24	0.840651066	0.951783982	24	9.74620777	0.045236763
ENSMUSG00000009995	Taz	tafazzin [Source:MGI Symbol;Acc:MGI:109626]	26.6064	26.9053	30.2052	27.4074	28.5873	26.5698	25.66	30.1142	28.8445	26.7146	25.8762	26.9081	29.0912	27.0623	0.20729371	0.698501896	12	12	24	0.209033657	0.768040178	24	7.314687369	0.051937466
ENSMUSG00000010021	Kif19a	kinesin family member 19A [Source:MGI Symbol;Acc:MGI:2447024]	30.227	28.8143	23.6994	32.017	40.6183	27.1269	33.4461	27.4042	36.6032	40.131	32.0281	33.4597	35.7434	25.4046	0.285193697	0.793714531	20	12	24	0.198326215	0.768040178	24	15.65944097	0.15204014
ENSMUSG00000010025	Aldh3a2	aldehyde dehydrogenase family 3, subfamily A2 [Source:MGI Symbol;Acc:MGI:1353452]	35.7679	33.3954	33.1461	33.8946	33.6261	33.995	33.2932	33.9983	30.5606	35.3877	34.0966	34.0794	34.9235	33.5226	0.203345222	0.689605535	24	12	24	0.261336457	0.768040178	24	20.12490738	0.033888174
ENSMUSG00000010045	Tmem115	transmembrane protein 115 [Source:MGI Symbol;Acc:MGI:1930765]	11.4264	9.01169	9.04003	8.92566	8.99597	10.1626	10.844	11.0557	9.44996	11.5256	9.46454	10.066	9.64668	9.08328	0.38940266	0.915479136	4	8	24	0.504877984	0.81121335	24	22.64321993	0.05805845
ENSMUSG00000010047	Hyal2	hyaluronoglucosaminidase 2 [Source:MGI Symbol;Acc:MGI:1196334]	7.01437	4.83483	6.18885	5.04459	4.84197	5.75167	5.31101	6.67493	6.40378	5.42767	5.3488	5.00978	5.6318	5.85632	0.38940266	0.915479136	12	8	24	0.207137066	0.768040178	24	4.170296266	0.098580119
ENSMUSG00000010048	Ifrd2	interferon-related developmental regulator 2 [Source:MGI Symbol;Acc:MGI:1316708]	3.72111	5.04454	5.40574	7.22938	4.10984	4.37365	3.44712	5.08046	5.73786	4.13165	4.04235	4.47104	4.21852	3.61154	0.097760727	0.501112642	12	16	24	0.065020235	0.663876076	24	10.01205554	0.200158272
ENSMUSG00000010051	Hyal1	hyaluronoglucosaminidase 1 [Source:MGI Symbol;Acc:MGI:96298]	2.18363	2.12215	2.63312	3.0979	2.47381	2.29416	2.43395	2.51567	2.96549	2.47089	2.30838	2.22347	2.417	2.46525	0.018640208	0.256345714	16	8	24	0.0237203	0.584955728	24	10.03146272	0.108642107
ENSMUSG00000010054	Tusc2	tumor suppressor 2, mitochondrial calcium regulator [Source:MGI Symbol;Acc:MGI:1931086]	56.521	44.1728	43.0971	45.2156	45.8372	54.1585	46.1337	50.0404	39.849	51.4934	47.916	49.9554	51.7751	48.6801	0.009640466	0.194720062	4	8	24	0.055628586	0.65534544	24	21.14570256	0.119033695
ENSMUSG00000010057	Nprl2	NPR2 like, GATOR1 complex subunit [Source:MGI Symbol;Acc:MGI:1914482]	18.6107	16.7154	17.1733	16.6859	16.587	16.2773	16.205	17.1255	17.454	16.2201	16.2167	15.7053	17.7505	16.7052	0.125855811	0.555618842	4	20	24	0.176635285	0.768040178	24	3.793244481	0.039920317
ENSMUSG00000010064	Slc38a3	solute carrier family 38, member 3 [Source:MGI Symbol;Acc:MGI:1923507]	2.86214	5.49719	7.71268	5.33315	4.49552	5.48373	5.88635	6.85599	6.84281	4.95309	3.32127	4.77099	3.08785	5.1033	0.059650003	0.406067671	12	8	24	0.09508294	0.71175955	24	7.884640111	0.29003948
ENSMUSG00000010066	Cacna2d2	calcium channel, voltage-dependent, alpha 2/delta subunit 2 [Source:MGI Symbol;Acc:MGI:1929813]	9.80236	8.70517	9.34537	8.71922	7.79184	8.91872	9.73925	8.65855	9.15036	9.60038	7.55761	9.01704	8.73037	9.60732	0.231491055	0.731037655	4	16	24	0.150530508	0.758865539	24	3.717039443	0.07311289
ENSMUSG00000010067	Rassf1	Ras association (RalGDS/AF-6) domain family member 1 [Source:MGI Symbol;Acc:MGI:1928386]	8.47197	9.84205	10.9103	12.0784	11.5896	11.2017	9.61399	11.1165	12.3607	9.79391	11.8384	10.4441	10.6347	9.91931	0.061928188	0.409814092	12	16	24	0.070882228	0.676359558	24	12.56823982	0.113007672
ENSMUSG00000010080	Epn3	epsin 3 [Source:MGI Symbol;Acc:MGI:1919139]	8.35036	5.86842	5.52705	7.44795	6.105	6.59328	7.74628	8.83089	5.31197	9.60806	6.1789	7.40209	6.97496	7.50529	0.643325306	0.966018836	4	8	24	0.812568171	0.94007069	24	23.0710184	0.058070759
ENSMUSG00000010086	Rnf112	ring finger protein 112 [Source:MGI Symbol;Acc:MGI:106611]	56.1163	48.8798	41.7792	46.8446	49.8631	51.6222	47.6605	51.9462	43.5636	50.6372	45.3831	52.5275	56.0916	52.3819	0.017495149	0.255326033	4	8	24	0.033684047	0.606793499	24	22.54756925	0.113398698
ENSMUSG00000010095	Slc3a2	solute carrier family 3 (activators of dibasic and neutral amino acid transport), member 2 [Source:MGI Symbol;Acc:MGI:96955]	61.9172	48.9702	52.5873	52.0816	55.6598	58.1783	57.5251	57.0859	47.6372	65.0631	48.8017	54.3844	51.8882	49.4342	0.622132989	0.966018836	24	12	24	0.47387522	0.805393068	24	19.45142543	0.065068605
ENSMUSG00000010097	Nxf1	nuclear RNA export factor 1 [Source:MGI Symbol;Acc:MGI:1858330]	39.2599	45.8438	46.6487	46.0832	45.377	42.542	47.8964	41.3978	50.0638	37.7537	48.1022	46.6554	42.5603	37.3795	0.960333395	0.967043964	12	20	24	0.698627666	0.899819721	24	14.60542576	0.043405873
ENSMUSG00000010110	Stx5a	syntaxin 5A [Source:MGI Symbol;Acc:MGI:1928483]	40.5709	46.0867	44.4189	45.1349	45.9997	41.7335	44.3212	40.9381	43.9725	39.3303	42.8271	40.5559	43.7194	41.6258	0.924770496	0.966018836	12	12	24	0.850986675	0.955422325	24	9.750470037	0.015680593
ENSMUSG00000010122	Slc47a1	solute carrier family 47, member 1 [Source:MGI Symbol;Acc:MGI:1914723]	10.7147	13.0898	8.66722	6.87543	9.8693	8.92021	10.5987	12.5888	14.7433	4.29372	8.52501	9.76753	7.84529	10.7932	0.004349449	0.140598674	8	8	24	0.100201139	0.71175955	24	2.441559231	0.301521453
ENSMUSG00000010136	Pifo	primary cilia formation [Source:MGI Symbol;Acc:MGI:1923670]	0.594309	1.04308	0.939929	0.963807	1.01004	0.85791	1.64744	0.773547	1.1325	1.39221	0.816722	1.17213	0.576111	0.96977	0.922029406	0.966018836	16	12	24	0.666806845	0.887459929	24	11.51362494	0.073641661
ENSMUSG00000010142	Tnfrsf13b	tumor necrosis factor receptor superfamily, member 13b [Source:MGI Symbol;Acc:MGI:1889411]	0.95191	1.23768	1.28273	0.782488	0.946143	0.850427	0.970763	1.14502	1.21671	0.917734	0.925601	1.28896	0.858501	0.730993	0.888379597	0.966018836	12	4	24	0.686705471	0.895971917	24	5.804377148	0.046810946
ENSMUSG00000010154	Spire2	spire type actin nucleation factor 2 [Source:MGI Symbol;Acc:MGI:2446256]	25.5301	20.1422	17.9318	18.8326	22.0142	21.2191	20.9325	22.1804	19.1522	26.5145	21.4963	22.7703	24.1988	21.928	0.300823756	0.809362476	4	8	24	0.319494308	0.768040178	24	20.95102986	0.088913413
ENSMUSG00000010205	Raver1	ribonucleoprotein, PTB-binding 1 [Source:MGI Symbol;Acc:MGI:1919016]	5.20588	5.65135	5.0129	4.99929	4.75999	5.56936	5.49491	6.21266	5.82124	8.43126	5.98898	6.00156	6.01704	5.74015	0.830949782	0.966018836	8	12	24	0.71004604	0.904113856	24	9.639961295	0.049932245
ENSMUSG00000010277	2610507B11Rik	RIKEN cDNA 2610507B11 gene [Source:MGI Symbol;Acc:MGI:1919753]	47.9092	40.4884	47.8765	48.19	43.9922	46.9969	42.9502	46.702	40.53	52.3412	44.0818	44.087	45.2518	45.6258	0.941579937	0.966018836	16	4	24	0.611525122	0.865117073	24	13.46506639	0.038505916
ENSMUSG00000010290	AI597479	expressed sequence AI597479 [Source:MGI Symbol;Acc:MGI:2138299]	13.005	12.975	12.0516	13.1018	12.4261	12.5007	13.201	11.6745	12.3617	12.093	12.2671	12.4917	11.8644	12.4952	0.891105762	0.966018836	4	8	24	0.597593118	0.858622259	24	20.94201856	0.018590578
ENSMUSG00000010307	Tmem86a	transmembrane protein 86A [Source:MGI Symbol;Acc:MGI:1915143]	8.48035	10.4078	9.87435	9.77061	8.84261	9.02378	6.76323	10.5815	11.8174	10.0724	8.68748	9.67432	8.75869	10.392	0.002094047	0.100255953	8	20	24	0.02294331	0.581965446	24	8.526448104	0.171234205
ENSMUSG00000010358	Ifi35	interferon-induced protein 35 [Source:MGI Symbol;Acc:MGI:1917360]	18.0438	16.4699	17.1631	16.6773	21.0429	18.0216	17.2315	17.4794	18.0785	13.2294	19.731	16.8362	17.0229	16.1228	0.251986462	0.75129457	20	12	24	0.336797729	0.771960538	24	19.16487898	0.083352138
ENSMUSG00000010362	Rdm1	RAD52 motif 1 [Source:MGI Symbol;Acc:MGI:1913849]	12.5332	16.5567	13.868	14.6719	17.9398	11.168	15.9768	14.463	15.1422	12.0464	15.9359	16.6858	17.304	12.9084	0.91241026	0.966018836	20	20	24	0.922659664	0.979411448	24	19.72166522	0.032953216
ENSMUSG00000010376	Nedd8	neural precursor cell expressed, developmentally down-regulated gene 8 [Source:MGI Symbol;Acc:MGI:97301]	184.98	199.477	183.699	185.331	203.653	189.623	173.818	179.772	202.218	149.546	197.564	178.622	192.999	177.344	0.952127548	0.965699234	20	8	24	0.876615818	0.965699234	24	20.88301968	0.024100784
ENSMUSG00000010392	Gosr1	golgi SNAP receptor complex member 1 [Source:MGI Symbol;Acc:MGI:1858260]	15.205	14.0755	14.9238	14.7279	13.8844	14.3767	15.2542	15.3346	12.0448	16.3839	13.436	14.2187	14.7753	14.302	0.622132989	0.966018836	4	16	24	0.845035598	0.953147907	24	23.64807607	0.022777215
ENSMUSG00000010406	Mrpl52	mitochondrial ribosomal protein L52 [Source:MGI Symbol;Acc:MGI:1916086]	62.8843	58.9353	50.8921	51.0291	58.3201	54.1095	59.2509	69.2809	79.8981	70.5392	84.3354	74.3398	80.5689	80.4307	0.881996584	0.966018836	4	12	24	0.723195823	0.909188168	24	3.685967758	0.046846345
ENSMUSG00000010453	Kansl3	KAT8 regulatory NSL complex subunit 3 [Source:MGI Symbol;Acc:MGI:1918055]	22.5149	19.7631	20.0581	22.3832	21.5178	22.6501	22.7499	21.1436	20.5547	25.0753	20.7791	21.7126	22.1776	20.9394	0.085992778	0.466404237	24	12	24	0.367711959	0.791015017	24	17.5953647	0.04767688
ENSMUSG00000010461	Eya4	EYA transcriptional coactivator and phosphatase 4 [Source:MGI Symbol;Acc:MGI:1337104]	0.610178	1.12056	1.0429	1.40859	0.9772	1.09966	1.16743	1.34736	1.65146	1.37809	1.00152	1.14196	0.922812	1.16557	0.211626905	0.701016641	16	4	24	0.034869746	0.608551791	24	9.515223884	0.15676943
ENSMUSG00000010476	Ebf3	early B cell factor 3 [Source:MGI Symbol;Acc:MGI:894289]	19.7498	18.5273	19.3884	19.6206	14.925	18.1226	16.8486	18.2969	17.1005	23.3763	16.0417	19.4017	19.1747	19.8042	0.503972924	0.966018836	16	4	24	0.543858777	0.831894202	24	6.805030643	0.067643499
ENSMUSG00000010492	Uckl1os	uridine-cytidine kinase 1-like 1, opposite strand [Source:MGI Symbol;Acc:MGI:3801877]	1.83486	1.86917	2.18115	2.42405	1.57443	1.42456	1.2065	3.17279	2.26848	2.40102	1.80421	2.04558	1.71775	2.26649	0.019088001	0.257576951	16	4	24	0.025534832	0.586541342	24	8.326473155	0.228702728
ENSMUSG00000010505	Myt1	myelin transcription factor 1 [Source:MGI Symbol;Acc:MGI:1100535]	18.1462	13.534	14.9997	15.4396	16.1201	18.3725	13.8466	15.0542	11.4733	15.64	16.1046	17.3153	15.1694	14.535	9.43E-04	0.072887999	24	8	24	0.013315563	0.529134128	24	19.04478979	0.170788437
ENSMUSG00000010517	Faf1	Fas-associated factor 1 [Source:MGI Symbol;Acc:MGI:109419]	18.8904	17.9845	18.8864	17.9169	16.1193	18.7927	17.0213	18.2286	15.4506	17.268	17.353	17.4897	18.291	17.749	0.55109836	0.966018836	4	16	24	0.341895937	0.776129793	24	0.193323235	0.043507891
ENSMUSG00000010529	Gm266	predicted gene 266 [Source:MGI Symbol;Acc:MGI:2685112]	0.452718	0.728984	0.943875	0.607021	0.424463	0.729684	0.644845	0.676506	0.850995	1.14639	0.961325	1.08077	0.77811	1.03913	0.941885246	0.966018836	16	12	24	0.264307786	0.768040178	24	9.426649281	0.090329117
ENSMUSG00000010538	Tsacc	TSSK6 activating co-chaperone [Source:MGI Symbol;Acc:MGI:1924177]	2.46056	2.72276	3.08861	3.55386	3.10656	3.50619	2.18543	2.82114	2.63349	2.67641	4.18119	2.70099	2.97596	2.86051	0.133764886	0.568189321	20	8	24	0.080881691	0.696856099	24	14.6442712	0.144138393
ENSMUSG00000010554	Mettl16	methyltransferase like 16 [Source:MGI Symbol;Acc:MGI:1914743]	9.07402	8.36204	7.27882	7.33844	9.67709	8.82206	11.0649	8.49224	7.89265	6.74151	8.26138	8.20842	9.19928	8.73399	7.21E-04	0.071344007	4	12	24	0.008087758	0.494192776	24	22.61117613	0.171325149
ENSMUSG00000010607	Pigyl	phosphatidylinositol glycan anchor biosynthesis, class Y-like [Source:MGI Symbol;Acc:MGI:1913518]	19.9314	25.8205	25.7313	18.4789	17.8608	18.5385	22.0169	23.5433	26.0046	20.3533	24.7547	23.8333	25.5735	25.4927	0.050447964	0.385880335	12	4	24	0.027583238	0.590220615	24	5.037749441	0.169607815
ENSMUSG00000010608	Rbm25	RNA binding motif protein 25 [Source:MGI Symbol;Acc:MGI:1914289]	39.7079	51.8346	47.3703	48.8742	52.3197	45.5493	44.8602	43.2063	53.7576	41.7337	45.4463	46.0633	46.2831	46.9245	0.55109836	0.966018836	12	16	24	0.466092187	0.805393068	24	10.18094197	0.059996498
ENSMUSG00000010609	Psen2	presenilin 2 [Source:MGI Symbol;Acc:MGI:109284]	18.3576	14.7623	13.2212	15.6552	16.3941	18.2677	14.7375	15.4643	13.4803	17.3331	16.0987	17.1169	16.1819	14.9981	0.010886922	0.200176658	24	12	24	0.01249093	0.525280095	24	19.23726293	0.141989145
ENSMUSG00000010651	Acaa1b	acetyl-Coenzyme A acyltransferase 1B [Source:MGI Symbol;Acc:MGI:3605455]	0.288224	0.183772	1.23254	1.92235	0.362348	0.510532	0.513858	1.13777	0.419634	1.17261	1.21276	0.8246	1.0162	0.90299	0.356926984	0.881188925	16	12	24	0.161064619	0.765941695	24	11.39192072	0.256892094
ENSMUSG00000010660	Plcd1	phospholipase C, delta 1 [Source:MGI Symbol;Acc:MGI:97614]	6.15622	6.77175	6.62551	6.42402	7.23214	6.73162	6.52303	7.40012	7.15537	6.71119	6.5906	6.33082	6.48965	6.30773	0.72778087	0.966018836	12	16	24	0.502123947	0.809328841	24	9.879695825	0.032328561
ENSMUSG00000010663	Fads1	fatty acid desaturase 1 [Source:MGI Symbol;Acc:MGI:1923517]	52.6251	51.2507	52.1699	50.3613	56.4528	56.4141	58.0712	51.1555	51.7166	60.492	47.9954	55.5188	51.7336	51.9548	0.55109836	0.966018836	24	8	24	0.581651989	0.850916398	24	18.52260644	0.038495663
ENSMUSG00000010721	Lmbr1	limb region 1 [Source:MGI Symbol;Acc:MGI:1861746]	14.6158	14.4568	14.4822	14.8388	13.6511	15.1161	13.3689	13.5446	12.8588	14.46	13.6031	14.3336	15.4592	14.4962	0.916556573	0.966018836	24	12	24	0.674539522	0.890827014	24	21.54500347	0.025545554
ENSMUSG00000010751	Tnfrsf22	tumor necrosis factor receptor superfamily, member 22 [Source:MGI Symbol;Acc:MGI:1930270]	1.74445	1.76645	1.55257	1.5647	1.10274	1.61018	1.52732	1.69464	1.7115	1.56994	1.66	1.37525	1.60728	1.25402	0.910436409	0.966018836	8	12	24	0.509967265	0.813985618	24	4.279338715	0.04981815
ENSMUSG00000010755	Cars	cysteinyl-tRNA synthetase [Source:MGI Symbol;Acc:MGI:1351477]	16.0552	10.159	16.3146	10.9514	12.0865	11.9447	15.5751	15.2407	16.1427	12.7363	12.6937	12.7514	12.6556	12.7986	0.308023687	0.814993005	12	4	24	0.481841099	0.805393068	24	4.24552098	0.097355621
ENSMUSG00000010760	Phlda2	pleckstrin homology like domain, family A, member 2 [Source:MGI Symbol;Acc:MGI:1202307]	0.552171	1.89391	0.364381	0.455404	0.582345	0.694436	1.25517	0.651492	0.290288	0.398762	1.23525	0.880564	0.760645	0.69507	0.047000349	0.375011045	8	4	24	0.216339801	0.768040178	24	22.78424896	0.21869454
ENSMUSG00000010803	Gabra1	gamma-aminobutyric acid (GABA) A receptor, subunit alpha 1 [Source:MGI Symbol;Acc:MGI:95613]	29.4435	25.1896	22.5107	26.3505	24.5547	25.8832	24.9528	23.773	25.1895	22.7773	27.8059	24.1368	29.0409	28.2139	0.43565473	0.966018836	4	8	24	0.276463022	0.768040178	24	22.74204107	0.073640576
ENSMUSG00000010825	Grid2ip	glutamate receptor, ionotropic, delta 2 (Grid2) interacting protein 1 [Source:MGI Symbol;Acc:MGI:2176213]	1.33967	1.02749	0.882071	0.792981	1.77304	1.07773	1.61916	1.30593	1.14915	1.21771	1.6089	0.896586	1.10364	1.27413	0.428730128	0.966018836	20	20	24	0.607407009	0.863866659	24	19.8554917	0.077802161
ENSMUSG00000010830	Kdelr3	KDEL (Lys-Asp-Glu-Leu) endoplasmic reticulum protein retention receptor 3 [Source:MGI Symbol;Acc:MGI:2145953]	13.3154	14.3542	11.5477	10.2373	13.721	11.1668	14.0504	14.4276	15.1258	10.9574	11.4558	11.6432	14.1572	12.3282	0.01457641	0.247599677	8	8	24	0.065636262	0.667622064	24	2.585468516	0.140049169
ENSMUSG00000010911	Apip	APAF1 interacting protein [Source:MGI Symbol;Acc:MGI:1926788]	20.3301	18.5657	20.0606	18.0704	17.9058	20.427	21.0134	19.9081	19.2761	15.8286	21.554	18.1637	20.5015	18.8306	0.059513104	0.406067671	4	12	24	0.162377646	0.76636332	24	23.55488787	0.080607152
ENSMUSG00000010914	Pdhx	pyruvate dehydrogenase complex, component X [Source:MGI Symbol;Acc:MGI:1351627]	16.6048	15.9875	15.9184	17.646	15.2035	17.3423	16.2133	17.885	14.563	17.3624	17.5289	16.1473	16.6502	17.0361	0.897425765	0.966018836	16	20	24	0.918086366	0.977931782	24	18.90132743	0.01393661
ENSMUSG00000010936	Vac14	Vac14 homolog (S. cerevisiae) [Source:MGI Symbol;Acc:MGI:2157980]	5.93494	5.34756	5.5202	6.27783	5.84155	6.36639	5.63346	5.75632	5.52625	7.92713	5.41296	6.2732	5.70815	6.15509	0.44861185	0.966018836	16	20	24	0.396168747	0.799546792	24	14.15239961	0.064324677
ENSMUSG00000011096	Akt1s1	AKT1 substrate 1 (proline-rich) [Source:MGI Symbol;Acc:MGI:1914855]	19.9274	14.7766	12.6777	16.7478	13.6535	14.1167	18.2497	20.5618	19.1349	23.0609	22.3454	23.106	22.1475	20.8658	0.891022863	0.966018836	24	12	24	0.847758249	0.954054905	24	0.144363938	0.046458457
ENSMUSG00000011114	Tbrg1	transforming growth factor beta regulated gene 1 [Source:MGI Symbol;Acc:MGI:1100877]	26.8849	25.6428	24.8709	22.3638	24.2319	25.0894	24.2152	27.7541	24.3517	26.1089	24.7303	25.4399	25.7203	25.6715	0.210154775	0.69936851	8	12	24	0.131427915	0.741500219	24	1.807355467	0.052937239
ENSMUSG00000011148	Adssl1	adenylosuccinate synthetase like 1 [Source:MGI Symbol;Acc:MGI:87947]	7.1972	16.1833	20.0604	21.9813	14.3993	19.4209	14.581	36.5719	26.0596	33.1586	14.665	15.4495	19.9838	17.3517	0.076247883	0.453790923	16	4	24	0.047659974	0.635670298	24	9.157051627	0.470805471
ENSMUSG00000011154	Cfap161	cilia and flagella associated protein 161 [Source:MGI Symbol;Acc:MGI:1922806]	0.338487	0.759078	0.552344	0.346237	0.594202	0.353749	0.785392	0.121449	0.540119	0.52012	0.443172	0.867257	0.320324	0.591426	0.823246279	0.966018836	24	16	24	0.954309054	0.988601783	24	18.36987836	0.025742269
ENSMUSG00000011158	Brf1	BRF1, RNA polymerase III transcription initiation factor 90 kDa subunit [Source:MGI Symbol;Acc:MGI:1919558]	14.1443	15.7572	14.3166	16.0538	15.6316	13.761	15.2039	15.1402	15.6655	16.8683	14.4198	14.8449	16.0224	14.8228	0.424718357	0.966018836	16	8	24	0.254645257	0.768040178	24	9.828962358	0.049662044
ENSMUSG00000011179	Odc1	ornithine decarboxylase, structural 1 [Source:MGI Symbol;Acc:MGI:97402]	15.9599	21.6833	22.3379	25.3673	24.7368	21.371	21.2406	20.1618	24.8494	21.3282	19.0275	21.812	18.0666	16.4024	0.029756552	0.323426482	12	16	24	0.05991038	0.658304792	24	12.60421705	0.161435085
ENSMUSG00000011254	Thg1l	tRNA-histidine guanylyltransferase 1-like (S. cerevisiae) [Source:MGI Symbol;Acc:MGI:1913878]	4.36573	3.92091	4.43682	4.22065	4.07495	3.87287	3.58156	4.37304	4.03604	3.84409	4.20522	4.1043	4.05882	4.24026	0.939103211	0.966018836	12	12	24	0.632708857	0.875112738	24	8.109962893	0.026961446
ENSMUSG00000011256	Adam19	a disintegrin and metallopeptidase domain 19 (meltrin beta) [Source:MGI Symbol;Acc:MGI:105377]	14.0422	9.21346	10.2533	9.45312	11.2157	12.8187	11.05	11.1284	7.63415	13.0232	11.1359	10.875	10.8019	11.2283	0.593560247	0.966018836	20	16	24	0.135773783	0.747972781	24	20.22571299	0.156923403
ENSMUSG00000011257	Pabpc4	poly(A) binding protein, cytoplasmic 4 [Source:MGI Symbol;Acc:MGI:2385206]	3.09638	3.23714	4.03358	4.27561	3.08035	3.1629	3.38352	4.09425	4.15391	5.19365	3.41645	3.51576	3.48346	3.31727	0.055569815	0.401074772	16	4	24	0.009096103	0.502965379	24	9.968581129	0.182766454
ENSMUSG00000011267	Zfp296	zinc finger protein 296 [Source:MGI Symbol;Acc:MGI:1926956]	1.38009	0.627852	0.595983	0.658518	0.865535	0.807317	0.659564	0.743763	0.48795	0.490321	0.884714	0.870512	0.936996	0.657085	0.009118941	0.192760771	4	8	24	0.009990463	0.503967859	24	21.08828289	0.184632838
ENSMUSG00000011305	Plin5	perilipin 5 [Source:MGI Symbol;Acc:MGI:1914218]	0.630157	0.740939	1.60799	3.63148	1.177	0.818335	0.770114	2.86942	1.50204	2.62628	1.67983	1.18697	2.64126	1.96455	0.435739761	0.966018836	16	12	24	0.05877035	0.658304792	24	9.976506953	0.389097053
ENSMUSG00000011306	Sugp1	SURP and G patch domain containing 1 [Source:MGI Symbol;Acc:MGI:1917866]	9.65841	11.2253	11.839	11.2573	10.7026	10.8658	9.99479	10.6781	12.9344	13.8563	10.2408	10.8348	11.0297	12.093	0.032514053	0.324908923	12	16	24	0.003881398	0.427431763	24	9.448005571	0.13875917
ENSMUSG00000011382	Dhdh	dihydrodiol dehydrogenase (dimeric) [Source:MGI Symbol;Acc:MGI:1919005]	5.03151	5.44769	5.21993	7.05356	5.61191	5.62985	5.84299	5.67933	5.78567	6.48201	6.12614	6.3734	6.92217	6.32647	0.907000607	0.966018836	16	20	24	0.403115255	0.799546792	24	13.43715639	0.048972854
ENSMUSG00000011427	Zfp790	zinc finger protein 790 [Source:MGI Symbol;Acc:MGI:1923431]	6.39673	5.01998	5.32461	6.06169	5.85498	5.58851	5.38554	5.65467	6.04328	5.35575	6.07963	6.50335	5.79505	5.5924	0.304473123	0.811201458	24	8	24	0.27528067	0.768040178	24	18.11134348	0.05686943
ENSMUSG00000011463	Cpb1	carboxypeptidase B1 (tissue) [Source:MGI Symbol;Acc:MGI:1923953]	2.26192	2.3504	1.78741	2.36803	2.60498	2.29234	1.85619	2.39555	2.38686	1.85186	2.41958	2.12368	2.19045	2.06294	0.883024237	0.966018836	20	8	24	0.651481516	0.882823265	24	17.31338387	0.042095654
ENSMUSG00000011589	Fsd1	fibronectin type 3 and SPRY domain-containing protein [Source:MGI Symbol;Acc:MGI:1934858]	21.0428	17.2003	15.5769	19.0761	15.9927	19.5033	20.3487	20.1502	16.1246	19.3049	16.9504	17.6539	18.1154	18.1874	0.060394272	0.407012001	4	8	24	0.231083623	0.768040178	24	23.21348709	0.086405659
ENSMUSG00000011658	Fuz	fuzzy planar cell polarity protein [Source:MGI Symbol;Acc:MGI:1917550]	6.62446	7.30617	8.0006	7.65365	8.12991	7.30008	8.41234	7.91923	7.51147	8.533	6.47697	8.17448	7.92122	8.22961	0.770162999	0.966018836	16	4	24	0.67880394	0.892520664	24	7.55360197	0.037031817
ENSMUSG00000011751	Sptbn4	spectrin beta, non-erythrocytic 4 [Source:MGI Symbol;Acc:MGI:1890574]	6.59645	6.25323	7.08898	5.70538	5.7646	6.7085	7.05416	6.68166	6.95712	8.95155	7.15668	8.66515	7.81968	8.31113	0.79256873	0.966018836	24	8	24	0.831337705	0.947661285	24	3.42145106	0.02864512
ENSMUSG00000011752	Pgam1	phosphoglycerate mutase 1 [Source:MGI Symbol;Acc:MGI:97552]	19.9498	16.0406	16.7473	16.6284	15.0847	18.5251	18.2856	19.1577	15.8692	23.1882	19.0102	19.0254	18.7552	21.5138	0.934110497	0.966018836	8	4	24	0.824253291	0.944824178	24	0.34782762	0.035698572
ENSMUSG00000011831	Evi5	ecotropic viral integration site 5 [Source:MGI Symbol;Acc:MGI:104736]	52.1359	54.7592	47.9074	50.9298	54.3444	45.419	48.3534	44.9067	53.837	44.1374	54.9751	51.2603	54.8736	51.4444	0.932671476	0.966018836	20	20	24	0.931146048	0.982264519	24	20.75113266	0.017707145
ENSMUSG00000011832	Evi5l	ecotropic viral integration site 5 like [Source:MGI Symbol;Acc:MGI:2442167]	27.7716	21.9301	23.9665	24.9799	25.2908	24.7921	27.5649	26.0763	24.6251	29.2175	21.5358	26.5204	25.9401	24.1681	0.446653834	0.966018836	4	8	24	0.807999684	0.938931097	24	21.64389656	0.031976738
ENSMUSG00000011837	Snapc2	small nuclear RNA activating complex, polypeptide 2 [Source:MGI Symbol;Acc:MGI:1914861]	9.80892	8.51786	10.6298	8.17992	9.60357	10.1414	9.67632	10.8996	8.72504	10.3237	8.38195	10.3785	9.80232	8.68621	0.922029406	0.966018836	24	20	24	0.658752005	0.884596426	24	22.75754076	0.046318028
ENSMUSG00000011877	Git1	GIT ArfGAP 1 [Source:MGI Symbol;Acc:MGI:1927140]	21.7363	19.3103	19.8262	23.2494	22.3577	19.1059	21.9801	20.2897	20.8452	27.4103	21.028	21.6378	20.8261	19.6533	0.031431762	0.324385159	16	16	24	0.135381191	0.747435826	24	13.70625546	0.094213489
ENSMUSG00000011884	Gltp	glycolipid transfer protein [Source:MGI Symbol;Acc:MGI:1929253]	17.5708	17.1629	17.9601	16.3574	16.9005	17.667	18.8046	17.0468	18.0084	18.4961	16.4829	17.7638	16.4333	16.6166	0.880992536	0.966018836	12	8	24	0.96644325	0.991974271	24	2.765992936	0.006356827
ENSMUSG00000011958	Bnip2	BCL2/adenovirus E1B interacting protein 2 [Source:MGI Symbol;Acc:MGI:109327]	27.5079	29.5712	30.4327	29.6124	31.1559	27.9227	31.2952	29.484	32.5407	28.763	32.2109	30.7589	31.2664	28.0441	0.916746631	0.966018836	20	12	24	0.643124458	0.878809617	24	13.0388237	0.026745735
ENSMUSG00000011960	Ccnt1	cyclin T1 [Source:MGI Symbol;Acc:MGI:1328363]	9.39994	7.66472	8.92647	8.5117	9.05578	8.43383	7.80697	7.00672	8.25946	6.95438	9.53256	7.45413	9.22331	8.17123	0.47028201	0.966018836	20	12	24	0.547575203	0.833732584	24	18.33020945	0.059311012
ENSMUSG00000012017	Scarf2	scavenger receptor class F, member 2 [Source:MGI Symbol;Acc:MGI:1858430]	1.77663	2.4278	2.3354	1.49008	1.63349	1.58042	1.96898	2.37903	3.21219	1.74532	2.56387	2.06437	2.88861	2.20462	0.248056532	0.746745468	12	4	24	0.103512615	0.715292223	24	5.273202081	0.167931836
ENSMUSG00000012076	Brms1l	breast cancer metastasis-suppressor 1-like [Source:MGI Symbol;Acc:MGI:1196337]	33.1617	32.7091	30.1053	31.1649	30.6699	32.9196	31.0677	30.4967	31.0847	30.1523	32.7981	33.1317	33.5193	33.5233	0.094000699	0.490854203	4	8	24	0.041392959	0.618736234	24	22.49222685	0.050610434
ENSMUSG00000012114	Med15	mediator complex subunit 15 [Source:MGI Symbol;Acc:MGI:2137379]	13.9349	12.7994	15.3848	12.2789	13.8569	13.9272	15.4561	14.4159	13.7962	16.6719	12.9739	13.997	13.7353	13.8133	0.826055781	0.966018836	4	16	24	0.910874447	0.974769498	24	6.777484093	0.019383776
ENSMUSG00000012117	Dhdds	dehydrodolichyl diphosphate synthase [Source:MGI Symbol;Acc:MGI:1914672]	20.4943	17.3182	18.3127	18.5774	18.4028	21.6448	19.3647	19.224	15.2713	23.5792	15.6663	18.0965	18.9844	20.1791	0.849179819	0.966018836	4	8	24	0.796104391	0.93482476	24	21.76730563	0.044220326
ENSMUSG00000012126	Ubxn11	UBX domain protein 11 [Source:MGI Symbol;Acc:MGI:1914836]	1.71174	1.87605	2.90119	1.79297	1.79104	2.1326	3.28559	2.32942	2.09019	1.68591	1.44487	2.18551	1.29246	1.98827	0.664568268	0.966018836	12	4	24	0.56335885	0.842513738	24	3.942333964	0.099400043
ENSMUSG00000012187	Mogat1	monoacylglycerol O-acyltransferase 1 [Source:MGI Symbol;Acc:MGI:1915643]	0.622982	0.68391	0.406244	0.771067	0.410562	0.53463	0.572916	0.778618	0.800128	0.625782	0.538571	0.514895	0.751053	0.9944	0.016024075	0.253395174	8	12	24	0.03457429	0.608551791	24	5.289442275	0.123198617
ENSMUSG00000012296	Tjap1	tight junction associated protein 1 [Source:MGI Symbol;Acc:MGI:1921344]	9.57724	10.1664	8.38508	11.271	9.99448	10.015	11.0667	10.2918	11.9878	12.157	10.9488	9.15003	10.7424	9.56381	0.657586167	0.966018836	16	8	24	0.470946443	0.805393068	24	11.76379647	0.063986272
ENSMUSG00000012405	Rpl15	ribosomal protein L15 [Source:MGI Symbol;Acc:MGI:1913730]	32.7645	35.7934	33.9224	32.8409	33.2409	32.4231	34.1006	34.3375	34.2341	30.9103	35.1317	33.745	34.5729	35.3043	0.030347909	0.324150946	8	8	24	0.165535608	0.767020009	24	3.281780793	0.037738138
ENSMUSG00000012422	Tmem167	transmembrane protein 167 [Source:MGI Symbol;Acc:MGI:1913324]	12.3915	12.7937	12.1566	12.8489	12.8462	12.0331	11.2982	11.1831	12.7257	10.1541	12.9388	11.0099	11.6929	12.061	0.503972924	0.966018836	20	4	24	0.900147382	0.971693683	24	12.9233572	0.016245683
ENSMUSG00000012428	Steap4	STEAP family member 4 [Source:MGI Symbol;Acc:MGI:1923560]	6.85121	7.02383	8.16281	9.70998	9.82652	7.05281	8.15081	8.77735	8.01636	6.97872	10.0084	7.08191	9.10213	7.48013	0.481530854	0.966018836	20	4	24	0.372224024	0.793651569	24	13.58811315	0.095951875
ENSMUSG00000012429	Mplkip	M-phase specific PLK1 intereacting protein [Source:MGI Symbol;Acc:MGI:1913558]	6.39921	8.02818	6.79845	6.82098	7.34314	7.25473	5.56117	7.60981	7.9802	5.82448	7.44666	7.27205	7.24267	7.71628	0.265774856	0.773510818	8	20	24	0.762876635	0.923505639	24	6.314830822	0.041822397
ENSMUSG00000012443	Kif11	kinesin family member 11 [Source:MGI Symbol;Acc:MGI:1098231]	1.15526	1.1564	1.47032	1.51131	1.30049	1.45295	1.29871	1.03679	1.065	0.805687	1.11021	1.35175	1.46456	1.05989	0.760245023	0.966018836	24	8	24	0.384855977	0.799546792	24	20.14092252	0.075232807

ENSMUSG00000013236	Ptprs	protein tyrosine phosphatase, receptor type, S [Source:MGI Symbol;Acc:MGI:97815]	47.0488	43.9904	46.3429	44.1263	45.9685	44.7637	50.5259	45.4965	47.7909	55.4898	48.3309	45.9518	48.9643	46.8317	0.93717134	0.966018836	4	4	24	0.816207746	0.94140005	24	11.97925356	0.020296949
ENSMUSG00000013275	Slc41a1	solute carrier family 41, member 1 [Source:MGI Symbol;Acc:MGI:2444823]	8.12612	9.70502	10.4591	10.0261	9.19696	9.82536	10.1679	10.2502	10.7745	10.9062	7.8051	9.44936	7.50043	9.37686	0.053674542	0.394331071	16	4	24	0.115454968	0.727799063	24	8.99480406	0.11999121
ENSMUSG00000013367	Iglon5	IgLON family member 5 [Source:MGI Symbol;Acc:MGI:2686277]	6.27846	5.63849	7.16636	5.572	5.69821	6.49228	6.55374	6.6134	6.49237	7.7185	6.23864	6.49819	5.58916	7.45633	0.885025617	0.966018836	12	8	24	0.45096392	0.805393068	24	7.822413612	0.064219544
ENSMUSG00000013419	Zfp651	zinc finger protein 651 [Source:MGI Symbol;Acc:MGI:2670992]	20.8978	19.9148	19.0618	21.5481	20.501	18.6343	21.0251	21.2702	23.1415	24.3358	21.423	19.9354	21.5999	18.9792	0.616047374	0.966018836	16	8	24	0.308645358	0.768040178	24	11.11178409	0.060629518
ENSMUSG00000013465	Nelfb	negative elongation factor complex member B [Source:MGI Symbol;Acc:MGI:1931035]	22.7172	18.3164	18.7507	19.6718	20.748	20.0191	19.4154	20.4564	19.6169	22.0577	19.0067	20.6324	20.2867	19.6675	0.483881213	0.966018836	24	12	24	0.576570538	0.848661503	24	19.22112769	0.035220143
ENSMUSG00000013483	Card14	caspase recruitment domain family, member 14 [Source:MGI Symbol;Acc:MGI:2386258]	1.62757	1.37901	1.32038	1.28389	1.56893	1.4906	1.52442	1.68206	1.21251	1.03342	1.33634	1.26824	1.45148	1.4558	0.057224139	0.401309455	4	12	24	0.023202883	0.581965446	24	23.55620208	0.099374257
ENSMUSG00000013495	Tmem175	transmembrane protein 175 [Source:MGI Symbol;Acc:MGI:1919642]	19.7216	17.8881	18.535	19.2476	20.9658	20.7113	19.27	19.4013	17.763	20.3904	19.9321	19.0236	18.3648	18.3464	0.018923071	0.256345714	20	16	24	0.007027823	0.477130707	24	17.19337179	0.075654282
ENSMUSG00000013523	Bcas1	brain enriched myelin associated protein 1 [Source:MGI Symbol;Acc:MGI:1924210]	70.3215	91.9334	89.2225	70.636	77.0733	72.6218	95.1752	78.0993	93.9524	75.2379	67.0308	80.3692	77.538	81.5734	0.083608421	0.463738799	12	4	24	0.084214783	0.702277994	24	4.980070684	0.13308324
ENSMUSG00000013539	Tango2	transport and golgi organization 2 [Source:MGI Symbol;Acc:MGI:101825]	73.9564	71.4281	67.9266	68.6239	74.8817	72.4861	66.2503	70.3898	69.7753	65.9612	74.6324	70.0631	69.421	72.6054	0.129903614	0.560263689	20	20	24	0.414638105	0.799824823	24	19.7156072	0.031533032
ENSMUSG00000013584	Aldh1a2	aldehyde dehydrogenase family 1, subfamily A2 [Source:MGI Symbol;Acc:MGI:107928]	6.46483	9.61288	6.54348	5.02068	5.85924	5.08979	6.38154	7.8927	10.4251	2.83805	5.63536	6.58174	5.00613	7.13154	0.0140901	0.241938013	12	4	24	0.102026289	0.713240751	24	3.900164599	0.298725993
ENSMUSG00000013593	Ndufs2	NADH:ubiquinone oxidoreductase core subunit S2 [Source:MGI Symbol;Acc:MGI:2385112]	106.434	102.489	111.331	129.332	112.586	120.974	113.968	138.749	103.7	126.621	113.485	109.427	121.439	117.981	0.865490576	0.966018836	16	20	24	0.845309219	0.953294887	24	11.36273047	0.028389264
ENSMUSG00000013622	Atraid	all-trans retinoic acid induced differentiation factor [Source:MGI Symbol;Acc:MGI:1918918]	49.6469	60.4819	61.9339	60.3638	62.2739	53.2186	53.975	59.2068	69.9842	53.3726	60.7639	60.5853	62.4548	56.8048	0.619356354	0.966018836	12	16	24	0.231787167	0.768040178	24	9.714501867	0.081955162
ENSMUSG00000013629	Cad	carbamoyl-phosphate synthetase 2, aspartate transcarbamylase, and dihydroorotase [Source:MGI Symbol;Acc:MGI:1916969]	2.90166	2.98865	4.28424	3.79601	3.17816	4.02695	4.04905	3.89196	3.51296	4.49109	3.94218	3.07883	3.34074	3.54067	0.685043116	0.966018836	16	12	24	0.244362166	0.768040178	24	11.10309411	0.100715802
ENSMUSG00000013646	Sh3bp5l	SH3 binding domain protein 5 like [Source:MGI Symbol;Acc:MGI:1933124]	28.7588	25.7265	22.6034	26.8678	24.9533	28.0286	25.7606	27.9089	23.0063	32.1379	25.2724	27.2779	26.794	27.1433	0.245061943	0.741580974	24	12	24	0.666846833	0.887459929	24	20.15831978	0.04619468
ENSMUSG00000013662	Atad1	ATPase family, AAA domain containing 1 [Source:MGI Symbol;Acc:MGI:1915229]	59.0165	64.4928	61.3377	59.9172	59.5704	56.2728	59.4722	59.6749	64.3464	54.63	63.3148	57.4991	59.0688	60.1064	0.010049466	0.194720062	12	12	24	0.321313764	0.768040178	24	6.659172446	0.039790187
ENSMUSG00000013663	Pten	phosphatase and tensin homolog [Source:MGI Symbol;Acc:MGI:109583]	32.2531	30.2523	30.1544	33.1482	31.6253	30.1224	28.7993	28.5945	29.6232	30.4149	32.3944	30.5361	32.0452	32.688	0.869721097	0.966018836	16	20	24	0.540978379	0.830298514	24	15.67618982	0.028727445
ENSMUSG00000013698	Pea15a	proliferation and apoptosis adaptor protein 15A [Source:MGI Symbol;Acc:MGI:104799]	351.581	360.982	338.627	333.905	395.945	350.469	384.472	346.288	370.093	319.184	348.485	352.581	357.643	322.609	0.305564675	0.811201458	4	12	24	0.32006824	0.768040178	24	20.88969881	0.051117034
ENSMUSG00000013701	Timm23	translocase of inner mitochondrial membrane 23 [Source:MGI Symbol;Acc:MGI:1858317]	9.37768	5.97057	9.15895	3.86445	2.77858	3.11621	5.79869	11.4013	13.4478	10.8539	11.6409	9.97432	13.1604	13.4077	0.519468828	0.966018836	12	12	24	0.062974916	0.66222475	24	5.508585084	0.485867914
ENSMUSG00000013707	Tnfaip8l2	tumor necrosis factor, alpha-induced protein 8-like 2 [Source:MGI Symbol;Acc:MGI:1917019]	0.910864	1.74709	1.93641	1.99721	2.11027	1.67107	1.76882	1.81816	2.06537	1.06671	1.62445	1.846	1.6534	1.60439	0.619356354	0.966018836	12	16	24	0.731123331	0.912382349	24	10.72251636	0.065099023
ENSMUSG00000013736	Trnt1	tRNA nucleotidyl transferase, CCA-adding, 1 [Source:MGI Symbol;Acc:MGI:1917297]	30.1753	31.3131	31.8334	28.9761	31.9225	29.7201	30.006	31.0535	31.0211	28.8009	33.9593	30.0297	30.4325	31.4822	0.280850951	0.792139038	12	12	24	0.966907852	0.992084942	24	9.347355882	0.006567882
ENSMUSG00000013787	Ehmt2	euchromatic histone lysine N-methyltransferase 2 [Source:MGI Symbol;Acc:MGI:2148922]	18.5249	21.0596	22.376	20.4552	18.9828	18.841	21.0601	21.7967	23.8792	21.4834	19.7378	20.9441	20.6793	22.8783	0.00486525	0.141782417	12	12	24	1.03E-04	0.114833119	24	7.115808995	0.120060461
ENSMUSG00000013822	Elof1	ELF1 homolog, elongation factor 1 [Source:MGI Symbol;Acc:MGI:1913376]	33.5593	37.1287	39.5695	39.1787	40.0493	36.0113	36.5031	34.4952	38.4716	32.74	39.9892	38.5892	37.9137	38.5721	0.133764886	0.568189321	20	8	24	0.586049544	0.853257144	24	13.45359426	0.036819446
ENSMUSG00000013833	Med16	mediator complex subunit 16 [Source:MGI Symbol;Acc:MGI:2158394]	8.31256	6.98974	7.52723	7.51644	7.39364	8.32232	8.24938	9.49547	7.15101	9.53486	7.30088	8.41134	8.06616	8.19367	0.649212555	0.966018836	24	20	24	0.769932895	0.92585938	24	23.95391866	0.034431191
ENSMUSG00000013846	St3gal1	ST3 beta-galactoside alpha-2,3-sialyltransferase 1 [Source:MGI Symbol;Acc:MGI:98304]	3.70719	3.28702	3.49658	3.27843	3.44597	3.47867	3.43408	3.70632	2.97384	3.81104	2.70542	3.22903	3.1906	3.30681	0.833301348	0.966018836	8	12	24	0.728544575	0.911275824	24	2.44173466	0.029127072
ENSMUSG00000013858	Tmem259	transmembrane protein 259 [Source:MGI Symbol;Acc:MGI:2177957]	24.2948	21.3368	23.3577	22.954	22.3809	21.8796	24.1429	26.3315	25.0697	30.5621	27.0044	28.2256	28.9278	26.7839	0.756529255	0.966018836	16	20	24	0.902402744	0.972751432	24	9.631198668	0.017846999
ENSMUSG00000013878	Rnf170	ring finger protein 170 [Source:MGI Symbol;Acc:MGI:1924983]	8.46213	9.90741	8.9522	9.60638	9.04523	8.76632	9.44605	9.25258	10.7424	9.50916	9.05257	9.33957	9.94589	10.1421	0.339274762	0.858712466	8	16	24	0.036815256	0.609176161	24	7.066432588	0.068861212
ENSMUSG00000013921	Clip3	CAP-GLY domain containing linker protein 3 [Source:MGI Symbol;Acc:MGI:1923936]	101.905	84.3763	85.4823	96.3108	98.1389	101.515	104.045	103.711	78.6534	165.374	86.4843	107.189	98.3496	108.887	0.46238066	0.966018836	24	12	24	0.823305024	0.94441254	24	16.06509995	0.060723045
ENSMUSG00000013936	Myl2	myosin, light polypeptide 2, regulatory, cardiac, slow [Source:MGI Symbol;Acc:MGI:97272]	15.2671	50.4063	79.3775	72.3746	40.2382	62.4541	38.3478	67.9956	45.378	53.2523	24.7162	9.76667	43.6091	51.396	0.056629114	0.401309455	16	4	24	0.078187271	0.688357494	24	8.324730433	0.70485908
ENSMUSG00000013973	Dedd	death effector domain-containing [Source:MGI Symbol;Acc:MGI:1333874]	15.74	14.6371	15.2925	15.3379	14.965	15.5417	17.2115	15.5906	15.3661	16.261	15.6481	14.9246	15.6979	16.3585	0.591351901	0.966018836	4	20	24	0.698877031	0.899901033	24	1.869188889	0.017881699
ENSMUSG00000013974	Mcemp1	mast cell expressed membrane protein 1 [Source:MGI Symbol;Acc:MGI:1916439]	1.25119	1.78825	3.50049	1.88572	1.36504	1.40871	1.6011	1.25655	1.71155	0.924818	1.47881	1.83753	1.76253	1.28335	0.895137379	0.966018836	12	20	24	0.708836343	0.903901793	24	7.599886956	0.094059674
ENSMUSG00000013997	Nit1	nitrilase 1 [Source:MGI Symbol;Acc:MGI:1350916]	19.2863	15.7472	16.8784	16.9681	17.4097	17.5727	18.5878	18.1488	15.5344	17.6741	17.2311	16.524	17.6666	16.5677	0.067104747	0.425837275	4	8	24	0.181092319	0.768040178	24	21.25296349	0.057828158
ENSMUSG00000014030	Pax5	paired box 5 [Source:MGI Symbol;Acc:MGI:97489]	0.325376	0.615626	0.726063	0.22996	0.687916	0.367037	0.903278	0.282515	1.08839	0.389411	0.307313	0.574645	0.7232	0.501799	0.94693335	0.966018836	12	4	24	0.619153757	0.869347961	24	4.441304158	0.090361863
ENSMUSG00000014039	Prdm15	PR domain containing 15 [Source:MGI Symbol;Acc:MGI:1930121]	2.80386	2.22248	2.61054	3.1034	2.90154	2.43936	3.30739	1.93425	2.37247	2.35886	3.30754	2.49135	2.30462	2.14586	0.004545736	0.140598674	20	12	24	0.067694797	0.671778447	24	16.81692379	0.140340874
ENSMUSG00000014074	Rnf168	ring finger protein 168 [Source:MGI Symbol;Acc:MGI:1917488]	14.8096	13.907	13.1388	13.6966	13.3641	13.7379	12.81	12.3016	12.0931	12.6698	13.8086	13.1661	13.7391	13.4838	0.559102416	0.966018836	4	8	24	0.151855135	0.758865539	24	20.67972825	0.051388892
ENSMUSG00000014075	Dynlt2b	dynein light chain Tctex-type 2B [Source:MGI Symbol;Acc:MGI:1913311]	14.4363	18.8732	17.7671	15.6805	16.6908	13.816	14.6566	15.2848	17.1169	14.8673	16.8794	15.7634	13.2076	16.5267	0.018194955	0.256345714	12	16	24	0.122743915	0.735412761	24	9.203472861	0.104124683
ENSMUSG00000014077	Chp1	calcineurin-like EF hand protein 1 [Source:MGI Symbol;Acc:MGI:1927185]	111.493	91.8421	92.9457	99.222	94.0329	107.89	99.1405	104.489	82.881	114.067	94.9837	100.371	100.976	100.127	0.321662653	0.83607295	24	12	24	0.401912986	0.799546792	24	20.72868351	0.065743564
ENSMUSG00000014158	Trpv4	transient receptor potential cation channel, subfamily V, member 4 [Source:MGI Symbol;Acc:MGI:1926945]	0.766991	0.982582	0.898335	0.860989	1.21201	0.842893	0.83698	1.06802	1.04334	0.995887	0.709926	1.00524	1.0733	0.960897	0.860993221	0.966018836	8	20	24	0.724703788	0.909665611	24	7.596422792	0.033118354
ENSMUSG00000014164	Klhl3	kelch-like 3 [Source:MGI Symbol;Acc:MGI:2445185]	2.77985	2.48022	2.30981	2.73157	2.79711	2.3518	3.11533	2.27092	2.54831	2.70469	2.32386	2.53307	2.38399	2.5528	0.945282252	0.966018836	4	4	24	0.804702178	0.937484836	24	19.08711761	0.025791721
ENSMUSG00000014177	Tvp23b	trans-golgi network vesicle protein 23B [Source:MGI Symbol;Acc:MGI:1914760]	18.9688	17.8989	16.9361	17.7924	18.2995	15.8822	19.5475	15.9175	18.1555	13.788	18.2909	16.3454	17.066	16.6196	0.957256673	0.966536401	4	12	24	0.552031191	0.836550142	24	22.86532767	0.049934798
ENSMUSG00000014195	Dnajc7	DnaJ heat shock protein family (Hsp40) member C7 [Source:MGI Symbol;Acc:MGI:1928373]	144.776	150.238	145.638	149.966	161.91	143.619	147.291	135.548	147.254	133.631	147.147	143.127	152.163	135.355	0.877082091	0.966018836	20	12	24	0.362635287	0.78733344	24	17.87367702	0.038873342
ENSMUSG00000014226	Cacybp	calcyclin binding protein [Source:MGI Symbol;Acc:MGI:1270839]	77.9831	68.8166	60.1557	66.1846	71.09	66.5109	70.1702	63.9432	63.5906	54.9313	78.6485	65.7675	68.2525	67.8403	0.041677362	0.34886238	20	20	24	0.088744762	0.707833669	24	21.16789614	0.109675679
ENSMUSG00000014232	Cluap1	clusterin associated protein 1 [Source:MGI Symbol;Acc:MGI:1924029]	9.66389	11.9507	11.416	9.75582	11.8217	10.2638	11.8302	10.8183	11.9506	10.2967	10.6405	12.1173	9.98365	10.8267	0.896799496	0.966018836	12	4	24	0.90842136	0.974606327	24	6.132917972	0.019670821
ENSMUSG00000014243	Zswim7	zinc finger SWIM-type containing 7 [Source:MGI Symbol;Acc:MGI:1916997]	12.7431	15.6745	14.7632	14.2418	14.9823	15.6368	11.4323	14.8834	17.3774	13.9277	17.4732	16.2547	16.1722	14.9925	0.932671476	0.966018836	20	20	24	0.552812075	0.837051098	24	12.28827147	0.060795714
ENSMUSG00000014245	Pigl	phosphatidylinositol glycan anchor biosynthesis, class L [Source:MGI Symbol;Acc:MGI:2681271]	3.99778	3.81892	3.6649	3.56486	3.37269	3.38371	4.18848	3.6275	3.39702	3.82892	3.9295	3.81406	3.43949	3.66098	0.94073613	0.966018836	4	8	24	0.641199099	0.878540546	24	23.63242478	0.028867679
ENSMUSG00000014294	Ndufa2	NADH:ubiquinone oxidoreductase subunit A2 [Source:MGI Symbol;Acc:MGI:1343103]	124.109	152.617	143.707	143.912	146.211	133.289	131.597	138.236	176.796	121.133	161.25	132.954	159.765	142.234	0.593560247	0.966018836	12	12	24	0.527285136	0.823319175	24	8.631500663	0.068689608
ENSMUSG00000014301	Pam16	presequence translocase-asssociated motor 16 homolog (S. cerevisiae) [Source:MGI Symbol;Acc:MGI:1913699]	3.14154	2.50939	2.63265	3.19927	2.19208	2.95116	3.01591	2.562	3.87817	1.62303	2.92629	3.45398	3.1931	2.99626	0.745469888	0.966018836	24	20	24	0.491885331	0.805393068	24	0.322404323	0.102095924
ENSMUSG00000014303	Glis2	GLIS family zinc finger 2 [Source:MGI Symbol;Acc:MGI:1932535]	5.12992	5.95445	5.54699	5.02481	7.18201	5.40282	6.11264	5.70376	6.83689	5.22971	6.21186	5.63677	5.41748	5.17261	0.939486266	0.966018836	20	20	24	0.773128981	0.927051312	24	14.59247083	0.038005671
ENSMUSG00000014313	Cox6c	cytochrome c oxidase subunit 6C [Source:MGI Symbol;Acc:MGI:104614]	341.436	406.788	398.858	403.411	401.517	339.372	347.689	367.429	440.148	323.171	418.156	365.154	426.056	408.219	0.787283372	0.966018836	12	12	24	0.403256367	0.799546792	24	8.349967246	0.075640304
ENSMUSG00000014329	Bicc1	BicC family RNA binding protein 1 [Source:MGI Symbol;Acc:MGI:1933388]	3.89161	5.2447	4.49402	4.47394	5.11444	3.74839	4.13099	4.90594	5.81656	3.98908	4.34972	4.65344	4.7504	4.4974	0.345459565	0.870418653	8	16	24	0.262013324	0.768040178	24	7.572288576	0.094640878
ENSMUSG00000014349	Ube2z	ubiquitin-conjugating enzyme E2Z [Source:MGI Symbol;Acc:MGI:1343160]	31.9962	26.1402	25.4962	27.67	27.6774	29.4607	26.9405	28.4849	24.6458	31.8873	26.4786	30.2295	28.6682	28.0631	0.025149041	0.292995596	24	12	24	0.26339645	0.768040178	24	20.13034085	0.070827251
ENSMUSG00000014353	Tmem87b	transmembrane protein 87B [Source:MGI Symbol;Acc:MGI:1919727]	25.524	25.5455	25.0239	26.5683	25.3147	23.9014	24.0957	23.9018	25.9944	26.6178	28.3705	26.5589	26.2441	26.4528	0.365996436	0.893596186	16	12	24	0.168997089	0.767020009	24	13.14855015	0.04262185
ENSMUSG00000014355	Anapc1	anaphase promoting complex subunit 1 [Source:MGI Symbol;Acc:MGI:103097]	17.9149	21.1754	21.2263	20.4823	19.9778	19.7888	18.5946	20.766	21.9948	17.3204	20.1813	19.722	21.6438	20.0405	0.133266423	0.567728672	12	12	24	0.430794815	0.805393068	24	6.691338972	0.050204554
ENSMUSG00000014361	Mertk	MER proto-oncogene tyrosine kinase [Source:MGI Symbol;Acc:MGI:96965]	7.35582	5.64293	7.55567	9.93484	7.68252	7.10891	6.15561	5.7845	6.88735	7.08115	6.51916	6.48029	5.79233	4.79291	0.002116007	0.100837868	16	16	24	0.002997297	0.414576087	24	14.29516988	0.200756151
ENSMUSG00000014402	Tsg101	tumor susceptibility gene 101 [Source:MGI Symbol;Acc:MGI:106581]	48.8337	47.6519	43.8784	43.9344	46.3486	48.1733	43.1977	44.4631	43.8652	43.2838	43.316	45.5046	44.2117	43.8631	0.718742371	0.966018836	24	16	24	0.042971975	0.625007356	24	21.66093329	0.045558652

ENSMUSG00000014956	Ppp1cb	protein phosphatase 1 catalytic subunit beta [Source:MGI Symbol;Acc:MGI:104871]	110.01	116.048	113.867	114.662	115.651	105.243	116.65	112.454	115.3	107.932	116.665	100.735	103.106	93.2471	0.893307667	0.966018836	20	4	24	0.372333057	0.793651569	24	12.4631818	0.041522313
ENSMUSG00000014959	Gorasp2	golgi reassembly stacking protein 2 [Source:MGI Symbol;Acc:MGI:2135962]	38.4573	31.5597	30.6287	31.7348	31.8176	37.9909	35.0954	35.6576	29.3164	40.9142	31.078	34.3241	32.0649	33.2497	0.091424683	0.483797062	24	12	24	0.460832493	0.805393068	24	20.96249036	0.069778793
ENSMUSG00000014980	Tsen15	tRNA splicing endonuclease subunit 15 [Source:MGI Symbol;Acc:MGI:1913887]	19.9568	19.341	17.7885	15.0761	21.7327	19.0864	19.7962	19.4555	19.4219	20.2984	19.1463	18.6114	20.7913	20.9187	0.745475226	0.966018836	4	20	24	0.393132208	0.799546792	24	23.68507227	0.06061223
ENSMUSG00000015002	Efr3a	EFR3 homolog A [Source:MGI Symbol;Acc:MGI:1923990]	23.3918	24.1685	24.3758	23.4318	23.8653	22.7053	24.5348	21.8181	22.3352	23.3524	23.8941	21.021	21.254	24.2895	0.922035832	0.966018836	12	12	24	0.635566656	0.876201318	24	9.739925895	0.025189179
ENSMUSG00000015013	Trappc2l	trafficking protein particle complex 2-like [Source:MGI Symbol;Acc:MGI:1916295]	42.4467	51.2615	49.3339	42.9055	48.1874	42.7059	42.4028	44.0553	51.2667	35.4661	49.9304	44.0751	47.9181	44.2592	0.433658121	0.966018836	12	16	24	0.82354107	0.94441254	24	5.316853091	0.036232999
ENSMUSG00000015016	Acsf3	acyl-CoA synthetase family member 3 [Source:MGI Symbol;Acc:MGI:2182591]	5.19797	5.55079	5.86329	7.62816	6.34709	6.32516	5.88484	6.13035	6.18923	8.00381	6.82524	6.07879	5.76466	6.46258	7.90E-04	0.071344007	16	12	24	0.001174938	0.336500758	24	12.68711196	0.15526949
ENSMUSG00000015023	Ddx19a	DEAD box helicase 19a [Source:MGI Symbol;Acc:MGI:99526]	5.71186	4.99365	5.57564	6.33221	5.16848	5.01214	5.32068	5.45614	5.26006	5.67526	4.9548	4.98804	4.92076	5.34682	0.142513744	0.585944911	16	4	24	0.172381578	0.768040178	24	9.791098619	0.058366139
ENSMUSG00000015027	Galns	galactosamine (N-acetyl)-6-sulfate sulfatase [Source:MGI Symbol;Acc:MGI:1355303]	1.48829	1.66682	1.97983	1.75509	1.85205	1.7474	1.55661	1.72088	1.93132	1.65997	1.45065	1.70033	1.50558	1.67642	0.009483698	0.194720062	12	16	24	0.105174962	0.717252017	24	9.820700913	0.066737007
ENSMUSG00000015053	Gata2	GATA binding protein 2 [Source:MGI Symbol;Acc:MGI:95662]	0.732348	1.05261	1.21349	1.26544	1.21078	1.16882	1.39503	1.30086	0.998719	1.83634	1.09344	1.34235	1.25415	2.62622	0.858514388	0.966018836	8	12	24	0.426174206	0.805393068	24	7.814995942	0.103973383
ENSMUSG00000015083	C8g	complement component 8, gamma polypeptide [Source:MGI Symbol;Acc:MGI:88237]	1.89887	1.90668	1.67453	2.00197	1.59844	1.40938	1.06929	1.48638	1.77387	1.85409	1.50312	1.70723	1.43889	1.59291	0.1982019	0.680119125	16	12	24	0.172797128	0.768040178	24	10.4531763	0.096560671
ENSMUSG00000015085	Entpd2	ectonucleoside triphosphate diphosphohydrolase 2 [Source:MGI Symbol;Acc:MGI:1096863]	19.1814	16.9268	18.1116	15.3912	17.2006	16.648	20.8193	19.6385	18.1508	17.1601	15.9986	16.2289	19.5266	16.9436	0.042936877	0.356048751	4	20	24	0.037406466	0.609717533	24	2.041873857	0.107688313
ENSMUSG00000015087	Rabl6	RAB, member RAS oncogene family-like 6 [Source:MGI Symbol;Acc:MGI:2442633]	34.8579	35.4918	36.3282	44.5689	42.4019	37.3411	38.2701	39.4935	39.1161	43.5946	36.4996	39.6159	39.2856	37.1128	0.217933169	0.704989686	16	16	24	0.064049653	0.663876076	24	13.13837103	0.086634854
ENSMUSG00000015090	Ptgds	prostaglandin D2 synthase (brain) [Source:MGI Symbol;Acc:MGI:99261]	1010.32	1290.09	762.027	736.033	811.623	811.871	808.038	975.19	1273.89	417.723	898.151	957.209	593.691	1022.65	0.053980818	0.395677346	8	8	24	0.3332968	0.769685083	24	2.719763527	0.234421819
ENSMUSG00000015092	Edf1	endothelial differentiation-related factor 1 [Source:MGI Symbol;Acc:MGI:1891227]	91.7745	73.7009	75.8277	70.6581	75.6032	77.8927	78.9369	89.3182	77.6697	83.4714	96.2789	88.1028	94.1323	88.8747	0.321662653	0.83607295	4	12	24	0.22590081	0.768040178	24	22.85076826	0.076875099
ENSMUSG00000015093	Clic3	chloride intracellular channel 3 [Source:MGI Symbol;Acc:MGI:1916704]	1.07285	0.50042	0.586653	1.1502	1.6178	0.776374	1.5435	1.13816	1.04944	1.00661	0.940968	0.726088	0.706585	1.00261	0.921572192	0.966018836	16	20	24	0.706422683	0.902918222	24	16.21567373	0.077828273
ENSMUSG00000015094	Npdc1	neural proliferation, differentiation and control 1 [Source:MGI Symbol;Acc:MGI:1099802]	126.334	99.3261	95.3471	107.364	108.377	116.726	108.691	112.629	98.1849	121.547	113.701	115.516	114.424	110.626	0.010049466	0.194720062	24	12	24	0.08258322	0.699403344	24	19.95789851	0.094731103
ENSMUSG00000015095	Fbxw5	F-box and WD-40 domain protein 5 [Source:MGI Symbol;Acc:MGI:1354731]	28.4225	24.2423	25.1075	26.8508	27.5502	26.1145	27.337	27.5848	23.4653	29.2744	24.2964	26.0963	26.2396	24.6603	0.942429894	0.966018836	4	8	24	0.529087126	0.824286178	24	18.92262876	0.04165208
ENSMUSG00000015112	Slc25a13	solute carrier family 25 (mitochondrial carrier, adenine nucleotide translocator), member 13 [Source:MGI Symbol;Acc:MGI:1354721]	2.11229	2.44253	2.22464	2.12629	2.66653	2.15433	2.90116	2.28021	2.52853	2.36663	1.96337	2.56301	2.32274	2.14212	0.759203569	0.966018836	4	12	24	0.847738471	0.954054905	24	23.57099078	0.025361059
ENSMUSG00000015120	Ube2i	ubiquitin-conjugating enzyme E2I [Source:MGI Symbol;Acc:MGI:107365]	12.7462	15.9331	16.1486	15.4244	12.8951	13.1909	13.1049	16.5253	16.8267	13.4346	14.2342	15.2	13.3069	14.8946	0.008389425	0.192760771	12	16	24	0.020297918	0.565108098	24	7.603840251	0.135709579
ENSMUSG00000015126	Tsr3	TSR3 20S rRNA accumulation [Source:MGI Symbol;Acc:MGI:1915577]	14.0659	15.5777	14.297	15.0146	12.6905	13.8039	12.8519	14.4747	13.7895	13.5415	15.8602	14.971	15.3246	13.8322	0.785563354	0.966018836	8	12	24	0.9769033	0.994118853	24	4.888552821	0.008116518
ENSMUSG00000015127	Unkl	unkempt family like zinc finger [Source:MGI Symbol;Acc:MGI:1921404]	7.61544	5.08162	5.30867	4.66852	4.97402	5.41254	4.40619	5.84956	5.00538	6.36168	5.7313	5.9026	5.43368	6.31202	0.873350015	0.966018836	8	4	24	0.785616094	0.931158434	24	23.98008557	0.048285706
ENSMUSG00000015133	Lrrk1	leucine-rich repeat kinase 1 [Source:MGI Symbol;Acc:MGI:2142227]	2.48174	3.08667	2.21448	2.24451	2.94864	2.31523	2.32439	2.40289	3.0482	2.18104	2.1005	2.7083	2.16238	2.25119	0.909604019	0.966018836	8	8	24	0.940062767	0.985398871	24	3.802754057	0.018737153
ENSMUSG00000015134	Aldh1a3	aldehyde dehydrogenase family 1, subfamily A3 [Source:MGI Symbol;Acc:MGI:1861722]	1.62898	2.68307	2.85998	2.25498	2.77827	2.18002	2.41969	2.22298	2.81372	2.49986	1.80994	2.01374	2.24289	2.58374	0.010049466	0.194720062	12	12	24	0.075440327	0.685464747	24	8.372597953	0.144290886
ENSMUSG00000015143	Actn1	actinin, alpha 1 [Source:MGI Symbol;Acc:MGI:2137706]	20.3139	18.3727	18.7479	19.596	20.6798	19.6841	18.3155	20.433	19.4824	21.7444	19.1016	20.6115	18.8704	19.2553	0.27817759	0.789849983	24	4	24	0.284567845	0.768040178	24	14.89367782	0.04124458
ENSMUSG00000015149	Sirt2	sirtuin 2 [Source:MGI Symbol;Acc:MGI:1927664]	102.633	101.032	99.1694	104.287	106.843	103.931	118.81	108.259	108.341	120.857	100.875	109.67	109.093	100.464	0.836635326	0.966018836	24	12	24	0.90861146	0.974606327	24	17.48348651	0.014889723
ENSMUSG00000015165	Hnrnpl	heterogeneous nuclear ribonucleoprotein L [Source:MGI Symbol;Acc:MGI:104816]	79.7479	87.132	87.2861	85.6196	87.5774	77.2156	80.8618	85.6805	95.454	78.0222	82.6924	83.8857	88.1777	81.8871	0.245061943	0.741580974	12	12	24	0.241484	0.768040178	24	7.854324993	0.05434077
ENSMUSG00000015176	Nolc1	nucleolar and coiled-body phosphoprotein 1 [Source:MGI Symbol;Acc:MGI:1918019]	14.9614	12.7541	14.0064	14.0381	13.0106	15.1947	14.7613	13.31	12.2331	16.1117	11.9973	14.5642	12.9374	14.6862	0.838532766	0.966018836	24	12	24	0.823869248	0.944567898	24	20.99023806	0.030982842
ENSMUSG00000015189	Casd1	CAS1 domain containing 1 [Source:MGI Symbol;Acc:MGI:2384865]	15.1918	15.1327	16.6448	16.2377	14.8021	14.9811	15.6807	14.2229	14.8052	13.8624	15.8563	14.7746	14.7059	14.7345	0.838244962	0.966018836	12	20	24	0.861534426	0.959461495	24	13.51587761	0.01316813
ENSMUSG00000015202	Cnksr3	Cnksr family member 3 [Source:MGI Symbol;Acc:MGI:2674130]	0.978051	1.10456	1.37361	1.66758	1.16743	1.16193	1.55789	1.36536	1.31506	1.05025	1.10844	0.99175	1.07456	1.00624	0.708020493	0.966018836	12	16	24	0.473284523	0.805393068	24	9.489229224	0.064378518
ENSMUSG00000015214	Mtmr1	myotubularin related protein 1 [Source:MGI Symbol;Acc:MGI:1858271]	11.6395	13.5183	12.8172	13.6819	12.1551	13.7467	10.652	11.5623	12.8898	13.0528	12.4098	12.4437	13.1846	12.0878	0.658895034	0.966018836	16	12	24	0.337059072	0.771960538	24	12.29908996	0.055130791
ENSMUSG00000015217	Hmgb3	high mobility group box 3 [Source:MGI Symbol;Acc:MGI:1098219]	4.89018	5.63907	5.76251	5.67925	5.74816	5.79779	5.35559	4.63272	6.01858	6.2097	5.58486	5.87354	5.4807	6.48208	0.219464938	0.708495385	12	16	24	0.192591521	0.768040178	24	11.78888523	0.067279854
ENSMUSG00000015222	Map2	microtubule-associated protein 2 [Source:MGI Symbol;Acc:MGI:97175]	47.0066	57.528	63.6653	50.9438	48.9085	51.478	59.168	45.4598	55.1625	48.237	48.9118	53.2644	44.4562	60.0603	0.912923872	0.966018836	12	8	24	0.400024819	0.799546792	24	6.486993184	0.085166951
ENSMUSG00000015224	Cyp2j9	cytochrome P450, family 2, subfamily j, polypeptide 9 [Source:MGI Symbol;Acc:MGI:1921769]	19.9299	21.7145	19.9402	16.9291	22.1457	20.5129	24.073	18.8454	18.6075	17.6978	21.7959	19.996	21.2798	19.6836	0.022000259	0.267229408	20	20	24	0.06783741	0.671868099	24	21.96596135	0.111749533
ENSMUSG00000015243	Abca1	ATP-binding cassette, sub-family A (ABC1), member 1 [Source:MGI Symbol;Acc:MGI:99607]	13.8117	14.4008	13.8542	13.3916	14.0545	14.267	13.3461	13.8908	13.3932	12.26	14.8619	11.6904	13.4773	12.3671	0.854540463	0.966018836	20	20	24	0.921951511	0.979411448	24	19.34527517	0.013056491
ENSMUSG00000015247	Nipsnap3b	nipsnap homolog 3B [Source:MGI Symbol;Acc:MGI:1913786]	23.5342	22.5724	21.4124	21.8762	24.574	24.3152	24.006	24.5973	21.568	21.7291	24.4537	22.3696	26.0795	22.4375	0.052138666	0.391073722	4	8	24	0.033551121	0.606793499	24	21.4164966	0.079953837
ENSMUSG00000015289	Lage3	L antigen family, member 3 [Source:MGI Symbol;Acc:MGI:1913442]	12.468	18.0908	16.6482	18.0267	16.4675	14.746	14.856	17.5569	21.8582	14.0165	16.7083	16.3181	17.7199	16.3261	0.302438464	0.811201458	12	12	24	0.157158017	0.763141258	24	8.438940575	0.134378555
ENSMUSG00000015290	Ubl4a	ubiquitin-like 4A [Source:MGI Symbol;Acc:MGI:95049]	36.6154	30.69	30.4028	30.0499	31.2888	35.5076	32.4743	32.0232	25.9742	32.7755	31.2604	32.669	32.6843	31.7985	0.004194713	0.140598674	24	12	24	0.014963308	0.537924552	24	21.25928305	0.113954876
ENSMUSG00000015291	Gdi1	guanosine diphosphate (GDP) dissociation inhibitor 1 [Source:MGI Symbol;Acc:MGI:99846]	217.426	190.173	191.583	207.37	197.735	220.925	203.828	192.751	174.136	224.511	188.896	202.687	199.138	194.002	0.071575474	0.440262583	24	12	24	0.188516268	0.768040178	24	19.03934751	0.070067748
ENSMUSG00000015305	Sash1	SAM and SH3 domain containing 1 [Source:MGI Symbol;Acc:MGI:1917347]	12.9033	12.2049	10.0902	8.34676	11.5788	11.0261	14.2643	12.2026	11.4182	11.2427	10.842	13.0945	12.4331	11.2787	0.001845448	0.100255953	4	12	24	0.008773819	0.502965379	24	23.61605817	0.173236922
ENSMUSG00000015312	Gadd45b	growth arrest and DNA-damage-inducible 45 beta [Source:MGI Symbol;Acc:MGI:107776]	5.2699	5.91915	5.44562	4.71902	4.42932	4.59276	4.41729	8.16655	7.59669	7.51102	6.20202	7.3162	6.59904	7.16384	0.443191651	0.966018836	8	20	24	0.072488239	0.679262305	24	6.680646116	0.184692297
ENSMUSG00000015335	Zdhhc12	zinc finger, DHHC domain containing 12 [Source:MGI Symbol;Acc:MGI:1913470]	9.43009	6.63187	6.7858	6.99435	6.80662	8.32497	7.5347	6.79901	6.10848	8.63812	6.05831	7.07863	7.13673	6.77034	0.117205961	0.538362324	4	8	24	0.309493871	0.768040178	24	21.16921888	0.094839633
ENSMUSG00000015337	Endog	endonuclease G [Source:MGI Symbol;Acc:MGI:1261433]	3.8973	4.01412	4.47142	4.27838	3.82614	4.02883	4.4296	4.25922	5.74694	6.35399	6.68857	5.87372	6.20397	6.47833	0.844448841	0.966018836	16	12	24	0.212638795	0.768040178	24	10.44959508	0.085835674
ENSMUSG00000015340	Cybb	cytochrome b-245, beta polypeptide [Source:MGI Symbol;Acc:MGI:88574]	1.92195	4.3167	5.45616	3.82915	4.29802	3.64503	3.70003	3.15608	4.23749	2.68059	3.34621	3.45523	3.91038	2.59627	0.956719163	0.96629412	12	12	24	0.588932511	0.853936396	24	10.74423867	0.117123555
ENSMUSG00000015341	Golga7	golgi autoantigen, golgin subfamily a, 7 [Source:MGI Symbol;Acc:MGI:1931029]	45.2157	45.3656	45.298	43.3844	43.013	43.9484	47.6817	44.8464	42.5433	44.2655	45.5842	41.2113	40.9963	42.9507	0.814075857	0.966018836	4	20	24	0.873257364	0.964375835	24	3.452204674	0.010559905
ENSMUSG00000015342	Xk	X-linked Kx blood group [Source:MGI Symbol;Acc:MGI:103569]	5.03918	4.6983	4.67956	4.734	3.77644	4.84018	4.66696	4.28792	3.70066	4.84246	4.33106	4.11825	3.84425	4.49245	0.849180783	0.966018836	16	4	24	0.92117153	0.979223729	24	0.60539329	0.016928117
ENSMUSG00000015354	Pcolce2	procollagen C-endopeptidase enhancer 2 [Source:MGI Symbol;Acc:MGI:1923727]	8.75757	7.57628	7.63464	9.41243	8.20996	9.08286	6.317	8.03869	5.51688	8.19828	8.9444	6.21705	8.12784	6.72388	0.243857167	0.741580974	16	20	24	0.278483207	0.768040178	24	16.66180978	0.118377367
ENSMUSG00000015355	Cd48	CD48 antigen [Source:MGI Symbol;Acc:MGI:88339]	1.70723	2.68315	2.89453	2.36298	2.24048	2.73643	2.55792	1.98269	3.26195	1.33284	2.06327	2.32225	2.37523	1.93584	0.879201926	0.966018836	12	4	24	0.869103503	0.96291905	24	4.433994992	0.047606359
ENSMUSG00000015357	Clpx	caseinolytic mitochondrial matrix peptidase chaperone subunit [Source:MGI Symbol;Acc:MGI:1346017]	13.719	13.4528	15.7817	16.0476	11.6997	13.1625	13.3329	14.179	12.7863	15.2753	14.2305	15.9791	15.1761	15.6013	0.907000607	0.966018836	16	4	24	0.646332346	0.880236216	24	8.07415175	0.047647501
ENSMUSG00000015363	Trabd	TraB domain containing [Source:MGI Symbol;Acc:MGI:1915226]	13.4579	12.1506	13.4062	14.4009	13.5443	13.2832	12.5569	13.3558	11.5442	14.366	13.4154	13.5277	12.5598	12.527	0.004182163	0.140598674	16	16	24	0.060070463	0.658304792	24	15.36942987	0.069207019
ENSMUSG00000015377	Dennd6b	DENN/MADD domain containing 6B [Source:MGI Symbol;Acc:MGI:1916690]	8.64044	10.4529	12.2283	10.0834	9.967	9.8804	9.72415	9.87882	11.5827	10.732	8.21189	10.6868	9.44934	10.8795	0.002087679	0.100255953	12	16	24	0.060367354	0.658854825	24	8.088329452	0.122973006
ENSMUSG00000015396	Cd83	CD83 antigen [Source:MGI Symbol;Acc:MGI:1328316]	3.33486	6.68143	9.00885	4.98958	4.15326	5.05388	6.33464	4.90786	7.92523	4.05278	3.68688	5.72593	4.14287	5.73487	0.071580004	0.440262583	12	8	24	0.117903706	0.728323355	24	6.637353309	0.277931943
ENSMUSG00000015401	Cltrn	collectrin, amino acid transport regulator [Source:MGI Symbol;Acc:MGI:1926234]	1.71263	3.12954	1.78841	3.07461	2.59769	1.59888	1.94995	1.84507	2.78211	1.13292	1.98857	1.78244	2.18848	2.15108	0.714044782	0.966018836	8	16	24	0.710008912	0.904099177	24	7.507414225	0.089820768
ENSMUSG00000015405	Ace2	angiotensin I converting enzyme (peptidyl-dipeptidase A) 2 [Source:MGI Symbol;Acc:MGI:1917258]	0.51096	0.933518	0.97796	1.06202	0.887571	0.728753	0.89078	0.808008	1.05389	0.867463	0.839146	0.877944	0.89425	0.984369	0.077301512	0.453790923	12	12	24	0.033054459	0.606585237	24	9.462871076	0.102907185
ENSMUSG00000015451	C4a	complement component 4A (Rodgers blood group) [Source:MGI Symbol;Acc:MGI:98320]	0.807107	0.979948	0.511395	1.28496	1.2616	0.777471	1.47035	0.710346	0.933441	0.553311	1.17318	0.678526	1.01683	0.796897	0.814576414	0.966018836	4	8	24	0.588343992	0.853936396	24	18.94207032	0.091098917
ENSMUSG00000015452	Ager	advanced glycosylation end product-specific receptor [Source:MGI Symbol;Acc:MGI:893592]	0.616003	0.974928	1.19868	0.981228	0.737003	0.564691	0.711185	0.934066	1.15601	0.815872	0.705774	0.678644	0.62879	1.01668	2.34E-05	0.019409398	12	12	24	1.43E-05	0.047905911	24	8.078117839	0.208403168
ENSMUSG00000015461	Atf6b	activating transcription factor 6 beta [Source:MGI Symbol;Acc:MGI:105121]	13.3028	10.5982	10.6565	9.9668	11.4899	10.7026	12.0034	13.1906	9.94024	13.617	12.6537	12.3795	12.7922	11.6767	0.43565473	0.966018836	4	8	24	0.405182122	0.799546792	24	21.76813558	0.072858735
ENSMUSG00000015467	Egfl8	EGF-like domain 8 [Source:MGI Symbol;Acc:MGI:1932094]	45.941	52.9721	48.5931	48.4758	57.8443	44.735	52.345	54.2212	53.6651	51.0003	50.5083	47.3484	53.7705	48.9792	0.913451828	0.966018836	8	16	24	0.650222908	0.882388586	24	8.288270388	0.038950107
ENSMUSG00000015468	Notch4	notch 4 [Source:MGI Symbol;Acc:MGI:107471]	0.869396	0.929905	1.12023	1.07914	1.45476	1.28441	1.01195	1.18952	1.03499	1.66628	1.36919	1.17268	1.27635	0.730536	0.030972684	0.324150946	20	12	24	0.018966723	0.561252866	24	15.20682751	0.15537969
ENSMUSG00000015474	Ppt2	palmitoyl-protein thioesterase 2 [Source:MGI Symbol;Acc:MGI:1860075]	11.9163	10.3676	11.812	12.0801	11.6836	11.7926	11.4731	11.9719	11.9151	13.3114	12.1201	12.4116	11.4511	11.8969	0.34708369	0.870418653	16	12	24	0.093326832	0.71175955	24	13.6517375	0.051663776
ENSMUSG00000015476	Prrt1	proline-rich transmembrane protein 1 [Source:MGI Symbol;Acc:MGI:1932118]	6.47982	7.06695	6.88017	5.29843	6.30134	6.40313	9.68574	7.57474	7.84875	6.27618	7.45812	7.614	7.15656	8.13469	0.074074737	0.448301997	8	8	24	0.069514253	0.673554016	24	1.97319203	0.136876385
ENSMUSG00000015478	Rnf5	ring finger protein 5 [Source:MGI Symbol;Acc:MGI:1860076]	33.2279	29.6032	27.6768	28.8357	29.7438	27.5362	29.2259	26.4497	28.1349	28.0289	28.5416	29.314	32.9481	30.8456	0.286642595	0.793714531	4	8	24	0.199867264	0.768040178	24	23.15055753	0.062408056
ENSMUSG00000015488	Cacfd1	calcium channel flower domain containing 1 [Source:MGI Symbol;Acc:MGI:1924317]	21.4094	18.185	17.5001	20.3729	19.6993	20.9304	19.4528	19.6679	18.2302	22.0169	18.4796	20.4688	20.4039	20.3039	0.077301512	0.453790923	24	12	24	0.346688304	0.778844143	24	19.62233245	0.053271341
ENSMUSG00000015501	Hivep2	human immunodeficiency virus type I enhancer binding protein 2 [Source:MGI Symbol;Acc:MGI:1338076]	9.74296	9.26296	9.49774	9.91311	9.25743	9.6705	9.45577	8.62097	8.57261	10.8659	8.54907	9.30222	8.76792	9.01885	0.91695334	0.966018836	16	16	24	0.493787161	0.806143905	24	14.65772334	0.037541853
ENSMUSG00000015522	Arnt	aryl hydrocarbon receptor nuclear translocator [Source:MGI Symbol;Acc:MGI:88071]	13.7329	14.18	14.2714	13.0872	15.3905	13.2681	15.2237	13.7741	16.0315	13.2403	14.7369	14.2619	14.6485	13.7209	0.939335547	0.966018836	20	20	24	0.989055849	0.997679907	24	1.926082779	0.004838569
ENSMUSG00000015533	Itga2	integrin alpha 2 [Source:MGI Symbol;Acc:MGI:96600]	1.81036	1.83845	1.49369	1.09233	1.9116	1.53798	1.85944	1.87535	1.43171	1.34201	1.80375	1.4251	1.93662	1.51192	0.008961721	0.192760771	4	[illegible]	24	0.073542798	0.682265445	24	23.43932578	0.123771818
ENSMUSG00000015536	Mocs2	molybdenum cofactor synthesis 2 [Source:MGI Symbol;Acc:MGI:1336894]	69.4145	77.5357	72.8016	69.6834	75.6483	68.2389	70.7935	69.0058	76.7787	63.0739	77.3452	70.1531	80.5998	73.1295	0.904831057	0.966018836	8	8	24	0.804165656	0.937374918	24	2.585863167	0.024326812
ENSMUSG00000015542	Nat9	N-acetyltransferase 9 (GCN5-related, putative) [Source:MGI Symbol;Acc:MGI:1913426]	17.4041	16.3127	15.9993	14.1016	15.7899	17.1926	17.7811	16.8903	14.642	15.5879	17.0585	16.8566	17.2385	16.4776	8.95E-06	0.009754434	4	12	24	8.67E-04	0.319044294	24	22.72160463	0.105119458
ENSMUSG00000015568	Lpl	lipoprotein lipase [Source:MGI Symbol;Acc:MGI:96820]	12.1221	16.693	29.8255	67.4969	20.5729	22.1843	18.9626	42.4662	27.4212	40.1277	30.155	18.1222	31.2053	33.1151	0.152782337	0.601788038	16	8	24	0.023863125	0.585033799	24	10.28943511	0.571879966
ENSMUSG00000015575	Atp6v0e	ATPase, H+ transporting, lysosomal V0 subunit E [Source:MGI Symbol;Acc:MGI:1328318]	58.0886	58.0506	52.4167	61.597	63.0511	49.6888	58.6645	53.4454	65.0987	44.9108	69.6595	50.2806	61.4975	53.7503	0.919571723	0.966018836	20	4	24	0.951814009	0.988339231	24	15.7562575	0.021661971
ENSMUSG00000015597	Zfp318	zinc finger protein 318 [Source:MGI Symbol;Acc:MGI:1889348]	8.35832	8.2161	8.30703	9.50264	8.59709	11.036	10.7347	8.50914	8.81528	9.75472	8.4373	9.93762	9.98182	10.0252	0.459019924	0.966018836	24	12	24	0.33626306	0.771737966	24	20.73704082	0.070398174
ENSMUSG00000015599	Ttbk1	tau tubulin kinase 1 [Source:MGI Symbol;Acc:MGI:2147036]	10.3979	8.10849	8.57581	8.42217	8.77097	9.48138	10.0693	9.29307	7.5633	13.2321	8.45892	10.2006	9.21436	10.478	0.44861185	0.966018836	4	8	24	0.81044025	0.939224719	24	20.55244891	0.048145344
ENSMUSG00000015605	Srf	serum response factor [Source:MGI Symbol;Acc:MGI:106658]	6.68825	5.53757	6.04699	7.39059	7.95206	6.93127	7.99418	7.00123	8.35887	7.67589	6.8146	6.38387	6.50785	5.76113	0.515451644	0.966018836	16	16	24	0.291547137	0.768040178	24	14.48002206	0.094324711
ENSMUSG00000015647	Lama5	laminin, alpha 5 [Source:MGI Symbol;Acc:MGI:105382]	5.39596	4.30934	4.25487	5.53473	6.0875	4.42797	5.64403	4.91707	6.57927	5.15894	5.21321	4.10391	7.533	4.48649	0.853711773	0.966018836	4	20	24	0.984796427	0.996934195	24	15.14467138	0.015101763
ENSMUSG00000015652	Steap1	six transmembrane epithelial antigen of the prostate 1 [Source:MGI Symbol;Acc:MGI:1917608]	1.26458	1.00241	1.33515	1.32525	2.28782	1.69283	1.36234	0.733692	1.16953	1.13019	1.58824	1.16013	0.940283	1.1904	0.033553741	0.324908923	20	12	24	0.011277386	0.517565995	24	16.52896983	0.220592011
ENSMUSG00000015653	Steap2	six transmembrane epithelial antigen of prostate 2 [Source:MGI Symbol;Acc:MGI:1921301]	4.96118	4.27823	5.39991	3.98368	4.21377	5.37334	5.11244	4.14675	3.74872	4.15957	4.03011	4.51998	4.22688	4.52027	0.202903295	0.689605535	4	12	24	0.169461938	0.767229759	24	22.71150117	0.092994426
ENSMUSG00000015656	Hspa8	heat shock protein 8 [Source:MGI Symbol;Acc:MGI:105384]	269.654	214.087	211.405	250.848	296.018	288.473	236.864	226.946	163.382	195.389	230.723	219.487	207.42	162.615	0.097779482	0.501112642	20	16	24	1.97E-04	0.154342346	24	18.85180038	0.269073065
ENSMUSG00000015659	Serac1	serine active site containing 1 [Source:MGI Symbol;Acc:MGI:2447813]	5.12392	6.07932	6.21297	5.88677	5.22837	4.77055	5.87503	5.43178	6.28281	5.01674	6.18379	5.94054	5.31094	5.25777	0.321662653	0.83607295	12	12	24	0.429431272	0.805393068	24	8.710078192	0.058908975
ENSMUSG00000015668	Pdzd11	PDZ domain containing 11 [Source:MGI Symbol;Acc:MGI:1919871]	45.0774	47.6661	40.7986	44.1358	48.6272	43.7669	44.4515	41.6914	43.8208	39.1441	45.5883	44.8451	46.4263	47.6061	0.549118437	0.966018836	20	20	24	0.332079807	0.768822984	24	21.96402762	0.051139019
ENSMUSG00000015671	Psma2	proteasome subunit alpha 2 [Source:MGI Symbol;Acc:MGI:104885]	46.5556	51.5035	47.8573	46.9289	48.9213	46.6123	46.5192	43.4787	48.0725	40.8684	49.2107	45.1812	47.3465	46.6298	0.939921059	0.966018836	20	4	24	0.912310637	0.975295827	24	23.23504362	0.013058579
ENSMUSG00000015672	Mrpl32	mitochondrial ribosomal protein L32 [Source:MGI Symbol;Acc:MGI:2137226]	24.7923	29.8352	25.8231	27.7483	28.0355	25.785	24.9312	26.2288	30.7493	22.7363	31.6588	25.9406	28.195	29.1281	0.798829513	0.966018836	20	8	24	0.808519296	0.939135535	24	8.713352349	0.034686478
ENSMUSG00000015697	Setdb1	SET domain, bifurcated 1 [Source:MGI Symbol;Acc:MGI:1934229]	8.98588	9.13132	8.40674	9.84793	9.31237	9.5094	9.46405	9.64327	9.25743	9.38553	9.16848	9.22163	8.46556	9.43589	0.874672901	0.966018836	16	12	24	0.737575926	0.91440753	24	14.27325166	0.01785283
ENSMUSG00000015702	Anxa9	annexin A9 [Source:MGI Symbol;Acc:MGI:1923711]	2.09405	1.78178	1.83213	1.60293	2.14407	1.88003	1.69226	1.66545	1.65104	1.41084	1.7332	1.55123	1.66855	1.54162	0.676344318	0.966018836	20	20	24	0.131181263	0.741500219	24	20.46992927	0.062841234
ENSMUSG00000015709	Arnt2	aryl hydrocarbon receptor nuclear translocator 2 [Source:MGI Symbol;Acc:MGI:107188]	11.4501	12.6943	14.759	12.7146	12.7461	13.2238	15.0831	13.5315	13.7179	15.7016	11.0957	13.1696	11.4707	13.2899	0.338172159	0.85758369	12	8	24	0.340828308	0.775204417	24	8.549940581	0.081731417
ENSMUSG00000015711	Prune1	prune exopolyphosphatase [Source:MGI Symbol;Acc:MGI:1925152]	14.4496	12.1305	12.7663	13.9299	13.0954	15.2845	12.9485	15.1927	11.8531	16.1971	12.0573	14.0921	14.0974	13.5153	0.601647512	0.966018836	24	12	24	0.766397598	0.92437333	24	19.87168378	0.039051413
ENSMUSG00000015714	Cers2	ceramide synthase 2 [Source:MGI Symbol;Acc:MGI:1924143]	109.441	110.556	104.989	105.317	126.542	106.387	125.311	112.232	110.702	111.309	106.106	108.3	108.937	98.7677	0.829428469	0.966018836	4	8	24	0.563509989	0.842665559	24	19.32262663	0.040214841
ENSMUSG00000015733	Capza2	capping protein (actin filament) muscle Z-line, alpha 2 [Source:MGI Symbol;Acc:MGI:106222]	120.25	121.43	117.77	118.244	117.133	114.814	114.618	112.22	122.162	110.701	119.22	113.529	116.84	119.246	0.801995186	0.966018836	12	12	24	0.659138609	0.884879972	24	5.973364254	0.015238286
ENSMUSG00000015745	Plekho1	pleckstrin homology domain containing, family O member 1 [Source:MGI Symbol;Acc:MGI:1914470]	21.0401	19.2067	18.4192	19.2297	20.223	20.5983	19.064	21.5436	18.1113	17.0265	21.1333	18.8204	18.119	18.9411	0.745469888	0.966018836	20	20	24	0.255855916	0.768040178	24	20.88530286	0.05920836
ENSMUSG00000015747	Vps45	vacuolar protein sorting 45 [Source:MGI Symbol;Acc:MGI:891965]	17.4876	18.2598	18.9521	16.8383	18.1394	19.2772	15.3053	18.5243	15.5289	16.9375	17.5832	16.2594	15.6342	16.9221	0.868022237	0.966018836	20	8	24	0.862610949	0.959941154	24	14.4547472	0.01923723
ENSMUSG00000015748	Prpf3	pre-mRNA processing factor 3 [Source:MGI Symbol;Acc:MGI:1918017]	9.8016	8.84636	9.38721	10.6123	11.6963	10.2775	10.4728	12.2046	10.7645	9.4358	10.4556	9.22826	9.6008	8.84115	0.945282252	0.966018836	20	12	24	0.667254038	0.887459929	24	15.19678698	0.045358003
ENSMUSG00000015749	Anp32e	acidic (leucine-rich) nuclear phosphoprotein 32 family, member E [Source:MGI Symbol;Acc:MGI:1913721]	33.2663	33.1353	34.4654	35.5467	35.8893	32.3329	33.1868	31.6604	36.5769	35.8435	30.6869	37.1256	35.595	32.6627	0.591351901	0.966018836	12	20	24	0.567492107	0.843902578	24	12.54201413	0.034379267
ENSMUSG00000015750	Aph1a	aph1 homolog A, gamma secretase subunit [Source:MGI Symbol;Acc:MGI:2385110]	2.40865	2.69154	3.22267	3.06234	3.324	3.06872	2.79687	3.15558	3.08043	3.4756	2.61466	2.48288	2.71277	3.25044	0.177969241	0.655480352	12	16	24	0.043616038	0.626787045	24	10.09964228	0.11234582
ENSMUSG00000015755	Tab2	TGF-beta activated kinase 1/MAP3K7 binding protein 2 [Source:MGI Symbol;Acc:MGI:1915902]	26.6597	25.3254	25.4742	24.6091	27.094	25.675	28.6153	26.8359	27.3703	29.2462	24.941	26.5598	27.7212	24.9902	0.837133694	0.966018836	4	4	24	0.916659456	0.977080301	24	22.91642126	0.012460866
ENSMUSG00000015757	Ppil4	peptidylprolyl isomerase (cyclophilin)-like 4 [Source:MGI Symbol;Acc:MGI:1914668]	9.51898	10.3281	9.69707	9.19523	9.81211	9.2594	10.5592	9.95622	11.1265	8.46807	11.6274	10.282	10.4369	10.2566	0.858269825	0.966018836	4	12	24	0.692852345	0.898196933	24	1.402374188	0.033980696
ENSMUSG00000015759	Cnih1	cornichon family AMPA receptor auxiliary protein 1 [Source:MGI Symbol;Acc:MGI:1277202]	123.263	114.965	102.287	109.67	111.051	112.815	113.211	109.337	99.8212	105.015	118.216	108.677	111.493	111.45	0.009118941	0.192760771	4	8	24	0.024181697	0.586541342	24	21.45089655	0.074852901
ENSMUSG00000015766	Eps8	epidermal growth factor receptor pathway substrate 8 [Source:MGI Symbol;Acc:MGI:104684]	2.56289	3.0456	4.64989	3.00168	2.79176	3.0971	5.01655	3.23196	3.3333	2.83246	2.63252	2.61413	2.41793	3.33477	0.115798555	0.537837134	12	16	24	0.348210436	0.779015211	24	5.875389104	0.134076404
ENSMUSG00000015776	Med22	mediator complex subunit 22 [Source:MGI Symbol;Acc:MGI:98446]	18.504	14.3786	15.9099	19.2609	18.6749	17.9915	17.2653	16.0963	15.8549	18.2736	15.9468	16.3429	15.1851	13.8184	0.050258003	0.385784509	16	16	24	0.016127664	0.544099157	24	16.33262854	0.122602091
ENSMUSG00000015790	Surf1	surfeit gene 1 [Source:MGI Symbol;Acc:MGI:98443]	59.4801	64.0381	60.705	67.4805	61.9467	64.2952	59.6073	63.5162	64.7287	61.4658	62.2618	64.3458	66.5301	65.0026	0.816361374	0.966018836	24	4	24	0.806522932	0.938308753	24	9.138762835	0.01393651
ENSMUSG00000015804	Med28	mediator complex subunit 28 [Source:MGI Symbol;Acc:MGI:1914249]	60.8654	58.4715	55.8822	55.0541	57.494	56.8975	54.2921	55.4274	61.7281	53.9295	72.7131	65.8759	75.1999	65.6128	0.664568268	0.966018836	20	20	24	0.481074986	0.805393068	24	21.74254171	0.059876135
ENSMUSG00000015806	Qdpr	quinoid dihydropteridine reductase [Source:MGI Symbol;Acc:MGI:97836]	107.534	109.937	127.931	102.128	100.174	120.406	115.519	100.481	101.416	101.435	95.2086	108.897	96.0594	108.827	0.897975397	0.966018836	24	20	24	0.628353615	0.872795898	24	1.757029206	0.042065391
ENSMUSG00000015812	Gnrh1	gonadotropin releasing hormone 1 [Source:MGI Symbol;Acc:MGI:95789]	1.35944	1.57833	1.01424	1.90857	1.47019	1.03569	0.551728	1.09698	1.45558	0.550895	1.24012	0.71972	0.605035	1.28156	0.628899575	0.966018836	8	20	24	0.451771632	0.805393068	24	10.20775263	0.129567774
ENSMUSG00000015829	Tnr	tenascin R [Source:MGI Symbol;Acc:MGI:99516]	3.20269	2.78464	3.7834	2.89393	2.91392	3.24588	3.33401	2.60408	3.02081	2.94483	2.89254	3.17303	2.79902	3.62634	0.880992536	0.966018836	12	8	24	0.895749322	0.970467191	24	3.491063326	0.021612286
ENSMUSG00000015837	Sqstm1	sequestosome 1 [Source:MGI Symbol;Acc:MGI:107931]	286.486	238.579	239.456	236.974	247.889	271.676	246.53	255.648	214.666	261.562	241.099	249.882	241.136	249.209	0.148073804	0.590732968	24	12	24	0.109253341	0.72082227	24	21.08468875	0.077384221
ENSMUSG00000015839	Nfe2l2	nuclear factor, erythroid derived 2, like 2 [Source:MGI Symbol;Acc:MGI:108420]	29.3486	27.644	28.1974	29.2347	29.2591	28.2469	28.8265	26.7202	26.747	29.3131	27.2987	24.3562	25.2788	25.1696	0.691437565	0.966018836	16	16	24	0.132031458	0.742323615	24	14.06971866	0.04360887
ENSMUSG00000015843	Rxrg	retinoid X receptor gamma [Source:MGI Symbol;Acc:MGI:98216]	7.34037	6.76394	6.15647	7.6125	6.59846	7.41775	7.60526	8.60007	6.7739	7.84922	6.76165	7.69866	8.47638	6.95248	0.455507782	0.966018836	4	16	24	0.417983977	0.802581477	24	23.27490542	0.059757916
ENSMUSG00000015846	Rxra	retinoid X receptor alpha [Source:MGI Symbol;Acc:MGI:98214]	5.64644	5.47085	5.76975	6.45599	5.65686	6.18943	5.90645	6.10423	5.99767	7.71585	5.1454	6.26734	6.34311	5.71407	0.918193129	0.966018836	16	16	24	0.603732244	0.861540092	24	11.7795934	0.046406127
ENSMUSG00000015850	Adamtsl4	ADAMTS-like 4 [Source:MGI Symbol;Acc:MGI:2389008]	3.73706	3.30907	4.19679	5.00331	3.96893	3.95371	3.34572	3.65391	4.43641	3.41794	4.14797	3.39642	3.21911	2.97749	0.005947452	0.162657213	12	20	24	0.020104112	0.565108098	24	12.95277463	0.14049058
ENSMUSG00000015852	Fcrls	Fc receptor-like S, scavenger receptor [Source:MGI Symbol;Acc:MGI:1933397]	3.45319	4.0217	5.08578	3.26793	4.67641	3.01086	4.38181	4.43546	3.9955	2.30535	4.19878	2.81206	3.78837	3.12662	0.601647512	0.966018836	12	12	24	0.709147964	0.903951515	24	4.739130361	0.078302643
ENSMUSG00000015869	Prpsap1	phosphoribosyl pyrophosphate synthetase-associated protein 1 [Source:MGI Symbol;Acc:MGI:1915013]	36.5873	32.0083	30.3775	37.0737	34.9531	35.7292	36.0129	35.4263	32.4386	37.6906	33.7662	32.7487	34.0912	33.3795	0.187699199	0.662239322	16	20	24	0.448308704	0.805393068	24	17.94350802	0.044615223

ENSMUSG00000015879	Fam184b	family with sequence similarity 184, member B [Source:MGI Symbol;Acc:MGI:2442958]	1.54816	1.34826	1.45851	1.11207	1.34548	1.32013	1.35333	1.22376	1.17626	1.3679	1.30353	1.14476	1.39446	1.49259	0.239653024	0.741580974	4	20	24	0.376147393	0.796215348	24	1.482380555	0.042741711
ENSMUSG00000015880	Ncapg	non-SMC condensin I complex, subunit G [Source:MGI Symbol;Acc:MGI:1930197]	0.429443	0.962914	1.44091	0.82991	0.582049	0.63308	0.674466	1.30707	0.642648	0.373315	1.05714	0.669139	1.48267	0.644594	0.833406028	0.966018836	8	8	24	0.536677695	0.827485499	24	4.983065528	0.121981074
ENSMUSG00000015882	Lcorl	ligand dependent nuclear receptor corepressor-like [Source:MGI Symbol;Acc:MGI:2651932]	8.49283	10.2324	9.15247	9.45925	8.78793	8.01853	9.68561	8.43222	9.29731	7.5036	9.26426	8.2158	8.17224	8.90313	0.601647512	0.966018836	12	12	24	0.509968282	0.813985618	24	6.174160003	0.047730933
ENSMUSG00000015889	Lta4h	leukotriene A4 hydrolase [Source:MGI Symbol;Acc:MGI:96836]	34.4508	32.634	32.4474	30.479	31.1257	32.7651	35.5995	34.5206	29.0142	30.8296	30.7325	32.5464	33.7021	31.1545	7.56E-04	0.071344007	4	12	24	0.00143632	0.354740824	24	23.72098754	0.088530236
ENSMUSG00000015932	Dstn	destrin [Source:MGI Symbol;Acc:MGI:1929270]	155.274	159.55	145.934	147.304	148.382	146.072	144.77	143.52	148.48	135.124	158.09	149.204	155.84	160.105	0.965163262	0.969272194	8	8	24	0.366972549	0.790573051	24	0.612877481	0.037402141
ENSMUSG00000015937	Macroh2a1	macroH2A.1 histone [Source:MGI Symbol;Acc:MGI:1349392]	32.2885	31.2152	33.292	32.3411	32.6728	32.9825	32.031	34.3177	35.0382	35.6143	33.6832	33.9269	34.4559	33.8095	0.896311169	0.966018836	12	16	24	0.154817723	0.760257003	24	10.21426591	0.027461848
ENSMUSG00000015942	Gtf2ird2	GTF2I repeat domain containing 2 [Source:MGI Symbol;Acc:MGI:2149780]	4.19348	3.75971	3.84605	3.59142	3.51604	3.87279	4.12185	3.96628	3.74136	4.66108	3.95121	3.8302	3.74289	3.73614	0.92721845	0.966018836	4	16	24	0.995456697	0.998760424	24	0.579649167	0.003162407
ENSMUSG00000015943	Bola1	bolA-like 1 (E. coli) [Source:MGI Symbol;Acc:MGI:1916418]	9.98482	10.3416	8.49895	7.75037	7.22041	8.4855	10.4635	12.7571	13.1096	11.8717	13.8215	11.3542	12.4956	12.8489	0.864782464	0.966018836	8	16	24	0.145095338	0.754043136	24	4.422790523	0.146634472
ENSMUSG00000015944	Castor2	cytosolic arginine sensor for mTORC1 subunit 2 [Source:MGI Symbol;Acc:MGI:1933384]	8.49439	7.19411	7.47759	7.95514	7.51763	8.131	9.35866	7.67822	7.42326	10.0309	7.04536	8.45473	7.86745	7.99163	0.27817759	0.789849983	24	12	24	0.827867672	0.946180429	24	20.28871151	0.032675257
ENSMUSG00000015947	Fcgr1	Fc receptor, IgG, high affinity I [Source:MGI Symbol;Acc:MGI:95498]	0.701987	0.846304	0.943094	0.509792	1.26726	0.952285	0.997443	0.888249	1.5345	0.784934	1.23479	0.72498	0.790781	1.12511	0.843639218	0.966018836	12	12	24	0.810497742	0.939224719	24	9.647860201	0.05080805
ENSMUSG00000015950	Ncf1	neutrophil cytosolic factor 1 [Source:MGI Symbol;Acc:MGI:97283]	2.81446	4.74591	5.47452	4.98557	4.13888	3.74831	3.69237	3.58084	4.96495	3.12299	3.82233	3.42692	4.58825	3.33307	0.300823756	0.809362476	12	16	24	0.251338533	0.768040178	24	9.285430792	0.146536486
ENSMUSG00000015957	Wnt11	wingless-type MMTV integration site family, member 11 [Source:MGI Symbol;Acc:MGI:101948]	1.20749	0.942862	1.14977	0.92059	0.941884	0.681668	0.867518	1.10492	1.04693	1.11539	1.0049	1.2196	1.26059	1.08555	0.908406451	0.966018836	4	16	24	0.515598243	0.817346854	24	5.269577339	0.05103745
ENSMUSG00000015961	Adss	adenylosuccinate synthetase, non muscle [Source:MGI Symbol;Acc:MGI:87948]	36.5307	40.0845	38.3454	38.0552	36.4169	37.6582	36.4101	35.5777	36.6576	35.8798	40.4865	38.5583	39.3648	40.326	0.784548857	0.966018836	8	8	24	0.954491386	0.988601783	24	1.202307232	0.007393127
ENSMUSG00000015966	Il17rb	interleukin 17 receptor B [Source:MGI Symbol;Acc:MGI:1355292]	2.99392	3.16822	2.36753	3.61085	3.61609	2.49189	3.48524	2.69985	3.79597	3.04403	3.24353	2.75095	3.25906	2.17081	0.774195442	0.966018836	16	16	24	0.525299846	0.822381894	24	14.69320015	0.080024462
ENSMUSG00000015968	Cacna1d	calcium channel, voltage-dependent, L type, alpha 1D subunit [Source:MGI Symbol;Acc:MGI:88293]	3.1767	3.8491	4.36544	3.48186	4.67994	3.71029	4.04205	3.38583	4.47038	3.48552	3.65604	3.86125	3.27902	5.11981	0.941901469	0.966018836	12	16	24	0.560355232	0.841060952	24	8.651359332	0.07215095
ENSMUSG00000015970	Chdh	choline dehydrogenase [Source:MGI Symbol;Acc:MGI:1860776]	4.55041	4.5082	4.15896	5.08798	5.4692	4.20677	5.75407	4.04641	4.66646	4.91859	5.07937	4.52429	4.22611	3.826	0.032263213	0.324385159	20	12	24	0.113330115	0.727393008	24	16.04851447	0.106179002
ENSMUSG00000015971	Actr8	ARP8 actin-related protein 8 [Source:MGI Symbol;Acc:MGI:1860775]	19.5192	21.0612	20.6126	22.4723	21.1253	22.3562	24.129	22.4138	20.6519	22.6788	19.1516	20.893	21.4252	22.4262	0.919571723	0.966018836	16	4	24	0.799877218	0.935884746	24	4.186540483	0.023046383
ENSMUSG00000015980	Lrrc27	leucine rich repeat containing 27 [Source:MGI Symbol;Acc:MGI:1923862]	4.15626	4.80692	4.21688	4.06578	3.4703	6.26931	4.73549	6.09518	3.11016	4.98052	4.17249	4.3163	4.74735	5.22482	0.147689593	0.590732968	8	4	24	0.31361543	0.768040178	24	0.560382127	0.130720466
ENSMUSG00000015981	Stk32c	serine/threonine kinase 32C [Source:MGI Symbol;Acc:MGI:2385336]	13.523	10.2661	11.1055	10.7204	11.0527	12.7652	11.1645	10.9232	8.57743	12.694	9.31913	11.1145	10.549	11.3326	0.718742371	0.966018836	24	12	24	0.349796663	0.779221593	24	21.20268721	0.088447494
ENSMUSG00000015994	Fnta	farnesyltransferase, CAAX box, alpha [Source:MGI Symbol;Acc:MGI:104683]	105.582	112.328	103.43	101.532	115.676	103.112	115.459	103.925	113.896	97.9941	109.75	103.072	116.22	107.701	0.117050591	0.538362324	4	12	24	0.500990586	0.80908345	24	0.293079304	0.036876272
ENSMUSG00000016018	Mtrex	Mtr4 exosome RNA helicase [Source:MGI Symbol;Acc:MGI:1919448]	32.5192	33.3002	32.2181	31.7039	33.705	30.2524	30.0985	30.5078	34.9649	27.0655	33.714	31.7258	34.4509	32.6896	0.919571723	0.966018836	12	12	24	0.877317645	0.965869843	24	2.574838164	0.019218467
ENSMUSG00000016024	Lbp	lipopolysaccharide binding protein [Source:MGI Symbol;Acc:MGI:1098776]	8.85718	11.7183	9.8999	9.54264	11.4584	9.47075	9.12165	11.5329	15.1421	6.44859	8.71896	9.92691	8.41969	9.88315	0.287843582	0.793714531	8	20	24	0.481700992	0.805393068	24	5.906042695	0.123930026
ENSMUSG00000016028	Celsr1	cadherin, EGF LAG seven-pass G-type receptor 1 [Source:MGI Symbol;Acc:MGI:1100883]	1.45257	1.29826	0.964587	1.27568	1.53609	1.18767	1.68376	1.11051	1.35871	1.77643	1.52711	1.01625	1.4483	0.953433	0.351840494	0.870418653	20	12	24	0.468965661	0.805393068	24	16.71759705	0.079442861
ENSMUSG00000016087	Fli1	Friend leukemia integration 1 [Source:MGI Symbol;Acc:MGI:95554]	2.52575	2.49037	2.33447	2.2877	1.99429	1.92553	1.91701	2.44754	2.4843	2.23623	2.26235	2.10038	2.34347	2.11672	0.154289127	0.606139807	8	16	24	0.096718357	0.71175955	24	6.511337679	0.075122865
ENSMUSG00000016128	Stard13	StAR-related lipid transfer (START) domain containing 13 [Source:MGI Symbol;Acc:MGI:2385331]	25.8715	23.5346	20.4985	21.0237	23.9802	23.2472	26.1292	24.5351	19.2812	25.8745	24.2052	24.4143	27.2736	25.3021	0.002171187	0.102623968	4	8	24	0.041425318	0.618736234	24	22.26888911	0.119569069
ENSMUSG00000016150	Tenm1	teneurin transmembrane protein 1 [Source:MGI Symbol;Acc:MGI:1345185]	1.42101	1.66498	1.54146	1.52828	1.62932	1.32961	1.55635	1.21145	1.82963	1.24451	1.59752	1.6649	1.37828	1.79065	0.896753532	0.966018836	12	4	24	0.843195643	0.952379736	24	8.424739053	0.026856448
ENSMUSG00000016179	Camk1g	calcium/calmodulin-dependent protein kinase I gamma [Source:MGI Symbol;Acc:MGI:2388073]	9.37427	8.36809	9.38316	7.56667	7.80624	9.62858	8.38604	9.42567	7.86226	11.6852	6.66995	8.21564	8.63626	9.04367	0.822170541	0.966018836	8	12	24	0.639777596	0.877957092	24	4.646150418	0.066238081
ENSMUSG00000016181	Utp25	UTP25 small subunit processome component [Source:MGI Symbol;Acc:MGI:2138080]	4.76683	3.98348	4.90399	4.5261	5.05424	4.63739	5.37344	4.2366	4.26239	4.02344	4.21074	4.25246	4.06236	3.76059	0.432054949	0.966018836	20	12	24	0.266778232	0.768040178	24	18.92118482	0.064640428
ENSMUSG00000016194	Hsd11b1	hydroxysteroid 11-beta dehydrogenase 1 [Source:MGI Symbol;Acc:MGI:103562]	3.26198	4.85014	9.90186	6.78701	3.68408	5.2268	6.56933	4.68662	6.46163	5.18766	6.77511	6.0807	5.45293	7.05665	0.851656735	0.966018836	12	16	24	0.241509598	0.768040178	24	9.011047793	0.221852616
ENSMUSG00000016200	Syt14	synaptotagmin XIV [Source:MGI Symbol;Acc:MGI:2444490]	3.87272	3.68072	3.34384	3.86667	3.77144	3.74784	3.74339	3.10071	3.23438	3.41449	3.81226	3.87395	4.09117	3.70773	0.024898193	0.291208026	24	12	24	0.029819648	0.596648309	24	19.96508409	0.086159795
ENSMUSG00000016206	H2-M3	histocompatibility 2, M region locus 3 [Source:MGI Symbol;Acc:MGI:95915]	5.87857	6.04387	5.85426	4.2231	5.65273	5.97034	5.54651	5.83304	5.63699	4.23222	5.83182	5.40058	6.28659	6.26304	0.024928639	0.291208026	8	8	24	0.034137771	0.608006956	24	0.794188018	0.136298641
ENSMUSG00000016252	Atp5e	ATP synthase, H+ transporting, mitochondrial F1 complex, epsilon subunit [Source:MGI Symbol;Acc:MGI:1855697]	308.493	332.223	336.287	341.514	314.467	278.043	304.35	359.187	416.566	307.305	371.732	342.862	405.084	377.056	0.801995186	0.966018836	12	12	24	0.100119836	0.71175955	24	6.928203925	0.114568464
ENSMUSG00000016253	Nelfcd	negative elongation factor complex member C/D, Th1l [Source:MGI Symbol;Acc:MGI:1926424]	18.0255	15.2776	20.7135	17.3566	19.4963	18.9624	19.2233	18.4634	16.0257	18.063	15.3528	16.9003	17.2435	16.1482	0.833301348	0.966018836	16	16	24	0.853800205	0.956375377	24	18.50484844	0.027645457
ENSMUSG00000016255	Tubb1	tubulin, beta 1 class VI [Source:MGI Symbol;Acc:MGI:107814]	0.510517	0.681652	0.902548	0.881005	0.383335	0.890387	0.545575	0.944716	0.69824	0.421518	0.703747	0.649468	0.59404	0.497983	0.937921962	0.966018836	12	16	24	0.78131108	0.929846332	24	7.222826862	0.046301923
ENSMUSG00000016256	Ctsz	cathepsin Z [Source:MGI Symbol;Acc:MGI:1891190]	47.8486	41.6551	43.7867	38.7757	44.7198	43.4089	45.5564	48.8658	40.3424	43.4113	42.973	42.9643	44.0259	41.4084	0.305564675	0.811201458	4	12	24	0.148767696	0.757304338	24	23.17200924	0.063558977
ENSMUSG00000016257	Prelid3b	PRELI domain containing 3B [Source:MGI Symbol;Acc:MGI:1913640]	33.2993	32.9246	34.6106	38.8841	32.1758	35.4203	36.2948	35.3661	33.0255	33.417	36.7182	35.6142	35.258	38.2977	0.852844413	0.966018836	16	20	24	0.998995729	0.999770694	24	2.379233971	0.001335446
ENSMUSG00000016262	Sertad4	SERTA domain containing 4 [Source:MGI Symbol;Acc:MGI:2443496]	9.53162	9.06609	8.97672	9.45707	8.27163	9.87397	8.24905	9.40949	8.32386	10.473	7.76385	9.64621	9.60983	10.0261	0.94499634	0.966018836	24	20	24	0.786523681	0.931349942	24	3.305846671	0.031039205
ENSMUSG00000016308	Ube2a	ubiquitin-conjugating enzyme E2A [Source:MGI Symbol;Acc:MGI:102959]	53.0456	47.1607	46.4444	46.2133	50.0676	49.8617	48.9295	45.9554	41.6745	49.107	50.5229	48.8592	46.9867	49.2072	0.033622862	0.324980923	20	16	24	0.030620996	0.597890721	24	19.83701842	0.078104427
ENSMUSG00000016319	Slc25a5	solute carrier family 25 (mitochondrial carrier, adenine nucleotide translocator), member 5 [Source:MGI Symbol;Acc:MGI:1353496]	127.509	129.968	131.309	126.948	121.884	128.79	124.227	127.385	121.832	119.417	137.522	121.113	124.959	130.422	0.953350544	0.966018836	8	8	24	0.92437087	0.979866138	24	3.174224863	0.008668208
ENSMUSG00000016344	Ppdpf	pancreatic progenitor cell differentiation and proliferation factor [Source:MGI Symbol;Acc:MGI:1913746]	40.2831	38.9891	32.559	34.9823	43.0076	43.2711	40.3542	41.2688	36.308	40.7288	39.6766	37.7191	41.4942	36.5228	0.085992778	0.466404237	24	12	24	0.058042581	0.656248849	24	20.44269758	0.102652305
ENSMUSG00000016346	Kcnq2	potassium voltage-gated channel, subfamily Q, member 2 [Source:MGI Symbol;Acc:MGI:1309503]	28.5572	25.4766	25.4714	27.0729	26.066	27.2177	28.386	26.2537	23.6381	30.0985	25.6555	26.7065	25.6249	28.7448	0.628286596	0.966018836	4	8	24	0.771910467	0.926423608	24	20.59690407	0.026587234
ENSMUSG00000016349	Eef1a2	eukaryotic translation elongation factor 1 alpha 2 [Source:MGI Symbol;Acc:MGI:1096317]	174.039	151.626	152.512	163.052	138.343	165.897	154.699	181.24	138.287	248.499	138.009	181.184	163.568	182.577	0.830364743	0.966018836	8	12	24	0.938974806	0.984940091	24	6.963662485	0.032141174
ENSMUSG00000016356	Col20a1	collagen, type XX, alpha 1 [Source:MGI Symbol;Acc:MGI:1920618]	0.591384	0.667547	0.79637	0.411854	0.851749	0.508507	0.578615	0.60359	0.829362	0.551858	0.607082	0.845477	0.596721	0.871182	0.796993551	0.966018836	12	4	24	0.745500021	0.916831595	24	6.040584513	0.039075035
ENSMUSG00000016382	Pls3	plastin 3 (T-isoform) [Source:MGI Symbol;Acc:MGI:104807]	107.424	105.928	100.652	100.748	100.314	102.276	97.5933	98.4166	95.0646	93.9514	107.159	102.837	108.853	107.002	0.235141005	0.73629392	4	8	24	0.173749505	0.768040178	24	22.7329469	0.048198159
ENSMUSG00000016386	Mpped2	metallophosphoesterase domain containing 2 [Source:MGI Symbol;Acc:MGI:1924265]	14.6769	12.677	11.7203	12.2869	12.166	14.199	11.4194	13.8547	11.2176	16.0801	11.6771	13.2189	13.3249	13.8614	0.760769894	0.966018836	24	12	24	0.834591952	0.949280815	24	22.67115656	0.034960427
ENSMUSG00000016409	Nkap	NFKB activating protein [Source:MGI Symbol;Acc:MGI:1914300]	5.31052	5.60637	5.75973	5.48226	5.62877	5.31785	5.57883	5.37236	5.49018	5.89602	5.61654	6.16568	5.3484	6.23281	0.588682825	0.966018836	20	8	24	0.656196738	0.8838564	24	10.30778052	0.020895524
ENSMUSG00000016427	Ndufa1	NADH:ubiquinone oxidoreductase subunit A1 [Source:MGI Symbol;Acc:MGI:1929511]	176.026	187.095	182.385	197.174	193.434	163.698	162.413	180.265	194.71	155.613	204.013	171.031	201.663	199.829	0.953760335	0.966018836	20	4	24	0.666768497	0.887459929	24	8.576131459	0.046927309
ENSMUSG00000016458	Wt1	Wilms tumor 1 homolog [Source:MGI Symbol;Acc:MGI:98968]	0.900773	1.00727	0.797978	1.2035	0.669994	0.839921	1.00516	0.733775	1.18924	1.06319	0.678827	0.912453	1.00583	1.11139	0.056268883	0.401074772	16	4	24	0.285714197	0.768040178	24	6.643563451	0.083193299
ENSMUSG00000016477	E2f3	E2F transcription factor 3 [Source:MGI Symbol;Acc:MGI:1096340]	5.7376	4.95664	5.19011	5.04075	4.84822	5.80154	5.5254	5.87476	4.09382	6.58551	4.88268	5.68096	5.2625	5.38158	0.644063931	0.966018836	24	20	24	0.594818445	0.85758266	24	21.5571294	0.057581786
ENSMUSG00000016481	Cr1l	complement component (3b/4b) receptor 1-like [Source:MGI Symbol;Acc:MGI:88513]	19.1709	16.2642	16.4832	16.5312	16.9605	17.6245	19.1115	17.6077	17.587	15.8285	16.9519	18.9397	20.1121	15.8067	0.003828753	0.140598674	4	12	24	0.011659344	0.521279312	24	22.30466866	0.10758836
ENSMUSG00000016487	Ppfibp1	PTPRF interacting protein, binding protein 1 (liprin beta 1) [Source:MGI Symbol;Acc:MGI:1914783]	22.0495	21.7218	19.04	22.0142	21.7258	21.5992	23.0134	23.3462	21.5373	21.2732	20.6417	22.4453	23.0422	21.2985	0.053683798	0.394331071	4	8	24	0.121063618	0.733023294	24	23.15588877	0.053678813
ENSMUSG00000016493	Cd46	CD46 antigen, complement regulatory protein [Source:MGI Symbol;Acc:MGI:1203290]	1.34761	2.42752	2.39531	1.99897	1.77888	1.84217	1.50996	2.21517	3.14439	1.84454	2.0924	2.14888	2.08356	2.20127	0.036657636	0.332441566	12	16	24	0.036645192	0.609176161	24	8.211153013	0.184011379
ENSMUSG00000016494	Cd34	CD34 antigen [Source:MGI Symbol;Acc:MGI:88329]	30.448	35.2264	27.5251	30.3342	32.7847	27.6568	29.5431	33.5505	37.4314	26.8626	30.3122	33.2821	33.3488	32.4687	0.33885934	0.858075318	8	8	24	0.405579891	0.799546792	24	3.964863641	0.07044564
ENSMUSG00000016495	Plgrkt	plasminogen receptor, C-terminal lysine transmembrane protein [Source:MGI Symbol;Acc:MGI:1915009]	23.9972	26.9132	26.7775	25.2539	26.9935	22.52	26.2664	27.9428	31.2314	23.0336	28.3833	26.3909	28.6314	27.7771	0.628286596	0.966018836	12	12	24	0.256353626	0.768040178	24	6.3784527	0.07500064
ENSMUSG00000016496	Cd274	CD274 antigen [Source:MGI Symbol;Acc:MGI:1926446]	3.66552	3.43254	3.40748	3.07525	3.44208	3.90823	3.00042	4.2912	2.94346	3.16996	3.52272	3.4208	3.22902	4.1965	0.3576519	0.882035609	8	4	24	0.476251799	0.805393068	24	0.934388202	0.061981927
ENSMUSG00000016503	Gtf3a	general transcription factor III A [Source:MGI Symbol;Acc:MGI:1913846]	10.5683	10.6031	11.548	12.384	10.8528	11.343	10.6848	11.1829	10.8595	12.34	11.7179	12.3784	12.9325	11.0163	0.459388416	0.966018836	16	16	24	0.30744776	0.768040178	24	14.59377671	0.045689571
ENSMUSG00000016510	Mtif3	mitochondrial translational initiation factor 3 [Source:MGI Symbol;Acc:MGI:1923616]	11.0594	10.8966	12.2554	10.4323	10.9862	12.2545	9.7619	12.4867	10.5665	9.70966	12.6939	12.0629	13.4858	11.546	0.664568268	0.966018836	24	16	24	0.54918617	0.834817817	24	22.57472649	0.055942937
ENSMUSG00000016520	Lnx2	ligand of numb-protein X 2 [Source:MGI Symbol;Acc:MGI:2155959]	3.1064	2.56205	2.10682	2.51645	2.24687	2.50237	3.1919	2.48628	2.58671	3.63333	2.51463	2.77376	3.20542	3.70207	0.5169953	0.966018836	4	16	24	0.414126431	0.799546792	24	1.837849167	0.080924985
ENSMUSG00000016526	Dyrk3	dual-specificity tyrosine-(Y)-phosphorylation regulated kinase 3 [Source:MGI Symbol;Acc:MGI:1330300]	1.24891	1.09294	1.34343	1.08079	1.95796	1.12329	1.04809	1.47477	1.02049	1.68773	1.07991	1.01432	1.34773	1.27869	0.860709018	0.966018836	20	4	24	0.679640214	0.892722891	24	12.60991382	0.05818807
ENSMUSG00000016528	Mapkapk2	MAP kinase-activated protein kinase 2 [Source:MGI Symbol;Acc:MGI:109298]	12.2226	13.9206	15.4967	15.7514	14.7027	13.9085	14.1524	18.262	14.9768	17.8237	13.5156	13.7102	14.995	13.5106	0.235187365	0.73629392	4	8	24	0.098525271	0.71175955	24	9.590689068	0.119751037
ENSMUSG00000016534	Lamp2	lysosomal-associated membrane protein 2 [Source:MGI Symbol;Acc:MGI:96748]	206.888	201.963	193.357	192.089	206.662	189.396	189.217	181.029	192.219	167.412	227.791	183.264	192.247	189.003	0.93357733	0.966018836	20	20	24	0.559964758	0.840850554	24	18.74078204	0.044277979
ENSMUSG00000016541	Atxn10	ataxin 10 [Source:MGI Symbol;Acc:MGI:1859293]	134.211	117.34	118.931	111.435	111.052	130.161	124.034	121.096	105.681	140.659	117.767	128.393	125.746	129.467	0.235187365	0.73629392	24	12	24	0.337804536	0.772605919	24	22.45974122	0.064927516
ENSMUSG00000016552	Foxred2	FAD-dependent oxidoreductase domain containing 2 [Source:MGI Symbol;Acc:MGI:106315]	1.49006	2.22204	2.88243	2.21266	1.88001	2.02512	2.33721	2.28351	2.54072	2.68991	1.6589	2.18196	2.0768	2.44691	0.004947794	0.141782417	12	8	24	0.019364442	0.563819767	24	7.960051606	0.173172345
ENSMUSG00000016554	Eif3d	eukaryotic translation initiation factor 3, subunit D [Source:MGI Symbol;Acc:MGI:1933181]	43.6152	36.6857	41.6757	39.9117	39.2254	44.0368	39.2963	40.3872	35.5385	43.5995	37.8877	40.3577	39.0373	40.223	0.8635847	0.966018836	24	12	24	0.573566227	0.847005958	24	19.35301549	0.038350871
ENSMUSG00000016559	H3f3b	H3.3 histone B [Source:MGI Symbol;Acc:MGI:1101768]	76.7843	98.3512	91.337	71.7723	76.7297	75.6233	76.7802	90.3464	91.2479	85.9711	71.8053	87.255	89.3566	87.663	0.003665833	0.140598674	12	8	24	0.01492254	0.537924552	24	5.30971766	0.145207281
ENSMUSG00000016619	Nup50	nucleoporin 50 [Source:MGI Symbol;Acc:MGI:1351502]	8.69934	7.2337	8.48194	8.23911	8.04828	8.67064	8.28135	7.86233	7.34661	9.03993	7.8966	7.94345	8.01921	8.05686	0.683024237	0.966018836	16	16	24	0.509656321	0.813985618	24	17.87079323	0.038828084
ENSMUSG00000016624	Phf21b	PHD finger protein 21B [Source:MGI Symbol;Acc:MGI:2443812]	1.20808	0.816664	0.830858	0.742086	0.629626	0.733023	0.801817	0.505132	1.02677	0.795361	0.55812	0.950493	1.04458	1.00458	0.606884664	0.966018836	4	20	24	0.708723065	0.903848183	24	3.981173474	0.052698466
ENSMUSG00000016637	Ift27	intraflagellar transport 27 [Source:MGI Symbol;Acc:MGI:1914292]	9.82759	10.2521	10.6668	12.2191	9.82931	10.8234	11.4677	10.8686	11.5256	13.3178	9.04167	11.0166	10.4689	12.2665	0.187699199	0.662239322	16	4	24	0.214805157	0.768040178	24	8.466446861	0.090661416
ENSMUSG00000016664	Pacsin2	protein kinase C and casein kinase substrate in neurons 2 [Source:MGI Symbol;Acc:MGI:1345153]	43.0805	33.956	35.4225	37.0654	38.3556	41.0284	42.0659	39.8751	31.1499	40.3729	36.1588	35.8918	36.4743	37.3239	0.182849367	0.659303873	4	8	24	0.170228549	0.767957732	24	20.92888466	0.090309179
ENSMUSG00000016757	Ttll12	tubulin tyrosine ligase-like family, member 12 [Source:MGI Symbol;Acc:MGI:3039573]	5.08923	3.51693	3.71004	4.02968	4.38953	4.53782	4.43966	4.7243	3.58471	4.82649	4.30505	4.09212	4.0939	4.01623	0.279136262	0.790764844	4	8	24	0.241961674	0.768040178	24	19.67812245	0.086663369
ENSMUSG00000016758	Bik	BCL2-interacting killer [Source:MGI Symbol;Acc:MGI:1206591]	0.611769	0.59498	0.776826	0.550187	0.669471	0.643636	0.684777	0.73167	0.73172	0.404567	0.491078	0.553286	0.951908	1.13271	0.048507657	0.379129806	12	4	24	0.033669006	0.606793499	24	3.41568718	0.133111213
ENSMUSG00000016763	Scube1	signal peptide, CUB domain, EGF-like 1 [Source:MGI Symbol;Acc:MGI:1890616]	5.23278	4.59451	3.83163	4.71771	6.68686	4.19961	5.76611	4.65543	4.92219	6.20412	4.49912	5.17478	4.93649	4.47748	0.530814489	0.966018836	4	4	24	0.462196467	0.805393068	24	17.20994854	0.090108367
ENSMUSG00000016831	Tox4	TOX high mobility group box family member 4 [Source:MGI Symbol;Acc:MGI:1915389]	12.3528	11.6867	12.2342	11.8994	11.9338	12.8079	12.2304	12.3049	10.8413	13.0256	11.0501	11.9895	12.0637	12.3003	0.939921059	0.966018836	4	8	24	0.713135769	0.905403147	24	22.47352991	0.022079404
ENSMUSG00000016833	Mrps18c	mitochondrial ribosomal protein S18C [Source:MGI Symbol;Acc:MGI:1915985]	33.0384	40.3093	38.4541	36.349	35.6401	34.6138	31.0624	33.1626	43.4329	29.8186	38.826	38.5157	39.2077	36.614	0.951082476	0.966018836	12	16	24	0.7454615	0.916816146	24	7.956881609	0.047120924
ENSMUSG00000016918	Sulf1	sulfatase 1 [Source:MGI Symbol;Acc:MGI:2138563]	13.9728	13.6634	13.5501	13.8828	15.8832	12.9887	12.6374	13.571	14.101	10.1265	15.6855	13.0341	13.0857	12.1439	0.664568268	0.966018836	20	4	24	0.62238108	0.871391591	24	17.75454232	0.055125881
ENSMUSG00000016921	Srsf6	serine and arginine-rich splicing factor 6 [Source:MGI Symbol;Acc:MGI:1915246]	56.4387	54.9184	55.3627	56.7741	59.1673	54.2245	61.3658	51.4543	55.4259	52.5608	59.4071	51.2577	52.2738	51.784	0.305564675	0.811201458	20	12	24	0.366807466	0.790399468	24	16.72273505	0.040858183
ENSMUSG00000016933	Plcg1	phospholipase C, gamma 1 [Source:MGI Symbol;Acc:MGI:97615]	13.3227	14.2006	13.9261	14.0242	13.2489	15.0074	16.2679	13.3433	14.3385	14.8564	12.8939	13.7723	13.7366	13.0445	0.919571723	0.966018836	16	4	24	0.935258914	0.983765461	24	23.11698625	0.012681304
ENSMUSG00000016940	Kctd2	potassium channel tetramerisation domain containing 2 [Source:MGI Symbol;Acc:MGI:1917632]	40.882	40.9124	39.9574	42.4886	43.5358	43.0894	42.8393	42.6079	39.9243	43.3974	42.7883	44.2613	46.2291	42.3102	0.010049466	0.194720062	24	12	24	0.035191635	0.608551791	24	19.45128173	0.043810348
ENSMUSG00000016946	Kctd5	potassium channel tetramerisation domain containing 5 [Source:MGI Symbol;Acc:MGI:1916509]	14.8797	14.043	14.0747	14.9752	14.3599	15.9814	14.4422	13.8265	13.5036	15.5997	13.3618	14.9604	15.0969	14.4885	0.133266423	0.567728672	24	12	24	0.331450946	0.768223159	24	19.47928397	0.04226929
ENSMUSG00000016984	Etaa1	Ewing tumor-associated antigen 1 [Source:MGI Symbol;Acc:MGI:1915395]	4.22913	3.85157	3.79004	3.53288	3.93024	3.65612	4.22194	3.85758	3.80664	3.25381	4.37233	3.56731	3.96046	3.66833	0.010037128	0.194720062	4	12	24	0.241151063	0.768040178	24	22.75629365	0.058421924
ENSMUSG00000016995	Matn4	matrilin 4 [Source:MGI Symbol;Acc:MGI:1328314]	6.88766	4.52863	4.50814	4.32176	6.06474	4.83398	6.57377	6.01555	6.01205	6.67463	6.64405	5.92391	6.59199	5.46291	0.503104368	0.966018836	4	8	24	0.462545484	0.805393068	24	20.92601036	0.092326419
ENSMUSG00000017002	Slpi	secretory leukocyte peptidase inhibitor [Source:MGI Symbol;Acc:MGI:109297]	1.65866	3.89269	3.42877	3.14784	2.98954	3.1703	3.74241	2.81322	3.14926	2.35644	3.02673	2.69501	3.76426	1.15425	0.881996584	0.966018836	4	12	24	0.882309238	0.966683041	24	16.32381945	0.065436001
ENSMUSG00000017009	Sdc4	syndecan 4 [Source:MGI Symbol;Acc:MGI:1349164]	22.1853	20.725	23.8828	22.5458	21.575	21.9408	24.0622	20.9013	20.9526	21.7378	20.7082	21.9233	20.3182	21.2206	0.90332508	0.966018836	4	4	24	0.9526103	0.988472181	24	16.82998706	0.008147256
ENSMUSG00000017057	Il13ra1	interleukin 13 receptor, alpha 1 [Source:MGI Symbol;Acc:MGI:105052]	4.51483	4.55261	4.73253	3.95193	4.68894	4.27685	3.54444	3.75004	4.46692	3.3642	4.7931	3.85324	3.66121	4.15942	0.787022682	0.966018836	20	8	24	0.834764816	0.949290995	24	13.5824219	0.029374431
ENSMUSG00000017119	Nbr1	NBR1, autophagy cargo receptor [Source:MGI Symbol;Acc:MGI:108498]	55.7323	54.8139	51.9182	54.3354	54.3564	55.56	53.0823	53.8062	51.2505	52.2395	53.7619	51.4207	53.2801	56.4724	0.253121224	0.753386477	8	4	24	0.437376485	0.805393068	24	23.14320243	0.021434587
ENSMUSG00000017132	Cyth1	cytohesin 1 [Source:MGI Symbol;Acc:MGI:1334257]	19.8932	17.6444	18.0114	18.0969	16.1405	17.7509	17.9218	17.1669	17.4533	20.7401	18.0861	19.3072	18.0098	19.0916	0.893609703	0.966018836	16	16	24	0.994203831	0.998755922	24	3.028513613	0.003701329
ENSMUSG00000017144	Rnd3	Rho family GTPase 3 [Source:MGI Symbol;Acc:MGI:1921444]	12.0699	11.4798	11.4563	12.1224	13.2556	11.155	12.6507	10.6267	11.9422	10.1778	13.2117	10.3358	11.4503	10.3697	0.702630284	0.966018836	20	12	24	0.367223442	0.790684479	24	17.03564037	0.060114826
ENSMUSG00000017146	Brca1	breast cancer 1, early onset [Source:MGI Symbol;Acc:MGI:104537]	0.861376	0.751965	0.725629	0.816579	0.695407	0.705262	0.824706	0.611361	0.637509	0.509222	0.655658	0.715015	0.821631	0.528638	0.081793634	0.462922111	4	12	24	0.076824591	0.687041208	24	21.82664112	0.067644959
ENSMUSG00000017167	Cntnap1	contactin associated protein-like 1 [Source:MGI Symbol;Acc:MGI:1858201]	20.7951	16.9115	17.826	18.1187	17.3219	19.3982	19.4237	18.6491	14.7856	25.2097	14.9995	18.455	16.9184	18.5851	0.644063931	0.966018836	4	8	24	0.949887093	0.988075055	24	20.29406239	0.024597576
ENSMUSG00000017176	Nt5c3b	5'-nucleotidase, cytosolic IIIB [Source:MGI Symbol;Acc:MGI:1915356]	23.0808	21.3366	20.2969	20.2012	22.2421	21.4959	21.9976	21.711	21.261	19.8398	21.0611	20.9925	21.3804	21.5012	0.004196064	0.140598674	4	12	24	0.012802365	0.526249719	24	23.07879163	0.054145958
ENSMUSG00000017188	Coa3	cytochrome C oxidase assembly factor 3 [Source:MGI Symbol;Acc:MGI:1098757]	65.182	61.6466	59.1494	60.0617	63.292	65.6203	58.8345	59.4039	59.9277	59.4591	61.6744	63.8181	67.3854	63.4533	0.039704107	0.338535904	24	12	24	0.033521618	0.606793499	24	21.27509245	0.058535837
ENSMUSG00000017210	Med24	mediator complex subunit 24 [Source:MGI Symbol;Acc:MGI:1344385]	15.7573	13.2372	12.015	12.2937	13.6542	14.6287	18.262	14.9772	11.8118	16.3502	13.2175	14.2107	13.8053	15.1493	0.018894761	0.256345714	4	8	24	0.153818473	0.760223561	24	22.73782575	0.121448558
ENSMUSG00000017221	Psmd3	proteasome (prosome, macropain) 26S subunit, non-ATPase, 3 [Source:MGI Symbol;Acc:MGI:98858]	36.8895	30.7822	32.6865	33.1924	33.1127	34.4668	33.6946	34.8171	32.0021	43.7215	31.4932	34.7214	33.6241	34.7668	0.836635326	0.966018836	4	8	24	0.903866541	0.973462584	24	14.2801512	0.021182334
ENSMUSG00000017264	Exosc10	exosome component 10 [Source:MGI Symbol;Acc:MGI:1355322]	12.3657	12.0544	13.8711	13.5595	12.844	13.2954	12.7083	12.99	13.882	13.7379	11.7563	12.7227	12.6546	12.901	0.009118941	0.192760771	12	16	24	0.100841229	0.712175739	24	9.806360804	0.055599818
ENSMUSG00000017286	Glod4	glyoxalase domain containing 4 [Source:MGI Symbol;Acc:MGI:1914451]	59.847	73.7704	64.4501	65.9446	69.9721	59.2804	68.0734	62.5314	79.0893	56.3258	69.6711	65.0283	65.4723	63.3407	0.922029406	0.966018836	20	4	24	0.752424036	0.919493971	24	8.033086583	0.040277252
ENSMUSG00000017288	Vps53	VPS53 GARP complex subunit [Source:MGI Symbol;Acc:MGI:1915549]	30.1828	22.52	23.0225	23.9946	21.6224	26.5126	25.3675	25.7043	19.501	28.7855	20.8183	25.0007	24.5028	24.9726	0.209352197	0.69936851	4	8	24	0.354644471	0.781612071	24	22.87610469	0.093715851
ENSMUSG00000017291	Taok1	TAO kinase 1 [Source:MGI Symbol;Acc:MGI:1914490]	17.1678	18.1274	17.3877	18.0355	17.6393	16.2049	16.4838	15.5079	17.5612	15.5677	18.4708	17.1098	16.4199	17.5243	0.522551072	0.966018836	20	4	24	0.674355404	0.890626495	24	12.84679202	0.0257228
ENSMUSG00000017299	Dnttip1	deoxynucleotidyltransferase, terminal, interacting protein 1 [Source:MGI Symbol;Acc:MGI:1923483]	10.996	11.3959	11.3487	12.0179	10.6382	11.3479	10.0943	10.8098	11.5313	12.3361	10.7671	10.9027	11.8945	10.9473	0.085045716	0.466404237	16	4	24	0.205055657	0.768040178	24	10.34280378	0.04918309
ENSMUSG00000017300	Tnnc2	troponin C2, fast [Source:MGI Symbol;Acc:MGI:98780]	103.615	328.857	355.077	454.2	276.702	392.99	262.567	753.738	639.443	611.573	268.532	314.296	400.186	263.03	0.287843582	0.793714531	12	16	24	0.089260143	0.708638675	24	9.504207051	0.564384086
ENSMUSG00000017307	Acot8	acyl-CoA thioesterase 8 [Source:MGI Symbol;Acc:MGI:2158201]	8.10861	7.1274	6.72402	7.68296	6.69489	8.07257	6.67417	6.91282	7.28874	7.31055	8.75075	7.51733	7.20582	5.94646	0.227302972	0.723724673	24	8	24	0.093756886	0.71175955	24	17.41661076	0.100101584
ENSMUSG00000017309	Cd300lg	CD300 molecule like family member G [Source:MGI Symbol;Acc:MGI:1289168]	1.61695	1.44886	2.23296	3.16408	1.70226	2.21383	1.77224	2.76663	2.22765	3.77037	1.94622	1.80416	2.52866	2.31513	0.401437339	0.933249185	16	4	24	0.085772663	0.703674543	24	10.398002	0.211658865
ENSMUSG00000017314	Mpp2	membrane protein, palmitoylated 2 (MAGUK p55 subfamily member 2) [Source:MGI Symbol;Acc:MGI:1858257]	3.55143	3.60812	4.61509	3.99818	3.68442	4.31382	4.21433	3.33273	3.6451	4.99966	3.3204	3.877	3.20394	4.10818	0.622132989	0.966018836	16	12	24	0.45612221	0.805393068	24	11.54137633	0.074013294
ENSMUSG00000017344	Vtn	vitronectin [Source:MGI Symbol;Acc:MGI:98940]	37.2349	49.99	44.8989	34.1473	34.5441	32.0234	39.6066	52.9984	59.5618	21.0381	32.1308	37.8981	33.7145	34.0819	0.05238827	0.391426791	12	4	24	0.08615133	0.704979776	24	4.429690959	0.285111234
ENSMUSG00000017376	Nlk	nemo like kinase [Source:MGI Symbol;Acc:MGI:1201387]	21.8576	19.8017	19.4383	21.9968	18.3578	20.3846	20.6444	18.46	18.0549	23.6849	21.0376	20.1063	18.1509	21.37	0.676344318	0.966018836	16	20	24	0.65353101	0.883312134	24	14.67518834	0.041090204
ENSMUSG00000017386	Traf4	TNF receptor associated factor 4 [Source:MGI Symbol;Acc:MGI:1202880]	2.9709	2.43746	2.89961	3.05833	3.06739	2.86404	3.26433	2.92684	3.4088	3.01266	3.54968	2.63018	3.40781	3.34009	0.90767227	0.966018836	20	4	24	0.804535443	0.93744342	24	11.54887689	0.026087233
ENSMUSG00000017390	Aldoc	aldolase C, fructose-bisphosphate [Source:MGI Symbol;Acc:MGI:101863]	75.4984	104.943	156.582	94.8655	74.1724	122.457	117.633	88.1778	105.102	101.151	70.5789	94.4014	62.6523	107.523	0.588682825	0.966018836	12	8	24	0.376928937	0.796956096	24	7.361742124	0.192254266
ENSMUSG00000017400	Stac2	SH3 and cysteine rich domain 2 [Source:MGI Symbol;Acc:MGI:2144518]	11.1584	10.566	11.0264	10.816	10.1526	12.1217	11.0119	11.3205	9.19554	15.335	9.02722	11.3759	10.7403	11.5391	0.935194469	0.966018836	24	20	24	0.996375854	0.999034817	24	10.09240939	0.005936782
ENSMUSG00000017404	Rpl19	ribosomal protein L19 [Source:MGI Symbol;Acc:MGI:98020]	13.073	10.8576	13.4718	10.0418	15.4551	11.8129	8.60099	11.9234	11.8487	8.20679	11.4909	8.75398	8.85496	9.30104	0.922035832	0.966018836	12	12	24	0.791037815	0.93306638	24	14.10809607	0.055917044

ENSMUSG00000017405	Nek8	NIMA (never in mitosis gene a)-related expressed kinase 8 [Source:MGI Symbol;Acc:MGI:1890646]	5.94056	6.75781	7.15991	6.424	6.46205	6.74989	6.48973	6.72699	7.57526	6.94568	6.12971	6.83209	7.60692	6.57397	0.016047374	0.966018836	12	8	24	0.28729272	0.768040178	24	6.984469274	0.052597743
ENSMUSG00000017412	Cacnb4	calcium channel, voltage-dependent, beta 4 subunit [Source:MGI Symbol;Acc:MGI:103301]	44.6535	44.1558	36.8163	46.1371	44.873	37.9888	38.9133	43.8299	43.2871	41.8512	48.5908	50.8575	47.3281	50.9439	0.935194469	0.966018836	20	16	24	0.926120632	0.98027178	24	19.69241858	0.019872004
ENSMUSG00000017417	Plxdc1	plexin domain containing 1 [Source:MGI Symbol;Acc:MGI:1919574]	1.96979	3.00047	3.38716	2.69538	3.02246	2.841	2.90717	2.80779	3.35463	2.80981	2.65976	3.16589	2.89915	2.91348	0.542718076	0.966018836	12	4	24	0.342778176	0.776697379	24	9.235975472	0.07662508
ENSMUSG00000017418	Arl5b	ADP-ribosylation factor-like 5B [Source:MGI Symbol;Acc:MGI:1923119]	5.84664	7.37131	5.89195	7.81917	7.01866	7.23428	6.00776	5.97746	6.31464	6.58936	7.26154	6.92656	7.68301	6.95775	0.800065124	0.966018836	16	20	24	0.384273729	0.799546792	24	16.28450738	0.066180087
ENSMUSG00000017421	Zfp207	zinc finger protein 207 [Source:MGI Symbol;Acc:MGI:1340045]	57.5132	58.3176	58.6549	58.852	62.3142	55.744	61.4318	51.9487	60.3926	49.6836	67.8221	56.1377	58.1179	55.5046	0.47028201	0.966018836	20	12	24	0.538712423	0.828741412	24	17.38189616	0.048389085
ENSMUSG00000017428	Psmd11	proteasome (prosome, macropain) 26S subunit, non-ATPase, 11 [Source:MGI Symbol;Acc:MGI:1916327]	15.6308	13.5407	14.7789	15.6998	15.8328	15.6594	15.7741	16.0692	14.6445	16.552	14.4528	14.9361	14.6542	15.1952	0.88067614	0.966018836	16	20	24	0.658645078	0.884596426	24	15.83085782	0.025930502
ENSMUSG00000017446	C1qtnf1	C1q and tumor necrosis factor related protein 1 [Source:MGI Symbol;Acc:MGI:1919254]	11.3825	9.98341	10.4238	11.2494	10.5228	12.0701	10.7447	12.0365	8.91907	13.6029	9.48739	10.3876	9.60825	11.0287	0.860709018	0.966018836	16	20	24	0.916554093	0.976997479	24	14.93813113	0.024387981
ENSMUSG00000017453	Pipox	pipecolic acid oxidase [Source:MGI Symbol;Acc:MGI:1197006]	3.12131	3.73481	4.1914	3.78277	4.06266	3.28986	2.80968	3.28541	4.19113	3.46437	3.45064	2.72039	2.71977	3.11649	3.44E-04	0.049770315	12	16	24	5.98E-04	0.293558874	24	10.64228396	0.173497399
ENSMUSG00000017466	Timp2	tissue inhibitor of metalloproteinase 2 [Source:MGI Symbol;Acc:MGI:98753]	162.165	125.945	126.053	141.217	132.251	151.028	147.355	152.727	118.798	186.101	127.14	147.587	141.86	142.464	0.321662653	0.83607295	24	12	24	0.706706543	0.903131193	24	20.44267838	0.058660645
ENSMUSG00000017478	Zc3h18	zinc finger CCCH-type containing 18 [Source:MGI Symbol;Acc:MGI:1923264]	6.5224	6.74568	7.09938	6.85562	6.31001	6.53751	7.06401	6.69278	6.9606	7.24919	6.41523	7.09107	6.49268	6.47649	0.369262716	0.893596186	16	4	24	0.41639607	0.801257196	24	8.86912658	0.030356567
ENSMUSG00000017485	Top2b	topoisomerase (DNA) II beta [Source:MGI Symbol;Acc:MGI:98791]	28.6949	29.987	30.2815	26.3575	28.2074	25.4646	24.641	27.4107	32.3321	24.8571	30.4497	32.335	29.5683	31.7068	0.95261238	0.966018836	12	4	24	0.646601721	0.880399382	24	5.655707833	0.048119223
ENSMUSG00000017491	Rarb	retinoic acid receptor, beta [Source:MGI Symbol;Acc:MGI:97857]	2.76983	2.16133	1.65819	1.9558	3.39701	2.20339	2.32659	2.67567	1.76693	1.40029	2.14876	2.16324	2.13879	2.08799	0.027063253	0.306445942	4	8	24	0.085175576	0.703021968	24	21.35149387	0.17738914
ENSMUSG00000017493	Igfbp4	insulin-like growth factor binding protein 4 [Source:MGI Symbol;Acc:MGI:96439]	29.4364	28.6217	27.2285	25.0854	26.3755	24.4747	31.8014	33.2029	34.5578	30.1694	31.6866	29.9298	30.0083	30.7479	0.570349842	0.966018836	8	16	24	0.152829749	0.759115906	24	4.812699283	0.091052199
ENSMUSG00000017499	Cdc6	cell division cycle 6 [Source:MGI Symbol;Acc:MGI:1345150]	0.264566	0.411918	0.818069	0.590333	0.510173	0.692861	0.633444	0.485281	0.72974	0.298001	0.662566	0.415063	0.740127	0.418746	0.919119843	0.966018836	12	20	24	0.895877762	0.970467191	24	11.26514746	0.03130134
ENSMUSG00000017548	Suz12	SUZ12 polycomb repressive complex 2 subunit [Source:MGI Symbol;Acc:MGI:1261758]	16.6561	16.8406	15.9859	15.6597	15.4728	15.3797	15.5107	15.6221	15.2467	15.2695	15.6382	16.4563	16.3144	16.3863	0.34708369	0.870418653	8	4	24	0.092836678	0.71175955	24	1.177338797	0.036250598
ENSMUSG00000017550	Atad5	ATPase family, AAA domain containing 5 [Source:MGI Symbol;Acc:MGI:2442925]	1.41767	2.08077	1.94245	1.94354	2.00524	1.77557	1.6679	1.7918	2.72867	1.69269	1.95322	1.95723	1.89846	2.05091	0.348367381	0.870418653	12	8	24	0.144138548	0.752803875	24	9.258724885	0.10251758
ENSMUSG00000017561	Crlf3	cytokine receptor-like factor 3 [Source:MGI Symbol;Acc:MGI:1860086]	14.0468	16.3195	17.9279	18.8376	19.4722	15.8415	15.1409	15.2092	20.5113	17.7561	18.8422	18.77	16.475	16.421	0.004014876	0.140598674	20	8	24	0.002315636	0.39629122	24	12.59564028	0.161053827
ENSMUSG00000017588	Krt27	keratin 27 [Source:MGI Symbol;Acc:MGI:1339999]	1.82934	1.46871	1.61719	1.34906	1.63217	1.80803	1.92044	1.20582	1.9552	2.02011	1.89219	2.14442	1.86191	1.61296	0.176754388	0.653256853	24	8	24	0.260867428	0.768040178	24	18.66187872	0.087482906
ENSMUSG00000017615	Tnfaip1	tumor necrosis factor, alpha-induced protein 1 (endothelial) [Source:MGI Symbol;Acc:MGI:104961]	17.1006	14.482	15.0923	15.3281	15.5297	16.4696	16.2664	16.2566	14.0747	18.4583	14.4907	15.9228	15.4908	15.5446	0.217827365	0.704989686	24	12	24	0.657314823	0.884466305	24	20.03964831	0.037041446
ENSMUSG00000017631	Abr	active BCR-related gene [Source:MGI Symbol;Acc:MGI:107771]	50.1131	40.2241	45.2392	40.9023	39.7515	47.6545	45.0829	42.6353	34.5963	53.3035	38.4719	44.2894	39.7123	43.8341	0.924553956	0.966018836	24	20	24	0.721559519	0.90866625	24	21.08371597	0.052970059
ENSMUSG00000017639	Rab11fip4	RAB11 family interacting protein 4 (class II) [Source:MGI Symbol;Acc:MGI:2442920]	13.4864	10.8427	13.5359	11.3908	10.8747	13.6886	13.0443	11.95	9.93398	15.1913	10.1954	13.2636	11.4886	14.6456	0.897570341	0.966018836	24	20	24	0.802607148	0.936640208	24	0.47547725	0.047736859
ENSMUSG00000017664	Slc35c2	solute carrier family 35, member C2 [Source:MGI Symbol;Acc:MGI:2385166]	18.6231	15.6393	15.6806	14.9843	15.7218	16.9752	17.5474	17.341	14.2567	16.7993	15.5278	16.4521	16.4451	15.8893	0.017495149	0.255326033	4	8	24	0.024599934	0.586541342	24	22.6599229	0.089913456
ENSMUSG00000017667	Zfp334	zinc finger protein 334 [Source:MGI Symbol;Acc:MGI:2388656]	2.71852	2.59924	2.72955	2.69373	2.63035	2.78597	2.72432	2.4293	2.0988	2.77535	2.53386	2.78487	2.43489	2.7302	0.648082424	0.966018836	24	12	24	0.343499906	0.777127004	24	19.0772899	0.046493141
ENSMUSG00000017670	Elmo2	engulfment and cell motility 2 [Source:MGI Symbol;Acc:MGI:2153045]	26.2208	23.1558	22.3098	23.7064	23.618	26.5627	24.7075	25.7919	21.4353	25.2799	20.7569	23.6129	22.4458	24.6886	0.636884864	0.966018836	24	12	24	0.354131362	0.781273395	24	23.08753505	0.057298906
ENSMUSG00000017677	Wsb1	WD repeat and SOCS box-containing 1 [Source:MGI Symbol;Acc:MGI:1926139]	49.2563	63.6088	65.1333	52.2782	57.5125	51.3744	57.4933	50.0422	62.0142	50.0869	53.6861	48.7644	53.4801	57.1297	0.085890569	0.466404237	12	4	24	0.150815215	0.758865539	24	7.213312925	0.101273493
ENSMUSG00000017679	Ttpal	tocopherol (alpha) transfer protein-like [Source:MGI Symbol;Acc:MGI:1923330]	17.9403	15.285	15.9577	17.6422	16.8841	16.114	15.9468	15.8633	15.0827	16.3341	17.455	16.8123	16.298	16.9269	0.133012827	0.567728672	16	20	24	0.183045387	0.768040178	24	17.59268686	0.050096212
ENSMUSG00000017686	Rhot1	ras homolog family member T1 [Source:MGI Symbol;Acc:MGI:1926078]	57.1032	55.6447	52.7524	64.0785	62.4454	56.0013	55.3711	52.7124	56.5353	54.4113	62.4384	57.3484	55.1638	50.5082	0.004196064	0.140598674	20	12	24	0.004384629	0.427431763	24	16.01422367	0.100725976
ENSMUSG00000017692	Rhbdl3	rhomboid like 3 [Source:MGI Symbol;Acc:MGI:2179276]	2.83759	2.97001	3.15243	2.61791	2.82633	3.22433	2.971	2.74779	3.2236	3.6988	2.44709	2.9425	2.63687	3.05373	0.97125725	0.971983696	12	8	24	0.57694233	0.848735805	24	8.553902376	0.049305301
ENSMUSG00000017697	Ada	adenosine deaminase [Source:MGI Symbol;Acc:MGI:87916]	2.65086	2.35378	2.2623	2.27476	2.46153	2.57243	1.96867	2.14674	2.18704	2.12849	2.11546	2.29591	2.89205	2.33318	0.539001179	0.966018836	4	12	24	0.288998336	0.768040178	24	22.08859639	0.064448476
ENSMUSG00000017707	Serinc3	serine incorporator 3 [Source:MGI Symbol;Acc:MGI:1349457]	156.213	153.408	148.259	156.023	148.876	151.82	144.825	152.247	140.627	151.263	156.78	145.663	149.797	144.565	0.942051135	0.966018836	16	20	24	0.468865815	0.805393068	24	17.34154703	0.021056928
ENSMUSG00000017713	Tha1	threonine aldolase 1 [Source:MGI Symbol;Acc:MGI:1919026]	2.16226	2.31341	2.94291	2.79731	2.73517	2.08222	2.73819	2.44069	2.95042	2.88687	2.71711	2.68022	2.87416	2.93552	0.004909071	0.141782417	12	12	24	0.026772661	0.586541342	24	9.942207446	0.100037845
ENSMUSG00000017715	Pgs1	phosphatidylglycerophosphate synthase 1 [Source:MGI Symbol;Acc:MGI:1921701]	28.7925	22.701	24.5179	24.1385	25.227	27.8953	27.7107	25.8569	20.5168	30.611	24.6017	26.2881	25.3565	24.053	0.080164531	0.458678242	24	12	24	0.193521545	0.768040178	24	19.74522778	0.101273614
ENSMUSG00000017716	Birc5	baculoviral IAP repeat-containing 5 [Source:MGI Symbol;Acc:MGI:1203517]	3.35669	4.44227	7.2699	5.58601	3.79208	4.28394	4.38526	3.26875	4.61421	3.40502	3.56447	4.91789	5.22412	3.61229	0.919571723	0.966018836	12	20	24	0.78320738	0.930229965	24	9.164266227	0.078035074
ENSMUSG00000017718	Afmid	arylformamidase [Source:MGI Symbol;Acc:MGI:2448704]	3.06388	2.84302	3.44019	2.834	2.44782	3.17808	1.84475	2.94809	2.18874	2.07025	2.40827	2.75995	2.45026	3.40338	0.722296281	0.966018836	8	8	24	0.682781542	0.894288096	24	3.482626371	0.065798784
ENSMUSG00000017721	Pigt	phosphatidylinositol glycan anchor biosynthesis, class T [Source:MGI Symbol;Acc:MGI:1926178]	7.79813	7.2443	7.8864	8.02712	6.85489	7.6031	8.19993	8.94704	8.20321	10.7379	6.52017	8.82758	7.10505	7.90311	0.369262716	0.893596186	16	4	24	0.522563261	0.820892304	24	8.375040957	0.074753294
ENSMUSG00000017723	Wfdc2	WAP four-disulfide core domain 2 [Source:MGI Symbol;Acc:MGI:1914951]	5.64086	5.33795	5.17984	7.31289	5.01011	6.52524	4.48495	6.22225	5.40029	5.9182	4.21359	6.87504	7.08331	6.27678	0.934818505	0.966018836	24	20	24	0.931911679	0.982514603	24	2.015451278	0.030078545
ENSMUSG00000017724	Etv4	ets variant 4 [Source:MGI Symbol;Acc:MGI:99423]	2.59837	3.65917	3.51961	3.258	3.99917	3.50632	4.06932	3.73559	4.09147	3.52336	4.51385	3.42236	3.9826	3.98382	0.90767227	0.966018836	20	4	24	0.725211334	0.909811519	24	10.71054826	0.040088783
ENSMUSG00000017733	Eppin	epididymal peptidase inhibitor [Source:MGI Symbol;Acc:MGI:1922776]	0.712138	0.920452	0.49261	1.03668	0.724733	0.696156	0.94693	0.925733	0.75072	0.556154	0.710686	1.11706	1.03665	0.971337	0.66832131	0.966018836	4	8	24	0.24835655	0.768040178	24	0.089258675	0.091806623
ENSMUSG00000017734	Dbndd2	dysbindin (dystrobrevin binding protein 1) domain containing 2 [Source:MGI Symbol;Acc:MGI:106562]	36.8709	43.9843	50.0202	38.5334	45.168	43.6914	39.5216	37.9947	40.9785	36.0423	38.3107	40.4285	38.0489	38.4156	0.909604019	0.966018836	12	16	24	0.907405386	0.974606327	24	11.45059583	0.020856113
ENSMUSG00000017737	Mmp9	matrix metallopeptidase 9 [Source:MGI Symbol;Acc:MGI:97011]	3.60045	6.01126	7.44609	8.0636	4.70021	4.95701	6.13872	5.25521	5.07573	4.12863	4.52421	4.56962	7.84146	3.48163	0.882263435	0.966018836	16	4	24	0.830917745	0.947661285	24	8.043001564	0.0816833
ENSMUSG00000017740	Slc12a5	solute carrier family 12, member 5 [Source:MGI Symbol;Acc:MGI:1862037]	2.912	8.10283	15.3594	6.02757	3.80591	8.58995	11.013	4.36352	10.4155	5.72763	2.60317	7.1305	2.69543	10.8685	0.173410058	0.648600527	12	8	24	0.278011611	0.768040178	24	6.755534159	0.44910134
ENSMUSG00000017747	Ghdc	GH3 domain containing [Source:MGI Symbol;Acc:MGI:1931556]	5.11297	4.87494	5.0161	5.8339	5.20162	5.04824	5.42508	4.91686	5.61214	5.19012	5.64109	5.37663	5.29373	[illegible]	0.156415999	0.611943293	16	20	24	0.126885477	0.739868076	24	15.34588873	0.049131615
ENSMUSG00000017754	Pltp	phospholipid transfer protein [Source:MGI Symbol;Acc:MGI:103151]	11.2949	15.0247	15.9976	12.1727	11.4121	12.0554	14.5627	18.5415	17.6873	15.7665	11.8417	13.3676	12.1556	13.0207	0.010153564	0.194720062	12	8	24	0.017244649	0.549489822	24	6.932483341	0.225515564
ENSMUSG00000017756	Slc12a7	solute carrier family 12, member 7 [Source:MGI Symbol;Acc:MGI:1342283]	7.70353	8.76059	7.47837	8.61469	9.79494	8.28957	7.55622	10.3718	8.9729	9.77346	8.97071	8.72481	6.62475	8.12832	0.08333408	0.462922111	20	8	24	0.20141938	0.768040178	24	12.32552787	0.106004546
ENSMUSG00000017760	Ctsa	cathepsin A [Source:MGI Symbol;Acc:MGI:97748]	52.5822	51.7747	53.3802	48.0183	53.6594	55.6156	59.2422	58.2699	49.7347	55.5488	50.8305	52.7041	52.7339	48.1038	0.772122688	0.966018836	4	8	24	0.407659427	0.799546792	24	22.24590243	0.047729037
ENSMUSG00000017764	Zswim1	zinc finger SWIM-type containing 1 [Source:MGI Symbol;Acc:MGI:1919221]	3.97086	3.39302	3.3389	3.35991	3.09629	3.85195	3.65524	3.82168	2.98552	5.20355	3.15197	3.7454	3.70049	4.0895	0.29905572	0.809362476	24	20	24	0.897348018	0.970734418	24	1.358478554	0.029337509
ENSMUSG00000017765	Slc12a4	solute carrier family 12, member 4 [Source:MGI Symbol;Acc:MGI:1309465]	12.569	11.4413	11.7177	12.1806	15.2045	11.8477	12.7635	12.1646	12.4702	13.3058	11.7323	12.1071	12.6344	11.5122	0.481530854	0.966018836	4	4	24	0.301677139	0.768040178	24	16.18294416	0.058677481
ENSMUSG00000017774	Myo1c	myosin IC [Source:MGI Symbol;Acc:MGI:106612]	17.2582	15.6213	15.8403	20.002	19.1822	15.3477	17.8502	18.2428	19.1644	18.9021	17.9081	15.8368	17.7443	16.335	0.078207999	0.457568093	16	8	24	0.181794691	0.768040178	24	12.00766616	0.082747795
ENSMUSG00000017776	Crk	v-crk avian sarcoma virus CT10 oncogene homolog [Source:MGI Symbol;Acc:MGI:88508]	57.2314	52.1261	52.7379	53.3171	53.9722	52.1082	52.5087	49.5702	50.2697	50.1372	56.1553	52.8405	54.7247	53.3677	0.347721987	0.870418653	20	16	24	0.17178997	0.768040178	24	20.00831705	0.042495535
ENSMUSG00000017778	Cox7c	cytochrome c oxidase subunit 7C [Source:MGI Symbol;Acc:MGI:103226]	34.8349	40.2175	36.4418	42.7208	46.7204	38.0136	39.4797	47.8539	34.4325	42.7931	36.1591	45.734	46.2427	[illegible]	0.869710197	0.966018836	12	12	24	0.631363685	0.87436603	24	7.357232316	0.060895529
ENSMUSG00000017781	Pitpna	phosphatidylinositol transfer protein, alpha [Source:MGI Symbol;Acc:MGI:99887]	145.849	114.715	118.691	120.219	113.856	130.702	127.319	124.163	102.665	139.915	116.148	127.005	126.051	127.891	0.173541845	0.648600527	4	8	24	0.289663502	0.768040178	24	21.95535528	0.079404064
ENSMUSG00000017801	Mlx	MAX-like protein X [Source:MGI Symbol;Acc:MGI:108398]	26.1185	28.2416	24.5962	27.1483	28.5643	26.1262	27.6818	24.5932	24.7345	22.8233	26.7471	26.8539	22.625	25.9863	0.835401638	0.966018836	20	16	24	0.378193824	0.797706855	24	19.14914851	0.051937704
ENSMUSG00000017802	Retreg3	reticulophagy regulator family member 3 [Source:MGI Symbol;Acc:MGI:1915248]	16.9654	16.6117	17.3606	18.2736	17.6584	17.5066	17.3906	17.7538	15.533	17.2571	17.1368	16.8409	16.7876	17.2292	0.929640773	0.966018836	16	20	24	0.526801064	0.827399772	24	16.52601064	0.022498947
ENSMUSG00000017817	Jph2	junctophilin 2 [Source:MGI Symbol;Acc:MGI:1891496]	1.54397	2.81665	3.64866	4.53731	3.39663	3.91768	2.93996	8.06689	6.22668	8.97594	3.08092	4.21977	4.84257	3.38486	0.595037318	0.966018836	16	12	24	0.0994262	0.71175955	24	9.893457861	0.380981172
ENSMUSG00000017830	Dhx58	DEXH (Asp-Glu-X-His) box polypeptide 58 [Source:MGI Symbol;Acc:MGI:1931560]	2.17209	1.80699	1.82959	1.6449	1.71188	1.89857	1.88389	2.28018	1.75579	1.80765	1.86093	1.94808	1.78153	1.89854	0.117105047	0.538362324	24	16	24	0.10268841	0.713699364	24	0.767879522	0.064450462
ENSMUSG00000017831	Rab5a	RAB5A, member RAS oncogene family [Source:MGI Symbol;Acc:MGI:105926]	58.9296	53.8574	53.6312	56.3215	53.372	54.0909	54.2479	54.0916	48.8228	58.6662	56.283	52.4035	52.0917	54.9657	0.885059765	0.966018836	16	20	24	0.722862145	0.909188168	24	16.82991987	0.021690997
ENSMUSG00000017837	Nkiras2	NFKB inhibitor interacting Ras-like protein 2 [Source:MGI Symbol;Acc:MGI:1919216]	10.6146	8.19596	8.97031	9.34123	10.0853	9.70143	8.86365	9.87097	7.91749	10.6236	9.83395	9.67664	10.3809	8.81881	0.272257525	0.783528688	20	16	24	0.131172861	0.741500219	24	18.46175469	0.092051879
ENSMUSG00000017843	Ppp2r5c	protein phosphatase 2, regulatory subunit B', gamma [Source:MGI Symbol;Acc:MGI:1349475]	68.4822	59.2179	62.8471	57.5657	57.1224	65.0867	61.6752	60.7986	54.8902	68.8543	56.8635	63.2502	61.5643	66.3388	0.718086778	0.966018836	24	20	24	0.487406944	0.805393068	24	23.87777198	0.047569603
ENSMUSG00000017858	Ift52	intraflagellar transport 52 [Source:MGI Symbol;Acc:MGI:2387217]	19.7029	17.4863	17.9085	18.0767	16.2488	18.4253	18.9239	18.5308	15.6414	19.4312	15.6852	17.0101	17.6618	19.5644	0.36081615	0.884073267	4	16	24	0.294501827	0.768040178	24	2.211832645	0.062227157
ENSMUSG00000017897	Eya2	EYA transcriptional coactivator and phosphatase 2 [Source:MGI Symbol;Acc:MGI:109341]	4.83698	4.76746	3.67893	4.22948	5.63707	3.56268	3.76739	4.17983	5.96827	2.75533	4.71083	5.57017	4.28015	3.38464	0.911685024	0.966018836	20	20	24	0.774536261	0.927605007	24	19.25582572	0.075243085
ENSMUSG00000017929	B4galt5	UDP-Gal:betaGlcNAc beta 1,4-galactosyltransferase, polypeptide 5 [Source:MGI Symbol;Acc:MGI:1927169]	13.733	11.793	12.0659	12.8181	12.197	12.8926	11.9301	12.5748	10.1416	15.1874	11.2857	12.0083	11.8856	12.265	0.897425765	0.966018836	16	20	24	0.81061987	0.939256342	24	17.20753653	0.032243074
ENSMUSG00000017943	Gdap1l1	ganglioside-induced differentiation-associated protein 1-like 1 [Source:MGI Symbol;Acc:MGI:2385163]	22.5332	17.7659	18.5606	16.5208	16.2304	20.0269	18.2241	20.7138	14.7266	22.2425	18.4606	19.8106	20.002	20.2788	0.608615915	0.966018836	4	8	24	0.331235193	0.768063804	24	23.30980205	0.095017714
ENSMUSG00000017969	Ptgis	prostaglandin I2 (prostacyclin) synthase [Source:MGI Symbol;Acc:MGI:1097156]	2.07812	2.71406	2.70501	1.99646	2.30932	1.89543	1.79286	3.18657	4.45453	1.81841	2.30531	2.38649	2.48406	2.40327	0.048507657	0.379129806	12	4	24	0.095578705	0.71175955	24	6.775786205	0.211038328
ENSMUSG00000017978	Cadps2	Ca2+-dependent activator protein for secretion 2 [Source:MGI Symbol;Acc:MGI:2443963]	12.1262	10.5877	10.3461	11.7795	10.1218	11.3033	10.2658	10.4717	9.47821	11.649	10.3482	11.2986	11.4136	11.8337	0.699015352	0.966018836	4	8	24	0.675326634	0.89119716	24	21.50971256	0.035493399
ENSMUSG00000017999	Ddx27	DEAD box helicase 27 [Source:MGI Symbol;Acc:MGI:2385884]	7.94568	8.11427	8.52019	8.25965	7.90018	8.94353	9.27688	9.26907	8.59619	8.98574	9.18216	8.96392	8.54848	9.14425	0.816361374	0.966018836	8	4	24	0.889123883	0.968820559	24	3.023494527	0.010697147
ENSMUSG00000018001	Cyth3	cytohesin 3 [Source:MGI Symbol;Acc:MGI:1335107]	20.376	16.4799	16.5286	17.3836	17.0404	17.8921	17.4511	17.0934	15.9348	18.9916	17.6731	18.7088	17.6965	18.1112	0.074438493	0.448301997	24	12	24	0.201021074	0.768040178	24	20.38319575	0.061360736
ENSMUSG00000018008	Cyth4	cytohesin 4 [Source:MGI Symbol;Acc:MGI:2441702]	4.89252	5.51689	6.02297	6.43156	7.14988	5.40623	5.33997	5.33725	6.11209	5.12548	6.02948	5.46678	5.23107	4.69741	0.026012493	0.29944494	20	8	24	0.021264731	0.571993737	24	13.73552879	0.125232396
ENSMUSG00000018012	Rac3	Rac family small GTPase 3 [Source:MGI Symbol;Acc:MGI:2180784]	31.7115	26.0667	25.2279	28.1289	27.5946	28.2235	27.1342	23.384	25.3604	28.2137	26.7155	31.2666	26.5425	27.2824	0.039704107	0.338535904	24	12	24	0.060824852	0.66037877	24	19.13072943	0.098902287
ENSMUSG00000018040	Rrp7a	ribosomal RNA processing 7 homolog A [Source:MGI Symbol;Acc:MGI:1922028]	21.8401	17.6506	21.8104	18.7303	20.9063	19.8096	21.718	22.9188	16.2983	19.3473	19.0354	18.6671	17.9875	16.9047	0.866861748	0.966018836	4	8	24	0.563606258	0.842665559	24	20.77210613	0.056029133
ENSMUSG00000018042	Cyb5r3	cytochrome b5 reductase 3 [Source:MGI Symbol;Acc:MGI:94893]	124.744	107.574	109.483	112.45	114.193	128.447	122.273	120.784	97.7791	130.89	101.538	108.605	106.085	104.101	0.787283372	0.966018836	24	12	24	0.477679972	0.805393068	24	20.0337717	0.060864075
ENSMUSG00000018068	Ints2	integrator complex subunit 2 [Source:MGI Symbol;Acc:MGI:1917672]	3.82565	4.59186	4.28791	4.53735	5.76385	4.61069	3.8976	3.94097	4.60662	4.02474	5.35437	5.34973	4.16017	5.2552	0.032515616	0.324908923	20	8	24	0.185746336	0.768040178	24	15.52896241	0.10426997
ENSMUSG00000018076	Med13l	mediator complex subunit 13-like [Source:MGI Symbol;Acc:MGI:2670178]	5.32656	5.00204	4.90452	5.6962	5.34627	4.97472	5.50749	5.17942	5.33292	6.07188	4.75549	5.23561	5.32861	5.0854	0.949152198	0.966018836	16	16	24	0.658296624	0.884596426	24	11.98616975	0.028142142
ENSMUSG00000018102	H2bc4	H2B clustered histone 4 [Source:MGI Symbol;Acc:MGI:1915274]	41.1379	31.6486	32.5705	29.1645	35.3091	32.6442	36.8465	37.7699	32.7869	34.7555	49.3508	40.6071	42.2483	36.1532	0.083354723	0.462922111	4	8	24	0.142953771	0.752803875	24	20.83154367	0.132277183
ENSMUSG00000018143	Mafk	v-maf musculoaponeurotic fibrosarcoma oncogene family, protein K (avian) [Source:MGI Symbol;Acc:MGI:99951]	5.20574	4.72464	5.48528	5.85076	5.7205	5.30485	5.74059	5.82661	5.49289	6.30661	5.87252	6.08027	5.91574	5.16911	0.210418232	0.69936851	16	16	24	0.08753042	0.707747429	24	14.90466102	0.06527324
ENSMUSG00000018160	Med1	mediator complex subunit 1 [Source:MGI Symbol;Acc:MGI:1100846]	7.3457	7.86443	7.8041	9.50051	8.94706	7.89701	6.90576	7.60441	7.86314	7.99856	7.53269	7.65192	7.85276	7.55549	0.016720774	0.253395174	16	12	24	0.033971198	0.606876472	24	13.01920427	0.08895938
ENSMUSG00000018166	Erbb3	erb-b2 receptor tyrosine kinase 3 [Source:MGI Symbol;Acc:MGI:95411]	16.1062	15.051	14.6952	17.8558	17.2884	15.3057	18.4205	14.4701	15.9336	18.5582	15.2012	15.2958	15.3456	14.393	0.086420527	0.467848941	16	16	24	0.260716954	0.768040178	24	15.00827291	0.073281452
ENSMUSG00000018167	Stard3	START domain containing 3 [Source:MGI Symbol;Acc:MGI:1929618]	16.0481	13.9331	12.979	14.1477	15.532	15.7306	17.5069	13.5933	14.761	14.8032	14.7737	14.3625	15.8194	13.4683	0.002268551	0.104113924	4	4	24	0.038941388	0.612479065	24	20.47203742	0.103152767
ENSMUSG00000018168	Ikzf3	IKAROS family zinc finger 3 [Source:MGI Symbol;Acc:MGI:1342542]	1.26896	0.978382	1.13328	1.22396	0.884573	1.04121	1.37212	0.78525	1.01157	0.493267	1.15488	1.02917	1.26198	0.752912	0.085045716	0.466404237	4	4	24	0.236997406	0.768040178	24	21.91375573	0.114426983
ENSMUSG00000018169	Mfng	MFNG O-fucosylpeptide 3-beta-N-acetylglucosaminyltransferase [Source:MGI Symbol;Acc:MGI:1095404]	1.15096	1.4665	1.59807	1.80614	1.66687	1.60862	1.39616	1.93236	1.70583	1.32682	1.65061	1.47088	1.77325	1.18562	0.833406028	0.966018836	12	16	24	0.608888846	0.864393298	24	12.32561713	0.052699894
ENSMUSG00000018171	Vmp1	vacuole membrane protein 1 [Source:MGI Symbol;Acc:MGI:1923159]	45.777	48.9418	53.5285	44.6534	46.9324	49.0609	47.8015	43.872	53.2818	41.337	47.6871	47.9712	45.8737	48.7916	0.369262716	0.893596186	12	4	24	0.736783791	0.914230051	24	5.218741422	0.031137287
ENSMUSG00000018189	Uchl5	ubiquitin carboxyl-terminal esterase L5 [Source:MGI Symbol;Acc:MGI:1914848]	27.8526	27.3962	24.987	24.8312	25.0015	26.0618	26.4775	25.1977	25.219	25.7901	26.8828	26.9927	27.6351	29.3486	0.021129232	0.264116537	4	8	24	0.040862257	0.616921492	24	0.37684991	0.058320677
ENSMUSG00000018196	Glrx2	glutaredoxin 2 (thioltransferase) [Source:MGI Symbol;Acc:MGI:1916617]	32.7299	35.9027	35.2836	35.5682	39.2484	34.7604	35.8258	33.881	35.5454	32.5181	35.3228	33.2778	37.8109	37.9277	0.890235289	0.966018836	8	16	24	0.983349942	0.996355202	24	5.607123937	0.005841769
ENSMUSG00000018199	Ro60	Ro60, Y RNA binding protein [Source:MGI Symbol;Acc:MGI:106652]	9.00225	10.1932	9.81888	9.97612	10.0468	8.40367	9.86845	8.07801	11.1426	7.91009	11.6251	9.19128	9.405	9.73257	0.849179819	0.966018836	20	4	24	0.747630038	0.917832057	24	11.5405104	0.044541309
ENSMUSG00000018209	Stk4	serine/threonine kinase 4 [Source:MGI Symbol;Acc:MGI:1929004]	11.3623	10.4551	10.7617	11.4827	10.8353	11.0423	11.2632	10.6425	9.92732	10.6208	11.0055	10.9689	10.6236	10.4394	0.124211791	0.552144108	4	4	24	0.039553113	0.615919442	24	19.0005388	0.044403712
ENSMUSG00000018217	Pmp22	peripheral myelin protein 22 [Source:MGI Symbol;Acc:MGI:97631]	3038.81	3235.04	2831.02	3103.4	3951.18	3019.5	3534.88	3032.75	3424.41	3225.74	3258.65	3016.7	3106.48	2669.82	0.939921059	0.966018836	20	4	24	0.30776607	0.768040178	24	16.32546713	0.0810188
ENSMUSG00000018238	Gdf9	growth differentiation factor 9 [Source:MGI Symbol;Acc:MGI:95692]	0.913149	0.68446	0.558222	1.03878	0.402152	1.08436	1.29432	0.567608	0.490333	0.545902	0.537597	0.762289	0.721726	0.627322	0.0140901	0.241938013	24	20	24	0.129686136	0.740162193	24	22.1546925	0.156228416
ENSMUSG00000018239	Zcchc10	zinc finger, CCHC domain containing 10 [Source:MGI Symbol;Acc:MGI:1196228]	7.99106	7.98833	7.35052	6.69391	8.57567	7.7108	7.50455	7.83238	7.88739	7.33139	8.12661	7.23545	7.30633	7.40767	0.91241026	0.966018836	20	20	24	0.743633565	0.916215545	24	20.44057583	0.024920939
ENSMUSG00000018286	Psmb6	proteasome (prosome, macropain) subunit, beta type 6 [Source:MGI Symbol;Acc:MGI:104880]	183.266	196.927	185.753	182.199	198.768	186.669	174.533	174.481	189.408	163.208	198.787	184.76	192.057	183.528	0.676344318	0.966018836	20	20	24	0.719830974	0.908178129	24	19.05965042	0.026586319
ENSMUSG00000018287	Spag7	sperm associated antigen 7 [Source:MGI Symbol;Acc:MGI:107380]	48.7143	46.7394	45.7299	47.4488	45.7285	46.9952	47.5423	42.6853	47.7125	44.5233	45.5218	46.7432	49.9057	47.9198	0.05211941	0.391073722	4	16	24	0.355143237	0.782076869	24	23.35335835	0.03115948
ENSMUSG00000018293	Pfn1	profilin 1 [Source:MGI Symbol;Acc:MGI:97549]	143.901	124.673	127.795	125.705	130.049	136.798	131.988	137.408	131.82	152.009	147.787	145.062	154.433	133.477	0.432699319	0.966018836	24	12	24	0.283453644	0.768040178	24	19.65591347	0.052765308
ENSMUSG00000018322	Tomm34	translocase of outer mitochondrial membrane 34 [Source:MGI Symbol;Acc:MGI:1914395]	36.1119	29.2933	31.1676	31.7348	29.8949	36.5943	36.4532	34.9441	26.9449	38.2649	29.7319	31.7781	33.9534	32.109	0.280850951	0.792139038	4	8	24	0.375761942	0.795959912	24	22.03496664	0.080422519
ENSMUSG00000018326	Ywhab	tyrosine 3-monooxygenase/tryptophan 5-monooxygenase activation protein, beta polypeptide [Source:MGI Symbol;Acc:MGI:1891917]	251.463	222.446	224.921	230.402	220.601	240.87	217.913	220.076	205.231	256.046	232.611	240.963	243.882	243.018	0.850398351	0.966018836	4	8	24	0.472554925	0.805393068	24	19.66845234	0.044125352
ENSMUSG00000018334	Ksr1	kinase suppressor of ras 1 [Source:MGI Symbol;Acc:MGI:105051]	7.40353	6.47016	6.81863	6.85211	7.15263	7.79024	8.14787	8.2713	7.44734	9.39076	8.412	7.20201	7.02474	7.20903	0.850906022	0.966018836	20	16	24	0.62942107	0.873335954	24	15.08656791	0.045890158
ENSMUSG00000018339	Gpx3	glutathione peroxidase 3 [Source:MGI Symbol;Acc:MGI:105102]	46.5387	38.9691	48.7985	68.9227	47.1917	51.5501	41.6592	63.4851	45.0065	75.3833	59.2433	49.9249	66.0363	58.4439	0.478724519	0.966018836	16	20	24	0.273934027	0.768040178	24	12.10092256	0.154001761
ENSMUSG00000018340	Anxa6	annexin A6 [Source:MGI Symbol;Acc:MGI:88255]	206.566	155.309	158.789	166.48	163.999	199.306	173.474	186.728	126.11	202.039	158.601	168.642	175.076	168.212	0.170659409	0.643496587	4	8	24	0.247644051	0.768040178	24	20.9832877	0.121366348
ENSMUSG00000018347	Zkscan6	zinc finger with KRAB and SCAN domains 6 [Source:MGI Symbol;Acc:MGI:1289293]	4.39415	4.40051	4.30828	4.63796	4.45893	4.69976	4.68637	4.17155	4.60588	4.44352	4.37776	4.66563	5.09165	4.80102	0.622132989	0.966018836	4	8	24	0.426989911	0.805393068	24	22.23110623	0.027202577
ENSMUSG00000018362	Kpna2	karyopherin (importin) alpha 2 [Source:MGI Symbol;Acc:MGI:103561]	1.59249	1.54716	1.42381	2.19494	1.92584	2.03149	1.36679	1.33205	1.42717	1.38775	1.77105	1.28545	1.52338	1.2149	0.34708369	0.870418653	20	12	24	0.019556788	0.564709807	24	16.17918797	0.139876356
ENSMUSG00000018363	Smurf2	SMAD specific E3 ubiquitin protein ligase 2 [Source:MGI Symbol;Acc:MGI:1913563]	12.1446	11.5523	10.9908	11.3466	11.624	10.4354	12.5816	11.1395	11.2293	10.1488	12.2258	10.831	11.3518	10.8191	0.580049522	0.966018836	4	12	24	0.460966724	0.805393068	24	22.44591734	0.038232396
ENSMUSG00000018372	Cep95	centrosomal protein 95 [Source:MGI Symbol;Acc:MGI:2443502]	6.84312	6.76577	6.00984	6.94349	6.05153	5.63811	7.67643	5.49991	6.98709	5.76691	6.52541	6.3742	6.01127	5.64305	0.877082091	0.966018836	4	4	24	0.933450124	0.983211997	24	21.77247438	0.018267662
ENSMUSG00000018377	Vezf1	vascular endothelial zinc finger 1 [Source:MGI Symbol;Acc:MGI:1313291]	10.5833	11.1235	11.6257	11.8411	10.7117	10.8612	11.9017	10.6406	11.1448	12.1558	10.2983	11.1067	10.6006	11.0505	0.082113105	0.462922111	16	4	24	0.284752801	0.768040178	24	9.707856753	0.041776835
ENSMUSG00000018378	Cuedc1	CUE domain containing 1 [Source:MGI Symbol;Acc:MGI:2144281]	13.7744	15.2871	16.4198	17.3323	14.5308	14.5005	14.1759	18.3446	19.9258	20.371	16.4112	15.8762	16.5187	16.359	0.017497334	0.255326033	16	12	24	8.95E-04	0.319044294	24	9.473494458	0.177998761
ENSMUSG00000018379	Srsf1	serine and arginine-rich splicing factor 1 [Source:MGI Symbol;Acc:MGI:98283]	77.2464	91.2744	85.6112	82.1659	86.6793	80.7598	85.8195	75.6721	84.7983	79.8586	87.0139	80.9891	75.3424	85.3139	0.909604019	0.966018836	20	8	24	0.602479318	0.861114219	24	11.09299424	0.032284462
ENSMUSG00000018381	Abi3	ABI family member 3 [Source:MGI Symbol;Acc:MGI:1913860]	0.486647	0.664501	1.48507	0.63371	1.15096	0.799853	0.321813	0.995642	1.02235	1.06408	0.899542	0.74004	0.734041	1.07329	0.018194955	0.256345714	12	16	24	0.05156389	0.64767123	24	10.31116572	0.207034597
ENSMUSG00000018387	Shroom1	shroom family member 1 [Source:MGI Symbol;Acc:MGI:1919024]	0.948741	1.49132	1.44709	1.23627	1.46472	1.10045	2.14067	1.09706	1.49663	1.37105	1.21932	1.45412	1.36921	1.27292	0.838244962	0.966018836	12	12	24	0.956851897	0.988639288	24	5.458901106	0.019940032
ENSMUSG00000018395	Kif3a	kinesin family member 3A [Source:MGI Symbol;Acc:MGI:107689]	72.5793	68.9141	66.7486	68.2535	68.6734	68.6493	67.3804	65.208	64.1215	70.6947	72.0137	73.2876	75.7958	74.9433	0.195343269	0.673387182	4	8	24	0.223691044	0.768040178	24	21.10597888	0.043520248
ENSMUSG00000018398	Septin8	septin 8 [Source:MGI Symbol;Acc:MGI:894310]	52.6464	49.1095	51.1008	49.3852	52.139	51.8068	57.8688	48.8515	50.0922	57.9307	47.5283	51.9943	50.5258	48.8205	0.46238066	0.966018836	4	4	24	0.737970342	0.91451866	24	19.15646537	0.027246119
ENSMUSG00000018401	Mtmr4	myotubularin related protein 4 [Source:MGI Symbol;Acc:MGI:2180699]	23.0977	17.2976	18.2354	19.3339	19.7695	21.6832	20.0864	18.9521	14.4394	23.1493	18.1041	19.0071	18.0052	18.2042	0.323424237	0.839647936	16	20	24	0.183306713	0.768040178	24	19.14101077	0.11719033
ENSMUSG00000018405	Mrm1	mitochondrial rRNA methyltransferase 1 [Source:MGI Symbol;Acc:MGI:2443470]	14.6976	12.6274	10.6173	12.6179	8.45362	11.9038	9.3908	10.2386	12.2794	9.66103	12.6929	8.88955	14.0597	10.1609	0.880631923	0.966018836	4	20	24	0.724961963	0.909665611	24	2.211592266	0.070763281

ENSMUSG00000018411	Mapt	microtubule-associated protein tau [Source:MGI Symbol;Acc:MGI:97180]	108.549	81.1448	81.681	79.8901	87.3591	103.113	91.8403	97.692	71.7638	105.821	88.9729	92.2953	92.9599	90.2327	0.064165315	0.414867207	4	8	24	0.135732509	0.747972781	24	21.0789485	0.128268622
ENSMUSG00000018412	Kansl1	KAT8 regulatory NSL complex subunit 1 [Source:MGI Symbol;Acc:MGI:1923969]	15.9529	12.4063	13.8146	14.1519	13.7575	14.9443	14.7039	13.5583	12.3549	14.9198	13.999	13.547	13.3996	13.0019	0.260026661	0.765037845	16	20	24	0.130619981	0.740728512	24	18.90756959	0.07204193
ENSMUSG00000018415	Gid4	GID complex subunit 4, VID24 homolog [Source:MGI Symbol;Acc:MGI:1914021]	15.3883	14.7149	14.5379	15.5753	15.4736	14.9233	17.118	15.8551	15.0466	17.526	14.5087	15.3928	16.0122	15.0477	0.774195442	0.966018836	16	20	24	0.9826568	0.996138716	24	20.7614746	0.005937584
ENSMUSG00000018417	Myo1b	myosin IB [Source:MGI Symbol;Acc:MGI:107752]	21.0773	23.2675	21.3087	25.357	26.3072	21.1207	24.1776	21.5046	23.3354	21.3869	23.7847	22.2862	22.7001	21.463	0.89563591	0.966018836	20	4	24	0.296324942	0.768040178	24	14.91591603	0.058452244
ENSMUSG00000018425	Dhx40	DEAH (Asp-Glu-Ala-His) box polypeptide 40 [Source:MGI Symbol;Acc:MGI:1914737]	11.4783	11.0959	10.5563	10.9897	10.2391	11.2921	11.754	11.8163	11.386	11.9817	11.0368	11.8012	11.753	11.5229	0.448396961	0.966018836	4	16	24	0.178591926	0.768040178	24	1.55796232	0.035972138
ENSMUSG00000018427	Ypel2	yippee like 2 [Source:MGI Symbol;Acc:MGI:1925114]	7.33763	6.42893	6.5355	7.18948	7.36153	6.58373	8.63379	6.86788	6.73359	7.47703	6.85502	6.57372	6.53609	6.42716	0.217933169	0.704989686	16	16	24	0.698059512	0.899652089	24	18.25611932	0.035647629
ENSMUSG00000018428	Akap1	A kinase (PRKA) anchor protein 1 [Source:MGI Symbol;Acc:MGI:104729]	7.36031	6.90154	7.22335	9.13893	6.8463	8.01348	7.71629	9.67084	6.80113	9.55615	8.1207	8.0252	9.54687	8.37438	0.864782464	0.966018836	16	20	24	0.979221533	0.994940912	24	9.623884769	0.012295343
ENSMUSG00000018433	Nol11	nucleolar protein 11 [Source:MGI Symbol;Acc:MGI:1916229]	9.44634	9.87049	9.75654	8.23454	9.79215	10.6793	8.50405	10.331	9.06706	9.39177	9.24752	9.9719	8.62066	10.0383	0.575387897	0.966018836	8	8	24	0.84099188	0.951840198	24	23.49483448	0.02363462
ENSMUSG00000018442	Derl2	Der1-like domain family, member 2 [Source:MGI Symbol;Acc:MGI:2151483]	16.8571	16.3641	16.6093	15.2488	16.1715	14.7262	17.0032	15.4578	15.9051	14.2109	18.0125	16.0358	16.1501	14.2118	0.702630284	0.966018836	4	12	24	0.563261699	0.84250706	24	20.66486438	0.039560714
ENSMUSG00000018446	C1qbp	complement component 1, q subcomponent binding protein [Source:MGI Symbol;Acc:MGI:1194505]	42.3463	41.6398	42.2428	42.706	40.2051	40.5737	38.4472	43.4014	38.5853	49.114	39.5014	41.9612	43.6239	46.2431	0.722296281	0.966018836	16	4	24	0.508839171	0.813704895	24	7.922064392	0.043695487
ENSMUSG00000018449	Rpain	RPA interacting protein [Source:MGI Symbol;Acc:MGI:1916973]	22.424	21.8689	23.6856	22.1138	23.2046	20.3868	22.2556	16.0484	22.174	19.1611	21.9536	20.0343	21.0891	19.8706	0.95247375	0.966018836	12	20	24	0.615622478	0.867203276	24	15.73840271	0.048395702
ENSMUSG00000018451	6330403K07Rik	RIKEN cDNA 6330403K07 gene [Source:MGI Symbol;Acc:MGI:1918001]	54.9549	58.6593	61.2786	58.4935	50.902	59.8968	56.878	53.2138	56.0255	60.3828	50.9984	63.2657	54.2736	63.1205	0.892555977	0.966018836	24	20	24	0.696337081	0.89922881	24	5.937083326	0.035700013
ENSMUSG00000018459	Slc13a3	solute carrier family 13 (sodium-dependent dicarboxylate transporter), member 3 [Source:MGI Symbol;Acc:MGI:2149635]	3.20811	3.96337	3.20832	3.45561	3.04137	3.0793	3.00034	3.88346	4.4376	2.4948	2.91269	3.36266	2.11963	3.68464	0.07600733	0.453790923	8	12	24	0.209806343	0.768040178	24	6.550459891	0.138917299
ENSMUSG00000018470	Kcnab3	potassium voltage-gated channel, shaker-related subfamily, beta member 3 [Source:MGI Symbol;Acc:MGI:1336208]	1.0835	3.43404	6.31011	2.64909	1.73685	4.12083	4.41499	1.97779	3.66174	2.90273	1.29563	3.05341	1.26526	4.49522	0.3964038	0.927127564	12	8	24	0.367592233	0.790948123	24	7.215105258	0.316780152
ENSMUSG00000018474	Chd3	chromodomain helicase DNA binding protein 3 [Source:MGI Symbol;Acc:MGI:1344395]	39.9073	34.1082	36.1188	33.8879	31.2696	37.6484	33.5589	38.597	33.4103	42.4175	36.063	40.4474	39.8582	41.3845	0.920620634	0.966018836	24	20	24	0.593223725	0.856770898	24	1.269537948	0.046885003
ENSMUSG00000018476	Kdm6b	KDM1 lysine (K)-specific demethylase 6B [Source:MGI Symbol;Acc:MGI:2448492]	4.25819	4.04912	4.79261	4.71566	4.29375	3.58233	5.39282	4.86363	5.81218	6.1345	4.22659	4.04119	4.33227	4.90321	0.042880436	0.356048751	12	12	24	0.030231425	0.596821382	24	8.518599878	0.168206305
ENSMUSG00000018481	Appbp2	amyloid beta precursor protein (cytoplasmic tail) binding protein 2 [Source:MGI Symbol;Acc:MGI:1914134]	17.1091	16.3227	17.5398	18.7872	16.318	16.0377	17.3434	15.1605	15.4134	17.5635	15.5034	15.8633	14.6465	16.0088	0.481811994	0.966018836	16	8	24	0.284245938	0.768040178	24	12.29138689	0.046883167
ENSMUSG00000018500	Adora2b	adenosine A2b receptor [Source:MGI Symbol;Acc:MGI:99403]	2.14362	1.75014	2.38391	2.307	2.05499	2.19017	1.75587	1.56556	1.82151	1.64915	2.63333	2.39458	1.89357	2.09587	0.143321297	0.585944911	20	12	24	0.162569945	0.76636332	24	16.65089277	0.109098336
ENSMUSG00000018501	Ncor1	nuclear receptor co-repressor 1 [Source:MGI Symbol;Acc:MGI:1349717]	45.2362	41.2534	42.3765	37.9558	42.8716	43.7032	47.3771	41.928	39.4107	45.1658	40.6452	44.8441	48.7853	45.2005	0.008360387	0.192760771	4	8	24	0.025240769	0.586541342	24	23.08509471	0.088306596
ENSMUSG00000018507	Trpv2	transient receptor potential cation channel, subfamily V, member 2 [Source:MGI Symbol;Acc:MGI:1341836]	23.9785	19.7745	18.7397	20.026	21.9423	24.0892	21.1893	21.775	15.6581	22.9447	21.8389	22.7845	20.1836	21.1437	0.004712708	0.140598674	24	12	24	0.028429823	0.593964798	24	19.822534	0.14973138
ENSMUSG00000018509	Cenpv	centromere protein V [Source:MGI Symbol;Acc:MGI:1920389]	9.0562	10.1936	9.84733	10.7469	10.6978	12.5284	11.9742	12.9839	11.6891	8.16808	9.75372	10.297	10.6124	8.92454	0.865514257	0.966018836	24	16	24	0.664452237	0.886705913	24	23.28844149	0.063411961
ENSMUSG00000018537	Pcgf2	polycomb group ring finger 2 [Source:MGI Symbol;Acc:MGI:99161]	8.23096	8.13473	9.91893	8.34573	8.13935	7.70457	8.88598	8.59635	10.4399	9.30609	8.38682	9.12381	7.77642	9.13614	0.079234726	0.458678242	12	12	24	0.039210846	0.614396028	24	8.536326896	0.106922756
ENSMUSG00000018541	Cwc25	CWC25 spliceosome-associated protein [Source:MGI Symbol;Acc:MGI:1914730]	7.88984	7.77056	7.79635	8.30215	7.05882	8.46875	8.05809	9.1884	7.18501	8.83436	7.94817	8.6282	8.90306	8.12326	0.969487593	0.970883022	24	12	24	0.667088838	0.887459929	24	0.070541642	0.032620395
ENSMUSG00000018547	Pip4k2b	phosphatidylinositol-5-phosphate 4-kinase, type II, beta [Source:MGI Symbol;Acc:MGI:1934234]	20.7288	17.111	18.0039	17.8196	17.8424	19.9801	18.1028	18.6368	15.0108	21.9444	16.5388	19.0161	18.2586	18.2278	0.326899754	0.845515883	24	12	24	0.508680026	0.813561894	24	20.29672448	0.061329501
ENSMUSG00000018548	Trim37	tripartite motif-containing 37 [Source:MGI Symbol;Acc:MGI:2153072]	41.7431	39.3048	39.7047	39.8498	38.3227	40.3292	41.3054	37.1833	36.4525	42.0019	38.5324	42.6979	42.24	43.3066	0.454881285	0.966018836	4	8	24	0.321623903	0.768040178	24	22.33564825	0.0423548
ENSMUSG00000018554	Ybx2	Y box protein 2 [Source:MGI Symbol;Acc:MGI:1096372]	1.53589	1.40437	2.41726	3.86859	1.60908	0.911231	2.00521	1.89765	2.03598	2.60417	3.01361	1.70168	2.82081	3.71355	0.275526571	0.789849983	16	8	24	0.09862957	0.71175955	24	9.53830402	0.282016661
ENSMUSG00000018559	Ctdnep1	CTD nuclear envelope phosphatase 1 [Source:MGI Symbol;Acc:MGI:1914431]	27.956	25.9566	30.5964	31.3969	34.3631	31.2045	26.858	32.8227	25.1521	33.7271	25.2092	30.068	28.3977	27.6136	0.559021844	0.966018836	16	20	24	0.410369073	0.799546792	24	14.7133652	0.075439027
ENSMUSG00000018565	Elp5	elongator acetyltransferase complex subunit 5 [Source:MGI Symbol;Acc:MGI:1859017]	44.4088	41.3203	38.9338	44.8965	43.997	41.3559	41.5002	40.8251	40.0053	39.5453	42.342	41.4204	40.5534	41.3209	0.066808003	0.425506559	20	16	24	0.171702029	0.768040178	24	18.5078448	0.040753294
ENSMUSG00000018566	Slc2a4	solute carrier family 2 (facilitated glucose transporter), member 4 [Source:MGI Symbol;Acc:MGI:95758]	2.96087	5.01308	7.5173	15.0123	8.09553	7.62579	6.37272	15.769	10.5551	14.8999	10.5419	7.24423	12.4813	7.94571	0.17953325	0.655480352	16	12	24	0.042461151	0.623698598	24	11.09492552	0.4639478
ENSMUSG00000018567	Gabarap	gamma-aminobutyric acid receptor associated protein [Source:MGI Symbol;Acc:MGI:1861742]	220.965	225.251	213.618	218.505	236.422	215.513	207.415	208.608	228.184	187.536	234.338	219.293	222.676	213.183	0.849179819	0.966018836	20	12	24	0.64805196	0.881049316	24	18.95625168	0.03199206
ENSMUSG00000018570	2810408A11Rik	RIKEN cDNA 2810408A11 gene [Source:MGI Symbol;Acc:MGI:1917669]	1.23977	1.69408	1.24158	1.3673	0.922505	1.04095	1.14184	1.35193	1.26744	1.11263	0.859812	1.37453	1.51424	1.23209	0.105345531	0.518002936	8	12	24	0.040877485	0.616921492	24	3.949166616	0.128052238
ENSMUSG00000018572	Phf23	PHD finger protein 23 [Source:MGI Symbol;Acc:MGI:1925496]	23.2226	20.0456	20.3924	23.0668	22.9949	22.7945	22.2197	21.9277	20.3265	22.7434	22.9403	23.2828	21.3151	22.1414	0.017595665	0.256116321	20	16	24	0.012430374	0.525280095	24	17.95836185	0.074314667
ENSMUSG00000018574	Acadvl	acyl-Coenzyme A dehydrogenase, very long chain [Source:MGI Symbol;Acc:MGI:895149]	52.5653	51.314	63.8392	90.2954	61.99	57.9898	60.0312	73.7551	59.3319	71.8719	75.1097	63.0414	79.6594	69.762	0.70641939	0.966018836	16	16	24	0.216677256	0.768040178	24	11.87402414	0.134234956
ENSMUSG00000018583	G3bp1	GTPase activating protein (SH3 domain) binding protein 1 [Source:MGI Symbol;Acc:MGI:1351465]	11.1284	9.9671	10.135	9.62	10.3238	11.1346	10.5014	10.98	8.69679	11.4566	9.33791	10.5218	10.8368	10.9318	0.480187506	0.966018836	24	12	24	0.259348975	0.768040178	24	22.8350698	0.065078393
ENSMUSG00000018585	Atox1	antioxidant 1 copper chaperone [Source:MGI Symbol;Acc:MGI:1333855]	149.751	147.965	143.393	144.283	156.518	144.938	134.85	137.836	150.264	112.975	164.967	132.764	154.758	133.821	0.959160181	0.967043964	20	12	24	0.690159758	0.897381208	24	19.87929239	0.046624941
ENSMUSG00000018593	Sparc	secreted acidic cysteine rich glycoprotein [Source:MGI Symbol;Acc:MGI:98373]	757.562	761.442	743.574	730.75	886.708	792.701	910.508	822.151	843.518	857.384	718.76	789.266	771.689	692.789	0.866861748	0.966018836	24	8	24	0.840195936	0.951645964	24	18.26414809	0.028778101
ENSMUSG00000018599	Mief2	mitochondrial elongation factor 2 [Source:MGI Symbol;Acc:MGI:2144199]	5.90937	6.17943	5.99374	8.07352	6.74088	7.09896	6.32397	8.66297	7.33506	8.50798	5.74967	6.68625	7.18224	7.12669	0.933421033	0.966018836	16	4	24	0.364144305	0.788767161	24	9.403801292	0.091409357
ENSMUSG00000018604	Tbx3	T-box 3 [Source:MGI Symbol;Acc:MGI:98495]	3.04341	2.18719	1.97992	2.72353	1.6633	1.89941	2.52213	3.52757	1.65423	3.74588	2.47776	2.76611	2.69674	2.5227	0.814576414	0.966018836	4	8	24	0.873082844	0.964303715	24	2.577044131	0.052314631
ENSMUSG00000018634	Crhr1	corticotropin releasing hormone receptor 1 [Source:MGI Symbol;Acc:MGI:88498]	0.637744	0.675609	0.862931	0.616338	0.584005	0.797333	0.884953	0.743613	0.767041	0.81958	0.607134	0.798701	0.629383	1.12261	0.351315142	0.870418653	12	8	24	0.202588418	0.768040178	24	4.95871898	0.074415752
ENSMUSG00000018648	Dusp14	dual specificity phosphatase 14 [Source:MGI Symbol;Acc:MGI:1927168]	5.24591	6.74239	5.88356	5.39592	6.92621	5.65396	7.07222	6.9731	7.62745	7.83331	5.91542	6.72791	6.2745	5.26309	0.880992536	0.966018836	16	8	24	0.72180281	0.90866625	24	10.06396605	0.05504897
ENSMUSG00000018651	Tada2a	transcriptional adaptor 2A [Source:MGI Symbol;Acc:MGI:2144471]	12.3626	12.3312	12.3624	11.76	12.0273	11.8916	15.3067	12.2865	14.5073	13.2289	11.361	12.6311	13.4443	12.6041	0.231491055	0.731037655	4	16	24	0.330967029	0.768040178	24	3.621169508	0.061326951
ENSMUSG00000018654	Ikzf1	IKAROS family zinc finger 1 [Source:MGI Symbol;Acc:MGI:1342540]	1.14574	1.35455	1.61522	1.51271	1.14114	1.21093	1.09322	0.997365	1.52621	1.19507	1.11043	1.1449	1.43712	1.24314	0.173410058	0.648600527	12	8	24	0.150387697	0.758865539	24	8.716006866	0.088954222
ENSMUSG00000018659	Pnpo	pyridoxine 5'-phosphate oxidase [Source:MGI Symbol;Acc:MGI:2144151]	12.4192	11.7202	13.5873	11.7673	10.6593	11.9844	11.0913	13.2069	10.7236	13.2153	10.4903	11.6946	10.8521	12.4473	0.136853693	0.578758359	8	12	24	0.308348644	0.768040178	24	6.44190162	0.068368349
ENSMUSG00000018661	Cog1	component of oligomeric golgi complex 1 [Source:MGI Symbol;Acc:MGI:1333873]	21.2555	17.9531	18.8755	21.2833	20.7199	22.8312	18.5486	21.5168	17.3482	21.8215	18.5942	19.1572	19.2036	19.8254	0.935194469	0.966018836	16	20	24	0.487220303	0.805393068	24	17.58046788	0.054262506
ENSMUSG00000018666	Cbx1	chromobox 1 [Source:MGI Symbol;Acc:MGI:105369]	19.9132	18.8122	17.5136	17.505	19.6765	17.2951	21.1461	19.2441	17.8188	17.6665	19.9003	19.6256	20.3605	19.3031	0.001925685	0.100255953	4	12	24	0.038587243	0.61140397	24	22.75290434	0.079158978
ENSMUSG00000018669	Cdk5rap3	CDK5 regulatory subunit associated protein 3 [Source:MGI Symbol;Acc:MGI:1933126]	12.5591	15.2598	13.6837	13.6484	13.7579	15.2591	11.5015	14.7318	15.6196	14.6275	14.2914	13.7682	13.1649	12.4734	0.360675801	0.883978426	12	16	24	0.311604551	0.768040178	24	11.42729593	0.06974657
ENSMUSG00000018672	Copz2	coatomer protein complex, subunit zeta 2 [Source:MGI Symbol;Acc:MGI:1929008]	15.7831	18.4583	16.7333	16.0341	19.1333	16.0711	18.1794	18.799	17.5463	15.0454	16.3935	16.3438	18.4409	15.1194	0.952127548	0.966018836	8	8	24	0.646789592	0.880587286	24	1.154446907	0.040526023
ENSMUSG00000018677	Slc25a39	solute carrier family 25, member 39 [Source:MGI Symbol;Acc:MGI:1196386]	38.865	30.2844	33.9225	36.9206	34.3667	36.9968	36.5493	38.1985	33.0571	43.096	36.0123	34.9973	37.5796	34.1876	0.581070934	0.966018836	4	8	24	0.637181304	0.876528137	24	17.03930195	0.044642604
ENSMUSG00000018678	Sp2	Sp2 transcription factor [Source:MGI Symbol;Acc:MGI:1926162]	3.01096	3.02313	2.90381	2.98042	3.26013	2.45528	2.99501	3.62218	3.52692	4.5184	2.94029	3.47321	3.40636	3.19743	0.896311169	0.966018836	8	16	24	0.291244031	0.768040178	24	9.287352161	0.089129288
ENSMUSG00000018697	Aatf	apoptosis antagonizing transcription factor [Source:MGI Symbol;Acc:MGI:1929608]	10.0571	7.90708	8.71865	9.21068	8.84941	9.20202	7.6755	9.74469	7.25266	8.83747	7.72716	9.40475	9.19236	8.23653	0.868638024	0.966018836	24	12	24	0.481498053	0.805393068	24	20.70025136	0.061887364
ENSMUSG00000018707	Dync1h1	dynein cytoplasmic 1 heavy chain 1 [Source:MGI Symbol;Acc:MGI:103147]	98.5298	66.181	73.0324	86.2179	99.9397	117.553	85.9007	101.972	54.554	112.314	73.6318	73.6327	71.9223	76.5043	0.44861185	0.966018836	16	20	24	0.306920795	0.768040178	24	18.38840594	0.187505508
ENSMUSG00000018727	Cpsf4l	cleavage and polyadenylation specific factor 4-like [Source:MGI Symbol;Acc:MGI:1277182]	1.38293	1.0616	0.957978	1.28864	1.60867	0.877796	1.02341	0.824927	0.874717	0.773992	1.30071	1.05457	0.945098	0.715763	0.295379186	0.805301811	20	12	24	0.034498677	0.608551791	24	17.48485919	0.143196785
ENSMUSG00000018733	Pex12	peroxisomal biogenesis factor 12 [Source:MGI Symbol;Acc:MGI:2144177]	4.43402	4.14807	4.3552	4.30014	4.30815	4.40254	5.37777	5.16992	4.96045	4.4521	4.68347	4.58018	4.20123	4.90144	0.826055781	0.966018836	8	8	24	0.49073153	0.805393068	24	3.770966645	0.042009348
ENSMUSG00000018736	Ndel1	nudE neurodevelopment protein 1 like 1 [Source:MGI Symbol;Acc:MGI:1932915]	54.9704	49.1855	48.1309	46.0576	44.2051	51.9776	49.1807	45.3518	39.683	51.8516	48.5407	49.2297	49.3214	48.5925	0.047000349	0.375011045	24	12	24	0.148927873	0.757588568	24	21.53255717	0.083966236
ENSMUSG00000018740	Slc25a35	solute carrier family 25, member 35 [Source:MGI Symbol;Acc:MGI:1919248]	3.6917	3.66208	4.4421	5.32809	5.13527	4.42273	4.61274	4.63256	4.51807	5.57408	4.96514	4.24023	5.48211	4.70525	0.271166436	0.780905461	16	16	24	0.054136396	0.654887447	24	13.05849489	0.113131247
ENSMUSG00000018750	Zbtb4	zinc finger and BTB domain containing 4 [Source:MGI Symbol;Acc:MGI:1922830]	25.4626	21.3326	21.896	23.3438	21.0361	22.931	23.0341	22.8401	20.5162	28.3693	21.3123	24.3009	23.6165	23.6344	0.418381424	0.963884303	4	8	24	0.922062042	0.979411448	24	20.35407143	0.019833928
ENSMUSG00000018752	Tnfsfm13	tumor necrosis factor (ligand) superfamily, membrane-bound member 13 [Source:MGI Symbol;Acc:MGI:3845075]	6.97131	7.61513	6.04957	5.2077	6.10068	4.57888	6.72124	6.33876	7.59373	6.06992	5.64997	6.3646	6.6375	5.79068	0.167343922	0.634848458	4	12	24	0.143026191	0.752803875	24	4.067401563	0.125185454
ENSMUSG00000018761	Mpdu1	mannose-P-dolichol utilization defect 1 [Source:MGI Symbol;Acc:MGI:1346040]	30.2513	28.4189	31.2779	30.9696	31.3433	32.4511	28.1913	33.1836	27.7477	31.2573	28.6738	27.1925	28.3452	29.3507	0.915082018	0.966018836	20	8	24	0.700435922	0.900629492	24	12.21172111	0.027345426
ENSMUSG00000018765	Fxr2	fragile X mental retardation, autosomal homolog 2 [Source:MGI Symbol;Acc:MGI:1346074]	58.1318	49.2981	48.3529	51.7852	53.2772	55.1315	52.3305	52.6921	46.2953	58.8705	47.6259	54.1344	57.2463	54.078	0.091424683	0.483797062	24	12	24	0.275089772	0.768040178	24	21.28350184	0.066820636
ENSMUSG00000018770	Atp5g3	ATP synthase, H+ transporting, mitochondrial F0 complex, subunit C3 (subunit 9) [Source:MGI Symbol;Acc:MGI:2442035]	337.628	320.46	328.607	337.252	301.319	323.973	327.215	367.539	330.041	373.818	321.663	342.042	359.879	360.538	0.510431574	0.966018836	16	4	24	0.195255944	0.768040178	24	5.393739846	0.05248888
ENSMUSG00000018774	Cd68	CD68 antigen [Source:MGI Symbol;Acc:MGI:88342]	3.15475	4.78566	5.79883	4.77473	4.93835	3.97371	4.11666	5.22862	6.17819	3.53511	4.5854	3.49627	4.11514	3.75684	0.020119233	0.259417302	12	12	24	0.076751833	0.687041208	24	9.03599792	0.194968683
ENSMUSG00000018796	Acsl1	acyl-CoA synthetase long-chain family member 1 [Source:MGI Symbol;Acc:MGI:102797]	17.7005	15.5964	23.1276	37.9339	18.8833	21.2543	19.105	30.1072	21.7989	34.2704	21.6674	21.4404	27.4513	26.939	0.287843582	0.793714531	16	12	24	0.126184617	0.739868076	24	10.39812613	0.253705702
ENSMUSG00000018800	Abca5	ATP-binding cassette, sub-family A (ABC1), member 5 [Source:MGI Symbol;Acc:MGI:2386607]	22.7571	25.0895	22.7829	24.6225	22.6545	22.6694	21.8796	20.3888	25.1926	20.4121	24.2013	23.8812	25.0015	25.6315	0.914360142	0.966018836	12	4	24	0.899993252	0.971572371	24	4.795330373	0.019129068
ENSMUSG00000018809	Smyd4	SET and MYND domain containing 4 [Source:MGI Symbol;Acc:MGI:2442796]	5.24119	3.84721	3.9134	4.00498	3.89651	4.62253	5.04726	4.47477	3.89476	4.67731	3.84334	4.33361	4.41227	4.46316	0.133766544	0.568189321	4	16	24	0.081202116	0.697074851	24	23.25046827	0.100704506
ENSMUSG00000018819	Lsp1	lymphocyte specific 1 [Source:MGI Symbol;Acc:MGI:96832]	11.6923	14.7409	15.7979	13.9392	14.2482	14.4175	14.1723	16.2161	17.2432	8.91252	11.5921	13.1342	13.4368	10.781	0.503972924	0.966018836	12	4	24	0.642457711	0.878809617	24	4.214782099	0.083208275
ENSMUSG00000018820	Zfyve27	zinc finger, FYVE domain containing 27 [Source:MGI Symbol;Acc:MGI:1919602]	32.4702	33.4937	33.5175	35.6465	33.362	32.8	33.4749	34.6955	33.2324	35.7042	34.0079	34.0487	31.9763	32.9177	0.008961721	0.192760771	16	12	24	0.037305461	0.609176161	24	11.83992195	0.040831278
ENSMUSG00000018821	Avpi1	arginine vasopressin-induced 1 [Source:MGI Symbol;Acc:MGI:1916784]	11.6778	11.3724	11.9159	10.911	8.91596	10.2709	11.9689	14.904	13.091	12.8384	13.1214	12.4049	12.9594	12.7141	0.920858634	0.966018836	8	16	24	0.062160597	0.661961741	24	5.33429204	0.123457627
ENSMUSG00000018822	Sfrp5	secreted frizzled-related sequence protein 5 [Source:MGI Symbol;Acc:MGI:1860298]	43.5639	31.2116	33.3334	36.6384	49.2015	42.5358	39.9687	41.1775	35.4941	44.1524	50.9998	46.1634	43.6207	37.0105	4.29E-04	0.054040373	20	16	24	0.001560541	0.36259991	24	18.11126133	0.219047326
ENSMUSG00000018830	Myh11	myosin, heavy polypeptide 11, smooth muscle [Source:MGI Symbol;Acc:MGI:102643]	1.92077	2.0623	2.21292	2.66436	1.84363	2.51966	2.17404	4.18184	1.97611	3.54041	1.56858	1.72209	2.29347	1.95736	0.369262716	0.893596186	16	4	24	0.488353725	0.805393068	24	7.532929891	0.137801555
ENSMUSG00000018841	Rad51d	RAD51 paralog D [Source:MGI Symbol;Acc:MGI:1261809]	10.9764	9.41959	8.95379	8.67639	9.02181	10.3554	8.86205	10.2801	8.04645	9.97183	11.204	9.20703	10.2536	9.58564	0.323424237	0.839647936	24	12	24	0.27291188	0.768040178	24	21.05721765	0.07941418
ENSMUSG00000018844	Fndc8	fibronectin type III domain containing 8 [Source:MGI Symbol;Acc:MGI:1926169]	0.692032	0.503186	0.322769	0.820685	0.520807	0.485314	0.724996	0.408613	0.541366	0.535387	0.641606	0.326438	0.538346	0.322176	0.671726933	0.966018836	16	16	24	0.547221898	0.833663107	24	16.9736777	0.060072264
ENSMUSG00000018845	Unc45b	unc-45 myosin chaperone B [Source:MGI Symbol;Acc:MGI:2443377]	1.27215	2.30211	2.37644	3.28315	2.62834	4.04745	1.98377	7.53228	3.65956	5.80769	2.44728	2.49947	2.87495	1.94839	0.47028201	0.966018836	16	12	24	0.376152761	0.796215348	24	10.46943668	0.272123141
ENSMUSG00000018846	Pank3	pantothenate kinase 3 [Source:MGI Symbol;Acc:MGI:2387464]	27.1489	27.6546	27.6772	30.6024	30.1445	25.8491	28.8465	24.7071	28.9073	25.169	29.8431	25.0794	24.6502	26.5894	0.184089873	0.662239322	20	4	24	0.203759552	0.768040178	24	13.21395068	0.065133847
ENSMUSG00000018848	Rars	arginyl-tRNA synthetase [Source:MGI Symbol;Acc:MGI:1914297]	28.9462	31.0398	29.4092	28.1932	30.73	28.7126	25.9536	28.1669	30.1247	27.6779	31.8223	28.884	32.0431	29.9123	0.890235289	0.966018836	20	20	24	0.973267235	0.993305115	24	13.04939926	0.007434224
ENSMUSG00000018849	Wwc1	WW, C2 and coiled-coil domain containing 1 [Source:MGI Symbol;Acc:MGI:2388637]	0.635572	1.09755	1.59692	0.794991	0.883171	1.01519	1.17395	0.941423	1.00497	0.950751	0.588568	0.918621	0.42648	1.33795	0.195950838	0.673646834	12	8	24	0.232321732	0.768040178	24	7.070905678	0.149420816
ENSMUSG00000018858	Mrpl58	mitochondrial ribosomal protein L58 [Source:MGI Symbol;Acc:MGI:1915822]	48.8636	48.9141	45.3595	45.8461	45.8654	44.6026	44.5174	51.0501	52.9286	44.4693	50.0135	45.9279	52.9593	48.3878	0.767183862	0.966018836	8	8	24	0.243523818	0.768040178	24	4.371869823	0.055064865
ENSMUSG00000018861	Fdxr	ferredoxin reductase [Source:MGI Symbol;Acc:MGI:104724]	9.84265	10.7303	10.5617	8.88924	9.55556	9.259	9.91589	8.7494	9.84737	9.18	10.4499	9.74757	9.33196	7.71594	0.872380105	0.966018836	12	4	24	0.757371047	0.921562129	24	17.69605706	0.030473046
ENSMUSG00000018865	Sult4a1	sulfotransferase family 4A, member 1 [Source:MGI Symbol;Acc:MGI:1888971]	222.696	167.729	165.247	171.708	170.278	201.756	182.462	192.645	140.328	222.328	174.032	194.516	187.5	192.384	0.085890569	0.466404237	24	12	24	0.267494881	0.768040178	24	21.2760492	0.113726736
ENSMUSG00000018882	Mrpl45	mitochondrial ribosomal protein L45 [Source:MGI Symbol;Acc:MGI:1914286]	12.0804	12.3417	12.5018	13.7289	12.3054	13.2692	12.6353	13.0384	12.6197	13.5429	13.4477	13.0489	14.4115	13.7429	0.833301348	0.966018836	16	20	24	0.827476397	0.946154971	24	12.93063903	0.011875705
ENSMUSG00000018893	Mb	myoglobin [Source:MGI Symbol;Acc:MGI:96922]	48.1756	149.505	183.531	183.304	134.886	160.359	85.2697	395.271	165.514	163.909	97.9487	69.5176	180.467	126.687	0.102109143	0.512431169	12	16	24	0.122381566	0.735058232	24	8.308212303	0.578038482
ENSMUSG00000018899	Irf1	interferon regulatory factor 1 [Source:MGI Symbol;Acc:MGI:96590]	9.95261	11.104	10.4958	10.2103	11.801	9.33063	8.4792	13.6961	10.8864	10.3137	11.9004	9.59037	9.88277	11.1301	0.079180494	0.458678242	8	20	24	0.308988902	0.768040178	24	8.949407006	0.096544617
ENSMUSG00000018900	Slc22a5	solute carrier family 22 (organic cation transporter), member 5 [Source:MGI Symbol;Acc:MGI:1329012]	8.95706	8.66931	9.97345	10.2054	10.6397	8.01272	10.4745	11.0505	9.10972	9.31117	8.65217	8.89793	9.85813	8.14412	0.928148816	0.966018836	16	8	24	0.849777175	0.954851064	24	9.2446829	0.030823897
ENSMUSG00000018906	P4ha2	procollagen-proline, 2-oxoglutarate 4-dioxygenase (proline 4-hydroxylase), alpha II polypeptide [Source:MGI Symbol;Acc:MGI:894286]	12.621	8.73926	6.69999	10.1481	10.1557	10.4769	10.5316	8.8669	7.35346	7.46929	10.7576	8.69186	9.40844	8.04009	0.008389425	0.192760771	4	8	24	0.005776614	0.455398301	24	20.28299902	0.241337202
ENSMUSG00000018909	Arrb1	arrestin, beta 1 [Source:MGI Symbol;Acc:MGI:99473]	52.8933	41.7548	47.2085	44.1997	49.0054	50.7759	47.3801	45.3139	35.7908	49.414	43.0578	43.6144	42.1725	43.0619	0.628899575	0.966018836	24	8	24	0.15829641	0.764753215	24	19.40337001	0.096189775
ENSMUSG00000018920	Cxcl16	chemokine (C-X-C motif) ligand 16 [Source:MGI Symbol;Acc:MGI:1932682]	4.27947	6.04518	4.78799	3.64762	4.61714	3.86849	4.26184	5.97384	7.3108	2.98867	5.00003	5.04077	4.49373	4.37271	0.265774856	0.773510818	12	4	24	0.320840094	0.768040178	24	4.725247692	0.157749786
ENSMUSG00000018921	Pelp1	proline, glutamic acid and leucine rich protein 1 [Source:MGI Symbol;Acc:MGI:1922523]	4.40179	3.79392	3.94894	4.42586	4.53873	4.40356	4.45099	4.83684	4.06447	5.95733	3.83639	4.59468	4.59931	4.57582	0.856752753	0.966018836	24	12	24	0.877813191	0.965913727	24	14.06676813	0.02574313
ENSMUSG00000018923	Med11	mediator complex subunit 11 [Source:MGI Symbol;Acc:MGI:1913422]	22.8788	22.0686	18.6511	20.9481	22.2991	22.3117	20.378	21.5243	22.958	17.2449	24.4663	21.1152	20.8953	21.7237	0.911371589	0.966018836	20	20	24	0.467880058	0.805393068	24	21.18509538	0.061164988
ENSMUSG00000018927	Ccl6	chemokine (C-C motif) ligand 6 [Source:MGI Symbol;Acc:MGI:98263]	7.9314	9.97995	12.3499	9.94293	9.51431	8.35291	9.08909	7.58474	10.8874	4.91873	8.71691	10.9511	9.75727	6.43468	0.915475528	0.966018836	12	20	24	0.972676456	0.993250534	24	21.36474216	0.028724697
ENSMUSG00000018931	Natd1	N-acetyltransferase domain containing 1 [Source:MGI Symbol;Acc:MGI:1344388]	2.78752	2.70838	2.93792	3.19686	3.11918	3.11516	2.96339	2.91523	2.60453	3.1416	2.88746	2.75444	2.90128	2.31342	0.030347909	0.324150946	16	16	24	0.035503249	0.609176161	24	15.96981754	0.077714564
ENSMUSG00000018932	Map2k3	mitogen-activated protein kinase kinase 3 [Source:MGI Symbol;Acc:MGI:1346868]	9.0065	10.2772	13.06	14.3659	12.1109	11.7447	10.6808	14.0471	13.6856	14.518	11.9366	11.0839	12.4941	10.6521	0.016720774	0.253395174	16	12	24	0.009253068	0.502965379	24	10.98938011	0.191469493
ENSMUSG00000018965	Ywhah	tyrosine 3-monooxygenase/tryptophan 5-monooxygenase activation protein, eta polypeptide [Source:MGI Symbol;Acc:MGI:109194]	748.738	589.829	587.235	625.921	619.655	734.723	643.376	645.892	449.423	740.016	576.851	613.18	612.216	622.363	0.649212555	0.966018836	4	8	24	0.239016637	0.768040178	24	20.42503025	0.122095457
ENSMUSG00000018974	Sart3	squamous cell carcinoma antigen recognized by T cells 3 [Source:MGI Symbol;Acc:MGI:1858230]	9.46876	8.83436	10.8791	10.348	10.2434	10.9563	9.46293	11.0108	9.21257	9.90746	9.72491	9.63493	9.9313	9.3932	0.814576414	0.966018836	16	16	24	0.63488691	0.875864536	24	14.8025832	0.033222309
ENSMUSG00000018983	E2f2	E2F transcription factor 2 [Source:MGI Symbol;Acc:MGI:1096341]	1.3868	1.81537	2.73545	2.31164	1.95612	1.70577	1.63856	2.03179	1.62248	1.64393	1.46432	2.19912	2.40795	1.77579	0.832293447	0.966018836	12	16	24	0.751429453	0.91921305	24	8.465558653	0.05952177
ENSMUSG00000018995	Nars2	asparaginyl-tRNA synthetase 2 (mitochondrial)(putative) [Source:MGI Symbol;Acc:MGI:2142075]	5.22733	5.49503	6.88887	6.11205	5.7382	5.74411	5.54831	5.02651	4.61419	5.47603	5.63472	5.61948	5.97746	6.39983	0.826055781	0.966018836	16	16	24	0.935188435	0.983765461	24	13.1641562	0.017881021
ENSMUSG00000018999	Slc35b4	solute carrier family 35, member B4 [Source:MGI Symbol;Acc:MGI:1931249]	17.1184	14.1511	15.0932	16.0144	14.6626	16.924	16.7578	16.1136	13.3381	17.9728	13.7941	16.8551	15.8927	17.8636	0.791413662	0.966018836	24	12	24	0.530962118	0.825104543	24	22.83462703	0.057438781
ENSMUSG00000019039	Dalrd3	DALR anticodon binding domain containing 3 [Source:MGI Symbol;Acc:MGI:1915039]	38.8978	37.632	37.3925	39.5566	36.9703	33.7639	36.7302	32.2991	37.3824	32.4313	38.5808	36.65	38.4561	34.9406	0.881996584	0.966018836	20	12	24	0.870903117	0.963364428	24	18.57007174	0.019454903
ENSMUSG00000019054	Fis1	fission, mitochondrial 1 [Source:MGI Symbol;Acc:MGI:1913687]	177.327	184.926	186.559	177.626	172.645	175.633	172.598	175.691	193.483	154.737	188.078	178.79	185.159	182.163	0.649212555	0.966018836	12	4	24	0.564492046	0.843170383	24	4.155103007	0.032793876
ENSMUSG00000019055	Plod1	procollagen-lysine, 2-oxoglutarate 5-dioxygenase 1 [Source:MGI Symbol;Acc:MGI:99907]	14.8469	12.3366	12.7992	13.0725	14.6961	13.4627	16.4798	14.2711	13.0157	13.9033	14.4181	12.0187	13.472	12.0425	0.321662653	0.83607295	4	4	24	0.448536369	0.805393068	24	19.85386542	0.061031167
ENSMUSG00000019066	Rab3d	RAB3D, member RAS oncogene family [Source:MGI Symbol;Acc:MGI:97844]	35.7434	25.0501	24.1193	25.4159	28.0098	31.7177	26.598	29.1819	20.3092	31.5811	27.0958	29.0615	28.4465	27.8148	0.020119233	0.259417302	24	12	24	0.077732838	0.687545294	24	20.65350316	0.163034392
ENSMUSG00000019080	Mfsd3	major facilitator superfamily domain containing 3 [Source:MGI Symbol;Acc:MGI:1916822]	3.83726	4.53079	7.15011	4.1861	5.01921	3.38974	3.37365	4.03381	4.11904	3.18395	4.97133	3.18209	3.74374	5.55626	0.071575474	0.440262583	12	12	24	0.230690845	0.768040178	24	8.521485812	0.18363388
ENSMUSG00000019082	Slc25a22	solute carrier family 25 (mitochondrial carrier, glutamate), member 22 [Source:MGI Symbol;Acc:MGI:1915517]	20.6103	16.7768	17.7767	20.7333	19.0545	20.4166	20.081	21.3558	17.2964	28.4561	17.836	20.8158	19.7983	20.1933	0.85447369	0.966018836	24	12	24	0.761465063	0.922782206	24	15.05952712	0.050521271
ENSMUSG00000019087	Atp6ap1	ATPase, H+ transporting, lysosomal accessory protein 1 [Source:MGI Symbol;Acc:MGI:109629]	154.042	135.624	139.193	140.89	140.824	157.863	147.117	142.879	120.676	143.989	134.799	139.539	139.448	138.823	0.351840494	0.870418653	24	12	24	0.053417323	0.652322075	24	20.89958522	0.077799426
ENSMUSG00000019088	Dnase1l1	deoxyribonuclease 1-like 1 [Source:MGI Symbol;Acc:MGI:109628]	13.2709	14.8504	14.915	13.324	14.8562	13.5565	12.8268	15.1332	18.3117	13.1038	13.6728	11.4455	14.0773	13.0137	0.133266423	0.567728672	12	12	24	0.113164221	0.727393008	24	7.917669214	0.115852383
ENSMUSG00000019122	Ccl9	chemokine (C-C motif) ligand 9 [Source:MGI Symbol;Acc:MGI:104533]	3.81071	4.52418	2.17814	3.71779	4.35622	3.51814	3.09728	2.77242	4.17398	1.41658	3.34236	2.7691	3.67909	2.68591	0.849180783	0.966018836	4	12	24	0.496553766	0.807549708	24	21.93647536	0.147132435
ENSMUSG00000019124	Scrn1	secernin 1 [Source:MGI Symbol;Acc:MGI:1917188]	52.2221	44.1103	48.283	48.2138	51.9274	51.5529	50.7455	43.0141	43.7903	53.2178	44.7401	45.9524	49.1024	48.371	0.291081671	0.797585788	4	4	24	0.322720673	0.768040178	24	18.41926325	0.060433509
ENSMUSG00000019132	BC005537	cDNA sequence BC005537 [Source:MGI Symbol;Acc:MGI:2441726]	76.1012	67.84	68.7421	78.1541	83.1843	71.509	68.1379	70.2402	68.543	76.1077	68.8189	68.4544	66.6574	67.8895	0.058566563	0.405213521	16	16	24	0.040340867	0.616921492	24	14.99671975	0.076553833
ENSMUSG00000019139	Isyna1	myo-inositol 1-phosphate synthase A1 [Source:MGI Symbol;Acc:MGI:1919030]	13.3091	13.4395	10.8092	9.74727	10.3174	11.3919	11.0487	15.5347	12.5656	10.8128	12.7607	12.4074	11.251	12.6619	0.031431762	0.324385159	8	8	24	0.105151622	0.717252017	24	2.626163513	0.127825349
ENSMUSG00000019143	Hars2	histidyl-tRNA synthetase 2 [Source:MGI Symbol;Acc:MGI:1918041]	16.1749	14.3903	15.678	14.9334	15.3292	15.0024	12.9979	15.0259	16.271	14.8298	16.8275	15.9672	14.4744	15.1973	0.622132989	0.966018836	20	8	24	0.407212682	0.799546792	24	13.46075837	0.044867114

ENSMUSG00000019146	Cacng2	calcium channel, voltage-dependent, gamma subunit 2 [Source:MGI Symbol;Acc:MGI:1316660]	2.33678	2.39541	3.41231	2.53445	1.51746	2.75744	2.79375	2.20889	2.37673	3.79276	1.88196	2.76919	2.26944	3.86984	0.923635729	0.966018836	16	4	24	0.323246636	0.768040178	24	6.568402047	0.155480856
ENSMUSG00000019158	Tmem160	transmembrane protein 160 [Source:MGI Symbol;Acc:MGI:1916344]	24.0577	21.5619	23.4873	18.8646	21.5954	20.9356	19.8493	26.5491	28.8256	25.0754	28.4258	26.4235	26.6544	25.6114	0.830949782	0.966018836	12	4	24	0.642271788	0.878809617	24	6.723088266	0.056874865
ENSMUSG00000019173	Rab5c	RAB5C, member RAS oncogene family [Source:MGI Symbol;Acc:MGI:105306]	59.138	55.2956	55.9976	52.3492	51.6466	55.9861	59.897	57.9325	51.6272	56.3402	54.386	55.8833	55.2027	54.001	0.328944792	0.847277612	4	16	24	0.052868412	0.650698269	24	0.034262954	0.055570498
ENSMUSG00000019179	Mdh2	malate dehydrogenase 2, NAD (mitochondrial) [Source:MGI Symbol;Acc:MGI:97050]	188.271	169.957	176.471	187.096	174.014	212.457	182.593	213.91	159.269	218.707	169.514	176.228	190.957	188.576	0.851656735	0.966018836	4	16	24	0.913281974	0.97565709	24	22.32420232	0.023896887
ENSMUSG00000019188	H13	histocompatibility 13 [Source:MGI Symbol;Acc:MGI:95886]	48.5331	37.5084	39.3672	38.5449	41.1468	43.5398	41.1817	46.1437	36.3426	46.7647	43.3053	43.7074	43.9436	42.2796	0.176754388	0.653256853	4	8	24	0.246974229	0.768040178	24	20.98230877	0.077845442
ENSMUSG00000019189	Rnf145	ring finger protein 145 [Source:MGI Symbol;Acc:MGI:1921565]	21.3291	20.7749	22.2054	21.2965	22.3258	22.023	22.5958	20.5621	20.7553	25.3971	21.2852	20.9404	21.3777	22.4676	0.915082018	0.966018836	16	16	24	0.474786242	0.805393068	24	13.09794026	0.035386498
ENSMUSG00000019194	Scn1b	sodium channel, voltage-gated, type I, beta [Source:MGI Symbol;Acc:MGI:98247]	81.9812	65.4129	71.8301	72.9599	65.2779	78.6091	71.5343	81.9105	60.728	102.903	58.7796	75.2188	69.3072	73.9503	0.921741413	0.966018836	24	20	24	0.993704013	0.998755922	24	3.606328345	0.009192539
ENSMUSG00000019210	Atp6v1e1	ATPase, H+ transporting, lysosomal V1 subunit E1 [Source:MGI Symbol;Acc:MGI:894326]	261.705	272.098	254.995	254.796	255.344	254.138	244.45	245.058	264.417	235.277	275.391	263.66	285.145	283.092	0.945282252	0.966018836	8	8	24	0.543989888	0.831967906	24	1.188915799	0.032665476
ENSMUSG00000019214	Chtf18	CTF18, chromosome transmission fidelity factor 18 [Source:MGI Symbol;Acc:MGI:2384887]	0.965409	0.596139	0.628049	1.17973	0.567729	0.614124	1.07662	0.773822	1.22762	0.789016	0.677741	0.679483	0.649961	0.763992	0.928892845	0.966018836	16	4	24	0.595970418	0.857839743	24	7.761745865	0.072253051
ENSMUSG00000019232	Etnppl	ethanolamine phosphate phospholyase [Source:MGI Symbol;Acc:MGI:1919010]	2.27655	1.7166	2.60221	3.36201	1.98436	3.17063	2.47767	1.0306	2.30321	1.73117	2.21201	3.41868	1.4638	1.87195	0.062631097	0.410322149	24	8	24	0.179091952	0.768040178	24	16.81653317	0.222018674
ENSMUSG00000019235	Rps6kl1	ribosomal protein S6 kinase-like 1 [Source:MGI Symbol;Acc:MGI:2443413]	5.22226	5.25645	5.39916	4.8239	5.36677	6.10064	4.97699	5.17327	4.79483	6.39684	5.25315	5.74856	5.5755	4.98625	0.46238066	0.966018836	24	12	24	0.307559595	0.768040178	24	17.73973684	0.061662856
ENSMUSG00000019254	Ppp1r12c	protein phosphatase 1, regulatory subunit 12C [Source:MGI Symbol;Acc:MGI:1924258]	46.7258	49.4655	45.626	44.9606	52.9616	45.8063	45.7572	43.8093	48.5011	46.0151	46.1465	46.0967	43.2317	46.9878	0.887862353	0.966018836	20	8	24	0.643663282	0.878958277	24	14.28603801	0.025388937
ENSMUSG00000019256	Ahr	aryl-hydrocarbon receptor [Source:MGI Symbol;Acc:MGI:105043]	6.07676	5.18551	5.06025	5.22625	6.09089	4.98341	6.05649	4.78652	4.37245	5.57053	5.52372	4.77367	4.73066	4.55615	0.287843582	0.793714531	20	12	24	0.115736019	0.727799063	24	18.18271877	0.090655394
ENSMUSG00000019261	Map1s	microtubule-associated protein 1S [Source:MGI Symbol;Acc:MGI:2443304]	8.66477	6.49999	5.73736	6.86902	7.22653	6.20297	7.29061	7.69018	8.01886	11.2355	8.81256	9.19784	9.00355	9.57289	0.829428469	0.966018836	4	8	24	0.964093129	0.991038325	24	15.27407656	0.019145688
ENSMUSG00000019278	Dpep1	dipeptidase 1 [Source:MGI Symbol;Acc:MGI:94917]	3.2476	3.24763	2.86918	4.38353	4.0547	3.52257	2.66185	3.48467	3.17765	2.54846	4.44517	3.83284	3.71673	2.84335	0.097779482	0.501112642	20	16	24	0.116446281	0.727799063	24	16.96499924	0.153084185
ENSMUSG00000019295	Tmem129	transmembrane protein 129 [Source:MGI Symbol;Acc:MGI:1915616]	16.0489	13.8738	14.6538	13.9424	13.1659	15.8776	14.1078	14.9814	12.5486	14.8914	13.6293	14.25	14.7183	14.1225	0.371875382	0.894537464	24	20	24	0.21495979	0.768040178	24	22.77487095	0.059811151
ENSMUSG00000019297	Nop9	NOP9 nucleolar protein [Source:MGI Symbol;Acc:MGI:1915092]	5.09578	4.47427	5.05631	5.00568	4.98694	5.06471	4.71803	5.69909	3.9101	4.87913	5.57855	4.01107	4.17332	5.14273	0.873350015	0.966018836	8	4	24	0.891305232	0.969291345	24	13.70471713	0.025416689
ENSMUSG00000019302	Atp6v0a1	ATPase, H+ transporting, lysosomal V0 subunit A1 [Source:MGI Symbol;Acc:MGI:103286]	59.6984	49.1275	51.9467	53.7091	50.9069	57.6493	56.4896	57.4599	46.9721	67.9464	52.9009	57.5063	51.6605	59.0586	0.939921059	0.966018836	24	12	24	0.836229213	0.949949931	24	19.55168317	0.03317428
ENSMUSG00000019303	Psmc3ip	proteasome (prosome, macropain) 26S subunit, ATPase 3, interacting protein [Source:MGI Symbol;Acc:MGI:1098610]	1.35341	2.40938	1.79004	1.08681	2.17094	2.1053	1.69922	1.71326	2.70939	1.20228	1.61847	1.30577	1.70007	1.26088	0.676344318	0.966018836	12	4	24	0.86629814	0.961349768	24	3.988487145	0.052406603
ENSMUSG00000019320	Noxo1	NADPH oxidase organizer 1 [Source:MGI Symbol;Acc:MGI:1919143]	0.579985	0.487414	1.06337	0.585557	0.807821	0.873689	0.825481	1.26804	1.1541	0.753236	0.859287	0.84628	1.1251	0.900564	0.893307667	0.966018836	12	4	24	0.551252375	0.83632237	24	5.943943221	0.067694601
ENSMUSG00000019326	Aoc3	amine oxidase, copper containing 3 [Source:MGI Symbol;Acc:MGI:1306797]	1.1304	0.999099	1.28139	2.29854	1.4089	1.21294	1.40536	1.9919	1.47839	2.75756	1.58903	1.44534	2.76398	1.86961	0.883024237	0.966018836	16	12	24	0.36099799	0.786486151	24	10.52643792	0.135163602
ENSMUSG00000019338	Zfp687	zinc finger protein 687 [Source:MGI Symbol;Acc:MGI:1925516]	5.42304	5.82077	5.99009	5.74029	5.48646	5.57467	5.97132	5.94564	5.50743	6.62231	4.93656	6.67707	5.6169	6.2538	0.839634479	0.966018836	8	12	24	0.668289622	0.887912848	24	5.991292427	0.035454898
ENSMUSG00000019359	Gdpd2	glycerophosphodiester phosphodiesterase domain containing 2 [Source:MGI Symbol;Acc:MGI:1918834]	3.82849	2.68632	4.49191	2.75098	2.93043	3.23642	3.82008	1.87285	2.88935	2.38021	3.01446	2.92944	2.5584	2.38708	0.338172159	0.85758369	24	8	24	0.6096685	0.864506849	24	19.6927345	0.086391169
ENSMUSG00000019362	D8Ertd738e	DNA segment, Chr 8, ERATO Doi 738, expressed [Source:MGI Symbol;Acc:MGI:1289231]	110.961	107.541	100.819	96.1754	95.0272	93.7577	97.8331	105.96	112.517	97.8149	118.737	111.552	115.946	115.017	0.658942124	0.966018836	4	20	24	0.319882862	0.768040178	24	2.925954013	0.058856724
ENSMUSG00000019370	Calm3	calmodulin 3 [Source:MGI Symbol;Acc:MGI:103249]	266.27	215.664	221.051	218.943	220.956	242.357	228.228	239.976	197.99	258.578	223.478	235.794	235.689	238.12	0.321662653	0.83607295	24	12	24	0.321214138	0.768040178	24	21.72451595	0.065755924
ENSMUSG00000019373	Cops3	COP9 signalosome subunit 3 [Source:MGI Symbol;Acc:MGI:1349409]	65.3801	70.347	67.056	64.7675	69.2143	64.1842	61.246	67.345	69.6485	59.3069	69.2983	66.6205	68.6819	68.094	0.560714538	0.966018836	8	16	24	0.794046012	0.933885455	24	4.782675139	0.01969885
ENSMUSG00000019428	Fkbp8	FK506 binding protein 8 [Source:MGI Symbol;Acc:MGI:1341070]	169.317	154.013	150.062	145.773	150.886	151.899	149.974	161.53	158.325	153.359	163.37	157.579	158.268	153.365	0.846840613	0.966018836	4	12	24	0.4233797	0.805346055	24	23.67899875	0.029310738
ENSMUSG00000019432	Ddx39b	DEAD box helicase 39b [Source:MGI Symbol;Acc:MGI:99240]	67.1837	64.6191	68.2234	67.386	68.5906	70.1169	70.2672	70.0811	67.8484	72.0906	62.1333	63.517	63.7642	62.3523	0.854540463	0.966018836	16	4	24	0.779141868	0.928962679	24	11.72987758	0.017850385
ENSMUSG00000019433	Gipc1	GIPC PDZ domain containing family, member 1 [Source:MGI Symbol;Acc:MGI:1926252]	26.6005	21.9806	21.1574	21.1013	20.6862	22.7523	22.4518	24.518	22.0779	27.4763	23.3294	25.2634	25.79	25.4769	0.481811994	0.966018836	4	16	24	0.439102831	0.805393068	24	0.313918261	0.056608711
ENSMUSG00000019437	Tlcd1	TLC domain containing 1 [Source:MGI Symbol;Acc:MGI:1915572]	14.2423	18.9463	17.4482	19.7289	16.7489	16.6516	16.6246	15.3109	19.587	13.5031	16.5544	18.2411	17.1804	14.5214	0.893609703	0.966018836	12	16	24	0.856482569	0.957486395	24	11.87252785	0.035289928
ENSMUSG00000019461	Plscr3	phospholipid scramblase 3 [Source:MGI Symbol;Acc:MGI:1917560]	20.8704	17.9227	16.6564	17.8552	18.6794	18.8456	18.6144	21.0059	18.1238	23.3165	18.2295	19.2519	18.9304	19.1104	0.43280837	0.966018836	4	8	24	0.949122438	0.987657816	24	21.1804138	0.015253643
ENSMUSG00000019464	Ptger1	prostaglandin E receptor 1 (subtype EP1) [Source:MGI Symbol;Acc:MGI:97793]	3.99112	3.35352	3.68989	4.18215	4.03677	4.17008	3.94948	4.65779	2.50321	3.34986	2.97255	3.02606	2.96775	3.11813	0.785563354	0.966018836	16	20	24	0.675371841	0.89119716	24	20.79093075	0.05961592
ENSMUSG00000019467	Arhgef25	Rho guanine nucleotide exchange factor (GEF) 25 [Source:MGI Symbol;Acc:MGI:1277173]	44.5374	39.4778	40.3365	41.1429	44.2417	42.4712	39.0531	41.1557	37.7473	43.4496	37.6077	38.9209	41.0022	42.9308	0.84680398	0.966018836	4	8	24	0.883472379	0.966818623	24	19.89703139	0.016126634
ENSMUSG00000019470	Xab2	XPA binding protein 2 [Source:MGI Symbol;Acc:MGI:1914689]	10.6864	9.7278	10.3956	10.2055	9.45781	10.5568	10.2474	11.4748	9.27747	11.5759	9.10033	10.0678	10.0329	10.3328	0.965971228	0.969272194	8	12	24	0.681614291	0.89381998	24	4.014977946	0.032402414
ENSMUSG00000019471	Cdc37	cell division cycle 37 [Source:MGI Symbol;Acc:MGI:109531]	77.9848	66.0111	68.0831	64.9251	61.324	71.2101	66.8286	73.2045	59.3161	65.2355	63.0081	67.3364	66.9023	64.6961	0.108029159	0.524035616	24	20	24	0.047926075	0.636814569	24	23.70617764	0.082622476
ENSMUSG00000019478	Rab4a	RAB4A, member RAS oncogene family [Source:MGI Symbol;Acc:MGI:105069]	22.2797	19.218	20.4476	22.3934	19.2547	23.1117	22.3579	21.0958	17.6282	28.591	18.938	22.5478	21.9716	21.5087	0.021795836	0.26698364	24	12	24	0.818768349	0.942446688	24	18.22686051	0.042007247
ENSMUSG00000019487	Trip10	thyroid hormone receptor interactor 10 [Source:MGI Symbol;Acc:MGI:2146901]	13.8472	13.9118	14.1654	18.4187	15.9892	15.5702	13.6064	15.4395	16.4097	19.9677	15.7193	15.753	15.1036	13.5485	8.95E-04	0.071344007	16	16	24	0.004101227	0.427431763	24	13.14684312	0.161869663
ENSMUSG00000019494	Cops6	COP9 signalosome subunit 6 [Source:MGI Symbol;Acc:MGI:1349439]	83.1775	78.3889	81.7903	81.676	80.7012	80.8729	78.7551	79.133	77.6964	81.1216	84.7597	83.7043	84.7838	83.7734	0.793403332	0.966018836	20	16	24	0.345204195	0.778298718	24	18.80869528	0.022174824
ENSMUSG00000019505	Ubb	ubiquitin B [Source:MGI Symbol;Acc:MGI:98888]	457.476	377.453	396.313	394.789	404.338	449.015	401.814	400.719	376.437	412.84	422.358	421.771	437.883	422.626	0.015345591	0.253245931	24	12	24	0.025785623	0.586541342	24	20.69091371	0.081602894
ENSMUSG00000019518	Ap4m1	adaptor-related protein complex AP-4, mu 1 [Source:MGI Symbol;Acc:MGI:1337063]	10.9481	11.9634	11.151	9.94067	11.4667	11.8232	10.2578	12.5331	12.2637	11.5028	10.3241	11.5593	12.4856	10.5862	0.89563591	0.966018836	8	8	24	0.571814267	0.846185279	24	3.095301994	0.041131539
ENSMUSG00000019528	Gyg	glycogenin [Source:MGI Symbol;Acc:MGI:1351614]	91.1272	84.7255	83.024	86.4845	81.8059	91.0151	78.7569	95.1295	77.4531	94.9332	87.0075	85.2533	91.4936	87.0879	0.948454836	0.966018836	8	4	24	0.894997434	0.970212381	24	21.88060799	0.017710534
ENSMUSG00000019539	Rcn3	reticulocalbin 3, EF-hand calcium binding domain [Source:MGI Symbol;Acc:MGI:1277122]	27.2092	33.2596	27.682	25.732	32.399	27.6534	30.6185	33.5706	39.3901	25.4857	28.6702	29.1264	30.2511	28.1009	0.338172159	0.85758369	8	8	24	0.45402582	0.805393068	24	4.653190574	0.082728081
ENSMUSG00000019558	Slc6a8	solute carrier family 6 (neurotransmitter transporter, creatine), member 8 [Source:MGI Symbol;Acc:MGI:2147834]	21.5782	24.127	28.0172	30.8644	26.2131	23.4988	26.0912	22.8049	29.9676	28.8503	22.099	23.3611	22.4424	24.7798	0.016024075	0.253395174	16	12	24	0.005486408	0.445165967	24	10.47177019	0.175415251
ENSMUSG00000019564	Arid3a	AT rich interactive domain 3A (BRIGHT-like) [Source:MGI Symbol;Acc:MGI:1328360]	1.81835	1.8859	1.85526	2.02193	1.90527	1.88919	1.80877	1.82234	1.63678	1.94421	1.98522	2.05322	2.20511	1.98792	0.640622311	0.966018836	16	20	24	0.412010196	0.799546792	24	18.80342825	0.032564647
ENSMUSG00000019577	Pdk4	pyruvate dehydrogenase kinase, isoenzyme 4 [Source:MGI Symbol;Acc:MGI:1351481]	8.80455	8.93192	14.901	20.4687	6.86038	14.3509	14.0394	54.8656	13.3428	18.8509	11.3423	19.2611	28.7919	25.8681	0.943920575	0.966018836	8	12	24	0.220532756	0.768040178	24	5.133451394	0.423065419
ENSMUSG00000019578	Ubxn6	UBX domain protein 6 [Source:MGI Symbol;Acc:MGI:1913780]	41.5824	34.6692	35.7779	36.4885	35.5068	35.2667	37.1837	38.193	40.5828	40.8581	40.7084	39.9321	42.1368	41.2314	0.833859682	0.966018836	4	20	24	0.829884291	0.947128979	24	2.780859464	0.018451481
ENSMUSG00000019579	Mydgf	myeloid derived growth factor [Source:MGI Symbol;Acc:MGI:2156020]	21.5294	23.4339	21.1979	18.5298	22.7689	21.4497	21.5433	22.0142	24.8482	17.6264	24.3209	20.9234	22.5399	21.8311	0.625825704	0.966018836	8	8	24	0.551443293	0.83632237	24	1.148606468	0.054551559
ENSMUSG00000019590	Cyb561	cytochrome b-561 [Source:MGI Symbol;Acc:MGI:103253]	17.7732	14.3975	17.4188	16.1831	16.6448	21.6688	20.4377	18.1394	12.7851	22.4513	18.9984	20.2164	14.7974	30.5915	0.846840613	0.966018836	24	12	24	0.861262414	0.959280176	24	21.12766804	0.062756346
ENSMUSG00000019647	Sema6a	sema domain, transmembrane domain (TM), and cytoplasmic domain, (semaphorin) 6A [Source:MGI Symbol;Acc:MGI:1203727]	2.4683	2.75154	3.59383	2.25324	3.1715	2.87954	3.18779	2.77208	2.61053	2.98849	2.98934	2.5745	2.62903	2.90914	0.882617464	0.966018836	20	8	24	0.916287579	0.976890292	24	10.62929186	0.021995174
ENSMUSG00000019659	Ccdc12	coiled-coil domain containing 12 [Source:MGI Symbol;Acc:MGI:1919904]	19.5105	21.6801	22.9392	22.9583	22.8759	21.5151	21.1477	23.5295	22.5053	18.315	24.6212	19.7999	22.7835	20.3968	0.718086778	0.966018836	20	4	24	0.825517985	0.9453534	24	11.22652421	0.028704378
ENSMUSG00000019689	Fmc1	formation of mitochondrial complex V assembly factor 1 [Source:MGI Symbol;Acc:MGI:1913367]	27.1522	30.2365	28.8812	34.7251	30.672	29.1454	33.4187	36.2236	40.9084	32.7793	38.2298	38.523	39.9469	40.0884	0.767868806	0.966018836	12	12	24	0.315095627	0.768040178	24	7.703504043	0.062030064
ENSMUSG00000019699	Akt3	thymoma viral proto-oncogene 3 [Source:MGI Symbol;Acc:MGI:1345147]	53.66	50.3883	46.9394	48.5982	49.5207	49.541	48.7051	47.1913	47.2107	52.8359	48.3358	52.1591	53.1302	53.6804	0.182849367	0.659303873	4	8	24	0.349538005	0.779170188	24	22.50165198	0.038584732
ENSMUSG00000019710	Mrpl24	mitochondrial ribosomal protein L24 [Source:MGI Symbol;Acc:MGI:1914957]	42.688	47.9828	45.9157	46.01	47.5217	42.4755	45.8093	43.3476	53.2983	39.8914	51.3899	45.1514	47.8515	46.922	0.928064146	0.966018836	12	12	24	0.77728646	0.928633759	24	8.819243018	0.031394809
ENSMUSG00000019715	Gle1	GLE1 RNA export mediator (yeast) [Source:MGI Symbol;Acc:MGI:1921662]	13.5151	12.6671	14.0808	13.2067	12.8253	14.7408	14.38	14.327	12.4973	13.7841	11.5785	14.5953	14.1848	13.8463	0.022000259	0.267229408	24	20	24	0.240981488	0.768040178	24	0.134480605	0.059929618
ENSMUSG00000019718	L3hypdh	L-3-hydroxyproline dehydratase (trans-) [Source:MGI Symbol;Acc:MGI:1914467]	1.44875	1.83349	2.18462	1.87289	1.39122	1.40176	1.54517	1.96463	1.89894	1.88645	2.34974	1.71445	1.97089	2.46143	0.268052211	0.774280302	12	12	24	0.019534457	0.564709807	24	7.958453742	0.148098573
ENSMUSG00000019726	Lyst	lysosomal trafficking regulator [Source:MGI Symbol;Acc:MGI:107448]	6.80893	8.19714	6.93872	6.49291	6.8963	8.07113	8.20034	6.26416	7.17202	6.40456	8.22776	9.66593	7.06619	6.67515	0.154830409	0.607427992	24	16	24	0.110614574	0.722405302	24	20.3130531	0.128739161
ENSMUSG00000019731	Slc35e1	solute carrier family 35, member E1 [Source:MGI Symbol;Acc:MGI:2142403]	14.819	12.1674	12.8054	13.1765	12.8705	14.432	14.5852	13.7956	10.8761	16.2104	12.2165	13.9988	13.3311	13.0426	0.106193509	0.519440352	4	8	24	0.397964514	0.799546792	24	20.37322153	0.073873566
ENSMUSG00000019732	Calr3	calreticulin 3 [Source:MGI Symbol;Acc:MGI:1920566]	2.70853	3.69773	3.20229	3.77604	3.2765	3.64761	2.83805	3.4593	3.1864	3.26468	3.49793	4.02863	3.28126	4.05307	0.843639218	0.966018836	24	4	24	0.800116124	0.935884746	24	12.09640936	0.032167191
ENSMUSG00000019734	Tmc4	transmembrane channel-like gene family 4 [Source:MGI Symbol;Acc:MGI:2669035]	4.93616	5.41989	4.27421	4.6627	5.18508	4.37623	5.34776	4.06994	5.16361	2.96572	4.66638	4.55128	3.78068	4.02915	0.81544087	0.966018836	20	20	24	0.577607021	0.848840315	24	22.13618639	0.070492209
ENSMUSG00000019737	Syne4	spectrin repeat containing, nuclear envelope family member 4 [Source:MGI Symbol;Acc:MGI:2141950]	11.5529	9.73786	8.79771	7.97627	9.8271	8.6105	10.0261	8.55978	8.24414	9.26271	9.15439	7.90481	9.79615	10.042	0.142596848	0.585944911	4	20	24	0.198516717	0.768040178	24	0.123971816	0.09208863
ENSMUSG00000019738	Polr2i	polymerase (RNA) II (DNA directed) polypeptide I [Source:MGI Symbol;Acc:MGI:1917170]	20.365	21.9423	22.5517	22.0315	26.0165	23.0203	19.4024	24.2481	24.1285	20.8864	24.3736	20.3656	23.4186	22.6017	0.743750765	0.966018836	20	8	24	0.484673672	0.805393068	24	11.89536566	0.053830868
ENSMUSG00000019761	Krt10	keratin 10 [Source:MGI Symbol;Acc:MGI:96685]	5.10258	4.06236	3.36368	4.22881	4.89182	4.42156	6.41704	5.22735	4.08653	4.28252	6.18046	4.27881	4.47152	4.2168	0.03452758	0.328311403	4	8	24	0.196728437	0.768040178	24	20.46877954	0.145321881
ENSMUSG00000019763	Rmnd1	required for meiotic nuclear division 1 homolog [Source:MGI Symbol;Acc:MGI:1913334]	15.0386	13.4573	14.0734	13.9356	16.31	15.7871	15.9892	15.4852	13.611	11.9877	19.2021	12.3796	15.8406	13.2956	0.248056532	0.746745468	20	20	24	0.304066959	0.768040178	24	19.62738122	0.105069352
ENSMUSG00000019767	Ccdc170	coiled-coil domain containing 170 [Source:MGI Symbol;Acc:MGI:2685067]	0.702992	0.538208	0.459497	0.487994	0.570271	0.447126	0.713555	0.38004	0.43368	0.352595	0.654682	0.590703	0.49433	0.563467	0.083354723	0.462922111	4	8	24	0.09097806	0.71175955	24	21.33857613	0.08756095
ENSMUSG00000019768	Esr1	estrogen receptor 1 (alpha) [Source:MGI Symbol;Acc:MGI:1352467]	3.1276	3.33416	2.95143	4.04005	3.06141	3.27308	3.00059	3.27161	3.32612	3.78932	2.8193	3.45288	3.16287	3.06756	0.745118544	0.966018836	16	12	24	0.399983767	0.799546792	24	11.57394042	0.057105224
ENSMUSG00000019772	Vip	vasoactive intestinal polypeptide [Source:MGI Symbol;Acc:MGI:98933]	0.464048	0.413134	0.694234	0.934766	0.414	1.30558	3.11859	0.338404	0.178545	1.45592	2.21983	2.05611	0.0385576	4.49092	0.917867465	0.966018836	24	12	24	0.831263523	0.947661285	24	18.7591527	0.167965863
ENSMUSG00000019773	Fbxo5	F-box protein 5 [Source:MGI Symbol;Acc:MGI:1914391]	0.958622	1.40684	1.6497	1.25891	1.5171	1.42458	1.2539	1.16146	1.58716	1.38916	1.27759	1.40198	1.56971	1.36633	0.773177465	0.966018836	12	16	24	0.428636299	0.805393068	24	10.64383298	0.057907992
ENSMUSG00000019774	Mtrf1l	mitochondrial translational release factor 1-like [Source:MGI Symbol;Acc:MGI:1918830]	5.58569	4.34546	4.31773	5.82346	5.59298	5.87709	6.09115	4.3032	4.99565	5.33065	5.24005	5.26382	5.58628	4.0043	0.015345591	0.253245931	4	4	24	0.012270515	0.525280095	24	18.2752755	0.167625929
ENSMUSG00000019775	Rgs17	regulator of G-protein signaling 17 [Source:MGI Symbol;Acc:MGI:1927469]	9.58	9.98395	10.7666	9.24504	9.81661	9.92526	9.88109	8.135	9.66716	8.0948	10.2437	9.38067	8.58146	10.1712	0.890235289	0.966018836	12	4	24	0.96619516	0.991892586	24	19.36229447	0.011838365
ENSMUSG00000019777	Hdac2	histone deacetylase 2 [Source:MGI Symbol;Acc:MGI:1097691]	60.6494	59.2909	55.0962	57.0081	60.9753	58.3797	54.8846	54.4873	54.7044	51.157	56.7555	55.2176	58.4372	55.0107	0.50311966	0.966018836	20	20	24	0.082922412	0.699403344	24	20.8865781	0.051187875
ENSMUSG00000019782	Rwdd1	RWD domain containing 1 [Source:MGI Symbol;Acc:MGI:1913771]	10.3803	11.9504	11.9698	10.6088	10.9342	10.2878	11.5442	11.1175	13.27	10.246	13.1592	11.6138	13.9085	12.7373	0.676344318	0.966018836	12	4	24	0.354441845	0.781577622	24	5.027301076	0.064282308
ENSMUSG00000019785	Clvs2	clavesin 2 [Source:MGI Symbol;Acc:MGI:2443223]	5.46737	5.90492	5.84018	5.44492	4.87575	6.19271	6.40254	5.61281	5.29146	5.06198	4.76475	6.104	5.41973	6.34102	0.042936877	0.356048751	24	20	24	0.050483789	0.644469829	24	1.521379853	0.100175008
ENSMUSG00000019787	Trdn	triadin [Source:MGI Symbol;Acc:MGI:1924007]	3.68148	11.6157	12.2365	14.1942	10.3056	10.9659	8.32202	19.463	41.3692	27.7127	12.6068	14.9261	17.7908	14.1518	0.34864256	0.870418653	16	12	24	0.009548836	0.502965379	24	9.541422197	0.661689476
ENSMUSG00000019789	Hey2	hairy/enhancer-of-split related with YRPW motif 2 [Source:MGI Symbol;Acc:MGI:1341884]	14.3244	12.417	11.8989	11.1965	11.8108	12.8629	13.5848	12.9031	9.76794	11.4262	12.4033	10.9011	11.2254	12.521	0.112015648	0.530777648	4	12	24	0.06331304	0.663715314	24	23.13475318	0.103851814
ENSMUSG00000019790	Stxbp5	syntaxin binding protein 5 (tomosyn) [Source:MGI Symbol;Acc:MGI:1926058]	12.7898	11.6872	13.0173	11.214	11.454	12.4352	12.162	11.2666	11.5513	12.4095	11.0221	12.6282	10.7877	11.9695	0.907000607	0.966018836	24	20	24	0.890407597	0.969022636	24	23.03301157	0.014823803
ENSMUSG00000019791	Hint3	histidine triad nucleotide binding protein 3 [Source:MGI Symbol;Acc:MGI:1914097]	29.3724	30.7642	28.2447	29.5384	28.6094	28.5268	29.7921	29.53	31.1656	24.6648	32.9138	30.0981	35.4689	31.0514	0.917867465	0.966018836	8	8	24	0.30065283	0.768040178	24	0.510502398	0.063903336
ENSMUSG00000019792	Trmt11	tRNA methyltransferase 11 [Source:MGI Symbol;Acc:MGI:1920931]	5.18283	5.82088	5.5418	5.63243	5.55649	5.53343	4.50945	4.9615	6.44444	5.47588	5.73916	5.38574	4.77473	5.5905	0.017495149	0.255326033	12	16	24	0.060301607	0.658544267	24	11.12165979	0.092992776
ENSMUSG00000019794	Katna1	katanin p60 (ATPase-containing) subunit A1 [Source:MGI Symbol;Acc:MGI:1344353]	9.0657	9.40326	8.90562	8.83658	9.69942	9.21183	9.47189	9.29606	9.04196	8.27262	10.0395	8.90286	9.58454	7.94347	0.125573493	0.555618842	20	20	24	0.232638181	0.768040178	24	19.64169367	0.053049831
ENSMUSG00000019795	Pcmt1	protein-L-isoaspartate (D-aspartate) O-methyltransferase 1 [Source:MGI Symbol;Acc:MGI:97502]	138.638	126.004	126.643	128.912	120.627	127.637	124.456	122.926	117.747	126.438	131.671	129.288	131.824	131.561	0.628286596	0.966018836	4	8	24	0.321955396	0.768040178	24	21.88707707	0.035459871
ENSMUSG00000019796	Lrp11	low density lipoprotein receptor-related protein 11 [Source:MGI Symbol;Acc:MGI:2442989]	64.784	50.7701	48.9727	52.0445	52.7567	60.2039	56.2484	56.9004	41.9345	64.6955	51.3275	58.724	57.1965	56.4437	0.019280932	0.257669847	24	12	24	0.147872359	0.756090042	24	21.12106131	0.123919105
ENSMUSG00000019797	Mtres1	mitochondrial transcription rescue factor 1 [Source:MGI Symbol;Acc:MGI:1915101]	30.251	32.9564	32.1252	33.3661	30.191	30.5681	29.6905	34.3573	31.6487	30.4466	32.8953	31.3222	34.2215	34.6337	0.151535314	0.599319068	8	16	24	0.128068089	0.740162193	24	6.099306723	0.05363804
ENSMUSG00000019802	Sec63	SEC63-like (S. cerevisiae) [Source:MGI Symbol;Acc:MGI:2155302]	30.5991	31.0861	26.6682	28.9478	30.5853	29.1914	25.1349	27.6253	28.7111	27.6517	31.057	29.4174	27.8784	26.1844	0.443191651	0.966018836	20	12	24	0.19624212	0.768040178	24	17.09362785	0.060548138
ENSMUSG00000019804	Snx3	sorting nexin 3 [Source:MGI Symbol;Acc:MGI:1860188]	76.0401	59.5752	60.6146	63.8457	63.6867	70.3578	70.0363	70.8107	58.7667	76.3806	66.3604	69.69	69.2334	64.4358	0.122530972	0.550724475	4	8	24	0.186662267	0.768040178	24	20.63042571	0.084954134
ENSMUSG00000019806	Aig1	androgen-induced 1 [Source:MGI Symbol;Acc:MGI:1913503]	33.7132	28.8759	28.4319	30.1696	25.6127	29.4478	26.0764	29.7228	27.3906	32.4081	29.067	31.6942	33.1466	33.1916	0.850398351	0.966018836	4	8	24	0.665331018	0.886947643	24	1.630782211	0.041524347
ENSMUSG00000019808	Adat2	adenosine deaminase, tRNA-specific 2 [Source:MGI Symbol;Acc:MGI:1914007]	1.62109	1.47934	1.72851	1.5631	1.76606	1.4464	1.72191	2.13247	1.94406	2.00866	1.60147	1.86737	2.32386	1.79741	0.854443826	0.966018836	4	20	24	0.421652325	0.804769987	24	5.575119076	0.053953975
ENSMUSG00000019809	Pex3	peroxisomal biogenesis factor 3 [Source:MGI Symbol;Acc:MGI:1929646]	15.0753	16.5921	15.788	16.5133	14.7083	15.2613	15.446	15.4502	16.4152	16.4681	15.4915	14.9389	15.8402	16.8456	0.032593161	0.324908923	16	4	24	0.00635455	0.470033096	24	7.669030809	0.06385041
ENSMUSG00000019810	Fuca2	fucosidase, alpha-L- 2, plasma [Source:MGI Symbol;Acc:MGI:1914098]	54.5774	50.9122	51.936	53.1263	55.4499	52.06	49.2543	52.7199	49.1155	46.5337	52.2039	50.9631	52.3509	52.419	0.921442568	0.966018836	20	16	24	0.471728935	0.805393068	24	20.78401229	0.030323375
ENSMUSG00000019813	Cep57l1	centrosomal protein 57-like 1 [Source:MGI Symbol;Acc:MGI:1915511]	3.05627	3.14018	3.68628	3.2151	3.16075	3.26671	3.18217	3.10188	3.22784	2.74911	3.5551	3.28979	2.90961	3.13184	0.302438464	0.811201458	24	4	24	0.803974597	0.93736298	24	13.92040175	0.020530384
ENSMUSG00000019814	Ltv1	LTV1 ribosome biogenesis factor [Source:MGI Symbol;Acc:MGI:2447810]	13.6229	13.5519	12.9448	12.6895	14.1876	13.8411	12.8377	13.1357	12.3851	12.4915	12.845	13.2898	13.5181	12.4747	0.079732457	0.458678242	24	16	24	0.012246854	0.525280095	24	20.60985153	0.054363081
ENSMUSG00000019817	Plagl1	pleiomorphic adenoma gene-like 1 [Source:MGI Symbol;Acc:MGI:1100874]	17.1552	16.4524	15.0848	15.095	15.3881	16.0224	14.1058	17.0634	15.4443	16.8494	15.8431	15.8501	15.5915	15.6757	0.96984707	0.97107529	8	4	24	0.927532658	0.981237035	24	2.455389909	0.011273813
ENSMUSG00000019818	Cd164	CD164 antigen [Source:MGI Symbol;Acc:MGI:1859568]	106.749	103.319	98.1041	105.772	98.9865	96.6679	99.004	98.6554	94.1892	108.665	104.502	98.4781	96.5803	99.758	0.893307667	0.966018836	16	20	24	0.552207525	0.83660198	24	13.81765965	0.025094572
ENSMUSG00000019820	Utrn	utrophin [Source:MGI Symbol;Acc:MGI:104631]	86.7248	76.964	70.9719	78.7663	80.942	78.7864	81.883	78.8934	70.7459	83.8135	72.5091	79.0753	79.0207	72.2428	0.033248781	0.324908923	4	8	24	0.133053891	0.743835554	24	20.46645368	0.066829746
ENSMUSG00000019822	Smpd2	sphingomyelin phosphodiesterase 2, neutral [Source:MGI Symbol;Acc:MGI:1278330]	6.5691	9.53937	9.66265	9.76513	9.90644	8.9014	8.95657	10.5365	11.1889	9.25898	8.1482	8.52593	8.69972	9.74059	0.043240321	0.357642487	12	12	24	0.034855222	0.608551791	24	8.764235899	0.155137925
ENSMUSG00000019823	Mical1	microtubule associated monooxygenase, calponin and LIM domain containing 1 [Source:MGI Symbol;Acc:MGI:2385847]	39.0346	36.8556	38.122	39.7898	41.7098	38.4825	33.5791	36.1932	40.8175	45.6794	35.6989	36.379	37.9837	35.3954	0.113947866	0.532950079	16	16	24	0.057365386	0.656248849	24	12.52852176	0.093194817
ENSMUSG00000019826	Zbtb24	zinc finger and BTB domain containing 24 [Source:MGI Symbol;Acc:MGI:3039618]	5.3545	5.83489	5.2651	5.74365	5.15047	5.39402	5.37437	5.54308	6.36559	5.38091	5.48273	5.84494	6.00712	6.25618	0.843639218	0.966018836	8	12	24	0.10008104	0.71175955	24	5.460902493	0.055311923
ENSMUSG00000019831	Wasf1	WASP family, member 1 [Source:MGI Symbol;Acc:MGI:1890563]	9.72723	7.71939	9.00292	8.32666	7.73779	9.01163	8.19308	8.05925	7.04584	12.0571	7.02671	8.2707	7.71169	8.45496	0.882263435	0.966018836	16	4	24	0.923826363	0.979691907	24	12.8348034	0.028486216
ENSMUSG00000019832	Rab32	RAB32, member RAS oncogene family [Source:MGI Symbol;Acc:MGI:1915094]	2.61106	2.58246	2.53919	2.75899	3.43529	2.52617	3.24169	2.74528	3.31839	2.56417	3.14116	2.3103	2.74028	2.51123	0.893307667	0.966018836	20	4	24	0.685075029	0.895310095	24	13.83222518	0.044762728
ENSMUSG00000019837	Gtf3c6	general transcription factor IIIC, polypeptide 6, alpha [Source:MGI Symbol;Acc:MGI:1914621]	32.3613	30.2911	27.3581	24.9707	29.6699	26.5593	24.986	27.5168	27.6323	24.4434	29.5774	27.0635	30.2604	27.651	0.25849765	0.761738864	4	12	24	0.345380776	0.778420274	24	23.5152196	0.064401026
ENSMUSG00000019838	Slc16a10	solute carrier family 16 (monocarboxylic acid transporters), member 10 [Source:MGI Symbol;Acc:MGI:1919722]	0.752331	0.979523	1.54239	2.09417	0.843603	0.96278	1.01094	1.34578	0.986395	1.32814	0.9066	1.42288	1.23192	1.18426	0.369262716	0.893596186	16	4	24	0.248804852	0.768040178	24	9.881889368	0.142789574
ENSMUSG00000019841	Rev3l	REV3 like, DNA directed polymerase zeta catalytic subunit [Source:MGI Symbol;Acc:MGI:1337131]	8.40868	9.66188	9.47927	8.92318	9.08756	9.18772	8.21638	9.66147	10.3649	8.15717	9.5342	9.01553	8.79467	8.84582	0.286642595	0.793714531	12	16	24	0.424314625	0.805393068	24	8.437611334	0.046955001
ENSMUSG00000019842	Traf3ip2	TRAF3 interacting protein 2 [Source:MGI Symbol;Acc:MGI:2143599]	3.8426	5.38579	6.23016	6.12747	5.73726	5.65254	5.98289	4.44816	6.46498	5.59226	6.27466	5.2176	5.13333	6.3066	0.097760727	0.501112642	12	16	24	0.087702221	0.707747429	24	11.41387945	0.138591817
ENSMUSG00000019843	Fyn	Fyn proto-oncogene [Source:MGI Symbol;Acc:MGI:95602]	27.1422	28.7099	29.7984	28.5908	27.8749	27.9923	30.5437	29.0009	29.9928	33.9179	25.4083	29.9363	28.3255	29.4997	0.184631358	0.662239322	12	8	24	0.310841006	0.768040178	24	8.020055285	0.055686877
ENSMUSG00000019845	Tube1	tubulin, epsilon 1 [Source:MGI Symbol;Acc:MGI:1919174]	1.35801	1.46463	1.43968	1.42487	1.45997	1.65426	1.86827	1.4267	1.69013	1.72443	1.42667	1.58661	1.5412	1.529	0.865490576	0.966018836	24	20	24	0.967765149	0.99226729	24	21.89717368	0.008110165
ENSMUSG00000019846	Lama4	laminin, alpha 4 [Source:MGI Symbol;Acc:MGI:109321]	15.7456	17.4505	14.4002	13.6786	18.8043	14.2294	17.5678	19.3471	14.7312	14.0921	14.0916	15.1978	15.1627	12.8762	0.47028201	0.966018836	4	12	24	0.443085075	0.805393068	24	23.70009146	0.07966311
ENSMUSG00000019848	Popdc3	popeye domain containing 3 [Source:MGI Symbol;Acc:MGI:1930153]	12.9607	10.4208	9.74282	10.7654	11.3388	12.113	10.1664	12.1652	9.14708	12.5008	11.0677	11.006	12.1595	10.7268	0.433658121	0.966018836	4	8	24	0.232081644	0.768040178	24	20.29674411	0.090644388
ENSMUSG00000019849	Prep	prolyl endopeptidase [Source:MGI Symbol;Acc:MGI:1270863]	6.23201	4.7508	4.90301	5.27081	5.25558	5.68034	4.89894	5.23045	4.25106	6.32755	4.96008	5.26444	5.14633	5.30922	0.44861185	0.966018836	16	20	24	0.443572128	0.805393068	24	18.8323485	0.064887633
ENSMUSG00000019850	Tnfaip3	tumor necrosis factor, alpha-induced protein 3 [Source:MGI Symbol;Acc:MGI:1196377]	1.39926	1.46931	1.36914	1.12278	1.38723	1.60532	1.50511	1.67862	1.36709	1.56807	1.15773	1.44587	1.44676	1.24436	0.327787265	0.846828588	24	20	24	0.333824451	0.769934824	24	0.418844285	0.055089657
ENSMUSG00000019851	Perp	PERP, TP53 apoptosis effector [Source:MGI Symbol;Acc:MGI:1929938]	6.66523	6.33333	6.43395	6.66072	6.62967	5.92548	6.08872	6.46453	7.06202	5.20643	6.95393	7.13139	5.92763	6.0559	0.915082018	0.966018836	20	8	24	0.906911385	0.974587616	24	17.03835716	0.018911373

ENSMUSG00000019852	Arfgef3	ARFGEF family member 3 [Source:MGI Symbol;Acc:MGI:106387]	15.8603	14.4319	14.0623	14.7018	14.6175	14.5326	13.9507	12.4851	12.8794	12.65	15.6328	13.9669	13.5433	15.2713	0.970950683	0.971732806	20	16	24	0.323767232	0.768040178	24	19.675952	0.058980497
ENSMUSG00000019853	Hebp2	heme binding protein 2 [Source:MGI Symbol;Acc:MGI:1860084]	29.7383	29.9457	25.8545	28.9399	26.9048	26.4917	28.4357	28.7951	24.9498	29.3943	28.7833	28.9148	30.8794	29.4316	0.278111325	0.789849983	8	4	24	0.379828318	0.799406242	24	23.67316695	0.045465267
ENSMUSG00000019854	Reps1	RalBP1 associated Eps domain containing protein [Source:MGI Symbol;Acc:MGI:1196373]	21.469	25.0266	22.8989	23.6858	22.4867	23.9082	22.1628	23.7935	21.7329	23.4444	20.3097	23.6949	23.5188	23.4331	0.962348911	0.967745676	8	12	24	0.596148778	0.857839743	24	4.103770285	0.030282975
ENSMUSG00000019856	Fam184a	family with sequence similarity 184, member A [Source:MGI Symbol;Acc:MGI:1923156]	7.46662	7.73072	7.08155	9.89134	8.04121	8.45642	9.44261	8.10255	8.07385	7.41092	7.2163	9.07698	7.83359	9.69772	0.892685629	0.966018836	24	20	24	0.864407592	0.960791419	24	23.48387435	0.030388291
ENSMUSG00000019857	Asf1a	anti-silencing function 1A histone chaperone [Source:MGI Symbol;Acc:MGI:1913653]	11.7308	14.5137	13.5834	14.5473	13.5762	11.2164	13.3583	11.3723	13.9938	10.9697	14.6418	13.3024	13.7999	13.2347	0.961261938	0.967043964	20	4	24	0.806194663	0.938114514	24	10.47749188	0.036520156
ENSMUSG00000019861	Gopc	golgi associated PDZ and coiled-coil motif containing [Source:MGI Symbol;Acc:MGI:2149946]	22.6076	20.1141	19.8374	20.0622	19.9036	20.147	19.2668	18.5113	18.5242	20.4578	19.7515	19.2423	19.0254	20.3792	0.81829516	0.966018836	16	20	24	0.677237847	0.89205558	24	19.78747152	0.022509249
ENSMUSG00000019863	Qrsl1	glutaminyl-tRNA synthase (glutamine-hydrolyzing)-like 1 [Source:MGI Symbol;Acc:MGI:1923813]	15.4588	14.9636	16.1472	13.7041	15.0195	14.4876	15.2954	14.6869	16.0264	14.7131	16.7911	15.4061	16.0377	16.1799	0.911135828	0.966018836	12	4	24	0.740264067	0.915274254	24	3.358241314	0.020618753
ENSMUSG00000019864	Rtn4ip1	reticulon 4 interacting protein 1 [Source:MGI Symbol;Acc:MGI:2178759]	10.0735	8.86365	9.74149	12.055	9.92687	10.0186	9.88543	10.4859	8.40397	11.3696	9.85407	9.99923	10.0284	10.5531	0.916860713	0.966018836	16	20	24	0.527809065	0.82357362	24	14.41051082	0.051197243
ENSMUSG00000019866	Crybg1	crystallin beta-gamma domain containing 1 [Source:MGI Symbol;Acc:MGI:109544]	0.973079	0.874949	0.854908	0.794073	0.995165	0.94097	0.757987	1.06032	1.03053	0.757507	0.966421	0.997824	0.808633	0.828341	0.934152439	0.966018836	20	20	24	0.854164563	0.956449829	24	19.71569215	0.018733793
ENSMUSG00000019868	Vta1	vesicle (multivesicular body) trafficking 1 [Source:MGI Symbol;Acc:MGI:1913451]	40.6386	40.5591	40.275	40.3246	40.0285	42.7653	38.517	42.3218	38.9811	38.5143	39.2263	41.822	41.3595	38.8132	0.289105414	0.794869797	24	16	24	0.253206951	0.768040178	24	21.82936496	0.031456099
ENSMUSG00000019872	Smpdl3a	sphingomyelin phosphodiesterase, acid-like 3A [Source:MGI Symbol;Acc:MGI:1931437]	25.4695	28.8505	27.5728	25.2655	25.1265	25.1171	24.4482	26.5203	28.8756	23.3404	29.6428	27.902	28.6403	29.0816	0.625825704	0.966018836	8	8	24	0.437241601	0.805393068	24	4.765562578	0.050979755
ENSMUSG00000019873	Reep3	receptor accessory protein 3 [Source:MGI Symbol;Acc:MGI:88930]	11.9079	12.0633	11.4102	11.51	11.4348	10.9855	12.8387	12.5481	11.7081	11.9627	11.4576	11.2034	11.3447	11.5952	0.037085178	0.334054683	8	16	24	0.186980237	0.768040178	24	4.158564869	0.039154687
ENSMUSG00000019874	Fabp7	fatty acid binding protein 7, brain [Source:MGI Symbol;Acc:MGI:101916]	411.796	394.604	204.417	95.7352	245.967	280.922	431.748	318.585	185.898	106.215	260.545	271.393	424.155	366.807	2.63E-07	0.001551842	4	12	24	7.52E-05	0.102884653	24	0.106261287	0.847176351
ENSMUSG00000019876	Pkib	protein kinase inhibitor beta, cAMP dependent, testis specific [Source:MGI Symbol;Acc:MGI:101937]	20.4763	14.826	24.9846	19.0757	22.5444	21.8804	19.2965	15.5529	16.7592	19.2005	24.7381	19.722	19.9356	15.5075	8.59E-04	0.071344007	20	12	24	0.025543473	0.586541342	24	16.70997369	0.214635307
ENSMUSG00000019877	Serinc1	serine incorporator 1 [Source:MGI Symbol;Acc:MGI:1926228]	431.809	439.35	419.705	414.003	418.01	400.374	404.748	391.264	393.214	379.678	441.548	389.062	384.671	418.799	0.882263435	0.966018836	20	4	24	0.985229043	0.996973154	24	19.92308672	0.004532563
ENSMUSG00000019878	Hsf2	heat shock factor 2 [Source:MGI Symbol;Acc:MGI:96239]	17.2049	16.3356	16.9756	16.3971	15.5348	16.3142	16.6297	14.5897	15.4572	14.9181	18.1694	16.1829	15.8478	16.1844	0.90332508	0.966018836	4	4	24	0.496575204	0.807549708	24	19.45915678	0.03686201
ENSMUSG00000019880	Rspo3	R-spondin 3 [Source:MGI Symbol;Acc:MGI:1920030]	0.799075	1.24123	0.987701	1.09429	1.16332	0.828201	0.794878	0.998624	1.50257	0.572412	0.870387	1.0483	0.835909	0.941266	0.267533088	0.774280302	12	16	24	0.520504313	0.820018348	24	7.983036038	0.07658382
ENSMUSG00000019883	Echdc1	enoyl Coenzyme A hydratase domain containing 1 [Source:MGI Symbol;Acc:MGI:1277169]	29.3157	26.2185	27.1942	34.8463	29.4667	27.2281	28.5808	28.1327	26.7776	28.4364	33.3082	29.948	33.1327	30.8003	0.251986462	0.75129457	20	16	24	0.408386004	0.799546792	24	16.4371372	0.059722366
ENSMUSG00000019888	Mgat4c	MGAT4 family, member C [Source:MGI Symbol;Acc:MGI:1914819]	1.9094	3.3702	2.94766	2.7052	2.3114	2.31363	3.29886	2.36306	2.80628	1.82051	1.82186	2.82862	1.93718	2.98045	0.163587268	0.626886304	8	12	24	0.267219585	0.768040178	24	4.842425363	0.137702061
ENSMUSG00000019889	Ptprk	protein tyrosine phosphatase, receptor type, K [Source:MGI Symbol;Acc:MGI:103310]	5.67546	4.13135	4.74704	5.13996	4.50867	5.62734	6.73477	4.53749	5.35594	4.99396	4.81593	5.10443	5.3503	4.79151	0.079180494	0.458678242	4	4	24	0.304724742	0.768040178	24	21.60497711	0.089937099
ENSMUSG00000019890	Nts	neurotensin [Source:MGI Symbol;Acc:MGI:1328351]	13.386	10.2108	25.5423	11.2674	19.3991	16.3569	13.8435	12.889	9.35083	6.76475	28.3663	9.47481	10.1215	9.3629	0.401437339	0.933249185	20	20	24	0.44740254	0.805393068	24	17.07255443	0.264229268
ENSMUSG00000019891	Dcbld1	discoidin, CUB and LCCL domain containing 1 [Source:MGI Symbol;Acc:MGI:1913936]	7.53894	5.98385	6.26819	6.47545	5.80696	7.21984	6.71462	6.57339	4.82743	7.88478	6.37181	6.53638	6.70251	6.06713	0.16456443	0.628002127	4	8	24	0.329856222	0.768040178	24	20.15983818	0.089608758
ENSMUSG00000019892	Lrriq1	leucine-rich repeats and IQ motif containing 1 [Source:MGI Symbol;Acc:MGI:1922228]	1.40953	0.644293	1.1307	1.48248	1.20308	1.31247	1.07421	0.505307	1.6793	1.2475	1.28211	1.08486	1.32046	0.905969	0.172865085	0.648600527	16	16	24	0.18624401	0.768040178	24	15.26392869	0.154769024
ENSMUSG00000019894	Slc6a15	solute carrier family 6 (neurotransmitter transporter), member 15 [Source:MGI Symbol;Acc:MGI:2143484]	65.4427	64.4749	60.7008	63.333	61.0235	60.6236	59.3782	55.0343	57.1083	60.6544	63.1992	60.8153	63.2837	64.7214	0.873350015	0.966018836	8	4	24	0.725153732	0.90980389	24	20.36170216	0.023112875
ENSMUSG00000019897	Ccdc59	coiled-coil domain containing 59 [Source:MGI Symbol;Acc:MGI:1289302]	9.9093	12.0692	11.2631	10.1688	10.3381	9.58936	9.70222	10.7511	13.2859	10.128	10.0893	11.3896	11.2167	10.022	0.321662653	0.83607295	12	12	24	0.213435484	0.768040178	24	7.021319464	0.085584578
ENSMUSG00000019899	Lama2	laminin, alpha 2 [Source:MGI Symbol;Acc:MGI:99912]	18.172	19.011	18.8094	16.445	18.3236	15.4341	20.2674	18.8182	19.6705	16.1078	14.7697	17.0344	20.5122	16.941	0.038580592	0.335882166	4	20	24	0.044341794	0.628432994	24	3.347216533	0.121252448
ENSMUSG00000019906	Lin7a	lin-7 homolog A (C. elegans) [Source:MGI Symbol;Acc:MGI:2135609]	12.2017	10.038	9.80539	9.81582	10.6591	9.54465	10.8698	8.18521	8.24902	8.73328	10.235	9.69613	9.33759	10.1032	0.671726933	0.966018836	4	8	24	0.095532834	0.71175955	24	20.81669915	0.105560957
ENSMUSG00000019907	Ppp1r12a	protein phosphatase 1, regulatory subunit 12A [Source:MGI Symbol;Acc:MGI:1309528]	29.0809	26.8513	25.744	27.5291	28.9295	26.0875	29.5722	25.4907	26.9938	25.031	29.3584	26.6283	25.8491	26.8274	0.725061903	0.966018836	20	12	24	0.294715016	0.768040178	24	19.30553685	0.047395117
ENSMUSG00000019916	P4ha1	procollagen-proline, 2-oxoglutarate 4-dioxygenase (proline 4-hydroxylase), alpha 1 polypeptide [Source:MGI Symbol;Acc:MGI:97463]	41.4062	26.0003	22.9569	26.074	35.3797	30.2753	37.7955	29.8622	23.0437	23.4058	32.0646	28.1943	29.7737	23.5403	0.004501001	0.140598674	4	8	24	0.002764599	0.414320197	24	20.8699221	0.29164366
ENSMUSG00000019917	Septin10	septin 10 [Source:MGI Symbol;Acc:MGI:1918110]	7.76104	8.94131	8.87079	8.2088	8.25985	8.08168	7.99713	8.14858	8.87918	7.3716	8.956	8.22066	9.07065	9.58779	0.94086481	0.966018836	8	20	24	0.311101909	0.768040178	24	5.471183502	0.052700765
ENSMUSG00000019920	Lims1	LIM and senescent cell antigen-like domains 1 [Source:MGI Symbol;Acc:MGI:1195263]	43.1742	39.4052	39.5343	41.6041	37.9745	40.7098	38.8441	37.1721	37.5856	37.3139	42.9653	37.0115	39.7044	39.7538	0.866861748	0.966018836	4	4	24	0.627957113	0.872795898	24	19.63350735	0.027577363
ENSMUSG00000019923	Zwint	ZW10 interactor [Source:MGI Symbol;Acc:MGI:1289227]	460.776	470.526	472.114	455.607	469.8	469.686	431.936	420.392	437.532	400.116	481.262	456.264	457.99	462.654	0.932671476	0.966018836	20	20	24	0.511616429	0.815032433	24	19.75839056	0.034853812
ENSMUSG00000019927	Ube2d1	ubiquitin-conjugating enzyme E2D 1 [Source:MGI Symbol;Acc:MGI:2384911]	31.4895	30.9018	31.8381	33.6564	31.8801	33.4785	32.3933	34.5923	32.6458	39.899	31.6262	31.2513	31.9022	31.8812	0.718720526	0.966018836	16	4	24	0.245709317	0.768040178	24	11.36254787	0.058308421
ENSMUSG00000019929	Dcn	decorin [Source:MGI Symbol;Acc:MGI:94872]	467.376	642.83	542.145	555.592	636.343	423.429	557.238	515.457	822.13	373.222	623.909	524.705	576.815	544.214	0.914502381	0.966018836	12	12	24	0.659577356	0.884879972	24	7.43318496	0.105177978
ENSMUSG00000019933	Mrln	myoregulin [Source:MGI Symbol;Acc:MGI:1916813]	1.39663	6.97929	4.66733	8.32183	3.5499	5.33773	5.9164	14.8575	10.6903	7.39632	4.31248	4.98352	7.10773	5.64341	0.137504104	0.580158868	16	4	24	0.100721883	0.712028712	24	7.6977593	0.501416569
ENSMUSG00000019935	Slc17a8	solute carrier family 17 (sodium-dependent inorganic phosphate cotransporter), member 8 [Source:MGI Symbol;Acc:MGI:3039629]	5.27021	3.88044	3.75118	3.757	3.86999	3.92086	4.09058	3.68038	2.91612	3.8026	3.37012	3.93162	4.54116	4.3204	3.44E-04	0.049770315	4	8	24	0.034855753	0.608551791	24	23.12099562	0.142709985
ENSMUSG00000019936	Epyc	epiphycan [Source:MGI Symbol;Acc:MGI:107942]	0.753103	0.849674	0.849549	1.10636	1.41968	0.887695	1.13168	1.08668	1.12427	0.69082	1.22079	0.770216	0.783685	0.774788	0.664568268	0.966018836	20	8	24	0.406237974	0.799546792	24	14.53703746	0.080678762
ENSMUSG00000019942	Cdk1	cyclin-dependent kinase 1 [Source:MGI Symbol;Acc:MGI:88351]	1.92168	2.37455	3.60023	3.21535	2.94951	3.53022	1.94253	2.58228	3.22933	1.66914	1.93434	2.53661	3.51542	1.84428	0.891742979	0.966018836	12	20	24	0.936900607	0.984453637	24	12.49320564	0.040536971
ENSMUSG00000019943	Atp2b1	ATPase, Ca++ transporting, plasma membrane 1 [Source:MGI Symbol;Acc:MGI:104653]	65.2166	63.027	60.837	62.841	61.4356	61.9602	57.5035	59.9642	60.8559	62.4882	61.737	65.0704	65.3311	68.2664	0.951945551	0.966018836	8	4	24	0.817653016	0.942028028	24	0.638327609	0.014643146
ENSMUSG00000019944	Rhobtb1	Rho-related BTB domain containing 1 [Source:MGI Symbol;Acc:MGI:1916538]	1.35186	1.94528	2.14246	1.59244	1.47003	1.38367	2.16831	2.20213	2.24789	1.93822	1.54549	1.63031	1.82713	1.50638	0.048889741	0.379129806	12	12	24	0.047975889	0.636997157	24	6.654240355	0.15283205
ENSMUSG00000019945	Cabcoco1	ciliary associated calcium binding coiled-coil 1 [Source:MGI Symbol;Acc:MGI:1920537]	0.371587	0.807919	0.471328	0.854256	0.521882	0.579168	0.66132	0.6286	0.50817	0.596276	0.323771	0.622335	0.68263	0.693076	0.192357053	0.669841558	8	12	24	0.569779912	0.844900526	24	5.011698169	0.058375559
ENSMUSG00000019947	Arid5b	AT rich interactive domain 5B (MRF1-like) [Source:MGI Symbol;Acc:MGI:2175912]	3.91801	3.31593	4.1928	5.37433	3.91912	3.72305	3.96919	3.49444	4.33776	5.6623	3.48253	3.68566	3.46062	3.49817	0.035655578	0.331814682	16	16	24	0.024127492	0.586541342	24	11.92643	0.177217036
ENSMUSG00000019948	Actr6	ARP6 actin-related protein 6 [Source:MGI Symbol;Acc:MGI:1914269]	12.9672	15.7886	14.4615	14.43	12.9104	13.232	13.9904	12.5255	14.0755	12.5302	15.9786	12.5912	13.8177	14.2753	0.834424969	0.966018836	12	12	24	0.698107914	0.899653191	24	8.034987896	0.038025919
ENSMUSG00000019951	Uhrf1bp1l	UHRF1 (ICBP90) binding protein 1-like [Source:MGI Symbol;Acc:MGI:2442888]	28.8098	24.7645	27.16	27.3082	22.2045	27.7314	26.4258	24.3446	22.1403	28.684	26.1283	27.1884	27.2441	29.1735	0.91695334	0.966018836	4	8	24	0.737958125	0.91451866	24	22.62516877	0.038945015
ENSMUSG00000019952	Poc1b	POC1 centriolar protein B [Source:MGI Symbol;Acc:MGI:1918511]	3.6339	2.82549	3.43887	3.87186	2.89351	3.64018	3.48001	3.63193	3.28802	3.84541	4.0249	3.48999	3.31844	3.1824	0.123856376	0.552144108	16	16	24	0.425010916	0.805393068	24	15.1461605	0.057848959
ENSMUSG00000019960	Dusp6	dual specificity phosphatase 6 [Source:MGI Symbol;Acc:MGI:1914853]	12.3331	9.57176	9.80121	8.36569	10.0307	9.50182	12.4008	9.15064	8.29069	10.4426	10.8878	8.83948	9.56233	8.26401	0.648082424	0.966018836	4	4	24	0.202205997	0.768040178	24	20.83773403	0.114511641
ENSMUSG00000019961	Tmpo	thymopoietin [Source:MGI Symbol;Acc:MGI:106920]	23.5935	26.9953	26.1011	30.7627	26.7992	26.1508	24.6654	26.7829	31.4973	24.7515	26.5486	27.1274	26.4664	22.2183	0.598711142	0.966018836	12	16	24	0.250800255	0.768040178	24	12.0101277	0.07990264
ENSMUSG00000019966	Kitl	kit ligand [Source:MGI Symbol;Acc:MGI:96974]	20.0275	18.9872	17.7226	19.892	19.2296	17.8501	17.94	17.067	16.5278	17.4357	21.5386	16.0934	18.1871	18.2081	0.941579937	0.966018836	20	16	24	0.500198848	0.808957913	24	16.81492051	0.048361068
ENSMUSG00000019969	Psen1	presenilin 1 [Source:MGI Symbol;Acc:MGI:1202717]	26.3127	20.442	23.4133	21.8646	21.7393	25.2889	26.4242	24.4986	18.5976	26.2639	21.2636	23.0792	22.5199	22.709	0.446967971	0.966018836	4	16	24	0.327307865	0.768040178	24	21.77470834	0.085295747
ENSMUSG00000019970	Sgk1	serum/glucocorticoid regulated kinase 1 [Source:MGI Symbol;Acc:MGI:1340062]	16.2796	18.5748	29.0756	38.3814	19.2593	23.05	22.5303	20.3305	33.3001	45.5841	17.2539	19.2589	14.497	13.9689	0.032263213	0.324385159	16	12	24	0.010665272	0.509710515	24	11.33203535	0.499514714
ENSMUSG00000019971	Cep290	centrosomal protein 290 [Source:MGI Symbol;Acc:MGI:2384917]	8.89665	9.6033	8.90828	7.93754	9.11935	7.56821	8.75494	7.39568	8.86461	7.57523	9.38578	8.81095	8.7362	8.965	0.90332508	0.966018836	20	4	24	0.87507558	0.964979482	24	0.390852741	0.022459142
ENSMUSG00000019975	Ikbip	IKBKB interacting protein [Source:MGI Symbol;Acc:MGI:1914704]	15.6395	17.4282	16.5263	14.5114	17.4154	13.3806	15.5307	13.7159	18.8619	13.0739	16.632	14.76	15.8637	16.4321	0.390686537	0.915479136	12	12	24	0.666056248	0.887215207	24	6.604669526	0.054414405
ENSMUSG00000019977	Hbs1l	Hbs1-like (S. cerevisiae) [Source:MGI Symbol;Acc:MGI:1891704]	27.942	25.2551	24.3612	25.1601	24.5298	26.3443	24.9449	25.9156	23.5753	25.7143	26.2524	26.7314	26.2567	24.5036	0.001971398	0.100255953	24	12	24	0.033334309	0.606585237	24	20.85237532	0.05914794
ENSMUSG00000019978	Epb41l2	erythrocyte membrane protein band 4.1 like 2 [Source:MGI Symbol;Acc:MGI:103009]	68.8691	79.677	70.6984	78.7115	89.1768	70.0437	84.1961	73.0906	85.2193	75.6664	74.2254	72.2424	76.4968	66.5093	0.892578749	0.966018836	20	4	24	0.611707166	0.865209198	24	13.36426305	0.047412528
ENSMUSG00000019979	Apaf1	apoptotic peptidase activating factor 1 [Source:MGI Symbol;Acc:MGI:1306796]	2.40844	3.21106	3.05431	4.10384	2.59773	2.42159	3.31656	2.53437	3.67591	2.76567	2.79057	2.59415	3.87583	3.28831	0.68887295	0.966018836	12	12	24	0.23836733	0.768040178	24	7.581199626	0.128366106
ENSMUSG00000019982	Myb	myeloblastosis oncogene [Source:MGI Symbol;Acc:MGI:97249]	0.552943	0.847386	1.13722	1.12539	0.84733	0.940333	0.770145	0.693308	0.903448	0.607074	0.916633	0.877505	0.925186	0.842805	0.586972936	0.966018836	12	20	24	0.508111959	0.813175565	24	12.0081953	0.060166559
ENSMUSG00000019984	Med23	mediator complex subunit 23 [Source:MGI Symbol;Acc:MGI:1917458]	5.52558	6.06575	6.51739	5.95385	6.41083	6.29982	5.59732	6.39093	5.77496	6.48677	5.36018	6.13145	5.95397	6.22557	0.510431574	0.966018836	12	16	24	0.56078004	0.841404418	24	9.447038277	0.03425989
ENSMUSG00000019986	Ahi1	Abelson helper integration site 1 [Source:MGI Symbol;Acc:MGI:87971]	19.5191	20.5932	26.8844	20.7548	18.1683	19.5223	23.3664	17.1182	22.093	20.4018	17.6369	21.9077	17.7902	22.4641	0.616047374	0.966018836	12	8	24	0.404334314	0.799546792	24	7.150781372	0.093517956
ENSMUSG00000019988	Nedd1	neural precursor cell expressed, developmentally down-regulated gene 1 [Source:MGI Symbol;Acc:MGI:97293]	2.04204	3.31001	3.25027	3.61423	3.21973	3.07908	2.93028	3.66255	3.71173	2.51042	3.10744	2.79685	2.90957	3.08628	0.030691182	0.324150946	12	16	24	0.205266569	0.768040178	24	9.058179691	0.118224523
ENSMUSG00000019989	Enpp3	ectonucleotide pyrophosphatase/phosphodiesterase 3 [Source:MGI Symbol;Acc:MGI:2143702]	0.589928	0.869275	0.503792	0.389104	0.424882	0.527044	0.417685	0.67727	0.949466	0.561737	0.417564	0.530499	0.464684	0.844417	0.032263213	0.324385159	8	12	24	0.022303173	0.578379975	24	5.451237814	0.156411578
ENSMUSG00000019990	Pde7b	phosphodiesterase 7B [Source:MGI Symbol;Acc:MGI:1352752]	4.62813	3.88888	4.53438	4.4033	4.51716	4.28616	4.91598	5.21857	4.46034	5.25088	4.01164	3.80405	4.19419	4.12256	0.945728192	0.966018836	16	8	24	0.646443897	0.880320244	24	8.314052287	0.045077617
ENSMUSG00000019992	Mtfr2	mitochondrial fission regulator 2 [Source:MGI Symbol;Acc:MGI:1919054]	0.455077	0.650684	0.915703	0.801104	0.505387	0.704819	0.503198	0.402859	0.815706	0.457984	0.403851	0.523034	0.748459	0.626285	0.658895034	0.966018836	12	8	24	0.386657803	0.799546792	24	7.893668056	0.082577141
ENSMUSG00000019996	Map7	microtubule-associated protein 7 [Source:MGI Symbol;Acc:MGI:1328328]	33.1498	33.6452	31.0307	30.3671	29.0351	32.7288	31.9281	31.6023	29.8498	31.7436	31.0882	35.2602	33.2375	35.4234	0.060746541	0.40906904	8	4	24	0.032001954	0.604838544	24	0.486531141	0.070411636
ENSMUSG00000019997	Ccn2	cellular communication network factor 2 [Source:MGI Symbol;Acc:MGI:95537]	11.2129	10.1038	10.5739	14.5787	9.69966	9.4461	10.9041	9.92097	15.9029	10.8976	11.7575	9.62456	7.1275	7.77258	0.226438798	0.722427815	16	8	24	0.081924731	0.699041389	24	11.52549974	0.209799672
ENSMUSG00000019998	Stx7	syntaxin 7 [Source:MGI Symbol;Acc:MGI:1858210]	107.397	92.2313	97.6405	93.0632	93.57	93.9574	94.0896	88.0503	80.9841	90.6549	95.0638	88.7859	96.0561	92.6013	0.226403091	0.722427815	4	4	24	0.197928247	0.768040178	24	21.37360983	0.056789644
ENSMUSG00000020000	Moxd1	monooxygenase, DBH-like 1 [Source:MGI Symbol;Acc:MGI:1921582]	19.1315	15.3512	14.1868	14.4512	14.0175	16.6903	13.5229	15.4644	13.9908	14.7068	16.2475	16.3838	16.2319	15.1244	0.041292272	0.346472636	24	12	24	0.099578726	0.71175955	24	21.75899248	0.102421679
ENSMUSG00000020003	Pex7	peroxisomal biogenesis factor 7 [Source:MGI Symbol;Acc:MGI:1321392]	18.5965	19.8842	19.3654	19.6008	17.9922	17.5918	16.8947	18.1509	18.7939	19.3874	20.6213	21.2662	20.7178	18.3558	0.881996584	0.966018836	16	12	24	0.718220739	0.9072289	24	13.47564926	0.028062177
ENSMUSG00000020009	Ifngr1	interferon gamma receptor 1 [Source:MGI Symbol;Acc:MGI:107655]	21.3715	21.746	21.764	23.0757	22.996	22.022	22.42	22.5909	22.5134	21.2488	22.5132	23.4295	23.8755	21.4824	0.919571723	0.966018836	24	8	24	0.465584599	0.805393068	24	18.67022057	0.02348573
ENSMUSG00000020015	Cdk17	cyclin-dependent kinase 17 [Source:MGI Symbol;Acc:MGI:97517]	10.266	9.89745	10.8759	10.1601	10.2414	9.89266	11.0463	9.22874	9.67078	10.0361	9.86198	9.27628	9.70085	9.4583	0.935593607	0.966018836	16	16	24	0.874271233	0.964836189	24	15.27618026	0.011801203
ENSMUSG00000020017	Hal	histidine ammonia lyase [Source:MGI Symbol;Acc:MGI:96010]	4.05745	3.87858	3.4561	2.32314	3.11535	3.7545	3.12766	3.73656	2.40677	3.41976	3.10787	3.32936	3.75754	3.29544	0.076942821	0.453790923	4	16	24	0.048575956	0.638992719	24	23.73454815	0.157013843
ENSMUSG00000020018	Snrpf	small nuclear ribonucleoprotein polypeptide F [Source:MGI Symbol;Acc:MGI:1917128]	3.10836	3.38496	3.68867	3.65015	3.69873	3.18231	2.87817	3.7356	4.37927	4.2785	6.79883	6.15592	7.25586	5.46128	0.860993221	0.966018836	20	8	24	0.753671443	0.920247814	24	15.36832034	0.062006885
ENSMUSG00000020019	Ntn4	netrin 4 [Source:MGI Symbol;Acc:MGI:1888978]	2.4536	3.17553	3.17777	3.14796	2.88547	2.65753	3.11157	3.31025	3.92138	2.97797	2.72462	3.15558	2.99215	2.92925	0.057264003	0.401309455	12	8	24	0.046345702	0.633507364	24	7.806192372	0.110153903
ENSMUSG00000020021	Fgd6	FYVE, RhoGEF and PH domain containing 6 [Source:MGI Symbol;Acc:MGI:1261419]	1.8088	1.54502	1.65482	2.01762	1.6836	1.44727	1.60172	1.53528	1.34414	1.61975	2.10346	1.76056	1.52348	1.42819	0.181558234	0.659020016	20	12	24	0.082397035	0.699403344	24	16.03144156	0.092081525
ENSMUSG00000020022	Ndufa12	NADH:ubiquinone oxidoreductase subunit A12 [Source:MGI Symbol;Acc:MGI:1913664]	39.145	40.7629	41.9876	38.8085	31.8821	34.1316	44.1964	48.4389	46.6411	37.8991	46.7978	42.9775	44.1297	44.4176	0.229694208	0.729069536	8	8	24	0.053915376	0.654390023	24	4.886284303	0.133394606
ENSMUSG00000020023	Tmcc3	transmembrane and coiled coil domains 3 [Source:MGI Symbol;Acc:MGI:2442900]	15.2122	14.9806	15.8527	15.1337	16.1311	16.4756	17.6833	14.9325	14.4139	15.5998	16.2094	17.9404	15.9178	16.7874	0.020957534	0.262822894	24	12	24	0.055471805	0.65534544	24	20.2249262	0.070913751
ENSMUSG00000020024	Cep83	centrosomal protein 83 [Source:MGI Symbol;Acc:MGI:1924298]	20.2057	20.2937	19.3158	18.9869	21.0149	18.3431	17.9754	17.9907	19.5363	17.6569	19.7282	18.5127	19.3735	18.2861	0.888379597	0.966018836	20	20	24	0.809309243	0.939184036	24	17.08263197	0.017097113
ENSMUSG00000020027	Socs2	suppressor of cytokine signaling 2 [Source:MGI Symbol;Acc:MGI:1201787]	13.9434	12.9026	16.0429	15.2565	15.5436	15.2615	12.2845	14.6355	15.8367	15.5835	17.5837	16.0638	15.7453	15.2952	0.146381309	0.590732968	20	8	24	0.024471102	0.586541342	24	13.56680227	0.103500744
ENSMUSG00000020029	Nudt4	nudix (nucleoside diphosphate linked moiety X)-type motif 4 [Source:MGI Symbol;Acc:MGI:1918457]	73.9879	70.3117	72.3355	66.609	68.5629	71.9879	70.7935	68.7294	62.6347	70.7466	72.3039	69.52	67.4223	70.7655	0.910436409	0.966018836	24	16	24	0.321857879	0.768040178	24	21.14826092	0.035346413
ENSMUSG00000020032	Nuak1	NUAK family, SNF1-like kinase, 1 [Source:MGI Symbol;Acc:MGI:1925226]	10.5552	8.58282	8.52239	8.38532	9.00167	10.0697	9.75488	9.80825	6.77503	10.8614	8.34287	10.0965	9.90762	10.1382	0.2679923	0.774280302	24	12	24	0.149862333	0.758419028	24	21.98785011	0.121293117
ENSMUSG00000020034	Tcp11l2	t-complex 11 (mouse) like 2 [Source:MGI Symbol;Acc:MGI:2444679]	14.2465	16.0599	15.959	15.788	14.2421	14.5825	13.9223	16.8181	15.259	16.5813	13.9069	14.9764	13.233	17.115	0.007049678	0.188070711	8	20	24	0.018318309	0.559812298	24	7.885612452	0.110407963
ENSMUSG00000020037	Rfx4	regulatory factor X, 4 (influences HLA class II expression) [Source:MGI Symbol;Acc:MGI:1918387]	0.262578	0.776822	1.37626	0.599467	0.565631	0.66497	1.05214	0.560289	0.817636	0.611218	0.471701	0.566646	0.151927	0.916945	0.289496388	0.794869797	12	8	24	0.231602371	0.768040178	24	8.046722658	0.17890875
ENSMUSG00000020038	Cry1	cryptochrome 1 (photolyase-like) [Source:MGI Symbol;Acc:MGI:1270841]	5.05929	3.06855	3.74645	4.94592	4.65214	4.79494	4.63101	3.00789	3.54043	4.36346	4.63263	4.45533	3.60172	3.22983	0.00455947	0.140598674	20	12	24	0.00105055	0.336500758	24	17.39702869	0.239848952
ENSMUSG00000020042	Btbd11	BTB (POZ) domain containing 11 [Source:MGI Symbol;Acc:MGI:1921257]	4.42644	3.71659	4.277	4.01279	3.66776	4.53406	4.69096	4.12716	3.57	4.47397	3.63201	4.05535	3.46245	4.15177	0.886572916	0.966018836	24	20	24	0.774967234	0.927711986	24	22.90156876	0.03221084
ENSMUSG00000020044	Timp3	tissue inhibitor of metalloproteinase 3 [Source:MGI Symbol;Acc:MGI:98754]	102.427	100.587	97.5826	105.123	117.454	96.1983	110.575	101.038	103.003	119.465	98.612	100.334	97.9102	89.8715	0.708020493	0.966018836	16	16	24	0.211635071	0.768040178	24	14.47700426	0.070706648
ENSMUSG00000020048	Hsp90b1	heat shock protein 90, beta (Grp94), member 1 [Source:MGI Symbol;Acc:MGI:98817]	225.585	185.091	166.384	162.151	207.842	185.134	198.729	180.076	166.792	141.685	214.5	193.486	200.429	180.135	0.001134165	0.079869094	20	20	24	0.007202491	0.47735824	24	21.50490398	0.191509083
ENSMUSG00000020053	Igf1	insulin-like growth factor 1 [Source:MGI Symbol;Acc:MGI:96432]	5.32334	7.00392	5.45408	5.25374	5.48752	4.91109	5.33192	6.89491	7.50294	3.52836	4.94686	4.81264	4.70513	4.32788	0.591351901	0.966018836	12	12	24	0.287192116	0.768040178	24	4.882827032	0.134172825
ENSMUSG00000020056	Washc3	WASH complex subunit 3 [Source:MGI Symbol;Acc:MGI:1914532]	29.2923	30.3587	28.1315	28.0485	31.5503	31.9318	27.3282	30.8035	31.1317	24.936	31.3417	30.1744	33.2583	29.6215	0.676344318	0.966018836	20	20	24	0.366436044	0.790241693	24	22.10541144	0.058335861
ENSMUSG00000020057	Dram1	DNA-damage regulated autophagy modulator 1 [Source:MGI Symbol;Acc:MGI:1918962]	1.25751	1.28803	1.48275	1.36475	1.7253	1.23521	1.78903	1.42663	1.554	1.27537	1.83066	1.44394	1.62972	1.55017	0.920992137	0.966018836	20	20	24	0.887741399	0.968424304	24	16.96143941	0.020891896
ENSMUSG00000020059	Sycp3	synaptonemal complex protein 3 [Source:MGI Symbol;Acc:MGI:109542]	0.366861	0.547315	0.626148	0.614364	0.623969	0.392377	0.282128	0.580461	0.381823	0.549506	0.706542	0.351506	0.539638	0.477504	0.014632027	0.248301935	20	4	24	0.096824796	0.71175955	24	11.56146329	0.099712289
ENSMUSG00000020061	Mybpc1	myosin binding protein C, slow-type [Source:MGI Symbol;Acc:MGI:1336213]	6.91902	19.7139	34.3937	36.4102	26.4428	29.9223	13.153	55.8298	26.4951	47.9499	12.417	12.9002	26.0528	20.35	0.10165277	0.511659635	16	12	24	0.081568516	0.697539186	24	9.65396719	0.666834126
ENSMUSG00000020063	Sirt1	sirtuin 1 [Source:MGI Symbol;Acc:MGI:2135607]	6.73148	7.5033	7.07835	7.86163	7.8487	7.0505	7.33224	6.52452	8.1017	6.53597	8.61659	6.99346	6.34812	6.91062	0.200625581	0.685057195	20	8	24	0.154387053	0.760257003	24	13.6961772	0.081179454
ENSMUSG00000020064	Herc4	hect domain and RLD 4 [Source:MGI Symbol;Acc:MGI:1914595]	17.6716	19.844	20.4136	20.87	21.8271	17.7486	17.9101	18.1553	19.2538	15.7359	21.317	18.9611	18.1605	19.7788	0.055842225	0.401074772	20	8	24	0.550995929	0.836129338	24	12.87195887	0.051885733
ENSMUSG00000020067	Mypn	myopalladin [Source:MGI Symbol;Acc:MGI:1916052]	0.4746	0.930742	1.18802	1.57319	1.1445	1.28056	0.989712	2.72347	1.85335	3.33898	0.828601	1.06058	1.23433	1.15418	0.217933169	0.704989686	16	12	24	0.078529285	0.689042744	24	9.726885912	0.330367006
ENSMUSG00000020069	Hnrnph3	heterogeneous nuclear ribonucleoprotein H3 [Source:MGI Symbol;Acc:MGI:1926462]	28.6694	30.0281	30.9873	27.4595	30.4482	26.8182	30.8711	30.3018	31.7321	26.3215	31.8478	29.5502	30.4196	28.1194	0.945282252	0.966018836	12	12	24	0.807404599	0.938693233	24	3.75224371	0.022555513
ENSMUSG00000020070	Rufy2	RUN and FYVE domain-containing 2 [Source:MGI Symbol;Acc:MGI:1917682]	54.1948	47.8671	43.5192	48.7956	45.6912	49.4902	50.4734	45.9579	45.2511	53.1223	48.8483	52.9871	50.3326	50.5223	0.030691182	0.324150946	4	8	24	0.163756111	0.767020009	24	20.95085006	0.06657804
ENSMUSG00000020072	Pbld2	phenazine biosynthesis-like protein domain containing 2 [Source:MGI Symbol;Acc:MGI:1914557]	1.92904	2.11282	1.85637	2.62161	1.59719	2.0476	2.32852	1.75335	1.63999	2.15104	2.1638	2.54606	1.89512	2.59017	0.880631923	0.966018836	16	20	24	0.924529296	0.979879533	24	19.5331861	0.025462003
ENSMUSG00000020074	Ccar1	cell division cycle and apoptosis regulator 1 [Source:MGI Symbol;Acc:MGI:1914750]	21.3646	21.1001	20.3724	20.5553	20.498	19.906	21.4276	18.4081	22.3759	18.6354	21.9225	21.1721	21.5533	21.1589	0.843639218	0.966018836	4	12	24	0.758033056	0.921733004	24	22.68742373	0.023555135
ENSMUSG00000020075	Ddx21	DExD box helicase 21 [Source:MGI Symbol;Acc:MGI:1860494]	9.33867	8.62753	9.47151	9.63345	9.93048	8.6238	9.16264	8.74549	8.36159	10.1252	8.19829	8.90901	8.77064	7.86589	0.350544145	0.870418653	16	16	24	0.203309745	0.768040178	24	14.56311917	0.056158324
ENSMUSG00000020076	Ddx50	DExD box helicase 50 [Source:MGI Symbol;Acc:MGI:2182303]	36.1742	38.3386	37.4669	35.9824	32.6049	31.4073	35.4409	31.5626	37.1156	31.6792	37.4175	30.4652	32.741	33.4392	0.085890569	0.466404237	12	12	24	0.323660703	0.768040178	24	7.607394222	0.058952663
ENSMUSG00000020077	Srgn	serglycin [Source:MGI Symbol;Acc:MGI:97756]	27.5076	36.5413	39.6395	29.1894	34.4519	28.4917	30.4643	35.7376	42.8107	23.9349	34.1462	29.0806	32.6841	30.1526	0.042880436	0.356048751	12	12	24	0.280430708	0.768040178	24	6.884876916	0.137971653
ENSMUSG00000020078	Vps26a	VPS26 retromer complex component A [Source:MGI Symbol;Acc:MGI:1353654]	31.219	30.9817	30.559	29.2664	29.9876	29.0205	29.3766	29.3043	28.9514	30.2044	31.5175	28.6661	29.1155	29.8484	0.867555223	0.966018836	20	4	24	0.92609263	0.98027178	24	11.1776145	0.00630275
ENSMUSG00000020079	Supv3l1	suppressor of var1, 3-like 1 (S. cerevisiae) [Source:MGI Symbol;Acc:MGI:2441711]	8.32142	7.53117	8.49542	8.41642	8.0186	8.4588	6.93386	8.23429	6.48919	8.70908	7.855	8.51862	7.37966	7.67596	0.325142889	0.842977406	24	4	24	0.334750734	0.770844856	24	16.00491569	0.062052607
ENSMUSG00000020083	Fam241b	family with sequence similarity 241, member B [Source:MGI Symbol;Acc:MGI:1917144]	19.0093	18.8639	20.826	17.1837	17.7741	18.45	16.6146	20.9889	18.3463	19.1509	21.653	22.022	20.4888	20.4775	0.823246279	0.966018836	8	8	24	0.917772511	0.977735567	24	3.314451855	0.017466457
ENSMUSG00000020085	Aifm2	apoptosis-inducing factor, mitochondrion-associated 2 [Source:MGI Symbol;Acc:MGI:1918611]	3.79827	4.68932	6.25214	8.26911	5.2455	4.60785	5.3054	6.99755	5.93916	7.56515	6.59773	5.92191	8.31367	6.51161	0.616047374	0.966018836	16	8	24	0.062605763	0.661961741	24	10.25544607	0.202329621
ENSMUSG00000020086	Macroh2a2	macroH2A.2 histone [Source:MGI Symbol;Acc:MGI:3037658]	5.99184	5.524	5.48232	4.88103	5.41765	6.38183	5.85572	6.3264	5.0497	6.14907	5.13903	6.32274	6.25776	5.6106	0.020646136	0.262705843	24	20	24	0.052564714	0.650526959	24	22.88974161	0.095890635
ENSMUSG00000020087	Tysnd1	trypsin domain containing 1 [Source:MGI Symbol;Acc:MGI:1919017]	6.35792	3.79793	3.87721	4.91744	4.21206	4.77416	4.81749	5.28509	4.64926	5.02587	5.61563	6.09228	5.5974	5.31737	0.348523784	0.870418653	4	8	24	0.198108814	0.768040178	24	21.15846326	0.116023104
ENSMUSG00000020088	Sar1a	secretion associated Ras related GTPase 1A [Source:MGI Symbol;Acc:MGI:98230]	73.2166	71.8671	71.6721	73.0232	75.2753	68.93	72.5086	68.8942	74.3144	68.1775	75.0769	69.8044	75.2821	70.5178	0.95269569	0.966018836	20	12	24	0.873048562	0.964303715	24	16.95703683	0.010479552
ENSMUSG00000020089	Ppa1	pyrophosphatase (inorganic) 1 [Source:MGI Symbol;Acc:MGI:97831]	58.7722	49.8406	53.3392	58.2003	49.4141	53.2624	51.5885	54.7229	42.963	53.166	56.6942	50.8175	54.7456	53.2035	0.890235289	0.966018836	4	8	24	0.651696428	0.882836756	24	19.97076104	0.042895268

ENSMUSG00000020091	Eif4ebp2	eukaryotic translation initiation factor 4E binding protein 2 [Source:MGI Symbol;Acc:MGI:109198]	7.78779	7.2988	8.13356	9.32467	8.02961	8.14367	9.48299	9.16699	8.58009	11.1583	7.95563	8.23169	9.01409	8.58206	0.885059765	0.966018836	16	4	24	0.445451245	0.805393068	24	10.42030315	0.065120692
ENSMUSG00000020092	Pald1	phosphatase domain containing, paladin 1 [Source:MGI Symbol;Acc:MGI:1351623]	4.43937	4.87399	5.20745	5.43333	5.42864	4.46322	5.80746	4.89347	5.70683	5.59926	5.18518	5.12334	5.43485	4.9615	0.313786068	0.824470524	12	20	24	0.207532264	0.768040178	24	10.98078012	0.065161354
ENSMUSG00000020097	Sgpl1	sphingosine phosphate lyase 1 [Source:MGI Symbol;Acc:MGI:1261415]	18.0557	17.1952	19.0196	18.8785	17.7022	17.0387	18.3003	18.0668	16.0316	19.702	17.6793	17.2886	17.3833	17.8596	0.424718357	0.966018836	16	8	24	0.529477944	0.824483137	24	11.46358582	0.031346026
ENSMUSG00000020098	Pcbd1	pterin 4 alpha carbinolamine dehydratase/dimerization cofactor of hepatocyte nuclear factor 1 alpha (TCF1) 1 [Source:MGI Symbol;Acc:MGI:94873]	41.1249	36.1361	37.3988	34.5575	36.6939	38.6417	34.8061	37.1583	30.554	36.518	37.146	38.6018	38.0544	39.9654	0.200364953	0.684570416	24	16	24	0.147180005	0.75567997	24	21.95091911	0.076328084
ENSMUSG00000020099	Unc5b	unc-5 netrin receptor B [Source:MGI Symbol;Acc:MGI:894703]	1.44685	2.01093	2.62553	1.5571	1.66778	2.05826	2.26572	2.13947	1.96658	2.48474	1.32298	1.87475	1.71439	2.16333	0.351315142	0.870418653	12	8	24	0.191921602	0.768040178	24	6.410258731	0.133829849
ENSMUSG00000020100	Slc29a3	solute carrier family 29 (nucleoside transporters), member 3 [Source:MGI Symbol;Acc:MGI:1918529]	5.91787	4.90512	4.9862	4.76889	5.20644	6.30683	5.66084	5.34641	4.05607	5.52939	5.00472	5.41683	4.86727	4.76016	0.077301512	0.453790923	24	12	24	0.020279396	0.565108098	24	20.44805933	0.128383287
ENSMUSG00000020101	Vsir	V-set immunoregulatory receptor [Source:MGI Symbol;Acc:MGI:1921298]	2.63736	3.73652	4.74181	3.38056	3.77357	3.77484	4.02943	3.20267	4.6927	2.79008	3.52863	3.16376	3.22503	2.62826	0.938993891	0.966018836	12	20	24	0.658084861	0.884596426	24	10.37441689	0.074760795
ENSMUSG00000020102	Slc16a7	solute carrier family 16 (monocarboxylic acid transporters), member 7 [Source:MGI Symbol;Acc:MGI:1330284]	4.75206	4.45371	3.38116	4.25244	5.46673	3.83111	4.30363	4.08425	3.83501	3.63185	4.11811	4.64725	5.1611	4.22381	0.055920623	0.401074772	4	8	24	0.118581096	0.729313257	24	21.0685262	0.122708257
ENSMUSG00000020105	Lrig3	leucine-rich repeats and immunoglobulin-like domains 3 [Source:MGI Symbol;Acc:MGI:2443955]	1.06647	1.19713	1.23513	1.32787	1.28353	1.19752	0.910001	1.14806	1.19539	1.10424	0.971831	1.08799	1.21253	1.03928	0.696393581	0.966018836	16	12	24	0.353125459	0.780651287	24	11.12601638	0.042770518
ENSMUSG00000020107	Anapc16	anaphase promoting complex subunit 16 [Source:MGI Symbol;Acc:MGI:1289325]	50.3278	45.3773	45.7562	49.3744	48.1478	48.6982	50.5111	52.54	49.8496	41.9268	53.7054	47.3623	51.0676	45.5648	0.883024237	0.966018836	4	12	24	0.437691209	0.805393068	24	21.52967489	0.048967699
ENSMUSG00000020108	Ddit4	DNA-damage-inducible transcript 4 [Source:MGI Symbol;Acc:MGI:1921997]	25.1905	21.7603	25.0545	26.5801	21.1856	23.748	26.0652	24.6544	29.1754	37.7637	28.9375	28.3928	21.0376	24.5371	0.226403091	0.722427815	16	16	24	0.135234026	0.746923165	24	12.01146072	0.153741203
ENSMUSG00000020109	Dnajb12	DnaJ heat shock protein family (Hsp40) member B12 [Source:MGI Symbol;Acc:MGI:1931881]	14.9144	13.8947	13.8903	13.9899	13.6762	14.8778	14.5687	14.182	13.2107	15.8284	12.8768	14.6518	13.9823	13.0196	0.338172159	0.85758369	24	12	24	0.567378745	0.843840591	24	20.11382669	0.033084556
ENSMUSG00000020111	Micu1	mitochondrial calcium uptake 1 [Source:MGI Symbol;Acc:MGI:2384909]	54.1332	46.4135	44.7266	47.6788	45.9095	49.46	46.4854	48.5046	43.7939	48.9471	45.4403	49.013	48.0451	48.7571	0.041292272	0.346472636	24	12	24	0.152600001	0.759088284	24	22.26917692	0.055464612
ENSMUSG00000020114	Cand1	cullin associated and neddylation disassociated 1 [Source:MGI Symbol;Acc:MGI:1261820]	32.5396	26.5301	29.3192	31.4015	29.3684	32.5415	31.5896	25.8787	24.8158	31.2248	27.8404	27.623	28.0451	30.7401	0.42627752	0.966018836	4	4	24	0.372354224	0.793651569	24	18.9344712	0.067630377
ENSMUSG00000020115	Tbk1	TANK-binding kinase 1 [Source:MGI Symbol;Acc:MGI:1929658]	28.8583	26.9078	28.6773	27.6169	27.0756	27.3764	27.4506	27.4702	24.9314	28.1238	26.3459	25.9353	27.9504	28.3633	0.643501552	0.966018836	4	20	24	0.675270113	0.89119716	24	2.374230982	0.018938732
ENSMUSG00000020116	Pno1	partner of NOB1 homolog [Source:MGI Symbol;Acc:MGI:1913499]	26.5549	24.5947	24.2698	23.7936	23.7409	23.1121	23.0404	24.337	22.7951	23.9678	27.368	22.6985	24.3619	24.0662	0.46238066	0.966018836	4	20	24	0.960610164	0.989784105	24	19.4632326	0.008804558
ENSMUSG00000020120	Plek	pleckstrin [Source:MGI Symbol;Acc:MGI:1860485]	4.34187	6.02322	6.58958	5.29821	5.1327	4.71435	4.54025	4.79797	5.74111	3.85939	4.84158	5.00984	5.73398	4.44908	0.44861185	0.966018836	12	16	24	0.548356601	0.834310879	24	7.111942168	0.075689207
ENSMUSG00000020121	Srgap1	SLIT-ROBO Rho GTPase activating protein 1 [Source:MGI Symbol;Acc:MGI:2152936]	7.3273	6.47402	6.33997	6.57547	7.25795	7.12225	6.87335	7.24919	5.42877	9.20334	6.58974	6.78731	6.88568	6.86086	0.277087842	0.789849983	4	8	24	0.624934844	0.872055388	24	17.72244671	0.056581067
ENSMUSG00000020122	Egfr	epidermal growth factor receptor [Source:MGI Symbol;Acc:MGI:95294]	1.42821	1.6019	1.52242	1.83776	1.76876	1.28189	1.67429	1.30235	2.09026	1.11585	1.46165	1.41637	1.46769	1.45216	0.601647512	0.966018836	12	12	24	0.682923467	0.894308158	24	9.322868792	0.049567078
ENSMUSG00000020124	Usp15	ubiquitin specific peptidase 15 [Source:MGI Symbol;Acc:MGI:101857]	48.7072	49.575	48.7547	48.5678	51.0674	49.1443	45.9886	46.6476	49.7522	43.4401	51.1068	49.8628	55.2367	51.6773	0.911685024	0.966018836	20	20	24	0.634913751	0.875864536	24	22.37896949	0.030152332
ENSMUSG00000020125	Elane	elastase, neutrophil expressed [Source:MGI Symbol;Acc:MGI:2679229]	2.37171	3.5668	8.21126	5.1636	2.75367	5.74302	3.42525	3.46734	3.90082	2.7821	2.42976	4.25871	5.64178	2.24078	0.934110497	0.966018836	12	20	24	0.953000652	0.988573174	24	8.465576664	0.057678277
ENSMUSG00000020128	Vps54	VPS54 GARP complex subunit [Source:MGI Symbol;Acc:MGI:2178798]	13.7612	13.8602	12.4199	14.0996	13.9139	13.3769	14.2881	12.1594	13.4973	12.1094	14.1814	13.3249	12.7528	11.5337	0.607427393	0.966018836	20	12	24	0.082746779	0.699403344	24	18.29470108	0.068873566
ENSMUSG00000020130	Tbc1d15	TBC1 domain family, member 15 [Source:MGI Symbol;Acc:MGI:1913937]	21.672	22.3642	22.483	27.7674	22.0514	20.259	22.1391	21.2813	24.3088	19.8595	21.0878	26.3576	26.4211	24.3727	0.887862353	0.966018836	12	8	24	0.907851933	0.974606327	24	5.707896101	0.025332238
ENSMUSG00000020131	Pcsk4	proprotein convertase subtilisin/kexin type 4 [Source:MGI Symbol;Acc:MGI:97514]	0.571096	0.824891	0.990672	0.992874	0.7561	0.84647	1.07098	1.24127	1.34412	1.45016	1.12823	1.18677	1.15383	1.09083	0.933543173	0.966018836	16	4	24	0.031620062	0.604305928	24	9.328038116	0.115763598
ENSMUSG00000020132	Rab21	RAB21, member RAS oncogene family [Source:MGI Symbol;Acc:MGI:894308]	27.3454	22.5175	23.3451	25.1221	23.0924	25.3383	24.8966	23.6065	20.3883	27.4963	22.4814	23.1781	22.8298	23.2347	0.840483986	0.966018836	24	12	24	0.568297413	0.844336151	24	19.17365711	0.046634382
ENSMUSG00000020133	2310011J03Rik	RIKEN cDNA 2310011J03 gene [Source:MGI Symbol;Acc:MGI:1913624]	17.7643	16.1497	16.6301	16.1255	16.8602	17.3646	18.0534	18.6783	17.1531	18.7096	18.2507	19.2596	18.4371	17.4187	0.59241921	0.966018836	24	12	24	0.231226566	0.768040178	24	21.19422976	0.039779688
ENSMUSG00000020134	Peli1	pellino 1 [Source:MGI Symbol;Acc:MGI:1914495]	7.7495	7.98973	9.28582	9.4297	7.99386	7.98467	7.35047	7.47926	8.07923	8.47563	8.04449	7.47976	6.82833	7.61132	1.16E-04	0.029372629	16	12	24	1.78E-04	0.142715477	24	11.50932298	0.116862792
ENSMUSG00000020135	Apc2	APC regulator of WNT signaling pathway 2 [Source:MGI Symbol;Acc:MGI:1346052]	15.0629	12.9641	13.9936	13.9165	13.1667	15.253	15.7901	15.7025	13.7315	20.243	14.0664	17.1033	15.9511	15.9818	0.787283372	0.966018836	12	24	24	0.944464635	0.986443184	24	21.04532217	0.019408498
ENSMUSG00000020137	Thap2	THAP domain containing, apoptosis associated protein 2 [Source:MGI Symbol;Acc:MGI:1914066]	7.39861	6.08018	6.4627	7.28155	7.19143	6.31046	7.22446	5.79784	6.64279	5.84943	7.57217	6.9471	6.11299	6.47621	0.192357053	0.669841558	20	12	24	0.162935057	0.766462333	24	17.64521225	0.081686867
ENSMUSG00000020140	Lgr5	leucine rich repeat containing G protein coupled receptor 5 [Source:MGI Symbol;Acc:MGI:1341817]	3.91623	3.53958	4.54451	3.76283	4.03799	4.04092	3.74388	3.86117	3.50428	4.10514	3.90563	3.25255	3.64451	3.45581	0.664568268	0.966018836	16	16	24	0.376534392	0.796552361	24	12.99464842	0.046439751
ENSMUSG00000020142	Slc1a4	solute carrier family 1 (glutamate/neutral amino acid transporter), member 4 [Source:MGI Symbol;Acc:MGI:2135601]	16.5211	12.1192	13.6652	12.7502	13.8938	15.497	14.2972	13.9494	9.97427	15.6076	13.0654	13.2459	12.2011	13.3869	0.865233232	0.966018836	24	12	24	0.24839136	0.768040178	24	19.95974817	0.10843123
ENSMUSG00000020143	Dock2	dedicator of cyto-kinesis 2 [Source:MGI Symbol;Acc:MGI:2149010]	0.759666	1.10758	1.11649	1.15775	0.926594	1.00167	0.890544	0.941357	1.25115	0.707458	0.851824	0.898747	1.14634	0.923578	0.455507782	0.966018836	12	8	24	0.396493767	0.799546792	24	7.014390089	0.064213083
ENSMUSG00000020149	Rab1a	RAB1A, member RAS oncogene family [Source:MGI Symbol;Acc:MGI:97842]	132.04	121.834	123.866	123.861	125.146	130.699	127.533	122.827	111.821	126.167	132.796	114.331	118.206	123.836	0.560714538	0.966018836	20	12	24	0.367082196	0.790611743	24	18.6964548	0.038545282
ENSMUSG00000020150	Gamt	guanidinoacetate methyltransferase [Source:MGI Symbol;Acc:MGI:1098221]	20.0829	13.9995	14.0632	13.7043	15.5827	17.5202	17.4866	15.9432	13.4186	18.076	14.9356	17.2863	17.7603	15.137	0.009118941	0.192760771	4	8	24	0.024496963	0.586541342	24	21.39233091	0.163815415
ENSMUSG00000020151	Ptprr	protein tyrosine phosphatase, receptor type, R [Source:MGI Symbol;Acc:MGI:109559]	17.1155	13.4554	13.5833	13.091	12.6456	15.0194	12.4773	13.5283	9.90678	14.8406	12.5915	13.2687	13.8882	14.5451	0.928892845	0.966018836	4	16	24	0.333787002	0.769898729	24	22.28937653	0.094960033
ENSMUSG00000020152	Actr2	ARP2 actin-related protein 2 [Source:MGI Symbol;Acc:MGI:1913963]	111.739	107.467	101.22	102.776	105.802	103.664	102.959	97.4033	96.4586	97.7722	108.558	102.01	104.501	105.654	0.277917941	0.789849983	4	8	24	0.116154832	0.727799063	24	21.07037367	0.046541653
ENSMUSG00000020153	Ndufs7	NADH:ubiquinone oxidoreductase core subunit S7 [Source:MGI Symbol;Acc:MGI:1922656]	78.4167	72.4255	80.3029	77.8218	63.0763	75.2587	77.7907	97.1413	82.6358	100.333	84.1752	80.2959	89.9335	78.6523	0.928127947	0.966018836	16	4	24	0.403379374	0.799546792	24	7.081203118	0.081437708
ENSMUSG00000020154	Ptprb	protein tyrosine phosphatase, receptor type, B [Source:MGI Symbol;Acc:MGI:97809]	2.12948	3.64705	3.59674	3.93402	3.38137	3.03	3.06855	4.28635	4.13285	3.80466	2.73758	3.28496	3.51636	2.91173	0.088482991	0.473415302	12	16	24	0.061164365	0.660703626	24	9.064028753	0.178097691
ENSMUSG00000020155	Kcnmb1	potassium large conductance calcium-activated channel, subfamily M, beta member 1 [Source:MGI Symbol;Acc:MGI:1334203]	7.58144	8.50184	7.48325	7.82656	6.91581	7.94163	7.37438	7.61532	6.87537	8.77414	7.60454	8.43792	8.51497	8.13079	0.935194469	0.966018836	24	12	24	0.841246351	0.951883465	24	0.265732041	0.021791733
ENSMUSG00000020156	Pwwp3a	PWWP domain containing 3A, DNA repair factor [Source:MGI Symbol;Acc:MGI:1915364]	17.5062	16.5567	16.4937	17.1606	15.5184	16.7329	18.3955	17.3554	16.9614	18.5458	16.7425	18.0688	17.4959	17.3416	0.33885934	0.858075318	4	16	24	0.623065317	0.871674425	24	1.52010528	0.022673011
ENSMUSG00000020160	Meis1	Meis homeobox 1 [Source:MGI Symbol;Acc:MGI:104717]	2.25848	2.23929	3.0724	2.97965	2.41445	2.77393	2.40214	2.14583	2.3105	2.52128	2.25316	2.73067	2.14217	3.29243	0.492524295	0.966018836	16	12	24	0.606427894	0.863212285	24	10.86948977	0.059137197
ENSMUSG00000020163	Uqcr11	ubiquinol-cytochrome c reductase, complex III subunit XI [Source:MGI Symbol;Acc:MGI:1913844]	77.196	77.0873	79.0492	81.055	72.8672	74.1337	78.9061	82.1689	89.757	71.2773	89.9698	85.4954	87.5822	76.0738	0.786317982	0.966018836	12	4	24	0.951259518	0.988338295	24	4.592741499	0.012874445
ENSMUSG00000020166	Cnot2	CCR4-NOT transcription complex, subunit 2 [Source:MGI Symbol;Acc:MGI:1919318]	25.1814	25.1815	23.2596	23.7231	23.1392	22.4507	25.3273	23.2748	23.6027	20.6905	23.7623	23.9718	23.2951	22.7961	0.648082424	0.966018836	4	12	24	0.114902734	0.726165588	24	0.310896602	0.053220006
ENSMUSG00000020167	Tcf3	transcription factor 3 [Source:MGI Symbol;Acc:MGI:98510]	7.16091	7.52009	7.80656	6.53339	6.64042	7.13502	7.44816	8.21591	8.89627	9.16873	8.05071	7.5482	8.84187	7.9706	0.714044782	0.966018836	12	8	24	0.107871881	0.72073763	24	6.724870315	0.086306683
ENSMUSG00000020170	Frs2	fibroblast growth factor receptor substrate 2 [Source:MGI Symbol;Acc:MGI:1100860]	10.7168	10.736	10.8666	10.9778	10.3477	10.494	11.0023	10.2663	11.1831	11.4475	10.2143	10.6612	10.3504	10.6964	0.036302654	0.33234124	16	4	24	0.204027345	0.768040178	24	9.112163216	0.031004598
ENSMUSG00000020171	Yeats4	YEATS domain containing 4 [Source:MGI Symbol;Acc:MGI:1927224]	16.4614	17.0033	14.0521	17.9094	14.888	15.5987	15.7715	15.8967	18.4584	16.7495	17.9884	18.5167	20.186	18.6033	0.873178365	0.966018836	8	12	24	0.866416279	0.961390246	24	2.525872853	0.022126762
ENSMUSG00000020173	Cobl	cordon-bleu WH2 repeat [Source:MGI Symbol;Acc:MGI:105056]	12.9378	11.5279	10.8306	12.12	10.792	14.2449	11.8057	14.0485	9.15548	16.1939	10.3743	13.7302	12.6245	12.2221	0.245061943	0.741580974	24	12	24	0.640757758	0.878446626	24	21.19472241	0.076617871
ENSMUSG00000020175	Rab36	RAB36, member RAS oncogene family [Source:MGI Symbol;Acc:MGI:1924127]	5.16111	7.08474	7.45299	6.36789	5.56598	6.56037	6.15278	7.41479	6.7834	7.35662	5.49696	7.32755	6.26276	7.39302	0.053432515	0.394331071	8	20	24	0.075549771	0.68592977	24	6.980368575	0.128068326
ENSMUSG00000020176	Grb10	growth factor receptor bound protein 10 [Source:MGI Symbol;Acc:MGI:103232]	10.5993	10.1634	12.3645	11.1372	10.5911	11.0071	11.1834	9.57635	10.1577	9.85441	10.6442	9.88758	9.50734	10.6376	0.830949782	0.966018836	12	20	24	0.72907466	0.911453005	24	12.69370068	0.025833023
ENSMUSG00000020178	Adora2a	adenosine A2a receptor [Source:MGI Symbol;Acc:MGI:99402]	1.59757	1.62169	1.43459	1.68944	1.56842	1.62564	1.47939	1.98913	1.47055	1.85895	1.28129	1.81298	1.56553	1.68487	0.851656735	0.966018836	8	12	24	0.822370507	0.946154971	24	4.228993175	0.024975911
ENSMUSG00000020180	Snrpd3	small nuclear ribonucleoprotein D3 [Source:MGI Symbol;Acc:MGI:1914582]	54.047	53.3665	52.4697	52.2997	58.7948	49.897	49.8413	43.9031	48.4805	42.5532	54.0879	52.675	53.6513	50.2563	0.265774856	0.773510818	20	20	24	0.261033687	0.768040178	24	19.32803163	0.075835777
ENSMUSG00000020181	Nav3	neuron navigator 3 [Source:MGI Symbol;Acc:MGI:2183703]	1.69259	2.68017	2.71487	2.24586	2.50856	2.38047	1.99876	2.47523	2.96372	2.8424	2.45148	2.66054	2.15175	2.66878	0.001837158	0.100255953	12	16	24	0.025440406	0.586541342	24	10.00809954	0.138358423
ENSMUSG00000020182	Ddc	dopa decarboxylase [Source:MGI Symbol;Acc:MGI:94876]	0.885113	1.78772	3.20124	3.63715	1.30157	4.4888	7.79044	1.2684	1.19303	5.20925	7.55654	9.60917	1.40576	19.8069	0.954584434	0.966018836	24	4	24	0.881244859	0.966661503	24	16.72303922	0.181934281
ENSMUSG00000020183	Cpm	carboxypeptidase M [Source:MGI Symbol;Acc:MGI:1917824]	0.955116	1.00398	1.4203	1.37642	0.812128	1.09635	1.05147	0.849484	0.840028	0.897171	0.614201	0.685857	0.72718	0.89342	0.79867683	0.966018836	16	4	24	0.318254193	0.768040178	24	8.103638042	0.078576768
ENSMUSG00000020184	Mdm2	transformed mouse 3T3 cell double minute 2 [Source:MGI Symbol;Acc:MGI:96952]	22.4067	22.6819	25.7333	25.4871	24.3393	23.5855	22.5952	24.3221	24.0008	21.9523	25.6738	23.4699	23.4222	23.4965	0.047000349	0.375011045	20	8	24	0.225668873	0.768040178	24	12.23656909	0.046146577
ENSMUSG00000020186	Csrp2	cysteine and glycine-rich protein 2 [Source:MGI Symbol;Acc:MGI:1202907]	23.2555	22.8269	21.684	24.0828	26.3244	18.0944	21.332	20.3196	23.4976	19.3113	25.3228	21.9865	20.8662	20.0571	0.446653834	0.966018836	20	12	24	0.400046117	0.799546792	24	14.29264752	0.072566582
ENSMUSG00000020189	Osbpl8	oxysterol binding protein-like 8 [Source:MGI Symbol;Acc:MGI:2443807]	16.185	17.121	16.5713	16.8591	16.3306	15.2646	16.3119	14.4308	15.2465	14.2907	16.8756	14.9839	14.8298	16.1317	0.8635847	0.966018836	20	4	24	0.945766038	0.986840136	24	13.88183537	0.009556617
ENSMUSG00000020190	Mknk2	MAP kinase-interacting serine/threonine kinase 2 [Source:MGI Symbol;Acc:MGI:894279]	17.5634	13.8956	16.155	23.4491	18.3261	15.5125	18.1052	21.1566	19.6552	24.274	22.5144	16.4315	21.2143	16.0553	0.128810631	0.560209134	16	16	24	0.141193923	0.751689872	24	12.63414538	0.163475497
ENSMUSG00000020196	Cabin1	calcineurin binding protein 1 [Source:MGI Symbol;Acc:MGI:1298375]	10.4993	11.0993	9.78231	11.2506	10.953	10.0953	9.31617	10.823	11.2116	13.0942	9.22435	12.2754	11.6721	10.7525	0.924553096	0.966018836	16	12	24	0.712703266	0.905277969	24	10.57158013	0.042177935
ENSMUSG00000020198	Ap3d1	adaptor-related protein complex 3, delta 1 subunit [Source:MGI Symbol;Acc:MGI:107734]	41.5282	33.0311	34.2848	37.0206	38.0723	41.548	39.1911	38.2266	32.6688	42.2543	34.4534	35.8355	35.8446	35.6039	0.210418232	0.69936851	24	12	24	0.278532552	0.768040178	24	19.42040716	0.075224659
ENSMUSG00000020205	Phlda1	pleckstrin homology like domain, family A, member 1 [Source:MGI Symbol;Acc:MGI:1096880]	6.23851	5.82858	4.83179	5.45982	6.30687	5.06127	7.62323	5.61864	6.20969	6.06964	8.0436	5.86695	7.87715	5.68806	0.216688884	0.704989686	20	16	24	0.448891985	0.805393068	24	20.6214018	0.091730563
ENSMUSG00000020211	Sf3a2	splicing factor 3a, subunit 2 [Source:MGI Symbol;Acc:MGI:104912]	6.28128	5.70402	6.03813	5.94617	5.92051	5.97671	6.29913	6.78432	6.31152	6.69464	5.5798	6.46895	6.60928	6.68591	0.47028201	0.966018836	8	12	24	0.590253522	0.854963454	24	9.318853184	0.067005424
ENSMUSG00000020212	Mdm1	transformed mouse 3T3 cell double minute 1 [Source:MGI Symbol;Acc:MGI:96951]	5.02345	4.89831	4.85355	4.9971	5.06007	4.82432	5.65693	4.27941	5.95775	4.43869	6.16014	5.58766	5.23551	4.58291	0.295379186	0.805301811	20	12	24	0.474575427	0.805393068	24	17.95118191	0.063090415
ENSMUSG00000020216	Jsrp1	junctional sarcoplasmic reticulum protein 1 [Source:MGI Symbol;Acc:MGI:1916700]	2.45752	4.78964	5.68726	5.71038	4.1608	4.88342	4.59537	14.3941	13.6236	16.3657	5.40618	6.84458	8.83282	6.58749	0.483374602	0.966018836	16	4	24	0.037203465	0.609176161	24	8.692265048	0.528369012
ENSMUSG00000020218	Wif1	Wnt inhibitory factor 1 [Source:MGI Symbol;Acc:MGI:1344332]	0.822241	1.04566	1.29251	1.26938	0.921063	1.50957	0.874255	1.62687	1.18203	1.20256	0.950728	0.777029	1.07107	0.936795	0.225060352	0.720143445	12	16	24	0.365517967	0.789741863	24	8.729634009	0.097198176
ENSMUSG00000020219	Timm13	translocase of inner mitochondrial membrane 13 [Source:MGI Symbol;Acc:MGI:1353432]	76.5431	68.9727	58.83	68.2172	62.4888	67.5926	65.4359	68.2315	62.9654	71.2151	76.2542	61.7434	71.2061	66.5926	0.948582958	0.966018836	4	4	24	0.692584974	0.898177673	24	21.01654915	0.038855891
ENSMUSG00000020220	Vps13d	vacuolar protein sorting 13D [Source:MGI Symbol;Acc:MGI:2448530]	35.6022	34.5468	33.4796	35.1491	37.1956	39.5664	36.4826	40.009	28.5079	41.171	30.6505	33.4301	37.1118	33.6966	0.832434677	0.966018836	4	8	24	0.658281053	0.884596426	24	21.04818647	0.05382671
ENSMUSG00000020224	Llph	LLP homolog, long-term synaptic facilitation (Aplysia) [Source:MGI Symbol;Acc:MGI:1913475]	7.8787	7.40012	7.72934	6.97947	7.18345	7.00393	7.3809	6.87391	8.02416	6.54955	8.59591	6.95257	8.41807	7.40288	0.949152198	0.966018836	4	12	24	0.817828901	0.942169199	24	1.180386224	0.025604462
ENSMUSG00000020225	Tmbim4	transmembrane BAX inhibitor motif containing 4 [Source:MGI Symbol;Acc:MGI:1915462]	73.0041	69.5684	70.0229	69.0196	63.4379	69.0294	65.605	71.3335	69.9464	65.401	75.2067	67.1413	73.8913	68.4911	0.951846915	0.966018836	4	12	24	0.633244064	0.875353057	24	2.367681122	0.025682893
ENSMUSG00000020227	Irak3	interleukin-1 receptor-associated kinase 3 [Source:MGI Symbol;Acc:MGI:1921164]	1.82885	2.04072	2.19658	2.14959	2.09668	2.32883	1.85966	1.99863	2.57628	1.87395	2.22244	2.29269	2.41066	1.89429	0.675497492	0.966018836	24	4	24	0.722263162	0.908924547	24	14.16408707	0.032253573
ENSMUSG00000020228	Helb	helicase (DNA) B [Source:MGI Symbol;Acc:MGI:2152895]	2.48817	2.89571	2.96952	3.06281	3.04728	2.68461	2.39086	3.41349	3.30031	2.84804	2.71571	2.71022	3.30386	2.87655	0.128989168	0.560209134	12	12	24	0.113493866	0.727440321	24	8.377183161	0.08852798
ENSMUSG00000020230	Prmt2	protein arginine N-methyltransferase 2 [Source:MGI Symbol;Acc:MGI:1316652]	49.7838	37.5894	37.9636	37.675	42.8095	45.3957	45.3016	43.165	33.4858	43.013	42.5669	40.7678	40.2686	39.466	0.073139747	0.447768907	24	12	24	0.02174962	0.574400012	24	20.69384256	0.128628362
ENSMUSG00000020231	Dip2a	disco interacting protein 2 homolog A [Source:MGI Symbol;Acc:MGI:2385920]	10.0641	11.5571	13.0774	12.3614	11.0219	12.3174	13.1363	10.5084	10.2901	11.7146	9.45145	10.4443	10.2453	10.8111	0.93717134	0.966018836	16	4	24	0.854285497	0.956491441	24	9.160587999	0.029065236
ENSMUSG00000020232	Hmg20b	high mobility group 20B [Source:MGI Symbol;Acc:MGI:1341190]	17.2277	16.6162	13.6704	14.5339	14.4491	16.7775	16.1631	15.1612	17.0618	19.0608	17.7025	17.6761	19.0373	15.087	0.57656008	0.966018836	24	12	24	0.457020896	0.805393068	24	20.56032052	0.06497204
ENSMUSG00000020235	Fzr1	fizzy and cell division cycle 20 related 1 [Source:MGI Symbol;Acc:MGI:1926790]	14.3189	11.3574	12.2819	12.0576	11.7331	13.252	12.7549	11.838	10.0481	15.4441	10.6879	12.4491	12.2397	11.713	0.277817749	0.789849983	4	4	24	0.608716722	0.864393296	24	20.05774454	0.059672857
ENSMUSG00000020238	Ncln	nicalin [Source:MGI Symbol;Acc:MGI:1926081]	18.5228	16.5756	14.4094	19.923	20.9925	18.0275	16.8731	17.5941	15.7748	18.95	17.5449	15.73	17.6674	18.4475	0.265774856	0.773510818	16	20	24	0.354577593	0.781596447	24	16.54997693	0.076282986
ENSMUSG00000020241	Col6a2	collagen, type VI, alpha 2 [Source:MGI Symbol;Acc:MGI:88460]	17.7478	19.7175	15.292	14.3889	17.953	14.5307	18.8981	21.0281	22.5649	13.2899	15.4291	15.5867	15.8922	16.3613	0.01518376	0.253245931	8	8	24	0.124550278	0.738497304	24	3.713626362	0.157177689
ENSMUSG00000020246	Hcfc2	host cell factor C2 [Source:MGI Symbol;Acc:MGI:1915183]	10.4637	10.0617	10.8157	10.8875	11.8436	10.1987	12.6615	9.93115	10.458	9.57473	9.92303	11.3442	10.124	9.95859	0.387380622	0.915479136	24	8	24	0.396419628	0.799546792	24	19.28153347	0.058938396
ENSMUSG00000020248	Nfyb	nuclear transcription factor-Y beta [Source:MGI Symbol;Acc:MGI:97317]	13.7425	14.1428	14.2252	15.3198	13.8859	11.8753	12.6104	11.0626	14.3429	13.1937	14.193	12.6824	13.2033	14.9464	0.130696419	0.562427886	12	12	24	0.220568885	0.768040178	24	10.43258866	0.080950253
ENSMUSG00000020250	Txnrd1	thioredoxin reductase 1 [Source:MGI Symbol;Acc:MGI:1354175]	40.3899	29.6331	31.297	32.1492	35.3912	37.7829	34.7635	34.2165	26.6336	37.7218	33.8341	33.8304	33.2161	32.1381	0.071575474	0.440262583	24	12	24	0.051431328	0.64767123	24	19.63536822	0.13354763
ENSMUSG00000020251	Glt8d2	glycosyltransferase 8 domain containing 2 [Source:MGI Symbol;Acc:MGI:1922032]	2.69021	2.60588	2.86731	2.50934	3.09304	2.07641	2.63752	2.47256	3.78918	1.92075	3.14996	2.95322	3.01803	3.41285	0.886572916	0.966018836	20	20	24	0.717247697	0.906810602	24	5.895966259	0.061039004
ENSMUSG00000020253	Ppm1m	protein phosphatase 1M [Source:MGI Symbol;Acc:MGI:1915155]	9.53345	11.8213	11.2883	12.0011	14.4499	10.1589	10.423	11.3073	12.6811	11.1616	11.1134	10.0191	9.95952	9.73645	0.030691182	0.324150946	12	16	24	0.038793461	0.611655116	24	11.88931839	0.131887884
ENSMUSG00000020256	Aldh1l2	aldehyde dehydrogenase 1 family, member L2 [Source:MGI Symbol;Acc:MGI:2444680]	1.49528	1.33805	1.57775	1.33373	1.55282	1.47618	1.22961	1.3653	1.32471	1.87965	1.61189	1.31361	1.33352	1.26101	0.351315142	0.870418653	20	8	24	0.123386735	0.736234617	24	15.46314406	0.051065207
ENSMUSG00000020257	Wdr82	WD repeat domain containing 82 [Source:MGI Symbol;Acc:MGI:1924555]	20.7259	18.1785	20.5079	19.4487	20.072	19.8075	20.3062	19.2689	18.2866	19.3405	18.4019	18.0268	18.5678	18.9473	0.860993221	0.966018836	4	24	24	0.852908589	0.956013379	24	20.33054527	0.012577045
ENSMUSG00000020258	Glyctk	glycerate kinase [Source:MGI Symbol;Acc:MGI:2444085]	0.676847	0.686933	0.713061	0.925802	0.721466	0.762946	0.901414	0.723739	0.67608	1.34337	0.719729	0.886701	0.811429	0.768905	0.503972924	0.966018836	16	20	24	0.385178316	0.799546792	24	13.47754084	0.065442739
ENSMUSG00000020260	Pofut2	protein O-fucosyltransferase 2 [Source:MGI Symbol;Acc:MGI:1916863]	10.8232	9.55417	9.4377	9.66227	10.2594	8.84333	10.5154	10.8505	10.0199	10.901	8.92455	9.32109	9.87939	10.0969	0.648082424	0.966018836	4	20	24	0.422385658	0.804917634	24	4.975020877	0.047314166
ENSMUSG00000020261	Slc36a1	solute carrier family 36 (proton/amino acid symporter), member 1 [Source:MGI Symbol;Acc:MGI:2445299]	39.3162	29.3249	28.6181	35.2353	32.3481	37.3577	33.6589	35.7123	25.2702	48.9207	31.539	37.1905	34.0713	36.4709	0.148073804	0.590732968	24	12	24	0.553154246	0.837286821	24	18.80066645	0.098010133
ENSMUSG00000020262	Adarb1	adenosine deaminase, RNA-specific, B1 [Source:MGI Symbol;Acc:MGI:891999]	10.6302	9.44545	11.9644	9.89402	9.12923	11.2875	12.0221	8.97524	9.0438	10.8184	8.70633	10.3634	8.01699	11.0502	0.838244962	0.966018836	24	20	24	0.95753862	0.988803933	24	1.259489223	0.018763398
ENSMUSG00000020263	Appl2	adaptor protein, phosphotyrosine interaction, PH domain and leucine zipper containing 2 [Source:MGI Symbol;Acc:MGI:2384914]	40.3115	51.8806	46.6123	46.1978	46.1519	43.8108	53.6985	47.4886	54.2292	43.2405	44.1651	45.7462	47.155	46.2535	0.117105047	0.538362324	8	16	24	0.252586322	0.768040178	24	5.589651856	0.07601028
ENSMUSG00000020264	Slc36a2	solute carrier family 36 (proton/amino acid symporter), member 2 [Source:MGI Symbol;Acc:MGI:1891430]	48.8635	46.3402	42.1222	56.8817	57.2653	42.7082	57.9127	48.1074	55.9579	55.4546	52.4201	48.7341	58.1744	45.0067	0.575202741	0.966018836	4	24	24	0.564326968	0.843102812	24	14.82361656	0.067584922
ENSMUSG00000020265	Sumo3	small ubiquitin-like modifier 3 [Source:MGI Symbol;Acc:MGI:1336201]	67.7706	67.9498	64.3247	68.3125	66.2522	64.4596	60.842	64.2861	64.3176	62.7546	64.2354	67.1506	65.6417	61.6192	0.853736882	0.966018836	24	12	24	0.627579706	0.872795898	24	17.90002732	0.017694705
ENSMUSG00000020267	Hint1	histidine triad nucleotide binding protein 1 [Source:MGI Symbol;Acc:MGI:1321133]	449.319	452.254	404.05	402.536	399.905	393.884	403.712	405.062	460.216	370.161	502.1	443.537	476.676	463.334	0.465004256	0.966018836	8	8	24	0.554739837	0.837958238	24	1.083977423	0.050665457
ENSMUSG00000020268	Lyrm7	LYR motif containing 7 [Source:MGI Symbol;Acc:MGI:1922780]	7.21076	4.42561	6.41246	4.91159	3.5146	4.03709	7.17582	5.05676	6.07661	4.38192	5.42879	4.51136	4.8846	5.32726	0.293067761	0.801049287	4	20	24	0.236006598	0.768040178	24	3.747232441	0.163524796
ENSMUSG00000020271	Fbxw11	F-box and WD-40 domain protein 11 [Source:MGI Symbol;Acc:MGI:2144023]	51.5744	41.8591	44.2527	43.7241	43.3288	47.7103	45.8381	45.1454	33.6165	52.5833	43.3908	45.2199	47.0321	44.9384	0.109194134	0.524035616	8	4	24	0.318025575	0.768040178	24	20.6035283	0.090248651
ENSMUSG00000020272	Stk10	serine/threonine kinase 10 [Source:MGI Symbol;Acc:MGI:1099439]	4.4323	4.02008	3.99452	4.07236	4.22995	3.70738	5.67984	3.97034	4.07303	4.64327	3.64465	3.68533	4.51526	3.54309	0.864782464	0.966018836	4	4	24	0.765383125	0.924119887	24	0.099698963	0.041709138
ENSMUSG00000020273	Papolg	poly(A) polymerase gamma [Source:MGI Symbol;Acc:MGI:2442119]	6.04792	6.33773	6.66199	6.01134	5.81945	6.22441	6.23341	5.49117	6.45495	4.93186	6.76274	5.90994	6.0154	6.194	0.849565901	0.966018836	12	4	24	0.884925521	0.967256182	24	1.058087759	0.019356073
ENSMUSG00000020275	Rel	reticuloendotheliosis oncogene [Source:MGI Symbol;Acc:MGI:97897]	1.41839	1.40912	1.29666	1.5582	1.48702	1.19302	1.5166	1.23767	1.56184	1.26903	1.5056	1.46492	1.38207	1.31784	0.893609703	0.966018836	12	20	24	0.702315281	0.901574817	24	14.9981043	0.024595777
ENSMUSG00000020277	Pfkl	phosphofructokinase, liver, B-type [Source:MGI Symbol;Acc:MGI:97547]	20.4379	17.252	20.2278	21.8097	18.7316	21.9821	19.9783	22.3052	18.7449	22.5307	21.0373	20.2942	21.2582	21.0367	0.897425765	0.966018836	16	20	24	0.798197046	0.935424343	24	15.60506611	0.026103656
ENSMUSG00000020280	Pus10	pseudouridylate synthase 10 [Source:MGI Symbol;Acc:MGI:1921717]	4.01403	5.54877	4.85942	4.37999	4.28711	4.35114	3.77262	4.66466	5.27809	4.29245	4.75242	5.08243	4.17249	4.69398	0.038363978	0.335328154	12	16	24	0.290085943	0.768040178	24	8.197508767	0.080661026
ENSMUSG00000020282	Rhbdf1	rhomboid 5 homolog 1 [Source:MGI Symbol;Acc:MGI:104328]	11.6561	10.1451	9.53225	11.2997	11.7641	10.663	10.3216	9.61949	11.7008	11.1504	10.3103	9.23837	10.919	8.67071	0.104315374	0.966018836	16	16	24	0.30472198	0.768040178	24	15.43882795	0.068453093
ENSMUSG00000020283	Pex13	peroxisomal biogenesis factor 13 [Source:MGI Symbol;Acc:MGI:1919379]	12.1878	11.9072	11.751	12.1587	11	11.2211	12.7676	13.1846	13.5034	11.849	13.0599	12.0548	13.0911	12.7423	0.418924655	0.964880223	4	20	24	0.101612863	0.713240751	24	4.887597278	0.057389063
ENSMUSG00000020284	Cfap410	cilia and flagella associated protein 410 [Source:MGI Symbol;Acc:MGI:1915134]	13.323	13.5424	14.1302	13.9522	12.1691	13.0197	13.3391	14.3292	12.843	16.3716	12.5763	13.8645	13.0368	13.4905	0.805045716	0.466404237	16	4	24	0.342076412	0.776293434	24	8.61165135	0.056833575
ENSMUSG00000020287	Mpg	N-methylpurine-DNA glycosylase [Source:MGI Symbol;Acc:MGI:97073]	5.34173	6.19483	7.11161	5.57283	5.79863	6.35582	6.37416	7.60784	6.77569	7.69788	5.85054	6.73181	6.65525	7.56788	0.454881285	0.966018836	12	8	24	0.044491199	0.628432994	24	6.605586571	0.112936771
ENSMUSG00000020288	Ahsa2	AHA1, activator of heat shock protein ATPase 2 [Source:MGI Symbol;Acc:MGI:1916133]	61.17	39.3721	41.1404	39.539	52.1018	49.4232	48.3998	39.3875	39.9288	35.1279	49.4489	41.3827	39.2535	36.1806	0.119598685	0.54287566	20	12	24	0.006865433	0.472731997	24	19.93237136	0.214858017
ENSMUSG00000020289	Nprl3	nitrogen permease regulator-like 3 [Source:MGI Symbol;Acc:MGI:109258]	19.9335	19.5679	19.7605	18.7709	19.8264	20.38	16.9898	18.8347	17.9317	22.4388	17.182	19.864	19.0091	19.4555	0.923635729	0.966018836	24	12	24	0.836124966	0.949866664	24	13.43136271	0.02318373
ENSMUSG00000020290	Xpo1	exportin 1 [Source:MGI Symbol;Acc:MGI:2144013]	44.5968	39.5521	38.0432	35.9464	43.1145	36.2884	41.3403	36.8225	39.0098	30.7915	44.1023	38.0097	41.333	39.2137	0.020204269	0.260320781	4	12	24	0.214076067	0.768040178	24	22.23503655	0.092866635
ENSMUSG00000020291	Nsg2	neuron specific gene family member 2 [Source:MGI Symbol;Acc:MGI:1202070]	74.3892	66.3269	70.878	63.654	61.8496	76.2375	70.9919	65.663	57.9673	78.1023	62.1462	70.5263	66.5431	75.9364	0.718086778	0.966018836	24	20	24	0.537299321	0.827923483	24	23.37464656	0.057310414
ENSMUSG00000020300	Cpeb4	cytoplasmic polyadenylation element binding protein 4 [Source:MGI Symbol;Acc:MGI:1914829]	21.4102	21.9901	23.7639	23.7508	21.0291	21.465	20.873	20.4937	19.2556	23.4768	20.0135	19.7393	18.8171	22.0218	0.374467935	0.89742288	16	12	24	0.159222289	0.765350752	24	10.40502596	0.06620103

ENSMUSG00000020305	Asb3	ankyrin repeat and SOCS box-containing 3 [Source:MGI Symbol;Acc:MGI:1929749]	8.51711	9.51805	8.52247	8.72718	9.41271	8.90024	8.80108	8.36929	9.77123	7.85967	9.9234	9.38494	10.9404	9.35253	0.932092277	0.966018836	20	20	24	0.642692866	0.878809617	24	22.26632366	0.038261852
ENSMUSG00000020307	Cdc34	cell division cycle 34 [Source:MGI Symbol;Acc:MGI:102657]	37.8067	37.0882	36.3121	41.8819	37.5233	41.0603	36.5748	46.4446	39.9731	41.5242	42.6652	38.3347	42.0616	35.4295	0.846840613	0.966018836	16	20	24	0.725609636	0.909996439	24	13.83652452	0.033831744
ENSMUSG00000020308	Tpgs1	tubulin polyglutamylase complex subunit 1 [Source:MGI Symbol;Acc:MGI:106618]	21.0897	17.1584	17.8182	13.417	13.0663	12.8884	13.188	24.3342	23.5004	27.1427	33.868	30.8172	33.927	28.9917	0.788254138	0.966018836	8	16	24	0.705631037	0.902918222	24	5.256723218	0.117830105
ENSMUSG00000020309	Chac2	ChaC, cation transport regulator 2 [Source:MGI Symbol;Acc:MGI:1915294]	20.9474	18.832	19.3434	19.5056	18.5667	19.893	18.7495	16.2709	15.0939	15.5909	18.8832	16.2977	16.7775	15.8609	0.793403332	0.966018836	4	4	24	0.035184209	0.608551791	24	19.62747297	0.090842997
ENSMUSG00000020311	Erlec1	endoplasmic reticulum lectin 1 [Source:MGI Symbol;Acc:MGI:1914003]	19.5686	20.4682	20.1756	20.816	18.9423	21.5004	21.6868	20.1547	20.1327	21.5197	20.4358	19.5434	20.9402	21.9056	0.933543173	0.966018836	8	12	24	0.651687302	0.882836756	24	4.874740741	0.020905051
ENSMUSG00000020312	Shc2	SHC (Src homology 2 domain containing) transforming protein 2 [Source:MGI Symbol;Acc:MGI:106180]	35.088	26.3915	26.1628	29.0212	29.0742	30.373	27.3794	28.9443	23.4351	35.8584	28.0633	31.4589	30.9297	28.533	0.042880436	0.356048751	24	12	24	0.26766884	0.768040178	24	19.31585718	0.101676873
ENSMUSG00000020315	Sptbn1	spectrin beta, non-erythrocytic 1 [Source:MGI Symbol;Acc:MGI:98388]	237.728	221.547	210.902	217.675	231.925	224.5	221.319	212.935	206.396	227.192	218.415	225.505	223.422	220.971	0.010049466	0.194720062	24	12	24	0.029886665	0.596648309	24	19.98247848	0.050964693
ENSMUSG00000020319	Wdpcp	WD repeat containing planar cell polarity effector [Source:MGI Symbol;Acc:MGI:2144467]	5.37702	5.94523	4.7715	4.64297	4.46713	4.75185	4.67398	5.09374	4.35928	4.06764	5.16888	5.3032	5.29243	4.5466	0.15076465	0.598311732	4	12	24	0.07114028	0.676901434	24	23.71352269	0.098566717
ENSMUSG00000020321	Mdh1	malate dehydrogenase 1, NAD (soluble) [Source:MGI Symbol;Acc:MGI:97051]	370.014	398.354	399.253	396.331	370.252	361.889	369.68	377.423	407.441	324.627	408.997	365.299	388.282	395.476	0.010886922	0.200176658	12	12	24	0.475514842	0.805393068	24	6.254204175	0.044174406
ENSMUSG00000020325	Fstl3	follistatin-like 3 [Source:MGI Symbol;Acc:MGI:1890391]	5.16463	4.31718	5.08188	6.78654	5.7375	5.33788	6.05616	4.99907	5.85191	5.85791	5.87299	5.17582	5.1437	4.29025	3.99E-04	0.052946481	16	16	24	0.03208948	0.605537343	24	14.65892437	0.139314551
ENSMUSG00000020326	Ccng1	cyclin G1 [Source:MGI Symbol;Acc:MGI:102890]	57.2171	56.6751	55.4273	59.761	57.6212	56.9714	53.3821	66.7289	61.9603	54.8545	63.2704	50.5749	56.1055	52.323	0.904387595	0.966018836	20	4	24	0.570895955	0.845581812	24	10.12615902	0.045380433
ENSMUSG00000020327	Fgf22	fibroblast growth factor 22 [Source:MGI Symbol;Acc:MGI:1914362]	0.744285	0.910724	0.907708	0.948046	0.451523	0.904996	1.20489	0.952369	0.955545	1.29258	0.580245	1.10059	0.636154	1.0796	0.718720526	0.966018836	16	4	24	0.393868812	0.799546792	24	6.042215576	0.100490078
ENSMUSG00000020328	Nudcd2	NudC domain containing 2 [Source:MGI Symbol;Acc:MGI:1277103]	16.9357	16.4146	20.7614	15.5532	16.8761	14.6269	12.7442	15.4271	17.2591	14.7318	17.49	16.1869	18.1654	16.7823	0.932671476	0.966018836	12	12	24	0.565187366	0.843471947	24	8.700571641	0.068751951
ENSMUSG00000020329	Polrmt	polymerase (RNA) mitochondrial (DNA directed) [Source:MGI Symbol;Acc:MGI:1915843]	7.92971	5.80468	7.20664	7.23298	7.04882	6.46467	5.89141	7.93031	8.39745	6.24891	7.04724	8.08831	5.78855	6.93463	0.92655264	0.966018836	12	16	24	0.762535101	0.923339085	24	10.94892843	0.047838678
ENSMUSG00000020331	Hcn2	hyperpolarization-activated, cyclic nucleotide-gated K+ 2 [Source:MGI Symbol;Acc:MGI:1298210]	10.4036	10.304	12.2217	10.0687	8.31504	11.6555	11.7098	10.8097	9.88901	15.583	8.34309	12.2045	10.2203	12.7743	0.894563866	0.966018836	8	12	24	0.612094827	0.865382448	24	5.785715277	0.087079285
ENSMUSG00000020333	Acsl6	acyl-CoA synthetase long-chain family member 6 [Source:MGI Symbol;Acc:MGI:894291]	27.4259	28.3339	32.6648	26.7331	23.2957	28.4606	29.4854	25.1669	24.7641	31.7791	22.8772	26.0974	23.5629	29.4321	0.481530854	0.966018836	16	4	24	0.457221025	0.805393068	24	6.379554245	0.077019252
ENSMUSG00000020334	Slc22a4	solute carrier family 22 (organic cation transporter), member 4 [Source:MGI Symbol;Acc:MGI:1353479]	3.04401	2.94829	4.06341	2.81898	2.58202	3.31995	3.95887	2.90543	3.41225	2.31636	2.57196	2.7045	2.49795	2.70001	0.435739761	0.966018836	12	4	24	0.409198269	0.799546792	24	3.528743466	0.084725101
ENSMUSG00000020335	Zfp354b	zinc finger protein 354B [Source:MGI Symbol;Acc:MGI:1351476]	1.53739	1.39447	1.63565	1.58082	1.8162	1.69917	1.75977	1.4176	1.58163	1.39177	1.73089	1.33498	1.19274	1.71755	0.934818505	0.966018836	20	8	24	0.572022015	0.846185279	24	14.86413352	0.043166707
ENSMUSG00000020340	Cyfip2	cytoplasmic FMR1 interacting protein 2 [Source:MGI Symbol;Acc:MGI:1924134]	55.5091	40.8531	49.2561	46.1802	44.1118	56.6795	50.18	49.3504	33.8457	65.0719	39.6286	47.6543	42.6543	48.2712	0.92655264	0.966018836	24	20	24	0.747575624	0.917832057	24	19.87916708	0.073574084
ENSMUSG00000020346	Mgat1	mannoside acetylglucosaminyltransferase 1 [Source:MGI Symbol;Acc:MGI:96973]	16.5713	14.7383	17.7737	17.2653	18.5211	18.3238	17.6392	17.4279	16.7294	18.0522	15.8756	15.3903	15.3305	15.0571	0.211626905	0.701016641	16	16	24	0.229808131	0.768040178	24	14.36945167	0.063453466
ENSMUSG00000020349	Ppp2ca	protein phosphatase 2 (formerly 2A), catalytic subunit, alpha isoform [Source:MGI Symbol;Acc:MGI:1321159]	49.6134	48.0914	48.209	47.8981	47.6405	47.9358	47.6972	46.0369	43.7685	46.1273	48.7594	48.5023	48.707	48.0661	0.451149628	0.966018836	4	8	24	0.075126144	0.684545305	24	20.89788547	0.037711321
ENSMUSG00000020354	Sgcd	sarcoglycan, delta (dystrophin-associated glycoprotein) [Source:MGI Symbol;Acc:MGI:1346525]	9.80509	12.2398	10.8264	11.7743	11.4578	9.42755	10.3773	9.90142	13.8221	7.69048	13.2288	13.0332	14.0744	12.6446	0.795556128	0.966018836	12	4	24	0.913953101	0.975882466	24	3.168297059	0.036224734
ENSMUSG00000020358	Hnrnpab	heterogeneous nuclear ribonucleoprotein A/B [Source:MGI Symbol;Acc:MGI:1330294]	56.901	49.8412	52.705	51.5305	51.4591	51.4145	52.6662	51.2525	45.9992	57.4917	51.7508	53.5936	55.9257	54.3087	0.85447369	0.966018836	4	8	24	0.553664281	0.837374348	24	21.18466757	0.035806206
ENSMUSG00000020359	Phykpl	5-phosphohydroxy-L-lysine phospholyase [Source:MGI Symbol;Acc:MGI:1920197]	5.97757	6.67083	5.84256	7.1854	5.60963	5.7856	5.356	7.2898	6.24612	7.46393	5.63474	6.55601	5.70909	5.48895	0.351315142	0.870418653	16	4	24	0.289466675	0.768040178	24	9.897211461	0.086585921
ENSMUSG00000020361	Hspa4	heat shock protein 4 [Source:MGI Symbol;Acc:MGI:1342292]	78.6702	67.2739	68.1325	63.2165	67.7933	69.7188	66.0742	67.1123	54.707	67.3905	65.5795	65.4992	66.7949	67.1242	0.873350015	0.966018836	8	4	24	0.097987738	0.71175955	24	21.85787146	0.081192578
ENSMUSG00000020362	Cnot6	CCR4-NOT transcription complex, subunit 6 [Source:MGI Symbol;Acc:MGI:2144529]	13.8136	17.0362	16.9109	16.7364	15.0567	13.4202	15.5703	14.3732	17.0448	14.4553	15.3524	14.8419	13.8347	15.3939	0.019280932	0.257669847	12	12	24	0.031757515	0.604316769	24	8.866820432	0.103427926
ENSMUSG00000020363	Gfpt2	glutamine fructose-6-phosphate transaminase 2 [Source:MGI Symbol;Acc:MGI:1338883]	1.79299	1.92637	1.61019	1.77245	1.90832	1.61042	1.77555	1.95643	2.61052	1.33369	1.7806	1.86038	1.94539	1.82031	0.350544145	0.870418653	8	8	24	0.521703616	0.820407334	24	4.444512591	0.061806125
ENSMUSG00000020364	Zfp354a	zinc finger protein 354A [Source:MGI Symbol;Acc:MGI:103172]	2.65354	3.0489	3.21078	2.88734	2.74045	2.56596	3.62447	2.23504	2.87415	2.34794	2.95851	2.57731	2.3437	2.64789	0.92769718	0.966018836	12	12	24	0.98148645	0.995695365	24	8.198735685	0.010617847
ENSMUSG00000020366	Mapk9	mitogen-activated protein kinase 9 [Source:MGI Symbol;Acc:MGI:1346862]	62.3999	54.7229	59.3644	58.1671	50.3061	59.7125	58.9705	54.5081	49.8134	66.6331	53.3234	58.5542	55.1014	61.2061	0.893609703	0.966018836	4	16	24	0.940233951	0.985398871	24	0.035356472	0.016536572
ENSMUSG00000020368	Canx	calnexin [Source:MGI Symbol;Acc:MGI:88261]	163.231	144.583	142.848	138.462	157.005	151.614	141.871	135.762	121.899	127.941	142.241	140.602	143.824	136.636	0.184505544	0.662239322	20	20	24	0.002563033	0.396968951	24	20.72247868	0.106654194
ENSMUSG00000020372	Rack1	receptor for activated C kinase 1 [Source:MGI Symbol;Acc:MGI:101849]	154.673	179.398	176.568	175.947	175.108	169.356	158.665	168.502	190.741	138.067	178.194	156.258	177.189	159.734	0.46238066	0.966018836	12	12	24	0.797518154	0.935337257	24	8.353184098	0.033295926
ENSMUSG00000020374	Rasgef1c	RasGEF domain family, member 1C [Source:MGI Symbol;Acc:MGI:1921813]	9.01375	9.15336	9.51004	10.9214	11.4871	10.3314	10.41	10.3305	9.54718	11.2227	9.76458	10.5142	9.56864	8.39232	0.02248335	0.27189101	16	16	24	0.024334244	0.586541342	24	15.47043759	0.109717967
ENSMUSG00000020375	Rufy1	RUN and FYVE domain containing 1 [Source:MGI Symbol;Acc:MGI:2429762]	7.08653	6.92755	7.57597	7.60135	7.10603	7.8515	7.36839	8.12225	6.71973	6.61561	6.96178	7.04398	7.49262	7.20272	0.881996584	0.966018836	4	12	24	0.548641028	0.834655201	24	0.371617924	0.031830718
ENSMUSG00000020376	Rnf130	ring finger protein 130 [Source:MGI Symbol;Acc:MGI:1891717]	46.3044	48.3257	49.0735	46.3696	48.8649	44.8244	52.982	45.882	49.8004	49.1129	45.7762	49.9287	50.3371	47.0818	0.911371589	0.966018836	4	16	24	0.832606626	0.948274776	24	3.299222129	0.01568722
ENSMUSG00000020377	Ltc4s	leukotriene C4 synthase [Source:MGI Symbol;Acc:MGI:107498]	0.492524	1.74071	1.23656	1.49773	1.03817	0.818472	0.899482	1.46573	1.65535	1.12308	1.34351	0.84467	1.38768	1.08415	0.038115943	0.334069441	12	12	24	0.036781352	0.609176161	24	8.617576284	0.228702818
ENSMUSG00000020380	Rad50	RAD50 double strand break repair protein [Source:MGI Symbol;Acc:MGI:109292]	5.88863	5.74263	5.41836	5.67743	5.78471	5.31793	5.11838	5.23587	6.17818	5.52128	6.1223	6.37404	6.45278	5.82701	0.881996584	0.966018836	20	12	24	0.843568389	0.952527283	24	17.81075519	0.0193693
ENSMUSG00000020381	Mrnip	MRN complex interacting protein [Source:MGI Symbol;Acc:MGI:1915317]	4.97319	5.78285	6.05202	5.6912	6.09884	5.19313	4.52488	5.72843	6.72246	5.25964	5.39088	6.18951	5.15594	5.81222	0.009848456	0.194720062	12	16	24	0.133911914	0.744007455	24	9.888927861	0.096564371
ENSMUSG00000020385	Clk4	CDC like kinase 4 [Source:MGI Symbol;Acc:MGI:1098551]	53.8832	63.9508	59.841	66.5477	63.5839	54.6448	62.0673	54.5828	66.6894	50.7785	68.6522	51.3622	50.8437	53.6685	0.575387897	0.966018836	20	8	24	0.275863222	0.768040178	24	11.86730972	0.090353473
ENSMUSG00000020386	Sar1b	secretion associated Ras related GTPase 1B [Source:MGI Symbol;Acc:MGI:1913647]	43.0554	56.463	59.3953	61.6503	52.5376	50.3076	49.9973	66.4715	70.4106	56.3029	55.0073	48.0555	48.9749	51.4153	1.13E-04	0.029372629	12	16	24	0.003091188	0.414576087	24	9.165741275	0.214405555
ENSMUSG00000020387	Jade2	jade family PHD finger 2 [Source:MGI Symbol;Acc:MGI:1924151]	5.2682	4.42886	4.60635	5.42083	4.8105	4.76494	4.90126	4.44677	4.37223	5.72527	4.52233	4.87701	4.41527	4.62976	0.265774856	0.773510818	16	20	24	0.279807145	0.768040178	24	15.17839074	0.059418002
ENSMUSG00000020388	Pdlim4	PDZ and LIM domain 4 [Source:MGI Symbol;Acc:MGI:1353470]	11.3859	11.4698	8.64115	8.83766	11.6122	10.6605	11.226	11.5854	11.7098	9.53783	14.4278	10.5103	12.71	11.0587	0.836635326	0.966018836	4	12	24	0.310703712	0.768040178	24	22.03144498	0.101412389
ENSMUSG00000020389	Cdkl3	cyclin-dependent kinase-like 3 [Source:MGI Symbol;Acc:MGI:2388268]	8.6752	8.0037	5.92938	6.72667	10.0804	7.08381	7.82191	6.92292	8.58857	7.1398	6.59917	8.53262	9.0279	9.78957	0.745199643	0.966018836	4	12	24	0.403979129	0.799546792	24	23.36375922	0.103270943
ENSMUSG00000020390	Ube2b	ubiquitin-conjugating enzyme E2B [Source:MGI Symbol;Acc:MGI:102944]	168.543	167.198	157.841	165.361	147.024	140.655	161.067	163.353	180.876	161.94	183.39	167.163	174.433	171.383	0.896773941	0.966018836	4	20	24	0.32078436	0.768040178	24	6.163918499	0.060309054
ENSMUSG00000020392	Cdkn2aipnl	CDKN2A interacting protein N-terminal like [Source:MGI Symbol;Acc:MGI:1261797]	21.2475	20.4584	20.176	21.4715	19.5055	20.0815	21.3219	20.4503	20.9401	19.1122	20.9521	20.9936	22.0267	21.1235	0.089177696	0.476253254	4	12	24	0.201748972	0.768040178	24	0.931942369	0.034977681
ENSMUSG00000020393	Kremen1	kringle containing transmembrane protein 1 [Source:MGI Symbol;Acc:MGI:1933988]	5.94436	6.13934	5.84048	6.3114	5.9712	6.01353	6.17783	7.27286	6.16611	7.50602	5.25849	6.7128	6.77931	5.83755	0.853736882	0.966018836	16	4	24	0.803451632	0.937199097	24	6.006301379	0.031355414
ENSMUSG00000020395	Itk	IL2 inducible T cell kinase [Source:MGI Symbol;Acc:MGI:96621]	0.92498	0.591104	0.723698	0.656592	0.972175	0.828498	0.577373	0.598054	0.475184	0.692909	0.712792	0.742446	0.58162	0.516917	0.12222587	0.550724475	24	8	24	0.002768497	0.414320197	24	17.96143622	0.123555839
ENSMUSG00000020396	Nefh	neurofilament, heavy polypeptide [Source:MGI Symbol;Acc:MGI:97309]	328.144	251.09	243.067	252.015	247.641	314.419	290.768	308.703	185.381	380.445	239.908	289.023	265.115	282.468	0.203305541	0.689605535	24	12	24	0.470307705	0.805393068	24	21.40793627	0.125206499
ENSMUSG00000020397	Med7	mediator complex subunit 7 [Source:MGI Symbol;Acc:MGI:1913463]	21.3396	20.9119	21.6682	18.9587	21.537	19.7153	19.6204	20.6787	19.0053	18.3093	21.1119	19.9603	21.9792	21.4521	0.784278077	0.966018836	4	12	24	0.320974822	0.768040178	24	0.422175877	0.046466037
ENSMUSG00000020400	Tnip1	TNFAIP3 interacting protein 1 [Source:MGI Symbol;Acc:MGI:1926194]	9.42572	8.05235	8.97508	8.53377	9.34075	10.9353	9.01159	9.98	7.44753	12.797	8.03341	9.90837	9.09016	9.18423	0.390686537	0.915479136	24	12	24	0.651758742	0.882836756	24	18.01592579	0.066942036
ENSMUSG00000020402	Vdac1	voltage-dependent anion channel 1 [Source:MGI Symbol;Acc:MGI:106919]	203.832	183.051	197.327	193.309	178.11	210.765	185.341	220.107	178.147	231.045	184.267	189.787	199.641	199.816	0.941955432	0.966018836	8	12	24	0.912212527	0.975295827	24	5.383181161	0.017785431
ENSMUSG00000020407	Upp1	uridine phosphorylase 1 [Source:MGI Symbol;Acc:MGI:1097668]	1.62686	1.01402	2.2806	1.92671	1.67145	1.32501	1.59402	1.966	2.43448	1.20731	1.33746	1.34065	1.21747	1.81047	0.591351901	0.966018836	12	20	24	0.325183588	0.768040178	24	9.749535395	0.136539032
ENSMUSG00000020409	Slu7	SLU7 splicing factor homolog (S. cerevisiae) [Source:MGI Symbol;Acc:MGI:2385598]	27.8985	31.2073	33.808	26.4195	29.4477	29.3669	30.4734	28.9344	29.5921	27.7863	32.3636	29.8423	31.4461	31.5066	0.960338592	0.967043964	12	4	24	0.633072613	0.875310914	24	3.313786965	0.033857037
ENSMUSG00000020412	Ascc2	activating signal cointegrator 1 complex subunit 2 [Source:MGI Symbol;Acc:MGI:1922702]	9.43032	8.04739	7.68592	8.78131	10.3102	9.29915	9.80446	8.63759	8.47185	8.97761	8.99888	8.87988	9.02601	8.06297	0.008047769	0.192760771	4	4	24	0.009476668	0.502965379	24	18.87421837	0.108907044
ENSMUSG00000020413	Hus1	HUS1 checkpoint clamp component [Source:MGI Symbol;Acc:MGI:1277962]	4.80247	4.85304	4.9394	5.53863	4.57494	4.89394	4.95109	4.88981	5.24585	4.75158	5.08902	5.12703	4.86812	5.14558	0.923635729	0.966018836	12	16	24	0.506260549	0.811994099	24	9.485915119	0.026616308
ENSMUSG00000020415	Pttg1	pituitary tumor-transforming gene 1 [Source:MGI Symbol;Acc:MGI:1353578]	47.3166	55.0745	48.6956	52.5427	49.0714	49.9532	45.0631	52.4131	62.6414	49.4876	47.9738	52.0689	58.0957	57.5876	0.542718076	0.966018836	8	12	24	0.106147116	0.71810271	24	6.439686254	0.095965223
ENSMUSG00000020420	Zfp607a	zinc finger protein 607A [Source:MGI Symbol;Acc:MGI:3584526]	0.292611	0.690344	0.545039	0.843774	0.674909	0.524653	0.432782	0.525774	0.58875	0.195306	0.510488	0.597496	0.558968	0.511007	0.917370979	0.966018836	16	12	24	0.895979621	0.970467191	24	11.83407999	0.029402934
ENSMUSG00000020422	Tns3	tensin 3 [Source:MGI Symbol;Acc:MGI:2443012]	31.061	27.2227	27.2586	33.1725	32.5598	28.367	34.5583	26.6698	27.5729	32.4822	28.0931	29.536	28.5065	25.0251	0.04160704	0.34886238	4	4	24	0.124423194	0.738426882	24	16.99851302	0.098529875
ENSMUSG00000020423	Btg2	BTG anti-proliferation factor 2 [Source:MGI Symbol;Acc:MGI:108384]	3.91227	4.47555	4.97622	5.15159	3.92899	4.33871	4.65175	5.255	5.11614	4.71134	4.27148	4.45917	5.50944	3.74517	0.756435587	0.966018836	16	4	24	0.436387788	0.805393068	24	7.719387762	0.074317548
ENSMUSG00000020424	Castor1	cytosolic arginine sensor for mTORC1 subunit 1 [Source:MGI Symbol;Acc:MGI:1919212]	2.5286	2.44425	2.5067	2.42589	2.72336	2.32174	2.36823	2.3454	2.47926	2.46811	2.38315	2.50063	2.27821	2.10666	0.664568268	0.966018836	12	20	24	0.08251315	0.699403344	24	15.14861993	0.040515595
ENSMUSG00000020427	Igfbp3	insulin-like growth factor binding protein 3 [Source:MGI Symbol;Acc:MGI:96438]	11.5436	13.3004	8.90116	7.46619	8.96264	9.65938	9.15303	13.3157	12.6258	6.28818	10.0622	10.0836	7.88354	9.12694	0.092593422	0.487919468	8	8	24	0.181138531	0.768040178	24	2.34651629	0.190250561
ENSMUSG00000020430	Pes1	pescadillo ribosomal biogenesis factor 1 [Source:MGI Symbol;Acc:MGI:1890613]	9.821	9.16437	10.4201	9.21668	10.265	10.6774	9.01231	9.82186	9.56814	8.42566	9.57471	9.50275	9.12829	8.9989	0.836635326	0.966018836	24	4	24	0.536386954	0.827399772	24	19.02901719	0.035274162
ENSMUSG00000020431	Adcy1	adenylate cyclase 1 [Source:MGI Symbol;Acc:MGI:99677]	14.0676	14.8168	14.581	15.5787	17.4079	13.9362	16.331	13.7288	17.8991	17.8444	13.0823	14.8403	14.7141	14.4012	0.708020493	0.966018836	12	20	24	0.297522009	0.768040178	24	11.26661639	0.082393076
ENSMUSG00000020432	Tcn2	transcobalamin 2 [Source:MGI Symbol;Acc:MGI:98534]	19.6218	19.2257	17.7937	19.1607	20.5223	20.228	22.383	22.7481	18.2421	19.8284	18.0914	19.3158	18.3747	17.1509	0.78898997	0.966018836	24	12	24	0.459472899	0.805393068	24	21.5260322	0.05470845
ENSMUSG00000020435	Osbp2	oxysterol binding protein 2 [Source:MGI Symbol;Acc:MGI:1921559]	6.62939	4.37257	5.68821	5.91101	4.66645	6.73131	4.54594	6.08381	4.10911	8.32851	3.54332	6.09149	5.81394	5.29395	0.891105762	0.966018836	24	12	24	0.976143108	0.994043662	24	20.02057308	0.025786577
ENSMUSG00000020436	Gabrg2	gamma-aminobutyric acid (GABA) A receptor, subunit gamma 2 [Source:MGI Symbol;Acc:MGI:95623]	40.5028	43.8363	39.4268	38.9217	40.201	37.5988	39.4022	37.6006	39.0051	35.0163	39.7299	39.586	41.3302	41.1971	0.351315142	0.870418653	8	8	24	0.224107399	0.768040178	24	1.253235135	0.047376233
ENSMUSG00000020437	Myo1g	myosin IG [Source:MGI Symbol;Acc:MGI:1927091]	0.779619	1.12037	1.7273	1.43849	1.50406	1.26961	1.61049	1.40022	1.4641	1.14212	1.13057	1.16093	1.02774	0.844765	0.46238066	0.966018836	12	20	24	0.362501219	0.78719863	24	11.60035572	0.097528197
ENSMUSG00000020439	Smtn	smoothelin [Source:MGI Symbol;Acc:MGI:1354727]	40.7215	45.9706	45.5444	54.1555	50.8808	40.1028	50.6486	44.0989	61.4587	58.3531	43.6109	42.8161	48.2622	37.9908	0.149354198	0.593798152	16	8	24	0.085719927	0.703577714	24	11.04898635	0.165221236
ENSMUSG00000020440	Arf5	ADP-ribosylation factor 5 [Source:MGI Symbol;Acc:MGI:99434]	62.2537	50.3838	55.6181	51.7379	49.2802	49.0103	53.1319	59.9687	56.6164	61.9745	58.5965	60.4313	60.8769	60.0576	0.932092277	0.966018836	4	16	24	0.561355031	0.841538771	24	4.348622014	0.045733136
ENSMUSG00000020441	2310033P09Rik	RIKEN cDNA 2310033P09 gene [Source:MGI Symbol;Acc:MGI:1915112]	5.33977	4.37015	4.92406	4.67578	4.48457	5.63521	5.09533	5.52013	5.30028	6.51727	5.2279	5.62281	6.18644	6.18223	0.948161972	0.966018836	24	20	24	0.744692596	0.916540741	24	1.851258061	0.030545293
ENSMUSG00000020444	Guk1	guanylate kinase 1 [Source:MGI Symbol;Acc:MGI:95871]	76.8121	83.9053	82.9693	82.0554	86.3378	83.4479	74.6041	76.5965	77.0312	69.6973	84.2398	80.6161	81.7288	76.8625	0.33885934	0.858075318	20	8	24	0.433563607	0.805393068	24	17.88413937	0.042416751
ENSMUSG00000020448	Rnf185	ring finger protein 185 [Source:MGI Symbol;Acc:MGI:1922078]	11.7357	9.96391	10.7039	10.6179	10.2942	11.5895	11.8103	11.805	9.14129	11.849	10.3069	10.8933	10.8936	10.7282	0.25849765	0.761738864	4	8	24	0.312585488	0.768040178	24	21.97678182	0.061061578
ENSMUSG00000020451	Limk2	LIM motif-containing protein kinase 2 [Source:MGI Symbol;Acc:MGI:1197517]	61.0973	54.1079	52.9352	54.1727	62.5385	58.5083	60.9021	54.3324	55.5873	56.2861	59.0954	54.2623	58.3439	56.5289	0.070873295	0.440262583	20	16	24	0.06169798	0.661743448	24	19.86704228	0.067120631
ENSMUSG00000020453	Patz1	POZ (BTB) and AT hook containing zinc finger 1 [Source:MGI Symbol;Acc:MGI:1891832]	8.56129	9.33272	9.99588	9.03125	9.94203	9.09127	9.94013	8.62295	9.78738	10.5314	8.79976	9.10112	9.19968	9.13988	0.57057742	0.966018836	12	12	24	0.271085356	0.768040178	24	11.01548903	0.050911857
ENSMUSG00000020454	Eif4enif1	eukaryotic translation initiation factor 4E nuclear import factor 1 [Source:MGI Symbol;Acc:MGI:1921453]	13.0867	12.9055	12.503	13.5014	13.776	14.1566	13.393	13.3851	10.551	14.3395	11.0256	14.7608	12.3904	12.8267	0.0444686	0.365196805	24	12	24	0.374039179	0.794557071	24	19.71808801	0.071049623
ENSMUSG00000020455	Trim11	tripartite motif-containing 11 [Source:MGI Symbol;Acc:MGI:2137355]	6.20029	5.88286	6.15067	6.03846	6.2225	5.83216	6.66138	7.01944	7.18991	6.69463	7.45659	7.04428	7.27007	6.44317	0.81544087	0.966018836	20	12	24	0.987807585	0.997530201	24	9.267905957	0.004679652
ENSMUSG00000020456	Ogdh	oxoglutarate (alpha-ketoglutarate) dehydrogenase (lipoamide) [Source:MGI Symbol;Acc:MGI:1098267]	50.2558	39.9829	46.5454	57.4256	46.797	54.1002	49.6694	57.1151	39.8487	72.8585	44.5003	49.0184	53.2323	52.4873	0.941955432	0.966018836	16	20	24	0.810053126	0.939224719	24	14.18553215	0.055605232
ENSMUSG00000020457	Drg1	developmentally regulated GTP binding protein 1 [Source:MGI Symbol;Acc:MGI:1343297]	77.2652	76.3888	77.3202	73.0148	76.6663	74.9626	67.0089	71.847	71.4775	71.4694	78.4262	78.5381	78.8428	78.482	0.833406028	0.966018836	24	16	24	0.761087598	0.92277295	24	19.85710571	0.021471878
ENSMUSG00000020458	Rtn4	reticulon 4 [Source:MGI Symbol;Acc:MGI:1915835]	344.266	337.282	339.79	345.754	343.056	340.885	328.472	349.978	324.888	353.158	341.623	331.143	341.363	351.176	0.784278077	0.966018836	16	8	24	0.780570557	0.929485273	24	10.6405716	0.010211241
ENSMUSG00000020459	Mtif2	mitochondrial translational initiation factor 2 [Source:MGI Symbol;Acc:MGI:1924034]	14.6056	15.5932	14.3735	14.388	14.7354	13.7209	13.8858	12.7623	15.0956	14.7577	15.4077	15.747	17.1825	16.4534	0.761979	0.966018836	8	4	24	0.95047548	0.988257443	24	1.267244513	0.011440357
ENSMUSG00000020460	Rps27a	ribosomal protein S27A [Source:MGI Symbol;Acc:MGI:1925544]	4.11819	5.24936	4.54566	6.07061	5.67237	4.13457	5.36375	5.35987	6.47404	4.8913	6.70095	5.03313	6.0413	5.53989	0.949152198	0.966018836	20	4	24	0.329698881	0.768040178	24	10.71023982	0.095285577
ENSMUSG00000020462	Cfap36	cilia and flagella associated protein 36 [Source:MGI Symbol;Acc:MGI:1913994]	59.5918	61.4736	57.6722	53.1359	59.5206	55.6259	56.299	55.4504	58.5667	52.9724	67.3392	57.3858	63.4873	64.5612	0.628286596	0.966018836	4	12	24	0.645637902	0.879878902	24	0.229315794	0.035547988
ENSMUSG00000020463	Ppp4r3b	protein phosphatase 4 regulatory subunit 3B [Source:MGI Symbol;Acc:MGI:2144474]	12.9074	14.3284	14.4125	14.4863	14.1828	12.5456	13.7357	12.5072	15.1944	12.1074	15.6321	13.0146	13.9436	14.0005	0.235187365	0.73629392	12	12	24	0.526722343	0.823075573	24	10.36968146	0.04699114
ENSMUSG00000020464	Pnpt1	polyribonucleotide nucleotidyltransferase 1 [Source:MGI Symbol;Acc:MGI:1918951]	18.383	19.905	18.8651	17.1969	19.211	17.5001	17.3618	17.2752	17.8197	15.6179	19.445	17.2632	18.4548	18.6505	0.851656735	0.966018836	8	8	24	0.765493774	0.924119887	24	0.872203688	0.024742931
ENSMUSG00000020467	Efemp1	epidermal growth factor-containing fibulin-like extracellular matrix protein 1 [Source:MGI Symbol;Acc:MGI:1339998]	25.6152	32.3111	26.0691	27.2731	29.8186	22.2935	28.2022	25.2729	30.9873	18.0991	25.9996	25.5888	25.8925	24.6291	0.912686312	0.966018836	12	12	24	0.68864298	0.897008658	24	3.279751176	0.064282111
ENSMUSG00000020471	Pold2	polymerase (DNA directed), delta 2, regulatory subunit [Source:MGI Symbol;Acc:MGI:1097163]	8.61202	7.05721	7.43341	6.06725	8.70299	10.2411	9.11919	10.2047	7.46962	8.47264	7.77851	7.31555	6.99443	6.50928	0.787283372	0.966018836	20	20	24	0.382462122	0.799546792	24	20.88938839	0.11212201
ENSMUSG00000020472	Zkscan17	zinc finger with KRAB and SCAN domains 17 [Source:MGI Symbol;Acc:MGI:2679270]	2.80787	2.92973	2.63352	3.05965	2.98499	2.48233	2.81444	3.0877	3.41764	3.552	2.78553	2.97085	2.93585	2.99022	0.211626905	0.701016641	16	8	24	0.080649541	0.696085161	24	9.439767628	0.080351453
ENSMUSG00000020473	Aebp1	AE binding protein 1 [Source:MGI Symbol;Acc:MGI:1197012]	16.1602	22.8226	16.8255	16.6087	18.0299	14.6247	17.1808	20.0631	26.1646	14.7301	16.4504	17.2639	13.9396	15.5466	0.608967886	0.966018836	12	16	24	0.246967803	0.768040178	24	7.042652676	0.153775539
ENSMUSG00000020474	Polm	polymerase (DNA directed), mu [Source:MGI Symbol;Acc:MGI:1860191]	3.40087	3.73331	3.85411	3.3311	3.7205	3.51549	3.53955	4.26649	3.82906	3.41543	3.25398	4.62501	4.01687	3.44977	0.61767505	0.966018836	12	4	24	0.560809219	0.841404418	24	1.461680015	0.047084026
ENSMUSG00000020475	Pgam2	phosphoglycerate mutase 2 [Source:MGI Symbol;Acc:MGI:1933118]	15.1625	36.2989	50.0283	59.2835	46.3674	56.6075	34.8266	106.276	74.9182	88.0339	36.2793	41.2778	50.7461	37.851	0.143321297	0.585944911	16	12	24	0.094918206	0.71175955	24	10.06391971	0.524067201
ENSMUSG00000020476	Dbnl	drebrin-like [Source:MGI Symbol;Acc:MGI:700006]	41.4944	33.4419	32.8442	32.5777	34.7289	35.6707	36.2553	39.2045	33.6143	41.8973	38.0395	40.6036	38.2047	36.6796	0.597596661	0.966018836	4	8	24	0.315012514	0.768040178	24	21.23942168	0.068248225
ENSMUSG00000020477	Mrps24	mitochondrial ribosomal protein S24 [Source:MGI Symbol;Acc:MGI:1928142]	30.9402	30.2671	32.6169	29.994	26.8736	29.2327	29.1053	31.5162	39.4826	28.6298	37.7861	31.7619	38.0728	32.4785	0.608967886	0.966018836	12	4	24	0.543302724	0.831565139	24	6.126267494	0.064200764
ENSMUSG00000020482	Ccdc117	coiled-coil domain containing 117 [Source:MGI Symbol;Acc:MGI:2144383]	8.7917	6.69288	6.76462	9.80834	7.4992	7.02742	8.45868	7.04163	7.35577	6.57118	8.82899	6.88306	7.02032	6.78953	0.588682825	0.966018836	20	12	24	0.46044189	0.805393068	24	16.99831895	0.077260266
ENSMUSG00000020483	Dynll2	dynein light chain LC8-type 2 [Source:MGI Symbol;Acc:MGI:1915347]	275.547	230.463	231.854	239.245	235.59	254.922	238.515	232.267	181.691	276.343	196.477	252.807	228.952	248.734	0.601647512	0.966018836	24	12	24	0.48301853	0.805393068	24	21.36015336	0.079032415
ENSMUSG00000020484	Xbp1	X-box binding protein 1 [Source:MGI Symbol;Acc:MGI:98970]	52.8099	36.492	36.0207	40.2684	46.6072	42.6095	49.9059	38.1633	35.3631	34.6628	47.2336	37.9716	45.2361	36.9937	0.009118941	0.192760771	4	8	24	0.012231258	0.525280095	24	20.54019523	0.201929407
ENSMUSG00000020485	Supt4a	SPT4A, DSIF elongation factor subunit [Source:MGI Symbol;Acc:MGI:107416]	21.8072	23.3555	25.0382	24.397	24.1718	22.5283	21.1951	23.9565	25.1891	19.8419	24.6449	22.0044	24.8954	22.1332	0.217827365	0.704989686	12	12	24	0.662035812	0.885885739	24	9.418343723	0.03774706
ENSMUSG00000020486	Septin4	septin 4 [Source:MGI Symbol;Acc:MGI:1270156]	25.8058	39.4685	55.8344	31.7364	29.6256	38.8484	41.6555	29.7851	38.7969	33.4684	27.1615	34.7164	26.9297	38.4078	0.616047374	0.966018836	12	8	24	0.333529391	0.769706681	24	7.055534162	0.174836537
ENSMUSG00000020491	2810021J22Rik	RIKEN cDNA 2810021J22 gene [Source:MGI Symbol;Acc:MGI:1917194]	2.92729	3.28051	2.90801	2.87469	3.11686	3.03616	2.99192	2.69076	2.77355	2.51155	3.15363	2.98791	3.0266	2.70712	0.082113105	0.462922111	20	20	24	0.113151271	0.727393008	24	20.36252836	0.058280114
ENSMUSG00000020492	Ska2	spindle and kinetochore associated complex subunit 2 [Source:MGI Symbol;Acc:MGI:1913390]	4.89614	5.98163	5.9925	5.78762	5.78644	5.21471	5.83988	4.95759	6.20485	4.47072	5.489	5.60222	5.3158	5.27667	0.46238066	0.966018836	12	12	24	0.865291041	0.960982718	24	8.763236696	0.024785905
ENSMUSG00000020495	Smg8	SMG8 nonsense mediated mRNA decay factor [Source:MGI Symbol;Acc:MGI:1921383]	6.08796	5.07176	4.65275	5.15867	4.81203	5.8636	5.33757	5.53287	3.79685	5.8252	5.07558	5.44805	4.86524	4.93126	0.148073804	0.590732968	24	12	24	0.13958146	0.74956518	24	20.60438783	0.1082702
ENSMUSG00000020496	Rnf187	ring finger protein 187 [Source:MGI Symbol;Acc:MGI:1914224]	87.2375	72.143	70.5787	74.0555	74.1833	81.8176	75.4103	81.829	67.7636	93.9907	74.0038	83.5655	77.9113	79.8136	0.148073804	0.590732968	24	12	24	0.541013936	0.830298514	24	20.46764129	0.056860428
ENSMUSG00000020513	Tubd1	tubulin, delta 1 [Source:MGI Symbol;Acc:MGI:1891826]	3.34844	3.39255	3.13789	2.96687	3.63727	3.31987	4.02143	3.19805	3.1869	2.92262	4.04377	3.49058	3.15804	3.53522	0.022000259	0.267229408	20	20	24	0.197458096	0.768040178	24	20.74836726	0.077568913
ENSMUSG00000020514	Mrpl22	mitochondrial ribosomal protein L22 [Source:MGI Symbol;Acc:MGI:1333794]	14.8526	18.0385	15.3685	15.5401	15.9135	14.1472	13.7416	14.2404	15.2257	13.3657	16.3414	13.3383	16.7152	15.4956	0.892044196	0.966018836	20	4	24	0.755991401	0.921194485	24	6.309969594	0.036287588
ENSMUSG00000020515	Cnot8	CCR4-NOT transcription complex, subunit 8 [Source:MGI Symbol;Acc:MGI:1916375]	25.3832	21.3359	21.7559	22.4481	22.624	22.3659	22.2409	23.7436	20.5086	21.9711	20.2187	20.7121	23.5371	21.1841	0.767183862	0.966018836	4	8	24	0.285275581	0.768040178	24	23.60094425	0.048159141
ENSMUSG00000020516	Rps6kb1	ribosomal protein S6 kinase, polypeptide 1 [Source:MGI Symbol;Acc:MGI:1270849]	14.4584	15.7568	15.4793	15.9264	15.2927	14.8855	13.971	14.5257	15.6373	15.02	15.7633	14.9653	15.2838	15.4442	0.028612069	0.316608413	12	16	24	0.062881961	0.662016894	24	11.00523686	0.044157095
ENSMUSG00000020519	Sap30l	SAP30-like [Source:MGI Symbol;Acc:MGI:1354709]	35.9627	29.5619	29.3268	31.8044	31.9326	32.1235	33.1583	29.5693	28.9163	35.4367	32.9193	35.0572	35.5382	31.879	0.002087679	0.100255953	4	8	24	0.051523332	0.64767123	24	19.69549835	0.092699771
ENSMUSG00000020520	Galnt10	polypeptide N-acetylgalactosaminyltransferase 10 [Source:MGI Symbol;Acc:MGI:1890480]	7.50727	5.83954	6.14461	5.90628	6.86531	6.4257	7.00038	6.36038	5.57551	6.82227	5.87425	6.29604	6.65254	5.52209	0.056400419	0.401074772	4	4	24	0.087542658	0.707747429	24	20.63796358	0.091020199
ENSMUSG00000020521	Rnft1	ring finger protein, transmembrane 1 [Source:MGI Symbol;Acc:MGI:1924142]	5.8267	5.56734	4.85162	4.47479	5.67898	5.33445	5.59387	5.46363	7.06373	5.73219	5.72472	6.06206	6.06897	5.98968	0.920872048	0.966018836	12	4	24	0.608661735	0.864393298	24	1.611363558	0.043927677
ENSMUSG00000020522	Mfap3	microfibrillar-associated protein 3 [Source:MGI Symbol;Acc:MGI:1924068]	9.66951	8.11823	7.70194	8.58613	9.42171	8.35753	9.12147	7.79408	8.13093	7.52096	9.64055	8.06483	8.49355	7.85892	0.112420845	0.530777648	20	16	24	0.050724414	0.644469829	24	19.30057005	0.096388249

ENSMUSG00000020523	Fam114a2	family with sequence similarity 114, member A2 [Source:MGI Symbol;Acc:MGI:1917629]	34.6987	31.212	29.9155	32.6132	32.3626	32.129	32.5022	31.1889	29.4487	29.7188	33.0018	31.1654	33.2946	31.2698	0.018894761	0.256345714	4	8	24	0.018419717	0.559812298	24	20.91990504	0.06482201
ENSMUSG00000020524	Gria1	glutamate receptor, ionotropic, AMPA1 (alpha 1) [Source:MGI Symbol;Acc:MGI:95808]	5.60912	6.64661	10.6133	6.38834	4.84534	7.38637	7.69526	5.5282	6.8716	6.47994	4.66701	6.71439	4.71024	8.09573	0.285027487	0.793714531	12	8	24	0.316382285	0.768040178	24	6.503605879	0.172313558
ENSMUSG00000020525	Ppm1d	protein phosphatase 1D magnesium-dependent, delta isoform [Source:MGI Symbol;Acc:MGI:1858214]	3.74668	3.46153	4.2355	4.49083	3.78147	3.68656	3.96182	3.81083	4.07616	4.01231	3.77813	3.7083	3.46606	3.97671	0.013161552	0.230545855	16	8	24	0.027768503	0.590220615	24	10.53948975	0.077954536
ENSMUSG00000020526	Znhit3	zinc finger, HIT type 3 [Source:MGI Symbol;Acc:MGI:3051596]	18.049	23.3889	22.2461	20.1548	22.7792	20.5271	20.5173	20.7283	23.8218	17.5806	25.1617	20.9675	21.7893	21.2319	0.625825704	0.966018836	20	8	24	0.880738806	0.966593118	24	10.39286954	0.02820027
ENSMUSG00000020527	Myo19	myosin XIX [Source:MGI Symbol;Acc:MGI:1913446]	1.80931	1.7816	1.52505	1.98743	2.05732	1.67258	2.41167	1.74358	1.68134	1.96556	1.93471	1.7025	1.51343	2.05302	0.945451136	0.966018836	16	20	24	0.834381172	0.949102201	24	16.70926674	0.029493358
ENSMUSG00000020528	Prpsap2	phosphoribosyl pyrophosphate synthetase-associated protein 2 [Source:MGI Symbol;Acc:MGI:2384838]	9.87696	10.3639	10.0445	10.0686	9.92053	10.1345	9.80041	9.81319	9.5167	8.65565	9.83933	9.10688	9.03523	8.47805	0.816406072	0.966018836	24	4	24	0.601253359	0.860450834	24	18.80980411	0.01972967
ENSMUSG00000020530	Ggnbp2	gametogenetin binding protein 2 [Source:MGI Symbol;Acc:MGI:2387356]	42.5876	46.2466	50.2173	43.999	44.0714	41.8888	41.4603	43.007	48.4976	45.4072	46.967	45.7769	45.335	48.2795	0.030691182	0.324150946	12	16	24	0.038791388	0.611655116	24	8.824202424	0.07391468
ENSMUSG00000020532	Acaca	acetyl-Coenzyme A carboxylase alpha [Source:MGI Symbol;Acc:MGI:108451]	19.289	15.8726	16.6109	22.5119	17.6836	15.9695	21.3109	17.1966	14.3373	18.8206	19.5674	22.1592	23.2111	21.7317	0.471214947	0.966018836	4	8	24	0.452173679	0.805393068	24	20.5707879	0.097096961
ENSMUSG00000020534	Shmt1	serine hydroxymethyltransferase 1 (soluble) [Source:MGI Symbol;Acc:MGI:98299]	0.846327	0.994488	1.3012	0.880003	1.00452	1.07547	1.32477	1.27344	1.03651	1.26121	1.19943	0.840773	1.3463	1.13723	0.929527443	0.966018836	4	20	24	0.527254821	0.823319175	24	5.891186795	0.053897665
ENSMUSG00000020536	Llgl1	LLGL1 scribble cell polarity complex component [Source:MGI Symbol;Acc:MGI:102682]	6.94515	7.54369	8.19397	7.60902	7.44173	7.48276	8.39475	7.90372	7.54892	8.57822	7.84069	7.50391	6.87094	7.43974	0.256878144	0.76168256	16	12	24	0.248323078	0.768040178	24	10.7117798	0.052572703
ENSMUSG00000020537	Drg2	developmentally regulated GTP binding protein 2 [Source:MGI Symbol;Acc:MGI:1342307]	29.0952	26.5934	27.5665	27.6598	29.7981	29.9818	28.4861	27.5788	26.2938	28.9795	28.4487	27.8124	28.3526	27.1623	0.009640466	0.194720062	20	16	24	0.005356296	0.4424469	24	18.50403466	0.059472762
ENSMUSG00000020538	Srebf1	sterol regulatory element binding transcription factor 1 [Source:MGI Symbol;Acc:MGI:107606]	19.4894	19.5559	22.9372	22.7444	24.928	19.2634	21.9422	20.405	21.07	30.968	23.6022	20.4168	24.5465	20.6645	0.101894436	0.512388497	20	4	24	0.053403945	0.652322075	24	12.76649542	0.148015556
ENSMUSG00000020541	Tom1l1	target of myb1-like 1 (chicken) [Source:MGI Symbol;Acc:MGI:1919193]	9.81898	10.7121	11.5755	9.168	9.68427	10.3495	10.9355	9.25605	10.7654	9.01657	10.7112	10.4709	10.6217	11.2759	0.087978327	0.472021905	12	4	24	0.39400271	0.799546792	24	2.516210847	0.053665025
ENSMUSG00000020544	Cox11	cytochrome c oxidase assembly protein 11, copper chaperone [Source:MGI Symbol;Acc:MGI:1917052]	6.45531	7.17005	8.12666	7.48779	6.61435	7.7408	7.22203	8.91398	7.31933	8.0764	7.81698	8.70673	8.50073	9.37206	0.885059765	0.966018836	8	12	24	0.250743614	0.768040178	24	5.738804527	0.062567795
ENSMUSG00000020546	Stxbp4	syntaxin binding protein 4 [Source:MGI Symbol;Acc:MGI:1342296]	2.90672	3.36134	2.95458	3.10558	3.02309	3.10239	2.9671	2.97785	2.93413	2.82971	3.07055	3.31956	3.46198	3.1347	0.718720526	0.966018836	8	4	24	0.334870925	0.770938748	24	22.96153207	0.035999841
ENSMUSG00000020547	Bzw2	basic leucine zipper and W2 domains 2 [Source:MGI Symbol;Acc:MGI:1914162]	15.5473	17.4215	17.249	15.0471	17.3122	15.7134	16.8015	18.3449	18.3574	17.882	17.7022	15.6113	17.6073	15.7787	0.483374602	0.966018836	8	4	24	0.35856185	0.785198873	24	8.110602182	0.052753778
ENSMUSG00000020549	Elac2	elaC ribonuclease Z 2 [Source:MGI Symbol;Acc:MGI:1890496]	8.8745	7.44524	7.55098	8.11243	7.75108	9.00242	8.0976	8.34681	7.21867	9.244	6.79999	8.5033	7.65106	8.30917	0.390686537	0.915479136	24	12	24	0.660420315	0.885470722	24	21.45510759	0.042680783
ENSMUSG00000020553	Pctp	phosphatidylcholine transfer protein [Source:MGI Symbol;Acc:MGI:107375]	7.91671	6.78551	5.0993	6.56557	5.98606	7.14628	7.35355	7.56182	4.91262	7.32146	6.08873	5.67166	6.79454	7.04595	0.11126948	0.53025242	4	8	24	0.207216419	0.768040178	24	23.4590399	0.123087795
ENSMUSG00000020561	Polr1f	RNA polymerase I subunit F [Source:MGI Symbol;Acc:MGI:106292]	4.75475	5.39596	4.56253	4.42126	4.55879	4.19736	4.63168	4.56003	5.24069	4.28644	4.8167	4.75709	4.64026	5.47376	0.480187506	0.966018836	8	8	24	0.108672021	0.72082227	24	4.72611957	0.074080689
ENSMUSG00000020562	Efcab10	EF-hand calcium binding domain 10 [Source:MGI Symbol;Acc:MGI:1922290]	3.83143	4.56275	4.24831	3.39049	3.31797	3.84025	3.90443	4.11638	5.11494	3.46254	4.1767	4.60602	5.11977	5.47635	0.128457847	0.560209134	8	8	24	0.01079425	0.51149922	24	3.752725004	0.156698771
ENSMUSG00000020564	Atxn7l1	ataxin 7-like 1 [Source:MGI Symbol;Acc:MGI:3584458]	8.10078	8.85075	9.38578	8.01035	9.73675	9.11768	10.0979	8.47274	6.98929	9.34254	7.42744	8.53738	8.04157	9.97129	0.81544087	0.966018836	8	4	24	0.832138948	0.948075545	24	23.71889072	0.035038582
ENSMUSG00000020566	Atp6v1c2	ATPase, H+ transporting, lysosomal V1 subunit C2 [Source:MGI Symbol;Acc:MGI:1916025]	1.34225	1.04174	0.993596	1.33478	1.16481	1.3356	0.995859	1.112	1.06028	0.864069	1.16661	1.20584	1.45123	1.37718	0.368981219	0.893596186	4	4	24	0.251575129	0.768040178	24	21.82688005	0.075329073
ENSMUSG00000020570	Sypl	synaptophysin-like protein [Source:MGI Symbol;Acc:MGI:108081]	34.2157	40.1615	39.324	40.5262	37.3102	35.9751	38.5922	39.1961	37.1497	35.8895	36.6538	30.2591	33.0627	31.4074	0.345459565	0.870418653	16	8	24	0.136985886	0.747972781	24	9.708711099	0.074208569
ENSMUSG00000020571	Pdia6	protein disulfide isomerase associated 6 [Source:MGI Symbol;Acc:MGI:1919103]	84.2268	62.6863	51.6422	57.46	73.8993	63.744	72.2448	62.0832	55.3396	50.273	79.5748	64.7046	71.6245	58.6032	0.004003768	0.140598674	20	20	24	0.007135	0.47735824	24	20.95646686	0.239860019
ENSMUSG00000020572	Nampt	nicotinamide phosphoribosyltransferase [Source:MGI Symbol;Acc:MGI:1929865]	18.141	20.9431	22.1958	26.4671	23.138	20.6272	18.9407	20.1059	23.3085	19.6445	24.5126	20.3695	20.3113	19.8653	0.01457641	0.247599677	20	8	24	0.012015398	0.525280095	24	12.59579515	0.144343987
ENSMUSG00000020573	Pik3cg	phosphatidylinositol-4,5-bisphosphate 3-kinase catalytic subunit gamma [Source:MGI Symbol;Acc:MGI:1353576]	0.620859	0.987395	1.09351	0.849001	1.01519	0.614102	0.795724	0.775151	0.95043	0.563637	0.667672	0.7707	0.780417	0.636562	0.396686537	0.915479136	12	12	24	0.439503252	0.805393068	24	8.37551769	0.063981746
ENSMUSG00000020576	Nbas	neuroblastoma amplified sequence [Source:MGI Symbol;Acc:MGI:1918419]	13.4193	11.7206	12.8788	11.7994	11.9337	14.0123	13.558	13.0826	9.75115	14.2591	10.8686	12.6629	12.6386	11.4985	0.549118437	0.966018836	24	4	24	0.324560371	0.768040178	24	21.44973363	0.082318201
ENSMUSG00000020577	Tspan13	tetraspanin 13 [Source:MGI Symbol;Acc:MGI:1913359]	61.724	52.2371	52.3382	49.6621	51.5501	57.9226	54.1873	55.2139	46.7476	58.8413	56.3989	57.3211	55.9607	58.8922	0.351617883	0.870418653	24	12	24	0.132063234	0.742323615	24	21.82889537	0.080597447
ENSMUSG00000020580	Rock2	Rho-associated coiled-coil containing protein kinase 2 [Source:MGI Symbol;Acc:MGI:107926]	17.0343	17.7052	16.7359	17.744	18.2879	16.2316	18.5588	15.7464	17.8956	14.9664	18.7412	16.8299	16.5833	17.0859	0.895686496	0.966018836	20	4	24	0.813589084	0.940453305	24	18.13019514	0.023024037
ENSMUSG00000020585	Laptm4a	lysosomal-associated protein transmembrane 4A [Source:MGI Symbol;Acc:MGI:108017]	213.491	228.066	226.713	204.029	207.9	191.78	219.359	219.586	230.016	183.71	216.652	198.75	204.266	211.313	0.011317069	0.204837764	12	4	24	0.085677332	0.703577714	24	5.10744286	0.07469337
ENSMUSG00000020589	Cyria	CYFIP related Rac1 interactor A [Source:MGI Symbol;Acc:MGI:1261783]	4.11891	4.85142	5.68984	4.55869	3.95326	4.3651	5.47231	3.88401	4.99575	3.95261	4.20881	5.0843	4.45185	4.88129	0.602156008	0.966018836	12	4	24	0.482305855	0.805393068	24	4.609523228	0.070201854
ENSMUSG00000020590	Snx13	sorting nexin 13 [Source:MGI Symbol;Acc:MGI:2661416]	26.0838	24.4859	23.4685	23.0986	25.28	24.3763	26.1656	21.3598	22.777	21.4077	25.1887	23.8558	25.1625	24.5105	0.017422135	0.255326033	4	8	24	0.031861078	0.604635642	24	21.45012139	0.084589888
ENSMUSG00000020591	Ntsr2	neurotensin receptor 2 [Source:MGI Symbol;Acc:MGI:108018]	3.19472	6.88809	13.015	5.23326	3.18135	7.20929	8.64727	4.67567	8.67326	5.73068	3.05897	6.08207	3.11875	7.89879	0.03782572	0.334069441	12	8	24	0.194354429	0.768040178	24	6.767237066	0.394212579
ENSMUSG00000020592	Sdc1	syndecan 1 [Source:MGI Symbol;Acc:MGI:1349162]	12.6015	9.34863	11.8968	10.2489	12.615	12.9796	9.16874	11.4122	9.30036	10.1782	12.9783	11.2683	9.87574	10.9657	0.207442359	0.698704663	24	4	24	0.149478467	0.758126113	24	17.80500426	0.125092849
ENSMUSG00000020593	Lpin1	lipin 1 [Source:MGI Symbol;Acc:MGI:1891340]	23.3488	16.1507	17.3757	23.5024	18.011	22.4165	20.3418	23.4154	14.8728	28.7421	17.9997	20.4984	20.1938	20.6172	0.916955334	0.966018836	16	20	24	0.792449405	0.93364006	24	18.22959207	0.067819451
ENSMUSG00000020594	Pum2	pumilio RNA-binding family member 2 [Source:MGI Symbol;Acc:MGI:1931751]	30.5272	31.2952	30.6583	31.2434	31.5715	27.9137	30.6332	27.2048	31.879	27.2321	32.4135	28.5479	30.6594	28.9416	0.676344318	0.966018836	20	4	24	0.857974571	0.958106625	24	13.30258496	0.017563516
ENSMUSG00000020598	Nrcam	neuronal cell adhesion molecule [Source:MGI Symbol;Acc:MGI:104750]	36.5221	37.9564	35.7456	35.7859	35.7994	37.1159	36.9321	34.8401	33.9959	36.3489	32.633	39.9237	35.2354	39.8247	0.446653834	0.966018836	24	12	24	0.197579713	0.796215348	24	10.43981799	0.041985364
ENSMUSG00000020599	Rgs9	regulator of G-protein signaling 9 [Source:MGI Symbol;Acc:MGI:1338824]	1.60168	2.25395	2.29963	2.19619	2.02152	1.98782	1.94074	1.65487	1.89548	2.53729	1.78755	2.176	1.77149	2.32395	0.117050591	0.538362324	16	12	24	0.189593955	0.768040178	24	10.74319465	0.092606285
ENSMUSG00000020600	Slc7a15	solute carrier family 7 (cationic amino acid transporter, y+ system), member 15 [Source:MGI Symbol;Acc:MGI:3045351]	0.760592	0.987898	0.647517	0.742395	0.895255	0.62438	0.934744	0.473349	0.595108	0.877858	1.01937	0.647503	0.774042	0.759658	0.850398351	0.966018836	20	16	24	0.582870101	0.851661806	24	17.14577794	0.055481365
ENSMUSG00000020601	Trib2	tribbles pseudokinase 2 [Source:MGI Symbol;Acc:MGI:2145021]	29.979	23.9127	22.3587	22.2082	26.6741	26.076	26.4538	26.0844	20.7539	28.2434	26.6542	23.9396	26.4001	24.8534	0.128989168	0.560209134	20	16	24	0.12044396	0.732894732	24	20.77529393	0.110531095
ENSMUSG00000020602	Arsg	arylsulfatase G [Source:MGI Symbol;Acc:MGI:1921258]	2.9513	4.90642	6.49123	4.19494	3.24314	4.29126	4.79139	4.45177	4.80306	4.29063	3.07562	4.07562	3.11376	4.72509	0.018923071	0.256345714	12	8	24	0.05321626	0.651696207	24	7.102777816	0.231083969
ENSMUSG00000020605	Hs1bp3	HCLS1 binding protein 3 [Source:MGI Symbol;Acc:MGI:1913224]	5.9013	5.33997	5.45334	5.87728	6.08316	5.86426	5.55549	5.42026	4.83603	6.32842	5.03702	5.62029	5.49514	5.15876	0.064405315	0.414867207	16	20	24	0.180792209	0.768040178	24	17.340157	0.061353757
ENSMUSG00000020607	Lratd1	LRAT domain containing 1 [Source:MGI Symbol;Acc:MGI:2145011]	0.501547	0.50852	1.01168	0.468193	0.283269	0.548467	0.701306	0.320188	0.596005	0.4766	0.273423	0.520358	0.209887	0.600091	0.424183557	0.966018836	12	8	24	0.391864248	0.799546792	24	6.455590176	0.099943536
ENSMUSG00000020608	Smc6	structural maintenance of chromosomes 6 [Source:MGI Symbol;Acc:MGI:1914491]	14.4959	15.1223	16.9543	19.5166	16.2653	13.4601	15.228	17.5577	19.0139	16.764	19.607	19.1057	17.3161	18.9951	0.541384199	0.966018836	16	12	24	0.046565385	0.633507364	24	10.43475224	0.121376954
ENSMUSG00000020610	Amz2	archaelysin family metallopeptidase 2 [Source:MGI Symbol;Acc:MGI:104837]	28.7098	26.3627	25.3392	27.2903	27.8326	28.4751	30.3439	28.2355	25.6758	29.1855	25.2087	25.4186	25.7511	27.4804	0.714062999	0.966018836	4	8	24	0.687181071	0.89619155	24	22.46755365	0.028846771
ENSMUSG00000020611	Gna13	guanine nucleotide binding protein, alpha 13 [Source:MGI Symbol;Acc:MGI:95768]	20.2426	17.7969	18.6163	19.9933	19.0385	18.9997	18.9727	19.078	17.8038	19.2305	19.274	20.0693	18.7069	19.238	0.43280837	0.966018836	16	20	24	0.199194798	0.768040178	24	18.47931373	0.037139972
ENSMUSG00000020612	Prkar1a	protein kinase, cAMP dependent regulatory, type I, alpha [Source:MGI Symbol;Acc:MGI:104878]	338.085	285.031	284.782	288.946	283.959	309.072	297.003	297.571	251.668	311.657	296.117	291.071	297.839	297.865	0.170659409	0.643496587	4	8	24	0.162455003	0.76636332	24	21.37183937	0.068228112
ENSMUSG00000020614	Fam20a	FAM20A, golgi associated secretory pathway pseudokinase [Source:MGI Symbol;Acc:MGI:2388266]	3.306	3.20829	3.49968	2.7411	2.85032	3.37735	3.407	3.30549	2.57565	3.02857	2.02363	3.34176	2.7471	2.65746	0.503972924	0.966018836	24	20	24	0.203948393	0.768040178	24	0.728909887	0.095523513
ENSMUSG00000020620	Abca8b	ATP-binding cassette, sub-family A (ABC1), member 8b [Source:MGI Symbol;Acc:MGI:1351668]	18.3306	19.4868	18.6699	21.4908	21.3565	18.7791	18.8698	16.3088	19.0921	18.9543	20.6122	18.8497	19.6061	17.6835	0.083354723	0.462922111	16	16	24	0.019922092	0.565108098	24	15.24474463	0.093014296
ENSMUSG00000020621	Rdh14	retinol dehydrogenase 14 (all-trans and 9-cis) [Source:MGI Symbol;Acc:MGI:1920402]	17.2519	16.7558	16.7718	17.1862	16.4411	16.432	16.9906	17.6976	16.2269	16.6653	18.8268	17.3085	17.7539	18.5628	0.853711773	0.966018836	8	4	24	0.758941971	0.922092694	24	0.743722963	0.015292031
ENSMUSG00000020623	Map2k6	mitogen-activated protein kinase kinase 6 [Source:MGI Symbol;Acc:MGI:1346870]	3.87982	3.75497	3.72754	3.85845	3.54652	4.35254	3.86146	4.15276	4.26175	4.94086	3.62094	3.92081	4.15559	3.55623	0.92721845	0.966018836	24	20	24	0.877087089	0.965814767	24	10.87823267	0.022009412
ENSMUSG00000020627	Klhl29	kelch-like 29 [Source:MGI Symbol;Acc:MGI:2683857]	6.7142	4.98643	4.69997	5.18262	4.83638	5.41889	5.39554	5.8671	4.44728	6.49078	5.25319	5.71957	6.04005	6.0304	0.035806379	0.331814682	4	8	24	0.32846341	0.768040178	24	23.02593588	0.083959557
ENSMUSG00000020628	Trappc12	trafficking protein particle complex 12 [Source:MGI Symbol;Acc:MGI:2445089]	16.884	14.6328	16.1102	16.1273	14.4712	17.5946	17.5405	16.8981	13.4036	19.0431	13.9524	15.777	14.79	16.0455	0.939103211	0.966018836	24	12	24	0.88235893	0.966683041	24	22.50606544	0.027703726
ENSMUSG00000020629	Adi1	acireductone dioxygenase 1 [Source:MGI Symbol;Acc:MGI:2144929]	10.9672	12.2945	12.801	11.7885	11.3842	11.8976	13.6554	13.2672	12.9184	12.5149	11.0146	13.0373	11.8345	11.6942	0.822170541	0.966018836	12	8	24	0.371612187	0.793362704	24	5.340518176	0.051096486
ENSMUSG00000020630	Rnaseh1	ribonuclease H1 [Source:MGI Symbol;Acc:MGI:1335073]	8.73344	9.25946	8.58568	8.38809	8.5276	8.91415	8.26943	9.14937	8.54566	8.50241	8.79362	9.5811	10.1325	9.49748	0.123511911	0.552144108	8	8	24	0.074319763	0.683293901	24	0.709428794	0.05371783
ENSMUSG00000020634	Ubxn2a	UBX domain protein 2A [Source:MGI Symbol;Acc:MGI:2442310]	28.4322	27.5494	27.3709	27.5202	25.5492	29.3744	27.0897	26.5344	27.2655	31.4131	29.9461	29.0911	27.2822	30.3334	0.933543173	0.966018836	16	20	24	0.78790709	0.931832121	24	14.63211971	0.019853197
ENSMUSG00000020635	Fkbp1b	FK506 binding protein 1b [Source:MGI Symbol;Acc:MGI:1336205]	118.638	95.1113	93.5555	98.0548	103.82	112.362	97.1515	100.506	80.4813	104.816	104.319	105.457	107.993	97.6266	0.004712708	0.140598674	24	12	24	0.012275861	0.525280095	24	20.1044472	0.138524823
ENSMUSG00000020638	Cmpk2	cytidine monophosphate (UMP-CMP) kinase 2, mitochondrial [Source:MGI Symbol;Acc:MGI:99830]	10.8377	10.4605	10.7382	9.21507	8.71617	10.3269	10.8244	9.86588	8.37749	11.219	10.1509	10.106	10.4861	11.1362	0.481721608	0.966018836	4	16	24	0.33341736	0.769685083	24	0.930736594	0.065937339
ENSMUSG00000020639	Pfn4	profilin family, member 4 [Source:MGI Symbol;Acc:MGI:1920121]	1.66114	2.69235	2.67268	2.17645	2.13518	2.37411	1.52663	2.18574	2.22269	2.31121	1.91233	2.23317	1.95493	2.30151	0.124028266	0.552144108	12	16	24	0.177282393	0.768040178	24	9.271791044	0.111520182
ENSMUSG00000020640	Itsn2	intersectin 2 [Source:MGI Symbol;Acc:MGI:1338049]	36.4447	34.5526	35.9681	33.9447	36.5239	35.073	34.1683	32.8687	35.9249	30.4171	36.0673	35.7017	35.8063	36.5336	0.905762334	0.966018836	20	20	24	0.530921937	0.825104543	24	22.19484982	0.032748942
ENSMUSG00000020641	Rsad2	radical S-adenosyl methionine domain containing 2 [Source:MGI Symbol;Acc:MGI:1929628]	3.1593	3.73252	4.7242	3.85029	3.42813	5.16305	3.19509	4.29697	3.66912	3.60223	4.50393	3.76463	4.31552	4.32091	0.88067614	0.966018836	12	16	24	0.946828667	0.986986655	24	10.29424829	0.022490273
ENSMUSG00000020642	Rnf144a	ring finger protein 144A [Source:MGI Symbol;Acc:MGI:1344401]	17.8434	13.4248	14.0121	15.2166	13.1803	15.2063	14.0913	15.3797	12.0842	18.2716	13.2647	15.4587	14.8884	15.1577	0.836635326	0.966018836	24	12	24	0.806936742	0.938616703	24	21.55976645	0.041863224
ENSMUSG00000020644	Id2	inhibitor of DNA binding 2 [Source:MGI Symbol;Acc:MGI:96397]	24.0848	30.6148	32.3841	25.1445	24.258	25.914	26.4682	24.8925	27.49	24.1391	26.6064	23.1116	24.137	28.3308	0.109194134	0.524035616	12	12	24	0.074475616	0.683293901	24	6.763616784	0.112461299
ENSMUSG00000020646	Mboat2	membrane bound O-acyltransferase domain containing 2 [Source:MGI Symbol;Acc:MGI:1914466]	17.4777	18.8383	19.4948	18.3439	20.1719	17.8083	19.4397	16.4346	19.1307	18.0864	16.7361	18.6271	18.7477	18.9022	0.911135828	0.966018836	12	12	24	0.929557229	0.981644008	24	8.936201333	0.012799313
ENSMUSG00000020647	Ncoa1	nuclear receptor coactivator 1 [Source:MGI Symbol;Acc:MGI:1276523]	12.3771	10.4163	11.3976	11.8722	11.0031	13.0112	12.4122	11.9607	9.13637	13.6228	10.1078	12.2109	10.8227	11.4898	0.390686537	0.915479136	24	12	24	0.526734718	0.823075573	24	19.98772688	0.0656299
ENSMUSG00000020648	Dus4l	dihydrouridine synthase 4-like (S. cerevisiae) [Source:MGI Symbol;Acc:MGI:1919166]	3.49711	3.53707	3.59413	3.50327	3.52492	3.50915	3.37171	3.29324	3.87695	2.8123	3.37729	2.98236	4.07781	3.93551	0.697196105	0.966018836	12	4	24	0.305034581	0.768040178	24	3.80822141	0.065666448
ENSMUSG00000020649	Rrm2	ribonucleotide reductase M2 [Source:MGI Symbol;Acc:MGI:98181]	1.34603	1.895	2.82468	2.40836	1.90475	1.93789	1.57025	1.5895	2.03551	1.49392	1.69718	2.18674	2.2544	1.25309	0.798829513	0.966018836	12	20	24	0.553420478	0.837326155	24	12.63421093	0.090401853
ENSMUSG00000020650	Bcap29	B cell receptor associated protein 29 [Source:MGI Symbol;Acc:MGI:101917]	38.2635	41.0574	39.407	40.6001	36.694	37.0293	36.6196	35.0717	41.1501	37.139	43.1425	37.2354	39.3246	42.6218	0.764889554	0.966018836	12	12	24	0.440412199	0.805393068	24	8.912376364	0.0458882
ENSMUSG00000020652	Cenpo	centromere protein O [Source:MGI Symbol;Acc:MGI:1923800]	3.36605	3.41982	3.71202	3.1856	3.29476	3.17793	3.35977	2.99444	3.67936	3.01529	3.26337	3.11041	3.09011	3.32755	0.338172159	0.85758369	12	12	24	0.348065914	0.779015211	24	7.096691471	0.039189265
ENSMUSG00000020653	Klf11	Kruppel-like factor 11 [Source:MGI Symbol;Acc:MGI:2653368]	2.93435	3.26074	3.56744	3.75009	3.34512	3.07939	3.194	4.05471	3.67479	3.76219	2.98379	3.24194	3.492	3.38313	0.023283676	0.278156767	16	8	24	0.016659906	0.544809764	24	8.485329097	0.107108791
ENSMUSG00000020654	Adcy3	adenylate cyclase 3 [Source:MGI Symbol;Acc:MGI:99675]	4.70169	3.73486	4.67127	8.18485	4.03488	4.74241	4.92349	4.98287	4.16277	5.61296	4.23501	4.38871	3.95924	4.88711	0.807012301	0.966018836	16	4	24	0.356440404	0.783126731	24	11.72046178	0.130628347
ENSMUSG00000020656	Grhl1	grainyhead like transcription factor 1 [Source:MGI Symbol;Acc:MGI:2182540]	0.599036	0.562014	0.534792	0.568341	0.626516	0.388991	0.600441	0.514121	0.673459	0.42327	0.490554	0.553572	0.569243	0.468846	0.934152439	0.966018836	4	20	24	0.836548805	0.949953371	24	4.207995116	0.017329223
ENSMUSG00000020657	Dnajc27	DnaJ heat shock protein family (Hsp40) member C27 [Source:MGI Symbol;Acc:MGI:2443036]	12.2886	11.9688	14.4605	12.9231	10.681	14.2794	14.0458	13.7827	11.5557	16.308	10.2673	13.2797	11.4955	13.5517	0.928064146	0.966018836	16	4	24	0.715743082	0.906323093	24	6.393779494	0.058332418
ENSMUSG00000020658	Efr3b	EFR3 homolog B [Source:MGI Symbol;Acc:MGI:2444851]	16.7725	16.1312	17.7759	16.3537	15.5852	17.6584	17.7177	16.8391	14.9832	18.6819	13.6717	16.0003	15.7904	17.0425	0.924553956	0.966018836	16	4	24	0.662681977	0.886154038	24	3.984155804	0.038679122
ENSMUSG00000020659	Cbll1	Casitas B-lineage lymphoma-like 1 [Source:MGI Symbol;Acc:MGI:2144842]	5.19818	4.93384	4.89026	4.78656	5.01669	4.76341	5.35521	4.49827	4.44326	5.02763	4.64745	4.5827	4.48713	4.77536	0.874054778	0.966018836	16	20	24	0.686071974	0.895740026	24	20.58048289	0.020698196
ENSMUSG00000020660	Pomc	pro-opiomelanocortin-alpha [Source:MGI Symbol;Acc:MGI:97742]	0.82995	0.570644	0.530904	0.727977	0.58545	0.582151	0.774343	0.874283	1.03362	0.580584	0.704972	0.622574	0.747931	0.765624	0.57057742	0.966018836	4	20	24	0.319962529	0.768040178	24	4.382284705	0.067435373
ENSMUSG00000020661	Dnmt3a	DNA methyltransferase 3A [Source:MGI Symbol;Acc:MGI:1261827]	6.41758	6.78079	7.09213	6.30309	7.28621	7.09078	7.9421	6.88283	7.15696	7.23585	6.58221	6.91091	6.87626	7.42303	0.731683656	0.966018836	12	4	24	0.787096104	0.931456435	24	3.032809987	0.020044712
ENSMUSG00000020664	Dld	dihydrolipoamide dehydrogenase [Source:MGI Symbol;Acc:MGI:107450]	79.0207	83.8775	84.8244	93.5746	81.0639	76.2877	78.8322	83.6604	89.3165	77.4316	91.2882	76.248	89.196	86.3022	0.08302755	0.462922111	12	12	24	0.183122908	0.768040178	24	8.975841985	0.069978326
ENSMUSG00000020668	Kif3c	kinesin family member 3C [Source:MGI Symbol;Acc:MGI:107979]	56.4927	40.8743	42.5138	49.3071	45.0487	52.1847	51.1709	49.6272	34.8786	61.3935	42.4428	48.3558	45.9195	46.3063	0.46238066	0.966018836	24	12	24	0.437876547	0.805393068	24	19.7997	0.105414202
ENSMUSG00000020669	Sh3yl1	Sh3 domain YSC-like 1 [Source:MGI Symbol;Acc:MGI:1346118]	7.91164	7.41826	7.10197	7.15812	7.15989	7.99885	6.75023	7.64471	6.85962	9.18426	7.35708	8.86826	7.52117	8.17744	0.245061943	0.741580974	24	12	24	0.784076419	0.930636207	24	18.51198147	0.031442723
ENSMUSG00000020671	Rab10	RAB10, member RAS oncogene family [Source:MGI Symbol;Acc:MGI:105066]	57.8228	49.2218	50.5742	51.5449	49.2876	53.9534	52.7009	54.789	45.3868	62.3136	51.6	51.6425	51.4449	51.6244	0.46238066	0.966018836	24	12	24	0.797637077	0.935414606	24	19.50715286	0.030162559
ENSMUSG00000020674	Pxdn	peroxidasin [Source:MGI Symbol;Acc:MGI:1916925]	12.6337	11.5327	11.3203	12.4802	12.7067	11.6365	13.2724	11.9596	10.7131	12.0068	14.0869	12.7752	10.9436	10.4314	0.027467587	0.308438477	20	16	24	0.026196691	0.586541342	24	17.73496397	0.107534151
ENSMUSG00000020676	Ccl11	chemokine (C-C motif) ligand 11 [Source:MGI Symbol;Acc:MGI:103576]	14.9521	27.504	26.7434	23.965	28.3974	13.8259	21.2553	20.3668	38.0595	18.6708	22.3335	17.7032	22.0073	21.9419	0.022634137	0.27189101	12	12	24	0.051162498	0.646727671	24	8.886640311	0.331774867
ENSMUSG00000020677	Ddx52	DExD box helicase 52 [Source:MGI Symbol;Acc:MGI:1925644]	11.0717	12.9949	11.6517	12.048	12.4153	11.2608	10.7822	10.9807	15.2386	10.2747	13.3305	11.7852	13.4694	13.1982	0.866861748	0.966018836	12	16	24	0.632169365	0.875020466	24	7.918819271	0.055143442
ENSMUSG00000020680	Taf15	TATA-box binding protein associated factor 15 [Source:MGI Symbol;Acc:MGI:1917689]	28.5454	26.2556	25.9712	27.1191	27.7321	25.1471	26.8717	25.1532	24.3962	24.1078	23.4933	25.5985	25.2116	23.531	0.917370979	0.966018836	4	8	24	0.14163815	0.751766185	24	21.13832602	0.040979925
ENSMUSG00000020681	Ace	angiotensin I converting enzyme (peptidyl-dipeptidase A) 1 [Source:MGI Symbol;Acc:MGI:87874]	2.82697	3.25752	3.38066	3.11168	3.09653	2.97503	3.01918	3.26736	3.92297	3.35466	2.46404	3.64993	2.52656	2.91004	0.033248781	0.324908923	12	16	24	0.279111115	0.768040178	24	8.802195197	0.088017279
ENSMUSG00000020682	Mmp28	matrix metallopeptidase 28 (epilysin) [Source:MGI Symbol;Acc:MGI:2153062]	2.49088	1.96721	2.0134	2.24072	2.22263	2.19945	2.40554	2.72724	2.00256	2.66374	2.37157	3.19461	2.56123	2.27285	0.649212555	0.966018836	4	8	24	0.236575812	0.768040178	24	20.3760498	0.082131475
ENSMUSG00000020684	Rasl10b	RAS-like, family 10, member B [Source:MGI Symbol;Acc:MGI:2685575]	11.1137	8.9742	9.99466	10.1935	9.45157	11.4037	10.4341	9.9854	8.18224	12.4048	9.31214	11.1116	9.24615	11.1976	0.939921059	0.966018836	24	12	24	0.654022885	0.883343578	24	19.29827017	0.056473514
ENSMUSG00000020687	Cdc27	cell division cycle 27 [Source:MGI Symbol;Acc:MGI:102685]	17.9226	19.7807	18.786	18.2235	19.1921	18.2566	17.7042	17.8321	19.6594	16.6726	19.913	19.0599	19.281	19.4708	0.912686312	0.966018836	20	8	24	0.971080566	0.993012791	24	3.944303666	0.006846731
ENSMUSG00000020689	Itgb3	integrin beta 3 [Source:MGI Symbol;Acc:MGI:96612]	4.50242	3.84826	4.23527	3.88596	4.28281	4.04567	4.40589	3.66553	3.38865	3.71632	3.81481	3.6075	4.50704	3.6135	0.128457847	0.560209134	4	8	24	0.106207286	0.71810271	24	21.48445488	0.078231391
ENSMUSG00000020691	Mettl2	methyltransferase like 2 [Source:MGI Symbol;Acc:MGI:1289171]	10.2369	10.2643	9.82205	10.4041	9.88022	10.2734	8.96874	10.2574	12.2068	10.4289	9.57988	10.3088	9.79034	11.3971	0.210566453	0.69936851	16	12	24	0.148397294	0.756728257	24	7.992093325	0.072391253
ENSMUSG00000020692	Nle1	notchless homolog 1 [Source:MGI Symbol;Acc:MGI:2429770]	5.33426	5.40852	6.37626	4.98694	6.08656	4.44767	4.83525	7.00068	6.04289	5.84543	5.46888	6.21924	6.23518	4.73597	0.896753532	0.966018836	12	12	24	0.631379506	0.87436603	24	8.430036115	0.063804433
ENSMUSG00000020694	Tlk2	tousled-like kinase 2 (Arabidopsis) [Source:MGI Symbol;Acc:MGI:1346023]	22.4787	22.0216	22.6406	19.2606	20.4832	21.4177	21.8685	22.0631	21.1338	23.2125	21.6571	22.9575	20.9058	23.226	0.868638024	0.966018836	8	12	24	0.431277932	0.805393068	24	2.939655013	0.035252428
ENSMUSG00000020695	Mrc2	mannose receptor, C type 2 [Source:MGI Symbol;Acc:MGI:107818]	3.80635	5.49678	4.51448	4.47672	5.86121	3.98157	4.31121	5.4853	6.60406	3.74328	4.90276	4.56624	4.11104	4.86939	0.372888979	0.89586062	8	20	24	0.38048062	0.799546792	24	8.357126148	0.114489128
ENSMUSG00000020696	Rffl	ring finger and FYVE like domain containing protein [Source:MGI Symbol;Acc:MGI:1914588]	6.79475	6.88484	7.39276	6.74279	6.8849	7.79696	7.38405	6.52694	6.71433	7.23892	6.73787	7.24988	6.14408	7.22804	0.922035832	0.966018836	24	8	24	0.774981058	0.927711986	24	17.45147722	0.02242604
ENSMUSG00000020697	Lig3	ligase III, DNA, ATP-dependent [Source:MGI Symbol;Acc:MGI:109152]	6.60186	7.02985	7.38719	7.0364	7.26647	7.82476	6.84497	7.38891	7.79503	7.1614	6.28803	7.69345	7.10024	6.91352	0.887027852	0.966018836	24	4	24	0.927820425	0.98127006	24	9.057407384	0.012806908
ENSMUSG00000020698	Cct6b	chaperonin containing Tcp1, subunit 6b (zeta) [Source:MGI Symbol;Acc:MGI:1329013]	0.780795	0.720131	0.743706	0.899986	0.513716	0.667712	0.506839	0.723083	0.634437	0.725192	0.488224	0.682175	0.74572	0.52558	0.676344318	0.966018836	16	4	24	0.714707785	0.905979936	24	7.30664252	0.033281632
ENSMUSG00000020700	Map3k3	mitogen-activated protein kinase kinase kinase 3 [Source:MGI Symbol;Acc:MGI:1346874]	9.8297	9.95724	11.5695	11.6079	11.0513	10.8704	9.80978	10.5328	10.7992	9.47691	9.94638	9.91348	10.5745	9.44733	0.29905572	0.809362476	12	20	24	0.39380634	0.799546792	24	12.2172438	0.044062733
ENSMUSG00000020701	Tmem132e	transmembrane protein 132E [Source:MGI Symbol;Acc:MGI:2685490]	22.7834	16.9354	15.5776	17.1467	19.1054	19.5753	17.6886	20.741	14.8731	25.7799	18.2451	20.2728	21.539	19.2433	0.074036902	0.448301997	4	8	24	0.435264017	0.805393068	24	20.27604626	0.103960652
ENSMUSG00000020702	Ccl1	chemokine (C-C motif) ligand 1 [Source:MGI Symbol;Acc:MGI:98258]	0.684466	1.15548	0.612806	0.905545	1.0119	0.426388	0.969722	0.877724	0.542659	1.01596	0.622349	1.24538	0.993938	0.63432	0.829428469	0.966018836	24	12	24	0.906330967	0.97449058	24	20.93743052	0.036951732
ENSMUSG00000020704	Asic2	acid-sensing (proton-gated) ion channel 2 [Source:MGI Symbol;Acc:MGI:1100867]	20.2025	15.4549	15.9231	18.3603	16.3541	18.6228	15.1757	17.7491	13.2169	22.3591	15.0275	17.585	16.9989	17.7832	0.893609703	0.966018836	24	12	24	0.788179815	0.931998618	24	18.20244439	0.052133187
ENSMUSG00000020705	Ddx42	DEAD box helicase 42 [Source:MGI Symbol;Acc:MGI:1919297]	22.0002	22.4215	23.4685	21.4699	20.2222	21.5183	20.7794	21.4418	21.4747	24.8191	20.2514	24.2444	22.1678	23.8155	0.628286596	0.966018836	8	12	24	0.515354339	0.817300608	24	6.683678668	0.040672179
ENSMUSG00000020706	Ftsj3	FtsJ RNA methyltransferase homolog 3 (E. coli) [Source:MGI Symbol;Acc:MGI:1860295]	12.3176	12.3951	13.0711	12.3554	14.5579	12.5346	12.6152	12.6854	12.3371	12.1876	12.1967	11.9924	12.5266	12.043	0.816406072	0.966018836	12	4	24	0.615719728	0.867203276	24	15.73225604	0.023539617
ENSMUSG00000020707	Rnf135	ring finger protein 135 [Source:MGI Symbol;Acc:MGI:1919206]	4.10863	3.70877	3.14849	4.06544	4.13366	4.21036	4.48964	4.37025	3.75704	3.6205	3.87385	4.28032	3.71544	4.25008	0.157616081	0.614233844	24	12	24	0.123541875	0.736492734	24	21.56384415	0.083753157
ENSMUSG00000020708	Psmc5	protease (prosome, macropain) 26S subunit, ATPase 5 [Source:MGI Symbol;Acc:MGI:105047]	139.176	126.272	122.952	122.843	119.593	133.317	124.912	123.491	119.766	133.267	127.616	133.033	133.095	132.462	0.106675896	0.521506614	24	12	24	0.136998532	0.747972781	24	22.13500849	0.05043065
ENSMUSG00000020709	Adap2	ArfGAP with dual PH domains 2 [Source:MGI Symbol;Acc:MGI:2663075]	0.872649	1.3407	1.72795	1.4042	1.44955	1.33755	1.06835	1.25051	1.58806	1.38571	1.31517	1.335	1.0875	1.25014	1.18E-04	0.029372629	12	16	24	0.007960979	0.490448898	24	11.02191769	0.14235408
ENSMUSG00000020715	Ern1	endoplasmic reticulum (ER) to nucleus signalling 1 [Source:MGI Symbol;Acc:MGI:1930134]	2.61688	2.43057	2.65864	2.65832	2.47122	2.50339	2.50079	2.65438	2.3616	2.74012	2.19474	2.48097	2.245	2.01255	0.892555977	0.966018836	16	4	24	0.599896318	0.860011178	24	13.3209107	0.027496262
ENSMUSG00000020716	Nf1	neurofibromin 1 [Source:MGI Symbol;Acc:MGI:97306]	16.0666	14.2036	14.3859	13.8832	14.1376	14.1679	15.9661	14.7893	13.3177	16.3033	13.6811	16.0606	15.5223	15.6499	0.196044629	0.673646834	4	16	24	0.219315604	0.768040178	24	23.67095574	0.060006928

ENSMUSG00000020717	Pecam1	platelet/endothelial cell adhesion molecule 1 [Source:MGI Symbol;Acc:MGI:97537]	6.96486	7.64099	8.32439	8.46012	7.2634	8.76806	7.89847	10.6701	7.11266	7.96343	6.99364	7.8949	9.12505	7.28754	0.85387321	0.966018836	8	12	24	0.703227739	0.901968234	24	3.20921609	0.05127422
ENSMUSG00000020718	Polg2	polymerase (DNA directed), gamma 2, accessory subunit [Source:MGI Symbol;Acc:MGI:1354947]	2.7879	2.97515	2.65415	2.5096	3.21448	2.72713	3.0946	2.80708	3.15819	2.85385	2.79211	3.27631	2.37725	3.21022	0.831272156	0.966018836	20	20	24	0.990556974	0.998244912	24	23.29695512	0.005804172
ENSMUSG00000020719	Ddx5	DEAD box helicase 5 [Source:MGI Symbol;Acc:MGI:105037]	244.296	242.281	241.884	266.221	259.631	226.502	257.278	221.622	250.797	229.826	271.898	229.409	228.777	230.86	0.351315142	0.870418653	20	4	24	0.197300913	0.768040178	24	14.23523667	0.060555596
ENSMUSG00000020720	Psmd12	proteasome (prosome, macropain) 26S subunit, non-ATPase, 12 [Source:MGI Symbol;Acc:MGI:1914247]	66.7179	67.6773	66.6186	66.302	64.5255	68.0033	62.2283	65.15	61.9488	68.0852	65.2656	65.6014	64.2139	68.836	0.890311217	0.966018836	8	20	24	0.970695358	0.99300601	24	10.39978038	0.004610067
ENSMUSG00000020721	Helz	helicase with zinc finger domain [Source:MGI Symbol;Acc:MGI:1925705]	3.35922	3.17113	3.23472	3.51837	3.09701	3.45529	3.5797	3.15874	3.33283	3.97456	3.20844	3.58684	3.53069	3.51481	0.475540699	0.966018836	4	4	24	0.925924441	0.980240357	24	15.47239793	0.010896388
ENSMUSG00000020722	Cacng1	calcium channel, voltage-dependent, gamma subunit 1 [Source:MGI Symbol;Acc:MGI:1206582]	0.913502	2.39705	2.62342	3.44431	1.78187	2.72364	2.6323	6.9792	4.796	5.43093	2.11419	1.93041	2.67386	1.93065	0.56005262	0.966018836	16	4	24	0.094504648	0.71175955	24	8.829926189	0.436286395
ENSMUSG00000020723	Cacng4	calcium channel, voltage-dependent, gamma subunit 4 [Source:MGI Symbol;Acc:MGI:1859167]	1.28055	1.53956	2.6537	1.27316	1.57283	1.54548	1.5659	1.18368	1.81913	1.73824	1.35224	1.62447	1.24307	1.48081	0.175612094	0.651716826	12	20	24	0.327361212	0.768040178	24	9.842127959	0.1101495
ENSMUSG00000020728	Cep112	centrosomal protein 112 [Source:MGI Symbol;Acc:MGI:1923673]	2.03919	3.54242	3.7805	3.19117	3.28732	2.27569	3.72399	2.42384	3.51323	3.3596	2.2405	2.90737	3.6895	3.24067	0.601647512	0.966018836	12	12	24	0.250162162	0.768040178	24	7.701199285	0.14926628
ENSMUSG00000020732	Rab37	RAB37, member RAS oncogene family [Source:MGI Symbol;Acc:MGI:1929945]	19.7122	19.2232	18.7902	19.2902	17.297	20.3789	17.1365	18.5716	18.124	20.9278	18.5773	20.8712	18.9688	19.9998	0.929527443	0.966018836	24	12	24	0.961968583	0.9903359	24	17.66320083	0.009651153
ENSMUSG00000020733	Slc9a3r1	solute carrier family 9 (sodium/hydrogen exchanger), member 3 regulator 1 [Source:MGI Symbol;Acc:MGI:1349482]	24.8221	21.4427	21.4577	23.7859	23.2037	24.4878	24.7835	22.195	22.2957	29.4021	25.7124	25.3089	23.0153	23.2649	0.034036381	0.327268552	16	20	24	0.096323653	0.71175955	24	16.65459461	0.089238991
ENSMUSG00000020736	Nt5c	5',3'-nucleotidase, cytosolic [Source:MGI Symbol;Acc:MGI:1354954]	52.7708	47.1822	45.3656	45.5219	48.9222	49.4234	47.6067	47.1834	50.9299	45.8308	58.1014	47.9205	54.3729	57.0181	0.339604637	0.858712466	20	20	24	0.536596012	0.827423907	24	22.58945082	0.045056119
ENSMUSG00000020737	Jpt1	Jupiter microtubule associated homolog 1 [Source:MGI Symbol;Acc:MGI:1096361]	70.5942	60.3308	64.1139	58.873	61.6112	68.567	61.111	65.2284	53.8403	61.1243	68.804	59.7329	64.8674	63.4207	0.60734371	0.966018836	4	12	24	0.172119768	0.768040178	24	21.30205191	0.074240606
ENSMUSG00000020738	Sumo2	small ubiquitin-like modifier 2 [Source:MGI Symbol;Acc:MGI:2158813]	0.552206	0.876971	0.976823	1.0805	0.88991	0.781868	0.589101	1.47447	1.39707	0.55667	1.25134	0.628247	1.2768	0.893218	0.57656008	0.966018836	12	12	24	0.314549885	0.768040178	24	8.140969384	0.138846471
ENSMUSG00000020739	Nup85	nucleoporin 85 [Source:MGI Symbol;Acc:MGI:3046173]	19.8059	20.3139	20.8743	22.6159	23.468	20.2432	19.888	21.3399	20.9009	18.0418	21.8568	19.2021	20.3384	19.1628	0.371875382	0.894537464	20	4	24	0.406812674	0.799546792	24	14.20360397	0.046320426
ENSMUSG00000020740	Gga3	golgi associated, gamma adaptin ear containing, ARF binding protein 3 [Source:MGI Symbol;Acc:MGI:2384159]	15.052	13.3891	15.9111	14.5873	15.5339	15.9296	14.7587	16.0806	17.0983	17.5765	13.4498	15.1637	15.9427	13.8379	0.890235289	0.966018836	12	20	24	0.668647855	0.888027111	24	10.79180543	0.040487886
ENSMUSG00000020741	Cluh	clustered mitochondria (cluA/CLU1) homolog [Source:MGI Symbol;Acc:MGI:1921398]	14.2182	12.682	14.2195	15.7204	14.2897	14.9717	13.9269	15.4696	12.7136	20.1232	12.5016	14.6807	15.3704	13.3556	0.879844076	0.966018836	16	4	24	0.710911541	0.904531083	24	12.00006447	0.052059
ENSMUSG00000020743	Mif4gd	MIF4G domain containing [Source:MGI Symbol;Acc:MGI:1916924]	11.487	12.3304	11.9737	13.9191	12.6567	12.8611	12.3185	15.0169	13.7716	13.9708	14.3642	14.0961	15.0344	13.879	0.860560779	0.966018836	16	20	24	0.501932859	0.809205492	24	10.82478185	0.033658821
ENSMUSG00000020744	Slc25a19	solute carrier family 25 (mitochondrial thiamine pyrophosphate carrier), member 19 [Source:MGI Symbol;Acc:MGI:1914533]	20.2183	17.4288	18.8276	25.2723	19.4816	22.4882	20.7621	21.5709	17.9975	21.2791	22.3531	20.3696	23.0415	21.1038	0.708665292	0.966018836	16	20	24	0.405735182	0.799546792	24	17.33520492	0.070258364
ENSMUSG00000020745	Pafah1b1	platelet-activating factor acetylhydrolase, isoform 1b, subunit 1 [Source:MGI Symbol;Acc:MGI:109520]	223.665	211.311	202.056	206.494	210.407	207.833	199.006	190.173	190.721	203.92	222.133	214.918	214.882	221.854	0.242077645	0.741580974	24	12	24	0.239273873	0.768040178	24	19.98591819	0.050062036
ENSMUSG00000020747	Tmem94	transmembrane protein 94 [Source:MGI Symbol;Acc:MGI:1919197]	7.76554	6.90551	8.06485	7.03913	7.64813	7.58099	7.10543	8.43229	7.16589	10.0866	6.94123	8.40693	7.69994	7.65433	0.764371375	0.966018836	24	20	24	0.809019801	0.939184036	24	11.43645774	0.033550941
ENSMUSG00000020752	Recql5	RecQ protein-like 5 [Source:MGI Symbol;Acc:MGI:2156841]	6.27758	6.99507	7.73795	6.0161	7.59533	6.72078	7.48827	6.35588	6.39844	6.82439	6.64935	6.57099	5.88247	6.50192	0.935593607	0.966018836	20	8	24	0.849813927	0.954851064	24	14.12731439	0.022449342
ENSMUSG00000020755	Sap30bp	SAP30 binding protein [Source:MGI Symbol;Acc:MGI:1927479]	12.7551	13.7504	16.301	13.9015	14.4538	13.7228	12.5579	12.7632	13.3535	14.7823	11.7102	14.4449	15.3952	17.6317	0.851656735	0.966018836	16	12	24	0.448393496	0.805393068	24	7.079510449	0.073362371
ENSMUSG00000020758	Itgb4	integrin beta 4 [Source:MGI Symbol;Acc:MGI:96613]	14.2358	13.7833	14.3386	14.8572	17.4791	13.467	16.3951	15.4707	18.0435	18.6974	15.1724	15.4013	15.6497	13.3381	0.830364743	0.966018836	16	16	24	0.253011033	0.768040178	24	12.62424486	0.088836911
ENSMUSG00000020766	Galk1	galactokinase 1 [Source:MGI Symbol;Acc:MGI:95730]	5.32852	6.05372	5.24172	5.24932	5.55836	4.39742	5.33884	5.72555	7.48426	6.28062	5.82791	6.25113	6.40715	5.99951	0.832742389	0.966018836	8	16	24	0.178513523	0.768040178	24	7.609295076	0.097162735
ENSMUSG00000020770	Unk	unkempt family zinc finger [Source:MGI Symbol;Acc:MGI:2442456]	4.87491	5.26946	5.78204	5.97976	6.19425	5.1972	5.07617	6.08548	6.39124	6.4545	5.25593	6.03767	5.89154	5.8453	0.064091035	0.414867207	16	12	24	0.020388092	0.565578297	24	10.44352319	0.099440896
ENSMUSG00000020773	Trim47	tripartite motif-containing 47 [Source:MGI Symbol;Acc:MGI:1917374]	3.01743	3.36868	2.61138	3.79851	3.35701	4.13034	3.94649	4.48156	3.85309	2.81349	3.36619	3.25898	2.87073	2.67351	0.817383384	0.966018836	24	16	24	0.847688727	0.954045905	24	20.31358508	0.04494551
ENSMUSG00000020774	Aspa	aspartoacylase [Source:MGI Symbol;Acc:MGI:87914]	53.2649	69.3082	58.523	53.2805	74.3816	54.8504	64.5957	58.9616	71.5895	46.9349	73.3848	59.0152	66.1557	56.382	0.890235289	0.966018836	24	20	24	0.954126138	0.988601783	24	20.84351407	0.025645951
ENSMUSG00000020775	Mrpl38	mitochondrial ribosomal protein L38 [Source:MGI Symbol;Acc:MGI:1926269]	20.582	15.4056	18.2126	17.2333	15.0836	19.1952	16.3434	19.5445	17.7465	24.1436	20.2416	19.4328	19.8123	18.5306	0.866861748	0.966018836	16	20	24	0.912287711	0.975295827	24	14.35809081	0.026702531
ENSMUSG00000020776	Fbf1	Fas (TNFRSF6) binding factor 1 [Source:MGI Symbol;Acc:MGI:1922033]	11.7857	10.6605	10.0241	11.2135	9.74718	11.3486	12.5029	11.4602	10.4378	11.9542	9.98481	10.6231	11.1983	10.2261	0.143673396	0.585944911	4	16	24	0.408040304	0.799546792	24	23.92202022	0.051897668
ENSMUSG00000020777	Acox1	acyl-Coenzyme A oxidase 1, palmitoyl [Source:MGI Symbol;Acc:MGI:1330812]	46.1722	38.374	42.9835	48.7067	41.524	47.9003	45.7189	48.1088	37.0771	55.7138	43.8255	44.8804	45.203	45.849	0.941955432	0.966018836	16	20	24	0.724975845	0.909665611	24	16.61573646	0.045699133
ENSMUSG00000020778	Ten1	TEN1 telomerase capping complex subunit [Source:MGI Symbol;Acc:MGI:1916785]	26.2947	23.0753	23.9385	26.2246	24.43	24.9825	21.9073	25.127	26.3741	24.06	24.262	26.9836	24.2206	26.1742	0.846643334	0.966018836	24	4	24	0.900659035	0.97190491	24	12.72766875	0.014689398
ENSMUSG00000020780	Srp68	signal recognition particle 68 [Source:MGI Symbol;Acc:MGI:1917447]	26.0873	22.409	23.7597	24.4295	22.7463	25.3907	24.4137	25.1846	21.5448	26.4572	21.6864	24.1606	22.5453	23.1228	0.891105762	0.966018836	24	12	24	0.788930745	0.93231806	24	20.90168485	0.025508228
ENSMUSG00000020781	Tsen54	tRNA splicing endonuclease subunit 54 [Source:MGI Symbol;Acc:MGI:1923515]	4.74155	3.97831	4.79743	5.00416	4.725	4.78214	5.25028	4.09546	4.18456	5.44688	4.9642	5.7091	5.19482	5.29487	0.669104912	0.966018836	24	8	24	0.193541227	0.768040178	24	17.86399295	0.078097093
ENSMUSG00000020782	Llgl2	LLGL2 scribble cell polarity complex component [Source:MGI Symbol;Acc:MGI:1918843]	0.822855	0.610571	0.735415	0.361976	0.368438	0.785991	1.01226	1.05836	0.799389	0.722478	0.57964	0.895186	0.893011	1.02198	0.016785747	0.253395174	8	8	24	0.001719841	0.374946468	24	2.076644047	0.186574409
ENSMUSG00000020783	Ncbp3	nuclear cap binding subunit 3 [Source:MGI Symbol;Acc:MGI:1914124]	3.1044	3.30698	3.12623	3.37059	3.25379	3.23071	3.28652	3.20607	3.16112	3.25391	3.18857	3.31665	3.16981	3.14172	0.595037318	0.966018836	16	20	24	0.41953195	0.803639585	24	16.0136214	0.013953974
ENSMUSG00000020785	Camkk1	calcium/calmodulin-dependent protein kinase kinase 1, alpha [Source:MGI Symbol;Acc:MGI:1891766]	8.34419	8.34444	10.7324	8.63198	7.25725	9.57428	9.10635	7.85284	7.85774	9.91085	7.43742	8.21233	7.44941	9.20892	0.602156008	0.966018836	16	4	24	0.623930981	0.871879816	24	7.823564503	0.060086511
ENSMUSG00000020788	Atp2a3	ATPase, Ca++ transporting, ubiquitous [Source:MGI Symbol;Acc:MGI:1194503]	1.83021	2.58338	3.49812	3.55174	2.69256	2.55587	2.26994	3.37139	3.50673	2.62562	2.47375	2.75872	3.01818	2.25345	0.180336821	0.657605589	12	16	24	0.077660334	0.687420473	24	9.75321719	0.174749482
ENSMUSG00000020790	Ankfy1	ankyrin repeat and FYVE domain containing 1 [Source:MGI Symbol;Acc:MGI:1337008]	17.3388	15.8835	15.5062	15.5154	15.9813	16.7671	14.9533	15.6341	14.6264	15.3407	15.8929	16.1941	16.9647	16.4304	0.018263889	0.256345714	4	8	24	0.052796054	0.650526959	24	21.74677061	0.058166215
ENSMUSG00000020792	Exoc7	exocyst complex component 7 [Source:MGI Symbol;Acc:MGI:1859270]	24.364	24.8955	24.7398	23.7101	24.0141	25.7257	21.2378	23.5437	22.0407	26.9289	19.3692	23.8065	22.1828	23.0529	0.880631923	0.966018836	16	12	24	0.909227683	0.974606327	24	9.831424912	0.019375508
ENSMUSG00000020793	Galr2	galanin receptor 2 [Source:MGI Symbol;Acc:MGI:1337018]	0.618241	0.43622	0.492645	0.440489	0.451123	0.520139	0.766662	0.474387	0.442766	0.531873	0.60821	0.597308	0.570344	0.448731	0.031295391	0.324385159	4	4	24	0.062590631	0.661961741	24	21.20546242	0.076588659
ENSMUSG00000020794	Ube2g1	ubiquitin-conjugating enzyme E2G 1 [Source:MGI Symbol;Acc:MGI:1914378]	14.0143	14.7423	15.3635	14.0591	14.4183	13.9335	15.342	16.1193	14.6408	16.8197	15.0428	14.0033	14.0235	15.3353	0.061697418	0.409814092	8	16	24	0.121526056	0.733843269	24	8.256043644	0.061507636
ENSMUSG00000020801	Med31	mediator complex subunit 31 [Source:MGI Symbol;Acc:MGI:1914529]	15.4805	15.8327	14.0335	14.2091	15.3291	13.4199	14.9852	15.337	17.0052	14.7237	15.9506	15.5715	16.2267	15.4509	0.935543173	0.966018836	4	12	24	0.565629499	0.843471947	24	4.124722508	0.033026358
ENSMUSG00000020802	Ube2o	ubiquitin-conjugating enzyme E2O [Source:MGI Symbol;Acc:MGI:2444266]	27.8517	25.2342	24.4972	25.806	24.1656	29.8575	25.895	27.3585	24.4575	35.4599	25.3414	30.1001	28.3574	30.9247	0.338172159	0.85758369	24	12	24	0.909517664	0.974606327	24	20.98463388	0.020015073
ENSMUSG00000020803	Txndc17	thioredoxin domain containing 17 [Source:MGI Symbol;Acc:MGI:1289248]	100.74	100.485	97.3847	94.2851	106.29	92.0724	88.0577	91.1782	97.3211	82.0728	108.792	97.361	104.018	98.5752	0.503972924	0.966018836	20	20	24	0.751632024	0.919304206	24	19.88372092	0.03424748
ENSMUSG00000020806	Rhbdf2	rhomboid 5 homolog 2 [Source:MGI Symbol;Acc:MGI:2442473]	3.33394	3.57085	3.35073	4.84726	3.44071	3.3794	3.78558	3.89575	4.56325	4.44623	3.64753	3.60566	3.45689	3.01888	0.435739761	0.966018836	16	12	24	0.08055023	0.695795041	24	11.26603088	0.127960486
ENSMUSG00000020807	4933427D14Rik	RIKEN cDNA 4933427D14 gene [Source:MGI Symbol;Acc:MGI:1921727]	1.43769	1.96298	2.36849	3.32759	1.36494	1.27588	1.00138	2.23185	2.00874	1.27112	1.95753	2.37312	2.1016	1.00103	0.920858634	0.966018836	12	16	24	0.672143879	0.889365603	24	11.82703669	0.128393835
ENSMUSG00000020810	Cygb	cytoglobin [Source:MGI Symbol;Acc:MGI:2149481]	13.3118	14.3345	17.4272	15.1658	11.2604	15.2978	15.1802	15.0743	16.287	17.6991	11.2333	14.4338	13.2841	16.0414	0.032211002	0.324385159	16	4	24	0.071258134	0.676901434	24	7.058698579	0.162217092
ENSMUSG00000020811	Wscd1	WSC domain containing 1 [Source:MGI Symbol;Acc:MGI:2448493]	1.36703	3.25104	5.51615	2.14292	1.78999	2.49676	3.40044	2.34936	3.57598	2.17542	1.1805	2.40515	1.00274	3.50636	0.036424767	0.332409444	12	8	24	0.094991601	0.71175955	24	6.883538673	0.380379738
ENSMUSG00000020812	Snhg16	small nucleolar RNA host gene 16 [Source:MGI Symbol;Acc:MGI:1913543]	2.02232	2.93438	2.73011	3.17045	2.89075	2.64054	2.48898	3.36811	2.93444	3.35451	3.88123	3.01878	3.54821	2.70868	0.718086778	0.966018836	16	12	24	0.145464297	0.754184974	24	12.30428958	0.107546297
ENSMUSG00000020814	Mxra7	matrix-remodelling associated 7 [Source:MGI Symbol;Acc:MGI:1914872]	13.4342	11.8997	11.4907	11.7734	12.1762	11.4822	13.4588	12.3232	12.1603	12.9411	11.1359	13.0065	13.5478	11.8419	0.052640391	0.391647859	4	16	24	0.188436028	0.768040178	24	23.62396806	0.059876358
ENSMUSG00000020817	Rabep1	rabaptin, RAB GTPase binding effector protein 1 [Source:MGI Symbol;Acc:MGI:1860236]	33.1764	33.6271	31.6099	32.4737	33.8987	31.9605	34.1085	30.1779	33.9357	31.1958	35.765	33.9682	35.745	34.3002	0.714044782	0.966018836	4	12	24	0.445715491	0.805393068	24	20.70332233	0.033274817
ENSMUSG00000020818	Mfsd11	major facilitator superfamily domain containing 11 [Source:MGI Symbol;Acc:MGI:1917150]	20.0495	16.3301	17.1691	13.9394	15.9076	18.4133	18.1577	19.3972	14.5808	22.6527	16.9936	17.2091	17.7759	16.943	0.313530643	0.824470524	4	8	24	0.60980744	0.864530314	24	22.9255158	0.068120149
ENSMUSG00000020821	Kif1c	kinesin family member 1C [Source:MGI Symbol;Acc:MGI:1098260]	11.9568	13.7042	15.474	15.3	13.8914	13.3864	15.9544	18.012	18.0712	21.1818	14.2263	14.5753	15.4119	14.3149	0.191659183	0.669841558	12	12	24	0.043012141	0.625007356	24	9.324099062	0.170374068
ENSMUSG00000020823	Sec14l1	SEC14-like lipid binding 1 [Source:MGI Symbol;Acc:MGI:1921386]	17.7321	15.8667	16.1109	19.0365	15.9038	16.5499	18.2034	17.3256	17.2415	19.5866	17.5518	16.7222	16.2513	16.8482	0.932092277	0.966018836	16	16	24	0.470069416	0.805393068	24	12.20237266	0.042690475
ENSMUSG00000020827	Mink1	misshapen-like kinase 1 (zebrafish) [Source:MGI Symbol;Acc:MGI:1355329]	49.5178	45.6444	44.4096	42.2798	48.1293	46.3103	46.0628	49.8881	45.3648	54.7843	42.5073	46.8145	46.8403	44.6114	0.668329321	0.966018836	4	8	24	0.954558672	0.988601783	24	23.3579142	0.011937201
ENSMUSG00000020828	Pld2	phospholipase D2 [Source:MGI Symbol;Acc:MGI:892877]	10.0645	7.4314	8.71373	8.04557	7.841	8.99336	8.93567	8.24916	8.59375	8.28418	7.8899	7.05009	7.76752	6.77634	0.920872048	0.966018836	12	20	24	0.813511196	0.940453305	24	21.28456201	0.028921188
ENSMUSG00000020829	Slc46a1	solute carrier family 46, member 1 [Source:MGI Symbol;Acc:MGI:1098733]	10.5302	7.13571	7.99494	8.03944	8.81073	10.1126	10.3064	9.18245	7.43761	10.7385	8.02203	9.24971	8.34271	8.70136	0.138543921	0.580158868	24	12	24	0.229673644	0.768040178	24	20.42059853	0.115736159
ENSMUSG00000020831	0610010K14Rik	RIKEN cDNA 0610010K14 gene [Source:MGI Symbol;Acc:MGI:1915609]	13.853	16.6279	19.6252	14.0226	16.6957	17.5028	16.9996	18.7232	17.215	15.0051	15.3266	16.7685	17.1402	16.371	0.369262716	0.893596186	12	4	24	0.453048339	0.805393068	24	4.244751948	0.066050943
ENSMUSG00000020832	Eral1	Era (G-protein)-like 1 (E. coli) [Source:MGI Symbol;Acc:MGI:1889295]	11.1811	10.5977	9.6405	10.0167	10.4816	11.8755	9.39781	10.4323	9.7088	10.881	11.2489	10.3705	10.6779	10.5841	0.308023687	0.814993005	24	12	24	0.20902905	0.768040178	24	19.25318767	0.059266874
ENSMUSG00000020834	Dhrs13	dehydrogenase/reductase (SDR family) member 13 [Source:MGI Symbol;Acc:MGI:1917701]	4.275	3.98213	4.11155	3.37474	3.91943	3.89153	3.90374	4.076	3.18596	3.62257	3.91848	4.04517	3.81899	3.94355	0.595952838	0.966018836	8	8	24	0.066807825	0.669146244	24	22.89586422	0.075213302
ENSMUSG00000020836	Coro6	coronin 6 [Source:MGI Symbol;Acc:MGI:2183448]	13.1877	12.2729	14.2338	13.8497	12.179	13.3595	12.6626	14.9516	14.4539	18.6805	12.5094	13.991	14.1185	16.2945	0.205280539	0.693330038	16	4	24	0.098453417	0.71175955	24	8.470101826	0.115427747
ENSMUSG00000020839	Tmigd1	transmembrane and immunoglobulin domain containing 1 [Source:MGI Symbol;Acc:MGI:1913851]	1.41781	1.68607	1.10442	1.34861	1.41093	1.6721	1.77275	1.23352	2.30217	1.52751	1.36336	1.25859	1.89513	1.89761	0.590547452	0.966018836	4	16	24	0.461110478	0.805393068	24	3.717173324	0.082206312
ENSMUSG00000020840	Blmh	bleomycin hydrolase [Source:MGI Symbol;Acc:MGI:1345186]	24.7781	24.1314	23.6827	22.868	24.6715	25.2752	23.8053	25.3472	21.9662	24.6027	23.5384	24.2325	25.2518	25.1768	0.152197148	0.600454138	8	4	24	0.125324814	0.739181992	24	22.81220702	0.044081495
ENSMUSG00000020841	Cpd	carboxypeptidase D [Source:MGI Symbol;Acc:MGI:107265]	30.2136	29.8436	29.255	28.3261	31.6908	29.357	30.3934	27.2697	28.1704	26.3041	31.1265	29.0872	28.6703	28.1849	0.048507657	0.379129806	20	20	24	0.072670648	0.679705968	24	19.66449409	0.057653715
ENSMUSG00000020843	Timm22	translocase of inner mitochondrial membrane 22 [Source:MGI Symbol;Acc:MGI:1929742]	23.3238	23.0837	22.9861	22.7577	20.5246	20.1699	24.5037	22.3604	18.5042	20.6551	20.8372	21.026	20.1126	20.685	0.891105762	0.966018836	4	20	24	0.36582746	0.789934347	24	1.22417451	0.046910818
ENSMUSG00000020844	Nxn	nucleoredoxin [Source:MGI Symbol;Acc:MGI:109331]	11.5997	9.8184	9.44608	10.4667	11.1839	11.4187	10.6272	12.8477	9.61512	10.7694	10.7886	10.3135	9.89764	10.1784	0.137763286	0.580158868	24	12	24	0.444997509	0.805393068	24	20.26625365	0.057055735
ENSMUSG00000020846	Rflnb	refilin B [Source:MGI Symbol;Acc:MGI:1923816]	4.85571	4.13288	4.08953	4.77433	3.9269	4.98457	4.83979	5.21718	3.77087	4.98535	4.00135	4.78097	5.37772	4.59234	0.108029159	0.524035616	4	8	24	0.225777369	0.768040178	24	23.28155683	0.087513446
ENSMUSG00000020847	Rph3al	rabphilin 3A-like (without C2 domains) [Source:MGI Symbol;Acc:MGI:1923492]	0.233412	0.297016	0.836287	0.523466	0.67432	0.510749	0.588103	0.566612	0.394753	0.537935	0.394875	0.469366	0.387499	0.791338	0.895493752	0.966018836	12	16	24	0.46625687	0.805393068	24	9.918533634	0.080626221
ENSMUSG00000020848	Doc2b	double C2, beta [Source:MGI Symbol;Acc:MGI:1100497]	0.310939	0.355084	0.553881	0.544188	0.277089	0.684421	0.760568	0.245825	0.484113	0.665755	0.403922	0.704985	0.343779	1.51692	0.873350015	0.966018836	24	4	24	0.822894844	0.944401121	24	5.795670305	0.060698601
ENSMUSG00000020849	Ywhae	tyrosine 3-monooxygenase/tryptophan 5-monooxygenase activation protein, epsilon polypeptide [Source:MGI Symbol;Acc:MGI:894689]	302.61	251.519	260.744	255.158	247.458	274.837	279.682	265.893	226.626	296.338	247.874	267.787	258.633	266.904	0.20729371	0.698501896	4	16	24	0.370085451	0.792235171	24	22.18960609	0.06031633
ENSMUSG00000020850	Prpf8	pre-mRNA processing factor 8 [Source:MGI Symbol;Acc:MGI:2179381]	23.2018	19.5564	21.5923	21.7058	19.7813	22.9143	23.2757	24.1714	17.9638	26.9889	17.2869	21.2788	20.1748	21.1461	0.935593607	0.966018836	4	16	24	0.906296659	0.97449058	24	1.35414677	0.028728497
ENSMUSG00000020857	Nme2	NME/NM23 nucleoside diphosphate kinase 2 [Source:MGI Symbol;Acc:MGI:97356]	33.2211	36.1142	33.9121	34.2943	40.793	33.6285	31.5624	38.9371	40.7578	34.2802	43.8214	34.3703	40.8857	33.4081	0.5169953	0.966018836	20	8	24	0.610502523	0.864861374	24	13.17810852	0.053956572
ENSMUSG00000020859	Spag9	sperm associated antigen 9 [Source:MGI Symbol;Acc:MGI:1918084]	83.2161	92.2341	88.7295	90.4212	89.8299	81.8706	91.4559	79.3288	93.9982	83.3141	87.5262	86.7106	87.451	90.0974	0.601647512	0.966018836	12	12	24	0.593524731	0.85692808	24	8.711505565	0.029904264
ENSMUSG00000020863	Luc7l3	LUC7-like 3 (S. cerevisiae) [Source:MGI Symbol;Acc:MGI:1914934]	48.5898	53.497	52.6654	50.3053	52.6177	47.3687	49.5026	45.0468	56.5235	44.5155	57.0395	46.8723	49.9758	52.3133	0.390686537	0.915479136	12	12	24	0.651903671	0.882906761	24	9.907053865	0.041254065
ENSMUSG00000020864	Ankrd40	ankyrin repeat domain 40 [Source:MGI Symbol;Acc:MGI:1918702]	41.6524	37.8455	42.2152	42.8583	42.1918	43.4637	44.145	44.0215	39.8154	51.1645	39.0431	43.3393	46.0335	40.6003	0.723119329	0.966018836	4	4	24	0.815438658	0.94108559	24	15.8955593	0.026120546
ENSMUSG00000020865	Abcc3	ATP-binding cassette, sub-family C (CFTR/MRP), member 3 [Source:MGI Symbol;Acc:MGI:1923658]	0.690834	0.787652	0.939983	1.12385	1.01152	0.547075	0.580304	0.632545	0.603059	0.485511	0.684911	0.748553	0.792169	0.639765	0.813775138	0.966018836	20	4	24	0.5669781	0.843665475	24	13.23714012	0.057371645
ENSMUSG00000020866	Cacna1g	calcium channel, voltage-dependent, T type, alpha 1G subunit [Source:MGI Symbol;Acc:MGI:1201678]	2.63013	3.24908	5.50433	2.02234	3.12279	2.79707	5.95007	3.5508	5.09896	2.50623	2.12611	2.40929	1.48888	4.44051	0.116913252	0.538362324	12	4	24	0.165406001	0.767020009	24	5.776856709	0.320780981
ENSMUSG00000020868	Xylt2	xylosyltransferase II [Source:MGI Symbol;Acc:MGI:2444797]	8.98881	9.19577	10.6382	9.39466	9.16468	9.82101	9.57777	9.92754	10.7604	11.6686	9.45234	11.1018	10.6854	10.7762	0.928127947	0.966018836	16	4	24	0.230277481	0.768040178	24	8.634604584	0.055667297
ENSMUSG00000020869	Lrrc59	leucine rich repeat containing 59 [Source:MGI Symbol;Acc:MGI:2138133]	52.4735	39.5516	39.3673	41.8065	41.2845	47.6096	42.2715	44.9791	32.0225	48.8044	41.4174	44.5119	43.411	42.6351	0.143321297	0.585944911	24	12	24	0.152187438	0.759088284	24	20.70706556	0.123728512
ENSMUSG00000020870	Cdc34b	cell division cycle 34B [Source:MGI Symbol;Acc:MGI:1858429]	1.11265	1.01844	1.33137	1.40679	1.25198	1.07614	1.13208	0.864643	0.861993	1.12699	0.919603	0.788604	0.775693	0.855738	0.454881285	0.966018836	16	16	24	0.042663053	0.624181317	24	13.21624713	0.085530035
ENSMUSG00000020873	Slc35b1	solute carrier family 35, member B1 [Source:MGI Symbol;Acc:MGI:1343133]	37.0623	34.202	33.4268	31.4471	35.4842	33.4411	32.72	33.8189	33.7729	30.3223	36.6682	32.7039	34.4329	32.4271	0.184631358	0.662239322	4	12	24	0.303726276	0.768040178	24	21.53604979	0.045811346
ENSMUSG00000020875	Hoxb9	homeobox B9 [Source:MGI Symbol;Acc:MGI:96190]	3.74196	5.14813	6.46627	3.64347	3.89182	5.09948	5.44654	4.39256	4.72546	4.99197	3.11728	4.78929	2.98571	5.65098	0.496745696	0.966018836	12	8	24	0.323706932	0.768040178	24	5.985884283	0.157896117
ENSMUSG00000020876	Snx11	sorting nexin 11 [Source:MGI Symbol;Acc:MGI:1921729]	28.4246	24.4463	25.0563	27.2287	26.9438	26.1062	24.7352	25.9671	26.4514	31.063	27.4563	27.4655	27.9102	25.1792	0.16079538	0.620479606	16	16	24	0.165627356	0.767020009	24	15.28553375	0.06160921
ENSMUSG00000020877	Scrn2	secernin 2 [Source:MGI Symbol;Acc:MGI:1343092]	3.03366	2.6057	2.66929	2.3526	2.70386	2.82642	2.68858	3.20552	3.03234	3.40552	2.82314	2.97654	2.42252	2.29635	0.865514257	0.966018836	24	8	24	0.852959791	0.956025132	24	14.64982242	0.027563728
ENSMUSG00000020878	Lrrc46	leucine rich repeat containing 46 [Source:MGI Symbol;Acc:MGI:1916547]	0.913114	0.825624	1.32449	0.933474	0.685391	1.11667	0.619495	0.879746	0.693718	1.36269	1.00205	1.26955	0.899654	0.880178	0.435037142	0.966018836	16	16	24	0.541623148	0.830485817	24	13.8217397	0.073587097
ENSMUSG00000020882	Cacnb1	calcium channel, voltage-dependent, beta 1 subunit [Source:MGI Symbol;Acc:MGI:102522]	13.7165	10.8825	11.1906	13.0732	11.3418	12.9146	12.2981	14.2748	12.5256	16.9846	11.7196	13.9399	11.8891	12.2457	0.916556573	0.966018836	24	12	24	0.845003131	0.953147907	24	13.65999078	0.035764909
ENSMUSG00000020883	Fbxl20	F-box and leucine-rich repeat protein 20 [Source:MGI Symbol;Acc:MGI:1919444]	11.0458	11.6698	11.7589	12.1354	11.7656	11.1191	12.1425	11.5765	11.3233	13.4481	11.705	12.5369	13.0299	11.8774	0.874607729	0.966018836	16	20	24	0.695010207	0.898633591	24	12.74364034	0.021775523
ENSMUSG00000020884	Asgr1	asialoglycoprotein receptor 1 [Source:MGI Symbol;Acc:MGI:88081]	2.3622	4.24263	2.45531	1.51293	2.20709	2.77751	2.43015	3.83975	3.74571	0.920929	1.81891	2.51492	1.90979	3.10549	0.002197392	0.103301163	8	8	24	0.04437099	0.628432994	24	3.035157543	0.3398149
ENSMUSG00000020886	Dlg4	discs large MAGUK scaffold protein 4 [Source:MGI Symbol;Acc:MGI:1277959]	30.7483	26.7775	29.3431	27.2736	27.2341	31.2412	31.0145	30.4281	27.4409	37.0577	27.4877	30.4166	26.7176	31.6335	0.84680398	0.966018836	24	20	24	0.998520608	0.999730606	24	8.949298184	0.002895734
ENSMUSG00000020887	A230052G05Rik	RIKEN cDNA A230052G05 gene [Source:MGI Symbol;Acc:MGI:3045239]	0.703468	0.537114	0.604423	0.640466	0.49236	0.771592	0.746735	1.03984	0.599828	0.976382	0.698698	0.696847	0.769582	0.528693	0.956794505	0.96629412	4	4	24	0.949180896	0.987684983	24	22.87282882	0.017034254
ENSMUSG00000020888	Dvl2	dishevelled segment polarity protein 2 [Source:MGI Symbol;Acc:MGI:106613]	4.0939	4.76391	5.8066	4.75055	4.89687	4.55568	4.88987	4.91377	6.60505	5.0983	4.96554	4.96394	4.66335	4.32981	0.017495149	0.255326033	12	16	24	0.068148133	0.672306079	24	9.885277964	0.122242043
ENSMUSG00000020889	Nr1d1	nuclear receptor subfamily 1, group D, member 1 [Source:MGI Symbol;Acc:MGI:2444210]	2.78206	14.5119	13.9528	8.65606	4.68234	2.61501	3.60936	17.5513	18.6817	14.7359	2.38981	2.91257	5.69215	14.7146	0.003384521	0.140598674	12	12	24	6.17E-06	0.027235575	24	7.512987134	1.323410839
ENSMUSG00000020893	Per1	period circadian clock 1 [Source:MGI Symbol;Acc:MGI:1098283]	4.56172	6.2502	10.8131	12.9741	7.51233	5.81062	4.94906	4.72174	14.6112	15.9783	5.53097	5.09827	3.41462	5.01577	3.19E-05	0.023823328	16	12	24	9.93E-05	0.114833119	24	11.03425532	0.749243059
ENSMUSG00000020894	Vamp2	vesicle-associated membrane protein 2 [Source:MGI Symbol;Acc:MGI:1313277]	138.516	119.493	124.54	132.176	130.583	135.894	120.803	131.767	118.631	173.272	124.043	135.635	129.534	135.834	0.745118544	0.966018836	24	12	24	0.559073981	0.840264473	24	14.44755726	0.05602426
ENSMUSG00000020895	Tmem107	transmembrane protein 107 [Source:MGI Symbol;Acc:MGI:1914160]	7.00493	5.91159	5.69031	6.50217	7.95819	6.8967	8.90124	6.45712	6.36043	5.61142	6.62318	6.48239	6.19336	6.31679	0.108215091	0.524035616	20	16	24	0.102190314	0.713413224	24	20.4961667	0.127228555
ENSMUSG00000020897	Aurkb	aurora kinase B [Source:MGI Symbol;Acc:MGI:107168]	0.261297	0.628302	0.903569	0.892805	0.494681	0.757853	0.738434	0.704011	1.31423	0.345318	0.757436	0.679906	0.860852	0.445789	0.9045879	0.966018836	12	20	24	0.614450119	0.866486296	24	9.029093838	0.091790449
ENSMUSG00000020898	Ctc1	CTS telomere maintenance complex component 1 [Source:MGI Symbol;Acc:MGI:1916214]	6.22474	8.74645	10.0896	7.46348	7.65513	7.62652	9.65191	6.54158	7.40242	9.56007	6.96225	8.15637	8.08793	6.98498	0.831272156	0.966018836	16	4	24	0.802297818	0.936565832	24	10.21860043	0.051740037
ENSMUSG00000020899	Pfas	phosphoribosylformylglycinamidine synthase (FGAR amidotransferase) [Source:MGI Symbol;Acc:MGI:2684864]	2.9923	4.40307	3.97266	3.763	4.08276	4.00878	3.88491	3.91062	4.39913	4.37446	3.60629	3.79714	3.88701	3.65427	0.714044782	0.966018836	12	16	24	0.249307918	0.768040178	24	9.837378951	0.073936102
ENSMUSG00000020900	Myh10	myosin, heavy polypeptide 10, non-muscle [Source:MGI Symbol;Acc:MGI:1930780]	29.6409	25.8742	26.6867	24.6575	23.7005	28.3849	28.9409	29.3894	22.5864	32.299	24.2559	27.801	26.0157	29.9308	0.397301892	0.927993503	8	12	24	0.440751386	0.805393068	24	1.071551099	0.071113459
ENSMUSG00000020901	Pik3r5	phosphoinositide-3-kinase regulatory subunit 5 [Source:MGI Symbol;Acc:MGI:2443588]	0.91279	0.98632	0.995942	0.803677	0.815312	0.905917	0.942926	0.820484	0.967985	0.901523	0.74502	0.865477	0.933818	0.791546	0.113424199	0.530777648	12	8	24	0.254275374	0.768040178	24	3.634959458	0.03564295
ENSMUSG00000020902	Ntn1	netrin 1 [Source:MGI Symbol;Acc:MGI:105088]	3.87994	4.66398	4.19302	3.23768	4.38234	3.74621	3.78286	4.60855	5.28288	2.36998	4.02698	4.40165	3.93744	4.21922	0.115745527	0.537734429	12	4	24	0.351518385	0.780345856	24	2.706132201	0.118697725
ENSMUSG00000020903	Stx8	syntaxin 8 [Source:MGI Symbol;Acc:MGI:1890156]	29.7026	34.8144	34.1889	30.5493	37.1372	31.1145	29.7664	32.6026	38.4284	26.4835	29.6796	33.4504	31.3417	29.3931	0.908797704	0.966018836	12	4	24	0.873000409	0.964303715	24	7.620511821	0.030215558
ENSMUSG00000020904	Cfap52	cilia and flagella associated protein 52 [Source:MGI Symbol;Acc:MGI:1919110]	0.950919	0.961555	0.987535	0.695256	0.674135	0.577393	0.546811	0.542275	0.699279	0.881041	0.658359	0.802801	0.49102	0.714288	0.612331511	0.966018836	12	16	24	0.485939531	0.805393068	24	9.297046691	0.059487129
ENSMUSG00000020910	Adprm	ADP-ribose/CDP-alcohol diphosphatase, manganese dependent [Source:MGI Symbol;Acc:MGI:1913608]	21.6093	21.2235	20.8497	21.5102	22.3176	19.743	20.2066	21.389	20.7184	16.7872	24.083	22.0823	22.6959	21.0455	0.338172159	0.85758369	20	16	24	0.578161375	0.849184181	24	21.16595641	0.048945637
ENSMUSG00000020911	Krt19	keratin 19 [Source:MGI Symbol;Acc:MGI:96693]	1.8667	2.70954	2.77107	1.16834	1.89489	2.38862	2.70046	3.21614	1.68895	0.741268	1.60098	2.81403	2.27662	2.03903	0.046535188	0.375011045	8	8	24	0.023794918	0.584955728	24	0.79924896	0.32983593
ENSMUSG00000020914	Top2a	topoisomerase (DNA) II alpha [Source:MGI Symbol;Acc:MGI:98790]	1.40917	3.22482	3.64047	3.33287	2.99108	3.95956	3.85853	2.84842	3.11387	1.83198	2.66003	2.18439	3.11224	1.73579	0.819898687	0.966018836	12	20	24	0.972884937	0.993305115	24	14.17846289	0.029537904
ENSMUSG00000020917	Acly	ATP citrate lyase [Source:MGI Symbol;Acc:MGI:103251]	184.173	138.598	153.111	175.636	154.985	181.004	168.437	174.636	122.346	185.578	182.956	172.914	183.091	171.932	0.287703522	0.793714531	16	20	24	0.182035693	0.768040178	24	19.56264584	0.124099702
ENSMUSG00000020918	Kat2a	K(lysine) acetyltransferase 2A [Source:MGI Symbol;Acc:MGI:1343101]	12.1234	10.6943	11.0466	10.9123	11.7962	11.9278	13.0592	11.6807	11.3899	12.6686	11.5503	12.0143	11.4647	11.9475	0.260713922	0.766800466	4	8	24	0.338574957	0.773151665	24	20.68547943	0.041221368

ENSMUSG00000020919	Stat5b	signal transducer and activator of transcription 5B [Source:MGI Symbol;Acc:MGI:103035]	10.3239	8.81126	9.16471	8.55121	8.78006	9.70455	9.92051	10.733	9.17663	11.7539	8.53259	8.92979	9.84853	9.6493	0.089506974	0.477279066	4	16	24	0.588104185	0.853883127	24	3.370951709	0.048991012
ENSMUSG00000020921	Tmem101	transmembrane protein 101 [Source:MGI Symbol;Acc:MGI:1923797]	12.1654	10.8279	9.91698	10.6228	10.1216	11.5599	10.989	12.8315	8.49127	11.4384	10.503	11.0196	10.9186	10.5597	0.082282215	0.462922111	24	12	24	0.18679374	0.768040178	24	22.49574119	0.090594483
ENSMUSG00000020922	Lsm12	LSM12 homolog [Source:MGI Symbol;Acc:MGI:1919592]	11.3349	9.79629	10.7524	10.4525	10.4106	11.8894	10.5858	10.8545	9.73205	12.1898	10.8211	11.4916	10.7672	11.8702	0.601647512	0.966018836	24	12	24	0.55463369	0.837869569	24	19.28291175	0.037971979
ENSMUSG00000020923	Ubtf	upstream binding transcription factor, RNA polymerase I [Source:MGI Symbol;Acc:MGI:98512]	28.873	24.4775	24.1346	25.5326	27.4585	26.3626	29.5058	25.7863	23.3387	25.1383	25.671	26.0153	25.7798	23.8898	0.002004172	0.100255953	4	8	24	0.005556428	0.44672648	24	20.61109964	0.098989359
ENSMUSG00000020925	Ccdc43	coiled-coil domain containing 43 [Source:MGI Symbol;Acc:MGI:1289318]	25.4759	25.0797	23.9298	24.4178	25.7064	25.942	23.9445	27.2145	25.039	25.4119	25.6666	26.17	27.7581	26.7327	0.160943355	0.620479606	24	12	24	0.299617612	0.768040178	24	23.51961464	0.02954005
ENSMUSG00000020926	Adam11	a disintegrin and metallopeptidase domain 11 [Source:MGI Symbol;Acc:MGI:1098667]	58.1445	52.7849	49.1657	54.4976	50.9543	52.5789	50.406	58.0466	52.9891	61.765	51.2813	57.9289	60.3917	57.6576	0.870491976	0.966018836	4	16	24	0.735211301	0.913659139	24	2.358290283	0.028759353
ENSMUSG00000020928	Higd1b	HIG1 domain family, member 1B [Source:MGI Symbol;Acc:MGI:1922939]	1.33311	2.05069	2.27726	1.83063	2.07722	2.2846	2.37437	2.56984	3.0155	1.5396	1.58072	1.64796	2.13863	2.46808	0.163947066	0.627573783	12	4	24	0.131814928	0.742139265	24	5.630296749	0.165826606
ENSMUSG00000020929	Eftud2	elongation factor Tu GTP binding domain containing 2 [Source:MGI Symbol;Acc:MGI:1336880]	17.0093	18.092	17.9722	16.1967	17.1903	18.242	16.8256	17.7947	17.3427	19.2383	19.3956	16.1207	18.4716	19.6075	0.885059765	0.966018836	8	16	24	0.633823054	0.875522631	24	7.709149973	0.031005096
ENSMUSG00000020930	Ccdc103	coiled-coil domain containing 103 [Source:MGI Symbol;Acc:MGI:1920543]	0.639063	0.797746	0.855104	0.652981	0.689258	0.922446	1.03938	0.865726	0.581692	0.768692	0.597616	0.672423	0.646063	0.72984	0.93447422	0.966018836	8	12	24	0.473915151	0.805393068	24	1.191698634	0.050388269
ENSMUSG00000020932	Gfap	glial fibrillary acidic protein [Source:MGI Symbol;Acc:MGI:95697]	17.9195	37.5521	72.1615	25.2594	23.6575	27.1582	40.3934	30.3168	52.8051	18.2508	19.0021	35.3699	11.7153	32.326	0.142513744	0.585944911	12	4	24	0.230946108	0.768040178	24	7.081686133	0.435235378
ENSMUSG00000020935	Dcakd	dephospho-CoA kinase domain containing [Source:MGI Symbol;Acc:MGI:1915337]	8.15628	8.91397	9.60636	9.11488	8.81593	10.3393	10.3148	8.75999	9.42548	10.3143	10.0355	10.1481	9.19574	9.00282	0.182849367	0.659303873	24	8	24	0.30180989	0.768040178	24	15.9107856	0.053635565
ENSMUSG00000020936	Nmt1	N-myristoyltransferase 1 [Source:MGI Symbol;Acc:MGI:102579]	38.2654	31.2721	31.3421	34.1675	35.5989	38.528	35.2967	36.3565	28.397	40.3023	33.6663	38.0622	34.1449	35.7025	0.085890569	0.466404237	24	12	24	0.132835802	0.742365977	24	19.54669139	0.103335793
ENSMUSG00000020937	Plcd3	phospholipase C, delta 3 [Source:MGI Symbol;Acc:MGI:107451]	2.63318	2.96416	3.36315	3.27998	2.66422	2.90184	2.35223	2.90115	2.84857	2.2843	3.08114	2.89744	2.70265	2.39879	0.767183862	0.966018836	12	16	24	0.583454176	0.851822355	24	12.07504494	0.047638123
ENSMUSG00000020940	Efcab15	EF-hand calcium binding domain 15 [Source:MGI Symbol;Acc:MGI:1916691]	0.612983	0.481351	0.500089	0.564358	0.477892	0.521255	0.710679	0.84899	0.733237	0.759238	0.652078	0.577124	0.746816	0.741908	0.787283372	0.966018836	8	12	24	0.15915847	0.765350752	24	5.031504793	0.060834675
ENSMUSG00000020941	Map3k14	mitogen-activated protein kinase kinase kinase 14 [Source:MGI Symbol;Acc:MGI:1858204]	1.46976	2.02491	2.06689	1.87623	2.13328	2.18817	2.12106	1.91071	2.28599	2.54698	2.0107	1.9702	2.24	1.70848	0.960269811	0.967043964	12	20	24	0.30681348	0.768040178	24	12.67369175	0.071798418
ENSMUSG00000020946	Gosr2	golgi SNAP receptor complex member 2 [Source:MGI Symbol;Acc:MGI:1927204]	35.0561	34.4114	34.4363	34.8085	35.1059	35.1	32.5653	35.0348	32.1967	33.43	33.3544	33.9568	32.6832	32.963	0.917867465	0.966018836	20	16	24	0.406952661	0.799546792	24	17.91249502	0.01785802
ENSMUSG00000020948	Klhl28	kelch-like 28 [Source:MGI Symbol;Acc:MGI:1913939]	9.59772	9.30605	8.63967	9.28137	9.31875	8.25928	8.79958	8.18205	8.26653	7.55074	10.0202	7.03873	7.44416	8.18889	0.904831057	0.966018836	20	4	24	0.769701083	0.925855377	24	14.7151075	0.030318745
ENSMUSG00000020949	Fkbp3	FK506 binding protein 3 [Source:MGI Symbol;Acc:MGI:1353460]	161.316	187.127	169.242	161.952	167.381	157.331	165.121	161.708	189.163	152.247	176.121	172.303	176.781	175.688	0.849179819	0.966018836	12	4	24	0.424882934	0.805393068	24	5.138511918	0.046199417
ENSMUSG00000020952	Scfd1	Sec1 family domain containing 1 [Source:MGI Symbol;Acc:MGI:1924233]	29.3755	33.4669	32.1838	32.867	33.7644	27.9473	27.5869	29.1566	35.8662	26.4282	35.1043	31.3865	34.5685	33.8221	0.939103211	0.966018836	12	12	24	0.65929356	0.884879972	24	9.292533918	0.051401716
ENSMUSG00000020953	Coch	cochlin [Source:MGI Symbol;Acc:MGI:1278313]	0.517993	1.06352	0.601109	0.896242	0.55902	0.345125	0.443248	0.806984	1.28501	0.296475	0.345834	0.616941	0.412544	0.587849	0.148133125	0.590834117	12	8	24	0.081218161	0.697074851	24	6.92995389	0.199356029
ENSMUSG00000020954	Strn3	striatin, calmodulin binding protein 3 [Source:MGI Symbol;Acc:MGI:2151064]	24.6544	27.6268	25.5168	27.9152	27.1284	25.1726	27.7774	26.8048	29.1056	27.3865	26.5004	26.1869	28.1853	26.9885	0.822170541	0.966018836	16	8	24	0.2432808	0.768040178	24	8.472234855	0.040553121
ENSMUSG00000020955	Ap4s1	adaptor-related protein complex AP-4, sigma 1 [Source:MGI Symbol;Acc:MGI:1337065]	27.353	28.8048	25.5796	27.0519	25.967	23.0413	26.6352	25.9823	31.9174	27.6663	30.5894	28.5532	29.608	28.6664	0.81544087	0.966018836	8	16	24	0.501417784	0.80908345	24	7.714393182	0.04980778
ENSMUSG00000020956	Dtd2	D-tyrosyl-tRNA deacylase 2 [Source:MGI Symbol;Acc:MGI:1923485]	4.00981	4.94986	4.72032	4.53811	3.95944	4.52612	4.74786	4.60055	4.73136	5.42848	5.4183	5.65148	5.67328	5.58873	0.858154388	0.966018836	8	12	24	0.725809385	0.909996439	24	6.126375997	0.028216525
ENSMUSG00000020961	Ston2	stonin 2 [Source:MGI Symbol;Acc:MGI:1918272]	5.67498	5.02299	5.55583	5.62409	5.15715	5.43254	5.36927	5.18918	5.43087	6.90584	4.55256	5.18248	5.36458	5.49456	0.187699199	0.662239322	16	4	24	0.529259315	0.824360771	24	9.515385783	0.051123525
ENSMUSG00000020962	Gtf2a1	general transcription factor II A, 1 [Source:MGI Symbol;Acc:MGI:1933277]	9.50867	10.1606	10.0089	9.63357	9.43333	9.35265	8.68858	9.36239	9.75763	9.54102	10.3916	9.70072	9.92337	10.2532	0.593560247	0.966018836	12	12	24	0.456258265	0.805393068	24	9.099979249	0.029981668
ENSMUSG00000020964	Sel1l	sel-1 suppressor of lin-12-like (C. elegans) [Source:MGI Symbol;Acc:MGI:1329016]	26.406	21.3628	21.5471	22.1246	21.8548	23.7837	26.0066	22.5894	19.4921	26.38	21.313	22.4347	22.0853	21.9891	0.170659409	0.643496587	4	8	24	0.333480258	0.769685083	24	21.19668213	0.072746666
ENSMUSG00000020973	Dnaaf2	dynein, axonemal assembly factor 2 [Source:MGI Symbol;Acc:MGI:1923566]	0.798135	1.06015	0.885933	1.18043	0.871211	0.733178	0.99397	0.823606	1.03911	1.39645	1.1952	0.940052	0.950517	1.08419	0.10951302	0.524035616	16	8	24	0.044384705	0.628432994	24	10.94014825	0.108186772
ENSMUSG00000020974	Pole2	polymerase (DNA directed), epsilon 2 (p59 subunit) [Source:MGI Symbol;Acc:MGI:1197514]	0.697981	0.94381	0.70596	0.952682	1.09384	1.10353	0.777653	1.19307	0.995487	0.853559	1.03439	1.05615	0.818595	0.986292	0.119293142	0.542554768	24	4	24	0.642300869	0.878809617	24	15.5182892	0.040901548
ENSMUSG00000020978	Klhdc2	kelch domain containing 2 [Source:MGI Symbol;Acc:MGI:1916804]	84.6106	77.6981	74.5509	75.4236	70.613	75.7353	75.1953	76.4646	74.9059	83.1456	82.1892	84.8603	85.1787	87.3387	0.46238066	0.966018836	8	4	24	0.424385526	0.805393068	24	0.367855629	0.03982973
ENSMUSG00000020982	Nemf	nuclear export mediator factor [Source:MGI Symbol;Acc:MGI:1918305]	27.7562	30.4235	28.3029	27.5103	28.3371	26.2693	27.6837	28.3265	32.6492	26.8195	30.9926	30.2596	31.0828	30.8805	0.865233232	0.966018836	12	4	24	0.416570049	0.801257196	24	5.58183741	0.042427933
ENSMUSG00000020986	Sec23a	SEC23 homolog A, COPII coat complex component [Source:MGI Symbol;Acc:MGI:1349635]	43.6505	41.1678	40.4546	41.0136	42.2309	40.6503	41.7167	42.0822	40.443	41.0054	44.6939	42.483	43.8029	43.0898	0.058157448	0.40386543	4	8	24	0.202245859	0.768040178	24	21.35609024	0.029110516
ENSMUSG00000020988	L2hgdh	L-2-hydroxyglutarate dehydrogenase [Source:MGI Symbol;Acc:MGI:2384968]	2.65291	2.78405	3.1046	3.62348	3.02236	2.91187	3.10689	3.38266	3.31829	3.15835	3.25383	3.19188	3.34726	3.07471	0.858269825	0.966018836	16	12	24	0.135401054	0.747435826	24	10.9582966	0.059336126
ENSMUSG00000020990	Cdkl1	cyclin-dependent kinase-like 1 (CDC2-related kinase) [Source:MGI Symbol;Acc:MGI:1918341]	5.22902	6.39689	6.56619	7.21952	5.35275	6.44746	5.07939	4.73759	6.68389	5.41792	5.47032	6.55011	5.77987	7.3596	0.643325306	0.966018836	12	16	24	0.53223986	0.825678486	24	9.247741785	0.078520002
ENSMUSG00000020993	Trappc6b	trafficking protein particle complex 6B [Source:MGI Symbol;Acc:MGI:1925482]	51.9112	51.9763	53.2657	50.5927	48.9229	51.7081	51.8856	51.4315	48.8147	52.2701	53.8769	52.1954	53.9624	52.862	0.842838536	0.966018836	4	12	24	0.551035788	0.836153898	24	0.188210539	0.017610077
ENSMUSG00000020994	Pnn	pinin [Source:MGI Symbol;Acc:MGI:1100514]	26.6548	29.2184	30.2374	27.8588	28.7818	26.5308	27.0882	26.1905	30.59	26.0522	28.6709	25.5646	26.4794	27.2322	0.046056765	0.374351104	12	12	24	0.103560046	0.715340058	24	9.457713956	0.066058251
ENSMUSG00000021000	Mia2	MIA SH3 domain ER export factor 2 [Source:MGI Symbol;Acc:MGI:2159614]	31.6817	25.9021	26.1444	31.4497	29.2296	30.4478	31.6797	28.7751	26.5567	29.8078	28.7342	27.9285	28.9195	26.9849	0.128989168	0.560209134	4	4	24	0.114417887	0.727799063	24	19.06192457	0.076728451
ENSMUSG00000021003	Galc	galactosylceramidase [Source:MGI Symbol;Acc:MGI:95636]	13.6713	11.234	11.0653	12.0507	11.8651	12.4385	13.6245	10.9545	10.2789	12.6911	11.875	12.7466	11.6708	11.9967	0.050760377	0.386400877	24	12	24	0.041969733	0.622498612	24	20.23435381	0.10074659
ENSMUSG00000021007	Spata7	spermatogenesis associated 7 [Source:MGI Symbol;Acc:MGI:2144877]	7.12849	7.58523	8.53873	7.87689	7.4291	7.98925	7.16118	7.46637	8.81807	8.02824	7.1983	7.97069	8.4179	9.30769	0.217827365	0.704989686	12	8	24	0.076966141	0.687041208	24	7.470164236	0.080349623
ENSMUSG00000021009	Ptpn21	protein tyrosine phosphatase, non-receptor type 21 [Source:MGI Symbol;Acc:MGI:1344406]	5.25946	5.26637	5.16434	5.73688	6.79603	5.14591	6.48455	5.70457	6.47911	6.15436	5.82534	5.63653	5.35689	5.20183	0.612141595	0.966018836	20	12	24	0.288838311	0.768040178	24	13.94848432	0.072315947
ENSMUSG00000021010	Npas3	neuronal PAS domain protein 3 [Source:MGI Symbol;Acc:MGI:1351610]	1.13132	0.690072	0.81382	1.01715	0.796787	0.551992	1.22904	0.491821	0.652983	0.609239	0.409714	0.545938	0.40148	0.787384	0.890648739	0.966018836	4	20	24	0.845564401	0.953396028	24	2.907326346	0.039833565
ENSMUSG00000021012	Zc3h14	zinc finger CCCH type containing 14 [Source:MGI Symbol;Acc:MGI:1919824]	40.9298	44.6227	43.2621	42.4397	45.142	43.4511	38.5187	42.374	43.221	40.0858	43.8251	45.072	43.1483	42.6177	0.25849765	0.761738864	24	4	24	0.715997405	0.906323093	24	15.41850646	0.020501927
ENSMUSG00000021013	Ttc8	tetratricopeptide repeat domain 8 [Source:MGI Symbol;Acc:MGI:1923510]	11.0715	12.2196	12.3987	11.6916	13.7729	10.5159	12.8326	10.8171	12.5351	10.1676	11.6807	11.9789	12.5894	11.3257	0.683907406	0.966018836	4	12	24	0.984255003	0.996767829	24	20.42775194	0.008427389
ENSMUSG00000021018	Polr2h	polymerase (RNA) II (DNA directed) polypeptide H [Source:MGI Symbol;Acc:MGI:2384309]	21.9668	22.0187	20.3736	22.4004	23.6213	21.4925	17.4149	18.7871	23.7087	17.004	22.0182	17.7508	20.8876	19.5655	0.767183862	0.966018836	20	8	24	0.747605744	0.917832057	24	13.43392976	0.042385773
ENSMUSG00000021022	Ppp2r3c	protein phosphatase 2, regulatory subunit B'', gamma [Source:MGI Symbol;Acc:MGI:1930009]	8.75537	9.31727	8.8316	10.12	9.75463	8.31114	7.48302	9.21053	9.49004	8.63077	9.28937	8.97108	9.79831	9.02057	0.83638114	0.966018836	20	4	24	0.347424308	0.778844143	24	11.06997206	0.056665744
ENSMUSG00000021023	Prorp	protein only RNase P catalytic subunit [Source:MGI Symbol;Acc:MGI:1913382]	3.72016	3.54747	4.10349	2.98371	3.98809	3.33658	3.31747	3.77228	3.13847	3.81102	3.13809	4.25469	4.1941	3.39996	0.904690479	0.966018836	24	16	24	0.676727359	0.891857599	24	22.87203	0.04685773
ENSMUSG00000021024	Psma6	proteasome subunit alpha 6 [Source:MGI Symbol;Acc:MGI:1347006]	137.653	142.976	137.825	143.737	142.877	133.337	128.608	130.224	139.544	127.031	143.44	140.284	149.818	142.382	0.859936714	0.966018836	20	8	24	0.978904646	0.994790651	24	17.73960658	0.005893779
ENSMUSG00000021025	Nfkbia	nuclear factor of kappa light polypeptide gene enhancer in B cells inhibitor, alpha [Source:MGI Symbol;Acc:MGI:104741]	14.122	14.1061	17.505	16.1491	16.3015	15.9275	15.4821	16.3558	20.0749	20.3735	16.2292	14.9789	14.253	13.3942	0.009913596	0.194720062	12	20	24	0.013581299	0.529134128	24	11.50498885	0.169789632
ENSMUSG00000021027	Ralgapa1	Ral GTPase activating protein, alpha subunit 1 [Source:MGI Symbol;Acc:MGI:1931050]	45.1975	42.7859	37.4454	40.6524	41.8693	41.3827	40.4465	40.9638	41.5238	37.7622	42.9156	42.6095	44.5407	43.5645	0.196044629	0.673646834	4	12	24	0.059209974	0.658304792	24	22.80860339	0.065547329
ENSMUSG00000021028	Mbip	MAP3K12 binding inhibitory protein 1 [Source:MGI Symbol;Acc:MGI:1918320]	9.46965	12.1735	12.4055	9.65384	10.7589	11.0279	9.30924	11.0471	12.9669	10.322	11.2673	11.725	10.3022	10.852	0.060394272	0.407012001	12	16	24	0.368025559	0.79131734	24	8.617122002	0.076239012
ENSMUSG00000021032	Ngb	neuroglobin [Source:MGI Symbol;Acc:MGI:2151886]	3.54651	2.89713	3.34584	3.51642	2.64479	3.45188	3.42323	2.98739	2.84405	3.66673	2.39075	3.67697	2.90588	3.2412	0.649212555	0.966018836	24	20	24	0.895748602	0.970467191	24	0.042490953	0.026975775
ENSMUSG00000021033	Gstz1	glutathione transferase zeta 1 (maleylacetoacetate isomerase) [Source:MGI Symbol;Acc:MGI:1341859]	24.3388	26.6562	27.8146	33.4583	28.0144	26.8821	25.6247	29.6207	30.1619	30.0638	31.3639	30.2844	29.5583	29.9933	0.017417473	0.255326033	16	12	24	0.006837349	0.472731997	24	11.6306137	0.10470237
ENSMUSG00000021036	Sptlc2	serine palmitoyltransferase, long chain base subunit 2 [Source:MGI Symbol;Acc:MGI:108074]	29.3168	39.7114	40.2131	37.0069	35.72	33.1188	32.9199	37.7908	50.4922	39.3756	34.5698	38.9261	39.3898	38.1922	0.029412383	0.322165609	12	16	24	0.013430527	0.529134128	24	8.517851063	0.173799156
ENSMUSG00000021037	Ahsa1	AHA1, activator of heat shock protein ATPase 1 [Source:MGI Symbol;Acc:MGI:2387603]	124.268	96.9112	91.3651	93.5058	101.889	104.88	101.563	102.597	80.2054	89.9458	98.8549	95.9567	100.037	98.2023	0.008389425	0.192760771	4	8	24	0.004530055	0.430087363	24	21.93172179	0.144592666
ENSMUSG00000021038	Vps33b	VPS33B interacting protein, apical-basolateral polarity regulator, spe-39 homolog [Source:MGI Symbol;Acc:MGI:2144805]	23.2069	23.8056	25.5332	25.4571	25.1443	24.8869	21.9699	23.7887	23.6308	24.8395	21.9338	24.1917	23.1034	23.4564	0.03484427	0.328311403	16	8	24	0.158012842	0.764630328	24	11.99410254	0.04594909
ENSMUSG00000021039	Snw1	SNW domain containing 1 [Source:MGI Symbol;Acc:MGI:1913604]	33.9446	32.458	31.8973	32.154	34.3565	31.9874	32.8033	31.2669	34.2805	29.1575	32.826	33.1052	32.4121	32.9667	0.926137994	0.966018836	20	20	24	0.546587361	0.833378191	24	21.96179606	0.025976275
ENSMUSG00000021040	Slirp	SRA stem-loop interacting RNA binding protein [Source:MGI Symbol;Acc:MGI:1916394]	71.7957	80.0176	68.033	66.9989	73.7843	66.6186	67.2684	67.7821	91.4407	60.1959	81.903	74.4701	85.4989	73.1969	0.904831057	0.966018836	12	4	24	0.814104773	0.940708369	24	2.898317595	0.040201637
ENSMUSG00000021044	Adck1	aarF domain containing kinase 1 [Source:MGI Symbol;Acc:MGI:1919363]	10.0278	9.47494	9.33931	8.5795	8.79277	10.7166	9.5928	10.8475	8.34364	9.89742	9.60155	10.1322	9.54094	10.0711	0.0444686	0.365196805	24	12	24	0.086078971	0.704714207	24	22.54787543	0.073672725
ENSMUSG00000021047	Nova1	NOVA alternative splicing regulator 1 [Source:MGI Symbol;Acc:MGI:104297]	1.47471	3.53303	5.38111	2.75123	2.41145	3.18738	4.79494	2.76129	2.88762	2.46082	2.63154	2.31554	1.32602	3.04561	0.29905572	0.809362476	8	20	24	0.459687457	0.805393068	24	8.018603376	0.1941215
ENSMUSG00000021048	Mthfd1	methylenetetrahydrofolate dehydrogenase (NADP+ dependent), methenyltetrahydrofolate cyclohydrolase, formyltetrahydrofolate synthase [Source:MGI Symbol;Acc:MGI:1342005]	11.004	11.8802	11.715	12.2561	12.0266	13.321	11.8401	14.0549	11.8346	14.4125	10.8759	12.6125	12.912	12.1447	0.908406451	0.966018836	24	12	24	0.920440082	0.978864885	24	8.911559089	0.017919056
ENSMUSG00000021051	Ppp2r5e	protein phosphatase 2, regulatory subunit B', epsilon [Source:MGI Symbol;Acc:MGI:1349473]	19.2826	16.9242	17.6266	17.1351	16.9672	17.1391	18.4677	17.625	15.6268	20.5051	17.6833	19.1515	18.9336	18.901	0.537381546	0.966018836	4	8	24	0.60822091	0.864328093	24	21.94398073	0.037137402
ENSMUSG00000021054	Sgpp1	sphingosine-1-phosphate phosphatase 1 [Source:MGI Symbol;Acc:MGI:2135760]	29.9446	28.555	26.941	28.1638	27.2108	27.2393	28.6204	27.2446	23.1871	28.4079	28.8434	24.239	23.6704	26.2225	0.907068194	0.966018836	4	8	24	0.758538701	0.921881031	24	17.5685455	0.027164054
ENSMUSG00000021057	Akap5	A kinase (PRKA) anchor protein 5 [Source:MGI Symbol;Acc:MGI:2685104]	0.487935	0.58091	0.863251	0.399335	0.608294	0.544865	0.526459	0.512806	0.577346	0.497436	0.504166	0.54551	0.472568	0.621628	0.496745696	0.966018836	12	4	24	0.505215731	0.811334839	24	7.067249863	0.044867851
ENSMUSG00000021061	Sptb	spectrin beta, erythrocytic [Source:MGI Symbol;Acc:MGI:98387]	16.7316	16.917	16.8298	18.3932	19.492	17.629	20.9003	17.7421	18.7152	23.6042	14.3666	19.3768	18.6856	17.364	0.928780444	0.966018836	16	16	24	0.892828618	0.969717376	24	12.62903326	0.030091422
ENSMUSG00000021062	Rab15	RAB15, member RAS oncogene family [Source:MGI Symbol;Acc:MGI:1916865]	56.6703	42.116	40.7771	42.7407	43.7498	48.3507	43.5064	47.4604	33.1669	52.93	43.2694	49.0213	49.9181	45.9299	0.018263889	0.256345714	4	8	24	0.150800548	0.758865539	24	21.17333512	0.138509694
ENSMUSG00000021065	Fut8	fucosyltransferase 8 [Source:MGI Symbol;Acc:MGI:1858901]	45.9701	51.3927	45.2223	55.2024	54.6479	49.5012	46.9937	51.2074	51.8547	52.2616	47.2533	52.6316	46.2303	46.5799	0.036134798	0.331852526	16	12	24	0.149199345	0.757645213	24	13.46864134	0.069029247
ENSMUSG00000021066	Atl1	atlastin GTPase 1 [Source:MGI Symbol;Acc:MGI:1921241]	131.348	132.479	131.215	115.518	125.156	129.966	113.451	130.187	123.422	133.822	141.341	131.435	144.603	143.655	0.891742979	0.966018836	8	4	24	0.794897353	0.93426902	24	1.854594703	0.023957125
ENSMUSG00000021067	Sav1	salvador family WW domain containing 1 [Source:MGI Symbol;Acc:MGI:1927144]	9.11337	9.84735	8.68125	10.4076	9.56995	8.86953	10.5273	9.72855	9.61361	9.6049	10.3569	9.1132	9.06461	9.03832	0.906657897	0.966018836	16	20	24	0.680197688	0.893034613	24	13.20860134	0.027989342
ENSMUSG00000021068	Nin	ninein [Source:MGI Symbol;Acc:MGI:105108]	6.58951	7.07827	6.56318	8.24378	7.26314	6.00585	7.16816	5.89156	6.85254	5.90918	6.88634	6.03508	6.53974	6.61819	0.882617464	0.966018836	20	4	24	0.647842872	0.881026758	24	12.0784635	0.041060544
ENSMUSG00000021069	Pygl	liver glycogen phosphorylase [Source:MGI Symbol;Acc:MGI:97829]	13.353	11.4906	16.4449	20.3493	13.64	14.655	16.441	16.5413	12.6835	17.7136	19.4595	17.3267	22.245	18.5457	0.911685024	0.966018836	16	20	24	0.751463116	0.91921305	24	14.69177599	0.061945315
ENSMUSG00000021070	Bdkrb2	bradykinin receptor, beta 2 [Source:MGI Symbol;Acc:MGI:102845]	1.24279	0.977035	0.973087	0.843473	1.04369	0.959951	1.15986	1.06883	0.896083	1.07265	0.844045	0.857318	0.998145	1.1759	0.084879076	0.466404237	4	20	24	0.115148962	0.727799063	24	1.770165991	0.068400835
ENSMUSG00000021071	Trim9	tripartite motif-containing 9 [Source:MGI Symbol;Acc:MGI:2137354]	12.6657	12.2176	12.8544	11.0005	10.1827	13.7122	12.6027	12.6621	11.1468	13.6115	11.3781	12.0082	11.7614	13.6907	0.745469888	0.966018836	8	12	24	0.311039341	0.768040178	24	2.706475301	0.069124189
ENSMUSG00000021072	Tmx1	thioredoxin-related transmembrane protein 1 [Source:MGI Symbol;Acc:MGI:1919986]	28.01	29.0228	29.0772	27.6009	28.8762	26.4362	27.826	26.4589	25.3186	23.6246	29.002	21.3625	21.7627	25.2068	0.924770496	0.966018836	20	4	24	0.73545121	0.91373222	24	10.99576756	0.03222604
ENSMUSG00000021076	Actr10	ARP10 actin-related protein 10 [Source:MGI Symbol;Acc:MGI:1891654]	109.604	106.206	105.528	109.349	110.606	106.422	109.55	104.937	108.097	90.1728	122.154	102.168	111.578	98.7212	0.291042071	0.797585788	20	12	24	0.454119461	0.805393068	24	19.65554195	0.04951414
ENSMUSG00000021079	Timm9	translocase of inner mitochondrial membrane 9 [Source:MGI Symbol;Acc:MGI:1353436]	34.7681	39.6232	39.2267	35.0367	35.65	33.3491	35.9691	32.7644	39.3501	31.6145	36.6563	35.1441	37.9788	36.3058	0.44861185	0.966018836	12	4	24	0.441825979	0.805393068	24	5.452029155	0.049435055
ENSMUSG00000021087	Rtn1	reticulon 1 [Source:MGI Symbol;Acc:MGI:1933947]	187.809	147.562	149.898	161.844	138.056	175.943	167.79	163.817	131.691	232.193	143.071	182.894	161.802	187.655	0.46694635	0.966018836	24	12	24	0.868512087	0.96247509	24	22.01752413	0.04630432
ENSMUSG00000021090	Lrrc9	leucine rich repeat containing 9 [Source:MGI Symbol;Acc:MGI:1925507]	0.646438	0.600395	0.47998	0.876717	0.602896	0.600417	0.807307	0.532654	0.570995	0.468322	0.601968	0.696185	0.504458	0.578508	0.336988702	0.85758369	20	16	24	0.490998482	0.805393068	24	19.25883398	0.048304835
ENSMUSG00000021091	Serpina3n	serine (or cysteine) peptidase inhibitor, clade A, member 3N [Source:MGI Symbol;Acc:MGI:105045]	78.9258	63.3932	69.7021	62.7839	81.4915	79.3727	66.081	65.7972	51.1397	67.304	76.3323	64.0195	61.6767	59.5854	0.114010995	0.532950079	20	12	24	0.010872628	0.51149922	24	18.51586359	0.176001114
ENSMUSG00000021094	Dhrs7	dehydrogenase/reductase (SDR family) member 7 [Source:MGI Symbol;Acc:MGI:1913625]	29.2092	31.564	32.9166	38.0998	28.4859	30.5896	29.1004	33.0645	30.0292	32.8664	32.7395	30.4201	35.6813	32.7964	0.559021844	0.966018836	16	4	24	0.332285495	0.768996731	24	9.304233997	0.066391826
ENSMUSG00000021096	Ppm1a	protein phosphatase 1A, magnesium dependent, alpha isoform [Source:MGI Symbol;Acc:MGI:99878]	144.515	141.516	140.058	144.266	136.166	143.322	134.423	138.377	133.411	150.946	138.925	144.119	148.111	148.152	0.882263435	0.966018836	16	20	24	0.990813109	0.99839802	24	21.82314243	0.002936319
ENSMUSG00000021097	Clmn	calmin [Source:MGI Symbol;Acc:MGI:2136957]	4.42491	4.55453	5.11061	4.82373	4.63143	4.2838	4.41756	3.98085	5.52255	4.66567	3.8008	3.8767	4.14049	4.06917	0.074438493	0.448301997	12	12	24	0.033294354	0.606585237	24	9.376902234	0.105252411
ENSMUSG00000021098	4930447C04Rik	RIKEN cDNA 4930447C04 gene [Source:MGI Symbol;Acc:MGI:1923051]	11.6325	14.924	13.3672	13.1103	12.6106	10.9818	12.7758	12.3551	15.1503	11.4004	13.9809	11.518	9.43925	12.9683	0.020119233	0.259417302	12	12	24	0.115723538	0.727799063	24	8.963230209	0.128413474
ENSMUSG00000021102	Glrx5	glutaredoxin 5 [Source:MGI Symbol;Acc:MGI:1920296]	24.9286	21.8921	23.1366	21.5444	18.7384	20.8402	24.2304	23.5003	23.3226	29.3908	25.1685	23.782	23.6001	24.1151	0.88067614	0.966018836	4	20	24	0.686624796	0.895935969	24	6.900350977	0.047590729
ENSMUSG00000021103	Mnat1	menage a trois 1 [Source:MGI Symbol;Acc:MGI:106207]	13.3121	16.3348	15.5856	14.6108	14.3814	15.01	15.0429	15.465	16.9748	15.7323	14.7341	15.3154	18.189	16.8351	0.132411359	0.567728672	12	8	24	0.038719727	0.61140397	24	5.704525463	0.080492821
ENSMUSG00000021108	Prkch	protein kinase C, eta [Source:MGI Symbol;Acc:MGI:97600]	1.9959	2.00158	1.86284	1.89527	1.66649	2.15772	2.10784	2.21885	1.77557	2.57261	1.67853	2.31235	1.95616	1.85259	0.369262716	0.893596186	24	20	24	0.835998477	0.949840573	24	23.60247027	0.028732691
ENSMUSG00000021109	Hif1a	hypoxia inducible factor 1, alpha subunit [Source:MGI Symbol;Acc:MGI:106918]	28.4475	28.7547	30.9645	28.0192	27.7783	26.176	27.9832	26.7676	26.7514	26.4153	26.368	23.3792	24.3078	24.8434	0.245061943	0.741580974	12	12	24	0.052308005	0.65045829	24	8.731495391	0.047920744
ENSMUSG00000021111	Papola	poly (A) polymerase alpha [Source:MGI Symbol;Acc:MGI:109301]	43.9471	37.8284	37.9272	37.606	40.211	37.1491	41.2828	34.8882	37.7052	37.8706	44.2009	39.2849	40.8084	39.6312	0.208438456	0.698704663	4	12	24	0.20968589	0.768040178	24	19.94134528	0.064658413
ENSMUSG00000021112	Pals1	protein associated with LIN7 1, MAGUK family member [Source:MGI Symbol;Acc:MGI:1927339]	13.618	16.2225	15.3563	14.4067	16.3127	14.1444	14.8285	13.8745	17.023	13.1154	16.9826	14.7164	15.5562	15.1251	0.850398351	0.966018836	12	12	24	0.855843552	0.957176686	24	10.77550116	0.02505679
ENSMUSG00000021113	Snapc1	small nuclear RNA activating complex, polypeptide 1 [Source:MGI Symbol;Acc:MGI:1922877]	13.4801	13.5787	13.5137	14.4739	13.94	13.5941	13.8754	14.1211	14.4388	14.3916	14.6394	14.0106	14.3245	15.0388	0.700065774	0.966018836	16	12	24	0.053102862	0.650879532	24	10.21394388	0.025954132
ENSMUSG00000021114	Atp6v1d	ATPase, H+ transporting, lysosomal V1 subunit D [Source:MGI Symbol;Acc:MGI:1921084]	83.8953	86.839	85.7776	82.842	81.121	83.0035	84.0052	75.2368	91.4973	78.792	88.1358	87.7617	86.0637	89.4503	0.911135828	0.966018836	12	4	24	0.916914568	0.977109697	24	3.997500672	0.012116632
ENSMUSG00000021115	Vrk1	vaccinia related kinase 1 [Source:MGI Symbol;Acc:MGI:1261847]	8.7086	9.35609	9.45714	9.56971	8.66165	8.81198	6.87562	7.94199	10.6936	9.85621	9.67441	8.97664	9.11141	8.88801	0.043240321	0.357642487	16	12	24	0.046590972	0.633507364	24	11.13174275	0.120619686
ENSMUSG00000021116	Eif2s1	eukaryotic translation initiation factor 2, subunit 1 alpha [Source:MGI Symbol;Acc:MGI:95299]	27.5254	27.6111	28.8186	24.5609	27.0887	23.8996	25.9188	25.4349	26.6365	23.2358	29.1023	25.4301	26.6958	24.7094	0.908797704	0.966018836	12	4	24	0.946614432	0.986959555	24	1.950164324	0.011928936
ENSMUSG00000021118	Plek2	pleckstrin 2 [Source:MGI Symbol;Acc:MGI:1351466]	2.65211	2.45713	2.49077	2.2177	2.66418	2.76847	2.25089	2.30547	2.69269	2.95027	2.90713	2.59115	3.52541	2.65272	0.801995186	0.966018836	20	12	24	0.675094957	0.891127362	24	18.83346781	0.040730714
ENSMUSG00000021120	Pigh	phosphatidylinositol glycan anchor biosynthesis, class H [Source:MGI Symbol;Acc:MGI:99463]	8.396	8.72238	8.65317	8.7125	8.35856	8.25246	8.87969	8.75597	8.38663	7.97522	9.32645	9.656	8.8446	9.23351	0.517917106	0.966018836	4	12	24	0.562581682	0.842381814	24	23.17686506	0.025369401
ENSMUSG00000021123	Rdh12	retinol dehydrogenase 12 [Source:MGI Symbol;Acc:MGI:1925224]	1.48374	1.32656	1.85971	1.84563	1.209	1.55026	1.6051	1.45825	1.33994	0.838927	1.64804	1.26947	1.3067	1.09139	0.806004771	0.966018836	12	20	24	0.946635	0.986959555	24	20.35686234	0.021308823
ENSMUSG00000021124	Vti1b	vesicle transport through interaction with t-SNAREs 1B [Source:MGI Symbol;Acc:MGI:1855688]	97.0223	96.8108	87.9248	94.4186	100.219	100.524	97.5091	87.1267	96.4656	82.7135	98.6767	94.1923	101.495	99.1307	0.127415507	0.560209134	20	20	24	0.126420101	0.739868076	24	21.34520794	0.068042189
ENSMUSG00000021125	Arg2	arginase type II [Source:MGI Symbol;Acc:MGI:1330806]	2.61193	2.60534	2.27572	2.39892	2.57411	2.10335	2.09683	2.37321	2.43715	1.74664	2.03075	2.34436	2.40168	2.66375	0.31548973	0.827946344	8	8	24	0.240338021	0.768040178	24	2.724277741	0.072893193
ENSMUSG00000021127	Zfp36l1	zinc finger protein 36, C3H type-like 1 [Source:MGI Symbol;Acc:MGI:107946]	10.0517	9.69363	8.63986	10.7864	8.38499	7.94813	11.642	10.4242	9.75958	9.88777	10.8052	10.6145	10.2576	10.1966	0.911371589	0.966018836	4	8	24	0.706729151	0.903131193	24	2.902802493	0.043003297
ENSMUSG00000021130	Galnt16	polypeptide N-acetylgalactosaminyltransferase 16 [Source:MGI Symbol;Acc:MGI:1917754]	9.02821	8.97817	11.1558	10.5118	11.3796	10.5803	11.2414	8.4771	9.15396	11.3916	9.17014	11.5762	8.51813	9.3007	0.074510812	0.448301997	24	8	24	0.108195622	0.72082227	24	15.53361493	0.119619385
ENSMUSG00000021131	Erh	ERH mRNA splicing and mitosis factor [Source:MGI Symbol;Acc:MGI:108089]	10.2955	12.4762	11.5538	10.3354	10.8993	10.6302	11.0725	10.5212	11.3345	9.51457	11.1106	10.4872	10.2356	9.10946	0.948161972	0.966018836	12	16	24	0.986113788	0.997339197	24	3.965209918	0.005930991
ENSMUSG00000021133	Susd6	sushi domain containing 6 [Source:MGI Symbol;Acc:MGI:2444661]	28.1935	20.2612	20.7838	22.3259	24.287	25.2099	23.6031	22.683	17.7492	27.4329	22.095	23.985	22.2683	21.2654	0.038115943	0.334069441	24	12	24	0.074806028	0.68394125	24	19.24628748	0.142628037
ENSMUSG00000021134	Srsf5	serine and arginine-rich splicing factor 5 [Source:MGI Symbol;Acc:MGI:98287]	101.069	130.158	128.845	122.369	110.504	103.666	111.03	109.017	138.011	118.85	106.264	108.45	103.082	117.022	0.002004172	0.100255953	12	16	24	0.001166434	0.336500758	24	8.612050951	0.160964068
ENSMUSG00000021136	Smoc1	SPARC related modular calcium binding 1 [Source:MGI Symbol;Acc:MGI:1929878]	4.67707	4.96057	4.6975	4.26911	4.24752	4.38447	4.09733	4.84507	4.80243	3.64662	4.42333	4.93956	4.31812	4.91166	0.05211941	0.391073722	8	8	24	0.164263724	0.767020009	24	3.163071384	0.073150273
ENSMUSG00000021139	Gm20498	predicted gene 20498 [Source:MGI Symbol;Acc:MGI:5141963]	24.7775	23.8863	24.3837	21.8482	24.4205	23.301	25.3948	23.8398	21.2539	19.5557	22.7319	23.0052	26.2741	22.6685	8.24E-04	0.071344007	4	12	24	0.010131356	0.503967859	24	23.42957967	0.105781324
ENSMUSG00000021140	Pcnx	pecanex homolog [Source:MGI Symbol;Acc:MGI:1891924]	19.1612	16.508	15.6927	15.6215	19.0596	16.6607	20.5358	13.1291	13.1933	20.2088	14.6365	16.9106	16.1341	17.285	0.66832131	0.966018836	4	8	24	0.345518647	0.778420274	24	19.88078817	0.11208808
ENSMUSG00000021143	Pacs2	phosphofurin acidic cluster sorting protein 2 [Source:MGI Symbol;Acc:MGI:1924399]	57.7615	52.8298	52.5832	57.5437	61.6539	56.1859	61.9246	54.0607	54.6961	62.4012	51.5612	55.8199	54.7255	51.2486	0.226787816	0.723408632	16	16	24	0.305330597	0.768040178	24	17.19967743	0.055696074
ENSMUSG00000021144	Mta1	metastasis associated 1 [Source:MGI Symbol;Acc:MGI:2150037]	10.4194	9.26429	9.08631	9.6036	9.28358	8.66968	9.26795	9.93804	10.3618	13.0811	9.21273	9.94135	10.1907	10.6994	0.918497267	0.966018836	16	4	24	0.395879972	0.799546792	24	8.722525641	0.068452128
ENSMUSG00000021147	Wdr37	WD repeat domain 37 [Source:MGI Symbol;Acc:MGI:1920393]	25.179	24.7232	26.8994	23.8669	24.4995	24.1177	23.7083	23.5378	22.9172	23.3609	23.4692	22.9299	21.764	24.2952	0.832293447	0.966018836	8	16	24	0.647054797	0.880719855	24	7.949103702	0.01906017

ENSMUSG00000021149	Gtpbp4	GTP binding protein 4 [Source:MGI Symbol;Acc:MGI:1916487]	19.0915	18.9198	17.5604	17.7606	19.3336	17.9527	16.9086	18.5883	18.8817	16.0683	21.2834	19.1674	18.9868	19.3472	0.187699199	0.662239322	20	20	24	0.708235528	0.903587413	24	20.66241874	0.031775085
ENSMUSG00000021156	Zmynd11	zinc finger, MYND domain containing 11 [Source:MGI Symbol;Acc:MGI:1913755]	39.1732	44.5274	38.6134	37.6593	39.6618	40.3793	43.037	40.1124	41.6698	45.7315	46.6373	38.6912	40.3028	46.2266	0.829428469	0.966018836	8	4	24	0.764572955	0.92403577	24	7.506028017	0.02868391
ENSMUSG00000021171	Esyt2	extended synaptotagmin-like protein 2 [Source:MGI Symbol;Acc:MGI:1261845]	22.9107	22.1499	22.4138	22.1161	23.259	23.5723	22.2334	22.2579	21.6627	20.6245	22.8584	22.1576	21.9764	21.583	0.23919737	0.741580974	20	20	24	0.023845616	0.585033799	24	19.86635885	0.041269902
ENSMUSG00000021175	Cdca7l	cell division cycle associated 7 like [Source:MGI Symbol;Acc:MGI:2384982]	0.432316	0.711933	0.533435	0.492543	0.473334	0.638732	0.737218	0.602583	0.443459	0.341497	0.480767	0.414255	0.726462	0.800815	0.110090633	0.526220521	8	8	24	0.028465594	0.593964798	24	1.98176172	0.120063933
ENSMUSG00000021176	Efcab11	EF-hand calcium binding domain 11 [Source:MGI Symbol;Acc:MGI:1926017]	1.07725	0.891752	0.834592	0.678286	0.936534	0.807172	0.524203	0.467612	0.577987	0.547278	0.70529	0.833759	1.14254	0.745144	0.70641939	0.966018836	4	12	24	0.253950839	0.768040178	24	21.34308649	0.106876676
ENSMUSG00000021177	Tdp1	tyrosyl-DNA phosphodiesterase 1 [Source:MGI Symbol;Acc:MGI:1920036]	5.6563	7.51935	5.60847	7.59376	6.67573	6.4306	7.43148	6.70709	6.83501	8.10905	7.69417	6.39072	7.0258	6.4007	0.481811994	0.966018836	16	8	24	0.425305382	0.805393068	24	12.84261308	0.068363859
ENSMUSG00000021178	Psmc1	protease (prosome, macropain) 26S subunit, ATPase 1 [Source:MGI Symbol;Acc:MGI:106054]	85.4956	88.4956	79.4571	91.5893	86.1571	89.5246	78.1296	82.086	84.1993	83.0976	89.065	83.4707	87.6243	90.8341	0.890176313	0.966018836	16	20	24	0.769850922	0.925855377	24	16.0713291	0.020138052
ENSMUSG00000021179	Nrde2	nrde-2 necessary for RNA interference, domain containing [Source:MGI Symbol;Acc:MGI:2670969]	3.62189	4.21024	4.69511	3.92361	4.60249	4.46709	3.6645	4.53475	4.5168	4.29006	3.81927	5.03019	4.27452	4.20612	0.595952838	0.966018836	12	16	24	0.7855154	0.931158434	24	11.09233802	0.031046016
ENSMUSG00000021180	Rps6ka5	ribosomal protein S6 kinase, polypeptide 5 [Source:MGI Symbol;Acc:MGI:1920336]	5.84668	7.14196	7.35597	7.6137	6.66283	6.10643	5.69738	5.65502	7.08148	6.96529	6.16147	6.43532	5.40891	6.10597	8.66E-04	0.071344007	12	16	24	0.001258215	0.346832852	24	11.0926043	0.143093668
ENSMUSG00000021182	Ccdc88c	coiled-coil domain containing 88C [Source:MGI Symbol;Acc:MGI:1915589]	0.857939	1.81131	3.16896	1.87087	1.43894	1.63012	1.75902	1.73575	2.59471	2.13205	1.20927	1.37545	1.51897	1.88102	0.002258173	0.104113924	12	8	24	0.006705584	0.471768573	24	8.493297918	0.312958254
ENSMUSG00000021185	Dglucy	D-glutamate cyclase [Source:MGI Symbol;Acc:MGI:2444813]	3.90387	4.59075	4.44212	4.68176	4.46685	4.926	4.58181	4.52109	4.61503	4.33568	4.50625	4.71396	4.42713	4.05736	0.495929335	0.966018836	24	4	24	0.488470393	0.805393068	24	16.00451277	0.032924358
ENSMUSG00000021186	Fbln5	fibulin 5 [Source:MGI Symbol;Acc:MGI:1346091]	37.9406	33.0559	31.7027	34.1757	37.7772	38.2717	36.7055	38.0528	30.5686	35.7186	33.1147	36.5921	34.9281	30.3979	0.077301512	0.453790923	24	12	24	0.017104748	0.547196997	24	20.02606193	0.113516165
ENSMUSG00000021187	Tc2n	tandem C2 domains, nuclear [Source:MGI Symbol;Acc:MGI:1921663]	0.532232	0.58984	0.351976	0.734158	0.495018	0.531798	0.489785	0.353973	0.537621	0.56326	0.429645	0.469487	0.58145	0.413984	0.865490576	0.966018836	16	20	24	0.678672279	0.892520664	24	15.71896761	0.033119549
ENSMUSG00000021188	Trip11	thyroid hormone receptor interactor 11 [Source:MGI Symbol;Acc:MGI:1924393]	7.84494	8.34276	8.05339	8.02077	8.36648	7.85645	8.20388	7.61675	8.40185	6.65726	8.66594	8.73049	8.54898	9.16099	0.879201926	0.966018836	20	20	24	0.569816868	0.844900526	24	23.83805574	0.039146178
ENSMUSG00000021189	Atxn3	ataxin 3 [Source:MGI Symbol;Acc:MGI:1099442]	9.49849	9.7673	9.2473	9.43261	9.73699	9.49408	8.92086	8.54231	9.28312	9.65903	10.4407	9.16318	10.3557	9.41732	0.510431574	0.966018836	20	16	24	0.382050257	0.799546792	24	16.48672065	0.036863474
ENSMUSG00000021190	Lgmn	legumain [Source:MGI Symbol;Acc:MGI:1330838]	144.981	113.529	126.664	113.036	129.403	143.044	125.373	130.551	91.823	128.043	121.928	115.778	112.351	116.789	0.937921962	0.966018836	24	12	24	0.142741981	0.752329696	24	20.24826838	0.117805212
ENSMUSG00000021192	Golga5	golgi autoantigen, golgin subfamily a, 5 [Source:MGI Symbol;Acc:MGI:1351475]	12.7247	13.0675	12.7471	12.9359	12.8166	13.6381	12.758	14.0678	12.6265	13.1407	13.3295	13.3451	13.4374	13.0164	0.245061943	0.741580974	24	12	24	0.637059553	0.876483853	24	21.8762987	0.01506972
ENSMUSG00000021193	Pitrm1	pitrilysin metallepetidase 1 [Source:MGI Symbol;Acc:MGI:1916867]	14.3161	16.2443	15.2113	16.2492	16.4024	16.548	16.5751	15.6627	16.6357	18.156	16.334	14.8926	14.8423	16.6791	0.725061903	0.966018836	16	12	24	0.12663594	0.739868076	24	11.47948629	0.062543949
ENSMUSG00000021194	Chga	chromogranin A [Source:MGI Symbol;Acc:MGI:88394]	59.2413	48.0887	55.8196	46.8684	41.9653	56.7806	54.161	53.3875	42.3767	64.8478	43.6671	55.7587	47.8885	57.7011	0.895493752	0.966018836	24	20	24	0.628939577	0.873061488	24	1.40452521	0.072749073
ENSMUSG00000021196	Pfkp	phosphofructokinase, platelet [Source:MGI Symbol;Acc:MGI:1891833]	124.67	98.3194	102.994	101.871	99.4564	119.589	103.715	107.529	79.0748	120.433	97.0724	106.838	102.006	110.195	0.625825704	0.966018836	24	20	24	0.340804136	0.775204417	24	21.17783989	0.094567303
ENSMUSG00000021198	Unc79	unc-79 homolog [Source:MGI Symbol;Acc:MGI:2684729]	3.63305	3.1261	4.14878	2.90568	3.56291	3.60029	3.36602	2.95164	2.78304	2.72175	3.25186	3.04806	2.89089	3.34417	0.628286596	0.966018836	4	12	24	0.589399995	0.854253633	24	21.39608065	0.049819756
ENSMUSG00000021200	Asb2	ankyrin repeat and SOCS box-containing 2 [Source:MGI Symbol;Acc:MGI:1929743]	2.36309	3.56203	2.80848	2.78078	2.86867	4.79553	3.75358	9.11114	4.84559	5.34418	3.41579	5.77677	6.98114	4.3073	0.915082018	0.966018836	8	8	24	0.416013926	0.800710663	24	2.892385158	0.181261112
ENSMUSG00000021203	Otub2	OTU domain, ubiquitin aldehyde binding 2 [Source:MGI Symbol;Acc:MGI:1915399]	5.73391	4.92088	5.16707	5.50193	5.53561	6.21117	7.03974	5.66863	4.38847	4.61085	5.97428	6.17955	5.43869	4.84442	0.002057111	0.100255953	24	12	24	0.004988437	0.442351542	24	20.53778516	0.17298498
ENSMUSG00000021209	Ppp4r4	protein phosphatase 4, regulatory subunit 4 [Source:MGI Symbol;Acc:MGI:1921771]	8.94275	8.59749	9.92301	7.89388	8.73755	8.66442	8.46854	7.22787	7.5905	8.27337	8.07063	7.74861	7.92634	9.14652	0.766867092	0.966018836	12	4	24	0.970232124	0.993003194	24	2.716948235	0.009792443
ENSMUSG00000021213	Akr1c13	aldo-keto reductase family 1, member C13 [Source:MGI Symbol;Acc:MGI:1351662]	0.605905	1.41552	1.06048	1.6468	1.44573	1.06274	0.990734	1.38318	1.40336	0.856089	1.04319	1.13816	1.01932	1.34823	0.243857167	0.741580974	8	20	24	0.306985112	0.768040178	24	9.538339334	0.117225124
ENSMUSG00000021215	Net1	neuroepithelial cell transforming gene 1 [Source:MGI Symbol;Acc:MGI:1927138]	5.45339	6.85901	8.7256	10.4526	6.28462	6.99796	6.16096	7.45314	7.34557	9.61872	6.46611	8.14636	6.82759	6.50361	0.004196064	0.140598674	16	12	24	0.028618687	0.594253518	24	11.10661745	0.217477152
ENSMUSG00000021217	Tshz3	teashirt zinc finger family member 3 [Source:MGI Symbol;Acc:MGI:2442819]	4.50146	3.98758	4.48662	4.05551	3.86237	4.47119	4.45551	4.06258	3.95879	5.23224	4.07344	4.02872	4.01834	4.38363	0.828910443	0.966018836	4	16	24	0.932234914	0.982622317	24	9.977888178	0.014978747
ENSMUSG00000021218	Gdi2	guanosine diphosphate (GDP) dissociation inhibitor 2 [Source:MGI Symbol;Acc:MGI:99845]	290.38	249.939	238.099	235.334	232.728	239.828	239.454	260.923	245.095	271.722	279.94	283.346	300.137	284.897	0.206597435	0.697236278	4	12	24	0.294692677	0.768040178	24	23.88084241	0.064468072
ENSMUSG00000021219	Rgs6	regulator of G-protein signaling 6 [Source:MGI Symbol;Acc:MGI:1354730]	7.76365	7.24111	5.70588	6.88379	6.15031	6.16263	7.44305	6.47723	6.11371	8.89889	5.75993	6.89177	6.76978	8.55664	0.243857167	0.741580974	4	8	24	0.600009118	0.860011178	24	3.395726407	0.068504334
ENSMUSG00000021221	Dpf3	D4, zinc and double PHD fingers, family 3 [Source:MGI Symbol;Acc:MGI:1917377]	3.97947	3.22651	2.77567	3.19301	3.45797	3.76796	3.38006	3.05071	2.68713	3.67777	3.12427	2.493	4.14538	4.1837	0.286642595	0.793714531	4	8	24	0.474898516	0.805393068	24	23.33347424	0.085990432
ENSMUSG00000021222	Dcaf4	DDB1 and CUL4 associated factor 4 [Source:MGI Symbol;Acc:MGI:1921078]	14.0974	14.7365	14.5642	13.5034	16.0965	15.2947	12.0605	15.6415	13.5016	15.4106	13.9754	15.1115	16.6829	15.2921	0.916556573	0.966018836	24	12	24	0.963574237	0.990884307	24	19.71195734	0.012583235
ENSMUSG00000021223	Papln	papilin, proteoglycan-like sulfated glycoprotein [Source:MGI Symbol;Acc:MGI:2386139]	1.45276	1.28794	1.22209	1.05948	1.26363	1.1835	1.21454	1.65869	1.0204	1.61641	1.15889	1.22008	1.48671	1.26895	0.313530643	0.824470524	8	4	24	0.528985938	0.824286178	24	1.898621549	0.051987653
ENSMUSG00000021224	Numb	NUMB endocytic adaptor protein [Source:MGI Symbol;Acc:MGI:107423]	19.2331	15.7386	16.9855	16.1103	18.5994	16.2318	18.3407	18.1471	14.9352	19.6595	16.0589	17.1074	18.3419	16.9932	0.43565473	0.966018836	4	8	24	0.611948263	0.865342826	24	21.48026898	0.045937121
ENSMUSG00000021226	Acot2	acyl-CoA thioesterase 2 [Source:MGI Symbol;Acc:MGI:2159605]	1.71422	1.89376	2.18358	2.26853	1.72999	1.91265	2.16667	2.433	2.12015	2.62446	2.11162	1.96669	2.1344	2.16398	0.142513744	0.585944911	16	4	24	0.04693455	0.633507364	24	8.870462894	0.097119811
ENSMUSG00000021234	Fam161b	family with sequence similarity 161, member B [Source:MGI Symbol;Acc:MGI:2443027]	4.71518	3.62906	4.36725	4.25767	3.60541	4.37517	4.28729	3.74689	3.2752	3.89863	4.0407	4.38577	4.22201	3.70068	0.117205961	0.538362324	24	8	24	0.104352264	0.716673794	24	20.55052165	0.093322557
ENSMUSG00000021235	Coq6	coenzyme Q6 monooxygenase [Source:MGI Symbol;Acc:MGI:1924408]	10.81	9.6611	9.95768	11.5969	9.35263	10.9937	11.5713	10.0179	9.98629	11.8832	13.0689	11.4197	11.4543	10.8541	0.256915694	0.76168256	16	20	24	0.257876235	0.768040178	24	17.42899251	0.070407791
ENSMUSG00000021236	Entpd5	ectonucleoside triphosphate diphosphohydrolase 5 [Source:MGI Symbol;Acc:MGI:1321385]	13.7228	12.5029	14.3887	15.1781	13.4246	13.2067	13.7058	13.3098	12.2092	15.7677	13.9143	13.2284	13.3394	13.2035	0.053980818	0.395677346	16	16	24	0.143419493	0.752803875	24	13.3213114	0.06646957
ENSMUSG00000021238	Aldh6a1	aldehyde dehydrogenase family 6, subfamily A1 [Source:MGI Symbol;Acc:MGI:1915077]	28.808	31.686	34.3964	39.4581	30.9305	30.1647	33.5855	32.3726	32.7159	36.4519	33.076	31.1515	32.6898	33.5851	0.019711532	0.258568924	16	8	24	0.019239457	0.562219749	24	10.36421763	0.107973626
ENSMUSG00000021240	Abcd4	ATP-binding cassette, sub-family D (ALD), member 4 [Source:MGI Symbol;Acc:MGI:1349217]	10.5271	9.22675	9.96608	10.8383	10.927	10.3704	9.91109	9.84893	9.3384	9.37353	10.3785	9.73509	10.5723	9.50045	0.160297544	0.620016786	20	12	24	0.062141795	0.661961741	24	18.08945917	0.063480618
ENSMUSG00000021241	Isca2	iron-sulfur cluster assembly 2 [Source:MGI Symbol;Acc:MGI:1921566]	56.8468	59.1018	58.6262	58.4593	56.4116	52.6635	65.1478	53.3325	57.8378	50.4803	62.1798	58.3092	63.5252	57.6508	0.93357733	0.966018836	4	12	24	0.554494796	0.837816048	24	23.82003344	0.042704475
ENSMUSG00000021242	Npc2	NPC intracellular cholesterol transporter 2 [Source:MGI Symbol;Acc:MGI:1915213]	45.25	47.7582	47.8316	48.4292	50.4013	47.5084	47.8549	48.5983	49.639	44.0844	50.9933	47.7967	50.53	46.2826	0.939335547	0.966018836	20	12	24	0.785966478	0.931158434	24	16.67544452	0.016310412
ENSMUSG00000021243	Fcf1	FCF1 rRNA processing protein [Source:MGI Symbol;Acc:MGI:1920986]	11.3371	12.9895	12.7038	12.4196	10.4811	9.73037	10.3207	10.5876	13.7777	9.61483	12.1816	11.4748	10.5702	10.721	0.203305541	0.689605535	12	12	24	0.243545122	0.768040178	24	8.405162721	0.095267743
ENSMUSG00000021244	Ylpm1	YLP motif containing 1 [Source:MGI Symbol;Acc:MGI:1926195]	15.8328	17.5349	16.9656	17.322	16.4141	16.6221	16.4102	15.9911	18.168	18.0751	17.2594	16.1008	15.2247	17.5085	0.004122392	0.140598674	12	16	24	0.009037843	0.502965379	24	10.21201819	0.074871411
ENSMUSG00000021245	Mlh3	mutL homolog 3 [Source:MGI Symbol;Acc:MGI:1353455]	6.32245	6.33464	5.5222	5.39139	5.62497	5.81224	6.17361	5.88297	6.79137	4.9619	6.58074	6.5988	6.53183	5.7029	0.212359178	0.701530532	4	12	24	0.186232804	0.768040178	24	23.46230159	0.077754719
ENSMUSG00000021248	Tmed10	transmembrane p24 trafficking protein 10 [Source:MGI Symbol;Acc:MGI:1915831]	53.3256	51.1785	53.0058	46.3857	49.5252	49.7484	53.0502	53.7666	52.0355	57.8067	49.2729	55.7657	52.1349	54.6905	0.651661131	0.966018836	8	12	24	0.366772429	0.790399468	24	3.678677406	0.040764006
ENSMUSG00000021250	Fos	FBJ osteosarcoma oncogene [Source:MGI Symbol;Acc:MGI:95574]	3.06003	2.46379	2.08243	2.5119	1.97671	2.17301	2.9219	3.98843	2.04889	2.47455	2.09465	2.46996	2.84419	2.14201	0.048396595	0.379129806	4	16	24	0.127610705	0.740162193	24	1.496854007	0.154101538
ENSMUSG00000021252	Erg28	ergosterol biosynthesis 28 [Source:MGI Symbol;Acc:MGI:1915571]	34.0452	30.2963	32.0192	28.6621	33.5206	29.4635	31.157	26.618	30.5369	29.6902	31.9516	26.5582	30.6054	28.6065	0.851656735	0.966018836	20	12	24	0.74882326	0.918296525	24	17.57332122	0.02893956
ENSMUSG00000021253	Tgfb3	transforming growth factor, beta 3 [Source:MGI Symbol;Acc:MGI:98727]	6.68489	5.35477	4.98897	4.73782	5.83242	5.90022	6.28072	6.84076	5.90826	5.96443	5.41	5.62789	6.04144	5.92159	0.064405315	0.414867207	4	12	24	0.087076238	0.706216588	24	0.287069628	0.098740023
ENSMUSG00000021254	Gpatch2l	G patch domain containing 2 like [Source:MGI Symbol;Acc:MGI:1917623]	7.07877	6.70035	6.92876	7.2083	6.4538	6.59215	7.83459	7.54655	6.4892	7.44996	6.10939	6.98076	6.46409	7.48065	0.17953325	0.655480352	8	12	24	0.344343453	0.7777007	24	4.346533009	0.052521178
ENSMUSG00000021255	Esrrb	estrogen related receptor, beta [Source:MGI Symbol;Acc:MGI:1346832]	0.676865	0.554431	0.779427	0.625217	0.421534	0.754444	0.863675	0.917646	0.502279	0.806562	0.528409	0.559755	0.637666	0.68999	0.522551072	0.966018836	8	12	24	0.350371522	0.779808031	24	3.437588501	0.07069842
ENSMUSG00000021256	Vash1	vasohibin 1 [Source:MGI Symbol;Acc:MGI:2442543]	10.7581	7.57802	8.03005	8.24935	8.12615	8.68851	8.78117	9.64126	6.94315	12.4171	9.60906	9.72625	9.28846	8.89796	0.44861185	0.966018836	4	8	24	0.603770848	0.861540092	24	19.05133797	0.077159842
ENSMUSG00000021257	Angel1	angel homolog 1 [Source:MGI Symbol;Acc:MGI:1915987]	14.0591	10.7118	11.1238	12.3199	11.5803	13.3475	11.373	13.5785	9.50172	16.2878	11.0094	12.711	13.1437	11.8752	0.648082424	0.966018836	24	12	24	0.670625441	0.888925819	24	19.5083375	0.067150265
ENSMUSG00000021258	Ccnk	cyclin K [Source:MGI Symbol;Acc:MGI:1276106]	6.34033	7.11087	7.22275	7.31721	6.72527	7.30564	7.78938	7.57017	8.54041	8.54179	6.96924	7.24048	7.54736	7.30307	0.676344318	0.966018836	16	4	24	0.120542592	0.732894732	24	8.660876	0.073595718
ENSMUSG00000021259	Cyp46a1	cytochrome P450, family 46, subfamily a, polypeptide 1 [Source:MGI Symbol;Acc:MGI:1341877]	5.1383	5.82881	7.45096	5.14131	5.08489	5.6617	6.54565	4.97453	6.21407	6.0873	4.50459	5.97801	4.12224	5.65751	0.616047374	0.966018836	12	8	24	0.447555569	0.805393068	24	7.787425209	0.094144251
ENSMUSG00000021260	Hhipl1	hedgehog interacting protein-like 1 [Source:MGI Symbol;Acc:MGI:1919265]	0.907486	1.11685	1.43637	1.18175	0.969798	1.07389	1.01936	0.902157	1.17289	1.20605	1.0651	1.16777	0.963148	1.22643	0.031969981	0.324385159	12	16	24	0.070555527	0.675489316	24	9.906725204	0.085290921
ENSMUSG00000021262	Evl	Ena-vasodilator stimulated phosphoprotein [Source:MGI Symbol;Acc:MGI:1194884]	47.7451	40.399	37.7191	39.4717	41.2107	44.0685	36.5847	42.0648	40.4483	47.9274	36.7066	49.4089	39.6645	45.1745	0.903207257	0.966018836	24	12	24	0.798439201	0.935424343	24	20.98716503	0.038817981
ENSMUSG00000021263	Degs2	delta(4)-desaturase, sphingolipid 2 [Source:MGI Symbol;Acc:MGI:1917309]	1.11691	1.43635	1.20139	0.88685	1.44738	1.53771	0.870753	1.16752	1.77211	1.55677	1.35739	0.891793	1.03761	0.673036	0.36081615	0.884073267	12	16	24	0.361419474	0.786774635	24	12.88845171	0.112177737
ENSMUSG00000021264	Yy1	YY1 transcription factor [Source:MGI Symbol;Acc:MGI:99150]	13.2014	11.3478	11.3367	12.7196	11.9166	11.4063	12.5604	11.9468	11.2948	13.1807	12.4062	12.5142	12.5423	12.5951	0.43565473	0.966018836	4	8	24	0.675560126	0.891275029	24	19.13097495	0.026188352
ENSMUSG00000021265	Slc25a29	solute carrier family 25 (mitochondrial carrier, palmitoylcarnitine transporter), member 29 [Source:MGI Symbol;Acc:MGI:2444911]	4.0484	3.41383	3.04363	4.21177	3.70704	3.80404	3.96155	3.89591	3.80997	4.59579	3.51281	4.0408	3.59231	3.6207	0.935316621	0.966018836	16	20	24	0.609834681	0.86453423	24	16.37306376	0.044980668
ENSMUSG00000021266	Wars	tryptophanyl-tRNA synthetase [Source:MGI Symbol;Acc:MGI:104630]	32.1068	24.6016	26.7793	24.5043	27.5698	30.8655	27.7817	29.8813	21.8771	33.9634	24.3661	27.3981	26.2615	27.04	0.66832131	0.966018836	24	12	24	0.51444321	0.816764397	24	20.88498919	0.078392574
ENSMUSG00000021268	Meg3	maternally expressed 3 [Source:MGI Symbol;Acc:MGI:1202886]	596.82	618.719	628.351	585.705	642.565	561.365	579.379	558.237	694.312	578.975	682.048	616.06	647.047	630.974	0.91724565	0.966018836	12	12	24	0.644538881	0.879359107	24	10.85061424	0.03540573
ENSMUSG00000021270	Hsp90aa1	heat shock protein 90, alpha (cytosolic), class A member 1 [Source:MGI Symbol;Acc:MGI:96250]	312.459	256.794	251.448	272.018	268.266	259.202	245.459	239.314	311.124	260.714	383.287	319.066	329.24	286.161	0.696669634	0.966018836	20	12	24	0.452535345	0.805393068	24	17.8981274	0.089136415
ENSMUSG00000021271	Zfp839	zinc finger protein 839 [Source:MGI Symbol;Acc:MGI:1920055]	2.55449	2.53497	2.43312	2.50213	2.49381	2.39916	2.24758	2.81768	2.52286	2.71656	2.50795	2.46919	2.66912	2.55463	0.182849367	0.659303873	8	16	24	0.426333554	0.805393068	24	7.480345524	0.02931374
ENSMUSG00000021273	Fdft1	farnesyl diphosphate farnesyl transferase 1 [Source:MGI Symbol;Acc:MGI:102706]	100.344	96.2735	95.5254	94.4179	96.0269	104.665	96.823	90.4629	94.9155	99.6265	100.944	102.352	104.853	101.328	0.020119233	0.259417302	24	12	24	0.050244761	0.644435324	24	20.23668284	0.050565123
ENSMUSG00000021275	Tecpr2	tectonin beta-propeller repeat containing 2 [Source:MGI Symbol;Acc:MGI:2144865]	8.55773	6.81642	6.74754	7.75828	7.16031	8.11923	7.27526	7.1793	5.90545	9.21743	6.84758	7.64454	7.25881	7.76764	0.14313801	0.585944911	24	12	24	0.49799695	0.807927978	24	19.01368982	0.068196762
ENSMUSG00000021276	Cinp	cyclin-dependent kinase 2 interacting protein [Source:MGI Symbol;Acc:MGI:1914486]	32.3041	29.9997	29.0486	28.7518	29.7872	27.8737	28.7422	26.832	27.7209	29.0947	31.6258	31.7528	29.336	33.2545	0.94086481	0.966018836	8	4	24	0.660641943	0.885496082	24	20.99280811	0.032362201
ENSMUSG00000021277	Traf3	TNF receptor-associated factor 3 [Source:MGI Symbol;Acc:MGI:108041]	6.82333	5.71204	6.83004	6.23392	5.75502	6.85327	6.51919	6.42835	4.9329	7.59581	5.87951	6.50364	6.61179	6.40249	0.483881213	0.966018836	24	20	24	0.736535882	0.914180155	24	20.94814789	0.042461297
ENSMUSG00000021279	Cdc42bpb	CDC42 binding protein kinase beta [Source:MGI Symbol;Acc:MGI:2136459]	19.3309	18.6969	19.7266	19.474	19.9755	16.4914	18.5999	21.7331	23.2727	23.226	19.4692	22.7707	19.3695	20.8024	0.152374472	0.60072565	12	16	24	0.113676431	0.727440321	24	9.268200774	0.095735218
ENSMUSG00000021280	Exoc3l4	exocyst complex component 3-like 4 [Source:MGI Symbol;Acc:MGI:1921363]	1.38604	1.33964	1.24853	1.70684	1.73696	1.79454	1.94033	1.28919	1.37274	1.98814	1.29979	1.46348	1.25424	1.88151	0.916746631	0.966018836	16	20	24	0.642626167	0.878809617	24	15.91486925	0.057532831
ENSMUSG00000021281	Tnfaip2	tumor necrosis factor, alpha-induced protein 2 [Source:MGI Symbol;Acc:MGI:104960]	1.12085	1.15157	2.23462	1.58495	1.4811	1.48962	1.15477	3.10073	1.62534	1.88862	1.32903	1.23496	1.55714	1.68495	0.053683798	0.394331071	12	16	24	0.113740077	0.727440321	24	8.10472608	0.207410836
ENSMUSG00000021282	Eif5	eukaryotic translation initiation factor 5 [Source:MGI Symbol;Acc:MGI:95309]	139.372	134.886	133.547	131.801	145.04	136.201	136.442	132.929	117.611	135.244	140.882	134.133	132.553	132.094	0.035023815	0.328311403	20	16	24	0.043372865	0.626520598	24	18.95338534	0.060642209
ENSMUSG00000021285	Ppp1r13b	protein phosphatase 1, regulatory subunit 13B [Source:MGI Symbol;Acc:MGI:1336199]	8.18052	7.18809	7.2138	7.57016	7.29046	8.17636	8.08494	7.51933	6.47693	9.30316	7.0429	8.36265	7.01176	7.41847	0.245061943	0.741580974	24	12	24	0.449484212	0.805393068	24	18.85443102	0.061006604
ENSMUSG00000021286	Zfyve21	zinc finger, FYVE domain containing 21 [Source:MGI Symbol;Acc:MGI:1915770]	19.8516	17.8529	18.2087	19.909	19.4873	16.5703	18.6755	17.2009	21.2395	17.8882	20.4364	18.303	19.8275	16.8663	0.478724519	0.966018836	20	12	24	0.572738696	0.846721006	24	13.88207511	0.042838599
ENSMUSG00000021287	Xrcc3	X-ray repair complementing defective repair in Chinese hamster cells 3 [Source:MGI Symbol;Acc:MGI:1921585]	2.6991	3.60484	3.89167	3.49621	3.84661	3.252	3.36333	3.52029	4.38838	3.4663	3.36838	3.33876	3.40761	3.70688	2.86E-04	0.049770315	12	12	24	0.021523576	0.574114605	24	9.204460827	0.119303987
ENSMUSG00000021288	Klc1	kinesin light chain 1 [Source:MGI Symbol;Acc:MGI:107978]	473.896	367.81	364.709	380.786	389.465	445.73	406.386	419.829	305.814	468.006	391.364	417.203	415.597	407.402	0.047644929	0.377556215	24	12	24	0.145407117	0.754184974	24	20.74307802	0.123228991
ENSMUSG00000021290	Atp5mpl	ATP synthase membrane subunit 6.8PL [Source:MGI Symbol;Acc:MGI:1917507]	583.842	657.953	590.946	622.453	599.813	556.051	594.292	604.755	714.037	524.447	683.049	634.481	733.439	721.209	0.945282252	0.966018836	8	8	24	0.370623779	0.79278606	24	4.454867964	0.070211232
ENSMUSG00000021294	Kif26a	kinesin family member 26A [Source:MGI Symbol;Acc:MGI:2447072]	1.0679	0.506832	0.605369	0.432981	0.472566	0.502506	0.744656	0.721719	0.537457	0.788721	0.619509	0.84205	0.702534	0.945683	0.334687844	0.85758369	4	12	24	0.164388416	0.767020009	24	0.939044393	0.10700186
ENSMUSG00000021301	Hecw1	HECT, C2 and WW domain containing E3 ubiquitin protein ligase 1 [Source:MGI Symbol;Acc:MGI:2444115]	24.5224	22.5963	19.7656	23.3826	20.1902	20.2737	19.4529	17.6266	20.9095	18.879	22.6516	22.5322	22.3771	23.3253	0.794347041	0.966018836	4	8	24	0.74361347	0.916215545	24	21.30368782	0.042430894
ENSMUSG00000021302	Ggps1	geranylgeranyl diphosphate synthase 1 [Source:MGI Symbol;Acc:MGI:1341724]	28.3795	31.2085	28.1621	30.7093	29.0634	31.1703	30.244	30.996	30.992	25.5075	34.0268	30.6854	32.0424	31.6431	0.783267549	0.966018836	8	8	24	0.574171837	0.847227916	24	23.10960034	0.038659751
ENSMUSG00000021303	Gng4	guanine nucleotide binding protein (G protein), gamma 4 [Source:MGI Symbol;Acc:MGI:102703]	3.46391	3.16661	3.57045	2.7414	2.73865	3.47212	3.16056	3.25647	3.01028	3.6565	3.11011	3.24632	2.77763	4.08359	0.772442842	0.966018836	8	12	24	0.484834841	0.805393068	24	4.557114515	0.060683658
ENSMUSG00000021306	Gpr137b	G protein-coupled receptor 137B [Source:MGI Symbol;Acc:MGI:1891463]	20.9903	17.1216	16.0752	18.0878	19.964	17.0507	19.338	17.5544	17.2747	19.8546	18.3659	17.9814	19.5557	17.1024	0.183755367	0.662021716	4	8	24	0.248587983	0.768040178	24	19.35296691	0.070162744
ENSMUSG00000021311	Mtr	5-methyltetrahydrofolate-homocysteine methyltransferase [Source:MGI Symbol;Acc:MGI:894292]	7.71339	7.38541	7.29571	8.15182	8.39568	7.49565	7.3994	7.47139	7.6854	8.10158	7.66327	8.10677	7.63701	6.77014	0.007831348	0.192760771	16	16	24	0.009697843	0.502965379	24	15.68786858	0.071129661
ENSMUSG00000021313	Ryr2	ryanodine receptor 2, cardiac [Source:MGI Symbol;Acc:MGI:99685]	9.94058	9.09149	11.3758	10.2123	8.513	10.3556	9.23436	9.1903	8.47673	10.2115	8.77431	9.75506	8.96532	11.7794	0.673567285	0.966018836	16	4	24	0.711871574	0.904842749	24	6.55393039	0.045682654
ENSMUSG00000021314	Amph	amphiphysin [Source:MGI Symbol;Acc:MGI:103574]	50.1572	41.9302	42.1642	42.1185	39.5188	47.9323	45.3735	44.4132	33.3327	51.6901	38.8094	44.1346	42.2518	44.7883	0.297978647	0.80820842	4	16	24	0.432773906	0.805393068	24	22.07640689	0.081149378
ENSMUSG00000021319	Sfrp4	secreted frizzled-related protein 4 [Source:MGI Symbol;Acc:MGI:892010]	23.7035	26.0407	26.0077	22.9321	29.0124	24.3623	18.728	27.1261	31.3423	13.2234	23.216	26.6212	18.7939	20.01	0.850398351	0.966018836	12	16	24	0.995524466	0.998760424	24	1.861751308	0.010937587
ENSMUSG00000021322	Aoah	acyloxyacyl hydrolase [Source:MGI Symbol;Acc:MGI:1350928]	0.589062	0.715738	1.04299	0.811886	0.940501	0.680347	0.601367	0.680168	0.99363	0.42029	0.834053	0.686764	0.585098	0.658269	0.009118941	0.192760771	12	16	24	0.334610209	0.770790723	24	11.1202942	0.080948013
ENSMUSG00000021326	Trim27	tripartite motif-containing 27 [Source:MGI Symbol;Acc:MGI:97904]	12.069	10.7779	10.4795	11.8113	12.6215	11.2245	10.2701	11.5401	10.2567	12.7148	12.0983	12.6197	10.0179	11.1137	0.073315272	0.447768907	16	20	24	0.044948298	0.628432994	24	15.92639017	0.098258884
ENSMUSG00000021327	Zkscan3	zinc finger with KRAB and SCAN domains 3 [Source:MGI Symbol;Acc:MGI:1919989]	20.6554	19.179	17.7838	22.1973	21.7234	18.1601	23.4714	16.592	20.4694	15.8137	22.8302	17.3828	18.8105	17.314	0.793403332	0.966018836	20	12	24	0.472095265	0.805393068	24	17.93314145	0.081623495
ENSMUSG00000021337	Scgn	secretagogin, EF-hand calcium binding protein [Source:MGI Symbol;Acc:MGI:2384873]	1.07962	1.53441	1.45094	1.79009	1.76443	1.53715	0.969552	1.13961	1.56348	1.27943	1.55394	1.37343	1.49003	0.849532	0.05960865	0.406067671	20	12	24	0.038705612	0.61140397	24	14.33811186	0.154466399
ENSMUSG00000021338	Carmil1	capping protein regulator and myosin 1 linker 1 [Source:MGI Symbol;Acc:MGI:1915982]	7.29002	6.5148	7.05218	6.45728	6.84277	6.80064	7.23772	7.90972	6.55275	7.55248	6.88552	6.53939	6.04369	7.09737	0.853736882	0.966018836	8	16	24	0.807006062	0.938616703	24	6.375281787	0.024123533
ENSMUSG00000021339	Mrs2	MRS2 magnesium transporter [Source:MGI Symbol;Acc:MGI:2685748]	18.4264	16.8517	19.4151	18.2499	18.2507	17.5259	18.9291	17.1536	14.7874	20.5415	16.8566	17.8698	18.2518	17.8663	0.909604019	0.966018836	4	4	24	0.803574845	0.937199097	24	17.25181245	0.028361242
ENSMUSG00000021340	Gpld1	glycosylphosphatidylinositol specific phospholipase D1 [Source:MGI Symbol;Acc:MGI:106604]	4.2188	4.26804	6.23915	3.9616	3.81032	6.29795	5.18118	4.24293	4.36428	4.46258	3.40338	4.22601	3.30137	4.68508	0.843639218	0.966018836	12	8	24	0.823490332	0.94441254	24	4.314919706	0.054002784
ENSMUSG00000021356	Irf4	interferon regulatory factor 4 [Source:MGI Symbol;Acc:MGI:1096873]	0.378383	0.51693	0.66463	0.61592	0.343707	0.403361	0.631053	0.626475	0.506807	0.545057	0.392327	0.512633	0.633334	0.616038	0.203532277	0.689899499	8	12	24	0.030289058	0.597625107	24	5.88599943	0.097865186
ENSMUSG00000021357	Exoc2	exocyst complex component 2 [Source:MGI Symbol;Acc:MGI:1913732]	17.7501	16.9202	17.173	17.7711	15.2436	17.7768	16.2634	16.8734	16.1128	18.998	16.1614	17.3248	17.598	18.1631	0.919119843	0.966018836	16	4	24	0.827738703	0.946180429	24	5.402079765	0.019605025
ENSMUSG00000021359	Tfap2a	transcription factor AP-2, alpha [Source:MGI Symbol;Acc:MGI:104671]	0.668533	0.455727	0.481276	0.438656	0.51189	0.446951	0.447961	0.559584	0.541882	0.587674	0.805717	0.410852	0.527872	0.58365	0.882617464	0.966018836	20	4	24	0.899510222	0.971453495	24	12.60308523	0.017527288
ENSMUSG00000021360	Gcnt2	glucosaminyl (N-acetyl) transferase 2, I-branching enzyme [Source:MGI Symbol;Acc:MGI:1100870]	102.413	81.7653	84.8105	81.8505	90.2349	96.3257	82.6325	86.2678	64.8611	89.3753	90.1697	86.8923	90.2664	82.707	0.045862815	0.374349041	24	12	24	0.03596139	0.609176161	24	20.05156381	0.138536553
ENSMUSG00000021361	Tmem14c	transmembrane protein 14C [Source:MGI Symbol;Acc:MGI:1913404]	59.9305	56.6233	57.7967	64.019	53.4567	58.1081	57.4092	62.5434	58.7729	59.2206	67.5638	57.6565	70.0998	62.3428	0.816300547	0.966018836	16	8	24	0.925075999	0.980131499	24	4.472147788	0.01456798
ENSMUSG00000021364	Elovl2	elongation of very long chain fatty acids (FEN1/Elo2, SUR4/Elo3, yeast)-like 2 [Source:MGI Symbol;Acc:MGI:1858960]	2.46002	2.47649	3.32617	2.24892	1.94663	2.98292	2.72364	2.46734	2.16122	2.90589	2.14952	2.13498	1.89887	2.94096	0.622132989	0.966018836	8	12	24	0.491681318	0.805393068	24	6.146022218	0.08384693
ENSMUSG00000021365	Nedd9	neural precursor cell expressed, developmentally down-regulated gene 9 [Source:MGI Symbol;Acc:MGI:97302]	6.8658	6.80323	6.52415	7.25068	5.90329	6.5895	6.37099	5.80983	7.01375	8.02159	5.67208	6.06173	6.69364	5.75781	0.187699199	0.662239322	16	4	24	0.49234676	0.805476481	24	10.23333689	0.057482202
ENSMUSG00000021366	Hivep1	human immunodeficiency virus type I enhancer binding protein 1 [Source:MGI Symbol;Acc:MGI:96100]	7.36855	4.89179	5.67942	5.54408	5.79594	6.17876	6.07705	6.27029	5.09634	7.58857	4.81435	5.78595	5.86539	6.20837	0.772442842	0.966018836	8	4	24	0.810492778	0.939224719	24	22.85379445	0.043431438
ENSMUSG00000021367	Edn1	endothelin 1 [Source:MGI Symbol;Acc:MGI:95283]	1.02219	1.29558	0.872499	0.924312	1.20666	1.29276	1.64131	1.40466	1.22101	1.44284	0.99631	1.29823	1.19795	0.890761	0.935316621	0.966018836	20	12	24	0.546485966	0.833354874	24	22.25587506	0.066823785
ENSMUSG00000021368	Tbc1d7	TBC1 domain family, member 7 [Source:MGI Symbol;Acc:MGI:1914296]	30.2429	32.8242	31.4206	30.7893	32.3533	30.6101	28.7822	29.6319	29.4115	31.5397	32.6605	27.6092	31.4845	31.0285	0.503972924	0.966018836	20	4	24	0.504464342	0.811028388	24	11.72926646	0.031450297
ENSMUSG00000021371	Mcur1	mitochondrial calcium uniporter regulator 1 [Source:MGI Symbol;Acc:MGI:1923387]	6.60725	7.58514	8.59672	9.28486	8.38199	7.727	7.96992	8.35479	8.56814	10.1364	8.45643	8.0145	9.06786	7.77801	0.022864519	0.273713312	16	16	24	0.008432655	0.498868519	24	11.56669945	0.126570409
ENSMUSG00000021373	Cap2	CAP, adenylate cyclase-associated protein, 2 (yeast) [Source:MGI Symbol;Acc:MGI:1914502]	4.72836	5.98181	7.19869	6.42565	5.51173	6.77501	6.51577	11.1542	7.87968	8.86013	4.2082	5.84205	6.50479	6.50059	0.251986462	0.75129457	12	8	24	0.105819119	0.717832234	24	7.002652841	0.247087121
ENSMUSG00000021374	Nup153	nucleoporin 153 [Source:MGI Symbol;Acc:MGI:2385621]	12.6652	11.6778	11.9931	13.7936	12.0988	12.5949	12.5562	11.259	14.2575	11.7666	13.6919	12.2987	13.444	14.5213	0.761468226	0.966018836	4	4	24	0.895170321	0.970250894	24	10.1598694	0.018828366
ENSMUSG00000021375	Kif13a	kinesin family member 13A [Source:MGI Symbol;Acc:MGI:1098264]	11.5912	10.5455	11.7859	13.2814	10.9696	11.9795	13.2014	13.4078	11.1512	15.0389	10.5142	10.8157	12.2627	11.8626	0.893307667	0.966018836	4	8	24	0.685366735	0.895382938	24	8.592003663	0.05076536
ENSMUSG00000021376	Tpmt	thiopurine methyltransferase [Source:MGI Symbol;Acc:MGI:98812]	9.84693	9.02962	9.2717	9.95204	9.02857	10.2131	8.8662	9.92237	8.48878	8.9547	10.4364	8.8075	7.40808	9.26858	0.941830768	0.966018836	20	8	24	0.539185646	0.829144433	24	15.3581034	0.04663134
ENSMUSG00000021377	Dek	DEK proto-oncogene (DNA binding) [Source:MGI Symbol;Acc:MGI:1926209]	20.7985	25.7284	27.6122	26.9879	25.0098	22.2756	22.8354	22.5729	26.7041	21.2478	22.3994	22.3184	23.6944	22.8711	0.0444686	0.365196805	12	12	24	0.087776552	0.707747429	24	9.192381047	0.100919113

ENSMUSG00000021379	Id4	inhibitor of DNA binding 4 [Source:MGI Symbol;Acc:MGI:99414]	19.6089	17.9703	18.6815	16.9186	16.9183	17.5215	18.4019	15.6687	15.6079	19.4623	17.5398	18.2554	16.3352	18.2764	0.853187563	0.966018836	4	8	24	0.799023706	0.935673715	24	20.02465082	0.027045532
ENSMUSG00000021384	Susd3	sushi domain containing 3 [Source:MGI Symbol;Acc:MGI:1913579]	0.574488	0.613677	0.797073	0.697231	1.09569	0.748586	1.54482	0.482031	0.938275	1.05095	0.662896	0.55746	0.546301	0.759455	0.336988702	0.85758369	12	20	24	0.741393532	0.915831442	24	13.4497979	0.065625638
ENSMUSG00000021385	Ippk	inositol 1,3,4,5,6-pentakisphosphate 2-kinase [Source:MGI Symbol;Acc:MGI:1922928]	6.89056	6.5185	6.23327	7.37916	6.63674	6.48598	6.65209	5.61493	5.93424	5.99667	7.18303	6.56183	6.12022	5.69609	0.166457038	0.633636735	20	12	24	0.016525398	0.544099157	24	17.38579846	0.091846521
ENSMUSG00000021388	Aspn	asporin [Source:MGI Symbol;Acc:MGI:1913945]	6.06783	7.96479	6.70124	6.65023	6.69987	6.19326	5.92901	8.44979	8.40114	4.96302	7.56139	6.22516	5.98203	6.05651	0.132887428	0.567728672	12	16	24	0.492075698	0.805393068	24	6.893062709	0.091217318
ENSMUSG00000021390	Ogn	osteoglycin [Source:MGI Symbol;Acc:MGI:109278]	285.388	324.841	275.576	284.307	371.214	262.979	327.476	282.444	375.717	192.203	354.248	261.87	280.624	245.492	0.895137379	0.966018836	20	4	24	0.941058642	0.985441727	24	19.54459016	0.036954429
ENSMUSG00000021391	Cenpp	centromere protein P [Source:MGI Symbol;Acc:MGI:1913586]	0.938141	1.33157	1.51489	0.873707	0.810351	1.14148	1.08188	0.973241	1.356	1.26439	1.32857	1.08694	0.937044	0.929754	0.43565473	0.966018836	12	16	24	0.417696773	0.802361287	24	9.102872543	0.078271866
ENSMUSG00000021392	Nol8	nucleolar protein 8 [Source:MGI Symbol;Acc:MGI:1918180]	8.28557	7.87203	7.36242	8.23847	7.94057	7.20515	7.97118	7.6044	9.04101	7.02069	9.02113	8.44643	8.91979	8.17431	0.908406451	0.966018836	4	12	24	0.907318688	0.974606327	24	22.4085902	0.01766633
ENSMUSG00000021395	Spin1	spindlin 1 [Source:MGI Symbol;Acc:MGI:109242]	35.5845	30.4527	30.8095	31.6537	30.6491	33.8612	34.5776	32.5676	27.1644	36.3175	30.7635	33.0407	31.7031	33.3681	0.336755135	0.85758369	4	8	24	0.334083483	0.770129916	24	21.4133675	0.062566075
ENSMUSG00000021396	Nxnl2	nucleoredoxin-like 2 [Source:MGI Symbol;Acc:MGI:1922374]	0.976077	1.06184	0.75618	1.09183	1.20956	1.25295	0.71415	0.719569	1.00882	1.2534	0.928379	1.27326	1.1718	1.25828	0.649212555	0.966018836	24	4	24	0.359605323	0.785405943	24	16.731696	0.07912823
ENSMUSG00000021400	Wrnip1	Werner helicase interacting protein 1 [Source:MGI Symbol;Acc:MGI:1926153]	18.4697	20.5677	18.1129	17.5278	17.7724	22.4329	20.4982	20.3913	16.0473	19.1691	20.084	19.8732	18.3448	17.2284	0.236893212	0.7385769	24	12	24	0.111754563	0.724576435	24	20.96947555	0.098046232
ENSMUSG00000021408	Ripk1	receptor (TNFRSF)-interacting serine-threonine kinase 1 [Source:MGI Symbol;Acc:MGI:108212]	4.00231	3.73294	4.38663	3.97557	3.99915	3.28569	3.87205	3.72498	4.15109	3.05119	3.88429	3.37791	3.62654	3.11498	0.390686537	0.915479136	12	12	24	0.775247795	0.927711986	24	9.142868257	0.030096431
ENSMUSG00000021411	Pxdc1	PX domain containing 1 [Source:MGI Symbol;Acc:MGI:1914145]	8.47886	8.17856	8.7281	9.09244	11.1392	8.49313	9.15855	8.61368	10.0357	8.49814	7.77604	8.29321	9.01452	7.17651	0.967855455	0.970142211	12	20	24	0.53938217	0.82930214	24	14.59952942	0.056808035
ENSMUSG00000021413	Prpf4b	pre-mRNA processing factor 4B [Source:MGI Symbol;Acc:MGI:109584]	33.0746	32.5621	30.3168	31.9318	33.4069	30.9291	33.3686	28.4581	35.0728	29.0644	31.6793	30.6522	35.3052	31.4718	0.777666236	0.966018836	4	12	24	0.706888649	0.903219612	24	23.03571754	0.030073756
ENSMUSG00000021417	Eci2	enoyl-Coenzyme A delta isomerase 2 [Source:MGI Symbol;Acc:MGI:1346064]	41.2271	42.2062	46.1827	49.9424	41.7806	37.6282	39.2568	40.1837	38.7495	41.4644	41.1859	38.9098	40.1474	41.6473	0.062036003	0.409814092	16	8	24	0.075975998	0.686372559	24	10.43737237	0.079890684
ENSMUSG00000021418	Rpp40	ribonuclease P 40 subunit [Source:MGI Symbol;Acc:MGI:1346084]	6.32492	8.43	8.6929	9.04029	6.67572	6.67105	5.44556	7.14732	8.59709	5.2634	8.16412	7.15587	8.12442	6.69149	0.277087842	0.789849983	12	16	24	0.466764501	0.805393068	24	8.892999288	0.108894315
ENSMUSG00000021420	Fars2	phenylalanine-tRNA synthetase 2 (mitochondrial) [Source:MGI Symbol;Acc:MGI:1917205]	18.3507	18.7926	19.7816	17.3625	18.3034	16.3992	15.5556	19.8191	17.0287	17.6121	19.7939	17.1621	24.2231	21.3829	0.643325306	0.966018836	8	16	24	0.390385767	0.799546792	24	4.500080601	0.079045787
ENSMUSG00000021423	Ly86	lymphocyte antigen 86 [Source:MGI Symbol;Acc:MGI:1321404]	111.585	89.622	101.319	87.9211	110.539	100.131	83.2218	85.4741	82.598	62.5422	113.894	84.1814	87.7556	82.9592	0.481530854	0.966018836	20	20	24	0.233053569	0.768040178	24	19.55944017	0.140175727
ENSMUSG00000021427	Ssr1	signal sequence receptor, alpha [Source:MGI Symbol;Acc:MGI:105082]	28.1385	27.2036	26.7976	27.0011	27.1541	26.096	26.7176	27.4548	28.6899	28.1911	27.8573	26.9493	28.759	27.7456	0.91724565	0.966018836	4	20	24	0.443313479	0.805393068	24	6.971294679	0.018987572
ENSMUSG00000021428	Riok1	RIO kinase 1 [Source:MGI Symbol;Acc:MGI:1918590]	7.40238	10.0848	8.77949	7.75397	8.57143	7.95129	7.5366	8.09494	9.39845	8.76017	8.74076	9.2537	10.2682	9.59329	0.696669634	0.966018836	8	20	24	0.429836127	0.805393068	24	6.450148625	0.062409703
ENSMUSG00000021431	Snrnp48	small nuclear ribonucleoprotein 48 (U11/U12) [Source:MGI Symbol;Acc:MGI:1915047]	18.3124	15.2034	13.1639	13.3238	15.4763	14.37	15.6711	13.691	13.9407	12.4651	17.8108	15.4028	17.4646	15.9714	0.008244783	0.192760771	4	12	24	0.031638725	0.604316769	24	22.02095155	0.150166524
ENSMUSG00000021432	Slc35b3	solute carrier family 35, member B3 [Source:MGI Symbol;Acc:MGI:1913978]	13.6869	15.963	15.7455	15.5997	13.984	12.3307	11.6409	14.4449	16.7684	12.6408	15.1525	14.9495	16.0742	15.3897	0.22872448	0.72665454	12	12	24	0.197801763	0.768040178	24	7.811336622	0.104926628
ENSMUSG00000021448	Shc3	src homology 2 domain-containing transforming protein C3 [Source:MGI Symbol;Acc:MGI:106179]	1.41198	1.34619	1.90483	1.42777	0.950589	1.55912	1.89768	1.30154	1.15223	1.5685	1.23406	1.57825	0.930022	1.8214	0.790218349	0.966018836	24	20	24	0.744317415	0.916430276	24	4.713571964	0.058663614
ENSMUSG00000021451	Sema4d	sema domain, immunoglobulin domain (Ig), transmembrane domain (TM) and short cytoplasmic domain, (semaphorin) 4D [Source:MGI Symbol;Acc:MGI:109244]	15.5629	15.2625	18.4018	16.2265	13.7047	17.4251	15.9727	17.902	15.5499	18.36	13.3247	16.2755	13.4101	16.4739	0.671726933	0.966018836	16	4	24	0.393216368	0.799546792	24	7.346397984	0.080567872
ENSMUSG00000021453	Gadd45g	growth arrest and DNA-damage-inducible 45 gamma [Source:MGI Symbol;Acc:MGI:1346325]	6.50614	6.20512	9.02022	6.55559	6.46246	6.20548	8.95188	9.49636	8.03048	11.7127	8.5358	7.45979	6.99879	7.19715	0.823657258	0.966018836	12	12	24	0.261704979	0.768040178	24	9.467178979	0.151652731
ENSMUSG00000021456	Fbp2	fructose bisphosphatase 2 [Source:MGI Symbol;Acc:MGI:95491]	2.2648	2.52406	3.18856	4.17558	2.57522	2.54099	2.60316	3.85421	2.77843	3.58602	3.48864	3.05132	3.27804	2.922	0.305564675	0.811201458	16	12	24	0.091974755	0.71175955	24	10.87294277	0.151251009
ENSMUSG00000021457	Syk	spleen tyrosine kinase [Source:MGI Symbol;Acc:MGI:99515]	1.19184	1.70854	2.4376	2.33585	1.92506	1.74862	1.99637	1.90558	2.09247	1.25012	1.6571	1.49334	1.96597	1.22628	0.714044782	0.966018836	12	12	24	0.593706818	0.856999712	24	10.15962628	0.083325833
ENSMUSG00000021458	Aopep	aminopeptidase O [Source:MGI Symbol;Acc:MGI:1919311]	71.1701	81.703	75.3725	77.1984	81.7287	72.2209	66.5572	72.0581	84.745	68.3891	79.2037	74.2839	75.6715	73.6219	0.321662653	0.83607295	20	8	24	0.435075396	0.805393068	24	11.08866756	0.050525959
ENSMUSG00000021460	Auh	AU RNA binding protein/enoyl-coenzyme A hydratase [Source:MGI Symbol;Acc:MGI:1338011]	40.7029	37.4897	38.9121	39.1487	39.9173	41.8766	40.7676	43.7438	34.1893	42.7664	35.3447	39.3595	40.885	39.2519	0.44861185	0.966018836	4	8	24	0.512433804	0.815745835	24	22.62942823	0.044450208
ENSMUSG00000021461	Fancc	Fanconi anemia, complementation group C [Source:MGI Symbol;Acc:MGI:95480]	6.43192	7.28998	6.73144	7.38037	6.91397	6.13617	6.03934	7.22103	8.2335	7.60392	6.557	6.69061	6.70122	6.53221	0.008673074	0.192760771	16	12	24	0.008779604	0.502965379	24	9.499917604	0.114555838
ENSMUSG00000021464	Ror2	receptor tyrosine kinase-like orphan receptor 2 [Source:MGI Symbol;Acc:MGI:1347521]	2.71688	2.16858	2.05228	2.42177	1.94787	2.20659	1.99368	2.09481	2.14985	2.64388	2.10046	2.77135	2.51722	2.19589	0.851656735	0.966018836	24	12	24	0.752486602	0.919493971	24	20.07284628	0.037254483
ENSMUSG00000021466	Ptch1	patched 1 [Source:MGI Symbol;Acc:MGI:105373]	10.1475	10.9239	10.547	8.99003	11.1963	9.89045	11.0537	10.4626	10.3119	10.2381	9.75492	10.2227	9.75459	9.98521	0.90332508	0.966018836	12	4	24	0.654215863	0.883406766	24	1.704411486	0.026620333
ENSMUSG00000021468	Sptlc1	serine palmitoyltransferase, long chain base subunit 1 [Source:MGI Symbol;Acc:MGI:1099431]	16.9983	16.9673	16.9962	16.4328	17.7043	17.1144	16.5776	15.9327	15.1963	16.6482	15.8141	17.1226	16.1557	17.2989	0.970092543	0.971116366	24	12	24	0.419136175	0.803638254	24	20.11179521	0.029104829
ENSMUSG00000021470	Ercc6l2	excision repair cross-complementing rodent repair deficiency, complementation group 6 like 2 [Source:MGI Symbol;Acc:MGI:1923501]	9.4771	11.3819	8.7392	10.5259	11.1232	9.10069	10.0645	9.89153	10.0585	9.69565	9.60871	10.4079	10.2257	10.4553	0.856752753	0.966018836	8	4	24	0.982723822	0.996149474	24	2.448962728	0.007074165
ENSMUSG00000021474	Sfxn1	sideroflexin 1 [Source:MGI Symbol;Acc:MGI:2137677]	11.5348	10.5852	11.6854	13.0784	11.239	11.8407	11.7019	11.7991	11.6269	12.6029	12.847	12.902	13.7051	13.7373	0.849180783	0.966018836	16	20	24	0.82003008	0.942893975	24	14.51859889	0.016671671
ENSMUSG00000021476	Habp4	hyaluronic acid binding protein 4 [Source:MGI Symbol;Acc:MGI:1891713]	41.5019	36.7493	39.1386	36.4654	34.1401	40.2327	40.3851	38.1338	35.1802	42.889	35.1245	40.6405	38.6022	43.5508	0.251986462	0.75129457	4	16	24	0.353375778	0.780711577	24	1.357750363	0.058029251
ENSMUSG00000021477	Ctsl	cathepsin L [Source:MGI Symbol;Acc:MGI:88564]	585.199	539.955	567.778	499.641	617.429	609.412	488.684	540.285	481.516	459.803	624.199	496.509	594.951	521.847	0.087978327	0.472021905	20	20	24	0.235466518	0.768040178	24	19.96724375	0.097863523
ENSMUSG00000021481	Zfp346	zinc finger protein 346 [Source:MGI Symbol;Acc:MGI:1349417]	8.69492	9.22119	10.3363	9.50757	9.16528	9.54433	9.00155	9.21381	10.4429	10.72	10.2995	9.75787	9.49952	9.77547	0.015486342	0.253395174	12	16	24	0.003253418	0.416319589	24	11.05755968	0.080107022
ENSMUSG00000021482	Prxl2c	peroxiredoxin like 2C [Source:MGI Symbol;Acc:MGI:1913379]	18.6424	15.0835	16.467	17.2978	19.6912	19.2496	17.3249	19.1478	15.5212	15.682	20.2385	16.5883	16.7217	17.1613	0.059650003	0.406067671	20	16	24	0.111710343	0.724455729	24	18.74983483	0.10271369
ENSMUSG00000021483	Cdk20	cyclin-dependent kinase 20 [Source:MGI Symbol;Acc:MGI:2145349]	6.16934	4.56245	4.59407	4.51571	5.72528	5.19187	4.36365	5.12355	3.95711	5.50042	4.76887	5.203	4.63101	4.2868	0.156274615	0.611802982	20	16	24	0.098872266	0.71175955	24	18.81288904	0.115878917
ENSMUSG00000021484	Lman2	lectin, mannose-binding 2 [Source:MGI Symbol;Acc:MGI:1914140]	20.3502	17.3795	18.2209	17.9578	18.3419	19.5575	20.702	19.3862	16.5935	17.5293	18.5178	18.7412	19.9483	17.9013	1.18E-04	0.029372629	4	8	24	0.002639658	0.40184247	24	22.07317967	0.098859821
ENSMUSG00000021485	Mxd3	Max dimerization protein 3 [Source:MGI Symbol;Acc:MGI:104987]	0.465512	0.557834	0.593228	0.321492	0.5905	0.513687	0.381593	0.675278	0.41952	0.350691	0.627607	0.447244	0.828905	0.57915	0.850398351	0.966018836	8	8	24	0.4326927	0.805393068	24	1.051707352	0.063368322
ENSMUSG00000021486	Prelid1	PRELI domain containing 1 [Source:MGI Symbol;Acc:MGI:1913744]	63.5183	53.605	55.8043	50.582	57.1574	59.4575	56.0629	57.9016	50.9863	58.6952	60.3104	60.3595	59.4237	57.4523	0.020119233	0.259417302	24	12	24	0.038092971	0.61140397	24	20.9409452	0.084251388
ENSMUSG00000021488	Nsd1	nuclear receptor-binding SET-domain protein 1 [Source:MGI Symbol;Acc:MGI:1276545]	16.2614	15.5316	16.7579	16.9495	15.5496	15.099	15.4666	14.0873	15.4836	14.7787	17.0559	16.2489	16.7205	16.6287	0.874054778	0.966018836	20	4	24	0.869173309	0.962936155	24	15.88177966	0.016545375
ENSMUSG00000021493	Pdlim7	PDZ and LIM domain 7 [Source:MGI Symbol;Acc:MGI:1914649]	63.1466	54.6479	49.3704	56.4118	49.9246	52.1971	58.5964	61.4052	62.0921	62.1687	58.7236	59.9582	57.1655	52.6472	0.823144114	0.966018836	4	8	24	0.912393322	0.975295827	24	2.042452553	0.019742803
ENSMUSG00000021494	Ddx41	DEAD box helicase 41 [Source:MGI Symbol;Acc:MGI:1920185]	9.13229	8.58456	9.00655	7.57263	8.99926	7.78051	8.7876	7.89636	8.85383	7.3258	8.11764	7.57343	8.91095	8.10813	0.305564675	0.811201458	4	12	24	0.340041325	0.774511771	24	1.96414339	0.051819328
ENSMUSG00000021495	Fam193b	family with sequence similarity 193, member B [Source:MGI Symbol;Acc:MGI:2385851]	28.3249	29.2843	27.7982	27.9858	33.9079	27.5059	35.2543	31.5418	31.6134	26.9325	32.7622	28.9201	29.456	29.6673	0.951846915	0.966018836	4	12	24	0.852794437	0.955976447	24	21.66227387	0.027131249
ENSMUSG00000021496	Pcbd2	pterin 4 alpha carbinolamine dehydratase/dimerization cofactor of hepatocyte nuclear factor 1 alpha (TCF1) 2 [Source:MGI Symbol;Acc:MGI:1919812]	15.6435	16.6189	15.0837	15.5016	15.2442	14.0219	16.8907	15.3152	15.7069	13.1965	17.4789	17.2742	16.6708	13.3046	0.542718076	0.966018836	20	20	24	0.429833225	0.805393068	24	21.25206106	0.064223884
ENSMUSG00000021497	Txndc15	thioredoxin domain containing 15 [Source:MGI Symbol;Acc:MGI:1916922]	36.1877	32.6042	33.7076	29.0558	32.1627	32.2995	34.5304	33.5815	34.7138	34.1626	37.106	35.5312	35.9543	34.261	0.636884864	0.966018836	4	12	24	0.339276624	0.773727252	24	23.48556352	0.044468429
ENSMUSG00000021500	Ddx46	DEAD box helicase 46 [Source:MGI Symbol;Acc:MGI:1920895]	16.2808	18.0889	18.5436	19.3373	16.5835	17.4767	16.2016	17.3411	18.2588	16.6407	18.3459	18.2369	18.576	20.0415	0.777867261	0.966018836	12	16	24	0.290917346	0.768040178	24	8.106731076	0.051652492
ENSMUSG00000021501	Caml	calcium modulating ligand [Source:MGI Symbol;Acc:MGI:104728]	37.5591	36.6352	33.7063	35.0129	40.4308	40.1673	34.1604	35.1685	33.5562	37.0749	40.8342	38.614	38.048	36.8096	5.51E-05	0.029372629	20	16	24	0.004600051	0.433238897	24	18.49748298	0.101122442
ENSMUSG00000021504	B4galt7	xylosylprotein beta1,4-galactosyltransferase, polypeptide 7 (galactosyltransferase I) [Source:MGI Symbol;Acc:MGI:2384987]	6.46105	5.84577	6.18904	6.01313	5.61411	6.66112	6.10029	6.93293	6.21116	7.11859	6.19205	6.0315	6.21991	5.79003	0.826055781	0.966018836	4	16	24	0.973674134	0.993305115	24	8.309386414	0.00818267
ENSMUSG00000021508	Cxcl14	chemokine (C-X-C motif) ligand 14 [Source:MGI Symbol;Acc:MGI:1888514]	0.690584	2.5313	3.80898	2.2222	1.53999	3.32752	3.4625	2.56022	2.7537	2.78754	1.6431	2.64759	1.71398	2.29917	0.793403332	0.966018836	12	8	24	0.525822428	0.822781291	24	8.221052266	0.183522026
ENSMUSG00000021510	Zfp729a	zinc finger protein 729a [Source:MGI Symbol;Acc:MGI:3036250]	4.65701	4.24028	4.06068	4.33867	4.58026	3.54995	5.54234	4.1782	4.53129	2.81197	5.68641	4.20261	4.1681	3.16214	0.892555977	0.966018836	20	12	24	0.52789115	0.82357362	24	20.3093043	0.099239338
ENSMUSG00000021514	Zfp369	zinc finger protein 369 [Source:MGI Symbol;Acc:MGI:2176229]	13.4009	12.9796	11.7954	12.2421	13.8487	12.2583	12.3073	12.4878	12.994	10.6685	13.5028	12.3638	11.6341	12.8248	0.920872048	0.966018836	20	20	24	0.691507133	0.897724829	24	20.84445998	0.031486925
ENSMUSG00000021518	Ptdss1	phosphatidylserine synthase 1 [Source:MGI Symbol;Acc:MGI:1276575]	27.0628	24.9566	27.0799	25.0372	27.0307	25.3727	26.9241	25.8591	21.3332	25.4378	24.1794	25.1333	24.7179	24.196	0.832293447	0.966018836	4	4	24	0.288797707	0.768040178	24	20.7437267	0.048517961
ENSMUSG00000021519	Mterf3	mitochondrial transcription termination factor 3 [Source:MGI Symbol;Acc:MGI:1913660]	22.3979	22.9398	19.3295	21.5014	21.5156	21.056	24.1218	18.2795	21.4788	18.385	23.2727	19.7189	22.634	19.5303	0.328944792	0.847277612	4	8	24	0.277289698	0.768040178	24	21.08475838	0.076151959
ENSMUSG00000021520	Uqcrb	ubiquinol-cytochrome c reductase binding protein [Source:MGI Symbol;Acc:MGI:1914780]	55.6548	68.2473	60.1774	80.1584	65.7871	63.2065	62.4481	66.3268	73.6573	60.5322	69.4262	65.0657	64.9767	60.4516	0.371875382	0.894537464	16	12	24	0.244106032	0.768040178	24	11.8493283	0.084123642
ENSMUSG00000021532	Fastkd3	FAST kinase domains 3 [Source:MGI Symbol;Acc:MGI:1916827]	7.76649	8.76316	7.48109	7.15489	8.24786	8.17727	6.8346	7.14006	7.68116	8.77554	8.90258	7.95118	7.74417	7.92853	0.277221318	0.789849983	20	8	24	0.38882181	0.799546792	24	15.31686803	0.0546051
ENSMUSG00000021534	1700001L19Rik	RIKEN cDNA 1700001L19 gene [Source:MGI Symbol;Acc:MGI:1916565]	3.18375	3.15189	2.12599	3.16411	3.74722	2.94037	2.23267	2.52783	2.57075	3.36233	2.94028	3.06255	2.67232	3.21989	0.745199643	0.966018836	16	20	24	0.261346641	0.768040178	24	16.22667296	0.106690991
ENSMUSG00000021536	Adcy2	adenylate cyclase 2 [Source:MGI Symbol;Acc:MGI:99676]	7.8661	7.68842	9.18015	8.45384	7.28683	8.40513	7.55304	7.82384	6.73429	8.76282	6.89136	7.33642	6.83918	8.17692	0.892555977	0.966018836	16	4	24	0.539060938	0.829144433	24	9.312398564	0.015141388
ENSMUSG00000021537	Cetn3	centrin 3 [Source:MGI Symbol;Acc:MGI:1097706]	55.8665	81.3791	76.1136	73.8949	78.558	65.5973	69.3613	70.1414	90.2579	63.6669	84.1909	65.1439	67.4549	68.5158	0.124028266	0.552144108	12	12	24	0.155581541	0.760951233	24	10.2808156	0.133968471
ENSMUSG00000021540	Smad5	SMAD family member 5 [Source:MGI Symbol;Acc:MGI:1328787]	7.5641	8.32504	8.46997	7.99252	8.39754	7.67516	8.2456	7.81982	8.7739	7.64651	8.22967	8.44018	7.3191	7.88727	0.286642595	0.793714531	12	16	24	0.437690731	0.805393068	24	10.90558194	0.034007179
ENSMUSG00000021541	Trpc7	transient receptor potential cation channel, subfamily C, member 7 [Source:MGI Symbol;Acc:MGI:1349470]	0.677673	0.669741	0.671191	0.398173	0.537207	0.661346	0.513345	0.374693	0.578319	0.642721	0.600899	0.53984	0.595842	[illegible]	0.834424969	0.966018836	20	16	24	0.267124573	0.768040178	24	21.94470525	0.057436466
ENSMUSG00000021546	Hnrnpk	heterogeneous nuclear ribonucleoprotein K [Source:MGI Symbol;Acc:MGI:99894]	79.2478	85.9319	85.7009	86.2432	79.7111	82.5413	80.4504	80.9731	80.6443	84.3428	80.9335	83.4588	79.9843	81.2629	0.064091035	0.414867207	16	12	24	0.208441078	0.768040178	24	10.35729035	0.027892706
ENSMUSG00000021548	Ccnh	cyclin H [Source:MGI Symbol;Acc:MGI:1913921]	20.8517	23.9688	23.2518	20.6166	20.1727	22.411	23.096	22.9507	23.8313	22.2993	22.2898	24.1163	23.2607	23.635	0.209185536	0.69936851	12	4	24	0.099149881	0.71175955	24	3.926148634	0.056289049
ENSMUSG00000021549	Rasa1	RAS p21 protein activator 1 [Source:MGI Symbol;Acc:MGI:97860]	33.8861	32.1302	29.5821	30.1924	32.3244	30.805	31.5218	29.0048	30.6943	28.06	33.9744	30.5262	31.0998	31.8315	0.327894132	0.846828588	20	20	24	0.210590957	0.768040178	24	21.13981248	0.052138988
ENSMUSG00000021550	2210016F16Rik	RIKEN cDNA 2210016F16 gene [Source:MGI Symbol;Acc:MGI:1917403]	4.61803	4.59783	4.37027	4.58681	4.41893	4.42103	4.25579	5.37227	4.87989	4.51566	4.9713	4.7383	5.12925	5.44088	0.622773557	0.966018836	8	16	24	0.097747293	0.71175955	24	5.112686312	0.061469617
ENSMUSG00000021552	Gkap1	G kinase anchoring protein 1 [Source:MGI Symbol;Acc:MGI:1891694]	14.9412	15.5704	14.0753	14.47	15.6312	14.936	14.8216	13.3135	15.1257	13.1007	16.9445	14.5872	13.3064	14.3817	0.823677655	0.966018836	20	20	24	0.344522162	0.777895009	24	17.52506723	0.053583637
ENSMUSG00000021555	Naa35	N(alpha)-acetyltransferase 35, NatC auxiliary subunit [Source:MGI Symbol;Acc:MGI:1925939]	37.7746	35.3424	38.0132	35.873	40.203	36.7806	29.7804	38.0891	45.7083	35.4592	44.2709	34.6382	50.2902	42.375	0.919571723	0.966018836	12	12	24	0.792029226	0.93349015	24	8.554943164	0.048514109
ENSMUSG00000021556	Golm1	golgi membrane protein 1 [Source:MGI Symbol;Acc:MGI:1917329]	4.26091	4.27203	3.88842	3.49685	4.7645	4.43386	4.31442	4.00695	3.55531	3.54949	4.54974	3.96676	4.10769	4.13063	0.002355803	0.106802459	20	20	24	0.035722875	0.609176161	24	20.87026358	0.103061191
ENSMUSG00000021557	Agtpbp1	ATP/GTP binding protein 1 [Source:MGI Symbol;Acc:MGI:2159437]	129.439	115.568	116.291	115.967	116.589	126.036	120.424	109.683	97.7431	118.385	117.903	118.986	118.067	125.545	0.033248781	0.324908923	4	8	24	0.083721085	0.700872524	24	20.96158532	0.081308621
ENSMUSG00000021559	Dapk1	death associated protein kinase 1 [Source:MGI Symbol;Acc:MGI:1916885]	17.7779	12.4363	12.6047	13.4671	13.4074	15.2285	13.0384	14.1109	10.5501	16.5614	12.3488	14.8405	13.4418	13.7803	0.390686537	0.915479136	24	12	24	0.298480099	0.768040178	24	20.45174274	0.109464748
ENSMUSG00000021565	Slc6a19	solute carrier family 6 (neurotransmitter transporter), member 19 [Source:MGI Symbol;Acc:MGI:1921588]	5.90763	4.54502	5.31489	4.20193	4.88467	5.21113	4.6998	4.63292	3.84231	5.43627	4.93993	4.72597	5.06478	4.82573	0.584233363	0.966018836	4	4	24	0.358317819	0.784922891	24	20.72008301	0.073773245
ENSMUSG00000021567	Nkd2	naked cuticle 2 [Source:MGI Symbol;Acc:MGI:1919543]	1.44766	1.71024	1.6907	1.22134	1.45499	1.28582	1.80997	1.51293	1.45064	1.3974	1.08231	1.59998	1.56484	1.65365	0.074074737	0.448301997	8	8	24	0.020785748	0.568066329	24	2.997790502	0.113773635
ENSMUSG00000021569	Trip13	thyroid hormone receptor interactor 13 [Source:MGI Symbol;Acc:MGI:1916966]	0.296851	0.463116	0.428896	0.488736	0.499425	0.452821	0.5936	0.71473	0.623546	0.461	0.544228	0.464701	0.570406	0.678324	0.870491976	0.966018836	8	16	24	0.157010239	0.763141258	24	6.156981515	0.061215655
ENSMUSG00000021572	Cep72	centrosomal protein 72 [Source:MGI Symbol;Acc:MGI:1921720]	0.578702	0.769304	1.25561	0.807372	0.695363	0.602423	0.844922	0.630592	0.882039	1.09541	0.940072	0.759532	0.786958	1.22978	0.121015203	0.548303839	12	12	24	0.033303949	0.606585237	24	9.029034692	0.146755372
ENSMUSG00000021573	Tppp	tubulin polymerization promoting protein [Source:MGI Symbol;Acc:MGI:1920198]	10.0417	13.5847	17.1008	10.7302	10.3141	12.7848	15.581	10.7688	13.0117	11.121	9.79749	11.7762	9.44605	14.1639	0.247023822	0.746090009	12	8	24	0.265542797	0.768040178	24	5.571609149	0.153325404
ENSMUSG00000021575	Ahrr	aryl-hydrocarbon receptor repressor [Source:MGI Symbol;Acc:MGI:1333776]	0.693042	0.60852	0.543242	0.61706	0.72971	0.892501	0.577079	0.651814	1.20809	0.692791	0.574764	0.837511	0.711791	0.723185	0.920620634	0.966018836	24	12	24	0.988273355	0.997559852	24	12.30538295	0.008101376
ENSMUSG00000021576	Pdcd6	programmed cell death 6 [Source:MGI Symbol;Acc:MGI:109283]	49.5078	51.1235	51.0165	49.421	50.7687	52.1623	53.339	48.4339	49.0399	47.8045	50.8733	46.3613	48.0781	49.1174	0.859936714	0.966018836	4	12	24	0.802180016	0.936565832	24	21.11041332	0.01284814
ENSMUSG00000021577	Sdha	succinate dehydrogenase complex, subunit A, flavoprotein (Fp) [Source:MGI Symbol;Acc:MGI:1914195]	114.545	115.138	129.967	146.331	116.28	126.017	116.077	130.16	117.661	139.829	121.907	123.621	131.018	131.308	0.581070934	0.966018836	16	12	24	0.18335352	0.768040178	24	10.73084531	0.07399236
ENSMUSG00000021578	Ccdc127	coiled-coil domain containing 127 [Source:MGI Symbol;Acc:MGI:1914683]	26.6675	22.2765	26.8331	25.9499	25.6637	24.7357	23.4241	22.6476	24.2983	23.7834	22.7678	22.3487	26.0088	26.0608	0.903465533	0.966018836	12	12	24	0.934568271	0.983690057	24	8.501775999	0.014399223
ENSMUSG00000021583	Erap1	endoplasmic reticulum aminopeptidase 1 [Source:MGI Symbol;Acc:MGI:1933403]	5.32478	6.50572	7.07969	5.22763	6.44029	6.24408	5.96783	5.84137	6.21714	5.12435	5.9589	6.5211	5.47257	6.21107	0.966321203	0.969386413	12	4	24	0.934025558	0.983539495	24	3.806364499	0.017513594
ENSMUSG00000021585	Cast	calpastatin [Source:MGI Symbol;Acc:MGI:1098236]	58.2278	54.6873	53.0576	53.3916	54.4323	56.1369	53.3779	56.0698	50.5644	59.6601	58.1732	57.3019	58.9321	57.7737	0.321662653	0.83607295	24	12	24	0.334280525	0.770433279	24	20.57828524	0.03715151
ENSMUSG00000021587	Pcsk1	proprotein convertase subtilisin/kexin type 1 [Source:MGI Symbol;Acc:MGI:97511]	16.9896	17.2304	15.0781	16.7147	16.1145	15.2488	15.1953	14.5793	14.8397	15.1756	17.0651	16.1893	15.4423	16.4087	0.843639218	0.966018836	20	16	24	0.618198653	0.86889287	24	18.67190769	0.030519906
ENSMUSG00000021589	Rhobtb3	Rho-related BTB domain containing 3 [Source:MGI Symbol;Acc:MGI:1920546]	27.1283	28.4347	26.8042	33.2761	35.1492	26.1444	31.1036	25.93	33.9217	29.1397	33.306	27.8201	26.8072	25.1325	0.06180207	0.409814092	20	12	24	0.043927487	0.628002763	24	13.94245412	0.13791302
ENSMUSG00000021590	Spata9	spermatogenesis associated 9 [Source:MGI Symbol;Acc:MGI:1922821]	1.61733	1.41945	0.966411	1.55209	1.99753	1.28724	1.58028	1.39456	1.77383	1.51127	1.95075	1.64313	1.66247	1.71183	0.784278077	0.966018836	20	12	24	0.389499261	0.799546792	24	17.46681703	0.076965918
ENSMUSG00000021591	Glrx	glutaredoxin [Source:MGI Symbol;Acc:MGI:2135625]	14.4008	18.36	20.5856	18.2178	19.0146	16.7657	15.4002	16.47	18.5755	16.6767	16.6685	15.1103	17.4195	18.8546	0.035787737	0.331814682	12	16	24	0.036817272	0.609176161	24	9.289268485	0.127992856
ENSMUSG00000021592	Arsk	arylsulfatase K [Source:MGI Symbol;Acc:MGI:1924291]	4.83086	6.29626	5.44342	5.69966	6.75644	5.89058	6.51549	5.50353	6.65211	5.07964	6.26639	5.3444	6.03155	5.643	0.852921792	0.966018836	20	20	24	0.941866191	0.985597432	24	15.1129637	0.017473714
ENSMUSG00000021594	Srd5a1	steroid 5 alpha-reductase 1 [Source:MGI Symbol;Acc:MGI:98400]	6.95956	5.94954	6.11316	6.36163	5.71642	6.39997	6.80789	5.78396	5.55542	7.89096	5.16974	6.45889	5.80368	5.742	0.360675801	0.883978426	4	8	24	0.901326274	0.97227778	24	19.08070378	0.025279641
ENSMUSG00000021595	Nsun2	NOL1/NOP2/Sun domain family member 2 [Source:MGI Symbol;Acc:MGI:107252]	31.5728	31.1578	31.2083	31.0044	32.3721	31.3997	31.7983	31.1862	28.4068	31.305	30.4703	32.1665	31.8637	31.9914	0.242077645	0.741580974	24	12	24	0.144452877	0.752809017	24	21.14576582	0.034155094
ENSMUSG00000021596	Mctp1	multiple C2 domains, transmembrane 1 [Source:MGI Symbol;Acc:MGI:1926021]	14.613	12.3667	10.9599	12.073	11.6755	12.8409	12.0499	11.0983	11.0709	12.3655	12.7872	12.4144	13.4873	12.9809	0.016795343	0.253395174	4	8	24	0.07584329	0.686372559	24	21.55365715	0.09119232
ENSMUSG00000021597	Slf1	SMC5-SMC6 complex localization factor 1 [Source:MGI Symbol;Acc:MGI:2145448]	6.19761	7.45555	7.54476	7.03458	6.37625	6.13691	5.23539	6.07012	6.39025	6.51129	7.17347	6.54517	6.13359	7.82885	0.151535314	0.599319068	12	16	24	0.174815905	0.768040178	24	9.46352828	0.098364904
ENSMUSG00000021598	Med10	mediator complex subunit 10 [Source:MGI Symbol;Acc:MGI:106331]	35.9929	34.3574	32.0683	37.289	35.3436	35.0374	33.2816	32.8144	31.1741	29.1549	33.9867	35.549	37.1878	34.469	0.181522804	0.659020016	24	12	24	0.103118545	0.714334878	24	21.10538549	0.077993876
ENSMUSG00000021606	Ndufs6	NADH:ubiquinone oxidoreductase core subunit S6 [Source:MGI Symbol;Acc:MGI:107932]	73.7387	74.9157	70.0629	75.3869	66.9853	62.8298	69.9969	71.9974	89.4695	68.4063	84.5925	76.5213	85.3919	83.5754	0.916556573	0.966018836	12	12	24	0.346435845	0.778844143	24	6.246807996	0.073352315
ENSMUSG00000021607	Mrpl36	mitochondrial ribosomal protein L36 [Source:MGI Symbol;Acc:MGI:2137228]	33.1124	33.4657	32.5722	32.4027	31.4363	32.5834	33.1863	34.8045	32.7674	32.714	38.6478	32.42	35.0477	32.3732	0.515451644	0.966018836	4	12	24	0.934456864	0.983641735	24	20.79046923	0.010663145
ENSMUSG00000021608	Lpcat1	lysophosphatidylcholine acyltransferase 1 [Source:MGI Symbol;Acc:MGI:2384812]	16.3345	14.0154	13.5288	14.2655	13.9166	14.7297	15.856	13.4443	11.7556	15.3126	14.5541	14.6479	14.4646	13.3039	0.01726379	0.255326033	4	4	24	0.037687186	0.611100708	24	20.26937771	0.101712544
ENSMUSG00000021610	Clptm1l	CLPTM1-like [Source:MGI Symbol;Acc:MGI:2442892]	43.3924	41.436	43.3804	41.7603	45.8141	44.8319	38.8793	43.1998	37.2709	44.5751	39.7294	39.8917	40.8983	39.8994	0.890176313	0.966018836	16	20	24	0.450024545	0.805393068	24	16.5235139	0.038045427
ENSMUSG00000021613	Hapln1	hyaluronan and proteoglycan link protein 1 [Source:MGI Symbol;Acc:MGI:1337006]	9.81136	9.09592	8.60045	9.76281	6.85446	9.091	8.01207	8.33811	8.38836	9.21703	9.07761	9.32345	8.97938	9.40676	0.858266942	0.966018836	16	4	24	0.892395951	0.969564004	24	3.223351946	0.022984447
ENSMUSG00000021614	Vcan	versican [Source:MGI Symbol;Acc:MGI:102889]	3.2894	3.73638	3.41519	4.05341	4.80804	3.40219	3.74924	5.33284	3.93108	3.03013	3.78039	3.32429	3.61392	4.0538	0.745118544	0.966018836	8	16	24	0.781779498	0.929907172	24	6.77700998	0.05123683
ENSMUSG00000021615	Xrcc4	X-ray repair complementing defective repair in Chinese hamster cells 4 [Source:MGI Symbol;Acc:MGI:1333799]	7.5951	9.17632	8.12765	8.15281	8.99273	7.82776	8.0461	10.6819	9.00422	8.0812	8.73123	8.11759	9.0834	9.2872	0.035806379	0.331814682	8	16	24	0.179921967	0.768040178	24	5.829465228	0.080046398
ENSMUSG00000021619	Atg10	autophagy related 10 [Source:MGI Symbol;Acc:MGI:1914045]	14.2718	14.9674	14.5009	16.2055	14.1265	13.2037	12.5561	14.123	15.5192	13.751	15.6674	15.9248	15.8115	13.9721	0.890176313	0.966018836	16	16	24	0.573803674	0.847011542	24	11.86940686	0.042505935
ENSMUSG00000021621	Zcchc9	zinc finger, CCHC domain containing 9 [Source:MGI Symbol;Acc:MGI:1916335]	10.8996	11.4376	8.7783	9.5843	11.9365	11.3453	10.6358	10.9185	11.078	9.04018	11.7	11.1627	10.9383	10.8499	0.054328576	0.395890765	20	20	24	0.111329633	0.723334499	24	21.3439017	0.097013562
ENSMUSG00000021622	Ckmt2	creatine kinase, mitochondrial 2 [Source:MGI Symbol;Acc:MGI:1923972]	13.427	31.6059	46.891	56.4302	44.2374	50.0974	25.1505	94.0041	52.7028	50.9956	27.172	22.28	44.989	36.8217	0.082282215	0.462922111	16	12	24	0.12103347	0.733023294	24	9.363246505	0.52966753
ENSMUSG00000021624	Cd180	CD180 antigen [Source:MGI Symbol;Acc:MGI:1194924]	0.573017	0.968932	1.28337	0.664974	0.754762	0.728932	0.670392	1.04522	1.09106	0.714774	0.817032	0.763348	1.18277	0.981744	0.187699199	0.662239322	12	4	24	0.043021015	0.625007356	24	6.288445096	0.146719636
ENSMUSG00000021629	Slc30a5	solute carrier family 30 (zinc transporter), member 5 [Source:MGI Symbol;Acc:MGI:1916298]	17.3164	17.3813	17.7649	17.4646	17.405	17.8689	16.6461	17.2595	14.9373	17.268	16.9308	16.1641	17.3703	17.4161	0.819898687	0.966018836	16	12	24	0.841496572	0.951983598	24	20.42839343	0.014791783
ENSMUSG00000021631	Mrps36-ps1	mitichondrial ribosomal protein S36, pseudogene 1 [Source:MGI Symbol;Acc:MGI:3809201]	9.56575	15.2651	10.9586	14.6539	12.3731	11.9993	11.545	14.2795	11.9369	10.9928	12.2349	13.6569	17.2343	14.4472	0.867488717	0.966018836	8	4	24	0.646152428	0.880190017	24	3.592863465	0.071778548
ENSMUSG00000021635	Rad17	RAD17 checkpoint clamp loader component [Source:MGI Symbol;Acc:MGI:1333807]	9.96047	9.34068	9.59034	9.73821	8.9902	8.54012	9.9518	9.46471	9.74394	8.96637	10.1503	9.27983	9.65648	10.4522	0.745475226	0.966018836	4	20	24	0.299882462	0.768040178	24	4.846696826	0.041626124

ENSMUSG00000021636	Marveld2	MARVEL (membrane-associating) domain containing 2 [Source:MGI Symbol;Acc:MGI:2446166]	1.23196	0.816671	0.867765	0.902991	0.823373	0.679298	0.504034	1.00163	0.657447	0.975686	0.898933	0.980329	0.756559	0.928708	0.828910443	0.966018836	16	12	24	0.960701867	0.989792995	24	11.58085566	0.016077908
ENSMUSG00000021638	Ocln	occludin [Source:MGI Symbol;Acc:MGI:106183]	1.50566	2.04951	2.10827	2.75884	2.10423	2.00903	1.53162	2.15813	2.28432	1.45574	1.63259	1.92476	1.47946	1.70225	0.062631097	0.410322149	12	16	24	0.221757618	0.768040178	24	10.63775036	0.115275869
ENSMUSG00000021639	Gtf2h2	general transcription factor II H, polypeptide 2 [Source:MGI Symbol;Acc:MGI:1345669]	31.4773	31.1817	31.2525	32.0238	32.5511	30.2984	32.0064	28.4664	34.0725	31.6476	37.1657	29.3211	34.9967	33.9537	0.953760335	0.966018836	20	4	24	0.702378236	0.901574817	24	12.67977408	0.030579667
ENSMUSG00000021640	Naip1	NLR family, apoptosis inhibitory protein 1 [Source:MGI Symbol;Acc:MGI:1298223]	0.687245	0.652353	0.842744	0.632941	0.792021	0.91404	0.65142	0.720486	0.625743	0.635044	0.731389	0.689657	0.669602	0.612205	0.22480491	0.719458436	20	20	24	0.270481282	0.768040178	24	18.20957192	0.044855928
ENSMUSG00000021643	Serf1	small EDRK-rich factor 1 [Source:MGI Symbol;Acc:MGI:1337114]	33.0088	37.5116	32.8433	33.737	32.8437	29.4739	32.7342	29.1916	36.0222	34.752	36.5102	36.1294	41.4368	39.2554	0.761468226	0.966018836	8	16	24	0.737572	0.91440753	24	5.494207196	0.038184632
ENSMUSG00000021645	Smn1	survival motor neuron 1 [Source:MGI Symbol;Acc:MGI:109257]	9.82031	8.77542	8.65509	8.53536	8.68858	8.75823	7.5443	9.63513	9.00755	8.95125	9.70363	9.64372	8.96278	9.7412	0.879901313	0.966018836	8	8	24	0.978842897	0.994790651	24	2.760561316	0.007352713
ENSMUSG00000021646	Mccc2	methylcrotonoyl-Coenzyme A carboxylase 2 (beta) [Source:MGI Symbol;Acc:MGI:1925288]	9.31294	10.5807	9.17105	12.4236	9.78691	9.3628	9.63472	11.0114	10.3702	9.65638	10.9187	9.48795	10.5251	10.8314	0.588208558	0.966018836	8	16	24	0.397690303	0.799546792	24	9.092341932	0.061476336
ENSMUSG00000021647	Cartpt	CART prepropeptide [Source:MGI Symbol;Acc:MGI:1351330]	11.7529	11.7217	10.6298	13.4939	10.798	13.7877	10.3955	12.7494	12.0122	13.8506	9.913	12.5055	12.0125	11.013	0.904831057	0.966018836	16	12	24	0.908845263	0.974606327	24	12.98159908	0.024950734
ENSMUSG00000021650	Ptcd2	pentatricopeptide repeat domain 2 [Source:MGI Symbol;Acc:MGI:1916177]	23.5578	20.2309	19.4772	20.7641	19.2175	22.7582	19.8904	22.2375	19.3732	19.7382	21.4141	20.6434	21.8404	21.3889	0.17395618	0.649730255	4	8	24	0.093373287	0.71175955	24	22.81229417	0.071791067
ENSMUSG00000021660	Btf3	basic transcription factor 3 [Source:MGI Symbol;Acc:MGI:1202875]	24.3699	28.8448	26.6195	27.6355	26.5099	27.8994	31.845	27.6376	30.8087	20.7439	26.6624	27.6485	27.5768	26.3229	0.921572192	0.966018836	24	20	24	0.450536049	0.805393068	24	1.306356593	0.069056883
ENSMUSG00000021661	Ankra2	ankyrin repeat, family A (RFXANK-like), 2 [Source:MGI Symbol;Acc:MGI:1915808]	16.3247	18.3434	17.5613	18.9901	19.0058	16.9782	16.4245	16.4182	19.4005	17.6649	19.9128	19.3339	18.835	18.8114	0.123856376	0.552144108	20	8	24	0.127356006	0.740162193	24	13.03408353	0.062820017
ENSMUSG00000021662	Arhgef28	Rho guanine nucleotide exchange factor (GEF) 28 [Source:MGI Symbol;Acc:MGI:1346016]	7.63077	7.04017	8.0461	7.93648	6.91314	8.34041	7.3426	6.75696	6.59325	8.54637	6.53367	8.42009	7.33578	9.24676	0.891742979	0.966018836	24	20	24	0.996146698	0.99900358	24	23.70303532	0.004846971
ENSMUSG00000021665	Hexb	hexosaminidase B [Source:MGI Symbol;Acc:MGI:96074]	55.7313	52.205	50.7504	48.2754	46.1166	51.9904	53.4829	52.6288	50.4103	51.4846	50.5536	55.9829	54.4614	55.6751	0.003680303	0.140598674	4	16	24	0.002934251	0.414576087	24	0.93470764	0.080224524
ENSMUSG00000021666	Gfm2	G elongation factor, mitochondrial 2 [Source:MGI Symbol;Acc:MGI:2444783]	12.9298	13.2006	13.5608	14.2759	11.9718	13.3734	12.9253	14.4093	13.8566	14.3819	13.5192	12.911	14.428	13.5181	0.205280539	0.693330038	16	4	24	0.13164552	0.741998858	24	7.739088821	0.053431665
ENSMUSG00000021668	Polk	polymerase (DNA directed), kappa [Source:MGI Symbol;Acc:MGI:1349767]	4.9217	5.09785	4.65671	4.98057	5.13561	4.76353	4.86666	4.92895	5.72373	4.58189	5.93727	5.15536	4.74346	4.83935	0.927004797	0.966018836	20	8	24	0.61460691	0.866603595	24	14.30009187	0.034148232
ENSMUSG00000021669	Cert1	ceramide transporter 1 [Source:MGI Symbol;Acc:MGI:1915268]	45.2587	44.613	43.0163	45.7274	43.6868	42.3289	43.1775	38.4197	40.5683	45.1189	45.1433	44.4083	44.2079	43.2694	0.369262716	0.893596186	16	20	24	0.26657796	0.768040178	24	16.92581788	0.04219588
ENSMUSG00000021670	Hmgcr	3-hydroxy-3-methylglutaryl-Coenzyme A reductase [Source:MGI Symbol;Acc:MGI:96159]	74.923	66.9114	70.4387	72.7166	74.3019	74.3923	73.8156	65.8585	69.0577	77.5979	70.2668	68.7358	71.2996	66.4246	0.064494584	0.4150273	16	16	24	0.072085324	0.678628941	24	16.80009353	0.058070507
ENSMUSG00000021671	Poc5	POC5 centriolar protein [Source:MGI Symbol;Acc:MGI:1914713]	10.3399	8.00112	9.28937	8.11959	8.55776	8.55578	10.288	7.74665	8.70727	9.3301	7.87308	8.98874	9.30421	10.2563	0.33885934	0.858075318	4	16	24	0.356114238	0.783018041	24	0.939535584	0.070814793
ENSMUSG00000021675	F2rl2	coagulation factor II (thrombin) receptor-like 2 [Source:MGI Symbol;Acc:MGI:1298208]	44.262	39.0668	39.7915	38.3866	41.7275	42.8735	36.0447	37.152	35.8551	38.643	44.858	40.1817	41.2513	37.604	0.087130946	0.470523339	20	16	24	0.029552021	0.596648309	24	18.63668034	0.095526486
ENSMUSG00000021676	Iqgap2	IQ motif containing GTPase activating protein 2 [Source:MGI Symbol;Acc:MGI:2449975]	5.94823	5.29079	5.39519	8.93904	7.55988	7.15775	5.72798	4.87076	6.16714	6.53151	7.41062	6.07	5.23715	4.65149	2.58E-05	0.02040759	20	12	24	5.80E-05	0.089877732	24	15.3499303	0.274683138
ENSMUSG00000021681	Aggf1	angiogenic factor with G patch and FHA domains 1 [Source:MGI Symbol;Acc:MGI:1913799]	15.7509	14.7445	14.6912	15.0156	15.3317	15.4781	15.8333	15.2465	14.0782	14.9752	14.3656	15.142	15.7798	15.5579	3.44E-04	0.049770315	4	8	24	0.033018179	0.606585237	24	22.49949998	0.04697133
ENSMUSG00000021684	Pde8b	phosphodiesterase 8B [Source:MGI Symbol;Acc:MGI:2443999]	8.08086	7.28873	10.6304	8.15676	8.3954	8.11704	8.36681	7.34129	7.19514	8.1677	6.73668	7.29559	6.73391	7.06304	0.865233232	0.966018836	16	16	24	0.56211051	0.842140451	24	11.66154912	0.05208197
ENSMUSG00000021686	Ap3b1	adaptor-related protein complex 3, beta 1 subunit [Source:MGI Symbol;Acc:MGI:1333879]	12.2669	12.663	13.7953	12.8817	13.328	13.0322	13.9207	13.1495	13.4219	12.1578	13.3112	12.4851	14.0301	12.8805	0.903027257	0.966018836	12	4	24	0.862823873	0.959967665	24	3.202718436	0.013007227
ENSMUSG00000021687	Scamp1	secretory carrier membrane protein 1 [Source:MGI Symbol;Acc:MGI:1349480]	66.6825	63.9014	61.8463	61.905	58.3827	63.263	60.3182	59.0732	55.3148	64.902	62.2079	61.7377	62.5604	65.4847	0.94086481	0.966018836	8	4	24	0.626338004	0.872596805	24	22.72893105	0.027459657
ENSMUSG00000021690	Jmy	junction-mediating and regulatory protein [Source:MGI Symbol;Acc:MGI:1913096]	7.98177	8.13867	8.57	9.05257	8.22718	7.52284	8.32166	7.84592	8.75703	8.67991	8.48229	8.93723	8.36589	8.89565	0.331157065	0.850959667	16	12	24	0.092712235	0.71175955	24	10.48063274	0.051702948
ENSMUSG00000021692	Dimt1	DIM1 dimethyladenosine transferase 1-like (S. cerevisiae) [Source:MGI Symbol;Acc:MGI:1913504]	3.24008	3.3189	3.21154	2.97682	2.77372	2.77336	5.24302	3.42816	3.49038	2.49031	3.93311	3.67893	3.45808	4.01217	0.444827951	0.966018836	4	12	24	0.149687131	0.758377578	24	1.261411918	0.1436617
ENSMUSG00000021693	Kif2a	kinesin family member 2A [Source:MGI Symbol;Acc:MGI:108390]	32.2797	29.8595	29.6153	28.5622	28.8515	29.5171	27.1065	27.0835	25.1553	30.307	27.503	28.8109	29.6345	29.157	0.872380105	0.966018836	4	8	24	0.529283419	0.824361959	24	21.27714554	0.036829468
ENSMUSG00000021694	Ercc8	excision repair-complementing rodent repair deficiency, complementation group 8 [Source:MGI Symbol;Acc:MGI:1919241]	3.42502	3.9949	4.19427	4.74871	4.00601	4.03049	3.90074	3.71433	4.58668	3.82782	4.20003	4.0299	4.4087	4.10133	0.557335707	0.966018836	12	20	24	0.19081484	0.768040178	24	11.10223907	0.06552013
ENSMUSG00000021696	Elovl7	ELOVL family member 7, elongation of long chain fatty acids (yeast) [Source:MGI Symbol;Acc:MGI:1921809]	63.6873	56.3047	50.4392	53.3188	58.1009	55.6637	57.1776	53.8067	50.4226	56.0727	58.6856	58.1305	61.3056	57.3452	0.004501001	0.140598674	4	8	24	0.011845174	0.525280095	24	21.22770809	0.096510026
ENSMUSG00000021699	Pde4d	phosphodiesterase 4D, cAMP specific [Source:MGI Symbol;Acc:MGI:99555]	8.10579	7.21573	8.71186	9.31421	6.52663	8.86367	8.14105	9.41815	7.59494	10.8975	6.98397	8.5683	8.81606	9.59452	0.893307667	0.966018836	16	4	24	0.545955036	0.833198211	24	6.92574158	0.077334842
ENSMUSG00000021700	Rab3c	RAB3C, member RAS oncogene family [Source:MGI Symbol;Acc:MGI:1914545]	58.0406	49.8801	51.1081	48.8526	52.0494	53.0496	50.6102	45.114	45.0522	50.6507	54.9806	55.3226	55.4417	57.7181	0.094000699	0.490854203	4	8	24	0.104100846	0.715480277	24	20.76190043	0.090060024
ENSMUSG00000021701	Plk2	polo like kinase 2 [Source:MGI Symbol;Acc:MGI:1099790]	12.3039	12.7624	11.8337	11.2447	11.7844	11.8517	10.55	12.1022	12.6678	11.609	11.4057	12.1986	11.6796	11.7364	0.100311279	0.511533473	4	12	24	0.114080268	0.727799063	24	1.275107565	0.060060924
ENSMUSG00000021702	Thbs4	thrombospondin 4 [Source:MGI Symbol;Acc:MGI:1101779]	5.89345	7.46052	8.20314	7.90184	10.55	6.56546	7.79647	7.09358	8.39276	6.15641	7.94769	6.65716	8.27193	5.78321	0.628286596	0.966018836	12	20	24	0.461519318	0.805393068	24	13.93285008	0.099140239
ENSMUSG00000021703	Serinc5	serine incorporator 5 [Source:MGI Symbol;Acc:MGI:2444223]	45.5096	43.8539	45.1975	41.6853	43.2664	46.1828	42.6687	45.5749	39.008	48.2597	40.6253	44.1075	43.5724	43.5059	0.908406451	0.966018836	24	20	24	0.789203984	0.932388659	24	22.74087188	0.021488079
ENSMUSG00000021704	Mtx3	metaxin 3 [Source:MGI Symbol;Acc:MGI:2686040]	12.7318	14.1732	13.1437	12.68	12.5543	12.2924	12.5824	12.5201	13.8782	12.0748	12.5357	12.8614	12.3076	14.0355	0.181151038	0.659020016	12	4	24	0.062530508	0.661961741	24	5.759578366	0.059796296
ENSMUSG00000021706	Zfyve16	zinc finger, FYVE domain containing 16 [Source:MGI Symbol;Acc:MGI:2145181]	7.97611	7.37969	7.92448	7.67662	8.14077	7.67857	9.59781	6.5424	7.81135	6.52248	9.13065	8.19689	8.61998	6.9053	0.073315272	0.447768907	4	4	24	0.151164462	0.758865539	24	19.82766964	0.109517727
ENSMUSG00000021707	Dhfr	dihydrofolate reductase [Source:MGI Symbol;Acc:MGI:94890]	1.60964	1.79523	1.69144	1.57522	1.56214	1.64121	1.6352	1.6212	1.90147	1.76308	1.72958	1.73218	1.77917	1.70712	0.93447422	0.966018836	12	8	24	0.289538558	0.768040178	24	6.90121584	0.029076532
ENSMUSG00000021708	Rasgrf2	RAS protein-specific guanine nucleotide-releasing factor 2 [Source:MGI Symbol;Acc:MGI:109137]	6.36643	6.24733	7.87684	5.91638	5.08695	6.39825	6.66204	5.68357	5.40714	6.94452	5.41209	6.31478	5.46305	7.27029	0.850398351	0.966018836	8	12	24	0.499381896	0.808768108	24	5.29635498	0.073699303
ENSMUSG00000021709	Erbin	Erbb2 interacting protein [Source:MGI Symbol;Acc:MGI:1890169]	11.1551	15.1839	16.5049	13.2889	13.4733	11.974	14.1023	12.7191	16.506	11.982	12.799	12.973	11.8991	12.9178	0.112800839	0.530777648	12	16	24	0.093116554	0.71175955	24	8.17937735	0.130632753
ENSMUSG00000021710	Nln	neurolysin (metallopeptidase M3 family) [Source:MGI Symbol;Acc:MGI:1923055]	4.22404	3.98914	4.74258	4.46756	4.28948	3.8476	4.0421	3.98225	3.98891	4.41455	3.92618	4.14183	3.69068	4.38477	0.277221318	0.789849983	16	12	24	0.098121172	0.71175955	24	10.4259866	0.060962006
ENSMUSG00000021711	Trappc13	trafficking protein particle complex 13 [Source:MGI Symbol;Acc:MGI:1914225]	27.1632	24.5389	25.7824	23.3482	24.7677	24.2309	24.7403	22.7095	24.3808	22.7738	28.339	24.891	27.3146	24.9525	0.47028201	0.966018836	4	12	24	0.380964454	0.799546792	24	21.17730343	0.051234485
ENSMUSG00000021712	Trim23	tripartite motif-containing 23 [Source:MGI Symbol;Acc:MGI:1933161]	21.502	23.0072	22.3643	21.9306	20.2785	19.9076	20.2998	20.0468	22.4107	18.7785	21.6323	18.73	19.4089	20.769	0.022634137	0.27189101	12	12	24	0.133310534	0.743843563	24	7.549631662	0.059015321
ENSMUSG00000021713	Ppwd1	peptidylprolyl isomerase domain and WD repeat containing 1 [Source:MGI Symbol;Acc:MGI:2443069]	4.65031	4.13949	4.02413	4.41021	4.26041	3.54913	4.22967	4.03136	4.35704	3.35107	4.48906	3.71482	4.08488	3.68562	0.908406451	0.966018836	20	4	24	0.971421061	0.993043186	24	23.65844123	0.009737971
ENSMUSG00000021714	Cenpk	centromere protein K [Source:MGI Symbol;Acc:MGI:1926210]	0.975439	2.53129	1.66421	1.41432	1.36564	1.59019	0.955095	1.12221	2.75224	1.59013	1.41498	1.6168	1.33721	1.21116	0.062631097	0.410322149	12	16	24	0.275871187	0.768040178	24	9.54002849	0.173329962
ENSMUSG00000021715	Cwc27	CWC27 spliceosome-associated protein [Source:MGI Symbol;Acc:MGI:1914535]	9.38306	11.5968	11.4126	10.912	9.9031	9.69698	10.3007	9.3431	12.5337	9.8317	10.3865	11.0401	10.4532	10.9394	0.542718076	0.966018836	16	12	24	0.184079521	0.768040178	24	8.133766408	0.081581392
ENSMUSG00000021716	Srek1ip1	splicing regulatory glutamine/lysine-rich protein 1 interacting protein 1 [Source:MGI Symbol;Acc:MGI:1914538]	5.47373	7.37255	7.73185	7.53296	6.74517	6.41118	6.68767	6.59542	8.22171	6.32469	7.97955	6.83972	7.02061	7.24885	0.143321297	0.585944911	12	12	24	0.089287109	0.708638675	24	9.746667094	0.108568039
ENSMUSG00000021719	Rgs7bp	regulator of G-protein signalling 7 binding protein [Source:MGI Symbol;Acc:MGI:106334]	92.8343	82.1344	73.8553	83.9644	79.0979	81.8192	79.4122	78.5618	72.851	92.2024	87.4192	86.495	89.1885	90.8993	0.628286596	0.966018836	4	8	24	0.471786772	0.805393068	24	20.43146057	0.053271495
ENSMUSG00000021720	Rnf180	ring finger protein 180 [Source:MGI Symbol;Acc:MGI:1919066]	5.0813	4.51303	4.29334	4.74229	4.24484	5.11679	4.7	4.78863	3.12308	5.06409	3.88647	4.95679	3.68828	3.69909	0.390686537	0.915479136	24	12	24	0.250558581	0.768040178	24	19.61946984	0.104316299
ENSMUSG00000021721	Htr1a	5-hydroxytryptamine (serotonin) receptor 1A [Source:MGI Symbol;Acc:MGI:96273]	1.54701	1.72951	1.56448	1.5544	1.39435	1.89537	1.5002	1.6965	1.40471	2.24258	1.31653	1.95014	1.52413	1.6696	0.826609618	0.966018836	8	12	24	0.999143658	0.999770694	24	17.71319803	0.002258103
ENSMUSG00000021725	Parp8	poly (ADP-ribose) polymerase family, member 8 [Source:MGI Symbol;Acc:MGI:1098713]	10.7733	10.2776	10.042	9.47047	10.8004	10.929	10.3388	9.56092	9.47706	10.5711	10.576	10.4803	10.0311	10.8445	0.114881734	0.535150745	24	12	24	0.098705677	0.71175955	24	19.82230352	0.054702813
ENSMUSG00000021728	Emb	embigin [Source:MGI Symbol;Acc:MGI:95321]	110.745	99.5099	96.9373	95.6093	101.882	104.402	97.1386	98.2104	92.5451	94.2518	110.811	102.284	105.407	104.608	0.02444487	0.287293295	20	20	24	0.038579981	0.61140397	24	20.98681809	0.07516407
ENSMUSG00000021730	Hcn1	hyperpolarization activated cyclic nucleotide gated potassium channel 1 [Source:MGI Symbol;Acc:MGI:1096392]	9.83252	8.7988	8.66101	8.90508	8.96271	8.89407	9.2034	8.55547	8.02005	10.1641	9.51483	8.5567	8.82091	9.18099	0.308023687	0.814993005	16	20	24	0.586065902	0.853257144	24	17.66320206	0.033174531
ENSMUSG00000021731	Mrps30	mitochondrial ribosomal protein S30 [Source:MGI Symbol;Acc:MGI:1926237]	6.78721	6.04222	5.89252	6.26494	6.04495	6.18116	6.28866	6.4727	6.24074	6.26369	6.66204	6.72818	7.21189	6.10424	0.062631097	0.410322149	4	8	24	0.131243555	0.741500219	24	21.65279376	0.049469828
ENSMUSG00000021733	Slc4a7	solute carrier family 4, sodium bicarbonate cotransporter, member 7 [Source:MGI Symbol;Acc:MGI:2443878]	2.36819	2.87666	2.78553	2.53306	2.81187	2.46773	2.57862	2.3995	2.55107	2.04923	2.75988	2.22821	1.99982	2.46515	0.625825704	0.966018836	12	16	24	0.733228418	0.91292127	24	10.89414401	0.031182969
ENSMUSG00000021737	Psmd6	proteasome (prosome, macropain) 26S subunit, non-ATPase, 6 [Source:MGI Symbol;Acc:MGI:1913663]	91.1384	94.5352	87.0234	90.3818	89.3596	83.357	89.4761	88.9982	100.238	91.9968	103.17	97.6562	105.54	103.607	0.830949782	0.966018836	4	20	24	0.670894592	0.888971832	24	5.188018872	0.026893884
ENSMUSG00000021738	Atxn7	ataxin 7 [Source:MGI Symbol;Acc:MGI:2179277]	6.04986	5.95559	6.26571	6.15653	6.46299	6.00008	6.43597	5.91998	7.78209	6.3775	6.98918	6.57562	6.47762	6.10254	0.064091035	0.414867207	20	12	24	0.37338761	0.794388449	24	12.36402744	0.046557873
ENSMUSG00000021741	Gm5457	predicted pseudogene 5457 [Source:MGI Symbol;Acc:MGI:3646665]	1.89648	5.96632	4.87692	7.1294	5.50166	5.73612	7.73566	6.24438	6.23769	5.17919	5.09613	5.88376	4.82367	5.75711	0.910436409	0.966018836	8	12	24	0.589567049	0.854319495	24	10.2885255	0.148119473
ENSMUSG00000021747	Cfap20dc	CFAP20 domain containing [Source:MGI Symbol;Acc:MGI:1926154]	3.86729	3.35859	3.44768	2.83861	3.44963	3.78846	3.6015	3.35038	2.70144	3.60444	2.84986	3.85338	3.66726	3.74522	0.038115943	0.334069441	24	12	24	0.042855786	0.624864512	24	22.80178341	0.118684065
ENSMUSG00000021748	Pdhb	pyruvate dehydrogenase (lipoamide) beta [Source:MGI Symbol;Acc:MGI:1915513]	73.4869	73.9133	75.116	87.1637	70.3301	69.7594	73.8634	80.5227	75.5278	75.9454	88.7143	77.1094	88.7343	82.6445	0.912686312	0.966018836	16	8	24	0.714252743	0.905831242	24	9.046928225	0.033855117
ENSMUSG00000021750	Fam107a	family with sequence similarity 107, member A [Source:MGI Symbol;Acc:MGI:3041256]	13.4817	14.8413	20.122	26.0609	23.1476	19.8481	18.9497	13.1672	18.9187	24.0939	18.3938	19.1452	13.0186	14.4832	0.001871852	0.100255953	16	16	24	4.51E-04	0.265389333	24	13.82132	0.364003421
ENSMUSG00000021752	Kctd6	potassium channel tetramerisation domain containing 6 [Source:MGI Symbol;Acc:MGI:1918643]	17.628	17.0355	17.447	17.8811	16.5174	19.0317	16.1321	17.0506	15.8481	20.4556	16.9194	18.3607	16.9472	18.8798	0.951846915	0.966018836	12	16	24	0.744928938	0.916569459	24	14.47027516	0.028531848
ENSMUSG00000021754	Map3k1	mitogen-activated protein kinase kinase kinase 1 [Source:MGI Symbol;Acc:MGI:1346872]	5.17527	4.88086	4.56038	4.73574	5.2948	5.31468	5.00509	5.56081	4.69807	5.13207	5.61184	5.38606	5.48709	4.69047	0.029825002	0.323426482	24	12	24	0.029072569	0.596621137	24	19.73876747	0.077421278
ENSMUSG00000021756	Il6st	interleukin 6 signal transducer [Source:MGI Symbol;Acc:MGI:96560]	87.1281	63.943	66.7822	67.476	72.1392	79.6698	73.1112	75.3234	55.3085	82.0658	66.3062	72.2283	72.3747	71.3477	0.105280783	0.518002936	4	8	24	0.165072418	0.767020009	24	20.87733689	0.119063606
ENSMUSG00000021758	Ddx4	DEAD box helicase 4 [Source:MGI Symbol;Acc:MGI:102670]	1.51783	1.11034	1.29429	1.52314	1.20573	1.13165	1.39627	0.945808	1.18913	1.09711	1.61053	1.0455	0.923152	1.05799	0.454605943	0.966018836	20	12	24	0.285747784	0.768040178	24	15.34252115	0.075845208
ENSMUSG00000021759	Plpp1	phospholipid phosphatase 1 [Source:MGI Symbol;Acc:MGI:108412]	54.21	47.7323	45.5587	45.2056	45.7733	46.156	48.052	51.3954	43.5953	53.3755	50.0602	52.5618	52.519	52.1989	0.105280783	0.518002936	4	8	24	0.269445375	0.768040178	24	23.44396098	0.058978603
ENSMUSG00000021760	Gpx8	glutathione peroxidase 8 (putative) [Source:MGI Symbol;Acc:MGI:1916840]	31.4858	42.6609	36.2029	38.4504	37.7035	31.2758	36.664	39.1419	43.5667	27.5058	38.7532	35.0515	40.1569	39.59	0.158469451	0.614860056	8	16	24	0.310846535	0.768040178	24	5.560664426	0.107388559
ENSMUSG00000021763	Cspg4b	chondroitin sulfate proteoglycan 4B [Source:MGI Symbol;Acc:MGI:3040697]	1.1812	1.74717	1.69092	1.20218	1.30246	1.46896	1.12843	1.6491	1.52953	1.85484	1.21128	1.57321	1.27335	1.56709	0.248056532	0.746745468	8	20	24	0.157174848	0.763141258	24	7.81316885	0.104970964
ENSMUSG00000021764	Ndufs4	NADH:ubiquinone oxidoreductase core subunit S4 [Source:MGI Symbol;Acc:MGI:1343135]	116.959	146.567	146.088	148.759	148.974	130.336	135.896	143.833	158.401	132.242	157.884	145.736	161.629	158.222	0.954744729	0.966018836	12	12	24	0.2658322	0.768040178	24	9.137273931	0.070176024
ENSMUSG00000021765	Fst	follistatin [Source:MGI Symbol;Acc:MGI:95586]	2.15422	2.21446	2.42516	1.5634	2.32118	2.52287	2.36305	2.25025	2.21202	2.65313	2.52291	2.46608	2.25045	3.68324	0.834424969	0.966018836	20	16	24	0.671141434	0.889065421	24	2.856794686	0.053070485
ENSMUSG00000021767	Kat6b	K(lysine) acetyltransferase 6B [Source:MGI Symbol;Acc:MGI:1858746]	5.03734	5.02511	5.25803	5.79002	5.10844	4.74875	5.62775	4.6405	5.51204	5.45544	4.9312	5.05521	4.70353	5.07238	0.152236561	0.600454138	16	16	24	0.161815832	0.76636332	24	10.96049336	0.058108449
ENSMUSG00000021768	Dusp13	dual specificity phosphatase 13 [Source:MGI Symbol;Acc:MGI:1351599]	1.51165	2.7752	2.78934	3.46512	2.34571	3.17607	1.72415	4.59948	3.3887	4.04594	2.22919	2.14031	2.89193	2.21309	0.059513104	0.406067671	16	12	24	0.099722637	0.71175955	24	9.564807839	0.26700439
ENSMUSG00000021770	Samd8	sterile alpha motif domain containing 8 [Source:MGI Symbol;Acc:MGI:1914880]	14.4473	14.5701	14.6477	14.4174	15.0561	14.0918	14.5213	13.2587	14.2718	15.746	14.7002	15.2959	15.0511	14.8925	0.833406028	0.966018836	20	12	24	0.358067112	0.784922891	24	15.31727834	0.028758768
ENSMUSG00000021771	Vdac2	voltage-dependent anion channel 2 [Source:MGI Symbol;Acc:MGI:106915]	99.0266	103.412	100.382	106.586	103.162	103.396	102.531	105.149	103.409	102.184	99.9936	99.5407	103.15	93.6581	0.933543173	0.966018836	16	12	24	0.668700042	0.888029633	24	13.65796995	0.014877143
ENSMUSG00000021772	Nkiras1	NFKB inhibitor interacting Ras-like protein 1 [Source:MGI Symbol;Acc:MGI:1916971]	58.1715	59.7279	58.3457	57.0827	59.1987	57.9652	55.7241	54.6588	55.3956	51.422	57.3169	53.6322	59.7321	57.7305	0.887010723	0.966018836	4	12	24	0.526687665	0.823075573	24	23.6949846	0.025916474
ENSMUSG00000021773	Comtd1	catechol-O-methyltransferase domain containing 1 [Source:MGI Symbol;Acc:MGI:1916406]	12.652	10.7682	11.527	10.978	11.683	11.7135	11.3938	13.0417	11.6072	12.4602	15.3156	12.7447	11.9979	10.7208	0.451149628	0.966018836	24	8	24	0.286063467	0.768040178	24	17.3443049	0.07491956
ENSMUSG00000021774	Ube2e1	ubiquitin-conjugating enzyme E2E 1 [Source:MGI Symbol;Acc:MGI:107411]	47.8531	49.6348	46.0864	45.2623	47.1531	44.4674	43.0114	46.0844	44.7021	42.8537	47.6106	45.0987	45.1181	48.297	0.46238066	0.966018836	8	8	24	0.704184877	0.902338136	24	3.143844924	0.019595561
ENSMUSG00000021775	Nr1d2	nuclear receptor subfamily 1, group D, member 2 [Source:MGI Symbol;Acc:MGI:2449205]	14.7646	32.2012	40.6389	43.1134	30.2675	16.8411	18.6662	27.478	49.5345	37.48	25.4411	17.3216	17.4571	29.9189	8.29E-05	0.029372629	12	12	24	4.15E-07	0.003664625	24	9.717934101	0.736240309
ENSMUSG00000021779	Thrb	thyroid hormone receptor beta [Source:MGI Symbol;Acc:MGI:98743]	2.51799	2.8392	3.35971	2.64279	1.82338	2.86062	2.78013	2.4466	2.58401	2.17699	2.05474	2.62474	2.61196	2.97949	0.421215102	0.966018836	8	12	24	0.070137986	0.675025526	24	3.873056011	0.133305652
ENSMUSG00000021782	Dlg5	discs large MAGUK scaffold protein 5 [Source:MGI Symbol;Acc:MGI:1918478]	8.05338	7.88263	7.15977	8.08388	7.05558	7.20336	7.31522	7.18704	6.49351	8.24849	6.9099	7.14831	6.7273	6.97893	0.874607729	0.966018836	16	4	24	0.887049304	0.968043979	24	13.86061096	0.01526043
ENSMUSG00000021785	Ngly1	N-glycanase 1 [Source:MGI Symbol;Acc:MGI:1913276]	25.8079	26.463	25.473	25.7212	25.9995	23.8103	25.0622	25.403	23.6063	22.8362	27.493	23.3371	24.5485	24.6191	0.896753532	0.966018836	20	4	24	0.977514671	0.994290879	24	20.26770172	0.005945713
ENSMUSG00000021786	Oxsm	3-oxoacyl-ACP synthase, mitochondrial [Source:MGI Symbol;Acc:MGI:1918397]	4.53156	4.8224	5.78763	5.41043	4.7064	5.66503	4.6173	5.43047	4.5506	5.40004	4.54251	5.15646	5.64721	5.3895	0.882263435	0.966018836	16	4	24	0.800669286	0.936185569	24	7.517685836	0.03075374
ENSMUSG00000021791	Dydc2	DPY30 domain containing 2 [Source:MGI Symbol;Acc:MGI:1918450]	1.45818	1.91318	1.58523	1.46041	0.862167	1.61737	1.40743	1.52111	1.07787	1.28472	1.58494	1.16417	1.37769	2.00322	0.295379186	0.805301811	8	12	24	0.186282229	0.768040178	24	3.697122263	0.125541246
ENSMUSG00000021792	Prxl2a	peroxiredoxin like 2A [Source:MGI Symbol;Acc:MGI:1917814]	57.2588	62.0975	68.2301	65.8304	59.9889	57.2887	66.4128	60.4951	66.4261	61.3262	64.9197	62.912	69.9927	65.1722	0.601647512	0.966018836	12	12	24	0.27760339	0.768040178	24	7.595931912	0.052088226
ENSMUSG00000021794	Glud1	glutamate dehydrogenase 1 [Source:MGI Symbol;Acc:MGI:95753]	60.0527	55.5741	64.0843	54.6404	57.8207	57.5036	55.2111	55.7594	47.2614	61.7987	48.145	51.8526	48.8462	51.9103	0.77722623	0.966018836	16	4	24	0.932851825	0.98297963	24	12.18171456	0.015358593
ENSMUSG00000021796	Bmpr1a	bone morphogenetic protein receptor, type 1A [Source:MGI Symbol;Acc:MGI:1338938]	20.996	22.0789	20.7287	20.5781	21.2409	18.4712	20.0475	20.4303	21.3179	17.6961	22.098	19.3055	19.8925	19.9068	0.338172159	0.85758369	12	12	24	0.804021482	0.93736298	24	6.129523624	0.022279657
ENSMUSG00000021798	Ldb3	LIM domain binding 3 [Source:MGI Symbol;Acc:MGI:1344412]	10.9757	19.3961	24.6984	30.2596	21.625	28.2405	20.3627	56.0193	35.3326	49.548	18.6935	20.8842	28.9699	20.9188	0.297978647	0.80820842	16	12	24	0.140964203	0.751014756	24	9.465790008	0.423519763
ENSMUSG00000021803	Cdhr1	cadherin-related family member 1 [Source:MGI Symbol;Acc:MGI:2157782]	9.10848	6.73558	7.88063	6.87156	7.09701	9.61052	7.91391	8.64666	5.18681	9.85539	7.90502	7.45396	7.37668	7.74965	0.868638024	0.966018836	24	12	24	0.449525916	0.805393068	24	20.29142892	0.108112954
ENSMUSG00000021806	Nid2	nidogen 2 [Source:MGI Symbol;Acc:MGI:1298229]	10.0288	11.2352	9.01262	10.5154	14.0767	9.98499	12.0768	12.9002	10.7579	10.7912	12.5466	9.97535	13.8718	10.2689	0.93717134	0.966018836	20	4	24	0.761338498	0.922782206	24	19.95025088	0.053893475
ENSMUSG00000021807	Rtraf	RNA transcription, translation and transport factor [Source:MGI Symbol;Acc:MGI:1915295]	18.0029	21.6828	20.2226	18.0229	22.091	18.8425	18.1878	18.9364	22.6547	16.659	20.8997	18.1822	20.5673	18.7037	0.840483986	0.966018836	12	12	24	0.870577645	0.963313607	24	8.378473068	0.027946646
ENSMUSG00000021809	Nudt13	nudix (nucleoside diphosphate linked moiety X)-type motif 13 [Source:MGI Symbol;Acc:MGI:1914975]	8.9463	9.95817	8.49317	11.7305	10.1979	9.63213	11.7153	11.6832	12.417	10.7221	10.2182	10.2451	12.0735	11.1693	0.887862353	0.966018836	4	20	24	0.573668612	0.847005958	24	6.749489162	0.055036368
ENSMUSG00000021810	Ecd	ecdysoneless cell cycle regulator [Source:MGI Symbol;Acc:MGI:1917851]	14.3675	12.6728	13.1534	12.7361	12.2795	14.0457	12.1908	13.6971	11.3658	14.3225	12.4295	12.517	12.9137	14.0548	0.935732159	0.966018836	8	12	24	0.785912259	0.931158434	24	0.771105114	0.026140661
ENSMUSG00000021811	Dnajc9	DnaJ heat shock protein family (Hsp40) member C9 [Source:MGI Symbol;Acc:MGI:1915326]	15.3172	16.8296	16.9791	14.7955	14.5866	14.6611	16.4099	14.9805	14.1054	15.3714	14.6972	15.2098	16.2828	14.5403	0.705189716	0.966018836	4	16	24	0.318000348	0.768040178	24	2.396188304	0.043978488
ENSMUSG00000021814	Anxa7	annexin A7 [Source:MGI Symbol;Acc:MGI:88031]	96.6212	86.9525	82.1597	83.6502	90.6906	93.1363	89.4255	91.6935	81.7021	92.6713	88.3932	89.3668	91.8453	90.0544	0.031969981	0.324385159	4	8	24	0.037328527	0.609176161	24	21.47537431	0.065144265
ENSMUSG00000021815	Mss51	MSS51 mitochondrial translational activator [Source:MGI Symbol;Acc:MGI:1922093]	1.90609	3.2251	3.29272	4.83134	2.00676	3.23439	2.15445	8.66614	4.02706	8.0098	4.06086	3.10333	6.28416	3.85142	0.351315142	0.870418653	16	8	24	0.162002462	0.76636332	24	8.640412074	0.360681199
ENSMUSG00000021816	Ppp3cb	protein phosphatase 3, catalytic subunit, beta isoform [Source:MGI Symbol;Acc:MGI:107163]	74.2521	68.2733	68.4185	69.1226	65.1891	69.1127	68.2869	69.0541	58.7713	74.1264	66.7465	61.2466	62.29	66.5954	0.847312766	0.966018836	16	4	24	0.993529342	0.998731933	24	17.76999287	0.003882988
ENSMUSG00000021819	Zswim8	zinc finger SWIM-type containing 8 [Source:MGI Symbol;Acc:MGI:1919156]	31.5246	30.7817	31.0653	29.3513	31.3227	29.9319	27.5409	31.2665	31.949	30.3855	29.4387	30.4309	30.5184	30.4125	0.676344318	0.966018836	12	4	24	0.527360467	0.8233276	24	7.473403236	0.024336731
ENSMUSG00000021820	Camk2g	calcium/calmodulin-dependent protein kinase II gamma [Source:MGI Symbol;Acc:MGI:88259]	92.5483	78.2722	86.1288	81.3121	75.2662	93.2953	79.9444	86.2176	64.9751	103.485	77.1458	82.9629	78.2423	84.3403	0.895137379	0.966018836	24	20	24	0.865606461	0.961156003	24	19.74841938	0.035832909
ENSMUSG00000021822	Plau	plasminogen activator, urokinase [Source:MGI Symbol;Acc:MGI:97611]	3.50715	3.72769	3.73785	3.33222	3.61147	4.0155	3.50958	3.88674	4.19233	3.87631	3.55137	4.2454	4.1988	3.68607	0.934818505	0.966018836	24	20	24	0.783368918	0.930282193	24	1.503051141	0.020776608
ENSMUSG00000021823	Vcl	vinculin [Source:MGI Symbol;Acc:MGI:98927]	21.3529	17.5102	18.1016	17.8936	20.7561	21.6694	20.4456	22.3263	15.4177	22.162	18.8287	19.9421	19.2304	17.9045	0.510031987	0.966018836	24	12	24	0.154041027	0.760223561	24	20.02019378	0.111197333
ENSMUSG00000021824	Ap3m1	adaptor-related protein complex 3, mu 1 subunit [Source:MGI Symbol;Acc:MGI:1929212]	21.1852	21.8081	20.536	22.2169	21.1418	20.7348	21.9623	20.647	23.0438	18.7969	23.0974	22.3699	23.489	21.0464	0.773770646	0.966018836	4	12	24	0.693451447	0.898256039	24	22.11966057	0.027899384
ENSMUSG00000021830	Txndc16	thioredoxin domain containing 16 [Source:MGI Symbol;Acc:MGI:1917811]	24.9746	28.0162	27.4487	29.699	30.3061	25.1686	27.4541	25.2153	30.6799	26.114	29.4582	27.1276	25.9699	25.4274	0.135099498	0.573291211	20	8	24	0.067081155	0.669636017	24	12.69122061	0.083968488
ENSMUSG00000021831	Ero1a	endoplasmic reticulum oxidoreductase 1 alpha [Source:MGI Symbol;Acc:MGI:1354385]	19.8916	16.9143	17.4537	20.0955	16.3242	18.3517	18.0191	18.2238	15.5843	18.0291	17.1978	19.2886	21.7125	18.0091	0.182320384	0.659303873	4	8	24	0.291481934	0.768040178	24	22.274279	0.073480267
ENSMUSG00000021832	Psmc6	proteasome (prosome, macropain) 26S subunit, ATPase, 6 [Source:MGI Symbol;Acc:MGI:1914339]	110.541	118.773	111.592	112.489	112.675	103.922	111.126	113.53	118.048	97.1622	132.141	108.298	119.321	118.111	0.640622311	0.966018836	8	16	24	0.8463497	0.953551806	24	4.33806764	0.023610718
ENSMUSG00000021835	Bmp4	bone morphogenetic protein 4 [Source:MGI Symbol;Acc:MGI:88180]	3.09317	3.82521	3.32397	2.98998	2.42114	2.07426	4.1234	3.86299	4.97583	2.34267	3.13776	3.33688	1.98885	3.69069	0.161145999	0.620813623	12	4	24	0.096437021	0.71175955	24	5.802733346	0.237178921
ENSMUSG00000021838	Samd4	sterile alpha motif domain containing 4 [Source:MGI Symbol;Acc:MGI:1921730]	3.49653	4.41947	4.62259	3.77641	4.0156	4.06843	5.43166	4.45898	4.80691	4.39929	3.58082	4.55346	4.04441	4.48431	0.251986462	0.75129457	12	8	24	0.250643019	0.768040178	24	5.026320735	0.088383657
ENSMUSG00000021840	Mapk1ip1l	mitogen-activated protein kinase 1 interacting protein 1-like [Source:MGI Symbol;Acc:MGI:2444022]	14.3818	12.29	12.0017	13.2497	12.8411	13.1395	13.4611	12.6477	10.9128	13.0869	13.3546	12.6725	12.0273	12.3832	0.119598685	0.54287566	20	16	24	0.065074528	0.663876076	24	19.41710913	0.073964174
ENSMUSG00000021843	Ktn1	kinectin 1 [Source:MGI Symbol;Acc:MGI:109153]	36.5253	39.0603	39.0202	39.2006	39.051	35.6211	36.8088	36.3416	39.7662	35.6727	38.6302	36.8095	38.8208	38.9063	0.118459043	0.541945448	12	12	24	0.283810101	0.768040178	24	8.8629015	0.035201504
ENSMUSG00000021846	Peli2	pellino 2 [Source:MGI Symbol;Acc:MGI:1891445]	11.9365	12.6338	13.1077	14.091	13.7719	10.3157	12.7348	12.0275	11.4129	14.2662	10.487	13.1651	10.8856	11.392	0.305564675	0.811201458	16	12	24	0.284178707	0.768040178	24	11.59178391	0.08249529

ENSMUSG00000021852	Slc35f4	solute carrier family 35, member F4 [Source:MGI Symbol;Acc:MGI:1922538]	8.37986	8.02858	8.24584	8.13032	6.52974	7.4572	6.99687	6.95193	8.2071	7.91981	7.93156	7.18213	9.1044	8.54766	0.483971878	0.966018836	4	20	24	0.337711902	0.772544138	24	5.985939699	0.068960231
ENSMUSG00000021866	Anxa11	annexin A11 [Source:MGI Symbol;Acc:MGI:108481]	23.9772	18.8908	18.6793	21.7574	20.0312	21.2089	19.7102	22.9992	20.5846	27.2736	19.7424	23.4503	23.3366	21.0784	0.742579234	0.966018836	24	12	24	0.912775935	0.97533472	24	18.08481414	0.024874842
ENSMUSG00000021868	Ppif	peptidylprolyl isomerase F (cyclophilin F) [Source:MGI Symbol;Acc:MGI:2145814]	8.44248	8.52222	9.04888	8.95931	7.5215	8.59668	8.01556	9.72897	8.36343	11.353	7.62285	8.94942	9.81714	8.91198	0.676344318	0.966018836	16	4	24	0.405887482	0.799546792	24	7.364518868	0.073258699
ENSMUSG00000021870	Slmap	sarcolemma associated protein [Source:MGI Symbol;Acc:MGI:1933549]	36.7037	35.4897	34.3347	36.292	36.3932	34.6242	34.2189	34.9437	34.1001	36.4443	34.0817	33.1567	34.0764	34.7962	0.941830768	0.966018836	16	8	24	0.573541427	0.847005958	24	12.18219143	0.015414657
ENSMUSG00000021876	Rnase4	ribonuclease, RNase A family 4 [Source:MGI Symbol;Acc:MGI:1926217]	29.0558	36.7388	36.261	31.2215	38.1653	31.0773	27.951	32.3016	40.8289	22.8673	36.3963	30.9066	31.6326	31.5503	0.203305541	0.689605535	12	12	24	0.800680143	0.936185569	24	8.85796955	0.057219766
ENSMUSG00000021877	Arf4	ADP-ribosylation factor 4 [Source:MGI Symbol;Acc:MGI:99433]	70.6667	62.1228	64.4194	62.9106	62.0725	63.3319	65.524	64.797	55.1222	61.585	62.2518	57.7671	57.2701	60.2666	0.891022863	0.966018836	4	12	24	0.455609066	0.805393068	24	22.25429353	0.033459652
ENSMUSG00000021880	Rnase6	ribonuclease, RNase A family, 6 [Source:MGI Symbol;Acc:MGI:1925666]	0.987285	1.10524	1.21453	0.496032	0.917607	0.971183	0.689808	1.1004	1.27144	0.576588	0.889066	1.17517	0.89715	1.04258	0.008177627	0.192760771	12	4	24	0.326249063	0.768040178	24	3.024973608	0.103711767
ENSMUSG00000021884	Hacl1	2-hydroxyacyl-CoA lyase 1 [Source:MGI Symbol;Acc:MGI:1929657]	8.68248	9.75548	8.82883	9.5932	10.773	8.58651	7.50426	9.4754	8.46004	10.2032	9.14971	8.04122	7.76543	8.62966	0.040414458	0.343246619	16	12	24	0.044014627	0.628008091	24	12.19083656	0.111378852
ENSMUSG00000021886	Gpr65	G-protein coupled receptor 65 [Source:MGI Symbol;Acc:MGI:108031]	0.976705	1.05712	1.28906	0.934261	1.15709	0.948792	0.939721	0.988111	1.19477	0.727403	1.01854	0.857853	0.924713	0.800582	0.396686537	0.915479136	12	12	24	0.703361515	0.901975795	24	9.005955484	0.032801475
ENSMUSG00000021890	Eaf1	ELL associated factor 1 [Source:MGI Symbol;Acc:MGI:1921677]	17.2334	13.2761	13.3984	13.5798	13.1849	14.9563	13.5609	15.5587	11.4904	15.7445	14.3412	13.4345	13.8575	14.2017	0.969174623	0.970883022	4	8	24	0.503156964	0.809997329	24	21.74861895	0.062473628
ENSMUSG00000021891	Mettl6	methyltransferase like 6 [Source:MGI Symbol;Acc:MGI:1914261]	8.82744	12.0268	8.85793	11.4089	10.7783	9.85784	9.0821	11.1482	13.7472	9.77778	10.807	10.4139	11.4096	11.2106	0.706051896	0.966018836	8	20	24	0.348169558	0.779015211	24	8.366356148	0.092205805
ENSMUSG00000021892	Sh3bp5	SH3-domain binding protein 5 (BTK-associated) [Source:MGI Symbol;Acc:MGI:1344391]	23.286	21.9907	21.8753	23.6607	24.6098	20.4515	26.163	23.3046	21.8899	23.6217	20.401	22.6304	22.498	19.0623	0.93717134	0.966018836	16	20	24	0.826817427	0.946041107	24	18.6617992	0.0270948
ENSMUSG00000021893	Capn7	calpain 7 [Source:MGI Symbol;Acc:MGI:1338030]	23.8754	24.5854	22.5139	23.1051	25.3318	23.5695	24.709	22.8083	24.3003	21.5459	24.5239	24.1216	25.07	24.5194	0.17847698	0.655480352	4	12	24	0.191065883	0.768040178	24	22.13713911	0.04419071
ENSMUSG00000021895	Arhgef3	Rho guanine nucleotide exchange factor (GEF) 3 [Source:MGI Symbol;Acc:MGI:1918954]	11.379	10.9288	11.0361	10.7754	9.71409	12.2565	11.7652	9.46773	9.44652	10.4593	9.64126	9.2554	10.637	11.5908	0.784278077	0.966018836	4	16	24	0.367178979	0.790684479	24	0.541766918	0.063024955
ENSMUSG00000021898	Asb14	ankyrin repeat and SOCS box-containing 14 [Source:MGI Symbol;Acc:MGI:2655107]	1.34177	2.58168	2.21271	2.46891	2.34196	2.66146	2.15314	4.62709	5.50042	4.84381	2.40801	2.41567	3.66771	2.31896	0.885955579	0.966018836	16	12	24	0.122943024	0.735700056	24	8.849678569	0.293402231
ENSMUSG00000021900	Btd	biotinidase [Source:MGI Symbol;Acc:MGI:1347001]	10.8345	10.6048	9.31327	10.0123	11.2111	9.95599	11.1541	9.84709	10.6208	10.0735	10.1481	11.0875	10.9707	10.1715	0.208747416	0.698797644	4	8	24	0.116245502	0.727799063	24	21.29095064	0.058312337
ENSMUSG00000021901	Bap1	Brca1 associated protein 1 [Source:MGI Symbol;Acc:MGI:1206586]	27.9018	22.1771	22.8943	24.854	22.9626	26.6066	25.7615	26.8877	21.1803	27.8319	22.0923	26.5596	25.5247	24.8254	0.210566453	0.69936851	24	12	24	0.301203033	0.768040178	24	21.96244198	0.077919174
ENSMUSG00000021902	Phf7	PHD finger protein 7 [Source:MGI Symbol;Acc:MGI:1919088]	1.96694	3.13354	2.7535	2.8778	3.15812	2.82109	2.17507	2.84284	3.65908	3.11414	2.56847	3.17191	3.10628	2.9638	0.850398351	0.966018836	12	16	24	0.159138361	0.765350752	24	10.23138047	0.114516986
ENSMUSG00000021903	Galnt15	polypeptide N-acetylgalactosaminyltransferase 15 [Source:MGI Symbol;Acc:MGI:1926004]	1.38583	1.74179	1.55196	1.89471	1.55044	1.28513	1.15371	1.77265	2.13074	1.73179	1.45567	1.45577	1.1021	1.35636	0.001920665	0.100255953	12	16	24	0.004450963	0.427431763	24	9.927003939	0.173935026
ENSMUSG00000021904	Sema3g	sema domain, immunoglobulin domain (Ig), short basic domain, secreted, (semaphorin) 3G [Source:MGI Symbol;Acc:MGI:3041242]	11.4856	9.0385	8.78466	11.9526	11.8436	9.5056	11.8038	10.0813	9.46568	13.7963	9.46647	10.5023	10.2924	8.74128	0.058209189	0.40386543	16	20	24	0.301113768	0.768040178	24	16.14222598	0.108715891
ENSMUSG00000021905	Dph3	diphthamine biosynthesis 3 [Source:MGI Symbol;Acc:MGI:1922658]	25.9949	30.7113	30.7363	27.6277	29.5318	26.5519	27.3585	30.7108	33.1382	26.1793	30.9641	27.2301	29.7515	28.111	0.039704107	0.338535904	12	12	24	0.203514453	0.768040178	24	7.823527871	0.074115126
ENSMUSG00000021906	Oxnad1	oxidoreductase NAD-binding domain containing 1 [Source:MGI Symbol;Acc:MGI:1916953]	8.88442	7.75097	8.90077	9.04363	9.60337	8.89864	11.5946	8.63918	9.36915	8.93324	9.7082	8.33873	8.93489	6.96705	0.018810871	0.256345714	20	12	24	0.378006437	0.797706855	24	17.45370879	0.081399077
ENSMUSG00000021908	Ncoa4-ps	nuclear receptor coactivator 4, pseudogene [Source:MGI Symbol;Acc:MGI:3648259]	24.7881	23.0188	21.9465	23.4253	23.6996	22.298	23.8263	23.1145	23.761	20.1555	24.1369	24.2748	25.0919	24.5079	0.038751555	0.336039716	4	12	24	0.133832828	0.744007455	24	23.24467007	0.058551668
ENSMUSG00000021910	Nisch	nischarin [Source:MGI Symbol;Acc:MGI:1928323]	83.596	80.8986	79.8933	84.0487	89.4732	86.7688	87.445	85.9471	85.3364	96.2383	84.5736	78.4603	81.8951	80.6355	0.700065774	0.966018836	16	20	24	0.294193074	0.768040178	24	13.99735088	0.044943526
ENSMUSG00000021911	Parg	poly (ADP-ribose) glycohydrolase [Source:MGI Symbol;Acc:MGI:1347094]	15.8284	14.2073	13.4728	14.2363	15.0209	13.9912	14.3643	13.4693	13.7703	15.4115	14.0654	14.3421	14.3557	14.5608	0.643325306	0.966018836	4	8	24	0.481708755	0.805393068	24	19.03488424	0.030933177
ENSMUSG00000021913	Ogdhl	oxoglutarate dehydrogenase-like [Source:MGI Symbol;Acc:MGI:3616088]	6.41233	6.41717	9.14545	6.65248	5.25248	8.34009	7.71822	7.217	6.2866	8.55694	5.47737	6.48023	5.61121	7.49657	0.89563091	0.966018836	16	4	24	0.524079417	0.821765089	24	6.711917239	0.099483602
ENSMUSG00000021916	Glt8d1	glycosyltransferase 8 domain containing 1 [Source:MGI Symbol;Acc:MGI:1923735]	44.0932	47.8057	46.0491	44.5666	49.8416	44.7621	48.649	44.3094	54.1239	41.5195	50.3095	43.9596	45.0297	42.4012	0.916860713	0.966018836	20	12	24	0.856540847	0.957486395	24	12.74386384	0.022953938
ENSMUSG00000021917	Spcs1	signal peptidase complex subunit 1 homolog (S. cerevisiae) [Source:MGI Symbol;Acc:MGI:1916269]	195.018	217.847	194.682	198.4	205.096	181.745	182.232	193.285	214.808	166.756	209.474	182.352	205.704	197.911	0.970092543	0.971116366	12	12	24	0.666046393	0.887215207	24	6.021955678	0.039108155
ENSMUSG00000021918	Nek4	NIMA (never in mitosis gene a)-related expressed kinase 4 [Source:MGI Symbol;Acc:MGI:1344404]	7.36577	8.39888	7.93207	6.86101	8.09908	7.61236	7.71844	7.42725	8.48038	7.20184	7.74515	7.88029	7.21262	7.77948	0.648082424	0.966018836	12	4	24	0.825284463	0.945239222	24	5.271946352	0.019409596
ENSMUSG00000021928	Ebpl	emopamil binding protein-like [Source:MGI Symbol;Acc:MGI:1915427]	2.64423	2.9575	2.62824	3.06889	2.71328	2.63174	2.56078	2.86673	2.60732	2.30384	2.9703	2.95526	2.95781	2.92024	0.873171835	0.966018836	20	16	24	0.851240956	0.955425185	24	23.70395886	0.019242026
ENSMUSG00000021929	Kpna3	karyopherin (importin) alpha 3 [Source:MGI Symbol;Acc:MGI:1100863]	28.1446	29.9162	29.3439	27.9505	28.8256	28.4354	26.8647	27.4588	28.6869	26.8246	30.2703	27.1908	26.5596	28.021	0.46238066	0.966018836	20	8	24	0.551331514	0.83632237	24	11.86743073	0.022483853
ENSMUSG00000021930	Spryd7	SPRY domain containing 7 [Source:MGI Symbol;Acc:MGI:1913924]	49.6482	47.1764	45.1371	44.9598	46.7204	45.1795	42.2935	42.0541	40.3483	40.8661	46.0919	40.3977	42.8657	42.9025	0.907068194	0.966018836	20	20	24	0.383428243	0.799546792	24	20.03343009	0.036342627
ENSMUSG00000021932	Rnaseh2b	ribonuclease H2, subunit B [Source:MGI Symbol;Acc:MGI:1914403]	9.91159	10.1131	10.36	11.3729	11.0643	9.38216	10.4059	9.33011	10.8673	8.08616	10.5027	11.2899	11.1567	8.60702	0.478724519	0.966018836	12	20	24	0.593029609	0.856634016	24	18.23232756	0.056433347
ENSMUSG00000021936	Mapk8	mitogen-activated protein kinase 8 [Source:MGI Symbol;Acc:MGI:1346861]	27.7361	27.6962	28.1839	26.9816	29.2802	25.1823	26.6706	25.8184	26.7472	22.1164	29.9937	27.4808	27.9111	29.7756	0.865514257	0.966018836	20	20	24	0.814790606	0.940847489	24	23.17546596	0.0281826
ENSMUSG00000021938	Pspc1	paraspeckle protein 1 [Source:MGI Symbol;Acc:MGI:1913896]	11.3304	13.2317	12.9874	10.4438	10.8112	11.7715	12.0739	11.5006	10.2679	13.0004	9.5361	11.7276	11.7524	11.6978	0.822170541	0.966018836	8	12	24	0.303681999	0.768040178	24	3.443006297	0.072450981
ENSMUSG00000021939	Ctsb	cathepsin B [Source:MGI Symbol;Acc:MGI:88561]	591.963	501.769	516.861	517.626	532.908	606.36	524.265	551.764	427.581	527.343	554.137	515.346	534.42	513.106	0.118761453	0.542330458	24	12	24	0.041476466	0.618736234	24	20.28583707	0.1076254
ENSMUSG00000021943	Gdf10	growth differentiation factor 10 [Source:MGI Symbol;Acc:MGI:95684]	0.352615	0.642524	0.861568	0.778625	0.53193	0.60522	0.527141	0.733283	0.853618	0.807186	0.612362	0.57125	0.602879	0.624686	1.13E-04	0.029372629	12	16	24	6.46E-04	0.296483534	24	9.505460938	0.150916915
ENSMUSG00000021945	Zmym2	zinc finger, MYM-type 2 [Source:MGI Symbol;Acc:MGI:1923257]	35.9245	36.1663	35.6447	32.6984	34.5917	34.7559	35.0218	31.7841	34.9374	25.3137	37.7917	34.336	40.0534	36.7265	0.117050591	0.538362324	4	12	24	0.199112342	0.768040178	24	23.67771296	0.101297817
ENSMUSG00000021947	Cryl1	crystallin, lambda 1 [Source:MGI Symbol;Acc:MGI:1915881]	6.40047	6.54278	6.75907	6.02527	6.27662	6.928	5.59827	7.17536	6.61646	6.3124	5.36544	6.61979	6.07388	7.22105	0.105345531	0.518002936	8	12	24	0.235395853	0.768040178	24	4.941818642	0.070363436
ENSMUSG00000021948	Prkcd	protein kinase C, delta [Source:MGI Symbol;Acc:MGI:97598]	152.131	114.331	114.28	126.773	130.542	140.09	122.609	125.643	98.4091	137.173	123.924	123.102	128.091	117.406	0.051610136	0.389629649	16	20	24	0.054999797	0.655263328	24	19.59389753	0.131604856
ENSMUSG00000021950	Anxa8	annexin A8 [Source:MGI Symbol;Acc:MGI:1201374]	1.29218	1.77312	1.07972	1.09013	1.076	1.18469	0.93051	1.63728	1.35799	0.990594	1.00321	1.26069	1.16498	1.0948	0.115745527	0.537734429	8	12	24	0.149856433	0.758419028	24	3.57575844	0.105342158
ENSMUSG00000021951	Eef1akmt1	EEF1A alpha lysine methyltransferase 1 [Source:MGI Symbol;Acc:MGI:1915293]	26.8401	29.1888	27.7025	23.8257	28.5764	25.9247	26.3839	25.9596	26.6276	19.4152	29.8774	24.9532	28.5809	27.234	0.724067924	0.966018836	8	8	24	0.396724261	0.799546792	24	0.053826452	0.078827105
ENSMUSG00000021952	Xpo4	exportin 4 [Source:MGI Symbol;Acc:MGI:1888526]	4.36877	4.78798	4.35093	4.51915	4.6024	4.28278	4.03248	4.22506	4.52805	3.91367	4.53673	4.6167	4.19347	4.70558	0.935593607	0.966018836	20	8	24	0.974548069	0.993631841	24	9.281467973	0.022549427
ENSMUSG00000021957	Tkt	transketolase [Source:MGI Symbol;Acc:MGI:105992]	117.886	101.729	118.112	140.264	120.205	122.32	126.391	132.087	100.368	149.127	133.041	124.082	149.615	129.307	0.893307667	0.966018836	16	20	24	0.579190689	0.849723241	24	16.33342367	0.062974082
ENSMUSG00000021958	Pinx1	PIN2/TERF1 interacting, telomerase inhibitor 1 [Source:MGI Symbol;Acc:MGI:1919650]	3.94655	3.37736	3.92881	4.11297	3.59764	4.27955	3.6024	3.46974	4.15037	4.04338	3.83352	4.02019	4.36684	4.09772	0.922035832	0.966018836	24	8	24	0.786443574	0.93134108	24	15.3977363	0.02284597
ENSMUSG00000021959	Lats2	large tumor suppressor 2 [Source:MGI Symbol;Acc:MGI:1354386]	14.0164	11.4657	10.0389	13.0841	13.092	12.0153	13.2729	12.3282	11.1956	14.5525	12.0234	12.9337	12.8002	11.9412	0.079180494	0.458678242	16	20	24	0.265412909	0.768040178	24	18.93890843	0.08246486
ENSMUSG00000021962	Dcp1a	decapping mRNA 1A [Source:MGI Symbol;Acc:MGI:1923151]	5.13611	4.92057	5.93373	5.27621	7.03167	5.03706	4.59077	5.69593	5.91044	5.77981	5.66358	5.04774	5.02538	4.6575	0.020551444	0.261884115	12	16	24	0.035128746	0.608551791	24	12.79536334	0.12492713
ENSMUSG00000021963	Sap18	Sin3-associated polypeptide 18 [Source:MGI Symbol;Acc:MGI:1277978]	9.0552	8.17023	8.01986	8.29667	8.2959	8.99617	8.36522	8.51486	7.5647	8.927	7.93318	8.63373	8.55376	8.27821	0.020119233	0.259417302	24	12	24	0.119239876	0.730243445	24	20.95741024	0.051602003
ENSMUSG00000021965	Ska3	spindle and kinetochore associated complex subunit 3 [Source:MGI Symbol;Acc:MGI:3041235]	0.586944	0.624781	0.780963	0.47907	0.810645	0.713527	1.03059	0.605768	0.579189	0.710541	0.495501	0.539537	0.655233	0.517366	0.887862353	0.966018836	4	12	24	0.732131174	0.912560748	24	22.26356877	0.039526256
ENSMUSG00000021967	Mrpl57	mitochondrial ribosomal protein L57 [Source:MGI Symbol;Acc:MGI:1915090]	29.5332	22.9107	24.1482	25.9668	21.6627	23.9822	27.2871	27.2052	28.9101	27.9464	27.7571	26.6238	27.6369	24.2643	0.935593607	0.966018836	4	20	24	0.907145405	0.974606327	24	2.82187865	0.022011263
ENSMUSG00000021969	Zdhhc20	zinc finger, DHHC domain containing 20 [Source:MGI Symbol;Acc:MGI:1923215]	16.6096	18.788	21.8521	18.3803	18.3716	17.9217	19.8014	17.6401	21.6587	16.3205	18.051	17.9447	14.2413	18.6064	0.122530972	0.550724475	12	16	24	0.260629055	0.768040178	24	8.877671197	0.093117602
ENSMUSG00000021972	Hmbox1	homeobox containing 1 [Source:MGI Symbol;Acc:MGI:2445066]	66.0488	53.8098	54.7088	58.431	57.3279	61.6401	53.9098	58.3038	43.316	73.6735	54.7343	59.7309	57.3216	57.6756	0.338172159	0.85758369	24	12	24	0.483905419	0.805393068	24	18.46112422	0.082378524
ENSMUSG00000021973	Micu2	mitochondrial calcium uptake 2 [Source:MGI Symbol;Acc:MGI:1915764]	20.0765	21.4843	20.5819	20.2638	22.6358	20.2183	21.4952	21.1673	22.3555	19.4409	21.3336	21.2264	22.2283	22.0044	0.915082018	0.966018836	8	8	24	0.698244484	0.899694264	24	2.410322867	0.019655461
ENSMUSG00000021974	Fgf9	fibroblast growth factor 9 [Source:MGI Symbol;Acc:MGI:104723]	8.66232	7.42137	8.38308	7.49369	6.71762	8.07272	7.54227	7.44164	5.58242	7.70414	7.47516	7.33943	7.06481	8.43756	0.874672901	0.966018836	4	16	24	0.588629111	0.853936396	24	23.28820632	0.056102729
ENSMUSG00000021975	Ints9	integrator complex subunit 9 [Source:MGI Symbol;Acc:MGI:1098533]	7.06455	6.30928	6.1662	6.03514	6.54971	7.74269	7.11298	6.73471	4.94643	7.23387	5.88614	6.74567	6.37551	6.65689	0.042880436	0.356048751	24	12	24	0.100384368	0.71175955	24	21.24486495	0.110827193
ENSMUSG00000021978	Extl3	exostosin-like glycosyltransferase 3 [Source:MGI Symbol;Acc:MGI:1860765]	21.1962	17.3149	17.2275	19.1195	18.2675	19.3962	18.0314	21.5009	14.5251	25.3191	17.8249	20.6728	19.2898	19.8565	0.830364743	0.966018836	24	12	24	0.784160418	0.930681482	24	19.31931961	0.05128512
ENSMUSG00000021981	Cab39l	calcium binding protein 39-like [Source:MGI Symbol;Acc:MGI:1914081]	103.105	101.386	87.9081	97.102	107.041	96.983	92.9804	92.2867	93.3367	97.6554	105.811	103.602	104.614	103.989	0.039226672	0.338446795	20	16	24	0.144684156	0.753524831	24	19.22822844	0.063452088
ENSMUSG00000021982	Cdadc1	cytidine and dCMP deaminase domain containing 1 [Source:MGI Symbol;Acc:MGI:1919141]	33.6177	34.7274	31.0329	31.1197	33.8968	29.9372	30.8459	29.2399	30.8507	30.4896	31.0084	31.9814	32.3531	33.2246	0.873350015	0.966018836	8	4	24	0.680277893	0.89309548	24	23.75657635	0.024332906
ENSMUSG00000021983	Atp8a2	ATPase, aminophospholipid transporter-like, class I, type 8A, member 2 [Source:MGI Symbol;Acc:MGI:1354710]	30.7112	26.938	24.8542	26.4398	24.7635	28.2388	27.6541	25.9309	22.1886	35.9573	29.4326	35.1444	33.368	33.0819	0.14313801	0.585949911	24	12	24	0.335331512	0.771377467	24	20.70196047	0.102054823
ENSMUSG00000021986	Amer2	APC membrane recruitment 2 [Source:MGI Symbol;Acc:MGI:1919375]	9.82951	8.12667	8.22902	8.72556	8.60147	8.54419	8.28191	7.65183	7.57842	8.39177	9.06965	8.93526	9.42888	9.07079	0.157813674	0.614233844	4	8	24	0.126265162	0.739868076	24	20.23738522	0.074669127
ENSMUSG00000021987	Mtmr6	myotubularin related protein 6 [Source:MGI Symbol;Acc:MGI:2145637]	144.455	137.157	125.291	135.185	128.38	134.917	127.04	128.774	117.491	139.154	135.351	135.681	138.979	139.064	0.773177465	0.966018836	4	8	24	0.368321568	0.791504785	24	21.15276561	0.044413454
ENSMUSG00000021990	Spata13	spermatogenesis associated 13 [Source:MGI Symbol;Acc:MGI:104838]	19.6393	17.3348	18.8809	19.1192	18.8267	20.609	17.6248	20.1635	14.6741	24.03	16.3385	19.4077	19.5615	17.7756	0.896545154	0.966018836	24	12	24	0.723359629	0.909188168	24	17.91851833	0.052552997
ENSMUSG00000021991	Cacna2d3	calcium channel, voltage-dependent, alpha2/delta subunit 3 [Source:MGI Symbol;Acc:MGI:1338890]	21.3617	18.2594	16.4793	17.9987	18.1904	19.9374	16.0735	16.5019	13.4584	19.9398	15.9302	19.6576	18.0789	18.8078	0.390685537	0.915479136	24	12	24	0.224373646	0.768040178	24	20.3710855	0.111357642
ENSMUSG00000021993	Mipep	mitochondrial intermediate peptidase [Source:MGI Symbol;Acc:MGI:1917728]	8.75728	8.35833	8.01133	7.71951	8.25886	9.18038	8.14395	8.60134	7.67272	8.98836	8.3213	8.52269	8.91306	8.53515	0.080164531	0.458678242	24	4	24	0.137088635	0.747972781	24	21.67034887	0.051886704
ENSMUSG00000021994	Wnt5a	wingless-type MMTV integration site family, member 5A [Source:MGI Symbol;Acc:MGI:98958]	5.48546	5.61792	4.33164	4.94101	4.11384	4.98444	4.98328	5.46143	4.75873	5.68138	4.68112	5.41377	5.5931	5.77461	0.036134798	0.331852526	8	12	24	0.100114317	0.71175955	24	2.302309381	0.093528341
ENSMUSG00000021996	Esd	esterase D/formylglutathione hydrolase [Source:MGI Symbol;Acc:MGI:95421]	53.2638	56.5608	54.6871	55.132	59.1445	54.5391	49.1761	53.9591	53.2044	45.483	60.8156	53.7295	56.3766	53.0747	0.586208558	0.966018836	20	8	24	0.672199157	0.889365603	24	18.21324553	0.036978843
ENSMUSG00000021998	Lcp1	lymphocyte cytosolic protein 1 [Source:MGI Symbol;Acc:MGI:104808]	53.0442	45.2525	44.9186	47.4601	43.6544	51.5345	49.6276	49.9484	35.7256	54.5858	42.3795	46.2756	47.1381	49.3007	0.445967571	0.966018836	4	16	24	0.436432682	0.805393068	24	22.33349421	0.078238379
ENSMUSG00000022000	Zc3h13	zinc finger CCCH type containing 13 [Source:MGI Symbol;Acc:MGI:1914552]	8.95539	7.11872	6.29463	8.99651	8.13371	7.9648	7.94032	8.72189	7.33957	8.03417	8.26582	8.20053	7.93083	7.56172	0.086705029	0.468805495	16	20	24	0.250052666	0.768040178	24	18.94916782	0.080160261
ENSMUSG00000022003	Slc25a30	solute carrier family 25, member 30 [Source:MGI Symbol;Acc:MGI:1914804]	11.4499	10.2574	10.3978	11.1232	10.591	10.7916	10.5825	10.0262	8.45038	12.1223	11.6762	11.0416	10.2638	10.1509	0.00424198	0.140598674	16	20	24	0.091934651	0.71175955	24	17.42267937	0.092417622
ENSMUSG00000022008	Gpalpp1	GPALPP motifs containing 1 [Source:MGI Symbol;Acc:MGI:1914717]	11.1314	11.4003	10.8247	11.5243	10.3429	10.7332	10.4777	10.4114	10.8724	11.6577	10.9957	11.5126	10.7857	11.3719	0.745118544	0.966018836	16	12	24	0.633621324	0.875436572	24	10.70344719	0.020388554
ENSMUSG00000022009	Nufip1	nuclear fragile X mental retardation protein interacting protein 1 [Source:MGI Symbol;Acc:MGI:1351474]	2.88575	2.89675	3.01701	3.04503	2.99729	3.03923	3.21175	2.89463	3.10022	3.21486	3.12887	3.29139	3.19544	3.25885	0.890235289	0.966018836	24	8	24	0.6124654	0.865550634	24	16.2971516	0.011422434
ENSMUSG00000022010	Tsc22d1	TSC22 domain family, member 1 [Source:MGI Symbol;Acc:MGI:109127]	185.972	188.194	170.955	165.929	163.582	161.707	172.101	177.96	172.746	162.237	177.806	171.792	172.641	171.311	0.097975419	0.501188411	8	8	24	0.066424032	0.668887716	24	2.406130555	0.053158555
ENSMUSG00000022012	Enox1	ecto-NOX disulfide-thiol exchanger 1 [Source:MGI Symbol;Acc:MGI:2444896]	16.5271	14.4602	12.3723	16.0606	13.5428	15.3241	14.4624	14.5386	12.0119	15.2913	14.5902	15.3869	15.2974	13.8599	0.021795836	0.26698364	24	12	24	0.117782642	0.728323355	24	20.27069485	0.098001931
ENSMUSG00000022013	Dnajc15	DnaJ heat shock protein family (Hsp40) member C15 [Source:MGI Symbol;Acc:MGI:1913398]	24.2037	28.6696	30.8387	33.9253	32.7854	31.5734	26.2157	32.0292	35.485	25.1065	35.5043	24.5172	32.0329	27.3883	0.304354943	0.811201458	20	8	24	0.323020799	0.768040178	24	11.62915822	0.110601166
ENSMUSG00000022014	Epsti1	epithelial stromal interaction 1 (breast) [Source:MGI Symbol;Acc:MGI:1915168]	0.861501	1.21183	1.03555	1.07936	1.13466	0.849673	1.16339	1.14655	1.57244	0.674497	0.993064	0.899483	1.00223	0.763732	0.791413662	0.966018836	12	12	24	0.574129162	0.847226836	24	6.843992452	0.065590879
ENSMUSG00000022016	Akap11	A kinase (PRKA) anchor protein 11 [Source:MGI Symbol;Acc:MGI:2684060]	44.1091	48.0512	45.2292	47.9392	49.58	42.4126	45.1298	39.7815	49.67	41.1355	48.1921	47.8066	47.952	48.3948	0.879901313	0.966018836	20	4	24	0.876996001	0.965814767	24	13.80806239	0.020351868
ENSMUSG00000022018	Rgcc	regulator of cell cycle [Source:MGI Symbol;Acc:MGI:1913464]	28.9103	32.5091	34.9286	44.1843	33.7954	33.6652	32.0528	32.5199	44.7265	40.9166	40.027	39.3734	37.4061	34.1325	0.008244783	0.192760771	16	12	24	0.004351148	0.427431763	24	12.06246179	0.178355741
ENSMUSG00000022019	Tdrd3	tudor domain containing 3 [Source:MGI Symbol;Acc:MGI:2444023]	14.9719	17.1547	15.9511	15.4643	15.6303	13.9548	16.8486	14.2748	17.1943	14.996	17.3866	15.109	14.7906	17.1687	0.591351901	0.966018836	12	12	24	0.446029577	0.805393068	24	7.937540502	0.052628326
ENSMUSG00000022020	Naa16	N(alpha)-acetyltransferase 16, NatA auxiliary subunit [Source:MGI Symbol;Acc:MGI:1914147]	3.72146	4.23965	3.71802	5.31199	4.09042	3.73324	4.75477	3.97487	5.55871	4.62258	5.77767	4.42706	4.59667	4.15836	0.800065124	0.966018836	16	16	24	0.20678129	0.768040178	24	12.38639288	0.107612994
ENSMUSG00000022021	Diaph3	diaphanous related formin 3 [Source:MGI Symbol;Acc:MGI:1927222]	7.60359	10.8289	8.5469	9.32747	11.326	8.25816	8.36287	8.56479	9.39139	8.26933	7.96262	9.46835	9.67569	9.40972	0.883870616	0.966018836	8	16	24	0.918268503	0.977931782	24	9.222258741	0.025836845
ENSMUSG00000022022	Mtrf1	mitochondrial translational release factor 1 [Source:MGI Symbol;Acc:MGI:2384815]	4.42387	4.95566	4.88825	4.93604	4.96598	4.37352	4.20535	4.6463	5.39283	4.8248	5.05293	5.02155	5.21817	6.05301	0.915082018	0.966018836	20	8	24	0.099355096	0.71175955	24	8.402200713	0.075197743
ENSMUSG00000022023	Wbp4	WW domain binding protein 4 [Source:MGI Symbol;Acc:MGI:109568]	33.4603	30.3097	31.2456	32.7932	30.7918	31.4179	29.0622	30.6142	28.7184	35.7385	33.5973	34.8749	36.1478	35.939	0.939103211	0.966018836	16	20	24	0.779896056	0.929265845	24	17.99559785	0.026979875
ENSMUSG00000022024	Sugt1	SGT1, suppressor of G2 allele of SKP1 (S. cerevisiae) [Source:MGI Symbol;Acc:MGI:1915205]	72.2818	73.8721	67.3139	71.402	78.148	67.5508	70.0278	65.5263	69.3608	54.3789	75.2304	73.3768	77.457	73.7598	0.435739761	0.966018836	20	20	24	0.328838579	0.768040178	24	21.84072186	0.07712094
ENSMUSG00000022025	Cnmd	chondromodulin [Source:MGI Symbol;Acc:MGI:1341171]	22.5	20.8123	16.5261	18.5801	25.5014	24.2689	22.9047	21.6597	18.2544	18.2952	22.006	19.9246	20.5659	15.8998	0.03753984	0.334069441	20	16	24	0.004487057	0.427431763	24	19.87452601	0.205273621
ENSMUSG00000022031	Elp3	elongator acetyltransferase complex subunit 3 [Source:MGI Symbol;Acc:MGI:1921445]	26.6402	26.1444	25.4347	25.9225	24.8157	28.1543	26.216	27.8291	23.872	28.6596	24.3413	26.3979	25.758	27.5845	0.481811994	0.966018836	24	20	24	0.663229061	0.88640646	24	0.828024124	0.027013178
ENSMUSG00000022032	Scara5	scavenger receptor class A, member 5 [Source:MGI Symbol;Acc:MGI:1918395]	9.28556	10.2894	8.23646	7.08207	11.176	7.84089	10.4093	9.22652	12.4323	5.16761	10.0946	10.0091	9.35425	8.63913	0.722296281	0.966018836	20	20	24	0.467214387	0.805393068	24	0.01188414	0.135129932
ENSMUSG00000022033	Pbk	PDZ binding kinase [Source:MGI Symbol;Acc:MGI:1289156]	0.432702	0.824635	0.941672	0.64497	0.716742	0.888114	0.942543	0.639929	0.840413	0.598421	0.825292	0.679377	0.676515	0.434289	0.916963526	0.966018836	12	20	24	0.83884967	0.950804194	24	14.91065203	0.033201257
ENSMUSG00000022035	Ccdc25	coiled-coil domain containing 25 [Source:MGI Symbol;Acc:MGI:1914429]	24.5207	23.6649	24.6099	24.0812	25.2189	23.955	22.7793	23.9037	23.6482	22.5213	25.4869	25.7283	26.1621	26.4948	0.86340968	0.966018836	20	20	24	0.663311522	0.88640646	24	21.37499065	0.023048913
ENSMUSG00000022037	Clu	clusterin [Source:MGI Symbol;Acc:MGI:88423]	165.062	145.891	153.495	128.629	124.914	163.589	166.309	173.061	132.527	158.098	122.021	150.437	124.904	149.432	0.351315142	0.870418653	4	16	24	0.194274767	0.768040178	24	1.746788625	0.114577412
ENSMUSG00000022040	Ephx2	epoxide hydrolase 2, cytoplasmic [Source:MGI Symbol;Acc:MGI:99500]	4.9936	5.41677	7.80605	13.4301	6.13012	6.11407	6.43219	9.04827	7.57412	9.55312	7.69739	7.12255	9.59857	8.99906	0.496745696	0.966018836	16	8	24	0.068355073	0.672656585	24	10.18621795	0.264581286
ENSMUSG00000022043	Trim35	tripartite motif-containing 35 [Source:MGI Symbol;Acc:MGI:1914104]	44.1046	38.1807	39.8213	41.4683	41.0061	43.9647	42.0566	45.966	35.8804	46.3893	39.3249	40.2308	41.2022	41.5132	0.892578749	0.966018836	4	8	24	0.720633194	0.908366535	24	20.87698967	0.033468491
ENSMUSG00000022044	Stmn4	stathmin-like 4 [Source:MGI Symbol;Acc:MGI:1931224]	13.54	21.6964	34.1558	17.8406	16.0022	18.8119	21.9727	16.2631	19.6807	15.6668	20.8557	18.0021	12.6588	21.9737	0.700065774	0.966018836	12	4	24	0.368040711	0.79131734	24	8.329064959	0.19172975
ENSMUSG00000022048	Dpysl2	dihydropyrimidinase-like 2 [Source:MGI Symbol;Acc:MGI:1349763]	42.0046	36.1675	35.3001	35.0259	37.8501	41.0372	38.6385	36.3122	32.9617	41.1715	36.8972	37.6897	36.6677	38.5986	0.068712455	0.432567661	24	12	24	0.112996858	0.727393008	24	20.51913622	0.076846336
ENSMUSG00000022051	Bnip3l	BCL2/adenovirus E1B interacting protein 3-like [Source:MGI Symbol;Acc:MGI:1332659]	41.7242	47.2544	45.9125	44.5934	45.2354	40.6915	42.8144	42.5922	49.9696	40.4143	46.9496	43.8256	46.0924	43.2208	0.798309651	0.966018836	12	12	24	0.457951588	0.805393068	24	8.697648885	0.044034504
ENSMUSG00000022052	Ppp2r2a	protein phosphatase 2, regulatory subunit B, alpha [Source:MGI Symbol;Acc:MGI:1919228]	30.3509	25.3601	26.7734	24.9107	25.9876	28.5248	26.8564	26.5069	23.9091	30.3962	25.6419	27.9413	27.1645	27.9834	0.34864256	0.870418653	24	12	24	0.392031156	0.799546792	24	21.53828663	0.053557831
ENSMUSG00000022053	Ebf2	early B cell factor 2 [Source:MGI Symbol;Acc:MGI:894332]	2.28655	3.50144	3.56078	2.826	3.3575	2.62125	3.15696	3.50436	3.98149	2.83126	3.57836	3.31161	3.23217	3.47603	0.052640391	0.391647859	12	12	24	0.160915874	0.765941695	24	7.919388673	0.1111287
ENSMUSG00000022054	Nefm	neurofilament, medium polypeptide [Source:MGI Symbol;Acc:MGI:97314]	586.092	432.508	424.474	425.513	397.7	513.846	483.983	511.359	314.207	688.754	401.395	496.986	448.695	499.358	0.302438464	0.811201458	24	20	24	0.633135298	0.875325783	24	22.11456034	0.10637695
ENSMUSG00000022055	Nefl	neurofilament, light polypeptide [Source:MGI Symbol;Acc:MGI:97313]	968.064	769.909	747.729	738.728	710.178	845.65	818.31	891.2	641.353	1034.31	852.771	894.267	877.428	888.842	0.509507891	0.966018836	4	8	24	0.423917646	0.805393068	24	22.1484991	0.093111603
ENSMUSG00000022064	Pibf1	progesterone immunomodulatory binding factor 1 [Source:MGI Symbol;Acc:MGI:1261910]	6.39834	7.53782	6.81496	5.54589	7.26696	6.31398	7.33418	7.01174	7.87812	6.37662	7.85015	7.8706	6.97807	7.06589	0.920992137	0.966018836	8	8	24	0.781094119	0.929846332	24	0.964965106	0.032984072
ENSMUSG00000022070	Bora	bora, aurora kinase A activator [Source:MGI Symbol;Acc:MGI:1924994]	1.44215	1.9355	2.19659	1.9141	1.69695	2.02543	1.68543	1.82052	1.7483	1.78598	1.97524	2.14503	1.74226	2.21825	0.591351901	0.966018836	24	4	24	0.655144363	0.883697438	24	9.979748337	0.039856076
ENSMUSG00000022075	Rhobtb2	Rho-related BTB domain containing 2 [Source:MGI Symbol;Acc:MGI:2180557]	10.3659	7.36328	7.1408	8.35716	8.23295	8.62494	8.82096	8.49228	6.6464	9.56449	8.05573	8.1821	8.40307	7.97799	0.060394272	0.407012001	4	8	24	0.180743865	0.768040178	24	20.33884986	0.105188966
ENSMUSG00000022089	Bin3	bridging integrator 3 [Source:MGI Symbol;Acc:MGI:1929883]	15.8274	16.0818	13.3608	14.3838	13.9612	13.484	13.1545	13.454	13.8889	12.3643	13.5485	13.3003	13.9174	15.6324	0.948582958	0.966018836	8	16	24	0.20901436	0.768040178	24	2.593445303	0.064625647
ENSMUSG00000022090	Pdlim2	PDZ and LIM domain 2 [Source:MGI Symbol;Acc:MGI:2384850]	27.1033	33.5202	38.9561	29.4389	28.9943	27.7667	35.7212	32.2445	42.6172	28.8638	28.7614	32.6569	33.1355	31.0375	0.125887884	0.555618842	12	8	24	0.083035402	0.699403344	24	6.313146852	0.154769699
ENSMUSG00000022091	Sorbs3	sorbin and SH3 domain containing 3 [Source:MGI Symbol;Acc:MGI:700013]	18.4186	21.004	19.8634	22.5602	25.0962	18.9769	22.9857	20.8853	25.61	28.6427	20.3056	20.7423	19.9846	18.5762	0.320283544	0.83607295	16	12	24	0.069076014	0.673520298	24	11.94897259	0.151573687
ENSMUSG00000022092	Ppp3cc	protein phosphatase 3, catalytic subunit, gamma isoform [Source:MGI Symbol;Acc:MGI:107162]	25.2161	24.7788	23.8293	25.624	24.3402	25.4932	21.671	22.7712	22.4735	25.0656	24.0194	24.8514	26.2421	25.362	0.96317972	0.967965568	24	12	24	0.66732147	0.887459929	24	18.07507604	0.028404875
ENSMUSG00000022094	Slc39a14	solute carrier family 39 (zinc transporter), member 14 [Source:MGI Symbol;Acc:MGI:2384851]	5.90986	5.41258	7.01565	6.14974	5.70848	6.06928	5.88194	6.11278	5.57455	5.90327	5.12331	5.15478	5.65411	5.59948	0.907068194	0.966018836	16	4	24	0.397339329	0.799546792	24	7.74121816	0.047558814
ENSMUSG00000022095	Fam160b2	family with sequence similarity 160, member B2 [Source:MGI Symbol;Acc:MGI:3036290]	6.58293	5.66348	6.18673	6.28061	6.06509	6.14368	5.56296	6.16995	5.38465	7.16182	5.42032	5.65244	5.95414	6.1461	0.907000607	0.966018836	16	4	24	0.800404178	0.936110827	24	11.28825947	0.0259437
ENSMUSG00000022096	Hr	lysine demethylase and nuclear receptor corepressor [Source:MGI Symbol;Acc:MGI:96223]	4.465	4.02051	4.03734	4.88384	3.84243	4.29352	5.38843	5.10431	4.41151	6.25242	4.05574	4.15528	4.46915	4.34066	0.718086778	0.966018836	16	4	24	0.760813169	0.92268731	24	8.236744415	0.049315605

ENSMUSG00000022098	Bmp1	bone morphogenetic protein 1 [Source:MGI Symbol;Acc:MGI:88176]	10.2278	10.3484	10.3753	10.7508	11.0693	9.47806	11.6681	9.28558	11.4394	8.49355	11.4724	9.17635	9.89182	8.8309	0.951945551	0.966018836	20	12	24	0.833931624	0.948922772	24	15.77332474	0.03031195
ENSMUSG00000022099	Dmtn	dematin actin binding protein [Source:MGI Symbol;Acc:MGI:99670]	58.8461	45.7423	46.5002	50.5164	48.0281	56.0288	53.6054	52.1718	39.8929	63.5485	45.6721	54.7677	50.2942	53.7744	0.245061943	0.741580974	24	12	24	0.425610354	0.805393068	24	20.74184412	0.090128227
ENSMUSG00000022100	Xpo7	exportin 7 [Source:MGI Symbol;Acc:MGI:1929705]	18.8079	16.5345	16.6063	18.2601	15.756	17.2699	18.2155	16.0702	14.4411	17.8304	15.8401	17.4335	17.2499	16.5527	0.302965859	0.811201458	4	8	24	0.302183024	0.768040178	24	21.24690773	0.058297041
ENSMUSG00000022102	Dok2	docking protein 2 [Source:MGI Symbol;Acc:MGI:1332623]	1.85651	1.94269	1.1625	1.91666	1.89448	1.6264	2.08862	2.30648	1.76447	0.872084	2.29077	1.45892	1.78317	1.70511	0.838532766	0.966018836	8	4	24	0.430640876	0.805393068	24	23.29834591	0.114826397
ENSMUSG00000022103	Gfra2	glial cell line derived neurotrophic factor family receptor alpha 2 [Source:MGI Symbol;Acc:MGI:1195462]	49.9422	33.5657	31.5243	36.6509	37.2352	42.6278	37.6288	39.6397	26.3808	48.7144	33.5327	37.4153	39.904	35.8804	0.016095537	0.253395174	4	8	24	0.239824955	0.768040178	24	20.55846406	0.156174593
ENSMUSG00000022105	Rb1	RB transcriptional corepressor 1 [Source:MGI Symbol;Acc:MGI:97874]	12.5381	13.7596	13.6831	13.4443	13.6719	12.4729	14.5138	13.3314	13.6711	12.4069	12.2604	13.5605	14.4039	14.1815	0.135859247	0.575392196	4	16	24	0.149241212	0.757748869	24	3.627144584	0.053585481
ENSMUSG00000022106	Rcbtb2	regulator of chromosome condensation (RCC1) and BTB (POZ) domain containing protein 2 [Source:MGI Symbol;Acc:MGI:1917200]	18.7307	18.1299	18.3979	17.5554	17.6538	16.5783	17.6172	17.4989	18.093	15.8929	18.488	20.1067	21.1936	20.6769	0.47028201	0.966018836	4	12	24	0.170413304	0.767986663	24	1.230697775	0.068232285
ENSMUSG00000022108	Itm2b	integral membrane protein 2B [Source:MGI Symbol;Acc:MGI:1309517]	453.469	438.503	437.73	426.745	412.6	463.734	467.707	471.377	432.002	444.449	433.973	445.114	438.007	438.424	0.05945106	0.406067671	24	20	24	0.079845513	0.694370548	24	0.564302415	0.043088205
ENSMUSG00000022109	Med4	mediator complex subunit 4 [Source:MGI Symbol;Acc:MGI:1914631]	15.9676	17.0545	16.9327	15.9887	15.3668	16.0898	14.7689	16.1229	17.5008	15.7658	16.8707	15.9429	18.6438	17.2556	0.221187015	0.712731927	12	4	24	0.256817683	0.768040178	24	5.750455912	0.049268883
ENSMUSG00000022110	Sucla2	succinate-Coenzyme A ligase, ADP-forming, beta subunit [Source:MGI Symbol;Acc:MGI:1306775]	130.747	136.851	133.705	158.029	131.901	129.699	128.984	140.908	135.642	145.365	153.164	145.692	164.926	155.332	0.919571723	0.966018836	16	12	24	0.656823925	0.884189656	24	10.29512873	0.034022529
ENSMUSG00000022111	Uchl3	ubiquitin carboxyl-terminal esterase L3 (ubiquitin thiolesterase) [Source:MGI Symbol;Acc:MGI:1355274]	11.6974	12.9356	13.5151	12.4674	12.5661	11.0817	13.8436	10.8879	11.345	9.2262	11.7013	10.16	12.0923	11.6243	0.90767227	0.966018836	4	20	24	0.603380732	0.861435876	24	2.469118456	0.050627099
ENSMUSG00000022112	Gpc5	glypican 5 [Source:MGI Symbol;Acc:MGI:1194894]	4.22623	3.84176	6.00536	3.57578	2.92847	4.86547	4.54125	3.55748	3.87748	4.61512	3.47791	4.5922	3.53973	4.69964	0.945282252	0.966018836	24	20	24	0.70272674	0.901733679	24	4.504284403	0.072580891
ENSMUSG00000022114	Spry2	sprouty RTK signaling antagonist 2 [Source:MGI Symbol;Acc:MGI:1345138]	8.95494	7.35518	8.33397	7.64157	8.14791	7.74266	7.42219	7.19464	6.80562	7.8584	6.97783	6.61525	6.86333	6.81701	0.896799496	0.966018836	16	16	24	0.634443165	0.87566973	24	16.4484212	0.026565409
ENSMUSG00000022119	Rbm26	RNA binding motif protein 26 [Source:MGI Symbol;Acc:MGI:1921463]	18.423	22.007	21.1399	19.9991	19.1789	18.2335	20.0828	19.0984	21.8674	18.2995	19.5086	18.5495	18.4223	19.0862	0.022634137	0.27189101	12	12	24	0.055601058	0.65534544	24	7.611205552	0.074700604
ENSMUSG00000022120	Obi1	ORC ubiquitin ligase 1 [Source:MGI Symbol;Acc:MGI:1919736]	11.4004	9.77655	8.34901	9.44816	10.2877	10.5255	9.58883	9.04915	8.44678	9.49389	9.8822	10.1083	11.0652	10.2178	0.001920665	0.100255953	4	8	24	0.006677194	0.470810671	24	21.0599406	0.127310206
ENSMUSG00000022122	Ednrb	endothelin receptor type B [Source:MGI Symbol;Acc:MGI:102720]	29.0192	27.2296	29.7394	25.5949	30.4961	31.4481	29.951	26.4968	23.4551	28.9182	32.0374	26.5948	24.5808	26.9311	0.217933169	0.704989686	20	16	24	0.112060274	0.725578172	24	17.96420877	0.09988353
ENSMUSG00000022123	Scel	sciellin [Source:MGI Symbol;Acc:MGI:1891228]	0.420272	0.667282	0.569495	0.887196	0.856977	0.590008	0.599371	0.44068	0.801412	0.636059	0.628891	0.633618	0.590567	0.51846	0.056973933	0.401309455	16	12	24	0.032998214	0.606585237	24	13.28784087	0.109603007
ENSMUSG00000022124	Fbxl3	F-box and leucine-rich repeat protein 3 [Source:MGI Symbol;Acc:MGI:1354702]	41.9423	44.8897	43.251	45.4096	44.2302	39.6807	40.8557	41.1273	47.2508	39.2381	43.6085	42.4848	40.1125	43.1891	0.128989168	0.560209134	12	12	24	0.230724092	0.768040178	24	9.694007742	0.050564984
ENSMUSG00000022125	Cln5	ceroid-lipofuscinosis, neuronal 5 [Source:MGI Symbol;Acc:MGI:2442253]	18.2174	16.9572	16.7114	16.6904	20.1326	18.5104	18.6851	16.9816	16.1647	15.7686	18.2618	17.4892	17.8427	16.5059	0.002094047	0.100255953	20	20	24	0.002657659	0.402846296	24	19.86583101	0.102458945
ENSMUSG00000022130	Tgds	TDP-glucose 4,6-dehydratase [Source:MGI Symbol;Acc:MGI:1923605]	3.70968	4.28428	4.20681	4.80116	5.184	3.84384	4.56491	4.68897	6.06928	4.64457	4.56618	4.42773	4.67498	4.76243	0.319330027	0.83607295	16	8	24	0.082613265	0.699403344	24	9.897689671	0.110693732
ENSMUSG00000022131	Gpr180	G protein-coupled receptor 180 [Source:MGI Symbol;Acc:MGI:1930949]	12.0803	12.3775	11.3325	12.7731	11.7313	12.6471	11.425	13.3858	13.2396	12.136	12.7444	12.5117	12.5296	12.9361	0.891022863	0.966018836	8	20	24	0.694629727	0.898526856	24	6.894636096	0.02167513
ENSMUSG00000022132	Cldn10	claudin 10 [Source:MGI Symbol;Acc:MGI:1913101]	0.803272	2.76287	5.87875	2.82265	1.54236	2.58728	3.63618	1.58349	3.22293	1.89788	1.77134	2.15844	0.82707	3.45118	0.321662653	0.83607295	12	8	24	0.218822097	0.768040178	24	8.436219177	0.363971719
ENSMUSG00000022136	Dnajc3	DnaJ heat shock protein family (Hsp40) member C3 [Source:MGI Symbol;Acc:MGI:107373]	32.986	26.9476	24.744	24.793	28.6847	26.6849	29.1324	24.8554	24.5647	23.3188	30.1634	26.6928	27.7487	26.4081	0.00455947	0.140598674	4	12	24	0.012875707	0.527834117	24	21.28632164	0.133578144
ENSMUSG00000022139	Mbnl2	muscleblind like splicing factor 2 [Source:MGI Symbol;Acc:MGI:2145597]	212.409	186.283	182.183	192.694	185.087	191.312	190.539	176.51	159.861	198.276	204.648	183.589	186.858	186.55	0.162672639	0.624917386	16	20	24	0.173337335	0.768040178	24	19.07738712	0.069336535
ENSMUSG00000022141	Nipbl	NIPBL cohesin loading factor [Source:MGI Symbol;Acc:MGI:1913976]	7.39391	7.66119	7.05071	7.33439	7.76529	6.79453	7.65651	6.37158	8.3996	6.28597	7.83651	7.75489	7.82893	7.51752	0.882617464	0.966018836	20	20	24	0.882635451	0.966732748	24	21.68220355	0.021667685
ENSMUSG00000022142	Nup155	nucleoporin 155 [Source:MGI Symbol;Acc:MGI:2181182]	6.22924	6.94691	6.71958	5.79328	7.06841	6.3434	6.32055	6.82318	7.80781	6.04956	6.6536	6.07934	7.13005	6.74871	0.371875382	0.894537464	12	4	24	0.341948747	0.776153333	24	5.664773247	0.057367264
ENSMUSG00000022146	Osmr	oncostatin M receptor [Source:MGI Symbol;Acc:MGI:1330819]	13.3423	11.9189	11.0371	12.6203	12.3704	12.8505	10.9807	11.3267	10.9695	11.8883	12.9751	11.7183	12.1502	11.9543	0.113424199	0.530777648	20	16	24	0.081174213	0.697074851	24	18.36748324	0.070825185
ENSMUSG00000022148	Fyb	FYN binding protein [Source:MGI Symbol;Acc:MGI:1346327]	1.32252	2.2253	2.52615	3.12515	2.95256	2.06784	2.80583	1.86178	2.65804	1.9693	2.86861	2.36074	2.48396	2.17866	0.22248906	0.716264058	20	12	24	0.196508971	0.768040178	24	13.28618254	0.147451135
ENSMUSG00000022150	Dab2	disabled 2, mitogen-responsive phosphoprotein [Source:MGI Symbol;Acc:MGI:109175]	6.86077	8.2068	6.6341	6.04469	8.20268	5.6432	6.94736	8.38154	10.0005	5.43549	7.05621	6.42898	7.27464	7.0704	0.085329704	0.466404237	8	8	24	0.295192245	0.768040178	24	5.225152193	0.128313974
ENSMUSG00000022151	Ttc33	tetratricopeptide repeat domain 33 [Source:MGI Symbol;Acc:MGI:1914765]	37.0693	39.9581	37.2312	37.1106	38.9482	37.1431	34.2254	35.0377	37.6667	32.6133	38.9305	35.4656	35.8725	37.2354	0.725061903	0.966018836	20	8	24	0.970708324	0.99300601	24	14.82294843	0.007030375
ENSMUSG00000022155	Mroh2b	maestro heat-like repeat family member 2B [Source:MGI Symbol;Acc:MGI:1921905]	4.04081	3.41259	3.19423	2.40465	3.37623	3.90026	2.1783	3.98725	2.54549	3.29602	2.67414	3.71169	3.2923	3.37265	0.27137637	0.781251337	24	16	24	0.423902231	0.805393068	24	23.14618278	0.108969483
ENSMUSG00000022157	Mcpt8	mast cell protease 8 [Source:MGI Symbol;Acc:MGI:1261780]	0.47297	1.96779	1.86622	1.48917	1.1324	1.0221	1.02828	1.11424	1.77934	0.839806	1.17725	1.21876	1.59329	0.732048	0.649212555	0.966018836	12	16	24	0.38960818	0.799546792	24	8.839259721	0.160999903
ENSMUSG00000022159	Rab2b	RAB2B, member RAS oncogene family [Source:MGI Symbol;Acc:MGI:1923588]	29.4927	30.3711	27.5714	28.2629	27.1831	29.9074	28.5668	28.0123	28.113	29.2771	28.9385	29.7032	30.2724	28.5428	0.124211791	0.552144108	24	12	24	0.15440308	0.760257003	24	22.57334474	0.035432626
ENSMUSG00000022160	Mettl3	methyltransferase like 3 [Source:MGI Symbol;Acc:MGI:1927165]	16.6634	16.0718	13.3779	13.9084	18.3062	16.0549	15.7075	15.3603	13.7387	14.5485	16.515	16.1302	15.5284	15.4685	0.001097088	0.07820797	20	16	24	0.012387949	0.525280095	24	20.28695215	0.120306446
ENSMUSG00000022174	Dad1	defender against cell death 1 [Source:MGI Symbol;Acc:MGI:101912]	105.63	107.541	101.088	100.594	113.013	109.457	100.08	100.136	101.783	91.4642	109.413	94.2785	102.458	96.4936	0.893307667	0.966018836	20	20	24	0.311644568	0.768040178	24	19.23354902	0.046672049
ENSMUSG00000022175	Lrp10	low-density lipoprotein receptor-related protein 10 [Source:MGI Symbol;Acc:MGI:1929480]	21.6761	16.3221	17.8358	16.717	17.3788	19.7508	18.8263	21.1747	16.2902	24.2601	17.2168	20.2616	19.2459	20.1998	0.649212555	0.966018836	4	8	24	0.704301245	0.902338136	24	22.91664489	0.0536181
ENSMUSG00000022176	Rem2	rad and gem related GTP binding protein 2 [Source:MGI Symbol;Acc:MGI:2155260]	6.98344	3.97374	3.69954	4.68168	3.53669	4.97073	5.85174	5.69595	4.09889	6.81101	3.7652	5.65188	5.42235	5.80526	0.143321297	0.585944911	4	16	24	0.293874516	0.768040178	24	0.326673992	0.160189653
ENSMUSG00000022177	Haus4	HAUS augmin-like complex, subunit 4 [Source:MGI Symbol;Acc:MGI:1261794]	8.32551	8.75773	8.52517	7.4929	7.7448	9.73972	9.83306	8.40513	8.23531	10.2531	8.86494	8.28336	10.2672	9.50669	0.481530854	0.966018836	4	8	24	0.422733788	0.805019467	24	0.512947806	0.059149466
ENSMUSG00000022178	Ajuba	ajuba LIM protein [Source:MGI Symbol;Acc:MGI:1341886]	2.99035	4.73113	4.10535	4.66393	4.72452	3.72924	4.56526	4.67921	5.38958	4.79551	4.18003	4.56989	4.38252	3.84126	0.261443857	0.768150062	16	12	24	0.163987662	0.767020009	24	10.30656635	0.118275169
ENSMUSG00000022179	4931414P19Rik	RIKEN cDNA 4931414P19 gene [Source:MGI Symbol;Acc:MGI:1921609]	2.42915	2.08486	2.13392	1.70271	2.36321	1.77199	1.24984	3.02931	1.8495	1.78399	1.43403	1.88891	1.87489	1.42748	0.887862353	0.966018836	8	8	24	0.80488417	0.937541099	24	4.290071503	0.053480368
ENSMUSG00000022180	Slc7a8	solute carrier family 7 (cationic amino acid transporter, y+ system), member 8 [Source:MGI Symbol;Acc:MGI:1355323]	4.73482	3.99166	3.64614	5.11322	4.33947	4.82968	4.54848	4.70106	3.70304	6.29222	3.74393	4.64469	3.90468	4.53435	0.195343269	0.673387182	16	20	24	0.706063437	0.902918222	24	15.77345357	0.059015766
ENSMUSG00000022184	Fbxo4	F-box protein 4 [Source:MGI Symbol;Acc:MGI:2146220]	4.50201	4.24548	4.31863	5.63853	5.19584	4.57054	6.12558	3.94196	5.11941	4.59369	5.03004	4.47336	5.54457	4.52145	0.249881062	0.749643185	20	12	24	0.574027254	0.847226836	24	17.21530876	0.063037834
ENSMUSG00000022185	Acin1	apoptotic chromatin condensation inducer 1 [Source:MGI Symbol;Acc:MGI:1891824]	56.4696	54.7541	55.1826	55.3597	57.5497	54.4575	55.8063	50.3183	57.9346	48.3848	58.6211	54.8872	55.9971	51.6475	0.192357053	0.669841558	20	12	24	0.431194728	0.805393068	24	18.74981425	0.039558875
ENSMUSG00000022186	Oxct1	3-oxoacid CoA transferase 1 [Source:MGI Symbol;Acc:MGI:1914291]	90.4972	88.5246	88.8574	88.953	92.7631	92.3451	86.5431	89.6942	79.8651	83.9148	86.8029	85.9625	85.6035	88.5052	0.906657897	0.966018836	20	16	24	0.234943249	0.768040178	24	20.36420511	0.033272076
ENSMUSG00000022191	Drosha	drosha, ribonuclease type III [Source:MGI Symbol;Acc:MGI:1261425]	13.8855	12.5204	12.1799	12.5552	13.106	13.752	13.4797	12.9894	11.6375	16.5568	11.2663	13.2309	12.8509	13.5033	0.321662653	0.83607295	24	12	24	0.89884108	0.971427194	24	20.16625293	0.024025591
ENSMUSG00000022193	Psmb5	proteasome (prosome, macropain) subunit, beta type 5 [Source:MGI Symbol;Acc:MGI:1194513]	65.482	62.9425	63.269	60.6672	59.7193	63.7843	60.8893	69.01	63.0348	62.6091	70.3575	63.1575	70.2855	64.1068	0.575387897	0.966018836	4	12	24	0.526903835	0.823139162	24	0.987181467	0.030133667
ENSMUSG00000022194	Pabpn1	poly(A) binding protein, nuclear 1 [Source:MGI Symbol;Acc:MGI:1859158]	88.2869	89.4896	97.0761	92.8431	94.1139	86.5316	90.4652	85.8558	101.755	89.1551	89.2842	88.1982	95.7885	90.0761	0.148073804	0.590732968	12	12	24	0.226731186	0.768040178	24	9.269918883	0.04664147
ENSMUSG00000022195	6030458C11Rik	RIKEN cDNA 6030458C11 gene [Source:MGI Symbol;Acc:MGI:1925127]	11.8345	10.105	10.2919	10.2532	10.1386	11.2122	10.7253	11.2284	9.97089	13.0916	10.6121	11.4298	11.4709	11.1152	0.257978335	0.761738864	4	8	24	0.721962071	0.908834346	24	21.15275766	0.031812954
ENSMUSG00000022197	Pdzd2	PDZ domain containing 2 [Source:MGI Symbol;Acc:MGI:1922394]	12.673	10.5856	9.65282	11.3307	13.9411	11.299	13.7961	13.5772	11.7499	15.9714	11.7719	13.295	13.3182	12.2966	0.784278077	0.966018836	4	8	24	0.625861311	0.872406749	24	18.92391389	0.062774408
ENSMUSG00000022199	Slc22a17	solute carrier family 22 (organic cation transporter), member 17 [Source:MGI Symbol;Acc:MGI:1926225]	57.0885	58.1543	68.9351	54.9505	55.0043	64.1161	67.71	55.3974	57.8069	62.2698	43.7322	52.1133	43.7108	51.6724	0.93447422	0.966018836	12	8	24	0.720496952	0.908336488	24	7.03352537	0.054611853
ENSMUSG00000022200	Golph3	golgi phosphoprotein 3 [Source:MGI Symbol;Acc:MGI:1913879]	49.4492	44.1998	42.917	46.5915	45.666	43.6899	46.7143	42.7376	38.9543	45.5192	47.3633	41.4317	41.0641	43.6247	0.581070934	0.966018836	20	16	24	0.323264619	0.768040178	24	18.12318658	0.048496761
ENSMUSG00000022201	Zfr	zinc finger RNA binding protein [Source:MGI Symbol;Acc:MGI:1341890]	66.6962	61.9336	59.4639	66.0713	66.4067	61.2531	61.4974	59.0975	53.3005	52.8239	61.9529	57.8146	58.4956	58.21	0.941579937	0.966018836	20	16	24	0.098052321	0.71175955	24	19.82296006	0.067596249
ENSMUSG00000022203	Efs	embryonal Fyn-associated substrate [Source:MGI Symbol;Acc:MGI:105311]	3.95653	3.56868	4.13535	3.02104	3.52687	3.43221	4.35036	3.84666	4.29338	3.89794	3.57402	3.01058	3.52693	2.55025	0.390686537	0.915479136	12	12	24	0.87788841	0.965956224	24	6.058716226	0.03262252
ENSMUSG00000022204	Ngdn	neuroguidin, EIF4E binding protein [Source:MGI Symbol;Acc:MGI:1916216]	18.8649	19.9122	20.6505	18.684	21.9865	18.8516	17.4391	19.7529	23.2111	18.0689	20.9931	18.4624	21.6523	18.8524	0.784278077	0.966018836	12	12	24	0.613521831	0.866162311	24	9.984857347	0.04619877
ENSMUSG00000022205	Sub1	SUB1 homolog, transcriptional regulator [Source:MGI Symbol;Acc:MGI:104811]	137.332	156.714	146.956	137.837	140.561	129.167	134.381	133.258	155.167	119.766	151.472	140.223	144.283	141.549	0.835401638	0.966018836	12	12	24	0.649972142	0.882325565	24	5.744249818	0.039511485
ENSMUSG00000022206	Npr3	natriuretic peptide receptor 3 [Source:MGI Symbol;Acc:MGI:97373]	1.41866	1.86948	1.84021	1.48783	1.89613	1.55666	1.55041	2.13722	1.78156	1.59445	1.67967	1.77072	1.63436	1.82468	0.125573493	0.555618842	8	20	24	0.327745224	0.768040178	24	6.720669108	0.060242367
ENSMUSG00000022208	Jph4	junctophilin 4 [Source:MGI Symbol;Acc:MGI:2443113]	25.9145	17.9485	18.5624	19.5563	21.0256	20.0931	23.2969	23.3827	20.0695	29.8709	25.512	25.5276	26.4781	25.5258	0.43565473	0.966018836	4	8	24	0.563110478	0.842499957	24	20.4156925	0.074405141
ENSMUSG00000022209	Dhrs2	dehydrogenase/reductase member 2 [Source:MGI Symbol;Acc:MGI:1918662]	4.07314	2.4836	2.61722	2.68343	4.06639	3.12963	3.68448	3.0593	2.46527	2.46695	4.27985	2.83416	3.52716	2.93426	0.011674605	0.209564579	20	20	24	0.037539248	0.610129395	24	20.10074193	0.207027286
ENSMUSG00000022210	Dhrs4	dehydrogenase/reductase (SDR family) member 4 [Source:MGI Symbol;Acc:MGI:90169]	12.9213	14.2576	16.5207	13.9604	14.0978	14.0347	13.8439	15.6452	12.6643	14.6746	14.0216	15.354	16.6006	14.3954	0.847846021	0.966018836	8	12	24	0.864211215	0.960637063	24	4.127402528	0.022694444
ENSMUSG00000022211	Carmil3	capping protein regulator and myosin 1 linker 3 [Source:MGI Symbol;Acc:MGI:2448573]	26.1252	22.3444	20.9003	21.6639	27.0824	23.8663	22.6262	22.0962	21.3535	24.6462	22.9401	23.8626	25.0039	23.9622	0.039486348	0.338535904	4	8	24	0.083566529	0.70038032	24	19.73195578	0.08964476
ENSMUSG00000022212	Cpne6	copine VI [Source:MGI Symbol;Acc:MGI:1334445]	102.246	75.7752	68.9069	84.6908	74.712	90.5745	81.882	90.2641	64.0638	107.617	78.5259	83.2462	85.0287	80.1558	0.217827365	0.704989686	24	12	24	0.489807988	0.805393068	24	20.4363963	0.097814313
ENSMUSG00000022214	Dcaf11	DDB1 and CUL4 associated factor 11 [Source:MGI Symbol;Acc:MGI:90168]	36.0789	32.4646	33.7728	36.9923	33.4547	35.5319	33.8529	39.1713	35.4952	40.7877	34.7778	36.3012	38.326	36.5863	0.874054778	0.966018836	16	4	24	0.810802182	0.93937513	24	9.159328985	0.021376669
ENSMUSG00000022215	Fitm1	fat storage-inducing transmembrane protein 1 [Source:MGI Symbol;Acc:MGI:1915930]	2.1597	3.87367	5.29521	6.03622	3.97586	4.38157	3.60053	9.68728	7.02046	8.83892	3.78777	3.38542	5.30513	3.63674	0.091669402	0.484208195	16	8	24	0.039587098	0.615919442	24	9.365582542	0.442311988
ENSMUSG00000022216	Psme1	proteasome (prosome, macropain) activator subunit 1 (PA28 alpha) [Source:MGI Symbol;Acc:MGI:1096367]	23.1304	28.2625	27.8977	27.5471	25.8876	26.0753	24.6287	30.014	26.9251	24.378	25.5049	26.4544	28.2729	27.423	0.202903295	0.689605535	8	12	24	0.175036298	0.768040178	24	6.281237493	0.069328488
ENSMUSG00000022217	Emc9	ER membrane protein complex subunit 9 [Source:MGI Symbol;Acc:MGI:1934682]	56.2241	50.1972	49.7471	47.0284	50.9915	45.4865	49.0487	50.023	50.5256	51.0092	56.6341	52.6321	57.0405	53.4601	0.94086481	0.966018836	4	8	24	0.673345219	0.890057965	24	23.45612316	0.031210526
ENSMUSG00000022219	Cideb	cell death-inducing DNA fragmentation factor, alpha subunit-like effector B [Source:MGI Symbol;Acc:MGI:1270844]	0.141475	0.585384	0.685528	0.702437	0.411685	0.477152	0.424376	0.667582	0.893608	0.321197	0.399463	0.388543	0.508571	0.405604	0.110277874	0.526825967	12	8	24	0.07675418	0.687041208	24	8.326563296	0.158192249
ENSMUSG00000022220	Adcy4	adenylate cyclase 4 [Source:MGI Symbol;Acc:MGI:99674]	0.97686	1.17112	0.933086	1.09222	1.10715	0.688112	1.05685	1.52494	1.25693	0.983752	1.0731	1.20131	1.09626	0.919699	0.801273005	0.966018836	8	20	24	0.522044185	0.820717128	24	6.415527053	0.061531421
ENSMUSG00000022221	Ripk3	receptor-interacting serine-threonine kinase 3 [Source:MGI Symbol;Acc:MGI:2154952]	0.608212	1.17876	1.39958	0.53412	0.671177	0.742829	0.688042	0.544495	0.752359	0.526786	0.699964	0.648883	0.689469	0.482276	0.897570341	0.966018836	12	4	24	0.695104386	0.898633591	24	6.18008284	0.060195073
ENSMUSG00000022223	Sdr39u1	short chain dehydrogenase/reductase family 39U, member 1 [Source:MGI Symbol;Acc:MGI:1916876]	31.5638	33.471	35.8835	36.3936	30.804	34.7072	33.5883	35.7781	31.9985	38.0392	30.3773	36.0594	34.4112	38.0382	0.503972924	0.966018836	16	4	24	0.379483002	0.798771123	24	6.991624437	0.055749176
ENSMUSG00000022225	Cma1	chymase 1, mast cell [Source:MGI Symbol;Acc:MGI:96941]	1.17706	1.81722	1.944	1.60163	1.9598	1.41201	1.6494	2.07017	2.1148	0.950409	2.15993	1.1191	1.45112	1.85294	0.074438493	0.448301997	12	12	24	0.406468896	0.799546792	24	7.691284354	0.121968757
ENSMUSG00000022228	Zscan26	zinc finger and SCAN domain containing 26 [Source:MGI Symbol;Acc:MGI:3531417]	22.4888	22.6964	21.017	22.7763	22.5804	21.3157	23.9885	20.3028	22.9016	19.6904	21.9938	21.1526	21.8207	21.5656	0.872380105	0.966018836	4	20	24	0.671565045	0.889193007	24	22.57944034	0.025009421
ENSMUSG00000022231	Sema5a	sema domain, seven thrombospondin repeats (type 1 and type 1-like), transmembrane domain (TM) and short cytoplasmic domain, (semaphorin) 5A [Source:MGI Symbol;Acc:MGI:107556]	19.9679	21.9815	20.4605	24.3743	25.1005	19.2847	23.7015	19.4147	23.8998	24.8186	21.1779	21.2706	21.154	20.5797	0.181151038	0.659020016	20	4	24	0.109021106	0.72082227	24	12.55479839	0.098539688
ENSMUSG00000022234	Cct5	chaperonin containing Tcp1, subunit 5 (epsilon) [Source:MGI Symbol;Acc:MGI:107185]	161.402	155.443	148.879	148.266	166.647	154.444	147.368	142.723	143.493	128.194	161.296	146.931	159.301	149.877	0.256915694	0.76168256	20	20	24	0.132556754	0.742700873	24	20.74572219	0.072045365
ENSMUSG00000022235	Cmbl	carboxymethylenebutenolidase-like (Pseudomonas) [Source:MGI Symbol;Acc:MGI:1916824]	25.3413	35.6169	29.2703	29.7397	32.1629	26.9738	28.714	30.6746	37.7982	30.9228	27.3929	30.9103	30.2498	30.8536	0.416876911	0.963884303	12	16	24	0.117887762	0.728323355	24	8.018379896	0.115269217
ENSMUSG00000022236	Ropn1l	ropporin 1-like [Source:MGI Symbol;Acc:MGI:2182357]	1.05122	1.01751	0.870951	0.975885	1.01143	1.36366	0.977119	1.47891	1.23063	0.926038	0.929207	0.9577	0.73364	1.12187	0.664568268	0.966018836	8	8	24	0.781276787	0.929846332	24	3.83361081	0.037935234
ENSMUSG00000022237	Ankrd33b	ankyrin repeat domain 33B [Source:MGI Symbol;Acc:MGI:1917904]	2.47523	2.46788	2.0402	2.56907	2.91876	2.55345	3.04223	3.09555	2.95488	3.07057	2.72418	2.94521	2.60873	2.70537	0.789670753	0.966018836	16	20	24	0.851153336	0.955425185	24	17.29712102	0.024374256
ENSMUSG00000022240	Ctnnd2	catenin (cadherin associated protein), delta 2 [Source:MGI Symbol;Acc:MGI:1195966]	8.67388	11.0461	10.3272	9.4537	9.45889	10.18	10.4023	9.64499	9.33083	12.1224	8.45278	9.65775	8.45107	10.641	0.939921059	0.966018836	8	12	24	0.446531884	0.805393068	24	8.155101748	0.070390499
ENSMUSG00000022241	Tars	threonyl-tRNA synthetase [Source:MGI Symbol;Acc:MGI:106314]	17.6006	19.3863	20.7603	17.1476	19.1845	19.614	20.2883	19.5807	15.8438	16.0922	21.1036	18.2622	19.2765	17.602	0.943890799	0.966018836	20	20	24	0.358712639	0.785236952	24	21.54544122	0.070267648
ENSMUSG00000022244	Amacr	alpha-methylacyl-CoA racemase [Source:MGI Symbol;Acc:MGI:1098273]	7.28677	6.61675	6.53513	7.53823	7.06679	7.21728	6.90611	8.37802	6.12409	6.41603	8.72019	6.6757	7.11856	6.61955	0.266025655	0.773510818	20	16	24	0.510486302	0.814224951	24	18.80284012	0.05985837
ENSMUSG00000022246	Rai14	retinoic acid induced 14 [Source:MGI Symbol;Acc:MGI:1922896]	7.00091	9.03153	8.55148	8.56611	9.42069	8.44813	8.00331	8.5674	9.78156	8.07087	8.36179	8.48296	8.52743	8.10654	0.43565473	0.966018836	12	16	24	0.300365968	0.768040178	24	10.73659936	0.061671748
ENSMUSG00000022247	Brix1	BRX1, biogenesis of ribosomes [Source:MGI Symbol;Acc:MGI:1915082]	10.6815	10.8256	10.0174	11.8563	10.5082	11.0696	12.559	9.51886	11.5367	10.5656	11.1289	12.0916	11.1135	12.1306	0.896311169	0.966018836	4	8	24	0.701405991	0.901104245	24	20.64733426	0.033639986
ENSMUSG00000022248	Rad1	RAD1 checkpoint DNA exonuclease [Source:MGI Symbol;Acc:MGI:1316678]	10.2914	12.2103	10.2491	13.145	12.707	10.9628	12.3228	11.4591	11.313	10.037	12.4128	11.5206	12.1332	10.8529	0.628286596	0.966018836	20	16	24	0.623641183	0.87183699	24	17.62906224	0.045064315
ENSMUSG00000022253	Nadk2	NAD kinase 2, mitochondrial [Source:MGI Symbol;Acc:MGI:1915896]	9.05777	11.0168	11.5277	10.0715	9.33718	9.2661	10.2602	9.96694	10.8353	7.79895	8.78021	9.94394	7.54658	9.90135	0.154830409	0.607427992	12	8	24	0.237335055	0.768040178	24	6.397961987	0.09564055
ENSMUSG00000022255	Mtdh	metadherin [Source:MGI Symbol;Acc:MGI:1914404]	27.5847	33.1621	31.8011	32.8948	31.799	31.4161	30.6057	31.1192	35.1463	34.3148	33.9488	33.3403	32.6968	34.2096	0.048771079	0.379129806	12	16	24	0.009680863	0.502965379	24	10.59343119	0.071576191
ENSMUSG00000022257	Laptm4b	lysosomal-associated protein transmembrane 4B [Source:MGI Symbol;Acc:MGI:1890494]	50.5575	46.6766	49.9608	47.8505	51.2135	50.9887	49.2091	48.4486	43.0892	49.5739	49.8162	47.1927	44.7197	47.8556	0.435739761	0.966018836	20	12	24	0.151637344	0.758865539	24	17.77045691	0.04870039
ENSMUSG00000022261	Sdc2	syndecan 2 [Source:MGI Symbol;Acc:MGI:1349165]	14.7844	16.0026	17.4374	14.8295	14.6004	13.9188	16.6695	14.7672	15.7331	12.7611	14.8772	13.1075	12.7833	14.2889	0.390686537	0.915479136	12	12	24	0.234594234	0.768040178	24	6.345931833	0.06936587
ENSMUSG00000022263	Trio	triple functional domain (PTPRF interacting) [Source:MGI Symbol;Acc:MGI:1927230]	33.324	33.6166	31.3221	33.9857	34.3367	32.4516	36.1949	35.1443	33.6848	35.8696	33.1621	34.063	33.9504	33.091	0.885059765	0.966018836	16	20	24	0.969776152	0.992917836	24	20.77558132	0.005438945
ENSMUSG00000022265	Ank	progressive ankylosis [Source:MGI Symbol;Acc:MGI:3045421]	51.777	46.1839	51.6529	49.3394	47.2719	52.1805	52.4022	51.8023	45.0829	58.1359	43.6483	49.2224	49.7632	47.8059	0.916746631	0.966018836	4	16	24	0.964009668	0.991038325	24	23.91338151	0.01143089
ENSMUSG00000022269	Marchf11	membrane associated ring-CH-type finger 11 [Source:MGI Symbol;Acc:MGI:3608327]	14.1554	11.1438	8.17947	10.0715	10.1437	8.80354	9.80045	9.22131	9.3777	8.81118	13.0549	11.0371	12.2729	10.1884	0.257978335	0.761738864	4	8	24	0.201905733	0.768040178	24	21.47415677	0.146936974
ENSMUSG00000022270	Retreg1	reticulophagy regulator 1 [Source:MGI Symbol;Acc:MGI:1913520]	54.8006	49.2158	46.6977	50.1306	49.6188	50.2312	51.6243	49.7472	42.7737	52.1253	48.2286	48.8645	51.1872	48.7821	0.018894761	0.256345714	4	8	24	0.119333454	0.730262024	24	21.26253482	0.062773272
ENSMUSG00000022272	Myo10	myosin X [Source:MGI Symbol;Acc:MGI:107716]	6.85221	5.67663	6.13984	5.75075	6.09616	6.65708	7.5669	6.93648	5.73458	7.71857	5.775	6.36165	6.88175	6.40386	0.176754388	0.653256853	4	8	24	0.433873424	0.805393068	24	23.31978787	0.063636442
ENSMUSG00000022280	Rnf19a	ring finger protein 19A [Source:MGI Symbol;Acc:MGI:1353623]	34.7896	31.7031	30.1969	33.5669	30.7499	32.7354	28.9881	29.787	28.3612	30.8626	32.6571	31.2651	32.4571	30.914	0.428730128	0.966018836	24	12	24	0.225895943	0.768040178	24	19.20503734	0.05241749
ENSMUSG00000022283	Pabpc1	poly(A) binding protein, cytoplasmic 1 [Source:MGI Symbol;Acc:MGI:1349722]	35.4823	32.8554	36.4459	38.6377	37.8713	35.2301	33.257	37.7776	40.6363	44.9763	36.2317	42.3106	39.1534	38.1862	0.515451644	0.966018836	16	12	24	0.083552114	0.70038032	24	12.05660392	0.083104516
ENSMUSG00000022285	Ywhaz	tyrosine 3-monooxygenase/tryptophan 5-monooxygenase activation protein, zeta polypeptide [Source:MGI Symbol;Acc:MGI:109484]	125.716	100.174	103.992	102.875	101.494	114.999	106.363	104.654	88.4728	128.736	106.299	111.624	104.195	108.613	0.245061943	0.741580974	24	12	24	0.405482737	0.799546792	24	19.68123023	0.073673833
ENSMUSG00000022292	Rrm2b	ribonucleotide reductase M2 B (TP53 inducible) [Source:MGI Symbol;Acc:MGI:2155865]	20.1176	20.4716	18.7213	19.334	18.8043	18.8696	20.4678	17.7043	18.2152	18.4119	21.3306	18.2792	19.1261	19.5306	0.978555823	0.978780908	4	8	24	0.596162788	0.857839743	24	21.1646476	0.030239654
ENSMUSG00000022295	Atp6v1c1	ATPase, H+ transporting, lysosomal V1 subunit C1 [Source:MGI Symbol;Acc:MGI:1913585]	93.0937	90.2247	90.634	84.5681	85.5199	93.2336	88.448	84.4272	82.2381	87.1886	87.6135	88.3304	91.1351	93.8852	0.177409177	0.65475392	4	12	24	0.099358145	0.71175955	24	23.41758184	0.046606092
ENSMUSG00000022296	Baalc	brain and acute leukemia, cytoplasmic [Source:MGI Symbol;Acc:MGI:1928704]	7.73584	9.04351	14.4879	7.65516	7.10216	10.0761	10.3316	8.15726	8.87699	8.89577	6.26429	9.63561	6.23403	10.6703	0.510431574	0.966018836	12	8	24	0.401793496	0.799546792	24	5.917531783	0.156457636
ENSMUSG00000022297	Fzd6	frizzled class receptor 6 [Source:MGI Symbol;Acc:MGI:108474]	1.75782	2.585	2.10328	1.66783	2.1607	2.1867	1.51878	3.15515	2.58979	2.47071	1.72719	1.9013	2.10171	2.20447	0.162672639	0.624917386	8	20	24	0.151905897	0.758865539	24	6.755758604	0.148723311
ENSMUSG00000022299	Slc25a32	solute carrier family 25, member 32 [Source:MGI Symbol;Acc:MGI:1917156]	6.51357	6.36532	6.71981	7.61518	6.124	6.39593	5.71711	5.77674	7.10488	6.37477	6.89381	6.5912	7.03635	6.74428	0.78898997	0.966018836	12	20	24	0.321302356	0.768040178	24	11.47305836	0.057061088
ENSMUSG00000022300	Dcaf13	DDB1 and CUL4 associated factor 13 [Source:MGI Symbol;Acc:MGI:2684929]	18.8558	21.227	20.9148	19.2187	19.7979	18.576	18.8342	17.9761	21.8328	17.4535	21.7836	19.2477	21.005	19.9364	0.953629935	0.966018836	12	12	24	0.782922891	0.930229965	24	7.807128846	0.028715111
ENSMUSG00000022304	Dpys	dihydropyrimidinase [Source:MGI Symbol;Acc:MGI:1928679]	0.516901	0.527443	0.824368	0.559341	0.636275	0.467903	0.63917	0.379117	0.480415	0.389477	0.789051	0.588919	0.581951	0.539644	0.969487593	0.970883022	20	12	24	0.686250352	0.895774045	24	16.41158313	0.040733241
ENSMUSG00000022305	Lrp12	low density lipoprotein-related protein 12 [Source:MGI Symbol;Acc:MGI:2443132]	9.3818	8.63406	9.85418	9.12064	8.29898	9.85422	9.7089	8.8377	7.44677	9.54963	8.40166	8.81787	8.03606	9.0318	0.854540463	0.966018836	24	20	24	0.828588281	0.946618078	24	21.09877502	0.025825164
ENSMUSG00000022306	Zfpm2	zinc finger protein, multitype 2 [Source:MGI Symbol;Acc:MGI:1334444]	0.644038	0.617018	0.782873	0.624227	0.549996	0.753631	0.638583	0.580302	0.641127	0.764286	0.563563	0.644652	0.402908	0.696267	0.759899911	0.966018836	12	16	24	0.543285271	0.831565139	24	10.20487185	0.038599661
ENSMUSG00000022307	Oxr1	oxidation resistance 1 [Source:MGI Symbol;Acc:MGI:2179326]	96.493	104.07	90.4457	98.9227	87.6132	87.3415	91.9617	87.9868	82.9135	99.1772	91.1024	96.8281	96.2027	101.746	0.944053021	0.966018836	8	12	24	0.789955127	0.932755012	24	2.983897782	0.026011494
ENSMUSG00000022309	Angpt1	angiopoietin 1 [Source:MGI Symbol;Acc:MGI:108448]	1.38578	2.69299	2.58886	1.99775	1.75936	1.76894	2.15865	1.97747	2.80706	1.62841	2.00739	2.08512	1.569	2.20168	0.1982019	0.680119125	12	4	24	0.099089601	0.71175955	24	7.460212934	0.164195022
ENSMUSG00000022311	Csmd3	CUB and Sushi multiple domains 3 [Source:MGI Symbol;Acc:MGI:2386403]	0.536757	0.781488	0.99739	0.522811	0.458414	0.723288	0.743868	0.534918	0.875651	0.582549	0.539135	0.71511	0.415545	0.833693	0.117335378	0.538362324	12	8	24	0.242750154	0.768040178	24	6.270338609	0.09874665
ENSMUSG00000022312	Eif3h	eukaryotic translation initiation factor 3, subunit H [Source:MGI Symbol;Acc:MGI:1915385]	53.8719	55.0239	55.8198	51.7843	47.449	49.0532	53.7122	53.3448	61.7117	52.957	58.6588	56.9891	59.5273	56.8884	0.823657258	0.966018836	12	4	24	0.175090052	0.768040178	24	5.486644727	0.064046705

ENSMUSG00000022313	Utp23	UTP23 small subunit processome component [Source:MGI Symbol;Acc:MGI:1925831]	4.43162	5.31113	4.58255	3.58983	3.96311	4.22139	3.23979	4.05098	4.97655	3.87759	4.41857	4.9984	3.91434	4.1487	0.960333395	0.967043964	12	4	24	0.844165036	0.952833645	24	5.845856364	0.037704627
ENSMUSG00000022314	Rad21	RAD21 cohesin complex component [Source:MGI Symbol;Acc:MGI:108016]	42.0878	42.2449	42.0754	42.6278	39.1799	39.5243	42.3337	40.5858	38.0681	44.7191	38.5609	39.0563	38.7491	41.2615	0.676344318	0.966018836	16	4	24	0.492219556	0.805393068	24	7.559305437	0.030609973
ENSMUSG00000022321	Cdh10	cadherin 10 [Source:MGI Symbol;Acc:MGI:107436]	6.17282	5.90494	5.91573	5.48497	5.51558	6.34446	6.32527	5.84311	5.00482	5.85004	5.46664	6.32722	6.0778	6.19895	0.004163919	0.140598674	24	20	24	0.014007517	0.530670295	24	23.02334945	0.086068373
ENSMUSG00000022322	Shcbp1	Shc SH2-domain binding protein 1 [Source:MGI Symbol;Acc:MGI:1338802]	0.750562	0.93504	1.11621	1.18715	0.9646	0.851145	0.772224	0.72513	0.937218	0.688626	0.85134	0.774024	0.900416	1.03377	0.474937444	0.966018836	12	16	24	0.284801781	0.768040178	24	9.741440167	0.069232777
ENSMUSG00000022323	Rida	reactive intermediate imine deaminase A homolog [Source:MGI Symbol;Acc:MGI:1095401]	42.7446	50.4303	48.5877	44.707	46.136	44.0013	49.8279	43.1939	49.6004	37.9727	47.7191	46.1207	45.6156	48.5656	0.843639218	0.966018836	12	4	24	0.53744752	0.827949555	24	3.46664732	0.047673137
ENSMUSG00000022324	Matn2	matrilin 2 [Source:MGI Symbol;Acc:MGI:109613]	50.7636	39.9664	43.6741	51.2864	61.8119	53.1204	45.1855	43.9638	41.7039	43.4831	54.3346	48.8915	42.4955	37.8148	7.90E-04	0.071344007	20	12	24	3.63E-05	0.067399697	24	17.22641546	0.23323349
ENSMUSG00000022325	Pop1	processing of precursor 1, ribonuclease P/MRP family, (S. cerevisiae) [Source:MGI Symbol;Acc:MGI:1914974]	3.40433	3.25124	3.61072	3.32784	3.5841	2.93085	2.67874	3.79933	3.06251	3.08507	2.90085	3.23889	3.01519	2.48023	0.896311169	0.966018836	12	16	24	0.719938431	0.908178129	24	11.66675332	0.034833035
ENSMUSG00000022329	Stk3	serine/threonine kinase 3 [Source:MGI Symbol;Acc:MGI:1928487]	10.2364	11.6448	11.0867	11.8077	12.5656	10.1911	10.3471	9.12485	10.6952	9.98619	11.2206	10.8453	11.1664	10.721	0.833406028	0.966018836	20	4	24	0.341801558	0.776129793	24	14.75386199	0.058325411
ENSMUSG00000022330	Osr2	odd-skipped related 2 [Source:MGI Symbol;Acc:MGI:1930813]	1.44031	2.10258	1.23078	1.49681	1.91326	1.34851	2.27931	1.44143	1.81457	1.62704	2.20101	1.41511	1.96966	1.65373	0.90332508	0.966018836	20	4	24	0.916877767	0.977109697	24	21.6873758	0.030505821
ENSMUSG00000022332	Khdrbs3	KH domain containing, RNA binding, signal transduction associated 3 [Source:MGI Symbol;Acc:MGI:1313312]	38.8434	32.1176	29.5652	30.3831	37.9285	34.8822	37.4504	35.4245	26.1282	32.5609	31.4576	29.9825	27.7387	32.2252	0.494171275	0.966018836	4	8	24	0.155848071	0.761411283	24	21.00261467	0.113547815
ENSMUSG00000022335	Zfat	zinc finger and AT hook domain containing [Source:MGI Symbol;Acc:MGI:2681865]	1.41688	1.07087	1.16363	0.997513	1.16756	1.24436	1.12832	0.962871	1.02602	1.18751	1.12622	1.158	1.14553	1.02589	0.109040811	0.524035616	24	8	24	0.057757058	0.656248849	24	19.97301027	0.065465525
ENSMUSG00000022336	Eif3e	eukaryotic translation initiation factor 3, subunit E [Source:MGI Symbol;Acc:MGI:99257]	36.3673	43.6203	40.5694	42.5363	41.798	35.6086	36.0627	38.2875	51.7152	35.7086	44.8243	41.5532	43.4453	41.8045	0.745475226	0.966018836	12	12	24	0.327409925	0.768040178	24	9.298213474	0.087168991
ENSMUSG00000022337	Emc2	ER membrane protein complex subunit 2 [Source:MGI Symbol;Acc:MGI:1913986]	92.2551	93.2252	89.0492	85.5365	91.3749	80.1793	83.8584	84.6316	94.6805	74.5893	93.7461	86.6592	96.2601	90.6172	0.916860713	0.966018836	12	4	24	0.627176505	0.872795898	24	2.561423169	0.038818375
ENSMUSG00000022338	Eny2	ENY2 transcription and export complex 2 subunit [Source:MGI Symbol;Acc:MGI:1919286]	42.6468	47.8725	43.3112	45.6854	45.4712	41.0531	39.4553	39.9999	48.8475	44.3416	44.9103	46.9493	49.5115	51.0455	0.858266942	0.966018836	8	20	24	0.536928939	0.827590998	24	8.349546117	0.04667945
ENSMUSG00000022339	Ebag9	estrogen receptor-binding fragment-associated gene 9 [Source:MGI Symbol;Acc:MGI:1859920]	10.8533	11.4077	10.7711	12.3895	10.7201	10.7014	10.2245	10.6301	9.58027	9.98613	10.6648	11.242	9.77036	10.5724	0.883907406	0.966018836	24	4	24	0.67937013	0.892679035	24	16.122462	0.026900656
ENSMUSG00000022340	Sybu	syntabulin (syntaxin-interacting) [Source:MGI Symbol;Acc:MGI:2442392]	31.0424	26.0688	27.7298	25.895	27.6692	29.798	25.1836	26.5532	22.2798	27.1268	26.3602	27.5884	26.2955	26.457	0.892578749	0.966018836	24	12	24	0.08880203	0.707896147	24	20.17832323	0.084852806
ENSMUSG00000022342	Kcnv1	potassium channel, subfamily V, member 1 [Source:MGI Symbol;Acc:MGI:1914748]	11.4936	9.06343	9.58056	7.63017	9.47375	8.96404	8.46155	8.55043	7.33287	7.21027	10.8603	8.30245	7.83607	7.83679	0.676344318	0.966018836	20	20	24	0.18634144	0.768040178	24	20.41176469	0.114768742
ENSMUSG00000022346	Myc	myelocytomatosis oncogene [Source:MGI Symbol;Acc:MGI:97250]	4.15193	3.82967	3.68807	3.6034	3.33457	3.76452	4.10365	3.58082	3.44525	4.51396	3.54892	3.59289	3.44599	4.56517	0.702630284	0.966018836	8	12	24	0.593550927	0.85692808	24	4.158511383	0.046406704
ENSMUSG00000022350	Washc5	WASH complex subunit 5 [Source:MGI Symbol;Acc:MGI:2146110]	25.1629	22.2922	21.8002	22.8838	22.718	24.6915	22.7618	22.8816	19.5726	24.6648	21.3249	23.8579	23.2279	22.8917	0.046056765	0.374351104	24	12	24	0.136476167	0.747972781	24	20.75462143	0.069394482
ENSMUSG00000022351	Sqle	squalene epoxidase [Source:MGI Symbol;Acc:MGI:109296]	95.7192	73.6529	73.6163	72.158	79.4231	87.5661	85.4163	82.2838	64.0953	91.8135	78.2699	80.6763	84.0463	80.9026	0.009118941	0.192760771	4	8	24	0.089699082	0.709708691	24	21.26520224	0.124085897
ENSMUSG00000022353	Mtss1	MTSS I-BAR domain containing 1 [Source:MGI Symbol;Acc:MGI:2384818]	20.8052	16.7457	18.0553	19.8464	18.6208	17.5289	20.8137	17.6445	17.6268	23.1288	18.9531	16.6531	19.3915	19.0832	0.708665292	0.966018836	4	20	24	0.816234648	0.94140005	24	13.41242926	0.032188345
ENSMUSG00000022354	Ndufb9	NADH:ubiquinone oxidoreductase subunit B9 [Source:MGI Symbol;Acc:MGI:1913468]	177.37	185.772	191.908	213.061	176.464	180.162	176.839	208.071	221.699	184.633	211.015	193.314	231.212	217.292	0.699015352	0.966018836	12	12	24	0.144095257	0.752803875	24	7.455460588	0.083481709
ENSMUSG00000022357	Klhl38	kelch-like 38 [Source:MGI Symbol;Acc:MGI:3045310]	0.995896	1.1312	1.04283	1.21927	1.00742	1.13	0.864196	1.60612	1.21392	1.21652	0.711192	1.13617	1.12497	0.997887	0.251986462	0.75129457	16	12	24	0.355285006	0.782134973	24	6.564991622	0.080417911
ENSMUSG00000022358	Fbxo32	F-box protein 32 [Source:MGI Symbol;Acc:MGI:1914981]	3.3945	4.43641	4.4162	4.6686	3.19634	4.20499	4.36546	6.32975	4.87844	5.27616	3.12291	4.86542	5.07177	4.77773	0.17847698	0.655480352	8	12	24	0.064617461	0.663876076	24	5.812356093	0.188020657
ENSMUSG00000022359	Wdyhv1	WDYHV motif containing 1 [Source:MGI Symbol;Acc:MGI:1924023]	18.4524	20.8397	17.9683	16.4705	19.1531	18.735	17.4768	18.7596	17.7212	16.4843	18.8774	18.4472	18.8725	19.3774	0.223774686	0.717349224	8	8	24	0.186949178	0.768040178	24	0.363602365	0.05906543
ENSMUSG00000022360	Atad2	ATPase family, AAA domain containing 2 [Source:MGI Symbol;Acc:MGI:1917722]	5.2384	5.81066	5.32842	4.82234	5.55157	4.9569	5.29171	4.04126	4.70905	3.73384	5.61232	4.28208	4.4615	4.52484	0.858891143	0.966018836	20	20	24	0.636116864	0.876354912	24	19.93018957	0.051103601
ENSMUSG00000022361	Zhx1	zinc fingers and homeoboxes 1 [Source:MGI Symbol;Acc:MGI:109271]	22.1311	22.7226	21.6496	21.8217	23.0182	20.879	22.5083	20.3946	23.0504	19.9864	23.4794	22.0004	23.0752	22.9476	0.890648739	0.966018836	20	20	24	0.864128857	0.960637063	24	22.7334549	0.015074868
ENSMUSG00000022362	Gm29394	predicted gene 29394 [Source:MGI Symbol;Acc:MGI:5580100]	1.53801	1.60948	1.71163	0.742248	1.13106	1.65104	1.43763	3.31769	1.4026	2.35268	0.690542	0.895762	1.17547	2.02285	0.032263213	0.324385159	8	12	24	0.080556824	0.695795041	24	4.950840125	0.304619184
ENSMUSG00000022364	Tbc1d31	TBC1 domain family, member 31 [Source:MGI Symbol;Acc:MGI:2684931]	3.19303	3.55426	3.84807	3.37902	3.38563	3.46741	4.2728	2.87943	3.61679	3.21793	3.89884	3.67682	3.5481	3.17343	0.959160181	0.967043964	24	8	24	0.713881595	0.905759775	24	18.63397776	0.038394637
ENSMUSG00000022365	Derl1	Der1-like domain family, member 1 [Source:MGI Symbol;Acc:MGI:1915069]	32.11	29.8887	30.003	30.5139	31.6402	30.5565	30.1214	31.899	29.8099	29.8549	31.3603	31.2312	32.879	30.2744	0.097760727	0.501112642	4	8	24	0.135121429	0.746923165	24	21.23174378	0.033913706
ENSMUSG00000022367	Has2	hyaluronan synthase 2 [Source:MGI Symbol;Acc:MGI:107821]	1.37875	1.06308	0.655312	0.571957	1.02493	0.966232	1.44871	1.15298	0.96445	0.634752	0.852567	0.898886	1.10788	0.91071	1.06E-04	0.029372629	4	12	24	0.00118828	0.336500758	24	23.76904016	0.213027448
ENSMUSG00000022369	Mtbp	Mdm2, transformed 3T3 cell double minute p53 binding protein [Source:MGI Symbol;Acc:MGI:2146005]	2.03639	2.58233	3.06992	2.20873	2.60173	1.80469	2.08442	1.74527	1.95624	2.18765	2.83272	2.23136	1.69137	1.91744	0.123856376	0.552144108	20	8	24	0.156514679	0.762206981	24	13.03208164	0.121238553
ENSMUSG00000022370	Mrpl13	mitochondrial ribosomal protein L13 [Source:MGI Symbol;Acc:MGI:2137218]	57.3339	56.7086	59.8114	51.9511	56.1083	54.0729	52.0542	54.6421	59.0637	50.4631	59.7906	53.9228	62.381	60.0264	0.665691261	0.966018836	12	4	24	0.494838507	0.806761911	24	3.789020283	0.041598858
ENSMUSG00000022371	Col14a1	collagen, type XIV, alpha 1 [Source:MGI Symbol;Acc:MGI:1341272]	16.6154	17.914	15.4388	14.4778	18.5625	16.4752	19.6583	19.4368	18.2832	17.8698	14.7808	17.4071	18.137	16.2045	0.636884864	0.966018836	4	12	24	0.301013042	0.768040178	24	1.419666725	0.076783184
ENSMUSG00000022372	Sla	src-like adaptor [Source:MGI Symbol;Acc:MGI:104295]	0.820953	1.86154	2.45546	2.24517	1.71041	1.54049	1.56017	1.59054	1.995	1.05538	1.61209	1.51208	1.38818	1.11457	0.031969981	0.324385159	12	16	24	0.239075759	0.768040178	24	10.57498524	0.159227947
ENSMUSG00000022375	Lrrc6	leucine rich repeat containing 6 (testis) [Source:MGI Symbol;Acc:MGI:1859553]	0.391748	0.619137	0.381449	0.380297	0.558521	0.41186	0.35279	0.500151	0.550381	0.558302	0.555112	0.654707	0.465576	0.672082	0.935316621	0.966018836	8	20	24	0.824753316	0.945021534	24	9.340728986	0.022840256
ENSMUSG00000022376	Adcy8	adenylate cyclase 8 [Source:MGI Symbol;Acc:MGI:1341110]	1.86592	2.02085	1.93811	1.57004	2.00928	2.04843	2.49766	1.9125	1.58676	1.99127	1.72138	1.96171	1.97353	2.08265	0.375495422	0.899637379	8	4	24	0.104436836	0.716673794	24	23.4449588	0.087506659
ENSMUSG00000022377	Asap1	ArfGAP with SH3 domain, ankyrin repeat and PH domain1 [Source:MGI Symbol;Acc:MGI:1342335]	46.4933	36.6093	36.1181	39.1901	39.2142	44.3916	40.5505	41.3476	29.8684	58.374	35.7773	44.3649	41.7658	44.2522	0.08302755	0.462922111	24	12	24	0.623515138	0.87183699	24	19.8768754	0.087128105
ENSMUSG00000022378	Cyrib	CYFIP related Rac1 interactor B [Source:MGI Symbol;Acc:MGI:1923520]	35.1198	33.504	36.3937	32.4752	31.6447	32.8621	33.5417	29.8842	29.4175	30.1808	31.5458	28.9848	28.1417	29.7145	0.715742619	0.966018836	4	20	24	0.958712394	0.989144435	24	20.98655348	0.006943033
ENSMUSG00000022383	Ppara	peroxisome proliferator activated receptor alpha [Source:MGI Symbol;Acc:MGI:104740]	1.02462	0.839829	2.0105	1.46151	0.942265	1.33577	1.14638	1.29287	0.998893	2.21103	0.907425	1.27919	1.96874	1.47371	0.911685024	0.966018836	16	4	24	0.580327364	0.850271896	24	8.949748296	0.106287454
ENSMUSG00000022386	Trmu	tRNA 5-methylaminomethyl-2-thiouridylate methyltransferase [Source:MGI Symbol;Acc:MGI:1919276]	8.54585	10.7381	10.5781	11.5321	10.9041	11.0112	12.5092	9.98511	10.4162	10.9691	8.91308	11.3607	10.5586	9.77989	0.7207001	0.966018836	24	8	24	0.913455428	0.975719942	24	15.18615611	0.022424399
ENSMUSG00000022387	Brd1	bromodomain containing 1 [Source:MGI Symbol;Acc:MGI:1924161]	7.82634	8.54658	8.39532	9.10509	7.60378	7.45726	8.65996	8.35634	8.73815	9.79103	8.59682	8.9551	8.4162	8.59262	0.374467935	0.89742288	16	12	24	0.100439426	0.71175955	24	9.586529604	0.069462366
ENSMUSG00000022389	Tef	thyrotroph embryonic factor [Source:MGI Symbol;Acc:MGI:98663]	28.9225	40.1683	54.2736	58.4452	44.3418	34.7691	31.4039	37.9576	60.9063	59.2594	37.983	35.1175	31.9917	41.2773	4.77E-06	0.009754434	16	12	24	7.54E-07	0.004440686	24	10.48273268	0.469793771
ENSMUSG00000022390	Zc3h7b	zinc finger CCCH type containing 7B [Source:MGI Symbol;Acc:MGI:1328310]	14.5051	13.8325	14.0462	13.8813	16.2541	14.0452	15.813	14.9784	15.4326	19.4043	13.4869	16.1764	14.8752	15.6039	0.710938687	0.966018836	16	20	24	0.749385632	0.918511706	24	12.24433876	0.036287447
ENSMUSG00000022391	Rangap1	RAN GTPase activating protein 1 [Source:MGI Symbol;Acc:MGI:103071]	63.8057	46.6009	49.9361	49.7781	51.7469	58.9016	52.9172	58.0032	41.4652	64.1835	50.5042	58.1697	53.9508	56.842	0.148073804	0.590732968	24	12	24	0.306805533	0.768040178	24	20.93141694	0.104873626
ENSMUSG00000022394	L3mbtl2	L3MBTL2 polycomb repressive complex 1 subunit [Source:MGI Symbol;Acc:MGI:2443584]	7.4163	5.93821	6.18352	5.73771	6.17001	7.06759	6.33488	7.18301	5.56605	7.28966	6.17612	6.40769	6.20723	6.49775	0.360033827	0.883787544	4	8	24	0.442216744	0.805393068	24	22.15470476	0.057659823
ENSMUSG00000022400	Rbx1	ring-box 1 [Source:MGI Symbol;Acc:MGI:1891829]	64.417	64.4439	64.6789	66.5463	64.0563	66.0794	62.2882	62.8138	62.9481	63.2938	73.0295	66.6374	67.7744	66.5498	0.801995186	0.966018836	24	4	24	0.268858354	0.768040178	24	17.13823813	0.034344597
ENSMUSG00000022401	Xpnpep3	X-prolyl aminopeptidase 3, mitochondrial [Source:MGI Symbol;Acc:MGI:2445217]	4.55951	4.16496	4.57245	3.68991	4.10848	4.31113	4.3997	4.28459	3.57618	4.80644	4.73013	4.48797	4.39836	4.01685	0.590547452	0.966018836	20	12	24	0.406536658	0.799546792	24	19.97511819	0.054804888
ENSMUSG00000022403	St13	suppression of tumorigenicity 13 [Source:MGI Symbol;Acc:MGI:1917606]	78.7758	63.7383	61.6028	59.4724	70.6166	70.1137	70.2683	65.9542	56.2123	63.8327	68.3577	64.6367	69.2093	63.5578	0.002087679	0.100255953	4	8	24	0.002177007	0.395705644	24	21.20978371	0.135736357
ENSMUSG00000022404	Slc25a17	solute carrier family 25 (mitochondrial carrier, peroxisomal membrane protein), member 17 [Source:MGI Symbol;Acc:MGI:1342248]	27.3865	30.6792	31.984	32.7158	32.0081	28.3643	28.4564	27.9545	32.2118	28.6215	31.4543	29.846	30.1073	29.5515	0.059082035	0.406067671	12	16	24	0.048767481	0.638992719	24	11.65819161	0.072778946
ENSMUSG00000022407	Adsl	adenylosuccinate lyase [Source:MGI Symbol;Acc:MGI:103202]	18.0898	16.3846	20.0003	19.3105	15.8596	19.5791	17.8575	19.3081	16.2298	20.0302	18.3456	17.0557	18.5413	18.1887	0.94073613	0.966018836	16	4	24	0.864676261	0.960819377	24	10.00256252	0.023865377
ENSMUSG00000022408	Fam83f	family with sequence similarity 83, member F [Source:MGI Symbol;Acc:MGI:2146227]	2.25597	1.78804	1.58025	1.20392	2.27236	1.69538	2.37459	1.8014	1.88338	1.8922	1.63472	1.83081	2.04094	1.52059	0.162391276	0.624504499	4	8	24	0.156926473	0.763141258	24	22.31926731	0.122028031
ENSMUSG00000022412	Mief1	mitochondrial elongation factor 1 [Source:MGI Symbol;Acc:MGI:2146020]	7.57545	7.01041	6.37497	6.99246	7.01538	7.80882	7.04818	7.47742	6.31661	8.51807	6.62315	7.84439	7.59097	7.17407	0.004712708	0.140598674	24	12	24	0.356283739	0.783018041	24	20.51020578	0.060216166
ENSMUSG00000022414	Tab1	TGF-beta activated kinase 1/MAP3K7 binding protein 1 [Source:MGI Symbol;Acc:MGI:1913763]	8.32693	8.20447	8.07034	7.4795	8.06702	8.47328	8.24017	9.61312	8.48385	8.41478	7.46708	8.41506	8.14113	8.39267	0.510431574	0.966018836	24	20	24	0.136508245	0.747972781	24	3.023456139	0.056761311
ENSMUSG00000022415	Syngr1	synaptogyrin 1 [Source:MGI Symbol;Acc:MGI:1328323]	83.0036	66.2093	66.3018	70.2095	66.8878	77.3306	72.6844	74.2018	58.9671	89.2445	60.0153	74.0211	72.4692	75.7147	0.628286596	0.966018836	24	12	24	0.616612489	0.867682235	24	22.50405754	0.064896362
ENSMUSG00000022416	Cacna1i	calcium channel, voltage-dependent, alpha 1I subunit [Source:MGI Symbol;Acc:MGI:2178051]	1.90062	1.02216	1.38717	1.16677	1.19227	1.38936	1.77722	1.13587	1.21941	1.81602	1.00798	1.34631	1.13086	1.49554	0.912686312	0.966018836	4	16	24	0.774777222	0.927711986	24	23.42516571	0.050372273
ENSMUSG00000022419	Deptor	DEP domain containing MTOR-interacting protein [Source:MGI Symbol;Acc:MGI:2146322]	28.7442	24.5256	24.7334	26.6636	25.4318	27.3578	25.8545	25.7379	24.0767	28.628	28.0698	29.6653	29.7851	27.5695	0.050760377	0.386400877	24	12	24	0.071539371	0.67819049	24	19.83361169	0.075042399
ENSMUSG00000022420	Dnal4	dynein, axonemal, light chain 4 [Source:MGI Symbol;Acc:MGI:1859217]	8.79144	7.61444	8.53796	7.14651	8.15755	9.06696	9.56721	9.06652	9.12194	10.4748	7.36934	9.62401	8.57207	8.68571	0.892685629	0.966018836	24	20	24	0.757461551	0.921562129	24	0.986287519	0.038867264
ENSMUSG00000022421	Nptxr	neuronal pentraxin receptor [Source:MGI Symbol;Acc:MGI:1920590]	19.1422	13.7586	15.2959	15.0779	18.1996	19.1964	14.6886	16.6882	12.8294	23.5466	14.4707	17.7945	15.38	16.3741	0.601647512	0.966018836	24	12	24	0.382795425	0.799546792	24	17.59552691	0.120779636
ENSMUSG00000022425	Enpp2	ectonucleotide pyrophosphatase/phosphodiesterase 2 [Source:MGI Symbol;Acc:MGI:1321390]	24.4163	38.0812	45.6578	32.4284	30.1255	29.4403	39.7243	30.3397	44.1761	26.887	27.6867	33.0266	25.0492	35.0896	0.200145651	0.684570416	12	8	24	0.139193788	0.748826538	24	7.228312999	0.212422367
ENSMUSG00000022426	Josd1	Josephin domain containing 1 [Source:MGI Symbol;Acc:MGI:1921408]	12.6418	10.2612	11.027	11.9469	12.1667	12.6522	11.6494	11.3477	9.7419	12.622	12.0591	11.3887	10.9603	11.4666	0.056291566	0.401074772	20	16	24	0.049234507	0.641244748	24	17.85837863	0.092526489
ENSMUSG00000022427	Tomm22	translocase of outer mitochondrial membrane 22 [Source:MGI Symbol;Acc:MGI:2450248]	52.8247	52.1962	49.4308	52.5064	49.7326	47.4055	50.5354	49.5169	48.149	44.35	50.0271	47.3985	47.6226	48.8367	0.94499634	0.966018836	4	20	24	0.535350044	0.82691856	24	1.298764267	0.022504532
ENSMUSG00000022428	Cby1	chibby family member 1, beta catenin antagonist [Source:MGI Symbol;Acc:MGI:1920989]	20.5177	19.7329	20.3437	19.8367	18.481	19.3813	19.9363	19.5217	18.1519	19.6964	19.193	18.9881	19.5381	19.4243	0.25849765	0.761738864	4	20	24	0.415942889	0.800710663	24	1.996508072	0.021090767
ENSMUSG00000022429	Dmc1	DNA meiotic recombinase 1 [Source:MGI Symbol;Acc:MGI:105393]	0.701738	0.684359	0.707042	1.09198	0.760107	0.682589	1.25156	0.699326	1.00731	0.597547	0.809753	1.07386	0.922979	0.958477	0.816406072	0.966018836	4	4	24	0.878890263	0.966097006	24	23.34186422	0.029466785
ENSMUSG00000022433	Csnk1e	casein kinase 1, epsilon [Source:MGI Symbol;Acc:MGI:1351660]	24.1305	21.5881	23.0169	23.9289	24.8821	25.713	24.9095	26.8924	23.1078	29.1802	24.0588	24.6812	23.8471	26.413	0.945282252	0.966018836	12	12	24	0.803782154	0.937296224	24	14.76794103	0.024670807
ENSMUSG00000022434	Fam118a	family with sequence similarity 118, member A [Source:MGI Symbol;Acc:MGI:1920475]	8.48998	7.85963	7.2181	6.71285	8.81229	8.46435	6.31359	7.25418	7.28076	7.22648	8.64481	7.38135	7.53916	7.45382	0.058209189	0.40386543	20	20	24	0.227142086	0.768040178	24	18.47837329	0.083829638
ENSMUSG00000022436	Sh3bp1	SH3-domain binding protein 1 [Source:MGI Symbol;Acc:MGI:104603]	1.45083	1.8483	1.99357	1.58055	1.43537	1.81303	2.09402	1.77276	2.66923	1.78417	1.85801	1.93702	1.53539	2.25302	0.446530834	0.966018836	12	8	24	0.129983879	0.740483001	24	6.766250389	0.120308391
ENSMUSG00000022437	Samm50	SAMM50 sorting and assembly machinery component [Source:MGI Symbol;Acc:MGI:1915903]	35.3618	32.0154	34.502	40.2463	34.5885	37.8976	35.944	37.1632	34.8462	34.8036	33.4495	33.9333	34.3391	37.9113	0.874607729	0.966018836	4	4	24	0.9496454	0.988056451	24	9.980353047	0.01126977
ENSMUSG00000022438	Parvb	parvin, beta [Source:MGI Symbol;Acc:MGI:2153063]	22.6631	16.3783	16.3394	17.8346	17.7641	19.1443	17.3613	20.474	13.7346	27.8432	15.9538	19.5664	19.7645	18.9722	0.44861185	0.966018836	4	8	24	0.807540775	0.938693233	24	20.13612503	0.0639869
ENSMUSG00000022439	Parvg	parvin, gamma [Source:MGI Symbol;Acc:MGI:2158329]	0.967096	1.23082	1.66659	1.05997	1.28979	1.26914	0.76849	1.1881	1.3133	0.998191	0.912999	1.11505	1.27328	0.817164	0.894563866	0.966018836	12	16	24	0.654674327	0.883584166	24	9.734295095	0.057026322
ENSMUSG00000022440	C1qtnf6	C1q and tumor necrosis factor related protein 6 [Source:MGI Symbol;Acc:MGI:1919959]	3.02901	3.14925	2.91872	2.69232	4.29927	3.21503	3.16633	3.56477	3.9142	2.99535	3.13198	2.60427	3.22162	2.4594	0.853736882	0.966018836	20	20	24	0.890722253	0.9692042	24	14.8546383	0.031188794
ENSMUSG00000022442	Ttll1	tubulin tyrosine ligase-like 1 [Source:MGI Symbol;Acc:MGI:2443047]	27.1184	20.007	21.635	22.2915	20.3909	27.1112	24.1607	25.3698	16.7676	27.525	20.9369	21.4128	21.9495	23.2409	0.762156552	0.966018836	4	8	24	0.483433901	0.805393068	24	21.59949755	0.093868261
ENSMUSG00000022443	Myh9	myosin, heavy polypeptide 9, non-muscle [Source:MGI Symbol;Acc:MGI:107717]	30.4165	23.0039	23.568	24.809	26.6485	28.417	26.8646	28.5243	21.1549	30.2508	24.8473	26.2568	25.473	24.3278	0.11436224	0.534017934	4	8	24	0.196093793	0.768040178	24	19.97661727	0.103478366
ENSMUSG00000022449	Adamts20	a disintegrin-like and metallopeptidase (reprolysin type) with thrombospondin type 1 motif, 20 [Source:MGI Symbol;Acc:MGI:2660628]	4.50157	5.19969	4.79096	4.88058	6.36618	4.80721	5.41866	4.44726	5.50104	4.80502	5.39829	5.09558	4.49849	4.51241	0.175272072	0.651010555	20	12	24	0.167681269	0.767020009	24	15.36854591	0.086048908
ENSMUSG00000022450	Ndufa6	NADH:ubiquinone oxidoreductase subunit A6 [Source:MGI Symbol;Acc:MGI:1914380]	158.166	174.461	172.388	197.372	161.842	158.231	166.863	181.927	213.566	173.862	215.279	185.111	205.342	197.179	0.843639218	0.966018836	12	16	24	0.175422495	0.768040178	24	9.1642157	0.088079761
ENSMUSG00000022451	Twf1	twinfilin actin binding protein 1 [Source:MGI Symbol;Acc:MGI:1100520]	21.126	20.1787	21.1564	19.6573	19.9567	20.8807	22.18	19.9154	19.2286	20.4059	19.413	19.5084	20.6583	18.7073	0.349109869	0.870418653	4	16	24	0.219621087	0.768040178	24	22.50625958	0.037138364
ENSMUSG00000022452	Smdt1	single-pass membrane protein with aspartate rich tail 1 [Source:MGI Symbol;Acc:MGI:1916279]	136.211	125.228	120.611	132.118	140.288	138.8	124.857	130.214	125.638	135.849	141.951	140.404	150.4	136.656	0.032221909	0.324385159	20	16	24	0.018556963	0.559812298	24	18.96599091	0.075463112
ENSMUSG00000022453	Naga	N-acetyl galactosaminidase, alpha [Source:MGI Symbol;Acc:MGI:1261422]	19.1627	19.6379	20.4664	18.9799	20.9957	20.1853	16.6902	22.9214	19.6473	21.1062	17.8243	19.8314	18.4754	17.7519	0.46238066	0.966018836	16	12	24	0.580572073	0.850320846	24	10.75522325	0.046049805
ENSMUSG00000022454	Nell2	NEL-like 2 [Source:MGI Symbol;Acc:MGI:1858510]	5.23069	4.43249	7.0558	4.1987	3.47827	6.0053	5.64469	5.03201	4.49346	5.3557	3.13086	4.50819	3.52185	6.06744	0.510431574	0.966018836	12	8	24	0.282921168	0.768040178	24	4.622800292	0.167777305
ENSMUSG00000022456	Sept3	septin 3 [Source:MGI Symbol;Acc:MGI:1345148]	46.2961	43.4441	43.2007	45.1804	47.1173	43.5341	48.1317	41.6672	45.349	51.5308	40.8346	43.1122	44.1402	41.7415	0.834424969	0.966018836	16	16	24	0.677816129	0.892251586	24	14.30899666	0.030410296
ENSMUSG00000022462	Slc38a2	solute carrier family 38, member 2 [Source:MGI Symbol;Acc:MGI:1915010]	80.0347	70.7287	68.7337	78.146	73.0972	71.8926	72.1019	65.7825	70.6429	69.4207	75.5134	65.2819	63.1239	65.5825	0.697196105	0.966018836	20	12	24	0.126609559	0.739868076	24	15.89946483	0.056482917
ENSMUSG00000022463	Srebf2	sterol regulatory element binding factor 2 [Source:MGI Symbol;Acc:MGI:107585]	47.0571	37.8107	39.1438	39.8316	39.0902	40.4445	43.5266	42.8337	37.2256	54.4885	38.3298	43.551	42.3817	43.0339	0.418381424	0.963884303	4	8	24	0.935417429	0.98380253	24	21.85859152	0.021401891
ENSMUSG00000022464	Slc38a4	solute carrier family 38, member 4 [Source:MGI Symbol;Acc:MGI:1916604]	0.73646	1.43246	0.799799	1.36304	0.72008	1.1404	1.65587	3.55007	1.89598	1.28229	0.87075	1.42446	1.88715	1.24059	0.295379186	0.805301811	8	12	24	0.143465946	0.752803875	24	4.36939509	0.261021796
ENSMUSG00000022466	Rpap3	RNA polymerase II associated protein 3 [Source:MGI Symbol;Acc:MGI:1277218]	9.17894	9.21493	9.57577	10.2456	8.76461	8.78235	8.86126	8.92177	9.37331	10.8499	9.07269	9.0126	8.704	9.44085	0.005378037	0.149433822	16	8	24	0.012769418	0.526249719	24	10.45760594	0.08390362
ENSMUSG00000022468	Endou	endonuclease, polyU-specific [Source:MGI Symbol;Acc:MGI:97746]	0.528786	0.564008	0.61424	0.802626	1.00547	0.499726	0.634595	0.70808	0.829582	0.881739	0.741456	0.727503	0.668109	0.496072	0.036868435	0.334054683	16	16	24	0.015753938	0.544099157	24	13.24611292	0.123244737
ENSMUSG00000022469	Rapgef3	Rap guanine nucleotide exchange factor (GEF) 3 [Source:MGI Symbol;Acc:MGI:2441741]	10.189	10.1097	12.023	14.1512	12.2375	12.0834	13.6283	9.25898	14.7699	13.0819	11.8075	12.9073	10.1823	9.72426	0.008810267	0.192760771	16	16	24	0.06270742	0.661961741	24	13.85963688	0.166033925
ENSMUSG00000022471	Xrcc6	X-ray repair complementing defective repair in Chinese hamster cells 6 [Source:MGI Symbol;Acc:MGI:95606]	10.0963	12.6639	12.8373	15.4977	13.7639	12.1251	12.6626	14.3995	12.4356	12.9421	14.0508	13.6179	13.7879	13.1462	0.664568268	0.966018836	20	8	24	0.218561192	0.768040178	24	12.12426471	0.081518747
ENSMUSG00000022472	Desi1	desumoylating isopeptidase 1 [Source:MGI Symbol;Acc:MGI:106313]	17.2657	17.3694	19.6197	16.5679	17.6426	18.0028	20.3603	17.3083	17.8714	19.1586	15.266	17.761	16.3274	18.3467	0.92721845	0.966018836	12	8	24	0.626319484	0.872596805	24	5.205455334	0.039690335
ENSMUSG00000022474	Pmm1	phosphomannomutase 1 [Source:MGI Symbol;Acc:MGI:1353418]	113.725	106.151	103.314	102.436	113.147	113.349	102.12	110.592	99.0967	104.442	118.112	111.249	119.404	111.335	0.062105896	0.409814092	20	16	24	0.04548819	0.629609444	24	20.58143088	0.072430681
ENSMUSG00000022475	Hdac7	histone deacetylase 7 [Source:MGI Symbol;Acc:MGI:1891835]	11.1796	11.4594	11.5824	10.9415	12.8493	10.7039	12.1802	10.1082	10.9651	10.0807	9.9778	9.49367	10.1318	8.84969	0.88067614	0.966018836	12	12	24	0.710188639	0.90416311	24	16.91285239	0.029286072
ENSMUSG00000022476	Polr3h	polymerase (RNA) III (DNA directed) polypeptide H [Source:MGI Symbol;Acc:MGI:1926179]	31.8137	37.5103	43.1185	42.6296	40.675	39.5857	35.471	43.0871	51.3592	43.5246	59.5845	39.8235	54.8087	48.1992	0.360033827	0.883787544	12	16	24	0.115119459	0.727799063	24	10.79481195	0.137497036
ENSMUSG00000022477	Aco2	aconitase 2, mitochondrial [Source:MGI Symbol;Acc:MGI:87880]	75.4395	74.0717	96.9271	123.612	86.0011	96.4106	87.9002	112.122	84.442	115.898	90.5095	86.5964	103.125	94.9784	0.718720526	0.966018836	16	12	24	0.232913795	0.768040178	24	11.21073818	0.136490783
ENSMUSG00000022483	Col2a1	collagen, type II, alpha 1 [Source:MGI Symbol;Acc:MGI:88452]	1.1586	1.32748	1.32764	1.28057	1.32603	2.3482	2.07728	1.87054	1.12426	2.34485	1.24517	1.08243	1.5223	1.02578	0.814221431	0.966018836	16	20	24	0.847137573	0.953831011	24	19.26481167	0.060841508
ENSMUSG00000022484	Hoxc10	homeobox C10 [Source:MGI Symbol;Acc:MGI:96192]	2.83978	4.06462	5.1853	3.65403	2.6454	4.45305	4.07606	3.97424	4.27024	5.1236	2.20718	4.10808	2.276	4.3058	0.321662653	0.83607295	12	8	24	0.229885455	0.768040178	24	7.541349307	0.210727829
ENSMUSG00000022485	Hoxc5	homeobox C5 [Source:MGI Symbol;Acc:MGI:96196]	1.70356	1.21924	1.26425	1.70164	1.02624	0.958701	1.26236	1.56007	1.52204	1.101	1.20576	1.24654	1.12307	1.23013	0.356926984	0.881188925	12	8	24	0.341276979	0.775525696	24	6.180630886	0.081557379
ENSMUSG00000022488	Nckap1l	NCK associated protein 1 like [Source:MGI Symbol;Acc:MGI:1926063]	1.9015	2.29704	3.22866	2.40904	2.44786	2.0884	2.36042	2.26064	2.64182	1.97187	2.15804	2.45466	1.818	1.63498	0.217827365	0.704989686	12	20	24	0.30962755	0.768040178	24	11.18956929	0.092052244
ENSMUSG00000022489	Pde1b	phosphodiesterase 1B, Ca2+-calmodulin dependent [Source:MGI Symbol;Acc:MGI:97523]	3.697	5.94319	9.44842	5.21132	3.76647	5.83482	6.82733	4.49562	6.24847	4.96231	3.12447	5.46547	3.14092	6.79624	0.113424199	0.530777648	12	8	24	0.207509652	0.768040178	24	6.741954659	0.264056983
ENSMUSG00000022490	Ppp1r1a	protein phosphatase 1, regulatory inhibitor subunit 1A [Source:MGI Symbol;Acc:MGI:1889595]	43.0787	39.9551	35.8227	41.2653	38.2647	45.4663	35.5375	42.9709	35.9511	40.0068	39.7	41.0939	40.8269	39.7569	0.245061943	0.741580974	24	12	24	0.30750507	0.768040178	24	20.68340521	0.06339289
ENSMUSG00000022494	Shisa9	shisa family member 9 [Source:MGI Symbol;Acc:MGI:1919805]	0.598615	0.950581	1.7894	0.658513	0.580329	0.972762	1.19693	0.47869	1.08375	0.696457	0.500646	0.986706	0.277344	1.36782	0.616047374	0.966018836	12	8	24	0.456320109	0.805393068	24	6.62334424	0.154466356
ENSMUSG00000022498	Txndc11	thioredoxin domain containing 11 [Source:MGI Symbol;Acc:MGI:1923620]	6.26234	6.11827	5.74663	6.15705	6.44251	5.975	6.69566	6.48156	6.66116	6.91761	5.89857	6.40334	6.44381	6.11051	0.873350015	0.966018836	4	20	24	0.981128059	0.995579529	24	5.89686038	0.005121695
ENSMUSG00000022500	Litaf	LPS-induced TN factor [Source:MGI Symbol;Acc:MGI:1929512]	15.631	16.49	17.6157	13.9041	17.1971	16.2944	18.178	19.1939	15.6753	15.4847	14.6608	17.225	18.1321	15.4318	0.285193697	0.793714531	4	12	24	0.139593207	0.74956518	24	0.962082693	0.09035049
ENSMUSG00000022503	Nubp1	nucleotide binding protein 1 [Source:MGI Symbol;Acc:MGI:1347073]	11.3546	9.85364	9.35164	9.83603	12.3581	11.1958	10.1199	11.2621	10.0846	12.7181	12.5023	9.41664	10.0078	8.93761	0.287843582	0.793714531	20	16	24	0.10965533	0.72082227	24	16.10850916	0.118180649
ENSMUSG00000022504	Ciita	class II transactivator [Source:MGI Symbol;Acc:MGI:108445]	0.959467	0.648521	0.664997	0.560429	0.7531	1.02573	1.01367	1.0979	0.884758	0.966151	0.911253	0.795469	0.798885	0.592953	0.838532766	0.966018836	4	4	24	0.433869473	0.805393068	24	21.21365285	0.072215881
ENSMUSG00000022505	Emp2	epithelial membrane protein 2 [Source:MGI Symbol;Acc:MGI:1098726]	38.4162	38.7063	35.4026	37.0676	42.9001	36.3802	44.7857	40.8836	40.1218	40.8345	34.6662	37.0146	39.7999	34.4413	0.873350015	0.966018836	4	20	24	0.874965768	0.964979482	24	23.4351808	0.023241068
ENSMUSG00000022507	1810013L24Rik	RIKEN cDNA 1810013L24 gene [Source:MGI Symbol;Acc:MGI:1916303]	35.3209	33.3064	33.7649	34.0147	33.4165	32.2197	32.8959	32.3919	32.3741	33.4082	34.009	32.8408	33.0078	34.1924	0.927004797	0.966018836	20	4	24	0.980564252	0.995264516	24	12.87983197	0.002720698
ENSMUSG00000022508	Bcl6	B cell leukemia/lymphoma 6 [Source:MGI Symbol;Acc:MGI:107187]	7.43467	6.84769	6.78675	7.19042	8.0465	6.52417	7.18733	6.43124	6.35759	10.1256	7.16339	6.83172	6.71659	6.50258	0.082113105	0.462922111	16	20	24	0.157042809	0.763141258	24	14.24221469	0.110013687
ENSMUSG00000022512	Cldn1	claudin 1 [Source:MGI Symbol;Acc:MGI:1276109]	7.5447	9.20841	10.7137	8.38541	10.6565	8.09395	8.0258	9.98247	9.57847	6.34815	9.79067	8.44556	8.67812	8.12662	0.325142889	0.842977406	12	16	24	0.839981399	0.9514886	24	9.135593378	0.045335449
ENSMUSG00000022514	Il1rap	interleukin 1 receptor accessory protein [Source:MGI Symbol;Acc:MGI:104975]	7.2692	9.97829	9.27353	7.84187	9.25468	8.03205	10.0393	8.66005	9.31899	7.13105	8.18223	7.67107	7.41087	8.04128	0.593560247	0.966018836	12	4	24	0.542948556	0.831275234	24	5.665611819	0.062743549

ENSMUSG00000022515	Anks3	ankyrin repeat and sterile alpha motif domain containing 3 [Source:MGI Symbol;Acc:MGI:1919865]	19.3709	21.4337	20.8917	21.5136	22.2276	19.9786	21.3448	21.1427	22.7362	19.6623	21.5615	21.7979	21.2453	19.852	0.742579234	0.966018836	20	8	24	0.691516662	0.897724829	24	12.48278556	0.022456437
ENSMUSG00000022516	Nudt16l1	nudix (nucleoside diphosphate linked moiety X)-type motif 16-like 1 [Source:MGI Symbol;Acc:MGI:1914161]	33.474	29.1318	31.5974	31.6293	32.9285	32.7128	31.2116	32.0817	29.3245	31.8607	32.5209	32.6388	32.249	29.8171	0.009640466	0.194720062	24	8	24	0.012703551	0.526249719	24	18.55692045	0.062821923
ENSMUSG00000022517	Mgrn1	mahogunin, ring finger 1 [Source:MGI Symbol;Acc:MGI:2447670]	19.7398	20.2539	21.9242	19.2044	19.5952	20.0462	22.8975	21.8239	21.4944	23.7128	15.9493	20.8955	18.6476	21.4137	0.285027487	0.793714531	12	8	24	0.244665008	0.768040178	24	5.859500401	0.089432768
ENSMUSG00000022519	Srl	sarcalumenin [Source:MGI Symbol;Acc:MGI:2146620]	6.32956	13.8906	15.5983	21.9501	14.4098	20.186	13.6223	39.2061	24.4369	31.8379	12.1399	15.3754	16.2091	15.1961	0.217933169	0.704989686	16	12	24	0.13355536	0.743843563	24	9.551640995	0.445314843
ENSMUSG00000022521	Crebbp	CREB binding protein [Source:MGI Symbol;Acc:MGI:1098280]	4.17265	3.74963	4.35408	3.76727	4.80383	3.95533	4.543	4.17399	5.36793	4.1625	4.19483	4.44678	3.65125	4.34315	0.920858634	0.966018836	12	16	24	0.713197279	0.905403147	24	10.72808962	0.040248161
ENSMUSG00000022523	Fgf12	fibroblast growth factor 12 [Source:MGI Symbol;Acc:MGI:109183]	247.046	238.352	214.066	233.24	215.665	223.981	208.66	201.433	217.467	201.367	242.515	225.252	251.607	238.045	0.668329321	0.966018836	4	8	24	0.475029385	0.805393068	24	22.33665763	0.05207175
ENSMUSG00000022525	Plaat1	phospholipase A and acyltransferase 1 [Source:MGI Symbol;Acc:MGI:1351473]	4.25582	5.49924	5.92176	4.85338	4.48318	4.42672	5.71092	5.44101	5.423	5.32478	4.42658	4.87103	4.93709	5.33483	0.025178621	0.293143865	12	8	24	0.006568555	0.470633744	24	6.420912252	0.137606972
ENSMUSG00000022526	Zfp251	zinc finger protein 251 [Source:MGI Symbol;Acc:MGI:1918841]	7.62211	6.68501	7.40845	6.40372	6.78409	7.07486	7.07157	6.56947	6.87487	7.83308	6.77356	7.52781	7.30309	6.99517	0.555368163	0.966018836	4	4	24	0.755997787	0.921194485	24	21.30286239	0.023278184
ENSMUSG00000022528	Hes1	hes family bHLH transcription factor 1 [Source:MGI Symbol;Acc:MGI:104853]	4.68253	4.82411	5.14654	4.49781	3.54217	4.3538	4.15153	5.26114	3.89028	4.57926	4.63254	4.05324	5.66284	4.10235	0.593560247	0.966018836	4	20	24	0.373554656	0.794388449	24	3.786227673	0.081562851
ENSMUSG00000022529	Zfp263	zinc finger protein 263 [Source:MGI Symbol;Acc:MGI:1921370]	7.24903	8.1878	7.81859	7.69211	7.38199	7.58226	7.29602	6.62702	8.44665	6.81395	7.89395	7.70945	7.63291	8.15623	0.954584434	0.966018836	12	16	24	0.794949802	0.93426902	24	7.626234371	0.024543673
ENSMUSG00000022533	Atp13a3	ATPase type 13A3 [Source:MGI Symbol;Acc:MGI:2685387]	13.4875	14.451	13.9217	14.4145	14.9954	12.5744	13.9821	12.2881	14.0818	12.7177	14.8646	13.1773	14.4451	13.1523	0.722296281	0.966018836	20	4	24	0.649440572	0.881952632	24	14.55083283	0.030897099
ENSMUSG00000022536	Glyr1	glyoxylate reductase 1 homolog (Arabidopsis) [Source:MGI Symbol;Acc:MGI:1921272]	44.7066	40.8543	42.6621	43.1488	39.9757	44.7251	41.3614	42.4946	38.101	48.2303	37.8701	42.0372	40.4315	42.6709	0.887862353	0.966018836	16	4	24	0.999123279	0.999770694	24	18.17729323	0.001566451
ENSMUSG00000022537	Tmem44	transmembrane protein 44 [Source:MGI Symbol;Acc:MGI:1924489]	2.71517	3.24226	4.02694	2.55304	2.8515	2.81693	4.37776	2.87238	3.72543	2.94515	2.48718	2.67155	2.26168	2.99293	0.106193509	0.519440352	12	8	24	0.272158652	0.768040178	24	6.099157534	0.125837923
ENSMUSG00000022538	Lsg1	large 60S subunit nuclear export GTPase 1 [Source:MGI Symbol;Acc:MGI:107236]	8.89704	9.0123	9.11908	7.69763	8.95636	8.86287	7.5107	11.1064	9.63267	9.70279	8.31542	9.42837	8.89653	8.4704	0.65208848	0.966018836	12	16	24	0.601498683	0.860529995	24	6.51914727	0.05246505
ENSMUSG00000022540	Rogdi	rogdi homolog [Source:MGI Symbol;Acc:MGI:1913299]	32.5816	33.0043	33.5672	34.7635	33.3728	35.9892	36.5285	33.9448	33.8432	32.2894	29.6647	35.3331	27.8984	31.7531	0.858366225	0.966018836	24	20	24	0.946752569	0.986968557	24	18.47639155	0.012915534
ENSMUSG00000022543	Dnaaf8	dynein axonemal assembly factor 8 [Source:MGI Symbol;Acc:MGI:1921934]	1.63799	2.26171	2.38021	2.6927	2.9058	3.77475	1.6861	2.22856	2.30019	2.45616	2.33154	2.49426	2.14227	2.65029	0.001971246	0.100255953	24	4	24	0.208646433	0.768040178	24	14.71178615	0.139926239
ENSMUSG00000022544	Eef2kmt	eukaryotic elongation factor 2 lysine methyltransferase [Source:MGI Symbol;Acc:MGI:1917761]	5.1825	4.66118	5.72446	5.5696	6.28428	5.65307	4.68518	6.14309	6.02161	5.10167	5.70182	4.92002	5.38992	3.95034	0.428730128	0.768040178	12	20	24	0.23433458	0.768040178	24	14.10474673	0.095084533
ENSMUSG00000022545	Ercc4	excision repair cross-complementing rodent repair deficiency, complementation group 4 [Source:MGI Symbol;Acc:MGI:1354163]	5.63236	5.15785	4.95147	5.15469	5.73223	5.61985	5.41403	5.30532	5.06646	6.32501	5.02945	5.9426	5.6068	6.4141	0.245061943	0.741580974	24	12	24	0.713688244	0.905681809	24	20.91705448	0.030453333
ENSMUSG00000022546	Gpt	glutamic pyruvic transaminase, soluble [Source:MGI Symbol;Acc:MGI:95802]	4.84657	4.58344	6.21597	6.23021	5.94071	5.19654	5.6096	5.57904	5.60725	6.5423	5.79743	4.18135	4.80038	5.82271	0.008360387	0.192760771	16	8	24	0.012405236	0.525280095	24	10.93205655	0.160093818
ENSMUSG00000022548	Apod	apolipoprotein D [Source:MGI Symbol;Acc:MGI:88056]	1353.87	1814.4	1640.52	1446.66	1866.46	1294.98	1630.82	1517.23	2261.21	1049.21	1874.3	1519.32	1715.29	1438.6	0.96317972	0.967965568	12	12	24	0.881633157	0.966661503	24	6.492310141	0.05748021
ENSMUSG00000022550	Adck5	aarF domain containing kinase 5 [Source:MGI Symbol;Acc:MGI:2679274]	8.60727	6.65254	5.96256	8.73226	7.96533	6.77716	7.07972	6.68653	6.86848	6.64916	7.14333	7.83828	7.41387	5.3397	0.128665061	0.560209134	20	12	24	0.067221453	0.670278734	24	18.14804917	0.137715186
ENSMUSG00000022551	Cyc1	cytochrome c-1 [Source:MGI Symbol;Acc:MGI:1913695]	91.7236	86.1554	100.528	127.019	95.6477	107.074	96.974	113.775	92.8713	108.556	96.2255	103.564	106.895	101.324	0.946423879	0.966018836	16	12	24	0.564853425	0.843459042	24	12.81969476	0.058192463
ENSMUSG00000022552	Sharpin	SHANK-associated RH domain interacting protein [Source:MGI Symbol;Acc:MGI:1913331]	26.8558	28.6967	26.4759	28.3058	30.5337	29.0942	30.5351	27.5415	26.7825	30.698	25.995	26.3255	27.6888	26.7009	0.895137379	0.966018836	16	20	24	0.705526436	0.902918222	24	16.86979148	0.026978848
ENSMUSG00000022553	Maf1	MAF1 homolog, negative regulator of RNA polymerase III [Source:MGI Symbol;Acc:MGI:1916127]	25.0792	25.6971	25.9534	26.5888	26.0876	24.9625	26.0723	25.7164	26.9745	25.7889	24.1146	27.7403	26.916	26.2238	0.862909389	0.966018836	12	8	24	0.742107756	0.916130449	24	5.949067671	0.014986229
ENSMUSG00000022554	Hgh1	HGH1 homolog [Source:MGI Symbol;Acc:MGI:1930628]	2.67484	2.47359	2.78367	2.68956	2.6149	2.37569	2.3023	2.68178	2.80303	2.31947	2.79461	2.7165	2.9131	2.65919	0.892044196	0.966018836	12	12	24	0.821085822	0.943551411	24	7.763189109	0.019838604
ENSMUSG00000022555	Dgat1	diacylglycerol O-acyltransferase 1 [Source:MGI Symbol;Acc:MGI:1333825]	14.2632	18.3511	19.9027	21.6376	19.6323	17.4819	21.9426	18.914	17.9901	19.9225	19.0942	17.9692	21.6396	19.6194	0.892578749	0.966018836	16	8	24	0.513572802	0.816307374	24	10.42000872	0.065959173
ENSMUSG00000022556	Hsf1	heat shock factor 1 [Source:MGI Symbol;Acc:MGI:96238]	16.4701	13.7791	14.8696	13.9443	16.3337	14.2056	16.0591	15.7042	13.0967	15.9899	14.8414	14.9724	14.6102	15.0175	0.85447369	0.966018836	4	8	24	0.478231411	0.805393068	24	20.49533351	0.046458463
ENSMUSG00000022557	Bop1	block of proliferation 1 [Source:MGI Symbol;Acc:MGI:1334460]	12.8232	10.2466	10.588	11.4048	11.0563	12.3639	13.0659	11.8421	9.59664	12.9307	11.2473	10.3459	10.5128	11.835	0.969174623	0.970883022	4	8	24	0.592485362	0.856568887	24	20.73560677	0.054105266
ENSMUSG00000022558	Mroh1	maestro heat-like repeat family member 1 [Source:MGI Symbol;Acc:MGI:2442558]	17.2603	16.7757	15.2902	16.6506	18.9039	18.6735	15.5105	17.84	16.6687	17.4518	17.9663	17.0301	18.3694	19.8025	0.43280837	0.966018836	20	16	24	0.649519322	0.88199334	24	19.55412683	0.034058467
ENSMUSG00000022559	Fbxl6	F-box and leucine-rich repeat protein 6 [Source:MGI Symbol;Acc:MGI:1354705]	8.18418	9.45988	8.98181	10.2584	10.6341	9.54064	9.01125	9.59447	10.7043	10.1067	8.61863	9.54039	8.44202	9.77536	0.037425327	0.334069441	16	12	24	0.077018421	0.687041208	24	11.20439429	0.089988691
ENSMUSG00000022560	Slc52a2	solute carrier protein 52, member 2 [Source:MGI Symbol;Acc:MGI:1289288]	3.53256	3.6541	3.86819	3.2766	3.57736	3.82141	2.89961	3.51752	2.90992	3.88512	3.10647	3.50548	3.52239	3.25468	0.830949782	0.966018836	16	12	24	0.914900278	0.976279382	24	16.62012594	0.017706638
ENSMUSG00000022561	Gpaa1	GPI anchor attachment protein 1 [Source:MGI Symbol;Acc:MGI:1202392]	18.3801	15.4693	13.2906	21.153	18.9903	16.4222	17.1735	14.8128	14.7921	18.1261	17.1721	14.7246	17.4863	15.6149	0.056268883	0.401074772	16	20	24	0.179183885	0.768040178	24	16.69503072	0.117856139
ENSMUSG00000022562	Oplah	5-oxoprolinase (ATP-hydrolysing) [Source:MGI Symbol;Acc:MGI:1922725]	7.50471	7.24519	8.13158	10.6656	8.18408	7.17617	6.88904	8.19726	7.91232	9.84325	7.14751	7.81951	8.05796	7.58461	0.050845331	0.38654007	16	12	24	0.033435125	0.606585237	24	11.38923667	0.145050871
ENSMUSG00000022564	Grina	glutamate receptor, ionotropic, N-methyl D-aspartate-associated protein 1 (glutamate binding) [Source:MGI Symbol;Acc:MGI:1913418]	158.625	131.692	146.014	142.555	142.939	168.267	151.417	154.805	129.871	190.25	137.482	153.281	144.416	148.276	0.581070934	0.966018836	24	12	24	0.697607174	0.899395227	24	18.30407079	0.049592983
ENSMUSG00000022565	Plec	plectin [Source:MGI Symbol;Acc:MGI:1277961]	22.5991	18.5892	17.9919	20.2607	20.3095	19.5751	20.534	24.2637	19.0843	33.315	20.8099	23.9196	23.2061	22.6488	0.834434272	0.966018836	4	8	24	0.911670815	0.975053334	24	14.13338281	0.032963671
ENSMUSG00000022568	Scrib	scribbled planar cell polarity [Source:MGI Symbol;Acc:MGI:2145950]	12.0848	14.7185	15.5817	14.2892	14.3966	12.0889	14.6816	13.1907	19.0204	16.4226	15.6461	13.9333	16.8417	13.6361	0.351840494	0.870418653	12	12	24	0.083749507	0.700872524	24	9.701684617	0.133713946
ENSMUSG00000022570	Gfus	GDP-L-fucose synthase [Source:MGI Symbol;Acc:MGI:98857]	23.3401	23.7616	23.2927	21.4958	26.1565	21.5477	21.5489	25.3484	23.9799	21.1835	22.1829	23.9533	21.6335	22.2115	0.581070934	0.966018836	8	8	24	0.953044226	0.988573174	24	5.177252326	0.011384321
ENSMUSG00000022571	Pycrl	pyrroline-5-carboxylate reductase-like [Source:MGI Symbol;Acc:MGI:1913444]	10.592	9.21305	9.3578	9.89291	10.5585	9.3715	11.6579	9.98537	8.69545	8.19977	10.3436	9.98	9.42209	8.6536	0.117105047	0.538362324	4	8	24	0.056049114	0.65534544	24	20.63443958	0.105064734
ENSMUSG00000022574	Naprt	nicotinate phosphoribosyltransferase [Source:MGI Symbol;Acc:MGI:2442664]	3.58776	3.89698	3.98041	4.21527	3.98401	4.1087	3.88944	4.76445	4.46125	4.76804	5.15193	4.7389	4.14584	3.29625	0.154948076	0.607427992	20	8	24	0.142834351	0.752591915	24	14.18030721	0.098504911
ENSMUSG00000022575	Gsdmd	gasdermin D [Source:MGI Symbol;Acc:MGI:1916396]	4.07586	6.24	6.09125	4.73241	5.17558	4.19457	5.84877	7.17172	6.64675	4.20905	5.46892	4.40578	4.73337	3.75807	0.474848197	0.966018836	12	12	24	0.315082348	0.768040178	24	6.951533897	0.145394701
ENSMUSG00000022577	Ly6h	lymphocyte antigen 6 complex, locus H [Source:MGI Symbol;Acc:MGI:1346030]	16.3474	15.8563	20.3432	11.7283	13.5258	14.0297	17.4218	16.5781	23.3965	20.5934	19.5762	19.0406	17.5926	22.4514	0.777666236	0.966018836	12	12	24	0.137822088	0.747972781	24	6.48739084	0.172948587
ENSMUSG00000022579	Gpihbp1	GPI-anchored HDL-binding protein 1 [Source:MGI Symbol;Acc:MGI:1915703]	3.08117	2.38691	4.19422	6.50587	2.87935	3.95871	2.88348	4.42884	3.30731	5.75709	4.62494	4.01305	4.58954	3.50204	0.351315142	0.870418653	16	16	24	0.11262872	0.726918602	24	12.64154837	0.235024096
ENSMUSG00000022580	Rhpn1	rhophilin, Rho GTPase binding protein 1 [Source:MGI Symbol;Acc:MGI:1098783]	9.43397	7.90464	7.77281	7.82405	9.2824	6.67672	9.89603	7.43243	8.21707	7.79798	10.1822	7.70948	8.40553	7.39215	0.632729413	0.966018836	20	12	24	0.590964187	0.855619585	24	18.7888481	0.066401055
ENSMUSG00000022582	Ly6g	lymphocyte antigen 6 complex, locus G [Source:MGI Symbol;Acc:MGI:109440]	0.796587	2.20831	3.77803	3.47905	3.2146	2.70019	2.30645	1.46697	2.40781	2.2854	3.63738	1.63338	2.38296	1.78086	0.048227303	0.379129806	12	20	24	0.038658265	0.61140397	24	12.99784601	0.350245487
ENSMUSG00000022584	Ly6c2	lymphocyte antigen 6 complex, locus C2 [Source:MGI Symbol;Acc:MGI:3712069]	3.92776	6.76361	11.7117	7.60348	7.15074	7.05441	5.97256	6.43386	8.38219	4.31897	5.19029	5.33208	7.11397	4.08663	0.43280837	0.966018836	12	16	24	0.487296331	0.805393068	24	9.557047563	0.172664193
ENSMUSG00000022587	Ly6e	lymphocyte antigen 6 complex, locus E [Source:MGI Symbol;Acc:MGI:106651]	39.493	42.0486	51.0644	42.5859	41.5972	45.1439	40.9397	55.3822	48.6647	44.6181	38.2362	41.0645	43.1346	42.267	0.114657038	0.534533508	12	8	24	0.08473267	0.70291653	24	7.1025281	0.12193463
ENSMUSG00000022591	Gm9747	predicted gene 9747 [Source:MGI Symbol;Acc:MGI:3710571]	4.53819	5.77977	5.04943	4.54435	5.39743	5.09773	5.84444	6.97173	6.12099	5.56921	6.00093	5.22373	6.42062	5.46314	0.870491976	0.966018836	8	16	24	0.348853269	0.779170188	24	4.471730849	0.073564002
ENSMUSG00000022594	Lynx1	Ly6/neurotoxin 1 [Source:MGI Symbol;Acc:MGI:1345180]	89.24	68.2191	73.1208	72.5721	71.8513	86.2674	77.3681	81.336	54.5631	89.7858	66.7045	74.8388	70.5203	76.4063	0.777666236	0.966018836	24	20	24	0.430023989	0.805393068	24	21.15640936	0.097174224
ENSMUSG00000022595	Lypd2	Ly6/Plaur domain containing 2 [Source:MGI Symbol;Acc:MGI:1915561]	11.1841	13.3251	11.4002	10.5082	12.6952	10.1968	9.87165	14.2228	15.4713	4.46331	10.5231	10.8229	9.0436	9.05229	0.625825704	0.966018836	12	4	24	0.620503599	0.870243288	24	2.644501421	0.136430269
ENSMUSG00000022601	Zbtb11	zinc finger and BTB domain containing 11 [Source:MGI Symbol;Acc:MGI:2443876]	11.5809	9.80215	9.85143	10.3391	9.93865	10.2052	11.0602	9.49772	9.0131	9.36108	11.5807	9.56975	10.0546	10.2042	0.267533088	0.774280302	4	8	24	0.164741814	0.767020009	24	20.57621778	0.072374252
ENSMUSG00000022602	Arc	activity regulated cytoskeletal-associated protein [Source:MGI Symbol;Acc:MGI:88067]	1.05382	1.15673	1.62379	1.05825	1.06196	1.09494	1.1546	1.00898	0.937272	1.14779	0.983702	0.998645	0.881012	0.809523	0.807813307	0.966018836	16	4	24	0.581379635	0.850792036	24	10.93754558	0.040398799
ENSMUSG00000022604	Cep97	centrosomal protein 97 [Source:MGI Symbol;Acc:MGI:1921451]	4.52617	5.07797	4.58585	5.37023	4.96752	4.62619	4.712	4.47819	4.64096	4.12776	5.25343	4.66141	5.2961	4.58941	0.945728192	0.966018836	20	16	24	0.722235685	0.908924547	24	17.85212492	0.029759468
ENSMUSG00000022607	Ptk2	PTK2 protein tyrosine kinase 2 [Source:MGI Symbol;Acc:MGI:95481]	15.1842	15.2329	17.9706	15.3621	17.2559	17.6076	18.1362	17.3123	17.9571	17.5131	18.64	16.3042	15.0778	16.0968	0.515451644	0.966018836	12	16	24	0.390182659	0.799546792	24	13.20089018	0.053701143
ENSMUSG00000022610	Mapk12	mitogen-activated protein kinase 12 [Source:MGI Symbol;Acc:MGI:1353438]	27.9774	27.8107	27.2298	28.944	32.4135	27.218	25.2027	28.3018	26.6988	26.9415	25.6026	27.1717	26.4309	25.9126	0.874672901	0.966018836	16	12	24	0.364766068	0.789112192	24	14.86450781	0.041356736
ENSMUSG00000022614	Lmf2	lipase maturation factor 2 [Source:MGI Symbol;Acc:MGI:2146015]	6.18094	5.82912	6.6456	6.38471	6.66332	6.37517	6.70479	6.98015	6.45312	8.05744	6.4313	6.49846	6.86962	6.27295	0.844511766	0.966018836	16	16	24	0.403216276	0.799546792	24	12.20123759	0.04577579
ENSMUSG00000022617	Chkb	choline kinase beta [Source:MGI Symbol;Acc:MGI:1328313]	29.5614	31.7944	33.3091	34.1597	34.1398	31.9066	29.1164	30.4246	36.2754	33.7719	32.9203	32.6321	33.7319	30.5947	0.007353096	0.192760771	12	20	24	0.012750893	0.526249719	24	12.17307798	0.086083037
ENSMUSG00000022619	Mapk8ip2	mitogen-activated protein kinase 8 interacting protein 2 [Source:MGI Symbol;Acc:MGI:1926555]	50.6086	37.2517	39.6034	39.2662	37.2813	46.1143	43.7863	47.4745	31.2737	58.2724	37.6598	46.2196	42.9609	47.4118	0.759899911	0.966018836	24	20	24	0.622072003	0.871320206	24	22.40896886	0.08717461
ENSMUSG00000022620	Arsa	arylsulfatase A [Source:MGI Symbol;Acc:MGI:88077]	15.1394	11.5801	12.5616	12.2645	13.1193	14.2329	13.2568	13.8501	10.863	15.0764	13.3848	13.5992	13.1441	13.7991	0.390686537	0.915479136	24	12	24	0.25413892	0.768040178	24	20.20843313	0.08109722
ENSMUSG00000022621	Rabl2	RAB, member RAS oncogene family-like 2 [Source:MGI Symbol;Acc:MGI:1915958]	13.6008	13.283	14.3674	14.2189	15.1886	14.1015	15.3996	13.3216	12.4545	12.6656	13.6132	15.5517	13.171	14.367	0.541384199	0.966018836	24	12	24	0.202326368	0.768040178	24	19.73548254	0.066033257
ENSMUSG00000022623	Shank3	SH3 and multiple ankyrin repeat domains 3 [Source:MGI Symbol;Acc:MGI:1930016]	4.96339	5.16359	5.24735	5.36866	4.84226	4.29897	5.76	4.94529	4.69181	7.27395	5.6518	5.83667	5.51232	5.35211	0.935194469	0.966018836	16	20	24	0.590209033	0.854963454	24	12.04353911	0.05718325
ENSMUSG00000022629	Kif21a	kinesin family member 21A [Source:MGI Symbol;Acc:MGI:109188]	192.708	159.967	158.68	164.879	158.993	186.13	160.178	172.316	145.707	183.042	174.286	183.935	193.913	174.348	0.010059617	0.194720062	4	8	24	0.091243207	0.71175955	24	21.05127985	0.098243258
ENSMUSG00000022634	Yaf2	YY1 associated factor 2 [Source:MGI Symbol;Acc:MGI:1914307]	28.2482	25.1804	26.3333	26.1057	22.5269	24.4698	27.0592	24.5969	21.4487	24.5409	26.7391	22.1202	20.6841	24.3163	0.773642901	0.966018836	4	20	24	0.990488277	0.998233149	24	23.27740981	0.006095121
ENSMUSG00000022635	Zcrb1	zinc finger CCHC-type and RNA binding motif 1 [Source:MGI Symbol;Acc:MGI:1914447]	54.2774	59.0379	61.4107	63.8251	57.5493	57.0894	63.0802	54.7761	74.5102	62.2601	71.9755	62.7654	66.2431	67.8751	0.937921962	0.966018836	12	12	24	0.200492332	0.768040178	24	10.29467782	0.07498432
ENSMUSG00000022636	Alcam	activated leukocyte cell adhesion molecule [Source:MGI Symbol;Acc:MGI:1313266]	57.4225	54.0622	50.9145	53.3489	49.6772	51.2972	51.8438	47.57	45.5163	49.719	52.9371	52.8645	53.4394	53.472	0.437217485	0.966018836	4	8	24	0.171119529	0.768040178	24	21.83804077	0.059003486
ENSMUSG00000022637	Cblb	Casitas B-lineage lymphoma b [Source:MGI Symbol;Acc:MGI:2146430]	6.05446	4.9125	5.17368	4.97158	5.32983	5.63149	5.37625	5.52474	4.73974	6.38433	5.31406	5.80854	5.5927	5.4303	0.039704107	0.338535904	24	12	24	0.325697681	0.768040178	24	20.09987347	0.05833834
ENSMUSG00000022639	Dubr	Dppa2 upstream binding RNA [Source:MGI Symbol;Acc:MGI:1915440]	34.9466	34.551	29.3553	32.7974	38.7924	30.2016	35.1928	28.8559	35.7637	28.753	35.8141	33.7758	36.5082	33.201	0.518444157	0.966018836	20	20	24	0.510627917	0.814299654	24	19.98178879	0.062890161
ENSMUSG00000022641	Bbx	bobby sox HMG box containing [Source:MGI Symbol;Acc:MGI:1917758]	7.29825	6.47716	6.36192	6.7225	7.01877	7.04044	6.35542	5.95051	6.05483	5.60809	7.55928	7.33922	6.96578	7.02275	0.033886887	0.326107552	24	12	24	0.040065395	0.616921492	24	19.69129691	0.107776052
ENSMUSG00000022651	Retnlg	resistin like gamma [Source:MGI Symbol;Acc:MGI:2667763]	17.6509	36.6923	56.6097	39.7552	30.6234	29.9669	31.1993	29.0445	34.0181	19.1604	25.5229	29.6838	36.9872	23.6758	0.892685629	0.966018836	12	12	24	0.605022933	0.862246789	24	8.206891115	0.169063606
ENSMUSG00000022656	Nectin3	nectin cell adhesion molecule 3 [Source:MGI Symbol;Acc:MGI:1930171]	15.0434	14.0966	15.1234	12.65	17.0082	14.333	13.5168	12.8175	12.4796	10.8288	17.6178	13.0699	13.7242	13.0437	0.085045716	0.466404237	20	20	24	0.238683583	0.768040178	24	18.96112971	0.110639551
ENSMUSG00000022657	Cd96	CD96 antigen [Source:MGI Symbol;Acc:MGI:1934368]	0.881807	0.787941	0.917015	0.587441	1.09134	0.648788	0.766433	0.704302	0.925531	0.711788	1.04178	0.871005	0.676671	0.798206	0.814576414	0.966018836	20	4	24	0.692690135	0.898177673	24	15.28417299	0.039671616
ENSMUSG00000022658	Tagln3	transgelin 3 [Source:MGI Symbol;Acc:MGI:1926784]	180.181	165.751	161.851	157.303	158.471	173.711	160.745	161.533	147.981	159.063	161.843	164.789	164.7	170.927	0.073852543	0.448301997	24	16	24	0.027118229	0.588305652	24	22.68625717	0.064117229
ENSMUSG00000022661	Cd200	CD200 antigen [Source:MGI Symbol;Acc:MGI:1196990]	54.3895	68.0917	71.1439	60.9652	61.5265	63.4845	58.3718	63.7379	77.1071	67.6007	65.8849	61.1216	58.4949	69.4827	3.15E-04	0.049770315	12	16	24	0.006633127	0.470810671	24	8.953537843	0.139649419
ENSMUSG00000022663	Atg3	autophagy related 3 [Source:MGI Symbol;Acc:MGI:1915091]	29.2228	25.3343	26.5192	25.2855	26.6098	27.1804	26.1406	24.6787	22.3624	25.3062	26.2947	25.5001	26.9068	25.2415	0.067450051	0.427872421	4	4	24	0.021583577	0.574198497	24	20.65532409	0.077915074
ENSMUSG00000022664	Slc35a5	solute carrier family 35, member A5 [Source:MGI Symbol;Acc:MGI:1921352]	35.3054	32.1127	32.9523	30.2247	32.2726	34.6695	33.2816	29.466	34.2827	30.0425	34.0424	34.2585	38.0505	33.3297	0.057224139	0.401309455	4	12	24	0.093362115	0.71175955	24	22.23305907	0.077044195
ENSMUSG00000022665	Ccdc80	coiled-coil domain containing 80 [Source:MGI Symbol;Acc:MGI:1915146]	3.75772	4.83482	4.288	4.62723	4.22919	5.00984	4.23425	5.11547	4.92991	4.31057	4.59061	4.87981	4.66521	4.61357	0.881355613	0.966018836	8	12	24	0.824633688	0.944988393	24	6.789805732	0.023862268
ENSMUSG00000022668	Gtpbp8	GTP-binding protein 8 (putative) [Source:MGI Symbol;Acc:MGI:1913317]	14.9987	12.7315	13.7385	13.8599	12.6308	13.4169	14.3205	12.8718	13.3544	13.8305	14.9213	15.0863	15.3745	16.3462	0.581070934	0.966018836	4	8	24	0.426933619	0.805393068	24	23.38196615	0.04425403
ENSMUSG00000022671	Mzt2	mitotic spindle organizing protein 2 [Source:MGI Symbol;Acc:MGI:1922845]	15.4295	13.5258	13.6776	12.2855	12.1918	14.2947	13.0127	13.5296	11.4256	15.5013	12.9327	14.8134	13.1174	13.3902	0.935732159	0.966018836	24	20	24	0.473252317	0.805393068	24	21.83420845	0.058582248
ENSMUSG00000022672	Prkdc	protein kinase, DNA activated, catalytic polypeptide [Source:MGI Symbol;Acc:MGI:104779]	2.94765	3.5753	3.09899	3.48186	3.30709	2.92443	3.28312	3.08709	3.58005	2.79184	3.6148	3.46396	3.51363	3.61774	0.909604019	0.966018836	8	16	24	0.859785324	0.958681059	24	6.221745909	0.021000453
ENSMUSG00000022673	Mcm4	minichromosome maintenance complex component 4 [Source:MGI Symbol;Acc:MGI:103199]	5.04669	4.84167	5.60887	4.6271	4.52637	4.89244	5.09812	5.161	4.83303	5.43347	4.79751	5.45694	4.82744	4.59959	0.917370979	0.966018836	24	20	24	0.940520711	0.985398871	24	5.192391864	0.01157285
ENSMUSG00000022674	Ube2v2	ubiquitin-conjugating enzyme E2 variant 2 [Source:MGI Symbol;Acc:MGI:1917870]	8.97164	10.4618	9.54372	9.25271	9.69135	8.97822	8.97149	8.20994	8.6154	8.31202	9.56424	7.76693	7.25769	9.42905	0.745118544	0.966018836	20	8	24	0.604272317	0.861869168	24	10.63947475	0.041869974
ENSMUSG00000022676	Snai2	snail family zinc finger 2 [Source:MGI Symbol;Acc:MGI:1096393]	0.53298	0.813763	0.696015	0.761778	0.68971	0.717783	0.468987	0.689479	0.481512	0.478725	0.750518	0.568705	0.807117	0.663472	0.794858476	0.966018836	20	8	24	0.989672799	0.997891892	24	19.21925282	0.006626978
ENSMUSG00000022677	Cep20	centrosomal protein 20 [Source:MGI Symbol;Acc:MGI:1913336]	44.5771	49.6142	46.4984	44.1253	47.972	40.9419	40.3333	42.6624	49.4266	39.4745	47.0217	43.0965	46.8099	48.7786	0.588208558	0.966018836	8	16	24	0.485911556	0.805393068	24	6.915848106	0.053509054
ENSMUSG00000022678	Nde1	nudE neurodevelopment protein 1 [Source:MGI Symbol;Acc:MGI:1914453]	14.7272	15.8594	15.2729	15.7736	16.0756	14.9321	16.7237	15.013	15.7889	15.9041	14.4799	14.6902	14.8963	13.5894	0.929527443	0.966018836	16	8	24	0.666945743	0.887459929	24	12.68591709	0.022851457
ENSMUSG00000022679	Mpv17l	Mpv17 transgene, kidney disease mutant-like [Source:MGI Symbol;Acc:MGI:2135951]	17.3257	15.8004	17.415	18.4106	16.6649	15.9224	16.0478	14.1063	15.5537	14.2577	15.8621	14.9529	14.6875	15.2294	0.951846915	0.966018836	20	12	24	0.441051695	0.805393068	24	14.8013628	0.037453329
ENSMUSG00000022680	Pdxdc1	pyridoxal-dependent decarboxylase domain containing 1 [Source:MGI Symbol;Acc:MGI:1920909]	47.5661	44.6722	42.6583	44.0541	46.0063	46.1852	44.64	43.8709	42.6028	42.4474	43.9225	44.2121	44.8517	43.0924	0.008024668	0.192760771	4	8	24	1.45E-04	0.134670799	24	20.98772962	0.052700507
ENSMUSG00000022681	Ntan1	N-terminal Asn amidase [Source:MGI Symbol;Acc:MGI:108471]	73.8791	78.6154	70.7688	77.2368	75.4627	67.8018	77.3299	72.0845	85.4613	61.1859	82.0914	76.0594	81.7625	77.7226	0.881996584	0.966018836	4	20	24	0.743810828	0.916218012	24	2.365124409	0.03650603
ENSMUSG00000022682	Rrn3	RRN3 RNA polymerase I transcription factor homolog (yeast) [Source:MGI Symbol;Acc:MGI:1925255]	23.5712	20.8801	21.2126	21.5574	20.3695	23.2705	20.8446	22.8526	18.2169	25.3209	20.7968	21.7168	21.8791	23.104	0.570349842	0.966018836	24	20	24	0.822358163	0.944152607	24	21.78040789	0.028539839
ENSMUSG00000022684	Bfar	bifunctional apoptosis regulator [Source:MGI Symbol;Acc:MGI:1914368]	20.9183	21.9149	20.1353	21.3022	21.0064	21.7411	20.6386	21.5652	20.5865	22.3348	20.849	21.584	22.1458	21.0222	0.8972237	0.966018836	24	12	24	0.797022	0.935133209	24	20.47702718	0.011364472
ENSMUSG00000022685	Parn	poly(A)-specific ribonuclease (deadenylation nuclease) [Source:MGI Symbol;Acc:MGI:1921358]	10.6805	11.341	11.8694	11.438	11.0924	10.5983	10.7905	10.8314	11.6028	11.7795	10.4663	11.6977	11.2912	11.2778	0.067929624	0.430600538	16	4	24	0.0854678	0.703577714	24	9.097452928	0.046018407
ENSMUSG00000022686	B3gnt5	UDP-GlcNAc:betaGal beta-1,3-N-acetylglucosaminyltransferase 5 [Source:MGI Symbol;Acc:MGI:2137302]	7.16961	5.61218	4.08408	5.75689	7.86569	4.52177	7.19421	4.33713	6.44446	4.42149	8.14544	4.64305	6.01041	4.67714	0.351840494	0.870418653	20	12	24	0.405149393	0.799546792	24	18.58703399	0.158288973
ENSMUSG00000022687	Boc	biregional cell adhesion molecule-related/down-regulated by oncogenes (Cdon) binding protein [Source:MGI Symbol;Acc:MGI:2151153]	5.34093	5.05468	4.64926	4.4007	4.60476	5.0123	5.2573	4.29049	4.57518	4.672	4.30526	5.36916	4.60153	4.9862	0.168929279	0.640164678	24	20	24	0.044417805	0.628432994	24	22.94999073	0.082500253
ENSMUSG00000022696	Sidt1	SID1 transmembrane family, member 1 [Source:MGI Symbol;Acc:MGI:2443155]	0.491512	0.633331	1.00722	0.448675	0.551219	0.68714	0.59534	0.678746	0.733033	0.574077	0.4195	0.677216	0.52523	0.758679	0.058456626	0.404936103	12	8	24	0.173044109	0.768040178	24	5.937039998	0.094209789
ENSMUSG00000022698	Naa50	N(alpha)-acetyltransferase 50, NatE catalytic subunit [Source:MGI Symbol;Acc:MGI:1919367]	24.8254	26.3326	26.5263	26.3728	26.6096	24.3505	26.2674	25.9765	26.855	26.0753	26.9136	22.7871	23.5969	25.6094	0.004163919	0.140598674	20	4	24	0.025286273	0.586541342	24	9.821038473	0.065333532
ENSMUSG00000022701	Ccdc191	coiled-coil domain containing 191 [Source:MGI Symbol;Acc:MGI:1922661]	4.84768	3.40409	3.33203	2.76426	2.71142	2.14302	2.58289	2.40963	3.17063	2.93186	2.59556	2.68171	2.64598	3.50804	0.257978335	0.761738864	8	16	24	0.324498523	0.768040178	24	4.784959517	0.120759394
ENSMUSG00000022702	Hira	histone cell cycle regulator [Source:MGI Symbol;Acc:MGI:99430]	11.8236	12.8099	13.8181	13.1586	11.6963	12.3147	13.4182	13.4173	12.8261	14.0151	11.5227	12.753	12.4216	12.4959	0.013400636	0.233323991	16	4	24	0.106825972	0.71909525	24	7.546029224	0.065843122
ENSMUSG00000022704	Qtrt2	queuine tRNA-ribosyltransferase accessory subunit 2 [Source:MGI Symbol;Acc:MGI:1922194]	7.23815	7.87077	9.4282	9.47267	8.71872	10.7311	10.4759	8.24664	8.4645	9.47155	8.44389	7.35358	10.6226	10.2583	0.819898687	0.966018836	16	16	24	0.984344115	0.996767829	24	7.480051173	0.012421278
ENSMUSG00000022706	Mrpl40	mitochondrial ribosomal protein L40 [Source:MGI Symbol;Acc:MGI:1332635]	25.7265	26.9177	28.7492	26.9304	28.4855	27.527	27.0624	26.4729	25.871	26.6824	27.6765	26.508	25.604	27.9315	0.350544145	0.870418653	20	8	24	0.416012185	0.800710663	24	13.31549559	0.025441006
ENSMUSG00000022707	Gbe1	glucan (1,4-alpha-), branching enzyme 1 [Source:MGI Symbol;Acc:MGI:1921435]	13.6671	12.2449	15.7467	19.5074	11.9706	15.4618	15.3902	14.2279	12.1207	15.6703	15.5059	13.9906	15.8466	15.3303	0.904831057	0.966018836	16	16	24	0.763422955	0.92366044	24	13.35281273	0.051452519
ENSMUSG00000022708	Zbtb20	zinc finger and BTB domain containing 20 [Source:MGI Symbol;Acc:MGI:1929213]	5.15071	5.80077	6.0217	6.16323	6.01058	5.4583	5.68551	5.52964	6.49469	4.69371	6.45162	6.23249	5.97548	5.5581	0.481811994	0.966018836	12	20	24	0.814216947	0.940708369	24	13.52097558	0.027718205
ENSMUSG00000022710	Usp7	ubiquitin specific peptidase 7 [Source:MGI Symbol;Acc:MGI:2182061]	42.0498	41.1539	41.3092	43.5379	40.9652	42.7784	39.2725	41.6129	41.4259	44.2405	44.0616	41.3431	42.8502	43.7803	0.951846915	0.966018836	16	20	24	0.271002166	0.768040178	24	12.58977145	0.027292599
ENSMUSG00000022711	Pmm2	phosphomannomutase 2 [Source:MGI Symbol;Acc:MGI:1859214]	17.1125	16.7899	18.7851	18.9574	17.741	18.8298	16.4878	18.7721	18.6129	17.7028	17.7981	17.0225	19.245	18.4828	0.920858634	0.966018836	12	16	24	0.387378154	0.799546792	24	9.378748171	0.037678422
ENSMUSG00000022718	Dgcr8	DGCR8, microprocessor complex subunit [Source:MGI Symbol;Acc:MGI:2151114]	8.24544	8.99367	8.39558	8.89396	8.69454	8.879	8.06429	8.17402	9.0229	8.88469	9.02103	8.70667	8.14483	8.55268	0.031333566	0.324385159	16	12	24	0.04007313	0.616921492	24	13.06655221	0.047486044
ENSMUSG00000022721	Trmt2a	TRM2 tRNA methyltransferase 2A [Source:MGI Symbol;Acc:MGI:96270]	21.0896	20.2719	23.1286	19.4661	21.4784	21.4218	21.5733	22.7791	20.9973	19.9781	18.0729	20.0649	21.2458	18.1454	0.851656735	0.966018836	12	4	24	0.560350675	0.841060952	24	2.179962222	0.037699784
ENSMUSG00000022722	Arl6	ADP-ribosylation factor-like 6 [Source:MGI Symbol;Acc:MGI:1927136]	25.4944	24.8325	23.2201	23.1825	25.2441	23.242	22.0898	22.6108	24.5982	21.2404	26.2487	24.7541	24.9073	24.5253	0.265774856	0.773510818	20	20	24	0.608146246	0.864256764	24	20.53268265	0.034695154
ENSMUSG00000022723	Crybg3	beta-gamma crystallin domain containing 3 [Source:MGI Symbol;Acc:MGI:2676311]	5.3476	6.29576	5.97544	5.61387	6.16801	5.12525	5.39699	7.84303	5.38475	6.36783	6.51937	6.24925	6.40628	5.1268	0.8635847	0.966018836	20	4	24	0.909482081	0.974606327	24	9.687001214	0.026472159
ENSMUSG00000022724	Riox2	ribosomal oxygenase 2 [Source:MGI Symbol;Acc:MGI:1914264]	8.15051	7.66368	7.70641	7.78824	8.55929	7.9904	7.37373	7.8103	7.49357	7.0115	8.01949	7.41261	9.63942	7.84265	0.593560247	0.966018836	20	20	24	0.409505982	0.799546792	24	21.83283739	0.05253412
ENSMUSG00000022742	Cpox	coproporphyrinogen oxidase [Source:MGI Symbol;Acc:MGI:104841]	10.8479	11.6412	13.0505	11.2631	11.0285	12.9833	12.5154	10.7294	11.9942	12.3968	9.98488	10.4799	11.256	10.4624	0.960269811	0.967043964	12	20	24	0.813991455	0.940696408	24	8.159426745	0.028257572
ENSMUSG00000022744	Cldnd1	claudin domain containing 1 [Source:MGI Symbol;Acc:MGI:2447860]	43.8435	46.7301	49.1275	47.3289	47.8563	46.1689	51.7882	46.5437	43.5323	47.9962	45.2775	45.273	42.9768	43.8602	0.745199643	0.966018836	16	12	24	0.743461416	0.916215545	24	13.67043332	0.020710076

ENSMUSG00000022747	St3gal6	ST3 beta-galactoside alpha-2,3-sialyltransferase 6 [Source:MGI Symbol;Acc:MGI:1888707]	21.7317	22.3731	20.0229	23.656	20.4713	20.5753	19.6996	24.877	26.7813	24.689	23.1515	20.7733	23.8206	20.3774	0.772122688	0.966018836	16	8	24	0.206653905	0.768040178	24	9.189576826	0.091338033
ENSMUSG00000022748	Cmss1	cms small ribosomal subunit 1 [Source:MGI Symbol;Acc:MGI:1913747]	10.4016	11.9621	11.8852	10.8458	10.4428	10.9732	9.13765	11.919	13.5028	11.0613	10.1883	11.5826	12.8721	11.6131	0.080148772	0.458678242	12	8	24	0.109540813	0.72082227	24	6.661010049	0.103659808
ENSMUSG00000022749	Tbc1d23	TBC1 domain family, member 23 [Source:MGI Symbol;Acc:MGI:1914831]	21.8718	24.6061	21.0915	21.8022	23.6254	19.9299	25.6118	22.2207	25.573	20.1379	22.0017	21.4426	22.8548	23.4366	0.348367381	0.870418653	4	20	24	0.30046929	0.768040178	24	3.942066163	0.065565765
ENSMUSG00000022750	Klhl22	kelch-like 22 [Source:MGI Symbol;Acc:MGI:1337995]	20.1645	20.4527	21.9771	20.949	18.3264	20.6836	21.9408	22.181	20.5049	20.4581	18.7055	20.7599	20.2835	21.0088	0.142513744	0.585944911	8	12	24	0.052353992	0.65045829	24	4.471746231	0.065276148
ENSMUSG00000022751	Nit2	nitrilase family, member 2 [Source:MGI Symbol;Acc:MGI:1261838]	9.60745	12.6707	10.8381	15.5151	12.7296	12.0154	11.1488	9.87714	14.0352	11.1116	11.8564	12.6734	14.2352	12.1518	0.890176313	0.966018836	16	16	24	0.499912862	0.808791684	24	12.94062898	0.080814933
ENSMUSG00000022752	Tomm70a	translocase of outer mitochondrial membrane 70A [Source:MGI Symbol;Acc:MGI:106295]	51.2344	50.409	48.3455	49.9826	49.1369	47.9657	45.818	46.2106	44.8431	48.5446	50.9491	49.2794	49.6099	50.9102	0.915835339	0.966018836	20	16	24	0.665163324	0.886947643	24	18.67139296	0.022079366
ENSMUSG00000022754	Tmem45a	transmembrane protein 45a [Source:MGI Symbol;Acc:MGI:1913122]	1.35067	2.98286	2.76851	1.70337	1.80125	1.09747	1.26535	2.5763	1.49992	0.758558	1.17686	2.14157	2.16979	2.5064	0.046535188	0.375011045	8	8	24	0.101839034	0.713240751	24	4.407402176	0.283778865
ENSMUSG00000022756	Slc7a4	solute carrier family 7 (cationic amino acid transporter, y+ system), member 4 [Source:MGI Symbol;Acc:MGI:2146512]	5.49627	5.19952	6.20876	5.19025	5.92645	6.03558	5.99286	4.51609	4.31353	6.24254	4.82149	4.93345	4.89159	5.71268	0.860993221	0.966018836	16	16	24	0.743053322	0.916215545	24	17.17969617	0.044450665
ENSMUSG00000022757	Tfg	Trk-fused gene [Source:MGI Symbol;Acc:MGI:1338041]	73.5937	65.1944	63.9557	57.1341	64.2034	66.8015	66.2661	65.6072	60.4147	71.4446	65.9654	64.719	70.3384	67.9849	0.035806379	0.331814682	4	8	24	0.169610598	0.767660404	24	23.19197573	0.065040278
ENSMUSG00000022758	P2rx6	purinergic receptor P2X, ligand-gated ion channel, 6 [Source:MGI Symbol;Acc:MGI:1337113]	9.27415	8.06313	7.60339	8.18745	7.80839	8.26355	8.22356	8.18993	7.55315	9.28556	8.01199	8.14971	8.64414	9.03308	0.109040811	0.524035616	4	8	24	0.600020054	0.860011178	24	23.7966222	0.033750085
ENSMUSG00000022760	Thap7	THAP domain containing 7 [Source:MGI Symbol;Acc:MGI:1916259]	13.7979	10.557	8.60261	10.0287	10.3078	9.54836	10.1131	10.7502	11.1225	13.3533	11.6004	12.7794	12.4721	13.8131	0.885059765	0.966018836	8	4	24	0.71136965	0.904655191	24	23.95685443	0.055059525
ENSMUSG00000022761	Lztr1	leucine-zipper-like transcriptional regulator, 1 [Source:MGI Symbol;Acc:MGI:1914113]	67.5667	57.3002	58.4359	66.7116	63.424	66.6656	61.4381	65.5104	56.8619	64.2776	63.8127	62.4886	62.8402	60.9526	0.125573493	0.555618842	16	20	24	0.119138726	0.730142553	24	18.60766014	0.062490637
ENSMUSG00000022762	Ncam2	neural cell adhesion molecule 2 [Source:MGI Symbol;Acc:MGI:97282]	4.80745	5.75702	6.18947	5.5324	4.91033	5.33	5.82409	4.11167	5.33	4.524	5.43988	5.38811	4.4443	5.72692	0.887862353	0.966018836	12	16	24	0.916171491	0.976814265	24	9.51399789	0.024366507
ENSMUSG00000022763	Aifm3	apoptosis-inducing factor, mitochondrion-associated 3 [Source:MGI Symbol;Acc:MGI:1919418]	11.5059	12.9193	12.6548	10.1592	10.4777	11.1855	13.0028	9.50175	11.9578	10.5651	9.70465	11.4529	8.97009	10.5417	0.914360142	0.966018836	12	8	24	0.653590902	0.883312134	24	3.615438903	0.048526156
ENSMUSG00000022765	Snap29	synaptosomal-associated protein 29 [Source:MGI Symbol;Acc:MGI:1914724]	15.7113	14.5031	14.5839	14.6341	16.0455	15.5249	14.6301	14.8777	13.4264	14.7592	16.6219	14.1462	15.406	15.36	0.03782572	0.334069441	20	16	24	0.168765938	0.767020009	24	18.87012039	0.054695026
ENSMUSG00000022766	Serpind1	serine (or cysteine) peptidase inhibitor, clade D, member 1 [Source:MGI Symbol;Acc:MGI:96051]	25.0498	30.4952	23.5162	24.8607	31.1917	23.0956	29.8458	27.6311	33.4143	23.292	24.519	26.489	26.3493	22.6168	0.858266942	0.966018836	12	4	24	0.878314069	0.965980735	24	3.589647059	0.035309473
ENSMUSG00000022769	Sdf2l1	stromal cell-derived factor 2-like 1 [Source:MGI Symbol;Acc:MGI:2149842]	9.7905	5.53274	4.57026	5.11507	7.11717	6.01874	7.84708	6.13775	4.45045	4.5247	8.44491	6.63805	7.32781	5.7313	0.002087679	0.100255953	4	8	24	0.004169573	0.427431763	24	21.20758815	0.33832574
ENSMUSG00000022770	Dlg1	discs large MAGUK scaffold protein 1 [Source:MGI Symbol;Acc:MGI:107231]	14.0324	15.6864	14.5025	11.9454	15.3142	13.1489	15.8857	14.2445	16.5165	13.2344	14.0154	14.229	14.4731	15.4072	0.076247883	0.453790923	12	4	24	0.198601065	0.768040178	24	3.467327967	0.078129835
ENSMUSG00000022771	Ppil2	peptidylprolyl isomerase (cyclophilin)-like 2 [Source:MGI Symbol;Acc:MGI:2447857]	33.811	30.489	30.9053	30.433	31.3776	32.3044	30.6177	31.5681	30.2528	31.1645	31.3094	30.7963	30.8098	28.9367	0.305259556	0.811201458	24	12	24	0.072433826	0.679262305	24	19.82347437	0.03680433
ENSMUSG00000022772	Senp5	SUMO/sentrin specific peptidase 5 [Source:MGI Symbol;Acc:MGI:2443596]	21.0672	18.9292	19.2847	18.8145	19.4334	19.7336	18.6508	19.2444	17.6312	19.765	19.2273	19.8176	19.8973	20.6347	0.575202741	0.966018836	4	8	24	0.275992207	0.768040178	24	21.970886	0.037925528
ENSMUSG00000022773	Ypel1	yippee like 1 [Source:MGI Symbol;Acc:MGI:1913303]	5.08054	5.55897	5.57814	5.52761	5.23406	6.19233	4.43433	5.88332	5.78898	6.19951	6.17379	6.26142	6.03391	6.16673	0.832506215	0.966018836	24	4	24	0.511140048	0.814670619	24	12.07251266	0.044527283
ENSMUSG00000022774	Ncbp2	nuclear cap binding protein subunit 2 [Source:MGI Symbol;Acc:MGI:1915342]	36.1606	36.4952	33.6336	32.8496	35.9776	33.5696	35.056	33.7166	32.2277	31.7008	35.9799	35.3349	36.9725	35.2993	0.033553741	0.324908923	4	12	24	0.035951788	0.609176161	24	22.48560018	0.063833789
ENSMUSG00000022779	Top3b	topoisomerase (DNA) III beta [Source:MGI Symbol;Acc:MGI:1333803]	16.0297	18.6026	19.2009	17.1289	18.8725	18.0701	20.2793	19.5806	17.9181	19.0749	18.6224	17.0772	17.7455	17.1782	0.92721845	0.966018836	12	12	24	0.790438808	0.932883394	24	9.335743357	0.024970699
ENSMUSG00000022781	Pak2	p21 (RAC1) activated kinase 2 [Source:MGI Symbol;Acc:MGI:1339984]	14.2752	12.5106	12.345	13.1177	13.8564	12.8381	14.7827	11.9196	11.7226	13.9135	13.0089	13.2493	13.4859	12.63	0.038123942	0.334069441	4	8	24	0.081210758	0.697074851	24	19.60294015	0.076573424
ENSMUSG00000022787	Wdr53	WD repeat domain 53 [Source:MGI Symbol;Acc:MGI:1916230]	8.6043	9.86418	9.88596	9.09444	9.64948	9.43258	9.20891	9.82899	9.23195	10.529	9.25692	10.7627	10.3795	8.92708	0.886002469	0.966018836	24	4	24	0.881037504	0.966661503	24	14.8665981	0.016188556
ENSMUSG00000022788	Fgd4	FYVE, RhoGEF and PH domain containing 4 [Source:MGI Symbol;Acc:MGI:2183747]	3.9727	4.73132	4.25515	4.50373	4.73867	3.8721	4.79711	3.64516	4.37782	3.29118	4.78126	4.17709	3.78844	3.99394	0.897425765	0.966018836	20	12	24	0.802569098	0.936626753	24	17.08224834	0.03452441
ENSMUSG00000022789	Dnm1l	dynamin 1-like [Source:MGI Symbol;Acc:MGI:1921256]	109.044	102.581	97.5978	98.3385	97.8767	100.339	99.7528	93.9837	91.4602	100.091	103.305	100.804	100.496	106.513	0.277087842	0.789849983	4	8	24	0.244654834	0.768040178	24	21.88604247	0.042702157
ENSMUSG00000022790	Igsf11	immunoglobulin superfamily, member 11 [Source:MGI Symbol;Acc:MGI:2388477]	8.97687	7.2495	7.72828	7.68313	9.13055	8.03302	9.6333	7.82433	6.80216	8.64798	7.62261	8.38429	7.3374	6.9514	0.215256468	0.704989686	20	16	24	0.071482202	0.67819049	24	19.17777034	0.111175008
ENSMUSG00000022791	Tnk2	tyrosine kinase, non-receptor, 2 [Source:MGI Symbol;Acc:MGI:1858308]	26.5484	26.1313	24.7787	24.3075	21.7913	25.2696	28.395	28.1258	28.117	37.1268	25.0846	31.7619	31.0162	31.3069	0.496745696	0.966018836	4	16	24	0.439404666	0.805393068	24	4.537436234	0.074897762
ENSMUSG00000022792	Yars2	tyrosyl-tRNA synthetase 2 (mitochondrial) [Source:MGI Symbol;Acc:MGI:1917370]	10.6165	9.1767	9.00256	9.40566	9.3101	9.46428	10.1516	8.96608	8.94095	8.77694	9.74229	9.96202	9.61	9.8486	0.004140921	0.140598674	4	8	24	0.012497617	0.525280095	24	21.83480139	0.073977056
ENSMUSG00000022793	B4galt4	UDP-Gal:betaGlcNAc beta 1,4-galactosyltransferase, polypeptide 4 [Source:MGI Symbol;Acc:MGI:1928387]	8.45108	7.50071	8.36942	7.97721	7.71934	8.81579	8.62253	7.52188	8.22467	7.95872	7.68732	7.74679	8.70883	7.59156	0.265774856	0.773510818	4	4	24	0.459110923	0.805393068	24	21.77382622	0.036715335
ENSMUSG00000022797	Tfrc	transferrin receptor [Source:MGI Symbol;Acc:MGI:98822]	45.7671	60.8664	66.8193	51.3864	57.0458	51.0404	47.249	56.0664	58.7974	45.3622	50.3447	50.9995	53.7383	59.9703	0.072609122	0.446119062	12	12	24	0.11047029	0.722224739	24	6.950157556	0.131041052
ENSMUSG00000022799	Arhgap31	Rho GTPase activating protein 31 [Source:MGI Symbol;Acc:MGI:1333857]	13.7261	12.0828	11.361	13.1675	15.1099	13.0747	14.372	13.2406	12.4708	16.2961	11.3421	13.423	12.8923	11.7752	0.569060064	0.966018836	16	20	24	0.466457489	0.805393068	24	17.35263284	0.069360168
ENSMUSG00000022800	Fyttd1	forty-two-three domain containing 1 [Source:MGI Symbol;Acc:MGI:1917955]	35.3905	39.5179	37.3501	35.892	36.4096	36.6762	38.0104	33.3943	40.4754	34.8487	40.4857	39.7642	45.5435	41.0582	0.897425765	0.966018836	4	12	24	0.609343676	0.864393298	24	0.912498597	0.037726107
ENSMUSG00000022801	Lrch3	leucine-rich repeats and calponin homology (CH) domain containing 3 [Source:MGI Symbol;Acc:MGI:1917394]	11.1786	9.99906	10.993	9.39834	10.8483	11.5074	11.5183	10.671	10.1633	10.7503	10.1138	12.3747	10.0488	10.42	0.256524739	0.76168256	24	16	24	0.119482822	0.730508014	24	21.02279797	0.075282443
ENSMUSG00000022802	Lmln	leishmanolysin-like (metallopeptidase M8 family) [Source:MGI Symbol;Acc:MGI:2444736]	4.59914	4.4535	4.17243	5.06323	4.20822	4.90724	4.3406	4.43832	3.67065	5.4072	3.94452	4.90533	3.92211	4.23216	0.300823756	0.809362476	16	20	24	0.533036708	0.825678486	24	16.58559244	0.05608873
ENSMUSG00000022803	Popdc2	popeye domain containing 2 [Source:MGI Symbol;Acc:MGI:1930150]	0.55283	0.823979	0.85386	1.22911	0.950635	1.27348	0.820405	2.07441	1.25241	1.75215	1.03855	1.15132	1.27797	1.13563	0.92769718	0.966018836	16	12	24	0.363222569	0.788075604	24	9.979185863	0.123649571
ENSMUSG00000022807	Osbpl11	oxysterol binding protein-like 11 [Source:MGI Symbol;Acc:MGI:2146553]	6.91031	6.43838	6.36326	7.43551	6.42946	6.70441	7.26039	6.92382	6.03336	7.17661	6.40843	6.58155	5.92503	6.69243	0.934152439	0.966018836	16	20	24	0.900698076	0.971917343	24	15.53423035	0.014900169
ENSMUSG00000022808	Snx4	sorting nexin 4 [Source:MGI Symbol;Acc:MGI:1916400]	26.6987	28.2535	27.818	29.2436	27.791	26.8951	26.5626	26.2785	29.0165	27.1732	28.3691	27.9912	27.6177	27.7961	0.076942821	0.453790923	12	16	24	0.094911793	0.71175955	24	11.47782215	0.035802065
ENSMUSG00000022811	Zfp148	zinc finger protein 148 [Source:MGI Symbol;Acc:MGI:1332234]	10.1178	10.4256	9.85269	9.27466	10.8636	9.80853	10.6366	8.4899	9.85245	9.07585	10.3272	10.1423	10.4433	10.257	0.17847698	0.655480352	4	12	24	0.272289104	0.768040178	24	20.93117706	0.056753039
ENSMUSG00000022812	Gsk3b	glycogen synthase kinase 3 beta [Source:MGI Symbol;Acc:MGI:1861437]	41.9559	33.3999	35.7798	36.5162	33.1895	38.1661	36.6946	33.2205	29.1847	43.8658	35.1133	39.2329	37.1356	37.7544	0.351617883	0.870418653	24	12	24	0.454116589	0.805393068	24	19.75341029	0.074899823
ENSMUSG00000022814	Umps	uridine monophosphate synthetase [Source:MGI Symbol;Acc:MGI:1298388]	5.92514	5.08063	5.19955	5.69446	5.5223	5.47525	5.31472	5.1559	4.93527	5.23887	5.32241	5.14505	5.28554	5.24556	0.130633892	0.562427886	20	16	24	0.122678438	0.735333681	24	18.89505259	0.040894758
ENSMUSG00000022816	Fstl1	follistatin-like 1 [Source:MGI Symbol;Acc:MGI:102793]	544.98	469.367	461.476	455.471	496.341	515.443	446.358	477.23	377.885	486.292	489.499	469.159	464.381	463.133	0.195343269	0.673387182	20	16	24	0.073098908	0.680291765	24	19.87220156	0.09895685
ENSMUSG00000022817	Itgb5	integrin beta 5 [Source:MGI Symbol;Acc:MGI:96614]	17.4344	16.4521	14.0347	14.204	16.6157	16.3449	19.8334	18.416	17.697	16.7108	13.7567	15.9617	16.9778	14.6282	0.223774686	0.717349224	4	16	24	0.167590822	0.767020009	24	0.569974621	0.103036218
ENSMUSG00000022820	Ndufb4	NADH:ubiquinone oxidoreductase subunit B4 [Source:MGI Symbol;Acc:MGI:1915444]	6.03326	9.92037	6.2875	7.06781	7.97341	6.69202	7.80784	7.71327	12.2585	7.97069	9.91904	8.64792	6.98582	9.32709	0.883024237	0.966018836	20	8	24	0.417146181	0.801870615	24	8.935475859	0.125835286
ENSMUSG00000022822	Abcc5	ATP-binding cassette, sub-family C (CFTR/MRP), member 5 [Source:MGI Symbol;Acc:MGI:1351644]	93.8195	71.7062	68.8731	77.3353	87.6287	79.0518	96.8825	77.4875	72.2036	80.5983	83.6855	81.1338	86.5204	74.911	0.001920665	0.100255953	4	8	24	0.019781822	0.565108098	24	20.45608821	0.143684513
ENSMUSG00000022827	Rabl3	RAB, member RAS oncogene family-like 3 [Source:MGI Symbol;Acc:MGI:1914907]	12.5787	12.7816	12.4476	11.9483	11.8256	11.9386	10.7564	11.3097	10.4149	12.428	12.658	12.1327	12.4209	12.2568	0.730442039	0.966018836	8	16	24	0.807088439	0.938616703	24	18.08950425	0.022168568
ENSMUSG00000022828	Gtf2e1	general transcription factor II E, polypeptide 1 (alpha subunit) [Source:MGI Symbol;Acc:MGI:1921447]	11.5838	10.3978	10.0984	10.6026	10.9218	10.7161	10.3333	10.1184	9.56397	9.47487	10.5304	10.6929	10.376	9.85664	0.028632001	0.316608413	24	12	24	8.89E-04	0.319044294	24	20.30366604	0.073434673
ENSMUSG00000022829	Stxbp5l	syntaxin binding protein 5-like [Source:MGI Symbol;Acc:MGI:2443815]	2.26152	3.02571	2.49018	2.71551	2.71891	2.55819	2.43397	2.0997	3.25257	1.8531	3.03742	3.36014	2.98873	3.22287	0.890235289	0.966018836	24	4	24	0.952568364	0.988472181	24	22.68980981	0.022118514
ENSMUSG00000022831	Hcls1	hematopoietic cell specific Lyn substrate 1 [Source:MGI Symbol;Acc:MGI:104568]	1.88099	3.44164	4.06862	3.33855	3.2896	2.7789	2.64826	3.45371	4.04571	2.0966	2.50848	2.72205	3.39196	2.50914	0.135537027	0.574447137	12	12	24	0.266632713	0.768040178	24	7.956050996	0.158985448
ENSMUSG00000022833	Ccdc14	coiled-coil domain containing 14 [Source:MGI Symbol;Acc:MGI:2443448]	1.0521	1.42452	1.26024	1.48395	1.34049	1.40856	1.21607	1.45068	1.60455	1.20802	1.30587	1.55605	1.51036	1.5699	0.912923872	0.966018836	8	8	24	0.604126154	0.861768549	24	7.272624835	0.035335493
ENSMUSG00000022836	Mylk	myosin, light polypeptide kinase [Source:MGI Symbol;Acc:MGI:894806]	4.02688	3.85983	3.98544	4.36801	4.19954	3.93894	3.91465	4.77477	4.47194	3.96589	3.52853	3.35542	3.82655	3.40495	0.506183531	0.966018836	12	12	24	0.344193211	0.777690541	24	8.49810392	0.060394744
ENSMUSG00000022837	Iqcb1	IQ calmodulin-binding motif containing 1 [Source:MGI Symbol;Acc:MGI:2443764]	19.4056	22.7969	20.0343	20.2851	19.6808	19.6268	20.1335	16.3861	22.5855	17.9192	21.9686	20.533	19.9562	20.5117	0.872380105	0.966018836	12	16	24	0.977982516	0.994453214	24	12.45052265	0.010066267
ENSMUSG00000022838	Eaf2	ELL associated factor 2 [Source:MGI Symbol;Acc:MGI:2146616]	0.763562	0.644663	0.76536	0.730702	0.82261	0.663873	0.424664	0.553966	0.896638	0.587021	1.03298	0.669816	0.683059	0.585143	0.109247218	0.524035616	20	12	24	0.153538487	0.76001013	24	13.93756794	0.091785937
ENSMUSG00000022840	Adcy5	adenylate cyclase 5 [Source:MGI Symbol;Acc:MGI:99673]	7.94804	7.19649	7.14371	7.32393	6.7992	7.57738	7.55844	8.05359	6.46981	9.3372	6.42532	8.36878	6.84858	8.09668	0.83638114	0.966018836	24	20	24	0.921400242	0.979266762	24	1.713167851	0.021514751
ENSMUSG00000022841	Ap2m1	adaptor-related protein complex 2, mu 1 subunit [Source:MGI Symbol;Acc:MGI:1298405]	42.1744	39.0782	37.9574	42.0588	41.3112	38.8739	37.4834	38.9526	36.3902	38.9406	39.5616	42.0204	39.7306	39.9265	0.593560247	0.966018836	20	16	24	0.207510998	0.768040178	24	18.6080323	0.043812606
ENSMUSG00000022842	Ece2	endothelin converting enzyme 2 [Source:MGI Symbol;Acc:MGI:1101356]	33.5829	31.4348	27.775	30.6948	31.9146	33.6676	30.7755	29.6853	25.4329	33.4427	27.8886	31.9229	31.0481	31.4807	0.021795836	0.26698364	24	12	24	0.12471557	0.738641389	24	20.6617028	0.088279846
ENSMUSG00000022843	Clcn2	chloride channel, voltage-sensitive 2 [Source:MGI Symbol;Acc:MGI:105061]	19.0461	21.6144	20.6046	26.5099	22.4398	18.8942	21.4808	19.4927	24.3123	22.5208	19.1182	18.5985	18.6069	18.9489	0.002466754	0.109177422	16	8	24	0.016232255	0.544099157	24	10.87415272	0.14293825
ENSMUSG00000022844	Pdia5	protein disulfide isomerase associated 5 [Source:MGI Symbol;Acc:MGI:1919849]	6.00643	5.40472	4.82343	4.99517	5.6606	5.74678	5.56764	5.97738	5.37079	6.12392	5.21986	5.77444	6.68251	5.87475	0.101520755	0.511659635	4	8	24	0.098457085	0.71175955	24	23.28023835	0.074758831
ENSMUSG00000022847	Thpo	thrombopoietin [Source:MGI Symbol;Acc:MGI:101875]	0.25549	0.636126	1.0182	0.490733	0.523741	0.762674	0.682181	0.541404	0.529163	0.593351	0.406015	0.360909	0.442063	0.525002	0.632729413	0.966018836	12	16	24	0.441768882	0.805393068	24	8.27927849	0.085154991
ENSMUSG00000022848	Slc49a4	solute carrier family 49 member 4 [Source:MGI Symbol;Acc:MGI:2387188]	25.4112	22.1	23.6636	23.6458	22.9071	24.6543	22.1383	26.1364	21.3131	27.4779	22.75	24.703	23.9692	24.1777	0.919571723	0.966018836	24	12	24	0.96299898	0.990726108	24	18.54380172	0.01107746
ENSMUSG00000022849	Hspbap1	Hspb associated protein 1 [Source:MGI Symbol;Acc:MGI:1913917]	4.59105	3.87688	3.74639	3.75738	3.76153	3.62586	3.85351	3.97798	3.05346	3.87563	3.94577	4.13709	4.02251	3.78017	0.062631097	0.410322149	4	8	24	0.12011577	0.731805836	24	21.82947064	0.077789254
ENSMUSG00000022853	Ehhadh	enoyl-Coenzyme A, hydratase/3-hydroxyacyl Coenzyme A dehydrogenase [Source:MGI Symbol;Acc:MGI:1277964]	2.89313	3.75399	4.29553	5.3166	3.70328	4.13316	3.29695	4.72279	3.94324	4.21068	3.93024	4.6604	5.30367	4.63176	0.933543173	0.966018836	16	4	24	0.45564977	0.805393068	24	9.789055341	0.086487733
ENSMUSG00000022855	Senp2	SUMO/sentrin specific peptidase 2 [Source:MGI Symbol;Acc:MGI:1923076]	28.6641	25.562	26.4571	24.9154	25.9348	27.0855	27.126	25.1259	24.4909	28.6548	26.6645	27.2804	25.9994	26.1056	0.144748194	0.589637022	24	12	24	0.229122871	0.768040178	24	19.78516127	0.045161758
ENSMUSG00000022856	Tmem41a	transmembrane protein 41a [Source:MGI Symbol;Acc:MGI:1919314]	17.4464	12.6841	13.1545	13.8863	15.2698	14.7705	14.3437	15.2354	11.0577	17.3256	15.8265	15.4493	13.7045	14.3654	0.101690661	0.511659635	20	16	24	0.162170334	0.76636332	24	18.69854569	0.121005313
ENSMUSG00000022858	Tra2b	transformer 2 beta [Source:MGI Symbol;Acc:MGI:106016]	17.1652	19.2541	20.1615	21.3095	18.9389	17.3877	18.422	17.2327	20.4735	17.0774	20.2877	15.8464	16.8287	17.6593	0.061755956	0.409814092	12	12	24	0.036817378	0.609176161	24	10.67293084	0.104634985
ENSMUSG00000022860	Chodl	chondrolectin [Source:MGI Symbol;Acc:MGI:2179069]	0.909544	1.24883	2.2336	1.17449	0.831905	1.479	2.135	1.20921	1.32964	1.20014	1.07503	0.948914	1.74605	[illegible]	0.152564468	0.704989686	12	8	24	0.305342929	0.768040178	24	5.680420979	0.152824127
ENSMUSG00000022861	Dgkg	diacylglycerol kinase, gamma [Source:MGI Symbol;Acc:MGI:105060]	9.70826	7.7378	7.9363	8.19796	7.05003	9.07929	7.91569	8.41411	6.16479	10.2973	7.0642	8.80269	9.08419	9.72891	0.590017078	0.966018836	12	8	24	0.54162515	0.830485817	24	23.56446096	0.080184475
ENSMUSG00000022863	Btg3	BTG anti-proliferation factor 3 [Source:MGI Symbol;Acc:MGI:109532]	1.24059	1.13874	0.871194	0.742871	0.667152	0.800719	0.869169	1.00218	0.703591	0.704601	1.26455	0.80387	0.872634	0.819454	0.19514372	0.673387182	4	12	24	0.308711291	0.768040178	24	0.151778137	0.063001318
ENSMUSG00000022864	D16Ertd472e	DNA segment, Chr 16, ERATO Doi 472, expressed [Source:MGI Symbol;Acc:MGI:1196400]	1.04914	1.2298	1.47539	1.32173	1.08537	1.189	1.35865	1.12628	1.79842	1.08013	1.32804	1.12089	1.35307	1.41573	0.136853693	0.578758359	12	4	24	0.127039098	0.739868076	24	7.498345033	0.095153716
ENSMUSG00000022865	Cxadr	coxsackie virus and adenovirus receptor [Source:MGI Symbol;Acc:MGI:1201679]	2.95188	4.2258	3.36434	2.8131	3.70843	3.19933	3.63926	3.63909	3.98547	2.25143	3.93103	3.49887	3.67147	4.30551	0.350544145	0.870418653	8	8	24	0.289077604	0.768040178	24	2.540710816	0.111956985
ENSMUSG00000022867	Usp25	ubiquitin specific peptidase 25 [Source:MGI Symbol;Acc:MGI:1353655]	20.7163	19.1265	18.461	18.4455	17.8369	19.5414	18.0161	19.7539	16.6874	20.0193	17.562	18.5541	19.4942	19.146	0.331157065	0.850959667	4	16	24	0.316493801	0.768040178	24	0.288536611	0.045957938
ENSMUSG00000022869	Samsn1	SAM domain, SH3 domain and nuclear localization signals, 1 [Source:MGI Symbol;Acc:MGI:1914992]	10.5531	10.7838	10.4778	11.0167	9.56417	10.619	9.65954	9.47946	9.62404	10.0276	11.1331	10.1855	10.5716	10.3731	0.858266942	0.966018836	16	20	24	0.801603402	0.936428041	24	17.09425433	0.019027558
ENSMUSG00000022878	Adipoq	adiponectin, C1Q and collagen domain containing [Source:MGI Symbol;Acc:MGI:106675]	7.37994	7.22045	14.0686	30.9521	9.96854	11.1407	10.771	20.8021	13.1665	25.6049	20.4583	16.0797	27.4654	21.8547	0.850398351	0.966018836	16	20	24	0.129921241	0.740483001	24	10.94160699	0.38009873
ENSMUSG00000022881	Rfc4	replication factor C (activator 1) 4 [Source:MGI Symbol;Acc:MGI:2146571]	5.89201	6.23694	6.29774	4.71599	4.70568	5.33802	6.07763	4.62141	5.90943	4.76017	5.18454	5.4977	6.91925	5.65076	0.437779403	0.966018836	8	12	24	0.072993479	0.6801051	24	1.962645354	0.172260029
ENSMUSG00000022883	Robo1	roundabout guidance receptor 1 [Source:MGI Symbol;Acc:MGI:1274781]	3.10131	3.26639	4.0348	2.92513	2.52839	3.23293	3.26499	3.18214	2.93294	3.62525	2.39791	3.39488	2.68111	3.40201	0.308023687	0.814993005	8	12	24	0.283751126	0.768040178	24	5.669483398	0.094026745
ENSMUSG00000022884	Eif4a2	eukaryotic translation initiation factor 4A2 [Source:MGI Symbol;Acc:MGI:106906]	495.825	543.087	492.989	510.984	490.523	473.51	487.003	465.306	519.614	440.946	523.42	493.62	524.43	511.968	0.896311169	0.966018836	8	16	24	0.738485087	0.914690529	24	3.268504197	0.024457519
ENSMUSG00000022885	St6gal1	beta galactoside alpha 2,6 sialyltransferase 1 [Source:MGI Symbol;Acc:MGI:108470]	39.5477	32.5089	29.3366	33.1841	39.0992	34.0605	40.4248	34.3635	30.3695	39.2116	32.6913	33.2396	35.8102	29.7445	0.018894761	0.256345714	4	8	24	0.147377468	0.756090042	24	19.8252616	0.113961101
ENSMUSG00000022887	Masp1	mannan-binding lectin serine peptidase 1 [Source:MGI Symbol;Acc:MGI:88492]	3.06225	3.67443	3.41955	3.89085	3.10603	3.8363	3.77392	3.65445	3.29999	3.88526	2.83993	4.00318	3.33723	3.96827	0.93717134	0.966018836	24	20	24	0.752040404	0.91939047	24	4.837994417	0.03685755
ENSMUSG00000022889	Mrpl39	mitochondrial ribosomal protein L39 [Source:MGI Symbol;Acc:MGI:1351620]	13.7838	12.5377	11.9414	11.8877	12.5153	11.5773	13.0064	12.8896	13.2297	13.3978	15.775	15.2016	15.3743	14.458	0.870491976	0.966018836	4	12	24	0.446298838	0.805393068	24	21.8508368	0.047952988
ENSMUSG00000022890	Atp5j	ATP synthase, H+ transporting, mitochondrial F0 complex, subunit F [Source:MGI Symbol;Acc:MGI:107777]	185.853	220.155	220.805	228.83	208.445	203.48	204.973	227.523	233.933	180.667	205.733	187.13	217.5	210.782	0.019280932	0.257669847	12	12	24	0.130542392	0.740728512	24	7.115362059	0.090030722
ENSMUSG00000022892	App	amyloid beta (A4) precursor protein [Source:MGI Symbol;Acc:MGI:88059]	228.077	190.582	211.184	189.249	188.057	227.04	227.963	211.735	171.799	256.578	171.481	210.561	183.043	210.723	0.891105762	0.966018836	4	16	24	0.777703561	0.928689964	24	23.23526759	0.048749472
ENSMUSG00000022893	Adamts1	a disintegrin-like and metallopeptidase (reprolysin type) with thrombospondin type 1 motif, 1 [Source:MGI Symbol;Acc:MGI:109249]	4.77275	3.58222	4.10374	4.32167	4.4722	3.85156	4.10886	5.34911	4.4883	4.17855	3.82565	3.81287	3.65347	3.55132	0.915475528	0.966018836	12	20	24	0.835447129	0.949499418	24	9.05287108	0.032114073
ENSMUSG00000022894	Adamts5	a disintegrin-like and metallopeptidase (reprolysin type) with thrombospondin type 1 motif, 5 (aggrecanase-2) [Source:MGI Symbol;Acc:MGI:1346321]	8.15263	9.60205	8.6494	8.02473	10.1771	9.32111	9.15071	9.59283	10.5632	9.07423	10.0115	10.7404	9.61412	9.18922	0.893307667	0.966018836	20	20	24	0.808270128	0.939135535	24	18.24211356	0.026258483
ENSMUSG00000022895	Ets2	E26 avian leukemia oncogene 2, 3' domain [Source:MGI Symbol;Acc:MGI:95456]	5.36827	5.99128	6.22185	5.68452	5.52465	5.83875	5.9541	6.09983	5.74298	6.77689	5.07378	5.1487	5.7247	5.62468	0.081336319	0.462794877	12	8	24	0.143361088	0.752803875	24	7.625365963	0.070872756
ENSMUSG00000022897	Dyrk1a	dual-specificity tyrosine-(Y)-phosphorylation regulated kinase 1a [Source:MGI Symbol;Acc:MGI:1330299]	11.5173	10.6156	11.9162	11.1715	11.3112	11.8799	11.6068	11.3888	10.7037	12.8145	10.3835	11.7512	11.0867	11.4076	0.94086481	0.966018836	24	20	24	0.92405751	0.979741358	24	15.89321261	0.011534574
ENSMUSG00000022898	Vps26c	VPS26 endosomal protein sorting factor C [Source:MGI Symbol;Acc:MGI:1206040]	21.1428	20.263	20.4568	22.1713	21.5211	20.5789	21.4499	18.1278	20.8418	18.1307	19.9477	19.901	21.1211	19.9125	0.939921059	0.966018836	4	4	24	0.574190141	0.847227916	24	19.59002579	0.032970029
ENSMUSG00000022899	Slc15a2	solute carrier family 15 (H+/peptide transporter), member 2 [Source:MGI Symbol;Acc:MGI:1890457]	6.32761	7.66411	9.71706	11.912	6.37221	7.98399	16.0887	8.01284	6.64425	5.99957	10.1724	7.08158	5.94381	8.43429	0.807813307	0.966018836	8	20	24	0.998199702	0.999671555	24	5.77456084	0.009281975
ENSMUSG00000022901	Cd86	CD86 antigen [Source:MGI Symbol;Acc:MGI:101773]	0.732089	0.77187	1.17757	1.22828	1.21542	0.760012	0.774024	1.26465	1.426	0.671195	0.949456	0.761454	0.885308	1.13179	0.071575474	0.440262583	12	12	24	0.087395983	0.707460769	24	9.001548317	0.151168322
ENSMUSG00000022905	Kpna1	karyopherin (importin) alpha 1 [Source:MGI Symbol;Acc:MGI:103560]	33.5878	33.4479	33.4305	33.2527	33.4922	32.8038	34.2717	32.6733	33.5378	33.0625	35.0309	33.9744	35.876	35.4496	0.305564675	0.811201458	4	12	24	0.456945467	0.805393068	24	0.031252914	0.015333588
ENSMUSG00000022906	Parp9	poly (ADP-ribose) polymerase family, member 9 [Source:MGI Symbol;Acc:MGI:1933117]	4.87309	4.73797	4.44858	5.02866	5.8083	4.77379	4.48704	5.00927	5.41632	3.93528	5.35848	4.77088	4.44475	5.06029	0.743750765	0.966018836	20	8	24	0.766803314	0.92458474	24	15.47516289	0.034108842
ENSMUSG00000022911	Arl13b	ADP-ribosylation factor-like 13B [Source:MGI Symbol;Acc:MGI:1915396]	6.09678	7.08614	5.91434	7.03755	8.13647	5.25044	7.45237	5.98221	6.40838	6.21933	6.05921	6.95006	6.93258	6.62944	0.880992536	0.966018836	20	16	24	0.960108837	0.989397471	24	18.411956	0.016887394
ENSMUSG00000022912	Pros1	protein S (alpha) [Source:MGI Symbol;Acc:MGI:1095733]	9.80734	10.4691	11.1758	9.37104	10.3894	9.20169	10.3224	10.2457	11.0477	8.56537	9.82008	8.98191	9.66782	9.80898	0.01130565	0.204837764	12	12	24	0.154930921	0.760294912	24	5.719651725	0.069023728
ENSMUSG00000022913	Psmg1	proteasome (prosome, macropain) assembly chaperone 1 [Source:MGI Symbol;Acc:MGI:1860263]	13.2053	11.6354	11.9427	12.1343	10.3952	9.81491	10.6507	11.2118	12.7284	10.1157	11.9443	10.5466	12.4492	12.4667	0.331157065	0.850959667	12	12	24	0.186650852	0.768040178	24	5.398330581	0.087750068
ENSMUSG00000022914	Brwd1	bromodomain and WD repeat domain containing 1 [Source:MGI Symbol;Acc:MGI:1890651]	15.8416	17.317	18.3315	16.9635	15.9232	16.3374	16.9803	16.7629	18.3527	14.4227	17.1337	17.3739	18.2499	16.9469	0.676344318	0.966018836	12	4	24	0.399941338	0.799546792	24	4.328539847	0.046747523
ENSMUSG00000022935	Grik1	glutamate receptor, ionotropic, kainate 1 [Source:MGI Symbol;Acc:MGI:95814]	100.842	83.9681	80.1004	83.1351	76.9769	94.8813	80.0187	90.4057	72.846	99.1027	77.24	94.3863	97.0022	94.0251	0.209352197	0.69936851	4	16	24	0.282610623	0.768040178	24	23.20170253	0.093310544
ENSMUSG00000022940	Pigp	phosphatidylinositol glycan anchor biosynthesis, class P [Source:MGI Symbol;Acc:MGI:1860433]	40.8474	49.1802	37.2884	47.2195	41.178	39.0965	37.4552	40.5496	47.6374	35.0214	43.176	35.4116	38.7791	36.6524	0.916556573	0.966018836	12	12	24	0.605760701	0.862797371	24	9.165744215	0.057785606
ENSMUSG00000022945	Chaf1b	chromatin assembly factor 1, subunit B (p60) [Source:MGI Symbol;Acc:MGI:1314881]	1.0617	0.838195	1.19605	1.09723	1.02918	1.09054	1.13319	0.922929	1.39305	0.951901	0.687912	0.814171	0.759989	0.894585	0.418381424	0.963884303	12	8	24	0.43277861	0.805393068	24	8.625438664	0.064338137
ENSMUSG00000022946	Dop1b	DOP1 leucine zipper like protein B [Source:MGI Symbol;Acc:MGI:1917278]	25.4139	23.2162	20.1422	21.7205	22.7433	25.6589	25.7651	24.4047	19.7513	27.5584	19.7425	23.9959	23.9589	23.0289	0.082282215	0.462922111	24	12	24	0.210314259	0.768040178	24	22.30387807	0.099041667
ENSMUSG00000022947	Cbr3	carbonyl reductase 3 [Source:MGI Symbol;Acc:MGI:1309992]	8.4359	6.59425	7.07495	5.38966	6.35501	6.59077	6.06353	6.4666	7.43598	6.27586	7.78969	6.85268	7.60716	6.4591	0.959160181	0.967043964	4	12	24	0.551552605	0.83632237	24	23.44581418	0.063108695
ENSMUSG00000022948	Setd4	SET domain containing 4 [Source:MGI Symbol;Acc:MGI:2136890]	5.02825	3.73002	4.91217	2.70429	4.78807	4.06719	5.3453	5.72222	5.23613	3.6498	5.19792	4.21612	4.65639	3.90003	0.702630284	0.966018836	4	12	24	0.467193696	0.805393068	24	0.589114803	0.111929626
ENSMUSG00000022949	Clic6	chloride intracellular channel 6 [Source:MGI Symbol;Acc:MGI:2146607]	0.484215	0.610823	0.694797	0.800612	0.588993	0.685867	0.726727	0.593912	0.83672	0.508701	0.760355	0.711902	0.649834	0.617927	0.874473208	0.966018836	12	20	24	0.635885417	0.876315013	24	12.23916061	0.03348455
ENSMUSG00000022951	Rcan1	regulator of calcineurin 1 [Source:MGI Symbol;Acc:MGI:1890564]	33.1029	27.0912	28.4364	31.3155	30.8452	32.7679	29.0742	29.8125	26.472	30.8952	30.6566	29.599	31.0626	28.614	0.108253011	0.524035616	16	20	24	0.032007567	0.604838544	24	18.7281299	0.08637212
ENSMUSG00000022952	Runx1	runt related transcription factor 1 [Source:MGI Symbol;Acc:MGI:99852]	13.7051	11.3933	10.737	12.0804	10.3454	11.8609	10.0916	11.5978	9.85502	16.4735	10.8032	12.2899	13.8552	12.582	0.883024237	0.966018836	4	8	24	0.987304749	0.997443552	24	20.69805482	0.012469012
ENSMUSG00000022956	Atp5o	ATP synthase, H+ transporting, mitochondrial F1 complex, O subunit [Source:MGI Symbol;Acc:MGI:106341]	129.901	152.473	154.544	152.872	150.675	144.746	130.274	152.967	163.602	130.119	163.434	141.189	164.489	151.687	0.46238066	0.966018836	12	12	24	0.469244574	0.805393068	24	9.021383103	0.058002051
ENSMUSG00000022957	Itsn1	intersectin 1 (SH3 domain protein 1A) [Source:MGI Symbol;Acc:MGI:1338069]	15.2292	12.9651	14.8674	15.0315	15.5203	13.4698	14.2439	14.2582	13.1838	15.1764	14.1277	13.21	14.6167	14.4388	0.849179819	0.966018836	16	8	24	0.616661897	0.867682235	24	13.53511595	0.030328275
ENSMUSG00000022960	Donson	downstream neighbor of SON [Source:MGI Symbol;Acc:MGI:1890621]	8.1156	6.74866	6.16439	6.38514	6.17422	5.83881	6.09281	6.08674	7.44252	6.60993	6.80149	6.31809	6.87885	8.18766	0.907000607	0.966018836	4	20	24	0.370974836	0.792839127	24	4.627705089	0.072804517
ENSMUSG00000022961	Son	Son DNA binding protein [Source:MGI Symbol;Acc:MGI:98353]	122.989	140.787	137.478	133.225	139.468	122.385	133.837	128.831	145.956	125.711	134.47	119.941	122.462	128.99	0.010468194	0.196621011	12	12	24	0.074275394	0.683293901	24	9.240559381	0.070896226

ENSMUSG00000022962	Gart	phosphoribosylglycinamide formyltransferase [Source:MGI Symbol;Acc:MGI:95654]	13.8445	12.5108	12.742	14.8461	13.8442	13.9005	14.0822	14.9356	10.4311	16.6937	11.3568	12.7143	13.2233	12.4166	0.920872048	0.966018836	16	20	24	0.848519693	0.954456288	24	17.37695973	0.036818189
ENSMUSG00000022964	Tmem50b	transmembrane protein 50B [Source:MGI Symbol;Acc:MGI:1925225]	75.5842	62.013	62.1604	65.2166	61.2955	68.9782	65.4989	69.0454	56.2138	72.3255	71.1681	65.1319	69.5543	66.4215	0.643325306	0.966018836	4	8	24	0.325685857	0.768040178	24	20.78165857	0.066343922
ENSMUSG00000022965	Ifngr2	interferon gamma receptor 2 [Source:MGI Symbol;Acc:MGI:107654]	26.3004	23.5542	26.9015	24.839	28.5276	26.1279	25.0266	24.4411	20.3405	24.9847	27.1562	22.9351	28.7055	24.6194	0.285193697	0.793714531	20	12	24	0.287349394	0.768040178	24	18.92508366	0.079161262
ENSMUSG00000022967	Ifnar1	interferon (alpha and beta) receptor 1 [Source:MGI Symbol;Acc:MGI:107658]	18.944	18.5795	17.1141	18.1643	18.0257	18.6251	17.643	18.3661	18.1496	19.2486	18.4233	19.9122	19.642	18.8032	0.148073804	0.590732968	24	12	24	0.288793377	0.768040178	24	21.15661207	0.029917704
ENSMUSG00000022969	Il10rb	interleukin 10 receptor, beta [Source:MGI Symbol;Acc:MGI:109380]	41.2173	36.2139	32.0998	33.9052	36.9657	39.6222	37.6602	39.1383	32.1004	39.0602	38.0765	38.0251	39.3132	37.1613	0.009640466	0.194720062	4	8	24	0.017075504	0.547196997	24	21.37444291	0.107253047
ENSMUSG00000022971	Ifnar2	interferon (alpha and beta) receptor 2 [Source:MGI Symbol;Acc:MGI:1098243]	17.5638	19.571	16.8882	17.1098	18.103	16.6612	17.3154	17.8117	20.0625	17.1044	19.6019	18.6419	18.9703	19.2844	0.952127548	0.966018836	8	8	24	0.636549968	0.876483853	24	5.146326243	0.029748248
ENSMUSG00000022972	Cfap298	cilia and flagella associate protien 298 [Source:MGI Symbol;Acc:MGI:1915251]	18.5056	15.019	14.7547	17.6533	12.9352	15.8717	16.0162	14.9753	18.6519	14.954	17.4323	18.4439	16.4115	16.3188	0.935593607	0.966018836	4	16	24	0.880272654	0.966381821	24	23.1276986	0.029153689
ENSMUSG00000022973	Synj1	synaptojanin 1 [Source:MGI Symbol;Acc:MGI:1354961]	47.2724	41.5662	47.0286	44.3084	40.45	47.5346	48.7531	45.8227	38.9981	58.9162	38.718	44.7964	41.6037	46.8516	0.92655264	0.966018836	4	16	24	0.971159406	0.993012791	24	6.544770606	0.015778166
ENSMUSG00000022974	Paxbp1	PAX3 and PAX7 binding protein 1 [Source:MGI Symbol;Acc:MGI:1914617]	22.2175	24.6672	25.0477	23.2899	24.5198	21.0477	24.8081	22.0772	27.6462	19.1405	25.2058	21.2894	23.1504	24.2986	0.148073804	0.590732968	12	12	24	0.502326541	0.809544549	24	6.824610269	0.062290251
ENSMUSG00000022978	Mis18a	MIS18 kinetochore protein A [Source:MGI Symbol;Acc:MGI:1913828]	4.55442	4.11154	3.81124	3.97362	3.86747	4.3816	3.92911	3.52851	4.37207	3.86472	5.35594	4.53842	4.73143	3.9785	0.246839642	0.745922296	4	4	24	0.189863783	0.768040178	24	19.16827568	0.087931916
ENSMUSG00000022982	Sod1	superoxide dismutase 1, soluble [Source:MGI Symbol;Acc:MGI:98351]	146.391	141.463	138.66	138.069	119.264	123.753	131.882	140.971	133.258	129.497	143.914	126.312	143.123	137.851	0.236246476	0.7385769	4	20	24	0.147144691	0.75567997	24	4.48152697	0.065185803
ENSMUSG00000022983	Scaf4	SR-related CTD-associated factor 4 [Source:MGI Symbol;Acc:MGI:2146350]	8.26127	9.10762	7.21701	9.81366	10.2472	8.33833	8.67564	8.41658	11.1001	9.78854	9.85443	9.89987	9.0666	9.83574	0.350544145	0.870418653	20	8	24	0.234893291	0.768040178	24	13.14227116	0.0822847
ENSMUSG00000022987	Zfp641	zinc finger protein 641 [Source:MGI Symbol;Acc:MGI:2442788]	3.71058	4.45888	4.2997	3.91967	4.23399	4.08005	4.12826	4.48184	4.8833	4.26008	4.35606	4.2165	4.10043	4.26009	0.031315549	0.324385159	8	20	24	0.103014643	0.713946463	24	8.27735694	0.059036467
ENSMUSG00000022992	Kansl2	KAT8 regulatory NSL complex subunit 2 [Source:MGI Symbol;Acc:MGI:1916862]	3.91595	3.58428	3.49237	4.91659	4.12394	3.81937	4.18044	3.37214	3.56976	4.35584	4.16806	4.33583	4.59288	4.10543	0.050447964	0.385880335	16	20	24	0.163743032	0.767020009	24	16.82569018	0.086417972
ENSMUSG00000022994	Adcy6	adenylate cyclase 6 [Source:MGI Symbol;Acc:MGI:87917]	10.2588	10.3485	10.1673	12.8177	11.6802	10.5085	12.6731	10.3482	10.4885	11.2019	10.2491	10.4012	10.9107	10.273	0.718086778	0.966018836	16	20	24	0.565351896	0.843471947	24	15.36616846	0.043589134
ENSMUSG00000022995	Enah	ENAH actin regulator [Source:MGI Symbol;Acc:MGI:108360]	24.6977	24.6076	24.4943	23.44	23.3888	23.1582	24.3872	25.049	20.5032	26.9485	22.891	24.3445	24.3189	26.6836	0.033553741	0.324908923	8	12	24	0.647596883	0.881007153	24	2.929356272	0.033771176
ENSMUSG00000022999	Lmbr1l	limb region 1 like [Source:MGI Symbol;Acc:MGI:1289247]	13.7371	16.147	14.9607	16.6437	18.8866	15.5894	14.3425	14.9845	18.8958	15.6873	16.633	16.0977	15.9101	15.5068	0.077161185	0.453790923	20	8	24	0.084262357	0.702277994	24	12.84699101	0.096741762
ENSMUSG00000023000	Dhh	desert hedgehog [Source:MGI Symbol;Acc:MGI:94891]	24.9074	19.9416	18.1008	20.2242	26.1218	21.1453	26.537	23.7562	20.9214	25.1582	24.5349	24.0148	24.1328	18.7194	0.079622943	0.458678242	20	16	24	0.085740939	0.703577714	24	19.31105609	0.14638409
ENSMUSG00000023004	Tuba1b	tubulin, alpha 1B [Source:MGI Symbol;Acc:MGI:107804]	201.203	178.674	169.893	182.734	191.626	180.895	187.246	179.41	154.932	173.924	179.217	177.078	170.782	170.936	0.187179226	0.662239322	20	16	24	0.015884436	0.544099157	24	20.14558509	0.078659052
ENSMUSG00000023007	Prpf40b	pre-mRNA processing factor 40B [Source:MGI Symbol;Acc:MGI:1925583]	22.5453	22.4978	22.8217	24.2559	23.4347	23.7986	22.2826	21.7287	27.0528	23.0297	24.7873	24.3024	25.1198	22.3904	0.082282215	0.462922111	20	12	24	0.347091663	0.778844143	24	13.98982406	0.043292061
ENSMUSG00000023008	Fmnl3	formin-like 3 [Source:MGI Symbol;Acc:MGI:109569]	2.88056	3.44666	3.73232	3.42273	3.77478	3.13158	3.79424	3.59771	3.87517	3.76724	2.91879	2.95573	3.33047	2.97249	0.351840494	0.870418653	12	12	24	0.163388577	0.767020009	24	9.105342666	0.088992506
ENSMUSG00000023009	Nckap5l	NCK-associated protein 5-like [Source:MGI Symbol;Acc:MGI:3609653]	3.69465	3.64631	4.79196	3.08613	3.12569	2.96138	3.61868	3.98404	4.99097	5.31215	3.66789	4.24372	4.01325	4.21364	0.361555248	0.885260695	12	8	24	0.049502565	0.642285999	24	7.65043538	0.173919345
ENSMUSG00000023010	Tmbim6	transmembrane BAX inhibitor motif containing 6 [Source:MGI Symbol;Acc:MGI:99682]	103.015	94.6102	101.516	100.007	99.8309	104.739	115.38	113.558	98.3819	124.35	93.7967	103.501	102.646	98.1565	0.665691261	0.966018836	24	20	24	0.993333185	0.998731933	24	1.652196564	0.005206162
ENSMUSG00000023011	Faim2	Fas apoptotic inhibitory molecule 2 [Source:MGI Symbol;Acc:MGI:1919643]	32.6873	30.9055	45.0571	32.4664	25.5446	38.6375	43.1181	32.5751	31.8012	41.8273	23.3614	34.2497	26.3405	39.963	0.858932988	0.966018836	12	8	24	0.48771971	0.805393068	24	5.531932839	0.135514672
ENSMUSG00000023015	Racgap1	Rac GTPase-activating protein 1 [Source:MGI Symbol;Acc:MGI:1349423]	2.62004	3.16889	3.56574	3.10512	2.50018	3.3026	3.26213	2.7455	2.87793	2.95223	3.11375	2.99545	2.77602	2.95175	0.860993221	0.966018836	24	4	24	0.854897614	0.956785307	24	8.873125265	0.024200488
ENSMUSG00000023017	Asic1	acid-sensing (proton-gated) ion channel 1 [Source:MGI Symbol;Acc:MGI:1194915]	21.4338	16.4909	16.395	18.2496	18.5157	18.0678	19.3823	20.6727	14.2423	24.0163	14.7419	18.9843	16.9726	18.507	0.793403332	0.966018836	4	8	24	0.829960542	0.947154789	24	21.52215137	0.048981941
ENSMUSG00000023018	Smarcd1	SWI/SNF related, matrix associated, actin dependent regulator of chromatin, subfamily d, member 1 [Source:MGI Symbol;Acc:MGI:1933623]	13.6143	13.8028	14.4204	15.6973	14.2071	14.3383	15.571	12.4237	13.3015	16.42	12.8407	15.6471	13.9137	15.1265	0.743750765	0.966018836	16	16	24	0.680309232	0.893103426	24	14.72703564	0.038200584
ENSMUSG00000023019	Gpd1	glycerol-3-phosphate dehydrogenase 1 (soluble) [Source:MGI Symbol;Acc:MGI:95679]	29.3502	23.157	50.282	121.6	50.5788	41.6323	50.9379	57.7695	50.5997	73.9482	96.8241	52.4978	80.3526	62.5042	0.823657258	0.966018836	16	20	24	0.049977408	0.643727973	24	12.92857548	0.439588946
ENSMUSG00000023020	Cox14	cytochrome c oxidase assembly protein 14 [Source:MGI Symbol;Acc:MGI:1913629]	20.8435	22.1397	19.927	19.8526	20.0133	20.5692	22.1307	22.5158	23.6712	19.9627	26.2768	22.9716	25.2008	23.4315	0.515451644	0.966018836	8	8	24	0.388441918	0.799546792	24	0.99950658	0.049954643
ENSMUSG00000023021	Cers5	ceramide synthase 5 [Source:MGI Symbol;Acc:MGI:1919199]	36.0576	37.3852	37.5127	36.9104	39.7494	37.8379	32.9412	37.6301	38.2549	35.0087	37.6804	38.212	39.8527	36.896	0.912923872	0.966018836	24	4	24	0.846023384	0.953396028	24	15.35169051	0.015716201
ENSMUSG00000023022	Lima1	LIM domain and actin binding 1 [Source:MGI Symbol;Acc:MGI:1920992]	8.99168	8.90553	9.37895	8.33726	9.58312	7.86303	8.99284	9.32207	10.777	7.57946	7.88375	7.94586	8.57951	8.74396	0.002786747	0.41072308	12	12	24	0.13754466	0.747972781	24	5.804355622	0.090556252
ENSMUSG00000023025	Larp4	La ribonucleoprotein domain family, member 4 [Source:MGI Symbol;Acc:MGI:2443114]	15.767	14.4464	14.6628	15.0167	14.5142	14.0294	14.3115	13.0046	12.7798	13.2174	16.3419	12.4773	12.5818	13.1698	0.977455189	0.977820713	20	4	24	0.313822344	0.768040178	24	16.47302086	0.054105568
ENSMUSG00000023026	Dip2b	disco interacting protein 2 homolog B [Source:MGI Symbol;Acc:MGI:2145977]	8.23142	7.46979	7.89751	7.97938	8.01881	7.98693	7.93506	7.05898	7.00216	8.87528	7.26531	7.40841	7.37086	7.51406	0.63897419	0.966018836	16	20	24	0.37492656	0.794931845	24	15.7875846	0.04246645
ENSMUSG00000023027	Atf1	activating transcription factor 1 [Source:MGI Symbol;Acc:MGI:1298366]	4.59096	5.10047	4.28584	5.14948	4.5477	4.56652	5.15024	4.60204	4.73582	4.41967	5.02691	5.15005	4.36736	4.21923	0.903207257	0.966018836	24	12	24	0.553084842	0.837286821	24	18.31365071	0.037967587
ENSMUSG00000023030	Slc11a2	solute carrier family 11 (proton-coupled divalent metal ion transporters), member 2 [Source:MGI Symbol;Acc:MGI:1345279]	11.6174	11.8018	10.8881	11.9072	11.1359	12.9079	10.7485	11.1203	11.5553	11.2207	10.554	11.2691	13.0186	12.0714	0.88565391	0.966018836	16	20	24	0.523102379	0.82106433	24	0.146205969	0.037288857
ENSMUSG00000023031	Cela1	chymotrypsin-like elastase family, member 1 [Source:MGI Symbol;Acc:MGI:95314]	8.47821	9.45068	9.79445	8.94019	8.11021	8.3049	8.78467	8.99399	9.27102	8.27774	9.59824	8.96429	9.31763	10.9722	0.088984621	0.475660262	12	4	24	0.046254625	0.633389716	24	5.748389627	0.087131809
ENSMUSG00000023032	Slc4a8	solute carrier family 4 (anion exchanger), member 8 [Source:MGI Symbol;Acc:MGI:1928745]	3.02649	3.03075	3.99746	3.04877	3.29576	3.45134	3.36614	3.69668	3.3141	3.42826	3.85307	3.80469	2.9698	5.2436	0.725061903	0.966018836	12	16	24	0.598223161	0.858819856	24	7.122016147	0.062820292
ENSMUSG00000023033	Scn8a	sodium channel, voltage-gated, type VIII, alpha [Source:MGI Symbol;Acc:MGI:103169]	16.126	14.2793	16.8935	15.069	13.4909	16.6047	16.2043	15.0648	12.4408	19.8854	12.426	15.485	14.3097	16.9844	0.843694678	0.966018836	16	4	24	0.9204306	0.978864885	24	4.148926042	0.028807505
ENSMUSG00000023034	Nr4a1	nuclear receptor subfamily 4, group A, member 1 [Source:MGI Symbol;Acc:MGI:1352454]	16.0748	14.2863	15.3621	17.6658	14.4358	14.6979	19.9202	18.1681	17.1675	22.6926	16.1807	16.9668	17.4858	14.035	0.912923872	0.966018836	16	16	24	0.30580732	0.768040178	24	11.4335093	0.10137609
ENSMUSG00000023036	Pcdhgc4	protocadherin gamma subfamily C, 4 [Source:MGI Symbol;Acc:MGI:1935203]	12.0057	10.9725	13.4128	12.4275	9.76177	13.5532	10.8709	10.5074	12.0152	13.6326	10.7524	13.2732	11.4796	13.1329	0.665691261	0.966018836	16	4	24	0.830717832	0.947596159	24	9.902647228	0.036368179
ENSMUSG00000023044	Csad	cysteine sulfinic acid decarboxylase [Source:MGI Symbol;Acc:MGI:2180098]	12.6177	13.5487	16.1018	18.8673	14.3364	14.0173	15.4223	17.0609	15.2022	18.8781	18.22	17.1168	16.5786	15.1418	0.115745527	0.537734429	16	12	24	0.039899495	0.616921492	24	12.14848036	0.132160361
ENSMUSG00000023046	Igfbp6	insulin-like growth factor binding protein 6 [Source:MGI Symbol;Acc:MGI:96441]	82.9295	85.9162	73.4519	61.0747	77.2072	65.1193	71.2447	88.416	125.745	49.2567	98.2626	89.1785	89.3142	83.1332	0.942429894	0.966018836	8	8	24	0.472761195	0.805393068	24	2.991258822	0.149504904
ENSMUSG00000023047	Amhr2	anti-Mullerian hormone type 2 receptor [Source:MGI Symbol;Acc:MGI:105062]	2.67523	2.11011	1.07091	3.34197	2.00187	2.50257	1.78193	2.57147	2.17365	2.09869	1.8452	1.17262	1.91139	1.75831	0.831226591	0.966018836	16	20	24	0.975355825	0.993762379	24	13.57364683	0.023957548
ENSMUSG00000023048	Prr13	proline rich 13 [Source:MGI Symbol;Acc:MGI:1913401]	106.822	80.5093	85.4197	83.3078	97.4393	104.025	91.9398	93.9764	67.5099	98.049	89.1245	89.4715	89.5544	86.1062	0.102109143	0.512431169	4	8	24	0.046080379	0.633009261	24	20.01744473	0.1499371
ENSMUSG00000023050	Map3k12	mitogen-activated protein kinase kinase kinase 12 [Source:MGI Symbol;Acc:MGI:1346881]	18.772	16.0993	17.327	16.3992	15.6201	18.154	16.9039	17.4498	14.9568	19.5543	16.1954	18.8213	17.6055	16.9939	0.300823756	0.809362476	24	20	24	0.490911971	0.805393068	24	21.37584992	0.05043947
ENSMUSG00000023051	Tarbp2	TARBP2, RISC loading complex RNA binding subunit [Source:MGI Symbol;Acc:MGI:103027]	8.52329	9.05877	8.86559	8.0463	7.73984	7.53588	8.59878	8.31651	8.27376	6.976	7.21731	7.59983	7.98902	8.98228	0.008747574	0.192760771	8	8	24	0.209499982	0.768040178	24	6.374671132	0.10862916
ENSMUSG00000023052	Npff	neuropeptide FF-amide peptide precursor [Source:MGI Symbol;Acc:MGI:1891708]	2.57897	3.42466	3.26747	3.49195	3.49828	2.33567	3.30305	3.13823	3.92127	2.52422	2.70977	1.9486	1.90161	3.08514	0.080164531	0.458678242	12	12	24	0.035387822	0.608560667	24	8.586001236	0.201448802
ENSMUSG00000023055	Calcoco1	calcium binding and coiled coil domain 1 [Source:MGI Symbol;Acc:MGI:1914738]	15.7926	17.1547	16.501	17.8472	15.7702	16.3016	16.2308	17.6674	17.5725	20.329	14.234	17.2266	15.9372	16.7825	0.020428812	0.261278501	16	4	24	0.166158905	0.767020009	24	8.739697619	0.082453819
ENSMUSG00000023064	Sncg	synuclein, gamma [Source:MGI Symbol;Acc:MGI:1298397]	2398.3	2365.87	2080.84	2146.49	2438.57	2368.31	2065.83	2076.22	2091.94	1764.92	2329.15	2168.33	2291.37	2168.11	0.256915694	0.76168256	20	20	24	0.088107009	0.707747429	24	21.05647702	0.099718674
ENSMUSG00000023066	Rttn	rotatin [Source:MGI Symbol;Acc:MGI:2179288]	1.05898	1.5154	1.58398	1.41754	1.57078	1.50928	1.89048	1.64911	1.5966	1.404	1.92532	1.59262	1.43711	1.33342	0.880631923	0.966018836	20	20	24	0.743762162	0.916218012	24	14.80633826	0.037219844
ENSMUSG00000023067	Cdkn1a	cyclin-dependent kinase inhibitor 1A (P21) [Source:MGI Symbol;Acc:MGI:104556]	73.1493	52.2538	46.0052	52.2962	65.5827	58.0748	75.3138	56.9651	51.307	58.8719	64.2116	61.5261	68.158	51.6891	3.29E-04	0.049770315	4	8	24	0.007986384	0.49654451	24	20.71277795	0.222485529
ENSMUSG00000023068	Nus1	NUS1 dehydrodolichyl diphosphate synthase subunit [Source:MGI Symbol;Acc:MGI:1196365]	27.8141	24.1198	23.7637	25.2673	25.9713	24.7642	24.2224	23.7631	22.3814	23.4327	26.1854	24.7292	26.4165	26.1542	0.080511813	0.4593055	4	8	24	0.121984246	0.734764914	24	20.66544151	0.064191472
ENSMUSG00000023072	Cep89	centrosomal protein 89 [Source:MGI Symbol;Acc:MGI:1919390]	6.07266	5.80805	4.83773	5.23629	6.74829	5.65211	4.86402	4.86969	5.15874	4.32814	5.70087	5.37111	5.73765	5.59242	0.332336445	0.853485917	20	20	24	0.185117138	0.768040178	24	20.27392336	0.097774828
ENSMUSG00000023074	Mospd1	motile sperm domain containing 1 [Source:MGI Symbol;Acc:MGI:1917630]	13.931	15.9626	14.517	15.5909	15.9376	14.4144	13.8684	15.3547	16.2029	13.4334	15.2875	13.6902	13.1648	14.4742	0.139992479	0.585202879	12	16	24	0.239171092	0.768040178	24	10.19850697	0.057940079
ENSMUSG00000023075	Akirin1	akirin 1 [Source:MGI Symbol;Acc:MGI:1915300]	32.8172	28.4019	29.0197	28.7693	29.4655	30.6436	27.2265	27.983	26.0376	29.3682	31.4241	29.3138	29.2582	29.2756	0.054878003	0.398010585	20	16	24	0.105363644	0.717252017	24	19.07375398	0.06421251
ENSMUSG00000023079	Gtf2ird1	general transcription factor II I repeat domain-containing 1 [Source:MGI Symbol;Acc:MGI:1861942]	6.79952	8.2246	7.12725	9.0123	8.25415	9.01433	7.98687	8.94844	6.50143	8.20975	7.65491	7.05182	7.63113	6.26227	0.623812175	0.966018836	16	20	24	0.455297144	0.805393068	24	16.57059534	0.072537037
ENSMUSG00000023084	Lrrc71	leucine rich repeat containing 71 [Source:MGI Symbol;Acc:MGI:1921735]	0.270767	0.592332	0.543066	0.552077	0.363827	0.32875	1.29358	0.486254	0.407682	0.388207	0.300818	0.972304	0.113366	0.662174	0.745118464	0.966018836	8	12	24	0.809441062	0.939184036	24	2.122432897	0.068971695
ENSMUSG00000023087	Noct	nocturnin [Source:MGI Symbol;Acc:MGI:109382]	5.12002	4.45025	5.01822	6.04784	5.27656	5.62195	5.63366	5.39787	4.93072	6.05313	5.69412	4.9146	5.03174	5.2416	0.129903614	0.560263689	16	20	24	0.131314218	0.741500219	24	14.60772424	0.076791475
ENSMUSG00000023088	Abcc1	ATP-binding cassette, sub-family C (CFTR/MRP), member 1 [Source:MGI Symbol;Acc:MGI:102676]	4.04338	3.68051	4.11638	4.54229	4.94385	4.56045	4.40143	4.13212	3.91753	5.44997	4.10376	4.51756	4.19293	4.15519	0.05945106	0.406067671	16	20	24	0.023869803	0.585033799	24	15.00421824	0.110297601
ENSMUSG00000023089	Ndufa5	NADH:ubiquinone oxidoreductase subunit A5 [Source:MGI Symbol;Acc:MGI:1915452]	189.154	216.801	203.09	212.081	212.996	187.193	201.742	199.647	239.872	189.27	222.144	197.223	222.621	227.236	0.601647512	0.966018836	12	12	24	0.262991217	0.768040178	24	7.845564801	0.067012752
ENSMUSG00000023092	Fhl1	four and a half LIM domains 1 [Source:MGI Symbol;Acc:MGI:1298387]	98.3198	115.601	131.957	126.734	116.082	117.756	101.901	158.341	120.553	138.167	104.468	101.425	116.421	110.983	0.057021923	0.386400877	12	16	24	0.064262043	0.663876076	24	8.834911208	0.166714949
ENSMUSG00000023094	Msrb2	methionine sulfoxide reductase B2 [Source:MGI Symbol;Acc:MGI:1923717]	26.9732	25.9103	23.2382	26.6456	26.9143	28.4087	26.363	26.2488	25.7424	24.0304	27.202	26.983	26.9462	28.838	0.004516345	0.140598674	24	12	24	0.079027497	0.690864635	24	21.39322542	0.064031403
ENSMUSG00000023104	Rfc2	replication factor C (activator 1) 2 [Source:MGI Symbol;Acc:MGI:1341868]	33.6516	32.1449	34.2561	31.9476	33.2827	32.4696	34.4596	31.7217	32.5087	28.7136	32.0782	32.3509	32.2792	31.7972	0.47028201	0.966018836	4	12	24	0.225080874	0.768040178	24	23.25031191	0.036986218
ENSMUSG00000023106	Denr	density-regulated protein [Source:MGI Symbol;Acc:MGI:1915434]	42.3435	42.7668	45.3795	42.3688	38.2991	41.0757	37.2532	37.5541	39.8851	40.3678	41.0474	40.0232	43.5766	40.6401	0.846643334	0.966018836	12	8	24	0.818806669	0.942446688	24	7.979879322	0.020456392
ENSMUSG00000023110	Prmt5	protein arginine N-methyltransferase 5 [Source:MGI Symbol;Acc:MGI:1351645]	20.7595	18.4206	20.9274	18.5916	20.6664	21.6729	20.7893	19.7345	17.6144	21.2531	18.0138	20.0045	19.4814	20.207	0.94086481	0.966018836	24	8	24	0.453964685	0.805393068	24	20.89794981	0.045757224
ENSMUSG00000023118	Sympk	symplekin [Source:MGI Symbol;Acc:MGI:1915438]	20.0555	18.155	17.9846	20.1484	19.6909	19.6799	21.7282	22.0944	19.0073	22.5659	19.6531	19.8275	18.6728	18.7397	0.94663657	0.966018836	16	20	24	0.796207701	0.93482476	24	16.74725221	0.02630545
ENSMUSG00000023132	Gzma	granzyme A [Source:MGI Symbol;Acc:MGI:109266]	0.278125	0.94792	0.423357	0.828444	0.457419	0.693105	0.542713	0.547435	1.12284	0.404289	0.76426	0.745539	0.889863	0.790795	0.882263435	0.966018836	8	20	24	0.722459726	0.908924547	24	6.946504578	0.064790736
ENSMUSG00000023143	Nagpa	N-acetylglucosamine-1-phosphodiester alpha-N-acetylglucosaminidase [Source:MGI Symbol;Acc:MGI:1351598]	6.14018	4.59541	4.31164	3.36742	4.94092	5.74529	4.47605	3.66759	3.57572	4.445	3.73389	3.99882	3.89095	3.5461	0.268242676	0.774280302	24	16	24	0.027927977	0.590904845	24	20.78250246	0.172167222
ENSMUSG00000023147	Get1	guided entry of tail-anchored proteins factor 1 [Source:MGI Symbol;Acc:MGI:2136882]	23.092	21.5659	22.6969	21.5818	21.7115	23.9622	21.9676	20.966	19.0012	21.8937	21.5065	21.8395	21.7476	23.5559	0.472013662	0.966018836	24	20	24	0.284678334	0.768040178	24	21.38101233	0.048441201
ENSMUSG00000023150	Ivns1abp	influenza virus NS1A binding protein [Source:MGI Symbol;Acc:MGI:2152389]	126.456	133.403	118.277	135.208	141.315	114.848	134.308	120.4	144.992	111.33	143.318	121.26	135.532	122.008	0.910436409	0.966018836	20	4	24	0.92851231	0.981515518	24	15.06966142	0.018978681
ENSMUSG00000023153	Tmem52	transmembrane protein 52 [Source:MGI Symbol;Acc:MGI:1916921]	0.737831	1.59903	1.62783	2.20629	1.74952	2.31131	1.45284	3.54973	2.13459	3.33816	1.21074	1.56163	1.40535	1.52131	0.018114172	0.256345714	16	12	24	0.207088331	0.768040178	24	10.11605703	0.251922111
ENSMUSG00000023156	Rpp14	ribonuclease P 14 subunit [Source:MGI Symbol;Acc:MGI:1914303]	33.0796	30.118	30.1144	29.9945	29.5244	29.6536	29.9546	29.4553	27.0355	29.1676	31.313	28.2707	33.1806	28.9604	0.510431574	0.966018836	4	8	24	0.24607983	0.768040178	24	22.07098334	0.049078359
ENSMUSG00000023169	Slc38a1	solute carrier family 38, member 1 [Source:MGI Symbol;Acc:MGI:2145895]	10.4613	9.88578	14.9271	9.58441	8.08376	10.9117	12.56	8.53439	9.80928	11.5186	8.82995	11.0625	8.14579	12.065	0.93357733	0.966018836	12	8	24	0.680956163	0.893402642	24	6.024432471	0.083532984
ENSMUSG00000023170	Gps2	G protein pathway suppressor 2 [Source:MGI Symbol;Acc:MGI:1891751]	9.31233	11.0291	12.7089	11.6353	13.1322	11.012	12.6242	11.2476	12.6866	11.3854	10.6815	11.5193	11.0468	9.93073	0.446277988	0.966018836	12	20	24	0.337507037	0.772425709	24	12.08647026	0.073012498
ENSMUSG00000023175	Bsg	basigin [Source:MGI Symbol;Acc:MGI:88208]	319.673	320.705	309.57	294.628	306.784	322.919	318.39	344.129	307.475	291.54	316.66	320.665	336.087	316.354	0.008244783	0.192760771	4	12	24	0.003677099	0.426426863	24	0.392031309	0.067634601
ENSMUSG00000023186	Vwa5a	von Willebrand factor A domain containing 5A [Source:MGI Symbol;Acc:MGI:1915026]	140.571	140.217	137.7	129.976	152.893	139.723	129.741	121.62	129.269	110.037	162.254	137.412	143.806	135.315	0.087978327	0.472021905	20	20	24	0.212984967	0.768040178	24	19.44526277	0.094095822
ENSMUSG00000023191	P3h3	prolyl 3-hydroxylase 3 [Source:MGI Symbol;Acc:MGI:1315208]	7.90951	9.70947	9.55262	7.60102	9.66562	8.57232	10.112	8.54807	11.5108	7.83427	9.97698	9.49786	8.23198	10.1981	0.951523604	0.966018836	12	4	24	0.733133626	0.91292127	24	6.211707188	0.050293471
ENSMUSG00000023206	Il15ra	interleukin 15 receptor, alpha chain [Source:MGI Symbol;Acc:MGI:104644]	3.96977	5.27324	6.12622	7.70517	4.86483	5.33345	4.73878	5.36321	6.51658	5.71301	5.55807	5.27322	5.08388	5.70106	1.57E-04	0.031913438	16	12	24	0.004748012	0.434006302	24	10.4103688	0.204794915
ENSMUSG00000023216	Epb42	erythrocyte membrane protein band 4.2 [Source:MGI Symbol;Acc:MGI:95402]	0.553308	1.24422	2.63738	1.56208	1.24789	1.50796	0.610087	0.974757	1.00383	0.840703	1.0906	0.830136	1.67311	0.833961	0.68887295	0.966018836	12	16	24	0.484163075	0.805393068	24	10.90613891	0.153709979
ENSMUSG00000023224	Serping1	serine (or cysteine) peptidase inhibitor, clade G, member 1 [Source:MGI Symbol;Acc:MGI:894696]	120.936	126.56	118.187	105.307	129.681	115.853	114.565	124.61	140.211	88.8832	124.535	114.544	115.21	111.218	0.961261938	0.967043964	12	4	24	0.690406716	0.897551603	24	1.456316877	0.051047227
ENSMUSG00000023232	Serinc2	serine incorporator 2 [Source:MGI Symbol;Acc:MGI:1919132]	13.3831	9.15915	9.07083	8.9288	11.0812	13.0285	10.9247	11.1607	8.60629	12.06	11.0206	10.5096	10.7497	9.45535	0.125855811	0.555618842	24	12	24	0.03058429	0.597890721	24	20.12151551	0.177676554
ENSMUSG00000023235	Ccl25	chemokine (C-C motif) ligand 25 [Source:MGI Symbol;Acc:MGI:1099448]	4.80128	5.3659	4.8156	5.68689	5.12809	4.41232	3.94334	4.9001	6.29531	4.73763	5.88659	5.52401	5.90334	5.65984	0.916556573	0.966018836	12	12	24	0.388723511	0.799546792	24	9.948560088	0.078683797
ENSMUSG00000023236	Scg5	secretogranin V [Source:MGI Symbol;Acc:MGI:98289]	63.5261	74.2361	76.0212	66.0086	63.9894	68.5637	70.2495	62.5233	75.0243	66.6868	65.5523	71.849	69.3252	78.5675	0.285027487	0.793714531	12	8	24	0.174049801	0.768040178	24	5.719435058	0.07272828
ENSMUSG00000023243	Kcnk5	potassium channel, subfamily K, member 5 [Source:MGI Symbol;Acc:MGI:1336175]	4.59528	4.26318	3.70814	3.91886	4.46983	3.70469	4.93053	4.2395	5.3733	3.75053	3.66451	4.78425	3.79735	4.12941	0.851656735	0.966018836	4	16	24	0.603685392	0.861540092	24	1.813738711	0.056479278
ENSMUSG00000023249	Parp3	poly (ADP-ribose) polymerase family, member 3 [Source:MGI Symbol;Acc:MGI:1891258]	16.071	14.0219	14.1488	13.7528	16.5375	12.9695	16.6758	18.2364	15.564	14.0341	14.7344	11.7128	14.7442	13.9098	0.675497492	0.966018836	4	20	24	0.578400161	0.849227023	24	4.250360021	0.062646749
ENSMUSG00000023259	Slc26a6	solute carrier family 26, member 6 [Source:MGI Symbol;Acc:MGI:2159728]	2.9039	2.57783	2.46794	2.81866	2.59574	2.56954	2.5756	2.74284	2.66639	2.39489	2.62499	2.72068	2.44115	2.76332	0.79625133	0.966018836	8	8	24	0.785743249	0.931158434	24	0.241748736	0.015958593
ENSMUSG00000023262	Acy1	aminoacylase 1 [Source:MGI Symbol;Acc:MGI:87913]	6.23569	5.34439	6.70858	5.07918	5.90804	5.92737	5.42521	6.48116	6.20671	5.06205	5.6859	5.70663	5.06627	4.51273	0.874672901	0.966018836	12	4	24	0.988431979	0.997559852	24	18.51715876	0.007679227
ENSMUSG00000023266	Frs3	fibroblast growth factor receptor substrate 3 [Source:MGI Symbol;Acc:MGI:2135965]	6.70887	6.60446	9.12649	4.30833	4.45812	6.14075	8.41656	6.99253	7.42478	7.27081	6.60577	7.23565	6.53819	7.13904	0.285027487	0.793714531	12	8	24	0.15253095	0.759088284	24	3.926494536	0.180980251
ENSMUSG00000023272	Creld2	cysteine-rich with EGF-like domains 2 [Source:MGI Symbol;Acc:MGI:1923987]	15.1343	7.68095	6.27745	7.35778	11.9558	8.07278	11.7582	8.96262	8.99605	6.46858	11.6114	6.9642	9.4064	7.32922	0.133766544	0.568189321	4	12	24	0.157067017	0.763141258	24	21.16045026	0.252895101
ENSMUSG00000023277	Twf2	twinfilin actin binding protein 2 [Source:MGI Symbol;Acc:MGI:1346078]	15.6007	15.0058	16.9195	15.5267	14.8252	15.3084	14.8399	21.2885	18.0904	17.2678	15.6874	16.0564	18.7686	16.1385	0.532553009	0.966018836	12	8	24	0.143654681	0.752803875	24	6.169679892	0.100546328
ENSMUSG00000023279	Bmp15	bone morphogenetic protein 15 [Source:MGI Symbol;Acc:MGI:1316745]	2.19937	1.84315	1.76943	1.81326	1.48213	1.62384	1.38635	1.56	1.62781	1.84936	1.4726	1.94141	1.67722	2.14127	0.93243477	0.966018836	8	12	24	0.615651557	0.867203276	24	4.255268293	0.049761451
ENSMUSG00000023284	Zfp605	zinc finger protein 605 [Source:MGI Symbol;Acc:MGI:2444933]	4.96781	4.71046	4.84366	5.37444	5.86694	4.7745	4.71088	4.87909	5.27713	4.00189	5.77037	4.59764	5.21863	4.96125	0.722296281	0.966018836	20	4	24	0.646163219	0.880190017	24	15.92778055	0.043589389
ENSMUSG00000023286	Ube2j2	ubiquitin-conjugating enzyme E2J 2 [Source:MGI Symbol;Acc:MGI:2153608]	13.7468	11.0047	12.1586	11.5341	10.1136	10.3985	10.8179	12.5709	14.0196	14.3047	12.6876	13.8125	11.8064	12.7827	0.93717134	0.966018836	16	4	24	0.464834666	0.805393068	24	8.132209405	0.072355399
ENSMUSG00000023307	Marchf5	membrane associated ring-CH-type finger 5 [Source:MGI Symbol;Acc:MGI:1915207]	33.2031	33.9943	36.5171	37.94	35.0527	35.1787	35.1773	34.7863	32.5083	36.9028	38.2042	33.0866	35.8985	37.549	0.530814489	0.966018836	20	4	24	0.286155147	0.768040178	24	12.26960282	0.043018789
ENSMUSG00000023328	Ache	acetylcholinesterase [Source:MGI Symbol;Acc:MGI:87876]	23.6563	20.2398	22.777	20.1805	16.9959	22.6748	24.6759	23.8398	19.9749	32.8667	18.9314	25.2068	22.5441	28.213	0.387380622	0.915479136	8	12	24	0.555573666	0.838702296	24	4.155156682	0.093013932
ENSMUSG00000023330	Dtwd1	DTW domain containing 1 [Source:MGI Symbol;Acc:MGI:1916435]	3.5671	4.50557	4.77169	3.40804	4.04084	3.62053	4.64342	3.77277	5.26834	3.41607	4.39981	3.68233	4.15333	3.92805	0.187699199	0.662239322	12	4	24	0.410818027	0.799546792	24	6.064259981	0.084400016
ENSMUSG00000023336	Wfdc1	WAP four-disulfide core domain 1 [Source:MGI Symbol;Acc:MGI:1915116]	5.21903	8.56818	7.03727	6.23362	7.56787	6.93636	6.33593	9.33971	10.1976	5.79669	7.31789	7.2169	7.66406	8.38249	0.215777641	0.704989686	8	8	24	0.187397488	0.768040178	24	6.204744324	0.157246076
ENSMUSG00000023341	Mx2	MX dynamin-like GTPase 2 [Source:MGI Symbol;Acc:MGI:97244]	2.05779	2.25457	3.41592	1.66011	2.70118	2.0536	2.2008	2.65277	2.63539	1.62396	3.28074	1.87598	2.7348	2.48911	0.338172159	0.85758369	12	12	24	0.764565784	0.92403577	24	6.112648889	0.069664768
ENSMUSG00000023345	Poc1a	POC1 centriolar protein A [Source:MGI Symbol;Acc:MGI:1917485]	1.35809	1.21437	0.766173	1.22265	1.10857	1.46173	1.2945	1.11451	1.09238	0.99475	1.07646	1.10467	1.12083	1.31147	0.093936711	0.490854203	4	8	24	0.081377743	0.697102286	24	22.27537206	0.098319751
ENSMUSG00000023348	Trip6	thyroid hormone receptor interactor 6 [Source:MGI Symbol;Acc:MGI:1343458]	6.48743	6.38345	6.2739	6.82368	6.81906	5.22858	6.62403	6.37443	7.63076	6.36618	6.20845	5.3593	6.73507	5.54855	0.913451828	0.966018836	4	20	24	0.299704258	0.768040178	24	8.86869151	0.076589104
ENSMUSG00000023353	Agap3	ArfGAP with GTPase domain, ankyrin repeat and PH domain 3 [Source:MGI Symbol;Acc:MGI:2183446]	64.6746	55.3916	54.5731	53.6213	53.3634	59.8036	60.6388	61.2007	54.5453	76.1031	50.5992	64.4063	57.7495	62.2853	0.128457847	0.560209134	4	16	24	0.79708357	0.935133209	24	0.818908303	0.039514891
ENSMUSG00000023367	Tmem176a	transmembrane protein 176A [Source:MGI Symbol;Acc:MGI:1913308]	81.5294	81.4168	73.9212	72.8363	94.9383	79.8917	80.449	87.9717	79.9308	71.8079	78.6078	73.2717	73.5756	70.6285	0.954744729	0.966018836	4	12	24	0.646928305	0.88064035	24	20.27748264	0.042010106
ENSMUSG00000023411	Nfatc4	nuclear factor of activated T cells, cytoplasmic, calcineurin dependent 4 [Source:MGI Symbol;Acc:MGI:1920431]	1.02248	1.76068	1.40068	1.39449	1.36829	1.16144	1.18381	1.78027	1.55289	1.17863	1.32011	1.27415	1.49042	1.68247	8.66E-04	0.071344007	8	16	24	0.029626161	0.596648309	24	6.314853894	0.131103925
ENSMUSG00000023439	Gnb3	guanine nucleotide binding protein (G protein), beta 3 [Source:MGI Symbol;Acc:MGI:95785]	1.29459	1.26243	1.04732	1.37554	1.4081	1.43806	1.56511	1.43397	1.16943	1.64993	1.63082	2.04192	1.1039	2.42665	0.390685537	0.915479136	24	12	24	0.813641637	0.940453305	24	19.45693739	0.044685128
ENSMUSG00000023452	Pisd	phosphatidylserine decarboxylase [Source:MGI Symbol;Acc:MGI:2445114]	20.4678	19.2181	20.7696	17.6073	21.7077	20.0818	24.0001	21.1538	20.4331	19.645	19.1949	18.5212	20.0007	18.6874	0.966321203	0.969386413	4	12	24	0.50393232	0.810542019	24	0.027509541	0.047573108
ENSMUSG00000023456	Tpi1	triosephosphate isomerase 1 [Source:MGI Symbol;Acc:MGI:98797]	257.135	260.726	268.115	270.769	277.993	286.187	248.793	295.362	274.812	262.764	271.709	257.947	271.689	261.555	0.5169953	0.966018836	20	8	24	0.745672072	0.91691176	24	12.0933123	0.020025473
ENSMUSG00000023460	Rab12	RAB12, member RAS oncogene family [Source:MGI Symbol;Acc:MGI:894284]	23.3644	27.4905	28.9346	29.7547	26.5025	25.5642	27.1187	30.7071	31.1338	30.9937	26.1233	27.5759	29.6551	26.9376	0.075513487	0.453550271	16	8	24	0.014192638	0.530991098	24	8.846023105	0.113793712
ENSMUSG00000023473	Celsr3	cadherin, EGF LAG seven-pass G-type receptor 3 [Source:MGI Symbol;Acc:MGI:1858236]	8.09412	8.4584	8.40545	9.06366	9.66426	7.04013	6.83009	8.35583	9.05579	8.50204	8.89998	7.65988	7.77846	7.98119	5.81E-04	0.070644749	20	4	24	0.013185441	0.529134128	24	11.32924667	0.12342325

ENSMUSG00000023484	Prph	peripherin [Source:MGI Symbol;Acc:MGI:97774]	674.735	537.302	516.081	531.659	553.729	598.194	527.159	579.544	475.767	602.188	620.049	607.428	636.69	587.758	0.045120335	0.368634624	24	12	24	0.075006845	0.68394125	24	20.80835388	0.114182852
ENSMUSG00000023495	Pcbp4	poly(rC) binding protein 4 [Source:MGI Symbol;Acc:MGI:1890471]	114.195	96.2653	98.0536	105.55	115.118	104.287	113.551	112.499	113.53	124.024	124.504	113.676	118.15	110.969	0.128262369	0.560209134	20	12	24	0.270257547	0.768040178	24	16.86114863	0.054690405
ENSMUSG00000023505	Cdca3	cell division cycle associated 3 [Source:MGI Symbol;Acc:MGI:1315198]	1.91436	1.85277	2.48062	2.48882	1.89243	2.32024	1.63467	1.92248	1.75791	1.38362	2.20382	1.56269	2.17984	1.81033	0.782779623	0.966018836	12	20	24	0.915979061	0.976753216	24	14.10122092	0.026079996
ENSMUSG00000023571	C1qtnf12	C1q and tumor necrosis factor related 12 [Source:MGI Symbol;Acc:MGI:1914639]	13.7105	14.4702	16.8379	16.8884	17.88	14.2846	15.7999	15.1286	15.7626	16.0579	12.0661	14.7517	13.8104	10.7325	0.174183691	0.650395613	16	12	24	0.257341803	0.768040178	24	12.90566176	0.099413297
ENSMUSG00000023572	Ccndbp1	cyclin D-type binding-protein 1 [Source:MGI Symbol;Acc:MGI:109595]	45.139	49.0333	47.7974	45.4767	45.2048	49.0839	45.2891	47.3882	43.646	48.3509	49.4251	48.4044	48.087	46.8626	0.835401638	0.966018836	24	12	24	0.765752725	0.924199664	24	19.37602027	0.016477909
ENSMUSG00000023707	Ogfod2	2-oxoglutarate and iron-dependent oxygenase domain containing 2 [Source:MGI Symbol;Acc:MGI:1913877]	13.8346	12.0714	12.7757	12.2109	13.2597	13.7097	14.9385	14.2827	11.8402	15.0851	14.2986	14.3041	13.6005	12.9428	0.212387017	0.701530532	24	12	24	0.166281257	0.767020009	24	19.90830349	0.073470522
ENSMUSG00000023723	Mrps23	mitochondrial ribosomal protein S23 [Source:MGI Symbol;Acc:MGI:1928138]	35.7473	35.7028	34.4054	33.3549	32.6895	32.8221	37.2573	40.6371	33.3714	36.9182	36.6178	36.1988	38.2116	39.5021	0.107430666	0.524035616	8	12	24	0.033235343	0.606585237	24	2.974715349	0.074112544
ENSMUSG00000023755	Rhebl1	Ras homolog enriched in brain like 1 [Source:MGI Symbol;Acc:MGI:1916409]	19.5736	18.3778	19.8064	19.2622	18.1954	18.9566	15.88	19.6038	18.9089	18.4053	18.49	19.2709	19.7483	19.0912	0.885025617	0.966018836	12	8	24	0.772525486	0.92687884	24	7.940438417	0.023175172
ENSMUSG00000023764	Sfi1	Sfi1 homolog, spindle assembly associated (yeast) [Source:MGI Symbol;Acc:MGI:1926137]	7.19817	6.07802	6.68268	7.82171	7.37905	6.99054	6.4889	7.07876	7.30414	5.47547	6.38782	5.62373	6.07465	5.6941	0.888482515	0.966018836	20	4	24	0.757188185	0.921562129	24	13.81930717	0.033433728
ENSMUSG00000023791	Pigx	phosphatidylinositol glycan anchor biosynthesis, class X [Source:MGI Symbol;Acc:MGI:1919334]	8.36683	11.4221	10.9447	10.4063	11.1331	9.10857	10.5841	9.7284	11.7029	9.22005	11.6751	11.3294	10.7047	9.65565	0.833406028	0.966018836	12	16	24	0.620327964	0.870154224	24	11.67898948	0.052776596
ENSMUSG00000023795	Pisd-ps2	phosphatidylserine decarboxylase, pseudogene 2 [Source:MGI Symbol;Acc:MGI:3612472]	3.88855	3.77009	2.90094	3.70168	4.83753	4.41621	3.83637	4.07559	2.89643	3.68701	3.85545	4.43244	3.90209	3.76154	7.55E-04	0.071344007	24	12	24	0.007413008	0.47735824	24	19.6748537	0.172729482
ENSMUSG00000023800	Tiam2	T cell lymphoma invasion and metastasis 2 [Source:MGI Symbol;Acc:MGI:1344338]	3.4776	3.2229	3.04818	3.11709	3.5098	3.54227	3.33371	4.08865	2.95678	3.47992	3.63513	3.19534	3.46672	2.77969	0.167216842	0.634643192	20	16	24	0.372483817	0.793806676	24	19.69315631	0.061790263
ENSMUSG00000023805	Synj2	synaptojanin 2 [Source:MGI Symbol;Acc:MGI:1201671]	10.5176	7.93282	8.28007	7.92667	7.23494	8.72187	7.58577	8.79285	7.69087	9.24028	7.2926	8.68219	8.35705	8.44733	0.481530854	0.966018836	24	20	24	0.458279316	0.805393068	24	0.880836071	0.062323799
ENSMUSG00000023806	Rsph3b	radial spoke 3B homolog (Chlamydomonas) [Source:MGI Symbol;Acc:MGI:3630308]	4.70301	3.11692	3.27776	3.00012	3.05422	3.2949	4.10233	4.1184	3.48379	4.13391	4.30465	4.2712	4.83922	4.48582	0.217827365	0.704989686	4	12	24	0.086717358	0.70594329	24	20.052628977	0.126712513
ENSMUSG00000023809	Rps6ka2	ribosomal protein S6 kinase, polypeptide 2 [Source:MGI Symbol;Acc:MGI:1342290]	24.1314	18.7334	18.6168	20.3857	20.1125	23.2432	20.303	22.4344	16.5114	28.1564	18.3307	22.648	22.1631	21.2596	0.14313801	0.585944911	24	12	24	0.559462606	0.840562399	24	20.25294163	0.082410997
ENSMUSG00000023826	Prkn	parkin RBR E3 ubiquitin protein ligase [Source:MGI Symbol;Acc:MGI:1355296]	5.27278	6.76439	7.50417	6.97598	6.13158	6.41718	6.14434	6.0203	7.30087	6.55356	6.23072	6.66823	6.1541	6.18445	0.008389425	0.192760771	12	16	24	0.042564757	0.623773613	24	10.10674536	0.102172445
ENSMUSG00000023827	Agpat4	1-acylglycerol-3-phosphate O-acyltransferase 4 (lysophosphatidic acid acyltransferase, delta) [Source:MGI Symbol;Acc:MGI:1915512]	30.8179	22.9445	26.186	20.5021	26.6294	30.8312	25.1697	23.8901	19.1575	23.1346	23.7946	25.5779	22.6248	23.4857	0.162786057	0.624917386	24	16	24	0.014993679	0.537924552	24	20.8257215	0.177503356
ENSMUSG00000023828	Slc22a3	solute carrier family 22 (organic cation transporter), member 3 [Source:MGI Symbol;Acc:MGI:1333817]	0.663192	0.578904	0.78635	1.05286	0.616166	0.665075	0.651677	0.958595	0.632688	1.12615	0.548433	0.826284	0.872245	1.01804	0.503972924	0.966018836	16	4	24	0.331840427	0.768621471	24	8.543761373	0.085786361
ENSMUSG00000023830	Igf2r	insulin-like growth factor 2 receptor [Source:MGI Symbol;Acc:MGI:96435]	4.38094	4.2426	4.16879	4.51439	4.15532	4.77331	4.45644	5.34548	3.69393	5.47257	4.06416	4.47973	4.26337	4.78547	0.949874888	0.966018836	24	12	24	0.970423983	0.993003194	24	3.418457225	0.012136158
ENSMUSG00000023832	Acat2	acetyl-Coenzyme A acetyltransferase 2 [Source:MGI Symbol;Acc:MGI:87871]	57.8735	43.2808	45.1495	51.7689	45.7358	53.459	43.7525	47.9732	42.3589	42.7765	49.056	46.1613	48.922	48.1199	0.278774299	0.790764844	24	12	24	0.210154601	0.768040178	24	20.75736862	0.087646697
ENSMUSG00000023845	Lnpep	leucyl/cystinyl aminopeptidase [Source:MGI Symbol;Acc:MGI:2387123]	28.9315	30.2894	28.1183	30.1777	29.9834	27.4405	27.4876	26.1797	28.2655	25.3445	31.2182	28.3313	29.0815	30.2107	0.868022237	0.966018836	20	4	24	0.886661256	0.967862704	24	17.78461111	0.016853422
ENSMUSG00000023852	Chd1	chromodomain helicase DNA binding protein 1 [Source:MGI Symbol;Acc:MGI:88393]	5.08339	4.9736	5.09385	4.66235	5.03289	4.60969	4.96272	4.48041	5.02152	4.44607	4.8479	5.3525	4.84627	5.19836	0.88067614	0.966018836	12	4	24	0.625097047	0.872055388	24	0.010704627	0.025586137
ENSMUSG00000023861	Mpc1	mitochondrial pyruvate carrier 1 [Source:MGI Symbol;Acc:MGI:1915240]	33.9635	34.7959	32.6408	38.8722	27.1727	24.8612	33.177	39.8412	40.2478	29.498	44.8849	29.6894	35.8884	36.6131	0.217827365	0.704989686	12	12	24	0.215132678	0.768040178	24	7.030964143	0.155898371
ENSMUSG00000023868	Pde10a	phosphodiesterase 10A [Source:MGI Symbol;Acc:MGI:1345143]	5.80095	4.75076	5.25134	5.07416	4.83904	5.50263	4.8568	5.13562	4.00818	6.18728	4.50159	5.06076	5.06599	5.20649	0.890235289	0.966018836	16	4	24	0.836611037	0.949953371	24	20.51243775	0.031653944
ENSMUSG00000023873	1700010I14Rik	RIKEN cDNA 1700010I14 gene [Source:MGI Symbol;Acc:MGI:1914181]	0.6221	0.668107	1.30436	0.729354	0.599479	0.855927	0.574028	0.673793	0.658286	0.573242	0.825089	0.817476	0.950101	0.808441	0.875755063	0.966018836	24	16	24	0.925842979	0.980197974	24	7.18726866	0.02470587
ENSMUSG00000023882	Zfp54	zinc finger protein 54 [Source:MGI Symbol;Acc:MGI:99201]	2.10075	1.83869	1.91911	2.05141	2.33157	1.92934	2.197	1.7325	2.11175	1.35877	2.45845	2.11527	1.91795	1.92091	0.036134798	0.331852526	20	12	24	0.284827669	0.768040178	24	19.05257594	0.084867332
ENSMUSG00000023883	Phf10	PHD finger protein 10 [Source:MGI Symbol;Acc:MGI:1919307]	19.5405	21.7851	19.6524	20.4087	22.1257	19.6046	22.3908	18.3686	21.1993	20.9652	22.6506	20.3087	20.1598	18.7837	0.173121348	0.648600527	20	12	24	0.252976836	0.768040178	24	15.78510436	0.056650337
ENSMUSG00000023885	Thbs2	thrombospondin 2 [Source:MGI Symbol;Acc:MGI:98738]	3.26407	4.42376	4.38726	2.7306	3.37401	3.31734	3.74249	4.67399	4.60117	2.69117	2.85343	3.30805	3.28956	3.6706	0.012443448	0.220221977	12	4	24	0.011978865	0.525280095	24	4.604068493	0.210702727
ENSMUSG00000023886	Smoc2	SPARC related modular calcium binding 2 [Source:MGI Symbol;Acc:MGI:1929881]	43.6349	58.12	53.9813	45.7617	62.2438	43.2608	57.8138	53.3252	70.0523	36.7275	50.9658	46.7747	54.3738	51.3961	0.44861185	0.966018836	12	4	24	0.506913915	0.812342247	24	5.210008746	0.110562283
ENSMUSG00000023892	Zfp51	zinc finger protein 51 [Source:MGI Symbol;Acc:MGI:99198]	4.00642	4.21285	4.09246	3.84537	4.35246	3.68584	3.89418	3.64558	4.47443	2.94948	4.6842	4.1061	4.38191	4.27672	0.883907406	0.966018836	20	20	24	0.85544667	0.957096509	24	23.48117046	0.029611083
ENSMUSG00000023903	Mmp25	matrix metallopeptidase 25 [Source:MGI Symbol;Acc:MGI:2443938]	11.97	9.84046	9.17713	10.6177	10.2774	11.0625	9.70693	10.6743	8.9828	14.5107	10.5246	11.6555	11.42	10.8399	0.148073804	0.590732968	24	12	24	0.521430997	0.820324275	24	17.86736686	0.071678856
ENSMUSG00000023904	Hcfc1r1	host cell factor C1 regulator 1 (XPO1-dependent) [Source:MGI Symbol;Acc:MGI:2663619]	137.902	124.25	123.361	126.139	129.671	134.984	126.665	134.933	122.174	145.551	127.35	130.369	136.422	132.006	0.074036902	0.448301997	4	8	24	0.638435407	0.877227735	24	20.43719664	0.027134619
ENSMUSG00000023905	Tnfrsf12a	tumor necrosis factor receptor superfamily, member 12a [Source:MGI Symbol;Acc:MGI:1351484]	7.52412	6.56043	7.38615	6.08232	9.03833	7.67676	6.67137	8.0672	6.89498	5.97379	8.76361	6.15852	7.77806	5.96565	0.369262716	0.893596186	20	20	24	0.437842329	0.805393068	24	18.89040398	0.091777946
ENSMUSG00000023908	Pkmyt1	protein kinase, membrane associated tyrosine/threonine 1 [Source:MGI Symbol;Acc:MGI:1923418]	2.37331	2.98529	3.45758	3.45022	2.61887	2.46125	2.83558	3.04318	2.76426	3.09505	2.92406	3.65475	2.97598	2.80984	0.700065774	0.966018836	16	12	24	0.36771291	0.791015017	24	10.27101081	0.074419631
ENSMUSG00000023909	Paqr4	progestin and adipoQ receptor family member IV [Source:MGI Symbol;Acc:MGI:1923748]	32.9122	23.1701	23.6937	25.9506	25.6302	28.2292	28.0675	27.8455	22.1795	33.4068	26.9445	31.0004	28.8804	27.4918	0.019280932	0.257668847	24	12	24	0.182572655	0.768040178	24	20.29413479	0.119983965
ENSMUSG00000023911	Flywch2	FLYWCH family member 2 [Source:MGI Symbol;Acc:MGI:1924167]	11.1095	12.5923	12.745	11.3652	7.74703	9.79983	12.429	10.6482	12.0136	9.62589	11.0114	9.42854	9.9842	12.0615	0.018263889	0.256345714	12	8	24	0.022320967	0.578379975	24	5.381571964	0.17647782
ENSMUSG00000023912	Slc25a27	solute carrier family 25, member 27 [Source:MGI Symbol;Acc:MGI:1921261]	22.6652	24.4972	20.7605	22.7091	23.1999	22.0782	25.5042	22.6068	25.1003	20.8604	23.347	22.7958	25.0024	22.6526	0.954584434	0.966018836	4	12	24	0.32309222	0.768040178	24	0.493267774	0.050250433
ENSMUSG00000023913	Pla2g7	phospholipase A2, group VII (platelet-activating factor acetylhydrolase, plasma) [Source:MGI Symbol;Acc:MGI:1351327]	174.906	169.995	163.95	161.1	159.478	168.528	158.469	162.684	162.41	159.075	169.155	168.704	177.86	171.548	0.250898337	0.75129457	4	12	24	0.097260287	0.71175955	24	23.80890384	0.04121675
ENSMUSG00000023915	Tnfrsf21	tumor necrosis factor receptor superfamily, member 21 [Source:MGI Symbol;Acc:MGI:2151075]	37.2922	28.7249	28.3635	29.934	30.4681	35.5188	31.2364	33.3383	22.5899	41.9874	29.2998	33.1985	30.2675	31.8306	0.148073804	0.590732968	24	12	24	0.418032631	0.802613561	24	19.82003894	0.109461471
ENSMUSG00000023919	Cenpq	centromere protein Q [Source:MGI Symbol;Acc:MGI:1933744]	4.78771	4.72285	4.5879	4.9381	4.5413	4.31756	4.60761	4.74954	5.40797	3.69896	4.72086	4.0872	4.84774	5.07477	0.208747416	0.698797644	8	16	24	0.221560496	0.768040178	24	5.035274486	0.073625072
ENSMUSG00000023921	Mmut	methylmalonyl-Coenzyme A mutase [Source:MGI Symbol;Acc:MGI:97239]	20.6434	21.5754	21.7822	23.4297	21.798	20.4386	21.2675	20.6308	23.0296	21.3028	23.2049	21.3833	23.5268	22.7499	0.640622311	0.966018836	20	4	24	0.269099708	0.768040178	24	10.48072304	0.039366404
ENSMUSG00000023923	Tbc1d5	TBC1 domain family, member 5 [Source:MGI Symbol;Acc:MGI:1919488]	7.56604	5.86794	6.50056	5.7088	5.99385	7.12395	6.47774	6.7188	5.54679	7.3883	5.56881	6.41624	6.21226	6.66086	0.65208848	0.966018836	4	16	24	0.442878261	0.805393068	24	23.40715799	0.062938043
ENSMUSG00000023926	Rhag	Rhesus blood group-associated A glycoprotein [Source:MGI Symbol;Acc:MGI:1202713]	0.304745	0.583556	0.871388	0.776491	0.342466	0.681303	0.600572	0.474253	0.585494	0.348511	0.615748	0.774028	1.11156	0.458031	0.896773941	0.966018836	12	20	24	0.953740237	0.988601783	24	0.326929374	0.024036138
ENSMUSG00000023927	Satb1	special AT-rich sequence binding protein 1 [Source:MGI Symbol;Acc:MGI:105084]	10.9779	10.9925	10.549	11.2727	11.6435	11.2975	12.4409	11.4269	10.765	14.5897	10.3788	11.4741	10.8905	11.1126	0.90767227	0.966018836	16	20	24	0.743763073	0.916218012	24	13.88643667	0.034186802
ENSMUSG00000023932	Cdc5l	cell division cycle 5-like (S. pombe) [Source:MGI Symbol;Acc:MGI:1918952]	12.8609	12.1881	12.3184	12.255	12.1864	12.621	11.8269	12.3939	11.6396	11.4617	12.4562	12.4144	13.1049	12.2657	0.132411359	0.567728672	24	16	24	0.076859734	0.687041208	24	22.19633549	0.040515508
ENSMUSG00000023935	Spats1	spermatogenesis associated, serine-rich 1 [Source:MGI Symbol;Acc:MGI:1918270]	1.79624	2.13144	1.50914	1.86637	2.03875	1.67477	1.17218	1.8506	1.95264	1.6814	2.00995	1.78255	1.46015	1.87536	0.326899754	0.845515883	20	8	24	0.468548709	0.805393068	24	11.81559819	0.068037158
ENSMUSG00000023938	Aars2	alanyl-tRNA synthetase 2, mitochondrial [Source:MGI Symbol;Acc:MGI:2681839]	6.83612	6.12176	6.44265	5.34456	5.19748	6.3538	6.8481	7.61435	6.72562	7.08097	6.54151	6.50667	7.06848	6.94057	0.27137637	0.781251337	8	12	24	0.040814523	0.616921492	24	3.087168237	0.103558691
ENSMUSG00000023939	Mrpl14	mitochondrial ribosomal protein L14 [Source:MGI Symbol;Acc:MGI:1333864]	29.5541	27.9769	30.1315	30.2187	29.8097	28.123	28.3198	29.9482	31.7846	26.723	28.7154	29.6678	33.9658	30.3669	0.895493752	0.966018836	12	12	24	0.560117443	0.840893245	24	3.939864002	0.03350615
ENSMUSG00000023942	Slc29a1	solute carrier family 29 (nucleoside transporters), member 1 [Source:MGI Symbol;Acc:MGI:1927073]	29.0657	25.436	25.2412	27.7959	27.6769	28.6798	26.7578	27.6053	24.3212	27.1483	27.7491	26.51	27.282	26.4366	0.073826349	0.448301997	20	16	24	0.041986434	0.622498612	24	19.520078	0.066582947
ENSMUSG00000023944	Hsp90ab1	heat shock protein 90 alpha (cytosolic), class B member 1 [Source:MGI Symbol;Acc:MGI:96247]	742.595	488.557	493.921	537.448	533.73	663.307	620.321	634.37	394.517	693.76	519.335	592.182	556.574	560.389	0.195343269	0.673387182	4	8	24	0.161780468	0.76636332	24	21.10875298	0.175777337
ENSMUSG00000023945	Slc5a7	solute carrier family 5 (choline transporter), member 7 [Source:MGI Symbol;Acc:MGI:1927126]	1.55451	2.06156	3.80112	2.04183	1.40892	2.03139	3.29556	1.50712	2.72284	1.86389	1.42248	1.97626	1.9	1.9	0.069194004	0.433403132	12	8	24	0.215035949	0.768040178	24	7.569186961	0.208007008
ENSMUSG00000023947	Nfkbie	nuclear factor of kappa light polypeptide gene enhancer in B cells inhibitor, epsilon [Source:MGI Symbol;Acc:MGI:1194908]	2.15782	2.02438	1.7153	2.05026	2.03397	2.19453	1.96783	2.51862	2.15728	2.85707	2.13254	2.41395	2.52389	2.32989	0.896753532	0.966018836	24	12	24	0.987636359	0.997530201	24	21.71781603	0.006606237
ENSMUSG00000023951	Vegfa	vascular endothelial growth factor A [Source:MGI Symbol;Acc:MGI:103178]	31.4996	34.1183	37.161	41.5876	36.3665	33.6107	34.0598	44.6702	33.0746	38.2981	33.67	32.4509	35.7509	35.6858	0.097779482	0.501112642	16	8	24	0.2025122	0.768040178	24	9.175210198	0.096240898
ENSMUSG00000023952	Gtpbp2	GTP binding protein 2 [Source:MGI Symbol;Acc:MGI:1860138]	38.1434	33.7406	34.4789	37.0985	38.5136	34.8656	37.0656	37.2338	35.7252	35.1519	35.6636	33.5773	35.4714	32.62	0.93447422	0.966018836	20	12	24	0.537570856	0.827958985	24	17.55688438	0.028202052
ENSMUSG00000023953	Polh	polymerase (DNA directed), eta (RAD 30 related) [Source:MGI Symbol;Acc:MGI:1891457]	3.59732	3.41483	4.15317	3.60123	3.40094	3.68412	3.59397	3.85628	3.23486	3.47218	3.30466	3.36992	3.97022	3.53438	0.833406028	0.966018836	4	16	24	0.432493768	0.805393068	24	3.183908992	0.04002816
ENSMUSG00000023959	Clic5	chloride intracellular channel 5 [Source:MGI Symbol;Acc:MGI:1917912]	1.85438	2.61517	3.15405	4.26472	2.73588	3.46688	2.9099	5.38113	3.94916	5.14912	3.36205	3.05152	4.38638	2.91691	0.658942124	0.966018836	16	12	24	0.172834173	0.768040178	24	10.11245927	0.208242869
ENSMUSG00000023960	Enpp5	ectonucleotide pyrophosphatase/phosphodiesterase 5 [Source:MGI Symbol;Acc:MGI:1933830]	70.6701	69.7925	69.2367	67.6937	65.6765	68.7919	67.7381	63.9832	66.5761	65.3451	65.5124	68.6491	67.6143	73.8253	0.27817759	0.789849983	8	8	24	0.250491503	0.768040178	24	1.889407428	0.033116814
ENSMUSG00000023961	Enpp4	ectonucleotide pyrophosphatase/phosphodiesterase 4 [Source:MGI Symbol;Acc:MGI:2682634]	15.926	16.0736	16.8043	16.3996	16.0694	15.9139	16.9937	15.8702	15.8088	14.704	16.6015	16.7017	15.9759	15.3956	0.910436409	0.966018836	4	4	24	0.518818047	0.818895951	24	20.5455842	0.023468019
ENSMUSG00000023963	Cyp39a1	cytochrome P450, family 39, subfamily a, polypeptide 1 [Source:MGI Symbol;Acc:MGI:1927096]	13.7956	18.8741	12.8645	11.2459	14.7521	14.1175	15.2882	15.5989	17.0407	12.2685	13.6575	15.5509	17.212	15.2419	0.01457641	0.247599677	8	8	24	0.047815896	0.636547989	24	1.775383666	0.160369892
ENSMUSG00000023965	Fbxl17	F-box and leucine-rich repeat protein 17 [Source:MGI Symbol;Acc:MGI:1354704]	11.1501	12.4857	11.3727	11.5456	12.6345	12.6139	11.1076	12.2775	11.8576	12.9401	12.174	11.9297	11.449	11.9886	0.217660303	0.704989686	20	8	24	0.3300636	0.768040178	24	13.67397164	0.036695942
ENSMUSG00000023966	Rsph9	radial spoke head 9 homolog (Chlamydomonas) [Source:MGI Symbol;Acc:MGI:1922814]	8.15265	9.45144	8.08614	8.84958	9.62243	7.97477	7.84905	6.21839	9.52835	7.48188	9.54774	8.55686	7.81682	8.31592	0.223774686	0.717349224	20	8	24	0.348498231	0.779170188	24	14.64727698	0.084746848
ENSMUSG00000023967	Mrps18a	mitochondrial ribosomal protein S18A [Source:MGI Symbol;Acc:MGI:1915815]	45.5377	38.0879	39.9023	38.9974	37.5054	44.2685	41.5215	44.1759	35.5972	44.0018	40.9393	42.5202	43.9395	44.3405	0.216688884	0.704989686	4	8	24	0.152058064	0.759065259	24	22.94550706	0.075915063
ENSMUSG00000023968	Crip3	cysteine-rich protein 3 [Source:MGI Symbol;Acc:MGI:2152434]	1.61066	1.26512	1.38363	1.99544	1.54838	1.31075	2.62435	1.26502	1.2857	1.51424	1.70061	1.25909	1.61631	1.15028	0.128457847	0.560209134	20	12	24	0.588865333	0.853936396	24	18.53874464	0.083523717
ENSMUSG00000023971	Rrp36	ribosomal RNA processing 36 [Source:MGI Symbol;Acc:MGI:2385053]	15.211	12.8879	12.0812	13.1567	12.3168	13.1191	13.4705	13.4963	12.5383	12.3903	14.1417	13.8744	12.9786	13.0799	0.188783717	0.664348865	24	12	24	0.078139168	0.688357494	24	21.86933214	0.069649987
ENSMUSG00000023972	Ptk7	PTK7 protein tyrosine kinase 7 [Source:MGI Symbol;Acc:MGI:1918711]	5.4764	4.35557	4.00088	4.3434	4.37093	4.67118	4.6394	4.75701	4.07894	6.10541	3.96223	4.56608	4.89159	4.84275	0.097760727	0.501112642	4	8	24	0.767836138	0.925038775	24	23.49873823	0.041634328
ENSMUSG00000023973	Cnpy3	canopy FGF signaling regulator 3 [Source:MGI Symbol;Acc:MGI:1919279]	19.2485	18.2667	19.4833	17.1171	19.601	19.4559	19.0448	20.3102	18.0234	18.4735	17.6494	18.8478	18.2533	19.0643	0.937921962	0.966018836	8	8	24	0.3153639	0.768040178	24	0.450021756	0.036616321
ENSMUSG00000023977	Ubr2	ubiquitin protein ligase E3 component n-recognin 2 [Source:MGI Symbol;Acc:MGI:1861099]	14.2048	13.2966	13.2221	14.3105	13.8068	14.207	14.7043	14.4699	12.7616	16.3234	12.8156	13.3382	13.2036	13.9229	0.917839756	0.966018836	16	20	24	0.943722612	0.986208939	24	13.83665601	0.011968088
ENSMUSG00000023979	Guca1b	guanylate cyclase activator 1B [Source:MGI Symbol;Acc:MGI:1194489]	0.741807	0.526882	0.460964	0.430899	0.63726	0.683609	0.942611	0.546297	0.633914	0.365277	0.696486	0.494188	0.568188	0.548814	0.01950757	0.258568924	4	12	24	0.065630685	0.667622064	24	22.16885624	0.112527758
ENSMUSG00000023980	Taf8	TATA-box binding protein associated factor 8 [Source:MGI Symbol;Acc:MGI:1926879]	7.63976	8.43778	8.973	8.98305	9.24917	8.71396	8.08907	8.02653	8.43051	8.53405	8.10504	8.58045	8.40093	7.54267	0.032059509	0.324385159	16	16	24	0.040512527	0.616921492	24	14.08787676	0.06484134
ENSMUSG00000023984	Gm20517	predicted gene 20517 [Source:MGI Symbol;Acc:MGI:5141982]	1.15204	1.50842	0.87714	1.89766	1.40987	1.00859	1.8117	0.990058	1.95555	0.964448	1.2984	0.913204	1.09407	1.60842	0.852844413	0.966018836	8	16	24	0.683173619	0.894420965	24	8.034990026	0.084494287
ENSMUSG00000023988	Bysl	bystin-like [Source:MGI Symbol;Acc:MGI:1858419]	5.02777	3.50187	3.29931	3.54458	3.43194	4.22392	4.02331	4.36982	3.1669	4.45311	3.49651	3.59938	3.75876	3.66532	0.060394272	0.407012001	4	8	24	0.267771218	0.768040178	24	22.6638301	0.095079698
ENSMUSG00000023990	Tfeb	transcription factor EB [Source:MGI Symbol;Acc:MGI:103270]	5.33681	5.39335	5.03643	6.04065	5.13643	5.60005	5.25689	5.70802	6.60198	6.59823	6.66054	5.75942	5.26862	4.67489	0.301918397	0.811201458	16	12	24	0.088872856	0.707896147	24	13.1832318	0.105350671
ENSMUSG00000023991	Foxp4	forkhead box P4 [Source:MGI Symbol;Acc:MGI:1921373]	2.79185	3.09418	3.20993	3.4773	2.869	2.48842	2.73028	3.26463	3.93745	4.29966	4.00875	3.29299	3.52301	3.31893	0.132411359	0.567728672	16	8	24	0.004747553	0.434006302	24	10.40752876	0.162059317
ENSMUSG00000023992	Trem2	triggering receptor expressed on myeloid cells 2 [Source:MGI Symbol;Acc:MGI:1913150]	1.42774	1.96451	3.53801	1.85255	2.28457	1.95768	1.37837	1.63446	2.93099	1.67616	2.09586	1.30847	1.25386	2.1917	0.004501001	0.140598674	12	16	24	0.032381242	0.606585237	24	9.779834206	0.268591071
ENSMUSG00000023993	Treml1	triggering receptor expressed on myeloid cells-like 1 [Source:MGI Symbol;Acc:MGI:1918576]	0.185097	0.715351	0.926202	0.639175	0.528785	0.674285	0.542397	0.73027	0.506118	0.366813	0.535445	0.565552	0.467931	0.30337	0.920858634	0.966018836	12	16	24	0.721996627	0.908845453	24	10.18175963	0.057516214
ENSMUSG00000023994	Nfya	nuclear transcription factor-Y alpha [Source:MGI Symbol;Acc:MGI:97316]	10.2989	8.3644	9.18635	9.47616	10.364	9.06487	10.9461	7.71209	8.46359	8.3197	9.8454	8.61647	9.58549	8.44539	0.05238827	0.391426791	4	4	24	0.098269778	0.71175955	24	19.24954012	0.104656793
ENSMUSG00000023995	Tspo2	translocator protein 2 [Source:MGI Symbol;Acc:MGI:1917276]	0.514686	1.10757	1.81943	1.20021	0.880159	1.07921	0.73321	0.981128	1.0889	0.808997	1.10185	0.934687	1.4718	0.960092	0.724067924	0.966018836	12	16	24	0.39336716	0.799546792	24	9.090443031	0.119627906
ENSMUSG00000024002	Brd4	bromodomain containing 4 [Source:MGI Symbol;Acc:MGI:1888520]	11.4818	10.6011	10.323	11.8318	11.0041	10.9312	11.58	10.2412	11.4002	16.125	13.1831	12.6311	11.5054	13.4323	0.522551072	0.966018836	16	20	24	0.235633977	0.768040178	24	13.58696883	0.087184319
ENSMUSG00000024006	Stk38	serine/threonine kinase 38 [Source:MGI Symbol;Acc:MGI:2442572]	27.4569	34.1238	35.5902	36.8364	34.5222	30.8704	28.2403	32.5016	40.5463	30.611	38.6705	33.9101	34.9946	36.045	0.158469451	0.614860056	12	16	24	0.063053849	0.662778521	24	10.42193904	0.12827205
ENSMUSG00000024007	Ppil1	peptidylprolyl isomerase (cyclophilin)-like 1 [Source:MGI Symbol;Acc:MGI:1916066]	10.5143	9.0031	9.84849	11.2249	9.34783	10.5965	10.5159	9.60962	8.04969	10.4568	9.64336	8.48468	9.28501	8.07934	0.446653834	0.966018836	16	16	24	0.318144853	0.768040178	24	17.17848369	0.069325328
ENSMUSG00000024008	Cpne5	copine V [Source:MGI Symbol;Acc:MGI:2385908]	1.48975	1.429	2.02276	1.48847	1.43308	1.85942	2.03099	1.56619	1.25708	2.07099	1.42504	1.32436	1.26917	1.89163	0.865514257	0.966018836	16	4	24	0.859930233	0.958681059	24	7.726051132	0.037452693
ENSMUSG00000024011	Pi16	peptidase inhibitor 16 [Source:MGI Symbol;Acc:MGI:1921366]	12.0162	13.0724	10.7339	10.3121	10.6305	9.55081	9.92167	9.44229	18.2836	5.28331	12.3584	12.5061	9.74031	11.1306	0.860993221	0.966018836	12	4	24	0.760445496	0.922513876	24	2.96579893	0.102301532
ENSMUSG00000024012	Mtch1	mitochondrial carrier 1 [Source:MGI Symbol;Acc:MGI:1929261]	218.337	197.16	197.701	196.003	202.077	217.11	206.668	211.905	181.621	213.201	187.705	198.696	196.011	192.194	0.649212555	0.966018836	4	8	24	0.225735416	0.768040178	24	21.40116135	0.049748318
ENSMUSG00000024013	Fgd2	FYVE, RhoGEF and PH domain containing 2 [Source:MGI Symbol;Acc:MGI:1347084]	1.69927	2.06993	4.10725	2.04708	2.50307	2.39136	2.04462	3.68909	3.94624	2.52711	2.43818	2.78629	2.33261	2.30397	0.018894761	0.256345714	12	16	24	0.140170127	0.749574609	24	8.736762066	0.211520822
ENSMUSG00000024014	Pim1	proviral integration site 1 [Source:MGI Symbol;Acc:MGI:97584]	5.97375	5.03068	7.17604	9.1828	5.99033	7.0953	5.96979	8.09765	5.55837	8.32606	6.43472	6.78946	8.24785	8.65322	0.920992137	0.966018836	16	12	24	0.630383478	0.873885049	24	10.24889752	0.082840217
ENSMUSG00000024018	Ccdc167	coiled-coil domain containing 167 [Source:MGI Symbol;Acc:MGI:1915847]	6.47123	7.14472	5.80968	6.13002	7.1322	7.02006	7.18486	7.10779	7.42929	5.29667	7.23708	6.69334	7.33718	6.72725	0.930944473	0.966018836	4	12	24	0.196177633	0.768040178	24	23.22827328	0.084204238
ENSMUSG00000024019	Cmtr1	cap methyltransferase 1 [Source:MGI Symbol;Acc:MGI:1921407]	35.1971	29.7723	34.7906	31.5695	32.8058	36.024	30.3668	37.4245	27.0604	34.9643	27.7513	32.8581	31.2602	32.5017	0.862909389	0.966018836	24	12	24	0.810944523	0.93944887	24	22.84495297	0.034887374
ENSMUSG00000024026	Glo1	glyoxalase 1 [Source:MGI Symbol;Acc:MGI:95742]	53.8332	51.088	50.657	48.0797	50.2473	50.2486	47.0944	48.5659	48.7163	45.1776	52.7848	49.4309	51.5583	49.6122	0.581070934	0.966018836	4	12	24	0.302183094	0.768040178	24	22.12900507	0.038435454
ENSMUSG00000024030	Abcg1	ATP binding cassette subfamily G member 1 [Source:MGI Symbol;Acc:MGI:107704]	10.8243	8.4164	8.69524	10.2922	8.79157	11.0776	9.27931	11.4906	7.22554	12.727	9.55971	10.2457	9.91	9.82494	0.581070934	0.966018836	24	12	24	0.630371104	0.873885049	24	19.09581326	0.074281458
ENSMUSG00000024033	Rsph1	radial spoke head 1 homolog (Chlamydomonas) [Source:MGI Symbol;Acc:MGI:1194909]	2.81916	3.29878	3.06617	3.01794	2.82419	3.05368	3.11237	2.55323	2.67136	3.23605	2.55294	3.71226	2.31236	3.85297	0.887862353	0.966018836	8	12	24	0.975257622	0.993747214	24	5.613885	0.014392266
ENSMUSG00000024036	Slc37a1	solute carrier family 37 (glycerol-3-phosphate transporter), member 1 [Source:MGI Symbol;Acc:MGI:2446181]	5.64707	5.49354	4.91467	6.71711	5.54347	7.02329	5.6219	5.16581	5.29605	6.98262	5.81064	6.72989	5.44655	5.78979	0.025223982	0.293279372	24	12	24	0.082034999	0.699158325	24	16.7564682	0.116368224
ENSMUSG00000024037	Wdr4	WD repeat domain 4 [Source:MGI Symbol;Acc:MGI:1889002]	10.4085	8.41586	10.5183	9.45995	10.1839	9.69252	9.18328	8.95551	10.3673	9.15196	9.55659	11.6836	10.6324	10.111	0.708020493	0.966018836	24	8	24	0.630642503	0.874000625	24	19.26127718	0.042313178
ENSMUSG00000024038	Ndufv3	NADH:ubiquinone oxidoreductase core subunit V3 [Source:MGI Symbol;Acc:MGI:1890894]	183.044	193.047	185.124	188.485	204.541	187.778	176.533	195.788	194.771	157.752	206.191	184.102	196.856	187.281	0.93357733	0.966018836	20	8	24	0.909723038	0.974611343	24	20.468147	0.017603627
ENSMUSG00000024039	Cbs	cystathionine beta-synthase [Source:MGI Symbol;Acc:MGI:88285]	0.672385	2.23221	2.29799	1.35015	1.61784	1.67814	2.79148	1.03565	1.99741	1.25282	0.685915	1.69309	0.836418	2.51303	0.664568268	0.966018836	12	8	24	0.482388449	0.805393068	24	5.87769497	0.190158272
ENSMUSG00000024042	Sik1	salt inducible kinase 1 [Source:MGI Symbol;Acc:MGI:104754]	1.42494	1.40976	1.90075	2.13478	1.33456	1.54704	1.40239	1.8699	2.15753	2.09866	1.46108	1.65392	1.5601	1.65533	0.032547207	0.324908923	16	4	24	0.00609712	0.469146176	24	9.519454056	0.166018665
ENSMUSG00000024043	Arhgap28	Rho GTPase activating protein 28 [Source:MGI Symbol;Acc:MGI:2147003]	27.9716	21.9211	21.6329	22.5909	22.9471	25.6798	21.2576	22.7187	18.1864	23.9331	23.3653	24.1515	25.3018	24.4826	0.047644929	0.377556215	24	12	24	0.079211443	0.691787772	24	20.86121002	0.120407588
ENSMUSG00000024044	Epb41l3	erythrocyte membrane protein band 4.1 like 3 [Source:MGI Symbol;Acc:MGI:103008]	267.658	211.008	212.17	217.003	202.398	232.911	228.269	226.706	176.154	275.551	204.046	229.285	214.061	228.847	0.338172159	0.85758369	24	12	24	0.606392465	0.863212285	24	21.65338416	0.066130956
ENSMUSG00000024045	Akap8	A kinase (PRKA) anchor protein 8 [Source:MGI Symbol;Acc:MGI:1928488]	35.889	40.9313	39.4505	43.595	40.1971	35.228	39.0846	35.9843	40.1459	35.1105	39.8655	38.1566	38.2422	38.2774	0.401437339	0.933249185	20	4	24	0.42761904	0.805393068	24	10.88974451	0.045058713
ENSMUSG00000024048	Myl12a	myosin, light chain 12A, regulatory, non-sarcomeric [Source:MGI Symbol;Acc:MGI:1914518]	36.3686	39.1632	42.6848	41.9026	38.6979	35.7433	40.8226	43.9928	44.8043	39.8437	41.2989	38.1598	44.5597	38.1492	0.047644929	0.377556215	12	12	24	0.099741365	0.71175955	24	7.988805863	0.081997565
ENSMUSG00000024049	Myom1	myomesin 1 [Source:MGI Symbol;Acc:MGI:1341430]	7.1617	13.0179	15.359	15.9277	12.3246	14.3863	9.54497	23.7731	17.846	24.6309	13.2248	12.9374	16.7561	12.0466	0.181522804	0.659020016	16	12	24	0.054961829	0.655263328	24	9.899762808	0.366377454
ENSMUSG00000024050	Wiz	widely-interspaced zinc finger motifs [Source:MGI Symbol;Acc:MGI:1332638]	7.98273	7.54372	6.90494	8.27752	7.88872	7.64639	7.97154	8.7237	8.03953	11.3482	8.0567	8.5551	8.88608	8.78437	0.844448841	0.966018836	16	20	24	0.678537312	0.892463655	24	11.59462246	0.043414356
ENSMUSG00000024052	Lpin2	lipin 2 [Source:MGI Symbol;Acc:MGI:1891341]	10.0207	8.85594	8.98789	9.53293	8.43794	9.88383	9.73458	8.23118	8.89477	9.12568	10.123	9.45925	9.57397	9.60449	0.278774299	0.790764844	4	4	24	0.223408477	0.768040178	24	20.17872779	0.055864599
ENSMUSG00000024053	Emilin2	elastin microfibril interfacer 2 [Source:MGI Symbol;Acc:MGI:2389136]	1.106	1.55454	1.8298	1.57839	1.54797	1.406	1.52826	1.62621	2.03273	1.00704	1.51452	1.812	1.1748	0.989891	0.286642595	0.793714531	12	16	24	0.737661331	0.914422817	24	11.3977932	0.057609349
ENSMUSG00000024054	Smchd1	SMC hinge domain containing 1 [Source:MGI Symbol;Acc:MGI:1921605]	12.0311	12.2738	11.5338	11.3517	11.5791	11.518	11.1117	10.3628	11.9182	9.66067	11.4512	10.8751	9.75002	10.2	0.852844413	0.966018836	12	4	24	0.829258604	0.946904474	24	18.06994818	0.017791087
ENSMUSG00000024055	Cyp4f13	cytochrome P450, family 4, subfamily f, polypeptide 13 [Source:MGI Symbol;Acc:MGI:2158641]	14.6605	13.8184	15.7457	16.9876	15.981	12.5692	16.3901	15.3499	17.254	14.0887	16.5245	15.5419	15.6481	13.781	0.336755135	0.85758369	16	20	24	0.486424394	0.805393068	24	11.5031732	0.058714453
ENSMUSG00000024056	Ndc80	NDC80 kinetochore complex component [Source:MGI Symbol;Acc:MGI:1914302]	0.448423	0.716221	0.816964	0.628896	0.680262	0.820447	0.412134	0.566986	0.715047	0.374833	0.554041	0.471336	0.580508	0.479623	0.742579234	0.966018836	12	16	24	0.757694817	0.921675291	24	10.52837062	0.036823516
ENSMUSG00000024059	Clip4	CAP-GLY domain containing linker protein family, member 4 [Source:MGI Symbol;Acc:MGI:1919100]	37.3633	30.9919	37.6869	35.1944	33.3638	39.6616	37.1006	36.0184	25.0495	42.4454	29.9721	31.0464	28.6179	31.5933	0.865514257	0.966018836	12	16	24	0.780992228	0.929788468	24	17.52695999	0.053296945
ENSMUSG00000024063	Lbh	limb-bud and heart [Source:MGI Symbol;Acc:MGI:1925139]	32.2482	24.5456	25.7329	23.4366	27.7521	28.8155	27.5411	27.6354	21.7395	26.2263	27.8423	27.2048	27.0143	26.7933	0.019555896	0.258568924	24	12	24	0.013512223	0.529134128	24	21.22346241	0.135131218

ENSMUSG00000024064	Galnt14	polypeptide N-acetylgalactosaminyltransferase 14 [Source:MGI Symbol;Acc:MGI:1918935]	31.6959	25.2352	24.178	24.6662	26.3708	28.9051	25.1585	25.8367	21.5175	30.0836	26.4942	26.3476	28.4479	27.4699	0.198504154	0.680216953	24	12	24	0.171390945	0.768040178	24	20.70148113	0.100508602
ENSMUSG00000024065	Ehd3	EH-domain containing 3 [Source:MGI Symbol;Acc:MGI:1928900]	51.0207	37.833	40.1867	42.3292	43.3448	50.419	43.5666	45.4308	30.5988	56.6418	38.5395	42.8849	40.2588	41.4215	0.601647512	0.966018836	24	12	24	0.392508952	0.799546792	24	19.22633676	0.115876646
ENSMUSG00000024066	Xdh	xanthine dehydrogenase [Source:MGI Symbol;Acc:MGI:98973]	3.00988	3.61902	4.21915	7.44319	5.6258	4.03739	3.84607	3.6524	4.73992	4.42014	5.20225	3.90686	3.63104	3.4365	5.97E-04	0.071344007	16	16	24	8.44E-04	0.319044294	24	13.31835348	0.313145472
ENSMUSG00000024067	Dpy30	dpy-30, histone methyltransferase complex regulatory subunit [Source:MGI Symbol;Acc:MGI:1913560]	55.1609	54.8805	51.1829	49.6448	51.3133	50.2898	50.8392	47.4018	58.6163	44.6254	57.6663	55.0559	58.5193	55.7665	0.951082476	0.966018836	4	12	24	0.533358221	0.825813837	24	0.28700688	0.049597504
ENSMUSG00000024068	Spast	spastin [Source:MGI Symbol;Acc:MGI:1858896]	13.6275	13.3507	13.3622	13.3395	12.9166	13.1243	12.2975	12.3213	12.6935	13.0207	13.6272	12.8353	12.1422	12.8851	0.742579234	0.966018836	20	8	24	0.232537501	0.768040178	24	14.11892961	0.027722475
ENSMUSG00000024069	Slc30a6	solute carrier family 30 (zinc transporter), member 6 [Source:MGI Symbol;Acc:MGI:2386741]	10.8745	11.2089	10.2853	10.1828	10.4705	10.6726	10.4774	9.59891	10.3277	9.75947	10.7447	9.46025	9.36144	8.7485	0.858514388	0.966018836	20	12	24	0.296650115	0.768040178	24	17.62079384	0.03548357
ENSMUSG00000024070	Prkd3	protein kinase D3 [Source:MGI Symbol;Acc:MGI:1922542]	5.05831	5.43381	5.95024	5.79881	5.76145	4.96648	6.6985	5.52237	6.28297	5.04894	5.24433	5.25717	5.62397	5.22952	0.390686537	0.915479136	12	12	24	0.583378011	0.851822355	24	6.803210816	0.045702827
ENSMUSG00000024072	Yipf4	Yip1 domain family, member 4 [Source:MGI Symbol;Acc:MGI:1915114]	47.7331	41.8011	46.203	41.0495	44.9026	42.5944	44.9515	44.094	39.3395	44.4977	39.6083	40.6551	45.3016	41.9746	0.215777641	0.704989686	4	12	24	0.399455526	0.799546792	24	0.267669524	0.040827994
ENSMUSG00000024073	Birc6	baculoviral IAP repeat-containing 6 [Source:MGI Symbol;Acc:MGI:1276108]	30.8019	31.6616	27.0471	30.0818	29.0296	29.674	27.8375	27.4061	20.5779	26.4865	29.1751	21.4285	20.4897	22.2983	0.868022237	0.966018836	20	16	24	0.347230449	0.778844143	24	17.14219143	0.079068544
ENSMUSG00000024074	Crim1	cysteine rich transmembrane BMP regulator 1 (chordin like) [Source:MGI Symbol;Acc:MGI:1354756]	4.16311	4.91432	6.11464	5.50121	4.29179	5.17795	4.75366	4.16122	4.5757	5.13958	3.77901	4.76291	3.38168	4.68579	0.446967971	0.966018836	16	12	24	0.238157048	0.768040178	24	10.21678607	0.108932074
ENSMUSG00000024076	Vit	vitrin [Source:MGI Symbol;Acc:MGI:1921449]	14.4513	16.8566	15.2589	15.3898	19.8459	13.9915	16.032	15.5319	20.3262	15.3447	16.1579	16.38	16.0752	15.2779	0.890176313	0.966018836	20	4	24	0.565807116	0.843471947	24	11.17472799	0.061203565
ENSMUSG00000024077	Strn	striatin, calmodulin binding protein [Source:MGI Symbol;Acc:MGI:1333757]	9.86549	9.99835	10.0041	9.59091	9.07171	9.58918	10.0427	8.68534	9.06195	9.73425	8.97169	10.0884	9.42323	9.93365	0.885955579	0.966018836	24	20	24	0.613554421	0.866162311	24	0.510887433	0.024724833
ENSMUSG00000024078	Ttc27	tetratricopeptide repeat domain 27 [Source:MGI Symbol;Acc:MGI:1921446]	9.6733	8.76678	9.7833	9.08847	7.62398	8.04671	7.62766	8.93226	6.96566	9.05225	8.28739	8.83718	8.13198	9.71908	0.690243289	0.966018836	16	4	24	0.681736771	0.893856974	24	5.585072987	0.046719027
ENSMUSG00000024079	Eif2ak2	eukaryotic translation initiation factor 2-alpha kinase 2 [Source:MGI Symbol;Acc:MGI:1353449]	10.0916	11.0245	11.164	9.42191	10.9223	10.9482	8.0747	10.4866	10.6356	9.29951	10.6672	11.6002	9.94372	11.7756	0.784278077	0.966018836	8	8	24	0.971202179	0.993012791	24	7.831035416	0.013417169
ENSMUSG00000024081	Cebpz	CCAAT/enhancer binding protein zeta [Source:MGI Symbol;Acc:MGI:109386]	14.716	14.083	13.0232	13.0172	15.3706	13.8472	13.7367	12.523	14.9799	13.9738	15.0653	15.9737	16.3202	16.0788	0.745469888	0.966018836	20	20	24	0.347287957	0.778844143	24	20.73461612	0.054439352
ENSMUSG00000024082	Ndufaf7	NADH:ubiquinone oxidoreductase complex assembly factor 7 [Source:MGI Symbol;Acc:MGI:1920944]	31.6933	29.3353	28.471	29.62	29.7021	27.1267	28.1557	30.4583	30.7875	25.6998	27.4196	29.899	29.0303	27.4618	0.920872048	0.966018836	12	4	24	0.48717782	0.805393068	24	1.724861227	0.03433051
ENSMUSG00000024083	Pja2	praja ring finger ubiquitin ligase 2 [Source:MGI Symbol;Acc:MGI:2159342]	141.373	142.07	137.191	135.296	137.825	136.042	135.11	129.674	130.608	130.421	140.136	133.955	135.205	142.674	0.897425765	0.966018836	8	8	24	0.579317593	0.849752419	24	22.73246267	0.018484828
ENSMUSG00000024084	Qpct	glutaminyl-peptide cyclotransferase (glutaminyl cyclase) [Source:MGI Symbol;Acc:MGI:1917786]	3.0276	4.24899	5.16711	3.29254	3.47696	3.36645	3.14748	3.01564	4.64279	2.79704	3.35617	3.25008	3.41846	3.86871	0.43280837	0.966018836	12	4	24	0.143237197	0.752803875	24	7.357406545	0.150927607
ENSMUSG00000024085	Man2a1	mannosidase 2, alpha 1 [Source:MGI Symbol;Acc:MGI:104669]	2.77369	2.70762	3.05751	3.0721	3.10221	2.59173	2.86885	2.91855	2.77759	2.48151	3.21471	2.78901	2.46135	3.02451	0.132887428	0.567728672	20	8	24	0.534311459	0.826328258	24	12.16177414	0.03888963
ENSMUSG00000024087	Cyp1b1	cytochrome P450, family 1, subfamily b, polypeptide 1 [Source:MGI Symbol;Acc:MGI:88590]	7.76057	8.10035	8.25846	7.39682	7.54626	7.87927	7.54093	7.04892	7.17147	6.31812	8.32627	7.64757	6.80979	6.91037	0.777666236	0.966018836	20	12	24	0.452958471	0.805393068	24	19.48084294	0.044163284
ENSMUSG00000024091	Vapa	vesicle-associated membrane protein, associated protein A [Source:MGI Symbol;Acc:MGI:1353561]	59.6443	56.6168	54.3553	60.4229	59.1128	60.7623	61.9184	57.2885	52.9814	61.6481	55.3131	61.4172	59.3066	55.5205	0.010886922	0.200176658	24	12	24	0.059254555	0.658304792	24	19.53837918	0.064722437
ENSMUSG00000024095	Hnrnpll	heterogeneous nuclear ribonucleoprotein L-like [Source:MGI Symbol;Acc:MGI:1919942]	19.4227	18.2894	18.0696	18.2522	16.2671	17.4699	17.0192	15.7689	16.1514	16.8668	17.5484	16.3466	16.7762	17.5602	0.813775138	0.966018836	4	20	24	0.882764641	0.966743616	24	0.170382005	0.013209414
ENSMUSG00000024096	Ralbp1	ralA binding protein 1 [Source:MGI Symbol;Acc:MGI:108466]	31.9093	24.3462	24.9709	27.8468	25.561	29.3219	29.0891	27.9102	24.7519	36.2353	28.2674	32.2008	31.1332	30.2755	0.080164531	0.458678242	24	12	24	0.428757541	0.805393068	24	19.93110136	0.076778316
ENSMUSG00000024097	Srsf7	serine and arginine-rich splicing factor 7 [Source:MGI Symbol;Acc:MGI:1926232]	61.7675	64.1393	63.9492	62.0524	64.6196	56.5352	63.5164	56.8327	62.3394	50.7528	64.4017	55.3205	57.9747	57.7603	0.893307667	0.966018836	20	4	24	0.993505662	0.998731933	24	5.611123621	0.004250734
ENSMUSG00000024098	Twsg1	twisted gastrulation BMP signaling modulator 1 [Source:MGI Symbol;Acc:MGI:2137520]	16.8877	19.0156	16.1051	15.4129	16.6534	15.5742	16.6501	17.8672	16.864	14.5455	15.533	16.5784	15.5296	16.0097	0.050258003	0.385784509	8	8	24	0.065255145	0.665048441	24	2.611579678	0.070096318
ENSMUSG00000024099	Ndufv2	NADH:ubiquinone oxidoreductase core subunit V2 [Source:MGI Symbol;Acc:MGI:1920150]	149.628	177.715	172.181	186.406	163.681	155.834	162.604	169.37	199.63	147.752	193.351	171.501	186.072	185.402	0.714062999	0.966018836	12	12	24	0.347316722	0.778844143	24	8.207293642	0.073484315
ENSMUSG00000024101	Washc1	WASH complex subunit 1 [Source:MGI Symbol;Acc:MGI:1916017]	15.5926	14.4918	15.6607	14.4364	14.024	15.326	16.2543	16.6819	15.9118	17.5522	14.609	16.5097	15.4367	16.7337	0.702630284	0.966018836	8	12	24	0.301042274	0.768040178	24	4.744630743	0.047320699
ENSMUSG00000024104	Washc2	WASH complex subunit 2` [Source:MGI Symbol;Acc:MGI:106463]	33.7515	31.9197	35.3272	34.1684	30.4415	33.6666	33.4675	32.2836	28.3957	34.3922	31.7927	33.4963	32.3912	33.247	0.93447422	0.966018836	16	16	24	0.903645277	0.973372694	24	21.27787284	0.014606541
ENSMUSG00000024107	Lhcgr	luteinizing hormone/choriogonadotropin receptor [Source:MGI Symbol;Acc:MGI:96783]	1.62814	1.71129	1.65493	1.46145	1.40972	1.11466	1.05841	1.10129	0.954821	1.54868	1.54188	1.57216	1.70878	1.69559	0.918193129	0.966018836	8	12	24	0.999643712	0.999970554	24	18.31741571	0.001865718
ENSMUSG00000024109	Nrxn1	neurexin I [Source:MGI Symbol;Acc:MGI:1096391]	40.2393	34.7603	32.9963	35.5512	33.3548	37.7837	35.3634	32.0157	32.7303	33.7054	34.7668	40.6437	39.9958	38.09	0.015984991	0.253395174	24	12	24	0.019149083	0.562219749	24	21.82473684	0.111362495
ENSMUSG00000024112	Cacna1h	calcium channel, voltage-dependent, T type, alpha 1H subunit [Source:MGI Symbol;Acc:MGI:1928842]	6.99083	6.63769	6.41563	6.73464	5.4025	7.84983	6.09563	6.18737	7.47795	8.55336	6.19353	6.43828	7.35109	6.60826	0.890235289	0.966018836	16	4	24	0.862636965	0.959941154	24	8.615092425	0.033454247
ENSMUSG00000024118	Tedc2	tubulin epsilon and delta complex 2 [Source:MGI Symbol;Acc:MGI:1919266]	4.96682	4.4629	4.44578	3.9705	3.84455	4.32792	4.27671	4.5438	4.4812	5.17321	4.25788	4.92519	4.32546	3.95035	0.865490576	0.966018836	24	20	24	0.921093011	0.979223729	24	0.898701036	0.016708938
ENSMUSG00000024120	Lrpprc	leucine-rich PPR-motif containing [Source:MGI Symbol;Acc:MGI:1919666]	25.0438	22.4744	23.8023	24.6035	21.8741	24.3803	22.3084	23.7632	21.1342	27.0383	23.6192	24.0915	25.2012	25.6688	0.896753532	0.966018836	16	4	24	0.993326481	0.998731933	24	17.56309753	0.004288138
ENSMUSG00000024121	Atp6v0c	ATPase, H+ transporting, lysosomal V0 subunit C [Source:MGI Symbol;Acc:MGI:88116]	52.7089	41.1178	50.1872	38.7749	40.5047	47.3082	48.6542	51.9224	39.4958	53.881	45.0319	45.2708	44.4494	51.9662	0.643501552	0.966018836	4	16	24	0.491747801	0.805393068	24	1.984127193	0.073694249
ENSMUSG00000024122	Pdpk1	3-phosphoinositide dependent protein kinase 1 [Source:MGI Symbol;Acc:MGI:1338068]	26.9327	23.9077	22.9306	25.112	24.1686	24.9027	24.2812	22.5694	22.425	24.361	25.9001	25.6107	25.3967	25.2679	0.068712455	0.432567661	24	12	24	0.047658424	0.635670298	24	19.84518182	0.067634792
ENSMUSG00000024127	Prepl	prolyl endopeptidase-like [Source:MGI Symbol;Acc:MGI:2441932]	122.632	106.207	117.941	108.483	95.3626	122.438	118.541	109.303	88.2327	131.312	100.651	113.167	104.064	117.172	0.893609703	0.966018836	24	20	24	0.732550489	0.912739737	24	23.05968119	0.049332769
ENSMUSG00000024130	Abca3	ATP-binding cassette, sub-family A (ABC1), member 3 [Source:MGI Symbol;Acc:MGI:1351617]	24.6502	18.3438	19.2963	19.6834	21.101	23.7968	21.7463	22.3617	15.7907	27.2003	18.5428	21.202	19.6053	19.9763	0.390686537	0.915479136	24	12	24	0.404163035	0.799546792	24	19.70256208	0.103648489
ENSMUSG00000024132	Eci1	enoyl-Coenzyme A delta isomerase 1 [Source:MGI Symbol;Acc:MGI:94871]	24.8434	27.0415	34.9389	49.3749	35.1347	31.2651	30.9667	40.3584	34.7427	39.3952	43.0398	32.9935	44.2385	36.9861	0.347721987	0.870418653	16	12	24	0.061516512	0.661180819	24	11.80180834	0.197154835
ENSMUSG00000024135	Srbd1	S1 RNA binding domain 1 [Source:MGI Symbol;Acc:MGI:1925836]	5.81353	5.70023	5.09632	5.17017	6.01011	6.01579	6.1742	6.22244	4.5468	4.49998	4.97405	5.12833	4.68073	5.23156	0.241950772	0.741580974	8	4	24	0.064258592	0.663876076	24	22.58345079	0.103976379
ENSMUSG00000024136	Dnase1l2	deoxyribonuclease 1-like 2 [Source:MGI Symbol;Acc:MGI:1913955]	3.01545	2.39481	2.37227	3.54982	2.45185	2.33201	3.48791	2.3973	2.60626	2.08679	3.7944	2.03611	2.17459	2.25264	0.90767227	0.966018836	20	4	24	0.715359204	0.90631377	24	16.15016749	0.068728319
ENSMUSG00000024137	E4f1	E4F transcription factor 1 [Source:MGI Symbol;Acc:MGI:109530]	10.3745	12.2431	11.5403	11.9886	15.3342	13.3585	11.4475	13.3314	11.7878	9.97834	10.1742	12.2828	12.4589	11.0331	0.945728192	0.966018836	24	16	24	0.735145166	0.91363828	24	20.0989379	0.050682833
ENSMUSG00000024140	Epas1	endothelial PAS domain protein 1 [Source:MGI Symbol;Acc:MGI:109169]	16.4225	15.9383	17.058	18.4314	15.8767	16.7853	16.9791	20.0297	16.2784	23.6739	15.507	16.8478	17.286	17.0991	0.265774856	0.773510818	16	4	24	0.393759509	0.799546792	24	9.611116285	0.082294602
ENSMUSG00000024142	Mlst8	MTOR associated protein, LST8 homolog (S. cerevisiae) [Source:MGI Symbol;Acc:MGI:1929514]	14.2801	12.8302	15.0807	13.1294	12.7608	15.5023	12.6398	14.8832	12.097	14.1105	12.7757	14.1854	14.0181	13.065	0.874607729	0.966018836	24	20	24	0.708855767	0.903901793	24	22.17518076	0.034839334
ENSMUSG00000024143	Rhoq	ras homolog family member Q [Source:MGI Symbol;Acc:MGI:1931553]	34.7565	31.2493	34.6081	33.2184	35.1573	35.4056	31.7635	33.8183	29.3983	35.4632	35.6594	32.8021	31.8426	33.0803	0.089612519	0.477695418	20	12	24	0.115554873	0.727799063	24	16.53166248	0.061855873
ENSMUSG00000024145	Pigf	phosphatidylinositol glycan anchor biosynthesis, class F [Source:MGI Symbol;Acc:MGI:99462]	10.6452	11.5193	9.89219	9.58567	9.63978	9.42975	9.61521	10.784	11.4962	9.38227	10.9376	10.9522	11.343	10.8073	0.231491055	0.731037655	8	8	24	0.160687788	0.765941695	24	3.350471204	0.069665883
ENSMUSG00000024146	Cript	cysteine-rich PDZ-binding protein [Source:MGI Symbol;Acc:MGI:1929655]	83.6382	84.5865	78.3626	78.6241	83.7761	77.3595	77.2771	73.2129	84.0724	67.0404	84.4039	80.0495	83.7242	83.2552	0.835668938	0.966018836	20	20	24	0.629268939	0.873262098	24	23.17594732	0.037055182
ENSMUSG00000024150	Mcfd2	multiple coagulation factor deficiency 2 [Source:MGI Symbol;Acc:MGI:2183439]	50.1874	44.6172	44.8386	45.5705	48.7553	50.059	46.6333	48.1234	41.9886	44.6875	48.104	46.0565	48.8192	48.7828	0.016795343	0.253395174	4	8	24	0.027054538	0.588305652	24	21.1942657	0.071451011
ENSMUSG00000024151	Msh2	mutS homolog 2 [Source:MGI Symbol;Acc:MGI:101816]	14.9277	14.3996	14.9174	14.1442	14.2476	15.0635	14.64	14.3136	12.9911	14.5813	14.6994	16.398	15.7882	15.1984	0.064422003	0.414867207	24	16	24	0.056117171	0.65534544	24	21.47703695	0.061503082
ENSMUSG00000024158	Hagh	hydroxyacyl glutathione hydrolase [Source:MGI Symbol;Acc:MGI:95745]	87.5622	84.4315	81.0848	86.0138	83.819	89.2107	77.2576	86.5179	77.2114	77.7448	90.897	86.2242	93.3355	86.6034	0.188783717	0.664348865	24	12	24	0.214687629	0.768040178	24	20.99823377	0.058042266
ENSMUSG00000024160	Spsb3	splA/ryanodine receptor domain and SOCS box containing 3 [Source:MGI Symbol;Acc:MGI:1891471]	15.8345	14.7689	15.1862	14.6763	14.4776	15.5701	15.2139	16.9763	13.9512	16.7597	13.0135	16.0595	14.5033	15.6378	0.581070934	0.966018836	8	12	24	0.557955576	0.839532165	24	2.450834597	0.040791823
ENSMUSG00000024163	Mapk8ip3	mitogen-activated protein kinase 8 interacting protein 3 [Source:MGI Symbol;Acc:MGI:1353598]	85.9617	71.7898	72.1281	78.5265	80.397	81.76	77.869	78.8124	67.954	90.8427	71.5888	80.5479	75.5231	76.1812	0.14313801	0.585944911	24	12	24	0.354239892	0.781403067	24	18.6872615	0.063964371
ENSMUSG00000024164	C3	complement component 3 [Source:MGI Symbol;Acc:MGI:88227]	6.7892	6.26325	8.11199	12.1965	9.60045	7.83414	8.09284	9.47967	6.39118	9.64147	7.16619	9.23484	8.77184	8.11575	0.348367381	0.870418653	16	20	24	0.327560396	0.768040178	24	14.1246118	0.134007685
ENSMUSG00000024165	Jpt2	Jupiter microtubule associated homolog 2 [Source:MGI Symbol;Acc:MGI:1196260]	5.52202	5.60827	6.17204	5.16073	6.12489	5.83818	6.40316	5.75318	5.14523	5.47632	5.43791	5.72616	5.64721	5.61924	0.724067924	0.966018836	24	16	24	0.338467318	0.773151665	24	22.33460438	0.04465769
ENSMUSG00000024168	Tmem204	transmembrane protein 204 [Source:MGI Symbol;Acc:MGI:3039635]	3.40731	3.47763	4.81611	4.32769	4.34186	4.03053	4.11205	3.85385	3.9562	4.52982	3.37062	3.53877	3.50449	3.62476	0.19514372	0.673387182	16	12	24	0.069732067	0.674183933	24	11.34903015	0.107610478
ENSMUSG00000024169	Ift140	intraflagellar transport 140 [Source:MGI Symbol;Acc:MGI:2146906]	9.2315	7.9993	8.98481	8.19439	8.63222	10.2681	9.37469	9.52842	7.41559	9.2697	8.05286	8.92114	8.60557	7.89475	0.595037318	0.966018836	24	12	24	0.175768494	0.768040178	24	20.80982283	0.082372938
ENSMUSG00000024170	Telo2	telomere maintenance 2 [Source:MGI Symbol;Acc:MGI:1918968]	9.77886	9.70239	10.4311	7.53823	9.5345	9.62186	9.16743	9.82314	9.93479	8.76871	8.74203	8.75289	8.79089	10.2717	0.002268551	0.104113924	12	4	24	0.129570849	0.740162193	24	3.794158193	0.082655924
ENSMUSG00000024172	St6gal2	beta galactoside alpha 2,6 sialyltransferase 2 [Source:MGI Symbol;Acc:MGI:2445190]	4.44182	4.70951	4.326	4.16475	4.65852	4.71179	4.07121	3.85354	3.99879	3.96698	4.41504	4.64012	4.58881	5.14369	0.44861185	0.966018836	24	16	24	0.382859781	0.799546792	24	21.74239308	0.053614184
ENSMUSG00000024174	Pot1b	protection of telomeres 1B [Source:MGI Symbol;Acc:MGI:1920086]	5.24114	5.7145	5.00379	5.43807	5.34132	5.19295	5.22335	4.8956	5.96061	4.77092	5.53352	5.4021	5.54031	4.8441	0.799229803	0.966018836	20	12	24	0.914332261	0.976006964	24	18.33812066	0.013790297
ENSMUSG00000024176	Sox8	SRY (sex determining region Y)-box 8 [Source:MGI Symbol;Acc:MGI:98370]	3.58698	4.81336	5.68242	4.68181	4.24628	4.36553	5.92229	4.363	5.06794	4.11676	4.45277	4.92831	4.20832	4.33487	0.888379597	0.966018836	12	16	24	0.696361073	0.89922881	24	7.349866134	0.055441222
ENSMUSG00000024177	Nme4	NME/NM23 nucleoside diphosphate kinase 4 [Source:MGI Symbol;Acc:MGI:1931148]	2.05282	2.07596	1.64671	2.22047	1.91833	1.73534	2.17833	1.9214	2.00704	1.57295	2.19671	1.69078	1.97736	2.08001	0.934110497	0.966018836	4	20	24	0.764051292	0.92403577	24	1.738470908	0.030752574
ENSMUSG00000024180	Pgap6	post-glycosylphosphatidylinositol attachment to proteins 6 [Source:MGI Symbol;Acc:MGI:1926283]	10.7405	9.28126	9.64436	10.4886	9.29606	10.5999	10.2607	9.95095	8.00708	10.7517	9.40547	10.0452	9.49971	9.72036	0.652290823	0.966018836	24	12	24	0.36737998	0.79073229	24	19.9001402	0.058081613
ENSMUSG00000024181	Mrpl28	mitochondrial ribosomal protein L28 [Source:MGI Symbol;Acc:MGI:1915861]	38.8059	35.7311	35.8388	35.9113	33.2301	36.5724	36.7638	38.3959	33.9286	39.0185	38.8159	36.5909	38.8647	36.4559	0.43565473	0.966018836	4	8	24	0.581793681	0.85096264	24	23.21929429	0.028652456
ENSMUSG00000024182	Axin1	axin 1 [Source:MGI Symbol;Acc:MGI:1096327]	5.57471	5.49446	6.1455	5.73935	5.55358	7.01304	5.39623	6.02012	6.18991	5.91462	4.92604	5.60527	4.96132	6.287	0.267533088	0.774280302	12	16	24	0.68521679	0.895310095	24	7.855433162	0.041737246
ENSMUSG00000024186	Rgs11	regulator of G-protein signaling 11 [Source:MGI Symbol;Acc:MGI:1354739]	31.3819	31.5466	26.1027	30.3934	32.2382	28.6565	28.3964	31.6324	33.904	30.4102	30.2138	29.4119	31.958	30.3266	0.916860713	0.966018836	8	16	24	0.920399567	0.978864885	24	6.534480217	0.015303747
ENSMUSG00000024187	Fam234a	family with sequence similarity 234, member A [Source:MGI Symbol;Acc:MGI:2146854]	18.4753	17.3222	16.1438	17.3502	19.5964	19.986	19.7936	19.0905	17.8712	20.8719	17.5138	18.6315	19.3181	17.7053	0.212387017	0.701530532	24	12	24	0.240124496	0.768040178	24	19.71257312	0.062972093
ENSMUSG00000024188	Luc7l	Luc7-like [Source:MGI Symbol;Acc:MGI:1914228]	28.7439	26.237	25.9856	26.9699	25.9196	25.1775	28.3972	24.894	26.9599	26.8174	29.3228	27.0663	28.1947	25.9894	0.503972924	0.966018836	4	4	24	0.563726147	0.842737781	24	19.66457236	0.030350109
ENSMUSG00000024190	Dusp1	dual specificity phosphatase 1 [Source:MGI Symbol;Acc:MGI:105120]	9.74094	9.44839	10.1882	11.2043	9.19146	9.11036	10.7841	14.0044	10.9384	12.7927	10.782	9.02578	10.5047	9.34764	0.069194004	0.433403132	16	8	24	0.231794912	0.768040178	24	8.625094137	0.116541464
ENSMUSG00000024191	Bnip1	BCL2/adenovirus E1B interacting protein 1 [Source:MGI Symbol;Acc:MGI:109328]	15.5409	14.3506	14.3557	14.6308	13.7092	13.4311	13.8959	13.3977	13.042	15.1045	12.8971	12.5213	14.4358	13.1539	0.933421033	0.966018836	4	20	24	0.862692723	0.959967665	24	6.064957617	0.015944755
ENSMUSG00000024193	Phf1	PHD finger protein 1 [Source:MGI Symbol;Acc:MGI:98647]	16.2742	16.226	14.4985	15.2801	16.762	14.4505	16.6001	15.8669	16.6006	17.433	17.3466	16.0955	16.6106	15.966	0.70641939	0.966018836	20	12	24	0.871374193	0.963499339	24	15.31947485	0.015055277
ENSMUSG00000024194	Cuta	cutA divalent cation tolerance homolog [Source:MGI Symbol;Acc:MGI:1914925]	51.432	44.2504	44.6378	46.9136	49.6774	47.702	51.1581	50.3788	46.3703	50.068	46.7146	46.4999	49.7638	44.4244	0.143321297	0.585944911	4	4	24	0.324065447	0.768040178	24	20.57724672	0.045469259
ENSMUSG00000024197	Plin3	perilipin 3 [Source:MGI Symbol;Acc:MGI:1914155]	11.9647	11.6241	10.6616	10.9161	12.7442	10.849	13.756	12.2739	13.2229	11.6527	12.9123	12.3809	13.1783	11.7108	0.767183862	0.966018836	4	12	24	0.537993517	0.828284875	24	22.52855413	0.04329417
ENSMUSG00000024201	Kdm4b	lysine (K)-specific demethylase 4B [Source:MGI Symbol;Acc:MGI:2442355]	10.8506	8.94773	9.31794	9.68758	9.47788	10.5144	9.78028	10.2095	8.92992	11.3431	9.61609	8.91087	10.5223	10.3068	0.7207001	0.966018836	4	8	24	0.858085055	0.95815511	24	22.09650881	0.022828016
ENSMUSG00000024205	Rpl36-ps2	ribosomal protein L36, pseudogene 2 [Source:MGI Symbol;Acc:MGI:3650081]	34.8145	38.8221	34.7304	36.8104	22.9706	22.1852	32.5071	37.1551	44.7099	33.8612	35.331	31.1955	35.1484	33.4633	0.038115943	0.334069441	12	12	24	0.020135575	0.565108098	24	6.668272194	0.259664708
ENSMUSG00000024206	Rfx2	regulatory factor X, 2 (influences HLA class II expression) [Source:MGI Symbol;Acc:MGI:106583]	0.633131	0.782621	0.687273	1.12379	0.838361	0.705092	1.0562	0.616525	0.931544	0.760318	0.834315	0.75798	0.747334	0.823962	0.478349072	0.966018836	16	16	24	0.508080277	0.813175565	24	12.56881549	0.052128108
ENSMUSG00000024208	Uqcc2	ubiquinol-cytochrome c reductase complex assembly factor 2 [Source:MGI Symbol;Acc:MGI:1914517]	124.464	137.993	125.039	124.729	120.699	115.798	122.783	129.323	146.792	109.231	143.18	133.849	143.606	133.202	0.708020493	0.966018836	12	4	24	0.462654895	0.805393068	24	4.000705202	0.056540532
ENSMUSG00000024210	Ip6k3	inositol hexaphosphate kinase 3 [Source:MGI Symbol;Acc:MGI:3045325]	0.381607	0.462493	0.514201	1.06883	0.984946	0.819702	0.621951	1.04517	0.870811	1.1881	0.564705	0.646039	0.6998	0.538113	0.151902463	0.600361608	16	12	24	0.103809405	0.715480277	24	12.59809999	0.160189585
ENSMUSG00000024211	Grm8	glutamate receptor, metabotropic 8 [Source:MGI Symbol;Acc:MGI:1351345]	5.22449	3.77985	3.89697	5.05276	3.71313	4.42284	4.66891	4.68688	3.73179	4.68046	4.21121	4.44927	4.54739	4.69277	0.465479825	0.966018836	4	16	24	0.589899817	0.854661432	24	22.79091291	0.05406502
ENSMUSG00000024212	Mllt1	myeloid/lymphoid or mixed-lineage leukemia; translocated to, 1 [Source:MGI Symbol;Acc:MGI:1927238]	7.02269	5.10274	6.04378	6.11672	6.4271	5.81149	6.91865	6.68185	5.64062	6.56743	5.67754	6.63554	5.21547	5.90528	0.90767227	0.966018836	4	8	24	0.721392328	0.90866625	24	19.16229028	0.041409213
ENSMUSG00000024213	Nudt3	nudix (nucleotide diphosphate linked moiety X)-type motif 3 [Source:MGI Symbol;Acc:MGI:1928484]	98.5866	89.1482	83.2883	83.6407	96.0415	94.7021	98.765	93.9349	83.0137	92.4024	91.5989	94.1108	89.8929	87.6128	0.005234097	0.147078973	4	8	24	0.005431651	0.445165967	24	20.91853897	0.092907189
ENSMUSG00000024217	Snrpc	U1 small nuclear ribonucleoprotein C [Source:MGI Symbol;Acc:MGI:109489]	3.64479	4.54213	2.977	4.69012	3.73513	4.20755	4.82971	4.47185	3.92003	4.7445	4.7325	3.86665	4.64455	4.00844	0.904690479	0.966018836	16	20	24	0.909003289	0.974606327	24	19.33497341	0.027760774
ENSMUSG00000024218	Taf11	TATA-box binding protein associated factor 11 [Source:MGI Symbol;Acc:MGI:1916026]	24.7189	31.487	33.2557	30.1878	26.1039	30.204	24.5402	30.8353	29.6035	26.5808	27.101	29.2856	28.9141	28.4495	0.243586913	0.741580974	12	16	24	0.254009247	0.768040178	24	7.649834383	0.083545317
ENSMUSG00000024219	Anks1	ankyrin repeat and SAM domain containing 1 [Source:MGI Symbol;Acc:MGI:2446180]	19.7345	15.9457	18.5521	18.8872	19.1353	17.861	18.5056	17.9723	17.8981	22.0829	18.8965	17.5533	18.9487	16.2302	0.115745527	0.537734429	20	12	24	0.171382906	0.768040178	24	14.78823312	0.07647566
ENSMUSG00000024220	Zfp523	zinc finger protein 523 [Source:MGI Symbol;Acc:MGI:2687278]	18.2573	17.3707	17.253	16.9716	12.8454	16.6715	17.3688	17.9976	16.3815	15.5852	14.8713	16.1834	15.3518	15.3363	0.080148772	0.458678242	4	16	24	0.065377898	0.665998448	24	3.281037853	0.095323743
ENSMUSG00000024222	Fkbp5	FK506 binding protein 5 [Source:MGI Symbol;Acc:MGI:104670]	13.0229	9.45167	10.6853	21.6251	11.2365	14.2319	10.3427	9.28198	9.17946	13.4313	10.6432	11.7294	9.96866	9.3662	0.01518376	0.253245931	16	16	24	0.080480764	0.695795041	24	15.41481017	0.24038155
ENSMUSG00000024227	Pdzph1	PDZ and pleckstrin homology domains 1 [Source:MGI Symbol;Acc:MGI:1916489]	9.85043	10.1456	8.26937	9.89969	11.8826	7.60818	11.5752	8.11084	12.057	7.56027	12.6903	9.55436	10.6963	9.29071	0.934818505	0.966018836	20	12	24	0.836689493	0.949953371	24	18.24323787	0.055799362
ENSMUSG00000024228	Nudt12	nudix (nucleoside diphosphate linked moiety X)-type motif 12 [Source:MGI Symbol;Acc:MGI:1915243]	10.4955	11.3342	10.3839	13.2861	10.0907	10.9106	10.8311	10.3991	9.77717	10.1047	12.3576	10.4536	9.95381	9.31667	0.92655264	0.966018836	16	20	24	0.253268747	0.768040178	24	15.52497375	0.072532126
ENSMUSG00000024231	Cul2	cullin 2 [Source:MGI Symbol;Acc:MGI:1918995]	41.8313	41.0391	37.3541	38.7727	38.601	40.2074	38.7814	39.9655	36.8636	37.5161	40.5394	40.6168	42.3404	40.8429	0.004501001	0.140598674	4	8	24	0.009989803	0.503967859	24	22.85246788	0.062177774
ENSMUSG00000024232	Bambi	BMP and activin membrane-bound inhibitor [Source:MGI Symbol;Acc:MGI:1915260]	1.57035	1.7554	1.65692	1.86088	1.69462	1.48351	1.81238	1.63761	1.78763	1.70167	1.59835	1.67452	1.60777	1.58132	0.335303345	0.85758369	16	8	24	0.267903354	0.768040178	24	9.668216648	0.035621257
ENSMUSG00000024234	Mtpap	mitochondrial poly(A) polymerase [Source:MGI Symbol;Acc:MGI:1914690]	11.2044	10.6652	10.8601	10.4722	10.747	10.3638	11.2802	10.5543	10.5971	10.6546	9.80665	10.9831	10.814	11.5623	0.127318615	0.560209134	4	16	24	0.087105237	0.706240303	24	2.307879576	0.042462622
ENSMUSG00000024235	Map3k8	mitogen-activated protein kinase kinase kinase 8 [Source:MGI Symbol;Acc:MGI:1346878]	1.24997	1.75224	1.45879	1.50037	1.55504	1.26108	0.949389	1.17985	1.61655	1.11358	1.26665	1.62914	1.34087	1.26009	0.93357733	0.966018836	12	16	24	0.693890605	0.898364349	24	11.1092601	0.047085849
ENSMUSG00000024236	Svil	supervillin [Source:MGI Symbol;Acc:MGI:2147319]	9.26981	11.2497	10.3414	12.727	11.8588	10.6363	11.4382	13.5425	13.077	13.3232	10.449	11.0647	11.2541	10.5484	0.801995186	0.966018836	16	8	24	0.128831332	0.740162193	24	10.01258194	0.111713809
ENSMUSG00000024238	Zeb1	zinc finger E-box binding homeobox 1 [Source:MGI Symbol;Acc:MGI:1344313]	19.5927	19.5296	20.3258	21.0184	19.5219	20.1254	20.4805	19.1772	19.3826	21.9074	19.7197	20.2782	18.7063	22.573	0.951846915	0.966018836	16	12	24	0.473115079	0.805393068	24	10.44297428	0.034184069
ENSMUSG00000024240	Epc1	enhancer of polycomb homolog 1 [Source:MGI Symbol;Acc:MGI:1278322]	7.89885	8.2328	9.12014	9.3927	7.84449	8.34051	8.75179	8.53574	9.36624	8.85031	9.09816	8.57518	7.61077	8.04366	0.028612069	0.316608413	12	16	24	0.03776834	0.61140397	24	11.02531181	0.080370266
ENSMUSG00000024241	Sos1	SOS Ras/Rac guanine nucleotide exchange factor 1 [Source:MGI Symbol;Acc:MGI:98354]	18.6543	16.5392	15.0136	16.4644	16.2835	16.7005	16.4736	15.6243	14.3808	16.6426	16.7406	16.1818	16.2367	16.6701	0.418381424	0.963884303	20	16	24	0.088409146	0.707747429	24	20.74947169	0.066163339
ENSMUSG00000024242	Map4k3	mitogen-activated protein kinase kinase kinase kinase 3 [Source:MGI Symbol;Acc:MGI:2154405]	15.7919	16.1735	15.5739	16.064	15.3656	16.2215	16.6278	14.8864	14.68	15.8461	15.2013	15.2252	14.1073	15.042	0.90332508	0.966018836	16	20	24	0.613401902	0.866162311	24	18.0618848	0.019475743
ENSMUSG00000024245	Tmem178	transmembrane protein 178 [Source:MGI Symbol;Acc:MGI:1915277]	0.878995	1.19705	1.32215	1.16123	0.777368	1.26765	1.04236	1.04456	1.09665	1.4514	0.891707	1.16657	1.2369	1.07413	0.718720526	0.966018836	16	4	24	0.500607302	0.808986978	24	7.88301418	0.061057731
ENSMUSG00000024246	Thumpd2	THUMP domain containing 2 [Source:MGI Symbol;Acc:MGI:1919417]	1.66082	1.83518	1.44581	1.55815	1.98416	1.78636	1.94246	1.89643	2.02943	1.22227	1.77362	2.04591	1.9264	1.9595	0.725061903	0.966018836	24	16	24	0.146147597	0.754849922	24	23.46436868	0.09274956
ENSMUSG00000024247	Pkdcc	protein kinase domain containing, cytoplasmic [Source:MGI Symbol;Acc:MGI:2147077]	10.2753	10.4135	11.2585	12.9962	11.7704	10.2063	12.6005	13.2666	13.5356	10.8014	10.5796	10.192	12.2516	10.224	0.085890569	0.466404237	12	12	24	0.409603179	0.799546792	24	8.090874717	0.078119785
ENSMUSG00000024248	Cox7a2l	cytochrome c oxidase subunit 7A2 like [Source:MGI Symbol;Acc:MGI:106015]	120.937	129.431	127.633	127.208	120.94	123.48	122.821	123.565	135.239	112.953	128.894	128.986	133.325	132.039	0.718720526	0.966018836	12	4	24	0.412804045	0.799546792	24	4.422175093	0.033848446
ENSMUSG00000024251	Thada	thyroid adenoma associated [Source:MGI Symbol;Acc:MGI:3039623]	2.20441	2.44216	2.72663	2.35158	2.63633	2.35215	2.56005	2.3998	2.30906	2.0887	2.43173	2.44959	2.62277	2.21122	0.924553956	0.966018836	20	20	24	0.818615972	0.942446688	24	21.12344917	0.020133842
ENSMUSG00000024253	Dync2li1	dynein cytoplasmic 2 light intermediate chain 1 [Source:MGI Symbol;Acc:MGI:1913996]	16.464	16.9025	15.6494	15.828	15.561	15.1103	17.0964	16.6499	15.2155	14.8737	15.6617	15.899	14.3663	15.9524	0.226403091	0.722427815	8	8	24	0.237975631	0.768040178	24	1.947126762	0.038628735
ENSMUSG00000024256	Adcyap1	adenylate cyclase activating polypeptide 1 [Source:MGI Symbol;Acc:MGI:105094]	44.8997	37.2897	48.7101	35.4422	46.6935	42.1913	37.3998	34.6293	30.7085	38.4295	42.4238	38.4109	36.7247	36.4699	0.109247218	0.524035616	20	12	24	0.174908387	0.768040178	24	18.02113912	0.11727466
ENSMUSG00000024258	Polr2d	polymerase (RNA) II (DNA directed) polypeptide D [Source:MGI Symbol;Acc:MGI:1916491]	14.5024	17.1417	17.7163	16.2789	15.7883	15.3144	15.7162	17.9075	15.8171	14.0325	15.6524	14.6788	15.8547	14.5758	0.321662653	0.83607295	12	12	24	0.297959003	0.768040178	24	6.397330767	0.057329157
ENSMUSG00000024259	Slc25a46	solute carrier family 25, member 46 [Source:MGI Symbol;Acc:MGI:1914703]	38.9699	36.3224	36.1807	36.0567	35.9126	35.73	35.4061	35.0053	32.3322	35.6186	38.2344	35.4656	37.1309	36.8201	0.928127947	0.966018836	20	16	24	0.324795711	0.768040178	24	20.79508575	0.036602249
ENSMUSG00000024260	Sap130	Sin3A associated protein [Source:MGI Symbol;Acc:MGI:1919782]	7.18479	7.13342	7.48374	6.96078	7.48637	7.75426	6.98512	7.92206	7.64083	8.69065	6.3448	7.19315	7.21156	7.0751	0.874672901	0.966018836	12	16	24	0.596427678	0.857839743	24	9.065103883	0.039203815
ENSMUSG00000024261	Syt4	synaptotagmin IV [Source:MGI Symbol;Acc:MGI:101759]	129.263	116.867	120.81	112.587	126.255	122.912	113.539	108.54	110.358	108.393	145.481	119.744	121.402	128.125	0.129903614	0.560263689	20	20	24	0.250855819	0.768040178	24	19.08036256	0.076732729
ENSMUSG00000024268	Celf4	CUGBP, Elav-like family member 4 [Source:MGI Symbol;Acc:MGI:1932407]	24.062	21.1594	24.1071	19.3438	17.2358	22.1907	22.426	22.1202	18.297	25.8976	20.7141	22.0346	18.6049	23.2096	0.924553956	0.966018836	16	4	24	0.731633002	0.912544394	24	3.582119799	0.051034548
ENSMUSG00000024269	Tpgs2	tubulin polyglutamylase complex subunit 2 [Source:MGI Symbol;Acc:MGI:1913898]	18.2141	15.6045	15.9366	16.5473	16.6337	17.5977	15.7973	15.7036	13.6723	15.9049	15.621	15.817	16.609	16.0565	0.413946331	0.958746003	24	12	24	0.049927548	0.643727973	24	20.4861663	0.076791632

ENSMUSG00000024270	Slc39a6	solute carrier family 39 (metal ion transporter), member 6 [Source:MGI Symbol;Acc:MGI:2147279]	28.1148	21.3588	22.1399	22.9751	21.9166	25.7341	24.1155	23.9786	18.5559	29.2059	23.0733	24.1509	24.2279	25.4544	0.143321297	0.585944911	24	12	24	0.453875257	0.805393068	24	20.85217785	0.08085712
ENSMUSG00000024271	Elp2	elongator acetyltransferase complex subunit 2 [Source:MGI Symbol;Acc:MGI:1889642]	43.5898	47.3071	47.2676	45.2497	47.0128	43.743	45.9881	46.6952	47.0078	41.8167	46.1373	46.7925	46.2398	48.197	0.150109238	0.595846665	8	20	24	0.381074365	0.799546792	24	4.804438593	0.029872367
ENSMUSG00000024273	2700062C07Rik	RIKEN cDNA 2700062C07 gene [Source:MGI Symbol;Acc:MGI:1915296]	5.96565	6.47436	6.69992	6.90668	7.07383	6.51436	6.11305	6.54527	7.46572	6.59814	7.4013	7.12009	6.88522	7.81859	0.33885934	0.858075318	20	8	24	0.096344161	0.71175955	24	11.08226611	0.058563354
ENSMUSG00000024274	Zscan30	zinc finger and SCAN domain containing 30 [Source:MGI Symbol;Acc:MGI:2685600]	2.59394	2.39956	2.71953	2.94908	3.08851	2.71066	3.95868	2.5627	2.85956	2.21917	2.73194	2.23943	2.30517	2.3409	0.91695334	0.966018836	20	12	24	0.846721669	0.953679112	24	18.36436682	0.03745104
ENSMUSG00000024276	Zfp397	zinc finger protein 397 [Source:MGI Symbol;Acc:MGI:1916506]	14.087	13.8911	13.074	13.4822	13.9883	12.5569	15.8281	12.7403	15.129	12.5814	17.1811	15.9949	15.2643	16.3002	0.895493752	0.966018836	20	20	24	0.609100055	0.864393298	24	22.07575901	0.047713623
ENSMUSG00000024277	Mapre2	microtubule-associated protein, RP/EB family, member 2 [Source:MGI Symbol;Acc:MGI:106271]	68.7717	51.8195	55.012	56.8675	52.7264	66.8584	62.4427	63.9765	43.8197	81.3413	49.493	59.612	56.1134	59.0387	0.46694635	0.966018836	24	12	24	0.717960257	0.907029631	24	21.16313864	0.074316028
ENSMUSG00000024283	Wac	WW domain containing adaptor with coiled-coil [Source:MGI Symbol;Acc:MGI:2387357]	26.5541	26.0814	26.5872	27.6004	25.6794	25.9704	27.5279	25.8933	23.2731	24.6075	24.2577	26.3762	24.938	23.5583	0.920858634	0.966018836	4	8	24	0.269208371	0.768040178	24	20.76555439	0.037081398
ENSMUSG00000024286	Ccny	cyclin Y [Source:MGI Symbol;Acc:MGI:1915224]	26.0311	24.5441	24.6245	26.5306	26.9777	25.5967	26.2667	27.5102	23.5532	24.327	23.9425	24.6012	25.8813	26.0821	0.930944473	0.966018836	8	4	24	0.477758149	0.805393068	24	23.30492511	0.031304641
ENSMUSG00000024287	Thoc1	THO complex 1 [Source:MGI Symbol;Acc:MGI:1919668]	25.9431	28.6373	28.1778	24.292	29.6615	24.5794	25.7393	22.9562	32.0083	23.5117	29.2445	27.7029	27.1472	31.1966	0.939103211	0.966018836	12	4	24	0.837639196	0.950322752	24	7.571875711	0.035471678
ENSMUSG00000024290	Rock1	Rho-associated coiled-coil containing protein kinase 1 [Source:MGI Symbol;Acc:MGI:107927]	11.7743	12.6263	11.7788	12.5202	12.0853	10.2508	12.12	10.7282	13.4228	10.0243	13.5406	10.8933	11.5471	9.95621	0.892685629	0.966018836	20	4	24	0.757743478	0.921675291	24	12.45647124	0.037860828
ENSMUSG00000024292	Cyp4f14	cytochrome P450, family 4, subfamily f, polypeptide 14 [Source:MGI Symbol;Acc:MGI:1927669]	3.19062	3.31531	3.37208	2.46479	3.95142	3.48724	3.45957	4.28685	3.24709	3.18884	3.45703	2.62994	3.25103	3.17421	0.903207257	0.966018836	20	20	24	0.784167267	0.930681482	24	2.010416574	0.042198028
ENSMUSG00000024293	Esco1	establishment of sister chromatid cohesion N-acetyltransferase 1 [Source:MGI Symbol;Acc:MGI:1925055]	6.28589	6.12692	6.55584	6.59258	6.35287	5.79767	6.5069	5.53972	6.43617	5.46057	7.41792	5.35548	5.75716	5.42335	0.384058496	0.915479136	20	12	24	0.416951476	0.801686744	24	14.03117681	0.055290581
ENSMUSG00000024294	Mib1	mindbomb E3 ubiquitin protein ligase 1 [Source:MGI Symbol;Acc:MGI:2443157]	33.2104	32.7727	32.0893	33.3047	34.2327	31.276	31.6302	28.441	32.3951	31.5966	35.1486	29.2363	29.0649	31.7585	0.256915694	0.76168256	20	4	24	0.128243959	0.740162193	24	13.82686052	0.058116445
ENSMUSG00000024295	Cyp4f41-ps	cytochrome P450, family 4, subfamily f, polypeptide 41 pseudogene [Source:MGI Symbol;Acc:MGI:1925125]	0.930362	0.41198	0.657224	1.13629	0.728439	0.492939	0.525608	0.509394	0.903321	0.377922	0.393908	0.332718	0.694477	0.821415	0.956719163	0.96629412	4	20	24	0.595548463	0.857839743	24	7.700818112	0.085853071
ENSMUSG00000024298	Zfp871	zinc finger protein 871 [Source:MGI Symbol;Acc:MGI:1921793]	14.0427	14.6858	13.3643	14.964	15.4857	13.1465	14.796	12.8185	15.393	11.4775	16.3968	13.8362	14.2458	14.373	0.920992137	0.966018836	20	4	24	0.851679021	0.955514806	24	17.97078568	0.028748307
ENSMUSG00000024299	Adamts10	a disintegrin-like and metallopeptidase (reprolysin type) with thrombospondin type 1 motif, 10 [Source:MGI Symbol;Acc:MGI:2449112]	9.47517	11.5793	10.738	10.0089	12.4913	10.2929	9.67026	11.0428	13.6378	8.5533	10.3832	10.4173	10.1166	10.2933	0.421132906	0.966018836	12	16	24	0.699771172	0.900387881	24	8.184002901	0.053987215
ENSMUSG00000024300	Myo1f	myosin IF [Source:MGI Symbol;Acc:MGI:107711]	1.10013	1.83183	2.39756	1.87166	2.08317	1.25779	1.82275	1.32294	2.25601	1.45199	1.92568	1.42739	1.42065	1.39599	0.049705476	0.384427323	12	16	24	0.075395079	0.685464747	24	10.79837189	0.175350978
ENSMUSG00000024301	Kifc5b	kinesin family member C5B [Source:MGI Symbol;Acc:MGI:2137414]	0.729162	0.831643	0.519094	0.817425	0.570997	0.771215	0.769302	0.54683	0.694965	0.659452	0.729057	0.672791	0.602467	0.868099	0.915475528	0.966018836	8	4	24	0.872799688	0.964262367	24	23.78664199	0.019884069
ENSMUSG00000024302	Dtna	dystrobrevin alpha [Source:MGI Symbol;Acc:MGI:106039]	30.0819	31.044	30.5673	31.9619	30.5141	31.0015	34.1474	27.0466	30.3398	32.829	30.2618	32.2924	30.026	31.0471	0.933543173	0.966018836	16	12	24	0.591321325	0.855808161	24	16.73155176	0.030793465
ENSMUSG00000024304	Cdh2	cadherin 2 [Source:MGI Symbol;Acc:MGI:88355]	16.2035	13.2166	13.3819	14.1141	13.874	15.3342	16.1653	13.3282	10.5125	17.4268	13.1839	14.6798	13.5465	13.7248	0.210418232	0.69936851	24	12	24	0.239808079	0.768040178	24	19.76654942	0.110352844
ENSMUSG00000024308	Tapbp	TAP binding protein [Source:MGI Symbol;Acc:MGI:1201689]	26.6526	23.7615	22.7066	24.155	23.1641	25.2724	26.0871	28.5995	26.0757	30.7373	24.0499	26.4726	25.9697	28.1893	0.822170541	0.966018836	8	12	24	0.553183997	0.837286821	24	4.057332989	0.044697861
ENSMUSG00000024309	Pfdn6	prefoldin subunit 6 [Source:MGI Symbol;Acc:MGI:95908]	45.0512	37.154	42.1219	45.3157	42.6254	45.953	43.3545	43.1607	41.8575	42.7925	39.5778	42.1248	43.0194	36.2122	0.441501717	0.966018836	4	4	24	0.272094015	0.768040178	24	18.06023552	0.059059535
ENSMUSG00000024312	Wdr46	WD repeat domain 46 [Source:MGI Symbol;Acc:MGI:1931871]	3.14273	3.36477	3.68322	3.72706	3.33234	3.24666	3.21694	3.71551	3.53781	4.39805	3.19087	3.58218	3.05801	3.38467	7.90E-04	0.071344007	16	12	24	0.020845	0.568066329	24	10.42181342	0.105430512
ENSMUSG00000024317	Rnf138	ring finger protein 138 [Source:MGI Symbol;Acc:MGI:1929211]	6.30809	7.40513	7.11377	6.57798	6.09671	6.81851	6.34731	6.31907	6.844	6.08147	6.40909	5.73006	5.20051	6.23563	0.496745696	0.966018836	12	16	24	0.17425589	0.768040178	24	8.357429323	0.062710338
ENSMUSG00000024319	Vps52	VPS52 GARP complex subunit [Source:MGI Symbol;Acc:MGI:1330304]	14.6748	13.8766	13.1527	12.5924	14.1565	13.6592	14.4814	13.7705	13.2187	16.8385	12.5424	17.4416	13.3741	14.0676	0.417963425	0.963884303	24	12	24	0.670669468	0.888925819	24	20.29242013	0.04721496
ENSMUSG00000024325	Ring1	ring finger protein 1 [Source:MGI Symbol;Acc:MGI:1101770]	11.8171	11.3053	10.2657	10.0665	10.5933	10.0452	11.7045	11.6468	10.5618	10.5736	11.3986	11.4996	12.1665	10.2578	0.008747574	0.192760771	4	12	24	0.052811593	0.650526959	24	23.53061617	0.075839028
ENSMUSG00000024327	Slc39a7	solute carrier family 39 (zinc transporter), member 7 [Source:MGI Symbol;Acc:MGI:95909]	43.7553	38.8254	38.2324	37.0865	40.2379	40.5023	36.1292	42.3149	35.348	41.8173	37.2947	41.0123	37.8316	38.9878	0.879901313	0.966018836	24	12	24	0.559536934	0.840567271	24	21.18461315	0.038565114
ENSMUSG00000024330	Col11a2	collagen, type XI, alpha 2 [Source:MGI Symbol;Acc:MGI:88447]	2.265	2.67437	3.07085	2.75945	1.93589	2.61906	2.67717	3.53843	3.30461	3.39092	2.43085	2.59787	2.54015	2.34121	0.112583132	0.530777648	16	4	24	0.069429569	0.673353451	24	7.828985058	0.153302351
ENSMUSG00000024335	Brd2	bromodomain containing 2 [Source:MGI Symbol;Acc:MGI:99495]	52.9786	47.0143	49.9154	51.207	52.6315	49.464	49.5443	49.2828	49.5277	58.5188	48.8161	50.6507	48.7143	53.1881	0.886952527	0.966018836	16	16	24	0.386281335	0.799546792	24	12.86192694	0.040445031
ENSMUSG00000024338	Psmb8	proteasome (prosome, macropain) subunit, beta type 8 (large multifunctional peptidase 7) [Source:MGI Symbol;Acc:MGI:1346527]	11.7795	12.3915	14.058	10.8055	13.2778	12.6003	11.7091	14.9094	14.3776	13.1229	15.5833	12.4465	13.9489	16.2044	0.625825704	0.966018836	8	20	24	0.325689439	0.768040178	24	7.338665001	0.077385313
ENSMUSG00000024339	Tap2	transporter 2, ATP-binding cassette, sub-family B (MDR/TAP) [Source:MGI Symbol;Acc:MGI:98484]	7.01486	6.69617	6.87402	5.78208	6.99252	6.52633	6.26662	8.74628	7.03122	7.66113	6.84404	6.39114	6.98751	7.00572	0.631736745	0.966018836	8	16	24	0.408676193	0.799546792	24	5.864239769	0.063949255
ENSMUSG00000024346	Pfdn1	prefoldin 1 [Source:MGI Symbol;Acc:MGI:1914449]	66.0231	73.067	66.823	65.0534	73.36	65.0882	62.7526	66.2934	75.4251	58.6155	74.7328	66.9867	70.5388	72.6828	0.720359655	0.966018836	12	12	24	0.914834381	0.976279382	24	5.927264406	0.018262202
ENSMUSG00000024347	Psd2	pleckstrin and Sec7 domain containing 2 [Source:MGI Symbol;Acc:MGI:1921252]	1.53225	3.0093	5.30655	3.04876	1.70309	3.17919	3.67766	2.36134	3.53606	2.44154	1.56844	2.64149	1.26031	3.51849	0.285027487	0.793714531	12	8	24	0.185838613	0.768040178	24	7.452392234	0.296110532
ENSMUSG00000024349	Sting1	stimulator of interferon response cGAMP interactor 1 [Source:MGI Symbol;Acc:MGI:1919762]	6.26813	4.17701	5.46902	3.40025	6.52005	4.68751	3.87162	3.93433	5.47077	2.62273	6.18804	3.14118	3.48135	2.91328	0.91695334	0.966018836	20	12	24	0.628390243	0.872795898	24	17.53096192	0.119361046
ENSMUSG00000024350	Dnajc18	DnaJ heat shock protein family (Hsp40) member C18 [Source:MGI Symbol;Acc:MGI:1923844]	16.979	15.9617	17.1697	16.125	15.696	17.6138	17.5623	17.7341	15.0269	19.6999	15.5942	16.6653	16.2581	16.4351	0.95032543	0.966018836	24	20	24	0.982611379	0.996130479	24	23.7092115	0.007175633
ENSMUSG00000024352	Spata24	spermatogenesis associated 24 [Source:MGI Symbol;Acc:MGI:1918492]	4.34109	6.88655	6.45949	6.81422	5.15198	3.77236	4.11893	5.11374	7.85804	5.05479	4.9362	4.7963	5.21073	6.3612	0.001971398	0.100255953	12	12	24	0.001947101	0.395705644	24	8.342621484	0.30316773
ENSMUSG00000024353	Mzb1	marginal zone B and B1 cell-specific protein 1 [Source:MGI Symbol;Acc:MGI:1917066]	0.461784	0.946701	0.572239	0.614911	0.610349	0.133158	0.785417	0.53312	0.587337	0.460891	0.502962	0.807954	0.231446	0.610746	0.896311169	0.966018836	8	16	24	0.622941565	0.871570345	24	7.003003517	0.081294476
ENSMUSG00000024357	Sil1	endoplasmic reticulum chaperone SIL1 homolog (S. cerevisiae) [Source:MGI Symbol;Acc:MGI:1932040]	15.1012	15.8522	14.2353	14.3804	13.4276	15.9946	15.669	17.3778	12.8898	16.4453	13.8312	15.5494	13.9278	17.4127	0.117050591	0.538362324	8	12	24	0.305613764	0.768040178	24	2.245779913	0.073835486
ENSMUSG00000024359	Hspa9	heat shock protein 9 [Source:MGI Symbol;Acc:MGI:96245]	86.8814	80.0196	87.5594	94.6105	84.4071	85.9005	85.222	90.8752	77.8425	92.9425	88.1477	87.4463	93.125	90.8407	0.829428469	0.966018836	16	20	24	0.868018098	0.962298123	24	14.57283893	0.016042128
ENSMUSG00000024360	Etf1	eukaryotic translation termination factor 1 [Source:MGI Symbol;Acc:MGI:2385071]	35.2566	32.4236	33.3379	33.8453	32.7914	33.2294	31.2038	31.832	31.0045	33.0148	32.6854	32.3593	33.2601	33.3284	0.826055781	0.966018836	16	16	24	0.740741452	0.915293122	24	18.91147183	0.01415768
ENSMUSG00000024366	Gfra3	glial cell line derived neurotrophic factor family receptor alpha 3 [Source:MGI Symbol;Acc:MGI:1201403]	13.7585	10.7505	13.0686	12.4473	11.6833	16.6877	12.195	16.0655	9.07638	16.69	12.225	13.8011	12.9689	11.9241	0.725061903	0.966018836	24	12	24	0.550272407	0.835462164	24	19.38864622	0.10204838
ENSMUSG00000024369	Nelfe	negative elongation factor complex member E, Rdbp [Source:MGI Symbol;Acc:MGI:102744]	13.8805	13.3845	12.7821	12.9787	13.4178	14.7921	16.7897	16.0344	14.9564	15.3578	13.354	15.0395	13.762	13.7579	0.435596482	0.966018836	24	20	24	0.523024631	0.82106433	24	0.135036838	0.04820287
ENSMUSG00000024370	Cdc23	CDC23 cell division cycle 23 [Source:MGI Symbol;Acc:MGI:1098815]	15.7985	15.7695	18.5662	14.8747	17.7729	17.7642	16.52	15.9037	16.9513	16.0799	16.6394	15.832	15.0411	14.9108	0.247964668	0.746745468	20	12	24	0.485000641	0.805393068	24	14.96910836	0.040869325
ENSMUSG00000024371	C2	complement component 2 (within H-2S) [Source:MGI Symbol;Acc:MGI:88226]	4.73604	6.08272	5.90703	5.30018	5.91766	5.39721	5.30903	6.40437	7.67122	4.21838	6.27891	6.11858	5.45153	6.41802	0.348367381	0.870418653	8	20	24	0.487267029	0.805393068	24	6.540382775	0.085048181
ENSMUSG00000024376	Epb41l4a	erythrocyte membrane protein band 4.1 like 4a [Source:MGI Symbol;Acc:MGI:103007]	3.25552	3.14543	3.40919	2.42397	3.22604	2.96926	3.7978	3.68306	3.13692	3.73463	3.77686	3.22274	3.3244	3.62676	0.916963526	0.966018836	4	20	24	0.627844571	0.872795898	24	2.636107719	0.045263139
ENSMUSG00000024378	Stard4	StAR-related lipid transfer (START) domain containing 4 [Source:MGI Symbol;Acc:MGI:2156764]	31.2798	28.7346	28.4015	28.1549	32.2801	32.0313	28.6677	26.0161	27.7402	27.6445	32.2493	30.795	32.6463	30.115	0.01148626	0.206824017	20	20	24	0.01002592	0.503967859	24	19.51144267	0.105191212
ENSMUSG00000024381	Bin1	bridging integrator 1 [Source:MGI Symbol;Acc:MGI:108092]	53.7398	51.2246	56.3613	51.5557	48.9595	55.8728	52.7795	62.7164	55.0267	65.2872	52.7759	56.2414	54.9867	57.0167	0.953629935	0.966018836	12	8	24	0.41969062	0.803639585	24	7.081836718	0.053653064
ENSMUSG00000024382	Ercc3	excision repair cross-complementing rodent repair deficiency, complementation group 3 [Source:MGI Symbol;Acc:MGI:95414]	15.304	12.4717	12.9414	13.6809	13.5157	15.3914	13.9572	14.6411	11.609	16.0843	11.9436	14.2886	13.385	13.5648	0.245061943	0.741580974	24	12	24	0.473502402	0.805393068	24	20.54785859	0.064080848
ENSMUSG00000024383	Map3k2	mitogen-activated protein kinase kinase kinase 2 [Source:MGI Symbol;Acc:MGI:1346873]	4.20421	4.5914	4.29507	4.72965	4.55233	3.91465	4.47251	4.04445	4.98125	3.41874	4.68489	4.3593	4.4999	4.38361	0.943920575	0.966018836	12	12	24	0.955264707	0.988639288	24	6.117094893	0.01377622
ENSMUSG00000024384	Iws1	IWS1, SUPT6 interacting protein [Source:MGI Symbol;Acc:MGI:1920723]	8.25599	9.18982	7.73356	7.46838	8.23513	7.48549	9.50981	7.62343	8.02433	7.02316	8.77894	9.31244	8.7711	8.55404	0.033553741	0.324908923	4	12	24	0.086710381	0.70594329	24	23.21769206	0.096022659
ENSMUSG00000024387	Csnk2b	casein kinase 2, beta polypeptide [Source:MGI Symbol;Acc:MGI:88548]	51.0324	46.3464	51.9321	49.1448	55.2477	62.503	51.3427	51.3836	47.9808	61.1685	49.5105	49.85	47.7831	51.7969	0.94397916	0.966018836	24	4	24	0.325421404	0.768040178	24	16.26501286	0.071977625
ENSMUSG00000024392	Bag6	BCL2-associated athanogene 6 [Source:MGI Symbol;Acc:MGI:1919439]	38.9663	33.9814	33.9142	34.7616	33.5362	33.8693	35.8525	40.4754	37.7235	46.5271	34.4419	39.5813	36.9948	39.546	0.891105762	0.966018836	8	12	24	0.640549886	0.878323188	24	6.684920718	0.046431075
ENSMUSG00000024393	Prrc2a	proline-rich coiled-coil 2A [Source:MGI Symbol;Acc:MGI:1915467]	41.8497	37.8255	38.6352	41.5526	38.5918	37.7592	35.7416	38.0993	40.5962	47.6562	39.2568	39.59	40.8003	40.1813	0.628286596	0.966018836	16	8	24	0.2197745	0.768040178	24	11.51827623	0.062084806
ENSMUSG00000024395	Lims2	LIM and senescent cell antigen like domains 2 [Source:MGI Symbol;Acc:MGI:2385067]	15.6286	14.9006	16.0837	20.2682	20.7948	15.2634	17.4499	17.0145	18.0778	18.8744	18.1702	16.717	15.529	14.7983	0.01457641	0.247599677	16	16	24	0.005092236	0.442351542	24	13.65393648	0.157706752
ENSMUSG00000024397	Aif1	allograft inflammatory factor 1 [Source:MGI Symbol;Acc:MGI:1343098]	3.77855	6.18113	7.22562	4.65827	4.57603	4.3208	4.75381	4.68916	6.33922	3.8438	5.09939	5.27756	4.63525	5.1158	0.143744387	0.585959661	12	4	24	0.185619376	0.768040178	24	7.367166018	0.159637322
ENSMUSG00000024399	Ltb	lymphotoxin B [Source:MGI Symbol;Acc:MGI:104796]	0.476633	0.677454	0.841676	0.73738	0.74641	0.584893	0.826319	1.00627	1.13202	0.456069	0.577021	0.688002	1.48633	0.458533	0.948161972	0.966018836	12	4	24	0.587541711	0.853591039	24	4.183987586	0.089772593
ENSMUSG00000024400	Wdr33	WD repeat domain 33 [Source:MGI Symbol;Acc:MGI:1921570]	16.9616	16.84	16.951	16.4823	15.6732	15.3325	16.1567	16.5004	15.7785	15.8911	16.0306	16.5869	17.0707	17.1833	0.070311451	0.4391373	8	12	24	0.057620895	0.656248849	24	3.628883077	0.041765201
ENSMUSG00000024403	Atp6v1g2	ATPase, H+ transporting, lysosomal V1 subunit G2 [Source:MGI Symbol;Acc:MGI:1913487]	44.2648	40.0869	43.8739	41.1015	36.143	46.0338	45.0467	39.4248	37.3899	53.9574	32.6745	44.0495	38.6904	47.2223	0.936399519	0.966018836	24	20	24	0.8407695	0.951824469	24	3.779774076	0.042501965
ENSMUSG00000024404	Riok3	RIO kinase 3 [Source:MGI Symbol;Acc:MGI:1914128]	43.8204	46.8057	48.58	47.3636	48.6624	41.7511	47.8388	45.1074	49.4022	44.5505	47.0362	45.5732	48.3922	42.6042	0.45229395	0.966018836	12	12	24	0.528458112	0.823973486	24	10.47800036	0.033764236
ENSMUSG00000024410	Rmc1	regulator of MON1-CCZ1 [Source:MGI Symbol;Acc:MGI:1916528]	21.3766	19.46	19.0126	18.4554	19.2414	19.9142	21.3811	20.8878	18.4509	19.1208	19.7501	19.2484	20.874	20.3999	3.72E-05	0.023983755	4	8	24	0.00152807	0.35978926	24	23.92896008	0.077846053
ENSMUSG00000024411	Aqp4	aquaporin 4 [Source:MGI Symbol;Acc:MGI:107387]	6.78052	15.0997	26.4101	11.5555	9.49961	13.1805	18.4639	11.7007	18.3671	9.05387	7.73407	11.2326	5.35984	15.1761	0.167216842	0.634643192	12	8	24	0.182036932	0.768040178	24	7.166098072	0.402805281
ENSMUSG00000024413	Npc1	NPC intracellular cholesterol transporter 1 [Source:MGI Symbol;Acc:MGI:1097712]	20.4696	18.6256	19.563	19.8528	20.5012	21.2927	22.7226	19.1557	19.4032	20.8578	18.6713	20.4944	18.8756	17.3908	0.035806379	0.331814682	24	8	24	0.138306604	0.747972781	24	18.75943551	0.067358566
ENSMUSG00000024414	Mrpl27	mitochondrial ribosomal protein L27 [Source:MGI Symbol;Acc:MGI:2137224]	41.9436	49.0184	45.9519	50.6175	51.3157	47.9143	46.4639	48.8103	49.8926	40.3741	50.9655	45.6411	53.2653	43.9737	0.897425765	0.966018836	20	8	24	0.899359316	0.971453495	24	16.68632606	0.021325194
ENSMUSG00000024420	Zfp521	zinc finger protein 521 [Source:MGI Symbol;Acc:MGI:95459]	8.47709	7.29637	7.07903	6.31583	7.54865	7.64161	7.31107	8.00651	6.31213	9.92004	5.64131	7.80609	7.91019	7.10685	0.27817759	0.789849983	4	8	24	0.694429585	0.898522833	24	23.59511427	0.06038288
ENSMUSG00000024421	Lama3	laminin, alpha 3 [Source:MGI Symbol;Acc:MGI:99909]	1.03506	1.02067	0.990804	1.06847	1.05168	0.877637	0.987519	1.00371	0.995709	0.804367	0.913806	0.89296	0.973335	1.00775	0.632729413	0.966018836	8	16	24	0.366958679	0.790573051	24	4.385925481	0.027471332
ENSMUSG00000024422	Dhx16	DEAH (Asp-Glu-Ala-His) box polypeptide 16 [Source:MGI Symbol;Acc:MGI:1916442]	12.4092	10.1157	7.81416	12.2086	9.886	12.0213	14.411	11.8719	13.3534	9.10186	11.3897	11.5052	10.9371	9.14563	0.588208558	0.966018836	4	12	24	0.339204105	0.773727252	24	21.66707457	0.129250671
ENSMUSG00000024423	Impact	impact, RWD domain protein [Source:MGI Symbol;Acc:MGI:1098233]	56.3869	62.397	68.3857	58.1191	58.3927	56.5954	58.4021	52.4158	64.1357	50.8891	59.8676	57.2064	54.8486	63.5591	0.300823756	0.809362476	12	16	24	0.422250077	0.804917634	24	7.362760257	0.059822338
ENSMUSG00000024424	Ttc39c	tetratricopeptide repeat domain 39C [Source:MGI Symbol;Acc:MGI:1919997]	24.1916	21.8489	23.2605	21.1146	24.6484	22.9838	21.0379	22.593	20.7705	24.6785	22.1182	23.0495	23.3875	23.3127	0.906657897	0.966018836	20	16	24	0.656126632	0.8838564	24	18.44943185	0.029634138
ENSMUSG00000024425	Ndfip1	Nedd4 family interacting protein 1 [Source:MGI Symbol;Acc:MGI:1929601]	485.341	428.975	419.239	406.85	396.783	435.738	432.412	415.189	368.567	437.936	429.079	415.657	400.409	437.6	0.895493752	0.966018836	4	8	24	0.276684766	0.768040178	24	22.50774616	0.054843809
ENSMUSG00000024426	Atat1	alpha tubulin acetyltransferase 1 [Source:MGI Symbol;Acc:MGI:1913869]	12.3067	13.9746	12.4585	15.0704	15.3425	12.1426	16.3597	12.2455	15.7385	14.2401	13.915	13.6088	13.5842	14.2371	0.745475226	0.966018836	16	8	24	0.579525581	0.849875019	24	11.82550953	0.053358302
ENSMUSG00000024427	Spry4	sprouty RTK signaling antagonist 4 [Source:MGI Symbol;Acc:MGI:1345144]	5.77939	3.6364	3.4411	3.64185	4.71581	4.20769	5.341	4.13044	3.33396	5.02779	4.68141	4.23749	4.83907	3.87277	0.009483698	0.194720062	4	8	24	0.054235391	0.654887447	24	20.47661314	0.179313528
ENSMUSG00000024429	Gnl1	guanine nucleotide binding protein-like 1 [Source:MGI Symbol;Acc:MGI:95764]	20.2346	18.9013	19.5426	19.2101	16.968	17.8025	20.0726	19.1436	19.4986	24.1539	16.1147	20.7262	18.6852	19.7405	0.858366225	0.966018836	16	4	24	0.501904689	0.809205492	24	6.587585465	0.062514876
ENSMUSG00000024430	Cabyr	calcium-binding tyrosine-(Y)-phosphorylation regulated (fibrousheathin 2) [Source:MGI Symbol;Acc:MGI:1918382]	2.32088	2.10191	1.82333	2.00124	1.86572	1.51932	2.00029	2.33501	1.84883	1.98508	1.66014	1.55863	1.86453	1.71779	0.143321297	0.585944911	4	20	24	0.149995949	0.758419028	24	4.820475238	0.079066624
ENSMUSG00000024431	Nr3c1	nuclear receptor subfamily 3, group C, member 1 [Source:MGI Symbol;Acc:MGI:95824]	13.8046	16.9272	16.1595	16.5765	17.6669	14.1786	16.7782	14.974	18.6804	15.3707	17.5599	16.1383	16.8083	16.4702	0.954744729	0.966018836	20	4	24	0.324218542	0.768040178	24	10.54243183	0.065050176
ENSMUSG00000024436	Mrps18b	mitochondrial ribosomal protein S18B [Source:MGI Symbol;Acc:MGI:1914223]	16.5221	14.4504	13.9791	11.8	12.0304	15.1301	12.5682	15.4401	12.6351	15.6174	15.3701	14.0371	13.0404	14.004	0.873350015	0.966018836	8	4	24	0.726905695	0.91035631	24	23.79856693	0.045615835
ENSMUSG00000024437	Gm8615	predicted pseudogene 8615 [Source:MGI Symbol;Acc:MGI:3647532]	4.78084	3.09263	3.56493	3.00712	3.37198	3.63185	4.71559	4.27228	2.59085	3.76825	4.09587	3.68697	3.41695	3.04676	0.238868829	0.741580974	4	8	24	0.123324758	0.736234617	24	21.50132375	0.154309112
ENSMUSG00000024442	Dele1	DAP3 binding cell death enhancer 1 [Source:MGI Symbol;Acc:MGI:1914089]	15.1653	14.0087	13.7391	16.3212	15.7447	15.2983	16.1142	19.3871	14.9511	17.6734	14.4402	15.9648	16.2008	14.1675	0.934152439	0.966018836	16	20	24	0.975444827	0.993762379	24	17.45682723	0.011597731
ENSMUSG00000024446	Rpp21	ribonuclease P 21 subunit [Source:MGI Symbol;Acc:MGI:1914926]	20.0958	21.9519	20.6319	20.6376	21.4185	21.7689	20.8664	20.0993	25.5792	16.7669	25.0395	18.654	21.2553	20.6556	0.854540463	0.966018836	20	20	24	0.984312557	0.996767829	24	8.524547565	0.010930849
ENSMUSG00000024451	Arap3	ArfGAP with RhoGAP domain, ankyrin repeat and PH domain 3 [Source:MGI Symbol;Acc:MGI:2147274]	3.60653	4.13821	5.67804	3.83628	4.39007	4.24436	4.18078	3.89112	4.79331	3.05508	3.91925	4.52716	4.21808	2.58508	0.664568268	0.966018836	12	20	24	0.917439952	0.977528712	24	16.52975715	0.034178824
ENSMUSG00000024454	Hdac3	histone deacetylase 3 [Source:MGI Symbol;Acc:MGI:1343091]	32.6621	32.931	37.5358	33.8537	33.1568	34.489	33.0475	33.1416	36.5622	36.429	31.5687	33.5019	33.9068	35.0944	0.16456443	0.628002127	12	16	24	0.057012919	0.65610184	24	8.707253366	0.06179786
ENSMUSG00000024456	Diaph1	diaphanous related formin 1 [Source:MGI Symbol;Acc:MGI:1194490]	5.81177	5.87618	5.72681	6.48375	5.96471	6.00873	5.77327	6.9302	5.98339	6.8252	5.45345	6.37091	6.1322	6.07976	0.917370979	0.966018836	16	4	24	0.666847278	0.887459929	24	8.880109885	0.030608776
ENSMUSG00000024457	Trim26	tripartite motif-containing 26 [Source:MGI Symbol;Acc:MGI:1337056]	16.9643	16.2493	14.7876	16.5059	16.2938	16.0865	15.0229	17.1067	15.8826	15.2359	16.2364	17.1381	18.3577	14.7559	0.631736745	0.966018836	4	8	24	0.288485146	0.768040178	24	21.16980015	0.053768182
ENSMUSG00000024459	H2-M5	histocompatibility 2, M region locus 5 [Source:MGI Symbol;Acc:MGI:95917]	1.15743	1.38875	1.83493	1.36459	1.80957	1.80043	1.43555	1.31544	2.03665	1.05638	0.726496	1.48243	0.793422	1.31972	0.643325306	0.966018836	12	16	24	0.78479677	0.930959336	24	8.882049088	0.064973129
ENSMUSG00000024462	Gabbr1	gamma-aminobutyric acid (GABA) B receptor, 1 [Source:MGI Symbol;Acc:MGI:1860139]	105.167	91.0663	97.1448	90.7902	89.1419	101.903	92.4885	95.8948	81.0796	109.518	86.2869	96.499	90.3674	94.9194	0.897425765	0.966018836	24	20	24	0.774731442	0.927711986	24	21.43000977	0.033119533
ENSMUSG00000024471	Myot	myotilin [Source:MGI Symbol;Acc:MGI:1889800]	6.92929	17.4141	22.2937	28.8501	18.8223	24.6885	14.6313	39.6133	31.0059	30.3689	15.9159	15.8872	24.5314	17.4451	0.086560674	0.468170511	12	16	24	0.085105763	0.70291653	24	9.708209636	0.475444578
ENSMUSG00000024472	Dcp2	decapping mRNA 2 [Source:MGI Symbol;Acc:MGI:1917890]	13.0856	12.7643	13.2829	13.1654	12.7949	12.6664	12.633	11.7778	12.3465	12.4405	13.488	13.7985	14.8443	15.4273	0.866861748	0.966018836	4	8	24	0.694701327	0.898526856	24	23.99959961	0.028340483
ENSMUSG00000024474	Ik	IK cytokine [Source:MGI Symbol;Acc:MGI:1345142]	68.2448	73.6784	69.0533	69.4578	72.5466	67.1908	67.2902	67.1324	72.5361	65.4129	68.7574	69.8656	70.6885	73.2288	0.920858634	0.966018836	8	16	24	0.642893568	0.878809617	24	5.664975807	0.020161227
ENSMUSG00000024477	Pggt1b	protein geranylgeranyltransferase type I, beta subunit [Source:MGI Symbol;Acc:MGI:1917514]	6.57019	8.16799	7.32486	8.06031	7.27236	6.75596	7.37994	6.73191	7.63534	6.44803	7.60581	7.49984	7.33966	7.53991	0.920858634	0.966018836	12	16	24	0.75120194	0.91921305	24	8.715396337	0.028928038
ENSMUSG00000024479	Mal2	mal, T cell differentiation protein 2 [Source:MGI Symbol;Acc:MGI:2146021]	94.1228	86.2474	76.9132	84.7163	81.618	90.2034	79.7299	78.5247	73.8395	76.7494	87.8094	86.7339	89.1137	84.4198	0.009630739	0.194720062	24	12	24	0.014882802	0.537924552	24	21.04035463	0.103643737
ENSMUSG00000024480	Ap3s1	adaptor-related protein complex 3, sigma 1 subunit [Source:MGI Symbol;Acc:MGI:1337062]	33.1874	30.4959	27.0946	29.0053	28.82	29.2509	31.9377	28.7088	24.4844	25.9097	30.1719	24.0061	22.4142	26.6316	0.941830768	0.966018836	4	8	24	0.533179884	0.825678486	24	21.08199125	0.053444006
ENSMUSG00000024483	Ankhd1	ankyrin repeat and KH domain containing 1 [Source:MGI Symbol;Acc:MGI:1921733]	14.3435	16.3145	14.449	16.8924	16.2504	15.293	14.7772	13.2985	14.336	13.0132	15.1709	14.8118	15.0394	14.656	0.480187506	0.966018836	20	16	24	0.47068424	0.805393068	24	17.59571208	0.045837686
ENSMUSG00000024486	Hbegf	heparin-binding EGF-like growth factor [Source:MGI Symbol;Acc:MGI:96070]	6.96289	6.51113	6.2107	7.05284	7.82433	7.13267	8.91006	7.24841	6.7325	7.7877	6.8457	7.08607	7.3677	6.32286	0.194424727	0.673387182	4	8	24	0.291025936	0.768040178	24	19.3497257	0.075352249
ENSMUSG00000024487	Yipf5	Yip1 domain family, member 5 [Source:MGI Symbol;Acc:MGI:1914430]	12.9673	12.383	12.0272	11.4024	12.8863	11.7422	12.3371	11.8467	13.4649	11.7765	12.9053	11.8645	12.1623	13.4778	0.539001179	0.966018836	8	8	24	0.715106142	0.906238356	24	4.296189577	0.023122088
ENSMUSG00000024491	Rbm27	RNA binding motif protein 27 [Source:MGI Symbol;Acc:MGI:2147194]	6.26376	6.08566	6.0376	6.56853	6.33824	5.93406	6.10905	5.45483	5.89207	5.91418	6.50492	5.69491	5.61298	5.87318	0.232647885	0.733359426	20	12	24	0.062982415	0.66222475	24	14.90844963	0.049062104
ENSMUSG00000024493	Lars	leucyl-tRNA synthetase [Source:MGI Symbol;Acc:MGI:1913808]	23.9037	26.196	26.4255	24.4682	26.8758	23.3142	21.597	24.0128	24.8401	24.6023	23.3563	24.6938	26.3698	26.1889	0.745199643	0.966018836	12	12	24	0.446319811	0.805393068	24	8.42269648	0.044536872
ENSMUSG00000024497	Pou4f3	POU domain, class 4, transcription factor 3 [Source:MGI Symbol;Acc:MGI:102523]	7.44189	5.63225	4.82008	6.60419	5.89707	6.68691	6.4737	5.44943	5.28315	8.1162	6.47258	7.20442	7.1649	6.28601	0.021795836	0.26698364	24	12	24	0.123469439	0.736432529	24	19.12049453	0.136764796
ENSMUSG00000024498	Tcerg1	transcription elongation regulator 1 (CA150) [Source:MGI Symbol;Acc:MGI:1926421]	21.4842	19.583	20.4594	19.2411	20.362	19.7736	20.8613	19.1224	20.1034	23.2807	20.5336	21.379	17.8598	19.7112	0.588208558	0.966018836	24	8	24	0.42913262	0.805393068	24	14.77297481	0.043444405
ENSMUSG00000024500	Ppp2r2b	protein phosphatase 2, regulatory subunit B, beta [Source:MGI Symbol;Acc:MGI:1920180]	69.236	61.233	61.0476	62.0655	58.5988	65.4701	60.4387	59.5352	53.3231	71.0772	62.793	64.7705	64.2619	70.6335	0.8972237	0.966018836	24	12	24	0.676782534	0.891872731	24	21.18253435	0.03993151
ENSMUSG00000024501	Dpysl3	dihydropyrimidinase-like 3 [Source:MGI Symbol;Acc:MGI:1349762]	120.598	92.3618	97.2374	96.7576	99.5324	111.521	99.6682	99.2152	71.1021	118.398	99.1427	101.993	99.3566	99.8591	0.039704107	0.338535904	24	12	24	0.194780781	0.768040178	24	19.84686086	0.126318291
ENSMUSG00000024502	Jakmip2	janus kinase and microtubule interacting protein 2 [Source:MGI Symbol;Acc:MGI:1923467]	23.9379	20.9463	22.9568	21.7503	21.2541	21.993	20.2098	18.4978	21.2859	18.7874	22.0663	23.7327	23.078	25.3173	0.834424969	0.966018836	4	12	24	0.603740274	0.861540092	24	22.57461868	0.049454764
ENSMUSG00000024505	Dtwd2	DTW domain containing 2 [Source:MGI Symbol;Acc:MGI:1916107]	1.60027	2.00501	1.93873	1.99538	2.34809	1.64049	1.89788	1.97974	2.01011	2.05978	2.12035	1.88945	2.14566	1.98591	0.056268883	0.401074772	20	4	24	0.178688157	0.768040178	24	11.47092438	0.062003473
ENSMUSG00000024507	Hsd17b4	hydroxysteroid (17-beta) dehydrogenase 4 [Source:MGI Symbol;Acc:MGI:105089]	27.3888	24.8978	25.8821	27.1916	23.6826	25.7975	25.0458	28.0751	23.4044	27.4874	25.7195	26.1763	28.4937	26.7516	0.965163262	0.969272194	4	16	24	0.694707358	0.898526856	24	1.678575618	0.027227328
ENSMUSG00000024511	Rab27b	RAB27B, member RAS oncogene family [Source:MGI Symbol;Acc:MGI:1931295]	37.3524	33.6656	30.4568	32.9178	34.4224	35.8795	29.453	32.6642	32.0229	30.3521	36.768	36.8059	38.9343	36.6942	0.088274239	0.473025915	4	8	24	0.116306446	0.727799063	24	21.13730402	0.093645191
ENSMUSG00000024513	Mbd2	methyl-CpG binding domain protein 2 [Source:MGI Symbol;Acc:MGI:1333813]	39.4562	38.2656	35.244	34.7905	40.4079	37.8347	39.9938	37.1139	32.8387	37.5663	35.8663	37.9959	36.3806	36.1484	0.055920623	0.401074772	8	4	24	0.021824911	0.574400012	24	21.03324449	0.077244657
ENSMUSG00000024515	Smad4	SMAD family member 4 [Source:MGI Symbol;Acc:MGI:894293]	14.3551	13.2034	12.8503	13.3423	12.5618	13.3705	13.8393	14.2855	12.2531	15.235	12.7458	14.4627	13.3767	13.1257	0.187179226	0.662239322	4	8	24	0.652038352	0.882906761	24	22.36830003	0.036767747
ENSMUSG00000024516	Sec11c	SEC11 homolog C, signal peptidase complex subunit [Source:MGI Symbol;Acc:MGI:1913536]	85.4283	102.4	88.3757	89.1552	93.5303	85.3066	96.9982	90.2978	105.615	75.7261	95.6451	90.4138	97.8588	103.896	0.446653834	0.966018836	8	8	24	0.267837741	0.768040178	24	3.811909726	0.07800803
ENSMUSG00000024517	Grp	gastrin releasing peptide [Source:MGI Symbol;Acc:MGI:95833]	0.345055	0.676564	1.12603	0.226211	0.68571	0.755679	0.782063	0.441483	0.875549	0.749276	0.644161	0.722702	0.502645	0.331449	0.675497492	0.966018836	12	20	24	0.717235362	0.906810602	24	13.80172428	0.072281702
ENSMUSG00000024521	Pmaip1	phorbol-12-myristate-13-acetate-induced protein 1 [Source:MGI Symbol;Acc:MGI:1930146]	1.16611	1.29148	1.46845	0.908523	0.781602	0.864521	1.17434	0.645543	0.878063	0.67292	1.12879	0.744589	0.754781	1.00102	0.939103211	0.966018836	12	4	24	0.615522499	0.867167562	24	4.041444406	0.060707607

ENSMUSG00000024524	Gnal	guanine nucleotide binding protein, alpha stimulating, olfactory type [Source:MGI Symbol;Acc:MGI:95774]	90.0018	65.8993	69.2966	72.5975	73.2679	81.0232	67.9778	70.9734	54.2548	86.9897	70.9433	79.0543	79.0412	75.0349	0.077301512	0.453790923	24	12	24	0.188246775	0.768040178	24	19.78166385	0.129586301
ENSMUSG00000024525	Impa2	inositol monophosphatase 2 [Source:MGI Symbol;Acc:MGI:2149728]	1.7294	1.51044	1.88633	1.97149	1.55543	1.62788	1.60406	1.94002	1.46354	2.43466	2.26351	1.73006	2.43766	2.95641	0.814723813	0.966018836	16	20	24	0.595383317	0.857839743	24	8.08872997	0.06664951
ENSMUSG00000024526	Cidea	cell death-inducing DNA fragmentation factor, alpha subunit-like effector A [Source:MGI Symbol;Acc:MGI:1270845]	9.84329	8.20612	28.2172	72.0916	23.5395	16.4302	22.1968	30.4257	24.7675	41.9918	53.2829	29.8086	57.7711	43.0208	0.850398351	0.966018836	16	12	24	0.066738202	0.669146244	24	12.15384876	0.569045609
ENSMUSG00000024527	Afg3l2	AFG3-like AAA ATPase 2 [Source:MGI Symbol;Acc:MGI:1916847]	26.7264	24.3305	25.7626	29.0426	25.3126	27.2441	25.2023	28.0861	24.0298	29.269	25.2768	26.0073	27.4115	27.7412	0.883051071	0.966018836	16	4	24	0.894033097	0.969984454	24	12.05879901	0.016323334
ENSMUSG00000024528	Srfbp1	serum response factor binding protein 1 [Source:MGI Symbol;Acc:MGI:1914472]	6.93796	6.34932	6.62672	6.73571	7.95816	6.2572	6.99273	5.19172	7.68359	5.37841	7.68187	6.30809	7.23034	6.03902	0.192357053	0.669841558	20	12	24	0.478537471	0.805393068	24	17.33168923	0.076977438
ENSMUSG00000024529	Lox	lysyl oxidase [Source:MGI Symbol;Acc:MGI:96817]	31.193	25.2154	22.9339	29.5367	36.3321	26.2025	31.0704	23.6901	24.403	27.8302	29.8878	25.829	23.0877	20.1647	0.029825002	0.323426482	20	12	24	0.005969795	0.46443032	24	17.18270421	0.21014886
ENSMUSG00000024530	Prelid3a	PRELI domain containing 3A [Source:MGI Symbol;Acc:MGI:2442865]	5.2317	5.70369	6.68618	7.59544	6.7058	5.53902	6.70766	4.6289	6.57521	6.85045	5.88888	5.50364	4.85875	5.45332	0.007831348	0.192760771	16	16	24	0.010338402	0.503967859	24	12.41349582	0.17334296
ENSMUSG00000024533	Spire1	spire type actin nucleation factor 1 [Source:MGI Symbol;Acc:MGI:1915416]	19.059	18.4841	19.6056	21.5498	20.4585	19.5392	22.1676	17.8916	18.9914	19.3671	18.3929	19.9959	18.6383	17.8029	0.085329704	0.466404237	16	16	24	0.264814745	0.768040178	24	16.81967169	0.053172845
ENSMUSG00000024534	Sncaip	synuclein, alpha interacting protein (synphilin) [Source:MGI Symbol;Acc:MGI:1915097]	0.884055	1.20262	0.932244	0.858523	0.993039	0.823189	1.10659	0.845727	1.20293	1.15542	0.555111	1.18632	0.687528	1.43634	0.449341601	0.966018836	12	8	24	0.321969481	0.768040178	24	5.939273116	0.104229749
ENSMUSG00000024535	Snx24	sorting nexing 24 [Source:MGI Symbol;Acc:MGI:1916476]	6.10552	5.84061	6.19817	6.11192	5.51362	5.91474	5.47095	5.43384	6.14543	5.73985	4.86664	5.11773	6.06603	6.24388	0.10951302	0.524035616	12	8	24	0.118553016	0.729313257	24	5.935832401	0.071405179
ENSMUSG00000024537	Psmg2	proteasome (prosome, macropain) assembly chaperone 2 [Source:MGI Symbol;Acc:MGI:1922901]	26.3183	24.8085	23.4123	22.7173	24.92	23.3222	23.9199	25.205	27.0127	22.2403	26.9329	23.8948	27.8013	25.7864	0.305564675	0.811201458	4	12	24	0.364318428	0.788789286	24	1.529421942	0.049865864
ENSMUSG00000024538	Ppic	peptidylprolyl isomerase C [Source:MGI Symbol;Acc:MGI:97751]	8.12414	10.9329	10.6481	9.89844	9.99077	8.30605	10.1176	11.7658	12.905	7.39449	9.61174	8.01608	9.48527	8.8668	0.039704107	0.338535904	12	12	24	0.128819205	0.740162193	24	6.797319695	0.157364168
ENSMUSG00000024539	Ptpn2	protein tyrosine phosphatase, non-receptor type 2 [Source:MGI Symbol;Acc:MGI:97806]	7.56169	6.87462	7.09031	6.94601	6.78698	6.49439	7.71546	6.57184	7.41314	6.18626	7.66993	7.48359	7.48599	6.89859	0.446653834	0.966018836	4	12	24	0.419291827	0.803639585	24	22.69274016	0.045498587
ENSMUSG00000024542	Cep192	centrosomal protein 192 [Source:MGI Symbol;Acc:MGI:1918049]	3.60927	3.24151	3.13018	3.24271	3.88514	3.15731	3.25499	4.08014	3.7116	2.6107	3.90574	2.83242	3.03381	3.19061	0.896753532	0.966018836	20	4	24	0.992159098	0.998516607	24	3.645737573	0.007050406
ENSMUSG00000024544	Ldlrad4	low density lipoprotein receptor class A domain containing 4 [Source:MGI Symbol;Acc:MGI:1277150]	3.73497	3.56636	3.91721	3.68176	3.4515	3.42408	3.49072	3.83012	3.8706	3.61022	3.74059	3.40527	3.69686	3.70644	7.90E-04	0.071344007	12	12	24	0.016041209	0.544099157	24	7.357274321	0.052920748
ENSMUSG00000024548	Setbp1	SET binding protein 1 [Source:MGI Symbol;Acc:MGI:1933199]	1.41322	1.36987	1.2397	1.35854	1.36233	1.21832	1.31402	1.17407	1.32275	1.41839	1.35785	1.30729	1.40545	1.28919	0.742579234	0.966018836	16	20	24	0.693097617	0.898239456	24	16.09482819	0.016029317
ENSMUSG00000024553	Galr1	galanin receptor 1 [Source:MGI Symbol;Acc:MGI:1096364]	1.66772	1.477	1.23558	1.76305	1.47817	1.6898	1.69638	1.86494	1.3628	1.65662	1.58812	1.68057	1.82303	1.63734	0.101520755	0.511659635	4	8	24	0.224019346	0.768040178	24	21.81675001	0.063522447
ENSMUSG00000024556	Me2	malic enzyme 2, NAD(+)-dependent, mitochondrial [Source:MGI Symbol;Acc:MGI:2147351]	17.5952	15.9616	15.5777	16.4143	15.5993	16.8029	15.0216	15.5979	14.2879	17.565	15.8559	16.9742	18.2036	16.8379	0.300823756	0.809362476	4	8	24	0.427368998	0.805393068	24	20.86765807	0.047482284
ENSMUSG00000024558	Mapk4	mitogen-activated protein kinase 4 [Source:MGI Symbol;Acc:MGI:2444559]	5.12342	4.37312	4.69847	4.28969	3.7082	4.77436	4.21058	4.3855	4.74793	4.85126	3.7855	4.8436	3.88917	4.14255	0.897570341	0.966018836	12	8	24	0.830421267	0.947343852	24	4.365921056	0.027942416
ENSMUSG00000024560	Cxxc1	CXXC finger 1 (PHD domain) [Source:MGI Symbol;Acc:MGI:1921572]	12.663	12.5903	14.179	13.9427	16.3445	12.8942	13.6251	13.3663	15.0402	12.4999	15.7251	12.5068	15.005	13.8389	0.636884864	0.966018836	20	4	24	0.452846595	0.805393068	24	13.24574373	0.05734901
ENSMUSG00000024561	Mbd1	methyl-CpG binding domain protein 1 [Source:MGI Symbol;Acc:MGI:1333811]	13.5316	12.4699	13.986	14.4082	14.4804	13.0619	12.9734	12.8011	13.9992	14.0521	13.1342	13.2462	13.317	13.264	0.046535188	0.375011045	16	16	24	0.017285327	0.549489822	24	12.65325506	0.05859168
ENSMUSG00000024563	Smad2	SMAD family member 2 [Source:MGI Symbol;Acc:MGI:108051]	18.0292	14.6705	14.9006	15.7959	15.7536	16.1425	16.1941	13.7544	12.7447	13.7269	14.7588	16.4819	16.9689	15.7939	0.002004172	0.100255953	4	8	24	0.007433802	0.47735824	24	21.33169435	0.134590585
ENSMUSG00000024566	Atp9b	ATPase, class II, type 9B [Source:MGI Symbol;Acc:MGI:1354757]	23.7881	22.4181	21.9152	22.9285	25.4895	25.6418	25.8703	24.5382	24.1924	26.2129	24.7667	24.6306	21.8132	23.9761	0.345575499	0.870418653	20	16	24	0.230032757	0.768040178	24	17.23660573	0.054748414
ENSMUSG00000024570	Rbfa	ribosome binding factor A [Source:MGI Symbol;Acc:MGI:1915981]	12.5036	13.6056	14.5131	14.0605	15.8547	14.9303	13.7056	14.4482	13.5877	13.6431	13.594	14.4599	14.7484	12.45	0.596159714	0.966018836	24	8	24	0.269689206	0.768040178	24	16.63698347	0.055407854
ENSMUSG00000024571	Naa12	N(alpha)-acetyltransferase 12, NatA catalytic subunit [Source:MGI Symbol;Acc:MGI:3833940]	87.5752	73.6491	75.715	75.8307	76.0908	86.2621	75.6444	83.8287	67.604	87.5851	79.3112	81.3377	81.5264	81.338	0.390686537	0.915479136	24	12	24	0.30649138	0.768040178	24	20.73257715	0.064593979
ENSMUSG00000024576	Csnk1a1	casein kinase 1, alpha 1 [Source:MGI Symbol;Acc:MGI:1934950]	113.573	109.76	106.664	111.797	105.944	103.954	104.972	103.961	107.125	117.741	113.414	112.78	111.844	115.961	0.858366225	0.966018836	16	8	24	0.778190113	0.928832041	24	11.94355115	0.015644625
ENSMUSG00000024579	Pcyox1l	prenylcysteine oxidase 1 like [Source:MGI Symbol;Acc:MGI:3606062]	19.1455	14.4453	14.1125	16.2608	17.6718	15.9649	17.5108	15.6227	15.6573	19.3432	17.3796	15.8929	16.6793	14.2288	0.055569815	0.401074772	16	20	24	0.137823365	0.747972781	24	17.7030061	0.103576815
ENSMUSG00000024580	Grpel2	GrpE-like 2, mitochondrial [Source:MGI Symbol;Acc:MGI:1334416]	8.46267	8.71246	7.60184	8.9588	8.65564	7.80567	8.45864	7.93688	8.7164	8.09829	8.06346	8.49134	9.29292	7.98649	0.826055781	0.966018836	4	20	24	0.883356218	0.966818623	24	21.45038449	0.015340102
ENSMUSG00000024581	Napg	N-ethylmaleimide sensitive fusion protein attachment protein gamma [Source:MGI Symbol;Acc:MGI:104561]	58.1749	56.5114	53.2168	55.3897	51.745	56.6952	53.8625	52.0275	51.0907	58.4228	55.2097	52.6772	53.0753	59.4226	0.881996584	0.966018836	8	4	24	0.95775231	0.9888878	24	0.96100911	0.008211161
ENSMUSG00000024583	Txnl1	thioredoxin-like 1 [Source:MGI Symbol;Acc:MGI:1860078]	81.3908	78.0235	74.7082	78.1566	78.1605	81.0433	75.8309	79.0127	78.9937	77.8565	82.0703	80.0159	83.6488	82.8019	0.29214565	0.798990791	4	8	24	0.257330212	0.768040178	24	21.82615732	0.025524787
ENSMUSG00000024587	Nars	asparaginyl-tRNA synthetase [Source:MGI Symbol;Acc:MGI:1917473]	150.378	134.787	135.624	129.417	134.162	144.431	133.599	132.011	112.664	135.619	141.553	131.552	131.08	135.542	0.831272156	0.966018836	20	20	24	0.109665271	0.72082227	24	20.50815049	0.072347447
ENSMUSG00000024588	Fech	ferrochelatase [Source:MGI Symbol;Acc:MGI:95513]	37.809	37.1331	38.942	40.1324	35.3087	41.2443	37.4774	39.1972	39.0132	43.4433	35.8569	39.9837	40.1945	36.251	0.868638024	0.966018836	16	16	24	0.849307834	0.954760051	24	11.75436534	0.01874369
ENSMUSG00000024589	Nedd4l	neural precursor cell expressed, developmentally down-regulated gene 4-like [Source:MGI Symbol;Acc:MGI:1933754]	74.5538	58.1642	61.8333	68.0044	59.6738	65.9893	66.0987	65.91	47.8723	80.0289	53.3661	73.6313	67.5674	62.1205	0.282728968	0.793714531	24	12	24	0.530203464	0.824795587	24	20.77985626	0.089003638
ENSMUSG00000024590	Lmnb1	lamin B1 [Source:MGI Symbol;Acc:MGI:96795]	2.01146	2.38352	3.29533	2.16447	1.91352	2.25304	2.6453	2.13776	2.48784	1.92137	2.02036	2.43659	2.55847	2.29384	0.205280539	0.693330038	12	4	24	0.281702005	0.768040178	24	4.505920238	0.092963504
ENSMUSG00000024592	C330018D20Rik	RIKEN cDNA C330018D20 gene [Source:MGI Symbol;Acc:MGI:1924672]	4.84825	4.85275	4.96823	5.11711	4.75539	4.48312	4.4527	4.90651	5.58484	4.22424	5.23713	5.20763	5.00553	4.97865	0.920872048	0.966018836	12	16	24	0.690012859	0.897381208	24	8.129639111	0.030718287
ENSMUSG00000024593	Megf10	multiple EGF-like-domains 10 [Source:MGI Symbol;Acc:MGI:2685177]	5.47515	5.6517	5.25229	4.60679	5.05096	5.21809	6.20442	5.33468	4.82932	5.1363	4.72545	5.19192	5.31027	5.48072	8.20E-04	0.071344007	8	8	24	0.00407241	0.427431763	24	1.074990087	0.097260786
ENSMUSG00000024594	Prrc1	proline-rich coiled-coil 1 [Source:MGI Symbol;Acc:MGI:1916106]	8.93391	8.0489	7.82323	7.5277	8.02125	7.86303	8.14099	8.63748	7.60558	9.7745	8.09196	8.01036	8.3263	7.96452	0.277087842	0.789849983	4	8	24	0.96685729	0.992082154	24	23.26134478	0.009391385
ENSMUSG00000024597	Slc12a2	solute carrier family 12, member 2 [Source:MGI Symbol;Acc:MGI:101924]	38.2889	44.3933	44.0842	42.8872	44.7435	39.8553	40.1373	40.3842	44.8024	40.2221	45.5495	41.5598	39.5259	40.6713	0.008397671	0.192760771	20	8	24	0.069284991	0.673520298	24	11.71088677	0.067046614
ENSMUSG00000024601	Isoc1	isochorismatase domain containing 1 [Source:MGI Symbol;Acc:MGI:1913557]	22.8006	22.4546	21.5755	22.3436	21.2286	22.9215	21.6181	22.5424	20.7175	25.2031	21.2468	24.1937	23.5525	25.0411	0.44861185	0.966018836	24	20	24	0.787879035	0.931832121	24	1.054639267	0.020779064
ENSMUSG00000024603	Dctn4	dynactin 4 [Source:MGI Symbol;Acc:MGI:1914915]	67.8422	66.45	62.9219	63.3151	64.1776	65.4165	60.9418	64.9585	60.0441	64.2283	69.1467	66.6018	66.7129	67.9216	0.289105414	0.794869797	8	4	24	0.359358836	0.785405943	24	21.17239469	0.032923711
ENSMUSG00000024604	Rbm22	RNA binding motif protein 22 [Source:MGI Symbol;Acc:MGI:1914060]	12.0875	12.6404	12.9302	12.6456	13.1617	13.0079	13.6217	13.7833	14.234	14.1379	12.4129	13.1304	13.5848	13.7266	0.777867261	0.966018836	12	8	24	0.166617984	0.767020009	24	7.254887774	0.040121514
ENSMUSG00000024608	Rps14	ribosomal protein S14 [Source:MGI Symbol;Acc:MGI:98107]	110.024	146.419	129.519	123.927	142.45	117.271	123.542	122.515	165.005	88.7699	132.484	127.423	135.545	125.668	0.951523604	0.966018836	12	12	24	0.803832757	0.937296224	24	5.194565003	0.055639453
ENSMUSG00000024610	Cd74	CD74 antigen (invariant polypeptide of major histocompatibility complex, class II antigen-associated) [Source:MGI Symbol;Acc:MGI:96534]	60.3812	72.8071	69.3705	63.4678	77.5479	55.9504	71.3767	77.8876	91.9335	52.9605	72.4396	66.7715	67.4102	61.2453	0.70641939	0.966018836	12	12	24	0.619795823	0.869755203	24	6.867587337	0.082996359
ENSMUSG00000024613	Tcof1	treacle ribosome biogenesis factor 1 [Source:MGI Symbol;Acc:MGI:892003]	3.14244	2.5699	2.94284	2.50802	3.6879	2.94956	3.29062	3.08087	2.88017	4.1722	3.09251	3.25774	3.53685	2.75205	0.22438807	0.718653303	4	4	24	0.3935845	0.799546792	24	17.39078984	0.078536291
ENSMUSG00000024614	Tmx3	thioredoxin-related transmembrane protein 3 [Source:MGI Symbol;Acc:MGI:2442418]	57.2592	60.9501	52.3642	57.686	58.244	54.6609	57.7248	53.2719	67.8606	51.1618	63.7551	59.9997	65.3387	62.1103	0.897425765	0.966018836	4	12	24	0.844551013	0.952844769	24	1.20062829	0.025459026
ENSMUSG00000024617	Camk2a	calcium/calmodulin-dependent protein kinase II alpha [Source:MGI Symbol;Acc:MGI:88256]	39.5143	31.2756	34.0628	37.2266	32.9698	40.7458	35.1221	40.5674	31.7604	48.8456	31.1852	36.9607	36.4851	36.6969	0.923635729	0.966018836	24	12	24	0.973100405	0.993305115	24	17.98678709	0.016938673
ENSMUSG00000024620	Pdgfrb	platelet derived growth factor receptor, beta polypeptide [Source:MGI Symbol;Acc:MGI:97531]	8.7026	8.7379	8.10297	8.7748	9.60773	9.17323	9.6577	9.5953	9.47539	10.6914	7.97407	9.38395	8.87795	8.23238	0.880631923	0.966018836	16	20	24	0.867656507	0.962111884	24	15.47292206	0.022355114
ENSMUSG00000024621	Csf1r	colony stimulating factor 1 receptor [Source:MGI Symbol;Acc:MGI:1339758]	7.04885	9.59701	10.072	8.27971	9.10238	7.61554	9.40974	9.43312	10.3678	6.42714	8.70642	8.14765	7.79722	8.09285	0.077301512	0.453790923	12	12	24	0.381291132	0.799546792	24	6.691437928	0.098017249
ENSMUSG00000024622	Hmgxb3	HMG box domain containing 3 [Source:MGI Symbol;Acc:MGI:2441817]	6.12061	5.15109	5.59236	5.86518	5.63078	5.79403	5.91265	6.02537	4.97292	6.52062	5.19297	6.06968	5.06571	5.84407	0.890311217	0.966018836	24	12	24	0.841120417	0.951862945	24	18.03091398	0.024239317
ENSMUSG00000024639	Gnaq	guanine nucleotide binding protein, alpha q polypeptide [Source:MGI Symbol;Acc:MGI:95776]	76.1752	61.9538	65.7998	65.0367	63.8899	70.4968	64.6148	63.4971	55.9442	83.2205	66.484	74.2323	71.6555	72.8156	0.455507782	0.966018836	24	12	24	0.511548163	0.814964722	24	19.51344223	0.066079571
ENSMUSG00000024640	Psat1	phosphoserine aminotransferase 1 [Source:MGI Symbol;Acc:MGI:2183441]	31.5345	33.4658	39.0658	31.6624	32.4171	32.3554	35.059	30.5738	34.035	27.4898	32.9446	30.1879	30.7418	31.1617	0.920992137	0.966018836	12	12	24	0.728079592	0.910948356	24	5.67674707	0.033368773
ENSMUSG00000024642	Tle4	transducin-like enhancer of split 4 [Source:MGI Symbol;Acc:MGI:104633]	12.9347	10.3544	9.9341	10.3644	9.61662	11.9627	11.1265	10.9874	8.13775	12.9699	9.6117	10.9817	9.98055	10.5296	0.243857167	0.741580974	24	12	24	0.378191437	0.797706855	24	21.40118288	0.090284418
ENSMUSG00000024644	Cndp2	CNDP dipeptidase 2 (metallopeptidase M20 family) [Source:MGI Symbol;Acc:MGI:1913304]	39.216	38.2356	35.085	33.6768	33.6992	39.4953	32.4348	35.1072	27.0588	37.5752	31.9267	33.3476	31.7755	31.8938	0.79625133	0.966018836	24	12	24	0.425036985	0.805393068	24	20.85558141	0.064198724
ENSMUSG00000024645	Timm21	translocase of inner mitochondrial membrane 21 [Source:MGI Symbol;Acc:MGI:1920595]	9.47206	11.897	11.7714	10.5632	10.3607	9.51264	10.5545	11.0146	12.2853	10.2947	10.1807	12.9613	11.9347	10.9947	0.399942636	0.932395417	12	8	24	0.315284483	0.768040178	24	6.034160958	0.071910459
ENSMUSG00000024646	Cyb5a	cytochrome b5 type A (microsomal) [Source:MGI Symbol;Acc:MGI:1926952]	124.885	134.599	118.114	131.072	144.989	119.427	129.185	116.225	138.625	103.943	146.899	124.253	139.125	126.45	0.897975397	0.966018836	20	20	24	0.762616314	0.923349658	24	18.9460324	0.041789765
ENSMUSG00000024647	Cbln2	cerebellin 2 precursor protein [Source:MGI Symbol;Acc:MGI:88282]	13.302	11.2925	10.6966	10.6013	10.9589	12.691	12.294	11.1066	10.2608	13.0815	11.3496	12.895	12.7656	12.884	0.035787737	0.331814682	4	8	24	0.048053974	0.637794156	24	22.05794345	0.103261172
ENSMUSG00000024650	Slc22a6	solute carrier family 22 (organic anion transporter), member 6 [Source:MGI Symbol;Acc:MGI:892001]	0.921675	1.5768	0.631288	0.609964	0.765125	0.716564	0.886533	1.06197	1.19886	0.471859	0.780374	0.886576	0.480932	1.03271	0.008810267	0.192760771	8	8	24	0.162933805	0.766462333	24	3.509286781	0.155186066
ENSMUSG00000024653	Scgb1a1	secretoglobin, family 1A, member 1 (uteroglobin) [Source:MGI Symbol;Acc:MGI:98919]	0.640556	1.94497	1.25128	1.1595	1.22869	1.07489	1.08901	1.70481	1.13064	0.677792	1.04571	0.578182	0.602227	1.76095	0.009118941	0.192760771	8	16	24	0.13858385	0.747972781	24	6.430652329	0.219147035
ENSMUSG00000024654	Aspgl1	asparaginase like 1 [Source:MGI Symbol;Acc:MGI:1913764]	17.1296	20.7239	23.1003	16.9657	18.3524	21.1848	21.7388	19.9461	21.3196	20.1026	17.6493	20.0697	18.8498	20.7389	0.093849599	0.490854203	12	8	24	0.179705203	0.768040178	24	5.456878392	0.089493741
ENSMUSG00000024659	Anxa1	annexin A1 [Source:MGI Symbol;Acc:MGI:96819]	19.7032	28.4741	29.9827	26.8506	25.8661	22.2987	21.3228	21.9826	34.5654	15.7825	24.3215	23.1483	24.1346	22.1721	0.035806379	0.331814682	12	16	24	0.476236016	0.805393068	24	8.40804508	0.130289129
ENSMUSG00000024660	Incenp	inner centromere protein [Source:MGI Symbol;Acc:MGI:1313288]	3.71178	4.63357	4.15497	4.29328	5.14104	4.09915	4.11624	4.42894	4.41782	4.55465	4.19036	4.10812	5.66502	4.43829	0.588208558	0.966018836	8	16	24	0.937388621	0.984507582	24	9.538557355	0.017750367
ENSMUSG00000024661	Fth1	ferritin heavy polypeptide 1 [Source:MGI Symbol;Acc:MGI:95588]	1581.14	1642.46	1642.54	1577.97	1690.93	1480.48	1725.44	1575.4	1909.9	1710.59	1802.46	2001.97	1914.91	1711.99	0.881896584	0.966018836	20	12	24	0.98573241	0.997214081	24	15.61677252	0.006420619
ENSMUSG00000024663	Rab3il1	RAB3A interacting protein (rabin3)-like 1 [Source:MGI Symbol;Acc:MGI:1922010]	4.78659	6.13156	6.34891	6.00324	6.35194	5.63287	6.18338	6.63356	7.19698	4.36485	6.20975	5.83583	5.69822	6.22961	0.138202217	0.580158868	12	12	24	0.554319588	0.837681708	24	6.709833529	0.069558635
ENSMUSG00000024664	Fads3	fatty acid desaturase 3 [Source:MGI Symbol;Acc:MGI:1928740]	20.0997	18.1428	17.9154	17.1088	20.0983	18.8488	17.7777	19.028	16.582	22.3187	18.3048	20.0291	19.3151	17.3575	0.266953862	0.774280302	24	12	24	0.307511899	0.768040178	24	18.30290622	0.06658069
ENSMUSG00000024665	Fads2	fatty acid desaturase 2 [Source:MGI Symbol;Acc:MGI:1930079]	50.3706	42.7127	46.6043	42.5578	49.3273	52.2917	48.9841	46.8515	39.2957	49.6241	41.538	47.4209	44.4477	45.7399	0.390686537	0.915479136	24	12	24	0.145259322	0.754184974	24	20.63289756	0.087850993
ENSMUSG00000024666	Tmem138	transmembrane protein 138 [Source:MGI Symbol;Acc:MGI:1920232]	3.5937	3.89803	4.82289	4.27893	4.51621	5.2418	4.45166	4.64758	4.70743	4.35356	4.63845	5.11803	4.87544	4.92006	0.871479522	0.966018836	24	16	24	0.857534036	0.957975236	24	16.39472475	0.021007601
ENSMUSG00000024667	Tmem216	transmembrane protein 216 [Source:MGI Symbol;Acc:MGI:1920020]	5.31843	5.43825	6.68478	5.24416	5.67741	5.0983	4.64531	5.1702	6.17457	5.32697	5.6645	5.34035	5.23567	5.19905	0.466646635	0.966018836	20	16	24	0.799182178	0.935782011	24	8.069128538	0.022447766
ENSMUSG00000024668	Sdhaf2	succinate dehydrogenase complex assembly factor 2 [Source:MGI Symbol;Acc:MGI:1913322]	39.236	37.0948	38.6675	39.1514	35.7516	38.3533	34.7244	36.2091	34.6781	32.6609	38.6008	35.5805	39.369	36.5206	0.88067614	0.966018836	4	20	24	0.620926305	0.870432734	24	22.03693467	0.030732286
ENSMUSG00000024672	Ms4a7	membrane-spanning 4-domains, subfamily A, member 7 [Source:MGI Symbol;Acc:MGI:1918846]	1.58815	1.92425	1.49357	2.41381	1.91171	1.38043	2.09235	2.00559	2.16365	1.34622	1.84653	1.58965	2.03937	1.81123	0.896799496	0.966018836	4	20	24	0.68457781	0.895095838	24	6.44211931	0.058299781
ENSMUSG00000024673	Ms4a1	membrane-spanning 4-domains, subfamily A, member 1 [Source:MGI Symbol;Acc:MGI:88321]	0.405245	0.736242	0.924592	0.904833	0.629166	0.794998	0.840033	0.794353	1.32615	0.427214	0.797732	0.812602	0.895064	0.842208	0.612141595	0.966018836	12	8	24	0.434873782	0.805393068	24	7.236461242	0.091678783
ENSMUSG00000024675	Ms4a4c	membrane-spanning 4-domains, subfamily A, member 4C [Source:MGI Symbol;Acc:MGI:1927656]	0.755918	1.20906	0.747753	0.842913	1.24965	0.690768	1.56241	0.990553	1.07721	0.789305	0.683226	0.60191	0.438947	0.37692	0.888482515	0.966018836	12	12	24	0.976630561	0.994103202	24	10.47871929	0.01909112
ENSMUSG00000024677	Ms4a6b	membrane-spanning 4-domains, subfamily A, member 6B [Source:MGI Symbol;Acc:MGI:1917024]	1.40394	1.54944	1.97756	2.35203	1.597	1.62633	1.90071	1.27191	2.4664	1.1171	1.77118	1.62789	1.07349	1.28625	0.253121224	0.753386477	12	20	24	0.343305377	0.77703533	24	11.91877362	0.12042057
ENSMUSG00000024678	Ms4a4d	membrane-spanning 4-domains, subfamily A, member 4D [Source:MGI Symbol;Acc:MGI:1913857]	0.61676	0.717166	0.607136	0.912712	0.882533	0.686094	0.394633	0.869841	1.08478	0.698363	1.03276	0.578922	0.995682	0.610728	0.640622311	0.966018836	20	4	24	0.280853561	0.768040178	24	11.82246542	0.1008709
ENSMUSG00000024679	Ms4a6d	membrane-spanning 4-domains, subfamily A, member 6D [Source:MGI Symbol;Acc:MGI:1916024]	1.29564	1.0609	0.72199	0.981672	1.32887	0.791904	0.463156	1.34125	1.33989	0.626206	1.11088	1.10551	0.777423	0.727999	0.88067614	0.966018836	20	20	24	0.924708385	0.979951713	24	14.54472642	0.034091624
ENSMUSG00000024680	Ms4a2	membrane-spanning 4-domains, subfamily A, member 2 [Source:MGI Symbol;Acc:MGI:95495]	0.445599	0.876691	0.854008	0.611138	0.787093	0.548922	0.436543	0.755088	0.591676	0.450744	0.731591	0.634071	0.513383	0.657124	0.125573493	0.555618842	8	20	24	0.501345233	0.80908345	24	9.208450821	0.061939688
ENSMUSG00000024681	Ms4a3	membrane-spanning 4-domains, subfamily A, member 3 [Source:MGI Symbol;Acc:MGI:2158468]	11.3539	11.5271	12.6416	9.27655	12.479	12.6398	11.5469	9.47552	9.08113	6.96668	10.7727	10.4203	11.5366	9.46821	0.217933169	0.704989686	24	16	24	0.065946958	0.668186543	24	21.68928205	0.173769211
ENSMUSG00000024683	Mrpl16	mitochondrial ribosomal protein L16 [Source:MGI Symbol;Acc:MGI:2137219]	16.2162	18.7888	19.1291	18.4942	18.3154	19.2383	18.1717	19.7079	18.0906	19.033	19.3215	19.6274	20.3192	20.9901	0.941885246	0.966018836	8	4	24	0.656319071	0.88391232	24	6.766621454	0.021965252
ENSMUSG00000024687	Osbp	oxysterol binding protein [Source:MGI Symbol;Acc:MGI:97447]	17.422	14.4148	15.3814	15.8888	15.4203	17.1838	16.61	17.0069	13.9652	19.162	14.4305	16.0911	15.1119	15.6878	0.838532766	0.966018836	24	12	24	0.776095754	0.928029314	24	19.59976725	0.035015805
ENSMUSG00000024691	Fam111a	family with sequence similarity 111, member A [Source:MGI Symbol;Acc:MGI:1915508]	4.21968	4.00502	4.3989	3.67923	4.55361	3.84014	3.49177	4.12714	4.31313	2.96652	4.83108	3.81718	4.0816	3.39462	0.883024237	0.966018836	20	12	24	0.851337412	0.955425185	24	18.08031867	0.033609066
ENSMUSG00000024695	Zfp91	zinc finger protein 91 [Source:MGI Symbol;Acc:MGI:104854]	30.4263	32.1044	32.6608	34.0857	32.2844	30.4327	31.9509	31.1224	35.3406	34.6211	34.1614	32.8517	32.8862	33.3693	0.076438025	0.453790923	16	12	24	0.001077554	0.336500758	24	10.82219655	0.065950686
ENSMUSG00000024696	Lpxn	leupaxin [Source:MGI Symbol;Acc:MGI:2147677]	0.990181	1.41864	1.7012	1.44045	1.15565	1.06964	0.923251	1.16977	1.51535	0.929366	1.13241	1.04335	1.12551	0.92802	0.017495149	0.255326033	12	16	24	0.063530206	0.663715314	24	9.144764965	0.123836858
ENSMUSG00000024697	Gna14	guanine nucleotide binding protein, alpha 14 [Source:MGI Symbol;Acc:MGI:95769]	46.7129	40.0248	41.1255	38.6954	40.1959	45.974	36.8492	39.4042	33.8337	36.932	42.6018	43.1357	47.39	41.644	0.064494584	0.4150273	24	16	24	0.036960873	0.609176161	24	21.38842839	0.127664508
ENSMUSG00000024712	Rfk	riboflavin kinase [Source:MGI Symbol;Acc:MGI:1914688]	63.3983	53.8609	52.7979	57.4627	55.8024	57.4517	56.5538	60.2278	55.2769	59.0539	54.7365	59.8788	58.0626	61.3615	0.560714538	0.966018836	4	8	24	0.341224452	0.775506126	24	23.43277934	0.040986036
ENSMUSG00000024713	Pcsk5	proprotein convertase subtilisin/kexin type 5 [Source:MGI Symbol;Acc:MGI:97515]	2.56647	2.46826	2.44575	2.2889	2.5212	2.57427	2.70588	2.95044	2.56835	2.47879	2.13228	2.35167	2.4459	2.43022	0.66832131	0.966018836	4	16	24	0.122518636	0.735333681	24	2.678561621	0.05769117
ENSMUSG00000024725	Ostf1	osteoclast stimulating factor 1 [Source:MGI Symbol;Acc:MGI:700012]	131.192	115.525	113.119	115.201	112.815	119.211	106.584	119.118	109.072	125.843	132.377	127.516	140.053	127.002	0.46238066	0.966018836	24	12	24	0.446752762	0.805393068	24	21.02573132	0.05261723
ENSMUSG00000024726	Carnmt1	carnosine N-methyltransferase 1 [Source:MGI Symbol;Acc:MGI:1914633]	1.032	1.97525	1.87404	1.486	1.92532	1.92085	1.00645	1.81471	1.37911	1.93564	1.2977	0.898321	1.96512	1.09312	0.858269825	0.966018836	16	8	24	0.541916768	0.830559643	24	10.47210325	0.10733397
ENSMUSG00000024727	Trpm6	transient receptor potential cation channel, subfamily M, member 6 [Source:MGI Symbol;Acc:MGI:2675603]	0.753918	0.802921	0.735485	0.655309	0.803876	0.648986	0.701205	0.629904	0.661764	0.531207	0.680982	0.647626	0.687151	0.634415	0.932092277	0.966018836	4	12	24	0.408221822	0.799546792	24	22.85811273	0.026614303
ENSMUSG00000024732	Ccdc86	coiled-coil domain containing 86 [Source:MGI Symbol;Acc:MGI:1277220]	1.86626	1.85419	1.57484	1.40394	1.72909	1.61959	1.64856	2.3527	1.74416	2.43994	1.71652	2.01616	1.93691	1.66788	0.873350015	0.966018836	4	8	24	0.864193914	0.960637063	24	3.451973362	0.029441485
ENSMUSG00000024735	Prpf19	pre-mRNA processing factor 19 [Source:MGI Symbol;Acc:MGI:106247]	82.8718	77.2009	81.3154	80.1228	77.0039	87.3409	78.1174	77.3963	69.5969	83.4592	71.5419	76.7776	73.6862	77.9747	0.842298032	0.966018836	16	4	24	0.722242678	0.908924547	24	19.7661171	0.026274597
ENSMUSG00000024736	Tmem132a	transmembrane protein 132A [Source:MGI Symbol;Acc:MGI:2147810]	4.16249	4.19115	4.90076	3.99656	4.0049	4.08314	4.33974	4.41862	4.55659	4.88019	4.56344	4.65692	4.55503	4.83058	0.850398351	0.966018836	12	8	24	0.129468616	0.740162193	24	7.502602012	0.053320259
ENSMUSG00000024737	Slc15a3	solute carrier family 15, member 3 [Source:MGI Symbol;Acc:MGI:1929691]	0.308494	0.729563	0.779815	0.640743	0.553215	0.575191	0.566642	0.655013	0.521768	0.399946	0.461413	0.581056	0.663619	0.541655	0.855823117	0.966018836	8	12	24	0.521241428	0.82025681	24	5.923294137	0.054400433
ENSMUSG00000024740	Ddb1	damage specific DNA binding protein 1 [Source:MGI Symbol;Acc:MGI:1202384]	80.9456	74.2857	74.2931	75.704	78.7175	85.3113	78.6447	81.3819	68.743	84.7416	68.7184	74.2232	74.5131	76.1101	0.919571723	0.966018836	24	12	24	0.609046878	0.864393298	24	21.14272814	0.037935339
ENSMUSG00000024742	Fen1	flap structure specific endonuclease 1 [Source:MGI Symbol;Acc:MGI:102779]	4.6272	4.98094	5.90269	5.29183	4.52929	5.50481	4.77935	5.23127	4.6421	5.03571	4.4644	4.99344	5.1053	4.19585	0.908406451	0.966018836	16	4	24	0.92564268	0.980131499	24	10.03161249	0.016664361
ENSMUSG00000024743	Syt7	synaptotagmin VII [Source:MGI Symbol;Acc:MGI:1859545]	43.2909	36.7012	36.9225	42.9068	39.4954	42.8485	34.6966	41.0454	36.2117	51.6673	39.417	43.4982	43.8727	42.8933	0.745118544	0.966018836	24	12	24	0.580183913	0.85017573	24	15.79813156	0.057207641
ENSMUSG00000024747	Aldh1a7	aldehyde dehydrogenase family 1, subfamily A7 [Source:MGI Symbol;Acc:MGI:1347050]	2.80986	3.18857	3.94125	5.18252	2.91295	2.92213	3.58675	4.13255	4.31652	4.34383	4.10931	4.16388	5.05942	4.256	0.328944792	0.847277612	16	4	24	0.086728805	0.70594329	24	9.15967761	0.154276982
ENSMUSG00000024750	Zfand5	zinc finger, AN1-type domain 5 [Source:MGI Symbol;Acc:MGI:1278334]	74.5912	68.615	65.4837	74.7988	66.3066	66.4992	68.2286	70.3399	62.6843	84.5969	69.4442	65.4093	65.7593	68.2376	0.930944473	0.966018836	16	8	24	0.592725164	0.856634016	24	12.41421534	0.043434859
ENSMUSG00000024754	Cemip2	cell migration inducing hyaluronidase 2 [Source:MGI Symbol;Acc:MGI:1890373]	7.15877	6.91355	6.55874	7.90455	7.17203	6.90651	6.85815	6.73868	6.49505	8.34126	6.18727	6.86749	7.0588	6.79382	0.129903614	0.560263689	16	20	24	0.559589168	0.840580553	24	13.4030084	0.040334175
ENSMUSG00000024758	Rtn3	reticulon 3 [Source:MGI Symbol;Acc:MGI:1339970]	495.274	426.995	426.055	422.048	407.237	477.671	449.154	446.073	352.687	519.757	420.959	457.635	454.66	469.338	0.121546923	0.548996929	24	20	24	0.384975479	0.799546792	24	21.83338973	0.075426903
ENSMUSG00000024759	Atl3	atlastin GTPase 3 [Source:MGI Symbol;Acc:MGI:1924270]	41.4773	41.2433	36.1509	39.2896	37.207	38.5892	36.7555	36.365	36.6734	37.7972	41.3589	40.1566	41.9651	39.896	0.182849367	0.659303873	4	8	24	0.318001359	0.768040178	24	21.47577051	0.045848903
ENSMUSG00000024761	Gm16437	predicted pseudogene 16437 [Source:MGI Symbol;Acc:MGI:3644508]	75.53	77.1436	69.8761	83.6049	73.4526	73.8607	71.3642	70.8422	71.4256	71.8483	77.1323	76.6076	75.962	79.5142	0.935194469	0.966018836	24	12	24	0.840119687	0.951614724	24	17.26534441	0.017369136
ENSMUSG00000024764	Naa40	N(alpha)-acetyltransferase 40, NatD catalytic subunit [Source:MGI Symbol;Acc:MGI:1918249]	6.52911	7.81004	8.35154	7.40058	7.8733	7.87936	7.61583	7.74462	8.88261	7.54541	7.22957	7.72146	7.85458	7.68746	0.648082424	0.966018836	12	4	24	0.243581039	0.768040178	24	8.378528912	0.058064345
ENSMUSG00000024766	Lipo3	lipase, member O3 [Source:MGI Symbol;Acc:MGI:2147592]	6.3943	6.82305	6.90636	7.26139	5.70845	6.32173	6.45897	6.2869	5.94784	5.50085	7.05508	5.81687	5.08632	5.80254	0.816300547	0.966018836	16	8	24	0.724276745	0.90965493	24	11.34205326	0.033266231
ENSMUSG00000024767	Otub1	OTU domain, ubiquitin aldehyde binding 1 [Source:MGI Symbol;Acc:MGI:2147616]	63.4118	56.57	62.6406	55.7489	56.8332	68.1899	60.4127	62.7628	50.8578	67.7329	53.8566	58.1219	56.8322	61.785	0.939103211	0.966018836	24	20	24	0.742575014	0.916215545	24	22.931766	0.037492714
ENSMUSG00000024769	Cdc42bpg	CDC42 binding protein kinase gamma (DMPK-like) [Source:MGI Symbol;Acc:MGI:2652845]	1.14014	1.16726	1.47646	1.16751	1.03838	0.989392	0.937199	1.5008	1.30548	1.24913	1.11149	1.13456	1.18261	0.960664	0.068712455	0.432567661	12	12	24	0.085730703	0.703577714	24	8.141474203	0.093695981
ENSMUSG00000024771	Lipk	lipase, family member K [Source:MGI Symbol;Acc:MGI:2679259]	0.783902	0.871444	1.01025	0.760945	0.822694	0.846898	0.907648	0.703967	0.91778	0.681212	1.01111	1.06118	1.08235	0.932238	0.914360142	0.966018836	4	12	24	0.616167688	0.867488256	24	22.51302822	0.035976683
ENSMUSG00000024772	Ehd1	EH-domain containing 1 [Source:MGI Symbol;Acc:MGI:1341878]	12.3737	10.5981	12.5192	13.329	13.0567	12.4842	11.8763	13.7499	10.811	13.152	12.2287	11.6059	12.7039	11.7063	0.745118544	0.966018836	16	16	24	0.501085531	0.80908345	24	15.17392292	0.046435618
ENSMUSG00000024773	Atg2a	autophagy related 2A [Source:MGI Symbol;Acc:MGI:1916291]	7.62274	6.17106	6.24783	7.39792	6.59329	6.71481	6.60564	7.68232	7.39116	8.22101	6.80011	6.11377	6.67734	6.85972	0.871479522	0.966018836	16	8	24	0.472513814	0.805393068	24	9.848518441	0.056448463
ENSMUSG00000024776	Stambpl1	STAM binding protein like 1 [Source:MGI Symbol;Acc:MGI:1923880]	13.0104	9.76297	9.77626	9.61175	11.6772	10.8411	12.2274	9.71259	7.96591	10.261	10.4565	11.0884	10.9235	9.92027	0.002004172	0.100255953	4	8	24	0.002246309	0.395705644	24	20.73280044	0.180463773

ENSMUSG00000024777	Ppp2r5b	protein phosphatase 2, regulatory subunit B', beta [Source:MGI Symbol;Acc:MGI:2388480]	73.7458	56.8244	56.3026	59.1716	59.2604	64.0719	60.3712	62.9472	52.003	79.7563	63.8359	67.9068	65.433	65.5071	0.085890569	0.466404237	24	12	24	0.457738732	0.805393068	24	19.68760946	0.078054168
ENSMUSG00000024778	Fas	Fas (TNF receptor superfamily member 6) [Source:MGI Symbol;Acc:MGI:95484]	2.8415	2.78399	3.10441	3.7313	2.78595	2.93508	3.3257	3.35971	2.82574	3.11341	2.65045	2.56294	3.09901	2.67493	0.676344318	0.966018836	16	4	24	0.632734413	0.875112738	24	8.417972595	0.043906245
ENSMUSG00000024780	Cdc37l1	cell division cycle 37-like 1 [Source:MGI Symbol;Acc:MGI:1914322]	34.5505	36.3413	36.7885	38.2932	37.4786	33.9789	40.1603	34.0193	37.8231	34.2818	38.1529	36.3204	36.2708	35.7312	0.595037318	0.966018836	20	4	24	0.798953287	0.935664328	24	13.4838572	0.018880995
ENSMUSG00000024781	Lipa	lysosomal acid lipase A [Source:MGI Symbol;Acc:MGI:96789]	35.6247	28.2271	29.241	29.4235	31.9631	33.871	33.7011	33.1774	25.0925	33.6072	31.9783	31.6612	34.6322	30.7245	0.001920665	0.100255953	4	8	24	0.033455289	0.606585237	24	20.89803144	0.125586259
ENSMUSG00000024782	Ak3	adenylate kinase 3 [Source:MGI Symbol;Acc:MGI:1860835]	54.7937	53.5618	53.014	56.5121	61.0042	55.2512	56.4585	54.5655	57.9468	59.4534	55.7249	59.1152	59.1576	53.4641	0.050712923	0.386400877	16	16	24	0.085526043	0.703577714	24	16.29515707	0.048752062
ENSMUSG00000024784	Gpha2	glycoprotein hormone alpha 2 [Source:MGI Symbol;Acc:MGI:2156541]	0.805785	0.499426	0.291772	0.917628	0.439938	0.426851	0.544826	0.676574	0.426645	0.234463	0.629836	0.746433	0.718664	0.608783	0.43565473	0.966018836	4	8	24	0.36385335	0.788687089	24	22.47236786	0.103426942
ENSMUSG00000024785	Rcl1	RNA terminal phosphate cyclase-like 1 [Source:MGI Symbol;Acc:MGI:1913275]	5.7585	6.11474	6.48608	5.75508	5.97198	6.59632	4.66509	6.6993	5.59853	6.04798	5.83382	5.66089	6.40041	6.04235	0.921572192	0.966018836	8	20	24	0.829757311	0.947045266	24	8.088030606	0.02866697
ENSMUSG00000024787	Snx15	sorting nexin 15 [Source:MGI Symbol;Acc:MGI:1916274]	17.0096	16.6657	13.6868	14.2527	16.3891	15.8979	16.0824	16.3076	16.928	16.06	15.7102	16.8968	16.5894	17.1103	0.208341949	0.698704663	8	8	24	0.129303579	0.740162193	24	23.82238876	0.062305348
ENSMUSG00000024789	Jak2	Janus kinase 2 [Source:MGI Symbol;Acc:MGI:96629]	10.1178	10.6161	10.4533	10.8921	11.8977	9.78921	10.5553	10.0191	10.7801	9.58884	11.6071	9.78556	10.0637	9.60486	0.369262716	0.893596186	20	4	24	0.213668042	0.768040178	24	14.42596994	0.05524725
ENSMUSG00000024790	Sac3d1	SAC3 domain containing 1 [Source:MGI Symbol;Acc:MGI:1913656]	6.63526	4.90511	4.92509	5.28023	4.8201	5.79276	5.98358	4.95232	4.55909	5.95975	7.03188	5.50187	6.55539	5.71334	0.035806379	0.331814682	4	8	24	0.131702615	0.741996858	24	20.39229818	0.122560911
ENSMUSG00000024792	Zfpl1	zinc finger like protein 1 [Source:MGI Symbol;Acc:MGI:1891017]	14.1976	11.2197	11.9402	12.358	11.7733	12.8312	14.2326	12.9639	12.1969	13.4978	11.9322	13.349	11.7379	13.9526	0.939921059	0.966018836	4	16	24	0.499416251	0.808768108	24	23.60471961	0.047951875
ENSMUSG00000024793	Tnfrsf25	tumor necrosis factor receptor superfamily, member 25 [Source:MGI Symbol;Acc:MGI:1934667]	5.36943	2.38478	2.15694	7.37686	5.94936	3.19423	6.68635	3.14635	3.96503	3.45882	4.69829	3.67904	3.79496	2.58651	0.156866314	0.612465245	20	12	24	0.180945998	0.768040178	24	17.42531064	0.308462859
ENSMUSG00000024795	Kif20b	kinesin family member 20B [Source:MGI Symbol;Acc:MGI:2444576]	0.504744	1.10474	0.937019	1.36937	0.778416	1.38419	1.19354	0.727363	1.23284	0.971661	0.899114	0.885069	1.58578	0.622086	0.905762334	0.966018836	16	16	24	0.912784092	0.97533472	24	14.94687092	0.038515877
ENSMUSG00000024797	Vps51	VPS51 GARP complex subunit [Source:MGI Symbol;Acc:MGI:1915755]	18.3124	18.9691	18.8506	21.8972	20.5595	18.9281	19.6949	21.0263	19.697	22.5135	19.0515	18.9375	19.6707	19.1609	0.185796491	0.662239322	16	8	24	0.088331306	0.707747429	24	11.50362464	0.068117046
ENSMUSG00000024798	Htr7	5-hydroxytryptamine (serotonin) receptor 7 [Source:MGI Symbol;Acc:MGI:99841]	4.17813	4.30326	4.29255	3.96206	3.50466	4.63555	4.25596	3.79832	3.2642	4.99637	3.57783	4.39896	4.10542	4.8919	0.932671476	0.966018836	24	20	24	0.721493134	0.90866625	24	0.854268061	0.045626811
ENSMUSG00000024799	Tm7sf2	transmembrane 7 superfamily member 2 [Source:MGI Symbol;Acc:MGI:1920416]	43.7317	37.2126	39.716	36.4914	44.1862	41.6933	39.7685	39.6264	37.2843	40.265	42.2984	44.5139	44.2523	42.7777	0.05005312	0.385784509	24	12	24	0.020037393	0.565108098	24	20.3953609	0.089468761
ENSMUSG00000024800	Rpp30	ribonuclease P/MRP 30 subunit [Source:MGI Symbol;Acc:MGI:1859683]	16.7674	17.8247	17.1819	16.646	18.0401	17.5209	17.361	16.9417	21.9658	15.3267	19.3869	15.8835	18.3598	17.208	0.90767227	0.966018836	20	20	24	0.832214116	0.948075545	24	7.778212117	0.030409203
ENSMUSG00000024805	Pcgf5	polycomb group ring finger 5 [Source:MGI Symbol;Acc:MGI:1923505]	2.80189	3.32955	3.53478	3.78228	3.54624	3.38558	2.9347	3.1933	3.52393	3.46714	4.02241	2.9624	3.57561	2.86013	0.04467378	0.365845448	20	8	24	0.019006128	0.561252866	24	12.9275421	0.115779831
ENSMUSG00000024807	Syvn1	synovial apoptosis inhibitor 1, synoviolin [Source:MGI Symbol;Acc:MGI:1921376]	13.1922	8.97043	9.0056	10.3243	10.3946	10.0808	12.8668	11.23	9.454	13.4433	11.0762	10.8222	10.4006	10.1254	0.496745696	0.966018836	4	8	24	0.583600883	0.851892977	24	19.89998478	0.074311965
ENSMUSG00000024810	Il33	interleukin 33 [Source:MGI Symbol;Acc:MGI:1924375]	6.68604	11.8078	13.8635	9.71502	11.2882	9.31518	10.6416	9.63684	14.9228	7.66413	8.81068	10.1607	7.68216	10.8216	0.156791576	0.61244839	12	16	24	0.202634339	0.768040178	24	8.254240935	0.205849118
ENSMUSG00000024811	Tnks2	tankyrase, TRF1-interacting ankyrin-related ADP-ribose polymerase 2 [Source:MGI Symbol;Acc:MGI:1921743]	26.723	27.9958	27.6312	28.5676	29.6646	26.0686	29.8291	27.4014	28.6482	27.0344	27.7099	27.2443	28.2362	27.6783	0.852844413	0.966018836	20	4	24	0.872596583	0.964128457	24	9.807027118	0.011383112
ENSMUSG00000024812	Tjp2	tight junction protein 2 [Source:MGI Symbol;Acc:MGI:1341872]	8.27582	8.31745	8.17831	8.19574	8.07335	7.87636	9.62075	8.52826	9.00071	9.90765	7.09264	9.93887	8.79988	8.43268	0.918497267	0.966018836	4	16	24	0.759136211	0.92214435	24	3.535847438	0.034243319
ENSMUSG00000024816	Frmd8	FERM domain containing 8 [Source:MGI Symbol;Acc:MGI:1914707]	9.67242	9.81778	9.60267	9.51691	10.3731	9.05132	11.335	9.17699	9.75202	10.1993	9.40166	9.87119	10.206	9.35446	0.92721845	0.966018836	4	4	24	0.815876717	0.941291601	24	21.14251554	0.020394409
ENSMUSG00000024817	Uhrf2	ubiquitin-like, containing PHD and RING finger domains 2 [Source:MGI Symbol;Acc:MGI:1923718]	23.25	20.8884	16.8081	22.4283	22.7475	19.2933	21.8769	17.3739	19.9145	16.9727	22.7959	18.6585	20.5003	19.5451	0.447817955	0.966018836	20	16	24	0.268325598	0.768040178	24	19.50697796	0.097159587
ENSMUSG00000024818	Slc25a45	solute carrier family 25, member 45 [Source:MGI Symbol;Acc:MGI:2147731]	3.77156	4.75004	3.92142	4.86794	4.88637	4.19161	4.26531	4.34024	5.33448	3.73124	4.3628	4.16182	4.279	3.71719	0.952127548	0.966018836	20	4	24	0.622207445	0.871331547	24	12.03159641	0.049412166
ENSMUSG00000024824	Rad9a	RAD9 checkpoint clamp component A [Source:MGI Symbol;Acc:MGI:1328356]	6.67446	5.19132	5.73314	6.37029	5.30811	6.47249	5.57796	6.20654	5.32894	5.67294	6.22764	5.49835	5.8078	5.21082	0.823657258	0.966018836	4	4	24	0.440493918	0.805393068	24	18.90761431	0.052151411
ENSMUSG00000024826	Dpf2	D4, zinc and double PHD fingers family 2 [Source:MGI Symbol;Acc:MGI:109529]	34.8109	28.7086	28.5739	31.5813	33.057	33.4313	33.4255	34.6548	29.8351	34.617	30.7352	30.6858	31.4943	29.8816	0.418381424	0.963884303	16	20	24	0.371964863	0.793398709	24	19.61204172	0.055522113
ENSMUSG00000024829	Mrpl21	mitochondrial ribosomal protein L21 [Source:MGI Symbol;Acc:MGI:2660674]	22.3458	23.0832	22.6748	23.2318	24.647	22.1606	22.0846	22.4739	26.0068	22.03	25.3806	24.3909	25.7383	25.0683	0.9143748	0.966018836	20	8	24	0.850480737	0.95526784	24	11.5296915	0.016167431
ENSMUSG00000024830	Rps6kb2	ribosomal protein S6 kinase, polypeptide 2 [Source:MGI Symbol;Acc:MGI:1927343]	11.8562	13.551	14.3126	13.2733	14.5134	14.0622	15.2224	14.9119	14.6116	14.5475	14.6172	13.3997	17.7683	11.4473	0.847312766	0.966018836	4	20	24	0.938724724	0.984934488	24	17.19275477	0.020580665
ENSMUSG00000024831	Ighmbp2	immunoglobulin mu binding protein 2 [Source:MGI Symbol;Acc:MGI:99954]	2.41491	2.41906	2.22434	2.37283	2.21169	2.54059	1.99328	2.33648	2.2745	2.89386	2.1244	2.57057	2.47477	2.63063	0.917839756	0.966018836	24	20	24	0.94100299	0.985441727	24	9.271984458	0.013567157
ENSMUSG00000024833	Pola2	polymerase (DNA directed), alpha 2 [Source:MGI Symbol;Acc:MGI:99690]	3.86162	5.33761	4.5868	4.67183	4.68105	5.81432	4.67972	5.72942	4.93915	6.04313	5.07694	4.46919	5.66492	4.72377	0.828830907	0.966018836	8	20	24	0.827624802	0.946180429	24	9.829478704	0.036001268
ENSMUSG00000024835	Coro1b	coronin, actin binding protein 1B [Source:MGI Symbol;Acc:MGI:1345963]	55.0369	49.9209	55.7849	51.2962	54.3886	48.9789	49.8975	53.9589	54.1201	54.8049	55.6481	60.8378	61.6536	59.1453	0.731683656	0.966018836	4	12	24	0.940416978	0.985398871	24	2.487264703	0.010802443
ENSMUSG00000024841	Eif1ad	eukaryotic translation initiation factor 1A domain containing [Source:MGI Symbol;Acc:MGI:1917110]	12.441	11.0479	10.6055	10.8052	10.2378	11.7508	10.8056	11.432	10.7713	12.392	10.1146	11.4853	11.3073	11.0367	0.125573493	0.555618842	24	20	24	0.524984933	0.822381703	24	0.12701399	0.036990042
ENSMUSG00000024843	Chka	choline kinase alpha [Source:MGI Symbol;Acc:MGI:107760]	45.7879	37.8881	37.2397	41.3921	40.1095	36.9606	41.7122	32.8422	39.0659	34.8052	39.6015	38.2126	38.1495	39.1623	0.843639218	0.966018836	4	4	24	0.447734018	0.805393068	24	20.43373578	0.055391296
ENSMUSG00000024844	Baiap1	BAF nuclear assembly factor 1 [Source:MGI Symbol;Acc:MGI:1346330]	45.9088	47.632	45.7754	40.3401	45.2838	50.0782	44.1734	49.3258	47.3512	48.5912	49.8056	44.3515	50.6031	48.4458	0.806004771	0.907440428	16	20	24	0.718516665	0.907440428	24	10.21595724	0.021922251
ENSMUSG00000024845	Tmem134	transmembrane protein 134 [Source:MGI Symbol;Acc:MGI:1914240]	34.118	33.5573	33.5943	34.1633	36.8905	32.5119	33.472	35.4464	42.8886	42.1045	42.2732	40.5116	43.5733	36.0614	0.93717134	0.966018836	20	4	24	0.311321599	0.768040178	24	12.99930669	0.063103036
ENSMUSG00000024846	Cst6	cystatin E/M [Source:MGI Symbol;Acc:MGI:1920970]	16.0878	14.0064	13.6986	16.9617	15.7082	11.9486	12.9529	18.4758	22.3763	15.9712	22.6809	17.3649	23.4071	18.6312	0.814221431	0.966018836	20	4	24	0.628168928	0.872795898	24	9.022115969	0.08999915
ENSMUSG00000024847	Aip	aryl-hydrocarbon receptor-interacting protein [Source:MGI Symbol;Acc:MGI:109622]	78.5113	66.2516	66.0667	63.5713	65.9199	71.6485	68.1411	75.25	59.5419	66.9	65.4772	68.8035	70.6514	67.5596	0.011641838	0.209408611	4	8	24	0.018938481	0.561252866	24	23.05929003	0.095349252
ENSMUSG00000024851	Pitpnm1	phosphatidylinositol transfer protein, membrane-associated 1 [Source:MGI Symbol;Acc:MGI:1197524]	12.1392	14.6486	15.9411	14.2621	11.9342	14.5943	15.3581	13.7411	13.7027	14.6461	12.1212	12.649	12.5708	14.9771	0.147689593	0.590732968	12	8	24	0.12569196	0.739632637	24	6.689708225	0.100994676
ENSMUSG00000024853	Sf3b2	splicing factor 3b, subunit 2 [Source:MGI Symbol;Acc:MGI:2441856]	53.2173	48.7604	49.9438	51.5814	50.2787	52.833	52.8942	51.8777	48.749	56.3862	45.7218	51.5197	49.8902	51.7103	0.834434272	0.966018836	4	8	24	0.922644979	0.979411448	24	23.35873716	0.011583523
ENSMUSG00000024854	Pold4	polymerase (DNA-directed), delta 4 [Source:MGI Symbol;Acc:MGI:1916995]	24.0457	24.2065	21.4736	21.0075	24.6603	23.3697	24.7186	23.7405	23.3907	25.3666	25.3423	24.6153	25.8531	24.4316	0.480187506	0.966018836	20	16	24	0.1707547	0.768040178	24	21.53051362	0.05019029
ENSMUSG00000024855	Pacs1	phosphofurin acidic cluster sorting protein 1 [Source:MGI Symbol;Acc:MGI:1277113]	4.25205	3.85104	4.73294	3.74147	4.10537	4.26346	4.50908	5.04918	4.03103	5.03698	3.90671	4.24847	3.94604	4.30811	0.933543173	0.966018836	8	12	24	0.678246376	0.892450912	24	6.087480173	0.04031544
ENSMUSG00000024856	Cdk2ap2	CDK2-associated protein 2 [Source:MGI Symbol;Acc:MGI:1098779]	25.4533	19.9292	18.6637	19.9981	24.4057	21.9222	25.3939	24.3496	20.8503	21.6694	25.2323	22.8059	22.2132	20.6143	0.039091182	0.324150946	4	8	24	0.053314657	0.652202934	24	20.39854595	0.126703653
ENSMUSG00000024858	Grk2	G protein-coupled receptor kinase 2 [Source:MGI Symbol;Acc:MGI:87940]	53.0694	41.3211	39.5632	44.0598	41.5703	53.3278	45.2269	52.957	46.0489	58.9073	45.4745	49.2771	46.9896	48.4894	0.881718085	0.966018836	24	12	24	0.788636259	0.932208289	24	21.27440417	0.043900748
ENSMUSG00000024862	Klc2	kinesin light chain 2 [Source:MGI Symbol;Acc:MGI:107953]	71.112	58.9767	59.1944	63.0198	54.2568	64.3255	62.8407	73.1424	58.1132	82.5444	59.4595	69.7858	67.4254	66.71	0.598711142	0.966018836	4	8	24	0.838827991	0.950804194	24	1.863735598	0.035516586
ENSMUSG00000024866	Acy3	aspartoacylase (aminoacylase) 3 [Source:MGI Symbol;Acc:MGI:1918920]	2.27411	2.55732	2.8543	3.49595	2.71368	2.9324	3.08495	3.61945	3.24139	3.76762	2.46431	3.0272	1.97765	2.56202	0.185945151	0.662239322	16	12	24	0.154286539	0.760257003	24	10.8701594	0.143123955
ENSMUSG00000024867	Pip5k1b	phosphatidylinositol-4-phosphate 5-kinase, type 1 beta [Source:MGI Symbol;Acc:MGI:107930]	3.28531	2.86827	3.21811	3.48689	2.75852	3.45279	2.89172	2.9974	2.35061	3.34122	2.92741	3.03394	2.78488	2.81991	0.708020493	0.966018836	16	16	24	0.463204319	0.805393068	24	17.09923215	0.052837518
ENSMUSG00000024869	Gm49405	predicted gene, 49405 [Source:MGI Symbol;Acc:MGI:6155029]	0.704992	0.591537	0.729676	0.817852	0.751168	0.765648	0.811417	0.574561	0.899783	0.583108	0.774265	0.725404	0.612514	0.815169	0.960269811	0.967043964	12	20	24	0.724585952	0.909665611	24	14.11869322	0.027143784
ENSMUSG00000024870	Rab1b	RAB1B, member RAS oncogene family [Source:MGI Symbol;Acc:MGI:1923558]	79.8419	74.4525	72.2793	74.0094	76.5131	77.8671	71.6187	82.0672	78.1883	83.8611	80.1285	81.3505	83.103	78.9706	0.874607729	0.966018836	24	12	24	0.861150357	0.959222172	24	19.45113025	0.013396233
ENSMUSG00000024871	Doc2g	double C2, gamma [Source:MGI Symbol;Acc:MGI:1926250]	1.19927	1.35551	1.43235	0.872002	1.41309	1.03414	1.74936	1.45361	1.30098	1.57228	1.52478	1.0032	1.39105	1.30563	0.841589986	0.966018836	20	4	24	0.689158419	0.897070579	24	5.275307431	0.05269435
ENSMUSG00000024873	Cnih2	cornichon family AMPA receptor auxiliary protein 2 [Source:MGI Symbol;Acc:MGI:1277225]	7.16892	6.02306	6.01107	5.39341	5.60428	5.99507	6.69031	7.21508	8.85601	6.73739	7.55866	6.61034	7.82853	6.89110576	0.891105762	0.966018836	12	8	24	0.65397709	0.883343578	24	3.906050121	0.052006156
ENSMUSG00000024875	Yif1a	Yip1 interacting factor homolog A (S. cerevisiae) [Source:MGI Symbol;Acc:MGI:1915340]	23.9048	24.121	23.9505	22.3139	22.8253	25.1914	23.5162	24.9761	21.6255	25.3593	20.7003	24.1956	22.9197	22.0552	0.90767227	0.966018836	24	20	24	0.619797241	0.869755203	24	23.78824271	0.031247616
ENSMUSG00000024878	Cbwd1	COBW domain containing 1 [Source:MGI Symbol;Acc:MGI:2385089]	14.9595	15.7797	14.7459	14.0926	15.3497	14.9318	14.4014	13.493	15.2647	13.073	15.8701	15.4553	16.8725	15.7923	0.718720526	0.966018836	20	20	24	0.385336	0.799546792	24	22.65229293	0.048810008
ENSMUSG00000024885	Aldh3b1	aldehyde dehydrogenase 3 family, member B1 [Source:MGI Symbol;Acc:MGI:1914939]	7.3564	7.64759	8.78872	7.28499	7.97022	7.08483	8.00509	8.00202	8.79897	7.85989	6.90918	8.10289	7.92022	7.22026	0.745118544	0.966018836	12	8	24	0.3153414	0.768040178	24	7.186813558	0.05767426
ENSMUSG00000024887	Asah2	N-acylsphingosine amidohydrolase 2 [Source:MGI Symbol;Acc:MGI:1859310]	8.97813	9.27571	9.80938	8.85661	7.93503	8.88898	8.73094	8.43864	8.0157	9.64224	8.11896	8.8272	8.32399	9.23952	0.401437339	0.933249185	16	4	24	0.526756248	0.823075573	24	6.307907847	0.038432845
ENSMUSG00000024889	Rce1	Ras converting CAAX endopeptidase 1 [Source:MGI Symbol;Acc:MGI:1336895]	14.1275	11.5353	9.54585	13.9073	14.2776	13.3713	14.2501	13.0936	11.7527	12.5981	13.3034	12.3918	12.5387	11.3889	0.097779482	0.501112642	20	16	24	0.053798853	0.654390023	24	19.06428077	0.133774524
ENSMUSG00000024891	Slc29a2	solute carrier family 29 (nucleoside transporters), member 2 [Source:MGI Symbol;Acc:MGI:1345278]	2.77432	2.60472	3.26811	2.32547	2.93015	4.24888	3.05203	3.31324	2.63313	3.13879	2.83709	2.20026	2.28084	2.60144	0.794347041	0.966018836	12	16	24	0.922923443	0.979439431	24	18.0627133	0.03030961
ENSMUSG00000024892	Pcx	pyruvate carboxylase [Source:MGI Symbol;Acc:MGI:97520]	12.0014	10.422	13.1123	17.3648	12.5888	12.9599	13.8319	15.9568	11.9696	18.4149	14.0616	13.8297	15.4103	14.1292	0.841589986	0.966018836	16	20	24	0.348746622	0.779170188	24	12.34900886	0.104408832
ENSMUSG00000024896	Minpp1	multiple inositol polyphosphate histidine phosphatase 1 [Source:MGI Symbol;Acc:MGI:1336159]	11.8574	9.81022	10.6919	10.4004	10.4	11.7108	11.3019	10.6762	9.63745	11.7138	10.2814	11.5293	10.0617	11.5976	0.347076276	0.870418653	24	20	24	0.197112756	0.768040178	24	21.50818139	0.06259962
ENSMUSG00000024897	Apba1	amyloid beta (A4) precursor protein binding, family A, member 1 [Source:MGI Symbol;Acc:MGI:1860297]	47.2575	34.0586	33.5421	38.4794	36.5852	43.2119	38.296	40.2562	28.4838	56.5123	34.7171	44.8418	41.3286	42.608	0.08302755	0.462922111	24	12	24	0.512815903	0.815912787	24	20.0761544	0.111637999
ENSMUSG00000024899	Papss2	3'-phosphoadenosine 5'-phosphosulfate synthase 2 [Source:MGI Symbol;Acc:MGI:1330223]	1.90137	2.32372	3.09259	2.69282	2.3123	2.51149	2.65863	2.36256	2.88455	2.53754	1.66698	2.22335	2.1178	2.43274	0.048889741	0.379129806	12	16	24	0.108286887	0.72082227	24	8.218226838	0.128996946
ENSMUSG00000024900	Cpt1a	carnitine palmitoyltransferase 1a, liver [Source:MGI Symbol;Acc:MGI:1098296]	32.9743	26.7019	27.7447	28.3054	26.8974	33.0626	31.525	32.6997	22.3664	36.9849	24.6096	29.1808	27.8375	29.3428	0.591351901	0.966018836	24	12	24	0.627217059	0.872795898	24	22.16201316	0.072552367
ENSMUSG00000024901	Peli3	pellino 3 [Source:MGI Symbol;Acc:MGI:1924963]	9.23383	6.89097	6.92294	7.29241	6.54194	8.25271	7.79655	8.14756	6.43706	9.99565	6.63426	8.37172	8.56655	9.02209	0.359479536	0.883787544	4	8	24	0.45649849	0.805393068	24	23.96214601	0.084998379
ENSMUSG00000024902	Mrpl11	mitochondrial ribosomal protein L11 [Source:MGI Symbol;Acc:MGI:2137215]	39.3577	42.3546	40.6278	37.8725	37.1731	37.2426	35.4897	36.3516	45.3252	37.8479	41.2776	39.5205	41.602	46.9478	0.522897078	0.966018836	12	12	24	0.16879731	0.767020009	24	6.453124905	0.079517726
ENSMUSG00000024906	Mus81	MUS81 structure-specific endonuclease subunit [Source:MGI Symbol;Acc:MGI:1918961]	7.00042	7.32056	7.8211	7.7888	8.4799	8.39048	9.27613	7.47858	7.23708	7.17459	8.60569	8.59336	7.99534	9.1496	0.441501717	0.966018836	20	12	24	0.287747708	0.768040178	24	20.0049294	0.066866739
ENSMUSG00000024907	Gal	galanin and GMAP prepropeptide [Source:MGI Symbol;Acc:MGI:95637]	29.6962	23.7117	33.5291	22.5597	29.2687	33.3237	23.8366	28.2898	20.416	24.7365	38.4409	27.1873	24.1936	28.1283	0.664568268	0.966018836	20	20	24	0.355925232	0.782855709	24	18.27546137	0.140595481
ENSMUSG00000024908	Ppp6r3	protein phosphatase 6, regulatory subunit 3 [Source:MGI Symbol;Acc:MGI:1921807]	26.3494	24.4332	24.6513	23.7772	23.0144	23.63	23.8425	20.6771	22.5921	20.605	22.4618	24.4486	23.2207	23.0144	0.522550172	0.966018836	24	20	24	0.165821073	0.767020009	24	22.82881126	0.052847299
ENSMUSG00000024909	Efemp2	epidermal growth factor-containing fibulin-like extracellular matrix protein 2 [Source:MGI Symbol;Acc:MGI:1891209]	9.65073	10.6452	10.2203	8.52918	10.7892	10.0961	10.2563	9.92436	11.1178	9.51465	9.2227	8.61848	9.46887	9.21935	0.913451828	0.966018836	16	12	24	0.608530315	0.864378097	24	5.233365664	0.039070159
ENSMUSG00000024911	Fibp	fibroblast growth factor (acidic) intracellular binding protein [Source:MGI Symbol;Acc:MGI:1926233]	29.1307	33.2707	34.4201	29.7986	28.8591	32.2857	30.0557	32.8493	31.5888	31.3332	30.1154	28.4107	30.3837	29.5594	0.162786057	0.624917386	12	4	24	0.104036451	0.715480277	24	6.875516784	0.062580386
ENSMUSG00000024913	Lrp5	low density lipoprotein receptor-related protein 5 [Source:MGI Symbol;Acc:MGI:1278315]	1.58402	1.72113	1.58187	1.49299	1.85434	1.70111	2.16104	2.27795	1.65247	1.91845	1.60457	1.66858	1.59689	1.85256	0.897425765	0.966018836	8	4	24	0.558134756	0.839603173	24	2.82792898	0.048211306
ENSMUSG00000024914	Drap1	Dr1 associated protein 1 (negative cofactor 2 alpha) [Source:MGI Symbol;Acc:MGI:1913806]	89.6097	97.3512	96.2075	98.1198	95.4978	96.5228	90.8838	95.0657	105.424	97.8829	95.1583	102.552	99.2176	92.0646	0.4776989	0.966018836	16	12	24	0.325394935	0.768040178	24	11.55565974	0.034905052
ENSMUSG00000024921	Smarca2	SWI/SNF related, matrix associated, actin dependent regulator of chromatin, subfamily a, member 2 [Source:MGI Symbol;Acc:MGI:99603]	46.4604	47.5387	48.4947	44.1586	45.9274	46.8851	47.12	42.9945	43.731	51.3496	41.8246	46.2165	50.6076	49.0685	0.763955526	0.966018836	4	16	24	0.701047283	0.900900643	24	2.653393295	0.027985826
ENSMUSG00000024924	Vldlr	very low density lipoprotein receptor [Source:MGI Symbol;Acc:MGI:98935]	6.10374	8.60171	9.29382	10.1722	7.88381	8.00003	7.9491	10.3458	10.6791	10.1676	8.47082	8.32187	8.44422	9.06997	0.008405716	0.192760771	12	16	24	8.49E-04	0.319044294	24	9.29016721	0.214578117
ENSMUSG00000024925	Rnaseh2c	ribonuclease H2, subunit C [Source:MGI Symbol;Acc:MGI:1915459]	20.4013	16.5131	20.2595	26.9313	18.5632	19.635	23.3057	18.6252	24.4835	16.4006	28.0859	18.5144	22.3724	15.1032	0.823677655	0.966018836	20	20	24	0.517397141	0.818298678	24	15.4270227	0.11544238
ENSMUSG00000024926	Kat5	K(lysine) acetyltransferase 5 [Source:MGI Symbol;Acc:MGI:1932051]	35.8303	36.011	33.2971	36.9628	35.514	33.1553	32.3623	30.3416	31.0866	33.8493	33.4868	31.8105	33.2149	33.8117	0.889025617	0.966018836	12	8	24	0.545837727	0.833198211	24	15.15668299	0.029681824
ENSMUSG00000024927	Rela	v-rel reticuloendotheliosis viral oncogene homolog A (avian) [Source:MGI Symbol;Acc:MGI:103290]	9.57046	9.33108	9.31485	9.88584	10.8531	9.41358	9.32616	12.127	11.7087	12.3754	10.8026	11.5547	10.3607	9.81445	0.725736046	0.966018836	16	12	24	0.265454947	0.768040178	24	11.99925154	0.07473728
ENSMUSG00000024935	Slc1a1	solute carrier family 1 (neuronal/epithelial high affinity glutamate transporter, system Xag), member 1 [Source:MGI Symbol;Acc:MGI:105083]	2.21695	3.16245	4.69744	2.3427	1.68913	3.24671	3.83477	2.21789	3.18554	2.93627	1.8408	2.72021	1.84023	3.45765	0.179603275	0.655480352	12	8	24	0.25082204	0.768040178	24	5.879269538	0.206887872
ENSMUSG00000024937	Ehbp1l1	EH domain binding protein 1-like 1 [Source:MGI Symbol;Acc:MGI:3612340]	9.64536	10.185	9.35785	10.0515	10.349	9.72825	9.75103	13.6526	10.6385	12.0186	10.0749	9.26571	10.5092	9.61873	0.959856266	0.967043964	16	8	24	0.463338969	0.805393068	24	8.060665792	0.069882684
ENSMUSG00000024939	Fam89b	family with sequence similarity 89, member B [Source:MGI Symbol;Acc:MGI:106595]	51.1141	38.7047	39.3451	40.6328	42.4029	46.7231	41.9591	45.9455	35.7273	51.1176	45.023	48.7097	47.2107	41.1313	0.021795836	0.26698364	24	12	24	0.096724972	0.71175955	24	19.87552699	0.124815444
ENSMUSG00000024940	Ltbp3	latent transforming growth factor beta binding protein 3 [Source:MGI Symbol;Acc:MGI:1101355]	22.0609	24.5657	22.1449	24.4435	21.9818	20.4971	24.6838	30.866	29.4941	29.0864	30.2022	27.7469	30.6165	28.8332	0.833406028	0.966018836	8	16	24	0.127114361	0.740096438	24	7.141668425	0.09949881
ENSMUSG00000024941	Scyl1	SCY1-like 1 (S. cerevisiae) [Source:MGI Symbol;Acc:MGI:1931787]	23.644	21.8275	23.529	22.0248	20.4512	24.9784	22.0969	24.5502	21.1476	26.9365	22.6546	21.7472	22.3115	22.5864	0.807813307	0.966018836	8	16	24	0.959267189	0.98927861	24	8.856024631	0.012267641
ENSMUSG00000024942	Capn1	calpain 1 [Source:MGI Symbol;Acc:MGI:88263]	48.7769	38.8213	34.9008	41.6458	41.9601	45.4317	39.3332	45.1678	33.7183	52.4163	41.204	45.5287	45.3212	43.4491	0.022634137	0.27189101	24	12	24	0.300889442	0.768040178	24	19.84775275	0.103655209
ENSMUSG00000024943	Smc5	structural maintenance of chromosomes 5 [Source:MGI Symbol;Acc:MGI:2385088]	13.6767	17.6906	16.246	16.9793	16.3732	15.4229	15.5861	14.6227	20.0503	12.0254	16.8151	15.4982	15.6017	16.1479	0.724067924	0.966018836	12	12	24	0.761588788	0.922794036	24	7.506892978	0.050195706
ENSMUSG00000024944	Arl2	ADP-ribosylation factor-like 2 [Source:MGI Symbol;Acc:MGI:1928393]	43.8075	36.8154	36.9333	40.8478	38.3994	42.7702	39.3338	43.5537	37.4027	43.3051	39.4307	39.9114	39.7282	40.746	0.960338592	0.967043964	24	12	24	0.644830712	0.879377986	24	20.69526603	0.032867508
ENSMUSG00000024947	Men1	multiple endocrine neoplasia 1 [Source:MGI Symbol;Acc:MGI:1316736]	18.2034	12.6288	13.3657	14.0876	14.8699	15.4818	15.6567	15.2537	12.0104	15.6255	14.4492	14.9552	14.3428	14.3938	0.064867922	0.416505219	4	8	24	0.074845427	0.68394125	24	20.62324531	0.117803552
ENSMUSG00000024948	Map4k2	mitogen-activated protein kinase kinase kinase kinase 2 [Source:MGI Symbol;Acc:MGI:1346883]	26.0178	28.2635	28.4622	31.2457	32.8906	29.1658	31.8378	27.0035	29.6064	28.4308	23.5014	26.7446	26.1963	27.2577	0.557335707	0.966018836	16	12	24	0.7347299	0.913487469	24	12.09649893	0.036404084
ENSMUSG00000024949	Sf1	splicing factor 1 [Source:MGI Symbol;Acc:MGI:1095403]	40.6071	47.6462	44.9437	47.4673	44.9303	39.3548	46.9382	41.0868	49.1456	47.438	41.9586	45.1631	44.5423	41.6577	0.260026661	0.765037845	12	12	24	0.206204266	0.768040178	24	9.832099244	0.068721844
ENSMUSG00000024953	Prdx5	peroxiredoxin 5 [Source:MGI Symbol;Acc:MGI:1859821]	158.506	170.773	169.914	174.262	172.754	175.345	166.644	166.768	167.573	147.388	176.42	148.276	161.24	155.182	0.5169953	0.966018836	20	8	24	0.785022411	0.931056644	24	14.4909572	0.020954166
ENSMUSG00000024955	Esrra	estrogen related receptor, alpha [Source:MGI Symbol;Acc:MGI:1346831]	15.5155	12.86	14.4081	16.1911	13.1471	12.0338	13.9372	15.1008	15.4037	17.7351	15.47	15.65	17.2156	14.9428	0.960132359	0.967043964	16	16	24	0.475531636	0.805393068	24	9.797302335	0.064584319
ENSMUSG00000024957	Kcnk4	potassium channel, subfamily K, member 4 [Source:MGI Symbol;Acc:MGI:1298234]	6.75141	5.18578	6.91767	7.78408	7.01404	6.87871	5.47606	4.97532	6.43943	7.66215	7.09606	6.11174	5.03264	5.09235	1.48E-05	0.01431811	16	16	24	7.57E-05	0.102884653	24	14.28274897	0.22986043
ENSMUSG00000024958	Gpr137	G protein-coupled receptor 137 [Source:MGI Symbol;Acc:MGI:2147529]	36.335	29.3214	29.5944	28.795	29.339	33.914	31.5544	33.0571	26.5127	35.8686	33.9387	34.5306	32.3361	34.0836	0.565721644	0.966018836	24	12	24	0.188638961	0.768040178	24	21.09641006	0.090172404
ENSMUSG00000024959	Bad	BCL2-associated agonist of cell death [Source:MGI Symbol;Acc:MGI:1096330]	57.852	46.2949	45.6835	47.2306	49.3107	50.1467	46.7127	49.1153	41.6984	55.1923	47.1815	46.6128	51.9624	47.9201	0.393470605	0.921010321	4	8	24	0.406963301	0.799546792	24	20.66341621	0.063121066
ENSMUSG00000024960	Plcb3	phospholipase C, beta 3 [Source:MGI Symbol;Acc:MGI:104778]	137.064	99.3507	94.1951	109.693	106.38	116.609	101.329	118.797	85.4824	142.158	100.507	113.961	116.695	110.617	0.300823756	0.809362476	4	8	24	0.531934177	0.82547989	24	20.34678836	0.09073124
ENSMUSG00000024962	Vegfb	vascular endothelial growth factor B [Source:MGI Symbol;Acc:MGI:106199]	16.2642	14.9699	15.8468	16.581	13.099	16.1453	15.6903	16.4427	17.5781	23.2539	17.1307	20.7137	20.3295	19.3867	0.860560779	0.966018836	16	16	24	0.811131826	0.93944887	24	8.601948608	0.039069718
ENSMUSG00000024963	Dnajc4	DnaJ heat shock protein family (Hsp40) member C4 [Source:MGI Symbol;Acc:MGI:1927346]	9.09558	9.79032	10.2962	9.68855	10.5595	9.48984	8.76964	10.9166	10.3799	9.08912	8.39691	8.46282	9.68211	9.12206	0.204218286	0.690546825	12	12	24	0.180295067	0.768040178	24	7.505521717	0.073106747
ENSMUSG00000024965	Fermt3	fermitin family member 3 [Source:MGI Symbol;Acc:MGI:2147790]	1.15246	2.38735	3.13229	2.28768	1.90124	1.86798	1.99821	2.14145	2.58618	1.64485	2.39311	2.23345	2.33514	1.82239	0.321662653	0.83607295	12	12	24	0.300954278	0.768040178	24	9.165473552	0.137964779
ENSMUSG00000024966	Stip1	stress-induced phosphoprotein 1 [Source:MGI Symbol;Acc:MGI:109130]	117.007	79.9494	79.1362	77.5166	86.9138	96.5127	94.8482	93.4902	62.3449	86.2731	85.8808	89.9659	89.1617	84.7602	7.57E-04	0.071344007	4	8	24	0.007954088	0.490448898	24	21.63452337	0.210471201
ENSMUSG00000024968	Rcor2	REST corepressor 2 [Source:MGI Symbol;Acc:MGI:1859854]	5.0717	5.45742	6.47216	4.97783	5.43404	5.68262	5.01125	5.15462	4.92511	5.99142	4.34672	4.78948	5.19191	5.32049	0.910436409	0.966018836	12	8	24	0.491955351	0.805393068	24	7.885269431	0.05539981
ENSMUSG00000024969	Mark2	MAP/microtubule affinity regulating kinase 2 [Source:MGI Symbol;Acc:MGI:99638]	16.6267	16.4499	16.1725	15.8503	18.05	14.9891	16.7782	18.0414	16.1541	19.6729	16.4116	18.6404	16.439	15.2259	0.882263435	0.966018836	16	20	24	0.65829207	0.884596426	24	14.76405464	0.037551956
ENSMUSG00000024970	Spindoc	spindlin interactor and repressor of chromatin binding [Source:MGI Symbol;Acc:MGI:2147611]	6.95601	5.64055	4.90356	5.89547	7.52608	6.74015	5.75636	5.43517	5.12593	5.75147	7.20472	5.42832	5.42953	5.30257	0.005378037	0.149433822	20	16	24	0.004876899	0.438275631	24	18.01864744	0.175693303
ENSMUSG00000024972	Lgals12	lectin, galactose binding, soluble 12 [Source:MGI Symbol;Acc:MGI:1929094]	0.514843	0.5241	1.44019	2.4179	0.762252	0.872288	1.05607	1.7585	0.810935	1.96572	1.78071	1.31532	2.05891	1.6971	0.850398351	0.966018836	16	12	24	0.243074857	0.768040178	24	11.02818843	0.209850837
ENSMUSG00000024974	Smc3	structural maintenance of chromosomes 3 [Source:MGI Symbol;Acc:MGI:1339795]	13.8069	15.1216	15.9414	15.0977	14.7189	13.7725	16.0585	13.7934	16.344	13.0856	15.1371	16.0059	16.0234	15.276	0.928892845	0.966018836	12	4	24	0.776171138	0.928029314	24	4.346871641	0.026813385
ENSMUSG00000024975	Pdcd4	programmed cell death 4 [Source:MGI Symbol;Acc:MGI:107490]	19.2337	19.9955	20.1529	20.3775	18.1688	18.4379	18.8744	19.1642	19.2437	22.5118	18.1288	19.2555	19.6435	22.1889	0.00789564	0.192760771	16	4	24	0.067269472	0.670376655	24	7.748229641	0.077434995
ENSMUSG00000024976	Shoc2	Shoc2, leucine rich repeat scaffold protein [Source:MGI Symbol;Acc:MGI:1927197]	33.5782	30.4698	30.5919	32.0937	32.5066	31.468	31.226	29.9899	30.9023	30.0963	34.2057	33.0096	35.5462	33.238	0.165096293	0.629893599	4	4	24	0.137822875	0.747972781	24	20.33597469	0.051890391
ENSMUSG00000024978	Gpam	glycerol-3-phosphate acyltransferase, mitochondrial [Source:MGI Symbol;Acc:MGI:109162]	18.609	19.5781	21.498	21.9035	20.1461	21.3777	19.7391	24.9097	19.1934	24.7253	20.7609	20.3607	19.5198	22.9694	0.192357053	0.669841558	16	12	24	0.246942509	0.768040178	24	9.793130923	0.079114862
ENSMUSG00000024981	Acsl5	acyl-CoA synthetase long-chain family member 5 [Source:MGI Symbol;Acc:MGI:1919129]	27.7843	22.4245	27.4702	32.6566	29.3002	31.0065	26.6542	25.9389	16.8487	23.7833	31.262	25.1061	25.8668	30.0294	0.664568268	0.966018836	20	16	24	0.239354034	0.768040178	24	18.31122147	0.156517853
ENSMUSG00000024982	Zdhhc6	zinc finger, DHHC domain containing 6 [Source:MGI Symbol;Acc:MGI:1914230]	12.7281	12.1436	12.8862	12.369	11.4564	13.3368	12.8089	12.5388	11.4233	12.7955	12.5778	11.7654	12.6807	12.6039	0.873567285	0.966018836	4	8	24	0.654116445	0.883343578	24	23.85991292	0.022323805
ENSMUSG00000024983	Vti1a	vesicle transport through interaction with t-SNAREs 1A [Source:MGI Symbol;Acc:MGI:1855699]	14.9397	13.3668	13.5373	13.8029	12.8452	14.4805	14.4422	14.2719	12.5653	17.0429	13.3458	14.5232	15.6247	15.6367	0.359479536	0.883787544	4	8	24	0.691774836	0.897792868	24	0.394652276	0.035371632

ENSMUSG00000024985	Tcf7l2	transcription factor 7 like 2, T cell specific, HMG box [Source:MGI Symbol;Acc:MGI:1202879]	1.93916	2.36531	2.21063	1.98006	2.12323	2.0042	2.47196	2.09053	2.76846	2.6693	2.30843	2.51024	2.38729	2.20618	0.832742389	0.966018836	12	20	24	0.537251798	0.827923483	24	8.775573717	0.044733982
ENSMUSG00000024986	Hhex	hematopoietically expressed homeobox [Source:MGI Symbol;Acc:MGI:96086]	1.03063	1.66431	1.4783	1.48517	1.16274	1.22181	1.42145	1.96339	1.65377	0.89069	1.22822	1.46163	1.40416	1.46953	0.059082035	0.406067671	8	12	24	0.082853608	0.699403344	24	4.901859412	0.138525023
ENSMUSG00000024989	Cep55	centrosomal protein 55 [Source:MGI Symbol;Acc:MGI:1921357]	0.359463	0.420849	0.640207	0.578048	0.460049	0.606545	0.454628	0.323055	0.455264	0.312772	0.640926	0.614512	0.618046	0.567655	0.648082424	0.966018836	20	12	24	0.588884239	0.853936396	24	17.47363635	0.046927929
ENSMUSG00000024990	Rbp4	retinol binding protein 4, plasma [Source:MGI Symbol;Acc:MGI:97879]	10.1178	17.8205	12.8985	15.1923	11.9491	8.30481	11.7148	13.6291	20.2404	7.65132	12.8886	14.8481	10.4518	13.1771	0.208071297	0.698704663	8	20	24	0.285103089	0.768040178	24	7.081866547	0.226221818
ENSMUSG00000024991	Eif3a	eukaryotic translation initiation factor 3, subunit A [Source:MGI Symbol;Acc:MGI:95301]	51.9844	45.6976	47.0912	47.6703	47.4344	49.5109	46.2518	46.1163	41.3949	48.3841	47.7536	49.1962	47.7104	48.8626	0.041292272	0.346472636	24	12	24	0.107661294	0.720284443	24	20.04752968	0.059129621
ENSMUSG00000024993	Dennd10	DENN domain containing 10 [Source:MGI Symbol;Acc:MGI:1915144]	25.5338	28.1389	26.5995	26.6511	25.8225	25.8997	24.9422	25.1219	31.9488	24.6493	30.027	28.7379	28.72	28.3598	0.844511766	0.966018836	12	16	24	0.768838881	0.925489148	24	8.502195256	0.029904257
ENSMUSG00000024997	Prdx3	peroxiredoxin 3 [Source:MGI Symbol;Acc:MGI:88034]	68.3592	76.5897	76.9662	86.6169	78.5026	72.9739	72.9646	79.2869	83.9596	72.8975	86.7099	75.3036	84.435	81.6679	0.714044782	0.966018836	20	8	24	0.190325053	0.768040178	24	10.38207789	0.066517213
ENSMUSG00000024998	Plce1	phospholipase C, epsilon 1 [Source:MGI Symbol;Acc:MGI:1921305]	6.8534	5.25428	5.35621	7.26128	6.74208	6.14487	6.96316	4.66921	5.41453	6.23237	6.48655	6.2914	5.77173	5.02308	0.018114172	0.256345714	20	12	24	0.006592996	0.470633744	24	17.43422915	0.172649283
ENSMUSG00000024999	Noc3l	NOC3 like DNA replication regulator [Source:MGI Symbol;Acc:MGI:1932610]	7.10497	6.63933	6.25508	6.95973	6.99079	6.93965	7.25564	6.61509	5.85452	6.26712	7.49333	6.91305	6.90193	7.11017	0.039226672	0.338446795	20	16	24	0.027371416	0.590220615	24	20.36188458	0.080966282
ENSMUSG00000025001	Hells	helicase, lymphoid specific [Source:MGI Symbol;Acc:MGI:106209]	2.78708	3.15872	1.81618	1.98422	2.29717	2.22833	2.72623	2.11383	2.53835	2.17655	2.84038	2.6962	3.92774	2.23062	0.056400419	0.401074772	4	12	24	0.09671281	0.71175955	24	22.98271587	0.166123605
ENSMUSG00000025006	Sorbs1	sorbin and SH3 domain containing 1 [Source:MGI Symbol;Acc:MGI:700014]	90.1581	70.9042	74.8016	74.1283	81.3859	81.9864	92.9706	84.3113	71.0452	93.2494	77.9106	78.9718	76.4413	80.5995	0.643325306	0.966018836	4	8	24	0.459099465	0.805393068	24	20.87030049	0.065794543
ENSMUSG00000025007	Aldh18a1	aldehyde dehydrogenase 18 family, member A1 [Source:MGI Symbol;Acc:MGI:1888908]	8.59714	5.67684	6.36659	4.65784	6.41948	7.69819	7.23692	7.04204	5.10388	6.4562	5.77891	6.00541	6.60963	6.00284	0.048771079	0.379129806	4	8	24	0.025026225	0.586541342	24	22.57016319	0.187859608
ENSMUSG00000025008	Tctn3	tectonic family member 3 [Source:MGI Symbol;Acc:MGI:1914840]	5.33074	5.19081	4.81842	5.83243	4.67397	5.39881	5.42194	5.12574	5.91125	5.54004	5.04243	5.17628	5.21049	5.36288	0.941885246	0.966018836	16	4	24	0.649156123	0.881796509	24	7.816307992	0.030058964
ENSMUSG00000025010	Ccnj	cyclin J [Source:MGI Symbol;Acc:MGI:2443297]	2.11109	2.0447	2.46283	2.24932	2.31742	2.05573	2.277	2.00658	2.03958	1.99366	2.18551	2.22398	1.96058	2.21444	0.922029406	0.966018836	20	12	24	0.642947608	0.878809617	24	14.12342715	0.023739085
ENSMUSG00000025016	Tm9sf3	transmembrane 9 superfamily member 3 [Source:MGI Symbol;Acc:MGI:1914262]	78.4126	76.2326	75.3416	77.8438	77.3023	73.7716	74.4512	72.0512	69.3914	73.6538	77.3561	72.4392	70.0232	72.2852	0.718720526	0.966018836	16	20	24	0.100489018	0.71180729	24	16.52556236	0.03279655
ENSMUSG00000025017	Pik3ap1	phosphoinositide-3-kinase adaptor protein 1 [Source:MGI Symbol;Acc:MGI:1933177]	1.19971	1.34337	1.44557	1.16369	1.22954	0.811931	1.24328	1.26884	1.50035	0.822458	1.1335	0.969714	1.14211	1.09555	0.005498159	0.152089577	12	12	24	0.0864457	0.705702571	24	6.115384941	0.10743042
ENSMUSG00000025019	Lcor	ligand dependent nuclear receptor corepressor [Source:MGI Symbol;Acc:MGI:2443930]	1.52557	1.84729	1.74412	2.09732	1.97034	1.48578	1.59186	1.57173	2.36952	0.964584	2.09762	2.02065	1.8755	2.0073	0.892578749	0.966018836	20	8	24	0.955957529	0.988639288	24	9.089237786	0.024293843
ENSMUSG00000025020	Slit1	slit guidance ligand 1 [Source:MGI Symbol;Acc:MGI:1315203]	13.2106	10.8339	10.7976	11.1615	11.784	11.8763	10.6078	13.3794	8.5746	17.124	9.90336	14.0334	11.7352	13.2586	0.928780444	0.966018836	24	12	24	0.896942407	0.970652406	24	20.02153428	0.043753461
ENSMUSG00000025024	Smndc1	survival motor neuron domain containing 1 [Source:MGI Symbol;Acc:MGI:1923729]	14.3667	15.2891	14.0642	14.9837	14.41	14.8553	13.9786	14.2592	13.1908	14.4283	13.4599	14.444	14.711	15.1401	0.924553956	0.966018836	8	4	24	0.653463454	0.883312134	24	0.311647122	0.020653636
ENSMUSG00000025025	Mxi1	MAX interactor 1, dimerization protein [Source:MGI Symbol;Acc:MGI:97245]	19.4259	18.6389	20.1972	22.9849	19.6396	20.0542	19.1843	19.8013	20.6882	25.3299	19.9119	20.6215	18.9607	19.5435	0.002086159	0.100255953	16	12	24	0.01326195	0.529134128	24	12.26368488	0.111070794
ENSMUSG00000025026	Add3	adducin 3 (gamma) [Source:MGI Symbol;Acc:MGI:1351615]	27.0246	27.9512	28.5382	25.9533	27.0743	25.339	28.427	26.389	27.5553	24.5436	25.7321	26.5963	25.3567	25.261	0.965521737	0.969272194	12	4	24	0.433777848	0.805393068	24	3.597211921	0.027220232
ENSMUSG00000025027	Xpnpep1	X-prolyl aminopeptidase (aminopeptidase P) 1, soluble [Source:MGI Symbol;Acc:MGI:2180003]	25.4259	25.0292	23.9311	24.9256	25.197	27.3174	23.4978	24.5253	24.1381	24.1792	24.3321	24.1145	23.7768	21.522	0.512510541	0.966018836	20	16	24	0.044652399	0.628432994	24	18.04342606	0.053075458
ENSMUSG00000025034	Trim8	tripartite motif-containing 8 [Source:MGI Symbol;Acc:MGI:1933302]	6.75053	5.57174	5.64658	5.39451	5.89318	6.18158	6.18767	5.83193	6.43777	7.81526	6.83587	7.2029	6.90075	6.63956	0.487435992	0.966018836	24	12	24	0.541182167	0.830298514	24	18.78874206	0.046916121
ENSMUSG00000025035	Arl3	ADP-ribosylation factor-like 3 [Source:MGI Symbol;Acc:MGI:1929699]	77.4651	69.7234	68.2419	64.0672	69.0949	67.5122	75.6322	67.0874	68.9989	65.3081	67.3428	69.5168	71.8702	74.2831	1.11E-04	0.029372629	4	12	24	0.006464807	0.470633744	24	0.442216182	0.080732197
ENSMUSG00000025036	Sfxn2	sideroflexin 2 [Source:MGI Symbol;Acc:MGI:2137678]	2.75666	4.11343	3.50429	3.47858	3.9725	3.60711	3.4538	3.09313	4.55956	3.74066	3.74463	4.11486	4.02169	3.3397	0.777666236	0.966018836	24	4	24	0.500827188	0.80908345	24	12.79633566	0.063680839
ENSMUSG00000025037	Maoa	monoamine oxidase A [Source:MGI Symbol;Acc:MGI:96915]	23.9675	26.6849	26.0581	25.8112	27.1586	25.5157	27.1613	23.6139	26.4482	24.3536	30.8532	32.996	24.3417	36.681	0.920872048	0.966018836	20	20	24	0.967388086	0.992187127	24	18.01336203	0.01637297
ENSMUSG00000025038	Efhc2	EF-hand domain (C-terminal) containing 2 [Source:MGI Symbol;Acc:MGI:1921655]	1.49016	1.62676	1.82009	1.68089	1.68321	1.53205	1.31568	1.36923	1.64378	1.93542	1.52109	1.68146	1.90158	1.96033	0.696393581	0.966018836	16	12	24	0.266764163	0.768040178	24	10.00723522	0.06526831
ENSMUSG00000025040	Fundc1	FUN14 domain containing 1 [Source:MGI Symbol;Acc:MGI:1919268]	65.5801	64.7971	61.8621	60.1821	60.1207	55.0522	63.0951	57.1009	60.498	51.7194	65.5519	53.453	56.9765	55.8289	0.960269811	0.967043964	4	20	24	0.843468559	0.952454249	24	1.932333345	0.020697984
ENSMUSG00000025041	Nt5c2	5'-nucleotidase, cytosolic II [Source:MGI Symbol;Acc:MGI:2178563]	19.3347	14.9009	18.0756	16.8053	16.3764	17.1832	15.4059	17.887	15.8783	18.1035	15.5541	16.5708	15.5081	17.3771	0.891742979	0.966018836	16	4	24	0.932003864	0.982523804	24	8.53724924	0.015978686
ENSMUSG00000025044	Msr1	macrophage scavenger receptor 1 [Source:MGI Symbol;Acc:MGI:98257]	0.554453	1.09001	0.964033	0.694988	0.782863	0.506292	0.637805	0.922409	0.997812	0.769702	0.725685	0.775523	0.568545	0.591207	0.053683798	0.394331071	12	16	24	0.057036474	0.65610184	24	8.504279801	0.128455096
ENSMUSG00000025047	Pdcd11	programmed cell death 11 [Source:MGI Symbol;Acc:MGI:1341788]	4.81537	4.10929	4.94643	4.93619	4.95754	4.81482	4.83624	5.00832	4.86915	5.06262	4.19798	4.59189	4.43108	4.85235	0.167216842	0.634643192	16	8	24	0.577558532	0.848840315	24	10.14117636	0.033022756
ENSMUSG00000025049	Taf5	TATA-box binding protein associated factor 5 [Source:MGI Symbol;Acc:MGI:2442144]	2.12101	1.99726	2.07227	2.12401	2.0316	2.01569	1.84358	2.25208	1.91642	1.99973	2.15459	2.03447	1.8908	2.10618	0.941830768	0.966018836	20	8	24	0.785063335	0.931056644	24	11.13668231	0.01474359
ENSMUSG00000025050	Pcgf6	polycomb group ring finger 6 [Source:MGI Symbol;Acc:MGI:1918291]	7.72954	8.10863	7.62964	6.88081	7.97963	7.57869	8.22855	6.98653	8.15968	6.55608	9.06293	8.08991	7.74033	7.45618	0.676344318	0.966018836	20	20	24	0.410173024	0.799546792	24	20.68592805	0.0578496
ENSMUSG00000025059	Gk	glycerol kinase [Source:MGI Symbol;Acc:MGI:106594]	10.8112	10.6841	11.1972	14.1071	11.4677	10.8058	10.6357	9.8628	11.0168	10.3917	11.9547	9.77658	10.0577	11.0174	0.14606006	0.590732968	20	4	24	0.06482564	0.663876076	24	12.76991286	0.097042946
ENSMUSG00000025060	Slk	STE20-like kinase [Source:MGI Symbol;Acc:MGI:103241]	20.8146	21.7988	20.9613	22.3597	21.9936	19.9783	19.2272	18.5333	21.8779	19.4299	21.752	20.8791	20.0341	20.8757	0.171553415	0.646447162	20	8	24	0.271373657	0.768040178	24	13.05900837	0.046777486
ENSMUSG00000025066	Sfr1	SWI5 dependent recombination repair 1 [Source:MGI Symbol;Acc:MGI:1915038]	64.8539	62.9375	62.8723	61.0717	61.7712	65.5995	64.2802	61.0011	56.5014	63.1991	64.414	60.0725	64.6591	65.0455	0.43565473	0.966018836	4	8	24	0.222362007	0.768040178	24	21.87200333	0.039163707
ENSMUSG00000025068	Gsto1	glutathione S-transferase omega 1 [Source:MGI Symbol;Acc:MGI:1342273]	29.9693	29.0347	29.6141	33.4555	25.3288	30.7411	28.3896	30.606	26.4716	32.7237	29.6362	28.2886	30.0235	29.0218	0.833859682	0.966018836	16	4	24	0.866650868	0.961559919	24	10.39328123	0.022523976
ENSMUSG00000025069	Gsto2	glutathione S-transferase omega 2 [Source:MGI Symbol;Acc:MGI:1915464]	0.844063	1.02355	1.62145	1.11967	1.47024	1.18588	1.08941	1.27701	1.0762	1.02601	1.17719	1.17998	1.27106	1.14978	0.919571723	0.966018836	24	4	24	0.674858714	0.891005773	24	12.1781623	0.044119723
ENSMUSG00000025076	Casp7	caspase 7 [Source:MGI Symbol;Acc:MGI:109383]	2.72576	2.18886	2.06859	3.0085	3.07622	2.35959	1.82264	2.81713	2.33168	3.0988	1.95063	2.59047	2.25394	2.24978	0.478724519	0.966018836	16	12	24	0.365810782	0.789934347	24	13.6490347	0.09451704
ENSMUSG00000025077	Dclre1a	DNA cross-link repair 1A [Source:MGI Symbol;Acc:MGI:1930042]	2.5303	2.99273	3.51324	5.17697	3.31277	2.86118	3.57076	3.16817	3.89569	2.97238	2.42079	2.82526	3.41242	3.42692	0.156791576	0.61244839	12	12	24	0.198598764	0.768040178	24	8.960328357	0.150696145
ENSMUSG00000025078	Nhlrc2	NHL repeat containing 2 [Source:MGI Symbol;Acc:MGI:1914116]	4.70474	4.56924	4.21313	4.72322	4.59504	4.08057	4.71126	4.40808	4.65144	4.61793	4.57786	4.23245	4.4568	4.28412	0.850398351	0.966018836	16	8	24	0.78304708	0.930229965	24	11.0166023	0.015526284
ENSMUSG00000025083	Afap1l2	actin filament associated protein 1-like 2 [Source:MGI Symbol;Acc:MGI:2147658]	18.3046	17.7896	14.7358	16.1087	20.6756	16.6276	19.76	19.5745	17.3217	19.384	17.0124	17.6174	19.1953	15.2304	0.643325306	0.966018836	4	8	24	0.471405701	0.805393068	24	20.57647595	0.069757953
ENSMUSG00000025085	Ablim1	actin-binding LIM protein 1 [Source:MGI Symbol;Acc:MGI:1194500]	42.413	35.7448	34.2018	37.9518	35.353	39.8679	38.1603	38.9732	32.2417	45.3812	34.9282	38.1358	38.721	39.3324	0.277087842	0.789849983	4	8	24	0.624060949	0.871879816	24	21.59572755	0.049728307
ENSMUSG00000025086	Trub1	TruB pseudouridine (psi) synthase family member 1 [Source:MGI Symbol;Acc:MGI:1919383]	7.15548	7.91696	8.82814	8.2243	7.02606	7.106	7.25762	7.96694	10.0025	8.11234	8.18481	8.7047	8.74754	9.01681	0.10560184	0.518002936	12	8	24	0.011514717	0.519508195	24	7.896962171	0.121094526
ENSMUSG00000025089	Gfra1	glial cell line derived neurotrophic factor family receptor alpha 1 [Source:MGI Symbol;Acc:MGI:1100842]	48.227	44.4769	43.8147	42.7121	49.2095	44.9026	45.7987	44.6906	43.7505	46.819	48.4755	44.6087	48.1909	44.3565	0.010153564	0.194720062	20	16	24	0.142736032	0.752329696	24	19.22468617	0.048873119
ENSMUSG00000025090	Ccdc172	coiled-coil domain containing 172 [Source:MGI Symbol;Acc:MGI:1922895]	6.83288	6.2863	4.98298	5.892	6.10045	5.60013	4.69515	5.34451	5.41761	4.90379	6.73856	5.95198	6.35787	5.37857	0.57057742	0.966018836	20	20	24	0.308337265	0.768040178	24	19.69188935	0.088550193
ENSMUSG00000025092	Hspa12a	heat shock protein 12A [Source:MGI Symbol;Acc:MGI:1920692]	147.097	128.207	126.472	135.158	140.192	142.51	131.388	129.054	117.992	157.094	121.362	135.33	127.36	124.713	0.14313801	0.585944911	24	12	24	0.261924358	0.768040178	24	17.75831357	0.069476819
ENSMUSG00000025094	Slc18a2	solute carrier family 18 (vesicular monoamine), member 2 [Source:MGI Symbol;Acc:MGI:106677]	3.35417	3.30177	3.85088	4.27286	3.37044	4.89773	5.95994	2.7188	2.57865	5.13655	6.18784	6.68616	2.86072	11.7608	0.481611551	0.966018836	24	12	24	0.852310251	0.955628938	24	18.56576544	0.105220254
ENSMUSG00000025102	3110040N11Rik	RIKEN cDNA 3110040N11 gene [Source:MGI Symbol;Acc:MGI:1914540]	16.7269	16.0334	15.8102	16.6094	18.4421	13.6717	14.3925	14.6001	17.4141	15.4507	15.7664	16.6661	17.9157	15.5698	0.859936714	0.966018836	20	12	24	0.765975494	0.924332582	24	12.9164415	0.033267272
ENSMUSG00000025103	Btbd1	BTB (POZ) domain containing 1 [Source:MGI Symbol;Acc:MGI:1933765]	24.4234	24.8965	23.8906	25.9968	25.3875	22.615	24.708	25.3082	24.0816	23.3745	24.9326	21.0716	23.335	22.6683	0.369262716	0.893596186	20	4	24	0.469724635	0.805393068	24	9.955437961	0.032951744
ENSMUSG00000025104	Hdgfl3	HDGF like 3 [Source:MGI Symbol;Acc:MGI:1352760]	17.4249	19.9513	18.8729	18.9742	18.5876	17.2846	19.21	16.7659	19.6231	17.8695	19.5047	18.8306	18.6603	19.7661	0.801561896	0.966018836	8	16	24	0.616644661	0.867682235	24	8.706618437	0.029521558
ENSMUSG00000025128	Bhlhe22	basic helix-loop-helix family, member e22 [Source:MGI Symbol;Acc:MGI:1930001]	0.945385	1.08308	1.44879	0.947815	0.862446	0.839045	1.01912	0.883756	1.03939	0.85431	0.851121	0.812745	0.833696	1.05732	0.001885685	0.100255953	12	12	24	0.024720969	0.586541342	24	6.723360409	0.103829204
ENSMUSG00000025129	Ppp1r27	protein phosphatase 1, regulatory subunit 27 [Source:MGI Symbol;Acc:MGI:1915951]	3.53651	4.72198	4.34247	3.9696	4.39824	5.00305	4.78937	11.7049	5.40504	3.63534	3.86386	3.34857	9.61366	6.04434	0.116174595	0.538362324	8	8	24	0.076972266	0.687041208	24	3.581193034	0.352636879
ENSMUSG00000025130	P4hb	prolyl 4-hydroxylase, beta polypeptide [Source:MGI Symbol;Acc:MGI:97464]	69.7561	63.7835	62.4964	59.9303	66.5046	65.5483	66.7578	65.2745	61.7809	63.4947	64.3318	65.9896	65.705	61.5614	0.004122392	0.140598674	4	8	24	0.001811877	0.383184938	24	21.53377657	0.064046006
ENSMUSG00000025132	Arhgdia	Rho GDP dissociation inhibitor (GDI) alpha [Source:MGI Symbol;Acc:MGI:2178103]	173.23	135.898	139.487	145.315	152.736	168.153	158.423	160.985	124.554	189.367	139.128	155.889	146.528	147.087	0.321662653	0.83607295	24	12	24	0.381590512	0.799546792	24	19.7008935	0.08809325
ENSMUSG00000025133	Ints4	integrator complex subunit 4 [Source:MGI Symbol;Acc:MGI:1917164]	18.07	17.763	16.662	18.1753	19.686	17.0157	16.5052	15.0544	15.1064	14.8964	18.3191	15.8739	17.5924	16.0452	0.496745696	0.966018836	20	16	24	0.117957121	0.728323355	24	18.38727776	0.082929569
ENSMUSG00000025134	Alyref	Aly/REF export factor [Source:MGI Symbol;Acc:MGI:1341044]	14.0582	13.6123	14.3539	13.9662	15.8467	15.06	15.7519	14.0581	15.2051	13.7772	15.7093	14.2369	17.2675	14.9473	0.360140629	0.883787544	20	20	24	0.401312764	0.799546792	24	20.54544833	0.045276276
ENSMUSG00000025135	Anapc11	anaphase promoting complex subunit 11 [Source:MGI Symbol;Acc:MGI:1913406]	52.7281	44.2466	43.0224	46.1498	48.3665	47.6528	49.7566	47.8436	46.8567	49.321	52.3708	50.1543	50.3458	46.7639	0.030691182	0.324150946	4	8	24	0.032733867	0.606585237	24	19.7997686	0.074471592
ENSMUSG00000025137	Pcyt2	phosphate cytidylyltransferase 2, ethanolamine [Source:MGI Symbol;Acc:MGI:1915921]	88.174	81.3056	84.1362	85.7124	94.0644	84.1539	93.2122	87.7628	91.3953	84.905	88.6284	81.4607	84.3888	80.4014	0.47028201	0.966018836	20	12	24	0.655433213	0.883697438	24	16.15253087	0.025522419
ENSMUSG00000025138	Sirt7	sirtuin 7 [Source:MGI Symbol;Acc:MGI:2385849]	17.3567	17.024	17.7386	17.4448	15.6864	15.2116	16.9729	16.226	19.2532	17.2841	18.09	17.1264	18.6558	17.2283	0.601647512	0.966018836	12	12	24	0.261465676	0.768040178	24	7.628342902	0.053313414
ENSMUSG00000025139	Tollip	toll interacting protein [Source:MGI Symbol;Acc:MGI:1891808]	40.4656	31.8311	34.6492	34.9774	33.6657	38.6561	35.1838	36.2537	28.6267	43.9667	32.625	36.1673	34.8302	36.4088	0.791413662	0.966018836	24	12	24	0.661049143	0.885520687	24	19.80313656	0.055612948
ENSMUSG00000025140	Pycr1	pyrroline-5-carboxylate reductase 1 [Source:MGI Symbol;Acc:MGI:2384795]	1.15269	1.36098	1.23498	1.36551	1.09364	1.28665	1.2711	1.82727	1.79802	1.23452	1.04489	1.13868	1.19682	1.52521	0.001925685	0.100255953	8	12	24	0.011484341	0.519508195	24	6.144072268	0.147815446
ENSMUSG00000025141	Myadml2	myeloid-associated differentiation marker-like 2 [Source:MGI Symbol;Acc:MGI:1915765]	4.18524	3.49735	5.43343	5.08706	4.87218	4.37572	4.47855	6.03556	6.66877	6.3579	3.11143	3.88281	5.32496	5.07059	0.05630976	0.401074772	12	12	24	0.075353062	0.685464747	24	8.149919332	0.224312414
ENSMUSG00000025142	Aspscr1	alveolar soft part sarcoma chromosome region, candidate 1 (human) [Source:MGI Symbol;Acc:MGI:1916188]	24.9877	25.0567	26.4886	27.0689	27.1747	27.5958	26.1721	28.7973	27.9085	28.0852	27.0231	26.3003	28.069	26.2825	0.745199643	0.966018836	16	12	24	0.339393894	0.773835607	24	11.73309793	0.029737009
ENSMUSG00000025144	Cenpx	centromere protein X [Source:MGI Symbol;Acc:MGI:894324]	11.8218	12.2352	12.0753	11.7173	13.5422	10.7331	12.035	11.722	14.2165	10.1975	11.7556	10.4767	11.0108	9.94674	0.601647512	0.966018836	12	12	24	0.693617608	0.898364349	24	10.16483035	0.041349357
ENSMUSG00000025145	Lrrc45	leucine rich repeat containing 45 [Source:MGI Symbol;Acc:MGI:2387183]	15.2935	16.389	15.4522	16.0096	17.0735	16.4982	17.5049	15.674	16.5445	15.8416	16.2904	15.4705	15.4266	15.73	0.948582958	0.966018836	20	8	24	0.682533142	0.89423365	24	16.86961362	0.019412797
ENSMUSG00000025147	Mob2	MOB kinase activator 2 [Source:MGI Symbol;Acc:MGI:1919891]	30.3732	28.5402	25.1619	27.5515	25.2621	28.1	26.5563	30.5436	26.9966	28.4243	27.5061	29.0336	29.538	28.0735	0.214861332	0.704989686	8	4	24	0.179913773	0.768040178	24	0.588638099	0.056351832
ENSMUSG00000025150	Cbr2	carbonyl reductase 2 [Source:MGI Symbol;Acc:MGI:107200]	5.32513	6.50637	5.7427	6.25998	7.57356	5.66044	5.47621	6.23785	8.71012	4.73389	6.1183	5.52981	5.22852	5.42049	0.176754388	0.653256853	12	16	24	0.484160087	0.805393068	24	10.46230764	0.093643453
ENSMUSG00000025151	Maged1	MAGE family member D1 [Source:MGI Symbol;Acc:MGI:1930187]	185.377	154.565	167.656	154.962	163.537	179.071	178.206	170.619	142.079	178.385	160.129	162.66	157.607	163.726	0.745475226	0.966018836	4	8	24	0.243375365	0.768040178	24	21.26991622	0.067395809
ENSMUSG00000025153	Fasn	fatty acid synthase [Source:MGI Symbol;Acc:MGI:95485]	75.5796	45.9515	86.7957	148.578	84.2631	85.4619	91.1592	101.716	65.099	127.72	147.408	89.811	134.787	111.249	0.91695334	0.966018836	16	20	24	0.24298473	0.768040178	24	14.52619372	0.254140218
ENSMUSG00000025154	Arhgap19	Rho GTPase activating protein 19 [Source:MGI Symbol;Acc:MGI:1918335]	35.2898	36.1541	34.4914	44.2393	44.53	32.8883	40.6001	31.8059	39.8184	38.7475	37.7439	36.9917	35.365	30.2807	0.048396595	0.379129806	16	16	24	0.050260835	0.644435324	24	14.5756443	0.134454984
ENSMUSG00000025155	Dus1l	dihydrouridine synthase 1-like (S. cerevisiae) [Source:MGI Symbol;Acc:MGI:1915980]	14.5753	13.2938	14.0549	13.6272	15.0474	14.5732	16.4197	15.5189	14.4481	16.0976	14.0255	15.8536	13.0047	13.7015	0.907068194	0.966018836	24	8	24	0.673530016	0.890201973	24	18.08414839	0.035996578
ENSMUSG00000025156	Gps1	G protein pathway suppressor 1 [Source:MGI Symbol;Acc:MGI:2384801]	7.273	8.36473	8.2538	7.65303	9.17811	8.3404	7.31458	8.63339	7.9371	6.52445	7.35623	6.63907	7.98567	6.55948	0.860560779	0.966018836	8	20	24	0.999273494	0.999783065	24	2.775325612	0.001810343
ENSMUSG00000025157	Zdhhc16	zinc finger, DHHC domain containing 16 [Source:MGI Symbol;Acc:MGI:1921418]	16.1364	15.0219	17.5871	17.0275	16.1069	16.114	17.0395	20.4372	17.8495	17.2846	16.6279	15.5128	17.2857	13.9636	0.245061943	0.741580974	12	12	24	0.655472965	0.883697438	24	8.918446376	0.046376439
ENSMUSG00000025158	Rfng	RFNG O-fucosylpeptide 3-beta-N-acetylglucosaminyltransferase [Source:MGI Symbol;Acc:MGI:894275]	12.3578	11.4947	11.3903	12.0456	12.725	13.5644	13.9571	13.089	11.5016	14.4736	12.7373	12.1899	13.1747	12.7668	0.300823756	0.809362476	4	8	24	0.394811516	0.799546792	24	19.57901385	0.050323045
ENSMUSG00000025159	Mms19	MMS19 cytosolic iron-sulfur assembly component [Source:MGI Symbol;Acc:MGI:1919449]	19.7076	18.4465	16.929	19.7499	20.4621	19.5371	18.6471	18.611	17.9786	20.0483	18.2682	18.7503	20.0912	18.2658	0.041131612	0.346472636	16	20	24	0.146805114	0.755260456	24	18.40179262	0.054972344
ENSMUSG00000025161	Slc16a3	solute carrier family 16 (monocarboxylic acid transporters), member 3 [Source:MGI Symbol;Acc:MGI:1933438]	2.27834	2.17595	3.24505	4.14131	3.00681	4.05764	3.31085	7.81074	4.39528	5.47806	3.20191	3.58561	3.94099	2.57563	0.798829513	0.966018836	16	16	24	0.553747922	0.837374348	24	10.29391629	0.164681959
ENSMUSG00000025162	Csnk1d	casein kinase 1, delta [Source:MGI Symbol;Acc:MGI:1355272]	48.0476	39.2222	39.9553	45.9534	44.8255	48.8664	45.6161	47.1619	34.6574	54.6328	38.3128	43.5973	42.9166	42.6746	0.338172159	0.85758369	24	12	24	0.526235156	0.823071475	24	19.09111408	0.076009145
ENSMUSG00000025169	Ogfod3	2-oxoglutarate and iron-dependent oxygenase domain containing 3 [Source:MGI Symbol;Acc:MGI:1913429]	5.90592	5.20297	4.51444	5.38558	5.82191	5.63013	6.39747	5.71793	6.0231	6.2613	6.40714	5.96217	6.202	5.46481	0.334531116	0.85758369	4	4	24	0.183857196	0.768040178	24	19.30422202	0.072734558
ENSMUSG00000025170	Rab40b	Rab40B, member RAS oncogene family [Source:MGI Symbol;Acc:MGI:2183451]	3.45338	3.41975	3.30088	3.49515	3.35181	3.11459	3.56415	3.87701	3.70446	3.89898	3.31372	3.29548	3.42575	3.02741	0.616047374	0.966018836	16	8	24	0.434330919	0.805393068	24	8.64842171	0.042971468
ENSMUSG00000025171	Ubtd1	ubiquitin domain containing 1 [Source:MGI Symbol;Acc:MGI:2385092]	7.86559	6.6618	7.20007	6.90252	6.89051	6.54921	6.2755	6.90328	5.83676	8.14762	7.30975	7.08653	6.71397	6.47851	0.787283372	0.966018836	16	16	24	0.472474544	0.805393068	24	15.70577425	0.051513585
ENSMUSG00000025172	Ankrd2	ankyrin repeat domain 2 (stretch responsive muscle) [Source:MGI Symbol;Acc:MGI:1861447]	1.22901	1.91748	5.06961	4.96396	2.50909	4.33568	2.17253	6.645	3.61953	5.9634	2.4209	1.9978	3.04629	2.93791	0.124211791	0.552144108	16	12	24	0.069610192	0.67392894	24	9.895603828	0.453969225
ENSMUSG00000025173	Wdr45b	WD repeat domain 45B [Source:MGI Symbol;Acc:MGI:1914090]	28.8237	27.1932	25.5998	25.7195	25.8018	26.5368	27.5411	26.0373	26.8522	25.8561	27.9733	27.4448	28.0942	27.453	0.001765212	0.100255953	4	12	24	0.02324757	0.581965446	24	23.17147499	0.049561824
ENSMUSG00000025175	Fn3k	fructosamine 3 kinase [Source:MGI Symbol;Acc:MGI:1926834]	7.89858	8.14993	9.07041	9.03935	9.09786	9.12366	9.9062	7.43539	7.46065	7.49755	6.21599	7.81673	7.84538	8.74174	0.886002469	0.966018836	4	4	24	0.784680086	0.930908103	24	0.105163182	0.040404053
ENSMUSG00000025176	Hoga1	4-hydroxy-2-oxoglutarate aldolase 1 [Source:MGI Symbol;Acc:MGI:1914682]	0.408726	0.620571	0.74872	0.524255	0.548447	0.646531	0.482629	0.60209	0.499914	0.73934	0.440611	0.55597	0.605362	0.475298	0.933543173	0.966018836	16	12	24	0.542466439	0.83094351	24	9.847812932	0.042502006
ENSMUSG00000025178	Pi4k2a	phosphatidylinositol 4-kinase type 2 alpha [Source:MGI Symbol;Acc:MGI:1934031]	32.5985	24.6446	27.2492	28.0872	26.5615	31.6819	28.4869	29.9672	21.5975	35.4001	26.8307	29.7142	27.8768	28.3807	0.591351901	0.966018836	24	12	24	0.454530646	0.805393068	24	19.65925983	0.086757562
ENSMUSG00000025184	R3hcc1l	R3H domain and coiled-coil containing 1 like [Source:MGI Symbol;Acc:MGI:1196316]	8.53222	7.63914	7.68268	7.8501	8.61797	7.97502	6.69417	7.39853	7.12252	5.95247	8.64019	8.0758	7.95203	7.04831	0.034640964	0.328311403	20	12	24	0.136740189	0.747972781	24	19.32835524	0.099119422
ENSMUSG00000025185	Loxl4	lysyl oxidase-like 4 [Source:MGI Symbol;Acc:MGI:1914823]	1.54932	0.763766	1.35411	0.76675	1.64032	1.32245	1.05559	1.06507	0.7277	1.07689	1.45285	1.03522	1.01326	1.0476	0.435739761	0.966018836	20	12	24	0.130386172	0.740714253	24	18.66345459	0.150709781
ENSMUSG00000025188	Hps1	HPS1, biogenesis of lysosomal organelles complex 3 subunit 1 [Source:MGI Symbol;Acc:MGI:2177763]	3.67794	3.72528	4.46319	3.29008	4.8849	3.51385	3.88515	4.53398	4.75597	4.39483	4.93003	3.88519	4.33754	4.93071	0.601395222	0.966018836	20	4	24	0.336047333	0.771666944	24	9.196842711	0.080127864
ENSMUSG00000025189	Cnnm1	cyclin M1 [Source:MGI Symbol;Acc:MGI:1891366]	4.46432	3.7932	5.03147	4.65351	4.37263	4.84081	6.55119	4.21247	3.39201	6.04628	3.62153	4.62216	3.98679	4.94734	0.831272156	0.966018836	16	4	24	0.940117841	0.985398871	24	20.07137277	0.032358487
ENSMUSG00000025190	Got1	glutamic-oxaloacetic transaminase 1, soluble [Source:MGI Symbol;Acc:MGI:95791]	77.6896	67.7585	78.3776	70.8675	65.1908	84.714	74.8231	83.1398	57.7858	88.6425	59.3123	68.9469	67.8837	78.6738	0.93447422	0.966018836	8	12	24	0.76538515	0.924119887	24	2.408051454	0.053106449
ENSMUSG00000025192	Entpd7	ectonucleoside triphosphate diphosphohydrolase 7 [Source:MGI Symbol;Acc:MGI:2135885]	2.15507	1.92091	2.31718	2.20394	2.38983	2.3139	2.75567	2.02991	2.04547	2.19396	2.18219	2.22466	2.51923	1.86741	0.054520405	0.397122249	4	4	24	0.179795403	0.768040178	24	19.34080528	0.075257446
ENSMUSG00000025193	Cutc	cutC copper transporter [Source:MGI Symbol;Acc:MGI:1913638]	2.82241	4.70426	4.01054	4.02843	5.31597	3.88255	3.17449	3.89391	4.98832	3.49272	4.19873	3.81549	3.90277	5.14886	0.157813674	0.614233844	20	8	24	0.304055821	0.768040178	24	9.888239418	0.123948884
ENSMUSG00000025195	Dnmbp	dynamin binding protein [Source:MGI Symbol;Acc:MGI:1917352]	3.0855	3.22241	3.08226	3.17249	3.47154	3.29134	3.33152	3.22017	3.53038	3.7477	2.97031	3.46841	3.20171	3.2106	0.881996584	0.966018836	16	12	24	0.612618611	0.865589526	24	12.74427462	0.026211755
ENSMUSG00000025196	Cpn1	carboxypeptidase N, polypeptide 1 [Source:MGI Symbol;Acc:MGI:2135874]	2.40722	1.55023	1.24313	1.64888	1.488	2.12058	1.44676	1.81512	1.21973	1.86289	1.66259	2.25407	1.85414	1.78398	0.010468194	0.196621011	24	12	24	0.058097971	0.656399277	24	21.00306808	0.163248074
ENSMUSG00000025198	Erlin1	ER lipid raft associated 1 [Source:MGI Symbol;Acc:MGI:2387613]	9.85203	9.20811	9.03006	9.22477	9.24694	8.60393	8.88667	8.21339	8.39797	8.13376	9.9833	8.51824	9.06982	9.08669	0.945728192	0.966018836	20	16	24	0.577278968	0.848840315	24	19.71277897	0.033194863
ENSMUSG00000025199	Chuk	conserved helix-loop-helix ubiquitous kinase [Source:MGI Symbol;Acc:MGI:99484]	31.2948	32.853	31.877	34.7819	33.4587	28.1518	33.8483	30.3209	33.1128	26.5838	33.8881	28.0692	28.104	31.4054	0.503972924	0.966018836	20	4	24	0.696895868	0.899309039	24	9.506535779	0.038808163
ENSMUSG00000025200	Cwf19l1	CWF19-like 1, cell cycle control (S. pombe) [Source:MGI Symbol;Acc:MGI:1919752]	8.25605	7.03505	7.54714	6.75656	6.78683	7.91382	7.70439	7.80967	5.80932	7.01617	7.30067	7.05438	8.40969	6.65697	0.015984991	0.253395174	4	12	24	0.034297874	0.608494889	24	22.46606348	0.115180143
ENSMUSG00000025201	Bloc1s2-ps	biogenesis of lysosomal organelles complex-1, subunit 2, pseudogene [Source:MGI Symbol;Acc:MGI:3702045]	35.6953	33.5201	36.6114	30.1421	31.5073	31.7949	33.8774	29.3471	28.755	27.3659	38.3994	32.2509	32.262	34.1764	0.47028201	0.966018836	4	12	24	0.455214137	0.805393068	24	22.24834186	0.066837837
ENSMUSG00000025203	Scd2	stearoyl-Coenzyme A desaturase 2 [Source:MGI Symbol;Acc:MGI:98240]	471.055	402.795	424.86	404.325	446.926	474.005	481.903	432.102	364.383	514.176	399.219	445.114	428.852	411.178	0.303361836	0.811201458	24	12	24	0.238265836	0.768040178	24	19.97504179	0.088220769
ENSMUSG00000025204	Ndufb8	NADH:ubiquinone oxidoreductase subunit B8 [Source:MGI Symbol;Acc:MGI:1914514]	321.484	328.284	327.751	338.885	296.059	289.269	291.749	326.906	359.846	301.189	359.92	334.945	382.918	348.494	0.92721845	0.966018836	12	12	24	0.405357556	0.799546792	24	6.440496071	0.059365907
ENSMUSG00000025207	Sema4g	sema domain, immunoglobulin domain (Ig), transmembrane domain (TM) and short cytoplasmic domain, (semaphorin) 4G [Source:MGI Symbol;Acc:MGI:1347047]	8.47754	12.2025	11.9899	9.41511	12.5432	9.73462	11.987	11.1253	13.746	12.8469	9.56218	9.43468	10.0356	10.1445	0.118459043	0.541945448	12	12	24	0.131285831	0.741500219	24	8.775480394	0.151359768
ENSMUSG00000025208	Mrpl43	mitochondrial ribosomal protein L43 [Source:MGI Symbol;Acc:MGI:2137229]	19.9644	19.3456	18.9524	17.4265	18.5679	19.5042	19.8346	21.9076	19.6695	23.1439	19.7293	21.9845	22.1489	22.3716	0.50155611	0.966018836	4	16	24	0.229288139	0.768040178	24	2.389576852	0.051713047
ENSMUSG00000025209	Twnk	twinkle mtDNA helicase [Source:MGI Symbol;Acc:MGI:2137410]	4.74527	4.36709	4.6037	5.07202	4.78695	4.84218	4.68924	4.56373	4.44729	5.09707	3.86453	4.53401	4.99924	4.25738	0.231491055	0.731037655	16	16	24	0.899550158	0.971453495	24	16.91902038	0.016238122
ENSMUSG00000025212	Sfxn3	sideroflexin 3 [Source:MGI Symbol;Acc:MGI:2137679]	35.296	32.4022	32.7817	32.4553	32.2545	35.6575	32.4247	33.0466	30.4508	42.5226	32.3918	35.233	33.9102	33.3176	0.195343269	0.673387182	24	20	24	0.690828964	0.897666745	24	16.74377116	0.03817048
ENSMUSG00000025213	Kazald1	Kazal-type serine peptidase inhibitor domain 1 [Source:MGI Symbol;Acc:MGI:2147606]	2.64163	3.48123	3.12	2.99884	2.98475	2.6353	2.91049	2.92489	3.57345	3.12256	3.25689	3.00758	3.27377	3.17537	0.364640768	0.892062098	12	12	24	0.05982528	0.658304792	24	8.291556193	0.081203764
ENSMUSG00000025217	Btrc	beta-transducin repeat containing protein [Source:MGI Symbol;Acc:MGI:1338871]	19.5554	16.2795	19.4751	17.6557	16.4257	19.1449	18.4829	18.0981	15.4796	19.4508	16.1214	19.7585	18.2488	19.1843	0.676344318	0.966018836	24	20	24	0.532822383	0.825678486	24	23.138961	0.051110566

ENSMUSG00000025218	Poll	polymerase (DNA directed), lambda [Source:MGI Symbol;Acc:MGI:1889000]	5.40542	4.5609	4.82596	5.55765	5.03467	5.36715	4.68113	5.467	4.55342	5.39712	4.94751	5.03707	5.09773	5.1007	0.907068194	0.966018836	16	20	24	0.628884657	0.873060183	24	16.99987346	0.031286792
ENSMUSG00000025220	Oga	O-GlcNAcase [Source:MGI Symbol;Acc:MGI:1932139]	55.3607	53.8271	50.6992	51.8449	52.1111	49.2432	53.7083	50.0253	52.3849	50.3824	50.6979	52.0548	54.6078	52.9278	0.036976155	0.334054683	4	12	24	0.1264959	0.739868076	24	1.075722881	0.036606797
ENSMUSG00000025221	Kcnip2	Kv channel-interacting protein 2 [Source:MGI Symbol;Acc:MGI:2135916]	69.7325	53.2011	51.3857	52.3016	54.5529	63.7518	54.0021	59.6831	44.0621	63.2942	54.5196	61.4594	60.3688	58.3179	0.004516345	0.140598674	24	12	24	0.086714846	0.70594329	24	21.34603955	0.136772289
ENSMUSG00000025223	Ldb1	LIM domain binding 1 [Source:MGI Symbol;Acc:MGI:894762]	33.2237	36.5469	38.5647	37.6418	33.079	36.2245	35.782	36.5863	41.5783	42.5171	33.3538	37.868	36.5315	37.4615	0.012443448	0.220221977	16	4	24	0.033217355	0.606585237	24	8.642174407	0.09655621
ENSMUSG00000025224	Gbf1	golgi-specific brefeldin A-resistance factor 1 [Source:MGI Symbol;Acc:MGI:1861607]	21.969	17.5748	19.2529	19.1362	17.3729	21.7229	20.8175	21.0276	16.8463	23.5208	17.5513	19.5803	19.8821	19.3661	0.217933169	0.704989686	4	16	24	0.627986355	0.872795898	24	22.6446561	0.053607697
ENSMUSG00000025225	Nfkb2	nuclear factor of kappa light polypeptide gene enhancer in B cells 2, p49/p100 [Source:MGI Symbol;Acc:MGI:1099800]	2.85888	2.45699	2.71219	2.43354	2.80845	2.28289	3.00274	2.80455	3.32222	2.96833	2.79462	2.63367	3.07342	2.38819	0.948161972	0.966018836	4	20	24	0.862893078	0.959990216	24	7.572034011	0.025433923
ENSMUSG00000025226	Fbxl15	F-box and leucine-rich repeat protein 15 [Source:MGI Symbol;Acc:MGI:1915681]	5.29628	4.15243	3.19914	3.32245	3.48815	3.37305	4.08896	5.69538	5.25379	7.06029	7.81767	7.31617	7.46767	7.25783	0.94086481	0.966018836	8	4	24	0.774179884	0.927465562	24	1.161380784	0.071249157
ENSMUSG00000025227	Mfsd13a	major facilitator superfamily domain containing 13a [Source:MGI Symbol;Acc:MGI:1922396]	6.25264	4.24825	5.91447	5.24103	5.04154	5.60217	5.53156	6.02378	4.58755	7.12827	5.37836	5.88371	5.75311	6.06741	0.865233232	0.966018836	4	8	24	0.958075539	0.989059113	24	19.44699962	0.018847203
ENSMUSG00000025228	Actr1a	ARP1 actin-related protein 1A, centractin alpha [Source:MGI Symbol;Acc:MGI:1858964]	138.967	118.094	121.929	123.211	123.325	142.312	125.057	125.652	99.2479	140.643	120.199	126.139	125.797	126.305	0.080164531	0.458678242	24	12	24	0.206159039	0.768040178	24	20.14483513	0.08883782
ENSMUSG00000025231	Sufu	SUFU negative regulator of hedgehog signaling [Source:MGI Symbol;Acc:MGI:1345643]	2.79296	2.74027	2.97365	3.10851	3.09361	2.72355	2.76484	2.63156	3.32006	3.48389	2.73602	2.96339	2.87822	3.25334	0.143268475	0.585944911	16	8	24	0.026607839	0.586541342	24	10.69299261	0.084610815
ENSMUSG00000025232	Hexa	hexosaminidase A [Source:MGI Symbol;Acc:MGI:96073]	37.4489	29.5567	28.8543	29.0649	32.3863	31.1959	34.2758	34.9797	27.8851	29.8152	31.3594	32.0296	34.0887	29.8603	3.29E-04	0.049770315	4	8	24	0.00835909	0.49701405	24	22.34331083	0.125712178
ENSMUSG00000025234	Arih1	ariadne RBR E3 ubiquitin protein ligase 1 [Source:MGI Symbol;Acc:MGI:1344363]	29.784	25.9327	25.6697	26.9159	27.0764	27.9719	27.8153	27.4226	25.515	26.2334	26.1881	28.9617	28.1993	29.2067	0.002004172	0.100255953	4	8	24	0.010321235	0.503967859	24	22.52318689	0.070493662
ENSMUSG00000025235	Bbs4	Bardet-Biedl syndrome 4 (human) [Source:MGI Symbol;Acc:MGI:2143311]	21.5761	24.2901	21.6811	22.1974	23.7351	22.2433	20.4959	21.3076	23.0251	20.1176	23.1899	22.0104	23.2079	24.3374	0.890176313	0.966018836	20	20	24	0.952314843	0.988407044	24	4.30577241	0.010274953
ENSMUSG00000025236	Adpgk	ADP-dependent glucokinase [Source:MGI Symbol;Acc:MGI:1919391]	8.85175	9.7726	11.7652	10.82	10.0221	9.79032	10.0465	10.2834	9.35372	10.1724	9.55566	9.79895	10.104	8.78127	0.851656735	0.966018836	16	16	24	0.321794938	0.768040178	24	11.59230379	0.055282228
ENSMUSG00000025237	Parp6	poly (ADP-ribose) polymerase family, member 6 [Source:MGI Symbol;Acc:MGI:1914537]	41.6302	34.7167	34.7302	36.4054	37.3174	39.957	36.9549	37.8781	30.7811	39.4894	35.7047	36.2975	36.3737	36.5879	0.44861185	0.966018836	4	8	24	0.13565966	0.747903031	24	20.35651776	0.077892293
ENSMUSG00000025239	Limd1	LIM domains containing 1 [Source:MGI Symbol;Acc:MGI:1352502]	6.09667	6.32584	6.43912	6.96833	7.35401	6.39843	7.09799	6.90889	6.68781	7.38777	7.5324	6.3445	6.70898	5.96086	0.057224139	0.401309455	20	12	24	0.05058385	0.644469829	24	14.19777817	0.079298068
ENSMUSG00000025240	Sacm1l	SAC1 suppressor of actin mutations 1-like (yeast) [Source:MGI Symbol;Acc:MGI:1933169]	72.9119	69.9376	67.4758	66.9249	66.8709	65.334	67.5227	63.538	61.4741	62.0687	71.6611	64.1204	67.7715	68.0283	0.4776989	0.966018836	4	12	24	0.325521888	0.768040178	24	22.19860788	0.039870726
ENSMUSG00000025241	Fyco1	FYVE and coiled-coil domain containing 1 [Source:MGI Symbol;Acc:MGI:107277]	5.42596	7.09596	7.39523	7.85571	7.12815	6.88885	7.56668	9.08454	8.3178	8.27569	6.27586	6.99826	7.17887	6.9372	0.122696414	0.550756498	16	4	24	0.084435109	0.702565919	24	8.643388704	0.132812387
ENSMUSG00000025245	Lztfl1	leucine zipper transcription factor-like 1 [Source:MGI Symbol;Acc:MGI:1934860]	9.11205	11.1897	9.15427	10.8845	10.957	8.885	9.83547	9.83944	11.3872	8.15036	11.8107	9.8655	10.0974	10.5677	0.954744729	0.966018836	20	4	24	0.871548826	0.96354675	24	10.16262809	0.030352824
ENSMUSG00000025246	Tbl1x	transducin (beta)-like 1 X-linked [Source:MGI Symbol;Acc:MGI:1336172]	16.8792	15.7043	15.8998	15.7768	15.7153	15.7422	17.065	15.8084	14.8373	16.5137	14.5822	16.9723	16.5069	17.1034	0.208341949	0.698704663	4	16	24	0.146721069	0.755260456	24	0.18077251	0.049156329
ENSMUSG00000025255	Zfhx4	zinc finger homeodomain 4 [Source:MGI Symbol;Acc:MGI:2137668]	2.32013	2.40105	2.33806	2.16774	2.53152	2.17086	2.34947	2.16403	2.4222	2.06873	2.37782	2.50899	2.56529	2.41851	0.956178643	0.96629412	20	20	24	0.503555681	0.810250042	24	22.89189497	0.029969033
ENSMUSG00000025260	Hsd17b10	hydroxysteroid (17-beta) dehydrogenase 10 [Source:MGI Symbol;Acc:MGI:1333871]	42.3987	43.0331	41.5375	45.1747	43.447	40.5117	41.9782	47.9007	45.6603	36.5915	43.8865	41.2467	47.5711	41.5909	0.834434272	0.966018836	8	16	24	0.591226452	0.855742807	24	3.370814374	0.039750513
ENSMUSG00000025261	Huwe1	HECT, UBA and WWE domain containing 1 [Source:MGI Symbol;Acc:MGI:1926884]	48.1948	40.5235	37.8798	44.1316	42.9162	42.8857	43.4053	41.8072	39.1268	43.7539	43.7864	43.7378	47.8621	44.839	0.277087842	0.789849983	4	8	24	0.088465016	0.707747429	24	21.0289371	0.075758626
ENSMUSG00000025262	Fam120c	family with sequence similarity 120, member C [Source:MGI Symbol;Acc:MGI:2387687]	5.57269	5.43017	5.27623	5.06277	5.614	5.63046	6.00281	5.19516	5.77939	4.74587	5.53701	5.96301	5.80874	5.54004	0.00486525	0.141782417	4	12	24	0.031415344	0.602100793	24	22.38336682	0.072936234
ENSMUSG00000025264	Tsr2	TSR2 20S rRNA accumulation [Source:MGI Symbol;Acc:MGI:1916749]	24.8824	22.8127	25.0884	24.0167	23.6197	24.2149	22.4376	20.2226	20.3085	23.762	23.4886	23.1857	22.7426	24.1404	0.942877366	0.966018836	16	16	24	0.383740505	0.799546792	24	17.39734146	0.047956915
ENSMUSG00000025265	Fgd1	FYVE, RhoGEF and PH domain containing 1 [Source:MGI Symbol;Acc:MGI:104566]	1.69599	1.9051	2.01451	2.17935	1.79041	2.04812	2.34554	1.83239	1.7954	2.18978	2.00491	1.92577	1.55254	1.96238	0.702630284	0.966018836	16	12	24	0.490376425	0.805393068	24	12.93552048	0.047997372
ENSMUSG00000025266	Gnl3l	guanine nucleotide binding protein-like 3 (nucleolar)-like [Source:MGI Symbol;Acc:MGI:2448557]	53.4935	46.8459	48.0541	47.8475	45.5493	51.3102	48.9443	46.3908	42.0814	53.2934	48.9327	50.8173	49.104	53.21	0.321662653	0.83607295	24	12	24	0.411329855	0.799546792	24	21.14963471	0.051147992
ENSMUSG00000025268	Maged2	MAGE family member D2 [Source:MGI Symbol;Acc:MGI:1933391]	35.2528	40.5962	39.1122	32.1638	40.1863	35.3704	37.133	41.1898	39.3586	38.9127	36.5649	33.713	37.0902	39.1508	0.018194955	0.256345714	8	16	24	0.142555823	0.752079087	24	6.256062746	0.078758073
ENSMUSG00000025269	Apex2	apurinic/apyrimidinic endonuclease 2 [Source:MGI Symbol;Acc:MGI:1924872]	2.77269	2.26213	2.856	2.92688	2.68022	2.64974	3.00485	2.87098	2.23529	2.13183	3.17084	2.91585	2.63711	2.2467	0.645341728	0.966018836	20	16	24	0.19773083	0.768040178	24	19.20195864	0.093050192
ENSMUSG00000025270	Alas2	aminolevulinic acid synthase 2, erythroid [Source:MGI Symbol;Acc:MGI:87990]	7.36792	18.3049	20.0282	18.4024	12.5535	11.8331	14.6155	16.2899	20.766	10.5808	13.0324	17.1418	20.9408	14.0844	0.496745696	0.966018836	12	8	24	0.266478822	0.768040178	24	7.243547839	0.253070919
ENSMUSG00000025271	Pfkfb1	6-phosphofructo-2-kinase/fructose-2,6-biphosphatase 1 [Source:MGI Symbol;Acc:MGI:107816]	1.63347	3.39658	3.66486	5.2867	3.83371	3.83815	2.49262	4.40901	5.74083	4.91857	3.59168	2.92255	3.61546	3.36936	0.008244783	0.192760771	16	12	24	0.004118667	0.427431763	24	10.6730111	0.371677042
ENSMUSG00000025272	Tro	trophinin [Source:MGI Symbol;Acc:MGI:1928994]	34.128	33.2733	33.6804	33.3274	32.3745	35.222	29.1798	30.5954	31.6718	32.0338	30.4258	34.1554	33.1067	36.5762	0.907068194	0.966018836	20	24	24	0.914712822	0.976258471	24	0.892295167	0.014398158
ENSMUSG00000025277	Abhd6	abhydrolase domain containing 6 [Source:MGI Symbol;Acc:MGI:1913332]	13.699	11.9495	13.0571	12.7495	14.3758	14.3877	13.8666	13.0347	12.1061	13.1755	14.5928	12.8473	13.2104	13.1535	0.027467567	0.308438477	20	16	24	0.020196405	0.565108098	24	18.54102445	0.081124443
ENSMUSG00000025278	Flnb	filamin, beta [Source:MGI Symbol;Acc:MGI:2446089]	12.6332	13.5668	12.6657	11.8696	15.1002	12.7379	14.4975	54.1797	14.2598	15.7516	12.2607	49.6145	13.3824	13.2014	0.81540067	0.966018836	8	8	24	0.803983773	0.93736298	24	0.169990682	0.176425301
ENSMUSG00000025279	Dnase1l3	deoxyribonuclease 1-like 3 [Source:MGI Symbol;Acc:MGI:1314633]	2.18292	1.50533	1.71525	1.63471	2.05619	2.5073	1.77289	1.66127	1.21769	1.82889	1.87941	1.9857	1.78563	1.75185	7.90E-04	0.071344007	24	12	24	0.003177707	0.414576087	24	19.50385149	0.178046749
ENSMUSG00000025280	Polr3a	polymerase (RNA) III (DNA directed) polypeptide A [Source:MGI Symbol;Acc:MGI:2681836]	6.66522	6.46564	6.70461	6.2286	6.15113	6.50574	6.32263	6.6417	6.106	6.11598	6.8239	6.91644	6.09864	6.18545	0.92655264	0.966018836	24	16	24	0.492658357	0.805558817	24	20.75479301	0.026857765
ENSMUSG00000025283	Sat1	spermidine/spermine N1-acetyl transferase 1 [Source:MGI Symbol;Acc:MGI:98233]	110.335	112.24	84.816	69.3259	96.2806	92.1979	121.615	101.251	92.193	60.7625	99.0325	97.9841	116.53	96.8307	1.11E-04	0.029372629	4	12	24	0.001345258	0.34751073	24	23.9213849	0.308621284
ENSMUSG00000025287	Acot9	acyl-CoA thioesterase 9 [Source:MGI Symbol;Acc:MGI:1928939]	18.7523	19.8857	19.6013	19.6153	19.8107	18.4184	17.2265	17.8506	20.3991	16.3977	21.5998	18.3645	19.4182	19.1312	0.522551072	0.966018836	20	4	24	0.774183483	0.927465562	24	12.25892186	0.027880903
ENSMUSG00000025289	Prdx4	peroxiredoxin 4 [Source:MGI Symbol;Acc:MGI:1859815]	33.1338	40.5031	36.6451	34.8817	36.382	30.8529	35.0906	35.3491	46.6742	28.8275	40.132	35.3896	38.0473	35.4325	0.446653834	0.966018836	12	12	24	0.504366205	0.810907526	24	6.649551999	0.078957322
ENSMUSG00000025290	Rps24	ribosomal protein S24 [Source:MGI Symbol;Acc:MGI:98147]	109.876	139.325	127.278	118.031	126.723	110.191	118.584	118.425	157.173	97.3288	136.366	125.581	132.787	127.151	0.649212555	0.966018836	12	4	24	0.59594778	0.857839743	24	6.304081051	0.078551879
ENSMUSG00000025314	Ptprj	protein tyrosine phosphatase, receptor type, J [Source:MGI Symbol;Acc:MGI:104574]	2.77746	2.72529	2.80724	2.86251	2.72147	3.02371	3.22396	2.51005	2.41313	2.82778	2.32219	2.6564	2.50465	2.68998	0.860560779	0.966018836	16	4	24	0.61385364	0.866162311	24	21.35209463	0.034057689
ENSMUSG00000025316	Banp	BTG3 associated nuclear protein [Source:MGI Symbol;Acc:MGI:1889023]	15.7561	8.0442	7.07005	8.74665	11.934	9.10108	14.9	6.50497	7.54031	9.33926	10.6447	7.52973	11.3855	5.73135	0.012922042	0.227495952	4	4	24	0.0543957	0.655234426	24	19.90212302	0.33789101
ENSMUSG00000025318	Jph3	junctophilin 3 [Source:MGI Symbol;Acc:MGI:1891497]	19.4044	13.9214	14.3989	14.8098	12.7618	15.0061	16.5631	17.3208	13.9117	24.1653	15.2873	21.2283	18.3448	19.7583	0.658942124	0.966018836	8	24	24	0.75210654	0.91939047	24	0.211774336	0.066911556
ENSMUSG00000025321	Itgb8	integrin beta 8 [Source:MGI Symbol;Acc:MGI:1338035]	22.2407	24.5674	25.7657	24.8425	30.9683	22.402	26.0831	22.1164	25.7534	19.941	31.7124	20.5543	19.8313	20.1345	0.496745696	0.966018836	20	12	24	0.216547429	0.768040178	24	14.46708721	0.130392365
ENSMUSG00000025323	Sp4	trans-acting transcription factor 4 [Source:MGI Symbol;Acc:MGI:107595]	3.6332	4.5644	4.02375	4.32499	4.01466	4.09116	4.36385	3.91119	4.7087	3.41233	4.62531	3.8847	3.5567	4.20593	0.939921059	0.966018836	12	16	24	0.754203659	0.920420346	24	9.112486501	0.035664635
ENSMUSG00000025324	Atp10a	ATPase, class V, type 10A [Source:MGI Symbol;Acc:MGI:1330809]	1.55747	1.33739	1.58085	1.93874	1.39616	1.567	1.30684	1.48272	1.40735	1.35138	1.38418	1.51192	1.17769	1.06626	0.593560247	0.966018836	12	20	24	0.137972897	0.747972781	24	14.51104876	0.071001548
ENSMUSG00000025326	Ube3a	ubiquitin protein ligase E3A [Source:MGI Symbol;Acc:MGI:105098]	21.8248	24.1618	22.4397	23.1026	24.4301	20.2042	22.326	21.2692	24.5215	18.3277	25.2568	21.1789	21.8198	23.4247	0.265774856	0.773510818	20	4	24	0.888323626	0.968626646	24	8.59299818	0.024400298
ENSMUSG00000025330	Padi4	peptidyl arginine deiminase, type IV [Source:MGI Symbol;Acc:MGI:1338898]	0.725386	1.30087	1.04533	0.966226	1.01573	1.11894	1.23014	0.875894	0.970498	0.601402	1.08905	1.13358	0.973073	0.55501	0.657586167	0.966018836	24	16	24	0.463563863	0.805393068	24	20.00656045	0.081574437
ENSMUSG00000025332	Kdm5c	lysine (K)-specific demethylase 5C [Source:MGI Symbol;Acc:MGI:99781]	9.78053	7.96715	7.01051	7.57882	8.50637	9.40204	8.31372	9.58536	7.64898	7.962	8.12294	8.20607	8.18618	9.45274	0.020551444	0.261884115	4	8	24	0.074749447	0.683760931	24	23.0392529	0.107021198
ENSMUSG00000025337	Sbds	SBDS ribosome maturation factor [Source:MGI Symbol;Acc:MGI:1913961]	60.5643	61.6696	61.8902	56.3533	63.6377	59.7717	61.3106	57.0195	62.7561	57.5697	60.9969	61.6211	60.8313	59.6714	0.933543173	0.966018836	12	4	24	0.70873695	0.903848183	24	21.1978197	0.017566075
ENSMUSG00000025340	Rabgef1	RAB guanine nucleotide exchange factor (GEF) 1 [Source:MGI Symbol;Acc:MGI:1929459]	18.8905	18.4017	18.849	19.9194	17.8112	20.0985	20.3349	20.7237	17.1395	20.7558	17.5872	20	19.8475	18.098	0.891105762	0.966018836	24	12	24	0.684031601	0.895068737	24	22.1519892	0.031522346
ENSMUSG00000025348	Itga7	integrin alpha 7 [Source:MGI Symbol;Acc:MGI:102700]	43.2318	38.4774	38.3056	34.6109	44.8157	41.7146	38.1206	40.4339	31.3632	39.7615	43.2958	36.5927	40.4508	36.0784	0.064422003	0.414867207	20	16	24	0.082462498	0.699403344	24	19.55736031	0.113612904
ENSMUSG00000025350	Rdh5	retinol dehydrogenase 5 [Source:MGI Symbol;Acc:MGI:1201412]	21.1293	17.7428	15.5233	15.9537	21.4097	15.7955	20.1158	18.4398	17.0238	15.791	19.3452	17.3742	19.8839	16.9457	0.026841899	0.305155548	4	12	24	0.126973041	0.739868076	24	21.80024781	0.119264881
ENSMUSG00000025351	Cd63	CD63 antigen [Source:MGI Symbol;Acc:MGI:99529]	16.0438	18.7143	16.5788	16.5527	21.3122	17.2619	18.4101	18.2205	18.0657	13.566	19.5183	15.444	17.4313	14.4873	0.91241026	0.966018836	20	20	24	0.694924256	0.898633591	24	19.10691901	0.055441304
ENSMUSG00000025352	Gdf11	growth differentiation factor 11 [Source:MGI Symbol;Acc:MGI:1338027]	5.97589	4.95999	5.27921	4.83949	5.6605	5.71508	5.76972	5.58558	4.26733	5.98291	5.00826	5.46231	4.65781	5.41824	0.764889554	0.966018836	24	12	24	0.352574291	0.780629136	24	20.34148771	0.069609777
ENSMUSG00000025353	Ormdl2	ORM1-like 2 (S. cerevisiae) [Source:MGI Symbol;Acc:MGI:1914094]	13.9212	10.8489	10.3425	7.65965	11.6864	10.0452	11.6337	9.96118	12.4924	7.27015	13.8378	11.3296	11.5612	9.75327	0.184631358	0.662239322	4	12	24	0.197917659	0.768040178	24	22.3303131	0.173281266
ENSMUSG00000025354	Dnajc14	DnaJ heat shock protein family (Hsp40) member C14 [Source:MGI Symbol;Acc:MGI:1921580]	27.0751	23.0339	23.6255	25.0916	25.4675	26.677	26.2645	24.0979	21.391	26.2505	23.1339	24.8341	25.1641	24.2332	0.0563097	0.401074772	4	8	24	0.0687625	0.673096105	24	20.16435977	0.078856253
ENSMUSG00000025355	Mmp19	matrix metallopeptidase 19 [Source:MGI Symbol;Acc:MGI:1927899]	1.72093	1.83717	1.50219	2.32746	2.47221	1.72527	1.73682	2.01952	2.1268	1.57285	2.19974	1.69803	1.99849	1.61195	0.941885246	0.966018836	20	4	24	0.360294635	0.78582634	24	14.77932533	0.080296324
ENSMUSG00000025357	Dgka	diacylglycerol kinase, alpha [Source:MGI Symbol;Acc:MGI:102952]	9.4586	10.025	9.42338	10.0477	11.2728	9.73725	10.0041	9.16874	10.0419	10.0913	9.84451	9.23987	10.2334	8.47328	0.510431574	0.966018836	16	16	24	0.190675849	0.768040178	24	15.17008448	0.058621865
ENSMUSG00000025358	Cdk2	cyclin-dependent kinase 2 [Source:MGI Symbol;Acc:MGI:104772]	2.83981	4.77001	5.3458	4.75459	4.37853	3.81382	4.71732	4.72878	5.09698	4.56437	4.17766	4.50527	4.64898	4.63353	0.001971398	0.100255953	12	12	24	0.043704894	0.626957533	24	8.598018976	0.156194293
ENSMUSG00000025362	Rps26	ribosomal protein S26 [Source:MGI Symbol;Acc:MGI:1351628]	42.9449	46.3755	45.4743	36.2493	39.9073	39.2643	41.0016	40.6615	50.2479	33.8596	47.7946	38.7437	45.1839	40.3021	0.187699199	0.662239322	12	4	24	0.477351978	0.805393068	24	3.816264113	0.073699286
ENSMUSG00000025364	Pa2g4	proliferation-associated 2G4 [Source:MGI Symbol;Acc:MGI:894684]	21.0464	20.4279	22.0871	21.3499	22.2119	20.9908	21.4025	20.92	20.9201	22.4185	20.6126	22.4661	21.4315	20.4171	0.108029159	0.524035616	24	8	24	0.237393548	0.768040178	24	15.35348339	0.029379613
ENSMUSG00000025366	Esyt1	extended synaptotagmin-like protein 1 [Source:MGI Symbol;Acc:MGI:1344426]	48.9051	36.4197	38.2507	41.1296	42.4379	47.9714	41.3066	45.2704	31.6576	54.9498	38.0063	42.8149	40.1648	40.0404	0.148073804	0.590732968	24	12	24	0.396714005	0.799546792	24	18.96168776	0.106380855
ENSMUSG00000025369	Smarcc2	SWI/SNF related, matrix associated, actin dependent regulator of chromatin, subfamily c, member 2 [Source:MGI Symbol;Acc:MGI:1915344]	27.8842	28.6836	30.9519	29.3079	29.8197	28.3404	33.2832	29.8657	31.4483	34.3974	27.5731	30.9172	31.1196	31.1859	0.602156008	0.966018836	16	4	24	0.433048585	0.805393068	24	7.785356785	0.043527188
ENSMUSG00000025370	Cdh9	cadherin 9 [Source:MGI Symbol;Acc:MGI:107433]	4.06162	4.04001	3.42734	2.76185	4.02197	3.96221	3.21119	3.73002	2.69328	2.95916	3.96007	3.73099	3.92017	3.17915	0.101491041	0.511659635	20	20	24	0.024590491	0.586541342	24	21.35181414	0.158735741
ENSMUSG00000025371	Chmp6	charged multivesicular body protein 6 [Source:MGI Symbol;Acc:MGI:1913682]	19.7188	15.7914	15.594	15.3229	15.5191	17.5572	15.9097	17.6467	15.6438	19.9492	15.2746	19.0485	17.6043	16.7009	0.093489599	0.490854203	4	16	24	0.472202777	0.805393068	24	22.25494928	0.063568969
ENSMUSG00000025372	Baiap2	brain-specific angiogenesis inhibitor 1-associated protein 2 [Source:MGI Symbol;Acc:MGI:2137336]	8.29699	7.294	7.54956	8.81178	8.19234	7.49661	6.49745	6.60683	7.97195	8.49602	8.17755	7.52332	8.15349	5.96645	0.030347509	0.324150946	16	16	24	0.199866317	0.768040178	24	16.66259875	0.098816378
ENSMUSG00000025373	Rnf41	ring finger protein 41 [Source:MGI Symbol;Acc:MGI:1914838]	39.1431	32.5721	35.1973	33.6722	31.2174	36.7631	36.2885	36.8602	31.3822	41.0037	35.7228	38.5441	38.06	37.8255	0.280850951	0.792139038	4	8	24	0.459804103	0.805393068	24	22.73832981	0.053888641
ENSMUSG00000025374	Nabp2	nucleic acid binding protein 2 [Source:MGI Symbol;Acc:MGI:1917167]	25.8218	23.2282	25.3579	25.694	26.2111	27.9833	27.3657	24.7933	23.1752	30.6748	24.5107	27.6482	26.313	28.6027	0.245061943	0.741580974	24	12	24	0.537309165	0.827923483	24	18.24029976	0.046114736
ENSMUSG00000025375	Aatk	apoptosis-associated tyrosine kinase [Source:MGI Symbol;Acc:MGI:1197518]	140.156	127.416	130.064	166.622	142.946	116.272	159.628	128.584	162.065	165.456	142.086	128.715	134.064	119.019	0.01579111	0.253395174	16	16	24	0.178379825	0.768040178	24	12.06877674	0.120477531
ENSMUSG00000025377	Tepsin	TEPSIN, adaptor related protein complex 4 accessory protein [Source:MGI Symbol;Acc:MGI:1926027]	6.71932	5.28581	5.07326	5.933	6.36325	5.29764	6.58423	6.56763	5.66143	6.41544	6.98158	6.76118	6.95446	5.52015	0.114010995	0.532950079	4	8	24	0.191831176	0.768040178	24	19.57959726	0.092311631
ENSMUSG00000025381	Cnpy2	canopy FGF signaling regulator 2 [Source:MGI Symbol;Acc:MGI:1928477]	34.0839	39.5344	38.2859	33.917	38.7138	31.6231	32.5363	37.5537	40.961	28.3447	37.2111	34.2707	34.6038	32.56	0.390686537	0.915479136	12	12	24	0.627800969	0.872795898	24	7.050630252	0.05489375
ENSMUSG00000025384	Faap100	Fanconi anemia core complex associated protein 100 [Source:MGI Symbol;Acc:MGI:1919135]	5.58991	3.8454	4.87207	4.42804	5.01331	4.13394	5.18073	5.36499	4.3223	6.0409	4.48854	4.13288	4.39231	4.30432	0.945728192	0.966018836	16	8	24	0.910035212	0.97464686	24	11.5433462	0.027473165
ENSMUSG00000025393	Atp5b	ATP synthase, H+ transporting mitochondrial F1 complex, beta subunit [Source:MGI Symbol;Acc:MGI:107801]	639.596	600.935	662.78	706.973	659.665	693.289	647.564	701.27	603.958	708.035	651.24	629.871	689.55	673.315	0.849179819	0.966018836	16	12	24	0.706421657	0.902918222	24	13.99015818	0.025379906
ENSMUSG00000025395	Prim1	DNA primase, p49 subunit [Source:MGI Symbol;Acc:MGI:97757]	6.09821	6.48602	6.81498	5.85885	6.47872	5.93059	5.37675	5.32366	6.7459	4.26881	6.20178	7.14527	5.98063	5.76798	0.835401638	0.966018836	12	20	24	0.871288389	0.963486859	24	21.17040367	0.034485538
ENSMUSG00000025400	Tac2	tachykinin 2 [Source:MGI Symbol;Acc:MGI:98476]	0.929116	3.6518	3.92192	1.81483	2.94004	2.12199	4.90367	1.79639	5.85655	3.26827	1.32571	2.75078	1.7587	2.7982	0.280810068	0.792139038	12	16	24	0.310394186	0.768040178	24	7.854701868	0.318928195
ENSMUSG00000025401	Myo1a	myosin IA [Source:MGI Symbol;Acc:MGI:107732]	8.83627	6.80732	6.2317	7.17381	7.06377	8.24143	6.45417	7.07142	6.30104	8.60602	6.61157	8.27087	7.94882	8.23592	0.148073804	0.590732968	24	12	24	0.348922289	0.779170188	24	20.91204476	0.088781129
ENSMUSG00000025402	Nab2	Ngfi-A binding protein 2 [Source:MGI Symbol;Acc:MGI:107563]	5.40042	7.05215	7.70325	8.61807	7.12582	6.39777	7.76797	6.74726	8.50614	7.75765	7.02248	6.38896	6.27333	7.29529	0.009067305	0.192760771	16	12	24	0.005122865	0.44237001	24	10.11568696	0.1741824
ENSMUSG00000025403	Shmt2	serine hydroxymethyltransferase 2 (mitochondrial) [Source:MGI Symbol;Acc:MGI:1277989]	9.52615	8.49003	7.63983	8.35111	9.07339	9.51952	9.94841	9.49919	9.88065	10.3154	9.68609	9.7051	8.6221	9.1562	0.833406028	0.966018836	24	12	24	0.543823295	0.83189324	24	19.1411688	0.044706187
ENSMUSG00000025404	R3hdm2	R3H domain containing 2 [Source:MGI Symbol;Acc:MGI:1919000]	15.9754	18.809	18.3201	17.9472	17.0324	17.9212	20.5929	18.2187	19.5834	24.0565	17.9036	21.0244	21.4585	19.5513	0.858932988	0.966018836	4	16	24	0.711680816	0.904745692	24	8.211165297	0.039061105
ENSMUSG00000025407	Gli1	GLI-Kruppel family member GLI1 [Source:MGI Symbol;Acc:MGI:95727]	4.7761	4.36562	4.37731	4.21853	4.94783	4.57509	4.7645	5.09139	5.68716	3.40531	5.26871	4.56338	4.17793	4.1638	0.874054778	0.966018836	20	20	24	0.883291994	0.966818623	24	22.01156998	0.030443708
ENSMUSG00000025408	Ddit3	DNA-damage inducible transcript 3 [Source:MGI Symbol;Acc:MGI:109247]	36.4314	31.5725	31.1237	29.0491	35.8629	35.4127	34.4692	29.2775	31.274	27.9571	37.0996	30.6772	31.537	30.1442	0.020428812	0.261278501	20	20	24	0.026835424	0.586541342	24	19.9334419	0.121631271
ENSMUSG00000025409	Mbd6	methyl-CpG binding domain protein 6 [Source:MGI Symbol;Acc:MGI:106378]	13.5245	15.5218	17.1759	16.4074	16.7085	14.1245	15.4171	17.2279	20.2193	19.7576	17.3401	16.4566	18.1832	17.5954	0.300218476	0.809362476	12	12	24	3.53E-04	0.227979988	24	9.64549042	0.150309764
ENSMUSG00000025410	Dctn2	dynactin 2 [Source:MGI Symbol;Acc:MGI:107733]	156.961	146.212	136.571	151.452	155.218	155.89	143.566	153.03	136.957	162.749	149.65	158.243	155.779	158.46	0.245061943	0.741580974	24	12	24	0.243162683	0.768040178	24	19.31155284	0.04889986
ENSMUSG00000025413	Ttc4	tetratricopeptide repeat domain 4 [Source:MGI Symbol;Acc:MGI:1919604]	27.3078	29.7572	28.122	30.1864	32.5416	28.7913	29.8496	28.5261	29.5799	24.756	30.7479	28.8361	28.0794	28.5236	0.542718076	0.966018836	20	8	24	0.628031223	0.872795898	24	17.58853382	0.034143948
ENSMUSG00000025417	Pip4k2c	phosphatidylinositol-5-phosphate 4-kinase, type II, gamma [Source:MGI Symbol;Acc:MGI:2152214]	21.1096	18.7758	17.1527	19.3022	19.7845	21.1715	19.9248	20.9471	17.8855	24.248	18.464	22.7231	21.6006	19.8508	0.004712708	0.140598674	24	12	24	0.302573297	0.768040178	24	20.1875927	0.075844808
ENSMUSG00000025420	Katnal2	katanin p60 subunit A-like 2 [Source:MGI Symbol;Acc:MGI:1924234]	0.900491	1.07809	1.18277	1.21378	1.06432	1.16608	1.21639	1.00686	1.01923	0.801922	0.934697	1.30996	1.02407	1.18005	0.833301348	0.966018836	24	20	24	0.765486891	0.924119887	24	23.01612415	0.029770704
ENSMUSG00000025421	Hdhd2	haloacid dehalogenase-like hydrolase domain containing 2 [Source:MGI Symbol;Acc:MGI:1924237]	41.9653	38.6711	40.3512	39.2925	37.7696	39.7601	40.2165	40.5098	37.0045	38.0338	38.0152	39.4274	38.5807	39.0683	0.31548973	0.827946344	4	16	24	0.060067087	0.658304792	24	0.13122272	0.036185871
ENSMUSG00000025422	Agap2	ArfGAP with GTPase domain, ankyrin repeat and PH domain 2 [Source:MGI Symbol;Acc:MGI:3580016]	3.16103	3.83557	4.87943	3.63719	3.09735	3.63592	3.9176	3.5312	4.35302	4.71543	2.98465	3.79485	2.85472	3.99089	0.035023815	0.328311403	12	8	24	0.059084449	0.658304792	24	8.417120463	0.165476288
ENSMUSG00000025423	Pias2	protein inhibitor of activated STAT 2 [Source:MGI Symbol;Acc:MGI:1096566]	8.71913	8.93802	10.269	9.77192	9.56253	9.31857	10.2621	8.59443	10.06	9.80089	7.9233	9.40479	8.84896	9.08863	0.111340266	0.530299176	12	20	24	0.519083975	0.819022865	24	9.76676905	0.044514891
ENSMUSG00000025425	St8sia5	ST8 alpha-N-acetyl-neuraminide alpha-2,8-sialyltransferase 5 [Source:MGI Symbol;Acc:MGI:109243]	5.17165	5.37794	5.48451	4.80581	5.60608	4.94763	6.64607	5.95084	6.40881	6.17982	4.61767	4.73483	5.22163	4.78849	0.435739761	0.966018836	12	12	24	0.424322322	0.805393068	24	6.45458822	0.076216326
ENSMUSG00000025427	Rnf165	ring finger protein 165 [Source:MGI Symbol;Acc:MGI:2444521]	0.401945	0.536746	0.967346	0.522197	0.539747	0.76936	0.858203	0.637299	0.77446	0.791706	0.61969	0.899452	0.521339	0.824797	0.911371589	0.966018836	12	16	24	0.721498053	0.90866625	24	8.793071507	0.046622698
ENSMUSG00000025428	Atp5a1	ATP synthase, H+ transporting, mitochondrial F1 complex, alpha subunit 1 [Source:MGI Symbol;Acc:MGI:88115]	309.762	295.436	317.312	350.094	289.218	324.088	305.153	356.785	297.122	359.548	321.497	318.541	346.383	340.223	0.843694678	0.966018836	16	4	24	0.69975943	0.900387881	24	8.758834884	0.033232089
ENSMUSG00000025429	Pstpip2	proline-serine-threonine phosphatase-interacting protein 2 [Source:MGI Symbol;Acc:MGI:1335088]	15.5662	11.5928	10.3289	12.4783	11.7213	13.94	11.3452	13.1637	10.3682	15.2213	10.9161	13.2597	13.6902	12.7539	0.148073804	0.590732968	24	12	24	0.390516752	0.799546792	24	21.36955731	0.096997357
ENSMUSG00000025432	Avil	advillin [Source:MGI Symbol;Acc:MGI:1333798]	211.796	141.988	146.722	160.946	152.15	192.823	164.962	179.906	113.287	211.831	150.156	168.745	161.739	160.768	0.148073804	0.590732968	24	12	24	0.319843828	0.768040178	24	20.49112803	0.143606393
ENSMUSG00000025436	Atp23	ATP23 metallopeptidase and ATP synthase assembly factor homolog [Source:MGI Symbol;Acc:MGI:1916984]	3.44392	2.8251	3.64661	2.72777	3.13206	3.32452	3.31663	2.92879	3.54146	3.40311	3.84289	3.15813	2.93608	2.63152	0.235141005	0.73629392	12	20	24	0.42774388	0.805393068	24	15.59025389	0.062986383
ENSMUSG00000025437	Usp33	ubiquitin specific peptidase 33 [Source:MGI Symbol;Acc:MGI:2159711]	49.6377	46.778	46.3731	45.0351	43.6132	46.2077	46.1354	44.9111	40.4029	43.1174	45.8521	46.3363	46.2572	48.604	0.096454606	0.501112642	4	12	24	0.051727837	0.648075114	24	23.47380874	0.0621025
ENSMUSG00000025439	Clns1a	chloride channel, nucleotide-sensitive, 1A [Source:MGI Symbol;Acc:MGI:109638]	29.3893	28.2934	27.9627	24.199	27.5079	29.654	31.5865	30.0966	26.7245	29.5618	27.678	29.5292	29.9489	31.4072	0.030486025	0.324150946	4	16	24	0.009563156	0.502965379	24	0.389366408	0.090922311
ENSMUSG00000025450	Gm9752	predicted gene 9752 [Source:MGI Symbol;Acc:MGI:3641896]	0.697539	1.33456	1.15519	0.918686	0.845767	0.909269	0.854826	0.945156	1.20973	0.869725	0.954435	0.940068	1.10839	0.869108	0.351840494	0.870418653	12	16	24	0.214965338	0.768040178	24	7.066350868	0.0845805
ENSMUSG00000025451	Paip1	polyadenylate binding protein-interacting protein 1 [Source:MGI Symbol;Acc:MGI:2384993]	43.6391	40.8085	39.0945	39.6747	41.6367	43.8086	42.1969	36.5336	36.3857	39.8986	38.2733	43.5028	39.0543	41.779	0.010468194	0.196621011	24	12	24	0.023055255	0.581965446	24	20.65239436	0.084147784
ENSMUSG00000025453	Nnt	nicotinamide nucleotide transhydrogenase [Source:MGI Symbol;Acc:MGI:109279]	0.954171	1.5106	1.81424	1.33136	1.83234	0.767884	1.62542	2.28277	1.9685	0.794274	1.78519	1.70823	1.77878	2.16261	0.279136262	0.790764844	8	16	24	0.274052085	0.768040178	24	5.749252991	0.169558012
ENSMUSG00000025464	Paox	polyamine oxidase (exo-N4-amino) [Source:MGI Symbol;Acc:MGI:1916983]	1.24151	1.3408	1.61991	1.78214	1.18858	1.45559	1.48949	2.4069	1.6642	2.23497	1.77002	1.76071	1.6059	1.4436	0.305564675	0.811201458	16	12	24	0.262949076	0.768040178	24	9.765577878	0.108280634
ENSMUSG00000025465	Echs1	enoyl Coenzyme A hydratase, short chain, 1, mitochondrial [Source:MGI Symbol;Acc:MGI:2136460]	40.9756	44.6395	48.0588	61.8267	46.5742	44.7859	47.2122	50.2323	51.5367	50.6526	53.8891	48.9794	60.4285	53.5502	0.619261897	0.966018836	16	8	24	0.213625367	0.768040178	24	10.53908954	0.090919558
ENSMUSG00000025466	Fuom	fucose mutarotase [Source:MGI Symbol;Acc:MGI:1916314]	14.3927	20.5782	18.9009	21.3842	18.2484	17.4622	16.3187	18.8501	20.5974	18.5895	21.7461	19.1363	21.044	21.8317	0.652290823	0.966018836	8	20	24	0.084238933	0.702277994	24	9.731541832	0.117603672
ENSMUSG00000025468	Caly	calcyon neuron-specific vesicular protein [Source:MGI Symbol;Acc:MGI:1915816]	6.98692	9.62953	13.1311	7.53272	6.21575	8.80002	9.87674	6.43655	9.68341	9.25139	6.81168	8.65224	5.75407	11.5	0.185796491	0.662239322	12	8	24	0.23565264	0.768040178	24	7.095823325	0.212044389
ENSMUSG00000025470	Zfp511	zinc finger protein 511 [Source:MGI Symbol;Acc:MGI:1917002]	10.3102	10.1293	9.98194	10.4308	11.329	9.51228	11.5286	9.30114	10.3907	9.10269	11.3144	9.49623	10.257	10.4777	0.873567285	0.966018836	20	4	24	0.802785006	0.93676746	24	18.69876185	0.026806813
ENSMUSG00000025473	Adam8	a disintegrin and metallopeptidase domain 8 [Source:MGI Symbol;Acc:MGI:107825]	4.33994	3.09707	4.33943	4.71924	5.25774	4.53308	4.05264	3.49808	3.13136	2.9409	4.67957	3.54191	4.75757	3.65597	0.137763286	0.580158868	20	12	24	0.111309522	0.723334499	24	18.38036605	0.167859275
ENSMUSG00000025474	Tubgcp2	tubulin, gamma complex associated protein 2 [Source:MGI Symbol;Acc:MGI:1921487]	10.9346	10.206	10.7648	10.681	9.55392	11.1422	9.90816	11.3121	9.75629	12.5301	9.8369	10.9542	9.84258	10.9618	0.941885246	0.966018836	8	12	24	0.840983956	0.951840198	24	9.045253881	0.024471261

ENSMUSG00000025475	Adgra1	adhesion G protein-coupled receptor A1 [Source:MGI Symbol;Acc:MGI:1277167]	1.208	1.26248	2.23062	2.45009	1.27297	1.39799	1.21068	0.944812	1.34134	1.32433	0.720204	1.18998	0.863582	1.57675	0.137604104	0.580158868	12	16	24	0.159313458	0.765350752	24	9.82597678	0.182579297
ENSMUSG00000025477	Inpp5a	inositol polyphosphate-5-phosphatase A [Source:MGI Symbol;Acc:MGI:2686961]	13.1048	11.8353	11.5302	12.1784	13.4014	12.2872	13.8468	14.9683	12.2605	14.2159	10.727	12.0356	12.861	11.7675	0.935732159	0.966018836	4	8	24	0.787142944	0.931456435	24	1.631776339	0.033226556
ENSMUSG00000025478	Dpysl4	dihydropyrimidinase-like 4 [Source:MGI Symbol;Acc:MGI:1349764]	4.81049	6.1847	7.11294	6.3264	6.11694	6.11011	6.10711	6.49038	6.35004	7.43883	5.9255	6.0085	5.89266	6.07336	0.004122392	0.140598674	12	16	24	0.019592695	0.564709807	24	10.15862378	0.119589241
ENSMUSG00000025479	Cyp2e1	cytochrome P450, family 2, subfamily e, polypeptide 1 [Source:MGI Symbol;Acc:MGI:88607]	1.58964	3.56837	6.01561	16.2895	2.77317	5.02346	2.26137	7.09829	4.52225	10.8077	4.50953	3.60317	4.56028	5.85833	0.036134798	0.331852526	16	12	24	0.020328765	0.565108098	24	10.56176743	0.689477981
ENSMUSG00000025484	Bet1l	Bet1 golgi vesicular membrane trafficking protein like [Source:MGI Symbol;Acc:MGI:1913128]	15.0285	17.0333	15.6601	15.2573	15.714	15.2341	17.378	15.2818	16.184	14.4954	16.6082	14.9486	15.6721	14.8167	0.903465533	0.966018836	4	12	24	0.809519932	0.939184036	24	1.086524315	0.018470848
ENSMUSG00000025485	Ric8a	RIC8 guanine nucleotide exchange factor A [Source:MGI Symbol;Acc:MGI:2141866]	46.9744	43.2672	42.0204	43.3664	45.9174	50.7243	43.6565	47.5548	43.1045	44.5186	40.5751	44.0131	42.2219	43.0384	0.601647512	0.966018836	24	12	24	0.331614343	0.768450584	24	21.16556703	0.04656279
ENSMUSG00000025486	Sirt3	sirtuin 3 [Source:MGI Symbol;Acc:MGI:1927665]	23.1914	21.8064	25.1179	24.0209	24.1226	24.8183	23.2149	22.8421	24.4651	22.5821	20.8175	22.3782	24.1816	23.0738	0.891742979	0.966018836	12	20	24	0.908201411	0.974606327	24	7.455748896	0.01309909
ENSMUSG00000025487	Psmd13	proteasome (prosome, macropain) 26S subunit, non-ATPase, 13 [Source:MGI Symbol;Acc:MGI:1345192]	72.9238	76.4709	70.7383	74.2116	74.9738	70.4569	65.9848	73.1975	73.1193	66.9104	77.5114	71.6858	74.9093	67.9279	0.847312766	0.966018836	20	4	24	0.809473293	0.939184036	24	16.018898	0.017888565
ENSMUSG00000025488	Cox8b	cytochrome c oxidase subunit 8B [Source:MGI Symbol;Acc:MGI:105958]	41.111	64.7512	94.8409	183.246	83.6076	74.6911	83.1562	160.091	127.018	127.521	149.583	103.302	176.665	120.944	0.602156008	0.966018836	16	12	24	0.067079612	0.669636017	24	10.48840077	0.405736609
ENSMUSG00000025491	Ifitm1	interferon induced transmembrane protein 1 [Source:MGI Symbol;Acc:MGI:1915963]	11.3614	13.6619	10.3828	11.0824	13.5581	10.6873	10.0353	12.6202	16.8372	6.91965	13.2729	10.4201	8.1571	9.47786	0.940053021	0.966018836	20	8	24	0.923606939	0.979525912	24	9.485852326	0.047183733
ENSMUSG00000025492	Ifitm3	interferon induced transmembrane protein 3 [Source:MGI Symbol;Acc:MGI:1913391]	61.0894	68.127	66.0463	58.1417	72.694	58.4028	65.9167	75.3101	72.9167	47.4373	68.1946	59.7312	56.9509	60.9773	0.560714538	0.966018836	12	4	24	0.72002871	0.908238012	24	4.3507079	0.055784229
ENSMUSG00000025494	Sigirr	single immunoglobulin and toll-interleukin 1 receptor (TIR) domain [Source:MGI Symbol;Acc:MGI:1344402]	1.03805	1.22402	1.33792	1.45297	1.46112	1.37902	1.48268	1.15017	1.39454	1.01042	1.05876	0.902291	1.11991	1.25881	0.912686312	0.966018836	12	12	24	0.739337772	0.914957371	24	9.174791732	0.037602369
ENSMUSG00000025495	Ptdss2	phosphatidylserine synthase 2 [Source:MGI Symbol;Acc:MGI:1351664]	22.642	18.4798	16.8808	17.1516	20.4349	17.3437	22.0808	18.2153	16.8708	20.5704	15.8749	15.597	19.7491	16.0462	0.337748746	0.85758369	4	8	24	0.443682124	0.805393068	24	22.87861822	0.078777729
ENSMUSG00000025498	Irf7	interferon regulatory factor 7 [Source:MGI Symbol;Acc:MGI:1859212]	3.23095	3.21172	3.40532	3.53544	3.91254	3.7939	3.36668	5.34738	4.0874	2.92629	4.15858	2.97367	2.61839	3.82095	0.593560247	0.966018836	20	8	24	0.714267378	0.905831242	24	8.790810618	0.069733309
ENSMUSG00000025499	Hras	Harvey rat sarcoma virus oncogene [Source:MGI Symbol;Acc:MGI:96224]	53.5468	43.9351	43.3544	47.4905	48.88	49.5766	49.1599	49.4309	41.4561	46.0603	46.5171	48.337	45.7406	46.5321	0.020119233	0.259417302	24	12	24	0.019535955	0.564709807	24	20.7098935	0.090922302
ENSMUSG00000025503	Taldo1	transaldolase 1 [Source:MGI Symbol;Acc:MGI:1274789]	100.309	99.2085	115.81	115.496	103.447	107.626	111.407	110.439	100.765	114.421	125.505	106.321	122.519	104.495	0.85447369	0.966018836	16	16	24	0.471477492	0.805393068	24	14.45353258	0.046719959
ENSMUSG00000025504	Eps8l2	EPS8-like 2 [Source:MGI Symbol;Acc:MGI:2138828]	10.5095	9.30309	8.15291	9.3902	11.7366	7.78704	13.1824	8.83779	11.9403	8.8488	11.858	7.69154	9.81861	8.33335	0.909604019	0.966018836	4	20	24	0.926846169	0.980775198	24	17.38181121	0.036806451
ENSMUSG00000025505	Tmem80	transmembrane protein 80 [Source:MGI Symbol;Acc:MGI:1918698]	23.6174	24.0688	23.6565	23.6178	26.5091	22.7813	23.9588	25.6947	24.2945	22.1882	25.9329	24.7495	25.603	26.5716	0.839634479	0.966018836	8	8	24	0.835350751	0.949499418	24	0.933208714	0.016685615
ENSMUSG00000025507	Pidd1	p53 induced death domain protein 1 [Source:MGI Symbol;Acc:MGI:1889507]	1.38378	1.19435	1.21109	1.21215	1.41809	1.15932	1.25138	1.51036	0.799322	1.65339	1.18264	1.25235	1.34655	1.29753	0.914502381	0.966018836	4	8	24	0.84686322	0.953747296	24	19.99626132	0.031279157
ENSMUSG00000025508	Rplp2	ribosomal protein, large P2 [Source:MGI Symbol;Acc:MGI:1914436]	14.2152	11.7032	11.8086	12.0438	10.8228	11.3728	12.0321	15.6987	13.0518	11.7408	14.6523	11.4401	13.4331	11.6824	0.834424969	0.966018836	4	20	24	0.607186245	0.863796174	24	3.257827576	0.058001184
ENSMUSG00000025509	Pnpla2	patatin-like phospholipase domain containing 2 [Source:MGI Symbol;Acc:MGI:1914103]	16.0135	14.7691	23.9533	45.6185	21.034	19.2429	22.7938	31.1942	22.935	48.5225	34.341	24.9501	36.4888	30.8783	0.478724519	0.966018836	16	12	24	0.056282182	0.65534544	24	11.6031393	0.361870095
ENSMUSG00000025510	Cd151	CD151 antigen [Source:MGI Symbol;Acc:MGI:1096360]	188.745	170.433	156.51	178.724	188.958	177.374	171.23	174.663	166.436	158.534	174.237	176.64	179.702	165.556	0.056761625	0.401309455	20	16	24	0.020306867	0.565108098	24	20.18989561	0.078881981
ENSMUSG00000025511	Tspan4	tetraspanin 4 [Source:MGI Symbol;Acc:MGI:1928097]	19.5274	21.0295	23.1787	27.5977	22.0843	20.0536	17.6625	20.115	29.152	26.2203	23.2324	21.9238	20.7762	21.1607	1.16E-04	0.029372629	16	12	24	2.47E-04	0.18167046	24	11.19876269	0.231662612
ENSMUSG00000025512	Chid1	chitinase domain containing 1 [Source:MGI Symbol;Acc:MGI:1915288]	23.7969	21.2199	23.0923	21.972	24.3613	23.4886	21.4978	23.466	21.6036	19.358	22.9658	20.1453	23.448	21.2838	0.879831094	0.966018836	20	20	24	0.541057544	0.830298514	24	21.25931285	0.039837599
ENSMUSG00000025521	Tmem192	transmembrane protein 192 [Source:MGI Symbol;Acc:MGI:1920317]	16.4026	14.7661	16.0767	15.126	16.1648	16.3751	15.7505	17.1414	14.7381	17.7251	16.0917	16.4293	16.0273	16.0859	0.759899911	0.966018836	24	12	24	0.695480025	0.898919068	24	18.29023463	0.023542668
ENSMUSG00000025525	Apool	apolipoprotein O-like [Source:MGI Symbol;Acc:MGI:1915367]	8.07445	8.87264	8.51491	9.23227	9.8607	8.69881	8.60573	8.91022	11.0878	7.13039	9.74774	8.94156	10.3658	9.82483	0.858514388	0.966018836	20	8	24	0.927167314	0.980909301	24	6.123084318	0.021798947
ENSMUSG00000025529	Zfp711	zinc finger protein 711 [Source:MGI Symbol;Acc:MGI:3045342]	1.38119	1.91938	2.03798	1.31364	1.53451	1.58788	1.90851	1.2573	1.61429	1.40615	1.6893	1.4847	1.2557	1.62179	0.93717134	0.966018836	12	4	24	0.853228139	0.956117714	24	6.24300538	0.031255133
ENSMUSG00000025531	Chm	choroidermia (RAB escort protein 1) [Source:MGI Symbol;Acc:MGI:892979]	15.1613	17.2457	14.6031	14.9953	15.0141	13.4197	14.1018	15.0893	15.5564	11.9288	17.3233	13.9263	14.7806	15.03	0.580049522	0.966018836	8	16	24	0.774277672	0.927520217	24	4.15159065	0.036028221
ENSMUSG00000025532	Crcp	calcitonin gene-related peptide-receptor component protein [Source:MGI Symbol;Acc:MGI:1100818]	31.0765	32.9375	33.2327	30.9771	30.0821	30.9233	28.8057	31.9025	30.2032	28.9553	33.9596	31.842	35.6893	32.7472	0.953350544	0.966018836	8	8	24	0.64043011	0.878305673	24	2.304882159	0.030486961
ENSMUSG00000025533	Asl	argininosuccinate lyase [Source:MGI Symbol;Acc:MGI:88084]	16.2756	14.853	17.1026	17.3677	16.9347	19.2008	16.332	16.9236	18.0496	20.3035	16.2107	18.1375	17.5468	19.858	0.888991156	0.966018836	16	12	24	0.515839005	0.817421075	24	12.0457724	0.043778131
ENSMUSG00000025534	Gusb	glucuronidase, beta [Source:MGI Symbol;Acc:MGI:95872]	12.0858	11.2499	12.5779	12.4469	12.2463	12.3751	11.546	12.6613	11.4582	12.3242	12.5324	12.3717	12.0639	11.4744	0.4274437	0.966018836	16	16	24	0.142404854	0.751766185	24	15.59254438	0.038774631
ENSMUSG00000025537	Phkg1	phosphorylase kinase gamma 1 [Source:MGI Symbol;Acc:MGI:97579]	3.18967	7.25625	9.0514	11.4656	6.68617	9.44737	8.07676	16.6104	15.0571	14.1179	6.46236	8.57804	7.97085	8.52217	0.156791576	0.61244839	12	16	24	0.054535641	0.655263328	24	9.184902477	0.449623488
ENSMUSG00000025538	Sumf2	sulfatase modifying factor 2 [Source:MGI Symbol;Acc:MGI:1915152]	7.8291	6.52035	9.52755	7.48403	7.6602	7.59746	7.4109	8.33776	6.74718	6.64363	8.38148	6.72084	6.89909	6.93389	0.85387321	0.966018836	20	12	24	0.896556771	0.970591958	24	13.22863245	0.024258042
ENSMUSG00000025544	Tm9sf2	transmembrane 9 superfamily member 2 [Source:MGI Symbol;Acc:MGI:1915309]	81.4198	76.2282	73.2746	76.0514	75.7742	75.6179	75.4747	73.7086	69.0342	78.2361	76.6907	73.8284	76.4967	76.9617	0.791413662	0.966018836	16	20	24	0.355086831	0.7820152	24	20.61416829	0.030411311
ENSMUSG00000025545	Clybl	citrate lyase beta like [Source:MGI Symbol;Acc:MGI:1916884]	14.2689	14.3796	13.4602	18.4081	14.3552	14.0796	15.7429	16.3927	14.5293	17.6242	14.9462	17.3619	17.956	17.3689	0.879206539	0.966018836	16	20	24	0.940751458	0.985421597	24	9.407602301	0.017027817
ENSMUSG00000025551	Fgf14	fibroblast growth factor 14 [Source:MGI Symbol;Acc:MGI:109189]	0.539781	1.63967	2.16659	1.05195	0.761848	1.16341	1.32021	1.30984	1.59386	0.963752	0.973002	1.54892	0.937291	1.63401	0.084879076	0.466404237	12	8	24	0.110323645	0.721828007	24	6.549535277	0.22047369
ENSMUSG00000025555	Farp1	FERM, RhoGEF (Arhgef) and pleckstrin domain protein 1 (chondrocyte-derived) [Source:MGI Symbol;Acc:MGI:2446173]	4.54175	5.31512	6.66412	6.10934	4.47976	5.24063	5.38253	5.00866	5.54083	5.68879	5.7424	4.70638	4.19573	5.38112	0.029985708	0.324150946	16	12	24	0.021988471	0.574400012	24	10.00634198	0.148454605
ENSMUSG00000025558	Dock9	dedicator of cytokinesis 9 [Source:MGI Symbol;Acc:MGI:106321]	30.22	33.8312	34.0503	34.1377	34.027	31.5172	31.5064	32.2739	37.1792	31.2823	31.1423	31.5461	32.2574	32.1657	0.045120335	0.368634624	12	12	24	0.053936501	0.654390023	24	9.044342042	0.067387502
ENSMUSG00000025571	Tnrc6c	trinucleotide repeat containing 6C [Source:MGI Symbol;Acc:MGI:2443265]	7.39826	9.06505	8.50523	7.88882	7.65569	7.6076	7.11123	8.31177	9.17452	9.94651	7.56946	9.8039	9.09347	9.92307	0.416142594	0.963576182	8	20	24	0.122388287	0.735058232	24	7.432335058	0.100620744
ENSMUSG00000025572	Tmc6	transmembrane channel-like gene family 6 [Source:MGI Symbol;Acc:MGI:1098686]	22.6165	24.4537	22.1547	24.8235	29.9184	26.3212	23.956	26.5225	23.3664	25.8633	24.1265	24.5117	27.8952	20.6644	0.43280837	0.966018836	20	16	24	0.214368277	0.768040178	24	17.51963992	0.090718396
ENSMUSG00000025574	Tk1	thymidine kinase 1 [Source:MGI Symbol;Acc:MGI:98763]	3.29325	3.58405	4.02943	3.73179	3.43844	4.15854	3.8706	3.99143	4.23371	3.54009	3.58939	3.68471	4.04116	3.32957	0.93447422	0.966018836	12	8	24	0.854070989	0.956445086	24	6.141448276	0.021807513
ENSMUSG00000025575	Cant1	calcium activated nucleotidase 1 [Source:MGI Symbol;Acc:MGI:1923275]	15.1715	13.4019	13.4878	13.7312	14.1094	14.7063	15.1059	14.7628	12.9894	16.5446	13.0177	14.9433	14.211	14.3249	0.182849367	0.659303873	4	8	24	0.575743106	0.848172813	24	21.2534747	0.039233513
ENSMUSG00000025576	Rbfox3	RNA binding protein, fox-1 homolog (C. elegans) 3 [Source:MGI Symbol;Acc:MGI:106368]	33.6127	26.0548	26.0702	26.7428	24.4207	25.5436	26.5731	28.659	23.5572	42.4299	27.4095	36.0236	33.2769	34.1965	0.94086481	0.966018836	4	8	24	0.923024561	0.979439431	24	23.16138523	0.035385503
ENSMUSG00000025577	Cbx2	chromobox 2 [Source:MGI Symbol;Acc:MGI:88289]	2.40829	2.09991	2.09122	2.52554	2.44282	2.09653	2.51744	2.25095	2.38966	2.34155	2.72803	2.28783	2.55966	2.01647	0.17953325	0.655480352	20	12	24	0.245937973	0.768040178	24	16.71987485	0.058600939
ENSMUSG00000025578	Cbx8	chromobox 8 [Source:MGI Symbol;Acc:MGI:1353589]	3.52094	2.69065	2.0282	3.05784	1.86739	2.36728	2.9121	2.95016	3.87261	2.79315	3.36478	2.32116	2.97323	3.08641	0.832506215	0.966018836	4	20	24	0.479800432	0.805393068	24	5.143877986	0.102501144
ENSMUSG00000025579	Gaa	glucosidase, alpha, acid [Source:MGI Symbol;Acc:MGI:95609]	53.1459	41.7084	45.2434	44.4718	44.0185	52.6751	49.6585	53.4212	41.4167	63.9882	38.2488	50.6714	46.1404	47.7037	0.718086778	0.966018836	24	20	24	0.85342626	0.956231172	24	22.80225831	0.043367444
ENSMUSG00000025580	Eif4a3	eukaryotic translation initiation factor 4A3 [Source:MGI Symbol;Acc:MGI:1923731]	13.8306	14.5187	11.1559	14.9671	12.8647	15.596	13.9109	13.7043	12.6387	13.3385	13.1482	13.5889	13.2785	11.9475	0.390686537	0.915479136	24	12	24	0.191397415	0.768040178	24	19.99107953	0.080503236
ENSMUSG00000025582	Nptx1	neuronal pentraxin 1 [Source:MGI Symbol;Acc:MGI:107811]	26.9572	17.4795	20.5723	19.4206	21.5842	23.0919	20.262	21.0316	15.4135	28.3519	19.9094	20.8093	19.0007	20.1643	0.66832131	0.966018836	24	12	24	0.420307112	0.803964828	24	18.38521597	0.112953434
ENSMUSG00000025583	Rptor	regulatory associated protein of MTOR, complex 1 [Source:MGI Symbol;Acc:MGI:1921620]	7.61953	6.6185	7.35455	7.28057	7.03206	7.37183	7.48626	7.65246	6.36938	9.25775	6.29604	7.90685	7.09313	7.95355	0.965971228	0.969272194	8	12	24	0.998156994	0.999660084	24	6.478983003	0.003173396
ENSMUSG00000025584	Pde8a	phosphodiesterase 8A [Source:MGI Symbol;Acc:MGI:1277116]	3.50368	3.81762	4.5562	3.80403	3.06924	4.10049	3.84228	4.2538	3.7681	4.67725	3.44206	3.90171	3.64086	4.79374	0.388056094	0.915479136	8	12	24	0.101146872	0.713208839	24	6.71180114	0.113378698
ENSMUSG00000025586	Cpeb1	cytoplasmic polyadenylation element binding protein 1 [Source:MGI Symbol;Acc:MGI:108442]	124.581	112.563	109.268	118.394	109.556	120.793	117.33	121.174	110.707	143.518	109.883	119.436	109.805	115.624	0.969487593	0.970883022	24	12	24	0.91824169	0.977931782	24	14.51548752	0.017432836
ENSMUSG00000025588	Nat1	N-acetyl transferase 1 [Source:MGI Symbol;Acc:MGI:97279]	0.493682	0.753192	0.826276	0.261886	0.541236	0.475302	0.602803	0.442672	0.623055	0.577051	0.480052	0.338514	0.555932	0.537236	0.08302755	0.462922111	12	12	24	0.223869515	0.768040178	24	5.610114033	0.090705098
ENSMUSG00000025591	Tma16	translation machinery associated 16 [Source:MGI Symbol;Acc:MGI:1913532]	2.61301	3.13454	3.17467	2.9549	2.68844	2.78111	2.5629	3.04625	3.47684	2.7171	2.83478	3.2062	2.82779	3.25737	0.058157448	0.40386543	12	16	24	0.05537519	0.65534544	24	7.365902062	0.087037418
ENSMUSG00000025597	Klhl4	kelch-like 4 [Source:MGI Symbol;Acc:MGI:2442829]	0.857058	1.87209	2.67493	1.40366	1.5232	1.44934	1.77201	1.3441	1.88427	1.08943	1.13498	1.28504	0.658969	1.47104	0.203345222	0.689605535	12	16	24	0.205608748	0.768040178	24	8.364384747	0.187213811
ENSMUSG00000025602	Zfp202	zinc finger protein 202 [Source:MGI Symbol;Acc:MGI:1933401]	1.31215	1.24156	1.31905	0.996419	1.40104	1.26087	1.28113	1.49188	1.25037	1.21205	1.24336	1.35444	1.18101	1.19719	0.908406451	0.966018836	24	16	24	0.553310908	0.837286821	24	23.40989542	0.032169565
ENSMUSG00000025607	Copg2	coatomer protein complex, subunit gamma 2 [Source:MGI Symbol;Acc:MGI:1858683]	42.6321	39.7753	42.0478	41.6209	38.0203	39.9852	38.8967	37.7553	36.312	39.9606	38.0516	42.9323	40.9092	42.5442	0.945544484	0.966018836	24	20	24	0.687111409	0.896182667	24	23.21204132	0.026015919
ENSMUSG00000025608	Podxl	podocalyxin-like [Source:MGI Symbol;Acc:MGI:1351317]	5.17835	5.61716	5.91479	5.06361	5.75023	5.23589	5.28601	7.57963	4.98168	6.61353	4.43607	4.58504	5.35541	5.65426	0.304473123	0.811201458	8	16	24	0.215108425	0.768040178	24	6.430441168	0.11691476
ENSMUSG00000025609	Mkln1	muskelin 1, intracellular mediator containing kelch motifs [Source:MGI Symbol;Acc:MGI:1351638]	71.0758	76.0676	75.9579	78.616	80.4153	71.2455	73.9582	73.8683	69.9944	66.1044	80.7602	71.2454	80.1241	73.1748	0.865490576	0.966018836	20	16	24	0.819590915	0.942798491	24	17.67649443	0.022109447
ENSMUSG00000025610	Map3k7cl	Map3k7 C-terminal like [Source:MGI Symbol;Acc:MGI:2446584]	0.479145	0.595925	0.608248	0.643724	0.560208	0.68287	0.84556	0.860338	0.746658	0.685686	0.403939	0.558343	0.725284	0.474089	0.920620634	0.966018836	12	8	24	0.531761783	0.82547989	24	4.524952274	0.053768098
ENSMUSG00000025612	Bach1	BTB and CNC homology 1, basic leucine zipper transcription factor 1 [Source:MGI Symbol;Acc:MGI:894680]	6.30204	6.3051	6.46823	6.81768	6.66365	6.05391	6.40447	5.91427	6.43572	6.45172	6.18081	5.52351	6.25938	5.54271	0.033622862	0.324908923	16	8	24	0.106694289	0.71885328	24	12.36997394	0.048159875
ENSMUSG00000025613	Cct8	chaperonin containing Tcp1, subunit 8 (theta) [Source:MGI Symbol;Acc:MGI:107183]	151.035	148.94	145.816	140.561	149.682	144.674	138.785	135.869	136.56	124.47	149.527	143.348	148.001	144.084	0.503972924	0.966018836	20	20	24	0.191141267	0.768040178	24	21.62480958	0.051572753
ENSMUSG00000025616	Usp16	ubiquitin specific peptidase 16 [Source:MGI Symbol;Acc:MGI:1921362]	23.0855	29.5248	24.3212	23.2661	32.071	25.8458	24.3725	24.6456	31.4857	23.6542	28.9644	24.0304	30.2078	27.2232	0.917839756	0.966018836	20	20	24	0.971045542	0.993012791	24	10.5569816	0.016194501
ENSMUSG00000025626	Phf6	PHD finger protein 6 [Source:MGI Symbol;Acc:MGI:1918248]	8.00499	7.77605	8.02422	8.16533	8.03766	7.38547	8.68993	6.98237	7.46846	6.70002	8.35789	7.54718	7.38312	7.74034	0.912686312	0.966018836	20	12	24	0.670694698	0.888925819	24	19.73665896	0.031560815
ENSMUSG00000025630	Hprt	hypoxanthine guanine phosphoribosyl transferase [Source:MGI Symbol;Acc:MGI:96217]	163.357	158.719	161.723	147.595	149.74	151.672	152.359	151.217	147.819	143.124	159.32	149.838	154.172	160.264	0.064405315	0.414867207	4	12	24	0.197557173	0.768040178	24	1.51441691	0.037304216
ENSMUSG00000025645	Ccdc51	coiled-coil domain containing 51 [Source:MGI Symbol;Acc:MGI:1913908]	5.26501	4.70582	5.41197	5.2902	4.3051	4.74037	4.65482	5.06484	4.35737	4.16927	5.19479	4.36492	4.89195	3.62901	0.768363194	0.966018836	4	4	24	0.893459284	0.969884586	24	18.91766921	0.022114845
ENSMUSG00000025646	Atrip	ATR interacting protein [Source:MGI Symbol;Acc:MGI:1925349]	6.33416	5.96205	7.46353	5.81707	5.91848	7.21183	6.18256	6.98681	6.18456	6.67794	6.96194	7.22405	6.33601	6.38733	0.905762334	0.966018836	24	16	24	0.910372782	0.974733097	24	18.94406014	0.018255337
ENSMUSG00000025647	Shisa5	shisa family member 5 [Source:MGI Symbol;Acc:MGI:1915044]	38.0363	30.6299	31.3294	30.6264	34.7351	35.1014	34.1936	34.7827	31.3638	33.3292	33.1717	33.1536	32.2579	32.8947	0.208071297	0.698704663	24	12	24	0.064183089	0.663876076	24	21.09866147	0.073964078
ENSMUSG00000025648	Pfkfb4	6-phosphofructo-2-kinase/fructose-2,6-biphosphatase 4 [Source:MGI Symbol;Acc:MGI:2687284]	7.87861	7.51417	7.99477	7.68266	8.82761	8.73415	6.99061	8.58823	7.20773	8.54156	8.57659	8.16875	8.11979	8.91693	0.560714538	0.966018836	20	16	24	0.492263264	0.805393068	24	16.27795533	0.043583719
ENSMUSG00000025650	Col7a1	collagen, type VII, alpha 1 [Source:MGI Symbol;Acc:MGI:88462]	4.2436	4.48113	3.87344	4.8156	5.59624	4.23209	5.40696	5.99891	4.11956	5.00922	5.25567	4.89109	4.36299	4.81899	0.777867261	0.966018836	20	16	24	0.755692693	0.921118175	24	17.23103882	0.045189242
ENSMUSG00000025651	Uqcrc1	ubiquinol-cytochrome c reductase core protein 1 [Source:MGI Symbol;Acc:MGI:107876]	88.2526	79.6515	90.9837	106.121	83.4664	96.8142	89.0889	101.876	79.9248	110.414	87.8221	90.8563	99.1362	96.5332	0.911135828	0.966018836	16	4	24	0.801339706	0.936384198	24	11.94338911	0.036642844
ENSMUSG00000025656	Arhgef9	CDC42 guanine nucleotide exchange factor (GEF) 9 [Source:MGI Symbol;Acc:MGI:2442233]	16.9405	17.5559	21.5524	17.1416	18.0954	18.0496	19.2545	16.342	18.1349	18.0399	14.7705	17.9702	16.3017	18.4564	0.920620634	0.966018836	12	16	24	0.579255426	0.849727189	24	6.940598124	0.049575724
ENSMUSG00000025658	Cnksr2	connector enhancer of kinase suppressor of Ras 2 [Source:MGI Symbol;Acc:MGI:2661175]	4.55611	4.59944	4.34869	4.46843	4.34423	4.4173	4.42445	3.80003	3.87312	4.69266	4.18595	5.21282	4.3695	5.13268	0.93447422	0.966018836	24	12	24	0.664310654	0.886686094	24	20.72093601	0.037984112
ENSMUSG00000025665	Rps6ka6	ribosomal protein S6 kinase polypeptide 6 [Source:MGI Symbol;Acc:MGI:1914321]	2.74404	2.68402	2.36843	2.32204	2.59389	2.63597	2.394	2.24398	2.35199	2.49362	2.78113	2.68157	2.86247	2.89731	0.29491602	0.805301811	4	8	24	0.172014988	0.768040178	24	21.46360069	0.059172571
ENSMUSG00000025666	Tmem47	transmembrane protein 47 [Source:MGI Symbol;Acc:MGI:2177570]	37.7778	38.3011	36.3052	36.3274	33.7555	33.8011	36.1066	32.3228	32.2908	34.6159	35.8701	30.8587	30.7264	36.7962	0.928892845	0.966018836	8	16	24	0.727829172	0.91070186	24	6.941877754	0.027726691
ENSMUSG00000025701	Alox5	arachidonate 5-lipoxygenase [Source:MGI Symbol;Acc:MGI:87999]	0.991231	0.989111	1.45593	0.631504	1.00656	0.826348	1.58432	0.992898	0.784442	0.766264	0.962976	1.02238	1.06172	0.607977	0.117050591	0.538362324	4	12	24	0.405745465	0.799546792	24	23.3198455	0.098338121
ENSMUSG00000025702	Marchf8	membrane associated ring-CH-type finger 8 [Source:MGI Symbol;Acc:MGI:1919029]	24.9247	24.6817	25.4466	24.0574	24.2689	22.841	27.5913	23.2173	25.5077	23.9966	24.4189	24.4423	24.0229	24.199	0.91724565	0.966018836	12	4	24	0.730499709	0.912198956	24	3.699056965	0.019709723
ENSMUSG00000025722	Wdr73	WD repeat domain 73 [Source:MGI Symbol;Acc:MGI:1919218]	9.95713	9.61077	9.5888	10.0487	9.27908	9.91477	9.1129	11.0471	9.36818	9.88136	8.85457	9.74348	9.93996	10.7088	0.117050591	0.538362324	8	12	24	0.199220779	0.768040178	24	4.338388619	0.052245353
ENSMUSG00000025723	Nmb	neuromedin B [Source:MGI Symbol;Acc:MGI:1915289]	188.868	174.151	158.834	163.56	166.718	172.94	153.317	169.407	172.235	148.855	188.481	179.633	183.739	169.305	0.219464938	0.708495385	24	16	24	0.238499692	0.768040178	24	21.78049724	0.068363762
ENSMUSG00000025724	Sec11a	SEC11 homolog A, signal peptidase complex subunit [Source:MGI Symbol;Acc:MGI:1929464]	34.5978	34.1162	35.7351	37.1089	37.9084	36.2582	34.087	35.3159	34.979	28.8678	36.8779	32.5033	34.7892	33.3828	0.351315142	0.870418653	20	8	24	0.733760436	0.9131851	24	17.95632587	0.028887796
ENSMUSG00000025728	Pigq	phosphatidylinositol glycan anchor biosynthesis, class Q [Source:MGI Symbol;Acc:MGI:1333114]	39.4053	34.9557	38.3919	39.222	37.8029	39.7687	39.5486	39.0196	33.966	41.3016	34.4638	37.9144	37.7227	36.2044	0.607427393	0.966018836	24	12	24	0.658988464	0.884814271	24	19.22985058	0.030748466
ENSMUSG00000025730	Rab40c	Rab40C, member RAS oncogene family [Source:MGI Symbol;Acc:MGI:2183454]	11.7721	10.1415	11.0601	11.0141	10.8285	10.9618	11.5199	10.173	11.0909	10.8937	10.5402	11.4972	11.4101	9.79494	0.082113105	0.462922111	4	4	24	0.239455981	0.768040178	24	19.52085519	0.046798773
ENSMUSG00000025731	Mettl26	methyltransferase like 26 [Source:MGI Symbol;Acc:MGI:1915597]	18.7726	18.3135	18.4464	16.0976	17.8244	18.4405	18.7303	19.1747	20.5044	18.5821	21.3436	20.1847	20.2791	19.222	0.912686312	0.966018836	12	8	24	0.581934835	0.851011325	24	23.96518236	0.030670409
ENSMUSG00000025732	Mcrip2	MAPK regulated corepressor interacting protein 2 [Source:MGI Symbol;Acc:MGI:1915491]	5.09743	4.8886	5.45244	11.3477	5.81529	5.91771	6.10157	7.39998	6.4869	8.35897	9.33794	6.93873	11.069	7.23444	0.95032543	0.966018836	16	20	24	0.346078407	0.778844143	24	13.09205439	0.159092219
ENSMUSG00000025733	Rhot2	ras homolog family member T2 [Source:MGI Symbol;Acc:MGI:2384892]	26.95	21.2874	20.2839	22.2161	23.8108	23.637	21.1301	20.7168	18.8392	25.1599	17.7751	20.3111	18.4611	19.4565	0.919119843	0.966018836	20	24	24	0.476769441	0.805393068	24	18.32955993	0.066815425
ENSMUSG00000025735	Rhbdl1	rhomboid like 1 [Source:MGI Symbol;Acc:MGI:2384891]	3.24157	2.39494	2.72439	2.62833	2.15289	2.96958	2.80559	3.31248	3.24284	2.9617	2.51231	2.55796	2.59614	3.07485	0.184044621	0.662239322	8	12	24	0.188711601	0.768040178	24	4.640810856	0.093209712
ENSMUSG00000025736	Jmjd8	jumonji domain containing 8 [Source:MGI Symbol;Acc:MGI:1919356]	11.1601	9.89105	10.696	10.4878	10.9525	10.6936	10.9949	10.0803	10.0535	10.1696	9.90201	9.62099	8.85845	8.83863	0.873171835	0.966018836	12	24	24	0.139897444	0.74956518	24	16.73097734	0.044655894
ENSMUSG00000025737	Wdr24	WD repeat domain 24 [Source:MGI Symbol;Acc:MGI:2446285]	6.00388	6.28175	6.08301	5.77181	6.05427	6.21373	5.96745	6.16774	5.46018	6.61808	5.83798	6.15233	5.82289	5.86717	0.875755063	0.966018836	24	12	24	0.841174313	0.951862945	24	18.72466973	0.013744828
ENSMUSG00000025738	Fbxl16	F-box and leucine-rich repeat protein 16 [Source:MGI Symbol;Acc:MGI:2448488]	21.4699	16.1317	18.6092	18.1768	16.5149	19.3039	18.9219	19.4678	17.1554	28.4588	17.6292	22.8788	18.9701	22.0503	0.96317972	0.967965568	24	20	24	0.987660781	0.997530201	24	14.96568208	0.012155641
ENSMUSG00000025739	Gng13	guanine nucleotide binding protein (G protein), gamma 13 [Source:MGI Symbol;Acc:MGI:1925616]	1.78826	5.18484	4.78233	2.59172	3.78796	3.21776	3.60528	2.36312	3.97184	3.28517	2.93002	2.80104	1.89241	3.25863	0.286642595	0.793714531	12	16	24	0.307381154	0.768040178	24	9.384526999	0.199651779
ENSMUSG00000025742	Prps2	phosphoribosyl pyrophosphate synthetase 2 [Source:MGI Symbol;Acc:MGI:97776]	12.2517	12.2958	11.4163	10.8874	11.0738	11.5319	12.1365	11.0902	12.0559	10.8715	12.0935	12.1188	10.5193	11.7142	0.371875382	0.894537464	4	12	24	0.527267385	0.823319175	24	23.86884615	0.030722288
ENSMUSG00000025743	Sdc3	syndecan 3 [Source:MGI Symbol;Acc:MGI:1349163]	37.2267	31.3613	33.9904	33.6741	35.166	35.4196	36.0917	35.0791	32.4652	45.3143	33.3951	36.4158	33.6138	35.3246	0.588682823	0.966018836	24	12	24	0.666801207	0.887459929	24	15.57996223	0.018283116
ENSMUSG00000025745	Hadha	hydroxyacyl-CoA dehydrogenase trifunctional multienzyme complex subunit alpha [Source:MGI Symbol;Acc:MGI:2135593]	75.6142	67.908	72.0227	94.6083	80.9134	78.451	84.8336	96.9443	69.1712	83.965	85.6168	73.4147	89.9512	83.1379	0.917839756	0.966018836	16	20	24	0.920626577	0.978829863	24	16.02517837	0.024900584
ENSMUSG00000025747	Tyms	thymidylate synthase [Source:MGI Symbol;Acc:MGI:98878]	2.01405	2.38898	2.95737	2.64314	1.7993	2.17124	1.99737	2.65999	1.71938	1.30135	2.30766	2.00892	1.61475	1.06189	0.874054778	0.966018836	12	16	24	0.950552057	0.988257443	24	15.20090745	0.026392117
ENSMUSG00000025757	Hspa4l	heat shock protein 4 like [Source:MGI Symbol;Acc:MGI:107422]	62.4916	50.7831	46.0139	48.4435	57.6084	51.6673	57.6448	48.2191	46.2062	42.8856	58.1658	52.829	54.8331	52.0071	0.006547696	0.177399733	4	8	24	0.005983232	0.46443032	24	21.17127565	0.164539782
ENSMUSG00000025758	Plk4	polo like kinase 4 [Source:MGI Symbol;Acc:MGI:101783]	2.72933	2.45791	2.87729	3.01931	2.6932	2.52963	3.24211	2.30028	3.30267	2.4632	2.63399	3.21193	3.30736	2.97242	0.924770496	0.966018836	4	4	24	0.90367355	0.973373462	24	0.81485094	0.022111097
ENSMUSG00000025759	Mfsd8	major facilitator superfamily domain containing 8 [Source:MGI Symbol;Acc:MGI:1919425]	6.88581	7.24998	6.7327	7.17608	7.84998	6.65448	7.66392	7.06733	7.10919	6.0938	7.5238	6.9658	8.05086	6.69094	0.930944473	0.966018836	4	12	24	0.479276154	0.805393068	24	21.38967635	0.04629274
ENSMUSG00000025762	Larp1b	La ribonucleoprotein domain family, member 1B [Source:MGI Symbol;Acc:MGI:1914604]	12.8551	11.9623	13.0934	15.4166	10.5871	12.1525	10.565	12.9434	11.129	13.9301	12.6141	12.4813	11.2662	14.7136	0.287843582	0.793714531	12	16	24	0.322218605	0.768040178	24	9.693755548	0.091944534
ENSMUSG00000025764	Jade1	jade family PHD finger 1 [Source:MGI Symbol;Acc:MGI:1925835]	5.85242	6.57842	7.80319	6.86254	6.18495	6.41896	7.28684	7.1031	7.39953	6.7057	6.58778	6.62075	6.75944	6.88145	0.019852054	0.259417302	12	8	24	0.039946499	0.616921492	24	7.493849744	0.085753409
ENSMUSG00000025766	D3Ertd751e	DNA segment, Chr 3, ERATO Doi 751, expressed [Source:MGI Symbol;Acc:MGI:1289213]	7.56331	9.44582	8.16396	8.37995	8.59424	8.13743	9.11545	8.08641	9.6045	7.7438	9.83366	9.30543	8.95962	9.01404	0.846643334	0.966018836	20	8	24	0.993808483	0.998755922	24	2.629137167	0.004341764
ENSMUSG00000025776	Crispld1	cysteine-rich secretory protein LCCL domain containing 1 [Source:MGI Symbol;Acc:MGI:1934666]	1.38009	1.8149	1.85497	1.88703	1.88919	1.7611	1.60874	2.18218	2.03742	1.77888	2.14104	1.70396	1.26347	2.03776	0.029078724	0.321139886	8	20	24	0.078139571	0.688357484	24	10.36573932	0.117901014
ENSMUSG00000025777	Gdap1	ganglioside-induced differentiation-associated-protein 1 [Source:MGI Symbol;Acc:MGI:1338002]	52.6929	52.5341	49.4729	51.2115	50.1361	50.6317	48.9691	45.6474	47.1278	50.2713	56.1614	52.8356	50.8555	54.5398	0.833406028	0.966018836	24	12	24	0.43003485	0.805393068	24	18.54495591	0.040109575
ENSMUSG00000025779	Ly96	lymphocyte antigen 96 [Source:MGI Symbol;Acc:MGI:1341909]	3.14054	4.43013	4.79768	4.93412	4.91466	5.06207	3.72944	5.15006	5.36296	3.87077	4.75651	4.2346	4.85481	4.23132	0.643325306	0.966018836	16	24	24	0.362939981	0.787656031	24	11.00052842	0.095930243
ENSMUSG00000025780	Itih5	inter-alpha (globulin) inhibitor H5 [Source:MGI Symbol;Acc:MGI:1925751]	18.5028	21.1897	19.8493	20.0954	22.3078	19.1558	20.9974	21.1986	22.0292	21.2027	19.1962	20.1503	19.8176	18.6753	0.360675881	0.883978426	16	12	24	0.448442326	0.805393068	24	10.4218747	0.041346417
ENSMUSG00000025781	Atp5c1	ATP synthase, H+ transporting, mitochondrial F1 complex, gamma polypeptide 1 [Source:MGI Symbol;Acc:MGI:1261437]	194.698	224.784	232.094	238.61	207.792	206.264	206.673	232.907	251.008	211.461	234.701	212.987	242.285	244.587	0.208071297	0.698704663	12	12	24	0.013232251	0.529134128	24	7.915935299	0.106144329
ENSMUSG00000025782	Taf3	TATA-box binding protein associated factor 3 [Source:MGI Symbol;Acc:MGI:2388097]	2.23102	1.9806	2.14752	2.18416	2.13444	2.40416	2.5967	2.35912	2.16712	3.06177	2.34034	2.42847	2.55265	2.59996	0.836635326	0.966018836	4	8	24	0.940929663	0.985441727	24	18.57329729	0.012876335
ENSMUSG00000025784	Clec3b	C-type lectin domain family 3, member b [Source:MGI Symbol;Acc:MGI:104540]	16.1053	18.6114	13.9177	11.0527	12.057	9.25286	13.9193	20.9467	25.3721	8.70861	14.9325	15.9822	14.8767	15.388	0.027096347	0.306445942	8	8	24	0.073713675	0.682388921	24	4.275342158	0.327758913
ENSMUSG00000025785	Exosc7	exosome component 7 [Source:MGI Symbol;Acc:MGI:1913696]	15.7997	16.2421	14.7807	16.0922	18.3592	16.5621	16.3398	16.4176	18.4116	13.5127	16.8404	15.1692	16.2365	16.8153	0.890648739	0.966018836	20	20	24	0.939448196	0.985166762	24	22.53361183	0.01583478
ENSMUSG00000025786	Zdhhc3	zinc finger, DHHC domain containing 3 [Source:MGI Symbol;Acc:MGI:1926134]	30.9278	28.5666	28.4151	28.8645	29.9929	31.211	33.1066	30.0192	27.8922	32.423	28.0535	30.262	28.7269	30.2811	0.143321297	0.585944911	24	12	24	0.382522072	0.799546792	24	21.20328822	0.041704249

ENSMUSG00000025787	Tgm4	transglutaminase 4 (prostate) [Source:MGI Symbol;Acc:MGI:3027002]	1.89588	2.46301	2.15324	2.00185	2.6556	1.7567	1.52808	2.68222	1.85853	2.00855	1.87023	2.6738	2.0941	1.89532	0.860993221	0.966018836	8	20	24	0.970963407	0.993012791	24	11.57995216	0.01674127
ENSMUSG00000025790	Slco3a1	solute carrier organic anion transporter family, member 3a1 [Source:MGI Symbol;Acc:MGI:1351867]	16.6896	13.8184	13.6917	13.8796	17.8807	17.1344	16.0981	15.7051	13.9186	16.3816	15.4298	15.705	14.5617	14.6471	0.046934151	0.375011045	24	12	24	0.022249889	0.578379975	24	19.08985211	0.1175751
ENSMUSG00000025791	Pgm1	phosphoglucomutase 1 [Source:MGI Symbol;Acc:MGI:97565]	47.2297	46.6009	51.8757	54.2683	43.9875	58.6804	49.4986	72.7595	54.4071	70.3835	49.1417	53.2724	54.0198	49.2779	0.943890799	0.966018836	16	4	24	0.596227112	0.857839743	24	8.854374853	0.083568891
ENSMUSG00000025792	Slc25a10	solute carrier family 25 (mitochondrial carrier, dicarboxylate transporter), member 10 [Source:MGI Symbol;Acc:MGI:1353497]	12.155	12.2184	13.1473	13.6707	14.2757	13.1267	13.3063	13.4483	13.8108	16.8944	13.4849	12.6916	13.127	11.0724	0.037038569	0.334054683	16	16	24	0.035756021	0.609176161	24	13.28274213	0.114589181
ENSMUSG00000025793	Hgs	HGF-regulated tyrosine kinase substrate [Source:MGI Symbol;Acc:MGI:104681]	21.0053	16.6922	16.9146	16.7318	16.6478	19.4376	21.496	20.068	16.1066	24.9157	17.6983	20.086	19.0371	19.2342	0.267533088	0.774280302	4	8	24	0.570729252	0.84547694	24	22.16117092	0.071183192
ENSMUSG00000025794	Rpl14	ribosomal protein L14 [Source:MGI Symbol;Acc:MGI:1914365]	121.781	121.441	120.575	112.174	124.174	123.204	126.105	125.087	143.998	114.725	135.052	125.779	124.351	120.222	0.435739761	0.966018836	20	4	24	0.962577427	0.990655056	24	0.281071871	0.009463906
ENSMUSG00000025795	Rassf3	Ras association (RalGDS/AF-6) domain family member 3 [Source:MGI Symbol;Acc:MGI:2179722]	21.3705	16.3032	15.5897	18.2469	16.6192	17.8046	18.7678	17.8067	16.1915	20.7977	16.4076	18.9494	20.0243	18.8671	0.033248781	0.324980923	4	8	24	0.36429202	0.788789286	24	22.38277837	0.074190065
ENSMUSG00000025804	Ccr1	chemokine (C-C motif) receptor 1 [Source:MGI Symbol;Acc:MGI:104618]	0.756033	0.950946	1.16173	0.804022	0.636503	0.846994	0.598101	0.959597	0.954202	0.646122	0.686217	0.705505	0.944583	0.679779	0.066808003	0.425506559	12	8	24	0.12941453	0.740162193	24	6.043083471	0.096133134
ENSMUSG00000025809	Itgb1	integrin beta 1 (fibronectin receptor beta) [Source:MGI Symbol;Acc:MGI:96610]	131.555	132.258	129.003	128.176	139.354	126.698	127.009	129.286	134.237	121.724	144.873	130.474	138.333	130.38	0.911685024	0.966018836	20	20	24	0.698666262	0.899819721	24	18.28465904	0.022049529
ENSMUSG00000025810	Nrp1	neuropilin 1 [Source:MGI Symbol;Acc:MGI:106206]	20.0188	17.2513	17.7798	17.8597	18.0018	18.6952	16.8383	18.6188	14.9762	19.6146	17.8727	18.599	20.295	18.7376	0.286642595	0.793714531	4	8	24	0.36893664	0.791959527	24	21.06174288	0.059725859
ENSMUSG00000025812	Pard3	par-3 family cell polarity regulator [Source:MGI Symbol;Acc:MGI:2135608]	5.23849	4.26032	3.97597	4.64769	4.8011	4.35808	5.43821	5.10229	4.41276	5.74005	4.70672	4.24489	4.54442	4.46939	0.941901469	0.966018836	4	8	24	0.921506344	0.979320587	24	18.65745738	0.021237793
ENSMUSG00000025813	Homer2	homer scaffolding protein 2 [Source:MGI Symbol;Acc:MGI:1347354]	9.40144	7.80888	7.64743	9.33942	7.68542	8.90238	7.74732	8.56142	7.93942	8.53941	8.30786	9.18524	8.73898	8.4157	0.390686537	0.915479136	24	12	24	0.473627169	0.805393068	24	19.88799843	0.047155364
ENSMUSG00000025815	Dhtkd1	dehydrogenase E1 and transketolase domain containing 1 [Source:MGI Symbol;Acc:MGI:2445096]	1.72439	1.89841	2.00558	2.29937	1.86487	1.99006	2.01006	1.93455	2.54745	1.92468	2.1923	2.2214	1.96235	2.16029	0.886952527	0.966018836	12	16	24	0.193673609	0.768040178	24	10.58925032	0.061871659
ENSMUSG00000025816	Sec61a2	Sec61, alpha subunit 2 (S. cerevisiae) [Source:MGI Symbol;Acc:MGI:1931071]	35.0051	32.4388	31.9949	32.5001	35.3898	31.2548	32.5569	29.6233	35.5812	29.6415	33.1319	35.7956	32.527	34.554	0.823657258	0.966018836	20	20	24	0.744903318	0.916569459	24	20.09423003	0.028185527
ENSMUSG00000025817	Nudt5	nudix (nucleoside diphosphate linked moiety X)-type motif 5 [Source:MGI Symbol;Acc:MGI:1858232]	17.173	15.3918	13.3771	15.3917	16.9896	15.7953	16.1755	14.4568	13.6677	13.5841	17.5115	15.3367	17.7957	16.7257	0.017495149	0.255326033	4	8	24	0.029437881	0.596648309	24	20.97050186	0.124546538
ENSMUSG00000025821	Zfp282	zinc finger protein 282 [Source:MGI Symbol;Acc:MGI:2141413]	2.91407	2.36951	2.59474	2.45954	2.812	2.45422	2.85379	2.58535	2.74476	2.84075	2.84277	2.67382	2.39288	2.55691	0.702630284	0.966018836	20	12	24	0.542470801	0.83094351	24	16.11678532	0.032696168
ENSMUSG00000025823	Pdia4	protein disulfide isomerase associated 4 [Source:MGI Symbol;Acc:MGI:104864]	35.5922	24.193	23.5696	22.7112	29.356	27.3423	31.6893	26.4785	20.5724	23.5173	29.3257	24.4154	26.4174	23.3575	0.019074173	0.257576951	4	8	24	0.007367718	0.47735824	24	21.07252287	0.213063553
ENSMUSG00000025825	Iscu	iron-sulfur cluster assembly enzyme [Source:MGI Symbol;Acc:MGI:1913633]	49.583	51.5774	51.2213	51.3642	49.3251	50.7802	48.6695	48.3408	48.9502	48.3949	49.3229	48.506	47.9899	45.2522	0.916963526	0.966018836	12	20	24	0.208387115	0.768040178	24	14.86701869	0.019644119
ENSMUSG00000025854	Fam20c	FAM20C, golgi associated secretory pathway kinase [Source:MGI Symbol;Acc:MGI:2136853]	12.4981	9.28136	9.04161	9.92969	11.764	10.9049	10.0338	9.6026	9.30357	10.4127	7.53363	8.36028	7.27593	9.79746	0.810949269	0.966018836	16	20	24	0.899479838	0.971453495	24	18.89724181	0.031135401
ENSMUSG00000025855	Prkar1b	protein kinase, cAMP dependent regulatory, type I beta [Source:MGI Symbol;Acc:MGI:97759]	183.834	131.631	141.059	142.152	129.535	172.089	151.741	152.05	108.764	179.863	126.498	155.146	140.587	156.435	0.636884864	0.966018836	20	20	24	0.393065204	0.799546792	24	21.84818501	0.113777314
ENSMUSG00000025856	Pdgfa	platelet derived growth factor, alpha [Source:MGI Symbol;Acc:MGI:97527]	21.8012	20.7771	19.3075	23.0996	24.6836	22.5164	20.711	21.7635	22.8144	24.685	19.2462	21.888	22.4576	19.7666	0.138719141	0.580158868	16	16	24	0.369088545	0.79209268	24	15.22767933	0.060157237
ENSMUSG00000025857	Dnaaf5	dynein, axonemal assembly factor 5 [Source:MGI Symbol;Acc:MGI:3616079]	2.4677	2.21271	2.10576	2.1264	2.16829	2.37661	2.57848	2.51164	2.19047	2.66823	2.1216	2.37275	2.21839	2.24195	0.196044629	0.673646834	4	16	24	0.515790185	0.817421075	24	23.36914149	0.036334475
ENSMUSG00000025858	Get4	golgi to ER traffic protein 4 [Source:MGI Symbol;Acc:MGI:1914854]	25.4564	24.2493	25.2606	24.7092	22.8304	23.695	24.7929	24.4891	23.9858	27.8816	23.5082	27.4343	25.1391	26.55	0.885025617	0.966018836	4	16	24	0.773721851	0.92732018	24	5.43695609	0.021738426
ENSMUSG00000025860	Xiap	X-linked inhibitor of apoptosis [Source:MGI Symbol;Acc:MGI:107572]	30.7053	32.5442	31.6157	32.9679	32.1887	28.3508	32.1483	28.6872	34.379	29.5327	34.7485	31.0148	31.3191	32.1954	0.44861185	0.966018836	20	4	24	0.521288601	0.82025681	24	10.77902869	0.038499926
ENSMUSG00000025862	Stag2	stromal antigen 2 [Source:MGI Symbol;Acc:MGI:1098583]	21.4457	25.9972	25.0492	25.6665	24.039	19.8974	23.6908	20.292	27.2216	20.5992	26.9603	22.7052	24.2173	24.7948	0.14313801	0.585944911	12	12	24	0.401525861	0.799546792	24	9.408942727	0.079353603
ENSMUSG00000025867	Cplx2	complexin 2 [Source:MGI Symbol;Acc:MGI:104726]	44.2892	33.0774	34.6154	36.5929	31.9569	42.2596	37.237	37.7586	27.0491	47.6174	31.1154	39.7544	36.3355	44.3453	0.658942124	0.966018836	24	12	24	0.629859226	0.873635005	24	22.77676346	0.082824677
ENSMUSG00000025868	Higd2a	HIG1 domain family, member 2A [Source:MGI Symbol;Acc:MGI:1914294]	94.8486	82.0039	87.4129	81.7001	90.261	92.0165	91.3477	91.413	83.8863	94.717	88.339	90.1728	94.3544	83.4203	0.133766544	0.568189321	4	4	24	0.132888215	0.74343239	24	20.529046	0.058605375
ENSMUSG00000025869	Nop16	NOP16 nucleolar protein [Source:MGI Symbol;Acc:MGI:107862]	14.6505	11.6408	12.7999	11.4252	12.073	13.6679	11.5445	12.3585	11.1738	12.2155	11.7498	11.4585	11.356	11.9017	0.718363231	0.966018836	8	4	24	0.419550207	0.803639585	24	21.66194532	0.047238358
ENSMUSG00000025870	Arl10	ADP-ribosylation factor-like 10 [Source:MGI Symbol;Acc:MGI:1930788]	8.94745	8.95364	9.81412	9.56019	10.8427	9.04585	11.267	10.7013	11.1151	10.9062	9.59454	10.6897	12.0099	9.27678	0.829428469	0.966018836	12	20	24	0.990287129	0.998186034	24	12.61489973	0.006365972
ENSMUSG00000025871	4833439L19Rik	RIKEN cDNA 4833439L19 gene [Source:MGI Symbol;Acc:MGI:1921162]	44.531	38.1048	39.9254	40.1263	42.894	42.6656	47.5663	49.3031	42.2318	52.9412	40.1804	44.7919	46.0019	43.9834	0.43565473	0.966018836	4	8	24	0.85920702	0.958530654	24	0.195710083	0.025844899
ENSMUSG00000025872	Thoc3	THO complex 3 [Source:MGI Symbol;Acc:MGI:1920916]	10.0283	9.81741	10.4863	10.3248	10.4336	10.4488	9.77743	10.3759	9.39419	11.9883	9.46738	10.2566	10.5206	10.3689	0.825856869	0.966018836	16	16	24	0.728791424	0.911292459	24	12.77366473	0.024443215
ENSMUSG00000025873	Faf2	Fas associated factor family member 2 [Source:MGI Symbol;Acc:MGI:1923827]	22.5012	19.8574	21.2912	19.8741	20.8095	23.2901	19.2687	21.9709	19.771	25.2428	20.9156	21.4022	23.2167	23.1465	0.919571723	0.966018836	24	12	24	0.958475529	0.989144435	24	20.11241474	0.012446542
ENSMUSG00000025875	Tspan17	tetraspanin 17 [Source:MGI Symbol;Acc:MGI:1921507]	157.26	132.21	131.413	130.233	167.178	149.874	137.586	138.05	123.716	154.796	143.449	137.339	146.379	129.109	0.059650003	0.406067671	20	16	24	0.036983988	0.609176161	24	18.55278497	0.115421657
ENSMUSG00000025876	Unc5a	unc-5 netrin receptor A [Source:MGI Symbol;Acc:MGI:894682]	1.83302	1.52748	1.94298	1.63343	1.69657	1.75823	1.69272	1.95611	1.65212	2.49731	1.63469	2.11592	1.73754	2.14334	0.842838536	0.966018836	24	20	24	0.790666368	0.932939823	24	9.380411219	0.033402543
ENSMUSG00000025877	Hk3	hexokinase 3 [Source:MGI Symbol;Acc:MGI:2670962]	0.768346	1.22564	1.85152	1.4775	1.37349	1.067	1.13064	1.27988	1.41476	1.03001	1.08821	1.00214	1.25531	0.873259	0.077301512	0.453790923	12	12	24	0.113190954	0.727393008	24	9.9814843	0.1347875
ENSMUSG00000025878	Uimc1	ubiquitin interaction motif containing 1 [Source:MGI Symbol;Acc:MGI:103185]	14.5945	15.4911	12.6985	12.5547	14.3741	12.4971	12.6046	12.7883	14.724	12.3707	14.7167	13.8967	14.2074	13.3961	0.850398351	0.966018836	8	8	24	0.819882591	0.942844597	24	23.52320548	0.02651021
ENSMUSG00000025880	Smad7	SMAD family member 7 [Source:MGI Symbol;Acc:MGI:1100518]	1.18545	1.99089	2.7773	1.9458	1.36445	1.49632	1.32099	1.52217	1.84203	1.57699	1.28873	1.04078	1.29242	1.94475	0.008047769	0.192760771	12	12	24	0.001807327	0.383184938	24	8.144218569	0.260386657
ENSMUSG00000025885	Myo5b	myosin VB [Source:MGI Symbol;Acc:MGI:106598]	0.514827	0.847528	1.26207	1.11753	0.871006	0.569496	0.808107	0.879583	0.885971	1.03587	0.982109	0.883503	0.850668	0.771916	0.004196064	0.140598674	16	12	24	0.00865189	0.502965379	24	10.61340467	0.157699306
ENSMUSG00000025887	Casp12	caspase 12 [Source:MGI Symbol;Acc:MGI:1312922]	9.8761	13.7006	14.0357	10.0536	12.5443	10.3409	11.8268	10.9555	13.8727	10.3976	14.6306	10.6642	11.4428	9.63094	0.699015352	0.966018836	12	12	24	0.616131099	0.867471324	24	10.4505708	0.076681011
ENSMUSG00000025888	Casp1	caspase 1 [Source:MGI Symbol;Acc:MGI:96544]	6.94336	6.79312	6.58417	5.60542	7.3308	6.32665	5.57429	6.72352	7.16906	5.39901	8.75681	5.86555	6.74994	6.77856	0.93447422	0.966018836	20	20	24	0.987486385	0.997530201	24	17.45705587	0.010617982
ENSMUSG00000025889	Snca	synuclein, alpha [Source:MGI Symbol;Acc:MGI:1277151]	42.4133	44.8571	45.5231	42.9624	43.2405	40.1936	44.7029	37.9318	44.1456	35.9569	41.0836	41.9971	39.2886	40.6357	0.905762334	0.966018836	12	12	24	0.918867574	0.978135058	24	3.298265799	0.013601177
ENSMUSG00000025892	Gria4	glutamate receptor, ionotropic, AMPA4 (alpha 4) [Source:MGI Symbol;Acc:MGI:95811]	23.3126	25.8187	27.7196	22.0091	22.071	23.531	25.4232	21.2124	19.8182	23.2684	21.9959	22.1062	19.3066	25.5897	0.934818505	0.966018836	8	12	24	0.716169231	0.906323093	24	4.651847144	0.043149477
ENSMUSG00000025893	Kbtbd3	kelch repeat and BTB (POZ) domain containing 3 [Source:MGI Symbol;Acc:MGI:1916399]	8.92613	10.6061	9.07289	9.21418	9.56312	8.61986	8.4549	8.75856	10.541	7.92086	9.98843	9.32777	10.1623	9.49928	0.90332508	0.966018836	12	4	24	0.770259932	0.926036042	24	5.532075223	0.032885759
ENSMUSG00000025894	Aasdhppt	aminoadipate-semialdehyde dehydrogenase-phosphopantetheinyl transferase [Source:MGI Symbol;Acc:MGI:1914868]	14.0027	14.8181	13.1802	14.0292	14.2254	12.9148	13.3975	12.9181	13.5925	11.8114	14.9772	12.503	13.0765	14.1324	0.893307667	0.966018836	20	4	24	0.991412704	0.998516607	24	2.353191119	0.004476908
ENSMUSG00000025898	Cwf19l2	CWF19-like 2, cell cycle control (S. pombe) [Source:MGI Symbol;Acc:MGI:1918023]	6.96896	8.18629	8.07695	7.54066	7.58246	6.88514	6.69369	6.8804	8.55756	6.95115	7.8932	7.28466	7.15699	7.94788	0.064867922	0.416505219	12	16	24	0.096456342	0.71175955	24	9.089042077	0.081677119
ENSMUSG00000025899	Alkbh8	alkB homolog 8, tRNA methyltransferase [Source:MGI Symbol;Acc:MGI:1914917]	4.99758	5.70662	5.35006	5.21929	5.45741	5.27125	5.37299	5.00194	5.78333	5.42724	5.93019	5.72665	5.20192	5.89473	0.743750765	0.966018836	20	8	24	0.566266122	0.843471947	24	11.50845748	0.026332595
ENSMUSG00000025902	Sox17	SRY (sex determining region Y)-box 17 [Source:MGI Symbol;Acc:MGI:107543]	2.76117	3.02378	3.04322	2.87559	3.05939	2.37566	3.40559	3.55275	2.85433	3.86048	4.58553	2.92356	3.46404	3.10082	0.874054778	0.966018836	20	4	24	0.645675529	0.879878902	24	11.77702705	0.060100876
ENSMUSG00000025903	Lypla1	lysophospholipase 1 [Source:MGI Symbol;Acc:MGI:1344588]	28.2716	27.3999	27.5112	29.7847	25.8899	26.6909	27.8958	26.0498	23.463	26.6039	28.4534	23.2554	23.8527	24.6707	0.88067614	0.966018836	16	20	24	0.570778626	0.845514618	24	14.56726707	0.03362171
ENSMUSG00000025905	Oprk1	opioid receptor, kappa 1 [Source:MGI Symbol;Acc:MGI:97439]	1.0255	1.16772	1.33926	1.02196	1.13101	1.0549	0.95978	1.03856	1.10943	0.98717	1.18379	1.14625	0.960142	1.35108	0.338172159	0.85758369	12	16	24	0.482731512	0.805393068	24	8.342385404	0.041854372
ENSMUSG00000025907	Rb1cc1	RB1-inducible coiled-coil 1 [Source:MGI Symbol;Acc:MGI:1341850]	50.7226	55.9553	50.2637	49.4231	52.1883	51.6367	47.3741	47.6395	52.2783	43.4066	58.2063	52.9406	53.0106	55.5844	0.932092277	0.966018836	20	20	24	0.78059964	0.929485273	24	22.05593052	0.030844099
ENSMUSG00000025909	Sntg1	syntrophin, gamma 1 [Source:MGI Symbol;Acc:MGI:1918346]	1.9432	1.84925	3.0798	1.39852	1.32275	1.99041	1.87704	1.84902	2.31775	1.74061	1.75887	2.01087	2.07414	1.95252	0.248056532	0.746745468	12	4	24	0.231487358	0.768040178	24	4.779057124	0.127543655
ENSMUSG00000025911	Adhfe1	alcohol dehydrogenase, iron containing, 1 [Source:MGI Symbol;Acc:MGI:1923437]	1.91685	3.15909	4.77696	7.36522	3.67863	3.47343	4.07998	4.41449	4.9167	5.88535	4.90755	4.70435	5.66713	4.78251	0.080148772	0.458678242	16	8	24	0.017496292	0.550995814	24	11.0386932	0.307996678
ENSMUSG00000025912	Mybl1	myeloblastosis oncogene-like 1 [Source:MGI Symbol;Acc:MGI:99925]	1.96917	1.73888	1.91352	1.74374	1.53113	1.61576	1.70954	1.28865	2.15882	1.48458	1.7941	1.4473	1.40637	1.83177	0.601647512	0.966018836	12	12	24	0.515443036	0.81731462	24	8.069522424	0.058350671
ENSMUSG00000025915	Sgk3	serum/glucocorticoid regulated kinase 3 [Source:MGI Symbol;Acc:MGI:2182368]	7.6393	9.40317	9.15995	8.42396	9.11767	7.96234	8.96258	8.337	9.36427	7.35886	9.06474	8.30199	7.92904	8.04548	0.300823756	0.809362476	12	16	24	0.702199343	0.901574817	24	9.268623409	0.034735807
ENSMUSG00000025917	Cops5	COP9 signalosome subunit 5 [Source:MGI Symbol;Acc:MGI:1349415]	118.768	129.83	120.113	123.195	130.133	118.945	111.824	114.385	131.285	104.498	128.484	112.59	120.871	118.67	0.767183862	0.966018836	20	4	24	0.867085488	0.961800416	24	11.41581842	0.019990402
ENSMUSG00000025920	Stau2	staufen double-stranded RNA binding protein 2 [Source:MGI Symbol;Acc:MGI:1352508]	48.4971	40.6934	41.9773	42.2723	37.4948	44.3001	41.2067	41.8201	37.9443	49.7493	41.3364	47.6447	47.5555	48.0498	0.939103211	0.966018836	24	12	24	0.562457638	0.842303209	24	23.21602114	0.052514808
ENSMUSG00000025921	Rdh10	retinol dehydrogenase 10 (all-trans) [Source:MGI Symbol;Acc:MGI:1924238]	3.88278	3.97124	4.01121	4.98619	5.1127	3.6972	4.10699	4.58364	4.3422	4.33324	4.68793	3.44815	3.65769	3.80564	0.004003768	0.140598674	20	4	24	0.033217092	0.606585237	24	12.27068615	0.123544388
ENSMUSG00000025922	Rpl7-ps8	ribosomal protein L7, pseudogene 8 [Source:MGI Symbol;Acc:MGI:3647181]	6.33599	8.09137	6.62038	7.67359	8.36034	6.13108	6.59949	6.54463	9.61122	5.97525	8.65591	7.62291	7.42609	6.73128	0.85447369	0.966018836	20	8	24	0.566073423	0.843471947	24	12.27029161	0.076594081
ENSMUSG00000025925	Terf1	telomeric repeat binding factor 1 [Source:MGI Symbol;Acc:MGI:109634]	8.21953	8.26858	9.23652	7.54619	8.31475	7.93801	8.25515	7.66024	9.5308	7.13897	8.04471	8.32481	10.0452	7.99479	0.581070934	0.966018836	4	12	24	0.538046741	0.828330709	24	2.649834913	0.051514468
ENSMUSG00000025930	Msc	musculin [Source:MGI Symbol;Acc:MGI:1333884]	2.48961	2.08186	3.20931	1.99767	2.85467	2.24667	2.80041	2.51009	2.59593	2.98326	2.87483	2.97856	2.62374	3.33424	0.822716578	0.966018836	12	16	24	0.8967033	0.970634157	24	9.536235685	0.026578895
ENSMUSG00000025931	Paqr8	progestin and adipoQ receptor family member VIII [Source:MGI Symbol;Acc:MGI:1921479]	16.9512	14.6316	18.3123	17.4391	16.0354	18.5659	17.1	14.0511	14.0801	17.7517	15.145	16.4544	12.617	15.5285	0.622773557	0.966018836	16	16	24	0.260412557	0.768040178	24	15.68251674	0.087871598
ENSMUSG00000025932	Eya1	EYA transcriptional coactivator and phosphatase 1 [Source:MGI Symbol;Acc:MGI:109344]	0.632064	1.07312	0.9571	1.25834	0.762745	1.16534	1.18059	1.15251	1.16538	0.974572	1.1181	1.53418	0.64065	2.53274	0.873350015	0.966018836	24	4	24	0.576494917	0.848661503	24	6.653828419	0.105628175
ENSMUSG00000025933	Tmem14a	transmembrane protein 14A [Source:MGI Symbol;Acc:MGI:1922962]	26.468	23.5338	24.8481	22.7478	23.803	23.6667	22.3472	21.6417	22.4715	20.3547	23.9391	22.1965	21.2189	20.6097	0.953350544	0.966018836	20	12	24	0.23786602	0.768040178	24	19.44060083	0.044019528
ENSMUSG00000025934	Gsta3	glutathione S-transferase, alpha 3 [Source:MGI Symbol;Acc:MGI:95856]	0.627053	1.01899	1.0378	1.73276	1.11241	1.44544	0.83372	0.81899	1.27658	0.979035	1.27326	1.17854	1.139	1.07425	0.172164153	0.648362388	24	4	24	0.088580247	0.707747429	24	13.76508451	0.14627502
ENSMUSG00000025935	Tram1	translocating chain-associating membrane protein 1 [Source:MGI Symbol;Acc:MGI:1919515]	23.7031	21.8591	19.7886	21.59	23.4938	21.664	22.8492	21.2395	21.5583	19.4301	23.5666	20.1063	19.8151	19.4064	0.431765373	0.966018836	20	16	24	0.164785828	0.767020009	24	19.08042908	0.064414051
ENSMUSG00000025937	Lactb2	lactamase, beta 2 [Source:MGI Symbol;Acc:MGI:2442551]	15.1415	14.8733	14.9929	18.6723	16.3951	17.291	16.1156	18.9568	17.5744	14.6847	21.0766	18.131	17.1376	18.7702	0.882263435	0.966018836	20	20	24	0.72179559	0.90866625	24	15.90500101	0.040224974
ENSMUSG00000025938	Slco5a1	solute carrier organic anion transporter family, member 5A1 [Source:MGI Symbol;Acc:MGI:2443431]	5.94871	5.68778	4.60465	6.3892	5.26334	5.29476	5.32325	5.44975	4.7731	6.07023	5.67475	5.62259	6.42273	6.02747	0.947524602	0.966018836	4	8	24	0.785431389	0.931158434	24	20.80924376	0.033814779
ENSMUSG00000025939	Ube2w	ubiquitin-conjugating enzyme E2W (putative) [Source:MGI Symbol;Acc:MGI:1914049]	31.8014	31.2327	29.0888	27.7484	31.1266	29.2554	29.6083	26.2822	30.6051	25.6517	31.938	29.8056	30.2459	29.9895	0.676344318	0.966018836	20	20	24	0.352212333	0.780629136	24	21.54158691	0.053532679
ENSMUSG00000025940	Tmem70	transmembrane protein 70 [Source:MGI Symbol;Acc:MGI:1915068]	22.1998	22.9513	22.1296	24.7776	22.2836	21.1523	23.3947	23.6897	21.8537	20.3952	25.171	22.7674	24.5836	23.168	0.935316621	0.966018836	8	4	24	0.891874417	0.969355921	24	23.91893604	0.015764329
ENSMUSG00000025949	Pikfyve	phosphoinositide kinase, FYVE type zinc finger containing [Source:MGI Symbol;Acc:MGI:1335106]	5.70758	5.79553	5.56404	6.19836	6.30057	5.76786	5.67348	5.2409	6.27359	5.24947	6.49957	5.9627	5.90821	6.00639	0.25849765	0.761738864	20	12	24	0.338010453	0.772742827	24	15.84234181	0.044852428
ENSMUSG00000025950	Idh1	isocitrate dehydrogenase 1 (NADP+), soluble [Source:MGI Symbol;Acc:MGI:96413]	86.3729	84.0937	87.5699	82.2069	90.8982	87.5541	79.3792	78.2109	79.4795	74.8669	90.5267	85.7022	86.7596	86.689	0.235141005	0.73629392	20	20	24	0.249007035	0.768040178	24	19.32903983	0.05490247
ENSMUSG00000025956	Mettl21a	methyltransferase like 21A [Source:MGI Symbol;Acc:MGI:1914349]	9.3391	9.47003	9.5498	8.89956	9.2283	9.19136	9.17613	9.30207	8.31588	8.76756	9.77027	8.87896	10.1235	9.73602	0.595952838	0.966018836	8	8	24	0.324533632	0.768040178	24	23.96434992	0.037806185
ENSMUSG00000025958	Creb1	cAMP responsive element binding protein 1 [Source:MGI Symbol;Acc:MGI:88494]	9.31485	8.80101	7.65677	8.96223	10.2195	7.5454	9.14808	7.2374	8.42303	6.88188	10.1659	7.51598	8.36663	7.74405	0.723119329	0.966018836	20	12	24	0.410927525	0.799546792	24	18.15946094	0.082178022
ENSMUSG00000025959	Klf7	Kruppel-like factor 7 (ubiquitous) [Source:MGI Symbol;Acc:MGI:1935151]	44.1864	34.4497	34.9312	38.0359	37.0534	41.8194	35.2732	37.9637	29.0539	48.3376	37.3498	40.8587	40.6958	40.8801	0.245061943	0.741580974	24	12	24	0.393819119	0.799546792	24	19.32885834	0.095323891
ENSMUSG00000025961	4933402D24Rik	RIKEN cDNA 4933402D24 gene [Source:MGI Symbol;Acc:MGI:1921676]	0.596277	0.910238	0.648837	0.915821	0.616442	0.736747	0.516071	0.580063	0.628988	0.639968	0.651369	0.694662	0.742847	0.737112	0.846643334	0.966018836	24	4	24	0.842084634	0.952050674	24	10.12431216	0.023359257
ENSMUSG00000025962	Fastkd2	FAST kinase domains 2 [Source:MGI Symbol;Acc:MGI:1922869]	12.6332	16.8085	15.2347	15.4829	15.4524	13.5672	15.8954	13.294	20.159	12.3556	18.1146	16.6269	15.983	15.2957	0.830949782	0.966018836	20	20	24	0.819501889	0.942798491	24	9.952703381	0.046859654
ENSMUSG00000025964	Adam23	a disintegrin and metallopeptidase domain 23 [Source:MGI Symbol;Acc:MGI:1345162]	61.4185	52.3917	51.6902	53.5065	51.831	57.8038	52.7032	53.5421	43.3807	65.6171	52.8491	52.4953	50.6196	56.668	0.879201926	0.966018836	16	20	24	0.71724198	0.906810602	24	19.05831783	0.046193016
ENSMUSG00000025967	Eef1b2	eukaryotic translation elongation factor 1 beta 2 [Source:MGI Symbol;Acc:MGI:1929520]	187.129	199.192	194.652	186.212	197.471	189.344	184.227	186.131	187.264	154.665	185.052	173.694	174.964	175.57	0.939103211	0.966018836	12	12	24	0.803731128	0.937296224	24	0.462753846	0.018738164
ENSMUSG00000025968	Ndufs1	NADH:ubiquinone oxidoreductase core subunit S1 [Source:MGI Symbol;Acc:MGI:2443241]	77.1482	83.263	83.6643	95.6971	81.5504	80.0402	79.3748	89.0547	84.8947	83.3093	87.2516	82.8115	87.5662	86.2565	0.274035549	0.788124063	8	20	24	0.123567958	0.736492734	24	9.872330693	0.058236349
ENSMUSG00000025969	Nrp2	neuropilin 2 [Source:MGI Symbol;Acc:MGI:1100492]	14.0079	11.4989	11.2975	11.8294	12.9344	12.1425	13.9924	12.4076	10.8268	14.4174	11.671	12.1625	12.4129	12.205	0.038363978	0.335328154	4	8	24	0.406640261	0.799546792	24	20.70572339	0.0621537
ENSMUSG00000025971	Maip1	matrix AAA peptidase interacting protein 1 [Source:MGI Symbol;Acc:MGI:1915365]	4.39934	4.87637	4.5758	4.65528	4.10721	4.13904	4.23635	5.086	4.86327	5.15856	4.85349	4.86328	4.89574	5.05003	0.173121348	0.648600527	8	12	24	0.027722045	0.590220615	24	7.396021342	0.074840532
ENSMUSG00000025978	Rftn2	raftlin family member 2 [Source:MGI Symbol;Acc:MGI:1921263]	4.76877	4.65486	4.61259	4.01686	3.79321	4.13231	5.80136	4.86711	4.59251	3.8109	3.61729	4.25538	5.13774	4.73405	8.17E-07	0.003551342	4	16	24	2.33E-04	0.177779102	24	2.346752321	0.182213794
ENSMUSG00000025979	Mob4	MOB family member 4, phocein [Source:MGI Symbol;Acc:MGI:104899]	53.1783	54.9971	48.0972	50.9263	50.4279	48.61	51.3009	45.3898	52.3053	42.9873	59.2723	51.4634	52.9457	52.2466	0.835401638	0.966018836	4	12	24	0.602560949	0.861114219	24	21.51087878	0.046485214
ENSMUSG00000025980	Hspd1	heat shock protein 1 (chaperonin) [Source:MGI Symbol;Acc:MGI:96242]	64.854	49.7162	52.2391	52.5694	57.8232	58.5562	61.4688	62.5661	46.1323	53.4491	61.6475	56.9066	61.8028	54.2366	0.002087679	0.100255953	4	8	24	0.016483714	0.544099157	24	21.21155306	0.142674057
ENSMUSG00000025981	Coq10b	coenzyme Q10B [Source:MGI Symbol;Acc:MGI:1915126]	15.4427	16.6117	18.0795	18.2191	16.3512	15.1355	14.8371	15.6114	18.6646	18.8487	18.1931	14.9076	15.6438	16.5921	1.51E-04	0.031913438	16	8	24	1.64E-05	0.047905911	24	10.63043772	0.153083301
ENSMUSG00000025982	Sf3b1	splicing factor 3b, subunit 1 [Source:MGI Symbol;Acc:MGI:1932339]	52.0412	58.0943	56.0513	58.1326	61.3297	50.9542	56.9024	48.4036	58.2209	48.6825	56.5546	52.5853	54.1852	52.7286	0.767183862	0.966018836	20	4	24	0.661456211	0.885525866	24	13.31653808	0.034853145
ENSMUSG00000025986	Slc39a10	solute carrier family 39 (zinc transporter), member 10 [Source:MGI Symbol;Acc:MGI:1914515]	19.192	17.6402	16.7038	15.7465	19.301	17.581	18.6026	15.7772	16.3236	15.4302	19.9858	17.3854	17.1774	15.6142	0.011964854	0.213452992	20	20	24	0.017066329	0.547196997	24	19.74458927	0.115053325
ENSMUSG00000025993	Slc40a1	solute carrier family 40 (iron-regulated transporter), member 1 [Source:MGI Symbol;Acc:MGI:1315204]	1.2892	3.2781	3.36194	3.12918	2.30271	2.55349	1.92348	3.38316	2.89009	2.17025	2.41425	2.43942	2.74148	2.10653	0.401437339	0.933249185	12	16	24	0.196146723	0.768040178	24	8.801501272	0.1858096
ENSMUSG00000025995	Wdr75	WD repeat domain 75 [Source:MGI Symbol;Acc:MGI:1920924]	15.0577	18.6618	17.4893	17.8813	16.459	16.7767	15.1335	18.1732	18.2277	14.8293	18.2141	16.3074	16.931	16.873	0.297978647	0.80820842	8	20	24	0.361349729	0.786774635	24	8.267920197	0.060208663
ENSMUSG00000025997	Ikzf2	IKAROS family zinc finger 2 [Source:MGI Symbol;Acc:MGI:1342541]	0.776314	0.986721	0.854639	0.927618	0.959049	0.811368	0.77465	0.850015	1.01927	0.744559	1.00035	0.917633	0.895417	0.951528	0.46238066	0.966018836	20	8	24	0.604192248	0.861793218	24	10.31817095	0.027787687
ENSMUSG00000026000	Lancl1	LanC (bacterial lantibiotic synthetase component C)-like 1 [Source:MGI Symbol;Acc:MGI:1336997]	68.6545	67.9895	63.6998	67.4442	66.3193	67.5058	61.4185	65.1061	60.541	68.0359	65.7434	63.594	64.0152	64.4542	0.904690479	0.966018836	16	20	24	0.489095944	0.805393068	24	16.93627644	0.023874668
ENSMUSG00000026003	Acadl	acyl-Coenzyme A dehydrogenase, long-chain [Source:MGI Symbol;Acc:MGI:87866]	64.8608	58.1608	69.4754	97.4543	63.6783	68.2959	66.0333	80.7507	60.7197	91.9294	78.3597	70.0162	85.075	82.4689	0.891022863	0.966018836	16	20	24	0.388628173	0.799546792	24	11.64933269	0.109802924
ENSMUSG00000026004	Kansl1l	KAT8 regulatory NSL complex subunit 1-like [Source:MGI Symbol;Acc:MGI:1915941]	6.66605	6.74895	5.80498	7.05453	6.72393	5.83437	7.14566	6.08333	7.53163	6.07987	6.74077	6.12893	6.27448	6.21818	0.852844413	0.966018836	4	20	24	0.904348996	0.973594617	24	10.27500301	0.018284024
ENSMUSG00000026005	Rpe	ribulose-5-phosphate-3-epimerase [Source:MGI Symbol;Acc:MGI:1913896]	18.6571	17.5809	16.2702	18.029	17.729	16.371	17.4898	16.586	15.2507	15.1556	19.8955	15.983	16.9205	17.0978	0.784278077	0.966018836	20	16	24	0.41395496	0.799546792	24	19.81405788	0.05389534
ENSMUSG00000026014	Raph1	Ras association (RalGDS/AF-6) and pleckstrin homology domains 1 [Source:MGI Symbol;Acc:MGI:1924550]	17.2882	14.4362	13.1617	14.652	15.5478	15.64	15.4682	14.0309	12.8415	15.478	15.8989	15.102	14.8069	14.9984	0.002363973	0.106802459	20	16	24	0.013621109	0.529134128	24	19.79647813	0.107319448
ENSMUSG00000026017	Carf	calcium response factor [Source:MGI Symbol;Acc:MGI:2182269]	3.52024	3.93346	3.53484	4.50057	3.39766	4.34098	3.86846	3.53565	4.69563	3.14007	4.43416	3.62836	4.2229	3.74908	0.74401906	0.966018836	12	20	24	0.985022405	0.996964449	24	14.4472407	0.009662211
ENSMUSG00000026018	Ica1l	islet cell autoantigen 1-like [Source:MGI Symbol;Acc:MGI:1917625]	7.97893	7.79973	7.87152	8.31562	7.14939	8.52281	7.62773	6.9755	7.51846	7.66722	7.47153	9.22074	7.7152	8.17664	0.893307667	0.966018836	24	20	24	0.537514504	0.827958985	24	19.9684482	0.042191514
ENSMUSG00000026019	Wdr12	WD repeat domain 12 [Source:MGI Symbol;Acc:MGI:1927241]	7.80348	7.80606	7.06529	7.65822	7.67541	7.45766	5.97504	7.53363	6.61765	6.57431	7.96521	7.03179	6.0569	5.9773	0.920620634	0.966018836	20	8	24	0.178488093	0.768040178	24	15.957759	0.073980735
ENSMUSG00000026020	Nop58	NOP58 ribonucleoprotein [Source:MGI Symbol;Acc:MGI:1933184]	32.857	35.0799	35.039	36.3446	34.245	32.5075	30.7945	32.4057	37.6342	26.088	38.535	36.6139	39.5957	34.4161	0.847846021	0.966018836	20	12	24	0.968302308	0.992326888	24	22.90361253	0.015365987
ENSMUSG00000026021	Sumo1	small ubiquitin-like modifier 1 [Source:MGI Symbol;Acc:MGI:1197010]	37.1604	43.135	40.2532	42.1372	42.0908	36.4059	36.2786	37.4445	44.7803	32.9181	46.0875	41.1307	43.998	43.2248	0.93447422	0.966018836	12	12	24	0.87367259	0.964653408	24	9.230356659	0.029396369
ENSMUSG00000026023	Cdk15	cyclin-dependent kinase 15 [Source:MGI Symbol;Acc:MGI:3583944]	24.1055	21.1208	18.1387	22.3877	19.7493	22.286	18.7621	19.1421	18.4685	25.1856	21.115	23.8901	25.4823	24.0555	0.44861185	0.966018836	4	8	24	0.524827648	0.822296127	24	20.22142175	0.07196084
ENSMUSG00000026024	Als2	alsin Rho guanine nucleotide exchange factor [Source:MGI Symbol;Acc:MGI:1921268]	10.6184	9.49338	8.95218	9.10919	10.4831	9.94016	10.0265	9.9129	9.18688	10.2218	9.11468	9.97413	9.50643	9.4645	0.073139747	0.447768907	24	12	24	0.11859239	0.729313257	24	20.77769363	0.056355957
ENSMUSG00000026027	Stradb	STE20-related kinase adaptor beta [Source:MGI Symbol;Acc:MGI:2144047]	9.80934	10.1356	11.9059	13.1572	10.5197	11.313	10.907	12.2545	13.2202	12.6589	11.8277	11.6827	12.5224	12.6623	0.140905066	0.585944911	12	16	24	0.007318645	0.47735824	24	9.718300174	0.108140199
ENSMUSG00000026028	Trak2	trafficking protein, kinesin binding 2 [Source:MGI Symbol;Acc:MGI:1918077]	29.7492	30.0457	30.989	30.7814	31.856	30.5554	32.9267	29.8445	29.4638	32.26	28.0212	29.3597	29.429	29.405	0.945728192	0.966018836	16	8	24	0.879333525	0.966161692	24	13.56553287	0.011516172
ENSMUSG00000026029	Casp8	caspase 8 [Source:MGI Symbol;Acc:MGI:1261423]	1.92043	2.87111	2.79624	2.74489	2.54684	2.31431	2.4467	2.67592	2.72152	2.18827	2.35257	2.45791	2.60945	2.494	0.108215091	0.524035616	12	12	24	0.169641458	0.767660404	24	7.698513132	0.081490564
ENSMUSG00000026031	Cflar	CASP8 and FADD-like apoptosis regulator [Source:MGI Symbol;Acc:MGI:1336166]	17.6843	15.49	14.1752	18.1596	17.0582	15.3569	16.6026	15.1367	14.3996	14.5189	17.1857	14.9521	16.2574	14.4673	0.10560184	0.518002936	20	16	24	0.123893902	0.737267876	24	18.70978511	0.08366372
ENSMUSG00000026032	Ndufb3	NADH:ubiquinone oxidoreductase subunit B3 [Source:MGI Symbol;Acc:MGI:1913745]	133.273	140.085	125.817	132.736	126.717	121.704	124.815	134.681	144.492	120.176	143.227	138.008	158.247	144.41	0.823657258	0.966018836	8	8	24	0.329341766	0.768040178	24	3.230321247	0.05641814
ENSMUSG00000026034	Clk1	CDC-like kinase 1 [Source:MGI Symbol;Acc:MGI:107403]	91.9745	102.527	101.052	118.675	113.128	94.326	119.954	91.1374	122.111	82.4907	122.087	87.251	83.104	90.0984	0.643501552	0.966018836	20	4	24	0.428789428	0.805393068	24	12.88208785	0.100932931

ENSMUSG00000026035	Ppil3	peptidylprolyl isomerase (cyclophilin)-like 3 [Source:MGI Symbol;Acc:MGI:1917475]	25.503	30.5519	31.6324	28.3537	31.1143	25.2871	26.6498	29.9295	29.9179	26.083	26.5767	25.7513	25.2731	28.4942	0.015345591	0.253245931	12	12	24	0.02946519	0.596648309	24	8.349630822	0.104881341
ENSMUSG00000026036	Nif3l1	Ngg1 interacting factor 3-like 1 (S. pombe) [Source:MGI Symbol;Acc:MGI:1929485]	16.2873	15.6049	15.1663	14.8804	16.2659	16.112	14.5731	14.7256	13.6558	14.342	16.9105	15.5915	16.7043	17.0205	0.435596482	0.966018836	20	20	24	0.170349626	0.767957732	24	20.85450765	0.066267414
ENSMUSG00000026037	Orc2	origin recognition complex, subunit 2 [Source:MGI Symbol;Acc:MGI:1328306]	15.3336	15.2094	14.5946	13.5337	14.5805	13.9817	13.1638	12.2424	15.7275	12.8217	16.8548	14.9733	14.8976	14.6677	0.865490576	0.966018836	20	20	24	0.842066542	0.952050674	24	18.26717152	0.028260534
ENSMUSG00000026039	Sgo2a	shugoshin 2A [Source:MGI Symbol;Acc:MGI:1098767]	1.65786	1.53505	1.49571	1.61587	1.65323	1.64093	1.41621	1.59306	1.49667	1.30005	1.67069	1.59533	1.69636	1.53305	0.110739523	0.528348425	20	16	24	0.208844735	0.768040178	24	20.43916891	0.044649996
ENSMUSG00000026042	Col5a2	collagen, type V, alpha 2 [Source:MGI Symbol;Acc:MGI:88458]	15.7568	19.3405	16.9644	16.7681	19.824	17.1738	20.0427	18.3316	19.3647	16.1745	17.0431	16.9568	17.6798	16.0465	0.825856869	0.966018836	12	4	24	0.921657169	0.979362996	24	2.122326534	0.017740755
ENSMUSG00000026043	Col3a1	collagen, type III, alpha 1 [Source:MGI Symbol;Acc:MGI:88453]	109.337	124.023	107.671	86.8601	112.912	97.8829	128.739	144.956	138.529	76.5093	110.224	93.1635	111.462	117.484	0.01518376	0.253245931	8	8	24	0.056335724	0.65534544	24	3.268949622	0.215590187
ENSMUSG00000026047	Poglut2	protein O-glucosyltransferase 2 [Source:MGI Symbol;Acc:MGI:1919300]	6.18472	6.75451	6.43105	5.57656	7.36185	6.24098	6.96774	6.43376	7.20322	5.48181	7.10173	5.8399	6.64699	6.17673	0.945282252	0.966018836	20	20	24	0.864568446	0.960819377	24	23.96726897	0.025076356
ENSMUSG00000026048	Ercc5	excision repair cross-complementing rodent repair deficiency, complementation group 5 [Source:MGI Symbol;Acc:MGI:103582]	9.34354	10.5093	9.50758	9.11591	9.74106	9.71075	10.1134	9.94324	10.0671	9.85958	9.60872	10.1033	10.1223	10.6642	0.089506974	0.477279066	8	8	24	0.047389692	0.633980733	24	3.108778037	0.042478081
ENSMUSG00000026049	Tex30	testis expressed 30 [Source:MGI Symbol;Acc:MGI:1922873]	6.28348	6.99904	7.02751	6.53556	6.78452	5.89488	7.34727	6.93691	6.43179	5.92823	7.68324	6.26068	6.4934	5.9184	0.888379597	0.966018836	20	4	24	0.996955613	0.999389144	24	11.95832005	0.003202005
ENSMUSG00000026051	Ecrg4	ECRG4 augurin precursor [Source:MGI Symbol;Acc:MGI:1926146]	17.2184	27.0644	19.892	15.7859	19.9984	12.7861	20.345	18.4899	37.3584	9.3501	18.2649	22.4879	14.8456	20.2003	0.503972924	0.966018836	4	4	24	0.406393218	0.799546792	24	5.048521709	0.23719278
ENSMUSG00000026058	Khdrbs2	KH domain containing, RNA binding, signal transduction associated 2 [Source:MGI Symbol;Acc:MGI:2159649]	3.11597	2.62536	3.36008	3.37344	1.9466	1.88035	3.11073	2.24255	2.52799	2.45314	2.95741	2.65775	2.75233	3.53515	0.835401638	0.966018836	4	20	24	0.410837561	0.799546792	24	5.946074105	0.110278834
ENSMUSG00000026062	Slc9a2	solute carrier family 9 (sodium/hydrogen exchanger), member 2 [Source:MGI Symbol;Acc:MGI:105075]	1.49042	2.15568	1.64421	1.87253	1.50079	1.86839	1.48427	2.33034	2.34128	1.40904	1.44487	1.76581	1.42613	1.66543	0.351840494	0.870418653	8	20	24	0.222023892	0.768040178	24	6.246980049	0.112717788
ENSMUSG00000026064	Ptp4a1	protein tyrosine phosphatase 4a1 [Source:MGI Symbol;Acc:MGI:1277096]	0.307804	0.81748	0.207019	1.74566	0.354563	0.303764	0.617042	0.777826	0.891648	0.45795	1.03983	1.67338	1.78498	1.31243	0.776152743	0.966018836	8	4	24	0.955565265	0.988639288	24	4.325791632	0.041948988
ENSMUSG00000026072	Il1r1	interleukin 1 receptor, type I [Source:MGI Symbol;Acc:MGI:96545]	3.10306	3.06854	3.3294	3.15159	2.86906	2.89789	2.59013	3.52119	3.31328	2.89238	2.89436	2.70716	2.93366	2.78495	0.114010995	0.532950079	12	12	24	0.049699355	0.642874369	24	7.691306314	0.078066616
ENSMUSG00000026073	Il1r2	interleukin 1 receptor, type II [Source:MGI Symbol;Acc:MGI:96546]	0.956298	0.54614	0.938579	0.581275	0.533722	0.90784	0.596138	0.453301	0.738789	0.625734	0.525378	1.21569	0.587859	0.633563	0.676344318	0.966018836	24	20	24	0.573007376	0.846884635	24	20.48607638	0.07694647
ENSMUSG00000026074	Map4k4	mitogen-activated protein kinase kinase kinase kinase 4 [Source:MGI Symbol;Acc:MGI:1349394]	50.0086	48.7113	45.9863	47.3354	54.7354	46.9108	55.7268	46.7455	48.4303	48.0495	47.0463	46.5769	49.4809	45.0737	0.628286596	0.966018836	4	20	24	0.396850302	0.799546792	24	20.34829591	0.046508216
ENSMUSG00000026077	Npas2	neuronal PAS domain protein 2 [Source:MGI Symbol;Acc:MGI:109232]	1.146	0.781436	0.708333	0.65062	0.55653	0.922034	1.25903	1.09461	0.751791	0.833717	0.786246	0.899528	1.0695	1.11177	0.002010614	0.100255953	4	16	24	8.89E-04	0.319044294	24	0.835144139	0.174370393
ENSMUSG00000026078	Pdcl3	phosducin-like 3 [Source:MGI Symbol;Acc:MGI:1916083]	51.5326	57.5077	57.8785	54.4477	55.2517	55.0444	53.2517	51.6258	58.5851	50.6959	52.6263	48.0695	54.1906	52.2288	0.46238066	0.966018836	12	12	24	0.265010553	0.768040178	24	8.054738166	0.045933034
ENSMUSG00000026080	Chst10	carbohydrate sulfotransferase 10 [Source:MGI Symbol;Acc:MGI:2138283]	4.71645	4.59281	6.08862	4.05605	4.17488	5.20331	5.46533	3.86298	4.34417	4.49799	3.91899	4.35138	3.69367	4.75067	0.915082018	0.966018836	4	16	24	0.790152211	0.932831789	24	3.310692321	0.042111868
ENSMUSG00000026082	Rev1	REV1, DNA directed polymerase [Source:MGI Symbol;Acc:MGI:1929074]	5.21491	6.90515	6.89623	6.99278	8.14356	6.34335	5.97483	4.89758	8.62057	7.59715	7.35318	6.86	6.3115	6.30961	0.007831348	0.192760771	20	8	24	0.013134258	0.529134128	24	12.73186659	0.191018137
ENSMUSG00000026083	Eif5b	eukaryotic translation initiation factor 5B [Source:MGI Symbol;Acc:MGI:2441772]	18.6662	19.3596	21.436	19.9136	18.8743	19.6995	16.2002	18.9442	18.4107	20.926	20.3889	21.9771	22.134	21.1521	0.801273005	0.966018836	24	4	24	0.712332364	0.905113511	24	12.3378146	0.034223498
ENSMUSG00000026087	Mrpl30	mitochondrial ribosomal protein L30 [Source:MGI Symbol;Acc:MGI:1333820]	71.9815	81.3039	78.3334	83.8595	80.3698	75.6909	67.312	74.3148	88.2818	62.7677	79.8182	67.015	82.2656	76.8728	0.593560247	0.966018836	12	12	24	0.566754533	0.843547308	24	8.601324869	0.05993829
ENSMUSG00000026088	Mitd1	MIT, microtubule interacting and transport, domain containing 1 [Source:MGI Symbol;Acc:MGI:1916278]	12.1596	13.0338	10.7896	13.1831	13.7505	10.7162	9.30889	10.7345	12.6312	9.02743	13.1604	11.1371	11.4239	11.9261	0.718086778	0.966018836	20	4	24	0.787254789	0.931463668	24	13.72976779	0.045962186
ENSMUSG00000026090	Cracdl	capping protein inhibiting regulator of actin like [Source:MGI Symbol;Acc:MGI:1919347]	1.06363	1.07767	1.18897	1.0332	0.829214	1.14689	1.15688	1.019	1.13629	1.15657	0.686779	1.35777	0.806345	1.39101	0.966749204	0.969592092	12	8	24	0.409158358	0.799546792	24	4.634919214	0.074224712
ENSMUSG00000026094	Stk17b	serine/threonine kinase 17b (apoptosis-inducing) [Source:MGI Symbol;Acc:MGI:2138162]	3.32253	4.3933	4.90082	4.76996	4.24157	3.72901	3.96475	4.08392	5.19806	3.4739	4.58206	4.08078	3.94657	3.68356	0.017495149	0.255326033	12	16	24	0.129847045	0.740383911	24	10.27609577	0.11672833
ENSMUSG00000026095	Asnsd1	asparagine synthetase domain containing 1 [Source:MGI Symbol;Acc:MGI:1917646]	26.9694	29.7539	28.2314	29.1905	30.0853	26.8478	27.5958	29.354	31.1151	26.0467	30.3637	29.0614	31.8121	28.408	0.891742979	0.966018836	20	4	24	0.896872993	0.970634157	24	7.392418025	0.015414274
ENSMUSG00000026096	Osgepl1	O-sialoglycoprotein endopeptidase-like 1 [Source:MGI Symbol;Acc:MGI:1919335]	8.98716	11.5046	10.8646	11.3864	9.87868	9.57204	9.711	11.1669	13.1582	9.71713	11.7613	11.0181	10.4296	11.6052	0.124211791	0.552144108	8	20	24	0.05637825	0.65534544	24	8.430967489	0.116532151
ENSMUSG00000026097	Ormdl1	ORM1-like 1 (S. cerevisiae) [Source:MGI Symbol;Acc:MGI:2181669]	10.4426	10.5731	10.5929	9.31909	10.6467	9.37438	9.94877	10.3475	10.1213	8.28253	11.4968	9.2283	10.1558	9.12773	0.908797704	0.966018836	20	4	24	0.851359518	0.955425185	24	23.27531421	0.024238446
ENSMUSG00000026098	Pms1	PMS1 homolog 1, mismatch repair system component [Source:MGI Symbol;Acc:MGI:1202302]	2.21731	2.51127	2.41372	2.93247	2.66123	2.23612	2.34057	2.71708	2.57736	2.18895	2.64285	2.56963	2.46094	2.60909	0.481811994	0.966018836	20	8	24	0.457937399	0.805393068	24	10.54023006	0.043426681
ENSMUSG00000026100	Mstn	myostatin [Source:MGI Symbol;Acc:MGI:95691]	0.373729	0.911679	0.601284	1.58113	0.37532	0.907389	0.856698	1.51188	1.68468	1.45353	0.860607	0.869648	0.775243	0.725744	0.446967971	0.966018836	16	12	24	0.129525149	0.740162193	24	9.310334836	0.237656864
ENSMUSG00000026102	Inpp1	inositol polyphosphate-1-phosphatase [Source:MGI Symbol;Acc:MGI:104848]	14.3898	14.5964	12.5382	14.5524	16.218	15.1585	15.865	14.7152	15.308	15.1187	15.1392	15.2364	13.9	16.0087	0.934110497	0.966018836	24	4	24	0.528684389	0.824046748	24	18.48049305	0.038179181
ENSMUSG00000026103	Gls	glutaminase [Source:MGI Symbol;Acc:MGI:95752]	103.209	99.0959	94.7644	96.5717	105.347	95.2429	102.284	91.0013	92.0843	92.6372	102.284	94.9812	98.2055	95.6555	0.1982019	0.680119125	4	8	24	0.086406215	0.705702571	24	19.75284377	0.052705753
ENSMUSG00000026104	Stat1	signal transducer and activator of transcription 1 [Source:MGI Symbol;Acc:MGI:103063]	26.9297	22.8162	23.6307	21.4186	26.4049	24.9059	24.6935	27.2105	23.0035	24.4815	25.7028	23.9289	23.7517	28.4012	0.7207001	0.966018836	8	4	24	0.478855512	0.805393068	24	23.56040246	0.050533975
ENSMUSG00000026107	Nabp1	nucleic acid binding protein 1 [Source:MGI Symbol;Acc:MGI:1923258]	5.32988	5.16698	5.56633	8.42513	6.50221	7.01467	7.07601	6.47267	6.02171	5.03372	10.431	6.73163	6.71237	5.82054	0.186921604	0.662239322	20	16	24	0.130746405	0.740728512	24	16.82728215	0.18352728
ENSMUSG00000026109	Tmeff2	transmembrane protein with EGF-like and two follistatin-like domains 2 [Source:MGI Symbol;Acc:MGI:1861735]	31.5301	25.228	26.323	26.1259	24.0771	27.6944	26.5547	25.0765	21.2954	29.1227	24.5411	28.4394	27.074	28.3391	0.313239838	0.824470524	24	20	24	0.296681875	0.768040178	24	22.06251273	0.080153805
ENSMUSG00000026110	Mgat4a	mannoside acetylglucosaminyltransferase 4, isoenzyme A [Source:MGI Symbol;Acc:MGI:2662992]	11.7815	10.3756	10.7535	11.4042	11.493	12.1788	10.1269	9.91659	9.62318	11.2031	11.0445	11.3373	10.689	11.1529	0.061697418	0.409814092	24	8	24	0.033196087	0.606585237	24	17.90429178	0.083350144
ENSMUSG00000026111	Unc50	unc-50 homolog [Source:MGI Symbol;Acc:MGI:1914637]	40.1675	37.2288	35.7699	37.9863	36.8235	35.2458	34.8461	39.4234	40.0874	34.8839	39.383	40.021	43.6699	39.3267	0.943890799	0.966018836	4	12	24	0.494911276	0.806761911	24	1.54885836	0.039316178
ENSMUSG00000026112	Coa5	cytochrome C oxidase assembly factor 5 [Source:MGI Symbol;Acc:MGI:1923428]	33.7125	30.1714	31.5021	33.1732	30.8889	33.1777	31.7949	33.9844	28.5196	35.5235	32.1278	31.9867	33.507	32.9843	0.953350544	0.966018836	4	8	24	0.808906151	0.939184036	24	19.83344996	0.020931267
ENSMUSG00000026113	Inpp4a	inositol polyphosphate-4-phosphatase, type I [Source:MGI Symbol;Acc:MGI:1931123]	24.1431	20.9817	21.259	22.7991	22.585	23.0245	22.6873	20.9723	19.3356	24.0743	21.9878	23.4585	23.2642	22.8307	0.114881734	0.535150745	24	12	24	0.090019486	0.7097999	24	19.481834	0.070929258
ENSMUSG00000026116	Tmem131	transmembrane protein 131 [Source:MGI Symbol;Acc:MGI:1927110]	11.3537	10.4306	10.1281	10.654	9.97161	11.3676	10.869	11.283	9.58897	12.6818	9.32268	10.6401	10.4832	11.2923	0.608967886	0.966018836	24	20	24	0.796767677	0.935045591	24	1.5697615	0.028198251
ENSMUSG00000026121	Sema4c	sema domain, immunoglobulin domain (Ig), transmembrane domain (TM) and short cytoplasmic domain, (semaphorin) 4C [Source:MGI Symbol;Acc:MGI:109252]	32.2714	37.2158	33.2361	38.721	45.3813	30.9514	37.8296	36.1251	46.7919	38.7088	38.9219	34.9274	40.8128	30.8649	0.4776989	0.966018836	20	4	24	0.320794613	0.768040178	24	12.31328364	0.104251249
ENSMUSG00000026123	Plekhb2	pleckstrin homology domain containing, family B (evectins) member 2 [Source:MGI Symbol;Acc:MGI:2385825]	75.7208	61.3721	69.3421	62.5194	59.2989	72.3058	67.7758	67.2093	54.2287	74.3418	60.5217	67.3019	63.5415	69.3067	0.839634479	0.966018836	24	20	24	0.504803641	0.8111308	24	22.9149877	0.06242596
ENSMUSG00000026126	Ptpn18	protein tyrosine phosphatase, non-receptor type 18 [Source:MGI Symbol;Acc:MGI:108410]	1.19606	1.4706	1.66006	1.48852	1.1667	1.41741	0.908463	1.0173	2.4427	0.9799	2.37039	2.26091	2.92326	1.60602	0.887862353	0.966018836	12	16	24	0.986441841	0.997339197	24	14.9167249	0.0191546
ENSMUSG00000026127	Imp4	IMP4, U3 small nucleolar ribonucleoprotein [Source:MGI Symbol;Acc:MGI:106572]	8.31548	7.20654	8.12369	6.21554	7.81937	7.44768	6.60159	7.63999	6.19164	6.03595	7.24985	6.32519	6.259	5.80017	0.941830768	0.966018836	20	20	24	0.442840908	0.805393068	24	20.8295441	0.056736557
ENSMUSG00000026131	Dst	dystonin [Source:MGI Symbol;Acc:MGI:104627]	196.554	140.202	144.101	150.115	137.047	159.144	159.652	170.668	119.369	197.543	149.108	156.486	155.112	156.405	0.421132906	0.966018836	4	8	24	0.595895111	0.857839743	24	21.88706131	0.079307246
ENSMUSG00000026134	Prim2	DNA primase, p58 subunit [Source:MGI Symbol;Acc:MGI:97758]	2.53597	3.48134	3.35449	2.69603	3.12325	2.71815	3.25701	2.94391	3.2603	2.55265	3.28638	3.70764	3.64333	3.33587	0.952127548	0.966018836	8	8	24	0.557821655	0.839489739	24	2.134478761	0.052869658
ENSMUSG00000026135	Zfp142	zinc finger protein 142 [Source:MGI Symbol;Acc:MGI:1924514]	3.62661	3.26797	3.87191	3.83636	3.51754	3.79895	3.84406	3.62186	3.47875	4.2947	3.76219	3.53007	3.42104	3.40449	0.148627651	0.59172107	16	16	24	0.162170384	0.76636332	24	13.7083081	0.05629544
ENSMUSG00000026141	Col19a1	collagen, type XIX, alpha 1 [Source:MGI Symbol;Acc:MGI:1095415]	0.666862	0.859438	0.923877	0.608856	0.777252	0.708936	0.681265	0.566856	1.01459	0.633015	0.634297	0.930783	0.847586	0.793977	0.503972924	0.966018836	12	4	24	0.718573373	0.907466097	24	5.314023185	0.037221107
ENSMUSG00000026142	Rhbdd1	rhomboid domain containing 1 [Source:MGI Symbol;Acc:MGI:1924117]	6.16516	5.49441	6.37301	5.77528	5.91076	6.06851	5.93009	6.06371	5.27512	6.2613	5.78807	6.39986	5.67513	5.72247	0.772122688	0.966018836	24	8	24	0.473636343	0.805393068	24	18.49328006	0.034107984
ENSMUSG00000026150	Mff	mitochondrial fission factor [Source:MGI Symbol;Acc:MGI:1922984]	138.075	144.138	141.309	144.271	137.316	131.945	135.164	134.525	151.412	132.055	146.456	145.25	146.404	146.564	0.891742979	0.966018836	12	12	24	0.555631456	0.838702296	24	7.512856105	0.026890089
ENSMUSG00000026153	Fam135a	family with sequence similarity 135, member A [Source:MGI Symbol;Acc:MGI:1915437]	10.8782	13.455	13.1377	12.8062	11.7255	10.1835	10.0353	11.4868	11.3189	10.4824	11.8879	9.86054	9.4202	11.5783	0.041131612	0.346472636	12	16	24	0.020763084	0.568066329	24	9.236554807	0.123435266
ENSMUSG00000026154	Sdhaf4	succinate dehydrogenase complex assembly factor 4 [Source:MGI Symbol;Acc:MGI:1915252]	46.005	48.5587	46.7429	48.6586	48.3982	42.6039	43.7068	47.8309	52.3785	38.1589	51.838	45.5493	48.9669	50.1305	0.601647512	0.966018836	12	12	24	0.679181259	0.892648327	24	6.306002838	0.042671911
ENSMUSG00000026155	Smap1	small ArfGAP 1 [Source:MGI Symbol;Acc:MGI:2138261]	36.9203	31.5079	31.2485	34.4444	30.932	35.0727	33.0389	35.2632	27.718	43.7108	30.2148	36.7352	34.4642	35.9435	0.46694635	0.966018836	24	12	24	0.869130004	0.96291905	24	20.68944081	0.034194413
ENSMUSG00000026156	B3gat2	beta-1,3-glucuronyltransferase 2 (glucuronosyltransferase S) [Source:MGI Symbol;Acc:MGI:2389490]	1.74525	2.05881	2.64924	1.93561	1.83163	1.7267	2.10149	1.70685	2.15202	1.82046	1.71524	1.87651	1.54041	1.89516	0.205280539	0.693330038	12	12	24	0.116429623	0.727799063	24	8.222520162	0.094682933
ENSMUSG00000026158	Ogfrl1	opioid growth factor receptor-like 1 [Source:MGI Symbol;Acc:MGI:1917405]	44.7682	37.968	36.2954	39.1792	37.6745	40.8264	38.4495	40.5142	37.6057	45.374	42.902	39.9522	45.8117	41.0727	0.286642595	0.793714531	4	8	24	0.533104346	0.825678486	24	20.80053081	0.044920876
ENSMUSG00000026159	Agfg1	ArfGAP with FG repeats 1 [Source:MGI Symbol;Acc:MGI:1333754]	43.7019	36.3756	34.2761	35.457	39.9554	41.1855	38.6819	35.7914	32.0481	34.8999	40.7042	37.147	37.1252	36.9593	7.55E-04	0.071344007	24	12	24	0.001125885	0.336500758	24	20.3491809	0.135034059
ENSMUSG00000026162	Nhej1	non-homologous end joining factor 1 [Source:MGI Symbol;Acc:MGI:1922820]	3.56581	3.1606	2.7583	3.27144	3.47498	3.31835	3.64146	3.27587	2.99191	3.05442	3.15076	3.18779	3.2402	3.03999	0.004140921	0.140598674	4	8	24	0.014168683	0.530991098	24	20.99524591	0.080550637
ENSMUSG00000026163	Sphkap	SPHK1 interactor, AKAP domain containing [Source:MGI Symbol;Acc:MGI:1924879]	8.00681	10.8795	10.3681	10.9759	8.89616	8.62194	9.71975	9.36488	9.90295	9.21824	7.86777	10.3324	8.84624	10.5473	0.104994359	0.518002936	12	8	24	0.125930841	0.739868076	24	7.205985905	0.111155955
ENSMUSG00000026170	Cyp27a1	cytochrome P450, family 27, subfamily a, polypeptide 1 [Source:MGI Symbol;Acc:MGI:88594]	6.39225	10.8598	11.6901	12.1584	11.726	9.48861	10.8737	11.1578	17.1212	10.0612	11.0367	10.2301	8.91172	9.04548	0.017495149	0.255326033	12	16	24	0.053711306	0.65435526	24	10.40311948	0.250764327
ENSMUSG00000026171	Rnf25	ring finger protein 25 [Source:MGI Symbol;Acc:MGI:1890215]	21.7249	20.8634	21.4521	19.4522	21.2744	21.1109	19.9442	18.3516	18.9127	21.3028	20.8242	20.63	18.1923	20.0099	0.891105762	0.966018836	20	12	24	0.220063104	0.768040178	24	16.79245564	0.048455337
ENSMUSG00000026172	Bcs1l	BCS1-like (yeast) [Source:MGI Symbol;Acc:MGI:1914071]	6.88936	5.23673	6.33358	6.43851	7.01036	7.82554	7.07595	6.97828	5.8882	7.30079	5.7317	6.03635	6.03549	7.14871	0.849180783	0.966018836	24	12	24	0.771737798	0.926421112	24	19.39159105	0.042201186
ENSMUSG00000026173	Plcd4	phospholipase C, delta 4 [Source:MGI Symbol;Acc:MGI:107469]	17.7933	17.5788	16.5752	17.2734	16.7106	18.4807	18.5124	17.6686	17.0733	19.0373	15.2719	16.7759	18.848	18.0886	0.048396595	0.379129806	4	16	24	0.282630892	0.768040178	24	1.877149053	0.049078293
ENSMUSG00000026174	Cnot9	CCR4-NOT transcription complex, subunit 9 [Source:MGI Symbol;Acc:MGI:1928902]	8.22729	7.10736	7.43875	7.69015	7.50622	8.1755	8.09209	8.35364	7.32142	8.27286	8.37028	8.23551	7.73075	7.7231	0.277221318	0.789849983	24	12	24	0.184264363	0.768040178	24	19.43982429	0.048054337
ENSMUSG00000026176	Ctdsp1	CTD (carboxy-terminal domain, RNA polymerase II, polypeptide A) small phosphatase 1 [Source:MGI Symbol;Acc:MGI:2654470]	11.487	12.3465	11.5387	12.4816	12.9137	10.9417	12.5238	11.6738	14.8441	14.1648	11.5811	11.71	12.4088	10.3968	0.718720526	0.966018836	16	16	24	0.22869428	0.768040178	24	11.06311155	0.08392197
ENSMUSG00000026177	Slc11a1	solute carrier family 11 (proton-coupled divalent metal ion transporters), member 1 [Source:MGI Symbol;Acc:MGI:1345275]	1.88937	2.38864	2.1593	1.92129	2.45063	1.55432	2.29492	1.4638	2.75077	1.61448	1.853	2.53644	2.1882	1.52584	0.856752753	0.966018836	12	20	24	0.975835289	0.993924858	24	17.39102308	0.018005027
ENSMUSG00000026179	Pnkd	paroxysmal nonkinesiogenic dyskinesia [Source:MGI Symbol;Acc:MGI:1930773]	32.731	32.5879	38.1557	33.5785	32.0477	38.4715	35.5725	33.3063	30.5243	39.8202	26.9545	34.0139	30.3406	34.8217	0.903207257	0.966018836	16	4	24	0.926102152	0.98027178	24	7.417738157	0.023703299
ENSMUSG00000026180	Cxcr2	chemokine (C-X-C motif) receptor 2 [Source:MGI Symbol;Acc:MGI:105303]	0.770273	1.12524	1.70333	1.3309	0.89983	1.23949	1.0574	0.805791	0.977671	0.634564	1.02444	0.897055	1.10234	0.669878	0.842838536	0.966018836	12	20	24	0.897542296	0.97082564	24	12.42536268	0.033179144
ENSMUSG00000026181	Ppm1f	protein phosphatase 1F (PP2C domain containing) [Source:MGI Symbol;Acc:MGI:1918464]	19.5215	16.7648	17.8226	16.6968	16.7601	18.727	19.1807	17.8936	15.0142	18.0547	16.2238	17.3918	17.6009	16.8722	0.085329704	0.466404237	4	16	24	0.062633535	0.661961741	24	22.45173978	0.079311148
ENSMUSG00000026185	Igfbp5	insulin-like growth factor binding protein 5 [Source:MGI Symbol;Acc:MGI:96440]	60.8343	59.4466	54.5648	40.3106	52.2347	50.0775	55.6472	64.3105	74.3846	34.892	46.2528	53.0755	47.1524	58.1112	0.003383846	0.140598674	8	8	24	0.056309517	0.65534544	24	3.034522655	0.23028257
ENSMUSG00000026187	Xrcc5	X-ray repair complementing defective repair in Chinese hamster cells 5 [Source:MGI Symbol;Acc:MGI:104517]	15.3145	16.6565	15.5845	15.198	16.4551	15.6274	14.3202	14.8693	16.3814	14.7598	16.3429	16.534	16.8087	16.3296	0.903207257	0.966018836	20	20	24	0.963237339	0.990872232	24	19.42826865	0.007797724
ENSMUSG00000026188	Tmem169	transmembrane protein 169 [Source:MGI Symbol;Acc:MGI:2442781]	8.37582	6.9854	7.17498	6.72602	6.00616	7.23443	6.69929	6.59802	6.19894	7.61801	6.60268	7.99631	7.13642	7.9663	0.676344318	0.966018836	24	20	24	0.407120556	0.799546792	24	0.003130033	0.064394909
ENSMUSG00000026189	Pecr	peroxisomal trans-2-enoyl-CoA reductase [Source:MGI Symbol;Acc:MGI:2148199]	4.46635	4.81762	5.47432	6.167	5.53233	5.17788	4.85702	5.58214	5.64237	5.35699	4.5087	5.43103	5.43282	5.81947	0.282728968	0.793714531	12	16	24	0.152899748	0.75924908	24	9.516408265	0.082641757
ENSMUSG00000026192	Atic	5-aminoimidazole-4-carboxamide ribonucleotide formyltransferase/IMP cyclohydrolase [Source:MGI Symbol;Acc:MGI:1351352]	19.2092	16.1235	16.0855	16.0536	18.2269	18.7763	17.1783	18.0904	14.0335	18.8471	16.9174	16.9458	16.4408	16.5498	0.185849533	0.662239322	24	12	24	0.120822652	0.732894732	24	19.75757968	0.090083177
ENSMUSG00000026193	Fn1	fibronectin 1 [Source:MGI Symbol;Acc:MGI:95566]	28.63	36.1039	26.8822	28.3353	37.1063	28.8928	28.1168	39.8449	35.9384	23.6586	27.1859	30.1158	25.7494	27.5556	0.912686312	0.966018836	8	8	24	0.76293394	0.923543354	24	4.384496965	0.062025116
ENSMUSG00000026197	Zfand2b	zinc finger, AN1 type domain 2B [Source:MGI Symbol;Acc:MGI:1916068]	18.4892	17.9505	18.2572	18.8273	19.7013	19.1491	17.4119	17.5511	17.6328	17.8818	18.5148	19.873	18.255	16.9503	0.001920665	0.100255953	24	8	24	0.001404429	0.354367925	24	17.46999718	0.0714614
ENSMUSG00000026198	Abcb6	ATP-binding cassette, sub-family B (MDR/TAP), member 6 [Source:MGI Symbol;Acc:MGI:1921354]	6.3651	7.6607	5.9736	6.26974	7.4187	7.43053	6.31537	6.67828	6.22852	9.49139	7.12914	6.42473	6.30019	6.7139	0.633249925	0.966018836	20	16	24	0.411673802	0.799546792	24	14.21707974	0.079729798
ENSMUSG00000026199	Ankzf1	ankyrin repeat and zinc finger domain containing 1 [Source:MGI Symbol;Acc:MGI:1098746]	8.58822	11.5203	11.3782	9.48627	10.7962	9.92425	10.7868	9.60978	11.2542	9.95436	9.71345	9.82623	9.84622	10.3222	0.560714538	0.966018836	12	12	24	0.319905176	0.768040178	24	7.852080462	0.065766909
ENSMUSG00000026200	Glb1l	galactosidase, beta 1-like [Source:MGI Symbol;Acc:MGI:1921827]	7.31318	9.11361	10.1164	6.99955	8.50166	7.20497	7.76658	9.83734	9.98684	8.22615	8.69775	7.79191	8.92689	7.66776	0.080164531	0.458678242	12	12	24	0.12367505	0.736492734	24	7.282386514	0.126075757
ENSMUSG00000026201	Stk16	serine/threonine kinase 16 [Source:MGI Symbol;Acc:MGI:1313271]	37.8388	34.5265	33.615	31.3763	37.7025	39.6323	37.8764	35.7346	34.5726	36.3905	38.6114	38.0627	39.195	37.6805	7.90E-04	0.071344007	24	12	24	0.001314411	0.346832852	24	21.06204502	0.098251249
ENSMUSG00000026202	Tuba4a	tubulin, alpha 4A [Source:MGI Symbol;Acc:MGI:1095410]	156.453	151.392	156.129	156.317	151.232	176.769	152.338	169.634	126.018	167.613	131.641	145.138	142.794	148.026	0.842298032	0.966018836	16	4	24	0.854733601	0.956691175	24	21.77016537	0.028701158
ENSMUSG00000026203	Dnajb2	DnaJ heat shock protein family (Hsp40) member B2 [Source:MGI Symbol;Acc:MGI:1928739]	48.7464	38.1108	40.2479	38.7065	43.5232	42.5024	46.2882	44.3498	36.9561	44.0424	45.8646	44.2167	44.1026	43.6648	0.017495149	0.255326033	4	8	24	0.051046839	0.645887282	24	21.06297726	0.09928502
ENSMUSG00000026204	Ptprn	protein tyrosine phosphatase, receptor type, N [Source:MGI Symbol;Acc:MGI:102765]	47.0334	35.1616	38.0407	42.3478	38.6126	41.9502	39.9513	38.5036	32.6849	49.4504	35.8036	40.0251	36.9378	40.6909	0.649212555	0.966018836	16	20	24	0.666666065	0.887459929	24	17.98935916	0.056874934
ENSMUSG00000026207	Speg	SPEG complex locus [Source:MGI Symbol;Acc:MGI:109282]	9.77773	10.4244	9.21557	9.70171	9.1575	7.53714	10.4372	11.8746	11.0552	11.6095	10.6202	10.0078	11.076	10.692	0.581865545	0.966018836	8	16	24	0.089423219	0.708706636	24	6.500359031	0.113334842
ENSMUSG00000026208	Des	desmin [Source:MGI Symbol;Acc:MGI:94885]	6.20404	15.0365	17.8497	24.3439	14.958	19.8291	14.0885	41.8684	24.5291	40.6396	14.1807	17.0629	23.1439	17.4477	0.192997592	0.670595308	16	4	24	0.101202295	0.713208839	24	9.550759332	0.483594459
ENSMUSG00000026209	Dnpep	aspartyl aminopeptidase [Source:MGI Symbol;Acc:MGI:1278328]	37.0379	29.8755	30.167	31.7638	32.525	32.5064	27.6696	32.4291	32.4194	33.2285	33.9935	34.5943	32.1752	32.6261	0.4776989	0.966018836	20	16	24	0.513382111	0.816307374	24	17.67409264	0.0447843
ENSMUSG00000026211	Obsl1	obscurin-like 1 [Source:MGI Symbol;Acc:MGI:2138628]	8.78048	6.10511	5.90767	7.84909	8.0101	6.83671	6.05276	7.73033	7.6508	8.85492	8.63976	5.87334	7.61489	6.44519	0.417963425	0.963884303	20	4	24	0.341488605	0.775806931	24	14.15080411	0.107926959
ENSMUSG00000026213	Stk11ip	serine/threonine kinase 11 interacting protein [Source:MGI Symbol;Acc:MGI:1918978]	11.4423	10.2409	10.7063	11.4162	12.6471	11.4734	10.7554	11.2508	10.3369	11.3009	10.2985	11.0653	11.6613	10.5539	0.215256468	0.704989686	4	4	24	0.192898541	0.768040178	24	18.10458173	0.054556676
ENSMUSG00000026219	Trip12	thyroid hormone receptor interactor 12 [Source:MGI Symbol;Acc:MGI:1309481]	53.9204	47.677	49.1282	50.057	50.2173	48.2131	44.8809	47.5827	47.3871	42.0056	48.699	48.7767	51.6224	48.6074	0.888991156	0.966018836	4	4	24	0.422354581	0.804917634	24	22.11204317	0.042818811
ENSMUSG00000026220	Slc16a14	solute carrier family 16 (monocarboxylic acid transporters), member 14 [Source:MGI Symbol;Acc:MGI:1919031]	1.64172	0.648673	0.904396	0.480213	0.852247	0.705876	0.604039	0.907605	0.49858	1.33718	0.729591	0.959066	0.62977	1.02191	0.782779623	0.966018836	8	4	24	0.882032739	0.966661503	24	22.45081912	0.048914146
ENSMUSG00000026222	Sp100	nuclear antigen Sp100 [Source:MGI Symbol;Acc:MGI:109561]	5.51978	8.03736	8.67735	6.35544	7.48048	6.73109	7.56731	8.70205	8.3799	6.25977	7.66243	7.50293	6.83695	7.48679	0.162672639	0.624917386	12	4	24	0.287708347	0.768040178	24	6.843865589	0.104603618
ENSMUSG00000026223	Itm2c	integral membrane protein 2C [Source:MGI Symbol;Acc:MGI:1927594]	117.239	119.034	119.77	105.599	104.713	114.542	114.134	118.941	118.278	104.376	109.191	120.086	111.627	120.703	0.009136573	0.192865964	8	8	24	0.009973422	0.503967859	24	2.866323725	0.077376706
ENSMUSG00000026229	Psmd1	proteasome (prosome, macropain) 26S subunit, non-ATPase, 1 [Source:MGI Symbol;Acc:MGI:1917497]	79.2979	71.4689	73.064	73.077	71.3201	76.7669	70.7754	71.579	62.5733	77.6146	71.5004	74.6787	75.3708	75.4069	0.95261238	0.966018836	24	12	24	0.321268445	0.768040178	24	20.72535887	0.049391441
ENSMUSG00000026234	Ncl	nucleolin [Source:MGI Symbol;Acc:MGI:97286]	68.4606	74.1929	77.4332	73.9891	71.4174	68.7712	66.0133	66.6455	71.739	68.1299	67.6671	70.4781	69.714	73.5588	0.277087842	0.789849983	12	16	24	0.162785846	0.766365035	24	8.757116289	0.047622733
ENSMUSG00000026235	Epha4	Eph receptor A4 [Source:MGI Symbol;Acc:MGI:98277]	1.92953	2.0933	2.22044	1.83474	1.59854	2.12329	1.92622	2.16701	2.24828	1.98952	1.90783	1.76795	1.52591	1.9539	0.019711532	0.258568924	12	8	24	0.114528401	0.727799063	24	7.023319411	0.083481001
ENSMUSG00000026237	Nmur1	neuromedin U receptor 1 [Source:MGI Symbol;Acc:MGI:1341898]	1.3946	0.99596	1.42016	1.25838	0.536115	0.837006	0.779848	0.809808	0.705978	1.22153	0.951684	0.917015	1.26115	1.21612	0.904831057	0.966018836	16	4	24	0.613832857	0.866162311	24	5.911449839	0.080622924
ENSMUSG00000026238	Ptma	prothymosin alpha [Source:MGI Symbol;Acc:MGI:97803]	69.7281	63.4423	63.5749	61.5942	58.2142	56.1747	61.9462	64.1291	59.5226	69.097	78.4147	56.6625	54.486	60.5127	0.83638114	0.966018836	16	8	24	0.610607656	0.864861374	24	11.74241149	0.05425322
ENSMUSG00000026239	Pde6d	phosphodiesterase 6D, cGMP-specific, rod, delta [Source:MGI Symbol;Acc:MGI:1270843]	44.3331	40.223	38.6047	40.3042	40.2634	44.6496	41.9679	43.7316	38.7376	44.4033	41.5927	44.8946	45.0752	40.0102	0.002057111	0.100255953	24	12	24	0.036797332	0.609176161	24	20.97244483	0.0734748
ENSMUSG00000026240	Cops7b	COP9 signalosome subunit 7B [Source:MGI Symbol;Acc:MGI:1349388]	10.36	9.97114	10.3719	10.5991	9.85138	11.0155	9.70778	9.91012	9.06306	10.6183	9.26881	8.57108	9.49856	8.94367	0.823677655	0.966018836	16	12	24	0.679790703	0.892805383	24	14.10225657	0.024875749
ENSMUSG00000026241	Nppc	natriuretic peptide type C [Source:MGI Symbol;Acc:MGI:97369]	0.632678	0.872886	1.37616	0.674295	0.643134	0.795812	1.04783	0.931087	1.48188	0.477689	0.746168	0.806396	0.33099	0.943969	0.058209189	0.40386543	12	4	24	0.209152034	0.768040178	24	6.83708468	0.165803271
ENSMUSG00000026245	Farsb	phenylalanyl-tRNA synthetase, beta subunit [Source:MGI Symbol;Acc:MGI:1346035]	52.7178	54.2797	52.1591	52.3286	55.7237	49.9773	50.0091	53.8849	55.0014	48.2342	56.249	52.8024	57.2673	51.5471	0.885059765	0.966018836	20	20	24	0.9784383	0.994576186	24	23.1016761	0.005947951
ENSMUSG00000026247	Ecel1	endothelin converting enzyme-like 1 [Source:MGI Symbol;Acc:MGI:1343461]	0.937952	0.851399	3.06675	0.793627	1.35413	1.99431	1.79651	1.05406	0.882409	0.660737	3.37493	0.832955	0.457812	1.42339	0.880247609	0.966018836	20	20	24	0.746536323	0.917587548	24	15.57182342	0.144322162
ENSMUSG00000026248	Mrpl44	mitochondrial ribosomal protein L44 [Source:MGI Symbol;Acc:MGI:1916413]	15.9767	14.6381	14.4487	14.2284	13.1311	14.938	14.4753	15.7181	13.3964	16.0588	15.1271	15.8754	16.2686	14.4424	0.347721987	0.870418653	4	8	24	0.34437245	0.777706131	24	22.95728921	0.050143633
ENSMUSG00000026249	Serpine2	serine (or cysteine) peptidase inhibitor, clade E, member 2 [Source:MGI Symbol;Acc:MGI:101780]	46.6846	57.9207	68.2229	53.6754	52.4645	57.544	63.0097	59.3922	59.739	52.4101	47.9339	50.8269	51.1668	53.3454	0.097779482	0.501112642	12	8	24	0.165095686	0.767020009	24	6.427427866	0.10714682
ENSMUSG00000026254	Eif4e2	eukaryotic translation initiation factor 4E member 2 [Source:MGI Symbol;Acc:MGI:1914440]	33.8658	28.2573	29.4267	27.3738	28.6883	33.4941	29.8938	31.4146	27.5031	31.81	32.1735	30.4715	29.3336	28.8039	0.279886222	0.792139038	24	12	24	0.150199634	0.758865539	24	20.35501725	0.073206333
ENSMUSG00000026255	Efhd1	EF hand domain containing 1 [Source:MGI Symbol;Acc:MGI:1921607]	19.9332	20.8968	19.3593	16.2582	20.5981	19.6407	25.1108	20.3135	20.2778	22.3912	16.6987	21.1161	20.786	17.7874	0.814576414	0.966018836	4	8	24	0.373869504	0.794450623	24	0.095027936	0.084084515
ENSMUSG00000026259	Ngef	neuronal guanine nucleotide exchange factor [Source:MGI Symbol;Acc:MGI:1858414]	1.97685	2.94234	3.66634	2.95871	1.91094	2.78574	2.9227	2.79535	3.30467	3.65953	1.53333	2.898	2.15966	3.44588	0.003242852	0.140341875	12	8	24	0.056921359	0.65610184	24	7.284075013	0.241823662
ENSMUSG00000026260	Ndufa10	NADH:ubiquinone oxidoreductase subunit A10 [Source:MGI Symbol;Acc:MGI:1914523]	120.425	106.813	118.472	118.835	102.116	124.713	118.858	124.961	91.7079	126.629	100.165	97.297	103.249	110.902	0.90703203	0.966018836	16	4	24	0.908523262	0.974606327	24	3.127485193	0.024605696

ENSMUSG00000026269	Rnpepl1	arginyl aminopeptidase (aminopeptidase B)-like 1 [Source:MGI Symbol;Acc:MGI:1914170]	11.6594	12.2446	10.8582	12.2257	13.694	11.121	14.1177	12.5018	14.124	13.6619	13.4093	12.0993	13.2313	11.3549	0.907068194	0.966018836	20	4	24	0.695090277	0.898633591	24	13.92168762	0.038655694
ENSMUSG00000026270	Capn10	calpain 10 [Source:MGI Symbol;Acc:MGI:1344392]	15.9704	13.9855	13.7463	15.6802	17.51	17.0381	15.6649	14.2782	13.8633	17.0965	15.4207	15.2533	14.3934	14.2344	0.001233377	0.085813454	16	20	24	0.003167126	0.414576087	24	17.28250596	0.122706113
ENSMUSG00000026271	Gpr35	G protein-coupled receptor 35 [Source:MGI Symbol;Acc:MGI:1929509]	14.5869	9.09895	8.82252	8.96536	11.1545	11.9046	9.71071	10.376	6.90892	9.06809	10.8971	10.4734	11.0185	9.68685	0.002057111	0.100255953	24	12	24	0.003879795	0.427431763	24	20.85906435	0.25181541
ENSMUSG00000026273	Mterf4	mitochondrial transcription termination factor 4 [Source:MGI Symbol;Acc:MGI:1918355]	16.7095	18.059	16.4242	17.3143	18.624	16.5472	16.6026	17.1147	17.2096	14.4865	17.1232	15.8625	17.2181	15.2435	0.8635847	0.966018836	20	4	24	0.893967113	0.969958849	24	15.1943584	0.017690769
ENSMUSG00000026274	Pask	PAS domain containing serine/threonine kinase [Source:MGI Symbol;Acc:MGI:2155936]	1.25701	1.627	1.21173	1.87243	1.67379	1.55049	2.10345	1.4911	1.6197	1.61017	1.85816	1.96166	1.32929	1.08298	0.334913244	0.765941695	20	12	24	0.160634234	0.765941695	24	16.76671511	0.122269185
ENSMUSG00000026275	Ppp1r7	protein phosphatase 1, regulatory subunit 7 [Source:MGI Symbol;Acc:MGI:1913635]	22.2228	21.8091	18.2132	18.7437	19.6981	20.5065	22.7623	20.7461	20.2182	26.8915	16.4522	23.2156	24.2735	23.7853	0.128810631	0.560209134	4	16	24	0.326737166	0.768040178	24	1.626744523	0.095703158
ENSMUSG00000026276	Septin2	septin 2 [Source:MGI Symbol;Acc:MGI:97298]	16.5432	16.1874	16.9896	15.6926	17.0402	13.5859	14.6669	15.5372	16.4173	13.1658	16.2581	16.0667	18.7003	15.074	0.912686312	0.966018836	12	12	24	0.831653419	0.947861607	24	2.044177732	0.030016527
ENSMUSG00000026277	Stk25	serine/threonine kinase 25 (yeast) [Source:MGI Symbol;Acc:MGI:1891699]	88.6562	82.5069	83.3737	86.0732	89.9365	90.5535	82.4166	85.977	77.392	82.7646	83.2969	79.9149	81.8231	79.7664	0.652290823	0.966018836	20	16	24	0.060132194	0.658304792	24	18.57659973	0.047734395
ENSMUSG00000026278	Bok	BCL2-related ovarian killer [Source:MGI Symbol;Acc:MGI:1858494]	7.89717	6.31047	6.6119	9.0916	9.04069	7.32697	7.49722	6.40791	8.17336	8.25989	10.0908	8.67652	8.62687	6.26626	1.06E-04	0.029372629	20	12	24	0.002855324	0.414576087	24	16.14279666	0.209637788
ENSMUSG00000026279	Thap4	THAP domain containing 4 [Source:MGI Symbol;Acc:MGI:1914276]	10.0496	11.1606	11.925	11.2657	10.9996	12.1723	13.0124	11.2039	10.3839	12.6638	10.778	12.2223	12.3182	10.7312	0.93717134	0.966018836	4	4	24	0.74026012	0.915274254	24	19.51208396	0.032813769
ENSMUSG00000026280	Atg4b	autophagy related 4B, cysteine peptidase [Source:MGI Symbol;Acc:MGI:1913865]	33.061	30.5125	29.4949	33.8585	31.4715	32.8281	29.9897	34.0482	27.1551	33.6191	29.0756	29.976	34.3858	28.5291	0.943890799	0.966018836	4	8	24	0.652999708	0.883278597	24	19.94293534	0.040028335
ENSMUSG00000026281	Dtymk	deoxythymidylate kinase [Source:MGI Symbol;Acc:MGI:108396]	27.7385	24.9737	26.5146	27.1319	27.4226	24.0569	27.8987	27.382	30.9893	24.3102	31.6096	25.9968	28.2129	25.1448	0.951846915	0.966018836	4	12	24	0.917616434	0.977606103	24	12.652327	0.018728737
ENSMUSG00000026283	Ing5	inhibitor of growth family, member 5 [Source:MGI Symbol;Acc:MGI:1922816]	3.90103	3.65048	3.86024	4.17908	3.91095	3.93983	3.6863	4.34027	4.19166	3.73529	4.10558	3.96843	3.78201	3.78925	0.598711142	0.966018836	12	16	24	0.547859204	0.833842569	24	12.12642376	0.026077138
ENSMUSG00000026288	Inpp5d	inositol polyphosphate-5-phosphatase D [Source:MGI Symbol;Acc:MGI:107357]	1.08359	1.51581	1.89692	1.53534	2.31671	1.48855	2.79607	2.19556	1.89929	1.49367	1.33702	1.3591	1.7515	1.22754	0.880247609	0.966018836	12	12	24	0.93783667	0.984646259	24	5.917558284	0.035865786
ENSMUSG00000026289	Atg16l1	autophagy related 16-like 1 (S. cerevisiae) [Source:MGI Symbol;Acc:MGI:1924290]	18.6058	18.1498	18.7487	18.6161	19.5669	18.8819	17.8634	18.1086	17.6231	17.3862	19.1032	18.0475	18.3497	18.3726	0.388056094	0.915479136	20	12	24	0.172033581	0.768040178	24	17.73875812	0.030391157
ENSMUSG00000026305	Lrrfip1	leucine rich repeat (in FLII) interacting protein 1 [Source:MGI Symbol;Acc:MGI:1342770]	10.4337	11.1418	11.8961	12.5193	12.0158	12.4073	10.8872	13.975	12.4971	14.7574	9.98181	11.0815	12.4821	12.9952	0.195175079	0.673387182	16	4	24	0.151374252	0.758865539	24	8.626620734	0.106008535
ENSMUSG00000026307	Scly	selenocysteine lyase [Source:MGI Symbol;Acc:MGI:1355310]	7.43348	7.91538	8.1226	7.40909	8.45785	8.33478	8.75852	8.6572	8.39979	8.69646	8.0492	9.30634	8.67191	7.9706	0.92655264	0.966018836	24	8	24	0.672874185	0.889786694	24	20.56202177	0.025290576
ENSMUSG00000026308	Klhl30	kelch-like 30 [Source:MGI Symbol;Acc:MGI:1918038]	0.500429	0.847014	0.824412	0.749345	0.774488	1.02031	0.661492	2.96745	1.13002	1.27723	0.710312	0.677885	1.52591	0.969971	0.648082424	0.966018836	8	20	24	0.288302812	0.768040178	24	5.836665473	0.207349777
ENSMUSG00000026309	Ilkap	integrin-linked kinase-associated serine/threonine phosphatase 2C [Source:MGI Symbol;Acc:MGI:1914694]	40.7161	41.9237	40.3408	38.6064	41.3035	37.162	41.3879	38.4803	44.0669	38.0036	39.975	39.9816	41.6659	37.669	0.83638114	0.966018836	12	4	24	0.807551365	0.938693233	24	3.820956106	0.017638449
ENSMUSG00000026311	Asb1	ankyrin repeat and SOCS box-containing 1 [Source:MGI Symbol;Acc:MGI:1929735]	16.9141	14.8787	14.952	15.5723	14.7419	16.2949	14.4179	15.7189	13.188	15.568	15.6586	16.6695	15.6716	16.7853	0.297978647	0.80820842	24	12	24	0.263359526	0.768040178	24	21.21459662	0.058641524
ENSMUSG00000026312	Cdh7	cadherin 7, type 2 [Source:MGI Symbol;Acc:MGI:2442792]	1.12874	1.10631	1.18148	0.920235	1.09778	0.904827	1.46137	0.838444	1.09206	0.95877	1.17784	1.01734	0.824774	1.5408	0.896311169	0.966018836	8	16	24	0.723102018	0.909188168	24	3.849409697	0.045619034
ENSMUSG00000026313	Hdac4	histone deacetylase 4 [Source:MGI Symbol;Acc:MGI:3036234]	6.79368	5.64061	6.10433	6.07186	5.85951	6.05962	6.35865	5.44429	5.44309	7.42768	5.56271	6.15532	6.74945	5.96502	0.321662653	0.83607295	4	4	24	0.768341066	0.925362993	24	19.61920541	0.03415932
ENSMUSG00000026315	Serpinb8	serine (or cysteine) peptidase inhibitor, clade B, member 8 [Source:MGI Symbol;Acc:MGI:894657]	5.56094	5.40682	4.33063	5.28536	6.25891	5.98384	6.07598	4.83454	4.16506	4.38239	6.64077	4.41524	5.5482	4.97068	0.002466754	0.109177422	20	16	24	0.048169191	0.63850599	24	19.72029358	0.165609402
ENSMUSG00000026317	Cln8	CLN8 transmembrane ER and ERGIC protein [Source:MGI Symbol;Acc:MGI:1349447]	8.19568	8.20126	7.40958	8.70211	7.35649	7.94216	8.68913	9.46651	7.6802	6.6643	7.71142	7.29386	6.47166	6.34478	0.819898687	0.966018836	8	12	24	0.853693524	0.95631654	24	0.271323869	0.027370603
ENSMUSG00000026319	Relch	RAB11 binding and LisH domain, coiled-coil and HEAT repeat containing [Source:MGI Symbol;Acc:MGI:1922832]	23.2218	22.2991	22.9782	21.345	21.5048	21.2457	19.2655	21.1406	21.4244	21.7693	21.9593	21.2967	21.9419	23.1933	0.914502381	0.966018836	8	16	24	0.610847884	0.864861374	24	7.171934204	0.026950872
ENSMUSG00000026321	Tnfrsf11a	tumor necrosis factor receptor superfamily, member 11a, NFKB activator [Source:MGI Symbol;Acc:MGI:1314891]	4.26622	4.17556	3.55768	4.45858	3.82694	4.22901	3.82022	4.2045	3.45107	4.85062	4.07777	5.02439	4.35856	4.26567	0.148073804	0.590732968	24	12	24	0.477860441	0.805393068	24	19.08262766	0.057420428
ENSMUSG00000026322	Htr4	5 hydroxytryptamine (serotonin) receptor 4 [Source:MGI Symbol;Acc:MGI:109246]	4.3933	3.36706	2.75024	3.89499	3.25751	4.09357	2.7134	3.26225	3.2021	4.38765	2.99481	4.24655	3.57323	3.79489	0.601647512	0.966018836	24	12	24	0.647382219	0.880898698	24	18.87780534	0.070926871
ENSMUSG00000026333	Gin1	gypsy retrotransposon integrase 1 [Source:MGI Symbol;Acc:MGI:2182036]	7.2895	7.09548	6.34537	7.34257	7.80239	6.41584	6.79255	6.83492	7.66436	5.69866	8.86199	6.25613	6.7968	7.14112	0.503972924	0.966018836	20	4	24	0.849365526	0.954760051	24	15.24797255	0.031621903
ENSMUSG00000026335	Pam	peptidylglycine alpha-amidating monooxygenase [Source:MGI Symbol;Acc:MGI:97475]	74.319	73.337	74.7645	70.8518	79.2687	79.0039	73.6222	72.0422	69.557	78.5845	67.6897	77.9907	72.8805	72.2144	0.379373376	0.907428562	24	12	24	0.315987958	0.768040178	24	18.8924268	0.041877123
ENSMUSG00000026339	Ccdc93	coiled-coil domain containing 93 [Source:MGI Symbol;Acc:MGI:1918079]	7.8636	7.3229	6.5104	8.05887	7.70922	7.49216	7.62397	6.30807	7.0209	6.41823	7.6845	7.45993	7.15064	7.0752	0.097779482	0.501112642	20	16	24	0.05065597	0.644469829	24	19.06234643	0.086027779
ENSMUSG00000026341	Actr3	ARP3 actin-related protein 3 [Source:MGI Symbol;Acc:MGI:1921367]	84.8045	80.803	79.4717	82.4338	84.9808	83.327	76.0423	76.3494	76.0485	84.2505	84.0044	86.1016	86.8019	83.1287	0.118761453	0.542330458	24	12	24	0.085316014	0.703523461	24	18.13596013	0.053655294
ENSMUSG00000026342	Slc35f5	solute carrier family 35, member F5 [Source:MGI Symbol;Acc:MGI:1921400]	18.0715	17.4648	15.727	18.928	18.2174	18.8311	17.188	18.28	16.9755	15.1614	17.6074	16.049	19.9589	15.8295	0.590547452	0.966018836	4	8	24	0.307425239	0.768040178	24	21.10954183	0.066301918
ENSMUSG00000026344	Lypd1	Ly6/Plaur domain containing 1 [Source:MGI Symbol;Acc:MGI:1919835]	8.54336	7.50883	9.93266	7.28694	7.64812	12.0908	7.84161	8.90048	6.69018	6.68263	7.57601	7.50295	8.65764	8.30337	0.322291998	0.837583597	4	12	24	0.292338885	0.768040178	24	22.80499198	0.123537345
ENSMUSG00000026347	Tmem163	transmembrane protein 163 [Source:MGI Symbol;Acc:MGI:1919410]	12.2213	9.78063	11.5261	8.95104	7.32103	11.1119	8.80663	10.6163	8.36523	11.8965	8.18823	12.3493	8.97595	11.3507	0.938903891	0.966018836	24	20	24	0.587449972	0.853591039	24	2.243663597	0.090151179
ENSMUSG00000026349	Ccnt2	cyclin T2 [Source:MGI Symbol;Acc:MGI:1920199]	16.9283	18.9992	18.4011	20.1979	20.3408	16.6965	19.7321	16.2553	20.2619	15.8485	19.7483	17.4175	18.0832	18.0393	0.669104912	0.966018836	4	4	24	0.645058107	0.879586202	24	12.48295208	0.043456048
ENSMUSG00000026353	Ubxn4	UBX domain protein 4 [Source:MGI Symbol;Acc:MGI:1915062]	37.9099	31.4532	32.0771	36.2192	33.4884	33.9776	33.4454	34.3817	34.4738	37.1338	41.4357	35.6531	38.6936	36.5091	0.838532766	0.966018836	16	20	24	0.428321577	0.805393068	24	17.22652635	0.047295353
ENSMUSG00000026355	Mcm6	minichromosome maintenance complex component 6 [Source:MGI Symbol;Acc:MGI:1298227]	4.11099	4.43752	4.63445	4.49723	4.53525	4.39339	3.56102	4.62115	4.49028	3.81463	4.19485	4.18761	4.906	4.13779	0.44861185	0.966018836	12	16	24	0.586293761	0.853300537	24	8.080724412	0.046146468
ENSMUSG00000026356	Dars	aspartyl-tRNA synthetase [Source:MGI Symbol;Acc:MGI:2442544]	42.5614	43.6376	43.522	39.9757	43.973	39.0589	39.7265	40.3899	42.6538	36.7746	43.5919	39.3788	41.7244	41.3839	0.581070934	0.966018836	12	12	24	0.859301569	0.958530654	24	4.879134344	0.016075409
ENSMUSG00000026357	Rgs18	regulator of G-protein signaling 18 [Source:MGI Symbol;Acc:MGI:1927498]	0.551324	1.23029	1.26918	1.41764	0.989149	0.907559	0.80337	1.18108	1.01592	0.616201	0.717228	0.983142	0.901362	0.785583	0.43565473	0.966018836	12	16	24	0.383144328	0.779546792	24	8.540942473	0.101208836
ENSMUSG00000026360	Rgs2	regulator of G-protein signaling 2 [Source:MGI Symbol;Acc:MGI:1098271]	8.23752	8.51585	9.30137	10.1485	8.00117	7.63906	9.29814	10.2011	9.08332	8.55107	8.88801	10.5365	14.147	8.40286	0.880992536	0.966018836	12	8	24	0.68260671	0.89423365	24	2.350819842	0.063967238
ENSMUSG00000026361	Cdc73	cell division cycle 73, Paf1/RNA polymerase II complex component [Source:MGI Symbol;Acc:MGI:2384876]	5.96184	5.66757	5.91574	5.95918	6.60031	5.75906	7.69982	5.25595	6.46851	5.1837	6.22805	6.194	6.08925	5.93026	0.295379186	0.805301811	20	12	24	0.510539521	0.814235033	24	20.64931366	0.057975345
ENSMUSG00000026365	Cfh	complement component factor h [Source:MGI Symbol;Acc:MGI:88385]	17.1159	22.845	19.7407	17.8077	19.3443	15.8173	16.2829	19.3345	24.6948	9.24204	17.9577	16.5894	13.2686	15.8581	0.133766544	0.568189321	12	12	24	0.623560779	0.87183699	24	5.597799235	0.116002356
ENSMUSG00000026374	Tsn	translin [Source:MGI Symbol;Acc:MGI:109263]	47.1025	42.4615	47.1582	46.4066	41.593	46.692	44.2986	45.5725	38.9285	43.4906	42.794	39.2393	46.4131	48.906	0.756435567	0.966018836	4	16	24	0.500007598	0.808809055	24	2.751665757	0.045159899
ENSMUSG00000026377	Nifk	nucleolar protein interacting with the FHA domain of MKI67 [Source:MGI Symbol;Acc:MGI:1915199]	16.2974	16.4121	14.5625	15.7114	17.2959	15.0065	13.8287	14.7825	16.0385	14.2961	16.7327	17.201	16.9257	16.8291	0.503972924	0.966018836	20	20	24	0.678205514	0.892450912	24	19.26657035	0.035879628
ENSMUSG00000026383	Epb41l5	erythrocyte membrane protein band 4.1 like 5 [Source:MGI Symbol;Acc:MGI:103006]	3.01372	2.90573	2.92848	2.49618	2.87831	3.15874	3.41432	2.80525	3.52505	2.46892	3.05361	3.51521	3.02287	2.84665	0.064422003	0.414867207	24	16	24	0.20709732	0.768040178	24	22.63726291	0.079274282
ENSMUSG00000026384	Ptpn4	protein tyrosine phosphatase, non-receptor type 4 [Source:MGI Symbol;Acc:MGI:1099792]	2.57727	3.8869	3.99385	3.88332	3.44227	3.18947	3.54884	3.24942	3.86351	2.86077	3.91905	3.12137	2.74761	3.5634	0.097760727	0.501112642	12	16	24	0.15518281	0.760294912	24	9.857271934	0.117145077
ENSMUSG00000026385	Dbi	diazepam binding inhibitor [Source:MGI Symbol;Acc:MGI:94865]	544.024	620.605	610.691	537.563	484.891	465.108	591.695	604.608	629.99	456.915	581.773	536.833	681.671	684.729	0.010230394	0.195117844	8	8	24	0.004872387	0.438275631	24	4.307662421	0.191488355
ENSMUSG00000026388	3110009E18Rik	RIKEN cDNA 3110009E18 gene [Source:MGI Symbol;Acc:MGI:1920353]	3.75007	3.52993	2.99535	3.97768	3.14731	3.14104	3.51213	3.87801	3.60246	2.32618	4.58163	3.24415	3.30818	3.72969	0.92655264	0.966018836	8	4	24	0.827509795	0.946154971	24	0.803805129	0.04327249
ENSMUSG00000026389	Steap3	STEAP family member 3 [Source:MGI Symbol;Acc:MGI:1915678]	14.5769	10.8372	12.1899	11.5299	13.3989	13.6053	12.4198	14.26	10.3413	14.2284	12.8492	11.9758	11.5406	10.4388	0.531498969	0.966018836	20	16	24	0.231467648	0.768040178	24	18.47019938	0.10026879
ENSMUSG00000026393	Nek7	NIMA (never in mitosis gene a)-related expressed kinase 7 [Source:MGI Symbol;Acc:MGI:1890645]	83.6075	80.6897	71.9182	79.4334	72.6697	72.9153	74.807	71.0699	71.4639	77.3354	81.5638	75.1675	80.3193	78.2623	0.92476132	0.966018836	4	8	24	0.744462678	0.916498249	24	21.5699519	0.024926739
ENSMUSG00000026395	Ptprc	protein tyrosine phosphatase, receptor type, C [Source:MGI Symbol;Acc:MGI:97810]	2.36211	3.42522	4.18066	3.65193	3.73297	2.97277	3.92234	3.06518	4.1073	2.44817	3.52921	3.05959	3.66669	2.46671	0.601647512	0.966018836	12	12	24	0.736322132	0.914122853	24	10.96145163	0.066474764
ENSMUSG00000026399	Cd55	CD55 molecule, decay accelerating factor for complement [Source:MGI Symbol;Acc:MGI:104850]	239.892	228.332	199.32	215.837	207.197	225.169	201.768	210.263	204.494	205.974	225.372	233.055	244.775	223.94	0.038650756	0.336039716	4	8	24	0.069777959	0.674183933	24	22.1341757	0.080791759
ENSMUSG00000026404	Ddx59	DEAD box helicase 59 [Source:MGI Symbol;Acc:MGI:1915247]	5.16056	4.5354	5.43416	5.03703	5.01591	4.95439	3.81058	4.82211	5.11475	5.36264	5.24353	5.4074	5.16528	5.29888	0.885025617	0.966018836	12	20	24	0.283310214	0.768040178	24	12.34684152	0.064273048
ENSMUSG00000026407	Cacna1s	calcium channel, voltage-dependent, L type, alpha 1S subunit [Source:MGI Symbol;Acc:MGI:88294]	2.25135	5.81093	5.95072	6.80806	4.90336	5.48097	4.64922	10.8521	9.80854	13.8976	4.52703	4.23156	7.38005	6.29076	0.074318596	0.448301997	16	4	24	0.01765522	0.5524966	24	8.957876445	0.522293391
ENSMUSG00000026409	Pfkfb2	6-phosphofructo-2-kinase/fructose-2,6-biphosphatase 2 [Source:MGI Symbol;Acc:MGI:107815]	12.7028	14.8212	14.1184	15.9626	15.8291	13.8611	14.0848	13.81	14.9651	13.3476	13.7914	14.8914	14.2782	14.3797	0.94086481	0.966018836	12	16	24	0.443336886	0.805393068	24	12.40194845	0.041833497
ENSMUSG00000026411	Tmem9	transmembrane protein 9 [Source:MGI Symbol;Acc:MGI:1913491]	34.4086	31.05	32.2595	28.5043	31.5377	34.992	33.1751	30.517	28.191	29.5447	30.2108	32.1514	30.2977	31.9422	0.064422003	0.414867207	24	16	24	0.007233395	0.47735824	24	22.09073067	0.091780814
ENSMUSG00000026414	Tnnt2	troponin T2, cardiac [Source:MGI Symbol;Acc:MGI:104597]	0.387646	0.601279	0.709763	0.676141	0.444591	0.693645	0.689899	1.13857	0.743284	0.868333	0.392138	0.493434	0.260888	0.514039	0.152236561	0.600454138	12	16	24	0.183504123	0.768040178	24	8.239714687	0.141740842
ENSMUSG00000026418	Tnni1	troponin I, skeletal, slow 1 [Source:MGI Symbol;Acc:MGI:105073]	4.22604	6.16504	14.0945	11.7394	8.31853	12.168	7.59848	11.4759	6.26048	9.38144	4.12293	3.37822	6.71089	10.1049	0.503104368	0.966018836	12	16	24	0.270732112	0.768040178	24	8.597972397	0.355929324
ENSMUSG00000026421	Csrp1	cysteine and glycine-rich protein 1 [Source:MGI Symbol;Acc:MGI:88549]	93.0902	98.8595	108.857	95.7562	93.5903	96.4172	101.996	96.2715	102.378	91.6254	86.3709	97.2562	90.945	94.2133	0.184531358	0.662239322	12	8	24	0.199094982	0.768040178	24	5.832922854	0.052753533
ENSMUSG00000026424	Gpr37l1	G protein-coupled receptor 37-like 1 [Source:MGI Symbol;Acc:MGI:1928503]	41.2727	44.4759	52.4075	36.9912	45.6022	47.3098	51.7124	45.5879	40.5072	42.6309	40.9899	41.2706	39.0172	41.1693	0.845386467	0.966018836	8	8	24	0.767247401	0.924784773	24	0.998322989	0.038757978
ENSMUSG00000026425	Srgap2	SLIT-ROBO Rho GTPase activating protein 2 [Source:MGI Symbol;Acc:MGI:109605]	14.876	17.9869	14.8117	14.5438	16.0451	15.6022	15.754	18.4525	18.7339	16.6728	13.4456	16.949	14.5026	14.833	0.914360142	0.966018836	8	12	24	0.434712204	0.805393068	24	5.789327233	0.070494783
ENSMUSG00000026426	Arl8a	ADP-ribosylation factor-like 8A [Source:MGI Symbol;Acc:MGI:1915974]	120.431	97.9351	101.011	107.024	113.002	117.609	107.597	107.178	91.3048	134.441	105.187	113.296	108.073	109.296	0.041292272	0.346472636	24	12	24	0.237373409	0.768040178	24	18.21307981	0.089138082
ENSMUSG00000026427	Eif2d	eukaryotic translation initiation factor 2D [Source:MGI Symbol;Acc:MGI:109342]	14.0279	14.9566	14.2526	14.6916	14.689	14.5998	14.204	15.0513	14.7521	15.4206	14.5109	14.3623	14.5688	14.8354	0.035058838	0.328311403	8	20	24	0.123246152	0.736234617	24	9.592849838	0.025545551
ENSMUSG00000026429	Ube2t	ubiquitin-conjugating enzyme E2T [Source:MGI Symbol;Acc:MGI:1914446]	1.42536	1.65794	2.01222	1.43264	1.80073	1.75979	1.77916	1.93904	1.48305	1.557	1.58114	1.90289	1.76448	1.47208	0.887862353	0.966018836	24	16	24	0.739866566	0.915196423	24	22.28307449	0.033679111
ENSMUSG00000026430	Rassf5	Ras association (RalGDS/AF-6) domain family member 5 [Source:MGI Symbol;Acc:MGI:1926375]	20.1055	14.9114	15.2576	16.3795	16.9751	19.7804	18.2301	19.2976	12.2487	22.3112	14.6806	16.9404	16.6824	16.6937	0.46238066	0.966018836	24	12	24	0.43118569	0.805393068	24	20.60858147	0.110767723
ENSMUSG00000026433	Rab29	RAB29, member RAS oncogene family [Source:MGI Symbol;Acc:MGI:2385107]	4.92499	7.44031	6.36193	6.16563	5.99917	6.36631	6.11763	6.88989	6.81442	5.16434	6.59398	6.78669	6.2715	5.47323	0.95032543	0.966018836	8	20	24	0.928060086	0.981362081	24	5.520162805	0.022714082
ENSMUSG00000026434	Nucks1	nuclear casein kinase and cyclin-dependent kinase substrate 1 [Source:MGI Symbol;Acc:MGI:1934811]	19.716	20.6316	22.5093	20.2658	19.8284	19.684	19.9685	18.8121	20.1675	22.4792	20.1043	22.13	20.8917	21.4032	0.79615867	0.966018836	24	12	24	0.533056686	0.825678486	24	10.55091572	0.030063201
ENSMUSG00000026435	Slc45a3	solute carrier family 45, member 3 [Source:MGI Symbol;Acc:MGI:1922082]	10.8761	7.75716	8.10892	7.74292	9.8048	11.2267	8.57775	9.5802	6.9146	8.13825	9.66573	8.92935	9.41039	8.16792	0.011207621	0.204559658	24	12	24	0.004683307	0.434006302	24	20.39285611	0.197824946
ENSMUSG00000026436	Elk4	ELK4, member of ETS oncogene family [Source:MGI Symbol;Acc:MGI:102853]	10.7269	10.8073	11.7232	11.7325	12.6018	10.9648	11.2838	12.9431	13.7727	11.7929	12.0315	12.1376	12.548	11.8679	0.942051135	0.966018836	20	8	24	0.221220977	0.768040178	24	9.841961524	0.055443733
ENSMUSG00000026437	Cdk18	cyclin-dependent kinase 18 [Source:MGI Symbol;Acc:MGI:97518]	1.68387	2.41973	3.17179	2.04331	1.34299	2.1878	2.36735	1.71702	1.60733	1.99397	1.43531	1.56437	1.43114	1.64237	0.328944792	0.847277612	12	8	24	0.39291361	0.799546792	24	6.563777816	0.109881652
ENSMUSG00000026439	Rbbp5	retinoblastoma binding protein 5, histone lysine methyltransferase complex subunit [Source:MGI Symbol;Acc:MGI:1918367]	23.2361	21.0336	20.6814	20.697	19.6795	22.3403	20.1306	19.6052	17.8049	20.7041	19.8226	20.2223	21.5905	20.0394	0.360140629	0.883787544	4	8	24	0.090528472	0.711456292	24	21.64708019	0.067344154
ENSMUSG00000026442	Nfasc	neurofascin [Source:MGI Symbol;Acc:MGI:104753]	33.2089	32.0804	32.8527	33.2399	35.8324	31.9786	36.9639	33.0298	35.2961	44.0898	29.4935	35.873	33.3761	33.8364	0.858514388	0.966018836	16	16	24	0.68296425	0.894309028	24	11.43848785	0.045173588
ENSMUSG00000026443	Lrrn2	leucine rich repeat protein 2, neuronal [Source:MGI Symbol;Acc:MGI:106037]	3.97496	3.70244	3.8387	4.21148	4.02784	4.29522	4.17898	4.15251	3.62722	5.73003	3.45094	4.37395	3.96577	4.84541	0.8972237	0.966018836	24	12	24	0.879510457	0.966193785	24	11.53757421	0.029192666
ENSMUSG00000026447	Pik3c2b	phosphatidylinositol-4-phosphate 3-kinase catalytic subunit type 2 beta [Source:MGI Symbol;Acc:MGI:2685045]	1.17909	1.59293	2.00137	1.1988	1.32921	1.67024	2.03716	1.518	1.628	1.62696	1.26207	1.54235	1.3286	1.30016	0.907068194	0.966018836	12	4	24	0.723648168	0.909368654	24	5.190800413	0.049697689
ENSMUSG00000026450	Chit1	chitinase 1 (chitotriosidase) [Source:MGI Symbol;Acc:MGI:1919134]	0.441808	0.489267	0.565573	0.508825	0.488397	0.518604	0.571546	0.63002	0.355614	0.463459	0.573682	0.478567	0.500823	0.520626	0.637736882	0.966018836	8	4	24	0.851427873	0.955425185	24	23.37469054	0.01497334
ENSMUSG00000026452	Syt2	synaptotagmin II [Source:MGI Symbol;Acc:MGI:99666]	45.404	33.9237	37.2966	35.3848	29.0503	40.4595	42.3531	37.2801	26.0032	57.065	32.5681	42.5395	41.2172	44.4463	0.387380622	0.915479136	4	16	24	0.650236292	0.882388586	24	23.27236932	0.102711621
ENSMUSG00000026455	Klhl12	kelch-like 12 [Source:MGI Symbol;Acc:MGI:2385619]	15.9593	15.0702	14.8935	14.8335	14.2684	15.6275	14.8452	14.8476	14.0828	16.302	14.6849	16.3333	15.2662	16.2443	0.503972924	0.966018836	24	20	24	0.541377514	0.830485817	24	22.67596054	0.02838053
ENSMUSG00000026456	Cyb5r1	cytochrome b5 reductase 1 [Source:MGI Symbol;Acc:MGI:1919267]	49.4132	35.918	41.4598	40.4889	40.753	48.2274	38.448	46.1104	30.2338	48.4904	38.3725	41.9149	41.9294	39.3838	0.835401638	0.966018836	24	12	24	0.371456678	0.793126592	24	19.98614147	0.105240085
ENSMUSG00000026457	Adipor1	adiponectin receptor 1 [Source:MGI Symbol;Acc:MGI:1919924]	55.1512	47.116	48.3258	52.1191	51.0409	55.0538	53.2146	55.2747	48.0503	60.6099	52.9642	53.607	54.9669	50.078	0.287843582	0.793714531	4	8	24	0.317919227	0.768040178	24	18.66699531	0.056571804
ENSMUSG00000026458	Ppfia4	protein tyrosine phosphatase, receptor type, f polypeptide (PTPRF), interacting protein (liprin), alpha 4 [Source:MGI Symbol;Acc:MGI:1915757]	12.7637	10.8753	11.3576	10.2206	11.7118	11.5867	11.2762	11.9661	11.3053	13.3202	10.0679	11.6487	12.1185	12.0437	0.952127548	0.966018836	4	16	24	0.623719635	0.87183699	24	1.653425267	0.038139797
ENSMUSG00000026463	Atp2b4	ATPase, Ca++ transporting, plasma membrane 4 [Source:MGI Symbol;Acc:MGI:88111]	13.5899	10.919	14.3014	12.2788	12.255	14.004	13.0893	13.2891	11.0252	16.118	10.7159	12.3777	11.8135	14.0228	0.852921792	0.966018836	16	4	24	0.959854182	0.989375125	24	7.749991527	0.019043577
ENSMUSG00000026466	Tor1aip1	torsin A interacting protein 1 [Source:MGI Symbol;Acc:MGI:3582693]	12.7292	12.7303	16.3615	13.6096	14.6334	11.5828	15.632	13.3284	16.1834	14.0334	14.0159	14.6639	13.6757	14.0484	0.773177465	0.966018836	12	12	24	0.275119331	0.768040178	24	9.314053039	0.082260135
ENSMUSG00000026469	Xpr1	xenotropic and polytropic retrovirus receptor 1 [Source:MGI Symbol;Acc:MGI:97932]	12.0336	11.1628	12.1345	11.1226	10.219	12.2402	12.0965	10.8274	10.3054	13.3101	11.3295	12.277	11.8253	12.8343	0.892685629	0.966018836	24	20	24	0.797114182	0.935133209	24	23.42863317	0.027215051
ENSMUSG00000026470	Stx6	syntaxin 6 [Source:MGI Symbol;Acc:MGI:1926235]	14.4209	13.1639	13.43	14.1709	13.1818	15.5501	17.4461	15.5007	12.393	14.046	14.4212	13.0982	15.633	13.442	0.062631097	0.410322149	4	8	24	0.157716437	0.764314876	24	22.46072241	0.091993099
ENSMUSG00000026471	Mr1	major histocompatibility complex, class I-related [Source:MGI Symbol;Acc:MGI:1195463]	4.54268	6.32139	7.00755	5.85574	5.49177	5.43464	6.34235	5.96273	7.06223	5.72559	5.70047	6.38583	6.02046	6.32628	0.215777641	0.704989686	12	8	24	0.077000243	0.687041208	24	7.496736366	0.113425112
ENSMUSG00000026473	Glul	glutamate-ammonia ligase (glutamine synthetase) [Source:MGI Symbol;Acc:MGI:95739]	123.27	121.032	164.964	179.979	125.218	148.577	142.602	127.257	141.918	168.454	129.841	132.61	107.726	133.968	0.059513104	0.406067671	16	12	24	0.034472202	0.608551791	24	11.60791698	0.183541303
ENSMUSG00000026475	Rgs16	regulator of G-protein signaling 16 [Source:MGI Symbol;Acc:MGI:108407]	1.1464	1.39866	1.54215	1.08912	1.15069	1.20527	1.2901	1.58628	1.2831	1.03351	0.955038	1.08592	1.05115	1.10466	0.09556163	0.498111776	12	4	24	0.030565853	0.597890721	24	4.948217988	0.100306864
ENSMUSG00000026478	Lamc1	laminin, gamma 1 [Source:MGI Symbol;Acc:MGI:99914]	23.6998	25.8408	22.2184	23.0582	28.665	25.5517	29.8524	30.3652	24.3596	25.8724	21.4199	23.6369	25.3955	22.1778	0.885059765	0.966018836	8	4	24	0.592753099	0.856634016	24	23.75007517	0.061743564
ENSMUSG00000026479	Lamc2	laminin, gamma 2 [Source:MGI Symbol;Acc:MGI:99913]	0.624078	0.884273	0.733732	0.908121	1.06876	1.09815	0.948385	1.01413	0.743465	0.709204	0.960466	0.881548	0.98765	0.873350015	0.966018836	24	4	24	0.856956653	0.957602831	24	2.27164394	0.028411968	
ENSMUSG00000026480	Ncf2	neutrophil cytosolic factor 2 [Source:MGI Symbol;Acc:MGI:97284]	2.90041	3.76404	3.36161	3.83822	4.44734	3.46255	3.74885	2.58825	3.37396	3.09227	3.50869	2.79619	3.11016	2.96677	0.496745696	0.966018836	20	8	24	0.19465723	0.768040178	24	14.90718532	0.100256581
ENSMUSG00000026482	Rgl1	ral guanine nucleotide dissociation stimulator,-like 1 [Source:MGI Symbol;Acc:MGI:107484]	12.737	11.338	11.4363	12.7065	12.6496	12.6694	12.121	12.4845	10.2265	14.1134	11.5557	12.7005	12.0994	11.549	0.05238827	0.391426791	16	20	24	0.218012913	0.768040178	24	17.83243859	0.069541165
ENSMUSG00000026483	Niban1	niban apoptosis regulator 1 [Source:MGI Symbol;Acc:MGI:2137237]	6.40276	7.04624	7.82108	6.22188	6.25647	7.11063	7.30682	7.62226	6.67561	8.24105	6.99635	6.86311	6.6623	7.44737	0.636977386	0.966018836	12	8	24	0.344944446	0.778248169	24	6.893693199	0.060744197
ENSMUSG00000026484	Rnf2	ring finger protein 2 [Source:MGI Symbol;Acc:MGI:1101759]	8.24776	9.57789	9.52445	9.69449	8.79606	8.25144	9.00282	8.29594	9.63979	8.17002	9.27702	8.20671	7.05941	8.28761	0.124211791	0.552144108	12	12	24	0.069973334	0.674670543	24	10.50899162	0.086530428
ENSMUSG00000026489	Coq8a	coenzyme Q8A [Source:MGI Symbol;Acc:MGI:1914676]	9.83712	14.0575	19.9095	25.2845	13.9481	16.9988	15.1851	32.9166	18.8391	33.5219	15.5738	15.1442	25.3694	21.3365	0.503972924	0.966018836	16	4	24	0.072766529	0.679705968	24	8.906717605	0.374619392
ENSMUSG00000026490	Cdc42bpa	CDC42 binding protein kinase alpha [Source:MGI Symbol;Acc:MGI:2441841]	35.6131	31.8434	32.1205	33.2672	32.5411	32.628	33.7079	30.1359	30.001	33.7357	30.4438	34.6945	32.2649	32.9069	0.277917941	0.789849983	24	12	24	0.249945206	0.768040178	24	20.53903215	0.046274809
ENSMUSG00000026491	Ahctf1	AT hook containing transcription factor 1 [Source:MGI Symbol;Acc:MGI:1915033]	12.7144	13.6616	12.6577	18.8188	15.5624	12.3711	12.9916	14.0126	15.9158	12.2338	14.6226	11.4414	11.9154	14.9797	0.110739523	0.528348425	12	12	24	0.127865205	0.740162193	24	10.66798934	0.139973634
ENSMUSG00000026492	Tfb2m	transcription factor B2, mitochondrial [Source:MGI Symbol;Acc:MGI:107937]	4.22475	4.48866	4.06354	5.20058	3.82367	4.18176	3.68774	4.84913	4.75589	4.93523	5.56305	5.07113	5.19328	5.69837	0.880247609	0.966018836	16	12	24	0.418411884	0.802992496	24	9.404233536	0.064708592
ENSMUSG00000026494	Kif26b	kinesin family member 26B [Source:MGI Symbol;Acc:MGI:2447076]	3.99964	2.94767	2.69906	2.9773	3.08989	3.53864	3.54833	3.32832	2.70656	4.19876	3.17626	3.63265	3.27638	3.52414	0.025149041	0.292995596	24	12	24	0.247528908	0.768040178	24	20.94186019	0.09536776
ENSMUSG00000026495	Efcab2	EF-hand calcium binding domain 2 [Source:MGI Symbol;Acc:MGI:1915476]	5.58754	6.38131	6.08816	6.16345	5.51948	5.44683	6.39398	5.46941	7.08482	6.49466	5.59915	6.02518	5.79289	7.08733	0.224668574	0.719154429	12	12	24	0.036420166	0.609176161	24	7.327918101	0.10181657
ENSMUSG00000026496	Parp1	poly (ADP-ribose) polymerase family, member 1 [Source:MGI Symbol;Acc:MGI:1340806]	28.1738	21.952	23.1535	22.5802	23.9906	26.3973	23.1688	26.3325	18.6706	31.1743	22.2913	24.4434	24.4182	24.1708	0.390686537	0.915479136	24	12	24	0.613772277	0.866162311	24	20.09397008	0.069808997
ENSMUSG00000026499	Acbd3	acyl-Coenzyme A binding domain containing 3 [Source:MGI Symbol;Acc:MGI:2181074]	21.7195	18.7211	19.0582	18.8489	18.8213	20.5862	19.1867	19.1986	15.4066	20.4008	19.8223	19.8905	19.4357	20.002	0.204218286	0.690546825	24	12	24	0.13981531	0.74956518	24	20.64862406	0.081494465
ENSMUSG00000026500	Cox20	cytochrome c oxidase assembly protein 20 [Source:MGI Symbol;Acc:MGI:1913609]	25.5107	28.5137	25.6642	25.7171	22.8002	21.7884	24.6132	28.962	27.8472	23.4411	27.6053	24.3449	26.6735	23.8535	0.158469451	0.614860056	8	16	24	0.153112091	0.759484836	24	5.796227052	0.089397082
ENSMUSG00000026502	Desi2	desumoylating isopeptidase 2 [Source:MGI Symbol;Acc:MGI:1926075]	4.22098	5.12389	4.53894	4.45927	4.99925	5.39113	4.5321	5.70901	5.13756	4.92732	5.41199	5.02063	4.72826	5.53053	0.801995186	0.966018836	8	20	24	0.902407495	0.972751432	24	7.921754295	0.017873455
ENSMUSG00000026504	Sdccag8	serologically defined colon cancer antigen 8 [Source:MGI Symbol;Acc:MGI:1924066]	10.7187	12.823	12.5093	11.2491	12.4897	12.2076	11.9087	10.6612	12.2908	10.4729	12.9231	12.9365	14.2871	13.4697	0.939486266	0.966018836	24	16	24	0.694069822	0.898415616	24	23.18024585	0.038136216
ENSMUSG00000026509	Capn2	calpain 2 [Source:MGI Symbol;Acc:MGI:88264]	102.141	86.0175	84.0712	88.4473	94.1462	94.7236	93.0034	91.818	74.8385	104.259	84.5989	92.1311	92.7539	87.0508	0.036527779	0.332441566	24	12	24	0.191764696	0.768040178	24	20.06542445	0.085445126
ENSMUSG00000026510	Trp53bp2	transformation related protein 53 binding protein 2 [Source:MGI Symbol;Acc:MGI:2138319]	3.75295	3.89638	4.985	4.29174	3.91518	4.55928	5.06659	4.5204	4.42402	5.28678	3.66353	4.4409	3.60655	4.48844	0.93717134	0.966018836	16	4	24	0.467637192	0.805393068	24	8.847976483	0.072465613
ENSMUSG00000026511	Srp9	signal recognition particle 9 [Source:MGI Symbol;Acc:MGI:1350930]	44.9296	51.9743	48.7126	46.947	47.2688	45.4339	44.5445	45.2935	52.4412	39.0881	51.9836	44.9008	49.5033	48.3533	0.601647512	0.966018836	12	12	24	0.712622906	0.905241009	24	5.866596002	0.037769242
ENSMUSG00000026514	Cnih3	cornichon family AMPA receptor auxiliary protein 3 [Source:MGI Symbol;Acc:MGI:1920228]	1.62767	1.41157	2.20155	1.568	1.60995	2.13073	1.31951	1.52	1.46431	1.76645	1.47372	1.85296	1.36368	1.2349	0.128989168	0.560209134	24	4	24	0.306689315	0.768040178	24	15.56660934	0.085556094

ENSMUSG00000026516	Nvl	nuclear VCP-like [Source:MGI Symbol;Acc:MGI:1914709]	13.8707	15.4145	17.3222	15.8279	14.8179	14.6872	14.7437	13.6772	19.4716	13.8809	16.4435	14.7388	14.8352	16.4014	0.143321297	0.585944911	12	12	24	0.130369734	0.740714253	24	9.134415265	0.102755247
ENSMUSG00000026519	Tmem63a	transmembrane protein 63a [Source:MGI Symbol;Acc:MGI:2384789]	10.945	11.2684	13.7001	11.1041	9.72689	11.4785	12.1929	11.3935	10.8401	10.993	8.50321	10.7224	8.81655	11.9919	0.192357053	0.669841558	8	12	24	0.152712146	0.759115906	24	5.668233166	0.112510377
ENSMUSG00000026520	Pycr2	pyrroline-5-carboxylate reductase family, member 2 [Source:MGI Symbol;Acc:MGI:1277956]	13.0627	11.3823	12.9074	11.4223	13.4498	13.3292	11.9305	13.7117	12.9466	14.1404	14.3685	14.184	14.7085	13.0082	0.331157065	0.850959667	20	16	24	0.351772082	0.780345856	24	18.08029106	0.048715945
ENSMUSG00000026525	Opn3	opsin 3 [Source:MGI Symbol;Acc:MGI:1338022]	3.79024	2.84659	2.49169	3.03538	2.75694	3.31709	3.27967	3.24245	2.79963	4.28462	2.85468	3.38663	3.18831	2.78573	0.071575474	0.440262583	24	12	24	0.555920603	0.838702296	24	20.09229164	0.070107679
ENSMUSG00000026526	Fh1	fumarate hydratase 1 [Source:MGI Symbol;Acc:MGI:95530]	46.4417	44.0503	51.8356	58.6763	47.7688	49.0627	43.9603	52.8102	54.6238	54.25	54.5345	50.3355	58.7796	57.4095	0.662399683	0.966018836	16	12	24	0.071953236	0.678388782	24	10.25033327	0.095967895
ENSMUSG00000026527	Rgs7	regulator of G protein signaling 7 [Source:MGI Symbol;Acc:MGI:1346089]	36.1601	31.413	30.0928	31.233	30.3669	33.8523	29.3053	31.6566	29.7765	33.4847	32.8357	34.4019	33.1927	34.2533	0.148073804	0.590732968	24	12	24	0.303780636	0.768040178	24	21.22791768	0.053235789
ENSMUSG00000026544	Dusp23	dual specificity phosphatase 23 [Source:MGI Symbol;Acc:MGI:1915690]	1.81286	2.10309	2.17991	2.15887	2.19712	2.15545	2.0976	3.19603	2.63174	3.1512	1.90718	2.42763	2.45307	2.19819	0.691437565	0.966018836	12	16	24	0.260976832	0.768040178	24	8.413026126	0.102751352
ENSMUSG00000026547	Tagln2	transgelin 2 [Source:MGI Symbol;Acc:MGI:1312985]	82.22	73.603	77.4671	80.7848	81.2869	86.5603	74.5843	81.2621	62.223	96.1346	79.3626	74.159	71.027	72.9469	0.481811994	0.966018836	16	20	24	0.227833763	0.768040178	24	16.11796001	0.092831804
ENSMUSG00000026548	Slamf9	SLAM family member 9 [Source:MGI Symbol;Acc:MGI:1923692]	4.79525	8.3691	8.66934	6.54572	7.14602	5.18416	5.77144	7.67381	10.8736	6.31229	6.39394	6.27826	7.15873	7.08158	0.005105434	0.144396606	12	12	24	0.010470952	0.506240926	24	7.961276235	0.282598001
ENSMUSG00000026553	Copa	coatomer protein complex subunit alpha [Source:MGI Symbol;Acc:MGI:1334462]	71.1637	64.8779	69.2635	67.8845	67.3163	73.2555	69.4	69.2609	59.1943	70.0072	63.7331	66.8986	66.4563	65.7895	0.652290823	0.966018836	24	12	24	0.279455922	0.768040178	24	20.67671872	0.045124429
ENSMUSG00000026554	Dcaf8	DDB1 and CUL4 associated factor 8 [Source:MGI Symbol;Acc:MGI:91860]	42.3581	43.9576	45.7519	46.4947	41.367	43.9414	45.0298	45.5504	43.3255	49.7397	43.8503	47.2115	45.9644	45.206	0.893307667	0.966018836	16	4	24	0.529118068	0.824286178	24	9.555836508	0.027727235
ENSMUSG00000026556	Vangl2	VANGL planar cell polarity 2 [Source:MGI Symbol;Acc:MGI:2135272]	0.896971	0.94135	1.24339	0.776053	0.697427	0.770171	1.00499	1.08983	1.01416	0.924659	0.676975	0.958851	0.577486	0.811997	0.035023815	0.328311403	12	8	24	0.120836534	0.732894732	24	6.326913748	0.102012325
ENSMUSG00000026558	Uck2	uridine-cytidine kinase 2 [Source:MGI Symbol;Acc:MGI:1931744]	28.0937	18.4538	20.0625	21.9223	21.567	24.0461	20.8873	21.2571	15.252	22.1384	20.9232	17.9915	19.8831	19.6707	0.623812175	0.966018836	16	20	24	0.198445428	0.768040178	24	19.81167997	0.125661586
ENSMUSG00000026563	Tada1	transcriptional adaptor 1 [Source:MGI Symbol;Acc:MGI:1196415]	13.2681	11.7795	13.7076	12.4086	11.6775	12.8013	12.7522	11.6189	11.9262	9.8136	13.1415	12.7088	13.1615	11.6668	0.369262716	0.893596186	4	4	24	0.258795837	0.768040178	24	22.05595438	0.071448506
ENSMUSG00000026564	Dusp27	dual specificity phosphatase 27 (putative) [Source:MGI Symbol;Acc:MGI:2685055]	0.379674	0.622722	0.580868	0.77035	0.754998	0.600234	0.398796	1.54918	0.745115	1.24708	0.546268	0.583905	0.644002	0.587127	0.17953325	0.655480352	16	12	24	0.205916182	0.768040178	24	9.613896128	0.164858222
ENSMUSG00000026565	Pou2f1	POU domain, class 2, transcription factor 1 [Source:MGI Symbol;Acc:MGI:101898]	2.54716	2.44285	2.42524	2.60364	2.22487	1.95078	2.40686	2.47276	2.58143	2.48137	2.4004	2.44908	2.2498	2.56521	0.073315272	0.447768907	16	4	24	0.074536739	0.683293901	24	7.54943611	0.066128232
ENSMUSG00000026566	Mpzl1	myelin protein zero-like 1 [Source:MGI Symbol;Acc:MGI:1915731]	16.3026	15.9327	14.4636	12.7097	15.9771	15.2029	18.7934	15.936	15.737	14.4002	17.6374	13.7271	18.1828	15.84	8.93E-04	0.071344007	4	12	24	0.130263924	0.740714253	24	23.92496502	0.106712703
ENSMUSG00000026568	Mpc2	mitochondrial pyruvate carrier 2 [Source:MGI Symbol;Acc:MGI:1917706]	95.5646	102.662	115.438	140.975	110.035	110.556	112.398	125.862	116.215	117.451	130.512	109.684	137.71	121.583	0.763955526	0.966018836	16	8	24	0.279385304	0.768040178	24	10.89938866	0.083157494
ENSMUSG00000026571	Dcaf6	DDB1 and CUL4 associated factor 6 [Source:MGI Symbol;Acc:MGI:1921356]	31.3141	30.9646	32.5379	30.8886	32.0409	31.3018	29.7358	31.8301	29.3519	34.2458	30.8015	31.6032	32.1457	29.0121	0.877082091	0.966018836	16	16	24	0.441858868	0.805393068	24	14.86560606	0.029751509
ENSMUSG00000026574	Dpt	dermatopontin [Source:MGI Symbol;Acc:MGI:1928392]	5.22986	8.59048	6.29852	6.28699	5.85947	4.35735	4.68392	9.164	10.4222	4.15848	5.44699	5.84048	6.74772	6.57281	0.031969981	0.324385159	8	16	24	0.043668146	0.626787045	24	6.134173383	0.311090974
ENSMUSG00000026575	Nme7	NME/NM23 family member 7 [Source:MGI Symbol;Acc:MGI:2449121]	8.31844	8.65546	9.33026	8.96429	9.39228	9.1125	8.96769	8.08693	9.72187	8.05147	8.68631	8.06836	8.27643	9.15781	0.869721097	0.966018836	12	12	24	0.530265348	0.824800121	24	10.27002927	0.036186881
ENSMUSG00000026576	Atp1b1	ATPase, Na+/K+ transporting, beta 1 polypeptide [Source:MGI Symbol;Acc:MGI:88108]	389.321	331.694	363.418	337.185	313.849	371.146	358.714	342.598	274.251	409.053	315.083	348.579	316.164	372.332	0.935593607	0.966018836	4	16	24	0.815222403	0.940912535	24	22.64204457	0.038767376
ENSMUSG00000026577	Blzf1	basic leucine zipper nuclear factor 1 [Source:MGI Symbol;Acc:MGI:1201607]	15.4239	15.624	16.3516	15.0319	15.7169	15.6046	15.7548	14.6484	14.695	14.6703	15.7102	15.1551	16.1909	15.9515	0.92721845	0.966018836	4	12	24	0.499964848	0.80880169	24	22.92676639	0.022999896
ENSMUSG00000026578	Ccdc181	coiled-coil domain containing 181 [Source:MGI Symbol;Acc:MGI:1922145]	17.7893	15.1846	14.667	15.4794	15.1897	17.4627	15.5556	15.9581	14.3014	16.08	17.3665	16.4467	16.7158	17.3578	0.026841899	0.305155548	24	12	24	0.063825424	0.663876076	24	21.08320481	0.078993156
ENSMUSG00000026581	Sell	selectin, lymphocyte [Source:MGI Symbol;Acc:MGI:98279]	1.16001	1.68912	2.93195	2.07387	1.71973	1.78102	1.76247	1.37332	2.41576	1.26467	1.46196	1.39488	1.98416	1.30867	0.675497492	0.966018836	12	20	24	0.371776919	0.793398709	24	9.28305264	0.134534713
ENSMUSG00000026584	Scyl3	SCY1-like 3 (S. cerevisiae) [Source:MGI Symbol;Acc:MGI:1921385]	8.71289	8.81213	8.41584	8.52139	10.7466	7.95803	10.7973	7.81596	9.89964	9.2314	8.20182	8.45105	8.22261	7.81993	0.935593607	0.966018836	16	16	24	0.761154291	0.92277295	24	15.13868011	0.040094944
ENSMUSG00000026585	Kifap3	kinesin-associated protein 3 [Source:MGI Symbol;Acc:MGI:107566]	95.1601	87.0701	88.8373	86.1111	85.5092	91.8003	85.0843	83.6204	77.1225	95.3068	90.876	91.5782	94.3815	95.6132	0.919571723	0.966018836	4	8	24	0.42640682	0.805393068	24	20.74014766	0.045725266
ENSMUSG00000026586	Prrx1	paired related homeobox 1 [Source:MGI Symbol;Acc:MGI:97712]	3.10206	4.23698	3.99336	3.11402	3.72525	2.95221	3.43629	4.13773	5.58961	2.8374	3.38928	3.74269	3.60918	3.82434	0.019643089	0.258568924	12	4	24	0.097890604	0.71175955	24	6.216516911	0.164230776
ENSMUSG00000026587	Astn1	astrotactin 1 [Source:MGI Symbol;Acc:MGI:1098567]	16.7715	15.1723	16.099	15.916	15.04	17.2883	15.9552	14.6293	12.858	17.2996	13.948	15.7277	15.0182	16.8473	0.888482515	0.966018836	24	20	24	0.679837958	0.892816607	24	21.07762378	0.04114339
ENSMUSG00000026589	Sec16b	SEC16 homolog B (S. cerevisiae) [Source:MGI Symbol;Acc:MGI:2148802]	0.692954	0.855457	0.623538	0.677475	0.694946	0.778709	0.713107	0.824643	1.11919	0.79859	0.568043	0.774903	0.711511	0.677934	0.93447422	0.966018836	8	12	24	0.432956462	0.805393068	24	6.22594476	0.054472639
ENSMUSG00000026594	Ralgps2	Ral GEF with PH domain and SH3 binding motif 2 [Source:MGI Symbol;Acc:MGI:1925505]	11.2942	11.0503	9.35353	12.2054	9.77189	12.3179	9.83434	10.1242	9.94331	10.8823	11.1985	11.4477	10.4842	11.621	0.245061943	0.741580974	24	12	24	0.505601559	0.81137931	24	18.74616049	0.054238041
ENSMUSG00000026596	Abl2	v-abl Abelson murine leukemia viral oncogene 2 (arg, Abelson-related gene) [Source:MGI Symbol;Acc:MGI:87860]	5.24313	5.13733	5.0271	5.17669	5.16063	5.16881	5.26366	5.01666	4.72479	6.02187	5.52977	5.64295	5.40627	5.54577	0.326899754	0.845515883	24	12	24	0.388382338	0.799546792	24	17.34173188	0.03444682
ENSMUSG00000026600	Soat1	sterol O-acyltransferase 1 [Source:MGI Symbol;Acc:MGI:104665]	6.54779	6.22644	6.38679	6.16199	6.61209	6.64196	6.94474	5.44879	5.96138	5.90847	6.44032	6.03959	5.74757	5.83133	0.287843582	0.793714531	24	8	24	0.058033465	0.656248849	24	18.74968164	0.063557862
ENSMUSG00000026603	Smyd2	SET and MYND domain containing 2 [Source:MGI Symbol;Acc:MGI:1915889]	39.1747	39.0933	42.442	40.6718	38.5037	43.1124	37.1941	39.8799	37.6468	42.4748	38.8321	38.0979	36.9003	37.8213	0.192357053	0.669841558	16	12	24	0.254574579	0.768040178	24	12.70158417	0.041397487
ENSMUSG00000026604	Ptpn14	protein tyrosine phosphatase, non-receptor type 14 [Source:MGI Symbol;Acc:MGI:102467]	4.55542	5.1672	4.05379	4.73814	5.23997	4.05727	5.44567	4.82642	5.36468	4.73487	4.7036	4.9219	5.06734	4.32818	0.806004771	0.966018836	4	20	24	0.969664834	0.992868519	24	0.370179268	0.011248545
ENSMUSG00000026608	Kctd3	potassium channel tetramerisation domain containing 3 [Source:MGI Symbol;Acc:MGI:2444629]	16.1487	14.6553	17.0881	15.4484	13.944	14.9317	16.5998	15.0984	13.5828	17.2244	14.07	14.5473	15.3462	16.057	0.700065774	0.966018836	16	4	24	0.61846101	0.868968106	24	4.946091304	0.040044848
ENSMUSG00000026610	Esrrg	estrogen-related receptor gamma [Source:MGI Symbol;Acc:MGI:1347056]	8.16662	7.62361	6.97771	7.03379	5.53563	5.88232	6.99734	6.31045	5.78198	7.42924	5.16332	5.25398	5.71541	6.5088	0.289828769	0.795531262	16	4	24	0.121324979	0.733729587	24	5.138171936	0.111893367
ENSMUSG00000026614	Slc30a10	solute carrier family 30, member 10 [Source:MGI Symbol;Acc:MGI:2685058]	0.611148	0.805281	0.950922	0.643262	0.556717	0.507261	0.708316	0.477573	0.986071	0.453161	0.401452	0.600938	0.34596	0.592275	0.179603275	0.655480352	12	8	24	0.116429633	0.727799063	24	7.119567098	0.117484741
ENSMUSG00000026615	Eprs	glutamyl-prolyl-tRNA synthetase [Source:MGI Symbol;Acc:MGI:97838]	42.45	42.7139	42.2238	41.6131	43.8019	43.5474	39.4688	41.43	40.4614	42.8538	44.7102	41.5036	45.0059	44.9852	0.93447422	0.966018836	20	16	24	0.769721513	0.925855377	24	17.65290729	0.015702816
ENSMUSG00000026617	Bpnt1	3'(2'), 5'-bisphosphate nucleotidase 1 [Source:MGI Symbol;Acc:MGI:1338800]	40.2658	31.1101	32.0207	32.9166	31.6175	36.3685	35.3122	36.1881	27.1348	38.1376	35.3133	32.6577	34.5822	36.7156	0.964321074	0.968944646	4	8	24	0.360223732	0.785733735	24	21.79367465	0.080136108
ENSMUSG00000026618	Iars2	isoleucine-tRNA synthetase 2, mitochondrial [Source:MGI Symbol;Acc:MGI:1919586]	23.2855	21.2913	20.8303	22.6358	21.941	23.1658	21.2206	22.2913	20.2663	25.15	21.8958	22.3372	22.8711	22.9206	0.581070934	0.966018836	24	12	24	0.597796703	0.858787489	24	17.87385363	0.030410828
ENSMUSG00000026620	Mark1	MAP/microtubule affinity regulating kinase 1 [Source:MGI Symbol;Acc:MGI:2664902]	10.3035	8.7914	9.12567	9.22115	8.50373	9.53314	9.47763	9.4444	7.26525	11.6895	8.11446	9.38908	8.55535	10.0935	0.570349842	0.966018836	4	8	24	0.935051189	0.983765461	24	23.07665865	0.022273198
ENSMUSG00000026623	Lpgat1	lysophosphatidylglycerol acyltransferase 1 [Source:MGI Symbol;Acc:MGI:2446186]	23.4629	22.5209	22.7526	22.2019	21.936	23.2532	22.8069	21.4212	20.9515	22.9196	22.5156	22.5449	22.3469	24.0892	0.745199643	0.966018836	4	8	24	0.477616904	0.805393068	24	22.34945375	0.024164549
ENSMUSG00000026626	Ppp2r5a	protein phosphatase 2, regulatory subunit B', alpha [Source:MGI Symbol;Acc:MGI:2388479]	9.82706	9.16613	12.4672	17.9112	11.2796	10.6442	12.4529	15.4488	15.6123	14.1922	19.3897	14.8192	20.2252	15.2465	0.906657897	0.966018836	16	8	24	0.292502515	0.768040178	24	11.32554402	0.142002965
ENSMUSG00000026627	Pacc1	proton activated chloride channel 1 [Source:MGI Symbol;Acc:MGI:1914200]	9.48656	6.67692	6.31046	7.58506	7.77003	9.07354	6.76207	7.29464	5.9734	6.99338	8.34884	7.60456	7.14515	7.16548	0.009067305	0.192760771	24	12	24	0.010919939	0.51149922	24	19.46820628	0.171659983
ENSMUSG00000026628	Atf3	activating transcription factor 3 [Source:MGI Symbol;Acc:MGI:109384]	7.03341	3.41968	10.1557	4.21629	12.2703	7.64106	5.83053	5.56136	3.17549	3.20321	15.2386	3.71378	4.68391	3.40582	0.09184219	0.484826421	20	20	24	0.162093288	0.76636332	24	17.42122917	0.457656203
ENSMUSG00000026630	Batf3	basic leucine zipper transcription factor, ATF-like 3 [Source:MGI Symbol;Acc:MGI:1925491]	0.499109	0.993359	0.339106	1.03691	0.780409	0.681856	1.01263	0.835012	1.13218	0.979181	1.01349	0.812927	0.812649	0.994283	0.685043116	0.966018836	16	8	24	0.689560231	0.897245516	24	10.39627939	0.058974287
ENSMUSG00000026632	Tatdn3	TatD DNase domain containing 3 [Source:MGI Symbol;Acc:MGI:1916222]	22.2008	24.1676	20.908	20.438	20.4601	18.5766	19.5185	22.3964	26.599	16.9584	24.2782	18.7797	24.1065	21.3411	0.243857167	0.741580974	8	16	24	0.328509556	0.768040178	24	5.024025072	0.100439117
ENSMUSG00000026634	Angel2	angel homolog 2 [Source:MGI Symbol;Acc:MGI:1196310]	11.6677	14.2119	13.6222	15.4709	13.259	13.2281	13.6736	12.5523	15.4884	13.5454	13.9096	14.2693	13.6559	13.7276	0.81872541	0.966018836	12	16	24	0.136824167	0.747972781	24	10.8534704	0.07220148
ENSMUSG00000026637	Traf5	TNF receptor-associated factor 5 [Source:MGI Symbol;Acc:MGI:107548]	1.32806	1.74442	1.47037	1.14902	1.71105	1.30384	1.56475	1.60353	2.17653	1.52575	1.75086	1.57946	1.75097	1.25975	0.858266942	0.966018836	20	20	24	0.877026545	0.965814767	24	7.895523503	0.03094116
ENSMUSG00000026638	Irf6	interferon regulatory factor 6 [Source:MGI Symbol;Acc:MGI:1859211]	2.60958	2.63044	2.46122	2.42776	2.87866	2.33229	2.44441	2.5655	2.40866	2.6088	2.74026	2.68119	2.28161	2.40987	0.505132703	0.966018836	20	16	24	0.255368663	0.768040178	24	15.89534431	0.042293703
ENSMUSG00000026639	Lamb3	laminin, beta 3 [Source:MGI Symbol;Acc:MGI:99915]	0.255629	0.297208	1.10205	0.440595	0.436922	0.44759	0.251653	0.685427	0.260442	0.28053	0.552711	1.06495	0.828509	0.613153	0.813775138	0.966018836	24	4	24	0.989611274	0.997887893	24	0.005396128	0.014361187
ENSMUSG00000026640	Plxna2	plexin A2 [Source:MGI Symbol;Acc:MGI:107684]	13.6187	12.6671	11.8539	12.4544	12.0444	12.696	12.597	13.4517	10.5464	14.1765	10.0523	13.0153	12.0351	12.8693	0.604964847	0.966018836	8	4	24	0.539990891	0.829752479	24	0.813875217	0.053360912
ENSMUSG00000026641	Usf1	upstream transcription factor 1 [Source:MGI Symbol;Acc:MGI:99542]	20.7592	23.0739	17.1535	15.5667	23.202	26.049	20.5531	22.7821	22.851	24.731	18.9602	21.0943	20.5237	18.3927	0.916556573	0.966018836	24	12	24	0.642507214	0.878809617	24	21.06417345	0.076807881
ENSMUSG00000026643	Nmt2	N-myristoyltransferase 2 [Source:MGI Symbol;Acc:MGI:1202298]	22.6271	20.4347	19.9929	20.7823	20.0903	20.7801	20.8625	19.2603	19.5849	18.8357	21.084	20.6876	20.5862	21.0994	0.339853834	0.859092804	4	8	24	0.061366315	0.661066697	24	21.83536375	0.053330779
ENSMUSG00000026644	Acbd7	acyl-Coenzyme A binding domain containing 7 [Source:MGI Symbol;Acc:MGI:1925495]	1.515	2.41341	1.76293	1.83655	2.08308	2.67394	1.58271	2.11483	1.98637	1.86179	2.36103	2.15879	1.45024	1.64592	0.032466698	0.324908923	24	4	24	0.38775194	0.799546792	24	15.83555985	0.093774793
ENSMUSG00000026646	Suv39h2	suppressor of variegation 3-9 2 [Source:MGI Symbol;Acc:MGI:1890396]	1.53102	1.58904	1.9683	2.0166	2.03314	1.89589	1.65485	1.6144	1.74161	1.5784	1.80312	1.46679	1.56641	1.47783	0.145153076	0.590732968	20	8	24	0.023311433	0.581965446	24	13.72052313	0.088244846
ENSMUSG00000026648	Dclre1c	DNA cross-link repair 1C [Source:MGI Symbol;Acc:MGI:2441769]	5.39985	5.90413	5.90495	5.74752	5.7551	5.72644	5.0831	5.59926	6.2995	4.86516	5.63954	5.86832	6.36377	5.53038	0.852921792	0.966018836	12	4	24	0.899941073	0.971572371	24	4.679655957	0.016614803
ENSMUSG00000026649	Cfap126	cilia and flagella associated protein 126 [Source:MGI Symbol;Acc:MGI:1922722]	2.34826	3.21599	3.24432	3.14094	3.08474	2.35567	3.16469	2.54949	2.87451	2.13793	3.24631	3.51299	1.90408	3.49679	0.915082018	0.966018836	20	8	24	0.815749072	0.941277631	24	10.65772545	0.053448071
ENSMUSG00000026650	Meig1	meiosis expressed gene 1 [Source:MGI Symbol;Acc:MGI:1202878]	1.28618	1.69393	1.80558	1.66784	1.30845	1.30198	1.54643	0.989892	2.17324	1.57449	0.868711	2.18768	1.21446	1.86683	0.43565473	0.966018836	12	16	24	0.470795909	0.805393068	24	7.642724327	0.116417873
ENSMUSG00000026655	Fam107b	family with sequence similarity 107, member B [Source:MGI Symbol;Acc:MGI:1913790]	21.918	20.563	20.3931	20.7014	19.3226	21.1693	18.4854	20.1031	18.2271	23.1624	18.6016	20.2479	21.8565	21.3785	0.929527443	0.966018836	4	16	24	0.933714334	0.983329045	24	2.093785241	0.014346313
ENSMUSG00000026656	Fcgr2b	Fc receptor, IgG, low affinity IIb [Source:MGI Symbol;Acc:MGI:95499]	5.56209	7.09089	7.77281	6.32676	7.84636	6.25769	5.82823	6.81345	7.56908	5.39622	7.73637	6.44524	6.05613	5.33378	0.133764886	0.568189321	20	8	24	0.385289937	0.799546792	24	12.45479131	0.08993744
ENSMUSG00000026657	Frmd4a	FERM domain containing 4A [Source:MGI Symbol;Acc:MGI:1919850]	21.2584	15.2612	17.5894	15.9315	22.7688	19.8326	20.9789	16.3859	13.6275	22.7801	16.8569	19.1364	17.5578	17.4172	0.192658608	0.670488962	20	16	24	0.095701368	0.71175955	24	18.90681878	0.174109835
ENSMUSG00000026659	Dusp12	dual specificity phosphatase 12 [Source:MGI Symbol;Acc:MGI:1890614]	9.03423	9.45481	9.40103	9.31957	9.538	9.16908	10.171	9.5857	9.4697	9.02316	10.0625	9.4032	10.2957	9.76086	0.47028201	0.966018836	4	12	24	0.491772676	0.805393068	24	0.528377254	0.02182546
ENSMUSG00000026662	Sephs1	selenophosphate synthetase 1 [Source:MGI Symbol;Acc:MGI:1923580]	8.56939	9.71071	10.0832	9.98842	8.83154	9.34524	8.68549	8.27308	9.22083	9.53993	8.01115	9.35269	8.14948	8.706	0.088377194	0.473285995	16	12	24	0.178251449	0.768040178	24	10.64075568	0.061793661
ENSMUSG00000026663	Atf6	activating transcription factor 6 [Source:MGI Symbol;Acc:MGI:1926157]	9.98057	8.75917	9.32222	9.20077	9.57689	9.652	9.35501	9.35673	8.01884	9.74904	9.05037	9.35654	8.92047	9.755	0.92721845	0.966018836	24	12	24	0.394005281	0.799546792	24	19.93202644	0.03990883
ENSMUSG00000026664	Phyh	phytanoyl-CoA hydroxylase [Source:MGI Symbol;Acc:MGI:891978]	50.215	57.476	59.5219	67.0752	51.2703	52.802	55.2057	64.6967	59.0194	65.773	54.991	55.8025	62.1692	61.9981	0.031295391	0.324385159	16	4	24	0.013705557	0.529134128	24	8.295891199	0.128207337
ENSMUSG00000026667	Uhmk1	U2AF homology motif (UHM) kinase 1 [Source:MGI Symbol;Acc:MGI:1341908]	97.6683	88.3337	87.7066	89.9184	89.373	92.4515	85.3553	82.9318	77.2902	91.4307	89.8175	94.7567	94.6525	95.2668	0.217827365	0.704989686	24	12	24	0.163064728	0.76696232	24	20.37689751	0.065655759
ENSMUSG00000026669	Mcm10	minichromosome maintenance 10 replication initiation factor [Source:MGI Symbol;Acc:MGI:1917274]	0.489216	1.08176	0.807689	1.90067	0.988529	0.879187	1.00786	0.874763	1.2207	1.07199	0.885132	0.913868	0.739352	0.933493	0.097542159	0.501112642	16	12	24	0.067868519	0.671987767	24	11.29430045	0.179662102
ENSMUSG00000026670	Uap1	UDP-N-acetylglucosamine pyrophosphorylase 1 [Source:MGI Symbol;Acc:MGI:1334459]	35.3555	32.3653	34.2867	34.2295	34.8028	33.3375	31.0284	33.5847	30.1689	34.9426	36.1516	31.6907	32.433	30.8879	0.25851124	0.761738864	20	12	24	0.083603848	0.70046678	24	15.47296876	0.058828101
ENSMUSG00000026672	Optn	optineurin [Source:MGI Symbol;Acc:MGI:1918898]	18.8726	18.7911	19.7156	20.0346	18.3041	19.5783	18.1431	19.5631	17.2335	22.7715	19.25	19.3015	20.5431	19.09	0.941955432	0.966018836	16	16	24	0.626421363	0.872596805	24	13.02622895	0.0332241
ENSMUSG00000026674	Ddr2	discoidin domain receptor family, member 2 [Source:MGI Symbol;Acc:MGI:1345277]	6.2076	7.38675	6.26038	5.86016	7.30233	5.22423	6.75231	6.68246	8.59616	4.81649	6.36801	6.38801	5.87828	6.40613	0.338172159	0.85758369	8	8	24	0.497093069	0.807576732	24	5.300441851	0.084635529
ENSMUSG00000026675	Hsd17b7	hydroxysteroid (17-beta) dehydrogenase 7 [Source:MGI Symbol;Acc:MGI:1330808]	22.9124	20.5621	21.701	22.2816	21.3888	23.0883	21.0516	20.4785	19.8085	23.3035	22.0748	20.9845	21.044	21.2936	0.124028266	0.552144108	16	16	24	0.14745739	0.756090042	24	16.70057091	0.048198369
ENSMUSG00000026676	Ccdc3	coiled-coil domain containing 3 [Source:MGI Symbol;Acc:MGI:1921436]	3.23691	3.49671	4.14175	3.22459	3.80543	3.08484	3.12721	3.09435	4.04351	2.51304	3.47957	3.38705	3.12346	3.53952	0.321662653	0.83607295	12	12	24	0.671076342	0.889065421	24	8.093020971	0.051745545
ENSMUSG00000026678	Rgs5	regulator of G-protein signaling 5 [Source:MGI Symbol;Acc:MGI:1098434]	75.0663	74.7373	68.7005	70.2719	72.2955	69.9129	67.6805	73.8423	72.6319	67.8757	70.9146	72.0708	82.8428	74.0589	0.203788871	0.689899499	8	8	24	0.160274184	0.76578501	24	1.273463691	0.051373514
ENSMUSG00000026679	Enkur	enkurin, TRPC channel interacting protein [Source:MGI Symbol;Acc:MGI:1918483]	0.881168	1.91401	1.96359	1.50022	1.49164	0.993659	1.99154	0.721298	1.28314	1.34493	1.23297	1.65855	0.957153	2.06497	0.891742979	0.966018836	8	20	24	0.689463221	0.897245516	24	8.642713607	0.097726857
ENSMUSG00000026683	Nuf2	NUF2, NDC80 kinetochore complex component [Source:MGI Symbol;Acc:MGI:1914227]	0.786662	1.19755	1.23281	0.814911	1.08918	1.21211	1.04186	1.06643	1.12426	0.6042	1.00472	1.01676	1.04671	1.10908	0.087978327	0.472021905	12	4	24	0.483879257	0.805393068	24	2.008048165	0.06270445
ENSMUSG00000026687	Aldh9a1	aldehyde dehydrogenase 9, subfamily A1 [Source:MGI Symbol;Acc:MGI:1861622]	21.5002	20.514	20.2753	19.5826	21.509	20.1735	22.0238	22.2048	21.5305	23.2165	20.4407	21.4505	23.1068	22.4353	0.54278565	0.966018836	4	20	24	0.33349824	0.769685083	24	2.752844492	0.033404466
ENSMUSG00000026688	Mgst3	microsomal glutathione S-transferase 3 [Source:MGI Symbol;Acc:MGI:1913697]	63.2223	71.0028	64.8789	69.5228	70.0002	62.803	61.8572	70.1771	78.4967	64.1522	73.6797	66.6564	73.4701	69.9164	0.622132989	0.966018836	8	20	24	0.326659007	0.768040178	24	8.834685958	0.056531901
ENSMUSG00000026692	Fmo4	flavin containing monooxygenase 4 [Source:MGI Symbol;Acc:MGI:2429497]	0.458874	0.973699	0.868517	0.787354	0.642025	0.577259	0.738644	0.817021	0.870337	1.04624	0.439368	0.924787	0.678279	0.875489	0.113258228	0.530777648	8	12	24	0.064082788	0.663876076	24	7.648631387	0.136103967
ENSMUSG00000026694	Eef1aknmt	EEF1A lysine and N-terminal methyltransferase [Source:MGI Symbol;Acc:MGI:1918699]	3.9829	3.67612	3.86672	3.74776	3.76403	3.53888	3.67777	3.85355	3.59909	4.36862	3.44468	4.41937	4.16844	3.75306	0.860993221	0.966018836	4	16	24	0.963764971	0.990924345	24	22.02430269	0.009446943
ENSMUSG00000026696	Vamp4	vesicle-associated membrane protein 4 [Source:MGI Symbol;Acc:MGI:1858730]	71.0912	65.3363	66.1665	64.425	62.874	64.63	64.2535	60.6648	57.6488	65.4568	68.0107	58.8874	60.6241	67.3252	0.795269577	0.966018836	20	4	24	0.939355534	0.985128092	24	20.03014539	0.01141838
ENSMUSG00000026697	Myoc	myocilin [Source:MGI Symbol;Acc:MGI:1202864]	16.4775	18.7875	19.8717	15.9126	20.0247	15.4367	18.8972	15.7906	29.3797	10.0611	17.8994	18.3358	15.7752	17.3667	0.745118544	0.966018836	12	12	24	0.77888322	0.928881414	24	5.281192754	0.089361786
ENSMUSG00000026698	Pigc	phosphatidylinositol glycan anchor biosynthesis, class C [Source:MGI Symbol;Acc:MGI:1914542]	13.7535	14.1452	13.5819	13.5496	13.6361	15.3695	14.4796	14.0171	12.4937	14.1229	13.9805	14.5335	14.1501	12.5211	0.002057111	0.100255953	24	12	24	0.024584793	0.586541342	24	19.98471051	0.071758818
ENSMUSG00000026701	Prdx6	peroxiredoxin 6 [Source:MGI Symbol;Acc:MGI:894320]	89.6889	89.6868	106.277	97.1187	87.6522	100.112	95.4841	89.8279	83.4531	96.0018	80.1479	86.248	84.2881	89.5336	0.897425765	0.966018836	16	4	24	0.802090371	0.936565832	24	8.922875791	0.026838741
ENSMUSG00000026705	Klhl20	kelch-like 20 [Source:MGI Symbol;Acc:MGI:2444855]	15.5011	13.7564	13.4491	13.5692	13.8299	13.7798	12.6746	14.8864	11.4311	14.9037	13.5797	13.1183	13.2542	13.5602	0.885059765	0.966018836	16	20	24	0.819377136	0.942753508	24	20.60626571	0.025302255
ENSMUSG00000026707	Nsun6	NOL1/NOP2/Sun domain family member 6 [Source:MGI Symbol;Acc:MGI:1921705]	4.52871	4.42746	4.70939	3.80162	4.63131	3.5218	3.81054	4.04996	4.38612	3.85562	3.94183	4.14498	4.95209	4.89937	0.37215823	0.894599261	8	8	24	0.262187938	0.768040178	24	4.488201648	0.077848252
ENSMUSG00000026708	Cenpl	centromere protein L [Source:MGI Symbol;Acc:MGI:1917704]	2.70526	3.16992	2.64502	2.48685	3.04969	2.2567	2.4714	3.43319	2.27967	2.13927	3.10347	2.30781	2.64009	2.50596	0.371875382	0.894537464	8	16	24	0.790300564	0.932883394	24	2.47201281	0.040016768
ENSMUSG00000026709	Dars2	aspartyl-tRNA synthetase 2 (mitochondrial) [Source:MGI Symbol;Acc:MGI:2442510]	14.7519	12.7825	12.4358	12.851	13.7674	13.5012	11.6349	12.9586	9.79807	10.273	13.5946	12.5813	12.6691	11.5449	0.185796491	0.662239322	20	16	24	0.030529549	0.597890721	24	20.38385795	0.130221383
ENSMUSG00000026712	Mrc1	mannose receptor, C type 1 [Source:MGI Symbol;Acc:MGI:97142]	4.42705	5.87939	5.3519	4.62444	5.64362	3.41263	5.39581	5.16943	6.35234	3.0978	4.98264	4.73014	5.32016	4.7845	0.300823756	0.809362476	12	4	24	0.394569714	0.799546792	24	4.842147936	0.124200122
ENSMUSG00000026715	Serpinc1	serine (or cysteine) peptidase inhibitor, clade C (antithrombin), member 1 [Source:MGI Symbol;Acc:MGI:88095]	2.1498	1.73023	1.70507	1.96142	1.99559	1.62529	1.97522	1.73026	1.70276	1.75872	2.0026	1.32792	1.64273	1.65921	0.369262716	0.893596186	20	4	24	0.803772358	0.937296224	24	15.0556348	0.02542186
ENSMUSG00000026718	Stam	signal transducing adaptor molecule (SH3 domain and ITAM motif) 1 [Source:MGI Symbol;Acc:MGI:1329014]	52.6961	45.246	44.2476	47.4247	45.8859	48.0087	48.0134	40.8806	43.198	47.6882	49.7842	44.604	46.4318	46.0639	0.115169104	0.536202194	4	8	24	0.141199435	0.751689872	24	18.99088585	0.066482677
ENSMUSG00000026721	Rabgap1l	RAB GTPase activating protein 1-like [Source:MGI Symbol;Acc:MGI:1352507]	55.3675	57.5456	50.8074	58.9115	52.0036	53.5442	46.9531	49.367	54.8813	52.2859	58.3851	58.3385	60.4146	59.1811	0.73379652	0.966018836	16	20	24	0.954135353	0.988601783	24	16.74940487	0.012999209
ENSMUSG00000026723	Trdmt1	tRNA aspartic acid methyltransferase 1 [Source:MGI Symbol;Acc:MGI:1274787]	3.67219	5.33611	4.73794	4.57617	3.84514	3.65512	4.27653	4.1949	5.43324	3.84699	4.24573	4.40211	4.14321	5.24603	0.059717207	0.406067671	12	12	24	0.018904905	0.561252866	24	6.836592347	0.156799015
ENSMUSG00000026727	Rsu1	Ras suppressor protein 1 [Source:MGI Symbol;Acc:MGI:103040]	78.4997	73.834	71.0787	70.3691	86.6234	75.2674	72.394	69.8573	74.0343	67.2833	76.0422	70.4983	75.4238	74.8588	0.125573493	0.555618842	20	20	24	0.31357172	0.768040178	24	19.6554102	0.052986287
ENSMUSG00000026728	Vim	vimentin [Source:MGI Symbol;Acc:MGI:98932]	313.084	288.942	308.39	321.857	386.742	347.27	331.678	338.996	301.364	356.55	326.997	308.58	291.015	278.731	0.097542159	0.501112642	20	12	24	0.030527694	0.597890721	24	16.0268323	0.116926928
ENSMUSG00000026730	Pter	phosphotriesterase related [Source:MGI Symbol;Acc:MGI:107372]	7.31653	9.93003	8.73184	8.51011	10.4272	7.44378	9.94157	8.47387	10.5512	8.58427	9.06728	8.11556	8.12597	8.5722	0.172322601	0.648362388	12	12	24	0.374815049	0.794819447	24	9.608413384	0.084065339
ENSMUSG00000026737	Pip4k2a	phosphatidylinositol-5-phosphate 4-kinase, type II, alpha [Source:MGI Symbol;Acc:MGI:1298206]	40.8345	38.6658	40.6051	37.8297	39.1635	39.5138	41.9215	38.141	38.1363	40.5541	35.9817	37.1459	36.7058	37.4986	0.886572916	0.966018836	4	16	24	0.851021804	0.955422325	24	1.825799733	0.011582511
ENSMUSG00000026739	Bmi1	Bmi1 polycomb ring finger oncogene [Source:MGI Symbol;Acc:MGI:88174]	12.7443	13.8021	14.5805	13.5663	13.8489	12.3407	12.3807	12.3385	13.7722	11.7593	12.9846	12.4931	10.8781	11.5439	0.337748746	0.85758369	12	16	24	0.078339544	0.68893025	24	11.42487312	0.065722774
ENSMUSG00000026740	Dnajc1	DnaJ heat shock protein family (Hsp40) member C1 [Source:MGI Symbol;Acc:MGI:103268]	7.69797	7.01613	6.21921	5.37584	7.40362	6.48357	8.18487	5.69754	7.3013	6.59171	7.29765	6.8813	6.42472	6.87978	0.745118544	0.966018836	4	12	24	0.359254008	0.785405943	24	21.68290361	0.082360005
ENSMUSG00000026743	Mllt10	myeloid/lymphoid or mixed-lineage leukemia; translocated to, 10 [Source:MGI Symbol;Acc:MGI:1329038]	10.4425	9.61515	9.45463	11.2139	8.75564	8.91062	10.0099	9.88097	9.51135	11.8972	10.5419	8.69647	9.06125	9.29898	0.285027487	0.793714531	16	8	24	0.260554364	0.768040178	24	10.90390394	0.077331362
ENSMUSG00000026748	Plxdc2	plexin domain containing 2 [Source:MGI Symbol;Acc:MGI:1914698]	11.8388	13.126	11.4858	12.2611	13.5037	11.6151	12.554	13.9017	14.0412	11.7791	12.3925	14.2823	14.2001	12.8024	0.935593607	0.966018836	8	4	24	0.636865211	0.876483853	24	1.910202756	0.034743477
ENSMUSG00000026749	Nek6	NIMA (never in mitosis gene a)-related expressed kinase 6 [Source:MGI Symbol;Acc:MGI:1891638]	10.5213	7.71069	9.68941	7.58324	9.73241	8.6981	9.52665	10.4963	8.39419	10.3402	7.84089	7.94557	7.80355	8.78859	0.776152743	0.966018836	4	20	24	0.883537905	0.966818623	24	4.277308254	0.031007404
ENSMUSG00000026750	Psmb7	proteasome (prosome, macropain) subunit, beta type 7 [Source:MGI Symbol;Acc:MGI:107637]	143.704	146.404	143.323	139.274	151.343	137.348	133.184	136.615	150.961	120.749	153.902	133.353	148.917	143.297	0.935194469	0.966018836	20	4	24	0.980840647	0.995440517	24	1.894156973	0.007331631
ENSMUSG00000026753	Ppp6c	protein phosphatase 6, catalytic subunit [Source:MGI Symbol;Acc:MGI:1915107]	31.8513	33.0994	31.9564	33.7436	33.4515	30.909	31.8722	33.0496	34.0876	28.6408	37.4139	31.9487	34.3539	32.74	0.927004797	0.966018836	20	4	24	0.982009876	0.995935219	24	14.67855495	0.006395943
ENSMUSG00000026754	Golga1	golgi autoantigen, golgin subfamily a, 1 [Source:MGI Symbol;Acc:MGI:1924149]	22.3447	22.3411	21.1775	22.3255	19.9011	21.045	19.3104	19.6655	21.7951	19.8998	21.6499	20.1703	23.2477	19.7694	0.788254138	0.966018836	4	4	24	0.986747351	0.997339197	24	3.036523771	0.00525513
ENSMUSG00000026755	Arpc5l	actin related protein 2/3 complex, subunit 5-like [Source:MGI Symbol;Acc:MGI:1921442]	46.1605	47.0948	44.9055	48.5309	41.9301	46.963	46.2066	49.467	50.8141	50.2703	54.943	51.2399	54.5394	52.6452	0.710703678	0.966018836	8	4	24	0.754258685	0.920455694	24	5.978203614	0.020521554
ENSMUSG00000026761	Orc4	origin recognition complex, subunit 4 [Source:MGI Symbol;Acc:MGI:1347043]	36.5184	37.3798	34.1839	40.9629	40.3592	33.8926	36.618	30.5915	36.84	31.255	44.6631	32.1225	35.2082	35.216	0.893307667	0.966018836	20	4	24	0.357466024	0.784480156	24	15.37002593	0.080835749

ENSMUSG00000026764	Kif5c	kinesin family member 5C [Source:MGI Symbol;Acc:MGI:1098269]	145.085	126.215	127.048	127.082	127.379	133.423	126.154	123.91	112.391	137.541	128.155	135.446	135.127	137.741	0.446967971	0.966018836	24	12	24	0.222208937	0.768040178	24	21.09215455	0.0605128
ENSMUSG00000026765	Lypd6b	LY6/PLAUR domain containing 6B [Source:MGI Symbol;Acc:MGI:1919147]	4.96031	4.9659	6.02653	5.01128	4.61698	5.42674	4.44582	5.08334	4.69078	4.60704	4.28285	5.17717	5.12617	5.97124	0.658942124	0.966018836	8	12	24	0.321631875	0.768040178	24	4.449480343	0.071001527
ENSMUSG00000026766	Mmadhc	methylmalonic aciduria (cobalamin deficiency) cblD type, with homocystinuria [Source:MGI Symbol;Acc:MGI:1923786]	46.5752	43.9126	43.818	45.798	46.0642	42.4494	42.6117	41.2179	42.6652	43.8312	45.3269	44.964	47.0834	42.9586	0.557756884	0.966018836	20	12	24	0.269537989	0.768040178	24	17.73536612	0.035871387
ENSMUSG00000026767	Mindy3	MINDY lysine 48 deubiquitinase 3 [Source:MGI Symbol;Acc:MGI:1914210]	99.0916	83.6139	81.1655	82.7744	78.5999	84.9788	82.653	81.6192	67.207	84.0092	86.3858	80.5343	82.6635	78.9006	0.649212555	0.966018836	4	8	24	0.127018201	0.739868076	24	21.04322306	0.083909915
ENSMUSG00000026768	Itga8	integrin alpha 8 [Source:MGI Symbol;Acc:MGI:109442]	0.580495	0.75901	0.783938	0.517716	0.608324	0.579319	0.478082	0.77466	0.794148	1.57912	0.64227	0.563268	0.514157	0.579199	0.017495149	0.255326033	12	16	24	0.094144335	0.71175955	24	10.15107254	0.165331967
ENSMUSG00000026771	Spopl	speckle-type BTB/POZ protein-like [Source:MGI Symbol;Acc:MGI:1924107]	6.47068	7.00923	7.36935	6.76017	6.43287	6.67966	6.81489	6.54507	7.15396	6.30787	7.3573	7.19924	6.55475	6.60299	0.935732159	0.966018836	12	16	24	0.847672545	0.954054905	24	10.51634164	0.015462973
ENSMUSG00000026773	Pfkfb3	6-phosphofructo-2-kinase/fructose-2,6-biphosphatase 3 [Source:MGI Symbol;Acc:MGI:2181202]	18.0071	17.6314	21.7057	23.146	19.0693	20.9361	17.3951	25.6041	20.2533	25.3592	18.8667	18.6085	20.1539	22.9941	0.109247218	0.524035616	16	12	24	0.073162118	0.68034895	24	9.317694321	0.147925969
ENSMUSG00000026775	Yme1l1	YME1-like 1 (S. cerevisiae) [Source:MGI Symbol;Acc:MGI:1351651]	35.9617	39.3575	36.451	36.6018	38.043	34.0293	33.9909	34.8416	38.5877	31.7293	40.0872	33.2063	36.5337	36.2669	0.503972924	0.966018836	20	4	24	0.791358699	0.933280278	24	8.871432775	0.026473336
ENSMUSG00000026778	Prkcq	protein kinase C, theta [Source:MGI Symbol;Acc:MGI:97601]	35.1351	34.0475	31.2146	31.5048	31.9948	35.9241	30.2896	34.2983	28.7772	34.6989	29.4419	33.8848	35.6302	33.5136	0.196044629	0.673646834	24	12	24	0.232070507	0.768040178	24	23.10539908	0.066896098
ENSMUSG00000026779	Mastl	microtubule associated serine/threonine kinase-like [Source:MGI Symbol;Acc:MGI:1914371]	0.605779	0.697717	0.916945	0.655071	0.782816	0.650421	0.686179	0.52456	0.839737	0.892878	0.745253	0.633336	0.634555	0.677621	0.030691182	0.324150946	12	16	24	0.029668152	0.596648309	24	11.29887205	0.088085985
ENSMUSG00000026781	Acbd5	acyl-Coenzyme A binding domain containing 5 [Source:MGI Symbol;Acc:MGI:1921409]	49.8786	47.4899	45.4774	46.4748	44.2303	44.9928	43.8016	42.3315	41.825	43.1142	46.7707	44.0069	47.4165	44.7699	0.919571723	0.966018836	4	8	24	0.357802339	0.78475603	24	21.79251701	0.036817522
ENSMUSG00000026782	Abi2	abl interactor 2 [Source:MGI Symbol;Acc:MGI:106913]	17.6493	17.0716	18.1922	16.2617	16.2624	17.3684	17.3407	15.5688	15.3479	18.049	16.3039	16.6532	14.8335	16.4145	0.785001719	0.966018836	24	8	24	0.857878017	0.958106625	24	16.24369494	0.016261978
ENSMUSG00000026784	Pdss1	prenyl (solanesyl) diphosphate synthase, subunit 1 [Source:MGI Symbol;Acc:MGI:1889278]	4.06145	5.09264	5.87879	5.60292	6.15223	4.51733	5.18145	4.60269	6.01494	5.34179	5.94764	4.18159	4.38895	5.65781	0.011964854	0.213452992	20	4	24	0.009129525	0.502965379	24	10.89199403	0.179463325
ENSMUSG00000026785	Pkn3	protein kinase N3 [Source:MGI Symbol;Acc:MGI:2388285]	1.51535	1.02765	0.938959	1.23052	1.14208	1.09463	0.847929	1.38269	1.16679	1.4022	1.56737	0.971367	1.23002	1.27615	0.921572192	0.966018836	20	4	24	0.78592915	0.931158434	24	12.76277091	0.03884701
ENSMUSG00000026786	Apbb1ip	amyloid beta (A4) precursor protein-binding, family B, member 1 interacting protein [Source:MGI Symbol;Acc:MGI:1861354]	0.569604	1.18272	1.40555	1.23636	1.16516	0.680358	1.13428	1.01034	1.14785	1.01985	1.03232	0.755255	0.832077	0.897422	0.019280932	0.257669847	12	12	24	0.01705019	0.547196997	24	9.718324832	0.171134199
ENSMUSG00000026787	Gad2	glutamic acid decarboxylase 2 [Source:MGI Symbol;Acc:MGI:95634]	0.821918	2.1851	4.50477	1.7522	0.833835	1.93799	2.52208	0.746012	2.45755	1.21647	0.725304	1.67253	0.399429	2.442	0.285027487	0.793714531	12	8	24	0.299530632	0.768040178	24	7.377988268	0.331426254
ENSMUSG00000026788	Zbtb43	zinc finger and BTB domain containing 43 [Source:MGI Symbol;Acc:MGI:1919084]	5.25222	5.28854	5.31363	6.13868	4.76687	5.41838	5.41041	4.61883	5.54468	5.72991	6.23385	5.352	4.87738	4.98369	0.046535188	0.375011045	16	16	24	0.09785238	0.71175955	24	13.74973559	0.082443436
ENSMUSG00000026790	Odf2	outer dense fiber of sperm tails 2 [Source:MGI Symbol;Acc:MGI:1098824]	24.2257	24.2289	25.3387	25.0639	27.8074	26.0794	23.2107	24.6725	25.4502	23.8081	25.5607	25.4547	25.3648	24.0846	0.053681914	0.394331071	20	12	24	0.106156982	0.71810271	24	16.00094999	0.048682017
ENSMUSG00000026791	Slc2a8	solute carrier family 2, (facilitated glucose transporter), member 8 [Source:MGI Symbol;Acc:MGI:1860103]	8.17863	7.39944	7.4433	8.14114	7.4616	7.924	6.35575	8.58635	8.59738	9.24141	6.82177	8.9987	8.56311	8.29204	0.897570341	0.966018836	16	4	24	0.722471684	0.908924547	24	8.461654517	0.043042503
ENSMUSG00000026792	Lrsam1	leucine rich repeat and sterile alpha motif containing 1 [Source:MGI Symbol;Acc:MGI:2684789]	34.7971	27.0945	24.3723	26.5511	25.3271	30.2646	26.6728	30.6772	22.0047	37.7227	26.5845	31.4358	29.3547	28.4479	0.14313801	0.585944911	24	12	24	0.499738757	0.808791684	24	21.13805737	0.094861633
ENSMUSG00000026796	Niban2	niban apoptosis regulator 2 [Source:MGI Symbol;Acc:MGI:2442910]	30.8314	24.7226	25.5953	26.4128	33.0756	28.4698	30.0458	28.0508	24.9294	28.888	28.0712	28.0078	27.8828	23.8037	0.05005312	0.385784509	20	12	24	0.014793072	0.537924552	24	18.79464293	0.128035201
ENSMUSG00000026797	Stxbp1	syntaxin binding protein 1 [Source:MGI Symbol;Acc:MGI:107363]	101.395	82.4231	89.7668	88.9217	78.6554	102.775	97.157	94.6319	73.2617	139.883	75.5062	99.6102	85.5784	102.725	0.649212555	0.966018836	24	20	24	0.981003079	0.995519545	24	22.3520183	0.019121583
ENSMUSG00000026798	Coq4	coenzyme Q4 [Source:MGI Symbol;Acc:MGI:1098826]	7.66001	6.66045	6.22673	6.73259	7.24061	6.44964	7.89522	7.54213	6.26026	6.94066	6.57277	7.82766	8.77406	5.80713	0.010213213	0.195026763	4	8	24	0.086067029	0.704714207	24	21.75982465	0.120301723
ENSMUSG00000026799	Med27	mediator complex subunit 27 [Source:MGI Symbol;Acc:MGI:1916225]	21.6984	15.9257	17.1998	15.4677	15.3625	17.7299	15.7432	16.9991	15.1792	18.5346	13.9557	15.7812	17.8162	15.4002	0.5169953	0.966018836	4	16	24	0.39058698	0.799546792	24	0.177082598	0.075122507
ENSMUSG00000026803	Ttf1	transcription termination factor, RNA polymerase I [Source:MGI Symbol;Acc:MGI:105044]	4.52056	4.81423	4.91015	4.6238	6.45493	4.35473	5.09725	5.50593	5.9875	4.53452	5.1945	4.99866	5.28396	5.57412	0.836635326	0.966018836	12	12	24	0.77368834	0.92732018	24	8.316961588	0.038963264
ENSMUSG00000026806	Ddx31	DEAD/H box helicase 31 [Source:MGI Symbol;Acc:MGI:2682639]	4.17477	3.21786	3.36529	2.68971	3.5857	3.40007	3.87383	3.41404	2.66402	3.97495	2.99129	3.01158	3.68473	3.26086	0.105280783	0.518002936	4	8	24	0.248621036	0.768040178	24	22.87000818	0.096689151
ENSMUSG00000026807	Ak8	adenylate kinase 8 [Source:MGI Symbol;Acc:MGI:1916120]	1.77888	1.20668	1.13673	1.17022	1.18812	1.04596	1.24177	1.16587	1.00442	1.10035	1.05147	1.25659	1.16359	1.15298	0.248423582	0.746945512	4	8	24	0.090036343	0.7097999	24	23.17684872	0.081570888
ENSMUSG00000026810	Dpm2	dolichol-phosphate (beta-D) mannosyltransferase 2 [Source:MGI Symbol;Acc:MGI:1330238]	39.7504	36.6111	37.1257	40.1433	42.113	41.968	35.1965	35.3999	33.579	32.9495	37.2078	36.3004	36.6819	35.29	0.327894132	0.846828588	20	16	24	0.022884559	0.581965446	24	18.61079362	0.090898521
ENSMUSG00000026811	St6galnac6	ST6 (alpha-N-acetyl-neuraminyl-2,3-beta-galactosyl-1,3)-N-acetylgalactosaminide alpha-2,6-sialyltransferase 6 [Source:MGI Symbol;Acc:MGI:1355316]	25.9202	23.5442	25.1053	22.5162	23.7327	24.2947	23.4114	25.0841	24.4818	25.2482	23.8192	24.7534	24.9169	25.039	0.945728192	0.966018836	12	8	24	0.421935507	0.804917634	24	2.743467694	0.026113493
ENSMUSG00000026812	Tsc1	TSC complex subunit 1 [Source:MGI Symbol;Acc:MGI:1929183]	9.15241	9.12964	9.5722	9.94977	9.85677	9.5514	9.02127	9.20383	9.10579	9.69951	8.81508	10.2888	9.72757	9.36849	0.560714538	0.966018836	16	16	24	0.401098341	0.799546792	24	15.88488276	0.029924732
ENSMUSG00000026814	Eng	endoglin [Source:MGI Symbol;Acc:MGI:95392]	4.53129	4.35002	4.24874	5.17183	4.43524	4.22942	4.79025	5.07606	5.16526	5.02008	3.86583	3.89763	4.54383	4.1156	0.212359178	0.701530532	16	4	24	0.243469537	0.768040178	24	8.101157906	0.077402714
ENSMUSG00000026816	Gtf3c5	general transcription factor IIIC, polypeptide 5 [Source:MGI Symbol;Acc:MGI:1917489]	8.39508	7.51046	7.14824	7.96939	9.89339	8.8508	8.39164	8.32458	7.74986	8.83516	7.95719	8.0839	7.64406	7.62066	0.076929733	0.453790923	24	12	24	0.056094007	0.65534544	24	17.59931304	0.093925542
ENSMUSG00000026817	Ak1	adenylate kinase 1 [Source:MGI Symbol;Acc:MGI:87977]	46.2859	51.89	51.7795	60.3506	46.2949	56.6906	53.3912	75.7109	60.266	80.3632	49.3248	54.3255	53.4773	56.3848	0.351315142	0.870418653	16	4	24	0.18766632	0.768040178	24	8.728992995	0.15803489
ENSMUSG00000026819	Slc25a25	solute carrier family 25 (mitochondrial carrier, phosphate carrier), member 25 [Source:MGI Symbol;Acc:MGI:1915913]	28.7469	20.9413	20.9052	22.3736	20.6977	26.5985	22.2803	22.9422	17.6614	24.653	20.7766	22.0517	22.1026	23.1684	0.368981219	0.893596186	4	8	24	0.192554335	0.768040178	24	21.58645856	0.113710752
ENSMUSG00000026820	Ptges2	prostaglandin E synthase 2 [Source:MGI Symbol;Acc:MGI:1917592]	16.6407	15.1016	17.2295	17.3018	18.3531	16.9924	15.2024	18.7223	14.4701	16.4699	16.1547	15.5654	16.3551	16.4962	0.881996584	0.966018836	16	12	24	0.759141146	0.92214435	24	15.01612804	0.028656833
ENSMUSG00000026821	Ralgds	ral guanine nucleotide dissociation stimulator [Source:MGI Symbol;Acc:MGI:107485]	20.3257	21.8404	23.8604	24.5264	24.1279	28.044	22.2238	24.1985	17.7744	24.9775	22.5599	20.8481	19.0392	21.2944	0.17953325	0.655480352	16	12	24	0.270624835	0.768040178	24	15.48707907	0.097123965
ENSMUSG00000026822	Lcn2	lipocalin 2 [Source:MGI Symbol;Acc:MGI:96757]	16.8357	29.6449	50.8062	37.687	29.9537	34.1347	25.1529	23.6087	28.4693	17.9985	26.5715	24.4008	33.5634	17.8573	0.823657258	0.966018836	12	20	24	0.665976867	0.887125207	24	12.31944651	0.146632173
ENSMUSG00000026824	Kcnj3	potassium inwardly-rectifying channel, subfamily J, member 3 [Source:MGI Symbol;Acc:MGI:104742]	8.53334	9.20989	9.18933	7.65179	8.57131	9.27154	8.34639	9.0636	8.4733	9.05282	7.76363	9.28292	8.23312	9.65832	0.522551072	0.966018836	8	12	24	0.374413855	0.794636296	24	3.379263445	0.04916744
ENSMUSG00000026825	Dnm1	dynamin 1 [Source:MGI Symbol;Acc:MGI:107384]	74.864	65.2902	73.7116	69.6021	59.3055	80.9746	72.0341	70.969	62.348	104.989	62.4064	73.8313	71.7176	77.6229	0.676344318	0.966018836	24	20	24	0.980320398	0.995255072	24	8.512343943	0.016603591
ENSMUSG00000026826	Nr4a2	nuclear receptor subfamily 4, group A, member 2 [Source:MGI Symbol;Acc:MGI:1352456]	6.4794	6.35189	6.40399	6.54345	7.79131	5.72687	7.19641	6.79532	7.62898	8.69495	7.05887	7.2194	7.08933	7.00113	0.722296281	0.966018836	16	16	24	0.239855061	0.768040178	24	11.89062638	0.074401463
ENSMUSG00000026827	Gpd2	glycerol phosphate dehydrogenase 2, mitochondrial [Source:MGI Symbol;Acc:MGI:99778]	13.6637	12.378	14.5536	19.488	13.4615	14.2029	14.6228	14.6021	13.5243	17.9967	14.7728	15.1279	16.9649	15.0539	0.915475528	0.966018836	16	20	24	0.325533692	0.768040178	24	12.71599264	0.0891726
ENSMUSG00000026828	Galnt5	polypeptide N-acetylgalactosaminyltransferase 5 [Source:MGI Symbol;Acc:MGI:2179403]	8.0535	7.51141	6.50888	7.65985	6.90201	6.90098	5.81198	6.71056	6.64221	8.01895	7.42463	7.87927	8.19518	8.04625	0.953350544	0.966018836	4	8	24	0.947405005	0.987174098	24	17.73419704	0.017079408
ENSMUSG00000026829	Gbgt1	globoside alpha-1,3-N-acetylgalactosaminyltransferase 1 [Source:MGI Symbol;Acc:MGI:2449143]	0.747002	1.53663	0.984291	1.09001	1.67792	0.90969	1.16041	1.1554	1.41688	0.604061	1.18751	1.15602	1.14809	0.998354	0.890235289	0.966018836	20	20	24	0.99899349	0.999770694	24	5.167911046	0.093643878
ENSMUSG00000026830	Ermn	ermin, ERM-like protein [Source:MGI Symbol;Acc:MGI:1925017]	2.45	6.10304	9.26587	3.829	3.89957	5.22754	8.02655	4.25343	6.71608	2.63376	2.77721	4.97249	1.3957	4.5926	0.503972924	0.966018836	12	4	24	0.524582361	0.82207924	24	6.629080874	0.255675223
ENSMUSG00000026831	1700017K13Rik	RIKEN cDNA 1700017K13 gene [Source:MGI Symbol;Acc:MGI:1916577]	0.674586	0.934331	1.5333	1.359	0.882987	0.764078	1.94383	0.439282	0.373907	0.607828	1.22476	1.33179	0.449915	1.00199	0.847846021	0.966018836	20	12	24	0.71549578	0.906323093	24	17.50429229	0.110793949
ENSMUSG00000026832	Cytip	cytohesin 1 interacting protein [Source:MGI Symbol;Acc:MGI:2183535]	0.9157	0.802377	0.624699	1.35595	0.610673	0.737438	0.674167	1.41543	1.14447	0.516062	0.638402	0.823144	0.683429	0.727298	0.745199643	0.966018836	8	12	24	0.514710116	0.816837918	24	6.083663345	0.095462783
ENSMUSG00000026833	Olfm1	olfactomedin 1 [Source:MGI Symbol;Acc:MGI:1860437]	94.9788	73.6461	75.0503	81.2268	81.5191	86.8983	81.1864	83.4522	68.7533	106.177	81.7957	91.8318	87.5676	91.1736	0.148073804	0.590732968	24	12	24	0.446621105	0.805393068	24	19.38541382	0.078488963
ENSMUSG00000026835	Fcnb	ficolin B [Source:MGI Symbol;Acc:MGI:1341158]	0.645903	1.16729	3.72554	2.38871	1.50747	1.9775	1.05871	1.31078	1.54197	1.20578	1.00425	1.48087	1.91491	0.993899	0.46238066	0.966018836	12	20	24	0.398022857	0.799546792	24	11.06867305	0.202399019
ENSMUSG00000026836	Acvr1	activin A receptor, type 1 [Source:MGI Symbol;Acc:MGI:87911]	7.01045	5.92649	6.28122	5.64525	5.85853	6.43205	6.58058	6.09943	5.28297	6.54842	6.0081	5.35895	5.35336	5.30378	0.839634479	0.966018836	4	8	24	0.566998237	0.843665475	24	20.39404938	0.040398632
ENSMUSG00000026837	Col5a1	collagen, type V, alpha 1 [Source:MGI Symbol;Acc:MGI:88457]	13.9752	15.9026	13.7874	13.1337	16.4484	12.0663	16.5854	16.9205	17.7058	16.1357	13.3748	14.0084	15.4349	12.7117	0.916746631	0.966018836	12	12	24	0.546443625	0.833354874	24	6.415996719	0.073593294
ENSMUSG00000026839	Upp2	uridine phosphorylase 2 [Source:MGI Symbol;Acc:MGI:1923904]	12.0489	12.3156	8.89546	10.4521	10.0428	14.395	9.8848	13.5905	10.4225	9.17276	10.916	12.7851	11.4193	12.0099	0.085508246	0.466404237	8	4	24	0.077202058	0.687185556	24	23.08546789	0.158509653
ENSMUSG00000026840	Lamc3	laminin gamma 3 [Source:MGI Symbol;Acc:MGI:1344394]	1.21928	1.74307	1.44226	1.81034	1.89689	1.49031	1.77643	1.85694	2.05893	1.44186	1.54133	1.70831	1.80948	1.2646	0.852844413	0.966018836	20	4	24	0.870458324	0.963313607	24	11.34014797	0.029805633
ENSMUSG00000026842	Abl1	c-abl oncogene 1, non-receptor tyrosine kinase [Source:MGI Symbol;Acc:MGI:87859]	7.93909	9.8656	10.7982	11.474	10.5744	9.27756	10.1899	10.283	11.7067	10.7378	10.1428	9.99545	9.35405	9.3662	0.001920665	0.100255953	12	16	24	0.004146454	0.427431763	24	10.91838712	0.140376819
ENSMUSG00000026843	Fubp3	far upstream element (FUSE) binding protein 3 [Source:MGI Symbol;Acc:MGI:2443699]	15.8448	16.3743	15.8297	16.8533	13.785	15.4711	16.4933	17.068	16.8173	17.7239	14.1529	16.1189	17.3794	16.9474	0.086560674	0.468170511	16	4	24	0.040291596	0.616921492	24	5.371659509	0.087434253
ENSMUSG00000026848	Tor1b	torsin family 1, member B [Source:MGI Symbol;Acc:MGI:1353605]	15.3681	14.9136	15.1916	14.3364	15.653	14.9	14.1379	15.814	14.1102	14.8875	14.7889	15.2597	15.5452	14.9477	0.843639218	0.966018836	8	4	24	0.604368561	0.861894646	24	22.36161239	0.019773962
ENSMUSG00000026849	Tor1a	torsin family 1, member A (torsin A) [Source:MGI Symbol;Acc:MGI:1353568]	19.1006	20.3544	21.6266	19.9296	21.2368	19.9576	18.8151	21.0413	17.6573	18.3172	22.0765	18.7113	18.1283	19.1454	0.5169953	0.966018836	20	8	24	0.600758616	0.860170376	24	14.49318824	0.035518223
ENSMUSG00000026851	BC005624	cDNA sequence BC005624 [Source:MGI Symbol;Acc:MGI:2385132]	21.8039	20.2096	20.3178	19.6287	19.832	20.1163	19.2803	18.3537	17.5529	19.0026	18.5395	18.8907	19.6278	20.3221	0.915082018	0.966018836	4	16	24	0.259371377	0.768040178	24	22.97943258	0.040740236
ENSMUSG00000026853	Crat	carnitine acetyltransferase [Source:MGI Symbol;Acc:MGI:109501]	13.7251	14.4971	15.148	25.0753	17.091	17.4841	15.8462	23.0024	16.5304	25.0346	16.7548	16.7481	21.1177	18.9451	0.47028201	0.966018836	16	12	24	0.266782846	0.768040178	24	11.148103	0.155127169
ENSMUSG00000026854	Usp20	ubiquitin specific peptidase 20 [Source:MGI Symbol;Acc:MGI:1921520]	35.5189	29.0014	25.6571	30.6965	29.7211	33.9675	29.1294	34.4296	25.3517	40.4682	27.5451	34.7472	35.2568	31.5855	0.143321297	0.585944911	24	12	24	0.470157602	0.805393068	24	20.95302682	0.092634885
ENSMUSG00000026856	Dolpp1	dolichyl pyrophosphate phosphatase 1 [Source:MGI Symbol;Acc:MGI:1914093]	15.2468	12.1568	11.8157	15.0117	12.3382	14.6975	13.3184	15.4984	12.6417	15.0663	14.1999	13.8473	12.5196	14.595	0.896753532	0.966018836	16	20	24	0.824511356	0.94493598	24	19.93975523	0.033204523
ENSMUSG00000026857	Ntmt1	N-terminal Xaa-Pro-Lys N-methyltransferase 1 [Source:MGI Symbol;Acc:MGI:1913867]	12.8011	12.849	12.8525	13.7629	11.566	12.2559	12.8127	14.4916	11.4257	13.2404	12.1301	12.2669	12.9627	12.8426	0.082113105	0.462922111	16	4	24	0.371709803	0.793398709	24	4.990783542	0.046561225
ENSMUSG00000026858	Miga2	mitoguardin 2 [Source:MGI Symbol;Acc:MGI:1922035]	14.0762	12.4258	13.068	14.1703	13.0867	14.8628	13.9999	15.0305	13.7955	17.4682	13.0629	14.1536	13.2341	14.0452	0.921741413	0.966018836	24	12	24	0.749091204	0.918462643	24	12.53289175	0.033202089
ENSMUSG00000026860	Sh3glb2	SH3-domain GRB2-like endophilin B2 [Source:MGI Symbol;Acc:MGI:2385131]	121.398	93.4896	96.4157	91.8604	93.3469	106.289	104.63	112.868	92.4764	111.312	96.5373	107.531	102.408	104.523	0.150959199	0.598641364	24	12	24	0.164936189	0.767020009	24	23.35515515	0.088296961
ENSMUSG00000026864	Hspa5	heat shock protein 5 [Source:MGI Symbol;Acc:MGI:95835]	238.944	141.098	120.097	125.273	175.691	144.622	185.162	142.934	120.912	123.935	197.948	153.815	177.018	142.809	9.02E-04	0.071344007	4	8	24	0.010835847	0.51149922	24	21.22024243	0.31257086
ENSMUSG00000026867	Gapvd1	GTPase activating protein and VPS9 domains 1 [Source:MGI Symbol;Acc:MGI:1913941]	27.2049	24.8533	23.4088	24.2043	25.1981	25.6452	26.4879	22.788	21.9275	25.1425	25.1056	24.9845	26.6255	25.7601	3.58E-04	0.050193498	4	8	24	0.018708052	0.560647889	24	21.2085699	0.084117963
ENSMUSG00000026869	Psmd5	proteasome (prosome, macropain) 26S subunit, non-ATPase, 5 [Source:MGI Symbol;Acc:MGI:1914248]	31.9719	31.0648	29.4276	31.7319	34.942	33.3983	30.7366	27.602	28.2608	23.4243	28.8359	31.8483	29.006	24.2737	0.14745801	0.590732968	24	8	24	0.009482664	0.502965379	24	19.53749154	0.138068106
ENSMUSG00000026872	Zeb2	zinc finger E-box binding homeobox 2 [Source:MGI Symbol;Acc:MGI:1344407]	22.9775	23.5663	21.7226	19.1136	24.2953	21.1703	24.4221	20.458	23.6792	19.544	23.3183	24.0266	23.7597	21.6113	0.243586913	0.741580974	4	12	24	0.189786483	0.768040178	24	22.27398561	0.080064765
ENSMUSG00000026873	Phf19	PHD finger protein 19 [Source:MGI Symbol;Acc:MGI:1921266]	0.320119	0.608191	0.740594	0.525275	0.607856	0.43673	0.468943	0.915568	0.556697	0.524216	0.62627	0.882932	0.515337	0.469929	0.496745696	0.966018836	12	16	24	0.738648351	0.914690529	24	9.885531222	0.046600459
ENSMUSG00000026875	Traf1	TNF receptor-associated factor 1 [Source:MGI Symbol;Acc:MGI:101836]	2.90742	4.24725	4.41546	4.35812	4.34771	4.41263	5.07897	4.29681	4.85859	3.96413	3.43356	3.74983	4.32916	4.17414	0.920992137	0.966018836	12	8	24	0.499860951	0.808791684	24	7.299321197	0.077699455
ENSMUSG00000026878	Rab14	RAB14, member RAS oncogene family [Source:MGI Symbol;Acc:MGI:1915615]	86.3784	77.433	76.6889	78.9602	75.9433	80.9621	81.5561	79.2511	71.1791	92.53	78.6622	82.0247	82.007	84.1932	0.170659409	0.643496587	4	8	24	0.698243596	0.899694264	24	21.19684	0.030775431
ENSMUSG00000026879	Gsn	gelsolin [Source:MGI Symbol;Acc:MGI:95851]	221.536	227.511	208.651	209.081	241.286	195.226	222.41	229.062	281.903	179.521	221.762	227.262	223.037	205.024	0.872380105	0.966018836	12	4	24	0.82320193	0.94441254	24	4.022342957	0.037643318
ENSMUSG00000026880	Stom	stomatin [Source:MGI Symbol;Acc:MGI:95403]	14.451	13.2704	15.9094	15.6136	13.2263	15.487	13.2415	16.4771	12.8312	13.3785	16.3738	14.3584	15.3698	14.6722	0.732668271	0.966018836	8	20	24	0.978312122	0.994576186	24	18.94589184	0.010401253
ENSMUSG00000026883	Dab2ip	disabled 2 interacting protein [Source:MGI Symbol;Acc:MGI:1916851]	12.7038	10.8424	9.85439	11.6023	10.7221	11.6263	12.28	13.3335	11.1854	17.1408	11.3344	13.763	12.8971	14.0676	0.708665292	0.966018836	8	4	24	0.959199592	0.98927861	24	1.32185362	0.01828551
ENSMUSG00000026885	Ttll11	tubulin tyrosine ligase-like family, member 11 [Source:MGI Symbol;Acc:MGI:1921660]	3.83731	2.97232	3.57716	3.9065	3.26184	4.21362	3.42296	3.16803	2.86064	4.1527	3.07399	3.36288	3.13241	4.5077	0.891742979	0.966018836	24	12	24	0.954759699	0.988639288	24	15.99561807	0.019837939
ENSMUSG00000026887	Mrrf	mitochondrial ribosome recycling factor [Source:MGI Symbol;Acc:MGI:1915121]	12.8037	10.2711	11.9749	9.24527	10.2689	10.8395	10.2687	12.5295	11.3635	10.6938	13.3331	10.97	11.8099	13.0889	0.79867683	0.966018836	8	8	24	0.460870973	0.805393068	24	2.756561083	0.065529251
ENSMUSG00000026888	Grb14	growth factor receptor bound protein 14 [Source:MGI Symbol;Acc:MGI:1355324]	28.0207	31.4686	28.6546	23.982	34.5462	28.5808	29.6319	31.9304	29.7551	26.2267	31.8332	28.4623	29.96	27.4883	0.924770496	0.966018836	20	20	24	0.677812772	0.892251586	24	22.18646745	0.046066073
ENSMUSG00000026889	Rbm18	RNA binding motif protein 18 [Source:MGI Symbol;Acc:MGI:1915139]	30.1423	28.9616	31.5781	30.7868	30.373	29.6384	28.6748	29.0285	27.492	29.1034	30.5623	30.4892	30.132	29.4545	0.241264473	0.741580974	20	12	24	0.311103201	0.768040178	24	16.79648494	0.029169297
ENSMUSG00000026893	Gca	grancalcin [Source:MGI Symbol;Acc:MGI:1918521]	13.8186	14.0153	15.9842	16.2752	16.0756	13.8824	15.8188	12.4889	13.7528	12.2561	14.5611	11.2147	11.033	11.0822	0.368981219	0.893596186	16	16	24	0.119510598	0.730508014	24	13.82418015	0.098183983
ENSMUSG00000026894	Morn5	MORN repeat containing 5 [Source:MGI Symbol;Acc:MGI:1922745]	0.650917	1.17471	0.943219	0.495677	1.078	0.858342	0.96926	1.09783	0.690261	1.00375	1.23923	0.786579	0.965844	1.19624	0.644063931	0.966018836	8	16	24	0.842731004	0.952251522	24	3.036503401	0.0377978
ENSMUSG00000026895	Ndufa8	NADH:ubiquinone oxidoreductase subunit A8 [Source:MGI Symbol;Acc:MGI:1915625]	84.2873	75.4137	77.079	82.3099	67.9352	77.6835	80.3383	88.958	70.4808	90.9761	76.4063	81.0171	84.2666	81.2634	0.725061903	0.966018836	4	16	24	0.66527937	0.886947643	24	2.774579355	0.040141667
ENSMUSG00000026896	Ifih1	interferon induced with helicase C domain 1 [Source:MGI Symbol;Acc:MGI:1918836]	2.96578	2.98085	2.94617	2.64619	3.13287	3.03645	2.82754	3.02164	3.24388	2.68838	3.13735	2.60871	2.88581	3.07764	0.579814065	0.966018836	12	12	24	0.792163197	0.933498508	24	5.261227499	0.019968856
ENSMUSG00000026904	Slc4a10	solute carrier family 4, sodium bicarbonate cotransporter-like, member 10 [Source:MGI Symbol;Acc:MGI:2150150]	7.4297	11.3041	9.82869	7.70564	8.30779	7.66435	8.60819	8.86113	12.934	4.94982	7.14497	8.4927	5.01601	9.08456	0.019643089	0.258568924	12	4	24	0.279817355	0.768040178	24	5.968662309	0.200932616
ENSMUSG00000026914	Psmd14	proteasome (prosome, macropain) 26S subunit, non-ATPase, 14 [Source:MGI Symbol;Acc:MGI:1913284]	97.9701	100.644	96.4109	92.0628	92.6043	95.4817	86.7815	91.5123	88.4831	91.2872	96.7663	96.4141	97.0719	96.6784	0.428730128	0.966018836	8	4	24	0.631206527	0.87436603	24	22.9277288	0.023142077
ENSMUSG00000026915	Strbp	spermatid perinuclear RNA binding protein [Source:MGI Symbol;Acc:MGI:104626]	22.1565	19.92	19.1793	19.1337	18.1949	18.3069	19.5927	18.1545	16.7304	20.4127	21.3454	20.5917	19.5532	19.514	0.708020493	0.966018836	4	8	24	0.413212184	0.799546792	24	20.51103502	0.05392484
ENSMUSG00000026917	Wdr5	WD repeat domain 5 [Source:MGI Symbol;Acc:MGI:2155884]	8.36927	8.06273	8.91744	8.77585	7.76299	8.4477	7.69582	8.01412	7.81909	8.43219	8.03673	8.01983	8.15296	7.83814	0.952127548	0.966018836	12	20	24	0.448515285	0.805393068	24	11.83659276	0.026275551
ENSMUSG00000026918	Brd3	bromodomain containing 3 [Source:MGI Symbol;Acc:MGI:1914632]	6.44741	6.0873	6.70459	6.68597	6.54176	6.69634	6.94662	6.99919	7.0874	8.10788	7.19199	7.24073	7.12409	7.10173	0.844511766	0.966018836	16	16	24	0.19059886	0.768040178	24	12.22017174	0.039161772
ENSMUSG00000026921	Egfl7	EGF-like domain 7 [Source:MGI Symbol;Acc:MGI:2449923]	37.5038	43.2163	39.5184	43.138	41.6875	38.7832	39.813	53.1345	42.6279	41.89	39.8055	35.6665	43.6675	42.0738	0.018894761	0.256345714	8	16	24	0.127668799	0.740162193	24	6.709807505	0.102216997
ENSMUSG00000026922	Agpat2	1-acylglycerol-3-phosphate O-acyltransferase 2 (lysophosphatidic acid acyltransferase, beta) [Source:MGI Symbol;Acc:MGI:1914762]	11.7028	10.3488	20.7361	47.4654	22.0062	16.104	20.4248	24.9628	17.7436	26.1876	50.5654	26.3	41.5788	29.4752	0.476029854	0.966018836	16	20	24	0.139978099	0.749574609	24	13.67083858	0.380923761
ENSMUSG00000026923	Notch1	notch 1 [Source:MGI Symbol;Acc:MGI:97363]	3.88898	3.17288	3.49738	3.78603	3.86916	3.69236	4.27434	3.44261	3.39382	3.96504	3.34133	3.31288	2.95681	3.10087	0.7207001	0.966018836	16	16	24	0.324976466	0.768040178	24	16.42558527	0.061076466
ENSMUSG00000026924	Sec16a	SEC16 homolog A, endoplasmic reticulum export factor [Source:MGI Symbol;Acc:MGI:2139207]	13.2274	12.7978	13.8802	12.5228	13.2949	12.4747	13.7462	13.4407	12.11	13.5976	10.5753	11.3386	11.5242	11.076	0.939103211	0.966018836	12	12	24	0.685752683	0.895588997	24	6.713277653	0.031022124
ENSMUSG00000026925	Inpp5e	inositol polyphosphate-5-phosphatase E [Source:MGI Symbol;Acc:MGI:1927753]	10.7559	10.3354	9.35929	10.5498	11.5826	11.9178	11.3085	12.4419	9.46749	12.0154	11.4224	10.1384	10.6871	9.25238	0.446653834	0.966018836	20	16	24	0.230396227	0.768040178	24	18.36103783	0.085408019
ENSMUSG00000026926	Pmpca	peptidase (mitochondrial processing) alpha [Source:MGI Symbol;Acc:MGI:1918568]	32.7315	30.4837	32.084	34.2665	32.4271	34.1024	32.5539	33.1739	29.7882	35.934	31.1954	33.3132	34.1322	31.9006	0.290796297	0.79743194	16	20	24	0.468956645	0.805393068	24	17.48309065	0.034221556
ENSMUSG00000026927	Entr1	endosome associated trafficking regulator 1 [Source:MGI Symbol;Acc:MGI:1915362]	48.3461	44.5267	42.0466	45.5941	50.0612	42.0662	49.6026	40.3012	43.212	42.6375	51.4285	42.824	47.5723	42.3312	0.133012827	0.567728672	20	12	24	0.178420369	0.768040178	24	18.69816143	0.079832564
ENSMUSG00000026928	Card9	caspase recruitment domain family, member 9 [Source:MGI Symbol;Acc:MGI:2685628]	0.994433	1.05723	1.06972	1.08076	1.12629	0.787747	0.94764	0.722421	1.19549	0.700981	1.77426	1.52234	1.34937	0.656558	0.192357053	0.669841558	20	12	24	0.31059289	0.768040178	24	17.28512548	0.129317856
ENSMUSG00000026930	Gpsm1	G-protein signalling modulator 1 (AGS3-like, C. elegans) [Source:MGI Symbol;Acc:MGI:1915089]	17.5952	18.7575	19.1109	19.656	18.6889	17.2917	18.8927	18.6204	19.9307	19.664	17.9778	17.6371	18.2134	19.1594	9.86E-04	0.074544544	16	8	24	4.31E-04	0.257978028	24	8.908397407	0.074461398
ENSMUSG00000026932	Nacc2	nucleus accumbens associated 2, BEN and BTB (POZ) domain containing [Source:MGI Symbol;Acc:MGI:1915241]	66.328	51.7549	53.9007	55.8518	55.6006	61.7598	62.0813	59.0123	46.9907	74.3733	51.2039	57.9351	58.1452	55.6208	0.105280783	0.518002936	4	8	24	0.559416678	0.840554	24	20.33293267	0.072766087
ENSMUSG00000026933	Camsap1	calmodulin regulated spectrin-associated protein 1 [Source:MGI Symbol;Acc:MGI:3036242]	16.6135	10.6399	13.9949	12.963	13.6564	18.9743	16.6828	14.1587	9.32351	16.4755	11.0887	13.9373	12.3748	13.7818	0.57057742	0.966018836	24	12	24	0.244504586	0.768040178	24	20.58399749	0.172269844
ENSMUSG00000026938	Fcna	ficolin A [Source:MGI Symbol;Acc:MGI:1340905]	0.75335	0.966257	0.855207	0.94192	0.737353	0.764664	0.680031	0.999063	1.32144	0.454345	0.474031	0.937656	1.0019	0.754803	0.326899754	0.845515883	12	8	24	0.222130679	0.768040178	24	4.662157472	0.118696295
ENSMUSG00000026939	Tmem141	transmembrane protein 141 [Source:MGI Symbol;Acc:MGI:1098773]	18.1278	20.5642	18.7678	18.5223	21.4291	18.8769	16.0821	19.2058	18.5339	17.4155	19.7737	17.956	19.9429	19.4019	0.952127548	0.966018836	8	8	24	0.931003299	0.982245205	24	12.72173545	0.014956003
ENSMUSG00000026941	Mamdc4	MAM domain containing 4 [Source:MGI Symbol;Acc:MGI:2685841]	1.02578	0.629238	0.755912	0.927745	0.673825	0.600269	0.875507	0.488722	0.914945	0.361738	0.798808	0.575708	0.743631	0.376189	0.93717134	0.966018836	4	4	24	0.791999713	0.93349015	24	19.79228574	0.044815103
ENSMUSG00000026942	Traf2	TNF receptor-associated factor 2 [Source:MGI Symbol;Acc:MGI:101835]	5.73678	4.88824	5.45168	4.86178	5.73482	5.92346	6.71093	5.28155	5.11822	5.36908	5.46608	5.61596	6.14347	4.78983	0.001134165	0.079869094	4	4	24	0.026886499	0.586541342	24	20.87202677	0.113487176
ENSMUSG00000026944	Abca2	ATP-binding cassette, sub-family A (ABC1), member 2 [Source:MGI Symbol;Acc:MGI:99606]	35.3666	29.3242	32.8327	34.5295	31.476	32.5264	36.7809	33.527	32.7644	41.1791	28.1738	33.4711	29.8444	28.1207	0.935932447	0.966018836	16	16	24	0.800131375	0.935884746	24	13.53908954	0.03811454
ENSMUSG00000026946	Nmi	N-myc (and STAT) interactor [Source:MGI Symbol;Acc:MGI:1928368]	9.40954	10.1506	9.47696	8.67092	10.5849	9.6922	9.10813	10.1014	11.8464	8.83246	10.5879	10.938	10.9137	9.85067	0.911135828	0.966018836	20	20	24	0.917623306	0.977606103	24	23.92175261	0.01809484
ENSMUSG00000026950	Neb	nebulin [Source:MGI Symbol;Acc:MGI:97292]	10.3985	28.2494	34.7828	45.6316	25.7463	38.5669	23.5346	69.3397	52.5854	67.6997	24.2457	29.6179	37.3964	28.2431	0.210566453	0.69936851	16	12	24	0.066323097	0.66882698	24	9.657490175	0.561513295
ENSMUSG00000026956	Uap1l1	UDP-N-acteylglucosamine pyrophosphorylase 1-like 1 [Source:MGI Symbol;Acc:MGI:2443318]	11.8929	12.6305	10.2565	13.3244	16.565	12.8545	11.4435	13.5793	12.8663	12.4469	11.4759	11.5342	10.7034	12.5379	0.777666236	0.966018836	16	12	24	0.519697733	0.819515315	24	14.22380495	0.068709966
ENSMUSG00000026958	Dpp7	dipeptidylpeptidase 7 [Source:MGI Symbol;Acc:MGI:1933213]	20.8563	19.0318	20.0602	18.769	22.0508	21.4454	20.2364	20.7531	18.456	17.0293	20.2587	20.0252	20.9321	20.1681	0.020428812	0.261278501	20	20	24	0.037509059	0.609919408	24	21.57168184	0.086329229
ENSMUSG00000026959	Grin1	glutamate receptor, ionotropic, NMDA1 (zeta 1) [Source:MGI Symbol;Acc:MGI:95819]	19.5069	19.2859	23.1182	19.7777	16.0642	21.4726	20.7184	19.8443	18.2923	26.5721	15.6037	21.7343	17.9807	24.8611	0.897975397	0.966018836	16	4	24	0.484451452	0.805393068	24	6.372987007	0.100519165
ENSMUSG00000026960	Arl6ip6	ADP-ribosylation factor-like 6 interacting protein 6 [Source:MGI Symbol;Acc:MGI:1929507]	4.27043	4.27474	4.29258	6.00544	5.18486	5.33777	4.3848	4.00026	5.87901	4.46155	5.26432	4.3841	4.45091	4.70816	0.121789249	0.549378164	16	16	24	0.069290542	0.673520298	24	13.67597982	0.127879261

ENSMUSG00000026965	Anapc2	anaphase promoting complex subunit 2 [Source:MGI Symbol;Acc:MGI:2139135]	34.9908	35.5495	35.4331	38.6447	37.5369	38.3446	38.9633	37.1341	35.4265	42.6441	34.1754	35.75	32.5173	32.706	0.496745696	0.966018836	16	16	24	0.238172138	0.768040178	24	14.04649804	0.062885449
ENSMUSG00000026966	Ssna1	SS nuclear autoantigen 1 [Source:MGI Symbol;Acc:MGI:1915725]	35.4938	39.14	37.9397	39.4806	41.9881	38.1567	34.443	40.0437	41.6824	34.5387	42.9858	38.4945	42.9175	39.2238	0.919571723	0.966018836	20	8	24	0.80231971	0.936565832	24	12.61691198	0.026100309
ENSMUSG00000026970	Rbms1	RNA binding motif, single stranded interacting protein 1 [Source:MGI Symbol;Acc:MGI:1861774]	59.4937	62.5075	57.6113	61.5371	56.5843	57.4706	54.0446	56.1676	57.3957	58.074	60.0553	55.4032	56.7133	57.9108	0.691437565	0.966018836	16	8	24	0.446833691	0.805393068	24	10.45646437	0.026138234
ENSMUSG00000026971	Itgb6	integrin beta 6 [Source:MGI Symbol;Acc:MGI:96615]	0.344812	0.759638	0.607324	0.81661	0.524796	0.65065	0.693612	1.02585	0.943449	1.00455	0.799764	0.630988	1.02377	0.806634	0.465197787	0.966018836	8	16	24	0.066292229	0.66876796	24	8.154464293	0.121678233
ENSMUSG00000026972	Arrdc1	arrestin domain containing 1 [Source:MGI Symbol;Acc:MGI:2446136]	17.6063	15.8976	16.0558	16.4842	16.9727	16.5104	14.5335	17.1289	17.9122	19.438	15.6768	17.9191	17.4361	16.9804	0.801280095	0.966018836	16	4	24	0.646867549	0.880609512	24	11.23489846	0.034763415
ENSMUSG00000026974	Zmynd19	zinc finger, MYND domain containing 19 [Source:MGI Symbol;Acc:MGI:1914437]	6.25133	6.3299	7.06219	5.80696	6.77675	7.08493	6.7666	6.95327	5.25178	6.39058	5.93365	6.27553	7.18604	7.04157	0.745199643	0.966018836	8	4	24	0.263643531	0.768040178	24	23.84863848	0.071324785
ENSMUSG00000026975	Dph7	diphthamine biosynethesis 7 [Source:MGI Symbol;Acc:MGI:1914478]	6.21516	7.44697	8.40768	6.57756	7.22901	7.52969	6.48148	7.45825	7.55294	7.36039	6.51108	7.58285	7.55951	7.44153	0.784278077	0.966018836	12	16	24	0.379969238	0.799461072	24	7.411207299	0.058012777
ENSMUSG00000026976	Pax8	paired box 8 [Source:MGI Symbol;Acc:MGI:97492]	0.858247	1.23984	1.21767	0.901848	0.957083	1.02642	1.36037	0.751351	1.12267	1.5496	0.559205	0.926762	0.797042	0.950532	0.921572192	0.966018836	16	4	24	0.558530482	0.839876509	24	7.848893876	0.080858339
ENSMUSG00000026977	Marchf7	membrane associated ring-CH-type finger 7 [Source:MGI Symbol;Acc:MGI:1931053]	25.9224	32.5068	28.9571	25.8593	30.6884	28.9908	29.0841	29.6994	31.7789	24.3646	28.7966	29.1683	29.7342	31.1106	0.05211941	0.391073722	8	8	24	0.350309731	0.779746585	24	3.035456125	0.062888799
ENSMUSG00000026979	Psd4	pleckstrin and Sec7 domain containing 4 [Source:MGI Symbol;Acc:MGI:2674093]	2.54943	1.8601	1.74487	2.23158	1.7114	2.00158	1.47897	2.62016	2.25935	1.61609	1.58159	2.48139	1.7899	1.71811	0.965971228	0.969272194	8	12	24	0.778193209	0.928832041	24	1.569464827	0.050435421
ENSMUSG00000026980	Ly75	lymphocyte antigen 75 [Source:MGI Symbol;Acc:MGI:106662]	1.70758	1.89836	1.81307	1.91904	1.77034	1.86007	1.78548	1.39955	1.54283	1.73802	1.76033	1.8946	1.91023	1.78377	0.923635729	0.966018836	24	12	24	0.365408477	0.789665091	24	18.58011902	0.045330329
ENSMUSG00000026981	Il1rn	interleukin 1 receptor antagonist [Source:MGI Symbol;Acc:MGI:96547]	0.423229	0.422133	0.544118	0.392717	0.460488	0.728896	0.451446	0.405434	0.569246	0.466316	0.513156	0.715976	0.685758	0.38647	0.037085178	0.334054683	24	8	24	0.230055049	0.768040178	24	19.44678932	0.072399812
ENSMUSG00000026986	Hnmt	histamine N-methyltransferase [Source:MGI Symbol;Acc:MGI:2153181]	9.07506	12.2092	10.7619	10.943	9.37839	10.1539	9.51211	10.2416	14.0627	9.36056	9.84171	10.8	10.8139	12.138	0.235883283	0.73833972	12	8	24	0.062470006	0.661961741	24	6.735534206	0.140215443
ENSMUSG00000026987	Baz2b	bromodomain adjacent to zinc finger domain, 2B [Source:MGI Symbol;Acc:MGI:2442782]	12.3185	12.4662	13.8544	13.1716	13.5445	12.8725	13.1567	11.495	13.2867	11.6935	13.0045	12.8609	14.5048	13.5403	0.856752753	0.966018836	12	20	24	0.972662789	0.993250534	24	20.10865108	0.008255997
ENSMUSG00000026988	Wdsub1	WD repeat, SAM and U-box domain containing 1 [Source:MGI Symbol;Acc:MGI:1919387]	17.0338	14.775	12.7788	14.7787	14.7359	14.7261	15.5233	15.582	14.754	14.6967	14.8943	16.9534	16.1815	13.6482	0.030691392	0.324150946	4	8	24	0.038067854	0.61140397	24	21.44415211	0.096631431
ENSMUSG00000026989	Dapl1	death associated protein-like 1 [Source:MGI Symbol;Acc:MGI:1923997]	5.94643	7.65906	3.81146	5.09954	6.43591	4.82517	4.26907	5.98806	6.56636	2.06223	5.393	6.69677	5.54717	6.42197	0.743750765	0.966018836	12	8	24	0.359834637	0.785502176	24	0.437437019	0.208523727
ENSMUSG00000026991	Pkp4	plakophilin 4 [Source:MGI Symbol;Acc:MGI:109281]	16.8125	20.6632	25.8316	22.2537	16.3832	20.6756	24.096	19.1937	20.7429	17.4522	15.4476	21.9378	15.2087	21.0526	0.791413662	0.966018836	12	8	24	0.446166997	0.805393068	24	6.233052742	0.113028597
ENSMUSG00000026994	Galnt3	polypeptide N-acetylgalactosaminyltransferase 3 [Source:MGI Symbol;Acc:MGI:894695]	2.97345	2.8248	3.07564	2.60447	2.97145	2.44684	3.51511	2.67974	2.15632	2.94916	3.6477	3.46293	3.72849	2.90503	0.226403091	0.722427815	4	8	24	0.23540813	0.768040178	24	20.72493474	0.107336968
ENSMUSG00000026999	Nup35	nucleoporin 35 [Source:MGI Symbol;Acc:MGI:1916732]	2.72459	3.06499	2.71753	3.02544	3.29689	2.47874	3.33787	3.65404	2.84846	2.43494	3.08811	2.79159	3.24723	2.73469	0.843639218	0.966018836	8	4	24	0.690520023	0.897566751	24	1.542108414	0.042872451
ENSMUSG00000027001	Dusp19	dual specificity phosphatase 19 [Source:MGI Symbol;Acc:MGI:1915332]	7.54084	7.51047	7.37498	8.65868	8.24314	7.56307	7.56672	7.56241	7.36784	7.1183	7.93793	7.21427	7.79719	7.62379	0.628286596	0.966018836	20	16	24	0.435303303	0.805393068	24	15.40594342	0.032821179
ENSMUSG00000027002	Nckap1	NCK-associated protein 1 [Source:MGI Symbol;Acc:MGI:1355333]	108.006	96.2911	96.6301	95.9928	99.3923	99.0507	96.5219	90.7463	85.7038	94.8974	97.1621	95.4042	95.3046	97.6864	0.562426145	0.966018836	20	20	24	0.06419361	0.663876076	24	20.44951917	0.058252483
ENSMUSG00000027004	Frzb	frizzled-related protein [Source:MGI Symbol;Acc:MGI:892032]	28.1209	23.1787	23.0309	25.9859	29.7849	28.0582	30.5746	29.9563	23.7207	32.308	22.5945	31.2009	28.5221	24.4679	0.595037318	0.966018836	24	12	24	0.276193002	0.768040178	24	20.0379655	0.110959167
ENSMUSG00000027006	Dnajc10	DnaJ heat shock protein family (Hsp40) member C10 [Source:MGI Symbol;Acc:MGI:1914111]	37.0019	40.2202	40.7355	38.7245	36.7201	36.4019	35.3915	35.7348	41.2392	36.3217	40.38	38.5573	38.7993	39.0001	0.217827365	0.704989686	12	12	24	0.279927929	0.768040178	24	8.912565159	0.046203817
ENSMUSG00000027007	Itprid2	ITPR interacting domain containing 2 [Source:MGI Symbol;Acc:MGI:1917849]	24.4397	24.7215	22.3128	24.2461	26.4032	23.663	27.8235	22.6355	24.4218	24.0849	24.1328	23.4337	25.1464	21.676	0.690243289	0.966018836	4	4	24	0.306321273	0.768040178	24	19.4509478	0.054121623
ENSMUSG00000027009	Itga4	integrin alpha 4 [Source:MGI Symbol;Acc:MGI:96603]	1.9883	2.35082	2.01098	2.10509	2.06647	1.90131	2.38862	2.00334	2.47558	1.91445	1.925	2.05014	2.237	2.05726	0.622132989	0.966018836	12	12	24	0.256032579	0.768040178	24	4.319999413	0.053599987
ENSMUSG00000027010	Slc25a12	solute carrier family 25 (mitochondrial carrier, Aralar), member 12 [Source:MGI Symbol;Acc:MGI:1926080]	49.9525	49.1629	53.5509	51.6378	48.158	56.6376	49.6651	56.5608	47.3276	59.2472	44.0846	49.01	50.7994	48.5435	0.911135828	0.966018836	16	4	24	0.87872968	0.966077307	24	7.691131544	0.024132357
ENSMUSG00000027011	Ube2e3	ubiquitin-conjugating enzyme E2E 3 [Source:MGI Symbol;Acc:MGI:107412]	28.7876	28.8693	29.4432	27.9768	30.9873	31.1368	29.3795	27.007	28.8463	29.0542	27.4114	31.7581	30.5629	29.6478	0.297978647	0.80820842	24	16	24	0.190753576	0.768040178	24	20.09160337	0.047036847
ENSMUSG00000027012	Dync1i2	dynein cytoplasmic 1 intermediate chain 2 [Source:MGI Symbol;Acc:MGI:107750]	192.452	171.575	166.952	170.905	174.541	181.737	165.421	166.8	149.938	170.535	176.662	170.012	178.643	173.233	0.140377635	0.585715181	24	12	24	0.050103423	0.644435324	24	20.55055398	0.071789708
ENSMUSG00000027014	Cwc22	CWC22 spliceosome-associated protein [Source:MGI Symbol;Acc:MGI:2136773]	7.04268	5.98035	6.82646	5.25151	6.25907	5.80136	5.1397	5.00245	6.15883	5.6233	5.18648	7.07744	7.00009	5.5971	0.94086481	0.966018836	24	8	24	0.653921207	0.883343578	24	22.32279926	0.057845512
ENSMUSG00000027015	Cybrd1	cytochrome b reductase 1 [Source:MGI Symbol;Acc:MGI:2654575]	1.58157	1.60712	1.65521	1.23595	1.70588	1.39012	1.71955	1.91127	1.21971	1.75572	1.37387	1.46269	1.12498	1.28017	0.788254138	0.966018836	8	4	24	0.985750552	0.997214081	24	2.409615566	0.008609767
ENSMUSG00000027016	Zfp385b	zinc finger protein 385B [Source:MGI Symbol;Acc:MGI:2444734]	11.3162	9.26291	8.87936	9.50239	9.79058	10.514	8.87744	10.1644	8.11047	13.0738	9.25507	11.0999	10.5891	10.7825	0.390686537	0.915479136	24	12	24	0.639673645	0.877957092	24	19.54189193	0.061600565
ENSMUSG00000027018	Hat1	histone aminotransferase 1 [Source:MGI Symbol;Acc:MGI:96013]	13.7389	16.8038	15.1289	13.9647	15.0346	14.0882	14.7263	12.9504	16.4265	12.8958	16.229	15.0275	13.6767	13.8765	0.943890799	0.966018836	12	16	24	0.915993071	0.976753216	24	12.53700162	0.01888667
ENSMUSG00000027022	Xirp2	xin actin-binding repeat containing 2 [Source:MGI Symbol;Acc:MGI:2685198]	3.68993	11.2667	11.7346	15.7215	8.87524	11.7134	8.56036	22.3461	19.3956	21.7317	11.0689	11.2917	14.2633	9.26176	0.162391276	0.624504499	12	16	24	0.050723808	0.644469829	24	9.798391615	0.501978264
ENSMUSG00000027030	Stk39	serine/threonine kinase 39 [Source:MGI Symbol;Acc:MGI:1858416]	60.7051	52.7951	49.826	49.613	50.7484	56.2554	56.8146	54.0423	46.4142	64.5731	53.5836	56.1559	56.2009	58.727	0.03452758	0.328311403	4	8	24	0.337942198	0.772742827	24	22.16777013	0.070496147
ENSMUSG00000027035	Cers6	ceramide synthase 6 [Source:MGI Symbol;Acc:MGI:2442564]	3.71872	3.36223	3.90705	3.90124	4.00387	3.9734	3.41708	3.21945	3.07027	3.32969	3.95086	4.04498	3.57364	3.65333	0.016095537	0.253395174	24	8	24	0.024849843	0.586541342	24	17.69985682	0.099013912
ENSMUSG00000027067	Ssrp1	structure specific recognition protein 1 [Source:MGI Symbol;Acc:MGI:107912]	33.2866	32.761	35.1288	31.3517	31.0633	32.9683	30.1541	31.7844	30.0244	33.1062	28.0845	30.5991	28.7945	32.8693	0.933543173	0.966018836	16	4	24	0.395687158	0.799546792	24	6.469095615	0.040553175
ENSMUSG00000027071	P2rx3	purinergic receptor P2X, ligand-gated ion channel, 3 [Source:MGI Symbol;Acc:MGI:1097160]	133.139	96.8337	104.294	101.148	115.622	123.924	102.081	109.853	78.1379	107.769	115.81	106.429	112.422	101.576	0.059229521	0.406067671	20	16	24	0.025704417	0.586541342	24	19.9523484	0.172931698
ENSMUSG00000027073	Prg2	proteoglycan 2, bone marrow [Source:MGI Symbol;Acc:MGI:103294]	5.71765	9.13166	16.4694	19.1671	8.41127	11.0209	7.83414	9.06711	9.97586	6.27075	6.31404	7.22654	10.1011	5.69488	0.895493752	0.966018836	12	20	24	0.401681119	0.799546792	24	10.29934593	0.238246714
ENSMUSG00000027074	Slc43a3	solute carrier family 43, member 3 [Source:MGI Symbol;Acc:MGI:1931054]	28.1038	24.952	23.6103	30.5806	32.4494	27.6249	23.2214	25.0818	25.631	27.2502	28.0347	27.2536	25.5485	23.2103	0.003846392	0.140598674	20	12	24	0.001607229	0.366220015	24	16.05166609	0.151407136
ENSMUSG00000027075	Slc43a1	solute carrier family 43, member 1 [Source:MGI Symbol;Acc:MGI:1931352]	1.18419	1.71609	2.14947	2.2585	1.54553	1.9169	1.21278	1.59881	2.25147	1.58308	1.31095	2.3485	1.81011	1.82357	0.351315142	0.870418653	12	16	24	0.381355472	0.799546792	24	9.912038382	0.113196637
ENSMUSG00000027076	Timm10	translocase of inner mitochondrial membrane 10 [Source:MGI Symbol;Acc:MGI:1353429]	39.1097	36.1632	34.28	34.0413	40.6226	33.2398	34.9229	39.9036	38.1004	30.4476	38.7635	35.2486	39.6651	39.0522	0.371875382	0.894537464	4	12	24	0.459555799	0.805393068	24	1.055307177	0.057753567
ENSMUSG00000027077	Smtnl1	smoothelin-like 1 [Source:MGI Symbol;Acc:MGI:1915928]	1.06632	2.877	2.96824	3.51359	1.90179	3.11117	2.0907	6.56913	2.44335	4.25842	2.2584	1.65458	3.80608	2.74053	0.459019924	0.966018836	16	4	24	0.214258439	0.768040178	24	7.874176899	0.313779654
ENSMUSG00000027078	Ube2l6	ubiquitin-conjugating enzyme E2L 6 [Source:MGI Symbol;Acc:MGI:1914500]	20.5026	25.5188	25.2571	23.9856	25.332	23.0229	22.5513	25.9202	27.9105	17.8974	24.0407	24.5912	26.2178	23.9412	0.787283372	0.966018836	12	4	24	0.651548042	0.882823265	24	5.001961097	0.057838873
ENSMUSG00000027079	Clp1	CLP1, cleavage and polyadenylation factor I subunit [Source:MGI Symbol;Acc:MGI:2138968]	4.1143	4.21812	4.06405	4.51603	4.77378	3.8625	4.5471	4.32749	4.28052	3.92026	3.95606	3.90111	4.43428	4.82707	0.337748746	0.85758369	8	16	24	0.569195959	0.844729091	24	5.047860699	0.037073401
ENSMUSG00000027080	Med19	mediator complex subunit 19 [Source:MGI Symbol;Acc:MGI:1914234]	14.5029	13.1188	11.8862	13.0063	13.8215	13.9695	12.673	12.1912	12.5369	15.8212	13.69	14.5987	14.5282	15.0184	0.245061943	0.741580974	24	12	24	0.343847209	0.777386111	24	18.75921627	0.059757977
ENSMUSG00000027082	Tfpi	tissue factor pathway inhibitor [Source:MGI Symbol;Acc:MGI:1095418]	7.41576	9.39667	7.94087	6.86161	7.5444	5.24136	6.7263	7.41688	8.67414	4.35	6.48164	6.88336	6.42174	6.24567	0.446653834	0.966018836	12	12	24	0.275618105	0.768040178	24	4.663947311	0.136061655
ENSMUSG00000027086	Fastkd1	FAST kinase domains 1 [Source:MGI Symbol;Acc:MGI:2444596]	10.2652	8.37052	9.55322	10.06	9.64897	9.00498	9.22963	9.34318	8.23071	10.5441	9.32115	9.38211	10.2181	10.1307	0.911685024	0.966018836	16	20	24	0.841430806	0.951974594	24	16.55028001	0.022669328
ENSMUSG00000027087	Itgav	integrin alpha V [Source:MGI Symbol;Acc:MGI:96608]	17.4046	22.9601	21.4309	19.7064	21.1524	16.9533	19.47	20.2699	23.6575	18.3214	18.7133	19.8536	20.4241	18.8807	0.22872448	0.72665454	12	12	24	0.14382549	0.752803875	24	7.52442673	0.100427366
ENSMUSG00000027088	Phospho2	phosphatase, orphan 2 [Source:MGI Symbol;Acc:MGI:1920623]	16.7279	16.6636	17.0717	16.6637	17.18	15.9276	17.4467	15.2112	16.7409	14.2594	21.3019	17.831	18.1492	17.6364	0.708020493	0.966018836	20	20	24	0.532066621	0.825565809	24	19.32066782	0.055475589
ENSMUSG00000027091	Zc3h15	zinc finger CCCH-type containing 15 [Source:MGI Symbol;Acc:MGI:1919747]	63.5788	64.1416	58.9133	62.1076	62.8439	62.3732	56.3702	63.1667	61.0723	58.3408	71.1644	65.2242	69.6315	61.1112	0.279136262	0.790764844	20	20	24	0.423965865	0.805393068	24	19.54987708	0.045909932
ENSMUSG00000027099	Mtx2	metaxin 2 [Source:MGI Symbol;Acc:MGI:1859652]	31.1502	35.2388	32.9833	33.6821	33.9639	34.357	32.9071	31.2212	35.5627	30.6825	36.1902	28.5772	31.5198	32.9144	0.581070934	0.966018836	20	8	24	0.56907689	0.844691814	24	10.94237269	0.03803972
ENSMUSG00000027102	Hoxd8	homeobox D8 [Source:MGI Symbol;Acc:MGI:96209]	10.6005	10.7941	11.4845	11.18	10.2537	11.1982	9.88999	12.3168	10.5642	12.8012	9.74531	10.1317	10.3835	12.0371	0.127318615	0.560209134	16	4	24	0.105290017	0.717252017	24	7.863355013	0.090187401
ENSMUSG00000027104	Atf2	activating transcription factor 2 [Source:MGI Symbol;Acc:MGI:109349]	28.2996	23.9773	24.6436	25.2298	24.6794	24.3044	23.6618	23.8313	21.4011	25.2167	25.0815	24.6094	24.217	25.3466	0.522551072	0.966018836	16	20	24	0.46540593	0.805393068	24	19.81734927	0.040207721
ENSMUSG00000027108	Ola1	Obg-like ATPase 1 [Source:MGI Symbol;Acc:MGI:1914309]	60.1537	55.3409	55.9195	54.0975	55.8346	58.859	52.9319	52.2592	50.2494	57.9381	54.1929	57.6982	57.2676	59.554	0.763955526	0.966018836	24	12	24	0.339622643	0.774107293	24	20.90115097	0.044271642
ENSMUSG00000027109	Sp3	trans-acting transcription factor 3 [Source:MGI Symbol;Acc:MGI:1277166]	16.2431	17.1726	17.4385	17.3583	17.3228	15.543	17.1104	13.6566	15.9055	13.8227	17.3083	13.9063	13.0335	14.7551	0.330576288	0.850598217	20	4	24	0.410893959	0.799546792	24	13.67133121	0.055568471
ENSMUSG00000027111	Itga6	integrin alpha 6 [Source:MGI Symbol;Acc:MGI:96605]	70.3239	68.1953	66.2884	63.8713	81.7552	63.6979	80.6773	69.595	66.9904	75.1192	64.9088	64.4848	68.6686	61.2022	0.941885246	0.966018836	4	20	24	0.798836242	0.935664328	24	18.81031771	0.033782166
ENSMUSG00000027115	Kif18a	kinesin family member 18A [Source:MGI Symbol;Acc:MGI:2446977]	0.567737	0.851637	0.8598	0.854202	0.835278	0.916812	0.811666	0.793345	0.960564	0.684666	0.895053	0.907766	0.728377	0.743551	0.115316704	0.536602128	24	4	24	0.493068016	0.805727364	24	13.27840649	0.039687124
ENSMUSG00000027122	Arl14ep	ADP-ribosylation factor-like 14 effector protein [Source:MGI Symbol;Acc:MGI:1926020]	13.3644	14.1022	13.2456	12.8475	12.3293	13.0957	12.5709	13.7673	13.3856	12.4059	13.4671	12.6309	12.9271	12.767	0.337071598	0.85758369	8	8	24	0.191457998	0.768040178	24	4.365791017	0.034608069
ENSMUSG00000027130	Slc12a6	solute carrier family 12, member 6 [Source:MGI Symbol;Acc:MGI:2135960]	28.9449	26.3541	26.5129	25.9674	25.545	26.3485	26.5026	26.0373	25.2329	28.0329	26.0265	29.5263	27.4926	28.6492	0.277428396	0.789849983	4	16	24	0.336497024	0.771914907	24	23.13373533	0.037987259
ENSMUSG00000027131	Emc4	ER membrane protein complex subunit 4 [Source:MGI Symbol;Acc:MGI:1915282]	70.0857	61.9426	61.4059	60.2455	59.6918	60.9849	62.383	65.5328	58.0867	64.4881	66.6219	65.6042	68.303	64.7227	0.083354723	0.462922111	4	8	24	0.16106948	0.765941695	24	23.08258298	0.053976728
ENSMUSG00000027132	Katnbl1	katanin p80 subunit B like 1 [Source:MGI Symbol;Acc:MGI:1919675]	6.06765	7.0152	7.2097	6.8126	6.1658	6.23111	6.34867	6.42348	6.65478	5.47818	7.58753	6.00718	5.83477	6.10706	0.383511101	0.915479136	12	12	24	0.585742339	0.853141382	24	10.00683785	0.044036883
ENSMUSG00000027133	Nop10	NOP10 ribonucleoprotein [Source:MGI Symbol;Acc:MGI:1913431]	51.7968	52.58	45.6541	49.747	53.4169	47.455	50.2091	48.373	54.869	40.5681	58.9364	47.5664	53.9221	47.3653	0.929527443	0.966018836	20	20	24	0.720440895	0.908336488	24	20.90408936	0.044233432
ENSMUSG00000027134	Lpcat4	lysophosphatidylcholine acyltransferase 4 [Source:MGI Symbol;Acc:MGI:2138993]	16.3648	13.8947	13.2059	13.8949	14.8675	14.4156	15.0839	15.9057	12.8344	18.0418	13.5202	14.6317	13.3473	13.4093	0.496745696	0.966018836	4	8	24	0.762246861	0.923059648	24	18.76211258	0.039321755
ENSMUSG00000027160	Ccdc34	coiled-coil domain containing 34 [Source:MGI Symbol;Acc:MGI:1915451]	3.9439	3.90668	5.06755	4.29932	4.07141	3.74016	3.96867	3.74122	4.7194	3.82429	4.33301	3.79682	4.67549	4.10057	0.085890569	0.466404237	12	12	24	0.271773881	0.768040178	24	8.913101119	0.072302033
ENSMUSG00000027162	Lin7c	lin-7 homolog C (C. elegans) [Source:MGI Symbol;Acc:MGI:1330839]	23.7399	25.9322	26.7344	25.3526	25.0075	22.2654	21.999	23.313	26.2314	21.6819	25.6251	22.233	22.3743	23.9869	0.010049466	0.194720062	12	12	24	0.084072189	0.701763292	24	9.467270661	0.078277976
ENSMUSG00000027163	Commd9	COMM domain containing 9 [Source:MGI Symbol;Acc:MGI:1923751]	24.0412	23.4438	22.9232	21.8208	25.1883	24.2553	23.0033	25.2982	21.0489	21.3831	24.3282	23.0059	21.0611	23.4056	0.265774856	0.773510818	20	20	24	0.36796353	0.79131734	24	20.75808164	0.0469912
ENSMUSG00000027164	Traf6	TNF receptor-associated factor 6 [Source:MGI Symbol;Acc:MGI:108072]	4.88403	4.50512	4.70956	4.83121	4.46651	4.57523	4.90706	4.79801	4.65588	4.65711	4.86447	4.66453	4.65518	4.51585	0.885059765	0.966018836	4	4	24	0.940676573	0.985398871	24	21.29498567	0.005434195
ENSMUSG00000027165	Iftap	intraflagellar transport associated protein [Source:MGI Symbol;Acc:MGI:1915420]	32.871	39.1706	38.7449	36.0864	37.9425	34.6287	36.0704	34.3118	41.698	32.2412	35.651	34.1898	38.0197	34.8702	0.245061943	0.741580974	12	12	24	0.444550502	0.805393068	24	7.634179073	0.052555981
ENSMUSG00000027166	Dnajc24	DnaJ heat shock protein family (Hsp40) member C24 [Source:MGI Symbol;Acc:MGI:1919522]	7.33911	8.54881	7.22385	6.41846	7.84907	5.95027	11.1097	7.16123	10.5679	7.02233	9.35295	9.83272	8.76928	8.54193	0.702630284	0.966018836	4	12	24	0.634516495	0.87566973	24	1.658140241	0.081842566
ENSMUSG00000027167	Elp4	elongator acetyltransferase complex subunit 4 [Source:MGI Symbol;Acc:MGI:1925016]	7.21867	7.66385	9.01198	8.2479	7.37669	7.70854	8.23181	8.36529	6.48687	7.25883	8.01398	8.07612	7.8947	8.70846	0.920872048	0.966018836	8	12	24	0.795576877	0.934624211	24	3.198853172	0.029258363
ENSMUSG00000027168	Pax6	paired box 6 [Source:MGI Symbol;Acc:MGI:97490]	0.629492	0.565084	0.753533	0.525605	0.258998	0.460472	0.443752	0.718028	0.807676	0.395381	0.497558	0.431844	0.229696	0.651703	0.010059617	0.194720062	12	8	24	0.030063528	0.596794384	24	6.57895324	0.155690327
ENSMUSG00000027170	Eif3m	eukaryotic translation initiation factor 3, subunit M [Source:MGI Symbol;Acc:MGI:1351744]	17.487	21.4086	19.2171	20.4744	19.5682	18.3604	18.3876	18.2436	22.3867	16.1587	21.7153	17.731	19.8383	18.6052	0.714044782	0.966018836	12	12	24	0.651062236	0.882725346	24	9.471054854	0.047985916
ENSMUSG00000027171	Prrg4	proline rich Gla (G-carboxyglutamic acid) 4 (transmembrane) [Source:MGI Symbol;Acc:MGI:2442211]	3.96752	5.09629	4.41464	4.87207	5.47642	3.41301	5.92782	4.24524	5.83107	4.27067	5.06305	4.6691	4.94515	4.73868	0.832742389	0.966018836	20	4	24	0.753369432	0.920101722	24	8.769200252	0.055423057
ENSMUSG00000027173	Depdc7	DEP domain containing 7 [Source:MGI Symbol;Acc:MGI:2139258]	9.29262	11.8961	8.7257	9.25502	11.4135	8.70463	9.60065	10.4207	11.522	7.95235	10.8231	9.64417	10.5849	9.84108	0.851656735	0.966018836	8	8	24	0.805277828	0.937619171	24	3.115463824	0.040888645
ENSMUSG00000027175	Tcp11l1	t-complex 11 like 1 [Source:MGI Symbol;Acc:MGI:2444263]	21.4766	21.0026	17.9412	21.3378	22.2262	21.5429	20.4723	22.0418	19.9384	23.5775	20.055	22.4131	22.0648	21.6309	0.390686537	0.915479136	24	12	24	0.499058211	0.808557177	24	19.40614186	0.041749797
ENSMUSG00000027176	Cstf3	cleavage stimulation factor, 3' pre-RNA, subunit 3 [Source:MGI Symbol;Acc:MGI:1351825]	29.0737	28.419	26.7803	29.7195	29.4716	29.1154	28.3654	25.4291	25.4639	25.9499	29.0485	27.9844	27.4767	26.21	0.097779482	0.501112642	20	16	24	0.002147744	0.395705644	24	18.65803343	0.080177867
ENSMUSG00000027177	Hipk3	homeodomain interacting protein kinase 3 [Source:MGI Symbol;Acc:MGI:1314882]	19.2589	18.7894	18.5247	20.9124	20.1778	18.9124	19.4962	19.1418	19.1635	19.9167	20.1528	18.1632	17.9942	18.3042	0.088274239	0.473025915	16	16	24	0.016271347	0.544099157	24	13.81960126	0.055209484
ENSMUSG00000027180	Fbxo3	F-box protein 3 [Source:MGI Symbol;Acc:MGI:1929084]	35.3973	34.6782	35.8162	35.5899	35.357	34.3615	34.29	31.7829	31.914	35.0194	34.1836	31.2932	30.9306	32.5504	0.481811994	0.966018836	16	8	24	0.068380946	0.672678756	24	13.93661036	0.040372842
ENSMUSG00000027184	Caprin1	cell cycle associated protein 1 [Source:MGI Symbol;Acc:MGI:1858234]	113.133	101.552	102.072	102.365	107.777	103.196	109.685	95.5152	88.6095	96.6784	109.801	96.9135	100.088	98.2659	0.158091814	0.614628746	4	4	24	0.040567967	0.616921492	24	19.82308841	0.079162178
ENSMUSG00000027185	Nat10	N-acetyltransferase 10 [Source:MGI Symbol;Acc:MGI:2138939]	8.34878	9.0072	10.6036	8.92937	8.20133	7.62656	6.96702	7.27259	8.21055	8.838	6.93168	7.59243	7.07585	8.72675	0.078380335	0.458114095	12	16	24	0.026190739	0.586541342	24	8.984194176	0.135526893
ENSMUSG00000027186	Elf5	E74-like factor 5 [Source:MGI Symbol;Acc:MGI:1335079]	0.631947	0.681973	0.722921	0.570456	0.687979	0.336926	1.05724	0.594899	0.552175	0.525496	0.587191	0.439439	0.56878	0.657591	0.45229395	0.966018836	8	16	24	0.440709557	0.805393068	24	4.060657922	0.06880575
ENSMUSG00000027187	Cat	catalase [Source:MGI Symbol;Acc:MGI:88271]	72.5489	62.4818	63.9431	69.8496	63.8594	70.8096	72.1389	68.2361	58.2146	81.2359	60.852	66.6573	68.5185	66.1596	0.267533088	0.774280302	4	8	24	0.761310313	0.922782206	24	20.20908699	0.037350536
ENSMUSG00000027188	Pamr1	peptidase domain containing associated with muscle regeneration 1 [Source:MGI Symbol;Acc:MGI:2445082]	3.27481	5.02249	4.57229	2.75956	4.01631	2.88032	3.39993	3.65241	5.48765	2.54722	3.30866	3.94127	3.16962	4.06523	0.032547207	0.324908923	12	4	24	0.19829459	0.768040178	24	5.735106032	0.177687987
ENSMUSG00000027189	Trim44	tripartite motif-containing 44 [Source:MGI Symbol;Acc:MGI:1931835]	50.6609	47.0646	45.3101	46.6582	46.6155	48.5978	46.5953	44.113	42.2014	50.5351	44.1607	49.923	47.2572	50.1125	0.601647512	0.966018836	24	12	24	0.364032627	0.788727838	24	21.0936105	0.045562266
ENSMUSG00000027193	Api5	apoptosis inhibitor 5 [Source:MGI Symbol;Acc:MGI:1888993]	25.8743	25.4875	23.841	25.2484	24.8107	24.7423	25.0911	23.7832	23.4894	22.6267	26.2671	24.2544	25.0886	24.9943	0.588208558	0.966018836	4	8	24	0.158281284	0.764753215	24	21.33165105	0.041191921
ENSMUSG00000027194	Ttc17	tetratricopeptide repeat domain 17 [Source:MGI Symbol;Acc:MGI:1921819]	8.43218	7.61799	7.47829	7.25574	8.40158	9.27896	7.63355	7.93097	6.05041	7.93444	5.87472	7.4563	6.9196	7.6513	0.865233232	0.966018836	24	12	24	0.314493919	0.768040178	24	21.69970986	0.085897537
ENSMUSG00000027195	Hsd17b12	hydroxysteroid (17-beta) dehydrogenase 12 [Source:MGI Symbol;Acc:MGI:1926967]	78.4625	74.6252	79.8019	79.8943	81.1126	78.5243	75.4748	76.7186	74.36	72.984	83.9598	78.049	84.8486	80.0327	0.581070934	0.966018836	20	16	24	0.424848424	0.805393068	24	18.8168829	0.032718716
ENSMUSG00000027198	Ext2	exostosin glycosyltransferase 2 [Source:MGI Symbol;Acc:MGI:108050]	13.6773	12.9693	13.6765	13.2542	14.9165	15.8451	14.218	14.4843	11.9008	16.8189	13.103	13.6952	14.9006	14.184	0.662399683	0.966018836	24	12	24	0.53592007	0.82731247	24	18.69110191	0.053308624
ENSMUSG00000027199	Gatm	glycine amidinotransferase (L-arginine:glycine amidinotransferase) [Source:MGI Symbol;Acc:MGI:1914342]	162.221	173.366	161.827	149.663	166.795	145.853	180.972	159.33	169.564	162.199	152.408	152.56	158.227	152.137	0.787283372	0.966018836	4	20	24	0.411132041	0.799546792	24	4.311541745	0.043248874
ENSMUSG00000027200	Sema6d	sema domain, transmembrane domain (TM), and cytoplasmic domain, (semaphorin) 6D [Source:MGI Symbol;Acc:MGI:2387661]	27.8809	29.3889	28.0544	24.2187	29.4065	27.5107	31.6489	28.1201	27.5437	29.3194	24.5527	28.8183	29.047	28.3786	0.612141595	0.966018836	4	12	24	0.175185581	0.768040178	24	1.085933107	0.067605598
ENSMUSG00000027201	Myef2	myelin basic protein expression factor 2, repressor [Source:MGI Symbol;Acc:MGI:104592]	19.3431	20.411	20.9637	17.8989	19.269	18.3654	18.1954	18.2842	17.9106	16.6206	19.5373	18.0941	17.0886	19.1648	0.935316621	0.966018836	8	8	24	0.814608597	0.940847489	24	4.083572743	0.019512514
ENSMUSG00000027203	Dut	deoxyuridine triphosphatase [Source:MGI Symbol;Acc:MGI:1346051]	14.3496	15.2288	15.9803	15.9004	14.4788	16.1512	14.9793	15.2243	14.118	14.4921	15.232	15.2655	14.9697	16.5395	0.887862353	0.966018836	24	4	24	0.929047698	0.981515518	24	4.362630754	0.01001114
ENSMUSG00000027204	Fbn1	fibrillin 1 [Source:MGI Symbol;Acc:MGI:95489]	5.89439	7.43832	5.59502	4.25697	4.94498	5.05502	6.35822	7.18026	6.41459	4.50963	4.20554	5.61219	6.26788	6.62341	4.34E-06	0.009754434	8	8	24	2.02E-05	0.047905911	24	3.047044875	0.282176015
ENSMUSG00000027206	Cops2	COP9 signalosome subunit 2 [Source:MGI Symbol;Acc:MGI:1330276]	94.3832	100.65	94.2464	96.849	100.436	86.9707	93.9331	89.2098	103.479	81.35	105.478	93.6383	99.4907	98.7413	0.923635729	0.966018836	20	4	24	0.972319012	0.993242403	24	6.069596901	0.010002886
ENSMUSG00000027207	Galk2	galactokinase 2 [Source:MGI Symbol;Acc:MGI:1917226]	13.1029	13.8864	13.0059	12.3552	13.1555	13.4953	11.3128	12.9011	12.1389	11.7231	13.5171	13.8459	13.534	13.4025	0.289105414	0.794869797	24	16	24	0.495548834	0.807241074	24	21.74412418	0.040412244
ENSMUSG00000027208	Fgf7	fibroblast growth factor 7 [Source:MGI Symbol;Acc:MGI:95521]	8.56691	10.4352	8.9638	9.56836	10.9839	7.26855	9.48326	7.64256	10.6008	8.34259	8.0336	8.99672	8.66771	8.3165	0.92769718	0.966018836	12	12	24	0.664694359	0.886778052	24	10.03299901	0.056389794
ENSMUSG00000027210	Meis2	Meis homeobox 2 [Source:MGI Symbol;Acc:MGI:108564]	6.92301	7.31465	8.24373	8.88525	6.32797	8.58398	7.88334	8.60222	6.17654	8.47945	5.24123	8.28081	6.14618	9.71528	0.725061903	0.966018836	12	8	24	0.612190888	0.865456875	24	5.515314884	0.090864541
ENSMUSG00000027215	Cd82	CD82 antigen [Source:MGI Symbol;Acc:MGI:104651]	54.9701	46.9287	44.2802	48.6105	51.3102	51.5502	50.2406	49.1867	44.7734	51.3672	46.311	51.8962	51.4831	45.7806	7.90E-04	0.071344007	24	12	24	0.014756966	0.537860185	24	20.33655151	0.09427744
ENSMUSG00000027217	Tspan18	tetraspanin 18 [Source:MGI Symbol;Acc:MGI:1917186]	9.41208	7.41181	8.1585	9.1849	8.49421	9.41081	9.03042	9.69149	7.2975	11.5686	9.80188	9.05405	9.24878	9.88809	0.817383384	0.966018836	16	20	24	0.577348374	0.848840315	24	17.12033265	0.059646365
ENSMUSG00000027219	Slc28a2	solute carrier family 28 (sodium-coupled nucleoside transporter), member 2 [Source:MGI Symbol;Acc:MGI:1913105]	0.443574	0.600312	0.826573	0.603735	0.684301	0.414405	0.606527	0.891608	0.725014	0.963128	0.531926	0.571503	0.874987	0.668352	0.138202217	0.580158868	12	12	24	0.047529009	0.635122786	24	8.1259004	0.125529943
ENSMUSG00000027220	Syt13	synaptotagmin XIII [Source:MGI Symbol;Acc:MGI:1933945]	1.22052	1.84774	3.01626	1.5237	0.878609	1.77925	2.48763	1.1176	1.85281	1.52483	0.912123	1.92471	1.01406	2.33153	0.185796491	0.662239322	12	8	24	0.316134778	0.768040178	24	5.55983441	0.201527264
ENSMUSG00000027221	Chst1	carbohydrate sulfotransferase 1 [Source:MGI Symbol;Acc:MGI:1924219]	2.2175	2.90634	4.65591	3.01219	2.57032	3.5847	3.57622	2.57309	3.80129	4.12326	2.2598	3.16394	2.08792	3.68825	0.105280783	0.518002936	16	16	24	0.211190541	0.768040178	24	9.048076129	0.188239316
ENSMUSG00000027222	Pex16	peroxisomal biogenesis factor 16 [Source:MGI Symbol;Acc:MGI:1338829]	14.2027	13.0758	14.6369	14.3236	14.9983	16.4795	14.1047	15.7796	14.3699	15.1735	14.2613	14.9729	14.3817	13.7485	0.397360907	0.927993503	16	16	24	0.476260424	0.805393068	24	14.90933457	0.028671495
ENSMUSG00000027223	Mapk8ip1	mitogen-activated protein kinase 8 interacting protein 1 [Source:MGI Symbol;Acc:MGI:1309464]	101.41	76.869	78.8867	85.0496	87.1788	82.2099	94.3871	91.0333	82.9182	113.187	95.7214	97.9168	95.5297	88.3746	0.27817759	0.789849983	4	4	24	0.513228597	0.816307374	24	18.50487015	0.065308791
ENSMUSG00000027227	Sord	sorbitol dehydrogenase [Source:MGI Symbol;Acc:MGI:98266]	14.7422	13.9587	15.0602	15.1456	14.5978	15.462	14.8143	16.2888	14.0179	17.2978	14.7902	16.8807	16.0576	15.3326	0.912686312	0.966018836	24	12	24	0.807733877	0.938776658	24	17.4045247	0.020105527
ENSMUSG00000027230	Creb3l1	cAMP responsive element binding protein 3-like 1 [Source:MGI Symbol;Acc:MGI:1347062]	4.57523	3.37808	2.0307	2.91998	3.27446	3.65135	3.55034	3.77726	2.56608	3.65365	3.58628	3.91293	4.09269	3.69736	0.009118941	0.192760771	4	8	24	0.0148409	0.537924552	24	21.8565392	0.217534048
ENSMUSG00000027236	Eif3j1	eukaryotic translation initiation factor 3, subunit J1 [Source:MGI Symbol;Acc:MGI:1925905]	5.37569	5.29463	5.12545	5.68928	6.14679	5.48065	5.30281	5.26946	5.33074	5.79502	5.68408	5.59954	5.94832	5.24349	0.002538391	0.111779175	20	12	24	0.016229213	0.544099157	24	16.48068	0.062904687
ENSMUSG00000027238	Frmd5	FERM domain containing 5 [Source:MGI Symbol;Acc:MGI:2442557]	2.37062	2.57184	4.90589	2.28914	2.22799	3.23742	3.83463	2.19089	3.04772	2.29428	2.29167	2.56006	2.18417	3.40838	0.745118544	0.966018836	12	8	24	0.520275959	0.819878026	24	5.250839434	0.124111594

ENSMUSG00000027239	Mdk	midkine [Source:MGI Symbol;Acc:MGI:96949]	14.9217	17.1816	17.4917	14.8878	14.2975	14.2971	16.6423	19.2137	20.792	14.7512	16.6439	17.0592	16.0498	15.6466	0.079174302	0.458678242	12	4	24	0.086215233	0.705339265	24	5.969561904	0.124404911
ENSMUSG00000027242	Wdr76	WD repeat domain 76 [Source:MGI Symbol;Acc:MGI:1926186]	1.16853	1.22284	1.48157	1.3084	1.30217	1.39646	1.13041	1.27712	1.13821	1.11069	1.24156	1.51055	1.32128	1.40059	0.911135828	0.966018836	24	4	24	0.869099108	0.96291905	24	19.78542538	0.01777552
ENSMUSG00000027243	Harbi1	harbinger transposase derived 1 [Source:MGI Symbol;Acc:MGI:2443194]	5.25547	4.31378	4.27499	4.41912	5.26458	5.00777	4.9835	5.06349	4.70516	3.80667	5.46261	4.80411	4.91802	4.33437	0.019643089	0.258568924	20	20	24	0.044483204	0.628432994	24	20.53874294	0.111587948
ENSMUSG00000027244	Atg13	autophagy related 13 [Source:MGI Symbol;Acc:MGI:1196429]	23.1402	20.759	20.5083	20.4368	19.3381	22.296	22.5798	22.2186	19.5906	26.3922	19.0617	22.5349	21.2425	21.6644	0.048396595	0.379129806	4	16	24	0.764295384	0.92403577	24	0.191595018	0.034651195
ENSMUSG00000027245	Hypk	huntingtin interacting protein K [Source:MGI Symbol;Acc:MGI:1914943]	14.548	14.0339	13.3183	14.1427	14.4808	14.2672	12.9268	11.1121	15.5949	14.1946	16.5259	13.1801	14.2867	14.9483	0.907000607	0.966018836	20	12	24	0.373276719	0.794388449	24	14.47030102	0.066909552
ENSMUSG00000027246	Ell3	elongation factor RNA polymerase II-like 3 [Source:MGI Symbol;Acc:MGI:2673679]	1.46874	1.28105	1.31222	1.26894	1.36006	1.34572	1.47395	1.51142	1.14869	1.47256	1.4142	1.45039	1.46417	1.48948	0.111340266	0.530299176	8	4	24	0.15744064	0.76387796	24	22.58292098	0.044810486
ENSMUSG00000027247	Arhgap1	Rho GTPase activating protein 1 [Source:MGI Symbol;Acc:MGI:2445003]	21.0521	17.2395	18.3636	18.5952	17.7135	21.0278	19.8551	21.4388	17.7594	23.4998	16.9935	19.142	20.0255	18.652	0.591351901	0.966018836	4	16	24	0.79815196	0.935424343	24	23.23161872	0.035339734
ENSMUSG00000027248	Pdia3	protein disulfide isomerase associated 3 [Source:MGI Symbol;Acc:MGI:95834]	155.234	122.51	117.966	115.057	133.627	136.346	134.007	125.859	100.312	114.792	128.377	132.605	135.643	125.632	1.57E-04	0.031913438	4	12	24	2.37E-04	0.177779102	24	21.51443878	0.181755362
ENSMUSG00000027253	Lrp4	low density lipoprotein receptor-related protein 4 [Source:MGI Symbol;Acc:MGI:2442252]	1.95247	2.10367	2.29212	1.94219	2.39175	1.99779	2.57202	2.25341	2.09394	1.9018	1.93688	1.97864	1.85301	2.03494	0.888991156	0.966018836	12	4	24	0.808726925	0.939182735	24	3.335309334	0.023572637
ENSMUSG00000027254	Map1a	microtubule-associated protein 1 A [Source:MGI Symbol;Acc:MGI:1306776]	92.0283	69.5632	72.2788	81.2829	73.7237	87.8356	82.6282	86.5432	55.8599	139.03	62.4737	86.395	76.1168	88.0867	0.774195442	0.966018836	24	12	24	0.934178992	0.983548589	24	18.59875588	0.046889049
ENSMUSG00000027255	Arfgap2	ADP-ribosylation factor GTPase activating protein 2 [Source:MGI Symbol;Acc:MGI:1924288]	16.1108	17.7475	17.58	17.9826	19.4207	17.2165	18.2282	18.2639	18.7374	18.2349	16.708	16.7172	17.8339	17.3908	0.628286596	0.966018836	16	8	24	0.288755899	0.768040178	24	9.874150337	0.042146194
ENSMUSG00000027257	Pacsin3	protein kinase C and casein kinase substrate in neurons 3 [Source:MGI Symbol;Acc:MGI:1891410]	15.9111	19.1256	20.5517	20.2938	20.338	18.2571	21.0576	24.3664	22.3554	22.0346	15.6473	18.2018	18.5231	17.8257	0.241950772	0.741580974	12	12	24	0.138921674	0.748341961	24	8.20750098	0.133630484
ENSMUSG00000027259	Adal	adenosine deaminase-like [Source:MGI Symbol;Acc:MGI:1923144]	13.8258	11.5943	10.4913	11.5534	10.412	11.6064	10.8865	10.8646	11.6707	10.5981	12.3033	11.4986	12.447	12.2703	0.591351901	0.966018836	4	12	24	0.268441352	0.768040178	24	23.45995049	0.065636929
ENSMUSG00000027263	Tubgcp4	tubulin, gamma complex associated protein 4 [Source:MGI Symbol;Acc:MGI:1196293]	22.3295	19.4762	17.7164	21.1386	19.8908	19.522	21.4698	19.4144	18.5777	20.3255	21.6667	19.7822	21.7053	20.1937	0.035806379	0.331814682	4	8	24	0.176940726	0.768040178	24	20.49546041	0.065028023
ENSMUSG00000027270	Lamp5	lysosomal-associated membrane protein family, member 5 [Source:MGI Symbol;Acc:MGI:1923411]	3.50196	8.65912	18.1693	5.70496	4.05586	10.6091	10.9747	5.14479	8.93987	6.04514	3.20946	6.52756	1.77899	9.25275	0.616047374	0.966018836	12	8	24	0.401202041	0.799546792	24	7.150811203	0.388648173
ENSMUSG00000027272	Ubr1	ubiquitin protein ligase E3 component n-recognin 1 [Source:MGI Symbol;Acc:MGI:1277977]	11.7122	12.1563	12.6842	12.0484	11.8063	11.3259	12.5157	11.7389	12.4259	11.5421	11.4508	12.9765	12.8272	12.7837	0.595037318	0.966018836	4	16	24	0.214231844	0.768040178	24	3.877485493	0.038632484
ENSMUSG00000027273	Snap25	synaptosomal-associated protein 25 [Source:MGI Symbol;Acc:MGI:98331]	553.133	547.95	554.147	511.711	484.601	544.474	545.734	495.516	459.886	569.78	477.406	514.966	498.353	573.647	0.823677655	0.966018836	8	12	24	0.569739114	0.844900526	24	2.39450288	0.041470418
ENSMUSG00000027274	Mkks	McKusick-Kaufman syndrome [Source:MGI Symbol;Acc:MGI:1891836]	13.2802	17.8781	13.8647	15.1857	14.4794	15.2847	16.5945	11.0304	13.763	11.6675	16.7117	11.8782	12.8387	14.4294	0.831272156	0.966018836	20	20	24	0.900615731	0.97190491	24	19.08821892	0.035045716
ENSMUSG00000027276	Jag1	jagged 1 [Source:MGI Symbol;Acc:MGI:1095416]	5.90206	5.01796	5.12453	5.23847	5.56468	5.15182	5.40443	5.3375	4.36447	4.44154	5.0605	4.78523	5.44212	5.15664	0.055920623	0.401074772	4	8	24	0.059483899	0.658304792	24	22.42356731	0.07985072
ENSMUSG00000027281	Slx4ip	SLX4 interacting protein [Source:MGI Symbol;Acc:MGI:1921493]	3.28591	2.94375	2.95124	2.87598	3.31699	2.78994	2.88169	2.50933	2.88528	2.32693	3.0033	3.22703	3.44847	3.05108	0.446653834	0.966018836	4	12	24	0.214401857	0.768040178	24	21.50108965	0.078491288
ENSMUSG00000027282	Mtch2	mitochondrial carrier 2 [Source:MGI Symbol;Acc:MGI:1929260]	70.109	75.2395	73.2461	80.5042	77.036	72.6907	71.3042	70.4124	75.0233	70.845	84.7151	70.1955	77.0166	73.6574	0.371875382	0.894537464	20	4	24	0.202129443	0.768040178	24	13.68734042	0.05326551
ENSMUSG00000027284	Cdan1	congenital dyserythropoietic anemia, type I (human) [Source:MGI Symbol;Acc:MGI:1916218]	4.77185	7.63354	7.21727	6.79831	6.95943	6.44262	5.30532	6.11226	7.11752	5.4353	6.76094	5.37983	5.28768	6.46879	8.66E-04	0.071344007	12	16	24	0.101859829	0.713240751	24	10.08684335	0.141778651
ENSMUSG00000027285	Haus2	HAUS augmin-like complex, subunit 2 [Source:MGI Symbol;Acc:MGI:1913546]	14.3797	16.115	15.3331	14.0243	14.4783	13.6052	13.6471	14.0784	15.0402	14.4706	15.2529	14.9326	15.0072	16.3037	0.403954478	0.937351147	8	16	24	0.240234816	0.768040178	24	6.543303484	0.048590819
ENSMUSG00000027286	Lrrc57	leucine rich repeat containing 57 [Source:MGI Symbol;Acc:MGI:1913856]	21.0831	20.9208	19.7791	20.9346	19.9192	19.7025	18.1068	19.8603	19.4118	19.5932	19.8941	20.1591	19.9866	19.1809	0.896311169	0.966018836	4	8	24	0.879161449	0.966161692	24	15.57804908	0.009765947
ENSMUSG00000027287	Snap23	synaptosomal-associated protein 23 [Source:MGI Symbol;Acc:MGI:109356]	10.8199	10.4421	9.87837	11.2349	11.8517	8.78819	11.1877	10.2257	12.394	8.23584	11.1526	9.03806	11.2553	8.90127	0.896773941	0.966018836	4	20	24	0.994718269	0.998755922	24	8.686206441	0.006901594
ENSMUSG00000027288	Zfp106	zinc finger protein 106 [Source:MGI Symbol;Acc:MGI:1270153]	23.7853	25.6319	26.1068	29.2321	23.6832	25.1814	24.937	27.1889	28.6568	28.6984	24.1488	26.1025	27.1544	27.1335	0.00789564	0.192760771	16	4	24	0.018110048	0.559589383	24	8.536836694	0.0930133
ENSMUSG00000027291	Vps39	VPS39 HOPS complex subunit [Source:MGI Symbol;Acc:MGI:2443189]	19.5412	19.5166	20.2455	19.6344	19.2975	21.2105	18.8469	19.4739	18.7952	19.8012	18.7866	20.1101	19.3306	18.6957	0.873337044	0.966018836	24	8	24	0.474992953	0.805393068	24	18.30380319	0.022315005
ENSMUSG00000027293	Ehd4	EH-domain containing 4 [Source:MGI Symbol;Acc:MGI:1919619]	17.2082	15.6616	15.4057	16.2275	18.8033	15.9114	18.0396	17.7477	15.8084	20.3803	16.7909	17.1709	17.3745	16.4632	0.496745696	0.966018836	4	8	24	0.547341476	0.833663107	24	16.86375147	0.045310637
ENSMUSG00000027298	Tyro3	TYRO3 protein tyrosine kinase 3 [Source:MGI Symbol;Acc:MGI:104294]	7.83735	6.10125	6.52502	6.68405	6.72666	7.12547	7.29382	7.33198	6.51481	8.77271	6.12052	7.04072	6.22965	7.35614	0.915475528	0.966018836	4	8	24	0.99729944	0.999506857	24	20.71613953	0.004012218
ENSMUSG00000027300	Ubox5	U box domain containing 5 [Source:MGI Symbol;Acc:MGI:2154658]	3.56056	2.682	2.93889	3.23947	3.15947	3.37919	3.48489	3.26727	2.74942	3.60351	2.951	2.99893	3.07518	3.21964	0.625825704	0.966018836	4	8	24	0.552709121	0.836966973	24	19.96499666	0.044841714
ENSMUSG00000027303	Ptpra	protein tyrosine phosphatase, receptor type, A [Source:MGI Symbol;Acc:MGI:97808]	61.4913	51.3793	51.1805	49.1596	50.4316	58.9259	54.0357	53.4852	46.7958	58.9056	49.7177	51.7421	53.6431	54.4182	0.058157448	0.40386543	4	8	24	0.219019714	0.768040178	24	22.48881317	0.073314949
ENSMUSG00000027304	Rtf1	RTF1, Paf1/RNA polymerase II complex component [Source:MGI Symbol;Acc:MGI:1309480]	10.5028	9.18402	10.5429	9.83649	8.64334	10.3443	9.87837	10.3175	9.49514	11.6396	9.94197	10.5481	10.1109	11.1191	0.842298032	0.966018836	24	20	24	0.814170054	0.940708369	24	5.639826447	0.02428351
ENSMUSG00000027305	Ndufaf1	NADH:ubiquinone oxidoreductase complex assembly factor 1 [Source:MGI Symbol;Acc:MGI:1916952]	22.5991	22.1775	22.011	22.3062	21.7531	21.3028	21.8894	23.0365	22.5847	23.7567	24.7014	21.5188	25.6609	23.6701	0.718720526	0.966018836	8	16	24	0.727813634	0.91070186	24	6.724265155	0.020359812
ENSMUSG00000027306	Nusap1	nucleolar and spindle associated protein 1 [Source:MGI Symbol;Acc:MGI:2675669]	1.37186	1.51905	2.13789	2.2805	1.51856	1.77214	1.7067	1.44763	2.00876	1.15304	1.75481	1.41906	2.06774	1.4687	0.895493752	0.966018836	12	20	24	0.773778513	0.92732018	24	9.998131643	0.05223236
ENSMUSG00000027309	Dnaaf9	dynein axonemal assembly factor 9 [Source:MGI Symbol;Acc:MGI:1923029]	17.5816	17.5258	18.1217	16.4201	17.0976	16.9036	19.4519	16.188	17.7976	15.5899	17.2026	17.2144	16.7102	18.1175	0.369262716	0.893596186	12	4	24	0.295601916	0.768040178	24	1.480566874	0.044219484
ENSMUSG00000027312	Atrn	attractin [Source:MGI Symbol;Acc:MGI:1341628]	17.7687	16.5149	17.8161	16.9795	16.2311	18.448	18.2624	16.4346	14.5149	20.082	15.3378	16.9744	16.567	17.584	0.891105762	0.966018836	24	20	24	0.88537146	0.967345379	24	21.01702725	0.02281699
ENSMUSG00000027313	Chac1	ChaC, cation transport regulator 1 [Source:MGI Symbol;Acc:MGI:1916315]	2.28573	2.06596	2.66263	2.75588	2.14233	2.94317	2.43112	5.67325	3.27461	2.75299	2.50392	2.08533	2.25174	2.315	0.38940266	0.915479136	12	16	24	0.412724973	0.799546792	24	7.214075907	0.156392279
ENSMUSG00000027314	Dll4	delta like canonical Notch ligand 4 [Source:MGI Symbol;Acc:MGI:1859388]	0.454921	0.719499	0.556498	0.632113	0.582607	0.593297	0.675058	0.734739	0.703189	0.845583	0.633348	0.53621	0.600566	0.579054	0.590017078	0.966018836	16	8	24	0.172541112	0.768040178	24	9.264004463	0.062117631
ENSMUSG00000027315	Spint1	serine protease inhibitor, Kunitz type 1 [Source:MGI Symbol;Acc:MGI:1338033]	4.11606	4.4412	2.77982	3.06564	4.62163	4.11781	5.08901	4.96514	3.49668	4.78998	3.66152	4.43484	4.93745	3.69802	0.170659409	0.643496587	4	8	24	0.115689024	0.727799063	24	22.40024247	0.16367796
ENSMUSG00000027316	Gfra4	glial cell line derived neurotrophic factor family receptor alpha 4 [Source:MGI Symbol;Acc:MGI:1341873]	9.87416	7.3671	7.91457	7.73866	7.63431	8.59634	8.99189	8.71301	7.67395	8.92926	8.07937	9.60053	8.05416	8.1135	0.235177833	0.73629392	4	8	24	0.151161955	0.758865539	24	21.58660323	0.086407003
ENSMUSG00000027318	Adam33	a disintegrin and metallopeptidase domain 33 [Source:MGI Symbol;Acc:MGI:1341813]	2.28398	2.47906	3.00038	3.35151	3.57225	2.34788	3.04798	2.7419	4.24909	2.49344	2.25899	2.38395	2.58764	1.68111	0.549437303	0.966018836	12	12	24	0.302851059	0.768040178	24	11.3487848	0.138655333
ENSMUSG00000027323	Rad51	RAD51 recombinase [Source:MGI Symbol;Acc:MGI:97890]	0.783718	0.878199	1.22774	1.38123	0.972691	1.31433	0.847544	0.924588	0.963969	1.03235	0.784774	0.817757	0.965029	0.98459	0.109247218	0.524035616	16	12	24	0.284402026	0.768040178	24	11.45977227	0.079876188
ENSMUSG00000027324	Rpusd2	RNA pseudouridylate synthase domain containing 2 [Source:MGI Symbol;Acc:MGI:1918066]	1.50989	1.32339	1.30454	1.34703	1.71167	1.48504	1.06569	1.44131	1.279	1.6139	1.40869	1.42249	1.28123	1.56606	0.669104912	0.966018836	20	8	24	0.385455266	0.799546792	24	14.95242566	0.052803094
ENSMUSG00000027327	1700037H04Rik	RIKEN cDNA 1700037H04 gene [Source:MGI Symbol;Acc:MGI:1914576]	58.8343	41.7366	45.0125	49.2528	46.766	53.7307	52.6961	52.6355	39.0949	60.7498	52.5882	49.5802	51.721	48.6305	0.11436224	0.534017934	4	8	24	0.273861484	0.768040178	24	19.60764571	0.10935659
ENSMUSG00000027329	Spef1	sperm flagellar 1 [Source:MGI Symbol;Acc:MGI:3513546]	2.27044	1.83839	2.43097	1.92678	2.11927	2.06795	2.00573	1.66617	2.31585	2.05116	2.43771	1.9776	1.916	2.05589	0.17882595	0.655480352	20	12	24	0.419624574	0.803639585	24	13.81192064	0.052947707
ENSMUSG00000027330	Cdc25b	cell division cycle 25B [Source:MGI Symbol;Acc:MGI:99701]	3.37971	4.57839	5.15728	4.06692	4.17064	3.99591	3.49745	5.11291	5.38425	3.9204	3.82689	4.09636	3.95658	4.63873	0.009483698	0.194720062	12	16	24	0.011697695	0.522142988	24	7.641882241	0.174238707
ENSMUSG00000027331	Knstrn	kinetochore-localized astrin/SPAG5 binding [Source:MGI Symbol;Acc:MGI:1289298]	1.23083	1.50511	1.6011	1.5503	1.90195	1.78728	1.40367	1.33162	1.45217	0.955791	1.75031	1.18942	1.18593	0.872543	0.217660303	0.704989686	20	8	24	0.133594687	0.743843563	24	16.3458538	0.120286813
ENSMUSG00000027332	Ivd	isovaleryl coenzyme A dehydrogenase [Source:MGI Symbol;Acc:MGI:1929242]	27.6874	29.5806	29.802	36.3341	35.158	31.6487	32.2966	30.3411	34.2943	31.8942	34.027	31.1844	32.6656	25.4348	0.015486342	0.253395174	16	16	24	0.030896836	0.599371601	24	14.73243917	0.122186574
ENSMUSG00000027333	Smox	spermine oxidase [Source:MGI Symbol;Acc:MGI:2445356]	25.2744	26.236	25.9683	26.0767	26.6114	27.6875	30.2051	34.2156	32.6712	30.1089	27.9513	25.7431	27.1815	20.4565	0.782779623	0.966018836	12	20	24	0.878364448	0.965980735	24	10.58012416	0.036623528
ENSMUSG00000027335	Adra1d	adrenergic receptor, alpha 1d [Source:MGI Symbol;Acc:MGI:106673]	0.707466	0.310605	0.62086	0.341875	0.283042	0.429639	0.529877	0.696936	0.499765	0.874212	0.58274	0.652403	0.664862	0.486973	0.883870616	0.966018836	4	16	24	0.871004127	0.963364428	24	3.392021955	0.031539649
ENSMUSG00000027339	Rassf2	Ras association (RalGDS/AF-6) domain family member 2 [Source:MGI Symbol;Acc:MGI:2442060]	9.01825	9.81813	10.509	9.3153	10.3069	9.0377	11.281	8.61643	10.8716	8.18139	9.40001	9.47634	8.41235	8.69353	0.911135828	0.966018836	12	20	24	0.972638563	0.993250534	24	10.51776483	0.011725108
ENSMUSG00000027340	Slc23a2	solute carrier family 23 (nucleobase transporters), member 2 [Source:MGI Symbol;Acc:MGI:1859682]	14.8059	16.6452	16.4004	16.0345	14.9263	14.0833	15.3948	14.4466	15.5576	16.5772	13.0596	16.4089	12.1765	17.1278	0.282647737	0.793714531	8	20	24	0.236400707	0.768040178	24	8.309081892	0.088617039
ENSMUSG00000027341	Tmem230	transmembrane protein 230 [Source:MGI Symbol;Acc:MGI:1917862]	27.15	25.5574	23.3902	24.8865	26.0707	23.9969	24.7114	23.8091	21.9972	26.4458	25.524	26.3519	26.5823	24.788	0.170659409	0.643496587	4	8	24	0.142186547	0.751766185	24	19.7838393	0.062405568
ENSMUSG00000027342	Pcna	proliferating cell nuclear antigen [Source:MGI Symbol;Acc:MGI:97503]	12.4445	10.8526	12.0561	9.64084	12.2442	10.7491	10.1356	11.3041	10.2162	8.16216	12.761	9.25277	9.31697	8.08553	0.896799496	0.966018836	20	20	24	0.549872784	0.835250398	24	18.82308117	0.070228021
ENSMUSG00000027346	Gpcpd1	glycerophosphocholine phosphodiesterase 1 [Source:MGI Symbol;Acc:MGI:104898]	34.5695	43.2481	43.4253	41.8628	38.7295	36.153	36.0059	41.2367	47.0678	40.0755	40.3978	35.5429	37.913	38.1326	1.13E-04	0.029372629	12	16	24	6.26E-04	0.296483534	24	9.038571004	0.150162751
ENSMUSG00000027347	Rasgrp1	RAS guanyl releasing protein 1 [Source:MGI Symbol;Acc:MGI:1314635]	50.4806	41.5944	38.5069	45.1452	45.4308	49.2722	39.5802	44.4802	35.3014	46.8448	43.4068	45.7466	46.859	44.6834	0.042880436	0.356048751	24	12	24	0.09366152	0.71175955	24	19.78958593	0.115036325
ENSMUSG00000027349	Fam98b	family with sequence similarity 98, member B [Source:MGI Symbol;Acc:MGI:1915465]	17.7173	17.3423	18.0193	15.9227	16.7038	17.5592	18.3204	16.295	14.8158	16.5164	14.6826	16.1787	16.4264	16.2497	0.894537464	0.966018836	4	12	24	0.092345206	0.71175955	24	23.92895463	0.060032752
ENSMUSG00000027350	Chgb	chromogranin B [Source:MGI Symbol;Acc:MGI:88395]	31.6726	26.822	30.7949	26.4437	23.8443	32.0297	30.9477	28.4874	22.8338	35.9697	21.9659	30.5586	26.2641	32.7803	0.89563591	0.966018836	24	20	24	0.621722368	0.871115984	24	1.498346603	0.078694746
ENSMUSG00000027351	Spred1	sprouty protein with EVH-1 domain 1, related sequence [Source:MGI Symbol;Acc:MGI:2150016]	18.8482	18.2693	17.9227	17.5006	19.3301	17.1806	19.031	15.8488	17.0632	16.7014	[illegible]	[illegible]	[illegible]	17.768	0.718720526	0.966018836	20	20	24	0.57859226	0.849323418	24	16.96105431	0.037823737
ENSMUSG00000027353	Mcm8	minichromosome maintenance 8 homologous recombination repair factor [Source:MGI Symbol;Acc:MGI:1913884]	2.55728	2.36278	2.9823	2.27188	2.31024	2.31238	2.46124	1.84236	2.23186	2.29081	2.87046	2.47306	2.42289	2.70181	0.92655264	0.966018836	4	12	24	0.92895947	0.981515518	24	16.84436977	0.018949104
ENSMUSG00000027357	Crls1	cardiolipin synthase 1 [Source:MGI Symbol;Acc:MGI:1913836]	17.2631	16.1138	17.8969	20.941	16.4008	16.448	16.4536	17.0752	17.5952	17.036	19.7503	18.0595	20.1698	18.4816	0.826055781	0.966018836	12	20	24	0.609160078	0.864393298	24	12.20977054	0.041236158
ENSMUSG00000027359	Slc27a2	solute carrier family 27 (fatty acid transporter), member 2 [Source:MGI Symbol;Acc:MGI:1347099]	0.359969	1.0354	3.41812	4.99074	0.792856	1.16465	1.53389	2.08494	3.21763	2.62423	1.27004	1.59466	1.37277	3.83599	0.081515548	0.462922111	16	4	24	0.003847175	0.427278365	24	9.071272828	0.656265953
ENSMUSG00000027360	Hdc	histidine decarboxylase [Source:MGI Symbol;Acc:MGI:96062]	0.932228	1.16648	1.74568	1.65455	1.66299	1.33107	1.63453	1.13654	0.933696	0.809084	1.35808	1.47846	1.23233	1.05708	0.658942124	0.966018836	12	8	24	0.425251491	0.805393068	24	17.38214462	0.099493427
ENSMUSG00000027361	Gabpb1	GA repeat binding protein, beta 1 [Source:MGI Symbol;Acc:MGI:95611]	8.24452	6.78036	6.56658	6.2172	7.17669	6.77055	6.63148	7.51512	6.73066	7.07622	6.51384	6.54336	6.51858	6.50747	0.847846021	0.966018836	4	8	24	0.525360647	0.822423089	24	23.76564093	0.037930247
ENSMUSG00000027363	Usp8	ubiquitin specific peptidase 8 [Source:MGI Symbol;Acc:MGI:1934029]	35.8821	29.9734	31.4613	31.2458	29.488	33.258	31.893	33.6001	27.0563	35.4274	31.4511	33.045	32.5821	33.2677	0.4776989	0.966018836	4	8	24	0.434154865	0.805393068	24	22.1302541	0.053461586
ENSMUSG00000027364	Usp50	ubiquitin specific peptidase 50 [Source:MGI Symbol;Acc:MGI:1922333]	3.6195	4.10819	3.83457	4.05397	4.02594	3.54425	3.74234	3.3926	4.27111	3.00037	3.8562	4.02657	4.25758	4.14638	0.888482515	0.966018836	12	4	24	0.851437009	0.955425185	24	2.982348554	0.025210194
ENSMUSG00000027365	Trpm7	transient receptor potential cation channel, subfamily M, member 7 [Source:MGI Symbol;Acc:MGI:1929996]	14.5972	18.8351	17.6331	18.3239	16.4135	17.1247	19.4568	18.1042	19.3959	18.0763	16.9356	16.7802	18.2016	18.8534	0.127318615	0.560209134	8	16	24	0.062712337	0.661961741	24	6.995689185	0.087716071
ENSMUSG00000027366	Sppl2a	signal peptide peptidase like 2A [Source:MGI Symbol;Acc:MGI:1913802]	17.2103	18.0454	16.7481	16.9557	17.2889	17.509	17.1022	17.981	19.8766	14.2044	18.0571	16.626	17.4781	16.4739	0.853187563	0.966018836	12	4	24	0.680967187	0.893402642	24	2.271626944	0.03401912
ENSMUSG00000027367	Stard7	START domain containing 7 [Source:MGI Symbol;Acc:MGI:2139090]	32.2107	36.7188	36.2011	39.993	37.0475	35.3287	34.026	36.8617	38.613	36.5645	33.3835	34.7476	35.1599	35.8885	0.026841899	0.305155548	16	12	24	0.019202712	0.562219749	24	9.823120306	0.078826203
ENSMUSG00000027371	Fahd2a	fumarylacetoacetate hydrolase domain containing 2A [Source:MGI Symbol;Acc:MGI:1915376]	8.89891	11.5936	12.1179	11.1597	9.13048	10.8616	11.802	10.5054	11.1706	10.9116	10.9476	12.3381	11.8156	13.7701	0.822170541	0.966018836	8	12	24	0.210196595	0.768040178	24	5.595679891	0.085604968
ENSMUSG00000027374	Mrps5	mitochondrial ribosomal protein S5 [Source:MGI Symbol;Acc:MGI:1924971]	20.7194	22.9655	18.9922	22.564	22.0208	22.6711	24.8624	25.267	20.599	25.3607	23.4688	19.2602	22.5238	23.0536	0.935316621	0.966018836	8	4	24	0.976495642	0.994103202	24	4.993059428	0.011111474
ENSMUSG00000027375	Mal	myelin and lymphocyte protein, T cell differentiation protein [Source:MGI Symbol;Acc:MGI:892970]	244.319	262.477	251.078	240.668	309.501	250.379	300.849	254.403	283.483	269.069	262.182	259.681	259.74	238.453	0.887862353	0.966018836	20	12	24	0.692861353	0.898196933	24	16.6632453	0.039044636
ENSMUSG00000027378	Nphp1	nephronophthisis 1 (juvenile) homolog (human) [Source:MGI Symbol;Acc:MGI:1858233]	5.0995	6.36657	6.31874	6.37286	6.72309	6.69297	7.56837	6.83989	7.92456	6.97235	5.91978	6.19576	8.04195	7.49317	0.94663657	0.966018836	4	20	24	0.304906137	0.768040178	24	6.116129343	0.078107683
ENSMUSG00000027379	Bub1	BUB1, mitotic checkpoint serine/threonine kinase [Source:MGI Symbol;Acc:MGI:1100510]	0.457881	0.688302	0.820759	0.597035	0.960698	0.485017	0.935643	0.448231	0.561841	0.530831	0.604206	0.574922	0.667749	0.393075	0.858269825	0.966018836	20	12	24	0.711550762	0.90471055	24	16.50350971	0.048421946
ENSMUSG00000027381	Bcl2l11	BCL2-like 11 (apoptosis facilitator) [Source:MGI Symbol;Acc:MGI:1197519]	1.47165	1.60742	1.55159	1.54407	1.66046	1.46985	1.82463	1.82413	1.80885	2.10683	1.73112	1.4269	1.93976	1.67055	0.887027852	0.966018836	4	20	24	0.361615101	0.786932131	24	8.282039704	0.053888087
ENSMUSG00000027384	Ndufaf5	NADH:ubiquinone oxidoreductase complex assembly factor 5 [Source:MGI Symbol;Acc:MGI:1916737]	26.2616	26.1609	24.1087	22.8341	23.0445	23.8271	23.9514	21.9096	23.5346	22.471	25.6155	23.24	25.2053	27.2923	0.115745527	0.537734429	4	12	24	0.266604022	0.768040178	24	1.295414057	0.056228472
ENSMUSG00000027386	Fbln7	fibulin 7 [Source:MGI Symbol;Acc:MGI:1917620]	2.78581	2.52051	2.41947	2.49871	3.1534	2.67217	3.10604	3.29636	2.52052	2.4879	2.55314	2.93082	2.73027	2.0174	0.129598979	0.560263689	4	8	24	0.192955581	0.768040178	24	20.57916497	0.094146651
ENSMUSG00000027387	Zc3h8	zinc finger CCCH type containing 8 [Source:MGI Symbol;Acc:MGI:1930128]	4.17817	3.75463	4.60523	5.31081	4.17998	4.43849	5.17074	4.35977	4.13901	3.54813	4.32259	3.81137	4.94047	4.32933	0.810949269	0.966018836	4	16	24	0.892840498	0.969717376	24	23.98534332	0.026337373
ENSMUSG00000027394	Ttl	tubulin tyrosine ligase [Source:MGI Symbol;Acc:MGI:1916987]	74.803	55.724	55.382	58.9597	56.1336	67.2315	62.0659	64.085	42.7869	72.9216	58.5738	65.5696	63.6592	64.8696	0.148073804	0.590732968	24	12	24	0.209021285	0.768040178	24	21.24515491	0.137025078
ENSMUSG00000027395	Polr1b	polymerase (RNA) I polypeptide B [Source:MGI Symbol;Acc:MGI:108014]	4.03474	3.49024	3.8831	3.47629	3.9488	4.10005	4.63761	4.43289	3.81007	4.26547	3.38427	3.61738	3.69458	3.95065	0.814576414	0.966018836	4	16	24	0.538491246	0.828653819	24	1.75045494	0.046747483
ENSMUSG00000027397	Slc20a1	solute carrier family 20, member 1 [Source:MGI Symbol;Acc:MGI:108392]	57.624	50.7384	51.295	50.8074	52.6473	57.8741	51.0275	49.2101	48.9908	49.5903	53.4809	50.5518	52.4016	52.1822	0.128408228	0.560209134	20	20	24	0.029374629	0.596648309	24	20.44411541	0.068012849
ENSMUSG00000027398	Il1b	interleukin 1 beta [Source:MGI Symbol;Acc:MGI:96543]	0.831401	0.541169	0.950509	0.458925	0.686984	0.83296	0.823229	1.08978	0.636603	0.351409	0.847788	0.737846	0.619122	0.642739	0.745118544	0.966018836	24	16	24	0.315767085	0.768040178	24	23.64197485	0.098413663
ENSMUSG00000027400	Pdyn	prodynorphin [Source:MGI Symbol;Acc:MGI:97535]	0.427472	0.634659	0.911092	0.451946	0.362053	0.539056	0.688225	0.512244	0.902337	0.294072	0.307518	0.576658	0.346575	0.606494	0.033404002	0.324908923	12	4	24	0.114131151	0.727799063	24	5.48223768	0.139905443
ENSMUSG00000027404	Snrpb	small nuclear ribonucleoprotein B [Source:MGI Symbol;Acc:MGI:98342]	54.7407	49.4576	50.5931	51.1458	46.5077	48.1052	49.8183	51.7491	49.9459	52.9608	48.8573	48.9222	51.6682	46.4394	0.56005262	0.966018836	16	4	24	0.764128681	0.92403577	24	4.716277128	0.01866472
ENSMUSG00000027405	Nop56	NOP56 ribonucleoprotein [Source:MGI Symbol;Acc:MGI:1914384]	36.3985	39.3858	43.0513	43.4413	41.3753	40.1466	41.3812	41.0397	42.8284	39.0679	41.1341	40.1973	38.1375	38.6655	0.107707354	0.524035616	12	16	24	0.077115581	0.687185556	24	11.44938656	0.05804779
ENSMUSG00000027406	Idh3b	isocitrate dehydrogenase 3 (NAD+) beta [Source:MGI Symbol;Acc:MGI:2158650]	111.991	119.183	132.571	142.353	119.874	123.506	122.564	134.684	122.62	130.54	131.161	122.621	136.416	127.523	0.833406028	0.966018836	16	12	24	0.187882633	0.768040178	24	10.29663378	0.06086242
ENSMUSG00000027408	Cpxm1	carboxypeptidase X 1 (M14 family) [Source:MGI Symbol;Acc:MGI:1934569]	8.88735	10.3103	11.6978	8.49328	10.5586	11.3717	10.5191	9.73648	12.7456	9.0229	10.9324	10.1386	9.9031	8.64986	0.711168999	0.966018836	12	20	24	0.951467965	0.988339231	24	13.46574465	0.019827499
ENSMUSG00000027411	Vps16	VSP16 CORVET/HOPS core subunit [Source:MGI Symbol;Acc:MGI:2136772]	32.7634	40.072	39.041	36.6294	37.7488	39.7825	31.9678	33.2481	36.1685	37.0112	33.5334	37.4195	35.1164	31.6156	0.495929335	0.966018836	24	4	24	0.353727225	0.781052712	24	13.86040571	0.058033569
ENSMUSG00000027412	Lpin3	lipin 3 [Source:MGI Symbol;Acc:MGI:1891342]	0.384282	0.966554	0.586017	0.687798	0.764997	0.61032	0.665438	0.622307	0.648668	0.563081	0.471697	0.595797	0.544041	0.44744	0.881996584	0.966018836	16	12	24	0.847581912	0.954054905	24	9.757314856	0.027840615
ENSMUSG00000027419	Pcsk2	proprotein convertase subtilisin/kexin type 2 [Source:MGI Symbol;Acc:MGI:97512]	42.2665	36.1531	37.0619	37.5581	33.7293	41.3886	38.8123	37.4934	29.2502	44.5086	35.1615	37.2518	34.6483	37.5145	0.850398351	0.966018836	24	12	24	0.628094224	0.872795898	24	20.60146007	0.057177852
ENSMUSG00000027420	Bfsp1	beaded filament structural protein 1, in lens-CP94 [Source:MGI Symbol;Acc:MGI:101770]	0.89546	0.956201	0.755006	0.868593	0.999747	0.777095	0.862335	1.20227	1.03267	0.988905	0.746603	0.726984	0.914449	0.958723	0.094000699	0.490854203	8	16	24	0.159449899	0.765591735	24	6.196262159	0.073256482
ENSMUSG00000027422	Rrbp1	ribosome binding protein 1 [Source:MGI Symbol;Acc:MGI:1932395]	42.7221	43.1745	42.9512	40.4586	44.4144	43.5408	43.3131	43.2927	43.7193	43.7	43.4558	41.7815	45.735	41.766	0.911685024	0.966018836	20	12	24	0.77267725	0.92687884	24	22.2552301	0.012061284
ENSMUSG00000027423	Snx5	sorting nexin 5 [Source:MGI Symbol;Acc:MGI:1916428]	34.0959	39.0512	40.3727	35.4755	38.4167	34.1454	39.0629	35.0692	40.7195	31.5034	38.4492	34.7211	35.3026	36.9897	0.148073804	0.590732968	12	12	24	0.555260832	0.838355251	24	6.956321719	0.046635532
ENSMUSG00000027424	Mgme1	mitochondrial genome maintenance exonuclease 1 [Source:MGI Symbol;Acc:MGI:1921778]	4.37469	3.84934	3.32671	4.15116	4.61024	3.59464	4.86577	4.18084	4.02206	4.02742	4.67276	4.03786	4.67784	3.85407	0.058157448	0.40386543	4	8	24	0.324973837	0.768040178	24	20.01326836	0.07400161
ENSMUSG00000027425	Kat14	lysine acetyltransferase 14 [Source:MGI Symbol;Acc:MGI:1917264]	7.02025	7.01789	6.94961	7.53677	6.81933	7.83417	8.6698	6.65786	6.85022	8.32594	6.14519	7.10746	6.25505	6.81505	0.851656735	0.966018836	16	16	24	0.91990475	0.978678675	24	17.12053493	0.020403253
ENSMUSG00000027427	Polr3f	polymerase (RNA) III (DNA directed) polypeptide F [Source:MGI Symbol;Acc:MGI:1924086]	8.22495	9.20706	9.07598	8.51934	9.2305	7.62177	8.0549	7.7902	9.3969	7.48897	8.64919	9.48151	8.27165	8.99178	0.93447422	0.966018836	12	16	24	0.810324287	0.939224719	24	9.103511179	0.028061618
ENSMUSG00000027428	Rbbp9	retinoblastoma binding protein 9, serine hydrolase [Source:MGI Symbol;Acc:MGI:1347074]	11.9519	12.7271	13.9587	11.7852	13.443	11.9569	13.2278	12.4909	13.9934	11.9055	12.4332	13.3822	13.2036	12.7352	0.893307667	0.966018836	12	4	24	0.691261345	0.897724829	24	5.688597027	0.027395476
ENSMUSG00000027429	Sec23b	SEC23 homolog B, COPII coat complex component [Source:MGI Symbol;Acc:MGI:1350925]	31.551	28.7039	27.0837	28.0128	30.6899	28.7103	30.5822	27.6327	25.4148	27.6752	29.328	27.7829	28.7855	29.0297	0.009483698	0.194720062	4	8	24	0.025894056	0.586541342	24	20.98400424	0.073995828
ENSMUSG00000027430	Dtd1	D-tyrosyl-tRNA deacylase 1 [Source:MGI Symbol;Acc:MGI:1913294]	26.2756	26.097	27.3156	25.8491	23.8277	26.8291	23.4564	24.8846	25.0578	26.8944	26.2684	25.2971	24.7206	28.1629	0.886952527	0.966018836	12	16	24	0.528061659	0.823775446	24	8.654373889	0.033507772
ENSMUSG00000027433	Xrn2	5'-3' exoribonuclease 2 [Source:MGI Symbol;Acc:MGI:894687]	13.1721	13.818	15.7485	14.5349	13.5876	14.3835	12.5162	13.8487	14.7651	13.8766	12.9825	13.5381	13.6882	14.7405	0.004501001	0.140598674	12	16	24	0.046483742	0.633507364	24	8.664243325	0.073602038
ENSMUSG00000027435	Cd93	CD93 antigen [Source:MGI Symbol;Acc:MGI:106664]	2.98033	2.75162	2.64328	2.52559	2.99063	2.68187	3.45269	4.09173	2.58293	2.89382	2.55711	2.3774	2.99072	2.34217	0.417653118	0.963884303	4	12	24	0.437340943	0.805393068	24	1.498437405	0.079651936
ENSMUSG00000027438	Napb	N-ethylmaleimide sensitive fusion protein attachment protein beta [Source:MGI Symbol;Acc:MGI:104562]	115.871	113.426	111.319	113.026	110.435	112.616	105.906	99.1007	104.458	109.314	117.48	114.88	116.617	121.557	0.823657258	0.966018836	4	8	24	0.601349318	0.860501201	24	19.52453941	0.031130577
ENSMUSG00000027439	Gzf1	GDNF-inducible zinc finger protein 1 [Source:MGI Symbol;Acc:MGI:1921783]	8.93374	9.31811	8.92108	9.22992	9.26951	8.86244	8.83712	8.72122	8.91743	8.45331	9.69434	9.15019	9.43345	9.51113	0.865514257	0.966018836	20	20	24	0.841812228	0.952050674	24	19.73357546	0.011672463
ENSMUSG00000027447	Cst3	cystatin C [Source:MGI Symbol;Acc:MGI:102519]	420.809	443.784	457.424	372.717	367.31	358.213	421.445	497.918	604.83	398.393	547.496	505.123	505.887	514.698	0.541384199	0.966018836	12	20	24	0.148850047	0.757433296	24	5.485222835	0.142755833
ENSMUSG00000027452	Acss1	acyl-CoA synthetase short-chain family member 1 [Source:MGI Symbol;Acc:MGI:1915988]	4.72526	4.62991	6.25753	6.57169	5.53657	5.16722	5.92352	5.99438	5.67093	6.60169	5.76674	5.33648	5.90374	5.71854	0.101690661	0.511659635	16	8	24	0.040728598	0.616921492	24	10.88202215	0.112432577
ENSMUSG00000027454	Gins1	GINS complex subunit 1 (Psf1 homolog) [Source:MGI Symbol;Acc:MGI:1916520]	2.1316	2.30339	2.38402	2.12202	2.09373	1.61131	2.16162	1.8044	2.14374	2.39282	1.80423	2.3133	2.79898	1.92256	0.928127947	0.966018836	16	4	24	0.787220203	0.931456435	24	5.820451551	0.039382031
ENSMUSG00000027455	Nsfl1c	NSFL1 (p97) cofactor (p47) [Source:MGI Symbol;Acc:MGI:3042273]	42.8297	43.8607	44.8411	44.3671	46.3326	44.7232	41.659	42.0607	44.4221	38.9018	47.8133	43.476	44.5527	40.4094	0.06180207	0.409814092	20	12	24	0.270914613	0.768040178	24	16.89868042	0.046504606
ENSMUSG00000027456	Sdcbp2	syndecan binding protein (syntenin) 2 [Source:MGI Symbol;Acc:MGI:2385156]	0.545508	0.84874	0.663301	0.812153	0.602345	0.840596	0.509479	0.863697	1.03136	0.614677	0.619264	0.938942	0.832133	0.834733	0.580409522	0.966018836	8	20	24	0.502271593	0.809492932	24	6.252715023	0.059514292

ENSMUSG00000027457	Snph	syntaphilin [Source:MGI Symbol;Acc:MGI:2139270]	31.8227	26.3582	27.9167	26.8128	25.6298	30.0132	30.9429	29.8168	26.246	37.6645	25.9519	31.9126	28.8614	30.6148	0.175272072	0.651010555	24	20	24	0.61862842	0.942446688	24	23.48811184	0.037280752
ENSMUSG00000027459	Fam110a	family with sequence similarity 110, member A [Source:MGI Symbol;Acc:MGI:1921097]	0.772142	0.680881	0.848015	1.02218	0.772381	0.932834	1.21735	1.17188	0.971459	1.01996	0.881985	1.15039	0.931679	0.968994	0.794858476	0.966018836	16	4	24	0.973289876	0.993305115	24	1.652606488	0.009426473
ENSMUSG00000027463	Slc52a3	solute carrier protein family 52, member 3 [Source:MGI Symbol;Acc:MGI:1916948]	1.95431	1.94579	1.65449	1.70998	1.90723	2.09728	1.90214	1.94485	2.06733	2.3522	1.68671	1.84959	1.69158	1.70888	0.768363194	0.966018836	16	4	24	0.9293133	0.981515518	24	14.3624678	0.014679169
ENSMUSG00000027465	Tbc1d20	TBC1 domain family, member 20 [Source:MGI Symbol;Acc:MGI:1914481]	14.292	13.5329	15.9731	17.0262	15.3115	15.4021	14.7287	15.4081	14.3569	16.4877	13.8786	14.47	14.4368	14.2422	0.231491055	0.731037655	16	16	24	0.103614125	0.715460676	24	12.34356369	0.068936771
ENSMUSG00000027466	Rbck1	RanBP-type and C3HC4-type zinc finger containing 1 [Source:MGI Symbol;Acc:MGI:1344372]	31.566	28.6552	28.9873	29.3237	28.621	29.836	31.4357	31.126	31.1479	30.3855	32.3438	30.8844	32.0604	29.697	0.347075276	0.870418653	4	12	24	0.3876081	0.799546792	24	22.40706255	0.028063045
ENSMUSG00000027469	Tpx2	TPX2, microtubule-associated [Source:MGI Symbol;Acc:MGI:1919369]	1.03506	1.27883	1.66202	1.26989	1.11342	1.55825	1.30846	1.24928	1.12973	0.951131	1.39825	1.46096	1.55264	1.16466	0.843639218	0.966018836	24	16	24	0.659168679	0.884879972	24	21.71238691	0.074747776
ENSMUSG00000027470	Mylk2	myosin, light polypeptide kinase 2, skeletal muscle [Source:MGI Symbol;Acc:MGI:2139434]	2.01579	5.55792	6.48954	9.24025	5.3045	8.14427	4.84247	15.2143	12.5792	18.0274	5.44382	7.0706	8.56427	6.53994	0.338172159	0.85758369	16	12	24	0.064958501	0.663876076	24	9.79524647	0.532997909
ENSMUSG00000027472	Pdrg1	p53 and DNA damage regulated 1 [Source:MGI Symbol;Acc:MGI:1915809]	95.1106	99.2805	94.2105	100.889	106.255	101.917	92.3762	95.5527	100.85	95.1687	105.708	97.2684	96.6083	84.2352	0.014841078	0.250870466	20	8	24	0.017274316	0.549489822	24	15.90693753	0.079362251
ENSMUSG00000027474	Ccm2l	cerebral cavernous malformation 2-like [Source:MGI Symbol;Acc:MGI:2385159]	1.44509	1.59427	2.45053	2.33998	1.71284	1.74577	1.63474	2.22713	1.76574	2.12431	1.69629	1.6869	1.87724	1.59116	0.091669402	0.484208195	12	16	24	0.060554596	0.659878804	24	10.19881244	0.132958367
ENSMUSG00000027475	Kif3b	kinesin family member 3B [Source:MGI Symbol;Acc:MGI:107688]	21.9188	19.1913	19.3853	19.3991	19.342	21.2916	18.8992	19.1779	16.2956	20.1856	18.8336	20.6184	20.0919	20.273	0.601647512	0.966018836	24	12	24	0.101269752	0.713208839	24	21.01915918	0.077936392
ENSMUSG00000027479	Mapre1	microtubule-associated protein, RP/EB family, member 1 [Source:MGI Symbol;Acc:MGI:891995]	55.1636	51.7525	50.5273	45.1795	51.4001	54.0504	47.2981	52.7094	48.2516	50.1325	52.3781	49.7063	58.5621	54.7394	0.140728644	0.585944911	4	12	24	0.135219569	0.746923165	24	23.80219486	0.069376859
ENSMUSG00000027487	Cdk5rap1	CDK5 regulatory subunit associated protein 1 [Source:MGI Symbol;Acc:MGI:1914221]	4.59833	3.44222	3.73835	3.74792	6.31855	4.76144	4.68769	4.29334	3.70816	3.795	4.81462	3.75688	5.20234	4.40764	0.009730499	0.194720062	20	16	24	0.108678003	0.72082227	24	19.62571446	0.159667901
ENSMUSG00000027488	Snta1	syntrophin, acidic 1 [Source:MGI Symbol;Acc:MGI:101772]	14.6623	12.2597	13.633	21.507	16.7174	16.3147	15.815	16.4243	16.1071	19.6133	16.2716	15.3992	14.6319	11.4307	0.007505042	0.192760771	16	16	24	0.0254854	0.586541342	24	14.46628226	0.206462138
ENSMUSG00000027489	Necab3	N-terminal EF-hand calcium binding protein 3 [Source:MGI Symbol;Acc:MGI:1861721]	29.6612	26.1211	24.1853	24.5472	24.49	29.6428	28.3003	29.9922	22.699	34.4768	25.01	28.984	30.5455	29.176	0.105280783	0.518002936	4	8	24	0.325987302	0.768040178	24	23.1077444	0.090830152
ENSMUSG00000027490	E2f1	E2F transcription factor 1 [Source:MGI Symbol;Acc:MGI:101941]	7.3561	5.2739	4.99593	5.71537	5.95802	5.89436	5.66212	6.22371	4.46072	6.41669	5.71574	5.90479	6.14486	5.75186	0.300823756	0.809362476	4	8	24	0.155289801	0.760445083	24	20.77006715	0.108277022
ENSMUSG00000027495	Fam210b	family with sequence similarity 210, member B [Source:MGI Symbol;Acc:MGI:1914267]	5.28064	4.0888	5.06233	4.04732	4.28249	4.59741	4.62252	4.45073	3.58557	4.50831	4.73487	4.82409	4.4766	4.28281	0.194424727	0.673387182	24	16	24	0.189467865	0.768040178	24	20.94167533	0.081711981
ENSMUSG00000027496	Aurka	aurora kinase A [Source:MGI Symbol;Acc:MGI:894678]	1.67925	1.77262	2.05549	1.74721	1.85907	2.55154	1.73828	1.81015	1.47945	2.11872	1.8186	1.88156	1.99796	1.45229	0.337748746	0.85758369	24	8	24	0.209524884	0.768040178	24	17.89232205	0.092697851
ENSMUSG00000027498	Cstf1	cleavage stimulation factor, 3' pre-RNA, subunit 1 [Source:MGI Symbol;Acc:MGI:1914587]	7.27062	6.03718	6.72995	4.68222	5.2702	7.34424	6.77971	6.64183	6.00148	6.83402	5.70566	6.70854	6.50647	6.12131	0.175185919	0.651010555	24	20	24	0.133008511	0.120307373	24	8.904609402	0.149631868
ENSMUSG00000027499	Pkia	protein kinase inhibitor, alpha [Source:MGI Symbol;Acc:MGI:104747]	43.4479	59.0469	58.189	58.7845	53.9597	53.8721	48.9929	61.6853	67.8463	55.5589	55.3351	52.1346	56.9442	55.2179	0.005024733	0.143276577	12	16	24	0.014828909	0.537924552	24	20.26681431	0.095624928
ENSMUSG00000027500	Stmn2	stathmin-like 2 [Source:MGI Symbol;Acc:MGI:98241]	736.22	636.741	649.881	629.471	649.348	730.769	628.557	638	521.006	658.103	686.876	632.303	653.437	658.124	0.846840613	0.966018836	24	12	24	0.093358923	0.71175955	24	21.59340722	0.063860446
ENSMUSG00000027502	Rtf2	replication termination factor 2 [Source:MGI Symbol;Acc:MGI:1913654]	41.5794	37.4035	38.2111	37.6427	37.5844	42.6219	39.23	40.4045	33.6913	41.5895	38.0469	39.962	40.5017	40.0509	0.279917941	0.789849983	24	12	24	0.145609909	0.754301054	24	21.59340722	0.063860446
ENSMUSG00000027506	Tpd52	tumor protein D52 [Source:MGI Symbol;Acc:MGI:107749]	143.008	117.514	115.289	119.477	122.501	131.822	119.78	120.079	97.7401	121.708	130.7	118.138	120.146	117.357	0.018923071	0.256345714	20	16	24	0.031555232	0.603393432	24	20.02828814	0.112123389
ENSMUSG00000027508	Pag1	phosphoprotein associated with glycosphingolipid microdomains 1 [Source:MGI Symbol;Acc:MGI:2443160]	4.98753	4.79352	5.5189	5.00339	5.19496	5.19288	5.66795	4.87393	4.87802	5.70838	4.92446	5.01672	5.24759	5.08484	0.791413662	0.966018836	4	4	24	0.883075038	0.966818623	24	14.9163763	0.013579193
ENSMUSG00000027509	Rae1	ribonucleic acid export 1 [Source:MGI Symbol;Acc:MGI:1913929]	15.376	13.5462	13.7468	15.1143	16.0644	13.7699	13.8093	14.2878	15.5927	16.4675	14.818	15.0629	14.3588	14.2795	0.143673396	0.585944911	16	16	24	0.074866582	0.68394125	24	13.79917204	0.067529199
ENSMUSG00000027510	Rbm38	RNA binding motif protein 38 [Source:MGI Symbol;Acc:MGI:1889294]	2.25612	3.22334	3.92961	4.94209	3.4472	3.13185	3.42417	6.38463	4.92675	5.08885	3.48037	3.10891	4.34203	3.2161	0.036424767	0.332409444	16	8	24	0.046763207	0.633507364	24	9.218655726	0.274745526
ENSMUSG00000027513	Pck1	phosphoenolpyruvate carboxykinase 1, cytosolic [Source:MGI Symbol;Acc:MGI:97501]	5.89782	3.37849	10.8096	27.4312	4.46021	7.01344	6.10768	18.8986	9.83392	26.9988	5.34999	12.6918	24.2314	24.0143	0.503972924	0.966018836	16	4	24	0.241821363	0.768040178	24	8.430127733	0.547348793
ENSMUSG00000027514	Zbp1	Z-DNA binding protein 1 [Source:MGI Symbol;Acc:MGI:1927449]	1.68349	2.20086	2.30831	1.6149	1.96982	1.47952	1.89839	2.26811	2.94285	1.12692	2.02628	1.64779	1.07281	2.00349	0.017958336	0.266982364	12	12	24	0.229088327	0.768040178	24	7.077205905	0.166038302
ENSMUSG00000027519	Rab22a	RAB22A, member RAS oncogene family [Source:MGI Symbol;Acc:MGI:105072]	9.57956	7.91535	7.97098	9.0651	8.77454	8.7463	9.84524	8.69365	8.12916	9.36884	8.53449	8.73341	8.34422	8.49736	0.230338208	0.730447182	16	20	24	0.319261208	0.768040178	24	19.46851519	0.051669968
ENSMUSG00000027522	Stx16	syntaxin 16 [Source:MGI Symbol;Acc:MGI:1923396]	27.3203	26.7295	26.8837	25.8531	29.06	25.6916	31.1647	26.1482	27.2868	26.6214	27.6891	26.5875	26.8246	25.5429	0.834424969	0.966018836	20	12	24	0.596467686	0.857839743	24	19.94055076	0.030807201
ENSMUSG00000027523	Gnas	GNAS (guanine nucleotide binding protein, alpha stimulating) complex locus [Source:MGI Symbol;Acc:MGI:95777]	797.418	685.718	749.082	769.696	711.095	829.876	783.538	773.085	678.527	975.249	738.354	805.828	758.259	882.66	0.601647512	0.966018836	24	12	24	0.946901699	0.987033652	24	17.00001207	0.017692989
ENSMUSG00000027524	Edn3	endothelin 3 [Source:MGI Symbol;Acc:MGI:95285]	1.08513	1.7257	2.30394	1.78526	1.55517	1.56957	1.93368	2.05994	2.27341	1.4734	2.06976	1.803	1.46949	2.487	0.271166436	0.780905461	12	16	24	0.074537331	0.683293901	24	7.862886879	0.163129981
ENSMUSG00000027525	Phactr3	phosphatase and actin regulator 3 [Source:MGI Symbol;Acc:MGI:1921439]	10.9635	11.077	11.6624	11.3348	10.768	11.5052	10.9774	10.2843	11.0017	12.4827	10.8827	12.0612	10.9862	13.5054	0.939921059	0.966018836	16	4	24	0.729996773	0.911943391	24	9.111539173	0.027889497
ENSMUSG00000027531	Impa1	inositol (myo)-1(or 4)-monophosphatase 1 [Source:MGI Symbol;Acc:MGI:1933158]	46.5252	44.8628	41.7207	41.9171	41.1777	43.2734	42.8965	42.0174	37.6908	39.0782	44.4874	38.0734	41.1668	40.8885	0.696669634	0.966018836	4	8	24	0.173753231	0.768040178	24	22.61968061	0.050304205
ENSMUSG00000027533	Fabp5	fatty acid binding protein 5, epidermal [Source:MGI Symbol;Acc:MGI:101790]	66.8116	66.919	69.7251	65.0438	67.1957	65.9056	57.3215	62.9336	61.0148	54.1137	63.0704	61.3512	63.1598	65.1117	0.823246279	0.966018836	8	8	24	0.871306608	0.963486859	24	0.933517736	0.017601125
ENSMUSG00000027534	Snx16	sorting nexin 16 [Source:MGI Symbol;Acc:MGI:1921968]	25.9319	24.8625	21.8221	21.4338	23.8068	23.0615	25.6033	22.941	19.9774	19.1708	26.7118	19.9284	21.9893	21.854	0.087599269	0.472021905	4	12	24	0.145012355	0.753947259	24	22.2001693	0.099268457
ENSMUSG00000027536	Chmp4c	charged multivesicular body protein 4C [Source:MGI Symbol;Acc:MGI:1913621]	0.660834	0.918949	0.500694	0.838832	0.815041	0.669697	0.689289	0.72987	0.820975	0.570529	1.01667	0.697964	0.688832	0.66963	0.916746631	0.966018836	20	8	24	0.722496962	0.908924547	24	15.8793219	0.036234181
ENSMUSG00000027540	Ptpn1	protein tyrosine phosphatase, non-receptor type 1 [Source:MGI Symbol;Acc:MGI:97805]	8.20437	6.51581	7.47525	7.32154	8.10811	7.19685	7.21158	6.8938	6.93226	7.76821	7.71275	6.97284	6.94184	6.83608	0.0043709	0.140598674	20	12	24	0.05772726	0.656248849	24	16.09462015	0.070740304
ENSMUSG00000027544	Nfatc2	nuclear factor of activated T cells, cytoplasmic, calcineurin dependent 2 [Source:MGI Symbol;Acc:MGI:102463]	1.04555	1.48709	1.73026	1.2866	1.3708	1.54318	1.75172	1.21202	1.96652	1.71699	1.16639	1.2805	1.1089	1.62188	0.277087842	0.789849983	12	16	24	0.188916427	0.768040178	24	8.548231901	0.119004654
ENSMUSG00000027546	Atp9a	ATPase, class II, type 9A [Source:MGI Symbol;Acc:MGI:1330826]	104.828	77.591	81.9512	83.2372	81.0804	95.7446	85.7821	94.1084	66.5293	111.727	76.4346	90.7058	86.2163	87.4269	0.581070934	0.966018836	24	12	24	0.569423724	0.844856622	24	21.05745277	0.082727695
ENSMUSG00000027550	Lrrcc1	leucine rich repeat and coiled-coil domain containing 1 [Source:MGI Symbol;Acc:MGI:1918960]	7.61056	9.20886	9.27654	8.41159	7.78364	7.82071	7.71949	8.19675	10.7902	7.83004	8.79789	8.56961	8.06392	9.35473	0.018194955	0.256345714	12	16	24	0.037111453	0.609176161	24	7.859966859	0.12051535
ENSMUSG00000027551	Zfp64	zinc finger protein 64 [Source:MGI Symbol;Acc:MGI:107342]	4.71044	4.62528	4.10337	4.0445	4.16482	4.49563	5.02842	4.51611	4.58217	5.19193	3.89433	5.03831	4.45368	4.98755	0.557335707	0.966018836	4	16	24	0.267799601	0.768040178	24	1.693514461	0.062295769
ENSMUSG00000027552	E2f5	E2F transcription factor 5 [Source:MGI Symbol;Acc:MGI:105091]	6.62062	7.59884	6.29708	7.51392	7.44882	6.88032	6.47178	6.67209	7.73489	6.70492	8.15472	6.9025	6.38646	6.78335	0.086420527	0.467848941	20	8	24	0.178734954	0.768040178	24	13.55099331	0.070160966
ENSMUSG00000027555	Car13	carbonic anhydrase 13 [Source:MGI Symbol;Acc:MGI:1931322]	7.87881	6.63372	6.51678	7.76051	9.27997	6.12847	7.18327	6.18071	7.32258	5.54614	8.99947	5.69657	6.22856	5.98439	0.581070934	0.966018836	20	12	24	0.282078617	0.768040178	24	15.88757548	0.116540346
ENSMUSG00000027556	Car1	carbonic anhydrase 1 [Source:MGI Symbol;Acc:MGI:88268]	0.615974	1.37029	3.43389	2.78708	0.866158	2.07938	0.810011	1.43453	1.89945	0.966427	1.3326	1.66676	2.22857	0.88863	0.700065774	0.966018836	12	16	24	0.503533651	0.810250042	24	10.27714443	0.204430418
ENSMUSG00000027559	Car3	carbonic anhydrase 3 [Source:MGI Symbol;Acc:MGI:88270]	49.2695	94.6098	122.235	223.581	69.5892	109.869	89.2982	244.116	161.92	253.47	142.6	134.22	209.021	139.04	0.287843582	0.793714531	16	12	24	0.061453304	0.661105021	24	9.562315353	0.511967939
ENSMUSG00000027560	Dok5	docking protein 5 [Source:MGI Symbol;Acc:MGI:1924079]	3.38248	3.22877	3.38512	3.12855	2.6732	3.37922	3.58996	3.50304	2.63541	3.70166	3.18546	2.99655	3.13322	3.97367	0.47028201	0.966018836	8	12	24	0.368959229	0.791959527	24	3.326329817	0.067966762
ENSMUSG00000027562	Car2	carbonic anhydrase 2 [Source:MGI Symbol;Acc:MGI:88269]	45.1787	53.5397	61.9446	47.9894	42.5558	51.2235	54.4638	46.5403	47.0616	43.2521	41.5154	44.7443	47.1201	47.7027	0.142596848	0.585944911	12	8	24	0.155515014	0.760753322	24	4.384589823	0.101825968
ENSMUSG00000027566	Psma7	proteasome subunit alpha 7 [Source:MGI Symbol;Acc:MGI:1347070]	158.42	174.449	173.012	164.261	167.531	152.203	151.977	156.084	187.143	155.985	188.319	173.882	189.722	178.713	0.887862353	0.966018836	12	12	24	0.654263287	0.883406766	24	8.618194691	0.039105373
ENSMUSG00000027569	Mrgbp	MRG/MORF4L binding protein [Source:MGI Symbol;Acc:MGI:1920497]	6.63147	7.44744	7.77688	7.38023	8.03841	7.94148	8.46101	8.03955	7.71897	6.86826	7.78623	7.10075	7.45537	6.2938	0.874607729	0.966018836	20	20	24	0.785740998	0.931158434	24	17.56700108	0.029112615
ENSMUSG00000027570	Col9a3	collagen, type IX, alpha 3 [Source:MGI Symbol;Acc:MGI:894686]	2.16227	3.03829	3.67897	2.18029	2.32999	2.93257	3.17387	2.34057	2.47663	2.7943	2.1374	2.62324	2.27387	2.64584	0.784278077	0.966018836	12	8	24	0.606346435	0.863192526	24	5.292390795	0.068849222
ENSMUSG00000027573	Gid8	GID complex subunit 8 [Source:MGI Symbol;Acc:MGI:1923675]	18.3635	17.9782	18.8257	19.2193	18.134	17.586	17.4655	17.6573	16.8364	18.0613	16.8255	18.9355	18.333	17.8892	0.860560779	0.966018836	16	16	24	0.967506118	0.992187127	24	16.50284669	0.005528415
ENSMUSG00000027574	Nkain4	Na+/K+ transporting ATPase interacting 4 [Source:MGI Symbol;Acc:MGI:1915372]	8.12292	10.8788	10.4742	8.50121	9.71507	10.1893	8.87101	10.1217	10.7984	8.41953	9.77484	10.5744	8.00804	9.556	0.4776989	0.966018836	12	16	24	0.793567819	0.933825946	24	8.193473675	0.040271747
ENSMUSG00000027575	Arfgap1	ADP-ribosylation factor GTPase activating protein 1 [Source:MGI Symbol;Acc:MGI:2183559]	19.2577	20.1957	20.2012	19.8303	20.5727	21.2232	19.3711	20.8886	20.6256	21.5336	17.5659	21.791	19.615	20.6579	0.9045879	0.966018836	24	4	24	0.885027008	0.967256182	24	7.558950279	0.015740712
ENSMUSG00000027577	Chrna4	cholinergic receptor, nicotinic, alpha polypeptide 4 [Source:MGI Symbol;Acc:MGI:87888]	0.817659	1.14726	1.76362	1.19273	0.826648	1.31059	1.39566	0.807517	1.05876	1.34686	0.753872	1.28047	0.706565	1.52464	0.943890799	0.966018836	12	8	24	0.554523185	0.837816048	24	8.089105653	0.097871535
ENSMUSG00000027580	Helz2	helicase with zinc finger 2, transcriptional coactivator [Source:MGI Symbol;Acc:MGI:2385169]	1.431	1.27671	1.23335	1.63043	1.50192	1.27961	1.43027	1.61682	1.70155	1.4431	1.50903	1.2471	1.41294	1.20013	0.619261897	0.966018836	16	16	24	0.422978262	0.805019467	24	11.63873254	0.048933598
ENSMUSG00000027581	Stmn3	stathmin-like 3 [Source:MGI Symbol;Acc:MGI:1277137]	599.823	571.457	530.104	503.572	506.357	496.241	517.463	581.631	598.997	514.666	678.05	589.145	643.828	617.656	0.622132989	0.966018836	4	12	24	0.427727883	0.805393068	24	2.292724651	0.063027913
ENSMUSG00000027582	Zgpat	zinc finger, CCCH-type with G patch domain [Source:MGI Symbol;Acc:MGI:2449939]	15.9117	14.5286	15.1795	12.7442	14.2495	14.3653	15.5385	14.0022	15.027	13.3144	14.7324	13.2992	13.8681	12.8623	0.98246092	0.982517406	4	12	24	0.41458702	0.799810051	24	23.80158244	0.041767731
ENSMUSG00000027583	Zbtb46	zinc finger and BTB domain containing 46 [Source:MGI Symbol;Acc:MGI:1919397]	3.66951	3.88846	3.4904	4.03121	3.93101	4.05045	3.72266	4.02717	3.6015	3.98372	4.15797	3.90315	3.56078	3.9012	0.077491245	0.454138381	16	20	24	0.204093777	0.768040178	24	15.91326655	0.041566059
ENSMUSG00000027584	Oprl1	opioid receptor-like 1 [Source:MGI Symbol;Acc:MGI:97440]	7.31624	8.65685	10.061	6.8603	6.89574	8.67757	8.72713	6.31924	7.45037	7.87795	5.64699	7.76187	6.98253	8.77814	0.664568268	0.966018836	12	8	24	0.418667044	0.803070657	24	4.109844828	0.09863084
ENSMUSG00000027589	Pcmtd2	protein-L-isoaspartate (D-aspartate) O-methyltransferase domain containing 2 [Source:MGI Symbol;Acc:MGI:1923927]	30.3297	30.0944	28.8926	31.198	31.167	27.1592	29.3494	27.8411	31.2628	25.5799	32.1308	26.7557	29.9659	28.7174	0.920992137	0.966018836	20	4	24	0.944437054	0.986443184	24	11.92855168	0.01246484
ENSMUSG00000027593	Raly	hnRNP-associated with lethal yellow [Source:MGI Symbol;Acc:MGI:97850]	80.3569	72.771	72.8661	74.9599	73.6308	80.726	75.1276	77.1319	69.4432	81.4131	78.3997	74.8409	79.3153	80.1686	0.46238066	0.966018836	24	12	24	0.399469333	0.799546792	24	20.82545549	0.036286218
ENSMUSG00000027597	Ahcy	S-adenosylhomocysteine hydrolase [Source:MGI Symbol;Acc:MGI:87968]	4.92198	4.90642	4.85191	4.79806	5.54924	5.64683	5.05148	5.63779	4.94056	5.06327	4.91028	4.65114	4.34862	4.40194	0.860560779	0.966018836	20	8	24	0.534047161	0.826231134	24	16.64898847	0.039271526
ENSMUSG00000027598	Itch	itchy, E3 ubiquitin protein ligase [Source:MGI Symbol;Acc:MGI:1202301]	26.6463	24.6976	23.3605	23.7614	25.3451	24.0869	26.5351	23.7765	22.1341	22.5261	25.2362	24.332	24.902	23.8813	0.002087679	0.100255953	4	8	24	0.003769558	0.426709186	24	21.58053216	0.080601459
ENSMUSG00000027599	Armc1	armadillo repeat containing 1 [Source:MGI Symbol;Acc:MGI:1921502]	26.6239	26.8904	24.6988	24.9901	26.5513	24.6442	25.3488	24.5143	24.8296	24.6675	27.0367	23.9204	26.4524	26.7092	0.939921059	0.966018836	8	16	24	0.769282321	0.925619883	24	23.64012363	0.017222327
ENSMUSG00000027601	Mtfr1	mitochondrial fission regulator 1 [Source:MGI Symbol;Acc:MGI:1914722]	11.8881	11.1448	11.4621	13.1928	12.1999	11.7059	13.5003	12.365	11.3783	11.6934	12.9624	11.8984	11.9356	11.5169	0.258307762	0.761738864	20	16	24	0.392040193	0.799546792	24	17.69807182	0.042291004
ENSMUSG00000027602	Map1lc3a	microtubule-associated protein 1 light chain 3 alpha [Source:MGI Symbol;Acc:MGI:1915661]	302.64	265.51	261.236	264.893	278.547	279.206	256.409	279.72	264.166	276.668	291.37	292.445	287.891	285.525	0.074438493	0.448301997	24	12	24	0.163809547	0.767020009	24	20.46170628	0.050065265
ENSMUSG00000027603	Ggt7	gamma-glutamyltransferase 7 [Source:MGI Symbol;Acc:MGI:1913385]	37.2292	30.5164	33.761	31.063	32.5087	36.2267	33.6886	32.3766	28.796	38.2439	29.4521	31.9999	32.7321	34.029	0.951945551	0.966018836	4	8	24	0.662173324	0.885891036	24	21.69011419	0.043365404
ENSMUSG00000027605	Acss2	acyl-CoA synthetase short-chain family member 2 [Source:MGI Symbol;Acc:MGI:1890410]	37.891	38.5813	41.2741	42.0727	41.447	44.004	41.2075	42.0769	37.3415	40.0481	45.4123	41.5407	46.4512	45.3285	0.846840613	0.966018836	24	12	24	0.508569476	0.813517676	24	19.4473581	0.036460267
ENSMUSG00000027606	Dnajc5b	DnaJ heat shock protein family (Hsp40) member C5 beta [Source:MGI Symbol;Acc:MGI:1913576]	0.866599	1.30255	1.1244	1.31923	1.20043	1.09898	0.527079	1.31644	0.833708	0.783891	1.25585	0.917559	1.08227	1.01671	0.664568268	0.966018836	8	20	24	0.700273752	0.900599679	24	11.75686571	0.058601859
ENSMUSG00000027610	Gss	glutathione synthetase [Source:MGI Symbol;Acc:MGI:95852]	14.5828	16.3376	14.9549	14.7249	16.7054	15.0855	15.2585	13.9523	16.234	14.1607	16.6122	16.0626	15.6518	15.6855	0.893307667	0.966018836	20	20	24	0.67364427	0.890227289	24	17.73250389	0.028259637
ENSMUSG00000027611	Procr	protein C receptor, endothelial [Source:MGI Symbol;Acc:MGI:104596]	1.84966	2.88013	2.85125	2.2504	2.88503	2.38257	2.3464	2.30641	3.49414	1.83466	2.96724	1.63215	1.96228	2.20782	0.360140629	0.883787544	12	12	24	0.296145472	0.768040178	24	9.687295619	0.133785039
ENSMUSG00000027612	Mmp24	matrix metallopeptidase 24 [Source:MGI Symbol;Acc:MGI:1341867]	9.0234	7.39034	7.12895	8.32171	7.49861	7.92036	7.93355	7.57134	6.54474	9.25337	7.45521	7.46655	7.34997	7.68112	0.503972924	0.966018836	16	20	24	0.639547761	0.877957092	24	18.24877245	0.044058542
ENSMUSG00000027613	Eif6	eukaryotic translation initiation factor 6 [Source:MGI Symbol;Acc:MGI:1196288]	28.4169	27.4579	28.22	28.7595	32.7188	33.8027	25.1013	30.1375	25.8488	28.193	27.0335	29.0237	26.9587	27.7944	0.495929335	0.966018836	24	4	24	0.224243003	0.768040178	24	17.51363174	0.074280231
ENSMUSG00000027615	Hps3	HPS3, biogenesis of lysosomal organelles complex 2 subunit 1 [Source:MGI Symbol;Acc:MGI:2153839]	24.5258	26.0244	23.175	23.4854	22.6963	22.4685	22.6705	22.381	22.6723	22.4844	25.2377	22.6391	24.1588	24.413	0.915475528	0.966018836	8	16	24	0.7004138	0.900629492	24	2.460664003	0.022572196
ENSMUSG00000027618	Nfs1	nitrogen fixation gene 1 (S. cerevisiae) [Source:MGI Symbol;Acc:MGI:1316706]	24.6563	23.6664	24.3379	26.4337	24.3857	24.0239	23.5382	26.8574	24.9802	25.7757	26.1585	25.2702	26.3545	25.7877	0.919571723	0.966018836	16	12	24	0.440328787	0.805393068	24	10.34271371	0.02607952
ENSMUSG00000027620	Rbm39	RNA binding motif protein 39 [Source:MGI Symbol;Acc:MGI:2157953]	99.7485	118.146	110.531	106.707	115.686	100.291	108.4	100.123	129.212	92.5023	111.846	107.739	113.725	109.108	0.960333395	0.967043964	12	12	24	0.736665927	0.914180155	24	7.091699492	0.039129967
ENSMUSG00000027624	Epb41l1	erythrocyte membrane protein band 4.1 like 1 [Source:MGI Symbol;Acc:MGI:103010]	32.7259	28.8796	35.2555	32.5791	29.0848	32.5563	33.8426	30.6386	24.5989	38.6835	27.0656	30.9629	29.8266	33.6909	0.896799496	0.966018836	16	4	24	0.99736004	0.999539227	24	6.092077349	0.004855014
ENSMUSG00000027628	Aar2	AAR2 splicing factor homolog [Source:MGI Symbol;Acc:MGI:1915545]	10.3002	10.6161	10.6131	11.1685	10.5932	11.5944	10.4345	11.3434	8.80247	12.3228	8.71564	10.5755	10.0467	11.258	0.745118544	0.966018836	16	12	24	0.989726345	0.997891892	24	5.33831304	0.007439735
ENSMUSG00000027630	Tbl1xr1	transducin (beta)-like 1X-linked receptor 1 [Source:MGI Symbol;Acc:MGI:2441730]	13.901	12.3695	12.8862	12.5943	12.0174	12.9035	11.4234	12.5213	12.0365	12.5711	12.5585	12.6889	13.9046	11.9508	0.935316621	0.966018836	4	4	24	0.613636804	0.866162311	24	21.7029576	0.02908558
ENSMUSG00000027634	Ndrg3	N-myc downstream regulated gene 3 [Source:MGI Symbol;Acc:MGI:1352499]	59.7609	52.9413	57.6444	58.474	48.2456	58.4901	60.4809	55.8177	48.572	61.1499	46.8089	51.8786	48.6706	59.497	0.843639218	0.966018836	4	16	24	0.652807721	0.883145019	24	4.039414578	0.048575775
ENSMUSG00000027635	Dsn1	DSN1 homolog, MIS12 kinetochore complex component [Source:MGI Symbol;Acc:MGI:1914184]	2.36294	2.99895	3.21062	2.49677	3.02848	2.79645	2.34922	2.71079	3.43987	2.66302	2.5399	2.53408	3.42917	2.68126	0.628286596	0.966018836	12	16	24	0.40366246	0.799546792	24	7.652897899	0.074522557
ENSMUSG00000027637	Rab5if	RAB5 interacting factor [Source:MGI Symbol;Acc:MGI:1914638]	33.7926	33.3303	36.4489	37.2646	35.8772	33.9134	33.3627	35.29	34.6971	32.8585	37.7021	32.0517	36.484	34.8233	0.912923872	0.966018836	20	4	24	0.41734134	0.802114794	24	11.58053137	0.036225722
ENSMUSG00000027639	Samhd1	SAM domain and HD domain, 1 [Source:MGI Symbol;Acc:MGI:1927468]	16.4863	16.7804	17.384	16.97	18.921	17.2773	16.5505	17.9284	17.0656	17.2079	16.1924	17.5688	15.287	15.5697	0.359479536	0.883787544	24	4	24	0.234038927	0.768040178	24	14.33754949	0.043970087
ENSMUSG00000027641	Rbl1	RB transcriptional corepressor like 1 [Source:MGI Symbol;Acc:MGI:103300]	1.171	1.17582	1.44977	1.22769	1.24817	0.974007	1.31922	1.39093	1.16594	1.11749	1.18218	0.979197	1.08978	0.984597	0.390686537	0.915479136	12	12	24	0.307357315	0.768040178	24	8.066705035	0.051650269
ENSMUSG00000027642	Rpn2	ribophorin II [Source:MGI Symbol;Acc:MGI:98085]	59.2685	49.0021	53.0717	52.1008	54.3674	58.6284	59.0467	59.4557	44.9222	59.2206	51.1052	51.539	51.6935	50.9896	0.922029406	0.966018836	4	8	24	0.361825718	0.787031962	24	20.78750183	0.068694409
ENSMUSG00000027646	Src	Rous sarcoma oncogene [Source:MGI Symbol;Acc:MGI:98397]	8.57661	7.63335	8.20386	8.1541	8.55559	8.35725	8.65235	8.59682	7.34669	9.99636	7.69382	8.15981	8.82043	8.1001	0.43565473	0.966018836	4	8	24	0.834325786	0.949069766	24	18.28653195	0.024090105
ENSMUSG00000027649	Ctnnbl1	catenin, beta like 1 [Source:MGI Symbol;Acc:MGI:1913892]	14.3921	12.7984	12.7776	12.4641	13.4587	13.7478	12.8669	13.6887	10.7402	13.1828	12.5767	12.3733	12.8377	12.9687	0.625825704	0.966018836	4	8	24	0.171130537	0.768040178	24	21.55205935	0.062674684
ENSMUSG00000027650	Tti1	TELO2 interacting protein 1 [Source:MGI Symbol;Acc:MGI:1922675]	5.92953	5.59879	5.96734	5.11933	5.17291	6.36143	6.35832	6.48756	5.00404	6.18783	5.4167	5.87445	6.0813	5.73103	0.296502765	0.807732044	4	8	24	0.126433149	0.739868076	24	0.089925703	0.078572821
ENSMUSG00000027651	Rprd1b	regulation of nuclear pre-mRNA domain containing 1B [Source:MGI Symbol;Acc:MGI:1917720]	5.20124	4.90256	6.05431	5.92242	4.74092	5.49572	5.30374	6.38053	4.56752	6.31643	5.28824	4.97294	4.93177	4.83924	0.88067614	0.966018836	16	16	24	0.542473087	0.83094351	24	10.6166325	0.05839776
ENSMUSG00000027652	Ralgapb	Ral GTPase activating protein, beta subunit (non-catalytic) [Source:MGI Symbol;Acc:MGI:2444531]	25.0139	21.796	22.3538	21.457	21.7849	23.5819	22.3821	21.839	18.5456	24.4538	20.931	23.1042	23.5819	23.6563	0.277917941	0.789849983	4	16	24	0.237898197	0.768040178	24	22.02653742	0.069163664
ENSMUSG00000027654	Fam83d	family with sequence similarity 83, member D [Source:MGI Symbol;Acc:MGI:1919128]	1.88308	1.82909	1.50923	2.20064	2.36293	2.1544	1.88401	1.62639	1.97462	2.70746	2.30617	2.48852	2.25189	1.69113	0.003669222	0.140598674	20	12	24	0.002263224	0.395705644	24	16.39878793	0.172866055
ENSMUSG00000027655	Dhx35	DEAH (Asp-Glu-Ala-His) box polypeptide 35 [Source:MGI Symbol;Acc:MGI:1918965]	7.58724	6.99112	6.18943	6.54558	7.9834	6.71959	6.40205	7.6012	6.50081	6.70306	6.63255	6.71695	6.87181	6.85254	0.119293142	0.542554768	8	4	24	0.570013299	0.845005229	24	21.58748887	0.038864757
ENSMUSG00000027656	Ccn5	cellular communication network factor 5 [Source:MGI Symbol;Acc:MGI:1328326]	1.68099	1.89676	2.29212	2.03683	2.2211	1.45227	1.54551	1.84673	2.30579	1.0252	1.96532	1.67716	1.17563	1.5931	0.029078724	0.321139886	12	12	24	0.418379569	0.802974112	24	10.22481028	0.102067262
ENSMUSG00000027660	Skil	SKI-like [Source:MGI Symbol;Acc:MGI:106203]	3.27573	3.65274	4.30325	3.67649	3.60021	3.06241	3.646	3.38488	3.84663	3.10745	3.55088	3.62262	3.36143	3.76079	0.020957534	0.262822894	12	12	24	0.198935764	0.768040178	24	7.902879434	0.070572914
ENSMUSG00000027661	Slc2a10	solute carrier family 2 (facilitated glucose transporter), member 10 [Source:MGI Symbol;Acc:MGI:2156687]	0.596823	0.762733	0.764501	0.581013	0.617518	0.628887	0.643963	0.89463	0.977874	0.517766	0.550035	0.645761	0.55907	0.674518	0.004163919	0.140598674	12	4	24	0.043623258	0.626787045	24	5.855822529	0.100877754
ENSMUSG00000027663	Zmat3	zinc finger matrin type 3 [Source:MGI Symbol;Acc:MGI:1195270]	11.0252	8.76144	9.65131	9.67939	8.93558	10.4793	10.5289	9.8405	7.75368	13.6404	9.41201	10.8995	10.1063	10.6804	0.310940564	0.820837787	24	12	24	0.731815803	0.912550805	24	19.61733116	0.055742281
ENSMUSG00000027665	Pik3ca	phosphatidylinositol-4,5-bisphosphate 3-kinase catalytic subunit alpha [Source:MGI Symbol;Acc:MGI:1206581]	26.1279	20.8328	21.1922	27.1561	27.3104	23.1959	27.2385	18.6779	23.7108	22.5372	23.2616	23.7278	22.1268	23.3362	0.285193697	0.793714531	20	12	24	0.170349109	0.767957732	24	17.52086544	0.109685875
ENSMUSG00000027667	Zfp639	zinc finger protein 639 [Source:MGI Symbol;Acc:MGI:1915028]	28.487	27.624	29.1208	27.926	26.9386	24.1393	25.5069	26.0271	27.8908	21.9008	28.4577	26.5078	28.0868	26.4809	0.887862353	0.966018836	12	12	24	0.792781987	0.93379199	24	4.003436602	0.028264239
ENSMUSG00000027668	Mfn1	mitofusin 1 [Source:MGI Symbol;Acc:MGI:1914664]	48.5457	51.1196	46.8225	51.6432	48.4415	45.0497	54.5621	45.2036	50.3205	48.9938	55.9876	49.9237	44.5444	51.6286	0.886572916	0.966018836	20	4	24	0.747512645	0.917832057	24	13.29798411	0.029514201
ENSMUSG00000027669	Gnb4	guanine nucleotide binding protein (G protein), beta 4 [Source:MGI Symbol;Acc:MGI:104581]	44.561	39.8396	37.7446	40.6955	41.5767	43.257	41.2995	41.2511	36.14	42.699	47.9584	44.1937	48.3276	42.4676	0.008047769	0.192760771	24	12	24	0.016360017	0.544099157	24	20.04738195	0.104729082
ENSMUSG00000027671	Actl6a	actin-like 6A [Source:MGI Symbol;Acc:MGI:1861453]	12.4363	11.6907	13.1277	12.4127	12.0032	10.5687	12.1069	11.4362	10.9456	11.3833	12.4964	12.0191	11.4785	11.4812	0.844448841	0.966018836	20	12	24	0.833962852	0.948922772	24	13.65830427	0.01721054
ENSMUSG00000027673	Ndufb5	NADH:ubiquinone oxidoreductase subunit B5 [Source:MGI Symbol;Acc:MGI:1913296]	130.457	148.822	163.837	157.299	132.038	130.402	140.658	159.971	177.991	130.56	154.295	144.391	163.103	160.736	0.020957534	0.262822894	12	12	24	0.022320522	0.578379975	24	6.997102856	0.138380795
ENSMUSG00000027674	Pex5l	peroxisomal biogenesis factor 5-like [Source:MGI Symbol;Acc:MGI:1916672]	56.3101	56.6215	58.4728	58.8376	56.5313	58.0118	55.2242	51.6542	52.1781	56.7204	52.325	55.5861	52.0511	57.0641	0.47028201	0.966018836	16	12	24	0.669861537	0.888925819	24	14.36133354	0.019954511
ENSMUSG00000027676	Ccdc39	coiled-coil domain containing 39 [Source:MGI Symbol;Acc:MGI:1289263]	8.16013	8.33327	8.20426	7.45782	8.04396	8.1422	7.32799	8.85973	8.51837	6.02733	8.36734	7.60404	6.74023	6.50582	0.960269811	0.967043964	12	20	24	0.478791628	0.805393068	24	17.49538413	0.068174317
ENSMUSG00000027677	Ttc14	tetratricopeptide repeat domain 14 [Source:MGI Symbol;Acc:MGI:1914370]	24.3	29.825	24.522	26.3024	27.9685	23.6691	26.6152	24.9391	31.113	20.7876	30.052	27.5601	27.6879	28.1593	0.916556573	0.966018836	12	20	24	0.907899551	0.974606327	24	3.571346958	0.026383866
ENSMUSG00000027678	Ncoa3	nuclear receptor coactivator 3 [Source:MGI Symbol;Acc:MGI:1276535]	8.13083	8.82978	7.72616	8.84931	9.55103	8.15382	8.33728	7.4513	9.07309	8.31606	10.1141	8.47928	7.50012	7.93402	0.035494145	0.331814682	20	12	24	0.039431398	0.615919442	24	14.89707701	0.10166521
ENSMUSG00000027679	Dnajc19	DnaJ heat shock protein family (Hsp40) member C19 [Source:MGI Symbol;Acc:MGI:1914963]	5.51835	6.33883	5.85344	5.4645	6.13351	5.18915	4.56598	5.218	7.10491	5.85717	6.72493	5.95789	5.88132	6.37628	0.70641939	0.966018836	20	8	24	0.229687972	0.768040178	24	10.37589486	0.090518378
ENSMUSG00000027680	Fxr1	fragile X mental retardation gene 1, autosomal homolog [Source:MGI Symbol;Acc:MGI:104860]	74.1278	73.7321	70.3168	69.7607	71.5583	71.5645	72.9114	74.8061	79.5515	66.7508	78.7714	75.1142	79.4659	74.945	0.371875382	0.894537464	4	8	24	0.310474452	0.768040178	24	0.990694795	0.038313083

ENSMUSG00000027684	Mecom	MDS1 and EVI1 complex locus [Source:MGI Symbol;Acc:MGI:95457]	1.00422	0.913829	1.36947	1.48327	0.917037	1.03849	0.947487	1.26845	1.24362	1.6117	0.605791	0.93277	0.535692	0.842869	0.112583132	0.530777648	16	4	24	0.040880435	0.616921492	24	9.959562384	0.188272897
ENSMUSG00000027692	Tnik	TRAF2 and NCK interacting kinase [Source:MGI Symbol;Acc:MGI:1916264]	12.537	15.0578	16.197	12.4133	13.1054	13.8477	13.7447	13.3571	15.8424	14.6978	13.3505	13.8032	13.6186	16.284	0.269657278	0.777976231	12	8	24	0.036958525	0.609176161	24	6.908698376	0.110460463
ENSMUSG00000027694	Gm8325	predicted pseudogene 8325 [Source:MGI Symbol;Acc:MGI:3644852]	26.1586	22.4712	20.5726	28.845	19.7738	25.8882	27.045	28.9079	21.8776	30.8498	24.3513	25.1985	24.5929	28.0702	0.943890799	0.966018836	8	12	24	0.934319525	0.983555797	24	2.410110506	0.026144086
ENSMUSG00000027695	Pld1	phospholipase D1 [Source:MGI Symbol;Acc:MGI:109585]	3.90281	4.03027	4.11786	4.7277	4.35634	3.71351	3.96466	4.29389	4.16947	4.0066	3.99317	4.20262	3.62574	3.56292	0.160297544	0.620016786	16	12	24	0.072926684	0.680039457	24	12.45668376	0.065857821
ENSMUSG00000027698	Nceh1	neutral cholesterol ester hydrolase 1 [Source:MGI Symbol;Acc:MGI:2443191]	21.9164	20.6266	21.7834	20.5222	19.9765	21.1825	20.4767	21.7695	19.1004	21.5511	20.0323	21.454	21.0112	21.7318	0.756435587	0.966018836	8	12	24	0.428110924	0.805393068	24	1.376525133	0.028520468
ENSMUSG00000027699	Ect2	ect2 oncogene [Source:MGI Symbol;Acc:MGI:95281]	1.38132	0.933589	0.970977	0.879138	0.871628	1.14749	0.792281	0.959955	0.797726	1.4074	0.74934	0.746568	0.707995	0.758096	0.951846915	0.966018836	16	12	24	0.930635717	0.982155977	24	14.55415976	0.021101434
ENSMUSG00000027702	Lrrc34	leucine rich repeat containing 34 [Source:MGI Symbol;Acc:MGI:1919077]	0.458549	0.653393	0.598791	0.501973	0.551829	0.693216	0.476298	0.453047	0.407941	0.439137	0.348097	0.713704	1.00523	1.01978	0.383843962	0.915479136	24	20	24	0.215197648	0.768040178	24	1.089848853	0.108532442
ENSMUSG00000027706	Sec62	SEC62 homolog (S. cerevisiae) [Source:MGI Symbol;Acc:MGI:1916526]	72.7467	76.3427	73.57	67.3867	73.0609	72.7958	75.4644	66.074	62.3286	61.6307	69.352	52.8893	52.4966	62.5707	0.823246279	0.966018836	8	8	24	0.99510605	0.998759942	24	14.80369508	0.004391947
ENSMUSG00000027708	Dcun1d1	DCN1, defective in cullin neddylation 1, domain containing 1 (S. cerevisiae) [Source:MGI Symbol;Acc:MGI:2150386]	10.2288	11.4007	10.3963	10.4784	10.8564	9.12876	10.4308	9.57874	12.1627	9.69172	12.0168	9.84137	11.3559	10.0767	0.913451828	0.966018836	20	4	24	0.718306389	0.907272193	24	9.510758792	0.038123299
ENSMUSG00000027709	Mccc1	methylcrotonoyl-Coenzyme A carboxylase 1 (alpha) [Source:MGI Symbol;Acc:MGI:1919289]	15.0172	15.9129	17.2152	19.5213	18.4071	16.7016	16.3861	15.5379	17.6343	15.9096	16.8532	18.0873	19.295	16.3434	0.241264473	0.741580974	20	12	24	0.316071569	0.768040178	24	14.66727128	0.061380155
ENSMUSG00000027710	Acad9	acyl-Coenzyme A dehydrogenase family, member 9 [Source:MGI Symbol;Acc:MGI:1914272]	15.782	16.2112	17.3482	17.132	17.8027	17.8194	16.3646	16.3445	15.9923	15.7598	15.5501	16.8204	16.4821	14.7015	0.657586167	0.966018836	24	8	24	0.180192037	0.768040178	24	17.18976992	0.046830345
ENSMUSG00000027712	Anxa5	annexin A5 [Source:MGI Symbol;Acc:MGI:106008]	595	531.22	491.974	526.131	510.462	568.737	507.681	527.471	449.832	512.597	520.89	514.319	552.072	536.923	0.112865	0.530777648	4	8	24	0.043514983	0.626787045	24	21.94130081	0.08575338
ENSMUSG00000027713	1810062G17Rik	RIKEN cDNA 1810062G17 gene [Source:MGI Symbol;Acc:MGI:1919532]	0.903649	0.891254	1.32937	1.34033	0.914384	0.87435	0.45386	0.808713	0.979857	1.26435	0.876698	1.05133	1.13352	0.516684	0.055670133	0.401074772	16	16	24	0.097786506	0.71175955	24	12.48158929	0.157145703
ENSMUSG00000027714	Exosc9	exosome component 9 [Source:MGI Symbol;Acc:MGI:1355319]	27.8789	29.713	28.2334	28.7898	27.9186	26.2569	24.2594	26.4184	28.9768	24.8216	28.2652	26.5325	29.2593	28.6368	0.649212555	0.966018836	8	16	24	0.592972498	0.856634016	24	7.283773303	0.036230141
ENSMUSG00000027715	Ccna2	cyclin A2 [Source:MGI Symbol;Acc:MGI:108069]	1.86076	2.58249	3.20482	2.75215	2.36126	2.81384	2.10713	2.04975	2.31552	2.17107	2.26582	2.26044	2.23175	1.76345	0.172164153	0.648362388	12	20	24	0.26768067	0.768040178	24	12.93258286	0.090241495
ENSMUSG00000027716	Trpc3	transient receptor potential cation channel, subfamily C, member 3 [Source:MGI Symbol;Acc:MGI:109526]	46.667	47.5613	45.2693	48.4022	49.1461	51.0026	40.9745	49.8373	35.1571	38.371	43.8756	43.8334	51.2078	44.9067	0.868638024	0.966018836	8	4	24	0.267718881	0.768040178	24	21.64690724	0.097080846
ENSMUSG00000027722	Spata5	spermatogenesis associated 5 [Source:MGI Symbol;Acc:MGI:1927170]	8.01633	8.68172	9.94644	7.8122	7.43078	7.39383	9.46863	7.0623	7.35032	5.45569	6.60634	9.18123	9.48669	10.3719	0.206597435	0.697236278	4	16	24	0.08002229	0.69470896	24	1.817377607	0.184867929
ENSMUSG00000027737	Slc7a11	solute carrier family 7 (cationic amino acid transporter, y+ system), member 11 [Source:MGI Symbol;Acc:MGI:1347355]	1.34485	2.52407	1.78223	1.15469	1.28089	0.990251	1.4805	1.78515	2.93763	0.626167	1.04213	1.30225	0.803797	1.23406	0.050447964	0.385880335	12	4	24	0.086829627	0.706113004	24	5.624938937	0.26809514
ENSMUSG00000027739	Rab33b	RAB33B, member RAS oncogene family [Source:MGI Symbol;Acc:MGI:1330805]	12.7041	14.0838	13.0416	12.9254	12.7843	13.7214	12.7296	12.8373	13.0347	13.1447	13.4447	14.0702	12.5279	13.7989	0.495929335	0.966018836	24	4	24	0.98163112	0.995786699	24	19.2121968	0.004259355
ENSMUSG00000027742	Cog6	component of oligomeric golgi complex 6 [Source:MGI Symbol;Acc:MGI:1914792]	20.7392	19.9538	18.6013	17.3802	18.4879	19.8417	19.7515	18.9293	17.138	18.9732	19.6966	19.3604	19.7782	19.1081	0.004140921	0.140598674	4	8	24	0.00522224	0.4424469	24	22.46018478	0.078336965
ENSMUSG00000027746	Ufm1	ubiquitin-fold modifier 1 [Source:MGI Symbol;Acc:MGI:1915140]	46.7353	40.3348	36.3814	40.3914	38.3663	40.4286	40.3219	40.2491	40.4701	42.2446	42.9875	42.1496	46.6248	44.2222	0.763955526	0.966018836	4	8	24	0.2001064	0.768040178	24	23.11831033	0.060892386
ENSMUSG00000027748	Trpc4	transient receptor potential cation channel, subfamily C, member 4 [Source:MGI Symbol;Acc:MGI:109525]	1.64932	1.32798	1.55932	1.79933	1.16483	1.45604	1.23836	1.26508	1.30551	1.71313	1.26024	1.50194	1.40578	1.35788	0.959160181	0.967043964	16	4	24	0.65519316	0.883697438	24	12.0818866	0.040337186
ENSMUSG00000027750	Postn	periostin, osteoblast specific factor [Source:MGI Symbol;Acc:MGI:1926321]	14.2325	16.2295	18.4699	14.5813	16.0158	14.3624	16.8145	23.931	15.6969	14.978	17.4732	14.9865	20.9552	17.8175	0.371875382	0.894537464	8	16	24	0.150106919	0.75876287	24	4.401638022	0.142672773
ENSMUSG00000027751	Supt20	SPT20 SAGA complex component [Source:MGI Symbol;Acc:MGI:1929651]	21.7301	23.0434	21.5355	22.1336	21.7141	22.5129	22.6057	19.7944	22.5418	20.8518	21.0998	20.2177	20.576	20.0398	0.757932695	0.966018836	12	20	24	0.895799063	0.970467191	24	16.18798287	0.009705237
ENSMUSG00000027752	Exosc8	exosome component 8 [Source:MGI Symbol;Acc:MGI:1916889]	10.751	13.4583	12.8025	12.9844	13.4892	12.3367	10.8979	10.3463	15.2984	10.231	12.591	13.3346	14.1832	12.5128	0.873350015	0.966018836	12	20	24	0.859584732	0.95862874	24	11.66497593	0.03586463
ENSMUSG00000027762	Sucnr1	succinate receptor 1 [Source:MGI Symbol;Acc:MGI:1934135]	1.43594	1.69027	1.37857	1.00471	1.8829	1.41206	1.5134	1.64275	2.27268	1.32038	1.5339	1.3604	1.51583	1.48411	0.591351901	0.966018836	8	8	24	0.705870466	0.902918222	24	4.851319849	0.054663024
ENSMUSG00000027763	Mbnl1	muscleblind like splicing factor 1 [Source:MGI Symbol;Acc:MGI:1928482]	77.0167	79.5922	76.3776	87.6424	79.3498	70.2055	76.0617	72.0296	76.1594	71.4611	81.7094	69.323	70.9162	75.3973	0.181442559	0.659020016	20	4	24	0.240196535	0.768040178	24	11.66695073	0.054093698
ENSMUSG00000027765	P2ry1	purinergic receptor P2Y, G-protein coupled 1 [Source:MGI Symbol;Acc:MGI:105049]	26.4994	22.4638	20.8693	23.3047	21.3741	23.0388	22.0747	21.0561	20.7379	20.9794	23.9266	23.5062	25.8548	22.5813	0.016095537	0.253395174	4	8	24	0.051925575	0.649400655	24	21.53304257	0.096411775
ENSMUSG00000027770	Dhx36	DEAH (Asp-Glu-Ala-His) box polypeptide 36 [Source:MGI Symbol;Acc:MGI:1919412]	22.4414	22.626	23.1641	22.3081	23.5862	21.5152	21.9252	22.1773	23.2859	20.5451	22.9124	22.8345	23.0764	23.0798	0.777666236	0.966018836	12	12	24	0.908884964	0.974606327	24	4.242335029	0.008970285
ENSMUSG00000027774	Gfm1	G elongation factor, mitochondrial 1 [Source:MGI Symbol;Acc:MGI:107339]	29.5595	24.553	25.2809	28.7697	24.2215	28.4736	26.3246	28.0883	20.9286	29.7329	24.2276	25.5011	27.3946	26.5066	0.843639218	0.966018836	4	8	24	0.661303546	0.885520687	24	21.48863667	0.050428484
ENSMUSG00000027775	Mfsd1	major facilitator superfamily domain containing 1 [Source:MGI Symbol;Acc:MGI:1914118]	21.2264	17.9717	19.1853	20.1032	16.9287	18.5636	19.2196	19.4495	16.2538	20.516	18.4264	19.6648	20.3386	19.1262	0.522551072	0.966018836	4	8	24	0.638417758	0.877227735	24	23.17549897	0.038566961
ENSMUSG00000027777	Schip1	schwannomin interacting protein 1 [Source:MGI Symbol;Acc:MGI:1353557]	47.1436	46.7588	45.2181	45.6917	48.8979	46.8967	46.2985	44.69	43.0542	40.5918	48.4208	41.2282	49.4447	43.117	0.163947066	0.627573783	20	20	24	0.284508841	0.768040178	24	20.86355644	0.051706297
ENSMUSG00000027778	Ift80	intraflagellar transport 80 [Source:MGI Symbol;Acc:MGI:1915509]	17.9212	16.6837	15.603	16.2067	16.8818	15.574	17.1493	16.7615	15.5043	14.6829	16.474	16.7229	18.0258	16.4831	7.56E-04	0.071344007	4	12	24	0.014017561	0.530670295	24	23.20090175	0.077086869
ENSMUSG00000027782	Kpna4	karyopherin (importin) alpha 4 [Source:MGI Symbol;Acc:MGI:1100848]	28.779	25.1696	25.9531	26.0696	26.0346	27.0452	28.0288	25.6881	21.72	23.5934	26.7384	24.1358	23.3761	23.6429	0.56005262	0.966018836	4	4	24	0.04233619	0.623698598	24	20.36364423	0.080394883
ENSMUSG00000027784	Ppm1l	protein phosphatase 1 (formerly 2C)-like [Source:MGI Symbol;Acc:MGI:2139740]	24.1115	21.3191	21.8496	22.673	24.5158	23.8204	23.7947	23.5552	20.9368	30.4584	21.3002	24.0416	23.567	23.1938	0.065849436	0.422183965	24	12	24	0.640608108	0.878333676	24	16.52679611	0.047735915
ENSMUSG00000027787	Nmd3	NMD3 ribosome export adaptor [Source:MGI Symbol;Acc:MGI:2140103]	27.6833	29.2333	27.6241	28.7636	28.202	26.2891	29.0754	28.5597	29.5801	23.1557	32.5525	28.0075	31.0024	24.0636	0.847846021	0.966018836	4	20	24	0.766270997	0.92437333	24	20.70745951	0.038014075
ENSMUSG00000027792	Bche	butyrylcholinesterase [Source:MGI Symbol;Acc:MGI:894278]	4.48579	5.24566	4.9476	5.9958	4.6571	4.58779	4.82327	4.43715	5.33707	4.9934	5.06784	4.85324	4.82653	5.40411	0.171124271	0.644969786	16	12	24	0.079152466	0.691443676	24	9.926116404	0.083200561
ENSMUSG00000027796	Smad9	SMAD family member 9 [Source:MGI Symbol;Acc:MGI:1859993]	9.0262	7.99078	7.07192	7.97139	6.84283	8.58117	7.98295	8.60749	6.48624	10.6361	6.74173	8.154	7.92666	8.6027	0.510431574	0.966018836	8	4	24	0.772759407	0.926880913	24	0.627459358	0.048457885
ENSMUSG00000027797	Dclk1	doublecortin-like kinase 1 [Source:MGI Symbol;Acc:MGI:1330861]	38.2939	31.6715	35.3013	31.437	34.0755	35.4603	31.9645	32.5609	26.7494	34.8979	32.0999	32.1195	29.4327	33.8283	0.854443826	0.966018836	24	12	24	0.51346749	0.816307374	24	19.73125787	0.053537517
ENSMUSG00000027799	Nbea	neurobeachin [Source:MGI Symbol;Acc:MGI:1347075]	22.3004	21.4559	22.9704	19.8481	18.585	21.354	20.6436	19.7793	20.1212	21.4155	20.8277	20.3021	19.9008	22.4899	0.916746631	0.966018836	12	8	24	0.412173163	0.799546792	24	4.480250546	0.041760157
ENSMUSG00000027800	Tm4sf1	transmembrane 4 superfamily member 1 [Source:MGI Symbol;Acc:MGI:104678]	20.6912	23.6772	24.2602	20.9592	24.2779	19.5456	21.1615	22.8742	30.467	19.0457	20.4866	23.3665	23.6071	23.6763	0.071569005	0.440262583	12	4	24	0.20668372	0.768040178	24	6.487149815	0.114709664
ENSMUSG00000027803	Wwtr1	WW domain containing transcription regulator 1 [Source:MGI Symbol;Acc:MGI:1917649]	13.4534	13.6168	11.8514	12.2773	13.8057	11.5116	14.4569	13.424	13.6579	11.3841	12.9306	12.549	12.8314	11.9416	0.816656735	0.966018836	4	12	24	0.416566417	0.801257196	24	0.409310745	0.049843176
ENSMUSG00000027804	Ppid	peptidylprolyl isomerase D (cyclophilin D) [Source:MGI Symbol;Acc:MGI:1914988]	26.5316	28.5145	27.2214	27.7078	26.6874	26.7489	27.1831	26.1478	26.6594	23.5852	28.146	23.6488	25.8308	25.2093	0.872380105	0.966018836	20	4	24	0.962947915	0.990726108	24	5.772520022	0.007409952
ENSMUSG00000027805	Pfn2	profilin 2 [Source:MGI Symbol;Acc:MGI:97550]	215.852	211.586	206.551	201.863	198.421	212.364	199.555	197.75	190.388	212.435	201.766	200.476	193.244	210.578	0.823246279	0.966018836	16	20	24	0.908134338	0.974606327	24	22.18324304	0.009393407
ENSMUSG00000027806	Tsc22d2	TSC22 domain family, member 2 [Source:MGI Symbol;Acc:MGI:1919283]	4.48222	4.87884	5.52843	4.63465	4.8358	4.47501	5.66959	4.87426	4.82431	4.20039	4.84816	4.40849	4.13434	4.47366	0.887010723	0.966018836	12	12	24	0.719209611	0.907893524	24	6.331888187	0.033166226
ENSMUSG00000027808	Serp1	stress-associated endoplasmic reticulum protein 1 [Source:MGI Symbol;Acc:MGI:92638]	17.0553	16.1852	17.0273	17.4733	18.5012	14.7812	16.7704	15.1526	17.0452	14.5682	18.5755	15.3914	15.9201	15.1015	0.338172159	0.85758369	20	12	24	0.382305756	0.799546792	24	14.71201764	0.05394204
ENSMUSG00000027809	Etfdh	electron transferring flavoprotein, dehydrogenase [Source:MGI Symbol;Acc:MGI:106100]	27.477	26.7648	29.7846	40.1464	28.9438	28.7294	29.0531	32.9366	29.0675	31.762	33.6963	30.1402	36.3344	33.4994	0.933543173	0.966018836	16	12	24	0.438985864	0.805393068	24	11.44012022	0.072299031
ENSMUSG00000027810	Eif2a	eukaryotic translation initiation factor 2A [Source:MGI Symbol;Acc:MGI:1098684]	29.2388	33.4716	32.052	32.5201	30.1575	29.1714	28.3371	30.6878	36.565	28.5943	31.9195	30.1667	33.6419	34.3971	0.223535563	0.717349224	12	12	24	0.070740125	0.675973954	24	7.228008871	0.090466443
ENSMUSG00000027811	4930579G24Rik	RIKEN cDNA 4930579G24 gene [Source:MGI Symbol;Acc:MGI:1923189]	1.64205	1.56196	1.99995	1.86969	1.56407	1.6006	1.49435	1.51545	1.25542	1.4323	1.48182	1.41891	1.6153	1.37748	0.838244962	0.966018836	4	4	24	0.896031314	0.970467191	24	13.86804398	0.015753454
ENSMUSG00000027820	Mme	membrane metallo endopeptidase [Source:MGI Symbol;Acc:MGI:97004]	16.6777	21.8437	20.6649	24.0712	24.3897	16.5001	23.8386	18.6273	28.3865	19.8714	26.1485	23.5852	25.6209	22.8821	0.951523604	0.966018836	20	4	24	0.4656899	0.805393068	24	11.18269158	0.099830951
ENSMUSG00000027822	Slc33a1	solute carrier family 33 (acetyl-CoA transporter), member 1 [Source:MGI Symbol;Acc:MGI:1332247]	13.819	12.888	12.2228	12.0003	12.2351	12.2622	13.0497	13.0623	11.3062	12.4523	13.2583	12.953	14.0386	12.6032	0.001837158	0.100255953	4	8	24	0.023368043	0.581965446	24	22.8960142	0.073267837
ENSMUSG00000027823	Gmps	guanine monophosphate synthetase [Source:MGI Symbol;Acc:MGI:2448526]	8.99502	9.12825	9.47865	9.00004	8.60272	8.78225	8.9275	8.11898	8.89899	9.0226	9.73612	9.38733	9.0966	9.9094	0.832293447	0.966018836	12	16	24	0.908820666	0.974606327	24	10.42669972	0.011075349
ENSMUSG00000027827	Kcnab1	potassium voltage-gated channel, shaker-related subfamily, beta member 1 [Source:MGI Symbol;Acc:MGI:109155]	68.9725	55.6969	58.6826	53.4011	57.2698	63.9879	53.9072	52.7645	42.9	55.42	54.5937	53.859	53.4113	56.065	0.882263435	0.966018836	20	20	24	0.098325592	0.71175955	24	20.8100434	0.110831613
ENSMUSG00000027828	Ssr3	signal sequence receptor, gamma [Source:MGI Symbol;Acc:MGI:1914687]	81.454	87.6307	85.7852	85.3585	87.8323	80.9942	83.3731	81.2064	91.651	82.0728	89.2763	83.8254	84.1316	85.7059	0.182320384	0.659303873	20	4	24	0.237667344	0.768040178	24	10.54697992	0.035443527
ENSMUSG00000027829	Ccnl1	cyclin L1 [Source:MGI Symbol;Acc:MGI:1922664]	28.3729	32.0927	27.5336	31.1447	29.0589	24.4509	30.2853	30.3891	31.62	24.5803	30.1325	25.756	26.9474	25.9101	0.217827365	0.704989686	12	12	24	0.525132713	0.822381894	24	7.044844498	0.055790818
ENSMUSG00000027832	Ptx3	pentraxin related gene [Source:MGI Symbol;Acc:MGI:104641]	0.490857	0.880388	1.14411	0.641354	0.972722	0.763039	0.778052	0.928353	0.954208	1.05269	0.784714	0.671555	0.862023	1.17528	0.036527779	0.332441566	12	12	24	0.039178678	0.614396028	24	8.227490713	0.13551637
ENSMUSG00000027833	Shox2	short stature homeobox 2 [Source:MGI Symbol;Acc:MGI:1201673]	5.11788	4.02683	2.88496	3.83634	3.97008	3.32682	3.52497	5.48337	2.80594	6.38433	3.58485	4.70089	4.45779	4.14568	0.939921059	0.966018836	8	4	24	0.890708472	0.9692042	24	22.50551596	0.053322926
ENSMUSG00000027834	Serpini1	serine (or cysteine) peptidase inhibitor, clade I, member 1 [Source:MGI Symbol;Acc:MGI:1194506]	44.0424	47.1319	43.7708	45.2629	45.5676	43.2749	42.0774	39.4998	43.9395	40.6955	46.3273	45.8167	47.5634	48.401	0.890235289	0.966018836	8	4	24	0.889723086	0.968953708	24	21.31275129	0.016487904
ENSMUSG00000027835	Pdcd10	programmed cell death 10 [Source:MGI Symbol;Acc:MGI:1928396]	21.0109	25.4094	24.4491	21.1751	21.1374	20.2894	21.503	20.7879	24.7092	18.9485	24.5829	22.1195	21.8529	24.3841	0.607422703	0.966018836	12	4	24	0.330905157	0.768040178	24	6.140423799	0.072721477
ENSMUSG00000027840	Wnt2b	wingless-type MMTV integration site family, member 2B [Source:MGI Symbol;Acc:MGI:1261834]	6.82281	4.58518	4.47781	5.28487	4.7258	5.69069	4.69285	5.18656	4.07051	6.36082	5.07946	5.53736	6.00928	5.53336	0.124211791	0.552144108	24	12	24	0.347510901	0.778844143	24	20.74215148	0.101676341
ENSMUSG00000027843	Ptpn22	protein tyrosine phosphatase, non-receptor type 22 (lymphoid) [Source:MGI Symbol;Acc:MGI:107170]	1.31602	1.77966	1.25069	1.89203	1.69807	0.93698	1.92927	0.841663	1.5912	0.9667	0.949507	1.38377	1.4404	1.25887	0.816406072	0.966018836	4	20	24	0.954077649	0.988601783	24	2.990800912	0.026541582
ENSMUSG00000027845	Dclre1b	DNA cross-link repair 1B [Source:MGI Symbol;Acc:MGI:2156057]	1.97873	2.32677	2.97741	2.52509	3.04113	2.79808	2.24978	2.03571	2.32146	2.62102	2.0868	2.20549	2.42252	1.99177	0.23195324	0.731832877	12	20	24	0.134173491	0.744382556	24	13.96257197	0.102660448
ENSMUSG00000027848	Olfml3	olfactomedin-like 3 [Source:MGI Symbol;Acc:MGI:1914877]	7.6067	9.13001	8.5951	7.87017	9.08165	7.66602	7.96974	8.74978	9.94135	6.99063	7.61672	7.84938	8.30679	7.61185	0.44861185	0.966018836	12	4	24	0.385193494	0.799546792	24	6.330451906	0.066065096
ENSMUSG00000027849	Syt6	synaptotagmin VI [Source:MGI Symbol;Acc:MGI:1859544]	22.7265	17.1799	17.1337	18.297	16.8681	21.7155	18.9117	19.9764	13.6686	22.7982	17.1661	20.6158	20.6139	19.5159	0.080164531	0.458678242	24	12	24	0.203742236	0.768040178	24	21.50763525	0.133783007
ENSMUSG00000027852	Nras	neuroblastoma ras oncogene [Source:MGI Symbol;Acc:MGI:97376]	32.1619	28.1109	27.3561	29.6131	30.8606	28.6979	27.7706	26.4444	27.0982	26.0214	29.2105	26.8057	27.469	25.7541	0.435739761	0.966018836	12	8	24	0.023378668	0.581965446	24	18.55677507	0.067774934
ENSMUSG00000027854	Sike1	suppressor of IKBKE 1 [Source:MGI Symbol;Acc:MGI:1913891]	15.9677	13.1769	12.8642	14.3546	14.9444	14.6745	15.3362	14.5027	15.6708	11.551	14.9952	13.4658	14.8995	13.9883	0.305564675	0.811201458	4	12	24	0.286912182	0.768040178	24	22.23911657	0.073406256
ENSMUSG00000027857	Tshb	thyroid stimulating hormone, beta subunit [Source:MGI Symbol;Acc:MGI:98848]	0.915751	0.808965	0.79343	0.771156	0.803293	0.828416	0.674285	0.902604	0.814506	0.904555	1.00131	0.787239	0.94533	[illegible]	0.839789452	0.966018836	4	4	24	0.839789452	0.951393228	24	17.03312095	0.016031641
ENSMUSG00000027858	Tspan2	tetraspanin 2 [Source:MGI Symbol;Acc:MGI:1917997]	33.4601	33.9147	38.8934	34.448	29.089	35.0274	31.661	30.4956	30.8972	32.3427	29.9826	31.3497	30.0648	33.9675	0.446653834	0.966018836	12	8	24	0.475689794	0.805393068	24	6.811710894	0.048489269
ENSMUSG00000027859	Ngf	nerve growth factor [Source:MGI Symbol;Acc:MGI:97321]	1.16553	0.92334	0.819386	0.793803	0.988378	1.11512	1.83902	1.58473	1.0997	0.889556	1.0244	0.845668	1.15016	0.83864	0.008961721	0.192760771	4	12	24	0.143238562	0.752803875	24	0.17029116	0.142383362
ENSMUSG00000027860	Vangl1	VANGL planar cell polarity 1 [Source:MGI Symbol;Acc:MGI:2159344]	3.6926	3.43813	3.19129	3.43265	3.52401	3.56604	3.52962	2.93343	2.84016	3.38897	2.7196	2.9574	2.75262	2.94922	0.917610614	0.966018836	4	8	24	0.366992615	0.790573051	24	19.30460553	0.044322841
ENSMUSG00000027861	Casq2	calsequestrin 2 [Source:MGI Symbol;Acc:MGI:1309469]	2.03657	3.46425	3.96057	3.702	3.42595	2.57447	2.79095	3.68791	3.26215	2.93897	2.21216	1.97466	3.41558	3.33777	0.038115943	0.334069441	12	12	24	0.02247886	0.579292623	24	7.75801233	0.239786264
ENSMUSG00000027863	Cd2	CD2 antigen [Source:MGI Symbol;Acc:MGI:88320]	0.55011	0.50971	0.610656	0.380933	0.297586	0.425764	0.886424	0.640752	0.711569	0.291395	0.542998	0.548668	0.595636	0.238978	0.172322601	0.648362388	4	12	24	0.309000343	0.768040178	24	1.452238412	0.098562875
ENSMUSG00000027864	Ptgfrn	prostaglandin F2 receptor negative regulator [Source:MGI Symbol;Acc:MGI:1277114]	7.19344	5.73788	6.24095	6.0356	6.63786	6.45044	6.37175	6.49826	5.59927	6.67001	6.2289	6.17544	5.50636	5.80171	0.917610614	0.966018836	16	20	24	0.242661978	0.768040178	24	18.33145577	0.055679284
ENSMUSG00000027865	Gdap2	ganglioside-induced differentiation-associated-protein 2 [Source:MGI Symbol;Acc:MGI:1338001]	16.2059	16.2468	16.6056	17.6995	15.2492	15.8002	14.7504	14.4137	15.3846	14.5826	18.3496	16.9064	14.2149	15.2208	0.371875382	0.894537464	24	4	24	0.22225342	0.768040178	24	15.06470802	0.066574142
ENSMUSG00000027868	Tbx15	T-box 15 [Source:MGI Symbol;Acc:MGI:1277234]	1.23243	3.10948	2.79695	4.24218	3.02131	3.04354	2.04735	5.68948	4.05343	5.91845	2.85295	2.64004	3.25572	2.50013	0.113148904	0.530777648	16	12	24	0.045291997	0.629029783	24	10.3146057	0.374249536
ENSMUSG00000027875	Hmgcs2	3-hydroxy-3-methylglutaryl-Coenzyme A synthase 2 [Source:MGI Symbol;Acc:MGI:101939]	28.485	25.9479	22.4244	20.5265	19.0724	22.7051	27.6202	32.5239	20.3149	21.8945	18.4007	22.2921	25.9813	24.572	1.38E-04	0.031913438	4	16	24	3.85E-04	0.23436643	24	1.996062363	0.260639994
ENSMUSG00000027878	Notch2	notch 2 [Source:MGI Symbol;Acc:MGI:97364]	4.84062	5.55104	4.93348	4.69929	6.1646	4.72029	6.02545	5.01905	5.75361	5.8224	4.71236	5.13718	5.36919	4.52789	0.788254138	0.966018836	4	4	24	0.966526815	0.992002385	24	13.43799191	0.013039812
ENSMUSG00000027879	Sec22b	SEC22 homolog B, vesicle trafficking protein [Source:MGI Symbol;Acc:MGI:1338759]	35.6706	33.5078	32.3537	32.9079	33.4689	33.1676	36.1398	33.7343	33.3721	32.6319	33.7718	32.9498	35.6485	33.861	8.93E-04	0.071344007	4	12	24	0.013420739	0.529134128	24	23.69764556	0.048338859
ENSMUSG00000027881	Prpf38b	PRP38 pre-mRNA processing factor 38 (yeast) domain containing B [Source:MGI Symbol;Acc:MGI:1914171]	26.7419	26.8134	28.6611	28.5921	29.8728	26.6904	26.8768	25.3631	27.6306	24.3974	26.4071	26.5568	27.2434	25.8211	0.919119843	0.966018836	12	20	24	0.596516387	0.85784298	24	15.11868545	0.025963404
ENSMUSG00000027882	Stxbp3	syntaxin binding protein 3 [Source:MGI Symbol;Acc:MGI:107362]	10.8385	12.9303	13.0589	11.7802	11.9701	11.3851	12.4245	10.9019	15.4836	10.5449	12.809	12.6523	12.4679	12.3918	0.834434272	0.966018836	12	4	24	0.56285819	0.842470381	24	7.652784581	0.057223953
ENSMUSG00000027883	Gpsm2	G-protein signalling modulator 2 (AGS3-like, C. elegans) [Source:MGI Symbol;Acc:MGI:1923373]	9.74881	8.58471	9.70016	9.73392	8.68718	8.58406	10.3089	9.48187	9.1572	8.65754	8.34737	11.4463	8.83102	11.0096	0.833406028	0.966018836	4	16	24	0.465581998	0.805393068	24	2.083395885	0.059847958
ENSMUSG00000027884	Clcc1	chloride channel CLIC-like 1 [Source:MGI Symbol;Acc:MGI:2385186]	13.6613	15.4977	14.2973	12.0828	12.428	13.2978	14.2674	13.7666	14.9461	15.8103	13.5258	13.878	14.8443	15.0998	0.083354723	0.462922111	12	8	24	0.05553903	0.65534544	24	5.025127177	0.087171381
ENSMUSG00000027887	Sypl2	synaptophysin-like 2 [Source:MGI Symbol;Acc:MGI:1328311]	0.893149	1.87113	2.37414	2.82716	1.95689	2.40754	2.21927	5.66121	3.96833	5.59371	1.88899	2.14631	2.50023	2.4419	0.369262716	0.893596186	16	4	24	0.06176083	0.661961741	24	9.122649482	0.396965898
ENSMUSG00000027889	Ampd2	adenosine monophosphate deaminase 2 [Source:MGI Symbol;Acc:MGI:88016]	15.1377	13.5175	16.5724	12.0529	12.4709	15.4003	13.6509	13.401	11.3261	15.7717	11.0736	13.4673	12.0057	13.4301	0.872380105	0.966018836	24	20	24	0.822090961	0.943977421	24	1.793418959	0.038489341
ENSMUSG00000027890	Gstm4	glutathione S-transferase, mu 4 [Source:MGI Symbol;Acc:MGI:95862]	7.37773	6.02434	6.75182	6.59939	6.33233	6.59306	6.25124	7.49844	6.26279	6.4531	5.95309	6.27252	6.69857	6.35843	0.935593607	0.966018836	4	16	24	0.448018757	0.805393068	24	2.412317506	0.039671062
ENSMUSG00000027893	Ahcyl1	S-adenosylhomocysteine hydrolase-like 1 [Source:MGI Symbol;Acc:MGI:2385184]	75.6106	82.6131	93.4167	82.2739	76.8472	81.1442	85.4644	77.2168	84.8629	84.6781	79.7492	81.29	80.3922	88.2444	0.173410058	0.648600527	12	8	24	0.111106224	0.723334499	24	7.886588264	0.065228295
ENSMUSG00000027894	Slc6a17	solute carrier family 6 (neurotransmitter transporter), member 17 [Source:MGI Symbol;Acc:MGI:2442535]	22.6808	20.2223	21.4501	23.9082	19.3247	25.5762	20.9877	22.6607	19.2758	30.7377	17.9449	23.8818	22.8951	24.4599	0.923635729	0.966018836	24	12	24	0.990488773	0.998233149	24	11.09730093	0.010635729
ENSMUSG00000027895	Kcnc4	potassium voltage gated channel, Shaw-related subfamily, member 4 [Source:MGI Symbol;Acc:MGI:96670]	13.3219	8.45643	10.4723	10.8977	10.1488	12.5636	11.2671	13.3495	8.4386	15.6425	10.6921	11.9618	12.6826	12.2238	0.636884864	0.966018836	4	8	24	0.671135599	0.889065421	24	20.62709896	0.081099
ENSMUSG00000027896	Slc16a4	solute carrier family 16 (monocarboxylic acid transporters), member 4 [Source:MGI Symbol;Acc:MGI:2385183]	1.75091	2.9396	2.45535	2.54338	2.93773	2.19188	2.27078	2.39632	2.97032	2.15462	2.7139	2.39447	2.25879	2.47049	0.130356961	0.561940267	12	16	24	0.235009816	0.768040178	24	10.58738138	0.096793618
ENSMUSG00000027900	Dram2	DNA-damage regulated autophagy modulator 2 [Source:MGI Symbol;Acc:MGI:1914421]	55.0292	53.7233	50.9712	55.0663	55.8509	48.4809	56.0655	52.5658	58.7819	45.4067	54.031	51.8109	53.807	51.6199	0.891742979	0.966018836	4	12	24	0.825215572	0.945190971	24	2.054752601	0.022368108
ENSMUSG00000027901	Dennd2d	DENN/MADD domain containing 2D [Source:MGI Symbol;Acc:MGI:2181193]	0.355932	0.672471	0.550365	0.620975	0.593159	0.724816	0.484602	0.564793	1.07869	0.61576	0.535384	0.614365	0.595277	0.567769	0.648082424	0.966018836	16	12	24	0.291497938	0.768040178	24	9.87191467	0.082371226
ENSMUSG00000027905	Ddx20	DEAD box helicase 20 [Source:MGI Symbol;Acc:MGI:1858415]	5.10421	5.54407	5.14009	5.30278	5.311	5.34029	5.24973	5.16691	5.67594	4.96128	5.43431	5.40706	5.35263	4.91049	0.933543173	0.966018836	20	12	24	0.849488854	0.954767108	24	17.64733061	0.011456313
ENSMUSG00000027907	S100a11	S100 calcium binding protein A11 [Source:MGI Symbol;Acc:MGI:1338798]	46.0293	47.8437	53.7166	47.4913	52.7569	46.7748	47.2663	47.5399	50.8259	39.1864	47.7508	43.4735	44.4532	40.9798	0.321662653	0.83607295	12	12	24	0.832298623	0.94810743	24	11.90346026	0.024867619
ENSMUSG00000027931	Npr1	natriuretic peptide receptor 1 [Source:MGI Symbol;Acc:MGI:97371]	2.1177	1.85154	1.99215	2.0075	1.78216	2.52255	1.91326	2.71931	2.70733	2.37334	2.55332	1.95157	2.18635	2.33612	0.859936714	0.966018836	12	16	24	0.631551148	0.874470672	24	8.043673568	0.051650509
ENSMUSG00000027932	Slc27a3	solute carrier family 27 (fatty acid transporter), member 3 [Source:MGI Symbol;Acc:MGI:1347358]	2.51852	2.27199	2.90502	2.95181	3.32705	1.96198	2.7982	2.70766	2.32976	2.682	2.72199	2.88986	2.01092	2.37431	0.347075276	0.870418653	20	8	24	0.186599595	0.768040178	24	13.59315179	0.104039992
ENSMUSG00000027933	Ints3	integrator complex subunit 3 [Source:MGI Symbol;Acc:MGI:2140050]	12.4929	9.64693	10.6821	10.9939	11.2232	11.8889	11.7711	11.5665	8.54424	11.6314	9.81567	10.4697	9.59367	10.742	0.913451828	0.966018836	4	8	24	0.373786699	0.794450623	24	20.33641482	0.073835972
ENSMUSG00000027935	Rab13	RAB13, member RAS oncogene family [Source:MGI Symbol;Acc:MGI:1927232]	6.93465	6.79699	6.09044	5.07645	6.29251	6.41653	6.57333	7.39624	6.05772	5.75969	6.07132	5.94075	6.8889	6.32833	7.56E-04	0.071344007	4	12	24	0.003687305	0.426426863	24	1.019512174	0.123785016
ENSMUSG00000027936	Crtc2	CREB regulated transcription coactivator 2 [Source:MGI Symbol;Acc:MGI:1921593]	12.6743	14.4884	14.2746	13.3846	14.8749	14.3708	13.5449	14.6948	15.9861	16.909	14.8456	14.2284	14.9886	14.8418	0.575202741	0.966018836	20	8	24	0.059976303	0.658304792	24	10.42868644	0.071068297
ENSMUSG00000027937	Jtb	jumping translocation breakpoint [Source:MGI Symbol;Acc:MGI:1346082]	25.2043	29.3097	29.0981	31.365	26.5442	25.6485	26.2151	29.046	33.294	29.5899	36.6439	32.9061	34.7457	32.1837	0.917867465	0.966018836	16	12	24	0.257473243	0.768040178	24	10.16021344	0.077953549
ENSMUSG00000027940	Tpm3	tropomyosin 3, gamma [Source:MGI Symbol;Acc:MGI:1890149]	43.9313	48.385	63.4633	53.9912	49.1316	51.1913	46.4993	53.1024	52.8728	52.8798	42.9473	40.0691	48.9937	51.3695	0.013420949	0.233443994	16	8	24	0.013033717	0.529134128	24	8.456831495	0.163417411
ENSMUSG00000027942	4933434E20Rik	RIKEN cDNA 4933434E20 gene [Source:MGI Symbol;Acc:MGI:1914027]	41.9426	51.0989	50.641	46.7639	50.8103	48.877	43.9192	48.1817	55.6842	45.7289	50.6623	43.3848	52.4155	47.1589	0.850398351	0.966018836	12	16	24	0.319063213	0.768040178	24	9.277299417	0.068065206
ENSMUSG00000027944	Hax1	HCLS1 associated X-1 [Source:MGI Symbol;Acc:MGI:1346319]	22.9215	26.6296	22.6873	29.4427	28.1016	23.989	21.8776	26.7418	19.7993	19.7369	23.7733	20.9978	24.3858	19.7841	0.828910443	0.966018836	20	16	24	0.693576241	0.898364349	24	17.34371951	0.055249847
ENSMUSG00000027947	Il6ra	interleukin 6 receptor, alpha [Source:MGI Symbol;Acc:MGI:105304]	1.81265	1.35496	1.46528	2.54662	1.97971	1.50305	2.02404	1.20731	1.77517	2.00902	1.68193	1.98029	1.25524	1.11764	0.008483961	0.192760771	16	16	24	0.027415404	0.590220615	24	15.23198018	0.19075202
ENSMUSG00000027950	Chrnb2	cholinergic receptor, nicotinic, beta polypeptide 2 (neuronal) [Source:MGI Symbol;Acc:MGI:87891]	15.2686	11.5814	11.7033	13.1626	12.5076	15.0983	12.8863	13.3937	9.65905	15.5734	11.1883	13.0747	12.4888	13.1787	0.954106009	0.966018836	24	12	24	0.376515641	0.796552361	24	20.39039862	0.097984315
ENSMUSG00000027951	Adar	adenosine deaminase, RNA-specific [Source:MGI Symbol;Acc:MGI:1889575]	15.5926	11.5396	14.1606	12.7575	12.6533	14.2489	15.6667	14.0628	13.2738	15.3102	12.6754	14.1087	12.3456	14.0127	0.907000607	0.966018836	4	16	24	0.798401444	0.935424343	24	23.15813801	0.033991899
ENSMUSG00000027952	Pmvk	phosphomevalonate kinase [Source:MGI Symbol;Acc:MGI:1915853]	68.7208	59.1333	55.6542	57.3809	67.104	65.6678	59.4931	62.2535	54.7495	57.0836	66.2349	62.9269	64.3321	59.3457	0.001051376	0.07619846	20	16	24	0.002106241	0.395705644	24	20.26480467	0.120878668
ENSMUSG00000027953	Slc50a1	solute carrier family 50 (sugar transporter), member 1 [Source:MGI Symbol;Acc:MGI:107417]	28.3564	34.127	31.6164	30.3071	30.7161	32.7062	33.0865	33.8697	34.3323	29.775	31.6308	30.271	31.0272	29.7821	0.893307667	0.966018836	12	8	24	0.529429358	0.824483137	24	5.365177549	0.037114311
ENSMUSG00000027954	Efna1	ephrin A1 [Source:MGI Symbol;Acc:MGI:103236]	15.6863	11.9006	10.81	14.2757	13.1619	13.6018	12.32	13.6447	10.914	13.2695	12.4543	12.6785	13.3102	12.256	0.125573493	0.555618842	16	20	24	0.215805427	0.768040178	24	19.57011792	0.08909625

ENSMUSG00000027955	Gask1b	golgi associated kinase 1B [Source:MGI Symbol;Acc:MGI:1915909]	11.7569	12.9005	11.0325	9.50896	12.8154	10.3734	12.5011	12.693	12.087	10.6384	10.316	10.9957	11.5018	12.3855	0.008157655	0.192760771	8	8	24	0.061228729	0.661066697	24	2.792824703	0.106722048
ENSMUSG00000027956	Tmem144	transmembrane protein 144 [Source:MGI Symbol;Acc:MGI:1917902]	0.925281	1.19481	1.21675	1.11083	0.893936	1.07686	1.82002	0.962818	1.32951	1.06307	0.932497	0.936598	0.835885	1.09878	0.10951302	0.524035616	12	8	24	0.509601039	0.813985618	24	5.488316973	0.073095305
ENSMUSG00000027957	Slc35a3	solute carrier family 35 (UDP-N-acetylglucosamine (UDP-GlcNAc) transporter), member 3 [Source:MGI Symbol;Acc:MGI:1917648]	12.4337	11.5116	10.5436	11.4961	12.5879	10.5774	11.4637	10.3013	11.1204	10.263	13.0374	10.7816	10.6546	11.0641	0.89563591	0.966018836	20	16	24	0.316709833	0.768040178	24	17.74272817	0.058515715
ENSMUSG00000027959	Sass6	SAS-6 centriolar assembly protein [Source:MGI Symbol;Acc:MGI:1920026]	3.15654	3.60033	3.92892	3.64282	3.83752	3.58739	3.72943	2.75198	3.36749	3.20757	3.98275	3.96248	4.56773	3.80585	0.832506215	0.966018836	20	12	24	0.539036469	0.829144433	24	18.81441838	0.060072087
ENSMUSG00000027961	Lrrc39	leucine rich repeat containing 39 [Source:MGI Symbol;Acc:MGI:1924557]	0.796914	1.48179	2.12099	2.0559	1.35838	1.80203	1.16431	2.77665	2.45799	2.09274	1.46777	1.46908	2.15983	1.5642	0.119434414	0.542554768	12	8	24	0.051640218	0.647896699	24	8.847551818	0.249741235
ENSMUSG00000027962	Vcam1	vascular cell adhesion molecule 1 [Source:MGI Symbol;Acc:MGI:98926]	3.94691	4.54927	6.67285	5.72941	4.18982	4.15036	4.62577	5.3188	5.48494	3.56357	4.00725	3.65248	3.56524	4.27136	0.071575474	0.440262583	12	12	24	0.025030835	0.586541342	24	8.072689989	0.201620886
ENSMUSG00000027963	Ext2	exostosin-like glycosyltransferase 2 [Source:MGI Symbol;Acc:MGI:1889574]	26.8672	28.6769	28.2229	26.2317	25.6113	27.419	25.3466	27.0737	25.6708	29.0227	25.6879	25.0376	25.0874	30.8596	0.114657038	0.534533508	8	20	24	0.163455211	0.767020009	24	6.781364681	0.065649971
ENSMUSG00000027965	Olfm3	olfactomedin 3 [Source:MGI Symbol;Acc:MGI:2387329]	1.46459	2.31175	3.31802	1.85912	2.36345	1.91072	2.72936	1.85263	2.13785	1.95746	1.7798	2.69186	1.86306	2.11006	0.847312766	0.966018836	12	16	24	0.851608661	0.955514806	24	8.073285551	0.050201392
ENSMUSG00000027966	Col11a1	collagen, type XI, alpha 1 [Source:MGI Symbol;Acc:MGI:88446]	9.8853	11.4879	12.38	10.4733	11.3446	12.6101	11.617	10.0044	12.0982	10.594	9.79212	10.6362	9.354	9.60999	0.956794505	0.96629412	12	16	24	0.779124081	0.928962679	24	12.47666725	0.032524378
ENSMUSG00000027968	Larp7	La ribonucleoprotein domain family, member 7 [Source:MGI Symbol;Acc:MGI:107634]	11.6309	11.9908	11.2337	11.3605	11.5892	12.2453	11.7105	11.5426	11.848	11.5479	12.225	12.3159	13.2834	13.0179	0.097975419	0.501188411	24	16	24	0.051023926	0.645887282	24	23.53542804	0.040665651
ENSMUSG00000027971	Ndst4	N-deacetylase/N-sulfotransferase (heparin glucosaminyl) 4 [Source:MGI Symbol;Acc:MGI:1932545]	9.75551	9.53846	7.01694	9.83858	7.68087	7.84069	9.98558	8.74732	7.02304	10.0437	8.8086	9.01389	9.08522	10.1171	0.595952838	0.966018836	4	8	24	0.622650012	0.87142241	24	23.97958831	0.064114239
ENSMUSG00000027977	Ndst3	N-deacetylase/N-sulfotransferase (heparan glucosaminyl) 3 [Source:MGI Symbol;Acc:MGI:1932544]	5.87759	5.43122	5.52636	5.62829	5.2976	5.50313	4.81935	5.14198	5.72834	5.14322	5.79395	5.96933	6.00236	5.70073	0.921572192	0.966018836	4	4	24	0.8937731	0.969947957	24	19.91483144	0.015124622
ENSMUSG00000027978	Prss12	protease, serine 12 neurotrypsin (motopsin) [Source:MGI Symbol;Acc:MGI:1100881]	11.5402	11.3613	9.57473	11.7034	13.1888	9.58641	14.0484	9.96497	12.2897	12.7122	10.6264	12.4008	13.2626	10.2069	0.648082424	0.966018836	4	4	24	0.7111491	0.904572639	24	18.86136391	0.057178651
ENSMUSG00000027981	Rnpc3	RNA-binding region (RNP1, RRM) containing 3 [Source:MGI Symbol;Acc:MGI:1914475]	16.0276	19.4719	17.8454	21.3055	18.3142	17.7327	18.2629	18.7458	22.7577	17.4543	21.4471	19.331	19.5383	18.766	0.890235289	0.966018836	12	16	24	0.271639061	0.768040178	24	10.66642207	0.075318458
ENSMUSG00000027983	Cyp2u1	cytochrome P450, family 2, subfamily u, polypeptide 1 [Source:MGI Symbol;Acc:MGI:1918769]	2.75409	1.96708	1.91499	2.43488	2.3281	2.45183	2.39677	2.63508	1.88929	2.48794	1.95046	2.32327	2.41603	2.47801	0.595952838	0.966018836	4	8	24	0.313796301	0.768040178	24	22.24210793	0.074352233
ENSMUSG00000027984	Hadh	hydroxyacyl-Coenzyme A dehydrogenase [Source:MGI Symbol;Acc:MGI:96009]	31.8249	35.0129	43.2457	60.5063	38.3811	36.449	38.6022	48.2216	44.9817	53.9938	48.9474	42.6561	55.7249	48.1259	0.510431574	0.966018836	16	8	24	0.038469867	0.61140397	24	10.60425266	0.202341443
ENSMUSG00000027985	Lef1	lymphoid enhancer binding factor 1 [Source:MGI Symbol;Acc:MGI:96770]	1.03775	0.840619	0.692276	1.0729	0.89368	0.79481	1.13898	1.20201	0.87774	0.908089	0.918599	0.824018	0.809738	0.764731	0.891742979	0.966018836	16	20	24	0.919120386	0.978264112	24	23.05991341	0.018201662
ENSMUSG00000027993	Trim2	tripartite motif-containing 2 [Source:MGI Symbol;Acc:MGI:1933163]	54.2829	49.1182	48.4068	49.179	48.8948	50.1385	49.5691	44.249	42.0946	47.3583	49.1695	48.52	48.6769	50.5043	0.286642595	0.793714531	4	8	24	0.088711419	0.707747429	24	20.83869158	0.067550576
ENSMUSG00000027994	Mcub	mitochondrial calcium uniporter dominant negative beta subunit [Source:MGI Symbol;Acc:MGI:1914065]	19.4551	14.7137	14.6292	16.5684	16.5092	15.9511	15.2419	16.4687	14.4605	15.9443	18.0842	16.6907	18.1668	15.0522	0.023858491	0.282503052	4	8	24	0.094343529	0.71175965	24	19.67261172	0.101018028
ENSMUSG00000027995	Tlr2	toll-like receptor 2 [Source:MGI Symbol;Acc:MGI:1346060]	0.444207	0.989466	1.19364	0.897215	0.920344	0.928133	0.950471	0.973567	1.22631	0.614194	0.997568	0.854315	0.838365	0.841667	0.286642595	0.793714531	12	16	24	0.416410187	0.801257196	24	8.91769695	0.085650303
ENSMUSG00000027997	Casp6	caspase 6 [Source:MGI Symbol;Acc:MGI:1312921]	4.60093	4.62362	4.67678	3.82887	5.57369	3.75897	3.4634	5.02867	4.34217	3.76385	4.48495	4.1494	4.10194	4.40381	0.532553009	0.966018836	8	16	24	0.881857621	0.966661503	24	8.068087703	0.030951486
ENSMUSG00000027998	Plrg1	pleiotropic regulator 1 [Source:MGI Symbol;Acc:MGI:1858197]	28.6816	30.8648	28.944	27.7256	28.7995	27.0889	26.5599	26.3869	29.4862	24.03	28.631	29.0604	29.0554	25.7818	0.879201926	0.966018836	12	4	24	0.817252434	0.941826666	24	22.58180365	0.022349011
ENSMUSG00000027999	Pla2g12a	phospholipase A2, group XIIA [Source:MGI Symbol;Acc:MGI:1913600]	10.9983	11.9052	11.7245	14.6174	11.3152	12.7824	11.3526	13.9723	12.9023	14.6882	12.3315	11.3357	13.3414	12.6238	0.226403091	0.722427815	16	4	24	0.12290774	0.735613551	24	9.924940565	0.09664347
ENSMUSG00000028004	Npy2r	neuropeptide Y receptor Y2 [Source:MGI Symbol;Acc:MGI:108418]	9.92901	8.16897	8.73625	7.53151	8.78989	9.83553	7.60846	8.2154	6.24665	7.95364	8.49613	8.04412	8.05197	8.15105	0.822170541	0.966018836	20	20	24	0.078753877	0.68925626	24	20.48170349	0.116959778
ENSMUSG00000028005	Gucy1b1	guanylate cyclase 1, soluble, beta 1 [Source:MGI Symbol;Acc:MGI:1860604]	2.11106	4.04633	4.67329	3.55292	2.94096	3.96034	4.1429	3.52363	4.17084	3.41902	3.07434	3.69394	2.41147	4.8617	0.166457038	0.633636735	12	8	24	0.211389755	0.768040178	24	7.676656026	0.181882837
ENSMUSG00000028007	Snx7	sorting nexin 7 [Source:MGI Symbol;Acc:MGI:1923811]	127.19	122.285	110.622	113.575	117.453	122.731	110.399	116.432	103.261	107.51	117.952	113.692	122.206	116.37	0.106781545	0.521876423	4	8	24	0.026523553	0.586541342	24	22.03243078	0.076499656
ENSMUSG00000028010	Gar1	GAR1 ribonucleoprotein [Source:MGI Symbol;Acc:MGI:1930948]	13.502	13.7856	14.7788	13.519	14.3847	14.1782	13.4485	13.5341	12.5035	13.4485	13.556	14.0062	12.2433	11.5796	0.521828922	0.966018836	24	4	24	0.071293389	0.676901434	24	16.79795466	0.057447106
ENSMUSG00000028013	Ppa2	pyrophosphatase (inorganic) 2 [Source:MGI Symbol;Acc:MGI:1922026]	33.4941	35.936	34.0193	34.2368	34.5455	34.4468	32.1599	33.6849	35.5501	35.3769	33.5634	36.8306	37.7134	38.9598	0.849180783	0.966018836	8	12	24	0.535537843	0.82702779	24	5.113162807	0.026335185
ENSMUSG00000028015	Ctso	cathepsin O [Source:MGI Symbol;Acc:MGI:2139628]	6.58351	9.36009	9.90368	7.82422	7.8355	7.08357	8.09026	7.70695	9.59891	7.77482	7.28799	7.91679	7.59164	8.95949	0.048889741	0.379129806	12	12	24	0.013515543	0.529134128	24	7.446443802	0.158794316
ENSMUSG00000028016	Ints12	integrator complex subunit 12 [Source:MGI Symbol;Acc:MGI:1919043]	5.14746	5.59017	4.88253	4.8024	5.57176	5.02467	4.85846	5.29761	4.95263	4.79914	5.35843	5.85495	5.42383	4.89876	0.142513744	0.585944911	20	20	24	0.229047047	0.768040178	24	20.70432624	0.053657437
ENSMUSG00000028017	Egf	epidermal growth factor [Source:MGI Symbol;Acc:MGI:95290]	0.929638	1.26133	0.99279	1.13953	1.08167	1.36297	1.34734	1.2975	1.87027	1.504	1.27792	1.564	1.12125	1.34212	0.883907406	0.966018836	24	4	24	0.664152643	0.886686094	24	9.729562469	0.047933724
ENSMUSG00000028018	Gstcd	glutathione S-transferase, C-terminal domain containing [Source:MGI Symbol;Acc:MGI:1914803]	2.33122	2.01352	2.15181	1.65704	2.30249	2.16757	1.83081	1.76858	1.86185	1.76014	1.68794	2.00073	2.08692	1.9825	0.351315142	0.870418653	24	16	24	0.190333838	0.768040178	24	22.33968993	0.070428249
ENSMUSG00000028019	Pdgfc	platelet-derived growth factor, C polypeptide [Source:MGI Symbol;Acc:MGI:1859631]	1.30014	1.40403	1.73605	1.60252	1.48691	1.62831	1.53102	1.43366	1.31353	1.66644	1.50199	1.59125	1.48101	1.3251	0.128810631	0.560209134	16	16	24	0.166859204	0.767020009	24	14.81769863	0.055235409
ENSMUSG00000028020	Glrb	glycine receptor, beta subunit [Source:MGI Symbol;Acc:MGI:95751]	66.2683	65.1567	66.0888	61.1132	57.0799	63.7361	65.2824	62.1759	56.2751	62.485	62.5241	58.79	59.6972	66.8158	0.418641555	0.964355874	8	12	24	0.257865312	0.768040178	24	2.51292014	0.047480506
ENSMUSG00000028024	Enpep	glutamyl aminopeptidase [Source:MGI Symbol;Acc:MGI:106645]	1.96251	2.02508	1.63699	1.60332	2.28794	1.63762	2.03661	2.35582	1.85283	1.88532	1.83622	1.62647	2.0252	2.11241	0.085992778	0.466404237	8	8	24	0.363761327	0.788663139	24	2.784558076	0.064912906
ENSMUSG00000028028	Alpk1	alpha-kinase 1 [Source:MGI Symbol;Acc:MGI:1918731]	2.71604	3.30639	3.56835	3.76143	3.97517	3.06217	2.71329	2.79445	4.18317	3.09453	3.30524	2.87987	3.06113	2.87602	0.004140921	0.140598674	12	16	24	0.013553576	0.529134128	24	11.6985943	0.1531623
ENSMUSG00000028029	Aimp1	aminoacyl tRNA synthetase complex-interacting multifunctional protein 1 [Source:MGI Symbol;Acc:MGI:102774]	44.0264	48.362	48.3337	47.0249	43.9221	45.1569	43.9242	47.007	54.4741	41.4157	53.5478	48.5565	49.8655	50.8796	0.849565901	0.966018836	12	4	24	0.48976086	0.805393068	24	6.997321506	0.04499963
ENSMUSG00000028030	Tbck	TBC1 domain containing kinase [Source:MGI Symbol;Acc:MGI:2445052]	5.21776	4.06607	4.45855	4.51521	4.71202	4.76849	5.17804	4.62462	3.92488	4.76873	4.52944	4.74039	4.76537	4.39494	0.004320961	0.140598674	4	8	24	0.03385609	0.606793499	24	20.55426618	0.088163461
ENSMUSG00000028032	Papss1	3'-phosphoadenosine 5'-phosphosulfate synthase 1 [Source:MGI Symbol;Acc:MGI:1330587]	35.9993	32.7086	34.9968	32.516	34.549	34.7711	36.2854	33.8963	30.4075	35.9333	30.2794	31.1662	31.8437	31.8197	0.904831057	0.966018836	4	16	24	0.747507571	0.917832057	24	22.26905018	0.023457909
ENSMUSG00000028033	Kcnq5	potassium voltage-gated channel, subfamily Q, member 5 [Source:MGI Symbol;Acc:MGI:1924937]	4.72931	3.61405	4.08423	3.7368	4.02676	4.26142	3.8935	3.92873	2.94786	4.77675	4.03946	4.05908	3.84038	4.17816	0.865233232	0.966018836	24	12	24	0.41004013	0.799546792	24	19.30422542	0.072531985
ENSMUSG00000028034	Fubp1	far upstream element (FUSE) binding protein 1 [Source:MGI Symbol;Acc:MGI:1196294]	32.6834	29.5844	30.1273	28.6855	28.935	26.4645	31.6452	26.5683	32.0642	24.8444	29.6253	30.7243	31.7843	28.7101	0.064091035	0.414867207	4	12	24	0.357231278	0.784327631	24	0.389541125	0.062086921
ENSMUSG00000028035	Dnajb4	DnaJ heat shock protein family (Hsp40) member B4 [Source:MGI Symbol;Acc:MGI:1914285]	33.9377	31.3862	29.1109	33.5345	31.8769	31.3731	33.6743	30.5201	29.0799	30.2193	35.6886	30.1705	31.5142	31.1701	0.058667689	0.405751803	4	8	24	0.18825469	0.768040178	24	19.26694005	0.060054499
ENSMUSG00000028036	Ptgfr	prostaglandin F receptor [Source:MGI Symbol;Acc:MGI:97796]	12.1221	13.3208	10.3962	10.5837	10.3699	9.87919	11.0368	10.3988	10.8871	9.14992	14.631	11.8734	13.4468	11.7548	0.305564675	0.811201458	4	12	24	0.418531196	0.803046926	24	23.33790937	0.089149071
ENSMUSG00000028037	Ifi44	interferon-induced protein 44 [Source:MGI Symbol;Acc:MGI:2443016]	1.08102	0.846585	1.39978	0.80929	1.15227	1.36119	1.20986	1.2905	0.99318	0.84892	2.01574	0.786234	0.774148	1.56724	0.866861748	0.966018836	8	20	24	0.977304069	0.994251213	24	15.72702458	0.019700006
ENSMUSG00000028039	Efna3	ephrin A3 [Source:MGI Symbol;Acc:MGI:106644]	1.08738	2.10696	3.02924	1.60001	1.28867	1.62282	1.85977	1.04476	1.85556	1.58882	1.40925	1.51689	0.956229	2.57313	0.300218476	0.809362476	12	8	24	0.190890277	0.768040178	24	7.895410424	0.213027779
ENSMUSG00000028041	Adam15	a disintegrin and metallopeptidase domain 15 (metargidin) [Source:MGI Symbol;Acc:MGI:1333882]	40.1521	37.1983	39.5806	29.6901	35.0932	34.7763	39.4799	33.9081	36.3605	34.5143	38.1699	41.9424	35.5303	32.2381	0.602156008	0.966018836	4	12	24	0.296386472	0.768040178	24	21.99697701	0.080877548
ENSMUSG00000028042	Zbtb7b	zinc finger and BTB domain containing 7B [Source:MGI Symbol;Acc:MGI:102755]	9.47728	8.14015	8.77096	9.54455	8.68016	8.47026	9.88515	9.77473	8.02736	8.35637	8.10473	8.90843	8.47058	8.65104	0.851656735	0.966018836	4	8	24	0.489977242	0.805393068	24	0.016904749	0.0413352
ENSMUSG00000028044	Cks1b	CDC28 protein kinase 1b [Source:MGI Symbol;Acc:MGI:1889208]	5.62533	6.61137	6.92106	6.50831	6.35059	5.79344	4.95015	5.3187	6.6888	4.90259	6.84805	6.77567	6.90623	5.94878	0.94086481	0.966018836	12	16	24	0.812549244	0.94007069	24	12.86389986	0.039093019
ENSMUSG00000028047	Thbs3	thrombospondin 3 [Source:MGI Symbol;Acc:MGI:98739]	12.7206	10.1405	11.4355	8.6582	11.6306	9.82606	11.1109	11.1589	12.4657	9.67032	10.4288	8.0492	10.8358	10.1022	0.433658121	0.966018836	12	4	24	0.439821236	0.805393068	24	4.045578959	0.08072928
ENSMUSG00000028048	Gba	glucosidase, beta, acid [Source:MGI Symbol;Acc:MGI:95665]	30.1519	26.1123	26.9482	24.6147	28.0638	27.986	26.0699	25.6476	22.8318	26.4482	24.1531	24.7484	25.4496	24.9918	0.904690479	0.966018836	4	8	24	0.112800595	0.727075283	24	21.20049083	0.06355159
ENSMUSG00000028049	Scamp3	secretory carrier membrane protein 3 [Source:MGI Symbol;Acc:MGI:1346346]	38.5379	30.2092	31.1798	29.5567	33.3337	37.2008	35.6681	36.7242	28.7572	38.9345	31.5693	34.4559	32.5294	29.7156	0.190044629	0.673646834	24	12	24	0.187852423	0.768040178	24	20.57450058	0.104331296
ENSMUSG00000028051	Hcn3	hyperpolarization-activated, cyclic nucleotide-gated K+ 3 [Source:MGI Symbol;Acc:MGI:1298211]	6.82717	4.31305	4.29922	5.1672	6.07055	5.58228	6.53403	5.66982	4.27049	6.78416	4.79747	5.9917	5.79139	4.84676	0.033248781	0.324908923	4	8	24	0.109157029	0.72082227	24	20.06911641	0.164705523
ENSMUSG00000028053	Ash1l	ASH1 like histone lysine methyltransferase [Source:MGI Symbol;Acc:MGI:2183158]	7.98727	8.282	8.25999	8.69849	8.05456	7.88608	8.39874	7.29446	8.54215	8.00223	7.93475	9.13912	8.47673	8.63949	0.816406072	0.966018836	16	4	24	0.994577216	0.998755922	24	14.53175141	0.002845801
ENSMUSG00000028057	Rit1	Ras-like without CAAX 1 [Source:MGI Symbol;Acc:MGI:108053]	61.1566	58.5043	57.2928	57.8584	61.2855	59.6859	55.9888	53.699	53.1841	48.0406	56.9835	54.357	56.5999	57.674	0.95032543	0.966018836	20	20	24	0.1655295	0.767020009	24	21.4073216	0.058552354
ENSMUSG00000028059	Arhgef2	rho/rac guanine nucleotide exchange factor (GEF) 2 [Source:MGI Symbol;Acc:MGI:103264]	6.80622	9.10372	10.0015	8.06978	7.83009	9.35229	11.6462	11.5851	11.5287	9.44071	9.76825	8.15147	7.8928	9.50377	0.443191651	0.966018836	12	16	24	0.216709065	0.768040178	24	6.991819396	0.143431027
ENSMUSG00000028060	Khdc4	KH domain containing 4, pre-mRNA splicing factor [Source:MGI Symbol;Acc:MGI:1921450]	14.9294	16.4984	17.063	16.8213	16.7592	15.3653	16.2603	15.2501	17.705	14.7603	16.8433	15.6398	16.0041	15.2147	0.193864198	0.67239758	12	16	24	0.337475837	0.772409896	24	11.08609248	0.0435168
ENSMUSG00000028062	Lamtor2	late endosomal/lysosomal adaptor, MAPK and MTOR activator 2 [Source:MGI Symbol;Acc:MGI:1932697]	64.4018	59.9677	66.2746	59.6873	60.8322	60.4807	60.6741	63.6139	61.1096	58.8762	65.0819	61.0169	68.1038	62.3508	0.47028201	0.966018836	4	12	24	0.52380041	0.821564372	24	1.581372302	0.02717072
ENSMUSG00000028063	Lmna	lamin A [Source:MGI Symbol;Acc:MGI:96794]	39.6654	32.0075	33.9091	33.1191	34.5081	31.8272	36.1853	38.8278	38.0674	42.0991	36.0976	37.8424	37.2478	34.9934	0.85387321	0.966018836	4	20	24	0.953143443	0.988579822	24	6.663993044	0.014147277
ENSMUSG00000028064	Sema4a	sema domain, immunoglobulin domain (Ig), transmembrane domain (TM) and short cytoplasmic domain, (semaphorin) 4A [Source:MGI Symbol;Acc:MGI:107560]	4.21305	5.40094	6.81908	5.55083	5.2779	4.96825	4.55393	5.74565	6.2889	5.52747	6.03671	4.93985	5.56721	4.85019	0.009118941	0.192760771	12	16	24	0.015549274	0.541053474	24	10.04747617	0.157520521
ENSMUSG00000028066	Pmf1	polyamine-modulated factor 1 [Source:MGI Symbol;Acc:MGI:1914287]	3.97603	5.99816	6.16695	5.05439	5.57755	5.79672	6.05987	7.37937	7.58965	6.25742	5.05946	5.55536	6.49914	6.17811	0.161023625	0.620479606	12	8	24	0.065090381	0.663876076	24	6.780686167	0.158964502
ENSMUSG00000028068	Iqgap3	IQ motif containing GTPase activating protein 3 [Source:MGI Symbol;Acc:MGI:3028642]	1.53955	1.31813	1.21591	1.20501	1.20711	1.52676	1.08398	1.52579	0.893001	1.60316	1.28874	1.43754	1.62466	1.21088	0.46238066	0.966018836	24	12	24	0.344904465	0.778238779	24	21.13997381	0.080204763
ENSMUSG00000028069	Gpatch4	G patch domain containing 4 [Source:MGI Symbol;Acc:MGI:1913864]	9.8318	9.43965	10.078	9.16097	9.16497	9.44164	10.2013	9.10749	9.21836	8.92827	8.41378	9.81089	9.30298	9.82613	0.048771079	0.379129806	4	16	24	0.077252448	0.687254899	24	1.521149495	0.053700147
ENSMUSG00000028070	Naxe	NAD(P)HX epimerase [Source:MGI Symbol;Acc:MGI:2180167]	48.8881	50.139	46.0777	52.7274	49.0559	49.4627	47.1316	48.8137	49.3727	48.3108	50.943	50.1931	50.647	50.6438	0.923635729	0.966018836	20	16	24	0.736821747	0.914236834	24	16.12443906	0.014386891
ENSMUSG00000028072	Ntrk1	neurotrophic tyrosine kinase, receptor, type 1 [Source:MGI Symbol;Acc:MGI:97383]	47.3582	35.1036	31.6265	41.4525	34.9139	42.623	41.8101	43.8026	27.2839	57.2575	40.6516	45.4336	37.9624	45.2124	0.390686537	0.915479136	24	12	24	0.545762164	0.833198211	24	19.74746073	0.11544307
ENSMUSG00000028073	Pear1	platelet endothelial aggregation receptor 1 [Source:MGI Symbol;Acc:MGI:1920432]	5.14755	6.08101	5.97033	6.30397	5.66089	5.46269	4.93479	6.78881	6.72463	5.3983	5.97917	5.8102	4.56376	5.96473	0.010214406	0.195026763	12	16	24	0.063934165	0.663876076	24	9.309789182	0.120579749
ENSMUSG00000028076	Cd1d1	CD1d1 antigen [Source:MGI Symbol;Acc:MGI:107674]	2.91757	3.59414	6.13042	9.48892	3.91552	3.68305	4.83173	5.70535	4.58221	6.09469	5.4018	4.80766	5.63887	5.86558	0.10951302	0.524035616	16	8	24	0.061420528	0.661066697	24	10.31890842	0.292040889
ENSMUSG00000028078	Dclk2	doublecortin-like kinase 2 [Source:MGI Symbol;Acc:MGI:1918012]	14.7271	13.0913	12.9195	13.6489	12.2694	13.2908	14.1788	12.4657	11.7767	16.8817	12.2591	13.4325	13.0751	13.6022	0.70641939	0.966018836	4	8	24	0.975511417	0.993775855	24	18.86575437	0.011462778
ENSMUSG00000028080	Lrba	LPS-responsive beige-like anchor [Source:MGI Symbol;Acc:MGI:1933162]	3.71768	4.51879	4.6146	4.09011	3.84408	3.66453	4.19909	4.44509	4.16408	4.49353	3.68599	4.00416	3.73508	4.08768	0.016811431	0.253395174	12	12	24	0.008843411	0.502965379	24	7.466028909	0.099156463
ENSMUSG00000028081	Rps3a1	ribosomal protein S3A1 [Source:MGI Symbol;Acc:MGI:1202063]	37.2015	48.6045	42.6851	44.6869	46.3001	41.2939	38.6579	42.097	53.1148	32.9968	46.8978	40.0318	43.0876	41.818	0.313786068	0.824470524	12	16	24	0.679380251	0.892679035	24	8.947300426	0.061214894
ENSMUSG00000028082	Sh3d19	SH3 domain protein D19 [Source:MGI Symbol;Acc:MGI:1350923]	13.0964	11.3733	10.5944	10.2853	13.2644	11.1009	13.0156	10.767	12.1284	12.3232	10.8537	10.8985	12.1001	11.4538	0.510431574	0.966018836	4	8	24	0.563228576	0.84250706	24	22.06202676	0.048956086
ENSMUSG00000028085	Gatb	glutamyl-tRNA(Gln) amidotransferase, subunit B [Source:MGI Symbol;Acc:MGI:2442496]	6.02745	5.6285	6.61082	6.08102	7.69204	5.0955	7.24834	7.13354	6.0675	6.48056	6.48693	5.99023	6.81954	6.72557	0.897425765	0.966018836	4	20	24	0.896895057	0.970634157	24	8.033532092	0.025805303
ENSMUSG00000028086	Fbxw7	F-box and WD-40 domain protein 7 [Source:MGI Symbol;Acc:MGI:1354695]	31.8403	28.2882	29.5014	29.0957	28.3952	30.8572	28.3816	27.7551	26.9644	26.4775	31.9212	30.2224	32.8298	29.9254	0.107220884	0.523582274	24	16	24	0.070361633	0.675025526	24	21.18700224	0.077952787
ENSMUSG00000028088	Fmo5	flavin containing monooxygenase 5 [Source:MGI Symbol;Acc:MGI:1310004]	4.33536	4.65978	4.30026	5.46326	4.87911	4.58098	5.58271	5.14145	4.62505	6.25525	4.42616	5.87995	5.49883	4.93515	0.907000607	0.966018836	16	20	24	0.87776879	0.965913727	24	15.67318917	0.026196257
ENSMUSG00000028089	Chd1l	chromodomain helicase DNA binding protein 1-like [Source:MGI Symbol;Acc:MGI:1915308]	3.54855	4.13951	3.80114	4.22324	3.9176	3.65413	3.77589	4.49457	3.87129	4.17123	3.6892	4.27076	4.00037	4.02215	0.483881213	0.966018836	16	12	24	0.410216312	0.799546792	24	7.93532665	0.04085029
ENSMUSG00000028093	Acp6	acid phosphatase 6, lysophosphatidic [Source:MGI Symbol;Acc:MGI:1931010]	9.6178	8.52017	8.90228	11.6919	9.92405	9.57074	10.1486	10.7391	9.49068	8.80419	11.1177	8.10116	8.62724	8.58876	0.918497267	0.966018836	20	4	24	0.443336417	0.805393068	24	14.23195717	0.068544455
ENSMUSG00000028096	Gpr89	G protein-coupled receptor 89 [Source:MGI Symbol;Acc:MGI:1914799]	11.8856	11.4981	12.0579	12.2255	12.6856	10.3181	10.1961	11.0818	10.6337	9.65364	11.722	11.6777	12.3889	10.8372	0.90767227	0.966018836	20	12	24	0.816910875	0.9417567	24	18.17274583	0.027864752
ENSMUSG00000028098	Rnf115	ring finger protein 115 [Source:MGI Symbol;Acc:MGI:1915095]	27.8002	27.2455	28.1062	27.7846	26.17	28.7128	27.9786	28.5302	25.7983	29.084	26.838	27.5303	27.4236	29.5169	0.93447422	0.966018836	8	12	24	0.628586264	0.872875873	24	3.18054123	0.01985586
ENSMUSG00000028099	Polr3c	polymerase (RNA) III (DNA directed) polypeptide C [Source:MGI Symbol;Acc:MGI:1921664]	8.31731	8.58934	8.83876	7.86712	8.22696	9.45186	7.46184	8.27157	7.60453	9.3769	8.28934	8.92418	8.48854	8.28778	0.830364743	0.966018836	24	12	24	0.663561753	0.886493109	24	17.34856532	0.03296529
ENSMUSG00000028100	Nudt17	nudix (nucleoside diphosphate linked moiety X)-type motif 17 [Source:MGI Symbol;Acc:MGI:1925623]	1.94831	2.14819	3.20827	2.28524	2.27438	2.81045	1.92704	2.81952	2.52828	2.25701	2.77064	3.27182	2.45067	2.8933	0.953760335	0.966018836	24	4	24	0.826282177	0.945708264	24	10.86898607	0.040084292
ENSMUSG00000028101	Pias3	protein inhibitor of activated STAT 3 [Source:MGI Symbol;Acc:MGI:1913126]	10.7183	11.939	12.1488	11.0763	13.6234	10.8522	12.0537	11.4569	12.7737	13.3881	11.2648	11.5286	12.0498	11.8502	0.718720526	0.966018836	12	12	24	0.25396801	0.768040178	24	10.57200529	0.061534892
ENSMUSG00000028102	Pex11b	peroxisomal biogenesis factor 11 beta [Source:MGI Symbol;Acc:MGI:1338882]	21.497	23.8604	23.6188	21.9039	21.9717	22.1833	23.0926	22.4135	23.2589	21.9735	20.1248	21.7375	21.542	25.8461	0.025980723	0.29944494	8	12	24	0.016196608	0.544099157	24	5.380644827	0.082852694
ENSMUSG00000028104	Polr3gl	polymerase (RNA) III (DNA directed) polypeptide G like [Source:MGI Symbol;Acc:MGI:1917120]	15.9451	16.7766	15.7538	16.1661	15.9492	14.6339	18.5476	14.7178	15.2926	16.739	15.8198	16.1913	14.9664	15.7969	0.874607729	0.966018836	16	20	24	0.988006111	0.997530201	24	19.79348018	0.005387358
ENSMUSG00000028106	Rprd2	regulation of nuclear pre-mRNA domain containing 2 [Source:MGI Symbol;Acc:MGI:1922387]	7.53657	7.59354	8.38769	8.47894	7.28098	7.38768	7.65043	7.58252	7.8269	9.80315	7.82614	8.17056	7.71805	8.48503	0.151535314	0.599319068	16	4	24	0.054288212	0.654887447	24	10.36621665	0.085138524
ENSMUSG00000028107	Tars2	threonyl-tRNA synthetase 2, mitochondrial (putative) [Source:MGI Symbol;Acc:MGI:1919057]	11.3541	10.398	10.5818	10.6129	11.4035	12.4215	11.1398	11.7335	10.7232	12.5565	10.4579	10.5207	11.5918	10.7806	0.888482515	0.966018836	24	12	24	0.728885739	0.91134586	24	19.48655749	0.027644552
ENSMUSG00000028108	Ecm1	extracellular matrix protein 1 [Source:MGI Symbol;Acc:MGI:103060]	13.8023	15.2276	15.568	13.2927	15.9332	12.1938	16.161	16.7262	19.6301	11.6578	16.9524	15.3714	15.5389	12.6447	0.919571723	0.966018836	12	12	24	0.836934866	0.950079249	24	5.526584552	0.04791359
ENSMUSG00000028111	Ctsk	cathepsin K [Source:MGI Symbol;Acc:MGI:107823]	9.5228	13.8307	15.4135	13.5993	11.5256	12.4928	9.65773	14.3762	16.4647	10.5027	12.8886	11.5497	12.9731	9.77897	0.053683798	0.394331071	12	16	24	0.197492176	0.768040178	24	9.194405447	0.16443768
ENSMUSG00000028114	Mettl14	methyltransferase like 14 [Source:MGI Symbol;Acc:MGI:2442926]	12.9931	11.6656	10.8603	11.6711	10.9948	12.4277	12.1277	11.6225	10.8569	12.4193	11.3501	12.5478	13.1977	12.7118	0.004501001	0.140598674	4	8	24	0.038125546	0.61140397	24	22.88920326	0.076071309
ENSMUSG00000028116	Myoz2	myozenin 2 [Source:MGI Symbol;Acc:MGI:1913063]	2.69924	8.23344	12.1304	11.4581	8.13947	9.67242	6.13908	14.656	8.5972	11.221	5.83204	2.63034	9.45546	7.91057	0.117105047	0.538362324	12	16	24	0.072162226	0.678728491	24	8.883103693	0.543209178
ENSMUSG00000028121	Bcar3	breast cancer anti-estrogen resistance 3 [Source:MGI Symbol;Acc:MGI:1352501]	13.3048	11.1705	9.15452	10.9671	12.1631	11.6083	13.5142	12.3868	9.90031	13.6774	11.3002	11.6534	11.6724	11.5041	0.03452758	0.328311403	4	8	24	0.219112543	0.768040178	24	21.06296401	0.102015061
ENSMUSG00000028124	Gclm	glutamate-cysteine ligase, modifier subunit [Source:MGI Symbol;Acc:MGI:104995]	48.8771	39.9148	40.1245	43.4505	43.0183	46.6632	48.095	43.7071	37.1315	47.3396	43.8797	48.6714	49.3187	46.7865	8.66E-04	0.071344007	4	8	24	0.01925411	0.562219749	24	21.12573096	0.116676821
ENSMUSG00000028126	Pip5k1a	phosphatidylinositol-4-phosphate 5-kinase, type 1 alpha [Source:MGI Symbol;Acc:MGI:107929]	6.80965	6.76566	7.62615	7.44874	6.8397	6.95037	6.25138	6.95577	6.75921	7.26211	7.15687	7.16054	7.57365	7.17304	0.865233232	0.966018836	16	12	24	0.345199404	0.778298718	24	11.28185015	0.037139948
ENSMUSG00000028127	Abcd3	ATP-binding cassette, sub-family D (ALD), member 3 [Source:MGI Symbol;Acc:MGI:1349216]	35.5498	32.7513	34.7909	38.0682	35.1842	32.7741	34.0557	33.3818	31.8595	35.915	35.6386	34.5494	34.9926	34.6486	0.039590247	0.338535904	16	16	24	0.230353998	0.768040178	24	14.71280002	0.041252585
ENSMUSG00000028128	F3	coagulation factor III [Source:MGI Symbol;Acc:MGI:88381]	15.5583	15.0512	15.0323	18.2001	14.2664	14.6833	16.2957	12.8257	13.8027	15.9089	14.2698	12.9667	12.4302	11.9953	0.743750765	0.966018836	16	16	24	0.146618168	0.755260456	24	13.92894722	0.083369018
ENSMUSG00000028132	Tlcd4	TLC domain containing 4 [Source:MGI Symbol;Acc:MGI:1923195]	25.4612	25.2512	23.672	25.8946	24.5007	23.2771	24.5934	22.158	23.1331	24.5059	26.1427	22.8332	23.7295	24.6962	0.93717134	0.966018836	20	4	24	0.609911366	0.864608237	24	14.91792763	0.025743301
ENSMUSG00000028133	Rwdd3	RWD domain containing 3 [Source:MGI Symbol;Acc:MGI:1920420]	3.05411	4.25136	3.90246	3.56372	3.30142	3.76909	3.04434	3.9637	3.86782	4.7945	3.50473	4.02177	4.5744	4.5646	0.581070934	0.966018836	8	12	24	0.155060434	0.760294912	24	7.375438624	0.10421962
ENSMUSG00000028134	Ptbp2	polypyrimidine tract binding protein 2 [Source:MGI Symbol;Acc:MGI:1860489]	54.533	62.2298	57.214	50.5357	55.5011	55.1528	54.2477	55.8409	62.4346	49.7663	57.5041	55.4211	55.5041	56.5833	0.112583132	0.530777648	12	4	24	0.336061195	0.771666944	24	4.128872709	0.050872464
ENSMUSG00000028136	Snx27	sorting nexin family member 27 [Source:MGI Symbol;Acc:MGI:1923992]	19.8475	17.4655	18.0233	18.5616	17.3514	19.0069	20.1801	17.6987	16.3526	21.3013	16.6393	19.2903	19.0901	19.1996	0.241264473	0.741580974	4	8	24	0.571861835	0.846185279	24	22.1704963	0.0443057
ENSMUSG00000028137	Celf3	CUGBP, Elav-like family member 3 [Source:MGI Symbol;Acc:MGI:1926034]	8.77355	6.79815	6.58375	8.20429	7.75042	8.60154	7.50432	9.48325	8.04263	9.33453	7.72464	9.412	6.0162	10.3879	0.881996584	0.966018836	8	12	24	0.972152727	0.993238187	24	13.19289425	0.018240207
ENSMUSG00000028138	Adh5	alcohol dehydrogenase 5 (class III), chi polypeptide [Source:MGI Symbol;Acc:MGI:87929]	49.5638	52.0946	50.5749	50.1623	54.1622	48.4105	45.1888	47.6676	52.8933	42.7509	51.8412	48.1624	51.2307	47.3165	0.767183862	0.966018836	20	4	24	0.921367066	0.979266762	24	14.46122019	0.014189167
ENSMUSG00000028139	Riiad1	regulatory subunit of type II PKA R-subunit (RIIa) domain containing 1 [Source:MGI Symbol;Acc:MGI:1913603]	0.953992	1.64729	1.9499	1.16877	1.08016	1.01179	1.72978	1.00737	2.09533	1.15713	0.965185	0.7669	0.689275	1.54791	0.010049466	0.194720062	12	12	24	0.026687531	0.586541342	24	7.367264045	0.257992805
ENSMUSG00000028140	Mrpl9	mitochondrial ribosomal protein L9 [Source:MGI Symbol;Acc:MGI:2137211]	27.3828	29.6859	29.6081	30.3817	26.9284	26.8951	27.6807	29.7179	28.6666	27.8399	30.689	30.2673	30.0981	28.5384	0.847846021	0.966018836	8	20	24	0.618615917	0.868995186	24	8.328952446	0.025671321
ENSMUSG00000028145	Them4	thioesterase superfamily member 4 [Source:MGI Symbol;Acc:MGI:1923028]	28.8078	27.9398	26.2578	25.9304	30.0126	26.1378	28.7125	25.4614	30.3195	25.2857	32.3762	28.0847	30.0687	27.3224	0.351315142	0.870418653	20	20	24	0.542598537	0.830991507	24	19.57182121	0.046928077
ENSMUSG00000028148	Them5	thioesterase superfamily member 5 [Source:MGI Symbol;Acc:MGI:1913448]	18.9397	18.2127	14.3709	13.8244	22.0051	17.147	19.5831	19.2401	16.8558	12.3472	18.2207	16.2398	18.0959	14.9358	0.059513104	0.406067671	4	12	24	0.075682967	0.68634302	24	22.09513363	0.178351247
ENSMUSG00000028149	Rap1gds1	RAP1, GTP-GDP dissociation stimulator 1 [Source:MGI Symbol;Acc:MGI:2385189]	153.313	114.833	117.509	122.972	124.673	139.447	134.021	131.533	98.5875	158.05	114.77	133.265	130.91	124.436	0.068712455	0.432567661	24	12	24	0.271014805	0.768040178	24	20.63984103	0.112653238
ENSMUSG00000028150	Rorc	RAR-related orphan receptor gamma [Source:MGI Symbol;Acc:MGI:104856]	2.21803	2.01938	3.12794	4.22648	3.2795	3.22969	3.05639	3.21199	2.84229	3.90668	4.2388	3.1845	3.45716	3.15803	0.037037369	0.334064683	16	20	24	0.006475095	0.470633744	24	13.9635008	0.202900314
ENSMUSG00000028152	Tspan5	tetraspanin 5 [Source:MGI Symbol;Acc:MGI:1928096]	34.3944	24.0053	23.8486	25.6672	25.5501	28.855	26.7737	26.9633	22.8887	31.2035	25.6576	28.8211	27.9958	27.2455	0.038115943	0.334064441	24	12	24	0.174533146	0.768040178	24	20.99808005	0.111289016
ENSMUSG00000028156	Eif4e	eukaryotic translation initiation factor 4E [Source:MGI Symbol;Acc:MGI:95305]	35.4786	35.3363	31.2091	33.4114	39.3386	34.6867	33.7805	32.1571	27.9211	26.3765	36.6693	23.14	25.1313	25.396	0.89563591	0.966018836	20	16	24	0.346563202	0.778844143	24	17.4792352	0.093190016

ENSMUSG00000028158	Mttp	microsomal triglyceride transfer protein [Source:MGI Symbol;Acc:MGI:106926]	2.31036	2.44216	2.5709	2.51656	2.06742	2.26835	2.4986	2.05396	2.15646	2.17408	2.25746	2.14813	2.04508	2.28649	0.897425765	0.966018836	16	4	24	0.723258978	0.909188168	24	7.300005706	0.022071828
ENSMUSG00000028159	Dapp1	dual adaptor for phosphotyrosine and 3-phosphoinositides 1 [Source:MGI Symbol;Acc:MGI:1347063]	0.905485	1.29088	1.73276	1.26713	1.06201	1.03642	1.45875	1.11277	1.26786	0.848814	0.927177	1.05762	0.905249	0.924662	0.588208558	0.966018836	12	16	24	0.347025533	0.778844143	24	7.137104448	0.083655507
ENSMUSG00000028161	Ppp3ca	protein phosphatase 3, catalytic subunit, alpha isoform [Source:MGI Symbol;Acc:MGI:107164]	196.34	169.435	173.129	177.735	162.568	190.219	170.256	171.652	153.632	208.599	175.704	191.42	192.425	186.858	0.622132989	0.966018836	24	12	24	0.532113652	0.825574717	24	20.30392494	0.053441524
ENSMUSG00000028163	Nfkb1	nuclear factor of kappa light polypeptide gene enhancer in B cells 1, p105 [Source:MGI Symbol;Acc:MGI:97312]	12.8002	12.5269	12.331	12.3972	13.5831	12.9124	16.2147	12.213	12.1501	12.6241	12.6051	12.5614	12.5859	12.021	0.023410118	0.278499043	4	4	24	0.239833458	0.768040178	24	20.85800983	0.066811665
ENSMUSG00000028164	Manba	mannosidase, beta A, lysosomal [Source:MGI Symbol;Acc:MGI:88175]	3.26336	3.51069	4.13008	3.38937	3.64386	3.64892	3.66296	3.89893	3.78605	3.3916	3.3191	3.61279	3.08826	3.78188	0.184044621	0.662239322	12	4	24	0.305575865	0.768040178	24	7.257692441	0.053622615
ENSMUSG00000028165	Cisd2	CDGSH iron sulfur domain 2 [Source:MGI Symbol;Acc:MGI:1914256]	19.67	19.1481	19.2718	20.1319	19.4679	19.0018	20.0329	18.5629	17.8224	18.4942	19.5347	16.8641	16.7807	17.7692	0.933543173	0.966018836	20	4	24	0.411802284	0.799546792	24	14.60802275	0.02845455
ENSMUSG00000028167	Bdh2	3-hydroxybutyrate dehydrogenase, type 2 [Source:MGI Symbol;Acc:MGI:1917022]	2.35887	3.47484	3.04505	2.19446	2.62439	2.32416	3.47655	2.93982	4.04784	1.64093	2.0765	3.01291	2.90144	3.78061	0.007049678	0.188070711	12	4	24	0.025337175	0.586541342	24	3.753224954	0.248028293
ENSMUSG00000028172	Tacr3	tachykinin receptor 3 [Source:MGI Symbol;Acc:MGI:892968]	0.362757	0.559765	0.801718	0.482372	0.389464	0.603395	0.766247	0.306514	0.572252	0.500876	0.379946	0.508931	0.291735	0.609116	0.930944473	0.966018836	12	8	24	0.685147497	0.895310095	24	7.286986333	0.053013739
ENSMUSG00000028173	Wls	wntless WNT ligand secretion mediator [Source:MGI Symbol;Acc:MGI:1915401]	20.0407	22.6906	20.5783	20.2166	18.9951	18.9179	21.2335	21.2514	21.5054	18.6674	19.367	20.7557	18.2248	20.9063	0.059513104	0.406067671	8	12	24	0.054718121	0.655263328	24	4.945773725	0.073417864
ENSMUSG00000028179	Cth	cystathionase (cystathionine gamma-lyase) [Source:MGI Symbol;Acc:MGI:1339968]	0.390405	0.592319	0.626398	0.794315	0.519825	0.518087	0.766705	0.517003	0.45437	0.427719	0.562702	0.416689	0.446182	0.602529	0.699015352	0.966018836	12	12	24	0.750983798	0.91921305	24	8.941533598	0.034690631
ENSMUSG00000028180	Zranb2	zinc finger, RAN-binding domain containing 2 [Source:MGI Symbol;Acc:MGI:1858211]	79.3038	90.3599	86.8884	79.7842	79.9259	78.6347	77.7388	75.9167	82.4013	72.9723	87.1417	73.0981	72.3776	82.4491	0.43280837	0.966018836	8	20	24	0.445476088	0.805393068	24	8.304960425	0.046124097
ENSMUSG00000028182	Lrriq3	leucine-rich repeats and IQ motif containing 3 [Source:MGI Symbol;Acc:MGI:1921685]	0.893388	1.06195	1.074	0.651176	0.901823	0.890079	1.013	0.629208	1.1158	0.878079	0.871158	0.799552	0.996167	0.824717	0.44861185	0.966018836	12	4	24	0.785610758	0.931158434	24	3.787055887	0.031357284
ENSMUSG00000028184	Adgrl2	adhesion G protein-coupled receptor L2 [Source:MGI Symbol;Acc:MGI:2139714]	7.25151	7.11132	8.13545	5.80255	6.22993	6.83205	8.21905	7.64298	7.18817	8.01843	6.38893	7.84541	6.74168	7.55924	0.615655369	0.966018836	12	8	24	0.197268243	0.768040178	24	3.671478261	0.090352901
ENSMUSG00000028187	Rpf1	ribosome production factor 1 homolog [Source:MGI Symbol;Acc:MGI:1917535]	10.84	11.1869	11.3326	9.84176	11.7849	10.5082	11.9349	10.6851	12.1633	9.32409	11.8515	11.6769	11.7589	11.4911	0.945282252	0.966018836	20	20	24	0.458957045	0.805393068	24	0.020299454	0.049032018
ENSMUSG00000028188	Spata1	spermatogenesis associated 1 [Source:MGI Symbol;Acc:MGI:1918201]	5.35853	3.77688	3.76442	4.86107	5.01855	3.65711	5.85091	4.69024	5.25714	4.26965	5.35005	4.33368	5.21051	3.11067	0.481811994	0.966018836	4	4	24	0.596489847	0.857839743	24	18.62506598	0.090200111
ENSMUSG00000028189	Ctbs	chitobiase [Source:MGI Symbol;Acc:MGI:1921495]	3.67766	4.69332	4.32267	3.91325	4.63803	3.90039	4.68736	4.57386	4.69212	4.1669	3.92531	3.88078	4.78378	4.63743	0.17882595	0.655480352	8	16	24	0.099666436	0.71175955	24	5.258153132	0.085172426
ENSMUSG00000028191	Bcl10	B cell leukemia/lymphoma 10 [Source:MGI Symbol;Acc:MGI:1337994]	10.3049	9.3738	9.25514	8.92233	8.89929	9.33182	9.7132	9.59729	9.21387	9.59449	10.4722	9.85114	10.6236	9.56158	0.026323104	0.301028315	4	8	24	0.115826791	0.727799063	24	22.7057673	0.051588001
ENSMUSG00000028194	Ddah1	dimethylarginine dimethylaminohydrolase 1 [Source:MGI Symbol;Acc:MGI:1916469]	26.6585	23.1291	24.3047	23.2556	27.188	25.3179	29.1365	23.5876	22.8337	28.1698	22.3835	24.1934	23.4773	22.7375	0.723119329	0.966018836	4	4	24	0.419949467	0.803803125	24	19.2953668	0.061942812
ENSMUSG00000028195	Ccn1	cellular communication network factor 1 [Source:MGI Symbol;Acc:MGI:88613]	4.52455	4.40992	4.59866	4.30499	4.04637	3.69021	4.40801	6.23117	5.06863	6.73758	4.52269	4.81212	4.94717	3.71632	0.904387595	0.966018836	12	12	24	0.436419943	0.805393068	24	8.699854886	0.10531891
ENSMUSG00000028197	Col24a1	collagen, type XXIV, alpha 1 [Source:MGI Symbol;Acc:MGI:1918605]	11.7193	12.6302	10.0826	13.3937	12.9423	10.1823	12.0174	11.4303	13.7175	10.7974	12.2955	11.24	11.0111	10.4144	0.909604019	0.966018836	20	4	24	0.615820204	0.867241036	24	12.49181597	0.050278027
ENSMUSG00000028199	Cryz	crystallin, zeta [Source:MGI Symbol;Acc:MGI:88527]	4.99671	5.13785	5.43816	6.61833	4.49053	5.27042	4.92946	4.78341	5.59853	4.83399	4.56358	5.02135	5.60444	5.52507	0.581070934	0.966018836	12	8	24	0.424717718	0.805393068	24	7.519489773	0.065527141
ENSMUSG00000028207	Asph	aspartate-beta-hydroxylase [Source:MGI Symbol;Acc:MGI:1914186]	38.806	39.7889	40.2917	41.7841	36.5325	40.2961	40.5056	41.6158	38.0899	42.4757	37.1958	38.6133	38.6377	40.7028	0.087978327	0.472021905	16	4	24	0.305137249	0.768040178	24	6.697065376	0.03867433
ENSMUSG00000028211	Trp53inp1	transformation related protein 53 inducible nuclear protein 1 [Source:MGI Symbol;Acc:MGI:1926609]	8.47739	10.2684	10.0049	10.8003	9.83132	8.53938	9.40971	9.45243	10.9202	8.71132	10.5423	8.98272	8.08634	8.69153	0.016795343	0.253395174	12	16	24	0.051463599	0.64767123	24	10.80733802	0.108171275
ENSMUSG00000028212	Ccne2	cyclin E2 [Source:MGI Symbol;Acc:MGI:1329034]	2.41022	2.87735	3.52294	3.35536	3.22806	2.93132	3.02316	2.99375	3.08815	2.80201	3.61538	3.68431	2.5084	2.26262	0.084879076	0.466404237	20	8	24	0.07423396	0.683293901	24	14.45983452	0.130208338
ENSMUSG00000028214	Gem	GTP binding protein (gene overexpressed in skeletal muscle) [Source:MGI Symbol;Acc:MGI:99844]	0.881219	0.847775	1.3566	0.723101	0.840852	0.851774	1.07696	1.27435	1.00029	0.908485	0.830804	1.13307	0.999302	1.13079	0.120632237	0.547424243	12	4	24	0.1032996	0.715079435	24	4.379790915	0.10012643
ENSMUSG00000028218	Cibar1	CBY1 interacting BAR domain containing 1 [Source:MGI Symbol;Acc:MGI:1915349]	30.0271	37.0594	33.189	33.017	36.5186	30.3677	34.442	31.2604	38.6092	29.3387	39.429	32.4127	35.0069	37.2577	0.800065124	0.966018836	12	12	24	0.760716337	0.922661478	24	8.490833281	0.040569771
ENSMUSG00000028221	Pip4p2	phosphatidylinositol-4,5-bisphosphate 4-phosphatase 2 [Source:MGI Symbol;Acc:MGI:1919769]	37.0488	30.7797	29.4886	31.2485	30.7671	34.1424	30.9625	30.8097	25.8988	31.2817	32.2702	26.9517	26.5809	30.5399	0.542718076	0.966018836	16	20	24	0.410611433	0.799546792	24	19.77918148	0.061646576
ENSMUSG00000028222	Calb1	calbindin 1 [Source:MGI Symbol;Acc:MGI:88248]	14.4817	13.8031	10.8951	12.652	10.7722	11.8884	12.1093	11.7653	11.0287	13.8353	11.2995	12.744	13.3244	13.3299	0.238201401	0.741459408	4	16	24	0.357540967	0.784480156	24	1.050001902	0.071865898
ENSMUSG00000028223	Decr1	2,4-dienoyl CoA reductase 1, mitochondrial [Source:MGI Symbol;Acc:MGI:1914710]	21.1759	21.7445	23.8963	30.4514	23.6847	21.7827	23.3657	26.9434	24.2198	24.8089	27.0567	23.8632	27.738	27.4074	0.668329321	0.966018836	16	8	24	0.214854442	0.768040178	24	10.26409031	0.087224885
ENSMUSG00000028224	Nbn	nibrin [Source:MGI Symbol;Acc:MGI:1351625]	10.6073	11.1397	10.0175	10.4251	10.7673	10.2945	10.5779	9.09894	10.2589	9.32176	11.6073	10.7299	11.1893	10.742	0.435739761	0.966018836	20	20	24	0.321817117	0.768040178	24	20.31156742	0.052860448
ENSMUSG00000028226	Mmp16	matrix metallopeptidase 16 [Source:MGI Symbol;Acc:MGI:1276107]	1.23516	1.6392	1.95742	1.6588	1.7366	1.6903	1.5517	1.23752	1.50338	1.2715	1.98731	1.88227	1.4515	1.77887	0.17882595	0.655480352	20	8	24	0.459544123	0.805393068	24	15.2852906	0.068296286
ENSMUSG00000028228	Cpne3	copine III [Source:MGI Symbol;Acc:MGI:1917818]	67.7075	60.9601	56.4543	58.676	59.9844	59.9574	56.0614	52.7637	52.1629	55.4392	62.2066	57.4537	62.3905	59.6133	0.257978335	0.761738864	4	8	24	0.107932443	0.72082227	24	20.72035337	0.078104932
ENSMUSG00000028229	Rmdn1	regulator of microtubule dynamics 1 [Source:MGI Symbol;Acc:MGI:1913552]	9.37686	10.5908	9.85193	9.61457	11.1628	9.19838	11.7947	11.2762	11.8469	10.8407	13.2072	11.8157	12.1001	12.4346	0.860560779	0.966018836	8	8	24	0.912329747	0.975295827	24	5.511177172	0.016323003
ENSMUSG00000028232	Tmem68	transmembrane protein 68 [Source:MGI Symbol;Acc:MGI:1919348]	12.5664	12.0215	11.2163	11.3354	12.5673	10.2685	11.4593	8.94516	10.2488	9.6485	11.9488	10.3882	9.85678	10.2573	0.628286596	0.966018836	20	12	24	0.304581713	0.768040178	24	18.04914384	0.067795702
ENSMUSG00000028233	Tgs1	trimethylguanosine synthase 1 [Source:MGI Symbol;Acc:MGI:2151797]	8.00906	8.11121	8.01968	8.74368	9.04987	7.84589	7.49511	7.78841	8.69903	7.06326	9.09372	8.01621	8.49694	8.562	0.251986462	0.75129457	20	8	24	0.666762273	0.887459929	24	14.0266192	0.032856069
ENSMUSG00000028234	Rps20	ribosomal protein S20 [Source:MGI Symbol;Acc:MGI:1914677]	79.2264	95.0622	89.5257	90.0405	90.9175	90.6152	77.4048	85.261	102.011	64.4267	98.8639	81.4912	89.1893	77.2569	0.65208848	0.966018836	20	8	24	0.951644172	0.988339231	24	12.73632152	0.021243489
ENSMUSG00000028243	Ubxn2b	UBX domain protein 2B [Source:MGI Symbol;Acc:MGI:1915303]	4.81505	4.44504	4.17238	4.3167	4.47564	4.60336	4.4941	4.09186	3.81796	3.3084	4.85056	4.32801	4.23718	3.98029	0.027439001	0.308438477	20	20	24	0.015377843	0.53936769	24	20.76422672	0.109498616
ENSMUSG00000028245	Nsmaf	neutral sphingomyelinase (N-SMase) activation associated factor [Source:MGI Symbol;Acc:MGI:1341864]	21.8968	27.7377	26.6162	27.2992	28.2204	25.9692	26.8908	27.2354	27.1297	21.8831	24.8465	26.1746	25.8064	24.9095	0.891742979	0.966018836	8	20	24	0.939174413	0.985025893	24	7.113515901	0.016358435
ENSMUSG00000028246	Faxc	failed axon connections homolog [Source:MGI Symbol;Acc:MGI:1923382]	24.1348	19.3639	18.8443	19.907	20.6305	21.8353	18.7007	18.4925	15.3949	23.5364	19.6608	22.2007	21.7681	22.0372	0.245061943	0.741580974	24	12	24	0.203912069	0.768040178	24	19.92487056	0.113377354
ENSMUSG00000028247	Coq3	coenzyme Q3 methyltransferase [Source:MGI Symbol;Acc:MGI:101813]	12.509	12.8957	12.3617	12.9773	11.8365	11.8599	10.9172	11.7366	11.8833	10.7753	13.5876	12.1108	12.6431	14.3133	0.845386467	0.966018836	8	16	24	0.861309634	0.959280176	24	5.156271313	0.022886736
ENSMUSG00000028248	Pnisr	PNN interacting serine/arginine-rich [Source:MGI Symbol;Acc:MGI:1913875]	70.5012	87.3075	83.184	80.8365	83.4911	74.0565	85.7989	70.2207	90.38	64.5957	86.2165	74.386	73.8106	81.2265	0.930944473	0.966018836	12	16	24	0.77880442	0.928873919	24	8.694781816	0.041842018
ENSMUSG00000028249	Sdcbp	syndecan binding protein [Source:MGI Symbol;Acc:MGI:1337026]	369.261	359.842	326.245	339.131	351.111	331.711	319.27	319.025	340.624	299.145	382.209	342.543	378.211	344.502	0.676344318	0.966018836	20	20	24	0.448623205	0.805393068	24	21.29498349	0.051551641
ENSMUSG00000028251	Tstd3	thiosulfate sulfurtransferase (rhodanese)-like domain containing 3 [Source:MGI Symbol;Acc:MGI:1924282]	20.4345	22.6772	19.5177	20.0234	18.8895	18.9777	19.8593	19.0893	19.964	20.1153	21.1332	22.2439	23.4754	21.6928	0.92655264	0.966018836	8	4	24	0.380826801	0.799546792	24	1.208729552	0.046444951
ENSMUSG00000028252	Ccnc	cyclin C [Source:MGI Symbol;Acc:MGI:1858199]	34.4999	33.6232	33.0028	31.0443	31.2053	29.0315	30.6574	27.4665	30.9779	25.7305	35.0151	30.8726	31.2084	33.4037	0.903465533	0.966018836	4	12	24	0.760246815	0.922505796	24	0.008638365	0.035072276
ENSMUSG00000028256	Odf2l	outer dense fiber of sperm tails 2-like [Source:MGI Symbol;Acc:MGI:1098600]	10.0556	11.9666	16.6772	13.6713	12.7635	11.7856	11.0608	12.6633	13.3772	11.7566	10.9225	11.2038	12.2553	11.7743	0.051446973	0.38924256	12	16	24	0.034302999	0.608494889	24	9.278574224	0.151843707
ENSMUSG00000028261	Ndufaf4	NADH:ubiquinone oxidoreductase complex assembly factor 4 [Source:MGI Symbol;Acc:MGI:1915743]	16.7803	19.2693	19.1153	19.2993	17.5716	17.2511	15.4545	17.4385	17.7865	15.2295	21.5844	16.9899	17.957	18.4991	0.360033827	0.883787544	20	8	24	0.648987789	0.881754555	24	10.92007979	0.047090105
ENSMUSG00000028266	Lmo4	LIM domain only 4 [Source:MGI Symbol;Acc:MGI:109360]	27.4835	27.8738	28.8814	26.8576	26.678	24.3302	26.5006	26.3289	34.361	27.4163	27.3693	30.8659	27.8957	28.6855	0.44861185	0.966018836	12	8	24	0.262565023	0.768040178	24	7.794083311	0.07096711
ENSMUSG00000028268	Gbp3	guanylate binding protein 3 [Source:MGI Symbol;Acc:MGI:1926263]	7.2881	8.69319	8.0544	7.60828	10.441	6.19574	5.93372	11.1145	9.90372	6.69321	8.20153	6.29131	6.13822	10.172	0.020428812	0.261278501	8	20	24	0.141355975	0.751766185	24	8.075831978	0.21486035
ENSMUSG00000028270	Gbp2	guanylate binding protein 2 [Source:MGI Symbol;Acc:MGI:102772]	8.25354	10.0212	8.03906	6.71558	14.215	6.51533	7.15779	14.7603	10.598	7.03361	9.69221	7.76405	7.04824	13.2233	0.163587268	0.626886304	8	16	24	0.521234298	0.82025681	24	6.478560054	0.173089493
ENSMUSG00000028271	Gtf2b	general transcription factor IIB [Source:MGI Symbol;Acc:MGI:2385191]	33.95	36.8998	36.1739	34.8846	34.969	33.9152	33.4177	34.7024	37.6156	31.5509	36.996	35.651	37.6359	34.3165	0.923635729	0.966018836	12	12	24	0.852126803	0.955622342	24	5.277404098	0.016388977
ENSMUSG00000028273	Pdlim5	PDZ and LIM domain 5 [Source:MGI Symbol;Acc:MGI:1927489]	20.3921	23.573	24.407	27.0169	24.0074	26.4696	23.5027	37.1752	27.6652	29.7352	22.9275	21.3458	25.3933	22.2803	0.251760751	0.75129457	16	12	24	0.286333748	0.768040178	24	8.748196811	0.134887341
ENSMUSG00000028274	Rngtt	RNA guanylyltransferase and 5'-phosphatase [Source:MGI Symbol;Acc:MGI:1329041]	9.55495	8.37858	8.45438	8.37392	8.03552	8.42047	8.43127	8.59846	7.07431	7.7385	8.42769	8.95226	8.99703	9.05996	0.016720774	0.253395174	4	12	24	0.033117504	0.606585237	24	23.08998651	0.087668193
ENSMUSG00000028277	Ube2j1	ubiquitin-conjugating enzyme E2J 1 [Source:MGI Symbol;Acc:MGI:1926245]	73.155	64.6464	65.7491	66.4322	63.2554	69.8708	67.8438	64.3526	59.1805	72.5049	68.7061	66.5012	66.3569	68.8399	0.588208558	0.966018836	24	12	24	0.407395801	0.799546792	24	20.11644738	0.042927631
ENSMUSG00000028278	Rragd	Ras-related GTP binding D [Source:MGI Symbol;Acc:MGI:1098604]	22.3368	20.1348	19.9882	21.0162	19.5465	21.8952	20.1375	22.3485	20.6749	23.4483	20.2686	22.3399	22.8422	22.2521	0.83638114	0.966018836	4	16	24	0.680013958	0.892958114	24	0.78932237	0.025138426
ENSMUSG00000028282	Casp8ap2	caspase 8 associated protein 2 [Source:MGI Symbol;Acc:MGI:1349399]	3.17853	4.23765	3.43334	3.53784	3.77459	3.23119	3.49482	2.82716	3.76366	2.73012	4.36826	3.36691	3.49379	3.61166	0.915475528	0.966018836	20	4	24	0.942202562	0.985597432	24	15.41301233	0.020372693
ENSMUSG00000028284	Map3k7	mitogen-activated protein kinase kinase kinase 7 [Source:MGI Symbol;Acc:MGI:1346877]	13.0275	14.3206	14.1492	13.3994	13.6428	13.5348	14.1785	13.9167	13.4326	13.5166	13.8331	13.2177	13.6731	13.3782	0.920858634	0.966018836	8	16	24	0.595724385	0.857839743	24	5.612547962	0.015117252
ENSMUSG00000028289	Epha7	Eph receptor A7 [Source:MGI Symbol;Acc:MGI:95276]	1.06091	1.31302	1.46011	1.50523	1.4104	1.44207	1.49781	1.54238	1.46842	1.43	1.36183	1.74737	1.01842	1.77464	0.951846915	0.966018836	8	20	24	0.553276027	0.837286821	24	10.37384956	0.053561889
ENSMUSG00000028291	Akirin2	akirin 2 [Source:MGI Symbol;Acc:MGI:1889364]	37.2725	32.1003	32.6346	32.0752	31.4545	34.9669	32.0593	29.793	28.3281	35.2294	32.8353	33.2661	34.5396	33.2634	0.499685403	0.966018836	24	12	24	0.194878594	0.768040178	24	20.42755284	0.06945798
ENSMUSG00000028292	Rars2	arginyl-tRNA synthetase 2, mitochondrial [Source:MGI Symbol;Acc:MGI:1923596]	11.2302	13.1796	10.6107	12.4425	11.6793	11.2277	11.6682	12.1145	11.2268	11.4018	13.239	12.5602	11.6067	12.2532	0.826609618	0.966018836	8	4	24	0.915582781	0.976610572	24	18.42336577	0.014790674
ENSMUSG00000028293	Slc35a1	solute carrier family 35 (CMP-sialic acid transporter), member 1 [Source:MGI Symbol;Acc:MGI:1345622]	13.5365	14.7748	13.9995	15.7341	14.193	13.0131	13.7289	11.195	14.8449	11.8289	15.2046	13.5449	14.2808	14.6269	0.608967886	0.966018836	20	4	24	0.836431693	0.949953371	24	12.97536976	0.030013125
ENSMUSG00000028294	Cfap206	cilia and flagella associated protein 206 [Source:MGI Symbol;Acc:MGI:1916579]	5.0301	5.12191	3.93955	5.22669	4.35649	4.90239	5.65136	4.54857	4.41634	4.55576	4.11054	5.08201	4.78841	5.11811	0.281427347	0.793378811	4	8	24	0.132330965	0.74244083	24	23.65127234	0.090302693
ENSMUSG00000028295	Smim8	small integral membrane protein 8 [Source:MGI Symbol;Acc:MGI:1913541]	21.9799	29.2694	27.2892	25.3957	27.9125	24.9343	26.7658	25.2158	29.8141	26.0349	27.6885	28.997	29.1503	28.3425	0.892044196	0.966018836	12	4	24	0.602950499	0.861140911	24	8.526875273	0.041866364
ENSMUSG00000028300	C9orf72	C9orf72, member of C9orf72-SMCR8 complex [Source:MGI Symbol;Acc:MGI:1920455]	40.7299	40.5512	37.5841	40.3788	38.2696	39.4033	35.9718	36.5107	36.1595	37.5797	39.4591	39.4684	41.8057	41.2528	0.773177465	0.966018836	4	8	24	0.570508667	0.845305671	24	21.64871875	0.030580259
ENSMUSG00000028309	Rnf20	ring finger protein 20 [Source:MGI Symbol;Acc:MGI:1925927]	25.0977	24.9834	24.018	23.6841	26.4314	23.377	25.3927	23.7284	24.3717	23.1181	25.5033	24.845	25.6524	25.5028	0.132887428	0.567728672	4	12	24	0.367483292	0.790832497	24	22.0057188	0.031238748
ENSMUSG00000028312	Smc2	structural maintenance of chromosomes 2 [Source:MGI Symbol;Acc:MGI:106067]	2.2431	2.58061	2.80717	3.13164	2.51213	2.40336	2.55681	1.83782	2.8564	2.03886	2.878	2.54697	2.65733	2.35638	0.628899575	0.966018836	12	20	24	0.497462043	0.807576732	24	13.52589339	0.066206608
ENSMUSG00000028318	Polr1e	polymerase (RNA) I polypeptide E [Source:MGI Symbol;Acc:MGI:1929022]	7.77167	7.50011	7.88128	8.20086	7.17583	6.89644	7.39169	8.90314	8.19561	8.06657	9.05588	7.99827	7.87539	7.61832	0.446069941	0.966018836	16	8	24	0.330181233	0.768040178	24	9.620053379	0.054691897
ENSMUSG00000028322	Exosc3	exosome component 3 [Source:MGI Symbol;Acc:MGI:1913612]	5.37457	5.55858	5.84434	5.72013	5.87752	5.36637	6.43973	6.00814	5.92149	5.13835	5.85742	5.66652	6.79436	5.87429	0.722296281	0.966018836	4	20	24	0.377379841	0.797290257	24	1.878186797	0.044533872
ENSMUSG00000028328	Tmod1	tropomodulin 1 [Source:MGI Symbol;Acc:MGI:98775]	63.7959	48.3214	48.0649	52.2498	48.8089	61.4805	53.6881	61.4637	41.5188	62.7013	53.9793	55.5051	56.1883	54.473	0.091424683	0.483797062	24	12	24	0.24795273	0.768040178	24	21.20281126	0.114012748
ENSMUSG00000028329	Xpa	xeroderma pigmentosum, complementation group A [Source:MGI Symbol;Acc:MGI:99135]	8.4913	11.2631	11.3862	10.024	7.91281	10.8059	7.98147	9.66993	13.9207	10.8808	9.06864	11.6004	11.0548	11.706	0.101690661	0.511659635	12	8	24	0.121719239	0.734072261	24	7.465115179	0.159659033
ENSMUSG00000028330	Ncbp1	nuclear cap binding protein subunit 1 [Source:MGI Symbol;Acc:MGI:1891840]	21.3913	19.6999	19.7681	18.776	21.9319	20.2567	19.682	19.8274	19.2296	17.972	21.7806	19.789	22.1731	19.9363	0.022000259	0.267229408	20	20	24	0.102745372	0.713699364	24	20.98406997	0.068674495
ENSMUSG00000028331	Trmo	tRNA methyltransferase O [Source:MGI Symbol;Acc:MGI:1922003]	2.23446	2.63131	2.32434	2.59229	2.30801	2.26472	2.44997	2.37976	2.26161	1.72724	2.62013	2.19365	2.15651	1.94267	0.831272156	0.966018836	8	20	24	0.876047758	0.965404684	24	20.18566434	0.021749312
ENSMUSG00000028332	Hemgn	hemogen [Source:MGI Symbol;Acc:MGI:2136910]	0.55795	1.28261	1.93706	2.203	1.21604	1.64655	1.3018	1.21317	1.24448	0.87958	1.16573	1.50821	2.03151	1.02591	0.875755063	0.966018836	24	8	24	0.856430548	0.957486395	24	12.77591103	0.062370682
ENSMUSG00000028333	Anp32b	acidic (leucine-rich) nuclear phosphoprotein 32 family, member B [Source:MGI Symbol;Acc:MGI:1914878]	21.7931	21.5286	20.9091	22.1617	23.0788	22.5244	22.2309	20.92	20.6201	23.9505	18.7137	21.8937	22.2811	20.938	0.069101108	0.433403132	16	20	24	0.751476968	0.91921305	24	18.61817211	0.025321185
ENSMUSG00000028334	Nans	N-acetylneuraminic acid synthase (sialic acid synthase) [Source:MGI Symbol;Acc:MGI:2149820]	16.8486	12.7193	13.9172	14.6282	15.7922	13.8383	12.2695	13	11.6261	12.1213	11.8758	13.0842	13.378	12.9618	0.895493752	0.966018836	24	12	24	0.314817594	0.768040178	24	19.98249053	0.072142369
ENSMUSG00000028337	Coro2a	coronin, actin binding protein 2A [Source:MGI Symbol;Acc:MGI:1345966]	30.5085	21.2401	21.7368	23.9598	24.4564	27.4399	22.7013	25.8681	17.2564	33.0486	21.9201	25.4016	23.9305	24.7042	0.245061943	0.741580974	24	12	24	0.456499607	0.805393068	24	19.39329502	0.113655163
ENSMUSG00000028339	Col15a1	collagen, type XV, alpha 1 [Source:MGI Symbol;Acc:MGI:88449]	28.9689	31.1177	23.971	21.942	32.7055	25.0086	32.5228	34.2934	30.7367	25.1099	27.6017	24.827	30.6774	25.4472	0.331157065	0.850959667	8	8	24	0.269928936	0.768040178	24	1.214226171	0.118651192
ENSMUSG00000028343	Erp44	endoplasmic reticulum protein 44 [Source:MGI Symbol;Acc:MGI:1923549]	20.3359	18.2816	17.8696	16.237	18.8521	16.5897	19.0504	19.266	17.4732	17.9752	19.4229	18.3936	18.7657	17.2101	0.217827365	0.704989686	4	12	24	0.293452504	0.768040178	24	22.77846387	0.052633368
ENSMUSG00000028344	Invs	inversin [Source:MGI Symbol;Acc:MGI:1335082]	3.95188	3.39193	3.13048	3.09816	3.59042	3.13536	3.63752	2.84189	3.45711	3.10778	3.23993	3.37388	3.15732	3.09684	0.351315142	0.870418653	4	4	24	0.249482211	0.768040178	24	21.02615969	0.057682817
ENSMUSG00000028345	Tex10	testis expressed gene 10 [Source:MGI Symbol;Acc:MGI:1344413]	12.3069	10.5342	11.3481	10.7757	11.4639	11.4492	10.0743	10.2971	10.3091	8.88993	12.1704	11.1861	12.2209	9.47952	0.215777641	0.704989686	4	4	24	0.100062107	0.71175955	24	19.94143815	0.102278377
ENSMUSG00000028347	Tmeff1	transmembrane protein with EGF-like and two follistatin-like domains 1 [Source:MGI Symbol;Acc:MGI:1926810]	15.16	15.348	16.7783	15.2836	14.3963	15.5603	15.778	13.9641	14.427	15.3255	13.316	15.1009	13.5061	16.603	0.722296281	0.966018836	12	8	24	0.584436351	0.852199457	24	5.792933629	0.03780744
ENSMUSG00000028348	Cavin4	caveolae associated 4 [Source:MGI Symbol;Acc:MGI:1915266]	0.733117	1.84428	2.41625	1.97991	1.37114	2.13974	1.63837	5.41958	3.35472	3.33394	1.27242	1.79591	2.71278	1.92435	0.153859465	0.605619266	12	8	24	0.087710448	0.707747429	24	7.48672778	0.373918639
ENSMUSG00000028351	Brinp1	bone morphogenic protein/retinoic acid inducible neural specific 1 [Source:MGI Symbol;Acc:MGI:1928478]	7.32017	6.67813	7.85804	6.54653	6.56502	8.2612	7.55661	6.95923	5.72409	8.54379	6.0003	7.34623	6.07858	7.68442	0.823246279	0.966018836	24	20	24	0.923069857	0.979439431	24	22.22142628	0.025589121
ENSMUSG00000028354	Fmn2	formin 2 [Source:MGI Symbol;Acc:MGI:1859252]	8.05683	8.8902	8.69507	8.74479	7.7444	9.76602	8.82658	11.0845	9.07965	9.18544	6.59043	9.72299	9.42345	8.28651	0.893307667	0.966018836	24	20	24	0.346306517	0.778844143	24	3.315088081	0.088313062
ENSMUSG00000028358	Zfp618	zinc finger protein 618 [Source:MGI Symbol;Acc:MGI:1919950]	0.724287	2.62871	0.868033	0.749149	0.843802	0.900665	0.994889	0.789029	0.804874	0.842251	0.80899	0.896893	0.799072	0.910918	0.937921962	0.966018836	8	8	24	0.492013195	0.805393068	24	3.365572489	0.11897261
ENSMUSG00000028364	Tnc	tenascin C [Source:MGI Symbol;Acc:MGI:101922]	1.43337	1.17753	1.81543	1.33593	1.15889	1.68197	1.44663	1.693	1.30162	1.36918	1.01195	1.22237	1.49729	1.05834	0.918193129	0.966018836	4	16	24	0.636834982	0.876483853	24	2.915986147	0.051664925
ENSMUSG00000028367	Txn1	thioredoxin 1 [Source:MGI Symbol;Acc:MGI:98874]	375.65	332.941	312.177	318.644	327.465	340.775	306.461	311.037	321.053	292.114	376.6	344.962	372.215	335.121	0.191123261	0.669841558	20	20	24	0.107742817	0.720556867	24	21.15873834	0.09103117
ENSMUSG00000028369	Svep1	sushi, von Willebrand factor type A, EGF and pentraxin domain containing 1 [Source:MGI Symbol;Acc:MGI:1928849]	0.552074	0.614792	0.817003	0.952888	0.593139	0.596154	0.682676	0.878327	0.829557	0.699584	0.644456	0.750033	0.635441	0.667271	0.15105645	0.598641364	16	4	24	0.058849584	0.658304792	24	9.17256404	0.088771621
ENSMUSG00000028370	Pappa	pregnancy-associated plasma protein A [Source:MGI Symbol;Acc:MGI:97479]	1.31711	0.945273	1.39439	0.879283	1.60121	1.28957	1.09951	0.92698	0.728146	0.819232	1.4326	0.922057	0.86115	0.887932	0.265774856	0.773510818	20	20	24	0.08732146	0.707460769	24	18.24483697	0.140058454
ENSMUSG00000028373	Astn2	astrotactin 2 [Source:MGI Symbol;Acc:MGI:1889277]	3.44645	3.15318	3.89107	3.92207	3.30075	3.83807	3.76613	3.83081	3.1836	4.45596	2.95146	3.42133	3.37593	4.36126	0.503972924	0.966018836	16	4	24	0.56194159	0.84198557	24	7.725599139	0.061110684
ENSMUSG00000028378	Ptgr1	prostaglandin reductase 1 [Source:MGI Symbol;Acc:MGI:1914353]	6.20055	6.52967	4.84901	5.69475	6.3444	5.5541	5.74109	6.02257	6.13827	4.86738	6.29438	5.91678	6.86603	5.78669	0.593560247	0.966018836	4	12	24	0.252498129	0.768040178	24	22.90233721	0.078505582
ENSMUSG00000028381	Ugcg	UDP-glucose ceramide glucosyltransferase [Source:MGI Symbol;Acc:MGI:1332243]	37.8656	32.4092	34.941	33.6436	33.6352	37.1805	33.594	31.7586	29.8848	37.1198	36.0948	35.0586	36.5252	35.1008	0.291637091	0.798132248	24	12	24	0.153238828	0.759484836	24	19.32164555	0.071345003
ENSMUSG00000028382	Ptbp3	polypyrimidine tract binding protein 3 [Source:MGI Symbol;Acc:MGI:1923334]	16.2429	17.7388	18.0512	17.976	17.5905	16.4389	16.0564	15.7247	16.9208	14.3375	18.7316	16.0337	16.456	16.6193	0.418381424	0.963884303	20	4	24	0.699974737	0.900387881	24	13.03100073	0.030199056
ENSMUSG00000028383	Hsdl2	hydroxysteroid dehydrogenase like 2 [Source:MGI Symbol;Acc:MGI:1919729]	41.2427	38.5034	39.1344	40.3544	35.9903	35.5379	41.9185	38.7024	34.4437	36.411	40.4303	35.2495	39.5713	40.4194	0.253121224	0.753386477	4	20	24	0.429763542	0.805393068	24	2.041086642	0.045192316
ENSMUSG00000028385	Snx30	sorting nexin family member 30 [Source:MGI Symbol;Acc:MGI:2443882]	2.55986	3.27243	3.52126	2.82474	2.97472	2.85763	3.38852	2.90712	3.40222	2.8549	2.47355	3.0728	2.49668	2.96917	0.212359178	0.701530532	12	4	24	0.252101092	0.768040178	24	6.919103746	0.078209529
ENSMUSG00000028389	Zfp37	zinc finger protein 37 [Source:MGI Symbol;Acc:MGI:99181]	6.04166	7.6987	7.48965	5.36213	6.51231	6.39737	6.38832	6.50102	7.72407	5.01392	6.37918	7.43995	7.17726	7.4147	0.036302654	0.33234124	12	4	24	0.180220234	0.768040178	24	3.041527916	0.113239202
ENSMUSG00000028391	Wdr31	WD repeat domain 31 [Source:MGI Symbol;Acc:MGI:1918604]	1.60436	1.70884	2.26261	1.69119	1.72668	1.48481	1.29392	1.33929	2.57131	1.76162	1.7099	1.57645	1.87397	1.84617	0.046056765	0.374351104	12	12	24	0.060708849	0.660111271	24	9.241546867	0.149250678
ENSMUSG00000028393	Alad	aminolevulinate, delta-, dehydratase [Source:MGI Symbol;Acc:MGI:96853]	13.0645	13.754	13.4742	14.5654	15.6812	13.6364	14.3037	15.0328	13.9381	16.018	13.5261	13.1895	16.2003	13.5671	0.912686312	0.966018836	16	8	24	0.719850848	0.908178129	24	12.0163321	0.031016844
ENSMUSG00000028394	Pole3	polymerase (DNA directed), epsilon 3 (p17 subunit) [Source:MGI Symbol;Acc:MGI:1933378]	19.464	21.5585	21.6735	19.183	21.5463	18.2523	20.0266	19.4741	21.8416	18.3267	19.9378	20.3861	21.2153	21.6338	0.069101108	0.433403132	12	4	24	0.322829759	0.768040178	24	5.543428867	0.051550626
ENSMUSG00000028396	2310002L09Rik	RIKEN cDNA 2310002L09 gene [Source:MGI Symbol;Acc:MGI:1916780]	1.30903	3.03359	4.93885	4.51031	3.26144	3.84378	2.36281	6.34263	8.40725	5.12455	2.84824	2.86128	4.48587	2.58063	0.031969981	0.324385159	12	16	24	0.037913483	0.61140397	24	9.406216061	0.475609347
ENSMUSG00000028397	Kdm4c	lysine (K)-specific demethylase 4C [Source:MGI Symbol;Acc:MGI:1924054]	6.30675	6.91492	6.3986	6.24216	6.09416	6.55747	6.42575	6.15	6.34415	5.85512	6.19976	6.46212	5.73249	6.37481	0.814077113	0.966018836	24	20	24	0.572128516	0.846236491	24	1.685734393	0.021492158
ENSMUSG00000028398	Dmac1	distal membrane arm assembly complex 1 [Source:MGI Symbol;Acc:MGI:1914178]	28.7388	26.1704	24.4195	24.97	21.9966	23.8908	26.1185	27.5181	27.257	28.101	30.2918	29.9668	29.211	29.9191	0.85447369	0.966018836	8	4	24	0.39885265	0.799546792	24	2.346679074	0.05495253
ENSMUSG00000028399	Ptprd	protein tyrosine phosphatase, receptor type, D [Source:MGI Symbol;Acc:MGI:97812]	5.78172	8.75516	11.3651	8.05665	7.12834	7.97643	9.40064	7.34493	9.97167	7.58646	6.6342	8.78845	7.45039	9.54828	0.064422003	0.414867207	12	8	24	0.158392306	0.764909967	24	7.095966356	0.170818443
ENSMUSG00000028402	Mpdz	multiple PDZ domain crumbs cell polarity complex component [Source:MGI Symbol;Acc:MGI:1343489]	40.1294	40.1175	39.3508	36.1425	41.6039	38.532	47.2304	38.5103	40.9072	40.5362	36.8091	38.2865	43.147	37.0959	0.588208558	0.966018836	4	12	24	0.382962582	0.799546792	24	0.065097219	0.053018375
ENSMUSG00000028403	Zdhhc21	zinc finger, DHHC domain containing 21 [Source:MGI Symbol;Acc:MGI:1915518]	19.4902	18.8256	17.4649	18.8559	18.9329	17.5845	17.8841	16.0955	17.6481	16.0821	19.2714	18.5396	19.0017	18.9141	0.349109869	0.870418653	4	8	24	0.376368924	0.796393126	24	20.51956582	0.048146835
ENSMUSG00000028405	Aco1	aconitase 1 [Source:MGI Symbol;Acc:MGI:87879]	11.043	9.56267	10.6693	11.2815	10.6596	11.4035	11.182	12.0026	10.2146	11.7796	10.4978	10.9879	11.3849	11.1764	0.891105762	0.966018836	4	8	24	0.890415415	0.969022636	24	20.32118645	0.015017263
ENSMUSG00000028407	Smim27	small integral membrane protein 27 [Source:MGI Symbol;Acc:MGI:1913684]	3.16323	4.25745	2.59874	3.62343	3.29297	2.75717	4.30134	4.57971	5.4463	3.0591	3.91222	4.11123	2.89577	2.78768	0.819898687	0.966018836	8	8	24	0.833620348	0.948756234	24	6.431469851	0.063974218
ENSMUSG00000028409	Smu1	smu-1 suppressor of mec-8 and unc-52 homolog (C. elegans) [Source:MGI Symbol;Acc:MGI:1915546]	29.3241	33.3249	31.4668	31.5827	32.4223	29.544	31.2669	30.3955	34.3067	28.1276	33.1922	30.7773	34.044	32.9028	0.938993891	0.966018836	12	12	24	0.64407476	0.879127822	24	6.1350424	0.031127782
ENSMUSG00000028410	Dnaja1	DnaJ heat shock protein family (Hsp40) member A1 [Source:MGI Symbol;Acc:MGI:1270129]	72.3843	61.854	56.8857	63.1879	65.6575	60.5903	63.5974	56.8401	56.0073	52.3178	70.2288	59.5906	61.0469	59.8072	0.119434414	0.542554768	20	16	24	0.069822499	0.674183933	24	20.21139511	0.103275546

ENSMUSG00000028411	Aptx	aprataxin [Source:MGI Symbol;Acc:MGI:1913658]	19.5976	18.9796	18.2682	18.418	17.9156	18.8971	18.3735	18.5784	18.3378	18.0831	18.393	19.3815	19.2387	18.3182	0.041677362	0.34886238	24	20	24	0.016520216	0.544099157	24	23.05178361	0.036791775
ENSMUSG00000028412	Slc44a1	solute carrier family 44, member 1 [Source:MGI Symbol;Acc:MGI:2140592]	78.4576	70.9456	73.2184	75.6146	78.0766	75.9395	77.2574	70.5042	63.7365	75.1305	70.422	72.8191	70.9885	65.9137	0.176949612	0.653256853	4	4	24	0.012632596	0.526249719	24	18.70627755	0.076625289
ENSMUSG00000028413	B4galt1	UDP-Gal:betaGlcNAc beta 1,4- galactosyltransferase, polypeptide 1 [Source:MGI Symbol;Acc:MGI:95705]	7.16171	7.73205	7.65437	9.19503	7.29211	7.68562	7.36949	8.02997	7.78903	8.50807	7.21456	7.21111	7.88062	8.03989	0.073315272	0.447768907	16	4	24	0.076225005	0.686372559	24	9.361952603	0.077068435
ENSMUSG00000028414	Fktn	fukutin [Source:MGI Symbol;Acc:MGI:2179507]	17.1457	15.5651	14.1031	13.9056	15.5797	16.0709	16.264	13.6715	13.5853	14.0156	14.9291	16.7459	16.1575	16.2558	7.57E-04	0.071344007	4	8	24	5.70E-04	0.293325979	24	21.91598815	0.131419548
ENSMUSG00000028416	Bag1	BCL2-associated athanogene 1 [Source:MGI Symbol;Acc:MGI:108047]	88.989	84.5614	81.2514	83.946	83.7458	89.1096	83.6712	85.0311	80.6104	82.4677	90.5994	91.938	98.6815	92.3199	0.039704107	0.338535904	24	12	24	0.015185953	0.537924552	24	22.01019562	0.070687478
ENSMUSG00000028419	Chmp5	charged multivesicular body protein 5 [Source:MGI Symbol;Acc:MGI:1924209]	90.5535	97.9203	91.2072	86.8968	97.0453	87.8821	91.149	84.6574	96.4976	74.9723	99.6336	87.734	95.1209	91.8505	0.921572192	0.966018836	20	20	24	0.7690944	0.925585646	24	23.4290565	0.030867033
ENSMUSG00000028420	Tmem38b	transmembrane protein 38B [Source:MGI Symbol;Acc:MGI:1098718]	4.7587	5.04479	5.77926	5.06789	5.02982	5.8455	6.13209	7.2732	5.58372	6.00668	5.59009	5.75924	6.50473	5.92525	0.763955526	0.966018836	8	12	24	0.328117734	0.768040178	24	3.754682508	0.065027885
ENSMUSG00000028423	Nfx1	nuclear transcription factor, X-box binding 1 [Source:MGI Symbol;Acc:MGI:1921414]	22.1228	23.4637	26.1344	23.7154	23.4213	21.3249	25.4911	25.0091	25.7597	22.9755	21.6268	22.3405	21.5705	24.668	0.004712708	0.140598674	12	12	24	0.011915264	0.525280095	24	7.078016469	0.099917793
ENSMUSG00000028426	Rad23b	RAD23 homolog B, nucleotide excision repair protein [Source:MGI Symbol;Acc:MGI:105128]	52.3649	44.3586	45.6962	45.5714	44.2507	47.0993	44.9059	45.4837	39.8532	53.5028	47.6555	49.5237	48.9284	48.0145	0.798829513	0.966018836	24	12	24	0.464775864	0.805393068	24	19.86155173	0.052869492
ENSMUSG00000028427	Aqp7	aquaporin 7 [Source:MGI Symbol;Acc:MGI:1314647]	0.35805	0.491064	0.740493	1.21545	0.568755	0.522085	0.656492	1.60864	0.653433	1.76285	0.851581	0.871791	1.96762	1.69386	0.890311217	0.966018836	16	8	24	0.23322534	0.768040178	24	7.820768406	0.187248654
ENSMUSG00000028430	Nol6	nucleolar protein family 6 (RNA-associated) [Source:MGI Symbol;Acc:MGI:2140151]	8.97806	8.67352	9.62071	10.4262	8.5717	9.9077	8.65015	9.99746	9.33619	12.4708	7.80676	10.2158	9.15198	9.79755	0.251986462	0.75129457	16	12	24	0.414059587	0.799546792	24	10.13841028	0.077636543
ENSMUSG00000028431	Elp1	elongator complex protein 1 [Source:MGI Symbol;Acc:MGI:1914544]	27.8985	25.1676	27.8392	27.7433	24.8901	26.3099	24.4437	28.9452	25.0715	28.0298	22.6718	27.7555	26.4137	27.5676	0.722296281	0.966018836	24	4	24	0.519053855	0.819011931	24	5.469440162	0.044494782
ENSMUSG00000028433	Ubap2	ubiquitin-associated protein 2 [Source:MGI Symbol;Acc:MGI:1916176]	16.1	15.3278	11.4597	11.9505	12.5913	13.1641	13.9993	15.7637	11.4359	23.0817	13.2732	14.3956	14.2683	14.2195	0.119293142	0.542554768	8	4	24	0.955869921	0.988639288	24	0.868575831	0.029047459
ENSMUSG00000028434	Epb41l4b	erythrocyte membrane protein band 4.1 like 4b [Source:MGI Symbol;Acc:MGI:1859149]	16.2856	14.6056	16.4756	15.5266	18.0224	14.1445	16.349	13.4207	14.8785	16.3795	14.8362	15.2851	15.8571	12.93	0.082685511	0.462922111	20	12	24	0.12026789	0.732348505	24	17.04652062	0.087663083
ENSMUSG00000028436	Dcaf12	DDB1 and CUL4 associated factor 12 [Source:MGI Symbol;Acc:MGI:1916220]	20.1518	18.1504	18.2445	20.5217	19.9078	19.9083	19.153	18.2972	16.8272	20.2642	18.8574	18.5537	19.0728	17.2563	0.011964854	0.213452992	16	20	24	0.02119563	0.571993737	24	17.53319036	0.075798084
ENSMUSG00000028437	Ubap1	ubiquitin-associated protein 1 [Source:MGI Symbol;Acc:MGI:2149543]	23.5	25.0791	22.3418	23.1586	23.5055	26.8317	23.842	25.6747	24.1558	27.8749	22.966	24.2875	23.4759	22.5316	0.874672901	0.966018836	24	12	24	0.919332842	0.978394423	24	16.94219473	0.015170269
ENSMUSG00000028439	Fam219a	family with sequence similarity 219, member A [Source:MGI Symbol;Acc:MGI:1919151]	40.5167	32.4728	32.7672	38.9529	35.3012	37.0858	36.0996	35.6163	31.2143	51.6456	34.7396	39.5835	36.0279	38.1898	0.245061943	0.741580974	24	12	24	0.5735083	0.847005958	24	16.04972241	0.071380376
ENSMUSG00000028441	1110017D15Rik	RIKEN cDNA 1110017D15 gene [Source:MGI Symbol;Acc:MGI:1920971]	1.64433	1.09175	0.623845	1.52491	1.45033	0.889254	0.488896	1.20552	0.708566	1.36081	1.55558	1.00043	0.784471	0.614663	0.229694208	0.729069536	16	20	24	0.118484817	0.729286647	24	15.63407264	0.203596305
ENSMUSG00000028443	Nudt2	nudix (nucleoside diphosphate linked moiety X)-type motif 2 [Source:MGI Symbol;Acc:MGI:1913651]	27.6511	31.35	30.5694	28.3898	29.6833	27.6343	26.9593	27.3395	30.3363	23.7057	29.6041	29.3871	30.5327	29.9662	0.70641939	0.966018836	12	4	24	0.727151151	0.91035631	24	3.520904181	0.032385569
ENSMUSG00000028444	Cntfr	ciliary neurotrophic factor receptor [Source:MGI Symbol;Acc:MGI:99605]	16.63	13.6847	15.0951	15.0963	14.9037	16.1701	14.5767	15.4632	14.4812	19.9162	15.9567	15.2901	14.5192	15.5791	0.767183862	0.966018836	16	20	24	0.391589834	0.799546792	24	14.24886988	0.062880718
ENSMUSG00000028445	Enho	energy homeostasis associated [Source:MGI Symbol;Acc:MGI:1916888]	19.1581	21.3283	23.7288	18.1894	18.3048	21.9882	20.1949	19.9477	22.1968	19.125	20.661	21.4635	20.1896	23.4636	0.187699199	0.662239322	12	4	24	0.264137343	0.768040178	24	4.709650367	0.071009791
ENSMUSG00000028447	Dctn3	dynactin 3 [Source:MGI Symbol;Acc:MGI:1859251]	120.261	107.908	96.3676	101.096	99.5262	109.608	105.274	107.973	98.4341	98.5292	110.444	108.128	110.724	106.746	0.008389425	0.192760771	4	8	24	0.004388065	0.427431763	24	22.64753743	0.094678297
ENSMUSG00000028452	Vcp	valosin containing protein [Source:MGI Symbol;Acc:MGI:99919]	52.5933	45.0718	45.4412	46.3876	46.3622	49.0837	45.6596	49.6602	43.6882	57.7378	48.9022	51.1968	51.4764	51.0395	0.217827365	0.704989686	24	12	24	0.723788698	0.909415818	24	19.57685255	0.033065801
ENSMUSG00000028453	Fancg	Fanconi anemia, complementation group G [Source:MGI Symbol;Acc:MGI:1926471]	6.77839	6.04974	6.28866	5.47959	4.98365	5.94367	6.0264	6.17388	6.84521	7.33672	5.49351	6.39879	5.98583	5.47094	0.581070934	0.966018836	12	8	24	0.605424276	0.86269185	24	5.633291619	0.052114786
ENSMUSG00000028454	Pigp	phosphatidylinositol glycan anchor biosynthesis, class P [Source:MGI Symbol;Acc:MGI:1861452]	5.39487	4.88962	6.42509	4.84531	4.87892	5.07229	4.81756	4.76014	4.54979	4.68363	4.36533	6.29845	5.7093	4.95464	0.349109869	0.870418653	24	20	24	0.596926517	0.858188176	24	23.42171706	0.057194136
ENSMUSG00000028455	Stoml2	stomatin (Epb7.2)-like 2 [Source:MGI Symbol;Acc:MGI:1913842]	44.1479	46.6002	47.8582	50.6549	52.1108	44.9232	44.2996	47.2905	45.5074	39.1849	45.9354	37.3765	46.0517	39.7238	0.036884864	0.966018836	20	4	24	0.690885041	0.897666745	24	12.09582166	0.037614768
ENSMUSG00000028456	Unc13b	unc-13 homolog B [Source:MGI Symbol;Acc:MGI:1342278]	11.8428	12.2632	11.7367	11.8825	14.3809	11.869	12.8718	11.6551	12.7115	9.85279	12.4926	12.9198	13.2762	12.3237	0.503972924	0.966018836	20	20	24	0.369911446	0.792235171	24	21.13019302	0.064497123
ENSMUSG00000028457	Atp8b5	ATPase, class I, type 8B, member 5 [Source:MGI Symbol;Acc:MGI:2444287]	0.462212	0.947325	0.696049	0.589866	0.66497	0.934463	0.542331	0.778034	0.403766	0.526309	0.813863	0.521453	0.737318	0.732749	0.945282252	0.966018836	8	4	24	0.832361561	0.948130119	24	23.07587951	0.036100142
ENSMUSG00000028458	Tesk1	testis specific protein kinase 1 [Source:MGI Symbol;Acc:MGI:1201675]	9.42297	10.0571	8.61145	9.73461	8.93513	8.77664	7.80354	10.7225	7.72961	11.8281	8.37558	9.93222	10.926	9.65543	0.868508291	0.966018836	8	4	24	0.922873181	0.979430367	24	5.79100692	0.026688291
ENSMUSG00000028459	Cd72	CD72 antigen [Source:MGI Symbol;Acc:MGI:88345]	1.72487	3.88916	4.07827	2.80229	3.1702	2.64345	3.24658	3.5751	4.57988	2.46013	2.81343	2.72621	3.55091	2.53554	0.128457847	0.560209134	12	12	24	0.197588604	0.768040178	24	7.356101805	0.190784838
ENSMUSG00000028461	Ccdc107	coiled-coil domain containing 107 [Source:MGI Symbol;Acc:MGI:1913423]	26.9832	24.865	21.6817	24.7261	25.2304	22.8371	24.9863	22.063	26.4565	22.3955	29.0264	28.6726	31.2303	26.8969	0.202982082	0.689605535	4	8	24	0.274727395	0.768040178	24	21.26994251	0.08574229
ENSMUSG00000028464	Tpm2	tropomyosin 2, beta [Source:MGI Symbol;Acc:MGI:98810]	25.374	57.5846	86.2376	95.9909	77.3476	93.1815	56.6807	178.228	109.22	179.065	50.1798	61.9767	88.0972	74.3713	0.185945151	0.662239322	16	12	24	0.0897064	0.709708691	24	9.70524363	0.566456909
ENSMUSG00000028465	Tln1	talin 1 [Source:MGI Symbol;Acc:MGI:1099832]	23.6999	21.0225	21.9372	22.7794	22.5052	23.4016	24.6162	24.3083	21.4451	29.8285	21.0358	25.2752	23.0319	22.4754	0.439800617	0.966018836	24	12	24	0.844077042	0.952782517	24	17.07679993	0.0294568
ENSMUSG00000028466	Creb3	cAMP responsive element binding protein 3 [Source:MGI Symbol;Acc:MGI:99946]	32.2241	29.6024	33.1263	28.7129	30.8383	29.6646	28.7861	30.2536	29.9567	26.2705	30.0532	28.5251	30.1978	29.8056	0.916860713	0.966018836	12	4	24	0.531152792	0.825104543	24	2.046105035	0.030634101
ENSMUSG00000028467	Gba2	glucosidase beta 2 [Source:MGI Symbol;Acc:MGI:2654325]	9.44913	9.72761	10.7377	10.5691	9.94711	10.805	9.83887	10.2752	9.98969	10.1906	9.07872	8.89116	9.992	10.0386	0.331157065	0.850996667	16	4	24	0.322509118	0.768040178	24	8.45824922	0.04366924
ENSMUSG00000028468	Rgp1	RAB6A GEF complex partner 1 [Source:MGI Symbol;Acc:MGI:1915956]	8.66116	10.114	11.1841	10.7421	11.0049	10.5257	13.0367	9.8333	9.99898	11.3327	9.49725	12.0175	10.6224	9.94451	0.892578749	0.966018836	16	16	24	0.789593552	0.932546317	24	16.81698592	0.037294322
ENSMUSG00000028469	Npr2	natriuretic peptide receptor 2 [Source:MGI Symbol;Acc:MGI:97372]	39.3913	35.467	37.3189	38.8901	37.1497	41.5837	36.8292	38.8738	35.4131	44.7027	37.0598	37.4697	40.0646	36.609	0.347075276	0.870418653	16	20	24	0.564139963	0.842999585	24	16.59579376	0.038318246
ENSMUSG00000028470	Hint2	histidine triad nucleotide binding protein 2 [Source:MGI Symbol;Acc:MGI:1916167]	27.552	34.8929	37.2326	36.347	35.5734	34.713	32.0199	37.2737	37.8906	34.2309	34.5605	34.4784	40.895	33.8467	0.643325306	0.966018836	12	16	24	0.310077329	0.768040178	24	9.461719187	0.072768951
ENSMUSG00000028475	Spaar	small regulatory polypeptide of amino acid response [Source:MGI Symbol;Acc:MGI:1918656]	0.441659	0.663155	0.737487	0.760977	0.589645	0.608715	0.623824	0.77949	0.614458	0.655236	0.735424	0.687033	0.854093	0.650362	0.887862353	0.966018836	8	16	24	0.556301095	0.838702296	24	9.018443083	0.035552941
ENSMUSG00000028476	Reck	reversion-inducing-cysteine-rich protein with kazal motifs [Source:MGI Symbol;Acc:MGI:1855698]	4.85622	5.37135	5.6852	5.71399	6.08537	5.36269	4.85364	6.33906	6.14625	5.74995	5.41887	6.14274	4.74086	4.27948	0.137504104	0.580158868	12	16	24	0.138354775	0.747972781	24	12.83266194	0.102366098
ENSMUSG00000028478	Clta	clathrin, light polypeptide (Lca) [Source:MGI Symbol;Acc:MGI:894297]	158.178	156.023	151.371	149.421	158.871	160.527	149.368	151.518	145.337	150.205	180.732	179.982	188.04	175.43	0.055738947	0.401074772	20	20	24	0.076225258	0.686372559	24	20.83606227	0.081560298
ENSMUSG00000028479	Gne	glucosamine (UDP-N-acetyl)-2-epimerase/N-acetylmannosamine kinase [Source:MGI Symbol;Acc:MGI:1354951]	15.5346	13.0091	12.9488	12.6804	12.3961	15.094	15.3425	15.4088	11.4435	15.9198	12.318	14.0663	13.6095	11.1985	0.505132703	0.966018836	4	8	24	0.274328126	0.768040178	24	21.60281101	0.098777108
ENSMUSG00000028480	Glipr2	GLI pathogenesis-related 2 [Source:MGI Symbol;Acc:MGI:1917770]	12.8503	11.8114	11.735	10.4682	12.7862	11.5159	12.626	10.8961	11.4049	8.45578	12.9557	11.1875	13.1456	10.7975	0.336134798	0.853152526	4	12	24	0.097511079	0.71175955	24	22.10403288	0.123064445
ENSMUSG00000028483	Snapc3	small nuclear RNA activating complex, polypeptide 3 [Source:MGI Symbol;Acc:MGI:1916338]	9.44765	9.60022	10.8232	10.0301	10.4356	9.72566	11.0321	9.35462	10.9713	10.1789	10.5889	8.85438	10.8405	9.37095	0.89563591	0.966018836	12	20	24	0.577195558	0.848840315	24	11.12710278	0.039178519
ENSMUSG00000028484	Psip1	PC4 and SFRS1 interacting protein 1 [Source:MGI Symbol;Acc:MGI:2142116]	70.2025	74.1018	71.4619	70.2324	74.4264	70.6507	72.5354	64.5196	71.4152	71.6531	74.9468	72.9764	70.6593	79.1957	0.937921962	0.966018836	20	8	24	0.893656902	0.969947957	24	15.3679551	0.011886419
ENSMUSG00000028487	Bnc2	basonuclin 2 [Source:MGI Symbol;Acc:MGI:2443805]	6.15216	4.43599	4.65494	4.03591	5.14313	3.93696	5.70604	3.09792	4.34143	4.4928	5.12672	5.7149	5.15156	4.34612	0.084879076	0.466404237	4	4	24	0.170112838	0.767957732	24	20.18465684	0.156554975
ENSMUSG00000028488	Sh3gl2	SH3-domain GRB2-like 2 [Source:MGI Symbol;Acc:MGI:700009]	30.1642	29.1547	32.9139	27.9035	24.5724	31.9646	33.7071	28.7317	25.9578	34.0485	24.9954	29.6001	27.1973	31.7104	0.894678274	0.966018836	4	16	24	0.534759903	0.82673015	24	3.061986759	0.065591085
ENSMUSG00000028494	Plin2	perilipin 2 [Source:MGI Symbol;Acc:MGI:87920]	34.939	27.4985	34.5214	42.7687	37.446	33.0218	32.9523	40.9908	28.3127	29.2297	53.4362	37.0492	41.0642	35.4828	0.784278077	0.966018836	20	16	24	0.365526124	0.789741863	24	17.29698788	0.135881587
ENSMUSG00000028495	Rps6	ribosomal protein S6 [Source:MGI Symbol;Acc:MGI:98159]	1.181	1.20286	1.54597	0.663707	0.848934	0.554763	1.02369	1.07066	1.51919	1.23513	1.93513	1.47059	1.3287	0.993565	0.935194469	0.966018836	12	12	24	0.763215704	0.923599528	24	8.02111231	0.073180123
ENSMUSG00000028496	Mllt3	myeloid/lymphoid or mixed-lineage leukemia; translocated to, 3 [Source:MGI Symbol;Acc:MGI:1917372]	11.9027	12.5813	11.3919	13.7498	10.9462	12.1297	12.6808	13.6929	14.9399	13.6485	12.3112	13.1868	13.6671	12.3304	0.834424969	0.966018836	16	4	24	0.357076165	0.784084555	24	7.372564775	0.060091026
ENSMUSG00000028497	Hacd4	3-hydroxyacyl-CoA dehydratase 4 [Source:MGI Symbol;Acc:MGI:1914025]	1.89541	2.97814	2.27347	2.14136	2.38268	1.99515	2.28369	2.68562	3.1139	1.40791	2.30561	2.17768	1.98039	2.2152	0.215426034	0.704989686	8	20	24	0.283060666	0.768040178	24	5.618848525	0.115016691
ENSMUSG00000028514	Usp24	ubiquitin specific peptidase 24 [Source:MGI Symbol;Acc:MGI:1919936]	6.14302	7.30982	7.43055	7.92322	7.39786	6.20818	7.36595	6.75757	8.12911	6.95613	7.04415	7.74	7.15335	7.3228	0.481811994	0.966018836	12	16	24	0.15451259	0.760257003	24	9.900102964	0.074318261
ENSMUSG00000028517	Plpp3	phospholipid phosphatase 3 [Source:MGI Symbol;Acc:MGI:1915166]	29.1514	29.6987	28.697	26.4381	29.918	28.7171	33.448	32.3188	30.2948	30.3199	25.3025	30.8706	27.8659	27.1657	0.890176313	0.966018836	20	20	24	0.436686504	0.805393068	24	1.612072343	0.05209021
ENSMUSG00000028518	Prkaa2	protein kinase, AMP-activated, alpha 2 catalytic subunit [Source:MGI Symbol;Acc:MGI:1336173]	10.9042	10.247	10.1346	11.6685	10.0635	10.8457	9.32472	10.8361	10.2248	12.1496	10.0739	11.1911	11.1291	10.964	0.891690964	0.966018836	20	20	24	0.690225045	0.897408416	24	12.218818	0.030434851
ENSMUSG00000028519	Dab1	disabled 1 [Source:MGI Symbol;Acc:MGI:108554]	0.842746	1.65606	2.24194	1.3496	0.965211	1.54125	1.17588	1.22106	1.65634	1.24751	1.09401	1.38455	1.1934	1.5221	0.027960311	0.313364468	12	8	24	0.126373085	0.739868076	24	7.62015901	0.162765776
ENSMUSG00000028521	Slc35d1	solute carrier family 35 (UDP-glucuronic acid/UDP-N-acetylgalactosamine dual transporter), member D1 [Source:MGI Symbol;Acc:MGI:2140361]	3.48647	3.35121	3.51128	3.64439	3.56724	3.24451	3.73154	3.05541	3.50232	3.09636	3.33905	3.22261	2.97915	3.2302	0.745469888	0.966018836	12	20	24	0.687608424	0.896350267	24	13.8996345	0.021100249
ENSMUSG00000028522	Mier1	MEIR1 treanscription regulator [Source:MGI Symbol;Acc:MGI:1918398]	14.6718	14.6789	14.1917	15.3821	14.4016	12.6128	15.0492	13.8449	15.3353	11.6395	13.3063	12.8005	12.7153	14.4015	0.723119329	0.966018836	12	8	24	0.328103631	0.768040178	24	5.633062003	0.059374012
ENSMUSG00000028523	Dynlt5	dynein light chain Tctex-type 5 [Source:MGI Symbol;Acc:MGI:1914594]	1.6241	1.88039	2.13788	2.73314	1.34205	1.88748	1.88869	2.23384	1.86626	1.28449	1.62119	2.02254	1.75198	2.21912	0.47028201	0.966018836	8	12	24	0.381953394	0.799546792	24	5.464832394	0.103553166
ENSMUSG00000028524	Sgip1	SH3-domain GRB2-like (endophilin) interacting protein 1 [Source:MGI Symbol;Acc:MGI:1920344]	13.2157	13.5853	12.1141	9.97306	11.945	13.8064	15.3129	12.9914	13.253	18.2968	9.33022	13.5292	13.0039	15.9053	0.476724519	0.966018836	8	12	24	0.363710711	0.788601811	24	3.179103359	0.122889148
ENSMUSG00000028525	Pde4b	phosphodiesterase 4B, cAMP specific [Source:MGI Symbol;Acc:MGI:99557]	6.93915	7.48908	8.96558	6.71205	6.25618	7.90487	8.30742	7.43998	7.65781	7.76006	7.63106	7.69132	7.03718	9.58101	0.956178643	0.96629412	12	8	24	0.205542405	0.768040178	24	5.226025909	0.092116721
ENSMUSG00000028527	Ak4	adenylate kinase 4 [Source:MGI Symbol;Acc:MGI:87979]	1.69584	1.50618	2.00705	1.81067	1.52395	1.62395	1.98979	1.23739	1.71553	1.68628	1.52694	1.71131	1.68753	1.51467	0.3576519	0.882035609	12	20	24	0.916159265	0.976814265	24	14.60698302	0.018034855
ENSMUSG00000028528	Dnajc6	DnaJ heat shock protein family (Hsp40) member C6 [Source:MGI Symbol;Acc:MGI:1919935]	46.8143	37.5926	39.6772	36.7865	34.7321	41.3983	39.6923	37.5138	30.0662	44.396	33.8677	38.3373	36.2456	39.7573	0.745118544	0.966018836	4	16	24	0.47131119	0.805393068	24	22.85307949	0.07409045
ENSMUSG00000028530	Jak1	Janus kinase 1 [Source:MGI Symbol;Acc:MGI:96628]	92.9085	82.5939	81.2203	85.3977	79.4666	87.7627	80.1975	82.9851	72.5488	97.9881	79.3031	84.3105	85.765	87.6363	0.636884864	0.966018836	24	12	24	0.839299037	0.951051187	24	20.72387278	0.026121151
ENSMUSG00000028531	Cachd1	cache domain containing 1 [Source:MGI Symbol;Acc:MGI:2444177]	6.22245	4.46498	5.24694	5.07975	6.64407	5.977	7.16165	5.20886	3.88381	6.17139	4.41363	5.37191	5.31209	4.96983	0.153754364	0.605479604	4	4	24	0.144822793	0.753662838	24	20.11261046	0.153869983
ENSMUSG00000028538	St3gal3	ST3 beta-galactoside alpha-2,3-sialyltransferase 3 [Source:MGI Symbol;Acc:MGI:1316659]	11.9437	11.4388	12.3819	12.4545	14.8709	12.1382	13.7423	12.9053	12.0137	13.4624	10.5885	12.9609	11.7666	12.0745	0.881718085	0.966018836	16	16	24	0.78125362	0.929846332	24	15.9764371	0.031658791
ENSMUSG00000028540	Dph2	DPH2 homolog [Source:MGI Symbol;Acc:MGI:1914978]	4.37972	4.48704	3.63399	4.26457	4.64794	4.63208	4.28185	4.11671	3.31648	4.34149	3.70933	3.68808	4.05293	4.04899	0.455507782	0.966018836	20	16	24	0.294275001	0.768040178	24	20.44641569	0.066754269
ENSMUSG00000028541	B4galt2	UDP-Gal:betaGlcNAc beta 1,4- galactosyltransferase, polypeptide 2 [Source:MGI Symbol;Acc:MGI:1858493]	7.00879	7.09817	7.60604	7.23888	7.69454	7.42424	7.76765	7.40524	7.21104	7.96926	6.57796	7.00052	6.607	7.47114	0.954744729	0.966018836	16	8	24	0.546300797	0.833341327	24	9.725416174	0.031788439
ENSMUSG00000028542	Slc6a9	solute carrier family 6 (neurotransmitter transporter, glycine), member 9 [Source:MGI Symbol;Acc:MGI:95760]	4.31352	8.83554	13.4721	5.87371	4.31216	7.53493	8.64068	6.09928	9.52198	6.51785	3.87584	6.36041	3.45563	8.29392	0.020499994	0.261611804	12	8	24	0.102551531	0.713699364	24	6.847681037	0.3854179
ENSMUSG00000028545	Bend5	BEN domain containing 5 [Source:MGI Symbol;Acc:MGI:1914871]	3.31589	2.27727	2.30612	2.35868	2.9308	3.01303	3.03032	2.4215	2.94336	2.6053	3.13084	3.83804	3.42288	3.42739	0.075245122	0.452094524	4	8	24	0.008557304	0.501317957	24	21.04624619	0.154234685
ENSMUSG00000028546	Elavl4	ELAV like RNA binding protein 4 [Source:MGI Symbol;Acc:MGI:107427]	109.614	92.5567	87.2738	90.0402	88.3228	96.5143	94.5153	85.9272	73.8658	91.9319	95.9867	88.6336	93.7166	96.591	0.058157448	0.40386543	4	8	24	0.081186214	0.697074851	24	21.54264638	0.102987762
ENSMUSG00000028549	Itgb3bp	integrin beta 3 binding protein (beta3-endonexin) [Source:MGI Symbol;Acc:MGI:1914983]	6.92335	9.19695	8.95665	8.19494	9.05469	7.28168	8.54455	7.49764	10.0899	5.7075	8.34393	8.57789	7.46132	7.25462	0.941901469	0.966018836	12	16	24	0.920344778	0.978864885	24	8.573841611	0.031313957
ENSMUSG00000028550	Atg4c	autophagy related 4C, cysteine peptidase [Source:MGI Symbol;Acc:MGI:2651854]	9.6281	9.08908	10.56	9.51179	9.29636	10.0132	10.1096	9.18419	9.25796	10.9083	9.01041	9.76865	9.18808	10.2651	0.896753532	0.966018836	16	4	24	0.822384242	0.944152607	24	9.869234302	0.020500478
ENSMUSG00000028551	Cdkn2c	cyclin dependent kinase inhibitor 2C [Source:MGI Symbol;Acc:MGI:105388]	4.05676	4.9795	5.49034	6.204	4.60695	3.7332	4.29056	5.95983	5.66544	5.49646	5.10126	5.21473	5.75134	5.68563	0.186921604	0.662239322	16	8	24	0.005047543	0.442351542	24	8.62193794	0.185414098
ENSMUSG00000028552	Eps15	epidermal growth factor receptor pathway substrate 15 [Source:MGI Symbol;Acc:MGI:104583]	90.1983	84.5012	87.9031	83.4259	85.1688	86.1391	83.8811	79.7341	77.6712	82.348	85.994	82.3902	80.7173	83.5386	0.966657897	0.966018836	20	20	24	0.247646801	0.768040178	24	19.32111506	0.03062824
ENSMUSG00000028555	Ttc39a	tetratricopeptide repeat domain 39A [Source:MGI Symbol;Acc:MGI:2444350]	6.969	6.00719	6.29366	5.8844	5.98275	6.43528	5.61967	6.37406	5.36487	6.90661	5.70873	6.85197	6.13698	6.64569	0.953760335	0.966018836	20	8	24	0.715127215	0.906238356	24	22.47823395	0.03389178
ENSMUSG00000028556	Dock7	dedicator of cytokinesis 7 [Source:MGI Symbol;Acc:MGI:1914549]	42.8553	44.4798	44.8735	43.0445	45.7231	38.6581	46.5351	41.3423	45.4933	44.7476	45.9292	43.217	46.0773	47.4368	0.888482515	0.966018836	20	4	24	0.51288457	0.815948524	24	7.77817229	0.033964166
ENSMUSG00000028557	Rnf11	ring finger protein 11 [Source:MGI Symbol;Acc:MGI:1352759]	94.7353	85.395	84.9486	87.584	85.1398	88.7217	84.1293	83.8311	73.5334	86.6185	83.2054	76.9189	74.4942	82.2717	0.904690479	0.966018836	20	20	24	0.62067872	0.870297169	24	18.39657452	0.028713447
ENSMUSG00000028558	Calr4	calreticulin 4 [Source:MGI Symbol;Acc:MGI:2140435]	1.08799	1.37953	1.33752	1.51015	1.30003	1.08106	1.09082	1.77221	1.51209	1.10771	1.03084	1.50029	1.27531	1.26454	0.176949612	0.653256853	12	8	24	0.244204109	0.768040178	24	7.02241569	0.089105134
ENSMUSG00000028559	Osbpl9	oxysterol binding protein-like 9 [Source:MGI Symbol;Acc:MGI:1923784]	70.1091	77.2582	70.3926	70.5345	73.5775	68.3953	63.6594	63.9823	75.1676	59.4985	67.2579	64.1065	68.6383	64.0281	0.923635729	0.966018836	12	12	24	0.919182699	0.978264112	24	7.792497724	0.015002743
ENSMUSG00000028560	Usp1	ubiquitin specific peptidase 1 [Source:MGI Symbol;Acc:MGI:2385198]	5.4118	6.63896	6.89367	6.55934	6.37383	6.08378	6.3238	5.99075	7.33597	5.53597	6.50242	5.73186	6.40192	6.05402	0.143321297	0.585944911	12	12	24	0.259826743	0.768040178	24	8.923665813	0.067797411
ENSMUSG00000028563	Tm2d1	TM2 domain containing 1 [Source:MGI Symbol;Acc:MGI:2137022]	20.0512	18.1767	15.3448	16.7613	15.1106	15.1562	16.4121	13.9987	12.2699	12.9894	17.7317	12.013	12.2483	14.4541	0.801280095	0.966018836	20	4	24	0.667784422	0.88767519	24	21.31345096	0.057943016
ENSMUSG00000028565	Nfia	nuclear factor I/A [Source:MGI Symbol;Acc:MGI:108056]	7.01293	6.6534	7.05201	7.64073	6.38198	7.39194	7.12091	6.88054	7.11994	9.12947	7.23515	7.0681	7.05498	7.52628	0.510431574	0.966018836	16	16	24	0.278205511	0.768040178	24	11.51823234	0.061645358
ENSMUSG00000028567	Txndc12	thioredoxin domain containing 12 (endoplasmic reticulum) [Source:MGI Symbol;Acc:MGI:1913323]	22.008	22.3418	21.8933	18.5641	22.695	21.2651	23.4452	22.8903	22.735	19.8854	22.2463	19.7763	22.9018	21.0284	0.117050591	0.538362324	4	12	24	0.208170355	0.768040178	24	1.469033363	0.061985637
ENSMUSG00000028568	Btf3l4	basic transcription factor 3-like 4 [Source:MGI Symbol;Acc:MGI:1915312]	8.03055	9.69628	11.368	9.75619	9.26477	8.80305	8.54373	8.88027	10.889	9.89386	10.3976	9.54455	9.06837	10.3628	3.44E-04	0.049770315	12	16	24	0.003473026	0.420609404	24	9.916719526	0.137280324
ENSMUSG00000028571	Cyp2j13	cytochrome P450, family 2, subfamily j, polypeptide 13 [Source:MGI Symbol;Acc:MGI:2385197]	1.87987	1.67537	1.17329	1.52148	1.89177	1.99846	1.80532	1.49287	1.13204	1.37319	1.64108	2.01345	1.22174	1.55803	1.03E-04	0.029372629	24	12	24	0.003510909	0.421762867	24	20.01710256	0.187515648
ENSMUSG00000028572	Hook1	hook microtubule tethering protein 1 [Source:MGI Symbol;Acc:MGI:1925213]	4.54807	6.63935	5.82327	5.56542	7.06286	5.24951	5.29822	5.38147	6.97695	4.06743	5.96826	5.37374	4.94604	6.1912	0.209352197	0.69936851	12	16	24	0.686974796	0.896157784	24	9.064276462	0.068745388
ENSMUSG00000028573	Fggy	FGGY carbohydrate kinase domain containing [Source:MGI Symbol;Acc:MGI:1922828]	11.2502	12.7136	11.3339	10.5575	12.683	12.001	12.3655	11.2737	12.9455	10.3975	11.4302	12.2373	12.1641	12.2991	0.745118544	0.966018836	8	8	24	0.338572324	0.773151665	24	0.338726123	0.051429261
ENSMUSG00000028576	Ift74	intraflagellar transport 74 [Source:MGI Symbol;Acc:MGI:1914944]	13.6863	14.5719	13.3243	12.696	13.8421	12.9158	14.7579	12.4616	17.1174	12.6255	15.9437	15.7573	16.0833	15.6749	0.953350544	0.966018836	4	12	24	0.706316346	0.902918222	24	1.269596853	0.039611933
ENSMUSG00000028577	Plaa	phospholipase A2, activating protein [Source:MGI Symbol;Acc:MGI:104810]	34.9854	36.1513	37.763	35.0032	35.1081	36.4082	32.8523	34.1011	34.8913	37.37	37.7751	36.3904	37.3749	36.2194	0.79867683	0.966018836	24	4	24	0.498200627	0.807983182	24	13.2909862	0.02560412
ENSMUSG00000028578	Caap1	caspase activity and apoptosis inhibitor 1 [Source:MGI Symbol;Acc:MGI:1915020]	4.52991	4.31019	3.7514	3.83538	4.00863	4.16807	4.16244	4.06891	3.25558	3.59618	4.40912	3.44846	3.29112	3.71945	0.669104912	0.966018836	20	16	24	0.296412839	0.768040178	24	20.86664383	0.062104091
ENSMUSG00000028580	Pum1	pumilio RNA-binding family member 1 [Source:MGI Symbol;Acc:MGI:1931749]	10.6971	8.65775	9.57972	10.2866	9.10765	9.73082	10.5653	9.34297	8.49609	11.7814	8.3398	9.22918	9.07225	9.66229	0.93447422	0.966018836	4	16	24	0.983300811	0.996355202	24	15.89461836	0.009308296
ENSMUSG00000028581	Laptm5	lysosomal-associated protein transmembrane 5 [Source:MGI Symbol;Acc:MGI:108046]	4.53214	7.06631	8.00103	6.63602	6.27419	5.82198	6.08656	5.99266	8.42845	5.27188	5.73803	5.9637	5.79077	5.26621	0.286642595	0.793714531	12	16	24	0.135898129	0.747972781	24	9.028488666	0.154527289
ENSMUSG00000028582	Cc2d1b	coiled-coil and C2 domain containing 1B [Source:MGI Symbol;Acc:MGI:2443076]	31.308	33.9046	29.0766	34.8993	33.803	34.3045	31.5264	32.2153	31.3832	31.0796	28.0691	27.5458	31.4211	30.0751	0.79256873	0.966018836	8	16	24	0.997567764	0.999623846	24	7.056372866	0.002587495
ENSMUSG00000028583	Pdpn	podoplanin [Source:MGI Symbol;Acc:MGI:103098]	15.4038	15.5492	12.6314	10.5156	15.4551	13.1431	14.3604	14.3285	12.9194	9.78836	15.0364	12.3644	12.846	12.8969	0.336755135	0.85758369	20	20	24	0.113438348	0.727440321	24	23.23531206	0.139976658
ENSMUSG00000028584	Lrrc38	leucine rich repeat containing 38 [Source:MGI Symbol;Acc:MGI:2442845]	0.219881	0.503658	0.558376	0.464899	0.537951	0.554501	0.604739	0.889211	0.865526	0.968093	0.379605	0.593281	0.656987	0.515497	0.923635729	0.966018836	12	8	24	0.206324876	0.768040178	24	8.515921943	0.117130157
ENSMUSG00000028597	Gpx7	glutathione peroxidase 7 [Source:MGI Symbol;Acc:MGI:1914555]	6.94161	7.30873	7.42814	8.38255	7.48129	6.59739	6.23892	7.73343	8.71381	5.37439	7.46038	6.80245	6.47043	6.81885	0.106193509	0.519440352	12	16	24	0.445449032	0.805393068	24	9.207125741	0.077350094
ENSMUSG00000028599	Tnfrsf1b	tumor necrosis factor receptor superfamily, member 1b [Source:MGI Symbol;Acc:MGI:1314883]	1.88016	1.71969	2.15618	1.79995	1.92566	1.94339	1.56195	2.27823	1.78683	1.44383	2.05021	1.79851	1.78816	1.67928	0.933543173	0.966018836	20	20	24	0.996997627	0.999402894	24	20.15794336	0.003610025
ENSMUSG00000028600	Podn	podocan [Source:MGI Symbol;Acc:MGI:2674939]	2.59456	3.03837	3.637	4.88893	4.48283	2.58607	3.4357	3.07815	5.21504	3.6856	3.20087	3.21312	3.07034	2.52832	0.025973358	0.29944494	12	20	24	0.018562037	0.559812298	24	11.64489881	0.249264639
ENSMUSG00000028601	Echdc2	enoyl Coenzyme A hydratase domain containing 2 [Source:MGI Symbol;Acc:MGI:1289238]	4.54767	6.17066	5.9158	4.7164	4.14704	4.61708	4.23683	6.18134	6.3825	5.16605	5.90778	4.87465	5.44182	5.46098	0.030966082	0.324150946	12	12	24	0.021881034	0.574400012	24	7.04650661	0.172342758
ENSMUSG00000028603	Scp2	sterol carrier protein 2, liver [Source:MGI Symbol;Acc:MGI:98254]	33.0179	42.9755	39.8727	47.7211	42.1649	39.3841	41.4241	43.3062	44.7336	36.8051	48.1703	41.5554	47.6979	44.3927	0.837934376	0.966018836	20	4	24	0.649131201	0.881796509	24	10.19738428	0.050046144
ENSMUSG00000028607	Cpt2	carnitine palmitoyltransferase 2 [Source:MGI Symbol;Acc:MGI:109176]	13.1211	9.98176	12.7192	17.7555	13.5207	13.9602	12.3795	16.0903	11.9765	17.0582	14.8692	14.2236	16.736	14.3194	0.369262716	0.893596186	16	20	24	0.35625954	0.783018041	24	14.37057576	0.104495556
ENSMUSG00000028608	Czib	CXXC motif containing zinc binding protein [Source:MGI Symbol;Acc:MGI:1921348]	50.1766	51.7923	50.1934	43.565	50.6506	46.8281	49.2066	50.2206	55.0099	39.5623	51.6739	49.6817	51.2138	50.6062	0.359479536	0.883787544	12	4	24	0.268826374	0.768040178	24	1.850626051	0.071000046
ENSMUSG00000028609	Magoh	mago homolog, exon junction complex core component [Source:MGI Symbol;Acc:MGI:1330312]	38.6591	47.1166	40.7828	39.5753	45.6899	36.8605	38.3703	40.2714	48.074	34.4154	42.386	40.5628	44.2174	44.684	0.483881213	0.966018836	12	12	24	0.530119877	0.824795587	24	5.716349515	0.061954008
ENSMUSG00000028613	Lrp8	low density lipoprotein receptor-related protein 8, apolipoprotein e receptor [Source:MGI Symbol;Acc:MGI:1340044]	12.3011	9.19826	10.8978	10.0112	10.983	10.8104	9.53202	7.66232	9.14315	9.60743	11.4302	10.5963	10.1644	10.1968	0.064091035	0.414867207	20	12	24	0.089136246	0.708638675	24	18.19567193	0.124621023
ENSMUSG00000028614	Ndc1	NDC1 transmembrane nucleoporin [Source:MGI Symbol;Acc:MGI:1920037]	8.43666	8.1876	8.4628	8.12338	7.89056	8.52069	7.94916	8.00177	7.52516	9.175	8.17217	8.19567	8.27304	8.86538	0.854443826	0.966018836	24	20	24	0.988059436	0.997530201	24	10.87329644	0.004037595
ENSMUSG00000028617	Lrrc42	leucine rich repeat containing 42 [Source:MGI Symbol;Acc:MGI:1925059]	12.3512	11.967	10.9881	10.2854	11.5398	11.4424	9.67199	11.7089	10.4648	10.9329	12.0209	12.1912	11.9888	10.9485	0.201875332	0.688108864	24	16	24	0.353955987	0.781118317	24	21.09040975	0.055829316
ENSMUSG00000028618	Tmem59	transmembrane protein 59 [Source:MGI Symbol;Acc:MGI:1929278]	178.221	177.651	170.569	182.455	166.417	175.435	169.251	173.797	161.144	170.13	167.662	169.41	176.686	171.373	0.371875382	0.894537464	8	12	24	0.541904258	0.830559643	24	23.91189054	0.018954243
ENSMUSG00000028619	Tceanc2	transcription elongation factor A (SII) N-terminal and central domain containing 2 [Source:MGI Symbol;Acc:MGI:1913776]	10.6303	9.36789	10.1394	9.53333	10.4974	9.6983	10.7273	9.33811	7.88241	10.3172	10.6817	9.99262	10.6018	10.0072	0.079234726	0.458678242	4	4	24	0.137717729	0.747972781	24	19.80757072	0.08329261

ENSMUSG00000028621	Cyb5rl	cytochrome b5 reductase-like [Source:MGI Symbol;Acc:MGI:1919657]	2.07028	2.57439	2.71422	2.28942	2.91874	3.23447	2.47139	2.716	2.38448	2.80288	2.11122	2.36974	1.75393	3.24075	0.893609703	0.966018836	8	20	24	0.787507171	0.93167113	24	9.806537366	0.050785345
ENSMUSG00000028622	Mrpl37	mitochondrial ribosomal protein L37 [Source:MGI Symbol;Acc:MGI:1926268]	25.0244	23.2089	22.5352	25.1168	22.5704	24.7774	24.6196	26.1911	23.3838	27.3678	23.5987	25.1943	23.9346	24.0883	0.8972237	0.966018836	24	12	24	0.979323115	0.994983268	24	19.30819964	0.006480007
ENSMUSG00000028626	Col9a2	collagen, type IX, alpha 2 [Source:MGI Symbol;Acc:MGI:88466]	5.71633	6.49181	5.82336	5.31046	5.10043	4.42318	7.38353	7.65282	10.2301	7.73378	6.56056	6.64472	6.38382	7.30655	0.313786068	0.824470524	12	8	24	0.031420486	0.602100793	24	6.543301427	0.221339742
ENSMUSG00000028629	Exo5	exonuclease 5 [Source:MGI Symbol;Acc:MGI:1920422]	4.57878	4.90196	4.15536	3.44439	4.93463	4.39847	4.25211	4.48441	4.31597	3.96439	4.77883	4.74805	4.98566	4.37385	0.032547207	0.324908923	20	20	24	0.114996663	0.727790063	24	22.40591549	0.090541712
ENSMUSG00000028630	Dyrk2	dual-specificity tyrosine-(Y)-phosphorylation regulated kinase 2 [Source:MGI Symbol;Acc:MGI:1330301]	4.7754	4.67289	4.61113	4.63324	4.57647	4.90564	5.00835	5.07724	4.38878	5.09706	4.4415	4.76848	4.83612	4.86136	0.238201401	0.741459408	8	4	24	0.336445025	0.771895895	24	0.60645324	0.031115655
ENSMUSG00000028631	Kcnq4	potassium voltage-gated channel, subfamily Q, member 4 [Source:MGI Symbol;Acc:MGI:1926803]	6.4986	4.58579	4.86128	6.1062	4.96271	5.52295	5.39701	5.84561	4.14127	7.68552	5.43189	5.63077	5.69505	5.60984	0.834434272	0.966018836	16	20	24	0.719416798	0.907983128	24	17.52517342	0.060978258
ENSMUSG00000028633	Ctps	cytidine 5'-triphosphate synthase [Source:MGI Symbol;Acc:MGI:1858304]	15.7125	16.0405	17.0664	16.4324	15.2998	17.2252	15.8939	15.5399	14.4952	16.6506	14.5673	15.1872	15.6198	15.5142	0.865514257	0.966018836	16	4	24	0.99422944	0.998755922	24	9.644721448	0.002890891
ENSMUSG00000028634	Hivep3	human immunodeficiency virus type I enhancer binding protein 3 [Source:MGI Symbol;Acc:MGI:106589]	7.44869	7.76486	6.99857	7.80797	8.43136	8.53291	6.64579	7.47094	7.47458	9.03475	7.80833	7.83738	7.92281	7.69293	0.121531327	0.548996929	24	4	24	0.110321774	0.721828007	24	15.27897938	0.075263716
ENSMUSG00000028636	Ppcs	phosphopantothenoylcysteine synthetase [Source:MGI Symbol;Acc:MGI:1915237]	16.3246	13.4929	13.3795	12.4475	13.5566	13.8553	14.0574	14.7426	12.9809	13.3469	14.788	13.9761	15.9016	16.6755	7.56E-04	0.071344007	4	12	24	0.018268798	0.559812298	24	23.93311003	0.10023435
ENSMUSG00000028637	Ccdc30	coiled-coil domain containing 30 [Source:MGI Symbol;Acc:MGI:1920582]	3.22131	3.48275	4.44503	4.119	3.68068	3.5203	3.7628	3.79792	3.94628	4.23249	3.41728	4.54212	3.33704	4.46442	0.152782337	0.601788038	12	16	24	0.149631481	0.758377578	24	9.67173582	0.094780278
ENSMUSG00000028639	Ybx1	Y box protein 1 [Source:MGI Symbol;Acc:MGI:99146]	79.8948	90.9005	78.3361	88.8621	90.7044	79.6124	83.4486	81.2774	105.618	86.4494	90.7295	90.9046	100.916	80.1905	0.807813307	0.966018836	20	4	24	0.759208955	0.922160315	24	12.193663	0.03635448
ENSMUSG00000028641	P3h1	prolyl 3-hydroxylase 1 [Source:MGI Symbol;Acc:MGI:1888921]	6.7523	6.37621	6.22959	6.49841	7.45701	6.61324	7.7571	6.66749	7.6068	6.78255	7.3128	7.87909	7.37833	7.83635	0.68441981	0.966018836	24	16	24	0.409837224	0.799546792	24	21.05285282	0.040191092
ENSMUSG00000028643	Svbp	small vasohibin binding protein [Source:MGI Symbol;Acc:MGI:1916466]	16.9414	18.5582	16.5603	14.8936	16.8003	15.1577	18.2672	16.8739	20.0828	14.3222	20.1684	18.7957	20.5615	17.4308	0.305564675	0.811201458	4	12	24	0.386098837	0.799546792	24	0.98715505	0.07680602
ENSMUSG00000028644	Ermap	erythroblast membrane-associated protein [Source:MGI Symbol;Acc:MGI:1349816]	0.441062	0.902053	1.93965	1.35694	0.795149	1.02135	1.03858	0.828959	0.9869	0.653778	0.728082	0.859936	1.33359	0.914937	0.934818505	0.966018836	12	8	24	0.521701975	0.820407334	24	8.080304	0.117504935
ENSMUSG00000028645	Slc2a1	solute carrier family 2 (facilitated glucose transporter), member 1 [Source:MGI Symbol;Acc:MGI:95755]	19.2013	18.0616	21.3032	20.1463	18.0743	20.2435	20.6084	19.3264	17.5614	22.1687	16.7177	17.1909	16.9074	16.8975	0.91724565	0.966018836	16	4	24	0.632175469	0.875020466	24	11.05447223	0.043398432
ENSMUSG00000028646	Rragc	Ras-related GTP binding C [Source:MGI Symbol;Acc:MGI:1858751]	45.7574	38.1908	38.6433	43.4531	39.9089	43.7551	38.9156	40.6052	33.5402	44.7869	40.1771	40.911	41.4265	41.3103	0.338172159	0.85758369	24	12	24	0.313487761	0.768040178	24	19.14375613	0.067539364
ENSMUSG00000028647	Mycbp	MYC binding protein [Source:MGI Symbol;Acc:MGI:1891750]	14.1845	16.7071	13.5385	13.7987	15.3165	13.992	16.9757	14.1096	13.8229	13.2804	15.5906	16.6444	19.481	17.1406	0.008603252	0.192760771	4	12	24	0.040682092	0.616921492	24	23.58745862	0.124786208
ENSMUSG00000028648	Ndufs5	NADH:ubiquinone oxidoreductase core subunit S5 [Source:MGI Symbol;Acc:MGI:1890889]	56.3089	52.0505	48.4129	57.882	60.4199	59.0083	64.5291	61.3332	55.6401	56.4476	66.7509	47.5775	62.7637	55.4519	0.431555283	0.966018836	20	16	24	0.632287018	0.875020466	24	19.22314115	0.055167703
ENSMUSG00000028649	Macf1	microtubule-actin crosslinking factor 1 [Source:MGI Symbol;Acc:MGI:108559]	44.0207	43.282	47.5794	43.1674	41.094	44.8342	48.7131	45.4305	42.0358	46.0159	41.5667	43.7854	44.3813	47.2135	0.235141005	0.73629392	4	16	24	0.182133747	0.768040178	24	3.487624201	0.050003138
ENSMUSG00000028651	Ppie	peptidylprolyl isomerase E (cyclophilin E) [Source:MGI Symbol;Acc:MGI:1917118]	11.7164	13.4066	12.4172	11.9108	12.6174	13.1643	11.9105	12.6041	13.8678	11.188	13.8872	11.8984	13.0339	12.0732	0.915082018	0.966018836	20	8	24	0.992360127	0.998516607	24	6.31738307	0.004578759
ENSMUSG00000028653	Trit1	tRNA isopentenyltransferase 1 [Source:MGI Symbol;Acc:MGI:1914216]	3.61059	4.80733	4.66739	5.8136	4.56719	4.42163	4.49122	5.71073	4.58115	4.73362	4.21696	5.02355	4.11868	5.25973	0.076247883	0.453790923	8	20	24	0.209021944	0.768040178	24	8.951711705	0.103776984
ENSMUSG00000028654	Mycl	v-myc avian myelocytomatosis viral oncogene lung carcinoma derived [Source:MGI Symbol;Acc:MGI:96799]	0.746212	1.05568	1.16969	1.21135	1.00034	1.04049	1.11302	0.807202	1.29866	1.24467	0.698977	1.32638	0.998489	1.29036	0.393470605	0.921010321	16	4	24	0.39451024	0.799546792	24	9.112507611	0.079694625
ENSMUSG00000028655	Mfsd2a	major facilitator superfamily domain containing 2A [Source:MGI Symbol;Acc:MGI:1923824]	15.1466	13.3563	14.1845	15.8378	17.6881	16.3343	17.4581	13.8883	14.1046	19.234	12.7358	13.4069	14.1744	11.3853	0.138719141	0.580158868	16	16	24	0.227513902	0.768040178	24	16.02375647	0.119188138
ENSMUSG00000028656	Cap1	CAP, adenylate cyclase-associated protein 1 (yeast) [Source:MGI Symbol;Acc:MGI:88262]	103.245	89.7695	88.1593	93.0303	94.9679	100.135	100.768	94.5109	80.2379	103.242	88.6074	97.8351	99.0215	92.7763	0.004140921	0.140598674	4	8	24	0.068103762	0.672292246	24	20.85411301	0.088804212
ENSMUSG00000028657	Ppt1	palmitoyl-protein thioesterase 1 [Source:MGI Symbol;Acc:MGI:1298204]	41.1854	44.1973	41.8594	41.0004	42.2277	42.999	42.2311	43.8526	41.8902	42.5556	40.9891	43.2692	43.2888	44.4486	0.015715881	0.253395174	8	12	24	0.073660468	0.688238921	24	2.713369258	0.029078095
ENSMUSG00000028661	Epha8	Eph receptor A8 [Source:MGI Symbol;Acc:MGI:109378]	0.599796	0.841131	0.955754	0.702955	0.589998	0.681231	0.722963	1.04019	1.17319	0.838056	0.643173	0.760546	0.627731	0.937048	0.005162915	0.145313511	12	8	24	0.001316845	0.346832852	24	7.100961412	0.163053338
ENSMUSG00000028664	Ephb2	Eph receptor B2 [Source:MGI Symbol;Acc:MGI:99611]	1.29598	1.10333	1.37354	1.37572	1.36978	1.33072	1.23022	1.12379	1.14219	1.17866	1.2667	1.25275	1.35411	1.31058	0.522551072	0.966018836	20	12	24	0.316958587	0.768040178	24	17.42957571	0.037093387
ENSMUSG00000028668	Eloa	elongin A [Source:MGI Symbol;Acc:MGI:1351315]	8.72837	8.59769	8.02584	8.32409	8.56717	9.13472	8.43205	8.59964	7.81214	8.87875	8.68383	8.9156	8.45077	8.31881	0.001971398	0.100255953	24	12	24	0.019587136	0.564709807	24	19.51553673	0.053710055
ENSMUSG00000028669	Pithd1	PITH (C-terminal proteasome-interacting domain of thioredoxin-like) domain containing 1 [Source:MGI Symbol;Acc:MGI:1913443]	30.811	30.9108	31.574	28.585	29.2538	30.7849	30.6839	28.6919	29.0451	33.386	27.1374	31.4252	29.9139	29.7447	0.874607729	0.966018836	24	20	24	0.891043023	0.969283641	24	1.651168581	0.013799309
ENSMUSG00000028670	Lypla2	lysophospholipase 2 [Source:MGI Symbol;Acc:MGI:1347000]	43.8518	29.8833	32.1639	33.2037	31.5984	35.1936	38.0479	38.3957	29.3807	44.7325	36.9247	37.996	35.9426	34.7993	0.286642595	0.793714531	4	8	24	0.479936517	0.805393068	24	20.63280563	0.087384085
ENSMUSG00000028671	Gale	galactose-4-epimerase, UDP [Source:MGI Symbol;Acc:MGI:1921496]	20.2136	17.5475	16.6309	19.0415	18.0703	19.8623	15.6864	18.6788	17.7868	19.2202	18.2363	18.4948	20.3324	17.7332	0.441501717	0.966018836	4	4	24	0.536379386	0.827399772	24	18.89996078	0.045578229
ENSMUSG00000028672	Hmgcl	3-hydroxy-3-methylglutaryl-Coenzyme A lyase [Source:MGI Symbol;Acc:MGI:96158]	13.8655	13.4604	14.7919	14.5709	15.2362	14.6513	16.141	15.3529	14.5648	13.4168	15.1506	14.8263	14.8917	15.7295	0.745469888	0.966018836	20	20	24	0.707600471	0.903565455	24	23.04594165	0.022428722
ENSMUSG00000028673	Fuca1	fucosidase, alpha-L- 1, tissue [Source:MGI Symbol;Acc:MGI:95593]	28.6577	31.8548	26.1967	32.3353	32.5753	30.8972	34.5838	27.8337	29.1959	29.1409	29.7767	26.8098	27.4465	29.9469	0.966657897	0.966018836	20	16	24	0.744053921	0.916233618	24	17.8458146	0.034362716
ENSMUSG00000028675	Pnrc2	proline-rich nuclear receptor coactivator 2 [Source:MGI Symbol;Acc:MGI:106512]	29.7166	34.5418	31.6541	33.3187	33.6401	29.9629	32.8512	31.2111	32.9632	26.5179	34.9224	27.8255	30.0861	32.4047	0.265774856	0.773510818	20	4	24	0.801563898	0.936422183	24	8.027414103	0.030602747
ENSMUSG00000028676	Srsf10	serine and arginine-rich splicing factor 10 [Source:MGI Symbol;Acc:MGI:1333805]	24.7928	24.1171	24.6398	24.778	23.7054	22.8164	25.2128	21.501	24.759	20.5758	25.9311	23.1893	21.9703	23.1349	0.890235289	0.966018836	20	12	24	0.915968067	0.976753216	24	17.27240502	0.014682542
ENSMUSG00000028677	Rnf220	ring finger protein 220 [Source:MGI Symbol;Acc:MGI:1913993]	30.9246	30.6057	31.4969	30.4942	28.8416	31.4265	28.9384	33.7391	32.4118	37.6755	26.8369	35.3318	31.2816	33.8281	0.784278077	0.966018836	8	12	24	0.462391515	0.805393068	24	6.944720702	0.058552177
ENSMUSG00000028680	Plk3	polo like kinase 3 [Source:MGI Symbol;Acc:MGI:109604]	4.03566	3.17589	3.4409	3.11249	3.51552	3.19379	3.39466	3.45584	3.07997	3.9198	3.47559	3.1032	3.49994	3.10091	0.708020493	0.966018836	20	16	24	0.842929758	0.952259721	24	19.0325259	0.023019058
ENSMUSG00000028681	Ptch2	patched 2 [Source:MGI Symbol;Acc:MGI:1095405]	0.50305	0.799871	0.967051	0.518447	0.836713	0.679135	0.497624	0.950756	1.0284	0.673617	0.925445	0.628479	1.34162	0.706129	0.46238066	0.966018836	12	12	24	0.573046294	0.846884635	24	7.06632963	0.07674012
ENSMUSG00000028683	Eif2b3	eukaryotic translation initiation factor 2B, subunit 3 [Source:MGI Symbol;Acc:MGI:1313286]	11.9797	13.6116	14.1387	13.859	13.5198	12.6527	11.9237	12.9173	14.7148	12.3374	13.1825	13.1217	13.9195	13.6241	0.074036902	0.448301997	12	16	24	0.116572975	0.727799063	24	9.042389763	0.066415177
ENSMUSG00000028684	Urod	uroporphyrinogen decarboxylase [Source:MGI Symbol;Acc:MGI:98916]	44.8887	48.3441	42.6098	44.9315	41.6356	41.0306	40.464	44.4907	41.3756	41.4373	43.1835	40.254	45.8725	44.6369	0.286642595	0.793714531	8	16	24	0.197194665	0.768040178	24	4.367592264	0.051265843
ENSMUSG00000028687	Mutyh	mutY DNA glycosylase [Source:MGI Symbol;Acc:MGI:1917853]	2.00836	1.39937	1.78987	1.50225	1.84447	1.3202	1.78622	1.61635	2.14652	1.566	2.02912	1.70281	1.75913	1.87812	0.912686132	0.966018836	12	12	24	0.923303621	0.979439431	24	7.568052484	0.02105506
ENSMUSG00000028688	Toe1	target of EGR1, member 1 (nuclear) [Source:MGI Symbol;Acc:MGI:1915526]	8.40568	8.35198	9.87388	8.79016	9.3935	8.51997	8.22742	8.44342	8.25599	7.77331	8.54319	8.49207	8.88373	8.67232	0.910436409	0.966018836	20	12	24	0.913696423	0.975865799	24	12.79969675	0.012594913
ENSMUSG00000028689	Ccdc163	coiled-coil domain containing 163 [Source:MGI Symbol;Acc:MGI:1915644]	10.5326	10.6088	11.4597	10.6078	10.4103	9.58374	14.3217	10.5278	10.1938	9.18722	11.4127	11.4628	10.986	12.751	0.657586167	0.966018836	4	12	24	0.302819823	0.768040178	24	1.248788007	0.085596509
ENSMUSG00000028690	Mmachc	methylmalonic aciduria cblC type, with homocystinuria [Source:MGI Symbol;Acc:MGI:1914346]	10.2173	10.057	9.59922	11.3818	9.05837	10.7598	10.4869	10.5999	8.2781	11.8997	9.0406	10.0459	10.0374	9.51768	0.956719163	0.96629412	16	20	24	0.923376413	0.979439431	24	19.27757468	0.020919659
ENSMUSG00000028691	Prdx1	peroxiredoxin 1 [Source:MGI Symbol;Acc:MGI:99523]	45.5643	59.9589	55.0857	54.3373	58.5783	47.2719	53.2866	53.7274	61.3347	42.0945	55.9037	49.0496	53.9708	51.5526	0.390686537	0.915479136	12	12	24	0.570935459	0.845581812	24	7.566477605	0.06587626
ENSMUSG00000028692	Akr1a1	aldo-keto reductase family 1, member A1 (aldehyde reductase) [Source:MGI Symbol;Acc:MGI:1929955]	322.156	305.036	298.232	295.225	306.646	312.411	299.375	311.222	285.015	295.968	310.175	311.139	313.398	304.901	0.004012334	0.140598674	4	12	24	0.00636151	0.470033096	24	21.71249604	0.048967664
ENSMUSG00000028693	Nasp	nuclear autoantigenic sperm protein (histone-binding) [Source:MGI Symbol;Acc:MGI:1355328]	11.3574	11.8978	11.7668	11.8869	11.5116	11.2407	11.3828	9.75171	12.5654	9.52517	13.0145	10.7704	11.9425	11.2309	0.896799496	0.966018836	12	12	24	0.940593143	0.985398871	24	15.83917912	0.015908984
ENSMUSG00000028696	Ipp	IAP promoted placental gene [Source:MGI Symbol;Acc:MGI:96581]	4.15307	4.68124	4.58582	4.36752	4.69547	4.64836	4.90259	4.47873	4.52054	4.62937	4.60146	5.27474	4.95115	4.81818	0.417653118	0.963884303	24	12	24	0.383581665	0.799546792	24	21.33781113	0.030022411
ENSMUSG00000028698	Pik3r3	phosphoinositide-3-kinase regulatory subunit 3 [Source:MGI Symbol;Acc:MGI:109277]	15.3855	14.4675	14.7559	14.5079	15.0906	14.3391	13.2955	12.8156	14.1553	14.2764	14.5783	14.8058	14.2355	15.7855	0.91241026	0.966018836	20	8	24	0.792191709	0.933498508	24	15.89284253	0.019782801
ENSMUSG00000028700	Pomgnt1	protein O-linked mannose beta 1,2-N-acetylglucosaminyltransferase [Source:MGI Symbol;Acc:MGI:1915523]	26.4436	25.2696	25.1436	26.9634	30.2863	26.7877	26.8423	25.293	26.7042	28.7762	27.2348	26.441	27.4106	25.8375	0.014316001	0.24509106	20	12	24	0.018543997	0.559812298	24	15.96913409	0.067881154
ENSMUSG00000028701	Lurap1	leucine rich adaptor protein 1 [Source:MGI Symbol;Acc:MGI:1915325]	6.83851	6.72121	6.68558	7.09673	7.51987	6.21066	7.9918	6.66051	7.36791	6.87904	6.41654	6.69477	7.67445	6.88313	0.920872048	0.966018836	4	20	24	0.859375321	0.95855262	24	3.083768945	0.019624224
ENSMUSG00000028702	Rad54l	RAD54 like (S. cerevisiae) [Source:MGI Symbol;Acc:MGI:894697]	0.539792	0.766382	0.86782	0.63072	0.809481	0.560678	0.601588	0.656943	0.75803	0.489315	0.806619	0.701719	0.82393	0.665583	0.953350544	0.966018836	12	12	24	0.837314902	0.950299676	24	8.794608837	0.025394829
ENSMUSG00000028703	Lrrc41	leucine rich repeat containing 41 [Source:MGI Symbol;Acc:MGI:2441984]	16.6616	14.4614	14.0205	14.9024	13.7207	15.8782	15.2562	15.6878	13.9701	15.6843	14.3469	15.2582	14.9135	14.3657	0.087592959	0.472021905	24	12	24	0.190883058	0.768040178	24	22.29644903	0.053455834
ENSMUSG00000028706	Nsun4	NOL1/NOP2/Sun domain family, member 4 [Source:MGI Symbol;Acc:MGI:1919431]	9.24106	8.59496	8.5821	9.00009	8.55267	9.53844	8.89884	8.59723	8.00553	9.94829	8.51691	8.08916	8.69986	8.1718	0.742579234	0.966018836	16	20	24	0.55837242	0.839674578	24	17.24239139	0.032864139
ENSMUSG00000028708	Mknk1	MAP kinase-interacting serine/threonine kinase 1 [Source:MGI Symbol;Acc:MGI:894316]	15.3379	17.625	18.9695	18.123	20.0361	17.7025	16.8191	17.7091	19.1498	18.3431	16.9072	15.23	16.1921	16.299	0.009118941	0.192760771	12	16	24	0.014020586	0.530670295	24	11.06708041	0.106791966
ENSMUSG00000028709	Mob3c	MOB kinase activator 3C [Source:MGI Symbol;Acc:MGI:2140623]	2.06551	2.14022	1.88404	2.32963	2.19153	2.08615	2.1519	2.68098	2.76908	2.43076	2.45223	2.27975	2.35567	2.12998	0.852772142	0.966018836	16	12	24	0.456550047	0.805393068	24	10.00081819	0.047891449
ENSMUSG00000028710	Atpaf1	ATP synthase mitochondrial F1 complex assembly factor 1 [Source:MGI Symbol;Acc:MGI:2180560]	7.98716	7.16506	8.37837	6.8248	5.99889	7.30871	7.56111	7.11573	6.58947	9.51621	7.79771	8.09685	8.44016	8.65214	0.896753532	0.966018836	4	16	24	0.782412446	0.930180373	24	3.744695895	0.039825962
ENSMUSG00000028713	Cyp4b1	cytochrome P450, family 4, subfamily b, polypeptide 1 [Source:MGI Symbol;Acc:MGI:103225]	1.51749	0.87803	1.83432	2.01741	2.1082	1.34299	1.69186	1.70968	1.06504	2.09885	2.16427	1.56258	1.40179	0.95124	0.016024075	0.253395174	20	12	24	0.021954923	0.574400012	24	15.15289022	0.236547142
ENSMUSG00000028716	Pdzk1ip1	PDZK1 interacting protein 1 [Source:MGI Symbol;Acc:MGI:1914432]	1.57721	2.14338	1.88928	2.14797	1.28529	2.16917	2.0031	2.02317	2.09032	1.49781	1.52463	2.59742	2.03171	1.66302	0.676344318	0.966018836	24	20	24	0.525652248	0.822686255	24	1.547375847	0.078410748
ENSMUSG00000028717	Tal1	T cell acute lymphocytic leukemia 1 [Source:MGI Symbol;Acc:MGI:98480]	0.410218	0.726189	0.99943	1.10557	0.581782	0.854649	0.748597	0.843506	0.829661	0.59337	0.668537	0.70604	0.945997	0.548234	0.90703203	0.966018836	12	8	24	0.612264632	0.865456875	24	9.359897628	0.064534585
ENSMUSG00000028719	Cmpk1	cytidine monophosphate (UMP-CMP) kinase 1 [Source:MGI Symbol;Acc:MGI:1913838]	48.548	46.4689	46.949	43.5227	44.8716	44.7008	42.8034	42.4489	42.3573	42.3149	50.1878	44.2575	45.379	46.3486	0.922029406	0.966018836	20	20	24	0.688343142	0.896921945	24	20.60388064	0.0266619
ENSMUSG00000028729	Ebna1bp2	EBNA1 binding protein 2 [Source:MGI Symbol;Acc:MGI:1916322]	27.7702	28.1908	28.9005	26.2937	28.2276	27.6705	29.427	27.4183	26.4931	24.1957	26.7703	27.485	30.49	26.3592	0.008961721	0.192760771	4	12	24	0.046173681	0.633283523	24	23.53947252	0.068915613
ENSMUSG00000028730	Cfap57	cilia and flagella associated protein 57 [Source:MGI Symbol;Acc:MGI:2686209]	0.533335	0.63198	0.759205	0.636683	0.595409	0.498555	0.514628	0.525892	0.456677	0.480872	0.574351	0.686404	0.415105	0.742464	0.921572192	0.966018836	8	20	24	0.768574466	0.925397739	24	9.23659779	0.028753864
ENSMUSG00000028737	Aldh4a1	aldehyde dehydrogenase 4 family, member A1 [Source:MGI Symbol;Acc:MGI:2443883]	7.62599	7.2209	7.00353	8.97641	7.39632	7.93593	8.07401	9.27068	7.91999	9.9759	7.82043	8.00669	8.12606	8.42391	0.919571723	0.966018836	16	4	24	0.575419056	0.848049166	24	10.38624643	0.048556565
ENSMUSG00000028741	Mrto4	mRNA turnover 4, ribosome maturation factor [Source:MGI Symbol;Acc:MGI:1917152]	11.3821	10.168	11.6254	10.3833	10.8008	10.3248	9.86788	10.5313	10.8515	10.6607	10.514	11.8332	9.57041	10.7867	0.920620634	0.966018836	12	16	24	0.753515266	0.920152554	24	12.52761233	0.023179654
ENSMUSG00000028743	Akr7a5	aldo-keto reductase family 7, member A5 (aflatoxin aldehyde reductase) [Source:MGI Symbol;Acc:MGI:107796]	11.4834	8.27071	8.5952	9.81481	9.36017	10.9775	10.197	11.4026	8.58084	11.6433	12.1194	9.50798	11.7166	8.67779	0.23371652	0.736061043	4	8	24	0.247698916	0.768040178	24	19.17959073	0.11628819
ENSMUSG00000028744	Slc66a1	solute carrier family 66 member 1 [Source:MGI Symbol;Acc:MGI:2384837]	6.46132	5.11689	5.96658	5.77902	5.31513	6.89209	5.15765	5.54752	4.98482	7.00738	6.20801	6.25639	5.45093	5.57204	0.360675801	0.883978426	24	8	24	0.213774368	0.768040178	24	16.9351237	0.091622857
ENSMUSG00000028745	Capzb	capping protein (actin filament) muscle Z-line, beta [Source:MGI Symbol;Acc:MGI:104652]	142.897	123.335	125.727	124.004	122.623	142.266	125.197	136.503	113.394	145.851	123.068	135.367	132.125	138.083	0.767183862	0.966018836	24	20	24	0.450656166	0.805393068	24	22.17080014	0.053309503
ENSMUSG00000028755	Cda	cytidine deaminase [Source:MGI Symbol;Acc:MGI:1919519]	33.4505	25.4107	26.1593	24.5543	25.1461	31.3958	29.0343	29.9672	21.8105	31.5609	24.4587	26.4294	27.7938	27.1909	0.119434414	0.542554768	4	16	24	0.188843725	0.768040178	24	22.70546764	0.115624472
ENSMUSG00000028756	Pink1	PTEN induced putative kinase 1 [Source:MGI Symbol;Acc:MGI:1916193]	112.563	118.295	115.901	118.378	106.809	123.942	117.024	123.287	120.477	138.389	105.912	127.978	122.826	128.103	0.945451136	0.966018836	8	12	24	0.503261177	0.810053779	24	5.985562591	0.043692015
ENSMUSG00000028757	Ddost	dolichyl-di-phosphooligosaccharide-protein glycotransferase [Source:MGI Symbol;Acc:MGI:1194508]	36.8333	32.3334	33.2132	30.5648	34.4795	35.5317	35.3714	35.3666	30.1708	34.2971	32.3869	34.1477	33.2112	31.6617	0.098024777	0.501188411	4	8	24	0.030421326	0.597890721	24	21.4977142	0.077066608
ENSMUSG00000028758	Kif17	kinesin family member 17 [Source:MGI Symbol;Acc:MGI:1098229]	1.26682	1.00844	1.4064	0.98251	1.04877	1.14112	1.20475	0.982671	1.08611	1.19976	0.895849	1.03891	0.870174	1.01435	0.885059765	0.966018836	12	20	24	0.89803432	0.970959341	24	6.997042163	0.016857992
ENSMUSG00000028759	Hp1bp3	heterochromatin protein 1, binding protein 3 [Source:MGI Symbol;Acc:MGI:109369]	65.2515	55.9185	60.7584	59.6113	56.233	62.5395	60.5069	59.036	56.2393	69.9452	55.6628	63.4715	62.655	63.5823	0.745118544	0.966018836	4	16	24	0.87340412	0.964477588	24	22.70312056	0.019976618
ENSMUSG00000028760	Eif4g3	eukaryotic translation initiation factor 4 gamma, 3 [Source:MGI Symbol;Acc:MGI:1923935]	28.9843	26.4271	24.381	25.1287	25.6628	27.8543	27.3573	28.896	22.4798	35.4917	23.0925	25.7938	27.0073	27.6004	0.15498249	0.607427992	4	8	24	0.874205648	0.964836189	24	0.283031516	0.032676149
ENSMUSG00000028763	Hspg2	perlecan (heparan sulfate proteoglycan 2) [Source:MGI Symbol;Acc:MGI:96257]	27.8478	29.3076	26.9066	25.6604	31.517	24.952	35.8175	36.4737	38.5226	47.4258	41.2798	39.0339	46.5669	32.8917	0.733896783	0.966018836	16	20	24	0.944184338	0.986326199	24	10.69340356	0.027814668
ENSMUSG00000028766	Alpl	alkaline phosphatase, liver/bone/kidney [Source:MGI Symbol;Acc:MGI:87983]	3.18866	3.14614	2.90423	2.85546	3.7289	4.04113	3.21777	3.6656	3.64845	3.34022	3.68967	3.38743	3.02393	3.03887	0.219644992	0.708945071	20	20	24	0.285970387	0.768040178	24	17.85047751	0.073144363
ENSMUSG00000028771	Ptpn12	protein tyrosine phosphatase, non-receptor type 12 [Source:MGI Symbol;Acc:MGI:104673]	23.0124	21.755	22.7927	23.8724	23.981	23.9333	26.6693	21.7668	19.4754	19.018	23.8112	18.4157	19.6608	19.2205	0.867888967	0.966018836	20	12	24	0.337473112	0.772409896	24	18.99921383	0.074321661
ENSMUSG00000028772	Zcchc17	zinc finger, CCHC domain containing 17 [Source:MGI Symbol;Acc:MGI:1919955]	23.5806	21.8511	23.6251	21.1912	23.5295	23.2979	21.1244	20.7451	24.9464	23.7656	23.6238	24.0149	26.356	24.8273	0.819898687	0.966018836	12	20	24	0.978543282	0.994595131	24	17.65559471	0.007113036
ENSMUSG00000028773	Fabp3	fatty acid binding protein 3, muscle and heart [Source:MGI Symbol;Acc:MGI:95476]	9.82803	10.9631	14.0273	17.8067	11.574	12.6365	10.0711	22.4281	16.0041	16.1358	13.9909	10.3584	15.199	14.0944	0.080511813	0.4593055	16	12	24	0.046289528	0.63341866	24	9.053956723	0.285289557
ENSMUSG00000028776	Tinagl1	tubulointerstitial nephritis antigen-like 1 [Source:MGI Symbol;Acc:MGI:2137617]	5.84028	4.95032	5.45711	7.17772	6.80043	5.34294	6.50561	6.58402	5.71437	6.68134	4.87309	5.48961	5.42564	4.66936	0.657586167	0.966018836	16	16	24	0.459349375	0.805393068	24	13.5931512	0.077487887
ENSMUSG00000028778	Hcrtr1	hypocretin (orexin) receptor 1 [Source:MGI Symbol;Acc:MGI:2385650]	2.35833	1.52069	2.04425	1.69116	2.19938	2.63204	2.63888	1.69491	1.35523	1.87148	2.22984	2.05003	2.32585	2.58711	0.016795343	0.253395174	4	8	24	0.038173642	0.61140397	24	21.01579616	0.183839025
ENSMUSG00000028779	Pef1	penta-EF hand domain containing 1 [Source:MGI Symbol;Acc:MGI:1915148]	62.7523	56.0609	56.9648	55.9293	57.3208	63.3981	56.9785	58.8886	51.8144	54.7864	57.8867	57.8754	57.8862	56.3276	0.01813461	0.256345714	24	16	24	0.005982297	0.46443032	24	21.19870076	0.075392119
ENSMUSG00000028780	Sema3c	sema domain, immunoglobulin domain (Ig), short basic domain, secreted, (semaphorin) 3C [Source:MGI Symbol;Acc:MGI:107557]	8.35691	8.40278	7.33411	7.70161	8.21624	7.04608	6.99349	7.25366	8.31586	6.37356	8.40261	7.58098	7.54155	8.12677	0.858266942	0.966018836	20	4	24	0.949975928	0.988090608	24	2.24168301	0.013786803
ENSMUSG00000028782	Adgrb2	adhesion G protein-coupled receptor B2 [Source:MGI Symbol;Acc:MGI:2451244]	2.73691	3.49713	5.34525	3.98098	2.74731	3.49201	4.26992	2.81766	3.84057	4.00851	3.23975	3.67008	2.9243	4.47479	0.287703522	0.793714531	16	4	24	0.201985752	0.768040178	24	8.574159803	0.160735024
ENSMUSG00000028785	Hpca	hippocalcin [Source:MGI Symbol;Acc:MGI:1336200]	16.71	18.5937	21.8692	15.2115	16.7036	18.7547	18.6785	17.3892	19.3091	18.7291	15.1824	17.39	16.9946	18.5687	0.117335378	0.538362324	12	8	24	0.184365487	0.768040178	24	5.675437096	0.093745813
ENSMUSG00000028786	Tmem54	transmembrane protein 54 [Source:MGI Symbol;Acc:MGI:1913510]	3.23078	2.97808	2.58087	2.83444	2.88218	3.11768	2.38877	2.88073	1.80389	2.28841	2.86524	2.77904	2.34452	2.95636	0.476029854	0.966018836	8	4	24	0.220362846	0.768040178	24	20.82340147	0.103053079
ENSMUSG00000028788	Ptp4a2	protein tyrosine phosphatase 4a2 [Source:MGI Symbol;Acc:MGI:1277117]	52.1015	53.7896	53.7238	55.6171	52.2172	52.5687	54.5796	60.2117	65.0118	61.5665	57.6014	60.9904	57.8885	56.8883	0.920858634	0.966018836	12	16	24	0.116278636	0.727799063	24	8.931732659	0.059786087
ENSMUSG00000028789	Azin2	antizyme inhibitor 2 [Source:MGI Symbol;Acc:MGI:2442093]	46.9575	35.8142	40.7388	37.032	40.8359	43.8821	36.9131	38.6533	32.4675	42.404	43.1175	40.1933	40.7473	39.4722	0.130356961	0.561940267	20	16	24	0.112645832	0.726918602	24	19.23793154	0.106000027
ENSMUSG00000028790	Khdrbs1	KH domain containing, RNA binding, signal transduction associated 1 [Source:MGI Symbol;Acc:MGI:893579]	24.3108	24.9527	24.5187	23.6956	24.2213	23.1551	23.434	24.3962	23.3207	27.2542	21.1072	23.5766	23.0478	25.8292	0.146541269	0.590732968	8	16	24	0.251538427	0.768040178	24	7.061191509	0.054067873
ENSMUSG00000028792	Ak2	adenylate kinase 2 [Source:MGI Symbol;Acc:MGI:87978]	20.9011	19.9395	22.2329	23.1189	20.7268	19.118	20.6102	22.3713	20.8418	22.9676	25.7278	22.108	24.9902	23.2025	0.908406451	0.966018836	20	4	24	0.491173584	0.805393068	24	11.31949286	0.044288104
ENSMUSG00000028793	Rnf19b	ring finger protein 19B [Source:MGI Symbol;Acc:MGI:1922484]	18.9438	15.1203	16.3067	15.7284	17.2489	18.6081	16.0147	17.5344	13.2199	19.9755	16.095	16.8847	16.1743	16.1648	0.764889554	0.966018836	24	12	24	0.312521976	0.768040178	24	18.98585769	0.085389578
ENSMUSG00000028794	A3galt2	alpha 1,3-galactosyltransferase 2 (isoglobotriaosylceramide synthase) [Source:MGI Symbol;Acc:MGI:2685279]	14.77	10.233	9.98349	11.8205	11.9577	13.5443	11.5297	12.3616	8.51708	14.243	12.1103	12.7869	12.7823	11.7992	0.010886922	0.200176658	24	12	24	0.079771651	0.694370548	24	19.76595676	0.16447667
ENSMUSG00000028795	Ccdc28b	coiled coil domain containing 28B [Source:MGI Symbol;Acc:MGI:1913514]	25.6338	28.0347	25.1734	25.6513	26.2149	26.9393	24.2854	23.5251	25.5307	22.6456	27.5587	25.5553	26.9504	21.2936	0.593560247	0.966018836	20	12	24	0.216049777	0.768040178	24	18.78309726	0.068379823
ENSMUSG00000028796	Phc2	polyhomeotic 2 [Source:MGI Symbol;Acc:MGI:1860454]	26.9334	22.6296	21.487	22.9828	25.4063	23.4701	26.7983	23.3661	21.4168	27.3627	23.6373	25.1825	26.0524	23.0619	0.018194955	0.256345714	4	8	24	0.109552051	0.72082227	24	20.25080817	0.091803313
ENSMUSG00000028797	Tmem234	transmembrane protein 234 [Source:MGI Symbol;Acc:MGI:1924049]	67.921	64.8939	68.4335	64.6133	68.6575	68.6458	66.4829	63.7055	66.594	64.544	74.3774	70.2877	69.7849	69.5027	0.079180494	0.458678242	20	20	24	0.143733267	0.752803875	24	18.82254093	0.041719382
ENSMUSG00000028798	Eif3i	eukaryotic translation initiation factor 3, subunit I [Source:MGI Symbol;Acc:MGI:1860763]	83.9368	91.3974	88.0382	86.2444	94.0039	88.3239	77.7821	89.8052	91.3313	73.8755	89.2824	84.8948	88.7266	85.436	0.772122688	0.966018836	20	8	24	0.994405343	0.998755922	24	5.290886382	0.004003741
ENSMUSG00000028799	Zfp362	zinc finger protein 362 [Source:MGI Symbol;Acc:MGI:2652839]	4.49877	4.94506	4.94266	4.3659	4.64882	4.31755	4.90924	5.10297	5.32258	5.90093	4.62933	4.70815	4.94591	4.98929	0.076438025	0.453790923	12	8	24	0.047295216	0.633507364	24	7.710156826	0.084627893
ENSMUSG00000028800	Hdac1	histone deacetylase 1 [Source:MGI Symbol;Acc:MGI:108086]	8.23807	10.167	10.0459	9.46352	11.088	8.58571	10.0519	8.41573	11.4597	9.42333	11.5364	9.99766	10.1223	9.50688	0.851656735	0.966018836	20	4	24	0.304145932	0.768040178	24	12.66927723	0.079007034
ENSMUSG00000028801	Stpg1	sperm tail PG rich repeat containing 1 [Source:MGI Symbol;Acc:MGI:1926056]	1.17161	1.19826	1.29663	1.3123	0.904112	1.04909	1.15939	1.03576	0.950057	1.57052	0.892507	1.11594	1.39474	1.38957	0.265774856	0.773510818	16	4	24	0.444354769	0.805393068	24	6.221360988	0.068145363
ENSMUSG00000028803	Nipal3	NIPA-like domain containing 3 [Source:MGI Symbol;Acc:MGI:1921802]	96.2941	64.2007	62.1486	73.1944	64.9503	82.865	73.2653	81.0301	51.4221	98.6584	62.2479	82.2232	76.66	77.2457	0.148073804	0.590732968	24	12	24	0.388390268	0.799546792	24	21.47658297	0.141788892
ENSMUSG00000028804	Csmd2	CUB and Sushi multiple domains 2 [Source:MGI Symbol;Acc:MGI:2386401]	3.71237	3.18493	3.28746	3.2794	3.23849	3.23255	3.08956	3.25791	3.24883	3.43426	3.36091	3.05326	2.76018	3.76498	0.32670903	0.845515883	16	8	24	0.664130421	0.886686094	24	8.829107058	0.031534523
ENSMUSG00000028807	Zbtb8a	zinc finger and BTB domain containing 8a [Source:MGI Symbol;Acc:MGI:1920930]	2.52402	3.06601	2.47562	3.18266	2.51961	3.19788	2.60175	2.83051	2.64467	2.70299	3.08206	3.08087	2.83575	3.02939	0.881718085	0.966018836	24	12	24	0.790488603	0.932883394	24	18.30877407	0.027004901
ENSMUSG00000028809	Srrm1	serine/arginine repetitive matrix 1 [Source:MGI Symbol;Acc:MGI:1858303]	45.3316	49.18	49.1944	49.9829	51.8588	43.3379	51.601	45.6208	56.2423	49.4512	52.3586	47.1668	46.5174	44.3648	0.097373374	0.501112642	20	4	24	0.128941281	0.740162193	24	11.67827317	0.078029508
ENSMUSG00000028811	Yars	tyrosyl-tRNA synthetase [Source:MGI Symbol;Acc:MGI:2147627]	29.0552	24.9212	28.0571	25.3822	24.8734	30.1022	26.3091	27.9975	22.1015	32.1851	25.5562	26.6887	25.8634	26.7597	0.891742979	0.966018836	24	20	24	0.801823994	0.936565832	24	19.88531281	0.036197901
ENSMUSG00000028820	Sfpq	splicing factor proline/glutamine rich (polypyrimidine tract binding protein associated) [Source:MGI Symbol;Acc:MGI:1918764]	46.0506	45.8607	41.872	49.1084	50.6065	44.6674	46.0242	36.4072	44.4104	39.2224	41.5635	43.3363	48.3662	42.7045	0.649148181	0.966018836	4	8	24	0.401043425	0.799546792	24	19.24953179	0.066178629
ENSMUSG00000028821	Syf2	SYF2 homolog, RNA splicing factor (S. cerevisiae) [Source:MGI Symbol;Acc:MGI:1915842]	40.8773	42.6218	42.1045	38.5085	40.9433	37.0603	41.5894	37.7287	45.8197	38.112	42.374	41.5726	41.976	40.79	0.897570341	0.966018836	12	12	24	0.693062763	0.898227246	24	5.605237682	0.028287141
ENSMUSG00000028822	Tmem50a	transmembrane protein 50A [Source:MGI Symbol;Acc:MGI:1919067]	236.313	234.681	207.465	219.56	225.419	210.176	216.28	215.651	243.358	210.396	242.571	226.462	259.718	234.819	0.923635729	0.966018836	4	12	24	0.613873724	0.866162311	24	0.787055875	0.034744976
ENSMUSG00000028825	Rhd	Rh blood group, D antigen [Source:MGI Symbol;Acc:MGI:1202882]	0.76304	1.71415	2.55447	2.39832	1.23463	2.04004	1.21887	1.68572	1.66611	1.02738	1.33552	1.99683	2.63366	1.24234	0.844448841	0.966018836	12	16	24	0.904094261	0.973559397	24	8.670624331	0.059971085

ENSMUSG00000028826	Maco1	macoilin 1 [Source:MGI Symbol;Acc:MGI:1913396]	17.6828	16.3453	18.7472	18.5764	18.2078	18.0402	15.5544	15.018	17.6378	20.1261	18.1614	18.3039	17.3136	17.066	1.45E-04	0.031913438	16	16	24	0.009548193	0.502965379	24	13.9880748	0.105383541
ENSMUSG00000028830	AU040320	expressed sequence AU040320 [Source:MGI Symbol;Acc:MGI:2140475]	13.3117	10.5939	11.4176	13.4168	11.5393	13.9666	11.8868	13.0501	11.008	16.0117	11.5241	13.0634	12.921	11.7051	0.133266423	0.567728672	24	12	24	0.532756698	0.825678486	24	17.00059329	0.066179505
ENSMUSG00000028832	Stmn1	stathmin 1 [Source:MGI Symbol;Acc:MGI:96739]	126.426	120.171	108.771	110.047	125.402	122.186	126.077	109.919	114.195	113.345	113.06	128.276	128.988	112.843	0.008389425	0.192760771	4	8	24	0.00128487	0.346832852	24	21.32451227	0.106684847
ENSMUSG00000028833	Ncdn	neurochondrin [Source:MGI Symbol;Acc:MGI:1347351]	71.3629	54.5471	67.3529	59.1161	53.9004	67.1748	68.0305	72.7364	54.1274	102.174	57.301	72.6144	61.9661	74.272	0.949874888	0.966018836	8	12	24	0.966992667	0.992134533	24	5.650500221	0.025478457
ENSMUSG00000028834	Trim63	tripartite motif-containing 63 [Source:MGI Symbol;Acc:MGI:2447992]	0.896787	2.61947	2.15191	3.89495	1.74253	2.75466	2.13552	7.49695	4.07552	3.87261	1.23774	3.53007	3.77295	2.74443	0.447817955	0.966018836	16	4	24	0.257034301	0.768040178	24	7.150392096	0.339573634
ENSMUSG00000028837	Psmb2	proteasome (prosome, macropain) subunit, beta type 2 [Source:MGI Symbol;Acc:MGI:1347045]	116.938	117.037	117.886	117.437	122.81	123.809	111.588	114.291	113.898	116.783	118.087	115.259	118.994	116.366	0.368981219	0.893596186	20	12	24	0.132643892	0.742819192	24	16.98880963	0.029324691
ENSMUSG00000028838	Extl1	exostosin-like glycosyltransferase 1 [Source:MGI Symbol;Acc:MGI:1888742]	1.29763	2.09426	2.36876	2.47869	2.40075	2.66586	2.47812	2.55912	2.9881	3.26277	1.78739	2.43058	2.0462	1.9906	0.47028201	0.966018836	16	12	24	0.177216956	0.768040178	24	11.09451342	0.154698571
ENSMUSG00000028840	Zfp593	zinc finger protein 593 [Source:MGI Symbol;Acc:MGI:1915290]	4.06854	3.66574	3.71557	3.11551	3.64152	3.65898	3.35782	3.51296	3.48427	4.10076	4.1011	3.95182	3.73228	3.99166	0.880631923	0.966018836	20	16	24	0.77813863	0.928832041	24	19.88164804	0.025937904
ENSMUSG00000028841	Cnksr1	connector enhancer of kinase suppressor of Ras 1 [Source:MGI Symbol;Acc:MGI:2670958]	0.240825	0.393468	0.528032	0.634015	0.385781	0.792461	0.461957	1.01894	0.823208	1.23486	0.441037	0.427922	0.479031	0.406796	0.481611551	0.966018836	16	4	24	0.18918728	0.768040178	24	10.20576195	0.165298934
ENSMUSG00000028842	Ago3	argonaute RISC catalytic subunit 3 [Source:MGI Symbol;Acc:MGI:2446634]	5.5723	5.26241	5.20559	5.18858	5.66126	5.22718	5.55146	4.94424	5.34286	5.04564	5.78919	5.52197	5.4071	5.72098	0.187699199	0.662239322	20	20	24	0.286032841	0.768040178	24	20.01757557	0.035858265
ENSMUSG00000028843	Sh3bgrl3	SH3 domain binding glutamic acid-rich protein-like 3 [Source:MGI Symbol;Acc:MGI:1920973]	407.897	301.978	307.86	307.849	307.638	407.789	339.178	346.599	247.071	360.843	316.813	316.563	319.712	307.488	0.243586913	0.741580974	4	8	24	0.088198615	0.707747429	24	20.93696847	0.151034794
ENSMUSG00000028845	Tekt2	tektin 2 [Source:MGI Symbol;Acc:MGI:1346335]	1.70205	1.63762	1.55206	0.928563	1.16084	1.61909	1.1045	0.80266	1.38908	1.13753	1.50072	1.59855	1.03358	1.55042	0.941830768	0.966018836	24	16	24	0.738699156	0.914690529	24	21.75280654	0.061175686
ENSMUSG00000028847	Trappc3	trafficking protein particle complex 3 [Source:MGI Symbol;Acc:MGI:1351486]	45.519	49.2152	48.3663	45.1271	46.0164	45.3016	43.6457	45.3411	48.5243	42.0108	44.5012	44.3404	46.8113	46.0096	0.168929279	0.640164678	12	4	24	0.161355251	0.76636332	24	5.321287245	0.042210301
ENSMUSG00000028848	Gpn2	GPN-loop GTPase 2 [Source:MGI Symbol;Acc:MGI:2140368]	5.19261	4.42119	4.61926	4.23098	5.06287	4.41219	4.03128	4.6697	4.47599	4.6831	5.35722	5.35216	6.06586	4.5853	0.796193867	0.966018836	20	12	24	0.360229754	0.785733735	24	19.89986517	0.07066448
ENSMUSG00000028849	Map7d1	MAP7 domain containing 1 [Source:MGI Symbol;Acc:MGI:2384297]	60.0021	49.3541	47.8676	48.6895	55.212	50.1727	55.3436	57.3749	52.1369	64.5869	59.5687	57.221	57.256	55.7151	0.787022682	0.966018836	4	8	24	0.632734407	0.875112738	24	19.46456093	0.043954111
ENSMUSG00000028850	Gpatch3	G patch domain containing 3 [Source:MGI Symbol;Acc:MGI:2442492]	1.67841	1.66317	2.55626	1.88822	2.5762	1.59341	1.50799	1.70096	1.54624	2.18592	1.66162	1.89017	1.93572	1.80724	0.762156552	0.966018836	16	12	24	0.227800881	0.768040178	24	12.63468329	0.10660891
ENSMUSG00000028851	Nudc	nudC nuclear distribution protein [Source:MGI Symbol;Acc:MGI:106014]	57.6659	54.1384	56.6123	46.9537	44.3559	51.5423	50.8475	54.2213	49.1766	51.3897	52.2626	54.1791	55.6109	49.9234	0.25849765	0.761738864	4	12	24	0.117348864	0.728323355	24	1.536284869	0.077891082
ENSMUSG00000028854	Slc9a1	solute carrier family 9 (sodium/hydrogen exchanger), member 1 [Source:MGI Symbol;Acc:MGI:102462]	7.87027	6.1606	8.20161	7.4544	7.07879	7.6508	7.13006	8.66322	7.1199	10.0826	6.62193	7.47645	6.46344	7.51145	0.908406451	0.966018836	16	4	24	0.498074268	0.807927978	24	10.48789685	0.076438541
ENSMUSG00000028857	Tmem222	transmembrane protein 222 [Source:MGI Symbol;Acc:MGI:1098568]	27.9599	28.0581	28.1625	26.7457	27.7349	29.0814	25.5118	27.7141	27.125	26.888	26.4969	25.6248	26.1737	27.324	0.883870616	0.966018836	8	20	24	0.827835631	0.946180429	24	7.046540154	0.01129387
ENSMUSG00000028859	Csf3r	colony stimulating factor 3 receptor (granulocyte) [Source:MGI Symbol;Acc:MGI:1339755]	1.84842	2.16465	3.70831	3.40203	2.45948	2.21124	2.22112	2.24691	2.69118	1.39987	2.30993	2.00697	2.92115	1.83024	0.649212555	0.966018836	12	20	24	0.651122179	0.882725346	24	9.818702407	0.094559545
ENSMUSG00000028861	Mrps15	mitochondrial ribosomal protein S15 [Source:MGI Symbol;Acc:MGI:1913657]	39.1984	38.8031	37.0259	37.2086	39.5796	39.9888	38.3096	37.0523	36.7121	40.4785	40.8881	41.3215	46.0357	40.9081	0.021795836	0.26698364	24	12	24	0.078619931	0.689042744	24	20.75528665	0.058445146
ENSMUSG00000028862	Map3k6	mitogen-activated protein kinase kinase kinase 6 [Source:MGI Symbol;Acc:MGI:1855691]	2.32292	1.72306	1.67577	3.81488	2.31575	2.00208	1.69667	2.01464	2.6453	2.5918	2.25369	2.34527	2.11164	2.40969	0.522551072	0.966018836	16	12	24	0.113824686	0.727440321	24	12.78403161	0.166528062
ENSMUSG00000028863	Meaf6	MYST/Esa1-associated factor 6 [Source:MGI Symbol;Acc:MGI:1917338]	26.768	23.2112	21.4582	24.4209	23.4266	25.9878	29.6474	24.5865	21.0931	26.1015	25.0656	25.3773	22.1315	25.8117	0.122530972	0.550724475	4	8	24	0.191964799	0.768040178	24	20.95158995	0.091975671
ENSMUSG00000028864	Hgf	hepatocyte growth factor [Source:MGI Symbol;Acc:MGI:96079]	4.28466	4.19816	3.67753	3.96681	4.45701	4.15486	3.61274	3.61649	3.52211	3.01024	4.85154	3.50098	4.19672	3.68803	0.602156008	0.966018836	20	20	24	0.222835584	0.768040178	24	19.55055237	0.093950359
ENSMUSG00000028865	Cd164l2	CD164 sialomucin-like 2 [Source:MGI Symbol;Acc:MGI:1916905]	1.92864	2.50408	2.82836	2.07293	2.185	1.93297	4.35927	3.79556	1.87951	2.12891	2.41896	2.29349	2.86895	3.06028	0.29905572	0.809362476	8	8	24	0.116544441	0.727799063	24	2.768068044	0.202477634
ENSMUSG00000028868	Wasf2	WASP family, member 2 [Source:MGI Symbol;Acc:MGI:1098641]	9.50445	9.64835	9.46363	9.89428	9.81747	9.38922	10.4876	10.2187	10.0428	12.7101	9.70638	10.3817	10.4715	9.3317	0.880631923	0.966018836	16	16	24	0.586676899	0.853342401	24	12.30201293	0.040231695
ENSMUSG00000028869	Gnl2	guanine nucleotide binding protein-like 2 (nucleolar) [Source:MGI Symbol;Acc:MGI:2385207]	36.3288	39.1845	41.2453	37.0872	39.1772	38.7807	36.446	38.3992	41.8215	36.4957	40.9351	39.6591	40.9024	41.0656	0.43565473	0.966018836	12	16	24	0.59565053	0.857839743	24	7.758238779	0.026893492
ENSMUSG00000028871	Rspo1	R-spondin 1 [Source:MGI Symbol;Acc:MGI:2183426]	6.22328	6.51104	7.57005	6.99516	7.18513	6.72099	6.58501	7.43413	7.09193	7.90258	8.71735	6.7214	8.35572	6.84047	0.224600164	0.719154429	20	8	24	0.188464612	0.768040178	24	12.41126621	0.074286139
ENSMUSG00000028873	Cdca8	cell division cycle associated 8 [Source:MGI Symbol;Acc:MGI:1196274]	1.20771	1.09072	1.30973	1.5918	1.06527	1.40086	1.32063	1.64002	1.76666	1.40264	1.16293	1.23275	1.42272	1.19712	0.814077113	0.966018836	16	4	24	0.318282812	0.768040178	24	8.066095775	0.076780864
ENSMUSG00000028874	Fgr	FGR proto-oncogene, Src family tyrosine kinase [Source:MGI Symbol;Acc:MGI:95527]	0.667327	0.86888	1.04249	0.756134	0.875731	0.897222	0.866251	0.567142	0.86106	0.725524	0.623869	0.858102	0.778023	0.686172	0.90703203	0.966018836	12	20	24	0.945177517	0.986693398	24	14.87757988	0.013300533
ENSMUSG00000028876	Epha10	Eph receptor A10 [Source:MGI Symbol;Acc:MGI:3586824]	1.96026	2.51383	3.55714	1.84342	2.04777	2.502	2.32737	1.70348	3.16934	2.30326	1.88692	2.4018	1.9639	2.93056	0.308023687	0.814993005	12	4	24	0.28333537	0.768040178	24	7.182657422	0.14140849
ENSMUSG00000028878	Fam76a	family with sequence similarity 76, member A [Source:MGI Symbol;Acc:MGI:2385211]	12.0592	11.8577	12.4577	13.5058	12.4404	10.7794	10.3603	11.3992	13.5999	14.6139	10.801	11.2651	10.8078	11.7997	0.004732672	0.140718127	16	8	24	0.002573925	0.396968951	24	10.44624503	0.148854299
ENSMUSG00000028879	Stx12	syntaxin 12 [Source:MGI Symbol;Acc:MGI:1931027]	83.7095	86.7573	82.5787	85.0219	84.5222	80.9624	81.0312	78.3051	79.0213	82.8384	86.4293	79.3906	78.6217	84.7907	0.886952527	0.966018836	20	4	24	0.485230227	0.805393068	24	13.2790038	0.022088524
ENSMUSG00000028882	Ppp1r8	protein phosphatase 1, regulatory subunit 8 [Source:MGI Symbol;Acc:MGI:2140494]	16.1457	15.6812	15.4174	15.9114	15.3589	16.3771	16.616	15.558	15.4463	16.1625	15.0238	15.8758	14.7949	15.3211	0.888991156	0.966018836	24	20	24	0.602207877	0.861006659	24	20.58667218	0.017157197
ENSMUSG00000028884	Rpa2	replication protein A2 [Source:MGI Symbol;Acc:MGI:1339939]	6.31069	7.52202	7.72984	7.28092	6.64318	6.94072	6.55173	7.21765	7.34437	7.33548	6.72017	7.06295	6.69415	6.86564	8.66E-04	0.071344007	12	16	24	0.014135377	0.530991098	24	8.962040311	0.075352795
ENSMUSG00000028885	Smpdl3b	sphingomyelin phosphodiesterase, acid-like 3B [Source:MGI Symbol;Acc:MGI:1916022]	0.451356	0.645378	0.537964	0.569784	0.582124	0.564175	0.630411	0.540626	0.500144	0.298096	0.48958	0.491451	0.399721	0.493726	0.849180783	0.966018836	8	8	24	0.707622035	0.903565455	24	0.112236961	0.02611099
ENSMUSG00000028886	Eya3	EYA transcriptional coactivator and phosphatase 3 [Source:MGI Symbol;Acc:MGI:109339]	17.5077	14.5341	14.966	15.5529	15.3186	17.6331	15.3077	16.5366	12.317	18.4052	14.8566	16.1898	15.359	15.3316	0.245061943	0.741580974	24	12	24	0.324754425	0.768040178	24	19.62655206	0.080943148
ENSMUSG00000028889	Yrdc	yrdC domain containing (E.coli) [Source:MGI Symbol;Acc:MGI:2387201]	9.767	10.1575	9.89055	12.0109	10.9924	10.5966	11.6887	12.9219	12.0205	9.73213	8.82863	9.98647	9.23329	10.3641	0.891105762	0.966018836	8	12	24	0.533619839	0.825955545	24	6.779799778	0.06573499
ENSMUSG00000028890	Mtf1	metal response element binding transcription factor 1 [Source:MGI Symbol;Acc:MGI:101786]	5.64086	5.50181	5.72256	5.86131	4.75761	5.43986	5.67821	4.87502	5.35425	5.40217	5.1231	4.95102	4.74064	6.3098	0.745469888	0.966018836	16	4	24	0.353523407	0.780895595	24	6.573946461	0.058324949
ENSMUSG00000028893	Sesn2	sestrin 2 [Source:MGI Symbol;Acc:MGI:2651874]	1.33802	1.24703	1.71653	1.43449	1.08948	1.40689	1.21252	1.45048	1.49738	1.46737	1.21381	1.32609	1.10186	1.61565	0.016811431	0.253395174	12	16	24	0.049896952	0.643630594	24	7.918662128	0.102061244
ENSMUSG00000028894	Inpp5b	inositol polyphosphate-5-phosphatase B [Source:MGI Symbol;Acc:MGI:103257]	11.8857	13.4565	13.2456	16.4457	13.8498	11.669	16.8369	14.9144	12.7294	13.6325	14.7063	12.7984	14.0278	15.0643	0.94086481	0.966018836	8	16	24	0.716088223	0.906323093	24	8.423507687	0.048986179
ENSMUSG00000028896	Rcc1	regulator of chromosome condensation 1 [Source:MGI Symbol;Acc:MGI:1913989]	4.58877	4.84532	5.14572	5.11358	4.86776	4.70518	4.64682	4.79138	4.40455	5.03463	4.90557	4.96343	4.91026	4.47844	0.216058318	0.704989686	16	16	24	0.191906067	0.768040178	24	14.07551711	0.037953775
ENSMUSG00000028898	Trnau1ap	tRNA selenocysteine 1 associated protein 1 [Source:MGI Symbol;Acc:MGI:1919037]	10.2477	13.5584	12.7024	11.8765	11.9268	11.8211	10.4341	13.5461	14.8341	10.4393	11.7939	13.4584	13.6101	12.4835	0.399942636	0.932395417	12	4	24	0.336537783	0.771941639	24	6.232249833	0.085858166
ENSMUSG00000028899	Taf12	TATA-box binding protein associated factor 12 [Source:MGI Symbol;Acc:MGI:1913714]	8.61823	7.69245	8.09274	7.2361	8.22042	7.19341	10.7161	8.27995	7.45046	7.13507	7.51194	8.32527	7.10819	7.42981	0.702630284	0.966018836	4	12	24	0.238922844	0.768040178	24	23.37853474	0.089269938
ENSMUSG00000028901	Gmeb1	glucocorticoid modulatory element binding protein 1 [Source:MGI Symbol;Acc:MGI:2135604]	5.90499	5.01289	5.86093	5.35514	6.28966	5.65753	5.57889	4.83871	5.24376	5.14288	6.26154	5.40505	5.52051	5.27314	0.009678659	0.194720062	20	12	24	0.045906638	0.632590964	24	17.58139274	0.085477347
ENSMUSG00000028902	Sf3a3	splicing factor 3a, subunit 3 [Source:MGI Symbol;Acc:MGI:1922312]	16.7258	18.4552	17.9276	17.5533	18.2241	16.7945	17.3087	16.9553	16.8227	16.4368	16.723	18.0304	16.3649	16.6874	0.829428469	0.966018836	12	16	24	0.908158803	0.974606327	24	14.87028116	0.008396591
ENSMUSG00000028906	Epb41	erythrocyte membrane protein band 4.1 [Source:MGI Symbol;Acc:MGI:95401]	26.4601	29.5906	27.7382	30.8373	26.8589	25.9748	26.7819	27.0578	28.8449	28.1727	27.8897	27.7758	30.5599	29.4784	0.29905572	0.809362476	16	8	24	0.193332654	0.768040178	24	8.070617076	0.051487364
ENSMUSG00000028907	Utp11	UTP11 small subunit processome component [Source:MGI Symbol;Acc:MGI:1914455]	17.9058	20.3931	18.7304	17.1427	21.5788	18.2032	19.3736	18.1638	21.8828	16.4918	20.4553	17.5056	20.188	18.883	0.920992137	0.966018836	12	4	24	0.950958789	0.988261248	24	3.823872278	0.015144297
ENSMUSG00000028909	Ptpru	protein tyrosine phosphatase, receptor type, U [Source:MGI Symbol;Acc:MGI:1321151]	3.50474	3.95681	2.79457	2.99707	4.32724	2.75822	2.76142	2.17713	3.51682	2.26171	2.89701	4.31383	3.1654	4.05414	0.813775138	0.966018836	20	20	24	0.75115545	0.91921305	24	21.44940618	0.076695747
ENSMUSG00000028910	Mecr	mitochondrial trans-2-enoyl-CoA reductase [Source:MGI Symbol;Acc:MGI:1349441]	23.7246	24.7503	28.2585	28.0498	27.4446	25.762	24.2701	27.0839	23.9677	29.8484	28.4518	27.0735	28.3435	28.6818	0.423253809	0.966018836	16	12	24	0.097754918	0.71175955	24	12.42029338	0.07228948
ENSMUSG00000028911	Srsf4	serine and arginine-rich splicing factor 4 [Source:MGI Symbol;Acc:MGI:1890577]	15.8574	14.6854	15.9277	15.0207	14.2693	14.8195	15.4689	15.8251	15.5328	16.237	14.4324	16.2819	14.486	13.7668	0.924770496	0.966018836	16	4	24	0.953324582	0.988601783	24	9.104819501	0.009115328
ENSMUSG00000028914	Casp9	caspase 9 [Source:MGI Symbol;Acc:MGI:1277950]	11.6998	10.2816	9.70897	10.5592	10.7931	10.9423	10.4491	10.3911	8.8146	12.5308	9.88323	11.3435	10.8015	10.7505	0.010468194	0.196621011	24	12	24	0.334862909	0.770938748	24	19.57748213	0.066560132
ENSMUSG00000028917	Plekhm2	pleckstrin homology domain containing, family M (with RUN domain) member 2 [Source:MGI Symbol;Acc:MGI:1916832]	19.9787	18.471	17.7647	18.0076	18.2484	17.0149	19.4195	20.0833	21.198	20.259	18.3364	19.6371	19.1557	17.8468	0.859936714	0.966018836	4	20	24	0.653638402	0.883312134	24	5.508869663	0.031680603
ENSMUSG00000028919	Arhgef19	Rho guanine nucleotide exchange factor (GEF) 19 [Source:MGI Symbol;Acc:MGI:1925912]	4.09092	4.10935	4.87384	3.43856	3.8247	3.95086	5.89664	4.30745	3.91929	4.98975	4.31875	4.71236	4.31006	4.0979	0.869755137	0.966018836	4	16	24	0.690492128	0.897566751	24	0.646114399	0.053128323
ENSMUSG00000028920	Fbxo42	F-box protein 42 [Source:MGI Symbol;Acc:MGI:1924992]	6.3805	5.17558	5.27388	5.5508	5.46384	6.14415	6.24421	5.72228	4.91561	7.21404	5.2754	5.86318	5.68028	5.77451	0.102109143	0.512431169	4	8	24	0.557319731	0.839072789	24	20.15638021	0.05483329
ENSMUSG00000028923	Necap2	NECAP endocytosis associated 2 [Source:MGI Symbol;Acc:MGI:1913397]	14.8809	16.5384	18.0768	18.3952	18.4504	15.9978	15.22	17.6868	18.8063	17.109	15.6969	15.7776	16.2909	15.4511	0.016095537	0.253395174	12	16	24	0.008160557	0.496066371	24	10.51738295	0.111173825
ENSMUSG00000028926	Cdk14	cyclin-dependent kinase 14 [Source:MGI Symbol;Acc:MGI:894318]	44.8192	37.4224	38.826	38.4353	34.379	40.8478	39.4745	37.238	30.1987	44.2086	36.0254	42.2165	38.9566	42.7888	0.313786068	0.824470524	24	20	24	0.431113301	0.805393068	24	22.28978102	0.077097828
ENSMUSG00000028927	Padi2	peptidyl arginine deiminase, type II [Source:MGI Symbol;Acc:MGI:1338892]	4.23428	5.84923	7.24332	6.37631	6.46769	6.44832	7.57311	8.98121	7.3475	8.48023	4.82773	5.70378	5.87072	5.50143	0.635666629	0.966018836	12	16	24	0.253810563	0.768040178	24	8.740491956	0.170356066
ENSMUSG00000028931	Kcnab2	potassium voltage-gated channel, shaker-related subfamily, beta member 2 [Source:MGI Symbol;Acc:MGI:109239]	187.046	131.029	128.58	149.534	134.911	159.152	146.408	159.948	112.219	193.511	133.986	159.703	149.426	155.744	0.390686537	0.915479136	24	12	24	0.521686812	0.820407334	24	20.97277019	0.098058249
ENSMUSG00000028932	Psmc2	proteasome (prosome, macropain) 26S subunit, ATPase 2 [Source:MGI Symbol;Acc:MGI:109555]	93.4064	90.0679	88.0452	90.0886	92.5167	93.2912	87.2106	89.6141	82.3453	90.3573	91.9363	90.1784	94.8856	93.1484	0.071569005	0.440262583	4	8	24	0.140586043	0.750453905	24	20.41862683	0.039602333
ENSMUSG00000028933	Xrcc2	X-ray repair complementing defective repair in Chinese hamster cells 2 [Source:MGI Symbol;Acc:MGI:1927345]	3.12905	3.10839	3.39386	3.14027	3.16976	3.23306	3.46112	2.80322	3.10709	3.49584	3.36764	3.03559	3.311	3.30082	0.935732159	0.966018836	16	16	24	0.71237624	0.905123001	24	14.33092666	0.020782553
ENSMUSG00000028936	Rpl22	ribosomal protein L22 [Source:MGI Symbol;Acc:MGI:99262]	106.361	124.778	121.239	112.35	117.69	100.599	116.447	109.421	142.969	91.922	126.615	121.186	125.456	126.621	0.888991156	0.966018836	12	4	24	0.529460242	0.824483137	24	5.56193408	0.07053326
ENSMUSG00000028937	Acot7	acyl-CoA thioesterase 7 [Source:MGI Symbol;Acc:MGI:1917275]	215.358	163.228	168.999	165.183	172.444	210.061	178.816	192.265	139.178	208.64	165.776	181.931	174.279	178.492	0.119434414	0.542554768	24	12	24	0.229868335	0.768040178	24	21.04450973	0.113966677
ENSMUSG00000028943	Espn	espin [Source:MGI Symbol;Acc:MGI:1861630]	0.345761	0.525658	0.493662	0.618367	0.379815	0.350937	0.497341	0.267591	0.692253	0.601538	0.496473	0.554871	0.553726	0.651699	0.55109836	0.966018836	12	16	24	0.146181985	0.754849922	24	9.434890141	0.0850101
ENSMUSG00000028944	Prkag2	protein kinase, AMP-activated, gamma 2 non-catalytic subunit [Source:MGI Symbol;Acc:MGI:1336153]	54.951	48.0115	47.1491	50.1202	49.4683	50.0939	48.0132	47.728	42.9153	51.7964	49.469	51.0853	50.6754	49.3637	0.038123942	0.334069441	24	12	24	0.109854031	0.72082227	24	19.80250787	0.063979982
ENSMUSG00000028945	Rheb	Ras homolog enriched in brain [Source:MGI Symbol;Acc:MGI:97912]	105.935	102.931	96.7116	97.9284	98.7384	100.744	95.8257	96.6363	91.7758	93.4791	102.052	97.6175	105.879	101.516	0.257978335	0.761738864	4	8	24	0.080043831	0.69470896	24	22.46410556	0.051359996
ENSMUSG00000028948	Nol9	nucleolar protein 9 [Source:MGI Symbol;Acc:MGI:1921285]	4.55848	3.78664	3.92969	4.22415	4.105	4.17076	4.22343	3.99991	4.19972	4.39045	4.30695	4.31632	4.1719	4.16953	0.149354198	0.593798152	24	8	24	0.251662133	0.768040178	24	18.08247133	0.034439764
ENSMUSG00000028949	Smarcd3	SWI/SNF related, matrix associated, actin dependent regulator of chromatin, subfamily d, member 3 [Source:MGI Symbol;Acc:MGI:1914243]	14.6541	16.7737	19.5674	17.4597	15.2909	16.8162	19.2878	19.0514	17.9049	19.0594	14.3201	16.0486	15.0746	16.6098	0.124028266	0.552144108	12	8	24	0.098780282	0.71175955	24	7.570661047	0.118602797
ENSMUSG00000028952	Zbtb48	zinc finger and BTB domain containing 48 [Source:MGI Symbol;Acc:MGI:2140248]	3.39765	5.89331	5.97419	3.90618	5.37056	5.65295	5.02153	5.73972	5.4867	5.49642	4.60282	4.41837	4.66615	5.26642	0.194398468	0.673387182	12	16	24	0.348177772	0.779015211	24	7.660203223	0.115187282
ENSMUSG00000028953	Abcf2	ATP-binding cassette, sub-family F (GCN20), member 2 [Source:MGI Symbol;Acc:MGI:1351657]	34.4546	30.347	33.1481	32.9149	31.6952	35.6286	32.5257	34.7863	29.1555	34.0765	30.7208	30.954	30.9121	32.313	0.852844413	0.966018836	24	20	24	0.821010214	0.943551411	24	21.04208199	0.020566109
ENSMUSG00000028954	Nub1	negative regulator of ubiquitin-like proteins 1 [Source:MGI Symbol;Acc:MGI:1889001]	31.0292	28.5705	30.7877	30.0102	29.6251	32.4944	29.3295	30.1372	27.6161	32.3927	28.8734	31.1083	30.3706	31.1256	0.942429894	0.966018836	24	12	24	0.628155937	0.872795898	24	19.38490486	0.02570504
ENSMUSG00000028955	Vamp3	vesicle-associated membrane protein 3 [Source:MGI Symbol;Acc:MGI:1321389]	26.266	30.1297	29.3766	31.0536	32.282	26.7852	30.7861	29.4458	34.9096	28.2397	31.2508	29.2902	28.6195	27.6115	0.371875382	0.894537464	20	4	24	0.212601536	0.768040178	24	11.12269531	0.071731287
ENSMUSG00000028957	Per3	period circadian clock 3 [Source:MGI Symbol;Acc:MGI:1277134]	4.46386	9.01771	14.093	16.3777	12.5852	6.81504	5.31013	7.35371	19.1065	15.2838	10.729	6.73983	5.0951	8.15594	4.77E-06	0.009754434	16	12	24	3.44E-07	0.003664625	24	11.07604371	0.802290996
ENSMUSG00000028958	Tmub1	transmembrane and ubiquitin-like domain containing 1 [Source:MGI Symbol;Acc:MGI:1923764]	8.41031	6.89883	8.43785	8.64369	8.70311	7.96735	7.69453	8.08055	8.1695	9.29108	6.96651	9.60778	7.73354	9.05602	0.636884864	0.966018836	16	12	24	0.676555019	0.891854664	24	12.78728293	0.042853723
ENSMUSG00000028959	Fastk	Fas-activated serine/threonine kinase [Source:MGI Symbol;Acc:MGI:1913837]	37.8974	35.5978	32.7103	38.8474	33.4729	36.1067	36.667	43.155	35.9848	39.9163	35.7297	34.8923	35.3263	42.525	0.47028201	0.966018836	8	12	24	0.341009345	0.775221917	24	5.470660004	0.064322388
ENSMUSG00000028960	Ube4b	ubiquitination factor E4B [Source:MGI Symbol;Acc:MGI:1927086]	27.6407	19.9354	21.0703	24.0325	20.2382	25.1991	26.3496	27.4384	23.678	32.4151	23.2354	27.6511	26.8191	22.8592	0.530814489	0.966018836	4	4	24	0.774886158	0.927711986	24	21.06460507	0.053443736
ENSMUSG00000028961	Pgd	phosphogluconate dehydrogenase [Source:MGI Symbol;Acc:MGI:97553]	36.6178	31.7752	36.5995	37.979	35.4396	36.7483	36.8606	39.096	31.2739	36.4153	41.4093	36.8965	40.346	36.7558	0.718720526	0.966018836	4	8	24	0.387356467	0.799546792	24	18.95284123	0.056045871
ENSMUSG00000028962	Slc4a2	solute carrier family 4 (anion exchanger), member 2 [Source:MGI Symbol;Acc:MGI:109351]	26.0901	23.9401	22.8874	23.767	23.8381	24.3158	25.7238	26.5212	24.459	27.8894	24.2515	24.4894	23.1368	22.629	0.879206539	0.966018836	4	8	24	0.95823939	0.989130462	24	17.83451338	0.010031601
ENSMUSG00000028963	Uts2	urotensin 2 [Source:MGI Symbol;Acc:MGI:1346329]	0.213383	2.81083	4.05466	2.90745	1.02716	3.11527	3.50236	2.372	1.6685	1.12865	0.946125	2.33195	0.714931	2.9969	0.834424969	0.966018836	8	12	24	0.549603848	0.835021453	24	6.64768991	0.276345368
ENSMUSG00000028964	Park7	Parkinson disease (autosomal recessive, early onset) 7 [Source:MGI Symbol;Acc:MGI:2135637]	126.95	125.596	130.007	128.528	119.476	128.536	120.281	135.532	130.891	130.995	124.607	122.179	129.058	127.648	0.078207999	0.457568093	12	8	24	0.069573371	0.673820536	24	7.158512799	0.043636963
ENSMUSG00000028967	Errfi1	ERBB receptor feedback inhibitor 1 [Source:MGI Symbol;Acc:MGI:1921405]	14.5192	13.8831	14.6666	15.1876	14.412	14.795	16.4906	12.9243	15.0953	17.321	13.9465	15.1528	13.2846	13.0424	0.029264055	0.322165609	16	16	24	0.286911882	0.768040178	24	14.57500441	0.066752723
ENSMUSG00000028969	Cdk5	cyclin-dependent kinase 5 [Source:MGI Symbol;Acc:MGI:101765]	58.9478	53.8695	51.0876	51.9154	57.9189	59.402	51.0008	52.9755	45.3468	52.5819	50.0634	47.1231	52.0562	48.8117	0.94086481	0.966018836	4	8	24	0.106658516	0.718749374	24	20.08869097	0.073849625
ENSMUSG00000028970	Abcb1b	ATP-binding cassette, sub-family B (MDR/TAP), member 1B [Source:MGI Symbol;Acc:MGI:97568]	1.89	1.58494	1.01281	2.04425	1.36156	1.51044	1.51252	1.42285	2.1027	1.43387	1.75292	1.67205	1.28572	1.45424	0.789670753	0.966018836	16	16	24	0.848862792	0.954659791	24	15.24191385	0.03833325
ENSMUSG00000028973	Abcb8	ATP-binding cassette, sub-family B (MDR/TAP), member 8 [Source:MGI Symbol;Acc:MGI:1351667]	16.2573	16.3525	18.643	21.1175	18.6935	19.2649	19.7603	18.7216	16.379	20.0274	18.7994	18.9885	19.5211	18.8146	0.676344318	0.966018836	16	20	24	0.287087537	0.768040178	24	15.26100438	0.060973905
ENSMUSG00000028974	Dffa	DNA fragmentation factor, alpha subunit [Source:MGI Symbol;Acc:MGI:1196227]	70.0879	54.9366	52.7311	55.978	55.3621	62.6955	57.2902	60.7251	47.5779	65.9796	55.5384	61.2282	60.1064	62.945	0.217827365	0.704989686	24	12	24	0.214964578	0.768040178	24	21.83875742	0.096253662
ENSMUSG00000028975	Pex14	peroxisomal biogenesis factor 14 [Source:MGI Symbol;Acc:MGI:1927868]	18.0625	15.1017	15.7962	15.8196	15.2468	16.0755	16.2556	16.697	14.4241	20.4852	15.1896	18.7186	17.6252	17.8738	0.46694635	0.966018836	4	8	24	0.808591593	0.939135535	24	22.35480581	0.033437147
ENSMUSG00000028976	Slc2a5	solute carrier family 2 (facilitated glucose transporter), member 5 [Source:MGI Symbol;Acc:MGI:1928369]	0.468714	0.397884	0.430214	0.715038	0.341227	0.428563	0.779131	0.766221	0.413006	0.717882	0.765141	0.536764	0.727544	0.511295	0.91724565	0.966018836	4	8	24	0.998363774	0.999730606	24	17.35843532	0.003255343
ENSMUSG00000028977	Casz1	castor zinc finger 1 [Source:MGI Symbol;Acc:MGI:1196251]	9.20269	7.01566	5.75394	6.71305	6.73633	7.82089	6.85731	8.41034	6.98558	12.6764	8.03305	9.68761	9.42894	9.00569	0.601647512	0.966018836	24	12	24	0.804156202	0.937374918	24	20.53674968	0.061753134
ENSMUSG00000028978	Nos3	nitric oxide synthase 3, endothelial cell [Source:MGI Symbol;Acc:MGI:97362]	1.22424	1.18705	1.46344	1.39663	1.1351	1.06999	1.18723	1.6808	1.40471	1.42967	1.18543	1.25399	1.40785	1.22842	0.117205961	0.538362324	12	8	24	0.045797593	0.631827882	24	7.547344397	0.092849286
ENSMUSG00000028980	H6pd	hexose-6-phosphate dehydrogenase (glucose 1-dehydrogenase) [Source:MGI Symbol;Acc:MGI:2140356]	3.44742	3.51867	3.90192	5.57652	4.28413	3.3975	3.84107	4.28885	4.60915	5.17281	4.57697	3.70531	4.29221	4.01565	0.010153564	0.194720062	16	8	24	0.003111639	0.414576087	24	11.46244232	0.183812368
ENSMUSG00000028982	Slc25a33	solute carrier family 25, member 33 [Source:MGI Symbol;Acc:MGI:1917806]	17.8092	16.0493	18.9357	18.9548	16.6762	17.5179	16.192	16.9449	15.267	18.9721	15.6916	17.0504	17.7786	18.1707	0.865490576	0.966018836	16	4	24	0.799849761	0.935884746	24	10.49118086	0.026768164
ENSMUSG00000028986	Klhl7	kelch-like 7 [Source:MGI Symbol;Acc:MGI:1196453]	32.4237	32.3282	32.7234	29.8471	31.1576	30.2125	30.3561	29.0588	29.9881	29.4067	33.1007	29.6435	30.0264	32.3314	0.890648739	0.966018836	20	20	24	0.961491168	0.990114152	24	2.964124633	0.00680609
ENSMUSG00000028988	Ctnnbip1	catenin beta interacting protein 1 [Source:MGI Symbol;Acc:MGI:1915756]	16.5213	13.804	14.7904	15.2638	15.2212	15.5143	15.3027	15.6172	14.0802	18.8542	13.8852	15.7176	15.3202	15.8988	0.920620634	0.966018836	4	8	24	0.94001254	0.985398871	24	16.46629789	0.015189861
ENSMUSG00000028989	Angptl7	angiopoietin-like 7 [Source:MGI Symbol;Acc:MGI:3605801]	13.7244	18.894	14.7416	22.0522	22.3014	16.6544	19.2506	14.7586	20.2434	14.2585	18.1146	21.1256	18.3208	15.5053	0.720359655	0.966018836	20	12	24	0.377870852	0.797706855	24	16.26734455	0.124505048
ENSMUSG00000028990	Lzic	leucine zipper and CTNNBIP1 domain containing [Source:MGI Symbol;Acc:MGI:1916401]	7.77062	7.82497	7.66728	10.9909	8.25809	7.06015	7.00411	6.67009	8.67621	8.39962	8.15876	7.69711	7.57588	9.66667	0.054935173	0.398010585	16	12	24	0.086501816	0.705702571	24	11.12667194	0.137737695
ENSMUSG00000028991	Mtor	mechanistic target of rapamycin kinase [Source:MGI Symbol;Acc:MGI:1928394]	13.5325	10.1941	11.4878	12.3241	9.52201	12.9163	13.2186	12.4778	9.35547	14.4323	9.18882	10.6306	10.331	12.1684	0.801561896	0.966018836	4	16	24	0.789082872	0.932379688	24	1.78505641	0.053025685
ENSMUSG00000028992	Nmnat1	nicotinamide nucleotide adenylyltransferase 1 [Source:MGI Symbol;Acc:MGI:1913704]	5.95667	4.61069	4.41302	5.58337	5.76081	6.27449	6.76459	5.68834	5.02753	5.1553	6.10414	4.57963	5.63633	4.41775	0.07600733	0.453790923	4	8	24	0.105982094	0.71810271	24	19.58669891	0.135832375
ENSMUSG00000028995	Fam126a	family with sequence similarity 126, member A [Source:MGI Symbol;Acc:MGI:2149839]	7.37649	8.3033	8.0327	7.92422	8.59575	7.31458	8.26784	7.77774	8.84581	7.28524	8.87282	8.51528	7.25528	7.75398	0.5169953	0.966018836	20	8	24	0.579427829	0.849867668	24	12.87834463	0.037982145
ENSMUSG00000028996	Rbp7	retinol binding protein 7, cellular [Source:MGI Symbol;Acc:MGI:1890409]	1.05058	1.28764	1.42664	1.39629	1.95188	1.62806	1.70502	1.40455	1.19225	1.21527	1.43704	1.17873	1.40521	1.7849	0.885059765	0.966018836	20	20	24	0.856500945	0.957486395	24	18.19254028	0.034232716
ENSMUSG00000028998	Tomm7	translocase of outer mitochondrial membrane 7 [Source:MGI Symbol;Acc:MGI:1913419]	47.0927	57.0959	50.9523	54.9895	56.0887	47.982	50.43	52.1552	58.242	39.2353	57.8434	52.1033	57.9891	53.8843	0.924770496	0.966018836	24	12	24	0.906567708	0.974544241	24	3.910672779	0.027266996
ENSMUSG00000028999	Rint1	RAD50 interactor 1 [Source:MGI Symbol;Acc:MGI:1916233]	10.313	9.02809	9.08729	10.3627	9.45165	8.88269	9.17223	9.47012	9.84288	8.89024	13.3052	9.05249	11.376	12.3665	0.882617464	0.966018836	4	20	24	0.997130882	0.999487828	24	9.593579652	0.004793701
ENSMUSG00000029001	Fbxo44	F-box protein 44 [Source:MGI Symbol;Acc:MGI:1354744]	31.9149	31.6953	33.8412	30.5346	27.855	34.5055	31.2777	29.7507	30.216	34.3358	29.771	31.9131	29.5553	35.0758	0.879206539	0.966018836	4	20	24	0.743330337	0.916215545	24	5.202236581	0.030248275
ENSMUSG00000029003	Mad2l2	MAD2 mitotic arrest deficient-like 2 [Source:MGI Symbol;Acc:MGI:1919140]	6.26153	6.27054	7.02648	6.87928	6.87156	5.41573	6.12342	6.11911	6.58647	6.39407	6.08206	6.46657	6.02705	5.92606	0.119434414	0.542554768	12	20	24	0.056933787	0.65610184	24	10.93262497	0.070486427
ENSMUSG00000029004	Kmt2e	lysine (K)-specific methyltransferase 2E [Source:MGI Symbol;Acc:MGI:1924825]	10.8254	11.3617	11.1417	11.3052	11.4411	10.5728	12.5866	11.0917	12.6031	12.8138	11.5233	11.5674	12.031	11.5407	0.859936714	0.966018836	16	8	24	0.457901601	0.805393068	24	9.275944555	0.034700317
ENSMUSG00000029007	Agtrap	angiotensin II, type I receptor-associated protein [Source:MGI Symbol;Acc:MGI:1339977]	10.5478	8.80852	9.51582	8.00993	9.7962	9.89166	9.51682	10.1566	8.57563	10.0214	9.31691	10.1446	9.7982	9.54485	0.200145651	0.684570416	24	12	24	0.124675966	0.738641389	24	21.93190464	0.074757059
ENSMUSG00000029009	Mthfr	methylenetetrahydrofolate reductase [Source:MGI Symbol;Acc:MGI:106639]	16.4821	17.0883	16.7618	15.5966	18.7275	16.8867	16.9843	19.1189	18.2045	17.303	18.2078	16.9226	18.784	16.6071	0.858266942	0.966018836	4	20	24	0.988786412	0.997581486	24	0.516972664	0.004595068
ENSMUSG00000029012	Orc5	origin recognition complex, subunit 5 [Source:MGI Symbol;Acc:MGI:1347044]	19.4599	21.1772	21.2048	21.2223	20.4778	18.1285	20.5019	18.2454	22.7676	18.1032	22.1095	20.1409	22.1189	21.3501	0.8972237	0.966018836	12	12	24	0.67039211	0.888925819	24	7.985830031	0.038371686
ENSMUSG00000029014	Dnajc2	DnaJ heat shock protein family (Hsp40) member C2 [Source:MGI Symbol;Acc:MGI:99470]	15.699	14.879	15.8373	16.0143	15.2856	15.631	14.4545	16.0164	15.8036	16.1413	17.1889	16.342	17.798	17.6844	0.761979	0.966018836	16	12	24	0.893292299	0.969822551	24	9.381560924	0.0110432

ENSMUSG00000029016	Clcn6	chloride channel, voltage-sensitive 6 [Source:MGI Symbol;Acc:MGI:1347049]	26.5954	22.7044	24.1176	24.1866	24.0223	26.0938	25.7267	25.0301	20.4711	31.4409	21.2391	25.2815	24.3438	24.4444	0.210418232	0.69936851	24	12	24	0.808585724	0.939135535	24	19.77521486	0.039038165
ENSMUSG00000029017	Pmpcb	peptidase (mitochondrial processing) beta [Source:MGI Symbol;Acc:MGI:1920328]	31.3256	28.6484	27.1769	31.9571	28.3812	27.9127	30.4448	29.5142	30.2759	28.6843	32.7457	28.6937	34.3211	32.0676	0.934818505	0.966018836	4	8	24	0.776178717	0.928029314	24	0.800492195	0.024509598
ENSMUSG00000029019	Nppb	natriuretic peptide type B [Source:MGI Symbol;Acc:MGI:97368]	16.75	17.8286	15.1263	17.2436	19.2328	17.9032	14.6059	13.8762	18.0614	13.0269	19.0955	18.1141	17.5793	16.2372	0.084464639	0.466404237	20	12	24	0.267772911	0.768040178	24	18.28175864	0.105294481
ENSMUSG00000029020	Mfn2	mitofusin 2 [Source:MGI Symbol;Acc:MGI:2442230]	45.0374	35.284	37.2601	41.8808	38.1857	47.5817	42.3981	47.5726	31.6296	58.6371	35.2537	42.5932	40.4054	42.2496	0.390686537	0.915479136	24	12	24	0.823841811	0.944567105	24	19.59885091	0.057019431
ENSMUSG00000029022	Miip	migration and invasion inhibitory protein [Source:MGI Symbol;Acc:MGI:106506]	8.9997	11.2685	11.8793	11.7298	12.6253	10.8294	11.1072	12.2439	11.6226	10.3309	11.9875	10.119	10.9825	10.5164	0.181442559	0.659020016	20	4	24	0.251350731	0.768040178	24	11.12612819	0.075992464
ENSMUSG00000029027	Dffb	DNA fragmentation factor, beta subunit [Source:MGI Symbol;Acc:MGI:1196287]	5.40297	5.75304	5.36771	5.47338	5.46423	4.9926	3.72079	5.15032	6.05206	5.10331	5.35997	5.73061	6.11569	5.52759	0.866861748	0.966018836	12	4	24	0.658470254	0.884596426	24	9.442829342	0.0533388
ENSMUSG00000029028	Lrrc47	leucine rich repeat containing 47 [Source:MGI Symbol;Acc:MGI:1920196]	9.26289	8.28367	8.23899	8.44994	7.71087	10.1801	8.37738	9.5443	8.06332	10.8824	7.5545	8.28215	8.44235	10.0313	0.702630284	0.966018836	8	12	24	0.770391814	0.926095918	24	4.042350606	0.041371436
ENSMUSG00000029029	Wrap73	WD repeat containing, antisense to Trp73 [Source:MGI Symbol;Acc:MGI:1891749]	11.1306	9.00288	9.44782	8.31879	8.79115	10.9838	10.1399	10.1672	9.21431	10.5092	9.04722	9.39998	9.2198	9.13035	0.505132703	0.966018836	24	20	24	0.280507777	0.768040178	24	22.91135483	0.06970514
ENSMUSG00000029030	Tprgl	transformation related protein 63 regulated like [Source:MGI Symbol;Acc:MGI:1915058]	166.021	152.714	143.471	154.988	150.225	155.936	156.229	156.084	158.153	169.632	163.826	171.549	175.503	166.908	0.708020493	0.966018836	4	8	24	0.306620749	0.768040178	24	21.69933196	0.03607194
ENSMUSG00000029032	Arhgef16	Rho guanine nucleotide exchange factor (GEF) 16 [Source:MGI Symbol;Acc:MGI:2446219]	1.55702	1.28172	0.926476	0.878429	1.48669	1.13417	1.9181	1.25678	0.962434	0.974597	1.40009	1.18034	1.97094	1.20559	3.97E-04	0.052946481	4	12	24	0.009580341	0.502965379	24	22.59809283	0.215233681
ENSMUSG00000029033	Acap3	ArfGAP with coiled-coil, ankyrin repeat and PH domains 3 [Source:MGI Symbol;Acc:MGI:2153589]	15.8383	16.5029	15.3666	14.4388	16.0494	14.5736	15.9135	13.1821	16.1445	16.526	13.2862	15.41	14.2473	14.063	0.874607729	0.966018836	12	20	24	0.962014782	0.9903359	24	11.24824136	0.010811302
ENSMUSG00000029034	Ints11	integrator complex subunit 11 [Source:MGI Symbol;Acc:MGI:1919207]	12.2619	10.6816	11.4664	10.6717	11.6341	11.1612	12.1892	12.313	10.7882	11.2927	11.6047	10.845	10.7894	10.5313	0.918497267	0.966018836	4	4	24	0.564936054	0.843471947	24	21.55545892	0.030090464
ENSMUSG00000029036	Atad3a	ATPase family, AAA domain containing 3A [Source:MGI Symbol;Acc:MGI:1919214]	13.191	12.9573	13.9582	13.8294	12.9908	13.7971	16.0877	13.7837	12.463	12.9642	14.0379	13.0538	14.0619	13.5829	0.664568268	0.966018836	4	8	24	0.455257104	0.805393068	24	22.94734948	0.041312935
ENSMUSG00000029038	Ssu72	Ssu72 RNA polymerase II CTD phosphatase homolog (yeast) [Source:MGI Symbol;Acc:MGI:1916241]	58.0981	62.9225	59.2344	59.3371	60.2632	55.3685	52.8308	57.8735	60.377	54.444	61.9048	60.0948	62.9354	59.1058	0.853736882	0.966018836	20	8	24	0.914987456	0.976279382	24	8.171300825	0.013156567
ENSMUSG00000029047	Pex10	peroxisomal biogenesis factor 10 [Source:MGI Symbol;Acc:MGI:2684988]	5.16665	4.61642	4.73023	4.71887	4.88931	4.84001	4.88021	5.55863	4.58375	5.63278	4.50389	4.50139	5.31102	4.2854	0.896773941	0.966018836	4	20	24	0.969207253	0.992631251	24	0.607452373	0.009868673
ENSMUSG00000029048	Rer1	retention in endoplasmic reticulum sorting receptor 1 [Source:MGI Symbol;Acc:MGI:1915080]	68.0284	63.8144	59.597	61.4557	66.0897	64.1518	62.967	64.4642	65.0427	59.7846	70.7244	63.897	68.4127	66.2045	0.487173827	0.966018836	20	20	24	0.212597488	0.768040178	24	21.56987757	0.046176292
ENSMUSG00000029049	Morn1	MORN repeat containing 1 [Source:MGI Symbol;Acc:MGI:1924116]	1.63785	2.19082	2.02766	2.08862	1.70357	1.44443	1.66099	1.46052	2.13337	1.88585	1.71298	1.57331	1.5157	1.84669	0.009630739	0.194720062	12	12	24	0.005429411	0.445165967	24	9.151772475	0.137106083
ENSMUSG00000029050	Ski	ski sarcoma viral oncogene homolog (avian) [Source:MGI Symbol;Acc:MGI:98310]	12.5703	12.1239	12.5378	13.3249	12.4079	12.0229	13.6531	13.4811	13.163	17.3633	12.1004	14.1515	13.6797	14.0821	0.915082018	0.966018836	4	16	24	0.510321585	0.814071261	24	9.49179238	0.053341503
ENSMUSG00000029053	Prkcz	protein kinase C, zeta [Source:MGI Symbol;Acc:MGI:97602]	14.4958	13.2294	13.2912	12.8432	12.0257	14.1982	14.026	15.04	11.8054	15.5748	12.1871	13.2746	12.8625	12.6416	0.839634479	0.966018836	4	16	24	0.674771009	0.89097199	24	0.626789945	0.039111823
ENSMUSG00000029055	Plch2	phospholipase C, eta 2 [Source:MGI Symbol;Acc:MGI:2443078]	1.26597	1.17578	1.62441	0.739104	0.953024	1.08248	1.56837	1.26534	1.46369	1.14737	0.929641	1.40407	1.22168	1.50612	0.050447964	0.385880335	12	4	24	0.03474058	0.608551791	24	3.454082924	0.153269224
ENSMUSG00000029056	Pank4	pantothenate kinase 4 [Source:MGI Symbol;Acc:MGI:2387466]	14.0812	12.772	13.0763	14.54	14.4139	13.8588	13.7536	13.5312	13.2812	13.8954	13.9971	13.0443	14.1543	13.5067	0.081793634	0.462922111	16	20	24	0.162743067	0.766365035	24	16.73579502	0.037818731
ENSMUSG00000029059	Prxl2b	peroxiredoxin like 2B [Source:MGI Symbol;Acc:MGI:1913719]	40.7094	45.3479	46.4247	40.1863	42.6175	39.6483	39.4768	47.4445	53.0893	42.1653	45.6135	41.5971	46.2054	42.6063	0.077301512	0.453790923	12	12	24	0.075415276	0.685464747	24	7.481008078	0.100577862
ENSMUSG00000029060	Mib2	mindbomb E3 ubiquitin protein ligase 2 [Source:MGI Symbol;Acc:MGI:2679684]	17.8761	15.1857	16.5602	18.4876	17.7344	17.3898	18.1468	16.67	16.1996	17.7998	17.1411	16.5381	16.9705	16.5019	0.108029159	0.524035616	16	20	24	0.145076391	0.754043136	24	16.92459125	0.053065739
ENSMUSG00000029061	Mmp23	matrix metallopeptidase 23 [Source:MGI Symbol;Acc:MGI:1347361]	3.66711	4.13754	3.7334	3.00345	3.66259	3.0462	3.48135	5.05689	4.62883	2.75759	3.58677	3.04974	2.77181	3.29554	0.150109238	0.595846665	12	4	24	0.17882405	0.768040178	24	5.64870669	0.140508637
ENSMUSG00000029062	Cdk11b	cyclin-dependent kinase 11B [Source:MGI Symbol;Acc:MGI:88353]	25.3168	24.5545	25.1739	26.5275	23.2152	25.7184	25.3107	26.5949	25.0469	30.7734	21.4355	25.3398	25.1865	26.0696	0.945282252	0.966018836	16	4	24	0.488130859	0.805393068	24	7.438088295	0.053663922
ENSMUSG00000029063	Nadk	NAD kinase [Source:MGI Symbol;Acc:MGI:2183149]	26.7195	22.8865	24.4416	28.6797	27.734	25.9702	27.4453	25.8351	23.1998	24.1353	27.2094	24.893	25.6742	22.8799	0.123511911	0.552144108	20	12	24	0.043343057	0.626520598	24	17.91087964	0.091006421
ENSMUSG00000029064	Gnb1	guanine nucleotide binding protein (G protein), beta 1 [Source:MGI Symbol;Acc:MGI:95781]	270.876	208.7	210.843	220.016	236.248	260.913	229.39	230.288	175.581	260.111	209.607	231.943	224.55	224.095	0.071575474	0.440262583	24	12	24	0.113859508	0.727440321	24	20.15607897	0.126226268
ENSMUSG00000029066	Mrpl20	mitochondrial ribosomal protein L20 [Source:MGI Symbol;Acc:MGI:2137221]	84.7732	93.0251	86.4817	88.7041	87.9418	81.2743	84.4008	78.9573	96.3191	74.4725	100.355	87.8134	95.4501	93.4678	0.849180783	0.966018836	12	12	24	0.973328078	0.993305115	24	5.353164843	0.011014865
ENSMUSG00000029068	Ccnl2	cyclin L2 [Source:MGI Symbol;Acc:MGI:1927119]	36.1031	33.9554	39.0939	42.2496	38.3649	32.8026	40.0228	34.4389	41.1124	32.7134	44.0086	30.617	34.3953	33.8514	0.649212555	0.966018836	20	4	24	0.303922448	0.768040178	24	11.86418283	0.090120003
ENSMUSG00000029070	Mxra8	matrix-remodelling associated 8 [Source:MGI Symbol;Acc:MGI:1922011]	34.274	40.1358	32.5525	35.8133	43.4452	37.8376	37.7483	43.8592	43.674	34.087	39.0182	38.1473	38.1815	34.1331	0.880247609	0.966018836	20	20	24	0.965478486	0.991618029	24	19.94197986	0.015386113
ENSMUSG00000029071	Dvl1	dishevelled segment polarity protein 1 [Source:MGI Symbol;Acc:MGI:94941]	24.6347	21.5429	22.6441	22.053	22.8044	21.6318	22.6653	22.2824	22.6246	23.78	24.803	24.1392	25.3127	21.5763	0.092972576	0.489012695	4	4	24	0.351562615	0.780345856	24	18.95245663	0.042206058
ENSMUSG00000029073	Cptp	ceramide-1-phosphate transfer protein [Source:MGI Symbol;Acc:MGI:1933107]	15.2068	12.6446	12.6547	13.6707	14.0655	15.3921	14.0207	15.2222	10.6401	16.8422	12.781	14.406	14.658	13.4888	0.148073804	0.590732968	24	12	24	0.301197595	0.768040178	24	20.08764012	0.094634773
ENSMUSG00000029075	Tnfrsf4	tumor necrosis factor receptor superfamily, member 4 [Source:MGI Symbol;Acc:MGI:104512]	0.974247	1.15713	1.38004	0.959712	0.74143	0.930325	1.11894	1.37049	1.38401	1.11689	0.905581	1.09187	0.937771	0.800521	0.00580828	0.159101132	12	8	24	0.096019099	0.71175955	24	6.556760255	0.117273697
ENSMUSG00000029076	Sdf4	stromal cell derived factor 4 [Source:MGI Symbol;Acc:MGI:108079]	129.083	132.336	129.568	123.639	130.446	128.474	119.372	124.889	121.611	114.41	120.635	122.787	125.205	123.01	0.887862353	0.966018836	8	8	24	0.352614255	0.780629136	24	23.88788936	0.025350765
ENSMUSG00000029082	Bst1	bone marrow stromal cell antigen 1 [Source:MGI Symbol;Acc:MGI:105370]	0.722444	0.600484	0.623887	1.24412	0.715608	0.546062	0.537509	0.689683	0.7676	0.311735	0.578579	0.701599	0.568758	0.684798	0.886952527	0.966018836	12	16	24	0.845259551	0.953278734	24	10.60466952	0.038296928
ENSMUSG00000029084	Cd38	CD38 antigen [Source:MGI Symbol;Acc:MGI:107474]	3.17374	3.85708	5.35052	3.55068	3.65695	3.9238	4.3097	3.64502	4.10346	3.30744	3.44413	3.1905	2.80933	3.46537	0.643325306	0.966018836	12	16	24	0.321272323	0.768040178	24	8.249020631	0.097606062
ENSMUSG00000029086	Prom1	prominin 1 [Source:MGI Symbol;Acc:MGI:1100886]	3.96504	5.66174	6.80493	5.89232	6.61136	5.76992	6.08873	5.36888	4.92699	6.29682	6.49549	6.03536	6.06799	5.31083	0.166709446	0.633685338	20	12	24	0.165556776	0.767020009	24	14.16541744	0.116539061
ENSMUSG00000029088	Kcnip4	Kv channel interacting protein 4 [Source:MGI Symbol;Acc:MGI:1933131]	119.902	105.824	97.3902	104.649	101.569	108.206	98.7623	93.6872	91.4723	94.945	110.012	100.541	103.717	104.421	0.417653118	0.963884303	20	16	24	0.080354405	0.695795041	24	20.71400291	0.083104208
ENSMUSG00000029089	5730480H06Rik	RIKEN cDNA 5730480H06 gene [Source:MGI Symbol;Acc:MGI:1917842]	9.78563	8.01509	9.12932	8.85418	8.35991	9.1458	8.85548	9.84229	9.62873	8.46021	7.467	10.097	9.14681	7.82859	0.369262716	0.893596186	24	20	24	0.579062368	0.849660354	24	0.370602552	0.048409402
ENSMUSG00000029090	Adgra3	adhesion G protein-coupled receptor A3 [Source:MGI Symbol;Acc:MGI:1917943]	7.80242	6.00707	6.42374	7.03332	6.87131	6.74678	6.63982	7.25446	5.4565	7.61944	6.30607	6.50746	6.33439	6.49848	0.893307667	0.966018836	16	20	24	0.632443736	0.875020483	24	18.70682283	0.044938972
ENSMUSG00000029093	Sorcs2	sortilin-related VPS10 domain containing receptor 2 [Source:MGI Symbol;Acc:MGI:1932289]	3.222	3.51338	4.04919	2.96775	4.13463	3.98165	3.81774	4.13641	3.21435	3.64831	3.76286	3.77878	3.60407	3.83005	0.907068194	0.966018836	20	20	24	0.773420442	0.927220663	24	21.22337546	0.033205589
ENSMUSG00000029094	Afap1	actin filament associated protein 1 [Source:MGI Symbol;Acc:MGI:1917542]	5.80782	5.87585	4.96019	4.96327	6.01223	5.2118	5.82907	4.68033	5.68743	5.22049	5.6978	5.19069	5.42125	4.75524	0.714044782	0.966018836	4	4	24	0.346902287	0.778844143	24	19.41321195	0.056675522
ENSMUSG00000029095	Ablim2	actin-binding LIM protein 2 [Source:MGI Symbol;Acc:MGI:2385758]	12.3067	14.0836	15.9256	10.8623	10.5044	13.2072	13.5984	16.2207	13.1531	14.2096	11.2481	12.5739	11.9117	14.6714	0.008961721	0.192760771	8	12	24	0.017409347	0.550995814	24	5.132314658	0.177595256
ENSMUSG00000029096	Htra3	HtrA serine peptidase 3 [Source:MGI Symbol;Acc:MGI:1925808]	10.409	8.92346	6.0385	6.83807	9.17252	7.49286	10.7957	9.55314	9.72187	4.96894	9.27664	8.81866	8.41265	7.8586	0.192357053	0.669841558	4	12	24	0.076300767	0.686372559	24	23.31121656	0.229882769
ENSMUSG00000029097	Trmt44	tRNA methyltransferase 44 [Source:MGI Symbol;Acc:MGI:1926140]	2.20949	1.67026	1.5638	1.87325	1.91194	1.66225	1.6706	1.83605	1.58629	1.63409	1.77517	1.82932	1.85906	1.61448	0.101520755	0.511659635	4	8	24	0.150363779	0.758865539	24	20.4194798	0.064451745
ENSMUSG00000029098	Acox3	acyl-Coenzyme A oxidase 3, pristanoyl [Source:MGI Symbol;Acc:MGI:1933156]	16.529	17.4269	17.5802	18.2071	17.492	18.5604	16.4428	18.6439	15.2759	21.0455	15.8458	18.5576	16.9389	16.8631	0.773177465	0.966018836	16	12	24	0.640988541	0.878514233	24	14.00133775	0.04098198
ENSMUSG00000029101	Rgs12	regulator of G-protein signaling 12 [Source:MGI Symbol;Acc:MGI:1918979]	6.21416	5.08227	5.77327	5.27201	5.48991	5.72215	4.90968	5.73199	5.93416	6.41297	4.75367	5.37933	5.99554	5.45624	0.915475528	0.966018836	12	8	24	0.772433058	0.926846189	24	6.909319774	0.032006636
ENSMUSG00000029103	Lrpap1	low density lipoprotein receptor-related protein associated protein 1 [Source:MGI Symbol;Acc:MGI:96829]	75.343	72.6086	70.4698	69.8017	72.5138	77.1211	72.7393	69.222	65.6269	71.599	68.9878	72.7429	70.3783	69.5941	0.020957534	0.262822894	24	12	24	0.00374903	0.426426863	24	20.66486056	0.058643746
ENSMUSG00000029104	Htt	huntingtin [Source:MGI Symbol;Acc:MGI:96067]	12.7737	11.2493	12.7872	11.7575	11.9069	11.9039	12.2157	11.0893	10.799	12.2682	11.2994	12.4735	11.6764	11.7817	0.772122688	0.966018836	24	8	24	0.480979258	0.805393068	24	19.71132139	0.033144304
ENSMUSG00000029106	Add1	adducin 1 (alpha) [Source:MGI Symbol;Acc:MGI:87918]	120.567	104.609	105.402	111.316	110.447	118.947	118.055	118.425	96.5115	137.513	98.7576	117.869	116.093	112.179	0.210566453	0.69936851	24	12	24	0.62903894	0.873082003	24	20.9619262	0.051683271
ENSMUSG00000029108	Pcdh7	protocadherin 7 [Source:MGI Symbol;Acc:MGI:1860487]	13.211	14.6966	12.7894	13.2779	13.7986	12.516	13.5989	11.1062	13.6847	14.5035	13.9611	14.4157	13.8537	15.3268	0.782779623	0.966018836	8	4	24	0.931989362	0.982523804	24	13.50095061	0.015047618
ENSMUSG00000029110	Rnf4	ring finger protein 4 [Source:MGI Symbol;Acc:MGI:1201691]	28.6247	27.7332	27.0325	28.3881	28.6937	27.4513	28.5692	26.9102	26.7768	27.1812	27.8143	29.3015	28.4761	26.6158	0.018773661	0.256345714	4	4	24	0.01057635	0.508211602	24	19.58254801	0.0447507
ENSMUSG00000029111	Nelfa	negative elongation factor complex member A, Whsc2 [Source:MGI Symbol;Acc:MGI:1346098]	4.61919	4.04632	4.72926	3.94009	4.13441	4.23973	4.49224	4.41945	4.35123	5.28442	4.08308	4.55482	4.36954	4.39681	0.904387595	0.966018836	4	16	24	0.836620656	0.949953371	24	6.892558798	0.021920479
ENSMUSG00000029119	Man2b2	mannosidase 2, alpha B2 [Source:MGI Symbol;Acc:MGI:1195262]	7.20028	6.62218	7.62198	7.37728	7.81442	7.19527	7.62553	7.13982	7.73683	9.17226	6.408	6.81792	6.43293	6.96016	0.397360907	0.927993503	12	20	24	0.162530134	0.76636332	24	11.35283651	0.085099377
ENSMUSG00000029120	Ppp2r2c	protein phosphatase 2, regulatory subunit B, gamma [Source:MGI Symbol;Acc:MGI:2442660]	81.4218	60.8872	63.1246	63.014	61.8913	72.5789	67.6761	65.9894	51.5686	80.227	61.5021	70.9408	67.423	69.4699	0.182320384	0.659303873	24	20	24	0.291587204	0.768040178	24	21.22198211	0.105254217
ENSMUSG00000029121	Crmp1	collapsin response mediator protein 1 [Source:MGI Symbol;Acc:MGI:107793]	39.1242	28.9993	31.9826	31.7823	34.734	34.5265	30.4284	31.5219	26.9647	37.8999	30.245	34.4426	30.6021	33.8472	0.814576414	0.966018836	16	20	24	0.372026707	0.793398709	24	18.62657922	0.080346628
ENSMUSG00000029122	Evc	EvC ciliary complex subunit 1 [Source:MGI Symbol;Acc:MGI:1890596]	5.63639	5.64321	5.52062	7.1531	6.66165	6.58899	6.44142	6.02635	7.64445	6.5444	5.63937	6.43867	6.73887	5.46374	0.725061903	0.966018836	16	16	24	0.467927013	0.805393068	24	14.11702327	0.061782355
ENSMUSG00000029125	Stx18	syntaxin 18 [Source:MGI Symbol;Acc:MGI:1918366]	33.5925	29.8818	27.8907	31.7231	31.0471	31.4582	31.2342	30.4428	28.5185	29.3021	30.9415	30.0271	33.1092	33.147	0.017495149	0.255326033	4	8	24	0.084661321	0.702884002	24	22.20825749	0.06387706
ENSMUSG00000029126	Nsg1	neuron specific gene family member 1 [Source:MGI Symbol;Acc:MGI:109149]	43.1182	46.205	56.7072	43.9195	39.5022	52.3049	51.469	44.343	45.1738	51.728	41.894	47.1726	38.4972	56.4627	0.70641939	0.966018836	12	8	24	0.514131355	0.816624526	24	6.509565664	0.082429503
ENSMUSG00000029127	Zbtb49	zinc finger and BTB domain containing 49 [Source:MGI Symbol;Acc:MGI:1922329]	3.76126	4.41891	4.99898	3.79497	3.63632	3.32193	3.74751	3.5846	5.56564	4.23054	4.06077	3.50894	3.5166	3.36533	0.004712708	0.140598674	12	12	24	0.025939825	0.586541342	24	9.016575815	0.166757532
ENSMUSG00000029128	Rab28	RAB28, member RAS oncogene family [Source:MGI Symbol;Acc:MGI:1917285]	31.335	32.3431	30.1977	31.895	30.5806	29.3853	31.7172	31.6783	31.4659	28.7995	31.5801	33.0318	32.5621	33.1525	0.371875382	0.894537464	8	4	24	0.174356281	0.768040178	24	1.900596132	0.03815263
ENSMUSG00000029130	Rnf32	ring finger protein 32 [Source:MGI Symbol;Acc:MGI:1861747]	5.71617	5.4113	4.30711	4.05372	5.23681	4.86335	4.89063	5.30858	4.34413	3.83034	4.63044	5.46509	5.29441	5.1411	0.008244783	0.192760771	4	12	24	0.001510956	0.358147394	24	23.34585586	0.165374422
ENSMUSG00000029131	Dnajb6	DnaJ heat shock protein family (Hsp40) member B6 [Source:MGI Symbol;Acc:MGI:1344381]	15.869	16.1945	17.5277	17.2404	16.8838	15.5159	17.523	14.8035	16.79	14.9587	18.4273	18.4417	17.196	16.9413	0.940625808	0.966018836	20	12	24	0.643701677	0.878958277	24	17.2419152	0.034755852
ENSMUSG00000029135	Fosl2	fos-like antigen 2 [Source:MGI Symbol;Acc:MGI:102858]	2.9802	2.00498	2.34984	3.06858	2.62093	2.25736	2.83751	2.43973	2.14534	2.62465	2.92918	2.59349	2.45979	1.96602	0.057707116	0.403601759	16	16	24	0.094959427	0.71175955	24	17.13807066	0.116651196
ENSMUSG00000029136	Rbks	ribokinase [Source:MGI Symbol;Acc:MGI:1918586]	3.29897	3.40425	4.34481	4.46464	3.2958	3.29564	3.11677	3.59611	4.14022	3.2389	3.41461	3.67567	3.87017	3.79571	0.347721987	0.870418653	12	8	24	0.097925352	0.71175955	24	8.699873271	0.107836052
ENSMUSG00000029141	Slc4a1ap	solute carrier family 4 (anion exchanger), member 1, adaptor protein [Source:MGI Symbol;Acc:MGI:1196608]	14.0689	12.927	13.9024	15.8837	15.0094	17.1832	14.6691	13.4717	14.1963	13.6862	15.204	15.5916	14.5719	15.5727	0.090240671	0.47972073	24	8	24	0.066191212	0.66830795	24	17.97446459	0.085445335
ENSMUSG00000029145	Eif2b4	eukaryotic translation initiation factor 2B, subunit 4 delta [Source:MGI Symbol;Acc:MGI:95300]	15.5782	14.5293	13.573	19.4999	16.7271	17.5273	13.7857	16.2096	15.3188	17.1354	14.2526	15.084	15.0191	16.7645	0.745118544	0.966018836	16	12	24	0.417511966	0.802268097	24	13.66439797	0.070533732
ENSMUSG00000029146	Snx17	sorting nexin 17 [Source:MGI Symbol;Acc:MGI:2387801]	32.9488	30.8877	34.1231	32.5146	32.3142	32.3236	31.8364	30.5856	30.0887	33.981	29.8372	32.8994	31.4605	30.0519	0.595952838	0.966018836	16	16	24	0.478527456	0.805393068	24	16.06844489	0.027311124
ENSMUSG00000029147	Ppm1g	protein phosphatase 1G (formerly 2C), magnesium-dependent, gamma isoform [Source:MGI Symbol;Acc:MGI:106065]	20.9821	19.4973	19.7106	19.5305	19.4298	21.637	19.2836	20.5572	18.0294	23.8719	18.5117	21.4352	20.8356	21.0638	0.44861185	0.966018836	24	20	24	0.831225814	0.947661285	24	20.81625507	0.024839203
ENSMUSG00000029148	Nrbp1	nuclear receptor binding protein 1 [Source:MGI Symbol;Acc:MGI:2183436]	56.5365	55.4678	51.5453	57.7878	55.5614	54.9003	53.2345	52.5097	50.6015	53.1748	52.6512	55.2409	52.4854	51.5576	0.136853693	0.578758359	16	20	24	0.053764751	0.654390023	24	18.57043273	0.041999658
ENSMUSG00000029149	Krtcap3	keratinocyte associated protein 3 [Source:MGI Symbol;Acc:MGI:1917065]	2.82749	2.68368	2.41127	4.02791	3.80777	3.25158	3.41312	2.94792	2.59262	2.94576	3.2524	3.52583	3.45394	3.24704	0.010153564	0.194720062	20	16	24	0.062196156	0.661961741	24	17.94569068	0.135940554
ENSMUSG00000029151	Slc30a3	solute carrier family 30 (zinc transporter), member 3 [Source:MGI Symbol;Acc:MGI:1345280]	0.434772	1.3099	1.86454	0.974664	0.561311	1.27536	1.60858	0.428729	1.48608	0.746993	0.312332	1.13333	0.177562	1.71575	0.285027487	0.793714531	12	8	24	0.434940303	0.805393068	24	6.765095902	0.221309259
ENSMUSG00000029152	Ociad1	OCIA domain containing 1 [Source:MGI Symbol;Acc:MGI:1915345]	220.634	206.561	214.553	203.048	192.557	211.732	215.592	199.851	199.147	214.544	207.738	209.617	224.396	228.99	0.077161185	0.453790923	4	16	24	0.166146106	0.767020009	24	1.335667606	0.046509583
ENSMUSG00000029153	Ociad2	OCIA domain containing 2 [Source:MGI Symbol;Acc:MGI:1916377]	2.98033	4.10803	3.90248	3.68128	4.0076	3.15898	4.1588	3.83996	4.1662	4.06869	3.61768	3.48733	3.55964	3.77136	0.039704107	0.338535904	12	12	24	0.06734675	0.670376655	24	8.713355649	0.100336016
ENSMUSG00000029156	Sgcb	sarcoglycan, beta (dystrophin-associated glycoprotein) [Source:MGI Symbol;Acc:MGI:1346523]	35.5437	34.3582	33.4608	35.0505	34.207	35.7809	33.4474	34.2182	32.9195	36.5478	34.0799	34.5126	33.8968	34.9001	0.44861185	0.966018836	16	20	24	0.551343815	0.83632237	24	17.18029024	0.017653121
ENSMUSG00000029158	Yipf7	Yip1 domain family, member 7 [Source:MGI Symbol;Acc:MGI:1922831]	1.74653	3.06789	3.87574	4.57608	2.81902	3.22903	2.27735	5.99859	6.60702	5.0911	3.06652	2.1417	3.47558	3.52618	0.017595665	0.256116321	12	16	24	0.00301102	0.414576087	24	8.996258905	0.476576068
ENSMUSG00000029161	Cgref1	cell growth regulator with EF hand domain 1 [Source:MGI Symbol;Acc:MGI:1915817]	20.8137	19.4773	17.6963	19.526	20.5686	21.7485	17.1267	17.5573	16.6755	17.7409	20.6584	19.448	20.0654	17.3123	0.048759831	0.379129806	20	16	24	0.003973195	0.427431763	24	18.9055985	0.128580575
ENSMUSG00000029162	Khk	ketohexokinase [Source:MGI Symbol;Acc:MGI:1096353]	28.5397	26.9637	28.1745	28.1578	27.7165	29.0946	25.4889	29.5981	30.0535	27.3253	26.4192	28.2216	30.0471	27.8808	0.959856266	0.967043964	12	8	24	0.725441485	0.90999235	24	5.067848707	0.021453368
ENSMUSG00000029163	Emilin1	elastin microfibril interfacer 1 [Source:MGI Symbol;Acc:MGI:1926189]	3.17285	3.36574	3.2622	2.00137	3.61595	3.78623	3.0087	4.38505	3.53818	2.03208	4.31074	3.52339	3.56582	3.20498	0.503972924	0.966018836	20	20	24	0.322474102	0.768040178	24	23.13054804	0.143406302
ENSMUSG00000029165	Agbl5	ATP/GTP binding protein-like 5 [Source:MGI Symbol;Acc:MGI:2441745]	2.54962	3.80363	3.54845	3.22643	2.9118	3.22557	3.67229	3.18836	3.98488	3.22247	3.04959	3.64214	2.73913	3.80164	0.247023822	0.746090009	12	8	24	0.199899028	0.768040178	24	7.135251667	0.102073505
ENSMUSG00000029166	Mapre3	microtubule-associated protein, RP/EB family, member 3 [Source:MGI Symbol;Acc:MGI:2140967]	70.4	65.597	62.4563	67.4959	64.9583	68.7383	73.6163	65.3984	66.3973	88.4026	65.1606	68.2969	71.487	69.5967	0.101520755	0.511659635	4	8	24	0.948960206	0.98757629	24	15.58807031	0.014998654
ENSMUSG00000029167	Ppargc1a	peroxisome proliferative activated receptor, gamma, coactivator 1 alpha [Source:MGI Symbol;Acc:MGI:1342774]	4.35578	5.29934	5.75037	5.46468	4.54514	4.88145	5.46041	5.2095	5.37792	6.15652	4.56691	5.10839	4.75329	5.89175	0.019643089	0.258568924	16	4	24	0.020072857	0.565108098	24	8.086359355	0.123616631
ENSMUSG00000029168	Dpysl5	dihydropyrimidinase-like 5 [Source:MGI Symbol;Acc:MGI:1929772]	22.3136	16.6408	17.0953	19.1446	18.8197	21.4056	18.693	18.9836	14.7903	24.4742	17.5608	19.8672	19.3129	18.6951	0.077301512	0.453790923	24	12	24	0.298908499	0.768040178	24	19.05732475	0.107589041
ENSMUSG00000029169	Dhx15	DEAH (Asp-Glu-Ala-His) box polypeptide 15 [Source:MGI Symbol;Acc:MGI:1099786]	54.0343	51.5896	49.9843	48.91	49.7171	49.3688	51.6748	48.8647	48.1726	45.8687	50.7074	48.3753	49.6713	47.7688	0.305564675	0.811201458	4	12	24	0.015260607	0.538738573	24	22.86259495	0.045208158
ENSMUSG00000029171	Pgm2	phosphoglucomutase 2 [Source:MGI Symbol;Acc:MGI:97564]	4.28326	4.52415	4.03791	4.76932	3.74298	4.06919	4.38574	4.10441	4.49052	3.75593	4.5694	4.17123	3.86905	3.7327	0.733896783	0.966018836	16	8	24	0.965931595	0.991740077	24	11.87935411	0.009338563
ENSMUSG00000029173	Sepsecs	Sep (O-phosphoserine) tRNA:Sec (selenocysteine) tRNA synthase [Source:MGI Symbol;Acc:MGI:1098791]	5.05956	5.76518	5.63008	5.26041	5.6447	5.26088	5.81023	5.55464	6.09956	5.42454	5.61495	5.90089	5.62754	5.60939	0.844448841	0.966018836	12	4	24	0.600064709	0.860011178	24	6.704816146	0.023031261
ENSMUSG00000029174	Tbc1d1	TBC1 domain family, member 1 [Source:MGI Symbol;Acc:MGI:1889508]	13.6562	10.1434	10.2143	10.7089	10.8902	11.2907	11.3651	11.9869	9.16942	12.479	11.1942	11.3502	10.7098	10.0675	0.162391276	0.624504499	4	8	24	0.211943494	0.768040178	24	20.17562898	0.090325017
ENSMUSG00000029175	Slc35f6	solute carrier family 35, member F6 [Source:MGI Symbol;Acc:MGI:1922169]	16.8568	15.8856	14.2592	15.7404	16.0432	19.275	14.6303	16.9107	14.0931	16.9455	15.5296	14.6423	15.7395	16.3441	0.888379597	0.966018836	8	4	24	0.518930365	0.819000028	24	20.2097873	0.05334731
ENSMUSG00000029176	Anapc4	anaphase promoting complex subunit 4 [Source:MGI Symbol;Acc:MGI:1098673]	47.2928	52.1616	51.4307	49.6194	52.0388	48.4081	42.0654	45.8132	46.8124	43.0787	45.5228	45.4837	46.5842	48.0146	0.92655264	0.966018836	8	20	24	0.758425608	0.921881031	24	9.800310533	0.024358806
ENSMUSG00000029177	Cenpa	centromere protein A [Source:MGI Symbol;Acc:MGI:88375]	2.1531	3.34609	3.99116	2.55129	3.03747	2.96792	3.87674	3.21187	3.07138	2.84079	3.16	3.46405	3.51868	2.7273	0.961536114	0.967151979	4	12	24	0.869944547	0.963168206	24	3.242251442	0.036835392
ENSMUSG00000029178	Klf3	Kruppel-like factor 3 (basic) [Source:MGI Symbol;Acc:MGI:1342773]	8.85455	8.47638	7.72906	8.41376	9.07366	8.12781	10.0121	8.6896	9.15651	8.28403	7.88096	8.48766	8.24094	8.22408	0.886572916	0.966018836	4	12	24	0.572855897	0.846815862	24	23.89728237	0.03601608
ENSMUSG00000029179	Zcchc4	zinc finger, CCHC domain containing 4 [Source:MGI Symbol;Acc:MGI:1926046]	5.17642	5.6046	5.79403	6.09155	5.96185	5.46107	5.85072	5.37518	6.06691	5.35965	5.90417	6.21742	6.90217	6.29974	0.710703678	0.966018836	20	20	24	0.997138955	0.999487828	24	4.530129542	0.002365532
ENSMUSG00000029185	Fam114a1	family with sequence similarity 114, member A1 [Source:MGI Symbol;Acc:MGI:1915553]	16.4016	17.0899	14.4526	14.1228	16.1384	14.8734	16.0778	16.1191	16.0077	13.77	15.0973	14.7507	15.1549	16.207	0.004014876	0.140598674	8	8	24	0.044877308	0.628432994	24	1.777755786	0.074144656
ENSMUSG00000029186	Pi4k2b	phosphatidylinositol 4-kinase type 2 beta [Source:MGI Symbol;Acc:MGI:1914323]	4.54135	5.48548	5.49793	5.64033	5.57286	4.79551	4.86692	5.48704	5.4251	4.85503	4.93476	5.11386	6.11033	5.26415	0.772122688	0.966018836	12	12	24	0.394288095	0.799546792	24	7.632878671	0.053800088
ENSMUSG00000029189	Sel1l3	sel-1 suppressor of lin-12-like 3 (C. elegans) [Source:MGI Symbol;Acc:MGI:1916941]	10.0042	7.54599	7.35015	8.13149	8.26834	9.55148	8.56574	9.39899	6.50081	10.3085	7.97444	8.404	8.27725	8.38711	0.091424683	0.483797062	24	12	24	0.329821384	0.768040178	24	20.44850413	0.096696062
ENSMUSG00000029190	D5Ertd579e	DNA segment, Chr 5, ERATO Doi 579, expressed [Source:MGI Symbol;Acc:MGI:1261849]	18.5438	20.3298	23.0509	20.8534	19.6957	20.5319	18.858	19.7059	19.0185	20.9819	19.1942	20.1303	18.0083	20.1951	0.00932019	0.194720062	16	12	24	0.114277016	0.727799063	24	10.63869965	0.064349959
ENSMUSG00000029191	Rfc1	replication factor C (activator 1) 1 [Source:MGI Symbol;Acc:MGI:97891]	17.4954	17.9191	15.4757	14.282	16.1917	17.8349	17.3935	17.7927	16.6701	16.7607	16.7852	17.1275	17.362	17.0439	0.009120508	0.192760771	8	4	24	0.01216519	0.525280095	24	23.96174342	0.083055965
ENSMUSG00000029192	Tbc1d14	TBC1 domain family, member 14 [Source:MGI Symbol;Acc:MGI:1098708]	20.6449	18.7479	18.6689	18.5104	21.2875	19.2634	20.5609	19.6118	16.6064	19.8464	18.5436	19.1897	18.4584	18.3948	0.43280837	0.966018836	20	16	24	0.105259796	0.717252017	24	19.98205541	0.065892833
ENSMUSG00000029193	Cckar	cholecystokinin A receptor [Source:MGI Symbol;Acc:MGI:99478]	12.3733	7.402	8.96701	8.64401	7.98962	9.16577	7.04782	9.22613	6.19952	9.21708	8.9341	9.22811	9.63076	9.34014	0.38940266	0.915479136	4	8	24	0.466576036	0.805393068	24	21.26524241	0.106529161
ENSMUSG00000029196	Tada2b	transcriptional adaptor 2B [Source:MGI Symbol;Acc:MGI:3035274]	7.61321	6.5404	7.24749	6.97334	6.44614	7.48532	7.22202	7.43175	5.50813	9.49422	6.00086	7.25936	6.81109	7.41165	0.944053021	0.966018836	24	20	24	0.982313623	0.996048133	24	22.4491532	0.01259128
ENSMUSG00000029198	Grpel1	GrpE-like 1, mitochondrial [Source:MGI Symbol;Acc:MGI:1334417]	13.9315	15.5318	16.6303	17.3827	14.5768	14.6985	14.2345	14.4753	16.1049	13.6808	16.2214	14.4012	15.118	15.3662	0.175808942	0.651752074	12	16	24	0.151858767	0.758865539	24	9.961943252	0.071585725
ENSMUSG00000029199	Lias	lipoic acid synthetase [Source:MGI Symbol;Acc:MGI:1934604]	20.1213	19.7872	21.0916	21.3539	18.6642	21.2777	18.7722	22.0618	21.242	20.9646	20.8714	21.2825	21.6091	22.5124	0.702630284	0.966018836	8	12	24	0.286905943	0.768040178	24	7.060426128	0.041234253
ENSMUSG00000029201	Ugdh	UDP-glucose dehydrogenase [Source:MGI Symbol;Acc:MGI:1306785]	14.0576	13.0582	13.5104	11.81	13.1054	12.4652	15.8702	11.9597	11.9016	11.2225	13.2994	11.7827	12.1243	12.9303	0.192357053	0.669841558	4	12	24	0.201319911	0.768040178	24	23.40114355	0.077006289
ENSMUSG00000029202	Pds5a	PDS5 cohesin associated factor A [Source:MGI Symbol;Acc:MGI:1918771]	26.4598	27.261	26.0124	26.6951	26.9083	24.4439	24.2236	22.1626	25.774	22.6242	28.1259	24.6858	24.7428	23.6246	0.702630284	0.966018836	20	12	24	0.400942907	0.799546792	24	15.85125043	0.046519885
ENSMUSG00000029203	Ube2k	ubiquitin-conjugating enzyme E2K [Source:MGI Symbol;Acc:MGI:1858216]	52.8151	44.8584	46.1855	43.0053	44.2313	50.4305	48.7959	44.7762	42.1366	48.5694	48.9941	47.2986	50.5326	47.485	0.008389425	0.192760771	4	8	24	0.034651177	0.608551791	24	21.6213437	0.087476769
ENSMUSG00000029206	Nsun7	NOL1/NOP2/Sun domain family, member 7 [Source:MGI Symbol;Acc:MGI:1918168]	0.603686	0.788079	1.05436	0.785284	0.704641	0.72887	0.843422	0.797998	0.739395	0.5652	0.615706	0.748902	0.640211	0.857918	0.167343922	0.634848458	8	12	24	0.218805022	0.768040178	24	5.838400765	0.068099292
ENSMUSG00000029207	Apbb2	amyloid beta (A4) precursor protein-binding, family B, member 2 [Source:MGI Symbol;Acc:MGI:108405]	13.5183	11.873	12.5849	11.5052	11.2528	13.7477	12.1902	12.7093	10.1494	14.2891	11.5842	11.9594	11.589	13.4293	0.838532766	0.966018836	24	20	24	0.764922919	0.92403577	24	23.44744454	0.036703428
ENSMUSG00000029208	Guf1	GUF1 homolog, GTPase [Source:MGI Symbol;Acc:MGI:2140726]	11.5848	15.4553	14.0629	14.4332	12.6984	12.2783	12.794	12.3805	15.6324	11.8159	13.5403	14.6016	13.2337	15.1985	0.454881285	0.966018836	12	16	24	0.253324004	0.768040178	24	7.528470698	0.087904609

ENSMUSG00000029247	Paics	phosphoribosylaminoimidazole carboxylase, phosphoribosylaminoribosylaminoimidazole, succinocarboxamide synthetase [Source:MGI Symbol;Acc:MGI:1914304]	73.4096	80.2277	77.3283	76.9595	80.6392	70.8233	74.6471	73.0141	84.3723	70.8083	77.5717	76.4618	78.0095	78.6018	0.798829513	0.966018836	12	12	24	0.491031599	0.805393068	24	8.271383619	0.034967464
ENSMUSG00000029249	Rest	RE1-silencing transcription factor [Source:MGI Symbol;Acc:MGI:104897]	2.71385	2.77374	2.52726	2.56227	2.81501	2.64723	2.58232	2.62674	2.77594	2.60246	2.62758	2.50952	2.57141	2.3529	0.823144114	0.966018836	20	4	24	0.696353376	0.89922881	24	16.57974387	0.014163044
ENSMUSG00000029250	Polr2b	polymerase (RNA) II (DNA directed) polypeptide B [Source:MGI Symbol;Acc:MGI:2388280]	22.396	23.4474	23.2995	22.0302	22.9384	21.3821	21.7957	21.4356	23.1306	21.5052	23.6674	22.5074	23.6813	22.9584	0.891742979	0.966018836	12	12	24	0.874735966	0.964976615	24	6.974891257	0.01112197
ENSMUSG00000029253	Cenpc1	centromere protein C1 [Source:MGI Symbol;Acc:MGI:99700]	11.2473	10.7411	10.4708	10.6176	9.80887	10.8743	9.63292	9.53473	10.3234	9.77587	11.0597	10.9436	11.553	10.7333	0.337071598	0.85758369	24	16	24	0.482812554	0.805393068	24	21.62125913	0.038950675
ENSMUSG00000029254	Stap1	signal transducing adaptor family member 1 [Source:MGI Symbol;Acc:MGI:1926193]	0.484186	0.635809	1.23042	0.711597	0.708943	0.631891	0.600431	0.715392	0.879067	0.25987	0.501407	0.644038	0.875253	0.901787	0.151191877	0.598641364	12	12	24	0.279953556	0.768040178	24	5.634634678	0.1226197
ENSMUSG00000029263	Pigg	phosphatidylinositol glycan anchor biosynthesis, class G [Source:MGI Symbol;Acc:MGI:3576484]	5.65043	4.6204	4.35455	4.24794	5.19437	4.8686	5.48475	4.70435	4.12291	4.50705	4.9986	4.62887	5.27714	4.61596	3.44E-04	0.049770315	4	8	24	0.003303695	0.416356043	24	21.60680113	0.127440632
ENSMUSG00000029265	Dr1	down-regulator of transcription 1 [Source:MGI Symbol;Acc:MGI:1100515]	15.7926	15.0119	15.1992	15.879	15.1831	14.6589	14.6388	14.1618	12.9196	14.5881	15.4883	12.0657	12.6057	13.9454	0.714044782	0.966018836	20	4	24	0.398228231	0.799546792	24	13.49508766	0.043167186
ENSMUSG00000029267	Mtf2	metal response element binding transcription factor 2 [Source:MGI Symbol;Acc:MGI:105050]	6.57053	8.62845	9.95167	8.50046	7.59797	7.73171	7.1591	6.94829	10.3619	6.79955	8.67582	8.31053	7.47169	8.07129	0.038363978	0.335328154	12	16	24	0.184405901	0.768040178	24	9.715338321	0.125097231
ENSMUSG00000029270	Dipk1a	divergent protein kinase domain 1A [Source:MGI Symbol;Acc:MGI:1914516]	8.5175	10.6219	11.3731	9.46641	8.08265	8.97912	10.9226	9.50896	10.5112	9.24465	8.4511	10.0983	10.2579	11.168	0.008360387	0.192760771	12	8	24	0.013213296	0.529134128	24	5.066306077	0.142952627
ENSMUSG00000029276	Glmn	glomulin, FKBP associated protein [Source:MGI Symbol;Acc:MGI:2141180]	10.2298	11.2426	11.3959	11.7697	9.78817	9.3299	8.40338	9.6843	12.2611	9.34747	11.1934	10.3846	11.0004	11.8563	0.119434414	0.542554768	12	12	24	0.15159613	0.758865539	24	8.462182689	0.110032479
ENSMUSG00000029279	Brdt	bromodomain, testis-specific [Source:MGI Symbol;Acc:MGI:1891374]	5.55226	5.89401	5.51301	5.44802	4.84672	6.02044	5.61919	5.12704	5.1453	5.58256	5.36922	5.39357	4.88439	6.09968	0.93447422	0.966018836	8	12	24	0.737738363	0.91445069	24	2.69287495	0.026275717
ENSMUSG00000029281	Smr2	submaxillary gland androgen regulated protein 2 [Source:MGI Symbol;Acc:MGI:102762]	9.37398	6.70212	9.44643	5.57222	9.14689	9.0696	7.56458	6.37142	6.87751	6.52073	9.9181	5.11875	5.46261	6.47348	0.93447422	0.966018836	20	12	24	0.55139446	0.83632237	24	17.29904253	0.112202866
ENSMUSG00000029283	Cdc7	cell division cycle 7 (S. cerevisiae) [Source:MGI Symbol;Acc:MGI:1309511]	4.27261	4.30446	4.83877	3.79189	4.39768	4.60621	4.69591	3.29669	4.26077	3.70964	4.891	3.56439	3.82981	3.63079	0.953350544	0.966018836	20	12	24	0.555937526	0.838702296	24	17.44890244	0.057642035
ENSMUSG00000029287	Tgfbr3	transforming growth factor, beta receptor III [Source:MGI Symbol;Acc:MGI:104637]	14.9775	14.9067	14.6101	14.7029	15.6918	13.317	16.3302	15.7546	15.6478	17.4509	13.2459	14.326	15.3954	13.9599	0.937921962	0.966018836	4	20	24	0.585998696	0.853257144	24	7.586550972	0.044121092
ENSMUSG00000029290	Zfp326	zinc finger protein 326 [Source:MGI Symbol;Acc:MGI:1927246]	13.4812	15.083	15.3793	14.5678	15.7206	12.5728	15.4352	12.029	15.1332	14.0051	16.2545	14.0073	13.5627	13.1482	0.281427347	0.793378811	20	12	24	0.257619414	0.768040178	24	13.35483172	0.073769607
ENSMUSG00000029291	Rufy3	RUN and FYVE domain containing 3 [Source:MGI Symbol;Acc:MGI:106484]	198.893	172.167	165.094	171.527	171.31	178.669	167.952	166.831	145.613	180.331	182.939	175.401	182.18	186.192	0.300823756	0.809362476	4	8	24	0.179918531	0.768040178	24	20.88070607	0.075129861
ENSMUSG00000029298	Gbp9	guanylate-binding protein 9 [Source:MGI Symbol;Acc:MGI:3605620]	1.82632	2.31009	2.08481	1.64274	2.01208	1.78274	1.88172	3.02331	2.46785	1.7848	1.91175	1.57319	1.71131	2.0493	0.008754183	0.192760771	8	16	24	0.051912361	0.649400655	24	6.010942513	0.153390506
ENSMUSG00000029304	Spp1	secreted phosphoprotein 1 [Source:MGI Symbol;Acc:MGI:98389]	266.78	304.903	268.614	277.491	319.948	256.742	286.484	274.48	313.853	223.594	286.044	255.967	273.982	258.123	0.913451828	0.966018836	20	4	24	0.976577412	0.994103202	24	4.683299768	0.011426348
ENSMUSG00000029306	Ibsp	integrin binding sialoprotein [Source:MGI Symbol;Acc:MGI:96389]	2.86928	3.4286	4.34439	5.00931	3.23843	5.69573	2.64277	4.25897	2.66119	4.69628	3.61699	4.05385	3.3832	2.92675	0.113148904	0.530777648	16	12	24	0.282145444	0.768040178	24	14.50765065	0.165979051
ENSMUSG00000029307	Dmp1	dentin matrix protein 1 [Source:MGI Symbol;Acc:MGI:94910]	0.304363	0.495847	0.861314	0.913133	0.401192	0.521663	0.57677	0.861922	0.468924	0.874346	0.241814	0.338004	0.313141	0.292677	0.351315142	0.870418653	16	4	24	0.093288233	0.71175955	24	9.493892395	0.174594927
ENSMUSG00000029309	Sparcl1	SPARC-like 1 [Source:MGI Symbol;Acc:MGI:108110]	202.173	266.144	359.023	235.28	212.062	266.667	292.837	226.215	266.83	240.405	194.565	240.379	194.103	268.253	0.285027487	0.793714531	12	8	24	0.255603105	0.768040178	24	6.782727419	0.157576819
ENSMUSG00000029310	Nudt9	nudix (nucleoside diphosphate linked moiety X)-type motif 9 [Source:MGI Symbol;Acc:MGI:1921417]	28.7954	28.1036	28.9542	29.2634	27.1588	29.2294	29.7671	29.5955	27.6145	27.7625	26.5578	29.196	28.7366	29.3518	0.162391276	0.624504499	4	16	24	0.094621983	0.71175955	24	1.735594668	0.038091218
ENSMUSG00000029311	Hsd17b11	hydroxysteroid (17-beta) dehydrogenase 11 [Source:MGI Symbol;Acc:MGI:2149821]	54.954	57.7173	56.8114	53.3628	58.9336	55.4931	56.7956	56.5867	56.0366	54.5316	53.2116	59.2679	55.8143	55.3373	0.952127548	0.966018836	8	8	24	0.55020723	0.835448041	24	23.52792387	0.019891603
ENSMUSG00000029312	Klhl8	kelch-like 8 [Source:MGI Symbol;Acc:MGI:2179430]	5.36428	5.12357	5.09787	5.41056	5.22607	5.54451	5.73017	4.95681	4.74761	5.33011	5.02641	4.65032	5.10594	4.63571	0.622132989	0.966018836	4	4	24	0.304066588	0.768040178	24	18.95882518	0.03987081
ENSMUSG00000029313	Aff1	AF4/FMR2 family, member 1 [Source:MGI Symbol;Acc:MGI:1100819]	6.48994	5.87692	6.08946	7.50425	7.19442	6.78688	6.61162	6.25514	6.01048	8.09394	6.51818	6.40651	6.29049	5.67489	4.15E-04	0.054040373	16	16	24	0.012916272	0.527834117	24	15.14619028	0.120121844
ENSMUSG00000029314	Gpat3	glycerol-3-phosphate acyltransferase 3 [Source:MGI Symbol;Acc:MGI:3603816]	1.75531	1.70681	2.0164	1.77888	1.28099	2.0043	2.2457	1.85963	1.39067	1.77472	1.29862	1.99722	2.43495	1.69702	0.132887428	0.567728672	4	16	24	0.120559338	0.732894732	24	0.621369458	0.131223832
ENSMUSG00000029319	Coq2	coenzyme Q2 4-hydroxybenzoate polyprenyltransferase [Source:MGI Symbol;Acc:MGI:1919133]	29.0468	29.6669	28.2101	30.8383	30.038	29.0056	28.7586	29.9597	30.6984	30.0307	31.4565	29.5762	31.5502	31.0626	0.953480735	0.966018836	20	4	24	0.534508214	0.82652194	24	11.31646367	0.016857243
ENSMUSG00000029321	Slc10a6	solute carrier family 10 (sodium/bile acid cotransporter family), member 6 [Source:MGI Symbol;Acc:MGI:1923000]	4.24131	3.00162	4.04425	4.26104	4.75712	4.17305	3.11027	3.66555	3.49753	4.33506	4.68499	3.43727	3.48734	3.15666	0.003846392	0.140598674	20	12	24	0.002332974	0.39629122	24	15.0844387	0.18631574
ENSMUSG00000029322	Plac8	placenta-specific 8 [Source:MGI Symbol;Acc:MGI:2445289]	4.66203	6.78711	11.1067	8.07155	7.41382	7.87099	5.347	7.59424	7.38673	3.39804	5.87667	5.87419	6.58949	4.68753	0.643325306	0.966018836	12	16	24	0.82225511	0.944114881	24	9.512014957	0.087885493
ENSMUSG00000029326	Enoph1	enolase-phosphatase 1 [Source:MGI Symbol;Acc:MGI:1915120]	20.2684	18.1585	17.718	17.2316	18.2006	19.4355	19.75	17.8691	17.1249	19.3433	17.353	18.888	18.4846	17.949	0.004501001	0.140598674	4	8	24	0.027613484	0.590220615	24	21.7748915	0.069384979
ENSMUSG00000029328	Hnrnpdl	heterogeneous nuclear ribonucleoprotein D-like [Source:MGI Symbol;Acc:MGI:1355299]	62.0485	84.6259	86.9637	72.6001	74.2175	69.6959	69.4323	75.4613	86.9276	70.3389	69.9666	73.4293	71.2917	75.9412	0.004320961	0.140598674	12	16	24	0.027461107	0.590220615	24	7.898525212	0.132208755
ENSMUSG00000029330	Cds1	CDP-diacylglycerol synthase 1 [Source:MGI Symbol;Acc:MGI:1921846]	15.8732	15.135	16.3732	16.0094	15.6178	15.8551	15.4491	15.8104	12.6462	19.3167	13.025	15.9292	14.7406	17.3121	0.891742979	0.966018836	16	12	24	0.964527347	0.991159965	24	8.804842248	0.016109914
ENSMUSG00000029334	Prkg2	protein kinase, cGMP-dependent, type II [Source:MGI Symbol;Acc:MGI:108173]	2.44482	2.56127	2.59499	2.56913	2.17274	2.48463	2.30556	2.36248	2.06956	2.49473	2.4883	2.45848	2.5861	2.92275	0.881718085	0.966018836	8	16	24	0.69058168	0.897604733	24	3.790175598	0.028662378
ENSMUSG00000029335	Bmp3	bone morphogenetic protein 3 [Source:MGI Symbol;Acc:MGI:88179]	0.606373	0.630875	0.862948	0.753955	0.66347	0.630846	0.583292	0.668794	0.742587	0.654255	0.586865	0.715766	0.681701	0.706278	0.101520755	0.511659635	12	16	24	0.083853769	0.701123914	24	8.980307875	0.054111645
ENSMUSG00000029337	Fgf5	fibroblast growth factor 5 [Source:MGI Symbol;Acc:MGI:95519]	0.795873	0.924673	1.24417	0.903492	0.941286	0.862188	0.768373	0.947163	0.591271	1.05107	0.584917	0.720143	1.17527	1.05729	0.575387897	0.966018836	8	16	24	0.505112798	0.811334839	24	5.898029158	0.071528076
ENSMUSG00000029338	Antxr2	anthrax toxin receptor 2 [Source:MGI Symbol;Acc:MGI:1919164]	14.6041	12.1415	12.5273	12.6337	14.2534	13.3329	13.2754	12.773	11.4024	13.215	13.3827	13.9744	14.0171	13.172	0.0032435	0.140341875	4	8	24	0.007516107	0.478114494	24	20.03625685	0.096064033
ENSMUSG00000029343	Crybb1	crystallin, beta B1 [Source:MGI Symbol;Acc:MGI:104992]	3.5414	2.78207	3.42984	3.32544	3.29976	3.54096	3.77173	3.50806	3.62519	3.26397	3.64807	3.60079	3.71717	3.7449	0.113148904	0.530777648	4	12	24	0.483926756	0.805393068	24	22.23691311	0.034798967
ENSMUSG00000029344	Tpst2	protein-tyrosine sulfotransferase 2 [Source:MGI Symbol;Acc:MGI:1309516]	9.95057	10.0731	10.3275	11.821	10.1021	9.94969	9.43342	10.0401	10.293	11.3021	10.7832	11.8622	10.1452	9.44323	0.029682156	0.323426482	16	12	24	0.03885814	0.611732283	24	13.93288144	0.084968149
ENSMUSG00000029345	Tfip11	tuftelin interacting protein 11 [Source:MGI Symbol;Acc:MGI:1930075]	11.7578	9.13055	9.40939	10.0767	8.58561	10.5536	10.5149	10.636	8.8212	11.7497	9.34746	10.5068	9.80414	10.3708	0.417653118	0.963884303	4	16	24	0.593875417	0.857116259	24	23.05829697	0.052097742
ENSMUSG00000029346	Srrd	SRR1 domain containing [Source:MGI Symbol;Acc:MGI:1917368]	8.23723	7.28265	9.14473	6.89547	7.10478	6.94164	7.46833	7.67554	8.73999	8.02786	9.21903	7.84708	7.55627	8.69961	0.895493752	0.966018836	12	12	24	0.353254375	0.780711577	24	7.991956837	0.070879331
ENSMUSG00000029348	Asphd2	aspartate beta-hydroxylase domain containing 2 [Source:MGI Symbol;Acc:MGI:1920148]	15.9077	13.343	15.8459	14.5317	14.2018	16.0486	15.4366	14.3529	12.1309	16.629	14.3603	15.2363	14.138	14.3267	0.625825704	0.966018836	24	8	24	0.38122363	0.799546792	24	18.88173169	0.062435956
ENSMUSG00000029352	Crybb3	crystallin, beta B3 [Source:MGI Symbol;Acc:MGI:102717]	1.68964	1.67678	1.69925	1.3957	1.82373	1.60351	2.06112	1.93172	1.52223	1.44418	1.97386	1.44446	2.20378	1.39265	0.113148904	0.530777648	4	12	24	0.299387091	0.768040178	24	22.81157697	0.083907754
ENSMUSG00000029359	Tesc	tescalcin [Source:MGI Symbol;Acc:MGI:1930803]	22.1265	15.9891	13.205	17.5757	15.4952	19.5882	17.6483	19.4208	13.5637	22.2043	16.0054	19.5614	17.5132	19.5305	0.148073804	0.590732968	24	12	24	0.338233864	0.772907855	24	21.61517335	0.127453676
ENSMUSG00000029361	Nos1	nitric oxide synthase 1, neuronal [Source:MGI Symbol;Acc:MGI:97360]	4.56589	3.29078	3.4741	3.82889	3.7785	4.88157	4.0867	5.07707	2.83868	6.3471	3.16542	4.26798	3.74883	3.67814	0.390686537	0.915479136	24	12	24	0.752565302	0.919532999	24	19.36494654	0.077772972
ENSMUSG00000029363	Rfc5	replication factor C (activator 1) 5 [Source:MGI Symbol;Acc:MGI:1919401]	3.26061	2.68085	2.76876	2.94384	2.51615	2.30487	2.36763	2.55074	2.95417	2.60156	2.44431	2.7223	2.76551	3.02673	0.29905572	0.809362476	12	8	24	0.301619852	0.768040178	24	5.967600142	0.063953792
ENSMUSG00000029364	Wsb2	WD repeat and SOCS box-containing 2 [Source:MGI Symbol;Acc:MGI:2144041]	59.3603	45.8022	45.2973	46.2043	56.3012	55.0065	51.7526	52.0916	40.4805	54.4803	45.7921	48.4878	49.5711	45.9798	0.069101108	0.433403132	4	8	24	0.064974557	0.663876076	24	20.24760394	0.127850636
ENSMUSG00000029366	Dck	deoxycytidine kinase [Source:MGI Symbol;Acc:MGI:102726]	10.8681	10.4062	10.1447	10.4655	9.61531	11.8554	10.2089	11.0106	8.02311	10.1934	9.76908	9.93975	9.95603	10.399	0.337748746	0.85758369	8	12	24	0.261991021	0.768040178	24	22.11989178	0.070737109
ENSMUSG00000029370	Rassf6	Ras association (RalGDS/AF-6) domain family member 6 [Source:MGI Symbol;Acc:MGI:1920496]	1.6972	2.01575	2.15575	1.99841	1.48544	2.29514	1.52158	1.58795	1.29986	2.1018	2.05221	2.0474	2.22199	1.99022	0.920858634	0.966018836	24	8	24	0.804981226	0.937541099	24	17.92840263	0.043519276
ENSMUSG00000029372	Ppbp	pro-platelet basic protein [Source:MGI Symbol;Acc:MGI:1888712]	5.39588	6.60517	14.2726	10.1605	5.36929	11.7503	4.95242	7.11676	7.639	4.19207	5.82239	7.17799	9.36797	4.92423	0.916963526	0.966018836	12	20	24	0.954462181	0.988601783	24	9.509470498	0.057217793
ENSMUSG00000029373	Pf4	platelet factor 4 [Source:MGI Symbol;Acc:MGI:1888711]	12.7493	19.8026	20.213	18.2356	14.2035	15.3633	14.0052	17.2546	24.2524	11.5403	15.6997	15.1672	19.3173	12.0967	0.446967971	0.966018836	12	12	24	0.388963987	0.799546792	24	7.376507643	0.161863812

ENSMUSG00000029442	Wdr66	WD repeat domain 66 [Source:MGI Symbol;Acc:MGI:1918495]	3.65617	3.57494	3.15458	4.81761	3.11033	2.87428	3.40867	2.51589	3.87169	2.73493	3.24769	4.43117	3.22876	3.44138	0.733896783	0.966018836	16	4	24	0.930099186	0.981952083	24	15.21648617	0.030102851
ENSMUSG00000029446	Psph	phosphoserine phosphatase [Source:MGI Symbol;Acc:MGI:97788]	12.2261	12.4426	11.3178	11.7825	14.3555	12.1135	10.8357	13.0744	13.7188	11.7853	15.0388	12.571	12.98	12.2588	0.891690964	0.966018836	20	8	24	0.477489736	0.805393068	24	15.384441	0.054871048
ENSMUSG00000029447	Cct6a	chaperonin containing Tcp1, subunit 6a (zeta) [Source:MGI Symbol;Acc:MGI:107943]	109.19	101.68	98.6646	96.6259	101.987	97.6688	99.1291	100.634	100.376	98.0222	117.426	111.667	113.743	110.189	0.147689593	0.590732968	20	20	24	0.258398721	0.768040178	24	21.63969047	0.051423883
ENSMUSG00000029449	Rhof	ras homolog family member F (in filopodia) [Source:MGI Symbol;Acc:MGI:1345629]	9.49113	7.15634	8.47646	7.93258	7.3185	9.5644	8.3316	8.99925	5.84805	9.69794	6.80798	7.96707	6.98616	8.18401	0.897570341	0.966018836	24	20	24	0.721682743	0.90866625	24	21.73085088	0.061084947
ENSMUSG00000029452	Tmem116	transmembrane protein 116 [Source:MGI Symbol;Acc:MGI:1924712]	1.30223	1.14797	1.11386	1.16252	1.22408	1.69961	1.08877	1.5743	1.75515	1.2014	1.77066	1.24978	1.3538	1.72062	0.885059765	0.966018836	24	4	24	0.982997817	0.996298481	24	5.854570283	0.01050446
ENSMUSG00000029454	Mapkapk5	MAP kinase-activated protein kinase 5 [Source:MGI Symbol;Acc:MGI:1333110]	27.409	30.9376	31.9456	29.4628	32.4025	27.7997	29.3428	29.9496	31.0339	30.2725	30.2334	26.9252	31.7119	30.8034	0.0444686	0.365196805	12	12	24	0.108069276	0.72082227	24	8.742906871	0.064662969
ENSMUSG00000029455	Aldh2	aldehyde dehydrogenase 2, mitochondrial [Source:MGI Symbol;Acc:MGI:99600]	80.0531	75.1298	77.4791	85.9673	80.2716	78.2124	85.1383	83.5186	80.3176	95.8841	82.412	86.9872	88.8532	80.0751	0.718086778	0.966018836	16	20	24	0.5128713	0.815948524	24	14.7099062	0.035184487
ENSMUSG00000029456	Acad10	acyl-Coenzyme A dehydrogenase family, member 10 [Source:MGI Symbol;Acc:MGI:1919235]	2.88571	2.86941	3.20526	3.461	3.31824	2.71132	2.89447	3.07476	3.20565	3.28814	3.84898	2.73422	3.70774	3.1305	0.007331665	0.192760771	20	4	24	0.15869831	0.765279481	24	11.90441081	0.080109948
ENSMUSG00000029458	Brap	BRCA1 associated protein [Source:MGI Symbol;Acc:MGI:1919649]	54.8794	43.3564	42.9704	48.2048	44.6099	50.7584	45.9376	52.0125	38.6033	58.9235	45.2569	48.0566	50.0953	49.1416	0.44861185	0.966018836	4	8	24	0.657232904	0.884447211	24	20.73042623	0.058119552
ENSMUSG00000029461	Fam168a	family with sequence similarity 168, member A [Source:MGI Symbol;Acc:MGI:2442372]	32.3091	28.1541	30.906	28.9	28.9416	30.8211	32.4029	29.5358	26.0482	35.4138	26.874	30.0724	27.9211	30.9927	0.935593607	0.966018836	4	16	24	0.918381	0.977957242	24	22.02724181	0.019429021
ENSMUSG00000029462	Vps29	VPS29 retromer complex component [Source:MGI Symbol;Acc:MGI:1928344]	125.013	127.352	115.756	113.193	124.326	113.471	118.829	111.495	126.499	98.6565	129.888	123.384	131.171	123.517	0.702630284	0.966018836	4	12	24	0.356165533	0.783018041	24	23.30332841	0.06184742
ENSMUSG00000029463	Fam216a	family with sequence similarity 216, member A [Source:MGI Symbol;Acc:MGI:1916198]	14.3554	15.2685	14.7949	12.5628	13.78	14.4425	14.2181	14.0029	13.2122	14.2735	13.4284	15.8841	15.4422	15.3698	0.154948076	0.607427992	24	20	24	0.06207821	0.661961741	24	0.534266837	0.072274167
ENSMUSG00000029464	Gpn3	GPN-loop GTPase 3 [Source:MGI Symbol;Acc:MGI:1289326]	14.8814	13.6561	13.9689	13.466	12.0249	14.1714	15.5849	16.1265	15.0036	14.236	13.3108	13.7748	15.4407	15.2947	0.00384759	0.140598674	4	16	24	0.002486672	0.39630666	24	3.183046594	0.109589398
ENSMUSG00000029465	Arpc3	actin related protein 2/3 complex, subunit 3 [Source:MGI Symbol;Acc:MGI:1928375]	179.428	173.188	171.334	158.623	180.432	175.854	158.951	171.867	163.058	156.234	177.546	171.25	186.077	175.387	0.082113105	0.462922111	20	20	24	0.182577168	0.768040178	24	22.21335643	0.054502521
ENSMUSG00000029466	Anapc7	anaphase promoting complex subunit 7 [Source:MGI Symbol;Acc:MGI:1929711]	14.1964	12.7989	14.3292	13.7028	12.1663	14.5861	14.1093	13.7093	11.8511	14.8487	12.3204	13.7644	13.713	13.6708	0.708665292	0.966018836	24	20	24	0.695876342	0.89909553	24	23.69537126	0.032063444
ENSMUSG00000029467	Atp2a2	ATPase, Ca++ transporting, cardiac muscle, slow twitch 2 [Source:MGI Symbol;Acc:MGI:88110]	68.811	59.3533	70.2405	64.7268	56.8698	70.2278	70.629	67.0471	47.6191	75.7339	52.4329	58.2505	59.6589	66.9279	0.94499634	0.966018836	4	16	24	0.794486094	0.934080484	24	1.41768959	0.048852791
ENSMUSG00000029468	P2rx7	purinergic receptor P2X, ligand-gated ion channel, 7 [Source:MGI Symbol;Acc:MGI:1339957]	16.6461	16.148	14.8637	17.9982	18.7452	13.6245	18.981	16.2501	17.8446	18.0211	17.8737	15.0844	17.4959	15.8925	0.416857905	0.963884303	20	4	24	0.627428556	0.872795898	24	12.25224585	0.050488476
ENSMUSG00000029469	Ift81	intraflagellar transport 81 [Source:MGI Symbol;Acc:MGI:1098597]	9.00269	10.4249	10.3671	9.87363	10.0282	8.82865	8.57546	8.57269	9.43697	7.25555	9.66775	9.2266	9.63487	10.4198	0.94397916	0.966018836	8	20	24	0.824365779	0.944830458	24	5.431686424	0.027220437
ENSMUSG00000029470	P2rx4	purinergic receptor P2X, ligand-gated ion channel 4 [Source:MGI Symbol;Acc:MGI:1338859]	22.0184	21.6373	19.0267	20.0278	22.2965	23.5383	21.1487	20.4081	19.9998	20.5636	22.573	20.3198	21.8891	20.7964	0.009730499	0.194720062	20	16	24	0.016923972	0.547196997	24	20.08722016	0.08108616
ENSMUSG00000029471	Camkk2	calcium/calmodulin-dependent protein kinase kinase 2, beta [Source:MGI Symbol;Acc:MGI:2444812]	8.10027	6.57306	8.04097	7.27526	7.39837	7.84375	7.30358	7.24107	7.10772	7.71079	7.43716	7.01203	6.44424	7.45042	0.880631923	0.966018836	20	12	24	0.610376354	0.864816145	24	14.44268294	0.03110384
ENSMUSG00000029472	Anapc5	anaphase-promoting complex subunit 5 [Source:MGI Symbol;Acc:MGI:1929722]	90.9631	89.2312	95.5474	97.6218	99.7755	93.9088	89.4609	98.4639	90.1362	94.72	90.021	91.6998	91.7503	89.5743	0.444754148	0.966018836	16	16	24	0.223129893	0.768040178	24	13.33118602	0.034605525
ENSMUSG00000029474	Rnf34	ring finger protein 34 [Source:MGI Symbol;Acc:MGI:2153340]	16.0178	13.6111	13.196	13.3271	15.249	14.0671	12.2268	13.3341	14.5976	13.8741	14.445	14.2966	15.4522	13.2516	0.459019924	0.966018836	20	12	24	0.409196158	0.799546792	24	18.88474786	0.053399172
ENSMUSG00000029475	Kdm2b	lysine (K)-specific demethylase 2B [Source:MGI Symbol;Acc:MGI:1354737]	3.0481	3.56981	2.93126	2.89045	2.94065	2.83632	2.80285	2.84313	3.68286	3.31694	4.09272	3.1185	3.29237	3.23032	0.93717134	0.966018836	4	20	24	0.61935653	0.869468249	24	10.68486482	0.047048692
ENSMUSG00000029477	Morn3	MORN repeat containing 3 [Source:MGI Symbol;Acc:MGI:1922140]	0.682647	0.897219	0.326619	0.831926	0.759541	0.459688	1.49881	1.02551	1.00774	0.32583	0.688547	0.7463	0.89052	0.482039	0.590017078	0.966018836	4	20	24	0.382020252	0.799546792	24	0.536962806	0.136022127
ENSMUSG00000029478	Ncor2	nuclear receptor co-repressor 2 [Source:MGI Symbol;Acc:MGI:1337080]	23.4229	22.2153	23.3955	23.7581	24.5862	20.9125	23.627	24.5813	24.2619	32.862	24.8584	23.3161	26.6483	25.4388	0.814077113	0.966018836	20	4	24	0.221230502	0.768040178	24	10.64356538	0.085546621
ENSMUSG00000029480	Dhx37	DEAH (Asp-Glu-Ala-His) box polypeptide 37 [Source:MGI Symbol;Acc:MGI:3028576]	4.85453	3.85519	3.95156	3.39551	4.53196	5.27013	5.62167	4.67765	4.35795	5.38079	2.92897	3.38818	5.23457	4.09165	0.428730128	0.966018836	4	16	24	0.387538887	0.799546792	24	0.651705306	0.125597721
ENSMUSG00000029482	Aacs	acetoacetyl-CoA synthetase [Source:MGI Symbol;Acc:MGI:1926144]	48.977	35.8542	37.5409	44.6775	43.2468	46.1355	44.2033	42.5589	35.6468	53.2862	49.069	45.4944	46.2762	46.3689	0.279136262	0.790764844	16	20	24	0.119606826	0.730969695	24	18.2705803	0.122709929
ENSMUSG00000029484	Anxa3	annexin A3 [Source:MGI Symbol;Acc:MGI:1201378]	18.803	27.9147	26.0874	22.1815	23.4721	18.4765	19.3521	21.7782	31.0327	17.7505	21.2041	20.2648	21.6819	21.73	0.010468194	0.196621011	12	12	24	0.08145681	0.697102286	24	7.680811506	0.186489484
ENSMUSG00000029486	Mrpl1	mitochondrial ribosomal protein L1 [Source:MGI Symbol;Acc:MGI:2137202]	6.27737	6.3752	6.90798	7.01929	6.67148	6.23937	5.58247	7.15169	7.5508	6.24478	6.97622	6.45473	7.06983	6.46573	0.245061943	0.741580974	12	12	24	0.160069709	0.76564405	24	9.676504308	0.070748781
ENSMUSG00000029490	Mfsd7a	major facilitator superfamily domain containing 7A [Source:MGI Symbol;Acc:MGI:2442629]	3.17715	3.33364	3.55083	4.14895	4.54489	3.23057	4.06876	3.39988	4.43989	3.18425	3.58837	3.25418	3.08037	2.79319	0.221187015	0.712731927	20	8	24	0.206040212	0.768040178	24	13.34891959	0.101728298
ENSMUSG00000029499	Pxmp2	peroxisomal membrane protein 2 [Source:MGI Symbol;Acc:MGI:107487]	5.73145	7.53032	8.03586	8.75557	7.72799	8.03941	6.34724	6.80768	8.18279	8.33055	7.43158	7.61543	6.97601	6.58149	3.11E-04	0.049770315	16	12	24	0.003591795	0.425687943	24	12.42909198	0.161400712
ENSMUSG00000029500	Pgam5	phosphoglycerate mutase family member 5 [Source:MGI Symbol;Acc:MGI:1919792]	16.4848	15.3964	17.1555	15.8743	14.7228	16.8319	16.0823	15.6273	13.9493	17.1734	15.5538	16.1765	15.5961	15.5758	0.909604019	0.966018836	24	8	24	0.794828389	0.93426902	24	19.65325037	0.02173569
ENSMUSG00000029501	Ankle2	ankyrin repeat and LEM domain containing 2 [Source:MGI Symbol;Acc:MGI:1261856]	8.08057	8.71278	10.0864	9.6753	8.14102	9.44661	9.96596	7.74991	7.64379	8.54398	8.31047	8.22668	8.31581	8.38227	0.813775138	0.966018836	16	4	24	0.937248719	0.984478515	24	15.67371173	0.015941764
ENSMUSG00000029502	Golga3	golgi autoantigen, golgin subfamily a, 3 [Source:MGI Symbol;Acc:MGI:96958]	15.3253	13.7318	13.3243	13.5776	13.5321	14.6685	13.6668	14.5398	11.4544	14.2667	12.6965	14.039	14.3742	13.8423	0.08464875	0.466404237	4	8	24	0.096214625	0.71175955	24	22.39631313	0.075777532
ENSMUSG00000029503	P2rx2	purinergic receptor P2X, ligand-gated ion channel, 2 [Source:MGI Symbol;Acc:MGI:2665170]	2.58452	2.32261	2.06431	2.33207	2.73953	2.67066	2.64135	2.26405	2.45	2.4499	2.30129	2.32026	2.40467	2.30249	0.356136148	0.880294466	4	4	24	0.084052094	0.701763292	24	19.62338631	0.065993054
ENSMUSG00000029504	Ddx51	DEAD box helicase 51 [Source:MGI Symbol;Acc:MGI:1916913]	6.74614	4.54014	7.36014	5.17594	6.10691	5.49343	7.01497	5.64999	6.34883	5.14242	6.05022	4.49229	5.90577	5.30083	0.834434272	0.966018836	4	12	24	0.935517316	0.983848988	24	3.797918961	0.026301984
ENSMUSG00000029505	Ep400	E1A binding protein p400 [Source:MGI Symbol;Acc:MGI:1276124]	8.7213	6.87863	8.03978	6.80335	6.95684	6.84627	6.72776	7.43143	7.45473	7.44044	8.41899	7.37458	6.55214	7.05535	0.801995186	0.966018836	12	16	24	0.883828064	0.966864521	24	12.50913123	0.021611565
ENSMUSG00000029507	Pus1	pseudouridine synthase 1 [Source:MGI Symbol;Acc:MGI:1929237]	7.52397	6.03767	6.47775	6.93768	6.30262	7.75752	7.58655	6.73735	6.16499	8.47982	6.07019	7.06694	7.61797	5.78481	0.196044629	0.673646834	4	4	24	0.40741285	0.799546792	24	19.76023744	0.079816333
ENSMUSG00000029510	Gpc2	glypican 2 (cerebroglycan) [Source:MGI Symbol;Acc:MGI:1919201]	0.544411	0.809255	0.95222	0.46857	0.867874	0.528628	0.87406	0.843897	0.61848	0.662513	0.85217	0.887476	0.491626	0.660331	0.859936714	0.966018836	20	8	24	0.985779409	0.99721471	24	10.29308996	0.010135212
ENSMUSG00000029512	Ulk1	unc-51 like kinase 1 [Source:MGI Symbol;Acc:MGI:1270126]	11.8966	12.5706	12.2041	14.8634	14.2456	12.386	11.7344	12.0977	13.349	16.0771	11.5797	12.2205	12.6502	13.0407	0.06180207	0.409814092	16	12	24	0.031245078	0.601042304	24	11.75355031	0.11958552
ENSMUSG00000029513	Prkab1	protein kinase, AMP-activated, beta 1 non-catalytic subunit [Source:MGI Symbol;Acc:MGI:1336167]	6.8019	6.08464	7.25405	6.62569	6.13018	5.80077	6.91801	6.63666	7.66804	6.28675	6.7104	6.11659	7.41374	7.43942	0.080164531	0.458678242	12	12	24	0.052800037	0.650526959	24	5.975193999	0.092900446
ENSMUSG00000029516	Cit	citron [Source:MGI Symbol;Acc:MGI:105313]	2.7398	3.07492	4.36144	2.69704	1.87521	3.53079	3.14791	2.59649	3.1689	3.45095	1.90714	3.0194	2.14409	4.17436	0.179603275	0.655480352	12	8	24	0.199812674	0.768040178	24	6.014063662	0.192000422
ENSMUSG00000029518	Rab35	RAB35, member RAS oncogene family [Source:MGI Symbol;Acc:MGI:1924657]	17.9209	16.0537	17.0093	17.9129	17.09	17.6596	17.102	17.8595	16.3196	20.3736	15.7974	18.4297	16.4433	16.7541	0.636884864	0.966018836	24	12	24	0.650953657	0.882698308	24	14.81124907	0.032907812
ENSMUSG00000029521	Chek2	checkpoint kinase 2 [Source:MGI Symbol;Acc:MGI:1355321]	1.21131	1.31681	1.15593	1.08058	1.15952	1.31121	1.09955	0.966292	1.20702	1.05204	1.64698	1.31692	1.2361	1.15685	0.093849599	0.490854203	24	16	24	0.257327968	0.768040178	24	18.49433844	0.066721884
ENSMUSG00000029524	Sirt4	sirtuin 4 [Source:MGI Symbol;Acc:MGI:1922637]	13.6488	11.5203	11.4154	12.0905	14.8421	12.551	14.1822	12.5378	14.1346	13.0931	15.1774	15.5035	13.8204	12.2524	0.039104	0.338446795	20	12	24	0.033998065	0.607049379	24	18.45969038	0.115608121
ENSMUSG00000029528	Pxn	paxillin [Source:MGI Symbol;Acc:MGI:108295]	23.1134	25.4279	25.0237	31.6159	27.566	22.5121	25.141	23.3322	32.0112	30.2862	23.3513	24.5436	23.2909	22.7528	0.062509846	0.410322149	16	8	24	0.010070252	0.503967859	24	11.21162383	0.174708701
ENSMUSG00000029534	St7	suppression of tumorigenicity 7 [Source:MGI Symbol;Acc:MGI:1927450]	5.96871	6.57177	6.21952	5.62862	6.79753	6.64075	6.18206	6.08908	5.6965	6.33957	6.578	6.04725	6.4258	6.89386	0.956178643	0.96629412	20	20	24	0.673134711	0.889936802	24	20.77478692	0.027714036
ENSMUSG00000029535	Triap1	TP53 regulated inhibitor of apoptosis 1 [Source:MGI Symbol;Acc:MGI:1916326]	39.8414	35.6841	34.9743	34.3593	38.0259	36.6224	34.1123	37.4303	33.6467	34.3186	39.7389	35.6664	36.8673	34.344	0.308023687	0.814993005	20	20	24	0.121505268	0.733843269	24	19.91406124	0.061618759
ENSMUSG00000029536	Gatc	glutamyl-tRNA(Gln) amidotransferase, subunit C [Source:MGI Symbol;Acc:MGI:1923776]	16.0779	14.5118	15.6218	15.6728	16.6485	15.5733	15.6751	15.9194	15.9796	16.5451	14.8416	16.8262	16.9494	16.6349	0.920858634	0.966018836	4	8	24	0.909886331	0.974625908	24	20.46621406	0.009912563
ENSMUSG00000029538	Srsf9	serine and arginine-rich splicing factor 9 [Source:MGI Symbol;Acc:MGI:104896]	20.0273	18.3224	18.1349	20.367	19.8197	18.486	22.0868	20.1929	19.9965	19.8199	19.5386	16.9405	22.9731	20.6787	0.05137861	0.38924256	4	20	24	0.608944958	0.864393298	24	3.00809238	0.041093625
ENSMUSG00000029544	Cabp1	calcium binding protein 1 [Source:MGI Symbol;Acc:MGI:1352750]	65.5109	48.5107	49.1265	54.1534	49.1739	55.3638	52.6972	52.9611	42.6069	69.8466	52.0105	54.5411	57.2827	54.8142	0.195343269	0.673387182	4	8	24	0.616413637	0.867682235	24	19.94652295	0.071051302
ENSMUSG00000029545	Acads	acyl-Coenzyme A dehydrogenase, short chain [Source:MGI Symbol;Acc:MGI:87868]	9.49574	9.03439	10.6865	14.9502	11.9763	9.53248	10.5856	13.7447	11.2938	14.9676	13.1337	10.5436	15.6222	13.1405	0.616047374	0.966018836	16	8	24	0.164962509	0.767020009	24	10.98340587	0.147449277
ENSMUSG00000029547	Ints1	integrator complex subunit 1 [Source:MGI Symbol;Acc:MGI:1915760]	9.56351	10.1233	9.60856	12.0414	11.6392	12.1051	9.67182	10.2371	10.9234	12.2819	9.89952	11.4455	9.38966	10.8067	0.004720572	0.140598674	16	12	24	0.060156799	0.658304792	24	14.10492685	0.107794455
ENSMUSG00000029550	Sppl3	signal peptide peptidase 3 [Source:MGI Symbol;Acc:MGI:1891433]	14.0024	12.8058	12.6605	12.1357	13.0354	14.3068	13.861	14.4381	12.7228	16.5431	11.8624	14.1839	13.2771	13.6203	0.290796297	0.79743194	24	20	24	0.822666043	0.944336753	24	23.31153598	0.028615528
ENSMUSG00000029551	Psmg3	proteasome (prosome, macropain) assembly chaperone 3 [Source:MGI Symbol;Acc:MGI:1913756]	21.8472	17.8881	16.3497	18.1045	19.2195	21.2823	17.3234	19.0646	15.5032	18.5559	18.2625	17.6821	18.1738	17.8077	0.290796297	0.79743194	20	16	24	0.047854566	0.63658289	24	20.3298984	0.108288246
ENSMUSG00000029552	Tes	testin LIM domain protein [Source:MGI Symbol;Acc:MGI:105081]	4.7284	4.27316	4.4756	4.71994	4.92249	4.19955	4.67622	4.45707	4.16648	3.83113	5.50044	4.87247	4.4809	4.33535	0.061981717	0.409814092	20	16	24	0.172851438	0.768040178	24	18.46998134	0.076481799
ENSMUSG00000029554	Mad1l1	MAD1 mitotic arrest deficient 1-like 1 [Source:MGI Symbol;Acc:MGI:1341857]	5.64143	5.39905	6.18411	4.91137	5.39709	6.80497	6.23132	5.42027	4.8684	6.26522	5.17714	5.17171	5.74908	5.42974	0.708020493	0.966018836	4	8	24	0.561213584	0.841538771	24	21.91436822	0.052855479
ENSMUSG00000029557	Mrm2	mitochondrial rRNA methyltransferase 2 [Source:MGI Symbol;Acc:MGI:1915267]	6.25828	5.4371	5.99987	5.13352	5.67725	5.75295	5.94107	5.33765	5.46663	5.54579	5.53142	5.79311	5.58213	5.45561	0.082113105	0.462922111	4	4	24	0.130541706	0.740728512	24	21.83385928	0.046843488
ENSMUSG00000029559	2210016L21Rik	RIKEN cDNA 2210016L21 gene [Source:MGI Symbol;Acc:MGI:1919607]	27.5525	22.4947	26.6355	20.9863	23.2109	25.7092	21.6573	24.9807	21.5395	25.9711	23.582	23.5322	23.8789	21.8427	0.850906022	0.966018836	24	16	24	0.773360196	0.927220663	24	20.7147218	0.034590037
ENSMUSG00000029560	Snx8	sorting nexin 8 [Source:MGI Symbol;Acc:MGI:2443816]	8.31011	7.63574	8.91996	8.70575	9.15731	7.42094	10.1138	7.84069	8.56289	8.82481	9.11709	8.71873	7.78602	8.02368	0.109247218	0.524035616	20	12	24	0.377923655	0.797706855	24	14.43064091	0.058977815
ENSMUSG00000029561	Oasl2	2'-5' oligoadenylate synthetase-like 2 [Source:MGI Symbol;Acc:MGI:1344390]	25.4149	19.4708	20.6649	16.0671	22.7235	24.4328	20.2651	25.0606	14.8133	20.1853	22.9098	17.954	17.8012	21.3202	0.521828922	0.966018836	20	20	24	0.32210203	0.768040178	24	21.47575399	0.132283742
ENSMUSG00000029563	Foxp2	forkhead box P2 [Source:MGI Symbol;Acc:MGI:2148705]	1.29757	1.51688	1.58333	0.986432	1.35287	1.203	1.40254	1.53055	1.83305	0.924373	1.45703	1.36925	1.04121	1.37652	0.021214536	0.264116537	12	4	24	0.39260166	0.799546792	24	4.960743456	0.083235237
ENSMUSG00000029569	Tmem168	transmembrane protein 168 [Source:MGI Symbol;Acc:MGI:1921794]	9.98259	11.1477	10.736	10.361	11.0206	10.0493	10.2988	10.2551	10.9105	9.90897	10.868	10.5764	10.5329	10.9399	0.652290823	0.966018836	8	20	24	0.645242687	0.879668078	24	8.230559749	0.01989161
ENSMUSG00000029570	Lfng	LFNG O-fucosylpeptide 3-beta-N-acetylglucosaminyltransferase [Source:MGI Symbol;Acc:MGI:1095413]	2.25194	3.62695	4.99114	4.07586	3.54376	3.61277	3.59065	2.76047	3.96561	3.44776	3.14143	2.91824	2.725	3.20638	0.031969981	0.324385159	12	16	24	0.047353254	0.63386192	24	10.55739955	0.190759971
ENSMUSG00000029571	Tmem106b	transmembrane protein 106B [Source:MGI Symbol;Acc:MGI:1919150]	30.7389	33.2106	33.8876	34.5606	33.2512	29.3866	33.7817	29.4719	32.9009	29.4288	33.5315	28.8727	30.9564	31.0793	0.198504154	0.680216953	12	12	24	0.400585335	0.799546792	24	10.37840595	0.044230273
ENSMUSG00000029575	Mmab	methylmalonic aciduria (cobalamin deficiency) cblB type homolog (human) [Source:MGI Symbol;Acc:MGI:1924947]	11.2756	9.21739	10.1757	10.2189	9.5057	11.0793	11.0225	10.6724	9.71932	11.6045	10.0222	11.4574	11.0672	10.3458	0.132887428	0.567728672	24	12	24	0.3051388	0.768040178	24	21.26193157	0.056098953
ENSMUSG00000029576	Radil	Ras association and DIL domains [Source:MGI Symbol;Acc:MGI:2443088]	5.32301	7.11426	6.57794	5.62217	6.01317	5.91928	8.67022	7.68686	9.61364	7.76158	6.59169	7.24515	7.15056	6.8272	0.852772142	0.966018836	12	8	24	0.205213387	0.768040178	24	6.180638446	0.129825415
ENSMUSG00000029577	Ube3b	ubiquitin protein ligase E3B [Source:MGI Symbol;Acc:MGI:1891295]	23.0449	18.2311	18.4078	23.1762	20.5458	23.1596	21.1156	23.808	17.1908	29.4151	18.5776	20.9585	21.3852	20.1678	0.649212555	0.966018836	16	20	24	0.739189798	0.914957371	24	17.19163049	0.061020587
ENSMUSG00000029578	Wipi2	WD repeat domain, phosphoinositide interacting 2 [Source:MGI Symbol;Acc:MGI:1923831]	26.4134	22.553	24.3513	24.8826	23.916	25.0063	23.0759	24.8532	22.0726	26.2323	22.0997	25.4542	25.6257	23.8597	0.496745696	0.966018836	4	8	24	0.673255537	0.890029909	24	20.55531132	0.030890603
ENSMUSG00000029580	Actb	actin, beta [Source:MGI Symbol;Acc:MGI:87904]	336.829	279.249	291.496	273.498	285.994	316.519	323.559	338.675	270.136	351.858	284.161	300.524	298.101	287.837	0.442397395	0.966018836	4	8	24	0.51532087	0.817300608	24	22.71204094	0.056872814
ENSMUSG00000029581	Fscn1	fascin actin-bundling protein 1 [Source:MGI Symbol;Acc:MGI:1352745]	25.9178	26.888	29.784	25.8744	32.983	26.7763	32.6278	27.9317	31.4307	40.7993	25.8752	31.3929	28.6483	28.5207	0.816361374	0.966018836	12	20	24	0.493391116	0.80595659	24	11.86308502	0.080428544
ENSMUSG00000029587	Zfp12	zinc finger protein 12 [Source:MGI Symbol;Acc:MGI:99157]	8.48331	6.94668	6.82151	7.01861	7.08267	6.66811	7.01996	6.12548	6.37028	6.70165	8.08569	6.65102	6.07899	5.97157	0.192357053	0.669841558	20	12	24	0.109883616	0.72082227	24	17.80443463	0.084805532
ENSMUSG00000029591	Ung	uracil DNA glycosylase [Source:MGI Symbol;Acc:MGI:109352]	0.457134	0.808188	0.633483	1.14387	0.59694	0.809129	0.541254	0.956048	0.751299	0.790407	0.601111	0.771683	0.647679	0.688733	0.305564675	0.811201458	16	12	24	0.307392919	0.768040178	24	10.16130048	0.087397907
ENSMUSG00000029592	Usp30	ubiquitin specific peptidase 30 [Source:MGI Symbol;Acc:MGI:2140991]	5.82221	6.47743	7.45846	6.80808	6.44255	7.02311	6.1898	6.73205	5.92206	7.05568	5.74979	6.53463	5.85809	6.91898	0.295379186	0.805301811	16	12	24	0.415652402	0.800637996	24	9.715515209	0.054675794
ENSMUSG00000029594	Rbm19	RNA binding motif protein 19 [Source:MGI Symbol;Acc:MGI:1921361]	4.00312	3.09045	3.99862	2.56117	3.3844	3.36615	3.0731	3.85327	2.62172	4.02945	3.1856	3.04934	2.54659	3.40744	0.832293447	0.966018836	8	20	24	0.972812121	0.993305115	24	5.731471828	0.016765322
ENSMUSG00000029596	Sdsl	serine dehydratase-like [Source:MGI Symbol;Acc:MGI:2182607]	3.50462	3.21144	3.08716	2.31691	2.59244	2.82291	3.59133	3.74991	3.71328	3.00973	3.75925	3.65607	4.12162	3.18458	0.185945151	0.662239322	4	12	24	0.064340398	0.663876076	24	1.413072041	0.131131715
ENSMUSG00000029597	Sds	serine dehydratase [Source:MGI Symbol;Acc:MGI:98270]	0.841955	1.08123	1.01547	0.842202	0.937552	0.447969	1.10263	0.586767	0.79909	0.78851	0.929743	0.671882	0.809804	0.73698	0.891690964	0.966018836	20	4	24	0.761823249	0.922863094	24	8.208990144	0.043567831
ENSMUSG00000029598	Plbd2	phospholipase B domain containing 2 [Source:MGI Symbol;Acc:MGI:1919022]	23.0489	19.6009	20.9208	20.8266	19.638	23.2191	19.9035	22.3155	17.2523	28.519	19.5606	22.2892	20.2859	22.0055	0.922029406	0.966018836	24	12	24	0.881777479	0.966661503	24	17.04332459	0.033182081
ENSMUSG00000029599	Ddx54	DEAD box helicase 54 [Source:MGI Symbol;Acc:MGI:1919240]	8.55227	5.75189	6.30228	5.89144	6.05553	6.62064	7.4494	7.60339	5.64872	9.07469	6.9376	7.49573	7.25532	6.83867	0.286642595	0.793714531	4	8	24	0.52048664	0.820018348	24	21.83560664	0.08255597
ENSMUSG00000029600	Rita1	RBPJ interacting and tubulin associated 1 [Source:MGI Symbol;Acc:MGI:1922021]	9.18831	8.02507	9.44112	8.38858	7.69243	9.34027	9.86543	8.33075	7.92626	7.4936	9.38778	8.44042	8.21176	8.54234	0.745199643	0.966018836	4	12	24	0.299230542	0.768040178	24	22.29402531	0.066209579
ENSMUSG00000029601	Iqcd	IQ motif containing D [Source:MGI Symbol;Acc:MGI:1922982]	0.856265	1.02712	0.881339	0.854107	0.588953	0.546917	0.890972	0.60398	0.572764	0.548463	0.751728	0.721962	0.547316	0.844027	0.745199643	0.966018836	8	16	24	0.449872822	0.805393068	24	3.692775394	0.059879326
ENSMUSG00000029603	Dtx1	deltex 1, E3 ubiquitin ligase [Source:MGI Symbol;Acc:MGI:1352744]	0.941535	2.5475	3.92205	2.04407	1.04116	2.12526	2.58914	1.94317	2.95139	1.52403	1.10253	1.79274	1.29059	2.76421	0.010576629	0.197206032	12	8	24	0.049871564	0.643630594	24	6.532422986	0.362661591
ENSMUSG00000029605	Oas1b	2'-5' oligoadenylate synthetase 1B [Source:MGI Symbol;Acc:MGI:97430]	1.57768	1.26603	1.53809	1.39869	1.51463	1.35769	1.51544	2.07591	1.66975	1.60615	1.96262	0.975106	1.41571	1.4758	0.601647512	0.966018836	12	12	24	0.490760213	0.805393068	24	8.718713492	0.076294171
ENSMUSG00000029608	Rph3a	rabphilin 3A [Source:MGI Symbol;Acc:MGI:102788]	58.7203	47.7834	50.9627	49.545	45.262	56.9437	55.5228	56.6587	44.2376	76.7551	44.3603	57.6095	52.3826	59.3404	0.570349842	0.966018836	24	20	24	0.890898765	0.969222457	24	1.049706798	0.039595322
ENSMUSG00000029610	Aimp2	aminoacyl tRNA synthetase complex-interacting multifunctional protein 2 [Source:MGI Symbol;Acc:MGI:2385237]	23.0964	22.1111	20.3595	23.7217	24.1231	22.973	25.5502	24.9821	22.7041	22.6024	21.3713	21.0356	21.1646	21.161	0.859936714	0.966018836	4	8	24	0.875165545	0.964979482	24	21.13582969	0.019431798
ENSMUSG00000029613	Eif2ak1	eukaryotic translation initiation factor 2 alpha kinase 1 [Source:MGI Symbol;Acc:MGI:1353448]	16.0918	13.9479	15.9593	14.493	14.4923	17.6401	16.0404	16.2683	12.513	16.5848	13.6851	15.5847	14.9064	15.5054	0.904387595	0.966018836	24	20	24	0.398853555	0.799546792	24	21.98502636	0.0662609
ENSMUSG00000029614	Rpl6	ribosomal protein L6 [Source:MGI Symbol;Acc:MGI:108057]	36.4975	37.1426	37.0191	34.567	49.3126	40.4298	34.5046	36.0341	35.7613	27.0129	36.1385	29.2679	33.2234	31.0087	0.832742389	0.966018836	20	20	24	0.558300362	0.839674578	24	18.45934211	0.0746764
ENSMUSG00000029616	Erp29	endoplasmic reticulum protein 29 [Source:MGI Symbol;Acc:MGI:1914647]	66.1152	63.1525	63.308	62.4904	69.3141	65.54	61.5689	66.6522	61.753	60.7182	63.6543	66.7034	64.4038	64.3722	0.090721279	0.481752771	24	16	24	0.176940613	0.768040178	24	20.47474377	0.038379189
ENSMUSG00000029617	Ccz1	CCZ1 vacuolar protein trafficking and biogenesis associated [Source:MGI Symbol;Acc:MGI:2141070]	21.478	22.7284	23.6689	22.2667	21.1761	22.2097	20.4645	21.4015	24.433	22.6317	22.7294	23.3124	23.0462	22.7142	0.643325306	0.966018836	12	16	24	0.201310921	0.768040178	24	9.247628901	0.043914927
ENSMUSG00000029621	Arpc1a	actin related protein 2/3 complex, subunit 1A [Source:MGI Symbol;Acc:MGI:1928896]	148.038	129.494	127.712	132.051	152.722	136.467	139.124	134.574	134.27	134.767	132.497	131.692	138.795	124.084	0.124211791	0.552144108	4	4	24	0.097715865	0.71175955	24	19.32093112	0.061372159
ENSMUSG00000029622	Arpc1b	actin related protein 2/3 complex, subunit 1B [Source:MGI Symbol;Acc:MGI:1343142]	155.586	112.46	123.426	113.756	130.574	128.792	127.277	133.56	107.016	122.327	136.001	118.162	125.247	113.998	0.176965982	0.653256853	20	20	24	0.103884399	0.715480277	24	20.64177286	0.107885002
ENSMUSG00000029623	Pdap1	PDGFA associated protein 1 [Source:MGI Symbol;Acc:MGI:2448536]	29.8959	28.546	33.4295	29.4884	29.8812	29.8586	28.4377	28.8692	33.6025	38.5202	30.669	37.8112	38.2893	36.5986	0.865514257	0.966018836	12	20	24	0.708332734	0.903587413	24	9.548924557	0.040151763
ENSMUSG00000029624	Ptcd1	pentatricopeptide repeat domain 1 [Source:MGI Symbol;Acc:MGI:1919049]	6.21576	5.90722	6.24693	6.47785	5.93813	6.35566	5.99932	6.73959	5.58609	6.34602	5.77956	6.34076	6.00792	5.80368	0.952551093	0.966018836	24	12	24	0.922602298	0.979411448	24	18.94132056	0.010549664
ENSMUSG00000029625	Cpsf4	cleavage and polyadenylation specific factor 4 [Source:MGI Symbol;Acc:MGI:1861602]	5.39978	5.3664	7.08109	5.65911	5.52126	4.96148	5.25307	6.29716	6.43671	5.53458	5.86179	5.92303	5.50348	5.68422	0.029412383	0.322165609	12	16	24	0.055189551	0.65534544	24	8.560113663	0.102195012
ENSMUSG00000029627	Zkscan14	zinc finger with KRAB and SCAN domains 14 [Source:MGI Symbol;Acc:MGI:1914485]	4.18436	4.4227	4.30193	4.58995	4.03243	3.78867	4.41437	4.06777	4.14099	4.12624	4.25932	4.07467	4.70041	4.0069	0.948161972	0.966018836	4	20	24	0.777911788	0.928772941	24	5.716610201	0.019653086
ENSMUSG00000029629	Phf14	PHD finger protein 14 [Source:MGI Symbol;Acc:MGI:1923539]	13.8444	20.4507	20.2185	21.1226	19.4145	18.2326	18.1662	17.891	18.3981	18.1687	19.5404	17.4336	16.412	16.0618	0.037982622	0.334069441	16	12	24	0.054016363	0.654752212	24	11.9801789	0.127785209
ENSMUSG00000029632	Ndufa4	Ndufa4, mitochondrial complex associated [Source:MGI Symbol;Acc:MGI:107686]	665.855	778.944	689.191	781.726	722.163	680.127	604.512	659.37	846.117	658.789	832.223	688.536	811.686	783.873	0.918193129	0.966018836	12	16	24	0.451922591	0.805393068	24	9.992388531	0.073182436
ENSMUSG00000029633	Gm5578	predicted pseudogene 5578 [Source:MGI Symbol;Acc:MGI:3645010]	35.5053	32.3864	35.1628	32.1388	31.9341	35.0987	31.655	33.8596	29.2769	37.9418	33.778	33.4539	34.6435	34.546	0.79625133	0.966018836	4	16	24	0.909333015	0.974606327	24	19.71033082	0.016318345
ENSMUSG00000029634	Rnf6	ring finger protein (C3H2C3 type) 6 [Source:MGI Symbol;Acc:MGI:1921382]	24.0202	20.7182	22.1608	23.6861	21.2269	22.9116	23.3944	22.2993	19.6374	23.2403	21.6799	22.3985	22.2079	22.6537	0.454881285	0.966018836	4	8	24	0.497324971	0.807576732	24	20.66404187	0.036657772
ENSMUSG00000029635	Cdk8	cyclin-dependent kinase 8 [Source:MGI Symbol;Acc:MGI:1196224]	10.7829	12.2106	11.9383	12.837	11.6943	12.8514	12.2575	10.7041	12.4921	12.3751	12.7617	11.6397	11.2574	12.3099	0.223125229	0.717349224	16	12	24	0.148930784	0.757588568	24	12.97840138	0.057837218
ENSMUSG00000029636	Wasf3	WASP family, member 3 [Source:MGI Symbol;Acc:MGI:2658986]	11.0866	11.1118	12.0995	9.70231	8.85023	11.7028	12.3422	9.2416	9.31615	12.6695	8.78908	10.7108	9.11756	11.6502	0.865490576	0.966018836	16	4	24	0.719016427	0.907779444	24	3.010045227	0.055581789
ENSMUSG00000029638	Glcci1	glucocorticoid induced transcript 1 [Source:MGI Symbol;Acc:MGI:2179717]	1.46483	1.5084	1.68782	1.2642	1.43848	1.12192	0.985008	1.31424	1.47188	1.63482	1.37847	1.2626	0.85874	1.37613	0.018263889	0.256345714	12	16	24	0.034550245	0.608551791	24	10.13130212	0.130592502
ENSMUSG00000029640	Usp12	ubiquitin specific peptidase 12 [Source:MGI Symbol;Acc:MGI:1270128]	29.2851	26.0558	25.726	26.2671	26.2736	28.4334	25.1228	25.0055	23.374	28.1519	26.8228	27.804	29.076	27.7389	0.052640391	0.391647859	24	12	24	0.136735898	0.747972781	24	20.33810927	0.068813463
ENSMUSG00000029641	Rasl11a	RAS-like, family 11, member A [Source:MGI Symbol;Acc:MGI:1916145]	1.60797	1.61404	1.32396	1.2125	1.35987	1.38293	1.58551	1.73004	0.9016	1.44883	1.3545	1.66149	1.48324	1.72051	4.55E-04	0.05689201	8	4	24	0.058729944	0.658304792	24	23.86548895	0.1194457
ENSMUSG00000029642	Polr1d	polymerase (RNA) I polypeptide D [Source:MGI Symbol;Acc:MGI:108403]	33.767	34.3648	31.3859	32.5883	32.3789	31.3399	35.3929	35.9623	33.5816	34.1758	39.1046	35.0374	37.9753	36.9478	0.470672946	0.966018836	8	4	24	0.396050816	0.799546792	24	1.048815735	0.036230178
ENSMUSG00000029647	Pan3	PAN3 poly(A) specific ribonuclease subunit [Source:MGI Symbol;Acc:MGI:1919837]	13.1892	12.4744	12.7175	13.0502	13.249	12.6617	12.8293	11.2462	13.4474	12.7561	12.3185	11.215	11.593	12.1609	0.417963425	0.963884303	12	20	24	0.259889114	0.768040178	24	12.34975584	0.040792175
ENSMUSG00000029648	Flt1	FMS-like tyrosine kinase 1 [Source:MGI Symbol;Acc:MGI:95558]	3.71874	4.05694	3.69669	3.93775	4.04251	4.36057	4.63131	5.12094	3.84771	5.22659	3.54846	3.84692	3.88084	3.58181	0.831272156	0.966018836	16	4	24	0.979209792	0.994940912	24	7.491697932	0.012630734
ENSMUSG00000029649	Pomp	proteasome maturation protein [Source:MGI Symbol;Acc:MGI:1913787]	173.504	175.6	149.408	154.075	162.05	156.661	149.278	156.546	174.733	135.517	171.058	158.324	162.809	165.476	0.953350544	0.966018836	8	8	24	0.52822909	0.82388535	24	0.865381853	0.046061952

ENSMUSG00000029650	Slc46a3	solute carrier family 46, member 3 [Source:MGI Symbol;Acc:MGI:1918956]	1.479	2.05608	2.75102	2.89152	1.77526	2.29339	3.25465	1.9494	3.07417	2.66486	2.32942	3.02797	2.30874	2.23129	0.675497492	0.966018836	12	20	24	0.503157919	0.809997329	24	10.85758701	0.101435638
ENSMUSG00000029651	Mtus2	microtubule associated tumor suppressor candidate 2 [Source:MGI Symbol;Acc:MGI:1915388]	5.99074	4.73808	6.52341	4.60193	4.68767	6.25129	5.49381	5.08614	4.00499	5.13829	6.42543	5.52344	5.53811	5.24983	0.285617913	0.793714531	24	16	24	0.276649063	0.768040178	24	20.63639689	0.109415246
ENSMUSG00000029655	N4bp2l2	NEDD4 binding protein 2-like 2 [Source:MGI Symbol;Acc:MGI:2687207]	11.9919	13.7524	16.4502	13.0655	17.1143	14.8266	13.2339	14.574	16.7609	11.4769	18.2125	16.8228	15.517	14.0312	0.239653024	0.741580974	20	20	24	0.63603263	0.876344329	24	15.75944147	0.068606131
ENSMUSG00000029657	Hsph1	heat shock 105kDa/110kDa protein 1 [Source:MGI Symbol;Acc:MGI:105053]	118.07	78.7121	72.6012	85.3857	90.9526	97.2656	96.1349	92.7556	60.7042	75.7206	97.9202	89.2847	78.9896	75.5801	0.009067305	0.192760771	24	12	24	0.003829387	0.427278365	24	20.42401133	0.252697155
ENSMUSG00000029659	Medag	mesenteric estrogen dependent adipogenesis [Source:MGI Symbol;Acc:MGI:1917967]	6.2562	6.58683	6.14875	5.9354	6.33181	5.02357	6.53797	5.61612	7.57056	4.98269	5.85225	6.15055	5.8992	5.78826	0.911135828	0.966018836	12	4	24	0.568761348	0.84461646	24	5.078865912	0.055530841
ENSMUSG00000029661	Col1a2	collagen, type I, alpha 2 [Source:MGI Symbol;Acc:MGI:88468]	107.265	114.997	107.131	107.98	127.881	108.93	123.476	129.601	140.128	125.351	113.298	113.649	124.793	97.7324	0.768123158	0.966018836	20	4	24	0.871004953	0.963364428	24	10.41185752	0.03027148
ENSMUSG00000029669	Tspan12	tetraspanin 12 [Source:MGI Symbol;Acc:MGI:1889818]	14.1224	12.7867	14.9918	13.735	13.7127	14.2787	12.8593	12.7555	10.1923	12.6827	15.1419	11.9948	11.9083	13.0852	0.881718085	0.966018836	20	12	24	0.362346648	0.787151161	24	17.24627949	0.071651851
ENSMUSG00000029670	Ing3	inhibitor of growth family, member 3 [Source:MGI Symbol;Acc:MGI:1919027]	7.60064	6.69141	7.23357	9.51219	7.18643	6.60919	7.06015	7.6286	7.76325	6.92749	7.32979	7.85073	7.89474	8.5533	0.831272156	0.966018836	16	4	24	0.634521437	0.87566973	24	8.772699491	0.047838888
ENSMUSG00000029671	Wnt16	wingless-type MMTV integration site family, member 16 [Source:MGI Symbol;Acc:MGI:2136018]	1.1242	1.29582	0.972367	0.959576	1.36463	1.05579	1.10744	1.66061	1.27004	0.753439	0.902392	1.15961	0.942057	1.28307	0.05211941	0.391073722	8	8	24	0.243030968	0.768040178	24	2.829376248	0.097658731
ENSMUSG00000029672	Fam3c	FAM3 metabolism regulating signaling molecule C [Source:MGI Symbol;Acc:MGI:107892]	24.2134	20.7274	18.9873	19.633	20.5375	21.2754	20.3058	20.4256	18.9864	21.0028	20.5158	19.6793	20.797	21.4333	0.247964668	0.746745468	4	8	24	0.134878817	0.74653384	24	22.50408067	0.062493916
ENSMUSG00000029673	Auts2	autism susceptibility candidate 2 [Source:MGI Symbol;Acc:MGI:1919847]	10.7339	8.96257	8.52318	9.33088	10.3569	9.00605	10.2195	8.93805	9.00634	12.2646	9.49093	9.61719	10.159	8.64495	0.107220884	0.523582274	4	4	24	0.382217932	0.799546792	24	17.32189991	0.072272976
ENSMUSG00000029674	Limk1	LIM-domain containing, protein kinase [Source:MGI Symbol;Acc:MGI:104572]	42.3097	33.7684	33.583	33.5123	35.3372	36.9959	35.7896	36.66	30.4069	47.0419	37.201	41.3758	37.3281	39.7997	0.217827365	0.704989686	24	12	24	0.500783992	0.80908345	24	19.82919002	0.072692869
ENSMUSG00000029675	Eln	elastin [Source:MGI Symbol;Acc:MGI:95317]	6.61568	5.62185	5.38199	4.23887	4.76348	5.06432	7.24933	8.68797	4.88666	3.90015	4.05825	4.05253	5.50534	4.64568	0.013006247	0.228055093	4	12	24	0.006488762	0.470633744	24	1.979229162	0.280233219
ENSMUSG00000029676	Pot1a	protection of telomeres 1A [Source:MGI Symbol;Acc:MGI:2141503]	8.96609	10.4784	10.4056	13.0456	10.7019	9.9764	10.662	10.1703	11.6387	10.3584	10.9034	11.8505	10.7926	11.0705	0.892578749	0.966018836	16	12	24	0.18661344	0.768040178	24	11.79582672	0.075587496
ENSMUSG00000029681	Bcl7b	B cell CLL/lymphoma 7B [Source:MGI Symbol;Acc:MGI:1332238]	18.9976	16.1694	16.8387	16.5288	15.8031	17.5289	15.4957	16.1516	15.4083	18.3751	17.2913	16.8658	17.3465	16.7099	0.791413662	0.966018836	24	12	24	0.611410737	0.865101735	24	19.10512381	0.03408592
ENSMUSG00000029683	Lmod2	leiomodin 2 (cardiac) [Source:MGI Symbol;Acc:MGI:2135672]	0.502543	3.29148	6.87171	6.3712	3.91207	3.81499	1.68225	9.28383	5.57775	10.7941	2.44314	2.10955	3.88951	3.49758	0.014066792	0.241938013	16	12	24	0.009459675	0.502965379	24	9.804874223	0.8272899
ENSMUSG00000029684	Wasl	WASP like actin nucleation promoting factor [Source:MGI Symbol;Acc:MGI:1920428]	34.9315	31.0734	31.068	30.3791	31.6436	32.183	31.9344	30.7164	27.2461	36.3712	32.1137	29.8927	30.7091	31.9817	0.924553956	0.966018836	20	16	24	0.662113114	0.885885739	24	18.81277424	0.035339885
ENSMUSG00000029685	Asb15	ankyrin repeat and SOCS box-containing 15 [Source:MGI Symbol;Acc:MGI:1926160]	0.225599	0.53136	0.76745	0.85574	0.588302	0.763441	0.494344	1.0317	1.19775	2.04838	0.467956	0.601982	0.860518	0.610872	0.156866314	0.612465245	16	4	24	0.04145668	0.618736234	24	10.16052088	0.274458668
ENSMUSG00000029686	Cul1	cullin 1 [Source:MGI Symbol;Acc:MGI:1349658]	56.2861	61.1986	61.3199	60.7873	58.5533	59.1614	57.8069	56.1839	57.2246	55.6096	61.6649	59.8067	58.4137	60.445	0.952127548	0.966018836	20	8	24	0.846365649	0.953551806	24	13.83269833	0.011480605
ENSMUSG00000029687	Ezh2	enhancer of zeste 2 polycomb repressive complex 2 subunit [Source:MGI Symbol;Acc:MGI:107940]	5.4948	8.56112	8.90396	7.16009	7.94185	6.77626	6.28619	8.04351	7.45971	7.41322	6.03183	8.83238	6.49157	6.51309	0.112800839	0.530777648	12	16	24	0.412164821	0.799546792	24	9.566770049	0.100999662
ENSMUSG00000029695	Aass	aminoadipate-semialdehyde synthase [Source:MGI Symbol;Acc:MGI:1353573]	0.682128	0.649142	0.559933	0.723113	0.708748	0.677727	0.560669	0.893644	0.765951	0.605522	0.694382	0.695036	0.600974	0.582551	0.714062999	0.966018836	20	8	24	0.91278494	0.97533472	24	12.61944044	0.013154463
ENSMUSG00000029699	Ssc4d	scavenger receptor cysteine rich family, 4 domains [Source:MGI Symbol;Acc:MGI:1924709]	1.4622	1.82853	1.42076	1.68394	2.30281	1.7299	1.97821	2.27193	1.98265	1.55313	1.275	1.78457	2.71466	1.95948	0.718742371	0.966018836	8	8	24	0.349797206	0.779221593	24	1.670666182	0.102114767
ENSMUSG00000029701	Rbm28	RNA binding motif protein 28 [Source:MGI Symbol;Acc:MGI:2655711]	19.3093	15.4819	15.9756	15.7669	19.1841	17.3592	20.479	16.8841	14.7124	12.3754	19.2293	15.5018	15.3525	14.9661	0.282728968	0.793714531	20	20	24	0.05492982	0.655253328	24	20.76535263	0.157113028
ENSMUSG00000029703	Lrwd1	leucine-rich repeats and WD repeat domain containing 1 [Source:MGI Symbol;Acc:MGI:1918985]	5.91011	5.7389	6.26513	5.74975	5.32677	5.89148	5.99979	6.35241	5.44579	5.60305	5.72548	6.05585	5.75839	5.44681	0.522551072	0.966018836	24	20	24	0.430894036	0.805393068	24	0.919582111	0.031238585
ENSMUSG00000029705	Cux1	cut-like homeobox 1 [Source:MGI Symbol;Acc:MGI:88568]	17.0594	15.6129	16.2634	16.3174	18.4323	17.4004	19.4057	17.7859	17.4822	20.9404	15.3135	17.6583	18.7912	17.547	0.919571723	0.966018836	4	8	24	0.953897946	0.988601783	24	21.03977096	0.014036216
ENSMUSG00000029708	Gcc1	golgi coiled coil 1 [Source:MGI Symbol;Acc:MGI:1921625]	5.34631	4.70468	5.19742	5.01842	5.25819	5.6087	5.58695	5.55548	5.40952	5.66202	5.35962	5.03548	5.8445	5.02462	0.741628485	0.966018836	4	4	24	0.742160413	0.916130449	24	20.31418852	0.022152673
ENSMUSG00000029710	Ephb4	Eph receptor B4 [Source:MGI Symbol;Acc:MGI:104757]	2.45332	2.74486	2.802	2.76537	2.90299	2.46308	2.62128	3.16881	3.03583	3.14519	2.42989	2.55418	2.65417	2.42831	0.208071297	0.698704663	16	8	24	0.069266415	0.673520298	24	9.252032661	0.086672748
ENSMUSG00000029712	Actl6b	actin-like 6B [Source:MGI Symbol;Acc:MGI:1933548]	21.9062	19.3	18.9516	17.2097	17.4337	21.0406	20.0825	19.8186	16.7491	23.22	19.725	21.9798	20.0584	21.9406	0.080164531	0.458678242	24	12	24	0.302800311	0.768040178	24	22.67649969	0.078416759
ENSMUSG00000029713	Gnb2	guanine nucleotide binding protein (G protein), beta 2 [Source:MGI Symbol;Acc:MGI:95784]	181.671	164.433	162.236	165.77	168.618	176.912	162.599	175.78	170.821	194.334	165.33	170.754	163.96	161.191	0.873337044	0.966018836	24	8	24	0.695771413	0.899065372	24	15.30846584	0.025527343
ENSMUSG00000029714	Gigyf1	GRB10 interacting GYF protein 1 [Source:MGI Symbol;Acc:MGI:1888677]	8.67628	10.6371	11.0143	9.42218	9.01557	8.80786	9.76748	11.3746	12.5459	10.9174	8.72316	10.4386	11.0785	10.0161	0.035023815	0.328311403	12	8	24	0.010275205	0.503967859	24	6.730880831	0.152015236
ENSMUSG00000029715	Pop7	processing of precursor 7, ribonuclease P family, (S. cerevisiae) [Source:MGI Symbol;Acc:MGI:1921347]	13.1197	11.6013	11.142	10.7558	10.9316	11.1811	11.5134	12.4811	9.93352	12.4649	9.81248	10.5622	10.689	11.4475	0.173121348	0.648600527	8	12	24	0.288392626	0.768040178	24	2.234780001	0.061534306
ENSMUSG00000029716	Tfr2	transferrin receptor 2 [Source:MGI Symbol;Acc:MGI:1354956]	1.80394	1.80683	1.96829	1.43952	1.74487	1.86382	1.40924	2.60539	1.72952	1.41934	1.8526	1.87395	1.73641	2.23067	0.350544145	0.870184653	8	8	24	0.289293474	0.768040178	24	3.411407867	0.093497664
ENSMUSG00000029718	Pcolce	procollagen C-endopeptidase enhancer protein [Source:MGI Symbol;Acc:MGI:105099]	40.7419	45.1973	35.4778	34.0362	43.2185	36.2843	42.4593	47.897	55.9764	28.804	37.8527	34.0846	37.0298	34.5141	0.338172159	0.85758369	8	8	24	0.389845357	0.799546792	24	3.663212515	0.124029171
ENSMUSG00000029720	Gm20605	predicted gene 20605 [Source:MGI Symbol;Acc:MGI:5306917]	6.72985	7.82728	6.77133	8.33112	5.77566	5.33229	6.67083	8.55782	9.5147	7.13895	8.19068	7.96137	8.09949	7.31443	0.459019924	0.966018836	12	12	24	0.12795444	0.740162193	24	7.408295987	0.147579026
ENSMUSG00000029722	Agfg2	ArfGAP with FG repeats 2 [Source:MGI Symbol;Acc:MGI:2443267]	18.129	14.8499	15.1351	15.5169	15.942	16.9326	15.5783	17.3503	14.3549	19.0965	16.8214	17.2907	16.8451	16.0161	0.148073804	0.590732968	24	12	24	0.338202536	0.772907855	24	19.19233813	0.062853729
ENSMUSG00000029723	Tsc22d4	TSC22 domain family, member 4 [Source:MGI Symbol;Acc:MGI:1926079]	80.5453	84.2577	87.7937	87.2884	96.3456	81.7542	95.1422	82.9907	95.2106	104.095	106.39	98.2486	97.7491	85.229	0.097542159	0.501112642	20	12	24	0.047634691	0.635670298	24	14.60841882	0.095037714
ENSMUSG00000029725	Ppp1r35	protein phosphatase 1, regulatory subunit 35 [Source:MGI Symbol;Acc:MGI:1922853]	10.7294	8.03782	9.39477	9.83683	6.74604	6.99707	7.41814	10.8095	10.4536	9.0909	13.6964	11.928	13.8167	10.4733	0.90703203	0.966018836	4	12	24	0.870018972	0.963168206	24	5.078676219	0.054434997
ENSMUSG00000029726	Mepce	methylphosphate capping enzyme [Source:MGI Symbol;Acc:MGI:106477]	13.4128	15.5883	14.8188	16.9509	14.6713	16.0318	15.58	14.5198	15.42	15.6624	16.2942	16.9132	15.3039	14.5066	0.105467177	0.518002936	16	16	24	0.155793734	0.761271595	24	15.12177223	0.060031024
ENSMUSG00000029727	Cyp3a13	cytochrome P450, family 3, subfamily a, polypeptide 13 [Source:MGI Symbol;Acc:MGI:88610]	1.44536	2.07778	2.29811	1.88072	2.24184	1.91097	1.71522	2.07675	2.41624	1.91835	1.8799	1.57342	1.52357	1.74992	3.29E-04	0.049770315	12	16	24	0.021170245	0.571993737	24	10.01906025	0.139362784
ENSMUSG00000029729	Zkscan1	zinc finger with KRAB and SCAN domains 1 [Source:MGI Symbol;Acc:MGI:1921820]	8.15104	8.23009	7.8449	8.31574	8.88743	8.14935	8.14583	7.74864	7.87844	7.79823	8.06807	9.01053	8.36724	8.36601	0.470566846	0.966018836	24	12	24	0.099860397	0.71175955	24	19.31614088	0.047048115
ENSMUSG00000029730	Mcm7	minichromosome maintenance complex component 7 [Source:MGI Symbol;Acc:MGI:1298398]	3.52973	4.0004	4.88039	4.61127	3.90155	3.58133	3.22284	4.28793	4.45543	4.11037	3.25548	4.5633	4.02007	3.42246	0.097760727	0.501112642	12	16	24	0.161272849	0.76636332	24	9.612925202	0.113537857
ENSMUSG00000029735	Tpk1	thiamine pyrophosphokinase [Source:MGI Symbol;Acc:MGI:1352500]	5.72606	5.72474	4.81909	4.7752	5.11105	5.53752	4.79114	5.24251	5.09292	5.54713	5.73126	6.99802	5.12859	5.69991	0.582084141	0.966018836	24	12	24	0.345204767	0.778298718	24	20.49344547	0.069717302
ENSMUSG00000029752	Asns	asparagine synthetase [Source:MGI Symbol;Acc:MGI:1350929]	81.5031	66.0993	71.2963	71.2405	68.5401	75.8539	63.746	68.7088	57.9729	67.811	75.359	69.0215	71.8652	71.5204	0.499268446	0.966018836	24	12	24	0.231224025	0.768040178	24	19.97539293	0.078143536
ENSMUSG00000029757	Dync1i1	dynein cytoplasmic 1 intermediate chain 1 [Source:MGI Symbol;Acc:MGI:107743]	65.5791	52.6143	54.331	53.6711	51.6503	64.6818	54.8235	57.0107	45.0269	63.3239	53.6158	56.1683	56.771	60.2295	0.313530643	0.824470524	24	20	24	0.3214696	0.768040178	24	21.69483049	0.085451893
ENSMUSG00000029759	Pon3	paraoxonase 3 [Source:MGI Symbol;Acc:MGI:106686]	2.84494	3.57845	4.55329	6.59113	3.51688	3.52232	3.68791	5.31636	4.99733	4.35587	5.62961	4.31602	5.34932	5.0744	0.590547452	0.966018836	16	8	24	0.061911134	0.661961741	24	9.92140159	0.206815643
ENSMUSG00000029761	Cald1	caldesmon 1 [Source:MGI Symbol;Acc:MGI:88250]	19.9022	22.1548	19.2706	17.1006	19.7237	18.4557	19.9357	21.4771	19.8996	17.8815	18.685	18.8261	19.5671	18.9162	0.007831348	0.192760771	8	8	24	0.026655978	0.586541342	24	2.565873979	0.082761826
ENSMUSG00000029762	Akr1b8	aldo-keto reductase family 1, member B8 [Source:MGI Symbol;Acc:MGI:107673]	4.9459	4.55845	4.08066	4.42889	4.65978	3.51527	3.89435	4.46664	5.10697	3.7713	4.32334	3.29443	4.10581	3.67713	0.601647512	0.966018836	12	12	24	0.449439871	0.805393068	24	7.905262133	0.067778237
ENSMUSG00000029763	Exoc4	exocyst complex component 4 [Source:MGI Symbol;Acc:MGI:1096376]	36.0909	29.3889	28.4279	31.9249	29.8957	32.9565	28.045	34.7889	23.4442	34.0486	27.0583	31.7459	34.2175	31.1004	0.625825704	0.966018836	4	8	24	0.398435991	0.799546792	24	22.1130036	0.088916889
ENSMUSG00000029765	Plxna4	plexin A4 [Source:MGI Symbol;Acc:MGI:2179061]	8.26945	6.45526	8.27915	6.87372	7.90922	8.84505	7.3319	7.30846	5.3418	8.95397	7.85914	7.37954	6.79082	7.95467	0.714044782	0.966018836	16	16	24	0.34949737	0.779170188	24	18.0253636	0.100087847
ENSMUSG00000029767	Calu	calumenin [Source:MGI Symbol;Acc:MGI:1097158]	74.8365	84.4426	76.5298	74.8395	84.5409	71.5415	78.8628	77.5508	85.5371	66.1332	78.0189	73.609	78.3185	77.4499	0.636884864	0.966018836	12	12	24	0.596858733	0.85816059	24	4.418829224	0.039476867
ENSMUSG00000029769	Ccdc136	coiled-coil domain containing 136 [Source:MGI Symbol;Acc:MGI:1918128]	27.711	28.5699	28.1715	25.9921	27.5558	31.1931	29.2054	27.7276	28.2112	32.5478	28.5313	30.2073	26.8443	28.5083	0.88067614	0.966018836	24	12	24	0.711564915	0.90471055	24	17.36388371	0.026915657
ENSMUSG00000029771	Irf5	interferon regulatory factor 5 [Source:MGI Symbol;Acc:MGI:1350924]	2.5965	2.89525	2.97825	3.13281	3.03067	2.5181	2.58038	2.74653	3.79942	3.36751	3.08475	2.99891	3.15731	3.19642	0.068072762	0.431350682	16	8	24	0.003064507	0.414576087	24	10.08705862	0.121514702
ENSMUSG00000029772	Ahcyl2	S-adenosylhomocysteine hydrolase-like 2 [Source:MGI Symbol;Acc:MGI:1921590]	26.6011	27.2266	25.5952	26.0945	26.8701	28.0417	30.1981	26.1087	25.2288	27.3558	25.1201	26.4675	25.0794	26.0515	0.917867465	0.966018836	24	12	24	0.33092559	0.768040178	24	21.53746943	0.039938815
ENSMUSG00000029775	Klhdc10	kelch domain containing 10 [Source:MGI Symbol;Acc:MGI:1924038]	19.4497	17.6062	19.6129	19.3716	17.8499	19.7829	18.8042	18.1542	17.0715	19.6614	17.088	18.569	17.7823	19.3953	0.888482515	0.966018836	24	12	24	0.988521425	0.997559852	24	20.96667064	0.004592602
ENSMUSG00000029776	Hibadh	3-hydroxyisobutyrate dehydrogenase [Source:MGI Symbol;Acc:MGI:1889802]	40.9348	39.169	38.8185	45.1378	37.7767	38.6735	41.7327	43.5635	40.8713	44.7564	41.5172	43.1482	46.9756	44.0707	0.92769718	0.966018836	16	4	24	0.709415309	0.903951515	24	5.747938988	0.025175443
ENSMUSG00000029777	Gars	glycyl-tRNA synthetase [Source:MGI Symbol;Acc:MGI:2449057]	89.8554	71.81	74.6648	79.2631	75.663	86.3056	76.6034	79.7515	61.415	89.2514	76.2501	77.0291	75.3454	77.0686	0.745118544	0.966018836	24	12	24	0.320373535	0.768040178	24	19.44442912	0.081781093
ENSMUSG00000029778	Adcyap1r1	adenylate cyclase activating polypeptide 1 receptor 1 [Source:MGI Symbol;Acc:MGI:108449]	2.10005	3.68329	6.9976	2.92145	2.11309	4.02488	4.93687	2.79099	4.62605	3.40948	2.10562	3.82351	1.69879	5.97314	0.3964038	0.927127564	12	8	24	0.227455942	0.768040178	24	6.694992933	0.321563155
ENSMUSG00000029780	Nt5c3	5'-nucleotidase, cytosolic III [Source:MGI Symbol;Acc:MGI:1927186]	29.1805	34.0511	32.8611	32.8228	33.1252	31.1172	29.9651	32.2068	35.5574	28.4866	32.719	33.637	34.0925	33.2282	0.849565901	0.966018836	12	4	24	0.63922027	0.877759779	24	7.493244985	0.033914112
ENSMUSG00000029781	Fkbp9	FK506 binding protein 9 [Source:MGI Symbol;Acc:MGI:1350921]	31.6359	34.1895	32.5575	35.5503	37.5594	31.1271	36.3174	35.0099	37.8348	32.4259	32.0263	33.677	33.8244	30.3638	0.911371589	0.966018836	12	12	24	0.781446731	0.929846332	24	10.93317638	0.027376122
ENSMUSG00000029782	Tmem209	transmembrane protein 209 [Source:MGI Symbol;Acc:MGI:1919899]	15.7918	15.2875	16.0861	14.9453	16.3273	15.744	15.1373	15.9701	15.6004	15.7304	16.3295	16.5716	15.9557	16.0923	0.888991156	0.966018836	20	20	24	0.544769671	0.832367521	24	18.20052923	0.016303889
ENSMUSG00000029787	Avl9	AVL9 cell migration associated [Source:MGI Symbol;Acc:MGI:1926187]	12.7059	12.1975	12.2002	12.3015	12.2567	12.249	11.1348	11.2691	11.2413	11.1827	11.9555	12.2721	11.936	12.5808	0.874607729	0.966018836	24	12	24	0.564671196	0.843293894	24	20.33330484	0.025903154
ENSMUSG00000029790	Cep41	centrosomal protein 41 [Source:MGI Symbol;Acc:MGI:1891414]	2.60035	2.57888	2.40861	2.20333	2.62803	2.71217	2.58855	2.81307	2.63207	2.9308	2.19852	2.50577	2.51634	2.99876	0.943890799	0.966018836	8	12	24	0.321558281	0.768040178	24	3.735571397	0.052800169
ENSMUSG00000029797	Sspo	SCO-spondin [Source:MGI Symbol;Acc:MGI:2674311]	0.469185	0.932146	0.757584	0.563391	0.458514	0.609481	0.627995	0.804234	0.879146	0.767405	0.894858	0.837173	1.34575	0.547014	0.831272156	0.966018836	12	4	24	0.676429523	0.891831571	24	4.521611372	0.059429616
ENSMUSG00000029798	Herc6	hect domain and RLD 6 [Source:MGI Symbol;Acc:MGI:1914388]	3.44664	2.47096	2.79657	2.91187	3.27997	2.43729	3.31925	2.9006	3.03962	2.91998	4.09873	3.18604	2.74869	3.6124	0.910436409	0.966018836	20	4	24	0.739338819	0.914957371	24	16.70165686	0.046259386
ENSMUSG00000029802	Abcg2	ATP binding cassette subfamily G member 2 (Junior blood group) [Source:MGI Symbol;Acc:MGI:1347061]	39.9069	47.3507	45.5494	48.5592	43.9089	44.6112	38.9235	39.805	50.1768	42.8665	52.4567	42.453	43.577	42.2746	0.074510812	0.448301997	20	8	24	0.070066885	0.67501971	24	12.20102662	0.104693584
ENSMUSG00000029804	Herc3	hect domain and RLD 3 [Source:MGI Symbol;Acc:MGI:1921248]	34.5603	30.6993	31.4443	32.3203	29.0341	33.7804	30.1308	33.4421	27.2921	36.6371	34.0239	33.7901	34.2689	32.8078	0.714044782	0.966018836	24	12	24	0.630728411	0.874071894	24	19.71693299	0.042516625
ENSMUSG00000029810	Tmem176b	transmembrane protein 176B [Source:MGI Symbol;Acc:MGI:1916348]	91.4892	86.8761	86.9813	81.7911	100.967	94.9292	84.1794	94.0576	81.3497	77.1879	93.4665	80.2826	82.6209	80.4488	0.893307667	0.966018836	20	20	24	0.258265707	0.768040178	24	19.35335945	0.067627712
ENSMUSG00000029814	Igf2bp3	insulin-like growth factor 2 mRNA binding protein 3 [Source:MGI Symbol;Acc:MGI:1890359]	1.23251	1.28289	0.889396	1.46088	1.34565	1.20027	1.57939	0.749813	0.922878	0.979387	1.28433	1.13694	1.42508	0.969665	0.166168222	0.633011401	4	8	24	0.082869972	0.699403344	24	19.49884397	0.138086981
ENSMUSG00000029815	Malsu1	mitochondrial assembly of ribosomal large subunit 1 [Source:MGI Symbol;Acc:MGI:1922843]	6.83354	9.45951	7.54761	9.69144	6.93393	8.28564	5.93751	7.88454	9.04869	7.10917	9.79877	7.43007	8.69916	8.26411	0.94086481	0.966018836	8	20	24	0.465329428	0.805393068	24	10.03965344	0.094597613
ENSMUSG00000029816	Gpnmb	glycoprotein (transmembrane) nmb [Source:MGI Symbol;Acc:MGI:1934765]	3.19963	2.1678	2.32094	2.31943	2.61051	2.82991	2.91801	3.27983	2.30568	2.73961	2.31536	2.55814	3.01873	2.25706	0.093849599	0.490854203	4	8	24	0.119651747	0.731117717	24	22.5373807	0.113588505
ENSMUSG00000029817	Tra2a	transformer 2 alpha [Source:MGI Symbol;Acc:MGI:1933972]	56.2167	54.2889	49.9897	52.2295	59.7147	51.3179	60.9793	45.2047	50.8245	40.2091	61.5678	48.3841	53.9411	47.7641	0.26148192	0.768150062	20	20	24	0.19546759	0.768040178	24	20.20558851	0.115309821
ENSMUSG00000029819	Npy	neuropeptide Y [Source:MGI Symbol;Acc:MGI:97374]	2.28149	2.46356	9.58789	4.96205	2.28039	7.97611	15.2027	2.38384	2.49688	7.27919	12.0567	12.1033	1.0627	27.1287	0.838244962	0.966018836	24	4	24	0.923875398	0.979691907	24	14.88591586	0.167568185
ENSMUSG00000029821	Gsdme	gasdermin E [Source:MGI Symbol;Acc:MGI:1889850]	6.58933	7.81745	8.3984	7.1384	6.75572	7.14722	7.21591	6.28961	7.3894	7.32076	6.51918	6.52254	6.76341	7.1762	0.097779482	0.501112642	12	8	24	0.115851861	0.727799063	24	8.06670431	0.074469964
ENSMUSG00000029822	Osbpl3	oxysterol binding protein-like 3 [Source:MGI Symbol;Acc:MGI:1918970]	81.1963	57.802	60.2622	62.1707	66.5232	72.0658	62.4328	67.0036	53.0479	79.0715	74.509	72.845	72.9472	72.7131	0.191659183	0.669841558	24	12	24	0.144231255	0.752803875	24	19.64763831	0.128737474
ENSMUSG00000029823	Luc7l2	LUC7-like 2 (S. cerevisiae) [Source:MGI Symbol;Acc:MGI:2183260]	63.1235	80.5687	77.5316	76.3485	73.6207	66.1795	69.2674	69.4802	84.0306	66.9573	70.6231	63.5773	65.8554	74.1798	0.001028555	0.076130598	12	16	24	0.010536854	0.507695221	24	8.605683756	0.129728584
ENSMUSG00000029826	Zc3hav1	zinc finger CCCH type, antiviral 1 [Source:MGI Symbol;Acc:MGI:1926031]	6.93223	6.55081	6.95132	6.54288	7.00325	6.52237	6.7899	7.22089	5.91482	6.13362	6.76135	6.61584	6.72896	6.32012	0.891105762	0.966018836	4	12	24	0.285464366	0.768040178	24	21.84597447	0.039838139
ENSMUSG00000029832	Nfe2l3	nuclear factor, erythroid derived 2, like 3 [Source:MGI Symbol;Acc:MGI:1339958]	1.34353	2.02616	1.75458	1.78248	1.46985	1.33787	1.69112	1.82314	1.70045	1.56035	1.52387	1.49305	1.47701	1.6919	0.004320961	0.140598674	8	16	24	0.009407359	0.502965379	24	6.997964645	0.112171594
ENSMUSG00000029833	Trim24	tripartite motif-containing 24 [Source:MGI Symbol;Acc:MGI:109275]	9.90919	11.0948	11.5803	11.0721	10.6525	11.3228	9.24385	10.8463	11.7907	11.1281	11.1912	12.1259	10.8114	11.2036	0.109040811	0.524035616	12	16	24	0.20623689	0.768040178	24	11.22835665	0.05884504
ENSMUSG00000029836	Cbx3	chromobox 3 [Source:MGI Symbol;Acc:MGI:108515]	2.55266	2.41739	2.76831	3.12618	2.35946	2.26983	2.93851	1.96646	3.49052	2.62176	3.08377	2.89471	2.16978	3.01809	0.435596482	0.966018836	12	20	24	0.310322102	0.768040178	24	11.10730625	0.099874491
ENSMUSG00000029838	Ptn	pleiotrophin [Source:MGI Symbol;Acc:MGI:97804]	109.916	122.811	112.968	96.9121	104.407	101.556	108.033	108.556	107.091	88.9637	108.638	92.0509	87.4445	104.028	0.441501717	0.966018836	12	4	24	0.270613568	0.768040178	24	4.511805803	0.068806653
ENSMUSG00000029840	Mtpn	myotrophin [Source:MGI Symbol;Acc:MGI:99445]	71.8266	65.5606	67.9687	66.5998	66.9688	68.3366	67.3007	62.5952	61.4529	70.988	70.2412	65.1991	64.2511	67.2654	0.700065774	0.966018836	20	12	24	0.282008911	0.768040178	24	17.32736966	0.038639529
ENSMUSG00000029843	Slc13a4	solute carrier family 13 (sodium/sulfate symporters), member 4 [Source:MGI Symbol;Acc:MGI:2442367]	4.43045	5.99383	3.83894	3.69427	4.11071	4.10242	4.00262	5.59144	6.3536	2.76151	4.09258	4.41483	3.40963	4.75223	0.053980818	0.395677346	8	8	24	0.182229313	0.768040178	24	4.090247502	0.179696457
ENSMUSG00000029844	Hoxa1	homeobox A1 [Source:MGI Symbol;Acc:MGI:96170]	0.760853	0.567858	0.414712	0.481131	0.542792	0.52009	0.412178	0.477394	0.476534	0.751612	0.366676	0.46963	0.629683	0.449417	0.859936714	0.966018836	4	8	24	0.888445183	0.96864266	24	22.43894413	0.02169751
ENSMUSG00000029851	Tcaf2	TRPM8 channel-associated factor 2 [Source:MGI Symbol;Acc:MGI:2385258]	1.30984	1.65061	1.33848	1.49702	1.57187	1.87256	1.67303	1.66918	1.56308	1.91903	1.32848	1.42307	1.53881	1.61161	0.886572916	0.966018836	8	12	24	0.972133311	0.993238187	24	7.353596539	0.010349307
ENSMUSG00000029860	Zyx	zyxin [Source:MGI Symbol;Acc:MGI:103072]	13.4549	12.8428	14.272	13.3797	14.0625	13.4404	13.4067	13.9489	12.5381	17.8451	13.0135	13.8871	13.4295	12.9295	0.917867465	0.966018836	16	16	24	0.35914049	0.785405943	24	13.0275689	0.06303153
ENSMUSG00000029861	Fam131b	family with sequence similarity 131, member B [Source:MGI Symbol;Acc:MGI:1923406]	32.8155	25.7473	27.7601	28.8193	27.468	31.9662	27.9756	28.3269	21.7716	35.31	25.1583	30.1445	27.651	29.3832	0.8972237	0.966018836	24	12	24	0.517793568	0.818405353	24	19.65238421	0.078673553
ENSMUSG00000029862	Clcn1	chloride channel, voltage-sensitive 1 [Source:MGI Symbol;Acc:MGI:88417]	1.64697	1.92228	2.25135	2.38473	1.81681	2.44862	1.12135	3.75379	3.54218	4.50115	1.20942	1.79357	2.38652	2.61013	0.192658608	0.670488962	16	4	24	0.062829541	0.66200925	24	8.495764097	0.343808772
ENSMUSG00000029863	Casp2	caspase 2 [Source:MGI Symbol;Acc:MGI:97295]	5.04774	5.64697	5.43937	5.7376	5.02551	5.01443	6.46884	5.86096	5.80334	4.66522	5.30893	5.25277	5.57768	5.73875	0.080148772	0.458678242	8	16	24	0.097895214	0.71175955	24	3.948477184	0.080981332
ENSMUSG00000029864	Gstk1	glutathione S-transferase kappa 1 [Source:MGI Symbol;Acc:MGI:1923513]	14.3493	18.3281	16.0487	16.481	15.9962	13.6024	16.6195	18.5432	20.1561	14.691	14.2515	15.0208	18.5201	18.7988	0.008754183	0.192760771	8	16	24	0.003823544	0.427278365	24	5.670520555	0.180927622
ENSMUSG00000029866	Kel	Kell blood group [Source:MGI Symbol;Acc:MGI:1346053]	0.717293	2.88653	3.94571	3.03104	1.59444	1.12584	2.40733	3.30626	2.99421	1.00823	2.34739	1.87896	4.78955	0.750905	0.787283372	0.966018836	12	12	24	0.578272774	0.849207394	24	6.991787969	0.242743948
ENSMUSG00000029869	Ephb6	Eph receptor B6 [Source:MGI Symbol;Acc:MGI:1096338]	13.6529	12.924	12.4087	13.4356	15.0695	12.8913	15.4693	14.8617	14.4135	15.2078	13.0167	14.2933	13.7407	13.1885	0.866861748	0.966018836	16	20	24	0.936280555	0.984153472	24	17.84300015	0.014122898
ENSMUSG00000029875	Ccdc184	coiled-coil domain containing 184 [Source:MGI Symbol;Acc:MGI:2146066]	4.05052	3.6376	4.62312	3.06788	2.68075	3.90596	4.3334	3.64197	3.51543	4.06807	3.04176	4.33687	3.32302	4.22238	0.522551072	0.966018836	24	20	24	0.236997835	0.768040178	24	2.886049042	0.115084297
ENSMUSG00000029878	Dbpht2	DNA binding protein with his-thr domain [Source:MGI Symbol;Acc:MGI:2679513]	2.8335	2.84349	2.9184	2.85924	2.7249	2.94492	2.78385	2.43971	2.59024	2.94525	2.83646	2.85166	2.39599	2.7623	0.249881062	0.749643185	16	12	24	0.227484281	0.768040178	24	15.31438118	0.041688397
ENSMUSG00000029910	Mad2l1	MAD2 mitotic arrest deficient-like 1 [Source:MGI Symbol;Acc:MGI:1860374]	5.91232	6.95676	5.96108	5.83972	6.4	6.37457	6.22587	5.4142	6.83795	5.13928	6.25073	6.14559	7.36842	6.26233	0.922035832	0.966018836	20	20	24	0.461285277	0.805393068	24	23.87175343	0.057147842
ENSMUSG00000029911	Ssbp1	single-stranded DNA binding protein 1 [Source:MGI Symbol;Acc:MGI:1920040]	11.448	13.4181	11.853	12.3721	12.2546	10.821	11.6536	13.284	14.8554	12.3722	14.8616	14.085	14.8214	12.9216	0.882263435	0.966018836	8	20	24	0.641161079	0.878540546	24	8.183451461	0.043798671
ENSMUSG00000029913	Prdm5	PR domain containing 5 [Source:MGI Symbol;Acc:MGI:1918029]	2.68013	3.29325	2.90679	2.64076	2.08609	2.58312	2.43283	3.04868	2.95345	2.46518	3.2425	3.06681	2.4798	2.91517	0.481530854	0.966018836	8	20	24	0.390710456	0.799546792	24	5.630259006	0.073633632
ENSMUSG00000029915	Clec5a	C-type lectin domain family 5, member a [Source:MGI Symbol;Acc:MGI:1345151]	0.666695	1.78877	1.73884	1.21048	1.2591	1.4581	1.42392	0.782134	1.6725	0.937327	1.16293	1.16118	1.13045	1.06858	0.915475528	0.966018836	12	8	24	0.7249988	0.909665611	24	9.440975207	0.072569023
ENSMUSG00000029916	Agk	acylglycerol kinase [Source:MGI Symbol;Acc:MGI:1917173]	5.00007	5.05689	5.22937	4.97948	5.02023	5.09192	4.70451	4.73324	4.44318	4.84086	4.53023	4.37238	4.45739	4.43364	0.807813307	0.966018836	16	12	24	0.501028444	0.80908345	24	13.66656438	0.016228003
ENSMUSG00000029917	Qrfpr	pyroglutamylated RFamide peptide receptor [Source:MGI Symbol;Acc:MGI:2441881]	0.935167	0.914254	0.710249	0.884071	0.577092	0.899768	0.624387	0.778025	0.777138	0.704805	0.695918	0.829027	0.939131	1.00703	0.059513104	0.406067671	8	8	24	0.215026592	0.768040178	24	2.293055833	0.069082713
ENSMUSG00000029918	Mrps33	mitochondrial ribosomal protein S33 [Source:MGI Symbol;Acc:MGI:1338046]	13.1615	15.5249	16.2586	15.4852	17.4897	14.3225	17.0748	16.8441	19.0026	12.6655	19.2195	14.7321	16.126	13.3111	0.891690964	0.966018836	20	4	24	0.815246177	0.940912535	24	12.22714334	0.04546362
ENSMUSG00000029919	Hpgds	hematopoietic prostaglandin D synthase [Source:MGI Symbol;Acc:MGI:1859384]	0.887878	1.19564	1.33359	0.818494	1.07739	0.82766	1.12939	0.991163	1.54736	0.747724	1.64647	1.089	0.930885	0.907246	0.676344318	0.966018836	8	4	24	0.784970075	0.931056644	24	9.831001933	0.052414504
ENSMUSG00000029920	Smarcad1	SWI/SNF-related, matrix-associated actin-dependent regulator of chromatin, subfamily a, containing DEAD/H box 1 [Source:MGI Symbol;Acc:MGI:95453]	8.62612	9.14354	9.5513	8.29353	9.39239	8.62793	9.4849	8.03705	8.61935	8.2149	9.17933	8.88486	7.75018	8.54396	0.951846915	0.966018836	20	20	24	0.736382473	0.914149062	24	16.89419168	0.024378518
ENSMUSG00000029922	Mkrn1	makorin, ring finger protein, 1 [Source:MGI Symbol;Acc:MGI:1859353]	37.4424	37.8066	38.2294	41.139	37.2314	38.5775	36.795	36.287	34.1937	40.485	36.9524	38.8227	38.7673	37.4984	0.581070934	0.966018836	16	16	24	0.430444058	0.805393068	24	15.63038024	0.032563117
ENSMUSG00000029924	Slc37a3	solute carrier family 37 (glycerol-3-phosphate transporter), member 3 [Source:MGI Symbol;Acc:MGI:1919394]	15.3477	14.1451	12.8795	12.2623	15.7925	15.0957	14.2384	13.0027	11.6777	13.8426	12.8803	13.072	13.1471	13.1979	0.097760727	0.501112642	4	8	24	0.030489576	0.597890721	24	20.54861146	0.102863098
ENSMUSG00000029925	Tbxas1	thromboxane A synthase 1, platelet [Source:MGI Symbol;Acc:MGI:98497]	0.780047	1.17971	1.56685	0.825091	1.04429	0.785505	1.0238	1.71691	1.44102	0.468456	0.827175	0.793723	1.16166	1.03698	0.036657636	0.332441566	12	12	24	0.053095955	0.650879532	24	5.16148139	0.211004258
ENSMUSG00000029992	Gfpt1	glutamine fructose-6-phosphate transaminase 1 [Source:MGI Symbol;Acc:MGI:95698]	14.2318	13.1733	13.6331	13.0001	13.8922	13.1904	15.2898	12.8164	13.5761	14.2503	13.6611	14.3779	13.9077	14.0254	0.46238066	0.966018836	4	4	24	0.435258668	0.805393068	24	21.41175271	0.031928797
ENSMUSG00000029993	Nfu1	NFU1 iron-sulfur cluster scaffold [Source:MGI Symbol;Acc:MGI:1913290]	30.7567	30.2647	29.5536	27.8046	26.1275	27.4061	30.2567	28.6259	30.6123	28.0415	28.5907	29.3492	31.1023	29.1929	0.008155765	0.192760771	4	16	24	0.016961162	0.547196997	24	2.931096096	0.067592476
ENSMUSG00000029994	Anxa4	annexin A4 [Source:MGI Symbol;Acc:MGI:88030]	17.0639	17.9446	15.7032	17.6492	18.8	15.9793	17.2416	17.9138	17.117	15.0017	18.1896	15.7396	16.2395	17	0.92769718	0.966018836	20	4	24	0.995613696	0.998763985	24	20.79621569	0.003435857

ENSMUSG00000029998	Pcyox1	prenylcysteine oxidase 1 [Source:MGI Symbol;Acc:MGI:1914131]	46.8499	39.7087	37.7797	42.4104	38.6476	45.0912	42.5453	43.9715	34.4745	47.4772	40.2243	43.4761	43.8096	43.4633	0.143321297	0.585944911	24	12	24	0.276176224	0.768040178	24	21.52759524	0.078618576
ENSMUSG00000029999	Tgfa	transforming growth factor alpha [Source:MGI Symbol;Acc:MGI:98724]	6.00009	6.42058	6.6106	5.49674	6.37684	6.20276	8.59094	7.68514	6.61714	7.70274	5.0637	6.47201	6.84189	6.3463	0.708665292	0.966018836	12	8	24	0.301937333	0.768040178	24	3.353483511	0.099973783
ENSMUSG00000030000	Add2	adducin 2 (beta) [Source:MGI Symbol;Acc:MGI:87919]	49.6479	36.9097	39.214	39.9099	40.141	46.3156	41.0808	42.4153	29.0167	52.3673	38.4646	43.9905	42.3279	43.4084	0.245061943	0.741580974	24	12	24	0.340497982	0.774852303	24	20.30884023	0.118085366
ENSMUSG00000030002	Dusp11	dual specificity phosphatase 11 (RNA/RNP complex 1-interacting) [Source:MGI Symbol;Acc:MGI:1919352]	46.7482	41.6538	40.7868	47.0213	54.9419	41.5587	53.0456	36.2407	44.1623	35.0095	58.6784	39.2685	45.4538	40.951	0.435739761	0.966018836	20	12	24	0.250085278	0.768040178	24	18.08392267	0.139162632
ENSMUSG00000030007	Cct7	chaperonin containing Tcp1, subunit 7 (eta) [Source:MGI Symbol;Acc:MGI:107184]	133.872	119.939	115.812	115.838	126.644	130.057	118.664	117.963	107.798	115.773	125.811	122.009	122.276	118.266	0.002326858	0.106508885	24	12	24	0.001004099	0.336500758	24	20.31693366	0.09214128
ENSMUSG00000030008	Pradc1	protease-associated domain containing 1 [Source:MGI Symbol;Acc:MGI:1920577]	10.0204	6.37977	7.75923	8.55829	8.76536	8.24159	7.81852	8.36583	8.67338	8.5344	10.132	8.46681	8.66978	8.57454	0.47028201	0.966018836	20	12	24	0.290662669	0.768040178	24	16.90080868	0.083628594
ENSMUSG00000030016	Zfp638	zinc finger protein 638 [Source:MGI Symbol;Acc:MGI:1203484]	37.8641	42.5758	37.6851	41.1718	44.4559	34.4414	41.0371	33.8176	41.5822	28.855	43.041	33.3782	38.4518	36.6583	0.921741413	0.966018836	20	4	24	0.987024244	0.997339197	24	17.49985082	0.010891022
ENSMUSG00000030019	Fbxl14	F-box and leucine-rich repeat protein 14 [Source:MGI Symbol;Acc:MGI:2141676]	5.76698	5.59165	5.41551	5.25287	5.01557	5.5005	5.72037	5.69362	5.36245	6.94476	5.9772	5.99985	5.69435	5.83235	0.665910519	0.966018836	24	12	24	0.995564889	0.998760424	24	11.46711161	0.003257184
ENSMUSG00000030020	Prickle2	prickle planar cell polarity protein 2 [Source:MGI Symbol;Acc:MGI:1925144]	8.85999	8.41005	8.66851	8.2147	9.00537	8.64581	9.4447	8.4731	8.38345	9.80954	7.70512	9.05256	8.64959	8.88375	0.851656735	0.966018836	4	16	24	0.871617961	0.963559632	24	22.91380543	0.016573627
ENSMUSG00000030022	Adamts9	a disintegrin-like and metallopeptidase (reprolysin type) with thrombospondin type 1 motif, 9 [Source:MGI Symbol;Acc:MGI:1916320]	3.38929	2.76764	2.70965	2.4763	3.40134	2.77038	2.7791	3.17135	2.51272	2.58796	2.77345	3.01453	3.52755	3.01804	0.007886314	0.192760771	4	12	24	0.059956466	0.658304792	24	22.69468963	0.107072614
ENSMUSG00000030029	Lrig1	leucine-rich repeats and immunoglobulin-like domains 1 [Source:MGI Symbol;Acc:MGI:107935]	7.7661	7.87673	10.454	8.84993	9.92637	8.7936	10.3623	8.59705	9.32779	8.06239	6.8753	7.44906	9.0716	8.07664	0.965521737	0.969272194	12	12	24	0.760845015	0.92268731	24	6.788160428	0.047746593
ENSMUSG00000030030	1700003E16Rik	RIKEN cDNA 1700003E16 gene [Source:MGI Symbol;Acc:MGI:1919087]	2.15764	1.55492	1.99349	1.62419	1.38601	1.8491	1.8963	1.67944	1.57853	2.19686	1.58294	2.30681	1.8955	1.9294	0.961261938	0.967043964	24	20	24	0.5910167	0.855623646	24	23.44790692	0.056128335
ENSMUSG00000030031	Kbtbd8	kelch repeat and BTB (POZ) domain containing 8 [Source:MGI Symbol;Acc:MGI:2661430]	2.13501	2.26962	2.45419	2.50803	2.33593	2.09028	2.14449	2.06046	2.33284	1.9739	2.6448	2.39709	2.2846	2.44116	0.635666629	0.966018836	20	8	24	0.515870048	0.817421075	24	12.65050507	0.037573344
ENSMUSG00000030032	Wdr54	WD repeat domain 54 [Source:MGI Symbol;Acc:MGI:1922909]	6.16159	6.50255	5.87905	6.57327	5.93617	6.59326	6.91874	6.25067	6.34051	7.65174	5.44196	6.04122	5.72858	6.48018	0.895137379	0.966018836	16	4	24	0.729158678	0.911525775	24	8.103994778	0.035226457
ENSMUSG00000030034	Ino80b	INO80 complex subunit B [Source:MGI Symbol;Acc:MGI:1917270]	4.82361	4.66976	4.43828	5.72431	4.27963	4.68916	5.25991	5.253	5.86718	6.94515	6.57089	7.09048	6.6824	6.61113	0.849180783	0.966018836	24	12	24	0.704742817	0.902490129	24	12.06729792	0.038765342
ENSMUSG00000030035	Wbp1	WW domain binding protein 1 [Source:MGI Symbol;Acc:MGI:104710]	20.6272	19.15	20.7472	20.9728	24.4877	23.2883	21.4775	18.7806	21.3815	20.8226	22.9407	20.703	21.0268	20.2271	3.11E-04	0.049770315	20	12	24	0.004824473	0.438022495	24	16.778205	0.105474612
ENSMUSG00000030036	Mogs	mannosyl-oligosaccharide glucosidase [Source:MGI Symbol;Acc:MGI:1929872]	8.94879	6.9701	7.56216	6.60548	7.13865	8.03372	7.88326	7.99911	6.16683	7.15697	7.51743	7.59966	7.53605	7.17486	0.031289434	0.324385159	4	12	24	0.013801239	0.529470441	24	22.21714155	0.116035626
ENSMUSG00000030037	Mrpl53	mitochondrial ribosomal protein L53 [Source:MGI Symbol;Acc:MGI:1915749]	46.7394	46.7323	46.0161	51.2146	42.8521	41.876	46.7652	45.6153	51.4497	41.7304	53.6409	46.6379	52.2336	45.6468	0.894678274	0.966018836	4	12	24	0.896826226	0.970634157	24	7.245957042	0.021734681
ENSMUSG00000030042	Pole4	polymerase (DNA-directed), epsilon 4 (p12 subunit) [Source:MGI Symbol;Acc:MGI:1914229]	17.8667	14.7752	15.1321	14.1419	17.8018	15.7976	17.8801	14.5809	14.4642	14.6037	17.1131	14.2366	15.3255	15.208	0.06180207	0.409814092	4	12	24	0.107009607	0.719758244	24	20.31767474	0.094541273
ENSMUSG00000030045	Mrpl19	mitochondrial ribosomal protein L19 [Source:MGI Symbol;Acc:MGI:1926274]	15.9897	14.4877	14.0686	11.5316	11.7515	15.3214	11.3628	14.7414	9.80696	12.7395	12.217	11.0895	13.1226	12.319	0.632729413	0.966018836	8	4	24	0.245965615	0.768040178	24	0.240877436	0.106670956
ENSMUSG00000030047	Arhgap25	Rho GTPase activating protein 25 [Source:MGI Symbol;Acc:MGI:2443687]	1.93758	1.90869	2.1851	1.73309	1.8211	2.09554	2.33958	2.14098	1.74915	2.18896	2.27715	1.98895	1.75986	1.76847	0.854443826	0.966018836	20	16	24	0.795951723	0.934803902	24	18.89840071	0.028737058
ENSMUSG00000030048	Gkn3	gastrokine 3 [Source:MGI Symbol;Acc:MGI:1916138]	1.49249	3.35332	3.43255	5.65388	6.22031	2.63517	2.16999	3.18349	7.83559	3.64122	3.78477	2.52857	2.64062	1.37513	0.007505042	0.192760771	20	8	24	0.011553215	0.520454643	24	12.05066434	0.540219977
ENSMUSG00000030051	Aplf	aprataxin and PNKP like factor [Source:MGI Symbol;Acc:MGI:1919353]	3.0828	3.22863	3.02552	3.30737	2.9501	3.43468	3.36876	3.13528	2.88793	3.03881	3.08199	3.39513	3.15906	3.3282	0.022634137	0.27189101	24	12	24	0.163947227	0.767020009	24	22.24188417	0.043595399
ENSMUSG00000030054	Gp9	glycoprotein 9 (platelet) [Source:MGI Symbol;Acc:MGI:1860137]	0.443132	0.98491	1.50565	1.20576	0.66257	1.09916	0.675548	0.842501	1.20994	0.475788	0.875232	1.09589	1.43991	0.372987	0.923635729	0.966018836	12	20	24	0.914995861	0.976279382	24	11.01654425	0.048305634
ENSMUSG00000030055	Rab43	RAB43, member RAS oncogene family [Source:MGI Symbol;Acc:MGI:1917084]	1.53858	2.4579	4.39267	1.40954	3.30241	3.21934	3.41926	3.92893	4.24417	4.72979	3.78049	5.26826	3.01529	3.05049	0.492524295	0.966018836	12	16	24	0.744288564	0.916430276	24	11.66266277	0.106371585
ENSMUSG00000030056	Isy1	ISY1 splicing factor homolog [Source:MGI Symbol;Acc:MGI:1923310]	4.94043	9.10116	13.4915	5.73642	8.80518	10.7167	9.63282	11.3249	9.07829	13.0036	8.96935	13.1686	9.72573	11.7624	0.910436409	0.966018836	12	8	24	0.755128322	0.92103958	24	7.811285258	0.101565441
ENSMUSG00000030057	Cnbp	cellular nucleic acid binding protein [Source:MGI Symbol;Acc:MGI:88431]	167.43	199.079	202.889	192.79	185.088	170.147	172.513	189.651	222.316	178.196	194.2	178.39	191.823	192.788	0.008047769	0.192760771	12	12	24	0.010363322	0.503967859	24	8.435811629	0.11607967
ENSMUSG00000030058	Copg1	coatomer protein complex, subunit gamma 1 [Source:MGI Symbol;Acc:MGI:1858696]	73.3754	64.4871	70.8458	67.4076	66.5956	76.8326	71.2387	69.3855	57.2207	75.5441	58.3676	66.2848	63.5828	67.6151	0.890311217	0.966018836	24	20	24	0.671099107	0.889065421	24	21.66981454	0.042879314
ENSMUSG00000030059	Tmf1	TATA element modulatory factor 1 [Source:MGI Symbol;Acc:MGI:2684999]	13.8191	13.798	14.0069	13.6061	13.3418	13.2072	13.5522	12.831	13.6361	12.7172	14.4796	14.3836	14.2637	15.3794	0.910436409	0.966018836	4	12	24	0.66137932	0.885520687	24	1.540813411	0.022159637
ENSMUSG00000030060	Hmces	5-hydroxymethylcytosine (hmC) binding, ES cell specific [Source:MGI Symbol;Acc:MGI:1914053]	6.81762	8.57773	8.66058	8.77205	8.80457	8.46496	8.29325	6.932	8.81745	7.50176	8.02392	7.91068	7.87732	7.70426	0.214861332	0.704989686	12	16	24	0.320387478	0.768040178	24	12.88576556	0.062087958
ENSMUSG00000030061	Uba3	ubiquitin-like modifier activating enzyme 3 [Source:MGI Symbol;Acc:MGI:1341217]	76.4003	76.4757	72.2553	70.5252	75.611	69.8086	70.8162	67.2582	68.9795	63.2615	77.387	64.3987	68.3062	69.5292	0.891022863	0.966018836	20	20	24	0.796947353	0.935133209	24	20.42768683	0.020967905
ENSMUSG00000030062	Rpn1	ribophorin I [Source:MGI Symbol;Acc:MGI:98084]	51.5439	44.6756	45.7101	44.2604	48.1678	49.5381	49.5638	43.4516	42.1472	42.668	44.9823	45.8006	47.0077	40.4006	0.006767691	0.181382464	4	4	24	1.04E-04	0.114833119	24	20.60636227	0.107783367
ENSMUSG00000030064	Frmd4b	FERM domain containing 4B [Source:MGI Symbol;Acc:MGI:2141794]	10.2957	9.96411	9.34448	9.43843	9.39117	10.1301	8.71156	9.54159	9.99518	10.2933	8.87083	10.6682	9.86195	10.0426	0.882617464	0.966018836	24	20	24	0.877568508	0.965881478	24	0.914840737	0.015464402
ENSMUSG00000030067	Foxp1	forkhead box P1 [Source:MGI Symbol;Acc:MGI:1914004]	4.14827	4.47969	4.41557	4.54037	4.80139	4.19559	4.24202	4.53693	5.33693	4.15756	4.80571	5.51169	4.98232	5.33472	0.858514388	0.966018836	20	8	24	0.870692978	0.963313607	24	7.792567213	0.019617952
ENSMUSG00000030074	Gxylt2	glucoside xylosyltransferase 2 [Source:MGI Symbol;Acc:MGI:2682940]	1.14521	1.28802	1.01705	0.750003	1.07526	0.830889	1.23898	1.16433	1.4698	0.641637	1.07397	0.641803	0.92125	0.868998	0.151902463	0.600361608	12	4	24	0.232361005	0.768040178	24	4.013207858	0.109392413
ENSMUSG00000030075	Cntn3	contactin 3 [Source:MGI Symbol;Acc:MGI:99534]	3.43616	3.53743	3.27169	3.35462	2.72818	3.25641	3.14535	3.38615	3.10708	3.52189	3.06404	3.69592	3.29663	4.13812	0.068668966	0.432567661	8	12	24	0.177397265	0.768040178	24	3.992574802	0.071677655
ENSMUSG00000030077	Chl1	cell adhesion molecule L1-like [Source:MGI Symbol;Acc:MGI:1098266]	26.1551	23.7483	27.7078	23.4779	30.3807	27.9966	26.2429	20.802	22.3683	21.622	33.1766	28.0169	25.4145	26.4139	0.021214536	0.264116537	20	20	24	0.047066816	0.633507364	24	18.43322534	0.166273602
ENSMUSG00000030079	Ruvbl1	RuvB-like protein 1 [Source:MGI Symbol;Acc:MGI:1928760]	3.04705	2.91383	2.80337	3.21625	3.34014	3.77056	3.65097	3.25457	3.13842	3.13371	2.64567	3.06445	3.17029	2.88163	0.632729413	0.966018836	24	12	24	0.348553978	0.779170188	24	20.79099571	0.061172568
ENSMUSG00000030082	Sec61a1	Sec61 alpha 1 subunit (S. cerevisiae) [Source:MGI Symbol;Acc:MGI:1858417]	38.6987	32.0656	33.8182	32.3938	35.5331	35.9029	36.0443	35.5708	30.7604	36.7509	33.5451	33.6559	34.3449	32.5364	0.277087842	0.789849983	4	8	24	0.144029747	0.752803875	24	20.5972981	0.064926947
ENSMUSG00000030083	Abtb1	ankyrin repeat and BTB (POZ) domain containing 1 [Source:MGI Symbol;Acc:MGI:1933148]	21.431	22.8445	23.1481	26.8481	21.6188	23.5464	20.6856	25.5	21.2956	26.1429	20.7187	22.9521	24.4708	23.042	0.510431574	0.966018836	16	4	24	0.480495213	0.805393068	24	9.583371418	0.05748988
ENSMUSG00000030084	Plxna1	plexin A1 [Source:MGI Symbol;Acc:MGI:107685]	7.75289	5.75681	7.49934	8.8375	8.0786	7.14936	7.79437	7.83615	5.67244	8.73398	6.61015	7.43642	7.83977	6.13132	0.503972924	0.966018836	16	20	24	0.348716071	0.779170188	24	16.60603788	0.100573619
ENSMUSG00000030086	Chchd6	coiled-coil-helix-coiled-coil-helix domain containing 6 [Source:MGI Symbol;Acc:MGI:1913348]	26.3612	26.1887	25.0945	22.8647	25.3084	26.2571	24.6392	24.232	24.3987	24.8396	27.9773	27.5394	27.7873	28.5573	0.304354943	0.811201458	24	16	24	0.129700059	0.740162193	24	22.24886053	0.059285259
ENSMUSG00000030087	Klf15	Kruppel-like factor 15 [Source:MGI Symbol;Acc:MGI:1929988]	5.67487	6.4555	8.6615	13.0013	7.56223	6.75814	8.24103	5.2762	10.2447	10.0171	6.78407	6.96	5.06153	5.51077	0.004078827	0.140598674	12	20	24	0.006188864	0.469371824	24	12.03124855	0.361994156
ENSMUSG00000030088	Aldh1l1	aldehyde dehydrogenase 1 family, member L1 [Source:MGI Symbol;Acc:MGI:1340024]	4.79186	6.01064	7.94671	6.25744	5.9311	6.05926	5.94871	5.36429	7.00226	8.11768	5.1112	6.37283	5.94425	5.85824	0.091669402	0.484208195	12	20	24	0.065830067	0.668087926	24	10.23881639	0.159053561
ENSMUSG00000030089	Slc41a3	solute carrier family 41, member 3 [Source:MGI Symbol;Acc:MGI:1918949]	7.25325	6.43385	6.00963	6.51815	6.44775	7.05127	5.42974	9.61582	6.96645	10.8498	6.62925	8.41206	8.87932	6.70579	0.858514388	0.966018836	24	12	24	0.96790826	0.99226729	24	10.31824633	0.023733383
ENSMUSG00000030091	Nup210	nucleoporin 210 [Source:MGI Symbol;Acc:MGI:1859555]	1.19864	2.51235	3.65339	2.74126	1.94665	2.12979	2.53178	2.5124	3.21837	2.70984	1.63116	2.90255	2.43654	2.67154	0.03782572	0.334069441	12	8	24	0.059619341	0.658304792	24	8.125966132	0.240634669
ENSMUSG00000030092	Cntn6	contactin 6 [Source:MGI Symbol;Acc:MGI:1858223]	2.79221	3.25725	3.83276	2.43463	2.89802	2.97494	2.66554	2.60959	3.11256	2.24902	2.80558	3.05761	2.68474	3.60051	0.503972924	0.966018836	12	4	24	0.438791675	0.805393068	24	4.541047917	0.078573138
ENSMUSG00000030093	Wnt7a	wingless-type MMTV integration site family, member 7A [Source:MGI Symbol;Acc:MGI:98961]	1.01266	1.01782	1.25973	0.728691	0.822512	1.03729	1.02917	1.26996	0.792601	1.23325	0.790698	1.29917	0.951312	1.15697	0.644063931	0.966018836	8	12	24	0.376504308	0.796552361	24	2.760754651	0.074581787
ENSMUSG00000030094	Xpc	xeroderma pigmentosum, complementation group C [Source:MGI Symbol;Acc:MGI:103557]	4.70105	5.15049	5.922	5.31857	4.56139	4.73059	4.82651	5.02877	5.06614	5.35371	4.59255	5.33937	5.10521	4.97839	0.054328576	0.395890765	16	4	24	0.111297396	0.723334499	24	8.117989058	0.069743303
ENSMUSG00000030095	Tmem43	transmembrane protein 43 [Source:MGI Symbol;Acc:MGI:1921372]	16.098	14.7238	15.9745	14.8439	15.6765	17.1924	16.5051	17.8624	14.2258	17.6552	15.2201	15.5828	15.2132	13.3989	0.917867465	0.966018836	24	12	24	0.598205627	0.858819856	24	18.96063232	0.045275536
ENSMUSG00000030096	Slc6a6	solute carrier family 6 (neurotransmitter transporter, taurine), member 6 [Source:MGI Symbol;Acc:MGI:98488]	7.05704	11.6924	14.0674	10.9034	10.488	9.89922	9.26914	12.3785	14.4144	15.3562	8.49057	11.0137	9.43719	11.5835	0.004501001	0.140598674	12	16	24	0.004080582	0.427431763	24	9.050042345	0.312101114
ENSMUSG00000030098	Grip2	glutamate receptor interacting protein 2 [Source:MGI Symbol;Acc:MGI:2681173]	2.30036	2.09126	2.90229	2.24614	2.15918	2.45173	2.5513	2.07987	2.39417	2.24366	2.37622	2.04865	2.09908	2.4886	0.868022237	0.966018836	12	12	24	0.67272766	0.88969839	24	8.345834431	0.03646981
ENSMUSG00000030101	Sumf1	sulfatase modifying factor 1 [Source:MGI Symbol;Acc:MGI:1889844]	17.0603	15.7091	15.3622	15.9027	16.2367	16.2138	15.1084	15.948	14.6146	16.5503	15.6763	16.5459	16.5498	17.0161	0.649212555	0.966018836	4	8	24	0.399025404	0.799546792	24	21.07689179	0.032625923
ENSMUSG00000030102	Itpr1	inositol 1,4,5-trisphosphate receptor 1 [Source:MGI Symbol;Acc:MGI:96623]	5.38329	6.63642	6.51073	6.19027	5.95981	6.4186	6.93975	6.62146	7.12849	5.7536	6.53782	6.84449	5.97465	5.73157	0.879201926	0.966018836	12	4	24	0.940968981	0.985441727	24	7.295943342	0.015062886
ENSMUSG00000030103	Bhlhe40	basic helix-loop-helix family, member e40 [Source:MGI Symbol;Acc:MGI:1097714]	8.97955	11.5155	15.2859	14.3882	11.5733	9.556	10.3706	11.9424	15.7359	16.3479	9.58807	9.08234	9.7532	11.0279	1.37E-04	0.031913438	16	8	24	1.95E-05	0.047905911	24	9.60522724	0.361089388
ENSMUSG00000030104	Edem1	ER degradation enhancer, mannosidase alpha-like 1 [Source:MGI Symbol;Acc:MGI:2180139]	11.5596	9.91032	8.76579	10.4445	10.8919	10.1378	10.7191	9.75139	9.19461	9.42839	11.7686	10.5328	10.606	9.73558	0.002363973	0.106802459	20	16	24	0.008993705	0.502965379	24	19.6770136	0.117818395
ENSMUSG00000030105	Arl8b	ADP-ribosylation factor-like 8B [Source:MGI Symbol;Acc:MGI:1914416]	73.7647	62.0981	64.4729	62.6519	63.2458	68.2376	64.9047	67.3962	54.6296	67.4341	65.5067	60.3924	65.2078	58.7582	0.801995186	0.966018836	4	8	24	0.20429572	0.768040178	24	20.65021266	0.067140216
ENSMUSG00000030107	Usp18	ubiquitin specific peptidase 18 [Source:MGI Symbol;Acc:MGI:1344364]	2.92835	3.10235	3.20067	2.85603	3.37199	3.94355	3.05566	4.46244	3.72105	2.46844	4.66529	2.81294	2.78007	4.07023	0.745199643	0.966018836	8	8	24	0.954222251	0.988601783	24	5.641456427	0.027012416
ENSMUSG00000030108	Slc6a13	solute carrier family 6 (neurotransmitter transporter, GABA), member 13 [Source:MGI Symbol;Acc:MGI:95629]	9.20811	12.4102	8.34785	5.89196	8.01805	7.76594	10.3799	10.4273	10.9299	3.66001	8.06987	9.1487	8.42179	8.7025	0.078487799	0.140598674	8	8	24	0.013367363	0.602082902	24	1.626708123	0.337921686
ENSMUSG00000030109	Slc6a12	solute carrier family 6 (neurotransmitter transporter, betaine/GABA), member 12 [Source:MGI Symbol;Acc:MGI:95628]	2.43974	2.7139	1.82284	1.69086	1.31882	1.80203	2.34495	2.15448	2.62445	0.714953	1.86156	1.64038	1.56447	1.94636	0.077161185	0.453790923	8	8	24	0.039922163	0.616921492	24	2.47429123	0.243337228
ENSMUSG00000030110	Ret	ret proto-oncogene [Source:MGI Symbol;Acc:MGI:97902]	129.956	81.5007	90.0794	93.8952	96.758	111.171	94.0015	108.286	63.6321	130.815	92.6664	101.438	97.4088	97.5582	0.245061943	0.741580974	24	12	24	0.351436552	0.780345856	24	19.88325961	0.15163575
ENSMUSG00000030111	A2m	alpha-2-macroglobulin [Source:MGI Symbol;Acc:MGI:2449119]	7.33711	9.26379	8.94564	7.2228	9.0172	7.10517	8.70146	8.2995	9.39952	6.26637	8.98859	8.21422	7.82536	7.36371	0.581070934	0.966018836	12	12	24	0.871517308	0.96354675	24	4.841313337	0.03326458
ENSMUSG00000030116	Mfap5	microfibrillar associated protein 5 [Source:MGI Symbol;Acc:MGI:1354387]	111.249	128.277	105.269	113.642	138.643	98.8631	129.754	112.731	148.107	100.194	114.865	108.439	119.319	105.232	0.912686312	0.966018836	12	12	24	0.916768232	0.977105913	24	6.386928477	0.029937593
ENSMUSG00000030120	Mlf2	myeloid leukemia factor 2 [Source:MGI Symbol;Acc:MGI:1353554]	114.009	99.3666	101.273	104.478	98.7754	109.666	107.732	107.154	97.222	119.313	105.568	110.902	108.673	104.38	0.020119233	0.259417302	24	4	24	0.414593275	0.799810051	24	20.1864391	0.043533783
ENSMUSG00000030122	Ptms	parathymosin [Source:MGI Symbol;Acc:MGI:1916452]	221.755	182.768	179.29	204.049	207.245	196.578	214.234	206.275	191.145	220.84	192.848	210.041	200.2	182.681	0.166168222	0.633011401	4	8	24	0.244226964	0.768040178	24	19.03074952	0.066443474
ENSMUSG00000030123	Plxnd1	plexin D1 [Source:MGI Symbol;Acc:MGI:2154244]	43.0872	33.0552	29.28	43.4806	35.0363	35.419	39.1375	37.0659	33.4471	47.1398	36.6234	41.0158	37.8229	34.9114	0.116913252	0.538362324	16	20	24	0.464767139	0.805393068	24	17.71976252	0.087967196
ENSMUSG00000030124	Lag3	lymphocyte-activation gene 3 [Source:MGI Symbol;Acc:MGI:106588]	0.603449	0.812976	1.07504	0.720376	0.490887	0.553409	0.558153	0.851214	0.708009	0.512417	0.624796	0.688793	0.672794	1.324	0.178384774	0.553395174	8	12	24	0.016110717	0.544099157	24	5.811299032	0.171384065
ENSMUSG00000030125	Lrrc23	leucine rich repeat containing 23 [Source:MGI Symbol;Acc:MGI:1315192]	1.07258	2.48829	2.87348	1.51168	2.40624	1.35768	1.81892	1.20794	1.85373	1.70032	1.23035	2.38216	1.17848	2.4581	0.286642595	0.793714531	12	16	24	0.533223897	0.825678486	24	8.710069605	0.140485499
ENSMUSG00000030126	Tmcc1	transmembrane and coiled coil domains 1 [Source:MGI Symbol;Acc:MGI:2442368]	21.8828	20.0985	21.5612	20.3299	19.4998	19.2377	21.1543	20.2333	21.6421	21.4102	22.3871	22.3383	22.4694	21.7221	0.910436409	0.966018836	4	12	24	0.827278245	0.946154971	24	3.895493708	0.015095434
ENSMUSG00000030127	Cops7a	COP9 signalosome subunit 7A [Source:MGI Symbol;Acc:MGI:1349400]	52.7332	43.7604	43.1138	44.0938	41.514	48.9782	48.4576	50.4028	43.397	52.8096	45.8547	48.2281	48.2134	46.2796	0.188831826	0.664348865	4	8	24	0.323890259	0.768040178	24	22.90486747	0.062333391
ENSMUSG00000030134	Rasgef1a	RasGEF domain family, member 1A [Source:MGI Symbol;Acc:MGI:1917977]	25.1753	22.3704	21.4718	22.4404	22.7653	23.9176	23.072	21.596	21.0257	24.6234	22.9294	24.0722	23.5867	23.2873	0.004712708	0.140598674	24	12	24	0.055313285	0.65534544	24	20.10304259	0.064840765
ENSMUSG00000030137	Tuba8	tubulin, alpha 8 [Source:MGI Symbol;Acc:MGI:1858225]	2.13476	2.92127	3.66837	3.82846	3.05778	4.00328	3.5265	6.03249	4.43659	5.03569	3.79365	2.80628	4.07713	3.57957	0.483971878	0.966018836	16	8	24	0.15331849	0.759559886	24	9.038909413	0.201262428
ENSMUSG00000030138	Bms1	BMS1, ribosome biogenesis factor [Source:MGI Symbol;Acc:MGI:2446132]	9.21046	8.73081	8.91215	8.1391	8.94316	8.49203	8.78236	8.8298	8.23295	9.14584	8.26965	8.53628	8.63841	9.27765	0.664568268	0.966018836	8	8	24	0.413357465	0.799546792	24	2.660379627	0.027715902
ENSMUSG00000030144	Clec4d	C-type lectin domain family 4, member d [Source:MGI Symbol;Acc:MGI:1298389]	1.01132	0.970755	1.42688	0.912573	1.15077	0.797205	1.09471	0.85851	0.797825	0.52829	1.17024	0.67627	0.807859	0.395271	0.919571723	0.966018836	20	4	24	0.707438193	0.903496858	24	17.18338882	0.055103091
ENSMUSG00000030145	Zfp248	zinc finger protein 248 [Source:MGI Symbol;Acc:MGI:1919970]	5.85022	5.56797	5.62037	6.13819	6.06775	5.63399	5.41403	5.10811	5.50226	5.52006	5.53557	6.03831	6.1109	5.88186	0.745475226	0.966018836	24	8	24	0.402752256	0.799546792	24	17.7873868	0.035819756
ENSMUSG00000030147	Clec4b1	C-type lectin domain family 4, member b1 [Source:MGI Symbol;Acc:MGI:1917060]	0.272349	0.838878	0.592036	0.975071	1.17344	0.434851	1.04573	0.591607	0.650032	0.539939	0.747079	0.591915	0.516923	0.460141	0.522551072	0.966018836	20	4	24	0.416330934	0.801219263	24	13.72363469	0.108326023
ENSMUSG00000030148	Clec4a2	C-type lectin domain family 4, member a2 [Source:MGI Symbol;Acc:MGI:1349412]	1.64724	3.16983	4.7951	3.37472	3.09371	2.74217	1.80635	1.78775	2.83117	1.60027	2.71951	2.31455	2.36525	1.84432	0.277087842	0.789849983	12	16	24	0.274623333	0.768040178	24	11.84390575	0.189929998
ENSMUSG00000030157	Clec2d	C-type lectin domain family 2, member d [Source:MGI Symbol;Acc:MGI:2135589]	15.3084	20.6812	18.9958	17.246	17.2657	16.105	19.1515	20.7743	22.1164	12.7842	19.1589	15.208	17.7547	19.8098	0.030964094	0.324150946	8	16	24	0.109475008	0.72082227	24	5.307152187	0.161197319
ENSMUSG00000030159	Clec1b	C-type lectin domain family 1, member b [Source:MGI Symbol;Acc:MGI:1913287]	0.448797	1.26499	1.33519	1.112	0.62865	1.32911	0.662873	0.617662	1.21991	0.424733	0.49586	1.01532	1.53226	0.688681	0.95032543	0.966018836	12	4	24	0.802131422	0.936565832	24	4.245638487	0.078224215
ENSMUSG00000030161	Gabarapl1	gamma-aminobutyric acid (GABA) A receptor-associated protein-like 1 [Source:MGI Symbol;Acc:MGI:1914980]	345.211	289.046	288.972	290.072	278.087	312.679	296.793	311.279	263.145	354.366	303.083	327.104	320.969	337.05	0.217827365	0.704989686	24	12	24	0.493358386	0.805944647	24	22.47749308	0.055879263
ENSMUSG00000030165	Klrd1	killer cell lectin-like receptor, subfamily D, member 1 [Source:MGI Symbol;Acc:MGI:1196275]	0.217894	0.607203	0.354409	0.594971	0.54844	0.680013	0.491406	0.328368	0.99854	0.435925	0.49725	0.352256	0.462797	0.46143	0.823657258	0.966018836	12	16	24	0.47779044	0.805393068	24	11.09731955	0.083893661
ENSMUSG00000030166	Rad52	RAD52 homolog, DNA repair protein [Source:MGI Symbol;Acc:MGI:101949]	11.6892	11.2643	12.8592	11.1843	9.94714	11.4969	10.1743	9.94785	11.506	9.77518	11.0902	11.2604	12.5681	10.3639	0.539040394	0.966018836	12	4	24	0.839237925	0.951051187	24	1.492066281	0.026304488
ENSMUSG00000030168	Adipor2	adiponectin receptor 2 [Source:MGI Symbol;Acc:MGI:93830]	25.7164	24.0061	28.2302	37.5498	25.1762	28.0336	26.3918	28.8987	27.2995	31.1968	28.8242	29.4678	29.7334	28.4893	0.784278077	0.966018836	16	16	24	0.192081319	0.768040178	24	12.28979124	0.099035309
ENSMUSG00000030170	Wnt5b	wingless-type MMTV integration site family, member 5B [Source:MGI Symbol;Acc:MGI:98959]	0.568117	1.02237	0.816998	0.561393	0.854929	0.660102	0.545565	1.18494	1.09715	0.67001	0.70038	0.515398	0.606369	1.0521	0.01790319	0.256345714	8	20	24	0.030678473	0.597890721	24	6.767476109	0.175109809
ENSMUSG00000030172	Erc1	ELKS/RAB6-interacting/CAST family member 1 [Source:MGI Symbol;Acc:MGI:2151013]	13.1978	9.1744	9.51676	9.815	10.785	11.845	11.612	11.038	8.06061	12.3009	9.83597	10.953	11.0938	11.3315	0.017495149	0.255326033	4	8	24	0.106298159	0.718239596	24	21.28474714	0.138406692
ENSMUSG00000030177	Ccdc77	coiled-coil domain containing 77 [Source:MGI Symbol;Acc:MGI:1914450]	3.97299	4.48078	3.84348	5.20978	4.6745	4.19583	4.78631	4.06814	5.67269	4.09599	5.21006	4.74102	4.76852	4.31458	0.916746631	0.966018836	20	12	24	0.525251907	0.822381894	24	13.93473644	0.057955825
ENSMUSG00000030180	Kdm5a	lysine (K)-specific demethylase 5A [Source:MGI Symbol;Acc:MGI:2136980]	7.95322	8.61392	9.13248	9.26872	8.4834	7.68686	7.76313	8.93893	11.4125	8.26522	10.3861	10.1111	9.11195	9.29641	0.588682585	0.966018836	12	16	24	0.186786893	0.768040178	24	9.968464218	0.09425574
ENSMUSG00000030187	Klra2	killer cell lectin-like receptor, subfamily A, member 2 [Source:MGI Symbol;Acc:MGI:101906]	0.847716	0.80498	0.817037	0.446451	0.677513	0.428227	0.621501	0.754053	0.845234	0.383925	0.701901	0.415519	0.627579	0.413114	0.338172159	0.85758369	12	12	24	0.340205866	0.774736654	24	4.426600833	0.080276218
ENSMUSG00000030188	Magohb	mago homolog B, exon junction complex core component [Source:MGI Symbol;Acc:MGI:1913691]	11.1094	10.4105	11.0157	9.76436	10.0266	9.64954	9.10223	10.7594	10.9098	9.25912	10.2795	9.83132	9.34868	11.061	0.277428396	0.789849983	8	20	24	0.172270892	0.768040178	24	6.116736816	0.065393282
ENSMUSG00000030189	Ybx3	Y box protein 3 [Source:MGI Symbol;Acc:MGI:2137670]	28.7797	37.2432	34.5399	43.5778	33.6396	35.4297	36.3719	54.997	49.0944	53.1862	36.0375	40.874	43.9504	33.6098	0.961261938	0.967043964	16	4	24	0.195850533	0.768040178	24	9.176412989	0.17658987
ENSMUSG00000030199	Etv6	ets variant 6 [Source:MGI Symbol;Acc:MGI:109336]	3.97123	3.3484	3.78172	3.78907	4.03433	3.67561	4.09879	3.73688	2.97994	4.36	3.54725	3.34527	3.62156	3.6365	0.93717134	0.966018836	16	20	24	0.675592598	0.891284576	24	17.26577991	0.038490532
ENSMUSG00000030200	Bcl2l14	BCL2-like 14 (apoptosis facilitator) [Source:MGI Symbol;Acc:MGI:1914063]	4.17715	3.81304	3.43404	3.71374	3.62199	4.20724	3.87398	3.17211	3.46742	3.41577	3.17598	4.06418	3.91971	3.88807	0.016811431	0.253395174	24	20	24	0.028990128	0.595626873	24	22.19939419	0.100003496
ENSMUSG00000030201	Lrp6	low density lipoprotein receptor-related protein 6 [Source:MGI Symbol;Acc:MGI:1298218]	13.823	15.3064	12.7133	14.9048	14.8037	13.2721	14.7333	13.3151	15.0034	13.7734	13.1233	13.5387	13.8195	14.3135	0.865233232	0.966018836	8	16	24	0.814115888	0.940708369	24	6.554518011	0.02059694
ENSMUSG00000030203	Dusp16	dual specificity phosphatase 16 [Source:MGI Symbol;Acc:MGI:1917936]	5.01133	5.29645	5.42068	5.48154	5.99517	5.30484	6.26223	5.34617	5.71646	5.85026	6.09549	5.51084	6.1524	5.26955	0.109247218	0.524035616	20	12	24	0.403680326	0.799546792	24	15.84478674	0.040340472
ENSMUSG00000030204	Ddx47	DEAD box helicase 47 [Source:MGI Symbol;Acc:MGI:1915005]	24.5723	23.4812	25.4801	23.9724	21.9312	25.4794	24.9505	24.9076	21.6751	25.5427	23.0791	23.681	24.1206	25.1878	0.882617464	0.966018836	16	4	24	0.562628001	0.842415325	24	2.119671866	0.031170577
ENSMUSG00000030207	Fam234b	family with sequence similarity 234, member B [Source:MGI Symbol;Acc:MGI:1921775]	21.4847	17.7956	20.4759	18.8279	17.6853	21.9248	21.0277	19.8905	15.7942	24.3641	15.957	19.9239	18.341	20.879	0.644063931	0.966018836	24	20	24	0.807105747	0.938616703	24	23.58012717	0.04447138
ENSMUSG00000030208	Emp1	epithelial membrane protein 1 [Source:MGI Symbol;Acc:MGI:107941]	17.1612	17.4265	14.1478	12.3915	17.1348	13.3671	19.1834	17.5813	17.0069	9.49421	15.2353	14.0838	15.5001	14.3904	0.02233689	0.270751126	4	12	24	0.074342923	0.683293901	24	0.859072732	0.195641508
ENSMUSG00000030213	Atf7ip	activating transcription factor 7 interacting protein [Source:MGI Symbol;Acc:MGI:1858965]	9.42559	8.88	9.59208	9.59454	8.51295	10.7698	9.38375	9.34934	9.09395	10.0336	8.54457	9.23976	8.87656	8.91369	0.893307667	0.966018836	16	4	24	0.953538349	0.988601783	24	17.58508474	0.010240199
ENSMUSG00000030214	Plbd1	phospholipase B domain containing 1 [Source:MGI Symbol;Acc:MGI:1914107]	2.96888	4.1915	5.74282	8.03825	4.38695	4.45836	3.98242	5.77013	5.53844	5.14339	6.12252	5.04952	6.80403	5.41232	0.521832658	0.966018836	12	16	24	0.07802038	0.688222295	24	10.64521208	0.224661663
ENSMUSG00000030216	Wbp11	WW domain binding protein 11 [Source:MGI Symbol;Acc:MGI:1891823]	13.2936	12.66	13.9476	15.2811	13.0673	12.7954	14.5393	12.9149	13.3958	17.2532	12.6536	14.0147	13.5955	13.1799	0.446653834	0.966018836	16	16	24	0.284048164	0.768040178	24	11.80570588	0.070724872
ENSMUSG00000030217	Art4	ADP-ribosyltransferase 4 [Source:MGI Symbol;Acc:MGI:1202710]	3.73363	4.41771	3.77071	3.78318	4.63502	3.72695	4.2764	4.65598	5.41191	4.43162	3.8218	5.04928	4.48841	4.19429	0.768363194	0.966018836	8	20	24	0.729748962	0.911863904	24	6.402784536	0.041828571
ENSMUSG00000030218	Mgp	matrix Gla protein [Source:MGI Symbol;Acc:MGI:96976]	202.137	214.092	182.961	194.775	222.963	194.724	173.349	194.526	301.135	100.12	227.847	199.542	160.616	176.458	0.843639218	0.966018836	20	8	24	0.981850035	0.995835378	24	23.86556417	0.026238934
ENSMUSG00000030220	Arhgdib	Rho, GDP dissociation inhibitor (GDI) beta [Source:MGI Symbol;Acc:MGI:101940]	13.1238	22.1344	26.6499	19.3292	17.4251	20.0648	17.201	17.2391	24.0862	12.8804	17.6922	17.3325	20.7872	15.3482	0.714044782	0.966018836	12	16	24	0.580149512	0.85017573	24	7.655242664	0.120930353
ENSMUSG00000030222	Rerg	RAS-like, estrogen-regulated, growth-inhibitor [Source:MGI Symbol;Acc:MGI:2665139]	1.0567	1.14961	2.02826	0.955022	0.949348	1.43865	1.59537	1.11895	1.21944	1.25894	0.801507	1.1714	0.935791	1.3551	0.616047374	0.966018836	12	8	24	0.418606991	0.803070657	24	5.213953092	0.100926483
ENSMUSG00000030223	Ptpro	protein tyrosine phosphatase, receptor type, O [Source:MGI Symbol;Acc:MGI:1097152]	4.81217	3.44251	4.16772	3.08144	4.22561	4.38671	3.92701	4.27534	3.23434	4.26343	3.75814	3.62645	3.63776	3.93034	0.896311169	0.966018836	4	8	24	0.431550809	0.805393068	24	21.68946446	0.073540399
ENSMUSG00000030224	Strap	serine/threonine kinase receptor associated protein [Source:MGI Symbol;Acc:MGI:1329037]	54.1338	52.0629	52.5142	51.8411	52.8461	52.3191	49.9741	50.3636	46.9836	49.7724	53.6187	49.1519	48.1322	51.0311	0.897975397	0.966018836	20	8	24	0.35330368	0.780711577	24	17.8222839	0.02742769
ENSMUSG00000030225	Dera	deoxyribose-phosphate aldolase (putative) [Source:MGI Symbol;Acc:MGI:1913762]	4.10137	3.73782	4.30442	4.5552	4.27027	3.89348	3.79809	3.30038	3.89558	3.95033	3.44017	4.52322	4.36465	3.76844	0.216058318	0.704989686	16	16	24	0.562798692	0.842470381	24	16.37158347	0.047176044
ENSMUSG00000030226	Lmo3	LIM domain only 3 [Source:MGI Symbol;Acc:MGI:102810]	1.41816	1.53031	2.47288	1.32558	0.877544	1.25233	1.57945	0.785754	1.82783	1.10075	0.711769	1.07703	0.768282	1.85671	0.021288455	0.264116537	12	8	24	0.106731791	0.718898247	24	6.357388897	0.228775655

ENSMUSG00000030231	Plekha5	pleckstrin homology domain containing, family A member 5 [Source:MGI Symbol;Acc:MGI:1923802]	53.1324	52.395	49.9532	51.6653	54.3079	49.7588	47.667	47.7751	55.3947	45.8002	54.8592	50.1971	56.983	51.8933	0.8635847	0.966018836	20	20	24	0.94455205	0.986443184	24	20.20479166	0.012791723
ENSMUSG00000030232	Aebp2	AE binding protein 2 [Source:MGI Symbol;Acc:MGI:1338038]	14.5323	14.1857	14.4427	16.5161	14.2576	13.7503	15.4032	12.8667	13.843	14.4984	14.6219	13.4071	14.0015	13.1164	0.231491055	0.731037655	16	16	24	0.264423922	0.768040178	24	14.35015905	0.047730417
ENSMUSG00000030235	Slco1c1	solute carrier organic anion transporter family, member 1c1 [Source:MGI Symbol;Acc:MGI:1889679]	2.93012	4.86057	5.21579	2.84095	4.02213	3.56704	4.60046	3.95789	5.41765	3.38623	2.72146	3.59356	4.01541	3.56523	0.125573493	0.555618842	12	4	24	0.14208586	0.751766185	24	5.393654421	0.186605972
ENSMUSG00000030237	Slco1a4	solute carrier organic anion transporter family, member 1a4 [Source:MGI Symbol;Acc:MGI:1351896]	8.93996	18.3343	20.6756	16.5797	14.7353	13.2633	13.7466	17.8644	21.7345	16.1218	14.2328	14.1872	13.8755	14.6009	9.53E-06	0.009754434	12	16	24	0.005495565	0.445165967	24	8.890630213	0.325201185
ENSMUSG00000030243	Recql	RecQ protein-like [Source:MGI Symbol;Acc:MGI:103021]	11.9434	11.8652	11.0539	11.7434	12.1663	11.3122	11.0657	12.1054	12.7894	13.007	11.4868	12.3429	12.4768	12.4323	0.816300547	0.966018836	16	8	24	0.536362514	0.827399772	24	8.856599535	0.027241042
ENSMUSG00000030244	Gys2	glycogen synthase 2 [Source:MGI Symbol;Acc:MGI:2385254]	0.301573	0.137366	0.92372	1.57491	0.274939	0.29921	0.310471	0.897602	0.661519	1.02882	1.40229	0.453299	0.748072	0.927685	0.173410058	0.648600527	16	8	24	0.028039909	0.590904845	24	10.80591539	0.324180992
ENSMUSG00000030245	Golt1b	golgi transport 1B [Source:MGI Symbol;Acc:MGI:1914214]	19.0029	18.435	18.9371	18.7517	19.0135	18.6027	16.8962	18.7598	18.4337	19.2486	19.1936	19.0538	19.3194	19.3428	0.865514257	0.966018836	20	16	24	0.548797631	0.834692497	24	12.72028088	0.01933859
ENSMUSG00000030246	Ldhb	lactate dehydrogenase B [Source:MGI Symbol;Acc:MGI:96763]	269.711	318.049	307.698	322.331	306.963	314.79	297.335	304.688	301.725	280.123	285.228	271.809	285.656	280.143	0.822170541	0.966018836	12	16	24	0.469991186	0.805393068	24	9.98134077	0.036418307
ENSMUSG00000030247	Kcnj8	potassium inwardly-rectifying channel, subfamily J, member 8 [Source:MGI Symbol;Acc:MGI:1100508]	1.03949	1.65882	1.84255	1.34545	1.41589	1.15024	1.15667	2.01086	2.20969	1.78391	1.44693	1.47501	1.6499	2.03345	0.018773661	0.256345714	12	12	24	1.49E-04	0.134670799	24	7.68858333	0.230123784
ENSMUSG00000030249	Abcc9	ATP-binding cassette, sub-family C (CFTR/MRP), member 9 [Source:MGI Symbol;Acc:MGI:1352630]	0.906017	1.77293	2.27011	2.69541	1.68879	2.11099	1.66868	3.2684	2.3841	3.43168	1.48873	1.72442	2.19467	1.87871	0.033799022	0.325496079	16	4	24	0.055666788	0.65534544	24	9.464356952	0.276099671
ENSMUSG00000030254	Rad18	RAD18 E3 ubiquitin protein ligase [Source:MGI Symbol;Acc:MGI:1890476]	3.22364	3.16706	3.4887	3.09386	3.13748	3.3998	3.42745	2.76279	3.19977	3.25288	3.47055	3.20313	3.83269	3.55439	0.94663657	0.966018836	4	12	24	0.725508583	0.90999235	24	22.20252916	0.026191595
ENSMUSG00000030255	Sspn	sarcospan [Source:MGI Symbol;Acc:MGI:1353511]	8.35018	8.54862	8.99035	9.51406	9.33713	8.42684	8.58536	10.1656	9.93754	11.7908	8.58004	9.23596	9.39076	9.26689	0.219464938	0.708495385	16	8	24	0.040631652	0.616921492	24	10.11138095	0.104049607
ENSMUSG00000030256	Bhlhe41	basic helix-loop-helix family, member e41 [Source:MGI Symbol;Acc:MGI:1930704]	6.28648	6.27454	8.21589	7.08848	5.90023	6.02516	6.2874	6.21171	7.20471	9.15422	6.55168	5.23678	5.69861	6.38245	0.005024733	0.143276577	16	8	24	0.004041536	0.427431763	24	9.951709744	0.200353851
ENSMUSG00000030257	Srgap3	SLIT-ROBO Rho GTPase activating protein 3 [Source:MGI Symbol;Acc:MGI:2152938]	26.5741	21.3052	21.2167	21.8845	22.0736	25.0624	24.4974	23.6321	18.0002	28.6026	20.1034	24.74	23.1491	24.9345	0.148073804	0.590732968	24	12	24	0.38822552	0.799546792	24	21.70896153	0.091705379
ENSMUSG00000030259	Rassf8	Ras association (RalGDS/AF-6) domain family (N-terminal) member 8 [Source:MGI Symbol;Acc:MGI:1918573]	8.31525	8.44846	8.7784	7.52033	8.29302	7.50598	8.12552	8.00274	8.51949	6.65292	7.54677	7.94578	7.75991	6.78698	0.187699199	0.662239322	12	4	24	0.600438156	0.860118739	24	1.720505659	0.033606953
ENSMUSG00000030263	Lrmp	lymphoid-restricted membrane protein [Source:MGI Symbol;Acc:MGI:108424]	1.37472	2.29238	3.93112	2.13806	1.99063	2.32466	2.30889	2.13163	2.74953	1.90711	1.99843	1.96807	2.62495	1.58443	0.865490576	0.966018836	12	8	24	0.453612277	0.805393068	24	8.475850204	0.127988762
ENSMUSG00000030264	Thumpd3	THUMP domain containing 3 [Source:MGI Symbol;Acc:MGI:1277973]	25.1851	25.0263	24.5694	22.2443	23.6984	22.7457	20.5324	22.9924	25.1453	21.8975	24.6558	22.8737	25.924	24.7621	0.46694635	0.966018836	4	12	24	0.58543574	0.852882894	24	4.631594523	0.038309364
ENSMUSG00000030265	Kras	Kirsten rat sarcoma viral oncogene homolog [Source:MGI Symbol;Acc:MGI:96680]	41.1379	41.3117	40.7971	43.0809	48.6954	37.7523	44.4319	37.8953	41.468	37.461	40.6347	36.8266	40.1475	39.3418	0.897425765	0.966018836	20	4	24	0.775516003	0.927742146	24	15.07077166	0.027278401
ENSMUSG00000030268	Bcat1	branched chain aminotransferase 1, cytosolic [Source:MGI Symbol;Acc:MGI:104861]	22.2963	19.7401	23.4937	19.5055	17.1671	23.9756	24.5157	21.5517	16.8152	26.0633	18.405	20.0272	18.1402	21.6431	0.915475528	0.966018836	4	16	24	0.833431572	0.948687906	24	1.617554188	0.045017606
ENSMUSG00000030269	Mtmr14	myotubularin related protein 14 [Source:MGI Symbol;Acc:MGI:1916075]	13.9526	13.5753	14.8462	13.2369	15.003	17.4879	12.8028	15.3003	15.1752	17.8403	13.5874	14.8011	16.9664	14.3348	0.846563228	0.966018836	24	4	24	0.87684453	0.96580091	24	14.93912323	0.026985009
ENSMUSG00000030270	Cpne9	copine family member IX [Source:MGI Symbol;Acc:MGI:2443052]	3.28456	2.74668	2.15518	2.76537	2.13898	2.32905	2.11558	2.18029	1.87575	3.06357	1.78526	2.96066	2.66921	3.37915	0.943890799	0.966018836	8	12	24	0.651268966	0.882725346	24	1.429827205	0.077368533
ENSMUSG00000030271	Ogg1	8-oxoguanine DNA-glycosylase 1 [Source:MGI Symbol;Acc:MGI:1097693]	4.96346	5.21266	5.24245	5.28488	4.42099	4.51149	3.77452	5.3896	4.84352	4.66609	4.45708	5.03619	4.96178	5.32572	0.018114172	0.256345714	8	12	24	0.149679374	0.758377578	24	6.728591556	0.088560894
ENSMUSG00000030272	Camk1	calcium/calmodulin-dependent protein kinase I [Source:MGI Symbol;Acc:MGI:1098535]	28.3983	25.9378	31.076	24.0427	27.7964	28.5524	26.6378	27.1535	25.7394	26.1845	30.1395	26.5119	27.5833	27.069	0.503972924	0.966018836	20	20	24	0.745364044	0.916764484	24	20.2613829	0.029446216
ENSMUSG00000030275	Etnk1	ethanolamine kinase 1 [Source:MGI Symbol;Acc:MGI:1922570]	107.874	94.5984	94.9162	97.8965	96.9208	100.033	96.4918	90.3977	82.6551	99.1543	97.027	95.5493	93.4178	98.4327	0.483047948	0.966018836	16	20	24	0.153800055	0.760223561	24	19.71984108	0.060811643
ENSMUSG00000030276	Ttll3	tubulin tyrosine ligase-like family, member 3 [Source:MGI Symbol;Acc:MGI:2141418]	6.73458	5.35246	6.07196	5.59473	5.53698	5.04826	5.44099	4.53442	5.77902	4.11431	5.88534	5.74911	4.1699	4.50059	0.928780444	0.966018836	12	20	24	0.611578023	0.865117073	24	17.82579909	0.058413117
ENSMUSG00000030278	Cidec	cell death-inducing DFFA-like effector c [Source:MGI Symbol;Acc:MGI:95585]	3.69304	2.45073	9.55562	27.927	7.12498	5.10822	5.31475	11.2611	8.16743	16.8418	15.21	7.65419	14.1673	11.4213	0.239653024	0.741580974	16	12	24	0.025262258	0.586541342	24	11.75482256	0.686513405
ENSMUSG00000030279	C2cd5	C2 calcium-dependent domain containing 5 [Source:MGI Symbol;Acc:MGI:1921991]	28.1682	27.7357	28.517	28.9514	30.9887	27.0726	29.3395	29.4334	27.1305	27.9644	28.7566	30.214	28.1204	28.1547	0.953226423	0.966018836	20	16	24	0.418134765	0.80272238	24	17.64168853	0.028373854
ENSMUSG00000030281	Il17rc	interleukin 17 receptor C [Source:MGI Symbol;Acc:MGI:2159336]	6.29431	3.44725	5.56837	3.77712	2.55408	3.13089	3.29446	4.12162	4.73521	4.95778	4.80578	4.88266	6.60957	4.34885	0.920872048	0.966018836	12	8	24	0.623376993	0.87183699	24	4.562630669	0.119963573
ENSMUSG00000030282	Cmas	cytidine monophospho-N-acetylneuraminic acid synthetase [Source:MGI Symbol;Acc:MGI:1337124]	70.5123	63.809	64.28	62.9059	63.6289	67.5328	60.8372	63.417	60.913	66.3825	65.5295	64.5105	66.9079	69.5293	0.801995186	0.966018836	4	8	24	0.528326995	0.823886696	24	22.24312092	0.02854035
ENSMUSG00000030283	St8sia1	ST8 alpha-N-acetyl-neuraminide alpha-2,8-sialyltransferase 1 [Source:MGI Symbol;Acc:MGI:106011]	11.3831	9.89326	9.90668	9.53625	9.56466	11.0892	10.4637	9.497	7.97291	11.5376	10.4973	11.0006	10.3501	11.1191	0.390686537	0.915479136	24	12	24	0.18260013	0.768040178	24	20.69990967	0.092268053
ENSMUSG00000030284	Creld1	cysteine-rich with EGF-like domains 1 [Source:MGI Symbol;Acc:MGI:2152539]	57.8701	43.2841	40.2654	46.6603	46.3826	56.3742	49.8218	51.3988	38.6772	54.1805	48.6955	50.8725	50.1377	46.7346	0.025149041	0.292995596	24	12	24	0.039064094	0.613457385	24	20.4899958	0.15508945
ENSMUSG00000030286	Emc3	ER membrane protein complex subunit 3 [Source:MGI Symbol;Acc:MGI:1913337]	47.6373	46.798	46.0873	49.9323	47.541	49.5382	48.0613	50.187	46.3568	47.8897	48.8179	50.9998	49.6227	49.4549	0.085890569	0.466404237	24	12	24	0.223309543	0.768040178	24	20.29276421	0.026236347
ENSMUSG00000030287	Itpr2	inositol 1,4,5-triphosphate receptor 2 [Source:MGI Symbol;Acc:MGI:99418]	0.950757	1.33547	1.68243	2.20754	1.15514	1.13296	1.38042	1.91089	1.56963	1.34542	1.27483	1.50382	1.43718	1.8938	0.154126887	0.606139807	12	8	24	0.044857723	0.628432994	24	8.018215893	0.175584976
ENSMUSG00000030291	Med21	mediator complex subunit 21 [Source:MGI Symbol;Acc:MGI:1347064]	62.6292	64.5002	55.9291	56.1654	63.1408	55.1197	59.7069	54.6849	59.2171	47.6454	68.0196	54.7715	57.3645	54.8748	0.883051071	0.966018836	20	20	24	0.576815102	0.848735805	24	20.91635533	0.052371686
ENSMUSG00000030298	Sec13	SEC13 homolog, nuclear pore and COPII coat complex component [Source:MGI Symbol;Acc:MGI:99832]	60.8125	64.7118	61.9001	62.5637	64.7801	62.8017	55.3803	61.5979	64.5818	55.1415	65.0029	60.4883	61.8244	61.7624	0.231491055	0.731037655	20	8	24	0.901433663	0.972353488	24	12.40839271	0.013377356
ENSMUSG00000030301	Ccdc91	coiled-coil domain containing 91 [Source:MGI Symbol;Acc:MGI:1914265]	25.0319	26.3511	25.1184	26.0739	25.6283	26.168	25.1506	26.2565	26.2306	26.2999	28.4125	26.2739	27.6342	26.7036	0.874607729	0.966018836	8	20	24	0.737599981	0.91440753	24	14.86807841	0.011196596
ENSMUSG00000030302	Atp2b2	ATPase, Ca++ transporting, plasma membrane 2 [Source:MGI Symbol;Acc:MGI:105368]	27.0546	21.4836	25.4846	23.7706	17.5301	26.5688	27.4344	22.9566	20.2756	37.0574	16.8894	22.7918	20.4265	26.4436	0.885025617	0.966018836	4	16	24	0.785889371	0.931158434	24	5.414121673	0.080022514
ENSMUSG00000030303	Far2	fatty acyl CoA reductase 2 [Source:MGI Symbol;Acc:MGI:2687035]	8.98193	8.25912	7.5931	7.56251	8.11799	8.80121	8.24687	7.96847	7.0326	7.55908	7.68569	8.17201	8.13332	7.64583	9.19E-04	0.072297567	4	12	24	8.70E-06	0.034160304	24	21.73466807	0.101454411
ENSMUSG00000030304	Ergic2	ERGIC and golgi 2 [Source:MGI Symbol;Acc:MGI:1914706]	37.8616	41.0606	44.2315	43.3531	42.4489	37.1364	43.1998	39.3189	47.5085	35.5301	49.545	45.3719	45.9054	40.8942	0.916963526	0.966018836	12	20	24	0.874797589	0.964976615	24	12.78354135	0.026876481
ENSMUSG00000030306	Tmtc1	transmembrane and tetratricopeptide repeat containing 1 [Source:MGI Symbol;Acc:MGI:3039590]	34.0431	30.3081	28.6736	32.0533	29.0588	31.2242	29.4013	29.974	27.3942	34.5732	30.0298	33.1898	31.9716	32.6661	0.591351901	0.966018836	24	12	24	0.618559681	0.868995186	24	20.38415959	0.038487123
ENSMUSG00000030307	Slc6a11	solute carrier family 6 (neurotransmitter transporter, GABA), member 11 [Source:MGI Symbol;Acc:MGI:95630]	3.14947	9.11382	18.5814	7.01041	3.68211	10.1095	11.612	5.09258	10.1387	7.61477	2.92854	7.81159	2.74849	11.1189	0.285027487	0.793714531	12	8	24	0.277999217	0.768040178	24	7.143945956	0.466233053
ENSMUSG00000030309	Caprin2	caprin family member 2 [Source:MGI Symbol;Acc:MGI:2448541]	14.0137	12.4434	11.9265	13.6156	11.0147	13.2441	11.7145	12.9876	12.7275	11.5285	15.1	12.5403	12.9078	11.9364	0.854443826	0.966018836	24	16	24	0.756661542	0.921470626	24	19.58510884	0.035230659
ENSMUSG00000030310	Slc6a1	solute carrier family 6 (neurotransmitter transporter, GABA), member 1 [Source:MGI Symbol;Acc:MGI:95627]	10.2127	14.8848	22.368	14.7723	10.2489	14.0413	16.999	10.8689	18.7082	13.3907	8.22726	10.9766	8.222	14.6101	0.066808003	0.425506559	12	8	24	0.081729734	0.698072736	24	7.640237099	0.321088512
ENSMUSG00000030313	Dennd5b	DENN/MADD domain containing 5B [Source:MGI Symbol;Acc:MGI:2444273]	3.93544	3.98529	4.27259	3.95959	3.58538	4.1402	4.55379	3.41627	3.88556	3.80012	4.03266	3.93397	3.32675	3.91779	0.79256873	0.966018836	24	8	24	0.963011136	0.990726108	24	18.21767572	0.010347659
ENSMUSG00000030314	Atg7	autophagy related 7 [Source:MGI Symbol;Acc:MGI:1921494]	10.0521	8.53989	8.76933	9.07226	9.73663	9.82526	8.94265	9.69473	8.36073	11.5818	8.98688	9.49108	9.21347	9.03852	0.236893212	0.7385769	24	12	24	0.423934924	0.805393068	24	16.89229004	0.055787736
ENSMUSG00000030315	Vgll4	vestigial like family member 4 [Source:MGI Symbol;Acc:MGI:2652840]	20.5141	17.8985	18.2295	18.4795	17.6379	17.1927	21.8966	18.4911	19.0594	20.4615	20.191	19.4302	19.7575	18.8403	0.46694635	0.966018836	4	20	24	0.772596509	0.92687884	24	23.18762596	0.026046712
ENSMUSG00000030316	Tamm41	TAM41 mitochondrial translocator assembly and maintenance homolog [Source:MGI Symbol;Acc:MGI:1916221]	8.38342	8.66431	9.16415	8.55082	9.08715	9.85622	8.7138	8.12141	8.14739	10.0239	8.77128	8.2197	9.1909	8.35678	0.439800617	0.966018836	16	16	24	0.322856329	0.768040178	24	15.82001401	0.050250623
ENSMUSG00000030317	Timp4	tissue inhibitor of metalloproteinase 4 [Source:MGI Symbol;Acc:MGI:109125]	1.01574	1.91707	3.21684	2.48277	1.21806	2.23443	2.37063	1.49155	2.25839	2.09192	1.69201	1.94635	1.28231	2.73122	0.277917941	0.789849983	12	16	24	0.190124734	0.768040178	24	8.83559442	0.218333435
ENSMUSG00000030319	Cand2	cullin-associated and neddylation-dissociated 2 (putative) [Source:MGI Symbol;Acc:MGI:1914338]	0.923316	1.27802	1.31318	1.35942	1.24129	1.32498	1.33884	2.31161	1.49991	2.09456	1.10721	1.37824	1.53092	1.33547	0.917839756	0.966018836	16	4	24	0.27565977	0.768040178	24	7.985552835	0.121021769
ENSMUSG00000030321	Efcab12	EF-hand calcium binding domain 12 [Source:MGI Symbol;Acc:MGI:2681834]	1.21482	1.29511	1.39606	1.50501	1.38078	1.43344	1.02385	1.35125	1.35922	1.34483	1.35932	1.40175	1.30118	1.45416	0.076593464	0.453790923	24	4	24	0.129145832	0.740162193	24	11.99805952	0.055615944
ENSMUSG00000030322	Mbd4	methyl-CpG binding domain protein 4 [Source:MGI Symbol;Acc:MGI:1333850]	3.2211	3.82565	3.82786	3.5592	3.41861	3.63444	3.19325	3.46768	4.45196	3.23245	4.33213	4.33003	3.58411	3.62121	0.286642595	0.793714531	12	16	24	0.6823524	0.894159472	24	11.83283536	0.042047989
ENSMUSG00000030323	Ift122	intraflagellar transport 122 [Source:MGI Symbol;Acc:MGI:1932386]	15.7891	12.452	12.6555	13.1616	12.9989	14.9348	16.1334	14.4083	11.8394	14.6518	12.3396	13.3739	13.3823	14.1663	0.031969981	0.324385159	4	8	24	0.128164098	0.740162193	24	22.77409733	0.097315577
ENSMUSG00000030324	Rho	rhodopsin [Source:MGI Symbol;Acc:MGI:97914]	1.47283	0.783053	1.25076	1.02133	1.50282	1.24179	1.04783	1.63566	0.735475	1.40085	1.04632	1.22321	1.06229	1.0785	0.849180783	0.966018836	24	12	24	0.586630546	0.853342401	24	18.82666331	0.074756436
ENSMUSG00000030327	Necap1	NECAP endocytosis associated 1 [Source:MGI Symbol;Acc:MGI:1914852]	71.5932	62.228	64.2451	62.5685	59.7465	68.0866	63.6938	63.3971	52.2485	68.1982	60.5941	65.3613	62.6216	66.432	0.777666236	0.966018836	24	20	24	0.309938999	0.768040178	24	21.93688844	0.063967197
ENSMUSG00000030329	Pianp	PILR alpha associated neural protein [Source:MGI Symbol;Acc:MGI:2441908]	3.75498	4.34749	5.85135	4.16759	3.43164	4.48691	5.26322	4.04064	4.94407	6.06962	2.9179	4.95456	4.30015	5.51893	0.338172159	0.85758369	12	8	24	0.218007741	0.768040178	24	6.498938064	0.164367552
ENSMUSG00000030330	Ing4	inhibitor of growth family, member 4 [Source:MGI Symbol;Acc:MGI:107307]	23.9854	24.6867	21.0505	23.9902	23.2036	23.359	23.0382	23.5326	23.9304	21.8285	22.22	23.3964	24.5661	22.5465	0.570349842	0.966018836	4	16	24	0.260548082	0.768040178	24	23.82148084	0.039120235
ENSMUSG00000030335	Mrpl51	mitochondrial ribosomal protein L51 [Source:MGI Symbol;Acc:MGI:1913743]	35.7597	40.5244	39.1031	42.6647	47.2369	44.4402	41.379	42.1739	39.9074	42.4025	46.6269	38.5358	43.1951	39.4895	0.089766625	0.477784783	20	16	24	0.083828594	0.701079395	24	15.45560334	0.084337175
ENSMUSG00000030336	Cd27	CD27 antigen [Source:MGI Symbol;Acc:MGI:88326]	0.690823	0.734303	0.625661	1.06514	0.876342	0.669854	0.750965	0.592435	1.08423	0.558991	0.782244	1.02092	0.883484	0.766021	0.881996584	0.966018836	12	20	24	0.731967845	0.912550805	24	15.17608519	0.041888558
ENSMUSG00000030337	Vamp1	vesicle-associated membrane protein 1 [Source:MGI Symbol;Acc:MGI:1313276]	151.576	142.651	158.811	145.489	127.543	161.744	157.335	145.562	120.255	172.249	121.611	145.646	133.038	153.429	0.890235289	0.966018836	24	20	24	0.832442311	0.948148785	24	2.152722076	0.036841555
ENSMUSG00000030339	Ltbr	lymphotoxin B receptor [Source:MGI Symbol;Acc:MGI:104875]	7.94227	6.94759	6.53639	9.25192	7.68092	6.32585	9.27194	8.11254	7.97459	7.79079	6.93799	7.78903	8.1173	6.26334	0.966095896	0.969272194	4	20	24	0.974052857	0.993499248	24	18.24802837	0.015286913
ENSMUSG00000030341	Tnfrsf1a	tumor necrosis factor receptor superfamily, member 1a [Source:MGI Symbol;Acc:MGI:1314884]	9.84937	9.95739	9.8615	9.82879	10.1563	8.77942	9.93376	10.9471	10.6448	10.4692	10.6813	10.0753	10.8187	10.4059	0.922029406	0.966018836	8	16	24	0.198086948	0.768040178	24	7.438560894	0.044436949
ENSMUSG00000030342	Cd9	CD9 antigen [Source:MGI Symbol;Acc:MGI:88348]	833.353	794.182	744.885	763.981	847.89	783.113	779.563	727.169	701.408	685.27	796.392	669.998	721.917	683.313	0.664568268	0.966018836	20	16	24	0.085724831	0.703577714	24	19.26216848	0.064916173
ENSMUSG00000030345	Dyrk4	dual-specificity tyrosine-(Y)-phosphorylation regulated kinase 4 [Source:MGI Symbol;Acc:MGI:1330292]	1.06236	1.02696	0.629629	0.541583	0.692639	0.954404	0.625605	0.883954	0.893426	0.793901	1.00427	1.12438	1.01479	1.00026	0.205904698	0.69530311	24	16	24	0.152543038	0.759088284	24	23.38751726	0.101146835
ENSMUSG00000030346	Rad51ap1	RAD51 associated protein 1 [Source:MGI Symbol;Acc:MGI:1098224]	1.79511	1.56139	1.87617	1.61546	1.35017	2.13509	1.62394	1.0584	1.85448	2.2351	1.55842	1.85236	1.68177	1.37158	0.321662653	0.83607295	12	20	24	0.546201549	0.833257583	24	15.7872835	0.074133417
ENSMUSG00000030347	D6Wsu163e	DNA segment, Chr 6, Wayne State University 163, expressed [Source:MGI Symbol;Acc:MGI:107893]	21.3203	21.0164	16.1831	18.834	19.0338	17.8797	16.9073	18.555	16.4733	16.5477	20.0581	18.3087	17.2045	17.9801	0.616047374	0.966018836	20	16	24	0.37772914	0.797551131	24	20.93928183	0.062922525
ENSMUSG00000030350	Prmt8	protein arginine N-methyltransferase 8 [Source:MGI Symbol;Acc:MGI:3043083]	11.8542	9.77572	8.74786	10.1142	9.46032	11.5516	9.34443	10.9353	8.11148	12.4034	8.49053	10.6751	9.7855	11.1733	0.745475226	0.966018836	24	12	24	0.652918523	0.883245411	24	22.64896327	0.062686718
ENSMUSG00000030351	Tspan11	tetraspanin 11 [Source:MGI Symbol;Acc:MGI:1915748]	1.74719	1.64341	1.5679	1.32764	1.56428	1.43637	1.77124	1.91205	2.12642	1.148	1.63645	1.14319	1.71342	1.61341	0.081336016	0.462794877	8	8	24	0.265222613	0.768040178	24	3.905527402	0.095007062
ENSMUSG00000030352	Tspan9	tetraspanin 9 [Source:MGI Symbol;Acc:MGI:1924558]	5.74008	5.8533	6.56241	4.72399	4.76645	5.69251	6.56373	6.03671	6.42914	6.1111	4.75107	5.65502	4.10618	5.71478	0.066808003	0.425506559	12	8	24	0.249411918	0.768040178	24	5.674636244	0.109896679
ENSMUSG00000030353	Tead4	TEA domain family member 4 [Source:MGI Symbol;Acc:MGI:106907]	1.50493	1.51262	1.22602	1.56918	1.52273	1.18166	1.5472	1.76319	1.40814	1.70231	1.27018	1.32538	1.4727	1.31161	0.944053021	0.966018836	16	8	24	0.714184845	0.905831242	24	6.792187638	0.034596283
ENSMUSG00000030357	Fkbp4	FK506 binding protein 4 [Source:MGI Symbol;Acc:MGI:95543]	153.024	118.706	111.26	119.614	133.38	139.474	129.572	130.066	99.9195	126.186	137.907	125.835	126.398	126.055	0.021949162	0.267229408	4	8	24	0.011866087	0.525280095	24	20.40600493	0.153657369
ENSMUSG00000030361	Klrb1a	killer cell lectin-like receptor subfamily B member 1A [Source:MGI Symbol;Acc:MGI:107540]	2.93222	3.66885	3.45777	3.14501	4.0465	2.92674	3.68807	3.1418	5.41777	3.5029	4.55156	3.57359	4.83681	4.20856	0.830949782	0.966018836	20	4	24	0.578152435	0.849184181	24	8.701271718	0.076823634
ENSMUSG00000030374	Strn4	striatin, calmodulin binding protein 4 [Source:MGI Symbol;Acc:MGI:2142346]	24.2504	23.8556	25.5216	29.0742	28.3481	25.3307	26.6082	24.3852	29.4098	26.3243	26.3616	26.0066	24.7345	24.9187	0.012445568	0.220221977	16	16	24	0.022251181	0.578379975	24	13.0846127	0.087661928
ENSMUSG00000030376	Slc8a2	solute carrier family 8 (sodium/calcium exchanger), member 2 [Source:MGI Symbol;Acc:MGI:107996]	0.779818	0.788746	1.28203	0.832484	0.54285	0.898498	0.832846	0.887467	0.858527	1.24445	0.459012	1.02711	0.713562	1.02568	0.432054949	0.966018836	12	8	24	0.262688013	0.768040178	24	6.967900537	0.116095927
ENSMUSG00000030380	Mzf1	myeloid zinc finger 1 [Source:MGI Symbol;Acc:MGI:107457]	0.9171	0.832093	0.949111	0.768781	1.00974	1.0447	0.813344	0.752362	0.801409	0.928591	1.02228	1.01578	0.919461	0.872701	0.017495149	0.255326033	24	8	24	0.0170191	0.547196997	24	17.95800224	0.074802548
ENSMUSG00000030386	Zfp606	zinc finger protein 606 [Source:MGI Symbol;Acc:MGI:1914620]	3.58184	4.56357	4.55283	3.92039	4.6044	3.84226	4.5895	4.28047	5.21212	3.84691	4.28563	4.30965	3.99567	4.51829	0.797413662	0.966018836	12	12	24	0.30123092	0.768040178	24	7.51521661	0.071497902
ENSMUSG00000030393	Zik1	zinc finger protein interacting with K protein 1 [Source:MGI Symbol;Acc:MGI:108070]	2.15623	2.63034	2.50725	2.47916	2.49038	2.29257	2.42125	2.15134	2.3432	1.86396	2.575	2.4673	2.02861	2.34535	0.798309651	0.966018836	20	8	24	0.887578749	0.968408461	24	15.58663922	0.018400727
ENSMUSG00000030397	Mark4	MAP/microtubule affinity regulating kinase 4 [Source:MGI Symbol;Acc:MGI:1920955]	11.7521	9.77646	9.96973	10.8295	10.6333	10.8649	11.0867	10.9837	9.88391	14.2272	11.4302	12.5562	11.8324	11.9271	0.46238066	0.966018836	24	12	24	0.50503179	0.811334839	24	17.9100185	0.053879671
ENSMUSG00000030399	Ckm	creatine kinase, muscle [Source:MGI Symbol;Acc:MGI:88413]	115.671	301.09	346.319	452.286	261.602	398.559	301.274	947.245	684.253	768.754	297.991	363.404	418.606	286.563	0.867888967	0.966018836	16	4	24	0.113322756	0.727393008	24	9.391603399	0.558087551
ENSMUSG00000030400	Ercc2	excision repair cross-complementing rodent repair deficiency, complementation group 2 [Source:MGI Symbol;Acc:MGI:95413]	9.31936	7.69246	7.40038	7.34897	7.70357	8.42913	7.99607	9.2361	7.47767	8.76853	10.2591	6.73032	8.16927	7.864	0.651661131	0.966018836	4	8	24	0.864840817	0.960819377	24	20.16361	0.033110086
ENSMUSG00000030401	Rtn2	reticulon 2 (Z-band associated protein) [Source:MGI Symbol;Acc:MGI:107612]	47.4615	44.9452	49.1871	55.3595	49.7053	57.797	44.7245	70.2658	54.8272	72.3751	44.1216	48.5069	54.7948	49.8625	0.581070934	0.966018836	16	12	24	0.455492479	0.805393068	24	9.701235415	0.109614624
ENSMUSG00000030402	Ppm1n	protein phosphatase, Mg2+/Mn2+ dependent, 1N (putative) [Source:MGI Symbol;Acc:MGI:2142330]	0.676114	0.550673	0.426127	1.48577	0.771628	0.396245	0.467	1.42137	0.954127	0.567413	0.889319	1.04566	0.515688	0.408989	0.762156552	0.966018836	16	12	24	0.520364897	0.819945009	24	12.45779059	0.126025428
ENSMUSG00000030403	Vasp	vasodilator-stimulated phosphoprotein [Source:MGI Symbol;Acc:MGI:109268]	25.6058	23.246	23.1641	23.5128	21.8187	21.003	21.1562	22.6064	20.5179	25.9226	24.3507	26.0623	26.193	23.0851	0.916860713	0.966018836	4	8	24	0.848642093	0.954502769	24	19.36819785	0.02708606
ENSMUSG00000030407	Qpctl	glutaminyl-peptide cyclotransferase-like [Source:MGI Symbol;Acc:MGI:1914619]	4.34721	4.1094	4.83371	4.0793	4.63593	4.63154	4.18183	4.86016	4.00567	5.24375	3.85439	4.51649	3.97561	4.16202	0.860709018	0.966018836	16	12	24	0.776499471	0.928225024	24	12.4582073	0.030249719
ENSMUSG00000030409	Dmpk	dystrophia myotonica-protein kinase [Source:MGI Symbol;Acc:MGI:94906]	14.236	12.3132	13.6321	17.1097	16.9482	13.4329	14.4807	19.4456	15.0808	16.4783	16.0826	12.5584	15.3612	12.8006	0.280810068	0.792139038	20	4	24	0.371823447	0.793398709	24	12.31408544	0.099291814
ENSMUSG00000030410	Dmwd	dystrophia myotonica-containing WD repeat motif [Source:MGI Symbol;Acc:MGI:94907]	24.5128	20.0861	22.028	21.487	21.8026	22.4477	23.8832	21.4433	20.4336	25.4567	21.2032	23.5536	22.0275	20.4257	0.087978327	0.472021905	4	4	24	0.320904868	0.768040178	24	19.0585465	0.059864573
ENSMUSG00000030411	Nova2	NOVA alternative splicing regulator 2 [Source:MGI Symbol;Acc:MGI:104296]	1.3051	1.24128	1.30029	1.50319	1.38475	1.17719	1.24312	1.20078	1.53465	1.66947	1.58269	1.53191	1.35391	1.54506	0.266953338	0.774280302	16	16	24	0.009875278	0.503967859	24	12.30170044	0.082347922
ENSMUSG00000030413	Pglyrp1	peptidoglycan recognition protein 1 [Source:MGI Symbol;Acc:MGI:1345092]	5.53479	9.36301	14.2119	11.8612	9.73083	9.93929	9.50914	9.75738	8.47771	6.78698	9.44219	8.26151	10.5428	6.00278	0.819898687	0.966018836	12	20	24	0.669208044	0.888537207	24	12.31235541	0.113303945
ENSMUSG00000030417	Pdcd5	programmed cell death 5 [Source:MGI Symbol;Acc:MGI:1913538]	9.15731	13.0221	9.82518	10.4042	11.3012	12.4191	11.4892	10.5109	12.4111	11.9431	12.2288	11.0316	11.442	10.6021	0.844448841	0.966018836	24	4	24	0.793133589	0.93379199	24	14.95726607	0.034386136
ENSMUSG00000030421	Uri1	URI1, prefoldin-like chaperone [Source:MGI Symbol;Acc:MGI:1342294]	15.8716	20.3431	20.9838	19.7399	18.105	20.1614	20.1031	15.6923	16.9181	19.0322	17.7175	17.0826	15.085	19.3838	0.85447369	0.966018836	16	12	24	0.542678971	0.831042618	24	11.33531046	0.0618097
ENSMUSG00000030423	Pop4	processing of precursor 4, ribonuclease P/MRP family, (S. cerevisiae) [Source:MGI Symbol;Acc:MGI:1913411]	18.8647	19.3205	19.881	17.4953	17.8428	18.2712	16.1966	18.3211	18.8171	16.5549	19.9594	17.6831	19.8217	18.941	0.943890799	0.966018836	12	4	24	0.678296048	0.892450912	24	4.592171253	0.031703652
ENSMUSG00000030424	Zfp939	zinc finger protein 939 [Source:MGI Symbol;Acc:MGI:3036240]	1.95228	1.9777	2.27781	1.89221	2.46771	1.96221	1.67619	2.06097	2.13506	2.1928	1.75215	2.08424	2.02016	2.32667	0.643325306	0.966018836	12	16	24	0.410225389	0.799546792	24	9.510539409	0.055174706
ENSMUSG00000030428	Ttyh1	tweety family member 1 [Source:MGI Symbol;Acc:MGI:1889007]	33.8324	37.7117	47.4254	36.1657	35.2727	41.3098	47.4827	33.7751	37.996	41.8574	33.5311	34.8895	29.3113	39.6441	0.760769894	0.966018836	12	16	24	0.636949092	0.876483853	24	8.511109449	0.073348899
ENSMUSG00000030431	Tmem238	transmembrane protein 238 [Source:MGI Symbol;Acc:MGI:1922935]	0.490899	1.27025	0.818981	1.26809	0.929912	1.58256	1.03495	0.587199	1.46488	1.283	0.496193	1.09996	0.885454	0.559545	0.622132989	0.966018836	16	12	24	0.643068714	0.878809617	24	13.47562107	0.09912373
ENSMUSG00000030432	Rpl28	ribosomal protein L28 [Source:MGI Symbol;Acc:MGI:101839]	5.16782	4.0714	4.35485	3.8519	5.27209	5.3481	4.30452	5.62182	4.27995	5.27138	3.70765	4.07295	4.09727	5.03579	0.826055781	0.966018836	8	8	24	0.877235179	0.965814767	24	1.173899751	0.03387408
ENSMUSG00000030433	Sbk2	SH3-binding domain kinase family, member 2 [Source:MGI Symbol;Acc:MGI:2685925]	0.437314	0.983656	1.09137	2.22949	1.27727	1.77942	0.980665	4.75859	1.58027	2.73988	1.02867	1.18551	1.12411	1.21114	0.03484427	0.328311403	16	12	24	0.318197961	0.768040178	24	9.840092352	0.290365154
ENSMUSG00000030435	U2af2	U2 small nuclear ribonucleoprotein auxiliary factor (U2AF) 2 [Source:MGI Symbol;Acc:MGI:98886]	28.9086	25.9272	26.4436	27.0551	29.4472	28.5423	31.0846	29.3827	27.6707	26.9749	27.498	26.8317	27.7263	25.5287	0.46238066	0.966018836	4	4	24	0.217174108	0.768040178	24	20.77868696	0.051668278
ENSMUSG00000030443	Zfp583	zinc finger protein 583 [Source:MGI Symbol;Acc:MGI:2682297]	2.84768	3.93992	2.76251	3.11306	4.10057	3.02842	3.46046	3.0365	3.3317	3.37844	4.78578	3.64351	2.84951	3.00474	0.208341949	0.698704663	20	8	24	0.173792329	0.768040178	24	15.91917726	0.125776192
ENSMUSG00000030446	Zfp273	zinc finger protein 273 [Source:MGI Symbol;Acc:MGI:3036278]	3.01736	3.67315	2.94305	3.0543	3.4733	2.85969	3.34283	2.89771	3.38147	2.68499	3.14787	2.8296	3.56488	3.16406	0.777666236	0.966018836	4	20	24	0.59339732	0.856916489	24	2.327303427	0.041719623
ENSMUSG00000030447	Cyfip1	cytoplasmic FMR1 interacting protein 1 [Source:MGI Symbol;Acc:MGI:1338801]	32.4816	31.9746	30.1034	30.7186	34.2718	32.0084	36.1544	30.6996	30.7445	31.5204	30.7599	31.0142	32.3499	29.4399	0.015647695	0.253395174	4	8	24	0.104593391	0.717030134	24	20.74695782	0.058894172
ENSMUSG00000030451	Herc2	HECT and RLD domain containing E3 ubiquitin protein ligase 2 [Source:MGI Symbol;Acc:MGI:103234]	19.2638	16.6689	18.2684	23.154	19.4819	23.9366	19.347	17.4927	14.2802	20.7409	15.6116	16.9848	16.3665	17.1521	0.718086778	0.966018836	16	20	24	0.280148724	0.768040178	24	17.12853644	0.113053968
ENSMUSG00000030452	Nipa2	non imprinted in Prader-Willi/Angelman syndrome 2 homolog (human) [Source:MGI Symbol;Acc:MGI:1913918]	13.4815	13.2227	12.684	14.064	13.3011	13.5212	11.5468	15.4747	14.5619	14.8975	14.5204	15.089	15.4587	14.0396	0.785001719	0.966018836	16	12	24	0.749506465	0.918524199	24	11.16236936	0.027914633
ENSMUSG00000030465	Psd3	pleckstrin and Sec7 domain containing 3 [Source:MGI Symbol;Acc:MGI:1918215]	16.9275	17.7208	16.762	16.6193	16.8996	15.3538	15.8673	14.5447	16.1808	15.2366	18.175	16.4027	16.7201	17.1666	0.882263435	0.966018836	20	4	24	0.939920511	0.985398871	24	16.60375886	0.011768103
ENSMUSG00000030468	Siglecg	sialic acid binding Ig-like lectin G [Source:MGI Symbol;Acc:MGI:2443630]	0.326964	0.671213	0.869316	0.542898	0.517647	0.628365	0.809215	0.601245	0.774474	0.537009	0.517072	0.654464	0.850366	0.498569	0.849179819	0.966018836	12	8	24	0.596548805	0.85785467	24	5.26869747	0.056434759
ENSMUSG00000030469	Zfp719	zinc finger protein 719 [Source:MGI Symbol;Acc:MGI:2444708]	5.54099	5.58235	5.53115	5.47531	5.82401	5.35308	5.51889	4.44106	5.63398	4.23871	5.72757	5.21728	5.33555	5.52464	0.962703749	0.967965568	20	4	24	0.74662459	0.917587548	24	20.18045912	0.034548043
ENSMUSG00000030470	Csrp3	cysteine and glycine-rich protein 3 [Source:MGI Symbol;Acc:MGI:1330824]	2.23534	7.31622	20.7936	10.3381	9.84604	7.65304	5.50945	19.2546	10.6904	12.79	7.37304	6.3674	10.157	7.1569	0.008024668	0.192760771	12	16	24	0.041666236	0.620283324	24	9.533024724	0.631703023
ENSMUSG00000030471	Zdhhc13	zinc finger, DHHC domain containing 13 [Source:MGI Symbol;Acc:MGI:1919227]	16.4877	16.5394	16.2399	15.5618	15.8648	16.2668	15.1053	16.0482	17.0542	14.2749	17.3227	16.93	18.4158	17.2021	0.850398351	0.966018836	8	8	24	0.37989141	0.799406242	24	0.628242799	0.043000977
ENSMUSG00000030474	Siglece	sialic acid binding Ig-like lectin E [Source:MGI Symbol;Acc:MGI:1932475]	0.634505	0.81163	0.843893	0.541537	0.646808	0.710455	0.918926	0.786742	0.627169	0.496672	0.794612	0.552425	0.889887	0.663015	0.105345531	0.518002936	4	12	24	0.143584259	0.752803875	24	1.42689855	0.082871356
ENSMUSG00000030486	Zfp108	zinc finger protein 108 [Source:MGI Symbol;Acc:MGI:1891198]	1.21632	1.78394	1.63485	1.58068	1.79069	1.47063	1.56377	1.49426	1.7093	1.14244	1.69548	1.56889	1.43704	1.20132	0.648082424	0.966018836	20	8	24	0.735925459	0.913953743	24	14.40661145	0.037109452

ENSMUSG00000030493	Faap24	Fanconi anemia core complex associated protein 24 [Source:MGI Symbol;Acc:MGI:2142208]	3.08419	1.86264	1.91689	1.77887	3.09095	2.72073	2.5151	2.16489	2.21537	2.66255	2.62324	2.69158	2.37773	2.14873	0.019918554	0.259417302	24	8	24	0.013311542	0.529134128	24	19.19070229	0.185892183
ENSMUSG00000030494	Rhpn2	rhophilin, Rho GTPase binding protein 2 [Source:MGI Symbol;Acc:MGI:1289234]	1.61039	2.0114	2.49134	1.63275	1.75264	1.92215	1.88604	1.79984	1.90744	1.76739	1.41822	2.12423	1.35449	1.91821	0.37215823	0.894599261	12	16	24	0.521482028	0.820324319	24	6.710873911	0.065892214
ENSMUSG00000030495	Slc7a10	solute carrier family 7 (cationic amino acid transporter, y+ system), member 10 [Source:MGI Symbol;Acc:MGI:1858261]	1.13518	3.72297	7.54018	2.5908	1.84635	3.67791	5.45493	2.47848	4.92499	3.1608	0.947015	2.32726	1.28608	4.96608	0.059650003	0.406067671	12	8	24	0.120961331	0.732894732	24	6.834545434	0.506583388
ENSMUSG00000030498	Gas2	growth arrest specific 2 [Source:MGI Symbol;Acc:MGI:95657]	8.88488	12.0849	11.2394	10.9075	11.1015	11.0086	10.344	10.4118	12.3861	9.81515	11.6334	11.6582	11.0901	11.4939	0.759899911	0.966018836	12	16	24	0.611912264	0.865342826	24	9.666564006	0.044321669
ENSMUSG00000030499	Kctd15	potassium channel tetramerisation domain containing 15 [Source:MGI Symbol;Acc:MGI:2385276]	8.40422	6.61845	7.04792	8.21791	7.73454	7.31506	7.94361	8.36533	7.25562	9.21207	7.38434	8.0918	8.09273	6.86312	0.745118544	0.966018836	4	4	24	0.596209635	0.857839743	24	16.90191246	0.047732195
ENSMUSG00000030500	Slc17a6	solute carrier family 17 (sodium-dependent inorganic phosphate cotransporter), member 6 [Source:MGI Symbol;Acc:MGI:2156052]	72.7984	64.2018	67.0452	63.2807	64.3585	67.5225	60.0433	58.2208	57.9431	65.1516	66.7446	60.4956	63.1415	65.2519	0.877082091	0.966018836	20	16	24	0.533674204	0.825955545	24	18.75903169	0.037774463
ENSMUSG00000030505	Prmt3	protein arginine N-methyltransferase 3 [Source:MGI Symbol;Acc:MGI:1919224]	18.8013	16.8084	16.4167	17.7887	16.3839	17.7052	16.5022	16.9704	14.7657	18.3005	17.8255	16.9429	18.7116	17.7268	0.894563866	0.966018836	4	8	24	0.43029586	0.805393068	24	20.53074254	0.046234918
ENSMUSG00000030509	Asb7	ankyrin repeat and SOCS box-containing 7 [Source:MGI Symbol;Acc:MGI:2152835]	4.47499	4.54689	4.89785	4.56694	4.43957	4.86346	4.55711	4.2987	4.26917	4.99266	4.3945	4.38114	4.58227	4.75162	0.85387321	0.966018836	16	4	24	0.848251668	0.954264952	24	10.95646902	0.013651675
ENSMUSG00000030512	Snrpa1	small nuclear ribonucleoprotein polypeptide A' [Source:MGI Symbol;Acc:MGI:1916231]	14.9649	15.0174	15.8806	15.2967	13.6883	14.4494	14.2775	15.3548	15.173	14.3868	13.2494	15.2632	14.4943	14.2159	0.10951302	0.524035616	12	8	24	0.131761633	0.742075803	24	5.737594636	0.047604335
ENSMUSG00000030513	Pcsk6	proprotein convertase subtilisin/kexin type 6 [Source:MGI Symbol;Acc:MGI:102897]	5.35397	4.96073	5.08746	6.24765	5.58538	4.72759	6.41933	5.84766	5.64659	6.34171	4.46051	6.06305	5.55576	5.23125	0.90767227	0.966018836	16	4	24	0.906415446	0.97449058	24	8.821139855	0.025324414
ENSMUSG00000030515	Tarsl2	threonyl-tRNA synthetase-like 2 [Source:MGI Symbol;Acc:MGI:2444486]	10.8946	10.4763	11.5419	11.3608	10.2368	11.4318	10.2587	10.6633	10.3441	12.6092	10.1794	11.1554	10.6526	11.7379	0.962855416	0.967965568	16	4	24	0.534844307	0.826752067	24	10.32150196	0.037742093
ENSMUSG00000030516	Tjp1	tight junction protein 1 [Source:MGI Symbol;Acc:MGI:98759]	21.1214	22.6445	21.9024	18.3786	20.6488	19.9892	22.9957	21.2245	22.4389	21.7197	21.0331	22.7429	19.5778	19.6893	0.90332508	0.966018836	12	4	24	0.779685779	0.929226662	24	2.794450847	0.025522103
ENSMUSG00000030518	Fam189a1	family with sequence similarity 189, member A1 [Source:MGI Symbol;Acc:MGI:1917888]	1.51578	1.36419	1.8673	1.35052	1.30156	2.00708	1.35731	1.69642	1.76514	1.69665	1.32584	1.51694	0.885401	1.49422	0.321662653	0.83607295	12	8	24	0.611593043	0.865117073	24	9.756703452	0.066536248
ENSMUSG00000030519	Apba2	amyloid beta (A4) precursor protein-binding, family A, member 2 [Source:MGI Symbol;Acc:MGI:1261791]	12.816	11.3586	15.3763	11.0314	10.581	13.9992	11.8818	13.1939	11.3693	15.543	10.6257	13.1107	11.5925	13.8856	0.95032543	0.966018836	24	20	24	0.689173113	0.897070579	24	5.986688845	0.061331431
ENSMUSG00000030521	Mphosph10	M-phase phosphoprotein 10 (U3 small nucleolar ribonucleoprotein) [Source:MGI Symbol;Acc:MGI:1915223]	10.8423	11.8184	12.0385	11.5759	12.1436	10.6692	10.6784	11.1956	12.6112	9.88071	13.5111	11.4135	12.7412	12.5321	0.960333395	0.967043964	12	12	24	0.826989369	0.946041107	24	8.914664026	0.0279386
ENSMUSG00000030522	Mtmr10	myotubularin related protein 10 [Source:MGI Symbol;Acc:MGI:2142292]	5.57806	5.5078	6.26479	5.62574	5.28901	5.00327	5.49192	5.18239	6.537	5.3828	5.24016	5.20765	5.02248	5.20426	0.01130565	0.204837764	12	12	24	0.033150345	0.606585237	24	8.578780425	0.083995954
ENSMUSG00000030525	Chrna7	cholinergic receptor, nicotinic, alpha polypeptide 7 [Source:MGI Symbol;Acc:MGI:99779]	17.3159	13.9534	13.7933	14.3183	15.2639	14.9085	14.0533	14.4738	12.8807	15.9205	15.9101	15.6454	14.9741	16.127	0.275526571	0.789849983	20	16	24	0.221660234	0.768040178	24	19.67272588	0.071184057
ENSMUSG00000030527	Crtc3	CREB regulated transcription coactivator 3 [Source:MGI Symbol;Acc:MGI:1917711]	6.2179	5.93652	5.95044	6.6278	7.17363	5.79304	6.18953	6.29296	6.98852	7.64773	6.40172	6.22184	6.43262	5.67333	0.046535188	0.375011045	16	16	24	0.050715781	0.644469829	24	12.97829283	0.090993699
ENSMUSG00000030528	Blm	Bloom syndrome, RecQ like helicase [Source:MGI Symbol;Acc:MGI:1328362]	1.99287	3.11414	2.13366	3.61535	2.92424	3.05106	2.41194	3.20072	2.8845	2.93048	2.90222	2.96158	3.17636	3.14089	0.942051135	0.966018836	8	4	24	0.620728311	0.870297169	24	12.05349485	0.060294218
ENSMUSG00000030530	Furin	furin (paired basic amino acid cleaving enzyme) [Source:MGI Symbol;Acc:MGI:97513]	5.91788	5.66879	5.50958	6.42571	5.55914	5.4621	5.61543	6.34941	5.90942	7.42571	6.10639	6.25557	6.64328	5.87675	0.873567285	0.966018836	16	20	24	0.492084471	0.805393068	24	11.32932348	0.045238438
ENSMUSG00000030532	Hddc3	HD domain containing 3 [Source:MGI Symbol;Acc:MGI:1915945]	5.42084	10.1769	9.35062	6.56114	6.79894	6.74514	10.1963	9.09539	8.44278	6.02419	6.8244	7.30239	10.0253	6.73274	0.387380622	0.915479136	4	12	24	0.139716691	0.74956518	24	3.779559006	0.199223566
ENSMUSG00000030533	Unc45a	unc-45 myosin chaperone A [Source:MGI Symbol;Acc:MGI:2142246]	18.2438	16.1467	15.7918	15.1332	15.7231	17.4224	15.439	19.1918	13.8597	21.0219	16.4224	17.3031	16.3558	17.7648	0.965521737	0.969272194	8	4	24	0.910148195	0.974669919	24	22.42244717	0.025597948
ENSMUSG00000030534	Vps33b	vacuolar protein sorting 33B [Source:MGI Symbol;Acc:MGI:2446237]	10.604	10.5506	10.5362	10.321	10.5727	10.5074	9.63314	8.91224	9.13222	9.65339	9.6179	9.77854	8.99653	9.279	0.834424969	0.966018836	24	8	24	0.049823354	0.643621507	24	17.15681633	0.046376887
ENSMUSG00000030536	Iqgap1	IQ motif containing GTPase activating protein 1 [Source:MGI Symbol;Acc:MGI:1352757]	12.1239	12.874	12.7109	12.2583	13.3559	11.8068	12.6176	11.5752	12.9877	12.2126	13.0255	12.8453	13.0378	11.8533	0.702630284	0.966018836	20	12	24	0.553230563	0.837286821	24	15.5871437	0.025265972
ENSMUSG00000030538	Cib1	calcium and integrin binding 1 (calmyrin) [Source:MGI Symbol;Acc:MGI:1344418]	17.7012	16.7047	19.5566	20.1426	18.0191	17.871	18.9752	17.4398	17.9189	16.5071	18.3304	18.7742	17.8262	17.9865	0.644063931	0.966018836	20	12	24	0.737290547	0.91440753	24	14.9501048	0.023029368
ENSMUSG00000030539	Sema4b	sema domain, immunoglobulin domain (Ig), transmembrane domain (TM) and short cytoplasmic domain, (semaphorin) 4B [Source:MGI Symbol;Acc:MGI:107559]	8.26297	7.28788	6.82078	7.85814	7.33596	7.48344	7.65053	8.08552	6.73718	9.7702	7.03209	6.99598	7.70704	7.062	0.643325306	0.966018836	4	8	24	0.943914649	0.986212398	24	14.76239391	0.016959564
ENSMUSG00000030541	Idh2	isocitrate dehydrogenase 2 (NADP+), mitochondrial [Source:MGI Symbol;Acc:MGI:96414]	16.3561	21.4029	27.3696	30.8715	22.365	22.1431	21.6908	29.1722	26.603	30.8474	21.4286	21.8872	26.6094	26.6167	0.082685511	0.462922111	16	12	24	0.006207753	0.469371824	24	9.373673737	0.253243138
ENSMUSG00000030545	Pex11a	peroxisomal biogenesis factor 11 alpha [Source:MGI Symbol;Acc:MGI:1338788]	4.84105	4.46924	4.63822	5.25484	4.54262	4.66629	5.53124	6.32506	4.46201	6.01194	5.22323	4.97036	6.17754	5.83048	0.893609703	0.966018836	8	4	24	0.584040439	0.85204429	24	3.861210134	0.05054986
ENSMUSG00000030546	Plin1	perilipin 1 [Source:MGI Symbol;Acc:MGI:1890505]	3.45151	1.78281	5.26357	12.7673	3.69562	3.82276	4.03612	9.53931	3.96944	12.6886	10.7818	7.43483	13.7205	10.6692	0.892555977	0.966018836	16	16	24	0.337664642	0.772537663	24	11.76488129	0.333614678
ENSMUSG00000030551	Nr2f2	nuclear receptor subfamily 2, group F, member 2 [Source:MGI Symbol;Acc:MGI:1352452]	19.5131	18.6037	16.2931	17.0823	20.4307	16.1406	19.4327	18.5006	18.4324	17.4199	20.174	15.9289	16.3652	14.613	0.885025617	0.966018836	20	16	24	0.628285721	0.872795898	24	17.10463266	0.048956099
ENSMUSG00000030554	Synm	synemin, intermediate filament protein [Source:MGI Symbol;Acc:MGI:2661187]	67.4634	52.9765	52.2226	57.7553	54.7596	61.5158	54.2661	60.872	46.702	72.0843	54.6489	58.5881	60.1078	58.6113	0.390686537	0.915479136	24	12	24	0.615694441	0.867203276	24	20.01798174	0.063356846
ENSMUSG00000030555	Ttc23	tetratricopeptide repeat domain 23 [Source:MGI Symbol;Acc:MGI:1914259]	4.34114	6.39911	5.58494	5.59898	6.12611	5.12187	6.04714	7.42426	6.01424	5.26145	5.35225	6.05698	4.76303	6.55678	0.125573493	0.555618842	8	20	24	0.316631827	0.768040178	24	6.747631633	0.101700822
ENSMUSG00000030556	Lrrc28	leucine rich repeat containing 28 [Source:MGI Symbol;Acc:MGI:1915689]	13.1131	10.6211	12.9545	9.5882	8.55937	13.0531	12.6012	13.3854	9.07146	11.5071	11.6862	11.6456	11.7163	12.9902	0.14635779	0.590732968	8	8	24	0.112834805	0.727075283	24	0.877338091	0.147099108
ENSMUSG00000030557	Mef2a	myocyte enhancer factor 2A [Source:MGI Symbol;Acc:MGI:99532]	34.3951	34.2131	32.4721	33.8795	30.6185	31.4208	30.6464	33.3203	30.8814	35.64	30.5198	31.8585	32.5813	34.2199	0.105345531	0.518002936	8	12	24	0.305920937	0.768040178	24	6.32862087	0.043978098
ENSMUSG00000030559	Rab38	RAB38, member RAS oncogene family [Source:MGI Symbol;Acc:MGI:1919683]	2.43911	2.63874	2.16582	1.77522	2.59119	1.98129	2.60295	2.18943	1.89864	1.48142	2.28143	1.90711	3.13051	2.12296	0.0043709	0.140598674	4	12	24	0.055129789	0.65534544	24	23.79851453	0.163826522
ENSMUSG00000030560	Ctsc	cathepsin C [Source:MGI Symbol;Acc:MGI:109553]	16.0238	15.393	14.8222	12.4355	15.1901	13.0749	15.3887	15.4748	14.6253	10.292	15.2595	12.193	13.0521	11.8276	0.522551072	0.966018836	4	12	24	0.385874494	0.799546792	24	0.517438279	0.080740236
ENSMUSG00000030562	Nox4	NADPH oxidase 4 [Source:MGI Symbol;Acc:MGI:1354184]	0.694964	1.13185	1.03165	1.03194	1.16422	0.814968	0.809502	0.941886	1.26061	0.82295	1.16904	0.97958	0.906937	1.05964	0.040123816	0.34094463	12	16	24	0.133493016	0.743843563	24	10.40389453	0.09129106
ENSMUSG00000030577	Cd22	CD22 antigen [Source:MGI Symbol;Acc:MGI:88322]	0.505056	0.57761	0.208407	0.63922	0.477571	0.38289	0.281284	0.317966	0.843274	0.272054	0.236671	0.681629	0.937698	0.755985	0.904387595	0.966018836	4	16	24	0.611353212	0.865079333	24	2.785361967	0.083413285
ENSMUSG00000030579	Tyrobp	TYRO protein tyrosine kinase binding protein [Source:MGI Symbol;Acc:MGI:1277211]	18.0252	35.6506	41.6072	29.4706	31.8786	26.8878	25.1326	27.2637	33.4577	14.9598	29.3385	26.1655	26.5456	23.8004	0.060394272	0.407012001	12	16	24	0.717679566	0.90689011	24	8.458926964	0.119885195
ENSMUSG00000030583	Sipa1l3	signal-induced proliferation-associated 1 like 3 [Source:MGI Symbol;Acc:MGI:1921456]	3.18374	3.48818	2.62601	3.37703	2.90749	2.6334	3.32904	2.61357	3.66374	4.64779	3.32219	3.24083	3.88171	3.02218	0.935593607	0.966018836	16	16	24	0.674781969	0.89097199	24	11.25141074	0.061145312
ENSMUSG00000030584	Dpf1	D4, zinc and double PHD fingers family 1 [Source:MGI Symbol;Acc:MGI:1352748]	2.20659	1.94395	2.4014	3.01711	2.16319	2.13517	3.00596	2.31331	2.46076	2.54199	1.65125	2.20785	2.07996	3.10501	0.265774856	0.773510818	16	4	24	0.375587317	0.795692691	24	6.900382864	0.10164719
ENSMUSG00000030587	2200002D01Rik	RIKEN cDNA 2200002D01 gene [Source:MGI Symbol;Acc:MGI:1919525]	1.63732	1.48435	1.70173	1.26408	1.24171	1.61835	1.52104	2.13563	2.67739	1.27154	1.49401	2.01009	2.32964	2.11147	0.112583132	0.530777648	12	4	24	0.035292649	0.608551791	24	3.676331209	0.175387336
ENSMUSG00000030588	Yif1b	Yip1 interacting factor homolog B (S. cerevisiae) [Source:MGI Symbol;Acc:MGI:1924504]	24.6207	21.9056	20.6841	22.7087	22.7105	22.923	21.9871	27.064	23.7393	25.4803	21.7207	24.8695	25.9911	25.2125	0.869721097	0.966018836	8	4	24	0.45405426	0.805393068	24	2.259257233	0.045949392
ENSMUSG00000030589	Rasgrp4	RAS guanyl releasing protein 4 [Source:MGI Symbol;Acc:MGI:2386851]	1.07109	1.17061	2.27312	1.2259	0.935421	1.29949	1.16877	0.943037	1.39368	0.779589	0.940669	1.16877	0.961498	0.969132	0.745118544	0.966018836	12	8	24	0.585106539	0.852625163	24	7.437263454	0.082135084
ENSMUSG00000030590	Fam98c	family with sequence similarity 98, member C [Source:MGI Symbol;Acc:MGI:1921083]	9.74293	9.66186	10.3182	9.05748	9.11927	10.2002	9.11495	8.93155	9.24687	10.2715	9.4076	11.0178	9.78314	9.57744	0.83638114	0.966018836	24	8	24	0.698726006	0.899821516	24	19.21642904	0.027916516
ENSMUSG00000030591	Psmd8	proteasome (prosome, macropain) 26S subunit, non-ATPase, 8 [Source:MGI Symbol;Acc:MGI:1888669]	70.5926	68.2414	70.5196	65.8047	71.9207	76.1302	66.9643	73.6886	71.5483	64.7541	71.9003	67.2722	72.3059	75.0796	0.622773557	0.966018836	8	8	24	0.534924432	0.826778911	24	0.595015594	0.030229885
ENSMUSG00000030592	Ryr1	ryanodine receptor 1, skeletal muscle [Source:MGI Symbol;Acc:MGI:99659]	1.14979	2.75039	3.2694	4.36576	3.06813	3.85787	2.82795	9.97902	5.90699	11.5198	2.57885	3.07808	5.0045	3.3339	0.541384199	0.966018836	16	4	24	0.122864952	0.735482095	24	9.378815587	0.481918555
ENSMUSG00000030595	Nfkbib	nuclear factor of kappa light polypeptide gene enhancer in B cells inhibitor, beta [Source:MGI Symbol;Acc:MGI:104752]	13.3877	10.972	12.2175	10.1594	11.0724	11.3916	11.5408	13.4223	14.5805	16.1448	13.7174	15.0058	14.3971	14.3021	0.722624967	0.966018836	12	8	24	0.757954053	0.921733004	24	6.223161056	0.041740205
ENSMUSG00000030598	Fbxo17	F-box protein 17 [Source:MGI Symbol;Acc:MGI:1354707]	1.27229	1.22137	1.5883	0.982277	1.08471	1.31664	1.042	1.80296	1.50338	1.37145	1.33804	1.30128	1.53219	1.51242	0.399942636	0.932395417	8	16	24	0.096389669	0.71175955	24	5.413209689	0.105062475
ENSMUSG00000030600	Lrfn1	leucine rich repeat and fibronectin type III domain containing 1 [Source:MGI Symbol;Acc:MGI:2136810]	6.19707	4.02265	3.79279	4.09178	3.66074	4.9957	4.18188	5.88591	4.69068	7.99935	4.46464	5.9094	6.34081	5.36464	0.625825704	0.966018836	4	16	24	0.798433684	0.935424343	24	0.495897512	0.066613566
ENSMUSG00000030602	Pak4	p21 (RAC1) activated kinase 4 [Source:MGI Symbol;Acc:MGI:1917834]	4.1719	3.9721	3.6652	3.25796	3.59174	3.62769	3.7882	4.02876	4.12087	4.45032	4.32281	4.79736	4.83294	3.9296	0.933543173	0.966018836	4	12	24	0.475834964	0.805393068	24	23.22647061	0.050270731
ENSMUSG00000030603	Psmc4	proteasome (prosome, macropain) 26S subunit, ATPase, 4 [Source:MGI Symbol;Acc:MGI:1346093]	72.4787	66.1723	70.0688	66.3911	67.3798	75.7266	65.0728	68.1457	58.3461	69.7789	66.4779	66.9153	65.5332	68.6107	0.846643334	0.966018836	24	4	24	0.34586565	0.778673045	24	20.13664912	0.047902618
ENSMUSG00000030604	Zfp626	zinc finger protein 626 [Source:MGI Symbol;Acc:MGI:1918413]	3.69747	4.61625	4.57522	4.22806	3.81678	3.88935	4.15739	4.3879	4.77816	3.54353	4.39958	4.26555	3.99584	4.60514	0.084943671	0.466404237	12	4	24	0.093117021	0.71175955	24	6.402009368	0.087278937
ENSMUSG00000030605	Mfge8	milk fat globule EGF and factor V/VIII domain containing [Source:MGI Symbol;Acc:MGI:102768]	8.58269	9.1998	10.0538	9.6423	9.89153	9.2792	9.42398	12.0132	13.3523	11.0043	9.35051	8.91855	9.5568	9.90428	0.009630739	0.194720062	12	12	24	0.019776156	0.565108098	24	8.520254096	0.155569684
ENSMUSG00000030606	Hapln3	hyaluronan and proteoglycan link protein 3 [Source:MGI Symbol;Acc:MGI:1914916]	1.15794	0.725273	1.35702	0.944465	0.653363	0.92298	0.689715	1.0032	1.01724	1.07135	1.03662	1.21756	0.854343	0.973212	0.886952527	0.966018836	12	16	24	0.694733276	0.898526856	24	9.637453948	0.05195251
ENSMUSG00000030609	Aen	apoptosis enhancing nuclease [Source:MGI Symbol;Acc:MGI:1915298]	12.9852	10.9837	11.8078	10.9671	11.6956	11.8788	12.28	11.3697	9.25201	13.3953	11.8863	11.3083	11.2051	11.0539	0.467283192	0.966018836	4	4	24	0.328660293	0.768040178	24	19.15099001	0.068571708
ENSMUSG00000030610	Det1	de-etiolated homolog 1 (Arabidopsis) [Source:MGI Symbol;Acc:MGI:1923625]	3.77122	3.62035	3.45881	3.72966	3.68847	3.75454	4.12645	4.2859	3.95876	4.28465	3.85669	3.75134	[illegible]	3.7418	0.274283096	0.789649983	20	12	24	0.229101454	0.768040178	24	15.91226376	0.048061541
ENSMUSG00000030611	Mrps11	mitochondrial ribosomal protein S11 [Source:MGI Symbol;Acc:MGI:1915244]	17.3422	18.553	16.8353	18.787	17.6302	17.9548	17.5057	18.0728	19.3415	18.8466	18.5012	17.7527	19.9172	17.7678	0.79256873	0.966018836	8	16	24	0.620470816	0.870243288	24	0.807593508	0.036430122
ENSMUSG00000030612	Mrpl46	mitochondrial ribosomal protein L46 [Source:MGI Symbol;Acc:MGI:1914558]	22.6115	23.0025	23.6603	22.1212	23.3561	21.6655	20.2142	22.4497	23.4438	20.5729	24.3106	23.6478	24.9084	23.0673	0.882263435	0.966018836	12	4	24	0.995733873	0.998816465	24	4.753527746	0.003013222
ENSMUSG00000030613	Ccdc90b	coiled-coil domain containing 90B [Source:MGI Symbol;Acc:MGI:1913615]	14.1452	14.7868	14.4359	13.7619	13.2176	12.126	13.3257	12.8699	14.6632	12.0907	14.6536	13.8322	13.3996	13.8176	0.913451828	0.966018836	12	4	24	0.574114516	0.847226836	24	6.242438378	0.037372684
ENSMUSG00000030614	Tmem126b	transmembrane protein 126B [Source:MGI Symbol;Acc:MGI:1915722]	27.8387	28.0071	27.0381	27.2195	27.6219	26.6895	27.8784	27.4192	29.6991	23.5595	29.4371	28.6251	29.9311	29.6563	0.691437565	0.966018836	4	12	24	0.33719455	0.772183698	24	1.045300468	0.045740177
ENSMUSG00000030615	Tmem126a	transmembrane protein 126A [Source:MGI Symbol;Acc:MGI:1913521]	21.2434	27.9595	25.1621	28.3092	24.6543	22.594	25.6652	28.1002	29.4624	22.854	24.2362	25.2967	26.6049	26.2472	0.125343232	0.555618842	12	8	24	0.084277635	0.702277994	24	6.985658307	0.109771794
ENSMUSG00000030616	Sytl2	synaptotagmin-like 2 [Source:MGI Symbol;Acc:MGI:1933366]	53.3798	50.9142	46.7794	49.8272	50.7193	50.7934	47.6819	48.2779	47.1537	51.0083	53.5186	49.5966	53.2075	54.2216	0.658895034	0.966018836	20	16	24	0.453612084	0.805393068	24	20.52184055	0.034077435
ENSMUSG00000030619	Eed	embryonic ectoderm development [Source:MGI Symbol;Acc:MGI:95286]	16.7176	17.6954	16.9913	19.9162	18.4155	16.4879	18.2063	18.1362	19.3095	15.0484	16.787	16.7464	16.4673	17.2556	0.588682825	0.966018836	12	12	24	0.632758197	0.875112738	24	8.190964936	0.037893672
ENSMUSG00000030621	Me3	malic enzyme 3, NADP(+)-dependent, mitochondrial [Source:MGI Symbol;Acc:MGI:1916679]	4.57339	6.09539	8.39847	6.94839	5.34092	6.36755	5.87997	6.78656	7.65633	9.23483	5.69582	5.49402	6.39923	7.13318	0.007331665	0.192760771	16	4	24	0.002493697	0.39630666	24	9.055763477	0.261260625
ENSMUSG00000030629	Zfand6	zinc finger, AN1-type domain 6 [Source:MGI Symbol;Acc:MGI:1929510]	77.2753	77.8654	67.7991	80.3102	78.3727	72.1425	78.949	82.8003	76.9326	67.7575	87.5362	74.4938	77.9542	76.1449	0.906657897	0.966018836	20	16	24	0.789246496	0.932388659	24	21.4195331	0.028283113
ENSMUSG00000030630	Fah	fumarylacetoacetate hydrolase [Source:MGI Symbol;Acc:MGI:95482]	20.0203	22.3663	20.1081	19.5757	20.761	20.1195	18.496	18.3358	15.7327	17.2955	18.7748	17.3868	18.8172	19.388	0.831272156	0.966018836	20	20	24	0.544701397	0.832363403	24	22.26040427	0.043542903
ENSMUSG00000030638	Sh3gl3	SH3-domain GRB2-like 3 [Source:MGI Symbol;Acc:MGI:700011]	6.25016	6.88734	7.26136	6.57688	5.91363	6.61783	6.96651	6.31862	7.47729	6.28532	5.54456	6.58028	6.2171	7.14396	0.005593158	0.153935753	12	8	24	0.043423022	0.626787045	24	5.628077297	0.093657575
ENSMUSG00000030641	Ddias	DNA damage-induced apoptosis suppressor [Source:MGI Symbol;Acc:MGI:1921291]	0.78548	0.956084	0.975232	0.821827	0.869452	0.813833	0.810413	0.795258	0.932635	0.860424	0.907633	1.08214	1.0628	0.861732	0.879206539	0.966018836	12	16	24	0.999176232	0.999770694	24	2.484426601	0.001081987
ENSMUSG00000030643	Rab30	RAB30, member RAS oncogene family [Source:MGI Symbol;Acc:MGI:1923235]	7.38727	8.5511	8.38722	8.09224	7.8857	7.34085	9.07445	7.25144	9.20103	6.53413	9.11038	9.00507	8.11359	7.39309	0.873350015	0.966018836	12	20	24	0.964170072	0.99108119	24	20.36731607	0.015452782
ENSMUSG00000030647	Ndufc2	NADH:ubiquinone oxidoreductase subunit C2 [Source:MGI Symbol;Acc:MGI:1344370]	138.965	150.684	154.958	148.514	121.268	120.765	141.424	140.353	168.941	137.788	169.322	149.695	164.228	163.519	0.833406028	0.966018836	12	12	24	0.130491378	0.740728512	24	6.751111762	0.108724301
ENSMUSG00000030649	Anapc15	anaphase promoting complex C subunit 15 [Source:MGI Symbol;Acc:MGI:1922680]	12.4773	14.0695	11.532	13.4211	12.3062	13.8846	15.7074	13.5528	13.0995	12.6695	15.5053	13.4339	13.9226	13.4024	0.441501717	0.966018836	4	8	24	0.353648142	0.780975743	24	21.76858253	0.06115796
ENSMUSG00000030650	Tmc5	transmembrane channel-like gene family 5 [Source:MGI Symbol;Acc:MGI:1921674]	0.757303	0.558282	0.894614	0.536936	0.787576	0.633177	0.602976	0.633487	0.442294	0.565967	0.885609	0.63302	0.849425	0.641979	0.94791825	0.966018836	20	20	24	0.519261874	0.819193768	24	19.5869599	0.056552646
ENSMUSG00000030652	Coq7	demethyl-Q 7 [Source:MGI Symbol;Acc:MGI:107207]	26.067	27.5504	27.9669	29.5069	22.1942	24.9077	25.4844	27.6546	26.8464	28.3518	27.9523	26.0015	28.4467	27.4783	0.064422003	0.414867207	16	8	24	0.113317136	0.727393008	24	7.54494943	0.079013065
ENSMUSG00000030654	Arl6ip1	ADP-ribosylation factor-like 6 interacting protein 1 [Source:MGI Symbol;Acc:MGI:1858943]	221.02	216.939	208.72	206.974	211.467	211.135	202.317	202.386	212.792	193.764	231.326	215.27	226.233	220.086	0.581070934	0.966018836	4	12	24	0.456963745	0.805393068	24	21.9618245	0.03364335
ENSMUSG00000030655	Smg1	SMG1 nonsense mediated mRNA decay associated PI3K related kinase [Source:MGI Symbol;Acc:MGI:1919742]	13.0357	13.2544	13.7129	15.1884	16.4971	13.073	13.8328	8.70414	13.0055	9.26539	12.492	12.3257	13.4075	13.3959	0.807813307	0.966018836	4	4	24	0.623790234	0.87183699	24	18.22614974	0.085506131
ENSMUSG00000030657	Xylt1	xylosyltransferase 1 [Source:MGI Symbol;Acc:MGI:2451073]	0.477578	0.702542	0.845529	0.696623	0.620522	0.723134	0.868207	0.74329	0.581175	0.68374	0.540438	0.596728	0.551731	0.743887	0.700065774	0.966018836	8	12	24	0.450602004	0.805393068	24	6.483889417	0.05030432
ENSMUSG00000030659	Nucb2	nucleobindin 2 [Source:MGI Symbol;Acc:MGI:1858179]	7.42842	9.52805	8.8495	7.61615	7.82333	7.61602	7.72895	8.59235	9.9915	7.69274	8.53594	8.5662	10.0929	8.60355	0.23919737	0.741580974	12	4	24	0.12999022	0.740483001	24	5.482764174	0.09622131
ENSMUSG00000030660	Pik3c2a	phosphatidylinositol-4-phosphate 3-kinase catalytic subunit type 2 alpha [Source:MGI Symbol;Acc:MGI:1203729]	11.0902	13.4895	13.3718	13.7839	12.895	10.8406	11.7493	11.8535	13.1318	10.8743	13.329	11.1273	11.5307	11.9513	0.020119233	0.259417302	12	12	24	0.091564543	0.71175955	24	9.959597118	0.092279871
ENSMUSG00000030662	Ipo5	importin 5 [Source:MGI Symbol;Acc:MGI:1917822]	28.5822	26.8612	28.4844	29.7835	28.8361	29.2803	26.9263	28.7068	26.6431	29.351	28.5582	27.6297	27.5344	27.7891	0.104170975	0.518002936	16	20	24	0.078558871	0.689042744	24	14.80028018	0.039185083
ENSMUSG00000030663	1110004F10Rik	RIKEN cDNA 1110004F10 gene [Source:MGI Symbol;Acc:MGI:1929274]	58.3145	58.5114	57.0257	54.876	53.7267	58.6927	53.0246	54.6825	55.8752	61.3395	61.487	57.8866	57.7899	56.2525	0.85447369	0.966018836	16	20	24	0.682284595	0.894159472	24	15.60015853	0.021815264
ENSMUSG00000030666	Calcb	calcitonin-related polypeptide, beta [Source:MGI Symbol;Acc:MGI:2151254]	156.46	140.548	134.96	117.011	137.737	148.913	133.8	146.344	124.049	105.185	147.439	137.559	155.634	134.993	0.016720774	0.253395174	4	12	24	0.008291306	0.49701405	24	22.89702826	0.164458718
ENSMUSG00000030669	Calca	calcitonin/calcitonin-related polypeptide, alpha [Source:MGI Symbol;Acc:MGI:2151253]	1468.32	1293.85	1368.24	1234.49	1471.36	1509.26	1235.81	1279.31	1115.24	1019.12	1526.13	1208.88	1252.45	1194.62	0.265774856	0.773510818	20	20	24	0.076234654	0.686372559	24	19.77926879	0.133954376
ENSMUSG00000030670	Cyp2r1	cytochrome P450, family 2, subfamily r, polypeptide 1 [Source:MGI Symbol;Acc:MGI:2449771]	0.324614	0.589239	0.518287	0.388409	0.523555	0.696158	0.336758	0.291601	0.713053	0.392784	0.704642	0.552464	0.427889	0.64101	0.718086778	0.966018836	24	4	24	0.67060896	0.888925819	24	13.69780109	0.04872987
ENSMUSG00000030671	Pde3b	phosphodiesterase 3B, cGMP-inhibited [Source:MGI Symbol;Acc:MGI:1333863]	4.74358	3.77398	4.98364	5.8105	4.62034	4.92988	4.23305	4.38462	4.07594	4.58533	5.05114	4.10101	5.45148	4.96552	0.881718085	0.966018836	16	16	24	0.573733578	0.847005958	24	14.64748158	0.058266524
ENSMUSG00000030672	Mylpf	myosin light chain, phosphorylatable, fast skeletal muscle [Source:MGI Symbol;Acc:MGI:97273]	109.623	384.145	399.972	490.701	305.788	429.03	288.374	760.75	806.716	560.875	314.434	346.744	413.54	277.233	0.203788871	0.689899499	12	16	24	0.083027083	0.699403344	24	9.50597252	0.571766866
ENSMUSG00000030674	Qprt	quinolinate phosphoribosyltransferase [Source:MGI Symbol;Acc:MGI:1914625]	1.65827	1.76346	1.41628	1.57276	1.48435	1.35343	1.53923	2.03716	2.42877	1.55422	1.739	1.90638	1.84025	1.99067	0.403251286	0.936219016	8	12	24	0.100083232	0.71175955	24	5.487104471	0.104900282
ENSMUSG00000030677	Kif22	kinesin family member 22 [Source:MGI Symbol;Acc:MGI:109233]	3.95266	5.25758	5.59343	5.77085	6.1158	6.26399	5.61807	5.19491	4.94643	4.27798	6.25277	4.05285	4.00282	5.82523	0.5169953	0.966018836	20	8	24	0.56334001	0.842513738	24	13.06418999	0.084934863
ENSMUSG00000030678	Maz	MYC-associated zinc finger protein (purine-binding transcription factor) [Source:MGI Symbol;Acc:MGI:1338823]	41.7558	37.7572	37.4673	40.7872	44.5403	42.1832	41.4169	41.165	38.6028	42.0942	39.2099	42.2192	43.3815	40.6025	0.004516345	0.140598674	24	12	24	0.052956035	0.650879532	24	19.41229569	0.062712037
ENSMUSG00000030680	Pagr1a	PAXIP1 associated glutamate rich protein 1A [Source:MGI Symbol;Acc:MGI:1914528]	11.0065	10.2512	3.38632	9.00441	11.2507	11.4694	7.03543	9.5299	1.54259	6.20857	1.48619	5.09859	0.125128	1.9701	0.911685024	0.966018836	24	12	24	0.514848319	0.816966796	24	18.72807973	0.3745237
ENSMUSG00000030681	Mvp	major vault protein [Source:MGI Symbol;Acc:MGI:1925638]	18.4141	15.7999	14.9738	14.816	18.1646	16.836	16.3187	18.5807	15.4301	18.5905	16.0403	16.434	16.5043	15.6443	0.483374602	0.966018836	24	12	24	0.496819645	0.807576732	24	20.39657434	0.052454218
ENSMUSG00000030682	Cdipt	CDP-diacylglycerol--inositol 3-phosphatidyltransferase (phosphatidylinositol synthase) [Source:MGI Symbol;Acc:MGI:105491]	102.004	78.7626	82.3244	82.7991	80.8878	95.9708	84.4741	90.7571	69.3977	99.0156	84.9079	92.6661	91.8276	90.5774	0.071575474	0.440262583	24	12	24	0.212367983	0.768040178	24	21.17859769	0.100403466
ENSMUSG00000030683	Sez6l2	seizure related 6 homolog like 2 [Source:MGI Symbol;Acc:MGI:2385295]	25.6861	26.1259	32.5026	27.3808	25.212	31.0691	30.6053	25.2596	23.6094	31.5626	22.4136	27.1379	20.6255	27.3781	0.928780444	0.966018836	16	4	24	0.872140034	0.963835087	24	11.25636729	0.037553405
ENSMUSG00000030685	Kctd13	potassium channel tetramerisation domain containing 13 [Source:MGI Symbol;Acc:MGI:1923739]	11.389	11.6943	11.563	11.948	8.69795	13.6029	12.8432	10.0293	10.9156	12.6089	9.75008	11.6328	9.42268	11.3428	0.921572192	0.966018836	24	20	24	0.945927818	0.986887534	24	2.195757266	0.021737573
ENSMUSG00000030688	Stard10	START domain containing 10 [Source:MGI Symbol;Acc:MGI:1860093]	38.1781	33.3514	35.813	32.8501	31.0935	33.1033	33.6874	35.1919	31.329	40.1999	36.5826	36.8073	34.6762	36.5075	0.838692453	0.966018836	4	8	24	0.982718041	0.996149474	24	1.620606524	0.007526002
ENSMUSG00000030689	Ino80e	INO80 complex subunit E [Source:MGI Symbol;Acc:MGI:2141881]	15.8075	12.0964	10.602	13.4358	12.0928	12.4371	12.6464	12.6597	13.345	13.8562	11.6717	14.1196	13.0152	11.727	0.836635326	0.966018836	4	4	24	0.570022371	0.845005229	24	20.94116213	0.057315992
ENSMUSG00000030691	Fchsd2	FCH and double SH3 domains 2 [Source:MGI Symbol;Acc:MGI:2448475]	13.6899	11.3872	10.9025	10.6	10.4118	11.9873	12.1264	11.6862	9.40827	13.0931	10.7502	12.0829	11.2411	12.2029	0.188783717	0.664348865	4	16	24	0.209806383	0.768040178	24	22.95190146	0.088038368
ENSMUSG00000030695	Aldoa	aldolase A, fructose-bisphosphate [Source:MGI Symbol;Acc:MGI:87994]	929.711	857.127	959.451	1037.45	886.494	1130.94	964.147	1390.02	978.378	1313.75	907.778	988.277	1041.27	924.885	0.90332508	0.966018836	16	4	24	0.841572412	0.952008406	24	9.190719542	0.049364101
ENSMUSG00000030697	Ppp4c	protein phosphatase 4, catalytic subunit [Source:MGI Symbol;Acc:MGI:1891763]	28.7409	28.1052	27.1933	33.1679	31.3877	26.6748	27.1679	31.8066	27.9078	25.4939	30.4855	28.1451	28.842	26.3144	0.841589986	0.966018836	20	16	24	0.651205275	0.882725346	24	15.11714481	0.038544845
ENSMUSG00000030699	Tbx6	T-box 6 [Source:MGI Symbol;Acc:MGI:102539]	1.2292	0.657553	1.04646	1.18463	1.14116	0.872918	1.02639	0.88734	1.16382	0.726249	1.22397	0.787611	0.67347	0.518451	0.192357053	0.669841558	20	12	24	0.176157691	0.768040178	24	15.1671284	0.110835912
ENSMUSG00000030701	Plekhb1	pleckstrin homology domain containing, family B (evectins) member 1 [Source:MGI Symbol;Acc:MGI:1351469]	275.061	329.276	304.974	282.697	326.82	276.711	343.208	288.536	359.546	255.44	275.279	301.3	293.586	279.116	0.932092277	0.966018836	12	24	24	0.679566212	0.892710167	24	4.152569464	0.048875631
ENSMUSG00000030703	Gdpd3	glycerophosphodiester phosphodiesterase domain containing 3 [Source:MGI Symbol;Acc:MGI:1915866]	9.29305	8.68182	7.6165	10.9831	9.48569	8.59322	8.84601	8.03987	11.485	8.48576	8.73384	8.66043	10.0913	9.65056	0.819898687	0.966018836	4	20	24	0.834827258	0.949290995	24	10.50177898	0.036411827
ENSMUSG00000030704	Rab6a	RAB6A, member RAS oncogene family [Source:MGI Symbol;Acc:MGI:894313]	208.988	181.124	182.38	177.723	178.217	192.356	185.232	175.856	146.168	194.87	178.796	173.496	171.006	185.122	0.858514388	0.966018836	4	8	24	0.351599986	0.780345856	24	20.88312277	0.062944257
ENSMUSG00000030706	Mrpl48	mitochondrial ribosomal protein L48 [Source:MGI Symbol;Acc:MGI:1289321]	14.3478	15.5862	15.1433	16.5709	17.4142	14.859	15.8463	15.7977	16.6425	14.4664	16.3151	15.3767	15.1427	14.6865	0.66832131	0.966018836	20	8	24	0.302994854	0.768040178	24	13.46607913	0.045847957
ENSMUSG00000030707	Coro1a	coronin, actin binding protein 1A [Source:MGI Symbol;Acc:MGI:1345961]	62.9045	63.1466	62.5972	59.4797	56.5309	60.3568	58.325	59.2951	60.4031	59.3165	62.4147	59.6944	64.9773	59.1577	0.910436409	0.966018836	4	12	24	0.496196054	0.807537609	24	2.259680811	0.024523002
ENSMUSG00000030711	Sult1a1	sulfotransferase family 1A, phenol-preferring, member 1 [Source:MGI Symbol;Acc:MGI:102896]	41.818	46.1069	44.9993	60.0993	53.9967	48.4896	46.8947	44.3388	65.1197	46.8782	50.3954	56.2654	49.0535	38.5059	0.385809259	0.820483168	20	12	24	0.12666273	0.739868076	24	14.02341839	0.189463698
ENSMUSG00000030714	Sgf29	SAGA complex associated factor 29 [Source:MGI Symbol;Acc:MGI:1922815]	14.68	14.5712	12.6121	15.2372	18.0337	17.2284	14.0829	15.3706	17.2783	19.0675	15.8721	16.4015	16.6072	15.4634	0.305259556	0.811201458	20	12	24	0.131734019	0.74203861	24	15.53983516	0.09813298

ENSMUSG00000030717	Nupr1	nuclear protein transcription regulator 1 [Source:MGI Symbol;Acc:MGI:1891834]	20.9676	27.7558	21.8921	22.2991	27.3228	19.538	25.2744	30.0813	30.4341	14.0316	24.5891	21.3762	23.7294	20.1429	0.791413662	0.966018836	12	12	24	0.597184846	0.858280068	24	3.436524491	0.110973163
ENSMUSG00000030718	Ppme1	protein phosphatase methylesterase 1 [Source:MGI Symbol;Acc:MGI:1919840]	58.1728	49.0883	52.0833	49.6274	48.8834	57.2114	53.7703	52.346	42.6599	56.9776	48.5777	52.1913	50.0599	51.276	0.297978647	0.80820842	24	20	24	0.264168427	0.768040178	24	21.32765065	0.072921774
ENSMUSG00000030720	Cln3	ceroid lipofuscinosis, neuronal 3, juvenile (Batten, Spielmeyer-Vogt disease) [Source:MGI Symbol;Acc:MGI:107537]	12.4319	11.6899	12.1466	11.1164	14.2847	14.267	11.7178	16.1126	12.0258	13.5178	13.5226	13.9263	13.0609	12.3118	0.151191877	0.598641364	24	12	24	0.50476856	0.811111334	24	19.04547017	0.062610282
ENSMUSG00000030722	Nfatc2ip	nuclear factor of activated T cells, cytoplasmic, calcineurin dependent 2 interacting protein [Source:MGI Symbol;Acc:MGI:1329015]	2.23301	2.42033	2.51023	2.18089	2.30241	2.7222	2.77571	2.27263	2.48924	2.37984	2.47625	2.54195	2.24705	2.89701	0.928127947	0.966018836	12	4	24	0.695106492	0.898633591	24	1.084550566	0.028311196
ENSMUSG00000030724	Cd19	CD19 antigen [Source:MGI Symbol;Acc:MGI:88319]	1.07013	1.6757	1.00819	1.21332	0.914882	0.943321	1.76383	1.04084	1.85255	0.740734	1.06903	0.966246	1.13854	0.819218	0.664568268	0.966018836	4	20	24	0.428565631	0.805393068	24	3.891322876	0.108910724
ENSMUSG00000030725	Lipt2	lipoyl(octanoyl) transferase 2 (putative) [Source:MGI Symbol;Acc:MGI:1914414]	1.86514	3.51294	2.52595	4.08044	3.05992	2.41203	2.63784	3.39601	2.98209	2.50786	3.01457	3.32817	2.67382	2.59045	0.93717134	0.966018836	8	20	24	0.437768848	0.805393068	24	11.24710992	0.105624382
ENSMUSG00000030726	Pold3	polymerase (DNA-directed), delta 3, accessory subunit [Source:MGI Symbol;Acc:MGI:1915217]	7.81911	8.02824	7.27182	7.80994	7.07609	7.6321	8.3239	8.67749	8.87604	7.96805	8.38046	7.75342	7.95574	7.45287	0.891105762	0.966018836	8	16	24	0.569653316	0.844000526	24	5.222231704	0.034425844
ENSMUSG00000030727	Rabep2	rabaptin, RAB GTPase binding effector protein 2 [Source:MGI Symbol;Acc:MGI:1917564]	8.57093	8.27166	8.25376	9.24122	8.7822	8.47585	10.3008	10.2069	8.73802	10.7083	8.17099	9.60731	8.85328	9.08985	0.882263435	0.966018836	16	4	24	0.952483459	0.988417782	24	6.798919542	0.014304945
ENSMUSG00000030729	Pgm2l1	phosphoglucomutase 2-like 1 [Source:MGI Symbol;Acc:MGI:1918224]	61.6581	57.5552	53.0971	55.0718	57.7806	56.2654	53.2386	51.5076	52.1078	53.6969	62.426	58.3586	61.1844	59.0607	0.108524038	0.524035616	20	16	24	0.125594933	0.739416874	24	20.304825	0.07146734
ENSMUSG00000030730	Atp2a1	ATPase, Ca++ transporting, cardiac muscle, fast twitch 1 [Source:MGI Symbol;Acc:MGI:105058]	28.2917	87.9911	108.025	145.487	110.222	139.257	74.9991	304.177	184.852	330.025	85.6847	110.235	125.64	98.2402	0.192357053	0.669841558	16	12	24	0.085663134	0.703577714	24	10.25936703	0.665040401
ENSMUSG00000030731	Syt3	synaptotagmin III [Source:MGI Symbol;Acc:MGI:99665]	8.89208	8.52275	10.1253	8.12938	7.04316	9.15606	10.0192	8.96747	8.64504	12.809	7.00724	9.41248	8.51727	10.7264	0.814077113	0.966018836	8	12	24	0.389933516	0.799546792	24	5.862653166	0.110921964
ENSMUSG00000030733	Sh2b1	SH2B adaptor protein 1 [Source:MGI Symbol;Acc:MGI:1201407]	13.6407	11.6992	12.2897	13.2309	13.1323	12.5154	12.4675	13.2485	12.2005	15.1168	11.5885	12.8812	12.4744	12.1993	0.581070934	0.966018836	16	16	24	0.654112906	0.883343578	24	13.99093673	0.033181033
ENSMUSG00000030735	Gm9755	predicted pseudogene 9755 [Source:MGI Symbol;Acc:MGI:3642279]	23.6739	19.2284	22.1015	24.3232	20.5423	25.5946	24.3636	27.5308	16.8245	27.7174	20.049	21.3654	23.3055	22.0261	0.920858634	0.966018836	4	8	24	0.839739237	0.95137802	24	21.38579351	0.047583894
ENSMUSG00000030737	Slco2b1	solute carrier organic anion transporter family, member 2b1 [Source:MGI Symbol;Acc:MGI:1351872]	3.24953	4.05081	3.99253	3.44368	4.02369	3.72655	4.2026	4.26718	4.82324	3.06272	3.88241	3.49288	3.80052	3.25772	0.849179819	0.966018836	12	4	24	0.697656482	0.899395227	24	5.565757677	0.04925098
ENSMUSG00000030738	Eif3c	eukaryotic translation initiation factor 3, subunit C [Source:MGI Symbol;Acc:MGI:1926966]	62.4704	58.9141	57.4884	57.5202	55.2871	60.0203	59.2881	62.1055	58.408	64.9379	66.5932	66.9519	67.7673	67.6996	0.244732441	0.741580974	4	8	24	0.313853446	0.768040178	24	23.37197471	0.037664136
ENSMUSG00000030739	Myh14	myosin, heavy polypeptide 14 [Source:MGI Symbol;Acc:MGI:1919210]	15.1578	13.0703	13.1344	14.0956	13.5813	14.9578	14.9094	17.3376	13.7084	20.8413	12.1538	14.6662	14.4538	14.2213	0.591351901	0.966018836	4	16	24	0.951541579	0.988339231	24	7.677997501	0.023731529
ENSMUSG00000030741	Spns1	spinster homolog 1 [Source:MGI Symbol;Acc:MGI:1920908]	17.895	14.5086	15.2998	13.601	14.5891	16.3868	16.5655	18.6022	17.7418	18.3395	15.9885	16.5488	17.1177	15.5166	0.722296281	0.966018836	4	16	24	0.549247028	0.834838463	24	1.683264878	0.052050533
ENSMUSG00000030742	Lat	linker for activation of T cells [Source:MGI Symbol;Acc:MGI:1342293]	0.731003	0.684679	1.09306	0.353166	0.471733	0.522092	0.470979	0.788352	0.921117	0.468377	0.821168	1.06896	0.805645	0.41839	0.908406451	0.966018836	12	4	24	0.830351199	0.947325117	24	3.211855239	0.050249534
ENSMUSG00000030744	Rps3	ribosomal protein S3 [Source:MGI Symbol;Acc:MGI:1350917]	282.03	348.545	328.149	323.214	340.277	287.493	302.897	301.649	357.429	236.04	326.586	297.41	328.442	309.621	0.390686537	0.915479136	12	12	24	0.773821371	0.92732018	24	6.350209872	0.045093446
ENSMUSG00000030745	Il21r	interleukin 21 receptor [Source:MGI Symbol;Acc:MGI:1890475]	0.499988	0.617365	0.534927	0.488764	0.530683	0.469944	0.49732	0.411302	0.70782	0.423306	0.550179	0.469092	0.75323	0.464215	0.91241026	0.966018836	12	12	24	0.810532599	0.939224719	24	4.293107889	0.024038754
ENSMUSG00000030747	Dgat2	diacylglycerol O-acyltransferase 2 [Source:MGI Symbol;Acc:MGI:1915050]	7.35893	7.2437	15.4196	28.4764	12.0563	10.06	9.68522	16.4216	14.3353	33.1642	16.6469	12.1336	20.4265	16.815	0.348367381	0.870418653	16	12	24	0.008717283	0.502965379	24	11.19395535	0.55856891
ENSMUSG00000030748	Il4ra	interleukin 4 receptor, alpha [Source:MGI Symbol;Acc:MGI:105367]	9.66789	8.08352	8.77644	8.28983	8.45513	9.69075	8.10109	8.97914	7.6603	8.89339	9.20182	8.28729	8.46799	8.28026	0.66832131	0.966018836	20	16	24	0.204094664	0.768040178	24	19.65377058	0.080089682
ENSMUSG00000030750	Nsmce1	NSE1 homolog, SMC5-SMC6 complex component [Source:MGI Symbol;Acc:MGI:1914961]	20.5375	23.2238	23.376	21.0144	23.4485	20.9909	18.2823	23.226	25.5168	16.1438	22.3899	20.4017	22.0642	19.2627	0.217827365	0.704989686	12	12	24	0.824005752	0.944693734	24	7.036487588	0.041395343
ENSMUSG00000030751	Psma1	proteasome subunit alpha 1 [Source:MGI Symbol;Acc:MGI:1347005]	116.306	125.861	121.007	126.509	125.473	111.755	112.03	111.37	125.288	110.698	132.553	115.185	127.308	122.46	0.865490576	0.966018836	20	4	24	0.589266489	0.854191861	24	11.46917607	0.035580644
ENSMUSG00000030752	Kdm8	lysine (K)-specific demethylase 8 [Source:MGI Symbol;Acc:MGI:1924285]	2.73924	2.85211	2.6715	3.09782	3.08	3.1081	2.67439	3.27714	2.82513	3.75903	2.60424	2.94713	2.7871	2.94935	0.702630284	0.966018836	16	12	24	0.420075734	0.803839449	24	12.02907741	0.054466851
ENSMUSG00000030753	Thap12	THAP domain containing 12 [Source:MGI Symbol;Acc:MGI:1920231]	20.8146	24.9996	24.5703	26.4336	24.55	22.3408	25.6048	25.5559	29.2606	23.2622	25.2342	24.2225	23.9967	24.657	0.313239838	0.824470524	12	12	24	0.08856589	0.707747429	24	8.959491547	0.089585937
ENSMUSG00000030754	Copb1	coatomer protein complex, subunit beta 1 [Source:MGI Symbol;Acc:MGI:1917599]	36.3302	37.4438	31.8751	33.8274	35.4188	33.653	37.8295	37.9253	36.5088	32.6427	37.0498	33.069	35.9805	35.613	0.149021625	0.593018107	8	8	24	0.197424235	0.768040178	24	1.561127135	0.064530015
ENSMUSG00000030757	Zkscan2	zinc finger with KRAB and SCAN domains 2 [Source:MGI Symbol;Acc:MGI:2444060]	2.76303	4.23088	3.94621	2.64729	2.94805	2.98826	2.40468	4.00897	3.97729	2.56328	2.76427	3.82216	3.09898	4.19948	0.016398461	0.253395174	8	8	24	0.063717571	0.663876076	24	5.40492355	0.194376841
ENSMUSG00000030759	Far1	fatty acyl CoA reductase 1 [Source:MGI Symbol;Acc:MGI:1914670]	76.974	80.1722	73.663	70.142	81.7439	68.6004	77.7195	64.8437	73.2657	58.4525	82.7295	64.0016	65.2479	67.6619	0.865490576	0.966018836	20	20	24	0.797435778	0.935271704	24	19.45668152	0.036475187
ENSMUSG00000030760	Acer3	alkaline ceramidase 3 [Source:MGI Symbol;Acc:MGI:1913440]	10.7138	11.1572	12.432	10.5835	10.4415	10.6094	9.69684	11	11.7191	9.19298	12.9204	10.7589	11.8886	12.4216	0.369262716	0.893596186	12	4	24	0.608093912	0.864217165	24	6.051659954	0.050591862
ENSMUSG00000030761	Myo7a	myosin VIIA [Source:MGI Symbol;Acc:MGI:104510]	8.39838	7.52048	7.00674	8.32573	10.2724	8.69317	8.72463	8.05434	8.37863	8.33717	8.3087	8.09811	8.30583	7.22117	0.150959199	0.598641364	20	12	24	0.033229873	0.606585237	24	17.68749873	0.110600819
ENSMUSG00000030763	Lcmt1	leucine carboxyl methyltransferase 1 [Source:MGI Symbol;Acc:MGI:1353593]	37.6384	34.2457	34.806	34.9328	33.6767	33.0678	31.7753	33.8823	34.5687	32.7244	35.0321	34.5601	35.1989	36.3426	0.859936714	0.966018836	4	20	24	0.727656096	0.910646611	24	3.548908313	0.018951164
ENSMUSG00000030766	Arhgap17	Rho GTPase activating protein 17 [Source:MGI Symbol;Acc:MGI:1917747]	16.7081	15.102	13.1881	15.9715	15.3481	14.8142	15.9086	17.3275	14.1842	19.7876	16.286	16.9817	17.3615	16.7018	0.774195442	0.966018836	4	8	24	0.839670975	0.95137802	24	19.79973064	0.029660365
ENSMUSG00000030768	Disp1	dispatched RND transporter family member 1 [Source:MGI Symbol;Acc:MGI:1916147]	7.13406	6.73806	6.18651	6.2053	6.91543	6.16047	6.57154	7.04774	6.80732	7.63172	6.27849	7.0093	7.88753	6.71585	0.920620634	0.966018836	4	8	24	0.646879887	0.880609512	24	1.562127777	0.032825449
ENSMUSG00000030769	Slc5a11	solute carrier family 5 (sodium/glucose cotransporter), member 11 [Source:MGI Symbol;Acc:MGI:1919316]	1.15074	1.0738	0.950934	1.71227	0.753626	2.7827	0.729349	1.75996	1.63531	1.50743	1.09259	1.65781	2.09736	1.18357	0.849180783	0.966018836	24	20	24	0.919461335	0.978472202	24	20.23993976	0.053319974
ENSMUSG00000030770	Parva	parvin, alpha [Source:MGI Symbol;Acc:MGI:1931144]	56.8422	51.5682	50.3935	55.4031	55.3038	58.3036	61.8288	54.3743	48.4063	66.1932	50.8346	59.5063	55.48	66.9075	0.217827365	0.704989686	24	12	24	0.693917914	0.898364349	24	21.6587658	0.045533902
ENSMUSG00000030772	Dkk3	dickkopf WNT signaling pathway inhibitor 3 [Source:MGI Symbol;Acc:MGI:1354952]	49.6842	45.3325	38.745	40.9222	39.3652	45.2163	47.0527	47.8997	40.0829	50.9604	38.1721	48.5194	47.7571	51.0885	0.148778801	0.59218729	8	12	24	0.074098124	0.683293901	24	0.977727457	0.117571236
ENSMUSG00000030774	Pak1	p21 (RAC1) activated kinase 1 [Source:MGI Symbol;Acc:MGI:1339975]	31.5296	33.5442	39.6544	32.3251	33.4882	36.5905	36.4723	33.6499	31.7037	35.8346	28.2495	35.4298	29.3064	36.699	0.919571723	0.966018836	8	12	24	0.778611513	0.928832041	24	6.051953855	0.037994666
ENSMUSG00000030779	Rbbp6	retinoblastoma binding protein 6, ubiquitin ligase [Source:MGI Symbol;Acc:MGI:894835]	19.6931	20.1348	21.0374	21.7362	19.8938	17.3274	19.1005	18.2797	22.3679	19.8772	20.1851	19.2053	20.7031	19.8437	0.038115943	0.334069441	12	12	24	0.056784626	0.65610184	24	9.547897125	0.080668098
ENSMUSG00000030780	Rusf1	RUS family member 1 [Source:MGI Symbol;Acc:MGI:2384572]	8.7423	7.13844	8.28485	7.15295	7.9342	7.94106	8.35277	8.16863	7.86926	9.18774	8.05333	8.1231	7.89123	7.62821	0.928780444	0.966018836	4	4	24	0.851470152	0.95543202	24	18.47466351	0.020495711
ENSMUSG00000030782	Tgfb1i1	transforming growth factor beta 1 induced transcript 1 [Source:MGI Symbol;Acc:MGI:102784]	14.7754	13.0929	12.3107	12.1018	13.4254	14.1441	13.7547	16.288	13.3941	18.2419	13.2185	15.3342	14.5219	14.1739	0.466710516	0.966018836	4	8	24	0.876274985	0.96555547	24	23.13894024	0.028497314
ENSMUSG00000030785	Cox6a2	cytochrome c oxidase subunit 6A2 [Source:MGI Symbol;Acc:MGI:104649]	11.6457	29.5478	43.999	41.81	32.5032	36.1	22.2626	65.3147	61.3866	47.8636	24.9856	24.2426	39.0029	31.2961	0.030691182	0.324150946	12	16	24	0.026861252	0.586541342	24	9.184072088	0.588783452
ENSMUSG00000030786	Itgam	integrin alpha M [Source:MGI Symbol;Acc:MGI:96607]	1.73053	3.12879	3.61512	2.80407	2.77891	2.44499	2.53689	2.41067	2.59078	1.6717	2.19667	2.23937	2.534	2.14414	0.772122668	0.966018836	12	12	24	0.48500132	0.805393068	24	8.367303136	0.090971999
ENSMUSG00000030787	Lyve1	lymphatic vessel endothelial hyaluronan receptor 1 [Source:MGI Symbol;Acc:MGI:2136348]	2.88506	2.24387	2.28826	2.59372	2.91605	2.4287	2.26934	2.59393	2.58124	1.79037	3.02514	2.45619	2.48095	2.18725	0.47028201	0.966018836	20	12	24	0.339716524	0.774150091	24	18.79437094	0.08028039
ENSMUSG00000030788	Rnf141	ring finger protein 141 [Source:MGI Symbol;Acc:MGI:1914400]	38.1692	40.0495	36.6305	35.7955	40.2681	35.2974	40.2337	37.1015	41.3213	33.1952	38.4911	38.0034	37.4725	36.6626	0.745118544	0.966018836	4	12	24	0.622618134	0.87142241	24	0.64955597	0.032047135
ENSMUSG00000030790	Adm	adrenomedullin [Source:MGI Symbol;Acc:MGI:108058]	1.26067	1.44362	1.6116	1.59901	1.3834	1.45425	1.26332	1.68206	2.04263	1.74787	1.20305	1.23377	1.11771	1.63382	0.016795343	0.253395174	12	16	24	0.002158362	0.395705644	24	8.765561078	0.169918207
ENSMUSG00000030792	Dkkl1	dickkopf-like 1 [Source:MGI Symbol;Acc:MGI:1354963]	0.872015	0.938321	0.972662	0.729122	0.667382	0.846148	1.18359	1.15535	0.824289	1.25699	1.12055	1.39845	0.995374	1.26139	0.850398351	0.966018836	8	12	24	0.497321815	0.807576732	24	2.079169316	0.05641507
ENSMUSG00000030793	Pycard	PYD and CARD domain containing [Source:MGI Symbol;Acc:MGI:1931465]	1.3651	3.48487	3.52886	2.51646	2.75135	2.0523	1.72841	2.98236	3.27404	1.82549	1.85157	2.16576	3.1594	2.5609	0.110888527	0.528581815	12	12	24	0.095400254	0.71175955	24	7.199033157	0.246530999
ENSMUSG00000030795	Fus	fused in sarcoma [Source:MGI Symbol;Acc:MGI:1353633]	83.9684	95.2852	101.205	95.2921	99.0768	89.3944	95.5689	92.0541	93.984	95.3748	86.828	95.9468	90.9789	94.337	0.745199643	0.966018836	16	12	24	0.249346909	0.768040178	24	9.747010407	0.046744135
ENSMUSG00000030796	Tead2	TEA domain family member 2 [Source:MGI Symbol;Acc:MGI:104904]	2.63643	2.58764	2.80584	2.87472	2.83056	2.4239	3.09482	2.37155	2.76433	2.21511	3.76612	2.59588	3.08393	2.83761	0.93447422	0.966018836	20	12	24	0.783049622	0.930229965	24	17.70199628	0.03959246
ENSMUSG00000030798	Cd37	CD37 antigen [Source:MGI Symbol;Acc:MGI:88330]	1.59142	3.02756	3.83595	2.67793	1.87006	2.31076	2.45069	2.93932	3.45574	1.76767	2.13677	2.79905	3.06454	2.09337	0.132887428	0.567728672	12	8	24	0.216656156	0.768040178	24	6.329780663	0.183612326
ENSMUSG00000030801	Kat8	K(lysine) acetyltransferase 8 [Source:MGI Symbol;Acc:MGI:1915023]	15.2377	13.3463	15.4484	15.6967	15.783	13.6307	15.288	14.8924	15.3745	14.4936	16.2619	15.1331	16.4692	15.0279	0.192357053	0.669841558	20	12	24	0.705571383	0.902918222	24	14.61850242	0.024941938
ENSMUSG00000030802	Bckdk	branched chain ketoacid dehydrogenase kinase [Source:MGI Symbol;Acc:MGI:1276121]	12.2963	13.0881	14.4313	16.8495	13.7725	15.4039	15.0729	16.9155	16.1695	17.5827	13.5135	14.1831	14.3758	13.9307	0.503972924	0.966018836	16	4	24	0.154729791	0.760257003	24	10.40599626	0.10325203
ENSMUSG00000030805	Stx4a	syntaxin 4A (placental) [Source:MGI Symbol;Acc:MGI:893577]	52.6043	54.7496	49.0211	53.4442	60.3891	55.3392	53.5867	53.1334	51.3215	52.9218	53.4937	50.3684	55.0941	52.611	0.275526571	0.789849983	20	16	24	0.298981508	0.768040178	24	18.60041341	0.042760547
ENSMUSG00000030806	Stx1b	syntaxin 1B [Source:MGI Symbol;Acc:MGI:1930705]	28.1472	26.5534	35.7173	27.263	22.4631	31.9896	32.0095	27.375	25.691	38.2789	20.4641	29.8054	23.0762	31.525	0.790218349	0.966018836	16	4	24	0.659496447	0.884879972	24	6.463699781	0.092640436
ENSMUSG00000030811	Fbxl19	F-box and leucine-rich repeat protein 19 [Source:MGI Symbol;Acc:MGI:3039600]	11.8987	9.6193	9.77194	9.41285	10.7195	10.975	10.2068	11.5278	9.22274	11.5674	11.2839	11.6741	11.7343	11.8809	0.170659409	0.643496587	4	8	24	0.125646736	0.739598573	24	21.50329944	0.083842882
ENSMUSG00000030814	Bcl7c	B cell CLL/lymphoma 7C [Source:MGI Symbol;Acc:MGI:1332237]	10.0028	9.76224	7.65464	9.45444	8.02801	8.22809	8.50029	8.64421	10.5755	8.9548	9.44266	7.91038	8.48595	8.13301	0.920620634	0.966018836	16	8	24	0.700942159	0.900900643	24	8.054856769	0.043457424
ENSMUSG00000030815	Phkg2	phosphorylase kinase, gamma 2 (testis) [Source:MGI Symbol;Acc:MGI:1916211]	41.5459	43.2032	42.0184	42.8886	44.1338	42.0716	41.2022	38.9066	40.4382	39.1409	42.9966	39.0813	42.026	41.3619	0.814077113	0.966018836	20	12	24	0.586471976	0.853300537	24	16.90182102	0.021059234
ENSMUSG00000030816	Rnf40	ring finger protein 40 [Source:MGI Symbol;Acc:MGI:2142048]	10.3548	9.77657	10.383	10.3228	9.73715	10.2801	11.4168	10.991	10.7174	10.6159	8.74156	11.454	11.0303	10.143	0.231491055	0.731037655	4	16	24	0.312229664	0.768040178	24	1.923874353	0.053369205
ENSMUSG00000030822	Prr14	proline rich 14 [Source:MGI Symbol;Acc:MGI:2384565]	15.4904	15.8534	14.1863	17.7105	16.9717	16.1425	13.0583	17.7756	17.4278	16.7969	15.837	15.8338	16.8324	15.0216	0.723119329	0.966018836	16	12	24	0.379373592	0.798714572	24	12.20246259	0.063623313
ENSMUSG00000030823	9130019O22Rik	RIKEN cDNA 9130019O22 gene [Source:MGI Symbol;Acc:MGI:1926171]	1.48586	1.16708	1.35757	1.2503	1.35597	1.45916	0.979304	1.05113	1.25391	1.22513	1.11164	1.03557	1.2159	1.23615	0.81872541	0.966018836	12	20	24	0.815070629	0.940891459	24	14.93470614	0.022472072
ENSMUSG00000030824	Nucb1	nucleobindin 1 [Source:MGI Symbol;Acc:MGI:97388]	60.462	50.6111	48.7689	43.6338	47.582	50.8606	54.5357	55.3962	42.8289	62.1808	42.0496	55.1902	49.6101	51.556	0.278343887	0.790064226	4	16	24	0.312604167	0.768040178	24	0.1574862	0.099550492
ENSMUSG00000030825	Hsd17b14	hydroxysteroid (17-beta) dehydrogenase 14 [Source:MGI Symbol;Acc:MGI:1913315]	2.44008	1.61714	2.26931	2.09114	2.40054	1.47358	2.24398	1.85311	1.96538	2.71646	2.77441	1.48198	2.89795	1.71027	0.431765373	0.966018836	20	12	24	0.638209165	0.877145054	24	13.67417509	0.082430261
ENSMUSG00000030826	Bcat2	branched chain aminotransferase 2, mitochondrial [Source:MGI Symbol;Acc:MGI:1276534]	17.1912	14.3281	14.1067	17.6733	17.3452	16.4609	16.6012	16.9386	15.2751	16.978	17.7348	15.6918	16.3788	16.1842	0.033404002	0.324908923	16	20	24	0.142053171	0.751766185	24	17.30229524	0.072434118
ENSMUSG00000030830	Itgal	integrin alpha L [Source:MGI Symbol;Acc:MGI:96606]	0.67027	0.843123	1.13126	0.968284	0.878424	0.916107	0.83326	0.973872	0.888721	0.643723	0.749551	0.685865	0.914197	0.749257	0.939921059	0.966018836	12	16	24	0.487023415	0.805393068	24	7.551705208	0.049718801
ENSMUSG00000030835	Nomo1	nodal modulator 1 [Source:MGI Symbol;Acc:MGI:2385850]	21.0581	16.4314	18.1611	19.228	18.3843	20.5281	19.7057	20.5888	15.4139	23.6192	16.2077	19.1448	18.4258	19.4328	0.759899911	0.966018836	4	8	24	0.802795762	0.93676746	24	20.52701059	0.04329285
ENSMUSG00000030839	Sergef	secretion regulating guanine nucleotide exchange factor [Source:MGI Symbol;Acc:MGI:1351630]	5.48643	6.62483	5.09688	5.49225	5.4537	6.33497	5.43487	5.79191	4.97673	5.91032	5.4229	6.4749	4.73073	6.44391	0.89563591	0.966018836	8	4	24	0.81345032	0.940453305	24	22.22824726	0.034064184
ENSMUSG00000030842	Lamtor1	late endosomal/lysosomal adaptor, MAPK and MTOR activator 1 [Source:MGI Symbol;Acc:MGI:1913758]	46.6564	45.6017	45.1224	44.8699	48.1201	48.4489	45.0143	50.4345	46.2936	46.1163	46.1348	46.0397	46.4597	45.7913	0.865233232	0.966018836	20	20	24	0.746118221	0.917113276	24	21.57256714	0.01455155
ENSMUSG00000030844	Rgs10	regulator of G-protein signalling 10 [Source:MGI Symbol;Acc:MGI:1915115]	199.544	179.038	158.762	171.468	163.177	180.213	156.461	175.299	171.174	168.789	181.072	186.929	199.434	188.658	0.208747416	0.698797644	4	8	24	0.180325953	0.768040178	24	23.2332445	0.073558083
ENSMUSG00000030846	Tial1	Tia1 cytotoxic granule-associated RNA binding protein-like 1 [Source:MGI Symbol;Acc:MGI:107913]	36.337	37.0508	34.9687	35.4713	37.0952	33.6379	36.4896	33.1023	38.0788	29.2177	35.9774	33.5898	36.3311	35.6008	0.948582958	0.966018836	4	12	24	0.619425491	0.869468249	24	1.267838932	0.035283448
ENSMUSG00000030847	Bag3	BCL2-associated athanogene 3 [Source:MGI Symbol;Acc:MGI:1352493]	43.3839	30.2929	28.4854	34.1531	33.1793	35.8644	35.0617	41.5331	28.2125	50.3489	38.1739	38.9001	37.8933	36.9992	0.321662653	0.83607295	24	12	24	0.564929031	0.843471947	24	19.58978456	0.094850144
ENSMUSG00000030849	Fgfr2	fibroblast growth factor receptor 2 [Source:MGI Symbol;Acc:MGI:95523]	3.56635	7.16048	8.77412	2.81255	3.71556	4.66926	7.54067	4.18384	6.65141	3.49617	2.99773	5.72826	2.98617	5.43725	0.129903614	0.560263689	12	4	24	0.20968149	0.768040178	24	4.511597951	0.301439632
ENSMUSG00000030850	Ate1	arginyltransferase 1 [Source:MGI Symbol;Acc:MGI:1333870]	22.1528	21.1722	21.1678	20.2677	20.7603	21.0844	21.7179	20.5539	20.6433	20.699	21.0933	22.2243	21.8508	22.2323	0.032263213	0.324385159	4	12	24	0.010216945	0.503967859	24	23.49855545	0.042107651
ENSMUSG00000030852	Tacc2	transforming, acidic coiled-coil containing protein 2 [Source:MGI Symbol;Acc:MGI:1928899]	20.088	21.1785	21.887	23.2095	20.3619	22.5479	21.4381	28.2547	23.0034	34.1525	18.8442	22.6079	22.9741	23.8651	0.187699199	0.662239322	16	4	24	0.268744509	0.768040178	24	8.621259543	0.128092657
ENSMUSG00000030854	Ptpn5	protein tyrosine phosphatase, non-receptor type 5 [Source:MGI Symbol;Acc:MGI:97807]	3.15939	3.63456	6.41509	3.01311	3.76787	4.81831	6.60323	3.29588	4.28009	3.76498	2.79491	4.03724	2.67401	4.65287	0.923635729	0.966018836	12	4	24	0.768680234	0.925424343	24	4.518878478	0.094691414
ENSMUSG00000030861	Acadsb	acyl-Coenzyme A dehydrogenase, short/branched chain [Source:MGI Symbol;Acc:MGI:1914135]	34.1786	38.6194	33.1269	37.7133	32.5442	34.9726	41.1687	37.6749	37.2501	31.1769	36.9828	31.9183	36.3984	33.7052	0.658942124	0.966018836	4	20	24	0.401591806	0.799546792	24	2.70049454	0.058812018
ENSMUSG00000030862	Cpxm2	carboxypeptidase X 2 (M14 family) [Source:MGI Symbol;Acc:MGI:1926006]	5.89279	7.3556	6.73601	5.70975	5.98655	4.47403	5.18945	6.71626	11.5768	2.98224	4.89341	5.8291	5.52024	5.30081	0.106193509	0.519440352	12	4	24	0.241793207	0.768040178	24	5.617521842	0.234311442
ENSMUSG00000030865	Chp2	calcineurin-like EF hand protein 2 [Source:MGI Symbol;Acc:MGI:1917511]	31.0065	30.3282	27.4273	29.2215	33.9925	28.1075	28.7318	26.7769	28.2326	29.7853	33.3836	30.4079	29.8098	28.5726	0.035023815	0.328311403	20	16	24	0.045372475	0.629400324	24	17.24199364	0.085912137
ENSMUSG00000030867	Plk1	polo like kinase 1 [Source:MGI Symbol;Acc:MGI:97621]	0.557362	0.57624	0.698557	0.784967	0.534941	0.710636	0.475488	0.506857	0.772246	0.590915	0.823239	0.663437	0.835418	0.432152	0.291478323	0.798044066	20	12	24	0.319230711	0.768040178	24	14.42162001	0.067765398
ENSMUSG00000030868	Dctn5	dynactin 5 [Source:MGI Symbol;Acc:MGI:1891689]	95.3225	86.3914	85.1779	85.6873	90.7955	96.7343	86.9042	86.7787	78.6542	89.0636	93.115	90.5153	91.2805	92.483	0.004712708	0.140598674	24	12	24	0.016122909	0.544099157	24	20.19524664	0.077947185
ENSMUSG00000030869	Ndufab1	NADH:ubiquinone oxidoreductase subunit AB1 [Source:MGI Symbol;Acc:MGI:1917566]	21.1852	20.8506	20.3531	21.5928	18.4563	18.8641	22.9629	22.0158	23.4305	21.0733	26.2215	24.5527	29.0425	25.6017	0.933543173	0.966018836	4	12	24	0.320673125	0.768040178	24	2.374369853	0.066943454
ENSMUSG00000030870	Ubfd1	ubiquitin family domain containing 1 [Source:MGI Symbol;Acc:MGI:107301]	21.0097	16.2835	17.6013	17.3948	17.2441	18.8131	18.7927	17.6085	14.0664	18.4366	17.047	17.4873	17.5751	18.1686	0.249881062	0.749643185	4	8	24	0.131323288	0.741500219	24	21.46595569	0.091864883
ENSMUSG00000030871	Ears2	glutamyl-tRNA synthetase 2, mitochondrial [Source:MGI Symbol;Acc:MGI:1914667]	2.21017	1.76443	2.03522	1.81481	2.25138	2.13684	2.19342	2.12311	1.70804	2.41073	2.027	2.16241	2.06032	2.01843	0.216027475	0.704989686	24	12	24	0.219611754	0.768040178	24	19.18743546	0.063914648
ENSMUSG00000030872	Gga2	golgi associated, gamma adaptin ear containing, ARF binding protein 2 [Source:MGI Symbol;Acc:MGI:1921355]	31.0893	25.2287	25.6149	27.4275	26.7255	29.8885	26.7269	28.6521	22.8076	30.2235	26.6898	29.602	28.1742	27.6083	0.12833063	0.560209134	24	12	24	0.142976649	0.752803875	24	20.30454906	0.08836519
ENSMUSG00000030876	Mettl9	methyltransferase like 9 [Source:MGI Symbol;Acc:MGI:1914862]	18.3834	21.3078	21.1764	19.0539	22.8766	18.4386	23.2378	19.0819	22.7623	18.6301	20.114	21.4822	20.1571	21.5164	0.890235289	0.966018836	12	4	24	0.873899472	0.96480494	24	5.481955112	0.024243517
ENSMUSG00000030878	Cdr2	cerebellar degeneration-related 2 [Source:MGI Symbol;Acc:MGI:1100885]	24.6052	21.7747	23.5509	23.288	22.2132	23.9846	23.3995	21.9506	19.1726	22.0244	22.6299	22.4446	22.4738	21.2189	0.175185919	0.651010555	4	4	24	0.049681454	0.642874369	24	20.09664088	0.068700992
ENSMUSG00000030879	Mrpl17	mitochondrial ribosomal protein L17 [Source:MGI Symbol;Acc:MGI:1351608]	51.0626	52.6428	51.7563	48.2408	51.0422	46.8526	50.115	58.2452	50.8892	55.7558	53.7026	60.6739	62.062	54.9529	0.935593607	0.966018836	8	8	24	0.486969456	0.805393068	24	2.269123059	0.041621918
ENSMUSG00000030880	Polr3e	polymerase (RNA) III (DNA directed) polypeptide E [Source:MGI Symbol;Acc:MGI:1349452]	9.19226	8.48137	8.64899	8.02839	8.66945	9.23617	9.34054	8.34476	8.18547	9.01441	8.79155	8.85611	7.54124	8.44068	0.289105414	0.794869797	24	8	24	0.279486517	0.768040178	24	19.30808577	0.047178828
ENSMUSG00000030881	Arfip2	ADP-ribosylation factor interacting protein 2 [Source:MGI Symbol;Acc:MGI:1924182]	29.3042	27.4225	28.8366	29.334	29.5809	31.6118	28.4425	29.1961	27.404	28.6009	30.6189	29.1551	28.941	28.287	0.01813461	0.256345714	20	16	24	0.011708966	0.522142988	24	18.0835194	0.054858782
ENSMUSG00000030882	Dnhd1	dynein heavy chain domain 1 [Source:MGI Symbol;Acc:MGI:1924755]	1.5433	2.57163	3.04059	3.10105	2.54849	2.46456	3.53256	2.66772	3.24714	2.65252	3.00724	3.1089	3.30467	2.12842	0.883907406	0.966018836	12	20	24	0.644360699	0.879332572	24	11.90414569	0.077044204
ENSMUSG00000030884	Uqcrc2	ubiquinol cytochrome c reductase core protein 2 [Source:MGI Symbol;Acc:MGI:1914253]	120.113	134.702	138.162	152.842	132.739	131.755	124.199	144.585	144.391	123.496	144.381	127.758	145.205	135.265	0.643501552	0.966018836	12	16	24	0.264613392	0.768040178	24	9.514914958	0.065976733
ENSMUSG00000030888	Rrp8	ribosomal RNA processing 8 [Source:MGI Symbol;Acc:MGI:1914251]	10.1366	8.57983	8.07928	11.5837	10.8271	9.41853	10.1763	8.75389	8.6723	12.2344	10.2502	9.01724	9.86547	8.26442	0.001270265	0.087678517	16	20	24	0.046666502	0.633507364	24	15.75900463	0.145825076
ENSMUSG00000030889	Vwa3a	von Willebrand factor A domain containing 3A [Source:MGI Symbol;Acc:MGI:3041229]	0.35143	0.763032	0.972474	0.530778	0.505837	0.545657	1.0455	0.926819	0.729414	0.507987	0.558736	0.826498	0.671928	0.774464	0.166587374	0.633636735	8	12	24	0.135269138	0.74694112	24	4.458062316	0.128004882
ENSMUSG00000030890	Ilk	integrin linked kinase [Source:MGI Symbol;Acc:MGI:1195267]	63.4692	60.6286	52.9604	63.9389	56.7819	61.8289	57.0944	58.7599	60.602	61.3521	59.5312	60.3767	55.9559	52.9439	0.916746631	0.966018836	16	16	24	0.385109418	0.799546792	24	16.94903606	0.043608615
ENSMUSG00000030894	Tpp1	tripeptidyl peptidase I [Source:MGI Symbol;Acc:MGI:1336194]	25.1484	23.9083	25.3607	24.5316	25.8351	24.2246	26.9008	26.21	24.6069	23.9929	22.2844	24.6803	23.4463	24.0499	0.847846021	0.966018836	12	4	24	0.587102975	0.853586751	24	2.106861669	0.024879426
ENSMUSG00000030905	Crym	crystallin, mu [Source:MGI Symbol;Acc:MGI:102675]	5.13265	3.47511	4.29065	3.21056	4.63296	4.64796	3.9663	3.5876	3.90469	4.10815	4.2384	4.4316	3.56391	4.16934	0.244732441	0.741580974	24	8	24	0.19156096	0.768040178	24	19.37614872	0.106920119
ENSMUSG00000030917	Tmem159	transmembrane protein 159 [Source:MGI Symbol;Acc:MGI:1925752]	11.6464	10.7964	10.4137	11.5994	12.0482	10.7784	10.5961	10.9574	10.7778	10.9996	11.9177	10.9962	10.6757	10.3901	0.032211002	0.324385159	20	12	24	0.018389749	0.559812298	24	16.24483265	0.060446792
ENSMUSG00000030921	Trim30a	tripartite motif-containing 30A [Source:MGI Symbol;Acc:MGI:98178]	1.60092	2.01016	2.35352	1.81072	2.60297	2.13961	2.60156	2.97911	2.62013	1.44819	2.04331	1.96191	2.33238	1.93619	0.924553956	0.966018836	12	4	24	0.672314207	0.889365603	24	3.263755518	0.06973351
ENSMUSG00000030922	Lyrm1	LYR motif containing 1 [Source:MGI Symbol;Acc:MGI:1921169]	15.0735	15.5344	14.0003	14.0724	15.0325	14.3672	14.6224	14.4122	15.502	13.4359	16.7718	14.808	15.1012	14.687	0.745118544	0.966018836	4	12	24	0.685621631	0.89558343	24	21.10787177	0.0262483
ENSMUSG00000030924	Rexo5	RNA exonuclease 5 [Source:MGI Symbol;Acc:MGI:1919402]	2.35049	3.63638	3.43505	1.56873	2.54651	1.87955	1.95495	2.06216	2.97998	1.77858	3.43791	1.96157	2.40017	1.77648	0.91695334	0.966018836	12	4	24	0.802904045	0.936800961	24	7.558316638	0.075963219
ENSMUSG00000030929	Eri2	exoribonuclease 2 [Source:MGI Symbol;Acc:MGI:1918401]	3.44869	4.32219	3.6146	4.05801	4.5757	3.59416	3.71671	3.49558	4.17788	2.91853	3.99641	3.89651	3.9035	3.71141	0.912686312	0.966018836	20	8	24	0.902028098	0.972579936	24	17.17903641	0.023498409
ENSMUSG00000030930	Chst15	carbohydrate sulfotransferase 15 [Source:MGI Symbol;Acc:MGI:1924840]	15.3182	11.109	11.0751	12.4256	11.5015	14.9909	10.9663	13.472	9.11027	14.9671	9.91882	12.5525	12.7717	11.7316	0.46238066	0.966018836	24	12	24	0.433846748	0.805393068	24	21.04352081	0.107862659
ENSMUSG00000030934	Oat	ornithine aminotransferase [Source:MGI Symbol;Acc:MGI:97394]	64.3547	67.2206	64.5973	62.0297	67.1096	61.8629	62.935	66.3697	65.871	56.8751	67.9938	58.3148	61.6994	59.8555	0.868638024	0.966018836	20	4	24	0.943742643	0.986208939	24	5.580412508	0.009875603
ENSMUSG00000030935	Acsm3	acyl-CoA synthetase medium-chain family member 3 [Source:MGI Symbol;Acc:MGI:99538]	0.93398	0.935447	2.45245	4.31416	1.45238	1.33472	2.67498	2.45285	2.26804	4.63173	3.57662	2.40174	4.0986	3.83333	0.70641939	0.966018836	16	8	24	0.083276567	0.699490063	24	10.72091618	0.323803759
ENSMUSG00000030942	Thumpd1	THUMP domain containing 1 [Source:MGI Symbol;Acc:MGI:2444479]	15.1423	15.1302	14.4639	14.1395	14.725	14.7833	14.1507	14.7949	13.4777	14.9593	14.9076	15.8429	15.4418	15.0323	0.181522804	0.659020016	24	12	24	0.143137284	0.752803875	24	21.46978621	0.040244854
ENSMUSG00000030946	Lhpp	phospholysine phosphohistidine inorganic pyrophosphate phosphatase [Source:MGI Symbol;Acc:MGI:1923679]	4.33273	4.77013	5.05133	4.56728	4.71494	4.80267	4.27785	4.68724	4.80962	4.37749	4.01894	3.94658	4.27258	3.83766	0.277087842	0.789849983	12	16	24	0.280871663	0.768040178	24	9.15396935	0.045840552
ENSMUSG00000030956	Fam53b	family with sequence similarity 53, member B [Source:MGI Symbol;Acc:MGI:1925188]	7.41834	6.10591	6.10815	9.05931	7.46228	6.71988	8.13512	7.32361	6.25443	9.84295	7.51544	7.56278	8.02177	7.05729	0.050447964	0.385880335	16	20	24	0.333447802	0.769685083	24	15.65939729	0.098237791
ENSMUSG00000030960	Eef1akmt2	EEF1A lysine methyltransferase 2 [Source:MGI Symbol;Acc:MGI:1919346]	15.0487	21.1633	18.143	18.7818	19.361	15.9306	19.3961	15.669	19.6157	14.6328	19.4164	16.3431	18.5317	17.8654	0.891742979	0.966018836	20	4	24	0.829245446	0.946904474	24	8.231567536	0.040064514

ENSMUSG00000030965	Abraxas2	BRISC complex subunit [Source:MGI Symbol;Acc:MGI:1926116]	20.26	19.9966	19.0916	19.4545	19.6944	19.7372	19.1532	19.1377	19.5531	19.0819	20.2823	20.6962	22.1813	20.9687	0.121015203	0.548303839	24	16	24	0.14199284	0.751766185	24	22.85931323	0.038234225
ENSMUSG00000030966	Trim21	tripartite motif-containing 21 [Source:MGI Symbol;Acc:MGI:106657]	4.47894	4.87926	5.04567	4.07876	4.86264	4.92115	4.89917	5.07572	4.71075	3.3539	4.95516	4.08887	4.41946	4.2825	0.94791825	0.966018836	12	4	24	0.482087752	0.805393068	24	0.619068775	0.060131195
ENSMUSG00000030967	Zranb1	zinc finger, RAN-binding domain containing 1 [Source:MGI Symbol;Acc:MGI:106441]	15.7065	19.1494	20.5536	19.8609	18.2878	17.1346	16.4563	18.4347	20.9392	17.5488	17.32	18.7277	18.7838	18.6245	0.030691182	0.324150946	12	16	24	0.026572121	0.586541342	24	8.871042425	0.108409824
ENSMUSG00000030970	Ctbp2	C-terminal binding protein 2 [Source:MGI Symbol;Acc:MGI:1201686]	6.86244	7.18328	7.3708	8.49526	10.6571	9.38168	7.29992	6.87308	8.15743	7.43875	8.02681	7.44579	6.59318	7.851	0.001953237	0.100255953	20	8	24	0.035665798	0.609176161	24	15.25889299	0.150714906
ENSMUSG00000030978	Rrm1	ribonucleotide reductase M1 [Source:MGI Symbol;Acc:MGI:98180]	6.90906	8.77499	9.43569	9.16436	9.26368	9.53985	7.7251	8.50716	11.0612	7.97157	9.25217	9.80436	10.3398	9.34973	0.873567285	0.966018836	24	4	24	0.558015741	0.839532165	24	10.95521555	0.026011772
ENSMUSG00000030979	Uros	uroporphyrinogen III synthase [Source:MGI Symbol;Acc:MGI:98917]	26.3292	25.0719	25.5587	26.7468	27.9805	26.7299	25.7893	23.6776	24.819	20.5444	29.0359	25.2114	25.9541	23.7737	0.008961721	0.192760771	20	12	24	0.12334174	0.736234617	24	18.90280193	0.087976842
ENSMUSG00000030980	Knop1	lysine rich nucleolar protein 1 [Source:MGI Symbol;Acc:MGI:1913606]	12.2292	12.8217	14.2622	12.8157	10.3507	12.655	11.6892	12.0371	15.4821	13.8635	13.5993	14.3482	13.4425	13.234	0.43565473	0.966018836	12	16	24	0.259489427	0.768040178	24	8.568323454	0.081696346
ENSMUSG00000030982	Vps35l	VPS35 endosomal protein sorting factor like [Source:MGI Symbol;Acc:MGI:1918767]	33.1027	27.3226	28.0052	28.5995	29.8836	32.7845	27.6631	30.1708	24.4389	30.5713	25.9478	28.6471	28.9183	28.3268	0.601647512	0.966018836	24	12	24	0.206341763	0.768040178	24	20.79111312	0.076772837
ENSMUSG00000030983	Bccip	BRCA2 and CDKN1A interacting protein [Source:MGI Symbol;Acc:MGI:1913415]	28.5033	28.5253	29.6063	26.1839	26.7727	28.15	28.5536	29.2698	29.5537	26.0277	28.799	27.6566	27.224	28.4315	0.013400636	0.233323991	12	4	24	0.13019566	0.740697538	24	3.630722106	0.043530815
ENSMUSG00000030986	Dhx32	DEAH (Asp-Glu-Ala-His) box polypeptide 32 [Source:MGI Symbol;Acc:MGI:2141813]	12.0187	10.9853	11.7447	11.751	12.8573	13.6852	11.236	12.5193	10.5437	12.0308	11.9922	12.1089	11.349	12.2559	0.236893212	0.7385769	24	12	24	0.160102668	0.76564405	24	18.27908562	0.065293395
ENSMUSG00000030987	Stim1	stromal interaction molecule 1 [Source:MGI Symbol;Acc:MGI:107476]	16.8192	17.856	19.165	20.4224	19.5145	17.8094	20.3546	21.8193	19.9291	25.355	18.4076	19.6011	18.232	19.8059	0.227302972	0.723724673	16	8	24	0.084089197	0.701763292	24	10.37084582	0.10956783
ENSMUSG00000030990	Pgap2	post-GPI attachment to proteins 2 [Source:MGI Symbol;Acc:MGI:2385286]	12.7731	12.7648	11.3278	11.8433	12.961	12.062	11.5451	12.424	12.5194	12.9707	12.1303	12.1911	11.5292	11.8868	0.91241026	0.966018836	20	8	24	0.733796604	0.9131851	24	14.37095147	0.018219453
ENSMUSG00000030994	D7Ertd443e	DNA segment, Chr 7, ERATO Doi 443, expressed [Source:MGI Symbol;Acc:MGI:1196431]	1.17231	1.18673	1.69926	1.22663	0.846545	1.00906	1.52912	1.31933	1.13544	1.25099	1.11721	1.03668	0.942473	1.67283	0.049012389	0.379911536	8	16	24	0.049401067	0.642159329	24	6.08707565	0.147146259
ENSMUSG00000030996	Art1	ADP-ribosyltransferase 1 [Source:MGI Symbol;Acc:MGI:107511]	1.31318	5.55955	7.2363	8.47625	4.02888	5.78651	3.35237	12.6197	9.46756	12.2264	4.07985	4.35865	6.90466	6.3567	0.070786393	0.440262583	16	4	24	0.012926665	0.527834117	24	9.049995098	0.670875163
ENSMUSG00000031004	Mki67	antigen identified by monoclonal antibody Ki 67 [Source:MGI Symbol;Acc:MGI:106035]	1.02239	1.73129	2.67621	2.46118	1.67304	2.17683	1.52932	1.44169	1.72708	0.949331	1.54753	1.94331	2.31419	1.21031	0.903465533	0.966018836	12	20	24	0.89999001	0.971572371	24	14.67907705	0.05062189
ENSMUSG00000031007	Atp6ap2	ATPase, H+ transporting, lysosomal accessory protein 2 [Source:MGI Symbol;Acc:MGI:1917745]	122.58	110.938	110.93	108.279	108.136	116.025	116.611	107.874	92.557	112.956	113.069	106.197	107.565	109.068	0.941955432	0.966018836	4	8	24	0.121763736	0.734072261	24	21.015189	0.06703449
ENSMUSG00000031010	Usp9x	ubiquitin specific peptidase 9, X chromosome [Source:MGI Symbol;Acc:MGI:894681]	41.077	41.9258	41.1095	44.1974	43.9129	39.3097	39.1224	39.5768	45.8837	38.1147	47.7583	45.1354	47.954	44.6876	0.825856869	0.966018836	20	8	24	0.861632225	0.95949572	24	14.9079044	0.020879971
ENSMUSG00000031012	Cask	calcium/calmodulin-dependent serine protein kinase (MAGUK family) [Source:MGI Symbol;Acc:MGI:1309489]	14.4503	16.1252	11.4698	13.0134	14.3145	11.9377	13.6591	12.2602	13.0783	15.0687	13.6242	14.5522	17.875	16.6528	0.648082424	0.966018836	8	4	24	0.490278555	0.805393068	24	0.825565358	0.073030027
ENSMUSG00000031015	Swap70	SWA-70 protein [Source:MGI Symbol;Acc:MGI:1298390]	7.02174	9.04823	7.7459	8.55958	9.13696	7.40691	8.47433	9.12938	9.74433	9.1178	7.64283	9.13256	8.79051	7.87418	0.943890799	0.966018836	12	16	24	0.43538173	0.805393068	24	9.242122225	0.064182299
ENSMUSG00000031016	Wee1	WEE 1 homolog 1 (S. pombe) [Source:MGI Symbol;Acc:MGI:103075]	2.45477	2.62985	3.62446	3.7321	2.69265	2.92467	2.77884	2.2842	3.02966	3.92422	2.81376	2.65172	2.04013	2.57767	0.001923196	0.100255953	16	16	24	0.002593171	0.398198373	24	11.86891235	0.211695423
ENSMUSG00000031021	Tmem9b	TMEM9 domain family, member B [Source:MGI Symbol;Acc:MGI:1915254]	56.9129	55.8033	53.8095	56.4655	53.168	54.4429	56.7477	54.2243	54.3574	54.185	57.8599	57.3772	59.4322	56.6341	0.090240671	0.47972073	4	8	24	0.169113013	0.767020009	24	22.64386183	0.030593217
ENSMUSG00000031023	Akip1	A kinase (PRKA) interacting protein 1 [Source:MGI Symbol;Acc:MGI:3041226]	7.68382	8.61353	6.10728	7.69508	8.02083	7.91997	8.01852	8.20699	7.51121	7.77702	9.29711	7.76976	7.69436	7.86676	0.595952838	0.966018836	20	16	24	0.451685391	0.805393068	24	19.58179021	0.05813472
ENSMUSG00000031024	Dennd2b	DENN domain containing 2B [Source:MGI Symbol;Acc:MGI:108517]	7.12685	8.42602	7.28447	5.8132	7.66393	6.64708	7.30067	8.74253	7.8545	7.56882	6.47342	8.24913	8.39944	8.11576	0.030347909	0.324150946	8	8	24	0.051567232	0.64767123	24	3.208833531	0.119574985
ENSMUSG00000031026	Trim66	tripartite motif-containing 66 [Source:MGI Symbol;Acc:MGI:2152406]	0.757585	0.864928	1.17323	0.779228	0.666537	0.926734	1.02532	0.849028	0.822254	0.8675	0.574922	0.768434	0.62602	0.934545	0.235141005	0.73629392	12	8	24	0.188884432	0.768040178	24	5.625561605	0.083851561
ENSMUSG00000031027	Stk33	serine/threonine kinase 33 [Source:MGI Symbol;Acc:MGI:2152419]	2.21961	1.71255	1.87099	1.52667	1.67096	1.64416	0.719977	1.14182	1.15519	1.20012	2.16013	1.31514	1.34908	1.68503	0.806523736	0.966018836	20	8	24	0.732517039	0.912739737	24	16.13701005	0.073566342
ENSMUSG00000031028	Tub	tubby bipartite transcription factor [Source:MGI Symbol;Acc:MGI:2651573]	4.17471	3.89859	6.03351	3.63637	2.93458	4.70936	5.90292	3.19842	4.15484	4.34163	3.68843	4.76654	2.73221	6.82061	0.928064146	0.966018836	12	8	24	0.63401179	0.875589926	24	4.700154153	0.121114333
ENSMUSG00000031029	Eif3f	eukaryotic translation initiation factor 3, subunit F [Source:MGI Symbol;Acc:MGI:1913335]	25.5161	27.4056	28.8524	28.7639	27.5966	28.7866	27.6883	28.6694	28.5002	33.3719	27.0657	31.3849	28.5059	28.5568	0.47028201	0.966018836	16	12	24	0.253047287	0.768040178	24	11.88169068	0.050381049
ENSMUSG00000031059	Ndufb11	NADH:ubiquinone oxidoreductase subunit B11 [Source:MGI Symbol;Acc:MGI:1349919]	70.0231	85.1196	83.2184	87.7763	73.7237	73.0135	76.4905	84.3494	96.3042	67.8467	89.9296	74.5746	85.1468	78.3797	0.570592509	0.966018836	12	12	24	0.230958146	0.768040178	24	7.905641593	0.099157674
ENSMUSG00000031060	Rbm10	RNA binding motif protein 10 [Source:MGI Symbol;Acc:MGI:2384310]	3.66855	3.51047	3.66607	3.34979	3.89775	3.62258	4.0365	3.53196	3.58539	2.96088	3.72656	3.48759	3.58802	3.45283	0.227302972	0.723724673	20	20	24	0.186549447	0.768040178	24	22.00820327	0.056070165
ENSMUSG00000031065	Cdk16	cyclin-dependent kinase 16 [Source:MGI Symbol;Acc:MGI:97516]	73.8003	59.1264	60.0175	65.5439	67.7335	69.4742	70.6782	65.481	57.0711	79.7121	65.917	64.4793	65.0376	67.7953	0.351840494	0.870418653	4	8	24	0.361890119	0.787031962	24	18.69451967	0.0715543
ENSMUSG00000031066	Usp11	ubiquitin specific peptidase 11 [Source:MGI Symbol;Acc:MGI:2384312]	9.83413	10.3062	11.8979	9.46024	8.47305	9.94091	12.0085	10.0066	11.1868	11.4152	7.38242	9.70415	8.69621	11.2845	0.021288455	0.264116537	12	8	24	0.071643694	0.67819049	24	5.707648439	0.154603818
ENSMUSG00000031068	Glrx3	glutaredoxin 3 [Source:MGI Symbol;Acc:MGI:1353653]	18.3611	20.0924	18.5246	19.9219	20.0666	19.0425	18.0258	19.3076	19.7617	17.2531	22.4347	18.3406	19.3112	18.6904	0.581070934	0.966018836	20	8	24	0.607282801	0.863811892	24	14.60107128	0.034218367
ENSMUSG00000031070	Mrgprf	MAS-related GPR, member F [Source:MGI Symbol;Acc:MGI:2384823]	2.92323	2.60889	2.41954	2.34943	2.67982	2.59655	2.71728	2.81127	2.7617	2.18893	2.62913	2.18329	2.21486	2.38232	0.798829513	0.966018836	4	12	24	0.551397833	0.83632237	24	0.942544293	0.036948145
ENSMUSG00000031072	LTO1	ABCE maturation factor [Source:MGI Symbol;Acc:MGI:1919534]	23.4028	19.1587	20.2866	21.03	23.5889	27.535	22.4326	23.0898	15.4734	19.3368	22.0441	20.7782	21.2752	20.8002	0.051610136	0.389629649	24	12	24	0.017465896	0.550995814	24	20.24372031	0.180448176
ENSMUSG00000031074	Fgf3	fibroblast growth factor 3 [Source:MGI Symbol;Acc:MGI:95517]	0.407844	0.594487	0.665801	0.491713	0.641923	0.412798	0.475863	0.316532	0.710684	0.550941	0.870504	0.435482	0.332354	0.576478	0.033248781	0.324908923	12	16	24	0.072583864	0.679437294	24	11.86434853	0.119964573
ENSMUSG00000031075	Ano1	anoctamin 1, calcium activated chloride channel [Source:MGI Symbol;Acc:MGI:2142149]	2.96808	2.9258	2.7844	3.0415	2.43899	2.70153	2.82965	3.34319	2.99454	3.82473	2.76655	2.61126	3.16569	3.01309	0.076247883	0.453790923	16	4	24	0.157075829	0.763141258	24	7.27359575	0.088101347
ENSMUSG00000031077	Fadd	Fas (TNFRSF6)-associated via death domain [Source:MGI Symbol;Acc:MGI:109324]	0.696761	0.947009	0.860672	0.610787	0.853248	0.849974	0.814957	1.00857	0.999898	0.873128	0.914062	0.925717	1.16376	0.742336	0.879206539	0.966018836	12	4	24	0.690927368	0.897666745	24	2.385531255	0.03311727
ENSMUSG00000031078	Cttn	cortactin [Source:MGI Symbol;Acc:MGI:99695]	46.3102	39.9806	42.4939	40.7904	45.4548	44.4391	42.5176	44.3378	39.0466	46.8348	40.8004	43.9848	40.893	41.6964	0.662399683	0.966018836	24	12	24	0.347080577	0.778844143	24	18.80898356	0.046446018
ENSMUSG00000031079	Zfp300	zinc finger protein 300 [Source:MGI Symbol;Acc:MGI:3045326]	0.98487	1.50031	1.24825	0.966942	1.08853	1.12588	1.12847	1.23831	1.56746	0.909067	1.20523	1.08418	0.930819	0.947261	0.503972924	0.966018836	12	4	24	0.462829327	0.805393068	24	6.695683254	0.064380984
ENSMUSG00000031090	Nadsyn1	NAD synthetase 1 [Source:MGI Symbol;Acc:MGI:1926164]	2.66686	3.18123	2.5431	3.75112	3.96572	3.44582	3.52108	2.62946	3.48844	2.83601	3.84919	3.23857	2.80309	2.88494	0.054935173	0.398010585	20	12	24	0.048501669	0.638992719	24	16.10505033	0.145272389
ENSMUSG00000031093	Dock11	dedicator of cytokinesis 11 [Source:MGI Symbol;Acc:MGI:1923224]	56.0999	53.6124	46.8944	51.1195	51.5145	50.6812	48.4925	47.9717	50.8438	47.8084	56.0346	56.0634	60.1697	56.3751	0.248423582	0.746945512	4	8	24	0.196679292	0.768040178	24	21.99186967	0.070439167
ENSMUSG00000031095	Cul4b	cullin 4B [Source:MGI Symbol;Acc:MGI:1919834]	16.2857	17.8047	18.1074	17.8319	16.9164	16.6917	16.2346	15.5303	17.037	17.022	17.5731	18.3657	17.3982	18.3072	0.952551093	0.966018836	12	16	24	0.569323429	0.844847263	24	11.50039384	0.02787327
ENSMUSG00000031097	Tnni2	troponin I, skeletal, fast 2 [Source:MGI Symbol;Acc:MGI:105070]	95.4851	321.931	329.461	424.507	254.293	332.471	253.477	638.456	686.062	538.639	277.664	308.436	377.686	267.919	0.151191877	0.598641364	12	16	24	0.049798848	0.643621507	24	9.412363562	0.592708365
ENSMUSG00000031099	Smarca1	SWI/SNF related, matrix associated, actin dependent regulator of chromatin, subfamily a, member 1 [Source:MGI Symbol;Acc:MGI:1935127]	12.2105	13.2346	12.2689	12.0789	12.2682	12.5997	11.1306	11.0629	13.226	11.0831	12.9555	12.5539	12.8058	12.8119	0.874607729	0.966018836	12	4	24	0.992807617	0.998516607	24	22.71172923	0.004112163
ENSMUSG00000031101	Sash3	SAM and SH3 domain containing 3 [Source:MGI Symbol;Acc:MGI:1921381]	1.18093	1.47494	1.38816	1.34438	1.24518	1.45718	1.36675	1.01914	1.3908	1.01905	1.17476	1.26909	1.58872	1.09499	0.886952527	0.966018836	4	12	24	0.665488174	0.886999446	24	22.72590593	0.039740233
ENSMUSG00000031103	Elf4	E74-like factor 4 (ets domain transcription factor) [Source:MGI Symbol;Acc:MGI:1928377]	0.681785	0.800802	0.810392	0.932587	0.797738	0.621026	0.799923	0.826513	0.962899	0.679891	0.692914	0.815791	0.793587	0.713639	0.432054949	0.966018836	12	12	24	0.241049343	0.768040178	24	8.262022759	0.052051601
ENSMUSG00000031104	Rab33a	RAB33A, member RAS oncogene family [Source:MGI Symbol;Acc:MGI:109493]	10.8921	10.2443	12.6721	9.85479	9.93135	10.5722	9.40005	10.2639	9.29819	9.62017	11.3996	10.6965	9.77068	10.2532	0.9143748	0.966018836	20	20	24	0.964482159	0.991159965	24	16.5948865	0.011610988
ENSMUSG00000031105	Slc25a14	solute carrier family 25 (mitochondrial carrier, brain), member 14 [Source:MGI Symbol;Acc:MGI:1330823]	25.3181	20.4275	20.432	19.9539	21.5878	21.414	21.5733	21.4344	18.725	18.1391	21.9183	21.7756	22.3766	22.3189	0.007886314	0.192760771	4	12	24	0.006229841	0.469371824	24	22.47020819	0.119954876
ENSMUSG00000031107	Rbmx2	RNA binding motif protein, X-linked 2 [Source:MGI Symbol;Acc:MGI:1919414]	3.9028	4.04849	4.24679	4.39727	4.35553	3.62367	3.30137	3.89295	4.793	4.17913	4.33232	3.94273	4.55877	4.36091	0.260026661	0.765037845	12	12	24	0.054900273	0.655263328	24	10.17504632	0.095518361
ENSMUSG00000031109	Enox2	ecto-NOX disulfide-thiol exchanger 2 [Source:MGI Symbol;Acc:MGI:2384799]	8.20816	8.85402	9.33544	8.31667	8.08099	8.74853	7.50816	9.14991	7.95808	8.40575	9.26598	8.88968	9.09355	8.41417	0.819898687	0.966018836	8	20	24	0.984928011	0.996964449	24	8.593024688	0.006094595
ENSMUSG00000031111	Igsf1	immunoglobulin superfamily, member 1 [Source:MGI Symbol;Acc:MGI:2147913]	0.532458	1.00668	1.93333	0.964016	0.416222	1.08522	1.62629	0.661981	1.13577	0.825345	0.406585	0.912525	0.284195	1.70021	0.10560184	0.518002936	12	8	24	0.248635668	0.768040178	24	6.361452446	0.241297389
ENSMUSG00000031112	Stk26	serine/threonine kinase 26 [Source:MGI Symbol;Acc:MGI:1917665]	3.19251	3.66382	3.59894	3.27096	3.5013	3.33444	3.0176	3.08234	3.17574	2.62604	3.6829	2.72545	2.91484	3.31336	0.909604019	0.966018836	20	8	24	0.881661903	0.966661503	24	10.14813547	0.021186382
ENSMUSG00000031119	Gpc4	glypican 4 [Source:MGI Symbol;Acc:MGI:104902]	2.30704	2.98075	2.90928	3.06562	3.03162	2.55396	3.11221	3.43264	3.23556	3.43591	3.12144	2.90814	3.12627	2.96638	0.616047374	0.966018836	16	8	24	0.051698057	0.648075114	24	9.499046064	0.090005059
ENSMUSG00000031129	Slc9a9	solute carrier family 9 (sodium/hydrogen exchanger), member 9 [Source:MGI Symbol;Acc:MGI:2679732]	83.3091	71.2606	67.213	72.1504	67.0052	77.3078	71.4821	73.3452	60.4815	79.2082	71.6255	75.481	79.3117	79.0963	0.16456443	0.628002127	4	8	24	0.178862736	0.768040178	24	22.28997074	0.08511794
ENSMUSG00000031133	Arhgef6	Rac/Cdc42 guanine nucleotide exchange factor (GEF) 6 [Source:MGI Symbol;Acc:MGI:1920591]	13.3618	13.7203	13.2915	13.4549	14.0012	14.7374	14.1367	14.6217	16.4396	15.5801	15.3839	15.2798	12.8038	13.1927	0.29905572	0.809362476	24	4	24	0.294815134	0.768040178	24	13.61329482	0.057871502
ENSMUSG00000031134	Rbmx	RNA binding motif protein, X chromosome [Source:MGI Symbol;Acc:MGI:1343044]	17.6625	23.962	23.3672	22.4656	21.5259	20.6185	18.7778	20.1237	25.6452	21.4345	20.5614	21.2128	19.637	21.9155	1.18E-04	0.029372629	12	16	24	0.01449127	0.534799026	24	9.560475286	0.137972782
ENSMUSG00000031137	Fgf13	fibroblast growth factor 13 [Source:MGI Symbol;Acc:MGI:109178]	275.883	223.703	228.23	239.925	233.897	269.51	225.374	242.715	184.318	271.627	226.558	247.269	260.266	245.811	0.390686537	0.915479136	24	12	24	0.243959874	0.768040178	24	20.54063651	0.100653689
ENSMUSG00000031138	F9	coagulation factor IX [Source:MGI Symbol;Acc:MGI:88384]	1.1855	1.32464	1.13949	1.24355	1.2524	1.0203	1.16701	1.13496	1.04368	1.52512	1.32954	1.02589	1.33877	1.21862	0.814077113	0.966018836	20	4	24	0.577145562	0.848822643	24	11.36721478	0.038043289
ENSMUSG00000031139	Mcf2	mcf.2 transforming sequence [Source:MGI Symbol;Acc:MGI:96932]	1.61278	1.91815	1.97654	1.89534	1.64924	1.69867	1.75157	1.67376	2.00851	1.32568	1.58365	1.78153	1.42178	2.17721	0.170143858	0.643496587	12	8	24	0.229681856	0.768040178	24	6.168688791	0.082451025
ENSMUSG00000031143	Ccdc22	coiled-coil domain containing 22 [Source:MGI Symbol;Acc:MGI:1859608]	6.51741	6.36072	6.66585	6.79618	6.68986	6.91974	7.227	6.62048	5.79689	6.63707	7.08496	6.7736	6.76666	6.45717	0.029412383	0.322165609	24	8	24	0.063487858	0.663715314	24	19.11688525	0.057929245
ENSMUSG00000031144	Syp	synaptophysin [Source:MGI Symbol;Acc:MGI:98467]	149.077	122.463	130.266	118.016	112.932	143.792	134.964	130.139	100.369	155.572	108.664	137.105	122.328	141.814	0.823657258	0.966018836	24	20	24	0.459730941	0.805393068	24	23.39578966	0.089199059
ENSMUSG00000031145	Prickle3	prickle planar cell polarity protein 3 [Source:MGI Symbol;Acc:MGI:1859635]	1.66895	1.84915	1.92253	2.27766	1.97456	2.05012	2.58623	2.43735	2.28179	2.55855	2.28837	2.12936	2.10801	2.06218	0.886572916	0.966018836	16	16	24	0.465785277	0.805393068	24	11.3603348	0.05248637
ENSMUSG00000031146	Plp2	proteolipid protein 2 [Source:MGI Symbol;Acc:MGI:1298382]	6.38849	5.48613	4.71376	5.001	4.8551	5.3229	5.83953	5.16842	5.5753	5.27597	5.06144	5.1276	6.35851	5.60684	0.001953237	0.100255953	4	16	24	0.019478103	0.564709807	24	0.706423632	0.106693662
ENSMUSG00000031147	Magix	MAGI family member, X-linked [Source:MGI Symbol;Acc:MGI:1859644]	1.45709	2.31897	1.58647	1.95331	2.12226	2.01559	2.02335	2.66627	2.73896	2.96393	1.84615	2.45788	2.86274	2.50045	0.910436409	0.966018836	16	4	24	0.386518217	0.799546792	24	7.065241794	0.088939471
ENSMUSG00000031148	Gpkow	G patch domain and KOW motifs [Source:MGI Symbol;Acc:MGI:1859610]	12.5577	12.5408	13.5375	12.5357	12.5659	12.2138	11.7089	12.9569	11.8739	11.4367	12.3877	11.736	12.4538	12.0911	0.922029406	0.966018836	8	16	24	0.662771361	0.886154038	24	5.980239344	0.019043296
ENSMUSG00000031149	Praf2	PRA1 domain family 2 [Source:MGI Symbol;Acc:MGI:1859607]	81.6325	62.2403	67.4784	64.4397	66.6624	76.0474	65.4163	68.6167	56.8549	71.1142	70.6855	66.301	69.8581	68.2506	0.628899575	0.966018836	24	12	24	0.14028392	0.749574609	24	20.58594205	0.093724643
ENSMUSG00000031150	Ccdc120	coiled-coil domain containing 120 [Source:MGI Symbol;Acc:MGI:1859619]	2.73321	1.74138	2.16229	2.04931	1.56014	1.91067	1.20825	2.60076	2.18351	2.51546	3.28336	2.74042	2.25554	3.12341	0.722624967	0.966018836	8	20	24	0.852225117	0.955628938	24	9.473807894	0.05567429
ENSMUSG00000031153	Gripap1	GRIP1 associated protein 1 [Source:MGI Symbol;Acc:MGI:1859616]	41.2891	38.8341	37.8036	38.5333	42.6655	40.4648	37.7987	40.1163	36.1546	35.6343	38.0379	37.3524	37.6571	38.6654	0.870491976	0.966018836	20	20	24	0.169788517	0.76790765	24	20.91241977	0.045195722
ENSMUSG00000031154	Otud5	OTU domain containing 5 [Source:MGI Symbol;Acc:MGI:1859615]	23.408	24.4812	25.8604	24.5671	25.9858	24.8569	26.7811	24.0678	22.5223	23.5667	22.6536	23.3431	22.1156	21.8799	0.847846021	0.966018836	20	12	24	0.65234282	0.88299052	24	17.55258635	0.024928149
ENSMUSG00000031155	Pim2	proviral integration site 2 [Source:MGI Symbol;Acc:MGI:97587]	8.35936	6.93732	7.18643	7.9084	7.55806	7.6109	8.38888	7.34176	6.21794	8.79756	7.62874	8.84732	8.02123	8.20552	0.128989168	0.560209134	24	12	24	0.200701083	0.768040178	24	19.82196475	0.083735764
ENSMUSG00000031156	Slc35a2	solute carrier family 35 (UDP-galactose transporter), member A2 [Source:MGI Symbol;Acc:MGI:1345297]	14.5403	12.695	13.783	11.6358	13.7191	16.6929	14.8702	13.1239	12.9027	14.7869	13.211	13.0544	14.8114	11.8392	0.221479046	0.71314449	4	4	24	0.138415486	0.747972781	24	20.52196606	0.101320418
ENSMUSG00000031157	Pqbp1	polyglutamine binding protein 1 [Source:MGI Symbol;Acc:MGI:1859638]	31.9347	29.2323	32.4624	26.5621	31.315	31.181	30.9617	28.5914	27.9539	31.7358	26.4002	31.3306	28.9032	29.8221	0.907068194	0.966018836	24	20	24	0.562651767	0.842415325	24	22.33077642	0.040122313
ENSMUSG00000031158	Timm17b	translocase of inner mitochondrial membrane 17b [Source:MGI Symbol;Acc:MGI:1343176]	16.4765	17.3365	16.4808	18.5368	18.0101	18.7574	15.7527	16.8667	16.731	13.628	16.2363	16.8459	15.5702	14.4668	0.885955579	0.966018836	24	4	24	0.345046804	0.778298718	24	18.21844267	0.058522313
ENSMUSG00000031161	Hdac6	histone deacetylase 6 [Source:MGI Symbol;Acc:MGI:1333752]	9.63268	9.2103	11.2114	9.60282	10.5897	10.6268	9.61847	9.06667	9.46137	10.5091	9.05787	9.29953	9.57229	10.4186	0.886572916	0.966018836	12	20	24	0.640765105	0.878446626	24	12.23727875	0.03342076
ENSMUSG00000031165	Was	Wiskott-Aldrich syndrome [Source:MGI Symbol;Acc:MGI:105059]	0.913522	0.929905	1.23718	1.19358	0.886022	0.897443	1.09689	0.893724	1.09027	0.777617	0.798848	1.00555	1.16589	0.872152	0.849179819	0.966018836	12	8	24	0.661792046	0.885615772	24	5.73615241	0.038917546
ENSMUSG00000031166	Wdr13	WD repeat domain 13 [Source:MGI Symbol;Acc:MGI:1914661]	35.285	33.5524	32.7019	36.0366	35.904	33.7049	37.1584	33.0178	32.7723	37.2647	33.706	34.423	36.6114	33.9376	0.079180494	0.458578242	16	20	24	0.44183277	0.805393068	24	18.45264609	0.033845643
ENSMUSG00000031167	Rbm3	RNA binding motif (RNP1, RRM) protein 3 [Source:MGI Symbol;Acc:MGI:1099460]	15.7124	16.9792	17.8812	17.9368	17.3303	15.0764	16.4262	15.2029	21.19	14.8832	18.9052	14.536	16.3889	16.8689	0.023472439	0.278499043	12	12	24	0.158935707	0.765350752	24	9.733281511	0.105122761
ENSMUSG00000031168	Ebp	phenylalkylamine Ca2+ antagonist (emopamil) binding protein [Source:MGI Symbol;Acc:MGI:107822]	26.8898	21.2448	21.8834	23.3764	25.8686	27.3219	24.2474	24.0108	20.5361	20.4184	26.249	21.2057	21.9863	21.0622	0.102932049	0.516411901	20	20	24	0.019179727	0.562219749	24	19.42116799	0.134999411
ENSMUSG00000031169	Porcn	porcupine O-acyltransferase [Source:MGI Symbol;Acc:MGI:1890212]	13.8651	13.3759	14.9374	15.1836	13.8812	14.908	14.6002	14.6171	13.226	15.7366	13.6668	14.7392	13.9901	15.4617	0.941955432	0.966018836	16	12	24	0.795098348	0.934298757	24	11.47599819	0.019644572
ENSMUSG00000031170	Slc38a5	solute carrier family 38, member 5 [Source:MGI Symbol;Acc:MGI:2148066]	0.790081	2.08706	1.83185	1.10637	1.08983	1.42462	1.22379	2.79669	1.77357	0.957324	0.808889	1.28439	1.55916	1.32778	0.032202658	0.324385159	8	12	24	0.042345738	0.623698598	24	4.972557678	0.275916415
ENSMUSG00000031171	Ftsj1	FtsJ RNA methyltransferase homolog 1 (E. coli) [Source:MGI Symbol;Acc:MGI:1859648]	15.0192	14.531	14.2046	13.0159	14.4579	14.258	14.485	14.2921	14.289	12.454	14.9869	14.27	14.3157	13.4936	0.032263213	0.324385159	4	8	24	0.079540569	0.693197702	24	22.53250977	0.056676052
ENSMUSG00000031174	Rpgr	retinitis pigmentosa GTPase regulator [Source:MGI Symbol;Acc:MGI:1344037]	4.33078	4.50592	4.19405	4.10821	4.63692	3.78835	3.83355	3.57758	4.52488	3.81864	4.65997	4.00893	3.57248	4.21013	0.522510172	0.966018836	20	4	24	0.426840065	0.805393068	24	12.73291342	0.051369758
ENSMUSG00000031176	Dynlt3	dynein light chain Tctex-type 3 [Source:MGI Symbol;Acc:MGI:1914367]	277.104	286.369	263.25	263.784	268.612	252.934	252.511	248.432	268.384	229.637	292.044	249.805	262.466	272.441	0.890648739	0.966018836	20	4	24	0.924850743	0.98002538	24	1.420581337	0.013850716
ENSMUSG00000031189	Aff2	AF4/FMR2 family, member 2 [Source:MGI Symbol;Acc:MGI:1202294]	2.36081	1.97331	2.1697	1.8831	2.02487	1.89608	1.97693	1.59801	1.98924	1.94826	1.96473	2.55607	2.29554	2.23194	0.47028201	0.966018836	4	12	24	0.449280078	0.805393068	24	21.75868434	0.056698193
ENSMUSG00000031196	F8	coagulation factor VIII [Source:MGI Symbol;Acc:MGI:88383]	1.23326	1.66002	1.32173	1.23663	1.46975	1.42198	1.28576	1.47832	1.50817	1.10607	1.40698	1.45519	1.46132	1.42314	0.350544145	0.870418653	8	8	24	0.496470795	0.807537659	24	1.789280806	0.039455654
ENSMUSG00000031197	Vbp1	von Hippel-Lindau binding protein 1 [Source:MGI Symbol;Acc:MGI:1333804]	89.4444	92.3976	89.0002	84.9442	87.5767	85.5488	83.6383	78.1886	83.801	74.0356	90.1629	81.668	81.7347	84.7866	0.84680398	0.966018836	20	20	24	0.778467978	0.928832041	24	21.61241634	0.021218258
ENSMUSG00000031198	Fundc2	FUN14 domain containing 2 [Source:MGI Symbol;Acc:MGI:1914641]	20.1325	25.3977	25.2346	24.7867	22.9645	22.2915	22.0497	25.1572	26.5036	22.4783	23.0433	19.7329	21.3769	22.7018	8.66E-04	0.071344007	12	16	24	0.002969869	0.414576087	24	8.601288284	0.134932434
ENSMUSG00000031200	Mtcp1	mature T cell proliferation 1 [Source:MGI Symbol;Acc:MGI:102699]	3.19606	3.62838	3.18106	3.90034	3.48771	2.968	3.72615	2.62548	4.5654	2.7073	3.39412	2.70217	2.91226	3.37943	0.868508291	0.966018836	12	16	24	0.500989236	0.80908345	24	9.155496954	0.081669307
ENSMUSG00000031201	Brcc3	BRCA1/BRCA2-containing complex, subunit 3 [Source:MGI Symbol;Acc:MGI:2389572]	21.6425	24.6821	23.8974	23.008	24.4447	20.2091	26.9415	20.068	26.0409	20.4018	26.2485	23.9971	22.0141	24.402	0.858266942	0.966018836	20	4	24	0.951645684	0.988339231	24	10.00172157	0.017903614
ENSMUSG00000031202	Rab39b	RAB39B, member RAS oncogene family [Source:MGI Symbol;Acc:MGI:1915040]	29.6034	26.5104	26.736	25.381	26.5333	26.6427	26.8382	24.4223	21.7626	26.6784	28.6077	25.0487	24.2633	25.9744	0.933543173	0.966018836	20	12	24	0.232826464	0.768040178	24	19.3214278	0.065056648
ENSMUSG00000031204	Asb12	ankyrin repeat and SOCS box-containing 12 [Source:MGI Symbol;Acc:MGI:1917642]	0.144954	0.571289	0.709539	0.708209	0.543772	0.907311	0.57522	2.27478	1.56073	1.38841	0.324509	0.738081	0.891938	0.620027	0.791413662	0.966018836	12	8	24	0.209210299	0.768040178	24	7.841354978	0.261145067
ENSMUSG00000031207	Msn	moesin [Source:MGI Symbol;Acc:MGI:97167]	117.545	87.441	93.3292	92.9026	98.8996	113.122	100.287	107.527	72.1765	113.525	96.014	97.5668	93.491	93.4119	0.196044629	0.673646834	24	12	24	0.182920777	0.768040178	24	20.06471815	0.127675684
ENSMUSG00000031209	Heph	hephaestin [Source:MGI Symbol;Acc:MGI:1332240]	0.400174	0.715515	1.07962	0.477238	0.422463	0.721651	0.880934	0.512019	0.628079	0.453894	0.430412	0.553821	0.406299	0.613301	0.481811994	0.966018836	12	8	24	0.422915077	0.805019467	24	5.083836456	0.086554631
ENSMUSG00000031210	Gpr165	G protein-coupled receptor 165 [Source:MGI Symbol;Acc:MGI:1923456]	1.05282	1.27208	1.68846	0.941852	0.971805	1.55	1.81664	0.927663	1.38545	1.25333	1.09705	1.31385	1.01761	1.48856	0.93717134	0.966018836	8	4	24	0.713150077	0.905403147	24	3.22437202	0.058716007
ENSMUSG00000031214	Ophn1	oligophrenin 1 [Source:MGI Symbol;Acc:MGI:2151070]	15.2733	14.7684	13.9317	13.4973	13.4799	14.3333	14.6934	12.8409	13.4318	14.3637	15.1828	14.836	13.912	13.5585	0.404482447	0.938325912	4	8	24	0.137827305	0.747972781	24	20.14773756	0.054347597
ENSMUSG00000031216	Stard8	START domain containing 8 [Source:MGI Symbol;Acc:MGI:2448556]	12.4689	11.1994	9.73012	10.0774	12.7515	10.6303	12.6722	9.46318	12.0098	11.3806	11.2757	11.6796	11.9372	9.21664	0.125573493	0.555618842	4	4	24	0.166476857	0.767020009	24	19.43864713	0.106418268
ENSMUSG00000031217	Efnb1	ephrin B1 [Source:MGI Symbol;Acc:MGI:102708]	6.86015	6.48217	5.17472	4.7731	6.13442	5.37901	6.15965	7.10673	5.79635	6.4218	7.18133	6.46424	7.61635	5.88789	0.147689593	0.590732968	4	8	24	0.223960294	0.768040178	24	23.2551979	0.101481882
ENSMUSG00000031221	Igbp1	immunoglobulin (CD79A) binding protein 1 [Source:MGI Symbol;Acc:MGI:1346500]	28.0911	30.8722	29.7531	29.41	29.9859	28.6707	28.5878	27.9526	32.1478	24.3309	29.6041	28.6097	30.2563	28.5366	0.929527443	0.966018836	12	20	24	0.84445628	0.952844769	24	3.662118471	0.020567351
ENSMUSG00000031224	Magee2	MAGE family member E2 [Source:MGI Symbol;Acc:MGI:2148316]	2.72197	2.34979	2.46199	2.05739	2.1321	2.33125	2.36051	2.19823	1.95153	2.18632	2.29879	2.3236	1.9661	2.28757	0.54278565	0.966018836	4	12	24	0.236036338	0.768040178	24	22.8214144	0.052008839
ENSMUSG00000031226	Pbdc1	polysaccharide biosynthesis domain containing 1 [Source:MGI Symbol;Acc:MGI:1914933]	8.9003	10.0465	8.43835	9.01424	9.63659	8.54665	9.28439	8.33211	8.9934	7.43811	10.3794	9.3325	10.3995	9.00789	0.602158008	0.966018836	20	20	24	0.399655495	0.799546792	24	21.44462242	0.064561531
ENSMUSG00000031227	Magee1	MAGE family member E1 [Source:MGI Symbol;Acc:MGI:2148149]	42.3811	33.5424	35.1335	34.9474	32.1196	39.0155	37.4694	37.4659	28.7081	49.3473	32.9114	39.2029	34.9651	39.4409	0.593560247	0.966018836	12	8	24	0.813334069	0.940400609	24	21.55605377	0.049747944
ENSMUSG00000031229	Atrx	ATRX, chromatin remodeler [Source:MGI Symbol;Acc:MGI:103067]	16.1136	22.1087	18.9001	19.6938	19.7461	15.6872	21.3253	17.0997	25.1843	14.5736	20.778	17.9211	20.17	20.3476	0.823677655	0.966018836	12	8	24	0.511317893	0.814808034	24	6.544683377	0.095606401
ENSMUSG00000031231	Cox7b	cytochrome c oxidase subunit 7B [Source:MGI Symbol;Acc:MGI:1913392]	219.013	320.608	254.632	324.664	323.643	242.157	237.22	288.059	317.894	234.928	303.718	271.008	305.526	283.451	0.575202741	0.966018836	20	8	24	0.454348997	0.805393068	24	10.36540869	0.098085359
ENSMUSG00000031232	Magt1	magnesium transporter 1 [Source:MGI Symbol;Acc:MGI:1914325]	19.2131	18.7723	17.5154	16.4568	20.0068	17.1752	18.5895	16.5533	18.5664	16.0549	22.428	18.7602	19.2784	18.0287	0.035008838	0.328311403	20	8	24	0.326390022	0.768040178	24	19.71887635	0.072491808
ENSMUSG00000031239	Itm2a	integral membrane protein 2A [Source:MGI Symbol;Acc:MGI:107706]	89.2584	116.511	101.899	98.455	112.694	86.6898	110.622	104.841	132.504	80.4749	100.42	85.9342	92.8666	89.7094	0.245061943	0.741580974	12	12	24	0.42177039	0.804844498	24	7.181897839	0.102088954
ENSMUSG00000031242	2610002M06Rik	RIKEN cDNA 2610002M06 gene [Source:MGI Symbol;Acc:MGI:1914278]	11.4404	11.2065	11.0902	10.0985	10.0814	10.8746	11.634	10.3949	10.1432	10.7084	11.5282	11.0095	10.2749	11.2189	0.423253809	0.966018836	12	12	24	0.324633394	0.768040178	24	23.33501441	0.039703849
ENSMUSG00000031245	Hmgn5	high-mobility group nucleosome binding domain 5 [Source:MGI Symbol;Acc:MGI:1355295]	2.16982	2.32233	2.94545	3.42861	2.57445	2.1441	2.37827	3.03405	2.92396	2.46724	2.76351	2.54353	2.61519	2.71911	0.073253108	0.447768907	12	8	24	0.025971339	0.586541342	24	9.742811873	0.133697904
ENSMUSG00000031246	Sh3bgrl	SH3-binding domain glutamic acid-rich protein like [Source:MGI Symbol;Acc:MGI:1930849]	65.7083	77.3492	71.4719	73.1421	75.1259	62.9133	68.3953	65.6603	72.7795	57.1705	76.5076	57.0266	57.5246	64.8187	0.265774856	0.773510818	20	4	24	0.409119881	0.799546792	24	10.39493503	0.069982049

ENSMUSG00000031250	Tnmd	tenomodulin [Source:MGI Symbol;Acc:MGI:1929885]	0.50001	1.06812	0.761548	0.958505	0.625576	0.588334	0.447794	1.0466	1.16245	0.374318	0.551301	0.487776	1.24139	0.669164	0.282213399	0.793714531	8	16	24	0.143954114	0.752803875	24	5.95717316	0.178245735
ENSMUSG00000031253	Srpx2	sushi-repeat-containing protein, X-linked 2 [Source:MGI Symbol;Acc:MGI:1916042]	0.966865	1.13767	1.09368	0.965342	0.7616	0.964127	1.09397	1.31849	1.00102	1.0131	0.769676	0.956475	0.896979	0.750072	0.166709446	0.633685338	8	12	24	0.15754253	0.76387796	24	4.610800619	0.076393436
ENSMUSG00000031255	Sytl4	synaptotagmin-like 4 [Source:MGI Symbol;Acc:MGI:1351606]	0.578945	0.736323	0.589795	0.60308	0.663231	0.60861	0.518364	0.864832	0.653892	0.624025	0.671928	0.840481	0.713108	0.691029	0.773177465	0.966018836	8	8	24	0.802825255	0.936770924	24	2.973798735	0.019978256
ENSMUSG00000031256	Cstf2	cleavage stimulation factor, 3' pre-RNA subunit 2 [Source:MGI Symbol;Acc:MGI:1343054]	20.2686	20.5096	22.468	19.8949	20.3691	21.0159	19.9716	19.5531	19.1341	21.3795	18.0319	19.1164	17.6352	19.7728	0.910436409	0.966018836	16	12	24	0.496890647	0.807576732	24	9.711525656	0.033555541
ENSMUSG00000031262	Cenpi	centromere protein I [Source:MGI Symbol;Acc:MGI:2147897]	0.542298	0.647795	0.663134	0.561237	0.639924	0.722596	0.528674	0.400639	0.872959	0.568882	0.722921	0.752656	0.792853	0.510685	0.4776989	0.966018836	24	8	24	0.576754648	0.848735805	24	16.23448943	0.046274897
ENSMUSG00000031264	Btk	Bruton agammaglobulinemia tyrosine kinase [Source:MGI Symbol;Acc:MGI:88216]	0.563033	1.08686	1.16014	1.09516	0.841031	0.899813	0.753338	0.763509	1.14033	0.637948	0.842747	1.03239	0.829264	0.616239	0.109040811	0.524035616	12	16	24	0.466289967	0.805393068	24	10.98524673	0.074728077
ENSMUSG00000031266	Gla	galactosidase, alpha [Source:MGI Symbol;Acc:MGI:1347344]	9.68574	9.17054	9.27375	8.05609	8.53097	9.40477	8.53401	9.24541	8.125	8.4764	9.25292	8.88351	9.48573	9.04986	0.06180207	0.409814092	4	12	24	0.064734863	0.663876076	24	23.58793359	0.064268831
ENSMUSG00000031274	Col4a5	collagen, type IV, alpha 5 [Source:MGI Symbol;Acc:MGI:88456]	2.32571	2.7854	2.63562	2.06041	2.60122	2.11059	3.23864	2.89561	2.86317	2.15677	1.73048	1.91032	1.84273	1.97464	0.296502765	0.807732044	8	16	24	0.178162414	0.768040178	24	4.963781517	0.122006055
ENSMUSG00000031278	Acsl4	acyl-CoA synthetase long-chain family member 4 [Source:MGI Symbol;Acc:MGI:1354713]	27.9794	24.2082	25.0359	24.9033	25.2814	26.1974	22.3925	22.5685	21.2896	22.1627	27.3326	23.8476	24.7912	24.2907	0.480187506	0.966018836	20	16	24	0.12235922	0.735058232	24	19.27464699	0.082865858
ENSMUSG00000031283	Chrdl1	chordin-like 1 [Source:MGI Symbol;Acc:MGI:1933172]	16.3347	20.0612	17.9534	16.1364	20.6258	16.4103	20.4995	17.4103	22.2688	17.0007	16.3916	17.2934	19.3569	15.5993	0.860709018	0.966018836	12	4	24	0.825487395	0.9453534	24	5.134657103	0.038757684
ENSMUSG00000031284	Pak3	p21 (RAC1) activated kinase 3 [Source:MGI Symbol;Acc:MGI:1339656]	10.2367	10.5789	10.4427	10.0018	10.0087	9.80706	10.1715	8.68093	10.265	8.5619	10.6862	10.9391	10.7068	11.694	0.892555977	0.966018836	8	8	24	0.602773939	0.861114219	24	23.98723308	0.042417191
ENSMUSG00000031285	Dcx	doublecortin [Source:MGI Symbol;Acc:MGI:1277171]	1.46183	1.47219	1.53108	1.32199	1.28781	1.5643	1.55723	1.38937	1.63768	1.25008	1.46816	1.71785	1.5651	1.83502	0.260026661	0.765037845	24	20	24	0.09077783	0.71175955	24	1.685528481	0.065984682
ENSMUSG00000031290	Lrch2	leucine-rich repeats and calponin homology (CH) domain containing 2 [Source:MGI Symbol;Acc:MGI:2147870]	3.50636	4.45728	4.75645	4.06663	3.64497	3.80184	4.11988	3.0235	3.95003	3.39658	3.25042	4.2505	3.739	4.21663	0.917867465	0.966018836	12	8	24	0.669391642	0.888647347	24	5.36818222	0.051824091
ENSMUSG00000031292	Cdkl5	cyclin-dependent kinase-like 5 [Source:MGI Symbol;Acc:MGI:1278336]	1.55666	2.11886	2.15297	1.95542	1.97492	1.77967	2.13387	1.6052	2.45878	1.69086	1.80565	2.03782	1.81832	2.04235	0.43280837	0.966018836	12	4	24	0.446573479	0.805393068	24	8.20507733	0.062658709
ENSMUSG00000031295	Phka2	phosphorylase kinase alpha 2 [Source:MGI Symbol;Acc:MGI:97577]	4.98174	6.7812	6.71016	6.40209	5.69728	5.5981	5.37553	5.51427	7.67814	6.49808	5.79597	6.74378	6.53709	6.78168	0.105280783	0.518002936	12	16	24	0.041021831	0.61771321	24	8.712032284	0.129751699
ENSMUSG00000031297	Slc7a3	solute carrier family 7 (cationic amino acid transporter, y+ system), member 3 [Source:MGI Symbol;Acc:MGI:1100521]	2.61991	2.46687	3.20485	2.60217	2.82654	2.63166	2.47286	2.5425	2.55323	2.48979	3.16426	3.08332	2.64089	2.82382	0.39533702	0.925130113	20	12	24	0.456974515	0.805393068	24	15.4932211	0.046682188
ENSMUSG00000031298	Adgrg2	adhesion G protein-coupled receptor G2 [Source:MGI Symbol;Acc:MGI:2446854]	12.2892	11.9012	9.89805	11.7679	10.811	11.4742	11.1571	11.3542	10.2303	13.3798	11.083	11.4767	12.2709	11.0761	0.421132906	0.966018836	4	8	24	0.698287348	0.899715901	24	20.40232295	0.035992416
ENSMUSG00000031299	Pdha1	pyruvate dehydrogenase E1 alpha 1 [Source:MGI Symbol;Acc:MGI:97532]	126.987	127.878	136.605	163.73	127.339	132.416	128.654	146.91	125.264	142.901	149.005	130.663	144.338	142.196	0.943920575	0.966018836	16	12	24	0.341527241	0.775809953	24	11.31784168	0.062684879
ENSMUSG00000031302	Nlgn3	neuroligin 3 [Source:MGI Symbol;Acc:MGI:2444609]	6.439	5.09187	5.55972	5.04695	5.60603	5.99476	6.66248	5.70622	5.57613	7.17873	5.04462	5.7803	5.66814	5.77167	0.260026661	0.765037845	4	16	24	0.745367579	0.916764484	24	22.68384148	0.039782191
ENSMUSG00000031304	Il2rg	interleukin 2 receptor, gamma chain [Source:MGI Symbol;Acc:MGI:96551]	1.86566	2.23981	2.29156	1.56365	2.59191	2.43025	2.03668	2.21198	1.78831	1.18589	2.01755	1.80565	1.82714	1.71764	0.927004797	0.966018836	20	20	24	0.378413103	0.797715859	24	21.98066506	0.095708829
ENSMUSG00000031309	Rps6ka3	ribosomal protein S6 kinase polypeptide 3 [Source:MGI Symbol;Acc:MGI:104557]	39.6035	36.3938	34.0226	37.3398	37.7322	34.2381	34.4398	33.178	33.3272	33.1018	37.6367	34.9267	36.2876	35.9733	0.591351901	0.966018836	20	16	24	0.328472246	0.768040178	24	19.57199746	0.045448459
ENSMUSG00000031310	Zmym3	zinc finger, MYM-type 3 [Source:MGI Symbol;Acc:MGI:1927231]	28.082	26.1212	28.8958	26.2768	28.344	29.3332	27.019	29.9686	28.674	28.9968	25.3297	27.1925	26.2982	29.2421	0.921572192	0.966018836	8	20	24	0.573705903	0.847005958	24	6.42505725	0.031758359
ENSMUSG00000031311	Nono	non-POU-domain-containing, octamer binding protein [Source:MGI Symbol;Acc:MGI:1855692]	50.2368	50.3774	48.1631	50.6212	53.8418	48.063	48.2422	44.4768	50.5914	44.5593	50.1068	48.7149	49.1987	47.2655	0.868508291	0.966018836	20	12	24	0.386558278	0.799546792	24	17.38570982	0.036988299
ENSMUSG00000031312	Itgb1bp2	integrin beta 1 binding protein 2 [Source:MGI Symbol;Acc:MGI:1353420]	1.82681	4.50405	5.70013	6.73507	4.65063	5.58745	3.74752	10.3537	8.94524	7.43495	3.91613	3.59426	5.54129	4.71099	0.053683798	0.394331071	12	16	24	0.035899141	0.609176161	24	9.117331739	0.468511409
ENSMUSG00000031314	Taf1	TATA-box binding protein associated factor 1 [Source:MGI Symbol;Acc:MGI:1336878]	19.0697	20.5902	20.8225	21.3721	22.7945	21.8029	21.4946	18.6087	18.9746	19.2383	20.3565	19.6408	20.7322	19.9469	0.531498969	0.966018836	20	12	24	0.197694166	0.768040178	24	17.63848683	0.055465865
ENSMUSG00000031320	Rps4x	ribosomal protein S4, X-linked [Source:MGI Symbol;Acc:MGI:98158]	72.3007	95.507	86.8073	90.4805	98.2289	78.1401	75.6857	77.1048	106.957	61.183	85.6471	76.9208	82.837	74.2679	0.43565473	0.966018836	12	16	24	0.677111604	0.891969863	24	10.29130204	0.071878873
ENSMUSG00000031323	Dmrtc1a	DMRT-like family C1a [Source:MGI Symbol;Acc:MGI:1918137]	1.98301	1.95451	1.90168	1.92717	1.88799	1.48283	1.929	1.82359	2.05869	1.59516	1.71467	1.75836	1.79503	1.81734	0.21107051	0.699706585	12	12	24	0.177329029	0.768040178	24	5.575117994	0.05423083
ENSMUSG00000031327	Chic1	cysteine-rich hydrophobic domain 1 [Source:MGI Symbol;Acc:MGI:1344694]	3.63461	4.39241	4.82239	4.08089	4.10366	3.96623	4.74135	3.68092	4.53054	3.59452	4.29315	3.75896	3.40614	4.09248	0.44861185	0.966018836	12	16	24	0.514280928	0.816624526	24	8.816500344	0.056065153
ENSMUSG00000031328	Flna	filamin, alpha [Source:MGI Symbol;Acc:MGI:95556]	59.887	69.0466	72.9678	61.4139	70.5808	61.292	62.4928	74.017	72.1618	61.6637	64.2329	64.0743	63.5716	63.8283	0.11436224	0.534017934	12	16	24	0.164958004	0.767020009	24	7.595759263	0.076875836
ENSMUSG00000031333	Abcb7	ATP-binding cassette, sub-family B (MDR/TAP), member 7 [Source:MGI Symbol;Acc:MGI:109533]	10.7771	11.7501	10.9346	11.3908	11.1865	10.4195	11.1699	10.2361	11.3594	9.70585	11.3895	10.53	10.8002	11.4475	0.883024237	0.966018836	8	16	24	0.828365789	0.946497021	24	5.681411766	0.017310993
ENSMUSG00000031337	Mtm1	X-linked myotubular myopathy gene 1 [Source:MGI Symbol;Acc:MGI:1099452]	6.67847	7.56428	7.55073	7.2676	6.6077	6.93384	7.44564	6.71099	7.53406	6.4181	7.66212	7.41154	7.0448	6.92036	0.85387321	0.966018836	12	4	24	0.922797557	0.979425536	24	6.676864891	0.01260989
ENSMUSG00000031340	Gabre	gamma-aminobutyric acid (GABA) A receptor, subunit epsilon [Source:MGI Symbol;Acc:MGI:1330235]	0.574026	0.491155	0.478094	0.562437	0.517623	0.393166	0.44372	0.540763	0.536326	0.542662	0.550627	0.546629	0.449691	0.430115	0.472013662	0.966018836	16	16	24	0.321967286	0.768040178	24	12.50176881	0.031324244
ENSMUSG00000031342	Gpm6b	glycoprotein m6b [Source:MGI Symbol;Acc:MGI:107672]	146.16	166.043	165.551	148.015	169.137	140.576	169.893	139.356	166.985	134.989	158.434	136.482	133.403	136.958	0.928780444	0.966018836	12	12	24	0.732981353	0.91287945	24	10.63037645	0.03768895
ENSMUSG00000031343	Gabra3	gamma-aminobutyric acid (GABA) A receptor, subunit alpha 3 [Source:MGI Symbol;Acc:MGI:95615]	5.73277	9.0447	10.9051	7.16805	6.47811	8.05086	9.50011	6.56989	9.24967	6.27962	6.41876	8.12683	6.44787	8.7096	0.142513744	0.585944911	12	4	24	0.321743942	0.768040178	24	5.893928717	0.149422165
ENSMUSG00000031347	Cetn2	centrin 2 [Source:MGI Symbol;Acc:MGI:1347085]	41.4502	49.9904	43.4017	44.4202	45.8371	40.9069	46.1776	40.696	50.1457	36.5229	49.0642	47.4534	50.3964	49.5168	0.897425765	0.966018836	12	4	24	0.701181619	0.900947082	24	2.23145426	0.044322102
ENSMUSG00000031349	Nsdhl	NAD(P) dependent steroid dehydrogenase-like [Source:MGI Symbol;Acc:MGI:1099438]	46.0551	39.6197	43.0565	39.8336	42.5355	46.0053	44.2326	43.3595	34.6074	43.0238	40.5843	40.0124	41.6627	40.5902	0.446069941	0.966018836	4	8	24	0.120980132	0.732894732	24	21.17095748	0.077851601
ENSMUSG00000031351	Zfp185	zinc finger protein 185 [Source:MGI Symbol;Acc:MGI:108095]	0.842668	1.01506	0.979029	0.901844	0.75216	0.853081	0.955116	0.786356	1.1418	0.931484	0.876435	0.768979	1.0597	0.956197	0.048889741	0.379129806	12	12	24	0.077654364	0.687420473	24	6.420187889	0.071569638
ENSMUSG00000031352	Hccs	holocytochrome c synthetase [Source:MGI Symbol;Acc:MGI:106911]	14.92	14.1704	13.9929	14.7677	13.0971	14.0901	14.009	13.6471	12.3494	13.6059	13.5156	12.329	11.6708	11.95	0.768363194	0.966018836	16	4	24	0.607618159	0.864031555	24	15.95622275	0.022663716
ENSMUSG00000031353	Rbbp7	retinoblastoma binding protein 7, chromatin remodeling factor [Source:MGI Symbol;Acc:MGI:1194910]	60.547	62.2566	59.613	59.7271	60.738	58.9528	54.5501	60.0624	61.934	60.9453	59.7175	60.5805	60.2242	61.6606	0.648082424	0.966018836	8	20	24	0.383105265	0.799546792	24	8.986012014	0.025329338
ENSMUSG00000031355	Arhgap6	Rho GTPase activating protein 6 [Source:MGI Symbol;Acc:MGI:1196332]	1.21822	1.56534	1.92778	1.16339	1.27336	1.1948	1.44731	1.43215	1.79574	1.41078	1.13949	1.45915	1.16309	1.36048	0.062036003	0.409814092	12	8	24	0.050959417	0.645887282	24	7.042587708	0.124638683
ENSMUSG00000031357	Syap1	synapse associated protein 1 [Source:MGI Symbol;Acc:MGI:1914293]	24.0684	26.3683	24.4831	28.1165	25.3501	23.4335	25.3864	25.4017	25.6748	23.1541	26.6972	23.4871	27.4877	26.7452	0.723119329	0.966018836	8	16	24	0.607994846	0.86414592	24	6.925746889	0.034821335
ENSMUSG00000031358	Msl3	MSL complex subunit 3 [Source:MGI Symbol;Acc:MGI:1341851]	13.8461	14.881	14.1592	13.884	14.9413	12.6962	14.6825	13.2362	15.5907	13.6388	14.9619	14.7659	15.2269	14.2047	0.882263435	0.966018836	20	20	24	0.952127923	0.988339231	24	8.095715342	0.010017239
ENSMUSG00000031360	Ctps2	cytidine 5'-triphosphate synthase 2 [Source:MGI Symbol;Acc:MGI:1933185]	46.134	50.5779	47.7987	50.4371	49.2285	47.1802	45.2198	41.4425	47.8485	41.8593	45.9241	50.7302	50.9869	47.7289	0.771670154	0.966018836	24	8	24	0.81625499	0.94140005	24	19.94366152	0.025123829
ENSMUSG00000031365	Zfp275	zinc finger protein 275 [Source:MGI Symbol;Acc:MGI:1350985]	8.65197	7.17212	8.2391	8.76833	8.37674	7.78895	8.12166	7.53134	7.95821	6.96023	8.42079	8.4264	8.2453	8.00485	0.416857905	0.963884303	20	12	24	0.406137002	0.799546792	24	18.69183327	0.047129978
ENSMUSG00000031367	Ap1s2	adaptor-related protein complex 1, sigma 2 subunit [Source:MGI Symbol;Acc:MGI:1889383]	82.3761	81.4873	75.2067	79.48	78.8969	79.6893	78.2088	74.3699	66.7849	77.4992	78.8217	71.6222	71.1344	76.1366	0.941955432	0.966018836	20	16	24	0.359768315	0.785405943	24	19.05803599	0.039243177
ENSMUSG00000031370	Zrsr2	zinc finger (CCCH type), RNA binding motif and serine/arginine rich 2 [Source:MGI Symbol;Acc:MGI:103287]	22.1656	17.9229	17.0022	18.2537	16.9549	16.9163	18.9592	20.1427	18.9229	19.7265	21.1554	24.2015	22.2058	24.3792	0.598711142	0.966018836	4	8	24	0.222500915	0.768040178	24	0.456262704	0.086400827
ENSMUSG00000031371	Haus7	HAUS augmin-like complex, subunit 7 [Source:MGI Symbol;Acc:MGI:1920988]	9.13339	11.2033	9.96986	10.2453	10.0392	8.63568	8.37896	6.3586	11.7017	10.3988	8.01963	8.37725	9.2449	11.2215	0.154126887	0.606139807	12	12	24	0.249773206	0.768040178	24	9.213634786	0.139897102
ENSMUSG00000031373	Car5b	carbonic anhydrase 5b, mitochondrial [Source:MGI Symbol;Acc:MGI:1926249]	2.51454	3.5776	4.39514	4.01975	3.50954	3.58049	3.8731	4.18585	3.51153	3.94372	3.84437	3.8502	4.32373	4.10175	0.742579234	0.966018836	8	12	24	0.291529763	0.768040178	24	8.902381603	0.083737622
ENSMUSG00000031375	Bgn	biglycan [Source:MGI Symbol;Acc:MGI:88158]	40.3946	44.5993	42.7373	39.0402	43.7823	41.3485	43.8382	50.1217	56.0232	30.858	37.6047	36.8374	35.5756	36.1419	0.622132989	0.966018836	12	4	24	0.413934996	0.799546792	24	5.09774364	0.103674463
ENSMUSG00000031376	Atp2b3	ATPase, Ca++ transporting, plasma membrane 3 [Source:MGI Symbol;Acc:MGI:1347353]	9.60829	7.67402	9.9661	8.49802	6.82779	9.56135	9.41049	8.51577	6.9305	11.614	6.23576	8.85834	7.89146	10.3376	0.873171835	0.966018836	4	16	24	0.707680025	0.903565455	24	3.954999422	0.075692899
ENSMUSG00000031377	Bmx	BMX non-receptor tyrosine kinase [Source:MGI Symbol;Acc:MGI:1101778]	1.20633	1.43929	1.68247	1.41517	1.53499	1.329	1.20906	1.60118	2.11662	1.30585	1.57807	1.42249	1.55267	1.08119	0.43565473	0.966018836	12	16	24	0.288538487	0.768040178	24	10.19326675	0.090180395
ENSMUSG00000031378	Abcd1	ATP-binding cassette, sub-family D (ALD), member 1 [Source:MGI Symbol;Acc:MGI:1349215]	5.00221	5.78836	5.57688	5.38573	5.85893	5.40855	5.3795	6.85621	6.60742	6.2822	5.58994	5.52577	5.85463	5.86157	0.181442559	0.659020016	8	20	24	0.055033964	0.655263328	24	8.016264594	0.087924595
ENSMUSG00000031379	Pir	pirin [Source:MGI Symbol;Acc:MGI:1916906]	19.2795	20.8328	18.1968	20.0718	18.4252	17.6384	17.8396	16.4284	19.5069	19.0189	20.7788	21.2316	21.1365	22.4507	0.740423399	0.966018836	8	4	24	0.992907071	0.998516607	24	6.277313625	0.005400068
ENSMUSG00000031380	Vegfd	vascular endothelial growth factor D [Source:MGI Symbol;Acc:MGI:108037]	1.81331	1.83607	2.13345	2.21426	1.33167	1.53627	1.36038	1.85422	1.76806	1.71872	1.67863	1.54874	1.71831	1.73679	0.01552245	0.253395174	12	8	24	0.040670299	0.616921492	24	8.060768935	0.117918573
ENSMUSG00000031381	Piga	phosphatidylinositol glycan anchor biosynthesis, class A [Source:MGI Symbol;Acc:MGI:99461]	3.07387	2.7908	3.59139	2.60453	3.08855	2.52682	3.86248	2.77964	3.1468	2.40554	2.88696	2.8059	2.55486	2.64687	0.907068194	0.966018836	12	4	24	0.783960091	0.930589812	24	2.517881653	0.03788948
ENSMUSG00000031382	Asb11	ankyrin repeat and SOCS box-containing 11 [Source:MGI Symbol;Acc:MGI:1916104]	1.22636	3.59657	3.83154	6.0074	2.3952	3.26145	2.54634	5.58616	7.21518	5.59439	3.47671	2.75256	3.71799	3.39012	0.014306035	0.24509106	16	8	24	0.004302838	0.427431763	24	9.422131834	0.528314265
ENSMUSG00000031385	Plxnb3	plexin B3 [Source:MGI Symbol;Acc:MGI:2154240]	10.366	9.58372	9.78832	10.1155	13.419	10.0811	11.3671	8.85922	9.74161	10.5956	10.237	8.46137	9.10253	7.62781	0.033553741	0.324908923	20	12	24	0.050889644	0.645589244	24	16.1545852	0.13369296
ENSMUSG00000031386	Hcfc1	host cell factor C1 [Source:MGI Symbol;Acc:MGI:105942]	14.9997	13.4534	14.2195	14.1213	13.875	15.265	15.2609	15.8042	12.9752	18.4879	12.6084	13.95	13.9333	14.6787	0.949382029	0.966018836	4	16	24	0.97700508	0.994118853	24	6.016606679	0.011601894
ENSMUSG00000031387	Renbp	renin binding protein [Source:MGI Symbol;Acc:MGI:105940]	3.7231	4.92298	4.40274	3.80783	3.58722	3.84767	3.97871	3.30055	3.75297	3.25355	3.96798	4.26979	3.54875	4.39457	0.907000607	0.966018836	12	4	24	0.640282721	0.878239964	24	2.965451387	0.048891939
ENSMUSG00000031388	Naa10	N(alpha)-acetyltransferase 10, NatA catalytic subunit [Source:MGI Symbol;Acc:MGI:1915255]	17.5403	16.9595	19.0293	17.8485	17.7713	18.503	16.5727	20.1497	17.5488	18.3045	18.4616	16.7643	18.4493	19.3921	0.826609618	0.966018836	8	20	24	0.393007325	0.799546792	24	7.7543428	0.041270655
ENSMUSG00000031389	Arhgap4	Rho GTPase activating protein 4 [Source:MGI Symbol;Acc:MGI:2159577]	2.35205	2.33005	2.96236	2.15922	2.47815	2.37896	2.56913	2.11494	2.28249	1.76837	2.23187	1.70487	2.23686	1.66521	0.912686312	0.966018836	4	12	24	0.96833236	0.992327132	24	23.19742202	0.012291791
ENSMUSG00000031391	L1cam	L1 cell adhesion molecule [Source:MGI Symbol;Acc:MGI:96721]	124.248	97.9008	93.8847	109.662	103.892	115.885	98.6407	110.179	87.2782	131.471	96.3472	106.614	109.924	104.33	0.390686537	0.915479136	24	12	24	0.546385169	0.833341327	24	19.41357436	0.07160263
ENSMUSG00000031392	Irak1	interleukin-1 receptor-associated kinase 1 [Source:MGI Symbol;Acc:MGI:107420]	19.7378	22.0923	22.293	21.5778	21.4518	20.4366	22.9816	22.7898	21.5399	24.3238	19.4934	20.688	22.722	22.3646	0.202982082	0.689605535	8	16	24	0.117489711	0.728323355	24	6.906971976	0.066566994
ENSMUSG00000031393	Mecp2	methyl CpG binding protein 2 [Source:MGI Symbol;Acc:MGI:99918]	10.0003	9.80652	9.8783	9.89335	9.61653	9.85151	9.95661	9.47636	9.57174	10.469	9.96869	10.1271	10.1423	10.622	0.953629935	0.966018836	4	8	24	0.986489692	0.997339197	24	0.230223138	0.002410165
ENSMUSG00000031398	Plxna3	plexin A3 [Source:MGI Symbol;Acc:MGI:107683]	12.3889	10.7439	10.1467	10.4775	11.8659	9.62403	11.2885	9.7629	9.86534	9.80996	10.1908	9.96618	10.9454	10.0302	0.397301892	0.927993503	4	8	24	0.229045933	0.768040178	24	21.67075819	0.06143145
ENSMUSG00000031399	Fam3a	FAM3 metabolism regulating signaling molecule A [Source:MGI Symbol;Acc:MGI:1913544]	13.164	14.1756	13.0304	13.5056	14.2872	12.5357	13.2887	13.2269	12.4369	14.3266	12.3049	12.9163	12.5656	12.5623	0.93447422	0.966018836	16	8	24	0.645160657	0.879668078	24	12.18034949	0.022959547
ENSMUSG00000031400	G6pdx	glucose-6-phosphate dehydrogenase X-linked [Source:MGI Symbol;Acc:MGI:105979]	22.1705	20.4695	23.4316	23.708	23.2975	24.3859	23.3652	22.4212	20.482	21.8408	23.3485	22.737	21.7929	21.5982	0.147689593	0.590732968	24	8	24	0.04713143	0.633507364	24	17.52460342	0.065287732
ENSMUSG00000031402	Mpp1	membrane protein, palmitoylated [Source:MGI Symbol;Acc:MGI:105941]	33.2204	30.9933	31.0954	32.3901	32.3769	32.9827	29.1811	31.9382	27.4461	29.9636	30.0178	31.1713	28.6931	30.6559	0.935316621	0.966018836	24	12	24	0.300929065	0.768040178	24	19.05356144	0.040496498
ENSMUSG00000031403	Dkc1	dyskeratosis congenita 1, dyskerin [Source:MGI Symbol;Acc:MGI:1861727]	11.4984	12.5357	11.9036	15.3671	11.9395	12.932	12.2085	14.0999	13.0421	12.8404	12.6155	11.8583	12.6311	12.1578	0.723016199	0.966018836	16	12	24	0.314931297	0.768040178	24	10.35687067	0.061287444
ENSMUSG00000031409	Tceal6	transcription elongation factor A (SII)-like 6 [Source:MGI Symbol;Acc:MGI:1923939]	6.59696	7.74778	9.44594	7.95997	6.16487	7.64224	8.1542	6.54803	6.07713	7.30944	4.88556	6.99465	5.9472	8.33953	0.628286596	0.966018836	8	12	24	0.38673394	0.799546792	24	5.618302667	0.112212799
ENSMUSG00000031422	Morf4l2	mortality factor 4 like 2 [Source:MGI Symbol;Acc:MGI:1927167]	161.72	150.592	146.223	135.509	152.262	145.959	146.975	138.907	145.496	124.717	160.474	145.186	158.177	149.065	0.037425327	0.334069441	4	12	24	0.116960129	0.727929292	24	22.47578703	0.077289076
ENSMUSG00000031425	Plp1	proteolipid protein (myelin) 1 [Source:MGI Symbol;Acc:MGI:97623]	337.451	473.746	609.829	388.372	410.514	426.242	508.286	398.677	508.848	357.555	352.572	411.978	310.505	425.602	0.308023687	0.814993005	12	4	24	0.305700052	0.768040178	24	7.118371467	0.15447561
ENSMUSG00000031428	Zcchc18	zinc finger, CCHC domain containing 18 [Source:MGI Symbol;Acc:MGI:1914245]	95.7264	87.3608	93.8239	84.3601	82.1923	98.0542	88.6031	86.8419	78.6512	93.6147	88.4345	92.5985	87.7708	96.0382	0.608967886	0.966018836	16	12	24	0.432334813	0.805393068	24	22.38556431	0.047453165
ENSMUSG00000031429	Psmd10	proteasome (prosome, macropain) 26S subunit, non-ATPase, 10 [Source:MGI Symbol;Acc:MGI:1858898]	15.5587	14.7074	15.3244	14.2405	15.1197	15.0446	15.1524	13.0898	15.0774	11.6962	15.1432	14.1951	15.0146	14.1629	0.664568268	0.966018836	20	24	24	0.322823385	0.768040178	24	22.02668885	0.058320387
ENSMUSG00000031431	Tsc22d3	TSC22 domain family, member 3 [Source:MGI Symbol;Acc:MGI:1196284]	105.269	92.1484	115.349	180.36	111.914	119.189	98.0575	89.627	125.443	156.54	116.256	108.072	87.4422	91.1437	1.45E-04	0.031913438	16	16	24	1.00E-03	0.336500758	24	13.08285749	0.302652821
ENSMUSG00000031432	Prps1	phosphoribosyl pyrophosphate synthetase 1 [Source:MGI Symbol;Acc:MGI:97775]	24.3577	24.9098	26.2982	25.3868	24.6408	25.8392	24.2334	24.4136	26.1291	29.2838	26.0887	27.8005	26.2996	26.187	0.658942124	0.966018836	16	12	24	0.150981966	0.758865539	24	12.53575869	0.045099196
ENSMUSG00000031433	Rbm41	RNA binding motif protein 41 [Source:MGI Symbol;Acc:MGI:2444923]	3.88486	3.78615	3.81028	3.94041	3.6747	3.18036	4.22998	2.74847	4.18616	2.96606	3.94106	3.74506	3.8374	3.60436	0.93717134	0.966018836	4	4	24	0.959849526	0.989375125	24	21.49793755	0.016796804
ENSMUSG00000031434	Morc4	microrchidia 4 [Source:MGI Symbol;Acc:MGI:1922996]	3.71729	3.34109	2.59213	3.86345	3.1106	3.58485	4.38098	3.5769	3.71742	3.79311	3.36477	3.55394	3.2973	3.27508	0.915082018	0.966018836	16	16	24	0.713213499	0.905403147	24	21.3427222	0.046032553
ENSMUSG00000031438	Rnf128	ring finger protein 128 [Source:MGI Symbol;Acc:MGI:1914139]	5.26343	6.39525	5.84577	5.27892	6.17721	5.70813	6.24795	6.89142	5.85663	6.8003	5.30641	5.42774	5.00699	5.74115	0.579092016	0.966018836	8	20	24	0.419284005	0.803639585	24	7.631627406	0.065080701
ENSMUSG00000031441	Atp11a	ATPase, class VI, type 11A [Source:MGI Symbol;Acc:MGI:1354735]	63.9104	48.0253	46.1421	52.0352	50.9469	54.9779	53.975	56.338	36.2603	66.8391	48.6509	48.979	51.2336	50.9056	0.43565473	0.966018836	4	8	24	0.499007483	0.808512079	24	20.38077138	0.099379402
ENSMUSG00000031442	Mcf2l	mcf.2 transforming sequence-like [Source:MGI Symbol;Acc:MGI:103263]	32.3351	30.2176	31.3664	31.6456	31.1371	32.458	32.4403	29.6868	29.8355	33.2172	31.1356	28.4079	30.4265	29.9189	0.33885934	0.858075318	16	16	24	0.517785222	0.818405353	24	15.87688652	0.025101963
ENSMUSG00000031445	Proz	protein Z, vitamin K-dependent plasma glycoprotein [Source:MGI Symbol;Acc:MGI:1860488]	0.593906	0.566408	0.538723	0.626905	0.497515	0.408597	0.603727	0.806454	0.458579	0.62915	0.461781	0.423827	0.40231	0.56655	0.39533702	0.925130113	8	16	24	0.20086983	0.768040178	24	6.567698793	0.068496857
ENSMUSG00000031446	Cul4a	cullin 4A [Source:MGI Symbol;Acc:MGI:1914487]	24.2435	23.5791	24.2483	24.1926	24.121	23.5927	24.1963	26.2156	23.253	25.9055	21.5699	24.1356	24.5691	23.3792	0.916746631	0.966018836	4	16	24	0.671827279	0.889340174	24	5.079204607	0.023311341
ENSMUSG00000031447	Lamp1	lysosomal-associated membrane protein 1 [Source:MGI Symbol;Acc:MGI:96745]	253.612	242.71	244.883	241.662	255.38	255.079	249.362	249.443	228.952	251.648	231.165	240.618	239.387	231.708	0.891105762	0.966018836	24	12	24	0.217399541	0.768040178	24	19.89615048	0.031014421
ENSMUSG00000031448	Adprhl1	ADP-ribosylhydrolase like 1 [Source:MGI Symbol;Acc:MGI:2442168]	1.45756	1.57075	2.33058	2.41741	1.70075	2.41643	1.84687	3.27201	1.6446	2.89872	1.85617	1.86794	2.36335	1.96688	0.94791825	0.966018836	16	12	24	0.602712774	0.861114219	24	9.716501615	0.093924201
ENSMUSG00000031451	Gas6	growth arrest specific 6 [Source:MGI Symbol;Acc:MGI:95660]	39.1905	36.031	39.4203	37.6661	36.2738	38.8051	38.3115	40.2998	35.6177	47.7949	34.901	38.255	36.5077	36.7019	0.865490576	0.966018836	16	4	24	0.787936193	0.931835336	24	10.93513448	0.030842924
ENSMUSG00000031453	Rasa3	RAS p21 protein activator 3 [Source:MGI Symbol;Acc:MGI:1197013]	10.3107	10.0183	10.397	10.3957	10.3653	9.57057	11.5399	9.29052	10.7577	10.4636	9.34916	9.50139	9.6154	9.3603	0.454881285	0.966018836	12	20	24	0.800094666	0.935884746	24	9.918172357	0.020975005
ENSMUSG00000031458	Coprs	coordinator of PRMT5, differentiation stimulator [Source:MGI Symbol;Acc:MGI:1913673]	34.5844	34.8149	34.0557	29.5896	37.9124	35.0186	33.663	36.587	33.6283	32.3383	36.275	36.438	38.1888	38.0533	0.183755367	0.662021716	20	20	24	0.146531047	0.755260456	24	23.17413978	0.065380977
ENSMUSG00000031461	Myom2	myomesin 2 [Source:MGI Symbol;Acc:MGI:1328358]	2.7602	5.90758	7.89406	10.9487	7.56039	11.0659	6.60026	20.3144	12.556	17.8952	7.34631	7.53677	9.06747	6.61617	0.310940564	0.820837787	16	12	24	0.14814825	0.756440638	24	10.44670441	0.443924356
ENSMUSG00000031465	Angpt2	angiopoietin 2 [Source:MGI Symbol;Acc:MGI:1202890]	7.67159	7.01011	5.70521	7.63422	7.73413	6.57883	6.49473	6.86143	5.8715	5.65317	6.3117	6.14638	6.7698	6.03228	0.700065774	0.966018836	4	8	24	0.243505298	0.768040178	24	20.51615334	0.077017362
ENSMUSG00000031467	Agpat5	1-acylglycerol-3-phosphate O-acyltransferase 5 (lysophosphatidic acid acyltransferase, epsilon) [Source:MGI Symbol;Acc:MGI:1196345]	15.2966	14.9778	14.8947	13.9458	15.907	14.8324	14.9927	13.908	13.8906	13.8945	15.3881	14.9023	13.9509	14.68	0.050447964	0.385880335	20	20	24	0.100528738	0.711832296	24	19.45651628	0.04713179
ENSMUSG00000031478	Nek3	NIMA (never in mitosis gene a)-related expressed kinase 3 [Source:MGI Symbol;Acc:MGI:1344371]	4.6689	5.22314	3.41361	4.21884	3.53368	4.04677	4.43321	3.89547	3.61965	4.09206	3.10014	4.67665	4.00245	3.87709	0.215777641	0.704989686	24	20	24	0.129454032	0.740162193	24	0.303842327	0.115063419
ENSMUSG00000031479	Vps36	vacuolar protein sorting 36 [Source:MGI Symbol;Acc:MGI:1917410]	13.4294	13.5847	13.5731	13.8351	13.3503	13.947	12.5815	14.6906	13.4268	12.8071	16.6879	14.9683	15.0294	14.1407	0.945728192	0.966018836	24	16	24	0.647161349	0.880787843	24	18.64353942	0.033614648
ENSMUSG00000031480	Thsd1	thrombospondin, type I, domain 1 [Source:MGI Symbol;Acc:MGI:1929096]	1.32629	1.50859	1.64958	1.8012	1.4503	1.62837	1.78789	1.42064	1.7012	1.45567	1.59113	1.67075	1.42269	1.42495	0.519468828	0.966018836	12	20	24	0.546742394	0.833499795	24	13.47049909	0.036282698
ENSMUSG00000031482	Slc25a15	solute carrier family 25 (mitochondrial carrier ornithine transporter), member 15 [Source:MGI Symbol;Acc:MGI:1342274]	3.96623	4.05364	4.33196	4.85798	4.58424	4.41475	4.209	4.01187	4.54083	4.75978	4.46978	4.31139	3.78978	3.88751	6.91E-05	0.029372629	16	12	24	2.03E-05	0.047905911	24	13.51282205	0.109148357
ENSMUSG00000031483	Erlin2	ER lipid raft associated 2 [Source:MGI Symbol;Acc:MGI:2387215]	12.0493	13.1794	12.8946	13.7765	13.3593	12.9183	14.331	14.2351	14.2649	15.8204	11.7692	16.1003	13.065	13.2368	0.8635847	0.966018836	16	4	24	0.784384917	0.930811164	24	10.48048392	0.032602173
ENSMUSG00000031485	Plpbp	pyridoxal phosphate binding protein [Source:MGI Symbol;Acc:MGI:1891207]	21.7594	21.1564	23.3333	25.8106	26.5597	24.7855	21.3784	22.5221	21.0295	24.2777	21.9749	24.0918	22.6398	22.1745	0.081483913	0.462922111	16	16	24	0.020614955	0.567777991	24	15.42135062	0.098397763
ENSMUSG00000031486	Adgra2	adhesion G protein-coupled receptor A2 [Source:MGI Symbol;Acc:MGI:1925810]	3.98205	4.10297	3.93107	3.96555	4.16463	3.84436	4.67464	4.75453	4.97413	4.3158	4.96163	5.04052	4.00071	4.03438	0.806004771	0.966018836	20	8	24	0.92660261	0.980664379	24	14.09952908	0.016595989
ENSMUSG00000031487	Brf2	BRF2, RNA polymerase III transcription initiation factor 50kDa subunit [Source:MGI Symbol;Acc:MGI:1913903]	5.85795	5.22194	4.67329	5.14596	5.32562	6.19706	6.28547	6.22345	4.75166	7.4104	4.58241	6.09893	5.27194	7.08497	0.590017078	0.966018836	8	4	24	0.652819157	0.883145019	24	0.213756747	0.064823869
ENSMUSG00000031489	Adrb3	adrenergic receptor, beta 3 [Source:MGI Symbol;Acc:MGI:87939]	0.961587	0.595614	1.52921	1.83206	0.419265	1.12016	1.96951	1.81555	0.789059	1.80307	1.99897	2.01872	4.18591	2.47516	0.935732159	0.966018836	4	8	24	0.641169885	0.878540546	24	1.61702531	0.13320386
ENSMUSG00000031490	Eif4ebp1	eukaryotic translation initiation factor 4E binding protein 1 [Source:MGI Symbol;Acc:MGI:103267]	5.6987	5.04999	8.56963	10.613	7.09758	7.20748	6.32484	8.15722	8.30232	8.80775	9.33484	7.7455	9.69516	6.75059	0.123856376	0.552144108	16	16	24	0.043662467	0.626787045	24	12.51404155	0.214710449
ENSMUSG00000031491	Chrna6	cholinergic receptor, nicotinic, alpha polypeptide 6 [Source:MGI Symbol;Acc:MGI:106213]	25.9866	25.6527	21.6102	25.3443	23.2614	27.6447	24.6981	25.2456	24.0597	24.2887	22.7705	27.4114	30.588	25.7377	0.019643089	0.258568924	24	20	24	0.037964047	0.61140397	24	23.03058412	0.105625304
ENSMUSG00000031492	Chrnb3	cholinergic receptor, nicotinic, beta polypeptide 3 [Source:MGI Symbol;Acc:MGI:106212]	3.32827	2.49973	2.63323	2.64704	2.73058	3.44359	2.82863	2.86548	1.90916	3.81877	2.24149	2.90983	3.14584	2.70917	0.038115943	0.334069441	24	12	24	0.371018077	0.792839127	24	20.69711593	0.10622616
ENSMUSG00000031494	Cd209a	CD209a antigen [Source:MGI Symbol;Acc:MGI:2157942]	0.962069	0.93288	1.07692	0.806185	0.812969	0.959121	1.27814	1.13309	1.1103	0.456704	0.561805	0.707339	0.815637	1.04946	0.018857365	0.256345714	12	4	24	0.014697254	0.537860185	24	3.056602907	0.168510085
ENSMUSG00000031497	Tnfsf13b	tumor necrosis factor (ligand) superfamily, member 13b [Source:MGI Symbol;Acc:MGI:1344376]	0.902137	1.44767	2.07217	1.29484	1.19347	1.40827	1.54403	1.34619	1.92374	1.05533	1.3558	1.48457	1.41967	1.22945	0.503972924	0.966018836	12	4	24	0.456494701	0.805393068	24	7.460301876	0.091902717
ENSMUSG00000031502	Col4a1	collagen, type IV, alpha 1 [Source:MGI Symbol;Acc:MGI:88454]	40.3554	30.0775	30.5135	31.9159	47.7656	34.2132	44.4522	42.1358	32.1223	43.9328	30.5512	36.7178	37.3558	31.0049	0.46238066	0.966018836	4	4	24	0.463324362	0.805393068	24	19.89607329	0.116421606
ENSMUSG00000031503	Col4a2	collagen, type IV, alpha 2 [Source:MGI Symbol;Acc:MGI:88455]	21.8208	19.2043	19.0756	18.6488	25.6066	21.453	26.4343	26.2303	20.7833	30.5084	19.9908	22.4176	23.1018	19.1393	0.662399683	0.966018836	4	8	24	0.789419183	0.932496294	24	18.76385406	0.058992596
ENSMUSG00000031504	Rab20	RAB20, member RAS oncogene family [Source:MGI Symbol;Acc:MGI:102789]	2.52986	2.0817	2.26484	1.7904	3.12293	1.5661	2.65934	1.72442	2.71958	2.37853	2.48326	2.12358	2.50744	2.18586	0.834424969	0.966018836	20	12	24	0.914315486	0.976006964	24	15.2239888	0.032113826

ENSMUSG00000031505	Naxd	NAD(P)HX dehydratase [Source:MGI Symbol;Acc:MGI:1913353]	32.6517	32.4096	33.005	33.5063	32.7597	34.6213	34.6911	34.2542	33.1857	35.0945	33.653	34.2506	34.979	32.0759	0.581070934	0.966018836	24	8	24	0.465481894	0.805393068	24	18.72010563	0.019698831
ENSMUSG00000031506	Ptpn7	protein tyrosine phosphatase, non-receptor type 7 [Source:MGI Symbol;Acc:MGI:2156893]	0.419667	0.581057	0.748702	0.49285	0.541209	0.571659	0.469952	0.521671	0.692758	0.390713	0.462299	0.392526	0.698643	0.413938	0.718720526	0.966018836	12	4	24	0.497469828	0.807576732	24	6.664565344	0.051884173
ENSMUSG00000031508	Ankrd10	ankyrin repeat domain 10 [Source:MGI Symbol;Acc:MGI:1921840]	17.892	18.4749	16.5334	18.488	19.1669	16.6196	18.3933	16.4566	18.3822	14.2998	18.4478	15.833	16.8554	16.6194	0.90767227	0.966018836	20	4	24	0.90187075	0.972475685	24	20.13018263	0.019242022
ENSMUSG00000031511	Arhgef7	Rho guanine nucleotide exchange factor (GEF7) [Source:MGI Symbol;Acc:MGI:1860493]	25.9067	18.9961	26.5496	18.7412	21.8243	28.0419	20.5372	23.7861	20.7459	19.4207	18.3438	17.4119	18.3879	26.8034	0.798829513	0.966018836	8	12	24	0.536460834	0.827423907	24	2.993398147	0.095164716
ENSMUSG00000031513	Leprotl1	leptin receptor overlapping transcript-like 1 [Source:MGI Symbol;Acc:MGI:1915442]	55.2012	47.3942	47.4416	49.4262	46.6446	53.6069	47.8922	50.1545	39.9861	55.0663	49.8498	48.5088	48.9221	50.4921	0.970092543	0.971116366	24	12	24	0.422797974	0.805019467	24	20.14514831	0.061027821
ENSMUSG00000031516	Dctn6	dynactin 6 [Source:MGI Symbol;Acc:MGI:1343154]	103.086	95.0059	93.0236	89.1861	92.8595	97.2981	93.7838	91.3015	90.9954	91.9093	95.9802	88.8998	93.4051	87.9018	0.454881285	0.966018836	4	12	24	0.088214307	0.707747429	24	21.33115013	0.041470262
ENSMUSG00000031517	Gpm6a	glycoprotein m6a [Source:MGI Symbol;Acc:MGI:107671]	37.8992	51.3946	58.6401	43.8094	43.1065	40.011	50.5745	39.3279	54.1583	36.7923	40.4027	38.8731	33.5912	44.7798	0.050760377	0.386400877	12	12	24	0.122168429	0.735058232	24	7.600104555	0.166410748
ENSMUSG00000031519	Asb5	ankyrin repeat and SOCS box-containing 5 [Source:MGI Symbol;Acc:MGI:1923544]	1.91421	5.0911	4.69905	6.61556	4.08676	5.35349	4.02985	9.67218	10.0039	6.27619	4.72133	3.82693	4.61405	3.66293	0.050721923	0.386400877	12	16	24	0.074037317	0.683293901	24	9.353354273	0.416658769
ENSMUSG00000031520	Vegfc	vascular endothelial growth factor C [Source:MGI Symbol;Acc:MGI:109124]	0.341471	0.528985	0.584344	0.419526	0.58688	0.480337	0.949582	0.435861	0.664018	0.594774	0.598092	0.673624	0.660592	0.628119	0.847846021	0.966018836	4	4	24	0.938885003	0.984935568	24	0.190101398	0.016418432
ENSMUSG00000031521	Aga	aspartylglucosaminidase [Source:MGI Symbol;Acc:MGI:104873]	12.396	12.516	13.363	11.9551	12.8271	11.6022	13.0328	13.1486	12.685	12.1175	11.3988	11.9353	12.5873	12.5751	0.074438493	0.448301997	12	12	24	0.036462218	0.609176161	24	4.982863279	0.056261443
ENSMUSG00000031523	Dlc1	deleted in liver cancer 1 [Source:MGI Symbol;Acc:MGI:1354949]	10.5756	10.5378	10.2419	10.8139	12.5833	10.9524	11.8428	12.2553	10.4938	14.2996	10.8041	11.5917	12.0349	11.8948	0.83638114	0.966018836	16	20	24	0.804061425	0.93736298	24	14.96316784	0.028677544
ENSMUSG00000031527	Eri1	exoribonuclease 1 [Source:MGI Symbol;Acc:MGI:1914526]	3.24585	3.19175	3.44184	3.38335	3.69884	3.4261	3.50417	3.62711	3.39214	2.94486	3.58718	3.31295	3.42599	3.12002	0.722296281	0.966018836	20	20	24	0.583566511	0.851892977	24	18.78877403	0.02910134
ENSMUSG00000031529	Tnks	tankyrase, TRF1-interacting ankyrin-related ADP-ribose polymerase [Source:MGI Symbol;Acc:MGI:1341087]	21.681	20.1784	19.2447	20.1241	18.8482	19.6693	19.8541	18.6874	18.4975	20.085	20.1135	21.879	21.8185	21.9351	0.608967886	0.966018836	4	8	24	0.179988073	0.768040178	24	22.7642743	0.054779422
ENSMUSG00000031530	Dusp4	dual specificity phosphatase 4 [Source:MGI Symbol;Acc:MGI:2442191]	2.33474	1.47013	1.839	1.63057	1.63127	2.14116	1.95301	2.33367	1.89318	2.78953	1.92069	2.16396	2.51834	2.24974	0.481530854	0.966018836	4	8	24	0.680533603	0.893265165	24	0.203552822	0.05168581
ENSMUSG00000031532	Saraf	store-operated calcium entry-associated regulatory factor [Source:MGI Symbol;Acc:MGI:1915137]	113.375	108.292	110.909	105.436	113.126	114.78	112.09	115.141	106.06	112.624	108.979	114.256	116.43	109.489	0.047481325	0.377556215	24	12	24	0.046432221	0.633507364	24	21.79097845	0.039540376
ENSMUSG00000031533	Mrps31	mitochondrial ribosomal protein S31 [Source:MGI Symbol;Acc:MGI:1913153]	14.6972	16.4025	15.9142	16.0942	15.8885	15.0939	14.1068	14.8753	17.9521	15.2687	17.1082	16.9775	17.983	17.265	0.887862353	0.966018836	12	16	24	0.545357267	0.832868977	24	9.503645257	0.03923226
ENSMUSG00000031534	Smim19	small integral membrane protein 19 [Source:MGI Symbol;Acc:MGI:2142501]	10.8727	12.0803	11.4439	11.523	10.5698	11.6022	12.4583	11.8621	11.0547	13.0263	10.4631	11.5116	12.2528	11.6659	0.25849765	0.761738864	8	12	24	0.414328081	0.799558474	24	4.315723788	0.042171717
ENSMUSG00000031536	Polb	polymerase (DNA directed), beta [Source:MGI Symbol;Acc:MGI:97740]	32.9536	35.85	34.7105	32.1872	32.8193	31.9072	36.3481	34.3368	38.1148	28.0542	37.8062	34.2814	38.0794	35.3527	0.336755135	0.85758369	12	4	24	0.293371411	0.768040178	24	2.435213571	0.067785778
ENSMUSG00000031537	Ikbkb	inhibitor of kappaB kinase beta [Source:MGI Symbol;Acc:MGI:1338071]	17.357	16.444	15.6688	17.2311	17.3235	16.212	17.7614	16.4191	15.67	16.1485	16.2551	17.0168	16.4073	15.5708	0.109040811	0.524035616	4	8	24	0.048302066	0.638992719	24	19.92899025	0.049118268
ENSMUSG00000031538	Plat	plasminogen activator, tissue [Source:MGI Symbol;Acc:MGI:97610]	14.3065	14.3762	12.9279	10.5824	12.3052	13.2029	16.4725	14.6273	12.5373	9.19067	10.4884	13.3902	12.2686	11.1045	0.037037369	0.334054683	4	12	24	0.002041849	0.395705644	24	0.428388259	0.21210472
ENSMUSG00000031539	Ap3m2	adaptor-related protein complex 3, mu 2 subunit [Source:MGI Symbol;Acc:MGI:1929214]	11.973	11.567	13.9371	11.3823	12.0635	13.1322	13.1955	10.9074	12.1013	12.473	11.1907	11.8445	11.5883	13.068	0.880247609	0.966018836	12	20	24	0.936865965	0.984446525	24	4.800698595	0.014067463
ENSMUSG00000031540	Kat6a	K(lysine) acetyltransferase 6A [Source:MGI Symbol;Acc:MGI:2442415]	6.69647	5.68821	6.24186	6.92375	6.49558	5.85894	6.82881	5.40122	5.7244	6.07957	5.50754	5.77953	5.62764	5.52108	0.498218846	0.966018836	16	16	24	0.458960325	0.805393068	24	16.65582234	0.041785969
ENSMUSG00000031543	Ank1	ankyrin 1, erythroid [Source:MGI Symbol;Acc:MGI:88024]	38.0998	34.3331	35.4945	38.3137	35.4632	39.7693	36.5674	49.6865	34.1674	51.7186	31.8374	37.7927	39.7144	38.8063	0.868638024	0.966018836	24	20	24	0.845035035	0.953147907	24	6.461887591	0.045091426
ENSMUSG00000031545	Gpat4	glycerol-3-phosphate acyltransferase 4 [Source:MGI Symbol;Acc:MGI:2142716]	26.3237	19.8219	24.264	23.0604	22.1536	23.4969	26.3244	24.0123	18.549	23.5741	20.2566	21.4955	21.1816	21.5268	0.888379597	0.966018836	4	16	24	0.492367397	0.805476481	24	22.11155332	0.062821646
ENSMUSG00000031546	Gins4	GINS complex subunit 4 (Sld5 homolog) [Source:MGI Symbol;Acc:MGI:1923847]	12.576	12.7076	12.5535	11.31	13.1311	11.673	13.2341	12.9015	12.5786	11.2234	13.2067	14.4277	12.723	12.111	0.136853693	0.578758359	20	20	24	0.254272278	0.768040178	24	22.09821055	0.057286464
ENSMUSG00000031548	Sfrp1	secreted frizzled-related protein 1 [Source:MGI Symbol;Acc:MGI:892014]	9.89581	8.38488	8.798	8.92309	8.16342	9.7235	8.82711	9.07176	7.70624	10.016	8.29125	9.68305	8.30389	9.5079	0.942429894	0.966018836	24	12	24	0.660749346	0.885520687	24	21.27129493	0.040052721
ENSMUSG00000031554	Adam5	a disintegrin and metallopeptidase domain 5 [Source:MGI Symbol;Acc:MGI:104730]	0.940222	1.01841	0.945858	1.04574	1.00425	0.814552	0.727468	0.725469	0.948601	0.788987	0.929611	0.928648	0.885277	0.937266	0.539001179	0.966018836	16	12	24	0.517060327	0.818088916	24	12.31317038	0.034294613
ENSMUSG00000031555	Adam9	a disintegrin and metallopeptidase domain 9 (meltrin gamma) [Source:MGI Symbol;Acc:MGI:105376]	33.3828	28.3365	27.5212	28.8107	29.6332	31.0669	30.4007	29.3225	24.0842	33.408	26.6673	29.0457	27.7073	27.832	0.390686537	0.915479136	24	12	24	0.278931887	0.768040178	24	20.14680814	0.073391304
ENSMUSG00000031556	Tm2d2	TM2 domain containing 2 [Source:MGI Symbol;Acc:MGI:1916992]	42.6764	42.8313	43.2892	41.5714	40.5388	41.4971	44.5068	44.2097	43.7512	40.9577	45.636	41.7294	43.4895	42.6803	0.25928362	0.763756019	12	4	24	0.346681192	0.778844143	24	3.463578004	0.026411863
ENSMUSG00000031557	Plekha2	pleckstrin homology domain-containing, family A (phosphoinositide binding specific) member 2 [Source:MGI Symbol;Acc:MGI:1928144]	6.2861	7.07707	6.71956	6.79859	6.72891	6.90397	6.61517	6.86692	6.27228	7.28533	6.09418	7.05381	6.87072	6.27285	0.897570341	0.966018836	24	12	24	0.956562525	0.988639288	24	16.31803372	0.00811459
ENSMUSG00000031558	Slit2	slit guidance ligand 2 [Source:MGI Symbol;Acc:MGI:1315205]	9.17178	12.0455	10.9127	12.4601	13.5983	11.6374	13.2427	10.2485	12.786	14.5016	11.4112	11.9112	12.1911	10.6934	0.510031987	0.966018836	16	16	24	0.161355308	0.76636332	24	13.5980905	0.110825465
ENSMUSG00000031561	Tenm3	teneurin transmembrane protein 3 [Source:MGI Symbol;Acc:MGI:1345183]	6.04429	4.86948	5.55754	5.61991	4.84584	5.55884	5.54581	5.39419	4.62499	7.37424	4.57895	5.79686	5.14665	5.86679	0.921572192	0.966018836	24	24	24	0.988986014	0.997679907	24	10.21752776	0.009472645
ENSMUSG00000031562	Dctd	dCMP deaminase [Source:MGI Symbol;Acc:MGI:2444529]	0.980932	0.828687	0.871652	0.789379	1.09154	0.891311	1.12735	1.00959	1.34477	1.19284	0.981735	1.01872	0.945678	0.828455	0.879844076	0.966018836	20	12	24	0.816216675	0.94140005	24	12.91010634	0.027468949
ENSMUSG00000031563	Wwc2	WW, C2 and coiled-coil domain containing 2 [Source:MGI Symbol;Acc:MGI:1261872]	3.73539	4.02716	4.07761	4.0424	3.89935	3.85576	4.47083	3.95888	4.26806	4.77402	3.4156	3.72183	3.82594	3.88674	0.024812831	0.290831795	16	4	24	0.256630685	0.768040178	24	8.293429767	0.027619515
ENSMUSG00000031565	Fgfr1	fibroblast growth factor receptor 1 [Source:MGI Symbol;Acc:MGI:95522]	47.5301	40.5859	40.873	43.5135	46.7577	41.6482	44.4985	45.4305	39.7762	52.0585	38.7935	41.0667	40.8403	40.7283	0.94663657	0.966018836	16	20	24	0.828608797	0.946618078	24	15.11779031	0.027824975
ENSMUSG00000031568	Rwdd4a	RWD domain containing 4A [Source:MGI Symbol;Acc:MGI:2681000]	9.66375	8.81854	8.72485	10.3888	9.45345	8.59286	8.92498	8.7802	8.54981	10.4481	10.1221	9.4431	9.96118	9.85202	0.058209189	0.40386543	16	20	24	0.294699775	0.768040178	24	14.7079182	0.052772351
ENSMUSG00000031570	Plpp5	phospholipid phosphatase 5 [Source:MGI Symbol;Acc:MGI:1919160]	13.8088	11.6928	12.0542	11.6002	13.3704	12.5867	12.6367	12.6585	10.472	12.6079	11.8987	12.5439	11.772	11.5606	0.424093452	0.966018836	4	8	24	0.048179921	0.63850599	24	20.10580983	0.07950884
ENSMUSG00000031574	Star	steroidogenic acute regulatory protein [Source:MGI Symbol;Acc:MGI:102760]	2.44504	2.1115	2.62831	2.45778	2.74973	2.26632	2.32986	2.12614	2.2611	2.43101	2.56375	2.03503	1.87217	2.12208	0.066875194	0.425513244	20	12	24	0.009645007	0.502965379	24	13.75265293	0.094332808
ENSMUSG00000031575	Ash2l	ASH2 like histone lysine methyltransferase complex subunit [Source:MGI Symbol;Acc:MGI:1344416]	21.8412	19.1578	20.4762	17.4767	20.3191	21.6498	19.6669	19.2423	17.5032	20.0814	18.5219	19.7635	20.3525	20.0986	0.372888979	0.89586062	4	12	24	0.085017417	0.70291653	24	22.03909223	0.075418691
ENSMUSG00000031576	Kcnu1	potassium channel, subfamily U, member 1 [Source:MGI Symbol;Acc:MGI:1202300]	8.11867	6.17968	5.46873	5.92692	5.16715	6.6583	4.88211	5.81684	5.29294	5.33915	5.62929	6.0341	6.32982	6.31107	0.191453468	0.669841558	24	20	24	0.169201339	0.767020009	24	23.3415324	0.111386885
ENSMUSG00000031577	Tti2	TELO2 interacting protein 2 [Source:MGI Symbol;Acc:MGI:2384576]	5.49407	5.46442	5.96348	5.50718	5.07985	5.20708	5.5038	5.92679	5.40826	5.9562	5.30781	5.71326	5.10227	5.80324	0.256686406	0.76168256	16	4	24	0.114747423	0.727799063	24	7.258913553	0.053845884
ENSMUSG00000031578	Mak16	MAK16 homolog [Source:MGI Symbol;Acc:MGI:1915170]	12.9654	12.8562	13.8822	11.8276	12.4837	12.4977	12.3442	12.5444	13.421	11.1603	12.6715	12.5087	12.2692	12.633	0.058209189	0.40386543	12	4	24	0.391505327	0.799546792	24	3.963283762	0.035295714
ENSMUSG00000031583	Wrn	Werner syndrome RecQ like helicase [Source:MGI Symbol;Acc:MGI:109635]	3.5574	4.27157	4.26984	3.8327	3.93921	3.79783	3.98769	3.87438	4.73768	3.57002	4.03295	4.05574	3.96516	4.11773	0.265774856	0.773510818	12	4	24	0.241890978	0.768040178	24	7.054851167	0.056853723
ENSMUSG00000031584	Gsr	glutathione reductase [Source:MGI Symbol;Acc:MGI:95804]	23.199	20.4493	22.1602	22.3996	21.6532	23.6943	21.8642	21.9323	17.8932	23.0694	21.3318	21.0886	21.6809	20.8849	0.777666236	0.966018836	16	16	24	0.243561233	0.768040178	24	19.16978175	0.06066561
ENSMUSG00000031585	Gtf2e2	general transcription factor II E, polypeptide 2 (beta subunit) [Source:MGI Symbol;Acc:MGI:1915403]	23.0179	19.4498	19.0297	20.045	22.6969	20.6083	17.2736	18.6487	18.4249	15.5776	21.629	18.6179	19.5957	19.5144	0.446653834	0.966018836	20	16	24	0.26052576	0.768040178	24	19.60395408	0.087324572
ENSMUSG00000031586	Rbpms	RNA binding protein gene with multiple splicing [Source:MGI Symbol;Acc:MGI:1334446]	2.68851	3.4164	3.35036	3.35465	3.16303	2.82097	2.1389	3.70321	3.44912	3.52123	2.97062	3.44609	3.12528	2.93025	0.052672766	0.391647859	12	16	24	0.090504612	0.711427083	24	9.914679806	0.125721848
ENSMUSG00000031590	Frg1	FSHD region gene 1 [Source:MGI Symbol;Acc:MGI:893597]	21.7212	20.6985	20.7385	20.7664	19.0373	20.9422	20.5859	22.4226	21.3907	22.8136	20.7615	20.9093	21.2271	21.1115	0.897425765	0.966018836	16	4	24	0.336046674	0.771666944	24	5.773980521	0.033484422
ENSMUSG00000031591	Asah1	N-acylsphingosine amidohydrolase 1 [Source:MGI Symbol;Acc:MGI:1277124]	66.5197	74.1044	68.1479	62.3294	76.1968	62.7221	72.8452	64.8355	67.5361	63.5229	69.6615	66.4014	64.0851	62.9467	0.91241026	0.966018836	20	20	24	0.86081747	0.959094969	24	19.71485501	0.019967917
ENSMUSG00000031592	Pcm1	pericentriolar material 1 [Source:MGI Symbol;Acc:MGI:1277958]	16.3536	18.2959	17.4577	18.0156	18.6564	16.2908	17.2048	15.9155	20.1832	14.5899	18.5598	19.3468	18.8699	18.5153	0.849180783	0.966018836	20	20	24	0.998598344	0.999730606	24	6.4332614	0.002592011
ENSMUSG00000031594	Fgl1	fibrinogen-like protein 1 [Source:MGI Symbol;Acc:MGI:102795]	0.663837	0.623827	1.17331	0.905383	0.97076	0.594494	0.455791	1.11548	0.510335	0.767449	0.746415	1.01009	0.945101	0.803901	0.881996584	0.966018836	16	12	24	0.742020717	0.916125557	24	11.23655021	0.055921809
ENSMUSG00000031595	Pdgfrl	platelet-derived growth factor receptor-like [Source:MGI Symbol;Acc:MGI:1916047]	3.11494	4.92578	4.18445	3.54783	4.53273	3.24695	4.63985	4.96223	5.81027	2.34003	3.84829	3.93087	3.30668	3.80659	0.399162533	0.931325702	12	4	24	0.415988465	0.800710663	24	5.250849428	0.139407109
ENSMUSG00000031596	Slc7a2	solute carrier family 7 (cationic amino acid transporter, y+ system), member 2 [Source:MGI Symbol;Acc:MGI:99828]	17.2856	17.241	18.6553	23.6937	19.2955	18.2821	16.6805	15.8035	19.751	18.6215	17.9347	18.412	15.8403	16.11	8.68E-04	0.071344007	12	20	24	0.002220697	0.395705644	24	13.21551324	0.150368325
ENSMUSG00000031600	Vps37a	vacuolar protein sorting 37A [Source:MGI Symbol;Acc:MGI:1261835]	20.3925	20.3166	20.2581	20.7384	20.4333	19.6431	20.8193	18.1135	19.6374	18.7864	21.5717	17.9883	18.5712	19.24	0.891105762	0.966018836	20	12	24	0.495687165	0.807333372	24	14.93355614	0.030201665
ENSMUSG00000031601	Cnot7	CCR4-NOT transcription complex, subunit 7 [Source:MGI Symbol;Acc:MGI:1298230]	42.1898	40.491	42.9492	40.8305	42.3729	40.7371	41.103	40.2568	37.1158	41.6181	43.246	39.6644	38.4034	41.2637	0.644063931	0.966018836	20	4	24	0.422095159	0.804917634	24	15.97954659	0.028530308
ENSMUSG00000031604	Msmo1	methylsterol monoxygenase 1 [Source:MGI Symbol;Acc:MGI:1913484]	224.392	219.509	203.811	190.461	229.33	212.748	213.261	191.684	225.619	175.125	240.095	204.168	232.196	204.118	0.187699199	0.662239322	20	20	24	0.322254243	0.768040178	24	21.33124958	0.076491845
ENSMUSG00000031605	Klhl2	kelch-like 2, Mayven [Source:MGI Symbol;Acc:MGI:1924363]	17.8005	14.5495	16.3633	17.1366	14.3984	16.7279	17.1653	14.6609	11.4982	15.8211	13.0434	14.399	13.1147	14.8015	0.945282252	0.966018836	4	16	24	0.601477828	0.860529995	24	21.05151868	0.058603393
ENSMUSG00000031608	Galnt7	polypeptide N-acetylgalactosaminyltransferase 7 [Source:MGI Symbol;Acc:MGI:1349449]	2.24754	2.64314	3.07246	2.47822	2.21511	2.63709	2.61413	2.43115	2.705	2.72035	2.80785	2.50589	2.29149	2.76173	0.286642595	0.793714531	12	16	24	0.173821404	0.768040178	24	9.019604216	0.069974648
ENSMUSG00000031609	Sap30	sin3 associated polypeptide [Source:MGI Symbol;Acc:MGI:1929129]	1.29056	1.8493	2.08443	2.06702	2.08213	1.86828	2.00521	1.78796	1.84432	1.78259	1.88496	1.46919	2.10787	1.74747	0.933543173	0.966018836	20	12	24	0.429326143	0.805393068	24	11.01170115	0.065149045
ENSMUSG00000031610	Scrg1	scrapie responsive gene 1 [Source:MGI Symbol;Acc:MGI:1328308]	0.742263	2.78769	4.92056	1.7799	2.10133	3.22887	3.07433	1.98785	3.28859	1.47168	1.83094	2.27996	1.33845	2.0659	0.887027852	0.966018836	12	4	24	0.621704403	0.871115984	24	8.278201826	0.17708004
ENSMUSG00000031613	Hpgd	hydroxyprostaglandin dehydrogenase 15 (NAD) [Source:MGI Symbol;Acc:MGI:108085]	5.00632	6.77947	6.24185	6.69654	6.01724	5.08416	6.1166	5.40792	7.47469	4.46047	6.61907	4.78933	5.19809	5.20424	0.041292272	0.346472636	12	12	24	0.323931598	0.768040178	24	9.354532347	0.10829024
ENSMUSG00000031616	Ednra	endothelin receptor type A [Source:MGI Symbol;Acc:MGI:105923]	2.27648	2.83232	2.36638	2.49712	2.49653	2.28095	2.83117	2.48544	2.28227	2.59163	2.46385	2.57557	2.33561	2.79251	0.94086481	0.966018836	8	16	24	0.717772786	0.90689011	24	4.203034033	0.026183982
ENSMUSG00000031617	Tmem184c	transmembrane protein 184C [Source:MGI Symbol;Acc:MGI:2384562]	72.2624	61.0752	56.0931	61.5689	60.2224	66.8336	63.9442	62.9641	49.8574	62.8723	60.3617	61.2195	64.6092	60.4763	0.004501001	0.140598674	4	8	24	0.020125234	0.565108098	24	21.49732299	0.116875999
ENSMUSG00000031618	Nr3c2	nuclear receptor subfamily 3, group C, member 2 [Source:MGI Symbol;Acc:MGI:99459]	4.3489	4.56807	4.56878	4.19627	4.05251	4.57111	4.89109	4.52759	4.32201	5.47024	4.39955	4.53531	4.04918	4.97973	0.880247609	0.966018836	8	12	24	0.661323046	0.885520687	24	7.156287961	0.035758393
ENSMUSG00000031622	Sin3b	transcriptional regulator, SIN3B (yeast) [Source:MGI Symbol;Acc:MGI:107158]	32.7227	36.5075	36.4576	39.6622	37.7815	36.5649	33.7234	35.2017	41.2677	36.2216	39.1132	36.4479	34.8864	33.343	0.010754404	0.199214173	20	8	24	0.009422127	0.502965379	24	12.58768296	0.094980361
ENSMUSG00000031626	Sorbs2	sorbin and SH3 domain containing 2 [Source:MGI Symbol;Acc:MGI:1924574]	14.4867	14.7812	15.7597	14.1186	13.9138	14.9802	15.1325	16.0373	14.3022	16.5068	13.2417	13.7503	11.8834	12.8804	0.793403332	0.966018836	16	4	24	0.497618201	0.8076691	24	9.31758616	0.050471299
ENSMUSG00000031627	Irf2	interferon regulatory factor 2 [Source:MGI Symbol;Acc:MGI:96591]	11.0816	9.26841	8.75367	10.4861	9.20945	9.18294	8.9558	8.50333	7.90707	11.4807	8.34952	9.08699	10.2686	9.16785	0.893307667	0.966018836	16	20	24	0.834161256	0.949034453	24	18.28970617	0.034930372
ENSMUSG00000031628	Casp3	caspase 3 [Source:MGI Symbol;Acc:MGI:107739]	6.42488	7.59109	8.31465	7.08368	7.317	7.69193	7.13413	7.46382	7.11845	6.26174	8.36958	7.78931	7.0585	7.5578	0.798829513	0.966018836	24	4	24	0.973275076	0.993305115	24	15.71303594	0.009829051
ENSMUSG00000031629	Cenpu	centromere protein U [Source:MGI Symbol;Acc:MGI:1919126]	0.83825	0.971795	0.812799	0.843728	0.590426	0.890373	0.881099	0.518979	0.896097	0.705942	0.992173	0.962059	0.978029	0.681729	0.798309651	0.966018836	24	8	24	0.598241296	0.858819856	24	20.54570275	0.051436545
ENSMUSG00000031631	Cfap97	cilia and flagella associated protein 97 [Source:MGI Symbol;Acc:MGI:1914006]	11.9799	12.0314	11.5425	12.1142	11.6563	11.8593	11.4617	11.4059	11.1875	10.4407	12.2918	11.6543	12.2795	12.6788	0.934110497	0.966018836	8	4	24	0.409309603	0.799546792	24	23.237694	0.033689482
ENSMUSG00000031633	Slc25a4	solute carrier family 25 (mitochondrial carrier, adenine nucleotide translocator), member 4 [Source:MGI Symbol;Acc:MGI:1353495]	479.179	483.503	487.754	493.17	455.203	482.536	444.998	552.767	507.943	521.335	503.197	476.435	512.553	508.191	0.250898337	0.75129457	8	16	24	0.102439275	0.713699364	24	7.593820046	0.060152226
ENSMUSG00000031634	Ufsp2	UFM1-specific peptidase 2 [Source:MGI Symbol;Acc:MGI:1913679]	40.5106	41.9767	40.7849	38.8635	39.0388	39.4158	39.5844	39.4037	41.0125	37.7053	42.7901	42.4116	42.8104	42.6965	0.371875382	0.894537464	4	12	24	0.312917833	0.768040178	24	1.248532614	0.031232695
ENSMUSG00000031636	Pdlim3	PDZ and LIM domain 3 [Source:MGI Symbol;Acc:MGI:1859274]	3.41925	6.14581	7.45749	7.88706	5.70714	8.29791	6.03038	15.1068	11.6762	15.1392	6.01574	7.81459	9.47424	5.94121	0.834424969	0.966018836	16	4	24	0.181853252	0.768040178	24	9.335506125	0.343860866
ENSMUSG00000031639	Tlr3	toll-like receptor 3 [Source:MGI Symbol;Acc:MGI:2156367]	3.10298	4.79401	4.73644	4.2295	4.06645	3.84906	3.56687	4.36743	4.91211	3.08901	3.50282	3.26314	2.9954	3.99104	0.018194955	0.256345714	12	16	24	0.043047621	0.625007356	24	7.949686075	0.16866701
ENSMUSG00000031641	Cbr4	carbonyl reductase 4 [Source:MGI Symbol;Acc:MGI:2384567]	17.0589	19.3389	17.5413	18.3546	17.5581	15.4165	16.7753	17.9102	18.4983	16.976	17.3687	16.8148	19.63	20.8812	0.010213213	0.195026763	8	16	24	0.031698883	0.604316769	24	5.964293985	0.095449055
ENSMUSG00000031642	Sh3rf1	SH3 domain containing ring finger 1 [Source:MGI Symbol;Acc:MGI:1913066]	1.6154	1.52455	1.66953	1.46009	1.54626	1.61298	1.71834	1.29604	1.45402	1.86844	1.25744	1.63628	1.3429	1.24892	0.849179819	0.966018836	24	8	24	0.639201901	0.877759779	24	16.72318494	0.037208577
ENSMUSG00000031644	Nek1	NIMA (never in mitosis gene a)-related expressed kinase 1 [Source:MGI Symbol;Acc:MGI:97303]	49.8558	53.6037	46.8187	49.699	53.6887	45.0108	51.315	45.1599	52.9057	43.6959	52.3225	48.2713	51.7616	49.4828	0.90332508	0.966018836	20	20	24	0.925417634	0.980131499	24	22.76550513	0.01568668
ENSMUSG00000031647	Mfap3l	microfibrillar-associated protein 3-like [Source:MGI Symbol;Acc:MGI:1918556]	25.5326	26.5113	25.0109	24.6116	29.369	23.2467	26.2175	22.3536	25.4916	22.6511	27.7818	22.5807	24.418	23.4825	0.93243477	0.966018836	20	4	24	0.592315874	0.85644831	24	16.34484488	0.043008055
ENSMUSG00000031652	N4bp1	NEDD4 binding protein 1 [Source:MGI Symbol;Acc:MGI:2136825]	6.39624	5.5792	5.85789	6.29981	5.96619	6.52325	6.6071	6.18098	5.6037	6.8329	5.66677	6.11416	5.93917	5.66333	0.208071297	0.698704663	4	4	24	0.430126108	0.805393068	24	18.76429655	0.042614304
ENSMUSG00000031654	Cbln1	cerebellin 1 precursor protein [Source:MGI Symbol;Acc:MGI:88281]	2.47271	3.01496	3.2597	2.48667	2.14027	3.1854	2.63381	2.4546	3.10978	3.1147	2.25484	3.18583	2.40066	3.10565	0.616047374	0.966018836	12	8	24	0.476003586	0.805393068	24	6.53319255	0.077038309
ENSMUSG00000031657	Heatr3	HEAT repeat containing 3 [Source:MGI Symbol;Acc:MGI:2444491]	12.8794	14.7593	14.1286	15.6349	14.7569	14.5286	14.4471	15.0023	15.9212	13.8295	14.358	14.3327	15.5101	14.3224	0.814221431	0.966018836	8	20	24	0.520848875	0.820180873	24	9.167057782	0.033102035
ENSMUSG00000031659	Adcy7	adenylate cyclase 7 [Source:MGI Symbol;Acc:MGI:102891]	3.12288	3.47727	3.28792	3.34772	3.28912	3.00682	3.53863	3.19552	3.27399	2.96934	3.03374	2.7542	2.93034	2.87547	0.9678483	0.970142211	12	12	24	0.404651065	0.799546792	24	7.106484508	0.031935843
ENSMUSG00000031660	Brd7	bromodomain containing 7 [Source:MGI Symbol;Acc:MGI:1349766]	18.2078	20.3679	21.247	22.7711	19.5721	19.238	17.205	22.315	18.7882	20.5506	18.7185	17.5927	18.5191	18.8831	0.008603252	0.192760771	16	12	24	0.03100049	0.59941366	24	9.675055498	0.103679137
ENSMUSG00000031661	Nkd1	naked cuticle 1 [Source:MGI Symbol;Acc:MGI:2135954]	3.65154	4.51597	4.39506	3.50431	4.64926	3.42923	4.569	4.28921	4.46581	5.60904	3.9639	4.25279	4.46939	3.70139	0.838244962	0.966018836	12	12	24	0.575263252	0.848005542	24	9.598211053	0.066321892
ENSMUSG00000031662	Snx20	sorting nexin 20 [Source:MGI Symbol;Acc:MGI:1918857]	0.52298	0.626943	0.419792	0.741577	0.633028	0.317641	0.712294	0.485434	0.958057	0.351647	0.788012	0.583486	0.900703	0.649074	0.852844413	0.966018836	4	20	24	0.896898061	0.970634157	24	5.744523808	0.031721696
ENSMUSG00000031665	Sall1	spalt like transcription factor 1 [Source:MGI Symbol;Acc:MGI:1889585]	0.325556	0.82114	1.41764	0.528417	0.555432	0.647482	0.910177	0.606807	0.663847	0.465778	0.289479	0.65844	0.164516	0.766692	0.297756231	0.80820842	12	4	24	0.316118018	0.768040178	24	6.788281169	0.151339757
ENSMUSG00000031666	Rbl2	RB transcriptional corepressor like 2 [Source:MGI Symbol;Acc:MGI:105085]	13.1216	13.2567	14.0938	15.0306	13.0943	13.531	15.0775	13.8624	14.6084	14.1778	14.4851	14.2293	13.12	14.3521	0.742579234	0.966018836	16	12	24	0.31322484	0.768040178	24	10.43547063	0.038737271
ENSMUSG00000031667	Aktip	AKT interacting protein [Source:MGI Symbol;Acc:MGI:3693832]	79.0515	72.0916	68.3577	67.5286	69.2343	71.2298	74.5235	70.2047	67.423	71.9376	76.3339	78.1608	77.2341	76.7311	0.004140921	0.140598674	4	8	24	0.011390962	0.519104533	24	22.38714322	0.07592297
ENSMUSG00000031668	Eif2ak3	eukaryotic translation initiation factor 2 alpha kinase 3 [Source:MGI Symbol;Acc:MGI:1341830]	12.2096	9.48945	10.1819	9.37258	11.4621	11.4292	10.9066	10.0995	8.30865	11.7642	9.26109	10.4166	9.15035	9.56385	0.649148181	0.966018836	24	12	24	0.148319546	0.756728257	24	19.50703995	0.105404675
ENSMUSG00000031669	Gins3	GINS complex subunit 3 (Psf3 homolog) [Source:MGI Symbol;Acc:MGI:1926083]	4.13374	3.77078	3.85964	4.31963	3.7491	3.86446	4.08103	4.51754	3.59167	3.52128	4.2205	4.35331	4.61906	4.21269	0.420039465	0.966018836	4	8	24	0.217642857	0.768040178	24	23.42025214	0.061630727
ENSMUSG00000031671	Setd6	SET domain containing 6 [Source:MGI Symbol;Acc:MGI:1913333]	10.4305	9.65107	10.3963	11.3069	10.703	10.879	9.82979	10.4204	9.89431	9.85142	9.6726	11.1945	9.89134	8.20527	0.257978335	0.761738864	24	8	24	0.08047968	0.695795041	24	17.09645364	0.078532902
ENSMUSG00000031672	Got2	glutamatic-oxaloacetic transaminase 2, mitochondrial [Source:MGI Symbol;Acc:MGI:95792]	32.9855	28.4604	30.0801	30.1297	28.8197	34.7741	30.983	37.1708	25.906	38.6718	25.7247	30.8944	28.8885	31.3968	0.838532766	0.966018836	24	20	24	0.902304218	0.972729171	24	0.838404443	0.030444076
ENSMUSG00000031673	Cdh11	cadherin 11 [Source:MGI Symbol;Acc:MGI:99217]	27.2554	29.0683	25.7959	24.5836	25.1795	28.1425	25.5068	26.8757	23.3964	26.1449	24.7781	27.1538	26.2908	28.1491	0.027055582	0.306445942	8	4	24	0.08089671	0.696856099	24	0.337729295	0.068070719
ENSMUSG00000031681	Smad1	SMAD family member 1 [Source:MGI Symbol;Acc:MGI:109452]	11.7611	9.54246	9.27126	10.5108	10.3226	10.0808	10.0133	9.85747	9.79315	12.5077	10.3022	10.1906	9.69673	9.82691	0.002443055	0.109177422	16	20	24	0.378358376	0.797715859	24	15.80433285	0.057466137
ENSMUSG00000031682	1700011L22Rik	RIKEN cDNA 1700011L22 gene [Source:MGI Symbol;Acc:MGI:1914937]	1.88548	1.46246	1.6187	1.36572	1.6339	2.084	0.908021	1.46599	1.69424	1.57353	1.26224	1.45547	1.61958	1.33424	0.832742389	0.966018836	24	8	24	0.897149807	0.970698391	24	17.40902355	0.030707458
ENSMUSG00000031683	Lsm6	LSM6 homolog, U6 small nuclear RNA and mRNA degradation associated [Source:MGI Symbol;Acc:MGI:1925901]	21.3461	22.112	23.5604	20.8695	23.0221	21.0099	20.5293	20.2283	23.3285	18.9444	21.1979	22.181	25.5646	21.0649	0.90703203	0.966018836	12	4	24	0.83871682	0.9507575	24	1.247242896	0.024687319
ENSMUSG00000031684	Slc10a7	solute carrier family 10 (sodium/bile acid cotransporter family), member 7 [Source:MGI Symbol;Acc:MGI:1924025]	5.87837	5.83825	5.61887	5.77472	5.92939	5.25209	6.21345	5.42421	5.1476	5.47981	5.64554	5.35303	5.85514	5.34611	0.43565473	0.966018836	4	8	24	0.482283727	0.805393068	24	21.53982954	0.029847951
ENSMUSG00000031688	Pou4f2	POU domain, class 4, transcription factor 2 [Source:MGI Symbol;Acc:MGI:102524]	5.93385	4.01514	3.73936	4.31116	3.89282	4.47743	3.7551	4.13073	3.09246	5.38802	3.9029	4.54038	4.17096	4.51158	0.245061943	0.741580974	24	12	24	0.536933579	0.827590998	24	20.62085788	0.085854736
ENSMUSG00000031691	Tnpo2	transportin 2 (importin 3, karyopherin beta 2b) [Source:MGI Symbol;Acc:MGI:2384849]	37.2162	28.8602	30.7199	32.1509	31.6047	36.676	34.5485	33.6311	24.1117	39.6071	28.8902	32.7068	30.5483	32.931	0.648082424	0.966018836	24	12	24	0.419512303	0.803639585	24	20.26513083	0.093502496
ENSMUSG00000031696	Vps35	VPS35 retromer complex component [Source:MGI Symbol;Acc:MGI:1890467]	108.524	115.071	108.144	107.846	116.44	103.826	103.934	104.188	114.703	94.1642	122.492	105.479	114.33	112.276	0.945282252	0.966018836	20	4	24	0.993400999	0.998731933	24	20.76462286	0.004586896
ENSMUSG00000031697	Orc6	origin recognition complex, subunit 6 [Source:MGI Symbol;Acc:MGI:1929285]	12.4336	15.7599	16.4823	15.5356	14.2263	12.5298	12.5645	15.0236	16.9376	12.8141	16.2322	14.4579	15.4936	14.6625	0.024605074	0.288980862	12	12	24	0.102570691	0.713699364	24	8.783237205	0.118405379
ENSMUSG00000031700	Gpt2	glutamic pyruvate transaminase (alanine aminotransferase) 2 [Source:MGI Symbol;Acc:MGI:1915391]	21.005	19.4729	19.5389	25.8165	21.7239	22.9906	21.1093	20.4188	19.3513	24.6243	19.58	20.6723	19.8096	17.7525	0.020428812	0.261278501	16	20	24	0.062491289	0.661961741	24	15.30048257	0.111945639
ENSMUSG00000031701	Dnaja2	DnaJ heat shock protein family (Hsp40) member A2 [Source:MGI Symbol;Acc:MGI:1931882]	66.1944	65.9588	67.9279	63.6256	64.2419	66.6064	63.3487	64.8257	60.893	64.4415	65.7205	66.389	65.9267	68.1517	0.759899911	0.966018836	24	16	24	0.533106015	0.825678486	24	23.47210159	0.018870633
ENSMUSG00000031703	Itfg1	integrin alpha FG-GAP repeat containing 1 [Source:MGI Symbol;Acc:MGI:106419]	133.229	133.275	124.529	130.537	129.33	128.864	126.884	122.323	124.142	120.382	135.001	130.479	134.251	134.879	0.892555977	0.966018836	20	20	24	0.245944412	0.768040178	24	21.64581437	0.035812998
ENSMUSG00000031706	Rfx1	regulatory factor X, 1 (influences HLA class II expression) [Source:MGI Symbol;Acc:MGI:105982]	3.39688	3.05763	3.08344	2.98538	2.9602	3.02307	3.55008	2.83286	3.30634	3.25023	3.73654	3.75275	3.45738	3.1478	0.456461936	0.966018836	4	4	24	0.26938782	0.768040178	24	20.06037726	0.057165749
ENSMUSG00000031708	Tecr	trans-2,3-enoyl-CoA reductase [Source:MGI Symbol;Acc:MGI:1915408]	330.125	288.692	317.363	301.074	294.305	365.762	316.224	341.93	247.57	335.981	270.48	271.076	274.646	273.72	0.826055781	0.966018836	4	16	24	0.72314875	0.909188168	24	20.88889057	0.04774313
ENSMUSG00000031709	Tbc1d9	TBC1 domain family, member 9 [Source:MGI Symbol;Acc:MGI:1918560]	27.2949	19.5995	20.876	22.4643	19.8098	24.6556	22.5602	23.0913	14.872	31.0537	20.3093	23.079	21.9804	23.1734	0.791413662	0.966018836	24	12	24	0.612968478	0.865918191	24	20.15542356	0.096228062
ENSMUSG00000031710	Ucp1	uncoupling protein 1 (mitochondrial, proton carrier) [Source:MGI Symbol;Acc:MGI:98894]	12.0284	8.77601	31.7391	114.896	22.9344	12.9004	31.4288	45.3443	35.7323	37.2707	49.3515	28.633	66.4359	65.4836	0.833406028	0.966018836	16	8	24	0.169104988	0.767020009	24	10.2503657	0.600707421
ENSMUSG00000031711	Zfp330	zinc finger protein 330 [Source:MGI Symbol;Acc:MGI:1353574]	11.1382	12.7986	12.5111	11.756	11.8967	11.0419	11.4813	11.4911	12.349	11.4897	12.1136	10.3429	10.995	10.6836	0.071575474	0.440262583	12	12	24	0.046684155	0.633507364	24	9.597953103	0.063141995

ENSMUSG00000031712	Il15	interleukin 15 [Source:MGI Symbol;Acc:MGI:103014]	1.87622	1.96087	2.86383	3.66277	2.29281	2.09996	2.07554	1.74103	3.31591	1.88556	2.79915	1.44042	2.05496	1.76625	0.027055582	0.306445942	12	20	24	0.040181718	0.616921492	24	11.38203938	0.234043756
ENSMUSG00000031714	Gab1	growth factor receptor bound protein 2-associated protein 1 [Source:MGI Symbol;Acc:MGI:108088]	18.0675	17.3632	17.1474	15.7514	20.0313	17.4916	21.5214	19.0762	18.1497	21.8923	17.6043	18.8593	18.6784	17.038	0.840483986	0.966018836	4	4	24	0.784989	0.931056644	24	19.89447698	0.03572074
ENSMUSG00000031715	Smarca5	SWI/SNF related, matrix associated, actin dependent regulator of chromatin, subfamily a, member 5 [Source:MGI Symbol;Acc:MGI:1935129]	6.40491	6.34136	7.04515	6.3589	6.17939	5.92391	5.43847	5.67878	5.32397	5.39667	6.41318	5.12174	5.01802	4.79608	0.836635326	0.966018836	20	12	24	0.136609498	0.747972781	24	13.82123756	0.059658191
ENSMUSG00000031722	Hp	haptoglobin [Source:MGI Symbol;Acc:MGI:96211]	10.1688	11.0476	23.0746	26.7106	15.8458	17.4444	13.8938	22.4283	13.7686	23.1073	27.3815	17.0214	29.9301	17.7422	0.575202741	0.966018836	16	12	24	0.20953571	0.768040178	24	12.58263624	0.272257972
ENSMUSG00000031723	Txnl4b	thioredoxin-like 4B [Source:MGI Symbol;Acc:MGI:2443724]	9.77275	9.68979	8.78737	9.25222	9.11039	9.48815	9.75576	10.5497	9.43517	10.0908	9.13493	9.83734	10.0124	9.82963	0.143268475	0.585944911	8	12	24	0.104871017	0.717030134	24	1.803891469	0.044976645
ENSMUSG00000031725	Ces1f	carboxylesterase 1F [Source:MGI Symbol;Acc:MGI:2142687]	0.157297	0.444615	0.86776	2.10042	0.287387	0.651379	0.113224	0.565157	0.630508	1.69298	1.05107	0.870745	1.34754	1.2017	0.101443845	0.511659635	16	12	24	0.057780962	0.656248849	24	11.33890446	0.342288558
ENSMUSG00000031728	Zfp821	zinc finger protein 821 [Source:MGI Symbol;Acc:MGI:1923121]	9.04827	8.09873	7.506	8.40809	9.22198	8.8199	8.43546	8.77243	7.38924	8.32809	8.05486	8.12771	8.2189	8.02579	0.122696414	0.550756498	24	12	24	0.055874878	0.65534544	24	19.9768808	0.070759254
ENSMUSG00000031729	Ist1	increased sodium tolerance 1 homolog (yeast) [Source:MGI Symbol;Acc:MGI:1919205]	47.4501	46.9774	46.4878	47.9045	48.6137	47.7562	44.7785	46.3809	46.7404	44.5422	49.3753	47.5129	48.6128	47.4159	0.035023815	0.328311403	20	16	24	0.342233184	0.776349899	24	18.18494597	0.02403647
ENSMUSG00000031730	Dhodh	dihydroorotate dehydrogenase [Source:MGI Symbol;Acc:MGI:1928378]	6.13817	5.4257	5.47724	6.03717	6.82386	5.74562	5.96138	5.72739	5.9469	6.76472	5.32233	6.14329	5.63088	6.22703	0.93717134	0.966018836	16	20	24	0.523157331	0.82106433	24	14.77581555	0.041190395
ENSMUSG00000031731	Ap1g1	adaptor protein complex AP-1, gamma 1 subunit [Source:MGI Symbol;Acc:MGI:101919]	32.6933	33.9905	31.9735	32.406	33.3533	32.8733	31.3922	32.2953	31.6173	33.6424	33.571	31.6224	34.0592	34.6868	0.893609703	0.966018836	8	4	24	0.984553003	0.996773483	24	5.842366115	0.003147729
ENSMUSG00000031732	Phlpp2	PH domain and leucine rich repeat protein phosphatase 2 [Source:MGI Symbol;Acc:MGI:2444928]	7.40632	6.91554	7.20567	7.36056	7.2292	7.51793	7.91181	6.61543	6.70519	7.74378	7.06597	7.37938	7.34243	7.41621	0.297978647	0.80820842	4	4	24	0.30663297	0.768040178	24	19.2503582	0.039081308
ENSMUSG00000031736	Crnde	colorectal neoplasia differentially expressed (non-protein coding) [Source:MGI Symbol;Acc:MGI:1918546]	0.364544	0.508296	1.59832	0.515581	0.714588	0.31581	0.558249	1.0708	0.663303	0.705386	0.674743	0.506811	0.633061	0.292059	0.27074138	0.780327352	20	4	24	0.219287102	0.768040178	24	9.246180102	0.179139621
ENSMUSG00000031740	Mmp2	matrix metallopeptidase 2 [Source:MGI Symbol;Acc:MGI:97009]	18.1037	19.5345	15.6461	15.1692	18.7809	16.3066	19.8264	19.5646	19.5051	15.1954	17.5392	16.4451	17.9342	16.146	0.088738238	0.474489058	4	12	24	0.209397968	0.768040178	24	1.120703245	0.089968585
ENSMUSG00000031748	Gnao1	guanine nucleotide binding protein, alpha O [Source:MGI Symbol;Acc:MGI:95775]	205.633	156.685	158.426	165.09	162.963	191.76	164.893	171.365	134.257	230.178	166.338	197.392	180.815	190.774	0.077301512	0.453790923	24	12	24	0.4332574	0.805393068	24	19.94191299	0.100837991
ENSMUSG00000031749	St3gal2	ST3 beta-galactoside alpha-2,3-sialyltransferase 2 [Source:MGI Symbol;Acc:MGI:99427]	13.6859	11.7238	12.1283	12.2723	12.0573	12.72	13.4825	13.3778	10.8991	16.494	11.7928	13.2493	12.8332	13.0316	0.286642595	0.793714531	4	8	24	0.908655368	0.974606327	24	20.67606127	0.023834593
ENSMUSG00000031750	Il34	interleukin 34 [Source:MGI Symbol;Acc:MGI:1923777]	26.2545	23.9764	20.9278	26.7181	30.6257	23.2258	25.8655	25.1829	25.1462	27.2694	27.1991	25.9698	25.1593	20.8837	0.016702001	0.253395174	16	20	24	0.05534765	0.65534544	24	16.59874275	0.123422736
ENSMUSG00000031751	Amfr	autocrine motility factor receptor [Source:MGI Symbol;Acc:MGI:1345634]	45.2257	38.8933	41.7375	41.5357	41.0159	42.5375	44.6314	44.1445	37.4737	49.6253	39.4955	43.9675	42.2505	41.8782	0.241264473	0.741580974	4	8	24	0.77358243	0.92732018	24	20.49318819	0.029795564
ENSMUSG00000031753	Cog4	component of oligomeric golgi complex 4 [Source:MGI Symbol;Acc:MGI:2142808]	40.047	36.2357	34.5389	36.822	38.0931	37.2768	33.2936	37.1109	33.8455	33.9634	36.0209	34.7941	36.3454	36.1783	0.759899911	0.966018836	24	12	24	0.345172498	0.778298718	24	20.97147225	0.038863473
ENSMUSG00000031754	Nudt21	nudix (nucleoside diphosphate linked moiety X)-type motif 21 [Source:MGI Symbol;Acc:MGI:1915469]	9.05437	8.30305	8.87479	8.57691	8.74873	8.32222	8.81533	7.47206	8.21445	8.01275	9.35131	8.79777	8.86399	9.6983	0.881355613	0.966018836	4	12	24	0.679579975	0.892710167	24	20.32134097	0.030609872
ENSMUSG00000031755	Bbs2	Bardet-Biedl syndrome 2 (human) [Source:MGI Symbol;Acc:MGI:2135267]	15.032	15.4403	16.0837	14.7897	14.9221	14.5512	14.7706	14.5432	16.7104	13.4386	13.6145	15.5411	16.5885	16.764	0.256915694	0.76168256	12	4	24	0.08923511	0.708638675	24	4.082640236	0.075325952
ENSMUSG00000031756	Cenpn	centromere protein N [Source:MGI Symbol;Acc:MGI:1919405]	1.49812	1.67488	1.55072	1.91551	1.66413	1.55491	1.66092	1.25551	1.22242	1.19251	1.74084	1.29364	1.60412	1.71277	0.887862353	0.966018836	20	16	24	0.79403073	0.933885455	24	19.01047798	0.035352126
ENSMUSG00000031758	Cdyl2	chromodomain protein, Y chromosome-like 2 [Source:MGI Symbol;Acc:MGI:1923046]	1.88457	1.28208	1.4185	1.34078	1.59125	1.76013	1.27027	1.61462	1.1214	2.2333	1.67863	1.78891	1.71898	1.21244	0.119434414	0.542554768	24	12	24	0.153739041	0.760223561	24	17.99762551	0.127675702
ENSMUSG00000031760	Mt3	metallothionein 3 [Source:MGI Symbol;Acc:MGI:97173]	227.147	191.592	193.803	199.055	219.362	220.249	211.67	184.287	190.303	198.047	199.389	209.633	194.11	199.408	0.010049466	0.194720062	24	12	24	0.002943182	0.414576087	24	19.52206112	0.09811117
ENSMUSG00000031762	Mt2	metallothionein 2 [Source:MGI Symbol;Acc:MGI:97172]	56.8431	60.3108	101.182	157.606	55.5633	67.0969	62.7829	48.15	86.7903	94.1929	57.3932	66.8746	41.2863	56.6529	0.046239714	0.375011045	12	20	24	0.011358523	0.518294984	24	11.43237518	0.475958012
ENSMUSG00000031765	Mt1	metallothionein 1 [Source:MGI Symbol;Acc:MGI:97171]	99.575	103.706	150.628	161.656	113.778	106.949	107.363	96.8455	136.031	121.274	114.728	109.124	81.2674	99.7492	3.87E-04	0.052628402	12	20	24	0.00110981	0.336500758	24	11.56692934	0.278559277
ENSMUSG00000031766	Slc12a3	solute carrier family 12, member 3 [Source:MGI Symbol;Acc:MGI:108114]	4.52765	3.61761	3.99627	4.32823	4.9479	3.83394	3.96384	3.61268	3.28254	3.34424	4.19294	3.81799	3.68348	3.47155	0.080511813	0.4593055	20	12	24	0.025945905	0.586541342	24	17.99307328	0.113047033
ENSMUSG00000031767	Nudt7	nudix (nucleoside diphosphate linked moiety X)-type motif 7 [Source:MGI Symbol;Acc:MGI:1914778]	24.9078	26.7023	27.3747	32.3107	27.8287	24.7842	26.5091	26.2019	28.5711	25.5247	35.0328	26.3026	33.1199	28.3225	0.676344318	0.966018836	20	4	24	0.494934886	0.806761911	24	12.72608414	0.065192866
ENSMUSG00000031770	Herpud1	homocysteine-inducible, endoplasmic reticulum stress-inducible, ubiquitin-like domain member 1 [Source:MGI Symbol;Acc:MGI:1927406]	30.7482	26.2099	27.6601	32.5936	27.5633	27.4042	29.8386	25.6246	31.0798	34.147	28.3784	27.1059	26.4178	26.4209	0.004516736	0.140598674	16	16	24	0.146357401	0.754936569	24	12.62214698	0.088740604
ENSMUSG00000031772	Cntnap4	contactin associated protein-like 4 [Source:MGI Symbol;Acc:MGI:2183572]	7.52146	6.94329	7.61092	7.31558	6.27739	8.10619	7.01142	7.18693	5.90531	8.7102	6.60695	7.8464	7.99162	7.96223	0.913451828	0.966018836	24	20	24	0.807239123	0.938686319	24	22.96858545	0.03529688
ENSMUSG00000031774	Psme3ip1	proteasome activator subunit 3 interacting protein 1 [Source:MGI Symbol;Acc:MGI:1919637]	19.5927	15.8695	14.7923	16.8911	15.6835	16.9536	15.7826	16.4928	14.5231	16.0271	13.2428	16.5801	16.0946	16.0855	0.17847698	0.655480352	24	12	24	0.159770338	0.76564405	24	23.20192065	0.081808477
ENSMUSG00000031775	Pllp	plasma membrane proteolipid [Source:MGI Symbol;Acc:MGI:1915051]	80.4542	87.5944	82.4409	95.1937	98.1846	84.6485	103.955	83.1131	95.9444	96.7109	82.2645	89.9845	83.1173	75.4553	0.760769894	0.966018836	16	16	24	0.37319584	0.794388449	24	14.39866546	0.070594678
ENSMUSG00000031776	Arl2bp	ADP-ribosylation factor-like 2 binding protein [Source:MGI Symbol;Acc:MGI:1349429]	63.4506	56.947	58.5176	56.2446	57.3032	63.9803	60.2719	62.3441	51.8031	65.1156	52.639	61.8654	56.9262	60.6895	0.745118544	0.966018836	24	20	24	0.428358408	0.805393068	24	22.6802504	0.051705414
ENSMUSG00000031778	Cx3cl1	chemokine (C-X3-C motif) ligand 1 [Source:MGI Symbol;Acc:MGI:1097153]	2.87484	4.84572	8.93297	3.59423	2.92332	4.9442	6.57366	3.63503	5.4888	4.35288	2.47022	4.96505	2.76483	5.31693	0.179603275	0.655480352	12	8	24	0.298870839	0.768040178	24	6.226641654	0.264906421
ENSMUSG00000031781	Ciapin1	cytokine induced apoptosis inhibitor 1 [Source:MGI Symbol;Acc:MGI:1922083]	37.6954	33.2426	34.5667	32.5029	32.3626	33.2792	31.6223	34.079	28.9531	34.8914	30.3167	31.9867	34.0183	33.8539	0.894563966	0.966018836	8	12	24	0.438949209	0.805393068	24	1.121473189	0.042308783
ENSMUSG00000031782	Coq9	coenzyme Q9 [Source:MGI Symbol;Acc:MGI:1915164]	20.1252	21.3639	26.0951	31.3551	26.5066	24.698	23.6033	30.6117	29.3081	29.9039	26.5783	24.1887	28.4051	25.8058	0.030972684	0.324150946	16	12	24	0.016530336	0.544099157	24	10.83706915	0.16633024
ENSMUSG00000031783	Polr2c	polymerase (RNA) II (DNA directed) polypeptide C [Source:MGI Symbol;Acc:MGI:109299]	10.8632	10.5432	11.998	12.2008	10.3073	11.4928	10.9432	10.4951	11.9815	10.6718	11.6583	9.64911	11.164	10.1672	0.176120994	0.651716826	12	20	24	0.306616719	0.768040178	24	10.91723617	0.053492628
ENSMUSG00000031785	Adgrg1	adhesion G protein-coupled receptor G1 [Source:MGI Symbol;Acc:MGI:1340051]	26.7491	20.1082	22.026	21.0183	21.8263	26.746	23.0407	27.2864	17.248	33.2502	19.8222	23.8823	22.1706	24.2227	0.625825704	0.966018836	24	20	24	0.811968481	0.939785122	24	21.07805102	0.061558919
ENSMUSG00000031787	Katnb1	katanin p80 (WD40-containing) subunit B 1 [Source:MGI Symbol;Acc:MGI:1921437]	8.41869	7.10511	7.53325	6.80682	7.7456	9.87286	8.19598	8.09002	7.24744	9.94495	6.46743	6.68031	8.14542	8.10403	0.474848197	0.966018836	4	16	24	0.814172018	0.940708369	24	23.46877028	0.04362996
ENSMUSG00000031788	Kifc3	kinesin family member C3 [Source:MGI Symbol;Acc:MGI:109202]	8.70781	8.82022	10.9044	8.52383	7.18889	8.49903	9.75619	9.93396	10.6367	8.44407	8.33227	8.88342	8.55168	9.2266	4.66E-04	0.0579004	12	8	24	0.018402019	0.559812298	24	5.666462238	0.140167388
ENSMUSG00000031790	Mmp15	matrix metallopeptidase 15 [Source:MGI Symbol;Acc:MGI:109320]	21.324	16.7215	15.7272	17.4497	22.2542	18.7921	22.6438	19.9541	16.6279	26.7421	17.5191	20.6617	20.2142	18.4297	0.055920623	0.401074772	4	8	24	0.409592094	0.799546792	24	19.18269716	0.107244499
ENSMUSG00000031791	Tmem38a	transmembrane protein 38A [Source:MGI Symbol;Acc:MGI:1921416]	40.2895	45.8338	46.4951	54.7235	46.3887	52.3404	46.2067	70.1274	58.0149	67.6958	45.4025	50.7914	55.0857	50.492	0.917867465	0.966018836	16	4	24	0.222740038	0.768040178	24	9.028631332	0.138284629
ENSMUSG00000031792	Usb1	U6 snRNA biogenesis 1 [Source:MGI Symbol;Acc:MGI:2142454]	5.84693	5.07445	5.49669	4.72778	5.1829	5.5079	6.02546	5.87977	5.71829	5.66064	5.04301	5.85894	5.55313	5.93792	0.10339906	0.517709628	4	16	24	0.059125387	0.658304792	24	1.688790373	0.070426316
ENSMUSG00000031796	Cfap20	cilia and flagella associated protein 20 [Source:MGI Symbol;Acc:MGI:107428]	34.3959	36.0935	33.4698	34.3469	40.9551	38.1589	33.7891	34.5336	32.901	35.1873	32.597	37.2571	34.4952	35.8684	0.601647512	0.966018836	24	20	24	0.237981893	0.768040178	24	18.77156052	0.058639623
ENSMUSG00000031799	Tpm4	tropomyosin 4 [Source:MGI Symbol;Acc:MGI:2449202]	60.0903	56.8932	56.9815	53.895	65.6108	55.4964	56.758	54.7561	59.192	45.8285	62.306	53.6998	58.0402	49.7057	0.745118544	0.966018836	20	12	24	0.423801752	0.805393068	24	19.60593232	0.063999863
ENSMUSG00000031805	Jak3	Janus kinase 3 [Source:MGI Symbol;Acc:MGI:99928]	1.9952	1.67788	1.80221	1.84683	1.80858	1.79447	1.4859	2.34394	1.76326	1.98714	2.23447	1.93787	2.04521	2.03911	0.838244962	0.966018836	8	4	24	0.950069906	0.988090608	24	10.99344907	0.013151458
ENSMUSG00000031807	Pgls	6-phosphogluconolactonase [Source:MGI Symbol;Acc:MGI:1913421]	20.7865	20.2666	20.6534	19.4827	20.7124	22.2942	19.7623	23.7832	21.0502	24.977	29.7786	26.2273	29.9567	28.3452	0.888482515	0.966018836	20	20	24	0.684503655	0.895095838	24	20.66335456	0.040808829
ENSMUSG00000031808	Slc27a1	solute carrier family 27 (fatty acid transporter), member 1 [Source:MGI Symbol;Acc:MGI:1347098]	39.2776	33.2665	33.6466	39.751	35.0162	31.2686	42.2622	42.4143	38.6521	40.3582	39.592	36.497	39.0816	34.8065	0.924553956	0.966018836	16	8	24	0.934958414	0.983765461	24	7.257972081	0.0201392
ENSMUSG00000031812	Map1lc3b	microtubule-associated protein 1 light chain 3 beta [Source:MGI Symbol;Acc:MGI:1914693]	134.819	107.037	109.91	109.765	108.564	136.454	117.357	124.928	88.0987	127.142	110.142	110.777	109.19	108.172	0.360140629	0.883787544	24	12	24	0.194356438	0.768040178	24	20.85549584	0.111280084
ENSMUSG00000031813	Mvb12a	multivesicular body subunit 12A [Source:MGI Symbol;Acc:MGI:1920961]	30.463	27.4486	29.9621	25.3142	26.4985	26.0371	25.1823	27.2136	30.2343	26.9011	28.9234	29.3022	33.8753	31.1617	0.475540699	0.966018836	12	4	24	0.315416414	0.768040178	24	3.360036932	0.06458706
ENSMUSG00000031816	Mthfsd	methenyltetrahydrofolate synthetase domain containing [Source:MGI Symbol;Acc:MGI:2679252]	34.1171	32.9393	32.0078	33.5045	30.5271	34.5321	28.8927	33.5371	30.1498	32.4766	30.3103	28.8605	29.1589	28.2468	0.801251296	0.966018836	16	12	24	0.829913084	0.947131234	24	12.75982707	0.016683043
ENSMUSG00000031818	Cox4i1	cytochrome c oxidase subunit 4I1 [Source:MGI Symbol;Acc:MGI:88473]	436.477	449.941	463.329	468.62	429.348	449.523	450.626	479.388	459.619	442.063	455.834	437.32	480.759	467.151	0.355803318	0.879596776	8	16	24	0.072632398	0.679671854	24	5.824721866	0.040588445
ENSMUSG00000031819	Emc8	ER membrane protein complex subunit 8 [Source:MGI Symbol;Acc:MGI:1343095]	19.155	16.5651	14.3362	20.4084	17.5699	18.0745	19.1849	15.5545	14.5807	16.0557	19.2873	16.6686	18.6635	16.7145	0.05280092	0.391647859	4	8	24	0.060698161	0.660111271	24	19.46925509	0.131985941
ENSMUSG00000031820	Babam1	BRISC and BRCA1 A complex member 1 [Source:MGI Symbol;Acc:MGI:1915501]	37.0102	36.3748	36.9659	35.8745	36.3421	39.2517	37.9714	38.1917	35.9111	41.5406	36.9302	39.792	40.6097	38.368	0.315627348	0.828057631	24	12	24	0.509552795	0.813985618	24	21.47127716	0.025361232
ENSMUSG00000031821	Gins2	GINS complex subunit 2 (Psf2 homolog) [Source:MGI Symbol;Acc:MGI:1921019]	2.37014	3.51184	3.33598	3.44155	3.39845	3.19355	3.04009	2.91496	3.33864	3.2268	3.08276	3.39931	2.67923	3.22128	0.064091035	0.414867207	16	12	24	0.104706528	0.717030134	24	11.62703288	0.092185344
ENSMUSG00000031822	Gse1	genetic suppressor element 1, coiled-coil protein [Source:MGI Symbol;Acc:MGI:1098275]	3.41387	3.05775	3.38949	2.68856	3.49996	3.08763	3.38886	3.11781	3.36288	5.77375	3.86131	3.14834	3.61388	3.08352	0.305564675	0.811201458	20	12	24	0.429682242	0.805393068	24	13.11918519	0.098582787
ENSMUSG00000031823	Zdhhc7	zinc finger, DHHC domain containing 7 [Source:MGI Symbol;Acc:MGI:2142662]	12.2644	9.82788	9.77038	13.3282	12.4852	11.7652	11.8913	12.4262	10.2632	12.8939	11.6943	10.2758	10.5172	11.2041	0.129903614	0.560263689	16	20	24	0.329797951	0.768040178	24	15.79348414	0.078557474
ENSMUSG00000031824	6430548M08Rik	RIKEN cDNA 6430548M08 gene [Source:MGI Symbol;Acc:MGI:2443793]	28.3828	21.3018	22.9448	23.4327	22.3674	27.088	24.8536	25.833	18.7116	31.1029	20.557	25.3613	23.0019	25.6562	0.791413662	0.966018836	24	12	24	0.657871158	0.884596426	24	21.45697264	0.070443675
ENSMUSG00000031825	Crispld2	cysteine-rich secretory protein LCCL domain containing 2 [Source:MGI Symbol;Acc:MGI:1926142]	11.9626	13.5253	12.3394	13.0971	17.7127	12.4286	14.3223	13.8045	16.1639	11.3179	16.0129	15.0948	13.1913	11.6185	0.722296281	0.966018836	20	20	24	0.421144006	0.804493443	24	16.44342939	0.093775954
ENSMUSG00000031826	Usp10	ubiquitin specific peptidase 10 [Source:MGI Symbol;Acc:MGI:894652]	19.487	20.4093	18.7287	18.6015	19.2027	20.7522	21.9799	21.4684	21.1459	21.1876	20.5819	22.7928	22.1984	21.0672	0.274035549	0.788124063	24	20	24	0.041200873	0.61831089	24	23.87087577	0.053689069
ENSMUSG00000031827	Cotl1	coactosin-like 1 (Dictyostelium) [Source:MGI Symbol;Acc:MGI:1919292]	17.8702	18.3384	19.775	15.4151	17.0044	16.019	19.8036	18.6986	18.6776	16.572	18.2385	17.691	17.7419	15.3596	0.369262716	0.893596186	12	4	24	0.509646367	0.813985618	24	2.941390294	0.049857577
ENSMUSG00000031828	Klhl36	kelch-like 36 [Source:MGI Symbol;Acc:MGI:2385305]	1.33449	1.38414	0.961096	1.24451	1.22589	1.05304	1.50653	1.26964	1.38365	1.36089	1.08294	1.34584	1.27717	1.45346	0.166587374	0.633636735	8	12	24	0.293880701	0.768040178	24	2.844967836	0.058448957
ENSMUSG00000031832	Taf1c	TATA-box binding protein associated factor, RNA polymerase I, C [Source:MGI Symbol;Acc:MGI:109576]	2.30835	2.26883	2.46981	2.12563	2.25061	2.23984	2.35143	2.39407	2.32456	2.8044	2.49053	2.53613	2.33815	2.26768	0.759825336	0.966018836	12	20	24	0.643082685	0.878809617	24	12.46183967	0.024251489
ENSMUSG00000031833	Mast3	microtubule associated serine/threonine kinase 3 [Source:MGI Symbol;Acc:MGI:2683541]	6.0149	5.98489	8.41112	7.15976	6.76701	6.40842	7.00397	6.83381	7.80062	8.38657	6.45811	7.46478	6.05894	6.72465	0.031315549	0.324385159	12	20	24	0.029243208	0.596648309	24	10.68231347	0.13515141
ENSMUSG00000031834	Pik3r2	phosphoinositide-3-kinase regulatory subunit 2 [Source:MGI Symbol;Acc:MGI:1098772]	17.7003	14.9131	16.6825	17.3085	15.455	16.4126	14.8091	17.8566	16.4009	21.2272	17.2816	17.3925	17.5281	17.2434	0.846643334	0.966018836	16	4	24	0.433742275	0.805393068	24	11.63148025	0.057873157
ENSMUSG00000031835	Mbtps1	membrane-bound transcription factor peptidase, site 1 [Source:MGI Symbol;Acc:MGI:1927235]	28.3392	25.1264	24.2238	26.5233	26.4747	25.7791	25.9433	27.6661	24.9738	31.751	27.2677	26.1235	25.0253	25.759	0.087978327	0.472021905	16	20	24	0.507486553	0.812725533	24	14.72159819	0.042923896
ENSMUSG00000031837	Necab2	N-terminal EF-hand calcium binding protein 2 [Source:MGI Symbol;Acc:MGI:2152211]	6.29274	5.69442	4.61161	5.49808	4.8878	6.06392	4.98596	5.17265	5.04081	7.41025	4.32989	6.19168	5.53512	5.29495	0.44861185	0.966018836	24	20	24	0.865643951	0.961167412	24	20.62519534	0.038962742
ENSMUSG00000031838	Ifi30	interferon gamma inducible protein 30 [Source:MGI Symbol;Acc:MGI:2137648]	12.4034	14.0407	13.3296	14.2274	15.5104	12.619	13.6496	14.4164	15.8459	11.1832	14.4827	12.9015	12.4085	11.1145	0.591351901	0.966018836	20	8	24	0.646316597	0.880236216	24	12.77670836	0.050754266
ENSMUSG00000031839	Hsbp1	heat shock factor binding protein 1 [Source:MGI Symbol;Acc:MGI:1915446]	125.326	127.699	126.081	122.355	128.545	122.628	122.408	122.765	129.446	113.399	127.546	119.894	125.933	121.084	0.855823117	0.966018836	12	12	24	0.904991045	0.973812495	24	1.739019488	0.00842213
ENSMUSG00000031840	Rab3a	RAB3A, member RAS oncogene family [Source:MGI Symbol;Acc:MGI:97843]	157.321	129.269	130.323	125.598	107.752	146.441	139.691	139.472	112.282	176.879	116.444	142.346	128.076	148.378	0.522551072	0.966018836	24	20	24	0.653187735	0.883305296	24	1.024807667	0.070200394
ENSMUSG00000031841	Cdh13	cadherin 13 [Source:MGI Symbol;Acc:MGI:99551]	3.61585	4.09634	6.52139	3.79488	3.4774	4.58219	5.28793	4.82675	5.50369	6.40215	3.56499	3.69615	4.11043	5.12429	0.01142276	0.206107348	12	8	24	0.05519892	0.65534544	24	7.442992831	0.23017816
ENSMUSG00000031842	Pde4c	phosphodiesterase 4C, cAMP specific [Source:MGI Symbol;Acc:MGI:99556]	11.9941	8.20911	7.98923	10.3173	12.0949	8.64209	8.75045	8.36283	8.50337	8.11934	11.1307	8.12809	9.77303	7.35981	0.118761453	0.542330458	20	12	24	0.076015595	0.686372559	24	17.76732754	0.16725975
ENSMUSG00000031843	Mphosph6	M phase phosphoprotein 6 [Source:MGI Symbol;Acc:MGI:1915783]	8.97429	10.6702	10.3143	8.24858	8.72928	9.59116	9.29787	7.9555	10.7503	8.58859	9.72504	9.6112	10.2609	9.55453	0.676344318	0.966018836	12	4	24	0.655353224	0.883697438	24	3.447100335	0.044321429
ENSMUSG00000031847	1700030J22Rik	RIKEN cDNA 1700030J22 gene [Source:MGI Symbol;Acc:MGI:1916778]	1.52696	1.22779	0.980111	0.941801	0.896328	0.976871	1.01493	1.01202	0.751511	1.35002	0.874938	1.16673	1.10596	1.12865	0.200364953	0.684570416	4	16	24	0.348048606	0.779015211	24	0.126165681	0.077139252
ENSMUSG00000031848	Lsm4	LSM4 homolog, U6 small nuclear RNA and mRNA degradation associated [Source:MGI Symbol;Acc:MGI:1354692]	22.1723	19.6214	17.4166	18.6094	18.0255	18.5532	20.3109	21.4593	22.9969	22.602	23.0683	22.982	25.1397	23.5624	0.910436409	0.966018836	4	12	24	0.329374408	0.768040178	24	1.870226085	0.060681526
ENSMUSG00000031849	Comp	cartilage oligomeric matrix protein [Source:MGI Symbol;Acc:MGI:88469]	5.48119	5.3318	4.92188	4.84143	4.00016	4.85288	4.60779	5.58659	8.47182	4.68021	4.61128	5.13229	5.23287	5.32584	0.003846392	0.140598674	8	12	24	0.10410706	0.715480277	24	5.801784903	0.166178782
ENSMUSG00000031851	Ntpcr	nucleoside-triphosphatase, cancer-related [Source:MGI Symbol;Acc:MGI:1913816]	12.2472	13.3374	13.7223	12.3387	12.2119	11.9837	12.2896	14.3808	11.9626	14.7039	12.7708	14.3334	13.203	13.7863	0.742579234	0.966018836	8	12	24	0.463693181	0.805393068	24	6.849149985	0.045598434
ENSMUSG00000031853	Map3k21	mitogen-activated protein kinase kinase kinase 21 [Source:MGI Symbol;Acc:MGI:2385307]	1.05833	0.881389	0.840761	0.958548	1.00335	1.07613	1.02339	1.0296	0.837835	0.972715	1.05842	1.14225	1.11451	1.07661	7.55E-04	0.071344007	24	12	24	5.77E-04	0.293325979	24	20.78758915	0.073991734
ENSMUSG00000031858	Mau2	MAU2 sister chromatid cohesion factor [Source:MGI Symbol;Acc:MGI:1921799]	20.7919	18.1486	18.4362	21.3119	22.0496	19.8682	20.6085	20.6827	20.4687	19.6791	18.6602	20.6138	19.9295	18.5662	0.582224696	0.966018836	4	4	24	0.369976892	0.792235171	24	17.92361551	0.045433133
ENSMUSG00000031860	Pbx4	pre B cell leukemia homeobox 4 [Source:MGI Symbol;Acc:MGI:1931321]	4.55826	4.40875	4.33716	4.64438	4.66904	4.22906	5.2698	4.03255	4.29084	5.60399	4.22575	5.25124	3.27131	4.37738	0.864782464	0.966018836	16	20	24	0.468724652	0.805393068	24	14.68276015	0.073408165
ENSMUSG00000031862	Atp13a1	ATPase type 13A1 [Source:MGI Symbol;Acc:MGI:2180801]	11.3301	10.3487	11.3232	11.2306	11.218	11.5732	11.655	10.1003	11.9811	11.7194	10.6949	10.1994	10.9222	11.9957	0.939103211	0.966018836	12	12	24	0.682018901	0.894118172	24	9.855452026	0.027302245
ENSMUSG00000031864	Ints10	integrator complex subunit 10 [Source:MGI Symbol;Acc:MGI:1918135]	15.9279	15.469	13.2607	13.887	15.8388	14.4464	15.8855	14.3002	13.5247	13.4688	16.1765	15.0765	16.434	15.6026	0.002004172	0.100255953	4	8	24	0.016603234	0.544474117	24	21.88958543	0.100658264
ENSMUSG00000031865	Dctn1	dynactin 1 [Source:MGI Symbol;Acc:MGI:107745]	93.2481	74.6494	81.3351	81.652	75.7661	83.4487	86.8718	92.4978	70.3495	110.386	76.8854	82.8751	79.1324	87.6171	0.885025617	0.966018836	4	8	24	0.98840394	0.997559852	24	3.060620681	0.009939268
ENSMUSG00000031870	Pgr	progesterone receptor [Source:MGI Symbol;Acc:MGI:97567]	1.1385	1.23738	1.06205	1.13521	0.888676	1.20173	1.30305	1.04398	1.16649	1.18879	1.13327	1.16195	1.03154	1.35816	0.918497267	0.966018836	8	12	24	0.419770459	0.803639585	24	3.344667815	0.040366597
ENSMUSG00000031871	Cdh5	cadherin 5 [Source:MGI Symbol;Acc:MGI:105057]	4.92428	5.15717	5.15809	5.23404	5.89949	5.57879	6.16103	6.64902	5.74215	6.0604	5.08867	5.22297	5.34898	5.00245	0.814075857	0.966018836	16	12	24	0.942688304	0.985829422	24	9.031070562	0.016100204
ENSMUSG00000031872	Bean1	brain expressed, associated with Nedd4, 1 [Source:MGI Symbol;Acc:MGI:1929597]	27.9831	19.3576	18.8636	22.6063	20.3267	25.4042	22.7995	24.0753	16.093	30.8146	21.9183	24.1749	22.5784	22.7372	0.08302755	0.462922111	24	12	24	0.367216827	0.790684479	24	19.8274521	0.12969514
ENSMUSG00000031875	Cmtm3	CKLF-like MARVEL transmembrane domain containing 3 [Source:MGI Symbol;Acc:MGI:2447162]	8.17445	9.74333	9.80099	7.74735	9.88105	8.25904	9.49565	9.95478	11.1961	8.36953	9.97741	10.0524	9.82966	9.70537	0.921572192	0.966018836	12	4	24	0.491962644	0.805393068	24	5.413881498	0.059017905
ENSMUSG00000031877	Ces2g	carboxylesterase 2G [Source:MGI Symbol;Acc:MGI:1919611]	0.885908	1.01248	1.17694	1.15997	1.27469	1.12808	1.04673	1.00482	1.16205	0.674566	0.978414	0.998748	0.938924	1.0432	0.643325306	0.966018836	12	16	24	0.941920067	0.985597432	24	13.234201	0.014541102
ENSMUSG00000031878	Nae1	NEDD8 activating enzyme E1 subunit 1 [Source:MGI Symbol;Acc:MGI:2384561]	56.0142	62.5834	56.7376	56.0508	59.3332	52.5672	55.888	55.1549	64.4938	49.5153	64.4507	56.8652	60.204	59.8593	0.919571723	0.966018836	12	12	24	0.816849543	0.9417567	24	5.56853003	0.027599013
ENSMUSG00000031879	Ciao2b	cytosolic iron-sulfur assembly component 2B [Source:MGI Symbol;Acc:MGI:1915773]	23.7878	21.8521	18.3014	20.0522	20.4065	21.8028	21.9043	22.1178	23.7645	21.5077	23.8758	22.1641	25.1267	23.1255	0.192357053	0.669841558	4	12	24	0.148734645	0.757245234	24	23.71463402	0.071114822
ENSMUSG00000031880	Rrad	Ras-related associated with diabetes [Source:MGI Symbol;Acc:MGI:1930943]	0.72893	0.4883	0.610089	0.258214	0.695198	0.463366	0.890481	0.729809	0.345208	0.480292	0.540292	0.570351	0.83853	0.603586	0.004545736	0.140598674	4	12	24	0.032978783	0.606585237	24	23.61896888	0.151125101
ENSMUSG00000031883	Car7	carbonic anhydrase 7 [Source:MGI Symbol;Acc:MGI:103100]	1.73084	1.66054	1.90603	2.08064	1.64357	1.90438	1.81035	2.66112	1.62817	2.26082	1.74024	2.13405	1.99034	2.18784	0.874672901	0.966018836	8	12	24	0.66389699	0.886635925	24	6.202584651	0.047210759
ENSMUSG00000031885	Cbfb	core binding factor beta [Source:MGI Symbol;Acc:MGI:99851]	97.3581	92.2862	85.3417	94.1046	90.177	85.3487	84.0183	80.742	73.5431	86.2468	94.3717	71.5218	77.0654	78.6659	0.658942124	0.966018836	20	4	24	0.360205879	0.785733735	24	15.82510518	0.055996279
ENSMUSG00000031887	Tradd	TNFRSF1A-associated via death domain [Source:MGI Symbol;Acc:MGI:109200]	10.6465	7.73097	8.84096	7.45057	8.77247	9.0739	7.45195	9.52132	7.92177	8.92944	8.9926	8.88112	9.52308	8.48984	0.669104912	0.966018836	4	8	24	0.439299058	0.805393068	24	21.5774485	0.068123585
ENSMUSG00000031889	Phaf1	phagosome assembly factor 1 [Source:MGI Symbol;Acc:MGI:2443049]	11.2725	10.8974	11.462	11.7441	9.85755	11.3416	12.0882	10.8549	11.0421	11.4323	10.9018	11.9034	11.6347	11.1486	0.628286596	0.966018836	4	4	24	0.690757409	0.897666745	24	0.791123419	0.022527125
ENSMUSG00000031896	Ctrl	chymotrypsin-like [Source:MGI Symbol;Acc:MGI:88558]	0.247391	0.320438	0.450108	0.91385	0.690101	0.357131	1.23148	0.509235	0.774406	0.403723	0.950697	0.538088	0.26466	0.268662	0.285521181	0.793714531	20	8	24	0.351726543	0.780345856	24	14.34405428	0.147943609
ENSMUSG00000031897	Psmb10	proteasome (prosome, macropain) subunit, beta type 10 [Source:MGI Symbol;Acc:MGI:1096380]	35.5628	39.849	37.9625	34.3903	43.1601	38.6575	38.5216	41.4018	42.1109	34.3261	37.407	37.4274	39.2429	37.1584	0.718086778	0.966018836	12	4	24	0.776966162	0.928500064	24	2.287198021	0.027648241
ENSMUSG00000031901	Dus2	dihydrouridine synthase 2 [Source:MGI Symbol;Acc:MGI:1913619]	5.18962	6.38399	7.05024	5.01595	7.12008	6.58768	5.95263	6.10241	6.11257	5.51348	6.32375	6.12449	6.23409	6.01158	0.849179819	0.966018836	20	20	24	0.961441312	0.99009168	24	18.83970111	0.014658618
ENSMUSG00000031902	Nfatc3	nuclear factor of activated T cells, cytoplasmic, calcineurin dependent 3 [Source:MGI Symbol;Acc:MGI:103296]	8.79941	7.80032	7.36197	8.32298	8.18528	8.3068	8.25194	7.82041	7.70538	8.24582	8.167	8.25891	8.10964	8.21476	0.022634137	0.27189101	24	12	24	0.049349837	0.641729576	24	19.76250166	0.050460292
ENSMUSG00000031903	Pla2g15	phospholipase A2, group XV [Source:MGI Symbol;Acc:MGI:2178076]	7.4782	5.66346	6.81528	5.94261	7.02157	6.86991	6.79397	7.63879	5.78028	6.40372	6.82174	6.66957	6.6891	6.57138	0.446069941	0.966018836	20	20	24	0.232829043	0.768040178	24	21.49779473	0.073565593
ENSMUSG00000031904	Slc7a6	solute carrier family 7 (cationic amino acid transporter, y+ system), member 6 [Source:MGI Symbol;Acc:MGI:2142598]	11.4401	10.1738	10.9154	11.2954	10.0579	11.4195	10.5398	9.9941	8.36282	10.9236	9.9795	9.63901	10.3717	10.2719	0.801995186	0.966018836	4	8	24	0.551864526	0.836477033	24	19.77279404	0.04415342
ENSMUSG00000031906	Smpd3	sphingomyelin phosphodiesterase 3, neutral [Source:MGI Symbol;Acc:MGI:1927578]	6.07873	4.55205	5.34331	5.58512	4.99871	6.11395	5.23126	6.17188	4.29909	7.04187	5.20581	5.8062	5.47745	6.23345	0.892685629	0.966018836	24	12	24	0.879424515	0.966193785	24	19.49912291	0.033454789
ENSMUSG00000031907	Zfp90	zinc finger protein 90 [Source:MGI Symbol;Acc:MGI:104786]	7.55931	8.41136	8.49674	7.56986	8.0653	7.62974	7.02164	7.95375	8.13589	7.63314	7.94243	8.56273	7.35513	8.46828	0.112800839	0.530777648	12	16	24	0.462992434	0.805393068	24	8.636468827	0.039195872
ENSMUSG00000031910	Has3	hyaluronan synthase 3 [Source:MGI Symbol;Acc:MGI:109599]	0.918322	0.828696	0.917177	0.953843	1.0177	0.987252	1.10341	0.743344	0.707686	0.816537	0.967343	0.754449	0.908756	0.814051	0.118761453	0.542330458	20	12	24	0.10941928	0.72082227	24	18.83934055	0.067080745
ENSMUSG00000031913	Vps4a	vacuolar protein sorting 4A [Source:MGI Symbol;Acc:MGI:1890520]	46.1925	39.6074	41.2614	42.2797	39.6154	45.6594	41.8898	41.5835	36.5099	46.6047	41.117	43.2855	44.1088	43.752	0.300218476	0.809362476	20	12	24	0.345439427	0.778420274	24	20.79339266	0.055510942
ENSMUSG00000031916	Cog8	component of oligomeric golgi complex 8 [Source:MGI Symbol;Acc:MGI:2142885]	10.7614	9.41718	10.4865	9.53577	9.22182	10.0955	9.77596	11.2467	8.75419	12.3194	9.82281	11.0105	10.7914	10.2452	0.916556573	0.966018836	24	20	24	0.919893758	0.978678675	24	23.85616753	0.018993939
ENSMUSG00000031917	Nip7	NIP7, nucleolar pre-rRNA processing protein [Source:MGI Symbol;Acc:MGI:1913414]	14.6411	15.3012	11.509	12.3629	14.3506	10.526	11.5535	11.4818	12.4619	8.80226	11.3421	10.8972	10.8636	11.6798	0.694910419	0.966018836	8	16	24	0.542819844	0.831118582	24	1.519404509	0.065487854
ENSMUSG00000031918	Mtmr2	myotubularin related protein 2 [Source:MGI Symbol;Acc:MGI:1924366]	27.4974	29.3608	31.4672	34.9526	29.5137	30.397	28.8533	29.2875	31.7399	31.0545	30.927	31.6043	29.1461	29.6886	8.93E-04	0.071344007	16	12	24	0.009718741	0.503293975	24	12.1141503	0.082083656
ENSMUSG00000031921	Terf2	telomeric repeat binding factor 2 [Source:MGI Symbol;Acc:MGI:1195972]	8.15684	8.49504	8.47138	9.68593	8.16395	9.25934	8.74411	9.40454	9.30902	9.47902	8.97205	9.43484	8.55675	8.3032	0.305564675	0.811201458	16	12	24	0.326952336	0.768040178	24	12.73504445	0.043803508

ENSMUSG00000031922	Cep57	centrosomal protein 57 [Source:MGI Symbol;Acc:MGI:1915551]	8.9548	11.1884	11.4433	10.1016	10.5553	9.70317	9.93902	8.96459	11.7132	9.02812	11.1398	9.19606	10.2715	9.51059	0.321662653	0.83607295	12	12	24	0.398239334	0.799546792	24	10.09649651	0.066577834
ENSMUSG00000031924	Cyb5b	cytochrome b5 type B [Source:MGI Symbol;Acc:MGI:1913677]	66.8912	59.4916	60.6753	59.7719	61.5576	65.1942	61.0625	63.3766	52.1724	62.7504	60.7705	61.4642	62.423	62.7082	0.300823756	0.809362476	4	8	24	0.105851698	0.717832234	24	21.33678316	0.064957031
ENSMUSG00000031925	Maml2	mastermind like transcriptional coactivator 2 [Source:MGI Symbol;Acc:MGI:2389460]	2.34854	2.95847	2.52088	2.38318	2.7223	2.664	2.20996	2.48543	2.76002	2.9399	2.28801	2.43419	2.48688	2.208	0.369262716	0.893596186	8	20	24	0.304948729	0.768040178	24	8.56682004	0.059680494
ENSMUSG00000031928	Mre11a	MRE11A homolog A, double strand break repair nuclease [Source:MGI Symbol;Acc:MGI:1100512]	6.04296	5.2935	5.01848	4.92829	5.99936	5.47029	6.28648	5.04155	6.01025	6.38073	5.60888	5.43414	6.64242	5.47441	0.432054949	0.966018836	4	12	24	0.56552744	0.843471947	24	20.94942527	0.04909616
ENSMUSG00000031930	Wwp2	WW domain containing E3 ubiquitin protein ligase 2 [Source:MGI Symbol;Acc:MGI:1914144]	16.2704	15.1057	15.8597	14.9603	15.972	16.6773	15.281	17.0856	14.9588	17.6158	13.3806	15.9988	15.9961	14.919	0.969487593	0.970883022	24	12	24	0.891296042	0.969291345	24	0.153026152	0.01798126
ENSMUSG00000031931	Ankrd49	ankyrin repeat domain 49 [Source:MGI Symbol;Acc:MGI:1930842]	8.13663	8.98422	9.0315	7.34976	9.65139	8.24367	8.19957	8.08804	8.75372	7.61624	9.26226	8.51092	8.19356	8.51918	0.745118544	0.966018836	20	8	24	0.938893079	0.984935568	24	17.48624208	0.014130073
ENSMUSG00000031932	Gpr83	G protein-coupled receptor 83 [Source:MGI Symbol;Acc:MGI:95712]	0.76287	0.888737	0.961148	1.04279	0.882917	0.94417	0.886313	1.02967	0.709948	1.03905	0.706366	0.912015	0.784905	0.75162	0.942429894	0.966018836	16	12	24	0.662163711	0.885891036	24	12.25690689	0.031749255
ENSMUSG00000031934	Panx1	pannexin 1 [Source:MGI Symbol;Acc:MGI:1860055]	40.4783	32.4146	38.2761	35.0536	36.1322	43.1654	33.5887	36.1444	29.2484	36.9759	42.3418	35.8438	35.5326	34.9135	0.388056094	0.915479136	20	12	24	0.097673103	0.71175955	24	18.46398377	0.118194385
ENSMUSG00000031935	Med17	mediator complex subunit 17 [Source:MGI Symbol;Acc:MGI:2182585]	14.963	15.9401	16.1774	15.746	15.7767	14.5568	15.1974	15.1559	14.6762	12.8267	15.1704	16.5747	16.4598	13.3617	0.881996584	0.966018836	24	16	24	0.541751026	0.830485817	24	21.21058651	0.043956211
ENSMUSG00000031937	Vstm5	V-set and transmembrane domain containing 5 [Source:MGI Symbol;Acc:MGI:1916387]	35.8421	26.4684	27.4966	25.6506	33.0454	33.0397	26.324	28.2654	22.81	29.9261	32.3829	30.3911	30.0103	27.6045	0.033622862	0.324908923	20	16	24	0.013821385	0.529470441	24	19.38632302	0.175311029
ENSMUSG00000031938	4931406C07Rik	RIKEN cDNA 4931406C07 gene [Source:MGI Symbol;Acc:MGI:1918234]	14.7141	18.8369	20.3231	24.3707	20.8352	17.2128	18.0731	17.4711	20.197	21.0344	21.8491	20.5998	21.1185	19.9919	0.024691579	0.28960575	16	12	24	0.007920654	0.490448898	24	12.22052285	0.162183204
ENSMUSG00000031939	Taf1d	TATA-box binding protein associated factor, RNA polymerase I, D [Source:MGI Symbol;Acc:MGI:1922566]	10.5809	11.5108	13.0209	14.4908	10.5131	11.6366	9.76151	11.9065	15.1968	10.9832	16.3581	11.6111	13.8114	14.1546	0.559102416	0.966018836	12	16	24	0.184633217	0.768040178	24	9.958368863	0.136180748
ENSMUSG00000031948	Kars	lysyl-tRNA synthetase [Source:MGI Symbol;Acc:MGI:1934754]	41.9459	36.4165	38.2971	36.648	37.7804	41.2927	37.8264	39.8323	33.4366	40.5046	37.4964	38.01	38.3982	38.8587	0.512510541	0.966018836	4	8	24	0.266037983	0.768040178	24	21.35723245	0.052033431
ENSMUSG00000031949	Adat1	adenosine deaminase, tRNA-specific 1 [Source:MGI Symbol;Acc:MGI:1353631]	3.6298	3.24714	3.3941	3.44608	3.65683	3.61336	3.6149	3.06784	3.05225	3.12345	3.5518	3.32125	3.5141	3.10092	0.032593161	0.324908923	4	4	24	0.001850892	0.384740371	24	19.31826036	0.082123286
ENSMUSG00000031950	Gabarapl2	gamma-aminobutyric acid (GABA) A receptor-associated protein-like 2 [Source:MGI Symbol;Acc:MGI:1890602]	43.4973	44.9356	41.8588	41.7606	43.3003	42.8325	40.8156	44.7218	42.1271	39.8885	45.6365	42.3572	43.7706	43.0615	0.326490454	0.845515883	8	8	24	0.575521317	0.848060327	24	23.55718834	0.022082962
ENSMUSG00000031951	Tmem231	transmembrane protein 231 [Source:MGI Symbol;Acc:MGI:2685024]	5.63707	4.47365	4.48239	4.59534	4.30464	5.07163	4.93258	4.76675	3.71688	5.23625	4.17969	5.01415	4.88222	4.53904	0.080164531	0.458678242	24	12	24	0.121571035	0.733843269	24	21.90255599	0.09630579
ENSMUSG00000031952	Chst5	carbohydrate (N-acetylglucosamine 6-O) sulfotransferase 5 [Source:MGI Symbol;Acc:MGI:1931825]	0.666916	0.738804	0.405595	0.565406	0.560972	0.806189	0.647319	0.934124	0.819034	0.906812	0.717782	0.871872	0.677345	0.828058	0.92655264	0.966018836	8	12	24	0.770130698	0.92597113	24	1.500494243	0.031241534
ENSMUSG00000031953	Tmem170	transmembrane protein 170 [Source:MGI Symbol;Acc:MGI:106426]	3.48221	2.89032	2.4459	3.27124	3.41989	2.81933	3.84176	2.60543	2.94695	3.07302	3.53361	2.76213	3.03799	2.74554	0.127415507	0.560209134	20	12	24	0.219242459	0.768040178	24	18.49332963	0.095541418
ENSMUSG00000031954	Cfdp1	craniofacial development protein 1 [Source:MGI Symbol;Acc:MGI:1344403]	64.6064	66.9326	63.8515	61.9206	64.4582	60.9636	64.5964	58.5821	62.787	51.23	62.8231	63.1254	62.7155	63.8118	0.702630284	0.966018836	4	12	24	0.265989109	0.768040178	24	23.93095605	0.054277105
ENSMUSG00000031955	Bcar1	breast cancer anti-estrogen resistance 1 [Source:MGI Symbol;Acc:MGI:108091]	7.34159	7.00261	8.55575	7.55981	7.26187	7.15252	7.82014	7.99005	8.47991	10.2378	7.85622	8.1482	7.65099	8.19541	0.216688884	0.704989686	12	12	24	0.052803951	0.650526959	24	9.921917424	0.100998102
ENSMUSG00000031958	Ldhd	lactate dehydrogenase D [Source:MGI Symbol;Acc:MGI:106428]	7.14951	6.25541	7.218	9.17828	7.63051	7.85327	7.86137	8.73353	6.94017	8.88322	8.08678	7.21764	8.63512	7.15761	0.371875382	0.894537464	16	20	24	0.404730581	0.799546792	24	14.54491873	0.070362942
ENSMUSG00000031959	Wdr59	WD repeat domain 59 [Source:MGI Symbol;Acc:MGI:2442115]	7.567	6.70859	7.46856	7.12766	7.39587	8.27733	7.57678	7.60256	6.31935	8.56049	6.83256	7.37527	7.30553	7.80708	0.887862353	0.966018836	24	12	24	0.711622592	0.904723135	24	19.76091583	0.03453471
ENSMUSG00000031960	Aars	alanyl-tRNA synthetase [Source:MGI Symbol;Acc:MGI:2384560]	25.4084	21.023	24.297	21.2665	23.3022	26.5915	23.6317	24.7994	17.9372	26.6195	22.1706	23.9251	21.9675	23.405	0.92655264	0.966018836	24	12	24	0.386001112	0.799546792	24	20.17131401	0.0811366
ENSMUSG00000031962	Cdh15	cadherin 15 [Source:MGI Symbol;Acc:MGI:106672]	1.6787	1.25275	1.36694	1.50889	1.35191	1.69928	1.34754	1.77465	1.14092	1.57399	1.4701	1.10557	1.50516	1.33561	0.860993221	0.966018836	16	20	24	0.854021359	0.956445086	24	20.91095935	0.027930607
ENSMUSG00000031963	Bmper	BMP-binding endothelial regulator [Source:MGI Symbol;Acc:MGI:1920480]	4.93798	3.52385	3.98838	3.58889	3.60048	3.77576	3.69391	3.64876	3.33986	4.09168	3.33188	4.06631	3.71152	3.79116	0.888991156	0.966018836	24	20	24	0.437108214	0.805393068	24	22.74967738	0.056998373
ENSMUSG00000031967	Afg3l1	AFG3-like AAA ATPase 1 [Source:MGI Symbol;Acc:MGI:1928277]	8.276	7.41441	7.78882	9.779	8.56714	8.17343	8.35465	9.19651	8.35316	8.40822	8.06647	8.12555	8.78328	9.61089	0.927804444	0.966018836	16	8	24	0.768983505	0.925568601	24	8.48592131	0.028247231
ENSMUSG00000031969	Acad8	acyl-Coenzyme A dehydrogenase family, member 8 [Source:MGI Symbol;Acc:MGI:1914198]	15.0182	17.3301	15.1367	20.2357	18.7618	17.821	18.0432	15.9157	17.804	17.1366	16.0621	17.2206	18.5567	17.3071	0.90332508	0.966018836	16	20	24	0.604041581	0.861768549	24	15.22509746	0.044388944
ENSMUSG00000031970	Dbndd1	dysbindin (dystrobrevin binding protein 1) domain containing 1 [Source:MGI Symbol;Acc:MGI:1919435]	12.3433	11.3885	11.2807	10.0204	9.72468	12.278	10.5795	11.3147	8.76277	11.0881	10.1969	12.6514	11.898	12.1284	0.011964854	0.213452992	24	20	24	0.059826436	0.658304792	24	23.34805842	0.120183494
ENSMUSG00000031971	Ccsap	centriole, cilia and spindle associated protein [Source:MGI Symbol;Acc:MGI:1920670]	2.4198	2.13609	2.28446	2.35943	1.9326	1.85826	2.02119	2.14467	2.0956	2.27789	2.35838	2.16788	1.90165	2.10891	0.800065124	0.966018836	16	8	24	0.365063994	0.789403671	24	10.39006704	0.044848481
ENSMUSG00000031972	Acta1	actin, alpha 1, skeletal muscle [Source:MGI Symbol;Acc:MGI:87902]	97.7251	237.174	302.03	382.851	262.9	354.235	245.89	848.424	536.249	820.996	249.206	312.964	372.096	266.274	0.496745696	0.966018836	16	12	24	0.107116571	0.71982646	24	9.793190563	0.580905404
ENSMUSG00000031974	Abcb10	ATP-binding cassette, sub-family B (MDR/TAP), member 10 [Source:MGI Symbol;Acc:MGI:1860508]	6.69812	6.66968	7.39795	8.30174	7.22584	7.63324	7.13077	7.28181	6.79951	8.02689	7.1493	7.61824	7.5759	7.85305	0.47028201	0.966018836	16	12	24	0.282793716	0.768040178	24	13.28684178	0.046632092
ENSMUSG00000031976	Urb2	URB2 ribosome biogenesis 2 homolog (S. cerevisiae) [Source:MGI Symbol;Acc:MGI:2681124]	3.03014	2.46886	2.6498	3.0758	2.56757	2.96152	2.92989	3.03148	2.15221	3.11375	2.51284	2.79987	2.68166	2.56539	0.888991156	0.966018836	16	20	24	0.561403068	0.841542955	24	19.83561	0.048973653
ENSMUSG00000031979	Cog2	component of oligomeric golgi complex 2 [Source:MGI Symbol;Acc:MGI:1923582]	10.2867	11.3433	11.0536	11.4053	10.8827	11.3057	10.3599	12.4396	11.5124	10.3237	10.7573	11.8615	11.5776	11.4966	0.834424969	0.966018836	12	24	24	0.502698027	0.809737567	24	4.870966689	0.032775362
ENSMUSG00000031980	Agt	angiotensinogen (serpin peptidase inhibitor, clade A, member 8) [Source:MGI Symbol;Acc:MGI:87963]	4.7689	7.60733	13.872	5.52171	4.69052	8.82869	10.1143	6.23968	9.13159	5.82991	3.73414	6.88941	3.51828	7.42741	0.179603275	0.655480352	12	8	24	0.3293918	0.768040178	24	6.068087522	0.278555468
ENSMUSG00000031981	Capn9	calpain 9 [Source:MGI Symbol;Acc:MGI:1920897]	2.64953	2.25414	1.88933	2.25484	3.18717	2.43796	1.80919	2.85907	1.70501	2.5068	2.37151	2.26738	2.59553	2.15093	0.588208558	0.966018836	20	16	24	0.33025641	0.768040178	24	18.69891869	0.104233758
ENSMUSG00000031982	Arv1	ARV1 homolog, fatty acid homeostasis modulator [Source:MGI Symbol;Acc:MGI:1916115]	5.66708	4.56151	4.94207	5.59503	5.36068	5.3458	5.25177	5.29581	5.89933	5.09347	5.06824	4.91501	5.32089	5.7102	0.830949782	0.966018836	12	12	24	0.922979039	0.979439431	24	7.41368402	0.013950259
ENSMUSG00000031983	2310022B05Rik	RIKEN cDNA 2310022B05 gene [Source:MGI Symbol;Acc:MGI:1916801]	12.9886	13.4064	14.0285	12.234	12.662	14.302	14.7127	14.2612	13.9987	16.3817	11.3165	14.9299	13.3713	14.1682	0.928064146	0.966018836	24	20	24	0.598205895	0.858819856	24	4.446020169	0.048777317
ENSMUSG00000031984	2810004N23Rik	RIKEN cDNA 2810004N23 gene [Source:MGI Symbol;Acc:MGI:1913773]	10.5854	9.83814	10.6569	9.77204	10.5135	10.1708	10.2673	11.498	9.82894	10.5192	10.1859	10.7827	10.5336	10.8332	0.66832131	0.966018836	8	8	24	0.507822368	0.81302223	24	1.564371476	0.025338545
ENSMUSG00000031985	Gnpat	glyceronephosphate O-acyltransferase [Source:MGI Symbol;Acc:MGI:1343460]	28.9894	29.8486	29.4063	34.7093	36.6829	30.5435	33.6656	36.8079	31.5366	34.1685	32.0832	30.5235	33.2671	31.0129	0.697196105	0.966018836	20	4	24	0.544677601	0.832363403	24	13.2327877	0.046459406
ENSMUSG00000031986	Sprtn	SprT-like N-terminal domain [Source:MGI Symbol;Acc:MGI:2685351]	4.88288	5.41089	5.18504	5.26555	5.48685	4.80864	5.0073	5.12883	5.45617	4.96977	5.24377	5.51552	5.5893	5.58006	0.828910443	0.966018836	8	8	24	0.663254846	0.88640646	24	7.419890937	0.02061331
ENSMUSG00000031987	Egln1	egl-9 family hypoxia-inducible factor 1 [Source:MGI Symbol;Acc:MGI:1932286]	19.7306	18.9605	19.0547	21.2909	21.0691	19.772	21.3216	21.9465	19.9753	24.9774	18.8113	21.123	21.7606	19.8029	0.859936714	0.966018836	16	4	24	0.761693879	0.922832987	24	13.08740929	0.029285712
ENSMUSG00000031988	Vps26b	VPS26 retromer complex component B [Source:MGI Symbol;Acc:MGI:1917656]	22.8737	19.3975	18.7754	20.539	20.3015	22.3264	21.5985	20.115	18.1265	24.7923	18.646	22.2896	21.8446	20.979	0.020119233	0.259417302	24	12	24	0.302631698	0.768040178	24	20.45271672	0.075271993
ENSMUSG00000031990	Jam3	junction adhesion molecule 3 [Source:MGI Symbol;Acc:MGI:1933825]	63.0131	63.5447	58.3437	63.7873	71.0394	61.2939	69.7178	59.9223	66.7461	58.5533	63.3271	61.746	62.3303	57.0222	0.937921962	0.966018836	20	12	24	0.398100752	0.799546792	24	18.52192815	0.048031612
ENSMUSG00000031993	Snx19	sorting nexin 19 [Source:MGI Symbol;Acc:MGI:1921581]	17.3784	14.7298	14.9095	16.3591	14.9239	18.3046	15.7941	18.2196	12.7703	20.412	14.8845	17.031	16.315	16.0959	0.390686537	0.915479136	24	12	24	0.65648664	0.883952697	24	19.92711413	0.060492132
ENSMUSG00000031994	Adamts8	a disintegrin-like and metallopeptidase (reprolysin type) with thrombospondin type 1 motif, 8 [Source:MGI Symbol;Acc:MGI:1353468]	1.53087	0.982326	1.16763	1.01252	1.59406	1.64089	1.30966	1.07597	0.93647	1.34004	1.29873	1.31181	1.27911	1.17834	0.004516345	0.140598674	24	12	24	0.004088507	0.427431763	24	19.26534473	0.15744546
ENSMUSG00000031996	Aplp2	amyloid beta (A4) precursor-like protein 2 [Source:MGI Symbol;Acc:MGI:88047]	243.569	191.339	205.778	217.799	192.427	229.773	221.518	223.874	171.155	251.534	204.074	216.524	212.289	212.851	0.658942124	0.966018836	24	12	24	0.535348375	0.82691856	24	20.67700797	0.066117584
ENSMUSG00000031997	Trpc6	transient receptor potential cation channel, subfamily C, member 6 [Source:MGI Symbol;Acc:MGI:109523]	7.01487	7.23571	7.19709	7.2597	6.49712	7.5713	6.51623	6.40987	7.55239	6.54283	7.31313	7.82947	8.516	8.071	0.906657897	0.966018836	24	20	24	0.677542549	0.892090954	24	0.809090838	0.033848159
ENSMUSG00000032000	Birc3	baculoviral IAP repeat-containing 3 [Source:MGI Symbol;Acc:MGI:1197007]	2.69387	3.63025	3.98899	3.60636	3.94789	3.52476	2.79047	3.62939	3.93041	3.46003	3.75878	2.81465	4.61483	3.62017	0.321662653	0.83607295	12	12	24	0.273190096	0.768040178	24	9.590295531	0.105596544
ENSMUSG00000032002	Dcun1d5	DCN1, defective in cullin neddylation 1, domain containing 5 (S. cerevisiae) [Source:MGI Symbol;Acc:MGI:1924113]	43.0525	41.5624	41.1244	43.5855	45.395	38.8504	46.1619	38.8907	46.7619	35.4762	46.22	38.6557	42.6899	37.9603	0.93447422	0.966018836	20	12	24	0.87711823	0.965814767	24	17.74373457	0.024914116
ENSMUSG00000032006	Pdgfd	platelet-derived growth factor, D polypeptide [Source:MGI Symbol;Acc:MGI:1919035]	2.19991	3.37957	4.35798	3.24488	2.38077	2.56961	3.82708	2.84461	3.45043	2.3309	2.40075	2.19432	2.06709	3.09551	0.118761453	0.542330458	12	12	24	0.043638372	0.626787045	24	7.037186164	0.217650616
ENSMUSG00000032009	Sesn3	sestrin 3 [Source:MGI Symbol;Acc:MGI:1922997]	11.2816	11.2739	11.0832	11.1658	11.347	10.7305	13.0541	10.0744	10.7665	10.7591	10.7146	11.7663	11.1213	11.7537	0.724067924	0.966018836	4	12	24	0.38688711	0.799546792	24	22.69310349	0.043750232
ENSMUSG00000032010	Usp2	ubiquitin specific peptidase 2 [Source:MGI Symbol;Acc:MGI:1858178]	2.86416	2.58787	4.73279	6.58997	2.84048	3.08558	3.43927	3.28842	5.07582	7.26708	3.11373	3.01782	2.33676	2.83098	0.050732383	0.386400877	16	12	24	0.003649709	0.426426863	24	10.99620814	0.44383788
ENSMUSG00000032011	Thy1	thymus cell antigen 1, theta [Source:MGI Symbol;Acc:MGI:98747]	303.368	261.055	269.256	290.434	253.362	323.976	281.409	288.629	226.694	321.892	284.891	275.895	254.338	278.567	0.913451828	0.966018836	16	20	24	0.49306828	0.805727364	24	18.62802188	0.066857217
ENSMUSG00000032012	Nectin1	nectin cell adhesion molecule 1 [Source:MGI Symbol;Acc:MGI:1926483]	3.80257	3.29968	3.26582	3.12248	3.22889	3.42915	3.62118	3.58881	2.79952	4.72342	3.32535	3.931	3.38702	3.84559	0.289496388	0.794869797	24	12	24	0.841163653	0.951862945	24	21.20199547	0.033496565
ENSMUSG00000032014	Oaf	out at first homolog [Source:MGI Symbol;Acc:MGI:94852]	10.2958	10.116	7.72934	10.2536	9.85755	7.55426	7.89775	9.69216	10.9958	9.65891	7.67042	8.06336	6.97458	7.05369	0.725736046	0.966018836	12	12	24	0.291844982	0.768040178	24	10.16341376	0.107461987
ENSMUSG00000032017	Grik4	glutamate receptor, ionotropic, kainate 4 [Source:MGI Symbol;Acc:MGI:95817]	11.4295	7.95989	7.91089	7.90114	8.07124	9.70703	8.12963	9.54328	6.78062	10.8564	8.338	10.0024	10.3933	9.58183	0.035787737	0.331814682	4	8	24	0.21820568	0.768040178	24	21.98548861	0.130116737
ENSMUSG00000032018	Sc5d	sterol-C5-desaturase [Source:MGI Symbol;Acc:MGI:1353611]	88.8025	82.7833	83.4329	84.9089	89.8147	86.6967	85.4967	76.9098	89.385	82.0953	92.3237	83.5295	92.1694	83.8142	0.109247218	0.524035616	20	12	24	0.260613804	0.768040178	24	18.11229031	0.046383538
ENSMUSG00000032020	Ubash3b	ubiquitin associated and SH3 domain containing, B [Source:MGI Symbol;Acc:MGI:1920078]	45.5978	35.4026	35.821	37.2196	37.1409	42.5933	35.9036	37.5434	29.1576	45.6808	38.5765	40.7831	40.2034	40.2087	0.085890569	0.466404237	24	12	24	0.225759912	0.768040178	24	19.85766463	0.110992711
ENSMUSG00000032023	Jhy	junctional cadherin complex regulator [Source:MGI Symbol;Acc:MGI:1918239]	6.78386	6.3633	5.72667	6.15116	5.29568	6.705	5.18625	5.84325	5.19666	6.95779	5.87855	6.38333	6.69905	6.17163	0.601647512	0.966018836	24	12	24	0.71017885	0.90416311	24	21.31366962	0.042397309
ENSMUSG00000032024	Clmp	CXADR-like membrane protein [Source:MGI Symbol;Acc:MGI:1918816]	12.3588	10.4137	9.60848	8.69541	9.47684	10.3465	10.0316	10.0634	8.08357	11.019	9.86124	10.263	10.6632	11.7198	0.076187236	0.453790923	8	4	24	0.116949846	0.727929292	24	23.5884306	0.110084099
ENSMUSG00000032026	Rexo2	RNA exonuclease 2 [Source:MGI Symbol;Acc:MGI:1888981]	102.604	92.6465	90.6627	88.6424	91.9276	97.9528	91.3512	88.7003	87.5173	92.016	101.155	96.0351	100.721	97.2113	0.061697418	0.409814092	4	8	24	0.036684567	0.609176161	24	21.09576174	0.069512591
ENSMUSG00000032028	Nxpe2	neurexophilin and PC-esterase domain family, member 2 [Source:MGI Symbol;Acc:MGI:1925502]	6.40006	6.85946	6.14939	7.26337	6.70871	6.09349	7.39771	6.4236	7.05438	5.25276	6.06441	6.84469	8.94533	6.22639	0.658895034	0.966018836	4	16	24	0.345926146	0.778673045	24	0.306839409	0.087149336
ENSMUSG00000032030	Cul5	cullin 5 [Source:MGI Symbol;Acc:MGI:1922967]	13.6179	14.2537	15.2446	14.3951	13.3004	13.5787	13.9244	14.5254	14.6356	13.4257	15.6723	14.7277	14.3316	14.9829	0.542718076	0.966018836	12	12	24	0.418643049	0.803070657	24	7.622693311	0.033487633
ENSMUSG00000032033	Barx2	BarH-like homeobox 2 [Source:MGI Symbol;Acc:MGI:109617]	3.00671	2.60243	2.1256	1.79588	2.45999	2.62972	2.22613	2.66146	2.28305	2.4931	2.57515	2.63475	3.14879	3.20419	0.025973358	0.29944494	8	4	24	0.033774215	0.606793499	24	0.057279645	0.132499998
ENSMUSG00000032035	Ets1	E26 avian leukemia oncogene 1, 5' domain [Source:MGI Symbol;Acc:MGI:95455]	4.29947	4.04796	4.3699	3.79482	3.70057	3.87357	4.44257	4.23581	3.71735	4.60507	3.83395	4.10328	4.28407	3.93845	0.231491055	0.731037655	4	16	24	0.448816525	0.805393068	24	2.505612995	0.040197124
ENSMUSG00000032036	Kirrel3	kirre like nephrin family adhesion molecule 3 [Source:MGI Symbol;Acc:MGI:1914953]	8.075	7.9506	7.78621	7.67447	7.05309	8.59958	7.60393	7.9625	6.7255	10.1545	5.69586	8.46692	7.11734	8.96545	0.745118544	0.966018836	8	12	24	0.803149758	0.936962113	24	4.223324879	0.047171188
ENSMUSG00000032038	St3gal4	ST3 beta-galactoside alpha-2,3-sialyltransferase 4 [Source:MGI Symbol;Acc:MGI:1316743]	12.7718	13.432	14.9657	12.3943	14.4009	13.8255	13.5165	12.8837	15.745	13.3554	14.7167	13.2288	13.081	14.0241	0.286642595	0.793714531	12	16	24	0.419484687	0.803639585	24	10.58289553	0.047352659
ENSMUSG00000032040	Dcps	decapping enzyme, scavenger [Source:MGI Symbol;Acc:MGI:1916555]	9.10716	9.10396	9.23871	8.13001	9.85734	9.71105	9.83194	8.6012	8.95133	7.44359	9.42663	9.22759	8.7367	8.06451	0.05238827	0.391426791	20	20	24	0.06658365	0.669018876	24	20.63164224	0.087998758
ENSMUSG00000032041	Tirap	toll-interleukin 1 receptor (TIR) domain-containing adaptor protein [Source:MGI Symbol;Acc:MGI:2152213]	3.20372	3.43157	3.17014	3.09697	3.28807	2.99199	3.28562	3.696	3.03309	3.35081	3.13872	3.13034	3.40702	3.10278	0.515451644	0.966018836	8	16	24	0.403444118	0.799546792	24	3.472864111	0.033038509
ENSMUSG00000032042	Srpr	signal recognition particle receptor ('docking protein') [Source:MGI Symbol;Acc:MGI:1914648]	25.9341	22.0163	21.2799	24.2686	23.155	23.8228	23.3227	26.7238	21.551	27.9235	22.8515	24.638	24.8	23.0083	0.43565473	0.966018836	4	8	24	0.766294584	0.92437333	24	19.86713128	0.03333223
ENSMUSG00000032044	Rpusd4	RNA pseudouridylate synthase domain containing 4 [Source:MGI Symbol;Acc:MGI:1919239]	8.14979	7.62182	7.6443	7.75968	8.39522	8.36916	7.56225	8.74362	7.0638	7.214	8.22481	7.24853	7.80169	7.16596	0.671726933	0.966018836	20	16	24	0.29691826	0.768040178	24	19.82342172	0.050359562
ENSMUSG00000032046	Abhd12	abhydrolase domain containing 12 [Source:MGI Symbol;Acc:MGI:1923442]	108.614	93.3541	92.5092	94.8672	88.905	103.252	97.5363	97.3333	84.3733	105.624	84.6357	92.3533	92.9205	90.8284	0.46238066	0.966018836	4	16	24	0.450032216	0.805393068	24	22.57440934	0.050784325
ENSMUSG00000032047	Acat1	acetyl-Coenzyme A acetyltransferase 1 [Source:MGI Symbol;Acc:MGI:87870]	42.3358	46.6793	47.8812	54.4758	44.7462	44.5308	41.5718	50.3615	48.6207	41.6057	51.9482	44.677	48.3954	51.7911	0.275983441	0.789849983	8	20	24	0.25818958	0.768040178	24	8.940026962	0.078244881
ENSMUSG00000032050	Rdx	radixin [Source:MGI Symbol;Acc:MGI:97887]	41.352	41.3348	40.9668	41.6007	41.2798	40.271	42.5986	39.9786	38.5403	40.8642	40.9131	39.833	39.0214	40.7763	0.874607729	0.966018836	16	20	24	0.775024559	0.927711986	24	18.32742417	0.009525266
ENSMUSG00000032051	Fdx1	ferredoxin 1 [Source:MGI Symbol;Acc:MGI:103224]	12.0286	12.5204	13.5849	15.456	14.502	13.9749	13.1906	14.0789	15.5789	11.3987	15.7443	14.3428	16.8622	16.127	0.819898687	0.966018836	20	20	24	0.977710139	0.994291995	24	10.17000462	0.011390662
ENSMUSG00000032053	Pou2af1	POU domain, class 2, associating factor 1 [Source:MGI Symbol;Acc:MGI:105086]	0.499773	0.650692	0.909515	0.6167	0.602049	0.578888	0.629056	0.779872	0.798158	0.307545	0.553413	0.682989	0.955224	0.572064	0.435739761	0.966018836	12	4	24	0.343983874	0.777470047	24	3.723084309	0.083584377
ENSMUSG00000032058	Ppp2r1b	protein phosphatase 2, regulatory subunit A, beta [Source:MGI Symbol;Acc:MGI:1920949]	2.18198	2.51318	3.1405	3.96393	2.47872	2.93996	3.65963	3.60381	3.2642	3.23855	3.07063	3.15531	2.9385	2.9663	0.696669634	0.966018836	16	12	24	0.366822518	0.790399468	24	9.95063313	0.09376965
ENSMUSG00000032059	Alg9	asparagine-linked glycosylation 9 (alpha 1,2 mannosyltransferase) [Source:MGI Symbol;Acc:MGI:1924753]	6.90365	5.95724	6.01602	6.1327	6.37524	6.61972	4.96606	6.48024	5.99319	5.57455	6.00647	5.91855	6.20017	6.30633	0.881996584	0.966018836	8	4	24	0.873738677	0.964696217	24	21.92475316	0.020745671
ENSMUSG00000032060	Cryab	crystallin, alpha B [Source:MGI Symbol;Acc:MGI:88516]	483.797	413.781	408.741	430.679	544.56	493.395	544.124	516.315	417.93	460.011	442.303	450.407	457.877	367.824	0.166168222	0.633011401	4	8	24	0.086141113	0.704979766	24	19.76078773	0.134972637
ENSMUSG00000032062	2310030G06Rik	RIKEN cDNA 2310030G06 gene [Source:MGI Symbol;Acc:MGI:1914202]	4.21819	4.20845	4.57714	5.7544	5.59277	3.32368	6.61032	4.26062	5.22306	4.51365	4.93906	4.33303	4.39444	3.92886	0.154289127	0.606139807	16	16	24	0.570363536	0.845183325	24	12.69120579	0.086576189
ENSMUSG00000032064	Dixdc1	DIX domain containing 1 [Source:MGI Symbol;Acc:MGI:2679721]	4.77726	6.03805	6.78433	5.7648	4.35661	5.57848	6.04277	4.42385	6.4871	4.97132	5.50676	5.87503	5.08713	6.28397	0.185796491	0.662239322	12	8	24	0.422602465	0.804954905	24	6.992031605	0.090498678
ENSMUSG00000032067	Pts	6-pyruvoyl-tetrahydropterin synthase [Source:MGI Symbol;Acc:MGI:1338783]	33.127	40.5872	38.1123	37.4249	35.3711	31.7299	34.3544	36.7465	46.5133	37.9873	51.2741	44.6988	50.3123	45.297	0.956719163	0.96629412	12	12	24	0.551700703	0.83632237	24	8.494855614	0.070602033
ENSMUSG00000032076	Cadm1	cell adhesion molecule 1 [Source:MGI Symbol;Acc:MGI:1889272]	107.727	93.1909	93.7401	100.581	97.2974	106.561	91.285	96.2685	81.4656	110.674	99.0651	101.401	102.333	101.884	0.560714538	0.966018836	24	12	24	0.270543818	0.768040178	24	18.86074382	0.072036256
ENSMUSG00000032077	Bud13	BUD13 homolog [Source:MGI Symbol;Acc:MGI:2443443]	2.87495	2.26887	2.87137	2.22482	3.07582	3.47863	2.36632	2.2548	2.85264	2.26956	2.15716	2.23142	1.90808	2.50282	0.819898687	0.966018836	12	4	24	0.792057855	0.93349015	24	17.35046934	0.04340972
ENSMUSG00000032078	Zpr1	ZPR1 zinc finger [Source:MGI Symbol;Acc:MGI:1330262]	20.7078	20.1379	18.3461	17.9267	19.5551	21.3218	19.0543	19.2154	18.6793	18.6005	18.921	17.7343	18.8096	19.6159	0.226403091	0.722427815	8	4	24	0.148725521	0.757245234	24	23.04958263	0.049564763
ENSMUSG00000032085	Tagln	transgelin [Source:MGI Symbol;Acc:MGI:106012]	40.7622	43.9328	40.3439	49.3063	46.3096	39.6452	38.5007	45.3473	56.9947	41.2528	46.0077	38.9618	44.0317	38.5004	0.201269978	0.686853052	20	4	24	0.158522391	0.764925912	24	10.55773823	0.115479678
ENSMUSG00000032086	Bace1	beta-site APP cleaving enzyme 1 [Source:MGI Symbol;Acc:MGI:1346542]	33.1957	28.5298	30.5355	31.0277	30.2377	34.3971	31.4551	30.1176	24.4558	36.883	27.8755	31.5293	29.4689	31.5281	0.593560247	0.966018836	24	12	24	0.513461443	0.816307374	24	19.15476304	0.064622923
ENSMUSG00000032087	Dscaml1	DS cell adhesion molecule like 1 [Source:MGI Symbol;Acc:MGI:2150309]	0.369093	0.484836	1.05243	0.51193	0.516793	0.458936	0.398701	0.661712	0.644361	0.606904	0.244191	0.532313	0.378005	0.618399	0.008389425	0.192760771	12	16	24	0.036017662	0.609176161	24	8.042890491	0.160551304
ENSMUSG00000032089	Il10ra	interleukin 10 receptor, alpha [Source:MGI Symbol;Acc:MGI:96538]	1.68475	2.00565	1.95005	1.86295	2.20865	1.81093	1.69741	1.83552	2.01525	1.52775	2.02067	1.85437	1.85789	2.09544	0.417653118	0.963884303	20	8	24	0.846117554	0.953396028	24	10.06791522	0.021691807
ENSMUSG00000032092	Mpzl2	myelin protein zero-like 2 [Source:MGI Symbol;Acc:MGI:1289160]	2.81127	3.57822	3.44271	3.93391	4.10478	3.13696	3.04106	3.21471	4.09568	2.55561	3.85705	3.53472	3.30471	3.49992	0.143673396	0.585944911	20	8	24	0.470246872	0.805393068	24	12.17215042	0.074646146
ENSMUSG00000032096	Arcn1	archain 1 [Source:MGI Symbol;Acc:MGI:2387591]	46.9205	44.5005	43.6615	45.8385	44.386	44.3664	44.137	43.5436	42.5423	42.6356	46.6286	43.3268	45.6272	44.2624	0.602156008	0.966018836	4	8	24	0.293224798	0.768040178	24	20.35482788	0.026319401
ENSMUSG00000032097	Ddx6	DEAD (Asp-Glu-Ala-Asp) box polypeptide 6 [Source:MGI Symbol;Acc:MGI:104976]	42.6801	39.787	39.9859	40.2683	40.2266	39.387	39.8656	36.8394	38.2286	39.7763	40.7535	40.1989	39.5512	40.7976	0.923635729	0.966018836	20	16	24	0.38366989	0.799546792	24	19.09065342	0.025581938
ENSMUSG00000032101	Ddx25	DEAD box helicase 25 [Source:MGI Symbol;Acc:MGI:1353582]	6.36491	5.98729	6.03938	5.77745	5.67502	6.23641	5.76458	5.50784	5.12579	5.94951	5.48162	5.62506	5.79671	6.24654	0.800065124	0.966018836	4	16	24	0.428550064	0.805393068	24	23.48302232	0.035447563
ENSMUSG00000032103	Pus3	pseudouridine synthase 3 [Source:MGI Symbol;Acc:MGI:1914299]	6.25862	6.71582	6.94508	6.08647	7.25996	5.65898	6.06408	5.77537	6.51469	5.7189	6.83573	6.44854	6.7179	6.19769	0.933543173	0.966018836	20	4	24	0.878207515	0.965980735	24	14.0738896	0.019423671
ENSMUSG00000032109	Nlrx1	NLR family member X1 [Source:MGI Symbol;Acc:MGI:2429611]	2.65014	2.70918	2.54295	2.56004	3.22716	2.93628	3.07638	3.71334	2.99168	3.70125	2.77525	3.09477	3.14613	3.13191	0.844448841	0.966018836	8	4	24	0.973591396	0.993305115	24	5.25193101	0.0105095
ENSMUSG00000032112	Trappc4	trafficking protein particle complex 4 [Source:MGI Symbol;Acc:MGI:1926211]	90.3527	80.7804	78.498	77.5295	87.594	82.4162	76.1081	78.135	69.5835	61.0586	85.4299	76.4171	81.3651	77.5172	0.265774856	0.773510818	20	20	24	0.069107834	0.673520298	24	21.33137819	0.112791115
ENSMUSG00000032113	Chek1	checkpoint kinase 1 [Source:MGI Symbol;Acc:MGI:1202065]	1.4213	1.4168	1.93701	1.62904	1.53958	1.79326	1.20756	1.27782	1.46196	1.24316	1.78586	1.43632	1.5893	1.4631	0.4776989	0.966018836	20	12	24	0.462436463	0.805393068	24	14.81527794	0.058570245
ENSMUSG00000032114	Slc37a4	solute carrier family 37 (glucose-6-phosphate transporter), member 4 [Source:MGI Symbol;Acc:MGI:1316650]	16.2194	16.2161	16.9303	15.9263	15.4384	14.326	12.8684	16.4535	18.3099	16.3753	14.7949	15.4681	14.7358	14.9415	0.008397671	0.192760771	12	12	24	0.027711583	0.590220615	24	8.946042278	0.108775718
ENSMUSG00000032115	Hyou1	hypoxia up-regulated 1 [Source:MGI Symbol;Acc:MGI:108030]	20.2109	13.3711	13.2965	14.9273	16.5345	17.1264	18.6698	15.8984	10.7505	17.0336	14.5278	14.7658	15.7146	14.512	0.017495149	0.255326033	4	8	24	0.04520463	0.628927515	24	20.77766887	0.192396689
ENSMUSG00000032116	Stt3a	STT3, subunit of the oligosaccharyltransferase complex, homolog A (S. cerevisiae) [Source:MGI Symbol;Acc:MGI:105124]	21.3845	21.4861	19.0004	17.7348	21.0109	17.3098	19.2717	19.743	18.1194	17.7695	19.6282	18.1052	20.6982	17.0306	0.652290823	0.966018836	4	12	24	0.360229929	0.785733735	24	23.33800655	0.055641118
ENSMUSG00000032118	Fez1	fasciculation and elongation protein zeta 1 (zygin I) [Source:MGI Symbol;Acc:MGI:2670976]	304.958	259.153	272.07	253.515	268.416	296.28	256.757	261.931	216.877	261.861	258.165	261.337	264.129	261.987	0.783267549	0.966018836	24	16	24	0.056591562	0.655587475	24	21.00946459	0.092855621
ENSMUSG00000032119	Hinfp	histone H4 transcription factor [Source:MGI Symbol;Acc:MGI:2429620]	7.36482	7.43861	8.01442	7.258	8.29836	7.98671	7.25254	6.92976	8.12029	6.9056	7.88848	7.66794	7.32862	7.8337	0.449428797	0.966018836	20	8	24	0.616898279	0.867879647	24	15.52817309	0.029095795
ENSMUSG00000032120	C2cd2l	C2 calcium-dependent domain containing 2-like [Source:MGI Symbol;Acc:MGI:1919014]	14.8537	13.4835	13.9266	13.7109	13.7943	15.6648	14.349	13.6411	11.0399	17.321	11.8469	12.8831	12.7796	15.4829	0.814221431	0.966018836	16	20	24	0.961057144	0.989941253	24	21.43665809	0.01825213
ENSMUSG00000032121	Tmem218	transmembrane protein 218 [Source:MGI Symbol;Acc:MGI:1913529]	5.43188	5.19409	4.84189	5.03951	5.47452	5.76364	5.43857	5.47685	4.51379	4.24718	5.57821	5.05353	5.76721	5.36657	0.024438424	0.287293295	4	8	24	0.013581108	0.529134128	24	21.97165136	0.110469775
ENSMUSG00000032122	Slc37a2	solute carrier family 37 (glycerol-3-phosphate transporter), member 2 [Source:MGI Symbol;Acc:MGI:1929693]	1.22332	1.16425	1.23868	1.22278	1.33637	1.36251	1.27232	1.69625	1.39303	1.21207	1.08511	1.42756	1.45834	1.12323	0.852772142	0.966018836	24	20	24	0.647235027	0.880854175	24	0.736911236	0.035823437
ENSMUSG00000032123	Dpagt1	dolichyl-phosphate (UDP-N-acetylglucosamine) acetylglucosaminephosphotransferase 1 (GlcNAc-1-P transferase) [Source:MGI Symbol;Acc:MGI:1196396]	14.3833	11.6289	10.4629	12.1535	12.5762	13.4526	12.6882	13.5097	9.48496	14.3351	12.5296	12.6332	12.1624	12.1005	0.056400419	0.401074772	24	12	24	0.14066169	0.750510107	24	20.02517527	0.115736041
ENSMUSG00000032125	Robo4	roundabout guidance receptor 4 [Source:MGI Symbol;Acc:MGI:1921394]	2.0981	2.19847	2.87122	2.47015	2.0413	2.34984	2.231	2.68627	2.91758	3.15556	2.44198	2.32795	2.62826	1.9375	0.177811964	0.655480352	12	20	24	0.103434705	0.715292223	24	10.01953494	0.118651206

ENSMUSG00000032126	Hmbs	hydroxymethylbilane synthase [Source:MGI Symbol;Acc:MGI:96112]	9.11712	10.7474	12.5198	12.4172	10.563	11.148	8.86912	11.1058	10.6842	8.85727	10.2854	10.4836	12.3987	9.12253	0.911371589	0.966018836	12	16	24	0.79536522	0.934545847	24	10.87456808	0.043861328
ENSMUSG00000032127	Vps11	VPS11, CORVET/HOPS core subunit [Source:MGI Symbol;Acc:MGI:1918982]	21.3061	18.5076	19.3442	20.3116	18.7294	21.4147	20.5222	21.3989	18.091	23.1424	18.7084	21.043	18.8916	19.6896	0.8972237	0.966018836	24	12	24	0.787976396	0.931851677	24	19.43384231	0.028797531
ENSMUSG00000032131	Abcg4	ATP binding cassette subfamily G member 4 [Source:MGI Symbol;Acc:MGI:1890594]	11.7035	10.6765	11.4738	11.3687	10.8327	12.6704	11.2331	11.4442	10.2533	13.4995	10.2708	12.8391	10.287	12.3797	0.919571723	0.966018836	24	12	24	0.883096103	0.966818623	24	17.5248602	0.02404709
ENSMUSG00000032135	Mcam	melanoma cell adhesion molecule [Source:MGI Symbol;Acc:MGI:1933966]	62.3059	52.1786	47.125	52.354	50.6279	59.4307	59.0314	58.6775	40.9305	64.2813	51.4124	54.8125	59.2036	62.2533	0.105280783	0.518002936	4	8	24	0.202811796	0.768040178	24	22.60539477	0.120382821
ENSMUSG00000032171	Pin1	protein (peptidyl-prolyl cis/trans isomerase) NIMA-interacting 1 [Source:MGI Symbol;Acc:MGI:1346036]	18.5014	17.8354	17.5651	16.1765	14.9036	16.6833	17.3407	17.0094	16.2372	16.2924	17.6354	17.5217	17.2106	17.2421	0.388833118	0.915479136	4	12	24	0.069132593	0.673520298	24	1.266007423	0.060612088
ENSMUSG00000032172	Olfm2	olfactomedin 2 [Source:MGI Symbol;Acc:MGI:3045350]	4.24309	5.36448	7.30493	4.93707	3.9137	5.21934	5.8835	5.04258	5.91285	6.09848	3.8776	5.17992	3.36386	6.75631	0.062036003	0.409814092	12	8	24	0.090695579	0.71175955	24	7.365583321	0.222770852
ENSMUSG00000032175	Tyk2	tyrosine kinase 2 [Source:MGI Symbol;Acc:MGI:1929470]	5.62255	5.3413	5.6659	5.02977	5.88868	5.39209	5.09881	5.26689	5.437	5.06241	5.60175	5.60152	5.53025	5.32919	0.503972924	0.966018836	20	20	24	0.506926845	0.812342247	24	19.25634616	0.027142119
ENSMUSG00000032177	Pde4a	phosphodiesterase 4A, cAMP specific [Source:MGI Symbol;Acc:MGI:99558]	2.58475	2.84401	4.20675	3.37387	2.59897	3.58167	3.91649	3.28248	3.78204	3.99305	2.79788	3.32224	2.90182	3.92871	0.243586913	0.741580974	12	8	24	0.187243837	0.768040178	24	8.021482832	0.129949554
ENSMUSG00000032178	Ilf3	interleukin enhancer binding factor 3 [Source:MGI Symbol;Acc:MGI:1339973]	17.9534	18.2058	20.3136	20.0526	17.518	18.0805	18.1056	18.5203	19.2481	19.0264	16.4012	18.2565	17.1685	16.7414	0.051610136	0.389629649	16	4	24	0.078765453	0.68925626	24	9.467883429	0.062957426
ENSMUSG00000032179	Bmp5	bone morphogenetic protein 5 [Source:MGI Symbol;Acc:MGI:88181]	3.47865	4.34005	3.67209	2.90925	3.35663	2.87879	4.36338	3.95824	4.53837	2.45673	3.49937	2.97701	3.91674	3.33512	0.108253011	0.524035616	12	4	24	0.055246906	0.65534544	24	3.489671996	0.171207131
ENSMUSG00000032180	Tmed1	transmembrane p24 trafficking protein 1 [Source:MGI Symbol;Acc:MGI:106201]	8.93941	9.55205	10.3888	9.71786	10.6744	9.03861	9.31088	11.4893	11.2267	9.54252	9.6763	9.58113	9.54743	10.2789	0.017495149	0.255326033	12	16	24	0.061807284	0.661961741	24	8.303508419	0.08811437
ENSMUSG00000032181	Scg3	secretogranin III [Source:MGI Symbol;Acc:MGI:103032]	401.305	288.892	301.286	291.338	315.101	366.374	305.698	318.328	229.526	337.426	308.176	321.937	329.668	310.109	0.038115943	0.334069441	24	12	24	0.048748526	0.638992719	24	20.81904721	0.16432274
ENSMUSG00000032182	Yipf2	Yip1 domain family, member 2 [Source:MGI Symbol;Acc:MGI:1922016]	11.123	10.4357	10.9639	10.8727	9.94057	12.3656	10.6101	10.6926	11.9504	11.8492	10.7	10.7477	11.0605	9.81457	0.591351901	0.966018836	12	20	24	0.82767635	0.946180429	24	14.4509231	0.021093928
ENSMUSG00000032184	Lysmd2	LysM, putative peptidoglycan-binding, domain containing 2 [Source:MGI Symbol;Acc:MGI:1917332]	32.6581	28.9615	31.8036	28.8676	29.0924	30.5182	30.5276	28.9077	27.0187	29.5883	30.9087	30.7059	31.243	29.2909	0.140377635	0.585715181	4	12	24	0.111659973	0.724377021	24	21.20430568	0.054429263
ENSMUSG00000032185	Carm1	coactivator-associated arginine methyltransferase 1 [Source:MGI Symbol;Acc:MGI:1913208]	13.8451	13.6794	13.9067	14.2781	13.6742	13.5445	12.4357	15.3393	15.3256	17.5141	12.0814	12.8592	11.9421	14.4757	0.030777129	0.324150946	16	4	24	0.041498808	0.618736234	24	9.157147703	0.128632664
ENSMUSG00000032186	Tmod2	tropomodulin 2 [Source:MGI Symbol;Acc:MGI:1355335]	62.929	59.3928	60.0448	62.7607	62.3886	60.0559	56.2984	49.0597	57.2425	53.7354	64.8625	62.8908	61.1099	60.718	0.208071297	0.698704663	20	16	24	0.193058045	0.768040178	24	17.92820092	0.074583947
ENSMUSG00000032187	Smarca4	SWI/SNF related, matrix associated, actin dependent regulator of chromatin, subfamily a, member 4 [Source:MGI Symbol;Acc:MGI:88192]	16.6401	12.3756	12.9444	12.893	13.409	14.9896	14.6325	14.9748	12.0526	19.8975	12.5549	15.5967	13.9395	15.0541	0.601647512	0.966018836	24	12	24	0.776306799	0.928057527	24	20.75693332	0.05394816
ENSMUSG00000032192	Gnb5	guanine nucleotide binding protein (G protein), beta 5 [Source:MGI Symbol;Acc:MGI:101848]	35.381	31.3343	30.6632	32.6046	30.9313	35.236	32.9621	34.9577	28.4439	36.5283	31.5127	34.131	31.984	33.8318	0.245061943	0.741580974	24	12	24	0.451665477	0.805393068	24	21.30914298	0.049520824
ENSMUSG00000032193	Ldlr	low density lipoprotein receptor [Source:MGI Symbol;Acc:MGI:96765]	38.7184	29.2974	31.8693	33.8168	35.9335	39.1117	36.0176	33.4583	30.0707	45.1849	31.7758	34.9976	34.1031	33.0873	0.071575474	0.440262583	24	12	24	0.288945893	0.768040178	24	17.96156143	0.099642961
ENSMUSG00000032194	Kank2	KN motif and ankyrin repeat domains 2 [Source:MGI Symbol;Acc:MGI:2384568]	7.89483	7.82141	6.85932	6.59593	8.19532	6.74661	8.29196	7.5566	7.83939	7.22366	7.54762	7.55713	7.56248	7.25525	0.059513104	0.406067671	4	12	24	0.386146063	0.799546792	24	23.50821827	0.047898902
ENSMUSG00000032198	Dock6	dedicator of cytokinesis 6 [Source:MGI Symbol;Acc:MGI:1914789]	9.20591	10.2843	9.19629	7.85347	9.61334	8.74331	9.20905	9.09498	12.8957	9.30353	9.31248	9.27828	10.2195	10.6091	0.920858634	0.966018836	8	16	24	0.201273197	0.768040178	24	5.681243776	0.09776566
ENSMUSG00000032199	Polr2m	polymerase (RNA) II (DNA directed) polypeptide M [Source:MGI Symbol;Acc:MGI:107282]	52.3667	48.1536	51.371	50.2046	47.2209	50.568	49.2834	52.7158	48.1477	56.1	46.8806	50.7566	49.8735	52.1694	0.894563866	0.966018836	8	12	24	0.657221202	0.884447211	24	6.06102357	0.025921346
ENSMUSG00000032202	Rab27a	RAB27A, member RAS oncogene family [Source:MGI Symbol;Acc:MGI:1861441]	11.6451	8.91868	9.58276	9.05018	11.1305	11.1236	9.17056	9.26085	7.00491	8.91679	11.6429	10.062	10.2848	8.5015	0.028632001	0.316608413	20	20	24	0.003018542	0.414576087	24	19.31665886	0.204923877
ENSMUSG00000032212	Sltm	SAFB-like, transcription modulator [Source:MGI Symbol;Acc:MGI:1913910]	22.2945	22.1205	21.4881	21.8033	23.3455	21.13	21.784	20.7047	24.6951	22.0724	23.5974	23.4984	23.4241	24.5407	0.79625133	0.966018836	20	12	24	0.902556433	0.972882286	24	11.43097529	0.011552289
ENSMUSG00000032215	Rsl24d1	ribosomal L24 domain containing 1 [Source:MGI Symbol;Acc:MGI:2681840]	12.5666	13.262	12.573	13.2944	13.0549	12.7569	12.9148	13.0529	13.2646	13.4917	11.9891	14.1461	12.6416	13.4334	0.47028201	0.966018836	16	12	24	0.840350017	0.951645964	24	7.909651706	0.013061826
ENSMUSG00000032216	Nedd4	neural precursor cell expressed, developmentally down-regulated 4 [Source:MGI Symbol;Acc:MGI:97297]	179.458	153.737	154.393	157.855	155.313	169.043	166.325	164.608	134.215	173.865	151.004	160.789	161.953	164.367	0.16456443	0.628002127	4	8	24	0.212476632	0.768040178	24	21.93030803	0.06948634
ENSMUSG00000032217	Rnf111	ring finger 111 [Source:MGI Symbol;Acc:MGI:1934919]	9.30738	8.25316	8.48251	8.05292	8.79723	9.06711	9.32926	8.61133	8.03246	8.51818	9.33728	7.31965	7.76824	8.20982	0.909604019	0.966018836	20	16	24	0.618122673	0.868871403	24	19.50075706	0.035417183
ENSMUSG00000032218	Ccnb2	cyclin B2 [Source:MGI Symbol;Acc:MGI:88311]	0.579635	1.35645	1.86044	1.43449	1.02336	1.6184	1.16829	1.43066	1.07049	0.861424	0.999193	1.12407	1.44485	1.0115	0.906657897	0.966018836	12	8	24	0.808336875	0.939135535	24	6.970875687	0.060062074
ENSMUSG00000032220	Myo1e	myosin IE [Source:MGI Symbol;Acc:MGI:106621]	29.9958	21.0021	20.3217	22.3316	20.1874	26.6753	24.38	26.9951	16.615	33.9306	19.1192	25.6389	25.0433	24.2612	0.173541845	0.648600527	24	12	24	0.491663213	0.805393068	24	22.26942147	0.126154075
ENSMUSG00000032221	Mns1	meiosis-specific nuclear structural protein 1 [Source:MGI Symbol;Acc:MGI:107933]	1.34894	1.75211	1.62594	2.21945	1.27477	1.24074	2.00784	1.06279	1.29915	1.114	1.68271	1.53446	1.39827	1.43754	0.705552097	0.966018836	4	20	24	0.976692987	0.994116341	24	15.39081963	0.015799763
ENSMUSG00000032224	Fam81a	family with sequence similarity 81, member A [Source:MGI Symbol;Acc:MGI:1924136]	11.0265	9.83609	9.41968	8.72164	9.97473	9.7173	10.8107	8.98618	8.8647	10.8023	9.41556	10.4502	10.1077	9.58961	0.033248781	0.324908923	4	8	24	0.106222011	0.71810271	24	21.37942118	0.079832197
ENSMUSG00000032228	Tcf12	transcription factor 12 [Source:MGI Symbol;Acc:MGI:101877]	11.8344	13.3492	14.5332	13.4191	13.0056	12.2127	13.7617	11.6259	14.5221	12.4973	13.5943	12.3479	12.597	12.2777	0.188831826	0.664348865	12	20	24	0.199361415	0.768040178	24	10.3773862	0.065116026
ENSMUSG00000032231	Anxa2	annexin A2 [Source:MGI Symbol;Acc:MGI:88246]	915.258	858.905	828.974	818.849	969.826	883.719	782.042	811.248	771.143	713.66	950.149	803.043	868.729	825.757	0.129903614	0.560263689	20	20	24	0.146456367	0.755124657	24	19.55348203	0.089190446
ENSMUSG00000032232	Cgnl1	cingulin-like 1 [Source:MGI Symbol;Acc:MGI:1915428]	18.792	16.423	15.7361	16.4175	16.2757	17.7919	16.5586	18.4768	13.6999	19.4876	15.3786	17.2071	16.5048	17.4871	0.268085731	0.774280302	24	12	24	0.558018428	0.839532165	24	22.25119824	0.053907705
ENSMUSG00000032235	Ice2	interactor of little elongation complex ELL subunit 2 [Source:MGI Symbol;Acc:MGI:2135947]	3.76816	3.93508	4.50887	3.91542	4.15767	3.91906	4.90543	3.78333	4.1577	4.33388	4.77895	4.03958	4.01632	4.27744	0.914360142	0.966018836	12	20	24	0.712090404	0.9049226	24	13.06750634	0.030138092
ENSMUSG00000032238	Rora	RAR-related orphan receptor alpha [Source:MGI Symbol;Acc:MGI:104661]	2.51804	3.29235	3.55321	3.5727	2.93078	2.82705	3.04933	3.07321	4.08682	2.92312	3.18112	3.22512	2.93208	3.62063	0.059229521	0.406067671	12	16	24	0.014460936	0.534237819	24	8.622981338	0.137653803
ENSMUSG00000032239	Rp9	retinitis pigmentosa 9 (human) [Source:MGI Symbol;Acc:MGI:2157166]	18.7067	16.2292	17.5735	18.629	18.5115	18.4238	17.2658	16.118	18.5879	20.0207	18.1288	22.0451	21.2421	22.775	0.919571723	0.966018836	24	8	24	0.75812396	0.921733004	24	18.51935292	0.03229109
ENSMUSG00000032243	Itga11	integrin alpha 11 [Source:MGI Symbol;Acc:MGI:2442114]	1.15209	0.920959	0.842096	0.827157	0.974427	1.00747	0.895804	1.2136	0.828782	0.847707	1.09833	0.881675	0.846041	0.941646	0.266953338	0.774280302	8	4	24	0.393596486	0.799546792	24	22.27990213	0.049341272
ENSMUSG00000032244	Fem1b	fem 1 homolog b [Source:MGI Symbol;Acc:MGI:1335087]	25.1014	22.8186	22.6473	23.533	23.5575	23.3059	22.7958	21.318	21.9956	21.4401	25.1582	24.5058	25.4237	24.6863	0.194398468	0.673387182	4	8	24	0.113254397	0.727393008	24	20.6320156	0.063793492
ENSMUSG00000032245	Cln6	ceroid-lipofuscinosis, neuronal 6 [Source:MGI Symbol;Acc:MGI:2159324]	6.71984	6.51606	5.73397	6.3681	5.75307	6.82271	6.40394	6.93657	5.90751	8.86459	5.65102	7.41314	7.08176	7.15288	0.398740961	0.9304669	24	20	24	0.874168523	0.964836189	24	1.2017619	0.029556605
ENSMUSG00000032246	Calml4	calmodulin-like 4 [Source:MGI Symbol;Acc:MGI:1922850]	12.1299	15.5817	12.7507	13.7622	15.6941	12.6066	12.5296	12.7422	16.6738	11.6632	13.9677	12.8145	13.5716	12.8495	0.628286596	0.966018836	20	8	24	0.653450018	0.883312134	24	10.33320263	0.053306703
ENSMUSG00000032249	Anp32a	acidic (leucine-rich) nuclear phosphoprotein 32 family, member A [Source:MGI Symbol;Acc:MGI:108447]	54.3458	61.6362	58.9481	58.156	57.1887	56.0833	59.8195	55.9924	56.0484	48.1611	54.1923	52.4457	59.5757	58.801	0.337071598	0.85758369	4	20	24	0.169646907	0.767660404	24	2.923298628	0.06109153
ENSMUSG00000032251	Irak1bp1	interleukin-1 receptor-associated kinase 1 binding protein 1 [Source:MGI Symbol;Acc:MGI:1929475]	5.01619	4.73195	4.52238	3.66343	4.00856	4.21513	4.00364	4.3223	4.70286	4.44208	4.99188	5.18222	4.23003	4.86339	0.911371589	0.966018836	8	8	24	0.730060803	0.911943391	24	1.596104649	0.036155772
ENSMUSG00000032252	Glce	glucuronyl C5-epimerase [Source:MGI Symbol;Acc:MGI:2136405]	17.4107	16.5145	15.1066	15.7804	15.1414	16.5219	14.3629	15.7475	14.1172	15.5587	16.3703	18.4728	18.3819	19.3415	0.130696419	0.562427886	24	12	24	0.198992538	0.768040178	24	23.10172046	0.080524861
ENSMUSG00000032253	Phip	pleckstrin homology domain interacting protein [Source:MGI Symbol;Acc:MGI:1932404]	5.59773	6.46604	6.21993	5.74008	6.33597	5.294	6.62019	5.27799	6.75206	4.33121	6.69999	5.00644	5.19596	5.86777	0.8972237	0.966018836	12	12	24	0.872126341	0.963835087	24	6.54774994	0.033770887
ENSMUSG00000032254	Kif23	kinesin family member 23 [Source:MGI Symbol;Acc:MGI:1919069]	6.15902	5.35467	4.09936	5.7892	4.79998	6.01322	5.37999	4.78419	6.15051	3.64182	6.35396	5.07137	6.76014	5.40418	0.628286596	0.966018836	4	12	24	0.272996073	0.768040178	24	22.27808739	0.131500949
ENSMUSG00000032258	Lca5	Leber congenital amaurosis 5 (human) [Source:MGI Symbol;Acc:MGI:1923032]	2.52725	2.72925	3.23032	2.87153	2.59725	3.13257	2.53531	2.5056	3.10073	2.72606	2.99315	2.99269	2.52709	3.77633	0.286642595	0.793714531	12	16	24	0.463345539	0.805393068	24	9.251969971	0.062295289
ENSMUSG00000032259	Drd2	dopamine receptor D2 [Source:MGI Symbol;Acc:MGI:94924]	0.415152	0.642473	0.849465	0.478926	0.412659	0.749301	1.36014	0.432871	0.852727	0.987007	0.458812	0.653487	0.562233	0.884757	0.91695334	0.966018836	12	8	24	0.620181666	0.870018116	24	5.489946669	0.086168139
ENSMUSG00000032261	Sh3bgrl2	SH3 domain binding glutamic acid-rich protein like 2 [Source:MGI Symbol;Acc:MGI:1915350]	86.9274	72.2011	67.752	71.4401	71.5735	78.8607	70.0945	71.5683	57.8593	75.7236	75.4084	74.6853	77.7722	74.3253	0.036527779	0.332441566	24	12	24	0.053083464	0.650879532	24	20.87403211	0.116427095
ENSMUSG00000032262	Elovl4	elongation of very long chain fatty acids (FEN1/Elo2, SUR4/Elo3, yeast)-like 4 [Source:MGI Symbol;Acc:MGI:1933331]	25.2408	22.1591	22.913	20.4407	21.2889	22.4902	22.626	21.1714	18.6827	23.3867	22.4242	22.2194	21.8047	22.8284	0.912686312	0.966018836	4	8	24	0.274903786	0.768040178	24	21.99587232	0.059836867
ENSMUSG00000032263	Bckdhb	branched chain ketoacid dehydrogenase E1, beta polypeptide [Source:MGI Symbol;Acc:MGI:88137]	29.9941	30.8498	27.4666	36.6522	32.8249	31.492	31.0536	29.8309	35.7434	34.4894	32.3406	28.6261	34.6281	32.5703	0.481611534	0.966018836	16	16	24	0.297708534	0.768040178	24	11.89009013	0.067972084
ENSMUSG00000032264	Zw10	zw10 kinetochore protein [Source:MGI Symbol;Acc:MGI:1349478]	9.10065	9.18266	8.33846	10.074	9.57594	9.64176	9.1275	8.54201	8.46057	7.9636	9.26716	8.8458	8.49797	8.34554	0.173410058	0.648600527	20	16	24	0.042026631	0.622498612	24	18.25614722	0.070809018
ENSMUSG00000032265	Tent5a	terminal nucleotidyltransferase 5A [Source:MGI Symbol;Acc:MGI:2670964]	1.87463	1.96259	2.12976	3.12271	2.1154	1.79217	2.1838	2.06176	2.59416	1.92131	2.47974	1.95686	1.96844	1.83371	0.280810068	0.792139038	12	12	24	0.089895221	0.7097999	24	11.71768122	0.124871361
ENSMUSG00000032267	Usp28	ubiquitin specific peptidase 28 [Source:MGI Symbol;Acc:MGI:2442293]	6.19518	7.27102	7.02067	7.57975	6.68618	6.31619	6.18083	7.9238	7.59721	7.88832	6.23045	7.32811	8.07501	7.21725	0.57057742	0.966018836	8	20	24	0.069898913	0.674363692	24	7.822147484	0.100144244
ENSMUSG00000032268	Tmprss5	transmembrane protease, serine 5 (spinesin) [Source:MGI Symbol;Acc:MGI:1933407]	6.40984	9.40289	8.43981	9.37012	9.62872	7.68842	7.70778	8.12796	10.0809	8.31137	8.12174	7.46869	7.37014	7.98908	0.002087679	0.100255953	12	16	24	0.017332587	0.550496676	24	10.48664973	0.155919897
ENSMUSG00000032269	Htr3a	5-hydroxytryptamine (serotonin) receptor 3A [Source:MGI Symbol;Acc:MGI:96282]	42.3748	42.9846	37.1377	45.6308	38.4813	43.0544	38.4179	40.0543	35.0612	51.8761	37.4	42.2398	38.3826	42.4131	0.952551093	0.966018836	16	20	24	0.799642449	0.935842908	24	14.29364325	0.037590178
ENSMUSG00000032271	Nnmt	nicotinamide N-methyltransferase [Source:MGI Symbol;Acc:MGI:1099443]	1.46728	2.08569	2.38621	2.67303	1.91214	1.85808	2.03819	2.75681	2.48114	1.73801	2.01871	1.36421	2.61639	3.23977	0.016095537	0.253395174	8	16	24	0.023475639	0.583060917	24	6.777938435	0.228609951
ENSMUSG00000032278	Paqr5	progestin and adipoQ receptor family member V [Source:MGI Symbol;Acc:MGI:1921340]	16.3831	13.4643	11.1569	14.1839	13.5729	16.3802	13.3021	14.1863	12.5446	16.3696	12.8399	16.8808	16.9232	13.8121	0.038115943	0.334069441	24	12	24	0.094185135	0.71175955	24	20.70144873	0.139627955
ENSMUSG00000032279	Idh3a	isocitrate dehydrogenase 3 (NAD+) alpha [Source:MGI Symbol;Acc:MGI:1915084]	84.2444	79.9134	91.9189	111.483	82.8974	89.7053	85.5305	101.221	76.7885	100.943	93.624	78.3488	85.9873	89.3735	0.602156008	0.966018836	16	12	24	0.285626938	0.768040178	24	11.46191024	0.093464991
ENSMUSG00000032280	Tle3	transducin-like enhancer of split 3 [Source:MGI Symbol;Acc:MGI:104634]	8.04729	6.46889	7.70498	7.99756	7.25372	6.38776	8.29312	7.31075	7.47539	9.55894	7.11014	7.56463	6.94429	6.29608	0.5169953	0.966018836	16	16	24	0.421417442	0.804563556	24	12.616345	0.073125237
ENSMUSG00000032281	Acsbg1	acyl-CoA synthetase bubblegum family member 1 [Source:MGI Symbol;Acc:MGI:2385656]	22.9901	24.1248	30.8112	22.4302	21.1694	27.3065	25.7475	23.2981	23.1543	24.1306	21.819	21.9436	19.3162	25.3958	0.510431574	0.966018836	12	8	24	0.514922131	0.816982023	24	6.624859474	0.071070758
ENSMUSG00000032285	Dnaja4	DnaJ heat shock protein family (Hsp40) member A4 [Source:MGI Symbol;Acc:MGI:1927638]	30.805	25.4028	24.3158	28.165	27.2142	28.499	28.4138	29.5586	24.3488	28.5359	27.9627	27.3878	28.6443	26.1501	0.067104747	0.425837275	4	8	24	0.100344886	0.71175955	24	20.5148921	0.080093279
ENSMUSG00000032288	Imp3	IMP3, U3 small nucleolar ribonucleoprotein [Source:MGI Symbol;Acc:MGI:1916119]	23.509	26.0669	24.3131	24.4337	22.705	24.3615	23.1326	24.0808	25.5039	23.0227	26.6421	25.3615	26.2121	25.2664	0.81544087	0.966018836	8	8	24	0.779669175	0.929226662	24	4.204120235	0.018569774
ENSMUSG00000032289	Thsd4	thrombospondin, type I, domain containing 4 [Source:MGI Symbol;Acc:MGI:2672033]	2.00133	2.68963	1.81213	2.1497	2.14604	2.06895	1.83182	2.58071	2.63355	1.77159	2.14836	2.58816	1.78517	2.28785	0.503972924	0.966018836	8	20	24	0.735586007	0.913798759	24	5.455470165	0.048066061
ENSMUSG00000032290	Ptpn9	protein tyrosine phosphatase, non-receptor type 9 [Source:MGI Symbol;Acc:MGI:1928376]	23.5617	18.5638	20.3215	19.806	21.4537	22.7536	19.5079	19.8957	15.6719	22.1246	21.5367	21.04	20.028	18.4865	0.07600733	0.453790923	24	8	24	0.021534026	0.574114605	24	18.71052381	0.13692239
ENSMUSG00000032291	Crabp1	cellular retinoic acid binding protein I [Source:MGI Symbol;Acc:MGI:88490]	2.98993	3.28989	3.11487	2.70856	2.09289	3.24482	3.26527	3.34112	2.67732	1.93457	2.13759	2.42739	2.82533	3.12867	9.36E-04	0.072887999	8	12	24	8.68E-04	0.319044294	24	2.520762017	0.208633106
ENSMUSG00000032293	Ireb2	iron responsive element binding protein 2 [Source:MGI Symbol;Acc:MGI:1928268]	25.1676	24.8163	23.6162	24.6035	25.547	22.8722	23.3364	22.0345	24.6839	21.5051	26.1638	23.2338	23.9954	24.5218	0.883870616	0.966018836	20	4	24	0.847511209	0.954020745	24	17.19495961	0.017972196
ENSMUSG00000032294	Pkm	pyruvate kinase, muscle [Source:MGI Symbol;Acc:MGI:97591]	295.806	278.721	304.982	300.702	290.898	336.993	293.904	338.25	264.724	329.919	297.179	288.159	289.091	288.323	0.88067614	0.966018836	16	20	24	0.808255616	0.939135535	24	16.8343732	0.026964567
ENSMUSG00000032295	Man2c1	mannosidase, alpha, class 2C, member 1 [Source:MGI Symbol;Acc:MGI:1920994]	52.3117	52.843	47.4217	53.4903	52.9878	47.4069	49.3601	52.1764	51.8427	50.9063	48.4447	48.9192	50.953	48.1973	0.904831057	0.966018836	16	8	24	0.800653911	0.936185569	24	8.750104232	0.01589554
ENSMUSG00000032297	Celf6	CUGBP, Elav-like family member 6 [Source:MGI Symbol;Acc:MGI:1923433]	18.1212	13.2736	14.7051	13.4458	13.4692	16.6902	15.0295	14.2711	11.0371	17.6257	14.0269	16.4814	16.6229	16.9256	0.29905572	0.809362476	4	8	24	0.199497209	0.768040178	24	21.81756838	0.128329081
ENSMUSG00000032298	Neil1	nei endonuclease VIII-like 1 (E. coli) [Source:MGI Symbol;Acc:MGI:1920024]	3.17823	3.62834	4.0602	3.05128	3.22523	3.65694	3.51053	3.65481	3.55705	3.58153	3.64833	3.5423	4.03791	3.25506	0.854540463	0.966018836	12	4	24	0.731303551	0.912429036	24	3.798729216	0.028863707
ENSMUSG00000032299	Commd4	COMM domain containing 4 [Source:MGI Symbol;Acc:MGI:1913449]	40.8849	50.0249	45.2366	43.3339	44.8759	45.2078	45.7015	42.4687	47.9539	39.0191	47.3253	46.5462	44.8265	47.2637	0.941885246	0.966018836	12	4	24	0.791433134	0.93328332	24	2.627113094	0.025032773
ENSMUSG00000032300	1700017B05Rik	RIKEN cDNA 1700017B05 gene [Source:MGI Symbol;Acc:MGI:1921461]	1.02782	1.3227	1.09532	1.6892	1.30503	1.27304	1.27602	1.27155	1.19456	1.37518	0.931372	1.2695	1.10082	1.07637	0.702630284	0.966018836	16	12	24	0.496282743	0.807537659	24	12.36943741	0.052597092
ENSMUSG00000032301	Psma4	proteasome subunit alpha 4 [Source:MGI Symbol;Acc:MGI:1347060]	42.8138	46.9628	46.9644	45.2329	44.8797	40.9919	43.8022	42.8374	55.0557	41.5415	51.7379	47.779	48.6916	48.9125	0.96317972	0.967965568	12	12	24	0.465390256	0.805393068	24	8.389144011	0.055512468
ENSMUSG00000032303	Chrna3	cholinergic receptor, nicotinic, alpha polypeptide 3 [Source:MGI Symbol;Acc:MGI:87887]	7.27285	5.13029	5.47564	7.068	5.95023	8.92985	7.24957	5.97227	3.88735	7.82305	6.67497	7.97567	5.13473	10.486	0.148073804	0.590732968	24	12	24	0.43163161	0.805393068	24	19.72747672	0.165078451
ENSMUSG00000032305	Fam219b	family with sequence similarity 219, member B [Source:MGI Symbol;Acc:MGI:1925573]	13.6468	11.4096	11.1339	11.8148	12.1643	13.6137	11.349	12.4127	10.9718	12.9795	12.2771	12.4267	12.7717	11.6183	0.021795836	0.26698364	24	12	24	0.064334585	0.663876076	24	19.71668381	0.082747005
ENSMUSG00000032306	Mpi	mannose phosphate isomerase [Source:MGI Symbol;Acc:MGI:97075]	26.9385	27.5621	26.6658	28.0591	27.3653	28.5489	25.6439	28.7597	27.9764	27.8628	24.9302	26.8619	26.1035	26.9045	0.760769894	0.966018836	16	12	24	0.575415731	0.848049166	24	8.563567706	0.023689065
ENSMUSG00000032307	Ube2q2	ubiquitin-conjugating enzyme E2Q family member 2 [Source:MGI Symbol;Acc:MGI:2388672]	23.4864	22.6093	22.0514	22.6263	22.015	23.1937	22.2323	21.8566	19.6425	22.9931	23.5947	21.7168	21.7175	22.5311	0.839634479	0.966018836	16	20	24	0.30533906	0.768040178	24	18.98173879	0.038007736
ENSMUSG00000032308	Ulk3	unc-51-like kinase 3 [Source:MGI Symbol;Acc:MGI:1918992]	15.6135	14.9776	14.7174	14.9946	14.723	15.4063	14.4346	13.5026	16.6028	14.4159	14.1197	15.0262	14.4823	14.4924	0.887862353	0.966018836	24	8	24	0.983332861	0.996355202	24	13.69765221	0.004683202
ENSMUSG00000032309	Fbxo22	F-box protein 22 [Source:MGI Symbol;Acc:MGI:1926014]	26.277	26.5222	26.2672	27.3328	24.9353	27.0017	25.0997	24.8154	25.6145	27.5367	26.4236	27.5292	27.0402	28.3848	0.883051071	0.966018836	16	20	24	0.942627987	0.985829422	24	12.73638491	0.007247664
ENSMUSG00000032311	Nrg4	neuregulin 4 [Source:MGI Symbol;Acc:MGI:1933833]	2.01423	2.0524	2.98382	5.63146	1.88686	2.44409	2.47331	2.78667	2.95798	4.41505	3.7242	2.81193	3.07602	3.12381	0.132411359	0.567728672	16	8	24	0.032685455	0.606585237	24	11.39859681	0.289381318
ENSMUSG00000032312	Csk	c-src tyrosine kinase [Source:MGI Symbol;Acc:MGI:88537]	23.786	21.7569	18.2578	23.2283	22.5905	24.7936	23.397	22.7955	17.7768	27.5673	18.2633	23.8248	25.6779	23.6019	0.020957534	0.262822894	24	12	24	0.367322173	0.790704242	24	21.40008898	0.101949014
ENSMUSG00000032313	Tmem266	transmembrane protein 266 [Source:MGI Symbol;Acc:MGI:2142980]	1.38966	0.996697	1.12397	1.00386	0.80846	1.20397	1.40449	0.94238	0.96565	1.17842	1.03064	1.18043	1.10586	1.21316	0.183755367	0.662021716	4	8	24	0.169065768	0.767020009	24	23.52587826	0.081819039
ENSMUSG00000032314	Etfa	electron transferring flavoprotein, alpha polypeptide [Source:MGI Symbol;Acc:MGI:106092]	43.8567	43.1997	44.1234	60.7284	42.4954	42.9167	44.9832	53.1402	43.9086	51.582	50.8996	43.7177	51.674	48.8555	0.446653834	0.966018836	16	8	24	0.382041308	0.799546792	24	10.38000299	0.08151219
ENSMUSG00000032316	Clk3	CDC-like kinase 3 [Source:MGI Symbol;Acc:MGI:1098670]	29.4263	28.3434	27.9463	27.8224	30.5133	34.8091	30.2089	25.694	32.2355	30.2129	35.4287	29.3645	31.0105	27.2216	0.030972684	0.324150946	20	12	24	0.061122686	0.660703626	24	17.48667942	0.107705141
ENSMUSG00000032318	Isl2	insulin related protein 2 (islet 2) [Source:MGI Symbol;Acc:MGI:109156]	65.588	48.3948	42.0683	49.1447	37.9714	46.967	48.1719	53.3459	43.4604	71.969	51.7492	60.2319	63.9333	56.6375	0.662399683	0.966018836	4	8	24	0.649020086	0.881754555	24	23.92637246	0.088422317
ENSMUSG00000032320	Rcn2	reticulocalbin 2 [Source:MGI Symbol;Acc:MGI:1349765]	27.8665	31.573	35.2659	26.8998	25.9171	29.0971	32.3309	27.6201	28.9153	26.8438	24.7328	28.0356	23.8592	30.17	0.217933169	0.704989686	12	8	24	0.156697057	0.762682854	24	5.023528071	0.098601188
ENSMUSG00000032322	Pstpip1	proline-serine-threonine phosphatase-interacting protein 1 [Source:MGI Symbol;Acc:MGI:1321396]	5.30884	5.62151	7.17118	6.3815	6.46625	5.30635	4.81864	5.04852	6.59102	5.04132	6.96519	5.64194	4.8718	5.53752	0.018194955	0.256345714	12	16	24	0.038049217	0.61140397	24	12.11365087	0.146955451
ENSMUSG00000032324	Tspan3	tetraspanin 3 [Source:MGI Symbol;Acc:MGI:1928098]	154.051	146.308	150.431	150.739	143.167	153.562	153.867	155.56	137.906	161.893	140.145	145.586	147.425	149.358	0.759899911	0.966018836	16	4	24	0.832824381	0.948359912	24	1.966197324	0.014749702
ENSMUSG00000032327	Stra6	stimulated by retinoic acid gene 6 [Source:MGI Symbol;Acc:MGI:107742]	3.68686	4.04017	4.63148	3.53841	5.64102	4.59126	3.82097	5.20884	5.29674	2.48944	4.79612	4.6475	3.51655	4.11355	0.46238066	0.966018836	20	8	24	0.970091412	0.993003194	24	19.56682863	0.02559671
ENSMUSG00000032328	Tmem30a	transmembrane protein 30A [Source:MGI Symbol;Acc:MGI:106402]	238.554	230.698	221.268	222.429	226.136	215.247	217.221	203.5	198.064	203.433	240.384	201.737	205.78	212.217	0.941830768	0.966018836	20	16	24	0.435671073	0.805393068	24	18.59177844	0.038781127
ENSMUSG00000032329	Hmg20a	high mobility group 20A [Source:MGI Symbol;Acc:MGI:1914117]	19.3285	19.1074	18.9045	19.3956	18.3112	19.3796	18.4894	19.2947	18.0604	19.1979	17.1989	18.3592	18.6802	19.2302	0.92655264	0.966018836	8	12	24	0.348169118	0.779015211	24	3.880366067	0.025046396
ENSMUSG00000032330	Cox7a2	cytochrome c oxidase subunit 7A2 [Source:MGI Symbol;Acc:MGI:1316715]	315.229	361.288	321.345	354.206	351.407	310.286	307.306	315.153	376.251	259.647	374.505	326.553	375.755	346.925	0.896799496	0.966018836	12	12	24	0.96553771	0.991637933	24	5.175137748	0.016232505
ENSMUSG00000032332	Col12a1	collagen, type XII, alpha 1 [Source:MGI Symbol;Acc:MGI:88448]	14.4331	13.1534	12.7927	10.7798	12.7743	11.1388	10.887	14.1819	16.0076	8.19416	11.1444	11.1901	10.06	10.9953	0.676344318	0.966018836	12	4	24	0.358936064	0.785405943	24	4.131320163	0.113415516
ENSMUSG00000032333	Stoml1	stomatin-like 1 [Source:MGI Symbol;Acc:MGI:1916356]	53.9232	45.5575	45.0723	43.693	45.5928	49.9797	46.7515	51.3118	42.322	50.3756	49.7204	51.2946	49.9931	49.0885	0.061697418	0.409814092	4	8	24	0.077692799	0.687535525	24	21.84393496	0.081842247
ENSMUSG00000032334	Loxl1	lysyl oxidase-like 1 [Source:MGI Symbol;Acc:MGI:106096]	1.83078	2.22439	1.48429	1.48676	1.5319	1.53002	1.76004	2.63426	2.36074	1.23401	1.71031	1.75407	2.26221	1.8292	0.001871852	0.100255953	8	8	24	0.024040761	0.586541342	24	3.081504446	0.184878496
ENSMUSG00000032336	Nptn	neuroplastin [Source:MGI Symbol;Acc:MGI:108077]	226.409	221.759	211.774	214.908	217.689	213.353	214.122	205.919	171.716	211.001	204.838	176.131	164.962	182.199	0.784548857	0.966018836	16	20	24	0.473453866	0.805393068	24	16.30207338	0.043821951
ENSMUSG00000032338	Hcn4	hyperpolarization-activated, cyclic nucleotide-gated K+ 4 [Source:MGI Symbol;Acc:MGI:1298209]	1.66004	1.39679	1.56144	1.41966	1.66818	1.58834	1.75444	1.63735	1.31946	2.28439	1.61672	1.77606	1.38273	1.71494	0.784278077	0.966018836	24	12	24	0.622401437	0.871391591	24	16.02790931	0.047388637
ENSMUSG00000032340	Neo1	neogenin [Source:MGI Symbol;Acc:MGI:1097159]	7.49193	7.34179	8.16449	6.9495	6.73285	7.91894	8.73256	7.54443	7.03398	8.82065	6.19655	7.99216	6.61201	7.93744	0.838244962	0.966018836	24	20	24	0.686498359	0.895899235	24	3.607132731	0.04588969
ENSMUSG00000032342	Mto1	mitochondrial tRNA translation optimization 1 [Source:MGI Symbol;Acc:MGI:1915541]	8.27499	8.75743	7.25854	8.56468	8.26406	8.75876	7.57608	7.40692	7.83326	9.01041	7.85639	7.5071	9.0448	9.58448	0.879206539	0.966018836	16	20	24	0.989502105	0.997867764	24	3.590952822	0.006382259
ENSMUSG00000032346	Ooep	oocyte expressed protein [Source:MGI Symbol;Acc:MGI:1915218]	1.5441	1.62284	1.855	1.6646	1.494	1.37354	1.34802	1.63961	1.7295	1.40385	1.59213	1.55358	1.81116	1.99038	0.122696414	0.550756498	12	12	24	0.043600921	0.626787045	24	6.448345755	0.090790776
ENSMUSG00000032348	Gsta4	glutathione S-transferase, alpha 4 [Source:MGI Symbol;Acc:MGI:1309515]	18.0149	27.6538	29.9964	26.4314	24.875	24.906	23.4907	23.7807	31.0753	21.7076	24.1371	24.7164	21.3324	26.9793	0.005041121	0.143276577	12	16	24	0.094621067	0.71175955	24	9.065080295	0.162119583
ENSMUSG00000032349	Elovl5	ELOVL family member 5, elongation of long chain fatty acids (yeast) [Source:MGI Symbol;Acc:MGI:1916051]	84.5656	67.7948	66.6872	65.7781	67.2082	78.5126	76.4805	75.6693	54.9169	74.4534	74.9276	71.1716	70.4724	71.8921	0.029412383	0.322165609	4	8	24	0.05395979	0.65444776	24	21.51201983	0.130740003
ENSMUSG00000032350	Gclc	glutamate-cysteine ligase, catalytic subunit [Source:MGI Symbol;Acc:MGI:104990]	17.5143	15.7322	16.1843	16.2832	14.8463	16.215	14.8863	14.8235	14.9551	16.8158	15.402	14.8589	13.9734	16.2002	0.941955432	0.966018836	16	12	24	0.75575188	0.921118175	24	11.06313495	0.022511009
ENSMUSG00000032352	Lrrc1	leucine rich repeat containing 1 [Source:MGI Symbol;Acc:MGI:2442313]	3.73023	2.9561	3.11696	3.24884	2.80002	3.24615	3.68675	3.01749	2.54607	2.83179	3.15627	3.48259	3.43137	3.06996	0.017495149	0.255326033	4	8	24	0.012160478	0.525280095	24	22.0466406	0.120819127
ENSMUSG00000032353	Tmed3	transmembrane p24 trafficking protein 3 [Source:MGI Symbol;Acc:MGI:1913361]	34.6897	34.6191	33.4644	31.3368	36.5077	32.6585	35.2385	36.1116	34.8018	27.2998	33.995	32.0622	32.4765	33.2268	0.664568268	0.966018836	8	8	24	0.301178976	0.768040178	24	0.778929673	0.058083634
ENSMUSG00000032355	Mlip	muscular LMNA-interacting protein [Source:MGI Symbol;Acc:MGI:1916892]	108.024	119.501	98.4665	106.691	136.989	96.4382	112.258	101.97	138.083	77.1699	115.799	103.689	117.917	102.721	0.904690479	0.966018836	4	12	24	0.923108975	0.979439431	24	23.14458378	0.034522528
ENSMUSG00000032356	Rasgrf1	RAS protein-specific guanine nucleotide-releasing factor 1 [Source:MGI Symbol;Acc:MGI:99694]	5.75781	9.5167	17.0115	7.84144	5.61313	10.8766	12.5978	6.27978	11.0502	8.63013	5.05746	8.56059	5.05576	11.5439	0.185796491	0.662239322	12	8	24	0.320120152	0.768040178	24	6.706915729	0.290913057

ENSMUSG00000032359	Ctsh	cathepsin H [Source:MGI Symbol;Acc:MGI:107285]	17.6466	22.5453	17.3282	14.8096	16.1463	12.6628	16.4748	20.7794	28.3906	9.50791	18.38	16.8005	17.8918	15.6306	0.25849765	0.761738864	12	4	24	0.238012113	0.768040178	24	4.326104934	0.224737926
ENSMUSG00000032360	Hcrtr2	hypocretin (orexin) receptor 2 [Source:MGI Symbol;Acc:MGI:2680765]	4.49665	4.4492	4.01127	4.21954	4.63623	4.31316	4.24326	4.19305	4.51252	4.14975	4.71709	3.96261	4.4799	4.38898	0.928064146	0.966018836	20	20	24	0.855723084	0.957163195	24	18.88700097	0.014258457
ENSMUSG00000032363	Adamts7	a disintegrin-like and metallopeptidase (reprolysin type) with thrombospondin type 1 motif, 7 [Source:MGI Symbol;Acc:MGI:1347346]	1.73069	1.91855	1.54918	1.65672	2.32833	1.5722	2.9	1.7379	2.29867	1.95317	2.17975	2.02649	1.9651	1.8465	0.951846915	0.966018836	20	12	24	0.815081134	0.940891459	24	19.84016121	0.043659964
ENSMUSG00000032366	Tpm1	tropomyosin 1, alpha [Source:MGI Symbol;Acc:MGI:98809]	219.192	396.231	378.466	463.676	287.847	427.794	343.954	712.81	702.47	658.138	367.625	418.323	475.887	346.089	0.664568268	0.966018836	16	4	24	0.108260163	0.72082227	24	8.88663574	0.361929963
ENSMUSG00000032368	Zic1	zinc finger protein of the cerebellum 1 [Source:MGI Symbol;Acc:MGI:106683]	3.62702	5.89834	4.93958	3.65256	3.8558	3.87443	4.15332	5.3284	6.60522	3.45043	4.38573	4.63469	3.54945	4.82758	0.048507657	0.379129806	12	4	24	0.078194979	0.688357494	24	6.140207904	0.202882342
ENSMUSG00000032369	Plscr1	phospholipid scramblase 1 [Source:MGI Symbol;Acc:MGI:893575]	2.32034	2.66167	2.65111	1.82268	2.04883	1.87412	2.22066	2.44695	2.26762	1.99872	2.04417	1.86325	2.39476	1.72553	0.200364953	0.684570416	12	4	24	0.10407489	0.715480277	24	4.247843817	0.096666258
ENSMUSG00000032370	Lactb	lactamase, beta [Source:MGI Symbol;Acc:MGI:1933395]	5.43266	5.16393	5.52356	5.76946	5.99312	6.06172	4.95617	6.16111	5.52424	6.4934	6.05985	5.64314	6.60532	5.73109	0.608804768	0.966018836	20	8	24	0.34203652	0.776252782	24	14.51649159	0.04927211
ENSMUSG00000032372	Plscr2	phospholipid scramblase 2 [Source:MGI Symbol;Acc:MGI:1270860]	7.12456	10.5578	10.7849	9.04746	9.7153	10.2015	9.10204	10.396	12.7787	9.42503	10.2851	9.46758	8.04453	9.70596	0.018894761	0.256345714	12	16	24	0.11714653	0.728210112	24	9.529092433	0.142100473
ENSMUSG00000032374	Plod2	procollagen lysine, 2-oxoglutarate 5-dioxygenase 2 [Source:MGI Symbol;Acc:MGI:1347007]	6.66273	8.16304	7.3542	7.61372	6.6243	6.1266	6.62355	7.04575	8.87867	6.13308	6.83547	7.08714	6.01612	6.90815	0.053683798	0.394331071	12	16	24	0.082875755	0.699403344	24	7.859302565	0.114529225
ENSMUSG00000032375	Aph1b	aph1 homolog B, gamma secretase subunit [Source:MGI Symbol;Acc:MGI:3522097]	9.23973	8.48276	8.00142	8.82736	9.0611	9.39939	8.70391	8.77823	8.05514	8.09961	9.18003	9.02165	8.83424	8.80885	7.90E-04	0.071344007	24	12	24	0.00213087	0.395705644	24	20.19175167	0.077283674
ENSMUSG00000032376	Usp3	ubiquitin specific peptidase 3 [Source:MGI Symbol;Acc:MGI:2152450]	6.26488	6.49229	8.074	8.01845	6.48521	7.04149	6.67943	9.20368	6.65373	6.05016	6.49613	6.56685	6.2881	8.01842	0.560714538	0.966018836	8	20	24	0.238999562	0.768040178	24	6.677485285	0.106573197
ENSMUSG00000032377	Plscr4	phospholipid scramblase 4 [Source:MGI Symbol;Acc:MGI:2143267]	9.77891	10.2109	10.7225	10.8373	11.6456	10.1759	10.4566	9.67505	11.561	11.3243	11.7888	10.3825	9.75823	10.4563	0.00384759	0.140598674	20	8	24	0.001103108	0.336500758	24	12.96264598	0.099092229
ENSMUSG00000032380	Dapk2	death-associated protein kinase 2 [Source:MGI Symbol;Acc:MGI:1341297]	8.38581	8.6106	8.6876	8.45519	7.27098	8.6662	7.74012	10.1471	7.35401	9.48368	8.08565	8.40035	10.4731	8.56087	0.772442842	0.966018836	8	12	24	0.468640889	0.805393068	24	3.315744964	0.063596909
ENSMUSG00000032381	Ciao2a	cytosolic iron-sulfur assembly component 2A [Source:MGI Symbol;Acc:MGI:1915500]	21.1869	24.0214	22.9269	25.243	22.8875	21.5514	20.065	21.1786	25.163	19.4965	26.6988	23.032	24.9294	22.5965	0.767183862	0.966018836	20	8	24	0.6513107	0.882727323	24	12.1996348	0.046716736
ENSMUSG00000032382	Snx1	sorting nexin 1 [Source:MGI Symbol;Acc:MGI:1928395]	58.7584	58.4568	55.5731	52.0759	58.9554	55.2696	55.0169	51.2824	52.9447	44.3901	58.975	56.979	58.3668	53.7394	0.035058838	0.328311403	20	20	24	0.09147368	0.71175955	24	21.67997373	0.089475289
ENSMUSG00000032383	Ppib	peptidylprolyl isomerase B [Source:MGI Symbol;Acc:MGI:97750]	156.376	168.608	157.247	149.947	169.85	156.509	152.59	154.312	161.856	131.518	156.573	151.989	153.701	151.169	0.93717134	0.966018836	20	20	24	0.661604117	0.885525866	24	23.55051506	0.028999493
ENSMUSG00000032384	Csnk1g1	casein kinase 1, gamma 1 [Source:MGI Symbol;Acc:MGI:2660884]	8.34595	7.48141	7.38267	7.58226	7.49172	7.92533	6.88577	6.30918	6.91904	7.69812	7.00798	7.48916	7.91939	7.61988	0.622132989	0.966018836	24	12	24	0.460130639	0.805393068	24	19.6571243	0.045347905
ENSMUSG00000032386	Trip4	thyroid hormone receptor interactor 4 [Source:MGI Symbol;Acc:MGI:1928469]	15.3475	17.0333	16.8844	15.038	17.353	17.4703	15.3596	16.0373	15.3522	14.7832	17.3748	16.7457	16.9584	17.198	0.187699199	0.662239322	20	20	24	0.526317326	0.823075573	24	20.81114402	0.038040006
ENSMUSG00000032387	Rbpms2	RNA binding protein with multiple splicing 2 [Source:MGI Symbol;Acc:MGI:1919223]	0.975695	1.04505	1.2094	1.83713	1.05376	1.29458	0.933445	1.19339	1.33915	1.94901	2.20038	1.27432	1.92258	1.50281	0.254637229	0.757509828	16	12	24	0.061542222	0.661255919	24	12.76332293	0.164516009
ENSMUSG00000032388	Spg21	SPG21, maspardin [Source:MGI Symbol;Acc:MGI:106403]	14.8373	15.454	13.071	14.7083	14.7565	13.3064	14.0279	16.0737	14.7635	14.8526	14.9535	14.5367	14.9	15.4346	0.062631097	0.410322149	8	16	24	0.518308541	0.818640537	24	5.229034049	0.033054985
ENSMUSG00000032392	Parp16	poly (ADP-ribose) polymerase family, member 16 [Source:MGI Symbol;Acc:MGI:2446133]	2.22081	2.16091	2.4497	2.57101	2.52396	2.40799	2.11685	2.22123	2.71906	3.09585	2.54848	2.33684	2.88871	2.40574	0.027096347	0.306445942	16	16	24	0.037182732	0.609176161	24	12.24985092	0.09261699
ENSMUSG00000032393	Dpp8	dipeptidylpeptidase 8 [Source:MGI Symbol;Acc:MGI:1921638]	55.8755	51.6981	52.6829	53.9625	51.3738	54.616	55.136	50.3996	46.6716	55.6268	50.8124	54.999	54.7271	56.5714	0.43565473	0.966018836	4	8	24	0.309242809	0.768040178	24	21.67497681	0.045782478
ENSMUSG00000032396	Dis3l	DIS3 like exosome 3'-5' exoribonuclease [Source:MGI Symbol;Acc:MGI:2143272]	11.5518	9.55224	10.5768	9.5448	9.76089	10.9531	10.8144	9.96225	9.14124	10.6314	9.3829	9.52959	9.72496	9.80549	0.787022682	0.966018836	4	16	24	0.31741714	0.768040178	24	22.45524388	0.049513211
ENSMUSG00000032397	Tipin	timeless interacting protein [Source:MGI Symbol;Acc:MGI:1921571]	11.0233	15.7677	15.4551	11.9744	13.1793	11.7888	13.3134	15.2336	15.9867	12.7652	12.0335	14.3808	14.2341	16.3669	0.038581842	0.335882166	12	4	24	0.006900819	0.472731997	24	5.861107915	0.177384338
ENSMUSG00000032398	Snapc5	small nuclear RNA activating complex, polypeptide 5 [Source:MGI Symbol;Acc:MGI:1914282]	31.4506	30.2237	26.5506	29.1674	28.1203	27.454	29.2544	25.9279	29.2679	23.508	29.4889	30.0071	31.1377	30.1496	0.101443845	0.511659635	4	12	24	0.151092291	0.758865539	24	23.29249312	0.081650206
ENSMUSG00000032399	Rpl4	ribosomal protein L4 [Source:MGI Symbol;Acc:MGI:1915141]	196.338	220.693	211.07	215.138	218.542	204.454	203.119	203.233	224.164	188.206	209.767	200.404	209.896	196.6	0.649212555	0.966018836	12	16	24	0.708036408	0.903565455	24	10.22387566	0.023255023
ENSMUSG00000032400	Zwilch	zwilch kinetochore protein [Source:MGI Symbol;Acc:MGI:1915264]	2.66197	3.17631	2.81364	4.05241	2.26373	2.92428	2.91155	2.55356	2.11511	2.27821	3.18114	2.15685	2.57204	2.39202	0.801273005	0.966018836	16	20	24	0.903410133	0.973208451	24	14.87790421	0.031029487
ENSMUSG00000032401	Lctl	lactase-like [Source:MGI Symbol;Acc:MGI:2183549]	13.1544	15.7547	13.117	15.7309	19.8972	14.3158	18.4973	16.1322	18.3467	15.458	16.2278	14.8215	17.9476	11.8619	0.922035832	0.966018836	20	12	24	0.661774554	0.885615772	24	16.22061341	0.072654783
ENSMUSG00000032402	Smad3	SMAD family member 3 [Source:MGI Symbol;Acc:MGI:1201674]	2.04686	2.66543	2.67392	2.57822	2.24005	2.46144	2.89629	3.41625	3.00271	2.4329	2.27595	2.22373	2.85901	2.5124	0.061112935	0.409814092	8	16	24	0.042263726	0.623698598	24	5.59949528	0.130277252
ENSMUSG00000032403	2300009A05Rik	RIKEN cDNA 2300009A05 gene [Source:MGI Symbol;Acc:MGI:1916728]	7.9071	7.78287	8.48858	9.05378	6.71399	7.06733	7.42781	8.81528	10.8723	8.77813	9.39397	8.408	8.52077	9.44463	0.24731677	0.746715482	12	12	24	0.012663028	0.526249719	24	8.361170221	0.151636482
ENSMUSG00000032405	Pias1	protein inhibitor of activated STAT 1 [Source:MGI Symbol;Acc:MGI:1913125]	11.0001	11.4392	11.2353	10.923	10.8487	11.0753	11.8573	9.85216	10.3281	10.2859	10.7076	10.2277	10.5907	11.4798	0.909604019	0.966018836	4	20	24	0.583671393	0.851892977	24	0.528952708	0.026253452
ENSMUSG00000032407	U2surp	U2 snRNP-associated SURP domain containing [Source:MGI Symbol;Acc:MGI:1915208]	15.2094	16.9407	16.8116	15.8078	16.282	15.647	16.5887	13.8942	18.6685	15.8535	18.3697	18.6407	18.7338	18.709	0.825856869	0.966018836	12	4	24	0.992411914	0.998516607	24	9.861319756	0.052190504
ENSMUSG00000032409	Atr	ataxia telangiectasia and Rad3 related [Source:MGI Symbol;Acc:MGI:108028]	6.01485	7.63423	5.52397	6.69384	6.76756	5.32768	5.84251	5.73314	7.28821	5.22904	6.71021	6.09981	6.91298	6.96005	0.919571723	0.966018836	8	16	24	0.740403421	0.915274254	24	6.011630184	0.047726093
ENSMUSG00000032410	Xrn1	5'-3' exoribonuclease 1 [Source:MGI Symbol;Acc:MGI:891964]	3.44757	4.89053	4.39849	4.28986	4.22168	4.05893	3.85507	4.16069	5.11751	3.53874	4.41764	4.5896	4.15722	4.57537	0.286642595	0.793714531	12	16	24	0.420596985	0.804127338	24	7.908223626	0.067934533
ENSMUSG00000032411	Tfdp2	transcription factor Dp 2 [Source:MGI Symbol;Acc:MGI:107167]	9.8914	10.647	10.6579	10.8505	9.30264	10.8301	11.6647	11.8073	10.7625	11.0788	11.0153	10.8172	10.9634	[illegible]	0.934818505	0.966018836	8	12	24	0.118960648	0.729851534	24	4.279489754	0.067874399
ENSMUSG00000032412	Atp1b3	ATPase, Na+/K+ transporting, beta 3 polypeptide [Source:MGI Symbol;Acc:MGI:107788]	227.008	192.579	180.313	187.92	205.359	207.369	190.731	189.993	172.786	185.403	200.288	197.808	205.522	189.942	0.001971398	0.100255953	24	12	24	0.001582734	0.362980479	24	20.75220931	0.112216759
ENSMUSG00000032413	Rasa2	RAS p21 protein activator 2 [Source:MGI Symbol;Acc:MGI:2149960]	9.04822	8.84818	8.08753	7.90687	8.43638	8.22107	8.88066	7.60758	8.2881	7.8169	8.84941	8.51809	8.62249	8.54662	0.010754065	0.199214173	4	12	24	0.089343148	0.708706636	24	22.26056577	0.055991708
ENSMUSG00000032415	Ube2cbp	ubiquitin-conjugating enzyme E2C binding protein [Source:MGI Symbol;Acc:MGI:1917598]	0.535806	0.545298	0.68905	0.673376	0.600696	0.708392	0.684462	0.459298	0.533279	0.595169	0.554543	0.572585	0.404532	0.614101	0.823657258	0.966018836	24	4	24	0.413833801	0.799546792	24	14.54925247	0.039468696
ENSMUSG00000032417	Rwdd2a	RWD domain containing 2A [Source:MGI Symbol;Acc:MGI:1916769]	3.8984	6.05732	8.84585	5.51907	4.31545	4.96172	5.6846	4.40064	5.70833	4.61961	4.74844	4.88616	4.41182	5.708	0.161023625	0.620479606	12	8	24	0.132670149	0.742819192	24	7.796792978	0.190929762
ENSMUSG00000032418	Me1	malic enzyme 1, NADP(+)-dependent, cytosolic [Source:MGI Symbol;Acc:MGI:97043]	31.0738	26.6471	28.9719	34.6884	29.0847	29.9212	30.8585	35.2549	25.9302	35.4129	34.926	31.2869	34.5185	31.748	0.911371589	0.966018836	16	20	24	0.706333903	0.902918226	24	16.41509006	0.044077989
ENSMUSG00000032419	Tbx18	T-box18 [Source:MGI Symbol;Acc:MGI:1923615]	0.777799	0.770687	0.619724	0.552751	0.712691	0.575828	0.920498	0.614627	0.809622	0.397769	0.65367	0.604726	0.73712	0.645942	0.036134798	0.331852526	4	12	24	0.090873208	0.71175955	24	0.824012599	0.08850878
ENSMUSG00000032420	Nt5e	5' nucleotidase, ecto [Source:MGI Symbol;Acc:MGI:99782]	17.8456	18.9873	18.723	17.9988	18.4031	17.1444	14.9683	15.4344	17.2067	18.8652	19.2281	18.3152	17.5121	18.8093	0.192909212	0.670595308	20	8	24	0.163922119	0.767020009	24	13.3900467	0.07108768
ENSMUSG00000032422	Snx14	sorting nexin 14 [Source:MGI Symbol;Acc:MGI:2155664]	23.0275	22.0413	18.9421	20.7714	20.65	21.407	22.5912	22.6194	20.1493	18.6813	20.9933	20.4221	22.629	21.3955	9.60E-04	0.07358601	4	12	24	0.002088384	0.395705644	24	23.87457914	0.096862403
ENSMUSG00000032423	Syncrip	synaptotagmin binding, cytoplasmic RNA interacting protein [Source:MGI Symbol;Acc:MGI:1891690]	18.4316	16.5847	17.0344	17.266	17.2	15.7012	18.2765	15.4146	16.4369	15.722	18.2174	16.1614	16.8965	16.4528	0.759899911	0.966018836	4	4	24	0.611083735	0.865062328	24	19.90753594	0.03085952
ENSMUSG00000032425	Zfp949	zinc finger protein 949 [Source:MGI Symbol;Acc:MGI:1918890]	9.92091	10.1919	9.87928	9.30639	10.8194	9.61178	9.19483	9.10585	10.3272	8.96599	9.46634	8.73337	9.24664	10.7437	0.869721097	0.966018836	12	12	24	0.660348872	0.885429061	24	7.012714634	0.032242817
ENSMUSG00000032431	Crtap	cartilage associated protein [Source:MGI Symbol;Acc:MGI:1891221]	14.4897	16.7556	16.2995	15.8623	18.9394	14.5596	17.4038	17.3666	18.4006	13.8196	15.5495	15.1311	15.5614	13.2024	0.891742979	0.966018836	12	12	24	0.905521939	0.974083049	24	9.925484955	0.025327832
ENSMUSG00000032434	Cmtm6	CKLF-like MARVEL transmembrane domain containing 6 [Source:MGI Symbol;Acc:MGI:2447165]	23.6674	22.6317	21.8028	20.8407	23.9792	22.1342	26.4463	24.1139	22.0845	25.0425	20.3155	23.1975	24.1608	20.5167	0.371875382	0.894537464	4	8	24	0.41411796	0.799546792	24	22.99436788	0.056402527
ENSMUSG00000032435	Dync1li1	dynein cytoplasmic 1 light intermediate chain 1 [Source:MGI Symbol;Acc:MGI:2135610]	69.4369	54.3107	55.5956	57.1685	54.7278	65.3752	58.8013	63.1577	46.8809	74.1822	59.0773	64.9162	62.5848	64.8272	0.245061943	0.741580974	24	12	24	0.420295365	0.803964828	24	20.95014466	0.086917925
ENSMUSG00000032436	Cmtm7	CKLF-like MARVEL transmembrane domain containing 7 [Source:MGI Symbol;Acc:MGI:2447166]	7.37744	8.49597	7.78951	7.50919	8.91408	7.7033	7.16881	9.28122	8.71655	5.49961	7.90839	6.85318	9.05051	7.01848	0.915082018	0.966018836	8	8	24	0.657818568	0.884588645	24	2.364569003	0.06289958
ENSMUSG00000032437	Stt3b	STT3, subunit of the oligosaccharyltransferase complex, homolog B (S. cerevisiae) [Source:MGI Symbol;Acc:MGI:1915542]	45.2982	38.1689	38.3491	37.1661	37.9985	41.773	37.6721	38.7685	33.0008	41.0741	40.296	40.0164	41.8391	39.1427	0.300823756	0.809362476	4	8	24	0.082929248	0.699403344	24	21.03776011	0.086939742
ENSMUSG00000032440	Tgfbr2	transforming growth factor, beta receptor II [Source:MGI Symbol;Acc:MGI:98729]	9.03498	9.06019	7.98742	8.44798	9.5059	8.8723	10.776	10.7883	9.04374	11.2426	9.13513	9.39696	9.83213	7.90967	0.911685024	0.966018836	4	8	24	0.622531843	0.944260674	24	20.57870716	0.035964862
ENSMUSG00000032443	Zcwpw2	zinc finger, CW type with PWWP domain 2 [Source:MGI Symbol;Acc:MGI:1921888]	0.637156	0.783282	0.490254	0.624858	0.368746	0.579866	0.651014	0.565283	0.58074	0.700601	0.678853	0.984454	0.856487	0.670387	0.801995186	0.966018836	4	8	24	0.363167878	0.788005352	24	23.55018936	0.065088316
ENSMUSG00000032449	Slc25a36	solute carrier family 25, member 36 [Source:MGI Symbol;Acc:MGI:1924909]	25.5374	26.1299	26.864	26.0588	26.2194	23.2425	25.631	23.0196	26.4812	23.7041	26.8965	24.0153	24.8896	24.3732	0.300823756	0.809362476	20	4	24	0.515638067	0.817346854	24	11.27261787	0.031823384
ENSMUSG00000032452	Clstn2	calsyntenin 2 [Source:MGI Symbol;Acc:MGI:1929897]	9.69568	7.35373	7.51675	8.14822	7.41684	8.73761	8.07805	8.16957	6.86497	9.7169	7.09012	8.9153	8.33927	8.66527	0.377663653	0.903463289	24	12	24	0.484639987	0.805393068	24	21.82295088	0.068149589
ENSMUSG00000032454	Rbp2	retinol binding protein 2, cellular [Source:MGI Symbol;Acc:MGI:97877]	2.97721	2.46478	2.42139	2.23496	2.91619	2.25572	2.48087	2.44684	2.08204	2.95159	2.57532	3.41127	2.19859	2.89124	0.649212555	0.966018836	20	16	24	0.504083421	0.810711214	24	19.01262927	0.071133712
ENSMUSG00000032456	Nmnat3	nicotinamide nucleotide adenylyltransferase 3 [Source:MGI Symbol;Acc:MGI:1921330]	0.514122	1.15727	1.09742	1.51227	0.892869	1.00079	0.892245	1.40355	1.68365	1.24341	1.05649	1.07907	1.6214	1.53302	0.128439072	0.560209134	8	20	24	0.014347964	0.533974075	24	8.148755426	0.195378348
ENSMUSG00000032458	Copb2	coatomer protein complex, subunit beta 2 (beta prime) [Source:MGI Symbol;Acc:MGI:1354962]	50.8217	51.1088	50.3481	48.2686	49.3874	48.1447	47.7253	47.0016	48.0541	46.167	50.4231	48.4733	49.4379	49.1549	0.880631923	0.966018836	20	20	24	0.670063455	0.888925819	24	23.38576483	0.014303592
ENSMUSG00000032459	Mrps22	mitochondrial ribosomal protein S22 [Source:MGI Symbol;Acc:MGI:1928137]	17.2677	18.507	16.5737	15.5823	17.6127	16.5458	18.2413	16.8023	20.16	16.3356	20.8271	20.2429	20.8104	21.0701	0.505132703	0.966018836	4	12	24	0.385804879	0.799546792	24	0.680066535	0.055962745
ENSMUSG00000032462	Pik3cb	phosphatidylinositol-4,5-bisphosphate 3-kinase catalytic subunit beta [Source:MGI Symbol;Acc:MGI:1922019]	6.13223	4.7031	5.25153	4.87567	4.76827	5.38004	5.09477	5.01119	4.04971	5.7493	4.94541	5.24069	4.77814	5.374	0.953356544	0.966018836	24	12	24	0.51215759	0.815453149	24	21.04626219	0.056111068
ENSMUSG00000032463	Faim	Fas apoptotic inhibitory molecule [Source:MGI Symbol;Acc:MGI:1344387]	31.3175	35.8972	34.2139	32.4045	33.2163	30.7737	33.9522	30.9689	35.728	29.6453	30.8917	35.3575	31.9406	32.3785	0.977455189	0.977820713	12	4	24	0.632613461	0.875112738	24	4.73337788	0.032586839
ENSMUSG00000032468	Armc8	armadillo repeat containing 8 [Source:MGI Symbol;Acc:MGI:1921375]	24.5176	24.833	24.1851	23.5591	23.604	24.0975	22.7575	22.2336	23.417	25.0784	23.1459	24.5763	25.24	25.558	0.890235289	0.966018836	8	12	24	0.893561108	0.969935496	24	2.527093243	0.010564488
ENSMUSG00000032469	Dbr1	debranching RNA lariats 1 [Source:MGI Symbol;Acc:MGI:1931520]	4.23008	4.0667	4.29194	3.76383	4.70746	3.50262	3.7642	3.62001	4.29741	3.5374	4.29225	3.69573	4.25821	3.74628	0.745118544	0.966018836	12	8	24	0.910700021	0.9747563	24	13.54311555	0.017798686
ENSMUSG00000032470	Mras	muscle and microspikes RAS [Source:MGI Symbol;Acc:MGI:1100856]	50.7424	49.7939	48.4743	48.6181	56.5416	49.5679	60.0957	47.9818	52.3299	53.3109	46.7734	49.5491	55.4783	48.7868	0.648082424	0.966018836	4	4	24	0.659433184	0.884879972	24	21.62405968	0.038523424
ENSMUSG00000032475	Nck1	non-catalytic region of tyrosine kinase adaptor protein 1 [Source:MGI Symbol;Acc:MGI:109601]	23.4978	22.8201	22.2278	21.5767	22.8575	21.6319	23.7914	21.0677	22.2775	17.7486	22.6877	21.3593	22.5187	20.0504	0.185945151	0.662239322	4	12	24	0.168882452	0.767020009	24	22.35290931	0.0679784
ENSMUSG00000032477	Cdc25a	cell division cycle 25A [Source:MGI Symbol;Acc:MGI:103198]	2.97862	2.88047	2.66155	2.95605	3.01841	2.99872	2.6293	3.08776	2.31254	3.18934	2.34499	2.99814	2.88982	3.42502	0.93447422	0.966018836	24	12	24	0.806411936	0.93822825	24	0.565778335	0.030569106
ENSMUSG00000032478	Nme6	NME/NM23 nucleoside diphosphate kinase 6 [Source:MGI Symbol;Acc:MGI:1861676]	6.97523	8.12351	7.39215	7.80338	7.81295	5.92504	7.08315	8.72916	8.91747	7.09816	8.30674	8.65564	7.41653	6.91031	0.94086481	0.966018836	12	16	24	0.491178705	0.805393068	24	9.251935463	0.069353285
ENSMUSG00000032479	Map4	microtubule-associated protein 4 [Source:MGI Symbol;Acc:MGI:97178]	181.623	154.765	163.555	164.545	177.82	182.416	180.334	144.84	219.783	152.98	177.402	165.869	171.676	[illegible]	0.16456443	0.628902127	4	8	24	0.837369273	0.950299676	24	19.97467435	0.03637048
ENSMUSG00000032480	Dhx30	DEAH (Asp-Glu-Ala-His) box polypeptide 30 [Source:MGI Symbol;Acc:MGI:1920081]	23.4665	22.2693	20.1768	25.1295	23.3937	24.2835	24.6117	27.8556	22.9086	25.8471	18.4523	21.6927	20.6389	21.2011	0.874607729	0.966018836	16	4	24	0.942230922	0.985597432	24	5.916907719	0.020927901
ENSMUSG00000032481	Smarcc1	SWI/SNF related, matrix associated, actin dependent regulator of chromatin, subfamily c, member 1 [Source:MGI Symbol;Acc:MGI:1203524]	12.7152	10.4878	11.1983	11.4056	11.6188	12.0972	12.0376	10.96	9.91336	13.5509	10.39	12.5409	11.4346	11.7026	0.074438493	0.448301997	24	12	24	0.364764009	0.789112192	24	19.49444816	0.065025945
ENSMUSG00000032482	Cspg5	chondroitin sulfate proteoglycan 5 [Source:MGI Symbol;Acc:MGI:1352747]	3.20476	5.1086	6.77325	4.32855	2.25711	4.23033	5.42592	4.09439	5.05135	4.31811	2.7512	4.0331	2.61378	5.65808	0.035023815	0.328311403	12	8	24	0.049189067	0.641092103	24	6.345752486	0.326404133
ENSMUSG00000032484	Ngp	neutrophilic granule protein [Source:MGI Symbol;Acc:MGI:105983]	27.2449	68.4897	110.067	82.6128	55.2653	65.5371	58.1548	51.2424	61.532	45.5756	52.9624	55.3954	75.7208	41.5373	0.906657897	0.966018836	12	8	24	0.524270258	0.821954853	24	10.37994667	0.214485499
ENSMUSG00000032485	Scap	SREBF chaperone [Source:MGI Symbol;Acc:MGI:2135958]	13.5416	12.4432	12.218	11.6714	12.671	13.9876	15.2407	13.3593	13.1644	17.773	12.687	15.4332	13.0658	13.923	0.280810068	0.792139038	24	12	24	0.80401501	0.93736298	24	20.19275812	0.037672722
ENSMUSG00000032489	Kif9	kinesin family member 9 [Source:MGI Symbol;Acc:MGI:1098237]	1.75119	2.30201	1.91147	1.98083	1.71729	2.01001	1.95389	1.68896	1.78037	1.9385	2.15943	2.22121	1.52836	1.89182	0.675497492	0.966018836	24	4	24	0.701294938	0.901054682	24	15.71790473	0.035749888
ENSMUSG00000032491	Nradd	neurotrophin receptor associated death domain [Source:MGI Symbol;Acc:MGI:1914419]	4.20206	4.699	4.50986	4.62316	4.45542	3.91127	5.12206	4.28546	4.71279	3.7678	4.80985	4.84905	4.67898	4.73686	0.890648739	0.966018836	4	12	24	0.698926302	0.899909544	24	2.121913756	0.032665956
ENSMUSG00000032492	Pth1r	parathyroid hormone 1 receptor [Source:MGI Symbol;Acc:MGI:97801]	5.09114	4.07493	4.71592	4.05612	3.53037	4.47039	4.84519	4.83331	3.6978	6.12127	4.6288	4.66554	4.87296	5.02477	0.216058318	0.704989686	4	16	24	0.769698688	0.925855377	24	1.947344622	0.045481737
ENSMUSG00000032495	Lrrc2	leucine rich repeat containing 2 [Source:MGI Symbol;Acc:MGI:1921499]	0.475584	1.32429	2.23509	3.07268	1.39346	1.90129	1.01592	3.09551	2.06499	2.96062	1.31134	0.993216	1.78912	1.77965	0.053674542	0.394331071	16	12	24	0.018530252	0.559812298	24	9.740743199	0.424725349
ENSMUSG00000032496	Ltf	lactotransferrin [Source:MGI Symbol;Acc:MGI:96837]	14.1826	36.0471	52.6306	43.8876	35.168	34.08	36.6662	27.6391	30.2621	19.2859	25.3766	22.7889	33.0179	17.9192	0.896773941	0.966018836	12	20	24	0.682613376	0.89423365	24	10.99313759	0.168391931
ENSMUSG00000032497	Lrrfip2	leucine rich repeat (in FLII) interacting protein 2 [Source:MGI Symbol;Acc:MGI:1918518]	24.289	23.0823	22.4459	23.4729	23.0706	22.8314	22.6279	23.2047	21.853	22.5312	22.3821	23.6005	24.509	23.4137	0.101520755	0.511659635	4	8	24	0.103978363	0.715480277	24	22.97570673	0.034923717
ENSMUSG00000032498	Mlh1	mutL homolog 1 [Source:MGI Symbol;Acc:MGI:101938]	9.9911	9.0424	9.58363	8.85782	9.41855	8.96572	7.76432	8.09015	10.3631	8.88457	9.08042	9.44671	9.67817	9.71544	0.832853014	0.966018836	12	4	24	0.876845037	0.96580091	24	9.66460247	0.021617101
ENSMUSG00000032500	Dclk3	doublecortin-like kinase 3 [Source:MGI Symbol;Acc:MGI:3039580]	12.9507	11.2731	9.95836	10.4953	12.6318	11.8333	12.3612	12.2206	11.5009	14.1621	11.1523	13.4916	13.1422	12.1195	0.143321297	0.585944911	24	12	24	0.246551079	0.768040178	24	21.1290702	0.07831244
ENSMUSG00000032501	Trib1	tribbles pseudokinase 1 [Source:MGI Symbol;Acc:MGI:2443397]	2.26425	1.88352	1.80786	2.58023	2.1899	2.26436	1.82326	2.00693	2.14126	2.85965	1.89364	2.15241	1.96648	2.07836	0.17847698	0.655480352	16	16	24	0.165154512	0.767020009	24	13.67144474	0.091977795
ENSMUSG00000032502	Stac	src homology three (SH3) and cysteine rich domain [Source:MGI Symbol;Acc:MGI:1201400]	24.9743	16.4005	18.0437	15.7235	20.7808	22.7461	18.6033	19.578	13.7771	21.4977	22.9792	20.1067	21.328	19.7498	0.057264003	0.401309455	20	16	24	0.040749783	0.616921492	24	20.06673237	0.206224178
ENSMUSG00000032503	Arpp21	cyclic AMP-regulated phosphoprotein, 21 [Source:MGI Symbol;Acc:MGI:107562]	8.6087	8.15149	8.28714	7.94011	8.03494	7.32216	6.63444	8.5016	7.02351	8.61023	6.44974	7.51544	6.55283	6.23984	0.910436409	0.966018836	16	4	24	0.585313593	0.852794576	24	10.9626466	0.045473303
ENSMUSG00000032504	Pdcd6ip	programmed cell death 6 interacting protein [Source:MGI Symbol;Acc:MGI:1333753]	39.0107	34.0852	30.0864	32.9991	40.4346	35.5853	37.2313	38.2289	34.9139	37.877	33.9269	35.8041	38.3104	33.2446	0.158469451	0.614860056	4	8	24	0.211573024	0.768040178	24	20.78268722	0.078745662
ENSMUSG00000032507	Fbxl2	F-box and leucine-rich repeat protein 2 [Source:MGI Symbol;Acc:MGI:1919429]	37.3299	30.7786	28.9768	34.2064	30.9687	32.6718	27.9375	31.6698	27.3315	34.7493	29.4463	35.8946	35.823	31.1809	0.245061943	0.741580974	24	12	24	0.384161632	0.799546792	24	20.22719396	0.077348996
ENSMUSG00000032508	Myd88	myeloid differentiation primary response gene 88 [Source:MGI Symbol;Acc:MGI:108005]	6.55003	5.45237	5.23818	4.8119	5.67138	5.77188	5.53074	5.63031	5.00663	5.16087	5.94995	5.44692	5.65118	5.35485	0.007331665	0.192760771	20	20	24	0.012041575	0.525280095	24	21.69124113	0.095586393
ENSMUSG00000032511	Scn5a	sodium channel, voltage-gated, type V, alpha [Source:MGI Symbol;Acc:MGI:98251]	0.856466	0.703747	0.767571	0.977991	0.74819	0.882079	0.762013	1.02714	0.825693	1.16873	0.683396	0.983925	0.823674	0.897274	0.896799496	0.966018836	16	4	24	0.742815045	0.916215545	24	10.46034146	0.031773027
ENSMUSG00000032512	Wdr48	WD repeat domain 48 [Source:MGI Symbol;Acc:MGI:1914811]	27.7935	27.0076	28.5062	26.7981	26.7121	25.6574	25.5782	27.2814	27.4754	24.6516	28.4037	28.5855	28.5212	27.0112	0.906657897	0.966018836	12	4	24	0.835250631	0.949499418	24	1.646409727	0.015040359
ENSMUSG00000032513	Gorasp1	golgi reassembly stacking protein 1 [Source:MGI Symbol;Acc:MGI:1921748]	8.41318	7.29107	8.14005	7.45487	7.14895	8.46216	8.18023	8.92475	7.24635	9.13492	7.73601	8.65341	8.10475	8.13384	0.913451828	0.966018836	24	20	24	0.650368551	0.882429682	24	23.55135544	0.035925508
ENSMUSG00000032515	Csrnp1	cysteine-serine-rich nuclear protein 1 [Source:MGI Symbol;Acc:MGI:2387989]	1.64308	1.30876	1.5628	2.33852	1.28668	1.02232	1.08221	1.63158	1.15602	1.74809	1.10805	1.45433	1.69792	1.31015	0.814077113	0.966018836	16	4	24	0.520093233	0.819699799	24	9.678414225	0.091511639
ENSMUSG00000032517	Mobp	myelin-associated oligodendrocytic basic protein [Source:MGI Symbol;Acc:MGI:108511]	39.3445	115.134	179.187	73.3272	53.847	98.2964	115.36	78.3706	113.516	74.2308	44.8583	82.1118	37.5404	99.6122	0.256524739	0.76168256	12	8	24	0.184720571	0.768040178	24	7.090905529	0.462391855
ENSMUSG00000032518	Rpsa	ribosomal protein SA [Source:MGI Symbol;Acc:MGI:105381]	117.201	136.779	133.919	131.893	149.146	129.021	132.651	123.115	150.212	103.892	124.876	127.786	128.541	104.647	0.896799496	0.966018836	12	20	24	0.861003568	0.959154979	24	15.94074506	0.031763605
ENSMUSG00000032519	Slc25a38	solute carrier family 25, member 38 [Source:MGI Symbol;Acc:MGI:2384782]	20.7796	17.0516	17.53	18.2125	17.4292	20.4843	18.9458	18.2033	15.875	20.5531	18.5754	18.7281	19.1798	17.9379	0.030691182	0.324150946	4	8	24	0.113334966	0.727393008	24	20.11143526	0.08400211
ENSMUSG00000032523	Hhatl	hedgehog acyltransferase-like [Source:MGI Symbol;Acc:MGI:1922020]	7.01984	7.35253	8.0177	6.82961	6.42872	8.15628	8.19633	9.19951	7.30381	11.3388	6.36469	7.34762	7.17454	8.36012	0.066380001	0.42386701	8	12	24	0.424695111	0.805393068	24	6.82612865	0.102416809
ENSMUSG00000032525	Nktr	natural killer tumor recognition sequence [Source:MGI Symbol;Acc:MGI:97346]	38.8728	40.4598	40.2846	43.3151	39.3776	35.5947	42.4097	35.2915	45.3634	31.731	42.1592	36.0336	34.3603	37.2237	0.81872541	0.966018836	12	12	24	0.728746132	0.911275869	24	9.831323077	0.043597053
ENSMUSG00000032526	Ss18l2	SS18, nBAF chromatin remodeling complex subunit like 2 [Source:MGI Symbol;Acc:MGI:1349474]	68.5672	75.1216	70.4465	73.4739	76.3662	66.6831	65.57	70.7136	86.3244	63.6145	82.362	66.7517	70.4606	66.5948	0.593560247	0.966018836	20	4	24	0.400383233	0.799546792	24	11.1367639	0.067101679
ENSMUSG00000032527	Pccb	propionyl Coenzyme A carboxylase, beta polypeptide [Source:MGI Symbol;Acc:MGI:1914154]	25.1533	23.2921	25.8964	29.565	23.7704	26.0109	25.4445	26.6919	25.6198	30.7663	26.3154	27.724	29.0414	27.551	0.849179819	0.966018836	16	12	24	0.447148463	0.805393068	24	11.74378609	0.046102526
ENSMUSG00000032531	Amotl2	angiomotin-like 2 [Source:MGI Symbol;Acc:MGI:1929286]	12.0136	12.9503	12.5859	16.9253	13.5309	11.6355	14.2599	13.2728	14.2263	15.0853	11.6265	11.2506	11.2824	10.6859	0.10560184	0.518002936	16	8	24	0.063605968	0.663836692	24	11.08638983	0.13805693
ENSMUSG00000032532	Cck	cholecystokinin [Source:MGI Symbol;Acc:MGI:88297]	0.827211	2.14338	4.14743	1.13179	1.95679	2.70056	5.03041	1.83081	3.96419	2.62062	1.62302	2.99528	0.806903	3.18743	0.791413662	0.966018836	12	8	24	0.65951893	0.884879972	24	7.550268247	0.205292334
ENSMUSG00000032534	Cep63	centrosomal protein 63 [Source:MGI Symbol;Acc:MGI:2158560]	17.2111	17.3918	16.6153	19.8397	16.2507	14.3086	17.8681	18.721	20.3478	14.9398	19.071	17.4319	18.976	17.816	0.907000607	0.966018836	12	12	24	0.435168848	0.805393068	24	6.385064019	0.070413833
ENSMUSG00000032536	Trak1	trafficking protein, kinesin binding 1 [Source:MGI Symbol;Acc:MGI:1914345]	37.2541	29.431	30.0361	31.773	31.5976	32.9393	31.8095	32.6808	28.1066	47.715	31.2043	35.3649	34.904	34.2321	0.143321297	0.585944911	24	12	24	0.786808404	0.931447579	24	17.2877722	0.049559882
ENSMUSG00000032537	Ephb1	Eph receptor B1 [Source:MGI Symbol;Acc:MGI:1096337]	1.50334	2.16967	2.76497	1.63927	1.67369	2.3792	2.20373	1.69401	2.48548	1.90342	1.4786	2.32678	1.38089	2.09742	0.602156008	0.966018836	12	16	24	0.607645274	0.864031555	24	6.964903717	0.085434611
ENSMUSG00000032540	Abhd5	abhydrolase domain containing 5 [Source:MGI Symbol;Acc:MGI:1914719]	20.3168	18.8601	19.5933	22.0593	22.2524	19.2547	20.0952	22.4664	18.873	19.3133	21.9399	18.2059	21.5419	16.5785	0.850398351	0.966018836	20	12	24	0.446152768	0.805393068	24	16.27915827	0.059164101
ENSMUSG00000032547	Ryk	receptor-like tyrosine kinase [Source:MGI Symbol;Acc:MGI:101766]	14.4274	13.5002	12.9246	12.9795	14.2333	13.4827	15.2698	13.1813	14.376	14.2205	14.0726	12.8987	13.4661	12.1495	0.93717134	0.966018836	4	4	24	0.582081264	0.851114094	24	18.73086789	0.033466916
ENSMUSG00000032548	Slco2a1	solute carrier organic anion transporter family, member 2a1 [Source:MGI Symbol;Acc:MGI:1346021]	0.931767	1.04936	1.12574	0.866854	1.08432	1.11839	0.86188	1.23979	1.16807	0.916084	0.892214	0.939749	0.911488	0.918152	0.46238066	0.966018836	12	4	24	0.538274643	0.828537104	24	6.781155298	0.039363247
ENSMUSG00000032549	Rab6b	RAB6B, member RAS oncogene family [Source:MGI Symbol;Acc:MGI:107283]	183.912	127.32	135.136	146.37	135.369	170.485	150.353	159.138	106.983	206.111	134.061	156.242	149.058	156.568	0.648082424	0.966018836	24	12	24	0.549694967	0.835052125	24	20.41845038	0.1051744
ENSMUSG00000032551	1110059G10Rik	RIKEN cDNA 1110059G10 gene [Source:MGI Symbol;Acc:MGI:1913452]	13.5691	15.5382	15.2483	15.8126	13.91	14.4396	15.8828	14.9699	15.1447	14.3148	15.9538	13.0035	14.2245	16.3121	0.628286596	0.966018836	8	16	24	0.164876998	0.767020009	24	7.55811347	0.06828318
ENSMUSG00000032553	Srprb	signal recognition particle receptor, B subunit [Source:MGI Symbol;Acc:MGI:102964]	13.7282	11.2973	12.1326	12.3063	12.62	13.181	13.9387	13.0388	11.2179	12.7396	11.7872	12.6897	12.8716	12.5311	0.004501001	0.140598674	4	8	24	0.061990847	0.661961741	24	21.79006721	0.075065306

ENSMUSG00000032554	Trf	transferrin [Source:MGI Symbol;Acc:MGI:98821]	76.4066	123.668	150.879	93.2133	105.039	111.695	134.776	110.883	140.331	71.192	92.6583	101.918	86.1621	105.79	0.129903614	0.560263689	12	4	24	0.394504164	0.799546792	24	5.57218881	0.16883389
ENSMUSG00000032555	Topbp1	topoisomerase (DNA) II binding protein 1 [Source:MGI Symbol;Acc:MGI:1920018]	6.62409	6.11227	6.50129	6.04852	6.81278	7.12773	5.90473	6.00723	6.69214	6.46831	7.92507	7.22685	6.6185	6.28193	0.014841078	0.250870466	24	8	24	0.026534141	0.586541342	24	17.15552696	0.093740139
ENSMUSG00000032556	Bfsp2	beaded filament structural protein 2, phakinin [Source:MGI Symbol;Acc:MGI:1333828]	4.24316	3.87881	3.9552	4.61678	5.06209	3.986	3.25792	3.89757	6.01452	3.45018	4.16206	3.21022	3.96845	3.28207	0.46694635	0.966018836	12	20	24	0.316435586	0.768040178	24	11.70450597	0.114887649
ENSMUSG00000032557	Uba5	ubiquitin-like modifier activating enzyme 5 [Source:MGI Symbol;Acc:MGI:1913913]	26.2978	24.2725	24.7667	23.6085	25.811	23.8788	26.8851	24.5108	25.4598	22.415	27.8104	25.4819	26.6324	25.2658	0.064091035	0.414867207	4	12	24	0.227376808	0.768040178	24	22.0108279	0.052780638
ENSMUSG00000032558	Nphp3	nephronophthisis 3 (adolescent) [Source:MGI Symbol;Acc:MGI:1921275]	4.105	4.59326	3.9331	3.27555	4.52688	3.40709	4.65913	3.95924	5.90942	3.68044	4.13284	4.25783	5.2981	5.18331	0.359479536	0.883787544	4	12	24	0.164200416	0.767020009	24	3.748760287	0.127977315
ENSMUSG00000032560	Dnajc13	DnaJ heat shock protein family (Hsp40) member C13 [Source:MGI Symbol;Acc:MGI:2676368]	29.6798	24.5211	28.3899	24.9553	27.0873	28.2847	25.4978	22.4159	24.5136	26.6253	24.5147	23.4787	27.0945	27.8889	0.897425765	0.966018836	4	4	24	0.829564322	0.947008621	24	21.23084087	0.028122604
ENSMUSG00000032561	Acpp	acid phosphatase, prostate [Source:MGI Symbol;Acc:MGI:1928480]	130.617	106.227	99.0397	110.805	98.2636	119.102	100.232	107.044	91.6949	125.459	107.316	121.728	129.819	120.773	0.286642595	0.793714531	4	8	24	0.294809152	0.768040178	24	21.73442256	0.096924777
ENSMUSG00000032562	Gnai2	guanine nucleotide binding protein (G protein), alpha inhibiting 2 [Source:MGI Symbol;Acc:MGI:95772]	94.9193	88.1853	91.33	91.1374	92.3693	90.6796	93.7961	98.1411	93.1985	106.234	93.4079	99.7873	96.9787	89.0973	0.92721845	0.966018836	4	4	24	0.757828869	0.921723259	24	15.56934841	0.019590536
ENSMUSG00000032563	Mrpl3	mitochondrial ribosomal protein L3 [Source:MGI Symbol;Acc:MGI:2137204]	27.9246	27.8049	30.1044	30.3627	29.6736	29.3908	30.4877	28.958	28.2083	30.8786	29.2557	31.4749	28.4133	30.1151	0.858269825	0.966018836	16	16	24	0.402410694	0.799546792	24	14.36752782	0.026381685
ENSMUSG00000032564	Cpne4	copine IV [Source:MGI Symbol;Acc:MGI:1921270]	26.0512	19.1447	19.7163	21.3084	18.1939	23.6276	20.3659	22.2095	14.0795	26.359	20.8214	20.5984	21.1013	21.4512	0.956178643	0.96629412	24	12	24	0.508528268	0.813517676	24	20.60281112	0.101537148
ENSMUSG00000032565	Nudt16	nudix (nucleoside diphosphate linked moiety X)-type motif 16 [Source:MGI Symbol;Acc:MGI:1922936]	9.97673	10.3007	10.0583	9.4851	9.11875	9.64982	9.31955	11.3277	10.2621	10.9977	9.38663	10.7809	11.9076	11.4069	0.036134798	0.331852526	8	12	24	0.026919992	0.586541342	24	4.340322387	0.086311701
ENSMUSG00000032567	Aste1	asteroid homolog 1 [Source:MGI Symbol;Acc:MGI:1913845]	3.72313	4.29856	3.48613	4.05539	3.84968	3.79246	3.94437	4.06934	4.88889	3.31415	4.881	4.38292	4.33119	4.43703	0.860993221	0.966018836	8	8	24	0.897658572	0.970873489	24	3.008934926	0.021844605
ENSMUSG00000032570	Atp2c1	ATPase, Ca++-sequestering [Source:MGI Symbol;Acc:MGI:1889008]	59.4332	58.8187	60.0369	57.4812	58.0306	58.3438	60.2072	55.7657	55.525	53.9972	56.4421	60.8826	58.1433	61.5221	0.191453468	0.669841558	4	12	24	0.118242047	0.728808483	24	23.6557116	0.041232028
ENSMUSG00000032571	Pik3r4	phosphoinositide-3-kinase regulatory subunit 4 [Source:MGI Symbol;Acc:MGI:1922919]	10.7207	9.45618	10.304	10.342	9.8015	11.6386	10.2887	10.9789	8.80446	11.8919	9.54226	11.0103	9.83721	10.1902	0.725061903	0.966018836	24	12	24	0.596893339	0.858175411	24	19.15153537	0.045691365
ENSMUSG00000032575	Manf	mesencephalic astrocyte-derived neurotrophic factor [Source:MGI Symbol;Acc:MGI:1922090]	91.0414	54.7714	47.0954	52.16	64.8983	59.061	71.8018	54.3688	54.924	51.3359	88.0095	65.1303	78.9085	63.8904	0.00455947	0.140598674	4	12	24	0.026048502	0.586541342	24	21.13791472	0.279279682
ENSMUSG00000032577	Mapkapk3	mitogen-activated protein kinase-activated protein kinase 3 [Source:MGI Symbol;Acc:MGI:2143163]	1.96699	3.03225	2.76311	4.59504	2.99804	3.12588	3.57946	2.81498	2.93044	2.99386	2.78786	2.5702	3.09884	2.32376	0.908406451	0.966018836	16	16	24	0.406988127	0.799546792	24	12.94435069	0.10829345
ENSMUSG00000032578	Cish	cytokine inducible SH2-containing protein [Source:MGI Symbol;Acc:MGI:103159]	2.54777	2.067	3.28662	3.03564	2.21416	2.82334	3.23055	3.06605	2.48217	2.68493	2.77774	1.73147	2.30858	2.98259	0.916746631	0.966018836	12	4	24	0.452805872	0.805393068	24	7.763874	0.09710017
ENSMUSG00000032579	Hemk1	HemK methyltransferase family member 1 [Source:MGI Symbol;Acc:MGI:1916786]	9.60995	9.58543	9.03761	9.95609	11.1295	11.9221	9.71789	10.1089	10.4992	9.21487	9.72455	8.26075	10.9633	11.808	0.844448841	0.966018836	20	20	24	0.894371282	0.970031575	24	0.662842755	0.025464601
ENSMUSG00000032580	Rbm5	RNA binding motif protein 5 [Source:MGI Symbol;Acc:MGI:1933204]	47.0444	49.7349	50.5584	53.0243	51.1216	46.779	51.1471	46.2412	57.9332	48.4144	53.0313	51.3818	50.7688	51.8568	0.697196105	0.966018836	12	20	24	0.253704073	0.768040178	24	10.69185236	0.051888469
ENSMUSG00000032582	Rbm6	RNA binding motif protein 6 [Source:MGI Symbol;Acc:MGI:1338037]	21.1381	18.8568	20.2979	19.3009	19.8846	18.93	19.0527	16.7217	20.8013	17.3453	21.6285	17.743	18.1235	17.3286	0.305564675	0.811201458	20	12	24	0.464006539	0.805393068	24	15.55064362	0.047449264
ENSMUSG00000032583	Mon1a	MON1 homolog A, secretory traffciking associated [Source:MGI Symbol;Acc:MGI:1920075]	10.6135	8.9766	7.58347	9.36103	8.20343	9.61784	9.1602	9.82633	7.66809	9.94867	10.8518	10.4764	10.5849	10.2655	0.118446606	0.541945448	4	8	24	0.11918343	0.730142553	24	21.25134148	0.110166706
ENSMUSG00000032584	Mst1r	macrophage stimulating 1 receptor (c-met-related tyrosine kinase) [Source:MGI Symbol;Acc:MGI:99614]	2.07463	1.93173	1.35343	1.56063	1.28603	1.68224	2.31722	1.75803	1.28777	1.59367	1.31697	1.23046	1.22775	1.36628	0.595952838	0.966018836	4	16	24	0.126564054	0.739868076	24	0.734708252	0.116394618
ENSMUSG00000032586	Traip	TRAF-interacting protein [Source:MGI Symbol;Acc:MGI:1096377]	1.59502	1.70388	1.61462	1.51316	1.70657	1.71118	1.48741	1.2411	1.62158	1.38564	1.36794	1.39213	1.68969	1.31955	0.887862353	0.966018836	24	8	24	0.702828651	0.901733679	24	19.99863214	0.028141442
ENSMUSG00000032589	Bsn	bassoon [Source:MGI Symbol;Acc:MGI:1277955]	8.31972	6.83503	6.59167	7.16647	7.42846	8.69622	7.84147	8.51079	5.8345	11.9746	7.80628	8.8945	8.3278	8.59418	0.047649229	0.377556215	24	12	24	0.547433475	0.833664411	24	18.98764798	0.090999303
ENSMUSG00000032590	Apeh	acylpeptide hydrolase [Source:MGI Symbol;Acc:MGI:88041]	20.1486	16.9224	17.7619	18.5205	22.2378	20.6337	17.7015	16.9917	15.9791	19.6246	18.7937	17.2767	18.5249	16.2909	0.008961721	0.192760771	20	12	24	0.004246162	0.427431763	24	17.5308154	0.134557392
ENSMUSG00000032593	Amigo3	adhesion molecule with Ig like domain 3 [Source:MGI Symbol;Acc:MGI:2444854]	19.1292	13.4723	13.9583	15.2746	12.9141	17.0538	14.7631	17.0898	12.448	21.0257	12.9717	16.4387	15.4267	15.3617	0.46694635	0.966018836	24	12	24	0.783065825	0.930229965	24	22.04986212	0.060256541
ENSMUSG00000032594	Ip6k1	inositol hexaphosphate kinase 1 [Source:MGI Symbol;Acc:MGI:1351633]	50.9356	47.7452	58.8267	51.5847	45.2597	50.3991	50.1781	47.0347	44.8191	54.4038	43.7958	49.2269	46.7934	47.4228	0.890235289	0.966018836	16	4	24	0.779207729	0.928978483	24	9.036382194	0.03069202
ENSMUSG00000032595	Cdhr4	cadherin-related family member 4 [Source:MGI Symbol;Acc:MGI:1916648]	0.847765	0.982085	0.26541	1.40261	0.64023	0.773171	1.56808	0.868243	0.415768	0.623372	0.373949	0.56808	0.496051	0.671249	0.495929335	0.966018836	8	4	24	0.569625298	0.844900526	24	23.44087951	0.115694501
ENSMUSG00000032596	Uba7	ubiquitin-like modifier activating enzyme 7 [Source:MGI Symbol;Acc:MGI:1349462]	8.07051	10.8546	9.46027	5.92241	9.47226	9.42789	8.17798	11.4503	10.335	7.49249	11.9176	8.2242	9.82683	9.48662	0.471214947	0.966018836	8	8	24	0.596104317	0.857839743	24	2.976642257	0.093654224
ENSMUSG00000032598	Nckipsd	NCK interacting protein with SH3 domain [Source:MGI Symbol;Acc:MGI:1931834]	10.6225	8.9909	9.59095	11.0587	9.40678	10.5396	10.6215	10.3117	8.23896	12.2025	9.52388	10.1809	8.71957	10.1053	0.959160181	0.967043964	16	20	24	0.655185068	0.883697438	24	16.22475387	0.049415851
ENSMUSG00000032599	Ip6k2	inositol hexaphosphate kinase 2 [Source:MGI Symbol;Acc:MGI:1923750]	44.1823	45.1522	49.4153	56.6441	51.6626	49.273	43.5227	39.9671	49.1898	50.8235	47.3312	49.8794	43.5984	45.9721	7.56E-04	0.071344007	16	12	24	0.001498518	0.358147394	24	13.69536484	0.138707198
ENSMUSG00000032601	Prkar2a	protein kinase, cAMP dependent regulatory, type II alpha [Source:MGI Symbol;Acc:MGI:108025]	66.2653	45.5998	52.7797	53.4951	45.9881	56.4576	50.0809	53.6934	40.5354	62.3702	47.9447	56.3212	51.9765	53.9637	0.92655264	0.966018836	24	12	24	0.60050941	0.860118739	24	21.08193309	0.075281551
ENSMUSG00000032602	Slc25a20	solute carrier family 25 (mitochondrial carnitine/acylcarnitine translocase), member 20 [Source:MGI Symbol;Acc:MGI:1928738]	15.7657	15.4041	19.3211	28.6814	16.1547	17.0269	18.1904	20.8944	17.1155	21.6475	21.9827	18.3086	22.3278	22.2855	0.928127947	0.966018836	16	8	24	0.252179377	0.768040178	24	10.86123406	0.141444314
ENSMUSG00000032604	Qars	glutaminyl-tRNA synthetase [Source:MGI Symbol;Acc:MGI:1915851]	27.7651	31.4915	28.8924	27.378	30.5961	28.5238	26.2573	29.9536	28.8595	29.9815	33.3875	35.0634	30.0756	34.0204	0.870491976	0.966018836	20	8	24	0.932150265	0.982590152	24	16.18414085	0.014583684
ENSMUSG00000032606	Nicn1	nicolin 1 [Source:MGI Symbol;Acc:MGI:1913507]	47.0262	38.9917	43.8535	39.7258	39.7361	47.2229	44.2293	43.5509	32.3077	46.69	40.1377	42.9366	43.5619	43.1865	0.27909437	0.790764844	4	8	24	0.239960012	0.768040178	24	21.64239564	0.093550375
ENSMUSG00000032607	Amt	aminomethyltransferase [Source:MGI Symbol;Acc:MGI:3646700]	7.98591	5.81363	6.03313	7.65398	7.60934	7.38619	7.21258	6.27577	6.98859	5.72457	7.50142	6.70815	6.13179	5.43881	0.101443845	0.511659635	20	12	24	0.022402738	0.578704263	24	18.04068434	0.142882869
ENSMUSG00000032609	Klhdc8b	kelch domain containing 8B [Source:MGI Symbol;Acc:MGI:1925517]	22.3316	22.0395	21.398	20.7541	22.3171	21.6702	22.4082	23.1329	25.1746	24.0502	23.0209	25.5135	23.304	22.8183	0.759203569	0.966018836	4	12	24	0.992711258	0.998516607	24	4.108967564	0.003045095
ENSMUSG00000032612	Usp4	ubiquitin specific peptidase 4 (proto-oncogene) [Source:MGI Symbol;Acc:MGI:98905]	24.2857	24.9795	25.5555	27.1641	26.7746	26.1593	22.9058	23.9169	26.4122	25.4873	25.4522	26.3098	24.0502	23.9616	0.011168445	0.204559658	16	16	24	0.003384772	0.417612737	24	13.88314905	0.074018856
ENSMUSG00000032615	Nt5m	5',3'-nucleotidase, mitochondrial [Source:MGI Symbol;Acc:MGI:1917127]	17.3737	15.9313	15.1366	15.1982	15.5459	17.8926	18.465	17.2261	14.483	19.5996	15.6924	17.5776	16.379	16.4575	0.010049466	0.194720062	24	12	24	0.296545709	0.768040178	24	21.51001261	0.071748686
ENSMUSG00000032621	Srek1	splicing regulatory glutamine/lysine-rich protein 1 [Source:MGI Symbol;Acc:MGI:2145245]	21.1781	24.663	25.4102	23.7449	26.2863	21.5841	23.0368	20.1193	26.7712	20.8283	28.5163	23.3799	23.9897	23.3064	0.368981219	0.893596186	20	8	24	0.399641476	0.799546792	24	12.9340798	0.073271682
ENSMUSG00000032624	Eml4	echinoderm microtubule associated protein like 4 [Source:MGI Symbol;Acc:MGI:1926048]	8.20093	8.34027	7.84253	8.36815	8.54571	8.42852	8.5195	7.34143	7.93134	8.27476	8.02549	8.46659	8.12098	8.25206	0.123394836	0.552144108	24	12	24	0.117477941	0.728323355	24	18.50801919	0.04149254
ENSMUSG00000032625	Thsd7a	thrombospondin, type I, domain containing 7A [Source:MGI Symbol;Acc:MGI:2685683]	9.86636	9.0663	9.43202	10.5906	9.21465	9.88827	9.06484	8.05321	7.91134	8.3877	10.1466	9.02136	8.78678	9.5629	0.800065124	0.966018836	20	16	24	0.239349655	0.768040178	24	17.81481712	0.070073959
ENSMUSG00000032633	Flcn	folliculin [Source:MGI Symbol;Acc:MGI:2442184]	11.729	10.5093	11.8664	14.8501	11.9888	11.2894	11.8972	11.8722	12.8208	12.8966	11.0443	10.8996	11.5159	11.29	0.062036003	0.409814092	16	8	24	0.069012574	0.673520298	24	11.22417232	0.096250231
ENSMUSG00000032637	Atxn2l	ataxin 2-like [Source:MGI Symbol;Acc:MGI:2446242]	28.8079	29.871	28.9516	27.5505	29.3312	28.6811	31.5098	29.8557	32.2887	33.296	30.3691	31.7658	31.2392	30.7265	0.706093972	0.966018836	4	20	24	0.783712302	0.93037654	24	6.492962739	0.016087091
ENSMUSG00000032640	Chsy1	chondroitin sulfate synthase 1 [Source:MGI Symbol;Acc:MGI:2681120]	3.86405	3.76986	3.50882	3.85959	3.63433	3.95746	3.65276	3.73322	3.13317	3.83393	3.45528	3.43782	3.09963	3.72755	0.882263435	0.966018836	16	20	24	0.862333912	0.959779249	24	18.35787686	0.016831298
ENSMUSG00000032641	Gpr19	G protein-coupled receptor 19 [Source:MGI Symbol;Acc:MGI:892973]	6.36507	7.39896	8.21275	7.15511	7.50061	7.57024	6.99591	7.80823	6.20701	7.45899	7.28849	7.06085	7.01142	6.92696	0.627735557	0.966018836	20	8	24	0.715635275	0.906323093	24	12.70132517	0.030100187
ENSMUSG00000032643	Fhl3	four and a half LIM domains 3 [Source:MGI Symbol;Acc:MGI:1341092]	4.72662	6.31459	6.16815	6.26422	7.52231	5.46453	6.45223	10.0519	8.40042	9.88885	6.33328	5.72861	8.34032	5.58432	0.834434272	0.966018836	16	8	24	0.236234863	0.768040178	24	9.266886523	0.183477578
ENSMUSG00000032648	Pygm	muscle glycogen phosphorylase [Source:MGI Symbol;Acc:MGI:97830]	19.8278	47.8348	56.2054	69.9157	46.1923	64.3972	42.0297	127.701	84.1372	122.237	42.9764	57.4353	57.0076	45.3445	0.192357053	0.669841558	16	12	24	0.106236263	0.71810271	24	9.882795605	0.507491465
ENSMUSG00000032649	Colgalt2	collagen beta(1-O)galactosyltransferase 2 [Source:MGI Symbol;Acc:MGI:2138232]	12.693	12.0154	11.0777	12.0427	11.2849	12.1711	11.1481	12.2264	10.773	13.2452	11.6217	13.1744	13.3208	13.3305	0.43280837	0.966018836	8	4	24	0.526468655	0.823075573	24	23.10212453	0.03999764
ENSMUSG00000032652	Crebl2	cAMP responsive element binding protein-like 2 [Source:MGI Symbol;Acc:MGI:1889385]	26.7876	26.8229	25.3489	25.9956	27.0174	25.3343	27.7379	23.5277	27.7229	24.4644	26.6383	26.5978	25.6501	24.7657	0.914502381	0.966018836	4	4	24	0.719850706	0.834817817	24	19.18065493	0.02943528
ENSMUSG00000032656	Marchf3	membrane associated ring-CH-type finger 3 [Source:MGI Symbol;Acc:MGI:2443667]	2.2798	2.77088	2.76139	3.13119	2.45857	2.64412	3.10632	2.38603	2.57065	2.42759	2.80157	2.38757	2.783	3.33903	0.809772024	0.966018836	8	16	24	0.658200501	0.884596426	24	6.400942773	0.045099914
ENSMUSG00000032657	Fam189b	family with sequence similarity 189, member B [Source:MGI Symbol;Acc:MGI:1915771]	121.21	90.8395	91.2538	101.495	99.4605	112.178	99.1239	117.65	75.2376	144.043	103.474	108.192	107.129	96.2238	0.138202217	0.580158868	24	12	24	0.447760846	0.805393068	24	18.82786011	0.112939433
ENSMUSG00000032666	1700025G04Rik	RIKEN cDNA 1700025G04 gene [Source:MGI Symbol;Acc:MGI:1916649]	25.2456	24.7542	21.9237	23.2	25.4201	25.2128	23.8074	24.4585	22.351	23.8372	26.4266	24.6232	25.7657	24.6203	0.01142276	0.206107348	20	16	24	0.019183765	0.562219749	24	20.39299232	0.071926799
ENSMUSG00000032667	Pon2	paraoxonase 2 [Source:MGI Symbol;Acc:MGI:106687]	16.6171	18.9472	19.0824	17.0681	18.3552	17.7256	19.9219	17.3594	18.3267	16.8686	18.3647	18.8846	18.4053	18.5499	0.8956591	0.966018836	12	4	24	0.666860423	0.887459929	24	1.300796511	0.024876244
ENSMUSG00000032673	Prorsd1	prolyl-tRNA synthetase domain containing 1 [Source:MGI Symbol;Acc:MGI:1915189]	7.81093	9.85636	8.64334	9.56962	8.75875	8.0683	9.02501	9.9675	11.1429	7.87481	10.3902	9.97513	10.9687	9.20477	0.831272156	0.966018836	12	4	24	0.635002911	0.875919107	24	6.125675231	0.05277195
ENSMUSG00000032679	Cd59a	CD59a antigen [Source:MGI Symbol;Acc:MGI:109177]	81.5303	82.4402	74.6725	81.0293	86.1979	74.7472	83.016	79.8501	87.2018	77.7656	89.8395	87.7001	90.39	81.3835	0.472013662	0.966018836	20	20	24	0.643286203	0.878809617	24	19.69146353	0.030604135
ENSMUSG00000032680	6820408C15Rik	RIKEN cDNA 6820408C15 gene [Source:MGI Symbol;Acc:MGI:3045333]	0.41692	0.755366	0.538713	0.870279	0.529702	0.522354	0.824732	0.561492	0.517036	0.61958	0.44937	0.662444	0.484834	0.696015	0.951846915	0.966018836	8	12	24	0.771295873	0.926252557	24	7.295779966	0.037288287
ENSMUSG00000032688	Malt1	MALT1 paracaspase [Source:MGI Symbol;Acc:MGI:2445027]	3.3265	4.46421	4.59281	3.72071	3.7778	2.97423	3.86691	3.52718	5.11397	3.09972	4.11536	3.64955	3.8429	3.94852	0.402347439	0.278499043	12	12	24	0.135598495	0.747707672	24	7.380077631	0.132104995
ENSMUSG00000032690	Oas2	2'-5' oligoadenylate synthetase 2 [Source:MGI Symbol;Acc:MGI:2180852]	1.46876	0.865876	0.839483	0.639227	1.15277	0.698175	1.0285	0.991216	0.871131	0.547484	1.11042	0.768056	0.709705	0.716593	0.581079034	0.966018836	4	12	24	0.333648089	0.76977941	24	22.14369645	0.097099603
ENSMUSG00000032698	Lmo2	LIM domain only 2 [Source:MGI Symbol;Acc:MGI:102811]	6.99405	8.93149	9.06543	7.517	6.77256	6.96244	8.17249	8.79715	8.3522	5.96164	8.36492	7.85444	8.71974	8.77235	0.016273603	0.253395174	8	8	24	0.063498719	0.663715314	24	4.232474161	0.133583312
ENSMUSG00000032702	Kank1	KN motif and ankyrin repeat domains 1 [Source:MGI Symbol;Acc:MGI:2147707]	5.22304	4.98673	4.53598	4.77517	5.49643	5.76163	6.6454	5.86386	4.79658	5.51152	4.87783	5.87481	6.15633	4.75985	0.008389425	0.192760771	4	8	24	0.029136103	0.596648309	24	21.71752451	0.130662223
ENSMUSG00000032705	Exd2	exonuclease 3'-5' domain containing 2 [Source:MGI Symbol;Acc:MGI:1922485]	21.6227	17.7394	17.9069	16.7841	21.076	19.8651	20.8467	17.4015	19.3498	17.1924	20.6132	18.413	18.9378	16.8845	0.108253011	0.524035616	20	20	24	0.035893499	0.609176161	24	20.00187811	0.111857752
ENSMUSG00000032712	Resf1	retroelement silencing factor 1 [Source:MGI Symbol;Acc:MGI:1914496]	4.0911	4.77979	4.90942	6.93396	5.67888	4.54735	5.20772	4.90485	6.24583	4.28427	5.63069	4.18083	4.76756	5.0633	0.175808942	0.651752074	20	4	24	0.137861394	0.747972761	24	11.10939008	0.138149556
ENSMUSG00000032714	Syde1	synapse defective 1, Rho GTPase, homolog 1 (C. elegans) [Source:MGI Symbol;Acc:MGI:1918959]	5.73612	6.76333	6.57452	5.97875	6.83226	6.29693	6.78175	6.8779	7.17199	7.41703	6.26586	6.94809	6.74735	5.96203	0.896311169	0.966018836	12	16	24	0.665348855	0.886947643	24	10.18370716	0.03346284
ENSMUSG00000032715	Trib3	tribbles pseudokinase 3 [Source:MGI Symbol;Acc:MGI:1345675]	1.6159	1.38481	1.06435	0.93129	1.84907	1.40745	1.35121	1.29885	1.30969	1.33711	2.19909	1.17197	1.41218	1.18156	0.080511813	0.4593055	20	20	24	0.204191668	0.768040178	24	18.60789631	0.129214246
ENSMUSG00000032717	Mdfi	MyoD family inhibitor [Source:MGI Symbol;Acc:MGI:107687]	0.561954	0.687891	0.530416	0.56663	0.673181	0.523515	0.737419	0.837081	0.981294	0.655899	0.935055	0.846757	0.985393	0.722297	0.854540463	0.966018836	4	12	24	0.796267457	0.935424343	24	3.478432801	0.02613842
ENSMUSG00000032718	Mansc1	MANSC domain containing 1 [Source:MGI Symbol;Acc:MGI:1914979]	2.84482	2.59568	2.66776	2.26993	2.72092	3.07525	3.01818	3.29639	2.48652	2.55775	3.02536	3.20305	2.69663	2.90058	0.542718076	0.966018836	12	12	24	0.600393117	0.860118739	24	22.57495138	0.056024813
ENSMUSG00000032719	Sbspon	somatomedin B and thrombospondin, type 1 domain containing [Source:MGI Symbol;Acc:MGI:2684952]	13.019	11.1155	10.2782	10.1309	15.2464	10.7771	13.0344	10.9717	11.8145	9.36773	12.9458	11.7694	11.8302	8.61253	0.083354723	0.462922111	20	20	24	0.087231504	0.707101738	24	19.46666131	0.161149175
ENSMUSG00000032724	Abtb2	ankyrin repeat and BTB (POZ) domain containing 2 [Source:MGI Symbol;Acc:MGI:2139365]	10.9104	8.42368	8.04968	9.45325	9.12127	10.1185	9.32816	10.7604	7.6069	13.4128	9.03714	10.7256	10.3771	10.0027	0.217827365	0.704989686	24	12	24	0.612099183	0.865382448	24	19.73261809	0.074748198
ENSMUSG00000032725	Folr2	folate receptor 2 (fetal) [Source:MGI Symbol;Acc:MGI:95569]	2.13076	2.03846	2.46722	1.73543	1.85695	1.63555	1.77408	2.46924	3.37815	1.53339	1.84843	2.22138	1.96864	1.66645	0.187699199	0.662239322	12	4	24	0.286333485	0.768040178	24	6.216829715	0.130452652
ENSMUSG00000032727	Mier3	MIER family member 3 [Source:MGI Symbol;Acc:MGI:2442317]	5.53611	6.03328	6.08946	6.65464	6.0786	5.45392	6.2887	5.53093	5.9609	5.35758	6.50113	5.15535	5.19445	5.48439	0.43280837	0.966018836	20	4	24	0.225319234	0.768040178	24	11.97977773	0.060556109
ENSMUSG00000032733	Snx33	sorting nexin 33 [Source:MGI Symbol;Acc:MGI:2443239]	1.91125	2.04266	2.13919	2.20786	2.08411	2.10435	2.05223	2.38958	2.6556	1.902	1.95444	2.11068	2.04754	2.23947	0.455507782	0.966018836	12	16	24	0.1320881	0.742323615	24	7.15465128	0.068451839
ENSMUSG00000032735	Ablim3	actin binding LIM protein family, member 3 [Source:MGI Symbol;Acc:MGI:2442582]	9.37985	6.94717	7.0616	8.60503	8.19927	9.16845	8.62445	8.54698	6.341	11.1136	7.04914	8.14311	8.48863	8.31434	0.496745696	0.966018836	4	8	24	0.565704526	0.843471947	24	19.29400046	0.081866033
ENSMUSG00000032737	Inppl1	inositol polyphosphate phosphatase-like 1 [Source:MGI Symbol;Acc:MGI:1333787]	10.8437	8.85986	9.11639	11.0265	10.3246	9.24441	10.4855	11.1695	10.1118	12.0809	9.81546	8.83646	10.1233	9.10978	0.850398351	0.966018836	16	8	24	0.619430989	0.869468249	24	11.92036573	0.049481434
ENSMUSG00000032739	Pram1	PML-RAR alpha-regulated adaptor molecule 1 [Source:MGI Symbol;Acc:MGI:3576625]	1.34598	1.61385	2.27561	1.68747	1.62917	1.78397	1.87658	1.45708	1.92458	1.41863	1.49572	1.49679	1.86962	1.16064	0.937421962	0.966018836	12	20	24	0.889959422	0.968974255	24	10.02571296	0.029326465
ENSMUSG00000032740	Ccdc88a	coiled coil domain containing 88A [Source:MGI Symbol;Acc:MGI:1925177]	8.8001	11.2898	10.4343	9.64524	11.2595	9.58893	10.4807	9.00372	10.4896	7.77457	10.0174	9.96197	8.48915	10.7609	0.72778087	0.966018836	8	20	24	0.961616897	0.990214752	24	6.072149451	0.016415551
ENSMUSG00000032741	Tpcn1	two pore channel 1 [Source:MGI Symbol;Acc:MGI:2182472]	11.0876	9.93403	10.418	10.5196	10.5588	10.5648	10.7004	11.2479	10.8021	12.6587	9.9889	9.85888	10.5029	11.0924	0.796193867	0.966018836	16	4	24	0.483636875	0.805393068	24	8.575774779	0.041308408
ENSMUSG00000032743	Katnip	katanin interacting protein [Source:MGI Symbol;Acc:MGI:2442760]	4.75281	4.01832	4.1619	4.38602	4.7412	4.14727	4.49369	4.33741	4.18202	4.47452	4.08675	4.52851	4.23053	4.16337	0.63897419	0.966018836	4	4	24	0.328324904	0.768040178	24	18.77146962	0.036988197
ENSMUSG00000032744	Heyl	hairy/enhancer-of-split related with YRPW motif-like [Source:MGI Symbol;Acc:MGI:1860511]	1.89887	2.3039	1.69317	1.62959	1.77662	2.06898	1.97971	2.37561	1.59212	2.08785	1.67574	2.20687	1.89056	2.02418	0.076593464	0.453790923	8	4	24	0.15179438	0.758865539	24	0.653831727	0.088423957
ENSMUSG00000032745	Gpbp1	GC-rich promoter binding protein 1 [Source:MGI Symbol;Acc:MGI:1920524]	36.7283	38.2894	36.6553	35.7764	38.555	34.5227	35.5139	33.1865	39.9929	31.2735	38.8421	36.359	38.9858	37.8378	0.912686312	0.966018836	12	12	24	0.925284887	0.980131499	24	1.270279105	0.01513286
ENSMUSG00000032750	Gab3	growth factor receptor bound protein 2-associated protein 3 [Source:MGI Symbol;Acc:MGI:2387324]	0.629894	0.588474	0.791023	0.691629	0.635085	0.615019	0.465113	0.720846	0.562806	0.664924	0.896088	0.645479	0.514953	0.621759	0.092596422	0.487919468	16	12	24	0.13408595	0.744355486	24	12.75206148	0.069651297
ENSMUSG00000032754	Slc8b1	solute carrier family 8 (sodium/lithium/calcium exchanger), member B1 [Source:MGI Symbol;Acc:MGI:2180781]	6.36779	5.84065	5.86779	6.6414	6.41199	6.25427	7.59468	6.72952	6.52621	6.28739	6.42452	6.64811	6.43072	5.96659	0.896545154	0.966018836	4	4	24	0.505133657	0.811334839	24	20.46375825	0.039534049
ENSMUSG00000032757	Bet1	Bet1 golgi vesicular membrane trafficking protein [Source:MGI Symbol;Acc:MGI:1343104]	25.4703	26.5796	28.7667	25.7742	28.33	24.0628	26.3495	27.5496	27.2332	22.752	29.8081	24.3455	23.2332	27.8898	0.220555749	0.710961211	12	12	24	0.570162764	0.845047627	24	8.862359738	0.049453844
ENSMUSG00000032763	Ilvbl	ilvB (bacterial acetolactate synthase)-like [Source:MGI Symbol;Acc:MGI:1351911]	18.6858	19.1825	20.1539	17.2408	17.9174	19.1242	19.9922	20.5081	18.2862	17.9469	19.0801	17.7777	17.823	18.2913	0.313239838	0.824470524	8	8	24	0.25481055	0.768040178	24	2.938859937	0.043566446
ENSMUSG00000032766	Gng11	guanine nucleotide binding protein (G protein), gamma 11 [Source:MGI Symbol;Acc:MGI:1913316]	18.8272	26.7476	23.1595	18.0844	24.0347	20.0174	23.5426	24.0517	29.8739	15.8793	23.5291	26.4039	21.7618	25.2798	0.184631358	0.662239322	8	8	24	0.443432737	0.805393068	24	3.747509521	0.115270179
ENSMUSG00000032769	Trpa1	transient receptor potential cation channel, subfamily A, member 1 [Source:MGI Symbol;Acc:MGI:3522699]	24.4145	19.8072	20.2358	21.5219	22.5098	25.4758	19.292	21.899	17.425	17.7913	23.4189	21.413	22.4689	21.2104	0.059229521	0.406067671	20	16	24	0.030038351	0.596794384	24	20.28871092	0.145515019
ENSMUSG00000032776	Mctp2	multiple C2 domains, transmembrane 2 [Source:MGI Symbol;Acc:MGI:2685335]	6.99634	6.32664	5.24578	5.90225	5.60362	5.75557	5.58518	5.13682	4.99793	5.5666	6.21492	5.79775	6.30501	5.85053	0.182849367	0.659303873	4	8	24	0.137523513	0.747972781	24	21.38196821	0.084710132
ENSMUSG00000032777	Gtf3c1	general transcription factor III C 1 [Source:MGI Symbol;Acc:MGI:107887]	11.7396	10.9996	11.8082	10.5598	11.972	12.1298	12.8321	12.606	10.8443	14.4809	9.83789	11.8906	11.4769	11.1781	0.939103211	0.966018836	24	20	24	0.954704717	0.988631361	24	23.74280611	0.015531841
ENSMUSG00000032782	Cntrob	centrobin, centrosomal BRCA2 interacting protein [Source:MGI Symbol;Acc:MGI:2443290]	4.10264	4.47651	5.62735	3.52032	5.17213	4.94344	5.45462	3.69943	4.53676	3.99379	4.38744	3.96247	4.70294	5.09362	0.890648739	0.966018836	12	4	24	0.793363897	0.933814107	24	0.734048403	0.04638035
ENSMUSG00000032786	Alas1	aminolevulinic acid synthase 1 [Source:MGI Symbol;Acc:MGI:87989]	29.4361	25.6662	29.3442	30.5679	28.1547	34.4547	31.6021	37.9685	28.1328	38.5269	26.28	32.8058	30.1554	28.9207	0.683024237	0.966018836	24	12	24	0.986446308	0.997339197	24	18.02756671	0.01162687
ENSMUSG00000032788	Pdxk	pyridoxal (pyridoxine, vitamin B6) kinase [Source:MGI Symbol;Acc:MGI:1351869]	8.62145	8.13505	9.48545	9.98706	9.27579	9.36982	9.25332	8.62145	8.85938	10.682	7.57186	8.88641	7.13821	8.81991	0.032263213	0.324385159	16	12	24	0.116868137	0.727929292	24	11.39880056	0.105228595
ENSMUSG00000032796	Lama1	laminin, alpha 1 [Source:MGI Symbol;Acc:MGI:99892]	0.385904	0.631178	0.738887	0.775782	0.50612	0.569391	0.393787	1.01283	0.927784	0.372451	0.344947	0.390844	0.285523	0.518237	0.07600733	0.453790923	12	16	24	0.041520043	0.618736234	24	7.827764403	0.180199979
ENSMUSG00000032802	Srxn1	sulfiredoxin 1 homolog (S. cerevisiae) [Source:MGI Symbol;Acc:MGI:104971]	88.1838	69.2919	77.0874	72.7047	82.927	86.9571	73.6628	79.2293	58.8053	84.9232	81.1979	81.0177	74.9392	76.5799	0.26715327	0.774280302	20	16	24	0.078107038	0.688357494	24	19.01251192	0.128286537
ENSMUSG00000032803	Cdv3	carnitine deficiency-associated gene expressed in ventricle 3 [Source:MGI Symbol;Acc:MGI:2448759]	33.0145	31.1705	32.667	33.0054	35.4307	31.9754	29.8175	31.0531	31.2233	32.6025	30.6341	31.4991	33.1547	30.8681	0.722296281	0.966018836	16	16	24	0.277288462	0.768040178	24	15.26732094	0.035690484
ENSMUSG00000032806	Slc10a3	solute carrier family 10 (sodium/bile acid cotransporter family), member 3 [Source:MGI Symbol;Acc:MGI:95048]	3.87937	4.51661	4.09841	3.9096	4.84622	3.9951	4.54174	4.39384	4.20155	3.73438	4.17999	4.32518	4.20407	3.90396	0.892555977	0.966018836	24	20	24	0.666152468	0.887215207	24	21.75562107	0.031315662
ENSMUSG00000032812	Arap1	ArfGAP with RhoGAP domain, ankyrin repeat and PH domain 1 [Source:MGI Symbol;Acc:MGI:1916960]	27.7012	24.2428	22.9073	22.3795	29.7651	26.6989	25.7996	23.8234	25.1896	19.5648	24.1602	22.211	22.0974	21.2799	0.893307667	0.966018836	24	20	24	0.127765009	0.740162193	24	20.41186358	0.102919421
ENSMUSG00000032815	Fanca	Fanconi anemia, complementation group A [Source:MGI Symbol;Acc:MGI:1341823]	2.52492	1.81364	1.98174	1.84431	1.32212	2.85435	0.965463	1.82203	1.60116	1.46324	1.41199	1.53128	1.16397	1.78085	0.933543173	0.966018836	8	20	24	0.95212151	0.988339231	24	0.976760964	0.028683913
ENSMUSG00000032816	Igdcc4	immunoglobulin superfamily, DCC subclass, member 4 [Source:MGI Symbol;Acc:MGI:1858497]	2.39894	3.07582	3.61323	2.78573	2.60307	2.74753	3.78193	3.13134	3.17106	3.32299	2.23948	2.69468	2.38211	3.19955	0.097779482	0.501112642	12	8	24	0.082565836	0.699403344	24	6.677151139	0.143521218
ENSMUSG00000032826	Ank2	ankyrin 2, brain [Source:MGI Symbol;Acc:MGI:88025]	246.84	209.886	209.488	206.295	207.922	232.873	208.192	213.682	181.113	244.573	208.541	213.674	218.803	228.328	0.28865292	0.794869797	4	8	24	0.458147574	0.805393068	24	20.40641295	0.057954505
ENSMUSG00000032827	Ppp1r9a	protein phosphatase 1, regulatory subunit 9A [Source:MGI Symbol;Acc:MGI:2442401]	11.6565	12.8263	12.6083	12.4361	12.7178	10.9317	13.5835	10.0453	13.9245	11.5989	11.7511	12.7422	13.1286	12.2117	0.910436409	0.966018836	4	8	24	0.942703921	0.985829422	24	7.579237484	0.016796774
ENSMUSG00000032834	Pwp2	PWP2 periodic tryptophan protein homolog (yeast) [Source:MGI Symbol;Acc:MGI:1341200]	6.77305	5.63791	6.54844	6.10962	6.00654	6.59923	6.18286	5.83333	5.18011	6.44479	5.5597	6.25699	6.01507	5.8861	0.360140629	0.883787544	24	8	24	0.319435383	0.768040178	24	20.10754996	0.052650375
ENSMUSG00000032839	Trpc1	transient receptor potential cation channel, subfamily C, member 1 [Source:MGI Symbol;Acc:MGI:109528]	9.71157	9.23957	9.23997	8.92896	8.77269	9.56746	10.4261	8.02446	8.12048	9.75211	9.5458	10.161	9.85927	10.449	0.257978335	0.761738864	4	8	24	0.190263573	0.768040178	24	21.57155904	0.072530123
ENSMUSG00000032840	Spring1	SREBF pathway regulator in golgi 1 [Source:MGI Symbol;Acc:MGI:1924042]	4.98094	4.92692	5.15568	4.8978	4.96997	5.18043	5.17564	4.85197	4.4401	5.51188	4.70636	4.87071	4.80458	5.18694	0.768363194	0.966018836	16	4	24	0.973720984	0.993324217	24	18.48968932	0.006068703
ENSMUSG00000032841	Prr5l	proline rich 5 like [Source:MGI Symbol;Acc:MGI:1919696]	1.15131	1.89718	3.68483	1.24381	0.850867	1.57236	2.97948	1.41461	1.97044	1.02008	0.771791	1.80038	0.545074	1.43881	0.161023625	0.620479606	12	8	24	0.347309179	0.778844143	24	5.290767713	0.237549506
ENSMUSG00000032842	Abcc10	ATP-binding cassette, sub-family C (CFTR/MRP), member 10 [Source:MGI Symbol;Acc:MGI:2386976]	2.54983	3.06605	2.71342	3.57988	3.0607	2.88917	2.88625	3.34367	3.31714	3.49714	2.71696	2.8279	2.70941	3.18465	0.00932019	0.194720062	16	12	24	0.039982087	0.616921492	24	9.804168743	0.105640506
ENSMUSG00000032845	Alpk2	alpha-kinase 2 [Source:MGI Symbol;Acc:MGI:2449492]	0.222974	0.237215	0.440547	0.786495	0.487709	0.466642	0.342222	1.45242	0.615529	1.07285	0.194756	0.447159	0.495071	0.584899	0.280810068	0.792139038	16	12	24	0.216014736	0.768040178	24	8.89768775	0.195966729
ENSMUSG00000032846	Zswim6	zinc finger SWIM-type containing 6 [Source:MGI Symbol;Acc:MGI:1914513]	8.36815	7.04929	6.77922	7.68877	8.11751	7.71946	7.64484	7.73686	7.0281	8.64525	7.62507	7.12571	7.67654	7.75702	0.865490576	0.966018836	16	20	24	0.513614034	0.816307374	24	17.88667514	0.040774309
ENSMUSG00000032849	Abcc4	ATP-binding cassette, sub-family C (CFTR/MRP), member 4 [Source:MGI Symbol;Acc:MGI:2443111]	5.74633	4.43847	4.36843	4.24171	4.15728	5.55336	4.7836	4.80055	3.47395	5.14412	4.35606	4.93162	4.27986	4.30728	0.326899754	0.845515883	24	12	24	0.08538915	0.703577714	24	21.153269	0.125671405

ENSMUSG00000032850	Rnft2	ring finger protein, transmembrane 2 [Source:MGI Symbol;Acc:MGI:2442859]	7.6761	5.8618	6.85198	7.2417	6.31359	7.21958	6.84293	7.23246	5.72078	8.05638	6.50547	7.06711	6.00716	6.99356	0.934818505	0.966018836	16	20	24	0.793035881	0.93379199	24	16.95811558	0.035155418
ENSMUSG00000032852	Rspo4	R-spondin 4 [Source:MGI Symbol;Acc:MGI:1924467]	1.27321	1.068	1.03439	1.34436	0.985526	1.4643	0.657614	1.09591	0.797982	1.40931	0.780207	1.18396	0.821543	1.04871	0.929527443	0.966018836	24	12	24	0.686147309	0.895740026	24	15.0222737	0.057699967
ENSMUSG00000032854	Ugt8a	UDP galactosyltransferase 8A [Source:MGI Symbol;Acc:MGI:109522]	113.408	127.173	114.814	113.763	150.904	103.292	145.516	117.112	137.151	109.228	122.66	105.159	111.04	103.105	0.907000607	0.966018836	12	12	24	0.932292067	0.982622317	24	12.80458285	0.025363958
ENSMUSG00000032855	Pkd1	polycystin 1, transient receptor potential channel interacting [Source:MGI Symbol;Acc:MGI:97603]	18.4153	17.0787	17.3934	21.9327	19.444	16.7408	19.8858	22.1909	20.4484	20.392	19.3459	19.6792	19.2691	18.3485	0.851656735	0.966018836	16	16	24	0.468954912	0.805393068	24	10.99803978	0.055455032
ENSMUSG00000032860	P2ry2	purinergic receptor P2Y, G-protein coupled 2 [Source:MGI Symbol;Acc:MGI:105107]	9.14657	7.56963	6.40638	8.21568	8.94385	7.68742	8.40741	8.49791	7.93447	9.70674	8.10496	7.90196	8.30656	7.66679	0.658942124	0.966018836	16	20	24	0.544300791	0.832119095	24	17.86485838	0.056909986
ENSMUSG00000032867	Fbxw8	F-box and WD-40 domain protein 8 [Source:MGI Symbol;Acc:MGI:1923041]	14.1706	12.8118	13.1315	13.093	13.0704	14.0257	12.9708	14.9818	12.0492	15.0482	12.9951	13.8809	13.8013	14.1507	0.888482515	0.966018836	24	12	24	0.767659499	0.924952565	24	23.20665267	0.024256648
ENSMUSG00000032869	Psmf1	proteasome (prosome, macropain) inhibitor subunit 1 [Source:MGI Symbol;Acc:MGI:1346072]	24.154	20.145	20.0231	18.6938	21.4124	22.3984	21.1023	22.3656	19.6353	24.2659	18.7363	21.9297	21.548	20.1345	0.202903295	0.689605535	24	12	24	0.358437007	0.785071516	24	22.06858117	0.064974422
ENSMUSG00000032870	Smap2	small ArfGAP 2 [Source:MGI Symbol;Acc:MGI:1917030]	24.7114	20.6041	23.3938	20.7268	19.9693	23.1329	23.9532	23.2655	17.8106	26.4266	19.6278	23.4846	20.6969	23.2755	0.846840613	0.966018836	4	16	24	0.666149665	0.887215207	24	23.58127458	0.053688113
ENSMUSG00000032872	Cyb5r4	cytochrome b5 reductase 4 [Source:MGI Symbol;Acc:MGI:2386848]	17.3451	17.0208	16.2684	17.8617	16.8111	15.7899	18.4533	15.4526	13.8832	15.8974	17.7838	14.5405	15.9062	16.6085	0.868638024	0.966018836	16	20	24	0.561258825	0.841538771	24	18.92518445	0.043079903
ENSMUSG00000032875	Arhgef17	Rho guanine nucleotide exchange factor (GEF) 17 [Source:MGI Symbol;Acc:MGI:2673002]	20.4832	17.2812	17.9714	19.3704	18.5885	19.7553	20.1954	19.601	17.606	25.0035	17.1678	20.4656	19.4069	17.6303	0.287703522	0.793714531	24	12	24	0.699013385	0.899922504	24	17.44947167	0.046818126
ENSMUSG00000032878	Ccdc85a	coiled-coil domain containing 85A [Source:MGI Symbol;Acc:MGI:2445069]	18.0598	14.3599	14.1596	15.8432	14.5434	15.9876	14.3745	14.3325	11.7141	15.0325	16.6808	13.6737	12.7814	13.777	0.308023687	0.814993005	16	20	24	0.1442787	0.752803875	24	18.29508483	0.099135368
ENSMUSG00000032883	Acsl3	acyl-CoA synthetase long-chain family member 3 [Source:MGI Symbol;Acc:MGI:1921455]	34.1576	38.7505	45.1143	39.4655	37.5835	38.3729	38.826	31.8944	40.2735	38.7918	36.3011	37.1434	33.7232	39.7753	0.055920623	0.401074772	12	16	24	0.165817185	0.767020009	24	10.36882906	0.086786971
ENSMUSG00000032889	Gm6685	predicted pseudogene 6685 [Source:MGI Symbol;Acc:MGI:3704119]	11.4127	8.76464	9.50822	7.72969	9.29271	8.20688	10.2869	9.16721	7.62235	9.30275	8.33906	9.83762	11.1811	11.739	0.105163243	0.518002936	4	12	24	0.047097133	0.633507364	24	0.644408747	0.153658576
ENSMUSG00000032890	Rims3	regulating synaptic membrane exocytosis 3 [Source:MGI Symbol;Acc:MGI:2443331]	33.1149	26.4664	27.0069	29.2596	28.8335	32.6912	28.2403	29.7512	22.2579	36.1599	25.6201	30.4196	28.1077	28.7444	0.390686537	0.915479136	24	12	24	0.419895091	0.803772782	24	19.35059324	0.089132738
ENSMUSG00000032897	Nfyc	nuclear transcription factor-Y gamma [Source:MGI Symbol;Acc:MGI:107901]	5.01531	6.76754	5.80832	6.07908	6.57089	5.54557	5.52245	5.74533	7.07126	5.82104	6.25831	5.59085	6.17724	5.42808	0.163947066	0.627573783	20	8	24	0.224431162	0.768040178	24	10.71253805	0.078165482
ENSMUSG00000032898	Fbxo21	F-box protein 21 [Source:MGI Symbol;Acc:MGI:1924223]	18.982	15.6005	16.782	17.0308	14.5705	17.6674	18.2209	18.5329	14.2412	21.7755	14.0692	17.1077	16.1561	18.1077	0.598711142	0.966018836	4	16	24	0.7728645	0.926922997	24	2.504758546	0.047380201
ENSMUSG00000032902	Slc16a1	solute carrier family 16 (monocarboxylic acid transporters), member 1 [Source:MGI Symbol;Acc:MGI:106013]	20.3386	21.169	21.1909	19.5602	20.7939	19.7899	22.0264	22.4965	20.0989	18.9141	21.614	21.7558	24.4231	22.5768	0.026012493	0.29944494	8	8	24	0.024823377	0.586541342	24	1.203104564	0.076718686
ENSMUSG00000032905	Atg12	autophagy related 12 [Source:MGI Symbol;Acc:MGI:1914776]	22.7242	20.7884	20.832	21.2048	21.9863	21.7613	21.3177	20.2138	20.3093	20.9508	24.6241	21.5426	22.6385	22.5394	0.063659307	0.414867207	20	16	24	0.121635384	0.733843269	24	19.07122778	0.055617197
ENSMUSG00000032908	Sgpp2	sphingosine-1-phosphate phosphatase 2 [Source:MGI Symbol;Acc:MGI:3589109]	42.3932	33.0183	33.3038	35.8244	32.7423	40.2275	37.3122	38.088	26.2629	47.1526	32.7	37.7371	37.8148	39.9821	0.441501717	0.966018836	4	8	24	0.52763841	0.823470321	24	21.61376504	0.09161523
ENSMUSG00000032911	Cspg4	chondroitin sulfate proteoglycan 4 [Source:MGI Symbol;Acc:MGI:2153093]	3.53885	4.17929	4.4183	3.04888	3.86186	3.65587	4.4522	4.88971	3.9335	4.60519	3.85215	3.98116	4.53662	4.2234	0.555368163	0.966018836	8	12	24	0.149657107	0.758377578	24	4.3008307	0.096719255
ENSMUSG00000032913	Lrig2	leucine-rich repeats and immunoglobulin-like domains 2 [Source:MGI Symbol;Acc:MGI:2443718]	14.2965	14.9341	13.6841	13.3299	13.3852	13.4834	13.9098	13.5776	13.8093	13.6919	14.7001	14.6167	14.7296	15.0996	0.29271558	0.80029783	8	8	24	0.165768067	0.767020009	24	1.296353836	0.037681773
ENSMUSG00000032925	Itgbl1	integrin, beta-like 1 [Source:MGI Symbol;Acc:MGI:2443439]	3.53128	5.03323	4.58816	3.6347	4.29239	2.94714	3.80424	3.98141	5.95054	2.46592	3.49024	3.8077	3.53343	4.43576	0.034356228	0.328311403	12	4	24	0.133161312	0.743835554	24	5.957567509	0.190729578
ENSMUSG00000032932	Hspa13	heat shock protein 70 family, member 13 [Source:MGI Symbol;Acc:MGI:1309463]	12.6502	13.6931	13.572	12.4538	14.1516	12.3523	13.5531	12.5412	14.0264	11.2874	14.6194	12.6414	12.9903	13.0627	0.745118544	0.966018836	12	12	24	0.999878977	0.999984043	24	6.373206547	5.94E-04
ENSMUSG00000032936	Camkv	CaM kinase-like vesicle-associated [Source:MGI Symbol;Acc:MGI:2384296]	1.10232	2.81342	4.54537	2.45965	1.86705	2.29216	2.74771	2.00011	4.09882	2.04673	1.27981	2.41519	1.12114	2.96891	0.033622862	0.324908923	12	8	24	0.079951876	0.694647074	24	7.956000024	0.357717502
ENSMUSG00000032939	Nup93	nucleoporin 93 [Source:MGI Symbol;Acc:MGI:1919055]	39.1004	32.8822	35.0557	33.339	36.7363	42.3686	32.6548	35.3305	28.7082	37.9704	38.734	36.0272	36.6793	35.474	0.059229521	0.406067671	20	16	24	0.05875981	0.658304792	24	19.07823781	0.118952584
ENSMUSG00000032940	Rbm11	RNA binding motif protein 11 [Source:MGI Symbol;Acc:MGI:2447622]	22.3219	20.3985	18.7658	20.5781	19.7447	20.5778	18.2638	18.0019	20.4403	21.0223	22.1468	21.1075	22.2416	22.6827	0.904831057	0.966018836	4	8	24	0.748090673	0.918016105	24	18.85275613	0.030045899
ENSMUSG00000032942	Ucp3	uncoupling protein 3 (mitochondrial, proton carrier) [Source:MGI Symbol;Acc:MGI:1099787]	2.07737	1.90336	2.76239	4.34055	2.25714	2.79142	2.52239	8.3835	4.03357	4.70242	2.79057	2.85987	3.90249	4.13998	0.722296281	0.966018836	16	4	24	0.154572459	0.760257003	24	7.868775529	0.305078233
ENSMUSG00000032946	Rasgrp2	RAS, guanyl releasing protein 2 [Source:MGI Symbol;Acc:MGI:1333849]	17.2755	19.4134	17.561	16.4231	19.5404	14.8936	18.3997	19.2147	17.5363	16.9302	15.9891	16.3801	17.6606	15.808	0.43280837	0.966018836	8	16	24	0.460775594	0.805393068	24	4.712878768	0.05150662
ENSMUSG00000032952	Ap4b1	adaptor-related protein complex AP-4, beta 1 [Source:MGI Symbol;Acc:MGI:1337130]	11.0683	11.1605	11.1165	11.153	11.1634	11.2622	10.0233	11.3807	11.381	11.7254	10.8167	11.2659	11.9268	11.195	0.844448841	0.966018836	16	4	24	0.652226759	0.88299052	24	9.5247806	0.01925742
ENSMUSG00000032959	Pebp1	phosphatidylethanolamine binding protein 1 [Source:MGI Symbol;Acc:MGI:1344408]	118.461	116.188	111.451	117.247	111.808	120.423	118.75	122.651	119.926	118.496	123.045	119.578	126.322	118.223	0.590017078	0.966018836	24	12	24	0.294664863	0.768040178	24	23.29938548	0.023706423
ENSMUSG00000032965	Ift57	intraflagellar transport 57 [Source:MGI Symbol;Acc:MGI:1921166]	12.8323	12.9522	11.6894	10.4838	12.515	11.8527	13.4116	12.3263	10.7912	13.0322	12.5752	11.7195	12.0196	13.1958	0.444754148	0.966018836	8	4	24	0.316016264	0.768040178	24	23.85383617	0.05730131
ENSMUSG00000032966	Fkbp1a	FK506 binding protein 1a [Source:MGI Symbol;Acc:MGI:95541]	188.781	159.376	157.39	163.268	171.88	178.24	168.041	171.285	151.399	181.679	170.17	172.555	170.018	168.236	0.020957534	0.262822894	24	12	24	0.071297167	0.676901434	24	19.97640769	0.072120869
ENSMUSG00000032968	Inha	inhibin alpha [Source:MGI Symbol;Acc:MGI:96569]	4.1708	4.4125	4.76798	5.39113	3.94998	3.58126	4.20849	4.80064	5.74655	5.62959	4.79234	4.90112	3.89793	4.51268	0.016024075	0.253395174	16	12	24	0.004254955	0.427431763	24	9.910967902	0.181933875
ENSMUSG00000032977	Fam207a	family with sequence similarity 207, member A [Source:MGI Symbol;Acc:MGI:1916334]	11.9346	9.68921	8.30556	10.0505	9.21933	10.0432	10.5568	10.3836	8.39124	10.9152	10.3086	11.3579	10.0393	10.1803	0.143321297	0.585944911	24	12	24	0.104864206	0.717030134	24	21.1705804	0.106999334
ENSMUSG00000032985	5730522E02Rik	RIKEN cDNA 5730522E02 gene [Source:MGI Symbol;Acc:MGI:1917876]	0.530863	0.512225	0.282087	0.527989	0.679931	0.621603	0.639271	0.47736	0.561063	0.737706	0.470358	0.4407	0.317725	0.979801	0.82502117	0.966018836	16	20	24	0.963377245	0.990884307	24	10.87923528	0.01759924
ENSMUSG00000032997	Chpf	chondroitin polymerizing factor [Source:MGI Symbol;Acc:MGI:106576]	18.584	13.0257	13.2737	12.9317	12.6511	13.9006	15.1468	17.2526	12.9671	19.3141	15.4784	16.5038	15.8449	15.5626	0.575387897	0.966018836	4	16	24	0.60789308	0.864036051	24	23.31309361	0.07218815
ENSMUSG00000032998	Foxj3	forkhead box J3 [Source:MGI Symbol;Acc:MGI:2443432]	12.2046	11.5756	12.5722	12.0271	11.7309	11.8497	11.8467	11.2259	11.2881	13.2628	10.3973	12.3766	12.3653	11.7196	0.796193867	0.966018836	16	16	24	0.995023917	0.998756742	24	9.523403989	0.003255451
ENSMUSG00000033004	Mycbp2	MYC binding protein 2, E3 ubiquitin protein ligase [Source:MGI Symbol;Acc:MGI:2179432]	58.3168	57.6515	57.5588	55.7513	57.1398	56.3636	58.7755	52.2213	54.1667	54.3459	56.2937	57.3288	56.7177	59.5261	0.157616081	0.614233844	4	12	24	0.295050032	0.768040178	24	22.52459158	0.030598767
ENSMUSG00000033006	Sox10	SRY (sex determining region Y)-box 10 [Source:MGI Symbol;Acc:MGI:98358]	40.8861	35.122	33.857	39.9227	39.368	32.919	47.1352	35.315	40.8678	43.1845	39.0997	39.6038	39.6101	34.4368	0.850398351	0.966018836	4	4	24	0.726263132	0.910096555	24	17.20271855	0.046538485
ENSMUSG00000033009	Ogfod1	2-oxoglutarate and iron-dependent oxygenase domain containing 1 [Source:MGI Symbol;Acc:MGI:2442978]	14.6916	11.7528	14.4991	13.4341	12.5196	14.3579	15.555	13.9093	11.4396	15.3751	11.6214	14.4813	12.0395	14.136	0.873337044	0.966018836	4	16	24	0.801477621	0.936422183	24	22.52356572	0.039268099
ENSMUSG00000033014	Trim33	tripartite motif-containing 33 [Source:MGI Symbol;Acc:MGI:2137357]	11.5124	12.4677	12.4151	13.8149	13.3471	12.9306	13.0279	11.6988	12.7359	11.6282	13.3919	13.2061	12.4113	13.6727	0.522715778	0.966018836	20	8	24	0.434321835	0.805393068	24	15.57444123	0.038728063
ENSMUSG00000033016	Nfatc1	nuclear factor of activated T cells, cytoplasmic, calcineurin dependent 1 [Source:MGI Symbol;Acc:MGI:102469]	2.24959	2.40164	2.89068	2.18545	2.48822	2.09109	2.80402	2.76974	2.55516	3.0476	2.15671	2.39746	2.52724	2.12691	0.471214947	0.966018836	12	12	24	0.40819021	0.799546792	24	7.762278588	0.06885996
ENSMUSG00000033020	Polr2f	polymerase (RNA) II (DNA directed) polypeptide F [Source:MGI Symbol;Acc:MGI:1349393]	43.7843	40.8524	40.1788	40.4064	45.404	42.3422	42.4411	42.1736	41.6121	32.1081	39.9298	37.0668	35.564	39.5276	0.880631923	0.966018836	20	20	24	0.575268174	0.848005542	24	22.7225794	0.044354143
ENSMUSG00000033021	Gmppa	GDP-mannose pyrophosphorylase A [Source:MGI Symbol;Acc:MGI:1916330]	23.1943	22.3992	23.0593	22.5601	21.6681	21.2464	20.0463	24.2417	22.4618	20.3684	18.4188	20.7607	21.791	18.5445	0.483881213	0.966018836	12	8	24	0.408872499	0.799546792	24	5.152004256	0.046622109
ENSMUSG00000033022	Cdo1	cysteine dioxygenase 1, cytosolic [Source:MGI Symbol;Acc:MGI:105925]	14.3117	12.2994	18.0094	35.8655	13.8928	15.1181	17.3804	20.6996	18.5531	26.8769	30.9096	23.9762	35.8152	27.4121	0.915082018	0.966018836	16	16	24	0.3582014	0.784922891	24	11.89503141	0.205914704
ENSMUSG00000033029	1700088E04Rik	RIKEN cDNA 1700088E04 gene [Source:MGI Symbol;Acc:MGI:1920774]	5.49409	4.73035	5.08895	4.68016	6.45804	4.25625	3.50196	3.16879	5.25335	3.31659	4.46821	4.60677	4.03196	3.85287	0.96984707	0.97107529	12	20	24	0.431937769	0.805393068	24	15.83903752	0.105568976
ENSMUSG00000033031	Cip2a	cell proliferation regulating inhibitor of protein phosphatase 2A [Source:MGI Symbol;Acc:MGI:2146335]	1.72708	2.0931	2.22112	2.00375	2.09319	1.74801	1.75259	2.1052	1.91839	1.80295	2.06983	1.88619	1.93035	2.10891	0.121243373	0.548623108	8	20	24	0.155797924	0.761271595	24	8.692730713	0.059482533
ENSMUSG00000033032	Afap1l1	actin filament associated protein 1-like 1 [Source:MGI Symbol;Acc:MGI:2147199]	4.11258	4.92386	5.00384	4.81683	5.18992	4.42754	5.21289	5.34469	5.47845	4.95916	4.48576	4.15858	4.4381	4.43452	0.041292272	0.346472636	12	12	24	0.090406858	0.711204167	24	8.754300332	0.091440689
ENSMUSG00000033033	Calhm2	calcium homeostasis modulator family member 2 [Source:MGI Symbol;Acc:MGI:1919941]	2.25254	1.7525	2.0945	1.77103	1.9224	1.77481	2.10887	1.94621	1.9669	1.39611	1.84682	1.83467	1.70169	1.88412	0.970223078	0.971116366	12	4	24	0.26039184	0.768040178	24	0.945037805	0.060754272
ENSMUSG00000033036	Gm7879	predicted pseudogene 7879 [Source:MGI Symbol;Acc:MGI:3645078]	0.325835	0.794146	0.425018	0.372659	0.875348	0.626935	1.03258	0.757082	0.800097	0.387572	0.536499	0.749665	0.56525	0.831186	0.743750765	0.966018836	8	8	24	0.462820542	0.805393068	24	0.989348872	0.090009295
ENSMUSG00000033039	Micall1	microtubule associated monooxygenase, calponin and LIM domain containing -like 1 [Source:MGI Symbol;Acc:MGI:105870]	8.57461	8.52031	10.1257	9.81392	9.18453	9.94629	10.4804	8.0164	9.50831	10.0108	9.82324	7.83041	8.17177	8.3756	0.371875382	0.894537464	12	20	24	0.15015128	0.75876965	24	13.20187995	0.088953018
ENSMUSG00000033044	Dhrs7c	dehydrogenase/reductase (SDR family) member 7C [Source:MGI Symbol;Acc:MGI:1915710]	1.71342	4.60464	5.01921	6.7952	4.37536	5.58978	3.92314	11.741	9.53833	9.05892	3.2864	4.69037	5.49543	3.75823	0.387380622	0.915479136	12	16	24	0.102292294	0.713699364	24	9.32902428	0.452083725
ENSMUSG00000033047	Eif3l	eukaryotic translation initiation factor 3, subunit L [Source:MGI Symbol;Acc:MGI:2386251]	52.2781	50.2429	50.1575	55.2966	52.4026	54.4108	49.3684	53.7365	47.97	53.0677	53.0131	51.0637	51.8863	52.2935	0.187699199	0.662239322	16	20	24	0.335859645	0.771482605	24	16.52511158	0.031945392
ENSMUSG00000033054	Npat	nuclear protein in the AT region [Source:MGI Symbol;Acc:MGI:107605]	4.23645	5.22582	5.23387	4.8494	4.32381	5.11205	4.51087	4.34336	5.30428	3.93429	4.95184	4.56309	4.49702	5.13598	0.326899754	0.845515883	12	16	24	0.553883407	0.837374348	24	6.931893834	0.050298718
ENSMUSG00000033055	Ankrd54	ankyrin repeat domain 54 [Source:MGI Symbol;Acc:MGI:2444209]	6.30437	5.05888	5.46348	6.0891	5.78268	6.10413	6.12485	5.0855	5.39784	5.76218	6.25102	5.82615	6.39279	4.6995	1.20E-04	0.029372629	4	4	24	0.015008572	0.537924552	24	18.42324562	0.117531911
ENSMUSG00000033059	Pygb	brain glycogen phosphorylase [Source:MGI Symbol;Acc:MGI:97828]	189.22	153.388	145.305	155.744	144.032	171.702	159.621	167.605	124.002	194.132	145.312	166.687	164.193	162.79	0.217933169	0.704989686	24	12	24	0.391803981	0.799546792	24	21.9123577	0.091060053
ENSMUSG00000033060	Lmo7	LIM domain only 7 [Source:MGI Symbol;Acc:MGI:1353586]	2.99731	3.34991	3.50594	3.53128	5.65601	4.26552	3.00009	5.33863	4.27749	4.20653	5.56847	3.43191	4.30068	4.10589	0.231491055	0.731037655	20	8	24	0.233202471	0.768040178	24	12.10614564	0.162733917
ENSMUSG00000033061	Resp18	regulated endocrine-specific protein 18 [Source:MGI Symbol;Acc:MGI:1098222]	107.682	119.127	123.217	111.61	99.1116	112.48	106.664	101.567	117.641	103.003	106.353	115.546	111.26	117.973	0.285027487	0.793714531	12	8	24	0.301866072	0.768040178	24	5.489893095	0.05707758
ENSMUSG00000033063	Cntnap3	contactin associated protein-like 3 [Source:MGI Symbol;Acc:MGI:3588199]	1.84878	1.63335	1.72718	2.07553	1.92991	1.83679	1.70158	1.40528	1.5079	1.71508	1.58563	1.58127	1.70543	1.87209	0.907000607	0.966018836	16	20	24	0.467338264	0.805393068	24	16.32924774	0.043926451
ENSMUSG00000033065	Pfkm	phosphofructokinase, muscle [Source:MGI Symbol;Acc:MGI:97548]	81.4264	85.4392	99.932	104.282	85.6538	112.399	91.5392	136.879	100.89	147.549	76.964	95.7383	94.3171	95.7259	0.184631358	0.662239322	16	12	24	0.387660226	0.799546792	24	9.061050058	0.144760207
ENSMUSG00000033066	Gas7	growth arrest specific 7 [Source:MGI Symbol;Acc:MGI:1202388]	15.2931	13.0177	11.7251	10.162	16.7515	13.4355	15.7334	12.9162	11.8919	11.8394	12.7789	12.3856	13.8932	11.1736	0.015984991	0.253395174	4	12	24	0.027076459	0.588305652	24	21.31841536	0.174425055
ENSMUSG00000033068	Entpd6	ectonucleoside triphosphate diphosphohydrolase 6 [Source:MGI Symbol;Acc:MGI:1202295]	7.51936	6.69596	7.83744	6.63968	6.01175	7.7397	7.7579	8.11306	6.31699	8.12751	6.53891	6.88883	6.77377	7.33436	0.886952527	0.966018836	4	16	24	0.478367891	0.805393068	24	3.063349632	0.058653878
ENSMUSG00000033075	Senp1	SUMO1/sentrin specific peptidase 1 [Source:MGI Symbol;Acc:MGI:2445054]	4.34165	5.82787	4.28398	6.13716	4.672	4.93548	4.98371	5.13687	4.89527	5.77789	4.26784	4.47358	4.6975	4.89128	0.503972924	0.966018836	16	4	24	0.396171967	0.799546792	24	9.052407875	0.076402569
ENSMUSG00000033082	Clec1a	C-type lectin domain family 1, member a [Source:MGI Symbol;Acc:MGI:2444151]	1.05609	1.57926	1.94533	1.87176	1.74354	1.47321	1.2724	1.68798	2.13754	1.34904	1.68004	1.42019	1.26808	1.6682	1.23E-04	0.029372629	12	16	24	0.012515115	0.525280095	24	10.03901805	0.172149515
ENSMUSG00000033083	Tbc1d4	TBC1 domain family, member 4 [Source:MGI Symbol;Acc:MGI:2429660]	7.13095	7.4469	8.1473	7.84208	6.79647	7.36307	6.92536	9.45939	7.31646	8.84332	7.00175	8.02571	8.33728	7.35706	0.718720526	0.966018836	16	4	24	0.384061873	0.799546792	24	7.21142285	0.068388439
ENSMUSG00000033088	Triobp	TRIO and F-actin binding protein [Source:MGI Symbol;Acc:MGI:1349410]	7.92417	6.65839	8.11944	8.08165	8.19664	6.2916	8.8601	7.08882	7.27363	7.74712	7.65191	7.04797	7.77006	6.3679	0.575202741	0.966018836	20	12	24	0.662700459	0.886154038	24	14.81917962	0.044536266
ENSMUSG00000033096	Apmap	adipocyte plasma membrane associated protein [Source:MGI Symbol;Acc:MGI:1919131]	29.1083	31.3297	31.6951	37.0109	30.8391	29.551	30.8424	29.9062	32.0842	32.6021	32.8227	33.2276	34.1199	33.2032	0.916746631	0.966018836	16	12	24	0.186284889	0.768040178	24	11.45922541	0.057017861
ENSMUSG00000033099	Nol12	nucleolar protein 12 [Source:MGI Symbol;Acc:MGI:2146285]	15.7233	14.1255	14.7234	14.962	15.4315	16.9581	15.526	15.0669	12.7205	13.0798	15.331	14.6795	15.1789	12.5976	0.037841083	0.334069441	4	8	24	0.00237597	0.39629122	24	19.98361483	0.12153919
ENSMUSG00000033102	Cdc14b	CDC14 cell division cycle 14B [Source:MGI Symbol;Acc:MGI:2441808]	9.21696	9.59728	10.693	9.63604	9.21124	9.88929	9.37044	8.20656	8.51495	9.87559	11.1002	10.5251	10.299	9.9756	0.833406028	0.966018836	24	8	24	0.378416997	0.797715859	24	16.94733171	0.058120517
ENSMUSG00000033105	Lss	lanosterol synthase [Source:MGI Symbol;Acc:MGI:1336155]	17.8075	21.4499	22.1827	24.657	21.1962	20.4048	19.0074	19.2177	27.4892	22.595	21.9431	20.5702	19.2177	22.4444	5.91E-05	0.029372629	16	12	24	0.001415301	0.354367925	24	10.54313197	0.177756396
ENSMUSG00000033106	Slc7a6os	solute carrier family 7, member 6 opposite strand [Source:MGI Symbol;Acc:MGI:1916951]	9.97484	9.72838	10.6188	11.4619	10.2643	10.3481	11.1359	10.7613	11.3326	11.4036	10.7041	10.6689	11.4603	11.4148	0.939335547	0.966018836	16	4	24	0.150804109	0.758865539	24	9.407375625	0.041033376
ENSMUSG00000033107	Rnf125	ring finger protein 125 [Source:MGI Symbol;Acc:MGI:1914914]	11.5541	10.0019	9.5098	10.6411	10.959	11.204	8.88942	11.1822	11.1985	13.065	11.9828	12.4752	11.9697	11.3467	0.593560247	0.966018836	24	12	24	0.346949593	0.778844143	24	15.915239	0.065082192
ENSMUSG00000033111	3830406C13Rik	RIKEN cDNA 3830406C13 gene [Source:MGI Symbol;Acc:MGI:1917937]	30.1518	32.3042	28.6711	29.7592	26.1193	28.0579	27.9317	28.6442	31.2624	27.0301	34.0788	33.3589	32.9652	30.7187	0.850906022	0.966018836	24	16	24	0.713106602	0.905403147	24	0.986766718	0.03496447
ENSMUSG00000033114	Slc35d2	solute carrier family 35, member D2 [Source:MGI Symbol;Acc:MGI:1917734]	3.19318	3.56441	3.06543	2.96854	3.2543	3.19872	1.77779	3.15324	2.96201	2.72504	2.47596	3.11979	2.88193	2.92428	0.860560779	0.966018836	8	8	24	0.916076101	0.976812262	24	8.010393099	0.02896341
ENSMUSG00000033124	Atg9a	autophagy related 9A [Source:MGI Symbol;Acc:MGI:2138446]	23.7308	17.6758	20.2365	20.3896	19.447	22.8144	23.1162	24.4052	17.6436	27.4798	17.4936	20.7435	20.3353	19.9319	0.83638114	0.966018836	4	8	24	0.859284284	0.958530654	24	22.15409316	0.04211071
ENSMUSG00000033126	Ybey	ybeY metallopeptidase [Source:MGI Symbol;Acc:MGI:2656825]	0.897743	0.856286	0.861885	0.968479	0.834797	1.0501	0.721364	0.905966	1.18195	0.930502	0.93352	1.16607	0.945995	0.875431	0.773177465	0.966018836	24	8	24	0.670651705	0.888925819	24	14.20055549	0.030699024
ENSMUSG00000033128	Gga1	golgi associated, gamma adaptin ear containing, ARF binding protein 1 [Source:MGI Symbol;Acc:MGI:2146207]	15.679	13.7448	15.0019	14.1025	13.8227	13.9203	14.1075	16.6431	14.6632	18.2661	13.8729	15.0328	15.2618	14.5449	0.671726933	0.966018836	4	16	24	0.646032725	0.880103669	24	7.835223235	0.042741146
ENSMUSG00000033147	Slc22a15	solute carrier family 22 (organic anion/cation transporter), member 15 [Source:MGI Symbol;Acc:MGI:3607704]	3.38393	3.0101	3.13157	3.15932	3.11123	3.20977	3.1567	3.00719	2.90735	3.20792	2.8435	3.12367	3.63905	3.10007	0.203305541	0.689605535	4	4	24	0.26526477	0.768040178	24	22.71030413	0.042696286
ENSMUSG00000033149	Phldb2	pleckstrin homology like domain, family B, member 2 [Source:MGI Symbol;Acc:MGI:2444981]	5.82486	7.15439	5.81909	5.61589	6.49459	5.95868	5.01107	6.18185	6.84701	5.53932	5.87896	5.90974	5.00962	6.51695	0.369262716	0.893596186	8	20	24	0.469779818	0.805393068	24	7.946440652	0.063648097
ENSMUSG00000033152	Podxl2	podocalyxin-like 2 [Source:MGI Symbol;Acc:MGI:2442488]	17.4066	20.771	26.013	18.7868	15.8618	19.5804	22.9621	20.068	22.6555	20.2231	16.0203	19.5607	17.084	23.2049	0.005162915	0.145313511	12	8	24	0.031145826	0.600113627	24	6.123129274	0.191615794
ENSMUSG00000033157	Abhd10	abhydrolase domain containing 10 [Source:MGI Symbol;Acc:MGI:2442422]	17.7206	16.865	16.3676	17.9871	15.8146	15.9039	14.9507	16.1646	15.9086	18.0484	17.5588	15.5445	17.3078	16.212	0.616047374	0.966018836	16	8	24	0.472140018	0.805393068	24	11.77033328	0.038783678
ENSMUSG00000033159	Cnppd1	cyclin Pas1/PHO80 domain containing 1 [Source:MGI Symbol;Acc:MGI:1916421]	20.6877	20.6984	20.843	21.2388	23.1302	21.5158	23.3794	23.4249	21.7181	25.5091	20.5706	22.2278	21.6234	23.011	0.807813307	0.966018836	4	8	24	0.828992948	0.946784793	24	10.46899023	0.020119706
ENSMUSG00000033161	Atp1a1	ATPase, Na+/K+ transporting, alpha 1 polypeptide [Source:MGI Symbol;Acc:MGI:88105]	562.137	421.386	409.155	460.185	460.004	513.354	461.42	492.597	339.22	602.519	428.914	482.608	461.383	465.143	0.245061943	0.741580974	24	12	24	0.36665208	0.790399468	24	19.88003072	0.115252389
ENSMUSG00000033166	Dis3	DIS3 homolog, exosome endoribonuclease and 3'-5' exoribonuclease [Source:MGI Symbol;Acc:MGI:1919912]	4.88115	6.67302	7.4101	5.63111	5.53305	5.46625	7.89318	6.05017	7.64085	5.32095	6.34912	5.64356	6.38195	5.51599	0.649212555	0.966018836	12	4	24	0.365835816	0.789934347	24	6.10235162	0.104014891
ENSMUSG00000033170	Card10	caspase recruitment domain family, member 10 [Source:MGI Symbol;Acc:MGI:2146012]	3.23313	3.24457	3.27427	3.25442	3.92866	3.35183	3.5303	3.86034	3.46798	3.79484	3.80009	3.74241	3.73147	3.26389	0.401437339	0.933249185	20	12	24	0.260829376	0.768040178	24	16.54109157	0.046735848
ENSMUSG00000033174	Mgll	monoglyceride lipase [Source:MGI Symbol;Acc:MGI:1346042]	21.7711	17.9574	19.7912	26.2662	20.6334	21.5926	21.3919	23.7129	19.3538	27.5396	21.7503	22.2933	24.5388	23.2027	0.919119843	0.966018836	16	20	24	0.621788988	0.871137783	24	13.96583364	0.056284461
ENSMUSG00000033177	Tmprss7	transmembrane serine protease 7 [Source:MGI Symbol;Acc:MGI:2686594]	1.21541	1.15099	1.04041	1.02199	1.03972	1.0974	1.26812	1.16301	1.04199	1.61855	1.38001	1.02622	0.934422	1.17102	0.823246279	0.966018836	4	20	24	0.747178345	0.917720337	24	12.69343687	0.034481487
ENSMUSG00000033182	Kbtbd12	kelch repeat and BTB (POZ) domain containing 12 [Source:MGI Symbol;Acc:MGI:1918481]	0.460512	0.534929	0.554584	0.720483	0.530083	0.607368	0.821038	0.720564	0.857174	0.877446	0.680131	0.469868	0.610983	0.616497	0.349109869	0.870418653	16	8	24	0.168211614	0.767020009	24	9.627928995	0.083858755
ENSMUSG00000033184	Tmed7	transmembrane p24 trafficking protein 7 [Source:MGI Symbol;Acc:MGI:1913926]	32.2939	34.2084	32.1633	33.4819	34.4371	30.7794	34.1813	30.5622	34.3902	29.8028	33.6174	31.4497	30.738	31.8625	0.914502381	0.966018836	20	4	24	0.887720945	0.968424304	24	12.3170346	0.012831536
ENSMUSG00000033186	Mzt1	mitotic spindle organizing protein 1 [Source:MGI Symbol;Acc:MGI:1924039]	34.7576	35.4845	34.928	36.1815	32.3044	34.1701	35.4489	34.5361	30.2096	34.8252	36.2314	27.7578	28.2851	31.0385	0.814077113	0.966018836	20	4	24	0.574341464	0.847247297	24	11.59714834	0.041660272
ENSMUSG00000033191	Tie1	tyrosine kinase with immunoglobulin-like and EGF-like domains 1 [Source:MGI Symbol;Acc:MGI:99906]	2.35552	2.88447	2.85931	2.99152	2.6706	2.94971	3.03981	4.43727	2.97807	3.74977	2.97211	2.92601	3.40501	2.41806	0.858891143	0.966018836	16	4	24	0.729816438	0.911863904	24	8.384466393	0.057583825
ENSMUSG00000033192	Lpcat2	lysophosphatidylcholine acyltransferase 2 [Source:MGI Symbol;Acc:MGI:3606214]	5.42857	7.30894	7.32417	4.79777	5.91765	5.08684	5.9917	6.29716	6.94042	5.50316	4.60267	5.89207	6.81483	6.74626	0.005499847	0.152089577	12	4	24	0.008554131	0.501317957	24	4.826515234	0.194294922
ENSMUSG00000033196	Myh2	myosin, heavy polypeptide 2, skeletal muscle, adult [Source:MGI Symbol;Acc:MGI:1339710]	3.24429	10.1739	15.2658	13.9162	8.61303	12.3174	7.18501	31.0762	10.9164	27.8351	6.89251	6.26479	15.1309	11.8556	0.184797469	0.662239322	16	4	24	0.09248378	0.71175955	24	8.661407704	0.625515762
ENSMUSG00000033207	Mamdc2	MAM domain containing 2 [Source:MGI Symbol;Acc:MGI:1918988]	2.56618	2.47972	2.7564	1.92607	2.40528	2.3795	2.50062	2.58325	2.40912	3.00599	2.58967	2.47216	2.62745	2.38402	0.874607729	0.966018836	4	20	24	0.902814054	0.97295148	24	4.377236166	0.018102384
ENSMUSG00000033208	S100b	S100 protein, beta polypeptide, neural [Source:MGI Symbol;Acc:MGI:98217]	477.143	509.302	460.674	467.497	513.072	460.364	483.497	461.25	499.989	443.817	495.238	494.382	504.114	482.091	0.943920575	0.966018836	20	20	24	0.639834216	0.877988377	24	22.02916267	0.024704401
ENSMUSG00000033209	Ttc28	tetratricopeptide repeat domain 28 [Source:MGI Symbol;Acc:MGI:2140873]	1.43392	1.85771	1.58067	1.38466	1.67984	1.57927	1.5051	1.83137	1.66765	2.19094	1.64555	1.83101	1.63769	1.7656	0.676344318	0.966018836	8	20	24	0.574141432	0.847226836	24	9.132225434	0.042506417
ENSMUSG00000033213	AA467197	expressed sequence AA467197 [Source:MGI Symbol;Acc:MGI:3034182]	0.253153	0.997294	1.6512	1.58857	0.886857	1.14864	1.08146	0.735549	1.19354	1.20998	1.06573	1.13299	1.4899	1.01716	0.175612094	0.651716826	12	20	24	0.211097538	0.768040178	24	11.13792699	0.174795608
ENSMUSG00000033214	Slitrk5	SLIT and NTRK-like family, member 5 [Source:MGI Symbol;Acc:MGI:2679448]	5.20427	4.52223	4.86959	4.51513	4.13654	5.65729	5.0044	4.97944	3.72898	6.12958	4.89117	5.29674	5.1458	4.62494	0.022634137	0.27189101	24	12	24	0.400905471	0.799546792	24	20.26991864	0.081543765
ENSMUSG00000033216	Eefsec	eukaryotic elongation factor, selenocysteine-tRNA-specific [Source:MGI Symbol;Acc:MGI:2137092]	4.69886	3.94317	4.02813	2.81692	4.10246	3.99969	4.1205	3.85693	3.48529	4.13345	3.37465	3.87069	4.64601	5.06936	0.249881062	0.749643185	4	12	24	0.052557546	0.650526959	24	0.957297439	0.141503026
ENSMUSG00000033220	Rac2	Rac family small GTPase 2 [Source:MGI Symbol;Acc:MGI:97846]	3.21937	3.20291	4.79746	2.76912	4.65039	4.64256	4.4317	4.92567	2.56679	3.07543	2.70421	3.35689	3.98084	2.4605	0.951082476	0.966018836	8	20	24	0.691471451	0.897724829	24	5.296676834	0.097132805
ENSMUSG00000033222	Ttf2	transcription termination factor, RNA polymerase II [Source:MGI Symbol;Acc:MGI:1921294]	3.67412	2.9148	2.75049	2.99355	3.04922	3.30781	3.33597	3.53857	2.07388	3.59156	3.04258	2.95151	2.65424	3.17158	0.894563866	0.966018836	4	8	24	0.436637706	0.805393068	24	20.86981475	0.081329061
ENSMUSG00000033227	Wnt6	wingless-type MMTV integration site family, member 6 [Source:MGI Symbol;Acc:MGI:98960]	5.31434	5.8367	4.63414	4.54676	4.7314	4.31014	5.96155	4.86773	6.53708	4.84436	6.95567	5.60313	6.3222	5.35834	0.904387595	0.966018836	12	12	24	0.705975489	0.902918222	24	1.599601476	0.054191931
ENSMUSG00000033228	Scaf11	SR-related CTD-associated factor 11 [Source:MGI Symbol;Acc:MGI:1919443]	20.5783	18.8197	19.1127	19.8496	17.9344	19.2349	18.7187	17.7705	17.6395	19.9338	18.4727	19.2341	19.1044	19.8245	0.868638024	0.966018836	16	20	24	0.863931503	0.960594787	24	21.38308046	0.0141612
ENSMUSG00000033233	Trim45	tripartite motif-containing 45 [Source:MGI Symbol;Acc:MGI:1918187]	4.48812	3.72887	3.955	3.26659	4.27657	4.52675	4.65114	4.22292	3.44602	3.96259	3.19619	4.16512	3.57937	3.24354	0.223535563	0.717349224	4	8	24	0.06982742	0.674183933	24	21.62645305	0.11882003
ENSMUSG00000033237	Arid2	AT rich interactive domain 2 (ARID, RFX-like) [Source:MGI Symbol;Acc:MGI:1924294]	5.45888	5.5267	5.56057	6.19476	5.74014	5.2401	5.07807	5.16351	6.01393	5.76357	5.81882	5.6023	5.39844	5.58417	0.001845448	0.100255953	16	12	24	0.003735981	0.426426863	24	11.99938261	0.074016504
ENSMUSG00000033249	Hsf4	heat shock transcription factor 4 [Source:MGI Symbol;Acc:MGI:1347058]	11.4927	8.68626	8.39435	9.2272	8.01841	9.64399	8.86985	9.76011	8.33208	11.7956	9.41951	10.0407	10.3859	10.1661	0.43565473	0.966018836	4	8	24	0.695467573	0.898919068	24	22.50718754	0.050650041
ENSMUSG00000033253	Szt2	SZT2 subunit of KICSTOR complex [Source:MGI Symbol;Acc:MGI:3033336]	8.42696	9.42267	9.62544	11.5608	10.3842	10.1272	10.1459	9.48848	12.532	9.37072	9.40973	8.88861	9.22924	9.54431	0.43565473	0.966018836	12	16	24	0.152595198	0.759088284	24	10.70075587	0.099822479
ENSMUSG00000033256	Shf	Src homology 2 domain containing F [Source:MGI Symbol;Acc:MGI:3613669]	13.2026	12.5532	12.5531	12.831	12.8914	12.7874	13.8881	12.263	9.93268	16.1103	13.2835	12.264	11.0099	14.3342	0.922029406	0.966018836	16	20	24	0.755786103	0.921118175	24	15.58182701	0.045345151

ENSMUSG00000033257	Ttll4	tubulin tyrosine ligase-like family, member 4 [Source:MGI Symbol;Acc:MGI:1914784]	5.03846	6.26929	6.367	6.42586	6.06944	5.08411	5.84848	5.03706	6.94789	5.46779	6.53693	5.85601	5.90215	5.04597	0.138350222	0.580158868	12	20	24	0.178107298	0.768040178	24	11.70188284	0.094202291
ENSMUSG00000033272	Slc35a4	solute carrier family 35, member A4 [Source:MGI Symbol;Acc:MGI:1915093]	17.6218	15.8964	17.1101	16.9986	16.9089	17.7249	18.8361	18.8264	15.3509	21.3937	15.8264	18.3298	18.3035	18.161	0.496745696	0.966018836	4	16	24	0.890478948	0.969061918	24	22.72522562	0.022230335
ENSMUSG00000033276	Stk36	serine/threonine kinase 36 [Source:MGI Symbol;Acc:MGI:1920831]	1.13941	0.990002	1.43343	1.04635	1.53531	0.906997	1.33013	1.20059	0.876956	0.986298	1.58099	0.781036	1.16041	0.93354	0.941955432	0.966018836	20	4	24	0.731914824	0.912550805	24	15.6532157	0.051556355
ENSMUSG00000033278	Ptprm	protein tyrosine phosphatase, receptor type, M [Source:MGI Symbol;Acc:MGI:102694]	8.99416	6.60924	7.82448	8.18143	7.32853	9.21824	8.23601	8.90161	6.72913	10.5512	6.17696	8.21901	7.42661	8.1586	0.888991156	0.966018836	24	12	24	0.952968022	0.988573174	24	21.40412229	0.024220945
ENSMUSG00000033282	Rpgrip1l	Rpgrip1-like [Source:MGI Symbol;Acc:MGI:1920563]	4.00326	4.09262	3.09809	3.96936	4.62442	3.35139	3.848	3.00397	3.23382	2.94839	3.72296	3.58923	3.49164	3.94895	0.922035832	0.966018836	20	20	24	0.387024895	0.799546792	24	19.92354213	0.081131205
ENSMUSG00000033285	Wdr3	WD repeat domain 3 [Source:MGI Symbol;Acc:MGI:2443143]	9.59554	10.9552	8.95823	13.0136	6.86417	9.85063	10.2412	8.64086	10.5758	8.19167	10.1103	8.73549	9.62063	8.31214	0.809772024	0.966018836	4	16	24	0.89776535	0.970873489	24	6.210323867	0.037564569
ENSMUSG00000033287	Kctd17	potassium channel tetramerisation domain containing 17 [Source:MGI Symbol;Acc:MGI:1920094]	58.7585	48.2165	49.0611	46.4044	48.5281	58.0173	57.53	57.0083	47.2809	61.0876	51.6879	57.7085	53.618	56.301	0.12833063	0.560209134	24	12	24	0.162051088	0.76636332	24	22.39583413	0.092578282
ENSMUSG00000033294	Noc4l	NOC4 like [Source:MGI Symbol;Acc:MGI:2140843]	6.3443	4.84841	5.22851	5.50813	5.10078	6.44233	6.74288	6.7606	4.77876	6.65308	5.31536	5.98136	5.85351	5.22047	0.628286596	0.966018836	4	8	24	0.291919827	0.768040178	24	21.65885205	0.095542006
ENSMUSG00000033295	Ptprf	protein tyrosine phosphatase, receptor type, F [Source:MGI Symbol;Acc:MGI:102695]	48.8927	38.3543	35.7869	43.8645	48.4138	43.0654	45.4567	43.1576	37.659	54.9993	43.1657	45.4774	44.9702	40.2095	0.048507657	0.379129806	16	20	24	0.134788377	0.746461599	24	18.00058159	0.121999592
ENSMUSG00000033306	Lpp	LIM domain containing preferred translocation partner in lipoma [Source:MGI Symbol;Acc:MGI:2441849]	1.93206	2.2782	2.16904	2.37179	2.34492	2.0106	2.13399	2.11219	2.51134	1.93301	2.05432	2.18838	2.22826	2.3463	0.596159714	0.966018836	12	12	24	0.331070176	0.768040178	24	8.144784113	0.046719939
ENSMUSG00000033307	Mif	macrophage migration inhibitory factor (glycosylation-inhibiting factor) [Source:MGI Symbol;Acc:MGI:96982]	36.8863	39.4611	38.0311	35.0946	36.8559	39.8105	34.9493	37.6798	32.511	37.4894	38.8441	33.8785	38.2292	30.5692	0.816300547	0.966018836	20	16	24	0.468622855	0.805393068	24	18.60611266	0.049121979
ENSMUSG00000033308	Dpyd	dihydropyrimidine dehydrogenase [Source:MGI Symbol;Acc:MGI:2139667]	4.37323	4.04033	4.40111	5.04885	4.17937	3.77981	3.92833	3.90495	3.92054	4.16622	4.68259	4.55578	4.28979	4.04467	0.371875382	0.894537464	20	12	24	0.211161523	0.768040178	24	14.43638739	0.062696055
ENSMUSG00000033313	Fbxl8	F-box and leucine-rich repeat protein 8 [Source:MGI Symbol;Acc:MGI:1354697]	1.25214	1.14761	1.04184	0.997613	1.30235	1.06607	1.44852	1.57654	0.9977	1.62597	1.69862	1.76596	1.46883	1.36824	0.455507782	0.966018836	24	12	24	0.33824293	0.772907855	24	20.10019576	0.077410624
ENSMUSG00000033316	Galnt9	polypeptide N-acetylgalactosaminyltransferase 9 [Source:MGI Symbol;Acc:MGI:2677965]	7.76585	5.82772	7.99339	6.74289	6.88792	7.63206	7.38747	6.93743	5.08239	7.97948	7.08217	7.67239	6.52575	6.67135	0.798309651	0.966018836	24	8	24	0.277909581	0.768040178	24	18.66098835	0.100952904
ENSMUSG00000033318	Gstt2	glutathione S-transferase, theta 2 [Source:MGI Symbol;Acc:MGI:106188]	5.4733	8.90871	12.2999	9.55093	8.26746	7.31954	6.30718	10.7429	12.3328	10.8613	8.42938	7.64972	9.25331	10.0218	3.29E-04	0.049770315	12	16	24	1.04E-04	0.114833119	24	8.960038235	0.375177662
ENSMUSG00000033319	Fem1c	fem 1 homolog c [Source:MGI Symbol;Acc:MGI:2444737]	7.63447	7.02012	7.08084	6.91648	6.83167	6.97981	7.26902	6.51834	6.80902	7.19572	7.41908	6.75867	6.8123	7.10845	0.866861748	0.966018836	4	8	24	0.781293601	0.929846332	24	19.6012753	0.014878593
ENSMUSG00000033323	Ctdp1	CTD (carboxy-terminal domain, RNA polymerase II, polypeptide A) phosphatase, subunit 1 [Source:MGI Symbol;Acc:MGI:1926953]	4.70578	4.21841	3.68945	4.18418	3.59062	4.56802	4.15471	4.29041	4.4277	5.63554	4.23937	4.87676	4.22564	4.39039	0.891022863	0.966018836	24	20	24	0.970738023	0.99300601	24	18.10875142	0.012630874
ENSMUSG00000033326	Kdm4a	lysine (K)-specific demethylase 4A [Source:MGI Symbol;Acc:MGI:2446210]	11.6215	11.0471	10.3255	12.0394	11.7985	12.1709	10.3823	11.8096	10.6904	13.3236	10.5933	12.2145	10.6677	11.2577	0.384058496	0.915479136	16	20	24	0.362100966	0.787054939	24	15.52375415	0.054913099
ENSMUSG00000033327	Tnxb	tenascin XB [Source:MGI Symbol;Acc:MGI:1932137]	5.93984	6.35747	5.80919	6.7001	8.1511	5.29294	6.63353	6.17904	8.89766	7.48755	6.72254	5.66004	6.39346	5.5942	0.175185919	0.651010555	20	4	24	0.136143568	0.747972781	24	11.51434185	0.138803737
ENSMUSG00000033335	Dnm2	dynamin 2 [Source:MGI Symbol;Acc:MGI:109547]	22.9759	21.8202	20.8306	23.1811	25.032	23.53	21.1672	23.7918	24.2244	23.2005	24.4085	22.8288	24.3608	22.6039	0.435739761	0.966018836	20	12	24	0.25769156	0.768040178	24	16.04536671	0.044497851
ENSMUSG00000033342	Plppr5	phospholipid phosphatase related 5 [Source:MGI Symbol;Acc:MGI:1923019]	1.26634	1.75858	2.14713	1.80767	1.43495	1.75529	1.93851	1.42777	1.73489	1.9277	1.5326	1.70015	1.4023	2.1298	0.328842658	0.847277612	12	16	24	0.227129471	0.768040178	24	8.640126614	0.099056615
ENSMUSG00000033350	Chst2	carbohydrate sulfotransferase 2 [Source:MGI Symbol;Acc:MGI:1891160]	7.04103	7.21225	6.87256	7.3287	8.49431	7.09065	8.03961	7.03266	7.20718	9.0289	7.11947	7.37847	7.19365	7.43688	0.967855455	0.970142211	16	20	24	0.34311	0.777029669	24	14.41587205	0.056142523
ENSMUSG00000033352	Map2k4	mitogen-activated protein kinase kinase 4 [Source:MGI Symbol;Acc:MGI:1346869]	65.3457	60.2201	63.0397	59.9237	56.9598	64.6395	59.1887	60.016	51.7856	67.4591	54.8289	59.8757	59.4148	64.4975	0.922029406	0.966018836	8	12	24	0.789061605	0.932379688	24	0.502397854	0.027712684
ENSMUSG00000033355	Rtp4	receptor transporter protein 4 [Source:MGI Symbol;Acc:MGI:1915025]	6.74211	7.7545	7.6447	6.79367	7.38624	6.69773	6.44598	9.25282	7.37918	6.15591	9.25168	6.79569	7.09827	7.70651	0.138543921	0.580158868	8	20	24	0.670135122	0.888925819	24	6.897869728	0.057139023
ENSMUSG00000033356	Pus7l	pseudouridylate synthase 7-like [Source:MGI Symbol;Acc:MGI:1926145]	1.53643	1.67588	1.60928	1.58678	1.66091	1.41079	1.62691	1.32929	1.53639	1.68745	1.79543	1.48603	1.46223	1.81664	0.676344318	0.966018836	20	4	24	0.442423701	0.805393068	24	11.50762733	0.039083653
ENSMUSG00000033361	Prrg3	proline rich Gla (G-carboxyglutamic acid) 3 (transmembrane) [Source:MGI Symbol;Acc:MGI:2685214]	28.2239	26.4943	25.9971	30.7106	35.9937	28.4422	32.5272	26.9266	30.2036	33.0661	28.0076	30.833	30.7318	25.2168	0.05282315	0.391647859	20	12	24	0.08765959	0.707747429	24	16.47177065	0.115379217
ENSMUSG00000033364	Usp37	ubiquitin specific peptidase 37 [Source:MGI Symbol;Acc:MGI:2442483]	5.24534	5.38547	5.92611	5.70041	6.00915	6.14608	5.42373	5.45458	5.63672	5.18643	5.91407	6.48735	5.9109	5.8949	0.082282215	0.462922111	24	4	24	0.23804543	0.768040178	24	17.99042459	0.048927311
ENSMUSG00000033365	Ipo13	importin 13 [Source:MGI Symbol;Acc:MGI:2385205]	26.0908	23.0089	26.0722	29.0456	24.0311	26.169	24.016	27.0463	24.8757	27.6589	20.9498	22.6779	21.5051	23.36	0.351315142	0.870418653	16	4	24	0.277207387	0.768040178	24	10.02686683	0.074007826
ENSMUSG00000033373	Fntb	farnesyltransferase, CAAX box, beta [Source:MGI Symbol;Acc:MGI:1861305]	21.2273	17.8788	16.6735	17.7178	21.1012	20.0802	23.4852	20.9737	18.0077	21.6733	17.0376	20.479	19.9364	18.3809	0.377567433	0.903463289	4	8	24	0.15605713	0.761695095	24	21.60176961	0.105654463
ENSMUSG00000033377	Palmd	palmdelphin [Source:MGI Symbol;Acc:MGI:2148896]	31.0372	27.3014	27.8405	28.6243	25.1208	28.9018	28.0568	29.3971	24.4428	33.4536	27.7541	30.012	29.5528	31.6646	0.598711142	0.966018836	4	8	24	0.786352638	0.93134108	24	0.414818379	0.031232481
ENSMUSG00000033379	Atp6v0b	ATPase, H+ transporting, lysosomal V0 subunit B [Source:MGI Symbol;Acc:MGI:1890510]	206.338	182.864	178.12	177.366	179.126	183.295	181.445	181.043	179.961	163.233	208.088	190.305	192.388	188.581	0.00486525	0.141782417	4	12	24	0.124116609	0.737882465	24	21.94296922	0.069593605
ENSMUSG00000033382	Trappc8	trafficking protein particle complex 8 [Source:MGI Symbol;Acc:MGI:2443008]	27.2555	25.2173	25.2916	25.3116	26.3254	26.7143	24.5201	24.4177	22.6397	24.318	25.3633	25.6991	26.7645	25.7923	0.053683798	0.394331071	4	8	24	0.03206869	0.605537343	24	20.71063619	0.063265024
ENSMUSG00000033386	Frrs1	ferric-chelate reductase 1 [Source:MGI Symbol;Acc:MGI:108076]	0.769679	0.550152	0.555326	0.494095	0.394225	0.589292	0.246919	0.448826	0.442143	0.453372	0.442833	0.64143	0.438589	0.53997	0.874672901	0.966018836	24	20	24	0.822671751	0.944336753	24	22.83525046	0.029520342
ENSMUSG00000033389	Arhgap44	Rho GTPase activating protein 44 [Source:MGI Symbol;Acc:MGI:2144423]	19.2698	17.8475	17.5955	17.9096	15.634	20.0341	17.7663	18.5875	14.6035	22.8835	15.0278	18.0254	16.8821	19.1246	0.892044196	0.966018836	24	12	24	0.901083182	0.972154687	24	1.361765689	0.029263441
ENSMUSG00000033392	Clasp2	CLIP associating protein 2 [Source:MGI Symbol;Acc:MGI:1923749]	69.2955	69.057	66.4334	64.7682	64.5443	66.0166	65.8361	61.6398	62.0341	63.9913	67.2133	67.5603	66.8709	69.8713	0.349109869	0.870418653	8	4	24	0.247863924	0.768040178	24	22.94588044	0.035403438
ENSMUSG00000033396	Spg11	SPG11, spatacsin vesicle trafficking associated [Source:MGI Symbol;Acc:MGI:2444989]	4.82728	4.24548	4.53599	4.53139	4.56166	4.48414	4.46379	4.6302	4.05971	4.66633	4.43481	4.45967	4.82599	4.30077	0.947524602	0.966018836	4	8	24	0.388912857	0.799546792	24	20.29382216	0.030881809
ENSMUSG00000033400	Agl	amylo-1,6-glucosidase, 4-alpha-glucanotransferase [Source:MGI Symbol;Acc:MGI:1924809]	26.3874	26.8852	26.7053	33.0235	24.5806	29.9529	26.5837	33.3591	27.3166	35.1583	27.2763	28.2507	30.1352	28.5059	0.959160181	0.967043964	16	4	24	0.543939379	0.831926684	24	9.817718185	0.062714784
ENSMUSG00000033405	Nudt15	nudix (nucleoside diphosphate linked moiety X)-type motif 15 [Source:MGI Symbol;Acc:MGI:2443366]	0.9845	0.819787	0.819498	0.775667	0.811892	0.821103	0.934796	1.15631	0.856785	1.03255	0.872643	0.866859	0.891471	0.924334	0.223362379	0.717349224	4	16	24	0.376348226	0.796393126	24	3.305485619	0.040144897
ENSMUSG00000033411	Ctdspl2	CTD (carboxy-terminal domain, RNA polymerase II, polypeptide A) small phosphatase like 2 [Source:MGI Symbol;Acc:MGI:1196405]	3.21894	4.09592	4.68954	3.94211	3.83838	3.4646	3.91565	3.44412	5.04406	2.8678	3.86658	3.54313	3.71433	3.80954	0.085890569	0.466404237	12	12	24	0.293347994	0.768040178	24	7.801194562	0.101871718
ENSMUSG00000033416	Gucd1	guanylyl cyclase domain containing 1 [Source:MGI Symbol;Acc:MGI:1916028]	13.9627	11.8856	13.3434	13.8487	13.1446	13.842	12.0539	14.2986	12.3457	14.3024	14.2584	14.2965	13.5163	13.3522	0.657586167	0.966018836	16	20	24	0.294521041	0.768040178	24	16.24162157	0.049911785
ENSMUSG00000033417	Cacul1	CDK2 associated, cullin domain 1 [Source:MGI Symbol;Acc:MGI:1926082]	20.1388	17.7658	17.6711	17.6999	17.406	19.0784	17.8397	18.0597	15.4101	19.6737	19.6604	18.5	17.9372	20.1348	0.595037318	0.966018836	24	12	24	0.460280912	0.805393068	24	20.51304867	0.049629816
ENSMUSG00000033419	Snap91	synaptosomal-associated protein 91 [Source:MGI Symbol;Acc:MGI:109132]	57.676	45.8736	49.1079	46.3988	44.6714	54.8005	53.0549	48.4592	36.406	61.7696	42.35	48.3764	43.6478	48.5542	0.893609703	0.966018836	4	16	24	0.617845706	0.868673353	24	21.14229948	0.075419573
ENSMUSG00000033420	Antxr1	anthrax toxin receptor 1 [Source:MGI Symbol;Acc:MGI:1916788]	6.90083	7.81945	7.74563	6.42442	7.61882	6.72366	7.54199	7.60352	7.94689	7.79627	6.05407	7.15451	7.59575	7.8989	0.016110955	0.253395174	12	12	24	0.045549649	0.629609444	24	5.408247386	0.096556766
ENSMUSG00000033423	Eri3	exoribonuclease 3 [Source:MGI Symbol;Acc:MGI:2153887]	45.4417	39.5667	50.8005	43.1289	46.6461	47.2798	34.6985	42.3879	40.3521	46.0115	38.6024	46.6775	35.9545	37.6604	0.10338739	0.517709628	24	4	24	0.211226748	0.768040178	24	14.31087158	0.097014476
ENSMUSG00000033427	Upb1	ureidopropionase, beta [Source:MGI Symbol;Acc:MGI:2143535]	0.986628	0.934245	0.7799	0.946846	0.694805	0.79992	0.653676	1.02822	0.84609	0.742553	0.85484	0.820184	0.898164	0.997237	0.109247218	0.524035616	8	12	24	0.216419418	0.768040178	24	4.340919831	0.060558058
ENSMUSG00000033429	Mcee	methylmalonyl CoA epimerase [Source:MGI Symbol;Acc:MGI:1920974]	36.9401	42.162	39.5794	43.0337	42.4908	39.3822	41.3618	38.9491	50.9321	38.5491	47.2928	46.2488	49.088	44.2847	0.826055781	0.966018836	20	8	24	0.875195812	0.964979482	24	11.42011426	0.023109809
ENSMUSG00000033430	Terf2ip	telomeric repeat binding factor 2, interacting protein [Source:MGI Symbol;Acc:MGI:1929871]	11.9504	11.4549	12.3304	10.4774	10.2444	11.7581	11.4372	10.8805	10.5836	13.1033	10.5275	12.299	11.8798	13.0552	0.881355613	0.966018836	24	20	24	0.507277686	0.812455891	24	2.819300397	0.047143255
ENSMUSG00000033434	Gtpbp6	GTP binding protein 6 (putative) [Source:MGI Symbol;Acc:MGI:1306825]	17.7534	16.4696	16.6719	15.6041	18.0898	14.2744	18.7941	16.144	16.4686	16.346	17.7527	18.1546	19.6142	17.5324	0.018810871	0.256345714	4	12	24	0.397949687	0.799546792	24	23.00537421	0.056292654
ENSMUSG00000033436	Armcx2	armadillo repeat containing, X-linked 2 [Source:MGI Symbol;Acc:MGI:1914666]	48.8478	42.9188	42.1479	42.8104	41.7974	46.864	42.4276	45.5031	40.5706	48.2776	40.8035	46.2296	44.4075	46.812	0.368981219	0.893596186	24	20	24	0.43873798	0.805393068	24	23.23363688	0.044466341
ENSMUSG00000033439	Trmt13	tRNA methyltransferase 13 [Source:MGI Symbol;Acc:MGI:1925219]	4.52098	6.65067	4.79583	6.92141	7.6278	5.37863	5.21665	5.29748	7.22341	5.06107	6.47012	5.96032	5.17839	6.48828	0.231491055	0.731037655	20	8	24	0.305454392	0.768040178	24	12.45524617	0.119526184
ENSMUSG00000033444	Specc1l	sperm antigen with calponin homology and coiled-coil domains 1-like [Source:MGI Symbol;Acc:MGI:1921642]	24.7703	21.0378	21.2338	22.8498	22.0578	23.0623	22.6039	23.0274	20.0085	23.5167	21.6894	23.1312	22.2859	23.1432	0.601647512	0.966018836	24	12	24	0.255997342	0.768040178	24	21.02187229	0.048903236
ENSMUSG00000033446	Lpar6	lysophosphatidic acid receptor 6 [Source:MGI Symbol;Acc:MGI:1914418]	1.08739	1.84991	1.86214	1.54278	1.5716	1.44728	1.62935	1.81008	1.82937	1.21169	1.39965	1.38183	1.89107	1.78357	0.094391858	0.492161865	12	4	24	0.066749558	0.669146244	24	5.672486517	0.127629402
ENSMUSG00000033453	Adamts15	a disintegrin-like and metallopeptidase (reprolysin type) with thrombospondin type 1 motif, 15 [Source:MGI Symbol;Acc:MGI:2449569]	5.55268	4.68588	3.82039	3.81774	4.82986	5.46058	4.33897	4.57786	3.72557	4.54714	4.19445	5.22478	5.37337	4.69631	0.008047769	0.192760771	24	12	24	0.002799324	0.414500244	24	21.75186432	0.178864734
ENSMUSG00000033454	Zbtb1	zinc finger and BTB domain containing 1 [Source:MGI Symbol;Acc:MGI:2442326]	3.75643	3.7452	3.38137	3.91057	3.84056	3.48718	3.77163	3.39967	3.52739	3.55667	4.0096	3.62276	3.52959	3.38712	0.059844417	0.406773654	20	16	24	0.076386995	0.686372559	24	16.92769376	0.050663767
ENSMUSG00000033458	Fan1	FANCD2/FANCI-associated nuclease 1 [Source:MGI Symbol;Acc:MGI:3045266]	2.33848	2.89531	3.09254	3.07615	3.19645	2.77119	2.70429	2.79863	3.09038	3.4386	2.97247	2.89652	2.81507	3.10074	0.004021228	0.140598674	16	12	24	3.41E-04	0.227979988	24	11.28108445	0.110683184
ENSMUSG00000033460	Armcx1	armadillo repeat containing, X-linked 1 [Source:MGI Symbol;Acc:MGI:1925498]	87.6936	80.0041	75.2626	74.5018	77.6802	81.1405	77.9644	75.9249	68.6392	83.58	78.5281	79.8769	83.877	84.835	0.055920623	0.401074772	4	8	24	0.138451531	0.747972781	24	22.19487467	0.067842738
ENSMUSG00000033467	Crlf2	cytokine receptor-like factor 2 [Source:MGI Symbol;Acc:MGI:1889506]	2.75315	1.96107	2.57277	1.612	1.401	1.94195	2.06158	3.20372	3.10512	3.85343	4.60557	4.788	4.27018	4.28234	0.826055781	0.966018836	8	8	24	0.729767372	0.911863904	24	3.741711752	0.084610391
ENSMUSG00000033470	Cysltr2	cysteinyl leukotriene receptor 2 [Source:MGI Symbol;Acc:MGI:1917336]	15.8573	14.8669	14.2755	15.4334	15.0478	13.0902	10.3091	13.6493	14.4987	13.2253	14.7952	13.2905	14.5536	13.4479	0.897425765	0.966018836	20	4	24	0.553358734	0.837286821	24	11.77899257	0.05880325
ENSMUSG00000033475	Tomm6	translocase of outer mitochondrial membrane 6 [Source:MGI Symbol;Acc:MGI:1913369]	84.4202	76.9017	73.7646	77.1782	66.0093	74.2177	80.1287	84.2061	76.4486	83.1024	80.812	78.5428	86.0453	81.4101	0.154289127	0.606139807	4	8	24	0.173476659	0.768040178	24	2.592108871	0.063743683
ENSMUSG00000033478	Fam160b1	family with sequence similarity 160, member B1 [Source:MGI Symbol;Acc:MGI:2147545]	11.2254	9.01927	9.18084	9.73651	9.08192	10.4561	9.79829	9.69736	7.80077	10.6013	8.75832	9.25064	8.95727	9.17807	0.850398351	0.966018836	24	12	24	0.363350513	0.788241076	24	20.71238072	0.064136679
ENSMUSG00000033486	Catsper2	cation channel, sperm associated 2 [Source:MGI Symbol;Acc:MGI:2387404]	2.78114	2.84066	2.35513	3.1166	2.65313	2.82628	2.94736	2.55462	2.88144	2.81365	2.95896	2.3113	2.86921	2.96437	0.85387321	0.966018836	16	8	24	0.961153446	0.989968428	24	10.04294962	0.01072764
ENSMUSG00000033487	Fndc3a	fibronectin type III domain containing 3A [Source:MGI Symbol;Acc:MGI:1196463]	11.6029	11.1391	11.149	10.4338	11.4053	10.4918	11.0887	10.3139	10.4175	9.28202	11.0853	10.2224	10.0839	10.2237	0.847846021	0.966018836	20	20	24	0.354118165	0.781273395	24	21.73729971	0.034825002
ENSMUSG00000033488	Cryzl2	crystallin zeta like 2 [Source:MGI Symbol;Acc:MGI:2448516]	6.55755	8.97541	9.3507	7.42102	9.31544	7.44778	7.88802	8.38566	10.8449	9.25188	8.0656	8.44986	8.27152	8.19129	0.067104747	0.425837275	12	16	24	0.061833292	0.661961741	24	9.393307906	0.137956447
ENSMUSG00000033491	Prss35	protease, serine 35 [Source:MGI Symbol;Acc:MGI:2444800]	11.6877	10.9459	10.3579	10.415	11.8705	11.6924	11.6531	11.8871	8.21218	13.0448	12.6362	9.16224	10.0085	10.7931	0.446653834	0.966018836	20	16	24	0.568579765	0.844453328	24	17.7860389	0.070712408
ENSMUSG00000033499	Larp4b	La ribonucleoprotein domain family, member 4B [Source:MGI Symbol;Acc:MGI:106330]	22.7504	21.6365	22.2037	21.6482	21.6892	22.4485	20.5181	21.6001	20.2724	23.8973	20.866	22.118	21.4713	22.7716	0.852844413	0.966018836	24	4	24	0.954239573	0.988601783	24	14.29561531	0.00742019
ENSMUSG00000033501	Crygs	crystallin, gamma S [Source:MGI Symbol;Acc:MGI:1298216]	2.90479	4.11485	3.52269	3.51969	3.42418	3.54685	3.54255	3.64828	5.1857	1.99313	3.26178	2.56707	3.57464	2.42441	0.465197787	0.966018836	12	4	24	0.672445686	0.889492013	24	5.312100678	0.088028435
ENSMUSG00000033502	Cdc14a	CDC14 cell division cycle 14A [Source:MGI Symbol;Acc:MGI:2442676]	2.22098	2.82741	2.98359	3.30897	2.63842	2.76882	2.80013	2.97207	3.30191	3.21911	2.84235	3.25514	2.89884	2.66515	0.097542159	0.501112642	16	12	24	0.061172797	0.660703626	24	10.85857551	0.093799556
ENSMUSG00000033508	Asprv1	aspartic peptidase, retroviral-like 1 [Source:MGI Symbol;Acc:MGI:1915105]	0.544266	0.699514	1.1532	0.923304	0.774805	0.701922	0.559887	0.539938	0.862154	0.491936	0.711107	0.710295	0.418891	0.68063	0.009483698	0.194720062	12	16	24	0.099925243	0.71175955	24	11.11654511	0.118227074
ENSMUSG00000033510	Otud7a	OTU domain containing 7A [Source:MGI Symbol;Acc:MGI:2158505]	5.7489	4.16902	5.02329	4.88866	4.47963	5.43633	4.98954	5.0593	3.68458	6.26222	4.58928	5.32392	4.96771	5.33538	0.904387595	0.966018836	24	20	24	0.637075061	0.876483853	24	20.28421849	0.062641
ENSMUSG00000033526	Ppip5k1	diphosphoinositol pentakisphosphate kinase 1 [Source:MGI Symbol;Acc:MGI:2443281]	24.4945	22.5272	23.6433	22.8461	23.1445	23.6978	24.0816	25.0296	21.591	27.0977	21.4202	23.1515	23.8969	25.327	0.325142889	0.842977406	8	12	24	0.704571994	0.902439904	24	3.697324155	0.028658858
ENSMUSG00000033530	Ttc7b	tetratricopeptide repeat domain 7B [Source:MGI Symbol;Acc:MGI:2144724]	23.8986	19.6367	19.3565	18.1216	18.0888	21.8147	21.3704	23.8723	19.2748	26.7126	18.5333	21.2537	21.028	22.093	0.217933169	0.704989686	4	16	24	0.489927789	0.805393068	24	1.758047468	0.072837301
ENSMUSG00000033538	Casp4	caspase 4, apoptosis-related cysteine peptidase [Source:MGI Symbol;Acc:MGI:107700]	3.18408	4.49047	3.42685	2.9544	4.51566	3.45226	3.35022	4.12077	5.27682	2.65489	3.81126	3.53083	3.65797	3.59182	0.800065124	0.966018836	8	8	24	0.7161224	0.906323093	24	5.1805963	0.068434416
ENSMUSG00000033540	Idua	iduronidase, alpha-L [Source:MGI Symbol;Acc:MGI:96418]	9.63075	8.37221	9.11228	8.53742	9.61483	7.96595	9.93627	8.68526	9.71373	8.52704	7.36251	8.53043	8.59118	8.22287	0.590017078	0.966018836	4	20	24	0.656544741	0.883952697	24	3.585689056	0.037057435
ENSMUSG00000033542	Arhgef5	Rho guanine nucleotide exchange factor (GEF) 5 [Source:MGI Symbol;Acc:MGI:1858952]	1.51399	1.65799	1.89908	1.70535	1.55414	1.46317	2.00393	1.63231	1.62267	1.40723	1.40685	1.57339	1.34484	1.10132	0.895493752	0.966018836	12	8	24	0.971519293	0.993114859	24	8.189038233	0.010475905
ENSMUSG00000033543	Gtf2a2	general transcription factor II A, 2 [Source:MGI Symbol;Acc:MGI:1933289]	42.6376	49.7354	46.9204	47.5447	46.952	44.4449	45.5623	42.4742	50.5686	43.6368	50.4334	47.0225	45.3916	47.7974	0.708020493	0.966018836	12	16	24	0.419663459	0.803639585	24	10.93403109	0.041997969
ENSMUSG00000033544	Angptl1	angiopoietin-like 1 [Source:MGI Symbol;Acc:MGI:1919963]	2.95816	3.20775	2.64914	3.32084	3.95751	2.7492	3.19755	3.03526	3.11311	1.96425	3.07748	2.86668	2.50087	2.36503	0.88067614	0.966018836	20	4	24	0.574931691	0.8477908	24	18.3300047	0.068384234
ENSMUSG00000033545	Znrf1	zinc and ring finger 1 [Source:MGI Symbol;Acc:MGI:2177308]	16.2404	15.4101	18.0365	16.8361	15.784	15.9719	18.7246	15.6848	15.8424	19.7957	16.1759	16.3942	16.9587	15.7848	0.446653834	0.966018836	16	16	24	0.713623264	0.905681809	24	12.09728249	0.033375146
ENSMUSG00000033554	Dph5	diphthamide biosynthesis 5 [Source:MGI Symbol;Acc:MGI:1916990]	10.2446	10.7001	10.2661	10.6134	10.1421	10.5571	9.15293	10.914	11.9509	9.95834	11.0517	9.85455	10.4497	9.75615	0.843639218	0.966018836	12	16	24	0.389085789	0.799546792	24	9.782462949	0.046503301
ENSMUSG00000033557	Fam20b	FAM20B, glycosaminoglycan xylosylkinase [Source:MGI Symbol;Acc:MGI:2443990]	23.657	20.6678	21.4638	21.9069	21.2431	22.9195	24.861	23.3	19.3217	25.6077	19.9798	23.3582	22.3185	23.3873	0.371875382	0.894537464	4	8	24	0.503261793	0.810053779	24	22.87909885	0.051813937
ENSMUSG00000033565	Rbfox2	RNA binding protein, fox-1 homolog (C. elegans) 2 [Source:MGI Symbol;Acc:MGI:1933973]	47.157	38.857	40.3043	38.7957	41.0997	44.7555	41.4641	40.7208	34.8934	48.0896	41.8754	45.377	41.3969	44.2988	0.038115943	0.334069441	24	12	24	0.218716449	0.768040178	24	20.03526148	0.082480712
ENSMUSG00000033569	Adgrb3	adhesion G protein-coupled receptor B3 [Source:MGI Symbol;Acc:MGI:2441837]	5.31291	6.27399	6.65295	5.78673	4.97923	6.04175	5.84907	5.44369	5.53456	6.14814	4.77964	6.08588	5.10216	6.33934	0.287843582	0.793714531	12	8	24	0.3346405	0.770810225	24	6.271341744	0.071614522
ENSMUSG00000033576	Apol6	apolipoprotein L 6 [Source:MGI Symbol;Acc:MGI:1919189]	1.10752	1.22119	1.18479	2.00244	1.66918	1.58505	1.02234	1.99426	1.38102	2.28199	1.25124	1.09757	1.6092	1.73755	0.117050591	0.538362324	16	12	24	0.194118239	0.768040178	24	10.82022497	0.150140572
ENSMUSG00000033577	Myo6	myosin VI [Source:MGI Symbol;Acc:MGI:104785]	11.5141	14.266	14.9529	12.672	12.7514	11.8203	14.2975	13.6239	15.0845	11.3759	13.8273	14.0741	11.6965	13.2435	0.369262716	0.893596186	12	4	24	0.378889659	0.798235801	24	7.025913989	0.073957814
ENSMUSG00000033578	Tmem35a	transmembrane protein 35A [Source:MGI Symbol;Acc:MGI:1914814]	30.2714	26.9162	27.4637	26.8202	25.6393	30.5224	29.2135	28.265	24.2231	29.7434	25.8928	29.6837	29.2855	31.4571	0.121243373	0.548623108	24	20	24	0.109122639	0.72082227	24	23.47631549	0.081503014
ENSMUSG00000033579	Fa2h	fatty acid 2-hydroxylase [Source:MGI Symbol;Acc:MGI:2443327]	43.1022	35.7073	35.1018	43.5879	53.3867	43.3791	52.7316	35.5736	40.8023	57.315	38.1928	43.0333	39.0773	32.0571	0.028180201	0.314814652	16	16	24	0.088983581	0.708139275	24	16.65162756	0.196010599
ENSMUSG00000033581	Igf2bp2	insulin-like growth factor 2 mRNA binding protein 2 [Source:MGI Symbol;Acc:MGI:1890358]	3.2227	2.40004	1.74035	3.00526	3.05954	2.65795	3.04157	2.2336	2.68377	2.68573	3.36416	2.29151	2.64126	2.29891	0.03484427	0.328311403	20	12	24	0.122821233	0.735469704	24	17.91624073	0.142133981
ENSMUSG00000033585	Ndn	necdin, MAGE family member [Source:MGI Symbol;Acc:MGI:97290]	40.0789	31.1465	35.8459	31.7894	31.0725	36.2341	34.1591	35.8573	28.5269	37.8093	35.4034	37.4502	34.6358	35.325	0.969487593	0.970883022	24	12	24	0.351149315	0.780345856	24	21.50055249	0.07433032
ENSMUSG00000033589	Reep4	receptor accessory protein 4 [Source:MGI Symbol;Acc:MGI:1919799]	4.47618	4.36587	4.15529	6.58748	5.25861	4.5528	5.04308	5.29362	5.22727	5.22703	5.71696	5.09015	4.84872	4.71865	0.176949612	0.653256853	16	20	24	0.08238911	0.699403344	24	13.72998014	0.112204052
ENSMUSG00000033594	Spata2l	spermatogenesis associated 2-like [Source:MGI Symbol;Acc:MGI:1926029]	1.62846	1.28968	1.18425	1.73228	1.45633	1.53814	1.28202	1.46058	1.26876	1.47357	1.41414	1.26871	1.36371	1.4375	0.676344318	0.966018836	16	20	24	0.522060681	0.820717128	24	16.69485095	0.040186438
ENSMUSG00000033595	Lgi3	leucine-rich repeat LGI family, member 3 [Source:MGI Symbol;Acc:MGI:2182619]	33.2279	30.046	34.1985	30.1564	26.5429	34.4001	35.151	32.8004	25.4789	36.979	26.829	31.938	28.7478	35.5171	0.94663657	0.966018836	8	12	24	0.583909626	0.852029424	24	2.113098656	0.067684996
ENSMUSG00000033596	Rfwd3	ring finger and WD repeat domain 3 [Source:MGI Symbol;Acc:MGI:2384584]	5.10721	5.08916	4.77831	5.06891	4.87378	5.29481	5.02107	4.71062	5.00645	5.11855	5.10809	5.55213	5.38322	4.85651	0.015345591	0.253245931	24	12	24	0.054519063	0.655263328	24	19.65430224	0.047326327
ENSMUSG00000033597	Caskin1	CASK interacting protein 1 [Source:MGI Symbol;Acc:MGI:2442952]	29.1825	24.2833	24.8113	22.827	24.0266	23.9955	25.1839	26.7075	27.0918	33.1081	23.4303	28.9401	28.9906	28.1341	0.5169953	0.966018836	4	16	24	0.647989423	0.881026758	24	3.663374828	0.049964231
ENSMUSG00000033610	Pank1	pantothenate kinase 1 [Source:MGI Symbol;Acc:MGI:1922985]	5.50973	5.12761	5.8721	9.00092	5.69335	6.14969	5.89031	6.76094	5.22183	7.26922	6.87389	5.99704	7.12415	8.10781	0.941885246	0.966018836	16	20	24	0.496512112	0.807543854	24	11.56919748	0.088074429
ENSMUSG00000033615	Cplx1	complexin 1 [Source:MGI Symbol;Acc:MGI:104727]	248.817	200.18	209.371	221.37	188.94	242.651	229.481	221.571	171.055	291.912	181.916	233.372	213.142	234.567	0.431765373	0.966018836	24	12	24	0.794275988	0.933999221	24	22.1658486	0.055869783
ENSMUSG00000033618	Map3k13	mitogen-activated protein kinase kinase kinase 13 [Source:MGI Symbol;Acc:MGI:2444243]	3.06967	2.1031	2.29767	2.51381	2.44196	2.64999	2.89416	2.27754	1.70608	2.88437	2.32726	2.40035	2.35076	2.34056	0.08464875	0.466404237	4	4	24	0.158458268	0.764909967	24	19.8846471	0.109509869
ENSMUSG00000033623	Pcgf3	polycomb group ring finger 3 [Source:MGI Symbol;Acc:MGI:1916837]	8.6949	7.32854	7.10804	7.65729	7.90465	8.11945	7.98909	7.66283	6.86995	8.15493	8.00362	7.3038	7.49134	7.85407	0.209352197	0.69936851	20	16	24	0.17540252	0.768040178	24	19.77261518	0.057894
ENSMUSG00000033624	Pdpr	pyruvate dehydrogenase phosphatase regulatory subunit [Source:MGI Symbol;Acc:MGI:2442188]	5.83101	6.80124	7.80573	8.64114	7.17071	6.84558	6.82298	7.57145	7.89176	8.54795	7.20771	7.71819	7.90504	7.47405	0.059513104	0.406067671	16	12	24	0.004875443	0.438275631	24	10.71842294	0.126805373
ENSMUSG00000033628	Pik3c3	phosphatidylinositol 3-kinase catalytic subunit type 3 [Source:MGI Symbol;Acc:MGI:2445019]	22.7702	20.279	19.8927	19.5733	18.5279	21.0885	20.8903	21.5695	17.821	23.4757	19.1316	21.4739	21.3131	22.3443	0.241950772	0.741580974	4	16	24	0.315263089	0.768040178	24	0.485192215	0.062618827
ENSMUSG00000033629	Hacd3	3-hydroxyacyl-CoA dehydratase 3 [Source:MGI Symbol;Acc:MGI:1889341]	103.888	98.0687	97.0142	95.4009	96.1508	103.44	93.2331	93.0298	87.1678	97.6748	100.393	99.4468	100.086	101.213	0.236893212	0.7385769	24	12	24	0.125720455	0.739647205	24	20.56824434	0.052762159
ENSMUSG00000033632	AW554918	expressed sequence AW554918 [Source:MGI Symbol;Acc:MGI:2147376]	3.6163	3.98979	3.66605	4.0331	3.79424	3.28993	3.52463	3.69234	4.10537	3.30186	3.63155	3.69581	3.90678	4.11573	0.335729873	0.85758369	8	16	24	0.195632891	0.768040178	24	6.481908888	0.0579574
ENSMUSG00000033633	Clec18a	C-type lectin domain family 18, member A [Source:MGI Symbol;Acc:MGI:2672935]	1.33988	1.12462	0.790749	1.47917	1.65847	1.38829	0.90299	1.08059	1.10224	1.07215	0.940318	0.964902	1.43349	1.04163	0.892044196	0.966018836	16	20	24	0.421356127	0.804555939	24	18.20771385	0.086221962
ENSMUSG00000033644	Piwil2	piwi-like RNA-mediated gene silencing 2 [Source:MGI Symbol;Acc:MGI:1930036]	0.873711	0.847565	0.760909	0.605652	0.640122	0.666573	0.966863	0.643876	0.826912	0.736287	0.799249	0.669965	0.767184	0.786878	0.081561283	0.462922111	4	12	24	0.190056977	0.768040178	24	2.300803978	0.057491369
ENSMUSG00000033653	Vps8	VPS8 CORVET complex subunit [Source:MGI Symbol;Acc:MGI:2146407]	16.4262	14.8965	13.6163	15.5255	13.3558	15.6582	12.9187	14.6758	12.7362	16.0114	13.1515	14.5734	15.4428	15.8216	0.815656735	0.966018836	4	8	24	0.749685097	0.918587922	24	0.243296952	0.035789794
ENSMUSG00000033658	Ddx19b	DEAD box helicase 19b [Source:MGI Symbol;Acc:MGI:2148251]	5.8675	5.34325	5.28647	5.06665	5.64832	6.01079	5.06251	5.40762	4.90284	5.3359	5.34813	5.62659	5.2937	5.49163	0.0444686	0.365196805	24	12	24	0.052053651	0.649509116	24	20.42221065	0.061810794
ENSMUSG00000033669	Zfp7	zinc finger protein 7 [Source:MGI Symbol;Acc:MGI:99208]	3.86158	3.87439	3.97076	4.03341	4.02079	4.28082	3.14675	3.9595	4.14811	3.88621	4.12964	4.28869	4.47501	4.54109	0.833013348	0.966018836	24	4	24	0.9093314	0.974606327	24	12.29486967	0.015554703
ENSMUSG00000033671	Cep350	centrosomal protein 350 [Source:MGI Symbol;Acc:MGI:1921331]	7.87489	7.74313	7.32247	7.39335	8.05136	7.21518	7.03203	6.22935	6.72252	6.93789	6.58394	7.34681	7.76447	7.07322	0.763955526	0.966018836	8	8	24	0.373247292	0.794388449	24	20.42693515	0.048054943
ENSMUSG00000033676	Gabrb3	gamma-aminobutyric acid (GABA) A receptor, subunit beta 3 [Source:MGI Symbol;Acc:MGI:95621]	20.0383	19.9869	20.7579	19.3219	18.9287	18.871	19.3808	17.9049	18.3335	20.1122	19.4965	19.6494	19.4498	21.3828	0.847846021	0.966018836	8	12	24	0.833345726	0.948650035	24	5.665156036	0.015907601
ENSMUSG00000033684	Qsox1	quiescin Q6 sulfhydryl oxidase 1 [Source:MGI Symbol;Acc:MGI:1330818]	13.7311	12.6302	12.2253	12.4553	13.6395	13.7366	11.3116	15.3363	12.827	14.5021	12.4071	13.1376	11.703	12.1484	0.890311217	0.966018836	24	4	24	0.813455911	0.940453305	24	13.87736059	0.028453847

ENSMUSG00000033685	Ucp2	uncoupling protein 2 (mitochondrial, proton carrier) [Source:MGI Symbol;Acc:MGI:109354]	55.8207	54.5413	56.1017	57.9566	56.4634	54.243	68.4669	65.5839	58.3476	60.5751	51.4273	55.4491	59.2644	50.1137	0.948454836	0.966018836	4	20	24	0.76607497	0.924332582	24	3.262942571	0.034718035
ENSMUSG00000033697	Arhgap39	Rho GTPase activating protein 39 [Source:MGI Symbol;Acc:MGI:107858]	25.4034	22.2627	20.9754	23.4541	25.0366	21.911	26.5905	22.3427	23.5415	24.5384	24.483	23.1235	25.6269	21.2547	0.129903614	0.560263689	4	4	24	0.28477343	0.768040178	24	19.35245622	0.06470446
ENSMUSG00000033701	Acbd6	acyl-Coenzyme A binding domain containing 6 [Source:MGI Symbol;Acc:MGI:1919732]	19.9064	16.1948	16.6622	16.2809	15.8879	15.8395	16.6169	20.1456	15.2339	20.6499	18.7815	18.3537	21.3459	20.6837	0.823677655	0.966018836	4	20	24	0.502797216	0.809737567	24	1.799227084	0.06369085
ENSMUSG00000033703	Fcsk	fucose kinase [Source:MGI Symbol;Acc:MGI:1916071]	2.65077	2.36119	2.37059	3.36472	2.98688	2.927	3.01849	3.05502	2.80302	3.06007	3.44252	3.11109	2.92495	2.79334	0.051295214	0.38924256	16	20	24	0.03013637	0.596794384	24	16.16797764	0.096811264
ENSMUSG00000033705	Stard9	START domain containing 9 [Source:MGI Symbol;Acc:MGI:3045258]	9.99233	11.6759	12.3563	12.4242	13.2603	10.7271	12.5316	8.95504	13.7701	11.1219	11.9948	10.4738	11.5369	10.0388	0.210934353	0.699706585	12	20	24	0.206629629	0.768040178	24	12.79792136	0.105083712
ENSMUSG00000033706	Smyd5	SET and MYND domain containing 5 [Source:MGI Symbol;Acc:MGI:108048]	15.3574	11.2805	10.5168	13.1219	12.7796	14.3057	11.4306	12.0804	10.9247	12.1762	11.0253	11.8233	11.6416	11.5621	0.14313801	0.585944911	24	12	24	0.116776411	0.727799063	24	19.89009977	0.104537827
ENSMUSG00000033707	Lrrc24	leucine rich repeat containing 24 [Source:MGI Symbol;Acc:MGI:3605040]	3.75829	2.83279	3.29605	1.95619	2.45421	3.93316	3.69842	3.04258	2.345	3.21486	2.58857	3.49051	2.80547	3.64757	0.112015648	0.530777648	24	20	24	0.061489538	0.661180819	24	23.59835129	0.189022441
ENSMUSG00000033712	Ccar2	cell cycle activator and apoptosis regulator 2 [Source:MGI Symbol;Acc:MGI:2444228]	11.9528	10.6801	12.1218	10.0242	11.5236	11.5739	11.6756	12.2086	11.4125	12.7183	10.4151	12.0197	11.2898	11.5146	0.833301348	0.966018836	24	20	24	0.870713525	0.963313607	24	3.752817588	0.0160242
ENSMUSG00000033713	Foxn3	forkhead box N3 [Source:MGI Symbol;Acc:MGI:1918625]	9.57744	8.75612	9.48381	9.46407	8.80305	9.11275	8.9766	10.6591	8.69887	10.4833	8.39168	9.57266	10.909	11.1683	0.371875382	0.894537464	8	12	24	0.208312338	0.768040178	24	4.272096099	0.071866409
ENSMUSG00000033715	Akr1c14	aldo-keto reductase family 1, member C14 [Source:MGI Symbol;Acc:MGI:2145458]	1.96858	2.09434	2.48384	2.01002	2.40497	2.27769	1.63907	2.30135	2.42122	1.90991	2.30982	1.86892	1.85082	1.88316	0.009483698	0.194720062	12	16	24	0.222053422	0.768040178	24	11.48418932	0.078167993
ENSMUSG00000033717	Adra2a	adrenergic receptor, alpha 2a [Source:MGI Symbol;Acc:MGI:87934]	2.89836	3.1918	3.55627	3.09137	3.29041	3.35009	3.23431	3.44013	3.66237	4.23525	2.72429	4.04096	3.06016	3.68185	0.580049522	0.966018836	12	16	24	0.438846166	0.805393068	24	9.124680315	0.066960505
ENSMUSG00000033720	Sfxn5	sideroflexin 5 [Source:MGI Symbol;Acc:MGI:2137681]	9.19742	10.4722	14.198	10.3347	9.5741	11.5806	12.1087	9.99037	10.5187	10.9396	9.35361	10.8109	9.33816	11.3139	0.801561896	0.966018836	12	8	24	0.556559736	0.838702296	24	7.35592648	0.069993081
ENSMUSG00000033721	Vav3	vav 3 oncogene [Source:MGI Symbol;Acc:MGI:1888518]	8.77231	7.50234	6.94372	7.1907	7.45785	7.60555	7.2077	7.07088	7.10488	6.90853	7.25105	7.93286	8.74297	8.13966	0.008012038	0.192760771	4	8	24	0.022815924	0.581965446	24	22.97816651	0.096744564
ENSMUSG00000033722	BC034090	cDNA sequence BC034090 [Source:MGI Symbol;Acc:MGI:2672904]	2.53427	3.82323	3.76773	2.66587	3.61915	3.7291	4.08224	3.31529	3.66617	2.99418	2.59964	3.57767	3.42243	3.93844	0.507179929	0.966018836	8	8	24	0.33252	0.769110818	24	3.006436445	0.099184564
ENSMUSG00000033728	Lrrc14	leucine rich repeat containing 14 [Source:MGI Symbol;Acc:MGI:2445060]	13.7292	11.4392	10.2202	13.2116	11.6339	14.4425	11.9615	16.4182	10.8726	12.6876	11.4598	12.7526	10.3696	12.3416	0.601647512	0.966018836	24	12	24	0.724318897	0.90965493	24	21.71605336	0.057889952
ENSMUSG00000033730	Egr3	early growth response 3 [Source:MGI Symbol;Acc:MGI:1306780]	2.30394	1.4438	1.22434	1.664	1.4976	1.50453	2.59184	1.5118	1.27615	1.52287	1.78292	1.28999	1.73487	1.21489	0.067104787	0.425837275	4	8	24	0.145405813	0.754184974	24	21.5413375	0.150777847
ENSMUSG00000033732	Sf3b3	splicing factor 3b, subunit 3 [Source:MGI Symbol;Acc:MGI:1289341]	15.0991	12.9065	14.2064	12.8377	13.4773	13.8609	14.1594	13.4418	12.1348	14.1854	12.0969	13.0225	12.4694	12.6912	0.858891143	0.966018836	4	16	24	0.544521494	0.832240355	24	22.16432956	0.033058983
ENSMUSG00000033735	Spr	sepiapterin reductase [Source:MGI Symbol;Acc:MGI:103078]	25.6157	22.1331	21.6293	21.5209	23.055	24.2054	21.1987	24.4108	20.4424	25.6786	25.5546	27.0123	28.025	25.5699	0.321662653	0.83607295	24	12	24	0.143896539	0.752803875	24	20.89426076	0.087474556
ENSMUSG00000033739	Fkbpl	FK506 binding protein-like [Source:MGI Symbol;Acc:MGI:1932127]	8.10469	8.40325	6.86263	5.56899	7.6996	8.65877	7.46378	7.63911	6.92133	7.43354	8.21033	8.83597	9.9367	8.24012	0.010059617	0.194720062	24	16	24	0.007313128	0.47735824	24	22.55125174	0.169083978
ENSMUSG00000033751	Gadd45gip1	growth arrest and DNA-damage-inducible, gamma interacting protein 1 [Source:MGI Symbol;Acc:MGI:1914947]	7.09097	6.61164	6.68059	6.82351	6.37108	6.40725	6.27783	8.36822	7.74345	7.33072	8.37605	8.33263	8.52085	8.54593	0.88067614	0.966018836	8	12	24	0.471873245	0.805393068	24	4.955122163	0.046836833
ENSMUSG00000033752	Mnd1	meiotic nuclear divisions 1 [Source:MGI Symbol;Acc:MGI:1924165]	1.0055	1.13513	0.850304	0.959914	0.918682	1.07672	0.885636	0.949553	0.915123	0.996565	0.920972	0.891342	1.22878	1.1286	0.773770646	0.966018836	8	4	24	0.285639285	0.768040178	24	1.413207175	0.046993116
ENSMUSG00000033760	Rbm4b	RNA binding motif protein 4B [Source:MGI Symbol;Acc:MGI:1913954]	9.83029	11.3958	11.472	11.5765	10.0531	10.4204	10.0331	9.99071	12.4082	8.98851	10.9629	9.85123	10.3979	10.5194	0.122530972	0.550724475	12	16	24	0.25690245	0.768040178	24	8.299729885	0.072075924
ENSMUSG00000033762	Recql4	RecQ protein-like 4 [Source:MGI Symbol;Acc:MGI:1931028]	1.22273	0.913205	1.1222	0.913149	0.818281	0.8352	0.989755	0.836275	0.852517	0.819899	0.931384	0.824538	1.04175	1.0563	0.135859247	0.575392196	4	20	24	0.202904438	0.768040178	24	2.348560067	0.060545379
ENSMUSG00000033763	Mtss2	MTSS I-BAR domain containing 2 [Source:MGI Symbol;Acc:MGI:3039591]	11.1127	11.2751	12.5795	10.4026	10.6635	11.5823	13.0711	10.5162	12.4005	14.3008	11.8073	11.5274	11.5457	12.3146	0.883907406	0.966018836	12	20	24	0.643455237	0.878927601	24	8.781830835	0.04365623
ENSMUSG00000033767	Tmem131l	transmembrane 131 like [Source:MGI Symbol;Acc:MGI:2443399]	17.5181	15.1897	13.3626	15.4738	17.271	16.5439	15.6263	16.4419	13.8199	12.5252	14.7097	14.3347	14.9319	14.1226	0.180646802	0.658184014	4	8	24	0.024900122	0.586541342	24	21.38205699	0.115039022
ENSMUSG00000033768	Nrxn2	neurexin II [Source:MGI Symbol;Acc:MGI:1096362]	24.0384	20.173	21.0495	22.8664	18.2252	22.5448	23.2236	26.2762	19.708	35.5064	21.735	27.915	23.9781	29.9341	0.823677655	0.966018836	8	4	24	0.895791217	0.970467191	24	5.411178134	0.041033019
ENSMUSG00000033769	Exoc6b	exocyst complex component 6B [Source:MGI Symbol;Acc:MGI:1923164]	20.0796	17.7807	19.4212	17.3816	19.296	19.2405	18.8738	17.585	16.0236	17.8208	18.1093	18.5914	18.3684	19.4464	0.432054049	0.966018836	4	12	24	0.1151175	0.727799063	24	21.40862869	0.063784017
ENSMUSG00000033773	Rpap2	RNA polymerase II associated protein 2 [Source:MGI Symbol;Acc:MGI:2141142]	12.4269	12.8307	10.9866	10.6639	12.809	12.9437	12.1141	12.4356	12.3	12.3279	13.044	11.3974	11.8398	11.9728	0.935316621	0.966018836	20	20	24	0.559276354	0.840399223	24	20.86516069	0.036201702
ENSMUSG00000033777	Tlr13	toll-like receptor 13 [Source:MGI Symbol;Acc:MGI:3045213]	0.477099	0.759889	1.0387	0.844121	0.711752	0.748615	0.670376	0.63007	0.980459	0.408897	0.603545	0.617964	0.686908	0.449878	0.700065774	0.966018836	12	16	24	0.536547378	0.827423907	24	9.516417042	0.066129704
ENSMUSG00000033781	Asb13	ankyrin repeat and SOCS box-containing 13 [Source:MGI Symbol;Acc:MGI:2145525]	14.64	13.409	14.7911	14.4643	13.0111	13.866	13.2491	14.1062	12.1903	14.6594	13.9973	13.1057	12.7828	14.3951	0.664568268	0.966018836	16	8	24	0.771308899	0.926252557	24	10.15291294	0.022994325
ENSMUSG00000033788	Dysf	dysferlin [Source:MGI Symbol;Acc:MGI:1349385]	13.5152	9.83316	10.6544	11.8263	11.7478	13.6844	10.8757	13.7636	10.8705	16.9091	10.0799	11.6345	11.6373	10.4645	0.965163262	0.969272194	16	20	24	0.763884909	0.923997781	24	16.13186549	0.059179509
ENSMUSG00000033790	Tubgcp5	tubulin, gamma complex associated protein 5 [Source:MGI Symbol;Acc:MGI:2178836]	15.2814	15.6825	14.6444	12.051	13.8401	13.8097	15.0898	13.9405	15.6963	12.3347	15.9224	15.5773	13.5717	15.8374	0.745118544	0.966018836	8	8	24	0.282394001	0.768040178	24	1.295816679	0.074405495
ENSMUSG00000033792	Atp7a	ATPase, Cu++ transporting, alpha polypeptide [Source:MGI Symbol;Acc:MGI:99400]	3.25835	3.87009	2.79051	3.63916	3.62184	3.75443	3.40835	3.26222	3.11207	3.64273	3.75419	4.389	3.38323	5.26345	0.148073804	0.590732968	24	12	24	0.778915906	0.928881414	24	20.84283325	0.044739982
ENSMUSG00000033793	Atp6v1h	ATPase, H+ transporting, lysosomal V1 subunit H [Source:MGI Symbol;Acc:MGI:1914864]	119.366	114.962	111.065	109.836	107.467	107.485	104.324	105.359	110.839	104.505	122.847	115.951	121.846	119.575	0.897425765	0.966018836	4	12	24	0.643233701	0.878809617	24	23.46349817	0.029934791
ENSMUSG00000033799	Tasor2	transcription activation suppressor family member 2 [Source:MGI Symbol;Acc:MGI:2145274]	6.61521	6.65786	6.91305	6.52997	7.14236	6.18494	8.16155	5.88477	6.99065	5.03158	8.07537	6.56752	6.32902	6.82265	0.921572192	0.966018836	20	4	24	0.753780428	0.920255919	24	20.91974162	0.047610314
ENSMUSG00000033805	Ephx4	epoxide hydrolase 4 [Source:MGI Symbol;Acc:MGI:2686228]	6.8628	6.02289	5.60813	4.78262	5.20225	6.4841	5.94763	6.37676	5.46417	7.18363	5.87829	6.78809	6.88147	7.9168	0.161408593	0.621687569	4	16	24	0.095587911	0.71175955	24	0.922874384	0.114797661
ENSMUSG00000033808	Tmem87a	transmembrane protein 87A [Source:MGI Symbol;Acc:MGI:2441844]	18.4948	20.4227	18.863	19.3119	19.7693	19.0283	18.5824	19.2984	19.322	15.9707	19.3253	18.5943	20.0823	18.3489	0.838244962	0.966018836	20	8	24	0.605288655	0.862555872	24	0.087225007	0.030937834
ENSMUSG00000033809	Alg3	asparagine-linked glycosylation 3 (alpha-1,3-mannosyltransferase) [Source:MGI Symbol;Acc:MGI:1098592]	5.67322	4.43055	4.33397	4.27442	5.29448	5.67584	6.31279	6.44598	3.36907	6.14427	4.30891	4.35689	4.76403	4.57922	0.267533088	0.774280302	4	8	24	0.359483178	0.785405943	24	21.92077332	0.127964781
ENSMUSG00000033813	Tcea1	transcription elongation factor A (SII) 1 [Source:MGI Symbol;Acc:MGI:1196624]	13.106	12.3656	12.4553	10.9804	12.0086	11.2502	11.357	11.247	13.2341	10.61	12.8772	11.4123	11.6388	11.4295	0.923635729	0.966018836	12	4	24	0.904538726	0.973594617	24	3.96970565	0.015954851
ENSMUSG00000033819	Ppp1r16a	protein phosphatase 1, regulatory subunit 16A [Source:MGI Symbol;Acc:MGI:1920312]	12.8233	11.2259	11.2815	11.6255	11.156	11.2175	13.1351	12.3524	12.6342	13.937	11.5248	13.4369	12.2796	12.2147	0.915082018	0.966018836	4	16	24	0.941077597	0.985441727	24	22.37664557	0.014322952
ENSMUSG00000033825	Tpsb2	tryptase beta 2 [Source:MGI Symbol;Acc:MGI:96942]	1.80336	1.70234	1.78797	1.24177	1.68559	1.26267	1.30059	2.19846	1.47007	0.828811	1.77089	1.32362	1.84315	1.40635	0.277917941	0.789849983	8	8	24	0.313366303	0.768040178	24	1.997277657	0.115865334
ENSMUSG00000033845	Mrpl15	mitochondrial ribosomal protein L15 [Source:MGI Symbol;Acc:MGI:1351639]	38.1078	36.7499	41.392	40.6894	35.6216	37.6862	36.1634	39.0483	42.6327	39.6778	42.1649	40.2548	39.5075	43.3992	0.43565473	0.966018836	12	16	24	0.058433445	0.658304792	24	9.0329936	0.066596719
ENSMUSG00000033849	B3galt2	UDP-Gal:betaGlcNAc beta 1,3-galactosyltransferase, polypeptide 2 [Source:MGI Symbol;Acc:MGI:1349461]	3.88414	4.93368	3.82044	3.80501	4.73255	3.56032	4.75411	4.13647	5.20485	3.25976	4.55924	4.12453	4.30849	4.00037	0.796193867	0.966018836	8	8	24	0.765855239	0.924276287	24	2.679379684	0.044803831
ENSMUSG00000033852	Gm28042	predicted gene, 28042 [Source:MGI Symbol;Acc:MGI:5547778]	1.22807	0.750785	1.28787	1.80547	0.749874	1.15088	1.13571	0.913432	1.4372	0.613874	1.20983	0.717561	0.827832	1.10367	0.888991156	0.966018836	12	12	24	0.677178019	0.892010043	24	9.497535283	0.079013
ENSMUSG00000033854	Kcnk10	potassium channel, subfamily K, member 10 [Source:MGI Symbol;Acc:MGI:1919508]	2.7967	2.12157	2.2189	2.26291	2.65732	2.47739	2.3237	2.02047	1.74867	2.50641	2.92366	2.60053	2.22946	2.31972	0.001051376	0.07619846	20	16	24	0.007173429	0.47735824	24	18.27470815	0.146398161
ENSMUSG00000033855	Ston1	stonin 1 [Source:MGI Symbol;Acc:MGI:1924307]	11.2285	12.3353	10.9566	12.5495	14.2883	10.7215	14.6933	11.6599	13.3209	14.4788	12.1626	12.8796	12.6561	10.4117	0.891105762	0.966018836	16	16	24	0.469595793	0.805393068	24	14.84847678	0.071052706
ENSMUSG00000033857	Engase	endo-beta-N-acetylglucosaminidase [Source:MGI Symbol;Acc:MGI:2443788]	2.95019	3.0623	3.16196	2.43185	2.50454	3.06901	3.2522	2.55023	2.78246	3.56936	2.51739	3.7531	3.23529	3.64423	0.676344318	0.966018836	24	20	24	0.408998879	0.799546792	24	1.346030102	0.074129472
ENSMUSG00000033860	Fgg	fibrinogen gamma chain [Source:MGI Symbol;Acc:MGI:95526]	1.23742	0.891607	0.971136	0.526002	1.10959	0.841458	0.866876	0.979746	0.541954	0.682288	1.38543	1.01295	0.94824	0.979867	0.187699199	0.662239322	20	20	24	0.165445887	0.767020009	24	21.20807715	0.134562733
ENSMUSG00000033862	Cdk10	cyclin-dependent kinase 10 [Source:MGI Symbol;Acc:MGI:2448549]	19.6015	21.1975	18.7091	20.0099	20.1038	19.2495	22.0639	17.461	19.9878	19.5393	17.4841	18.748	20.0793	16.6125	0.895733497	0.966018836	4	4	24	0.765265189	0.924116523	24	20.96391943	0.028576674
ENSMUSG00000033863	Klf9	Kruppel-like factor 9 [Source:MGI Symbol;Acc:MGI:1333856]	25.0964	25.1786	29.7455	35.2488	24.4037	25.9423	22.9189	25.2498	28.3268	36.1834	25.4728	25.7276	21.9665	26.8846	2.84E-04	0.049770315	16	12	24	0.002502304	0.39630666	24	11.06822459	0.222465994
ENSMUSG00000033871	Ppargc1b	peroxisome proliferative activated receptor, gamma, coactivator 1 beta [Source:MGI Symbol;Acc:MGI:2444934]	0.781546	0.767096	1.11343	1.1687	0.876317	0.977871	0.80337	1.11409	1.04737	1.4361	1.06481	1.08478	1.11226	1.23863	0.401437339	0.933249185	16	12	24	0.01208365	0.525280095	24	10.60496118	0.108832625
ENSMUSG00000033880	Lgals3bp	lectin, galactoside-binding, soluble, 3 binding protein [Source:MGI Symbol;Acc:MGI:99554]	24.5701	22.153	22.8539	23.0215	22.0768	26.9904	22.8841	29.8288	20.4528	26.6263	20.8006	24.42	19.6997	22.872	0.890648739	0.966018836	24	12	24	0.952870497	0.988573174	24	22.96891166	0.020090749
ENSMUSG00000033882	Rbm46	RNA binding motif protein 46 [Source:MGI Symbol;Acc:MGI:3645057]	0.719155	0.818758	0.747281	0.701747	0.534731	0.57964	0.720778	0.819482	0.709423	0.828794	0.684345	0.78426	0.647645	0.641404	0.542718076	0.966018836	8	12	24	0.263439203	0.768040178	24	6.331256357	0.049013457
ENSMUSG00000033883	Zfp267	zinc finger protein 267 [Source:MGI Symbol;Acc:MGI:1098769]	4.27166	4.79737	4.61655	4.64877	4.71458	4.45787	4.58776	4.42386	5.1065	4.19706	5.17228	4.84164	5.62067	5.17187	0.813775138	0.966018836	20	20	24	0.84127886	0.951889754	24	3.436896249	0.018698931
ENSMUSG00000033885	Pxk	PX domain containing serine/threonine kinase [Source:MGI Symbol;Acc:MGI:1289230]	14.123	12.5436	13.2788	13.2638	13.0929	14.6834	13.4764	13.413	12.0998	15.6203	13.8954	14.327	13.1324	13.4921	0.148073804	0.590732968	24	12	24	0.211188711	0.768040178	24	17.76764674	0.05883634
ENSMUSG00000033900	Map9	microtubule-associated protein 9 [Source:MGI Symbol;Acc:MGI:2442208]	16.1128	15.3482	15.0649	15.117	15.9337	15.1774	14.611	13.7655	15.5674	14.7457	18.3626	16.7681	16.9209	17.9858	0.772122688	0.966018836	20	20	24	0.583187223	0.851793019	24	18.93957687	0.04063461
ENSMUSG00000033902	Mapkbp1	mitogen-activated protein kinase binding protein 1 [Source:MGI Symbol;Acc:MGI:1347004]	6.45785	7.22813	8.11683	8.16785	8.73243	6.47411	8.18299	7.26362	8.49473	7.14519	7.96914	6.42751	6.85812	6.35998	0.27074138	0.780327352	20	4	24	0.091866239	0.71175955	24	11.62311352	0.111995992
ENSMUSG00000033904	Ccp110	centriolar coiled coil protein 110 [Source:MGI Symbol;Acc:MGI:2141942]	40.9259	36.58	36.746	36.1936	37.1358	36.3045	34.19	32.0233	31.5172	31.5807	37.4558	33.502	33.3463	32.5496	0.328944792	0.847277612	20	16	24	0.058430833	0.658304792	24	19.22587043	0.070314427
ENSMUSG00000033906	Zdhhc15	zinc finger, DHHC domain containing 15 [Source:MGI Symbol;Acc:MGI:1915336]	6.95341	7.14698	6.30546	6.39362	7.02514	6.69159	6.75893	6.15056	6.64455	6.1204	7.12672	7.07106	7.20702	7.29412	0.244223134	0.741580974	8	4	24	0.133796948	0.744007455	24	22.03283561	0.056262396
ENSMUSG00000033909	Usp36	ubiquitin specific peptidase 36 [Source:MGI Symbol;Acc:MGI:1919594]	4.78537	5.39615	5.70982	5.19038	5.65142	4.97935	5.04735	5.60373	5.28756	5.5429	4.88585	5.42077	5.49478	5.57543	0.418381424	0.963884303	8	20	24	0.169957938	0.767957732	24	8.045258937	0.050306181
ENSMUSG00000033910	Gucy1a1	guanylate cyclase 1, soluble, alpha 1 [Source:MGI Symbol;Acc:MGI:1926562]	1.76836	2.12581	2.98077	2.64105	1.92915	2.4066	3.02967	2.42667	2.00965	2.21273	2.02854	2.48122	1.76943	3.60892	0.951523604	0.966018836	8	12	24	0.497175516	0.807576732	24	6.227677434	0.103202622
ENSMUSG00000033916	Chmp2a	charged multivesicular body protein 2A [Source:MGI Symbol;Acc:MGI:1916203]	78.9106	86.365	80.76	80.3184	88.3789	78.622	86.5865	73.6934	90.5342	71.268	81.5843	77.8496	83.0264	81.506	0.904831057	0.966018836	12	12	24	0.940610211	0.985398871	24	2.323394071	0.013206446
ENSMUSG00000033917	Gde1	glycerophosphodiester phosphodiesterase 1 [Source:MGI Symbol;Acc:MGI:1891827]	111.098	93.4201	100.736	104.753	97.5325	114.157	99.9864	97.5553	82.2573	96.1453	104.386	97.8754	97.0251	94.2774	0.267533088	0.774280302	24	8	24	0.035265522	0.608551791	24	19.4618047	0.100940774
ENSMUSG00000033918	Parl	presenilin associated, rhomboid-like [Source:MGI Symbol;Acc:MGI:1277152]	16.1945	20.1723	17.9518	19.5774	17.9674	19.8746	18.9054	18.4392	17.7463	19.7623	18.9434	19.2301	20.1058	19.3926	0.922035832	0.966018836	24	12	24	0.962486295	0.990591773	24	14.48717476	0.008410009
ENSMUSG00000033931	Rbm34	RNA binding motif protein 34 [Source:MGI Symbol;Acc:MGI:1098653]	8.53306	8.7696	7.75004	8.47734	8.31275	8.50145	8.15081	8.49649	9.42418	9.68593	8.9614	9.89726	9.77104	9.51822	0.830949782	0.966018836	24	12	24	0.998660715	0.999758959	24	5.298999067	0.001359237
ENSMUSG00000033933	Vhl	von Hippel-Lindau tumor suppressor [Source:MGI Symbol;Acc:MGI:103223]	11.1131	10.1282	10.373	9.6596	10.3385	9.93518	10.4	9.85391	10.9832	9.4358	9.74641	10.4043	9.76352	9.82214	0.446653834	0.966018836	4	12	24	0.490753654	0.805393068	24	0.565860801	0.026897081
ENSMUSG00000033938	Ndufb7	NADH:ubiquinone oxidoreductase subunit B7 [Source:MGI Symbol;Acc:MGI:1914166]	108.757	109.633	116.863	108.244	102.757	106.996	108.828	115.17	108.467	103.841	115.842	107.875	117.872	106.97	0.330576288	0.850598217	4	20	24	0.476718424	0.805393068	24	3.628159082	0.029098079
ENSMUSG00000033940	Brk1	BRICK1, SCAR/WAVE actin-nucleating complex subunit [Source:MGI Symbol;Acc:MGI:1915406]	115.256	110.946	105.244	102.55	110.286	109.335	107.703	109.768	106.207	102.206	112.135	108.575	113.734	107.93	0.030972684	0.324150946	4	12	24	0.012581255	0.526249719	24	22.64131049	0.051432192
ENSMUSG00000033943	Mga	MAX gene associated [Source:MGI Symbol;Acc:MGI:1352483]	8.53573	8.67786	8.93339	9.72881	10.1186	7.91575	8.42669	8.05795	10.3396	7.62032	10.2784	9.60967	9.05461	8.88524	0.4776989	0.966018836	20	12	24	0.396142423	0.799546792	24	13.7921286	0.066621326
ENSMUSG00000033948	Zswim5	zinc finger SWIM-type containing 5 [Source:MGI Symbol;Acc:MGI:1921714]	4.34931	3.25586	3.72592	3.46577	3.5506	4.50121	3.60617	3.97947	2.75501	4.56334	3.13935	3.83937	3.47503	3.30695	0.723119329	0.966018836	24	12	24	0.400266804	0.799546792	24	19.95176187	0.087384742
ENSMUSG00000033949	Trim36	tripartite motif-containing 36 [Source:MGI Symbol;Acc:MGI:106264]	110.266	89.2089	83.6364	90.3148	94.1074	95.4111	87.6915	84.4214	73.2393	89.5711	100.657	88.9744	93.8812	87.8499	0.010153564	0.194720062	20	16	24	0.024337513	0.586541342	24	19.80346249	0.129543721
ENSMUSG00000033953	Ppp3r1	protein phosphatase 3, regulatory subunit B, alpha isoform (calcineurin B, type I) [Source:MGI Symbol;Acc:MGI:107172]	116.669	110.731	118.482	106.726	104.896	113.796	109.181	106.319	100.262	115.218	108.339	108.522	102.734	113.036	0.816300547	0.966018836	12	8	24	0.964237504	0.991092813	24	0.37294464	0.007143897
ENSMUSG00000033955	Tnks1bp1	tankyrase 1 binding protein 1 [Source:MGI Symbol;Acc:MGI:2446193]	19.1276	15.4876	14.3152	16.3745	16.6018	16.2757	16.8521	18.9378	15.5823	20.9943	16.2054	17.7307	17.58	18.7114	0.643325306	0.966018836	4	8	24	0.845520198	0.953396028	24	23.12252067	0.030482398
ENSMUSG00000033960	Jcad	junctional cadherin 5 associated [Source:MGI Symbol;Acc:MGI:2685174]	8.70347	8.06605	7.75194	9.32079	8.83414	9.18643	9.03185	7.76869	8.03107	8.20835	8.25627	8.75279	7.31248	7.03818	0.030691182	0.324150946	24	8	24	0.004987745	0.442351542	24	17.52952819	0.103927259
ENSMUSG00000033961	Zfp446	zinc finger protein 446 [Source:MGI Symbol;Acc:MGI:2442185]	3.57135	3.90586	3.80613	4.02915	3.97268	3.39156	4.1339	3.8314	3.84636	3.80692	3.56918	4.00049	3.70833	3.73163	0.888379597	0.966018836	16	8	24	0.709516553	0.903951515	24	8.694816558	0.021272168
ENSMUSG00000033964	Zbtb41	zinc finger and BTB domain containing 41 [Source:MGI Symbol;Acc:MGI:2444487]	5.66007	6.05671	5.64299	5.99297	5.95038	5.14627	5.50864	5.16114	5.60954	4.92887	6.28814	5.09359	5.50628	5.47511	0.829428469	0.966018836	20	4	24	0.779318915	0.929016959	24	12.92018122	0.023215388
ENSMUSG00000033965	Slc16a2	solute carrier family 16 (monocarboxylic acid transporters), member 2 [Source:MGI Symbol;Acc:MGI:1203732]	5.76157	5.59036	4.84694	6.12771	5.32487	5.79607	5.23925	6.19844	5.70626	7.11668	5.44807	5.61544	5.66429	5.49453	0.904831057	0.966018836	16	4	24	0.648351437	0.881219014	24	11.78734794	0.040640905
ENSMUSG00000033966	Cdkl4	cyclin-dependent kinase-like 4 [Source:MGI Symbol;Acc:MGI:3587025]	0.796592	1.60969	1.12054	1.42445	0.735472	0.872709	1.22941	0.887382	1.42442	1.64024	0.886672	1.43028	0.824652	1.37014	0.142596848	0.585944911	16	4	24	0.169025358	0.767020009	24	8.478966218	0.159620226
ENSMUSG00000033970	Rfc3	replication factor C (activator 1) 3 [Source:MGI Symbol;Acc:MGI:1916513]	4.45243	5.41873	6.34843	5.25254	5.0893	4.4189	5.13311	4.66508	6.90734	4.68257	6.4459	6.15356	7.20586	5.99843	0.934152439	0.966018836	12	12	24	0.581132985	0.850543068	24	7.842001248	0.074971056
ENSMUSG00000033972	Zfp944	zinc finger protein 944 [Source:MGI Symbol;Acc:MGI:2442394]	9.07619	8.65717	8.4487	8.27753	8.97508	7.32527	8.54855	8.10541	8.82085	6.76177	9.73441	8.29913	8.12944	8.10971	0.924553956	0.966018836	20	12	24	0.91162662	0.975053334	24	21.41409354	0.020412746
ENSMUSG00000033981	Gria2	glutamate receptor, ionotropic, AMPA2 (alpha 2) [Source:MGI Symbol;Acc:MGI:95809]	7.78409	10.1935	12.7902	9.20071	8.58476	8.9462	10.7844	7.87817	12.3948	8.34193	8.58307	9.66127	7.16573	10.6151	0.374467935	0.89742288	12	8	24	0.213558247	0.768040178	24	7.852913844	0.1563051
ENSMUSG00000033983	Coil	coilin [Source:MGI Symbol;Acc:MGI:104842]	7.66837	7.34571	8.13324	6.64046	8.17056	7.84934	7.27939	8.17053	7.6512	7.04457	8.31866	7.60438	7.96963	7.24728	0.369262716	0.893596186	20	20	24	0.685735536	0.895588997	24	20.79790351	0.030184993
ENSMUSG00000033985	Tesk2	testis-specific kinase 2 [Source:MGI Symbol;Acc:MGI:2385204]	6.83216	6.96136	7.62035	9.13565	8.76686	7.78027	6.94387	6.77429	8.31184	8.66905	6.87511	7.8424	7.04153	6.85829	0.004014876	0.140598674	16	16	24	0.004254359	0.427431763	24	13.25727177	0.143458444
ENSMUSG00000033991	Ttc37	tetratricopeptide repeat domain 37 [Source:MGI Symbol;Acc:MGI:2679923]	7.18301	7.34603	7.35665	7.48441	7.52932	6.8574	7.72037	7.79446	7.28989	6.17899	7.99016	7.83905	7.25381	7.08219	0.915082018	0.966018836	20	8	24	0.706148649	0.902918222	24	21.74884377	0.028423538
ENSMUSG00000033998	Kcnk1	potassium channel, subfamily K, member 1 [Source:MGI Symbol;Acc:MGI:109322]	41.6969	37.2087	37.205	43.3512	37.143	39.012	39.846	39.2519	34.4375	46.079	36.5049	35.9383	37.3666	35.8311	0.870491976	0.966018836	16	20	24	0.703631184	0.902090877	24	14.33922102	0.035744096
ENSMUSG00000034006	Slc66a2	solute carrier family 66 member 2 [Source:MGI Symbol;Acc:MGI:1914193]	61.5098	47.345	46.8637	50.5351	53.6552	53.2817	48.7581	53.4889	41.9509	53.9241	60.3762	50.9899	51.8644	50.6097	0.03782572	0.334069441	20	16	24	0.10199414	0.713240751	24	19.06955882	0.113978702
ENSMUSG00000034007	Scaper	S phase cyclin A-associated protein in the ER [Source:MGI Symbol;Acc:MGI:1925976]	11.8608	11.7532	10.977	11.4123	11.3833	11.3951	10.0456	10.3499	11.059	11.5842	11.8898	12.0613	12.9156	12.9558	0.924553956	0.966018836	8	4	24	0.889226336	0.968820559	24	20.80907824	0.017432834
ENSMUSG00000034009	Rxfp1	relaxin/insulin-like family peptide receptor 1 [Source:MGI Symbol;Acc:MGI:2682211]	0.281876	0.577059	0.724237	0.750778	0.426383	0.568159	0.814337	0.290159	0.736475	0.5789	0.889293	1.22395	0.364886	1.59102	0.90767227	0.966018836	16	12	24	0.815611074	0.941210649	24	10.27677355	0.065717155
ENSMUSG00000034021	Pds5b	PDS5 cohesin associated factor B [Source:MGI Symbol;Acc:MGI:2140945]	18.229	17.6122	17.194	17.8139	17.5428	15.9513	17.9687	15.1192	17.0857	13.2989	17.4411	15.2243	16.0194	16.0386	0.886572916	0.966018836	4	20	24	0.774310714	0.927520217	24	22.7722899	0.029108889
ENSMUSG00000034022	Cpsf1	cleavage and polyadenylation specific factor 1 [Source:MGI Symbol;Acc:MGI:2679722]	13.709	11.5749	13.9318	13.7562	12.8393	10.9764	11.4333	11.647	11.144	12.4023	10.1904	10.1917	12.2033	11.0303	0.581070934	0.966018836	16	8	24	0.480748753	0.805393068	24	9.187141544	0.05291717
ENSMUSG00000034023	Fancd2	Fanconi anemia, complementation group D2 [Source:MGI Symbol;Acc:MGI:2448480]	0.53037	0.582624	0.812169	0.605629	0.560953	0.711481	0.395224	0.524187	0.660782	0.636309	0.797185	0.650388	0.683072	0.62957	0.948568958	0.966018836	12	16	24	0.402215889	0.799546792	24	10.97587058	0.053594558
ENSMUSG00000034024	Cct2	chaperonin containing Tcp1, subunit 2 (beta) [Source:MGI Symbol;Acc:MGI:107186]	100.645	90.0411	90.9471	89.0523	90.6655	98.7215	94.6675	96.0789	82.392	95.5189	93.8901	94.0046	94.9942	95.771	0.137758149	0.580158868	4	8	24	0.055030789	0.655263328	24	21.85923398	0.063162422
ENSMUSG00000034032	Rpap1	RNA polymerase II associated protein 1 [Source:MGI Symbol;Acc:MGI:1916175]	3.1409	2.90276	3.07821	3.60491	3.43157	2.88106	3.05335	3.61321	3.00524	3.6118	2.94541	3.30765	3.20564	2.89665	0.892555977	0.966018836	16	16	24	0.405689804	0.799546792	24	12.78163679	0.049032021
ENSMUSG00000034035	Ccdc17	coiled-coil domain containing 17 [Source:MGI Symbol;Acc:MGI:1915667]	1.20893	1.99057	1.85817	1.5073	1.44625	1.66493	1.76582	2.21737	1.8616	1.66383	1.50943	1.57219	1.36005	1.64192	0.079174302	0.458678242	8	12	24	0.092908469	0.71175955	24	6.629400611	0.117841779
ENSMUSG00000034037	Fgd5	FYVE, RhoGEF and PH domain containing 5 [Source:MGI Symbol;Acc:MGI:2443369]	2.41567	2.87152	3.41113	2.61106	3.20504	2.64032	3.24922	3.57053	2.72348	3.24339	3.02329	2.31602	3.04207	2.56714	0.941579937	0.966018836	12	12	24	0.547565276	0.833732584	24	8.652731776	0.064851797
ENSMUSG00000034040	Galnt17	polypeptide N-acetylgalactosaminyltransferase 17 [Source:MGI Symbol;Acc:MGI:2137594]	13.9685	9.9579	10.5611	11.8966	11.0624	12.1946	10.789	12.9569	9.9169	17.4916	11.0819	13.4497	13.014	12.6295	0.390686537	0.915479136	24	12	24	0.829530135	0.947008621	24	18.01704548	0.049597742
ENSMUSG00000034041	Lyl1	lymphoblastomic leukemia 1 [Source:MGI Symbol;Acc:MGI:96891]	1.13418	2.44633	2.35185	2.06359	2.27597	1.47402	2.09118	2.67034	2.66544	1.69543	2.07628	1.97483	2.17351	1.39244	0.351840494	0.870416653	12	12	24	0.362197522	0.787064638	24	8.813796188	0.135332044
ENSMUSG00000034042	Gpbp1l1	GC-rich promoter binding protein 1-like 1 [Source:MGI Symbol;Acc:MGI:1924360]	11.9153	14.0608	14.344	17.0203	16.0566	15.1014	15.8101	16.1489	14.8281	15.5373	16.4669	13.2423	11.6854	15.0397	0.092972576	0.489012695	20	8	24	0.081668894	0.697886011	24	12.18609661	0.126153449
ENSMUSG00000034055	Phka1	phosphorylase kinase alpha 1 [Source:MGI Symbol;Acc:MGI:97576]	7.96438	8.7958	9.93538	10.7868	9.0711	10.9623	9.22916	14.0806	11.4326	13.1536	8.69966	9.94554	9.72519	9.13898	0.591351901	0.966018836	12	8	24	0.263553907	0.768040178	24	9.544018731	0.134518679
ENSMUSG00000034057	Myrfl	myelin regulatory factor-like [Source:MGI Symbol;Acc:MGI:2685085]	1.55601	2.02764	1.52145	1.59826	1.93756	1.42442	1.88647	1.84981	1.96873	1.69808	1.52182	1.79861	1.82895	1.6085	0.851656735	0.966018836	8	16	24	0.588140138	0.853898438	24	4.4368418	0.042267303
ENSMUSG00000034059	Ypel4	yippee like 4 [Source:MGI Symbol;Acc:MGI:3605071]	19.2311	17.4498	18.1957	16.5283	16.3659	17.2839	17.9896	16.9191	15.8118	18.2611	15.6468	19.0569	17.5457	18.1796	0.5169953	0.966018836	4	16	24	0.206920426	0.768040178	24	0.309701693	0.058727495
ENSMUSG00000034063	4930590J08Rik	RIKEN cDNA 4930590J08 gene [Source:MGI Symbol;Acc:MGI:2685917]	0.720428	0.92323	0.808546	1.11879	0.832866	0.873856	0.906961	1.0667	0.78223	1.1361	0.827176	0.761227	0.926006	1.24801	0.360140629	0.883787544	16	8	24	0.240539393	0.768040178	24	7.888177455	0.074479434
ENSMUSG00000034064	Pofut1	protein O-glucosyltransferase 1 [Source:MGI Symbol;Acc:MGI:2444232]	37.4993	39.4184	36.8112	36.8981	39.5179	37.3145	37.9602	36.8509	37.9742	33.9186	39.1476	37.0421	39.9969	38.9155	0.561701903	0.966018836	4	12	24	0.378856558	0.798235801	24	23.43416752	0.032563782
ENSMUSG00000034066	Farp2	FERM, RhoGEF and pleckstrin domain protein 2 [Source:MGI Symbol;Acc:MGI:2385126]	4.16716	3.24767	3.08265	4.07131	4.09513	4.03171	4.70232	3.86263	3.40401	4.37688	3.71073	3.88538	3.92923	3.63734	0.110778815	0.528348425	16	20	24	0.141296534	0.751766185	24	19.3250678	0.100225289
ENSMUSG00000034071	Zfp551	zinc finger protein 551 [Source:MGI Symbol;Acc:MGI:3588205]	1.38947	1.49654	1.67681	2.20892	1.54136	1.49595	1.31559	1.24934	1.76432	1.18525	1.50842	1.58441	1.46513	1.61695	0.176754388	0.653256653	12	16	24	0.364081487	0.788727838	24	11.53736102	0.07547841
ENSMUSG00000034075	Zdhhc5	zinc finger, DHHC domain containing 5 [Source:MGI Symbol;Acc:MGI:1923573]	14.1992	12.2533	12.7489	13.1831	13.7698	13.6281	12.8486	13.2412	11.5399	14.8389	12.2253	13.2787	13.0482	13.2841	0.581070934	0.966018836	12	24	24	0.492234398	0.805393068	24	18.48414062	0.041626518
ENSMUSG00000034083	Ccdc174	coiled-coil domain containing 174 [Source:MGI Symbol;Acc:MGI:2444652]	9.83452	12.1147	10.032	10.3747	11.02	10.1274	10.3148	10.5013	12.5174	10.0645	11.7439	11.0835	12.59	10.9568	0.826055781	0.966018836	4	20	24	0.838004926	0.950408078	24	5.352973272	0.025078409
ENSMUSG00000034088	Hdlbp	high density lipoprotein (HDL) binding protein [Source:MGI Symbol;Acc:MGI:99256]	86.5509	67.9594	70.7871	75.1166	71.9052	80.257	74.6525	76.7829	60.6809	80.3092	75.0763	76.1851	73.1207	76.5404	0.601647512	0.966018836	8	12	24	0.197006078	0.768040178	24	20.33441747	0.084878171

ENSMUSG00000034098	Fstl5	follistatin-like 5 [Source:MGI Symbol;Acc:MGI:2442179]	8.25588	9.68053	9.80546	9.21463	8.72177	8.39954	9.07724	7.7879	9.40914	8.31267	9.99492	9.18509	9.09464	10.9436	0.85447369	0.966018836	12	16	24	0.56319057	0.84250706	24	7.778087716	0.049329277
ENSMUSG00000034101	Ctnnd1	catenin (cadherin associated protein), delta 1 [Source:MGI Symbol;Acc:MGI:105100]	29.0829	26.2384	25.3957	27.3026	28.5554	28.692	30.7615	27.1484	23.4094	27.4232	27.8708	28.9949	27.0943	26.8735	7.90E-04	0.071344007	24	12	24	0.003479088	0.420609404	24	20.2071116	0.104561773
ENSMUSG00000034105	Meak7	MTOR associated protein , eak-7 homolog [Source:MGI Symbol;Acc:MGI:1921597]	6.1479	5.20071	5.5537	6.09783	5.52458	5.72264	6.13288	5.80056	4.77111	5.61564	5.50362	5.28754	5.58859	5.96563	0.515451644	0.966018836	4	8	24	0.556702924	0.838702296	24	22.2305395	0.037456305
ENSMUSG00000034108	Ccs	copper chaperone for superoxide dismutase [Source:MGI Symbol;Acc:MGI:1333783]	17.2739	18.6897	19.698	18.4277	20.4692	17.7452	18.1264	18.638	16.9238	18.3542	19.3701	19.7261	19.0177	19.4803	0.937921962	0.966018836	20	8	24	0.659942707	0.88511968	24	15.44805616	0.025836806
ENSMUSG00000034109	Golim4	golgi integral membrane protein 4 [Source:MGI Symbol;Acc:MGI:1920374]	21.782	22.2239	20.773	19.3388	19.8493	20.0564	20.697	19.9056	17.1846	19.7836	18.9232	19.2642	17.548	21.0875	0.241264473	0.741580974	8	12	24	0.386769664	0.799546792	24	1.269462377	0.044361857
ENSMUSG00000034110	Kctd7	potassium channel tetramerisation domain containing 7 [Source:MGI Symbol;Acc:MGI:2442265]	6.04646	5.90902	5.6844	5.6152	6.07662	6.33962	6.79328	6.27935	5.50616	6.42096	6.58064	6.5957	6.73906	6.48488	0.030691182	0.324150946	4	8	24	0.00660734	0.470633744	24	21.43815425	0.072957028
ENSMUSG00000034111	Tmed8	transmembrane p24 trafficking protein 8 [Source:MGI Symbol;Acc:MGI:1923480]	16.1234	13.296	13.8409	14.5945	14.1019	14.9816	13.5499	13.3188	11.849	13.9863	13.5211	14.7093	13.8319	13.8216	0.030691182	0.324150946	24	8	24	0.053668111	0.654054635	24	19.84259762	0.081752065
ENSMUSG00000034115	Scn11a	sodium channel, voltage-gated, type XI, alpha [Source:MGI Symbol;Acc:MGI:1345149]	101.534	69.6965	71.815	75.6249	71.5723	93.1749	78.0905	86.719	54.347	99.6115	71.3874	82.512	84.7031	81.1309	0.347075276	0.870418653	4	8	24	0.274748399	0.768040178	24	21.37609548	0.149346598
ENSMUSG00000034116	Vav1	vav 1 oncogene [Source:MGI Symbol;Acc:MGI:98923]	0.851517	1.18784	1.61132	1.92098	1.024	1.18299	1.42536	1.21934	1.31138	1.0244	1.23185	1.05735	1.42724	0.846143	0.894563866	0.966018836	12	20	24	0.514216026	0.816624526	24	10.51445379	0.083648326
ENSMUSG00000034118	Tpst1	protein-tyrosine sulfotransferase 1 [Source:MGI Symbol;Acc:MGI:1298231]	26.4724	23.3478	23.4756	23.3664	22.9667	25.3735	24.8093	24.4123	21.4394	24.1344	22.1566	25.5155	23.4034	23.0676	0.076247883	0.453790923	24	20	24	0.029697924	0.596648309	24	21.94884351	0.072909111
ENSMUSG00000034120	Srsf2	serine and arginine-rich splicing factor 2 [Source:MGI Symbol;Acc:MGI:98284]	83.8321	82.4409	83.4	83.0008	80.5568	77.3673	83.7846	75.5699	82.772	86.7855	88.6222	77.9727	79.3358	76.0619	0.33885934	0.858075318	16	16	24	0.200475014	0.768040178	24	13.20508877	0.044354399
ENSMUSG00000034121	Mks1	MKS transition zone complex subunit 1 [Source:MGI Symbol;Acc:MGI:3584243]	2.99722	3.00267	3.29585	3.88693	3.59666	3.04812	4.2894	3.32898	3.45964	3.3452	3.29267	3.50224	3.57705	3.39267	0.907068194	0.966018836	16	16	24	0.91527929	0.976474646	24	14.42422319	0.018019432
ENSMUSG00000034126	Pomt2	protein-O-mannosyltransferase 2 [Source:MGI Symbol;Acc:MGI:2444430]	7.91985	7.29453	7.24438	7.60589	7.64818	7.65356	8.33086	7.17527	6.63977	8.38484	7.26419	7.3927	7.68603	7.13424	0.030197136	0.324150946	4	4	24	0.2667564	0.768040178	24	19.37914472	0.051188545
ENSMUSG00000034127	Tspan8	tetraspanin 8 [Source:MGI Symbol;Acc:MGI:2384918]	68.4155	71.6659	67.0756	63.0175	70.3395	66.4555	60.6638	64.2983	67.4413	59.4711	78.8461	69.0197	71.776	77.1909	0.893307667	0.966018836	20	20	24	0.856801189	0.957538975	24	22.6562377	0.025285549
ENSMUSG00000034135	Sik3	SIK family kinase 3 [Source:MGI Symbol;Acc:MGI:2446296]	5.52491	7.21363	9.26613	6.71994	5.52447	7.01973	7.63203	6.31646	7.62567	7.935	5.44113	6.74544	5.48712	7.56168	0.071580004	0.440262583	12	8	24	0.103497726	0.715292223	24	7.73589509	0.168748749
ENSMUSG00000034145	Tmem63c	transmembrane protein 63c [Source:MGI Symbol;Acc:MGI:2444386]	25.056	19.0481	19.9691	21.0666	21.599	24.2831	19.4771	22.2631	15.6995	24.8938	19.8801	22.0414	20.9428	20.0491	0.390686537	0.915479136	24	12	24	0.20318822	0.768040178	24	19.27868723	0.118093318
ENSMUSG00000034152	Exoc3	exocyst complex component 3 [Source:MGI Symbol;Acc:MGI:2443972]	34.3748	34.0956	32.225	32.6363	34.4956	33.7871	34.0061	33.5184	32.5207	32.0167	32.9747	35.3257	34.6199	35.1411	0.019088001	0.257576951	4	8	24	0.004379147	0.427431763	24	22.73399433	0.047420331
ENSMUSG00000034154	Ino80	INO80 complex subunit [Source:MGI Symbol;Acc:MGI:1915392]	4.78374	5.00974	4.77683	5.05627	5.01624	4.80278	4.89855	5.09367	5.51922	5.04816	4.63438	4.85351	4.95554	4.84946	0.330576288	0.850598217	16	8	24	0.160801919	0.765941695	24	8.152379921	0.037598207
ENSMUSG00000034156	Tspoap1	TSPO associated protein 1 [Source:MGI Symbol;Acc:MGI:2450877]	8.3582	12.0128	16.9583	12.5305	8.3588	11.6933	12.0286	9.93424	14.7732	12.502	8.7295	12.7192	8.66577	13.3786	0.048889741	0.379129806	12	16	24	0.093950086	0.71175955	24	8.147022343	0.246352055
ENSMUSG00000034157	Cipc	CLOCK interacting protein, circadian [Source:MGI Symbol;Acc:MGI:1919185]	23.7976	23.3211	24.7734	26.2654	24.7686	26.3585	24.8901	24.2682	24.3434	28.337	23.7985	24.1839	22.3188	24.0914	0.05945106	0.406067671	16	12	24	0.077603725	0.687420473	24	13.16881829	0.065952753
ENSMUSG00000034158	Lrrc58	leucine rich repeat containing 58 [Source:MGI Symbol;Acc:MGI:2443542]	17.6502	15.4577	15.2598	16.5609	15.9822	15.5507	17.2247	14.4784	14.6559	16.1822	16.8034	15.8864	14.9355	15.9497	0.277221318	0.789849983	16	20	24	0.22163663	0.768040178	24	18.67806775	0.053101966
ENSMUSG00000034160	Ogt	O-linked N-acetylglucosamine (GlcNAc) transferase (UDP-N-acetylglucosamine:polypeptide-N-acetylglucosaminyl transferase) [Source:MGI Symbol;Acc:MGI:1339639]	70.2196	86.2774	75.9729	80.2705	80.7965	69.4914	81.7769	71.6117	92.8854	64.3742	85.0354	80.2143	79.489	80.4235	0.928780444	0.966018836	12	12	24	0.840480354	0.951645964	24	7.145753663	0.034261961
ENSMUSG00000034161	Scx	scleraxis [Source:MGI Symbol;Acc:MGI:102934]	3.25239	3.12674	2.55957	3.09796	2.42916	2.39084	2.2891	3.58989	3.03887	3.09839	3.66617	3.77189	4.38572	3.87054	0.832506215	0.966018836	8	4	24	0.531613205	0.82545228	24	3.463692605	0.076046812
ENSMUSG00000034163	Zfc3h1	zinc finger, C3H1-type containing [Source:MGI Symbol;Acc:MGI:2446143]	34.0392	34.9311	35.2133	31.9466	35.7406	32.6104	35.5933	31.0344	34.7208	30.9591	38.9774	32.4921	31.2742	32.4478	0.722296281	0.966018836	20	20	24	0.676710227	0.891857599	24	16.38448626	0.032295173
ENSMUSG00000034164	Emid1	EMI domain containing 1 [Source:MGI Symbol;Acc:MGI:2155091]	13.4175	14.9139	13.455	14.5412	17.699	14.2619	15.5026	17.2804	16.971	17.935	15.8599	14.9435	17.0544	12.8151	0.823657258	0.966018836	16	16	24	0.533221642	0.825678486	24	13.55539636	0.0634565
ENSMUSG00000034165	Ccnd3	cyclin D3 [Source:MGI Symbol;Acc:MGI:88315]	13.2981	13.3114	16.6503	16.1078	14.3501	13.9636	14.838	15.5448	16.245	13.145	15.2368	14.3246	15.0327	12.2003	0.297978647	0.80820842	12	12	24	0.490791471	0.805393068	24	11.16035398	0.058054786
ENSMUSG00000034168	Irf2bpl	interferon regulatory factor 2 binding protein-like [Source:MGI Symbol;Acc:MGI:2442463]	7.72062	5.78488	5.61388	6.1537	6.01045	5.70308	6.42998	6.75532	6.3975	10.0373	7.80508	8.25537	8.8167	8.14552	0.93447422	0.966018836	4	8	24	0.943136409	0.986048125	24	19.3954263	0.025083222
ENSMUSG00000034171	Faah	fatty acid amide hydrolase [Source:MGI Symbol;Acc:MGI:109609]	6.64989	7.17307	6.96985	6.80352	6.88132	7.14553	7.94635	7.4632	7.01038	8.31846	6.32989	6.63474	6.34443	7.58444	0.295379186	0.805301811	8	12	24	0.443659945	0.805393068	24	6.904441169	0.052616715
ENSMUSG00000034173	Zbed5	zinc finger, BED type containing 5 [Source:MGI Symbol;Acc:MGI:1919220]	1.27535	1.58521	1.61075	1.43218	1.47283	1.30165	1.72554	1.65153	1.7933	1.35893	1.17446	1.44692	1.33534	1.80024	0.079174302	0.458678242	8	16	24	0.015976154	0.544099157	24	5.773273451	0.116471652
ENSMUSG00000034175	Rhbdd3	rhomboid domain containing 3 [Source:MGI Symbol;Acc:MGI:2444684]	8.14973	8.20568	7.65709	6.93318	8.38904	8.04151	6.05045	8.53819	8.27039	7.21868	6.82106	7.21181	7.1349	7.45421	0.40165019	0.933249185	8	20	24	0.729379218	0.911672396	24	6.092683294	0.038544676
ENSMUSG00000034187	Nsf	N-ethylmaleimide sensitive fusion protein [Source:MGI Symbol;Acc:MGI:104560]	108.465	86.6826	100.369	87.5116	79.1504	113.045	104.683	100.356	67.4313	122.203	73.8881	93.6671	85.0541	97.5698	0.893609703	0.966018836	24	20	24	0.649945898	0.882325565	24	23.50549743	0.088169002
ENSMUSG00000034189	Hsdl1	hydroxysteroid dehydrogenase like 1 [Source:MGI Symbol;Acc:MGI:1919802]	27.7978	22.4788	22.745	23.7315	24.2211	25.5675	25.0712	26.3491	20.5086	30.1328	22.7832	25.3482	24.8871	24.0419	0.143321297	0.585944911	24	12	24	0.522631429	0.820916024	24	20.34522842	0.062516936
ENSMUSG00000034190	Chmp7	charged multivesicular body protein 7 [Source:MGI Symbol;Acc:MGI:1913922]	26.5246	24.3102	25.1583	23.054	23.7272	25.8451	25.5003	25.054	22.8893	27.6053	24.8248	27.6726	25.9773	26.943	0.277428396	0.789849983	24	12	24	0.275024357	0.768040178	24	22.53537746	0.048671087
ENSMUSG00000034192	Lsm3	LSM3 homolog, U6 small nuclear RNA and mRNA degradation associated [Source:MGI Symbol;Acc:MGI:1914928]	28.6342	37.3738	30.3037	34.1414	33.9055	30.5466	29.0582	28.2776	37.34	26.5036	35.4329	31.5001	36.0691	34.2256	0.858266942	0.966018836	8	20	24	0.898915038	0.97144767	24	8.789566453	0.030143348
ENSMUSG00000034194	R3hcc1	R3H domain and coiled-coil containing 1 [Source:MGI Symbol;Acc:MGI:1919093]	8.9618	9.19367	9.61438	8.0403	8.65603	9.32153	9.2987	9.48358	9.09925	9.93898	8.33128	9.20989	8.41295	9.26686	0.883024237	0.966018836	12	8	24	0.42959688	0.805393068	24	5.004231906	0.039819687
ENSMUSG00000034201	Gas2l1	growth arrest-specific 2 like 1 [Source:MGI Symbol;Acc:MGI:1926176]	22.5885	17.7941	17.5326	17.5369	18.5487	17.7762	21.5654	22.8716	19.7216	29.7959	21.4717	23.6924	23.503	21.2646	0.612331511	0.966018836	4	8	24	0.950180814	0.988176855	24	22.40395972	0.02342936
ENSMUSG00000034203	Chchd4	coiled-coil-helix-coiled-coil-helix domain containing 4 [Source:MGI Symbol;Acc:MGI:1919420]	5.61734	5.98659	6.03451	6.11224	5.73618	5.69156	5.82869	5.44961	5.1739	5.41358	6.01195	5.86034	5.91629	6.10368	0.761979	0.966018836	16	12	24	0.868366462	0.96247509	24	20.5400608	0.013816446
ENSMUSG00000034205	Loxl2	lysyl oxidase-like 2 [Source:MGI Symbol;Acc:MGI:2137913]	1.77282	1.79409	1.88818	1.51636	1.82927	1.51048	1.81172	2.1546	2.05595	1.37842	1.58169	1.44004	1.66448	1.56914	0.084464639	0.466404237	12	12	24	0.109476305	0.72082227	24	4.821085358	0.089776225
ENSMUSG00000034206	Polq	polymerase (DNA directed), theta [Source:MGI Symbol;Acc:MGI:2155399]	0.593887	0.761028	0.874925	1.00475	0.818573	0.86433	0.65008	0.671445	1.05634	0.682917	0.931638	0.883344	0.756229	0.709601	0.041677362	0.34886238	12	16	24	0.075014024	0.68394125	24	12.82351168	0.0902123
ENSMUSG00000034209	Rasl10a	RAS-like, family 10, member A [Source:MGI Symbol;Acc:MGI:1922918]	1.47286	1.05723	0.684122	0.722305	0.902528	1.14083	1.48422	0.864233	0.687645	1.08031	1.44426	1.20191	1.39546	1.2813	1.18E-04	0.029372629	4	8	24	0.00656677	0.470633744	24	21.87332682	0.210505597
ENSMUSG00000034210	Efcab14	EF-hand calcium binding domain 14 [Source:MGI Symbol;Acc:MGI:2442397]	17.2323	16.7565	17.5878	16.7878	16.5961	16.4088	18.0693	17.2906	16.381	16.3216	15.8041	17.3412	15.423	16.2431	0.890176313	0.966018836	24	20	24	0.628063653	0.872795898	24	1.810143558	0.019154389
ENSMUSG00000034211	Mrps17	mitochondrial ribosomal protein S17 [Source:MGI Symbol;Acc:MGI:1913508]	46.7538	47.8614	41.3371	45.5339	47.7896	44.8728	40.3407	43.2834	48.8659	40.0359	50.6864	46.77	48.4751	45.0221	0.879201926	0.966018836	20	20	24	0.69966829	0.900387881	24	18.79983976	0.03625893
ENSMUSG00000034216	Vps18	VPS18 CORVET/HOPS core subunit [Source:MGI Symbol;Acc:MGI:2443626]	8.46101	6.91657	7.59122	7.70281	6.98264	7.97257	8.00691	8.60502	6.37001	10.2311	7.32055	8.80806	7.96903	8.10755	0.791413662	0.966018836	24	12	24	0.860207192	0.958819744	24	20.62350884	0.033707466
ENSMUSG00000034218	Atm	ataxia telangiectasia mutated [Source:MGI Symbol;Acc:MGI:107202]	6.26785	5.02403	5.58791	3.46435	7.29026	5.43357	3.48533	6.67311	6.13367	2.71773	7.17397	5.59426	5.05027	8.49112	0.911685024	0.966018836	20	20	24	0.753496507	0.920152554	24	0.888399818	0.112761136
ENSMUSG00000034220	Gpc1	glypican 1 [Source:MGI Symbol;Acc:MGI:1194891]	24.9388	20.9042	22.7402	23.1319	21.5875	24.4852	22.0579	26.7149	20.6903	29.5759	21.5619	25.6486	23.7446	23.6023	0.979945083	0.980057772	24	12	24	0.972579351	0.993250534	24	18.7270873	0.013559428
ENSMUSG00000034226	Rhov	ras homolog family member V [Source:MGI Symbol;Acc:MGI:2444227]	10.2168	8.31499	8.56882	7.6471	8.70571	10.3006	7.89217	8.68818	6.69083	10.8954	7.74036	9.55155	8.73843	9.68002	0.390686537	0.915479136	24	12	24	0.476558219	0.805393068	24	20.89657869	0.086945725
ENSMUSG00000034227	Foxj1	forkhead box J1 [Source:MGI Symbol;Acc:MGI:1347474]	4.79544	3.51443	3.71308	4.66001	3.75686	3.66766	4.32804	3.52957	3.30546	5.51552	3.8795	4.984	3.52518	4.77439	0.503972924	0.966018836	16	20	24	0.72340193	0.909188168	24	16.14132592	0.060501369
ENSMUSG00000034235	Usp54	ubiquitin specific peptidase 54 [Source:MGI Symbol;Acc:MGI:1926037]	6.21815	6.32727	9.42479	7.54456	6.47527	7.81985	7.88492	5.67604	7.44347	5.55366	6.29486	6.81209	4.86082	5.69409	0.928148816	0.966018836	12	20	24	0.681357044	0.893635751	24	12.83124848	0.066268446
ENSMUSG00000034243	Golgb1	golgi autoantigen, golgin subfamily b, macrogolgin 1 [Source:MGI Symbol;Acc:MGI:1099447]	18.555	17.334	15.304	17.7995	17.3925	17.1244	14.5068	15.9234	16.8525	14.7127	17.1851	16.6417	16.3013	16.2917	0.885025617	0.966018836	20	16	24	0.545915304	0.833198211	24	18.76890696	0.040992125
ENSMUSG00000034245	Hdac11	histone deacetylase 11 [Source:MGI Symbol;Acc:MGI:2385252]	27.0426	26.5138	29.5375	26.0451	22.2853	27.9124	26.5873	24.9228	24.5503	28.4199	22.0718	26.9607	23.6432	27.3415	0.888379597	0.966018836	24	20	24	0.5459367	0.833198211	24	4.922225151	0.052258187
ENSMUSG00000034247	Plekhm1	pleckstrin homology domain containing, family M (with RUN domain) member 1 [Source:MGI Symbol;Acc:MGI:2443207]	3.53158	2.93095	3.39081	3.65711	3.97978	3.55604	3.87305	3.23534	3.35552	4.27247	3.53629	3.36191	3.4149	3.4498	0.026194135	0.300938431	16	16	24	0.09185632	0.71175955	24	15.30035299	0.082584016
ENSMUSG00000034248	Slc25a37	solute carrier family 25, member 37 [Source:MGI Symbol;Acc:MGI:1914962]	8.2067	10.3998	10.982	11.9133	10.2644	9.42532	10.3425	9.5519	10.4316	8.06715	10.3843	10.1315	11.426	8.54172	0.895493752	0.966018836	12	12	24	0.857398867	0.957854533	24	12.81958997	0.034075888
ENSMUSG00000034252	Senp6	SUMO/sentrin specific peptidase 6 [Source:MGI Symbol;Acc:MGI:1922075]	53.1372	57.2546	50.8022	50.5621	50.6001	51.3449	50.6721	49.9592	50.6285	48.8742	53.794	52.6061	56.4042	53.9855	0.14606006	0.590732968	4	12	24	0.179479325	0.768040178	24	0.458831299	0.045567286
ENSMUSG00000034254	Agpat1	1-acylglycerol-3-phosphate O-acyltransferase 1 (lysophosphatidic acid acyltransferase, alpha) [Source:MGI Symbol;Acc:MGI:1932075]	31.1519	24.9571	26.138	29.1181	31.2699	31.6783	31.9219	31.6211	25.7844	35.337	26.5474	29.9703	29.1649	28.8611	0.319330027	0.83607295	24	12	24	0.405594863	0.799546792	24	19.04900421	0.075266963
ENSMUSG00000034255	Arhgap27	Rho GTPase activating protein 27 [Source:MGI Symbol;Acc:MGI:1916903]	6.05599	5.14053	5.30724	5.12818	4.84371	5.62686	5.28926	5.73902	5.06904	6.57735	5.3036	6.2726	5.10753	6.03745	0.879844076	0.966018836	24	20	24	0.922337098	0.979411448	24	23.52609833	0.017858718
ENSMUSG00000034258	Flvcr2	feline leukemia virus subgroup C cellular receptor 2 [Source:MGI Symbol;Acc:MGI:2384974]	0.967509	1.13298	1.22873	1.12475	0.975188	1.00214	1.13903	1.26117	1.01875	0.87044	0.785666	0.866776	0.914762	0.679223	0.560714538	0.966018836	12	8	24	0.431390654	0.805393068	24	5.611015788	0.047121152
ENSMUSG00000034259	Exosc4	exosome component 4 [Source:MGI Symbol;Acc:MGI:1923576]	6.13658	6.17371	7.3405	6.48376	5.30074	6.17641	6.87384	7.57991	6.7762	8.44327	6.40165	6.95219	7.28233	6.29588	0.91695334	0.966018836	16	4	24	0.327255237	0.768040178	24	7.445310163	0.081373449
ENSMUSG00000034263	Ints14	integrator complex subunit 14 [Source:MGI Symbol;Acc:MGI:1917132]	5.27744	5.38644	5.36406	5.83112	5.94953	5.446	5.62782	5.46294	5.38892	5.42964	5.60947	5.86089	5.67929	5.4183	0.10560184	0.518002936	20	16	24	0.06190773	0.661961741	24	16.93782148	0.03856924
ENSMUSG00000034265	Zdhhc14	zinc finger, DHHC domain containing 14 [Source:MGI Symbol;Acc:MGI:2653229]	6.51658	5.39159	5.12771	5.04581	5.16567	6.01304	5.56092	5.98172	4.92764	7.03788	5.56321	6.70978	6.55922	6.27353	0.038115943	0.334069441	24	12	24	0.243888046	0.768040178	24	22.30016535	0.085046103
ENSMUSG00000034269	Setd5	SET domain containing 5 [Source:MGI Symbol;Acc:MGI:1920145]	23.249	22.5037	22.4991	22.9804	22.2963	23.3289	22.2872	21.7829	21.4261	24.2768	21.1193	22.7491	22.6001	24.9094	0.932092277	0.966018836	24	12	24	0.924488913	0.979866138	24	3.446454137	0.009768061
ENSMUSG00000034271	Jdp2	Jun dimerization protein 2 [Source:MGI Symbol;Acc:MGI:1932093]	8.78541	6.84808	7.69245	7.51917	8.12964	6.92633	7.80428	5.96458	6.85679	8.40257	7.36173	6.91841	6.43539	6.93461	0.350544145	0.870418653	16	16	24	0.246655729	0.768040178	24	15.57091917	0.077583516
ENSMUSG00000034274	Thoc5	THO complex 5 [Source:MGI Symbol;Acc:MGI:1351333]	14.6068	13.4373	15.5726	14.6558	13.7836	15.0994	15.4038	14.4787	13.3901	15.5086	13.9465	14.6879	14.0917	15.0592	0.858266942	0.966018836	24	20	24	0.994055489	0.998755992	24	21.2569207	0.003172504
ENSMUSG00000034275	Igsf9b	immunoglobulin superfamily, member 9B [Source:MGI Symbol;Acc:MGI:2685354]	4.84558	3.74796	3.96664	4.11761	4.76588	4.13864	4.48218	[illegible]	3.6151	3.86037	5.04503	4.11953	4.15091	4.16745	0.078380335	0.458114095	4	4	24	0.046124753	0.633283523	24	18.61092863	0.10665522
ENSMUSG00000034278	Dnajc17	DnaJ heat shock protein family (Hsp40) member C17 [Source:MGI Symbol;Acc:MGI:1916658]	4.35246	4.9147	4.56325	4.15203	3.39535	4.35298	4.93921	4.51696	4.12135	4.39802	3.2847	4.64506	4.36579	4.18193	0.162391276	0.624504499	4	16	24	0.04047175	0.616921492	24	2.783633915	0.119397894
ENSMUSG00000034285	Nipsnap1	nipsnap homolog 1 [Source:MGI Symbol;Acc:MGI:1278344]	45.7916	41.4906	40.486	39.7188	38.4246	45.5401	44.3361	42.42	34.936	43.6671	40.6166	42.176	42.8034	42.7524	0.008754183	0.192760771	4	8	24	0.050380133	0.644469829	24	22.51774736	0.089857634
ENSMUSG00000034290	Nek9	NIMA (never in mitosis gene a)-related expressed kinase 9 [Source:MGI Symbol;Acc:MGI:2387995]	25.5011	23.8126	23.1044	25.2666	25.0896	26.0967	25.2555	25.8561	21.7157	27.2486	22.0773	24.5935	25.33	23.4555	0.496745696	0.966018836	24	12	24	0.477146099	0.805393068	24	20.33875604	0.044752184
ENSMUSG00000034292	Traf3ip1	TRAF3 interacting protein 1 [Source:MGI Symbol;Acc:MGI:1921269]	4.05874	4.00398	4.16256	3.72591	4.34905	4.67534	4.17715	4.05062	3.84165	4.28698	3.86974	3.81597	3.7887	3.99257	0.854443826	0.966018836	24	12	24	0.593606565	0.856966636	24	18.91092316	0.029346069
ENSMUSG00000034295	Fhod3	formin homology 2 domain containing 3 [Source:MGI Symbol;Acc:MGI:1925847]	4.15579	2.95443	3.02104	4.26362	3.66528	3.45879	3.19921	4.2381	3.0292	3.95684	2.60726	3.12088	4.26688	4.95229	0.935593607	0.966018836	8	4	24	0.620571963	0.87025454	24	3.814759466	0.080792531
ENSMUSG00000034297	Med13	mediator complex subunit 13 [Source:MGI Symbol;Acc:MGI:3029632]	6.32735	7.49532	7.47821	7.71156	7.36244	6.55903	7.07508	6.62097	7.77058	6.52544	7.11783	6.77784	6.43122	6.90164	0.031969981	0.324385159	12	16	24	0.071277719	0.676901434	24	10.26286762	0.072283311
ENSMUSG00000034300	Fam53c	family with sequence similarity 53, member C [Source:MGI Symbol;Acc:MGI:1913556]	17.1474	13.6633	14.7874	15.0131	15.5268	16.2615	15.8553	15.5332	13.0712	18.4253	17.2584	16.3525	15.464	15.6575	0.046934151	0.375011045	24	12	24	0.12693902	0.739868076	24	18.1702436	0.092196226
ENSMUSG00000034303	Ccdc15	coiled-coil domain containing 15 [Source:MGI Symbol;Acc:MGI:2444488]	0.71137	0.664102	0.680322	0.688162	0.638376	0.660074	0.737666	0.753029	0.711854	0.582656	0.868175	0.621797	0.619591	0.774007	0.787022682	0.966018836	24	12	24	0.859873515	0.958681059	24	4.645533368	0.013912114
ENSMUSG00000034308	Sdr42e1	short chain dehydrogenase/reductase family 42E, member 1 [Source:MGI Symbol;Acc:MGI:1921282]	1.53362	2.40907	2.08007	1.70995	1.87671	1.68002	1.96698	2.07896	1.73231	1.85747	1.19949	1.82352	2.02636	2.26075	0.015345591	0.253245931	8	12	24	0.035308523	0.608551791	24	4.566822205	0.145797895
ENSMUSG00000034312	Iqsec1	IQ motif and Sec7 domain 1 [Source:MGI Symbol;Acc:MGI:1196356]	30.1107	28.7241	33.2412	27.0716	30.7944	28.9849	37.0814	31.1825	28.6257	42.1823	26.4112	32.0747	30.6306	28.9586	0.880631923	0.966018836	4	16	24	0.98034774	0.995255072	24	8.110889935	0.01466902
ENSMUSG00000034317	Trim59	tripartite motif-containing 59 [Source:MGI Symbol;Acc:MGI:1914199]	1.83917	3.40435	4.54682	2.91239	2.34148	2.85955	3.69624	2.75013	3.16329	1.98661	2.52187	3.17456	2.271	2.93495	0.602156008	0.966018836	12	4	24	0.45902268	0.805393068	24	6.210240562	0.12961228
ENSMUSG00000034320	Slc26a2	solute carrier family 26 (sulfate transporter), member 2 [Source:MGI Symbol;Acc:MGI:892977]	2.17683	3.58544	2.996	2.4773	3.04268	2.49241	2.31826	3.27353	4.03271	1.99513	2.44595	2.9194	2.48422	3.15772	0.160013844	0.619746337	8	20	24	0.173984051	0.768040178	24	6.394434578	0.155284704
ENSMUSG00000034321	Exosc1	exosome component 1 [Source:MGI Symbol;Acc:MGI:1913833]	10.7043	11.695	12.0965	13.0889	11.7475	11.5995	11.4603	10.8954	14.0543	11.2416	13.2499	11.4659	11.3445	12.099	0.053683798	0.394331071	12	16	24	0.066128986	0.668186543	24	11.21008526	0.087004033
ENSMUSG00000034324	Tmem132c	transmembrane protein 132C [Source:MGI Symbol;Acc:MGI:2443061]	3.51436	2.45804	2.5102	2.191	2.12679	2.53375	2.39743	2.47079	1.82391	2.85454	2.00803	2.7401	2.47965	2.52533	0.676344318	0.966018836	24	20	24	0.215656901	0.768040178	24	23.44599463	0.107754994
ENSMUSG00000034327	Kctd9	potassium channel tetramerisation domain containing 9 [Source:MGI Symbol;Acc:MGI:2145579]	37.7251	35.8936	36.3418	33.9897	31.8505	36.9367	37.2211	33.9511	32.1969	37.3582	32.7482	36.6023	36.5023	38.8565	0.231491055	0.731037655	4	16	24	0.146773638	0.755260456	24	0.995282492	0.064452809
ENSMUSG00000034330	Plcg2	phospholipase C, gamma 2 [Source:MGI Symbol;Acc:MGI:97616]	1.06184	1.56378	1.57243	1.55798	1.29113	1.44987	1.54433	1.30809	1.50724	1.21917	1.28314	1.24953	1.29457	1.29991	0.314193491	0.825291692	12	8	24	0.396676274	0.799546792	24	8.189031155	0.052329142
ENSMUSG00000034333	Zbed4	zinc finger, BED type containing 4 [Source:MGI Symbol;Acc:MGI:2682302]	1.0221	1.00011	1.00634	1.05945	0.997738	1.01911	1.07438	1.14138	1.0453	0.893276	0.959276	1.04809	1.0318	1.03726	0.40165019	0.933249185	8	12	24	0.136474762	0.747972781	24	2.029231528	0.02988261
ENSMUSG00000034334	Fam151b	family with sequence similarity 151, member B [Source:MGI Symbol;Acc:MGI:1921192]	3.58703	5.09842	5.50708	5.01053	4.82928	4.32796	5.2332	4.2451	5.01714	4.4711	5.32641	3.97964	4.40236	5.27523	0.245061943	0.741580974	12	12	24	0.151009026	0.758865539	24	9.451294305	0.112256856
ENSMUSG00000034336	Ina	internexin neuronal intermediate filament protein, alpha [Source:MGI Symbol;Acc:MGI:96568]	10.7127	10.2453	16.1708	11.1133	8.91571	15.0312	13.2406	10.0499	10.2906	13.9373	9.05365	12.1154	8.65112	14.3202	0.928127947	0.966018836	16	4	24	0.851269767	0.955425185	24	7.139300741	0.065245839
ENSMUSG00000034341	Wbp2	WW domain binding protein 2 [Source:MGI Symbol;Acc:MGI:104709]	108.524	90.7396	91.4286	95.9986	89.2321	108.253	105.221	108.944	84.572	140.498	85.7706	107.733	97.9177	103.957	0.46694635	0.966018836	24	20	24	0.906617013	0.974544241	24	21.8872893	0.034670317
ENSMUSG00000034342	Cbl	Casitas B-lineage lymphoma [Source:MGI Symbol;Acc:MGI:88279]	12.7189	10.5278	10.8383	11.7802	11.449	11.6483	10.9914	10.0584	10.0425	11.7885	11.5015	12.3868	12.0064	11.5215	0.098022314	0.501188411	24	12	24	0.051798097	0.648725249	24	19.00960339	0.085375994
ENSMUSG00000034343	Ube2f	ubiquitin-conjugating enzyme E2F (putative) [Source:MGI Symbol;Acc:MGI:1915171]	17.6626	16.8391	15.858	18.0766	17.462	16.7435	18.2661	16.0565	17.4854	17.0893	19.2672	18.8055	17.6443	17.5228	0.206977373	0.698112164	20	16	24	0.102972829	0.713936861	24	18.13048604	0.055641749
ENSMUSG00000034345	Gtf2h5	general transcription factor IIH, polypeptide 5 [Source:MGI Symbol;Acc:MGI:107227]	61.4808	63.3765	55.045	56.0386	62.356	58.887	57.3379	55.9166	61.6951	52.3813	63.9234	56.4252	60.4672	62.3484	0.967855455	0.970142211	20	20	24	0.6202157	0.870031302	24	22.98662683	0.034590005
ENSMUSG00000034349	Smc4	structural maintenance of chromosomes 4 [Source:MGI Symbol;Acc:MGI:1917349]	13.448	13.6338	14.4523	13.5734	14.2282	12.9789	13.949	12.7714	15.0355	11.7284	14.8004	15.5892	15.1206	14.8108	0.879206539	0.966018836	24	16	24	0.83398162	0.948922772	24	23.15903508	0.024064958
ENSMUSG00000034353	Ramp1	receptor (calcitonin) activity modifying protein 1 [Source:MGI Symbol;Acc:MGI:1858418]	2.282	4.55019	6.18873	3.22191	3.21243	3.85979	4.43969	4.27468	5.40354	3.61298	3.38361	4.38037	2.66388	5.12667	0.036424767	0.332409444	12	8	24	0.105696941	0.717832234	24	7.24378755	0.254951144
ENSMUSG00000034354	Mtmr3	myotubularin related protein 3 [Source:MGI Symbol;Acc:MGI:1921552]	17.8204	16.8172	17.1761	16.8139	16.0967	18.7041	18.0563	17.6449	14.776	19.4563	14.4868	17.6301	17.6193	16.246	0.249881062	0.749643185	24	20	24	0.506904443	0.812342247	24	22.69595043	0.052036729
ENSMUSG00000034361	Cpne2	copine II [Source:MGI Symbol;Acc:MGI:2387578]	18.4822	15.1107	14.4064	15.3284	16.9065	16.7412	15.9504	14.8445	15.2694	19.9216	15.3631	16.1441	17.0707	15.844	0.054224213	0.395890765	24	12	24	0.414602254	0.799810051	24	18.53534264	0.064850434
ENSMUSG00000034371	Tkfc	triokinase, FMN cyclase [Source:MGI Symbol;Acc:MGI:2385084]	4.2482	3.88454	4.20027	4.32327	4.06479	4.54817	3.79045	4.24371	3.04961	5.39669	3.50627	4.75866	4.19658	4.51619	0.9678483	0.970142211	24	12	24	0.821638169	0.943784856	24	18.04809243	0.040920788
ENSMUSG00000034377	Tulp4	tubby like protein 4 [Source:MGI Symbol;Acc:MGI:1916092]	29.2709	31.7395	32.6804	32.5342	32.2529	30.2039	30.1772	31.1319	31.0029	33.0052	29.4226	31.7823	32.1034	32.5892	0.17953325	0.655480352	16	12	24	0.118335253	0.728974286	24	9.422143287	0.043609314
ENSMUSG00000034379	Wdr5b	WD repeat domain 5B [Source:MGI Symbol;Acc:MGI:1916794]	2.04514	2.45082	2.62578	1.95036	2.6767	2.44047	1.97406	2.33707	2.318	2.3152	2.03983	2.51675	2.51315	2.27431	0.880992536	0.966018836	24	16	24	0.995394733	0.998759942	24	7.910009392	0.004290103
ENSMUSG00000034390	Cmip	c-Maf inducing protein [Source:MGI Symbol;Acc:MGI:1921690]	29.8848	20.6765	23.292	22.926	22.883	25.7502	24.563	28.0043	20.3079	43.1889	24.4397	30.0081	25.6901	29.881	0.601647512	0.966018836	24	12	24	0.928683013	0.981515518	24	17.56725764	0.040216135
ENSMUSG00000034394	Lif	leukemia inhibitory factor [Source:MGI Symbol;Acc:MGI:96787]	0.909695	0.638178	0.367265	0.653554	0.516879	0.713258	1.09508	0.567796	0.6964	0.627609	0.751376	0.88351	0.903609	0.305395	0.007049678	0.188070711	4	4	24	0.057333255	0.656248849	24	20.9513416	0.168030243
ENSMUSG00000034401	Spata6	spermatogenesis associated 6 [Source:MGI Symbol;Acc:MGI:1915196]	13.1201	15.4139	13.8747	13.2479	13.5009	13.0329	13.7574	12.5867	13.952	13.687	13.6204	12.9766	13.4697	13.2344	0.085890569	0.466404237	12	12	24	0.527284381	0.823319175	24	7.469299402	0.028443108
ENSMUSG00000034402	Kcnh5	potassium voltage-gated channel, subfamily H (eag-related), member 5 [Source:MGI Symbol;Acc:MGI:3584508]	0.853125	0.858547	0.630177	0.762445	0.627888	0.702776	0.596196	0.657802	0.779204	0.724322	0.828545	0.669806	0.653572	0.862711	0.924770496	0.966018836	8	20	24	0.754945847	0.920912425	24	6.923392382	0.024661213
ENSMUSG00000034403	Pja1	praja ring finger ubiquitin ligase 1 [Source:MGI Symbol;Acc:MGI:1101765]	81.5683	70.8646	69.3664	67.1126	71.0186	76.6264	71.0528	70.2512	63.0259	68.5828	71.3795	69.8601	72.5841	72.0877	0.001980441	0.100255953	4	12	24	0.006890599	0.472731997	24	21.92833097	0.087465907
ENSMUSG00000034412	Tbc1d10a	TBC1 domain family, member 10a [Source:MGI Symbol;Acc:MGI:2144164]	14.943	13.2626	14.9977	14.0934	14.9305	14.235	14.1373	13.9787	13.7469	15.0938	15.2361	12.6597	13.6061	12.8326	0.109247218	0.524035616	20	12	24	0.130043093	0.740637625	24	14.38565423	0.054992562
ENSMUSG00000034422	Parp14	poly (ADP-ribose) polymerase family, member 14 [Source:MGI Symbol;Acc:MGI:1919489]	4.37788	4.47775	4.23055	4.12396	5.13375	4.20459	4.40287	4.98004	4.80688	3.91274	4.93603	4.14739	4.35288	4.57706	0.327174535	0.845975006	8	8	24	0.975638554	0.993814468	24	4.843578225	0.008707745
ENSMUSG00000034424	Gcsh	glycine cleavage system protein H (aminomethyl carrier) [Source:MGI Symbol;Acc:MGI:1915383]	45.5429	42.3148	42.2012	40.569	41.6451	42.3706	40.5279	43.3406	39.9615	44.9598	47.6471	45.3793	47.1905	45.4634	0.398157656	0.929472831	4	8	24	0.463858208	0.805393068	24	21.12586203	0.0353874
ENSMUSG00000034429	Zfp707	zinc finger protein 707 [Source:MGI Symbol;Acc:MGI:1916270]	2.12604	2.80724	2.85665	2.9931	2.7299	2.9819	2.46072	3.06687	2.94679	2.85815	2.28055	3.45693	2.42301	2.52599	0.185945151	0.662239322	16	12	24	0.575074649	0.847865121	24	11.41507841	0.057877855
ENSMUSG00000034430	Zxdc	ZXD family zinc finger C [Source:MGI Symbol;Acc:MGI:1933108]	5.86905	5.9778	5.82687	6.31129	7.02892	7.14778	7.09087	6.94741	7.00376	7.29898	6.55608	6.15505	6.40478	6.04481	0.622132989	0.966018836	16	20	24	0.468828877	0.805393068	24	15.27290943	0.04737512
ENSMUSG00000034432	Cops8	COP9 signalosome subunit 8 [Source:MGI Symbol;Acc:MGI:1915363]	50.9349	50.3953	49.2806	58.4547	55.2901	55.1935	53.4231	52.3721	45.0263	52.7945	46.5162	47.123	45.7267	46.9397	0.676344318	0.966018836	16	20	24	0.334764664	0.770844856	24	16.10884022	0.054034462
ENSMUSG00000034435	Tmem30b	transmembrane protein 30B [Source:MGI Symbol;Acc:MGI:2442082]	0.545788	0.705389	0.533136	0.51686	0.622063	0.647774	0.427771	0.654323	0.594184	0.563748	0.618879	0.541635	0.427246	0.600194	0.891105762	0.966018836	8	20	24	0.777680747	0.928689964	24	10.49106948	0.021434334
ENSMUSG00000034437	Gm9761	predicted gene 9761 [Source:MGI Symbol;Acc:MGI:3708640]	27.7713	29.1041	27.499	26.7616	29.2374	25.3052	26.4857	26.0604	32.4291	26.0167	28.6906	27.5409	29.51	28.3211	0.879844076	0.966018836	12	12	24	0.646442782	0.880320244	24	7.482058748	0.034846009
ENSMUSG00000034442	Trmt5	TRM5 tRNA methyltransferase 5 [Source:MGI Symbol;Acc:MGI:1923607]	7.76355	8.49579	9.04535	8.12215	9.12786	9.34006	9.26641	7.94721	8.07465	7.21923	9.32497	9.10267	9.07666	7.27837	0.055569815	0.401074772	24	8	24	0.094289219	0.71175955	24	19.32957371	0.098838801

ENSMUSG00000034445	Cyb561a3	cytochrome b561 family, member A3 [Source:MGI Symbol;Acc:MGI:2686925]	5.18725	4.73915	4.35434	5.01778	4.89595	5.10391	4.76743	5.40789	4.55538	5.33715	4.25586	4.6539	5.07271	4.31728	0.94086481	0.966018836	4	8	24	0.770113541	0.92597113	24	21.37201685	0.027455191
ENSMUSG00000034449	Dhrs11	dehydrogenase/reductase (SDR family) member 11 [Source:MGI Symbol;Acc:MGI:2652816]	4.26669	5.53596	6.67247	6.96866	4.5699	5.49576	5.28684	5.53513	5.69477	5.90004	4.43365	5.95507	5.67237	5.34889	0.089766625	0.477784783	16	4	24	0.19702672	0.768040178	24	8.842185125	0.121210763
ENSMUSG00000034453	Polr3b	polymerase (RNA) III (DNA directed) polypeptide B [Source:MGI Symbol;Acc:MGI:1917678]	9.81186	9.01521	9.25763	8.99327	8.75941	9.10659	8.94757	8.62356	8.21358	9.91327	9.11571	9.30861	9.45175	9.30975	0.94086481	0.966018836	4	8	24	0.670189715	0.888925819	24	20.34845628	0.023754327
ENSMUSG00000034459	Ifit1	interferon-induced protein with tetratricopeptide repeats 1 [Source:MGI Symbol;Acc:MGI:99450]	16.7361	11.4625	13.0482	11.0105	17.5195	17.5698	10.5214	15.7299	11.6007	8.2317	19.0641	10.4905	9.15966	15.2836	0.893307667	0.966018836	20	20	24	0.635531523	0.876201318	24	19.36359919	0.138692303
ENSMUSG00000034460	Six4	sine oculis-related homeobox 4 [Source:MGI Symbol;Acc:MGI:106034]	9.04783	6.74187	6.09063	7.46891	7.23471	7.27708	7.63122	7.10176	5.65361	8.21664	7.43366	7.10734	7.49072	7.21549	0.087592959	0.472021905	4	8	24	0.169432457	0.767229759	24	20.20608535	0.106561749
ENSMUSG00000034462	Pkd2	polycystin 2, transient receptor potential cation channel [Source:MGI Symbol;Acc:MGI:1099818]	12.488	13.2701	12.7919	12.5681	13.2535	11.4366	13.6747	12.6584	14.3541	11.8024	11.9262	11.9251	12.1892	11.7395	0.245061943	0.741580974	12	12	24	0.374632697	0.794671327	24	6.562638222	0.045541033
ENSMUSG00000034463	Scara3	scavenger receptor class A, member 3 [Source:MGI Symbol;Acc:MGI:2444418]	6.86685	9.62157	9.11767	7.27703	7.12981	6.48173	7.97453	10.2693	13.321	6.42148	6.60211	8.17725	7.46124	8.68385	0.009855766	0.194720062	12	4	24	0.013648501	0.529134128	24	6.030684089	0.27567832
ENSMUSG00000034467	Dynlrb2	dynein light chain roadblock-type 2 [Source:MGI Symbol;Acc:MGI:1922715]	0.227499	2.34281	2.07623	1.65989	2.32107	1.20108	2.61472	0.390983	1.52593	1.11897	1.08528	1.73679	0.985174	2.23693	0.789670753	0.966018836	8	20	24	0.783667812	0.93037654	24	10.94728294	0.134858367
ENSMUSG00000034471	Caskin2	CASK-interacting protein 2 [Source:MGI Symbol;Acc:MGI:2157062]	14.6071	12.8592	11.6135	12.5063	14.5357	11.5257	15.9585	14.9289	16.9613	18.9913	13.3282	14.764	15.2904	13.1053	0.814303179	0.966018836	4	20	24	0.922512988	0.979411448	24	8.816211705	0.030365739
ENSMUSG00000034472	Rasd2	RASD family, member 2 [Source:MGI Symbol;Acc:MGI:1922391]	1.42055	1.05813	1.51362	1.36317	1.239	1.40967	1.55396	1.37124	1.32087	1.83674	0.875069	1.31894	1.07192	1.52628	0.928064146	0.966018836	16	4	24	0.675230961	0.89119716	24	7.68024304	0.055523412
ENSMUSG00000034473	Sec22a	SEC22 homolog A, vesicle trafficking protein [Source:MGI Symbol;Acc:MGI:2447876]	7.99766	10.2593	10.1286	8.81187	8.72305	7.91741	7.34663	8.75651	10.7725	8.00249	9.0943	8.93998	9.06895	7.18783	0.643325306	0.966018836	12	16	24	0.313623307	0.768040178	24	9.357639217	0.092928324
ENSMUSG00000034480	Diaph2	diaphanous related formin 2 [Source:MGI Symbol;Acc:MGI:1858500]	4.57235	4.7327	4.59884	4.40387	4.75348	4.21683	4.76975	4.43977	4.44254	4.55703	4.28461	4.29587	4.33522	4.41207	0.591351901	0.966018836	12	12	24	0.613257384	0.866078878	24	5.725879487	0.01616731
ENSMUSG00000034484	Snx2	sorting nexin 2 [Source:MGI Symbol;Acc:MGI:1915054]	43.1266	42.9534	44.3777	42.6992	44.2436	40.2236	40.5526	40.2468	40.5344	36.107	42.6813	39.1199	40.6076	39.6191	0.897570341	0.966018836	12	12	24	0.959215388	0.98927861	24	18.73747986	0.007388114
ENSMUSG00000034485	Uaca	uveal autoantigen with coiled-coil domains and ankyrin repeats [Source:MGI Symbol;Acc:MGI:1919815]	32.3537	31.9457	29.2016	29.2988	33.7622	30.9379	30.4701	34.7178	31.7013	34.3598	28.0907	31.7439	34.3554	30.6112	0.916963526	0.966018836	8	4	24	0.692269284	0.898055706	24	1.524208748	0.031138258
ENSMUSG00000034487	Poglut3	protein O-glucosyltransferase 3 [Source:MGI Symbol;Acc:MGI:1923765]	7.83553	6.51473	7.36557	6.81528	6.60906	7.00264	7.06472	6.53606	6.12253	6.95411	6.76043	7.07579	6.48165	7.26856	0.858266942	0.966018836	4	4	24	0.547300857	0.833663107	24	21.81763192	0.03451184
ENSMUSG00000034488	Edil3	EGF-like repeats and discoidin I-like domains 3 [Source:MGI Symbol;Acc:MGI:1329025]	73.8144	73.1148	69.9097	69.5833	71.6361	67.5466	68.5528	63.7114	59.349	67.9462	76.0498	59.7719	61.6505	66.7195	0.895733497	0.966018836	20	4	24	0.548084931	0.834077197	24	16.28238796	0.03979367
ENSMUSG00000034501	Pcnx4	pecanex homolog 4 [Source:MGI Symbol;Acc:MGI:1914958]	7.04681	6.34461	6.69263	6.2289	5.8181	6.05369	6.37311	6.38247	5.72475	6.30124	5.9961	6.25677	6.21602	6.50348	0.054935173	0.398010585	8	12	24	0.139393065	0.749556072	24	2.288046511	0.046116805
ENSMUSG00000034509	Mad2l1bp	MAD2L1 binding protein [Source:MGI Symbol;Acc:MGI:1913841]	9.0521	8.00601	9.45797	8.72052	9.04718	10.262	9.48029	9.4014	8.24314	9.40653	8.67587	8.1976	9.54367	8.86452	0.912923872	0.966018836	4	4	24	0.68878537	0.897070579	24	20.83984986	0.03246847
ENSMUSG00000034518	Hmgxb4	HMG box domain containing 4 [Source:MGI Symbol;Acc:MGI:1918073]	8.8127	10.791	8.41647	9.1854	9.6931	9.45476	8.88506	10.3649	9.42383	9.28252	8.67757	9.89549	9.80814	9.2089	0.89563591	0.966018836	8	4	24	0.618083511	0.868871403	24	1.892145645	0.034515202
ENSMUSG00000034520	Gjc1	gap junction protein, gamma 1 [Source:MGI Symbol;Acc:MGI:95718]	2.14737	2.10074	2.36396	2.603	2.56619	2.52472	3.03353	2.76182	2.46225	2.41403	2.36317	2.36523	2.68512	1.94179	0.890235289	0.966018836	4	4	24	0.741699007	0.915984529	24	18.49848566	0.038004218
ENSMUSG00000034522	Zfp395	zinc finger protein 395 [Source:MGI Symbol;Acc:MGI:2682318]	3.52666	3.48361	3.03943	3.31893	3.49533	3.14533	3.60512	3.39574	3.65213	3.52692	3.21518	3.70675	3.40765	3.52011	0.88067614	0.966018836	4	16	24	0.687395029	0.89619155	24	1.138544147	0.021060849
ENSMUSG00000034525	Ice1	interactor of little elongation complex ELL subunit 1 [Source:MGI Symbol;Acc:MGI:2385865]	19.4365	20.1383	19.6664	18.9952	18.844	18.6245	18.8267	18.7481	19.3936	19.2	18.1943	18.1068	17.4955	19.5001	0.015345591	0.253245931	12	12	24	0.01744782	0.550995814	24	7.241447221	0.042051823
ENSMUSG00000034532	Fbxo16	F-box protein 16 [Source:MGI Symbol;Acc:MGI:1354706]	4.10866	4.45461	3.98174	3.17768	3.49379	4.0634	3.32025	4.35257	3.76481	3.73918	3.90937	4.53202	3.60635	4.26343	0.086420527	0.467848941	8	8	24	0.353313782	0.780711577	24	1.972489174	0.068687908
ENSMUSG00000034533	Scn10a	sodium channel, voltage-gated, type X, alpha [Source:MGI Symbol;Acc:MGI:108029]	150.326	100.403	104.459	111.881	123.54	137.368	110.729	127.376	78.6015	140.503	110.689	119.79	115.085	110.353	0.133266423	0.567728672	24	12	24	0.138771338	0.748341961	24	19.7616714	0.177463409
ENSMUSG00000034538	Zfp418	zinc finger protein 418 [Source:MGI Symbol;Acc:MGI:2444763]	1.61983	1.38436	1.47805	1.62134	1.82517	1.5874	1.15122	1.17989	1.2272	1.28507	1.80272	1.70552	1.46546	1.33722	0.008244783	0.192760771	20	12	24	0.007205646	0.47735824	24	17.20164305	0.137578881
ENSMUSG00000034543	Morc2a	microrchidia 2A [Source:MGI Symbol;Acc:MGI:1921772]	9.21051	9.04868	10.8937	10.213	9.90204	9.18978	9.14926	9.5592	9.40245	9.63682	8.84754	9.14257	9.09669	8.63682	0.219371727	0.708495385	12	20	24	0.051349379	0.64767123	24	11.16566426	0.060435546
ENSMUSG00000034544	Rsrc1	arginine/serine-rich coiled-coil 1 [Source:MGI Symbol;Acc:MGI:1914130]	20.1427	19.1229	19.1571	20.5998	18.9344	19.5133	19.5837	19.1187	19.032	21.1512	20.1886	19.8223	20.7437	21.5628	0.896753532	0.966018836	16	20	24	0.971074197	0.993012791	24	10.92260741	0.004962317
ENSMUSG00000034551	Hdx	highly divergent homeobox [Source:MGI Symbol;Acc:MGI:2685226]	1.05561	1.69835	1.41257	1.2264	1.44122	1.27819	1.18165	1.36117	1.51057	1.0222	1.56224	1.36461	1.12972	1.41905	0.239653024	0.741580974	12	16	24	0.707205781	0.903330697	24	8.527367916	0.042348772
ENSMUSG00000034557	Zfyve9	zinc finger, FYVE domain containing 9 [Source:MGI Symbol;Acc:MGI:2652838]	30.3951	28.5419	27.363	27.101	26.7409	28.8461	27.577	26.1366	25.5959	27.9429	26.4748	28.0336	29.546	29.8139	0.092596422	0.487919468	4	8	24	0.084914285	0.70291653	24	23.54104016	0.058502871
ENSMUSG00000034560	Washc4	WASH complex subunit 4 [Source:MGI Symbol;Acc:MGI:2441787]	26.9404	26.4579	26.6838	26.1048	27.553	22.8098	26.8566	21.9411	27.7266	21.2762	30.7295	28.5366	29.2363	28.8816	0.893307667	0.966018836	20	20	24	0.832273254	0.94810743	24	21.33187376	0.037413051
ENSMUSG00000034563	Ccpg1	cell cycle progression 1 [Source:MGI Symbol;Acc:MGI:1196419]	69.1921	65.8867	62.6208	65.393	61.899	65.5248	63.9682	61.7754	59.2753	64.0893	61.4261	67.113	69.3813	66.3186	0.097760727	0.501112642	4	8	24	0.073781985	0.682388921	24	22.87060366	0.055287767
ENSMUSG00000034566	Atp5h	ATP synthase, H+ transporting, mitochondrial F0 complex, subunit D [Source:MGI Symbol;Acc:MGI:1918929]	59.0976	82.6728	76.1162	76.5161	75.5006	73.224	68.0203	72.6246	74.4746	61.6662	86.5739	63.5045	67.7705	63.5896	0.297978647	0.80820842	20	8	24	0.439572807	0.805393068	24	12.11152484	0.077845753
ENSMUSG00000034570	Inpp5j	inositol polyphosphate 5-phosphatase J [Source:MGI Symbol;Acc:MGI:2158663]	9.37036	7.56424	7.4313	7.44871	7.2644	8.60589	7.94399	8.6904	7.05007	10.8655	7.00202	8.01154	7.93429	8.22086	0.332284134	0.853485917	24	20	24	0.888036089	0.968493323	24	0.010697295	0.030707146
ENSMUSG00000034573	Ptpn13	protein tyrosine phosphatase, non-receptor type 13 [Source:MGI Symbol;Acc:MGI:103293]	9.66941	8.82255	7.59679	8.906	10.1575	8.69866	9.99265	9.38861	9.11329	8.51806	9.17576	8.83308	8.59652	8.05267	0.745118544	0.966018836	20	16	24	0.234389219	0.768040178	24	19.82890817	0.06775708
ENSMUSG00000034574	Daam1	dishevelled associated activator of morphogenesis 1 [Source:MGI Symbol;Acc:MGI:1914596]	17.7635	14.921	15.9504	15.1059	15.1079	16.3731	15.6151	15.6629	12.8783	17.3946	14.4268	15.6846	15.512	16.2276	0.95032543	0.966018836	24	20	24	0.500073779	0.808829718	24	22.06726514	0.050339993
ENSMUSG00000034575	Tent4a	terminal nucleotidyltransferase 4A [Source:MGI Symbol;Acc:MGI:2682295]	5.9016	5.1369	5.76173	5.8767	5.15336	5.86386	6.366	5.0868	5.23307	6.31611	5.41948	5.77364	4.67744	5.25658	0.591351901	0.966018836	16	16	24	0.537232798	0.827923483	24	16.09685074	0.046570209
ENSMUSG00000034579	Pla2g3	phospholipase A2, group III [Source:MGI Symbol;Acc:MGI:2444945]	11.7144	9.3133	8.75903	7.75568	10.1381	11.7712	9.84855	9.5332	7.18784	9.25434	9.6946	9.1322	9.43169	8.56037	0.027063253	0.306445942	4	8	24	0.003158072	0.414576087	24	20.91613881	0.189691597
ENSMUSG00000034586	Hid1	HID1 domain containing [Source:MGI Symbol;Acc:MGI:2445087]	24.7841	22.2163	22.3941	22.5695	23.2455	25.1483	24.6036	24.4995	23.0962	29.1785	20.2745	22.6995	22.6258	23.1771	0.939921059	0.966018836	24	12	24	0.995831966	0.998858123	24	6.346783708	0.004446948
ENSMUSG00000034587	8430429K09Rik	RIKEN cDNA 8430429K09 gene [Source:MGI Symbol;Acc:MGI:1918773]	3.52045	4.43377	3.78426	3.20544	4.20026	3.5222	3.95676	3.47902	3.98665	4.58742	3.96895	4.11945	3.87345	3.91442	0.833406028	0.966018836	20	8	24	0.889047314	0.968820559	24	11.6928902	0.021441968
ENSMUSG00000034591	Slc41a2	solute carrier family 41, member 2 [Source:MGI Symbol;Acc:MGI:2442940]	11.6175	9.58583	9.44054	9.1453	9.54958	10.5343	10.089	9.95519	7.68118	10.9297	10.0265	10.2372	9.73598	10.5769	0.128989168	0.560209134	24	12	24	0.166652425	0.767020009	24	21.34557574	0.093088099
ENSMUSG00000034593	Myo5a	myosin VA [Source:MGI Symbol;Acc:MGI:105976]	60.7831	56.3928	59.4795	61.7004	57.6375	63.2421	56.4497	53.2282	48.9659	59.499	58.9747	62.8316	62.0872	64.2832	0.390686537	0.915479136	24	12	24	0.273087162	0.768040178	24	19.52914762	0.067227277
ENSMUSG00000034595	Ppp1r18	protein phosphatase 1, regulatory subunit 18 [Source:MGI Symbol;Acc:MGI:1923698]	11.6844	10.7719	10.8333	13.0829	11.4696	10.8028	11.7362	11.4862	10.4675	11.7352	12.1518	11.4021	12.0167	10.4965	0.175185919	0.651010555	16	20	24	0.263111807	0.768040178	24	16.47899913	0.052566874
ENSMUSG00000034601	2700049A03Rik	RIKEN cDNA 2700049A03 gene [Source:MGI Symbol;Acc:MGI:1924217]	2.0784	2.36149	2.45607	2.20239	2.42563	2.14955	2.45547	2.19156	2.24249	2.62992	2.15575	2.4165	2.39515	2.29812	0.916963526	0.966018836	12	20	24	0.712300447	0.905113511	24	10.45957978	0.022180175
ENSMUSG00000034602	Mon2	MON2 homolog, regulator of endosome to Golgi trafficking [Source:MGI Symbol;Acc:MGI:1914324]	18.0989	18.6083	16.6474	19.6928	19.8323	20.9564	22.0679	19.5844	17.3652	19.386	19.3819	18.0214	17.9923	17.8139	0.193864198	0.67239758	20	16	24	0.211755243	0.768040178	24	18.98003786	0.072309572
ENSMUSG00000034610	Tut4	terminal uridylyl transferase 4 [Source:MGI Symbol;Acc:MGI:2445126]	7.03081	9.44601	8.56779	8.54577	8.53483	8.6677	10.107	12.0289	10.7723	8.97736	9.77154	8.12887	9.37237	9.10111	0.424589909	0.966018836	8	16	24	0.221763083	0.768040178	24	6.568728035	0.108814188
ENSMUSG00000034612	Chst11	carbohydrate sulfotransferase 11 [Source:MGI Symbol;Acc:MGI:1927166]	8.36767	6.67991	7.42397	7.88003	7.30744	8.18352	7.23221	8.4585	5.8595	8.37815	6.53172	7.28094	7.41261	7.67766	0.858514388	0.966018836	16	4	24	0.770734152	0.926171026	24	21.62832755	0.039822999
ENSMUSG00000034613	Ppm1h	protein phosphatase 1H (PP2C domain containing) [Source:MGI Symbol;Acc:MGI:2442087]	13.4144	9.40377	9.96237	9.65503	8.84767	11.629	10.9395	10.7174	7.82114	12.7378	9.80152	10.3258	10.1172	11.4894	0.313786068	0.824470524	4	8	24	0.411728013	0.799546792	24	22.59576406	0.099349923
ENSMUSG00000034614	Pik3ip1	phosphoinositide-3-kinase interacting protein 1 [Source:MGI Symbol;Acc:MGI:1917016]	35.5629	43.1661	42.7009	42.7364	36.904	38.7011	37.9613	43.8968	44.8075	50.1505	39.4007	44.4846	42.7041	44.3696	0.140905066	0.585944911	12	16	24	0.013449493	0.529134128	24	8.627050381	0.119647655
ENSMUSG00000034616	Ssh3	slingshot protein phosphatase 3 [Source:MGI Symbol;Acc:MGI:2683546]	21.4633	19.161	19.2257	22.6533	21.1604	18.6081	20.7886	19.2771	21.4526	19.0019	21.1835	19.0417	18.6832	19.4796	0.933421033	0.966018836	20	4	24	0.423327907	0.805346055	24	13.4195666	0.042473616
ENSMUSG00000034617	Mtrr	5-methyltetrahydrofolate-homocysteine methyltransferase reductase [Source:MGI Symbol;Acc:MGI:1891037]	6.1738	4.25517	4.1243	4.75066	4.87922	4.89424	4.71737	4.98171	4.38368	4.73793	4.29951	4.73768	5.48455	3.93668	0.243586913	0.741580974	4	8	24	0.101824872	0.713240751	24	21.2406084	0.110999566
ENSMUSG00000034620	Rxylt1	ribitol xylosyltransferase 1 [Source:MGI Symbol;Acc:MGI:2384919]	18.2421	20.4665	18.9719	18.4187	19.7188	18.6794	18.5303	19.0073	18.3742	17.2917	18.7317	19.0099	20.1861	18.8385	0.839634479	0.966018836	8	8	24	0.385792643	0.799546792	24	0.414246981	0.031433104
ENSMUSG00000034621	Gpatch8	G patch domain containing 8 [Source:MGI Symbol;Acc:MGI:1918667]	14.1345	14.5118	13.6643	14.0844	14.0281	14.5397	15.1963	13.4102	14.7808	14.7753	14.6531	14.7444	14.4082	14.1307	0.826609618	0.966018836	24	8	24	0.489596911	0.805393068	24	18.99104842	0.021406716
ENSMUSG00000034634	Ly6d	lymphocyte antigen 6 complex, locus D [Source:MGI Symbol;Acc:MGI:96881]	1.02839	2.61043	2.90617	3.29131	1.78954	2.09376	2.00512	1.71483	2.85184	1.31391	1.45742	1.75349	2.60834	2.11103	0.347721987	0.870418653	12	8	24	0.286069473	0.768040178	24	7.796468419	0.198889412
ENSMUSG00000034636	Zyg11b	zyg-11 family member B, cell cycle regulator [Source:MGI Symbol;Acc:MGI:2685277]	27.2622	25.7772	25.7847	26.2148	24.9485	25.9818	26.1397	24.1688	24.4019	25.724	26.0627	27.9732	27.7945	28.6346	0.4274437	0.966018836	4	8	24	0.237301433	0.768040178	24	22.6198499	0.042389856
ENSMUSG00000034639	Setmar	SET domain without mariner transposase fusion [Source:MGI Symbol;Acc:MGI:1921979]	3.33748	3.59092	3.45283	4.18928	4.11174	4.13213	3.20839	3.70825	3.85518	3.32466	3.58481	4.1214	4.07258	4.14223	0.914502381	0.966018836	24	4	24	0.738139038	0.91451866	24	16.59379082	0.032414756
ENSMUSG00000034640	Tiparp	TCDD-inducible poly(ADP-ribose) polymerase [Source:MGI Symbol;Acc:MGI:2159210]	18.3642	19.894	18.5115	18.4506	21.8507	17.832	19.898	18.4673	21.6084	18.6333	21.8132	20.6224	23.1216	19.1147	0.911135828	0.966018836	20	20	24	0.787428875	0.931609708	24	18.221116	0.029187025
ENSMUSG00000034641	Cd300ld	CD300 molecule like family member d [Source:MGI Symbol;Acc:MGI:2442358]	0.622017	0.844424	0.815875	0.754456	0.924894	0.573978	0.896924	0.659513	1.11389	0.431845	0.738176	0.698432	0.942273	0.849599	0.92655264	0.966018836	12	12	24	0.558624508	0.839921589	24	5.065070883	0.062376961
ENSMUSG00000034647	Ankrd12	ankyrin repeat domain 12 [Source:MGI Symbol;Acc:MGI:1914357]	10.8441	11.0584	10.2127	12.6257	12.0535	10.6617	10.3229	9.74215	14.1026	11.5625	12.3755	11.1254	12.4971	11.3257	0.352925135	0.87272957	16	16	24	0.200461568	0.768040178	24	12.56355676	0.08597612
ENSMUSG00000034648	Lrrn1	leucine rich repeat protein 1, neuronal [Source:MGI Symbol;Acc:MGI:106038]	96.6547	76.0964	71.9596	74.0367	73.1947	85.1811	78.68	76.7542	58.2058	81.5101	75.6507	81.8885	81.8744	77.6124	7.90E-04	0.071344007	24	12	24	0.02797506	0.590904845	24	21.50108154	0.152247355
ENSMUSG00000034652	Cd300a	CD300A molecule [Source:MGI Symbol;Acc:MGI:2443411]	0.520909	0.685216	0.634037	0.550503	0.752596	0.560315	0.519439	0.810581	0.755711	0.496151	0.573943	0.516118	0.609166	0.495205	0.850398351	0.966018836	12	12	24	0.53054586	0.824858059	24	7.507438228	0.042443929
ENSMUSG00000034653	Ythdc2	YTH domain containing 2 [Source:MGI Symbol;Acc:MGI:2448561]	7.73765	7.72823	6.68844	7.85885	7.03967	6.80017	7.24542	6.85312	7.01498	6.18495	8.47698	6.47476	6.20901	7.04663	0.844448841	0.966018836	20	4	24	0.9006519	0.97190491	24	16.10475315	0.019778146
ENSMUSG00000034656	Cacna1a	calcium channel, voltage-dependent, P/Q type, alpha 1A subunit [Source:MGI Symbol;Acc:MGI:109482]	19.4016	17.5525	16.6281	18.5787	20.7936	18.9074	20.2059	18.6568	18.5158	21.52	20.4773	18.4404	17.9983	17.419	0.036415739	0.332409444	20	16	24	0.063165364	0.663164648	24	16.58319092	0.086034118
ENSMUSG00000034659	Tmem109	transmembrane protein 109 [Source:MGI Symbol;Acc:MGI:1915789]	21.0375	19.0743	19.5812	22.1342	22.5893	23.7927	21.2099	28.9678	21.9506	27.4303	19.3626	21.9959	23.469	21.2287	0.90332508	0.966018836	16	20	24	0.962947306	0.990726108	24	10.52723355	0.018335317
ENSMUSG00000034663	Bmp2k	BMP2 inducible kinase [Source:MGI Symbol;Acc:MGI:2155456]	3.6393	4.04463	4.32891	4.27069	3.96959	3.83733	4.50641	3.61185	4.40778	4.0722	3.8539	4.02231	3.4798	3.70549	0.085329704	0.466404237	12	20	24	0.245780882	0.768040178	24	11.10894488	0.056925314
ENSMUSG00000034664	Itga2b	integrin alpha 2b [Source:MGI Symbol;Acc:MGI:96601]	5.08592	5.96574	6.85013	6.324	5.12611	4.52196	6.98875	5.30948	6.66198	5.79659	6.08865	6.94074	5.97707	5.73052	0.916860713	0.966018836	12	20	24	0.497445305	0.807576732	24	8.253913814	0.076894673
ENSMUSG00000034667	Xpot	exportin, tRNA (nuclear export receptor for tRNAs) [Source:MGI Symbol;Acc:MGI:1920442]	19.7248	17.0108	19.4079	19.2597	16.1071	17.8179	20.3019	16.8315	15.5443	18.4473	17.5991	17.0916	17.279	16.9472	0.762156552	0.966018836	4	4	24	0.821579698	0.943750464	24	21.35309231	0.02596926
ENSMUSG00000034673	Pbx2	pre B cell leukemia homeobox 2 [Source:MGI Symbol;Acc:MGI:1341793]	22.5242	18.5704	17.4291	16.5051	16.8233	18.7727	19.8726	17.9749	15.7581	21.1545	17.045	18.4328	18.981	19.8985	0.030691182	0.324150946	4	8	24	0.159228146	0.765350752	24	23.76598743	0.097930572
ENSMUSG00000034674	Tdg	thymine DNA glycosylase [Source:MGI Symbol;Acc:MGI:108247]	6.06426	6.74222	6.04057	5.13756	6.08064	5.91678	5.55659	6.63112	6.87295	6.65475	5.71233	6.90228	6.0873	5.94776	0.906657897	0.966018836	12	4	24	0.623738774	0.87183699	24	5.12563158	0.041659802
ENSMUSG00000034675	Dbn1	drebrin 1 [Source:MGI Symbol;Acc:MGI:1931838]	36.9095	33.6003	32.8012	32.104	37.2905	34.8561	36.5499	34.6397	38.0814	43.5497	36.3186	38.3219	38.5227	37.3414	0.826609618	0.966018836	4	4	24	0.795414468	0.934545847	24	16.99124799	0.0247311
ENSMUSG00000034681	Rnps1	RNA binding protein with serine rich domain 1 [Source:MGI Symbol;Acc:MGI:97960]	17.6854	17.7101	19.3701	17.8175	17.1757	17.4008	18.4217	17.5141	17.936	18.4003	16.869	17.3348	18.4606	19.8977	0.035023815	0.328311403	12	8	24	0.128366086	0.740162193	24	6.256560422	0.037870037
ENSMUSG00000034683	Ppp1r1c	protein phosphatase 1, regulatory inhibitor subunit 1C [Source:MGI Symbol;Acc:MGI:1923185]	307.49	264.277	243.013	251.94	254.452	268.781	242.374	234.115	213.23	255.206	267.209	256.136	263.452	255.488	0.071575474	0.440262583	24	12	24	0.065204734	0.664809696	24	20.58902646	0.100213902
ENSMUSG00000034684	Sema3f	sema domain, immunoglobulin domain (Ig), short basic domain, secreted, (semaphorin) 3F [Source:MGI Symbol;Acc:MGI:1096347]	6.35383	6.93454	5.46681	8.65728	6.22199	5.28457	6.97347	8.00254	6.14464	7.56402	6.53112	6.11908	6.50901	6.00135	0.767183862	0.966018836	16	8	24	0.605516278	0.862720716	24	9.500377441	0.071162942
ENSMUSG00000034685	Fam171a2	family with sequence similarity 171, member A2 [Source:MGI Symbol;Acc:MGI:2448496]	5.19615	4.48076	3.99409	4.85318	5.32441	4.36646	4.97797	6.56806	3.92336	6.15534	6.61642	6.45468	6.05562	4.90101	0.173410058	0.648600527	20	16	24	0.327639337	0.768040178	24	18.85699319	0.113932044
ENSMUSG00000034686	Prr7	proline rich 7 (synaptic) [Source:MGI Symbol;Acc:MGI:3487246]	1.69147	1.14709	1.44327	1.06815	1.0955	0.69869	1.17461	1.51739	1.88233	3.08899	2.90268	3.14395	3.15395	2.46578	0.784688048	0.966018836	4	12	24	0.920878981	0.979223729	24	10.14828943	0.049024745
ENSMUSG00000034706	Dnai2	dynein axonemal intermediate chain 2 [Source:MGI Symbol;Acc:MGI:2685574]	1.75797	2.12692	1.99992	1.91574	3.0257	2.23515	1.87368	2.33643	2.2877	2.18082	1.76501	2.12138	1.94021	2.4567	0.503972924	0.966018836	8	20	24	0.779833794	0.929226662	24	11.34990851	0.041237993
ENSMUSG00000034707	Gns	glucosamine (N-acetyl)-6-sulfatase [Source:MGI Symbol;Acc:MGI:1922862]	28.5547	23.8433	24.2558	26.9331	25.6793	26.8226	26.1791	26.9147	23.9457	30.2247	24.8011	26.1615	25.395	25.0362	0.549118437	0.966018836	16	20	24	0.570493088	0.845305671	24	17.67721466	0.040198536
ENSMUSG00000034708	Grn	granulin [Source:MGI Symbol;Acc:MGI:95832]	25.7215	25.616	26.143	27.1228	26.3049	25.8902	23.4773	28.8282	26.193	29.0812	22.4316	24.7749	26.9868	23.807	0.328944792	0.847277612	16	8	24	0.454702025	0.805393068	24	9.003179229	0.050960732
ENSMUSG00000034709	Ppp1r21	protein phosphatase 1, regulatory subunit 21 [Source:MGI Symbol;Acc:MGI:1921075]	28.1785	26.7409	25.6547	24.296	25.8049	26.1868	25.3765	26.9855	23.2391	24.7645	24.9295	25.1304	24.7553	25.4894	0.126560612	0.558021618	8	8	24	0.035071109	0.608551791	24	23.58782257	0.0524988
ENSMUSG00000034714	Ttyh2	tweety family member 2 [Source:MGI Symbol;Acc:MGI:2157091]	6.47739	6.11144	6.97296	5.49904	7.31393	6.26921	7.96445	6.70368	6.82601	6.52624	5.97804	5.98913	5.2404	5.72836	0.85387321	0.966018836	12	12	24	0.999281209	0.999783065	24	7.643679961	0.002119316
ENSMUSG00000034723	Tmx4	thioredoxin-related transmembrane protein 4 [Source:MGI Symbol;Acc:MGI:106558]	41.1496	39.8983	42.4815	39.7708	40.0237	40.6129	38.9518	36.1917	36.8947	37.4838	39.3116	39.4702	37.9698	42.0587	0.773642901	0.966018836	24	16	24	0.840960565	0.951840198	24	20.60828704	0.015523145
ENSMUSG00000034724	Cnot6l	CCR4-NOT transcription complex, subunit 6-like [Source:MGI Symbol;Acc:MGI:2443154]	5.09287	6.15151	5.74233	6.14956	6.01192	5.28465	8.54398	5.05687	6.77574	4.96749	6.09749	5.26035	4.65802	5.4476	0.935732159	0.966018836	12	16	24	0.963433712	0.990884307	24	9.544345439	0.021648884
ENSMUSG00000034729	Mrps10	mitochondrial ribosomal protein S10 [Source:MGI Symbol;Acc:MGI:1928139]	22.3378	23.0834	22.4161	19.2018	20.8084	20.1122	21.6368	23.6603	24.0609	22.4256	24.8935	23.9889	26.4111	27.0551	0.671726933	0.966018836	8	8	24	0.03464332	0.608551791	24	3.749360932	0.08736864
ENSMUSG00000034730	Adgrb1	adhesion G protein-coupled receptor B1 [Source:MGI Symbol;Acc:MGI:1933736]	8.52239	8.07465	11.3719	7.90236	6.58736	8.06553	11.26	8.91895	10.271	10.5704	8.16441	9.07889	8.29991	9.93237	0.179603275	0.655480352	12	8	24	0.141427394	0.751766185	24	6.184986563	0.151261399
ENSMUSG00000034731	Dgkh	diacylglycerol kinase, eta [Source:MGI Symbol;Acc:MGI:2444188]	58.2057	59.4079	56.1145	63.4409	57.1969	54.3923	52.2203	50.1645	56.643	58.5144	59.5324	57.3326	59.8347	60.2763	0.928064146	0.966018836	16	8	24	0.44898564	0.805393068	24	12.76208631	0.042401276
ENSMUSG00000034738	Nostrin	nitric oxide synthase trafficker [Source:MGI Symbol;Acc:MGI:3606242]	1.49124	1.38348	1.43106	1.60954	1.1356	1.19523	1.05959	0.843648	1.09687	1.16148	1.13023	1.10195	1.00823	1.16155	0.935316621	0.966018836	16	12	24	0.543286035	0.831565139	24	12.28243546	0.043943491
ENSMUSG00000034744	Nagk	N-acetylglucosamine kinase [Source:MGI Symbol;Acc:MGI:1860418]	49.7042	44.5441	41.046	47.0787	47.5751	46.4902	44.7644	46.8873	40.171	50.3587	48.8669	50.8807	52.7218	49.2832	0.191659183	0.669841558	24	12	24	0.108968209	0.72082227	24	19.92096797	0.080384978
ENSMUSG00000034748	Sirt6	sirtuin 6 [Source:MGI Symbol;Acc:MGI:1354161]	7.07066	5.86097	4.98215	6.12438	5.8295	6.30269	5.87907	6.14968	6.18182	6.94434	6.46915	6.38996	6.24669	6.56136	0.93447422	0.966018836	24	12	24	0.612465406	0.865550634	24	19.9691696	0.040770721
ENSMUSG00000034751	Mast4	microtubule associated serine/threonine kinase family member 4 [Source:MGI Symbol;Acc:MGI:1918885]	2.28431	2.51864	3.72587	2.87713	2.28802	2.89657	3.03717	2.54176	2.42165	3.30033	1.89501	2.87565	1.77926	2.46083	0.891105762	0.966018836	16	4	24	0.517782757	0.818405353	24	9.89891798	0.092870597
ENSMUSG00000034755	Pcdh11x	protocadherin 11 X-linked [Source:MGI Symbol;Acc:MGI:2442849]	0.603617	1.24376	1.03412	0.786022	0.940862	0.89223	0.882044	0.532229	1.15455	0.833418	0.802431	0.98588	0.722441	0.761421	0.773177465	0.966018836	12	16	24	0.659261762	0.884879972	24	11.04764865	0.055193636
ENSMUSG00000034757	Tmub2	transmembrane and ubiquitin-like domain containing 2 [Source:MGI Symbol;Acc:MGI:1919303]	32.7399	29.8036	29.4378	30.5289	29.7099	33.7306	32.3037	32.8767	26.1494	35.3223	29.5688	31.1197	31.165	31.2108	0.280768839	0.792139038	4	8	24	0.42766255	0.805393068	24	21.16999866	0.054433252
ENSMUSG00000034758	Tle6	transducin-like enhancer of split 6 [Source:MGI Symbol;Acc:MGI:2149593]	5.56412	5.39364	2.99074	4.05872	4.25605	4.04561	3.21081	3.7857	4.91036	4.28778	3.46624	3.34358	4.33122	4.07017	0.88067614	0.966018836	4	20	24	0.733544693	0.913033708	24	3.435451812	0.064143599
ENSMUSG00000034761	Map4k5	mitogen-activated protein kinase kinase kinase kinase 5 [Source:MGI Symbol;Acc:MGI:1925503]	14.3643	14.1636	15.1314	14.513	14.7694	13.661	14.4553	13.6997	14.8114	13.3881	15.3409	13.9505	13.7954	13.6155	0.510431574	0.966018836	12	20	24	0.420957138	0.80434587	24	13.26157553	0.027241879
ENSMUSG00000034765	Dusp5	dual specificity phosphatase 5 [Source:MGI Symbol;Acc:MGI:2685183]	10.6263	5.99388	6.09974	7.43356	7.12352	7.62507	6.91403	7.03988	5.22171	7.85504	7.09068	7.20207	8.40864	6.9001	0.062105896	0.409814092	24	12	24	0.109428019	0.72082227	24	20.62031361	0.170086005
ENSMUSG00000034768	Asb16	ankyrin repeat and SOCS box-containing 16 [Source:MGI Symbol;Acc:MGI:2654437]	0.295375	0.736863	0.900189	1.1078	0.667505	0.666631	0.469471	1.56681	0.960752	1.74581	0.418357	0.684119	0.68618	1.02676	0.018857365	0.256345714	16	4	24	0.018584688	0.559812298	24	8.96802435	0.300517655
ENSMUSG00000034771	Tle2	transducin-like enhancer of split 2 [Source:MGI Symbol;Acc:MGI:104635]	10.7826	13.9388	11.7613	9.36267	10.8011	12.2349	12.7657	11.7335	12.3158	11.5972	12.3621	9.98584	11.6914	11.1423	0.961261938	0.967043964	12	4	24	0.408372714	0.799546792	24	3.36818708	0.069960821
ENSMUSG00000034773	Hrob	homologous recombination factor with OB-fold [Source:MGI Symbol;Acc:MGI:2387601]	2.03575	1.79777	2.67916	1.45357	1.80226	1.71325	2.2807	1.99852	1.95376	1.48468	1.83272	2.20168	1.69579	1.45301	0.893609703	0.966018836	4	12	24	0.642215469	0.878809617	24	0.731787314	0.059714595
ENSMUSG00000034780	B3galt1	UDP-Gal:betaGlcNAc beta 1,3-galactosyltransferase, polypeptide 1 [Source:MGI Symbol;Acc:MGI:1349403]	22.829	21.816	20.3325	21.1203	18.8489	19.9563	20.0792	21.1072	24.338	24.1731	21.7292	23.5607	25.4114	27.1094	0.767183862	0.966018836	8	12	24	0.135463831	0.747573586	24	5.128759924	0.082729784
ENSMUSG00000034781	Gna11	guanine nucleotide binding protein, alpha 11 [Source:MGI Symbol;Acc:MGI:95766]	22.3584	19.2775	20.1401	20.6747	20.2157	21.2201	21.818	21.3767	19.6207	23.6247	19.2391	22.4016	20.4309	21.4626	0.591351901	0.966018836	24	12	24	0.745883691	0.917063753	24	21.32560136	0.026437956
ENSMUSG00000034786	Gpsm3	G-protein signalling modulator 3 (AGS3-like, C. elegans) [Source:MGI Symbol;Acc:MGI:2146785]	4.59453	5.28361	5.45336	6.21953	4.21337	6.40055	4.65065	5.26169	5.94509	6.37769	5.5829	5.72009	5.72688	5.49378	0.315232163	0.827645017	16	12	24	0.346243557	0.778844143	24	11.04479214	0.081487699
ENSMUSG00000034789	Rab24	RAB24, member RAS oncogene family [Source:MGI Symbol;Acc:MGI:105065]	38.8935	33.2697	33.9427	36.5275	39.1762	36.3453	35.6155	35.1339	35.0657	38.8949	40.2518	38.3801	38.2568	37.2013	0.002569536	0.112580623	20	16	24	0.013083057	0.529134128	24	17.484844	0.076061375
ENSMUSG00000034793	G6pc3	glucose 6 phosphatase, catalytic, 3 [Source:MGI Symbol;Acc:MGI:1915651]	27.8869	24.6882	27.4232	28.3606	26.0034	29.6095	27.684	29.0322	24.1374	32.9963	23.6578	26.2577	24.4736	25.7191	0.90332508	0.966018836	16	20	24	0.838750396	0.950765045	24	13.71032603	0.029669236
ENSMUSG00000034795	Ccdc122	coiled-coil domain containing 122 [Source:MGI Symbol;Acc:MGI:1918358]	4.36391	5.29547	4.50387	4.8042	4.76929	4.875	4.06309	4.02331	5.07479	5.21066	5.08591	4.77389	5.01887	5.64482	0.951846915	0.966018836	16	12	24	0.466841603	0.805393068	24	11.00470781	0.052412835
ENSMUSG00000034796	Cpne7	copine VII [Source:MGI Symbol;Acc:MGI:2142747]	0.291976	0.920035	1.16335	1.16426	1.09225	0.649159	0.870543	0.792225	1.6336	0.812753	0.976806	0.855484	0.522008	1.03796	0.004320961	0.140598674	12	16	24	0.013662591	0.529134128	24	10.23381962	0.250754819
ENSMUSG00000034799	Unc13a	unc-13 homolog A [Source:MGI Symbol;Acc:MGI:3051532]	20.709	15.7835	13.3923	15.3766	17.7811	18.6076	15.0374	15.5236	12.5722	17.9005	17.5095	16.9271	17.0214	16.4418	0.035787737	0.331814682	20	16	24	0.0262343	0.586541342	24	19.7531078	0.173243739
ENSMUSG00000034800	Zfp661	zinc finger protein 661 [Source:MGI Symbol;Acc:MGI:1919430]	2.54947	2.30437	2.50056	3.06953	2.6579	2.44583	2.83497	2.39746	2.81654	2.10392	2.42207	2.18642	2.33664	2.12646	0.904831057	0.966018836	12	20	24	0.74144131	0.915858429	24	13.43308968	0.030972081
ENSMUSG00000034801	Sos2	SOS Ras/Rho guanine nucleotide exchange factor 2 [Source:MGI Symbol;Acc:MGI:98355]	12.4978	11.5159	12.5054	12.4411	12.3695	12.8738	14.242	11.0868	10.6039	13.2971	11.736	11.4565	11.2974	10.8021	0.580049522	0.966018836	16	16	24	0.253733938	0.768040178	24	17.87045434	0.066342579
ENSMUSG00000034807	Colgalt1	collagen beta(1-O)galactosyltransferase 1 [Source:MGI Symbol;Acc:MGI:1924348]	23.5671	25.3314	25.2407	24.512	23.9838	25.0924	22.6258	22.6913	25.0966	25.4574	20.791	24.6474	24.2725	23.3692	0.373702442	0.896700272	12	16	24	0.656860342	0.884189656	24	8.691811297	0.029180244
ENSMUSG00000034810	Scn7a	sodium channel, voltage-gated, type VII, alpha [Source:MGI Symbol;Acc:MGI:102965]	102.145	108.14	91.1324	106.242	104.243	91.4571	95.8891	88.7281	109.796	82.6695	112.445	102.323	110.604	103.849	0.81829516	0.966018836	20	20	24	0.913798401	0.975875087	24	21.13297396	0.023948539

ENSMUSG00000034813	Grip1	glutamate receptor interacting protein 1 [Source:MGI Symbol;Acc:MGI:1921303]	10.828	8.67192	10.2001	8.76391	8.26816	10.3153	9.10813	10.7137	8.79393	11.9506	8.9782	10.2996	9.84968	11.308	0.951846915	0.966018836	8	12	24	0.656476923	0.883952697	24	3.355823983	0.051362784
ENSMUSG00000034818	Celf5	CUGBP, Elav-like family member 5 [Source:MGI Symbol;Acc:MGI:2442333]	4.23197	3.61159	3.97191	3.77093	3.50038	3.64673	3.73063	3.70013	4.5469	3.76894	3.78484	3.6888	3.74517	4.2541	0.390686537	0.915479136	12	12	24	0.163136167	0.766991901	24	6.370415391	0.062414824
ENSMUSG00000034820	Cpsf7	cleavage and polyadenylation specific factor 7 [Source:MGI Symbol;Acc:MGI:1917826]	23.3899	23.8781	24.3897	22.2084	23.376	22.4668	25.0605	22.4217	24.5343	24.5395	24.0574	23.291	22.5712	24.7083	0.95261238	0.966018836	12	12	24	0.49670739	0.807576732	24	7.451792551	0.027123004
ENSMUSG00000034825	Nrip3	nuclear receptor interacting protein 3 [Source:MGI Symbol;Acc:MGI:1925843]	49.8959	45.5168	42.8193	45.0685	45.667	45.9244	44.0512	43.975	38.6759	50.6407	46.7301	48.918	47.1892	49.2176	0.236893212	0.7385769	24	12	24	0.31168098	0.768040178	24	19.9469819	0.06007037
ENSMUSG00000034826	Nup54	nucleoporin 54 [Source:MGI Symbol;Acc:MGI:1920460]	7.03487	8.71635	6.96418	7.04724	9.89076	7.48373	8.45985	9.05112	7.87737	7.81236	6.6649	7.45408	9.17138	9.23858	0.313239838	0.824470524	8	12	24	0.39065787	0.799546792	24	2.707679481	0.082970403
ENSMUSG00000034832	Tet3	tet methylcytosine dioxygenase 3 [Source:MGI Symbol;Acc:MGI:2446229]	3.78007	3.66646	4.14753	4.1998	4.0522	3.59854	3.96736	3.84913	4.37622	4.82912	4.05329	3.97413	3.73163	3.98766	0.004196064	0.140598674	16	12	24	0.001988184	0.395705644	24	11.20401769	0.09796706
ENSMUSG00000034839	Larp6	La ribonucleoprotein domain family, member 6 [Source:MGI Symbol;Acc:MGI:1914807]	7.33775	6.85976	7.66439	6.45814	5.94365	7.10985	6.61048	6.42311	6.48788	8.46317	6.74912	8.21201	7.16896	7.73474	0.8635847	0.966018836	24	20	24	0.965857301	0.991740077	24	3.612271662	0.012967963
ENSMUSG00000034842	Art3	ADP-ribosyltransferase 3 [Source:MGI Symbol;Acc:MGI:1202729]	133.612	142.024	126.73	144.289	145.136	132.198	135.432	136.35	145.936	131.712	135.141	125.29	132.215	116.746	0.927004797	0.966018836	20	4	24	0.468410327	0.805393068	24	13.39880847	0.03735219
ENSMUSG00000034845	Pivap	plasmalemma vesicle associated protein [Source:MGI Symbol;Acc:MGI:1890497]	2.4541	3.32688	2.52882	2.70335	2.10765	2.20582	3.23801	3.35558	3.52267	2.72525	2.72823	2.80028	2.85619	3.18662	0.057224139	0.401309455	8	12	24	0.006261214	0.46954479	24	5.249750532	0.168885228
ENSMUSG00000034848	Ttc21b	tetratricopeptide repeat domain 21B [Source:MGI Symbol;Acc:MGI:1920918]	4.55157	4.04166	4.00736	4.26135	4.49096	4.30403	4.78788	3.44791	3.97525	4.11407	4.13363	4.39053	4.2785	4.77767	0.017495149	0.255326033	4	8	24	0.258952768	0.768040178	24	20.53543334	0.06436154
ENSMUSG00000034850	Tmem127	transmembrane protein 127 [Source:MGI Symbol;Acc:MGI:1916720]	26.7445	22.3937	23.8158	23.5541	23.8984	24.7815	24.9989	24.4122	20.8936	26.9766	22.9721	24.4274	23.5734	23.8821	0.321662653	0.83607295	24	12	24	0.423206692	0.805321221	24	20.25773103	0.048295445
ENSMUSG00000034853	Acot11	acyl-CoA thioesterase 11 [Source:MGI Symbol;Acc:MGI:1913736]	4.21576	4.46528	7.20304	8.23479	5.87929	6.13482	6.47326	5.35387	5.11971	6.3146	7.79399	5.98303	6.18097	6.67615	0.640622311	0.966018836	16	16	24	0.097267826	0.71175955	24	13.46283442	0.173882269
ENSMUSG00000034854	Mfsd12	major facilitator superfamily domain containing 12 [Source:MGI Symbol;Acc:MGI:3604804]	4.66796	4.2422	4.27867	4.24414	3.95928	4.68727	4.65653	4.88688	4.16084	5.63671	4.92095	4.92431	4.9385	4.99961	0.92721845	0.966018836	24	12	24	0.832587221	0.948274776	24	22.55755419	0.022246568
ENSMUSG00000034855	Cxcl10	chemokine (C-X-C motif) ligand 10 [Source:MGI Symbol;Acc:MGI:1352450]	2.14859	4.66381	5.30769	3.23592	5.09142	2.32941	2.77132	6.67766	4.22021	3.89254	4.73654	2.08613	1.7807	4.01256	0.036657636	0.332441566	12	16	24	0.040358684	0.616921492	24	9.191669272	0.416291178
ENSMUSG00000034858	Fam214a	family with sequence similarity 214, member A [Source:MGI Symbol;Acc:MGI:2387648]	5.30502	7.19306	8.18222	6.9425	6.20955	6.28809	5.85863	8.53806	7.27446	7.28511	5.95268	7.59807	6.13829	7.19445	0.033248781	0.324908923	12	16	24	0.041505558	0.618736234	24	8.065928404	0.158728712
ENSMUSG00000034863	Ano8	anoctamin 8 [Source:MGI Symbol;Acc:MGI:2687327]	9.97969	8.33071	8.75873	9.18312	9.49876	9.0359	9.71228	9.86352	8.93838	12.3104	10.787	10.6043	10.8737	10.6075	0.70641939	0.966018836	4	8	24	0.559744181	0.840699339	24	16.94192453	0.043671786
ENSMUSG00000034867	Ankrd27	ankyrin repeat domain 27 (VPS9 domain) [Source:MGI Symbol;Acc:MGI:2444103]	22.0798	18.7728	16.9235	19.7518	22.7348	22.4585	22.5021	18.9422	16.4804	21.9877	19.1299	20.4974	19.8299	20.1502	0.020119233	0.259417302	24	12	24	0.026369026	0.586541342	24	19.5576556	0.139204389
ENSMUSG00000034868	Myl12b	myosin, light chain 12B, regulatory [Source:MGI Symbol;Acc:MGI:107494]	305.725	267.914	269.2	243.359	278.458	309.592	267.004	289.762	237.963	287.982	280.818	262.155	288.38	294.553	0.711168999	0.966018836	8	4	24	0.187691231	0.768040178	24	22.18877877	0.078758389
ENSMUSG00000034875	Nudt19	nudix (nucleoside diphosphate linked moiety X)-type motif 19 [Source:MGI Symbol;Acc:MGI:94203]	28.5468	30.4013	30.9732	32.3008	30.1675	28.3225	29.1605	27.273	31.9122	27.7769	29.351	31.3832	29.6715	30.6397	0.631736745	0.966018836	12	16	24	0.517543352	0.818310252	24	10.38265063	0.033534116
ENSMUSG00000034880	Mrpl34	mitochondrial ribosomal protein L34 [Source:MGI Symbol;Acc:MGI:2137227]	23.1144	19.6115	18.954	21.7122	14.9655	17.8323	21.1255	24.6027	21.0018	24.7323	29.5776	23.7076	27.2544	26.3001	0.874054778	0.966018836	4	8	24	0.668217541	0.887912848	24	3.984610313	0.069409371
ENSMUSG00000034881	Tbxa2r	thromboxane A2 receptor [Source:MGI Symbol;Acc:MGI:98496]	2.90706	2.55934	2.3166	2.96343	3.22772	2.51597	3.30567	3.01424	3.58526	3.89813	2.90129	3.10654	3.25776	2.80201	0.887862353	0.966018836	16	16	24	0.660601891	0.885496082	24	13.05427567	0.049139358
ENSMUSG00000034889	Cactin	cactin, spliceosome C complex subunit [Source:MGI Symbol;Acc:MGI:1917562]	7.12147	4.92489	4.6171	6.21716	4.73473	4.38898	5.17534	5.61576	5.00619	5.414	5.29884	5.56088	5.40136	5.32706	0.920858634	0.966018836	4	8	24	0.854764792	0.956691175	24	0.343437325	0.033783066
ENSMUSG00000034891	Sncb	synuclein, beta [Source:MGI Symbol;Acc:MGI:1889011]	29.1494	28.841	37.0069	25.6992	25.881	31.4465	31.647	29.9624	30.6579	40.527	24.8438	35.1066	26.2798	36.3846	0.959836008	0.967043964	12	8	24	0.505550875	0.811334839	24	6.761273688	0.099433891
ENSMUSG00000034892	Rps29	ribosomal protein S29 [Source:MGI Symbol;Acc:MGI:107681]	1.63312	1.90888	3.13699	2.04916	2.67122	1.85746	2.21814	1.61016	1.57093	2.08123	2.29056	2.7095	2.10118	2.02381	0.305564675	0.811201458	20	12	24	0.370479369	0.792546812	24	15.43956939	0.109725229
ENSMUSG00000034893	Cog3	component of oligomeric golgi complex 3 [Source:MGI Symbol;Acc:MGI:2450151]	13.0326	13.6984	13.3211	13.6498	12.8237	12.4826	12.7629	13.0519	13.2608	12.6931	13.1502	13.0922	12.667	13.1939	0.073139747	0.447768907	12	12	24	0.110586751	0.722405302	24	8.052710047	0.028070349
ENSMUSG00000034898	Filip1	filamin A interacting protein 1 [Source:MGI Symbol;Acc:MGI:1917848]	23.838	29.6805	24.7273	23.4071	33.1867	24.1545	27.7361	27.0791	30.3355	29.4225	24.3689	29.8142	27.996	29.6365	0.858514388	0.966018836	8	20	24	0.878994836	0.966150244	24	6.877453755	0.03066072
ENSMUSG00000034902	Pip5k1c	phosphatidylinositol-4-phosphate 5-kinase, type 1 gamma [Source:MGI Symbol;Acc:MGI:1298224]	27.4161	22.3917	22.5597	24.2278	22.6305	24.9478	24.9532	27.2111	21.4002	32.3561	23.2379	26.8377	24.5062	26.3132	0.643325306	0.966018836	4	8	24	0.921615921	0.979362996	24	21.2025034	0.024812002
ENSMUSG00000034903	Cobll1	Cobl-like 1 [Source:MGI Symbol;Acc:MGI:2442894]	4.97905	5.95202	6.84423	5.58025	6.11661	5.01599	6.64034	7.08068	6.27822	6.23708	5.23341	5.31943	6.56651	4.99231	0.390686537	0.915479136	12	12	24	0.285599592	0.768040178	24	6.965791842	0.096968084
ENSMUSG00000034906	Ncaph	non-SMC condensin I complex, subunit H [Source:MGI Symbol;Acc:MGI:2444777]	0.312675	0.740502	1.11211	0.711016	0.880985	0.587479	0.994659	0.778807	0.908414	0.53095	0.754192	0.590045	0.950183	0.460944	0.924770496	0.966018836	12	12	24	0.705387502	0.902869416	24	9.050279657	0.066420641
ENSMUSG00000034908	Sidt2	SID1 transmembrane family, member 2 [Source:MGI Symbol;Acc:MGI:2446134]	21.2751	22.9079	24.7787	31.1402	29.1993	24.3789	23.8108	25.489	27.4475	29.6308	23.0653	23.7719	23.4961	21.1389	0.021406186	0.26465279	16	12	24	0.006073736	0.468868574	24	12.47961776	0.173211698
ENSMUSG00000034910	Pygo1	pygopus 1 [Source:MGI Symbol;Acc:MGI:1919385]	2.35078	2.6546	3.28102	2.64674	2.48978	2.53366	3.08167	2.57952	3.22889	2.63644	2.35312	2.70463	2.58662	2.93373	0.03782572	0.334069441	12	8	24	0.076607854	0.687041208	24	6.848381939	0.097159368
ENSMUSG00000034911	Ushbp1	USH1 protein network component harmonin binding protein 1 [Source:MGI Symbol;Acc:MGI:1922920]	2.47619	3.48852	3.62192	3.79583	3.36863	3.29414	3.91521	4.73072	3.92521	4.14002	3.79075	3.06006	3.84467	3.45083	0.175808942	0.651752074	16	8	24	0.120524413	0.732894732	24	8.748850852	0.127212394
ENSMUSG00000034912	Mdga2	MAM domain containing glycosylphosphatidylinositol anchor 2 [Source:MGI Symbol;Acc:MGI:2444706]	4.58213	4.14851	3.94869	3.62429	3.71527	3.91842	3.51827	3.99119	4.19482	4.0011	3.94885	4.84631	4.52085	4.77539	0.671726933	0.966018836	24	20	24	0.243856727	0.768040178	24	1.495296336	0.066336263
ENSMUSG00000034919	Ttc22	tetratricopeptide repeat domain 22 [Source:MGI Symbol;Acc:MGI:3045307]	0.520788	0.438299	0.633373	0.362919	0.491118	0.749399	0.332126	0.446207	0.351428	0.560167	0.491303	0.483493	0.685999	0.516682	0.896311169	0.966018836	24	8	24	0.618859302	0.869185487	24	20.17807294	0.047046975
ENSMUSG00000034923	Ly6g6f	lymphocyte antigen 6 complex, locus G6F [Source:MGI Symbol;Acc:MGI:3616082]	1.25843	2.33223	1.8545	1.02588	1.61291	1.36174	1.83348	2.14668	2.24712	1.28723	1.81648	1.31853	2.18833	1.33076	0.232647885	0.733359426	12	4	24	0.231984052	0.768040178	24	4.330961687	0.15275036
ENSMUSG00000034926	Dhcr24	24-dehydrocholesterol reductase [Source:MGI Symbol;Acc:MGI:1922004]	91.0829	70.5515	69.6352	77.8643	90.6815	88.1245	85.793	85.6717	67.6258	109.216	71.5575	88.9532	85.1501	78.5289	0.0444686	0.365196805	24	12	24	0.300103326	0.768040178	24	19.07260462	0.11745633
ENSMUSG00000034928	Rnf44	ring finger protein 44 [Source:MGI Symbol;Acc:MGI:2145310]	18.245	15.0496	15.13	16.5394	16.2021	16.2184	18.6805	16.397	15.6022	21.4222	17.9371	16.17	17.7791	15.8148	0.162391276	0.624504499	4	8	24	0.586457927	0.853300537	24	17.32636735	0.054056201
ENSMUSG00000034930	Rtkn	rhotekin [Source:MGI Symbol;Acc:MGI:107371]	55.8756	40.8407	45.095	49.391	47.9167	46.691	53.6114	46.6667	46.114	59.3233	47.7535	47.8059	42.714	39.2444	0.08333408	0.462922111	16	16	24	0.297092164	0.768040178	24	16.35544141	0.094176401
ENSMUSG00000034931	Dhx8	DEAH (Asp-Glu-Ala-His) box polypeptide 8 [Source:MGI Symbol;Acc:MGI:1306823]	8.63911	6.81468	7.40764	7.04778	7.35976	8.98279	8.25564	8.71845	5.61222	8.74232	6.86692	7.74641	7.6787	7.34331	0.463629316	0.966018836	24	12	24	0.202388486	0.768040178	24	21.47397244	0.113854209
ENSMUSG00000034932	Mrpl54	mitochondrial ribosomal protein L54 [Source:MGI Symbol;Acc:MGI:1913297]	48.3855	54.4108	53.9682	48.9007	49.9944	48.5291	48.1938	54.7775	60.8276	50.2427	63.3084	55.5412	68.1616	62.4804	0.904831057	0.966018836	8	20	24	0.328907479	0.768040178	24	5.532293121	0.069491925
ENSMUSG00000034936	Arl4d	ADP-ribosylation factor-like 4D [Source:MGI Symbol;Acc:MGI:1933155]	6.21474	6.23936	6.69039	6.47537	5.86298	6.04015	6.4376	6.53887	6.3858	7.73735	6.90684	6.58224	6.2313	6.65128	0.316183628	0.829266891	16	8	24	0.124817085	0.738693118	24	10.37292818	0.060116479
ENSMUSG00000034940	Synrg	synergin, gamma [Source:MGI Symbol;Acc:MGI:1354742]	15.8136	13.3621	14.1895	14.8989	13.5291	16.117	14.7295	14.145	12.2179	18.9283	14.5997	15.9377	15.4032	16.1284	0.390686537	0.915479136	24	12	24	0.606481728	0.863245947	24	18.57460728	0.056240753
ENSMUSG00000034947	Tmem106a	transmembrane protein 106A [Source:MGI Symbol;Acc:MGI:1922056]	6.06075	3.90676	4.9587	4.92247	5.66661	5.86726	4.2338	5.35647	3.90881	5.22994	5.45369	5.50423	4.92835	5.71566	0.220555749	0.710961211	24	12	24	0.202579236	0.768040178	24	18.61248442	0.119306884
ENSMUSG00000034949	Zfr2	zinc finger RNA binding protein 2 [Source:MGI Symbol;Acc:MGI:2143792]	10.7178	8.48158	9.54873	7.73341	7.93106	8.79808	10.8889	9.88294	8.70675	9.79962	8.99951	9.56225	10.2967	9.5769	0.01457641	0.247599677	4	16	24	0.044692531	0.628432994	24	0.981526244	0.115815985
ENSMUSG00000034951	Cog7	component of oligomeric golgi complex 7 [Source:MGI Symbol;Acc:MGI:2685013]	12.8317	10.2381	10.9433	10.3055	10.59	11.6053	11.6669	11.8741	9.80579	14.5727	9.16389	11.625	11.176	11.6481	0.447817955	0.966018836	4	16	24	0.780564071	0.929485273	24	0.403598913	0.043326944
ENSMUSG00000034957	Cebpa	CCAAT/enhancer binding protein (C/EBP), alpha [Source:MGI Symbol;Acc:MGI:99480]	3.4487	3.39863	4.44361	5.52964	3.41568	3.40534	3.90003	5.60951	5.03328	7.26463	4.91143	5.67556	9.60798	7.35383	0.865514257	0.966018836	16	4	24	0.214960326	0.768040178	24	7.602027341	0.174266448
ENSMUSG00000034958	Atcay	ataxia, cerebellar, Cayman type [Source:MGI Symbol;Acc:MGI:2448730]	23.0866	19.0043	19.7188	18.8657	17.4303	22.7506	21.2335	21.4836	15.9458	23.697	17.7322	21.5998	19.3372	21.8414	0.608967886	0.966018836	24	20	24	0.364855992	0.789167625	24	23.18243707	0.090442536
ENSMUSG00000034959	Rubcnl	RUN and cysteine rich domain containing beclin 1 interacting protein like [Source:MGI Symbol;Acc:MGI:2685590]	1.41153	1.09653	1.19647	1.48198	1.24329	1.34861	1.2044	1.09072	1.37972	0.909662	1.42054	1.20707	1.34981	1.0826	0.285193697	0.793714531	20	12	24	0.412693032	0.799546792	24	18.53229149	0.056458443
ENSMUSG00000034973	Dop1a	DOP1 leucine zipper like protein A [Source:MGI Symbol;Acc:MGI:1289294]	18.9429	21.2002	20.7307	21.3676	18.7973	16.993	19.387	19.0841	21.0759	18.011	20.984	18.1754	19.6104	19.4263	0.074438493	0.448301997	12	12	24	0.111984294	0.725433106	24	8.174600507	0.076259722
ENSMUSG00000034974	Dapk3	death-associated protein kinase 3 [Source:MGI Symbol;Acc:MGI:1203520]	18.066	18.9359	15.8612	16.6481	18.0102	20.7647	17.645	14.983	15.1611	20.544	16.2248	16.858	16.6568	17.1213	0.595037318	0.966018836	24	12	24	0.320240653	0.768040178	24	18.83270854	0.08080199
ENSMUSG00000034981	Parm1	prostate androgen-regulated mucin-like protein 1 [Source:MGI Symbol;Acc:MGI:2443349]	114.502	81.9274	81.3062	85.3044	85.7286	104.43	90.8583	100.546	62.9807	116.739	82.4577	94.9971	94.8247	92.1389	0.085890569	0.466404237	24	12	24	0.288841689	0.768040178	24	21.15328831	0.142407082
ENSMUSG00000034987	Hrh2	histamine receptor H2 [Source:MGI Symbol;Acc:MGI:108482]	1.00591	0.899446	0.905919	1.00355	0.799993	1.00027	0.953635	0.949277	0.871541	1.16122	0.933139	0.973814	1.07631	0.898986	0.251797405	0.75129457	24	12	24	0.909928124	0.974634733	24	19.4835477	0.011779026
ENSMUSG00000034990	Otoa	otoancorin [Source:MGI Symbol;Acc:MGI:2149209]	3.53766	2.98632	2.92541	2.65676	2.69378	3.66503	3.01295	3.06927	3.23341	3.36667	3.12521	3.37985	3.72093	3.29971	0.121789249	0.549378164	24	20	24	0.118817776	0.729829751	24	23.15854201	0.080255691
ENSMUSG00000034993	Vat1	vesicle amine transport 1 [Source:MGI Symbol;Acc:MGI:1349450]	63.6678	49.5919	49.4211	51.7979	52.8936	58.8242	56.052	56.1556	45.3298	68.7054	55.6613	61.6101	57.5505	59.4958	0.047644929	0.377556215	24	12	24	0.261333752	0.768040178	24	20.25687818	0.097775437
ENSMUSG00000034994	Eef2	eukaryotic translation elongation factor 2 [Source:MGI Symbol;Acc:MGI:95288]	266.381	221.797	244.044	242.819	225.649	273.855	255.527	264.115	211.874	291.858	211.319	248.722	240.362	242.806	0.960338592	0.967043964	24	20	24	0.762145996	0.92297519	24	22.32440909	0.041587815
ENSMUSG00000034997	Htr2a	5-hydroxytryptamine (serotonin) receptor 2A [Source:MGI Symbol;Acc:MGI:109521]	4.38098	3.63374	3.75694	3.39173	3.74321	3.65411	3.11037	3.04009	3.28545	3.22389	3.53755	3.61517	3.33922	3.09392	0.510431574	0.966018836	24	8	24	0.132819365	0.743265977	24	19.20169998	0.068402619
ENSMUSG00000034998	Foxn2	forkhead box N2 [Source:MGI Symbol;Acc:MGI:1347478]	5.12638	5.71244	5.432	6.73824	5.75035	4.90443	5.64121	5.1332	5.91709	5.1095	5.7811	4.69362	4.78025	4.87569	0.082341274	0.462922111	20	4	24	0.061325319	0.661066697	24	11.50317727	0.094284529
ENSMUSG00000035000	Dpp4	dipeptidylpeptidase 4 [Source:MGI Symbol;Acc:MGI:94919]	1.87382	3.12359	1.68736	1.36055	1.99838	1.67951	1.75295	2.44875	3.1056	0.956584	2.09136	2.01116	1.58486	2.13861	0.143673396	0.585944911	8	8	24	0.291269545	0.768040178	24	3.698446559	0.172480909
ENSMUSG00000035004	Igsf6	immunoglobulin superfamily, member 6 [Source:MGI Symbol;Acc:MGI:1891393]	1.18101	1.94361	2.78479	2.51913	1.62951	2.1236	1.63596	1.50334	2.41176	1.33896	1.61723	1.58736	1.68621	1.39951	0.773177465	0.966018836	12	16	24	0.244329842	0.768040178	24	10.20169382	0.147631718
ENSMUSG00000035007	Rundc1	RUN domain containing 1 [Source:MGI Symbol;Acc:MGI:2144506]	6.39998	5.36582	5.19189	5.47772	5.7689	6.05777	5.67514	6.0288	5.0248	6.18953	5.61735	6.42641	5.74023	5.68361	0.004909071	0.141782417	24	12	24	0.05659103	0.655587475	24	20.38128709	0.080866631
ENSMUSG00000035011	Zbtb7a	zinc finger and BTB domain containing 7a [Source:MGI Symbol;Acc:MGI:1335091]	11.4409	8.29766	8.45659	9.46292	7.77942	9.58259	10.1033	10.6948	10.1697	14.6865	12.179	13.2707	12.8365	12.3216	0.916556573	0.966018836	24	12	24	0.88205413	0.966661503	24	21.3301748	0.036778222
ENSMUSG00000035021	Baz1a	bromodomain adjacent to zinc finger domain 1A [Source:MGI Symbol;Acc:MGI:1309478]	1.76186	2.21997	1.83267	2.04527	2.01088	2.15421	1.66438	1.95812	3.19183	2.23768	3.55875	2.99437	2.33504	1.90099	0.522551072	0.966018836	24	4	24	0.267423828	0.768040178	24	14.43782059	0.12709228
ENSMUSG00000035024	Ncapd3	non-SMC condensin II complex, subunit D3 [Source:MGI Symbol;Acc:MGI:2142989]	7.46405	9.78776	10.4622	9.90253	8.81865	8.92956	8.13314	7.74458	9.84176	8.29611	9.07913	8.40252	7.86929	8.87915	0.004320961	0.140598674	12	16	24	0.045244296	0.628927515	24	10.45856827	0.116753866
ENSMUSG00000035027	Map2k2	mitogen-activated protein kinase kinase 2 [Source:MGI Symbol;Acc:MGI:1346867]	39.1303	31.0825	36.1645	35.5339	39.4056	38.9028	38.1079	39.206	35.8203	42.6253	37.0537	39.1033	37.7384	36.1181	0.505728924	0.966018836	20	16	24	0.254805924	0.768040178	24	17.57548098	0.063779626
ENSMUSG00000035032	Nek11	NIMA (never in mitosis gene a)-related expressed kinase 11 [Source:MGI Symbol;Acc:MGI:2442276]	1.05129	1.01095	1.00109	0.814544	1.00088	0.968423	1.05362	0.824706	1.07089	0.797244	1.08699	1.01541	0.781404	1.01347	0.802253124	0.966018836	20	20	24	0.770755382	0.926171026	24	21.58836825	0.024334277
ENSMUSG00000035042	Ccl5	chemokine (C-C motif) ligand 5 [Source:MGI Symbol;Acc:MGI:98262]	1.08447	1.7031	0.941988	1.96954	0.976234	0.791379	1.05471	1.44633	1.91689	1.48393	0.66563	0.767054	1.2356	1.2086	0.012443448	0.220221977	16	4	24	0.025306851	0.586541342	24	7.549592009	0.258082508
ENSMUSG00000035045	Zc3h12b	zinc finger CCCH-type containing 12B [Source:MGI Symbol;Acc:MGI:2442133]	1.31265	1.58994	1.87696	1.64493	1.50099	1.71813	1.90628	1.37343	1.57404	1.48337	1.54612	1.87302	1.3181	1.88909	0.816361374	0.966018836	24	4	24	0.979352792	0.994983268	24	9.834231288	0.009975003
ENSMUSG00000035047	Kri1	KRI1 homolog [Source:MGI Symbol;Acc:MGI:2384899]	15.8021	10.3385	11.4852	13.4984	16.3417	14.4783	12.49	12.7585	12.8376	12.5032	12.6638	12.6374	11.6151	10.5745	0.113148904	0.530777648	20	12	24	0.026846127	0.586541342	24	17.66004155	0.158525824
ENSMUSG00000035048	Anapc13	anaphase promoting complex subunit 13 [Source:MGI Symbol;Acc:MGI:1916260]	43.1834	55.5222	50.5558	57.6319	58.5086	52.3386	51.1258	55.29	57.8024	48.7023	54.3709	51.6371	57.9278	49.6767	0.891742979	0.966018836	20	8	24	0.576022909	0.84837269	24	11.85630642	0.049868135
ENSMUSG00000035049	Rrp12	ribosomal RNA processing 12 homolog [Source:MGI Symbol;Acc:MGI:2147437]	5.91863	4.91421	5.23681	5.28327	5.51251	6.52655	5.70979	6.09133	4.32054	6.89742	4.93036	5.33806	4.93138	5.39894	0.95269569	0.966018836	24	12	24	0.621323082	0.870720844	24	19.11138049	0.060623365
ENSMUSG00000035051	Dhx57	DEAH (Asp-Glu-Ala-Asp/His) box polypeptide 57 [Source:MGI Symbol;Acc:MGI:2147067]	19.1005	16.2686	15.9023	15.8326	15.8118	17.8483	17.6471	16.5993	14.69	18.3923	16.4713	16.5476	16.9895	17.6562	0.097760727	0.501112642	4	8	24	0.177141705	0.768040178	24	22.33841216	0.068482459
ENSMUSG00000035062	Zc4h2	zinc finger, C4H2 domain containing [Source:MGI Symbol;Acc:MGI:2679294]	12.6657	13.2647	11.8018	11.327	12.6127	11.3025	12.5719	11.9943	13.4619	10.024	13.3659	12.7272	13.1614	12.8517	0.947524602	0.966018836	8	8	24	0.306994881	0.768040178	24	0.828429977	0.064053713
ENSMUSG00000035064	Eef2k	eukaryotic elongation factor-2 kinase [Source:MGI Symbol;Acc:MGI:1195261]	9.27012	7.20869	9.05245	8.67423	8.50133	7.43088	8.00532	7.60542	9.63679	10.9644	8.59458	8.60783	8.66562	8.55579	0.163904621	0.627573783	12	20	24	0.140110107	0.749574609	24	11.48511481	0.102725279
ENSMUSG00000035067	Xkr6	X-linked Kx blood group related 6 [Source:MGI Symbol;Acc:MGI:2447765]	0.882548	1.19436	1.24545	1.15999	1.24464	1.11925	1.16382	1.12647	1.49874	1.07739	1.50635	0.994133	1.18352	1.30374	0.14313801	0.585944911	12	12	24	0.164493018	0.767020009	24	10.0838398	0.077085258
ENSMUSG00000035069	Oma1	OMA1 zinc metallopeptidase [Source:MGI Symbol;Acc:MGI:1914263]	4.30089	3.58238	4.03614	4.56019	4.32021	4.19271	4.55948	4.73244	3.97742	4.26453	4.10437	3.95028	4.30185	3.65399	0.93447422	0.966018836	4	4	24	0.685186748	0.895310095	24	17.72928351	0.032762402
ENSMUSG00000035078	Mtmr9	myotubularin related protein 9 [Source:MGI Symbol;Acc:MGI:2442842]	55.7382	44.4708	45.5978	47.1589	47.3677	53.2423	49.3634	50.4958	37.9022	56.7701	47.5423	50.6152	49.9621	48.1257	0.020957534	0.262822894	24	12	24	0.183579634	0.768040178	24	20.24713505	0.103306968
ENSMUSG00000035085	1700020L24Rik	RIKEN cDNA 1700020L24 gene [Source:MGI Symbol;Acc:MGI:1913580]	2.09725	2.51321	1.15799	1.34368	1.25949	1.9096	2.1614	2.61499	2.48075	2.82637	3.80744	2.99956	3.3586	3.15246	0.934818505	0.966018836	8	4	24	0.357542619	0.784480156	24	0.810479074	0.134132546
ENSMUSG00000035086	Becn1	beclin 1, autophagy related [Source:MGI Symbol;Acc:MGI:1891828]	91.725	79.0448	80.1386	83.2429	87.3841	90.6419	82.7355	80.1467	73.4743	80.3039	84.6777	85.006	83.8533	78.6517	7.90E-04	0.071344007	24	12	24	1.21E-04	0.122523627	24	19.57470317	0.10229919
ENSMUSG00000035093	Secisbp2l	SECIS binding protein 2-like [Source:MGI Symbol;Acc:MGI:1917604]	111.379	122.504	108.942	108.052	131.909	104.925	131.335	109.686	125.04	123.386	105.735	116.563	119.751	104.261	0.809772024	0.966018836	4	20	24	0.987856705	0.997530201	24	18.96034522	0.007831132
ENSMUSG00000035095	Fam167a	family with sequence similarity 167, member A [Source:MGI Symbol;Acc:MGI:3606565]	3.03384	2.14912	2.18054	2.78538	2.4928	2.6694	2.49737	1.94197	1.6653	2.69044	2.85056	2.41702	2.10293	2.2191	0.075951231	0.453790923	16	20	24	0.02014832	0.565108098	24	17.82003927	0.159006692
ENSMUSG00000035104	Eva1a	eva-1 homolog A (C. elegans) [Source:MGI Symbol;Acc:MGI:2385247]	0.597491	0.851647	2.35685	1.07742	0.610708	1.05609	1.17288	0.980883	1.54938	1.17044	0.764175	0.735067	0.621416	0.930974	0.070795672	0.440262583	12	16	24	0.046794747	0.633507364	24	8.609706224	0.263477458
ENSMUSG00000035105	Egln3	egl-9 family hypoxia-inducible factor 3 [Source:MGI Symbol;Acc:MGI:1932288]	3.23145	3.42727	3.5516	3.4725	3.29268	2.98612	3.44707	4.28615	3.74192	3.04416	3.3375	3.46831	3.46997	3.69155	0.061112935	0.409814092	8	16	24	0.035598066	0.609176161	24	5.504932149	0.091813663
ENSMUSG00000035107	Dcbld2	discoidin, CUB and LCCL domain containing 2 [Source:MGI Symbol;Acc:MGI:1920629]	12.1218	11.25	10.6029	11.1704	12.9078	11.7788	12.7432	9.87248	11.2947	10.6921	11.8034	11.4816	11.0191	10.7049	0.053432515	0.394331071	20	16	24	0.01980554	0.565108098	24	19.04250159	0.09427795
ENSMUSG00000035109	Shc4	SHC (Src homology 2 domain containing) family, member 4 [Source:MGI Symbol;Acc:MGI:2655364]	28.7215	31.2128	26.4098	27.8768	34.009	24.9944	33.2834	27.6616	33.7067	31.1531	28.7547	31.3279	33.203	26.7405	0.912686312	0.966018836	20	12	24	0.96593428	0.991740077	24	18.83218559	0.015529265
ENSMUSG00000035112	Wnk4	WNK lysine deficient protein kinase 4 [Source:MGI Symbol;Acc:MGI:1917097]	3.39965	4.51821	2.41035	5.97712	2.98168	3.3727	2.69042	3.6358	5.66732	3.22789	4.55986	3.68131	3.08808	2.73485	0.960829223	0.967043964	16	12	24	0.421170747	0.804493443	24	11.75497789	0.16092252
ENSMUSG00000035121	Neil2	nei like 2 (E. coli) [Source:MGI Symbol;Acc:MGI:2686058]	2.44157	2.36815	2.55413	2.81222	2.69136	2.92828	2.28176	2.80478	2.77855	3.01542	2.64839	2.71559	2.81532	2.60578	0.515451644	0.966018836	16	12	24	0.118026856	0.72837192	24	13.32139833	0.057951746
ENSMUSG00000035125	Gcfc2	GC-rich sequence DNA binding factor 2 [Source:MGI Symbol;Acc:MGI:2141656]	1.64786	3.10303	2.44893	3.09344	2.49246	2.38828	1.48885	2.64171	3.72097	2.64656	2.47624	2.60267	2.23809	3.19382	0.008747574	0.192760771	8	20	24	0.023091275	0.581965446	24	9.506807067	0.235836656
ENSMUSG00000035126	Dnai4	dynein axonemal intermediate chain 4 [Source:MGI Symbol;Acc:MGI:2385328]	2.94253	2.46496	2.63969	2.35716	3.14504	2.57099	2.99626	2.40785	2.35827	2.16506	3.04879	2.72154	2.47401	2.56441	0.004163919	0.140598674	20	20	24	0.064412934	0.663876076	24	19.90362524	0.102402936
ENSMUSG00000035129	Gm6781	predicted gene 6781 [Source:MGI Symbol;Acc:MGI:3644510]	81.3533	74.773	75.6142	72.2069	73.1431	81.7357	77.237	77.5331	75.2987	82.6752	82.4306	86.4543	85.3892	85.3985	0.387380622	0.915479136	24	16	24	0.079700599	0.69400043	24	22.08419012	0.052727972
ENSMUSG00000035131	Brinp3	bone morphogenetic protein/retinoic acid inducible neural specific 3 [Source:MGI Symbol;Acc:MGI:2443035]	0.881653	1.61329	2.09055	1.26223	1.26871	1.40845	1.48613	0.946761	1.57798	1.17237	0.894798	1.25517	0.897403	1.98927	0.258307762	0.761738864	12	8	24	0.233819948	0.768040178	24	7.24808479	0.157763859
ENSMUSG00000035133	Arhgap5	Rho GTPase activating protein 5 [Source:MGI Symbol;Acc:MGI:1332637]	23.3737	28.7259	26.7446	26.205	26.4425	23.0744	27.6632	25.2462	29.9681	21.0771	28.368	25.4686	25.2063	27.9854	0.377663653	0.903463289	12	12	24	0.492962636	0.805703845	24	6.333531927	0.064703674
ENSMUSG00000035139	Secisbp2	SECIS binding protein 2 [Source:MGI Symbol;Acc:MGI:1922670]	5.01384	4.65789	4.97809	5.35801	4.70232	5.18689	5.48567	5.53342	5.56492	5.29576	4.99432	4.62146	5.16874	5.0517	0.888991156	0.966018836	16	4	24	0.429433609	0.805393068	24	7.107376783	0.039329759
ENSMUSG00000035142	Nubpl	nucleotide binding protein-like [Source:MGI Symbol;Acc:MGI:1924076]	1.95392	1.852	1.91884	2.12056	1.86532	1.9029	2.17029	1.98681	2.02465	1.99906	2.11984	1.93164	2.52487	2.11353	0.92721845	0.966018836	4	20	24	0.68999354	0.897381208	24	1.647254136	0.022518236
ENSMUSG00000035150	Eif2s3x	eukaryotic translation initiation factor 2, subunit 3, structural gene X-linked [Source:MGI Symbol;Acc:MGI:1349431]	5.25556	5.24608	4.46055	4.40154	4.61906	4.65461	4.73317	4.10519	4.40826	5.00997	4.67036	5.77037	4.30804	5.2275	0.887010723	0.966018836	24	12	24	0.536864315	0.827590998	24	21.23766176	0.052186938
ENSMUSG00000035151	Elmod2	ELMO/CED-12 domain containing 2 [Source:MGI Symbol;Acc:MGI:2445165]	11.9227	12.2483	11.7679	12.0841	12.7166	10.9289	12.2808	11.1399	12.0948	9.87673	13.5826	11.7079	12.045	11.7765	0.93357733	0.966018836	20	4	24	0.802865043	0.936786401	24	19.51234672	0.027304718
ENSMUSG00000035152	Ap2b1	adaptor-related protein complex 2, beta 1 subunit [Source:MGI Symbol;Acc:MGI:1919020]	60.8736	49.3783	50.797	51.9897	50.6816	60.1974	53.1052	56.5827	40.7832	64.0559	46.7865	53.8344	50.6071	54.4796	0.969487593	0.970883022	24	12	24	0.536582086	0.827423907	24	21.06200745	0.074541648
ENSMUSG00000035158	Mitf	melanogenesis associated transcription factor [Source:MGI Symbol;Acc:MGI:104554]	2.76332	3.27006	3.0243	2.767	3.17881	2.83746	3.57689	2.84395	3.45008	2.96602	2.78417	2.95238	2.90485	3.13369	0.777867261	0.966018836	12	4	24	0.479248884	0.805393068	24	5.103606159	0.043871316
ENSMUSG00000035161	Ints6	integrator complex subunit 6 [Source:MGI Symbol;Acc:MGI:1202397]	6.10488	4.65178	4.64902	6.28402	4.8846	5.05813	4.52181	5.12569	4.23624	5.58824	6.1162	4.9768	3.98028	3.86132	0.224031297	0.717774981	16	20	24	0.055039492	0.655263328	24	15.66319682	0.146373565
ENSMUSG00000035164	Zc3h12c	zinc finger CCCH type containing 12C [Source:MGI Symbol;Acc:MGI:3026959]	2.46183	2.34007	2.37366	2.73327	2.3411	2.21682	2.75505	2.18332	2.67201	2.09625	2.656	2.44852	2.26011	2.30239	0.883870616	0.966018836	20	12	24	0.882638088	0.966732748	24	15.11845465	0.017729644
ENSMUSG00000035168	Tanc1	tetratricopeptide repeat, ankyrin repeat and coiled-coil containing 1 [Source:MGI Symbol;Acc:MGI:1914110]	16.4381	12.2384	13.7575	12.6391	12.915	14.6081	14.3533	14.5387	10.1985	16.758	12.7021	13.4792	13.9222	13.8339	0.349109869	0.870418653	4	8	24	0.474150957	0.805393068	24	21.69296895	0.082653281
ENSMUSG00000035171	1110059E24Rik	RIKEN cDNA 1110059E24 gene [Source:MGI Symbol;Acc:MGI:1913456]	6.77047	7.09548	6.00152	8.15379	7.17174	7.79047	6.61587	7.01795	7.90719	6.22704	7.88111	6.68883	8.72604	7.77009	0.7866493	0.966018836	20	8	24	0.938205556	0.984659033	24	20.18752315	0.01945635
ENSMUSG00000035172	Plekhh3	pleckstrin homology domain containing, family H (with MyTH4 domain) member 3 [Source:MGI Symbol;Acc:MGI:2384950]	6.00191	5.21575	6.09691	5.27965	5.02241	5.108	4.96246	5.28826	6.28159	7.07364	5.9426	5.35129	5.80676	5.53717	0.369326376	0.893596186	12	12	24	0.175631751	0.768040178	24	9.886324704	0.088831846
ENSMUSG00000035173	Ccdc186	coiled-coil domain containing 186 [Source:MGI Symbol;Acc:MGI:2445022]	14.8295	15.5799	14.1545	15.1554	15.4157	13.6535	15.1564	14.351	14.9231	13.2755	16.2128	15.3146	16.2249	15.6765	0.714044782	0.966018836	20	20	24	0.663972472	0.886640273	24	23.01396403	0.028416204
ENSMUSG00000035181	Heatr5a	HEAT repeat containing 5A [Source:MGI Symbol;Acc:MGI:2444133]	13.8048	11.8578	10.8955	11.89	13.1337	11.7323	14.5363	12.6268	10.3125	12.5665	11.7839	12.6938	12.1793	11.5514	0.035806379	0.331814682	4	8	24	0.056089912	0.65534544	24	21.1955583	0.105501144
ENSMUSG00000035183	Slc24a5	solute carrier family 24, member 5 [Source:MGI Symbol;Acc:MGI:2677271]	1.75695	1.89848	1.68459	2.51658	1.79559	1.24861	1.55986	1.39389	1.66467	1.46462	1.51766	1.12577	1.50999	1.32419	0.387380622	0.915479136	24	8	24	0.17637647	0.768040178	24	10.49903504	0.100247461
ENSMUSG00000035184	Fam124a	family with sequence similarity 124, member A [Source:MGI Symbol;Acc:MGI:3645930]	5.53553	4.45457	5.19503	4.68312	4.99232	5.5709	4.24699	4.38058	4.54552	6.97002	5.89325	5.50482	6.65596	5.57465	0.767183862	0.966018836	24	8	24	0.441162572	0.805393068	24	16.73983888	0.083495891
ENSMUSG00000035189	Ano4	anoctamin 4 [Source:MGI Symbol;Acc:MGI:2443344]	14.6601	14.0602	13.8211	13.8172	12.2838	13.6373	13.4864	13.7218	11.4661	15.1328	13.9767	13.8046	13.5412	15.1456	0.920858634	0.966018836	8	16	24	0.832897455	0.948359912	24	1.289653316	0.023707105
ENSMUSG00000035198	Tubg1	tubulin, gamma 1 [Source:MGI Symbol;Acc:MGI:101834]	9.99287	7.80531	8.14064	8.37581	8.15849	9.48865	8.80447	9.93523	7.00631	10.1279	7.64173	8.38366	7.5271	7.81568	0.95261238	0.966018836	8	12	24	0.673325007	0.890057965	24	20.65035053	0.053505037

ENSMUSG00000035199	Arl6ip5	ADP-ribosylation factor-like 6 interacting protein 5 [Source:MGI Symbol;Acc:MGI:1929501]	143.898	135.671	127.243	130.828	140.877	146.002	130.768	134.714	119.936	129.824	134.931	124.177	134.489	132.374	0.214543022	0.704989686	20	16	24	0.073410022	0.681536373	24	20.71865624	0.06214383
ENSMUSG00000035200	Chrnb4	cholinergic receptor, nicotinic, beta polypeptide 4 [Source:MGI Symbol;Acc:MGI:87892]	2.05595	1.35816	1.24601	2.16877	1.18118	2.68781	2.26958	1.85923	1.1875	3.04557	1.44424	2.13562	1.63691	2.73424	0.784278077	0.966018836	24	12	24	0.781461014	0.929846332	24	21.28422358	0.08522685
ENSMUSG00000035202	Lars2	leucyl-tRNA synthetase, mitochondrial [Source:MGI Symbol;Acc:MGI:2142973]	68.2365	42.6607	45.2711	44.6977	45.1189	68.687	36.0271	49.9924	31.8711	32.9053	36.2376	46.9891	36.7686	47.6525	0.4776989	0.966018836	24	16	24	0.13062028	0.740728512	24	21.95406622	0.232472912
ENSMUSG00000035203	Epn1	epsin 1 [Source:MGI Symbol;Acc:MGI:1333763]	38.8349	34.3067	36.2128	40.5507	37.2714	36.8021	40.9399	43.6369	36.3051	52.2069	38.9147	40.6405	39.6994	37.9805	0.946423879	0.966018836	16	20	24	0.659112305	0.884879972	24	13.07442538	0.048964023
ENSMUSG00000035206	Sppl2b	signal peptide peptidase like 2B [Source:MGI Symbol;Acc:MGI:1920468]	9.89133	8.8462	9.30982	9.34064	10.5349	9.69738	11.4329	9.93466	9.59143	10.2815	10.1947	10.3187	9.32137	8.91294	0.117977321	0.54116496	20	12	24	0.149337036	0.757987548	24	18.3841597	0.069089362
ENSMUSG00000035208	Slfn8	schlafen 8 [Source:MGI Symbol;Acc:MGI:2672859]	0.6154	0.788564	1.20635	0.779581	0.792475	0.85121	0.975908	1.30252	1.14695	0.675985	0.933442	0.671414	0.703757	0.680514	0.784278077	0.966018836	12	12	24	0.303427294	0.768040178	24	7.629674666	0.100589913
ENSMUSG00000035212	Leprot	leptin receptor overlapping transcript [Source:MGI Symbol;Acc:MGI:2687005]	23.6567	25.3212	25.2215	22.1031	23.7188	22.823	23.1327	22.9096	24.5808	21.5512	24.111	23.8773	25.5748	24.3074	0.503972924	0.966018836	12	4	24	0.286712257	0.768040178	24	2.879393721	0.042271353
ENSMUSG00000035215	Lsm7	LSM7 homolog, U6 small nuclear RNA and mRNA degradation associated [Source:MGI Symbol;Acc:MGI:1913344]	3.44824	4.33553	4.47716	3.78234	4.92956	4.03503	3.65644	3.51246	4.50794	4.67113	4.1135	4.33621	4.19657	4.61597	0.226403091	0.722427815	20	8	24	0.188706504	0.768040178	24	11.76864103	0.084619426
ENSMUSG00000035226	Rims4	regulating synaptic membrane exocytosis 4 [Source:MGI Symbol;Acc:MGI:2674366]	1.12905	1.20459	1.46236	1.00927	1.0753	1.56526	1.47167	1.05295	1.20264	1.44756	1.11133	1.27481	1.08233	1.7157	0.929527443	0.966018836	24	20	24	0.782528288	0.930180373	24	3.631915126	0.037699116
ENSMUSG00000035227	Spcs2	signal peptidase complex subunit 2 homolog (S. cerevisiae) [Source:MGI Symbol;Acc:MGI:1913874]	50.7284	47.5188	46.1594	42.5706	46.798	43.114	48.6333	45.2695	51.4245	42.1929	53.4901	53.6654	57.2716	53.4867	0.113148904	0.530777648	4	12	24	0.173881176	0.768040178	24	0.142975162	0.081454139
ENSMUSG00000035228	Ccdc106	coiled-coil domain containing 106 [Source:MGI Symbol;Acc:MGI:2385900]	2.20828	2.71734	4.11415	2.27257	1.26161	3.43792	2.26089	4.53969	3.05961	3.35666	1.94914	3.49251	2.64822	3.80636	0.083608421	0.463738799	8	12	24	0.097321607	0.71175955	24	5.457103512	0.275927881
ENSMUSG00000035232	Pdk3	pyruvate dehydrogenase kinase, isoenzyme 3 [Source:MGI Symbol;Acc:MGI:2384308]	14.7712	13.4283	12.9446	12.3741	12.6575	13.0228	13.6052	13.1438	13.0131	12.421	14.6941	14.6246	14.6069	14.0639	0.001845448	0.100255953	4	12	24	0.024603213	0.586541342	24	22.90031306	0.077647656
ENSMUSG00000035234	Abraxas1	BRCA1 A complex subunit [Source:MGI Symbol;Acc:MGI:1917931]	3.44965	3.68975	3.74385	3.90353	3.90937	3.79225	3.72054	4.4109	4.35101	3.86992	3.96297	4.26209	3.52641	4.2519	0.278111325	0.789849983	20	8	24	0.286717815	0.768040178	24	9.397476582	0.048534177
ENSMUSG00000035235	Trim13	tripartite motif-containing 13 [Source:MGI Symbol;Acc:MGI:1913847]	12.9197	14.4689	13.2485	13.1945	15.3553	11.7277	14.2462	12.4774	16.5018	10.9501	14.2111	12.9937	14.2683	13.2432	0.924770496	0.966018836	12	12	24	0.907951913	0.974606327	24	5.811413233	0.025961918
ENSMUSG00000035236	Scai	suppressor of cancer cell invasion [Source:MGI Symbol;Acc:MGI:2443716]	3.73417	4.97703	4.26841	4.39655	4.4534	3.55612	3.71106	3.41299	4.44334	3.07464	4.55067	3.51409	3.19813	3.6813	0.943890799	0.966018836	20	4	24	0.430718868	0.805393068	24	11.10092808	0.076071351
ENSMUSG00000035237	Lcat	lecithin cholesterol acyltransferase [Source:MGI Symbol;Acc:MGI:96755]	3.39827	3.42255	4.28262	3.40538	3.58637	4.13753	3.94458	3.28982	4.69324	3.51156	3.4082	3.16828	2.77463	3.45251	0.595952838	0.966018836	12	16	24	0.441161509	0.805393068	24	9.776091817	0.074879866
ENSMUSG00000035239	Neu3	neuraminidase 3 [Source:MGI Symbol;Acc:MGI:1355305]	1.05834	0.969988	1.00292	0.991832	1.08846	1.07471	1.0962	1.08614	1.16828	1.08534	0.894951	1.27161	0.950156	1.21477	0.844448841	0.966018836	24	20	24	0.856893868	0.957562969	24	2.156592484	0.015075593
ENSMUSG00000035242	Oaz1	ornithine decarboxylase antizyme 1 [Source:MGI Symbol;Acc:MGI:109433]	43.6574	38.9097	38.4106	39.8184	30.7418	37.791	32.2752	40.3568	39.0211	42.415	37.9806	38.8837	40.711	35.1065	0.935194469	0.966018836	16	4	24	0.764064329	0.92403577	24	6.454082165	0.041489885
ENSMUSG00000035245	Eogt	EGF domain-specific O-linked N-acetylglucosamine (GlcNAc) transferase [Source:MGI Symbol;Acc:MGI:2141669]	10.0157	10.2085	9.28768	10.3926	11.5853	9.3173	9.25862	9.78576	9.73363	9.23222	10.5928	9.29237	8.82794	10.5862	0.743750765	0.966018836	20	8	24	0.506194702	0.811942842	24	13.52117774	0.043374192
ENSMUSG00000035246	Pcyt1b	phosphate cytidylyltransferase 1, choline, beta isoform [Source:MGI Symbol;Acc:MGI:2147987]	21.6773	17.8565	18.8442	19.0391	18.3994	20.8305	19.5657	18.4456	15.6048	22.5568	17.7355	19.1944	18.4083	18.6894	0.560714538	0.966018836	24	12	24	0.420017623	0.803834751	24	19.52016481	0.065878802
ENSMUSG00000035247	Hectd1	HECT domain E3 ubiquitin protein ligase 1 [Source:MGI Symbol;Acc:MGI:2384768]	17.8908	17.4742	17.926	18.3242	18.0836	17.7307	17.4091	17.1241	17.301	17.5819	18.5619	19.1314	19.3868	19.4512	0.772122688	0.966018836	24	8	24	0.583049982	0.851725163	24	19.81863486	0.020237846
ENSMUSG00000035248	Tut7	terminal uridylyl transferase 7 [Source:MGI Symbol;Acc:MGI:2387179]	19.4832	21.0712	21.8811	21.0272	21.3245	20.1954	21.0854	19.4813	22.152	22.3815	20.1179	21.1842	22.0285	22.7362	0.892578749	0.966018836	12	16	24	0.147052403	0.755652082	24	8.875253881	0.046759257
ENSMUSG00000035258	Abi3bp	ABI family member 3 binding protein [Source:MGI Symbol;Acc:MGI:2444583]	5.55278	6.86971	6.21516	6.61022	6.23593	5.0887	6.19669	6.01126	7.20483	5.39949	5.99486	5.15351	5.36176	5.61361	0.004712708	0.140598674	12	12	24	0.054914359	0.655263328	24	8.483902256	0.109167854
ENSMUSG00000035266	Helq	helicase, POLQ-like [Source:MGI Symbol;Acc:MGI:2176740]	5.29866	7.57319	5.82953	6.18409	9.15814	7.14713	7.06158	6.317	6.94261	5.06034	6.61322	6.25171	6.68529	6.60828	0.935194469	0.966018836	20	20	24	0.725711525	0.909996439	24	19.64140318	0.062140127
ENSMUSG00000035268	Pkig	protein kinase inhibitor, gamma [Source:MGI Symbol;Acc:MGI:1343086]	34.5219	29.4297	28.9752	31.1053	31.789	32.7601	32.5328	34.7111	31.6607	32.6024	32.724	30.8091	32.5411	26.9564	0.725736046	0.966018836	4	8	24	0.328703015	0.768040178	24	19.50784616	0.057030447
ENSMUSG00000035273	Hpse	heparanase [Source:MGI Symbol;Acc:MGI:1343124]	1.28608	1.43306	1.22488	1.63691	1.12563	1.33222	1.06473	1.08418	1.37619	1.63717	1.19625	1.26322	1.25358	1.26544	0.351315142	0.870418653	16	4	24	0.179016484	0.768040178	24	11.23507317	0.076378398
ENSMUSG00000035275	Raver2	ribonucleoprotein, PTB-binding 2 [Source:MGI Symbol;Acc:MGI:2443623]	3.15368	3.05446	2.88437	3.18248	3.63892	3.09944	3.40171	2.83329	3.26951	3.46536	3.00284	3.33819	2.84312	2.54649	0.089766625	0.477784783	16	16	24	0.05674064	0.65610184	24	16.2514329	0.085644918
ENSMUSG00000035278	Plekhj1	pleckstrin homology domain containing, family J member 1 [Source:MGI Symbol;Acc:MGI:1925920]	29.0014	30.3236	30.0148	32.9761	27.0097	27.251	27.7268	27.0645	31.6713	29.7853	32.2158	30.9405	30.3285	28.5802	0.832506215	0.966018836	12	20	24	0.262880179	0.768040178	24	11.36231076	0.057666576
ENSMUSG00000035279	Ssc5d	scavenger receptor cysteine rich family, 5 domains [Source:MGI Symbol;Acc:MGI:3606211]	1.89054	1.99124	1.94841	1.87875	2.01086	1.79301	2.59943	1.85033	2.54645	1.56031	1.88255	1.62635	1.82497	1.79034	0.960829223	0.967043964	12	12	24	0.630804953	0.874088564	24	4.423050124	0.051493403
ENSMUSG00000035284	Vps13c	vacuolar protein sorting 13C [Source:MGI Symbol;Acc:MGI:2444207]	14.9466	15.2155	14.9228	16.5745	15.1273	14.4998	13.8015	15.6807	14.5946	15.854	15.5596	15.1046	16.384	15.3278	0.628286596	0.966018836	16	8	24	0.441715904	0.805393068	24	11.22521934	0.032347458
ENSMUSG00000035285	Nat14	N-acetyltransferase 14 [Source:MGI Symbol;Acc:MGI:3039561]	6.15891	6.82563	6.83133	4.21167	5.15115	5.78917	6.12055	5.92799	6.66148	7.20606	6.46283	6.83751	6.02983	6.81025	0.93447422	0.966018836	12	8	24	0.412840216	0.799546792	24	4.325776014	0.085180454
ENSMUSG00000035293	G2e3	G2/M-phase specific E3 ubiquitin ligase [Source:MGI Symbol;Acc:MGI:2444298]	3.18257	3.52118	3.4778	3.68135	3.45445	2.8968	3.37855	3.14981	3.83765	2.57056	3.91933	2.76107	2.96457	3.20721	0.128457847	0.560209134	12	12	24	0.490129131	0.805393068	24	9.624618774	0.063258474
ENSMUSG00000035296	Sgcg	sarcoglycan, gamma (dystrophin-associated glycoprotein) [Source:MGI Symbol;Acc:MGI:1346524]	0.927599	2.40805	2.5124	3.32954	1.87083	2.77304	1.96015	5.34731	3.77193	3.73273	1.52527	1.79181	2.71776	2.37311	0.184505544	0.662239322	16	4	24	0.069377282	0.673520298	24	8.27907743	0.373549877
ENSMUSG00000035297	Cops4	COP9 signalosome subunit 4 [Source:MGI Symbol;Acc:MGI:1349414]	67.328	67.7801	64.6109	63.1575	65.431	62.9968	62.1688	66.2179	63.3433	57.2536	68.2578	60.9217	67.5765	64.1791	0.622773557	0.966018836	8	8	24	0.402706814	0.799546792	24	8.865001142	0.035115867
ENSMUSG00000035299	Mid1	midline 1 [Source:MGI Symbol;Acc:MGI:1100537]	1.11884	0.889894	0.798549	1.23326	0.966267	0.874217	0.971891	0.952712	0.93716	0.933253	1.05193	0.864221	0.783473	0.923377	0.914360142	0.966018836	20	4	24	0.49269124	0.805558817	24	14.43075612	0.038225621
ENSMUSG00000035305	Ror1	receptor tyrosine kinase-like orphan receptor 1 [Source:MGI Symbol;Acc:MGI:1347520]	0.52264	0.492816	0.622795	0.530501	0.51148	0.472838	0.596096	0.521165	0.522636	0.494349	0.46407	0.353664	0.576137	0.422193	0.44861185	0.966018836	4	20	24	0.484108509	0.805393068	24	6.266532633	0.029314033
ENSMUSG00000035311	Gnptab	N-acetylglucosamine-1-phosphate transferase, alpha and beta subunits [Source:MGI Symbol;Acc:MGI:3643902]	17.329	15.8054	17.2988	16.5381	17.9253	17.1923	16.1097	14.6815	13.6549	17.2444	14.8413	17.2108	15.8695	15.6187	0.313530643	0.824470524	20	12	24	0.131421756	0.741500219	24	18.20921142	0.076739554
ENSMUSG00000035314	Gdpd5	glycerophosphodiester phosphodiesterase domain containing 5 [Source:MGI Symbol;Acc:MGI:2686926]	15.3803	12.1901	10.4207	10.6028	14.4038	14.183	12.4588	13.5289	10.57	14.2907	11.9586	12.9018	13.6558	12.0832	0.036527779	0.332441566	24	12	24	0.057400186	0.656248849	24	20.892275	0.149219919
ENSMUSG00000035325	Sec31a	Sec31 homolog A (S. cerevisiae) [Source:MGI Symbol;Acc:MGI:1916412]	36.7736	30.9848	31.8415	31.3681	34.0436	35.941	33.5223	36.1653	29.8185	38.4683	29.8954	32.1317	31.6728	33.5903	0.923635729	0.966018836	4	8	24	0.769102496	0.925585646	24	21.60122585	0.033417389
ENSMUSG00000035329	Fbxo33	F-box protein 33 [Source:MGI Symbol;Acc:MGI:1917861]	9.34411	10.5115	10.0243	9.86401	9.34996	9.83132	9.39247	8.84572	10.7426	7.09009	7.983	9.14454	7.82632	8.91309	0.83638114	0.966018836	12	16	24	0.583293702	0.851793019	24	4.514578899	0.051003753
ENSMUSG00000035337	Uchl4	ubiquitin carboxyl-terminal esterase L4 [Source:MGI Symbol;Acc:MGI:1890440]	5.24532	5.31371	4.97066	4.88489	5.12461	4.94866	4.85096	5.20046	4.23667	4.09509	4.7331	4.5042	4.83917	4.61736	0.800065124	0.966018836	8	8	24	0.074337743	0.683293901	24	23.50576593	0.059599158
ENSMUSG00000035342	Lzts2	leucine zipper, putative tumor suppressor 2 [Source:MGI Symbol;Acc:MGI:2385095]	11.9497	11.7644	11.2239	14.9765	12.3918	10.6498	14.3054	13.4352	15.0736	14.7161	15.5272	14.7751	14.5351	12.506	0.725061903	0.966018836	16	16	24	0.409896436	0.799546792	24	12.716328	0.072318857
ENSMUSG00000035351	Nup37	nucleoporin 37 [Source:MGI Symbol;Acc:MGI:1919964]	4.46046	5.05938	5.1225	5.15916	6.41259	4.24977	4.82326	5.76765	5.57314	4.17347	5.44907	4.84763	5.16248	4.69165	0.849179819	0.966018836	20	4	24	0.717629875	0.90689011	24	10.99132947	0.047370591
ENSMUSG00000035352	Ccl12	chemokine (C-C motif) ligand 12 [Source:MGI Symbol;Acc:MGI:108224]	3.53033	3.05451	4.21658	1.94338	3.24811	1.71484	3.51178	3.99561	3.20043	1.5561	3.13783	2.13202	2.53879	2.84281	0.265774856	0.773510818	12	4	24	0.193950494	0.768040178	24	3.986508031	0.212020006
ENSMUSG00000035354	Uvrag	UV radiation resistance associated gene [Source:MGI Symbol;Acc:MGI:1925860]	7.94096	8.1898	7.33893	7.57005	7.11841	8.1053	8.44107	7.91966	7.16037	10.2058	7.91395	8.53933	8.8251	8.11724	0.157813674	0.614233844	4	8	24	0.827660074	0.946180429	24	21.97678912	0.028070666
ENSMUSG00000035356	Nfkbiz	nuclear factor of kappa light polypeptide gene enhancer in B cells inhibitor, zeta [Source:MGI Symbol;Acc:MGI:1931595]	1.74161	2.14822	2.56926	1.92309	2.31407	1.84351	2.30417	1.87257	2.0324	1.70452	2.17431	1.65284	1.62981	1.77576	0.417653118	0.963884303	12	12	24	0.542563287	0.830991507	24	10.66362764	0.053689709
ENSMUSG00000035357	Pdzrn3	PDZ domain containing RING finger 3 [Source:MGI Symbol;Acc:MGI:1933157]	2.21492	2.55894	2.23858	1.91771	2.22495	2.31458	2.65987	3.12151	2.68735	2.76171	2.16687	2.39639	2.36322	2.2458	0.688644847	0.966018836	8	12	24	0.268672347	0.768040178	24	4.403121703	0.078514514
ENSMUSG00000035367	Rmi1	RecQ mediated genome instability 1 [Source:MGI Symbol;Acc:MGI:1921636]	5.17144	5.59282	4.70116	4.90074	4.85823	4.23694	5.11609	4.84831	5.7386	4.81402	5.30826	5.31888	5.50354	5.30888	0.939921059	0.966018836	8	8	24	0.374410517	0.794636296	24	4.397025509	0.050708069
ENSMUSG00000035370	Gm49322	predicted gene, 49322 [Source:MGI Symbol;Acc:MGI:6121503]	0.901835	0.871196	0.77793	1.25304	1.03492	1.02388	0.914871	0.993187	0.967909	0.784857	1.25445	0.772615	1.00994	0.825435	0.339604637	0.858712466	20	16	24	0.31892307	0.768040178	24	15.77669428	0.06571657
ENSMUSG00000035372	1810055G02Rik	RIKEN cDNA 1810055G02 gene [Source:MGI Symbol;Acc:MGI:1919306]	5.28132	5.64014	5.47558	5.0006	6.08333	5.12007	6.2178	5.91741	5.8443	6.52468	4.60211	4.59929	5.10456	4.81923	0.918497267	0.966018836	12	12	24	0.547732294	0.833771137	24	7.974867527	0.0592144
ENSMUSG00000035373	Ccl7	chemokine (C-C motif) ligand 7 [Source:MGI Symbol;Acc:MGI:99512]	2.86988	3.54853	3.1876	2.49862	3.44701	1.34234	3.8028	3.90409	3.70306	1.69027	3.38268	1.83341	3.24597	3.64818	0.033248781	0.324908923	8	16	24	0.129668226	0.740162193	24	5.056587514	0.263437197
ENSMUSG00000035376	Hacd2	3-hydroxyacyl-CoA dehydratase 2 [Source:MGI Symbol;Acc:MGI:1918007]	6.98003	6.42229	7.33464	8.65808	6.70233	7.17129	6.60595	7.51071	6.61912	7.72787	7.57668	7.26167	6.99287	7.46423	0.101443845	0.511659635	16	12	24	0.178180784	0.768040178	24	12.42594951	0.06860332
ENSMUSG00000035378	Shq1	SHQ1 homolog (S. cerevisiae) [Source:MGI Symbol;Acc:MGI:1919421]	2.03771	1.38962	1.52171	1.61498	1.70125	1.61846	1.45581	1.43775	1.17285	1.56123	1.64941	1.49966	1.60244	1.64022	0.097779482	0.501112642	20	16	24	0.17163542	0.768040178	24	19.58537463	0.077697096
ENSMUSG00000035382	Pcsk7	proprotein convertase subtilisin/kexin type 7 [Source:MGI Symbol;Acc:MGI:107421]	7.52503	8.28521	7.80729	7.76827	8.66271	8.52947	7.727	7.97905	8.25554	8.252	7.68447	7.85259	7.54589	7.30336	0.481811994	0.966018836	24	4	24	0.289509653	0.768040178	24	14.63837379	0.03659791
ENSMUSG00000035385	Ccl2	chemokine (C-C motif) ligand 2 [Source:MGI Symbol;Acc:MGI:98259]	4.82552	5.21312	5.18292	2.89704	6.50729	4.03442	5.12888	4.95982	3.82593	2.30616	4.9382	2.69853	3.77285	3.3707	0.928892845	0.966018836	8	8	24	0.56640957	0.843471947	24	23.65576377	0.125940881
ENSMUSG00000035390	Brsk1	BR serine/threonine kinase 1 [Source:MGI Symbol;Acc:MGI:2685946]	32.4152	27.0381	28.4267	25.9422	24.3969	30.283	31.0767	28.7189	24.3868	38.0912	24.6343	33.119	29.3842	34.7118	0.359479536	0.883787544	24	20	24	0.55493792	0.838149909	24	0.787969992	0.078828713
ENSMUSG00000035392	Dennd1a	DENN/MADD domain containing 1A [Source:MGI Symbol;Acc:MGI:2442794]	6.7065	7.59749	7.8132	7.78516	8.22886	7.94193	8.75301	7.7917	8.85287	8.77947	7.52354	8.76354	8.5209	7.83247	0.880631923	0.966018836	12	20	24	0.715366868	0.90631377	24	12.31085502	0.027661246
ENSMUSG00000035397	Klf16	Kruppel-like factor 16 [Source:MGI Symbol;Acc:MGI:2153049]	4.19339	4.1412	4.23809	3.71057	4.21493	3.92646	4.93388	4.13812	4.29612	6.16326	4.53039	4.83233	4.80327	4.98004	0.746444581	0.966018836	4	4	24	0.954976726	0.988639288	24	9.435031299	0.015497456
ENSMUSG00000035399	Oser1	oxidative stress responsive serine rich 1 [Source:MGI Symbol;Acc:MGI:1913930]	3.22831	3.62693	4.1909	3.17687	4.58911	3.73166	2.55924	2.8575	3.84916	3.31256	4.3716	3.2478	3.89449	3.29891	0.235141005	0.73629392	20	8	24	0.246011857	0.768040178	24	14.1041257	0.115976247
ENSMUSG00000035401	Emsy	EMSY, BRCA2-interacting transcriptional repressor [Source:MGI Symbol;Acc:MGI:1924203]	10.7189	11.2988	10.8701	10.7043	11.3846	10.7906	13.0795	10.0258	11.2093	9.88308	10.9005	10.9975	10.3532	10.341	0.970223078	0.971116366	20	20	24	0.523266349	0.821146401	24	21.40053422	0.040434215
ENSMUSG00000035407	Kank4	KN motif and ankyrin repeat domains 4 [Source:MGI Symbol;Acc:MGI:3043381]	21.1331	19.9393	19.4085	21.4257	21.4373	19.9826	20.8139	20.5802	19.9245	25.0231	18.9478	21.2206	21.8395	19.7445	0.265774856	0.773510818	16	20	24	0.697481432	0.899366528	24	14.7290043	0.031515545
ENSMUSG00000035413	Tmem98	transmembrane protein 98 [Source:MGI Symbol;Acc:MGI:1923457]	3.1009	3.56697	4.69432	3.60169	3.44515	3.48913	3.39308	3.48583	3.32043	3.50852	2.97568	3.34224	2.95678	3.2219	0.109247218	0.524035616	16	12	24	0.159515146	0.765591735	24	9.209213566	0.082450166
ENSMUSG00000035431	Sstr1	somatostatin receptor 1 [Source:MGI Symbol;Acc:MGI:98327]	0.599202	0.501164	0.838386	0.665993	0.543246	0.708289	0.610873	0.604905	0.496254	0.857885	0.36934	0.665418	0.582765	1.04681	0.887862353	0.966018836	16	4	24	0.545524709	0.833016631	24	6.6522325	0.065520072
ENSMUSG00000035437	Rabgap1	RAB GTPase activating protein 1 [Source:MGI Symbol;Acc:MGI:2385139]	28.415	27.1022	27.2611	27.9444	27.2983	27.806	26.1236	25.3651	26.5032	26.1608	27.0064	27.5176	28.215	27.8918	0.868638024	0.966018836	4	8	24	0.452117219	0.805393068	24	20.24747542	0.023552532
ENSMUSG00000035439	Haus8	4HAUS augmin-like complex, subunit 8 [Source:MGI Symbol;Acc:MGI:1923728]	14.0932	12.4757	12.5165	14.0859	16.8352	14.4276	13.8476	14.3298	12.7815	11.8488	14.174	12.9837	14.8456	11.7374	0.180336821	0.657605589	20	16	24	0.070212024	0.675025526	24	18.9538104	0.112511258
ENSMUSG00000035441	Myo1d	myosin ID [Source:MGI Symbol;Acc:MGI:107728]	8.03682	6.95001	7.04125	8.56306	7.95885	7.6521	8.27472	8.1032	6.91881	12.2969	6.79828	8.63931	7.83576	7.44846	0.649212555	0.966018836	16	20	24	0.580096228	0.850153156	24	14.43245924	0.078263534
ENSMUSG00000035443	Thyn1	thymocyte nuclear protein 1 [Source:MGI Symbol;Acc:MGI:1925112]	44.2461	48.4259	49.0879	46.5237	50.3921	43.6679	43.4438	41.1604	50.1925	39.4722	48.2441	45.861	48.6371	46.9623	0.823657258	0.966018836	12	12	24	0.907735451	0.974606327	24	10.93340048	0.019045245
ENSMUSG00000035455	Fignl1	fidgetin-like 1 [Source:MGI Symbol;Acc:MGI:1890648]	1.10156	0.855755	1.06842	1.05843	0.856282	0.822236	0.989696	0.730016	0.868286	0.684537	1.10629	0.908644	1.02793	0.875905	0.917867465	0.966018836	4	4	24	0.743917855	0.916218012	24	20.42020099	0.032440138
ENSMUSG00000035456	Prdm8	PR domain containing 8 [Source:MGI Symbol;Acc:MGI:1924880]	3.16774	2.19538	2.28093	2.74968	2.02684	2.73186	2.29044	2.54709	1.97495	3.21163	2.60014	2.74867	2.70266	2.42727	0.195343269	0.673387182	16	20	24	0.567218553	0.843774904	24	19.18793886	0.067166481
ENSMUSG00000035458	Tnni3	troponin I, cardiac 3 [Source:MGI Symbol;Acc:MGI:98783]	1.9503	1.97252	2.59993	2.25315	2.50553	1.56216	2.99256	1.72079	1.93988	1.81532	1.19194	1.5996	2.33725	2.50401	0.714044782	0.966018836	4	20	24	0.40794587	0.799546792	24	4.641042772	0.121109352
ENSMUSG00000035469	Rcbtb1	regulator of chromosome condensation (RCC1) and BTB (POZ) domain containing protein 1 [Source:MGI Symbol;Acc:MGI:1918580]	18.6672	19.3552	21.9302	19.0997	18.9392	20.6414	20.4551	19.06	19.0001	20.141	17.985	19.5895	19.9319	21.0098	0.911135828	0.966018836	8	12	24	0.52088386	0.820180873	24	4.802578197	0.033144563
ENSMUSG00000035473	Galm	galactose mutarotase [Source:MGI Symbol;Acc:MGI:2442420]	7.90603	6.70002	6.45025	8.63901	7.01491	7.29899	7.48013	8.38613	6.14473	8.62792	7.55606	7.52306	8.19424	7.63925	0.895493752	0.966018836	16	20	24	0.801426442	0.936422183	24	18.89074884	0.036117552
ENSMUSG00000035476	Tab3	TGF-beta activated kinase 1/MAP3K7 binding protein 3 [Source:MGI Symbol;Acc:MGI:1913974]	7.69009	8.51019	8.31515	8.09214	7.91945	7.97619	7.56607	8.29932	8.5484	8.19497	8.08609	9.3946	9.26518	9.42268	0.313239838	0.824470524	8	20	24	0.328497856	0.768040178	24	4.769128692	0.039208503
ENSMUSG00000035478	Mbd3	methyl-CpG binding domain protein 3 [Source:MGI Symbol;Acc:MGI:1333812]	29.89	25.6864	27.8804	28.6918	28.287	28.401	29.0076	26.388	25.3972	27.8763	27.6775	27.8389	25.7019	26.6807	0.40165019	0.933249185	4	4	24	0.055775114	0.65534544	24	18.11469474	0.056191313
ENSMUSG00000035486	Plk5	polo like kinase 5 [Source:MGI Symbol;Acc:MGI:3026984]	10.2701	6.80876	6.74543	14.4391	16.039	9.96956	10.7995	7.09446	9.54839	9.75083	16.6328	12.2463	9.92515	6.15189	1.16E-04	0.029372629	20	12	24	3.81E-04	0.23436643	24	16.67596703	0.498268244
ENSMUSG00000035493	Tgfbi	transforming growth factor, beta induced [Source:MGI Symbol;Acc:MGI:99959]	28.1697	33.7164	31.3716	28.7441	35.1024	25.7439	32.5344	32.8065	42.5591	23.6704	31.6764	29.4041	31.8283	27.2556	0.91695334	0.966018836	12	12	24	0.647437649	0.880898698	24	6.252576981	0.077354449
ENSMUSG00000035495	Tstd2	thiosulfate sulfurtransferase (rhodanese)-like domain containing 2 [Source:MGI Symbol;Acc:MGI:3039624]	9.17454	8.9282	8.09905	7.04064	7.63663	8.13211	8.36582	7.81177	8.28962	9.10316	8.37242	8.2844	9.63284	8.18631	0.297978647	0.80820842	4	12	24	0.259669523	0.768040178	24	0.654729404	0.064054892
ENSMUSG00000035504	Reep6	receptor accessory protein 6 [Source:MGI Symbol;Acc:MGI:1917585]	1.24741	1.42449	1.36227	1.32708	1.57758	1.4754	1.34521	1.35188	1.71707	1.84852	1.54309	1.37261	1.64515	1.35812	0.398157656	0.929472831	20	8	24	0.164752908	0.767020009	24	12.46225008	0.063937703
ENSMUSG00000035505	Cox18	cytochrome c oxidase assembly protein 18 [Source:MGI Symbol;Acc:MGI:2448532]	11.0055	8.67044	9.38533	9.02794	10.1723	10.0509	10.7384	11.2095	8.61264	10.582	10.9287	9.20099	9.30692	8.82663	0.851656735	0.966018836	4	8	24	0.370686369	0.792823907	24	19.36203204	0.070967994
ENSMUSG00000035509	Fbxl21	F-box and leucine-rich repeat protein 21 [Source:MGI Symbol;Acc:MGI:2442921]	3.67409	3.92796	3.523	3.5122	3.44022	3.25285	3.63578	3.49925	4.09356	4.33422	3.74579	3.71873	4.06214	3.61263	0.874607729	0.966018836	4	20	24	0.442499338	0.805393068	24	8.033835772	0.043759324
ENSMUSG00000035513	Ntng2	netrin G2 [Source:MGI Symbol;Acc:MGI:2159341]	7.42435	5.53474	5.46261	6.38576	6.30869	6.23183	6.89616	6.55933	5.418	8.77041	6.1596	6.10263	7.14344	6.70189	0.176754388	0.653256853	4	8	24	0.811090278	0.93944887	24	19.69907013	0.043255903
ENSMUSG00000035517	Tdrd7	tudor domain containing 7 [Source:MGI Symbol;Acc:MGI:2140279]	27.2377	26.0903	24.7666	23.5147	25.4447	26.5125	27.8099	27.9914	21.4735	27.8285	26.4039	22.3367	25.0963	26.5522	0.628899575	0.966018836	8	4	24	0.557330782	0.839072789	24	23.82347481	0.048747789
ENSMUSG00000035521	Gnptg	N-acetylglucosamine-1-phosphotransferase, gamma subunit [Source:MGI Symbol;Acc:MGI:2147006]	30.9761	28.7009	28.2742	26.9318	29.155	30.5388	30.2959	29.6723	29.4569	29.8043	30.8908	29.3905	29.1562	29.7967	0.379902653	0.908319827	4	8	24	0.150722876	0.758865539	24	21.72596481	0.037804283
ENSMUSG00000035529	Prdm4	PR domain containing 4 [Source:MGI Symbol;Acc:MGI:1920093]	13.2745	11.6271	12.1588	13.0214	11.789	13.3853	12.0262	11.9141	10.0036	14.0653	11.7737	12.9234	12.1481	11.5907	0.157813674	0.614233844	24	8	24	0.239472608	0.768040178	24	18.2995078	0.073597173
ENSMUSG00000035530	Eif1	eukaryotic translation initiation factor 1 [Source:MGI Symbol;Acc:MGI:105125]	94.9058	91.0991	84.5206	89.2885	92.2686	85.0539	93.0991	93.4891	99.6192	100.031	103.366	105.996	110.833	105.681	0.941830768	0.966018836	4	8	24	0.702868457	0.901733679	24	23.94686454	0.022616911
ENSMUSG00000035545	Leng8	leukocyte receptor cluster (LRC) member 8 [Source:MGI Symbol;Acc:MGI:2142195]	62.975	79.4072	80.2447	81.285	76.5365	65.0357	68.3915	74.9244	95.3092	81.6674	80.3152	75.8561	77.9489	79.538	0.001130596	0.079869094	16	12	24	7.23E-04	0.319044294	24	9.645512833	0.167440255
ENSMUSG00000035547	Capn5	calpain 5 [Source:MGI Symbol;Acc:MGI:1100859]	36.4274	25.7361	27.494	28.9994	29.8605	35.8495	29.8386	33.135	21.5812	40.3326	29.8187	30.8354	28.6333	29.1942	0.080164531	0.458678242	24	12	24	0.30834234	0.768040178	24	19.01100593	0.133316694
ENSMUSG00000035559	Mpv17l2	MPV17 mitochondrial membrane protein-like 2 [Source:MGI Symbol;Acc:MGI:2681846]	13.3189	13.3911	13.2034	13.8417	16.2243	15.1452	14.1759	14.0857	13.8701	17.1901	16.0456	15.5309	16.1381	12.9675	0.031018002	0.324235048	20	12	24	0.00777702	0.486810697	24	16.57827267	0.117541904
ENSMUSG00000035561	Aldh1b1	aldehyde dehydrogenase 1 family, member B1 [Source:MGI Symbol;Acc:MGI:1919785]	1.11291	1.25562	1.15056	1.02679	0.921699	1.39782	0.929792	1.35331	0.914164	1.1269	1.23861	1.36594	1.33004	1.12604	0.387380622	0.915479136	24	12	24	0.40346165	0.799546792	24	22.60284676	0.059778353
ENSMUSG00000035566	Pcdh17	protocadherin 17 [Source:MGI Symbol;Acc:MGI:2684924]	3.56158	3.42922	4.35007	3.73732	3.21962	4.00014	3.89864	3.1302	3.11881	4.33761	3.44093	3.61168	2.93439	3.64016	0.935593607	0.966018836	16	16	24	0.620702924	0.870297169	24	13.03504932	0.050893975
ENSMUSG00000035569	Ankrd11	ankyrin repeat domain 11 [Source:MGI Symbol;Acc:MGI:1924337]	35.7616	34.3858	34.1538	36.0374	33.8794	36.1159	33.1563	34.4074	34.2048	40.4564	31.7452	36.3219	35.2679	37.2998	0.945282256	0.966018836	24	20	24	0.852053144	0.955622342	24	9.758427536	0.018873666
ENSMUSG00000035572	Dcaf10	DDB1 and CUL4 associated factor 10 [Source:MGI Symbol;Acc:MGI:2140179]	5.32954	5.32134	5.80896	5.77515	5.38621	5.79375	5.59208	5.25044	4.8969	5.41399	5.21697	5.48873	5.5378	5.19629	0.607427393	0.966018836	24	8	24	0.448790886	0.805393068	24	18.49617513	0.028654113
ENSMUSG00000035575	Utp6	UTP6 small subunit processome component [Source:MGI Symbol;Acc:MGI:2445193]	16.8653	17.4456	17.2001	15.5364	18.4049	16.5268	15.2382	15.1696	16.15	15.7998	17.2117	17.3513	17.7119	16.6878	0.893307667	0.966018836	20	20	24	0.511369152	0.814822274	24	18.87761357	0.038207004
ENSMUSG00000035576	L3mbtl1	L3MBTL1 histone methyl-lysine binding protein [Source:MGI Symbol;Acc:MGI:2676663]	4.38698	3.88598	3.84724	4.08381	4.12308	4.12554	4.52932	3.12022	3.50808	4.9431	4.26231	3.69761	4.25256	4.39212	0.643325306	0.966018836	4	8	24	0.543817911	0.83189324	24	17.54082267	0.059042106
ENSMUSG00000035578	Iqcg	IQ motif containing G [Source:MGI Symbol;Acc:MGI:1916957]	6.37956	7.11445	5.8247	6.16722	6.72273	5.83485	6.50829	5.79099	5.46859	5.94135	6.14745	6.13441	6.71284	7.23483	0.3576519	0.882035609	8	4	24	0.368953398	0.791959527	24	0.177986753	0.056073207
ENSMUSG00000035580	Kcnh8	potassium voltage-gated channel, subfamily H (eag-related), member 8 [Source:MGI Symbol;Acc:MGI:2445160]	1.53124	1.46554	1.389	1.31847	1.87877	1.54378	1.87696	1.44969	1.3774	1.94742	1.86953	1.6161	1.76152	1.55868	0.039226672	0.338446795	20	16	24	0.128386713	0.740162193	24	18.31944395	0.084247527
ENSMUSG00000035585	Tsen34	tRNA splicing endonuclease subunit 34 [Source:MGI Symbol;Acc:MGI:1913328]	22.7302	19.2941	21.1841	22.8041	21.1197	21.4752	21.5108	24.3533	20.7898	21.5664	21.8779	23.0305	22.6515	20.7154	0.777666236	0.966018836	4	4	24	0.677402098	0.892060593	24	20.05333522	0.028691801
ENSMUSG00000035595	Fam174c	family with sequence similarity 174, member C [Source:MGI Symbol;Acc:MGI:1917020]	13.3173	10.9616	11.0217	11.3102	10.8378	10.6664	12.5421	10.6401	10.8689	7.98297	11.2951	10.8557	11.3798	10.5515	0.327894132	0.846828588	4	4	24	0.060797842	0.660288491	24	23.2235314	0.118602528
ENSMUSG00000035596	Mboat7	membrane bound O-acyltransferase domain containing 7 [Source:MGI Symbol;Acc:MGI:1924832]	28.0621	27.0519	28.6474	26.4609	27.2196	30.2452	28.5603	28.9646	25.5327	33.3407	25.0507	29.4956	28.2925	28.0701	0.888379597	0.966018836	24	20	24	0.916188611	0.976814265	24	21.66607975	0.01747981
ENSMUSG00000035597	Prpf39	pre-mRNA processing factor 39 [Source:MGI Symbol;Acc:MGI:104602]	15.6104	19.6618	19.0931	18.4886	18.855	15.8319	17.9314	17.2909	23.1958	15.5597	18.9944	16.9745	17.3024	18.5638	0.085890569	0.466404237	12	12	24	0.148040145	0.756225271	24	8.494682267	0.111338948
ENSMUSG00000035601	Trmt10b	tRNA methyltransferase 10B [Source:MGI Symbol;Acc:MGI:1917184]	9.21852	10.6309	10.674	11.8237	12.8429	10.4645	10.905	10.0078	11.8827	9.6172	10.8728	11.2274	10.8276	11.5939	0.800065124	0.966018836	20	8	24	0.476797097	0.805393068	24	13.14193464	0.05377893
ENSMUSG00000035606	Ky	kyphoscoliosis peptidase [Source:MGI Symbol;Acc:MGI:96709]	0.125629	0.614085	1.91314	1.13557	0.699251	0.960308	0.813435	2.14392	1.33229	2.11691	0.366349	0.715631	0.693304	0.55032	0.288043872	0.793763484	12	16	24	0.066103533	0.668186543	24	9.280590409	0.388273359
ENSMUSG00000035614	Togaram1	TOG array regulator of axonemal microtubules 1 [Source:MGI Symbol;Acc:MGI:2684313]	16.3096	14.356	13.8703	14.9737	13.9124	13.3763	15.1167	13.5296	11.654	12.3811	16.2075	13.0371	13.6364	13.8217	0.918497267	0.966018836	4	8	24	0.324709789	0.768040178	24	20.89120899	0.070702234
ENSMUSG00000035615	Frmpd1	FERM and PDZ domain containing 1 [Source:MGI Symbol;Acc:MGI:2446274]	17.2327	11.9204	11.6137	13.5942	13.4734	14.6728	14.0351	14.8909	10.1579	19.609	12.5836	14.3134	14.3141	13.4157	0.122530972	0.550724475	4	8	24	0.484877015	0.805393068	24	19.7257739	0.108028978
ENSMUSG00000035620	Ric8b	RIC8 guanine nucleotide exchange factor B [Source:MGI Symbol;Acc:MGI:2682307]	21.9617	19.8966	19.597	20.0679	19.1139	21.7998	20.8255	19.731	17.8776	21.1398	20.195	21.3632	22.3902	21.3438	0.004501004	0.140598674	4	8	24	0.040017158	0.616921492	24	21.88946897	0.076488424
ENSMUSG00000035621	Midn	midnolin [Source:MGI Symbol;Acc:MGI:1890222]	8.96717	7.98319	8.55323	7.9519	8.96219	7.79375	7.81803	9.05757	7.94684	11.2138	8.68154	8.34384	9.73373	9.00933	0.911371589	0.966018836	8	16	24	0.756121758	0.921194485	24	10.82297009	0.037258397
ENSMUSG00000035623	Rsf1	remodeling and spacing factor 1 [Source:MGI Symbol;Acc:MGI:2682305]	5.29213	6.3171	5.75945	6.04252	5.37568	5.66282	5.80247	5.01595	5.7652	4.98306	6.21893	6.28874	5.85028	5.63348	0.853736882	0.966018836	24	16	24	0.848181557	0.954244184	24	19.50098624	0.022675049
ENSMUSG00000035629	Rubcn	RUN domain and cysteine-rich domain containing, Beclin 1-interacting protein [Source:MGI Symbol;Acc:MGI:1915160]	21.4465	19.4821	20.0287	18.8555	22.0538	20.5846	20.4662	20.6249	20.5242	21.2257	20.3719	21.2675	21.2152	20.6145	0.454881285	0.966018836	24	12	24	0.262132219	0.768040178	24	20.17203005	0.034626467

ENSMUSG00000035632	Cnot3	CCR4-NOT transcription complex, subunit 3 [Source:MGI Symbol;Acc:MGI:2385261]	7.74404	6.70119	7.70747	9.38848	8.6334	8.11284	11.9235	6.82953	7.40584	7.95804	7.92863	7.45584	6.76103	6.32584	0.008360387	0.192760771	16	16	24	0.291475524	0.768040178	24	17.21002603	0.122506909
ENSMUSG00000035637	Grhpr	glyoxylate reductase/hydroxypyruvate reductase [Source:MGI Symbol;Acc:MGI:1923488]	4.54678	5.54624	7.14877	5.84039	4.71783	6.77302	6.76545	7.43733	6.31844	6.95228	5.39694	5.62902	6.29868	6.65955	0.622132989	0.966018836	12	8	24	0.160414675	0.765941695	24	6.544268732	0.131237961
ENSMUSG00000035640	Cbarp	calcium channel, voltage-dependent, beta subunit associated regulatory protein [Source:MGI Symbol;Acc:MGI:1354170]	24.5889	19.7513	23.1924	19.8766	19.7849	21.442	20.958	25.1214	19.3755	29.6353	18.7326	27.2363	23.0089	23.8553	0.949382029	0.966018836	24	20	24	0.860140656	0.958819744	24	2.404727904	0.040372618
ENSMUSG00000035642	Aamdc	adipogenesis associated Mth938 domain containing [Source:MGI Symbol;Acc:MGI:1913523]	35.7095	42.9794	37.3363	45.0351	42.3581	34.8346	38.2111	44.3595	44.4072	34.4958	43.007	36.64	39.9594	39.6003	0.313239838	0.824470524	12	12	24	0.381087578	0.799546792	24	8.70897384	0.075690546
ENSMUSG00000035649	Zcchc7	zinc finger, CCHC domain containing 7 [Source:MGI Symbol;Acc:MGI:2442912]	19.3873	24.1359	19.9888	22.835	23.4426	20.7422	22.3885	18.288	22.9183	18.7033	23.8812	20.395	20.7213	21.5445	0.939335547	0.966018836	20	8	24	0.724203782	0.90965493	24	14.76999609	0.039801458
ENSMUSG00000035653	Lrfn5	leucine rich repeat and fibronectin type III domain containing 5 [Source:MGI Symbol;Acc:MGI:2144814]	19.5857	18.8308	18.1577	18.5512	17.057	19.2878	16.9099	18.015	17.1318	20.1498	18.386	21.3914	20.5065	21.0824	0.960333395	0.967043964	24	12	24	0.646566673	0.880385602	24	22.90454004	0.03562409
ENSMUSG00000035666	Gtf3c4	general transcription factor IIIC, polypeptide 4 [Source:MGI Symbol;Acc:MGI:2138937]	6.77478	6.51182	6.93636	6.37661	6.70305	6.71167	6.76976	6.35272	5.96756	6.3743	6.21588	6.65886	6.16256	6.56351	0.914360142	0.966018836	4	12	24	0.326828625	0.768040178	24	21.44729037	0.028095215
ENSMUSG00000035671	Zswim4	zinc finger SWIM-type containing 4 [Source:MGI Symbol;Acc:MGI:2443726]	3.14683	3.02011	3.00105	3.04133	2.98808	3.12342	2.88386	3.73239	2.85421	3.71694	2.93602	3.31027	3.19282	3.27258	0.965971228	0.969272194	8	4	24	0.865272315	0.960982718	24	5.784507814	0.019664768
ENSMUSG00000035673	Sbno2	strawberry notch 2 [Source:MGI Symbol;Acc:MGI:2448490]	10.0199	8.43676	10.1627	10.1472	11.0777	9.2525	8.83847	9.4891	9.11763	10.7637	10.6517	9.33165	9.99748	9.17769	0.038580592	0.335882166	20	12	24	0.022759857	0.581965446	24	14.46325775	0.098245499
ENSMUSG00000035674	Ndufa3	NADH:ubiquinone oxidoreductase subunit A3 [Source:MGI Symbol;Acc:MGI:1913341]	187.157	215.595	214.865	226.648	217.979	211.636	202.953	218.51	243.84	184.157	239.548	202.047	224.327	219.561	0.694784292	0.966018836	12	12	24	0.531029346	0.825104543	24	9.6547864	0.051678721
ENSMUSG00000035681	Kcnc2	potassium voltage gated channel, Shaw-related subfamily, member 2 [Source:MGI Symbol;Acc:MGI:96668]	8.81172	7.52579	8.5403	6.8353	7.95123	8.3114	7.94057	6.88296	7.34932	7.61968	7.39726	7.52623	7.88551	8.08281	0.658942124	0.966018836	4	12	24	0.311394215	0.768040178	24	22.61110714	0.055294821
ENSMUSG00000035686	Thrsp	thyroid hormone responsive [Source:MGI Symbol;Acc:MGI:109126]	26.1597	18.2468	48.5899	119.154	39.8483	40.8838	51.0735	67.3293	39.3711	61.5228	122.449	66.1189	102.545	74.2142	0.911371589	0.966018836	16	20	24	0.281067048	0.768040178	24	13.74173438	0.362538504
ENSMUSG00000035692	Isg15	ISG15 ubiquitin-like modifier [Source:MGI Symbol;Acc:MGI:1855694]	10.2596	11.4257	10.9893	8.17597	12.7582	10.1335	8.09254	13.3764	11.622	7.41467	11.8857	9.96556	9.16835	10.3814	0.399942636	0.932395417	8	20	24	0.905876987	0.97423153	24	4.3693292	0.04133749
ENSMUSG00000035696	Rnf38	ring finger protein 38 [Source:MGI Symbol;Acc:MGI:1920719]	15.3133	17.4988	16.9777	17.936	16.2355	15.9157	17.3346	15.5369	16.4813	18.3287	17.2632	17.2607	15.9341	16.4954	0.192357053	0.669841558	16	12	24	0.113347317	0.727393008	24	12.05424634	0.056889259
ENSMUSG00000035697	Arhgap45	Rho GTPase activating protein 45 [Source:MGI Symbol;Acc:MGI:1917969]	3.08437	3.75113	3.85121	4.32442	3.75005	3.48032	4.33249	3.43389	4.26027	2.82342	4.15386	3.26418	4.03847	2.7502	0.934110497	0.966018836	12	20	24	0.865823517	0.961251055	24	13.9762662	0.036131032
ENSMUSG00000035699	Slc51a	solute carrier family 51, alpha subunit [Source:MGI Symbol;Acc:MGI:2146634]	1.20334	1.13123	0.906225	1.06897	1.07943	1.42568	1.25244	0.971076	0.987176	0.737729	0.834751	1.2881	1.35788	1.24832	0.002363973	0.106802459	24	16	24	0.004291162	0.427431763	24	22.82627594	0.153936291
ENSMUSG00000035704	Alg8	asparagine-linked glycosylation 8 (alpha-1,3-glucosyltransferase) [Source:MGI Symbol;Acc:MGI:2141959]	5.33656	4.93935	5.01445	3.57956	5.10714	4.92574	4.98377	4.6133	4.55074	4.01387	4.75541	4.32216	4.23106	4.54295	0.894563866	0.966018836	4	12	24	0.17789543	0.768040178	24	23.31578723	0.081834971
ENSMUSG00000035711	Dok3	docking protein 3 [Source:MGI Symbol;Acc:MGI:1351490]	3.36911	2.5838	3.40678	3.33425	2.89093	2.39616	3.35645	3.08959	3.43144	2.55473	3.83553	2.83591	3.53954	2.70128	0.956719163	0.96629412	12	12	24	0.966299385	0.991934487	24	8.982980373	0.01724093
ENSMUSG00000035713	Usp35	ubiquitin specific peptidase 35 [Source:MGI Symbol;Acc:MGI:2685339]	19.1971	16.2465	14.5943	15.7466	16.696	17.8196	15.3043	17.3903	15.0755	18.4719	16.8997	17.425	19.0123	17.3783	0.056400419	0.401074772	24	12	24	0.18875766	0.768040178	24	21.24965005	0.079922853
ENSMUSG00000035722	Abca7	ATP-binding cassette, sub-family A (ABC1), member 7 [Source:MGI Symbol;Acc:MGI:1351646]	20.2867	16.6569	15.3717	17.4404	17.0433	17.9059	18.3741	18.7006	14.8272	24.3791	17.7507	19.4724	19.067	19.2436	0.112800839	0.530777648	4	8	24	0.662085964	0.885885739	24	19.98227426	0.06005088
ENSMUSG00000035725	Prkx	protein kinase, X-linked [Source:MGI Symbol;Acc:MGI:1309999]	22.9103	16.5567	17.3737	17.919	17.9235	19.9994	18.4174	18.5947	15.2941	22.386	19.4043	20.1444	19.2705	19.0253	0.021795836	0.26698364	24	12	24	0.203393829	0.768040178	24	19.76368266	0.103498336
ENSMUSG00000035726	Supt16	SPT16, facilitates chromatin remodeling subunit [Source:MGI Symbol;Acc:MGI:1890948]	16.869	18.813	19.2981	19.0723	19.4284	18.6854	17.7479	17.4715	18.0421	18.2506	18.4203	19.199	19.1523	19.412	0.942051135	0.966018836	20	8	24	0.50537273	0.811334839	24	13.21875436	0.026189842
ENSMUSG00000035735	Dagla	diacylglycerol lipase, alpha [Source:MGI Symbol;Acc:MGI:2677061]	3.60903	3.0278	4.18538	3.00021	3.85493	3.45648	4.04933	3.52547	3.39001	5.18913	3.37212	3.47294	3.30625	3.37473	0.743370164	0.966018836	12	20	24	0.640393945	0.878305673	24	12.57067577	0.060953104
ENSMUSG00000035754	Wdr18	WD repeat domain 18 [Source:MGI Symbol;Acc:MGI:2158400]	12.0548	11.9097	11.5267	10.8048	10.9321	12.5949	11.8985	13.0962	8.5832	13.0556	12.6302	11.0841	10.5251	10.7973	0.845386467	0.966018836	20	16	24	0.588686682	0.853936396	24	19.91113109	0.061649616
ENSMUSG00000035757	Selenoo	selenoprotein O [Source:MGI Symbol;Acc:MGI:1919007]	12.3026	11.4482	11.5363	11.9835	11.4306	12.1839	12.3504	12.1237	11.8356	12.966	11.5958	13.1984	13.4678	12.4181	0.210418232	0.69936851	4	8	24	0.284157749	0.768040178	24	22.69777845	0.034749291
ENSMUSG00000035759	Bbs10	Bardet-Biedl syndrome 10 (human) [Source:MGI Symbol;Acc:MGI:1919019]	2.97927	2.77472	2.89162	2.62294	2.86932	2.59947	2.87763	2.72408	2.61291	2.54671	3.15823	2.82492	3.0875	3.04596	0.192357053	0.669841558	4	12	24	0.346414549	0.778844143	24	23.08837064	0.040417431
ENSMUSG00000035762	Tmem161b	transmembrane protein 161B [Source:MGI Symbol;Acc:MGI:1919995]	7.12432	8.06286	8.18452	7.59921	8.10552	7.56646	7.4776	7.18947	9.3324	7.13619	7.86503	6.67608	9.18637	9.16058	0.390686537	0.915479136	12	12	24	0.233373808	0.768040178	24	6.646451452	0.086323454
ENSMUSG00000035764	Fbxo45	F-box protein 45 [Source:MGI Symbol;Acc:MGI:2447775]	23.2851	19.4672	19.1072	19.7614	19.2869	20.9161	19.3945	19.9104	16.5178	22.1603	19.1975	20.4366	19.2961	20.7335	0.588208558	0.966018836	24	12	24	0.400703864	0.799546792	24	20.91364747	0.059531841
ENSMUSG00000035765	Dym	dymeclin [Source:MGI Symbol;Acc:MGI:1918480]	34.2613	26.8581	26.1773	28.5544	26.0866	33.6636	28.554	30.6943	22.3841	38.6822	24.373	30.3713	28.4714	30.0126	0.581070934	0.966018836	24	12	24	0.647153665	0.880787843	24	21.30496096	0.077186129
ENSMUSG00000035769	Xylb	xylulokinase homolog (H. influenzae) [Source:MGI Symbol;Acc:MGI:2142985]	0.93587	0.939242	1.24851	1.22211	0.863527	1.06955	0.969584	0.966174	1.13791	0.886275	0.973327	0.997405	0.867136	0.902026	0.45229395	0.966018836	12	16	24	0.269138661	0.768040178	24	10.27303845	0.052737535
ENSMUSG00000035770	Dync1li2	dynein, cytoplasmic 1 light intermediate chain 2 [Source:MGI Symbol;Acc:MGI:107738]	110.079	101.744	105.054	99.9717	99.9661	104.39	102.051	95.296	89.004	115.302	102.244	102.73	100.555	102.693	0.919571723	0.966018836	24	12	24	0.581530093	0.850877448	24	18.39834177	0.036316374
ENSMUSG00000035772	Mrps2	mitochondrial ribosomal protein S2 [Source:MGI Symbol;Acc:MGI:2153089]	8.80155	9.32786	9.38864	9.16875	8.2197	10.0579	9.64851	9.75559	7.48851	11.2113	9.19718	9.68932	9.58516	9.2495	0.745118544	0.966018836	12	24	24	0.873207578	0.964375835	24	20.34515482	0.026221081
ENSMUSG00000035773	Kiss1r	KISS1 receptor [Source:MGI Symbol;Acc:MGI:2148793]	0.976131	0.825262	0.965369	0.838938	0.67234	0.813553	0.658561	0.763834	0.937888	1.10254	0.810107	0.906472	0.841147	0.954848	0.602156008	0.966018836	16	4	24	0.322744948	0.768040178	24	8.719950511	0.056920178
ENSMUSG00000035776	Cd99l2	CD99 antigen-like 2 [Source:MGI Symbol;Acc:MGI:2177151]	63.577	56.5187	54.4852	53.0639	57.174	59.4377	56.8739	56.8787	50.5368	55.8472	54.5112	56.8384	58.0388	57.8427	0.004140921	0.140598674	4	8	24	0.005307005	0.4424469	24	22.38416606	0.081143516
ENSMUSG00000035778	Ggta1	glycoprotein galactosyltransferase alpha 1, 3 [Source:MGI Symbol;Acc:MGI:95704]	12.492	12.9019	11.6169	12.0175	13.2161	12.8861	12.6441	12.7183	12.1416	11.6945	13.0247	12.658	13.3687	12.8899	0.112583132	0.530777648	20	20	24	0.030213159	0.596794384	24	21.8257324	0.053096335
ENSMUSG00000035781	R3hdm4	R3H domain containing 4 [Source:MGI Symbol;Acc:MGI:1924814]	26.0716	26.4849	27.2924	25.0372	27.2732	25.3576	28.7122	30.9818	26.5951	32.1308	26.0183	25.8789	27.0791	26.2559	0.587179814	0.966018836	12	12	24	0.553368038	0.837286821	24	7.490910275	0.045015509
ENSMUSG00000035783	Acta2	actin, alpha 2, smooth muscle, aorta [Source:MGI Symbol;Acc:MGI:87909]	12.7746	10.4167	12.2405	10.4747	9.42415	13.1142	13.1264	21.1009	10.813	13.2255	9.76196	8.00105	13.5144	11.3988	0.17882595	0.655480352	4	16	24	0.195698467	0.768040178	24	3.828802181	0.208290671
ENSMUSG00000035790	Cep19	centrosomal protein 19 [Source:MGI Symbol;Acc:MGI:1914244]	24.4132	24.6371	24.4527	21.533	24.9532	24.5923	23.2447	24.6608	22.3506	24.2192	23.6993	23.3082	25.6959	25.8875	0.337748746	0.85758369	8	8	24	0.264623038	0.768040178	24	0.853323094	0.042726361
ENSMUSG00000035798	Zdhhc17	zinc finger, DHHC domain containing 17 [Source:MGI Symbol;Acc:MGI:2445110]	45.3629	46.5477	42.6982	43.8551	45.7494	43.9089	43.8188	41.5153	40.8884	42.8812	45.014	44.1132	44.9923	47.3597	0.479398357	0.966018836	8	4	24	0.384321732	0.799546792	24	21.63209841	0.032615326
ENSMUSG00000035799	Twist1	twist basic helix-loop-helix transcription factor 1 [Source:MGI Symbol;Acc:MGI:98872]	1.94808	2.42282	1.85471	1.74467	1.66744	1.71195	1.55359	2.57472	2.88753	1.48327	2.38867	1.75426	2.13979	2.18881	0.081336319	0.462794877	8	8	24	0.138611525	0.747972781	24	5.564634879	0.147213564
ENSMUSG00000035805	Mlc1	megalencephalic leukoencephalopathy with subcortical cysts 1 homolog (human) [Source:MGI Symbol;Acc:MGI:2157910]	10.3789	11.8479	19.1018	10.838	9.5786	13.408	14.7209	10.0186	11.9464	11.3186	8.99297	12.544	8.11887	14.3183	0.871479522	0.966018836	12	8	24	0.531858282	0.82547989	24	6.188296749	0.133723416
ENSMUSG00000035824	Tk2	thymidine kinase 2, mitochondrial [Source:MGI Symbol;Acc:MGI:1913266]	12.8737	13.4789	14.265	12.8654	12.2438	13.5953	11.9904	14.3271	13.606	14.4641	12.1442	15.2032	13.9401	14.4375	0.772442842	0.966018836	8	12	24	0.359660287	0.785405943	24	5.867540192	0.0523465
ENSMUSG00000035828	Pim3	proviral integration site 3 [Source:MGI Symbol;Acc:MGI:1355297]	14.2644	14.5624	19.6298	18.5853	13.3878	13.4898	13.2509	18.9157	18.1982	23.3261	15.4018	15.5086	15.9116	16.0498	0.018212384	0.256345714	16	4	24	0.003190547	0.414576087	24	9.425730429	0.255470707
ENSMUSG00000035829	Ppp1r26	protein phosphatase 1, regulatory subunit 26 [Source:MGI Symbol;Acc:MGI:2685193]	3.37048	2.99736	2.58287	2.88265	2.91207	2.53408	3.77385	2.81763	3.04182	3.09421	2.66358	2.6392	2.63163	2.69975	0.911135828	0.966018836	4	20	24	0.714677516	0.905979936	24	1.682613099	0.037158553
ENSMUSG00000035831	Krt25	keratin 25 [Source:MGI Symbol;Acc:MGI:1918060]	0.932831	0.794923	0.684629	1.00014	0.620176	0.84175	0.636288	0.857464	0.593888	0.845329	0.883455	0.936987	0.850148	0.852039	0.922035832	0.966018836	24	12	24	0.738738924	0.914695741	24	19.69580664	0.033520683
ENSMUSG00000035834	Polr3g	polymerase (RNA) III (DNA directed) polypeptide G [Source:MGI Symbol;Acc:MGI:1914736]	40.453	37.1716	35.1472	35.5462	38.6159	36.9332	32.0363	32.5462	33.5837	34.6338	40.6406	37.1659	38.992	37.1313	0.416876911	0.963884303	20	16	24	0.202362585	0.768040178	24	18.96681419	0.074153683
ENSMUSG00000035835	Plppr3	phospholipid phosphatase related 3 [Source:MGI Symbol;Acc:MGI:2388640]	4.58515	5.09376	6.80823	6.18987	5.20327	5.47843	5.79837	4.97713	5.76665	6.38427	5.18153	5.79928	5.37207	6.07714	0.268085731	0.774280302	12	20	24	0.085569869	0.703577714	24	10.26644668	0.107652762
ENSMUSG00000035840	Lysmd3	LysM, putative peptidoglycan-binding, domain containing 3 [Source:MGI Symbol;Acc:MGI:1915906]	6.36468	7.08655	6.93042	7.93412	5.82898	5.76297	5.78218	6.33308	6.72201	5.19239	7.86379	5.50531	7.36462	6.59086	0.777666236	0.966018836	12	12	24	0.599154954	0.859466291	24	7.948793575	0.067780986
ENSMUSG00000035842	Ddx11	DEAD/H box helicase 11 [Source:MGI Symbol;Acc:MGI:2443590]	1.40941	0.771718	0.737049	0.491158	0.881616	0.87288	0.981339	0.960152	1.05512	0.723637	1.32007	0.964175	1.04982	1.02143	0.03484427	0.328311403	4	12	24	0.152830571	0.759115906	24	22.81729806	0.126091936
ENSMUSG00000035845	Alg12	asparagine-linked glycosylation 12 (alpha-1,6-mannosyltransferase) [Source:MGI Symbol;Acc:MGI:2385025]	6.87847	5.56061	5.65708	5.2677	5.98595	6.37985	5.5738	5.85659	4.93937	6.37055	5.08856	5.78872	5.49053	5.98195	0.895493752	0.966018836	24	12	24	0.350844174	0.780345856	24	21.76341863	0.063035576
ENSMUSG00000035847	Ids	iduronate 2-sulfatase [Source:MGI Symbol;Acc:MGI:96417]	111.816	85.4193	91.4278	90.5065	89.8665	103.564	95.7062	91.0153	64.1341	102.263	83.9725	82.6697	74.0176	85.0764	0.819898687	0.966018836	16	20	24	0.36970852	0.792235171	24	19.70731957	0.100318313
ENSMUSG00000035849	Krt222	keratin 222 [Source:MGI Symbol;Acc:MGI:2442728]	17.4299	16.6737	17.2937	16.0554	15.2534	17.3114	16.1758	15.2398	15.1979	17.7517	15.7518	16.4862	16.7173	18.3095	0.909604019	0.966018836	4	16	24	0.724410437	0.909665611	24	2.058172904	0.025875504
ENSMUSG00000035851	Ythdc1	YTH domain containing 1 [Source:MGI Symbol;Acc:MGI:2443713]	22.5977	21.9485	23.9421	23.4932	24.0984	22.0165	23.8614	22.5741	20.5409	20.972	24.7259	21.5976	20.5306	21.5527	0.70641939	0.966018836	20	12	24	0.340283586	0.774739261	24	16.24035233	0.043801309
ENSMUSG00000035852	Misp	mitotic spindle positioning [Source:MGI Symbol;Acc:MGI:1926156]	1.36258	0.805825	0.852153	1.24584	1.01974	1.11892	1.2282	1.05917	0.762673	0.664557	1.48509	1.13391	1.27459	1.08737	0.055920623	0.401074772	4	8	24	0.055403859	0.65534544	24	20.64718741	0.151744004
ENSMUSG00000035860	Cdhr3	cadherin-related family member 3 [Source:MGI Symbol;Acc:MGI:1916014]	0.358938	0.699979	1.00011	0.866092	0.429981	0.485278	0.686315	0.233714	0.278506	0.463774	0.332398	0.824387	0.0958365	0.781561	0.94397916	0.966018836	16	12	24	0.788212335	0.932005868	24	10.39519313	0.07090928
ENSMUSG00000035863	Palm	paralemmin [Source:MGI Symbol;Acc:MGI:1261814]	41.3545	34.3156	32.6837	38.9984	38.7205	36.6585	36.5474	36.8212	38.8108	46.5363	40.0932	44.8471	42.9296	39.0202	0.590017078	0.966018836	16	20	24	0.268274352	0.768040178	24	17.1756114	0.072502475
ENSMUSG00000035864	Syt1	synaptotagmin I [Source:MGI Symbol;Acc:MGI:99667]	82.0571	68.1185	70.3035	71.4913	66.9397	80.1416	69.6431	67.0537	59.6778	82.9274	69.7807	77.127	70.7037	86.9478	0.93447422	0.966018836	24	12	24	0.635068851	0.87594009	24	21.04766797	0.057469651
ENSMUSG00000035868	Zfp983	zinc finger protein 983 [Source:MGI Symbol;Acc:MGI:1920479]	9.5661	8.96457	7.33884	8.4772	8.8529	8.15252	9.71395	8.27733	7.76931	6.1368	10.6733	8.6073	9.1594	8.26097	0.016795343	0.253395174	4	8	24	0.078623995	0.689042744	24	21.55084822	0.146237787
ENSMUSG00000035873	Pawr	PRKC, apoptosis, WT1, regulator [Source:MGI Symbol;Acc:MGI:2149961]	1.50298	1.5168	1.52642	1.84667	1.56316	1.69985	1.19367	1.95693	1.474	1.65447	1.74549	1.60775	1.41475	1.72801	0.113148904	0.530777648	16	12	24	0.381784967	0.799546792	24	11.83516279	0.058102751
ENSMUSG00000035877	Zhx3	zinc fingers and homeoboxes 3 [Source:MGI Symbol;Acc:MGI:2444772]	4.19576	3.90065	4.76897	4.59701	3.90968	4.68014	4.74725	4.07831	4.03289	4.78324	3.8223	4.51059	4.12187	4.09174	0.773770646	0.966018836	16	16	24	0.895808946	0.970467191	24	15.11871601	0.01824636
ENSMUSG00000035878	Hykk	hydroxylysine kinase 1 [Source:MGI Symbol;Acc:MGI:2443139]	1.17418	1.17943	1.43789	1.16685	1.26438	1.32901	1.05789	1.21397	1.22088	1.05015	1.02248	1.20502	1.02329	1.2734	0.581070934	0.966018836	12	16	24	0.737211202	0.914374898	24	7.371003013	0.02411887
ENSMUSG00000035885	Cox8a	cytochrome c oxidase subunit 8A [Source:MGI Symbol;Acc:MGI:105959]	297.632	287.763	284.037	320.169	295.6	294.625	290.555	305.882	316.313	332.264	527.194	488.139	559.837	478.06	0.928127947	0.966018836	16	20	24	0.498108999	0.807927978	24	19.97259048	0.101230918
ENSMUSG00000035890	Rnf126	ring finger protein 126 [Source:MGI Symbol;Acc:MGI:1917544]	7.97821	6.74299	7.62623	7.94499	7.77239	6.68367	7.82087	8.74521	9.2298	11.6155	12.4286	12.5132	12.4626	11.6978	0.79256873	0.966018836	4	8	24	0.69149724	0.897724829	24	13.87905362	0.050541439
ENSMUSG00000035891	Cerk	ceramide kinase [Source:MGI Symbol;Acc:MGI:2386052]	28.461	21.2933	21.8423	24.3415	22.6349	26.5591	25.5771	23.1816	19.089	29.4512	22.8888	23.0735	23.5867	23.6931	0.277087842	0.789849983	4	8	24	0.366378093	0.790213221	24	19.65647232	0.090968669
ENSMUSG00000035898	Uba6	ubiquitin-like modifier activating enzyme 6 [Source:MGI Symbol;Acc:MGI:1913894]	18.3161	16.233	14.2527	14.5978	17.0245	14.6882	16.4792	15.0041	14.5844	11.5102	18.5589	15.3009	16.6001	15.4843	0.010754404	0.199214173	20	20	24	0.09778874	0.71175955	24	21.88670636	0.131548162
ENSMUSG00000035900	Gramd4	GRAM domain containing 4 [Source:MGI Symbol;Acc:MGI:2676308]	12.3636	10.9739	9.99592	10.7694	10.5331	11.1693	11.2568	11.7183	9.74303	13.1162	10.2796	12.2092	12.572	11.4686	0.060394272	0.407012001	4	8	24	0.3066432	0.768040178	24	22.72140615	0.068720297
ENSMUSG00000035901	Dennd5a	DENN/MADD domain containing 5A [Source:MGI Symbol;Acc:MGI:1201681]	37.3478	38.41	41.5015	38.5646	38.0581	40.6347	45.278	38.844	38.1001	39.5069	37.4266	39.1557	36.1272	37.4243	0.910436409	0.966018836	16	12	24	0.95366606	0.988601783	24	22.43357192	0.009774617
ENSMUSG00000035910	Dcdc2a	doublecortin domain containing 2a [Source:MGI Symbol;Acc:MGI:2652818]	1.49811	1.27068	1.90231	0.985221	1.21754	0.913874	1.83879	1.59403	1.01656	0.414913	0.609141	0.947314	0.871411	1.77931	0.110739523	0.528348425	8	8	24	0.025521961	0.586541342	24	3.064934861	0.274314366
ENSMUSG00000035914	Cd276	CD276 antigen [Source:MGI Symbol;Acc:MGI:2183926]	1.62313	1.93955	1.86638	1.5413	1.94309	1.8106	1.77656	2.02456	2.2427	2.455	1.53782	2.03445	2.12825	1.83857	0.912686312	0.966018836	12	8	24	0.493352125	0.805944647	24	7.537697994	0.058289611
ENSMUSG00000035919	Bbs9	Bardet-Biedl syndrome 9 (human) [Source:MGI Symbol;Acc:MGI:2442833]	8.14717	7.8999	7.2837	7.54223	7.62699	7.85112	8.83054	7.37723	7.41216	7.8617	8.30246	7.59854	8.82611	8.89857	0.037085178	0.334054683	4	8	24	0.105813445	0.717832234	24	23.51513677	0.063295651
ENSMUSG00000035923	Myf6	myogenic factor 6 [Source:MGI Symbol;Acc:MGI:97253]	1.08219	1.50427	1.88358	2.15646	2.0299	2.60696	1.62971	3.62311	2.86065	2.76414	2.48489	1.40051	2.18363	1.20785	0.658942124	0.966018836	16	12	24	0.278311711	0.768040178	24	11.52527708	0.203623739
ENSMUSG00000035929	H2-Q4	histocompatibility 2, Q region locus 4 [Source:MGI Symbol;Acc:MGI:95933]	5.84656	8.26238	8.78464	6.4614	7.54035	7.12664	6.8807	10.1706	9.21986	8.89309	7.76299	7.74145	8.00103	9.18517	0.059717207	0.406067671	8	20	24	0.012895289	0.527834117	24	7.442532325	0.180310952
ENSMUSG00000035933	Cog5	component of oligomeric golgi complex 5 [Source:MGI Symbol;Acc:MGI:2145130]	23.5806	24.4689	21.3241	24.1347	24.3889	21.5069	22.7972	21.6772	22.6311	19.6054	23.6284	22.6699	24.9441	23.2259	0.759899911	0.966018836	4	8	24	0.533372202	0.825813837	24	22.39349661	0.04143785
ENSMUSG00000035934	Pknox2	Pbx/knotted 1 homeobox 2 [Source:MGI Symbol;Acc:MGI:2445415]	16.7246	14.3534	13.9939	14.9121	15.1812	16.7187	14.3936	15.7825	12.1667	18.4	13.7759	16.1162	14.6875	14.6108	0.080164531	0.458678242	24	12	24	0.380433702	0.799546792	24	19.14125788	0.077490382
ENSMUSG00000035936	Aldh5a1	aldehyde dehydrogenase family 5, subfamily A1 [Source:MGI Symbol;Acc:MGI:2441982]	4.54769	5.3149	6.74745	5.78939	4.17386	5.72028	5.93272	4.9349	5.24913	5.68648	4.14695	5.36752	4.62058	6.05735	0.338172159	0.85758369	12	8	24	0.281305508	0.768040178	24	6.883560374	0.111164907
ENSMUSG00000035941	Ibtk	inhibitor of Bruton agammaglobulinemia tyrosine kinase [Source:MGI Symbol;Acc:MGI:1918677]	5.9834	6.31362	5.89844	6.85101	6.13197	6.99653	6.29723	7.10836	6.77479	8.00386	6.52579	6.7067	5.78303	6.70174	0.117050591	0.538362324	16	12	24	0.264119663	0.768040178	24	11.71938787	0.065640084
ENSMUSG00000035944	Ttc38	tetratricopeptide repeat domain 38 [Source:MGI Symbol;Acc:MGI:2146198]	4.57746	4.63044	5.37004	5.11961	4.14992	5.16977	4.14741	5.051	4.35566	5.86361	4.21288	5.05791	4.9702	5.79812	0.581070934	0.966018836	16	12	24	0.438800655	0.805393068	24	7.786384053	0.070493373
ENSMUSG00000035948	Acss3	acyl-CoA synthetase short-chain family member 3 [Source:MGI Symbol;Acc:MGI:2685720]	1.43676	2.09099	2.09335	4.39002	1.88911	1.81559	2.31319	2.06054	2.95289	2.07406	3.13436	2.48229	2.51679	2.41635	0.894563866	0.966018836	16	12	24	0.16796333	0.767020009	24	11.48784523	0.190636646
ENSMUSG00000035949	Fbxw2	F-box and WD-40 domain protein 2 [Source:MGI Symbol;Acc:MGI:1353435]	39.2966	40.3732	40.3256	37.8599	38.9726	38.6583	38.546	39.2212	37.8591	39.7578	39.109	40.1337	40.3639	41.5718	0.397301892	0.927993503	8	8	24	0.298085004	0.768040178	24	3.045020369	0.021077411
ENSMUSG00000035953	Pip4p1	phosphatidylinositol-4,5-bisphosphate 4-phosphatase 1 [Source:MGI Symbol;Acc:MGI:2448501]	53.7425	48.8138	44.9269	53.2098	49.9086	51.8071	52.8114	50.7979	44.6214	56.7832	46.3569	51.5076	54.2007	51.6635	0.062631097	0.410322149	4	8	24	0.43714563	0.805393068	24	21.15254121	0.052638136
ENSMUSG00000035954	Dock4	dedicator of cytokinesis 4 [Source:MGI Symbol;Acc:MGI:1918006]	6.48986	5.28151	5.44877	5.3767	5.12855	5.80617	5.20012	5.17696	4.5626	5.49926	5.24844	5.49677	5.09238	5.67344	0.858514388	0.966018836	24	12	24	0.289229617	0.768040178	24	21.47049438	0.057684866
ENSMUSG00000035958	Tdp2	tyrosyl-DNA phosphodiesterase 2 [Source:MGI Symbol;Acc:MGI:1860486]	14.8699	15.5033	13.9277	16.3247	14.3468	14.7788	14.8584	14.3284	15.2835	14.1842	15.3446	14.3519	15.4848	15.1743	0.885059765	0.966018836	8	4	24	0.974415969	0.993545331	24	5.842020893	0.005614506
ENSMUSG00000035960	Apex1	apurinic/apyrimidinic endonuclease 1 [Source:MGI Symbol;Acc:MGI:88042]	26.7318	27.3288	29.4419	26.9227	28.4862	26.7232	24.8041	26.751	26.9333	23.6431	29.1503	24.7878	28.1974	28.8144	0.390686537	0.915479136	12	12	24	0.848630997	0.954502769	24	7.372151384	0.021823701
ENSMUSG00000035963	Odf3l2	outer dense fiber of sperm tails 3-like 2 [Source:MGI Symbol;Acc:MGI:2686003]	0.219443	0.52077	0.238713	1.30332	0.0548772	0.915652	0.49122	1.18067	1.1501	1.18383	0.453458	0.488872	1.26518	0.59879	0.369262716	0.893596186	16	4	24	0.479836971	0.805393068	24	7.928569998	0.173403147
ENSMUSG00000035964	Tmem59l	transmembrane protein 59-like [Source:MGI Symbol;Acc:MGI:1915187]	55.9796	49.2578	53.0513	50.5482	45.9868	56.152	53.2842	55.0647	45.1319	66.9943	48.4593	56.6563	52.1695	57.514	0.888991156	0.966018836	24	20	24	0.913261208	0.97565709	24	0.497388848	0.024263876
ENSMUSG00000035967	Ints6l	integrator complex subunit 6 like [Source:MGI Symbol;Acc:MGI:2442593]	18.9562	23.3734	23.8651	23.5197	22.8578	20.9703	22.625	19.7949	26.0515	19.6141	21.1633	21.1081	22.094	19.8338	0.210566453	0.69936851	12	12	24	0.401556521	0.799546792	24	9.911486177	0.066565205
ENSMUSG00000035969	Rusc2	RUN and SH3 domain containing 2 [Source:MGI Symbol;Acc:MGI:2140371]	21.3539	16.4794	18.4201	19.0746	19.8956	18.829	18.3796	19.9051	17.9325	25.3071	16.3372	20.8175	20.4562	19.6069	0.956178643	0.96629412	24	12	24	0.920203613	0.978790242	24	15.85316766	0.024572416
ENSMUSG00000035983	Gm7008	predicted gene 7008 [Source:MGI Symbol;Acc:MGI:3647211]	0.777259	0.555044	1.01841	0.948405	0.519882	0.489045	0.944263	1.63647	1.56262	0.91663	0.898103	1.48648	1.07391	0.785998	0.668329321	0.966018836	12	8	24	0.390432328	0.799546792	24	6.72574984	0.134145706
ENSMUSG00000035984	Nme5	NME/NM23 family member 5 [Source:MGI Symbol;Acc:MGI:1922783]	3.89774	5.53093	5.2148	6.73678	5.14086	4.5641	6.30307	5.34508	4.94432	4.59968	3.94725	5.50121	4.86698	6.31984	0.628286596	0.966018836	8	12	24	0.481044907	0.805393068	24	6.292953557	0.096917593
ENSMUSG00000035992	Fnip1	folliculin interacting protein 1 [Source:MGI Symbol;Acc:MGI:2444668]	5.61915	6.86534	7.02383	7.14126	6.354	5.73663	6.26442	6.43716	7.3873	5.76653	6.43554	5.98607	5.82389	6.33154	0.004194713	0.140598674	12	12	24	0.017632074	0.5524966	24	8.890290061	0.107874023
ENSMUSG00000036002	Fam214b	family with sequence similarity 214, member B [Source:MGI Symbol;Acc:MGI:2441854]	16.9042	14.903	16.6186	14.57	15.3689	14.7493	15.7446	16.7668	13.4127	17.3328	15.3038	16.383	14.3997	15.0982	0.740423399	0.966018836	4	8	24	0.943297859	0.98607097	24	20.55674566	0.014190314
ENSMUSG00000036006	Ripor2	RHO family interacting cell polarization regulator 2 [Source:MGI Symbol;Acc:MGI:2444879]	1.79916	2.55668	2.62662	2.23549	2.44099	2.08527	2.97451	2.54539	2.75063	1.82239	2.17383	2.27015	2.4373	2.32631	0.248056532	0.746745468	12	4	24	0.326174395	0.768040178	24	4.848143603	0.084703232
ENSMUSG00000036009	Mettl25	methyltransferase like 25 [Source:MGI Symbol;Acc:MGI:3041259]	4.01257	4.26809	3.9969	4.33536	3.88891	3.81028	3.46805	4.58089	4.21848	4.37603	3.85397	4.18904	5.23488	4.459	0.219464938	0.708495385	8	16	24	0.28158222	0.768040178	24	5.845147603	0.066029895
ENSMUSG00000036019	Tmtc2	transmembrane and tetratricopeptide repeat containing 2 [Source:MGI Symbol;Acc:MGI:1914057]	8.78997	8.39747	9.89733	9.32597	9.34216	9.00807	8.21914	8.5778	8.24678	9.74456	7.90156	8.48072	8.64378	9.43212	0.851656735	0.966018836	16	12	24	0.338638353	0.773156803	24	10.65240829	0.051115314
ENSMUSG00000036022	Fam122b	family with sequence similarity 122, member B [Source:MGI Symbol;Acc:MGI:1926005]	7.49155	6.55278	5.57142	5.99425	5.70379	6.11294	6.00949	6.47868	6.232	6.38647	6.52872	6.39125	6.88842	6.45977	0.141084603	0.585944911	8	4	24	0.165568146	0.767020009	24	0.249270095	0.066076866
ENSMUSG00000036023	Parp2	poly (ADP-ribose) polymerase family, member 2 [Source:MGI Symbol;Acc:MGI:1341112]	14.4211	12.1205	12.2728	11.6645	13.5747	12.1138	9.1125	16.5999	14.122	11.6004	13.7698	12.8984	11.7927	12.6514	0.915082018	0.966018836	8	8	24	0.883386771	0.966818623	24	8.330370609	0.038765622
ENSMUSG00000036026	Tmem63b	transmembrane protein 63b [Source:MGI Symbol;Acc:MGI:2387609]	51.0018	39.8907	40.7956	42.4944	39.5667	44.4599	46.1791	43.6836	38.0378	56.967	41.3467	42.9759	42.9352	45.2122	0.286642595	0.793714531	4	4	24	0.900957413	0.972105389	24	21.12979608	0.028713693
ENSMUSG00000036036	Zfp57	zinc finger protein 57 [Source:MGI Symbol;Acc:MGI:99204]	3.58404	3.93832	3.6921	3.82358	4.23324	2.88873	3.81251	3.58944	5.01643	3.44921	3.85256	3.96124	3.63168	3.58476	0.944053021	0.966018836	20	8	24	0.422522391	0.804917634	24	9.414672333	0.073430939
ENSMUSG00000036040	Adamtsl2	ADAMTS-like 2 [Source:MGI Symbol;Acc:MGI:1925044]	4.24866	4.11342	3.27688	3.14632	3.5669	2.97666	5.12195	2.91977	2.65711	3.88209	3.95709	3.21399	2.79517	2.98399	0.928127947	0.966018836	20	16	24	0.529545198	0.824494657	24	20.52493056	0.091965853
ENSMUSG00000036046	5031439G07Rik	RIKEN cDNA 5031439G07 gene [Source:MGI Symbol;Acc:MGI:2444899]	17.216	17.105	20.5108	17.0113	18.5025	18.2651	19.6574	17.0339	19.0102	19.3882	18.1559	17.575	16.2649	18.3278	0.648082424	0.966018836	12	20	24	0.388474239	0.799546792	24	10.98410092	0.048831383
ENSMUSG00000036052	Dnajb5	DnaJ heat shock protein family (Hsp40) member B5 [Source:MGI Symbol;Acc:MGI:1930018]	7.0463	7.66145	9.0346	9.44107	7.4767	9.11338	8.21198	9.81048	8.58009	10.1656	7.41294	8.04093	7.46086	7.01033	0.285193697	0.793714531	16	12	24	0.211444519	0.768040178	24	10.7441634	0.108645021
ENSMUSG00000036053	Fmnl2	formin-like 2 [Source:MGI Symbol;Acc:MGI:1918659]	25.821	24.785	22.596	24.2249	26.1031	22.289	25.1866	23.0405	23.5646	23.5445	25.3595	23.6111	24.3861	22.1349	0.302438464	0.811201456	20	16	24	0.285588814	0.768040178	24	18.81677827	0.044592473
ENSMUSG00000036054	Sugp2	SURP and G patch domain containing 2 [Source:MGI Symbol;Acc:MGI:2678085]	19.6362	17.47	16.9632	18.2781	17.7247	20.2145	20.0643	17.2745	17.1673	19.4284	18.1282	18.6148	17.9528	17.4586	0.004319982	0.140598674	12	4	24	0.041494794	0.618736234	24	19.81359743	0.076117242
ENSMUSG00000036057	Ptpn23	protein tyrosine phosphatase, non-receptor type 23 [Source:MGI Symbol;Acc:MGI:2144837]	10.9321	10.9607	11.3997	12.3434	14.2432	12.0909	14.2082	13.3531	11.429	12.101	11.8005	11.2357	11.7499	12.9858	0.906657897	0.966018836	16	20	24	0.942012577	0.985597432	24	17.38294389	0.016696012
ENSMUSG00000036061	Smug1	single-strand selective monofunctional uracil DNA glycosylase [Source:MGI Symbol;Acc:MGI:1918976]	7.65011	6.56175	7.41957	6.85318	7.61029	7.80969	6.54386	7.35481	7.21506	6.83562	7.527	7.21972	7.73234	8.05461	0.891105762	0.966018836	24	16	24	0.738675525	0.914600529	24	21.20881864	0.025351529
ENSMUSG00000036062	Phf24	PHD finger protein 24 [Source:MGI Symbol;Acc:MGI:2140712]	371.822	272.514	265.338	295.929	277.972	334.521	280.983	306.771	221.035	367.383	270.496	317.948	322.992	305.213	0.071575474	0.440262583	24	12	24	0.299596547	0.768040178	24	20.90218894	0.123769713
ENSMUSG00000036067	Slc2a6	solute carrier family 2 (facilitated glucose transporter), member 6 [Source:MGI Symbol;Acc:MGI:2443286]	17.0541	13.7315	13.2919	13.5197	15.3066	17.3116	13.1638	14.6107	10.8762	14.8285	13.9639	13.6138	13.9911	13.4673	0.1982019	0.680119125	24	4	24	0.053098544	0.650879632	24	19.97762612	0.133150367
ENSMUSG00000036073	Galt	galactose-1-phosphate uridyl transferase [Source:MGI Symbol;Acc:MGI:95638]	16.3885	14.8495	15.1743	12.1627	13.6625	15.2165	12.0345	15.1659	15.1725	14.8044	12.4953	14.0825	14.3706	14.692	0.338172159	0.85758369	12	8	24	0.313173188	0.768040178	24	3.753307874	0.072732346

ENSMUSG00000036078	Sigmar1	sigma non-opioid intracellular receptor 1 [Source:MGI Symbol;Acc:MGI:1195268]	41.1373	28.8395	30.8797	30.9569	30.885	37.7408	30.9704	31.6503	24.5637	36.9349	31.1055	31.3613	31.4897	31.948	0.195343269	0.673387182	4	8	24	0.217054012	0.768040178	24	20.23109943	0.118035413
ENSMUSG00000036086	Zranb3	zinc finger, RAN-binding domain containing 3 [Source:MGI Symbol;Acc:MGI:1918362]	2.26914	2.29455	2.03306	2.4078	2.34801	2.435	1.98862	2.29007	2.41406	2.14297	2.26479	2.24774	2.38107	2.3241	0.746444581	0.966018836	24	4	24	0.896404929	0.970591958	24	16.71496011	0.011649355
ENSMUSG00000036087	Slain2	SLAIN motif family, member 2 [Source:MGI Symbol;Acc:MGI:1923241]	15.0684	15.1205	13.9169	15.8298	14.9751	14.2928	15.4128	15.1519	15.0532	15.2891	15.3254	14.7762	14.7384	13.961	0.95247375	0.966018836	16	20	24	0.587516998	0.853591039	24	14.95851094	0.020027865
ENSMUSG00000036091	Hyal3	hyaluronoglucosaminidase 3 [Source:MGI Symbol;Acc:MGI:1330288]	0.716907	0.815311	0.80803	0.876321	1.00098	0.832746	0.580884	0.853881	0.601794	0.969672	0.803664	0.891011	1.27945	0.793114	0.559021844	0.966018836	16	20	24	0.671210798	0.889066748	24	17.008738	0.045551885
ENSMUSG00000036093	Arl5a	ADP-ribosylation factor-like 5A [Source:MGI Symbol;Acc:MGI:1922673]	26.9048	25.8384	25.3561	25.3318	26.3051	24.8923	25.0991	23.0506	25.4367	23.3208	26.7915	23.4088	23.3467	24.583	0.927004797	0.966018836	20	12	24	0.618758546	0.86909184	24	16.34753261	0.023284649
ENSMUSG00000036095	Dgkb	diacylglycerol kinase, beta [Source:MGI Symbol;Acc:MGI:2442474]	3.81726	4.33939	4.89907	3.77181	3.8945	4.52902	4.56288	3.50355	4.29908	3.91731	4.21758	4.98074	3.94745	6.00014	0.959856266	0.967043964	24	16	24	0.709764396	0.903951515	24	3.26132642	0.051086749
ENSMUSG00000036097	Slf2	SMC5-SMC6 complex localization factor 2 [Source:MGI Symbol;Acc:MGI:1924968]	9.36893	9.2604	9.223	9.44882	9.14172	8.70969	9.21046	7.67496	9.20967	8.06041	9.45546	8.35821	8.09526	8.87013	0.90703203	0.966018836	20	4	24	0.728737611	0.911275869	24	14.15011033	0.024063211
ENSMUSG00000036098	Myrf	myelin regulatory factor [Source:MGI Symbol;Acc:MGI:2684944]	0.963398	1.74938	2.98357	1.42256	1.04957	1.92342	1.78439	1.4768	1.86919	1.57203	0.756603	1.66155	0.763097	2.03163	0.173410058	0.648600527	12	8	24	0.204887688	0.768040178	24	7.005819487	0.227249906
ENSMUSG00000036099	Vezt	vezatin, adherens junctions transmembrane protein [Source:MGI Symbol;Acc:MGI:2143698]	25.0126	26.9366	24.8192	25.7621	25.9821	25.468	25.2961	23.8859	28.1112	24.5119	25.5783	25.5958	25.3897	25.0695	0.718742371	0.966018836	20	12	24	0.865260756	0.960982718	24	11.06425266	0.012071973
ENSMUSG00000036103	Colec12	collectin sub-family member 12 [Source:MGI Symbol;Acc:MGI:2152907]	4.27722	6.44995	5.83078	4.73547	4.8739	4.57824	4.97983	6.12346	8.51207	3.61676	5.00361	5.45531	5.05563	4.88778	0.116913252	0.538362324	12	4	24	0.197109509	0.768040178	24	6.038042754	0.175097066
ENSMUSG00000036104	Rab3gap1	RAB3 GTPase activating protein subunit 1 [Source:MGI Symbol;Acc:MGI:2445001]	18.4763	18.4448	19.345	17.5011	18.368	19.713	18.353	18.292	17.7902	18.2523	17.7349	18.0834	17.7788	18.4799	0.767183862	0.966018836	8	8	24	0.66715522	0.887459929	24	23.50290512	0.015030932
ENSMUSG00000036106	Prr5	proline rich 5 (renal) [Source:MGI Symbol;Acc:MGI:1924714]	10.0095	7.82813	6.48486	8.71234	8.65464	8.55363	8.09062	9.19482	8.06833	9.42178	8.6268	8.25453	8.8337	8.03048	0.369262716	0.893596186	16	20	24	0.436601598	0.805393068	24	19.5358059	0.068812139
ENSMUSG00000036111	Lmo1	LIM domain only 1 [Source:MGI Symbol;Acc:MGI:102812]	12.1232	9.69665	9.38539	9.26469	12.9053	9.90945	13.3581	11.1232	10.7723	12.3161	16.8597	13.4237	13.8342	12.0327	0.075079679	0.451256373	20	16	24	0.100593041	0.711862888	24	19.31501903	0.153249909
ENSMUSG00000036112	Metap2	methionine aminopeptidase 2 [Source:MGI Symbol;Acc:MGI:1929701]	67.4358	67.9033	64.6576	68.2022	68.2414	66.3907	66.5266	62.9076	69.7603	62.9019	68.7931	68.0311	71.4337	71.6695	0.789670753	0.966018836	20	20	24	0.761819986	0.922863094	24	23.09786867	0.01601627
ENSMUSG00000036114	Rpp25l	ribonuclease P/MRP 25 subunit-like [Source:MGI Symbol;Acc:MGI:1917211]	12.6861	11.0805	12.482	11.5708	11.7543	12.3625	11.9	12.4259	12.4586	11.7202	12.5371	12.3863	12.5908	11.6027	0.581070934	0.966018836	4	12	24	0.621323594	0.870720844	24	21.03202431	0.021984518
ENSMUSG00000036120	Rfxank	regulatory factor X-associated ankyrin-containing protein [Source:MGI Symbol;Acc:MGI:1333865]	6.78171	6.26813	5.78838	6.4028	7.29043	6.29948	8.15041	6.36906	6.67309	5.99924	6.49957	5.84571	6.89364	6.77437	0.210566453	0.69936851	4	12	24	0.407061558	0.799546792	24	22.89255226	0.060423912
ENSMUSG00000036123	Slc9a3	solute carrier family 9 (sodium/hydrogen exchanger), member 3 [Source:MGI Symbol;Acc:MGI:105064]	1.25465	1.22662	1.42562	2.71367	1.57746	1.33483	1.41921	1.20433	2.08777	2.05479	1.06593	1.74867	1.96186	1.16206	0.087806047	0.472021905	16	16	24	0.228794461	0.768040178	24	12.15294461	0.16093853
ENSMUSG00000036131	Frmd7	FERM domain containing 7 [Source:MGI Symbol;Acc:MGI:2686379]	1.95087	2.08927	1.81346	2.1554	1.8962	1.98337	2.16081	2.13619	1.88462	1.94605	2.09036	2.02609	2.46974	2.03221	0.182849367	0.659303873	4	8	24	0.266922274	0.768040178	24	23.72955012	0.04373949
ENSMUSG00000036138	Acaa1a	acetyl-Coenzyme A acyltransferase 1A [Source:MGI Symbol;Acc:MGI:2148491]	25.7495	26.9921	26.6297	27.3942	32.237	26.6626	29.7417	27.2605	28.8998	29.7496	29.3087	29.828	30.1994	28.2248	0.607427393	0.966018836	20	12	24	0.207544439	0.768040178	24	16.00761172	0.049756557
ENSMUSG00000036139	Hoxc9	homeobox C9 [Source:MGI Symbol;Acc:MGI:96199]	0.877502	1.07567	1.09146	1.09468	0.984198	0.991922	1.00697	1.22318	1.38538	1.31278	1.31095	1.33013	1.18902	1.14008	0.651661131	0.966018836	12	16	24	0.048435964	0.638992719	24	10.25816732	0.068021122
ENSMUSG00000036144	Meox2	mesenchyme homeobox 2 [Source:MGI Symbol;Acc:MGI:103219]	3.5144	2.6788	3.62871	3.34652	3.92111	2.87121	3.45876	3.51476	4.15796	2.9518	3.72142	3.2837	2.93174	3.04193	0.417963425	0.963884303	20	4	24	0.502610331	0.809706321	24	12.41002897	0.065019292
ENSMUSG00000036151	Tm6sf2	transmembrane 6 superfamily member 2 [Source:MGI Symbol;Acc:MGI:1933210]	1.10148	0.456654	0.814353	0.783818	1.45149	0.545472	1.02326	0.495672	0.542309	0.789213	0.974808	0.473638	0.619536	0.657808	0.305564675	0.811201458	20	12	24	0.243436901	0.768040178	24	16.53030093	0.138924993
ENSMUSG00000036155	Mgat5	mannoside acetylglucosaminyltransferase 5 [Source:MGI Symbol;Acc:MGI:894701]	5.65756	4.8354	5.16324	4.90208	4.87399	5.42244	5.51047	5.46203	4.38154	6.26725	4.61864	5.11126	5.13213	5.72922	0.591351901	0.966018836	4	16	24	0.697148882	0.899338675	24	0.859880897	0.038456432
ENSMUSG00000036158	Prickle1	prickle planar cell polarity protein 1 [Source:MGI Symbol;Acc:MGI:1916034]	3.57053	3.48322	3.93068	2.76086	3.35308	3.95674	4.31701	3.5319	3.46212	3.69401	2.77743	3.55569	3.20578	3.53854	0.718086778	0.966018836	24	20	24	0.273133028	0.768040178	24	1.372113517	0.082728744
ENSMUSG00000036160	Surf6	surfeit gene 6 [Source:MGI Symbol;Acc:MGI:98447]	5.97143	6.41312	6.23915	5.97572	6.8752	6.22823	6.71375	6.45956	5.86015	7.109	6.23155	6.76096	6.22789	5.94198	0.745118544	0.966018836	20	16	24	0.433175651	0.805393068	24	17.10684592	0.038308033
ENSMUSG00000036167	Pphln1	periphilin 1 [Source:MGI Symbol;Acc:MGI:1917029]	18.0179	15.179	16.2073	14.1519	15.0494	16.318	15.9687	17.9989	14.7042	17.8277	17.446	16.9145	17.7377	17.2893	0.619261897	0.966018836	4	8	24	0.326914932	0.768040178	24	23.67179689	0.05861581
ENSMUSG00000036169	Sostdc1	sclerostin domain containing 1 [Source:MGI Symbol;Acc:MGI:1913292]	22.5314	21.597	21.3717	18.3668	22.9819	22.3056	24.748	22.0809	17.7465	22.6132	18.9086	19.219	19.6888	19.6526	0.643325306	0.966018836	4	8	24	0.328037936	0.768040178	24	22.43872251	0.073211417
ENSMUSG00000036180	Gatad2a	GATA zinc finger domain containing 2A [Source:MGI Symbol;Acc:MGI:2384585]	9.20645	7.99974	7.94876	7.97074	8.77499	8.6905	9.14276	9.16416	7.66932	10.7883	8.32185	8.85767	8.54131	8.84014	0.182849367	0.659303873	4	8	24	0.747158489	0.917720337	24	19.70743614	0.034944669
ENSMUSG00000036181	H1f2	H1.2 linker histone, cluster member [Source:MGI Symbol;Acc:MGI:1931526]	38.6793	38.3312	38.3179	35.3476	33.1778	37.4709	34.5627	46.9705	38.8625	40.0369	41.0563	42.7775	45.7597	40.643	0.879901313	0.966018836	8	8	24	0.25314604	0.768040178	24	3.244569485	0.072448457
ENSMUSG00000036185	Sapcd1	suppressor APC domain containing 1 [Source:MGI Symbol;Acc:MGI:2388100]	2.09118	2.17892	1.54846	1.39481	1.42978	2.146	1.57512	2.33466	2.40313	1.8511	1.7869	1.6336	1.62012	1.70188	0.722296281	0.966018836	8	8	24	0.355225266	0.782111212	24	3.814568629	0.093957586
ENSMUSG00000036186	Dipk1b	divergent protein kinase domain 1B [Source:MGI Symbol;Acc:MGI:1927576]	24.0922	19.5209	19.364	18.7591	19.8404	22.2258	21.5654	20.9921	17.5518	20.2325	20.0532	20.0338	18.9036	21.3012	0.162786057	0.624917386	4	8	24	0.051965252	0.649509116	24	22.25647081	0.095418654
ENSMUSG00000036188	Ankmy2	ankyrin repeat and MYND domain containing 2 [Source:MGI Symbol;Acc:MGI:2144755]	12.5072	13.6021	12.6291	12.393	12.6031	13.9997	12.9185	12.4179	12.7655	12.6012	12.7277	13.7563	12.931	13.8035	0.117335378	0.538362324	24	16	24	0.287500417	0.768040178	24	22.88084232	0.033924333
ENSMUSG00000036196	Slc26a8	solute carrier family 26, member 8 [Source:MGI Symbol;Acc:MGI:2385046]	0.73011	0.840397	1.09347	0.93593	0.477194	1.17485	0.555936	1.02257	1.11234	0.777087	0.893207	0.796083	1.01927	1.30561	0.27817759	0.789849983	12	8	24	0.18337239	0.768040178	24	6.368715924	0.12391735
ENSMUSG00000036197	Gxylt1	glucoside xylosyltransferase 1 [Source:MGI Symbol;Acc:MGI:2684933]	3.62869	4.11874	4.04554	4.64847	4.12078	3.73262	4.11573	3.77621	4.71571	3.78082	4.32227	4.01635	4.34656	3.96001	0.597596661	0.966018836	12	12	24	0.30719781	0.768040178	24	10.83304617	0.054559475
ENSMUSG00000036198	Arhgap36	Rho GTPase activating protein 36 [Source:MGI Symbol;Acc:MGI:1922654]	6.67409	5.32882	6.34897	6.23896	5.06998	7.25775	6.08662	6.41028	4.55174	8.27762	5.60281	6.69291	5.38117	6.17873	0.979633649	0.979802639	24	12	24	0.811269022	0.939484534	24	18.02334536	0.051135974
ENSMUSG00000036199	Ndufa13	NADH:ubiquinone oxidoreductase subunit A13 [Source:MGI Symbol;Acc:MGI:1914434]	87.3882	101.993	96.3714	98.1506	93.6639	88.0343	94.8102	95.8612	105.916	81.2892	95.9184	88.4946	99.4286	89.594	0.148073804	0.590732968	12	12	24	0.388994437	0.799546792	24	6.384627978	0.055207189
ENSMUSG00000036202	Rif1	replication timing regulatory factor 1 [Source:MGI Symbol;Acc:MGI:1098622]	4.90375	5.61021	5.63882	5.66098	5.73962	4.9066	5.24958	4.87003	6.14114	4.3027	6.08766	4.94913	4.49952	5.18727	0.217933169	0.704989686	20	8	24	0.391186597	0.799546792	24	11.27800871	0.06807157
ENSMUSG00000036206	Sh3bp4	SH3-domain binding protein 4 [Source:MGI Symbol;Acc:MGI:2138297]	4.63264	3.785	3.84031	4.02844	3.95328	4.26104	4.01442	4.71329	3.40383	4.91832	3.76404	4.15111	3.94681	4.09066	0.920620634	0.966018836	24	12	24	0.811668839	0.93956283	24	21.42356497	0.030848345
ENSMUSG00000036208	Nepro	nucleolus and neural progenitor protein [Source:MGI Symbol;Acc:MGI:2384836]	3.78264	3.3179	3.33534	4.0079	3.92431	3.96207	2.85545	3.56195	3.18603	3.37941	3.96638	3.92987	3.27626	3.66006	0.070873295	0.440262583	20	16	24	0.075915065	0.686372559	24	16.65168344	0.097268
ENSMUSG00000036214	Polr1has	RNA polymerase I subunit H, antisense [Source:MGI Symbol;Acc:MGI:1923666]	5.73861	6.93397	6.79517	5.8104	6.69753	5.74053	7.47075	6.86646	7.40722	6.27349	6.20825	6.63439	8.18674	6.39087	0.483047948	0.966018836	4	20	24	0.243594999	0.768040178	24	3.780173481	0.079623587
ENSMUSG00000036225	Kctd1	potassium channel tetramerisation domain containing 1 [Source:MGI Symbol;Acc:MGI:1918269]	7.6024	5.52176	6.40727	6.72942	6.15028	7.12904	7.32844	5.71651	5.34253	7.70643	6.94584	6.74687	5.41122	6.48589	0.313786068	0.824470524	4	4	24	0.21267813	0.768040178	24	17.70099598	0.105948876
ENSMUSG00000036241	Ube2r2	ubiquitin-conjugating enzyme E2R 2 [Source:MGI Symbol;Acc:MGI:1914865]	39.3847	34.0204	36.3345	36.2068	38.3175	35.459	37.1165	37.3191	33.4886	41.5741	32.0319	35.2344	35.103	34.3536	0.649180783	0.966018836	16	20	24	0.931870135	0.982500133	24	16.09758784	0.019375716
ENSMUSG00000036242	Armh4	armadillo-like helical domain containing 4 [Source:MGI Symbol;Acc:MGI:1914669]	57.4825	53.5907	51.1503	51.0955	53.1791	54.8071	48.7817	51.4041	46.3707	48.7429	56.2876	55.4221	55.9669	54.4431	0.033341689	0.324908923	24	16	24	0.052338809	0.65045829	24	21.03886255	0.061155394
ENSMUSG00000036246	Gmip	Gem-interacting protein [Source:MGI Symbol;Acc:MGI:1926066]	7.03385	6.4698	5.94996	7.89534	8.16993	7.273	5.38981	6.61244	7.07228	6.13947	7.41646	6.27517	5.15395	6.50689	0.08333408	0.462922111	20	8	24	0.07401	0.683293901	24	14.44320334	0.129025285
ENSMUSG00000036249	Rbm43	RNA binding motif protein 43 [Source:MGI Symbol;Acc:MGI:1918934]	13.7042	11.7973	13.0323	10.9631	12.3181	14.0711	12.5387	11.6978	11.308	10.935	14.1548	12.4448	12.7133	11.4752	0.023283676	0.278156767	24	16	24	0.029154233	0.596648309	24	20.46013874	0.111268451
ENSMUSG00000036251	Trpm8	transient receptor potential cation channel, subfamily M, member 8 [Source:MGI Symbol;Acc:MGI:2181435]	8.09345	7.34873	6.72385	8.0816	7.47073	7.6905	6.82388	7.36089	6.32579	8.99288	7.08492	7.45841	8.24922	7.92212	0.608967886	0.966018836	16	20	24	0.783302935	0.930268241	24	18.06356389	0.033813621
ENSMUSG00000036256	Igfbp7	insulin-like growth factor binding protein 7 [Source:MGI Symbol;Acc:MGI:1352480]	44.4564	49.2895	46.4051	44.428	49.6219	46.468	49.2008	57.0911	54.337	42.364	56.0088	47.7971	50.2373	44.7759	0.891690964	0.966018836	20	20	24	0.923566391	0.979525912	24	3.050270322	0.020114832
ENSMUSG00000036257	Pnpla8	patatin-like phospholipase domain containing 8 [Source:MGI Symbol;Acc:MGI:1914702]	38.0647	39.1871	37.9961	43.7144	38.9445	36.2262	38.4184	36.5618	40.8642	37.0045	40.9721	36.7827	35.6531	38.4565	0.188118386	0.663314657	20	4	24	0.104487741	0.716673794	24	11.497869	0.059704035
ENSMUSG00000036270	Edc4	enhancer of mRNA decapping 4 [Source:MGI Symbol;Acc:MGI:2446249]	8.94261	9.30168	9.63291	9.31813	8.84971	9.85487	8.92377	9.58575	9.2282	10.42	8.98092	9.38266	9.60018	8.99493	0.943890799	0.966018836	16	4	24	0.62753337	0.872795898	24	10.56782495	0.023358483
ENSMUSG00000036273	Lrrk2	leucine-rich repeat kinase 2 [Source:MGI Symbol;Acc:MGI:1913975]	20.6715	18.0692	16.5342	17.6506	22.1484	19.1215	18.6151	19.9891	14.1693	16.2795	16.5486	16.0859	18.4177	18.0835	0.195950838	0.673646834	4	8	24	0.125488588	0.739416874	24	21.75163022	0.114803636
ENSMUSG00000036275	9530068E07Rik	RIKEN cDNA 9530068E07 gene [Source:MGI Symbol;Acc:MGI:2654705]	50.2618	46.0934	44.918	48.3081	47.2252	47.6445	49.1776	51.2559	44.9438	55.6508	45.3897	52.0988	48.96	49.3526	0.515451644	0.966018836	4	8	24	0.846708145	0.953679112	24	21.1972716	0.020009415
ENSMUSG00000036278	Macrod1	mono-ADP ribosylhydrolase 1 [Source:MGI Symbol;Acc:MGI:2147583]	6.22305	5.91211	6.60725	8.35189	6.84837	5.9996	5.6416	11.3936	9.0682	11.7171	8.28974	8.32609	10.6602	8.95891	0.72778087	0.966018836	16	12	24	0.121585159	0.733843269	24	9.307202097	0.190147071
ENSMUSG00000036281	Snapc4	small nuclear RNA activating complex, polypeptide 4 [Source:MGI Symbol;Acc:MGI:2443935]	11.7074	13.2125	13.9748	14.3985	12.707	11.4117	14.7213	11.0216	17.321	14.4401	11.851	13.2087	10.5729	12.6736	0.117086232	0.538362324	12	16	24	0.094165481	0.71175955	24	9.938911207	0.146886574
ENSMUSG00000036282	Naa30	N(alpha)-acetyltransferase 30, NatC catalytic subunit [Source:MGI Symbol;Acc:MGI:1922259]	11.7204	12.2789	12.8661	11.8813	11.9479	11.7948	11.6644	11.0467	10.8545	11.2981	11.9879	11.2617	11.1284	12.0019	0.915082018	0.966018836	20	8	24	0.893125405	0.96974576	24	12.25547067	0.010191586
ENSMUSG00000036285	Noa1	nitric oxide associated 1 [Source:MGI Symbol;Acc:MGI:1914306]	7.37008	4.80335	7.4286	6.44893	6.59453	6.92023	5.23286	6.48447	4.77632	7.41169	5.81297	6.13168	5.88545	7.10054	0.826055781	0.966018836	16	16	24	0.769747661	0.925855377	24	15.18204958	0.054226107
ENSMUSG00000036291	Ap5m1	adaptor-related protein complex 5, mu 1 subunit [Source:MGI Symbol;Acc:MGI:1921635]	10.1099	10.0589	9.8978	9.63364	9.74317	9.30268	9.36109	9.5764	9.99715	8.86025	10.859	9.77206	9.8878	9.8378	0.935194469	0.966018836	12	4	24	0.976326171	0.994103202	24	23.43109119	0.005510015
ENSMUSG00000036292	Gramd1c	GRAM domain containing 1C [Source:MGI Symbol;Acc:MGI:2443024]	2.52212	2.60406	1.95134	2.14971	2.05782	2.27952	2.15615	2.59557	2.35753	2.01166	2.60127	2.42338	2.17684	2.36343	0.126560612	0.558021618	8	8	24	0.343927428	0.777417177	24	0.614952365	0.055864022
ENSMUSG00000036295	Lrrn3	leucine rich repeat protein 3, neuronal [Source:MGI Symbol;Acc:MGI:106036]	14.961	13.6154	14.1453	15.2289	17.5864	14.2989	13.2516	11.4773	13.403	13.3586	17.4789	15.0317	13.4284	13.3577	0.004196064	0.140598674	20	12	24	0.003150532	0.414576087	24	16.44509227	0.165256626
ENSMUSG00000036298	Slc2a13	solute carrier family 2 (facilitated glucose transporter), member 13 [Source:MGI Symbol;Acc:MGI:2146030]	2.36612	2.46807	2.60074	2.53843	2.11099	2.31139	2.67909	1.97108	2.55712	2.31507	2.02141	2.32785	1.92553	2.81984	0.266025655	0.773510818	12	8	24	0.353809714	0.781118317	24	6.598807796	0.068159496
ENSMUSG00000036299	BC031181	cDNA sequence BC031181 [Source:MGI Symbol;Acc:MGI:3039614]	141.023	144.443	139.498	128.605	136.257	128.925	129.796	124.867	144.337	120.073	151.995	134.922	144.667	144.476	0.920620634	0.966018836	4	12	24	0.807493618	0.938693233	24	2.067068567	0.025172978
ENSMUSG00000036304	Zdhhc23	zinc finger, DHHC domain containing 23 [Source:MGI Symbol;Acc:MGI:2685625]	3.98102	3.69332	3.32041	4.10927	3.4173	3.8803	3.32219	3.72273	3.1465	4.653	3.14871	3.94038	4.10635	4.06335	0.961261938	0.967043964	16	20	24	0.979756627	0.995083382	24	22.60000361	0.01097024
ENSMUSG00000036305	Rpl39-ps	ribosomal protein L39, pseudogene [Source:MGI Symbol;Acc:MGI:3705781]	634.51	834.444	833.01	714.004	812.317	661.645	639.134	700.727	855.775	470.171	773.03	682.066	802.958	682.763	0.217827365	0.704989686	12	12	24	0.82691134	0.946041107	24	5.688501808	0.057896979
ENSMUSG00000036306	Lzts1	leucine zipper, putative tumor suppressor 1 [Source:MGI Symbol;Acc:MGI:2684762]	6.12519	5.02657	5.17814	5.19288	4.61905	5.75042	4.37721	5.77256	4.04969	6.1796	4.52441	5.1205	5.17281	5.24987	0.885059765	0.966018836	16	4	24	0.871851644	0.963715083	24	23.58441223	0.031708519
ENSMUSG00000036309	Skp1	S-phase kinase-associated protein 1 [Source:MGI Symbol;Acc:MGI:103575]	799.017	784.283	719.755	685.122	752.607	710.317	691.32	707.836	702.017	686.677	829.633	765.271	807.569	827.101	0.325142889	0.842977406	20	20	24	0.378144709	0.797706855	24	23.00670343	0.054530338
ENSMUSG00000036315	Polr1h	RNA polymerase I subunit H [Source:MGI Symbol;Acc:MGI:1913386]	15.8593	19.6611	18.202	16.4219	20.7283	17.7805	20.1023	20.0026	20.3927	13.8245	21.5237	17.5867	18.5547	17.8855	0.93717134	0.966018836	20	20	24	0.870325782	0.963313607	24	0.385590354	0.034971295
ENSMUSG00000036323	Srp72	signal recognition particle 72 [Source:MGI Symbol;Acc:MGI:1333795]	39.0911	38.6359	38.2885	38.4746	37.8928	38.0925	35.8346	36.5124	37.8201	37.6023	40.7817	39.045	41.1164	40.191	0.919571723	0.966018836	4	8	24	0.878048852	0.965978948	24	19.12635771	0.010757699
ENSMUSG00000036327	Qsox2	quiescin Q6 sulfhydryl oxidase 2 [Source:MGI Symbol;Acc:MGI:2387194]	7.59154	7.19896	7.33942	7.6066	7.77803	7.88046	7.83283	8.1559	6.88138	8.55214	7.51666	8.34624	7.87954	8.14959	0.390686537	0.915479136	24	12	24	0.503815967	0.810465603	24	19.64095499	0.031890787
ENSMUSG00000036330	Slc18a1	solute carrier family 18 (vesicular monoamine), member 1 [Source:MGI Symbol;Acc:MGI:106684]	0.764124	0.810745	0.627447	0.820119	0.589114	0.942378	0.901533	0.614783	0.486584	0.840992	0.713321	0.799468	0.685042	1.06908	0.953350544	0.966018836	24	12	24	0.527545754	0.823409555	24	22.37178254	0.057749631
ENSMUSG00000036333	Kidins220	kinase D-interacting substrate 220 [Source:MGI Symbol;Acc:MGI:1924730]	78.2112	67.2871	68.9363	66.0868	64.2364	71.534	70.537	65.929	57.9814	69.6061	66.6713	68.5464	67.2394	69.4772	0.29214565	0.798990791	4	8	24	0.099516597	0.71175955	24	22.14418843	0.073212895
ENSMUSG00000036334	Igsf10	immunoglobulin superfamily, member 10 [Source:MGI Symbol;Acc:MGI:1923481]	0.797004	0.93307	0.9726	0.645625	0.804995	0.845298	0.91248	1.02474	0.879715	0.79028	0.89893	0.854952	0.763939	0.945747	0.050258003	0.385784509	8	8	24	0.158114146	0.764630328	24	4.006047572	0.055323077
ENSMUSG00000036339	Tmem260	transmembrane protein 260 [Source:MGI Symbol;Acc:MGI:2443219]	7.00197	6.78078	6.4193	6.80143	6.62404	6.65283	7.17215	6.78337	6.39294	6.44247	6.55102	7.06953	6.55109	6.60975	0.031969981	0.324385159	4	8	24	0.029815541	0.596648309	24	22.2742667	0.042255315
ENSMUSG00000036352	Ubac1	ubiquitin associated domain containing 1 [Source:MGI Symbol;Acc:MGI:1920995]	43.5955	38.3228	39.3826	41.4994	41.3064	40.9256	35.2802	42.698	38.0637	40.4776	39.3359	37.6857	42.0136	40.1311	0.844511766	0.966018836	4	8	24	0.983899209	0.996654399	24	16.37848505	0.005784183
ENSMUSG00000036353	P2ry12	purinergic receptor P2Y, G-protein coupled 12 [Source:MGI Symbol;Acc:MGI:1918089]	3.51283	5.84074	6.79049	4.32291	4.51579	4.5057	4.97438	4.87995	6.06769	4.22053	4.53085	4.39706	3.86272	4.91394	0.130696419	0.562427886	12	4	24	0.060078726	0.658304792	24	7.624146448	0.186000932
ENSMUSG00000036356	Csgalnact1	chondroitin sulfate N-acetylgalactosaminyltransferase 1 [Source:MGI Symbol;Acc:MGI:2442354]	8.38717	8.39287	8.35844	8.57853	9.49492	7.91543	8.87245	7.74479	7.8361	7.91811	7.72792	7.99076	8.67432	7.69248	0.801995186	0.966018836	4	4	24	0.518113116	0.818566127	24	19.16081565	0.034028231
ENSMUSG00000036362	P2ry13	purinergic receptor P2Y, G-protein coupled 13 [Source:MGI Symbol;Acc:MGI:1921441]	0.318055	1.05668	0.87453	0.579324	0.680684	0.793557	0.450206	0.707196	1.13465	0.431163	0.450934	0.775636	0.488975	0.681327	0.243586913	0.741580974	12	16	24	0.346751727	0.778844143	24	7.430985851	0.120284178
ENSMUSG00000036368	Rmdn2	regulator of microtubule dynamics 2 [Source:MGI Symbol;Acc:MGI:2147043]	19.1172	19.1828	16.5147	18.3424	18.0918	16.0041	16.911	16.7236	17.4511	15.1537	15.2317	18.5328	18.2617	16.8076	0.7207001	0.966018836	4	12	24	0.303683462	0.768040178	24	0.482726546	0.058384894
ENSMUSG00000036371	Serbp1	serpine1 mRNA binding protein 1 [Source:MGI Symbol;Acc:MGI:1914120]	88.8151	95.7586	98.0872	90.0948	91.104	94.0968	94.4476	88.4584	101.005	87.2766	93.0166	89.9827	105.553	94.4035	0.503972924	0.966018836	12	4	24	0.446289097	0.805393068	24	3.954224513	0.037585852
ENSMUSG00000036372	Tmem258	transmembrane protein 258 [Source:MGI Symbol;Acc:MGI:1916288]	68.35	74.5227	64.5293	67.2622	74.984	66.0527	57.7323	63.032	73.8068	50.4558	69.6711	62.0371	65.8511	65.5808	0.915082018	0.966018836	20	8	24	0.987613193	0.997530201	24	23.78313393	0.009181606
ENSMUSG00000036376	Abt1	activator of basal transcription 1 [Source:MGI Symbol;Acc:MGI:1353636]	2.2942	2.48217	2.3169	2.4479	2.49424	2.63498	2.74954	2.40385	2.07663	2.32415	2.56103	2.55876	2.36531	2.36772	0.041292272	0.346472636	24	12	24	0.038964095	0.612479065	24	19.88830077	0.066484961
ENSMUSG00000036377	Cracd	capping protein inhibiting regulator of actin [Source:MGI Symbol;Acc:MGI:2444817]	11.1159	8.39466	10.0532	8.23162	9.08822	9.31699	10.5857	8.65221	7.14113	12.837	8.09103	8.51605	8.77563	9.08664	0.885025617	0.966018836	4	8	24	0.90950844	0.974606327	24	20.58795202	0.035283771
ENSMUSG00000036381	P2ry14	purinergic receptor P2Y, G-protein coupled, 14 [Source:MGI Symbol;Acc:MGI:2155705]	2.32343	3.05747	4.061	2.77616	2.83511	2.65959	2.69214	2.90174	3.02116	2.13144	2.59352	3.05544	2.87227	2.77104	0.265774856	0.773510818	12	4	24	0.458381074	0.805393068	24	6.496479061	0.079426709
ENSMUSG00000036390	Gadd45a	growth arrest and DNA-damage-inducible 45 alpha [Source:MGI Symbol;Acc:MGI:107799]	12.2369	9.51743	12.8574	9.26929	14.3726	13.6673	10.4679	12.368	8.87388	12.9704	15.2697	13.1455	11.9395	11.908	0.016811431	0.253395174	20	12	24	0.092262493	0.71175955	24	17.98015786	0.176667447
ENSMUSG00000036391	Sec24a	Sec24 related gene family, member A (S. cerevisiae) [Source:MGI Symbol;Acc:MGI:1924621]	5.21365	4.94969	4.98993	5.78428	5.29428	4.98085	5.06888	5.09498	5.06869	4.80907	5.1433	4.35709	4.33417	4.92661	0.822170541	0.966018836	20	4	24	0.196013032	0.768040178	24	11.74058483	0.048594821
ENSMUSG00000036395	Glb1l2	galactosidase, beta 1-like 2 [Source:MGI Symbol;Acc:MGI:2388283]	3.53496	3.9307	3.53893	4.2599	3.31381	3.50619	3.06813	3.5786	3.18385	4.60922	3.21431	3.88075	3.82529	3.79351	0.94791825	0.966018836	16	4	24	0.584276233	0.852087247	24	10.32016708	0.054738616
ENSMUSG00000036398	Ppp1r11	protein phosphatase 1, regulatory inhibitor subunit 11 [Source:MGI Symbol;Acc:MGI:1923747]	87.2964	77.9881	78.8893	70.6186	81.276	80.6506	73.879	74.4565	81.6256	65.2262	85.7274	77.268	74.6531	68.1386	0.510431574	0.966018836	20	20	24	0.289378066	0.768040178	24	20.07361036	0.069154576
ENSMUSG00000036402	Gng12	guanine nucleotide binding protein (G protein), gamma 12 [Source:MGI Symbol;Acc:MGI:1336171]	22.7838	20.1196	19.4767	21.1851	20.3304	18.7549	23.6098	20.7776	21.1557	19.8976	20.4336	21.3303	20.6285	20.3926	0.568601606	0.966018836	4	8	24	0.396698347	0.799546792	24	0.007669449	0.04299981
ENSMUSG00000036403	Cep135	centrosomal protein 135 [Source:MGI Symbol;Acc:MGI:2681869]	1.76726	2.33176	2.30902	2.32823	2.4302	2.49946	2.52019	2.22335	2.2922	1.82547	2.51569	2.14708	2.0384	2.34154	0.935593607	0.966018836	20	8	24	0.88396101	0.966884566	24	15.48458716	0.021885112
ENSMUSG00000036411	9530077C05Rik	RIKEN cDNA 9530077C05 gene [Source:MGI Symbol;Acc:MGI:1915533]	9.94061	11.3097	9.85082	10.5761	8.68526	10.5043	8.55904	9.77448	10.031	11.1095	10.8852	10.7208	10.0253	10.5186	0.47028201	0.966018836	16	12	24	0.686678592	0.895969947	24	10.40827555	0.037795867
ENSMUSG00000036412	Arsi	arylsulfatase i [Source:MGI Symbol;Acc:MGI:2670959]	0.675568	0.809727	0.566812	0.324874	0.636202	0.329749	0.722748	0.746629	0.838522	0.288128	0.593225	0.455429	0.616505	0.603628	0.026012493	0.29944494	8	8	24	0.067986282	0.671988997	24	3.314063489	0.138478229
ENSMUSG00000036422	Pcdh8	protocadherin 8 [Source:MGI Symbol;Acc:MGI:1306800]	0.647379	0.506141	0.66623	0.503169	0.282401	0.418919	0.515161	0.472205	0.502557	0.741392	0.422631	0.690191	0.377996	0.596009	0.864782464	0.966018836	16	4	24	0.555665492	0.838702296	24	6.810160901	0.055508929
ENSMUSG00000036427	Gpi1	glucose-6-phosphate isomerase 1 [Source:MGI Symbol;Acc:MGI:95797]	117.504	90.2408	95.1319	104.968	87.6995	105.856	110.85	115.582	87.0096	132.291	104.104	100.638	110.067	109.81	0.286642595	0.793714531	4	8	24	0.848734957	0.954576817	24	23.26741275	0.038616246
ENSMUSG00000036430	Tbcc	tubulin-specific chaperone C [Source:MGI Symbol;Acc:MGI:1919976]	16.2199	13.9735	14.5801	12.2562	12.221	13.2829	11.5705	16.9566	14.121	17.4477	15.3723	16.9955	18.3148	17.8707	0.920872048	0.966018836	8	12	24	0.415534911	0.800637996	24	3.793798967	0.087778473
ENSMUSG00000036432	Siah2	siah E3 ubiquitin protein ligase 2 [Source:MGI Symbol;Acc:MGI:108062]	4.87289	3.92415	4.12904	4.33746	5.19275	4.57428	5.19623	4.13744	3.40563	4.82157	4.84563	4.56993	4.45657	4.00806	0.010153564	0.194720062	20	16	24	0.008309578	0.49701405	24	18.733291	0.147429121
ENSMUSG00000036435	Exoc1	exocyst complex component 1 [Source:MGI Symbol;Acc:MGI:2445020]	33.4518	37.0235	38.4145	34.2534	34.5462	35.7982	33.6909	37.3329	35.5232	35.0933	37.203	37.1628	37.513	41.6922	0.588208558	0.966018836	8	8	24	0.175536681	0.768040178	24	5.560300528	0.050736162
ENSMUSG00000036437	Npy1r	neuropeptide Y receptor Y1 [Source:MGI Symbol;Acc:MGI:104963]	7.8165	7.63569	9.27217	7.48319	9.13374	8.81895	7.32295	6.71518	6.19661	7.03758	8.76248	7.0263	6.7352	7.00792	0.47028201	0.966018836	20	12	24	0.089989149	0.7097999	24	16.83458818	0.115583587
ENSMUSG00000036438	Calm2	calmodulin 2 [Source:MGI Symbol;Acc:MGI:103250]	809.285	841.823	794.325	784.56	780.62	750.423	763.8	712.254	797.48	677.118	865.634	728.008	751.537	785.449	0.939921059	0.966018836	12	12	24	0.994669581	0.998755922	24	3.504974373	0.003679728
ENSMUSG00000036442	Thap11	THAP domain containing 11 [Source:MGI Symbol;Acc:MGI:1930964]	9.14015	7.78032	8.87855	8.35271	8.22284	8.52952	9.1513	9.34742	9.00507	8.80231	9.79054	9.4256	8.75891	7.8599	0.882263435	0.966018836	4	4	24	0.635928716	0.876340489	24	18.51147301	0.033711921
ENSMUSG00000036446	Lum	lumican [Source:MGI Symbol;Acc:MGI:109347]	46.783	64.5821	61.5715	63.3562	69.7959	47.2795	59.8153	58.3375	74.6754	43.3116	67.614	44.5233	46.9004	49.8741	0.188831826	0.664348865	20	4	24	0.271344519	0.768040178	24	10.16012946	0.158761
ENSMUSG00000036450	Hif1an	hypoxia-inducible factor 1, alpha subunit inhibitor [Source:MGI Symbol;Acc:MGI:2442345]	14.5028	12.9472	12.1127	12.8736	13.296	14.5489	13.1849	14.6389	11.1683	15.4076	12.3536	13.7238	13.926	13.0749	0.148073804	0.590732968	24	12	24	0.320419758	0.768040178	24	21.10038709	0.070065122
ENSMUSG00000036452	Arhgap26	Rho GTPase activating protein 26 [Source:MGI Symbol;Acc:MGI:1918552]	14.2939	12.0483	12.6248	14.2204	12.5892	15.0006	12.1175	12.771	10.0734	16.1089	12.2968	13.6204	13.1522	13.6658	0.44861185	0.966018836	16	20	24	0.475904454	0.805393068	24	17.94549092	0.07534817
ENSMUSG00000036459	Wtip	WT1-interacting protein [Source:MGI Symbol;Acc:MGI:2141920]	5.11774	6.14839	5.95953	6.7291	6.67555	5.78379	7.36424	7.1958	7.97668	6.24428	6.39757	6.89947	7.94719	6.17864	0.715742619	0.966018836	4	20	24	0.77341083	0.927220663	24	7.109423359	0.040022548
ENSMUSG00000036461	Elf1	E74 like ETS transcription factor 1 [Source:MGI Symbol;Acc:MGI:107180]	8.90618	8.15046	8.38196	9.26605	8.29812	9.20019	9.11664	8.41618	7.92607	8.87574	8.82274	8.98226	8.49883	8.72105	0.039704107	0.338535904	24	12	24	0.138541677	0.747972781	24	18.8320292	0.047546889
ENSMUSG00000036466	Megf11	multiple EGF-like-domains 11 [Source:MGI Symbol;Acc:MGI:1920951]	3.31992	3.23708	3.2751	3.30563	2.65543	2.95983	2.9401	2.88778	2.61742	2.93002	2.87397	2.75927	2.40422	3.13993	0.93717134	0.966018836	16	4	24	0.592270782	0.856446446	24	6.862247134	0.035027641
ENSMUSG00000036469	Marchf1	membrane associated ring-CH-type finger 1 [Source:MGI Symbol;Acc:MGI:1920175]	0.723413	1.65629	2.26477	1.13582	1.06968	1.23079	1.41269	1.27535	1.62612	1.1046	1.08173	1.24651	0.885913	1.50014	0.018923071	0.256345714	12	8	24	0.083400912	0.699822624	24	7.427358241	0.193590572
ENSMUSG00000036473	Tbc1d24	TBC1 domain family, member 24 [Source:MGI Symbol;Acc:MGI:2443456]	4.78881	5.28444	5.4617	5.41732	5.95056	5.60971	5.21678	5.0326	5.85053	5.08615	5.62455	5.51571	4.91709	5.57984	0.016398461	0.253395174	20	8	24	0.160636522	0.765941695	24	13.49189164	0.055073757
ENSMUSG00000036478	Btg1	BTG anti-proliferation factor 1 [Source:MGI Symbol;Acc:MGI:88215]	11.2612	10.5232	12.9732	12.2911	12.7536	12.9627	10.6008	13.2242	11.8751	11.9436	10.8506	13.007	11.0215	12.5147	0.92721845	0.966018836	24	4	24	0.638617786	0.877410058	24	12.29108236	0.041183138
ENSMUSG00000036499	Eea1	early endosome antigen 1 [Source:MGI Symbol;Acc:MGI:2442192]	21.2065	20.7284	18.8309	20.0235	20.2976	18.7877	19.2932	17.7033	20.3186	18.4117	22.3323	19.9625	20.9063	21.8322	0.924770496	0.966018836	24	20	24	0.85431704	0.956491441	24	20.72416267	0.021242455
ENSMUSG00000036501	Fam13b	family with sequence similarity 13, member B [Source:MGI Symbol;Acc:MGI:2447834]	48.2768	40.2878	39.4537	41.6491	41.616	43.6276	41.1816	36.4924	35.3983	43.5088	38.1762	42.8232	40.9063	41.6466	0.077301512	0.453790923	24	12	24	0.115945148	0.727799063	24	20.23052057	0.085308229

ENSMUSG00000036502	Tmem255a	transmembrane protein 255A [Source:MGI Symbol;Acc:MGI:3045722]	75.799	64.5004	67.2209	66.6012	59.7385	75.2886	63.3275	65.3672	47.9276	73.5163	60.1155	63.1728	60.8226	62.8619	0.880247609	0.966018836	16	4	24	0.494556651	0.806517607	24	20.52721013	0.074693539
ENSMUSG00000036503	Rnf13	ring finger protein 13 [Source:MGI Symbol;Acc:MGI:1346341]	95.2466	111.808	106.212	99.9407	103.713	91.5755	97.6236	96.8014	112.661	88.6851	106.28	97.3386	99.9085	106.075	0.138202217	0.580158868	12	12	24	0.285045071	0.768040178	24	7.262983669	0.064270826
ENSMUSG00000036504	Phpt1	phosphohistidine phosphatase 1 [Source:MGI Symbol;Acc:MGI:1922704]	25.396	27.971	29.7666	29.0181	28.273	27.9591	27.108	28.7914	29.0582	27.45	27.6844	27.5641	29.739	27.0599	0.43565473	0.966018836	12	16	24	0.263182287	0.768040178	24	9.761733174	0.03792716
ENSMUSG00000036510	Cdh8	cadherin 8 [Source:MGI Symbol;Acc:MGI:107434]	6.00369	5.97491	5.59929	5.55269	4.80012	5.47955	6.00135	5.35614	5.52412	5.65943	5.20926	6.22147	6.76216	6.341	0.016398461	0.253395174	4	16	24	0.023142938	0.581965446	24	1.721416972	0.095190683
ENSMUSG00000036513	Commd2	COMM domain containing 2 [Source:MGI Symbol;Acc:MGI:1098806]	12.756	14.8294	13.7774	13.4366	15.0717	13.3556	14.4592	14.1001	14.714	9.58775	14.9419	13.1321	15.6075	12.2674	0.907000607	0.966018836	20	20	24	0.603718745	0.861540092	24	23.25894427	0.06583025
ENSMUSG00000036523	Greb1	gene regulated by estrogen in breast cancer protein [Source:MGI Symbol;Acc:MGI:2149712]	2.48802	1.84693	1.93078	2.002	2.04534	2.45309	2.50074	1.72026	1.66342	2.28092	2.22889	1.99933	1.88729	1.82144	0.047000349	0.375011045	4	4	24	0.025286059	0.586541342	24	19.17526247	0.125406919
ENSMUSG00000036526	Card11	caspase recruitment domain family, member 11 [Source:MGI Symbol;Acc:MGI:1916978]	3.2251	1.69872	1.47649	1.33855	1.49014	2.13473	2.00824	1.99906	1.45636	1.75467	1.37444	2.11426	2.52116	1.92065	0.001320294	0.090771496	4	12	24	0.002236415	0.395705644	24	23.4453839	0.26794087
ENSMUSG00000036528	Ppfibp2	PTPRF interacting protein, binding protein 2 (liprin beta 2) [Source:MGI Symbol;Acc:MGI:894649]	4.62045	5.03614	4.9912	5.41486	6.29793	5.6928	5.75246	5.81606	5.7304	5.55046	5.36639	4.99577	5.03492	6.10533	0.90332508	0.966018836	8	20	24	0.616605822	0.867682235	24	11.60364715	0.040324643
ENSMUSG00000036529	Sbf1	SET binding factor 1 [Source:MGI Symbol;Acc:MGI:1925230]	26.8964	27.231	27.912	31.9988	28.3151	27.8638	27.8728	28.9945	27.3338	32.1282	25.1558	28.1598	27.6358	25.5766	0.25849765	0.761738864	16	12	24	0.257304706	0.768040178	24	12.20332514	0.059110183
ENSMUSG00000036533	Cdc42ep3	CDC42 effector protein (Rho GTPase binding) 3 [Source:MGI Symbol;Acc:MGI:2384718]	9.80634	12.0114	8.32503	10.5523	12.8771	11.1308	11.6772	10.6622	12.2316	12.4577	12.0737	12.4775	14.1672	12.0427	0.773770646	0.966018836	20	16	24	0.509085685	0.813832486	24	19.24308968	0.063891656
ENSMUSG00000036534	Slc38a7	solute carrier family 38, member 7 [Source:MGI Symbol;Acc:MGI:2679005]	13.7868	10.5093	11.3153	11.2984	11.1749	12.1458	10.4918	11.8461	9.9435	13.0111	12.4728	11.4341	12.484	12.5921	0.919119843	0.966018836	4	8	24	0.634294001	0.875590992	24	20.11860001	0.047554253
ENSMUSG00000036537	Rnf113a1	ring finger protein 113A1 [Source:MGI Symbol;Acc:MGI:1917192]	2.77058	2.18945	2.60654	1.97946	2.21864	2.61987	2.37702	3.28985	2.40572	2.91843	2.39808	2.66941	2.93927	2.70333	0.532553009	0.966018836	4	16	24	0.283105503	0.768040178	24	2.11249492	0.077247277
ENSMUSG00000036545	Adamts2	a disintegrin-like and metallopeptidase (reprolysin type) with thrombospondin type 1 motif, 2 [Source:MGI Symbol;Acc:MGI:1347356]	3.03254	3.55324	3.52915	3.0116	3.23022	3.07927	3.23841	3.89205	4.58641	3.06264	3.46227	3.32472	2.99534	3.14342	0.217933169	0.704989686	12	16	24	0.178668617	0.768040178	24	7.466595353	0.098473658
ENSMUSG00000036550	Cnot1	CCR4-NOT transcription complex, subunit 1 [Source:MGI Symbol;Acc:MGI:2442402]	30.2407	26.2616	27.589	26.9469	25.6484	28.2733	24.8688	28.7479	26.5085	24.6439	25.6186	24.9965	30.2978	22.7838	0.941885246	0.966018836	4	16	24	0.471411688	0.805393068	24	23.4706002	0.050308709
ENSMUSG00000036552	Ermard	ER membrane associated RNA degradation [Source:MGI Symbol;Acc:MGI:1917317]	6.88757	8.83441	7.51915	8.76989	9.21813	8.19986	7.92502	7.39643	8.71771	7.86573	8.43829	7.86961	7.57884	7.7705	0.192909212	0.670595308	20	8	24	0.129248298	0.740162193	24	13.51409371	0.07731566
ENSMUSG00000036553	Sh3tc1	SH3 domain and tetratricopeptide repeats 1 [Source:MGI Symbol;Acc:MGI:2678949]	0.66401	0.701371	1.17013	1.3007	0.423782	0.385179	0.525353	0.549044	0.838955	0.57746	0.675594	0.595389	0.722567	0.703675	0.080164531	0.458678242	12	12	24	0.064760157	0.663876076	24	8.845755915	0.176502599
ENSMUSG00000036555	Iqce	IQ motif containing E [Source:MGI Symbol;Acc:MGI:1921489]	11.4376	12.0634	11.4315	11.9664	12.7731	11.0496	12.8392	9.40478	12.727	12.4047	12.2827	11.253	11.3479	10.2102	0.462767035	0.966018836	20	12	24	0.192493583	0.768040178	24	14.77397028	0.078227459
ENSMUSG00000036560	Lgi4	leucine-rich repeat LGI family, member 4 [Source:MGI Symbol;Acc:MGI:2180197]	48.0689	47.4995	40.4871	40.9761	52.5016	46.058	51.0222	47.5235	44.3004	45.8408	45.4486	41.7397	44.2531	40.9079	0.588208558	0.966018836	4	8	24	0.307573095	0.768040178	24	20.80833521	0.067291209
ENSMUSG00000036561	Ppp6r2	protein phosphatase 6, regulatory subunit 2 [Source:MGI Symbol;Acc:MGI:1918724]	23.7086	22.3682	23.4808	24.6551	23.9592	24.5838	23.697	24.5202	23.0273	27.6347	21.9813	24.098	24.536	24.4767	0.960338592	0.967043964	16	20	24	0.780076888	0.929318742	24	12.30102208	0.020541626
ENSMUSG00000036564	Ndrg4	N-myc downstream regulated gene 4 [Source:MGI Symbol;Acc:MGI:2384590]	749.057	595.808	596.7	623.753	577.996	702.429	643.757	662.87	490.848	806.148	601.777	674.038	623.973	663.168	0.390686537	0.915479136	24	12	24	0.521017798	0.820180873	24	20.78283901	0.081619894
ENSMUSG00000036565	Ttyh3	tweety family member 3 [Source:MGI Symbol;Acc:MGI:1925589]	13.9504	10.9279	12.5427	12.497	11.3274	12.1169	12.7099	12.1486	11.9059	15.1113	13.7393	12.7714	12.1418	12.4007	0.834424969	0.966018836	16	16	24	0.510104195	0.81402698	24	14.3868245	0.049891705
ENSMUSG00000036568	Bicral	BRD4 interacting chromatin remodeling complex associated protein like [Source:MGI Symbol;Acc:MGI:2673855]	9.53535	7.84276	8.1624	7.94734	8.69022	7.64989	9.83039	7.55112	7.19521	9.46709	8.43969	8.89954	7.17111	8.0273	0.745469888	0.966018836	4	4	24	0.406629921	0.799546792	24	18.60305207	0.070701016
ENSMUSG00000036570	Fxyd1	FXYD domain-containing ion transport regulator 1 [Source:MGI Symbol;Acc:MGI:1889273]	118.749	130.304	128.076	129.685	140.339	138.407	128.023	142.602	155.042	124.166	120.638	129.342	136.158	115.618	0.828910443	0.966018836	12	16	24	0.8996806	0.971531812	24	9.472282015	0.021848076
ENSMUSG00000036572	Upf3b	UPF3 regulator of nonsense transcripts homolog B (yeast) [Source:MGI Symbol;Acc:MGI:1915384]	13.8217	15.3633	14.9926	14.5373	13.8581	13.9092	14.2972	13.2172	16.8209	13.9686	15.5926	16.2419	15.3589	15.7858	0.883907406	0.966018836	12	16	24	0.68983399	0.897368576	24	7.770036028	0.030922152
ENSMUSG00000036578	Fxyd7	FXYD domain-containing ion transport regulator 7 [Source:MGI Symbol;Acc:MGI:1889006]	137.965	105.916	91.9738	101.641	94.1224	116.501	120.307	121.918	103.761	169.088	113.138	135.009	115.017	125.502	0.28865292	0.794869797	4	8	24	0.741980059	0.916124847	24	22.17597615	0.068878897
ENSMUSG00000036580	Spg20	spastic paraplegia 20, spartin (Troyer syndrome) homolog (human) [Source:MGI Symbol;Acc:MGI:2139806]	25.2514	24.3594	24.6429	25.7956	25.8422	24.4896	27.972	24.7395	24.4483	29.1154	23.4793	25.3681	23.688	23.6452	0.743750765	0.966018836	16	16	24	0.638731858	0.877476407	24	14.4487911	0.031654683
ENSMUSG00000036585	Fgf1	fibroblast growth factor 1 [Source:MGI Symbol;Acc:MGI:95515]	176.52	141.167	139.93	146.705	152.482	162.347	162.007	153.006	120.372	180.149	143.289	154.449	154.931	153.175	0.009640466	0.194720062	4	8	24	0.214245649	0.768040178	24	20.59469478	0.100703801
ENSMUSG00000036591	Arhgap21	Rho GTPase activating protein 21 [Source:MGI Symbol;Acc:MGI:1918685]	32.431	34.1063	34.6822	36.2853	30.8235	30.9444	31.8904	29.5718	35.0593	32.7884	28.9517	36.0715	33.7522	38.7342	0.251986462	0.75129457	12	8	24	0.30723245	0.768040178	24	6.58043525	0.07479865
ENSMUSG00000036594	H2-Aa	histocompatibility 2, class II antigen A, alpha [Source:MGI Symbol;Acc:MGI:95895]	54.5259	73.6431	65.3696	56.4502	67.484	50.4087	60.0651	72.5111	90.8908	40.4563	71.3363	62.0069	63.436	66.0441	0.151191877	0.598641364	12	12	24	0.393207615	0.799546792	24	5.593825849	0.149421655
ENSMUSG00000036596	Cpz	carboxypeptidase Z [Source:MGI Symbol;Acc:MGI:88487]	0.532568	0.580881	0.5796	0.504451	0.415173	0.575512	0.395225	0.734942	0.833292	0.263791	0.328335	0.495139	0.442051	0.408447	0.137763286	0.580158868	12	4	24	0.212436454	0.768040178	24	4.870402202	0.092375703
ENSMUSG00000036599	Chst12	carbohydrate sulfotransferase 12 [Source:MGI Symbol;Acc:MGI:1929064]	13.516	10.1465	8.1498	9.46922	9.14317	10.9253	10.5805	11.0787	8.96772	15.8327	9.52592	12.4753	11.1028	10.9828	0.066633212	0.425327737	4	8	24	0.593548756	0.85692808	24	21.73001794	0.094752157
ENSMUSG00000036606	Plxnb2	plexin B2 [Source:MGI Symbol;Acc:MGI:2154239]	12.2465	13.3615	13.7643	12.3042	11.9383	10.6592	12.1617	12.5318	15.0145	12.9472	14.0069	12.8131	13.2581	11.4477	0.245061943	0.741580974	12	12	24	0.20675922	0.768040178	24	8.957883574	0.081634449
ENSMUSG00000036611	Eepd1	endonuclease/exonuclease/phosphatase family domain containing 1 [Source:MGI Symbol;Acc:MGI:1914734]	45.1654	36.3993	34.82	34.9153	39.011	43.5667	36.9568	42.4796	28.8616	42.6421	36.2012	39.2993	40.1719	37.8033	0.033552374	0.324908923	24	12	24	0.106657155	0.718749374	24	21.19868216	0.132015694
ENSMUSG00000036613	Eipr1	EARP complex and GARP complex interacting protein 1 [Source:MGI Symbol;Acc:MGI:1289332]	14.6715	14.8743	17.7115	14.2911	15.8631	14.9537	12.03	14.309	13.6424	14.6459	14.3934	13.6768	14.5296	13.705	0.838692453	0.966018836	20	8	24	0.408935459	0.799546792	24	12.17112962	0.051760853
ENSMUSG00000036615	Rfxap	regulatory factor X-associated protein [Source:MGI Symbol;Acc:MGI:2180854]	14.8532	15.8706	13.4102	17.3246	17.7489	17.429	14.9621	13.8006	11.2317	14.94	16.0898	15.2665	15.2041	14.6436	0.00109088	0.07820797	20	16	24	0.020913133	0.568598943	24	18.19067695	0.156475462
ENSMUSG00000036617	Etl4	enhancer trap locus 4 [Source:MGI Symbol;Acc:MGI:95454]	18.2318	14.5464	15.174	16.0253	15.2458	16.893	15.1207	17.4498	14.0844	21.4458	13.8775	15.3955	14.3879	18.8306	0.921572192	0.966018836	16	20	24	0.882414507	0.966689896	24	14.18358283	0.03188259
ENSMUSG00000036620	Mgat4b	mannoside acetylglucosaminyltransferase 4, isoenzyme B [Source:MGI Symbol;Acc:MGI:2143974]	20.3226	16.7334	15.7544	19.6322	19.0802	18.9243	18.2869	19.9254	17.3053	25.3509	19.5611	20.1559	19.7896	20.5573	0.833301348	0.966018836	16	20	24	0.531044933	0.825104543	24	16.2123042	0.061383427
ENSMUSG00000036622	Atp13a2	ATPase type 13A2 [Source:MGI Symbol;Acc:MGI:1922022]	40.2378	29.0759	31.0097	30.8564	30.4102	31.5598	33.2189	34.9627	27.8533	41.3619	35.1968	38.6444	36.5612	37.3379	0.66832131	0.966018836	4	8	24	0.528540523	0.824000718	24	21.47476062	0.07315459
ENSMUSG00000036632	Alg5	asparagine-linked glycosylation 5 (dolichyl-phosphate beta-glucosyltransferase) [Source:MGI Symbol;Acc:MGI:1913498]	15.9187	19.3874	17.5328	18.5018	17.3	16.8819	15.2179	16.9964	19.7292	16.5384	17.8385	17.02	17.3175	17.0304	0.097760727	0.501112642	12	16	24	0.129289132	0.740162193	24	9.609541961	0.073715766
ENSMUSG00000036634	Mag	myelin-associated glycoprotein [Source:MGI Symbol;Acc:MGI:96912]	44.907	59.3401	78.914	50.6832	55.9732	59.166	76.6624	54.4688	64.894	59.2236	45.0598	55.6418	44.6631	55.2282	0.43565473	0.966018836	12	16	24	0.476202819	0.805393068	24	7.350823757	0.122318212
ENSMUSG00000036636	Clcn7	chloride channel, voltage-sensitive 7 [Source:MGI Symbol;Acc:MGI:1347048]	11.956	11.2496	11.0034	12.5256	11.7252	12.8211	11.7114	12.3307	11.4687	13.2578	10.5623	11.3565	11.8508	11.6957	0.924553956	0.966018836	16	20	24	0.951465978	0.988339231	24	14.85157107	0.010269674
ENSMUSG00000036639	Nudt1	nudix (nucleoside diphosphate linked moiety X)-type motif 1 [Source:MGI Symbol;Acc:MGI:109280]	6.2007	8.14804	9.20753	8.6522	8.35134	7.95421	8.81319	7.82445	8.93994	6.83097	7.61891	7.63277	8.07998	9.84259	0.277221318	0.789849983	12	12	24	0.285049387	0.768040178	24	7.264973711	0.095533017
ENSMUSG00000036641	Ccdc148	coiled-coil domain containing 148 [Source:MGI Symbol;Acc:MGI:3039583]	1.61427	1.5905	1.81703	1.49435	1.40774	1.3898	1.26384	1.35157	1.48789	1.62509	1.32064	1.51755	1.31003	1.86002	0.336256288	0.850598217	12	16	24	0.170972279	0.768040178	24	7.609986785	0.076025466
ENSMUSG00000036644	Tbc1d9b	TBC1 domain family, member 9B [Source:MGI Symbol;Acc:MGI:1924045]	45.4325	36.6711	39.3668	40.6716	39.6379	43.9585	44.2197	45.1397	35.7036	51.5388	36.5857	42.9179	42.3914	39.9894	0.277087842	0.789849983	4	8	24	0.692145446	0.898026996	24	20.9152994	0.050735968
ENSMUSG00000036646	Man1b1	mannosidase, alpha, class 1B, member 1 [Source:MGI Symbol;Acc:MGI:2684954]	32.4895	26.1789	29.5335	29.0345	31.3193	31.6685	32.2897	29.7272	27.0657	34.1517	28.1253	32.0887	30.3005	28.9297	0.135537027	0.574447137	4	4	24	0.181248297	0.768040178	24	19.14343535	0.07645386
ENSMUSG00000036655	Colec11	collectin sub-family member 11 [Source:MGI Symbol;Acc:MGI:1918943]	0.598027	0.63171	0.565356	0.456884	0.744456	0.322828	0.499757	0.59307	0.624232	0.473838	0.520702	0.644039	0.39753	0.507502	0.866861748	0.966018836	8	20	24	0.853887863	0.956382569	24	8.71126465	0.023978261
ENSMUSG00000036661	Dennd3	DENN/MADD domain containing 3 [Source:MGI Symbol;Acc:MGI:2146009]	0.708211	1.26741	1.58628	1.08634	0.930621	1.30771	0.809967	0.976207	1.16855	1.35924	1.54503	1.10091	1.29764	0.733183	0.360675801	0.883978426	12	20	24	0.2337428	0.768040178	24	12.59983376	0.124025633
ENSMUSG00000036667	Tcaf1	TRPM8 channel-associated factor 1 [Source:MGI Symbol;Acc:MGI:1914665]	65.5943	56.8651	58.7757	54.6436	55.2937	62.2223	56.374	56.7247	47.7325	60.7667	54.6533	57.8502	57.1219	60.5846	0.612141595	0.966018836	24	20	24	0.252964592	0.768040178	24	22.24796069	0.067729697
ENSMUSG00000036672	Cenpt	centromere protein T [Source:MGI Symbol;Acc:MGI:2443939]	4.03306	5.09964	6.02182	4.41509	5.35212	5.65841	5.21595	7.11664	4.71416	5.75648	5.15067	5.19118	5.76598	4.95754	0.733896783	0.966018836	24	16	24	0.864930656	0.96088896	24	6.082894396	0.036655972
ENSMUSG00000036676	Tmtc3	transmembrane and tetratricopeptide repeat containing 3 [Source:MGI Symbol;Acc:MGI:3036255]	8.17887	7.56542	6.77577	7.54713	8.12612	7.03653	6.91403	7.17421	7.37637	6.43066	7.89902	7.284	7.36827	7.10384	0.924770496	0.966018836	20	16	24	0.405986847	0.799546792	24	19.4720477	0.045310284
ENSMUSG00000036678	Aaas	achalasia, adrenocortical insufficiency, alacrimia [Source:MGI Symbol;Acc:MGI:2443767]	7.38182	7.2388	6.61657	7.53263	7.26569	7.24346	7.97204	8.14933	6.59037	7.30334	8.53802	7.39986	6.35928	7.24062	0.581070934	0.966018836	20	16	24	0.578436133	0.849227023	24	18.16489604	0.04410129
ENSMUSG00000036686	Cc2d1a	coiled-coil and C2 domain containing 1A [Source:MGI Symbol;Acc:MGI:2384831]	6.83125	6.01386	6.43064	5.66462	6.63528	6.4521	7.15612	6.01216	6.30762	7.8841	6.29293	6.69187	6.32559	6.38555	0.616919373	0.966018836	4	4	24	0.723814728	0.909416171	24	18.28999006	0.033353026
ENSMUSG00000036693	Nop14	NOP14 nucleolar protein [Source:MGI Symbol;Acc:MGI:1922666]	10.4768	10.6346	11.596	10.2895	9.2463	11.0683	10.8448	11.4517	10.1721	12.3619	11.5547	10.7143	11.2384	11.4015	0.816300547	0.966018836	16	8	24	0.609029429	0.864393298	24	6.438552308	0.035634672
ENSMUSG00000036698	Ago2	argonaute RISC catalytic subunit 2 [Source:MGI Symbol;Acc:MGI:2446632]	8.00347	6.34203	6.74789	7.11578	6.92597	7.0853	7.91241	7.04562	6.13162	8.52568	7.05643	7.3616	6.81124	6.90289	0.175808942	0.651752074	16	20	24	0.433375511	0.805393068	24	18.80378916	0.058960712
ENSMUSG00000036699	Zcchc12	zinc finger, CCHC domain containing 12 [Source:MGI Symbol;Acc:MGI:1919943]	7.3252	11.2491	15.2642	9.53825	7.28839	10.7217	10.9509	8.40672	11.2138	9.56512	8.43489	11.2075	7.83999	12.9213	0.10560184	0.518002936	12	8	24	0.255298019	0.768040178	24	6.928397455	0.188312981
ENSMUSG00000036707	Cab39	calcium binding protein 39 [Source:MGI Symbol;Acc:MGI:107438]	102.125	95.6999	95.9665	95.2672	95.6606	97.4785	93.6756	93.0231	87.9357	95.0499	100.739	94.418	93.5582	97.7541	0.93447422	0.966018836	20	16	24	0.289628879	0.768040178	24	19.5409692	0.031650367
ENSMUSG00000036712	Cyld	CYLD lysine 63 deubiquitinase [Source:MGI Symbol;Acc:MGI:1921506]	14.8656	15.3647	14.1546	14.8468	14.1408	13.2501	14.4348	13.3522	14.7036	12.5094	14.8669	13.5722	14.0536	14.5382	0.94086481	0.966018836	8	16	24	0.689778467	0.897368576	24	3.813651905	0.024692551
ENSMUSG00000036718	Micall2	MICAL-like 2 [Source:MGI Symbol;Acc:MGI:2444818]	3.51099	3.42458	2.77599	2.74017	3.38404	3.17528	3.32428	3.05855	3.23602	3.43872	3.21464	2.65749	3.74121	3.10294	0.591351901	0.966018836	4	20	24	0.534903748	0.826778911	24	23.7872222	0.048421793
ENSMUSG00000036721	Zscan12	zinc finger and SCAN domain containing 12 [Source:MGI Symbol;Acc:MGI:1099444]	3.09002	3.24831	3.48565	3.01819	3.37155	2.9966	2.61009	2.81931	3.42887	2.38547	3.2096	2.68184	3.13812	2.85326	0.390686537	0.915479136	12	12	24	0.831513836	0.947752995	24	9.268305302	0.027968783
ENSMUSG00000036733	Rbm42	RNA binding motif protein 42 [Source:MGI Symbol;Acc:MGI:1915285]	18.5285	16.4322	16.5525	15.4441	17.9936	17.4938	19.0183	20.855	17.7348	22.861	21.3309	21.7445	23.3462	22.2649	0.826609618	0.966018836	4	8	24	0.41419649	0.799546792	24	22.83171971	0.05398022
ENSMUSG00000036737	Oxsr1	oxidative-stress responsive 1 [Source:MGI Symbol;Acc:MGI:1917378]	21.7766	20.9499	20.7511	20.3953	20.259	22.3359	21.2292	20.746	19.8919	21.1945	19.2883	22.1772	21.6799	21.0879	0.001179532	0.082396678	24	20	24	0.04481557	0.628432994	24	22.862337	0.051075329
ENSMUSG00000036745	Ttll7	tubulin tyrosine ligase-like family, member 7 [Source:MGI Symbol;Acc:MGI:1918142]	199.37	188.166	173.599	186.761	186.138	183.332	171.164	168.516	174.016	167.26	199.625	188.644	197.765	198.554	0.714044782	0.966018836	20	20	24	0.324420401	0.768040178	24	20.90681009	0.055562744
ENSMUSG00000036748	Cuedc2	CUE domain containing 2 [Source:MGI Symbol;Acc:MGI:1914366]	112.726	103.925	102.513	101.123	105.868	110.987	108.206	109.599	97.2959	107.347	103.753	108.372	107.087	103.768	0.008047769	0.192760771	24	12	24	0.008337059	0.49701405	24	21.62413139	0.059785726
ENSMUSG00000036751	Cox6b1	cytochrome c oxidase, subunit 6B1 [Source:MGI Symbol;Acc:MGI:107460]	455.269	535.203	496.161	521.267	504.467	462.737	469.792	467.57	523.336	394.507	536.322	477.387	542.756	540.623	0.912686312	0.966018836	8	16	24	0.785045661	0.931056644	24	5.035209402	0.036522304
ENSMUSG00000036752	Tubb4b	tubulin, beta 4B class IVB [Source:MGI Symbol;Acc:MGI:1915472]	300.65	247.742	253.74	252.584	262.492	301.818	261.233	272.793	202.401	287.98	247.851	255.746	255.999	251.488	0.446277988	0.966018836	24	12	24	0.153436869	0.759640117	24	20.41960745	0.104192083
ENSMUSG00000036760	Kcnk9	potassium channel, subfamily K, member 9 [Source:MGI Symbol;Acc:MGI:3521816]	0.661104	1.29736	2.6203	0.949011	0.715424	1.31473	1.64559	0.759386	1.43745	1.11603	0.70766	1.08316	0.509523	1.49573	0.424718357	0.966018836	12	8	24	0.287373784	0.768040178	24	7.311804612	0.210124974
ENSMUSG00000036764	Dnajc12	DnaJ heat shock protein family (Hsp40) member C12 [Source:MGI Symbol;Acc:MGI:1353428]	12.4276	13.1259	14.202	14.6852	13.651	12.4182	11.2513	13.4089	13.0147	12.9653	15.9768	14.1824	13.2788	14.0764	0.313239838	0.824470524	20	8	24	0.116425238	0.727799063	24	12.53392013	0.08399727
ENSMUSG00000036766	Dner	delta/notch-like EGF repeat containing [Source:MGI Symbol;Acc:MGI:2152889]	25.5704	20.4949	20.4969	21.4793	18.6893	22.9334	22.8174	21.2547	17.0535	27.8057	18.763	21.0211	19.7963	23.6301	0.723119329	0.966018836	4	8	24	0.831244516	0.947661285	24	23.52835756	0.043939292
ENSMUSG00000036768	Kif15	kinesin family member 15 [Source:MGI Symbol;Acc:MGI:1098258]	1.15599	1.26639	1.88588	1.77787	1.63894	1.41704	1.74227	1.68727	2.66078	0.894695	1.33102	1.20129	1.14954	1.17108	0.286642595	0.793714531	12	16	24	0.395654771	0.799546792	24	8.800694898	0.129870201
ENSMUSG00000036769	Wdr44	WD repeat domain 44 [Source:MGI Symbol;Acc:MGI:1919654]	12.6347	11.1885	10.7509	10.9684	11.0372	11.0685	10.5386	10.8622	10.0234	10.3979	10.7514	11.0736	10.6775	10.5708	0.390686537	0.915479136	24	12	24	0.038441182	0.61140397	24	21.53470836	0.05280565
ENSMUSG00000036775	Decr2	2-4-dienoyl-Coenzyme A reductase 2, peroxisomal [Source:MGI Symbol;Acc:MGI:1347059]	12.7921	11.8815	11.4045	13.6643	13.0927	12.6631	12.1095	12.4991	10.6924	14.6357	11.652	12.3928	12.661	13.0293	0.187699199	0.662239322	16	20	24	0.586107834	0.853257144	24	16.20925062	0.042853352
ENSMUSG00000036777	Anln	anillin, actin binding protein [Source:MGI Symbol;Acc:MGI:1920174]	5.46939	8.24461	10.708	6.08283	7.38917	7.15796	8.7473	7.28147	9.33626	4.90766	5.8858	6.98831	6.53422	5.18978	0.676344318	0.966018836	12	4	24	0.641893391	0.878809617	24	5.41200852	0.103617331
ENSMUSG00000036779	Tent4b	terminal nucleotidyltransferase 4B [Source:MGI Symbol;Acc:MGI:1917820]	8.26822	7.61156	7.81658	9.27577	7.52619	7.51388	8.19672	7.10372	7.92225	7.37224	7.89971	6.90095	6.94634	7.40787	0.954106009	0.966018836	12	20	24	0.428395154	0.805393068	24	11.97172782	0.042284654
ENSMUSG00000036781	Rps27l	ribosomal protein S27-like [Source:MGI Symbol;Acc:MGI:1915191]	75.2444	90.1368	84.755	91.6661	84.2367	70.933	79.9858	84.1134	106.205	68.3373	100.707	83.2553	92.3548	85.177	0.601647512	0.966018836	12	12	24	0.497456817	0.807576732	24	8.513430213	0.083303078
ENSMUSG00000036782	Klhl13	kelch-like 13 [Source:MGI Symbol;Acc:MGI:1914705]	11.0892	13.2883	12.9101	9.76968	12.3944	11.5072	13.6833	10.4283	11.855	10.3305	11.2019	11.0641	11.0191	12.5885	0.81705122	0.966018836	12	4	24	0.395705687	0.799546792	24	2.265657	0.069092607
ENSMUSG00000036790	Slitrk2	SLIT and NTRK-like family, member 2 [Source:MGI Symbol;Acc:MGI:2679449]	2.99017	2.62731	2.8972	3.06837	3.00079	2.72005	2.97136	2.62937	2.42691	2.38287	2.93203	2.62263	2.34514	2.4488	0.916746631	0.966018836	20	12	24	0.120764947	0.732894732	24	17.47053162	0.059669621
ENSMUSG00000036792	Mbd5	methyl-CpG binding domain protein 5 [Source:MGI Symbol;Acc:MGI:2138934]	9.40858	11.6881	11.8522	10.9748	11.3367	10.6726	11.0491	9.82657	10.2117	12.5228	10.3605	12.6936	10.3472	11.3544	0.932671476	0.966018836	16	12	24	0.481478475	0.805393068	24	12.82134886	0.054002734
ENSMUSG00000036810	Cnep1r1	CTD nuclear envelope phosphatase 1 regulatory subunit 1 [Source:MGI Symbol;Acc:MGI:1921981]	27.5904	29.4886	29.4671	27.8221	28.0593	27.707	28.2268	27.1873	29.0474	26.401	28.7328	26.107	26.2968	28.8347	0.138202217	0.580158868	12	12	24	0.249391962	0.768040178	24	7.520050937	0.034814249
ENSMUSG00000036814	Slc6a20a	solute carrier family 6 (neurotransmitter transporter), member 20A [Source:MGI Symbol;Acc:MGI:2143217]	5.50731	8.49727	4.98472	4.2699	4.85779	5.23107	4.71274	6.41238	6.55575	2.97827	4.96149	5.73196	3.5451	6.43252	0.031431762	0.324385159	8	8	24	0.199432531	0.768040178	24	3.317117561	0.208833102
ENSMUSG00000036815	Dpp10	dipeptidylpeptidase 10 [Source:MGI Symbol;Acc:MGI:2442409]	55.9432	55.7766	48.5175	49.0931	51.2382	48.7504	52.3241	48.5747	51.4836	47.5768	48.6511	57.6168	58.6239	59.8424	0.057224139	0.401309455	4	12	24	0.032587621	0.606585237	24	0.751296623	0.09934877
ENSMUSG00000036817	Sun1	Sad1 and UNC84 domain containing 1 [Source:MGI Symbol;Acc:MGI:1924303]	30.6774	30.5789	29.0088	30.6514	28.9506	28.3925	28.8461	30.3295	30.5675	31.1622	26.6729	29.5784	30.4463	28.5582	0.265774856	0.773510818	16	4	24	0.343856556	0.777386111	24	6.045650249	0.034065738
ENSMUSG00000036819	Jmjd4	jumonji domain containing 4 [Source:MGI Symbol;Acc:MGI:2144404]	5.90636	5.38308	5.80695	5.44744	5.49788	5.83097	5.24612	5.61444	4.92417	6.22911	5.17955	5.91073	5.65834	5.97734	0.849180783	0.966018836	24	12	24	0.898088366	0.970959341	24	21.55958117	0.015264321
ENSMUSG00000036820	Amdhd2	amidohydrolase domain containing 2 [Source:MGI Symbol;Acc:MGI:2443978]	8.76113	10.9642	11.1404	11.9981	11.6225	12.0139	10.6435	10.749	12.305	11.2517	10.7536	10.1977	11.5453	11.8658	0.321662653	0.83607295	16	12	24	0.15095005	0.758865539	24	10.53951356	0.081432425
ENSMUSG00000036822	Topors	topoisomerase I binding, arginine/serine-rich [Source:MGI Symbol;Acc:MGI:2146189]	6.12754	6.0333	5.92382	6.24741	6.60592	6.04589	5.50223	5.531	6.14916	5.89975	6.45023	6.00564	5.87773	6.25201	0.148627651	0.59172107	20	8	24	0.102509775	0.713699364	24	14.69250854	0.048770895
ENSMUSG00000036825	Ssx2ip	synovial sarcoma, X 2 interacting protein [Source:MGI Symbol;Acc:MGI:2139150]	27.5821	22.3186	22.8026	23.2124	22.4736	25.33	22.6381	22.5254	17.8172	26.0367	23.0284	22.7611	23.202	23.6863	0.838532766	0.966018836	16	20	24	0.299280094	0.768040178	24	20.22056226	0.083723313
ENSMUSG00000036826	Igflr1	IGF-like family receptor 1 [Source:MGI Symbol;Acc:MGI:3655979]	1.84715	0.978975	0.506988	0.667975	2.29577	1.39531	1.43713	1.24124	1.83629	2.23772	0.598958	1.49208	2.23354	1.34407	0.579092016	0.966018836	4	4	24	0.599965593	0.860011178	24	21.71240009	0.151829784
ENSMUSG00000036832	Lpar3	lysophosphatidic acid receptor 3 [Source:MGI Symbol;Acc:MGI:1929469]	24.4674	17.4477	16.3859	19.2519	16.531	21.8051	17.3738	19.7488	14.0301	25.215	18.6221	22.2918	23.5778	20.5155	0.217827365	0.704989686	24	12	24	0.315930636	0.768040178	24	20.97405516	0.139438294
ENSMUSG00000036833	Pnpla7	patatin-like phospholipase domain containing 7 [Source:MGI Symbol;Acc:MGI:2385325]	3.62427	5.38074	6.34266	6.41216	4.72565	5.46682	4.36294	6.32265	5.99626	6.47762	4.46115	5.40838	4.33133	4.79373	2.98E-04	0.049770315	16	12	24	0.019102648	0.562219749	24	9.981521161	0.217991762
ENSMUSG00000036834	Plch1	phospholipase C, eta 1 [Source:MGI Symbol;Acc:MGI:2683547]	1.05943	1.66908	1.85627	1.11064	1.45898	1.20136	1.86618	1.58555	1.84196	1.12738	1.04764	1.50082	1.08976	1.2686	0.212359178	0.701530532	12	4	24	0.278739118	0.768040178	24	5.533767265	0.115362318
ENSMUSG00000036835	Psenen	presenilin enhancer gamma secretase subunit [Source:MGI Symbol;Acc:MGI:1913590]	7.11848	5.80699	6.56057	6.45729	7.91988	6.58499	6.69826	5.14379	5.33391	5.72496	6.43022	5.216	5.36959	4.76152	0.431765373	0.966018836	20	12	24	0.005361778	0.4424469	24	17.2958587	0.141857718
ENSMUSG00000036840	Siah1a	siah E3 ubiquitin protein ligase 1A [Source:MGI Symbol;Acc:MGI:108064]	15.5603	16.4299	15.7629	14.9235	14.3967	15.2023	15.3952	15.2985	14.5394	15.292	14.739	16.1988	15.7731	15.3703	0.046535188	0.375011045	4	16	24	0.097733799	0.71175955	24	1.447812134	0.040312282
ENSMUSG00000036845	Lin37	lin-37 homolog (C. elegans) [Source:MGI Symbol;Acc:MGI:1922910]	11.0188	13.5856	12.3288	9.3474	16.2905	15.5104	12.1805	10.3077	10.454	13.2741	12.5035	9.96858	11.9445	9.80546	0.839634479	0.966018836	20	12	24	0.250602427	0.768040178	24	17.61713376	0.140794273
ENSMUSG00000036850	Mrpl41	mitochondrial ribosomal protein L41 [Source:MGI Symbol;Acc:MGI:1333816]	78.2775	81.9801	74.2047	74.5867	81.6461	73.0126	67.2008	70.3841	84.1102	61.598	89.1671	76.0891	83.4655	80.1518	0.882263435	0.966018836	20	20	24	0.956778304	0.988639288	24	21.3584015	0.017532698
ENSMUSG00000036854	Hspb6	heat shock protein, alpha-crystallin-related, B6 [Source:MGI Symbol;Acc:MGI:2685325]	6.83271	12.0085	20.0664	18.3664	16.6834	19.3691	11.4152	36.0302	17.9288	32.1252	11.5723	12.4233	15.4017	13.9961	0.091424683	0.483797062	16	12	24	0.121296362	0.733729587	24	9.96158267	0.448040093
ENSMUSG00000036856	Wnt4	wingless-type MMTV integration site family, member 4 [Source:MGI Symbol;Acc:MGI:98957]	1.57011	1.85402	1.93351	1.4168	1.21344	1.29643	1.10413	2.39236	2.24527	1.01738	1.22125	1.64277	1.21087	1.67423	0.046934151	0.375011045	12	4	24	0.036713815	0.609176161	24	5.712545089	0.214667992
ENSMUSG00000036860	Mrpl55	mitochondrial ribosomal protein L55 [Source:MGI Symbol;Acc:MGI:1914462]	24.814	24.4256	25.969	25.1482	24.6046	27.417	26.0562	28.436	26.3986	26.8625	25.3076	25.329	27.2071	24.8987	0.844448841	0.966018836	24	20	24	0.836637943	0.949953371	24	4.152396797	0.014810164
ENSMUSG00000036862	Dchs1	dachsous cadherin related 1 [Source:MGI Symbol;Acc:MGI:2685011]	0.459068	0.723929	0.931198	0.627603	0.691675	0.79403	0.615382	0.720928	0.598557	0.756313	0.454546	0.583603	0.549556	0.644438	0.480187506	0.966018836	12	16	24	0.36433319	0.788789286	24	8.750298932	0.062269804
ENSMUSG00000036863	Syde2	synapse defective 1, Rho GTPase, homolog 2 (C. elegans) [Source:MGI Symbol;Acc:MGI:3036264]	1.41728	1.78363	1.85514	2.3512	2.15726	1.47219	1.96732	1.46139	2.158	2.11324	1.69549	1.9538	1.73083	1.38024	0.046535188	0.375011045	16	16	24	0.042873032	0.624864512	24	12.73909117	0.14185672
ENSMUSG00000036864	Proser3	proline and serine rich 3 [Source:MGI Symbol;Acc:MGI:2681861]	3.09736	2.01944	1.96026	3.05033	3.00881	2.22482	1.931	2.19209	1.75698	1.8011	3.26043	1.48935	2.47421	2.095	0.616047374	0.966018836	20	16	24	0.384378975	0.799546792	24	17.13862605	0.127932702
ENSMUSG00000036867	Smad6	SMAD family member 6 [Source:MGI Symbol;Acc:MGI:1336883]	0.887948	0.638659	0.644326	0.488005	0.361027	0.523517	0.648251	0.823729	0.859021	0.912156	0.788846	0.661763	0.579968	0.781672	0.928127947	0.966018836	12	8	24	0.257284368	0.768040178	24	5.738682151	0.090411732
ENSMUSG00000036872	Abcc12	ATP-binding cassette, sub-family C (CFTR/MRP), member 12 [Source:MGI Symbol;Acc:MGI:2441679]	0.77881	0.558391	1.06729	0.86468	0.618598	0.808625	0.860754	0.557105	0.701246	0.599011	0.497252	0.605953	0.620599	0.537384	0.964321074	0.968944646	12	20	24	0.974349201	0.993545331	24	9.503700796	0.010697567
ENSMUSG00000036873	2410004B18Rik	RIKEN cDNA 2410004B18 gene [Source:MGI Symbol;Acc:MGI:1913671]	21.5402	21.9083	21.0622	23.1529	21.1244	20.7474	22.4138	21.2108	20.4639	18.2375	23.0049	19.8165	20.2722	22.0623	0.903465533	0.966018836	8	16	24	0.905727947	0.974160235	24	0.700545142	0.014883291
ENSMUSG00000036875	Dna2	DNA replication helicase/nuclease 2 [Source:MGI Symbol;Acc:MGI:2443732]	1.13988	1.41147	1.76866	1.17493	1.54332	1.43053	1.2008	1.44218	1.45902	1.37369	1.56346	1.31157	1.31217	1.61293	0.101520755	0.511659635	12	16	24	0.301926856	0.768040178	24	9.564944436	0.064341927
ENSMUSG00000036879	Phkb	phosphorylase kinase beta [Source:MGI Symbol;Acc:MGI:97578]	22.1873	23.8381	22.5357	24.972	22.9661	22.76	22.9252	30.2835	29.23	25.6027	25.1385	24.232	26.3165	21.0016	0.886952527	0.966018836	20	16	24	0.47234526	0.805393068	24	8.738841656	0.070406952
ENSMUSG00000036880	Acaa2	acetyl-Coenzyme A acyltransferase 2 (mitochondrial 3-oxoacyl-Coenzyme A thiolase) [Source:MGI Symbol;Acc:MGI:1098623]	44.7475	49.6429	58.7932	89.4839	57.1056	50.9258	53.4077	69.9327	64.862	73.1842	74.9817	60.297	81.4053	69.5756	0.602156008	0.966018836	12	8	24	0.051605285	0.647688506	24	10.94001897	0.208850678
ENSMUSG00000036882	Arhgap33	Rho GTPase activating protein 33 [Source:MGI Symbol;Acc:MGI:2673998]	13.3834	10.8121	11.5402	11.3946	13.2151	11.2364	10.3951	12.1909	12.9311	11.7123	14.0394	11.9379	12.1122	10.634	0.702630284	0.966018836	20	16	24	0.321674048	0.768040178	24	15.73993504	0.070212935
ENSMUSG00000036885	Arhgef26	Rho guanine nucleotide exchange factor (GEF) 26 [Source:MGI Symbol;Acc:MGI:1918053]	3.98312	4.26879	4.59714	4.98811	5.35002	5.10649	5.37855	4.94074	4.45716	5.12474	4.57028	4.38933	4.32168	4.0244	0.215414708	0.704989686	20	12	24	0.195606424	0.768040178	24	15.15551716	0.080191655
ENSMUSG00000036887	C1qa	complement component 1, q subcomponent, alpha polypeptide [Source:MGI Symbol;Acc:MGI:88223]	22.421	24.1974	24.1379	17.7275	25.7839	19.1944	24.4869	25.8459	29.6743	14.6628	25.0224	20.9569	20.1766	17.7743	0.961261938	0.967043964	12	4	24	0.787669622	0.931762722	24	2.751057604	0.069883193

ENSMUSG00000036890	Gtdc1	glycosyltransferase-like domain containing 1 [Source:MGI Symbol;Acc:MGI:2444269]	17.0493	19.5475	22.0865	19.4904	19.109	17.6657	21.8063	19.0014	23.3518	21.7996	17.973	21.8085	21.5908	20.6764	0.373702442	0.896700272	12	8	24	0.163123175	0.766991901	24	7.708503505	0.090605192
ENSMUSG00000036893	Ehmt1	euchromatic histone methyltransferase 1 [Source:MGI Symbol;Acc:MGI:1924933]	9.80637	10.6543	9.53048	10.3524	10.1981	10.4144	9.66118	8.43344	10.0473	10.4419	9.51005	9.39631	9.85433	9.82034	0.951082476	0.966018836	16	12	24	0.533122975	0.825678486	24	14.36323887	0.032854799
ENSMUSG00000036894	Rap2b	RAP2B, member of RAS oncogene family [Source:MGI Symbol;Acc:MGI:1921262]	2.20058	1.9615	2.19447	2.2126	2.11488	2.09864	2.02806	2.09863	2.03881	2.15008	2.12422	2.16188	2.1553	2.15985	0.92769718	0.966018836	16	16	24	0.577926961	0.849052597	24	14.8111444	0.013758025
ENSMUSG00000036896	C1qc	complement component 1, q subcomponent, C chain [Source:MGI Symbol;Acc:MGI:88225]	15.8408	18.5597	19.8886	15.701	17.7196	13.3888	15.9217	20.8273	26.2794	11.9789	21.8058	16.2898	16.0329	17.1566	0.12833063	0.560209134	12	12	24	0.331027734	0.768040178	24	7.247951223	0.164604377
ENSMUSG00000036898	Zfp157	zinc finger protein 157 [Source:MGI Symbol;Acc:MGI:1919404]	4.91925	5.0391	5.15583	5.15174	5.05436	4.8533	4.75958	4.59541	5.22351	4.22009	5.11741	4.92658	4.94002	5.1185	0.074036902	0.448301997	12	16	24	0.973585971	0.993305115	24	7.82495805	0.006582643
ENSMUSG00000036902	Neto2	neuropilin (NRP) and tolloid (TLL)-like 2 [Source:MGI Symbol;Acc:MGI:1921763]	7.77952	7.1935	7.5566	6.63722	7.38316	8.11141	7.88472	6.69646	6.68434	5.9554	8.04895	6.93671	6.79134	7.11922	0.056712099	0.401309455	20	20	24	0.08311101	0.699403344	24	21.06115163	0.090248567
ENSMUSG00000036904	Fzd8	frizzled class receptor 8 [Source:MGI Symbol;Acc:MGI:108460]	2.26601	1.99983	1.68165	1.30541	1.87378	1.83017	2.74685	2.24744	1.53867	1.87374	1.97684	2.05306	2.27901	1.93331	1.57E-04	0.031913438	4	12	24	0.004378838	0.427431763	24	23.45696753	0.183602373
ENSMUSG00000036905	C1qb	complement component 1, q subcomponent, beta polypeptide [Source:MGI Symbol;Acc:MGI:88224]	21.2087	26.8449	28.6239	23.6128	28.6964	22.154	22.1127	25.0433	31.8279	12.6497	27.5956	21.1428	21.0629	21.8291	0.105280783	0.518002936	12	16	24	0.868024622	0.962298123	24	7.018194231	0.066071758
ENSMUSG00000036908	Unc93b1	unc-93 homolog B1, TLR signaling regulator [Source:MGI Symbol;Acc:MGI:1859307]	9.27255	10.2757	10.9206	9.24983	9.36058	7.9965	11.1092	11.0089	11.5432	8.40743	10.0777	8.38399	10.4887	10.0813	0.05282315	0.391647859	12	12	24	0.032223475	0.606585237	24	5.506194185	0.14248938
ENSMUSG00000036913	Trim67	tripartite motif-containing 67 [Source:MGI Symbol;Acc:MGI:3045323]	9.32869	6.87661	6.71283	8.21452	7.8295	7.87788	7.17664	7.75278	6.17246	10.4286	7.55857	7.90428	8.35978	7.76527	0.175185919	0.651010555	16	20	24	0.489666257	0.805393068	24	17.8443093	0.081459043
ENSMUSG00000036916	Zfp280c	zinc finger protein 280C [Source:MGI Symbol;Acc:MGI:2387585]	11.7357	14.5082	13.5967	12.3538	14.3225	11.8171	11.5904	11.8833	14.5461	10.5078	13.5027	11.1161	11.278	12.3112	0.068712455	0.432567661	12	12	24	0.406783374	0.799546792	24	9.540570559	0.071872253
ENSMUSG00000036918	Ttc7	tetratricopeptide repeat domain 7 [Source:MGI Symbol;Acc:MGI:1920999]	3.70801	3.58964	5.13993	4.674	4.24027	3.73172	4.10162	4.04681	4.34264	3.9218	3.19862	3.48996	3.54814	3.79567	0.071575474	0.440262583	12	12	24	0.052564205	0.650526959	24	9.202913251	0.117797575
ENSMUSG00000036928	Stag3	stromal antigen 3 [Source:MGI Symbol;Acc:MGI:1355311]	0.428466	0.78814	0.710241	0.60492	0.575483	0.222502	0.256366	0.479865	0.369407	0.977939	0.385147	0.522406	0.530901	0.391423	0.499268446	0.966018836	16	8	24	0.155184677	0.760294912	24	10.1621205	0.140301934
ENSMUSG00000036931	Nfkbid	nuclear factor of kappa light polypeptide gene enhancer in B cells inhibitor, delta [Source:MGI Symbol;Acc:MGI:3041243]	0.733138	0.928248	0.932118	1.14389	0.762533	0.824122	0.922334	0.865114	0.885471	0.683296	1.11046	0.801947	1.06283	0.997253	0.888482515	0.966018836	12	12	24	0.798520496	0.935456643	24	7.559736898	0.029082404
ENSMUSG00000036932	Aifm1	apoptosis-inducing factor, mitochondrion-associated 1 [Source:MGI Symbol;Acc:MGI:1349419]	15.776	16.4784	15.8855	19.5786	18.4181	16.3277	16.2528	15.4253	16.2684	16.2951	17.7632	15.732	17.7701	16.3667	0.435596482	0.966018836	20	4	24	0.123065574	0.736059265	24	14.61058182	0.070473333
ENSMUSG00000036934	4921524J17Rik	RIKEN cDNA 4921524J17 gene [Source:MGI Symbol;Acc:MGI:1913964]	9.07638	8.21035	9.10817	9.43234	8.01196	8.94678	9.1181	7.34749	9.09501	7.5067	9.87258	8.63457	8.67826	7.58051	0.492524295	0.966018836	20	12	24	0.438353435	0.805393068	24	17.56327878	0.05904638
ENSMUSG00000036940	Kdm1a	lysine (K)-specific demethylase 1A [Source:MGI Symbol;Acc:MGI:1196256]	22.0052	23.2706	24.1418	21.3028	20.8935	23.069	22.5616	24.3781	21.2985	23.9811	20.1379	22.8136	22.2674	23.4151	0.06180207	0.409814092	8	12	24	0.131040166	0.741427049	24	4.369993688	0.060327413
ENSMUSG00000036941	Elac1	elaC ribonuclease Z 1 [Source:MGI Symbol;Acc:MGI:1890495]	7.02481	6.77832	8.06822	6.90912	6.17294	6.55992	7.01254	7.04602	6.67822	7.60989	6.40521	7.30599	7.23473	7.75471	0.421215102	0.966018836	8	12	24	0.145883922	0.754579257	24	5.80720201	0.06936726
ENSMUSG00000036943	Rab8b	RAB8B, member RAS oncogene family [Source:MGI Symbol;Acc:MGI:2442982]	15.3273	15.4046	16.1295	16.4772	16.9111	15.075	16.2378	14.7013	16.5129	14.8417	16.6134	14.5187	14.3691	14.0579	0.109058178	0.524035616	20	12	24	0.072451763	0.679262305	24	13.60712908	0.059871081
ENSMUSG00000036944	Tmem71	transmembrane protein 71 [Source:MGI Symbol;Acc:MGI:2146049]	0.378255	1.04138	1.06741	0.856958	0.580504	0.832592	0.599516	0.995123	1.05532	0.712373	0.648577	0.817763	0.762425	0.756223	0.029825002	0.323426482	12	16	24	0.075217549	0.684673558	24	7.753944324	0.145517644
ENSMUSG00000036948	Trappc14	trafficking protein particle complex 14 [Source:MGI Symbol;Acc:MGI:2385896]	24.2891	20.0262	20.6837	21.584	22.1153	22.2918	23.2346	22.3705	19.2352	24.6182	21.998	21.8742	20.6454	21.3266	0.379902653	0.908319827	24	12	24	0.299133447	0.768040178	24	18.90769634	0.058869432
ENSMUSG00000036949	Slc39a12	solute carrier family 39 (zinc transporter), member 12 [Source:MGI Symbol;Acc:MGI:2139274]	1.46552	4.31401	7.91029	2.62013	2.11256	4.23432	5.50987	2.42239	4.89941	2.14469	1.67645	3.21228	0.993743	3.84814	0.285027487	0.793714531	12	8	24	0.385455171	0.799546792	24	6.777448254	0.326836639
ENSMUSG00000036955	Kifbp	kinesin family binding protein [Source:MGI Symbol;Acc:MGI:1919570]	45.2556	43.2047	43.1269	42.752	42.3932	45.1262	42.4499	42.2282	38.8422	45.6045	40.9939	44.2096	42.4479	42.8934	0.922029406	0.966018836	24	20	24	0.37207834	0.793398709	24	20.42110447	0.03283706
ENSMUSG00000036957	Lrfn3	leucine rich repeat and fibronectin type III domain containing 3 [Source:MGI Symbol;Acc:MGI:2442512]	2.10759	1.73164	1.69281	2.08	1.94903	2.11784	1.93706	2.23469	1.98072	3.15801	2.28975	2.71155	2.35393	2.62289	0.868508291	0.966018836	24	12	24	0.603274062	0.861388046	24	15.6765312	0.048291919
ENSMUSG00000036959	Bcorl1	BCL6 co-repressor-like 1 [Source:MGI Symbol;Acc:MGI:2443910]	2.4861	2.23531	2.51507	2.05823	2.75534	2.66316	2.71583	1.68457	2.02084	2.8763	1.92421	2.02361	2.52415	2.49265	0.93447422	0.966018836	16	16	24	0.74840667	0.918047827	24	19.40959332	0.050722027
ENSMUSG00000036964	Trim17	tripartite motif-containing 17 [Source:MGI Symbol;Acc:MGI:1861440]	1.10951	0.954337	0.968036	0.891771	0.846937	0.935377	0.986101	1.1689	0.812983	1.03593	1.00797	0.881185	1.02461	1.10176	0.202982082	0.689605535	8	4	24	0.171548083	0.768040178	24	2.521903509	0.052255458
ENSMUSG00000036966	Spryd3	SPRY domain containing 3 [Source:MGI Symbol;Acc:MGI:2446175]	47.4685	37.8009	39.4704	39.9427	37.9529	44.8776	40.2387	42.5374	34.0448	48.5116	36.9364	43.6828	41.1152	43.1686	0.446653834	0.966018836	24	12	24	0.513849892	0.816381038	24	21.748041	0.065469231
ENSMUSG00000036968	Cnpy4	canopy FGF signaling regulator 4 [Source:MGI Symbol;Acc:MGI:1913705]	10.984	10.9624	10.1621	9.93734	12.6541	10.9894	12.7036	10.7423	10.6242	8.52371	11.4491	11.4524	11.2962	9.82852	0.002006795	0.100255953	20	20	24	0.040416252	0.616921492	24	21.13516833	0.12559313
ENSMUSG00000036972	Zic4	zinc finger protein of the cerebellum 4 [Source:MGI Symbol;Acc:MGI:107201]	1.4888	2.93445	2.09773	1.96955	2.60434	1.6156	1.40326	2.45902	3.5785	0.922376	2.26493	2.06906	1.76601	2.00125	0.235187365	0.73629392	12	16	24	0.552462907	0.836737636	24	7.537188643	0.142125425
ENSMUSG00000036975	Tmem177	transmembrane protein 177 [Source:MGI Symbol;Acc:MGI:1913593]	4.02168	3.39813	2.98165	3.85233	3.49995	4.03548	3.71491	4.17661	2.91645	4.16048	3.86378	4.09127	3.61886	3.85598	0.148073804	0.590732968	24	12	24	0.211686581	0.768040178	24	19.95657061	0.087882736
ENSMUSG00000036977	Anapc10	anaphase promoting complex subunit 10 [Source:MGI Symbol;Acc:MGI:1916249]	12.1123	12.876	12.2687	14.1622	14.4786	12.9552	12.8026	12.904	14.6082	10.4908	12.2136	12.2988	10.3819	12.204	0.671726933	0.966018836	12	16	24	0.68517002	0.895310095	24	12.24993726	0.042760988
ENSMUSG00000036980	Taf6	TATA-box binding protein associated factor 6 [Source:MGI Symbol;Acc:MGI:109129]	12.9491	11.4401	12.5585	11.625	12.5285	13.8843	13.1625	12.972	11.0456	13.1946	13.7376	13.4639	11.844	12.075	0.038115943	0.334069441	24	12	24	0.048749955	0.638992719	24	18.97821663	0.086396603
ENSMUSG00000036983	Tfb1m	transcription factor B1, mitochondrial [Source:MGI Symbol;Acc:MGI:2146851]	10.9858	10.4516	9.81977	10.0151	10.4345	10.8644	10.4621	10.0149	9.41631	9.92132	10.7804	11.0996	11.6043	10.778	1.57E-04	0.031913438	24	12	24	7.41E-04	0.319044294	24	21.49339928	0.085280758
ENSMUSG00000036985	Zdhhc9	zinc finger, DHHC domain containing 9 [Source:MGI Symbol;Acc:MGI:2444393]	19.9188	16.0811	17.8376	17.3929	18.5547	20.239	18.9694	18.8221	14.399	22.4929	16.0281	18.5898	17.9104	17.6109	0.204218286	0.690546825	24	12	24	0.445417661	0.805393068	24	19.55012311	0.078490983
ENSMUSG00000036986	Pml	promyelocytic leukemia [Source:MGI Symbol;Acc:MGI:104662]	3.13002	3.92942	3.14959	3.24265	4.19885	3.87589	4.24351	3.66918	3.61648	3.85458	4.1233	3.39361	3.84033	3.37777	0.616047374	0.966018836	20	16	24	0.482398718	0.805393068	24	18.51525879	0.057263305
ENSMUSG00000036989	Trim3	tripartite motif-containing 3 [Source:MGI Symbol;Acc:MGI:1860040]	32.7452	34.6275	35.4161	38.3233	33.3141	34.3938	33.4352	33.6252	34.9301	37.0316	31.7973	32.7072	30.5441	35.6206	0.006570511	0.177465007	16	4	24	0.018466207	0.559812298	24	9.916649141	0.080691153
ENSMUSG00000036990	Otud4	OTU domain containing 4 [Source:MGI Symbol;Acc:MGI:1098801]	13.0652	14.2759	13.6252	15.1995	14.0855	12.819	12.5533	12.4346	14.5794	12.6042	14.4977	12.9656	13.0211	13.8239	0.216688884	0.704989686	20	4	24	0.132365842	0.742518551	24	11.33281835	0.064675346
ENSMUSG00000036992	Nxt1	NTF2-related export protein 1 [Source:MGI Symbol;Acc:MGI:1929619]	8.37028	8.77	8.31351	8.21036	8.04107	7.43697	7.83826	8.01405	8.75437	6.57641	9.82497	9.05327	9.77451	9.24504	0.915082018	0.966018836	8	8	24	0.613555966	0.866162311	24	1.135606797	0.050543634
ENSMUSG00000036995	Asap3	ArfGAP with SH3 domain, ankyrin repeat and PH domain 3 [Source:MGI Symbol;Acc:MGI:2684986]	3.37916	3.43672	3.27564	3.05944	3.54646	3.51989	3.6444	3.52173	3.52231	2.82946	3.68292	3.53209	3.1849	3.00634	0.129903614	0.560263689	20	20	24	0.227418379	0.768040178	24	20.93987526	0.058956314
ENSMUSG00000037001	Zfp39	zinc finger protein 39 [Source:MGI Symbol;Acc:MGI:99183]	1.63465	1.91887	2.18381	1.81423	1.73645	1.66309	1.8433	2.18648	1.80319	1.72432	1.48228	1.70982	1.65494	1.80353	0.049157914	0.380869821	8	16	24	0.018236573	0.559812298	24	6.232195563	0.094357605
ENSMUSG00000037003	Tns2	tensin 2 [Source:MGI Symbol;Acc:MGI:2387586]	14.0873	18.7523	21.2018	26.3047	17.7845	16.2466	13.9248	19.2942	27.1649	18.9135	17.6334	14.4561	14.9223	15.9333	9.10E-06	0.009754434	12	16	24	3.55E-04	0.227979988	24	10.05836026	0.345175828
ENSMUSG00000037007	Zfp113	zinc finger protein 113 [Source:MGI Symbol;Acc:MGI:1929116]	2.62821	2.77963	1.84034	2.34997	2.37704	2.67036	2.55679	2.9485	2.76941	2.41729	2.42826	3.20538	2.64875	2.56512	0.143744387	0.585959661	8	8	24	0.15878891	0.765350752	24	23.2248096	0.090279201
ENSMUSG00000037010	Apln	apelin [Source:MGI Symbol;Acc:MGI:1353624]	0.702404	2.07157	2.81296	1.36151	1.00859	2.31778	2.07421	1.20365	1.68818	1.59285	0.805945	1.80768	0.958661	2.20448	0.70641939	0.966018836	12	8	24	0.482282742	0.805393068	24	6.322742511	0.176431202
ENSMUSG00000037012	Hk1	hexokinase 1 [Source:MGI Symbol;Acc:MGI:96103]	65.0972	47.7449	54.2322	50.5914	51.9303	60.5278	56.7929	59.239	39.7897	75.7084	46.4062	56.7186	49.1424	56.7968	0.916746631	0.966018836	24	12	24	0.773064301	0.927005228	24	20.62026841	0.067350851
ENSMUSG00000037013	Ss18	SS18, nBAF chromatin remodeling complex subunit [Source:MGI Symbol;Acc:MGI:107708]	15.2414	15.7636	15.5237	15.1989	16.6182	13.8936	17.9888	14.0456	16.4556	14.6298	16.7324	15.0778	15.6304	15.0721	0.933543173	0.966018836	20	4	24	0.962903494	0.990726108	24	17.50466244	0.010893472
ENSMUSG00000037014	Sstr4	somatostatin receptor 4 [Source:MGI Symbol;Acc:MGI:105372]	0.566102	0.783885	0.650112	0.506598	0.448726	0.806057	0.691422	1.01589	0.774293	0.581667	0.997206	0.90017	0.878252	0.747862	0.644063931	0.966018836	24	16	24	0.315558839	0.768040178	24	1.190691006	0.07274561
ENSMUSG00000037016	Frem2	Fras1 related extracellular matrix protein 2 [Source:MGI Symbol;Acc:MGI:2444465]	0.672812	0.762704	0.975017	0.720414	0.5164	0.752395	0.505081	0.694912	0.554587	0.978756	0.85758	0.736892	0.57519	0.665058	0.305564675	0.811201458	16	12	24	0.305406868	0.768040178	24	11.48997087	0.075115356
ENSMUSG00000037017	Zscan21	zinc finger and SCAN domain containing 21 [Source:MGI Symbol;Acc:MGI:99182]	12.6322	14.082	13.9937	12.9212	12.7642	12.7441	12.3048	12.4175	14.0655	12.5525	12.1578	12.6768	12.6455	13.5372	0.176754388	0.653256853	12	16	24	0.032463378	0.606585237	24	7.046283448	0.063010998
ENSMUSG00000037020	Wdr62	WD repeat domain 62 [Source:MGI Symbol;Acc:MGI:1923696]	2.41484	1.74059	2.27024	2.85121	3.11622	3.33135	2.46523	2.46712	2.69453	2.89125	3.0409	2.74818	2.94363	3.41005	0.194763766	0.673387182	20	16	24	0.085640272	0.703577714	24	16.31722462	0.117983889
ENSMUSG00000037022	Mmaa	methylmalonic aciduria (cobalamin deficiency) type A [Source:MGI Symbol;Acc:MGI:1923805]	9.60157	9.30471	9.05117	9.04546	10.3967	9.27125	10.2548	9.36586	9.28613	8.58643	10.9075	9.54528	10.3353	9.54254	0.00432407	0.140598674	20	20	24	0.135892568	0.747972781	24	20.39212075	0.064625349
ENSMUSG00000037029	Zfp146	zinc finger protein 146 [Source:MGI Symbol;Acc:MGI:1347092]	15.6233	16.4364	16.5583	15.7862	16.5514	15.6486	14.8412	15.1723	17.4407	14.1429	15.3016	14.7406	16.1308	14.1652	0.830364743	0.966018836	12	12	24	0.795986936	0.934814172	24	9.157263757	0.020821519
ENSMUSG00000037031	Tspan15	tetraspanin 15 [Source:MGI Symbol;Acc:MGI:1917673]	26.8135	24.82	21.7798	27.982	30.1621	24.6439	25.2111	26.3424	24.3902	24.4228	26.7912	24.2863	26.2114	22.887	0.424718357	0.966018836	20	16	24	0.162929521	0.766462333	24	17.56675053	0.082175329
ENSMUSG00000037032	Apbb1	amyloid beta (A4) precursor protein-binding, family B, member 1 [Source:MGI Symbol;Acc:MGI:107765]	76.9752	68.4377	67.4213	65.7058	64.2952	74.2196	71.8546	69.3087	59.2387	79.4889	61.4991	71.6851	66.691	71.9657	0.371875382	0.894537464	24	20	24	0.438031836	0.805393068	24	22.96325245	0.059764644
ENSMUSG00000037035	Inhbb	inhibin beta-B [Source:MGI Symbol;Acc:MGI:96571]	1.04401	0.89103	1.23119	1.0384	1.02043	1.08914	1.04037	1.14791	0.953584	1.3852	1.11616	0.901052	0.923441	1.09231	0.851656735	0.966018836	16	12	24	0.238520597	0.768040178	24	11.11875083	0.060308209
ENSMUSG00000037049	Smpd1	sphingomyelin phosphodiesterase 1, acid lysosomal [Source:MGI Symbol;Acc:MGI:98325]	29.4898	25.4661	27.517	26.2034	24.2756	28.1439	26.4952	30.7135	25.4912	31.0316	25.319	27.6677	26.3735	27.665	0.89563591	0.966018836	24	20	24	0.70962471	0.903951515	24	3.627533835	0.033863285
ENSMUSG00000037058	Paip2	polyadenylate-binding protein-interacting protein 2 [Source:MGI Symbol;Acc:MGI:1915119]	198.483	207.989	197.323	208.499	209.784	202.402	196.997	189.685	192.971	172.013	206.453	186.004	191.35	189.082	0.869721097	0.966018836	20	12	24	0.536719322	0.827513555	24	18.47859125	0.029849095
ENSMUSG00000037060	Cavin3	caveolae associated 3 [Source:MGI Symbol;Acc:MGI:1923422]	26.4033	27.4479	25.081	22.8642	27.6024	22.7755	27.4823	26.5084	30.9027	24.0521	30.5366	29.1426	30.3495	26.7773	0.903207257	0.966018836	20	20	24	0.686253228	0.895774045	24	0.817793089	0.042934235
ENSMUSG00000037062	Sh3glb1	SH3-domain GRB2-like B1 (endophilin) [Source:MGI Symbol;Acc:MGI:1859730]	49.0618	46.8577	52.966	56.4456	49.3576	50.545	48.9733	53.7619	51.7542	54.5269	52.8714	48.1113	53.9627	50.234	0.203788871	0.689899499	16	8	24	0.088216341	0.707747429	24	10.91981672	0.061731291
ENSMUSG00000037070	Rbmxl1	RNA binding motif protein, X-linked like-1 [Source:MGI Symbol;Acc:MGI:1343045]	9.21753	9.1421	9.65136	8.57554	8.88515	8.8953	9.3153	8.3709	9.18448	8.13054	8.66287	9.19287	8.43053	9.04121	0.510431574	0.966018836	12	4	24	0.526635418	0.823075573	24	1.56292984	0.025511088
ENSMUSG00000037071	Scd1	stearoyl-Coenzyme A desaturase 1 [Source:MGI Symbol;Acc:MGI:98239]	281.488	205.686	330.208	458.313	281.85	316.876	337.907	382.137	235.937	391.91	484.573	363.6	513.787	379.862	0.783267549	0.966018836	16	20	24	0.550845156	0.836129338	24	15.99802738	0.135420241
ENSMUSG00000037072	Selenof	selenoprotein F [Source:MGI Symbol;Acc:MGI:1927947]	252.286	278.329	265.065	276.465	280.139	248.375	251.354	245.818	303.602	214.808	284.085	245.869	254.715	241.761	0.83638114	0.966018836	20	4	24	0.736036352	0.913997413	24	11.40572815	0.038069283
ENSMUSG00000037075	Rnf139	ring finger protein 139 [Source:MGI Symbol;Acc:MGI:1923091]	12.6623	13.7074	13.5244	13.4694	13.7385	12.9649	11.9653	12.8428	13.8413	11.4017	13.2009	12.5201	13.23	12.9007	0.217827365	0.704989686	12	12	24	0.767661767	0.924952565	24	8.877043183	0.021649006
ENSMUSG00000037085	Trmt12	tRNA methyltransferase 12 [Source:MGI Symbol;Acc:MGI:1915510]	2.07404	1.88714	1.82629	1.44813	1.83506	1.75044	1.64599	1.97374	1.64819	1.78349	1.76552	1.99303	1.92004	1.66771	0.485187247	0.966018836	4	12	24	0.176073224	0.768040178	24	23.14570083	0.061656297
ENSMUSG00000037087	Prr32	proline rich 32 [Source:MGI Symbol;Acc:MGI:1916050]	0.796673	0.468968	0.592874	0.481537	0.502809	0.667341	0.358257	0.526055	0.343822	0.526299	0.808077	0.400638	0.418637	0.218759	0.657586167	0.966018836	20	12	24	0.150490293	0.758865539	24	17.42098292	0.103941422
ENSMUSG00000037089	Slc35b2	solute carrier family 35, member B2 [Source:MGI Symbol;Acc:MGI:1921086]	16.3971	17.0824	18.6615	16.4014	18.0131	16.9663	14.9883	18.8318	18.6167	17.9985	17.0182	17.987	17.0068	17.8917	0.019594567	0.258568924	12	16	24	0.141215876	0.751689872	24	9.259671818	0.064114553
ENSMUSG00000037095	Lrg1	leucine-rich alpha-2-glycoprotein 1 [Source:MGI Symbol;Acc:MGI:1924155]	2.19805	1.53935	2.04479	2.78538	1.45912	1.66907	1.20611	3.00109	1.8735	2.75649	2.32742	1.80341	3.15603	2.05972	0.928127947	0.966018836	8	4	24	0.529607555	0.824503202	24	9.031040658	0.123456069
ENSMUSG00000037096	Gm9762	predicted pseudogene 9762 [Source:MGI Symbol;Acc:MGI:3704220]	109.743	92.7061	95.1816	102.898	101.787	102.444	101.086	94.0391	87.6082	107.465	99.6346	99.694	102.251	104.727	0.659334214	0.966018836	16	20	24	0.225543989	0.768040178	24	19.1407074	0.055938045
ENSMUSG00000037098	Rab11fip3	RAB11 family interacting protein 3 (class II) [Source:MGI Symbol;Acc:MGI:2444431]	12.5195	11.2488	11.0032	11.9304	11.6488	12.1395	12.6601	11.847	11.4124	14.7718	11.0835	12.3025	11.4462	11.7407	0.321662653	0.83607295	24	12	24	0.796031985	0.934818454	24	16.50296077	0.027341492
ENSMUSG00000037101	Ttc29	tetratricopeptide repeat domain 29 [Source:MGI Symbol;Acc:MGI:1920551]	1.23153	1.24641	0.970206	1.00377	0.873335	0.994282	0.71955	0.879467	1.16976	0.791143	0.937502	1.2516	0.881994	1.27837	0.917867465	0.966018836	8	12	24	0.62550779	0.872249537	24	3.378051848	0.051175552
ENSMUSG00000037103	Dcaf15	DDB1 and CUL4 associated factor 15 [Source:MGI Symbol;Acc:MGI:2684420]	6.08748	6.1396	5.7343	5.84566	6.07925	6.66805	5.67461	6.78221	6.57943	6.61034	5.71103	6.65112	6.11873	6.16354	0.865233232	0.966018836	24	20	24	0.97040928	0.993003194	24	0.508165437	0.007749627
ENSMUSG00000037104	Socs5	suppressor of cytokine signaling 5 [Source:MGI Symbol;Acc:MGI:2385459]	21.8092	19.6772	20.7954	19.7078	18.8497	20.248	21.0345	19.8965	18.8114	20.6381	20.6502	20.5368	21.4902	21.0043	0.05688647	0.401309455	4	12	24	0.16876088	0.767020009	24	23.59946343	0.042909801
ENSMUSG00000037106	Fer1l6	fer-1-like 6 (C. elegans) [Source:MGI Symbol;Acc:MGI:3645398]	0.223805	0.420309	0.571893	0.418104	0.349601	0.377312	0.342245	0.672723	1.13294	0.873157	0.785693	0.521523	1.12992	0.882078	0.496745696	0.966018836	12	16	24	0.010618375	0.508846343	24	8.155508145	0.181500817
ENSMUSG00000037108	Zcwpw1	zinc finger, CW type with PWWP domain 1 [Source:MGI Symbol;Acc:MGI:2685899]	7.94821	10.9523	9.12834	9.88054	9.76762	8.84878	10.2615	9.13324	9.84506	8.3724	9.45379	9.05681	10.355	9.44275	0.917839756	0.966018836	4	20	24	0.737689985	0.914422817	24	4.958532262	0.035312719
ENSMUSG00000037110	Ralgapa2	Ral GTPase activating protein, alpha subunit 2 (catalytic) [Source:MGI Symbol;Acc:MGI:3036245]	4.28157	4.20166	4.31334	4.44035	4.46333	4.38085	4.09018	4.44015	4.01914	4.87887	4.51323	5.13609	5.02937	5.00258	0.496745696	0.966018836	12	16	24	0.669700266	0.888856595	24	18.40745137	0.024496967
ENSMUSG00000037111	Setd7	SET domain containing (lysine methyltransferase) 7 [Source:MGI Symbol;Acc:MGI:1920501]	34.7876	28.396	27.7933	30.8728	30.6914	32.3036	33.4798	31.2787	25.9198	36.8166	29.0463	31.0538	29.523	30.108	0.138543921	0.580158868	24	12	24	0.361860973	0.787031962	24	19.64918026	0.074057636
ENSMUSG00000037112	Sik2	salt inducible kinase 2 [Source:MGI Symbol;Acc:MGI:2445031]	1.9653	2.08732	2.59013	3.51701	2.28363	2.27916	2.93537	2.6899	2.43085	3.30696	2.47401	3.67245	3.66041	2.77789	0.872380105	0.966018836	16	16	24	0.792171753	0.933498508	24	13.14232113	0.047998302
ENSMUSG00000037119	Fam91a1	family with sequence similarity 91, member A1 [Source:MGI Symbol;Acc:MGI:1277178]	30.6909	29.488	28.9225	30.0809	30.9146	28.1942	27.3797	26.9783	27.9791	27.474	30.7944	28.2286	27.7464	29.1399	0.522551072	0.966018836	20	12	24	0.292617848	0.768040178	24	15.93205551	0.03609398
ENSMUSG00000037126	Psd	pleckstrin and Sec7 domain containing [Source:MGI Symbol;Acc:MGI:1920978]	7.27497	7.38205	8.56524	5.49309	6.2625	7.33748	6.53809	6.99894	9.37399	8.84961	6.59249	9.07607	8.61493	9.25296	0.581070934	0.966018836	12	8	24	0.248168939	0.768040178	24	5.266306681	0.119634536
ENSMUSG00000037134	Prmt9	protein arginine methyltransferase 9 [Source:MGI Symbol;Acc:MGI:2142651]	12.8466	13.9728	13.3157	13.8121	13.9697	12.3949	12.5262	11.5318	14.1586	12.2131	14.2746	13.0377	14.24	13.0778	0.941955432	0.966018836	20	4	24	0.665295541	0.886947643	24	13.2431688	0.031817441
ENSMUSG00000037138	Aff3	AF4/FMR2 family, member 3 [Source:MGI Symbol;Acc:MGI:106927]	9.97268	8.25091	8.15283	8.60879	8.87954	9.30384	8.75723	9.33162	7.3363	11.1432	8.1811	9.34301	8.84093	8.50908	0.041292272	0.346472636	24	12	24	0.495448281	0.807162937	24	19.25019338	0.063697526
ENSMUSG00000037139	Myom3	myomesin family, member 3 [Source:MGI Symbol;Acc:MGI:2685280]	0.712576	1.31721	2.00489	2.14983	1.18656	1.63964	1.3381	3.92155	1.108	2.54997	1.24487	0.838526	1.79842	1.77734	0.435739761	0.966018836	16	4	24	0.209836789	0.768040178	24	8.079957627	0.276761931
ENSMUSG00000037143	Cfap61	cilia and flagella associated protein 61 [Source:MGI Symbol;Acc:MGI:1926024]	3.9648	5.57264	3.87398	6.78225	3.68166	4.7378	4.49741	3.49131	4.51291	4.05319	4.40241	5.71704	4.51794	4.76062	0.849180783	0.966018836	24	20	24	0.924468698	0.979866138	24	15.88165532	0.03495192
ENSMUSG00000037148	Arhgap10	Rho GTPase activating protein 10 [Source:MGI Symbol;Acc:MGI:1925764]	4.15863	4.18719	4.27446	4.6561	4.52978	4.17698	4.78329	5.20124	5.04341	4.96672	4.10532	4.80005	5.09526	4.41202	0.928127947	0.966018836	16	4	24	0.566860656	0.843567381	24	6.936433123	0.038992568
ENSMUSG00000037149	Ddx1	DEAD box helicase 1 [Source:MGI Symbol;Acc:MGI:2144727]	66.2467	61.445	63.3324	63.7534	61.702	62.5768	57.0753	63.4552	51.5244	57.8627	55.1322	56.4786	59.4077	61.491	0.875755063	0.966018836	8	12	24	0.600653752	0.860170376	24	0.483707877	0.032271073
ENSMUSG00000037151	Lrrc20	leucine rich repeat containing 20 [Source:MGI Symbol;Acc:MGI:2387182]	7.71216	6.87017	7.67503	9.11213	7.50113	8.80857	7.46482	10.5252	7.27022	11.3841	7.16603	7.33463	8.21442	8.2114	0.858932988	0.966018836	16	4	24	0.603094013	0.861235418	24	10.20518699	0.078221689
ENSMUSG00000037152	Ndufc1	NADH:ubiquinone oxidoreductase subunit C1 [Source:MGI Symbol;Acc:MGI:1913627]	99.6276	100.011	101.54	91.9799	70.004	92.7458	82.9372	124.547	116.3	117.669	118.315	102.209	121.464	111.412	0.868638024	0.966018836	12	8	24	0.138371288	0.747972781	24	6.276035151	0.152675669
ENSMUSG00000037161	Mgarp	mitochondria localized glutamic acid rich protein [Source:MGI Symbol;Acc:MGI:1914999]	0.42428	0.592502	0.321967	0.64366	0.693782	0.718089	0.770524	0.904503	0.673122	0.318779	0.588083	0.500813	0.729122	0.818225	0.17882595	0.655480352	8	8	24	0.390913674	0.799546792	24	0.96732377	0.08220461
ENSMUSG00000037166	Ppp1r14a	protein phosphatase 1, regulatory inhibitor subunit 14A [Source:MGI Symbol;Acc:MGI:1931139]	0.735708	2.17425	3.51971	0.942621	0.904972	1.94998	2.44675	2.21719	2.22592	1.15516	1.35411	2.55465	1.48381	2.7254	0.175185919	0.651010555	12	4	24	0.148663985	0.757169638	24	4.601128169	0.300627621
ENSMUSG00000037169	Mycn	v-myc avian myelocytomatosis viral related oncogene, neuroblastoma derived [Source:MGI Symbol;Acc:MGI:97357]	1.84578	1.48251	1.4166	1.31706	1.84524	1.55771	1.53943	1.63718	1.30158	1.69627	2.10023	2.06672	1.89363	2.08183	0.121546923	0.548996929	20	16	24	0.09601148	0.71175955	24	20.38474846	0.102015822
ENSMUSG00000037172	Dennd11	DENN domain containing 11 [Source:MGI Symbol;Acc:MGI:2444256]	5.49144	5.78083	7.35084	5.08751	5.59656	6.02385	6.37795	4.82687	5.82374	5.42445	5.31587	5.84603	4.91436	6.02557	0.911371589	0.966018836	12	4	24	0.895667455	0.970467191	24	5.8818926	0.025661402
ENSMUSG00000037174	Elf2	E74-like factor 2 [Source:MGI Symbol;Acc:MGI:1916507]	9.95087	10.6326	11.5906	11.3257	11.1546	11.1009	10.781	10.6197	10.0608	11.4615	10.7259	10.6078	11.2544	10.6632	0.437217485	0.966018836	16	16	24	0.282968825	0.768040178	24	13.3142326	0.036340663
ENSMUSG00000037185	Krt80	keratin 80 [Source:MGI Symbol;Acc:MGI:1921377]	1.57654	1.4484	1.09649	1.04873	1.34885	1.09933	1.29775	1.40957	1.39878	0.736366	1.43848	1.37624	1.13166	1.11767	0.359479536	0.883787544	4	12	24	0.230412961	0.768040178	24	23.41135197	0.095160607
ENSMUSG00000037188	Grhl3	grainyhead like transcription factor 3 [Source:MGI Symbol;Acc:MGI:2655333]	3.10504	1.62684	1.54215	1.68714	1.70304	2.17015	2.13767	1.69388	1.53787	2.4288	1.81197	1.92256	2.15104	1.7281	0.004140921	0.140598674	4	8	24	0.125011857	0.738693118	24	21.18604359	0.152391463
ENSMUSG00000037190	Cyb561d2	cytochrome b-561 domain containing 2 [Source:MGI Symbol;Acc:MGI:1929280]	16.4482	15.8498	14.5917	15.3195	17.043	17.4224	14.82	15.8804	13.6057	14.1094	15.3467	16.1497	15.3254	16.5052	0.077301512	0.453790923	24	12	24	0.050110845	0.644435324	24	20.89411403	0.086391994
ENSMUSG00000037196	Pacrg	PARK2 co-regulated [Source:MGI Symbol;Acc:MGI:1916560]	5.26491	7.56919	8.25497	5.46069	4.97265	7.16045	6.09122	5.58718	5.40689	6.28148	6.75505	5.92176	5.45009	7.00774	0.916746631	0.966018836	8	20	24	0.712464611	0.905177113	24	6.496420362	0.065174954
ENSMUSG00000037197	Rbm17	RNA binding motif protein 17 [Source:MGI Symbol;Acc:MGI:1924188]	26.0246	30.7513	29.805	27.721	31.0906	31.9235	30.3005	27.9685	30.0601	27.8329	28.4594	30.7258	27.624	27.8273	0.675497492	0.966018836	24	4	24	0.657208625	0.884447211	24	17.76007501	0.030995456
ENSMUSG00000037204	Atg101	autophagy related 101 [Source:MGI Symbol;Acc:MGI:1915368]	24.3886	22.9373	20.8608	22.3351	23.4252	22.2461	19.7827	25.3987	23.7062	22.6243	25.1442	23.2925	24.984	23.7524	0.916963526	0.966018836	8	4	24	0.972743017	0.993273328	24	23.84418871	0.008744612
ENSMUSG00000037206	Islr	immunoglobulin superfamily containing leucine-rich repeat [Source:MGI Symbol;Acc:MGI:1349645]	41.6351	49.366	43.4208	36.3243	46.1482	37.0363	44.0603	49.104	61.3813	26.9654	46.1342	45.055	43.3765	40.223	0.91695334	0.966018836	12	4	24	0.486273293	0.805393068	24	3.186192197	0.123092576
ENSMUSG00000037210	Fam193a	family with sequence homology 193, member A [Source:MGI Symbol;Acc:MGI:2447768]	7.13369	6.63792	5.87227	6.6135	7.1823	6.87327	7.35183	6.45228	6.2123	8.22452	6.40271	7.08449	6.92967	7.01543	0.170659409	0.643496587	4	8	24	0.426748996	0.805393068	24	19.25174398	0.053975055
ENSMUSG00000037211	Spry1	sprouty RTK signaling antagonist 1 [Source:MGI Symbol;Acc:MGI:1345139]	1.60929	1.67364	2.16582	1.67663	1.98038	1.38402	2.15762	2.11518	1.77705	1.46278	1.58573	1.70238	2.05636	1.52698	0.892555977	0.966018836	12	4	24	0.540578532	0.830021415	24	3.888440598	0.060594804
ENSMUSG00000037214	Thap1	THAP domain containing, apoptosis associated protein 1 [Source:MGI Symbol;Acc:MGI:1921004]	6.89156	6.37391	6.36663	6.94334	6.32493	5.92266	5.77549	6.10867	6.12861	5.90087	6.61008	6.20948	5.34878	5.7946	0.575387897	0.966018836	20	8	24	0.206655074	0.768040178	24	13.71756177	0.045510411
ENSMUSG00000037216	Lipt1	lipoyltransferase 1 [Source:MGI Symbol;Acc:MGI:3645211]	2.57587	2.79825	2.49082	3.53606	2.81838	2.75344	2.80835	3.23992	3.01716	2.78342	3.8646	2.63214	3.03509	2.67993	0.945282252	0.966018836	20	4	24	0.380749809	0.799546792	24	13.36685457	0.070510082
ENSMUSG00000037217	Syn1	synapsin I [Source:MGI Symbol;Acc:MGI:98460]	41.4555	34.3622	35.4361	37.7062	34.1141	39.4505	36.9052	42.4762	32.6668	65.67	36.2954	47.1662	42.5272	47.5599	0.830364743	0.966018836	24	12	24	0.982500638	0.996130479	24	13.10770615	0.017303051
ENSMUSG00000037221	Mospd3	motile sperm domain containing 3 [Source:MGI Symbol;Acc:MGI:1916179]	4.74131	4.97293	6.72907	6.47816	6.3806	5.78743	5.63448	5.48895	8.39906	8.43759	5.04467	6.14429	5.72198	4.70494	9.37E-04	0.072887999	12	20	24	0.033789063	0.606793499	24	11.54141845	0.212565543
ENSMUSG00000037224	Zfyve28	zinc finger, FYVE domain containing 28 [Source:MGI Symbol;Acc:MGI:2684992]	17.4062	15.1163	14.2863	15.0602	15.2133	16.4239	14.8181	15.9754	13.3775	19.5499	13.9524	16.6905	15.696	15.6901	0.148073804	0.590732968	24	12	24	0.700007091	0.900387881	24	20.1009912	0.046166401
ENSMUSG00000037225	Fgf2	fibroblast growth factor 2 [Source:MGI Symbol;Acc:MGI:95516]	6.69954	7.09758	6.45911	6.81711	7.86669	6.27107	7.49501	6.21564	6.96513	7.22948	6.68419	6.19369	6.13309	6.69543	0.814077113	0.966018836	20	4	24	0.512811449	0.815912787	24	12.85445018	0.041097498
ENSMUSG00000037234	Hook3	hook microtubule tethering protein 3 [Source:MGI Symbol;Acc:MGI:2443554]	9.22601	9.9328	9.05553	9.53724	10.1921	8.91675	10.1346	8.49649	9.97635	7.67994	10.5001	8.80212	8.91435	9.61455	0.865490576	0.966018836	20	4	24	0.941572459	0.985544192	24	19.6185306	0.016045141
ENSMUSG00000037235	Mxd4	Max dimerization protein 4 [Source:MGI Symbol;Acc:MGI:104991]	33.3686	29.8295	30.1331	37.0115	34.5219	31.6278	36.9619	31.9066	34.9094	31.0682	32.7593	28.1603	30.6618	28.4968	0.851656735	0.966018836	16	16	24	0.593344289	0.856874943	24	14.63447524	0.04538118

ENSMUSG00000037236	Matr3	matrin 3 [Source:MGI Symbol;Acc:MGI:1298379]	160.142	168.333	166.381	164.899	168.316	148.147	156.935	147.01	167.653	140.943	181.537	137.707	137.361	149.391	0.503972924	0.966018836	20	4	24	0.332516101	0.769110818	24	11.80902213	0.062102894
ENSMUSG00000037239	Spred3	sprouty-related EVH1 domain containing 3 [Source:MGI Symbol;Acc:MGI:2142186]	6.90661	5.96092	6.90351	5.57975	6.65025	6.37289	7.0131	5.88615	5.88075	5.57116	6.10914	6.29976	6.37836	6.6541	0.004895408	0.141782417	4	12	24	0.101418427	0.713208839	24	23.06082349	0.076066137
ENSMUSG00000037242	Clic4	chloride intracellular channel 4 (mitochondrial) [Source:MGI Symbol;Acc:MGI:1352754]	37.1217	32.9704	32.5653	34.1824	36.8702	33.6944	39.2554	35.9877	32.5444	34.6515	33.4517	34.3913	33.8726	32.569	0.060394272	0.407012001	4	8	24	0.182865402	0.768040178	24	21.04613069	0.056208812
ENSMUSG00000037243	Zfp692	zinc finger protein 692 [Source:MGI Symbol;Acc:MGI:2144276]	8.32347	8.40726	7.58239	7.74711	9.3082	7.99745	8.40965	7.67614	8.75373	7.62687	8.22586	8.86922	8.44818	7.92915	0.24731677	0.746715482	20	20	24	0.263276358	0.768040178	24	19.86812681	0.052045831
ENSMUSG00000037251	Pomk	protein-O-mannose kinase [Source:MGI Symbol;Acc:MGI:1921903]	9.07123	8.01775	7.71467	7.23006	8.41931	8.87598	7.96557	8.33416	7.1009	8.92863	7.14225	8.3356	8.53748	8.12649	0.188783717	0.664348865	24	12	24	0.143668484	0.752803875	24	22.06995665	0.079739921
ENSMUSG00000037253	Mex3c	mex3 RNA binding family member C [Source:MGI Symbol;Acc:MGI:2652843]	20.1079	16.2013	16.2867	18.1336	17.56	17.6601	17.0248	15.2096	14.4858	16.6108	18.5128	16.0375	16.7849	15.8702	0.048889741	0.379129806	20	12	24	0.02830142	0.5921502	24	18.69343743	0.101715236
ENSMUSG00000037254	Itih2	inter-alpha trypsin inhibitor, heavy chain 2 [Source:MGI Symbol;Acc:MGI:96619]	4.31179	5.94297	3.75461	3.20401	4.22344	4.08137	3.42687	4.7253	5.85962	1.69955	3.93864	4.1888	2.48927	4.06452	0.350544145	0.870418653	8	8	24	0.50896775	0.813786183	24	2.835237189	0.152617217
ENSMUSG00000037257	Aagab	alpha- and gamma-adaptin binding protein [Source:MGI Symbol;Acc:MGI:1914189]	14.4851	13.7451	12.2196	13.0024	13.8541	14.6979	15.0434	14.9589	13.7574	12.9353	13.1554	14.272	13.389	13.0759	0.029985708	0.324150946	4	12	24	0.022893326	0.581965446	24	22.48277242	0.080678354
ENSMUSG00000037259	Dzank1	double zinc ribbon and ankyrin repeat domains 1 [Source:MGI Symbol;Acc:MGI:2139080]	25.1625	25.5784	30.0009	25.0273	21.444	26.6104	26.4383	21.6144	24.0139	26.5471	21.7048	26.592	23.5876	28.2556	0.911135828	0.966018836	24	20	24	0.627996591	0.872795898	24	5.040450988	0.054673976
ENSMUSG00000037260	Hgsnat	heparan-alpha-glucosaminide N-acetyltransferase [Source:MGI Symbol;Acc:MGI:1196297]	39.612	37.2045	36.1605	37.1164	35.3308	38.7181	36.073	36.7453	31.2902	39.895	33.97	34.2894	37.938	37.9794	0.843639218	0.966018836	4	16	24	0.625536161	0.872249537	24	23.6506432	0.035057011
ENSMUSG00000037262	Kin	Kin17 DNA and RNA binding protein [Source:MGI Symbol;Acc:MGI:96676]	10.186	10.2856	9.23336	9.7209	10.2865	8.92019	10.0419	10.1178	10.9562	8.30946	11.0337	9.60453	10.9176	10.2379	0.814576414	0.966018836	8	8	24	0.594679292	0.857513003	24	1.843626967	0.041567874
ENSMUSG00000037266	Rsrp1	arginine/serine rich protein 1 [Source:MGI Symbol;Acc:MGI:106498]	199.447	281.808	256.307	264.808	246.499	223.743	231.011	244.983	315.461	265.084	219.367	260.49	256.337	275.904	0.09856758	0.503519672	12	16	24	0.01723092	0.549489822	24	8.255554166	0.163648229
ENSMUSG00000037270	4932438A13Rik	RIKEN cDNA 4932438A13 gene [Source:MGI Symbol;Acc:MGI:2444631]	23.0477	24.8864	23.8807	24.8337	23.7285	22.5062	24.0639	21.1351	25.8663	20.6539	24.8706	24.1086	23.945	24.0121	0.823246279	0.966018836	12	16	24	0.981701089	0.995786699	24	8.782236033	0.007055987
ENSMUSG00000037275	Gemin5	gem nuclear organelle associated protein 5 [Source:MGI Symbol;Acc:MGI:2449311]	4.8819	4.37357	4.25016	4.16571	4.23489	4.8392	5.04255	4.59056	3.56256	5.05151	3.86304	4.15873	4.39234	4.36395	0.138719141	0.580158868	4	16	24	0.277104238	0.768040178	24	22.8887016	0.071810489
ENSMUSG00000037278	Tmem97	transmembrane protein 97 [Source:MGI Symbol;Acc:MGI:1916321]	11.2252	9.65458	9.39944	9.29847	9.31198	9.89509	10.1519	9.98239	9.53339	9.38191	8.75104	9.03563	9.05313	9.51226	0.027096327	0.306445942	4	16	24	0.036406635	0.609176161	24	1.105840254	0.059795361
ENSMUSG00000037280	Galnt6	polypeptide N-acetylgalactosaminyltransferase 6 [Source:MGI Symbol;Acc:MGI:1891640]	1.11013	1.61876	2.66711	1.4711	1.53423	2.04226	1.81012	1.75268	2.07995	1.56689	1.45931	1.58065	1.11946	1.92218	0.156791576	0.61244839	12	8	24	0.267119768	0.768040178	24	8.293363435	0.137144881
ENSMUSG00000037286	Stag1	stromal antigen 1 [Source:MGI Symbol;Acc:MGI:1098658]	8.55284	9.52797	9.19115	8.83002	9.22364	7.76511	9.2064	8.43542	10.7021	8.02503	9.12251	8.5063	9.36624	8.58072	0.601647512	0.966018836	12	12	24	0.417198337	0.801927226	24	7.230300031	0.055903603
ENSMUSG00000037287	Tbcel	tubulin folding cofactor E-like [Source:MGI Symbol;Acc:MGI:1925543]	13.1074	11.5756	12.2024	12.1995	11.544	12.9047	12.0483	12.8302	11.6991	15.3934	11.4505	13.0492	12.222	13.4289	0.89563591	0.966018836	24	20	24	0.965885002	0.991740077	24	8.578990712	0.011469827
ENSMUSG00000037295	Ldlrap1	low density lipoprotein receptor adaptor protein 1 [Source:MGI Symbol;Acc:MGI:2140175]	1.72754	1.7049	2.16636	1.95116	2.26893	2.11054	2.50161	2.48364	2.42091	1.89451	1.6761	1.62321	1.49589	1.69251	0.939103211	0.966018836	12	12	24	0.671530005	0.889179947	24	8.905898649	0.058712604
ENSMUSG00000037296	Lsm1	LSM1 homolog, mRNA degradation associated [Source:MGI Symbol;Acc:MGI:1914457]	9.19904	8.87228	7.94246	8.50706	8.36948	9.92909	9.26311	10.072	11.6653	8.38226	11.7372	10.7004	12.0414	9.58924	0.699015352	0.966018836	24	16	24	0.454038434	0.805393068	24	22.10411725	0.070119907
ENSMUSG00000037300	Ttc13	tetratricopeptide repeat domain 13 [Source:MGI Symbol;Acc:MGI:2384573]	18.9864	21.2576	21.8196	20.178	19.1383	19.9578	18.8732	18.8624	22.1098	20.6694	24.1673	20.0581	19.9411	21.2866	0.018894761	0.256345714	12	16	24	0.157306955	0.763469431	24	10.761669	0.069899702
ENSMUSG00000037306	Man1c1	mannosidase, alpha, class 1C, member 1 [Source:MGI Symbol;Acc:MGI:2446214]	9.18853	7.5872	7.36963	8.01785	7.90045	8.60906	8.8284	8.06315	7.24762	9.75013	7.71631	8.33855	8.10477	8.52441	0.060394272	0.407012001	4	8	24	0.459951816	0.805393068	24	20.69803837	0.054912018
ENSMUSG00000037313	Tacc3	transforming, acidic coiled-coil containing protein 3 [Source:MGI Symbol;Acc:MGI:1341163]	3.34393	3.2271	3.63483	3.62299	2.45687	3.14793	2.35269	3.19857	3.03967	2.81722	3.01026	3.40719	3.63043	2.53786	0.865490576	0.966018836	12	8	24	0.958297275	0.989144435	24	6.612772267	0.018755334
ENSMUSG00000037315	Jade3	jade family PHD finger 3 [Source:MGI Symbol;Acc:MGI:2148019]	1.63146	1.74742	1.6204	1.72939	1.80758	1.77038	1.92344	1.73273	1.79594	1.51869	1.81442	1.79345	1.48377	1.84397	0.917839756	0.966018836	20	20	24	0.867140465	0.961815241	24	20.43819155	0.015093575
ENSMUSG00000037316	Bag4	BCL2-associated athanogene 4 [Source:MGI Symbol;Acc:MGI:1914634]	8.01886	7.99264	8.58039	8.4852	7.48104	7.78334	7.58244	8.01307	7.79404	8.34736	7.63051	8.17699	7.60734	8.0736	0.248056532	0.746745468	16	4	24	0.148218362	0.756579871	24	9.002694547	0.04101332
ENSMUSG00000037318	Traf3ip3	TRAF3 interacting protein 3 [Source:MGI Symbol;Acc:MGI:2441706]	1.60554	2.11688	2.63295	2.43893	2.35529	2.07547	1.40928	1.94491	2.91429	1.83242	2.43383	2.45732	2.46919	2.04672	0.337748746	0.85758369	12	16	24	0.176793142	0.768040178	24	11.49551229	0.133256246
ENSMUSG00000037321	Tap1	transporter 1, ATP-binding cassette, sub-family B (MDR/TAP) [Source:MGI Symbol;Acc:MGI:98483]	2.51265	3.23776	2.81982	2.34207	3.16904	3.07094	3.1594	4.1723	3.58191	3.07122	2.93914	2.99178	3.20004	3.93369	0.050258003	0.385784509	8	8	24	0.074562962	0.683293901	24	4.563263843	0.121301222
ENSMUSG00000037325	Bbs7	Bardet-Biedl syndrome 7 (human) [Source:MGI Symbol;Acc:MGI:1918742]	9.64391	10.7883	8.8354	9.18816	9.55701	9.39251	8.84932	8.84841	8.65683	8.60743	9.44337	9.25107	9.90253	10.1777	0.163904621	0.627573783	8	4	24	0.318338435	0.768040178	24	23.93875616	0.049803514
ENSMUSG00000037326	Capn15	calpain 15 [Source:MGI Symbol;Acc:MGI:1355075]	6.84589	6.6615	6.54595	6.59683	6.4733	6.57494	6.89463	7.25489	6.95087	7.32261	6.35203	6.66355	6.19719	6.17647	0.888379597	0.966018836	16	4	24	0.711296687	0.904655191	24	8.317706398	0.021722795
ENSMUSG00000037331	Larp1	La ribonucleoprotein domain family, member 1 [Source:MGI Symbol;Acc:MGI:1890165]	24.857	18.2362	19.3613	21.417	20.468	23.2326	22.2856	22.6483	16.5846	31.3643	19.0469	21.968	20.7394	22.8123	0.649212555	0.966018836	4	8	24	0.792973307	0.93379199	24	18.73001366	0.059715414
ENSMUSG00000037336	Mfsd2b	major facilitator superfamily domain containing 2B [Source:MGI Symbol;Acc:MGI:3583946]	1.29544	1.57944	1.9778	2.10623	1.52054	1.33188	1.54122	1.54938	1.738	1.32216	1.26908	1.42247	1.61902	1.16958	0.289105414	0.794869797	12	8	24	0.223055127	0.768040178	24	9.317553594	0.092879727
ENSMUSG00000037337	Map4k1	mitogen-activated protein kinase kinase kinase kinase 1 [Source:MGI Symbol;Acc:MGI:1346882]	0.945876	1.0972	1.53296	0.891798	0.814906	0.794783	0.623972	1.30762	1.02175	0.724944	0.990045	1.05144	0.82077	0.721447	0.560714538	0.966018836	12	16	24	0.383186479	0.799546792	24	7.445687585	0.092651081
ENSMUSG00000037339	Fam53a	family with sequence similarity 53, member A [Source:MGI Symbol;Acc:MGI:1919225]	5.10223	4.54569	4.67852	5.61124	4.27621	4.2534	5.74889	6.05127	6.15604	6.40995	4.70997	5.5177	4.71263	5.70224	0.503972924	0.966018836	16	4	24	0.170090459	0.767957732	24	7.547991384	0.11664333
ENSMUSG00000037341	Slc9a7	solute carrier family 9 (sodium/hydrogen exchanger), member 7 [Source:MGI Symbol;Acc:MGI:2444530]	5.45027	7.03779	5.98549	6.82787	7.76162	6.80802	6.24708	5.39377	7.66878	5.11701	7.66065	8.89684	8.38309	10.1911	0.914502381	0.966018836	20	20	24	0.860897766	0.959130799	24	21.18769978	0.047639235
ENSMUSG00000037343	Taf2	TATA-box binding protein associated factor 2 [Source:MGI Symbol;Acc:MGI:2443028]	10.1935	11.4492	11.6535	9.93441	11.1826	10.5545	11.0997	9.40313	10.7301	10.9573	10.7349	11.1972	9.4873	10.0506	0.933543173	0.966018836	20	12	24	0.545970326	0.833198211	24	14.33255579	0.036529881
ENSMUSG00000037344	Slc12a9	solute carrier family 12 (potassium/chloride transporters), member 9 [Source:MGI Symbol;Acc:MGI:1933532]	4.58744	5.25523	5.93118	5.80929	6.14255	5.2807	5.86005	5.93127	6.76774	7.08694	5.38378	6.01042	5.61169	5.86879	0.152782337	0.601788038	12	16	24	0.016051898	0.544099157	24	10.52443791	0.120079345
ENSMUSG00000037348	Paqr7	progestin and adipoQ receptor family member VII [Source:MGI Symbol;Acc:MGI:1919154]	26.9253	21.9433	23.3793	20.8126	23.2416	24.9447	25.9074	23.4032	19.491	26.5433	22.0796	22.8087	24.0737	21.9793	0.170659409	0.643496587	4	8	24	0.170928533	0.768040178	24	21.44933222	0.092243886
ENSMUSG00000037349	Nudt22	nudix (nucleoside diphosphate linked moiety X)-type motif 22 [Source:MGI Symbol;Acc:MGI:1915573]	8.71819	8.53546	8.33952	8.71486	9.08392	8.20537	9.33611	9.16108	8.01752	8.61498	7.6868	9.20205	7.52589	7.66106	0.891742979	0.966018836	24	12	24	0.732029025	0.912550805	24	19.51648452	0.027669179
ENSMUSG00000037351	Actr1b	ARP1 actin-related protein 1B, centractin beta [Source:MGI Symbol;Acc:MGI:1917446]	88.9351	74.7376	70.074	72.7644	75.7143	85.573	81.1004	83.3479	66.566	94.8737	72.8593	83.28	83.6665	81.8932	0.033552374	0.324908923	24	12	24	0.24421303	0.768040178	24	21.84287246	0.091450836
ENSMUSG00000037353	Letmd1	LETM1 domain containing 1 [Source:MGI Symbol;Acc:MGI:1915864]	23.4842	18.6184	21.0858	27.0249	22.5418	24.2868	22.8345	23.0588	19.0299	25.8133	25.5297	22.8154	25.3656	22.7328	0.054328576	0.395890765	16	20	24	0.139806968	0.74956518	24	16.92649227	0.107934679
ENSMUSG00000037355	Uvssa	UV stimulated scaffold protein A [Source:MGI Symbol;Acc:MGI:2781351]	2.33326	3.51687	3.1205	3.46415	3.01084	2.58727	2.7781	3.1201	4.50631	3.10777	3.23519	3.45435	3.1026	3.7831	0.054126506	0.395890765	12	16	24	0.018316379	0.559812298	24	8.701258838	0.164088014
ENSMUSG00000037358	Dipk2b	divergent protein kinase domain 2B [Source:MGI Symbol;Acc:MGI:1923155]	0.60686	0.691076	0.596222	0.553667	0.625744	0.611369	0.450156	0.648146	0.692513	0.697286	0.495856	0.552964	0.684925	0.518644	0.760769894	0.966018836	16	4	24	0.549941945	0.835266054	24	7.752002487	0.032262937
ENSMUSG00000037361	Sf3b6	splicing factor 3B, subunit 6 [Source:MGI Symbol;Acc:MGI:1913305]	50.8028	63.4514	59.7377	57.8426	63.692	52.8974	45.9204	50.2705	65.5488	43.9095	60.5037	55.7669	54.9128	53.4469	0.371875382	0.894537464	12	16	24	0.614605977	0.866603595	24	11.4655825	0.067069527
ENSMUSG00000037362	Ccn3	cellular communication network factor 3 [Source:MGI Symbol;Acc:MGI:109185]	15.9576	18.2965	14.6465	12.1363	13.9382	12.2603	15.0574	17.7925	17.5379	13.3684	12.1876	15.0261	13.6981	16.832	3.67E-04	0.050643729	8	8	24	0.002951454	0.414576087	24	4.200374473	0.210212873
ENSMUSG00000037363	Letm2	leucine zipper-EF-hand containing transmembrane protein 2 [Source:MGI Symbol;Acc:MGI:2444979]	10.6101	9.93549	10.8672	10.8878	10.1448	8.96228	9.9582	8.81977	9.37519	9.54933	10.4763	9.17321	11.483	7.86663	0.722296281	0.966018836	16	16	24	0.647425709	0.880898698	24	16.3516775	0.0456181
ENSMUSG00000037364	Srrt	serrate RNA effector molecule homolog (Arabidopsis) [Source:MGI Symbol;Acc:MGI:1933527]	52.1302	49.0469	53.3729	52.7592	54.6327	53.1207	54.0876	47.6684	51.4583	45.4105	47.9799	51.9484	48.1891	53.286	0.832293447	0.966018836	24	8	24	0.718182612	0.907213187	24	20.8800934	0.02611024
ENSMUSG00000037366	Pafah2	platelet-activating factor acetylhydrolase 2 [Source:MGI Symbol;Acc:MGI:2140321]	8.1458	6.71884	6.60848	7.50312	7.96753	7.59311	7.77976	8.31355	6.78633	7.33535	7.42464	7.07634	7.46941	7.20205	0.182849367	0.659303873	4	8	24	0.237049462	0.768040178	24	20.62874081	0.060270117
ENSMUSG00000037369	Kdm6a	lysine (K)-specific demethylase 6A [Source:MGI Symbol;Acc:MGI:1095419]	8.22762	10.5887	9.54751	9.40724	11.1358	8.97779	9.58322	9.17965	10.54	8.81414	9.2599	10.0025	9.56865	10.2849	0.890176313	0.966018836	8	20	24	0.751885773	0.919370646	24	8.86874667	0.033303566
ENSMUSG00000037370	Enpp1	ectonucleotide pyrophosphatase/phosphodiesterase 1 [Source:MGI Symbol;Acc:MGI:97370]	4.71573	5.34149	4.97842	5.10935	4.76953	4.0653	5.10325	5.25327	5.39925	5.09351	4.73491	4.26862	4.66738	4.86506	0.001885685	0.100255953	12	12	24	0.003091632	0.414576087	24	7.515099892	0.10794027
ENSMUSG00000037373	Ctbp1	C-terminal binding protein 1 [Source:MGI Symbol;Acc:MGI:1201685]	70.771	51.7191	57.2619	57.9764	51.7654	58.4895	61.1805	58.6362	50.332	73.164	51.3195	61.8777	61.4172	60.1002	0.163587268	0.626886304	4	8	24	0.715726233	0.906323093	24	23.00075803	0.053182069
ENSMUSG00000037375	Hhat	hedgehog acyltransferase [Source:MGI Symbol;Acc:MGI:2444681]	0.712895	0.858748	0.813655	0.726234	0.936448	0.923255	0.916301	1.06433	0.941607	1.16823	0.76403	1.09672	0.958807	0.792848	0.814221431	0.966018836	24	20	24	0.977101404	0.994159581	24	15.72304941	0.008251851
ENSMUSG00000037376	Trmt6	tRNA methyltransferase 6 [Source:MGI Symbol;Acc:MGI:1914176]	8.2842	11.2389	12.0591	12.0968	11.0359	10.8318	9.93208	10.4074	12.5575	10.6059	10.2369	10.8695	10.8452	11.008	0.016795343	0.253395174	12	16	24	0.042187695	0.623424695	24	10.00013284	0.121046204
ENSMUSG00000037379	Spon2	spondin 2, extracellular matrix protein [Source:MGI Symbol;Acc:MGI:1923724]	8.92515	9.69101	6.95731	6.59531	8.52663	6.77067	9.44019	9.73145	7.07014	8.01984	5.46565	6.62876	8.65382	7.91498	0.033136984	0.324908923	8	8	24	0.029641828	0.596648309	24	2.404935133	0.197226941
ENSMUSG00000037386	Rims2	regulating synaptic membrane exocytosis 2 [Source:MGI Symbol;Acc:MGI:2152972]	17.2122	17.595	17.4539	16.5554	14.8618	17.5786	17.5512	15.8281	14.9918	17.7543	15.3441	16.837	16.3521	18.5239	0.702630284	0.966018836	8	12	24	0.377937043	0.797706855	24	2.333598046	0.050078399
ENSMUSG00000037395	Rcor3	REST corepressor 3 [Source:MGI Symbol;Acc:MGI:2441920]	14.424	16.5549	17.7002	15.7984	16.7663	16.0622	13.7436	17.4805	17.6815	15.8883	17.6961	16.0163	15.7233	17.3682	0.009483698	0.194720062	12	16	24	0.076335917	0.686372559	24	9.710190076	0.087036183
ENSMUSG00000037400	Atp11b	ATPase, class VI, type 11B [Source:MGI Symbol;Acc:MGI:1923545]	25.1633	31.0998	31.1044	26.6846	28.2615	26.1037	25.0971	24.7601	31.4033	25.634	25.6015	25.1922	25.6698	29.2293	0.019594567	0.258568924	12	16	24	0.058900809	0.658304792	24	7.980604824	0.108002271
ENSMUSG00000037405	Icam1	intercellular adhesion molecule 1 [Source:MGI Symbol;Acc:MGI:96392]	1.83918	1.73711	1.78001	1.30553	1.75218	1.26918	1.72716	2.33148	2.01322	1.19524	1.48076	1.4353	1.15771	1.70628	0.27074138	0.780327352	12	4	24	0.129601779	0.740162193	24	4.894293325	0.133379924
ENSMUSG00000037406	Htra4	HtrA serine peptidase 4 [Source:MGI Symbol;Acc:MGI:3036260]	1.39349	1.37914	1.02213	1.33568	1.28396	1.1456	1.07994	1.33847	1.87865	1.64662	1.28787	1.33193	1.28935	1.32097	0.532538925	0.966018836	16	4	24	0.303733119	0.768040178	24	9.513808114	0.077619219
ENSMUSG00000037408	Cnnm4	cyclin M4 [Source:MGI Symbol;Acc:MGI:2151060]	2.41795	2.1387	1.92502	2.32416	2.44249	2.52829	2.39451	2.72734	1.86663	2.80608	2.22091	2.42544	2.35203	2.16516	0.08302755	0.462922111	24	12	24	0.313045406	0.768040178	24	19.29095981	0.070754996
ENSMUSG00000037410	Tbc1d2b	TBC1 domain family, member 2B [Source:MGI Symbol;Acc:MGI:1914266]	3.77477	3.65159	4.12703	4.00914	3.84883	3.87273	3.6472	3.77294	3.71519	4.21261	3.7262	3.58553	3.70629	3.83832	0.097542159	0.501112642	16	12	24	0.033932693	0.606793499	24	11.20375696	0.049207392
ENSMUSG00000037411	Serpine1	serine (or cysteine) peptidase inhibitor, clade E, member 1 [Source:MGI Symbol;Acc:MGI:97608]	0.541567	0.597072	0.680645	0.962499	0.50258	0.437292	0.324619	0.875078	0.984862	1.1363	0.672177	0.371315	0.405114	0.521942	8.83E-04	0.071344007	16	8	24	7.89E-04	0.319044294	24	10.02686543	0.256319179
ENSMUSG00000037415	Ranbp10	RAN binding protein 10 [Source:MGI Symbol;Acc:MGI:1921584]	8.50098	7.79171	8.34268	9.06877	8.30003	9.25886	8.60648	8.66159	7.47176	8.91518	7.44762	8.5463	8.12349	7.91114	0.823677655	0.966018836	24	12	24	0.533736295	0.825979252	24	18.5786479	0.038485029
ENSMUSG00000037416	Dmxl1	Dmx-like 1 [Source:MGI Symbol;Acc:MGI:2443926]	7.04497	8.26254	7.05562	7.96371	8.1787	6.88722	7.86866	6.92733	8.2772	5.63785	8.05164	6.85123	7.12471	7.33852	0.649212555	0.966018836	20	4	24	0.992913527	0.998516607	24	4.855172905	0.006403201
ENSMUSG00000037418	Best1	bestrophin 1 [Source:MGI Symbol;Acc:MGI:1346332]	0.86778	0.756218	0.607098	0.690725	0.761793	0.672141	0.833315	0.612571	0.721232	0.586762	0.613323	0.590472	0.699829	0.780643	0.109247218	0.524035616	4	12	24	0.146241345	0.754849922	24	0.613651177	0.053164768
ENSMUSG00000037419	Endod1	endonuclease domain containing 1 [Source:MGI Symbol;Acc:MGI:1919196]	103.154	79.3628	81.0678	87.8064	84.854	95.6245	88.9235	91.3379	65.4874	106.822	83.0087	89.0067	84.5997	84.3933	0.138202217	0.580158868	24	12	24	0.322523538	0.768040178	24	19.67323895	0.102256062
ENSMUSG00000037426	Depdc5	DEP domain containing 5 [Source:MGI Symbol;Acc:MGI:2141101]	20.5902	15.876	15.5031	15.9049	15.6416	16.0872	17.5892	15.4859	12.1934	18.4858	13.1754	16.9074	17.3458	17.927	0.060394272	0.407012001	4	8	24	0.222188375	0.768040178	24	23.22413785	0.11954185
ENSMUSG00000037428	Vgf	VGF nerve growth factor inducible [Source:MGI Symbol;Acc:MGI:1343180]	18.5921	9.45824	14.2944	10.0713	14.3912	13.36	11.5825	13.8187	9.47063	18.1203	22.6653	16.9005	15.2273	15.8362	0.504585278	0.966018836	20	16	24	0.282472234	0.768040178	24	18.27587224	0.206845945
ENSMUSG00000037432	Fer1l5	fer-1-like 5 (C. elegans) [Source:MGI Symbol;Acc:MGI:3616091]	1.49823	1.17634	1.18945	1.08271	1.29781	1.07209	1.08587	1.4368	0.973604	1.2584	1.0161	1.21094	1.14749	0.922803	0.795269577	0.966018836	4	8	24	0.642363066	0.878809617	24	22.084527	0.037099641
ENSMUSG00000037434	Slc30a1	solute carrier family 30 (zinc transporter), member 1 [Source:MGI Symbol;Acc:MGI:1345281]	4.91661	5.34995	6.04723	4.71001	4.70695	4.80399	4.56795	5.72478	5.37751	5.54826	4.12453	4.85307	4.48485	5.28757	0.004947794	0.141782417	12	8	24	0.009104831	0.502965379	24	6.977872768	0.132449726
ENSMUSG00000037437	Adam32	a disintegrin and metallopeptidase domain 32 [Source:MGI Symbol;Acc:MGI:2653822]	0.865642	0.54142	0.459404	0.431717	0.958777	0.295211	0.496347	0.995052	0.701034	0.541972	0.752783	0.261453	0.918197	0.563944	0.796193867	0.966018836	8	16	24	0.811008362	0.93944887	24	4.326471462	0.056384927
ENSMUSG00000037438	Uqcrh-ps1	ubiquinol-cytochrome c reductase hinge protein, pseudogene 1 [Source:MGI Symbol;Acc:MGI:3641869]	6.43816	8.90723	6.95528	6.65286	8.90782	7.17008	8.20889	9.22116	12.7145	10.0356	9.80788	4.25631	7.00626	10.3301	0.208071297	0.698704663	12	12	24	0.108535636	0.72082227	24	8.415221196	0.274904502
ENSMUSG00000037443	Cep85	centrosomal protein 85 [Source:MGI Symbol;Acc:MGI:1917262]	6.87445	6.57502	8.87258	7.6482	6.84464	7.91347	5.75414	8.57629	6.18336	7.82442	6.0559	6.30597	7.84663	7.09072	0.928127947	0.966018836	16	4	24	0.566284489	0.843471947	24	8.068313286	0.074325038
ENSMUSG00000037447	Arid5a	AT rich interactive domain 5A (MRF1-like) [Source:MGI Symbol;Acc:MGI:2443039]	5.71359	3.82364	5.32242	4.1606	4.95633	5.12742	4.94341	6.83986	5.13668	5.34272	5.37015	4.81699	4.94852	5.51905	0.854540463	0.966018836	8	16	24	0.796792913	0.935045591	24	3.397619142	0.042888277
ENSMUSG00000037455	Slc18b1	solute carrier family 18, subfamily B, member 1 [Source:MGI Symbol;Acc:MGI:1923556]	5.10103	5.9369	4.67743	6.54538	7.39881	6.27114	7.41097	7.5277	6.92682	6.00895	6.43831	6.36577	5.71966	5.63306	0.915082018	0.966018836	20	8	24	0.722763482	0.909130303	24	16.72658349	0.055705228
ENSMUSG00000037458	Azin1	antizyme inhibitor 1 [Source:MGI Symbol;Acc:MGI:1859169]	84.5495	82.217	80.6948	89.4784	92.2818	81.8579	79.2092	77.9144	77.6382	82.8114	82.0897	75.332	77.6815	78.8758	0.303361836	0.811201458	16	20	24	0.030165829	0.596794384	24	14.72313581	0.060296842
ENSMUSG00000037461	Ints7	integrator complex subunit 7 [Source:MGI Symbol;Acc:MGI:1924315]	6.05914	6.6461	6.98372	6.98795	6.75756	6.97616	6.20359	7.14128	6.65368	7.0047	5.99407	7.41362	6.95483	6.83148	0.810607738	0.966018836	16	12	24	0.751357964	0.91921305	24	9.524212364	0.023997359
ENSMUSG00000037463	Fbxo27	F-box protein 27 [Source:MGI Symbol;Acc:MGI:2685007]	3.75776	4.15294	3.46597	4.35476	5.00208	4.20364	4.73196	4.17211	4.03304	4.29111	4.00776	4.43727	4.7311	3.73114	0.246839642	0.745922296	24	12	24	0.195784656	0.768040178	24	18.71869167	0.081822877
ENSMUSG00000037465	Klf10	Kruppel-like factor 10 [Source:MGI Symbol;Acc:MGI:1101353]	8.214	7.04176	7.45481	7.97318	6.99916	7.65506	7.4149	7.7062	6.50477	8.71875	7.77121	6.97493	7.21404	6.91632	0.718086778	0.966018836	16	20	24	0.584271829	0.852087247	24	15.47988164	0.039490385
ENSMUSG00000037466	Tedc1	tubulin epsilon and delta complex 1 [Source:MGI Symbol;Acc:MGI:2144738]	1.51859	1.4774	1.47042	1.22006	1.20416	1.23658	1.33298	0.979231	1.29631	1.14357	1.40595	1.06693	1.26256	1.03623	0.934818505	0.966018836	4	12	24	0.934807576	0.983765461	24	21.74016654	0.013200423
ENSMUSG00000037469	Acp7	acid phosphatase 7, tartrate resistant [Source:MGI Symbol;Acc:MGI:2142121]	0.872849	0.843836	0.625351	0.635778	0.838746	0.701063	0.588211	0.881185	0.433674	0.832243	0.52109	0.684215	0.493629	0.799614	0.843694678	0.966018836	8	4	24	0.862815674	0.959967665	24	0.61838824	0.026919137
ENSMUSG00000037470	Uggt1	UDP-glucose glycoprotein glucosyltransferase 1 [Source:MGI Symbol;Acc:MGI:2443162]	16.2219	13.597	13.08	13.7124	13.5768	15.4817	14.5729	14.713	12.2076	14.8034	14.3329	15.8659	15.2072	13.4273	0.001885685	0.100255953	24	12	24	0.006539642	0.470633744	24	20.94274088	0.117291808
ENSMUSG00000037475	Thoc2	THO complex 2 [Source:MGI Symbol;Acc:MGI:2442413]	12.975	15.0111	13.6807	13.3474	14.8326	11.9289	13.3772	11.7954	14.6351	11.4012	14.7874	11.1223	12.0614	12.4295	0.649212555	0.966018836	20	4	24	0.589693739	0.854432964	24	10.65297938	0.051954045
ENSMUSG00000037486	Asxl2	ASXL transcriptional regulator 2 [Source:MGI Symbol;Acc:MGI:1922552]	5.6084	6.13381	5.1984	5.9814	6.44956	5.63656	5.58716	5.16315	6.92528	5.00742	6.21927	6.62622	5.67013	5.87366	0.885025617	0.966018836	24	4	24	0.685248002	0.895310095	24	16.65114047	0.041253634
ENSMUSG00000037487	Ubr5	ubiquitin protein ligase E3 component n-recognin 5 [Source:MGI Symbol;Acc:MGI:1918040]	49.7461	50.3627	54.3874	54.3197	51.71	53.0811	52.8395	49.2779	48.7618	49.5534	49.1196	51.1	50.6411	48.9741	0.66832131	0.966018836	24	8	24	0.560869799	0.841459562	24	16.73581119	0.020702192
ENSMUSG00000037490	Slc2a12	solute carrier family 2 (facilitated glucose transporter), member 12 [Source:MGI Symbol;Acc:MGI:3052471]	0.63259	1.00805	0.652575	0.548265	0.653953	0.671041	0.839057	0.902294	0.768208	0.659174	0.55955	0.687941	0.63027	0.675492	0.054623609	0.397541027	8	8	24	0.052690566	0.650526959	24	3.435003973	0.09031735
ENSMUSG00000037492	Zmat4	zinc finger, matrin type 4 [Source:MGI Symbol;Acc:MGI:2443497]	13.7512	13.1938	12.2989	13.876	12.9829	12.9338	13.0096	11.6343	11.9456	12.9423	12.9068	13.0059	13.0871	13.2112	0.43565473	0.966018836	4	8	24	0.309431892	0.768040178	24	19.16418934	0.038809871
ENSMUSG00000037493	Cib2	calcium and integrin binding family member 2 [Source:MGI Symbol;Acc:MGI:1929293]	15.2984	15.47	17.2568	23.5698	14.9115	17.0023	17.1247	22.7225	19.0918	22.6671	18.1866	19.4232	20.7611	19.6525	0.664568268	0.966018836	16	4	24	0.189082867	0.768040178	24	9.45313372	0.126758877
ENSMUSG00000037499	Nenf	neuron derived neurotrophic factor [Source:MGI Symbol;Acc:MGI:1913458]	65.5645	68.2812	63.485	62.3569	68.9034	63.34	60.1108	62.8689	75.5774	53.2397	71.8012	70.2983	71.361	67.1727	0.891022863	0.966018836	20	20	24	0.844481464	0.952844769	24	23.27135212	0.029356145
ENSMUSG00000037503	Fam168b	family with sequence similarity 168, member B [Source:MGI Symbol;Acc:MGI:2448487]	43.219	36.7939	40.578	39.8314	37.5701	43.1685	42.3653	39.4218	31.0195	48.5427	33.6778	39.9438	36.6041	39.344	0.914360142	0.966018836	24	12	24	0.778128978	0.928832041	24	20.22149628	0.045773467
ENSMUSG00000037509	Arhgef4	Rho guanine nucleotide exchange factor (GEF) 4 [Source:MGI Symbol;Acc:MGI:2442507]	16.9342	16.307	20.7094	15.6421	14.0238	19.0945	18.224	15.5571	14.8308	20.3938	13.4496	16.6333	14.6431	19.2854	0.873171835	0.966018836	12	8	24	0.670855907	0.888971832	24	5.319351578	0.068749259
ENSMUSG00000037514	Pank2	pantothenate kinase 2 [Source:MGI Symbol;Acc:MGI:1921700]	13.137	12.7742	13.9797	12.6053	13.9944	13.7603	12.467	10.7739	12.7651	13.1793	13.0186	12.8747	12.7409	12.2297	0.06180207	0.409814092	20	12	24	0.095757448	0.71175955	24	16.14815162	0.066311653
ENSMUSG00000037519	Ppfia1	protein tyrosine phosphatase, receptor type, f polypeptide (PTPRF), interacting protein (liprin), alpha 1 [Source:MGI Symbol;Acc:MGI:1924750]	11.0382	11.0163	11.3604	13.2727	12.1432	12.4524	11.8338	11.5044	11.9858	13.2253	12.5584	11.2248	11.1793	10.3925	3.35E-04	0.049770315	16	16	24	0.002363403	0.39629122	24	14.12704466	0.103930065
ENSMUSG00000037523	Mavs	mitochondrial antiviral signaling protein [Source:MGI Symbol;Acc:MGI:2444773]	4.36814	4.46951	6.07117	6.26832	5.61813	4.92634	6.06228	6.66009	5.27089	6.25874	4.87858	4.50065	5.24286	4.85184	0.247023822	0.746090009	16	8	24	0.19712604	0.768040178	24	10.0065261	0.121111582
ENSMUSG00000037525	Bcdin3d	BCDIN3 domain containing [Source:MGI Symbol;Acc:MGI:1922534]	4.32407	3.71762	3.5053	4.382	4.2651	4.10531	3.65435	4.21835	3.97154	3.65763	4.04078	4.36187	4.38992	4.46409	0.834424969	0.966018836	4	8	24	0.46994595	0.805393068	24	20.75283054	0.044679094
ENSMUSG00000037526	Atg14	autophagy related 14 [Source:MGI Symbol;Acc:MGI:1261775]	5.53723	5.1116	4.91061	5.04242	5.57687	5.40194	5.34934	5.73644	5.78385	5.4393	5.59752	5.57143	5.01586	5.77261	0.915082018	0.966018836	20	8	24	0.980050047	0.995152883	24	20.40855691	0.005227232
ENSMUSG00000037531	Mrpl47	mitochondrial ribosomal protein L47 [Source:MGI Symbol;Acc:MGI:1921850]	15.3792	16.9294	18.3198	17.7297	19.4214	17.2188	16.3999	17.4367	21.3411	15.0555	20.2827	17.5965	21.7414	18.3947	0.94086481	0.966018836	12	16	24	0.844228632	0.952833645	24	10.75451113	0.032779429
ENSMUSG00000037533	Rapgef6	Rap guanine nucleotide exchange factor (GEF) 6 [Source:MGI Symbol;Acc:MGI:2384761]	16.4264	18.487	17.3681	18.6739	19.1258	16.1417	17.4337	16.6348	20.4827	16.1535	19.9137	18.6911	19.1098	20.2205	0.914502381	0.966018836	20	4	24	0.670461466	0.888925819	24	9.800827203	0.037918109
ENSMUSG00000037536	Fbxo34	F-box protein 34 [Source:MGI Symbol;Acc:MGI:1926188]	8.48749	7.3663	9.29743	7.72628	7.51446	9.62024	8.41058	8.0824	6.8139	9.63753	8.24772	8.46795	8.69645	8.18163	0.622132989	0.966018836	24	8	24	0.608072959	0.864217165	24	19.45870125	0.052989278
ENSMUSG00000037537	H2-M11	histocompatibility 2, M region locus 11 [Source:MGI Symbol;Acc:MGI:2676637]	0.70698	0.383275	1.28141	0.811165	1.16457	1.04379	0.862659	0.928636	0.643868	0.342523	1.49117	0.540808	0.721936	0.492526	0.47028201	0.966018836	20	12	24	0.36619238	0.790132267	24	16.68409316	0.146565554
ENSMUSG00000037541	Shank2	SH3 and multiple ankyrin repeat domains 2 [Source:MGI Symbol;Acc:MGI:2671987]	0.705188	0.925511	1.5395	1.08142	0.760041	0.968865	1.44584	0.739043	1.14748	1.25815	1.12061	0.992909	0.876875	1.12924	0.326490454	0.845515883	12	16	24	0.32731401	0.768040178	24	9.732463946	0.103358412
ENSMUSG00000037544	Dlgap5	DLG associated protein 5 [Source:MGI Symbol;Acc:MGI:2183453]	0.738602	0.769993	0.691933	0.454329	0.720486	0.60997	0.434528	0.612345	0.28789	0.450721	0.62595	0.799653	0.636339	0.987272	0.522551072	0.966018836	8	4	24	0.377292887	0.797219305	24	23.39295701	0.08857808
ENSMUSG00000037552	Plekhg2	pleckstrin homology domain containing, family G (with RhoGef domain) member 2 [Source:MGI Symbol;Acc:MGI:2141874]	15.9347	22.2666	20.8874	22.5039	25.7881	17.8873	20.1076	21.9386	31.3341	26.134	20.8373	19.7646	20.8579	17.4839	0.026323104	0.301028315	16	8	24	0.029520885	0.596648309	24	10.79050355	0.227925741
ENSMUSG00000037553	Zdhhc18	zinc finger, DHHC domain containing 18 [Source:MGI Symbol;Acc:MGI:3527792]	24.1668	21.2061	20.5238	20.8001	25.1448	26.1361	23.4153	23.3826	21.371	32.9377	20.0712	22.7271	20.5461	23.9193	0.93447422	0.966018836	24	12	24	0.748417316	0.918047827	24	15.95164597	0.055707882
ENSMUSG00000037563	Rps16	ribosomal protein S16 [Source:MGI Symbol;Acc:MGI:98118]	0.675369	0.910703	0.913082	0.388446	0.638351	0.674998	0.589572	0.678362	1.20968	0.475236	1.17191	1.06547	0.84272	0.5755	0.880247609	0.966018836	12	4	24	0.976052362	0.993979912	24	0.784447048	0.017809865
ENSMUSG00000037568	Vash2	vasohibin 2 [Source:MGI Symbol;Acc:MGI:2444826]	0.784008	0.922564	1.37241	0.75859	0.830656	1.02385	0.971559	0.991124	0.959322	1.04944	0.970962	0.994397	0.850516	0.978333	0.924553956	0.966018836	12	16	24	0.518267969	0.818613065	24	8.126899363	0.04975261
ENSMUSG00000037570	Mcrs1	microspherule protein 1 [Source:MGI Symbol;Acc:MGI:1858420]	6.29009	6.15074	5.18796	5.93563	6.08274	5.93208	6.07399	6.31159	5.80746	5.34483	5.86263	6.30209	5.61744	5.50165	0.393470605	0.921010321	24	12	24	0.095490236	0.71175955	24	21.51087038	0.060963152
ENSMUSG00000037572	Wdhd1	WD repeat and HMG-box DNA binding protein 1 [Source:MGI Symbol;Acc:MGI:2443514]	2.98399	4.73936	5.60588	4.0061	4.32997	3.96624	3.28811	3.21602	4.48134	2.81998	3.76665	3.82419	4.27148	3.58988	0.92476132	0.966018836	12	16	24	0.675003279	0.891050122	24	9.215296448	0.077494581
ENSMUSG00000037573	Tob1	transducer of ErbB-2.1 [Source:MGI Symbol;Acc:MGI:1349721]	10.1186	10.1492	12.3913	14.6987	10.1696	10.2137	12.0721	14.3679	11.685	14.2142	11.4394	9.4293	11.8042	11.8794	0.062036003	0.409814092	16	8	24	0.048856033	0.638992719	24	8.993912272	0.172049187

ENSMUSG00000037578	Pkd2l1	polycystic kidney disease 2-like 1 [Source:MGI Symbol;Acc:MGI:1352448]	0.438549	0.823016	1.66394	0.810059	0.311607	0.684897	1.02654	0.394389	0.841002	0.521289	0.333335	0.723303	0.0748322	1.26881	0.101690661	0.511659635	12	8	24	0.242950314	0.768040178	24	7.109042895	0.226294692
ENSMUSG00000037580	Gch1	GTP cyclohydrolase 1 [Source:MGI Symbol;Acc:MGI:95675]	1.50874	1.34091	1.79102	1.88304	1.41322	1.97531	2.4849	1.63055	1.34469	1.84823	2.71805	2.45588	1.44646	4.13278	0.858269825	0.966018836	24	12	24	0.957128079	0.988639288	24	21.41207782	0.033279653
ENSMUSG00000037594	Clba1	clathrin binding box of aftiphilin containing 1 [Source:MGI Symbol;Acc:MGI:2443738]	3.19671	3.11889	2.99876	3.02338	2.869	3.45136	2.72135	3.77349	2.70058	3.11593	3.18001	3.58571	3.08443	3.36562	0.247964668	0.746745468	24	12	24	0.588939995	0.853936396	24	22.94318413	0.042732968
ENSMUSG00000037601	Nme1	NME/NM23 nucleoside diphosphate kinase 1 [Source:MGI Symbol;Acc:MGI:97355]	76.8912	72.6048	64.6317	66.6348	77.5332	75.8934	68.0448	69.8568	66.8771	68.2512	77.972	70.058	73.8032	67.4085	0.002466754	0.109177422	20	16	24	0.016408492	0.544099157	24	19.64898984	0.092027015
ENSMUSG00000037605	Adgrl3	adhesion G protein-coupled receptor L3 [Source:MGI Symbol;Acc:MGI:2441950]	13.9302	13.7385	12.7017	13.2859	12.9103	15.0662	15.2252	12.2216	12.2711	15.1267	12.5898	17.673	12.1338	18.6212	0.928780444	0.966018836	24	12	24	0.636501464	0.876483853	24	22.23901215	0.067488463
ENSMUSG00000037606	Osbpl5	oxysterol binding protein-like 5 [Source:MGI Symbol;Acc:MGI:1930265]	22.7588	21.5942	20.9671	24.2558	26.9448	23.8214	26.9722	23.1344	24.9425	25.7762	22.4713	25.2468	24.0834	19.2105	0.231389127	0.731037655	24	8	24	0.159092456	0.765350752	24	17.19447464	0.09766637
ENSMUSG00000037608	Bclaf1	BCL2-associated transcription factor 1 [Source:MGI Symbol;Acc:MGI:1917580]	31.9086	39.8377	39.5216	39.9165	38.5307	33.0125	34.8196	33.9568	43.9453	33.9214	39.9894	36.5179	37.5418	38.6964	0.106193059	0.519440352	12	16	24	0.100309993	0.71175955	24	9.759907825	0.103880813
ENSMUSG00000037610	Kcnmb2	potassium large conductance calcium-activated channel, subfamily M, beta member 2 [Source:MGI Symbol;Acc:MGI:1919663]	2.43867	3.38069	2.8361	4.47819	2.61959	2.73116	2.94983	3.58898	2.89834	2.5934	2.48091	4.85197	2.18299	2.4918	0.911135828	0.966018836	12	16	24	0.905700005	0.974159848	24	12.91887775	0.04565801
ENSMUSG00000037613	Tnfrsf23	tumor necrosis factor receptor superfamily, member 23 [Source:MGI Symbol;Acc:MGI:1930269]	1.29837	1.15474	1.20365	0.934933	1.18116	1.33391	1.16593	0.850798	1.0525	1.20626	0.907467	0.908862	1.25355	1.41511	0.941830768	0.966018836	4	16	24	0.540640475	0.830061866	24	1.065294819	0.052426819
ENSMUSG00000037617	Spag1	sperm associated antigen 1 [Source:MGI Symbol;Acc:MGI:1349387]	3.25031	3.97038	4.88537	3.8316	3.44115	3.92475	4.15442	3.2369	3.84421	2.8877	4.19342	3.18852	3.19308	3.74483	0.907000603	0.966018836	12	12	24	0.754446363	0.920525691	24	8.387992974	0.048147468
ENSMUSG00000037621	Atoh8	atonal bHLH transcription factor 8 [Source:MGI Symbol;Acc:MGI:1918343]	1.01953	0.681315	0.627501	1.12917	0.91326	1.06831	0.974109	0.711068	1.08044	0.822729	0.963425	0.754983	0.767962	0.998915	0.935593607	0.966018836	16	16	24	0.562239438	0.842208433	24	16.57816051	0.054369576
ENSMUSG00000037622	Wdtc1	WD and tetratricopeptide repeats 1 [Source:MGI Symbol;Acc:MGI:2685541]	12.6881	10.1519	10.3568	11.5357	11.1267	12.3168	12.5512	13.8913	10.6128	16.4421	10.7861	12.7242	12.6398	12.6165	0.830364743	0.966018836	4	8	24	0.922385301	0.979411448	24	21.67658563	0.027064363
ENSMUSG00000037624	Kcnk2	potassium channel, subfamily K, member 2 [Source:MGI Symbol;Acc:MGI:109366]	6.07492	7.1502	6.9629	6.04842	5.39455	7.07684	7.07341	7.09023	6.44275	6.2857	4.96485	6.76827	6.0093	6.78095	0.004196064	0.140598674	8	12	24	0.047898806	0.636814569	24	3.441832571	0.119507584
ENSMUSG00000037625	Cldn11	claudin 11 [Source:MGI Symbol;Acc:MGI:106925]	17.7791	32.4815	45.1599	24.471	20.6683	27.0232	32.2767	25.5159	35.8008	19.3611	19.573	24.4777	12.1193	28.6331	0.056291566	0.401074772	12	8	24	0.189864294	0.768040178	24	7.305833407	0.314399516
ENSMUSG00000037628	Cdkn3	cyclin-dependent kinase inhibitor 3 [Source:MGI Symbol;Acc:MGI:1919641]	5.93844	6.02308	6.3164	5.33979	6.23581	5.59901	5.59285	5.08695	5.13865	3.91852	6.92095	4.7105	6.11846	5.03423	0.919571723	0.966018836	20	12	24	0.601341659	0.860501201	24	20.948198	0.06881003
ENSMUSG00000037636	Slc25a43	solute carrier family 25, member 43 [Source:MGI Symbol;Acc:MGI:2684854]	1.2578	0.834036	0.671465	0.882743	1.02937	1.20878	0.9792	1.00423	0.591986	1.1007	1.39086	0.86898	1.43156	1.54404	0.277087842	0.789849983	4	8	24	0.207957748	0.768040178	24	21.27990508	0.126300372
ENSMUSG00000037638	Zbtb42	zinc finger and BTB domain containing 42 [Source:MGI Symbol;Acc:MGI:3644133]	1.16218	0.988273	0.805008	1.02854	0.995079	1.12512	0.861368	1.3064	0.736128	1.16654	0.965229	1.06769	1.06978	1.24501	0.495929335	0.966018836	8	4	24	0.600826388	0.860188349	24	22.9153291	0.046525878
ENSMUSG00000037640	Zfp60	zinc finger protein 60 [Source:MGI Symbol;Acc:MGI:99207]	10.4673	10.5436	10.3419	9.83043	10.7809	9.61157	10.4124	8.97795	10.9438	9.14938	12.0419	11.0859	10.8847	11.0669	0.873171835	0.966018836	20	20	24	0.759110896	0.92214435	24	19.64316836	0.031381808
ENSMUSG00000037643	Prkci	protein kinase C, iota [Source:MGI Symbol;Acc:MGI:99260]	15.8394	14.6571	14.336	14.8377	15.6264	14.6395	14.9524	14.6216	12.8283	15.4177	14.1183	14.618	15.1212	15.2972	0.286642595	0.793714531	4	8	24	0.361185327	0.786651663	24	21.00952524	0.040127011
ENSMUSG00000037646	Vps13b	vacuolar protein sorting 13B [Source:MGI Symbol;Acc:MGI:1916380]	5.83225	5.96827	5.95982	5.98584	5.81411	5.77338	5.81198	5.25472	5.8454	5.25202	5.84182	5.91661	6.2613	6.1429	0.896311169	0.966018836	8	16	24	0.683186884	0.894420965	24	23.76599711	0.021182493
ENSMUSG00000037649	H2-DMa	histocompatibility 2, class II, locus DMa [Source:MGI Symbol;Acc:MGI:95921]	24.2921	22.6505	19.259	21.6264	26.2328	22.6839	26.2196	24.3726	23.9399	25.3053	20.8097	23.9849	23.3922	19.4211	0.85447369	0.966018836	4	8	24	0.435983784	0.805393068	24	20.0523997	0.07222129
ENSMUSG00000037652	Phc3	polyhomeotic 3 [Source:MGI Symbol;Acc:MGI:2181434]	7.93151	8.07771	7.91234	8.05158	8.06987	7.22846	8.19791	6.92381	8.32304	6.70148	8.00759	7.99987	7.6476	8.04492	0.819898687	0.966018836	12	12	24	0.958680752	0.989144435	24	0.277441024	0.009763416
ENSMUSG00000037653	Kctd8	potassium channel tetramerisation domain containing 8 [Source:MGI Symbol;Acc:MGI:2443804]	18.6392	15.7596	14.8188	15.2276	15.5553	16.8465	15.4684	15.9297	12.9292	16.7224	18.5113	17.6831	17.8534	17.2511	0.035787737	0.331814682	4	8	24	0.053047784	0.650879532	24	20.71145978	0.117609927
ENSMUSG00000037656	Slc20a2	solute carrier family 20, member 2 [Source:MGI Symbol;Acc:MGI:97851]	15.6677	16.3555	16.7996	14.4053	14.139	16.4685	18.0104	18.5697	15.9642	18.7441	13.1277	17.8778	16.5879	15.8673	0.700065774	0.966018836	8	12	24	0.240492367	0.768040178	24	3.148471492	0.089800429
ENSMUSG00000037661	Gpr160	G protein-coupled receptor 160 [Source:MGI Symbol;Acc:MGI:1919112]	4.59591	3.53039	4.26953	4.35488	3.72204	4.90485	3.35026	3.50917	3.28722	2.92529	3.78678	3.66496	3.65463	3.59451	0.435596482	0.966018836	20	16	24	0.283746381	0.768040178	24	19.88413224	0.089100408
ENSMUSG00000037664	Cdkn1c	cyclin-dependent kinase inhibitor 1C (P57) [Source:MGI Symbol;Acc:MGI:104564]	6.58983	8.45707	9.72702	9.25747	7.19404	6.30479	6.9855	9.00314	12.6239	8.21668	8.76011	9.20727	9.86583	8.47389	0.210566453	0.69936851	12	12	24	0.0229424	0.581965446	24	8.43074574	0.220427734
ENSMUSG00000037669	Ldah	lipid droplet associated hydrolase [Source:MGI Symbol;Acc:MGI:1916082]	31.5327	30.2599	30.9995	31.137	31.7407	30.3302	30.0673	28.2033	30.0211	25.7095	32.0012	31.5891	33.7076	29.0095	0.06180207	0.409814092	20	12	24	0.265924801	0.768040178	24	20.85660922	0.058141076
ENSMUSG00000037674	Rfx7	regulatory factor X, 7 [Source:MGI Symbol;Acc:MGI:2442675]	5.0783	6.15788	5.46214	5.85061	5.76386	5.22165	5.28803	5.58588	5.74443	5.32139	5.9394	5.52433	5.35616	5.9093	0.230338208	0.730447182	8	20	24	0.268106041	0.768040178	24	9.605083133	0.044564024
ENSMUSG00000037679	Inf2	inverted formin, FH2 and WH2 domain containing [Source:MGI Symbol;Acc:MGI:1917685]	20.5399	19.6641	19.3552	20.1724	23.1585	18.7159	23.4369	19.9572	22.1437	24.2946	20.8992	20.6914	21.81	18.6447	0.371875382	0.894537464	20	12	24	0.527332587	0.8233276	24	14.92201953	0.050385687
ENSMUSG00000037681	Esyt3	extended synaptotagmin-like protein 3 [Source:MGI Symbol;Acc:MGI:1098699]	1.2247	1.02952	1.0619	1.12618	0.864116	1.1493	0.946607	1.13837	0.916633	1.04576	1.05878	1.38666	1.22489	1.35037	0.373664134	0.896700272	24	20	24	0.306854409	0.768040178	24	23.92565196	0.057166986
ENSMUSG00000037685	Atp8a1	ATPase, aminophospholipid transporter (APLT), class I, type 8A, member 1 [Source:MGI Symbol;Acc:MGI:1330848]	63.7723	59.9584	57.8496	57.782	61.8514	60.5358	61.0813	55.506	58.3841	56.462	63.1079	63.4292	65.1014	61.1066	0.016720774	0.253395174	4	12	24	0.010332654	0.503967859	24	20.9715764	0.070958315
ENSMUSG00000037686	Aspg	asparaginase [Source:MGI Symbol;Acc:MGI:2144822]	1.09325	2.01625	3.78585	5.31451	1.71424	2.38964	2.68526	5.29706	3.4171	3.27598	3.7238	2.41491	4.33499	5.02374	0.383511101	0.915479136	8	16	24	0.045538838	0.629609444	24	8.376729757	0.381408202
ENSMUSG00000037692	Ahdc1	AT hook, DNA binding motif, containing 1 [Source:MGI Symbol;Acc:MGI:2444218]	4.04898	3.20553	3.83267	3.11539	3.63998	3.78884	3.82561	3.57961	4.52713	4.89153	4.1349	3.91536	3.40341	4.62973	0.042838536	0.966018836	12	20	24	0.633343029	0.875353057	24	10.12674027	0.053137563
ENSMUSG00000037697	Ddhd1	DDHD domain containing 1 [Source:MGI Symbol;Acc:MGI:2150302]	30.3231	29.9988	28.3418	27.8132	33.4848	30.6019	31.632	27.2288	28.0375	27.8754	31.7099	28.6371	30.451	27.9703	0.006767691	0.181382464	20	20	24	0.023286244	0.581965446	24	19.4549863	0.085132157
ENSMUSG00000037703	Lzts3	leucine zipper, putative tumor suppressor family member 3 [Source:MGI Symbol;Acc:MGI:2656976]	8.64022	7.12815	7.83623	8.07556	7.49645	7.86048	8.55296	7.90837	7.52056	9.07708	7.69292	8.78789	8.33141	8.3353	0.455607782	0.966018836	4	8	24	0.702194928	0.901574817	24	21.00140063	0.028897596
ENSMUSG00000037705	Tecta	tectorin alpha [Source:MGI Symbol;Acc:MGI:109575]	1.58926	1.08962	1.6078	1.06152	1.60969	1.91301	1.17526	1.41383	1.19057	1.71813	1.69483	1.39484	1.4025	1.42318	0.607427393	0.966018836	12	20	24	0.25522629	0.768040178	24	17.50146735	0.097716493
ENSMUSG00000037706	Cd81	CD81 antigen [Source:MGI Symbol;Acc:MGI:1096398]	112.165	125.876	134.481	109.101	124.331	123.081	135.203	123.87	134.237	111.082	112.467	119.969	112.8	115.057	0.187699199	0.662239322	12	4	24	0.535495632	0.827019069	24	4.482362992	0.04661394
ENSMUSG00000037708	Spag6	sperm associated antigen 6 [Source:MGI Symbol;Acc:MGI:3040687]	0.740819	0.498065	0.420794	0.75959	0.708764	0.635579	0.625908	0.529767	0.461803	0.862018	0.609135	0.72304	0.460328	0.727304	0.129903614	0.560263689	16	20	24	0.209734643	0.768040178	24	16.59288776	0.081303481
ENSMUSG00000037709	Fam13a	family with sequence similarity 13, member A [Source:MGI Symbol;Acc:MGI:1889842]	5.45393	4.7976	5.88781	7.77426	4.79836	5.84054	6.08547	5.68976	4.57963	6.68774	5.26772	5.30208	5.26383	5.99337	0.858891143	0.966018836	4	12	24	0.629648443	0.873514169	24	11.83183032	0.066391108
ENSMUSG00000037710	Cisd1	CDGSH iron sulfur domain 1 [Source:MGI Symbol;Acc:MGI:1261855]	98.3134	100.942	93.9373	99.4647	98.5781	98.9239	98.73	101.845	96.459	100.044	119.675	106.563	118.694	112.553	0.718720526	0.966018836	8	4	24	0.467109877	0.805393068	24	21.22716454	0.037480864
ENSMUSG00000037712	Fermt2	fermitin family member 2 [Source:MGI Symbol;Acc:MGI:2385001]	64.7485	58.7053	58.7498	56.1268	64.0293	58.8642	65.8544	63.2759	55.9606	60.2952	62.9629	57.6015	63.3592	58.459	0.031969981	0.324385159	4	8	24	0.160385605	0.765941695	24	21.86278718	0.057305033
ENSMUSG00000037716	Ccdc33	coiled-coil domain containing 33 [Source:MGI Symbol;Acc:MGI:1922464]	0.568697	1.37653	1.9017	0.954724	1.09415	0.839813	0.503465	1.36962	1.11538	0.831046	0.982992	1.48668	0.620772	1.52696	0.056400419	0.401074772	8	20	24	0.208084214	0.768040178	24	8.448351164	0.198402379
ENSMUSG00000037720	Tmem33	transmembrane protein 33 [Source:MGI Symbol;Acc:MGI:1915128]	37.602	33.7961	31.3935	32.0632	36.1653	34.6023	37.8573	34.4258	32.1806	31.3589	41.4326	33.4143	36.1568	33.6269	0.005982427	0.163356881	4	12	24	0.055140527	0.65534544	24	20.68422527	0.101735685
ENSMUSG00000037722	Gnpnat1	glucosamine-phosphate N-acetyltransferase 1 [Source:MGI Symbol;Acc:MGI:1858963]	5.33682	5.31693	6.28957	4.72329	6.29498	4.32946	5.40381	3.8372	5.19648	4.23837	5.04159	4.36633	5.15427	4.5874	0.933543173	0.966018836	4	12	24	0.963356829	0.990884307	24	13.78601606	0.016981492
ENSMUSG00000037725	Ckap2	cytoskeleton associated protein 2 [Source:MGI Symbol;Acc:MGI:1931797]	1.39735	1.58078	1.77609	1.47775	1.46313	1.41801	0.938635	1.54204	1.56025	1.04734	1.59018	1.35108	1.41262	1.36004	0.105280783	0.518002936	12	16	24	0.596087514	0.857839743	24	8.992921105	0.054766791
ENSMUSG00000037730	Mynn	myoneurin [Source:MGI Symbol;Acc:MGI:1931415]	5.23813	5.98371	7.97499	6.82529	8.00158	6.51811	5.6375	6.48829	6.53836	5.86857	7.22368	5.84263	5.42694	5.80428	3.67E-04	0.050643729	20	8	24	0.030388212	0.597890721	24	12.70896793	0.148390501
ENSMUSG00000037731	Themis2	thymocyte selection associated family member 2 [Source:MGI Symbol;Acc:MGI:2446213]	0.800722	0.995029	1.03405	0.751913	1.13504	0.744586	0.97905	1.0617	1.18966	0.8335	1.00347	0.680218	0.954723	0.852905	0.011724377	0.210241052	12	12	24	0.333588948	0.769755361	24	7.59555897	0.069624704
ENSMUSG00000037735	2810032G03Rik	RIKEN cDNA 2810032G03 gene [Source:MGI Symbol;Acc:MGI:1919919]	3.41991	2.63488	2.73918	2.14371	2.80608	3.00691	2.62639	2.67002	2.22035	2.76956	3.25637	3.15737	3.37327	3.02334	0.124070509	0.552144108	4	8	24	0.030029832	0.596794384	24	21.42161549	0.137178178
ENSMUSG00000037736	Limch1	LIM and calponin homology domains 1 [Source:MGI Symbol;Acc:MGI:1924819]	54.0482	57.1744	53.3489	58.4682	64.2427	51.9527	66.1611	52.4338	64.6398	63.7087	54.4652	61.2174	60.2016	52.9176	0.965971228	0.969272194	16	16	24	0.683119302	0.89440331	24	14.45910024	0.042452159
ENSMUSG00000037740	Mrps26	mitochondrial ribosomal protein S26 [Source:MGI Symbol;Acc:MGI:1333830]	21.0853	18.4345	18.3662	18.8394	17.2048	18.4619	16.2355	19.8087	19.8197	19.8995	19.3272	21.0897	21.3831	21.3289	0.908406451	0.966018836	4	16	24	0.646197807	0.880190017	24	3.703169868	0.036918213
ENSMUSG00000037742	Eef1a1	eukaryotic translation elongation factor 1 alpha 1 [Source:MGI Symbol;Acc:MGI:1096881]	570.769	607.208	586.554	623.087	657.116	619.827	587.989	571.132	568.249	510.862	567.532	543.717	553.351	504.841	0.828830907	0.966018836	20	12	24	0.209843289	0.768040178	24	17.46133371	0.052414622
ENSMUSG00000037747	Phyhipl	phytanoyl-CoA hydroxylase interacting protein-like [Source:MGI Symbol;Acc:MGI:1918161]	33.0503	30.6876	35.6746	30.5624	27.8071	35.6049	36.1952	28.2187	25.7352	35.5057	24.4776	30.6542	24.7694	32.8649	0.9143748	0.966018836	4	16	24	0.898019794	0.970959341	24	1.39065946	0.033546303
ENSMUSG00000037750	Fam222b	family with sequence similarity 222, member B [Source:MGI Symbol;Acc:MGI:2384939]	8.23309	6.01763	6.46417	6.36135	6.55179	7.51649	8.42652	7.595	6.06252	8.68806	6.61623	6.95884	6.85523	6.91208	0.176754388	0.653256853	4	8	24	0.442380097	0.805393068	24	21.77887121	0.078458458
ENSMUSG00000037752	Xkr8	X-linked Kx blood group related 8 [Source:MGI Symbol;Acc:MGI:2685877]	2.53656	1.78686	1.73	1.63976	1.60746	1.66265	2.31501	1.65635	1.40733	1.93649	1.32659	1.87104	1.50428	1.66754	0.5169953	0.966018836	4	16	24	0.167390841	0.767020009	24	23.51709342	0.106280093
ENSMUSG00000037754	Ppp1r16b	protein phosphatase 1, regulatory subunit 16B [Source:MGI Symbol;Acc:MGI:2151841]	5.15141	5.79676	7.18206	6.0401	6.02323	6.09197	7.05358	5.30301	6.80586	6.66033	5.28732	5.96192	5.40849	6.23106	0.302965859	0.811201458	12	16	24	0.314090643	0.768040178	24	9.501312197	0.082004203
ENSMUSG00000037759	Ptger2	prostaglandin E receptor 2 (subtype EP2) [Source:MGI Symbol;Acc:MGI:97794]	0.615514	0.678675	0.640543	0.716625	0.597654	0.593709	0.561888	0.730908	0.781108	0.60531	0.478396	0.725264	0.658489	0.792255	0.030972684	0.324150946	8	12	24	0.056609008	0.655587475	24	5.929619576	0.067453976
ENSMUSG00000037761	Actr5	ARP5 actin-related protein 5 [Source:MGI Symbol;Acc:MGI:1924748]	2.95974	2.10564	2.41198	2.59792	2.99699	1.90968	2.72526	2.00135	2.76779	2.38615	2.2002	2.90349	2.50136	2.90928	0.831272156	0.966018836	4	4	24	0.978413219	0.994576186	24	19.07262973	0.013606158
ENSMUSG00000037762	Slc16a9	solute carrier family 16 (monocarboxylic acid transporters), member 9 [Source:MGI Symbol;Acc:MGI:1914109]	4.62934	4.75666	4.57208	4.78227	5.29641	4.9146	4.50091	5.19299	5.20713	4.60233	5.54947	5.19682	4.46637	4.6782	0.018220512	0.256345714	20	8	24	0.230136594	0.768040178	24	15.23350199	0.053529457
ENSMUSG00000037771	Slc32a1	solute carrier family 32 (GABA vesicular transporter), member 1 [Source:MGI Symbol;Acc:MGI:1194488]	0.976764	3.3356	6.01898	1.94213	1.09796	3.09081	4.13374	1.44556	3.42654	2.49087	1.01743	2.53647	0.807921	4.10127	0.285027487	0.793714531	12	8	24	0.264896379	0.768040178	24	6.774391801	0.397385883
ENSMUSG00000037772	Mrpl23	mitochondrial ribosomal protein L23 [Source:MGI Symbol;Acc:MGI:1196612]	0.875436	1.06739	1.42648	0.5663	1.14826	0.957596	1.1611	0.728317	1.55204	1.11753	1.20794	1.18926	1.35	1.10752	0.928148816	0.966018836	12	20	24	0.921248849	0.979223729	24	7.01550965	0.030066675
ENSMUSG00000037773	Pced1a	PC-esterase domain containing 1A [Source:MGI Symbol;Acc:MGI:2442177]	7.31644	5.7866	5.69902	6.75339	6.59006	6.51993	6.79953	5.71192	5.89857	6.86643	6.42549	6.29451	6.2761	6.40205	0.076247883	0.453790923	16	20	24	0.130100164	0.740637625	24	18.54298171	0.073244416
ENSMUSG00000037784	Dzip1l	DAZ interacting protein 1-like [Source:MGI Symbol;Acc:MGI:1919757]	1.09274	1.48807	1.93167	2.12807	1.44378	1.30491	1.37038	1.22169	1.80227	1.25689	1.62019	1.53078	1.23016	1.77082	0.030691182	0.324150946	12	16	24	0.055320596	0.65534544	24	10.35865554	0.150800949
ENSMUSG00000037787	Coa8	cytochrome c oxidase assembly factor 8 [Source:MGI Symbol;Acc:MGI:1915270]	19.9744	20.1438	19.6125	20.229	16.3692	18.737	18.5258	19.2867	20.7577	19.5779	20.0676	20.195	22.281	20.2922	0.909604019	0.966018836	12	8	24	0.264995378	0.768040178	24	4.79934059	0.055890763
ENSMUSG00000037788	Vopp1	vesicular, overexpressed in cancer, prosurvival protein 1 [Source:MGI Symbol;Acc:MGI:2141658]	25.8948	20.8982	20.7503	20.9599	21.4743	23.2304	20.9118	22.1606	18.3387	22.5832	20.5441	23.6656	23.9529	23.6252	0.031969981	0.324385159	4	8	24	0.067517153	0.670954081	24	22.19552115	0.102691273
ENSMUSG00000037791	Phf12	PHD finger protein 12 [Source:MGI Symbol;Acc:MGI:1924057]	8.09372	10.3787	9.81948	10.9457	9.12163	9.89271	9.41115	10.0455	12.8056	9.60916	10.3788	10.1305	10.1385	9.38599	0.801995186	0.966018836	12	16	24	0.162015047	0.76636332	24	9.705635064	0.096540997
ENSMUSG00000037795	N4bp2	NEDD4 binding protein 2 [Source:MGI Symbol;Acc:MGI:2684414]	1.15721	1.40816	1.3995	1.1666	1.71353	1.01156	1.40771	1.12875	2.16551	1.22578	1.27706	1.1394	1.04105	1.36361	0.14313801	0.585944911	12	12	24	0.237489426	0.768040178	24	9.232604613	0.11656936
ENSMUSG00000037805	Rpl10a	ribosomal protein L10A [Source:MGI Symbol;Acc:MGI:1343877]	11.7254	12.1362	11.658	10.1471	10.1679	9.07409	10.1069	13.9266	15.7423	14.0007	16.4974	13.2548	13.4303	16.0152	0.79867683	0.966018836	8	16	24	0.104807211	0.717030134	24	7.454355901	0.158393889
ENSMUSG00000037808	Fam76b	family with sequence similarity 76, member B [Source:MGI Symbol;Acc:MGI:1920076]	7.29993	9.49502	9.49813	9.54318	8.56337	7.96256	8.43809	7.93222	9.58014	7.19146	8.92934	7.77694	7.57243	8.45578	0.031969981	0.324385159	12	16	24	0.147397568	0.756090042	24	9.395384212	0.095385834
ENSMUSG00000037813	D630003M21Rik	RIKEN cDNA D630003M21 gene [Source:MGI Symbol;Acc:MGI:3606579]	5.52179	6.01081	5.72344	5.77457	6.6461	5.7269	6.71405	6.49823	6.59683	6.5925	5.48569	6.06615	6.2033	5.10081	0.761468226	0.966018836	4	4	24	0.939032156	0.98496444	24	12.41857095	0.01520363
ENSMUSG00000037815	Ctnna1	catenin (cadherin associated protein), alpha 1 [Source:MGI Symbol;Acc:MGI:88274]	80.9385	72.2653	68.0385	73.0384	81.1305	74.1875	79.4474	74.976	66.1002	75.048	72.5791	72.5434	74.2865	67.993	0.170659409	0.643496587	4	8	24	0.02812927	0.590935816	24	20.04666738	0.081479448
ENSMUSG00000037816	Fbxw17	F-box and WD-40 domain protein 17 [Source:MGI Symbol;Acc:MGI:1923584]	26.5672	28.7723	28.2817	23.4382	28.8279	30.4074	25.8378	35.3407	25.9166	25.5037	27.0629	29.2132	28.0986	26.709	0.285027487	0.793714531	24	16	24	0.411451614	0.799546792	24	0.195092243	0.070455128
ENSMUSG00000037818	Abhd18	abhydrolase domain containing 18 [Source:MGI Symbol;Acc:MGI:1915468]	3.00854	3.47262	3.4671	3.60331	3.56054	2.78448	3.248	3.91371	4.18765	2.78547	3.52062	2.86885	3.17016	3.25759	0.010049466	0.194720062	12	12	24	0.106636101	0.718749374	24	8.076152035	0.110542298
ENSMUSG00000037820	Tgm2	transglutaminase 2, C polypeptide [Source:MGI Symbol;Acc:MGI:98731]	8.29401	8.6484	7.75891	7.8132	9.74678	7.9947	8.19867	12.2402	8.66374	7.79862	7.83426	7.33142	7.86176	7.22681	0.914502381	0.966018836	8	8	24	0.774136241	0.927465562	24	4.679628227	0.049323097
ENSMUSG00000037822	Smim14	small integral membrane protein 14 [Source:MGI Symbol;Acc:MGI:1915802]	96.5839	95.3445	90.0905	91.7114	89.7875	95.9438	90.6093	87.7914	93.3465	93.6334	92.8191	95.7141	96.7909	95.2274	0.180646802	0.658184014	24	20	24	0.345875915	0.778673045	24	22.26207441	0.025113405
ENSMUSG00000037824	Tspan14	tetraspanin 14 [Source:MGI Symbol;Acc:MGI:1196325]	29.16	23.1182	21.5449	24.1101	27.4752	27.73	27.9214	24.8649	20.7996	24.3732	25.1568	25.9512	27.1356	22.9161	3.88E-05	0.024132722	4	8	24	1.68E-04	0.141648277	24	20.47657745	0.17737506
ENSMUSG00000037826	Ppm1k	protein phosphatase 1K (PP2C domain containing) [Source:MGI Symbol;Acc:MGI:2442111]	4.2718	6.2059	5.16897	5.40078	5.7737	4.79885	5.09416	4.80797	6.11345	4.91672	5.6981	5.24449	5.92883	6.01983	0.835401638	0.966018836	12	12	24	0.462143287	0.805393068	24	8.637731646	0.06396164
ENSMUSG00000037827	Gm5884	predicted pseudogene 5884 [Source:MGI Symbol;Acc:MGI:3644646]	18.621	15.4963	17.8008	17.9621	16.8157	21.0791	18.9174	19.712	16.1774	21.2356	15.8913	18.2223	17.9477	17.9952	0.893609703	0.966018836	24	12	24	0.803620655	0.937203616	24	20.75813158	0.03617408
ENSMUSG00000037833	Sh2d4b	SH2 domain containing 4B [Source:MGI Symbol;Acc:MGI:1925182]	3.80657	3.09609	2.91949	3.84901	3.7108	2.68438	2.99879	3.23695	3.25863	3.44438	3.20216	2.57767	3.31719	3.41054	0.424718357	0.966018836	16	8	24	0.55501339	0.838228049	24	10.27469156	0.056862347
ENSMUSG00000037843	Vstm2l	V-set and transmembrane domain containing 2-like [Source:MGI Symbol;Acc:MGI:2685537]	15.4698	14.2571	14.6362	13.7637	12.6805	13.7203	14.5731	14.6192	14.5563	20.6117	16.128	18.5269	17.3215	17.62	0.809772024	0.966018836	4	16	24	0.962808696	0.990726108	24	6.892837186	0.015923297
ENSMUSG00000037845	Fdxacb1	ferredoxin-fold anticodon binding domain containing 1 [Source:MGI Symbol;Acc:MGI:3584513]	3.37906	3.4151	3.5365	3.28854	4.27374	3.621	3.42257	3.7889	3.48492	3.16142	3.61052	2.98434	3.29241	3.50252	0.904387595	0.966018836	8	8	24	0.910366381	0.974733097	24	14.46432244	0.016059348
ENSMUSG00000037847	Nmrk1	nicotinamide riboside kinase 1 [Source:MGI Symbol;Acc:MGI:2147434]	9.43872	9.83462	9.66476	10.1078	10.0268	9.0941	9.06713	9.81797	10.7823	8.63513	9.77282	9.45336	10.1808	9.05851	0.916556573	0.966018836	12	12	24	0.753641371	0.92024291	24	10.707538	0.025268648
ENSMUSG00000037851	Iars	isoleucine-tRNA synthetase [Source:MGI Symbol;Acc:MGI:2145219]	22.5249	18.2519	18.9409	18.7565	18.3453	22.2377	18.588	20.2406	14.7249	22.37	18.0232	19.1604	18.793	19.4135	0.91695334	0.966018836	4	20	24	0.410750221	0.799546792	24	20.75800586	0.080310195
ENSMUSG00000037852	Cpe	carboxypeptidase E [Source:MGI Symbol;Acc:MGI:101932]	370.775	325.167	330.187	324.852	307.612	356.344	343.779	319.133	258.594	350.098	290.029	310.584	297.787	316.874	0.93447422	0.966018836	4	16	24	0.365846201	0.789934347	24	21.84594089	0.066747696
ENSMUSG00000037855	Zfp365	zinc finger protein 365 [Source:MGI Symbol;Acc:MGI:2143676]	17.6631	14.5129	14.7898	14.7986	13.1492	16.2139	15.2698	15.4936	11.6708	18.885	13.5438	14.3281	13.5855	16.7439	0.883024237	0.966018836	12	8	24	0.855518554	0.957116295	24	0.396359465	0.037872902
ENSMUSG00000037857	Nufip2	nuclear fragile X mental retardation protein interacting protein 2 [Source:MGI Symbol;Acc:MGI:1915814]	16.8205	16.4256	14.4061	15.0484	17.0021	16.0209	15.8302	13.7782	14.1939	12.9281	19.1598	14.9081	14.746	14.7279	0.004947794	0.141782417	20	16	24	0.028039647	0.590904845	24	18.74429611	0.130284711
ENSMUSG00000037860	Aim2	absent in melanoma 2 [Source:MGI Symbol;Acc:MGI:2686159]	1.11945	1.63334	1.92328	1.05613	0.854638	0.856125	0.919535	1.62496	2.80349	0.858352	0.64468	1.41088	0.975288	1.46887	5.29E-05	0.029372629	12	8	24	0.012264565	0.525280095	24	6.292385382	0.338484382
ENSMUSG00000037868	Egr2	early growth response 2 [Source:MGI Symbol;Acc:MGI:95296]	11.2158	11.7286	10.0224	8.96139	13.5648	10.5267	13.0694	12.5624	12.6547	20.7096	9.25437	11.2905	13.6956	8.37542	0.81829516	0.966018836	4	4	24	0.941020164	0.985441727	24	13.10037559	0.042542729
ENSMUSG00000037872	Ackr1	atypical chemokine receptor 1 (Duffy blood group) [Source:MGI Symbol;Acc:MGI:1097689]	51.6983	42.1187	43.3146	49.3169	49.2695	54.113	47.225	50.4909	42.5465	63.0439	43.2574	47.1742	46.6139	47.2902	0.976909628	0.977527818	16	20	24	0.574347211	0.847247297	24	16.69798837	0.06488693
ENSMUSG00000037876	Jmjd1c	jumonji domain containing 1C [Source:MGI Symbol;Acc:MGI:1918614]	12.5078	13.8366	13.4451	13.1752	13.4819	11.2544	13.8461	11.4342	14.638	11.197	14.4549	13.3297	13.4418	13.0502	0.912686312	0.966018836	12	12	24	0.946672197	0.986968557	24	8.623756645	0.016343994
ENSMUSG00000037885	Stk35	serine/threonine kinase 35 [Source:MGI Symbol;Acc:MGI:1914583]	6.00403	4.69953	4.91213	5.65507	5.30775	5.44139	5.21825	5.30746	4.2923	6.424	5.46236	5.88219	4.76472	4.91974	0.033799022	0.325496079	16	20	24	0.115916907	0.727799063	24	17.21103135	0.102148543
ENSMUSG00000037887	Dusp8	dual specificity phosphatase 8 [Source:MGI Symbol;Acc:MGI:106626]	6.68983	6.10618	6.41983	6.76379	5.88719	6.88357	7.47346	7.06183	6.27053	9.55538	5.96973	7.50996	7.04899	7.63441	0.888991156	0.966018836	24	20	24	0.943820053	0.986212398	24	6.193060681	0.020685418
ENSMUSG00000037890	Wdr19	WD repeat domain 19 [Source:MGI Symbol;Acc:MGI:2443231]	5.95485	5.58317	4.79968	4.53329	4.8696	5.38835	5.1898	4.75532	5.35231	5.21593	4.85609	5.74584	5.28258	5.12857	0.191123261	0.669841558	24	20	24	0.08477031	0.70291653	24	23.15809836	0.077081293
ENSMUSG00000037892	Pcdh18	protocadherin 18 [Source:MGI Symbol;Acc:MGI:1920423]	2.56494	2.08609	2.47738	1.94463	2.30803	2.05923	2.3099	2.21227	2.04213	1.9935	2.08471	1.93866	1.98205	1.78086	0.890648739	0.966018836	4	12	24	0.628271489	0.872795898	24	22.6077081	0.027767005
ENSMUSG00000037894	H2az1	H2A.Z variant histone 1 [Source:MGI Symbol;Acc:MGI:1888388]	7.80201	9.67353	9.60598	8.62052	9.51835	7.97489	8.06191	8.10781	10.2424	7.66646	9.34224	8.81869	8.61095	7.36459	0.636884864	0.966018836	12	16	24	0.449887278	0.805393068	24	11.57416029	0.064038894
ENSMUSG00000037896	Rcor1	REST corepressor 1 [Source:MGI Symbol;Acc:MGI:106340]	7.28433	6.06669	5.62201	6.50556	6.08932	7.18041	6.9476	6.60014	5.46279	7.42076	6.11813	6.37301	6.02829	6.16906	0.390686537	0.915479136	24	12	24	0.273650079	0.768040178	24	20.18281744	0.074973814
ENSMUSG00000037902	Sirpa	signal-regulatory protein alpha [Source:MGI Symbol;Acc:MGI:108563]	20.2572	20.1559	20.4613	18.6774	20.6895	21.0084	21.6493	20.0707	20.6755	22.3261	18.054	21.4453	20.2162	21.508	0.896311169	0.966018836	24	8	24	0.66628112	0.88724859	24	1.934847044	0.026987397
ENSMUSG00000037904	Ankrd9	ankyrin repeat domain 9 [Source:MGI Symbol;Acc:MGI:1921501]	3.90721	3.57851	3.67697	4.40996	4.05888	4.25491	4.77107	5.46448	4.96494	7.08951	4.73242	5.24532	5.65881	4.64637	0.801280095	0.966018836	16	20	24	0.702905685	0.901733679	24	11.88519702	0.052965006
ENSMUSG00000037905	Bri3bp	Bri3 binding protein [Source:MGI Symbol;Acc:MGI:1924059]	5.39562	5.18652	5.92238	5.09131	4.6191	5.70574	5.7413	4.86945	5.06545	6.21584	4.45596	5.66416	4.66544	5.83946	0.91724565	0.966018836	16	4	24	0.799280678	0.935782011	24	5.392579259	0.034691615
ENSMUSG00000037907	Ankrd13b	ankyrin repeat domain 13b [Source:MGI Symbol;Acc:MGI:2144501]	23.482	18.3488	17.145	19.095	22.0717	20.4813	20.1347	20.5718	19.0966	25.4117	22.8192	21.7866	21.7064	19.6642	0.03782572	0.334069441	20	16	24	0.120615194	0.732894732	24	18.05638593	0.10840962
ENSMUSG00000037916	Ndufv1	NADH:ubiquinone oxidoreductase core subunit V1 [Source:MGI Symbol;Acc:MGI:107851]	90.5576	83.063	93.507	103.375	81.359	93.8168	91.4048	98.8616	85.4863	103.502	94.4778	93.3299	98.843	95.73	0.873171835	0.966018836	16	12	24	0.801277036	0.936384198	24	10.77432997	0.026585293
ENSMUSG00000037921	Ddx60	DExD/H box helicase 60 [Source:MGI Symbol;Acc:MGI:2384570]	1.1884	1.48732	1.60569	1.08603	1.41278	1.19597	1.04605	1.61078	1.61844	1.09146	1.5312	1.2543	1.08867	1.6249	0.054328576	0.395890765	8	20	24	0.185686734	0.768040178	24	7.409628235	0.103202945
ENSMUSG00000037922	Bank1	B cell scaffold protein with ankyrin repeats 1 [Source:MGI Symbol;Acc:MGI:2442120]	1.07445	1.34681	0.940393	1.07535	1.02111	1.12827	1.03902	1.51714	1.30626	0.960189	1.20175	1.20831	1.35052	1.24681	0.143673396	0.585944911	8	8	24	0.123774803	0.736807431	24	2.946288786	0.077839565
ENSMUSG00000037926	Ssh2	slingshot protein phosphatase 2 [Source:MGI Symbol;Acc:MGI:2679255]	6.19256	5.92734	5.82815	6.43804	6.6815	6.02712	6.44338	5.93399	6.2995	6.08717	6.28782	6.20553	6.50364	5.83121	0.101443845	0.511659635	20	12	24	0.134120086	0.744355886	24	17.47840339	0.040657994
ENSMUSG00000037933	Bicd2	BICD cargo adaptor 2 [Source:MGI Symbol;Acc:MGI:1924145]	25.5441	18.7936	18.4218	19.3647	23.3716	21.3724	25.0643	20.1033	16.4768	19.8254	21.9224	19.8154	20.1535	18.5263	0.004140921	0.140598674	4	8	24	0.007521586	0.478114494	24	20.32587864	0.175809244
ENSMUSG00000037935	Smarce1	SWI/SNF related, matrix associated, actin dependent regulator of chromatin, subfamily e, member 1 [Source:MGI Symbol;Acc:MGI:1927347]	10.0525	10.8854	9.65702	9.51928	10.1911	9.03905	9.56452	10.0734	8.75779	8.48525	10.5059	8.74802	9.35945	8.67135	0.90332508	0.966018836	20	20	24	0.770815768	0.926171026	24	22.26116331	0.024904261
ENSMUSG00000037936	Scarb1	scavenger receptor class B, member 1 [Source:MGI Symbol;Acc:MGI:893578]	7.62762	8.33108	8.00095	9.36373	9.56356	8.30551	9.3112	9.68904	10.8866	11.5035	8.64764	8.36132	9.08874	9.17069	0.327894132	0.846828588	16	8	24	0.064075448	0.663876076	24	10.28199196	0.118341806
ENSMUSG00000037938	Chchd5	coiled-coil-helix-coiled-coil-helix domain containing 5 [Source:MGI Symbol;Acc:MGI:1913420]	6.74822	6.93677	6.874	6.4825	6.96119	6.91488	7.14792	6.04841	6.89758	7.24492	6.56002	6.2139	6.93315	7.0391	0.801273005	0.966018836	4	20	24	0.946340833	0.986887534	24	7.820293458	0.009168237
ENSMUSG00000037940	Inpp4b	inositol polyphosphate-4-phosphatase, type II [Source:MGI Symbol;Acc:MGI:2158925]	0.7275	1.12586	1.27558	0.844639	0.965319	0.883103	1.449	0.878992	1.16939	0.749635	0.83881	0.775683	0.866178	1.04387	0.369262716	0.893596186	12	4	24	0.261994125	0.768040178	24	5.289744766	0.095532923

ENSMUSG00000037946	Fgd3	FYVE, RhoGEF and PH domain containing 3 [Source:MGI Symbol;Acc:MGI:1353657]	0.823364	1.46087	2.20209	1.02674	0.965172	1.22493	1.56083	1.11322	1.18697	0.811694	0.899542	1.07641	0.93842	1.25843	0.173541845	0.648600527	12	4	24	0.252308606	0.768040178	24	5.367870891	0.137856066
ENSMUSG00000037949	Ano10	anoctamin 10 [Source:MGI Symbol;Acc:MGI:2143103]	22.8867	20.6875	21.9425	19.7294	21.6251	22.6431	20.9289	21.9376	18.5099	22.7946	20.2894	21.1045	21.6756	21.2857	0.919119843	0.966018836	4	8	24	0.364752573	0.789112192	24	21.65424147	0.045897018
ENSMUSG00000037957	Wdr20	WD repeat domain 20 [Source:MGI Symbol;Acc:MGI:1916891]	10.3	9.50148	9.27829	9.53053	9.15129	9.16513	9.27295	8.80559	9.68392	10.0956	9.54239	9.19409	9.41347	10.0168	0.930944473	0.966018836	16	8	24	0.803174811	0.936962113	24	8.132982223	0.015737358
ENSMUSG00000037958	Nsrp1	nuclear speckle regulatory protein 1 [Source:MGI Symbol;Acc:MGI:2144305]	5.36358	6.87297	6.16614	6.39607	7.38193	5.22921	6.65415	5.66773	6.76169	5.73079	7.10524	6.4113	6.87165	6.73463	0.919571723	0.966018836	20	4	24	0.797350586	0.935271704	24	11.51746912	0.034694752
ENSMUSG00000037960	Card19	caspase recruitment domain family, member 19 [Source:MGI Symbol;Acc:MGI:1915730]	55.3159	45.2323	45.1384	47.4493	47.2043	50.2363	46.9784	53.4864	45.2259	55.8001	56.8184	49.7491	54.0329	46.5388	0.082685511	0.462922111	20	16	24	0.384133321	0.799546792	24	18.93554661	0.065369653
ENSMUSG00000037962	Rflna	refilin A [Source:MGI Symbol;Acc:MGI:1920371]	2.97849	2.67208	2.82753	2.03668	2.24245	2.7777	3.29444	3.94357	2.84552	2.82673	3.20974	2.72422	3.37233	3.37195	0.074510812	0.448301997	4	12	24	0.017661494	0.5524966	24	2.38561632	0.154151294
ENSMUSG00000037965	Zc3h7a	zinc finger CCCH type containing 7 A [Source:MGI Symbol;Acc:MGI:2445044]	34.2509	38.1217	35.1275	41.678	43.47	36.0204	41.8572	36.5339	40.691	33.951	45.1299	37.8932	36.4406	35.4623	0.70641939	0.966018836	20	12	24	0.259686759	0.768040178	24	15.49052642	0.081401309
ENSMUSG00000037966	Ninj1	ninjurin 1 [Source:MGI Symbol;Acc:MGI:1196617]	21.842	20.7407	22.3005	21.6288	21.679	20.3394	18.8997	18.9223	20.1788	18.0565	21.0927	18.8174	19.1769	17.5418	0.951846915	0.966018836	20	12	24	0.187135131	0.768040178	24	15.10859918	0.046679999
ENSMUSG00000037971	1110032A03Rik	RIKEN cDNA 1110032A03 gene [Source:MGI Symbol;Acc:MGI:1915971]	44.3233	52.0227	50.02	47.3743	47.8228	42.4568	46.3836	45.2965	56.9213	38.176	47.9519	45.779	47.5685	47.3538	0.148073804	0.590732968	12	12	24	0.35875845	0.785239895	24	6.433768896	0.074935003
ENSMUSG00000037972	Snn	stannin [Source:MGI Symbol;Acc:MGI:1276549]	41.8587	43.738	43.3025	41.4438	42.8688	45.1467	46.6227	41.6276	41.9704	48.7443	39.9546	43.9158	43.6669	43.8484	0.892555977	0.966018836	24	20	24	0.95176795	0.988339231	24	23.27097424	0.009218275
ENSMUSG00000037977	6430571L13Rik	RIKEN cDNA 6430571L13 gene [Source:MGI Symbol;Acc:MGI:2445137]	3.24402	3.06978	3.23678	4.34066	2.71959	4.07203	3.47671	4.14866	3.42707	4.79058	2.94361	3.64431	3.50273	3.28263	0.912923872	0.966018836	16	4	24	0.760211422	0.922505796	24	10.52845843	0.053942443
ENSMUSG00000037979	Ccdc92	coiled-coil domain containing 92 [Source:MGI Symbol;Acc:MGI:106485]	58.7387	50.84	48.9386	51.6293	49.6638	58.0998	55.5776	56.1318	36.8014	76.1461	46.4896	55.5983	51.7279	52.6843	0.217933169	0.704989686	24	12	24	0.682306442	0.894159472	24	19.98666826	0.081628491
ENSMUSG00000037989	Wnk2	WNK lysine deficient protein kinase 2 [Source:MGI Symbol;Acc:MGI:1922857]	4.25784	5.07416	5.1432	4.88407	4.25272	4.60873	4.99445	4.48591	5.34792	7.43425	5.15103	5.02371	4.25543	4.97606	0.132411359	0.567728672	12	16	24	0.07389415	0.682862099	24	10.72491295	0.142258728
ENSMUSG00000037990	Sh3rf3	SH3 domain containing ring finger 3 [Source:MGI Symbol;Acc:MGI:2444637]	0.781082	0.390482	0.686431	0.538187	0.372953	0.553487	0.587212	0.541615	0.476211	0.678778	0.585458	0.48216	0.636774	0.386027	0.960829223	0.967043964	4	4	24	0.932544104	0.982858629	24	20.89435682	0.01762752
ENSMUSG00000037992	Rara	retinoic acid receptor, alpha [Source:MGI Symbol;Acc:MGI:97856]	2.24723	2.2925	2.23555	2.3124	1.89152	1.84766	2.39856	2.35423	2.54221	2.40361	2.0448	2.40503	2.55768	2.07134	0.892555977	0.966018836	4	16	24	0.219154853	0.768040178	24	5.458367555	0.066385478
ENSMUSG00000037993	Dhx38	DEAH (Asp-Glu-Ala-His) box polypeptide 38 [Source:MGI Symbol;Acc:MGI:1927617]	5.9101	5.22179	5.39748	5.57656	5.73901	5.78171	5.61752	6.13746	5.33383	6.06959	5.28096	5.6926	5.74584	5.66285	0.822170541	0.966018836	4	8	24	0.734492254	0.913383989	24	21.65264947	0.01954499
ENSMUSG00000037994	Slc9b2	solute carrier family 9, subfamily B (NHA2, cation proton antiporter 2), member 2 [Source:MGI Symbol;Acc:MGI:2140077]	2.99621	3.2942	2.35089	1.92315	2.10266	2.45546	3.25631	2.91298	3.18603	1.69307	2.50922	2.56564	2.7545	2.84651	8.58E-04	0.071344007	8	8	24	0.002052524	0.395705644	24	1.884387003	0.225299735
ENSMUSG00000037995	Igsf9	immunoglobulin superfamily, member 9 [Source:MGI Symbol;Acc:MGI:2135283]	2.51278	3.77178	3.17773	2.89196	4.54779	3.00216	3.89676	2.91032	4.59688	3.43961	3.46541	3.42147	2.96841	2.35379	0.78898997	0.966018836	20	12	24	0.453199022	0.805393068	24	13.7051596	0.106653299
ENSMUSG00000037996	Slc24a2	solute carrier family 24 (sodium/potassium/calcium exchanger), member 2 [Source:MGI Symbol;Acc:MGI:1923626]	44.5757	45.2244	44.1148	45.81	40.6423	41.9779	42.3278	38.9828	43.0326	46.8761	46.7195	49.6984	49.6526	52.3116	0.768363194	0.966018836	16	4	24	0.974758625	0.993631841	24	3.970581761	0.008830596
ENSMUSG00000037997	Parp11	poly (ADP-ribose) polymerase family, member 11 [Source:MGI Symbol;Acc:MGI:2141505]	11.564	10.2895	12.7273	9.26578	11.11	11.9396	10.65	10.7769	10.6637	10.6733	11.1109	11.8297	10.926	12.9076	0.871479522	0.966018836	24	16	24	0.635686759	0.87621219	24	0.242033944	0.042160266
ENSMUSG00000037999	Arap2	ArfGAP with RhoGAP domain, ankyrin repeat and PH domain 2 [Source:MGI Symbol;Acc:MGI:2684416]	9.44431	10.0972	8.25913	9.10467	8.84288	8.2359	9.79107	8.01528	9.03577	8.55012	9.68755	8.32657	8.47582	10.0506	0.923635729	0.966018836	8	16	24	0.785813598	0.931158434	24	3.283518255	0.028517042
ENSMUSG00000038000	Acd	adrenocortical dysplasia [Source:MGI Symbol;Acc:MGI:87873]	7.21664	6.96112	7.68925	8.05076	7.81609	7.84295	7.37813	7.15795	7.40978	7.8161	7.06662	7.46841	6.2628	7.24853	0.015486342	0.253395174	16	16	24	0.03615644	0.609176161	24	13.48664662	0.068440092
ENSMUSG00000038002	Cramp1	cramped chromatin regulator 1 [Source:MGI Symbol;Acc:MGI:1930190]	5.79548	5.37462	5.68747	6.07592	5.87669	5.679	5.79243	5.78338	5.88917	6.37125	5.61486	5.45275	5.56806	5.78621	0.232647885	0.733359426	16	8	24	0.088822588	0.707896147	24	11.49318865	0.043034452
ENSMUSG00000038005	Hpf1	histone PARylation factor 1 [Source:MGI Symbol;Acc:MGI:1919862]	28.5154	34.0481	32.7633	29.7648	30.6998	30.6632	28.7656	30.288	33.4818	30.3556	34.1358	31.6424	33.8544	33.3964	0.920858634	0.966018836	12	16	24	0.459415874	0.805393068	24	7.760995653	0.041209166
ENSMUSG00000038007	Acer2	alkaline ceramidase 2 [Source:MGI Symbol;Acc:MGI:1920932]	1.70366	1.97678	2.45509	2.98169	1.61439	1.73306	1.49409	1.65517	2.34462	2.07091	1.85756	1.59424	1.2928	1.55806	9.60E-04	0.07358601	16	12	24	5.50E-04	0.293325979	24	10.69577544	0.228660258
ENSMUSG00000038009	Dnajc22	DnaJ heat shock protein family (Hsp40) member C22 [Source:MGI Symbol;Acc:MGI:1920028]	0.580858	0.704658	0.631223	0.390908	0.886574	0.596665	0.380244	0.76198	0.531431	0.448564	0.487836	0.521712	0.613718	0.916388	0.050258003	0.385784509	8	8	24	0.545443439	0.83296456	24	3.561917703	0.063808531
ENSMUSG00000038010	Ccdc138	coiled-coil domain containing 138 [Source:MGI Symbol;Acc:MGI:1923388]	3.64733	3.48557	3.33956	3.03014	3.70232	3.70467	2.29215	3.66257	3.19502	3.2389	3.9006	4.18201	4.0373	3.52678	0.02672151	0.305155548	24	16	24	0.58343985	0.851822355	24	19.26122899	0.067385224
ENSMUSG00000038013	Wipf2	WAS/WASL interacting protein family, member 2 [Source:MGI Symbol;Acc:MGI:1924462]	4.38221	3.95849	4.37457	4.34156	3.95659	4.41004	4.75887	4.27965	4.25564	5.5672	4.24878	4.54791	4.5691	4.53439	0.446653834	0.966018836	4	16	24	0.919763983	0.978615091	24	11.74124343	0.015082457
ENSMUSG00000038014	Fam120a	family with sequence similarity 120, member A [Source:MGI Symbol;Acc:MGI:2446163]	31.4899	26.716	27.6511	29.9017	27.6984	28.8067	29.5209	28.9032	24.6725	34.3375	27.8539	27.8364	26.3641	28.4088	0.941885246	0.966018836	16	20	24	0.725345158	0.909947085	24	16.18812339	0.035468478
ENSMUSG00000038020	Rapgefl1	Rap guanine nucleotide exchange factor (GEF)-like 1 [Source:MGI Symbol;Acc:MGI:3611446]	0.808031	2.13993	2.76741	1.1959	1.10438	1.72618	1.64345	1.44539	2.27393	1.62292	0.913262	1.44287	0.855488	1.61048	0.082282215	0.462922111	12	8	24	0.095075243	0.71175955	24	7.445602922	0.258195129
ENSMUSG00000038022	Mindy4	MINDY lysine 48 deubiquitinase 4 [Source:MGI Symbol;Acc:MGI:3583959]	2.56793	2.84496	2.83912	1.70985	2.7829	2.94308	2.49995	2.97908	2.78275	2.7746	3.19072	2.58995	3.23717	3.28962	0.743750765	0.966018836	8	8	24	0.448540685	0.805393068	24	1.826743645	0.07066798
ENSMUSG00000038023	Atp6v0a2	ATPase, H+ transporting, lysosomal V0 subunit A2 [Source:MGI Symbol;Acc:MGI:104855]	16.1335	13.2973	14.4761	13.6126	14.4961	15.4796	16.2353	15.374	12.2937	14.5235	14.2612	14.5592	14.082	13.8539	0.090240671	0.47972073	4	8	24	0.045096038	0.628927515	24	21.40260912	0.091348944
ENSMUSG00000038024	Dennd4c	DENN/MADD domain containing 4C [Source:MGI Symbol;Acc:MGI:1914769]	2.59246	2.86511	2.63464	2.85916	2.76293	2.50053	3.16141	2.5692	3.26817	2.30646	2.78814	2.82589	3.10241	2.70873	0.903465533	0.966018836	4	12	24	0.703096488	0.90183264	24	2.396318181	0.033106498
ENSMUSG00000038025	Phf2	PHD finger protein 2 [Source:MGI Symbol;Acc:MGI:1338034]	5.63774	5.0438	5.1361	5.40015	5.39978	4.9702	5.72542	5.22667	5.72806	6.95566	5.2177	5.67091	5.04782	5.22322	0.952127548	0.966018836	16	16	24	0.427110655	0.805393068	24	12.34905968	0.053656546
ENSMUSG00000038026	Kcnj9	potassium inwardly-rectifying channel, subfamily J, member 9 [Source:MGI Symbol;Acc:MGI:108007]	0.52477	1.05395	1.61202	0.769424	0.581298	0.78865	1.07629	0.579281	1.05913	1.06676	0.466423	0.920679	0.421803	1.1856	0.275526571	0.789849983	12	8	24	0.143969378	0.752803875	24	7.636944562	0.196091722
ENSMUSG00000038028	Tigar	Trp53 induced glycolysis regulatory phosphatase [Source:MGI Symbol;Acc:MGI:2442752]	14.2467	11.4305	12.483	13.5168	11.9272	14.5593	12.8029	14.1708	11.584	15.2879	12.9213	13.8991	13.3286	14.1474	0.8972237	0.966018836	24	12	24	0.730961526	0.912358609	24	19.91436336	0.037760634
ENSMUSG00000038034	Igsf8	immunoglobulin superfamily, member 8 [Source:MGI Symbol;Acc:MGI:2154090]	25.4739	20.4649	24.2928	22.5976	19.346	22.7204	24.4533	23.3597	22.8479	29.2161	20.8333	24.2625	22.423	23.9383	0.942877366	0.966018836	4	16	24	0.77515869	0.927711986	24	6.280150281	0.041108477
ENSMUSG00000038039	Gcc2	GRIP and coiled-coil domain containing 2 [Source:MGI Symbol;Acc:MGI:1917547]	19.3262	20.0352	20.4146	20.6231	21.706	19.1466	20.0784	21.473	20.8088	18.4702	20.0428	19.4456	19.0158	20.8403	0.417653118	0.963884303	8	20	24	0.466902154	0.805393068	24	8.19559328	0.032627536
ENSMUSG00000038042	Ptpdc1	protein tyrosine phosphatase domain containing 1 [Source:MGI Symbol;Acc:MGI:2145430]	29.6338	31.3867	30.8788	39.3573	38.3673	31.0625	31.2494	29.9171	37.4996	35.1645	36.0462	32.067	31.6284	27.0357	0.007178736	0.191220422	16	16	24	0.001776284	0.382759815	24	13.68797278	0.169762989
ENSMUSG00000038046	Mrm3	mitochondrial rRNA methyltransferase 3 [Source:MGI Symbol;Acc:MGI:1914640]	6.68089	6.50951	7.21803	8.29786	7.07364	6.82915	6.98614	5.5576	5.72036	7.05119	7.45477	6.61813	5.75268	6.35728	0.008157655	0.192760771	16	16	24	0.017991249	0.557381523	24	14.98766423	0.124122744
ENSMUSG00000038047	Haus6	HAUS augmin-like complex, subunit 6 [Source:MGI Symbol;Acc:MGI:1923389]	4.13775	4.36265	4.6549	4.42914	3.88564	3.95272	3.76741	4.0444	4.31808	4.07634	4.3437	4.36972	4.09225	4.18195	0.503104368	0.966018836	12	16	24	0.184656466	0.768040178	24	9.396507787	0.047879428
ENSMUSG00000038048	Cntnap5c	contactin associated protein-like 5C [Source:MGI Symbol;Acc:MGI:3646013]	0.881916	1.04255	0.845726	0.982127	1.14768	0.810496	0.731859	0.746082	0.855821	0.40905	1.13227	1.12522	1.05875	1.11683	0.435739761	0.966018836	20	20	24	0.639416557	0.877927579	24	20.8389902	0.065494145
ENSMUSG00000038055	Dexi	dexamethasone-induced transcript [Source:MGI Symbol;Acc:MGI:1926236]	22.2098	19.5567	19.1238	22.68	21.1535	21.6986	22.4307	24.2214	21.287	28.0237	21.0013	22.9819	21.7662	23.4406	0.921741413	0.966018836	8	12	24	0.866066369	0.961251055	24	12.58663804	0.025070237
ENSMUSG00000038056	Kmt2c	lysine (K)-specific methyltransferase 2C [Source:MGI Symbol;Acc:MGI:2444959]	12.5865	13.683	12.9036	13.4325	13.1272	12.3845	14.1292	12.1609	13.5921	13.4215	13.2097	13.1826	13.2491	13.1975	0.864782464	0.966018836	16	8	24	0.801741844	0.936527813	24	9.883348883	0.014707355
ENSMUSG00000038057	Dbil5	diazepam binding inhibitor-like 5 [Source:MGI Symbol;Acc:MGI:108039]	1.72084	1.7931	1.3498	1.84875	2.19938	1.18337	1.27934	2.29832	1.95158	1.01144	1.95926	1.22719	2.30349	1.30243	0.948161972	0.966018836	20	4	24	0.946273004	0.986887534	24	4.893334529	0.032999892
ENSMUSG00000038058	Nod1	nucleotide-binding oligomerization domain containing 1 [Source:MGI Symbol;Acc:MGI:1341839]	6.87551	6.94553	5.97736	6.2316	8.24196	6.09403	8.79217	8.14263	6.84964	7.49735	7.27723	6.21423	7.34726	6.88063	0.838532766	0.966018836	4	8	24	0.826788721	0.946041107	24	0.066005915	0.037009897
ENSMUSG00000038059	Smim3	small integral membrane protein 3 [Source:MGI Symbol;Acc:MGI:1917088]	4.22256	3.6165	4.65111	4.02337	4.35102	4.2868	3.28874	4.03406	3.5391	3.4045	4.84504	3.36704	3.47608	2.86593	0.522551072	0.966018836	20	12	24	0.129326417	0.740162193	24	15.63373938	0.109470842
ENSMUSG00000038060	Dlec1	deleted in lung and esophageal cancer 1 [Source:MGI Symbol;Acc:MGI:2443671]	1.23069	0.677501	0.886554	0.86487	0.769798	1.09418	0.658434	1.01825	1.01293	0.76032	0.933047	1.10862	0.650327	0.546297	0.941955432	0.966018836	24	8	24	0.488785628	0.805393068	24	18.16383492	0.073186216
ENSMUSG00000038065	Mturn	maturin, neural progenitor differentiation regulator homolog (Xenopus) [Source:MGI Symbol;Acc:MGI:1915485]	56.7375	51.7887	49.4934	53.0285	52.7155	52.7775	53.0003	50.5995	47.2753	55.432	53.4614	54.1585	54.7429	52.5589	0.04066816	0.345232782	4	8	24	0.070281048	0.675025526	24	19.85947378	0.057505216
ENSMUSG00000038068	Rnf144b	ring finger protein 144B [Source:MGI Symbol;Acc:MGI:2384986]	2.4954	2.54682	2.8046	3.08164	2.4161	2.67953	2.82796	2.87811	2.51099	3.14167	2.48481	2.51973	2.7267	2.38242	0.369262716	0.893596186	16	4	24	0.487509231	0.805393068	24	10.45398793	0.04471484
ENSMUSG00000038069	Cdkn2aip	CDKN2A interacting protein [Source:MGI Symbol;Acc:MGI:1918175]	4.56088	4.25162	4.23581	4.15733	3.68747	3.39	4.26407	4.1179	3.87026	3.66989	4.08761	4.10283	3.70683	4.12178	0.227809102	0.725203425	4	20	24	0.272982204	0.768040178	24	3.761382615	0.052766346
ENSMUSG00000038070	Cntln	centlein, centrosomal protein [Source:MGI Symbol;Acc:MGI:2443104]	9.15754	11.4012	10.2082	11.1374	10.4353	8.80509	8.74544	9.01204	11.7745	9.75233	10.3902	10.4048	10.5347	10.0005	0.530814489	0.966018836	12	16	24	0.161603009	0.76636332	24	10.22464541	0.091243921
ENSMUSG00000038071	Npy6r	neuropeptide Y receptor Y6 [Source:MGI Symbol;Acc:MGI:1098590]	0.345821	0.561674	0.555617	0.778911	0.694451	0.404458	0.664343	0.444072	0.902004	0.652518	0.55737	0.45989	0.306099	0.389932	0.057224139	0.401309455	16	12	24	0.017822226	0.555066465	24	11.44954384	0.152756433
ENSMUSG00000038072	Galnt11	polypeptide N-acetylgalactosaminyltransferase 11 [Source:MGI Symbol;Acc:MGI:2444392]	13.6098	14.2578	13.5514	12.8862	14.0921	14.4371	14.7924	14.4821	12.7717	13.9471	13.3396	13.8912	13.5545	13.9001	0.253121224	0.753386477	8	4	24	0.130697755	0.740728512	24	23.04060315	0.043638089
ENSMUSG00000038074	Fkbp14	FK506 binding protein 14 [Source:MGI Symbol;Acc:MGI:2387639]	18.46	18.3151	16.9247	15.9826	18.7396	15.2179	16.4194	15.9491	18.3239	14.8521	18.4186	16.7315	16.6889	16.6935	0.905275015	0.966018836	20	20	24	0.968184974	0.992322419	24	23.18236546	0.010438133
ENSMUSG00000038077	Kcna6	potassium voltage-gated channel, shaker-related, subfamily, member 6 [Source:MGI Symbol;Acc:MGI:96663]	40.864	35.5759	35.0399	35.2571	39.153	43.3982	35.9205	36.5305	25.7891	40.2129	31.5201	37.2763	36.1309	37.1324	0.085890569	0.466404237	24	12	24	0.158114065	0.764630328	24	20.55058779	0.129158028
ENSMUSG00000038079	Tmem237	transmembrane protein 237 [Source:MGI Symbol;Acc:MGI:2138365]	21.9841	20.9483	17.6074	20.8762	18.5764	18.7045	19.0759	19.3243	19.3217	18.7036	19.5941	18.0712	21.0761	18.5994	0.478724519	0.966018836	4	20	24	0.527391964	0.823340379	24	0.584254486	0.037858798
ENSMUSG00000038080	Kdm1b	lysine (K)-specific demethylase 1B [Source:MGI Symbol;Acc:MGI:2145261]	5.58151	5.46651	4.61098	5.58346	5.10179	4.76263	6.0029	5.08427	6.01203	4.79018	5.73794	5.25496	4.87016	5.10923	0.782779623	0.966018836	4	20	24	0.971327612	0.993017774	24	0.465131327	0.010170483
ENSMUSG00000038084	Opa1	OPA1, mitochondrial dynamin like GTPase [Source:MGI Symbol;Acc:MGI:1921393]	40.5962	37.8178	38.9006	38.9776	38.349	39.336	36.9351	37.353	36.2104	39.7242	38.3007	39.9963	39.4217	40.0015	0.939921059	0.966018836	24	12	24	0.507153626	0.812385664	24	19.45980589	0.022439635
ENSMUSG00000038085	Cnbd2	cyclic nucleotide binding domain containing 2 [Source:MGI Symbol;Acc:MGI:1918123]	1.85876	2.17111	1.84407	1.6083	1.81898	2.06489	2.06798	1.90962	2.13696	1.80301	2.87777	1.97541	2.021	1.92087	0.478724519	0.966018836	4	12	24	0.751018026	0.91921305	24	19.9500962	0.039793903
ENSMUSG00000038086	Hspb2	heat shock protein 2 [Source:MGI Symbol;Acc:MGI:1916503]	20.2392	22.555	20.4813	27.1732	26.2348	20.7916	25.7077	25.7496	31.1765	24.279	27.2641	25.7124	28.1105	20.8847	0.891105762	0.966018836	20	8	24	0.489176578	0.805393068	24	12.84017397	0.081106858
ENSMUSG00000038094	Atp13a4	ATPase type 13A4 [Source:MGI Symbol;Acc:MGI:1924456]	0.796384	1.46005	2.3157	1.31286	0.803537	0.800734	2.94143	0.777525	1.26409	1.52219	1.00023	0.748412	0.28603	1.70078	0.268242676	0.774280302	12	12	24	0.299822781	0.768040178	24	7.806119976	0.256495665
ENSMUSG00000038095	Sbno1	strawberry notch 1 [Source:MGI Symbol;Acc:MGI:2384298]	17.6556	16.4915	17.221	17.4772	17.1029	16.1697	18.4152	15.9209	18.8294	15.3191	17.2883	17.778	18.3502	18.6742	0.914360142	0.966018836	4	12	24	0.566420635	0.843471947	24	1.900331581	0.034104272
ENSMUSG00000038102	Trappc11	trafficking protein particle complex 11 [Source:MGI Symbol;Acc:MGI:2444585]	18.6056	16.6637	17.3077	15.9542	15.7743	18.0753	17.4184	17.7711	14.9293	19.3682	15.9747	16.9001	16.052	17.1234	0.760769494	0.966018836	4	16	24	0.697293294	0.899338675	24	23.58577133	0.032842186
ENSMUSG00000038112	AW551984	expressed sequence AW551984 [Source:MGI Symbol;Acc:MGI:2143322]	2.1823	2.59624	3.36569	2.47032	2.14688	2.8507	3.18348	2.4923	2.66275	2.79345	2.35547	3.26279	1.77053	3.37246	0.843639218	0.966018836	12	8	24	0.704228973	0.902338136	24	7.123799238	0.06841413
ENSMUSG00000038116	Phf20	PHD finger protein 20 [Source:MGI Symbol;Acc:MGI:2444148]	19.2908	18.2522	18.6654	17.7539	17.5686	19.4857	17.9301	16.8955	16.9466	18.6402	17.6777	19.6772	19.4668	19.0823	0.085045716	0.466404237	24	20	24	0.175763859	0.768040178	24	21.6996841	0.050833536
ENSMUSG00000038119	Cdon	cell adhesion molecule-related/down-regulated by oncogenes [Source:MGI Symbol;Acc:MGI:1926387]	3.0109	3.29239	2.70972	2.5085	3.11872	2.644	3.29934	3.10912	3.56275	2.514	2.40915	2.94526	2.9154	2.95794	0.192909212	0.670595308	8	8	24	0.078396587	0.68893025	24	2.869892748	0.101167509
ENSMUSG00000038121	Fam210a	family with sequence similarity 210, member A [Source:MGI Symbol;Acc:MGI:1914000]	5.72019	5.84815	5.92864	7.68608	5.63882	6.01727	6.24525	6.18261	6.70003	5.88723	6.53155	6.15317	6.82577	6.65076	0.847312766	0.966018836	16	4	24	0.564237115	0.843073421	24	9.513826439	0.043169837
ENSMUSG00000038122	Tbc1d32	TBC1 domain family, member 32 [Source:MGI Symbol;Acc:MGI:2442827]	4.24177	4.64299	4.21262	4.11353	4.38832	4.16393	4.11423	3.81652	4.92248	3.78648	5.0655	4.39879	4.68629	4.7809	0.858891143	0.966018836	20	20	24	0.986133073	0.997339197	24	2.546907423	0.006648696
ENSMUSG00000038126	Mphosph9	M-phase phosphoprotein 9 [Source:MGI Symbol;Acc:MGI:2443138]	3.45822	4.18934	3.6603	4.85141	3.84245	3.61523	3.08789	3.80216	4.41688	4.06923	4.06326	4.44365	4.30003	4.5074	0.702630284	0.966018836	16	12	24	0.183899535	0.768040178	24	10.45170881	0.090568697
ENSMUSG00000038127	Ccdc50	coiled-coil domain containing 50 [Source:MGI Symbol;Acc:MGI:1914751]	14.0593	16.1024	15.7557	16.6698	15.5125	13.9606	15.5898	14.6393	18.2118	14.2104	15.9435	15.1191	14.6818	15.8173	0.12833063	0.560209134	12	12	24	0.115088918	0.727799063	24	9.17237973	0.078992925
ENSMUSG00000038128	Camk4	calcium/calmodulin-dependent protein kinase IV [Source:MGI Symbol;Acc:MGI:88258]	4.11029	3.18443	3.04974	3.70672	3.7443	3.40441	3.62481	2.81458	2.89732	2.3541	3.8742	3.87198	3.88686	3.67367	0.083562154	0.463738799	4	8	24	0.042596822	0.623773613	24	20.78105731	0.161989066
ENSMUSG00000038132	Rbm24	RNA binding motif protein 24 [Source:MGI Symbol;Acc:MGI:3610364]	0.701688	1.49749	1.56807	2.43787	1.29554	1.59881	1.41941	3.33344	3.05821	3.64385	1.27588	1.60312	1.87216	1.43954	0.256915694	0.76168256	16	4	24	0.046696231	0.633507364	24	9.392002958	0.356291847
ENSMUSG00000038135	Crygn	crystallin, gamma N [Source:MGI Symbol;Acc:MGI:2449167]	0.0658183	1.56339	0.680208	0.075268	0.147332	0.983688	0.16044	0.54334	1.78341	0.272155	0.721317	0.552613	0.435719	2.58367	0.413946331	0.958746003	8	20	24	0.145452252	0.754184974	24	5.745318537	0.38457064
ENSMUSG00000038141	Tmem181a	transmembrane protein 181A [Source:MGI Symbol;Acc:MGI:1924356]	8.69525	7.46517	7.68386	7.51774	7.48779	8.39231	8.17541	8.60478	7.00463	9.46759	7.28188	8.18844	8.26006	8.94364	0.560714538	0.966018836	4	16	24	0.529578722	0.824494657	24	0.549270291	0.047738199
ENSMUSG00000038143	Stox2	storkhead box 2 [Source:MGI Symbol;Acc:MGI:1918319]	5.49661	4.96448	5.63091	4.92433	4.87842	5.27559	5.96779	5.1149	4.79579	5.82008	4.57725	5.14513	5.09521	5.3283	0.5169953	0.966018836	4	16	24	0.566573726	0.843471947	24	2.036981409	0.038155359
ENSMUSG00000038145	Snrk	SNF related kinase [Source:MGI Symbol;Acc:MGI:108104]	19.4664	15.852	17.3087	21.2563	19.169	18.131	20.8314	20.1101	15.6142	24.2596	17.8357	19.6866	19.0764	19.7711	0.941955432	0.966018836	16	20	24	0.693235767	0.898256039	24	16.21555132	0.05317365
ENSMUSG00000038146	Notch3	notch 3 [Source:MGI Symbol;Acc:MGI:99460]	1.67374	1.8439	1.82795	1.95594	1.70772	1.46774	2.20606	1.75118	1.94646	2.73944	1.85199	1.75445	1.68278	1.68421	0.059650003	0.406067671	16	8	24	0.198619284	0.768040178	24	10.58109925	0.09916867
ENSMUSG00000038147	Cd84	CD84 antigen [Source:MGI Symbol;Acc:MGI:1336885]	0.854202	1.6491	2.18519	1.97485	1.42067	1.39219	1.4765	1.80136	1.91437	1.21132	1.51444	1.25659	1.59868	1.33146	0.0444686	0.365196805	12	12	24	0.082586655	0.699403344	24	8.802742509	0.170572928
ENSMUSG00000038150	Ormdl3	ORM1-like 3 (S. cerevisiae) [Source:MGI Symbol;Acc:MGI:1913862]	21.0262	17.1153	19.108	22.3228	18.6855	19.0833	19.4243	21.041	19.3288	25.1067	20.9845	20.8419	22.3432	21.8709	0.914502381	0.966018836	16	4	24	0.593429131	0.856919867	24	12.23450061	0.045951254
ENSMUSG00000038152	5033430I15Rik	RIKEN cDNA 5033430I15 gene [Source:MGI Symbol;Acc:MGI:1923250]	2.93106	2.69844	2.43946	2.88057	2.53593	3.22471	2.29368	3.11752	3.44659	3.15206	3.03056	2.99218	2.31386	3.1559	0.725061903	0.966018836	16	12	24	0.575061759	0.847865121	24	10.90933457	0.056945254
ENSMUSG00000038156	Spon1	spondin 1, (f-spondin) extracellular matrix protein [Source:MGI Symbol;Acc:MGI:2385287]	2.35208	2.72871	4.02215	2.22743	2.05245	2.82903	3.09359	3.20164	2.62967	2.9618	1.84085	2.63016	2.51398	3.02335	0.057224139	0.401309455	8	12	24	0.069172505	0.673520298	24	5.154198519	0.177562798
ENSMUSG00000038160	Atg5	autophagy related 5 [Source:MGI Symbol;Acc:MGI:1277186]	26.5234	26.8032	25.0018	25.6599	24.5522	25.3052	24.5431	24.2381	25.46	22.8049	29.1181	25.1488	27.2631	27.13	0.860993221	0.966018836	4	12	24	0.699052779	0.899922504	24	23.1745039	0.028696172
ENSMUSG00000038168	P3h2	prolyl 3-hydroxylase 2 [Source:MGI Symbol;Acc:MGI:2146663]	3.44432	3.14774	2.3992	3.15465	3.72036	3.06738	4.13434	3.19584	3.31505	2.55079	3.25228	3.45768	3.03556	2.4929	0.291694073	0.798132248	20	20	24	0.096430004	0.71175955	24	20.46856084	0.134440479
ENSMUSG00000038170	Pde4dip	phosphodiesterase 4D interacting protein (myomegalin) [Source:MGI Symbol;Acc:MGI:1891434]	48.6697	52.541	51.3644	60.7666	51.7029	61.1537	54.8065	80.3179	64.4571	88.545	49.4687	53.1647	56.8948	62.1766	0.708020493	0.966018836	16	4	24	0.225135978	0.768040178	24	8.705662176	0.164081888
ENSMUSG00000038172	Ttc39b	tetratricopeptide repeat domain 39B [Source:MGI Symbol;Acc:MGI:1917113]	11.0758	11.8735	12.8113	12.2513	11.3941	10.9029	11.8648	11.1737	11.3411	10.5338	12.1097	12.3319	12.034	13.1488	0.814221431	0.966018836	8	20	24	0.67812167	0.892430843	24	5.466098302	0.029402971
ENSMUSG00000038173	Enpp6	ectonucleotide pyrophosphatase/phosphodiesterase 6 [Source:MGI Symbol;Acc:MGI:2445171]	0.503763	1.53555	1.76642	1.08335	0.86343	1.20927	2.11383	1.08148	1.98987	0.630215	0.725969	1.17922	0.671192	1.30022	0.308023687	0.814993005	12	4	24	0.304956059	0.768040178	24	5.7739392	0.200827424
ENSMUSG00000038174	Fam126b	family with sequence similarity 126, member B [Source:MGI Symbol;Acc:MGI:1098784]	15.1851	15.5954	13.957	15.5267	14.1217	13.6361	14.3159	12.3583	15.2869	11.9705	17.8933	14.1464	13.664	14.7083	0.807012301	0.966018836	20	4	24	0.859519045	0.958585753	24	16.62199656	0.030232209
ENSMUSG00000038175	Mylip	myosin regulatory light chain interacting protein [Source:MGI Symbol;Acc:MGI:2388271]	6.27308	6.98495	6.64991	6.21744	6.76641	6.08671	6.96981	6.35186	7.64873	7.09648	6.84145	6.3679	6.74255	5.11201	0.949874888	0.966018836	12	12	24	0.630385905	0.873885049	24	12.32227418	0.044085278
ENSMUSG00000038178	Slc43a2	solute carrier family 43, member 2 [Source:MGI Symbol;Acc:MGI:2442746]	11.1807	9.80247	10.6611	9.24653	11.2794	11.0725	12.2293	10.9178	10.4991	13.5822	10.3491	11.5301	11.0985	11.1648	0.40165019	0.933249185	4	8	24	0.767626204	0.924592565	24	20.32870024	0.034867891
ENSMUSG00000038180	Spag4	sperm associated antigen 4 [Source:MGI Symbol;Acc:MGI:2444120]	2.17315	0.548799	1.22129	1.65182	1.14412	1.1209	0.68799	1.44768	1.10225	0.889683	1.03471	1.09672	1.14182	0.928256	0.790218349	0.966018836	12	20	24	0.896547652	0.970591958	24	17.61960104	0.047696409
ENSMUSG00000038181	Chpf2	chondroitin polymerizing factor 2 [Source:MGI Symbol;Acc:MGI:1917522]	16.0493	15.2762	13.4167	15.1165	15.1162	15.668	15.0826	18.0423	14.8748	19.8899	14.6067	16.5736	15.2462	15.9626	0.725061903	0.966018836	8	4	24	0.997249568	0.999495538	24	9.791800302	0.003879133
ENSMUSG00000038187	Btbd10	BTB (POZ) domain containing 10 [Source:MGI Symbol;Acc:MGI:1916065]	20.1089	20.0997	18.8486	19.7289	20.2285	20.3203	19.4636	19.4163	18.1461	22.6183	18.5919	20.5664	20.636	20.3253	0.148073804	0.590732968	24	12	24	0.726067636	0.910047737	24	19.01157217	0.0243167
ENSMUSG00000038188	Scarf1	scavenger receptor class F, member 1 [Source:MGI Symbol;Acc:MGI:2449455]	0.93683	1.0905	1.1652	1.48892	1.20635	1.43177	1.14672	1.40763	1.55834	1.42147	1.40562	1.19613	1.15725	1.30194	0.00932019	0.194720062	16	12	24	0.027745468	0.590220615	24	11.55567698	0.09920937
ENSMUSG00000038195	Rilp	Rab interacting lysosomal protein [Source:MGI Symbol;Acc:MGI:2144271]	1.78034	1.89098	2.44057	3.40189	2.1661	2.02836	2.74185	3.03426	2.53464	3.10784	2.45597	2.13063	2.92083	1.93513	0.328944792	0.847277612	16	8	24	0.248768675	0.768040178	24	10.74911872	0.133721108
ENSMUSG00000038201	Kcna7	potassium voltage-gated channel, shaker-related subfamily, member 7 [Source:MGI Symbol;Acc:MGI:96664]	0.353073	0.708852	0.811189	1.19866	0.60526	0.906962	0.623345	2.50275	1.31583	2.22817	0.812583	0.685042	1.34166	1.08955	0.404482447	0.938325912	16	4	24	0.093112434	0.71175955	24	8.591173263	0.292290227
ENSMUSG00000038204	Asb10	ankyrin repeat and SOCS box-containing 10 [Source:MGI Symbol;Acc:MGI:2152836]	0.384258	0.897949	0.812454	1.28725	0.867794	1.08657	0.893731	2.15013	1.61591	2.14237	0.891104	0.801556	1.15464	0.828578	0.793403332	0.966018836	16	8	24	0.125738768	0.739647205	24	9.583592567	0.244239904
ENSMUSG00000038205	Prkab2	protein kinase, AMP-activated, beta 2 non-catalytic subunit [Source:MGI Symbol;Acc:MGI:1336185]	29.0332	29.1907	27.9404	30.7388	30.8074	30.4643	30.156	31.7914	30.7743	35.9977	29.1332	29.766	29.6568	28.4707	0.664568268	0.966018836	16	16	24	0.322698898	0.768040178	24	12.76667964	0.049015061
ENSMUSG00000038206	Fbxo8	F-box protein 8 [Source:MGI Symbol;Acc:MGI:1354696]	13.5432	18.5906	18.1787	16.1321	15.0384	17.413	14.2464	17.1433	19.0149	17.7764	17.5592	18.2173	18.0554	16.6192	0.43565473	0.966018836	12	16	24	0.259942566	0.768040178	24	9.15126271	0.084856417
ENSMUSG00000038208	Pgap3	post-GPI attachment to proteins 3 [Source:MGI Symbol;Acc:MGI:2444461]	3.94182	2.63266	2.61583	3.01262	2.6001	3.0344	3.1349	3.0751	2.91299	3.48691	3.13173	2.81542	2.71112	3.2087	0.920872048	0.966018836	4	8	24	0.890289196	0.969016997	24	22.61083627	0.025510059
ENSMUSG00000038212	Mfsd14b	major facilitator superfamily domain containing 14B [Source:MGI Symbol;Acc:MGI:1913881]	20.7788	22.3496	21.7592	21.79	21.3827	20.395	21.263	20.882	21.9348	18.9459	20.6353	20.2495	19.7662	20.2118	0.390686537	0.915479136	12	12	24	0.457641545	0.805393068	24	7.017352134	0.024568133
ENSMUSG00000038213	Tapbpl	TAP binding protein-like [Source:MGI Symbol;Acc:MGI:2384853]	7.83235	6.43608	6.16201	6.67576	6.99934	7.71549	6.63328	7.00644	6.47646	7.38263	7.24518	7.51018	6.70168	7.17753	0.020957534	0.262822894	24	12	24	0.062753707	0.661987884	24	19.44541334	0.079668808
ENSMUSG00000038214	Bend3	BEN domain containing 3 [Source:MGI Symbol;Acc:MGI:2677212]	1.21716	1.31334	1.26541	1.04702	1.31084	1.30868	1.1246	1.51236	1.08548	1.40647	1.15301	1.45546	1.29809	1.31299	0.96984707	0.97107529	8	4	24	0.710799133	0.904485814	24	0.119651464	0.027858402
ENSMUSG00000038215	Cep44	centrosomal protein 44 [Source:MGI Symbol;Acc:MGI:3525111]	7.09179	9.02026	8.93156	8.27408	7.31577	7.22047	7.60934	7.85819	9.65406	7.47042	8.57499	8.0276	8.25952	8.8398	0.038115943	0.334069441	12	12	24	0.026161661	0.586541342	24	7.556195801	0.116853476
ENSMUSG00000038217	Tlcd2	TLC domain containing 2 [Source:MGI Symbol;Acc:MGI:1917141]	1.92849	2.3282	2.11164	2.42935	2.49296	2.00051	2.03709	2.21526	2.23457	2.13052	1.92202	1.64608	2.57378	2.41572	0.688644847	0.966018836	8	16	24	0.307858119	0.768040178	24	7.503410517	0.074221527
ENSMUSG00000038225	Primpol	primase and polymerase (DNA-directed) [Source:MGI Symbol;Acc:MGI:3603756]	2.60036	3.04654	2.97843	2.78247	2.38932	3.02502	2.91696	2.72337	2.79534	3.04645	2.70096	2.81921	2.48283	2.94509	0.590017078	0.966018836	8	12	24	0.490426368	0.805393068	24	7.119013677	0.039458643
ENSMUSG00000038227	Hoxa9	homeobox A9 [Source:MGI Symbol;Acc:MGI:96180]	14.0079	11.4835	12.2143	9.72984	10.7293	13.3123	12.3413	11.3307	11.2441	12.7967	10.047	13.8231	9.92497	12.4554	0.503972924	0.966018836	24	20	24	0.403674634	0.799546792	24	23.28996587	0.08462952
ENSMUSG00000038233	Gask1a	golgi associated kinase 1A [Source:MGI Symbol;Acc:MGI:3041196]	1.24726	1.52339	1.51132	1.15733	1.42266	1.20722	1.19893	1.25731	1.32136	0.93516	1.12407	1.17508	1.37924	1.29602	0.05238827	0.391426791	12	4	24	0.225696942	0.768040178	24	3.536408994	0.062368566

ENSMUSG00000038235	F11r	F11 receptor [Source:MGI Symbol;Acc:MGI:1321398]	4.91603	5.83499	6.58257	4.93493	5.82271	6.17941	6.37108	6.70724	5.15951	5.65716	4.76126	5.78016	7.32215	6.65639	0.076942821	0.453790923	8	8	24	0.109004704	0.72082227	24	2.422888373	0.119565188
ENSMUSG00000038236	Hoxa7	homeobox A7 [Source:MGI Symbol;Acc:MGI:96179]	11.7518	11.1332	12.6975	10.5998	9.18004	11.4427	11.0049	11.5323	12.2004	13.2616	9.77705	12.7123	11.705	12.2883	0.616047374	0.966018836	12	8	24	0.21384712	0.768040178	24	5.377594929	0.089533542
ENSMUSG00000038239	Hrc	histidine rich calcium binding protein [Source:MGI Symbol;Acc:MGI:96226]	3.31451	7.02445	10.2052	12.9388	8.18258	10.3783	6.23661	21.5878	13.2433	17.8676	5.88262	7.48315	10.3128	8.72083	0.203305541	0.689605535	16	12	24	0.071974228	0.6784056	24	9.366375381	0.513733197
ENSMUSG00000038240	Pdss2	prenyl (solanesyl) diphosphate synthase, subunit 2 [Source:MGI Symbol;Acc:MGI:1918615]	4.67019	4.49103	4.95852	5.40513	4.55024	5.59406	4.80211	5.97818	4.19629	5.96002	4.28743	4.74559	5.08565	5.7516	0.874054778	0.966018836	8	12	24	0.795670037	0.934659561	24	6.418118179	0.039655041
ENSMUSG00000038241	Cep250	centrosomal protein 250 [Source:MGI Symbol;Acc:MGI:108084]	7.75574	7.37146	7.43264	7.92053	9.13193	7.9669	8.65961	8.05119	7.60993	9.17273	7.19974	8.02823	8.006	7.89536	0.888991156	0.966018836	16	20	24	0.58039091	0.850271896	24	16.72364673	0.039157104
ENSMUSG00000038242	Aox4	aldehyde oxidase 4 [Source:MGI Symbol;Acc:MGI:1919122]	1.49846	1.81253	1.03428	0.915042	1.21075	1.19971	0.985571	1.09453	0.737409	2.20137	1.20213	1.10609	1.19816	1.25117	0.891690964	0.966018836	20	16	24	0.983551334	0.996530668	24	19.34648295	0.017253176
ENSMUSG00000038244	Mical2	microtubule associated monooxygenase, calponin and LIM domain containing 2 [Source:MGI Symbol;Acc:MGI:2444947]	9.04918	7.06384	8.52537	10.3828	8.06219	8.45449	8.72962	8.62464	7.67646	11.2494	7.24765	9.28931	6.63582	8.95465	0.892044196	0.966018836	16	20	24	0.460455988	0.805393068	24	12.52455641	0.090275991
ENSMUSG00000038248	Sobp	sine oculis binding protein [Source:MGI Symbol;Acc:MGI:1924427]	3.42802	2.83749	2.89517	3.01923	2.8775	3.00615	3.07615	3.11012	2.74872	4.49993	3.09281	3.70196	3.38032	3.75088	0.949152198	0.966018836	4	8	24	0.968303727	0.992326888	24	17.19802761	0.013830685
ENSMUSG00000038250	Usp38	ubiquitin specific peptidase 38 [Source:MGI Symbol;Acc:MGI:1922091]	8.28652	7.96634	8.29813	8.17509	8.11354	8.03619	7.90487	7.72636	7.67427	8.01964	7.8397	8.06387	8.13871	7.98832	0.921572192	0.966018836	4	4	24	0.611136207	0.865079333	24	18.87193256	0.011312153
ENSMUSG00000038252	Ncapd2	non-SMC condensin I complex, subunit D2 [Source:MGI Symbol;Acc:MGI:1915548]	5.23341	8.39477	9.24656	7.60515	7.81901	6.81789	6.10051	8.3328	8.19018	7.59176	6.59732	7.01598	6.84435	5.45029	0.017495149	0.255326033	12	16	24	0.081401607	0.697102286	24	10.09951663	0.16975114
ENSMUSG00000038253	Hoxa5	homeobox A5 [Source:MGI Symbol;Acc:MGI:96177]	3.42105	3.9877	3.89885	3.942	3.33796	3.30973	4.01103	3.7993	4.52403	3.51134	3.90819	4.11181	4.02319	4.45548	0.652290823	0.966018836	12	8	24	0.038584444	0.61140397	24	6.051172065	0.090947599
ENSMUSG00000038256	Bcl9	B cell CLL/lymphoma 9 [Source:MGI Symbol;Acc:MGI:1924828]	5.65075	6.45676	5.27745	6.15994	6.85295	6.2886	7.24139	6.4524	8.3244	7.66469	5.76642	6.43554	7.10108	7.81822	0.833406028	0.966018836	8	12	24	0.485550878	0.805393068	24	6.757531484	0.067085201
ENSMUSG00000038260	Trpm4	transient receptor potential cation channel, subfamily M, member 4 [Source:MGI Symbol;Acc:MGI:1915917]	12.7162	14.3195	13.1793	13.1472	16.7092	15.8488	14.0361	15.3637	13.4441	14.6882	15.6031	14.15	15.3512	14.1178	0.251986462	0.75129457	20	16	24	0.235147062	0.768040178	24	18.0445305	0.068651133
ENSMUSG00000038264	Sema7a	sema domain, immunoglobulin domain (Ig), and GPI membrane anchor, (semaphorin) 7A [Source:MGI Symbol;Acc:MGI:1306826]	18.3048	14.5871	12.4516	14.4157	13.5682	16.7003	18.1253	17.6223	12.1842	20.3793	12.5743	16.4096	16.2711	15.3336	0.060394272	0.407012001	4	8	24	0.292927362	0.768040178	24	22.93887269	0.132376172
ENSMUSG00000038267	Slc22a23	solute carrier family 22, member 23 [Source:MGI Symbol;Acc:MGI:1920352]	9.60652	9.04209	10.1571	8.9546	9.49644	9.42904	10.4102	10.7759	8.8454	11.8846	8.48924	9.6047	10.2783	9.82699	0.590017078	0.966018836	4	16	24	0.71624078	0.90635549	24	4.609990914	0.037762103
ENSMUSG00000038268	Ovca2	candidate tumor suppressor in ovarian cancer 2 [Source:MGI Symbol;Acc:MGI:2179725]	9.07154	8.77431	8.5811	8.69429	8.25718	9.33227	8.39039	9.14541	8.13205	8.31222	8.50153	9.33044	9.31101	8.94794	0.020428812	0.261278501	24	20	24	0.041575604	0.619302898	24	23.14957276	0.056082081
ENSMUSG00000038271	Iffo1	intermediate filament family orphan 1 [Source:MGI Symbol;Acc:MGI:2444516]	22.3441	21.6595	21.6578	21.6919	21.5675	22.1315	19.0988	24.5329	22.2818	24.6644	21.2037	22.8977	20.8562	23.2328	0.725061903	0.966018836	8	20	24	0.433013874	0.805393068	24	9.125432495	0.046547434
ENSMUSG00000038274	Fau	Finkel-Biskis-Reilly murine sarcoma virus (FBR-MuSV) ubiquitously expressed (fox derived) [Source:MGI Symbol;Acc:MGI:102547]	3.63696	4.0818	2.76839	2.64881	3.92018	3.51583	2.99119	2.66327	5.03115	3.08836	5.23665	3.90306	3.9134	3.4152	0.893307667	0.966018836	20	20	24	0.688937978	0.897070579	24	17.98356934	0.083484382
ENSMUSG00000038276	Asic3	acid-sensing (proton-gated) ion channel 3 [Source:MGI Symbol;Acc:MGI:2159339]	35.9438	27.6435	25.7732	30.7829	29.1945	35.2571	32.2198	33.7729	25.5933	40.6537	28.2749	32.119	30.0334	30.6527	0.217827365	0.704989686	24	12	24	0.487539027	0.805393068	24	19.99655935	0.089651313
ENSMUSG00000038279	Nop2	NOP2 nucleolar protein [Source:MGI Symbol;Acc:MGI:107891]	7.1217	5.68424	6.07175	6.3422	6.28334	7.55082	7.09831	7.26612	5.75702	6.68089	6.14541	6.60579	5.71155	6.07728	0.725061903	0.966018836	24	12	24	0.232911394	0.768040178	24	20.52291968	0.078247867
ENSMUSG00000038280	Ostm1	osteopetrosis associated transmembrane protein 1 [Source:MGI Symbol;Acc:MGI:2655574]	25.2696	25.6004	24.7555	24.7538	23.5362	24.7196	23.2407	23.6816	23.9474	23.8016	27.0813	24.6081	24.8243	25.2102	0.866861748	0.966018836	8	16	24	0.9260597	0.98027178	24	18.43934291	0.009135981
ENSMUSG00000038286	Bphl	biphenyl hydrolase-like (serine hydrolase, breast epithelial mucin-associated antigen) [Source:MGI Symbol;Acc:MGI:1915271]	15.9296	19.1776	16.3583	15.4144	15.2349	16.5508	16.52	18.6944	18.3056	15.8068	17.7901	18.2831	17.1488	16.1316	0.496745696	0.966018836	8	8	24	0.366425092	0.790241693	24	3.173344154	0.054969418
ENSMUSG00000038290	Smg6	SMG6 nonsense mediated mRNA decay factor [Source:MGI Symbol;Acc:MGI:2144117]	7.63805	6.51154	7.42539	6.76447	6.62603	7.26926	7.493	8.51867	6.75386	9.06297	7.00871	7.39127	6.93477	8.03699	0.92769718	0.966018836	8	12	24	0.728817926	0.911293334	24	5.849549798	0.038688174
ENSMUSG00000038291	Snx25	sorting nexin 25 [Source:MGI Symbol;Acc:MGI:2142610]	19.0497	17.6265	17.3076	18.4744	17.0642	18.7201	17.6625	16.701	15.4876	18.3228	16.7603	18.4671	18.3807	19.4971	0.387380622	0.915479136	24	12	24	0.354509794	0.781577622	24	21.9556541	0.048726468
ENSMUSG00000038295	Atg9b	autophagy related 9B [Source:MGI Symbol;Acc:MGI:2685420]	1.1257	0.971995	1.16145	1.06403	0.96387	1.05659	1.46356	1.28975	0.858414	1.09414	1.21303	0.936008	1.1205	0.981804	0.874473208	0.966018836	4	20	24	0.625031081	0.872055388	24	23.57998077	0.041228187
ENSMUSG00000038296	Galnt18	polypeptide N-acetylgalactosaminyltransferase 18 [Source:MGI Symbol;Acc:MGI:2446239]	11.9922	8.54844	8.11887	9.78231	8.80357	10.0687	9.03739	10.7492	8.07325	11.6863	9.46162	10.0216	10.3264	10.7434	0.43565473	0.966018836	4	8	24	0.592142318	0.856434841	24	21.85899509	0.066877997
ENSMUSG00000038299	Wdr36	WD repeat domain 36 [Source:MGI Symbol;Acc:MGI:1917819]	13.4937	12.0891	13.1802	13.6832	12.721	12.9234	12.8203	12.2218	11.2831	12.2909	13.0836	12.5509	11.9188	13.2493	0.882263435	0.966018836	16	20	24	0.52210713	0.820717128	24	17.82920441	0.031340304
ENSMUSG00000038301	Snx10	sorting nexin 10 [Source:MGI Symbol;Acc:MGI:1919232]	104.356	92.561	92.9807	97.6191	92.5288	93.925	87.4009	89.3289	82.2478	91.5713	105.748	90.4341	96.6849	95.8554	0.93447422	0.966018836	20	16	24	0.430380249	0.805393068	24	18.76221837	0.04923407
ENSMUSG00000038302	Afg1l	AFG1 like ATPase [Source:MGI Symbol;Acc:MGI:2148801]	7.13049	6.92828	6.72025	7.98538	7.19602	6.92208	7.65933	7.47843	6.51487	7.56279	8.60426	7.14475	8.60734	7.8261	0.591351901	0.966018836	16	20	24	0.714103777	0.905831242	24	18.53988317	0.031789646
ENSMUSG00000038305	Spats2l	spermatogenesis associated, serine-rich 2-like [Source:MGI Symbol;Acc:MGI:1914448]	13.9088	11.3086	10.7339	10.2654	11.6455	12.2016	12.2424	10.6811	9.02469	12.3644	12.3561	11.823	12.8508	12.4295	0.031969981	0.324385159	4	8	24	0.033135792	0.606585237	24	21.27853238	0.13637592
ENSMUSG00000038312	Edem2	ER degradation enhancer, mannosidase alpha-like 2 [Source:MGI Symbol;Acc:MGI:1915540]	12.3308	11.2967	11.8569	10.7095	12.0843	10.8748	11.3932	11.2714	11.325	11.6044	11.9254	11.7754	11.1823	11.0084	0.702630284	0.966018836	20	12	24	0.597683459	0.858717127	24	18.10615647	0.022213535
ENSMUSG00000038319	Kcnh2	potassium voltage-gated channel, subfamily H (eag-related), member 2 [Source:MGI Symbol;Acc:MGI:1341722]	16.4037	12.8832	13.3033	13.1523	11.6885	13.0127	16.0431	14.2483	13.1205	19.8604	13.8145	14.5525	15.6473	15.1054	0.310940564	0.820837787	4	16	24	0.819655164	0.942827679	24	2.362478509	0.043048688
ENSMUSG00000038323	1700066M21Rik	RIKEN cDNA 1700066M21 gene [Source:MGI Symbol;Acc:MGI:1920717]	19.9905	18.4473	17.708	18.5674	19.2296	18.6389	18.012	17.4782	16.465	16.5999	19.952	17.6802	17.4598	18.632	0.424718357	0.966018836	20	16	24	0.136817111	0.747972781	24	19.64289146	0.059323086
ENSMUSG00000038324	Trpc4ap	transient receptor potential cation channel, subfamily C, member 4 associated protein [Source:MGI Symbol;Acc:MGI:1930751]	50.5726	43.3603	46.0625	45.8139	46.3388	51.0632	46.9993	51.0487	42.3463	52.6126	46.541	48.935	48.1707	48.5044	0.245061943	0.741580974	24	12	24	0.441303979	0.805393068	24	20.57306908	0.045465748
ENSMUSG00000038332	Sesn1	sestrin 1 [Source:MGI Symbol;Acc:MGI:2155278]	30.7303	35.7765	35.1201	42.7907	39.4687	35.8552	32.3988	31.5716	41.2791	36.3255	37.479	37.8811	32.895	32.4049	0.004122392	0.140598674	20	8	24	0.003722266	0.426426863	24	13.23665141	0.151978825
ENSMUSG00000038335	Tsr1	TSR1 20S rRNA accumulation [Source:MGI Symbol;Acc:MGI:2144566]	10.1229	10.1829	10.1898	9.51772	10.8107	10.3418	9.68057	9.70319	9.22119	10.2904	9.79131	9.52114	10.138	10.5406	0.838692453	0.966018836	20	8	24	0.854050182	0.956445086	24	19.94647286	0.013767453
ENSMUSG00000038342	Mlxip	MLX interacting protein [Source:MGI Symbol;Acc:MGI:2141183]	7.81255	7.46898	7.4004	9.5635	7.3012	8.12898	8.47369	8.09362	7.81742	9.52086	6.954	8.1284	8.05776	7.42572	0.708665292	0.966018836	16	20	24	0.761578117	0.922794036	24	12.37925843	0.035297073
ENSMUSG00000038344	Txlng	taxilin gamma [Source:MGI Symbol;Acc:MGI:3590652]	7.50452	6.83725	6.09925	7.03151	7.04076	6.64616	7.30883	6.35427	6.53382	5.82945	7.684	6.84202	6.72004	6.73683	0.17847698	0.655480352	20	16	24	0.10353555	0.715310751	24	20.40334315	0.077472907
ENSMUSG00000038346	Zfp384	zinc finger protein 384 [Source:MGI Symbol;Acc:MGI:2443203]	9.20809	9.27557	9.03316	9.47519	8.94628	9.50196	9.41306	8.79344	9.04885	9.63337	9.6322	8.57179	8.93632	8.30222	0.143673396	0.585944911	16	16	24	0.189464547	0.768040178	24	15.26155548	0.03603536
ENSMUSG00000038347	Tcte2	t-complex-associated testis expressed 2 [Source:MGI Symbol;Acc:MGI:98641]	2.37804	3.04489	2.25625	1.91283	2.23936	2.40396	2.54427	1.99854	2.96286	2.162	1.99328	1.80573	2.42949	2.13964	0.278111325	0.789849983	4	12	24	0.249115794	0.768040178	24	3.818585264	0.093099541
ENSMUSG00000038349	Plcl1	phospholipase C-like 1 [Source:MGI Symbol;Acc:MGI:3036262]	3.2842	3.54332	5.49293	3.15485	3.23108	3.90511	3.72349	2.98902	3.24885	2.89691	3.006	3.42874	3.51766	3.50966	0.235147982	0.73629392	4	12	24	0.696892862	0.899309039	24	3.630211192	0.05920612
ENSMUSG00000038351	Sgsm2	small G protein signaling modulator 2 [Source:MGI Symbol;Acc:MGI:2144695]	8.20472	7.62753	8.00536	7.33331	8.00931	8.0242	7.90542	8.27839	7.46088	8.87205	7.72765	8.64402	7.85367	7.71837	0.662399683	0.966018836	24	12	24	0.622549373	0.87142241	24	19.3540539	0.027013242
ENSMUSG00000038352	Arl5c	ADP-ribosylation factor-like 5C [Source:MGI Symbol;Acc:MGI:3028577]	7.21805	7.00216	5.59058	6.15201	8.82571	6.37876	9.10931	5.25578	6.2443	3.26517	7.23445	5.7513	6.28171	5.52287	0.20866337	0.698797644	20	20	24	0.146458007	0.755124657	24	21.26581842	0.218230394
ENSMUSG00000038354	Ankrd35	ankyrin repeat domain 35 [Source:MGI Symbol;Acc:MGI:2442590]	2.18887	2.11705	2.45723	1.97339	1.89328	2.09107	2.16774	2.33858	2.41834	2.5427	2.18711	2.46077	1.96134	2.75949	0.504585278	0.966018836	12	8	24	0.121777263	0.734072261	24	6.872559843	0.079319162
ENSMUSG00000038357	Camp	cathelicidin antimicrobial peptide [Source:MGI Symbol;Acc:MGI:108443]	21.2848	49.6493	91.7415	63.7478	41.6934	51.9135	42.7445	37.7357	51.8109	28.3526	40.767	48.8207	63.8745	29.7946	0.911371589	0.966018836	12	20	24	0.739307912	0.914957371	24	10.59990866	0.166592504
ENSMUSG00000038365	Fbxo25	F-box protein 25 [Source:MGI Symbol;Acc:MGI:1914072]	36.0109	33.728	32.468	32.2606	35.8735	34.3053	35.2678	33.9734	33.0444	32.5973	33.4538	34.0236	36.4553	35.2071	1.02E-04	0.029372629	4	12	24	0.005465164	0.445165967	24	22.72617378	0.06041146
ENSMUSG00000038366	Lasp1	LIM and SH3 protein 1 [Source:MGI Symbol;Acc:MGI:109656]	25.9653	20.1798	20.8347	21.0286	21.0883	22.5515	25.7051	23.7334	18.6733	27.3408	20.7017	24.041	23.6405	23.8138	0.105280783	0.518002936	4	8	24	0.325578322	0.768040178	24	22.54497912	0.088124191
ENSMUSG00000038368	Focad	focadhesin [Source:MGI Symbol;Acc:MGI:2676921]	7.55661	7.11287	8.03848	9.05974	8.51364	7.58681	7.44106	6.91212	7.65735	7.98469	8.79164	7.1672	7.19153	8.30959	0.040741957	0.345521986	16	16	24	0.011943201	0.525280095	24	13.22544076	0.106594921
ENSMUSG00000038369	Ncoa6	nuclear receptor coactivator 6 [Source:MGI Symbol;Acc:MGI:1929915]	9.37021	9.30815	8.63824	9.18048	8.71849	9.33448	9.49219	8.32815	8.68067	9.64183	8.89863	9.48998	8.53312	8.69754	0.361555248	0.885260695	24	12	24	0.301342217	0.768040178	24	18.60297144	0.037140407
ENSMUSG00000038370	Pcp4l1	Purkinje cell protein 4-like 1 [Source:MGI Symbol;Acc:MGI:1913675]	101.574	88.7044	91.9099	86.3402	79.4633	93.9354	99.3914	90.9996	79.9156	111.569	88.647	101.825	94.3253	105.066	0.446653834	0.966018836	4	16	24	0.535257368	0.82691856	24	0.164368182	0.059349599
ENSMUSG00000038371	Sbf2	SET binding factor 2 [Source:MGI Symbol;Acc:MGI:1921831]	18.8761	19.4161	18.8669	17.9123	19.9977	17.2665	19.6413	17.5571	19.9217	17.4647	17.5495	18.9881	18.9831	17.6264	0.910436409	0.966018836	4	12	24	0.797818558	0.935424343	24	1.17427253	0.018722483
ENSMUSG00000038372	Gmds	GDP-mannose 4, 6-dehydratase [Source:MGI Symbol;Acc:MGI:1891112]	7.23585	6.83752	6.71735	6.32531	6.37741	6.52795	6.65407	6.6348	6.31129	7.03014	6.30693	6.94663	7.51984	7.31275	0.077161185	0.453790923	4	16	24	0.066668125	0.669134584	24	1.508678384	0.058170011
ENSMUSG00000038374	Rbm8a	RNA binding motif protein 8a [Source:MGI Symbol;Acc:MGI:1913129]	16.3995	15.5941	15.3393	16.8449	15.6482	15.8193	16.6964	15.2172	14.7032	14.0427	17.6208	15.8848	16.0417	14.34	0.1642793	0.628002127	4	4	24	0.082987771	0.699403344	24	19.35313697	0.070296537
ENSMUSG00000038375	Trp53inp2	transformation related protein 53 inducible nuclear protein 2 [Source:MGI Symbol;Acc:MGI:1915978]	35.5447	38.8498	44.7567	42.1772	37.9013	42.6703	41.8272	46.0899	41.5351	52.2807	36.6336	39.825	39.6066	41.2001	0.356953959	0.881188925	16	4	24	0.157682943	0.764314876	24	9.161930393	0.103276662
ENSMUSG00000038383	Pigu	phosphatidylinositol glycan anchor biosynthesis, class U [Source:MGI Symbol;Acc:MGI:3039607]	18.0659	19.6129	17.5072	18.0552	18.8102	19.035	18.711	18.708	17.4473	17.3352	19.6565	18.0296	19.6438	18.6508	0.15105645	0.598641364	20	20	24	0.165088285	0.767020009	24	22.05744162	0.04344288
ENSMUSG00000038384	Setd1b	SET domain containing 1B [Source:MGI Symbol;Acc:MGI:2652820]	3.08527	2.71966	2.79465	3.21691	2.91207	2.39736	2.89341	2.94589	3.96304	3.99671	3.18704	3.35423	3.32598	2.76421	0.350544145	0.870418653	16	16	24	0.201411037	0.768040178	24	11.08867661	0.098976482
ENSMUSG00000038387	Rras	related RAS viral (r-ras) oncogene [Source:MGI Symbol;Acc:MGI:98179]	34.8453	30.3162	31.3254	32.2035	38.185	34.1939	33.1574	34.0802	32.4894	31.3266	35.6285	32.6697	33.1919	29.8428	0.036084337	0.331852526	20	12	24	0.028397254	0.593803552	24	18.22295864	0.087849121
ENSMUSG00000038388	Pals2	protein associated with LIN7 2, MAGUK family member [Source:MGI Symbol;Acc:MGI:1927340]	13.06	15.5817	13.8249	14.622	15.9181	14.187	14.0628	14.4783	16.8111	12.4275	14.6562	13.5583	13.0929	13.9364	0.588208558	0.966018836	20	8	24	0.636642835	0.876483853	24	10.00128758	0.042111492
ENSMUSG00000038390	Gpr162	G protein-coupled receptor 162 [Source:MGI Symbol;Acc:MGI:1315214]	8.47053	8.29221	11.6204	7.65192	7.66083	9.46356	8.41692	8.18259	8.99548	9.3482	6.60227	8.78812	8.18741	10.1173	0.966749204	0.969592092	12	8	24	0.267093828	0.768040178	24	5.845446114	0.112714929
ENSMUSG00000038393	Txnip	thioredoxin interacting protein [Source:MGI Symbol;Acc:MGI:1889549]	34.1764	49.2695	48.7806	55.8886	37.3267	41.1067	48.7476	74.4655	57.9027	57.9258	43.5602	52.8499	54.1609	52.651	0.221479046	0.71314449	16	4	24	0.032661543	0.606585237	24	7.222485785	0.244095707
ENSMUSG00000038398	Upf3a	UPF3 regulator of nonsense transcripts homolog A (yeast) [Source:MGI Symbol;Acc:MGI:1914281]	11.6274	11.6757	13.0115	13.5211	12.4842	11.6076	12.0548	12.2966	14.0431	13.609	12.9995	12.9013	13.3849	12.5571	0.038471411	0.335882166	16	8	24	0.004049926	0.427431763	24	10.92856508	0.080069519
ENSMUSG00000038400	Pmepa1	prostate transmembrane protein, androgen induced 1 [Source:MGI Symbol;Acc:MGI:1929600]	9.93125	7.98857	7.74122	9.14022	8.93927	8.2726	10.3928	8.83445	8.48771	9.73589	9.62893	8.24195	8.46004	8.67272	0.742579234	0.966018836	16	20	24	0.653986904	0.883343578	24	18.35932859	0.043134396
ENSMUSG00000038403	Hjv	hemojuvelin BMP co-receptor [Source:MGI Symbol;Acc:MGI:1916835]	1.86903	5.7506	6.65662	8.49504	5.32122	7.09033	5.26925	14.253	11.9199	14.2376	5.13129	5.49022	7.87145	6.24765	0.168929279	0.640164678	16	4	24	0.043211303	0.625533005	24	9.332603308	0.53640398
ENSMUSG00000038406	Scaf1	SR-related CTD-associated factor 1 [Source:MGI Symbol;Acc:MGI:2141980]	23.3711	20.0955	21.1948	21.9198	20.0788	19.8421	20.3652	22.1084	21.1642	29.22	20.7705	22.5282	23.0259	22.7612	0.94073613	0.966018836	4	20	24	0.613255195	0.866078878	24	9.840360262	0.052790305
ENSMUSG00000038412	Higd1a	HIG1 domain family, member 1A [Source:MGI Symbol;Acc:MGI:1930666]	23.5885	23.0358	22.9106	23.3136	22.8948	23.5317	22.7814	22.29	21.2818	21.9493	23.7507	23.6201	22.76	23.4201	0.1982019	0.680119125	24	12	24	0.077848138	0.687802873	24	20.0926288	0.037020465
ENSMUSG00000038416	Cdc16	CDC16 cell division cycle 16 [Source:MGI Symbol;Acc:MGI:1917207]	21.8639	22.7566	24.3564	22.1426	23.2058	21.5685	21.5851	22.2374	24.4369	20.443	23.928	23.4187	23.5834	24.2426	0.251986462	0.75129457	12	12	24	0.631364908	0.87436603	24	7.054649294	0.028808137
ENSMUSG00000038417	Fig4	FIG4 phosphoinositide 5-phosphatase [Source:MGI Symbol;Acc:MGI:2143585]	25.7671	21.9246	22.3439	20.5777	22.8307	24.9171	21.4703	22.8982	19.9152	23.3832	22.3936	23.0498	23.6825	23.4069	0.119434414	0.542554768	24	12	24	0.085954845	0.704351094	24	21.58537337	0.078997193
ENSMUSG00000038418	Egr1	early growth response 1 [Source:MGI Symbol;Acc:MGI:95295]	3.67789	3.6749	3.77834	3.38643	2.501	3.25114	3.48636	5.25161	3.53721	4.00252	2.70339	3.64449	5.03726	3.05549	0.191659183	0.669841558	8	12	24	0.11121807	0.723334499	24	3.728365182	0.175325455
ENSMUSG00000038421	Fcrla	Fc receptor-like A [Source:MGI Symbol;Acc:MGI:2138647]	0.418471	1.04761	0.778631	0.335479	0.432376	0.881621	0.923277	0.756498	1.02907	0.599624	0.792376	0.525196	0.753599	0.869218	0.25849765	0.761738864	12	4	24	0.1454395	0.754184974	24	4.187092173	0.141992983
ENSMUSG00000038422	Hdhd3	haloacid dehalogenase-like hydrolase domain containing 3 [Source:MGI Symbol;Acc:MGI:1919998]	2.47585	2.6341	3.23179	2.9766	2.68539	2.99	2.27866	2.80028	2.49942	2.51618	3.01655	2.93423	3.0665	2.9209	0.908406451	0.966018836	24	4	24	0.794680611	0.934196011	24	13.63618552	0.028120649
ENSMUSG00000038425	Poli	polymerase (DNA directed), iota [Source:MGI Symbol;Acc:MGI:1347081]	4.18084	3.15673	3.82112	3.91384	3.72548	3.28573	3.4856	3.773	3.07554	3.27728	3.311	3.65084	3.18174	3.55176	0.759825336	0.966018836	20	12	24	0.911093072	0.974779246	24	20.17649338	0.017111893
ENSMUSG00000038429	Usp5	ubiquitin specific peptidase 5 (isopeptidase T) [Source:MGI Symbol;Acc:MGI:1347343]	76.0483	62.5442	61.2652	67.3858	67.0921	72.9303	65.1679	71.5719	56.8978	81.1393	61.5716	68.8172	64.5661	68.2495	0.245061943	0.741580974	24	12	24	0.605709925	0.862773266	24	19.48751403	0.055459817
ENSMUSG00000038437	Mllt6	myeloid/lymphoid or mixed-lineage leukemia; translocated to, 6 [Source:MGI Symbol;Acc:MGI:1935145]	10.9165	9.86219	10.8805	10.705	11.1282	9.89497	12.3681	10.3231	11.0052	12.1378	11.8533	11.3798	11.2042	11.0199	0.483881213	0.966018836	20	12	24	0.553880227	0.837374348	24	15.32165243	0.0342219
ENSMUSG00000038446	Cdc40	cell division cycle 40 [Source:MGI Symbol;Acc:MGI:1918963]	8.2106	8.54717	8.36742	8.3141	8.21271	8.1343	8.56416	8.02753	9.34068	7.61516	8.55278	8.44058	8.36984	8.48955	0.919119843	0.966018836	12	4	24	0.653434649	0.883312134	24	5.100626133	0.021549699
ENSMUSG00000038451	Spsb2	splA/ryanodine receptor domain and SOCS box containing 2 [Source:MGI Symbol;Acc:MGI:1315199]	6.10929	5.59569	6.15302	5.57334	6.24299	5.50672	5.65502	6.21467	6.16711	5.9002	7.90769	7.31826	6.72957	5.57395	0.954584434	0.966018836	20	20	24	0.31365164	0.768040178	24	17.38307819	0.073393223
ENSMUSG00000038453	Srcin1	SRC kinase signaling inhibitor 1 [Source:MGI Symbol;Acc:MGI:1933179]	5.53181	5.90174	7.20454	5.88153	5.27088	5.68427	6.61008	5.26977	6.34985	6.48628	5.83855	6.0911	5.7852	6.76643	0.455507782	0.966018836	12	8	24	0.222315138	0.768040178	24	7.678718204	0.078191572
ENSMUSG00000038456	Dennd2a	DENN/MADD domain containing 2A [Source:MGI Symbol;Acc:MGI:2444961]	4.19804	3.45591	3.54359	3.42803	4.39574	3.8439	4.7839	3.35972	3.59105	3.95517	3.90624	3.78687	3.94104	3.92956	0.217827365	0.704989686	4	4	24	0.12263301	0.735333681	24	20.28692649	0.091237879
ENSMUSG00000038457	Tmem255b	transmembrane protein 255B [Source:MGI Symbol;Acc:MGI:2685533]	0.977821	0.638996	1.08528	0.857083	0.711407	0.651237	0.81357	0.867554	0.603422	0.947639	0.698831	0.733309	0.737635	1.11357	0.521828922	0.966018836	8	12	24	0.427486821	0.805393068	24	6.442198539	0.067932679
ENSMUSG00000038459	Abhd17c	abhydrolase domain containing 17C [Source:MGI Symbol;Acc:MGI:1917428]	8.33997	7.08985	7.1864	7.07969	7.38131	7.50529	7.11475	7.20272	5.94419	7.73884	7.0896	7.0506	7.45162	7.41944	0.798309651	0.966018836	4	8	24	0.272727499	0.768040178	24	21.0961741	0.058096592
ENSMUSG00000038462	Uqcrfs1	ubiquinol-cytochrome c reductase, Rieske iron-sulfur polypeptide 1 [Source:MGI Symbol;Acc:MGI:1913944]	85.15	90.3343	94.1461	118.513	94.297	90.4148	90.9507	110.934	93.5084	102.513	106.153	92.9921	108.127	102.894	0.496745696	0.966018836	16	8	24	0.2248039	0.768040178	24	10.56156724	0.083719031
ENSMUSG00000038463	Olfml2b	olfactomedin-like 2B [Source:MGI Symbol;Acc:MGI:2443310]	11.6077	9.7104	10.6072	12.0896	12.397	10.6296	11.2058	10.7775	10.4451	12.9066	10.894	9.05131	10.2653	9.38038	0.138719141	0.580158868	16	16	24	0.094567266	0.71175955	24	13.9329524	0.099007675
ENSMUSG00000038467	Chmp4b	charged multivesicular body protein 4B [Source:MGI Symbol;Acc:MGI:1922858]	114.417	99.1202	101.833	99.2822	100.475	111.351	107.088	107.051	96.8	114.606	104.084	113.039	113.818	112.857	0.097760727	0.501112642	4	8	24	0.100159356	0.71175955	24	22.2420451	0.064421632
ENSMUSG00000038473	Nos1ap	nitric oxide synthase 1 (neuronal) adaptor protein [Source:MGI Symbol;Acc:MGI:1917979]	2.41281	3.11807	4.21392	3.55502	2.53267	3.12377	3.49114	3.22444	2.71318	3.03457	3.41103	3.84653	2.39456	4.07555	0.940625808	0.966018836	12	16	24	0.641186107	0.878540546	24	8.75047409	0.07798903
ENSMUSG00000038481	Cdk19	cyclin-dependent kinase 19 [Source:MGI Symbol;Acc:MGI:1925584]	9.21619	10.1692	10.469	10.8416	9.73506	9.24246	10.374	9.74851	10.5851	10.6454	9.85921	9.60162	9.81839	9.96392	0.001005664	0.075485393	16	8	24	0.004755661	0.434006302	24	9.482672303	0.073973079
ENSMUSG00000038482	Tfdp1	transcription factor Dp 1 [Source:MGI Symbol;Acc:MGI:101934]	11.029	10.7646	11.4228	10.3626	11.4281	10.5663	10.6108	11.0433	10.7542	10.7863	10.5511	10.0393	9.5976	9.24695	0.939921059	0.966018836	12	12	24	0.30350251	0.768040178	24	13.00662959	0.032006381
ENSMUSG00000038485	Socs7	suppressor of cytokine signaling 7 [Source:MGI Symbol;Acc:MGI:2651588]	15.1853	12.9017	12.0919	14.5765	13.5361	14.3415	14.9241	14.0955	12.3057	16.1125	12.954	15.1778	13.6448	14.2875	0.14313801	0.585944911	24	12	24	0.389883668	0.799546792	24	19.99793658	0.062810898
ENSMUSG00000038486	Sv2a	synaptic vesicle glycoprotein 2 a [Source:MGI Symbol;Acc:MGI:1927139]	14.5758	17.5049	25.5821	15.5832	13.4871	19.2852	19.6181	15.1708	17.2715	19.7668	12.139	17.7083	12.7866	21.4284	0.496745696	0.966018836	12	8	24	0.327694585	0.768040178	24	6.667545649	0.171570385
ENSMUSG00000038489	Polr2l	polymerase (RNA) II (DNA directed) polypeptide L [Source:MGI Symbol;Acc:MGI:1913741]	2.0791	1.69525	1.7982	2.39419	2.18374	2.43124	1.98649	1.67513	2.0971	1.9366	1.76501	2.28722	1.5411	1.78025	0.035806379	0.331814682	24	8	24	0.071630117	0.67819049	24	16.60305761	0.109822178
ENSMUSG00000038495	Otud7b	OTU domain containing 7B [Source:MGI Symbol;Acc:MGI:2654703]	15.912	15.5616	17.774	21.9283	19.1935	17.5782	15.3035	14.0035	17.1141	18.9843	17.1799	17.8517	15.0502	14.9172	1.51E-04	0.031913438	16	16	24	9.04E-05	0.114031042	24	14.04485204	0.19316619
ENSMUSG00000038496	Slc19a3	solute carrier family 19, member 3 [Source:MGI Symbol;Acc:MGI:1931307]	0.551465	1.26507	0.876439	1.05728	1.14816	0.994225	0.894945	0.966819	0.887802	0.682122	1.04784	0.874544	0.843496	0.988408	0.787283372	0.966018836	20	8	24	0.851229402	0.955425185	24	12.13650794	0.031551283
ENSMUSG00000038497	Tmco3	transmembrane and coiled-coil domains 3 [Source:MGI Symbol;Acc:MGI:2444946]	20.7995	20.1613	19.0097	21.6569	20.3823	19.6178	18.8148	21.5291	17.9001	19.6539	18.4201	19.8137	20.8834	18.9496	0.890648739	0.966018836	8	4	24	0.777473354	0.928654033	24	22.08455663	0.02219919
ENSMUSG00000038500	Prr3	proline-rich polypeptide 3 [Source:MGI Symbol;Acc:MGI:1922460]	7.59065	8.91589	8.83125	8.18742	7.76428	8.59596	9.02561	8.59335	8.2968	8.17089	7.40287	7.43713	8.76595	7.83233	0.14606006	0.590732968	8	12	24	0.158130696	0.764630328	24	4.4436492	0.062168525
ENSMUSG00000038502	Ptov1	prostate tumor over expressed gene 1 [Source:MGI Symbol;Acc:MGI:1933946]	93.6584	80.8689	79.3861	77.1745	79.8688	75.9816	83.7201	89.0612	82.4861	97.1941	81.3902	86.9174	82.4362	88.0331	0.628899575	0.966018836	4	20	24	0.688451544	0.896969506	24	3.951631432	0.034179535
ENSMUSG00000038503	Mesd	mesoderm development LRP chaperone [Source:MGI Symbol;Acc:MGI:1891421]	24.9178	24.0404	24.0886	20.6636	26.5941	28.1105	22.8306	25.5078	23.5683	25.865	25.6072	26.244	29.5856	23.1656	0.207677584	0.698704663	24	8	24	0.142514304	0.752027519	24	20.35897734	0.092415224
ENSMUSG00000038506	Dcun1d2	DCN1, defective in cullin neddylation 1, domain containing 2 (S. cerevisiae) [Source:MGI Symbol;Acc:MGI:2142792]	24.9217	24.0469	23.8123	24.0572	23.4863	24.0838	23.3684	24.9346	23.2139	24.2408	25.7487	22.8211	24.6694	23.8242	0.896311169	0.966018836	4	8	24	0.98228785	0.996048133	24	20.68278336	0.003588565
ENSMUSG00000038507	Parp12	poly (ADP-ribose) polymerase family, member 12 [Source:MGI Symbol;Acc:MGI:2143990]	8.78562	10.6527	11.1756	10.0646	9.8254	9.53923	9.39674	11.7316	11.3362	9.18816	10.8692	9.48632	9.83783	11.1182	0.025767171	0.298199713	8	20	24	0.029721403	0.596648309	24	7.472143938	0.112928539
ENSMUSG00000038510	Rpf2	ribosome production factor 2 homolog [Source:MGI Symbol;Acc:MGI:1914489]	11.2519	13.696	13.0052	15.012	13.5068	12.6116	12.6279	14.2447	15.3477	11.7803	13.0236	13.2843	14.0258	14.621	0.346246696	0.870418653	8	20	24	0.193812484	0.768040178	24	7.848227594	0.081120039
ENSMUSG00000038515	Grtp1	GH regulated TBC protein 1 [Source:MGI Symbol;Acc:MGI:1914040]	2.6191	2.89805	3.2874	2.8806	2.00365	2.86249	2.30191	2.75144	2.61725	2.62172	2.37659	2.89989	2.54074	2.99999	0.351840494	0.870418653	8	12	24	0.22552611	0.768040178	24	6.049987247	0.087895505
ENSMUSG00000038517	Tbkbp1	TBK1 binding protein 1 [Source:MGI Symbol;Acc:MGI:1920424]	9.7643	8.08899	8.73286	8.53792	6.50322	8.16587	9.01371	6.56526	7.94446	9.24261	8.5272	8.52217	8.27569	9.42805	0.858514388	0.966018836	4	16	24	0.897778959	0.970873489	24	1.914349489	0.028183262
ENSMUSG00000038518	Jarid2	jumonji, AT rich interactive domain 2 [Source:MGI Symbol;Acc:MGI:104813]	5.47159	5.06801	5.65129	5.12073	5.5182	5.28119	5.22963	5.52927	5.09648	6.71782	4.61593	5.30691	4.9823	5.79246	0.933543173	0.966018836	16	12	24	0.550475439	0.835734495	24	9.068016824	0.048022361
ENSMUSG00000038520	Tbc1d17	TBC1 domain family, member 17 [Source:MGI Symbol;Acc:MGI:2449973]	17.8822	14.9759	17.763	13.7868	17.3415	17.4583	17.4919	23.3489	16.3822	21.4451	18.0483	17.8934	17.4809	17.03	0.854443826	0.966018836	20	16	24	0.954064087	0.988601783	24	1.861260971	0.020545551
ENSMUSG00000038521	C1s1	complement component 1, s subcomponent 1 [Source:MGI Symbol;Acc:MGI:1355312]	9.26935	12.8725	11.4463	11.7177	13.1197	9.55353	12.5841	11.7766	12.6777	9.199	13.4602	10.2192	11.9056	10.6705	0.920872048	0.966018836	20	4	24	0.805914119	0.938090863	24	9.201266079	0.047790886
ENSMUSG00000038522	Mfsd4b1	major facilitator superfamily domain containing 4B1 [Source:MGI Symbol;Acc:MGI:2143575]	0.678331	0.697354	0.815078	0.924418	0.637622	0.568202	0.59027	0.762677	0.698552	0.823502	0.731318	0.763314	0.891992	0.991325	0.338172159	0.85758369	16	8	24	0.104723842	0.717030134	24	8.123320892	0.07504625
ENSMUSG00000038524	Fchsd1	FCH and double SH3 domains 1 [Source:MGI Symbol;Acc:MGI:2441771]	6.08706	5.31325	4.1851	6.11701	6.03737	6.03207	6.17822	6.45138	5.54342	6.96731	5.40102	6.32282	6.80397	7.7347	0.895493752	0.966018836	8	4	24	0.579872795	0.850060717	24	23.06814482	0.060731189
ENSMUSG00000038525	Armc10	armadillo repeat containing 10 [Source:MGI Symbol;Acc:MGI:1914461]	8.56823	8.19758	8.60458	8.02748	8.59359	8.15779	8.38863	8.42381	8.94277	8.95278	8.21252	8.46842	8.85215	8.45346	0.943920575	0.966018836	12	12	24	0.591704352	0.856116931	24	7.441326787	0.017694699
ENSMUSG00000038526	Car14	carbonic anhydrase 14 [Source:MGI Symbol;Acc:MGI:1344341]	4.58635	6.52358	8.04169	7.10727	5.7961	5.94162	8.16488	6.41891	8.76747	6.19167	4.69151	5.06539	4.64526	5.10382	0.061697418	0.409814092	12	16	24	0.097942517	0.71175955	24	8.446477523	0.210916802
ENSMUSG00000038530	Rgs4	regulator of G-protein signaling 4 [Source:MGI Symbol;Acc:MGI:108409]	573.777	467.598	439.661	445.739	474	524.484	449.247	463.618	381.817	436.949	502.898	491.924	515.449	487.235	0.00604487	0.16480324	24	12	24	0.006923458	0.472731997	24	21.30544376	0.153242383
ENSMUSG00000038533	Cbfa2t2	CBFA2/RUNX1 translocation partner 2 [Source:MGI Symbol;Acc:MGI:1333833]	5.11852	4.87501	5.90519	5.80061	5.37011	5.67886	5.09161	5.75941	6.49662	6.0865	5.02125	5.87481	5.45283	5.90492	0.097760727	0.501112642	12	16	24	0.078499964	0.688981542	24	9.632337349	0.081020482

ENSMUSG00000038534	Osbpl7	oxysterol binding protein-like 7 [Source:MGI Symbol;Acc:MGI:1918490]	4.78409	5.14528	4.55204	5.49333	5.18713	5.35263	5.38579	5.26819	5.37581	5.49986	5.01332	4.48262	4.58032	3.91919	0.542718076	0.966018836	16	16	24	0.318703193	0.768040178	24	14.08179596	0.062550404
ENSMUSG00000038535	Zfp280d	zinc finger protein 280D [Source:MGI Symbol;Acc:MGI:2384583]	14.5249	15.8374	16.3711	14.8471	17.0532	14.4597	14.631	14.099	17.526	13.2672	15.8392	14.0242	13.6492	14.9079	0.14313801	0.585944911	12	12	24	0.391123595	0.799546792	24	10.48119416	0.058281396
ENSMUSG00000038538	Ubn2	ubinuclein 2 [Source:MGI Symbol;Acc:MGI:2444236]	6.17359	7.61975	7.19149	7.39234	7.45292	6.15331	6.94886	6.39463	8.31979	5.65506	7.20668	6.82624	6.74772	7.3654	0.423605651	0.966018836	12	12	24	0.463724879	0.805393068	24	8.342815414	0.065344036
ENSMUSG00000038539	Atf5	activating transcription factor 5 [Source:MGI Symbol;Acc:MGI:2141857]	32.7057	24.9577	23.2145	22.8707	27.5947	22.9192	26.1425	28.1485	25.1986	26.1209	27.5742	28.0067	32.6338	27.9102	0.047000349	0.375011045	4	12	24	0.128846891	0.740162193	24	23.31878197	0.113569593
ENSMUSG00000038540	Tmc3	transmembrane channel-like gene family 3 [Source:MGI Symbol;Acc:MGI:2669033]	1.33454	1.32699	0.939268	1.10067	1.49599	1.74002	1.24104	1.29442	0.933135	1.22698	1.01608	1.59184	1.55051	1.7195	0.010468194	0.196621011	24	12	24	0.037657901	0.611100708	24	21.98451205	0.150378026
ENSMUSG00000038541	Srd5a2	steroid 5 alpha-reductase 2 [Source:MGI Symbol;Acc:MGI:2150380]	1.18205	2.45943	2.45267	3.63986	1.67332	3.16504	2.11574	1.0858	2.12501	3.3876	1.17545	3.18043	1.49814	1.69359	0.17953325	0.655480352	16	12	24	0.366206328	0.790132267	24	13.30017949	0.215054821
ENSMUSG00000038542	Pcid2	PCI domain containing 2 [Source:MGI Symbol;Acc:MGI:2443003]	10.5949	8.90364	9.18145	8.83858	7.7695	8.58598	8.64835	9.28876	7.52514	10.2848	7.46267	8.43259	8.20146	7.78219	0.644063931	0.966018836	8	12	24	0.795923958	0.934803902	24	2.731168723	0.043095283
ENSMUSG00000038543	BC028528	cDNA sequence BC028528 [Source:MGI Symbol;Acc:MGI:2385885]	6.94026	6.89959	8.31729	7.35796	8.23607	6.14906	6.16704	9.07186	8.5224	5.40259	7.71236	6.83314	6.50933	6.76583	0.245061943	0.741580974	12	12	24	0.469303323	0.805393068	24	8.564938877	0.09140577
ENSMUSG00000038544	Inip	INTS3 and NABP interacting protein [Source:MGI Symbol;Acc:MGI:1913459]	12.6471	11.5046	11.7794	11.56	10.8327	11.8276	11.5208	11.0922	10.6866	12.6265	12.0588	11.8735	11.9924	11.9935	0.683024237	0.966018836	4	8	24	0.694557246	0.898526856	24	20.61873387	0.023451646
ENSMUSG00000038545	Cul7	cullin 7 [Source:MGI Symbol;Acc:MGI:1913765]	10.8725	9.37372	9.99047	9.21686	9.47228	10.0088	10.5109	9.32378	8.95278	10.9733	9.0019	10.158	9.98876	10.991	0.612141595	0.966018836	4	8	24	0.445373727	0.805393068	24	0.023304021	0.046853844
ENSMUSG00000038546	Ranbp9	RAN binding protein 9 [Source:MGI Symbol;Acc:MGI:1928741]	24.9359	23.4028	23.3133	29.0718	25.2506	24.688	24.6257	22.4911	23.5693	24.8676	24.7854	26.3294	22.8409	23.0928	0.007505042	0.192760771	16	16	24	0.022891475	0.581965446	24	15.83429862	0.086070793
ENSMUSG00000038550	Ciart	circadian associated repressor of transcription [Source:MGI Symbol;Acc:MGI:2684975]	1.54309	2.99267	5.04665	5.55167	1.97612	1.54331	1.55908	2.67341	7.80686	8.84581	1.20774	1.41817	0.927464	2.72325	0.001051376	0.07619846	16	8	24	1.21E-04	0.122523627	24	9.524178374	0.946444387
ENSMUSG00000038552	Fndc4	fibronectin type III domain containing 4 [Source:MGI Symbol;Acc:MGI:1917195]	14.9339	17.7773	22.9739	15.5915	14.0713	17.4322	18.7733	17.275	17.1782	14.1297	14.5774	15.1396	15.9377	15.6145	0.057707116	0.403601759	12	8	24	0.145721618	0.754301054	24	4.899964038	0.126270898
ENSMUSG00000038555	Reep2	receptor accessory protein 2 [Source:MGI Symbol;Acc:MGI:2385070]	42.1107	33.3694	33.9412	34.9335	34.8027	40.4609	34.4356	38.35	27.8346	47.0463	33.235	38.5126	35.7198	38.0087	0.390686537	0.915479136	24	12	24	0.595180066	0.857704078	24	20.01505086	0.074862636
ENSMUSG00000038563	Efl1	elongation factor like GTPase 1 [Source:MGI Symbol;Acc:MGI:2141969]	4.28394	4.07478	4.38337	3.16858	3.91533	4.3656	4.42481	4.58326	4.00156	4.75451	4.52672	4.24896	4.16013	4.32637	0.710703678	0.966018836	8	4	24	0.606275668	0.863126574	24	0.658935627	0.042078906
ENSMUSG00000038564	Ift172	intraflagellar transport 172 [Source:MGI Symbol;Acc:MGI:2682064]	24.2803	21	22.0346	20.6369	21.3882	24.1738	23.3885	23.8683	19.307	20.6337	24.0232	24.1754	24.2422	23.4251	0.003102478	0.1355892	4	12	24	0.003192653	0.414576087	24	22.12322231	0.116153745
ENSMUSG00000038569	Rad9b	RAD9 checkpoint clamp component B [Source:MGI Symbol;Acc:MGI:2385231]	2.55546	2.72765	3.13955	3.36005	2.7031	1.98169	3.24382	2.24068	3.03438	2.59515	2.96185	3.32368	2.43437	3.1626	0.873350015	0.966018836	12	20	24	0.472603125	0.805393068	24	10.01602338	0.080716321
ENSMUSG00000038570	Saxo2	stabilizer of axonemal microtubules 2 [Source:MGI Symbol;Acc:MGI:1914618]	4.51771	4.82715	3.90002	4.9994	4.41351	4.4605	5.32748	4.2317	5.48805	3.8206	5.32145	4.89767	3.34046	4.84134	0.706093972	0.966018836	24	4	24	0.944991805	0.986644414	24	14.95085155	0.022550302
ENSMUSG00000038576	Susd4	sushi domain containing 4 [Source:MGI Symbol;Acc:MGI:2138351]	24.6539	18.8654	21.2865	19.5866	17.5058	23.2951	22.5249	22.8914	16.3439	29.2178	17.4527	21.7477	21.1615	22.9059	0.612141595	0.966018836	4	16	24	0.696209242	0.89922881	24	0.682927461	0.073475239
ENSMUSG00000038578	Susd1	sushi domain containing 1 [Source:MGI Symbol;Acc:MGI:3651543]	1.54563	1.62134	2.10351	2.18743	1.09707	1.68625	2.3195	1.65393	1.26012	1.6233	1.83428	2.03617	1.49494	2.24587	0.785001719	0.966018836	8	12	24	0.849362006	0.954760051	24	3.506578174	0.045081317
ENSMUSG00000038582	Pptc7	PTC7 protein phosphatase homolog [Source:MGI Symbol;Acc:MGI:2444593]	14.2112	13.0021	13.9833	15.2184	12.1419	14.0968	14.0711	14.4823	13.1613	15.9289	12.2061	13.5448	13.8086	13.6989	0.676344318	0.966018836	16	4	24	0.695230349	0.898727918	24	7.320086841	0.035667546
ENSMUSG00000038583	Pln	phospholamban [Source:MGI Symbol;Acc:MGI:97622]	2.45379	2.01009	3.10089	2.53415	1.57596	2.26968	2.60605	3.18842	2.19821	2.8342	3.28093	1.86956	2.75525	2.50856	0.873567285	0.966018836	16	8	24	0.499440298	0.808768108	24	7.122852124	0.101295456
ENSMUSG00000038587	Akap12	A kinase (PRKA) anchor protein (gravin) 12 [Source:MGI Symbol;Acc:MGI:1932576]	177.847	133.899	144.01	142.207	142.216	169.363	145.569	155.088	105.749	190.44	135.668	153.794	149.207	147.944	0.432054049	0.966018836	24	12	24	0.416617294	0.801290071	24	20.20184603	0.108461737
ENSMUSG00000038593	Tctn1	tectonic family member 1 [Source:MGI Symbol;Acc:MGI:3603820]	5.50786	6.55914	6.05152	5.06655	6.09237	5.01502	5.34765	5.53575	5.39303	6.71579	4.85027	5.88771	5.68805	5.90266	0.723119329	0.966018836	8	16	24	0.513582898	0.816307374	24	6.967200736	0.056428109
ENSMUSG00000038594	Cep85l	centrosomal protein 85-like [Source:MGI Symbol;Acc:MGI:3642684]	6.20105	6.46419	5.862	6.60783	6.21173	6.17641	6.74003	6.10079	6.15954	6.07901	6.83606	5.82037	6.14077	5.84336	0.800065124	0.966018836	20	12	24	0.619175684	0.869347961	24	17.17269631	0.024369647
ENSMUSG00000038602	Slc35f1	solute carrier family 35, member F1 [Source:MGI Symbol;Acc:MGI:2139810]	14.2207	13.7135	13.102	13.159	13.8218	13.9551	14.2347	13.2868	12.5783	14.6705	13.3774	14.0375	12.8694	13.9022	0.148073804	0.590732968	24	12	24	0.337006196	0.771960538	24	19.92423995	0.034983552
ENSMUSG00000038604	Ripor1	RHO family interacting cell polarization regulator 1 [Source:MGI Symbol;Acc:MGI:1922937]	8.80404	8.10381	8.01196	7.97954	8.92858	8.51574	8.96442	8.74072	8.21529	11.0713	8.63801	9.01014	8.7943	8.67286	0.23734581	0.739722813	24	12	24	0.64057218	0.878323188	24	16.29467754	0.036931347
ENSMUSG00000038605	Samd10	sterile alpha motif domain containing 10 [Source:MGI Symbol;Acc:MGI:2443872]	13.6296	11.0991	11.9998	12.3207	11.2123	13.9079	11.5372	11.9665	10.5092	13.9935	11.2399	13.3778	12.4756	13.1282	0.601647512	0.966018836	24	12	24	0.534233709	0.826328258	24	20.47987962	0.05660142
ENSMUSG00000038607	Gng10	guanine nucleotide binding protein (G protein), gamma 10 [Source:MGI Symbol;Acc:MGI:1336169]	24.0767	26.8607	24.5131	23.9488	25.6543	22.4497	22.6173	22.0198	24.5556	19.8296	24.2611	19.7202	20.7659	21.9525	0.742579234	0.966018836	12	12	24	0.665557443	0.887035358	24	9.14569707	0.034971472
ENSMUSG00000038608	Dock10	dedicator of cytokinesis 10 [Source:MGI Symbol;Acc:MGI:2146320]	15.3075	18.908	16.7719	17.2298	19.0513	15.9584	17.7884	16.105	19.972	14.5461	17.4771	17.8342	18.5471	18.7295	0.890311217	0.966018836	12	4	24	0.817054138	0.941826666	24	5.128108644	0.031600399
ENSMUSG00000038611	Phrf1	PHD and ring finger domains 1 [Source:MGI Symbol;Acc:MGI:2141847]	12.1393	11.3647	12.2064	11.9688	12.8835	13.4444	13.3436	11.0445	11.0902	12.2379	11.6804	12.1388	10.9278	12.7493	0.783267549	0.966018836	24	8	24	0.269691351	0.768040178	24	18.67653301	0.05747666
ENSMUSG00000038612	Mcl1	myeloid cell leukemia sequence 1 [Source:MGI Symbol;Acc:MGI:101769]	33.0159	31.3818	33.3446	36.1531	34.8136	32.5632	32.94	31.9884	33.2282	34.4699	34.1281	29.3626	30.0871	29.845	0.01518376	0.253245931	16	16	24	0.002130083	0.395705644	24	13.11175512	0.08104037
ENSMUSG00000038615	Nfe2l1	nuclear factor, erythroid derived 2,-like 1 [Source:MGI Symbol;Acc:MGI:99421]	131.855	105.282	108.186	115.334	111.069	126.579	113.849	124.809	94.021	141.565	108.668	123.283	124.644	122.973	0.321662653	0.83607295	24	12	24	0.48076511	0.805393068	24	20.95306837	0.073652745
ENSMUSG00000038618	Rassf7	Ras association (RalGDS/AF-6) domain family (N-terminal) member 7 [Source:MGI Symbol;Acc:MGI:1914235]	3.69414	4.03189	3.7206	4.27862	4.84343	3.65442	3.63315	4.42628	5.26688	4.40985	4.31753	5.17472	3.83916	3.9054	0.215777641	0.704989686	20	8	24	0.191555058	0.768040178	24	12.69093753	0.096997714
ENSMUSG00000038619	Ensa	endosulfine alpha [Source:MGI Symbol;Acc:MGI:1891189]	70.627	68.0965	65.0771	66.4454	67.1736	69.8457	66.5019	68.6837	66.1748	65.2011	68.5276	70.0067	68.7891	69.8606	0.009067305	0.192760771	24	12	24	0.010912066	0.51149922	24	22.42328316	0.039347783
ENSMUSG00000038622	Med30	mediator complex subunit 30 [Source:MGI Symbol;Acc:MGI:1917040]	17.6534	20.4091	21.9117	19.715	20.9375	18.6859	18.2666	18.9723	21.4797	16.5148	22.4585	20.1923	20.9157	19.938	0.522551072	0.966018836	12	16	24	0.775713323	0.927819125	24	10.82151358	0.034510545
ENSMUSG00000038623	Tm6sf1	transmembrane 6 superfamily member 1 [Source:MGI Symbol;Acc:MGI:1933209]	18.7765	19.6984	19.7217	19.3156	20.633	20.0368	18.8227	22.2447	17.6705	17.4126	19.4479	18.709	19.5379	18.9066	0.880631923	0.966018836	8	8	24	0.702942688	0.901733679	24	23.21953613	0.027794389
ENSMUSG00000038628	Polr3k	polymerase (RNA) III (DNA directed) polypeptide K [Source:MGI Symbol;Acc:MGI:1914255]	16.5302	18.4427	18.2676	17.0248	17.0119	16.3304	16.0721	16.6057	18.2005	14.5463	18.6543	16.2935	17.1848	16.7511	0.245061943	0.741580974	12	12	24	0.723398721	0.909188168	24	7.279850521	0.029964336
ENSMUSG00000038630	Zkscan16	zinc finger with KRAB and SCAN domains 16 [Source:MGI Symbol;Acc:MGI:3510405]	0.737171	2.09862	2.81643	1.94434	1.54116	1.59014	1.91289	1.40663	3.34804	1.26176	1.3512	2.40577	1.65763	2.08088	0.251986462	0.75129457	12	8	24	0.242446791	0.768040178	24	8.023317643	0.22229435
ENSMUSG00000038633	Degs1	delta(4)-desaturase, sphingolipid 1 [Source:MGI Symbol;Acc:MGI:1097711]	162.734	143.19	137.578	138.624	139.426	155.808	150.909	144.236	118.613	148.036	142.276	140.497	146.873	138.005	0.002004172	0.100255953	4	8	24	0.023398577	0.581965446	24	21.41710554	0.099165411
ENSMUSG00000038637	Lrrc56	leucine rich repeat containing 56 [Source:MGI Symbol;Acc:MGI:1917802]	2.08459	2.26002	2.42047	2.11117	2.0974	2.15266	2.35633	2.41747	2.17468	2.56852	2.19544	2.44869	2.37594	2.06122	0.801280095	0.966018836	16	4	24	0.898102151	0.970959341	24	7.072962666	0.013619197
ENSMUSG00000038642	Ctss	cathepsin S [Source:MGI Symbol;Acc:MGI:107341]	15.5075	21.3264	22.4822	14.6214	19.2355	14.4943	18.5725	18.0309	24.5525	10.4413	17.1075	16.6198	13.942	15.7605	0.17847698	0.655480352	12	12	24	0.447189064	0.805393068	24	5.671471281	0.145235798
ENSMUSG00000038644	Pold1	polymerase (DNA directed), delta 1, catalytic subunit [Source:MGI Symbol;Acc:MGI:97741]	3.74842	3.38255	3.05067	3.02225	3.50768	4.02949	4.71829	4.6223	4.33963	3.00042	4.35927	3.65453	3.64279	2.90995	0.435739761	0.966018836	4	12	24	0.349120572	0.779170188	24	22.28116669	0.108832374
ENSMUSG00000038646	Ramac	RNA guanine-7 methyltransferase activating subunit [Source:MGI Symbol;Acc:MGI:1914398]	34.6069	34.0063	29.8562	31.5422	26.8513	29.0901	32.917	26.6952	26.1554	25.2555	31.2697	22.3125	25.8864	27.0184	0.91241026	0.966018836	4	20	24	0.639492581	0.877957092	24	1.093418416	0.047659261
ENSMUSG00000038648	Creb3l2	cAMP responsive element binding protein 3-like 2 [Source:MGI Symbol;Acc:MGI:2442695]	5.49723	5.15826	5.09022	5.55684	6.41083	5.34576	6.32614	5.46013	5.20799	5.95799	5.93199	5.27334	5.37321	5.21278	0.039590247	0.338535904	20	16	24	0.144206395	0.752803875	24	16.90066456	0.068998197
ENSMUSG00000038650	Rnh1	ribonuclease/angiogenin inhibitor 1 [Source:MGI Symbol;Acc:MGI:1195456]	45.5344	41.9591	44.3844	42.5894	46.0169	47.695	41.4258	48.4439	41.5163	43.2863	45.8198	42.6819	42.3619	42.5646	0.566932234	0.966018836	24	4	24	0.514326858	0.816624526	24	18.37050443	0.033677464
ENSMUSG00000038658	Ric1	RAB6A GEF complex partner 1 [Source:MGI Symbol;Acc:MGI:1924893]	7.81931	8.23726	8.95061	8.81641	8.75591	7.88135	8.93747	8.08485	8.70415	7.74958	8.60833	8.32055	7.01737	8.08133	0.450844752	0.966018836	12	16	24	0.271933697	0.768040178	24	11.8683671	0.05167277
ENSMUSG00000038663	Fsd2	fibronectin type III and SPRY domain containing 2 [Source:MGI Symbol;Acc:MGI:2444310]	0.828038	2.05562	2.06188	3.06018	1.75575	2.50962	1.75948	5.48397	3.60887	4.02579	1.66727	2.29733	2.72614	1.98024	0.356953959	0.881188925	16	4	24	0.156223162	0.761842768	24	8.90951605	0.322832746
ENSMUSG00000038664	Herc1	HECT and RLD domain containing E3 ubiquitin protein ligase family member 1 [Source:MGI Symbol;Acc:MGI:2384589]	20.7157	24.2371	21.9513	23.7875	21.5378	22.3358	20.2798	20.6424	22.8629	22.7649	21.7875	23.4534	23.9453	24.1569	0.934152439	0.966018836	8	12	24	0.618225886	0.868896567	24	8.962456691	0.032290626
ENSMUSG00000038665	Dgki	diacylglycerol kinase, iota [Source:MGI Symbol;Acc:MGI:2443430]	11.7087	12.4543	11.2209	11.4549	12.4408	11.9329	10.8943	10.4024	13.1974	8.42054	14.63	17.3955	16.6461	15.2092	0.745118544	0.966018836	24	16	24	0.32806826	0.768040178	24	21.99013995	0.133738508
ENSMUSG00000038668	Lpar1	lysophosphatidic acid receptor 1 [Source:MGI Symbol;Acc:MGI:108429]	31.921	30.5963	30.5872	25.0263	29.8183	29.0294	30.5111	30.072	26.9452	29.0623	29.117	25.8769	27.1437	28.2154	0.664568268	0.966018836	4	20	24	0.374794598	0.794819447	24	1.10403847	0.046190704
ENSMUSG00000038670	Mybpc2	myosin binding protein C, fast-type [Source:MGI Symbol;Acc:MGI:1336170]	6.24224	17.1312	17.5617	28.2875	13.9855	25.0279	16.3333	56.7836	38.1264	53.6938	16.1805	23.6601	24.2874	17.2206	0.351315142	0.870418653	16	4	24	0.15396762	0.760223561	24	9.604502305	0.538959484
ENSMUSG00000038671	Arfrp1	ADP-ribosylation factor related protein 1 [Source:MGI Symbol;Acc:MGI:1923938]	20.2911	20.7021	21.4052	21.5965	23.1852	20.6548	19.9103	21.6137	19.7063	21.5139	21.0129	21.4612	21.2608	21.5812	0.522551072	0.966018836	16	12	24	0.324282788	0.768040178	24	14.22268019	0.032257291
ENSMUSG00000038679	Trps1	transcriptional repressor GATA binding 1 [Source:MGI Symbol;Acc:MGI:1927616]	0.942391	1.1744	1.3979	1.26384	1.10077	1.0901	1.22821	1.04966	1.34496	0.937966	1.08441	1.09451	0.981821	1.11452	0.300823756	0.809362476	12	16	24	0.221882117	0.768040178	24	8.743685989	0.062425482
ENSMUSG00000038683	Pak1ip1	PAK1 interacting protein 1 [Source:MGI Symbol;Acc:MGI:1915333]	60.4303	54.4713	57.6897	50.3224	57.7416	55.2702	51.2232	51.6449	49.0464	48.725	60.2314	55.2842	56.3331	56.4813	0.369262716	0.893596186	20	20	24	0.231235663	0.768040178	24	20.8415543	0.068815224
ENSMUSG00000038685	Rtel1	regulator of telomere elongation helicase 1 [Source:MGI Symbol;Acc:MGI:2139369]	13.5452	16.4153	13.5072	13.5052	14.8442	13.8187	15.0161	15.4584	18.6029	13.666	12.6974	14.3467	13.3389	15.5944	0.157744865	0.614233844	8	12	24	0.128516139	0.740162193	24	5.665994548	0.106281522
ENSMUSG00000038690	Atp5j2	ATP synthase, H+ transporting, mitochondrial F0 complex, subunit F2 [Source:MGI Symbol;Acc:MGI:1927558]	164.906	196.129	182.205	197.765	182.319	171.832	175.185	183.193	219.447	156.315	216.581	172.639	207.923	189.971	0.838532766	0.966018836	12	12	24	0.584864415	0.852600138	24	8.322785077	0.060263161
ENSMUSG00000038692	Hoxb4	homeobox B4 [Source:MGI Symbol;Acc:MGI:96185]	1.99342	2.1386	1.90064	1.9766	1.86091	1.92903	2.4168	2.19167	2.52666	2.01277	2.46938	2.17493	2.55918	2.40153	0.664568268	0.966018836	4	16	24	0.167397075	0.767020009	24	3.180978535	0.058479138
ENSMUSG00000038695	Josd2	Josephin domain containing 2 [Source:MGI Symbol;Acc:MGI:1913374]	51.7343	43.592	42.9215	41.6144	49.1535	44.0521	55.8885	48.6628	49.8527	45.333	50.3335	47.7646	50.6759	44.9863	0.03484427	0.328311403	4	12	24	0.183465042	0.768040178	24	22.36509692	0.082609698
ENSMUSG00000038696	Mapkap1	mitogen-activated protein kinase associated protein 1 [Source:MGI Symbol;Acc:MGI:2444554]	28.027	23.0838	20.3253	23.0659	20.1388	22.7933	24.9934	24.4909	22.9919	24.4756	24.0575	26.8385	25.777	25.2071	0.027960311	0.313364468	4	16	24	0.077338632	0.687324958	24	23.67190579	0.099708961
ENSMUSG00000038697	Taf5l	TATA-box binding protein associated factor 5 like [Source:MGI Symbol;Acc:MGI:1919039]	5.06607	4.8228	4.89237	4.86838	4.70696	5.05126	4.82573	4.75491	4.58852	5.58267	4.97641	4.76334	5.49784	5.32717	0.920872048	0.966018836	4	8	24	0.97122263	0.993012791	24	0.757224355	0.006322904
ENSMUSG00000038700	Hoxb5	homeobox B5 [Source:MGI Symbol;Acc:MGI:96186]	5.02785	4.9819	4.75135	3.58429	4.22253	4.30275	5.58612	4.9815	4.84932	6.01492	4.61173	6.15868	5.98529	5.99135	0.283573096	0.793714531	4	16	24	0.083301863	0.699490063	24	1.854158427	0.121183934
ENSMUSG00000038702	Dsel	dermatan sulfate epimerase-like [Source:MGI Symbol;Acc:MGI:2442948]	6.67899	7.67098	7.45936	6.97209	8.60529	6.77635	7.01793	6.97357	7.97344	7.04154	9.14894	7.69433	7.93765	7.63673	0.885025617	0.966018836	20	8	24	0.423455855	0.805346055	24	14.19415242	0.055188169
ENSMUSG00000038704	Aspdh	aspartate dehydrogenase domain containing [Source:MGI Symbol;Acc:MGI:1915602]	0.232739	0.526038	0.820221	0.381566	0.715713	0.571886	0.532499	0.615537	0.504785	0.748262	0.248947	0.514074	0.353092	0.681993	0.664568268	0.966018836	12	16	24	0.352826303	0.780629136	24	8.800930184	0.101374337
ENSMUSG00000038705	Gmeb2	glucocorticoid modulatory element binding protein 2 [Source:MGI Symbol;Acc:MGI:2652836]	5.26624	5.06029	5.58538	4.88086	5.10451	4.40571	4.87326	5.02804	5.6184	5.21407	4.69757	4.91697	4.86867	5.90432	0.020119233	0.259417302	12	12	24	0.013562934	0.529134128	24	6.999942172	0.095838143
ENSMUSG00000038708	Golga4	golgi autoantigen, golgin subfamily a, 4 [Source:MGI Symbol;Acc:MGI:1859646]	27.5206	29.3069	30.0246	28.4267	29.0559	28.9246	28.6351	30.4869	28.6568	31.4736	27.7946	29.2703	29.6671	30.6635	0.521072381	0.966018836	8	12	24	0.16511083	0.767020009	24	7.386598672	0.03606511
ENSMUSG00000038712	Mindy1	MINDY lysine 48 deubiquitinase 1 [Source:MGI Symbol;Acc:MGI:1922257]	32.5843	30.3104	31.5503	31.007	31.0106	34.4147	28.5233	32.5431	27.6267	34.1903	29.3101	32.1885	30.5872	30.3214	0.9678483	0.970142211	24	12	24	0.607052385	0.863710114	24	18.31862102	0.035772423
ENSMUSG00000038717	Atp5l	ATP synthase, H+ transporting, mitochondrial F0 complex, subunit G [Source:MGI Symbol;Acc:MGI:1351597]	3.7517	5.91634	3.90004	4.8228	5.08136	3.14785	3.00454	2.64006	4.94355	4.30404	6.36805	3.49137	6.44115	6.75099	0.896753532	0.966018836	20	4	24	0.627116995	0.872795898	24	10.04092992	0.134571271
ENSMUSG00000038718	Pbx3	pre B cell leukemia homeobox 3 [Source:MGI Symbol;Acc:MGI:97496]	6.67986	6.50393	6.43195	5.61885	5.461	6.42914	6.94495	5.99054	5.95797	6.49766	5.92499	7.04348	6.62913	6.67631	0.00384759	0.140598674	4	16	24	0.037260971	0.609176161	24	0.492275636	0.084075461
ENSMUSG00000038721	Hoxb7	homeobox B7 [Source:MGI Symbol;Acc:MGI:96188]	7.08867	7.19931	8.32641	5.94514	5.80693	5.78061	7.85996	7.08293	8.17484	6.77585	7.28151	7.56481	7.31397	7.96861	0.181442559	0.659020016	12	4	24	0.039584031	0.615919442	24	4.849430359	0.132947324
ENSMUSG00000038722	Bud31	BUD31 homolog [Source:MGI Symbol;Acc:MGI:2141291]	70.1403	76.2919	70.9541	71.6938	76.1694	69.1916	70.2858	67.7092	77.4163	54.2219	80.7382	68.6942	76.5686	71.9057	0.880631923	0.966018836	20	4	24	0.841809699	0.952050674	24	23.21518994	0.032335529
ENSMUSG00000038729	Pakap	paralemmin A kinase anchor protein [Source:MGI Symbol;Acc:MGI:5141924]	14.7496	12.8944	13.1154	12.4474	13.1637	13.3518	14.5086	13.2002	11.4247	17.3066	12.2487	14.0115	14.1908	13.6786	0.03452758	0.328311403	4	8	24	0.737297759	0.91440753	24	21.51178673	0.042924869
ENSMUSG00000038732	Mboat1	membrane bound O-acyltransferase domain containing 1 [Source:MGI Symbol;Acc:MGI:2387184]	16.1995	17.7945	15.9947	15.2754	20.2769	15.3168	19.5824	17.264	19.5099	19.0573	15.6788	17.2617	19.1027	16.0429	0.897425765	0.966018836	4	20	24	0.970527019	0.993003194	24	4.543656904	0.013568665
ENSMUSG00000038733	Wdr26	WD repeat domain 26 [Source:MGI Symbol;Acc:MGI:1923825]	24.5534	28.0091	27.1209	27.8258	26.9071	26.0241	24.7051	25.7319	26.9204	26.695	25.4301	25.5012	26.2775	27.4435	0.047000349	0.375011045	8	20	24	0.035044032	0.608551791	24	9.323979974	0.054632842
ENSMUSG00000038736	Nudcd1	NudC domain containing 1 [Source:MGI Symbol;Acc:MGI:1914679]	6.94498	7.43975	6.91357	7.31984	7.18626	6.5112	6.1932	6.93168	7.39261	6.32808	7.59063	7.00938	7.53264	7.43767	0.8635847	0.966018836	8	16	24	0.756354155	0.921325655	24	8.162837147	0.025425885
ENSMUSG00000038738	Shank1	SH3 and multiple ankyrin repeat domains 1 [Source:MGI Symbol;Acc:MGI:3613677]	2.21948	3.08418	3.73579	2.7888	2.86787	2.97701	2.80205	2.77681	3.82689	3.0241	2.89425	2.84179	2.69277	4.11208	0.020294373	0.261094911	12	16	24	0.026558545	0.586541342	24	8.158035798	0.163353817
ENSMUSG00000038740	Mvb12b	multivesicular body subunit 12B [Source:MGI Symbol;Acc:MGI:1919793]	20.0344	18.3057	18.3397	17.8609	23.3794	19.8304	22.2203	20.965	19.3221	25.1271	18.0472	20.5623	20.3648	18.3841	0.648583058	0.966018836	4	8	24	0.639009856	0.877675589	24	17.82208992	0.054626272
ENSMUSG00000038742	Angptl6	angiopoietin-like 6 [Source:MGI Symbol;Acc:MGI:1917976]	4.28476	3.13967	2.57573	3.68985	3.62258	3.62161	4.24704	3.66043	3.1054	3.28839	3.78619	2.82029	3.70923	2.94324	0.037085178	0.334054683	4	8	24	0.170931187	0.768040178	24	20.68967009	0.120176864
ENSMUSG00000038754	Elovl3	elongation of very long chain fatty acids (FEN1/Elo2, SUR4/Elo3, yeast)-like 3 [Source:MGI Symbol;Acc:MGI:1195976]	0.138605	0.0938887	0.878354	4.64408	0.674971	0.450632	0.575295	0.945326	0.70213	0.335208	3.26762	1.04773	1.41168	1.41895	0.858514388	0.966018836	20	8	24	0.264578721	0.768040178	24	13.14341423	0.390101976
ENSMUSG00000038756	Ttll6	tubulin tyrosine ligase-like family, member 6 [Source:MGI Symbol;Acc:MGI:2683461]	2.5539	3.5094	2.81238	3.13994	3.25055	2.9299	3.63838	2.68726	4.05961	2.89419	2.96033	3.04419	3.14621	3.10354	0.755349378	0.966018836	12	12	24	0.761004518	0.92277295	24	7.703736362	0.041494069
ENSMUSG00000038759	Nup205	nucleoporin 205 [Source:MGI Symbol;Acc:MGI:2141625]	5.99305	6.24684	6.07664	6.12644	6.35441	6.01478	6.24724	6.05158	6.13668	5.37359	6.66288	5.98247	6.72553	6.33879	0.933543173	0.966018836	4	12	24	0.585114561	0.852625163	24	23.01267252	0.027196361
ENSMUSG00000038762	Abcf1	ATP-binding cassette, sub-family F (GCN20), member 1 [Source:MGI Symbol;Acc:MGI:1351658]	22.1452	18.766	20.2628	20.9112	21.1345	20.2767	20.0198	22.6911	19.2765	24.1931	19.2979	20.9558	21.1646	19.8896	0.904831057	0.966018836	4	4	24	0.885908164	0.967665755	24	15.02119943	0.01875765
ENSMUSG00000038763	Alpk3	alpha-kinase 3 [Source:MGI Symbol;Acc:MGI:2151224]	0.422865	0.613345	0.984962	1.47272	0.898915	1.08294	0.880698	1.96104	1.08271	1.67865	0.652269	0.476504	1.25067	0.738082	0.496745696	0.966018836	16	12	24	0.239030762	0.768040178	24	9.538770052	0.208849701
ENSMUSG00000038764	Ptpn3	protein tyrosine phosphatase, non-receptor type 3 [Source:MGI Symbol;Acc:MGI:105307]	18.5843	14.3777	15.5855	15.9454	14.4946	17.0554	13.7148	15.9911	12.849	20.1584	15.4237	16.6093	16.9149	15.2225	0.745475226	0.966018836	24	12	24	0.645170421	0.879668078	24	18.17026101	0.061723999
ENSMUSG00000038766	Gabpb2	GA repeat binding protein, beta 2 [Source:MGI Symbol;Acc:MGI:95612]	5.60363	6.50706	6.10916	6.6562	6.39065	5.65435	5.74927	6.2045	7.21799	5.1199	6.1058	5.93367	6.0541	6.03889	0.119434414	0.542554768	12	12	24	0.371540634	0.793257896	24	8.332204134	0.058917878
ENSMUSG00000038773	Kdm3b	KDM3B lysine (K)-specific demethylase 3B [Source:MGI Symbol;Acc:MGI:1923356]	7.82397	7.69192	7.92893	7.58962	7.77236	7.98219	8.45463	8.18975	8.37413	8.02554	7.46479	8.06992	7.68648	7.72201	0.928127947	0.966018836	24	20	24	0.609633366	0.864491738	24	3.430442659	0.018423948
ENSMUSG00000038774	Ascc3	activating signal cointegrator 1 complex subunit 3 [Source:MGI Symbol;Acc:MGI:1925237]	5.87445	6.52803	6.08239	6.68007	5.65397	6.34235	6.44753	6.06699	5.59697	4.69973	6.4753	6.51229	6.66318	4.20873	0.935316621	0.966018836	24	12	24	0.277028837	0.768040178	24	20.22025081	0.101091393
ENSMUSG00000038775	Vill	villin-like [Source:MGI Symbol;Acc:MGI:1201781]	2.36884	2.55973	2.17391	1.88781	2.48179	2.27924	1.44331	2.54334	3.11737	1.46141	2.37021	2.34079	2.16797	2.06668	0.921572192	0.966018836	8	20	24	0.907767209	0.974606327	24	4.614222584	0.036176656
ENSMUSG00000038776	Ephx1	epoxide hydrolase 1, microsomal [Source:MGI Symbol;Acc:MGI:95405]	54.2727	48.11	47.6547	50.2341	49.2911	53.7165	50.4958	59.5986	46.3552	57.479	48.2553	50.7565	51.8881	49.2964	0.195343269	0.673387182	4	8	24	0.764222122	0.92403577	24	22.5380142	0.030944035
ENSMUSG00000038777	Sema6c	sema domain, transmembrane domain (TM), and cytoplasmic domain, (semaphorin) 6C [Source:MGI Symbol;Acc:MGI:1338032]	23.6731	37.7446	31.47	38.9137	36.8034	31.7469	31.7939	36.1471	45.2274	42.277	31.3209	36.0419	34.4903	31.7896	0.285193697	0.793714531	16	12	24	0.050568532	0.644469829	24	10.49249218	0.191691063
ENSMUSG00000038780	Smurf1	SMAD specific E3 ubiquitin protein ligase 1 [Source:MGI Symbol;Acc:MGI:1923038]	8.65683	8.61591	8.99084	9.91626	9.13984	9.01823	9.80402	10.3525	9.59227	11.3965	8.69717	10.7838	9.29642	9.5756	0.934818505	0.966018836	16	12	24	0.575269403	0.848005542	24	11.02560544	0.041849564
ENSMUSG00000038781	Stap2	signal transducing adaptor family member 2 [Source:MGI Symbol;Acc:MGI:2147039]	1.82633	1.83655	1.78617	1.77963	1.35939	1.61441	1.66164	2.26935	2.06943	1.63074	1.77466	1.37211	1.71576	1.41637	0.043240321	0.357642487	12	12	24	0.13817203	0.747972781	24	6.165350833	0.097533175
ENSMUSG00000038784	Cnot4	CCR4-NOT transcription complex, subunit 4 [Source:MGI Symbol;Acc:MGI:1859026]	11.6737	9.95572	10.3956	10.5956	10.1652	10.7703	11.1203	9.82983	9.49283	10.7392	11.3356	10.7364	10.681	10.0822	0.031295391	0.324385159	4	4	24	0.042803131	0.624864512	24	19.49038952	0.06993186
ENSMUSG00000038793	Lefty1	left right determination factor 1 [Source:MGI Symbol;Acc:MGI:107405]	0.544129	0.983445	1.28967	0.783142	0.633827	0.651315	0.664763	1.12346	1.944	0.949052	1.35094	0.760948	1.20554	0.988844	0.077301512	0.453790923	12	12	24	0.023768107	0.584955728	24	8.21427857	0.237683144
ENSMUSG00000038797	Zscan2	zinc finger and SCAN domain containing 2 [Source:MGI Symbol;Acc:MGI:99176]	2.50012	1.57923	2.24033	1.89802	1.66043	1.97124	1.85008	1.99902	1.76128	1.90682	1.47435	1.84942	1.70647	1.85706	0.921741413	0.966018836	12	8	24	0.587001921	0.853586751	24	2.524637906	0.04689687
ENSMUSG00000038803	Ost4	oligosaccharyltransferase complex subunit 4 (non-catalytic) [Source:MGI Symbol;Acc:MGI:1914945]	37.893	40.3331	37.8195	40.9235	43.166	38.6885	41.1696	39.1238	44.992	30.8442	43.7264	38.5817	42.7142	35.8753	0.923635729	0.966018836	20	12	24	0.830839951	0.94763783	24	20.215123	0.03463757
ENSMUSG00000038806	Sde2	SDE2 telomere maintenance homolog (S. pombe) [Source:MGI Symbol;Acc:MGI:2384788]	7.24096	6.30654	6.9908	6.64635	7.18836	6.88683	6.92898	7.14124	6.68949	6.7344	6.43431	7.07005	7.02063	7.08136	0.632729413	0.966018836	12	12	24	0.38941739	0.799546792	24	23.08119148	0.028512535
ENSMUSG00000038807	Rap1gap2	RAP1 GTPase activating protein 2 [Source:MGI Symbol;Acc:MGI:3028623]	31.0469	21.855	23.28	23.2204	23.1615	27.4546	24.2663	25.9192	18.5324	31.3335	23.7357	26.7548	26.2888	25.1156	0.123398436	0.552144108	24	12	24	0.304094959	0.768040178	24	20.67361305	0.117610532
ENSMUSG00000038811	Gngt2	guanine nucleotide binding protein (G protein), gamma transducing activity polypeptide 2 [Source:MGI Symbol;Acc:MGI:893584]	2.8889	3.61224	4.43103	4.51991	4.19062	4.18232	2.58685	4.01843	5.25438	3.19839	5.46071	3.94111	5.19131	4.29557	0.371875382	0.894537464	12	16	24	0.329636469	0.768040178	24	11.55930944	0.134590746
ENSMUSG00000038812	Trmt112	tRNA methyltransferase 11-2 [Source:MGI Symbol;Acc:MGI:1914924]	2.74483	1.97814	2.13274	2.63687	1.62466	1.88053	2.15584	2.90711	2.30657	2.68333	2.32181	2.20106	2.68818	2.54168	0.887010723	0.966018836	4	20	24	0.314393166	0.768040178	24	5.564960629	0.095422706
ENSMUSG00000038816	Ctnnal1	catenin (cadherin associated protein), alpha-like 1 [Source:MGI Symbol;Acc:MGI:1859649]	69.1808	91.2891	70.3775	69.5248	95.7578	69.0306	79.0898	78.4802	98.4351	68.5486	80.7495	76.9914	86.7574	74.9251	0.916565673	0.966018836	8	12	24	0.920992582	0.979223729	24	5.386193673	0.029840821
ENSMUSG00000038822	Hace1	HECT domain and ankyrin repeat containing, E3 ubiquitin protein ligase 1 [Source:MGI Symbol;Acc:MGI:2446110]	14.2112	14.534	13.6001	13.8103	15.7288	13.4343	13.1787	13.1692	13.9372	13.0025	13.8515	13.7443	14.0325	13.2002	0.879844076	0.966018836	8	20	24	0.476931088	0.805393068	24	17.48168311	0.030219229
ENSMUSG00000038827	Abitram	actin binding transcription modulator [Source:MGI Symbol;Acc:MGI:2677850]	3.65391	3.44	3.33965	3.51052	3.84949	3.84619	3.72194	3.38374	3.26914	3.8824	3.24679	3.83883	3.39852	3.59053	0.08302755	0.462922111	24	12	24	0.138333578	0.747972781	24	18.93907053	0.056134368
ENSMUSG00000038828	Tmem214	transmembrane protein 214 [Source:MGI Symbol;Acc:MGI:1916046]	23.7184	21.3142	23.6222	21.826	26.1366	21.4563	23.1847	22.3358	21.2265	22.3525	23.3627	20.6571	22.2698	20.997	0.810607738	0.966018836	20	12	24	0.41074307	0.799546792	24	16.55238198	0.04222531
ENSMUSG00000038831	Ralgps1	Ral GEF with PH domain and SH3 binding motif 1 [Source:MGI Symbol;Acc:MGI:1922008]	6.51097	6.2931	6.38122	7.09892	6.10121	6.49565	6.93525	5.95018	6.62475	6.3762	5.84169	6.72877	6.43967	6.95579	0.934818505	0.966018836	4	16	24	0.819196359	0.94266835	24	3.465609185	0.018194618
ENSMUSG00000038836	Agbl3	ATP/GTP binding protein-like 3 [Source:MGI Symbol;Acc:MGI:1923473]	7.14196	9.57639	10.0269	8.53616	8.11302	7.99864	8.55229	8.56342	9.88616	6.52521	8.28838	8.3895	8.09064	10.1452	0.025980723	0.29944494	12	4	24	0.110328821	0.721828007	24	6.023614539	0.129404942
ENSMUSG00000038838	Vars2	valyl-tRNA synthetase 2, mitochondrial [Source:MGI Symbol;Acc:MGI:1916165]	5.45997	5.00296	6.15302	6.46618	5.65848	6.39091	5.5607	5.26459	5.39323	6.93888	5.52976	6.16607	5.86049	5.7651	0.01518376	0.253245931	16	8	24	0.077758581	0.68760079	24	14.54081923	0.018856342
ENSMUSG00000038843	Gcnt1	glucosaminyl (N-acetyl) transferase 1, core 2 [Source:MGI Symbol;Acc:MGI:95676]	0.860445	0.969709	1.11325	0.989903	0.899515	0.976687	0.958145	1.01226	1.08525	0.687504	1.05227	0.920013	0.971652	0.849999	0.830364743	0.966018836	12	16	24	0.859126913	0.958530654	24	5.923479116	0.018573983

ENSMUSG00000038844	Kif16b	kinesin family member 16B [Source:MGI Symbol;Acc:MGI:1098240]	10.6288	11.3088	9.96151	10.3798	10.5071	11.2631	10.9315	11.6596	9.20636	11.7993	10.0245	9.7629	9.52535	11.136	0.9045879	0.966018836	8	4	24	0.884899698	0.967256182	24	2.635753824	0.019712785
ENSMUSG00000038845	Phb	prohibitin [Source:MGI Symbol;Acc:MGI:97572]	13.7132	12.1665	13.6128	13.3464	14.0408	15.244	13.7289	13.724	12.0526	13.999	13.9073	12.3434	14.961	12.9642	0.154305339	0.606139807	20	16	24	0.263543355	0.768040178	24	19.09305905	0.061273111
ENSMUSG00000038848	Ythdf1	YTH N6-methyladenosine RNA binding protein 1 [Source:MGI Symbol;Acc:MGI:1917431]	18.7839	15.44	14.971	16.8394	15.7292	17.302	16.5775	16.26	14.6129	19.1823	16.7428	16.404	15.9955	17.782	0.44861185	0.966018836	16	20	24	0.625580265	0.872249537	24	19.28603639	0.042808808
ENSMUSG00000038855	Itpkb	inositol 1,4,5-trisphosphate 3-kinase B [Source:MGI Symbol;Acc:MGI:109235]	31.3037	26.656	27.425	26.1708	30.6257	30	31.8346	28.8714	26.1382	34.508	26.5517	31.3675	30.2473	27.8593	0.073139747	0.447768907	24	12	24	0.243592909	0.768040178	24	20.26963837	0.080514946
ENSMUSG00000038859	Baiap2l1	BAI1-associated protein 2-like 1 [Source:MGI Symbol;Acc:MGI:1914148]	10.0981	6.40906	5.6005	6.41366	5.88842	7.92802	6.55818	7.27018	5.5737	9.16959	5.622	7.43971	7.70769	7.7523	0.11436224	0.534017934	4	8	24	0.321020693	0.768040178	24	23.02134999	0.138973751
ENSMUSG00000038860	Garnl3	GTPase activating RANGAP domain-like 3 [Source:MGI Symbol;Acc:MGI:2139309]	28.8771	26.8367	28.5146	29.8698	26.0385	28.5267	26.8556	29.0761	26.7279	31.3104	28.8713	29.6846	29.938	31.1359	0.90332508	0.966018836	16	20	24	0.940225882	0.985398871	24	9.408696481	0.010698268
ENSMUSG00000038861	Pi4kb	phosphatidylinositol 4-kinase beta [Source:MGI Symbol;Acc:MGI:1334433]	20.1506	16.5244	19.4762	16.1779	16.7739	19.6412	19.1685	18.0347	14.0745	18.8716	15.0396	16.1008	14.0883	16.4661	0.887862353	0.966018836	4	16	24	0.70243743	0.90159202	24	22.40830186	0.047980676
ENSMUSG00000038866	Zcchc2	zinc finger, CCHC domain containing 2 [Source:MGI Symbol;Acc:MGI:2444114]	4.7867	3.29482	3.98639	4.62679	4.38196	3.95573	4.74617	3.99159	3.96211	3.96864	3.97579	3.58344	3.48783	3.82241	0.919571723	0.966018836	20	12	24	0.642729413	0.878809617	24	16.58294252	0.044435722
ENSMUSG00000038871	Bpgm	2,3-bisphosphoglycerate mutase [Source:MGI Symbol;Acc:MGI:1098242]	13.287	19.7792	22.9861	20.024	18.3363	18.1841	19.6019	22.8615	23.2442	18.9242	15.5978	20.1161	18.4401	18.07	0.085329704	0.466404237	12	8	24	0.101048845	0.713198062	24	8.00119898	0.16881022
ENSMUSG00000038872	Zfhx3	zinc finger homeobox 3 [Source:MGI Symbol;Acc:MGI:99948]	3.51804	2.5815	2.35799	2.77958	2.91404	3.01628	2.98219	3.10491	2.48386	3.89112	3.18023	3.1513	3.15983	3.25691	0.43565473	0.966018836	4	8	24	0.39824139	0.799546792	24	19.67345108	0.073926878
ENSMUSG00000038876	Rnf146	ring finger protein 146 [Source:MGI Symbol;Acc:MGI:1915281]	27.098	25.0883	26.4508	26.5271	26.271	23.898	26.0141	24.7058	28.766	25.435	26.227	27.3857	28.2901	27.0972	0.854540463	0.966018836	12	20	24	0.816898657	0.9417567	24	6.625691697	0.017639202
ENSMUSG00000038879	Nipal2	NIPA-like domain containing 2 [Source:MGI Symbol;Acc:MGI:1924488]	10.8158	8.02694	6.90416	7.2064	8.23072	8.5521	9.29288	9.08711	8.09974	8.49071	8.17984	11.2362	10.1474	10.662	0.008754183	0.192760771	4	8	24	0.003404373	0.417612737	24	23.19263425	0.187717206
ENSMUSG00000038880	Mrps34	mitochondrial ribosomal protein S34 [Source:MGI Symbol;Acc:MGI:1930188]	19.3153	19.0065	17.8538	19.1395	18.9714	21.4779	17.8607	21.8362	20.2481	24.4076	20.8108	22.8142	21.4711	23.1975	0.875755063	0.966018836	24	12	24	0.930944451	0.982245205	24	13.65871168	0.0138544
ENSMUSG00000038884	Shfl	shiftless antiviral inhibitor of ribosomal frameshifting [Source:MGI Symbol;Acc:MGI:2441788]	9.09424	12.9534	16.5613	9.94221	12.4102	12.7085	14.2619	13.0813	13.6636	13.6506	11.3323	13.4236	11.8648	14.4594	0.285027487	0.793714531	12	8	24	0.324476342	0.768040178	24	6.927192572	0.118691931
ENSMUSG00000038886	Man2a2	mannosidase 2, alpha 2 [Source:MGI Symbol;Acc:MGI:2150656]	24.3003	24.4698	26.8742	24.5299	25.0309	26.1936	27.6666	28.2081	25.0991	32.0401	22.6117	26.4953	24.3241	26.5843	0.89563591	0.966018836	16	4	24	0.650392747	0.882429682	24	7.980261274	0.04532063
ENSMUSG00000038888	Ctu1	cytosolic thiouridylase subunit 1 [Source:MGI Symbol;Acc:MGI:2385277]	1.78093	1.60396	1.56555	1.71851	1.79224	1.77015	1.67468	2.00088	1.86412	2.01301	1.5967	2.15222	1.93269	1.90104	0.849180763	0.966018836	24	12	24	0.899738313	0.971534724	24	22.93486248	0.013640616
ENSMUSG00000038893	Fam117a	family with sequence similarity 117, member A [Source:MGI Symbol;Acc:MGI:2144564]	3.63232	4.42093	5.04276	4.37042	4.65312	4.627	3.99977	3.70304	4.75092	5.17124	3.97542	4.56272	4.41563	4.06728	0.057707116	0.403601759	12	16	24	0.106464944	0.718721872	24	12.14996752	0.097827397
ENSMUSG00000038894	Irs2	insulin receptor substrate 2 [Source:MGI Symbol;Acc:MGI:109334]	6.48319	4.73417	5.72332	5.36361	5.63715	5.85919	6.38369	6.22168	4.89541	7.9643	6.03396	6.25716	5.80634	5.69762	0.289105414	0.794869797	4	8	24	0.592594386	0.856632634	24	17.90851415	0.06377021
ENSMUSG00000038895	Zfp653	zinc finger protein 653 [Source:MGI Symbol;Acc:MGI:2442362]	4.21515	4.02099	4.15787	3.88587	3.79035	4.1061	3.76383	4.32569	4.85776	4.22814	4.26023	4.12597	4.48112	3.96433	0.888482515	0.966018836	12	8	24	0.496177809	0.807537659	24	6.954318044	0.036587682
ENSMUSG00000038900	Rpl12	ribosomal protein L12 [Source:MGI Symbol;Acc:MGI:98002]	4.57721	4.42243	4.88122	4.83188	5.41378	4.92101	4.34897	5.15762	6.35028	3.83253	7.53886	5.28277	5.24885	5.21626	0.664568268	0.966018836	20	20	24	0.648634616	0.881434297	24	14.7550581	0.068377143
ENSMUSG00000038902	Pogz	pogo transposable element with ZNF domain [Source:MGI Symbol;Acc:MGI:2442117]	7.53966	7.83899	8.73995	8.51404	8.45833	8.44799	8.07506	8.63371	8.9079	8.74395	7.9071	8.25802	8.06878	7.53472	0.009797283	0.194720062	12	20	24	0.077000411	0.687041208	24	11.5028574	0.058146554
ENSMUSG00000038903	Ccdc68	coiled-coil domain containing 68 [Source:MGI Symbol;Acc:MGI:3612676]	23.3683	22.3172	20.0132	20.7917	21.7277	21.4263	20.1105	19.8145	21.7266	19.8464	24.341	22.2499	25.3064	23.4906	0.496745696	0.966018836	4	12	24	0.340246514	0.774739261	24	21.89124688	0.059448722
ENSMUSG00000038909	Kat7	K(lysine) acetyltransferase 7 [Source:MGI Symbol;Acc:MGI:2182799]	34.3715	27.51	27.8664	29.4788	27.1607	33.7124	29.3621	30.2238	23.5427	33.6427	27.2222	32.295	30.2576	27.7132	0.133266423	0.567728672	24	12	24	0.152839226	0.759115906	24	20.57834096	0.111113229
ENSMUSG00000038910	Plcl2	phospholipase C-like 2 [Source:MGI Symbol;Acc:MGI:1352756]	4.18979	4.49768	4.72148	4.56526	3.87092	4.58353	3.95732	5.15819	4.50733	4.76627	4.07314	4.87631	4.69121	4.1187	0.957256673	0.966536401	16	4	24	0.582731418	0.851636365	24	6.885796333	0.042712634
ENSMUSG00000038914	Dido1	death inducer-obliterator 1 [Source:MGI Symbol;Acc:MGI:1344352]	8.65475	10.1469	10.7434	9.88038	10.7255	9.49568	10.1899	9.68206	10.5134	9.98442	10.2089	10.4525	9.99957	9.83214	0.349109869	0.870418653	12	20	24	0.230267617	0.768040178	24	11.55818014	0.046885238
ENSMUSG00000038916	Soga3	SOGA family member 3 [Source:MGI Symbol;Acc:MGI:1914662]	52.8617	35.0391	35.239	33.5194	38.8552	43.7287	39.4101	37.3021	23.8654	40.4687	37.4594	43.455	40.3719	37.6064	0.001971398	0.100255953	24	12	24	0.013723508	0.529134128	24	20.94509052	0.25413132
ENSMUSG00000038930	Rccd1	RCC1 domain containing 1 [Source:MGI Symbol;Acc:MGI:2444156]	3.4974	4.52455	5.12605	3.73839	5.64272	5.69926	3.51118	4.70854	5.18823	3.55583	3.86043	5.04678	3.95787	4.99034	0.3576519	0.882035609	24	4	24	0.97599044	0.993976564	24	11.30465739	0.019202957
ENSMUSG00000038936	Sccpdh	saccharopine dehydrogenase (putative) [Source:MGI Symbol;Acc:MGI:1924486]	63.1718	66.9286	63.247	56.9069	64.3093	56.9669	64.0685	60.4955	66.2977	59.876	62.5518	63.4555	65.3049	64.9259	0.235187365	0.73629392	8	8	24	0.240778456	0.768040178	24	3.348410564	0.045853668
ENSMUSG00000038943	Prc1	protein regulator of cytokinesis 1 [Source:MGI Symbol;Acc:MGI:1858961]	2.68378	2.63637	4.18834	2.93284	3.17158	3.91296	2.42827	2.88623	3.32995	2.50153	3.59764	2.83715	2.58662	2.88729	0.088377194	0.473285995	20	8	24	0.413125699	0.799546792	24	13.27347789	0.093398843
ENSMUSG00000038949	Cnst	consortin, connexin sorting protein [Source:MGI Symbol;Acc:MGI:2445141]	41.2754	34.199	33.2224	35.0869	35.3264	36.9373	34.9428	35.1734	27.9107	39.8767	35.5372	33.4873	35.6171	36.3408	0.455507782	0.966018836	4	8	24	0.37622753	0.796215348	24	20.26802378	0.071254717
ENSMUSG00000038954	Supt3	SPT3, SAGA and STAGA complex component [Source:MGI Symbol;Acc:MGI:1923723]	11.2031	10.0964	10.5901	9.57404	11.3031	11.6215	9.82019	10.9265	8.52135	11.6655	10.0281	11.7952	11.4613	11.7706	0.245061943	0.741580974	24	12	24	0.282111605	0.768040178	24	20.93681983	0.078774723
ENSMUSG00000038957	Edc3	enhancer of mRNA decapping 3 [Source:MGI Symbol;Acc:MGI:2142951]	5.75061	5.5661	5.89405	5.99561	5.81071	5.82074	5.45862	5.92096	5.42049	6.26838	5.47865	5.53424	5.32409	5.65065	0.117050591	0.538362324	16	12	24	0.15466096	0.760257003	24	11.84638862	0.038553616
ENSMUSG00000038963	Slco4a1	solute carrier organic anion transporter family, member 4a1 [Source:MGI Symbol;Acc:MGI:1351866]	1.17438	1.53892	1.48087	0.955001	1.52836	1.53061	1.07815	1.95061	1.88603	1.35842	1.13863	1.67808	1.51851	1.39391	0.745118544	0.966018836	8	8	24	0.518972962	0.819011931	24	4.871744233	0.077152626
ENSMUSG00000038965	Ube2l3	ubiquitin-conjugating enzyme E2L 3 [Source:MGI Symbol;Acc:MGI:109240]	42.8756	36.3147	37.7795	37.551	35.717	43.2765	42.2268	43.8372	32.8733	50.1755	35.7379	39.8422	39.0347	40.1064	0.570349842	0.966018836	24	20	24	0.748168923	0.918016105	24	22.58856249	0.047743272
ENSMUSG00000038967	Pdk2	pyruvate dehydrogenase kinase, isoenzyme 2 [Source:MGI Symbol;Acc:MGI:1343087]	13.6056	12.881	16.826	17.7459	14.1341	16.9518	16.661	20.0077	16.9208	26.3665	14.1129	16.8177	16.2091	16.2984	0.295379186	0.805301811	16	12	24	0.271980559	0.768040178	24	10.30060404	0.145790685
ENSMUSG00000038970	Lmtk2	lemur tyrosine kinase 2 [Source:MGI Symbol;Acc:MGI:3036247]	4.11701	3.82193	4.86117	4.42595	3.47472	4.63094	5.06089	4.30553	3.64254	5.27281	3.29559	4.41738	3.97278	4.32747	0.917839756	0.966018836	16	4	24	0.862288417	0.959779249	24	4.64585238	0.035978047
ENSMUSG00000038973	Cldnd2	claudin domain containing 2 [Source:MGI Symbol;Acc:MGI:1921526]	0.665951	0.557471	0.346022	0.272458	0.651756	0.374741	0.925975	0.928827	0.271205	0.566021	0.714978	0.866725	0.26599	0.573527	0.459028788	0.966018836	8	4	24	0.378302085	0.797706855	24	21.90546908	0.12312585
ENSMUSG00000038975	Rabggtb	Rab geranylgeranyl transferase, b subunit [Source:MGI Symbol;Acc:MGI:99537]	38.1435	39.8783	38.5845	37.0365	36.5511	37.9153	36.4502	36.486	38.4812	34.1383	38.9078	37.3468	41.423	39.4006	0.832506215	0.966018836	8	8	24	0.25824579	0.768040178	24	1.871694276	0.041299701
ENSMUSG00000038976	Ppp1r9b	protein phosphatase 1, regulatory subunit 9B [Source:MGI Symbol;Acc:MGI:2387581]	30.6694	24.5047	28.0837	28.4964	27.8718	29.9537	30.9384	28.3062	25.2948	33.6828	26.164	30.4916	28.3083	29.318	0.289496388	0.794869797	24	12	24	0.572222382	0.846269005	24	19.3889864	0.050958165
ENSMUSG00000038982	Bloc1s5	biogenesis of lysosomal organelles complex-1, subunit 5, muted [Source:MGI Symbol;Acc:MGI:2178598]	7.59026	6.75135	5.75282	4.9847	5.55255	6.16643	5.57055	7.25132	7.20003	6.86437	7.93205	6.6959	7.50441	7.92408	0.665691261	0.966018836	8	8	24	0.311253092	0.768040178	24	2.613243333	0.092624602
ENSMUSG00000038984	Tspyl5	testis-specific protein, Y-encoded-like 5 [Source:MGI Symbol;Acc:MGI:2442458]	6.13903	4.9014	5.4178	4.69109	5.39584	5.12778	5.39502	4.80082	4.1912	4.76012	5.40296	5.41897	4.67315	5.19554	0.519468828	0.966018836	20	20	24	0.081400957	0.697102286	24	20.70130391	0.092168861
ENSMUSG00000038987	Cfap157	cilia and flagella associated protein 157 [Source:MGI Symbol;Acc:MGI:2447809]	0.884688	0.507705	1.10331	2.39439	1.68482	1.21383	0.299582	2.06514	0.527268	1.53869	1.37231	1.221	0.349583	0.473884	0.001845448	0.100255953	16	12	24	0.034639407	0.608551791	24	13.99416341	0.394828255
ENSMUSG00000038990	Cables2	CDK5 and Abl enzyme substrate 2 [Source:MGI Symbol;Acc:MGI:2182335]	6.20105	5.77518	6.1681	6.74785	5.8024	6.89479	6.78558	6.49401	5.70181	8.37543	5.87514	6.26777	6.06738	6.47559	0.944053021	0.966018836	24	12	24	0.767533825	0.924952565	24	13.12913453	0.036245195
ENSMUSG00000038991	Txndc5	thioredoxin domain containing 5 [Source:MGI Symbol;Acc:MGI:2145316]	34.0265	33.6057	30.4523	32.6024	33.8217	34.8864	34.0464	35.9604	32.1952	33.6289	30.6919	32.6593	32.4465	32.344	0.217827365	0.704989686	24	12	24	0.31081368	0.768040178	24	23.19208189	0.037274042
ENSMUSG00000039000	Ube3c	ubiquitin protein ligase E3C [Source:MGI Symbol;Acc:MGI:2140998]	30.8	26.9939	26.8356	27.5324	26.9574	29.0033	27.9826	27.197	23.797	27.2401	28.2894	28.0002	28.7746	27.8808	9.43E-04	0.072887999	4	8	24	0.017637678	0.5524966	24	21.22618948	0.079350022
ENSMUSG00000039001	Rps21	ribosomal protein S21 [Source:MGI Symbol;Acc:MGI:1913731]	270.298	327.504	295.866	284.786	309.143	261.717	266.268	269.494	371.27	214.249	321.94	275.582	339.412	311.658	0.914360142	0.966018836	12	12	24	0.665072639	0.886947643	24	5.44151045	0.072228179
ENSMUSG00000039004	Bmp6	bone morphogenetic protein 6 [Source:MGI Symbol;Acc:MGI:88182]	3.04367	3.48738	2.46518	2.746	2.55997	2.00244	3.13617	3.57598	3.4309	2.25599	2.80977	2.78747	2.96196	3.23043	0.001871852	0.100255953	8	8	24	0.023615303	0.584818005	24	4.073752699	0.169322369
ENSMUSG00000039005	Tlr4	toll-like receptor 4 [Source:MGI Symbol;Acc:MGI:96824]	1.62809	1.91425	1.92009	1.94253	2.22286	1.74374	1.73589	1.73124	2.13674	1.44829	2.2636	1.59397	2.05752	1.86194	0.369262716	0.893596186	20	4	24	0.699233087	0.900024462	24	12.19577691	0.041328249
ENSMUSG00000039007	Cpq	carboxypeptidase Q [Source:MGI Symbol;Acc:MGI:1889205]	16.2359	18.7967	17.4317	15.4762	15.8895	15.3745	16.7989	17.6794	21.4308	14.15	16.3794	16.5993	16.0302	17.7008	0.004163919	0.140598674	12	4	24	0.056615375	0.655587475	24	5.371394071	0.120056021
ENSMUSG00000039016	Timm8b	translocase of inner mitochondrial membrane 8B [Source:MGI Symbol;Acc:MGI:1353424]	131.723	147.081	138.843	122.672	144.497	123.855	131.037	123.273	173.143	103.279	158.192	135.251	146.018	137.476	0.932092277	0.966018836	12	4	24	0.907001268	0.974606327	24	4.111115442	0.032100355
ENSMUSG00000039018	Mtg1	mitochondrial ribosome-associated GTPase 1 [Source:MGI Symbol;Acc:MGI:2685015]	16.3944	15.6199	12.6235	16.0292	16.9496	14.7375	14.1284	16.1343	15.2579	12.8681	14.6238	15.2229	15.7133	15.1051	0.917839756	0.966018836	8	4	24	0.476263878	0.805393068	24	22.02831779	0.056530933
ENSMUSG00000039021	Ttc16	tetratricopeptide repeat domain 16 [Source:MGI Symbol;Acc:MGI:2443048]	0.518635	0.564714	0.53295	0.399616	0.655087	0.398082	0.683967	0.384373	0.441488	0.474868	0.497807	0.688009	0.455523	0.481904	0.840483986	0.966018836	20	20	24	0.407762797	0.799546792	24	20.30404198	0.05273092
ENSMUSG00000039031	Arhgap18	Rho GTPase activating protein 18 [Source:MGI Symbol;Acc:MGI:1921160]	3.39702	4.92564	3.74163	3.47971	4.03371	3.14861	4.01642	3.85669	4.39032	2.68176	3.65312	3.5986	3.98913	3.85451	0.334687844	0.85758369	8	16	24	0.211021068	0.768040178	24	3.760035329	0.109405276
ENSMUSG00000039033	Tasp1	taspase, threonine aspartase 1 [Source:MGI Symbol;Acc:MGI:1923062]	6.10994	7.40018	8.03042	7.26993	6.74617	6.70866	6.29195	6.77975	6.9653	7.54646	7.19384	6.81446	6.83845	7.02632	0.007697866	0.192760771	16	12	24	0.008865474	0.502965379	24	10.12228024	0.094049778
ENSMUSG00000039037	St6galnac5	ST6 (alpha-N-acetyl-neuraminyl-2,3-beta-galactosyl-1,3)-N-acetylgalactosaminide alpha-2,6-sialyltransferase 5 [Source:MGI Symbol;Acc:MGI:1349471]	7.37128	5.1328	5.13225	5.60877	5.3255	5.71282	5.21594	5.27184	4.12807	6.23364	5.83129	6.64243	6.32538	6.47881	0.128989168	0.560209134	24	12	24	0.185625328	0.768040178	24	20.88928984	0.125127306
ENSMUSG00000039041	Adrm1	adhesion regulating molecule 1 [Source:MGI Symbol;Acc:MGI:1929289]	8.21057	6.76614	5.96217	7.78995	5.85549	4.53857	7.49187	5.95712	7.16772	6.79374	6.99272	6.97867	7.82828	8.8174	0.675497492	0.966018836	4	20	24	0.460681717	0.805393068	24	4.598929281	0.100644549
ENSMUSG00000039043	Arpin	actin-related protein 2/3 complex inhibitor [Source:MGI Symbol;Acc:MGI:1917670]	10.8269	9.26765	9.72418	9.24525	9.63022	10.6197	9.1127	10.7394	8.24516	10.1812	8.80853	10.3587	10.1246	9.83353	0.396283915	0.927127564	24	20	24	0.245191965	0.768040178	24	22.35907139	0.068713733
ENSMUSG00000039046	Usp6nl	USP6 N-terminal like [Source:MGI Symbol;Acc:MGI:2138893]	17.958	18.1561	16.7636	17.5641	19.7333	16.2549	19.9765	16.2627	19.1993	18.4573	16.1551	17.9314	19.5991	17.1083	0.790218349	0.966018836	4	20	24	0.925582584	0.980131499	24	22.96761099	0.016453732
ENSMUSG00000039047	Pigk	phosphatidylinositol glycan anchor biosynthesis, class K [Source:MGI Symbol;Acc:MGI:1913863]	36.9222	36.2378	32.8499	35.1741	35.2728	36.0118	33.1157	35.6627	33.4516	36.2508	36.9101	33.0042	37.2015	35.8386	0.643325306	0.966018836	4	8	24	0.811382531	0.939523565	24	20.80969382	0.016903069
ENSMUSG00000039048	Foxred1	FAD-dependent oxidoreductase domain containing 1 [Source:MGI Symbol;Acc:MGI:2446262]	15.7367	16.8338	20.645	18.5089	16.7647	16.776	16.2207	17.1941	18.2151	17.3143	18.3857	17.2413	18.2147	17.9505	0.060394272	0.407012001	12	16	24	0.045940325	0.632654269	24	9.766141769	0.083144678
ENSMUSG00000039050	Osbpl2	oxysterol binding protein-like 2 [Source:MGI Symbol;Acc:MGI:2442832]	21.3629	19.8016	20.4646	20.8282	19.7634	21.9178	20.2321	20.3504	19.7482	23.3447	18.5541	21.4672	20.7369	21.3359	0.446653834	0.966018836	24	12	24	0.98631317	0.997339197	24	21.19628618	0.00535637
ENSMUSG00000039057	Myo16	myosin XVI [Source:MGI Symbol;Acc:MGI:2685951]	0.415909	0.655391	1.00287	0.615493	0.477589	0.632292	0.538056	0.429369	0.627858	0.801925	0.379649	0.581895	0.449744	0.706989	0.210418232	0.69936851	12	16	24	0.115102643	0.727799063	24	8.880759455	0.11815519
ENSMUSG00000039058	Ak5	adenylate kinase 5 [Source:MGI Symbol;Acc:MGI:2677491]	22.6035	19.7396	17.9091	18.9087	20.8866	20.6136	20.022	22.5242	18.0103	24.3506	16.5386	21.2127	20.0949	19.0119	0.351617883	0.870418653	24	12	24	0.660259081	0.885409508	24	22.24923205	0.053013233
ENSMUSG00000039059	Hrh3	histamine receptor H3 [Source:MGI Symbol;Acc:MGI:2139279]	1.44901	0.808846	1.44591	1.11	0.973714	1.16775	1.17475	0.939826	1.16012	1.45897	0.953247	1.40774	1.03954	1.3748	0.833859682	0.966018836	12	20	24	0.982914338	0.996256812	24	10.19075152	0.011312127
ENSMUSG00000039062	Anpep	alanyl (membrane) aminopeptidase [Source:MGI Symbol;Acc:MGI:5000466]	2.83668	3.89384	4.4475	3.01685	3.80699	3.23671	4.10352	4.47222	4.42586	2.38103	4.10971	3.60288	4.55306	3.7553	0.590017078	0.966018836	12	4	24	0.34922142	0.779170188	24	3.758750109	0.119746362
ENSMUSG00000039063	Echdc3	enoyl Coenzyme A hydratase domain containing 3 [Source:MGI Symbol;Acc:MGI:1915106]	0.602126	1.26708	1.05577	2.14614	1.04787	1.06954	1.20852	1.99171	1.48306	1.47082	1.49892	1.35987	1.79054	1.30253	0.909604019	0.966018836	16	12	24	0.272684005	0.768040178	24	9.65808369	0.142927193
ENSMUSG00000039065	Atpsckmt	ATP synthase C subunit lysine N-methyltransferase [Source:MGI Symbol;Acc:MGI:1915323]	8.09657	6.57828	6.47201	7.4777	7.08316	7.59317	7.94578	7.13225	7.10021	6.9561	8.37255	7.45652	8.46981	7.15806	0.008754183	0.192760771	4	8	24	0.036508892	0.609176161	24	20.49740613	0.094968762
ENSMUSG00000039067	Psmd7	proteasome (prosome, macropain) 26S subunit, non-ATPase, 7 [Source:MGI Symbol;Acc:MGI:1351511]	85.2771	81.356	81.7127	83.7903	85.786	88.168	84.1612	85.0863	77.3382	89.632	83.1158	81.7791	86.0057	88.9126	0.834434272	0.966018836	16	20	24	0.658661474	0.884596426	24	19.89241982	0.021045291
ENSMUSG00000039068	Zzz3	zinc finger, ZZ domain containing 3 [Source:MGI Symbol;Acc:MGI:1920453]	12.7352	13.7798	13.3192	13.4486	14.2901	12.5306	13.8198	12.6314	14.4535	12.1379	14.1033	13.4571	12.8967	13.3047	0.93717134	0.966018836	20	4	24	0.80668514	0.938416757	24	12.48730836	0.018547527
ENSMUSG00000039069	Mtg2	mitochondrial ribosome associated GTPase 2 [Source:MGI Symbol;Acc:MGI:106565]	9.27975	8.47147	7.68804	9.01749	9.02793	8.30764	8.43017	8.96966	8.7818	7.9868	9.92043	8.58416	8.77362	8.38464	0.965553553	0.969272194	20	16	24	0.453916677	0.805393068	24	18.50623296	0.042297451
ENSMUSG00000039081	Zfp503	zinc finger protein 503 [Source:MGI Symbol;Acc:MGI:1353644]	2.43997	1.71472	1.66802	1.86003	1.48649	1.46802	1.82625	1.82624	1.82524	2.97754	2.38876	2.12892	2.20785	2.44359	0.896773941	0.966018836	4	20	24	0.922542669	0.979411448	24	8.894623725	0.029736404
ENSMUSG00000039084	Chad	chondroadherin [Source:MGI Symbol;Acc:MGI:1096866]	3.08291	2.83148	2.21741	2.43718	2.74943	3.5153	3.03243	3.33092	6.73875	3.24414	2.34288	2.45755	2.48268	2.83025	0.920872048	0.966018836	4	20	24	0.557816594	0.839489739	24	6.931657539	0.139662415
ENSMUSG00000039086	Ss18l1	SS18, nBAF chromatin remodeling complex subunit like 1 [Source:MGI Symbol;Acc:MGI:2444061]	4.42869	4.03842	5.32576	4.38317	3.89594	4.30822	4.58307	4.04378	4.29151	4.53666	3.76218	4.13417	3.68489	4.4027	0.814077113	0.966018836	16	4	24	0.337974374	0.772742827	24	7.84411802	0.06088971
ENSMUSG00000039087	Rreb1	ras responsive element binding protein 1 [Source:MGI Symbol;Acc:MGI:2443664]	1.75967	1.52353	1.62168	1.90434	1.79099	2.07353	1.63508	2.24195	2.27919	2.55447	1.76455	2.12389	1.93548	1.69165	0.786665292	0.966018836	16	16	24	0.446335762	0.805393068	24	12.66629198	0.06563496
ENSMUSG00000039089	L3mbtl3	L3MBTL3 histone methyl-lysine binding protein [Source:MGI Symbol;Acc:MGI:2143628]	3.41913	3.45806	3.0617	3.70475	4.16928	3.45381	3.98318	3.62644	3.63899	3.62847	4.42528	3.61252	3.57469	3.3185	0.105345531	0.518002936	20	12	24	0.078689583	0.68925626	24	16.72832974	0.08636645
ENSMUSG00000039096	Rsad1	radical S-adenosyl methionine domain containing 1 [Source:MGI Symbol;Acc:MGI:3039628]	3.66348	4.10445	4.21605	4.08431	4.10261	3.5492	3.30313	3.59967	4.16447	3.92003	3.64618	3.33356	2.97211	4.23316	0.004140921	0.140598674	12	20	24	0.005170014	0.4424469	24	9.694662265	0.126698657
ENSMUSG00000039100	Marchf6	membrane associated ring-CH-type finger 6 [Source:MGI Symbol;Acc:MGI:2442773]	43.7261	42.5217	41.9265	43.197	41.5215	43.1528	43.1281	42.8475	40.1928	45.9129	40.153	43.5938	42.3654	45.0227	0.745199643	0.966018836	24	20	24	0.812732926	0.940101979	24	1.726895887	0.013398365
ENSMUSG00000039103	Nexn	nexilin [Source:MGI Symbol;Acc:MGI:1916060]	16.8203	21.3768	22.2641	21.1182	18.089	23.2496	19.1409	28.348	32.6638	32.316	20.5852	22.6266	28.1589	22.3249	0.671726933	0.966018836	12	8	24	0.093589126	0.71175955	24	8.295545427	0.205011282
ENSMUSG00000039105	Atp6v1g1	ATPase, H+ transporting, lysosomal V1 subunit G1 [Source:MGI Symbol;Acc:MGI:1913540]	90.8017	92.4961	84.1916	85.8603	92.2873	86.9911	85.4104	84.0542	89.6588	80.0335	103.419	85.0167	89.6825	90.3592	0.940625808	0.966018836	20	4	24	0.70191911	0.901483676	24	18.95850884	0.030675037
ENSMUSG00000039106	Htr5a	5-hydroxytryptamine (serotonin) receptor 5A [Source:MGI Symbol;Acc:MGI:96283]	0.933095	0.822965	0.949734	0.788904	0.766778	1.08077	1.02997	0.914721	0.790044	0.876634	0.960127	1.03872	0.910693	0.99669	0.03782572	0.334069441	24	16	24	0.074112532	0.683293001	24	22.31241708	0.060949511
ENSMUSG00000039108	Lsm14b	LSM family member 14B [Source:MGI Symbol;Acc:MGI:3040677]	21.2066	17.2638	18.6934	18.4181	19.49	19.3409	19.6718	19.4998	16.6287	23.3302	18.0586	19.4681	17.5317	20.1801	0.919119843	0.966018836	4	8	24	0.812077634	0.939835431	24	17.7512813	0.031857604
ENSMUSG00000039109	F13a1	coagulation factor XIII, A1 subunit [Source:MGI Symbol;Acc:MGI:1921395]	5.06112	6.71193	5.65924	6.78092	7.66777	5.25049	6.0717	5.88791	7.90181	3.86914	7.10546	5.80227	5.93888	4.92011	0.95603254	0.966018836	20	20	24	0.83603198	0.949648082	24	13.73713372	0.055250478
ENSMUSG00000039110	Mycbpap	MYCBP associated protein [Source:MGI Symbol;Acc:MGI:2388726]	2.06571	1.91031	1.72016	1.55475	1.65165	2.07906	2.72233	2.01639	1.85331	1.40023	1.23722	1.58463	1.58247	1.59076	0.080148772	0.458678242	4	16	24	0.020546315	0.56691777	24	0.863716443	0.158067059
ENSMUSG00000039114	Nrn1	neuritin 1 [Source:MGI Symbol;Acc:MGI:1915654]	359.136	296.62	283.671	286.654	290.492	307.531	284.245	297.097	271.577	305.468	332.693	309.075	328.549	317.007	0.064165315	0.414867207	4	8	24	0.151280039	0.758865539	24	21.49591113	0.080386093
ENSMUSG00000039115	Itga9	integrin alpha 9 [Source:MGI Symbol;Acc:MGI:104756]	1.2916	1.31467	1.47261	1.40689	1.33416	1.25739	1.38251	1.7127	1.28018	1.34781	1.4976	1.16935	1.02089	1.39947	0.200364953	0.684570416	16	8	24	0.302951228	0.768040178	24	8.941852425	0.05983493
ENSMUSG00000039116	Adgrg6	adhesion G protein-coupled receptor G6 [Source:MGI Symbol;Acc:MGI:1916151]	10.2681	10.1575	8.90886	10.2116	10.625	9.24967	10.6655	9.39266	9.41505	10.763	9.09429	9.0024	10.0075	8.65038	0.891105762	0.966018836	16	20	24	0.764319137	0.92403577	24	17.55468736	0.027012844
ENSMUSG00000039117	Taf4	TATA-box binding protein associated factor 4 [Source:MGI Symbol;Acc:MGI:2152346]	3.90684	4.68764	3.61049	4.23937	3.76612	3.96513	4.9317	3.52143	4.58266	4.89089	4.68975	4.4598	3.87267	3.50931	0.777666236	0.966018836	16	12	24	0.61814595	0.868871403	24	15.28394824	0.055513494
ENSMUSG00000039126	Prune2	prune homolog 2 [Source:MGI Symbol;Acc:MGI:1925004]	232.885	186.964	185.013	195.724	197.843	212.208	195.464	192.793	159.594	224.212	202.445	203.403	204.872	206.99	0.042880436	0.356048751	24	12	24	0.159158243	0.765350752	24	19.9783786	0.097521289
ENSMUSG00000039128	Cdc123	cell division cycle 123 [Source:MGI Symbol;Acc:MGI:2138811]	78.1293	81.4375	74.9582	74.8711	79.253	78.0866	76.7685	73.7293	72.0512	60.7213	73.7072	66.1971	74.0815	66.7208	0.93447422	0.966018836	8	4	24	0.199678587	0.768040178	24	22.54541611	0.05806005
ENSMUSG00000039130	Zc3hc1	zinc finger, C3HC type 1 [Source:MGI Symbol;Acc:MGI:1916023]	10.4124	9.57506	10.1915	9.55471	9.90145	10.3297	9.46499	10.7042	8.73034	9.61581	9.5792	10.2568	10.086	10.0815	0.423605651	0.966018836	24	16	24	0.22498688	0.768040178	24	22.66470877	0.045779429
ENSMUSG00000039131	Gipc2	GIPC PDZ domain containing family, member 2 [Source:MGI Symbol;Acc:MGI:1889209]	3.61737	2.92081	3.20472	2.37735	4.80153	2.95132	2.08791	2.68421	2.97507	2.00969	2.46273	3.22763	3.19931	[illegible]	0.814303714	0.966018836	12	4	24	0.843380249	0.952436645	24	22.25626831	0.060533937
ENSMUSG00000039137	Whrn	whirlin [Source:MGI Symbol;Acc:MGI:2682003]	5.19621	5.08888	5.20472	4.93193	4.40028	4.39771	5.03051	5.32207	4.69338	5.97273	4.5626	4.81442	4.20392	5.53273	0.083354723	0.462922111	8	16	24	0.136569496	0.747972781	24	7.194418846	0.090717279
ENSMUSG00000039145	Camk1d	calcium/calmodulin-dependent protein kinase ID [Source:MGI Symbol;Acc:MGI:2442190]	11.2177	10.1154	10.9907	9.41019	9.32875	11.0735	11.1544	9.83978	9.11748	13.5088	9.85203	11.2369	10.4157	12.1626	0.636884864	0.966018836	24	20	24	0.870047482	0.963168206	24	0.618664541	0.029821187
ENSMUSG00000039148	Sart1	squamous cell carcinoma antigen recognized by T cells 1 [Source:MGI Symbol;Acc:MGI:1309453]	26.9353	24.9867	25.7102	24.2944	22.92	25.3638	22.9979	24.5415	23.7028	22.7405	17.5491	22.9743	23.219	23.4791	0.142596848	0.585944911	12	8	24	0.095368798	0.71175955	24	3.203494476	0.089154528
ENSMUSG00000039153	Runx2	runt related transcription factor 2 [Source:MGI Symbol;Acc:MGI:99829]	1.09593	1.08351	1.18119	1.37309	1.20903	1.24344	1.46258	1.2818	0.916905	1.45862	1.03447	1.22884	0.968689	1.02405	0.954584434	0.966018836	16	20	24	0.57064028	0.845416058	24	15.88423017	0.046632497
ENSMUSG00000039154	Shd	src homology 2 domain-containing transforming protein D [Source:MGI Symbol;Acc:MGI:1099461]	9.94105	7.96313	9.70454	7.64324	8.55589	8.56925	8.12022	8.61687	10.049	9.66421	7.19536	10.0957	8.2062	9.73567	0.537381546	0.966018836	12	8	24	0.222667294	0.768040178	24	4.74241411	0.132118896
ENSMUSG00000039156	Stim2	stromal interaction molecule 2 [Source:MGI Symbol;Acc:MGI:2151156]	15.5061	14.523	14.6621	13.4948	15.5077	14.985	15.9533	15.6783	14.3173	16.9921	13.3292	16.1261	12.6559	12.8648	0.888482515	0.966018836	12	8	24	0.741626289	0.91592675	24	18.09006986	0.037116236
ENSMUSG00000039157	Fam102a	family with sequence similarity 102, member A [Source:MGI Symbol;Acc:MGI:2138935]	14.3141	13.3659	13.3214	14.4322	14.693	14.2449	14.7665	14.6934	13.7368	17.0899	14.0267	14.8809	14.2213	13.7964	0.124028266	0.556711408	24	12	24	0.35264908	0.780629136	24	15.6120733	0.044827298
ENSMUSG00000039158	Akna	AT-hook transcription factor [Source:MGI Symbol;Acc:MGI:2140346]	0.99926	1.37406	1.28562	1.36456	1.26195	1.13913	1.4167	1.42369	1.51971	1.25469	1.3403	1.07012	1.39579	1.11192	0.601647512	0.966018836	24	8	24	0.244335505	0.768040178	24	8.401219417	0.065745608
ENSMUSG00000039159	Ube2h	ubiquitin-conjugating enzyme E2H [Source:MGI Symbol;Acc:MGI:104632]	16.6074	14.5484	14.7314	14.6714	14.6886	15.8622	15.2975	15.1053	13.3947	17.5617	14.3271	15.7031	15.3484	15.6048	0.22872448	0.72665454	24	12	24	0.549742449	0.835088337	24	20.95750471	0.040532297
ENSMUSG00000039163	Cmc1	COX assembly mitochondrial protein 1 [Source:MGI Symbol;Acc:MGI:1915149]	23.628	24.0673	20.3991	23.5069	20.9761	20.5357	21.1649	22.4319	22.652	20.3818	22.5229	23.3183	24.0432	23.0441	0.320283544	0.83607295	8	4	24	0.363108503	0.787924926	24	1.961483563	0.04674884
ENSMUSG00000039164	Naif1	nuclear apoptosis inducing factor 1 [Source:MGI Symbol;Acc:MGI:1918504]	0.99845	0.722761	0.789754	0.977581	1.03982	0.8471	1.13202	0.83273	0.697276	1.02897	1.12522	0.824613	0.946862	0.785086	0.035023815	0.328311403	20	16	24	0.084711806	0.70291653	24	17.67908472	0.090375192
ENSMUSG00000039166	Akap7	A kinase (PRKA) anchor protein 7 [Source:MGI Symbol;Acc:MGI:1859150]	17.4896	20.7198	20.9264	18.8277	15.8491	17.6423	19.9968	17.6885	21.1193	19.7061	18.4859	20.04	19.3294	23.5447	0.059650003	0.406067671	12	8	24	0.024468728	0.586541342	24	6.07646217	0.123549801
ENSMUSG00000039167	Adgrl4	adhesion G protein-coupled receptor L4 [Source:MGI Symbol;Acc:MGI:2655562]	6.4097	5.16423	6.68666	5.91499	6.16442	4.89751	6.17914	6.69342	6.15426	5.26343	4.49988	5.32268	4.38337	4.84536	0.895493752	0.966018836	24	12	24	0.452285279	0.805393068	24	7.318511477	0.072930188
ENSMUSG00000039168	Dap	death-associated protein [Source:MGI Symbol;Acc:MGI:1918190]	14.4587	14.4024	12.9529	13.9012	13.3315	13.9788	14.682	16.0028	14.8255	14.9187	13.2174	14.6126	14.7914	13.8321	0.640052099	0.966018836	4	16	24	0.224376367	0.768040178	24	2.706258213	0.048603722
ENSMUSG00000039176	Polg	polymerase (DNA directed), gamma [Source:MGI Symbol;Acc:MGI:1196389]	20.7299	19.5969	16.9022	22.2475	23.9205	20.9547	18.2584	17.3332	19.3255	18.3377	18.5125	17.5402	18.4933	17.2138	0.287843582	0.793714531	20	16	24	0.071687739	0.67819049	24	16.69349368	0.103244287
ENSMUSG00000039178	Tbc1d19	TBC1 domain family, member 19 [Source:MGI Symbol;Acc:MGI:1914499]	52.4475	44.8733	39.9829	42.0531	52.0735	43.0801	50.2372	40.5543	42.5112	37.6517	51.9562	39.9728	44.0436	41.1413	0.20729371	0.698501896	4	12	24	0.126345034	0.739868076	24	20.19717619	0.118613562
ENSMUSG00000039179	Tekt5	tektin 5 [Source:MGI Symbol;Acc:MGI:1917676]	1.21068	1.42004	2.05811	0.58454	1.17485	1.60006	1.68285	1.25849	1.62021	1.30855	0.661655	1.30591	0.802621	1.72934	0.090721279	0.481752771	12	8	24	0.259893157	0.768040178	24	4.234361344	0.173546008

ENSMUSG00000039182	AW209491	expressed sequence AW209491 [Source:MGI Symbol;Acc:MGI:2145422]	12.2956	12.2177	12.0086	10.7926	12.4016	11.8952	11.1488	11.8989	11.5715	9.66243	12.479	11.541	12.562	11.513	0.503972924	0.966018836	20	20	24	0.238927227	0.768040178	24	22.93442169	0.060494989
ENSMUSG00000039183	Nubp2	nucleotide binding protein 2 [Source:MGI Symbol;Acc:MGI:1347072]	24.6029	23.4042	22.6202	22.3103	23.6668	25.7366	23.3502	25.5594	21.8749	22.7136	22.9706	23.9734	22.6502	22.4862	0.020119233	0.259417302	24	12	24	0.052019831	0.649509116	24	21.61440535	0.059796559
ENSMUSG00000039191	Rbpj	recombination signal binding protein for immunoglobulin kappa J region [Source:MGI Symbol;Acc:MGI:96522]	4.8658	5.08917	4.75165	4.72897	5.07849	4.89345	5.17959	5.17454	5.12541	4.84785	4.60717	4.62081	4.60555	5.09776	0.348367381	0.870418653	8	16	24	0.208164714	0.768040178	24	4.676362552	0.035527291
ENSMUSG00000039194	Rlbp1	retinaldehyde binding protein 1 [Source:MGI Symbol;Acc:MGI:97930]	2.97307	3.18751	2.03811	2.07917	2.74702	2.88542	3.57135	2.93657	2.25292	2.12469	2.58863	2.31854	3.22084	2.76272	1.65E-04	0.032908473	4	12	24	0.001671176	0.374946468	24	23.67205243	0.21070366
ENSMUSG00000039195	Bbln	bublin coiled coil protein [Source:MGI Symbol;Acc:MGI:1920987]	120.492	97.4085	96.5842	78.4828	78.3361	82.2806	97.7923	117.269	107.101	112.458	138.436	120.313	124.155	115.891	0.591351901	0.966018836	4	12	24	0.449942356	0.805393068	24	2.178292696	0.104082963
ENSMUSG00000039196	Orm1	orosomucoid 1 [Source:MGI Symbol;Acc:MGI:97443]	1.56143	1.25533	2.33376	4.39022	1.74785	2.02502	2.69617	3.54666	1.99697	1.97766	5.71332	3.11841	3.90678	4.24658	0.73726975	0.966018836	16	20	24	0.847969442	0.954100589	24	13.51766404	0.084128011
ENSMUSG00000039197	Adk	adenosine kinase [Source:MGI Symbol;Acc:MGI:87930]	181.584	173.793	173.817	163.603	162.979	168.93	156.327	155.306	159.644	157.626	174.442	170.476	186.541	173.847	0.886952527	0.966018836	4	12	24	0.396690228	0.799546792	24	22.92673446	0.044281056
ENSMUSG00000039199	Zdhhc1	zinc finger, DHHC domain containing 1 [Source:MGI Symbol;Acc:MGI:1918046]	14.0957	12.7105	12.8639	13.4509	14.6591	14.0204	13.9807	14.1287	15.376	14.584	14.5722	14.1991	13.6867	13.3356	0.105345531	0.518002936	20	12	24	0.266568622	0.768040178	24	15.69505539	0.041512426
ENSMUSG00000039201	Tbc1d25	TBC1 domain family, member 25 [Source:MGI Symbol;Acc:MGI:2444862]	4.67451	4.24822	4.18221	4.18219	5.05135	5.53655	4.60852	4.57649	3.86414	5.40952	3.93047	4.43826	4.25546	4.02771	0.148073804	0.590732968	24	12	24	0.213819071	0.768040178	24	18.59702574	0.090196771
ENSMUSG00000039202	Abhd2	abhydrolase domain containing 2 [Source:MGI Symbol;Acc:MGI:1914344]	30.4739	24.3973	24.7461	27.8307	25.5284	29.9816	26.279	27.5565	20.1635	35.245	24.2938	27.5854	24.8617	26.575	0.581070934	0.966018836	24	12	24	0.562927078	0.842470381	24	18.42686137	0.079689787
ENSMUSG00000039205	Ciz1	CDKN1A interacting zinc finger protein 1 [Source:MGI Symbol;Acc:MGI:1920234]	23.5141	21.9182	20.2474	20.095	21.6546	20.7191	22.1358	20.4033	20.0827	21.8077	21.1374	17.7977	19.1827	19.5203	0.853736882	0.966018836	4	8	24	0.941335443	0.985472945	24	20.92481884	0.010967914
ENSMUSG00000039206	Daglb	diacylglycerol lipase, beta [Source:MGI Symbol;Acc:MGI:2442032]	3.78737	3.58909	4.30204	3.94629	3.79774	4.17293	3.81933	4.13051	3.4998	4.16028	3.34229	3.9651	3.83176	3.46296	0.920858634	0.966018836	24	8	24	0.945138393	0.986693398	24	15.8391284	0.011505333
ENSMUSG00000039208	Metrnl	meteorin, glial cell differentiation regulator-like [Source:MGI Symbol;Acc:MGI:2384806]	28.1144	27.0532	24.8188	30.5765	36.1093	27.4995	35.446	26.6707	29.5481	29.2565	28.5578	28.9182	29.7967	22.1538	0.104170975	0.518002936	20	12	24	0.140241215	0.749574609	24	17.56372198	0.130457166
ENSMUSG00000039210	Gpatch2	G patch domain containing 2 [Source:MGI Symbol;Acc:MGI:1915019]	4.66162	4.69746	4.80817	4.52969	4.8154	4.83747	4.46341	4.5147	4.83749	4.87003	4.9483	5.2558	4.93203	4.64688	0.11524339	0.536404476	24	8	24	0.189088277	0.768040178	24	16.93329435	0.032901107
ENSMUSG00000039217	Il18	interleukin 18 [Source:MGI Symbol;Acc:MGI:107936]	7.29333	9.95013	9.44332	5.79799	8.01465	7.92766	8.31592	8.37727	10.3934	6.36131	7.90405	10.0059	8.29281	8.10487	0.036302654	0.33234124	12	4	24	0.341981777	0.776178419	24	2.756939916	0.118660552
ENSMUSG00000039218	Srrm2	serine/arginine repetitive matrix 2 [Source:MGI Symbol;Acc:MGI:1923206]	142.154	167.241	170.301	185.974	188.352	161.6	155.635	156.138	189.55	183.581	164.714	159.353	155.929	166.747	1.06E-04	0.029372629	16	12	24	5.21E-04	0.282966379	24	11.54094768	0.144378952
ENSMUSG00000039219	Arid4b	AT rich interactive domain 4B (RBP1-like) [Source:MGI Symbol;Acc:MGI:2137512]	10.3043	13.2129	12.7812	11.3389	12.2328	10.657	11.5894	10.1324	13.9731	11.3723	12.5485	11.9692	12.1666	12.1454	0.46238066	0.966018836	12	12	24	0.339280649	0.773727252	24	9.379284066	0.071473952
ENSMUSG00000039220	Ppp1r10	protein phosphatase 1, regulatory subunit 10 [Source:MGI Symbol;Acc:MGI:1289273]	6.80055	5.79944	6.30717	7.15123	6.74776	7.40401	6.73402	5.68918	5.99534	7.74227	5.74046	6.56812	6.08182	6.83069	0.581070934	0.966018836	16	16	24	0.378285927	0.797706855	24	16.19049186	0.066064725
ENSMUSG00000039221	Rpl22l1	ribosomal protein L22 like 1 [Source:MGI Symbol;Acc:MGI:1915278]	28.3782	39.5007	38.3354	36.2983	38.2877	31.6301	35.2123	35.1903	53.4281	32.049	42.6639	38.6382	43.2596	41.0876	0.93447422	0.966018836	12	12	24	0.27443621	0.768040178	24	8.454653221	0.126153333
ENSMUSG00000039230	Tbcd	tubulin-specific chaperone d [Source:MGI Symbol;Acc:MGI:1919686]	37.0411	33.5513	32.4767	35.2505	35.3171	41.9715	32.2198	34.4627	28.9801	35.7422	34.2246	35.6635	34.074	34.9299	0.080164531	0.458678242	24	12	24	0.065922824	0.668186543	24	19.33413266	0.100339889
ENSMUSG00000039231	Suv39h1	suppressor of variegation 3-9 1 [Source:MGI Symbol;Acc:MGI:1099440]	7.80715	6.31	7.02295	7.77111	6.191	7.11063	6.83752	6.80477	7.00595	7.53004	6.49577	8.03937	7.29835	7.85185	0.920872048	0.966018836	24	20	24	0.941561955	0.985544192	24	1.750328916	0.014136741
ENSMUSG00000039232	Stx11	syntaxin 11 [Source:MGI Symbol;Acc:MGI:1921982]	1.71112	1.29924	1.59074	2.14723	1.65951	1.6289	1.68111	1.8997	1.7698	1.754	1.6935	2.12783	2.25035	2.59801	0.854540463	0.966018836	8	12	24	0.849226328	0.954760051	24	4.266726613	0.027583242
ENSMUSG00000039233	Tbce	tubulin-specific chaperone E [Source:MGI Symbol;Acc:MGI:1917680]	41.8371	52.7422	48.5202	45.9828	49.9394	44.8622	45.0758	43.8249	55.1669	41.5959	49.2286	46.8173	47.5186	48.9499	0.588682825	0.966018836	12	16	24	0.532868126	0.825678486	24	8.368086992	0.052983111
ENSMUSG00000039234	Sec24d	Sec24 related gene family, member D (S. cerevisiae) [Source:MGI Symbol;Acc:MGI:1916858]	8.50332	8.04906	8.2375	6.57866	7.40609	7.01703	7.58848	6.6827	6.71087	6.15568	7.51686	5.48401	5.72896	5.52985	0.856752753	0.966018836	12	12	24	0.944107421	0.986282661	24	21.55066891	0.014157276
ENSMUSG00000039236	Isg20	interferon-stimulated protein [Source:MGI Symbol;Acc:MGI:1928895]	4.3733	6.24375	6.69519	6.46093	4.50769	3.91526	5.39391	5.9768	6.70612	3.30996	4.95168	5.22142	5.57892	4.24112	0.05280092	0.391647859	12	12	24	0.257281129	0.768040178	24	6.354302629	0.165018289
ENSMUSG00000039239	Tgfb2	transforming growth factor, beta 2 [Source:MGI Symbol;Acc:MGI:98726]	2.01494	2.10511	2.14681	1.59323	1.70857	2.28743	2.21529	2.11202	2.06889	2.60792	2.07318	2.0016	1.82968	2.4089	0.688991156	0.966018836	8	12	24	0.587103725	0.853586751	24	5.001359924	0.049904898
ENSMUSG00000039242	B3galnt2	UDP-GalNAc:betaGlcNAc beta 1,3-galactosaminyltransferase, polypeptide 2 [Source:MGI Symbol;Acc:MGI:2145517]	7.66956	9.81898	9.12224	10.1378	9.47627	8.04725	8.709	9.72451	10.918	7.91377	8.47494	8.72484	9.22528	8.59789	0.435596482	0.966018836	12	12	24	0.166624357	0.767020009	24	8.190056546	0.096986478
ENSMUSG00000039244	E130309D02Rik	RIKEN cDNA E130309D02 gene [Source:MGI Symbol;Acc:MGI:2442621]	5.0924	3.9796	4.76077	3.95839	4.37587	4.42238	4.62539	4.88486	4.11085	4.76639	4.69671	4.84654	4.49807	4.0369	0.910436409	0.966018836	4	4	24	0.568301547	0.844336151	24	20.86000522	0.043760146
ENSMUSG00000039246	Lyplal1	lysophospholipase-like 1 [Source:MGI Symbol;Acc:MGI:2385115]	5.62639	5.98517	7.63929	6.22017	7.13261	6.98893	6.26863	6.76005	6.69101	5.84745	7.68466	6.2128	6.35816	7.27884	0.801561896	0.966018836	20	8	24	0.612652161	0.865589526	24	11.94356255	0.047205834
ENSMUSG00000039252	Lgi2	leucine-rich repeat LGI family, member 2 [Source:MGI Symbol;Acc:MGI:2180196]	19.9841	16.8139	18.3043	17.5109	14.8917	18.7642	19.285	17.026	14.8582	22.5573	14.4516	18.9913	17.5387	19.9191	0.591351901	0.966018836	4	16	24	0.697065687	0.899338675	24	1.414402262	0.058361129
ENSMUSG00000039253	Fn3krp	fructosamine 3 kinase related protein [Source:MGI Symbol;Acc:MGI:2679256]	9.6413	9.99558	9.04914	9.53313	9.67564	9.3631	9.67124	10.4173	9.07352	10.3162	10.0354	9.97905	10.0804	9.40952	0.882617464	0.966018836	8	4	24	0.751013714	0.91921305	24	20.68030886	0.017505229
ENSMUSG00000039254	Pomt1	protein-O-mannosyltransferase 1 [Source:MGI Symbol;Acc:MGI:2138994]	7.17091	6.77909	7.42488	6.85651	7.48233	7.21557	9.04581	7.54141	6.7014	6.47797	7.93749	7.5182	7.58658	7.55221	0.156791576	0.61244839	4	12	24	0.146939954	0.755442533	24	22.26229554	0.077665531
ENSMUSG00000039257	Vstm2b	V-set and transmembrane domain containing 2B [Source:MGI Symbol;Acc:MGI:1914525]	2.54693	3.03855	3.03807	2.08094	2.35159	2.67498	3.07299	3.0563	2.79069	4.11564	2.4758	2.74482	3.00008	3.19281	0.030972684	0.324150946	8	12	24	0.23042734	0.768040178	24	5.566694089	0.109765563
ENSMUSG00000039262	Prrc2b	proline-rich coiled-coil 2B [Source:MGI Symbol;Acc:MGI:1923304]	17.9027	16.8803	17.912	18.7853	18.4341	18.9425	18.5781	19.2068	17.5671	28.189	15.3846	20.077	18.5306	20.7184	0.887862353	0.966018836	24	12	24	0.68217155	0.894159472	24	10.84250564	0.063566817
ENSMUSG00000039263	Npepl1	aminopeptidase-like 1 [Source:MGI Symbol;Acc:MGI:2448523]	20.9959	23.8712	23.1186	23.9524	26.7485	21.5173	23.2611	25.1415	29.1544	24.8348	23.4833	21.7189	25.6376	21.5747	0.43280837	0.966018836	20	4	24	0.192353823	0.768040178	24	9.962268483	0.09018239
ENSMUSG00000039264	Gimap3	GTPase, IMAP family member 3 [Source:MGI Symbol;Acc:MGI:1932723]	0.346232	0.608865	0.508755	0.715443	0.481205	0.389293	0.407454	0.716432	0.940472	0.510172	0.402701	0.574994	0.573567	0.616296	0.062704268	0.410492299	8	20	24	0.021896801	0.574400012	24	7.86162022	0.140441587
ENSMUSG00000039270	Megf9	multiple EGF-like-domains 9 [Source:MGI Symbol;Acc:MGI:1918264]	43.2016	41.2492	39.5007	47.877	49.5333	40.2418	44.3962	37.7526	41.7508	44.2961	45.4486	42.6992	40.8081	38.9737	0.008603252	0.192760771	20	12	24	0.007553653	0.478114494	24	15.68516471	0.111182448
ENSMUSG00000039275	Foxk2	forkhead box K2 [Source:MGI Symbol;Acc:MGI:1916087]	13.0188	9.99875	10.5585	11.4563	12.0826	12.8464	12.0325	11.858	9.99494	12.6982	11.787	11.6949	10.8681	11.7377	0.184044621	0.662239322	24	12	24	0.094491013	0.71175955	24	18.96577026	0.093391936
ENSMUSG00000039278	Pcsk1n	proprotein convertase subtilisin/kexin type 1 inhibitor [Source:MGI Symbol;Acc:MGI:1353431]	13.3829	9.74098	9.95743	8.05279	5.93175	6.94916	11.8508	14.3742	15.1033	27.791	25.6328	31.2164	29.5244	26.9455	0.816406072	0.966018836	4	16	24	0.722561372	0.908973198	24	3.578174944	0.146590108
ENSMUSG00000039285	Azi2	5-azacytidine induced gene 2 [Source:MGI Symbol;Acc:MGI:1351332]	47.954	45.3834	45.4428	45.7954	48.5555	45.9781	48.2007	44.158	44.0409	43.0315	49.2458	46.5143	49.8597	46.1454	0.026841899	0.305155548	20	20	24	0.038000639	0.61140397	24	20.5351776	0.058774933
ENSMUSG00000039286	Fndc3b	fibronectin type III domain containing 3B [Source:MGI Symbol;Acc:MGI:1919257]	6.5063	5.44229	5.10695	5.72864	6.11744	5.71449	6.17259	5.80477	5.76316	5.14997	5.91988	6.09215	5.4383	5.16544	0.187550294	0.662239322	4	8	24	0.037342613	0.609176161	24	20.16179579	0.083389995
ENSMUSG00000039294	Cybc1	cytochrome b 245 chaperone 1 [Source:MGI Symbol;Acc:MGI:2384959]	26.0155	24.841	22.1558	23.2671	25.8312	24.9544	24.4352	24.0157	21.8396	22.2173	26.1957	23.6642	24.7735	23.8577	0.016273603	0.253395174	20	16	24	0.011298584	0.517565995	24	20.71772712	0.085714963
ENSMUSG00000039298	Cdk5rap2	CDK5 regulatory subunit associated protein 2 [Source:MGI Symbol;Acc:MGI:2384875]	17.9529	13.648	12.5504	8.86859	8.16762	8.4322	11.7122	15.7821	18.3814	18.3815	20.6415	20.1591	20.9748	20.3452	0.847846021	0.966018836	4	12	24	0.309292244	0.768040178	24	4.095239182	0.218786756
ENSMUSG00000039304	Tnfsf10	tumor necrosis factor (ligand) superfamily, member 10 [Source:MGI Symbol;Acc:MGI:107414]	1.08904	1.4429	1.51537	1.20146	1.40189	1.14768	1.16176	1.7859	1.70542	1.23279	1.30805	1.11088	1.10796	1.2936	0.004320961	0.140598674	12	16	24	0.030713407	0.597890721	24	7.899810201	0.12885092
ENSMUSG00000039307	Hexdc	hexosaminidase (glycosyl hydrolase family 20, catalytic domain) containing [Source:MGI Symbol;Acc:MGI:3605542]	5.92558	7.09262	7.46879	6.63243	6.4719	6.42133	6.6458	6.70928	8.33556	7.18312	6.71941	7.05348	6.39277	7.18451	0.002004172	0.100255953	12	16	24	0.010121828	0.503967859	24	8.651295261	0.107214809
ENSMUSG00000039308	Ndst2	N-deacetylase/N-sulfotransferase (heparan glucosaminyl) 2 [Source:MGI Symbol;Acc:MGI:97040]	6.0383	6.35715	6.3548	7.05132	6.77655	6.18396	6.4389	7.10007	6.74616	6.00542	5.27143	7.0676	4.86397	5.47908	0.865490576	0.966018836	16	12	24	0.720858615	0.908547187	24	11.53105383	0.041771141
ENSMUSG00000039313	Minar1	membrane integral NOTCH2 associated receptor 1 [Source:MGI Symbol;Acc:MGI:2667167]	7.95434	7.36882	6.95555	8.46234	5.9227	7.70056	7.13676	7.877	6.25449	10.1567	6.37532	7.72417	7.61757	8.46897	0.893609703	0.966018836	8	12	24	0.838197602	0.95041487	24	6.05174328	0.043482873
ENSMUSG00000039316	Rftn1	raftlin lipid raft linker 1 [Source:MGI Symbol;Acc:MGI:1923688]	1.28304	2.09341	2.66728	1.55249	1.40888	1.53912	2.04735	2.04995	2.12807	1.95063	1.12381	1.88451	1.48149	1.69211	0.002466754	0.109177422	12	8	24	0.048426252	0.638992719	24	6.581438715	0.192897497
ENSMUSG00000039318	Rab3gap2	RAB3 GTPase activating protein subunit 2 [Source:MGI Symbol;Acc:MGI:1916043]	20.8255	18.0005	20.4905	18.7863	18.569	19.6127	18.2643	19.6288	15.9438	21.2394	17.6338	19.9199	18.4925	18.6536	0.854443826	0.966018836	16	4	24	0.804427574	0.937432708	24	19.71318384	0.027985219
ENSMUSG00000039323	Igfbp2	insulin-like growth factor binding protein 2 [Source:MGI Symbol;Acc:MGI:96437]	18.0555	24.049	15.7296	15.5706	13.5346	11.0581	13.0965	21.2053	26.6641	8.51761	12.6597	15.7385	12.1654	17.7667	0.047504581	0.377556215	8	12	24	0.05105944	0.645887282	24	5.043949767	0.360535942
ENSMUSG00000039328	Rnf122	ring finger protein 122 [Source:MGI Symbol;Acc:MGI:1916117]	7.99763	7.12655	7.3241	7.58802	8.24183	8.15099	8.46839	9.16319	7.14216	8.94166	7.31036	8.11898	8.03436	7.70172	0.777666236	0.966018836	24	12	24	0.634403076	0.87566973	24	20.94210388	0.039445915
ENSMUSG00000039345	Mettl22	methyltransferase like 22 [Source:MGI Symbol;Acc:MGI:2384301]	13.5733	15.9659	14.5704	15.6718	16.3909	16.8731	14.1984	16.2502	19.2714	14.6526	16.2619	14.1063	18.5538	14.4841	0.921442568	0.966018836	20	8	24	0.824773456	0.945021534	24	10.07949319	0.035432138
ENSMUSG00000039347	Atp6v0e2	ATPase, H+ transporting, lysosomal V0 subunit E2 [Source:MGI Symbol;Acc:MGI:1923502]	153.196	129.757	131.504	126.602	125.351	152.271	140.037	142.892	106.096	155.721	120.774	136.107	127.676	135.794	0.522551072	0.966018836	24	20	24	0.362024504	0.787054939	24	22.06362738	0.083937839
ENSMUSG00000039349	C130074G19Rik	RIKEN cDNA C130074G19 gene [Source:MGI Symbol;Acc:MGI:2444831]	7.55134	9.18379	7.16942	6.91852	8.08762	8.33155	9.43516	9.5158	7.49275	8.18083	7.61751	8.34932	9.52934	8.0695	0.049705476	0.384427323	4	8	24	0.035336221	0.608551791	24	0.414645533	0.119388738
ENSMUSG00000039354	Smarcal1	SWI/SNF related matrix associated, actin dependent regulator of chromatin, subfamily a-like 1 [Source:MGI Symbol;Acc:MGI:1859183]	16.1236	13.2189	13.8107	12.9604	14.5206	15.7749	14.1459	14.9655	12.4826	16.0042	13.1946	14.9894	14.687	14.3647	0.14313801	0.585944911	24	12	24	0.193025637	0.768040178	24	21.23309937	0.077398267
ENSMUSG00000039356	Exosc2	exosome component 2 [Source:MGI Symbol;Acc:MGI:2385133]	12.2124	12.9656	13.7998	14.1949	14.6615	14.1071	11.3446	13.4082	12.2584	13.7114	14.0331	12.2534	13.6865	13.1602	0.013361709	0.233323991	20	8	24	0.095952613	0.71175955	24	13.57424604	0.077163372
ENSMUSG00000039357	Fut11	fucosyltransferase 11 [Source:MGI Symbol;Acc:MGI:1920318]	10.7695	9.21845	8.65867	9.86681	9.90097	9.75849	10.0773	8.90154	8.85097	9.52297	10.5423	10.0673	10.6766	9.38896	3.29E-04	0.049770315	4	8	24	0.006356356	0.470033096	24	19.79735594	0.099105515
ENSMUSG00000039361	Picalm	phosphatidylinositol binding clathrin assembly protein [Source:MGI Symbol;Acc:MGI:2385902]	91.0858	89.5248	88.1595	93.0025	90.0001	85.9662	87.8446	85.2274	82.8904	87.2772	86.1356	84.8704	84.7311	86.348	0.921442568	0.966018836	16	8	24	0.583039071	0.851725163	24	15.30154929	0.012874398
ENSMUSG00000039364	Sectm1b	secreted and transmembrane 1B [Source:MGI Symbol;Acc:MGI:1929083]	0.930947	0.465925	1.3987	0.69583	0.846146	0.867194	0.428188	1.62202	0.44624	1.12499	0.879262	0.684133	0.922371	0.626502	0.757932695	0.966018836	16	8	24	0.869340037	0.962936155	24	10.89639691	0.056711246
ENSMUSG00000039367	Sec24c	Sec24 related gene family, member C (S. cerevisiae) [Source:MGI Symbol;Acc:MGI:1919746]	25.9136	19.8539	20.5703	24.2452	22.9063	22.7155	23.3239	22.6089	18.8457	25.4458	21.4847	21.4051	20.9182	22.2661	0.187699199	0.662239322	16	20	24	0.470689144	0.805393068	24	18.15458562	0.060908281
ENSMUSG00000039372	Marchf4	membrane associated ring-CH-type finger 4 [Source:MGI Symbol;Acc:MGI:2683550]	3.89225	2.6613	3.30533	3.19526	3.39036	3.63844	3.18464	3.518	2.40725	4.50977	3.45765	3.61033	3.28115	3.56596	0.390686537	0.915479136	24	12	24	0.407322454	0.799546792	24	17.91941223	0.08877466
ENSMUSG00000039375	Wdr17	WD repeat domain 17 [Source:MGI Symbol;Acc:MGI:1924662]	7.39629	9.06579	7.0495	7.30523	8.22878	7.37676	6.97597	6.76208	8.64178	8.84375	7.6628	7.97078	8.81665	8.59224	0.795556128	0.966018836	16	12	24	0.791521816	0.933325571	24	8.842538445	0.034256629
ENSMUSG00000039376	Synpo2l	synaptopodin 2-like [Source:MGI Symbol;Acc:MGI:1916010]	0.335945	0.886893	0.973392	1.65999	0.945737	1.11211	0.890902	2.18029	1.39419	2.73762	0.890981	0.848573	1.13672	0.836735	0.23195324	0.731832877	16	12	24	0.074685832	0.683760931	24	10.34725584	0.305753668
ENSMUSG00000039377	Hlx	H2.0-like homeobox [Source:MGI Symbol;Acc:MGI:96109]	0.644813	0.599283	1.02217	0.640567	0.683876	0.811353	0.780565	0.673	0.642153	0.755149	0.991635	0.925769	1.2169	0.882762	0.823657258	0.966018836	24	16	24	0.701931057	0.901483676	24	20.78004779	0.040432813
ENSMUSG00000039382	Wdr45	WD repeat domain 45 [Source:MGI Symbol;Acc:MGI:1859606]	29.749	30.1209	26.5752	30.5835	30.9319	28.1385	26.4075	28.6605	29.9535	27.8934	28.8262	30.1381	29.2387	28.4211	0.917867465	0.966018836	20	8	24	0.682156388	0.894159472	24	16.09268348	0.023836857
ENSMUSG00000039384	Dusp10	dual specificity phosphatase 10 [Source:MGI Symbol;Acc:MGI:1927070]	1.44536	2.05422	2.13032	1.94582	1.81451	2.58057	2.09842	2.76126	2.37118	2.98281	1.99713	2.09233	2.49834	2.19383	0.917867465	0.966018836	8	12	24	0.458155194	0.805393068	24	8.280149774	0.080289678
ENSMUSG00000039385	Cdh6	cadherin 6 [Source:MGI Symbol;Acc:MGI:107435]	3.03273	2.75248	2.6027	2.50666	2.84413	2.76809	2.96687	2.3119	2.40778	2.74613	2.82681	2.44852	2.7809	2.76505	0.060394272	0.407012001	4	8	24	0.202830414	0.768040178	24	20.81736248	0.058202556
ENSMUSG00000039395	Mreg	melanoregulin [Source:MGI Symbol;Acc:MGI:2151839]	1.25087	1.8246	2.09283	2.38981	1.77073	1.81208	1.93975	2.0804	1.98099	2.53997	1.74042	1.96767	1.71076	1.7694	0.01950757	0.258568924	16	12	24	0.024696215	0.586541342	24	10.78238069	0.148175183
ENSMUSG00000039396	Neil3	nei like 3 (E. coli) [Source:MGI Symbol;Acc:MGI:2384588]	0.510965	0.747558	0.715456	0.786557	0.328016	0.519226	0.324232	0.425657	0.518812	0.437966	0.583578	0.300753	0.587371	0.266571	0.843639218	0.966018836	16	8	24	0.42409306	0.805393068	24	9.718943992	0.073494369
ENSMUSG00000039405	Prss23	protease, serine 23 [Source:MGI Symbol;Acc:MGI:1923703]	22.1321	19.6679	16.7966	18.4863	21.1611	19.8793	20.2422	22.7184	17.7007	18.9846	18.3213	19.0182	15.9498	15.4238	0.834434272	0.966018836	4	8	24	0.307293837	0.768040178	24	20.10211971	0.08198402
ENSMUSG00000039410	Prdm16	PR domain containing 16 [Source:MGI Symbol;Acc:MGI:1917923]	2.8921	2.40655	2.43827	2.28479	2.59638	2.92902	2.63494	3.40279	2.7228	3.06591	2.89677	2.47752	3.42538	2.27466	0.865233232	0.966018836	4	4	24	0.686300184	0.895774045	24	22.37512387	0.049038972
ENSMUSG00000039414	Heatr5b	HEAT repeat containing 5B [Source:MGI Symbol;Acc:MGI:2444098]	14.5684	11.7187	13.6573	12.3553	12.3021	13.3426	14.3349	13.5323	10.2927	14.9374	11.8293	12.7688	12.2658	13.2377	0.890648739	0.966018836	4	16	24	0.66117575	0.885520687	24	22.49567083	0.049120804
ENSMUSG00000039419	Cntnap2	contactin associated protein-like 2 [Source:MGI Symbol;Acc:MGI:1914047]	49.9599	43.6668	40.3793	45.5932	42.9631	44.8746	44.6844	42.1216	35.1797	50.206	40.6551	46.8497	45.3411	48.8197	0.321662653	0.83607295	24	12	24	0.419910855	0.803772782	24	21.38616713	0.071565541
ENSMUSG00000039427	Alg1	asparagine-linked glycosylation 1 (beta-1,4-mannosyltransferase) [Source:MGI Symbol;Acc:MGI:2384774]	15.7312	12.4413	12.7694	14.2092	15.5411	15.8834	14.2598	14.7421	15.1803	15.4927	13.5555	15.0607	15.1559	13.8372	0.277087842	0.789849983	24	8	24	0.239978085	0.768040178	24	18.86554585	0.068516614
ENSMUSG00000039428	Tmem135	transmembrane protein 135 [Source:MGI Symbol;Acc:MGI:1920009]	19.9191	18.1588	18.892	20.3189	18.0448	20.1175	18.7795	20.8059	16.3263	20.8956	18.5988	18.2492	19.883	21.0497	0.935732159	0.966018836	8	12	24	0.892422748	0.969564004	24	1.557862669	0.018735783
ENSMUSG00000039431	Mtmr7	myotubularin related protein 7 [Source:MGI Symbol;Acc:MGI:1891693]	46.3123	41.103	39.8601	40.992	40.2458	43.4843	40.1013	38.6552	34.827	44.2028	40.3825	42.6334	43.1499	44.2214	0.46238066	0.966018836	24	12	24	0.235644667	0.768040178	24	21.08013476	0.067227533
ENSMUSG00000039449	Prpf18	pre-mRNA processing factor 18 [Source:MGI Symbol;Acc:MGI:1914479]	26.5747	26.0004	25.0617	25.6184	25.9072	26.0349	28.1886	25.4867	24.4699	25.3835	24.7103	24.9323	27.8667	25.9162	0.001920665	0.100255953	4	8	24	0.033386069	0.606585237	24	23.51121376	0.052744747
ENSMUSG00000039450	Dcxr	dicarbonyl L-xylulose reductase [Source:MGI Symbol;Acc:MGI:1915130]	5.48195	4.91575	5.35415	4.91098	6.25668	5.58506	4.55177	6.48381	6.83164	5.65258	5.46027	6.18954	5.26374	5.97078	0.879201926	0.966018836	12	16	24	0.719277213	0.907919194	24	10.30144624	0.044091607
ENSMUSG00000039452	Snx22	sorting nexin 22 [Source:MGI Symbol;Acc:MGI:2685966]	1.10635	1.54871	1.73874	1.35267	1.42382	1.2434	1.63067	0.985499	1.28766	1.08409	1.04105	1.27403	1.12503	1.34455	0.879201926	0.966018836	12	8	24	0.780008134	0.929305332	24	6.29953461	0.03772299
ENSMUSG00000039456	Morc3	microrchidia 3 [Source:MGI Symbol;Acc:MGI:2136841]	10.381	12.9237	12.7902	11.8404	16.1911	10.6312	11.0594	10.7774	12.0712	10.1002	11.7052	10.9096	10.8166	11.2762	0.310940564	0.820837787	12	12	24	0.453174265	0.805393068	24	12.74953619	0.075048112
ENSMUSG00000039457	Ppl	periplakin [Source:MGI Symbol;Acc:MGI:1194898]	0.594365	0.617787	0.546368	0.942761	0.77527	0.571064	0.499546	0.523142	1.0562	0.792848	0.686589	0.585905	0.628822	0.629622	0.097542159	0.501112642	16	12	24	0.019168415	0.562219749	24	11.63473687	0.130210097
ENSMUSG00000039458	Mtmr12	myotubularin related protein 12 [Source:MGI Symbol;Acc:MGI:2443034]	5.81859	6.27922	6.24853	6.77305	5.98875	6.66953	6.00554	6.29676	6.39033	6.96029	5.64901	6.07229	5.81979	6.4497	0.058456626	0.404936103	16	12	24	0.158585234	0.764943091	24	9.872874628	0.056431584
ENSMUSG00000039461	Tcta	T cell leukemia translocation altered gene [Source:MGI Symbol;Acc:MGI:1918829]	49.6015	48.9188	47.5408	47.8399	48.0832	47.9391	44.9634	47.8919	46.7157	52.3358	51.5886	51.9553	49.4362	48.6705	0.308023687	0.814993005	24	12	24	0.34551104	0.778420274	24	16.03780051	0.031562583
ENSMUSG00000039463	Slc9a8	solute carrier family 9 (sodium/hydrogen exchanger), member 8 [Source:MGI Symbol;Acc:MGI:1924281]	11.8418	11.0208	12.9376	12.3045	11.8619	12.4193	11.3232	11.9621	11.3035	13.1874	10.3161	12.2481	11.1109	11.1648	0.351315142	0.870418653	16	16	24	0.526126978	0.822969183	24	12.53470168	0.038559849
ENSMUSG00000039470	Zdhhc2	zinc finger, DHHC domain containing 2 [Source:MGI Symbol;Acc:MGI:1923452]	136.943	120.267	109.072	120.613	124.983	119.775	114.111	109.129	101.33	112.787	131.134	111.185	118.448	116.451	0.064422003	0.414867207	20	16	24	0.085048047	0.70291653	24	19.42197242	0.09048162
ENSMUSG00000039473	Ubn1	ubinuclein 1 [Source:MGI Symbol;Acc:MGI:1891307]	12.5642	10.7572	12.7036	13.02	13.2765	12.0519	11.4102	12.8631	11.475	11.9135	11.3853	11.4328	11.407	10.7452	0.687668716	0.966018836	16	16	24	0.258391848	0.768040178	24	14.38578502	0.049582231
ENSMUSG00000039474	Wfs1	wolframin ER transmembrane glycoprotein [Source:MGI Symbol;Acc:MGI:1328355]	11.5881	8.50742	9.27654	10.16	9.1544	11.1697	10.0772	11.8481	8.05155	15.3443	8.7024	10.2892	9.90381	9.94638	0.883024237	0.966018836	24	12	24	0.933350952	0.983206815	24	18.02897314	0.033631926
ENSMUSG00000039476	Prrx2	paired related homeobox 2 [Source:MGI Symbol;Acc:MGI:98218]	1.75256	2.28671	1.47565	1.35109	2.00497	1.48167	2.18221	1.68147	1.86691	1.45298	2.08414	1.34567	1.49948	1.74916	0.598711142	0.966018836	8	8	24	0.723270844	0.909188168	24	1.721294654	0.051372334
ENSMUSG00000039477	Tnrc18	trinucleotide repeat containing 18 [Source:MGI Symbol;Acc:MGI:3648294]	8.31013	6.19489	7.32957	7.07765	6.62918	5.91548	7.93772	7.57437	7.63883	9.44654	8.66597	8.27244	9.14635	8.39777	0.879844076	0.966018836	4	20	24	0.930967249	0.982245205	24	6.793926568	0.022256588
ENSMUSG00000039478	Micu3	mitochondrial calcium uptake family, member 3 [Source:MGI Symbol;Acc:MGI:1925756]	61.7946	55.2669	53.8448	52.9591	52.5761	54.2206	53.0336	48.4454	50.8725	54.2083	60.453	53.7267	54.9209	59.607	0.865233232	0.966018836	4	8	24	0.618459722	0.868968106	24	20.93289413	0.038102192
ENSMUSG00000039480	Nt5dc1	5'-nucleotidase domain containing 1 [Source:MGI Symbol;Acc:MGI:2442446]	2.71298	3.2359	3.1165	3.05051	3.37744	2.88237	2.41458	3.78829	3.05322	2.70718	3.02857	2.80941	3.00723	2.85105	0.210566453	0.69936851	8	20	24	0.527121975	0.82330352	24	8.734109914	0.054346025
ENSMUSG00000039483	Asb6	ankyrin repeat and SOCS box-containing 6 [Source:MGI Symbol;Acc:MGI:1919573]	15.3971	13.1561	15.4538	12.2525	14.9808	15.3156	12.7005	14.9194	13.4656	14.5692	14.7267	15.5754	14.0672	13.8968	0.560714538	0.966018836	24	8	24	0.489237065	0.805393068	24	19.17952205	0.051360709
ENSMUSG00000039485	Tspyl4	TSPY-like 4 [Source:MGI Symbol;Acc:MGI:106393]	163.872	164.57	175.422	158.624	136.37	167.709	157.404	151.798	134.337	165.522	148.567	158.859	147.099	167.662	0.886572916	0.966018836	8	12	24	0.731817824	0.912550805	24	2.786596	0.034476201
ENSMUSG00000039488	Cntn5	contactin 5 [Source:MGI Symbol;Acc:MGI:3042287]	0.647176	0.642136	0.645197	0.782482	0.68988	0.749541	0.946641	0.483018	0.71082	0.599329	0.521561	0.76377	0.397339	0.927127	0.832293447	0.966018836	24	20	24	0.991050781	0.998487558	24	22.3822211	0.007447721
ENSMUSG00000039496	Cdnf	cerebral dopamine neurotrophic factor [Source:MGI Symbol;Acc:MGI:3606576]	3.3605	4.13972	4.76499	4.84912	2.9817	4.00609	3.10097	3.92872	3.31885	4.12663	3.31314	3.25988	4.54879	3.36785	0.265774856	0.773510818	16	4	24	0.403089331	0.799546792	24	8.37673669	0.100803022
ENSMUSG00000039497	Dse	dermatan sulfate epimerase [Source:MGI Symbol;Acc:MGI:2443455]	5.97652	5.34868	4.42205	4.38955	5.42367	4.33095	4.82157	4.87129	4.96942	4.02364	4.91203	4.74188	4.48912	5.02405	0.482528947	0.966018836	8	8	24	0.292204116	0.768040178	24	0.138042768	0.070501079
ENSMUSG00000039501	Znfx1	zinc finger, NFX1-type containing 1 [Source:MGI Symbol;Acc:MGI:2138982]	19.8715	13.3915	13.0898	17.2199	16.6688	19.3594	15.5784	17.8729	11.2207	19.1669	13.5957	15.9151	14.1525	14.9478	0.245061943	0.741580974	24	12	24	0.260712756	0.768040178	24	19.3415922	0.147543306
ENSMUSG00000039508	Calhm4	calcium homeostasis modulator family member 4 [Source:MGI Symbol;Acc:MGI:2685489]	2.42293	2.0741	1.88495	2.1761	2.12292	2.04021	1.66726	1.70504	1.85601	1.84512	2.23535	1.8902	2.00907	1.95473	0.725061903	0.966018836	20	16	24	0.312007191	0.768040178	24	17.6824885	0.05616545
ENSMUSG00000039509	Nup133	nucleoporin 133 [Source:MGI Symbol;Acc:MGI:2442620]	6.2444	6.15258	5.93569	6.62547	5.81574	5.69716	6.59943	6.64049	5.93421	7.63993	5.93019	5.6542	5.98502	6.74378	0.018894761	0.256345714	8	16	24	0.276351975	0.768040178	24	8.020730857	0.066778462
ENSMUSG00000039512	Uhrf1bp1	UHRF1 (ICBP90) binding protein 1 [Source:MGI Symbol;Acc:MGI:3041238]	2.64143	2.58167	3.06337	3.12611	2.72613	2.6476	2.78043	2.52472	2.38907	3.36947	2.3442	2.69394	2.46927	2.78442	0.700065774	0.966018836	16	12	24	0.276571013	0.768040178	24	11.4508665	0.066865896
ENSMUSG00000039515	Ptpa	protein phosphatase 2 protein activator [Source:MGI Symbol;Acc:MGI:1346006]	64.6786	59.0049	59.009	64.6648	61.959	65.783	62.4824	63.6601	57.926	67.562	60.4812	61.7877	63.2553	64.1641	0.791413662	0.966018836	16	20	24	0.595036369	0.857661186	24	19.04860655	0.026519165
ENSMUSG00000039519	Cyp7b1	cytochrome P450, family 7, subfamily b, polypeptide 1 [Source:MGI Symbol;Acc:MGI:104978]	6.22825	7.32637	7.40216	6.77062	7.39546	6.27523	7.32246	6.96418	7.53851	6.37241	7.35799	6.34044	6.84092	6.75102	0.02431074	0.286685431	12	12	24	0.352224438	0.780629136	24	8.462230458	0.051115909
ENSMUSG00000039523	Cep104	centrosomal protein 104 [Source:MGI Symbol;Acc:MGI:2687282]	10.3351	8.03474	7.81138	9.47085	8.85668	9.21399	11.1131	9.83199	7.79114	11.6419	8.39356	9.05144	8.69205	8.07096	0.193864198	0.67239758	4	8	24	0.586167119	0.853257144	24	19.474329	0.071182015
ENSMUSG00000039529	Atp8b1	ATPase, class I, type 8B, member 1 [Source:MGI Symbol;Acc:MGI:1859665]	0.895546	0.912353	1.04539	0.90618	1.11487	0.735208	0.900063	0.931513	1.06622	0.752666	0.842738	0.913769	0.811873	0.774226	0.648082424	0.966018836	12	8	24	0.586080491	0.853257144	24	9.853421401	0.033555786
ENSMUSG00000039531	Zup1	zinc finger containing ubiquitin peptidase 1 [Source:MGI Symbol;Acc:MGI:1919830]	4.90288	5.67952	4.66669	4.74117	5.23262	4.78203	5.71024	4.87255	5.91609	4.38266	5.5155	5.59881	5.58248	5.88408	0.33885934	0.858075318	8	8	24	0.299923671	0.768040178	24	1.379769175	0.064134554
ENSMUSG00000039533	Mmd2	monocyte to macrophage differentiation-associated 2 [Source:MGI Symbol;Acc:MGI:1922354]	23.4161	22.0403	22.8192	18.7401	22.6445	24.8141	25.2012	21.6666	18.3763	21.5121	23.6002	21.3551	20.4814	20.8265	0.372888979	0.89586062	20	20	24	0.060091897	0.658304792	24	20.87986397	0.109847606
ENSMUSG00000039536	Stau1	staufen double-stranded RNA binding protein 1 [Source:MGI Symbol;Acc:MGI:1338864]	24.3629	21.2578	19.9361	20.6016	20.767	23.5144	23.0413	23.0685	19.8338	25.2963	20.9156	23.5747	21.5014	21.4361	0.020119233	0.259417302	24	12	24	0.264513694	0.768040178	24	21.24679991	0.068331783
ENSMUSG00000039539	Sgcz	sarcoglycan zeta [Source:MGI Symbol;Acc:MGI:2388820]	1.63956	1.71129	1.56444	1.54042	1.64922	1.20634	1.54693	1.40578	1.74005	1.36756	1.58934	1.50609	1.41849	1.67543	0.601647512	0.966018836	12	12	24	0.435439233	0.805393068	24	7.010905397	0.044936351

ENSMUSG00000039542	Ncam1	neural cell adhesion molecule 1 [Source:MGI Symbol;Acc:MGI:97281]	30.5898	25.5213	30.3972	25.8041	25.4428	33.209	30.667	29.2018	22.0056	33.1027	22.3095	28.2695	24.0952	28.7935	0.914502381	0.966018836	24	20	24	0.709753417	0.903951515	24	23.12253845	0.061792911
ENSMUSG00000039543	Cfap70	cilia and flagella associated protein 70 [Source:MGI Symbol;Acc:MGI:1923920]	0.975465	0.873683	1.69916	1.03433	0.773967	0.902604	0.781241	0.702806	0.872273	0.660486	0.429027	1.28189	0.508094	0.818299	0.923635729	0.966018836	12	8	24	0.818771489	0.942446688	24	7.496516308	0.054808447
ENSMUSG00000039546	Ajap1	adherens junction associated protein 1 [Source:MGI Symbol;Acc:MGI:2685419]	0.616211	1.36834	1.80599	0.823806	0.53253	0.57231	0.772726	0.653045	1.54531	0.577015	0.313709	0.84437	0.273117	0.973689	0.005378037	0.149433822	12	8	24	0.026251577	0.586541342	24	7.094572354	0.307981344
ENSMUSG00000039552	Rsph4a	radial spoke head 4 homolog A (Chlamydomonas) [Source:MGI Symbol;Acc:MGI:3027894]	0.419809	0.635533	0.765742	0.53716	0.624326	0.402199	0.790875	0.316358	0.442981	0.591914	0.494105	0.647662	0.240903	0.679071	0.794858476	0.966018836	12	16	24	0.751448113	0.91921305	24	11.13738379	0.047511163
ENSMUSG00000039556	Ppp1r3f	protein phosphatase 1, regulatory subunit 3F [Source:MGI Symbol;Acc:MGI:1859617]	3.95847	4.77035	3.59133	3.62989	3.80729	3.80931	4.20143	4.45424	4.26201	6.05686	5.2297	4.48583	4.03225	4.3705	0.886572916	0.966018836	8	4	24	0.717555813	0.90689011	24	11.23151791	0.051366112
ENSMUSG00000039563	2210406O10Rik	RIKEN cDNA 2210406O10 gene [Source:MGI Symbol;Acc:MGI:1923960]	2.10626	2.12447	1.69989	2.7126	2.72042	1.98756	2.61551	2.11342	2.1921	2.16368	2.53353	2.24848	2.00163	2.534	0.724067924	0.966018836	20	8	24	0.443689848	0.805393068	24	15.88951725	0.067138183
ENSMUSG00000039568	Ubald1	UBA-like domain containing 1 [Source:MGI Symbol;Acc:MGI:1916255]	17.3024	13.3046	14.0774	14.6772	11.8255	14.2146	15.5839	16.0957	15.5193	21.5975	14.3641	17.2452	16.0754	15.5003	0.969487593	0.970883022	4	16	24	0.904292448	0.973594617	24	5.714301414	0.033196727
ENSMUSG00000039577	Nphp4	nephronophthisis 4 (juvenile) homolog (human) [Source:MGI Symbol;Acc:MGI:2384210]	1.60101	1.7077	1.79446	1.62614	1.82185	1.57682	1.90637	1.69874	1.90046	2.02028	1.7327	1.77594	1.7265	1.68494	0.65208848	0.966018836	12	20	24	0.32439112	0.768040178	24	10.59422039	0.037216138
ENSMUSG00000039578	Ccser1	coiled-coil serine rich 1 [Source:MGI Symbol;Acc:MGI:3045354]	2.29229	2.11473	3.15084	1.9225	1.90745	2.7932	2.78986	2.42665	2.25589	1.95722	2.81584	2.41792	2.77265	3.71576	0.636977386	0.966018836	24	16	24	0.225066036	0.768040178	24	2.194023612	0.121445643
ENSMUSG00000039579	Grin3a	glutamate receptor ionotropic, NMDA3A [Source:MGI Symbol;Acc:MGI:1933206]	4.56306	4.42801	5.13682	4.14922	4.21709	4.96798	4.22266	3.92149	3.99631	4.50016	4.10118	4.50381	4.27724	4.47418	0.938993891	0.966018836	24	20	24	0.870557332	0.963313607	24	20.93892559	0.019287207
ENSMUSG00000039585	Myo9a	myosin IXa [Source:MGI Symbol;Acc:MGI:107735]	21.0476	23.1562	21.9314	23.6606	24.67	21.5312	24.1441	19.1866	24.5818	16.7813	26.3264	23.0022	24.7155	22.4954	0.953629935	0.966018836	20	12	24	0.704617555	0.902439904	24	19.51249983	0.056195388
ENSMUSG00000039599	Fam149b	family with sequence similarity 149, member B [Source:MGI Symbol;Acc:MGI:2145567]	20.848	16.2773	17.6573	19.1341	18.8421	19.0861	19.4048	18.4676	18.2559	16.3311	19.053	17.5576	18.8381	18.1361	0.321662653	0.83607295	4	4	24	0.206891508	0.768040178	24	20.91630688	0.062840442
ENSMUSG00000039601	Rcan2	regulator of calcineurin 2 [Source:MGI Symbol;Acc:MGI:1858219]	116.877	93.6175	96.3416	95.6747	89.145	106.633	99.326	100.728	76.5	116.154	88.9931	104.384	101.633	105.925	0.289105414	0.794869797	24	20	24	0.374382533	0.794636296	24	22.44532023	0.088922434
ENSMUSG00000039607	Rbms3	RNA binding motif, single stranded interacting protein [Source:MGI Symbol;Acc:MGI:2444477]	74.1886	67.2505	60.7627	69.5955	65.4148	68.2998	65.2557	64.6506	61.3368	74.8076	70.5331	75.2835	71.4105	74.5557	0.236893212	0.7385769	24	12	24	0.343633535	0.777219607	24	19.88470096	0.056063178
ENSMUSG00000039611	Pgap4	post-GPI attachment to proteins GalNAc transferase 4 [Source:MGI Symbol;Acc:MGI:1914313]	21.2821	17.5143	19.1886	19.2478	17.7913	21.6847	20.7672	21.9782	15.2081	23.202	19.5372	20.7359	20.0399	20.3329	0.46238066	0.966018836	24	12	24	0.452527131	0.805393068	24	21.09571754	0.07545832
ENSMUSG00000039615	Stub1	STIP1 homology and U-Box containing protein 1 [Source:MGI Symbol;Acc:MGI:1891731]	56.6016	47.0569	49.4176	48.0284	48.0425	54.426	50.3868	51.0831	43.5387	54.7128	48.8167	53.0866	51.8358	49.5373	0.071575474	0.440262583	24	12	24	0.12199619	0.734764914	24	20.99495707	0.077700048
ENSMUSG00000039616	Mocos	molybdenum cofactor sulfurase [Source:MGI Symbol;Acc:MGI:1915841]	2.9781	2.88042	2.71743	2.92031	3.04656	2.79402	2.52021	3.2938	2.62192	2.41121	3.0078	2.91902	3.4159	3.13843	0.760769894	0.966018836	8	4	24	0.431028484	0.805393068	24	23.93997345	0.051783192
ENSMUSG00000039617	Gm7488	predicted gene 7488 [Source:MGI Symbol;Acc:MGI:3647006]	27.473	26.4849	25.1974	23.2346	24.3906	22.6009	23.6755	21.7865	23.4164	20.5345	25.1086	23.7588	24.5379	22.5229	0.943920575	0.966018836	4	12	24	0.325993399	0.768040178	24	22.86221262	0.053669885
ENSMUSG00000039620	Trmt9b	tRNA methyltransferase 9B [Source:MGI Symbol;Acc:MGI:2442328]	3.07307	3.61674	3.6506	3.41429	3.36281	3.25481	2.67056	3.86257	2.87976	3.87751	2.73748	4.03475	3.78312	4.44189	0.917867465	0.966018836	8	12	24	0.510874545	0.814511881	24	5.884682864	0.074812917
ENSMUSG00000039621	Prex1	phosphatidylinositol-3,4,5-trisphosphate-dependent Rac exchange factor 1 [Source:MGI Symbol;Acc:MGI:3040696]	5.31851	5.68881	5.63891	5.45393	5.52546	6.61373	6.27103	6.21102	5.45824	6.40744	4.86498	5.81428	4.90914	5.75139	0.924553956	0.966018836	24	20	24	0.958490685	0.989144435	24	2.115327042	0.013129776
ENSMUSG00000039623	Ap5z1	adaptor-related protein complex 5, zeta 1 subunit [Source:MGI Symbol;Acc:MGI:1924908]	7.4916	6.62485	6.75676	7.02444	6.97892	7.33497	7.76346	7.78666	7.45441	8.45147	8.23982	8.04847	8.4112	7.40171	0.839634479	0.966018836	4	4	24	0.405254358	0.799546792	24	19.43002894	0.037951069
ENSMUSG00000039628	Hs3st6	heparan sulfate (glucosamine) 3-O-sulfotransferase 6 [Source:MGI Symbol;Acc:MGI:3580487]	0.901128	0.649156	0.490119	0.388396	0.733008	0.529496	0.396424	0.656447	0.46198	0.978814	0.70264	0.75226	0.711309	0.698744	0.622132989	0.966018836	20	16	24	0.77856355	0.928832041	24	19.55210886	0.04508207
ENSMUSG00000039629	Strip2	striatin interacting protein 2 [Source:MGI Symbol;Acc:MGI:2444363]	3.46583	3.10925	3.94703	3.37996	3.01938	3.43863	3.43342	2.78065	2.88812	3.28891	3.08555	3.00325	3.03785	3.60496	0.850906022	0.966018836	12	8	24	0.941837068	0.985597432	24	6.55543352	0.014745293
ENSMUSG00000039630	Hnrnpu	heterogeneous nuclear ribonucleoprotein U [Source:MGI Symbol;Acc:MGI:1858195]	107.052	130.123	139.248	123.723	127.358	124.748	118.484	121.123	137.17	111.545	113.57	118.881	114.182	117.026	0.112800839	0.530777648	12	16	24	0.214083126	0.768040178	24	8.878634264	0.071997061
ENSMUSG00000039632	Odad3	outer dynein arm docking complex subunit 3 [Source:MGI Symbol;Acc:MGI:1924859]	0.753435	0.77008	0.611693	0.58615	1.04273	0.84213	0.870669	0.726105	0.674321	0.610278	0.751798	1.17834	0.652091	0.794297	0.005593158	0.153935753	24	16	24	0.043660657	0.626787045	24	20.20576918	0.120648537
ENSMUSG00000039633	Lonrf1	LON peptidase N-terminal domain and ring finger 1 [Source:MGI Symbol;Acc:MGI:3609241]	8.12838	6.74361	6.59613	6.58564	6.44457	7.68074	7.02515	7.14161	6.02831	8.37734	7.31092	7.65221	7.21817	7.34665	0.148073804	0.590732968	24	12	24	0.291765038	0.768040178	24	20.76548038	0.07172624
ENSMUSG00000039634	Zfp189	zinc finger protein 189 [Source:MGI Symbol;Acc:MGI:2444707]	3.12578	2.70084	3.25669	3.40708	3.86117	3.3093	3.21219	2.92234	3.27849	2.74238	3.70807	3.16362	2.98046	3.11648	8.59E-04	0.071344007	20	12	24	0.056080061	0.65534544	24	16.12705886	0.095113573
ENSMUSG00000039637	Coro7	coronin 7 [Source:MGI Symbol;Acc:MGI:1926135]	24.4434	18.5425	19.9825	22.1847	21.7484	20.489	20.5216	21.8189	21.5872	25.1041	18.7637	21.9146	22.2946	20.0443	0.849179819	0.966018836	4	4	24	0.890230197	0.969000219	24	15.59911792	0.02328864
ENSMUSG00000039640	Mrpl12	mitochondrial ribosomal protein L12 [Source:MGI Symbol;Acc:MGI:1926273]	33.0971	32.6528	31.6503	31.7731	33.6538	32.0212	30.0339	35.566	33.1031	33.0737	35.9065	32.8687	35.6528	35.6535	0.882263435	0.966018836	20	20	24	0.900925693	0.972103557	24	5.551729899	0.011664986
ENSMUSG00000039646	Vasn	vasorin [Source:MGI Symbol;Acc:MGI:2177651]	4.86542	4.57072	3.78128	4.04327	4.07903	4.28182	4.40544	5.01686	5.75874	4.97823	5.46501	4.56073	5.98361	4.82905	0.915082018	0.966018836	8	8	24	0.595973196	0.857839743	24	3.273367826	0.052181948
ENSMUSG00000039648	Kyat1	kynurenine aminotransferase 1 [Source:MGI Symbol;Acc:MGI:1917516]	5.70425	5.45494	6.37098	6.04231	5.79857	5.5861	5.52704	6.30898	5.07083	5.57464	5.91307	5.563	5.42636	5.90899	0.725061903	0.966018836	16	12	24	0.75802842	0.921733004	24	10.13382309	0.022854003
ENSMUSG00000039652	Cpeb3	cytoplasmic polyadenylation element binding protein 3 [Source:MGI Symbol;Acc:MGI:2443075]	8.87481	7.40971	8.10496	8.96186	6.45545	7.9524	9.01647	7.81614	6.87199	10.2209	6.37556	7.86558	6.98952	8.43216	0.940625808	0.966018836	16	4	24	0.835483136	0.949499418	24	5.959494516	0.043263879
ENSMUSG00000039656	Rxrb	retinoid X receptor beta [Source:MGI Symbol;Acc:MGI:98215]	18.7852	16.005	17.7266	17.1627	17.5279	17.4993	19.1625	17.2948	17.2129	18.7492	16.9968	17.6402	17.4073	17.976	0.70641939	0.966018836	4	16	24	0.770933791	0.926171026	24	21.51133115	0.018945658
ENSMUSG00000039660	Spout1	SPOUT domain containing methyltransferase 1 [Source:MGI Symbol;Acc:MGI:106544]	11.7688	10.6648	10.9623	11.6018	11.2407	11.509	10.0556	11.4668	10.4256	12.1426	9.68794	11.1147	10.1669	10.2755	0.918497267	0.966018836	16	12	24	0.644001807	0.879114352	24	14.03114522	0.029793181
ENSMUSG00000039661	Dusp26	dual specificity phosphatase 26 (putative) [Source:MGI Symbol;Acc:MGI:1914209]	107.339	89.8138	92.7868	93.0527	93.8345	105.12	94.9495	93.2288	79.2757	90.7279	81.798	93.7024	90.2719	86.907	0.124211791	0.552144108	24	12	24	0.021944504	0.574400012	24	21.344301	0.097428685
ENSMUSG00000039662	Icmt	isoprenylcysteine carboxyl methyltransferase [Source:MGI Symbol;Acc:MGI:1888594]	33.7838	25.0678	24.9926	27.9002	26.029	31.4687	28.4741	29.8995	21.6277	33.546	25.051	29.2496	29.2272	28.6517	0.124211791	0.552144108	24	12	24	0.282085306	0.768040178	24	21.35994884	0.109825923
ENSMUSG00000039670	Oxld1	oxidoreductase like domain containing 1 [Source:MGI Symbol;Acc:MGI:1913681]	4.07387	5.96919	5.52465	6.62098	5.36041	5.70221	5.74336	5.7231	6.4592	6.04026	6.36159	5.82725	6.83709	6.1777	0.924553956	0.966018836	16	8	24	0.200310892	0.768040178	24	10.17210083	0.085637257
ENSMUSG00000039671	Zmynd8	zinc finger, MYND-type containing 8 [Source:MGI Symbol;Acc:MGI:1918025]	15.5966	13.1944	13.3818	13.8754	14.5808	14.7381	16.5995	14.9706	14.0285	14.9163	15.5864	15.8204	13.9914	14.9568	0.162786057	0.624917386	4	8	24	0.092562816	0.71175955	24	20.45131098	0.070697199
ENSMUSG00000039678	Tbc1d13	TBC1 domain family, member 13 [Source:MGI Symbol;Acc:MGI:2385326]	26.333	23.031	24.992	21.9813	23.5863	23.7788	25.0618	25.1696	22.3752	28.7844	22.6749	24.4154	24.6173	23.5245	0.297978647	0.80820842	4	12	24	0.887187926	0.96813543	24	0.291629341	0.01944674
ENSMUSG00000039680	Mrps6	mitochondrial ribosomal protein S6 [Source:MGI Symbol;Acc:MGI:2153111]	63.1329	56.6679	52.875	53.366	53.2443	56.344	63.4129	56.0525	52.3456	54.1957	65.0623	63.7026	68.234	62.7606	0.004140921	0.140598674	4	8	24	0.004088767	0.427431763	24	22.57215841	0.125600234
ENSMUSG00000039682	Lap3	leucine aminopeptidase 3 [Source:MGI Symbol;Acc:MGI:1914238]	33.854	31.1993	32.9841	31.513	31.9916	38.4109	33.7987	35.6734	30.9017	31.6297	33.1936	35.1936	34.001	33.4413	0.004653717	0.140598674	24	12	24	0.035912922	0.609176161	24	21.75126161	0.077690344
ENSMUSG00000039683	Sdk1	sidekick cell adhesion molecule 1 [Source:MGI Symbol;Acc:MGI:2444413]	2.9858	2.82186	2.87906	2.74877	3.32623	3.05322	3.81633	3.09946	3.17363	4.39769	2.60546	3.32591	3.47343	2.92947	0.834424969	0.966018836	4	4	24	0.95904004	0.989178508	24	18.68969317	0.01797983
ENSMUSG00000039684	Gm5422	predicted pseudogene 5422 [Source:MGI Symbol;Acc:MGI:3643411]	11.8689	11.2856	12.6151	13.2937	12.734	14.5648	12.6003	13.136	9.81173	13.4644	11.2803	11.0533	10.7031	11.2999	0.864782464	0.966018836	16	12	24	0.465307082	0.805393068	24	16.36205378	0.064707574
ENSMUSG00000039686	Zer1	zyg-11 related, cell cycle regulator [Source:MGI Symbol;Acc:MGI:2442511]	17.4847	17.5408	18.2264	17.8884	18.4797	18.2188	18.9372	18.427	19.0912	20.8869	16.4584	18.5181	17.0685	19.0253	0.834424969	0.966018836	16	4	24	0.403072037	0.799546792	24	9.327707951	0.042348093
ENSMUSG00000039693	Msantd3	Myb/SANT-like DNA-binding domain containing 3 [Source:MGI Symbol;Acc:MGI:1913915]	6.13097	5.49288	5.06246	6.30589	5.81807	6.4052	5.59811	6.76714	5.6493	5.78048	6.69198	7.09702	5.85798	5.73024	0.020957534	0.262822894	24	12	24	0.1284963	0.740162193	24	18.81847989	0.085869079
ENSMUSG00000039697	Ncoa7	nuclear receptor coactivator 7 [Source:MGI Symbol;Acc:MGI:2444847]	93.1156	78.6855	79.4177	77.3495	81.1299	85.8852	77.5904	76.1698	59.8347	76.1472	77.9951	70.5257	71.8409	76.271	0.882263435	0.966018836	20	20	24	0.136335153	0.747972781	24	20.51598494	0.091698062
ENSMUSG00000039701	Usp53	ubiquitin specific peptidase 53 [Source:MGI Symbol;Acc:MGI:2139607]	4.12366	5.23944	4.81788	4.91624	5.06065	4.72053	4.89254	4.70709	5.14638	4.11928	4.75401	4.57327	3.85284	4.48738	0.643325306	0.966018836	12	16	24	0.540840168	0.830298514	24	10.70024459	0.043951815
ENSMUSG00000039703	Nploc4	NPL4 homolog, ubiquitin recognition factor [Source:MGI Symbol;Acc:MGI:2679787]	24.4771	20.2925	21.2184	23.0673	21.6684	23.9536	20.9214	24.2954	18.7831	30.1304	19.5299	22.7547	21.2329	21.9135	0.914502381	0.966018836	24	12	24	0.834554107	0.94926834	24	15.51866337	0.039339965
ENSMUSG00000039704	Lmbrd2	LMBR1 domain containing 2 [Source:MGI Symbol;Acc:MGI:2444173]	18.8679	19.6217	18.1504	18.7974	19.0705	17.2467	17.9256	16.8747	18.2451	15.8668	20.382	17.5978	18.3422	18.9399	0.896753532	0.966018836	20	4	24	0.952042343	0.988339231	24	20.39132828	0.011494323
ENSMUSG00000039706	Ldb2	LIM domain binding 2 [Source:MGI Symbol;Acc:MGI:894670]	53.4662	40.1074	38.3695	42.3017	41.0002	49.4575	41.9374	46.3995	32.9897	50.4202	39.8772	51.68	49.2575	44.7644	0.02431074	0.286685431	24	12	24	0.095651601	0.71175955	24	21.27404643	0.15718086
ENSMUSG00000039713	Plekhg5	pleckstrin homology domain containing, family G (with RhoGef domain) member 5 [Source:MGI Symbol;Acc:MGI:2652860]	5.69206	7.66566	6.53162	6.75522	7.06698	6.23403	7.77769	7.48864	8.48304	8.84755	7.72944	7.1631	7.68176	6.37555	0.915475528	0.966018836	16	8	24	0.331283895	0.768063804	24	10.21486905	0.084231709
ENSMUSG00000039715	Dync2i2	dynein 2 intermediate chain 2 [Source:MGI Symbol;Acc:MGI:1919070]	5.86131	6.01352	6.8702	6.77003	5.79238	5.78121	6.37141	5.87415	7.06335	6.67983	6.55695	7.09533	6.65812	6.07052	0.346699241	0.870418653	12	20	24	0.248243441	0.768040178	24	10.93022935	0.057465436
ENSMUSG00000039716	Dock3	dedicator of cyto-kinesis 3 [Source:MGI Symbol;Acc:MGI:2429763]	19.3681	15.1254	16.4733	15.1327	11.5134	15.6689	14.7939	14.8543	15.0781	18.9301	13.674	15.9343	16.6851	17.8462	0.850398351	0.966018836	4	16	24	0.35956158	0.785405943	24	3.844657578	0.099308576
ENSMUSG00000039717	Ralyl	RALY RNA binding protein-like [Source:MGI Symbol;Acc:MGI:1924147]	67.4845	59.4988	59.7191	51.501	53.2598	58.3103	45.6448	57.3617	45.2667	64.7284	51.1207	58.2563	58.8084	60.8589	0.865233232	0.966018836	8	12	24	0.766244562	0.92437333	24	0.70184929	0.048635606
ENSMUSG00000039725	Trp53rka	transformation related protein 53 regulating kinase A [Source:MGI Symbol;Acc:MGI:1918294]	3.81184	4.29065	3.67613	4.68112	4.23245	4.71047	4.00943	4.26745	4.94633	4.24889	4.24024	4.48217	4.13603	3.87884	0.722296281	0.966018836	24	4	24	0.413215297	0.799546792	24	14.31884261	0.0500006
ENSMUSG00000039728	Slc6a5	solute carrier family 6 (neurotransmitter transporter, glycine), member 5 [Source:MGI Symbol;Acc:MGI:105090]	1.38933	4.24767	9.46098	2.96635	1.39432	4.93767	5.93722	1.74281	4.49341	3.31074	1.00154	3.20874	0.747951	5.18443	0.424718357	0.966018836	12	8	24	0.35158067	0.780345856	24	6.998656417	0.43611395
ENSMUSG00000039735	Fnbp1l	formin binding protein 1-like [Source:MGI Symbol;Acc:MGI:1925642]	10.6526	11.9597	13.3908	12.1264	12.2325	11.1579	10.1291	10.5167	12.6818	10.5574	11.5231	11.5723	10.5592	11.5389	0.018894761	0.256345714	12	16	24	0.087050154	0.706216588	24	10.70946707	0.086036284
ENSMUSG00000039737	Prkrip1	Prkr interacting protein 1 (IL11 inducible) [Source:MGI Symbol;Acc:MGI:1914051]	18.7346	21.4329	17.1815	15.1402	20.1597	20.8283	19.3676	18.724	20.8484	17.6494	21.5904	21.738	18.1355	16.6603	0.133012827	0.567728672	20	20	24	0.268898018	0.768040178	24	20.25220897	0.097839501
ENSMUSG00000039738	Slx4	SLX4 structure-specific endonuclease subunit homolog (S. cerevisiae) [Source:MGI Symbol;Acc:MGI:106299]	3.16166	4.11914	4.72653	4.50919	4.451	3.7353	4.43497	3.73885	4.28142	4.09027	4.23328	4.3337	3.91696	3.73434	0.210934353	0.699706585	12	20	24	0.114768551	0.727799063	24	12.21307357	0.092897944
ENSMUSG00000039740	Alg2	asparagine-linked glycosylation 2 (alpha-1,3-mannosyltransferase) [Source:MGI Symbol;Acc:MGI:1914731]	41.3799	31.0431	33.3782	32.4996	30.0481	41.3544	36.7166	37.0069	26.3785	42.7213	31.1166	35.3762	33.711	37.5338	0.591351901	0.966018836	4	16	24	0.419780036	0.803639585	24	22.27510337	0.102176491
ENSMUSG00000039741	Bahcc1	BAH domain and coiled-coil containing 1 [Source:MGI Symbol;Acc:MGI:2679272]	2.31229	2.15455	2.16879	1.80714	2.44611	1.97555	2.5327	2.22371	2.77881	2.74247	2.16008	2.14618	2.67105	1.97762	0.940101201	0.966018836	4	20	24	0.934128057	0.983548589	24	6.098825911	0.019841563
ENSMUSG00000039745	Htatip2	HIV-1 Tat interactive protein 2 [Source:MGI Symbol;Acc:MGI:1859271]	39.1727	33.2103	30.7504	32.5656	33.3015	37.2132	32.3595	35.3599	28.6623	33.3814	36.5834	36.3501	36.9695	34.0836	0.009630739	0.194720062	24	12	24	0.010458477	0.506240926	24	21.00835298	0.123255363
ENSMUSG00000039747	Orai2	ORAI calcium release-activated calcium modulator 2 [Source:MGI Symbol;Acc:MGI:2443195]	10.29	9.00058	9.32821	8.45994	8.18018	9.35395	8.71049	8.75242	7.44881	10.3538	8.08226	9.22163	8.78655	9.38261	0.95032543	0.966018836	24	20	24	0.643612011	0.878958277	24	23.70612965	0.043348459
ENSMUSG00000039753	Fbxl5	F-box and leucine-rich repeat protein 5 [Source:MGI Symbol;Acc:MGI:2152883]	32.4847	35.9957	34.1698	33.5316	34.0566	30.6161	31.9101	30.1118	35.036	28.912	32.8405	30.592	29.4894	30.5276	0.649212555	0.966018836	12	16	24	0.458256439	0.805393068	24	9.722011604	0.037803087
ENSMUSG00000039754	Alkbh4	alkB homolog 4, lysine demethylase [Source:MGI Symbol;Acc:MGI:1919291]	3.93706	3.55717	3.2501	3.20038	3.46918	3.78142	3.76681	3.95607	3.90622	3.94211	4.45451	4.06487	3.62144	3.90387	0.914502381	0.966018836	24	12	24	0.543596672	0.831743353	24	20.82694617	0.03895035
ENSMUSG00000039756	Dnttip2	deoxynucleotidyltransferase, terminal, interacting protein 2 [Source:MGI Symbol;Acc:MGI:1923173]	15.4476	15.548	15.116	15.1394	16.192	15.0127	15.8933	14.9216	17.3263	12.8124	16.8949	16.1884	16.3942	15.797	0.934818505	0.966018836	20	20	24	0.701481227	0.901135342	24	22.60203396	0.032924368
ENSMUSG00000039759	Thap3	THAP domain containing, apoptosis associated protein 3 [Source:MGI Symbol;Acc:MGI:1917126]	12.2045	15.3575	14.6035	12.9496	12.7637	12.3906	12.4021	15.893	17.259	14.7792	13.1226	14.1748	14.021	15.3823	0.018851976	0.256345714	8	20	24	0.00118041	0.336500758	24	7.100726319	0.181837785
ENSMUSG00000039763	Dnajc28	DnaJ heat shock protein family (Hsp40) member C28 [Source:MGI Symbol;Acc:MGI:2181053]	2.98303	3.16477	3.16166	4.34399	3.18204	3.51922	2.85328	3.48393	3.74102	3.33368	3.79155	3.63207	3.62844	3.4899	0.462767035	0.966018836	24	12	24	0.176613533	0.768040178	24	12.49647715	0.079335953
ENSMUSG00000039765	Cc2d2a	coiled-coil and C2 domain containing 2A [Source:MGI Symbol;Acc:MGI:1924487]	3.03044	4.13976	4.42283	3.81566	3.81669	3.88428	3.71301	3.85687	4.87496	3.97444	3.4539	4.12228	3.46225	4.8362	0.031969981	0.324385159	12	16	24	0.04880417	0.638992719	24	8.038306233	0.126465062
ENSMUSG00000039768	Dnajc11	DnaJ heat shock protein family (Hsp40) member C11 [Source:MGI Symbol;Acc:MGI:2443386]	21.0828	20.906	23.2971	23.8482	21.435	22.728	21.4049	21.0106	19.2698	22.4961	20.2462	20.5497	21.016	20.9919	0.929527443	0.966018836	16	16	24	0.499896112	0.808791684	24	13.46050376	0.033133693
ENSMUSG00000039770	Ypel5	yippee like 5 [Source:MGI Symbol;Acc:MGI:1916937]	77.2341	75.1657	73.1046	71.334	72.9031	74.9895	70.4161	71.095	68.2977	68.8778	78.7974	73.2217	76.6137	74.0852	0.200994225	0.686181266	4	8	24	0.14206967	0.751766185	24	21.2186903	0.046323432
ENSMUSG00000039771	Polr2j	polymerase (RNA) II (DNA directed) polypeptide J [Source:MGI Symbol;Acc:MGI:109582]	36.9032	38.7376	33.9198	36.4052	42.3104	40.2149	34.0698	38.0508	37.2876	30.5047	39.03	36.947	42.4023	38.5968	0.265774856	0.773510818	20	20	24	0.335065326	0.770984245	24	21.49194512	0.073344571
ENSMUSG00000039774	Galnt12	polypeptide N-acetylgalactosaminyltransferase 12 [Source:MGI Symbol;Acc:MGI:2444664]	0.51784	0.726209	0.653995	0.764027	0.653824	0.628412	0.986338	0.569364	0.79091	0.38152	0.628978	0.591167	0.50847	0.706671	0.887010723	0.966018836	12	16	24	0.805016238	0.937541099	24	3.908258535	0.032997482
ENSMUSG00000039781	Cep131	centrosomal protein 131 [Source:MGI Symbol;Acc:MGI:107440]	2.9459	4.40718	5.0135	4.0685	4.16569	4.19784	3.46865	3.15715	3.57342	4.56098	2.39613	3.21619	4.69517	4.32688	0.891105762	0.966018836	16	4	24	0.469292444	0.805393068	24	7.732234542	0.11850875
ENSMUSG00000039782	Cpeb2	cytoplasmic polyadenylation element binding protein 2 [Source:MGI Symbol;Acc:MGI:2442640]	26.2755	24.6192	24.3412	25.1642	25.7296	24.0408	25.0945	22.1834	21.249	24.2779	24.5769	21.2631	21.85	21.6082	0.945728192	0.966018836	20	16	24	0.125908982	0.739868076	24	16.95903437	0.052979959
ENSMUSG00000039787	Cercam	cerebral endothelial cell adhesion molecule [Source:MGI Symbol;Acc:MGI:2139134]	2.55344	3.07439	3.1169	2.49921	3.2008	2.71052	3.83512	3.49571	4.10367	3.09249	2.546	2.67992	3.04016	3.00236	0.289105414	0.794869797	12	4	24	0.170166531	0.767957732	24	5.816184878	0.119874121
ENSMUSG00000039789	Zfp597	zinc finger protein 597 [Source:MGI Symbol;Acc:MGI:1918313]	7.93684	6.69568	6.32583	7.12554	7.51985	6.61355	7.90414	6.92546	7.53572	7.11339	7.68588	7.41281	8.06291	7.35386	0.098459729	0.503116486	4	8	24	0.321978989	0.768040178	24	21.18407767	0.052181284
ENSMUSG00000039795	Zfand1	zinc finger, AN1-type domain 1 [Source:MGI Symbol;Acc:MGI:1913611]	8.81118	10.6416	10.163	10.1422	9.60117	8.85355	8.51908	10.3953	9.49191	8.76711	8.81093	10.0779	9.65326	9.72842	0.211626905	0.701016641	8	20	24	0.258975973	0.768040178	24	6.686918179	0.061817151
ENSMUSG00000039798	2600006K01Rik	RIKEN cDNA 2600006K01 gene [Source:MGI Symbol;Acc:MGI:1917569]	0.414795	0.766266	1.11264	0.734171	0.447671	0.749211	0.740418	0.707008	0.72342	0.747865	0.56126	0.872723	0.598453	0.888956	0.560714538	0.966018836	12	8	24	0.21805031	0.768040178	24	7.285821355	0.103442425
ENSMUSG00000039801	Cplane1	ciliogenesis and planar polarity effector 1 [Source:MGI Symbol;Acc:MGI:1920942]	5.57571	5.88647	5.19989	4.98567	5.14273	5.13678	4.69713	5.00764	5.47403	4.98646	5.49479	5.92353	5.35312	5.62502	0.892578749	0.966018836	8	8	24	0.756859567	0.921470626	24	0.950135427	0.024225503
ENSMUSG00000039804	Ncoa5	nuclear receptor coactivator 5 [Source:MGI Symbol;Acc:MGI:2385165]	13.3306	11.1894	12.2563	10.6575	11.6609	11.1585	11.8599	11.4473	12.1457	11.8529	11.9699	12.0751	12.7034	11.1916	0.702630284	0.966018836	4	12	24	0.530651992	0.824858059	24	23.08193289	0.034847947
ENSMUSG00000039809	Gabbr2	gamma-aminobutyric acid (GABA) B receptor, 2 [Source:MGI Symbol;Acc:MGI:2386030]	26.2306	18.0282	19.1025	20.3119	19.2339	23.1546	20.4234	22.5319	15.4254	30.7577	19.324	22.8059	20.8713	22.6514	0.245061943	0.741580974	24	12	24	0.689089231	0.897070579	24	19.86873337	0.082975058
ENSMUSG00000039810	Zc3h10	zinc finger CCCH type containing 10 [Source:MGI Symbol;Acc:MGI:2143670]	3.68346	2.7568	2.69286	2.89959	2.95325	4.02953	3.4124	3.6561	3.81805	3.43049	2.9449	3.15325	3.65363	3.55892	0.619261897	0.966018836	4	12	24	0.298797147	0.768040178	24	0.281409784	0.081097055
ENSMUSG00000039813	Tbc1d2	TBC1 domain family, member 2 [Source:MGI Symbol;Acc:MGI:2652885]	0.991182	0.827048	1.07772	1.26911	1.04185	0.858743	1.25786	1.1581	1.08027	0.883277	1.23947	1.31756	1.13729	1.20615	0.77722623	0.966018836	16	20	24	0.980400887	0.995255072	24	18.27312039	0.008354486
ENSMUSG00000039824	Myl6b	myosin, light polypeptide 6B [Source:MGI Symbol;Acc:MGI:1917789]	5.41682	5.40397	6.6619	5.55346	5.77403	6.05372	5.46685	6.63083	6.03641	7.28435	5.20931	5.00763	5.89545	5.44872	0.398157656	0.929472831	16	4	24	0.176985145	0.768040178	24	9.141483781	0.09529162
ENSMUSG00000039826	Trub2	TruB pseudouridine (psi) synthase family member 2 [Source:MGI Symbol;Acc:MGI:2442186]	11.0249	10.2859	10.6019	11.0556	9.6845	11.6036	8.57708	11.2794	10.2579	11.9422	9.24366	11.0686	10.1065	11.3308	0.965971228	0.969272194	16	12	24	0.747948554	0.917956946	24	8.666987775	0.038901491
ENSMUSG00000039828	Wdr70	WD repeat domain 70 [Source:MGI Symbol;Acc:MGI:1921020]	21.73	18.4669	18.9159	17.433	19.494	18.9412	19.2356	19.4346	16.5541	19.4884	17.5384	18.3543	19.1837	19.4915	0.45229395	0.966018836	4	8	24	0.137600458	0.747972781	24	23.374177	0.064898536
ENSMUSG00000039830	Olig2	oligodendrocyte transcription factor 2 [Source:MGI Symbol;Acc:MGI:1355331]	0.434865	1.0643	1.67474	0.366595	0.453403	0.93618	1.34947	0.586318	1.00772	0.762152	0.473659	0.870523	0.255384	1.25009	0.435739761	0.966018836	12	4	24	0.333890103	0.769985678	24	5.649511701	0.192988774
ENSMUSG00000039831	Arhgap29	Rho GTPase activating protein 29 [Source:MGI Symbol;Acc:MGI:2443818]	14.3812	18.4885	14.9524	14.1023	17.1883	13.6781	16.1998	17.3013	19.334	12.4542	16.8088	15.4274	15.9132	16.106	0.287843582	0.793714531	8	8	24	0.432472592	0.805393068	24	4.486211776	0.083455436
ENSMUSG00000039834	Zfp335	zinc finger protein 335 [Source:MGI Symbol;Acc:MGI:2682313]	4.25504	3.92367	4.16751	3.62764	4.41435	3.82129	4.36693	4.11143	4.3187	4.54632	4.17719	4.19579	4.10078	3.85443	0.928148816	0.966018836	20	12	24	0.890923859	0.969222457	24	15.6492849	0.013943122
ENSMUSG00000039835	Nhsl1	NHS-like 1 [Source:MGI Symbol;Acc:MGI:106390]	1.9868	1.90908	2.21946	1.68943	1.5561	1.95307	2.13096	2.03697	1.76848	1.75783	1.49658	1.76224	1.48248	1.95965	0.097542159	0.501112642	8	12	24	0.068915362	0.673520298	24	3.747123313	0.090313888
ENSMUSG00000039838	Slc45a1	solute carrier family 45, member 1 [Source:MGI Symbol;Acc:MGI:2653235]	11.765	10.0169	10.9039	11.5973	10.4381	12.728	11.268	11.0185	9.22952	14.4145	9.41884	10.9667	10.3687	11.3184	0.960333395	0.967043964	24	12	24	0.858874104	0.958441518	24	16.95651321	0.034577974
ENSMUSG00000039840	Epg5	ectopic P-granules autophagy protein 5 homolog (C. elegans) [Source:MGI Symbol;Acc:MGI:1918673]	5.35703	4.46331	4.59974	4.87508	4.6373	5.31958	4.87339	4.70591	4.0355	5.25227	4.27559	4.88113	5.00002	4.84159	0.039704107	0.338535904	24	12	24	0.231803485	0.768040178	24	21.12692444	0.065211167
ENSMUSG00000039841	Zfp800	zinc finger protein 800 [Source:MGI Symbol;Acc:MGI:1889334]	4.73239	4.57544	4.17442	4.33288	4.52879	4.61588	4.18261	3.28011	4.49419	3.52231	4.99321	4.6431	4.15686	4.42041	0.23371652	0.736061043	20	20	24	0.268394566	0.768040178	24	18.98459636	0.084466132
ENSMUSG00000039842	Mcph1	microcephaly, primary autosomal recessive 1 [Source:MGI Symbol;Acc:MGI:2443308]	3.47282	3.36572	3.6247	3.60633	3.39862	3.76614	3.17559	3.19819	2.71604	3.72352	3.16575	3.27725	3.33726	3.29666	0.935593607	0.966018836	16	16	24	0.500584122	0.808986978	24	16.93305311	0.040849583
ENSMUSG00000039844	Rapgef1	Rap guanine nucleotide exchange factor (GEF) 1 [Source:MGI Symbol;Acc:MGI:104580]	11.8891	11.2242	12.3805	12.2878	11.6676	12.6885	12.5692	13.7556	11.2387	15.9428	11.1159	12.9378	12.8054	12.2707	0.922029406	0.966018836	24	20	24	0.925583465	0.980131499	24	10.76792586	0.019681873
ENSMUSG00000039849	Pcif1	phosphorylated CTD interacting factor 1 [Source:MGI Symbol;Acc:MGI:2443858]	11.9372	13.1226	12.9805	12.0756	12.3801	10.6513	9.86112	14.8262	13.3874	12.7283	12.1157	11.9448	12.2837	12.6381	0.003210246	0.139597556	8	20	24	0.038725598	0.61140397	24	7.890880576	0.122178115
ENSMUSG00000039850	Endov	endonuclease V [Source:MGI Symbol;Acc:MGI:2444688]	4.83739	5.48947	5.04629	5.47401	5.20711	5.21837	5.24459	5.49067	5.12363	6.08369	4.91815	5.65798	6.19709	5.33843	0.922029406	0.966018836	8	12	24	0.945581376	0.986808796	24	6.490767584	0.010929195
ENSMUSG00000039852	Rere	arginine glutamic acid dipeptide (RE) repeats [Source:MGI Symbol;Acc:MGI:2683486]	14.481	13.6839	16.0134	16.6154	15.1751	14.0225	15.01	14.6724	16.9904	19.3164	14.7009	14.5118	13.1905	15.5176	0.004021228	0.140598674	16	12	24	0.003368378	0.417612737	24	10.835243	0.147261552
ENSMUSG00000039853	Trim14	tripartite motif-containing 14 [Source:MGI Symbol;Acc:MGI:1921985]	3.10156	2.66349	2.96106	3.20967	3.26699	2.75946	3.6195	3.49358	2.38787	3.26801	3.12696	3.92255	2.8665	3.84176	0.852921792	0.966018836	24	12	24	0.70603616	0.902918222	24	20.88005731	0.049713915
ENSMUSG00000039860	Srrm3	serine/arginine repetitive matrix 3 [Source:MGI Symbol;Acc:MGI:1920309]	13.647	10.5888	9.70103	10.9776	11.8373	10.6467	11.8066	11.2745	11.6582	11.2835	13.1885	12.9249	13.2028	12.5752	0.158469451	0.614860056	4	8	24	0.126853047	0.739868076	24	21.20705428	0.091021597
ENSMUSG00000039873	Neurl2	neuralized E3 ubiquitin protein ligase 2 [Source:MGI Symbol;Acc:MGI:3043305]	1.11778	1.31786	1.8873	1.51188	0.990448	1.45595	1.57101	2.13257	2.42962	2.54827	1.35246	1.39963	1.76874	1.79063	0.024253829	0.286402646	12	8	24	0.009031465	0.502965379	24	7.838448937	0.239787019
ENSMUSG00000039879	Heca	hdc homolog, cell cycle regulator [Source:MGI Symbol;Acc:MGI:2685715]	9.87313	10.35	9.80224	10.7714	10.1653	9.55117	11.348	9.51199	10.0025	11.3099	10.4725	9.67253	9.72284	10.37	0.424718357	0.966018836	16	8	24	0.497384235	0.807576732	24	11.80676496	0.035690966
ENSMUSG00000039883	Lrrc17	leucine rich repeat containing 17 [Source:MGI Symbol;Acc:MGI:1921761]	0.576859	0.850452	1.10404	1.05496	0.763458	0.719571	0.656433	1.00088	1.14372	1.03783	0.933873	0.952212	0.792591	0.839796	1.18E-04	0.029372629	12	16	24	6.44E-04	0.296483534	24	10.00886963	0.15686264
ENSMUSG00000039886	Tmem120a	transmembrane protein 120A [Source:MGI Symbol;Acc:MGI:2686991]	12.4333	10.8298	12.3504	13.7636	11.4399	12.6991	11.6357	13.3503	13.1648	14.3871	12.7745	13.1836	13.3272	13.3685	0.745118544	0.966018836	16	12	24	0.298132572	0.768040178	24	10.96481159	0.052173602
ENSMUSG00000039887	Alg14	asparagine-linked glycosylation 14 [Source:MGI Symbol;Acc:MGI:1914039]	25.4989	29.6523	25.0355	27.2758	29.5398	25.7156	29.4946	24.6839	26.2308	22.865	31.2238	26.9749	26.918	27.8741	0.602156008	0.966018836	20	20	24	0.574297382	0.847244567	24	19.82539518	0.051981324
ENSMUSG00000039891	Txlnb	taxilin beta [Source:MGI Symbol;Acc:MGI:2671945]	1.39559	3.61935	3.99403	6.15195	2.9919	4.70179	2.76162	8.68813	6.39766	8.21945	2.94245	3.07266	4.31652	3.69837	0.130696419	0.562427886	16	12	24	0.049519115	0.642285999	24	9.381875693	0.484977522
ENSMUSG00000039899	Fgl2	fibrinogen-like protein 2 [Source:MGI Symbol;Acc:MGI:103266]	21.2599	19.3567	17.7963	15.5899	21.703	16.6804	18.2046	19.8001	17.0369	13.0229	20.3624	14.729	14.8294	16.8356	0.947524602	0.966018836	20	20	24	0.652527364	0.883020861	24	23.08777806	0.066846563

ENSMUSG00000039901	Armh3	armadillo-like helical domain containing 3 [Source:MGI Symbol;Acc:MGI:1918867]	7.03696	6.02152	7.06128	6.33322	6.63852	7.61747	6.8202	6.85321	5.37669	8.50464	5.95686	6.81774	6.81363	7.03979	0.858266942	0.966018836	24	20	24	0.795615173	0.934626199	24	19.66462126	0.039478449
ENSMUSG00000039903	Eva1c	eva-1 homolog C (C. elegans) [Source:MGI Symbol;Acc:MGI:1918217]	6.33954	4.88363	4.80145	5.58214	5.91339	5.57068	4.78443	5.65805	4.59465	5.9402	4.87908	5.36713	5.33712	5.13457	0.503972924	0.966018836	16	20	24	0.376996022	0.796956096	24	18.58134409	0.065155225
ENSMUSG00000039904	Gpr37	G protein-coupled receptor 37 [Source:MGI Symbol;Acc:MGI:1313297]	3.08478	4.41433	6.73939	3.41316	3.03276	4.66047	5.04831	3.34632	4.37079	3.78478	2.71375	4.0713	2.31206	4.43749	0.616047374	0.966018836	12	8	24	0.421662286	0.804769987	24	6.59719248	0.163839566
ENSMUSG00000039908	Slc26a11	solute carrier family 26, member 11 [Source:MGI Symbol;Acc:MGI:2444589]	4.78509	4.5586	3.9195	5.69567	4.8026	4.70576	5.06782	4.98069	5.06197	5.59634	4.95437	4.83253	4.3863	3.87981	0.138719141	0.580158868	16	16	24	0.192103434	0.768040178	24	14.73097646	0.088751768
ENSMUSG00000039910	Cited2	Cbp/p300-interacting transactivator, with Glu/Asp-rich carboxy-terminal domain, 2 [Source:MGI Symbol;Acc:MGI:1306784]	3.5556	4.08763	4.60639	3.69574	3.59657	3.33572	4.10726	5.37203	4.5235	5.05971	4.54698	4.53964	3.20207	4.2192	0.170659409	0.643496587	12	16	24	0.12077728	0.732894732	24	8.689143931	0.139906
ENSMUSG00000039911	Spsb1	splA/ryanodine receptor domain and SOCS box containing 1 [Source:MGI Symbol;Acc:MGI:1921896]	8.12659	6.63575	7.4343	9.21054	6.92799	7.15905	8.29385	7.99241	6.93494	10.5283	6.10445	6.93227	7.37691	7.45769	0.369262716	0.893596186	16	4	24	0.640918863	0.878468414	24	9.643161546	0.069699076
ENSMUSG00000039913	Pak5	p21 (RAC1) activated kinase 5 [Source:MGI Symbol;Acc:MGI:1920334]	0.852637	1.03075	1.18074	0.877865	0.711592	1.00813	1.06803	0.747113	1.06012	0.763721	0.73553	0.992599	0.596303	0.973807	0.285027487	0.793714531	12	8	24	0.542813313	0.831118582	24	5.536097916	0.055575204
ENSMUSG00000039914	Coq10a	coenzyme Q10A [Source:MGI Symbol;Acc:MGI:2684847]	24.8163	28.8102	30.8101	36.9687	30.2978	32.8261	29.5551	39.2255	32.8307	37.0331	28.91	28.0128	32.0789	31.8859	0.287843582	0.793714531	16	12	24	0.107347425	0.71991576	24	9.642773406	0.134837338
ENSMUSG00000039917	Rhbdd2	rhomboid domain containing 2 [Source:MGI Symbol;Acc:MGI:1915612]	10.5436	10.4856	12.1864	12.3552	11.1183	13.1183	12.3913	11.9059	9.63658	12.9803	10.6695	11.2341	10.2586	10.4387	0.570349842	0.966018836	16	16	24	0.498178261	0.807983182	24	15.6260318	0.05808251
ENSMUSG00000039929	Urb1	URB1 ribosome biogenesis 1 homolog (S. cerevisiae) [Source:MGI Symbol;Acc:MGI:2146468]	1.78386	1.80543	1.81008	1.78677	1.72288	1.8433	2.5097	1.92913	1.56575	1.90098	1.71401	2.01206	1.69765	1.85625	0.892685629	0.966018836	24	20	24	0.361650228	0.786932131	24	23.07042278	0.058579061
ENSMUSG00000039934	Gsap	gamma-secretase activating protein [Source:MGI Symbol;Acc:MGI:2442259]	4.07241	4.40589	4.24507	3.67711	4.6191	4.21107	4.2161	3.75511	4.01391	3.89165	5.20476	3.42563	4.18096	4.14064	0.91241026	0.966018836	20	20	24	0.771349967	0.926270379	24	16.87004788	0.034685824
ENSMUSG00000039936	Pik3cd	phosphatidylinositol-4,5-bisphosphate 3-kinase catalytic subunit delta [Source:MGI Symbol;Acc:MGI:1098211]	10.6974	8.62718	8.73014	9.92831	10.0777	10.1576	9.8131	10.192	8.52877	12.5331	9.09308	10.2514	10.033	9.55311	0.080164531	0.458678242	24	12	24	0.466147589	0.805393068	24	17.86401788	0.064883053
ENSMUSG00000039942	Ptger4	prostaglandin E receptor 4 (subtype EP4) [Source:MGI Symbol;Acc:MGI:104311]	2.32016	2.56056	2.34428	2.16507	2.99156	2.56896	2.33845	2.61229	2.2264	2.83849	2.64576	2.50033	2.62313	2.53977	0.616047374	0.966018836	20	16	24	0.452797326	0.805393068	24	17.17720349	0.044980173
ENSMUSG00000039943	Plcb4	phospholipase C, beta 4 [Source:MGI Symbol;Acc:MGI:107464]	22.3989	22.6887	22.4736	24.4322	22.0785	22.8413	21.7712	21.9464	22.0088	22.1184	23.5395	24.9155	24.352	24.9722	0.890311217	0.966018836	24	12	24	0.861850324	0.959610017	24	19.67734877	0.013729305
ENSMUSG00000039952	Dag1	dystroglycan 1 [Source:MGI Symbol;Acc:MGI:101864]	48.8297	42.2244	40.7814	45.459	56.197	47.4081	58.3477	53.2384	42.5925	70.1785	43.853	49.9626	49.8598	43.2479	0.27817759	0.789849983	4	8	24	0.57705188	0.848822643	24	17.65394276	0.090252608
ENSMUSG00000039953	Clstn1	calsyntenin 1 [Source:MGI Symbol;Acc:MGI:1929895]	123.312	101.329	108.83	108.555	116.95	117.451	123.47	113.629	95.9357	153.118	95.9108	122.088	110.412	111.867	0.321662653	0.83607295	24	12	24	0.722390143	0.908924547	24	18.75876164	0.057455015
ENSMUSG00000039954	Stk32a	serine/threonine kinase 32A [Source:MGI Symbol;Acc:MGI:2442403]	3.24949	2.76748	3.83359	3.7544	2.89407	4.13756	3.33304	2.82976	2.61936	3.73486	3.5776	3.79963	2.88084	4.54223	0.836635326	0.966018836	24	12	24	0.720243508	0.908336488	24	15.94911653	0.059514753
ENSMUSG00000039956	Mrap	melanocortin 2 receptor accessory protein [Source:MGI Symbol;Acc:MGI:1924287]	1.8483	1.57913	3.25739	5.57793	3.1071	3.17188	2.76463	3.71425	2.47961	4.50343	6.0115	3.45008	6.57693	4.53522	0.931321934	0.966018836	16	20	24	0.207038442	0.768040178	24	13.7606475	0.234954165
ENSMUSG00000039958	Etfbkmt	electron transfer flavoprotein beta subunit lysine methyltransferase [Source:MGI Symbol;Acc:MGI:2443575]	4.37948	5.94899	6.24059	9.27122	6.12312	5.47829	4.91216	7.09961	7.54819	6.79127	6.47271	6.94604	6.66075	7.43271	0.101443845	0.511659635	16	12	24	0.014019045	0.530670295	24	10.27994824	0.216738871
ENSMUSG00000039959	Hip1	huntingtin interacting protein 1 [Source:MGI Symbol;Acc:MGI:1099804]	14.3724	12.2422	11.5245	13.9729	14.5113	14.0294	13.2988	13.1319	12.101	15.1068	12.8384	13.0959	12.6761	13.0339	0.056268883	0.401074772	16	20	24	0.13614598	0.747972781	24	17.39061219	0.077696702
ENSMUSG00000039960	Rhou	ras homolog family member U [Source:MGI Symbol;Acc:MGI:1916831]	19.7518	15.4037	18.0271	16.7195	17.9499	19.5164	17.7275	15.9785	14.4683	19.0581	19.3101	17.5261	16.0501	15.3842	0.048771079	0.379129806	24	8	24	0.014596739	0.536706227	24	17.87795951	0.134122975
ENSMUSG00000039963	Ccdc40	coiled-coil domain containing 40 [Source:MGI Symbol;Acc:MGI:2443893]	7.63168	5.74992	7.05978	5.86771	5.64242	7.76141	6.78404	7.15624	4.5413	7.57472	6.81258	5.55461	5.86124	6.87316	0.79256873	0.966018836	4	8	24	0.738708816	0.914690529	24	21.87961614	0.061316958
ENSMUSG00000039967	Zfp292	zinc finger protein 292 [Source:MGI Symbol;Acc:MGI:1353423]	3.33179	4.11441	3.58315	3.75874	4.08569	3.34543	3.71442	3.41335	4.36866	2.72851	4.29421	3.82966	4.06125	3.95845	0.915082018	0.966018836	20	8	24	0.971587441	0.993121308	24	2.824586315	0.013465724
ENSMUSG00000039968	Rsbn1l	round spermatid basic protein 1-like [Source:MGI Symbol;Acc:MGI:3036237]	7.22219	8.37608	7.09444	7.75871	7.61169	6.95399	7.5889	7.4679	7.45351	7.04732	8.3202	7.09604	7.20764	7.27425	0.887027852	0.966018836	20	4	24	0.891806758	0.969342045	24	11.04631421	0.014260029
ENSMUSG00000039976	Tbc1d16	TBC1 domain family, member 16 [Source:MGI Symbol;Acc:MGI:2652878]	5.00571	4.35322	4.88541	4.71884	4.15087	4.94055	4.30441	4.79438	3.87874	6.21685	3.83568	5.1232	4.46352	4.88929	0.922029406	0.966018836	24	20	24	0.983733947	0.996593965	24	10.00355175	0.011340962
ENSMUSG00000039982	Dtx4	deltex 4, E3 ubiquitin ligase [Source:MGI Symbol;Acc:MGI:2672905]	7.35805	7.10509	5.88435	5.99272	7.30566	6.64533	8.56045	6.50138	5.82264	5.58601	6.83895	6.62037	7.31526	6.25012	3.97E-04	0.052946481	4	12	24	0.003192839	0.414576087	24	22.17923864	0.161666931
ENSMUSG00000039983	Ccdc32	coiled-coil domain containing 32 [Source:MGI Symbol;Acc:MGI:2685477]	19.1239	18.6088	17.3869	17.6718	18.0606	19.6342	18.3464	19.6492	16.494	18.7395	17.8798	19.9921	19.4541	18.854	0.01130565	0.204837764	24	12	24	0.020540199	0.56691777	24	22.2886687	0.071264753
ENSMUSG00000039987	Phtf2	putative homeodomain transcription factor 2 [Source:MGI Symbol;Acc:MGI:1916020]	7.2322	8.3465	7.61987	10.2676	8.13109	8.69308	7.72641	12.2086	9.61938	9.43886	8.40639	7.4334	8.42003	7.89199	0.588208558	0.966018836	16	12	24	0.252946745	0.768040178	24	9.223357846	0.122565705
ENSMUSG00000039988	Ankrd13c	ankyrin repeat domain 13c [Source:MGI Symbol;Acc:MGI:2139746]	19.0183	16.6884	17.1187	16.605	18.663	16.2058	18.3931	15.5206	16.1616	17.7194	18.6524	16.6732	17.2695	16.9008	0.313239838	0.824470524	20	12	24	0.268840325	0.768040178	24	18.35681553	0.054009139
ENSMUSG00000039989	Cbx4	chromobox 4 [Source:MGI Symbol;Acc:MGI:1195985]	6.61368	4.31558	4.28577	4.13882	3.30025	3.84415	5.58444	5.42252	5.09915	6.58985	5.08581	6.2779	5.47853	5.49194	0.138719141	0.580158868	4	16	24	0.433747529	0.805393068	24	2.087528449	0.113776541
ENSMUSG00000039990	Edrf1	erythroid differentiation regulatory factor 1 [Source:MGI Symbol;Acc:MGI:1919831]	6.76758	6.69304	5.61618	7.25483	8.27815	6.35709	8.2557	7.02667	6.72887	6.80018	8.36733	7.86273	8.65411	7.28805	0.167216842	0.634643192	20	16	24	0.128573907	0.740162193	24	19.58458595	0.105582644
ENSMUSG00000039994	Timeless	timeless circadian clock 1 [Source:MGI Symbol;Acc:MGI:1321393]	15.2255	10.4843	11.343	11.5769	14.8725	14.431	11.4959	11.8315	8.38614	12.5237	14.1689	12.6518	10.7433	11.3826	0.018923071	0.256345714	20	16	24	0.007449854	0.477520755	24	18.5723965	0.225254362
ENSMUSG00000039997	Ifi203	interferon activated gene 203 [Source:MGI Symbol;Acc:MGI:96428]	2.95664	3.10585	2.99159	2.93786	3.73144	2.50748	3.25647	3.14739	3.55757	2.62494	3.26966	2.55416	3.0248	2.79919	0.745118544	0.966018836	12	12	24	0.77717115	0.928587932	24	9.086678338	0.03683118
ENSMUSG00000040003	Magi2	membrane associated guanylate kinase, WW and PDZ domain containing 2 [Source:MGI Symbol;Acc:MGI:1354953]	5.90904	7.00399	7.06325	6.1777	6.84694	6.74695	6.65867	6.4118	7.63362	8.91069	5.37608	8.35377	6.21106	8.10696	0.722296281	0.966018836	12	16	24	0.495709549	0.807333372	24	8.60592439	0.081605223
ENSMUSG00000040006	Ginm1	glycoprotein integral membrane 1 [Source:MGI Symbol;Acc:MGI:2384905]	20.7564	25.2929	25.4785	25.5295	23.7181	21.6947	24.1983	24.8889	27.6388	24.6065	24.7484	24.1272	24.0576	26.1165	0.004012334	0.140598674	12	8	24	0.001086747	0.336500758	24	8.578895968	0.111407252
ENSMUSG00000040007	Bahd1	bromo adjacent homology domain containing 1 [Source:MGI Symbol;Acc:MGI:2139371]	9.91856	6.60102	6.14816	7.68226	7.46549	7.87246	7.53791	7.85472	6.48116	10.0141	7.54652	8.48714	8.45597	8.24214	0.148073804	0.590732968	24	12	24	0.382411021	0.799546792	24	20.13211765	0.099055314
ENSMUSG00000040009	Gnaz	guanine nucleotide binding protein, alpha z subunit [Source:MGI Symbol;Acc:MGI:95780]	14.7906	11.1729	12.2073	11.8376	11.3598	12.8812	12.1484	12.6128	8.70789	15.0576	12.5819	13.6195	12.0967	13.0071	0.917867465	0.966018836	24	12	24	0.422195032	0.804917634	24	20.16490143	0.094563843
ENSMUSG00000040010	Slc7a5	solute carrier family 7 (cationic amino acid transporter, y+ system), member 5 [Source:MGI Symbol;Acc:MGI:1298205]	10.4234	9.8308	12.2436	13.4679	12.5426	12.6807	11.4682	11.0372	11.6573	19.4959	9.73423	12.1835	9.65132	9.96754	0.013006247	0.228055093	16	12	24	0.064493999	0.663876076	24	12.99976602	0.206681832
ENSMUSG00000040016	Ptger3	prostaglandin E receptor 3 (subtype EP3) [Source:MGI Symbol;Acc:MGI:97795]	18.7602	13.9103	13.2001	16.3399	14.7365	16.6304	14.89	14.9869	12.638	16.7549	17.8236	19.2608	18.5085	19.3681	0.143321297	0.585944911	24	12	24	0.097383381	0.71175955	24	20.49472255	0.139881104
ENSMUSG00000040018	Cox15	cytochrome c oxidase assembly protein 15 [Source:MGI Symbol;Acc:MGI:1920112]	8.77297	8.11341	8.65096	7.82809	6.85041	10.0274	7.50094	8.51228	7.4919	9.95896	7.96578	8.93079	8.0882	8.5213	0.892685629	0.966018836	20	20	24	0.948121794	0.987280175	24	21.81003599	0.018521833
ENSMUSG00000040021	Lats1	large tumor suppressor [Source:MGI Symbol;Acc:MGI:1333883]	8.17006	8.28588	8.08831	8.37994	8.26087	7.53474	8.46373	7.8585	8.02491	7.92544	8.49742	7.44992	7.44552	7.93945	0.893307667	0.966018836	12	4	24	0.45282241	0.805393068	24	10.09982268	0.025476154
ENSMUSG00000040022	Rab11fip2	RAB11 family interacting protein 2 (class I) [Source:MGI Symbol;Acc:MGI:1922248]	12.0363	11.9303	11.1885	10.8522	10.606	10.6861	10.189	10.5005	9.61077	12.2836	11.3949	11.6981	11.3672	12.2167	0.835668938	0.966018836	8	4	24	0.945905304	0.986887534	24	23.19590815	0.013263276
ENSMUSG00000040025	Ythdf2	YTH N6-methyladenosine RNA binding protein 2 [Source:MGI Symbol;Acc:MGI:2444233]	17.316	14.1136	14.5908	15.7661	15.069	15.5304	13.7675	14.5187	10.3484	16.5989	15.5076	16.5949	15.6696	14.1557	0.235187365	0.73629392	24	8	24	0.119185868	0.730142553	24	18.96126609	0.132522465
ENSMUSG00000040028	Elavl1	ELAV (embryonic lethal, abnormal vision)-like 1 (Hu antigen R) [Source:MGI Symbol;Acc:MGI:1100851]	11.5728	11.5934	11.6137	12.446	12.2392	11.9648	13.1367	11.4229	11.1902	11.4734	12.088	12.0328	10.2473	10.5708	0.636884864	0.966018836	20	12	24	0.373794698	0.794450623	24	15.27118332	0.042093014
ENSMUSG00000040029	Ipo8	importin 8 [Source:MGI Symbol;Acc:MGI:2444611]	20.3999	16.8471	18.0708	18.5769	16.5863	17.9886	17.6903	16.0368	15.6725	17.7152	17.7086	18.9393	17.8358	17.5991	0.387380622	0.915479136	24	8	24	0.224771187	0.768040178	24	20.16258739	0.062242092
ENSMUSG00000040033	Stat2	signal transducer and activator of transcription 2 [Source:MGI Symbol;Acc:MGI:103039]	21.7192	18.361	19.3543	18.9809	22.7033	21.3332	19.0486	21.668	17.8136	21.8272	21.9174	20.598	20.6407	22.2159	0.424718357	0.966018836	20	16	24	0.29120864	0.768040178	24	18.61196024	0.064683185
ENSMUSG00000040034	Nup43	nucleoporin 43 [Source:MGI Symbol;Acc:MGI:1917162]	5.61112	6.45308	5.9079	5.33565	5.40563	4.8025	5.831	6.06995	6.50305	4.69494	6.21091	5.44437	5.74631	5.62015	0.033799022	0.325496079	12	4	24	0.168889026	0.767020009	24	4.894010926	0.085154138
ENSMUSG00000040035	Disp2	dispatched RND tramsporter family member 2 [Source:MGI Symbol;Acc:MGI:2388733]	37.9066	31.271	38.7346	33.9314	30.6062	42.5831	38.0458	36.2923	26.5262	49.4111	27.4956	33.924	29.3015	38.0133	0.852921792	0.966018836	16	4	24	0.993799806	0.998755922	24	0.924151567	0.010836829
ENSMUSG00000040037	Negr1	neuronal growth regulator 1 [Source:MGI Symbol;Acc:MGI:2444846]	7.00929	8.40737	10.2613	7.95147	7.0272	8.04418	8.58175	6.59714	8.71619	7.95493	7.02575	8.18215	6.0813	8.60761	0.275526571	0.789849983	12	8	24	0.310485984	0.768040178	24	8.477168793	0.104641892
ENSMUSG00000040040	Ift88	intraflagellar transport 88 [Source:MGI Symbol;Acc:MGI:98715]	9.7981	10.673	10.029	10.0824	9.5267	10.2787	8.14742	10.1679	10.2959	9.72304	9.83027	10.0208	10.9682	10.2919	0.305564675	0.811201458	8	12	24	0.652653328	0.883085974	24	6.717106471	0.033987712
ENSMUSG00000040043	Rbms2	RNA binding motif, single stranded interacting protein 2 [Source:MGI Symbol;Acc:MGI:1861776]	7.6945	7.85795	8.0348	9.00806	9.1425	7.86062	7.80076	9.13873	8.31126	7.99358	8.11763	7.9899	8.7427	8.39752	0.454881285	0.966018836	20	8	24	0.551481081	0.83632237	24	10.73282316	0.034052133
ENSMUSG00000040044	Orc3	origin recognition complex, subunit 3 [Source:MGI Symbol;Acc:MGI:1354944]	22.7639	29.4951	27.481	25.9408	26.8904	24.6014	26.3068	24.2108	28.7935	23.9995	26.6439	25.8536	28.237	28.0541	0.628286596	0.966018836	8	16	24	0.40390435	0.799546792	24	6.93977715	0.056357305
ENSMUSG00000040048	Ndufb10	NADH:ubiquinone oxidoreductase subunit B10 [Source:MGI Symbol;Acc:MGI:1915592]	138.297	141.602	153.888	154.627	141.928	151.194	148.373	157.948	143.095	148.44	140.448	141.41	155.798	151.317	0.718720526	0.966018836	16	4	24	0.408534472	0.799546792	24	6.37282025	0.033768105
ENSMUSG00000040054	Baz2a	bromodomain adjacent to zinc finger domain, 2A [Source:MGI Symbol;Acc:MGI:2151152]	10.9603	10.3399	9.88265	10.7625	11.0754	10.7233	10.285	10.9146	9.99536	11.3565	10.7969	10.57	10.4717	10.6586	0.087978327	0.472021905	16	20	24	0.253412614	0.768040178	24	16.94371382	0.032676817
ENSMUSG00000040055	Gjb6	gap junction protein, beta 6 [Source:MGI Symbol;Acc:MGI:107588]	9.88471	18.4205	14.1147	8.79408	10.675	11.7919	13.9774	9.50741	15.9886	7.46997	8.42072	11.2231	6.40513	10.7714	0.369262716	0.893596186	12	4	24	0.406054882	0.799546792	24	4.710746784	0.185091434
ENSMUSG00000040061	Plcb2	phospholipase C, beta 2 [Source:MGI Symbol;Acc:MGI:107465]	0.638924	0.584994	1.00036	1.20188	0.708564	0.592413	1.21934	0.656152	0.87613	0.515044	0.692626	0.493921	0.731079	0.483283	0.868638024	0.966018836	12	12	24	0.746662969	0.917587548	24	9.934955805	0.056892293
ENSMUSG00000040078	Ptges3-ps	prostaglandin E synthase 3, pseudogene [Source:MGI Symbol;Acc:MGI:3704271]	148.987	123.923	111.318	124.232	138.974	132.612	133.784	121.312	91.4527	117.263	128.469	119.107	118.123	111.791	0.027439001	0.308438477	4	8	24	0.003653769	0.426426863	24	20.07122177	0.170755935
ENSMUSG00000040084	Bub1b	BUB1B, mitotic checkpoint serine/threonine kinase [Source:MGI Symbol;Acc:MGI:1333889]	1.55772	1.70229	2.14542	2.25078	2.94481	1.70872	2.49208	1.39092	2.01003	2.30255	1.56184	1.87354	1.79186	2.40488	0.914360142	0.966018836	16	16	24	0.428687472	0.805393068	24	12.5702929	0.103960733
ENSMUSG00000040093	Bmf	BCL2 modifying factor [Source:MGI Symbol;Acc:MGI:2176433]	1.59834	2.11819	2.03796	2.04213	2.32633	2.21398	1.99808	2.42994	2.769	2.88108	1.59983	2.41075	1.92653	2.15168	0.285193697	0.793714531	16	12	24	0.250335913	0.768040178	24	9.781808425	0.107525903
ENSMUSG00000040097	Flywch1	FLYWCH-type zinc finger 1 [Source:MGI Symbol;Acc:MGI:2442638]	90.6697	71.8544	84.2969	76.251	70.8007	89.7264	80.7241	82.1441	64.9066	100.794	71.3853	90.2953	77.8641	83.8468	0.960338592	0.967043964	24	20	24	0.730811812	0.912268461	24	21.30099328	0.056566168
ENSMUSG00000040102	Klhl42	kelch-like 42 [Source:MGI Symbol;Acc:MGI:2444786]	5.64876	5.11087	5.33861	5.19569	5.76057	5.77936	5.57063	4.87376	4.46539	5.14156	5.32798	5.17736	5.03916	5.0715	0.076942821	0.453790923	24	8	24	0.005002729	0.442351542	24	19.24330107	0.085040804
ENSMUSG00000040105	Plpp6	phospholipid phosphatase 6 [Source:MGI Symbol;Acc:MGI:1921661]	5.2411	4.75368	4.82574	5.6417	4.47895	5.30982	5.49249	5.0025	4.54402	6.3794	4.73602	5.78394	4.83175	5.70986	0.942429894	0.966018836	24	12	24	0.957316727	0.988678477	24	16.42891928	0.014777472
ENSMUSG00000040111	Gramd1b	GRAM domain containing 1B [Source:MGI Symbol;Acc:MGI:1925037]	40.8034	35.1714	33.6249	33.8693	35.7019	36.0989	37.4917	35.7201	27.5738	41.5985	31.6621	35.3496	36.1065	34.0549	0.018894761	0.256345714	4	8	24	0.318362363	0.768040178	24	21.40530758	0.083830322
ENSMUSG00000040112	Mrps35	mitochondrial ribosomal protein S35 [Source:MGI Symbol;Acc:MGI:2385255]	17.4171	20.0177	17.2098	21.0478	17.7603	17.3419	17.3529	17.192	21.1113	18.4557	18.2517	18.4217	23.701	22.2592	0.844511766	0.966018836	8	16	24	0.374840866	0.794826471	24	6.356106355	0.073902226
ENSMUSG00000040118	Cacna2d1	calcium channel, voltage-dependent, alpha2/delta subunit 1 [Source:MGI Symbol;Acc:MGI:88295]	47.7137	45.7889	49.7504	48.9037	51.7259	47.8448	41.1157	43.3716	46.0633	44.3361	54.7548	45.6361	44.4651	45.8027	0.046535188	0.375011045	20	8	24	0.041412793	0.618736234	24	14.7798738	0.090727691
ENSMUSG00000040121	Rep15	RAB15 effector protein [Source:MGI Symbol;Acc:MGI:1913782]	0.978318	0.659209	0.863115	0.761234	0.854591	0.813987	0.742998	0.688427	0.976836	0.768416	0.69306	0.773068	0.720417	1.05951	0.814075857	0.966018836	12	20	24	0.763106977	0.923594538	24	6.398640218	0.029114723
ENSMUSG00000040123	Zmym5	zinc finger, MYM-type 5 [Source:MGI Symbol;Acc:MGI:3041170]	17.0835	17.9046	17.7777	19.5506	19.1162	16.6022	17.8527	16.361	19.9596	15.9689	19.8237	18.0872	18.6117	17.7722	0.850398351	0.966018836	20	12	24	0.52069382	0.82006084	24	13.31773304	0.042934696
ENSMUSG00000040124	Gorab	golgin, RAB6-interacting [Source:MGI Symbol;Acc:MGI:2138271]	5.46062	5.47083	5.21515	4.69388	5.485	4.87169	4.53331	4.7833	4.84035	4.5472	5.1022	4.96368	4.71055	5.49736	0.944053021	0.966018836	8	8	24	0.87438468	0.964836189	24	1.354755813	0.016918925
ENSMUSG00000040125	Gpr26	G protein-coupled receptor 26 [Source:MGI Symbol;Acc:MGI:2441758]	1.67007	1.76685	1.81452	1.80694	1.85444	1.74791	1.73381	1.49105	1.95677	1.78908	1.66881	1.74732	1.67916	1.84178	0.060394272	0.407012001	12	16	24	0.251265157	0.768040178	24	11.27552421	0.037513226
ENSMUSG00000040128	Pnrc1	proline-rich nuclear receptor coactivator 1 [Source:MGI Symbol;Acc:MGI:1917838]	22.1024	20.6463	17.3964	19.9167	19.292	19.1241	21.0471	21.7468	19.775	22.6145	20.5059	24.1614	22.6086	23.0716	0.532538925	0.966018836	8	4	24	0.223981607	0.768040178	24	23.78138861	0.065326699
ENSMUSG00000040133	Gpr176	G protein-coupled receptor 176 [Source:MGI Symbol;Acc:MGI:2685858]	3.88618	2.76622	2.77794	2.98383	2.81794	3.72386	3.37987	3.18132	2.35736	3.46129	2.65728	3.36436	2.96467	3.67656	0.196044629	0.673646834	24	12	24	0.159876689	0.76564405	24	22.35081901	0.117176442
ENSMUSG00000040136	Abcc8	ATP-binding cassette, sub-family C (CFTR/MRP), member 8 [Source:MGI Symbol;Acc:MGI:1352629]	2.51648	2.33417	2.41796	1.82094	2.11773	2.97864	2.29368	2.43553	2.28148	3.51395	2.3791	2.2229	2.25005	2.33617	0.830949782	0.966018836	8	12	24	0.977290651	0.994251213	24	15.1640618	0.01366493
ENSMUSG00000040138	Ndp	Norrie disease (pseudoglioma) (human) [Source:MGI Symbol;Acc:MGI:102570]	9.07859	9.26313	9.36973	8.38034	10.3024	9.35406	9.89514	9.41713	9.44939	8.55938	9.8991	9.23846	10.3719	8.96082	0.676344318	0.966018836	20	20	24	0.301694328	0.768040178	24	21.51969838	0.049043464
ENSMUSG00000040139	9430038I01Rik	RIKEN cDNA 9430038I01 gene [Source:MGI Symbol;Acc:MGI:1924502]	2.92763	3.23256	3.77984	3.69012	2.88928	2.78686	2.14931	3.55979	4.65975	3.47559	3.5417	3.34146	3.61617	3.50012	0.020119233	0.259417302	12	12	24	0.01401812	0.530670295	24	9.150897297	0.195535764
ENSMUSG00000040140	Tdrd6	tudor domain containing 6 [Source:MGI Symbol;Acc:MGI:2679727]	2.58839	3.24931	2.62305	1.9706	2.98749	2.29921	2.82381	3.69307	3.01986	2.42554	2.972	2.87042	3.58139	3.39109	0.048396595	0.379129806	8	8	24	0.046064728	0.633009261	24	2.879874419	0.145818914
ENSMUSG00000040146	Rgl3	ral guanine nucleotide dissociation stimulator-like 3 [Source:MGI Symbol;Acc:MGI:1918996]	1.90584	2.12009	1.87261	1.58169	2.08988	2.05215	1.61206	3.21867	2.97128	2.35426	2.64089	2.11409	2.91637	1.78344	0.801280095	0.966018836	8	20	24	0.817014073	0.941826666	24	6.896884669	0.05506981
ENSMUSG00000040147	Maob	monoamine oxidase B [Source:MGI Symbol;Acc:MGI:96916]	2.6067	3.84625	5.86754	5.72877	3.23554	4.16397	5.68393	4.6539	4.4253	5.00165	4.91424	6.00403	4.23313	8.91729	0.933543173	0.966018836	16	12	24	0.293215944	0.768040178	24	7.952667957	0.184976654
ENSMUSG00000040151	Hs2st1	heparan sulfate 2-O-sulfotransferase 1 [Source:MGI Symbol;Acc:MGI:1346049]	12.9021	10.9291	11.3954	10.918	11.4396	11.4722	11.4756	11.2379	9.62036	12.9466	11.9407	11.6839	11.0039	11.3426	0.206977373	0.698112164	20	16	24	0.331973203	0.768777758	24	18.88380279	0.057367143
ENSMUSG00000040152	Thbs1	thrombospondin 1 [Source:MGI Symbol;Acc:MGI:98737]	6.5619	7.0197	6.31541	4.22002	5.03746	4.41941	7.58093	7.28609	6.87205	3.27944	4.79135	5.21235	4.86218	5.5466	0.007505042	0.192760771	8	8	24	0.009866508	0.503967859	24	2.902656139	0.283344512
ENSMUSG00000040158	Tax1bp3	Tax1 (human T cell leukemia virus type I) binding protein 3 [Source:MGI Symbol;Acc:MGI:1923531]	5.87797	5.93736	4.51466	4.68759	6.38581	5.27992	6.21934	5.8986	5.47179	4.38483	6.44757	5.72017	6.53017	5.37684	0.066380001	0.42386701	4	12	24	0.050022597	0.643877315	24	22.17625217	0.141590257
ENSMUSG00000040164	Kcns1	K+ voltage-gated channel, subfamily S, 1 [Source:MGI Symbol;Acc:MGI:1197019]	11.7245	7.92954	6.71014	8.60071	6.72533	8.88042	9.20791	9.80019	6.92225	11.8552	7.86367	9.73681	10.5143	10.3391	0.097760727	0.501112642	4	8	24	0.320242649	0.768040178	24	23.76714859	0.139005684
ENSMUSG00000040167	Ikzf5	IKAROS family zinc finger 5 [Source:MGI Symbol;Acc:MGI:1914393]	4.43028	4.55859	4.62717	4.92357	5.22563	4.53987	5.5572	4.62435	4.20863	4.07806	4.61991	3.97518	3.86318	3.91909	0.93717134	0.966018836	20	4	24	0.581436179	0.850792036	24	17.07760445	0.043376428
ENSMUSG00000040170	Fmo2	flavin containing monooxygenase 2 [Source:MGI Symbol;Acc:MGI:1916776]	3.67798	4.21656	7.86064	13.7701	11.9767	7.16007	3.95414	3.53023	10.3925	9.78487	9.77067	6.57543	3.73144	3.72324	3.51E-04	0.049770315	16	16	24	5.82E-07	0.004113811	24	13.4588342	0.800540024
ENSMUSG00000040174	Alkbh3	alkB homolog 3, alpha-ketoglutarate-dependent dioxygenase [Source:MGI Symbol;Acc:MGI:1916363]	34.0791	39.6284	36.8852	38.7767	32.347	37.6059	27.8248	35.9914	37.3325	40.792	34.9752	34.8465	39.3232	33.1466	0.446653834	0.966018836	16	12	24	0.326120862	0.768040178	24	10.09729081	0.084037375
ENSMUSG00000040177	2310057M21Rik	RIKEN cDNA 2310057M21 gene [Source:MGI Symbol;Acc:MGI:1915527]	7.02803	7.6022	7.78647	7.12612	6.43587	8.23727	7.52939	8.38045	6.15888	8.26894	6.34369	8.11043	8.45794	8.70492	0.305564675	0.811201458	8	12	24	0.224267145	0.768040178	24	2.072574692	0.089925579
ENSMUSG00000040181	Fmo1	flavin containing monooxygenase 1 [Source:MGI Symbol;Acc:MGI:1310002]	31.3456	27.904	37.6491	54.4872	45.6378	31.6013	29.8671	32.1823	39.1254	39.6462	56.8058	32.4278	40.1281	29.8543	7.56E-04	0.071344007	20	12	24	0.002920921	0.414576087	24	13.6820931	0.33915027
ENSMUSG00000040183	Ankrd6	ankyrin repeat domain 6 [Source:MGI Symbol;Acc:MGI:2154278]	8.03586	6.62898	7.27233	6.35036	6.70246	7.34502	7.17564	6.33404	5.54987	6.8273	6.78928	7.65073	7.0545	7.15964	0.083646462	0.463801902	24	16	24	0.038029789	0.61140397	24	21.45607445	0.10725034
ENSMUSG00000040187	Arntl2	aryl hydrocarbon receptor nuclear translocator-like 2 [Source:MGI Symbol;Acc:MGI:2684845]	3.89735	5.13161	4.48521	4.97349	5.76697	4.5176	4.32897	4.06295	5.27421	4.18758	5.71128	5.28586	5.43176	5.17535	0.480187506	0.966018836	20	12	24	0.436532961	0.805393068	24	14.93063299	0.070830187
ENSMUSG00000040188	Scamp2	secretory carrier membrane protein 2 [Source:MGI Symbol;Acc:MGI:1346518]	13.2956	12.6753	13.9079	12.8689	14.5459	13.7555	13.2781	14.5818	13.9713	13.5425	11.7974	12.8516	13.4124	11.9224	0.833301348	0.966018836	12	20	24	0.972138904	0.993238187	24	8.945018278	0.008163197
ENSMUSG00000040189	Odad1	outer dynein arm docking complex subunit 1 [Source:MGI Symbol;Acc:MGI:2446120]	1.43949	2.02439	1.75948	1.72929	1.85523	1.44667	1.79105	1.90113	2.22222	1.87499	1.58568	2.13214	1.50117	1.71701	0.43565473	0.966018836	12	16	24	0.263849953	0.768040178	24	8.761797062	0.081311099
ENSMUSG00000040195	Nemp1	nuclear envelope integral membrane protein 1 [Source:MGI Symbol;Acc:MGI:2446113]	3.48837	3.18106	2.84459	3.19306	3.31888	3.19923	3.17532	3.19325	2.94773	3.35273	3.03172	3.02375	3.89647	2.74491	0.581070934	0.966018836	4	8	24	0.310676811	0.768040178	24	20.93197598	0.05917181
ENSMUSG00000040204	Pclaf	PCNA clamp associated factor [Source:MGI Symbol;Acc:MGI:1915276]	0.5391	0.818847	1.38024	1.12408	1.05104	0.889711	0.95269	1.01492	1.00591	0.698935	0.705573	0.840789	0.849514	0.59972	0.729086532	0.966018836	12	16	24	0.391097291	0.799546792	24	10.51984672	0.087579569
ENSMUSG00000040209	Zfp704	zinc finger protein 704 [Source:MGI Symbol;Acc:MGI:2180715]	2.08739	2.75558	2.97383	2.80784	2.76445	2.29858	2.76971	2.20658	3.12212	2.45604	2.66092	2.73077	2.48398	2.77053	0.091424683	0.483797062	12	12	24	0.171258732	0.768040178	24	10.0624922	0.08583388
ENSMUSG00000040212	Emp3	epithelial membrane protein 3 [Source:MGI Symbol;Acc:MGI:1098729]	37.3444	40.5137	43.9139	37.9187	47.7468	42.5286	39.3848	39.4724	43.1125	30.6053	42.6037	36.2548	39.5442	36.6318	0.633249925	0.966018836	12	16	24	0.891541089	0.969321764	24	18.00873855	0.028710746
ENSMUSG00000040213	Kyat3	kynurenine aminotransferase 3 [Source:MGI Symbol;Acc:MGI:2677849]	4.49825	4.5806	4.44987	6.34365	4.17081	4.17451	5.56568	5.07781	5.20049	5.05652	5.25004	5.02857	5.17483	5.16054	0.904690479	0.966018836	16	8	24	0.55684722	0.838702296	24	9.034748861	0.057854603
ENSMUSG00000040219	Ttc12	tetratricopeptide repeat domain 12 [Source:MGI Symbol;Acc:MGI:2444588]	3.91894	4.2945	4.01166	4.19536	3.74817	3.9243	4.12322	4.08217	4.04036	3.64716	3.90704	3.99068	3.91027	3.89259	0.708665292	0.966018836	8	12	24	0.222535884	0.768040178	24	3.742545757	0.030001088
ENSMUSG00000040220	Gas8	growth arrest specific 8 [Source:MGI Symbol;Acc:MGI:1202386]	10.6589	8.68736	8.39005	9.45243	9.98607	10.5028	10.2174	9.64952	8.29514	10.3156	8.76765	9.12144	8.86138	9.49081	0.143321297	0.585944911	24	12	24	0.201254289	0.768040178	24	20.09496807	0.076703129
ENSMUSG00000040225	Prrc2c	proline-rich coiled-coil 2C [Source:MGI Symbol;Acc:MGI:1913754]	20.572	19.5816	21.109	20.8889	21.2409	20.7441	22.2805	19.8502	19.343	22.6965	18.7971	21.391	20.2168	20.0132	0.417653118	0.963884303	16	16	24	0.670166489	0.888925819	24	17.18588758	0.026348738
ENSMUSG00000040229	Gpr34	G protein-coupled receptor 34 [Source:MGI Symbol;Acc:MGI:1346334]	1.05048	2.3244	2.21166	1.23663	1.855	1.32881	1.53474	1.43599	2.29727	0.754067	1.59196	1.58123	1.29	1.67622	0.287843582	0.793714531	12	16	24	0.49444571	0.806413922	24	6.29864547	0.126004057
ENSMUSG00000040234	Tm7sf3	transmembrane 7 superfamily member 3 [Source:MGI Symbol;Acc:MGI:1914873]	23.6928	22.9304	23.1208	23.2054	23.7094	22.6314	25.8759	23.8622	22.0301	26.8959	19.8088	23.1458	22.2021	21.4852	0.858514388	0.966018836	16	20	24	0.994284628	0.998755922	24	4.78504851	0.004354447
ENSMUSG00000040236	Trappc5	trafficking protein particle complex 5 [Source:MGI Symbol;Acc:MGI:1913932]	12.6615	10.4622	10.627	9.97524	9.90981	11.1922	11.62	12.318	10.898	13.1681	12.3324	12.1784	12.4076	11.5183	0.664568268	0.966018836	4	8	24	0.461734145	0.805393068	24	23.18628533	0.052429058
ENSMUSG00000040242	Fgfr1op2	FGFR1 oncogene partner 2 [Source:MGI Symbol;Acc:MGI:1914779]	47.8757	46.2201	43.4245	45.3119	47.9667	45.2038	47.8326	43.9353	42.9391	42.047	50.0692	45.1873	46.87	46.4233	0.032211002	0.324385159	20	20	24	0.074190745	0.683293901	24	20.5850655	0.059968595
ENSMUSG00000040247	Tbc1d10c	TBC1 domain family, member 10c [Source:MGI Symbol;Acc:MGI:1922072]	0.597166	1.53354	1.75903	1.23171	1.02962	1.75134	1.23345	1.7913	1.68458	1.67558	0.922856	1.60279	1.02825	1.13867	0.286642595	0.793714531	12	16	24	0.360841817	0.786242909	24	8.653945837	0.142109245
ENSMUSG00000040249	Lrp1	low density lipoprotein receptor-related protein 1 [Source:MGI Symbol;Acc:MGI:96828]	30.8184	29.1585	32.833	29.7701	27.518	27.78	32.6143	31.719	35.2494	30.5255	27.5506	31.1884	29.9901	30.1173	0.021288455	0.264116537	12	8	24	0.062883774	0.662016894	24	5.881339964	0.08677783
ENSMUSG00000040250	Ints13	integrator complex subunit 13 [Source:MGI Symbol;Acc:MGI:1918427]	20.1914	19.8882	19.4439	23.8345	20.9205	18.3419	19.5267	20.1504	21.1741	15.8425	21.055	17.3684	20.8066	18.5702	0.942055135	0.966018836	20	4	24	0.924129415	0.979741358	24	9.782900257	0.021341214
ENSMUSG00000040253	Gbp7	guanylate binding protein 7 [Source:MGI Symbol;Acc:MGI:2444421]	5.11937	5.25751	5.16635	4.73975	6.25563	4.98355	4.74706	6.26055	4.91495	4.65935	6.21982	4.35512	4.2648	5.56373	0.46238066	0.966018836	8	20	24	0.85004672	0.955046946	24	11.15371397	0.035478009
ENSMUSG00000040254	Sema3d	sema domain, immunoglobulin domain (Ig), short basic domain, secreted, (semaphorin) 3D [Source:MGI Symbol;Acc:MGI:1860118]	2.91878	3.98182	3.31568	3.21294	3.18526	2.60139	2.95945	2.98285	3.23242	2.41378	2.91589	3.03884	2.6632	3.25762	0.073826349	0.448301997	12	16	24	0.25715651	0.768040178	24	6.060770377	0.079482864
ENSMUSG00000040258	Nxph4	neurexophilin 4 [Source:MGI Symbol;Acc:MGI:1336197]	0.52426	0.866929	1.56874	0.45282	0.512295	1.01622	1.18496	0.5413	0.878759	1.02149	0.415879	1.01628	0.522575	1.18645	0.424718357	0.966018836	12	8	24	0.520607377	0.82006084	24	5.519437961	0.125181615

ENSMUSG00000040260	Daam2	dishevelled associated activator of morphogenesis 2 [Source:MGI Symbol;Acc:MGI:1923691]	9.2585	10.3813	11.0884	11.1583	10.8727	10.7021	10.8976	10.1621	10.3199	10.8147	9.42813	10.4055	9.30876	9.97894	0.173121348	0.648600527	16	12	24	0.215296822	0.768040178	24	11.86408015	0.053003885
ENSMUSG00000040263	Klhdc4	kelch domain containing 4 [Source:MGI Symbol;Acc:MGI:2384569]	7.85842	9.282	9.42537	9.25636	9.16212	8.94704	7.85066	9.79008	9.13764	8.92224	8.11069	7.99573	9.40726	8.22632	0.215777641	0.704989686	8	20	24	0.185993294	0.768040178	24	8.734689627	0.070253601
ENSMUSG00000040265	Dnm3	dynamin 3 [Source:MGI Symbol;Acc:MGI:1341299]	70.5185	60.2328	57.6293	61.4292	55.6166	64.1798	61.0414	58.4333	51.3688	69.1058	56.9337	63.9504	62.3161	64.6745	0.210418232	0.69936851	24	12	24	0.393211913	0.799546792	24	22.02018626	0.066198587
ENSMUSG00000040268	Plekha1	pleckstrin homology domain containing, family A (phosphoinositide binding specific) member 1 [Source:MGI Symbol;Acc:MGI:2442213]	41.8376	44.2047	45.3607	44.2009	43.3072	39.9573	43.6773	37.8914	44.0146	44.1006	42.661	40.0342	40.6084	41.818	0.029825002	0.323426482	12	12	24	0.081980805	0.699041389	24	10.71994375	0.055736834
ENSMUSG00000040269	Mrps28	mitochondrial ribosomal protein S28 [Source:MGI Symbol;Acc:MGI:1913480]	17.9798	23.2488	22.8253	19.7591	19.9379	17.3732	19.4898	27.0889	26.9826	20.0284	23.5281	21.8708	25.1902	22.9747	0.248423582	0.746945512	8	16	24	0.034527161	0.608551791	24	6.566188272	0.156156321
ENSMUSG00000040270	Bach2	BTB and CNC homology, basic leucine zipper transcription factor 2 [Source:MGI Symbol;Acc:MGI:894679]	5.25566	4.25696	4.46758	4.6962	4.59789	4.18747	4.63932	4.60544	4.43338	6.33764	4.37949	4.91305	5.47509	4.76085	0.917867465	0.966018836	4	8	24	0.966150288	0.991892586	24	12.73380924	0.01356777
ENSMUSG00000040272	Accs	1-aminocyclopropane-1-carboxylate synthase (non-functional) [Source:MGI Symbol;Acc:MGI:1919717]	4.38211	4.52512	4.56878	4.80309	4.27052	4.991	4.04339	5.68898	4.91032	4.8879	3.79961	4.46632	4.87711	5.48387	0.185945151	0.662239322	8	12	24	0.102042297	0.713240751	24	5.840016471	0.101933502
ENSMUSG00000040274	Cdk6	cyclin-dependent kinase 6 [Source:MGI Symbol;Acc:MGI:1277162]	2.17337	1.63267	1.43957	1.51451	1.68352	1.44894	1.63017	1.36241	1.40676	1.40288	1.61296	1.43407	1.64598	1.50134	0.176754388	0.653256853	4	8	24	0.117552256	0.728323355	24	21.89964277	0.077547547
ENSMUSG00000040276	Pacsin1	protein kinase C and casein kinase substrate in neurons 1 [Source:MGI Symbol;Acc:MGI:1345181]	42.8015	32.3849	35.4056	35.5547	31.79	37.8578	35.1023	37.8461	28.9171	50.616	30.7343	39.4482	35.754	40.2569	0.823677655	0.966018836	24	12	24	0.940484581	0.985398871	24	23.15253009	0.029087594
ENSMUSG00000040280	Ndufa4l2	Ndufa4, mitochondrial complex associated like 2 [Source:MGI Symbol;Acc:MGI:3039567]	1.18589	1.17898	1.6933	1.12313	1.04706	0.789856	1.31598	1.42458	1.76031	1.46485	1.09866	1.24978	1.33979	1.07897	0.069194004	0.433403132	12	8	24	0.04182154	0.621655363	24	7.442551746	0.153123367
ENSMUSG00000040282	Cdan1	CDAN1 interacting nuclease 1 [Source:MGI Symbol;Acc:MGI:3026886]	1.79675	1.60619	1.95984	1.79356	1.59632	1.59141	1.73102	1.71561	1.68973	1.76499	1.61037	1.61971	1.64282	1.82152	0.112865	0.530777648	16	4	24	0.089262504	0.708638675	24	7.316970776	0.047050303
ENSMUSG00000040287	Stac3	SH3 and cysteine rich domain 3 [Source:MGI Symbol;Acc:MGI:3606571]	3.43474	6.32327	6.76137	11.0104	7.39949	8.5412	7.71961	17.3778	13.4877	12.3599	5.40455	6.41748	8.59713	7.33346	0.756435587	0.966018836	16	4	24	0.14335144	0.752803875	24	8.940977515	0.387136682
ENSMUSG00000040289	Hey1	hairy/enhancer-of-split related with YRPW motif 1 [Source:MGI Symbol;Acc:MGI:1341800]	8.86148	7.99107	8.91523	8.17704	7.24867	8.492	9.03035	8.13305	7.46998	9.46689	7.94158	8.083	7.90682	8.80572	0.891690964	0.966018836	16	4	24	0.775223357	0.927711986	24	3.76544008	0.02791894
ENSMUSG00000040296	Ddx58	DEAD/H box helicase 58 [Source:MGI Symbol;Acc:MGI:2442858]	10.8575	9.43834	10.0437	9.17676	10.6185	11.0018	10.1584	10.9517	8.08794	8.67595	12.0974	9.12107	8.8718	9.42898	0.503972924	0.966018836	20	20	24	0.208853555	0.768040178	24	19.76245904	0.09929181
ENSMUSG00000040297	Suco	SUN domain containing ossification factor [Source:MGI Symbol;Acc:MGI:2138346]	17.52	19.1655	18.2842	19.7156	19.7262	16.4367	19.129	18.0045	21.1553	17.2629	20.2794	17.9476	18.0887	18.987	0.265774856	0.773510818	20	4	24	0.307330325	0.768040178	24	10.01321221	0.057167441
ENSMUSG00000040302	Rbm48	RNA binding motif protein 48 [Source:MGI Symbol;Acc:MGI:2442653]	4.72052	4.75317	5.31387	5.37636	4.91702	3.97574	4.14841	4.7712	4.4462	4.04869	4.37269	4.94919	4.75895	4.65196	0.865514257	0.966018836	12	16	24	0.660339149	0.885429061	24	8.38440471	0.039937064
ENSMUSG00000040312	Cchcr1	coiled-coil alpha-helical rod protein 1 [Source:MGI Symbol;Acc:MGI:2385321]	2.29018	2.05237	3.52688	1.45105	2.80582	2.31068	3.95512	2.95354	2.89189	2.02511	2.89378	2.32794	2.28632	2.16163	0.93243477	0.966018836	20	20	24	0.790084234	0.932831789	24	1.425582153	0.071188254
ENSMUSG00000040321	Zfp770	zinc finger protein 770 [Source:MGI Symbol;Acc:MGI:2445100]	0.852444	1.34707	3.6804	2.22773	1.52468	2.52776	1.15579	1.35718	1.74675	1.0325	1.27027	1.17741	2.05008	0.777764	0.823657258	0.966018836	12	20	24	0.61588121	0.867292367	24	11.70684835	0.145306148
ENSMUSG00000040322	Slc25a24	solute carrier family 25 (mitochondrial carrier, phosphate carrier), member 24 [Source:MGI Symbol;Acc:MGI:1917160]	7.55071	7.42803	7.90836	7.59173	8.05159	7.53237	7.71439	7.30286	7.83649	7.17887	8.68475	7.70217	8.43671	7.65318	0.47028201	0.966018836	20	12	24	0.517466523	0.818298678	24	17.59963875	0.029586982
ENSMUSG00000040323	Gm15429	predicted pseudogene 15429 [Source:MGI Symbol;Acc:MGI:3642423]	6.52602	6.26293	6.4183	5.84314	6.54267	6.16699	5.77413	5.64049	5.25754	5.65281	6.42691	5.7984	6.07376	6.15632	0.664568268	0.966018836	20	20	24	0.340718586	0.775204417	24	19.82235597	0.044622758
ENSMUSG00000040325	Dcaf1	DDB1 and CUL4 associated factor 1 [Source:MGI Symbol;Acc:MGI:2445220]	12.598	12.4469	15.0597	14.8471	13.0573	14.3739	13.6021	13.0004	13.3001	16.9418	11.7017	11.3336	13.2551	12.0315	0.241264473	0.741580974	12	20	24	0.186962513	0.768040178	24	10.97363115	0.099091512
ENSMUSG00000040327	Cul9	cullin 9 [Source:MGI Symbol;Acc:MGI:1925559]	6.5697	8.09075	6.95152	11.5496	7.88499	9.6168	7.32517	9.10952	7.23498	7.78729	8.03049	6.75195	8.4489	8.16781	0.873350015	0.966018836	8	4	24	0.694744079	0.898526856	24	12.67947051	0.066171148
ENSMUSG00000040329	Il7	interleukin 7 [Source:MGI Symbol;Acc:MGI:96561]	7.83125	8.52819	5.91541	7.51716	7.90231	8.69093	7.87076	7.74787	7.40624	8.3597	6.50596	7.88945	7.73842	7.09532	0.148073804	0.590732968	24	12	24	0.345651921	0.778604155	24	21.36822759	0.076585971
ENSMUSG00000040331	Nsmce4a	NSE4 homolog A, SMC5-SMC6 complex component [Source:MGI Symbol;Acc:MGI:1915122]	2.22261	2.32963	2.37707	2.39652	2.77346	2.28145	2.57202	1.91166	2.29895	1.97812	2.616	2.45014	2.57409	2.45587	0.287843582	0.793714531	20	12	24	0.330949001	0.768040178	24	18.61339636	0.061015589
ENSMUSG00000040339	Fam102b	family with sequence similarity 102, member B [Source:MGI Symbol;Acc:MGI:3036259]	28.2455	31.7187	27.8938	30.2424	30.1816	28.3405	27.6158	30.0881	31.4653	25.8778	29.4466	29.0649	32.3733	34.3222	0.745118544	0.966018836	8	16	24	0.274143549	0.768040178	24	4.331043283	0.060481717
ENSMUSG00000040345	Arhgap9	Rho GTPase activating protein 9 [Source:MGI Symbol;Acc:MGI:2143764]	57.135	47.623	49.2549	50.8654	55.3268	55.012	49.3098	46.3939	41.1452	50.115	57.4593	50.3351	50.2608	50.0347	0.001097088	0.07820797	20	16	24	0.008843908	0.502965379	24	18.60169643	0.131546601
ENSMUSG00000040350	Trim7	tripartite motif-containing 7 [Source:MGI Symbol;Acc:MGI:2137353]	1.9065	3.62198	2.88507	4.01543	3.37425	3.34757	2.34634	4.82123	3.75362	3.4453	3.0022	3.77526	2.71447	3.0476	0.066380001	0.42386701	16	12	24	0.240845989	0.768040178	24	10.45411874	0.164344641
ENSMUSG00000040351	Ankib1	ankyrin repeat and IBR domain containing 1 [Source:MGI Symbol;Acc:MGI:1918047]	15.56	14.0336	14.1043	15.3993	15.1894	13.3827	15.6592	12.8681	13.4534	13.1281	14.3044	13.6805	13.5654	13.8029	0.93717134	0.966018836	4	4	24	0.440535118	0.805393068	24	19.04229596	0.039678328
ENSMUSG00000040354	Mars1	methionine-tRNA synthetase 1 [Source:MGI Symbol;Acc:MGI:1345633]	15.4127	13.4717	11.7495	13.2184	16.1394	16.3039	16.2389	16.7569	15.073	16.1506	15.9673	15.0593	14.3887	14.6609	0.865233232	0.966018836	24	12	24	0.232139863	0.768040178	24	19.75407735	0.087499747
ENSMUSG00000040356	Skiv2l	superkiller viralicidic activity 2-like (S. cerevisiae) [Source:MGI Symbol;Acc:MGI:1099835]	16.8773	16.0151	15.5873	16.6269	16.536	17.7225	15.6839	18.5973	15.1023	19.3381	16.5433	16.9769	15.7968	15.8886	0.300823756	0.809362476	16	20	24	0.58453291	0.852273999	24	16.11874449	0.039235579
ENSMUSG00000040359	Ufl1	UFM1 specific ligase 1 [Source:MGI Symbol;Acc:MGI:1914740]	14.3437	14.9135	11.9182	14.0181	13.3978	13.7252	15.5114	13.325	14.4195	12.5935	14.4395	14.4068	13.819	15.1264	0.593560247	0.966018836	4	12	24	0.201312549	0.768040178	24	23.65101193	0.064195213
ENSMUSG00000040363	Bcor	BCL6 interacting corepressor [Source:MGI Symbol;Acc:MGI:1918708]	7.93125	6.36435	6.19738	6.25666	5.59961	6.88448	6.6845	7.51554	6.20671	8.85354	6.24837	7.44476	7.50639	7.61665	0.210154775	0.69936851	4	16	24	0.506735948	0.812342247	24	1.565175816	0.06715421
ENSMUSG00000040365	Trim41	tripartite motif-containing 41 [Source:MGI Symbol;Acc:MGI:2384814]	13.6691	12.3072	12.8864	11.6694	11.6683	14.2303	11.5684	13.4625	11.3351	13.6688	11.0682	12.6577	12.5427	11.882	0.893307667	0.966018836	24	20	24	0.601674392	0.860619632	24	23.33916724	0.041941707
ENSMUSG00000040370	Etfrf1	electron transfer flavoprotein regulatory factor 1 [Source:MGI Symbol;Acc:MGI:1914886]	26.7276	30.4503	31.4269	36.251	29.6203	26.549	24.9634	29.1592	31.7086	29.5818	33.048	29.7004	30.0534	33.5839	0.117233015	0.538362324	16	8	24	0.019973499	0.565108098	24	10.34584156	0.133093065
ENSMUSG00000040372	Gpr63	G protein-coupled receptor 63 [Source:MGI Symbol;Acc:MGI:2135884]	0.753514	1.04475	0.937235	1.21385	0.994755	1.10641	1.093	1.03458	0.906626	0.912326	1.12096	1.40094	0.777788	1.26173	0.773177465	0.966018836	24	20	24	0.733761316	0.9131851	24	16.24720413	0.038498587
ENSMUSG00000040373	Cacng5	calcium channel, voltage-dependent, gamma subunit 5 [Source:MGI Symbol;Acc:MGI:2157946]	3.24659	2.81872	2.75674	3.23628	2.43499	3.22198	3.4012	3.20082	2.24268	4.145	2.46729	3.08125	2.94636	3.05671	0.43280637	0.966018836	4	8	24	0.885395739	0.967345379	24	23.19183646	0.034306918
ENSMUSG00000040374	Pex2	peroxisomal biogenesis factor 2 [Source:MGI Symbol;Acc:MGI:107486]	20.5871	25.6264	28.1844	26.5693	27.0478	22.934	24.6852	23.8226	28.6326	22.0853	25.1248	24.5235	26.2941	24.3541	0.291478323	0.798044066	12	12	24	0.264297375	0.768040178	24	9.753011681	0.082469999
ENSMUSG00000040383	Aqr	aquarius [Source:MGI Symbol;Acc:MGI:1276102]	10.3451	8.4549	9.30496	9.13249	8.50687	9.79309	10.0396	8.92407	7.1638	9.60745	8.22929	9.29809	9.53135	8.84292	0.105163243	0.518002936	4	8	24	0.127778179	0.740162193	24	21.78634604	0.093496892
ENSMUSG00000040385	Ppp1ca	protein phosphatase 1 catalytic subunit alpha [Source:MGI Symbol;Acc:MGI:103016]	114.097	103.091	113.098	113.158	115.371	116.185	111.144	110.168	102.569	113.068	108.375	109.035	108.296	106.765	0.23371652	0.736061043	24	8	24	0.085295914	0.703521975	24	17.50283385	0.043267434
ENSMUSG00000040387	Klhl32	kelch like 32 [Source:MGI Symbol;Acc:MGI:3612790]	1.91541	1.69948	1.73	1.4815	1.45493	1.9665	1.59728	1.4726	1.49103	1.65028	1.97089	1.69341	1.57461	2.19856	0.890648739	0.966018836	24	16	24	0.642016182	0.878809617	24	22.83566809	0.044504574
ENSMUSG00000040389	Wdr47	WD repeat domain 47 [Source:MGI Symbol;Acc:MGI:2139593]	42.8369	37.2609	35.9583	35.2676	40.036	40.579	40.0743	35.6696	33.1702	37.9749	37.1703	38.0307	38.7869	41.4734	0.009483698	0.194720062	4	8	24	0.02560551	0.586541342	24	21.83319577	0.09641994
ENSMUSG00000040390	Map3k10	mitogen-activated protein kinase kinase kinase 10 [Source:MGI Symbol;Acc:MGI:1346879]	17.2316	13.8291	14.2959	13.4012	13.1855	15.2929	12.7905	15.3507	13.1477	16.4296	15.172	16.1294	16.2151	14.0998	0.814576414	0.966018836	4	4	24	0.509893932	0.813985618	24	21.86347916	0.058622225
ENSMUSG00000040396	Abhd13	abhydrolase domain containing 13 [Source:MGI Symbol;Acc:MGI:1916154]	5.57252	6.50525	6.89146	6.31811	6.71898	6.07257	6.43532	6.24583	6.55432	5.78936	6.36379	6.00861	6.22265	6.22738	0.08302755	0.462922111	12	12	24	0.397485855	0.799546792	24	9.288678953	0.038292305
ENSMUSG00000040407	Akap9	A kinase (PRKA) anchor protein (yotiao) 9 [Source:MGI Symbol;Acc:MGI:2178217]	18.9392	18.9229	15.7022	15.0383	17.7369	20.1667	16.5095	19.0416	20.5235	18.123	16.3674	17.7103	18.2542	18.1888	0.530814489	0.966018836	8	8	24	0.398525351	0.799546792	24	1.351219298	0.06435508
ENSMUSG00000040410	Fbxl4	F-box and leucine-rich repeat protein 4 [Source:MGI Symbol;Acc:MGI:2140367]	12.5621	15.9477	16.1304	15.2409	14.596	13.6439	13.2392	15.4304	15.6145	14.2539	15.3893	14.9388	15.1405	16.0406	0.01662439	0.253395174	8	20	24	0.032095982	0.605537343	24	8.494127763	0.094033513
ENSMUSG00000040412	Elapor1	endosome-lysosome associated apoptosis and autophagy regulator 1 [Source:MGI Symbol;Acc:MGI:1923930]	2.54382	2.25656	2.36957	2.19905	1.97283	2.20552	2.28043	2.12019	1.80058	2.5131	1.95826	2.24731	2.45819	2.51048	0.216058318	0.704989686	4	16	24	0.362395319	0.787151161	24	1.473483608	0.056252356
ENSMUSG00000040414	Slc25a28	solute carrier family 25, member 28 [Source:MGI Symbol;Acc:MGI:2180509]	15.835	15.8862	15.6877	15.8019	15.3573	13.7865	14.1478	15.6995	16.7965	17.3467	15.8906	15.3041	15.7134	15.2206	0.173410058	0.648600527	16	8	24	0.033857143	0.606793499	24	9.665210247	0.074473494
ENSMUSG00000040415	Dtx3	deltex 3, E3 ubiquitin ligase [Source:MGI Symbol;Acc:MGI:2135752]	45.8498	46.2474	45.9779	41.4978	48.5361	45.8906	47.811	47.0169	46.0766	45.2795	46.4212	44.8563	45.313	44.0808	0.8635847	0.966018836	20	20	24	0.580080339	0.850153156	24	22.18534563	0.022272607
ENSMUSG00000040420	Cdh18	cadherin 18 [Source:MGI Symbol;Acc:MGI:1344366]	8.67593	9.33598	8.90314	8.61704	7.96298	8.65672	8.62148	8.53938	8.03879	8.99798	8.56747	9.40636	10.066	9.17959	0.446653834	0.966018836	4	16	24	0.263733809	0.768040178	24	1.16442042	0.046432939
ENSMUSG00000040423	Rc3h1	RING CCCH (C3H) domains 1 [Source:MGI Symbol;Acc:MGI:2685397]	10.6485	10.2324	10.0535	10.8922	10.5835	9.69403	9.88354	9.43544	9.71159	9.45266	10.9406	10.1692	10.2402	10.7499	0.912686313	0.966018836	20	16	24	0.672651255	0.889663971	24	17.3157227	0.024120922
ENSMUSG00000040424	Hipk4	homeodomain interacting protein kinase 4 [Source:MGI Symbol;Acc:MGI:2685008]	7.24706	6.08516	7.39736	6.81386	5.84357	7.26466	7.40587	6.81251	6.41683	8.18312	5.48538	6.6607	6.25125	6.70011	0.90767227	0.966018836	16	4	24	0.824873096	0.94504389	24	5.497621122	0.033807735
ENSMUSG00000040428	Plekha4	pleckstrin homology domain containing, family A (phosphoinositide binding specific) member 4 [Source:MGI Symbol;Acc:MGI:1916467]	140.967	144.724	131.281	153.533	169.257	123.573	164.858	144.076	182.623	160.883	153.576	146.015	164.911	124.446	0.774195442	0.966018836	20	12	24	0.60963075	0.864491738	24	12.91249713	0.064868692
ENSMUSG00000040429	Mterf1a	mitochondrial transcription termination factor 1a [Source:MGI Symbol;Acc:MGI:1918240]	2.33425	2.61255	2.08768	2.54578	2.33063	2.27065	2.28022	2.09592	2.72385	2.03864	2.80811	2.10914	3.19791	2.60733	0.891742979	0.966018836	4	12	24	0.885152958	0.967289588	24	2.469416708	0.025771937
ENSMUSG00000040430	Pitpnc1	phosphatidylinositol transfer protein, cytoplasmic 1 [Source:MGI Symbol;Acc:MGI:1919045]	56.027	40.6903	41.5398	42.9366	43.5937	49.4993	43.1959	45.77	33.0806	54.6274	42.9282	47.0388	46.8631	46.5663	0.047644929	0.377556215	24	12	24	0.264871425	0.768040178	24	20.55253401	0.119492704
ENSMUSG00000040438	Zbtb38	zinc finger and BTB domain containing 38 [Source:MGI Symbol;Acc:MGI:2442866]	11.7375	11.3578	11.0492	10.8498	12.2415	12.5051	12.008	10.6412	9.82554	10.8009	12.0721	10.7445	11.3294	10.9478	0.051961445	0.391073722	20	16	24	0.00830796	0.49701405	24	19.72981829	0.091172311
ENSMUSG00000040435	Ppp1r15a	protein phosphatase 1, regulatory subunit 15A [Source:MGI Symbol;Acc:MGI:1927072]	12.7111	16.4103	16.0778	15.3381	15.8867	16.7287	15.77	16.707	15.095	17.1992	13.2192	16.6954	17.1935	13.9904	0.830949782	0.966018836	24	12	24	0.986882713	0.997339197	24	9.167706961	0.008871723
ENSMUSG00000040441	Slc26a10	solute carrier family 26, member 10 [Source:MGI Symbol;Acc:MGI:2143920]	0.462835	0.56799	0.706061	1.31843	1.07333	0.623664	0.587494	0.985626	0.816022	1.24625	0.787996	0.533743	0.761409	0.843656	0.016024075	0.253395174	16	12	24	0.007330681	0.47735824	24	11.46037065	0.211391431
ENSMUSG00000040446	Rprd1a	regulation of nuclear pre-mRNA domain containing 1A [Source:MGI Symbol;Acc:MGI:2385066]	16.412	17.8398	16.3369	18.4124	18.517	16.7816	17.6283	17.885	16.8078	18.2133	17.8718	17.1021	17.6082	0.151902463	0.600361608	20	12	24	0.250717823	0.768040178	24	15.00801185	0.040973739
ENSMUSG00000040447	Spns2	spinster homolog 2 [Source:MGI Symbol;Acc:MGI:2384936]	8.40519	8.45074	10.3165	9.57666	7.64911	9.98244	9.44523	10.4384	9.07708	11.1397	8.57307	9.01096	8.90303	9.02936	0.369262716	0.893596186	16	4	24	0.394355742	0.799546792	24	8.51341458	0.072362755
ENSMUSG00000040451	Sgms1	sphingomyelin synthase 1 [Source:MGI Symbol;Acc:MGI:2444110]	14.8959	17.9402	17.6216	16.5572	17.3689	15.0833	17.0575	15.8851	16.4491	15.525	13.8986	13.8218	13.4045	13.8434	0.46238066	0.966018836	12	12	24	0.122531161	0.735333681	24	8.986822684	0.072970617
ENSMUSG00000040452	Cdh12	cadherin 12 [Source:MGI Symbol;Acc:MGI:109503]	0.809107	1.40826	1.03823	1.18552	1.13331	0.818494	0.963258	1.02995	0.989226	0.977995	1.43322	0.851766	0.905638	1.52194	0.078207999	0.457568093	8	20	24	0.320028626	0.768040178	24	8.955214308	0.094577273
ENSMUSG00000040455	Usp45	ubiquitin specific petidase 45 [Source:MGI Symbol;Acc:MGI:101850]	17.6927	16.2941	15.3282	16.0152	14.8976	15.8601	15.3229	13.9463	13.7761	13.7769	15.8142	14.5674	14.6122	15.2177	0.883907406	0.966018836	4	8	24	0.176145201	0.768040178	24	21.51583985	0.055623425
ENSMUSG00000040459	Arglu1	arginine and glutamate rich 1 [Source:MGI Symbol;Acc:MGI:2442985]	45.6066	60.8642	59.1696	53.8132	51.6889	49.3963	48.9486	51.0317	63.8828	51.0573	55.0457	50.8996	50.2556	55.8949	3.29E-04	0.049770315	12	16	24	0.016829777	0.547196997	24	8.606429286	0.13456387
ENSMUSG00000040462	Os9	amplified in osteosarcoma [Source:MGI Symbol;Acc:MGI:1924301]	42.5601	37.023	38.6948	36.2482	40.3006	41.8199	40.0124	40.9336	33.459	44.6039	34.9506	40.9114	38.6796	39.5289	0.601647512	0.966018836	12	12	24	0.409112533	0.799546792	24	21.32122141	0.060145693
ENSMUSG00000040463	Mybbp1a	MYB binding protein (P160) 1a [Source:MGI Symbol;Acc:MGI:106181]	13.5376	12.2124	13.8928	13.398	13.8635	14.2435	13.341	16.1476	12.3728	14.887	11.5376	13.2829	12.1232	13.6527	0.912686312	0.966018836	16	12	24	0.908747575	0.974606327	24	7.250515548	0.02162863
ENSMUSG00000040464	Gtpbp10	GTP-binding protein 10 (putative) [Source:MGI Symbol;Acc:MGI:2385599]	3.3999	4.26587	3.84061	3.35346	3.85016	3.30133	3.11782	3.8639	4.76835	3.39665	3.53555	3.57143	3.47701	3.64012	0.113393908	0.530777648	8	20	24	0.109262546	0.72082227	24	7.644148805	0.103173012
ENSMUSG00000040466	Blvrb	biliverdin reductase B (flavin reductase (NADPH)) [Source:MGI Symbol;Acc:MGI:2385271]	26.6569	29.6615	28.2816	23.8814	29.59	27.7192	24.4876	27.6271	28.5722	23.4493	28.4372	28.391	30.3061	26.9404	0.911135828	0.966018836	20	20	24	0.632863388	0.875223946	24	23.69550535	0.042977715
ENSMUSG00000040472	Rabggta	Rab geranylgeranyl transferase, a subunit [Source:MGI Symbol;Acc:MGI:1860443]	10.9755	10.6521	12.1452	11.2069	10.8891	12.3071	12.0503	12.9136	10.3952	13.1724	11.4044	12.1304	11.6946	12.1485	0.828830907	0.966018836	24	12	24	0.990168775	0.998166998	24	1.105808592	0.004957067
ENSMUSG00000040473	Cfap69	cilia and flagella associated protein 69 [Source:MGI Symbol;Acc:MGI:2443778]	5.59898	5.84852	6.37738	5.49301	5.45375	4.97831	5.76034	4.3398	6.60005	6.50633	4.83315	6.25127	5.08715	5.20867	0.302965859	0.811201458	12	8	24	0.459606819	0.805393068	24	10.32157493	0.074535
ENSMUSG00000040479	Dgkz	diacylglycerol kinase zeta [Source:MGI Symbol;Acc:MGI:1278339]	229.563	204.386	183.307	216.604	214.111	213.549	180.986	210.785	192.358	233.737	198.626	217.911	222.3	208.675	0.649212555	0.966018836	16	20	24	0.579545556	0.849875019	24	18.29018374	0.047223856
ENSMUSG00000040481	Bptf	bromodomain PHD finger transcription factor [Source:MGI Symbol;Acc:MGI:2444008]	12.8261	12.4213	13.2314	12.5939	12.2525	12.0127	13.6803	11.3576	12.2317	13.8284	12.1714	13.4108	12.6828	12.0678	0.904387595	0.966018836	16	16	24	0.844811304	0.953077545	24	16.61524707	0.017594971
ENSMUSG00000040482	Dxo	decapping exoribonuclease [Source:MGI Symbol;Acc:MGI:1890444]	10.5769	10.2104	10.5874	9.4407	11.8793	9.77636	10.8096	8.74929	11.5382	10.3023	9.91222	11.5422	9.85004	9.364	0.591351901	0.966018836	20	12	24	0.501312925	0.80908345	24	16.85041196	0.053655122
ENSMUSG00000040483	Xaf1	XIAP associated factor 1 [Source:MGI Symbol;Acc:MGI:3772572]	27.8499	28.6507	26.9382	26.897	34.949	26.8959	27.2942	28.7525	28.1764	23.6152	31.7155	27.5508	24.5194	26.3525	0.917839756	0.966018836	20	20	24	0.432156074	0.805393068	24	16.87685847	0.066682506
ENSMUSG00000040488	Ltbp4	latent transforming growth factor beta binding protein 4 [Source:MGI Symbol;Acc:MGI:1321395]	10.5661	11.247	12.5209	11.2429	11.7539	10.973	11.5349	13.485	15.2996	12.8025	10.9677	11.1355	10.5511	10.3568	0.101520755	0.511659635	12	16	24	0.066292011	0.66876796	24	9.073768277	0.127814162
ENSMUSG00000040490	Lrfn2	leucine rich repeat and fibronectin type III domain containing 2 [Source:MGI Symbol;Acc:MGI:1917780]	1.86206	1.06743	1.1589	1.21754	1.2037	1.32476	1.47199	1.3583	0.936376	1.7198	1.37681	1.4704	1.12336	1.56903	0.913451828	0.966018836	4	8	24	0.515242134	0.817300608	24	20.79543378	0.073799534
ENSMUSG00000040495	Chrm4	cholinergic receptor, muscarinic 4 [Source:MGI Symbol;Acc:MGI:88399]	1.67695	1.011	1.04738	1.07199	0.932876	1.30894	1.78272	1.39328	1.44421	1.82561	1.2202	1.52084	1.48658	1.66134	0.46238066	0.966018836	4	16	24	0.319879259	0.768040178	24	1.060097574	0.09225801
ENSMUSG00000040502	Marchf9	membrane associated ring-CH-type finger 9 [Source:MGI Symbol;Acc:MGI:2446144]	3.22223	2.38374	2.56301	3.06746	2.57296	2.91952	2.37398	2.68684	2.39526	3.88296	2.55961	3.10151	2.67768	3.25812	0.90332508	0.966018836	16	20	24	0.733503639	0.913014785	24	14.416041	0.047734251
ENSMUSG00000040506	Ambra1	autophagy/beclin 1 regulator 1 [Source:MGI Symbol;Acc:MGI:2443564]	9.12843	8.6834	8.4797	9.27365	8.9692	9.27367	9.13741	9.33391	8.70911	10.6061	7.78827	9.38736	8.67575	9.00536	0.8972237	0.966018836	24	12	24	0.966746769	0.992082154	24	12.8815154	0.009304712
ENSMUSG00000040511	Pvr	poliovirus receptor [Source:MGI Symbol;Acc:MGI:107741]	6.67041	6.38645	5.11906	6.3151	7.3942	7.13048	8.41365	5.70816	5.4154	6.81408	6.36936	6.50816	6.23535	5.54828	0.010049466	0.194720062	24	12	24	0.017392861	0.550995814	24	19.48892567	0.165929606
ENSMUSG00000040520	Manea	mannosidase, endo-alpha [Source:MGI Symbol;Acc:MGI:2444484]	13.6208	14.4864	15.0088	14.2757	14.4194	13.1898	14.6118	12.9969	13.5424	12.0534	14.2576	12.8745	12.9856	13.7033	0.93447422	0.966018836	12	12	24	0.945188626	0.986693398	24	7.709065905	0.010080508
ENSMUSG00000040521	Tsfm	Ts translation elongation factor, mitochondrial [Source:MGI Symbol;Acc:MGI:1913649]	12.5404	10.8376	12.5484	11.3202	9.59784	10.5038	12.7574	14.7081	11.9546	12.4927	10.0872	10.1505	12.1864	11.0467	0.048781137	0.379129806	12	8	24	0.04892163	0.639457489	24	4.907488825	0.137497306
ENSMUSG00000040524	Zfp609	zinc finger protein 609 [Source:MGI Symbol;Acc:MGI:2674092]	3.46418	3.0472	3.0696	3.3613	3.32898	3.27679	3.88071	3.50598	3.26214	4.15205	3.02473	3.3299	3.36073	3.39721	0.708665292	0.966018836	4	16	24	0.997489903	0.999623846	24	0.045133823	0.002776109
ENSMUSG00000040528	Milr1	mast cell immunoglobulin like receptor 1 [Source:MGI Symbol;Acc:MGI:2685731]	0.911334	1.43197	1.31593	1.66506	1.55096	1.25372	1.07022	1.05418	1.56837	0.586465	1.29566	0.747402	1.35607	1.19344	0.539040394	0.966018836	12	12	24	0.739675836	0.915107718	24	9.520995964	0.067513068
ENSMUSG00000040532	Abhd11	abhydrolase domain containing 11 [Source:MGI Symbol;Acc:MGI:1916008]	20.5745	21.743	22.9877	23.2348	25.9991	25.0649	20.9116	22.1804	21.9479	25.2226	23.0326	21.4306	24.0159	22.3737	0.042812373	0.356048751	20	8	24	0.06343179	0.663715314	24	14.39842486	0.082160365
ENSMUSG00000040536	Necab1	N-terminal EF-hand calcium binding protein 1 [Source:MGI Symbol;Acc:MGI:1916602]	33.1285	32.5393	30.5297	30.692	30.7274	31.059	29.704	29.5373	25.8236	31.1178	31.8934	27.9514	28.1875	31.9022	0.880247609	0.966018836	8	4	24	0.845822986	0.953396028	24	19.72895152	0.020728949
ENSMUSG00000040537	Adam22	a disintegrin and metallopeptidase domain 22 [Source:MGI Symbol;Acc:MGI:1340046]	46.6694	40.0872	40.0246	42.3228	42.2332	42.0147	37.4848	37.8332	37.3357	37.3033	43.9383	42.0074	43.0293	41.116	0.251986462	0.75129457	20	16	24	0.107340021	0.71991576	24	19.65607073	0.077247859
ENSMUSG00000040540	Ctdsp2-ps	CTD (carboxy-terminal domain, RNA polymerase II, polypeptide A) small phosphatase 2, pseudogene [Source:MGI Symbol;Acc:MGI:3708545]	17.013	19.4953	20.7651	19.9628	23.5289	19.6171	20.292	22.6787	21.1204	25.8931	19.7303	21.5774	18.229	18.6889	0.105345531	0.518002936	16	12	24	0.081963619	0.699041389	24	12.30111374	0.120071502
ENSMUSG00000040543	Pitpnm3	PITPNM family member 3 [Source:MGI Symbol;Acc:MGI:2685726]	2.1263	2.05668	2.35254	1.76867	1.94381	2.03931	2.40117	1.65136	2.24659	2.14633	1.76978	2.05329	2.47785	2.66595	0.206596984	0.697236278	4	16	24	0.215421759	0.768040178	24	3.204768365	0.084819051
ENSMUSG00000040548	Tex2	testis expressed gene 2 [Source:MGI Symbol;Acc:MGI:102465]	54.0087	43.3348	40.0488	43.6245	45.3004	48.8452	48.0616	47.4767	37.4997	52.5017	41.2182	46.9355	46.2597	46.5483	0.042945285	0.356048751	4	8	24	0.164327118	0.767020009	24	21.62433414	0.104084442
ENSMUSG00000040549	Ckap5	cytoskeleton associated protein 5 [Source:MGI Symbol;Acc:MGI:1923036]	81.6636	79.7778	77.33	78.5449	75.178	80.6531	71.9198	72.8642	72.3795	78.2246	78.2664	86.3652	85.6193	80.1705	0.14313801	0.585944911	24	12	24	0.306044267	0.768040178	24	20.71842972	0.048707711
ENSMUSG00000040550	Otud6b	OTU domain containing 6B [Source:MGI Symbol;Acc:MGI:1919451]	33.1651	34.132	32.6193	33.2601	34.2966	31.582	30.8534	32.0436	34.5327	28.8375	35.7082	32.3403	35.2372	32.5017	0.897425765	0.966018836	20	4	24	0.978285159	0.994576186	24	20.77006718	0.007031692
ENSMUSG00000040552	C3ar1	complement component 3a receptor 1 [Source:MGI Symbol;Acc:MGI:1097680]	0.725794	1.10398	1.22671	0.728035	1.13773	0.759212	1.05117	1.07392	1.17104	0.706299	1.05647	0.974924	0.804571	0.966229	0.17847698	0.655480352	12	12	24	0.532969605	0.825678486	24	6.942537726	0.063222134
ENSMUSG00000040557	Mettl27	methyltransferase like 27 [Source:MGI Symbol;Acc:MGI:1933146]	8.18669	10.3006	9.07278	9.53067	8.35736	9.09573	8.39971	10.7371	10.2809	12.2042	7.58105	8.82527	9.09802	8.91103	0.073315272	0.447768907	16	4	24	0.086338703	0.705702571	24	8.123822537	0.137712488
ENSMUSG00000040560	Wdr7	WD repeat domain 7 [Source:MGI Symbol;Acc:MGI:1860197]	29.8604	23.8272	26.0223	24.7792	24.3184	28.3675	27.1182	25.6133	19.3323	31.9252	22.6426	25.2471	23.7978	26.4712	0.922035832	0.966018836	4	16	24	0.664208648	0.886686094	24	20.95019897	0.063223728
ENSMUSG00000040562	Gstm2	glutathione S-transferase, mu 2 [Source:MGI Symbol;Acc:MGI:95861]	5.80014	7.48732	6.28025	6.53813	6.61272	5.84279	4.57274	7.934	7.86278	4.78382	6.25545	5.58832	6.3582	6.53362	0.087592959	0.472021905	8	16	24	0.237483018	0.768040178	24	6.870095994	0.134657543
ENSMUSG00000040563	Plppr2	phospholipid phosphatase related 2 [Source:MGI Symbol;Acc:MGI:2384575]	9.74927	7.87374	8.14699	7.8002	8.63513	8.59539	8.39755	9.54371	9.38805	11.4041	9.7822	9.68562	9.75781	10.19	0.828910443	0.966018836	20	16	24	0.990214996	0.998185102	24	11.70560111	0.006111016
ENSMUSG00000040564	Apoc1	apolipoprotein C-I [Source:MGI Symbol;Acc:MGI:88053]	12.8652	11.7773	27.614	55.6243	18.8348	16.3814	20.109	30.0519	22.5114	27.1484	43.0713	28.2272	43.0041	38.5108	0.910436409	0.966018836	16	12	24	0.199093958	0.768040178	24	11.34985701	0.356238339
ENSMUSG00000040565	Btaf1	B-TFIID TATA-box binding protein associated factor 1 [Source:MGI Symbol;Acc:MGI:2147538]	7.68654	6.8908	6.0737	6.57366	5.73498	5.29256	6.31968	5.0692	6.23661	4.95331	6.44656	5.4774	5.55341	6.991	0.887027852	0.966018836	4	20	24	0.570541837	0.845305671	24	3.082852473	0.062379564
ENSMUSG00000040569	Slc26a7	solute carrier family 26, member 7 [Source:MGI Symbol;Acc:MGI:2384791]	2.61148	2.91697	2.25508	2.24294	2.65971	1.92809	2.90692	2.2488	3.12036	1.53834	2.57809	2.54797	1.98222	2.40148	0.95032543	0.966018836	12	4	24	0.665123881	0.886947643	24	1.967960693	0.065304464
ENSMUSG00000040570	Rundc3b	RUN domain containing 3B [Source:MGI Symbol;Acc:MGI:2685286]	6.94457	7.1667	8.39136	6.42098	6.263	7.37739	6.4408	6.91568	6.75903	7.53372	6.22838	7.46872	6.94898	7.96465	0.628286596	0.966018836	8	12	24	0.334686361	0.770815317	24	5.465165135	0.06620177
ENSMUSG00000040584	Abcb1a	ATP-binding cassette, sub-family B (MDR/TAP), member 1A [Source:MGI Symbol;Acc:MGI:97570]	6.97603	7.91283	6.6464	6.67243	7.6471	7.16112	7.45793	7.96069	6.63856	6.41524	6.58699	7.4263	5.88609	5.6469	0.916963526	0.966018836	8	4	24	0.510087927	0.81402698	24	20.71159584	0.052431446
ENSMUSG00000040586	Ofd1	OFD1, centriole and centriolar satellite protein [Source:MGI Symbol;Acc:MGI:1350328]	2.79369	3.5216	3.35233	3.41881	2.99656	3.18529	2.99955	3.25162	3.80877	3.68349	2.85608	3.33808	3.16819	3.59492	0.026841899	0.305155548	16	4	24	0.010696459	0.510510179	24	8.255922606	0.10663989
ENSMUSG00000040591	1110051M20Rik	RIKEN cDNA 1110051M20 gene [Source:MGI Symbol;Acc:MGI:1915079]	61.3532	49.7251	52.8953	50.1287	52.5841	58.369	54.9319	58.4553	50.3812	58.4236	51.5646	54.9267	55.6911	55.8323	0.441246538	0.966018836	4	8	24	0.220126018	0.768040178	24	22.86535474	0.062709807
ENSMUSG00000040592	Cd79b	CD79B antigen [Source:MGI Symbol;Acc:MGI:96431]	1.42268	2.71792	3.17903	3.04826	2.13446	1.81554	2.95816	2.68949	4.46891	1.98177	2.70181	2.86495	2.8919	2.26214	0.586828825	0.966018836	12	12	24	0.189894543	0.768040178	24	8.245062684	0.202289534
ENSMUSG00000040594	Ranbp17	RAN binding protein 17 [Source:MGI Symbol;Acc:MGI:1929706]	3.02077	3.26496	3.63557	3.10989	3.12179	3.12049	2.63732	2.80117	3.26662	3.16434	3.4593	3.7275	3.2119	3.58574	0.870491976	0.966018836	12	16	24	0.588928854	0.853936396	24	11.72121649	0.041099459
ENSMUSG00000040596	Pogk	pogo transposable element with KRAB domain [Source:MGI Symbol;Acc:MGI:1918842]	13.9976	11.5773	12.7905	11.8798	12.8569	13.2768	13.9128	11.9397	10.3456	12.2606	12.0118	11.9984	11.8207	11.6652	0.244223134	0.741580974	4	4	24	0.035043628	0.608551791	24	20.61520954	0.091091448
ENSMUSG00000040599	Mis12	MIS12 kinetochore complex component [Source:MGI Symbol;Acc:MGI:1914389]	13.7989	11.9949	13.0173	12.5337	12.6528	12.5505	11.9392	11.5276	11.0088	11.774	12.1787	13.5236	12.6448	12.3932	0.330576288	0.850598217	24	8	24	0.118209945	0.728789843	24	20.24273912	0.06295874

ENSMUSG00000040605	Bace2	beta-site APP-cleaving enzyme 2 [Source:MGI Symbol;Acc:MGI:1860440]	9.83197	9.75343	11.2508	9.53286	11.2174	10.0047	12.0653	11.11	10.5905	11.6721	9.82184	10.1307	9.50911	9.75703	0.896753532	0.966018836	12	20	24	0.786124436	0.931220622	24	10.26714676	0.030368984
ENSMUSG00000040606	Kazn	kazrin, periplakin interacting protein [Source:MGI Symbol;Acc:MGI:1918779]	14.2595	14.1927	14.6206	14.4601	16.1144	15.2242	16.7288	13.9908	14.3768	18.2126	14.8112	16.0328	14.4519	14.279	0.008047769	0.192760771	20	12	24	0.1840126	0.768040178	24	16.09297839	0.071798686
ENSMUSG00000040610	Tlx3	T cell leukemia, homeobox 3 [Source:MGI Symbol;Acc:MGI:1351209]	8.50815	6.10981	5.60465	5.79621	5.19631	5.19442	6.3632	9.53247	8.33365	13.9728	11.3941	12.6594	12.9845	12.335	0.890176313	0.966018836	4	16	24	0.831150578	0.947661285	24	4.210669778	0.072805016
ENSMUSG00000040612	Ildr2	immunoglobulin-like domain containing receptor 2 [Source:MGI Symbol;Acc:MGI:1196370]	3.19643	3.75379	3.20994	2.64159	3.25874	3.13763	3.10137	3.05012	3.73679	2.20526	2.96223	3.27918	2.42281	3.36398	0.371875382	0.894537464	12	4	24	0.47397238	0.805393068	24	2.831992084	0.073062708
ENSMUSG00000040613	Apobec1	apolipoprotein B mRNA editing enzyme, catalytic polypeptide 1 [Source:MGI Symbol;Acc:MGI:103298]	4.63085	7.42773	5.50124	4.22531	6.42486	5.39856	6.28488	7.00367	7.06616	4.38773	6.67728	5.40647	6.71268	5.54577	0.223774686	0.717349224	8	8	24	0.450365912	0.805393068	24	2.730036268	0.108895409
ENSMUSG00000040616	Tmem51	transmembrane protein 51 [Source:MGI Symbol;Acc:MGI:2384874]	6.66498	5.35849	4.93593	4.63992	5.91786	6.98363	5.00551	5.48736	5.24198	6.11338	6.13242	5.76286	5.78036	6.00485	0.205280539	0.693330038	24	12	24	0.126000007	0.739868076	24	20.11183964	0.110223716
ENSMUSG00000040618	Pck2	phosphoenolpyruvate carboxykinase 2 (mitochondrial) [Source:MGI Symbol;Acc:MGI:1860456]	17.6634	16.9191	15.8944	17.0167	16.8544	18.0553	15.6657	17.6425	15.5534	18.1218	16.3082	18.4433	19.0312	17.833	0.148073804	0.590732968	24	12	24	0.348811573	0.779170188	24	21.54729804	0.046740097
ENSMUSG00000040620	Dhx33	DEAH (Asp-Glu-Ala-His) box polypeptide 33 [Source:MGI Symbol;Acc:MGI:2445102]	8.42489	8.30905	8.5237	9.33871	9.08814	8.6323	8.48681	8.64809	7.57795	9.00314	8.27771	7.99074	7.78057	7.9615	0.336755135	0.85758369	16	12	24	0.086474665	0.705702571	24	14.50652549	0.05307735
ENSMUSG00000040621	Gemin8	gem nuclear organelle associated protein 8 [Source:MGI Symbol;Acc:MGI:2384300]	2.10354	2.05822	2.24404	2.17902	2.40185	2.13594	2.67016	2.20403	1.85027	2.25207	2.03327	2.35987	1.83552	2.34322	0.844448841	0.966018836	24	12	24	0.786377507	0.93134108	24	19.50376446	0.028853883
ENSMUSG00000040624	Plekhg1	pleckstrin homology domain containing, family G (with RhoGef domain) member 1 [Source:MGI Symbol;Acc:MGI:2676551]	6.209	6.41373	5.31609	4.81235	6.06236	5.88095	7.20023	6.2786	5.39696	6.37053	5.61945	6.35056	6.1947	5.77336	0.195175079	0.673387182	4	8	24	0.04720245	0.633507364	24	23.1684744	0.109075232
ENSMUSG00000040631	Dok4	docking protein 4 [Source:MGI Symbol;Acc:MGI:2148865]	124.124	100.746	101.353	114.096	107.704	113.61	98.2918	107.647	89.9918	129.438	109.152	111.934	109.866	106.855	0.082113105	0.462922111	16	20	24	0.315368558	0.768040178	24	17.60253211	0.079362372
ENSMUSG00000040640	Erc2	ELKS/RAB6-interacting/CAST family member 2 [Source:MGI Symbol;Acc:MGI:1098749]	19.0907	16.8105	16.9651	17.1128	17.9427	18.8253	16.3985	17.6202	15.6514	19.7108	18.2414	17.6568	18.2334	18.0575	0.287843582	0.793714531	24	12	24	0.294683502	0.768040178	24	18.47621784	0.05243905
ENSMUSG00000040648	Ppip5k2	diphosphoinositol pentakisphosphate kinase 2 [Source:MGI Symbol;Acc:MGI:2142810]	24.3605	26.9606	24.1464	26.1937	26.8143	23.7979	23.5505	24.3518	26.3199	23.1244	25.7976	24.4926	25.423	24.9698	0.90332508	0.966018836	8	20	24	0.748936208	0.918339374	24	10.99901218	0.021125825
ENSMUSG00000040649	Rimklb	ribosomal modification protein rimK-like family member B [Source:MGI Symbol;Acc:MGI:1918325]	18.3477	25.0006	19.4172	20.4747	23.576	20.1059	30.1916	18.1359	24.9133	18.2824	17.7666	19.0371	17.0538	17.6812	0.795269577	0.966018836	12	20	24	0.960572206	0.98977387	24	2.347202318	0.024651002
ENSMUSG00000040651	Tasor	transcription activation suppressor [Source:MGI Symbol;Acc:MGI:1921694]	12.1557	11.787	11.6185	12.6432	12.8599	9.75921	11.3156	10.3967	10.9005	8.90246	13.2142	8.38481	8.53844	9.63492	0.369262716	0.893596186	20	4	24	0.447613758	0.805393068	24	12.97825296	0.076383634
ENSMUSG00000040652	Oaz2	ornithine decarboxylase antizyme 2 [Source:MGI Symbol;Acc:MGI:109492]	51.1083	47.9925	48.2334	47.1849	47.2815	48.7007	47.5203	50.8256	46.0879	55.7146	51.1859	53.0077	52.8833	52.4323	0.764889554	0.966018836	24	12	24	0.845846392	0.953396028	24	21.44224362	0.015770276
ENSMUSG00000040653	Ppp1r14c	protein phosphatase 1, regulatory inhibitor subunit 14C [Source:MGI Symbol;Acc:MGI:1923392]	7.08873	7.92106	7.43593	7.75229	7.55625	8.10152	7.77368	9.21711	8.23754	9.59127	6.97843	7.60877	7.65742	8.34947	0.17953325	0.655480352	8	12	24	0.225144998	0.768040178	24	7.663527993	0.07565117
ENSMUSG00000040658	Dnph1	2'-deoxynucleoside 5'-phosphate N-hydrolase 1 [Source:MGI Symbol;Acc:MGI:3039376]	1.31406	1.15947	1.30325	0.710581	1.24632	1.47281	0.990863	1.45524	1.18874	1.17443	1.27746	1.50106	1.20867	1.12238	0.285027487	0.793714531	24	16	24	0.417009587	0.801695312	24	21.88487242	0.074278585
ENSMUSG00000040659	Efhd2	EF hand domain containing 2 [Source:MGI Symbol;Acc:MGI:106504]	15.7272	14.8936	17.7189	14.3095	14.6222	16.4527	16.4316	14.8565	14.9833	17.3423	14.338	16.4663	14.5668	16.1482	0.880247609	0.966018836	24	20	24	0.952371507	0.988407044	24	4.588905996	0.012472203
ENSMUSG00000040661	Rad54l2	RAD54 like 2 (S. cerevisiae) [Source:MGI Symbol;Acc:MGI:1933196]	4.18144	3.71375	4.10521	4.07947	3.75977	4.01086	3.92529	4.20393	3.65705	4.95096	3.61129	4.06941	4.08851	4.11667	0.838532766	0.966018836	8	12	24	0.872884513	0.964303715	24	8.898251921	0.019986534
ENSMUSG00000040663	Clcf1	cardiotrophin-like cytokine factor 1 [Source:MGI Symbol;Acc:MGI:1930088]	0.940639	1.28315	1.45968	1.03076	1.2108	0.968169	0.717075	1.48132	1.60849	0.990934	1.16535	0.925139	1.26469	1.11708	0.020957534	0.262822894	12	12	24	0.058686891	0.658304792	24	7.913475193	0.149544753
ENSMUSG00000040666	Sh3bgr	SH3-binding domain glutamic acid-rich protein [Source:MGI Symbol;Acc:MGI:1354740]	23.6418	40.2036	34.8831	46.4712	35.4656	41.1671	29.525	62.8449	55.801	48.9229	34.2414	33.4617	43.3381	32.7378	0.235141005	0.73629392	12	16	24	0.128076785	0.740162193	24	9.18284639	0.280098133
ENSMUSG00000040667	Nup88	nucleoporin 88 [Source:MGI Symbol;Acc:MGI:104900]	30.0269	32.7826	33.251	30.4127	32.3544	30.8089	28.7407	30.3264	30.424	30.2492	29.9083	32.122	31.7237	30.2063	0.873567285	0.966018836	12	16	24	0.931367501	0.982348071	24	10.77463244	0.008764201
ENSMUSG00000040669	Phc1	polyhomeotic 1 [Source:MGI Symbol;Acc:MGI:103248]	3.59506	4.1935	4.66327	3.75922	3.95464	4.01149	4.8242	3.93121	4.85588	4.05464	3.61064	4.23423	3.52979	4.08676	0.46238066	0.966018836	12	8	24	0.396275397	0.799546792	24	6.912520338	0.066535052
ENSMUSG00000040675	Mthfd1l	methylenetetrahydrofolate dehydrogenase (NADP+ dependent) 1-like [Source:MGI Symbol;Acc:MGI:1924836]	6.04119	5.49246	6.07074	5.75324	5.91766	6.29592	5.53695	5.83578	5.29926	7.72824	5.74392	5.58153	5.71286	5.46451	0.499762123	0.966018836	16	16	24	0.329902479	0.768040178	24	14.0383829	0.065363313
ENSMUSG00000040681	Hmgn1	high mobility group nucleosomal binding domain 1 [Source:MGI Symbol;Acc:MGI:96120]	22.8921	22.0433	22.5494	22.0684	24.2064	21.1863	23.3043	21.5261	24.41	19.534	23.9405	21.2793	23.2058	20.5895	0.851656735	0.966018836	20	12	24	0.882060146	0.966661503	24	19.07910376	0.018486283
ENSMUSG00000040687	Madd	MAP-kinase activating death domain [Source:MGI Symbol;Acc:MGI:2444672]	25.7126	20.8401	25.9088	22.2953	22.8057	24.4064	24.5816	23.1435	20.2822	26.0459	20.8317	23.8296	21.8172	25.3577	0.860560779	0.966018836	16	4	24	0.883481707	0.966818623	24	23.43572763	0.024226637
ENSMUSG00000040688	Tbl3	transducin (beta)-like 3 [Source:MGI Symbol;Acc:MGI:2384863]	11.9991	12.4526	13.6092	12.6005	12.5413	14.9192	11.4836	13.9248	12.6057	13.0197	12.0627	12.783	11.5134	13.7001	0.17847698	0.655480352	12	16	24	0.773664164	0.92732018	24	9.467173165	0.029734345
ENSMUSG00000040690	Col16a1	collagen, type XVI, alpha 1 [Source:MGI Symbol;Acc:MGI:1095396]	21.2639	23.2438	23.1419	20.6493	23.2624	20.6821	20.4476	22.4196	24.543	20.3955	23.6775	21.0162	24.2929	20.8951	0.922029406	0.966018836	12	4	24	0.715392017	0.90631377	24	7.693097523	0.032426531
ENSMUSG00000040694	Apobec2	apolipoprotein B mRNA editing enzyme, catalytic polypeptide 2 [Source:MGI Symbol;Acc:MGI:1343178]	5.93151	18.2152	21.0073	26.3178	16.3476	21.4193	13.6799	40.5101	28.8696	29.1137	14.9839	12.2816	22.1675	20.2573	0.090761402	0.481752771	16	12	24	0.038584763	0.61140397	24	8.975310747	0.568693823
ENSMUSG00000040697	Dnajc16	DnaJ heat shock protein family (Hsp40) member C16 [Source:MGI Symbol;Acc:MGI:2442146]	6.1564	5.14704	5.29435	5.43004	5.68543	6.02311	5.81594	5.93491	5.49753	6.5797	5.04815	5.60868	5.66252	6.0479	0.892578749	0.966018836	4	8	24	0.837939084	0.950394443	24	23.50030645	0.021504926
ENSMUSG00000040699	Limd2	LIM domain containing 2 [Source:MGI Symbol;Acc:MGI:1915053]	14.0437	13.7104	13.4434	13.0738	12.3947	14.1732	12.515	14.821	11.8152	13.4375	12.6118	13.0724	14.395	12.9124	0.496745696	0.966018836	4	16	24	0.256077711	0.768040178	24	0.648685625	0.053398878
ENSMUSG00000040701	Ap1g2	adaptor protein complex AP-1, gamma 2 subunit [Source:MGI Symbol;Acc:MGI:1328307]	4.562	4.20338	6.9923	5.91161	5.80978	3.803	4.19918	4.08232	4.48816	4.11158	5.25455	4.49789	5.81502	7.01319	0.912686312	0.966018836	12	12	24	0.521441757	0.820324275	24	9.454847289	0.11514456
ENSMUSG00000040703	Cyp2s1	cytochrome P450, family 2, subfamily s, polypeptide 1 [Source:MGI Symbol;Acc:MGI:1921384]	1.37988	1.57868	1.58679	1.45582	1.56157	1.10378	1.44731	1.53995	1.50717	1.57393	1.60204	1.42258	1.47582	1.39469	0.079180494	0.458678242	20	4	24	0.139187975	0.748826538	24	9.404709805	0.061116692
ENSMUSG00000040705	A930016O22Rik	RIKEN cDNA A930016O22 gene [Source:MGI Symbol;Acc:MGI:3605623]	0.357569	0.552269	0.626016	0.829375	0.575063	0.556677	0.563798	1.23829	0.825569	0.958033	0.602266	0.548469	0.542061	0.485842	0.008244783	0.192760771	16	12	24	0.08696422	0.706216588	24	9.639485243	0.154045345
ENSMUSG00000040710	St8sia4	ST8 alpha-N-acetyl-neuraminide alpha-2,8-sialyltransferase 4 [Source:MGI Symbol;Acc:MGI:106018]	2.5163	3.06728	2.84183	2.57456	2.56235	2.39191	2.77162	2.41717	3.08964	2.05357	3.11888	2.35731	2.2605	2.2589	0.949874888	0.966018836	12	12	24	0.80942837	0.939184036	24	8.877003777	0.034053239
ENSMUSG00000040711	Sh3pxd2b	SH3 and PX domains 2B [Source:MGI Symbol;Acc:MGI:2442062]	7.08629	5.8947	6.00779	6.62949	6.83118	6.68071	7.57422	6.11272	5.6731	7.37851	6.45823	6.4973	6.69257	5.93855	0.048880363	0.379129806	4	4	24	0.085597221	0.703577714	24	18.91419221	0.091358031
ENSMUSG00000040712	Camta2	calmodulin binding transcription activator 2 [Source:MGI Symbol;Acc:MGI:2135957]	12.8257	11.3059	11.9445	12.6637	11.8354	11.7861	13.5527	12.3814	12.4377	17.9962	11.8362	12.622	12.8461	13.6562	0.855823117	0.966018836	4	16	24	0.635070186	0.87594009	24	10.34297063	0.055314547
ENSMUSG00000040713	Creg1	cellular repressor of E1A-stimulated genes 1 [Source:MGI Symbol;Acc:MGI:1344382]	14.4668	19.4526	25.0279	24.5259	20.4207	17.7066	21.1958	22.0859	23.4354	18.0737	19.0894	18.4754	18.5893	18.1533	0.038115943	0.334069441	12	12	24	0.057788762	0.656248849	24	9.515887075	0.179579429
ENSMUSG00000040715	Rsc1a1	regulatory solute carrier protein, family 1, member 1 [Source:MGI Symbol;Acc:MGI:3526447]	2.59985	1.70077	2.13899	1.93978	1.58916	1.77864	1.63991	2.47089	1.91553	1.08062	0.802412	2.1651	2.21966	2.07512	0.123856376	0.552144108	4	16	24	0.045256327	0.628927515	24	2.3461513	0.233531129
ENSMUSG00000040717	Il17rd	interleukin 17 receptor D [Source:MGI Symbol;Acc:MGI:2159727]	1.52301	1.44033	1.09591	1.32879	1.24677	1.36325	1.43001	1.36039	1.3584	1.48681	1.30608	1.68108	1.52514	1.44518	0.05282315	0.391647859	4	16	24	0.113076464	0.727393008	24	22.74466738	0.06151483
ENSMUSG00000040720	Virma	vir like m6A methyltransferase associated [Source:MGI Symbol;Acc:MGI:1913435]	9.10241	7.90756	8.45587	7.98046	8.3768	8.46362	8.848	8.28237	7.85663	7.85707	8.42996	8.60393	8.7265	8.11541	7.90E-04	0.071344007	4	12	24	0.003795857	0.427278365	24	21.7641298	0.065680282
ENSMUSG00000040721	Zfhx2	zinc finger homeobox 2 [Source:MGI Symbol;Acc:MGI:2686934]	4.99546	4.6459	4.9953	4.16299	5.08387	4.72179	5.15008	4.83211	5.12685	5.8567	5.19547	5.2548	5.61341	5.07152	0.801561896	0.966018836	4	4	24	0.942613215	0.985829422	24	20.12839152	0.011668775
ENSMUSG00000040722	Scamp5	secretory carrier membrane protein 5 [Source:MGI Symbol;Acc:MGI:1928948]	64.9515	56.1818	56.2009	57.4165	56.4635	67.3648	66.9337	61.5088	51.9675	69.0275	54.6222	60.3636	58.5014	61.4085	0.310940564	0.820837787	24	12	24	0.33180551	0.768621471	24	21.87868116	0.072860014
ENSMUSG00000040723	Rcsd1	RCSD domain containing 1 [Source:MGI Symbol;Acc:MGI:2676394]	6.25177	7.77581	8.22845	8.20563	7.76468	7.24004	7.2835	9.48422	9.16502	9.67707	7.22654	7.25123	7.87357	6.80309	0.076779403	0.453790923	16	8	24	0.027957724	0.590904845	24	9.588838743	0.155139318
ENSMUSG00000040724	Kcna2	potassium voltage-gated channel, shaker-related subfamily, member 2 [Source:MGI Symbol;Acc:MGI:96659]	53.6191	54.2101	49.7536	50.5744	50.4838	50.4576	52.5553	49.1212	50.5744	53.3713	48.1185	56.9229	57.13	58.0504	0.518444157	0.966018836	4	16	24	0.14163552	0.751766185	24	0.99364587	0.056374791
ENSMUSG00000040725	Hnrnpul1	heterogeneous nuclear ribonucleoprotein U-like 1 [Source:MGI Symbol;Acc:MGI:2443517]	16.2813	15.2924	16.1355	16.0255	15.8236	15.1364	16.1355	17.7651	17.0882	20.3308	15.8041	17.1791	17.4423	16.6678	0.796193867	0.966018836	8	12	24	0.386027966	0.799546792	24	8.999040479	0.050472097
ENSMUSG00000040729	Cep126	centrosomal protein 126 [Source:MGI Symbol;Acc:MGI:2680221]	6.86862	7.99413	6.19137	6.54402	8.08006	7.69583	8.1509	5.44997	6.24181	6.24746	7.97347	6.77765	6.79606	7.60031	0.196044629	0.673646834	20	16	24	0.171789371	0.768040178	24	19.90054436	0.116806367
ENSMUSG00000040731	Eif4h	eukaryotic translation initiation factor 4H [Source:MGI Symbol;Acc:MGI:1341822]	104.979	89.3933	91.1633	98.1891	92.3436	95.9422	97.1494	99.0589	87.5483	111.234	87.055	100.83	95.1884	96.8334	0.70641939	0.966018836	24	12	24	0.870967295	0.963364428	24	20.85091698	0.021731492
ENSMUSG00000040732	Erg	ETS transcription factor [Source:MGI Symbol;Acc:MGI:95415]	1.28781	1.89878	1.51664	1.98252	1.41189	1.60361	1.37971	2.0644	1.9577	1.84536	1.74332	1.58192	1.68693	1.74349	0.080148772	0.458678242	8	20	24	0.046792429	0.633507364	24	8.713450558	0.115962687
ENSMUSG00000040734	Ppp1r13l	protein phosphatase 1, regulatory subunit 13 like [Source:MGI Symbol;Acc:MGI:3525053]	0.866964	0.884343	0.890375	0.960192	0.858802	0.801571	0.662194	0.963422	1.32765	1.43672	1.23309	1.15554	1.03943	1.05425	0.400729905	0.933249185	16	12	24	0.02894787	0.595626873	24	11.10454948	0.117892076
ENSMUSG00000040738	Ints8	integrator complex subunit 8 [Source:MGI Symbol;Acc:MGI:1919906]	18.8097	14.7169	15.2925	15.5652	14.762	15.2528	14.1135	14.5413	15.5545	12.063	17.891	14.8129	15.3834	14.1481	0.895137379	0.966018836	20	12	24	0.491510355	0.805393068	24	20.78644442	0.06607392
ENSMUSG00000040740	Slc25a34	solute carrier family 25, member 34 [Source:MGI Symbol;Acc:MGI:2686215]	0.505274	0.321536	0.601499	0.582977	0.344867	0.47806	0.694935	1.10715	0.57301	0.625654	0.250878	0.450938	0.492625	0.619123	0.122696414	0.550756498	16	4	24	0.138605066	0.747972781	24	5.298712154	0.137602894
ENSMUSG00000040746	Rnf167	ring finger protein 167 [Source:MGI Symbol;Acc:MGI:1917760]	29.2568	29.3277	28.925	31.5275	28.7044	29.7737	31.0352	32.9726	31.8379	33.5179	26.4259	30.8195	31.1988	29.8417	0.369262716	0.893596186	16	4	24	0.422633156	0.80496995	24	6.508705779	0.045190036
ENSMUSG00000040747	Cd53	CD53 antigen [Source:MGI Symbol;Acc:MGI:88341]	3.56383	5.63179	6.52696	5.66616	5.47201	4.49382	5.24375	4.98049	6.02741	3.41471	4.74428	4.51351	4.6359	4.50517	0.047644929	0.377556215	12	12	24	0.370095052	0.792235171	24	8.442909073	0.120859991
ENSMUSG00000040749	Siah1b	siah E3 ubiquitin protein ligase 1B [Source:MGI Symbol;Acc:MGI:108063]	2.07377	2.59503	2.21033	2.1075	3.01842	2.31416	2.44069	2.69019	2.55654	3.07376	2.30359	2.45741	2.3843	2.59768	0.887010723	0.966018836	8	20	24	0.637549654	0.876726967	24	10.76713923	0.044681301
ENSMUSG00000040751	Lat2	linker for activation of T cells family, member 2 [Source:MGI Symbol;Acc:MGI:1926479]	2.53453	2.53508	2.87769	2.34866	3.05456	2.19622	2.93979	1.26129	2.69016	1.84878	2.40654	2.48631	2.01189	2.0344	0.890648739	0.966018836	20	12	24	0.551709497	0.83632237	24	17.02221594	0.089557866
ENSMUSG00000040752	Myh6	myosin, heavy polypeptide 6, cardiac muscle, alpha [Source:MGI Symbol;Acc:MGI:97255]	0.834518	0.666779	0.683475	1.41707	1.11255	0.796271	0.559793	0.714891	0.83429	0.585472	0.994458	0.430609	0.662065	0.564399	0.334687844	0.85758369	20	4	24	0.077499142	0.687420473	24	13.82283263	0.146709751
ENSMUSG00000040759	Cmtm5	CKLF-like MARVEL transmembrane domain containing 5 [Source:MGI Symbol;Acc:MGI:2447164]	53.6874	55.5158	58.801	49.6423	66.4987	54.1655	60.0718	56.0158	60.1444	43.2887	58.9724	47.8413	53.025	44.7563	0.916860713	0.966018836	12	12	24	0.868534435	0.96247509	24	19.32931822	0.034976728
ENSMUSG00000040760	Appl1	adaptor protein, phosphotyrosine interaction, PH domain and leucine zipper containing 1 [Source:MGI Symbol;Acc:MGI:1920243]	18.2635	19.5752	18.3291	18.3133	19.6931	18.0716	19.6491	18.0602	20.0271	16.5836	20.49	18.1988	19.9039	20.0907	0.873567285	0.966018836	12	12	24	0.751637371	0.919304206	24	1.441481312	0.023930041
ENSMUSG00000040761	Spen	spen family transcription repressor [Source:MGI Symbol;Acc:MGI:1891706]	2.80285	3.14784	2.865	3.77943	3.47689	3.07186	3.66505	3.77435	4.21355	4.64574	3.06537	4.00551	3.61958	3.90828	0.745118544	0.966018836	12	16	24	0.237625531	0.768040178	24	9.816825836	0.090606344
ENSMUSG00000040767	Snrnp25	small nuclear ribonucleoprotein 25 (U11/U12) [Source:MGI Symbol;Acc:MGI:1925622]	22.7548	25.4502	25.5239	26.9294	25.0724	22.9953	22.2683	24.351	27.7645	19.8734	28.1914	25.445	26.6141	24.3969	0.625825704	0.966018836	12	16	24	0.821730021	0.943800354	24	10.55904416	0.03384637
ENSMUSG00000040770	Il25	interleukin 25 [Source:MGI Symbol;Acc:MGI:2155888]	0.440821	0.537796	1.15065	0.253028	0.462352	0.876628	0.322795	0.789873	0.722815	0.698936	0.727966	0.730302	0.557613	0.756132	0.883024237	0.966018836	12	4	24	0.675351982	0.89119716	24	7.956848001	0.07456868
ENSMUSG00000040771	Oard1	O-acyl-ADP-ribose deacylase 1 [Source:MGI Symbol;Acc:MGI:2146818]	13.9627	17.1256	16.0237	17.4628	16.7572	16.5157	15.3351	17.0629	17.8107	15.4117	16.4703	15.312	17.0737	17.0925	0.121015203	0.548303839	8	20	24	0.144522745	0.753062008	24	8.815026832	0.067901912
ENSMUSG00000040774	Cept1	choline/ethanolaminephosphotransferase 1 [Source:MGI Symbol;Acc:MGI:2139793]	43.332	45.6117	39.8354	37.5288	42.4511	37.6051	42.8602	40.9727	41.87	35.9655	47.5187	41.6478	45.4761	43.0062	0.06180207	0.409814092	4	12	24	0.279534228	0.768040178	24	0.147808164	0.067986234
ENSMUSG00000040782	Cop1	COP1, E3 ubiquitin ligase [Source:MGI Symbol;Acc:MGI:1347046]	8.68121	8.81629	8.10659	9.24761	9.58275	9.89869	8.84472	9.13534	9.54398	8.36474	11.2271	9.34184	9.824	8.25851	0.100794585	0.511659635	20	12	24	0.089960606	0.7097999	24	17.37955722	0.091436953
ENSMUSG00000040785	Ttc3	tetratricopeptide repeat domain 3 [Source:MGI Symbol;Acc:MGI:1276539]	144.901	146.048	150.33	137.938	136.379	139.424	142.895	136.086	147.956	142.703	157.121	156.137	158.301	164.99	0.921572192	0.966018836	12	4	24	0.549379235	0.834967545	24	2.877105969	0.028899045
ENSMUSG00000040794	C1qtnf4	C1q and tumor necrosis factor related protein 4 [Source:MGI Symbol;Acc:MGI:1914695]	1.23218	1.62928	1.9915	0.647663	0.396277	0.841542	1.63357	1.54714	2.86423	2.02321	2.18286	3.00215	2.43532	2.77603	0.873171835	0.966018836	12	4	24	0.10372126	0.715474111	24	4.987625444	0.282889003
ENSMUSG00000040795	Iqcc	IQ motif containing C [Source:MGI Symbol;Acc:MGI:2446212]	12.0536	14.1684	12.8089	12.6622	15.5403	13.7512	14.2428	13.8701	15.2975	10.5958	15.9084	13.0825	13.2592	13.6344	0.920872048	0.966018836	20	20	24	0.912472394	0.975295827	24	19.89672566	0.025899946
ENSMUSG00000040797	Iqsec3	IQ motif and Sec7 domain 3 [Source:MGI Symbol;Acc:MGI:2677208]	6.37034	6.16552	8.51141	5.8736	5.5204	7.00789	7.29702	5.79038	6.16269	7.82917	5.14749	6.94487	5.51937	7.70423	0.951523604	0.966018836	12	8	24	0.619960586	0.869915296	24	6.11673066	0.076203491
ENSMUSG00000040809	Chil3	chitinase-like 3 [Source:MGI Symbol;Acc:MGI:1330860]	6.94385	11.5506	21.2388	14.2273	12.6042	11.9362	11.4744	10.1374	11.1747	7.68854	11.7769	10.1489	14.0282	7.33841	0.840483986	0.966018836	12	20	24	0.680723765	0.893322401	24	12.10473102	0.131369479
ENSMUSG00000040811	Eml2	echinoderm microtubule associated protein like 2 [Source:MGI Symbol;Acc:MGI:1919455]	49.0904	47.772	50.2793	47.1713	45.926	50.8682	45.8741	46.9406	42.846	62.9512	40.2125	52.2497	44.5502	51.1479	0.910436409	0.966018836	12	20	24	0.910316615	0.974732058	24	10.62283308	0.026871555
ENSMUSG00000040813	Tex264	testis expressed gene 264 [Source:MGI Symbol;Acc:MGI:1096570]	50.9898	43.0225	43.6324	41.323	43.8992	46.1437	43.795	47.2586	40.2164	46.5903	48.7388	46.3982	46.7614	44.5395	0.311445561	0.821921422	4	8	24	0.123201439	0.736234617	24	21.08711757	0.070744972
ENSMUSG00000040818	Dennd6a	DENN/MADD domain containing 6A [Source:MGI Symbol;Acc:MGI:2442980]	13.0602	11.8974	10.984	12.6659	12.8208	12.2565	12.0794	11.7973	11.248	11.9087	13.0008	11.7897	11.511	12.5631	0.117335378	0.538362324	20	16	24	0.168386936	0.767020009	24	18.32728734	0.051946756
ENSMUSG00000040820	Hlcs	holocarboxylase synthetase (biotin- [propriony-Coenzyme A-carboxylase (ATP-hydrolysing)] ligase) [Source:MGI Symbol;Acc:MGI:894646]	6.99636	6.05086	5.77221	6.37255	5.31274	7.04531	5.79379	6.7025	5.26835	8.07894	5.91885	6.64505	6.22113	6.92162	0.601647512	0.966018836	24	12	24	0.930763134	0.982191975	24	22.17437657	0.022996982
ENSMUSG00000040822	1700123O20Rik	RIKEN cDNA 1700123O20 gene [Source:MGI Symbol;Acc:MGI:1920893]	15.9184	14.4063	15.9958	15.9103	17.2082	14.0929	15.4284	14.5889	14.7844	12.3566	16.2461	14.616	15.8742	13.188	0.192357053	0.669841558	20	12	24	0.454505357	0.805393068	24	18.49558899	0.056692524
ENSMUSG00000040824	Snrpd2	small nuclear ribonucleoprotein D2 [Source:MGI Symbol;Acc:MGI:98345]	13.8221	15.7452	13.2021	12.6655	15.6848	14.2393	12.9645	13.5624	14.3776	10.5861	15.9131	12.6269	13.6597	13.4675	0.890235289	0.966018836	20	20	24	0.727461995	0.910611797	24	21.52781592	0.046356115
ENSMUSG00000040828	Catsperd	cation channel sperm associated auxiliary subunit delta [Source:MGI Symbol;Acc:MGI:2147030]	0.582621	0.817314	0.598522	0.650466	0.438228	0.58025	0.816905	0.491583	0.718885	0.703205	0.752045	0.632711	0.676056	0.821639	0.668329321	0.966018836	8	16	24	0.465802593	0.805393068	24	5.062843793	0.049211794
ENSMUSG00000040829	Zmynd15	zinc finger, MYND-type containing 15 [Source:MGI Symbol;Acc:MGI:3603821]	0.703508	1.18373	0.646132	1.01787	0.873611	0.73639	1.14538	1.14727	0.934462	0.65792	1.05692	0.870409	0.894897	0.943438	0.348367381	0.870418653	8	4	24	0.624961387	0.872055388	24	2.987750234	0.052695566
ENSMUSG00000040836	Gpr161	G protein-coupled receptor 161 [Source:MGI Symbol;Acc:MGI:2685054]	6.39795	5.26703	5.52602	5.84634	5.94976	6.08721	5.6244	5.30674	4.13339	6.13043	5.47928	5.54721	5.47162	5.46909	0.064303071	0.414867207	24	8	24	0.102498374	0.713699364	24	18.94431335	0.098041794
ENSMUSG00000040840	4930579G18Rik	RIKEN cDNA 4930579G18 gene [Source:MGI Symbol;Acc:MGI:1923163]	0.888664	1.48026	0.96637	0.962589	1.07157	1.17696	1.2649	1.23769	1.55097	1.07639	1.39176	0.944449	1.18392	0.774361	0.849180783	0.966018836	12	12	24	0.951168995	0.98830325	24	7.849478808	0.020641284
ENSMUSG00000040841	Six5	sine oculis-related homeobox 5 [Source:MGI Symbol;Acc:MGI:106220]	3.71549	3.5	3.02933	3.16404	4.01544	2.80715	4.37273	3.51597	3.65939	3.08773	4.00009	3.75238	4.24835	3.01486	0.210789085	0.699573621	4	8	24	0.335494843	0.771419717	24	21.54431216	0.091620954
ENSMUSG00000040842	Szrd1	SUZ RNA binding domain containing 1 [Source:MGI Symbol;Acc:MGI:1098672]	12.29	11.4458	11.3753	10.8932	12.2689	11.9034	13.6625	12.7333	13.049	13.3176	12.3196	12.6409	12.3769	11.051	0.895137379	0.966018836	4	4	24	0.743039236	0.916215545	24	20.49826515	0.028512161
ENSMUSG00000040843	Tiprl	TIP41, TOR signalling pathway regulator-like (S. cerevisiae) [Source:MGI Symbol;Acc:MGI:1915087]	35.7178	40.0968	34.9285	36.8324	41.6556	37.1174	35.909	38.1437	38.3461	37.2766	45.9289	39.0519	42.85	42.526	0.932671476	0.966018836	20	20	24	0.757879486	0.921733004	24	17.33686534	0.027815813
ENSMUSG00000040848	Sft2d2	SFT2 domain containing 2 [Source:MGI Symbol;Acc:MGI:1917362]	10.6777	10.9405	11.069	11.3559	12.5298	10.3576	12.5588	11.5475	12.0601	11.6483	10.676	11.3214	10.9379	9.91516	0.851656735	0.966018836	16	8	24	0.677743892	0.892246589	24	13.2336161	0.031245916
ENSMUSG00000040850	Psme4	proteasome (prosome, macropain) activator subunit 4 [Source:MGI Symbol;Acc:MGI:2143994]	26.2431	27.8604	26.7454	29.725	28.5987	26.691	26.1475	27.3441	28.759	28.9313	30.1642	27.7328	28.7359	28.0717	0.027467587	0.308438477	20	8	24	0.011761372	0.523158883	24	12.51054689	0.057015791
ENSMUSG00000040852	Plekhh2	pleckstrin homology domain containing, family H (with MyTH4 domain) member 2 [Source:MGI Symbol;Acc:MGI:2146813]	1.49873	1.9841	2.04028	1.84044	1.92481	1.73819	1.91481	1.769	2.28285	1.74467	1.69873	1.90467	1.71894	1.61746	0.286642595	0.793714531	12	16	24	0.295830662	0.768040178	24	9.534332824	0.060931353
ENSMUSG00000040855	Reps2	RALBP1 associated Eps domain containing protein 2 [Source:MGI Symbol;Acc:MGI:2663511]	16.7706	17.1336	15.5838	16.6638	16.4872	16.2133	16.4566	14.5725	15.1121	14.3235	16.9613	15.769	15.7712	17.032	0.93447422	0.966018836	8	4	24	0.400936569	0.799546792	24	21.13048094	0.041672801
ENSMUSG00000040856	Dlk1	delta like non-canonical Notch ligand 1 [Source:MGI Symbol;Acc:MGI:94900]	1.69491	2.04315	3.07119	1.32446	2.36108	1.93187	1.85006	1.69837	1.75751	1.48909	2.00557	1.44205	1.77334	1.76334	0.880631923	0.966018836	20	20	24	0.927841714	0.98127006	24	7.540776072	0.031172968
ENSMUSG00000040857	Erf	Ets2 repressor factor [Source:MGI Symbol;Acc:MGI:109637]	5.10106	4.35807	5.69461	5.38549	4.08749	4.38119	5.14337	5.41099	6.08661	7.05965	4.65551	5.20116	5.04301	5.03071	0.116913252	0.538362324	16	4	24	0.080171089	0.695354253	24	8.809916188	0.143454932
ENSMUSG00000040859	Bsdc1	BSD domain containing 1 [Source:MGI Symbol;Acc:MGI:1913466]	16.7311	17.2849	17.5707	17.9764	17.0078	18.6629	16.8503	19.7515	17.659	20.8128	16.2473	17.1476	17.7694	18.375	0.470374729	0.966018836	16	4	24	0.336765943	0.771960538	24	8.499486853	0.053534286
ENSMUSG00000040860	Crocc	ciliary rootlet coiled-coil, rootletin [Source:MGI Symbol;Acc:MGI:3529431]	3.99573	3.89928	4.6248	4.14068	3.83754	3.91038	5.30355	3.65934	4.21035	3.3754	3.87043	4.40415	4.14915	4.5039	0.480187506	0.966018836	4	16	24	0.409821572	0.799546792	24	1.412293731	0.066766356
ENSMUSG00000040865	Ino80d	INO80 complex subunit D [Source:MGI Symbol;Acc:MGI:3027003]	4.41676	4.48389	4.11151	4.76384	4.88874	4.084	4.5798	3.75954	4.6386	3.71779	5.07312	4.51435	4.66808	4.3929	0.305564675	0.811201458	20	12	24	0.467273583	0.805393068	24	17.80380812	0.053616247
ENSMUSG00000040867	Begain	brain-enriched guanylate kinase-associated [Source:MGI Symbol;Acc:MGI:3044626]	3.3707	4.76707	7.17577	4.05749	3.25504	4.73314	5.63398	4.10863	6.3377	5.00246	3.96069	5.42736	3.52017	6.63074	0.064422003	0.414867207	12	8	24	0.145662287	0.754301054	24	7.02658261	0.227713906
ENSMUSG00000040875	Osbpl10	oxysterol binding protein-like 10 [Source:MGI Symbol;Acc:MGI:1921736]	15.8062	11.1595	10.3568	11.3567	11.7741	13.2707	11.4325	13.4551	10.1699	16.0255	11.5456	13.9677	13.0827	13.1622	0.085890569	0.466404237	24	12	24	0.41019335	0.799546792	24	21.15502769	0.100997474
ENSMUSG00000040877	Wdr25	WD repeat domain 25 [Source:MGI Symbol;Acc:MGI:3045255]	3.656	3.26567	3.27533	3.04608	3.10445	3.66852	2.79414	3.61338	2.65744	3.9963	3.04337	3.94165	3.47541	4.64049	0.934818505	0.966018836	24	12	24	0.699856841	0.900387881	24	0.970354013	0.052497486
ENSMUSG00000040883	Tmem205	transmembrane protein 205 [Source:MGI Symbol;Acc:MGI:3045495]	41.0456	41.1541	39.2759	43.2053	45.3782	40.0628	40.766	41.8289	50.2245	36.065	51.1334	42.5502	47.6284	43.7432	0.829428469	0.966018836	20	4	24	0.929838832	0.981794609	24	15.49825921	0.019381005
ENSMUSG00000040888	Gfer	growth factor, augmenter of liver regeneration [Source:MGI Symbol;Acc:MGI:107757]	7.21588	7.45662	6.6955	8.19272	8.58455	7.94057	7.14706	7.65306	8.39178	6.95673	8.86017	8.81251	8.35452	9.0788	0.774195442	0.966018836	20	20	24	0.488708623	0.805393068	24	17.44140982	0.048381055
ENSMUSG00000040896	Kcnd3	potassium voltage-gated channel, Shal-related family, member 3 [Source:MGI Symbol;Acc:MGI:1928743]	35.6055	29.4923	26.6766	29.1379	28.3836	30.139	26.5617	28.3407	23.5564	30.7123	27.1049	31.4819	32.9271	31.0535	0.195343269	0.673387182	4	8	24	0.165281301	0.767020009	24	22.00337687	0.10550049
ENSMUSG00000040901	Kcnk18	potassium channel, subfamily K, member 18 [Source:MGI Symbol;Acc:MGI:2685627]	2.18953	1.24283	1.25027	1.63006	1.46084	1.91516	1.37529	1.5818	1.34395	2.42562	1.24873	1.71515	1.57988	1.49432	0.597596661	0.966018836	24	12	24	0.627615753	0.872795898	24	18.53252232	0.074974866
ENSMUSG00000040904	Gm21988	predicted gene 21988 [Source:MGI Symbol;Acc:MGI:5439457]	2.89875	1.8764	1.7397	2.31439	1.87407	1.86339	2.54147	2.07407	1.90008	2.64261	2.48555	2.92298	2.18232	2.33187	0.649212555	0.966018836	4	8	24	0.409858147	0.799546792	24	20.14597638	0.090554002
ENSMUSG00000040907	Atp1a3	ATPase, Na+/K+ transporting, alpha 3 polypeptide [Source:MGI Symbol;Acc:MGI:88107]	80.5883	79.4657	112.11	76.7943	66.0765	100.244	99.026	81.1723	74.4637	116.349	56.4187	86.7873	68.2253	102.101	0.922035832	0.966018836	16	4	24	0.586404382	0.853300537	24	5.705425671	0.126262469
ENSMUSG00000040913	Fbxw4	F-box and WD-40 domain protein 4 [Source:MGI Symbol;Acc:MGI:1354698]	12.5446	9.05756	11.5218	9.17656	12.5192	13.3451	9.19812	9.32033	9.65976	8.77528	10.683	6.48012	8.82487	7.61463	0.919571723	0.966018836	20	12	24	0.425995395	0.805393068	24	17.33459651	0.107254929
ENSMUSG00000040918	Slc19a2	solute carrier family 19 (thiamine transporter), member 2 [Source:MGI Symbol;Acc:MGI:1928761]	7.8912	6.36024	7.61286	6.69108	6.78139	7.5137	7.86066	6.83499	6.55961	7.64688	6.82273	6.83854	6.57562	7.0354	0.94791825	0.966018836	4	4	24	0.731771541	0.912550805	24	21.14396388	0.029558468
ENSMUSG00000040928	S100pbp	S100P binding protein [Source:MGI Symbol;Acc:MGI:1921898]	10.1899	10.434	11.4915	11.944	10.479	11.1406	11.1776	10.4077	10.8305	11.1824	9.97275	12.0747	10.073	11.8665	0.777666236	0.966018836	16	12	24	0.6927478	0.898177673	24	10.60138323	0.029870797
ENSMUSG00000040929	Rfx3	regulatory factor X, 3 (influences HLA class II expression) [Source:MGI Symbol;Acc:MGI:106582]	1.57776	1.92661	1.76717	1.7403	1.84158	1.42677	1.99462	1.35723	1.85956	1.30791	1.84698	1.6392	1.43795	1.57793	0.828910443	0.966018836	12	12	24	0.956176401	0.988639288	24	12.48443764	0.013984069
ENSMUSG00000040936	Ulk4	unc-51-like kinase 4 [Source:MGI Symbol;Acc:MGI:1921622]	0.534638	0.760832	0.705887	0.885919	1.07555	0.871612	0.756646	0.693605	0.807186	0.686236	0.628259	0.702472	0.685737	0.721027	0.774195442	0.966018836	16	12	24	0.335489431	0.771419717	24	14.25069489	0.058557477
ENSMUSG00000040938	Slc16a11	solute carrier family 16 (monocarboxylic acid transporters), member 11 [Source:MGI Symbol;Acc:MGI:2663709]	2.02884	4.17961	3.87641	2.1712	2.79565	2.05202	3.37441	3.79204	5.16042	2.40075	2.43649	3.5945	2.4933	3.38565	0.048507657	0.379129806	12	4	24	0.055529713	0.65534544	24	6.093394288	0.283245773

ENSMUSG00000040940	Arhgef1	Rho guanine nucleotide exchange factor (GEF) 1 [Source:MGI Symbol;Acc:MGI:1353510]	31.04	32.5036	28.5837	29.2089	30.4343	27.9376	34.7057	32.8217	37.8406	33.9409	36.3848	32.6651	32.6659	32.5551	0.816361374	0.966018836	4	20	24	0.69345956	0.898256039	24	6.853887984	0.037496365
ENSMUSG00000040943	Tet2	tet methylcytosine dioxygenase 2 [Source:MGI Symbol;Acc:MGI:2443298]	4.02019	5.51817	5.69464	5.16398	5.10458	4.50932	4.77336	5.05567	6.8846	4.35174	5.31971	5.3298	5.53743	5.39812	0.280810068	0.792139038	12	4	24	0.153201817	0.759484836	24	8.04347829	0.115429662
ENSMUSG00000040945	Rcc2	regulator of chromosome condensation 2 [Source:MGI Symbol;Acc:MGI:1919784]	17.8712	13.8016	14.184	15.6934	15.3999	16.1812	15.599	16.6572	14.0759	19.3336	14.9873	16.4723	16.519	15.6288	0.628286596	0.966018836	4	8	24	0.579052075	0.849660354	24	19.31805828	0.0531052
ENSMUSG00000040950	Mgl2	macrophage galactose N-acetyl-galactosamine specific lectin 2 [Source:MGI Symbol;Acc:MGI:2385729]	4.85894	6.34761	4.33663	3.79384	4.8228	3.30984	5.36848	5.94834	6.23514	2.91146	5.07911	4.52719	5.21864	4.74055	0.046535188	0.375011045	8	8	24	0.111665793	0.724377021	24	3.164511687	0.204348463
ENSMUSG00000040952	Rps19	ribosomal protein S19 [Source:MGI Symbol;Acc:MGI:1333780]	130.043	135.713	135.414	128.839	130.997	120.934	117.594	129.786	159.82	103.672	147.135	124.578	134.524	125.017	0.390686537	0.915479136	12	12	24	0.751742846	0.919351611	24	7.357913224	0.045806837
ENSMUSG00000040957	Cables1	CDK5 and Abl enzyme substrate 1 [Source:MGI Symbol;Acc:MGI:1927065]	2.08496	1.64692	2.38052	3.29121	1.89062	2.01979	2.09627	1.82896	1.78018	2.4327	2.50878	1.66814	1.83138	1.87001	0.08333408	0.462922111	16	16	24	0.059231347	0.658304792	24	12.98075265	0.157198216
ENSMUSG00000040964	Arhgef10l	Rho guanine nucleotide exchange factor (GEF) 10-like [Source:MGI Symbol;Acc:MGI:1920004]	16.256	14.0332	12.2169	14.2772	13.655	14.1476	14.8901	15.8187	12.2966	18.0929	13.3324	16.1468	15.0931	15.1053	0.347075276	0.870418653	4	8	24	0.577600356	0.848840315	24	22.1205215	0.060998774
ENSMUSG00000040966	Slc22a2	solute carrier family 22 (organic cation transporter), member 2 [Source:MGI Symbol;Acc:MGI:1335072]	1.1553	1.19672	1.19413	1.07864	1.39001	1.01242	1.02431	1.28548	1.41151	0.971627	1.05714	0.940517	1.0742	0.923754	0.148073804	0.590732968	12	12	24	0.502793808	0.809737567	24	8.478733799	0.04517373
ENSMUSG00000040972	Igsf21	immunoglobulin superfamily, member 21 [Source:MGI Symbol;Acc:MGI:2681842]	3.42472	3.19722	3.13344	3.34376	2.69127	3.23615	3.38559	2.74332	3.17988	5.1288	2.71012	3.96268	3.54981	4.07371	0.745118544	0.966018836	4	16	24	0.909124995	0.974606327	24	7.808012071	0.033801103
ENSMUSG00000040978	Gm11992	predicted gene 11992 [Source:MGI Symbol;Acc:MGI:3651127]	0.973479	1.21632	1.1558	0.959485	0.586324	1.01073	0.865441	0.874909	0.727043	0.876681	1.05124	0.955053	0.693336	0.968243	0.846643334	0.966018836	8	20	24	0.793604504	0.933825946	24	4.800784824	0.03459998
ENSMUSG00000040987	Mill2	MHC I like leukocyte 2 [Source:MGI Symbol;Acc:MGI:2179989]	5.27995	5.97768	4.62649	4.35822	4.94301	4.72617	5.1175	5.47763	5.79577	4.56684	5.20726	5.36918	5.25895	5.87316	0.001006819	0.075485393	8	8	24	0.036695605	0.609176161	24	2.82019482	0.101797687
ENSMUSG00000040990	Sh3kbp1	SH3-domain kinase binding protein 1 [Source:MGI Symbol;Acc:MGI:1889583]	12.0969	10.9322	11.8054	11.3168	12.4752	11.4261	12.557	11.7122	10.9327	11.6495	10.9136	10.7177	13.1875	11.3616	0.336755135	0.85758369	4	20	24	0.480755384	0.805393068	24	23.175056	0.039172448
ENSMUSG00000040997	Abhd4	abhydrolase domain containing 4 [Source:MGI Symbol;Acc:MGI:1915938]	51.7802	49.3338	48.1908	47.499	50.1055	53.1059	55.6697	55.2653	46.3663	55.3163	46.5546	48.7271	51.3243	48.7098	0.16456443	0.628002127	4	8	24	0.368871403	0.791915398	24	23.38351649	0.049895601
ENSMUSG00000040998	Npnt	nephronectin [Source:MGI Symbol;Acc:MGI:2148811]	0.789868	1.17688	0.963258	0.953617	0.826176	0.970699	1.00988	1.68344	1.17301	1.07567	0.821559	0.90723	1.11999	0.987784	0.008244783	0.192760771	8	12	24	0.054751044	0.655263328	24	5.412870645	0.129470079
ENSMUSG00000041000	Trim62	tripartite motif-containing 62 [Source:MGI Symbol;Acc:MGI:1914775]	4.40211	3.59927	3.59725	4.15502	3.93264	4.45686	4.05286	3.80157	3.32065	5.40431	4.01511	4.21397	3.81353	3.94013	0.010468194	0.196621011	24	12	24	0.22480953	0.768040178	24	17.10357507	0.091085756
ENSMUSG00000041012	Cmtm8	CKLF-like MARVEL transmembrane domain containing 8 [Source:MGI Symbol;Acc:MGI:2447167]	2.60084	2.77861	2.20541	2.73271	2.0923	2.63191	2.47476	2.84354	2.37927	2.34315	2.57821	2.76167	3.12027	3.41991	0.046240749	0.375011045	8	4	24	0.051267083	0.64758613	24	1.987744143	0.10306847
ENSMUSG00000041014	Nrg3	neuregulin 3 [Source:MGI Symbol;Acc:MGI:1097165]	6.84904	5.53673	6.30406	5.98473	6.25511	6.62384	5.47295	5.60544	5.20913	7.69426	5.40846	5.72077	5.62935	6.8712	0.842298032	0.966018836	16	20	24	0.823481717	0.94441254	24	13.8615158	0.035011966
ENSMUSG00000041020	Map7d2	MAP7 domain containing 2 [Source:MGI Symbol;Acc:MGI:1917474]	168.864	147.409	141.508	148.217	144.007	153.902	142.14	140.427	120.439	180.72	157.438	155.972	152.453	159.603	0.338172159	0.85758369	24	12	24	0.469236731	0.805393068	24	18.77316804	0.068316476
ENSMUSG00000041025	Iffo2	intermediate filament family orphan 2 [Source:MGI Symbol;Acc:MGI:2140675]	3.01416	2.6328	2.64379	3.02248	2.99201	2.84594	3.45766	2.53648	2.40623	2.89789	2.69467	3.06449	2.83372	2.6631	0.036084337	0.331852526	24	12	24	0.056322924	0.65534544	24	19.64481746	0.089098632
ENSMUSG00000041028	Ghitm	growth hormone inducible transmembrane protein [Source:MGI Symbol;Acc:MGI:1913342]	318.084	314.302	315.714	329.961	302.447	306.166	308.638	308.693	305.041	306.887	341.36	320.402	347.055	342.935	0.896773941	0.966018836	8	4	24	0.891936368	0.969383816	24	1.265739003	0.01129294
ENSMUSG00000041035	-	deleted in primary ciliary dyskinesia [Source:NCBI gene (formerly Entrezgene);Acc:226162]	33.9464	34.9697	29.401	34.1717	30.6248	32.0275	33.9382	29.4927	28.3839	27.8497	32.9249	31.1689	33.1246	30.8923	0.158469451	0.614860056	4	8	24	0.08592656	0.704313881	24	21.96728201	0.081216242
ENSMUSG00000041037	Irgq	immunity-related GTPase family, Q [Source:MGI Symbol;Acc:MGI:2667176]	16.899	12.3495	13.6489	14.3114	13.1018	16.3137	15.3768	16.1938	10.0048	21.3339	12.8993	16.2401	14.4097	15.3633	0.390686537	0.915479136	24	12	24	0.664450566	0.886705913	24	20.20443279	0.089700637
ENSMUSG00000041040	Fam117b	family with sequence similarity 117, member B [Source:MGI Symbol;Acc:MGI:1920000]	12.5932	12.7239	12.7757	13.2045	12.1377	13.284	11.7026	12.9366	11.6451	15.4111	12.4253	13.6564	13.229	13.8745	0.919571723	0.966018836	24	12	24	0.769311073	0.925619883	24	11.99288002	0.026550383
ENSMUSG00000041046	Ramp3	receptor (calcitonin) activity modifying protein 3 [Source:MGI Symbol;Acc:MGI:1860292]	1.21311	1.45543	1.27516	1.36418	1.12811	1.34831	1.35851	1.45162	1.31837	1.37201	1.2602	1.60542	1.23451	1.34216	0.895493752	0.966018836	8	12	24	0.763060396	0.923594538	24	3.548917161	0.020858905
ENSMUSG00000041057	Wdr43	WD repeat domain 43 [Source:MGI Symbol;Acc:MGI:1919765]	14.1767	17.4439	15.9427	14.8006	16.2319	14.7298	12.8372	16.4173	18.0533	13.9488	16.3989	15.439	15.2885	16.4977	0.079180494	0.458678242	8	20	24	0.270906976	0.768040178	24	8.051111203	0.080534494
ENSMUSG00000041058	Wwp1	WW domain containing E3 ubiquitin protein ligase 1 [Source:MGI Symbol;Acc:MGI:1861728]	15.471	17.3932	17.1309	17.218	17.5516	15.4692	16.752	16.8998	19.1345	15.8893	16.9763	15.3149	15.1096	15.7609	0.020957534	0.262822894	12	12	24	0.051325657	0.64767123	24	9.680773148	0.078402651
ENSMUSG00000041073	Nacad	NAC alpha domain containing [Source:MGI Symbol;Acc:MGI:3603030]	44.396	38.2997	37.2866	44.3231	44.2755	45.7552	37.2858	42.9969	35.2769	52.4975	35.5936	46.362	43.5401	47.047	0.601647512	0.966018836	24	12	24	0.736335043	0.914122853	24	18.00686633	0.05314565
ENSMUSG00000041075	Fzd7	frizzled class receptor 7 [Source:MGI Symbol;Acc:MGI:108570]	3.37684	3.66536	3.55775	2.92824	3.22232	2.84292	3.69821	4.17022	3.97555	3.25812	3.01103	3.11685	3.36302	3.81045	0.001920665	0.100255953	8	16	24	4.77E-04	0.276229484	24	4.996281962	0.153987802
ENSMUSG00000041078	Grid1	glutamate receptor, ionotropic, delta 1 [Source:MGI Symbol;Acc:MGI:95812]	1.2809	1.6781	2.51482	1.46373	1.49412	1.94636	2.09423	1.5561	1.74066	2.14272	1.33382	1.7772	1.17043	2.15913	0.696669634	0.966018836	12	16	24	0.444838775	0.805393068	24	8.013457817	0.106610764
ENSMUSG00000041079	Rwdd2b	RWD domain containing 2B [Source:MGI Symbol;Acc:MGI:1858215]	4.78268	5.28371	5.20287	5.01858	4.50206	5.34892	5.00717	4.60441	4.99066	5.01412	5.68405	5.28352	5.22862	5.70479	0.880247609	0.966018836	24	12	24	0.986957144	0.997339197	24	1.916657443	0.004802334
ENSMUSG00000041084	Ostc	oligosaccharyltransferase complex subunit (non-catalytic) [Source:MGI Symbol;Acc:MGI:1913607]	72.7138	76.6308	66.9308	63.5826	74.3576	66.8524	68.7817	65.4609	71.9008	54.0479	81.7561	63.1817	72.3904	66.3858	0.722296281	0.966018836	20	20	24	0.588105416	0.853883127	24	22.36217611	0.05921172
ENSMUSG00000041096	Tspyl2	TSPY-like 2 [Source:MGI Symbol;Acc:MGI:106244]	25.6866	24.318	27.5433	25.949	26.9105	26.2827	26.5813	22.6645	24.9833	24.6476	22.668	23.5539	21.9554	25.675	0.886002469	0.966018836	12	20	24	0.767284903	0.924784773	24	12.04418411	0.023416595
ENSMUSG00000041112	Elmo1	engulfment and cell motility 1 [Source:MGI Symbol;Acc:MGI:2153044]	20.6533	16.3556	17.7554	17.9104	16.4249	20.8656	19.4767	17.7272	13.6906	23.5676	14.9028	18.564	16.3015	19.6911	0.953350544	0.966018836	12	24	24	0.760453594	0.922513876	24	21.18066247	0.0605151
ENSMUSG00000041115	Iqsec2	IQ motif and Sec7 domain 2 [Source:MGI Symbol;Acc:MGI:3528396]	35.9454	31.1085	29.3853	32.5357	30.7034	36.8859	29.4021	33.6197	30.8054	37.1847	31.2372	33.0393	35.426	34.0573	0.601647512	0.966018836	12	12	24	0.673536925	0.890201973	24	20.99706784	0.040454176
ENSMUSG00000041117	Ccdc8	coiled-coil domain containing 8 [Source:MGI Symbol;Acc:MGI:3612184]	1.02155	1.08387	1.08427	0.816289	1.20057	0.896045	1.02038	1.26379	1.28892	1.14114	1.03619	0.9229	0.863939	1.11246	0.068712455	0.432567661	12	12	24	0.181508123	0.768040178	24	7.64971241	0.074325267
ENSMUSG00000041119	Pde9a	phosphodiesterase 9A [Source:MGI Symbol;Acc:MGI:1277179]	1.53136	2.60338	3.35299	2.28108	2.39976	2.54223	4.017	2.38756	3.02224	2.24479	2.32579	2.49483	2.11618	3.33746	0.503972924	0.966018836	12	4	24	0.448523537	0.805393068	24	6.291186361	0.123511631
ENSMUSG00000041120	Nbl1	NBL1, DAN family BMP antagonist [Source:MGI Symbol;Acc:MGI:104591]	101.409	92.7202	83.0069	85.7646	93.4371	89.8613	89.6163	98.4116	102.054	88.5362	93.3548	104.992	94.5418	93.6289	0.566932234	0.966018836	24	16	24	0.342868808	0.776697379	24	23.32809593	0.052724261
ENSMUSG00000041124	Msantd4	Myb/SANT-like DNA-binding domain containing 4 with coiled-coils [Source:MGI Symbol;Acc:MGI:1925350]	30.3077	29.9658	29.0155	27.814	30.5256	30.1175	29.0424	28.386	27.2873	26.0159	33.3234	30.0038	29.9289	31.3005	0.01387923	0.240214262	20	20	24	0.158479367	0.764909967	24	20.96477633	0.063384634
ENSMUSG00000041126	H2az2	H2A.Z histone variant 2 [Source:MGI Symbol;Acc:MGI:1924855]	22.2136	23.085	19.637	25.1707	22.4175	21.0311	27.0498	23.0563	26.1369	20.8563	23.8706	24.1756	24.072	19.2601	0.860993221	0.966018836	4	4	24	0.830992176	0.947661285	24	20.13038293	0.035862674
ENSMUSG00000041130	Zfp598	zinc finger protein 598 [Source:MGI Symbol;Acc:MGI:2670965]	9.94266	10.0413	10.8079	10.4005	9.66609	9.66053	10.6823	10.7837	10.9841	9.99359	10.3888	9.51503	10.7173	10.2874	8.24E-04	0.071344007	12	12	24	0.031802581	0.604522913	24	6.519629919	0.058734238
ENSMUSG00000041132	N4bp2l1	NEDD4 binding protein 2-like 1 [Source:MGI Symbol;Acc:MGI:2140872]	8.38091	10.9757	10.6	12.0224	8.92723	10.2245	11.4523	10.9915	12.2839	11.3821	9.64477	12.1891	10.2736	11.2384	0.602156008	0.966018836	16	4	24	0.203947682	0.768040178	24	8.050192019	0.098850437
ENSMUSG00000041133	Smc1a	structural maintenance of chromosomes 1A [Source:MGI Symbol;Acc:MGI:1344345]	30.9064	30.1697	29.8373	29.7907	31.7382	31.7096	29.9081	29.0888	29.1982	27.4076	31.5802	30.6962	31.3064	30.3846	0.002094047	0.100255953	20	20	24	0.027734097	0.590220615	24	20.34710877	0.055270347
ENSMUSG00000041134	Cyyr1	cysteine and tyrosine-rich protein 1 [Source:MGI Symbol;Acc:MGI:2152187]	1.49973	1.11927	1.27464	1.05697	1.14036	1.21022	1.15911	1.63885	1.05569	1.51107	1.28487	1.2758	1.40216	1.12229	0.833406028	0.966018836	4	8	24	0.746039675	0.917098266	24	23.94931589	0.034931572
ENSMUSG00000041135	Ripk2	receptor (TNFRSF)-interacting serine-threonine kinase 2 [Source:MGI Symbol;Acc:MGI:1891456]	3.80824	4.23692	4.20292	3.47868	4.17331	3.72644	3.39	3.63811	4.18927	4.1448	4.44343	4.08585	4.03565	3.88549	0.522551072	0.966018836	12	16	24	0.466491673	0.805393068	24	12.29019743	0.044086119
ENSMUSG00000041141	Pnmal1	PNMA-like 1 [Source:MGI Symbol;Acc:MGI:1918941]	3.75738	3.80118	5.16478	3.6881	3.39488	4.48764	4.27486	3.68811	4.27829	3.69184	3.29414	4.06904	3.20776	4.77678	0.424718357	0.966018836	12	8	24	0.388563665	0.799546792	24	5.59791038	0.088603077
ENSMUSG00000041143	Tmco4	transmembrane and coiled-coil domains 4 [Source:MGI Symbol;Acc:MGI:1924306]	3.45686	3.34033	3.96865	4.13263	4.77983	3.44789	4.16732	3.52966	4.099	4.75519	4.35279	4.02829	3.92151	3.21722	0.001845448	0.100255953	20	12	24	0.007310355	0.47735824	24	14.22378264	0.147052577
ENSMUSG00000041144	Dnah7b	dynein, axonemal, heavy chain 7B [Source:MGI Symbol;Acc:MGI:2684953]	1.17955	1.99824	1.54025	1.36983	1.50012	1.28254	1.21082	1.61718	1.92872	1.49466	1.26094	1.75495	0.795322	1.47731	0.029104594	0.321221647	12	16	24	0.2264234	0.768040178	24	8.860008443	0.128219369
ENSMUSG00000041147	Brca2	breast cancer 2, early onset [Source:MGI Symbol;Acc:MGI:109337]	2.73116	3.54945	3.78298	3.28736	3.17207	3.61312	2.72959	3.41543	3.62298	3.94277	3.51408	4.58862	3.21041	3.8737	0.227302972	0.723724673	12	16	24	0.372902935	0.794259532	24	11.23541979	0.076248193
ENSMUSG00000041153	Osgin2	oxidative stress induced growth inhibitor family member 2 [Source:MGI Symbol;Acc:MGI:2384798]	11.999	11.5392	10.2358	10.3843	11.7259	11.894	11.7901	9.14101	10.8733	8.26415	10.9251	10.6405	10.96	9.75294	0.054224213	0.395890765	4	12	24	0.02795087	0.590904845	24	21.16726071	0.125759163
ENSMUSG00000041161	Otud3	OTU domain containing 3 [Source:MGI Symbol;Acc:MGI:1920412]	4.66134	2.96129	2.60765	3.42708	3.48394	3.54327	4.08626	3.59392	3.06295	4.29635	3.65132	3.78287	3.62264	3.45884	0.018194955	0.256345714	4	8	24	0.156550468	0.762206981	24	20.31639394	0.124723573
ENSMUSG00000041164	Zmiz2	zinc finger, MIZ-type containing 2 [Source:MGI Symbol;Acc:MGI:106374]	17.5018	15.1678	15.9267	16.1805	16.4689	16.4678	17.4577	17.9148	15.6857	22.6005	16.4017	17.0584	16.2969	17.453	0.834434272	0.966018836	4	8	24	0.815449412	0.94108559	24	12.80895679	0.031949263
ENSMUSG00000041168	Lonp1	lon peptidase 1, mitochondrial [Source:MGI Symbol;Acc:MGI:1921392]	17.9093	15.2972	17.4138	18.4527	17.1165	18.3779	16.6053	18.0925	16.7672	19.9067	15.8073	17.4619	17.0859	17.4755	0.883024237	0.966018836	16	12	24	0.643841471	0.879013224	24	13.05869567	0.033484204
ENSMUSG00000041180	Hectd2	HECT domain E3 ubiquitin protein ligase 2 [Source:MGI Symbol;Acc:MGI:2442663]	1.72659	1.83703	1.64109	1.81418	1.68469	1.82235	1.44871	1.49725	1.93807	1.68236	1.87014	1.746	1.39347	1.69827	0.45229395	0.966018836	20	8	24	0.206350253	0.768040178	24	13.0474938	0.057424337
ENSMUSG00000041187	Prkd2	protein kinase D2 [Source:MGI Symbol;Acc:MGI:2141917]	1.85599	2.11965	2.75725	2.11991	1.99205	2.15997	1.24733	2.99963	2.79975	2.21138	1.89685	2.2102	2.56566	1.44642	0.588682825	0.966018836	12	16	24	0.411532987	0.799546792	24	9.048762421	0.125944885
ENSMUSG00000041189	Chrnb1	cholinergic receptor, nicotinic, beta polypeptide 1 (muscle) [Source:MGI Symbol;Acc:MGI:87890]	2.56653	2.20497	1.83563	1.81087	2.67485	2.25823	2.36614	3.23788	2.53311	2.64819	2.60578	2.25166	2.16405	2.43779	0.879206539	0.966018836	20	4	24	0.918463455	0.977999647	24	0.349805748	0.024976409
ENSMUSG00000041193	Pla2g5	phospholipase A2, group V [Source:MGI Symbol;Acc:MGI:101899]	2.67846	3.12618	3.41441	2.9427	3.1724	2.97011	2.91055	2.96376	3.67862	2.58308	3.09029	2.34548	3.76479	2.93084	0.601647512	0.966018836	12	12	24	0.437725512	0.805393068	24	6.893323447	0.072810718
ENSMUSG00000041199	Rpusd1	RNA pseudouridylate synthase domain containing 1 [Source:MGI Symbol;Acc:MGI:1919186]	8.10856	8.17592	9.88168	8.53943	7.72511	9.57105	10.3195	9.31109	8.45079	10.3284	8.55411	8.79539	8.92144	9.17229	0.82502117	0.966018836	16	4	24	0.799459062	0.935842205	24	5.860328939	0.031300875
ENSMUSG00000041202	Pla2g2d	phospholipase A2, group IID [Source:MGI Symbol;Acc:MGI:1341796]	0.509318	0.60352	0.666722	0.345463	0.608127	0.565363	0.829828	0.84517	1.14322	0.503767	0.450271	0.702712	0.524977	0.468382	0.503972924	0.966018836	12	4	24	0.446619436	0.805393068	24	4.557385147	0.086699216
ENSMUSG00000041203	Trir	telomerase RNA component interacting RNase [Source:MGI Symbol;Acc:MGI:1922833]	37.9342	35.3478	35.162	36.6899	34.7256	35.0016	36.096	32.6943	35.485	35.054	42.2976	39.5513	45.5051	37.3423	0.662399683	0.966018836	4	12	24	0.324093927	0.768040178	24	20.42448245	0.064884127
ENSMUSG00000041205	Map6d1	MAP6 domain containing 1 [Source:MGI Symbol;Acc:MGI:3607784]	1.72653	2.63083	3.48226	1.89056	1.78898	2.45992	2.63895	2.06638	2.65185	2.1797	1.59681	2.43209	1.55009	2.65562	0.062036003	0.409814092	12	8	24	0.221877427	0.768040178	24	6.314346633	0.158713889
ENSMUSG00000041215	Yeats2	YEATS domain containing 2 [Source:MGI Symbol;Acc:MGI:2447762]	7.71783	8.10249	8.52275	9.17938	9.96278	9.02164	9.56142	9.0392	9.56974	7.67753	8.37222	7.86203	7.0674	7.3894	0.760769894	0.966018836	20	8	24	0.461362906	0.805393068	24	13.782478	0.061112296
ENSMUSG00000041216	Clvs1	clavesin 1 [Source:MGI Symbol;Acc:MGI:1921688]	18.4366	18.1207	18.2862	18.6279	17.7829	17.7444	17.7341	16.0227	18.9936	17.0843	19.3019	17.5769	15.8525	17.6044	0.37215823	0.894599261	12	20	24	0.28209403	0.768040178	24	13.34318948	0.043477798
ENSMUSG00000041219	Arhgap11a	Rho GTPase activating protein 11A [Source:MGI Symbol;Acc:MGI:2444300]	1.25883	1.54718	1.54393	1.64537	1.34078	1.39951	1.31103	1.2676	1.46419	1.41102	1.24413	1.40105	1.53148	1.25221	0.673447313	0.966018836	16	4	24	0.424310901	0.805393068	24	9.978598809	0.039495657
ENSMUSG00000041220	Elovl6	ELOVL family member 6, elongation of long chain fatty acids (yeast) [Source:MGI Symbol;Acc:MGI:2156528]	46.7862	40.5051	43.0273	48.2478	49.4742	45.4747	49.4557	44.0932	40.0823	45.7391	48.7824	46.5914	49.3029	45.409	0.010153564	0.194720062	20	16	24	0.029186338	0.596648309	24	18.66095175	0.092655866
ENSMUSG00000041225	Arhgap12	Rho GTPase activating protein 12 [Source:MGI Symbol;Acc:MGI:1922665]	27.6492	27.0595	24.4039	26.9357	25.9639	24.1853	25.7407	24.8032	24.8686	22.9249	28.2591	27.3826	30.1066	26.825	0.351315142	0.870418653	4	8	24	0.265818402	0.768040178	24	22.32995272	0.062987972
ENSMUSG00000041229	Phf8	PHD finger protein 8 [Source:MGI Symbol;Acc:MGI:2444341]	6.10839	5.34709	4.79018	6.19846	5.0511	6.56952	6.27932	5.51582	5.03084	6.27929	5.60367	5.65437	5.2228	5.64537	0.08302755	0.462922111	24	12	24	0.350468301	0.779902933	24	19.59514459	0.070045661
ENSMUSG00000041231	Ublcp1	ubiquitin-like domain containing CTD phosphatase 1 [Source:MGI Symbol;Acc:MGI:1933105]	43.1161	47.5312	44.2236	44.9314	42.5452	39.1428	38.8676	37.2671	44.0516	39.4522	41.612	44.4497	41.8823	46.3035	0.892578749	0.966018836	8	20	24	0.64475806	0.879359107	24	7.684947203	0.039030143
ENSMUSG00000041235	Chd7	chromodomain helicase DNA binding protein 7 [Source:MGI Symbol;Acc:MGI:2444748]	1.63245	2.14174	2.34595	1.35165	1.41853	1.95112	2.54765	2.07408	2.32452	1.52124	2.00891	2.25529	1.35217	2.07125	0.187699199	0.662239322	12	4	24	0.380041639	0.799474855	24	3.615173835	0.109822878
ENSMUSG00000041236	Vps41	VPS41 HOPS complex subunit [Source:MGI Symbol;Acc:MGI:1929215]	92.0883	87.8	84.3173	84.1957	85.9483	85.7506	83.9816	82.4958	80.6805	80.2215	86.0703	85.9472	89.8162	87.4209	0.181522804	0.659020016	4	12	24	0.030121533	0.596794384	24	22.5929298	0.05063902
ENSMUSG00000041238	Rbbp8	retinoblastoma binding protein 8, endonuclease [Source:MGI Symbol;Acc:MGI:2442995]	3.75374	4.04337	3.888	4.56666	3.82112	4.18345	4.11611	3.42444	4.03564	3.4654	4.14618	4.01205	3.87417	4.17591	0.842298032	0.966018836	24	4	24	0.809362339	0.939184036	24	16.74919558	0.02309494
ENSMUSG00000041241	Mul1	mitochondrial ubiquitin ligase activator of NFKB 1 [Source:MGI Symbol;Acc:MGI:1915600]	9.43359	7.74745	8.69129	9.84883	8.0588	10.0732	9.01377	12.5162	7.2277	9.79088	8.23968	10.6435	8.80284	10.0545	0.601647512	0.966018836	24	12	24	0.735312749	0.913720909	24	23.53264934	0.057826893
ENSMUSG00000041245	Wnk3	WNK lysine deficient protein kinase 3 [Source:MGI Symbol;Acc:MGI:2652875]	1.62192	2.09576	2.35229	2.02173	1.85363	1.88853	2.21342	1.65019	2.21402	1.36275	1.96638	1.93037	1.77599	2.21199	0.393470605	0.921010321	12	12	24	0.627979137	0.872795898	24	5.802368717	0.054797058
ENSMUSG00000041258	Zfp236	zinc finger protein 236 [Source:MGI Symbol;Acc:MGI:1926950]	8.45033	7.42192	7.07137	7.67355	7.35651	7.40672	7.85225	7.20868	6.9116	8.07964	6.7696	6.97115	7.73897	7.3238	0.16942967	0.641781289	4	8	24	0.557026243	0.838828843	24	23.31929891	0.032717154
ENSMUSG00000041261	Car8	carbonic anhydrase 8 [Source:MGI Symbol;Acc:MGI:88253]	4.92103	5.24112	5.66022	5.24961	4.9625	5.35191	4.78559	5.55477	4.40632	5.03374	4.98057	5.18102	5.33481	5.17994	0.910436409	0.966018836	8	12	24	0.890896323	0.969222457	24	2.640004287	0.01467208
ENSMUSG00000041263	Rusc1	RUN and SH3 domain containing 1 [Source:MGI Symbol;Acc:MGI:1919546]	48.2299	41.2997	40.9747	39.1657	43.8925	46.4137	41.8019	46.0255	36.9144	52.2306	40.028	42.8109	44.9107	42.7232	0.321662653	0.83607295	24	12	24	0.566229197	0.843471947	24	21.04559584	0.056024577
ENSMUSG00000041264	Uspl1	ubiquitin specific peptidase like 1 [Source:MGI Symbol;Acc:MGI:2442342]	16.3434	16	15.3897	15.3027	16.0751	15.5768	14.9306	15.0381	16.3427	13.8997	17.8224	15.7389	15.5137	15.2536	0.722296281	0.966018836	20	20	24	0.58874472	0.853936396	24	18.62599791	0.032393945
ENSMUSG00000041268	Dmxl2	Dmx-like 2 [Source:MGI Symbol;Acc:MGI:2444630]	44.0478	44.158	42.0552	41.6251	43.1318	41.4074	40.9266	39.2417	40.7134	39.3615	42.8504	39.9151	40.1995	43.9707	0.858514388	0.966018836	8	8	24	0.904546645	0.973594617	24	23.98164491	0.009659546
ENSMUSG00000041272	Tox	thymocyte selection-associated high mobility group box [Source:MGI Symbol;Acc:MGI:2181659]	1.2268	1.32604	1.61125	0.926257	1.19241	1.33837	1.54946	1.31181	1.73545	1.49945	1.1065	1.38706	1.56442	1.35035	0.251986462	0.75129457	12	8	24	0.231648641	0.768040178	24	4.437182856	0.085108889
ENSMUSG00000041278	Ttc1	tetratricopeptide repeat domain 1 [Source:MGI Symbol;Acc:MGI:1914077]	55.3653	56.2011	53.6704	56.2121	54.4695	52.9981	52.3122	53.9647	53.6751	54.7004	56.612	57.9419	58.7619	59.1085	0.81829516	0.966018836	8	4	24	0.90032096	0.971777625	24	0.693740385	0.008530616
ENSMUSG00000041297	Cdk13	cyclin-dependent kinase 13 [Source:MGI Symbol;Acc:MGI:1916812]	11.1341	11.2494	11.8058	12.6748	10.5169	10.4134	11.2191	10.4775	10.8368	10.1772	11.3865	11.4115	10.3522	10.7762	0.877082091	0.966018836	12	16	24	0.687425142	0.89619155	24	11.65726742	0.025497255
ENSMUSG00000041298	Katnal1	katanin p60 subunit A-like 1 [Source:MGI Symbol;Acc:MGI:2387638]	18.5588	13.7863	14.4507	14.4574	14.1235	15.595	16.9025	15.3494	13.4761	18.03	13.9871	16.9899	16.8476	16.1837	0.101520755	0.511659635	4	8	24	0.205462697	0.768040178	24	22.59756979	0.095959632
ENSMUSG00000041303	Gtf3c3	general transcription factor IIIC, polypeptide 3 [Source:MGI Symbol;Acc:MGI:2138383]	13.6856	13.9709	13.1442	13.5788	13.6901	13.5982	12.8344	12.7234	12.693	12.2406	13.8801	13.8349	13.828	13.6478	0.209352197	0.69936851	20	20	24	0.192716628	0.768040178	24	20.64744361	0.040046821
ENSMUSG00000041308	Sntb2	syntrophin, basic 2 [Source:MGI Symbol;Acc:MGI:101771]	4.65129	4.32912	4.2017	4.68982	4.98405	4.1415	4.87768	4.02347	4.66471	4.45439	4.52335	4.10091	4.12603	4.19038	0.339604637	0.858712466	20	4	24	0.354280877	0.781421173	24	14.88285596	0.042445221
ENSMUSG00000041309	Nkx6-2	NK6 homeobox 2 [Source:MGI Symbol;Acc:MGI:1352738]	0.349491	0.690401	1.08973	0.401065	0.315732	0.632315	0.595665	0.502764	0.785715	0.551929	0.671937	0.95231	0.53393	0.673427	0.542718076	0.966018836	12	16	24	0.622713721	0.871448778	24	6.901430698	0.073864911
ENSMUSG00000041313	Slc7a1	solute carrier family 7 (cationic amino acid transporter, y+ system), member 1 [Source:MGI Symbol;Acc:MGI:88117]	15.5119	10.4266	11.0277	11.1501	11.5135	14.0068	12.7576	12.9801	8.10012	16.7488	10.6338	12.1403	11.337	11.9828	0.321662653	0.83607295	24	12	24	0.435750593	0.805393068	24	20.47691575	0.125541869
ENSMUSG00000041319	Thoc6	THO complex 6 [Source:MGI Symbol;Acc:MGI:2677480]	8.33225	7.38912	7.24407	6.34192	8.81096	8.39537	8.29463	8.81654	6.94705	6.97574	7.97798	7.25554	8.41371	7.0356	0.062105896	0.409814092	4	12	24	0.042751244	0.624436905	24	21.82202102	0.121667525
ENSMUSG00000041323	Ak7	adenylate kinase 7 [Source:MGI Symbol;Acc:MGI:1926051]	0.91199	1.35111	1.62132	0.840144	0.721162	0.664342	1.06604	0.637553	0.631176	0.854516	0.740212	1.20347	0.270196	1.19586	0.880247609	0.966018836	8	12	24	0.674291543	0.890626495	24	6.829632514	0.092383061
ENSMUSG00000041324	Inhba	inhibin beta-A [Source:MGI Symbol;Acc:MGI:96570]	0.342894	0.507779	0.698207	0.505253	0.517409	0.450611	0.706842	0.497179	0.626551	0.301906	0.448019	0.438672	0.509527	0.595028	0.338172159	0.85758369	12	12	24	0.309951369	0.768040178	24	5.479622809	0.068045676
ENSMUSG00000041328	Pcf11	PCF11 cleavage and polyadenylation factor subunit [Source:MGI Symbol;Acc:MGI:1919579]	15.3034	15.7122	15.8419	17.7652	14.7579	13.1347	13.7248	14.7838	16.6656	13.8191	14.5769	14.6879	13.9462	15.6197	0.019280932	0.257669847	12	12	24	0.049471632	0.642285999	24	8.702780756	0.096274547
ENSMUSG00000041329	Atp1b2	ATPase, Na+/K+ transporting, beta 2 polypeptide [Source:MGI Symbol;Acc:MGI:88109]	27.9562	40.5218	64.0641	33.1543	29.2255	46.8457	49.0641	36.532	42.2505	46.0012	23.6113	35.4622	24.5119	44.2601	0.424718357	0.966018836	12	8	24	0.26440319	0.768040178	24	6.991621625	0.257449988
ENSMUSG00000041341	Atg2b	autophagy related 2B [Source:MGI Symbol;Acc:MGI:1923809]	8.59497	8.6569	8.70481	8.7701	7.84062	9.25687	8.66851	8.78623	8.21761	9.81319	8.11277	9.02587	8.1293	8.50662	0.93717134	0.966018836	16	4	24	0.964305027	0.991133372	24	11.47791265	0.008264093
ENSMUSG00000041343	Ankrd42	ankyrin repeat domain 42 [Source:MGI Symbol;Acc:MGI:1921095]	5.23563	4.04243	4.97822	4.55501	4.0153	4.24467	4.49866	3.9621	4.19126	4.65497	4.14499	4.2535	4.18395	4.55461	0.937921962	0.966018836	16	4	24	0.908670489	0.974606327	24	5.926755245	0.01683579
ENSMUSG00000041346	Wrap53	WD repeat containing, antisense to Trp53 [Source:MGI Symbol;Acc:MGI:2384933]	4.07493	3.45442	3.79829	4.01583	4.31842	5.12642	4.39884	4.21899	4.0401	4.56992	3.8374	4.23632	3.5872	3.70541	0.286642595	0.793714531	24	8	24	0.171236445	0.768040178	24	17.5865038	0.087189718
ENSMUSG00000041351	Rap1gap	Rap1 GTPase-activating protein [Source:MGI Symbol;Acc:MGI:109338]	34.9982	31.2614	33.9161	34.9569	33.7475	33.0619	36.5689	31.653	31.8735	44.633	33.9115	34.1421	32.9861	33.7484	0.223774686	0.717349224	16	16	24	0.365949966	0.789975236	24	13.89391781	0.066243555
ENSMUSG00000041353	Tmem29	transmembrane protein 29 [Source:MGI Symbol;Acc:MGI:1923420]	22.0826	25.8787	25.8869	25.4616	24.7378	23.8347	21.7038	21.8242	25.5848	20.05	25.7374	23.4771	23.977	20.2136	0.45229395	0.966018836	12	16	24	0.525737545	0.822757825	24	13.81330327	0.051868968
ENSMUSG00000041354	Rgl2	ral guanine nucleotide dissociation stimulator-like 2 [Source:MGI Symbol;Acc:MGI:107483]	23.2822	24.9704	24.0847	23.4673	22.3174	23.3081	22.3322	22.2646	26.4144	22.9718	21.3971	21.2637	24.158	21.3609	0.308023687	0.814993005	12	12	24	0.217241378	0.768040178	24	7.017739997	0.054893326
ENSMUSG00000041355	Ssr2	signal sequence receptor, beta [Source:MGI Symbol;Acc:MGI:1913506]	60.7489	57.0566	57.5021	55.2542	60.8448	58.4036	54.6083	60.5391	55.8495	50.2137	63.8544	54.5956	59.4763	55.1884	0.893307667	0.966018836	20	20	24	0.467372779	0.805393068	24	20.88784632	0.04334451
ENSMUSG00000041360	Pum3	pumilio RNA-binding family member 3 [Source:MGI Symbol;Acc:MGI:106253]	20.0036	21.7994	27.4917	21.2818	25.314	24.5703	23.4106	19.0623	25.556	19.0173	19.4426	20.4518	24.0876	18.7506	0.9045879	0.966018836	12	20	24	0.975261118	0.993747214	24	14.07616517	0.015087765
ENSMUSG00000041361	Myzap	myocardial zonula adherens protein [Source:MGI Symbol;Acc:MGI:2142908]	1.30173	1.75729	1.57382	2.14037	1.76952	1.20544	1.35528	1.49584	1.93459	1.61709	1.32792	1.35485	1.21799	1.16688	0.046820236	0.375011045	16	8	24	0.006080237	0.468868574	24	10.75998831	0.159118919
ENSMUSG00000041362	Shtn1	shootin 1 [Source:MGI Symbol;Acc:MGI:1918903]	14.3008	16.05	14.5283	13.7955	15.7714	13.8561	15.1945	13.9587	16.1391	13.3901	14.484	14.2231	14.7742	14.4026	0.671726933	0.966018836	12	4	24	0.714687954	0.905979936	24	3.859254266	0.025321945
ENSMUSG00000041372	B4galnt3	beta-1,4-N-acetyl-galactosaminyl transferase 3 [Source:MGI Symbol;Acc:MGI:3041155]	0.484133	0.605378	0.502539	0.661333	0.661756	0.611804	0.585324	0.640494	0.609241	0.774356	0.505588	0.490381	0.754497	0.651716	0.869721097	0.966018836	16	8	24	0.602641853	0.861114219	24	9.224316141	0.032377315
ENSMUSG00000041375	Ccdc9	coiled-coil domain containing 9 [Source:MGI Symbol;Acc:MGI:1921443]	25.8203	30.1119	29.1873	28.4753	29.4613	27.5947	30.511	26.5213	30.6675	31.7072	25.9525	31.0605	28.8214	30.7395	0.910436409	0.966018836	16	16	24	0.535069922	0.826859366	24	8.602289918	0.041811321
ENSMUSG00000041378	Cldn5	claudin 5 [Source:MGI Symbol;Acc:MGI:1276112]	12.8189	14.9247	10.3307	11.0341	12.0027	14.2049	17.976	19.4251	12.2514	13.5059	13.4391	18.1596	20.6454	13.1722	0.070244838	0.438878845	4	8	24	0.011918145	0.525280095	24	23.61957473	0.259890496
ENSMUSG00000041380	Htr2c	5-hydroxytryptamine (serotonin) receptor 2C [Source:MGI Symbol;Acc:MGI:96281]	0.58156	2.41417	4.30095	1.5862	1.06957	2.01051	2.55528	1.29482	2.83393	1.33749	0.685955	1.65792	0.434964	2.34789	0.10560184	0.518002936	12	8	24	0.179667564	0.768040178	24	7.349453196	0.382522533
ENSMUSG00000041390	Mdfic	MyoD family inhibitor domain containing [Source:MGI Symbol;Acc:MGI:104611]	6.58403	6.16762	5.15229	4.82084	6.37708	5.28884	6.27029	6.4045	5.62271	4.09106	6.26817	5.39838	5.94036	5.70313	0.017417473	0.255326033	4	12	24	0.067567013	0.671260695	24	23.7233714	0.134984845
ENSMUSG00000041396	Mettl18	methyltransferase like 18 [Source:MGI Symbol;Acc:MGI:1917212]	2.84801	3.54709	3.72219	2.96141	3.86398	3.17654	2.89446	2.73535	3.83804	2.67841	3.27148	3.01959	3.44645	3.07317	0.767183862	0.966018836	12	16	24	0.783178797	0.930229965	24	10.72441424	0.038820942
ENSMUSG00000041406	BC055324	cDNA sequence BC055324 [Source:MGI Symbol;Acc:MGI:3590554]	0.565997	0.758024	0.649763	1.40569	0.787991	1.0064	0.972882	0.773391	0.885797	0.774828	0.909609	0.765845	0.944991	0.644958	0.301918397	0.811201458	24	8	24	0.378743179	0.79816526	24	14.95804782	0.082640173
ENSMUSG00000041408	Wapl	WAPL cohesin release factor [Source:MGI Symbol;Acc:MGI:2675859]	13.4001	12.9084	13.2793	14.2809	13.161	13.0245	12.5006	13.3414	13.6409	11.3355	13.5045	13.199	14.4697	13.4319	0.809772024	0.966018836	12	12	24	0.856806377	0.957538975	24	1.370731185	0.017253262

ENSMUSG00000041415	Dicer1	dicer 1, ribonuclease type III [Source:MGI Symbol;Acc:MGI:2177178]	10.0301	8.8998	9.38743	8.57799	8.85118	8.07213	7.21972	8.49548	8.31246	8.85382	7.60946	9.75389	8.50583	9.65049	0.860993221	0.966018836	8	12	24	0.79406699	0.933885455	24	5.021174112	0.033214902
ENSMUSG00000041417	Pik3r1	phosphoinositide-3-kinase regulatory subunit 1 [Source:MGI Symbol;Acc:MGI:97583]	52.4674	44.7184	44.4582	52.9376	49.1003	51.5896	49.4119	41.1844	41.6355	47.5853	50.6798	50.1585	48.0702	45.0183	0.015345591	0.253245931	24	12	24	0.002166695	0.395705644	24	18.38565711	0.13092475
ENSMUSG00000041420	Meis3	Meis homeobox 3 [Source:MGI Symbol;Acc:MGI:108519]	13.4327	12.081	12.436	14.5009	13.7338	12.9673	12.3282	12.5838	13.0403	15.6184	13.4953	14.5815	13.347	14.3424	0.081336319	0.462794877	16	16	24	0.080526599	0.695795041	24	14.17994114	0.072208343
ENSMUSG00000041423	Paqr6	progestin and adipoQ receptor family member VI [Source:MGI Symbol;Acc:MGI:1916207]	27.057	29.3015	27.9717	27.7502	30.6602	28.0259	30.8425	26.9236	31.7649	28.106	25.8047	26.9347	28.8496	23.7857	0.858514388	0.966018836	12	20	24	0.955391182	0.988639288	24	14.2268195	0.011943424
ENSMUSG00000041426	Hibch	3-hydroxyisobutyryl-Coenzyme A hydrolase [Source:MGI Symbol;Acc:MGI:1923792]	23.4831	23.7449	24.9955	29.5466	25.8801	22.6481	23.1043	24.4763	25.2601	25.4532	27.396	23.7921	27.2452	26.4969	0.082113105	0.462922111	20	4	24	0.078867007	0.689973975	24	11.48114011	0.082684152
ENSMUSG00000041429	Nthl1	nth (endonuclease III)-like 1 (E.coli) [Source:MGI Symbol;Acc:MGI:1313275]	4.30495	4.67935	3.92468	4.78539	5.17	4.42594	4.79919	4.45873	5.75418	3.5796	4.61604	4.43372	5.07701	3.47677	0.838692453	0.966018836	20	12	24	0.819920565	0.942856593	24	18.9153014	0.042274043
ENSMUSG00000041438	Utp4	UTP4 small subunit processome component [Source:MGI Symbol;Acc:MGI:1096573]	9.7545	8.62868	9.37687	9.21622	8.43855	10.7942	9.43278	10.2194	8.6278	9.77356	9.89155	8.69667	9.07789	9.51456	0.860993221	0.966018836	24	12	24	0.833785402	0.948852355	24	21.56399004	0.023391445
ENSMUSG00000041439	Mfsd6	major facilitator superfamily domain containing 6 [Source:MGI Symbol;Acc:MGI:1922925]	54.898	45.0642	43.5351	46.3623	46.2308	52.7057	48.3732	46.2806	37.1739	55.7429	44.7523	49.0618	48.583	49.9144	0.071575474	0.440262583	24	12	24	0.211450952	0.768040178	24	20.67007881	0.101743154
ENSMUSG00000041440	Gk5	glycerol kinase 5 (putative) [Source:MGI Symbol;Acc:MGI:2443336]	2.39768	2.8199	2.54121	2.68503	3.18547	2.57123	2.76838	2.5525	3.19907	2.36738	2.82574	2.91413	3.09491	2.80844	0.880247609	0.966018836	20	20	24	0.965379355	0.991608289	24	17.72073513	0.010622064
ENSMUSG00000041444	Arhgap32	Rho GTPase activating protein 32 [Source:MGI Symbol;Acc:MGI:2450166]	8.85762	7.89745	9.45731	7.68175	7.49033	8.3347	8.95844	7.59374	7.79208	8.93674	7.02603	8.32917	8.47115	8.77863	0.148627651	0.59172107	4	16	24	0.394933963	0.799546792	24	2.843557703	0.057517621
ENSMUSG00000041445	Mmrn2	multimerin 2 [Source:MGI Symbol;Acc:MGI:2385618]	2.42901	2.32586	1.92094	2.67183	2.88058	2.23193	2.34852	2.75457	2.03363	2.94775	1.68643	2.12624	2.52742	2.01818	0.885059765	0.966018836	16	20	24	0.9635935	0.990884307	24	15.66201898	0.017683523
ENSMUSG00000041449	Serpina3h	serine (or cysteine) peptidase inhibitor, clade A, member 3H [Source:MGI Symbol;Acc:MGI:2182839]	0.522678	0.834245	1.05343	0.770866	0.893157	0.828352	0.455802	0.636027	0.662232	0.710956	1.00115	0.831814	1.0144	0.637271	0.350544145	0.870418653	20	8	24	0.337008151	0.771960538	24	14.31561866	0.084331505
ENSMUSG00000041453	Rpl21	ribosomal protein L21 [Source:MGI Symbol;Acc:MGI:1278340]	23.5056	30.3284	28.525	30.1513	29.5873	26.6139	26.4481	22.5341	27.4499	15.5982	27.4617	20.6126	21.9312	18.4988	0.648583058	0.966018836	12	16	24	0.825725077	0.945421318	24	15.80429905	0.053953687
ENSMUSG00000041459	Tardbp	TAR DNA binding protein [Source:MGI Symbol;Acc:MGI:2387629]	119.993	118.211	111.096	116.873	125.032	109.075	113.466	107.925	99.2458	88.3571	116.152	99.4244	107.187	105.945	0.928780444	0.966018836	20	16	24	0.37697382	0.796956096	24	21.02358412	0.059848578
ENSMUSG00000041471	Shld2	shieldin complex subunit 2 [Source:MGI Symbol;Acc:MGI:1922948]	2.54866	3.42391	3.34863	3.04474	3.35197	2.5608	3.85161	3.26896	3.48221	3.36451	2.96107	3.72899	3.74171	3.61459	0.912923872	0.966018836	4	16	24	0.351869727	0.780400886	24	5.60186282	0.070116488
ENSMUSG00000041476	Smpx	small muscle protein, X-linked [Source:MGI Symbol;Acc:MGI:1913356]	8.74315	21.2992	19.9032	25.7824	16.9775	21.187	17.289	33.6381	31.9282	26.9984	16.6272	13.6443	23.5208	18.0378	0.105687008	0.518128471	16	4	24	0.051139489	0.646668262	24	8.768578744	0.426024225
ENSMUSG00000041477	Dcp1b	decapping mRNA 1B [Source:MGI Symbol;Acc:MGI:2442404]	4.04106	4.28168	4.53476	5.08785	4.19598	4.3908	4.86036	4.77019	5.44241	5.07158	4.69181	4.49099	5.14878	4.90887	0.539040394	0.966018836	16	4	24	0.041874029	0.62217373	24	8.67624108	0.078379246
ENSMUSG00000041479	Syt15	synaptotagmin XV [Source:MGI Symbol;Acc:MGI:2442166]	1.29743	1.02645	1.00522	0.823641	1.31372	1.04368	1.16842	1.24657	1.06206	1.10854	0.852675	1.10523	1.20561	1.15794	0.173121348	0.648600527	4	12	24	0.143218997	0.752803875	24	0.301981284	0.075780201
ENSMUSG00000041481	Serpina3g	serine (or cysteine) peptidase inhibitor, clade A, member 3G [Source:MGI Symbol;Acc:MGI:105046]	73.792	62.9382	63.9512	58.1545	73.3394	67.2894	53.0111	54.4065	51.568	49.8463	72.1831	54.3448	61.2871	53.8702	0.676344318	0.966018836	20	20	24	0.097320776	0.71175955	24	18.93858386	0.140941326
ENSMUSG00000041482	Piezo2	piezo-type mechanosensitive ion channel component 2 [Source:MGI Symbol;Acc:MGI:1918781]	96.3581	80.7921	78.9153	82.3003	83.4367	89.5893	76.8632	85.6791	66.3451	89.3095	78.8457	78.5113	81.8739	81.6841	0.817383384	0.966018836	16	20	24	0.363102445	0.787924926	24	20.52949598	0.068287752
ENSMUSG00000041483	Zfp281	zinc finger protein 281 [Source:MGI Symbol;Acc:MGI:3029290]	5.89516	6.20739	7.13966	6.47551	6.10945	5.87686	6.12522	5.98822	6.85978	5.52434	6.42056	5.75582	5.37619	6.25428	0.004319982	0.140598674	12	12	24	0.08444509	0.702565919	24	9.161569879	0.076264547
ENSMUSG00000041488	Stx3	syntaxin 3 [Source:MGI Symbol;Acc:MGI:103077]	10.9683	14.1057	13.4791	13.8317	11.8615	11.2519	10.6568	11.6099	14.4097	10.9679	12.6832	12.9071	12.4686	12.3907	0.182849367	0.659303873	12	16	24	0.176113718	0.768040178	24	9.017462979	0.097277141
ENSMUSG00000041491	Cep78	centrosomal protein 78 [Source:MGI Symbol;Acc:MGI:1924386]	5.67939	5.93741	6.04692	5.51054	6.2702	5.44436	5.35407	5.43548	5.63513	5.11536	6.55172	5.92167	6.09837	6.36388	0.890235289	0.966018836	20	20	24	0.951169822	0.98830325	24	18.84942755	0.011654157
ENSMUSG00000041506	Rrp9	ribosomal RNA processing 9, U3 small nucleolar RNA binding protein [Source:MGI Symbol;Acc:MGI:2384313]	7.12497	7.23277	9.15633	8.06973	7.30685	7.32874	6.66273	8.01946	8.44364	7.28751	7.16353	7.59798	6.98955	8.14542	0.001920665	0.100255953	12	16	24	0.019005135	0.561252866	24	8.630974423	0.11099724
ENSMUSG00000041515	Irf8	interferon regulatory factor 8 [Source:MGI Symbol;Acc:MGI:96395]	1.237	2.10205	2.67407	1.9719	1.72123	1.79825	1.72357	1.84302	2.11664	1.41162	1.92281	1.62031	1.54866	1.48832	0.033248781	0.324908923	12	16	24	0.189547622	0.768040178	24	9.395043563	0.12266202
ENSMUSG00000041528	Rnf123	ring finger protein 123 [Source:MGI Symbol;Acc:MGI:2148796]	20.1171	18.7326	19.5527	21.6772	20.4508	22.519	20.1992	19.0504	17.7767	23.6464	18.1918	18.2993	18.5836	16.1101	0.446653834	0.966018836	16	16	24	0.119856694	0.731610562	24	15.8011241	0.094866525
ENSMUSG00000041530	Ago1	argonaute RISC catalytic subunit 1 [Source:MGI Symbol;Acc:MGI:2446630]	6.52246	7.52332	8.29831	6.92901	7.46044	7.67633	8.06456	6.91457	6.99894	7.49063	6.79819	7.62598	6.73927	6.94168	0.907068194	0.966018836	24	4	24	0.941476566	0.985509295	24	14.4708799	0.012380771
ENSMUSG00000041538	H2-Ob	histocompatibility 2, O region beta locus [Source:MGI Symbol;Acc:MGI:95925]	0.897527	1.11345	1.33553	0.793321	0.993424	1.0985	1.06703	1.22639	1.8829	0.568748	0.910331	1.15414	0.961011	1.0742	0.01387923	0.240214262	12	4	24	0.317547748	0.768040178	24	4.877264661	0.118315259
ENSMUSG00000041540	Sox5	SRY (sex determining region Y)-box 5 [Source:MGI Symbol;Acc:MGI:98367]	0.999779	1.14492	1.51462	1.64759	0.68401	0.776918	1.05557	1.23702	1.51317	1.44972	0.665423	1.52114	0.952504	1.34035	0.007331665	0.192760771	16	4	24	0.04710502	0.633507364	24	7.795288753	0.209091244
ENSMUSG00000041544	Disp3	dispatched RND transporter family member 3 [Source:MGI Symbol;Acc:MGI:2444403]	3.0825	2.55878	2.92676	2.75812	3.77597	2.66089	3.04355	3.02396	2.30251	3.38224	2.67195	2.73938	2.73916	2.4806	0.885025617	0.966018836	16	20	24	0.345236942	0.778314715	24	16.77548817	0.075911986
ENSMUSG00000041548	Hspb8	heat shock protein 8 [Source:MGI Symbol;Acc:MGI:2135756]	156.561	131.399	124.765	133.672	132.23	148.371	132.134	151.07	119.165	148.455	144.066	142.589	145.488	139.802	0.321662653	0.83607295	24	12	24	0.165770675	0.767020009	24	21.09671288	0.081687252
ENSMUSG00000041552	Ptchd1	patched domain containing 1 [Source:MGI Symbol;Acc:MGI:2685233]	0.625508	0.547527	0.794187	0.665746	0.638933	0.691452	0.631619	0.478663	0.749147	0.635028	0.690571	0.780878	0.616264	0.969658	0.591351901	0.966018836	12	20	24	0.78788851	0.931832121	24	10.48860597	0.026168333
ENSMUSG00000041556	Fbxo2	F-box protein 2 [Source:MGI Symbol;Acc:MGI:2446216]	100.479	89.6984	88.3193	88.4412	83.5246	90.1043	89.8775	95.2074	81.9325	102.133	98.2331	99.2262	98.9012	97.5724	0.217827365	0.704989686	24	12	24	0.503773546	0.810434276	24	22.42394137	0.043294344
ENSMUSG00000041559	Fmod	fibromodulin [Source:MGI Symbol;Acc:MGI:1328364]	7.0395	11.0183	8.52577	6.11734	5.97975	6.40193	6.66007	11.1557	11.3375	4.50732	5.49346	7.08112	6.30591	6.68855	0.00448422	0.140598674	12	4	24	0.023073391	0.581965446	24	4.740963992	0.327801338
ENSMUSG00000041560	Nop53	NOP53 ribosome biogenesis factor [Source:MGI Symbol;Acc:MGI:2154441]	25.3203	24.5654	23.2855	23.9443	18.6901	22.2385	24.0772	29.017	31.1159	33.0427	30.0906	39.9737	42.1665	36.1586	0.90703203	0.966018836	4	16	24	0.287749806	0.768040178	24	3.60218829	0.114942752
ENSMUSG00000041565	L3mbtl4	L3MBTL4 histone methyl-lysine binding protein [Source:MGI Symbol;Acc:MGI:2444889]	3.01851	3.26688	2.89099	3.07557	2.60286	2.88756	2.78881	2.89493	2.89414	2.91877	3.36101	2.88471	2.7268	3.20144	0.65208848	0.966018836	8	20	24	0.721350007	0.90866625	24	7.736053983	0.02564109
ENSMUSG00000041570	Camsap2	calmodulin regulated spectrin-associated protein family, member 2 [Source:MGI Symbol;Acc:MGI:1922434]	41.5906	38.5087	38.8626	41.2848	40.0188	38.9336	36.9261	33.442	35.2061	36.3697	39.7574	39.1729	37.9562	38.8205	0.248372848	0.746945512	20	12	24	0.144225929	0.752803875	24	17.96149179	0.060264014
ENSMUSG00000041571	Selenow	selenoprotein W [Source:MGI Symbol;Acc:MGI:1100878]	398.444	374.327	364.598	377.248	384.292	408.373	369.201	425.998	395.034	411.856	382.351	388.984	385.8	368.832	0.890648739	0.966018836	24	12	24	0.915915606	0.976753216	24	18.34166349	0.011569946
ENSMUSG00000041577	Prelp	proline arginine-rich end leucine-rich repeat [Source:MGI Symbol;Acc:MGI:2151110]	9.73072	10.7662	10.2803	8.6684	10.7815	8.46622	11.9274	10.8034	12.921	7.7276	8.84935	9.78465	10.3538	9.98678	0.175272072	0.651010555	12	4	24	0.139886508	0.74956518	24	3.613032882	0.13314179
ENSMUSG00000041592	Sdk2	sidekick cell adhesion molecule 2 [Source:MGI Symbol;Acc:MGI:2443847]	2.36279	2.09325	2.31275	2.3098	2.85374	2.40054	2.24832	2.3937	2.23325	3.26985	2.65502	2.73004	2.2631	2.17958	0.015327376	0.253245931	20	12	24	0.013120385	0.529134128	24	15.23038097	0.123235058
ENSMUSG00000041594	Tmtc4	transmembrane and tetratricopeptide repeat containing 4 [Source:MGI Symbol;Acc:MGI:1921050]	10.9538	10.9725	11.9085	10.4985	11.8828	11.8806	10.39	10.0813	8.72689	10.4418	11.5275	10.5919	12.2574	9.812	0.455507782	0.966018836	20	12	24	0.120158051	0.731805836	24	19.05963015	0.094900309
ENSMUSG00000041596	Nlrp5-ps	NLR family, pyrin domain containing 5, pseudogene [Source:MGI Symbol;Acc:MGI:5010941]	0.793331	0.522458	0.278288	0.841185	0.573367	0.668335	0.411573	0.405006	0.720327	0.468249	0.701183	0.508106	0.554293	0.496324	0.588208558	0.966018836	16	16	24	0.388081043	0.799546792	24	16.93784369	0.079913452
ENSMUSG00000041598	Cdc42ep4	CDC42 effector protein (Rho GTPase binding) 4 [Source:MGI Symbol;Acc:MGI:1929760]	15.597	13.9838	13.7782	15.3757	14.8813	13.3574	15.6063	13.5214	15.9099	14.9514	15.9785	15.0042	15.3614	13.311	0.581070934	0.966018836	20	12	24	0.453529597	0.805393068	24	16.26323325	0.044663168
ENSMUSG00000041607	Mbp	myelin basic protein [Source:MGI Symbol;Acc:MGI:96925]	2320.12	2660.6	2644.91	2268.38	2746.61	2434.26	2994.42	2550.65	2896.33	2589.98	2290.51	2610.36	2440.37	2295.31	0.882617464	0.966018836	12	4	24	0.870783021	0.963360317	24	5.455184108	0.027485057
ENSMUSG00000041608	Entpd3	ectonucleoside triphosphate diphosphohydrolase 3 [Source:MGI Symbol;Acc:MGI:1321386]	37.8257	27.9998	27.3778	30.5028	27.5777	35.4644	32.4466	33.2968	22.1778	37.7378	26.9515	31.9985	30.1012	30.7404	0.203305541	0.689605535	24	12	24	0.296458841	0.768040178	24	21.43852515	0.126282511
ENSMUSG00000041609	Bicdl1	BICD family like cargo adaptor 1 [Source:MGI Symbol;Acc:MGI:1922915]	27.4784	21.6334	23.7637	23.7487	25.6001	25.0958	25.65	25.9905	21.4523	29.9049	26.1162	26.5927	24.278	24.0258	0.191123261	0.669841558	24	12	24	0.216188493	0.768040178	24	18.01435756	0.083142077
ENSMUSG00000041617	Ccdc74a	coiled-coil domain containing 74A [Source:MGI Symbol;Acc:MGI:1919565]	37.7962	29.7543	29.0415	31.2667	30.9114	32.3847	29.8871	33.4676	30.0028	36.7943	33.6868	33.9702	35.8389	33.4437	0.44861185	0.966018836	4	8	24	0.501144731	0.80908345	24	20.67323279	0.053115429
ENSMUSG00000041623	D11Wsu47e	DNA segment, Chr 11, Wayne State University 47, expressed [Source:MGI Symbol;Acc:MGI:106356]	5.57674	5.01975	4.8809	4.71353	5.10544	4.42252	4.92236	5.19139	4.5015	5.04178	5.06339	5.07882	4.99789	4.71495	0.760769894	0.966018836	20	16	24	0.565053535	0.843471947	24	22.27059645	0.030351543
ENSMUSG00000041624	Gucy1a2	guanylate cyclase 1, soluble, alpha 2 [Source:MGI Symbol;Acc:MGI:2660877]	0.853843	1.07428	0.824343	1.22729	1.24142	0.887075	1.03014	0.727334	1.19639	0.920522	1.14187	0.980724	1.19875	1.13635	0.823677655	0.966018836	20	4	24	0.566035696	0.843471947	24	13.95225746	0.048819455
ENSMUSG00000041625	Ggact	gamma-glutamylamine cyclotransferase [Source:MGI Symbol;Acc:MGI:2385008]	28.4731	28.3776	25.3386	26.3544	27.0245	25.9975	25.5349	26.9071	27.8605	24.648	28.0498	28.1592	27.2007	26.6462	0.643501552	0.966018836	4	12	24	0.504632273	0.811111334	24	22.89217298	0.029739982
ENSMUSG00000041629	Fam104a	family with sequence similarity 104, member A [Source:MGI Symbol;Acc:MGI:106351]	11.3071	10.2682	9.78684	9.78367	9.96873	10.1295	9.92911	9.66074	10.7463	10.9305	10.5007	10.7424	10.9362	10.5571	0.93717134	0.966018836	4	4	24	0.743940878	0.916218012	24	22.16830615	0.01963079
ENSMUSG00000041632	Mrps27	mitochondrial ribosomal protein S27 [Source:MGI Symbol;Acc:MGI:1919064]	16.8924	14.4211	15.0437	15.5222	15.2849	15.7607	15.976	15.7763	13.247	16.0196	15.3399	15.8283	16.1611	15.1137	0.004861081	0.141782417	4	8	24	0.081957962	0.699041389	24	20.71560746	0.066800549
ENSMUSG00000041633	Kctd12b	potassium channel tetramerisation domain containing 12b [Source:MGI Symbol;Acc:MGI:2444667]	1.05025	1.7891	1.93341	1.72629	1.72985	1.4177	1.93204	2.12058	1.92582	1.65633	1.6919	1.34292	1.53488	1.43523	0.080164531	0.458678242	12	12	24	0.11625348	0.727799063	24	8.742723675	0.128498797
ENSMUSG00000041638	Gcn1	GCN1 activator of EIF2AK4 [Source:MGI Symbol;Acc:MGI:2444248]	7.44927	7.36361	7.26152	9.05123	8.46919	7.91471	8.2372	9.0923	7.64809	9.50607	7.91183	7.8975	8.06794	7.90807	0.265774856	0.773510818	16	20	24	0.340326485	0.774739261	24	13.09643062	0.057831118
ENSMUSG00000041642	Kif21b	kinesin family member 21B [Source:MGI Symbol;Acc:MGI:109234]	1.80511	2.04751	2.62643	2.05614	1.89759	2.05812	2.4739	2.1813	2.69954	2.36277	1.5786	1.96154	1.87058	2.21367	0.019711532	0.258568924	12	8	24	0.045248714	0.628927515	24	7.23953965	0.135421262
ENSMUSG00000041645	Ddx24	DEAD box helicase 24 [Source:MGI Symbol;Acc:MGI:1351337]	47.4879	40.3163	43.366	41.1819	40.2044	46.7227	43.918	45.9272	36.1065	53.9025	36.5455	41.8983	40.542	43.5704	0.83638114	0.966018836	4	16	24	0.91194857	0.975149099	24	0.052007377	0.025684128
ENSMUSG00000041649	Klf8	Kruppel-like factor 8 [Source:MGI Symbol;Acc:MGI:2442430]	5.23895	3.89482	5.46578	3.36886	4.51644	4.41056	3.52168	3.4912	4.54403	4.2568	4.12097	5.0091	4.31316	3.66627	0.834424969	0.966018836	24	8	24	0.740776335	0.915293122	24	18.24299928	0.056202541
ENSMUSG00000041650	Pcca	propionyl-Coenzyme A carboxylase, alpha polypeptide [Source:MGI Symbol;Acc:MGI:97499]	10.6548	19.8949	11.3852	21.1635	16.6875	16.4597	20.4586	22.2908	22.5495	18.6747	14.14	20.2147	24.2982	16.009	0.890176313	0.966018836	4	16	24	0.851878997	0.955545499	24	5.660961697	0.07285005
ENSMUSG00000041653	Pnpla3	patatin-like phospholipase domain containing 3 [Source:MGI Symbol;Acc:MGI:2151796]	10.7734	11.7781	15.0349	19.3321	13.8992	14.9204	13.8947	15.6387	14.4636	16.6836	19.4936	16.8944	20.2642	14.6602	0.50155611	0.966018836	16	16	24	0.117571003	0.728323355	24	13.87318984	0.147282732
ENSMUSG00000041654	Slc39a11	solute carrier family 39 (metal ion transporter), member 11 [Source:MGI Symbol;Acc:MGI:1917056]	6.43679	5.7815	6.35925	5.48361	6.11127	6.5001	5.48244	5.3574	4.54231	6.97814	6.64078	6.02008	6.19102	5.95914	0.208438456	0.698704063	20	12	24	0.231400387	0.768040178	24	17.95019145	0.090465427
ENSMUSG00000041658	Rragb	Ras-related GTP binding B [Source:MGI Symbol;Acc:MGI:3038613]	24.7001	25.9647	29.1563	23.8988	22.2883	25.8	24.6386	22.7203	22.5864	24.8765	24.1993	24.8737	24.0998	27.2296	0.933543173	0.966018836	8	12	24	0.638851115	0.877547951	24	4.52984286	0.038399442
ENSMUSG00000041660	Bbox1	butyrobetaine (gamma), 2-oxoglutarate dioxygenase 1 (gamma-butyrobetaine hydroxylase) [Source:MGI Symbol;Acc:MGI:1891372]	0.427853	1.22609	2.11395	1.04952	0.615305	1.04007	1.02405	0.576701	1.46553	0.667994	0.628723	1.14632	0.354035	1.38758	0.03452758	0.328311403	12	16	24	0.230371547	0.768040178	24	8.05956854	0.22948432
ENSMUSG00000041669	Prima1	proline rich membrane anchor 1 [Source:MGI Symbol;Acc:MGI:1926097]	4.51818	4.57932	4.27519	3.56517	5.1947	3.46979	5.31809	3.50991	3.93786	6.0019	4.75986	4.52027	4.21341	3.62086	0.892555977	0.966018836	20	12	24	0.522582346	0.820892304	24	16.29887875	0.089990989
ENSMUSG00000041670	Rims1	regulating synaptic membrane exocytosis 1 [Source:MGI Symbol;Acc:MGI:2152971]	17.4281	15.305	15.2684	16.0976	12.838	15.3506	15.4128	14.296	12.1107	16.1406	13.6463	15.8238	14.3488	16.6116	0.83638114	0.966018836	4	16	24	0.561338312	0.841538771	24	0.174604391	0.057051836
ENSMUSG00000041671	Pyroxd1	pyridine nucleotide-disulphide oxidoreductase domain 1 [Source:MGI Symbol;Acc:MGI:2676395]	8.77936	10.3499	9.49045	8.4467	9.13547	8.05285	9.16166	8.64477	10.7251	8.04147	9.34615	9.06143	9.21741	9.75073	0.175185919	0.651010555	12	4	24	0.194170968	0.768040178	24	5.590512063	0.076278395
ENSMUSG00000041679	Lrrc29	leucine rich repeat containing 29 [Source:MGI Symbol;Acc:MGI:2443262]	0.579161	1.06513	1.20911	1.04931	0.572928	1.02482	0.592569	1.04738	1.53071	1.48244	0.842656	0.926092	0.916917	1.00011	0.006570511	0.177465007	12	16	24	0.00995039	0.503967859	24	9.008324424	0.221741241
ENSMUSG00000041684	Bivm	basic, immunoglobulin-like variable motif containing [Source:MGI Symbol;Acc:MGI:2179809]	10.6221	9.91421	10.426	11.7363	11.1476	10.2784	8.52719	8.68457	9.37488	9.30032	10.7618	10.1539	9.62493	10.0379	0.214861332	0.704989686	20	12	24	0.077628979	0.687420473	24	15.31974455	0.091799517
ENSMUSG00000041685	Fcho2	FCH domain only 2 [Source:MGI Symbol;Acc:MGI:3505790]	17.4175	18.1774	18.4667	20.0105	19.2156	16.0315	19.0389	15.873	19.6542	14.4337	20.136	15.5922	15.2065	15.9177	0.481530854	0.966018836	20	4	24	0.471355393	0.805393068	24	12.60818694	0.066270343
ENSMUSG00000041688	Amot	angiomotin [Source:MGI Symbol;Acc:MGI:108440]	3.49569	3.63767	4.13425	4.08879	3.66655	3.99319	4.36751	4.2284	4.08094	4.03196	3.29946	3.86075	3.46648	3.72085	0.591351901	0.966018836	12	8	24	0.483185833	0.805393068	24	7.726334607	0.047080633
ENSMUSG00000041695	Kcnj2	potassium inwardly-rectifying channel, subfamily J, member 2 [Source:MGI Symbol;Acc:MGI:104744]	2.68084	2.99392	3.22825	3.04578	2.95421	2.88352	3.39818	4.36647	4.29953	2.48228	3.14368	2.85761	2.79617	3.34388	0.187179226	0.662239322	8	16	24	0.131448712	0.741500219	24	6.191854515	0.13768588
ENSMUSG00000041696	Rasl12	RAS-like, family 12 [Source:MGI Symbol;Acc:MGI:1918034]	3.33861	3.37683	3.51963	4.66204	4.70357	3.62006	4.13323	4.3037	3.78959	4.58396	5.21676	4.09499	5.06348	4.24459	0.27817759	0.789849983	20	4	24	0.103245939	0.714848084	24	14.8057659	0.104341618
ENSMUSG00000041697	Cox6a1	cytochrome c oxidase subunit 6A1 [Source:MGI Symbol;Acc:MGI:103099]	295.317	260.879	264.217	312.192	286.102	311.368	294.349	287.225	263.609	300.761	354.405	333.606	359.465	323.2	0.133266423	0.567728672	24	12	24	0.027271604	0.589821493	24	19.16688372	0.107840208
ENSMUSG00000041700	Lhfpl1	lipoma HMGIC fusion partner-like 1 [Source:MGI Symbol;Acc:MGI:1891214]	12.3478	12.6608	11.3931	11.7198	12.424	11.2001	10.3397	11.1645	9.91273	10.6907	12.999	9.84627	11.6221	10.5041	0.911135828	0.966018836	20	4	24	0.625101175	0.872055388	24	17.48030887	0.041214151
ENSMUSG00000041702	Btbd7	BTB (POZ) domain containing 7 [Source:MGI Symbol;Acc:MGI:1917858]	7.08597	7.06358	6.93122	7.55701	7.05447	6.96036	7.30711	6.70229	7.11174	6.77897	7.58903	6.97504	6.67184	6.94671	0.916746631	0.966018836	20	12	24	0.292219312	0.768040178	24	15.15185735	0.028617172
ENSMUSG00000041707	Tmem273	transmembrane protein 273 [Source:MGI Symbol;Acc:MGI:1916319]	0.358888	0.738984	0.325345	0.344149	0.845627	0.786064	1.1887	0.817165	0.368145	0.556738	0.535402	0.495871	0.964568	0.399802	0.235177833	0.73629392	4	8	24	0.159326982	0.765350752	24	22.12643153	0.171291087
ENSMUSG00000041708	Mpped1	metallophosphoesterase domain containing 1 [Source:MGI Symbol;Acc:MGI:106316]	1.96449	2.71149	3.66567	2.42982	1.78799	2.55942	2.57371	2.15666	2.38706	2.52195	1.53398	2.47626	1.65858	2.63895	0.227302972	0.723724673	12	8	24	0.209182049	0.768040178	24	6.946301408	0.152512734
ENSMUSG00000041712	Ubr7	ubiquitin protein ligase E3 component n-recognin 7 (putative) [Source:MGI Symbol;Acc:MGI:1913872]	11.6188	11.5627	11.6103	12.4122	11.2307	11.2547	10.8348	11.7527	11.3493	11.9884	11.3189	12.1722	12.0938	11.8576	0.907000607	0.966018836	16	4	24	0.672023271	0.889365603	24	9.52960789	0.017714566
ENSMUSG00000041716	Gm20604	predicted gene 20604 [Source:MGI Symbol;Acc:MGI:5305180]	8.47358	7.41313	8.11018	8.58347	6.5785	9.0236	8.1005	8.39027	8.04503	8.72247	8.12741	8.11425	7.68982	8.31088	0.93717134	0.966018836	16	20	24	0.961900183	0.9903359	24	6.11799635	0.011541058
ENSMUSG00000041718	Alg13	asparagine-linked glycosylation 13 [Source:MGI Symbol;Acc:MGI:1914824]	4.23488	4.84498	4.71453	4.58554	4.82171	4.37443	4.85074	4.6731	4.93567	4.53178	5.37687	4.68272	4.46833	4.81941	0.70641939	0.966018836	20	4	24	0.450587704	0.805393068	24	11.14650222	0.033468552
ENSMUSG00000041720	Pi4ka	phosphatidylinositol 4-kinase alpha [Source:MGI Symbol;Acc:MGI:2448506]	40.5735	40.8637	44.6538	44.9344	41.0793	44.4024	42.3539	41.1922	37.7252	44.3621	35.1856	38.6776	41.9705	38.7618	0.883870616	0.966018836	16	4	24	0.949759139	0.988062071	24	9.859748388	0.01221003
ENSMUSG00000041729	Coro2b	coronin, actin binding protein, 2B [Source:MGI Symbol;Acc:MGI:2444283]	31.7427	25.1695	24.4433	28.8313	26.9779	30.2469	29.4912	28.9776	23.632	39.9787	25.1796	31.2288	28.1066	29.0737	0.046056765	0.374351104	24	12	24	0.667624095	0.887585935	24	18.72354608	0.068765455
ENSMUSG00000041730	Prrxl1	paired related homeobox protein-like 1 [Source:MGI Symbol;Acc:MGI:2148204]	37.3032	23.718	24.1467	25.4984	25.4409	28.7591	27.9169	30.737	20.9817	35.3918	26.613	30.4113	30.3232	28.8625	0.170659409	0.643496587	4	8	24	0.291482938	0.768040178	24	21.59510842	0.134302737
ENSMUSG00000041731	Pgm5	phosphoglucomutase 5 [Source:MGI Symbol;Acc:MGI:1925668]	1.36364	1.7489	1.59633	1.39596	1.41575	1.32585	1.32134	2.49987	1.62114	1.17236	1.19134	1.44449	1.48544	1.52363	0.010754404	0.199214173	8	8	24	0.048647488	0.638992719	24	4.738303633	0.150460239
ENSMUSG00000041733	Coq5	coenzyme Q5 methyltransferase [Source:MGI Symbol;Acc:MGI:1098643]	11.8924	10.3716	13.0958	12.5275	10.6335	11.9409	11.5728	13.1177	10.6609	12.3656	10.2495	10.92	12.5621	11.902	0.542718076	0.966018836	16	4	24	0.515634775	0.817346854	24	5.575572322	0.052661524
ENSMUSG00000041734	Kirrel	kirre like nephrin family adhesion molecule 1 [Source:MGI Symbol;Acc:MGI:1891396]	5.28873	5.49205	5.00331	4.9801	6.20229	5.5106	6.20875	5.8226	5.68839	6.04652	5.63992	5.55189	5.93158	5.18947	0.851656735	0.966018836	20	16	24	0.567135771	0.843774904	24	19.67347958	0.037521092
ENSMUSG00000041736	Tspo	translocator protein [Source:MGI Symbol;Acc:MGI:88222]	23.9083	27.4358	32.0895	35.2818	24.9878	23.8471	27.5683	33.1546	37.961	31.0105	37.4945	34.5438	43.2991	34.7075	0.916746631	0.966018836	12	12	24	0.137526786	0.747972781	24	8.505022898	0.14778237
ENSMUSG00000041737	Tmem45b	transmembrane protein 45b [Source:MGI Symbol;Acc:MGI:2384574]	116.38	105.087	98.9495	107.313	104.815	118.99	93.6088	105.42	87.9245	103.682	111.498	108.95	118.966	104.956	0.010049466	0.194720062	24	12	24	0.08136609	0.697102286	24	20.21334223	0.104352417
ENSMUSG00000041740	Rnf10	ring finger protein 10 [Source:MGI Symbol;Acc:MGI:1859162]	47.7045	42.8365	44.7867	50.912	47.8805	48.4862	42.4625	49.6093	41.2263	54.1572	43.7197	46.173	46.2979	46.8778	0.676344318	0.966018836	16	20	24	0.496954302	0.807576732	24	14.38935714	0.049115531
ENSMUSG00000041741	Pde3a	phosphodiesterase 3A, cGMP inhibited [Source:MGI Symbol;Acc:MGI:1860764]	0.562219	0.626637	0.620401	0.612704	0.54966	0.593143	0.706812	0.611566	0.663662	0.587246	0.621793	0.809317	0.643616	0.957899	0.630352198	0.966018836	8	8	24	0.229545104	0.768040178	24	3.386827078	0.045525117
ENSMUSG00000041747	Utp15	UTP15 small subunit processome component [Source:MGI Symbol;Acc:MGI:2145443]	6.71663	7.24066	7.61007	7.13573	6.9731	6.78113	5.94218	6.65351	7.40766	7.47811	7.42832	7.25383	7.09433	7.14162	0.008754183	0.192760771	12	16	24	0.039787252	0.616921492	24	11.01560141	0.072305351
ENSMUSG00000041754	Trem3	triggering receptor expressed on myeloid cells 3 [Source:MGI Symbol;Acc:MGI:1930003]	0.525981	1.31229	1.664	1.31655	1.18133	1.276	1.16687	1.02769	1.04509	0.821365	0.822108	1.18888	1.46494	0.535474	0.832293447	0.966018836	24	8	24	0.931531209	0.982348071	24	13.77013914	0.034984409
ENSMUSG00000041757	Plekha6	pleckstrin homology domain containing, family A member 6 [Source:MGI Symbol;Acc:MGI:2388662]	60.6422	49.4713	43.1344	53.2432	52.4068	52.8778	53.9737	49.8447	46.5975	61.3213	54.064	53.9388	55.9079	52.2737	0.05630976	0.401074772	4	8	24	0.170480079	0.767986663	24	19.43717641	0.094967098
ENSMUSG00000041762	Gpr155	G protein-coupled receptor 155 [Source:MGI Symbol;Acc:MGI:1915776]	22.1046	19.0987	20.8209	20.2036	21.7471	22.4378	20.6217	18.337	17.5332	21.8674	19.2445	19.8528	19.1689	18.6564	0.146541269	0.590732968	24	8	24	0.035232069	0.608551791	24	18.03337409	0.088070053
ENSMUSG00000041763	Tpp2	tripeptidyl peptidase II [Source:MGI Symbol;Acc:MGI:102724]	26.7216	26.449	26.7965	25.8401	25.8906	26.521	26.0843	25.3692	25.5319	27.1224	28.3898	26.3788	27.5767	27.4263	0.874607729	0.966018836	4	8	24	0.779207568	0.928978483	24	18.48862231	0.011789502
ENSMUSG00000041765	Ubac2	ubiquitin associated domain containing 2 [Source:MGI Symbol;Acc:MGI:1916139]	10.7631	9.80353	10.4045	10.3523	9.38178	10.9776	10.5828	11.3454	9.72692	12.0722	10.4751	10.6277	10.4089	11.5057	0.948454836	0.966018836	8	4	24	0.860720943	0.959078188	24	4.245957168	0.018402688
ENSMUSG00000041769	Ppp2r2d	protein phosphatase 2, regulatory subunit B, delta [Source:MGI Symbol;Acc:MGI:1289252]	34.4636	31.5396	33.7936	32.2838	32.1837	34.9357	31.3791	33.4707	32.4635	36.1791	33.3444	35.1119	33.2898	37.1427	0.858266942	0.966018836	24	20	24	0.998787437	0.999770694	24	10.72028086	0.001280118
ENSMUSG00000041771	Slc24a4	solute carrier family 24 (sodium/potassium/calcium exchanger), member 4 [Source:MGI Symbol;Acc:MGI:2447362]	0.565415	0.638916	1.21075	0.754262	0.397441	0.787614	0.907402	0.531788	0.765277	0.776899	0.417213	0.824604	0.601192	0.671715	0.944053021	0.966018836	12	8	24	0.579519133	0.849875019	24	6.867294044	0.074435516
ENSMUSG00000041773	Enc1	ectodermal-neural cortex 1 [Source:MGI Symbol;Acc:MGI:109610]	2.74976	2.37363	3.52996	2.42746	2.2498	2.91344	3.44777	2.45067	2.81392	2.70652	2.47548	2.98781	2.48356	2.76442	0.745199643	0.966018836	24	20	24	0.757440038	0.921562129	24	2.312682759	0.042447297
ENSMUSG00000041774	Ydjc	YdjC homolog (bacterial) [Source:MGI Symbol;Acc:MGI:1916351]	8.30357	5.62835	5.4796	5.4181	6.73087	7.30794	6.53787	7.47261	6.34787	8.38323	9.66567	9.83754	8.04518	8.44191	0.360140629	0.883787544	24	12	24	0.076945255	0.687041208	24	19.94812762	0.162620589
ENSMUSG00000041775	Mapk1ip1	mitogen-activated protein kinase 1 interacting protein 1 [Source:MGI Symbol;Acc:MGI:1916796]	20.6575	18.9079	19.645	21.9879	21.2412	20.5636	23.0264	19.4965	19.3	19.5784	21.1822	20.0901	19.1379	19.694	0.184044621	0.662239322	20	12	24	0.140880562	0.751014756	24	17.90288642	0.058752965
ENSMUSG00000041777	Cir1	corepressor interacting with RBPJ, 1 [Source:MGI Symbol;Acc:MGI:1914185]	4.21192	4.35792	4.31051	4.16802	4.58311	4.0927	3.85846	4.63359	4.87158	4.4511	5.04105	5.03082	5.39904	5.73933	0.860993221	0.966018836	8	8	24	0.587538161	0.853591039	24	6.016746076	0.034612477
ENSMUSG00000041779	Tram2	translocating chain-associating membrane protein 2 [Source:MGI Symbol;Acc:MGI:1924817]	0.962111	1.26967	1.25147	1.29248	1.56022	1.15486	1.23041	1.26509	1.45551	1.39023	1.24894	1.31457	1.24291	1.21232	0.057224139	0.401309455	16	12	24	0.052704311	0.650526959	24	11.96867133	0.074977379
ENSMUSG00000041781	Cpsf2	cleavage and polyadenylation specific factor 2 [Source:MGI Symbol;Acc:MGI:1861601]	13.3566	12.692	12.9405	12.7595	12.4549	12.7785	12.1351	12.474	11.6777	12.619	13.4139	13.0989	13.7817	13.4355	0.85447369	0.966018836	4	8	24	0.45730032	0.805393068	24	21.55846811	0.029093636
ENSMUSG00000041789	2700046A07Rik	RIKEN cDNA 2700046A07 gene [Source:MGI Symbol;Acc:MGI:1919803]	0.325595	0.655744	0.885906	0.590334	0.515594	0.534319	0.675876	0.788537	0.662924	0.377244	0.468976	0.516184	0.6009	0.345568	0.243586913	0.741580974	12	8	24	0.430743412	0.805393068	24	6.418640064	0.075812951
ENSMUSG00000041794	Myrip	myosin VIIA and Rab interacting protein [Source:MGI Symbol;Acc:MGI:2384407]	2.00961	1.72843	2.8372	2.0219	1.84895	2.04168	2.57189	1.72562	1.85097	2.34915	1.60883	2.11365	1.51548	2.47825	0.906657897	0.966018836	16	4	24	0.746054428	0.917098266	24	7.839901187	0.057155436
ENSMUSG00000041797	Abca9	ATP-binding cassette, sub-family A (ABC1), member 9 [Source:MGI Symbol;Acc:MGI:2386796]	7.40893	7.96607	6.78938	7.88143	9.56622	6.48888	9.15008	7.80798	9.67334	5.78221	8.81813	8.18167	8.4976	7.00285	0.896799496	0.966018836	20	20	24	0.862355595	0.959779249	24	20.65646912	0.044106975
ENSMUSG00000041798	Gck	glucokinase [Source:MGI Symbol;Acc:MGI:1270854]	0.426415	0.966133	0.783782	0.519409	0.559283	0.781764	0.374489	1.07107	0.899661	1.00228	1.15916	0.929541	0.870637	0.743784	0.885025617	0.966018836	8	20	24	0.500990474	0.80908345	24	9.693572922	0.086197946
ENSMUSG00000041801	Phlda3	pleckstrin homology like domain, family A, member 3 [Source:MGI Symbol;Acc:MGI:1351485]	32.2632	33.3915	40.1553	49.1156	38.2162	37.1031	37.9965	39.2976	41.3772	44.053	43.4965	37.04	37.2747	34.8961	0.004021228	0.140598674	16	12	24	0.001695868	0.374946468	24	12.32458937	0.17512061

ENSMUSG00000041809	Efhc1	EF-hand domain (C-terminal) containing 1 [Source:MGI Symbol;Acc:MGI:1919127]	2.22489	2.19921	1.89098	2.34869	2.86909	2.23355	2.24577	2.27139	1.53112	2.07087	2.31499	2.67795	2.44242	2.1598	0.005378037	0.149433822	20	16	24	0.025481607	0.586541342	24	19.32192373	0.142046417
ENSMUSG00000041815	Poldip3	polymerase (DNA-directed), delta interacting protein 3 [Source:MGI Symbol;Acc:MGI:1921076]	20.7014	19.3746	20.5125	21.3337	21.482	20.8627	21.4508	20.6324	22.1352	23.1153	18.9138	21.3718	20.2641	20.2084	0.231491055	0.731037655	16	16	24	0.500255777	0.808975895	24	12.37331775	0.032699001
ENSMUSG00000041817	Fam169a	family with sequence similarity 169, member A [Source:MGI Symbol;Acc:MGI:2444268]	13.2177	12.8701	12.9546	12.4028	12.2948	12.2779	12.0718	11.1774	11.3729	11.5947	12.5216	11.4411	10.5633	11.7591	0.854540463	0.966018836	20	12	24	0.625657032	0.872249537	24	15.95947321	0.020570031
ENSMUSG00000041827	Oasl1	2'-5' oligoadenylate synthetase-like 1 [Source:MGI Symbol;Acc:MGI:2180849]	0.960417	1.04786	0.759384	0.631468	0.792406	0.870697	1.03231	0.863185	0.726416	0.979401	0.828032	0.615848	0.612885	0.966718	0.593560247	0.966018836	8	4	24	0.492744844	0.805558817	24	2.730776157	0.054025721
ENSMUSG00000041828	Abca8a	ATP-binding cassette, sub-family A (ABC1), member 8a [Source:MGI Symbol;Acc:MGI:2386846]	74.7502	65.469	64.1017	75.3675	80.2756	68.6255	70.8369	61.2263	72.6156	71.083	76.5191	64.4756	68.9936	63.8427	0.008603252	0.192760771	20	12	24	0.033223057	0.606585237	24	15.85684336	0.102388823
ENSMUSG00000041831	Sytl3	synaptotagmin-like 3 [Source:MGI Symbol;Acc:MGI:1933367]	11.3297	7.78219	8.32802	8.75199	8.96892	9.81783	9.2296	8.61275	7.38427	9.97502	9.2569	8.62	9.09388	9.02556	0.082341274	0.462922111	4	8	24	0.136312911	0.747972781	24	19.94043494	0.10599933
ENSMUSG00000041836	Ptpre	protein tyrosine phosphatase, receptor type, E [Source:MGI Symbol;Acc:MGI:97813]	14.5973	14.3624	13.4825	13.542	15.1219	13.8258	15.6386	14.5712	14.1238	18.1578	12.3724	14.0107	14.6003	13.325	0.671726933	0.966018836	4	8	24	0.991591272	0.998516607	24	10.21372606	0.006554752
ENSMUSG00000041837	Pdcd7	programmed cell death 7 [Source:MGI Symbol;Acc:MGI:1859170]	6.77528	6.13751	6.32239	6.82183	5.86678	6.37439	5.66376	6.82991	6.11291	6.8847	6.47794	6.84049	6.665	7.2777	0.910436409	0.966018836	8	12	24	0.807953032	0.938907778	24	6.994117789	0.021922957
ENSMUSG00000041840	Haus1	HAUS augmin-like complex, subunit 1 [Source:MGI Symbol;Acc:MGI:2385076]	12.9283	16.095	12.8018	13.7742	13.718	13.7267	13.52	14.0251	15.5956	12.6713	13.2391	14.2273	13.4608	14.0641	0.628286596	0.966018836	8	8	24	0.453536685	0.805393068	24	4.851006703	0.045971673
ENSMUSG00000041841	Rpl37	ribosomal protein L37 [Source:MGI Symbol;Acc:MGI:1914531]	6.04734	6.27804	6.9607	7.0124	6.24039	6.30019	5.04409	5.66892	8.16977	6.40985	7.2058	7.74637	5.94173	6.06218	0.020061908	0.259417302	12	16	24	0.099514381	0.71175955	24	12.42006087	0.124016902
ENSMUSG00000041842	Fhdc1	FH2 domain containing 1 [Source:MGI Symbol;Acc:MGI:2684972]	3.33408	2.73823	2.82066	2.69686	3.20703	3.03116	3.49139	3.2539	2.25017	3.28416	3.00839	2.77431	3.06452	3.04678	0.112800839	0.530777648	4	8	24	0.232477333	0.768040178	24	21.49664073	0.081486264
ENSMUSG00000041845	Rhod	ras homolog family member D [Source:MGI Symbol;Acc:MGI:108446]	1.32408	1.41503	1.35372	1.1543	1.67314	1.44483	1.44505	1.57482	1.31805	0.925916	1.52647	1.21624	0.934242	1.22703	0.676344318	0.966018836	20	20	24	0.780606565	0.929485273	24	20.24916412	0.038156618
ENSMUSG00000041846	Ppp4r3a	protein phosphatase 4 regulatory subunit 3A [Source:MGI Symbol;Acc:MGI:1915984]	10.4854	11.4899	10.8263	10.5772	10.7221	10.1986	11.2867	9.45772	11.8671	9.91233	11.3251	10.6525	10.3607	10.3882	0.905762334	0.966018836	12	12	24	0.946181868	0.986887534	24	10.65918516	0.010891767
ENSMUSG00000041849	Card6	caspase recruitment domain family, member 6 [Source:MGI Symbol;Acc:MGI:3032959]	0.789684	0.810699	0.694057	1.05126	0.450134	0.628502	0.814838	0.585768	0.659924	0.773044	0.552613	0.621348	0.535098	0.770663	0.503972924	0.966018836	16	4	24	0.453649509	0.805393068	24	7.131289288	0.061910082
ENSMUSG00000041852	Tcf20	transcription factor 20 [Source:MGI Symbol;Acc:MGI:108399]	9.06678	8.21131	8.01597	8.09927	7.98972	8.56887	8.66179	8.55271	7.39797	9.68046	8.52979	9.02395	8.62146	9.38683	0.16456443	0.628002127	4	8	24	0.508882024	0.813722792	24	22.09058301	0.039914697
ENSMUSG00000041859	Mcm3	minichromosome maintenance complex component 3 [Source:MGI Symbol;Acc:MGI:101845]	1.63109	2.88005	2.68352	3.14154	2.71527	2.62649	3.11102	2.51476	2.85672	2.04076	2.04433	2.39793	2.92314	2.03759	0.768363194	0.966018836	16	4	24	0.908739462	0.974606327	24	8.013855627	0.035820664
ENSMUSG00000041870	Ankrd13a	ankyrin repeat domain 13a [Source:MGI Symbol;Acc:MGI:1915670]	13.9106	13.3113	16.1611	14.4293	15.2338	15.3093	13.396	14.2468	15.3319	14.9026	13.501	13.1246	13.1107	12.3987	0.041102888	0.346472636	12	20	24	0.064746118	0.663876076	24	12.24362558	0.076784381
ENSMUSG00000041878	8430432A02Rik	RIKEN cDNA 8430432A02 gene [Source:MGI Symbol;Acc:MGI:1918774]	0.65833	0.557968	0.437641	0.546552	0.658526	0.582687	0.49122	0.426353	0.775069	0.531516	0.47865	0.65183	0.53682	0.571572	0.883907406	0.966018836	24	8	24	0.836922624	0.950079249	24	17.92849731	0.021758625
ENSMUSG00000041879	Ipo9	importin 9 [Source:MGI Symbol;Acc:MGI:1918944]	20.2038	19.5615	21.6495	19.8503	18.2937	20.9191	22.8721	21.1543	19.472	22.6908	17.9403	21.7468	19.4496	21.3224	0.847312766	0.966018836	24	20	24	0.546752588	0.833499795	24	3.542382831	0.044015826
ENSMUSG00000041881	Ndufa7	NADH:ubiquinone oxidoreductase subunit A7 [Source:MGI Symbol;Acc:MGI:1913666]	158.981	166.756	164.065	155.919	152.674	156.857	161.054	160.852	197.89	146.283	181.19	167.791	186.471	177.448	0.503972924	0.966018836	12	4	24	0.371440622	0.793126592	24	4.588224373	0.058091429
ENSMUSG00000041889	Shisa4	shisa family member 4 [Source:MGI Symbol;Acc:MGI:1924802]	24.0239	24.0696	29.1362	23.2303	25.3441	27.9166	25.5082	28.0049	25.3267	31.9091	23.4288	25.7127	23.8088	26.0544	0.892578749	0.966018836	12	8	24	0.560142395	0.840893245	24	9.601783463	0.056559352
ENSMUSG00000041890	Git2	GIT ArfGAP 2 [Source:MGI Symbol;Acc:MGI:1347053]	14.3959	16.0506	13.6579	13.4921	15.4771	14.3837	17.6811	14.744	16.8149	14.4573	14.2279	13.9698	14.1704	14.3999	0.767183862	0.966018836	8	8	24	0.596016878	0.857839743	24	2.697147144	0.042674305
ENSMUSG00000041891	Lman1	lectin, mannose-binding, 1 [Source:MGI Symbol;Acc:MGI:1917611]	15.7343	13.8402	14.9238	14.3514	15.0675	13.6198	13.874	13.8177	13.7093	13.3959	15.166	13.9529	14.3646	13.8739	0.643501552	0.966018836	20	12	24	0.537553656	0.827958985	24	18.39993122	0.027402873
ENSMUSG00000041895	Wipi1	WD repeat domain, phosphoinositide interacting 1 [Source:MGI Symbol;Acc:MGI:1261864]	13.0887	13.9128	14.0905	12.8103	13.9208	14.7235	15.7476	15.6217	14.5888	15.9507	13.2063	15.5733	13.3042	14.182	0.881996584	0.966018836	12	8	24	0.641999358	0.878809617	24	5.986894464	0.036580867
ENSMUSG00000041907	Gpr45	G protein-coupled receptor 45 [Source:MGI Symbol;Acc:MGI:2135882]	12.5144	10.1619	10.4055	9.9878	9.72662	10.3703	9.78285	11.1449	9.13825	12.017	10.0943	10.7687	10.6545	11.1079	0.708665292	0.966018836	8	4	24	0.679638704	0.892722891	24	0.444576464	0.038941909
ENSMUSG00000041912	Tdrkh	tudor and KH domain containing protein [Source:MGI Symbol;Acc:MGI:1919884]	21.8898	23.3943	20.8326	20.0853	21.3465	21.872	22.0965	20.1093	19.636	20.7573	21.438	19.8786	23.1775	22.083	0.023770386	0.281841912	4	8	24	0.116994069	0.727976837	24	23.77032754	0.058339078
ENSMUSG00000041915	Ammecr1l	AMME chromosomal region gene 1-like [Source:MGI Symbol;Acc:MGI:2442711]	12.8582	11.951	11.8204	12.3423	12.6866	12.6692	13.0834	10.823	11.4468	12.6979	10.7878	11.799	11.0615	12.1434	0.745116544	0.966018836	4	4	24	0.485004631	0.805393068	24	20.2080479	0.03736049
ENSMUSG00000041920	Slc16a6	solute carrier family 16 (monocarboxylic acid transporters), member 6 [Source:MGI Symbol;Acc:MGI:2144585]	5.64005	5.15423	4.70432	4.27206	5.01914	5.65699	4.49938	5.35076	4.63156	4.85068	4.83793	4.80245	4.49622	4.98859	0.70641939	0.966018836	8	4	24	0.312044569	0.768040178	24	22.66427522	0.057759066
ENSMUSG00000041921	Metap1d	methionyl aminopeptidase type 1D (mitochondrial) [Source:MGI Symbol;Acc:MGI:1913809]	16.1786	21.5375	20.4066	21.1953	20.5935	19.4198	19.0945	17.6703	23.5672	18.533	22.8682	18.8205	19.2869	19.6626	0.163587268	0.626886304	12	16	24	0.128925145	0.740162193	24	11.36766187	0.103370708
ENSMUSG00000041923	Nol4	nucleolar protein 4 [Source:MGI Symbol;Acc:MGI:2441684]	4.58528	3.7959	4.76089	3.44082	3.4051	3.3503	5.27719	3.44286	3.69727	3.27981	3.43839	3.96028	2.96307	4.58238	0.336988702	0.85758369	4	16	24	0.288369953	0.768040178	24	2.672349446	0.113572383
ENSMUSG00000041926	Rnpep	arginyl aminopeptidase (aminopeptidase B) [Source:MGI Symbol;Acc:MGI:2384902]	11.6832	12.4187	11.639	10.8537	13.1903	11.5702	11.4653	13.3275	12.7563	12.4168	12.2619	12.8265	13.5445	15.0343	0.830364743	0.966018836	8	8	24	0.239166156	0.768040178	24	4.157716426	0.054382316
ENSMUSG00000041930	Fam222a	family with sequence similarity 222, member A [Source:MGI Symbol;Acc:MGI:3605543]	2.5414	1.18703	1.61915	1.17204	1.57324	1.19331	1.8013	1.44038	1.33212	1.7286	1.9865	1.52472	1.48097	1.24816	0.652290823	0.966018836	20	12	24	0.518444783	0.818673182	24	20.05784058	0.092927241
ENSMUSG00000041935	AW549877	expressed sequence AW549877 [Source:MGI Symbol;Acc:MGI:2146232]	32.6089	33.0467	30.716	33.6868	34.5984	30.0741	32.4612	29.3156	32.168	27.6488	35.4898	29.5644	31.7038	31.0836	0.922035832	0.966018836	20	4	24	0.697602905	0.899395227	24	17.25834851	0.031981228
ENSMUSG00000041936	Agrn	agrin [Source:MGI Symbol;Acc:MGI:87961]	28.9446	37.0342	36.1204	34.5749	37.1651	30.1577	33.5795	34.3083	40.6313	38.2789	32.451	32.6821	33.0615	34.1166	0.004516345	0.140598674	12	12	24	0.006178454	0.469371824	24	9.417969588	0.139211358
ENSMUSG00000041939	Mvk	mevalonate kinase [Source:MGI Symbol;Acc:MGI:107624]	27.1594	21.8377	23.9798	21.6424	22.2918	25.9945	24.1339	24.3939	21.3168	27.2463	23.8264	25.4164	24.329	24.2682	0.266853213	0.7385769	24	12	24	0.316259282	0.768040178	24	21.24348965	0.065852957
ENSMUSG00000041945	Mfsd9	major facilitator superfamily domain containing 9 [Source:MGI Symbol;Acc:MGI:2443548]	4.3396	4.01528	3.96406	4.52087	3.96817	4.77952	3.84654	4.8987	3.56313	4.73122	3.66166	4.11469	4.26989	4.02939	0.93447422	0.966018836	24	12	24	0.930771369	0.982191975	24	21.18072339	0.018192064
ENSMUSG00000041949	Tango6	transport and golgi organization 6 [Source:MGI Symbol;Acc:MGI:2142786]	1.15819	1.15893	0.882268	1.15141	1.14749	1.25214	1.0511	1.48607	1.15704	1.5502	1.17875	1.1728	1.22358	1.19222	0.908406451	0.966018836	24	12	24	0.959712735	0.989375125	24	12.75679308	0.011374175
ENSMUSG00000041954	Tnfrsf18	tumor necrosis factor receptor superfamily, member 18 [Source:MGI Symbol;Acc:MGI:894675]	2.49182	3.03007	2.47692	2.20411	2.85105	2.01829	2.42611	2.7094	2.94648	1.71762	2.55396	2.52647	2.55432	2.19796	0.952127548	0.966018836	8	8	24	0.578682834	0.849385767	24	2.770603948	0.061871434
ENSMUSG00000041957	Pkp2	plakophilin 2 [Source:MGI Symbol;Acc:MGI:1914701]	1.30731	1.46282	1.17275	1.02793	1.14719	1.21171	1.3876	1.22707	1.19659	0.901276	1.43929	1.22147	1.4779	1.17912	0.001925685	0.100255953	4	12	24	0.033473997	0.606585237	24	23.75954342	0.099130356
ENSMUSG00000041958	Pigs	phosphatidylinositol glycan anchor biosynthesis, class S [Source:MGI Symbol;Acc:MGI:2687325]	21.5055	15.3265	16.7896	17.3458	16.5502	19.4198	18.5476	19.6037	15.1528	24.7927	15.6842	19.4224	16.9977	18.0206	0.628286596	0.966018836	24	12	24	0.812687509	0.940101979	24	20.06931818	0.051074308
ENSMUSG00000041959	S100a10	S100 calcium binding protein A10 (calpactin) [Source:MGI Symbol;Acc:MGI:1339468]	728.684	748.253	685.769	683.425	761.889	678.266	660.995	649.491	746.87	564.535	815.432	721.089	763.731	735.866	0.897975397	0.966018836	20	20	24	0.695801357	0.899071171	24	21.3209202	0.046253464
ENSMUSG00000041961	Znrf3	zinc and ring finger 3 [Source:MGI Symbol;Acc:MGI:3039616]	1.25701	1.45726	1.43997	1.37186	1.30586	1.34052	1.36159	1.61869	1.57029	1.64571	1.3259	1.39534	1.13827	1.52582	0.044627045	0.365845448	8	20	24	0.025305719	0.586541342	24	8.400832462	0.084044003
ENSMUSG00000041966	Dcaf17	DDB1 and CUL4 associated factor 17 [Source:MGI Symbol;Acc:MGI:1923013]	2.63067	3.03007	3.69996	3.5595	3.56705	2.90579	3.78655	3.03808	3.54668	3.31807	2.87369	3.34189	3.66863	3.00153	0.916556573	0.966018836	12	8	24	0.589481278	0.854265306	24	10.47741137	0.05211002
ENSMUSG00000041974	Spidr	scaffolding protein involved in DNA repair [Source:MGI Symbol;Acc:MGI:1924834]	4.007	3.88733	3.42461	3.2422	4.26773	3.52363	3.83356	3.43141	3.17843	2.81736	3.6343	3.63848	3.57148	3.21598	0.050721923	0.386400877	20	20	24	0.014808865	0.537924552	24	21.30164076	0.111740468
ENSMUSG00000041975	Mettl8	methyltransferase like 8 [Source:MGI Symbol;Acc:MGI:2385142]	5.27569	6.47175	6.29276	6.2691	6.32811	6.40748	6.37372	5.83534	6.03083	5.35069	7.30938	6.11086	6.60761	6.10602	0.840483986	0.966018836	20	20	24	0.67553308	0.891272641	24	17.55641409	0.036280616
ENSMUSG00000041977	Arhgef11	Rho guanine nucleotide exchange factor (GEF) 11 [Source:MGI Symbol;Acc:MGI:2441869]	29.4603	22.4419	21.2107	24.9533	23.3334	28.1462	26.3413	26.7561	19.1226	34.8575	21.6219	26.9402	25.3927	25.5383	0.08302755	0.462922111	24	12	24	0.525481456	0.82250284	24	20.52230568	0.098685955
ENSMUSG00000041986	Elmod1	ELMO/CED-12 domain containing 1 [Source:MGI Symbol;Acc:MGI:3583900]	7.06234	10.9168	16.3883	8.95248	7.66683	10.3324	12.4783	7.35235	10.6532	8.86813	6.76004	9.79334	5.87883	11.2616	0.424718357	0.966018836	12	8	24	0.383210374	0.799546792	24	7.107444743	0.196709049
ENSMUSG00000041992	Rapgef5	Rap guanine nucleotide exchange factor (GEF) 5 [Source:MGI Symbol;Acc:MGI:2444365]	17.3401	16.1161	15.5936	15.6033	16.0713	15.4092	16.385	15.7773	13.8828	16.1904	15.9955	16.2463	15.9206	17.4039	0.247964668	0.746745468	4	8	24	0.279360975	0.768040178	24	22.79568997	0.04567474
ENSMUSG00000041995	Zbed3	zinc finger, BED type containing 3 [Source:MGI Symbol;Acc:MGI:1919364]	2.19721	2.24795	1.9676	2.16353	2.64325	2.16745	2.01376	2.4439	2.60674	2.50904	2.37423	2.2181	2.6541	2.26277	0.832742389	0.966018836	20	4	24	0.718274169	0.907263943	24	12.38945585	0.028722229
ENSMUSG00000041997	Tlk1	tousled-like kinase 1 [Source:MGI Symbol;Acc:MGI:2441683]	28.1872	26.3675	25.8083	26.1939	24.1362	25.6102	25.1968	26.2349	24.6399	29.0124	25.6608	27.219	26.5567	28.2675	0.84369218	0.966018836	8	12	24	0.737668227	0.914422817	24	3.671536273	0.02231679
ENSMUSG00000042010	Acacb	acetyl-Coenzyme A carboxylase beta [Source:MGI Symbol;Acc:MGI:2140940]	2.12255	2.39467	4.88059	9.89609	4.02239	4.65532	4.42805	7.06074	4.60993	8.35052	7.98414	4.9882	6.74473	6.00631	0.305564675	0.811201458	16	12	24	0.043118667	0.625007356	24	12.05449669	0.382552722
ENSMUSG00000042015	Wdr41	WD repeat domain 41 [Source:MGI Symbol;Acc:MGI:2445123]	13.6922	13.2927	13.5982	12.8028	12.0391	14.4512	13.3564	12.6403	11.8419	14.2453	13.1343	13.5603	13.5309	14.305	0.649215555	0.966018836	24	20	24	0.534628526	0.826570085	24	22.8830605	0.035660058
ENSMUSG00000042029	Ncapg2	non-SMC condensin II complex, subunit G2 [Source:MGI Symbol;Acc:MGI:1923294]	2.99775	3.54775	3.71172	3.77877	3.81042	3.35007	3.82622	3.29426	4.04029	3.14104	3.5844	3.49976	3.34873	4.01909	0.891105762	0.966018836	12	16	24	0.446631835	0.805393068	24	9.011637992	0.051553139
ENSMUSG00000042032	Mat2b	methionine adenosyltransferase II, beta [Source:MGI Symbol;Acc:MGI:1913667]	52.8156	54.2101	49.5692	54.3322	50.9282	48.9694	49.4199	50.6757	55.4169	52.2698	51.2289	53.3528	55.8716	55.7123	0.891105762	0.966018836	8	16	24	0.383271679	0.799546792	24	6.197301878	0.033863969
ENSMUSG00000042035	Igsf3	immunoglobulin superfamily, member 3 [Source:MGI Symbol;Acc:MGI:1926158]	10.8641	9.2101	9.2127	9.1387	9.40324	10.1733	8.97196	10.2846	8.27737	12.4244	9.15821	10.9216	10.3093	10.1765	0.601647512	0.966018836	24	12	24	0.721662404	0.90866625	24	20.25755039	0.044920405
ENSMUSG00000042041	2010003K11Rik	RIKEN cDNA 2010003K11 gene [Source:MGI Symbol;Acc:MGI:1917111]	0.395385	0.24124	0.559092	1.81705	0.563279	0.494656	0.530941	0.602518	0.486407	0.783574	1.06169	0.654606	0.763009	0.736217	0.306258944	0.812796472	16	20	24	0.089184621	0.708638675	24	13.27484511	0.215289563
ENSMUSG00000042042	Csgalnact2	chondroitin sulfate N-acetylgalactosaminyltransferase 2 [Source:MGI Symbol;Acc:MGI:1926002]	7.60757	8.93741	8.36309	8.48542	8.14497	7.44428	7.62369	8.56256	8.87863	6.96673	7.90693	7.2172	6.95828	7.51086	0.089717696	0.476253254	12	12	24	0.081609379	0.697719691	24	8.126090823	0.078784232
ENSMUSG00000042043	Tbca	tubulin cofactor A [Source:MGI Symbol;Acc:MGI:107549]	145.855	155.607	136.689	149.498	142.019	133.212	137.28	134.962	154.312	113.985	157.853	126.086	147.385	134.194	0.85387321	0.966018836	20	4	24	0.928510522	0.981515518	24	3.583881764	0.019159271
ENSMUSG00000042045	Sln	sarcolipin [Source:MGI Symbol;Acc:MGI:1913652]	0.480876	1.67704	1.04627	1.69848	0.618709	1.66194	0.922781	2.15662	0.58712	1.04369	0.40233	0.152993	0.868093	1.09845	0.109247218	0.524035616	8	12	24	0.292059576	0.768040178	24	6.139815494	0.249362222
ENSMUSG00000042046	Dstyk	dual serine/threonine and tyrosine protein kinase [Source:MGI Symbol;Acc:MGI:1925064]	12.0079	11.858	12.2005	12.229	11.6727	11.7372	12.8517	11.6581	12.0743	11.7354	11.9891	12.2467	11.8982	11.9412	0.90703203	0.966018836	4	4	24	0.838978734	0.95084077	24	0.250300271	0.008203911
ENSMUSG00000042050	Dync2i1	dynein 2 intermediate chain 1 [Source:MGI Symbol;Acc:MGI:2445085]	5.6869	5.87798	5.62241	5.74555	5.54663	4.78894	5.12163	5.48185	5.72743	5.34902	5.62686	5.62113	5.20901	5.45035	0.542718076	0.966018836	12	12	24	0.227589974	0.768040178	24	9.010259442	0.04351315
ENSMUSG00000042055	Wdr11	WD repeat domain 11 [Source:MGI Symbol;Acc:MGI:1920230]	14.0973	13.7511	13.7469	13.6611	14.1977	14.9107	12.4307	14.3443	12.9678	14.955	13.1678	13.1163	13.143	13.2078	0.912686312	0.966018836	16	12	24	0.58155585	0.850879883	24	14.32773067	0.027932917
ENSMUSG00000042063	Zfp386	zinc finger protein 386 (Kruppel-like) [Source:MGI Symbol;Acc:MGI:1930708]	16.456	20.0393	18.0649	16.7457	19.1661	16.8821	15.362	16.8917	20.9554	15.1355	18.7403	17.955	19.1566	19.4697	0.475540699	0.966018836	12	4	24	0.659282195	0.884879972	24	6.79722364	0.050129292
ENSMUSG00000042066	Tmcc2	transmembrane and coiled-coil domains 2 [Source:MGI Symbol;Acc:MGI:1916125]	16.9857	16.865	18.7907	17.5649	15.3501	17.6296	19.767	16.4124	15.7848	19.4455	15.2348	18.5078	18.3109	18.1035	0.745118544	0.966018836	4	16	24	0.687388224	0.89619155	24	2.144649467	0.038646557
ENSMUSG00000042073	Abhd14b	abhydrolase domain containing 14b [Source:MGI Symbol;Acc:MGI:1923741]	15.3477	17.2402	15.5528	15.9039	16.3762	16.1843	15.36	17.2117	16.6715	17.9789	15.9092	18.1885	16.526	16.6371	0.891105762	0.966018836	8	20	24	0.825737907	0.945421318	24	9.961769856	0.016834605
ENSMUSG00000042078	Svop	SV2 related protein [Source:MGI Symbol;Acc:MGI:1915916]	16.307	12.2423	13.594	13.1952	12.2491	16.4286	14.6036	13.9911	9.65143	19.333	11.5166	14.5685	13.5913	14.0251	0.290796297	0.79743194	24	20	24	0.592083646	0.856385053	24	20.80133714	0.096350859
ENSMUSG00000042079	Hnrnpf	heterogeneous nuclear ribonucleoprotein F [Source:MGI Symbol;Acc:MGI:2138741]	21.4913	20.2542	19.1765	19.1881	18.5139	19.0204	19.4293	18.1971	18.3388	17.8284	20.7583	18.2405	19.4885	17.9328	0.591351901	0.966018836	4	12	24	0.262545525	0.768040178	24	21.69642876	0.043331364
ENSMUSG00000042082	Arsb	arylsulfatase B [Source:MGI Symbol;Acc:MGI:88075]	11.9952	11.6462	13.1239	11.3778	10.367	13.0535	11.5732	12.4332	10.7894	13.7847	9.70842	12.1495	11.3764	12.9303	0.933421033	0.966018836	8	12	24	0.510234479	0.814051013	24	4.711503673	0.060152891
ENSMUSG00000042096	Dao	D-amino acid oxidase [Source:MGI Symbol;Acc:MGI:94859]	0.620181	0.932227	2.80413	0.8256	0.349908	1.61219	1.74096	0.466103	1.02523	0.940018	0.453247	1.01901	0.26861	1.69534	0.616047374	0.966018836	12	8	24	0.591700221	0.856116931	24	6.281494694	0.189130244
ENSMUSG00000042097	Zfp239	zinc finger protein 239 [Source:MGI Symbol;Acc:MGI:1306812]	4.82989	4.24979	4.12989	3.99869	4.65574	4.65448	3.99971	3.88197	3.93405	4.73027	3.22356	4.48863	3.87919	5.46572	0.768363194	0.966018836	24	20	24	0.818498263	0.942446688	24	1.070661994	0.038190051
ENSMUSG00000042099	Kank3	KN motif and ankyrin repeat domains 3 [Source:MGI Symbol;Acc:MGI:1098615]	11.9896	14.7455	14.6823	16.7542	15.5104	15.6466	13.8091	13.0859	16.7445	16.3496	13.8606	14.8128	12.6963	10.7379	0.00759188	0.192760771	16	12	24	0.016335016	0.544099157	24	13.38095968	0.164291706
ENSMUSG00000042104	Uggt2	UDP-glucose glycoprotein glucosyltransferase 2 [Source:MGI Symbol;Acc:MGI:1913685]	6.89666	8.93359	7.70004	9.76385	7.43369	7.04096	8.77858	7.83945	10.383	6.12714	8.49264	8.60745	9.94692	7.27098	0.869755137	0.966018836	12	8	24	0.832809588	0.948359912	24	5.439970998	0.048912568
ENSMUSG00000042105	Inpp5f	inositol polyphosphate-5-phosphatase F [Source:MGI Symbol;Acc:MGI:2141867]	36.3099	39.671	41.6403	37.282	35.6965	37.7076	35.7862	33.7289	36.4242	36.4706	35.8205	36.5492	36.2065	39.8079	0.708020493	0.966018836	12	8	24	0.452084153	0.805393068	24	7.157446475	0.038291359
ENSMUSG00000042106	Inka1	inka box actin regulator 1 [Source:MGI Symbol;Acc:MGI:1915426]	6.15796	5.04481	4.51098	3.01467	4.16644	4.7902	4.78038	5.07751	4.0158	3.68524	4.97736	4.43558	6.22352	5.09639	0.001925685	0.100255953	4	12	24	0.002123081	0.395705644	24	0.210545732	0.246680849
ENSMUSG00000042109	Csdc2	cold shock domain containing C2, RNA binding [Source:MGI Symbol;Acc:MGI:2146027]	17.4105	15.2632	17.1347	14.8224	14.7038	18.5109	17.2994	16.2913	13.875	20.6839	14.2315	16.8252	14.203	17.0638	0.907000607	0.966018836	24	20	24	0.943708683	0.986208939	24	22.09148215	0.022333144
ENSMUSG00000042111	Ccdc115	coiled-coil domain containing 115 [Source:MGI Symbol;Acc:MGI:1916918]	12.0478	11.3489	11.4387	11.0516	11.7922	11.3233	11.416	12.0202	10.943	11.0615	12.0811	12.0811	12.4829	11.3321	0.110778815	0.528348425	4	12	24	0.063385825	0.663715314	24	21.6857241	0.046482397
ENSMUSG00000042115	Klhdc8a	kelch domain containing 8A [Source:MGI Symbol;Acc:MGI:2442630]	5.86695	4.51354	4.7926	5.1409	4.52544	5.52083	5.05453	5.40799	4.1511	6.43158	4.86239	5.2962	5.15743	5.18672	0.601647512	0.966018836	24	12	24	0.702172033	0.901574817	24	20.18621815	0.047397077
ENSMUSG00000042116	Vwa1	von Willebrand factor A domain containing 1 [Source:MGI Symbol;Acc:MGI:2179729]	47.9349	37.504	35.7234	41.1293	53.0763	43.6912	48.5502	40.8921	40.7356	42.2712	52.8311	50.3328	46.6608	34.9126	5.26E-04	0.064393065	20	12	24	0.001161122	0.336500758	24	18.65757375	0.227827582
ENSMUSG00000042121	Ssh1	slingshot protein phosphatase 1 [Source:MGI Symbol;Acc:MGI:2686240]	6.96997	6.16227	5.79706	7.38764	7.71234	6.60883	7.34975	6.40729	7.14819	8.50026	7.58596	6.24249	6.55183	6.30928	0.025415961	0.29519482	16	16	24	0.065404975	0.666082155	24	14.64500788	0.111492419
ENSMUSG00000042129	Rassf4	Ras association (RalGDS/AF-6) domain family member 4 [Source:MGI Symbol;Acc:MGI:2386853]	19.6192	17.3427	17.5936	23.7492	19.8877	18.5887	18.0052	16.4624	17.687	19.3257	17.0311	18.5632	17.4581	16.5115	0.008483961	0.192760771	16	16	24	0.062947963	0.66222475	24	15.12655995	0.099938756
ENSMUSG00000042133	Ppig	peptidyl-prolyl isomerase G (cyclophilin G) [Source:MGI Symbol;Acc:MGI:2445173]	13.6755	15.0531	14.9481	14.8949	13.9066	12.8095	15.2915	13.0004	15.8368	14.4	17.4669	15.0224	15.1639	16.8217	0.890311217	0.966018836	12	12	24	0.53292049	0.825678486	24	9.208897209	0.047810976
ENSMUSG00000042138	Msantd2	Myb/SANT-like DNA-binding domain containing 2 [Source:MGI Symbol;Acc:MGI:2384579]	14.0289	14.1553	12.4189	15.5315	15.8864	13.9151	15.6841	11.8159	14.7582	11.8094	16.0116	11.968	12.9292	13.9492	0.961536114	0.967151979	20	12	24	0.558264983	0.839674578	24	16.72859544	0.06332751
ENSMUSG00000042148	Cox10	heme A:farnesyltransferase cytochrome c oxidase assembly factor 10 [Source:MGI Symbol;Acc:MGI:1917633]	5.65956	4.48113	4.8996	5.35544	4.66959	5.16901	4.94104	5.42663	4.68786	6.21131	4.21327	5.15585	5.21173	4.86047	0.858269825	0.966018836	4	16	24	0.996179961	0.999008571	24	3.088630319	0.004231624
ENSMUSG00000042155	Klhl23	kelch-like 23 [Source:MGI Symbol;Acc:MGI:2683536]	2.8772	4.38239	4.72911	3.79566	3.85981	3.50492	4.17247	4.38548	5.08111	3.88195	3.32089	4.54176	3.84136	3.7212	0.193864198	0.67239758	12	8	24	0.158066917	0.764630328	24	7.549679045	0.128593633
ENSMUSG00000042156	Dzip1	DAZ interacting protein 1 [Source:MGI Symbol;Acc:MGI:1914311]	17.0925	14.4194	15.5053	15.1103	14.7684	14.6512	17.5707	13.4771	16.7029	13.2797	16.4262	15.6205	14.6984	16.504	0.94663657	0.966018836	4	12	24	0.650435085	0.882429682	24	23.40459706	0.04329467
ENSMUSG00000042165	Gm9774	predicted pseudogene 9774 [Source:MGI Symbol;Acc:MGI:3642386]	22.9455	18.9266	20.434	20.1594	19.0211	21.4467	19.786	22.3813	19.4427	27.3284	21.9978	23.0951	21.03	22.5719	0.912686312	0.966018836	24	12	24	0.88855667	0.968671311	24	14.99224161	0.024379254
ENSMUSG00000042167	Tent2	terminal nucleotidyltransferase 2 [Source:MGI Symbol;Acc:MGI:2140950]	5.96122	6.53529	6.88458	5.98214	5.61336	5.47942	6.07209	5.75425	6.7112	5.57135	5.66041	5.19686	5.2846	5.16342	0.041292272	0.346472636	12	12	24	0.027868616	0.590904845	24	7.766962444	0.083191397
ENSMUSG00000042178	Armc5	armadillo repeat containing 5 [Source:MGI Symbol;Acc:MGI:2384586]	4.01519	3.44094	3.59051	3.86842	3.37292	3.71983	4.03224	4.07738	3.59929	5.29937	3.80788	3.84952	4.33148	3.68003	0.718086778	0.966018836	4	8	24	0.956121494	0.988639288	24	12.48975869	0.015030715
ENSMUSG00000042182	Bend6	BEN domain containing 6 [Source:MGI Symbol;Acc:MGI:2444572]	51.1104	57.037	60.8183	52.471	47.7205	54.731	55.9053	49.6979	56.3939	54.2804	52.0018	57.4207	54.1301	63.057	0.510431574	0.966018836	12	8	24	0.196336014	0.768040178	24	5.377793894	0.071926582
ENSMUSG00000042185	Nfrkb	nuclear factor related to kappa B binding protein [Source:MGI Symbol;Acc:MGI:2442410]	5.14577	6.34289	6.40373	6.12656	5.93997	5.66762	6.04044	6.33695	6.64237	6.91627	5.84028	6.33388	6.03556	6.19513	0.011156761	0.204559658	12	16	24	0.010206353	0.503967859	24	9.074535538	0.088035244
ENSMUSG00000042190	Cmklr1	chemokine-like receptor 1 [Source:MGI Symbol;Acc:MGI:109603]	1.4411	1.41497	1.22857	1.12552	1.29395	1.11454	1.45938	1.49213	1.69445	1.0382	1.31754	0.960603	0.898521	1.26703	0.368981219	0.893596186	8	16	24	0.279726669	0.768040178	24	5.660585917	0.083985056
ENSMUSG00000042195	Slc35f2	solute carrier family 35, member F2 [Source:MGI Symbol;Acc:MGI:1919272]	3.78647	3.6112	3.37224	3.5758	3.64031	3.42779	2.93005	3.41964	3.11793	3.43048	2.96977	3.44878	3.76291	3.67637	0.85447369	0.966018836	4	8	24	0.755753777	0.921118175	24	1.208488325	0.026557074
ENSMUSG00000042197	Zfp451	zinc finger protein 451 [Source:MGI Symbol;Acc:MGI:2137896]	9.94561	10.4216	9.66001	9.34279	9.32248	9.83044	9.33538	9.66209	9.14665	9.46257	9.93066	9.07239	9.35482	9.16997	0.846563228	0.966018836	8	4	24	0.736592417	0.914180155	24	0.062076529	0.013170862
ENSMUSG00000042198	Chchd7	coiled-coil-helix-coiled-coil-helix domain containing 7 [Source:MGI Symbol;Acc:MGI:1913683]	25.7818	26.2824	30.5292	29.7246	27.8076	25.2192	25.9199	30.3451	31.1248	26.7029	31.3074	29.0146	29.4831	28.598	0.124211791	0.552144108	12	12	24	0.108335841	0.72082227	24	9.774893226	0.078088637
ENSMUSG00000042200	Cdrt4	CMT1A duplicated region transcript 4 [Source:MGI Symbol;Acc:MGI:1913588]	1.06728	1.9142	1.70126	0.731617	1.90936	1.0055	1.47378	1.58851	1.69784	0.940444	1.60953	1.45638	1.85265	0.855606	0.921741413	0.966018836	12	4	24	0.84445291	0.952844769	24	1.359014027	0.060164044
ENSMUSG00000042202	Slc35e2	solute carrier family 35, member E2 [Source:MGI Symbol;Acc:MGI:2444240]	7.1624	6.2593	6.85835	7.2698	6.58538	6.71824	7.4628	6.43264	6.67832	7.14351	7.27836	6.85628	7.01085	7.10302	0.708020493	0.966018836	4	4	24	0.679100145	0.892648327	24	17.11640735	0.022308056
ENSMUSG00000042203	Tbc1d22b	TBC1 domain family, member 22B [Source:MGI Symbol;Acc:MGI:2681867]	5.71445	4.82902	5.54569	6.05236	5.11603	5.64724	5.42643	6.17246	4.93611	6.73154	5.02931	5.64064	5.38912	5.7595	0.895137379	0.966018836	16	4	24	0.840394483	0.951645964	24	10.68628882	0.027162837
ENSMUSG00000042207	Kdm5b	lysine (K)-specific demethylase 5B [Source:MGI Symbol;Acc:MGI:1922855]	7.48589	5.59105	6.0257	5.86158	6.62328	6.68055	6.3717	5.66652	5.50461	6.09658	5.97852	6.48287	5.92562	6.84589	0.220555749	0.710961211	24	12	24	0.095411011	0.71175955	24	20.76979304	0.089873753
ENSMUSG00000042208	Sanbr	SANT and BTB domain regulator of CSR [Source:MGI Symbol;Acc:MGI:1918925]	32.7988	33.5941	33.1838	32.3083	33.9706	32.3247	31.0099	29.5376	32.2974	30.6038	37.4466	33.5833	34.1927	34.2388	0.911685024	0.966018836	20	20	24	0.504156477	0.810754871	24	17.40074916	0.037271148
ENSMUSG00000042210	Abhd14a	abhydrolase domain containing 14A [Source:MGI Symbol;Acc:MGI:1915894]	24.2505	21.9184	19.6613	24.3998	22.2982	22.8925	23.6754	21.1569	21.3458	23.3227	20.208	21.7406	22.4035	20.2762	0.287843582	0.793714531	4	8	24	0.352575368	0.780629136	24	19.6135835	0.051563887
ENSMUSG00000042211	Fbxo38	F-box protein 38 [Source:MGI Symbol;Acc:MGI:2444639]	13.3762	13.4181	11.988	13.735	12.426	13.8027	12.2653	12.4497	12.6684	14.0074	12.7952	14.0312	14.2965	13.8079	0.8972237	0.966018836	24	12	24	0.684663901	0.895095838	24	20.35342138	0.026355272
ENSMUSG00000042213	Zfand4	zinc finger, AN1-type domain 4 [Source:MGI Symbol;Acc:MGI:1914742]	0.962666	1.63323	1.61263	1.44855	0.874648	0.96269	1.1892	1.10543	1.26784	0.96722	0.874143	1.05179	0.993265	1.55645	0.004301626	0.140598674	12	8	24	0.009828909	0.503967859	24	6.432519643	0.1837351
ENSMUSG00000042215	Bag2	BCL2-associated athanogene 2 [Source:MGI Symbol;Acc:MGI:1891254]	14.881	10.8557	11.3083	11.8935	10.3529	12.4095	11.8212	12.5549	9.29605	13.2383	11.7406	13.6867	12.937	10.7839	0.124211791	0.552144108	24	12	24	0.160849943	0.765941695	24	21.06275972	0.121488133
ENSMUSG00000042216	Sgsm1	small G protein signaling modulator 1 [Source:MGI Symbol;Acc:MGI:107320]	11.1765	8.92465	9.23402	9.74014	8.50427	11.1887	10.7427	10.2676	7.94194	12.5435	8.40998	10.0765	8.73511	10.679	0.636884864	0.966018836	24	12	24	0.778458284	0.928832041	24	22.53221159	0.050565797
ENSMUSG00000042225	Ammecr1	Alport syndrome, mental retardation, midface hypoplasia and elliptocytosis chromosomal region gene 1 [Source:MGI Symbol;Acc:MGI:1860206]	1.01227	1.07313	1.12649	1.19567	1.15949	0.982468	1.11894	1.1403	1.1508	1.01503	1.31722	1.08848	0.925008	1.07921	0.036657636	0.332441566	20	8	24	0.167680764	0.767020009	24	12.28924363	0.047763307
ENSMUSG00000042228	Lyn	LYN proto-oncogene, Src family tyrosine kinase [Source:MGI Symbol;Acc:MGI:96892]	3.05324	3.2543	3.84549	3.39779	3.42401	3.24111	3.01729	3.55383	3.55758	3.32697	3.15372	3.0043	2.83285	2.82904	0.004501001	0.140598674	12	16	24	0.010571341	0.508211602	24	10.42522119	0.088462671
ENSMUSG00000042229	Rabif	RAB interacting factor [Source:MGI Symbol;Acc:MGI:2138605]	21.477	17.9624	18.5222	18.5547	18.6982	20.1339	18.51	17.8453	16.3644	19.5696	19.3897	18.7199	19.4934	19.4427	0.130696419	0.562427886	24	12	24	0.112737011	0.727075283	24	20.26793331	0.069922825
ENSMUSG00000042246	Tmc7	transmembrane channel-like gene family 7 [Source:MGI Symbol;Acc:MGI:2443317]	5.69978	4.76832	5.50131	4.98297	4.79267	5.51108	5.3426	4.89676	4.11545	5.4604	5.0233	4.37082	4.16591	4.45028	0.866861748	0.966018836	4	4	24	0.599371299	0.859498784	24	17.569322	0.042464424

ENSMUSG00000042249	Grk3	G protein-coupled receptor kinase 3 [Source:MGI Symbol;Acc:MGI:87941]	5.95812	6.20938	7.51903	7.70754	6.15829	6.97913	6.5716	6.13519	6.14813	6.65918	6.65207	7.06283	6.49222	9.1093	0.914360142	0.966018836	16	12	24	0.662119198	0.885885739	24	8.921976148	0.052026963
ENSMUSG00000042254	Cilp	cartilage intermediate layer protein, nucleotide pyrophosphohydrolase [Source:MGI Symbol;Acc:MGI:2444507]	1.48712	1.89833	1.51092	1.76342	0.852148	1.13965	1.37011	2.16722	1.57562	1.80691	1.02816	1.16933	1.60481	1.59631	0.001765212	0.100255953	8	12	24	0.004319864	0.427431763	24	5.886091623	0.234774208
ENSMUSG00000042258	Isl1	ISL1 transcription factor, LIM/homeodomain [Source:MGI Symbol;Acc:MGI:101791]	40.1758	33.7922	31.0033	34.5536	32.7788	35.4588	32.1093	32.9004	31.6443	36.9121	37.1186	34.8166	35.6057	38.1849	0.46238066	0.966018836	24	12	24	0.520005989	0.819667105	24	20.56200979	0.048961707
ENSMUSG00000042269	Cibar2	CBY1 interacting BAR domain containing 2 [Source:MGI Symbol;Acc:MGI:3588213]	0.170374	0.598015	0.463805	0.580175	0.552307	0.354184	0.425903	0.459625	0.344422	0.499152	0.841678	0.518584	0.434293	0.76122	0.223774686	0.717349224	20	8	24	0.325889532	0.768040178	24	11.94380434	0.08521045
ENSMUSG00000042271	Nxt2	nuclear transport factor 2-like export factor 2 [Source:MGI Symbol;Acc:MGI:2147914]	19.156	21.0803	20.6389	19.8235	18.6039	17.6828	20.3415	17.8002	20.5654	16.0717	20.3317	17.6704	17.5154	18.4193	0.245061943	0.741580974	12	12	24	0.613953572	0.866235709	24	6.586838129	0.039969607
ENSMUSG00000042272	Sestd1	SEC14 and spectrin domains 1 [Source:MGI Symbol;Acc:MGI:1916262]	17.224	17.4388	17.4448	17.7745	17.2946	16.9795	15.4297	15.8091	16.3961	17.0782	16.3002	17.3383	17.9056	17.523	0.804027134	0.966018836	16	4	24	0.889748505	0.968953708	24	13.15954739	0.01168369
ENSMUSG00000042275	Pelo	pelota mRNA surveillance and ribosome rescue factor [Source:MGI Symbol;Acc:MGI:2145154]	5.44072	4.48203	4.86926	4.82229	4.73026	4.96758	5.49303	5.43327	5.2139	6.1469	5.29131	5.31085	5.29402	5.5156	0.759899911	0.966018836	4	16	24	0.959820892	0.989375125	24	4.006170315	0.009321566
ENSMUSG00000042284	Itga1	integrin alpha 1 [Source:MGI Symbol;Acc:MGI:96599]	2.19887	2.54682	2.56993	2.74472	2.56842	2.41755	2.64231	2.47333	2.64211	2.48527	2.99338	2.77315	2.6512	3.06884	0.823246279	0.966018836	20	8	24	0.485231601	0.805393068	24	11.06853492	0.031902784
ENSMUSG00000042286	Stab1	stabilin 1 [Source:MGI Symbol;Acc:MGI:2178742]	10.8525	11.5881	11.0653	10.3879	16.0323	11.6844	11.6655	10.3667	17.5385	8.47488	14.7529	9.94581	11.4303	5.9449	0.702630284	0.966018836	20	12	24	0.439074858	0.805393068	24	16.03243311	0.168641818
ENSMUSG00000042289	Hsd3b7	hydroxy-delta-5-steroid dehydrogenase, 3 beta- and steroid delta-isomerase 7 [Source:MGI Symbol;Acc:MGI:2141879]	9.13091	9.51153	8.50573	10.096	11.5948	8.63992	9.72226	9.79636	10.9578	9.65068	10.0696	8.34365	9.43393	10.1555	0.125573493	0.555618842	20	4	24	0.382760376	0.799546792	24	10.90181022	0.065875642
ENSMUSG00000042292	Mrtfa	myocardin related transcription factor A [Source:MGI Symbol;Acc:MGI:2384495]	4.54969	4.16513	5.51839	4.13044	3.92883	4.84698	4.27655	4.28452	4.84029	6.79394	4.29089	5.30676	5.43664	4.37635	0.896773941	0.966018836	12	20	24	0.758355863	0.921856281	24	10.90501653	0.052511171
ENSMUSG00000042293	Gm5617	predicted gene 5617 [Source:MGI Symbol;Acc:MGI:3643566]	2.82737	2.91971	4.84093	3.20913	3.94468	3.23509	3.43717	4.44746	5.10101	4.81732	5.11699	5.734	7.1427	6.77351	0.766867092	0.966018836	12	4	24	0.291990204	0.768040178	24	7.602337678	0.10926395
ENSMUSG00000042298	Ttc19	tetratricopeptide repeat domain 19 [Source:MGI Symbol;Acc:MGI:1920045]	18.5603	18.2296	18.5124	17.5247	17.7281	18.4308	18.1243	17.9036	15.9564	17.645	17.2922	18.0096	19.2702	18.7346	0.021406186	0.26465279	4	12	24	0.072238182	0.679008169	24	23.63149471	0.051407915
ENSMUSG00000042302	Ehbp1	EH domain binding protein 1 [Source:MGI Symbol;Acc:MGI:2667252]	23.301	21.2678	19.2542	21.0567	21.9616	21.0929	22.5644	20.592	18.5941	21.3019	20.2109	21.9997	20.7915	20.5372	0.016095537	0.253395174	4	8	24	0.015867209	0.544099157	24	20.88301968	0.080703644
ENSMUSG00000042303	Sgsm3	small G protein signaling modulator 3 [Source:MGI Symbol;Acc:MGI:1916329]	14.5833	10.8216	13.7519	12.072	12.9295	14.2206	13.8914	14.6825	10.6069	13.8879	11.255	13.2689	11.5189	11.6779	0.835401638	0.966018836	4	8	24	0.490020927	0.805393068	24	20.79619792	0.071969035
ENSMUSG00000042305	Tmem183a	transmembrane protein 183A [Source:MGI Symbol;Acc:MGI:1914729]	32.454	28.3243	29.7715	28.5445	30.8555	30.3026	30.8276	29.1643	25.5951	30.7065	32.6216	31.4943	32.1696	30.4147	0.027439001	0.308438477	4	8	24	0.028512254	0.593964798	24	20.04835135	0.086080418
ENSMUSG00000042308	Setd1a	SET domain containing 1A [Source:MGI Symbol;Acc:MGI:2446244]	6.55287	6.9651	7.17266	6.73003	6.67694	6.95263	6.56105	6.56239	7.1955	8.405	6.34696	7.0631	6.95545	7.09769	0.085992778	0.466404237	12	8	24	0.234934263	0.768040178	24	9.84211219	0.056218364
ENSMUSG00000042312	S100a13	S100 calcium binding protein A13 [Source:MGI Symbol;Acc:MGI:109581]	120.055	127.811	123.022	111.903	137.897	111.873	109.063	117.126	126.63	92.51	140.324	113.843	134.632	125.317	0.93717134	0.966018836	20	20	24	0.909361907	0.974606327	24	23.60084278	0.027924462
ENSMUSG00000042320	Prox2	prospero homeobox 2 [Source:MGI Symbol;Acc:MGI:1920672]	1.66755	2.08593	2.04012	2.87945	1.61666	2.1966	2.21743	2.74702	2.69449	2.65127	2.3906	2.09056	2.50621	2.07607	0.85447369	0.966018836	16	4	24	0.180690316	0.768040178	24	9.23322111	0.117681814
ENSMUSG00000042323	Pbrm1	polybromo 1 [Source:MGI Symbol;Acc:MGI:1923998]	16.0893	17.5379	16.6707	17.6188	17.5242	17.2844	15.6898	14.9035	16.8593	15.6291	17.3055	17.4643	17.0549	18.2915	0.935593607	0.966018836	20	8	24	0.717222054	0.906810602	24	15.82953252	0.025203908
ENSMUSG00000042328	Hps4	HPS4, biogenesis of lysosomal organelles complex 3 subunit 2 [Source:MGI Symbol;Acc:MGI:2177742]	7.11636	6.21014	6.25373	6.48461	7.07146	6.57841	8.50516	8.02317	6.34898	6.20059	6.69435	5.91189	6.87539	6.28078	0.235141005	0.73629392	4	8	24	0.313982405	0.768040178	24	23.69409299	0.076138917
ENSMUSG00000042331	Specc1	sperm antigen with calponin homology and coiled-coil domains 1 [Source:MGI Symbol;Acc:MGI:2442356]	2.84265	3.83724	5.82692	3.37337	3.27612	4.28508	4.93645	3.44415	4.17155	3.6992	2.74166	3.85305	3.26894	4.44028	0.424718357	0.966018836	12	8	24	0.360135783	0.785733735	24	5.927591349	0.136167016
ENSMUSG00000042333	Tnfrsf14	tumor necrosis factor receptor superfamily, member 14 (herpesvirus entry mediator) [Source:MGI Symbol;Acc:MGI:2675303]	0.999202	1.69465	2.50668	1.71732	1.50414	1.37016	1.8027	1.58994	1.40048	1.5356	1.37189	2.01417	1.13827	1.66292	0.881718085	0.966018836	12	16	24	0.445108656	0.805393068	24	9.241263545	0.104075612
ENSMUSG00000042340	Ctf1	cardiotrophin 1 [Source:MGI Symbol;Acc:MGI:105115]	1.7407	1.82137	1.66969	1.56341	0.953306	1.21065	1.95086	2.07734	2.04371	1.48486	2.07027	1.71731	1.63885	1.24937	0.50659994	0.966018836	8	8	24	0.426675753	0.805393068	24	4.65814502	0.101023079
ENSMUSG00000042348	Arl15	ADP-ribosylation factor-like 15 [Source:MGI Symbol;Acc:MGI:2442308]	9.88966	9.80033	10.0168	9.63322	9.29387	10.5081	8.55541	8.91583	7.86382	9.08831	9.55895	9.85671	10.1258	10.1958	0.800065124	0.966018836	24	16	24	0.417552654	0.80230263	24	20.93179517	0.054803951
ENSMUSG00000042349	Ikbke	inhibitor of kappaB kinase epsilon [Source:MGI Symbol;Acc:MGI:1929612]	1.87608	2.22661	1.57622	1.56753	1.89162	1.6182	2.28921	2.26679	1.95135	1.47082	1.99423	1.63387	1.8223	1.5733	0.371875382	0.894537464	4	12	24	0.245774749	0.768040178	24	1.267461055	0.087415711
ENSMUSG00000042350	Arel1	apoptosis resistant E3 ubiquitin protein ligase 1 [Source:MGI Symbol;Acc:MGI:1915747]	19.3718	16.1279	17.9151	18.0476	16.7915	19.5954	17.3742	17.6978	14.2833	20.4034	16.2382	17.2069	16.4558	19.0138	0.888482515	0.966018836	16	20	24	0.843809382	0.952738483	24	19.45327374	0.03133633
ENSMUSG00000042354	Gnl3	guanine nucleotide binding protein-like 3 (nucleolar) [Source:MGI Symbol;Acc:MGI:1353651]	21.3953	22.5838	23.0103	20.1885	23.8507	21.6782	20.8016	22.5476	23.2314	20.4129	22.4545	22.3972	22.2078	24.6206	0.268085731	0.774280302	12	4	24	0.607089807	0.863728561	24	5.081665054	0.030682288
ENSMUSG00000042357	Gjb5	gap junction protein, beta 5 [Source:MGI Symbol;Acc:MGI:95723]	0.330677	0.595375	0.442855	0.527364	0.650179	0.53665	0.279712	0.479638	0.974613	0.470412	0.579088	0.40413	0.388745	0.528357	0.050258003	0.385784509	20	8	24	0.11076001	0.722405302	24	10.59536721	0.120375525
ENSMUSG00000042359	Osbpl6	oxysterol binding protein-like 6 [Source:MGI Symbol;Acc:MGI:2139014]	10.5473	8.90707	9.29473	9.71218	7.9183	9.3164	9.55893	8.90574	8.1014	11.5122	7.92581	10.3955	9.94808	10.8358	0.846840613	0.966018836	4	16	24	0.702634738	0.901733679	24	1.862801902	0.049429154
ENSMUSG00000042363	Lgalsl	lectin, galactoside binding-like [Source:MGI Symbol;Acc:MGI:1916114]	16.9964	16.617	17.7697	20.2375	17.0637	17.6895	17.051	16.6487	17.7795	17.1602	17.1008	16.971	16.0998	16.2866	0.00169935	0.100255953	12	20	24	0.041249595	0.618621321	24	12.78569185	0.061103903
ENSMUSG00000042364	Snx18	sorting nexin 18 [Source:MGI Symbol;Acc:MGI:2137642]	14.3386	13.4163	11.788	12.534	13.9247	13.2966	14.9372	13.118	11.4244	13.2239	12.7267	12.9955	13.2238	12.4167	0.004501001	0.140598674	4	8	24	0.012968594	0.528286959	24	21.36930834	0.095707803
ENSMUSG00000042369	Rbm45	RNA binding motif protein 45 [Source:MGI Symbol;Acc:MGI:2387367]	10.4354	10.0634	10.0886	10.3148	10.3394	10.4866	10.1851	9.45507	9.81354	9.60682	10.779	10.1254	10.5424	10.6641	0.304483943	0.811201458	20	16	24	0.212101366	0.768040178	24	19.81886747	0.034833183
ENSMUSG00000042379	Esm1	endothelial cell-specific molecule 1 [Source:MGI Symbol;Acc:MGI:1918940]	1.10589	0.948513	0.936057	0.613837	0.970854	0.701307	0.9261	0.956779	0.841044	0.500438	0.7069	0.832362	0.802457	0.622014	0.305564675	0.811201458	4	12	24	0.064873291	0.663876076	24	0.405697161	0.098630616
ENSMUSG00000042380	Smim12	small integral membrane protein 12 [Source:MGI Symbol;Acc:MGI:1933141]	39.3581	33.9208	33.4983	34.8691	32.1064	35.7232	33.2976	37.9449	31.2135	37.002	35.0267	37.025	36.5878	35.7553	0.397301892	0.927993503	4	8	24	0.361319781	0.786774635	24	22.82313006	0.051184367
ENSMUSG00000042388	Dlgap3	DLG associated protein 3 [Source:MGI Symbol;Acc:MGI:3039563]	6.34233	4.79181	4.75418	4.8957	4.57989	4.55574	4.84409	6.58217	4.78111	9.10434	6.05944	7.42199	6.57145	6.84591	0.891742979	0.966018836	8	4	24	0.992049178	0.998516607	24	5.163206745	0.011529549
ENSMUSG00000042389	Tsen2	tRNA splicing endonuclease subunit 2 [Source:MGI Symbol;Acc:MGI:2141599]	7.98391	7.31116	8.48274	6.47267	6.85491	7.86881	6.60341	7.04632	7.03299	7.06105	7.1156	7.01133	7.5471	7.04099	0.933543173	0.966018836	4	12	24	0.652369484	0.88299052	24	1.3198958	0.032850392
ENSMUSG00000042390	Gatad2b	GATA zinc finger domain containing 2B [Source:MGI Symbol;Acc:MGI:2443225]	8.67766	8.37794	8.70809	8.83009	8.40393	9.12068	9.03399	8.53002	8.44883	9.83456	8.37066	8.85749	7.92108	8.86484	0.890176313	0.966018836	24	20	24	0.62275598	0.871448778	24	13.36233839	0.025362098
ENSMUSG00000042396	Rbm7	RNA binding motif protein 7 [Source:MGI Symbol;Acc:MGI:1914260]	20.3858	25.8526	24.9976	25.8946	22.8469	19.7379	21.8792	22.7249	24.5201	19.2185	22.9112	20.031	18.3069	21.663	0.071575474	0.440262583	12	12	24	0.045947058	0.632654269	24	9.028528912	0.123896758
ENSMUSG00000042401	Crtac1	cartilage acidic protein 1 [Source:MGI Symbol;Acc:MGI:1920082]	8.06108	7.71743	9.15944	7.86245	8.43013	10.162	8.05882	7.13791	5.62675	10.0731	7.70912	7.22966	5.96091	7.99626	0.917867465	0.966018836	16	16	24	0.389449955	0.799546792	24	15.91259604	0.107420375
ENSMUSG00000042404	Dennd4b	DENN/MADD domain containing 4B [Source:MGI Symbol;Acc:MGI:2446201]	4.92113	5.23869	5.67061	5.83947	5.30133	5.38667	5.36481	6.54056	6.33095	7.49222	4.91983	6.40398	6.23829	5.90866	0.954744729	0.966018836	16	4	24	0.179929198	0.768040178	24	8.846238196	0.093455257
ENSMUSG00000042406	Atf4	activating transcription factor 4 [Source:MGI Symbol;Acc:MGI:88096]	93.6368	77.7195	79.2968	80.7978	86.8694	87.4399	84.5742	84.0138	79.2726	82.7052	93.2509	83.5629	90.5443	84.7912	0.016795343	0.253395174	4	8	24	0.030982555	0.59941366	24	20.1697326	0.079063079
ENSMUSG00000042408	Zmym6	zinc finger, MYM-type 6 [Source:MGI Symbol;Acc:MGI:106505]	13.963	12.8408	11.7928	11.6706	15.0349	13.1837	14.8408	12.2947	15.1416	10.3375	14.9372	13.412	11.352	11.8296	0.265774856	0.773510818	20	20	24	0.375534245	0.795627983	24	19.7492816	0.091379134
ENSMUSG00000042410	Agps	alkylglycerone phosphate synthase [Source:MGI Symbol;Acc:MGI:2443065]	18.9593	16.7839	15.8038	16.3465	16.8096	15.5023	17.964	14.5925	16.5339	14.5211	16.7067	16.2119	16.4115	14.9122	0.588208558	0.966018836	4	12	24	0.151396231	0.758865539	24	21.38853493	0.067811894
ENSMUSG00000042419	Nfkbil1	nuclear factor of kappa light polypeptide gene enhancer in B cells inhibitor like 1 [Source:MGI Symbol;Acc:MGI:1340031]	3.54257	3.78721	3.5304	3.1634	2.60805	3.65811	3.64489	4.33374	4.01044	4.73384	4.4274	4.94559	5.11063	4.58636	0.882263435	0.966018836	24	20	24	0.26951495	0.768040178	24	3.024016489	0.083721065
ENSMUSG00000042423	Fbrs	fibrosin [Source:MGI Symbol;Acc:MGI:104648]	15.7614	15.1459	14.6385	13.7003	17.9148	15.6288	18.2348	15.8704	16.5794	18.6704	16.3822	15.7489	14.408	15.2933	0.910436409	0.966018836	20	12	24	0.649008468	0.881754555	24	16.4010857	0.045217259
ENSMUSG00000042425	Frmpd3	FERM and PDZ domain containing 3 [Source:MGI Symbol;Acc:MGI:3646547]	1.86319	1.9261	2.17421	2.04114	1.56276	2.10848	2.18723	2.03596	1.72485	2.87309	1.68169	2.27097	1.96347	2.3266	0.865490576	0.966018836	16	4	24	0.769292605	0.925619883	24	6.970098593	0.043626939
ENSMUSG00000042426	Dhx29	DEAH (Asp-Glu-Ala-His) box polypeptide 29 [Source:MGI Symbol;Acc:MGI:2145374]	9.00663	7.78326	8.57202	7.97794	8.28002	8.81844	8.958	8.06521	6.58434	8.86636	8.54115	8.60109	8.66204	9.12283	0.16456443	0.628002127	4	8	24	0.187824671	0.768040178	24	20.98131063	0.077265698
ENSMUSG00000042428	Mgat3	mannoside acetylglucosaminyltransferase 3 [Source:MGI Symbol;Acc:MGI:104532]	38.7703	31.4136	30.0497	32.5689	34.7833	35.1381	36.7701	33.6646	28.9264	41.1898	30.3873	35.7823	35.1114	32.7584	0.009640466	0.194720062	4	8	24	0.249090828	0.768040178	24	20.40705561	0.093197107
ENSMUSG00000042429	Adora1	adenosine A1 receptor [Source:MGI Symbol;Acc:MGI:99401]	23.985	18.1857	21.7629	18.9152	18.4043	24.2576	21.1221	21.5772	15.4387	25.6927	17.956	21.3429	20.4906	20.8908	0.94693335	0.966018836	24	20	24	0.540012261	0.829752479	24	21.7084687	0.085502562
ENSMUSG00000042436	Mfap4	microfibrillar-associated protein 4 [Source:MGI Symbol;Acc:MGI:1342276]	7.70236	8.59712	7.1409	6.19811	5.86114	5.4828	6.73982	11.9862	10.5769	2.84241	4.74396	5.24442	4.69047	5.46873	0.101894436	0.512388497	12	4	24	0.066804282	0.669146244	24	4.268256408	0.344306404
ENSMUSG00000042439	Zfp532	zinc finger protein 532 [Source:MGI Symbol;Acc:MGI:3036282]	11.4208	10.4102	9.50005	9.51896	12.0215	9.84205	10.6378	9.61263	10.2872	10.5709	10.3894	9.65898	10.3034	8.86448	0.349109869	0.870418653	4	4	24	0.241886245	0.768040178	24	18.46155385	0.064200271
ENSMUSG00000042444	Mindy2	MINDY lysine 48 deubiquitinase 2 [Source:MGI Symbol;Acc:MGI:2443086]	23.4955	22.9846	21.9841	23.7285	24.032	22.2888	23.5045	20.5035	22.3517	21.7078	24.8514	22.8714	23.2441	23.0165	0.266025655	0.773510818	20	16	24	0.176671041	0.768040178	24	18.01741781	0.048017758
ENSMUSG00000042446	Zmym4	zinc finger, MYM-type 4 [Source:MGI Symbol;Acc:MGI:1915035]	10.3539	12.1792	12.4587	11.3787	11.7132	11.9028	14.2672	12.0465	12.3832	11.1498	11.6549	12.8586	12.0143	13.772	0.111887343	0.530777648	12	4	24	0.322743769	0.768040178	24	3.117021463	0.057193554
ENSMUSG00000042447	Mios	meiosis regulator for oocyte development [Source:MGI Symbol;Acc:MGI:2182066]	6.48326	7.62592	7.61105	7.16633	7.22129	6.56807	6.93052	6.90903	7.94208	6.53836	7.11096	7.50565	7.21243	7.36915	0.416857905	0.963884303	12	16	24	0.262049458	0.768040178	24	7.723470106	0.050792261
ENSMUSG00000042448	Hoxd1	homeobox D1 [Source:MGI Symbol;Acc:MGI:96201]	7.92875	4.9486	4.23786	5.7233	5.1563	6.01269	5.87026	5.90635	4.11719	7.30049	6.13126	6.47437	7.62205	6.05122	0.051446973	0.38924256	4	8	24	0.124900338	0.738693118	24	21.02159681	0.181599428
ENSMUSG00000042451	Mybph	myosin binding protein H [Source:MGI Symbol;Acc:MGI:1858196]	0.724953	0.888398	0.647623	0.957335	0.875398	0.78775	1.15303	3.00571	1.74657	1.31743	0.67112	0.730384	0.772942	0.421898	0.896545154	0.966018836	16	4	24	0.495936443	0.807537659	24	7.126110299	0.182241181
ENSMUSG00000042453	Reln	reelin [Source:MGI Symbol;Acc:MGI:103022]	41.2448	43.7864	39.959	49.5316	47.8828	42.2035	47.9132	40.2911	42.1389	46.4048	40.1435	39.3136	35.2384	34.2282	0.688644847	0.966018836	16	16	24	0.081377076	0.697102286	24	14.39142956	0.103283575
ENSMUSG00000042460	C1galt1	core 1 synthase, glycoprotein-N-acetylgalactosamine 3-beta-galactosyltransferase, 1 [Source:MGI Symbol;Acc:MGI:2151071]	6.1891	7.23199	6.99923	7.26814	7.52211	6.56442	6.36328	6.5301	7.19287	6.01128	7.7131	5.93915	6.38231	6.57497	0.026194135	0.300938431	20	8	24	0.21041243	0.768040178	24	11.85506811	0.068390652
ENSMUSG00000042462	Dctpp1	dCTP pyrophosphatase 1 [Source:MGI Symbol;Acc:MGI:1913672]	12.2038	14.3178	13.5145	14.4588	14.0628	11.9365	11.0765	15.0514	16.526	11.4565	15.5978	13.437	15.5276	13.7546	0.777666236	0.966018836	12	12	24	0.430421763	0.805393068	24	8.652750476	0.084280551
ENSMUSG00000042472	Zfp410	zinc finger protein 410 [Source:MGI Symbol;Acc:MGI:1289280]	20.536	20.2828	17.9441	20.0839	21.3198	20.8665	20.2854	21.2224	20.2083	20.0177	22.0779	20.4105	20.1095	19.398	0.01891286	0.256345714	20	20	24	0.113606834	0.727440321	24	18.65816197	0.052616583
ENSMUSG00000042473	Tbc1d8b	TBC1 domain family, member 8B [Source:MGI Symbol;Acc:MGI:1918101]	2.93547	4.16973	3.02973	3.52133	3.61748	3.13395	4.02208	3.36297	4.14323	2.89168	3.84379	3.29983	4.01106	3.59383	0.92721845	0.966018836	8	8	24	0.714790977	0.906007311	24	3.621565792	0.045879189
ENSMUSG00000042474	Fcmr	Fc fragment of IgM receptor [Source:MGI Symbol;Acc:MGI:1916419]	0.363208	0.547016	0.841855	0.688064	0.311019	0.716994	0.574979	0.371841	1.10977	0.419432	0.541829	0.570683	0.900969	0.98051	0.424718357	0.966018836	12	8	24	0.257795121	0.768040178	24	6.45379042	0.129594492
ENSMUSG00000042476	Abcb4	ATP-binding cassette, sub-family B (MDR/TAP), member 4 [Source:MGI Symbol;Acc:MGI:97569]	3.29449	3.3419	3.86377	4.50028	3.80154	3.70215	3.073	4.12907	4.07682	4.88239	3.05593	3.71698	3.78467	3.21597	0.054935173	0.398010585	16	12	24	0.068127695	0.672292246	24	11.13439939	0.135870641
ENSMUSG00000042485	Mustn1	musculoskeletal, embryonic nuclear protein 1 [Source:MGI Symbol;Acc:MGI:1913425]	6.10248	8.06839	6.63828	7.60958	6.21481	6.48105	6.23165	9.02038	8.64653	6.66975	7.56567	6.45064	7.77511	6.1935	0.446277988	0.966018836	12	16	24	0.236391523	0.768040178	24	7.402754512	0.114472317
ENSMUSG00000042487	Leo1	Leo1, Paf1/RNA polymerase II complex component [Source:MGI Symbol;Acc:MGI:2685031]	12.0564	9.95551	10.3388	9.84401	9.60655	13.3639	11.894	11.9804	8.29972	12.2334	10.0324	12.3557	12.5386	12.696	0.106193509	0.519440352	24	20	24	0.057199642	0.656248849	24	22.72290835	0.153269797
ENSMUSG00000042492	Tbc1d10b	TBC1 domain family, member 10b [Source:MGI Symbol;Acc:MGI:1915699]	17.4789	17.4629	19.1183	16.1377	19.8414	19.7655	17.4237	21.4207	17.6323	20.301	17.2905	20.441	17.268	18.479	0.838244962	0.966018836	24	4	24	0.957189043	0.988639288	24	17.0818881	0.01377455
ENSMUSG00000042496	Prdm10	PR domain containing 10 [Source:MGI Symbol;Acc:MGI:2682952]	4.30028	3.39599	3.54254	3.44963	3.86892	3.854	4.16964	3.25972	3.18931	3.40143	4.08004	4.08731	3.418	3.58575	0.039486348	0.338535904	4	8	24	0.004114892	0.427431763	24	19.90874662	0.124615537
ENSMUSG00000042498	Radx	RPA1 related single stranded DNA binding protein, X-linked [Source:MGI Symbol;Acc:MGI:2147848]	0.586855	0.738466	0.602486	0.894992	0.867815	0.626676	0.717563	0.587445	0.948056	0.665203	0.589373	0.625005	0.609664	0.580779	0.45229395	0.966018836	16	16	24	0.217466714	0.768040178	24	11.76048172	0.067590191
ENSMUSG00000042499	Hoxd11	homeobox D11 [Source:MGI Symbol;Acc:MGI:96203]	0.31016	0.763107	1.17047	0.946938	0.591305	0.864801	0.873682	0.458502	0.955461	1.11747	0.453485	1.03283	0.513983	0.785835	0.141084603	0.585944911	12	16	24	0.244468428	0.768040178	24	10.58924733	0.148252248
ENSMUSG00000042500	Ago4	argonaute RISC catalytic subunit 4 [Source:MGI Symbol;Acc:MGI:1924100]	2.52724	2.39242	1.98551	2.45916	2.48191	2.13367	2.09437	2.27802	2.31662	2.00712	2.30675	2.44239	2.29546	2.60681	0.858514388	0.966018836	8	4	24	0.789554854	0.932546317	24	22.98594868	0.023993466
ENSMUSG00000042502	Cd2bp2	CD2 cytoplasmic tail binding protein 2 [Source:MGI Symbol;Acc:MGI:1917483]	25.172	23.4327	23.3886	22.476	23.5071	25.8373	23.9254	23.2042	22.4897	26.8275	21.1964	24.6224	23.1506	24.3593	0.44861185	0.966018836	24	20	24	0.695221302	0.898727918	24	21.80614531	0.029681303
ENSMUSG00000042505	Sdhaf3	succinate dehydrogenase complex assembly factor 3 [Source:MGI Symbol;Acc:MGI:1913288]	5.50808	6.61776	5.61918	5.30485	6.00105	5.3913	5.29123	5.41258	5.35562	5.42878	5.91868	4.80046	5.22316	5.49833	0.669104912	0.966018836	20	4	24	0.772753842	0.926880913	24	8.791617428	0.024608385
ENSMUSG00000042506	Usp22	ubiquitin specific peptidase 22 [Source:MGI Symbol;Acc:MGI:2144157]	41.5311	35.4028	38.7815	37.9719	35.8252	41.1235	39.3066	39.6329	33.0826	48.187	33.6176	38.6663	35.4668	38.0359	0.924553956	0.966018836	24	20	24	0.968079795	0.992305273	24	18.22940248	0.014479397
ENSMUSG00000042507	Mideas	mitotic deacetylase associated SANT domain protein [Source:MGI Symbol;Acc:MGI:2685106]	2.75408	2.68882	2.78802	3.12492	2.92601	2.8838	2.78685	3.75464	2.97495	3.10905	2.5363	3.06328	2.91178	3.06406	0.702630284	0.966018836	16	12	24	0.519518941	0.819306539	24	6.861228641	0.046011615
ENSMUSG00000042508	Dmtf1	cyclin D binding myb-like transcription factor 1 [Source:MGI Symbol;Acc:MGI:1344415]	24.01	26.3809	23.5527	25.7585	26.3949	24.8877	26.5671	23.916	25.3179	21.1561	27.8018	24.66	23.9013	24.9413	0.942429894	0.966018836	20	16	24	0.633351424	0.875353057	24	19.43125668	0.036956184
ENSMUSG00000042510	AA986860	expressed sequence AA986860 [Source:MGI Symbol;Acc:MGI:2138143]	1.33253	1.49947	1.52233	1.46997	1.76569	1.70312	1.65176	1.52373	1.69547	1.90658	1.11486	1.83751	1.64982	1.6208	0.742579234	0.966018836	24	8	24	0.989637616	0.997887893	24	12.12865486	0.00671874
ENSMUSG00000042515	Pwwp3b	PWWP domain containing 3B [Source:MGI Symbol;Acc:MGI:2445062]	0.877259	0.977201	1.10694	0.67078	0.794054	0.787927	0.999147	0.699506	0.916981	0.617336	0.845636	1.09782	0.705075	0.981486	0.700065774	0.966018836	12	4	24	0.507005339	0.812342247	24	1.745453118	0.05556606
ENSMUSG00000042520	Ubap2l	ubiquitin-associated protein 2-like [Source:MGI Symbol;Acc:MGI:1921633]	37.7711	30.2781	27.3741	30.8983	30.732	36.342	34.0985	35.1214	28.1254	37.9517	27.7011	30.8388	30.0536	36.7355	0.723119329	0.966018836	4	8	24	0.493639305	0.806143905	24	23.63096923	0.077091399
ENSMUSG00000042523	Dnal1	dynein, axonemal, light chain 1 [Source:MGI Symbol;Acc:MGI:1921462]	9.90333	10.928	10.9568	10.5184	11.0758	10.1523	10.3207	9.78947	11.3685	10.2786	10.0111	10.7113	9.92717	11.1442	0.182849367	0.659303873	12	16	24	0.255134543	0.768040178	24	9.227683961	0.043175919
ENSMUSG00000042524	Sun2	Sad1 and UNC84 domain containing 2 [Source:MGI Symbol;Acc:MGI:2443011]	15.2764	15.6641	16.1495	18.1338	15.8679	16.3653	16.7881	19.3597	15.7924	19.0521	14.9302	16.1119	17.2904	14.8321	0.88067614	0.966018836	16	4	24	0.713232883	0.905403147	24	9.797631097	0.039096837
ENSMUSG00000042529	Kcnj12	potassium inwardly-rectifying channel, subfamily J, member 12 [Source:MGI Symbol;Acc:MGI:108495]	12.4861	9.52866	9.23481	10.1287	9.60941	11.5911	10.099	11.8561	8.23225	12.5985	9.47513	11.3535	11.874	10.3901	0.182849367	0.659303873	4	8	24	0.268745112	0.768040178	24	21.78701936	0.108054269
ENSMUSG00000042532	Golga7b	golgi autoantigen, golgin subfamily a, 7B [Source:MGI Symbol;Acc:MGI:1918396]	15.7133	12.1891	13.1834	13.4449	14.2123	15.0141	12.1707	12.9628	12.1558	15.9289	14.9787	15.0604	14.5561	15.4849	0.54278565	0.966018836	24	12	24	0.208231452	0.768040178	24	17.98069979	0.086421797
ENSMUSG00000042535	Gtpbp1	GTP binding protein 1 [Source:MGI Symbol;Acc:MGI:109443]	6.52584	6.09687	7.05506	6.22483	7.12653	6.17234	6.40825	8.51439	7.39504	7.93549	5.80206	6.83386	6.62085	6.29973	0.916556573	0.966018836	12	12	24	0.373652236	0.794450623	24	8.20474893	0.077373612
ENSMUSG00000042541	Sem1	SEM1, 26S proteasome complex subunit [Source:MGI Symbol;Acc:MGI:109238]	52.6322	61.5143	52.1419	54.155	53.5759	49.9323	51.5221	54.1256	63.1157	44.2808	60.929	52.9901	61.6349	59.2971	0.841143063	0.966018836	8	16	24	0.454048446	0.805393068	24	4.275619645	0.068314673
ENSMUSG00000042548	Asxl1	ASXL transcriptional regulator 1 [Source:MGI Symbol;Acc:MGI:2684063]	7.32576	7.47739	7.37797	7.7019	6.98353	7.86237	7.47709	8.04997	7.24046	8.49386	6.71886	7.79271	7.5358	7.70716	0.969375968	0.970883022	8	12	24	0.729955632	0.911943391	24	5.679671009	0.024015255
ENSMUSG00000042549	Map2k3os	mitogen-activated protein kinase kinase 3, opposite strand [Source:MGI Symbol;Acc:MGI:1344334]	1.96974	1.89616	1.45403	1.41551	2.03242	1.82173	2.10458	1.38803	2.02001	1.69612	1.91437	1.85788	1.75467	1.83158	0.61767505	0.966018836	20	20	24	0.25706685	0.768040178	24	20.86430519	0.0812107
ENSMUSG00000042557	Sin3a	transcriptional regulator, SIN3A (yeast) [Source:MGI Symbol;Acc:MGI:107157]	8.23631	7.33811	8.09898	7.29619	7.42569	7.47281	7.41724	7.68451	7.00861	7.9757	7.87388	7.45166	7.12999	7.22031	0.881996584	0.966018836	20	12	24	0.80711788	0.938616703	24	16.12834541	0.015292449
ENSMUSG00000042558	Adprs	ADP-ribosylserine hydrolase [Source:MGI Symbol;Acc:MGI:2140364]	8.99837	8.95281	9.72209	8.4859	8.7146	8.40757	8.96078	9.03863	8.88894	8.88144	8.64523	9.70103	8.87376	9.25835	0.143609806	0.585944911	8	12	24	0.358899187	0.785401914	24	4.356830414	0.030638474
ENSMUSG00000042564	Fam227a	family with sequence similarity 227, member A [Source:MGI Symbol;Acc:MGI:1922979]	2.12252	2.37027	2.20045	2.10523	2.45265	2.53599	2.40594	1.96822	2.74117	1.63592	2.38451	2.93603	2.46412	1.91247	0.050845331	0.38654007	24	8	24	0.274662006	0.768040178	24	20.25898102	0.097717623
ENSMUSG00000042569	Dhrs7b	dehydrogenase/reductase (SDR family) member 7B [Source:MGI Symbol;Acc:MGI:2384931]	14.4365	13.5887	14.5893	14.4157	15.9958	13.4927	14.0424	15.134	14.2868	13.2724	16.8991	13.6576	15.8719	14.999	0.924770496	0.966018836	20	4	24	0.876890792	0.96580091	24	16.00903002	0.019315173
ENSMUSG00000042570	Mier2	MIER family member 2 [Source:MGI Symbol;Acc:MGI:1917677]	8.81246	8.10437	7.86757	9.01069	8.51064	7.64342	8.90731	8.70191	8.90724	9.22452	8.91305	9.49903	8.4699	7.73981	0.223774686	0.717349224	16	16	24	0.480175634	0.805393068	24	15.41122058	0.040387117
ENSMUSG00000042572	Ube2q1	ubiquitin-conjugating enzyme E2Q family member 1 [Source:MGI Symbol;Acc:MGI:1917343]	35.6207	31.357	32.3899	35.144	33.1628	35.3236	32.0378	31.852	31.4061	36.1915	31.3399	33.668	34.6685	32.6248	0.335107163	0.85758369	16	20	24	0.388161814	0.799546792	24	17.84894681	0.040074876
ENSMUSG00000042579	4632404H12Rik	RIKEN cDNA 4632404H12 gene [Source:MGI Symbol;Acc:MGI:1921284]	2.73101	3.16964	3.11868	3.10117	2.79304	2.78189	2.46195	2.9002	3.21588	2.82884	2.73526	2.92787	2.84983	3.00706	0.008389425	0.192760771	12	16	24	0.027991657	0.590904845	24	8.363275873	0.074952169
ENSMUSG00000042581	Thsd7b	thrombospondin, type I, domain containing 7B [Source:MGI Symbol;Acc:MGI:2443925]	3.18995	2.79219	3.43358	2.93901	2.4749	2.96594	2.45285	2.99377	2.34696	3.40237	2.48809	3.02487	2.90566	3.10789	0.853736882	0.966018836	16	4	24	0.79194635	0.93349015	24	6.035276574	0.036748381
ENSMUSG00000042589	Cux2	cut-like homeobox 2 [Source:MGI Symbol;Acc:MGI:107321]	2.11492	2.15849	2.65905	2.33486	2.32912	1.95541	2.53443	1.40992	2.3722	3.0567	1.58719	2.57231	1.99077	3.36114	0.635666629	0.966018836	16	4	24	0.592040184	0.85635726	24	8.939642903	0.095973309
ENSMUSG00000042590	Ipo11	importin 11 [Source:MGI Symbol;Acc:MGI:2442377]	19.5375	20.8101	18.6651	19.3284	19.7732	19.5197	20.4492	20.1216	18.7273	18.5065	20.8574	20.3661	20.9497	20.7212	0.057707116	0.403601759	8	4	24	0.048568342	0.638992719	24	23.11081639	0.049652233
ENSMUSG00000042594	Sh2b3	SH2B adaptor protein 3 [Source:MGI Symbol;Acc:MGI:893598]	3.24694	3.89961	4.10324	4.37578	4.66332	3.99457	3.74899	3.81233	4.18044	4.89884	4.04996	3.68318	4.06263	3.32654	0.008603252	0.192760771	16	12	24	0.004954944	0.442351542	24	12.93768509	0.137596344
ENSMUSG00000042595	Fam199x	family with sequence similarity 199, X-linked [Source:MGI Symbol;Acc:MGI:2384304]	4.26144	4.54189	4.27976	4.18491	4.58021	4.1891	4.10167	3.91458	4.48209	3.6256	4.94194	4.08896	3.86301	4.75928	0.891105762	0.966018836	20	8	24	0.948545617	0.987442414	24	12.92159702	0.012908589
ENSMUSG00000042599	Kdm7a	lysine (K)-specific demethylase 7A [Source:MGI Symbol;Acc:MGI:2443388]	6.00294	6.65781	6.49946	6.97626	6.65934	5.83884	6.87698	5.98297	6.96912	5.18529	7.00628	6.32827	6.05925	6.11307	0.767183862	0.966018836	20	4	24	0.908117078	0.974606327	24	12.30383081	0.018682369
ENSMUSG00000042604	Kcna4	potassium voltage-gated channel, shaker-related subfamily, member 4 [Source:MGI Symbol;Acc:MGI:96661]	5.39138	4.46019	5.30256	5.33367	4.84434	5.52308	4.48366	6.11701	3.59998	6.29249	4.05855	5.09192	4.99868	4.73117	0.916556573	0.966018836	16	20	24	0.963912474	0.990989455	24	18.7005446	0.020456664
ENSMUSG00000042605	Atxn2	ataxin 2 [Source:MGI Symbol;Acc:MGI:1277223]	50.2227	44.6594	42.7186	47.4459	46.6467	55.1952	51.266	47.142	43.6171	56.1707	46.0546	48.2293	43.3321	49.016	0.245061943	0.741580974	24	12	24	0.402525673	0.799546792	24	18.71096982	0.06529169
ENSMUSG00000042606	Hirip3	HIRA interacting protein 3 [Source:MGI Symbol;Acc:MGI:2142364]	7.78851	7.24682	8.12112	7.48904	8.3007	7.09334	7.18157	7.02369	6.72168	6.86451	5.77885	5.9613	5.91438	7.42788	0.880247609	0.966018836	12	12	24	0.510109437	0.81402698	24	7.711564716	0.048686005
ENSMUSG00000042608	Stk40	serine/threonine kinase 40 [Source:MGI Symbol;Acc:MGI:1921428]	8.48751	6.94914	7.78171	9.43703	7.99904	7.95233	8.37157	8.63722	7.79872	10.7106	9.2715	7.98363	9.37662	8.52008	0.676344318	0.966018836	16	20	24	0.342546669	0.776661954	24	13.58305031	0.070915493
ENSMUSG00000042613	Pbxip1	pre B cell leukemia transcription factor interacting protein 1 [Source:MGI Symbol;Acc:MGI:2441670]	8.23572	10.9189	12.2597	11.674	10.3212	10.5959	11.9233	14.6665	12.8758	13.422	9.0672	10.4512	10.5621	10.8456	0.016811431	0.253395174	16	4	24	0.048758418	0.638992719	24	8.035451847	0.182294249
ENSMUSG00000042616	Oscp1	organic solute carrier partner 1 [Source:MGI Symbol;Acc:MGI:1916308]	9.58854	10.7608	10.0341	8.91644	9.03634	10.8505	8.86861	9.81019	9.54566	9.51889	7.77192	9.83816	9.45277	10.9962	0.06180207	0.409814092	8	12	24	0.141473745	0.751766185	24	3.424438573	0.087912278
ENSMUSG00000042622	Maff	v-maf musculoaponeurotic fibrosarcoma oncogene family, protein F (avian) [Source:MGI Symbol;Acc:MGI:96910]	1.73764	1.51629	1.3608	1.96094	2.03945	1.85187	1.76938	1.75245	1.75443	2.10872	2.57062	1.59127	2.12561	1.72501	0.054014579	0.395758043	20	12	24	0.062313384	0.661961741	24	16.05988782	0.116678833
ENSMUSG00000042625	Safb2	scaffold attachment factor B2 [Source:MGI Symbol;Acc:MGI:2146808]	30.6131	30.3566	35.2408	40.7529	35.7923	29.8218	37.8371	33.6964	39.9453	31.0398	33.0295	31.7229	31.7818	32.115	0.287843582	0.793714531	12	12	24	0.227606429	0.768040178	24	10.40071826	0.095821042

ENSMUSG00000042626	Shc1	src homology 2 domain-containing transforming protein C1 [Source:MGI Symbol;Acc:MGI:98296]	54.1484	33.6716	33.0182	34.4676	43.1771	41.004	39.6763	39.4108	28.645	35.0353	46.0317	38.3343	38.6123	35.2322	0.004947794	0.141782417	20	16	24	0.009544228	0.502965379	24	20.28667772	0.233753709
ENSMUSG00000042628	Zfyve1	zinc finger, FYVE domain containing 1 [Source:MGI Symbol;Acc:MGI:3026685]	13.657	11.7447	11.7302	13.3591	11.8984	13.279	14.092	13.468	11.857	14.5311	12.0426	13.8544	13.7381	13.0535	0.097760727	0.501112642	4	8	24	0.416885476	0.801631254	24	21.85829583	0.051418764
ENSMUSG00000042631	Xkr7	X-linked Kx blood group related 7 [Source:MGI Symbol;Acc:MGI:3526711]	1.19622	0.980707	0.625624	0.699082	1.00627	0.860878	1.04461	1.15917	0.87227	1.35424	1.09469	1.16336	1.09309	0.94238	0.612331511	0.966018836	4	8	24	0.392694297	0.799546792	24	21.34897579	0.075393438
ENSMUSG00000042632	Pla2g6	phospholipase A2, group VI [Source:MGI Symbol;Acc:MGI:1859152]	13.149	9.40619	8.96742	10.4007	12.0289	13.2724	11.5943	13.2591	9.44115	12.7885	11.9137	11.4953	11.3648	11.3342	0.128262369	0.560209134	24	12	24	0.098530649	0.71175955	24	19.97808658	0.141004331
ENSMUSG00000042642	Flad1	flavin adenine dinucleotide synthetase 1 [Source:MGI Symbol;Acc:MGI:2443030]	13.0749	11.7745	11.6259	13.1658	11.8401	13.6716	11.0468	12.3667	10.8071	13.663	11.5713	11.5704	12.5991	12.2049	0.911135828	0.966018836	16	20	24	0.745585519	0.916904814	24	17.39858454	0.031084728
ENSMUSG00000042644	Itpr3	inositol 1,4,5-triphosphate receptor 3 [Source:MGI Symbol;Acc:MGI:96624]	13.1083	11.354	9.85407	11.6263	15.1314	11.3428	14.4974	12.7875	12.0127	14.0288	11.8331	12.0205	13.1157	10.387	0.542718076	0.966018836	4	8	24	0.370783546	0.792839127	24	18.95204041	0.093028502
ENSMUSG00000042647	Acad12	acyl-Coenzyme A dehydrogenase family, member 12 [Source:MGI Symbol;Acc:MGI:2443320]	4.00702	2.86074	3.35817	2.84986	4.00162	3.91344	3.47023	3.22333	4.23641	4.17351	4.12165	3.53465	3.73087	3.72651	0.397301892	0.927993503	20	12	24	0.447138355	0.805393068	24	16.84687094	0.068928652
ENSMUSG00000042650	Alkbh5	alkB homolog 5, RNA demethylase [Source:MGI Symbol;Acc:MGI:2144489]	9.80971	8.77854	9.02447	10.9417	9.66679	9.9651	11.4164	10.5983	9.40813	12.0819	9.03014	10.3198	10.491	9.98535	0.956794505	0.96629412	4	8	24	0.938759148	0.984935568	24	17.33199237	0.017106248
ENSMUSG00000042659	Arrdc4	arrestin domain containing 4 [Source:MGI Symbol;Acc:MGI:1913662]	8.34788	6.9339	5.89967	7.49352	6.47621	6.8628	6.73073	6.19566	6.07229	6.48143	7.78387	7.10514	7.3633	7.14579	0.182849367	0.659303873	4	8	24	0.161714718	0.76636332	24	20.55164737	0.0927227
ENSMUSG00000042660	Wdr55	WD repeat domain 55 [Source:MGI Symbol;Acc:MGI:1915186]	8.88942	9.57486	8.58718	8.6567	8.91611	9.72572	7.615	9.93663	7.47337	8.73789	9.21858	8.17628	8.8478	9.43854	0.253121224	0.753386477	8	4	24	0.896775565	0.970634157	24	23.60173916	0.020794768
ENSMUSG00000042662	Dusp15	dual specificity phosphatase-like 15 [Source:MGI Symbol;Acc:MGI:1934928]	91.2492	90.2932	81.7246	105.181	120.089	81.8073	108.714	93.1485	119.657	103.342	117.176	96.9705	108.678	88.5565	0.305564675	0.811201458	20	12	24	0.289375294	0.768040178	24	14.4188242	0.108921143
ENSMUSG00000042670	Immp1l	IMP1 inner mitochondrial membrane peptidase-like (S. cerevisiae) [Source:MGI Symbol;Acc:MGI:1913791]	4.46648	5.30448	4.80238	5.23701	5.63321	4.04431	5.02042	4.31315	6.16873	3.91251	5.5893	5.1208	5.18202	4.46154	0.951846915	0.966018836	20	12	24	0.801921532	0.936565832	24	12.6015437	0.042211473
ENSMUSG00000042671	Rgs8	regulator of G-protein signaling 8 [Source:MGI Symbol;Acc:MGI:108408]	14.531	9.08594	10.1441	10.3048	10.639	12.2934	11.4844	11.0353	7.83444	12.5235	9.46147	11.4153	11.0925	10.7432	0.026841899	0.305155548	24	12	24	0.122118241	0.735058232	24	21.08778224	0.155517718
ENSMUSG00000042672	Dcst1	DC-STAMP domain containing 1 [Source:MGI Symbol;Acc:MGI:1925022]	1.30807	1.1003	1.1287	1.47077	0.946532	0.991037	0.97957	1.24111	1.59539	1.44055	1.23873	1.05057	1.17007	1.20165	0.124028266	0.552144108	16	8	24	0.044633589	0.628432994	24	9.208405905	0.115901118
ENSMUSG00000042675	Ypel3	yippee like 3 [Source:MGI Symbol;Acc:MGI:1913340]	172.45	147.739	145.928	158.22	135.266	160.559	163.419	158.075	145.556	171.275	151.037	151.62	154.148	148.988	0.170659409	0.643496587	4	8	24	0.676053891	0.891626697	24	22.87858787	0.034147626
ENSMUSG00000042677	Zc3h12a	zinc finger CCCH type containing 12A [Source:MGI Symbol;Acc:MGI:2385891]	1.33652	1.53429	1.56943	2.00043	1.68504	1.56697	1.46501	2.33377	1.25689	1.64465	1.69482	0.932694	1.49867	1.59004	0.109058178	0.524035616	20	4	24	0.466768444	0.805393068	24	9.315632497	0.090548366
ENSMUSG00000042680	Garem1	GRB2 associated regulator of MAPK1 subtype 1 [Source:MGI Symbol;Acc:MGI:2685790]	3.02414	3.01266	3.13851	3.177	3.08275	3.26998	3.23125	2.84073	2.89715	4.0762	2.9358	3.30253	3.21275	2.9931	0.060394272	0.407012001	24	8	24	0.411751625	0.799546792	24	14.94130022	0.050120399
ENSMUSG00000042682	Selenok	selenoprotein K [Source:MGI Symbol;Acc:MGI:1931466]	68.8248	66.0949	62.4565	59.7126	67.3743	60.9507	62.0228	58.6805	62.5928	47.2382	63.7754	56.7789	56.8219	53.1513	0.886572916	0.966018836	20	20	24	0.370750392	0.792839127	24	21.42037704	0.060066615
ENSMUSG00000042684	Npl	N-acetylneuraminate pyruvate lyase [Source:MGI Symbol;Acc:MGI:1921341]	3.85705	4.015	3.29176	2.84567	4.16964	3.2919	4.39825	4.32357	4.38116	2.79725	3.7356	3.32258	3.94305	3.59043	0.175272072	0.651010555	4	12	24	0.151557032	0.758865539	24	1.510656018	0.12214392
ENSMUSG00000042686	Jph1	junctophilin 1 [Source:MGI Symbol;Acc:MGI:1891495]	1.35355	2.28046	2.44655	3.45202	2.18977	2.82626	2.13446	5.14708	3.59847	4.85124	2.2723	2.2074	2.50712	2.3569	0.128989168	0.560209134	16	12	24	0.073918753	0.682883214	24	9.643113608	0.311654225
ENSMUSG00000042688	Mapk6	mitogen-activated protein kinase 6 [Source:MGI Symbol;Acc:MGI:1354946]	9.64031	11.598	13.6517	10.0174	10.2183	10.7232	10.6946	11.2877	11.3077	10.6266	9.57988	10.9521	10.3772	11.4758	0.018311724	0.256345714	12	8	24	0.058357785	0.658304792	24	6.402158214	0.105928266
ENSMUSG00000042694	Stn1	STN1, CST complex subunit [Source:MGI Symbol;Acc:MGI:1915581]	7.62539	7.95681	8.52185	8.66804	7.84074	9.54672	8.2732	8.193	8.07771	7.81881	7.49788	8.02641	8.2652	7.98259	0.9143748	0.966018836	24	20	24	0.945660924	0.986808796	24	21.33182293	0.010821415
ENSMUSG00000042699	Dhx9	DEAH (Asp-Glu-Ala-His) box polypeptide 9 [Source:MGI Symbol;Acc:MGI:108177]	43.0607	38.6529	40.2474	38.2739	39.6035	39.5754	39.1154	36.5083	34.1428	36.7389	37.0487	37.5403	38.7848	39.5113	0.826609618	0.966018836	4	12	24	0.099418116	0.71175955	24	22.19781639	0.05479282
ENSMUSG00000042700	Sipa1l1	signal-induced proliferation-associated 1 like 1 [Source:MGI Symbol;Acc:MGI:2443679]	10.7469	11.7786	10.7498	11.5569	13.7421	11.4346	13.5381	11.5124	12.3776	12.5017	11.7046	12.4466	12.4127	11.3064	0.798829513	0.966018836	20	12	24	0.486825027	0.805393068	24	17.42747476	0.046176476
ENSMUSG00000042705	Commd10	COMM domain containing 10 [Source:MGI Symbol;Acc:MGI:1916706]	14.6237	15.2003	16.8753	16.7301	12.7813	15.6027	15.8116	16.7283	15.4916	15.7736	14.5251	14.1678	14.3414	16.5203	0.028632001	0.316608413	16	4	24	0.040654094	0.616921492	24	6.808448242	0.099621737
ENSMUSG00000042707	Dnali1	dynein, axonemal, light intermediate polypeptide 1 [Source:MGI Symbol;Acc:MGI:1922813]	0.341772	0.746972	0.809525	0.625809	0.529519	0.550808	0.442293	0.505774	0.522919	0.438159	0.388633	0.676637	0.320431	0.625447	0.43565473	0.966018836	12	16	24	0.389445346	0.799546792	24	8.5607975	0.074262695
ENSMUSG00000042708	Shcbp1l	Shc SH2-domain binding protein 1-like [Source:MGI Symbol;Acc:MGI:1919086]	0.468779	0.607244	0.94314	0.766815	0.716894	0.833903	0.842658	0.690844	0.874831	0.443753	0.83063	0.896005	0.739452	0.64883	0.773177465	0.966018836	24	8	24	0.883776637	0.966864521	24	16.19456867	0.026197769
ENSMUSG00000042709	Atpaf2	ATP synthase mitochondrial F1 complex assembly factor 2 [Source:MGI Symbol;Acc:MGI:2180561]	13.8383	13.1241	13.0783	15.5549	14.3396	14.6197	14.4621	14.5129	13.068	15.0176	13.2888	13.1194	14.9255	14.2304	0.369262716	0.893596186	16	20	24	0.864177997	0.960637063	24	16.6689245	0.017554751
ENSMUSG00000042712	Tceal9	transcription elongation factor A like 9 [Source:MGI Symbol;Acc:MGI:109567]	85.5499	88.5566	84.4718	82.4813	80.7767	79.6907	78.6275	73.6673	76.8843	68.0278	92.1748	66.3847	69.1078	79.0009	0.891742979	0.966018836	20	4	24	0.947017809	0.987061058	24	11.67224941	0.015442201
ENSMUSG00000042717	Ppp1r3a	protein phosphatase 1, regulatory subunit 3A [Source:MGI Symbol;Acc:MGI:2153588]	0.569648	1.98667	2.24653	3.40633	1.96054	2.18577	1.64967	3.8557	4.59656	3.88044	1.8296	1.98925	1.90029	1.70673	0.007663688	0.192760771	12	16	24	0.015138273	0.537924552	24	10.05644548	0.46550102
ENSMUSG00000042719	Naa25	N(alpha)-acetyltransferase 25, NatB auxiliary subunit [Source:MGI Symbol;Acc:MGI:2442563]	15.9578	13.5748	13.5843	13.1741	14.0913	14.5634	14.6693	14.9201	12.483	14.5462	14.1986	15.1933	14.9174	14.7344	0.008754183	0.192760771	4	8	24	0.014003198	0.530670295	24	22.13772378	0.085834433
ENSMUSG00000042724	Map3k9	mitogen-activated protein kinase kinase kinase 9 [Source:MGI Symbol;Acc:MGI:2449952]	9.07488	8.10255	7.37465	8.90933	8.04179	8.3965	7.88449	8.62326	7.67241	12.3261	8.05439	9.48065	9.58603	9.16655	0.784278077	0.966018836	24	12	24	0.867080418	0.961800416	24	14.9518176	0.033047791
ENSMUSG00000042726	Trafd1	TRAF type zinc finger domain containing 1 [Source:MGI Symbol;Acc:MGI:1923551]	21.6999	22.8855	24.4442	22.1448	21.5537	22.6964	22.9231	22.7733	24.9738	24.1589	21.9674	24.8293	21.2075	23.4753	0.143321297	0.585944911	12	8	24	0.240860169	0.768040178	24	8.427674187	0.048951684
ENSMUSG00000042729	Wdr74	WD repeat domain 74 [Source:MGI Symbol;Acc:MGI:2147427]	21.0886	20.2166	18.9817	20.2491	20.1001	22.2019	20.1094	19.7139	17.9694	20.1185	19.6597	19.4346	20.3757	19.421	0.16456443	0.628002127	4	8	24	0.025473313	0.586541342	24	20.42596157	0.062652284
ENSMUSG00000042734	Ttc9	tetratricopeptide repeat domain 9 [Source:MGI Symbol;Acc:MGI:1916730]	96.575	71.2484	69.3196	80.3153	78.1465	86.0122	80.978	77.0568	58.7529	97.9249	73.6991	83.7753	82.5126	77.4697	0.010468194	0.196621011	24	12	24	0.171400426	0.768040178	24	19.84751511	0.137450587
ENSMUSG00000042737	Dpm3	dolichyl-phosphate mannosyltransferase polypeptide 3 [Source:MGI Symbol;Acc:MGI:1915813]	39.1582	37.141	34.1964	35.5847	30.4755	28.8418	38.6092	35.4132	45.45	30.8749	38.7615	37.4603	44.7208	39.5959	0.328944792	0.847277612	4	20	24	0.137977707	0.747972781	24	3.677072417	0.130256129
ENSMUSG00000042742	Bmt2	base methyltransferase of 25S rRNA 2 [Source:MGI Symbol;Acc:MGI:2141466]	5.69317	5.98071	6.65568	7.09209	6.33108	5.67347	6.41201	5.983	6.28176	5.34512	6.53728	5.20644	4.84617	5.45012	0.25851124	0.761738864	20	4	24	0.109566091	0.72082227	24	11.52326785	0.086823259
ENSMUSG00000042743	Sgtb	small glutamine-rich tetratricopeptide repeat (TPR)-containing, beta [Source:MGI Symbol;Acc:MGI:2444615]	24.604	22.4505	22.6119	20.9739	22.5367	23.2657	20.6392	21.4682	20.8253	21.7708	24.2283	23.6018	24.245	24.678	0.549437303	0.966018836	4	8	24	0.256186892	0.768040178	24	21.56705235	0.055248332
ENSMUSG00000042744	Hectd4	HECT domain E3 ubiquitin protein ligase 4 [Source:MGI Symbol;Acc:MGI:3647820]	12.4072	10.8666	10.1821	12.3486	12.7451	12.1177	13.1755	11.8994	11.5263	15.7677	10.7194	12.6795	11.4835	12.6124	0.888991156	0.966018836	16	20	24	0.698972722	0.899909544	24	16.27172761	0.047107675
ENSMUSG00000042745	Id1	inhibitor of DNA binding 1, HLH protein [Source:MGI Symbol;Acc:MGI:96396]	8.87259	9.69147	9.97812	10.4052	9.08749	7.43602	10.3515	9.95689	13.6616	13.4055	12.1131	11.3722	10.9512	12.5254	0.591351901	0.966018836	16	12	24	0.012342487	0.525280095	24	9.194580978	0.176309629
ENSMUSG00000042747	Krtcap2	keratinocyte associated protein 2 [Source:MGI Symbol;Acc:MGI:1913309]	43.9928	43.9453	46.6309	44.5119	43.5726	47.3215	46.6331	46.1332	42.3917	37.4494	45.6611	40.4757	44.5661	39.6409	0.893609703	0.966018836	4	12	24	0.45799398	0.805393068	24	22.35528324	0.043162905
ENSMUSG00000042750	Bex2	brain expressed X-linked 2 [Source:MGI Symbol;Acc:MGI:1338017]	155.883	136.777	142.619	137.202	131.844	147.315	135.537	139.881	132.063	149.398	154.562	158.434	168.414	161.098	0.652290823	0.966018836	4	8	24	0.256874296	0.768040178	24	22.36437011	0.060903917
ENSMUSG00000042751	Nmnat2	nicotinamide nucleotide adenylyltransferase 2 [Source:MGI Symbol;Acc:MGI:2444155]	25.2091	20.1131	22.3798	21.6451	20.1424	24.3266	22.5869	22.4477	15.9163	26.9034	18.6376	22.609	20.4406	23.0248	0.649212555	0.966018836	24	20	24	0.667395756	0.887459929	24	21.52385954	0.066979919
ENSMUSG00000042757	Tmem108	transmembrane protein 108 [Source:MGI Symbol;Acc:MGI:1932411]	2.95346	2.14769	2.26774	2.28094	1.78209	2.17046	2.88232	2.20907	1.85117	3.11655	1.56579	2.70413	2.17214	2.98674	0.664568268	0.966018836	4	16	24	0.442934057	0.805393068	24	2.041982819	0.103934182
ENSMUSG00000042759	Apobr	apolipoprotein B receptor [Source:MGI Symbol;Acc:MGI:2176230]	0.933029	1.23668	1.44098	1.03892	1.20136	1.00784	1.15434	1.28556	0.996822	1.03714	0.99905	1.30049	1.32446	0.776533	0.718363231	0.966018836	12	4	24	0.96363054	0.990884307	24	0.231185999	0.013639322
ENSMUSG00000042761	Mrap2	melanocortin 2 receptor accessory protein 2 [Source:MGI Symbol;Acc:MGI:3609239]	3.38612	4.0066	4.58054	3.74724	2.97434	4.53338	4.27099	3.48725	3.6725	3.95408	3.149	4.30525	3.24491	4.15019	0.951523604	0.966018836	24	20	24	0.724321292	0.90965493	24	4.354765412	0.05104309
ENSMUSG00000042763	Maneal	mannosidase, endo-alpha-like [Source:MGI Symbol;Acc:MGI:2684896]	19.591	14.8859	14.249	15.943	14.8139	18.3097	16.3451	17.8758	11.5964	21.2164	14.4195	17.2333	16.7855	17.3817	0.310940564	0.820837787	24	12	24	0.441224168	0.805393068	24	21.51425358	0.107087171
ENSMUSG00000042766	Trim46	tripartite motif-containing 46 [Source:MGI Symbol;Acc:MGI:2673000]	17.228	12.3454	12.3919	12.8418	14.876	13.9088	12.9674	13.4425	13.5152	14.8334	14.6991	14.8547	14.6994	13.43	0.104994359	0.518002936	24	8	24	0.123680231	0.736492734	24	19.35865369	0.096355338
ENSMUSG00000042770	Hebp1	heme binding protein 1 [Source:MGI Symbol;Acc:MGI:1333880]	5.47459	7.74968	8.89681	9.14682	8.05167	6.85387	6.48432	8.03642	7.93845	6.43369	7.27168	7.51911	8.49438	7.65713	0.503192174	0.966018836	12	16	24	0.223857158	0.768040178	24	9.472933065	0.119668397
ENSMUSG00000042772	Smg7	SMG7 nonsense mediated mRNA decay factor [Source:MGI Symbol;Acc:MGI:2682334]	15.9916	12.5963	13.1087	12.7122	13.543	13.5152	13.2403	12.8848	12.0328	14.3853	11.4934	12.3133	12.4932	12.6555	0.832293447	0.966018836	4	16	24	0.628848362	0.873055798	24	22.0295254	0.036940041
ENSMUSG00000042787	Exog	endo/exonuclease (5'-3'), endonuclease G-like [Source:MGI Symbol;Acc:MGI:2143333]	6.39605	4.97486	5.69872	5.13186	5.47779	6.34836	5.89038	5.96336	4.61584	6.09876	5.40123	5.88197	6.04913	6.45453	0.238868829	0.741580974	4	8	24	0.152202442	0.759088284	24	22.47390629	0.088882169
ENSMUSG00000042788	Fam166b	family with sequence similarity 166, member B [Source:MGI Symbol;Acc:MGI:2445194]	0.383488	0.697883	0.770392	0.30834	0.521871	0.323715	0.530889	0.309581	0.457239	0.332827	0.443268	0.983018	0.178616	0.817656	0.891742979	0.966018836	12	4	24	0.905686135	0.974159848	24	4.740592037	0.039423716
ENSMUSG00000042790	Rnf214	ring finger protein 214 [Source:MGI Symbol;Acc:MGI:2444451]	18.0591	15.4161	16.7469	17.5874	17.2665	19.4198	18.5678	16.7965	14.7959	20.508	15.086	18.9196	17.0907	17.2859	0.020957534	0.262822894	24	12	24	0.360643126	0.786233489	24	19.19884126	0.074627261
ENSMUSG00000042797	Aqp11	aquaporin 11 [Source:MGI Symbol;Acc:MGI:1913583]	3.08017	2.68664	2.74706	2.93217	3.18677	2.91161	3.30848	3.72399	3.03473	3.37211	3.02942	3.11162	2.73611	3.05034	0.81829516	0.966018836	16	20	24	0.991080464	0.998487558	24	13.84531296	0.005098766
ENSMUSG00000042804	Gpr153	G protein-coupled receptor 153 [Source:MGI Symbol;Acc:MGI:1916157]	9.61788	7.59677	7.99755	7.33093	7.89188	8.94863	8.24845	8.31142	6.66336	10.4052	8.35759	8.33362	7.55775	7.48943	0.432699319	0.966018836	24	12	24	0.376966542	0.796956096	24	18.80610303	0.082516529
ENSMUSG00000042807	Hecw2	HECT, C2 and WW domain containing E3 ubiquitin protein ligase 2 [Source:MGI Symbol;Acc:MGI:2685817]	3.18873	3.23308	3.39199	3.00231	3.20513	2.9844	3.05187	2.68424	3.08241	2.92309	2.95095	3.07744	3.0661	2.82743	0.879844076	0.966018836	12	4	24	0.901500289	0.972353488	24	17.49987906	0.009888772
ENSMUSG00000042808	Gpx2	glutathione peroxidase 2 [Source:MGI Symbol;Acc:MGI:106609]	3.19291	3.74397	2.24459	2.74497	3.05826	2.61131	3.09087	2.31063	2.27967	2.42156	2.87505	2.63131	3.71958	2.94379	0.010577971	0.197206032	4	8	24	0.129053281	0.740162193	24	22.80476595	0.136563648
ENSMUSG00000042810	Krba1	KRAB-A domain containing 1 [Source:MGI Symbol;Acc:MGI:1925077]	5.63882	7.44504	7.68433	7.35555	7.09829	6.57705	6.7315	6.97088	8.73628	8.02519	6.81481	7.20392	7.41089	7.21217	0.015345591	0.253245931	12	12	24	0.005019844	0.442351542	24	9.46703082	0.13308692
ENSMUSG00000042814	Mcts2	malignant T cell amplified sequence 2 [Source:MGI Symbol;Acc:MGI:1913655]	9.76754	11.4899	11.0247	10.5867	10.9183	10.2976	10.4248	9.54908	11.96	8.59306	12.2574	10.0588	12.3457	12.8995	0.920858634	0.966018836	8	16	24	0.716313473	0.90635549	24	4.793157457	0.047166866
ENSMUSG00000042816	Gpr151	G protein-coupled receptor 151 [Source:MGI Symbol;Acc:MGI:2441887]	1.50231	0.877241	2.17073	1.10646	2.44435	1.61519	1.2037	1.03945	0.758322	0.731979	3.44942	0.938971	1.14543	0.96809	0.092972576	0.489012695	20	20	24	0.14696347	0.755442533	24	16.93290356	0.292536022
ENSMUSG00000042817	Flt3	FMS-like tyrosine kinase 3 [Source:MGI Symbol;Acc:MGI:95559]	6.8817	5.23552	5.42676	5.82121	5.09397	5.87753	5.98136	5.40448	4.45093	6.4027	4.8641	5.47072	5.3048	5.9671	0.718086778	0.966018836	4	8	24	0.554093159	0.837447029	24	22.63649079	0.058841155
ENSMUSG00000042821	Snai1	snail family zinc finger 1 [Source:MGI Symbol;Acc:MGI:98330]	0.734048	0.67778	0.414142	0.626084	0.69469	0.573385	0.797138	0.829817	0.78115	0.502792	0.787542	0.48389	0.668525	0.726419	0.519468828	0.966018836	8	8	24	0.507989298	0.813142043	24	1.792198779	0.051806656
ENSMUSG00000042826	Fgf11	fibroblast growth factor 11 [Source:MGI Symbol;Acc:MGI:109167]	6.10557	5.57474	6.06659	4.87568	5.79694	5.95509	6.02334	6.02123	4.70434	5.84358	5.48886	5.19977	4.97407	5.86882	0.793403332	0.966018836	4	16	24	0.576559071	0.848661503	24	23.66247235	0.043227698
ENSMUSG00000042828	Trim72	tripartite motif-containing 72 [Source:MGI Symbol;Acc:MGI:3612190]	0.536094	1.49631	1.47265	2.48464	1.33982	1.60439	1.37549	4.13047	1.89394	3.68852	1.14773	1.32329	2.49275	2.01772	0.323424237	0.839647936	16	4	24	0.133149106	0.743835554	24	8.522663314	0.329370945
ENSMUSG00000042831	Alkbh6	alkB homolog 6 [Source:MGI Symbol;Acc:MGI:2142037]	19.5451	18.8254	20.3428	19.3727	19.116	20.8759	20.4236	20.7927	19.7416	22.1974	22.8827	22.9721	23.1254	22.2053	0.793403332	0.966018836	12	16	24	0.387684534	0.799546792	24	19.6112539	0.028592088
ENSMUSG00000042834	Nrep	neuronal regeneration related protein [Source:MGI Symbol;Acc:MGI:99444]	23.3104	31.2308	31.0318	30.6833	28.0701	27.5921	33.3425	36.4432	37.9257	31.5069	26.2056	28.7727	30.2965	29.8365	0.057264003	0.401309455	12	12	24	0.033763707	0.606793499	24	7.18733909	0.163977552
ENSMUSG00000042842	Serpinb6b	serine (or cysteine) peptidase inhibitor, clade B, member 6b [Source:MGI Symbol;Acc:MGI:894688]	5.36004	5.84349	4.85849	4.81059	6.30626	5.17689	5.42562	5.47238	7.24657	4.61859	5.58638	5.78618	5.71292	5.5119	0.892685429	0.966018836	20	20	24	0.879121881	0.966161692	24	1.952549972	0.027969589
ENSMUSG00000042846	Lrrtm3	leucine rich repeat transmembrane neuronal 3 [Source:MGI Symbol;Acc:MGI:2389177]	6.52538	6.00045	6.50696	4.31765	4.68464	6.05692	6.34777	6.90959	5.81873	6.02091	5.87737	6.37474	5.88229	5.87615	0.258066659	0.761738864	4	12	24	0.07343757	0.681536373	24	1.84100582	0.125722009
ENSMUSG00000042851	Zc3h6	zinc finger CCCH type containing 6 [Source:MGI Symbol;Acc:MGI:1926001]	1.54477	2.22413	2.46881	1.85779	1.59232	1.82522	1.48661	1.81011	2.26886	2.39703	1.45477	2.28936	1.80395	2.24209	0.033248781	0.324908923	12	16	24	0.070671414	0.675829204	24	8.067123091	0.152320766
ENSMUSG00000042854	Trp53rkb	transformation related protein 53 regulating kinase B [Source:MGI Symbol;Acc:MGI:1914050]	1.03948	0.762309	1.04309	0.94672	0.728272	1.09039	0.841459	0.885594	0.8031	0.973455	0.701627	0.894101	0.80656	0.758483	0.947524602	0.966018836	24	20	24	0.956530267	0.988639288	24	20.02372327	0.011068837
ENSMUSG00000042857	Gm9776	predicted gene 9776 [Source:MGI Symbol;Acc:MGI:3641792]	1.24808	1.22716	1.51784	1.25953	1.02651	1.35958	1.20872	1.29486	1.23151	1.43565	1.17216	1.55852	1.30207	1.29311	0.897570341	0.966018836	24	20	24	0.886093512	0.967667196	24	5.96446038	0.017474739
ENSMUSG00000042870	Tom1	target of myb1 trafficking protein [Source:MGI Symbol;Acc:MGI:1338026]	1.16461	0.652707	0.877419	0.93168	0.925676	0.742362	0.579431	1.96219	1.03007	0.426967	0.869991	0.667857	0.762495	0.315531	0.846643334	0.966018836	12	12	24	0.9505849	0.988257443	24	4.10688883	0.034106953
ENSMUSG00000042873	Lhfpl4	lipoma HMGIC fusion partner-like protein 4 [Source:MGI Symbol;Acc:MGI:3057108]	66.4193	47.9543	48.1758	53.8971	53.7084	59.1778	50.7852	53.5355	37.0169	68.7491	47.7251	55.0371	52.3422	52.5818	0.338172159	0.85758369	12	12	24	0.338526647	0.773151665	24	19.4558736	0.12615591
ENSMUSG00000042895	Abra	actin-binding Rho activating protein [Source:MGI Symbol;Acc:MGI:2444891]	0.983801	1.13773	2.01926	2.10869	1.89531	1.91144	1.47781	3.29819	2.40612	2.25534	1.63148	0.900641	1.45512	0.966504	0.130633892	0.562427886	12	16	24	0.134864697	0.74653384	24	19.49727069	0.261578212
ENSMUSG00000042901	Aida	axin interactor, dorsalization associated [Source:MGI Symbol;Acc:MGI:1919737]	26.0849	25.0753	25.7376	22.3411	25.1426	24.0866	25.496	24.5164	24.9383	24.5768	25.702	25.0251	25.5293	24.7932	0.305564675	0.811201458	4	12	24	0.331787438	0.768621471	24	20.04351199	0.090458102
ENSMUSG00000042903	Foxo4	forkhead box O4 [Source:MGI Symbol;Acc:MGI:1891915]	12.6895	13.7758	12.6251	14.5144	14.0258	12.2201	15.8635	14.6027	16.0784	14.6901	14.1851	13.5485	15.0239	13.9712	0.830364743	0.966018836	12	12	24	0.458463284	0.805393068	24	7.877717137	0.050928094
ENSMUSG00000042918	Mamstr	MEF2 activating motif and SAP domain containing transcriptional regulator [Source:MGI Symbol;Acc:MGI:1921740]	0.981417	1.33326	1.56334	1.23458	1.21019	0.956484	0.999611	2.33658	2.37174	1.92312	1.56843	1.35126	1.30354	1.12826	0.051446973	0.38924256	12	16	24	0.030206973	0.596794384	24	8.968993108	0.241604256
ENSMUSG00000042938	Gm14117	predicted gene 14117 [Source:MGI Symbol;Acc:MGI:3651328]	20.4779	20.3574	19.0193	19.0937	18.3168	17.9685	19.9759	20.5126	15.8916	20.7992	17.4772	18.193	20.2467	21.1183	0.033553741	0.324908923	8	12	24	0.215086338	0.768040178	24	2.627501037	0.073481561
ENSMUSG00000042942	Greb1l	growth regulation by estrogen in breast cancer-like [Source:MGI Symbol;Acc:MGI:3576497]	1.13845	1.06124	1.17235	1.36435	1.23503	1.14537	0.960487	1.14406	0.953748	1.18502	1.10056	1.20857	0.930885	1.21416	0.113148904	0.530777648	16	12	24	0.178356009	0.768040178	24	13.73534358	0.055919182
ENSMUSG00000042961	Egflam	EGF-like, fibronectin type III and laminin G domains [Source:MGI Symbol;Acc:MGI:2146149]	2.60031	2.94838	3.01989	2.91545	3.37193	3.1843	3.1655	3.79165	2.87568	3.80435	2.72895	2.50354	2.36467	2.9263	0.953226423	0.966018836	16	12	24	0.398787022	0.799546792	24	10.27370377	0.081394645
ENSMUSG00000042962	Gm5436	predicted pseudogene 5436 [Source:MGI Symbol;Acc:MGI:3643291]	57.1088	63.8444	60.9643	69.184	62.3842	58.0413	55.362	61.5273	57.5845	48.9012	58.3104	49.6941	55.4574	60.1828	0.275983441	0.789849983	8	20	24	0.507969827	0.813142043	24	8.089679599	0.054307788
ENSMUSG00000042978	Sbk1	SH3-binding kinase 1 [Source:MGI Symbol;Acc:MGI:2135937]	12.7959	8.98747	8.99667	10.3914	10.1888	10.2291	10.8929	11.6051	8.85808	14.8119	11.6657	12.1175	12.3764	11.1417	0.286642595	0.793714531	4	8	24	0.489338035	0.805393068	24	19.34782328	0.087541461
ENSMUSG00000042992	Borcs5	BLOC-1 related complex subunit 5 [Source:MGI Symbol;Acc:MGI:1915024]	11.6827	9.1842	9.6277	10.2414	9.09999	10.8678	9.91977	11.3632	8.89829	13.9739	9.82937	11.2122	11.4509	9.98677	0.43565473	0.966018836	4	8	24	0.834869687	0.949290995	24	19.79293861	0.040058176
ENSMUSG00000042997	Nhlrc3	NHL repeat containing 3 [Source:MGI Symbol;Acc:MGI:2444520]	2.61749	3.43278	3.26457	2.71591	2.84395	2.37164	2.77962	3.05096	2.94021	3.20501	2.49778	2.92744	2.87043	2.95295	0.028380812	0.316608413	8	16	24	0.029594272	0.596648309	24	6.913704165	0.104959338
ENSMUSG00000043003	Rasef	RAS and EF hand domain containing [Source:MGI Symbol;Acc:MGI:2448565]	0.973845	0.973278	1.17221	0.989122	1.07958	0.817098	0.832829	0.73378	0.863667	0.81256	1.389	0.852718	0.936656	0.723034	0.305564675	0.811201458	20	12	24	0.185851619	0.768040178	24	14.9644147	0.085277972
ENSMUSG00000043004	Gng2	guanine nucleotide binding protein (G protein), gamma 2 [Source:MGI Symbol;Acc:MGI:102705]	135.235	130.019	125.159	126.703	127.588	128.713	123.928	123.444	115.806	128.809	128.691	126.069	130.441	127.789	0.643325306	0.966018836	4	8	24	0.192942296	0.768040178	24	20.69353558	0.035257407
ENSMUSG00000043008	Klhl6	kelch-like 6 [Source:MGI Symbol;Acc:MGI:2686922]	1.31626	1.82897	1.76164	1.62071	1.58018	1.47279	1.47853	1.54471	1.85095	1.29561	1.84153	1.49995	1.65337	1.42201	0.46238066	0.966018836	12	12	24	0.526903353	0.823139162	24	9.361673446	0.048184434
ENSMUSG00000043013	Onecut1	one cut domain, family member 1 [Source:MGI Symbol;Acc:MGI:1196423]	0.94235	0.815911	0.525005	0.98152	0.569843	0.800039	0.900742	0.717195	0.891974	1.27994	0.923143	1.16301	1.30585	1.11113	0.935316621	0.966018836	4	8	24	0.83804295	0.95041487	24	22.45095937	0.034138253
ENSMUSG00000043015	Nemp2	nuclear envelope integral membrane protein 2 [Source:MGI Symbol;Acc:MGI:2444113]	2.3528	2.8362	3.41676	2.80291	3.28722	2.85106	3.15222	3.08386	3.77569	3.06188	3.09589	3.42439	3.68648	3.39847	0.883907406	0.966018836	12	4	24	0.384911774	0.799546792	24	8.398019061	0.057302791
ENSMUSG00000043017	Ptgir	prostaglandin I receptor (IP) [Source:MGI Symbol;Acc:MGI:99535]	18.7443	11.7754	12.4992	12.8926	14.8667	15.9463	13.1826	14.1313	8.46241	15.0288	15.8668	13.6058	12.3797	12.3505	0.048759831	0.379129806	20	16	24	0.043302267	0.626294375	24	19.23639964	0.224177787
ENSMUSG00000043019	Edem3	ER degradation enhancer, mannosidase alpha-like 3 [Source:MGI Symbol;Acc:MGI:1914217]	12.1951	11.9773	11.6956	12.4258	12.0448	11.7162	12.359	10.9228	11.3324	11.2682	12.0408	12.0653	11.7582	11.6675	0.341022016	0.86129475	4	4	24	0.134940387	0.74653384	24	19.21776387	0.035921908
ENSMUSG00000043024	Gm16433	predicted gene 16433 [Source:MGI Symbol;Acc:MGI:3645626]	5.67766	5.51333	5.56312	5.91702	5.64599	6.04494	5.4499	5.41295	5.56811	5.45948	6.23119	5.91379	5.88076	5.65838	0.080148772	0.458678242	24	8	24	0.035340045	0.608551791	24	17.70543044	0.048000241
ENSMUSG00000043051	Disc1	disrupted in schizophrenia 1 [Source:MGI Symbol;Acc:MGI:2447658]	0.724097	0.486549	0.315957	0.474201	0.668708	0.439026	0.328457	0.627348	0.558947	0.585745	0.802327	0.658445	0.985738	0.612644	0.581070934	0.966018836	20	16	24	0.569195144	0.844729091	24	20.09756955	0.06250574
ENSMUSG00000043059	Zfp513	zinc finger protein 513 [Source:MGI Symbol;Acc:MGI:2141255]	5.02182	4.64082	5.26222	4.79581	4.359	4.397	4.72709	5.00749	5.23433	6.31545	5.46313	5.28932	5.76486	5.50978	0.820042705	0.966018836	16	4	24	0.272121722	0.768040178	24	8.637886226	0.063865897
ENSMUSG00000043061	Tmem18	transmembrane protein 18 [Source:MGI Symbol;Acc:MGI:2387176]	19.0003	21.9199	23.3807	21.7878	21.1657	21.4272	20.0642	19.5913	21.2822	19.8781	21.3736	20.9346	19.4811	20.7685	0.062631097	0.410322149	12	16	24	0.180283646	0.768040178	24	11.42483766	0.052236802
ENSMUSG00000043065	Spice1	spindle and centriole associated protein 1 [Source:MGI Symbol;Acc:MGI:1196252]	9.73971	8.02975	7.09293	7.38815	7.97563	9.07353	8.46796	7.44	7.31451	7.37817	8.97184	7.27626	8.27695	6.30064	0.031969981	0.324385159	4	8	24	0.010478003	0.506240926	24	20.27065421	0.145008765
ENSMUSG00000043067	Dpy19l1	dpy-19-like 1 (C. elegans) [Source:MGI Symbol;Acc:MGI:1915685]	9.68943	11.0574	13.0422	10.4368	9.95969	11.2529	10.7515	10.317	10.3572	9.64645	9.9234	10.3871	9.36527	10.2928	0.940625808	0.966018836	12	16	24	0.592959166	0.856634016	24	7.176936075	0.041633682
ENSMUSG00000043068	Fam89a	family with sequence similarity 89, member A [Source:MGI Symbol;Acc:MGI:1916877]	26.874	20.3135	19.4662	23.0642	21.7939	25.7743	19.79	24.1064	18.7215	26.1368	22.7521	24.1014	23.6428	26.2342	0.658895034	0.966018836	24	12	24	0.464206669	0.805393068	24	20.234229	0.081726628
ENSMUSG00000043079	Synpo	synaptopodin [Source:MGI Symbol;Acc:MGI:1099446]	6.74826	6.49415	6.87793	7.01191	7.32194	6.86565	6.58546	7.74068	6.69998	8.85197	6.18351	6.50779	6.81718	6.68572	0.636884864	0.966018836	16	12	24	0.3637095	0.788601811	24	10.81665888	0.063726461
ENSMUSG00000043090	Zfp866	zinc finger protein 866 [Source:MGI Symbol;Acc:MGI:3584369]	9.78975	10.1774	10.5323	10.3235	11.5803	9.91982	10.592	9.21306	10.1917	8.63643	10.9489	9.78743	10.248	10.0732	0.702630284	0.966018836	20	12	24	0.59752867	0.858564588	24	17.55436993	0.037881037
ENSMUSG00000043091	Tuba1c	tubulin, alpha 1C [Source:MGI Symbol;Acc:MGI:1095409]	23.96	23.15	22.5088	21.3945	23.9546	26.4853	2.66141	13.192	18.6998	21.5513	10.2193	19.9473	20.5862	18.1405	0.875755063	0.966018836	24	8	24	0.691433191	0.897724829	24	12.09272469	0.254283672
ENSMUSG00000043099	Hic1	hypermethylated in cancer 1 [Source:MGI Symbol;Acc:MGI:1338010]	0.85302	1.13641	0.935307	0.977286	1.19857	0.579197	1.31748	1.56139	1.506	0.813175	1.73431	1.33552	1.92632	1.08618	0.907068194	0.966018836	4	12	24	0.778450322	0.928832041	24	3.170221413	0.060338651
ENSMUSG00000043110	Lrrn4	leucine rich repeat neuronal 4 [Source:MGI Symbol;Acc:MGI:2445154]	0.623744	0.550161	0.739591	0.565922	0.705266	0.738742	0.531216	0.61112	0.534052	0.661974	0.669851	0.777059	0.894204	0.707736	0.622773557	0.966018836	24	8	24	0.37930392	0.798714572	24	19.72624286	0.045453723
ENSMUSG00000043122	A530016L24Rik	RIKEN cDNA A530016L24 gene [Source:MGI Symbol;Acc:MGI:2443020]	0.272124	0.25855	0.700754	1.48592	0.74263	0.583801	0.615325	1.23121	0.482682	1.38586	2.29357	1.0589	1.73196	1.22363	0.483374602	0.966018836	16	20	24	0.154319724	0.760257003	24	13.73680197	0.220668603
ENSMUSG00000043131	Mob1a	MOB kinase activator 1A [Source:MGI Symbol;Acc:MGI:2442631]	18.2429	18.5645	18.0276	18.5532	19.7588	16.8726	20.0952	18.175	19.3176	16.0569	19.4049	18.0379	18.8675	17.6401	0.835401638	0.966018836	4	12	24	0.857671497	0.958007588	24	22.26678083	0.018963702
ENSMUSG00000043140	Tmem186	transmembrane protein 186 [Source:MGI Symbol;Acc:MGI:1913940]	5.62637	5.48629	6.96492	6.96273	6.50358	6.54464	5.63588	5.91984	5.66694	6.5972	6.27778	5.91714	6.07602	6.06085	0.033136984	0.324908923	16	16	24	0.021190973	0.571993737	24	13.33325878	0.093426683
ENSMUSG00000043153	Crppa	CDP-L-ribitol pyrophosphorylase A [Source:MGI Symbol;Acc:MGI:1923097]	11.4334	11.2359	10.171	10.4351	11.5392	11.2079	12.1198	10.8046	10.3068	10.4021	10.8083	10.7472	11.1948	11.2747	0.004320961	0.140598674	4	8	24	0.007400739	0.47735824	24	22.44968373	0.069856615
ENSMUSG00000043154	Ppp2r3a	protein phosphatase 2, regulatory subunit B'', alpha [Source:MGI Symbol;Acc:MGI:2442104]	6.33871	9.30649	9.75209	9.60066	7.69324	8.63767	8.31357	11.5306	12.5897	10.9562	7.84316	9.16638	9.25722	9.53158	0.004947794	0.141782417	12	8	24	0.006549935	0.470633744	24	8.174926341	0.233515811

ENSMUSG00000043155	Hpdl	4-hydroxyphenylpyruvate dioxygenase-like [Source:MGI Symbol;Acc:MGI:2444646]	1.13488	0.835533	0.735709	0.918004	0.926022	1.12928	0.973665	0.975813	1.05745	1.18015	1.08297	1.12	1.15506	1.37878	0.840483986	0.966018836	4	8	24	0.458754612	0.805393068	24	21.9208516	0.042018315
ENSMUSG00000043157	Arl11	ADP-ribosylation factor-like 11 [Source:MGI Symbol;Acc:MGI:2444054]	0.390809	0.596419	1.01394	0.691944	0.49678	0.513793	0.236928	0.511418	0.843009	0.41699	0.68395	0.421096	0.439803	0.669916	0.002004172	0.100255953	12	16	24	0.023272162	0.581965446	24	9.433473802	0.178112532
ENSMUSG00000043162	Pyurf	Pigy upstream reading frame [Source:MGI Symbol;Acc:MGI:1913709]	5.97765	6.69062	6.70542	6.6905	6.25458	6.32106	6.59737	6.3689	6.36088	6.40378	6.98646	6.77237	7.38987	7.09947	0.807813307	0.966018836	8	12	24	0.794913935	0.93426902	24	4.69073475	0.015090194
ENSMUSG00000043164	Tmem212	transmembrane protein 212 [Source:MGI Symbol;Acc:MGI:2685410]	0.216181	1.86539	1.33136	0.987785	1.12783	0.529443	1.22912	0.128689	0.687757	0.904838	0.691462	1.75176	0.0962295	1.3543	0.858932988	0.966018836	8	20	24	0.708267571	0.903587413	24	11.61143954	0.150737531
ENSMUSG00000043183	Simc1	SUMO-interacting motifs containing 1 [Source:MGI Symbol;Acc:MGI:2442599]	2.45497	3.07189	2.57915	3.06215	3.04887	3.23356	2.83465	3.21979	2.74584	3.94731	3.84509	3.2649	2.78946	3.83218	0.347075276	0.870418653	16	12	24	0.252282013	0.768040178	24	13.45826573	0.081492874
ENSMUSG00000043190	Rfesd	Rieske (Fe-S) domain containing [Source:MGI Symbol;Acc:MGI:2145198]	18.6323	21.3872	17.7715	23.2324	20.1857	17.7235	22.8912	20.9286	21.93	17.3969	20.7357	21.3127	20.3	19.2306	0.788254138	0.966018836	8	16	24	0.968187276	0.992322419	24	3.048343756	0.013241075
ENSMUSG00000043192	Gpi-ps	glucose-6-phosphate isomerase, pseudogene [Source:MGI Symbol;Acc:MGI:3037698]	126.786	99.7671	112.404	114.454	105.867	135.082	116.799	128.401	89.8922	137.746	100.967	107.453	103.62	107.446	0.919571723	0.966018836	24	12	24	0.740450382	0.915274625	24	19.88003954	0.056411807
ENSMUSG00000043207	Zmpste24	zinc metallopeptidase, STE24 [Source:MGI Symbol;Acc:MGI:1890508]	26.1528	26.2962	26.4254	26.4057	25.9831	24.9827	25.0063	23.9813	24.9023	22.808	27.0516	23.818	25.2839	25.2759	0.829428469	0.966018836	20	4	24	0.963336812	0.990884307	24	17.20818664	0.006650592
ENSMUSG00000043219	Hoxa6	homeobox A6 [Source:MGI Symbol;Acc:MGI:96178]	0.895047	0.852551	1.21644	1.15351	1.00361	0.951379	1.02334	1.20112	0.840045	0.850128	0.883848	1.32107	0.969509	1.42325	0.890176313	0.966018836	8	8	24	0.763085508	0.923594538	24	4.197712102	0.037179025
ENSMUSG00000043241	Upf2	UPF2 regulator of nonsense transcripts homolog (yeast) [Source:MGI Symbol;Acc:MGI:2449307]	10.3341	11.1387	10.9747	10.305	10.4969	10.3535	11.6505	9.20977	11.1144	9.23114	11.2161	10.94	10.7485	11.0231	0.841589986	0.966018836	12	4	24	0.708001601	0.903565455	24	23.38752473	0.030474102
ENSMUSG00000043243	Niban3	niban apoptosis regulator 3 [Source:MGI Symbol;Acc:MGI:3686743]	1.37322	2.04135	3.06472	2.26589	2.03243	1.94773	1.94238	2.50926	1.58874	1.31546	2.07528	1.74512	2.1498	1.83011	0.896799496	0.966018836	12	12	24	0.767449737	0.924920314	24	7.260086184	0.063807803
ENSMUSG00000043251	Exoc3l	exocyst complex component 3-like [Source:MGI Symbol;Acc:MGI:3041195]	3.2461	2.76077	2.91705	3.56535	2.31851	3.40405	3.05527	3.33392	2.15321	3.34681	3.72874	3.91531	2.72096	2.45293	0.12833063	0.560209134	24	12	24	0.255529409	0.768040178	24	18.18546467	0.127243919
ENSMUSG00000043252	Tmem64	transmembrane protein 64 [Source:MGI Symbol;Acc:MGI:2140359]	15.7787	16.1653	14.177	14.5987	15.0428	14.2816	14.8441	17.844	15.7217	14.8177	15.2618	14.4606	14.7159	16.1471	0.018894761	0.256345714	8	16	24	0.101426614	0.713208839	24	4.532304775	0.067459582
ENSMUSG00000043257	Pigy	phosphatidylinositol glycan anchor biosynthesis, class Y [Source:MGI Symbol;Acc:MGI:2442480]	2.94427	2.8475	2.86109	2.621	3.83673	3.45033	4.46007	3.70335	3.71354	4.20585	4.31355	3.60758	4.38378	3.9755	0.912923872	0.966018836	4	8	24	0.544743409	0.832363403	24	20.2140131	0.058497591
ENSMUSG00000043259	Fam13c	family with sequence similarity 13, member C [Source:MGI Symbol;Acc:MGI:1918971]	5.51724	4.95403	5.65933	5.55912	5.58859	5.70118	6.05441	5.28503	4.96631	5.67663	5.37607	5.08977	4.98644	5.1046	0.588208558	0.966018836	16	16	24	0.33194346	0.768759286	24	16.84133226	0.041170032
ENSMUSG00000043262	Uevld	UEV and lactate/malate dehyrogenase domains [Source:MGI Symbol;Acc:MGI:1860490]	5.93445	6.0293	5.56473	5.4075	5.5634	5.79962	5.59552	5.90013	5.90281	5.64058	6.17038	5.75783	5.80218	5.89552	0.33885934	0.858075318	8	8	24	0.446509402	0.805393068	24	1.246101198	0.021423494
ENSMUSG00000043263	Ifi209	interferon activated gene 209 [Source:MGI Symbol;Acc:MGI:2138243]	0.628058	0.882942	1.04267	0.829147	0.710018	0.780212	0.856618	0.738642	1.11677	0.414748	0.709575	0.822083	0.665195	0.76777	0.297978647	0.80820842	12	8	24	0.468800602	0.805393068	24	6.005195284	0.068816618
ENSMUSG00000043279	Trim56	tripartite motif-containing 56 [Source:MGI Symbol;Acc:MGI:2685298]	5.01539	4.32075	4.07916	4.76707	4.77957	4.23754	4.50169	4.86978	4.00105	4.2736	4.84519	4.76069	4.56508	4.74794	0.424718357	0.966018836	20	16	24	0.315158698	0.768040178	24	20.57547913	0.051685452
ENSMUSG00000043284	Tmem11	transmembrane protein 11 [Source:MGI Symbol;Acc:MGI:2144726]	10.6068	10.2334	10.5322	11.2855	9.7134	10.0239	10.3268	11.5538	10.4448	11.0643	10.9312	10.5725	11.6176	11.2111	0.912923872	0.966018836	16	4	24	0.293126397	0.768040178	24	6.337581046	0.03862554
ENSMUSG00000043290	Zfp784	zinc finger protein 784 [Source:MGI Symbol;Acc:MGI:3606042]	1.7638	2.0712	1.88948	1.89125	2.19994	2.14055	1.84017	1.96754	2.16336	2.09585	2.12549	1.65642	2.06574	2.02844	0.350544145	0.870418653	20	8	24	0.431094619	0.805393068	24	11.42060358	0.040747255
ENSMUSG00000043298	Smco3	single-pass membrane protein with coiled-coil domains 3 [Source:MGI Symbol;Acc:MGI:2443451]	36.0396	42.7147	34.0905	35.0989	47.3221	31.4102	42.7314	36.2511	48.5322	31.1186	42.3354	37.8777	41.3204	37.4015	0.922029406	0.966018836	20	20	24	0.964929587	0.99132528	24	1.873366328	0.020903935
ENSMUSG00000043300	B3galnt1	UDP-GalNAc:betaGlcNAc beta 1,3-galactosaminyltransferase, polypeptide 1 [Source:MGI Symbol;Acc:MGI:1349405]	20.8354	18.376	20.2703	17.7	18.6323	19.9741	19.2341	18.4507	16.2372	16.3697	20.3199	18.4536	17.873	20.2065	0.718720526	0.966018836	20	20	24	0.200613555	0.768040178	24	22.18424862	0.073014322
ENSMUSG00000043301	Kcnj6	potassium inwardly-rectifying channel, subfamily J, member 6 [Source:MGI Symbol;Acc:MGI:104781]	0.251694	0.681679	0.675201	0.368243	0.398485	0.465535	0.367074	0.449038	0.571765	0.4559	0.467368	0.746891	0.425369	0.81177	0.46238066	0.966018836	8	20	24	0.336620594	0.771945551	24	6.777830086	0.084688083
ENSMUSG00000043311	D17H6S53E	DNA segment, Chr 17, human D6S53E [Source:MGI Symbol;Acc:MGI:90673]	3.70912	3.71559	4.1734	4.75073	4.60025	5.15095	4.96522	4.8016	4.21452	4.37202	3.97246	3.63351	5.61791	3.8661	0.833301348	0.966018836	4	4	24	0.880394789	0.966395747	24	19.98955342	0.033941896
ENSMUSG00000043313	Pcdhb19	protocadherin beta 19 [Source:MGI Symbol;Acc:MGI:2136757]	0.867277	0.814217	0.925681	0.661754	0.872562	0.830577	0.748898	0.673445	0.839017	0.714904	0.854863	0.772349	0.849495	0.84888	0.923635729	0.966018836	20	20	24	0.859030892	0.958530654	24	22.48195963	0.014607555
ENSMUSG00000043323	Fbrsl1	fibrosin-like 1 [Source:MGI Symbol;Acc:MGI:1920907]	4.28451	4.33118	4.84402	4.31365	4.08793	3.93516	4.43033	5.05787	6.07061	6.83729	5.9154	5.78824	6.01451	5.74116	0.916746631	0.966018836	12	12	24	0.086791333	0.706014767	24	9.192117808	0.113593948
ENSMUSG00000043329	Gm8849	predicted gene 8849 [Source:MGI Symbol;Acc:MGI:3647326]	1.26474	1.28019	1.23534	0.858747	0.746668	1.20299	2.15038	0.56111	1.37394	0.92004	0.946524	1.22286	1.01903	1.255	0.149354198	0.593798152	4	12	24	0.331840001	0.768621471	24	0.728171077	0.136786219
ENSMUSG00000043336	Filip1l	filamin A interacting protein 1-like [Source:MGI Symbol;Acc:MGI:1925999]	2.4685	3.05158	3.13368	3.57736	2.91518	3.04479	3.81641	3.94904	4.16385	3.10619	3.44296	2.68493	2.99152	2.96307	0.204218286	0.690546825	12	12	24	0.221685732	0.768040178	24	8.525754709	0.112391871
ENSMUSG00000043340	6530409C15Rik	RIKEN cDNA 6530409C15 gene [Source:MGI Symbol;Acc:MGI:1923474]	0.422668	0.543896	0.611824	0.647449	0.784207	0.512771	0.756404	0.681405	1.00162	0.57717	0.562635	0.734922	0.773766	0.795748	0.891742979	0.966018836	12	12	24	0.435325863	0.805393068	24	7.686391289	0.057270123
ENSMUSG00000043342	Hoxd9	homeobox D9 [Source:MGI Symbol;Acc:MGI:96210]	2.8103	3.05486	3.25501	3.12429	2.4623	3.36858	2.9932	3.28253	2.87168	3.83136	2.59157	3.90065	3.23941	3.70232	0.945451136	0.966018836	8	12	24	0.66949011	0.888703971	24	4.887961322	0.048808565
ENSMUSG00000043346	Gm6741	predicted gene 6741 [Source:MGI Symbol;Acc:MGI:3643504]	1.15947	0.761774	1.18611	0.935563	0.457815	0.925803	0.748044	0.426505	0.837191	0.811303	0.982217	0.959376	0.773869	0.717033	0.349109869	0.870418653	12	20	24	0.893761047	0.969947957	24	16.63648441	0.033711936
ENSMUSG00000043366	Olfr78	olfactory receptor 78 [Source:MGI Symbol;Acc:MGI:2157548]	0.535676	0.540778	0.681055	0.818313	0.529211	0.884984	1.09895	0.419574	0.694576	0.809465	0.900351	0.972812	0.397959	1.48697	0.904831057	0.966018836	24	4	24	0.937453706	0.984507582	24	14.6904368	0.030564575
ENSMUSG00000043372	Hexim2	hexamethylene bis-acetamide inducible 2 [Source:MGI Symbol;Acc:MGI:1918309]	3.11885	3.49621	3.77237	2.88278	2.87902	3.4664	2.82194	2.86965	3.08693	3.69705	2.7313	3.50142	3.63159	2.76757	0.916963526	0.966018836	12	20	24	0.989504639	0.997867764	24	4.431666656	0.007743064
ENSMUSG00000043384	Gprasp1	G protein-coupled receptor associated sorting protein 1 [Source:MGI Symbol;Acc:MGI:1917418]	81.7314	84.3194	85.2152	79.589	75.8069	80.8914	84.1037	72.3137	79.4266	77.8681	75.2071	83.8834	80.2632	88.7119	0.268242676	0.774280302	8	12	24	0.329282885	0.768040178	24	2.527627277	0.045102793
ENSMUSG00000043388	Tmem130	transmembrane protein 130 [Source:MGI Symbol;Acc:MGI:3607706]	43.836	36.3233	42.2497	33.7288	32.1734	45.2516	40.5161	38.6964	27.9345	46.5795	30.089	38.8258	32.8949	41.3435	0.924553956	0.966018836	24	20	24	0.601596109	0.860592628	24	0.09536874	0.088010156
ENSMUSG00000043391	2510009E07Rik	RIKEN cDNA 2510009E07 gene [Source:MGI Symbol;Acc:MGI:1919440]	38.8167	32.816	31.7193	33.4833	31.4883	36.797	31.6261	34.2675	28.2711	38.1226	31.9227	35.9076	35.0702	35.426	0.390686537	0.915479136	24	12	24	0.366689856	0.790399468	24	21.43603646	0.069480237
ENSMUSG00000043398	Gpr135	G protein-coupled receptor 135 [Source:MGI Symbol;Acc:MGI:2676315]	4.78269	3.76401	4.38387	4.00671	4.12286	4.11155	3.59625	3.64548	3.27543	4.14701	4.77912	4.10163	3.97403	4.0255	0.407577742	0.945128283	20	12	24	0.212333507	0.768040178	24	17.63114487	0.080914751
ENSMUSG00000043410	Hfm1	HFM1, ATP-dependent DNA helicase homolog [Source:MGI Symbol;Acc:MGI:3036246]	1.24906	1.33449	1.22436	1.56551	1.38954	1.04613	0.945959	1.09331	1.58685	1.02385	1.47398	1.11004	1.25682	1.36549	0.416857905	0.963884303	20	4	24	0.24552165	0.768040178	24	10.61256607	0.084472892
ENSMUSG00000043411	Usp48	ubiquitin specific peptidase 48 [Source:MGI Symbol;Acc:MGI:2158502]	41.1655	38.9879	41.3855	39.6677	38.3849	36.3552	36.6906	33.6613	43.4914	38.1601	37.7784	35.9465	35.6695	36.7648	0.039704107	0.338535904	12	12	24	0.15482294	0.760257003	24	10.17945341	0.062390131
ENSMUSG00000043415	Otud1	OTU domain containing 1 [Source:MGI Symbol;Acc:MGI:1918448]	3.85573	3.37486	2.90541	4.07295	3.69757	3.19436	3.66247	3.39985	3.78777	4.2993	3.55532	3.76411	3.76991	3.45502	0.5169953	0.966018836	16	16	24	0.517812051	0.818405353	24	14.79593776	0.049101589
ENSMUSG00000043419	Rnf227	ring finger protein 227 [Source:MGI Symbol;Acc:MGI:1915359]	5.90189	9.23418	13.8399	6.48189	5.36132	8.13288	10.4959	6.41161	10.1167	7.98953	5.94205	8.80409	4.99221	11.5902	0.10560184	0.518002936	12	8	24	0.195718717	0.768040178	24	6.434613041	0.281859665
ENSMUSG00000043421	Hilpda	hypoxia inducible lipid droplet associated [Source:MGI Symbol;Acc:MGI:1916823]	6.79296	7.86619	7.17609	6.28933	8.7533	6.72108	6.45287	7.95091	8.38408	6.12285	7.4739	7.61038	8.53203	7.42748	0.935194469	0.966018836	8	8	24	0.807446839	0.938693233	24	2.827776949	0.037130167
ENSMUSG00000043424	Eif3j2	eukaryotic translation initiation factor 3, subunit J2 [Source:MGI Symbol;Acc:MGI:3704486]	9.89871	9.19529	9.06194	9.87115	9.67176	8.94799	8.69282	9.2174	10.0916	10.6437	11.9694	11.7739	12.8123	11.5071	0.850906022	0.966018836	20	16	24	0.707513692	0.903508876	24	17.17022087	0.035604527
ENSMUSG00000043432	Leng9	leukocyte receptor cluster (LRC) member 9 [Source:MGI Symbol;Acc:MGI:2444509]	1.72918	1.42504	1.65099	1.22459	1.72046	1.89705	1.93564	1.92046	1.66147	1.6659	1.75492	2.0931	1.79499	1.56188	0.028632001	0.316608413	24	12	24	0.055169498	0.65534544	24	21.46427117	0.10096047
ENSMUSG00000043441	Gpr149	G protein-coupled receptor 149 [Source:MGI Symbol;Acc:MGI:2443628]	7.41572	6.94952	7.40683	6.86909	5.92852	6.40839	4.92991	6.54621	5.35296	5.30199	4.62457	7.09783	7.05011	7.00143	0.517849107	0.966018836	4	16	24	0.321587828	0.768040178	24	2.360624791	0.108581515
ENSMUSG00000043445	Pgp	phosphoglycolate phosphatase [Source:MGI Symbol;Acc:MGI:1914328]	22.0326	18.8965	21.1503	20.0521	19.9972	20.1336	20.1564	20.3473	20.2966	24.016	22.8551	23.6674	24.1798	21.1166	0.860993221	0.966018836	4	4	24	0.534062817	0.826231134	24	17.76884294	0.039973488
ENSMUSG00000043448	Gjc2	gap junction protein, gamma 2 [Source:MGI Symbol;Acc:MGI:2153060]	0.863751	1.75615	2.74696	0.970105	0.780491	1.50788	1.93716	1.42778	2.06261	0.930411	0.810579	1.65613	0.721084	1.86014	0.03782572	0.334069441	12	8	24	0.138324953	0.747972781	24	5.452870125	0.268574506
ENSMUSG00000043456	Zfp536	zinc finger protein 536 [Source:MGI Symbol;Acc:MGI:1926102]	8.79111	8.89835	7.72969	8.85794	9.00782	8.55123	9.71573	8.25759	9.02675	10.6822	8.16683	9.45699	9.08322	8.7406	0.914360142	0.966018836	4	4	24	0.844296206	0.952833645	24	17.17980228	0.023633494
ENSMUSG00000043458	Pcdhb12	protocadherin beta 12 [Source:MGI Symbol;Acc:MGI:2136747]	1.2523	0.899966	0.801649	0.609964	0.918613	1.01192	0.848984	0.774763	0.712692	1.17344	1.04094	0.73558	0.85897	0.816387	0.593560297	0.966018836	20	16	24	0.429150692	0.805393068	24	19.7821821	0.070369197
ENSMUSG00000043460	Elfn2	leucine rich repeat and fibronectin type III, extracellular 2 [Source:MGI Symbol;Acc:MGI:3608416]	0.868703	1.15249	1.76069	0.827208	0.776171	1.20609	1.34399	0.848429	1.45268	1.42576	0.678788	1.12522	0.698842	1.44131	0.03782572	0.334069441	12	8	24	0.220272253	0.768040178	24	7.062801179	0.158723451
ENSMUSG00000043461	Sptssb	serine palmitoyltransferase, small subunit B [Source:MGI Symbol;Acc:MGI:1913433]	2.26073	2.80782	2.63176	1.80434	1.9117	2.24632	2.48223	2.33591	2.43668	1.42725	1.57978	2.42792	1.59396	2.41234	0.022000259	0.267729408	12	4	24	0.049442196	0.642285999	24	2.998255315	0.164922394
ENSMUSG00000043463	Rab9b	RAB9B, member RAS oncogene family [Source:MGI Symbol;Acc:MGI:2442454]	17.7329	16.1604	15.5915	14.914	15.9084	16.1383	16.87	13.8164	13.78	13.2635	17.2104	15.3269	16.5708	16.2149	0.004021228	0.140598674	4	12	24	0.030509864	0.597890721	24	21.74660294	0.111456724
ENSMUSG00000043467	Zbtb37	zinc finger and BTB domain containing 37 [Source:MGI Symbol;Acc:MGI:2444467]	1.40754	1.82932	1.55023	1.80601	1.84027	1.40286	2.58357	1.84232	2.17927	1.53813	1.96533	1.81131	1.96791	1.80177	0.885059765	0.966018836	4	12	24	0.807417716	0.938693233	24	3.849690918	0.038493372
ENSMUSG00000043483	Gm6863	predicted gene 6863 [Source:MGI Symbol;Acc:MGI:3647386]	242.902	258.407	247.402	248.979	252.518	225.543	243.641	227.936	284.211	203.439	264.859	250.877	272.499	248.235	0.858266942	0.966018836	20	4	24	0.881956558	0.966661503	24	3.480487476	0.024027349
ENSMUSG00000043484	Gm5867	predicted gene 5867 [Source:MGI Symbol;Acc:MGI:3644922]	18.8099	16.4299	15.7787	15.6846	15.3472	16.3549	16.656	13.5193	13.7531	13.9118	16.7367	15.9528	16.4502	15.9159	0.016131606	0.253395174	4	8	24	0.028170372	0.590935816	24	21.54908771	0.110818823
ENSMUSG00000043487	Acot6	acyl-CoA thioesterase 6 [Source:MGI Symbol;Acc:MGI:1921287]	3.88436	3.51031	3.2878	2.97829	3.38536	3.34122	3.06429	3.77337	3.30794	3.45017	3.17412	3.69553	3.3621	3.5362	0.328944792	0.847277612	8	4	24	0.261553199	0.768040178	24	1.106426837	0.052324695
ENSMUSG00000043496	Tril	TLR4 interactor with leucine-rich repeats [Source:MGI Symbol;Acc:MGI:1914123]	1.5129	2.24958	3.40703	1.70178	1.31934	2.33975	2.77391	2.01308	2.53158	2.40104	1.643	2.46347	1.70658	2.6881	0.185796491	0.662239322	12	8	24	0.216792951	0.768040178	24	6.051991258	0.178072732
ENSMUSG00000043505	Gimap5	GTPase, IMAP family member 5 [Source:MGI Symbol;Acc:MGI:2442232]	0.614913	1.0301	1.1042	1.76475	0.914546	0.764384	1.10204	1.02755	1.37117	1.23718	1.19853	0.594341	0.792721	0.888419	4.29E-04	0.054040373	16	8	24	0.004236843	0.427431763	24	10.36049861	0.235837874
ENSMUSG00000043510	Hscb	HscB iron-sulfur cluster co-chaperone [Source:MGI Symbol;Acc:MGI:2141135]	9.41924	10.3689	8.75731	12.3645	11.8796	10.0725	10.4235	9.61063	11.0186	10.6956	9.61446	10.4391	10.9401	11.184	0.909604019	0.966018836	16	20	24	0.55511056	0.838303107	24	13.46477295	0.05073933
ENSMUSG00000043518	Rai2	retinoic acid induced 2 [Source:MGI Symbol;Acc:MGI:1344378]	2.67632	1.95249	1.94273	2.37152	2.23435	2.62895	2.30119	2.71648	2.26032	2.83858	1.9668	2.2867	2.4066	2.43127	0.885025617	0.966018836	4	8	24	0.847004442	0.953798522	24	22.77937571	0.029002033
ENSMUSG00000043531	Sorcs1	sortilin-related VPS10 domain containing receptor 1 [Source:MGI Symbol;Acc:MGI:1929666]	17.1932	18.5955	17.2307	18.335	19.8815	16.0984	19.4663	17.5788	18.3526	19.0431	16.7534	17.6421	17.8565	16.7607	0.890648739	0.966018836	16	8	24	0.738118589	0.91451866	24	11.55201078	0.02586581
ENSMUSG00000043535	Setx	senataxin [Source:MGI Symbol;Acc:MGI:2443480]	6.95636	8.90944	8.42873	10.9035	8.89484	8.56106	8.2921	7.93585	10.1436	8.61006	7.9622	8.88171	8.53584	9.00167	0.192357053	0.669841558	16	12	24	0.112504474	0.726529814	24	10.78280852	0.107936932
ENSMUSG00000043541	Casc1	cancer susceptibility candidate 1 [Source:MGI Symbol;Acc:MGI:2444480]	0.696936	0.852802	1.13113	0.974365	1.04254	0.793618	0.644789	0.794869	0.777413	0.791122	0.853728	0.860881	0.621873	0.844529	0.030347909	0.324150946	20	8	24	0.037323596	0.609176161	24	12.00824859	0.09463937
ENSMUSG00000043542	Zc2hc1a	zinc finger, C2HC-type containing 1A [Source:MGI Symbol;Acc:MGI:1914556]	43.3253	42.3562	38.9557	43.345	40.9767	40.0278	40.0036	36.9617	36.2062	38.2058	41.8851	38.6864	38.1038	42.0033	0.880631923	0.966018836	16	20	24	0.57707881	0.848822643	24	19.0667972	0.0322856
ENSMUSG00000043556	Fbxl7	F-box and leucine-rich repeat protein 7 [Source:MGI Symbol;Acc:MGI:3052506]	0.938143	0.951038	0.847842	0.807706	1.03451	0.774571	1.06081	0.859972	0.679087	1.02275	0.844387	1.0381	0.909965	0.895278	0.43565473	0.966018836	4	8	24	0.374616436	0.794671327	24	20.75640601	0.048816315
ENSMUSG00000043557	Mdga1	MAM domain containing glycosylphosphatidylinositol anchor 1 [Source:MGI Symbol;Acc:MGI:1922012]	2.49143	1.92329	2.44582	1.97246	2.65769	2.54001	2.19004	3.2402	2.02359	3.49333	1.98631	2.43622	2.36987	2.36992	0.814221431	0.966018836	24	12	24	0.980403053	0.995255072	24	12.6541751	0.01462671
ENSMUSG00000043572	Pars2	prolyl-tRNA synthetase (mitochondrial)(putative) [Source:MGI Symbol;Acc:MGI:2386296]	2.2384	1.9508	2.70277	2.71444	1.64898	2.30582	2.48223	1.82533	1.89923	2.28347	2.01409	2.2529	2.31245	2.29171	0.946633657	0.966018836	16	4	24	0.767406717	0.924900028	24	10.51770428	0.038237094
ENSMUSG00000043587	Pxylp1	2-phosphoxylose phosphatase 1 [Source:MGI Symbol;Acc:MGI:2442444]	1.7895	1.69141	1.70485	1.43058	1.36213	2.00688	1.20676	1.5413	1.70511	1.42417	1.09645	1.83445	1.73414	1.29529	0.129903614	0.560263689	24	20	24	0.53023563	0.824795587	24	0.137541044	0.064900461
ENSMUSG00000043602	Zfp3	zinc finger protein 3 [Source:MGI Symbol;Acc:MGI:99177]	3.21379	3.38816	3.72454	3.34382	3.09334	3.31093	3.62172	3.63722	3.07095	3.55591	3.51508	3.35318	3.6175	3.88077	0.46238066	0.966018836	8	12	24	0.222649476	0.768040178	24	4.668040258	0.047327364
ENSMUSG00000043614	Vps37d	vacuolar protein sorting 37D [Source:MGI Symbol;Acc:MGI:2159402]	2.58372	2.77494	2.9986	2.74288	1.62025	2.30006	2.70032	2.96322	3.20086	3.20458	3.06836	3.10294	2.86232	3.70779	0.322884702	0.838998583	12	8	24	0.03017843	0.596794384	24	6.367978882	0.161829471
ENSMUSG00000043618	Eif5al3-ps	eukaryotic translation initiation factor 5A-like 3, pseudogene [Source:MGI Symbol;Acc:MGI:3643585]	33.3625	30.5263	30.52	37.504	31.2163	32.9765	28.6486	29.4299	26.4643	26.9292	31.8707	27.939	27.9472	26.433	0.729086532	0.966018836	20	12	24	0.111164213	0.723334499	24	16.97990839	0.082687917
ENSMUSG00000043629	1700019D03Rik	RIKEN cDNA 1700019D03 gene [Source:MGI Symbol;Acc:MGI:1914330]	7.66869	5.47955	4.89813	6.91993	6.94858	6.99275	6.2491	5.28933	4.91005	4.53311	6.19935	6.75114	6.11754	4.88143	0.019280932	0.257669847	24	12	24	3.81E-04	0.23436643	24	19.68898387	0.240910435
ENSMUSG00000043631	Ecm2	extracellular matrix protein 2, female organ and adipocyte specific [Source:MGI Symbol;Acc:MGI:3039578]	7.20455	9.42113	8.00979	7.49448	8.51861	6.80028	8.08616	8.38992	11.578	5.19807	8.19811	7.93725	7.35608	7.94275	0.372888979	0.89586062	12	12	24	0.480341073	0.805393068	24	5.414533927	0.109147058
ENSMUSG00000043633	Fam221b	family with sequence similarity 221, member B [Source:MGI Symbol;Acc:MGI:2441678]	10.7193	11.7159	10.8505	10.4949	11.1291	10.1538	10.5693	11.8786	11.8922	9.40994	12.2075	10.7994	12.616	11.3295	0.745118544	0.966018836	8	8	24	0.423853732	0.805393068	24	3.237217166	0.051431783
ENSMUSG00000043635	Adamts3	a disintegrin-like and metallopeptidase (reprolysin type) with thrombospondin type 1 motif, 3 [Source:MGI Symbol;Acc:MGI:3045353]	2.24788	1.721	2.03399	1.73571	1.56635	1.90232	1.65471	1.74682	1.52218	2.19034	1.41876	1.92707	1.94863	1.96975	0.894563866	0.966018836	4	16	24	0.641628133	0.878809617	24	1.79573336	0.046638283
ENSMUSG00000043639	Rbm20	RNA binding motif protein 20 [Source:MGI Symbol;Acc:MGI:1920963]	0.573879	0.768714	0.747046	0.895521	0.72228	0.797576	0.711782	1.10597	1.29427	1.34179	0.642919	0.696385	0.947166	0.733443	0.168929279	0.640164678	16	4	24	0.064908974	0.663876076	24	9.028164474	0.148499124
ENSMUSG00000043644	0610009L18Rik	RIKEN cDNA 0610009L18 gene [Source:MGI Symbol;Acc:MGI:1914088]	2.71399	3.01929	2.66306	2.49337	2.53744	2.6641	2.3444	3.66743	3.33831	3.64582	2.63458	2.75038	3.33737	3.56979	0.101443845	0.511659635	8	12	24	0.025832499	0.586541342	24	6.267136851	0.140584613
ENSMUSG00000043664	Tmem221	transmembrane protein 221 [Source:MGI Symbol;Acc:MGI:3525074]	0.357614	0.48985	0.538766	0.53844	0.744215	0.376304	0.638524	0.592664	0.545736	0.563254	0.512246	0.542101	0.566764	0.530894	0.814221431	0.966018836	20	4	24	0.560916764	0.841464579	24	11.30049548	0.037616214
ENSMUSG00000043668	Tox3	TOX high mobility group box family member 3 [Source:MGI Symbol;Acc:MGI:3039593]	4.49331	3.55186	3.59639	4.10639	3.61223	4.36032	3.24876	4.44204	3.03016	5.06804	3.52877	3.87477	4.2355	4.41194	0.875185226	0.966018836	8	4	24	0.964495357	0.991159965	24	21.33366477	0.018605465
ENSMUSG00000043670	Diras1	DIRAS family, GTP-binding RAS-like 1 [Source:MGI Symbol;Acc:MGI:2183442]	46.0036	35.5047	34.9412	35.7721	35.0643	40.0725	36.9949	39.1308	27.9096	45.4419	36.0548	41.8304	39.5613	40.7953	0.071575474	0.440262583	24	12	24	0.280515044	0.768040178	24	21.47813467	0.112522007
ENSMUSG00000043671	Dpy19l3	dpy-19-like 3 (C. elegans) [Source:MGI Symbol;Acc:MGI:2443952]	1.59653	1.97077	2.12016	2.13741	1.26046	1.67214	2.66906	1.59697	1.99303	2.0324	1.5351	1.68658	1.39533	2.06543	0.129903614	0.560263689	16	4	24	0.261998311	0.768040178	24	6.773735421	0.120505966
ENSMUSG00000043673	Kcns3	potassium voltage-gated channel, delayed-rectifier, subfamily S, member 3 [Source:MGI Symbol;Acc:MGI:1098804]	24.1373	21.8103	21.1241	23.9927	22.2576	24.2252	20.6828	21.4979	19.4445	23.5913	21.1706	21.0013	21.274	23.2862	0.911685024	0.966018836	16	20	24	0.660430085	0.885470722	24	17.77801016	0.033676975
ENSMUSG00000043683	Fem1a	fem 1 homolog a [Source:MGI Symbol;Acc:MGI:1335089]	6.59974	6.35912	7.4509	7.59364	6.35047	7.74198	6.28087	9.45121	7.33053	9.36477	6.93058	7.96138	7.82871	7.33878	0.880631923	0.966018836	16	12	24	0.49856215	0.808235473	24	9.485671962	0.072676113
ENSMUSG00000043687	1190005I06Rik	RIKEN cDNA 1190005I06 gene [Source:MGI Symbol;Acc:MGI:1916168]	2.76105	3.32932	2.98369	5.03402	2.91909	3.50478	2.42207	4.27309	4.89383	3.81157	3.65079	3.61971	3.76422	3.98108	0.17953325	0.655480352	16	12	24	0.085088256	0.70291653	24	9.729375864	0.182781828
ENSMUSG00000043702	Pde12	phosphodiesterase 12 [Source:MGI Symbol;Acc:MGI:2443226]	3.36172	3.15576	3.25579	3.23331	2.9314	3.17522	2.71182	3.00232	2.85187	3.13463	3.45787	3.18387	3.26217	3.37822	0.747854804	0.966018836	20	20	24	0.941994196	0.985597432	24	16.60325936	0.010490615
ENSMUSG00000043716	Rpl7	ribosomal protein L7 [Source:MGI Symbol;Acc:MGI:98073]	62.0877	80.0501	82.5332	87.7857	82.1818	67.7044	69.4134	67.5828	88.3142	56.1803	73.075	73.3391	75.1207	75.6943	0.143321297	0.585944911	12	12	24	0.481452632	0.805393068	24	9.440224523	0.090439087
ENSMUSG00000043733	Ptpn11	protein tyrosine phosphatase, non-receptor type 11 [Source:MGI Symbol;Acc:MGI:99511]	32.1787	29.2139	30.551	32.0706	30.4947	32.75	32.1513	30.6965	28.1807	35.2745	28.4129	31.7331	30.2684	30.5089	0.607427393	0.966018836	24	12	24	0.644827659	0.879377986	24	18.04157257	0.031878255
ENSMUSG00000043747	1520401A03Rik	RIKEN cDNA 1520401A03 gene [Source:MGI Symbol;Acc:MGI:2443785]	0.450424	0.840151	0.610802	0.280208	1.18391	0.727641	0.660934	0.80176	1.38907	0.614877	0.501355	0.815811	0.999045	0.649603	0.888379597	0.966018836	24	16	24	0.867296568	0.961883504	24	3.598614445	0.048939377
ENSMUSG00000043770	Gm12481	predicted gene 12481 [Source:MGI Symbol;Acc:MGI:3650743]	28.7691	25.6327	27.9511	32.0551	31.0003	28.118	21.9836	28.1346	25.5014	24.3129	33.2918	26.5682	26.2959	21.9444	0.258307762	0.761738864	20	12	24	0.043870858	0.627808337	24	15.557345	0.145148268
ENSMUSG00000043773	1700048O20Rik	RIKEN cDNA 1700048O20 gene [Source:MGI Symbol;Acc:MGI:1920637]	2.30239	4.06817	3.04093	2.73456	2.64451	2.24989	1.21151	2.70028	4.02755	1.59694	2.64698	2.50573	2.25056	2.53825	0.018194955	0.256345714	12	16	24	0.292006317	0.768040178	24	8.069768577	0.19327016
ENSMUSG00000043795	Prr33	proline rich 33 [Source:MGI Symbol;Acc:MGI:3642289]	1.81544	2.81607	2.75301	3.90397	3.62009	3.45194	3.05779	5.8935	5.05427	5.084	2.63357	3.03182	3.50041	2.71202	0.767183862	0.966018836	16	12	24	0.200563329	0.768040178	24	9.920520559	0.234562189
ENSMUSG00000043801	Oaz1-ps	ornithine decarboxylase antizyme 1, pseudogene [Source:MGI Symbol;Acc:MGI:108188]	209.888	197.557	195.795	199.21	198.109	208.844	210.344	228.15	205.345	212.217	207.914	206.468	214.237	200.121	0.286642595	0.793714531	4	8	24	0.455604179	0.805393068	24	23.9519947	0.027437365
ENSMUSG00000043811	Rtn4r	reticulon 4 receptor [Source:MGI Symbol;Acc:MGI:2136886]	2.87912	2.5329	2.08874	2.31489	2.16573	2.17747	1.99105	2.04487	2.19948	3.40505	2.63406	2.75042	2.20898	2.31592	0.890235289	0.966018836	16	20	24	0.564790054	0.843417971	24	15.00379432	0.066238929
ENSMUSG00000043822	Adamtsl5	ADAMTS-like 5 [Source:MGI Symbol;Acc:MGI:1913798]	14.7375	11.6947	9.52936	13.5285	15.6626	11.9256	12.3464	12.0385	14.9013	14.8621	14.7555	13.0849	14.3496	8.77087	0.064091035	0.414867207	20	12	24	0.12434399	0.738204916	24	16.59845398	0.176687719
ENSMUSG00000043831	Lysmd4	LysM, putative peptidoglycan-binding, domain containing 4 [Source:MGI Symbol;Acc:MGI:1922349]	14.3837	14.9191	15.382	15.208	16.2643	15.4346	13.9527	14.3346	15.8344	12.5842	14.1896	13.5272	13.897	14.1587	0.644063931	0.966018836	12	16	24	0.822705067	0.944336753	24	11.65527034	0.020107622
ENSMUSG00000043832	Clec4a3	C-type lectin domain family 4, member a3 [Source:MGI Symbol;Acc:MGI:1920399]	1.25628	1.3339	1.21473	2.28533	1.57286	1.3529	1.69684	1.24487	1.95639	1.15353	1.69175	1.4646	1.64791	1.36066	0.897975397	0.966018836	20	12	24	0.556209679	0.838702296	24	13.84044292	0.073629238
ENSMUSG00000043833	2900005J15Rik	RIKEN cDNA 2900005J15 gene [Source:MGI Symbol;Acc:MGI:1914511]	1.08997	1.80986	1.91012	1.28133	1.36773	1.31587	1.01956	1.58372	2.12919	1.77962	1.36083	1.60697	1.28908	1.40592	0.002254694	0.104113924	12	16	24	0.026195048	0.586541342	24	8.943210075	0.178424873
ENSMUSG00000043843	Tmem145	transmembrane protein 145 [Source:MGI Symbol;Acc:MGI:3607779]	2.44687	2.80264	3.57069	3.01313	2.10802	2.53434	2.88489	2.63702	2.94053	3.30076	2.47684	3.31429	2.54078	3.31199	0.093849599	0.490854203	12	8	24	0.131548238	0.741602131	24	7.814781459	0.121192495
ENSMUSG00000043850	Clrn1	clarin 1 [Source:MGI Symbol;Acc:MGI:2388124]	7.10047	7.07294	5.89371	7.43418	7.14307	5.62547	6.37076	6.78668	6.76677	6.05932	6.91742	6.45015	7.46612	7.70124	0.92655264	0.966018836	8	16	24	0.714573869	0.905979936	24	4.34649972	0.038279705
ENSMUSG00000043857	Mgat5b	mannoside acetylglucosaminyltransferase 5, isoenzyme B [Source:MGI Symbol;Acc:MGI:3606200]	1.477	2.16783	1.97063	1.53424	1.45358	1.59919	1.82087	1.43169	2.65565	1.93585	1.03112	1.51788	0.879828	1.81701	0.10951302	0.524035616	12	8	24	0.087082316	0.706216588	24	7.648169002	0.1991265
ENSMUSG00000043866	Taf10	TATA-box binding protein associated factor 10 [Source:MGI Symbol;Acc:MGI:1346320]	6.07085	7.92882	8.32612	6.38092	10.0556	7.7347	6.02464	8.19555	7.72745	5.44097	7.43248	6.25534	8.07778	4.30658	0.866861748	0.966018836	12	16	24	0.755752815	0.921118175	24	16.25762849	0.076474183
ENSMUSG00000043870	Gm5809	predicted pseudogene 5809 [Source:MGI Symbol;Acc:MGI:3647324]	4.7019	4.32056	4.74891	5.33622	4.37077	4.05043	3.32313	5.24944	5.42633	5.78387	5.21944	4.35981	4.69071	3.98758	0.027467587	0.308438477	16	8	24	0.030719166	0.597890721	24	10.98507327	0.164722168
ENSMUSG00000043872	Zmym1	zinc finger, MYM domain containing 1 [Source:MGI Symbol;Acc:MGI:1915560]	10.2997	10.1581	8.92197	8.96671	11.3246	8.36112	8.81782	10.8964	9.41186	8.24371	11.7966	9.20693	9.66532	8.2631	0.932092277	0.966018836	20	20	24	0.756525362	0.921381638	24	18.51440858	0.046943643
ENSMUSG00000043881	Kbtbd7	kelch repeat and BTB (POZ) domain containing 7 [Source:MGI Symbol;Acc:MGI:2685141]	7.46104	7.12843	6.40487	7.42492	7.31487	6.7463	7.26246	6.76146	5.90011	6.7048	7.66016	5.89168	5.93548	6.05827	0.885025617	0.966018836	20	16	24	0.235639979	0.768040178	24	17.0252103	0.063274596
ENSMUSG00000043885	Slc36a4	solute carrier family 36 (proton/amino acid symporter), member 4 [Source:MGI Symbol;Acc:MGI:2442595]	12.2861	12.6151	13.165	12.3493	12.3714	13.0199	12.2333	11.447	11.9634	13.056	12.1567	13.2999	12.0207	13.0006	0.891105762	0.966018836	24	4	24	0.71680722	0.906600572	24	15.66523054	0.019015772
ENSMUSG00000043889	Gm8399	predicted gene 8399 [Source:MGI Symbol;Acc:MGI:3647971]	89.9845	69.8699	71.2547	75.0957	84.4778	89.4556	71.0228	83.9616	53.6402	82.8716	63.3701	68.0274	67.5109	65.4172	0.95247375	0.966018836	24	12	24	0.281004729	0.768040178	24	19.58068078	0.118919276
ENSMUSG00000043895	S1pr2	sphingosine-1-phosphate receptor 2 [Source:MGI Symbol;Acc:MGI:99569]	1.21696	1.27968	1.03999	1.27278	1.26927	1.45303	1.38514	1.28785	1.60903	1.14694	1.57339	1.28659	1.4901	1.08746	0.691437565	0.966018836	24	16	24	0.522901308	0.821039867	24	19.21768865	0.04845531
ENSMUSG00000043903	Zfp469	zinc finger protein 469 [Source:MGI Symbol;Acc:MGI:2684868]	0.734725	0.552258	0.518078	0.767466	0.593642	0.442099	0.763921	0.622454	0.763709	0.932598	0.599049	0.56968	0.632163	0.524318	0.718086778	0.966018836	16	8	24	0.514623621	0.816837918	24	10.67446386	0.052909902

ENSMUSG00000043909	Trp53bp1	transformation related protein 53 binding protein 1 [Source:MGI Symbol;Acc:MGI:1351320]	27.9787	24.0942	24.91	24.03	25.7594	27.7973	27.1951	25.9399	23.187	24.2533	24.1351	28.3254	23.3645	23.9497	0.071575474	0.440262583	24	12	24	0.010346926	0.503967859	24	20.88371759	0.099468651
ENSMUSG00000043923	Ccdc84	coiled-coil domain containing 84 [Source:MGI Symbol;Acc:MGI:2685960]	9.3189	12.0188	13.2771	11.7159	12.8355	10.7082	11.9433	11.7842	13.1843	10.557	12.883	11.8493	11.4857	12.8034	0.091424683	0.483797062	12	12	24	0.227780254	0.768040178	24	9.522593993	0.085636643
ENSMUSG00000043924	Ncmap	noncompact myelin associated protein [Source:MGI Symbol;Acc:MGI:2444888]	87.7435	71.7567	67.5587	92.2094	99.8558	82.4877	87.8337	71.8049	82.8628	92.3911	86.1009	87.3441	78.055	66.7757	0.01579111	0.253395174	16	16	24	0.004489982	0.427431763	24	16.63921844	0.190082135
ENSMUSG00000043929	Klhl15	kelch-like 15 [Source:MGI Symbol;Acc:MGI:1923400]	2.90445	3.70626	3.0974	3.29569	3.35948	3.05303	3.39809	3.1972	3.59956	3.09507	3.93639	3.06061	3.74191	3.663	0.948582958	0.966018836	8	16	24	0.733489047	0.913014785	24	7.066096071	0.031171887
ENSMUSG00000043940	Wdfy3	WD repeat and FYVE domain containing 3 [Source:MGI Symbol;Acc:MGI:1096875]	19.4483	14.4199	16.1862	16.62	15.2261	17.114	17.5591	17.5099	13.0195	22.252	14.1423	15.9316	15.2302	16.8299	0.729086532	0.966018836	4	16	24	0.959880317	0.989375125	24	20.83407805	0.022246229
ENSMUSG00000043943	Naalad2	N-acetylated alpha-linked acidic dipeptidase 2 [Source:MGI Symbol;Acc:MGI:1919810]	2.32557	2.78576	2.15671	1.98194	2.50849	2.23776	2.5643	2.451	2.75152	2.03642	2.39652	2.19944	2.52544	2.56265	0.028180201	0.314814652	8	8	24	0.124678706	0.738641389	24	2.651486398	0.079029179
ENSMUSG00000043953	Ccrl2	chemokine (C-C motif) receptor-like 2 [Source:MGI Symbol;Acc:MGI:1920904]	0.673553	0.664555	0.967949	0.591722	0.413174	0.648515	0.554552	0.848376	0.998026	0.95535	0.386413	0.776985	0.820294	0.721219	0.00580828	0.159101132	12	8	24	0.054630484	0.655263328	24	6.406491365	0.14660242
ENSMUSG00000043962	Thrap3	thyroid hormone receptor associated protein 3 [Source:MGI Symbol;Acc:MGI:2442637]	28.7402	29.0923	30.1571	26.2985	26.9199	25.3622	29.8719	27.4283	29.6894	31.1732	26.5181	28.3308	28.2562	29.0758	0.173410058	0.648600527	12	8	24	0.112413048	0.726478319	24	6.17682001	0.065708182
ENSMUSG00000043964	Orai3	ORAI calcium release-activated calcium modulator 3 [Source:MGI Symbol;Acc:MGI:3039586]	8.882	6.84551	7.05338	7.72154	8.11284	9.01582	9.08525	8.81368	7.24389	8.70637	7.34467	8.88092	8.19613	8.42108	0.019057972	0.257576951	4	8	24	0.087864592	0.707747429	24	21.38706992	0.103041341
ENSMUSG00000043987	Cep164	centrosomal protein 164 [Source:MGI Symbol;Acc:MGI:2384878]	6.15885	7.60954	8.60089	7.18748	7.9421	6.87698	5.58973	7.03362	7.9601	8.29733	6.80946	7.32825	7.34623	7.72219	0.016095537	0.253395174	12	16	24	0.023048322	0.581965446	24	10.08599039	0.141388441
ENSMUSG00000043991	Pura	purine rich element binding protein A [Source:MGI Symbol;Acc:MGI:103079]	6.33343	6.8583	6.49373	6.95632	6.69844	5.7058	7.28799	5.98823	6.93029	6.26864	7.1847	6.6834	6.49956	7.08616	0.832742389	0.966018836	20	4	24	0.804542217	0.93744342	24	9.296962996	0.023910829
ENSMUSG00000043993	2900052L18Rik	RIKEN cDNA 2900052L18 gene [Source:MGI Symbol;Acc:MGI:1924085]	1.39093	2.36358	2.11879	1.95371	1.88139	1.42695	1.83516	2.32236	2.94965	2.95825	2.41731	2.61736	2.53303	2.54005	0.772442842	0.966018836	12	16	24	0.010736337	0.511032276	24	8.783637621	0.18384814
ENSMUSG00000043998	Mgat2	mannoside acetylglucosaminyltransferase 2 [Source:MGI Symbol;Acc:MGI:2384966]	17.0558	14.6439	15.2234	14.609	14.669	15.1858	14.5917	15.3026	13.5027	15.7008	16.1998	14.5663	14.9568	14.9497	0.814075857	0.966018836	20	16	24	0.560788989	0.841404418	24	20.16144836	0.032755305
ENSMUSG00000043999	Gpr75	G protein-coupled receptor 75 [Source:MGI Symbol;Acc:MGI:2441843]	4.73619	3.79455	3.60159	3.68404	4.30499	4.4118	4.20239	3.67056	3.5117	4.02509	4.42414	3.77191	3.80946	3.61222	0.004947794	0.141782417	20	16	24	0.005332265	0.4424469	24	19.34312019	0.115315671
ENSMUSG00000044005	Gls2	glutaminase 2 (liver, mitochondrial) [Source:MGI Symbol;Acc:MGI:2143539]	5.25648	5.5734	6.53848	4.71596	4.91416	5.54003	5.48766	5.94223	3.87781	4.58782	4.70169	4.49725	4.70681	5.46646	0.280850951	0.792139038	8	8	24	0.247946333	0.768040178	24	2.504566398	0.092729237
ENSMUSG00000044017	Adgrd1	adhesion G protein-coupled receptor D1 [Source:MGI Symbol;Acc:MGI:3041203]	1.74185	1.3777	1.35258	1.05097	1.31981	1.5517	1.4246	1.47323	1.25631	1.60345	1.21135	1.37615	1.34827	1.44799	0.246839642	0.745922296	4	16	24	0.182074088	0.768040178	24	23.98826627	0.071867371
ENSMUSG00000044018	Mrpl50	mitochondrial ribosomal protein L50 [Source:MGI Symbol;Acc:MGI:107329]	22.0463	23.6403	23.4623	26.0716	23.999	23.104	24.0919	22.7626	26.2479	21.961	25.5771	24.5072	25.4134	25.3537	0.860993221	0.966018836	12	20	24	0.700328228	0.900629492	24	10.84149297	0.02549202
ENSMUSG00000044022	Pcdhb21	protocadherin beta 21 [Source:MGI Symbol;Acc:MGI:2136759]	0.899665	0.882271	0.772781	0.835629	1.09329	0.735968	0.860018	0.711245	0.860295	0.636533	0.89866	0.840354	0.743101	0.917756	0.91241026	0.966018836	20	20	24	0.728721174	0.911275869	24	18.79360752	0.028466097
ENSMUSG00000044024	Rell2	RELT-like 2 [Source:MGI Symbol;Acc:MGI:1918044]	33.9774	24.3959	24.6567	24.2208	23.6181	29.7342	30.7357	29.5753	23.5053	37.4071	26.7064	32.1583	29.7629	31.3889	0.231389127	0.731037655	4	8	24	0.349097303	0.779170188	24	22.57651728	0.111697129
ENSMUSG00000044026	Slc35g1	solute carrier family 35, member G1 [Source:MGI Symbol;Acc:MGI:2444789]	2.05657	2.75819	2.14845	2.09528	2.10947	1.74004	2.04749	2.25157	2.71799	1.47698	2.47887	1.9534	1.7529	2.00121	0.220555749	0.710961211	12	12	24	0.46932419	0.805393068	24	6.572096117	0.079328578
ENSMUSG00000044030	Irf2bp1	interferon regulatory factor 2 binding protein 1 [Source:MGI Symbol;Acc:MGI:2442159]	4.12614	4.1542	4.0385	3.855	4.20944	3.80855	4.72452	4.84433	4.49098	5.51076	4.83951	5.47143	5.4266	5.0681	0.782779623	0.966018836	8	4	24	0.775012229	0.927711986	24	2.113594604	0.024034487
ENSMUSG00000044033	Ccdc141	coiled-coil domain containing 141 [Source:MGI Symbol;Acc:MGI:1919735]	23.2287	21.7291	18.2074	20.0586	20.8809	21.8014	20.0135	19.5751	18.8592	17.3207	22.6888	23.2795	26.9021	21.9946	0.011207621	0.204559658	24	12	24	0.011845786	0.525280095	24	21.95427894	0.158032142
ENSMUSG00000044034	Npb	neuropeptide B [Source:MGI Symbol;Acc:MGI:2387153]	0.738864	0.74846	0.816012	0.833661	0.403607	0.301322	0.641702	0.872523	0.343243	0.425853	0.648114	0.621375	0.641998	0.320428	0.760769894	0.966018836	16	8	24	0.730480055	0.912198956	24	3.563222359	0.053851902
ENSMUSG00000044037	Als2cl	ALS2 C-terminal like [Source:MGI Symbol;Acc:MGI:2447532]	2.07729	2.34662	3.74915	2.49652	2.08935	2.43219	3.7301	2.53278	2.71717	2.55682	3.32849	2.09994	2.01922	2.12058	0.248423582	0.746945512	12	16	24	0.616856076	0.867869295	24	9.971442773	0.089563145
ENSMUSG00000044042	Fmn1	formin 1 [Source:MGI Symbol;Acc:MGI:101815]	14.006	12.1866	11.0418	12.5126	13.1605	12.2269	11.1045	10.689	10.4336	11.0728	13.9518	12.9016	12.9369	13.2755	0.10951302	0.524035616	20	16	24	0.104493711	0.716673794	24	19.52927048	0.107839815
ENSMUSG00000044043	Pcdhb14	protocadherin beta 14 [Source:MGI Symbol;Acc:MGI:2136749]	0.722822	0.594779	0.615435	0.616018	0.657428	0.647226	0.623532	0.700699	0.58109	0.590481	0.625141	0.566642	0.599074	0.560412	0.70641939	0.966018836	20	16	24	0.369805638	0.792235171	24	21.36220765	0.019623752
ENSMUSG00000044052	Ccr10	chemokine (C-C motif) receptor 10 [Source:MGI Symbol;Acc:MGI:1096320]	0.380327	0.61927	0.469024	0.759316	0.533731	0.491596	0.588612	0.500871	0.659277	0.73685	0.839617	0.725091	0.640208	0.583077	0.301918397	0.811201458	16	12	24	0.062053588	0.661961741	24	13.22956666	0.082666216
ENSMUSG00000044058	Mthfr-ps1	5,10-methylenetetrahydrofolate reductase, pseudogene 1 [Source:MGI Symbol;Acc:MGI:2384077]	0.273554	0.184946	0.792403	0.207623	1.08352	0.718891	0.665795	0.731131	0.806929	0.482213	0.685658	0.818882	0.427036	0.206773	0.268085731	0.774280302	20	12	24	0.361894758	0.787031962	24	16.30025035	0.146780781
ENSMUSG00000044066	Cep68	centrosomal protein 68 [Source:MGI Symbol;Acc:MGI:2667663]	7.63818	6.84118	6.78274	6.72107	7.24453	7.43383	7.56848	7.34357	6.46794	7.88454	7.39453	7.83751	7.13706	6.66708	0.020957534	0.262822894	24	12	24	0.037206962	0.609176161	24	19.52093882	0.075554546
ENSMUSG00000044067	Gpr22	G protein-coupled receptor 22 [Source:MGI Symbol;Acc:MGI:1920260]	2.9639	3.88509	4.02799	3.57839	4.13277	3.5881	4.09202	3.20175	3.71098	3.03175	3.64163	4.11667	3.70609	3.78992	0.846643334	0.966018836	20	20	24	0.925698077	0.980131499	24	19.25979127	0.020249936
ENSMUSG00000044068	Zrsr1	zinc finger (CCCH type), RNA binding motif and serine/arginine rich 1 [Source:MGI Symbol;Acc:MGI:98885]	32.5595	28.2421	29.3013	28.5241	31.4085	31.8889	27.8895	27.4971	26.186	29.2139	28.332	30.5814	29.2293	30.1131	0.352925135	0.87272957	24	12	24	0.053883409	0.654390023	24	19.78607363	0.075522573
ENSMUSG00000044071	Tafa2	TAFA chemokine like family member 2 [Source:MGI Symbol;Acc:MGI:2143691]	17.4838	17.3631	15.8381	17.2129	15.1163	15.123	16.3448	16.7356	13.7622	18.5842	16.242	16.4353	16.9126	16.9583	0.549118437	0.966018836	8	12	24	0.891732585	0.969342045	24	2.483668449	0.019540649
ENSMUSG00000044072	Eml6	echinoderm microtubule associated protein like 6 [Source:MGI Symbol;Acc:MGI:2442895]	4.38964	5.53744	5.15128	5.42139	4.8558	5.24415	5.01297	4.61798	5.0764	4.50583	4.77731	5.52561	4.37559	4.52043	0.9045879	0.966018836	24	4	24	0.736595476	0.914180155	24	14.54092376	0.029435812
ENSMUSG00000044080	S100a1	S100 calcium binding protein A1 [Source:MGI Symbol;Acc:MGI:1338917]	40.232	55.5484	57.6308	59.4404	51.7014	46.5113	52.4395	57.527	61.529	44.8398	55.8695	49.0595	55.9071	50.235	0.0444686	0.365196805	12	12	24	0.165085219	0.767020009	24	8.566608382	0.125580077
ENSMUSG00000044086	Lmod3	leiomodin 3 (fetal) [Source:MGI Symbol;Acc:MGI:2444169]	0.907053	3.00586	3.25549	4.9179	2.71624	3.43738	2.12299	8.44534	4.05568	6.92086	2.11873	2.1322	4.4726	3.44435	0.221479046	0.71314449	16	4	24	0.084534608	0.702664096	24	8.870615947	0.480013037
ENSMUSG00000044098	Rsbn1	rosbin, round spermatid basic protein 1 [Source:MGI Symbol;Acc:MGI:2444993]	5.42458	5.86686	5.64513	5.6568	5.78016	5.28082	5.50097	4.94121	5.09295	4.71787	6.14169	4.83918	4.55249	5.23147	0.924553956	0.966018836	20	4	24	0.547347915	0.833663107	24	14.6445581	0.038500108
ENSMUSG00000044117	Bmerb1	bMERB domain containing 1 [Source:MGI Symbol;Acc:MGI:1914504]	47.7208	37.1954	41.6507	36.1233	36.5994	48.4396	43.6143	43.5725	31.2085	46.9956	33.9981	40.8031	37.5582	41.942	0.208071297	0.698704663	24	20	24	0.335050712	0.770984245	24	22.89461372	0.107497516
ENSMUSG00000044122	Proca1	protein interacting with cyclin A1 [Source:MGI Symbol;Acc:MGI:1918274]	2.86473	2.91399	4.24686	4.03976	2.94889	2.80746	3.86195	3.34102	3.70062	3.77013	3.95318	3.62754	4.49318	4.22109	0.724067924	0.966018836	12	12	24	0.212522629	0.768040178	24	8.517668249	0.102738152
ENSMUSG00000044125	9530080O11Rik	RIKEN cDNA 9530080O11 gene [Source:MGI Symbol;Acc:MGI:2441751]	0.549811	0.376561	0.540811	0.626067	0.340526	0.475849	0.523286	0.690007	0.674043	0.6578	0.627722	0.704096	0.550117	0.604916	0.882263435	0.966018836	16	4	24	0.49972415	0.808791684	24	8.772443242	0.043502693
ENSMUSG00000044134	Pheta1	PH domain containing endocytic trafficking adaptor 1 [Source:MGI Symbol;Acc:MGI:2442708]	4.02268	3.70254	3.69246	3.96484	3.82086	3.66592	4.20001	3.83091	4.68157	3.83498	4.58936	4.39051	4.13206	4.44999	0.761468226	0.966018836	4	20	24	0.993080088	0.998593587	24	7.585032616	0.003747436
ENSMUSG00000044139	Prss53	protease, serine 53 [Source:MGI Symbol;Acc:MGI:2652890]	0.553389	0.681905	0.640891	0.857146	0.946351	0.703299	1.08613	0.380833	0.728986	0.535931	0.75163	0.691092	0.693926	0.73677	0.745475226	0.966018836	20	12	24	0.479235333	0.805393068	24	17.60026706	0.07142467
ENSMUSG00000044145	1810024B03Rik	RIKEN cDNA 1810024B03 gene [Source:MGI Symbol;Acc:MGI:1925560]	0.287826	0.622597	0.567718	0.601142	0.635965	0.523306	0.483623	0.630019	0.504793	0.427715	0.484257	0.502835	0.692984	0.386733	0.748433002	0.966018836	8	20	24	0.908241512	0.974606327	24	11.00224082	0.019286238
ENSMUSG00000044147	Arf6	ADP-ribosylation factor 6 [Source:MGI Symbol;Acc:MGI:99435]	20.3294	17.1391	17.9029	17.6643	16.0121	16.5277	16.8855	18.4264	16.4178	19.8447	18.4833	19.2876	17.4909	18.1668	0.807813067	0.966018836	8	12	24	0.983978762	0.996706396	24	0.909301343	0.007068407
ENSMUSG00000044148	1810030O07Rik	RIKEN cDNA 1810030O07 gene [Source:MGI Symbol;Acc:MGI:1916405]	15.0626	14.9392	13.9649	14.2638	14.4483	13.7856	14.0831	14.6213	13.8086	14.3076	15.5491	14.3436	15.1583	15.3808	0.495929335	0.966018836	8	4	24	0.649739213	0.882162033	24	0.454595423	0.01891054
ENSMUSG00000044149	Nkrf	NF-kappaB repressing factor [Source:MGI Symbol;Acc:MGI:1924536]	11.676	11.3118	11.987	10.017	10.5199	11.298	11.0471	10.1097	9.71821	10.4239	10.2652	10.7986	10.2208	11.1115	0.652290823	0.966018836	4	12	24	0.25240857	0.768040178	24	23.93317466	0.046649416
ENSMUSG00000044150	Bclaf3	Bclaf1 and Thrap3 family member 3 [Source:MGI Symbol;Acc:MGI:2685992]	6.7232	6.11268	7.09074	6.87307	6.46772	6.50776	7.14232	6.22037	6.6167	6.0297	7.66414	5.83502	5.91955	5.74407	0.954237589	0.966018836	12	20	24	0.42135648	0.804555939	24	14.83820325	0.049884937
ENSMUSG00000044155	Lsm8	LSM8 homolog, U6 small nuclear RNA associated [Source:MGI Symbol;Acc:MGI:1923772]	21.6731	26.8729	22.549	23.5134	22.9691	21.4542	23.7782	20.4304	22.5804	20.4098	24.439	19.0852	20.9968	22.0643	0.9143748	0.966018836	20	4	24	0.790248168	0.932883394	24	8.376019164	0.030696849
ENSMUSG00000044164	Rnf182	ring finger protein 182 [Source:MGI Symbol;Acc:MGI:3045355]	1.97771	2.36387	2.05087	1.66197	1.99681	1.92791	2.32239	1.82387	1.82956	2.24541	1.49642	1.70718	1.65392	2.36525	0.46238066	0.966018836	8	12	24	0.261087212	0.768040178	24	4.403636751	0.085453519
ENSMUSG00000044165	Bcl2l15	BCL2-like 15 [Source:MGI Symbol;Acc:MGI:2685412]	0.892639	0.916282	1.37586	1.50161	0.857388	1.11	1.69574	0.863884	0.737732	0.340149	1.61401	0.875155	1.25474	1.09323	0.917839756	0.966018836	4	8	24	0.711441246	0.904655191	24	21.69954015	0.091932113
ENSMUSG00000044167	Foxo1	forkhead box O1 [Source:MGI Symbol;Acc:MGI:1890077]	3.35252	3.16203	3.33609	3.74678	3.49876	3.30076	3.3562	3.66654	3.37775	4.09269	2.96943	3.2439	3.30192	3.10345	0.424718357	0.966018836	16	8	24	0.285631932	0.768040178	24	11.24938525	0.055771682
ENSMUSG00000044176	Spink10	serine peptidase inhibitor, Kazal type 10 [Source:MGI Symbol;Acc:MGI:3584533]	1.40763	1.82882	1.89694	2.31571	2.39699	2.25958	2.33993	2.47473	2.02322	1.69003	2.09885	2.09887	1.72146	2.09884	0.874607729	0.966018836	24	4	24	0.748624305	0.918203377	24	14.76991232	0.045164448
ENSMUSG00000044197	Gpr146	G protein-coupled receptor 146 [Source:MGI Symbol;Acc:MGI:1933113]	5.45647	5.30082	5.64909	5.06132	5.477	4.86254	5.99048	5.93045	5.91694	6.44591	4.24696	4.85306	5.41611	5.18076	0.435596482	0.966018836	12	12	24	0.176593867	0.768040178	24	6.395740754	0.093182128
ENSMUSG00000044211	Gm7887	predicted gene 7887 [Source:MGI Symbol;Acc:MGI:3648251]	49.1037	48.6714	50.1811	50.8923	49.3554	50.6973	49.9453	47.3765	47.3519	48.0267	50.3512	49.3116	52.7628	48.8963	0.216404185	0.704989686	24	8	24	0.257841403	0.768040178	24	19.55884403	0.027474389
ENSMUSG00000044216	Kcnj4	potassium inwardly-rectifying channel, subfamily J, member 4 [Source:MGI Symbol;Acc:MGI:104743]	1.43364	1.17562	0.953465	1.4922	1.24374	1.35252	1.31419	1.84275	1.18576	1.86735	1.20976	1.59316	1.52067	1.48957	0.924553956	0.966018836	24	12	24	0.935666913	0.983894024	24	22.5037654	0.020996625
ENSMUSG00000044221	Grsf1	G-rich RNA sequence binding factor 1 [Source:MGI Symbol;Acc:MGI:106479]	57.098	56.0466	55.5984	56.2773	51.3803	56.4983	55.9972	56.2006	52.7622	63.2139	54.2834	57.3775	57.6453	63.0472	0.481721608	0.966018836	24	20	24	0.577468794	0.848840315	24	4.611662921	0.030713885
ENSMUSG00000044224	Dnajc21	DnaJ heat shock protein family (Hsp40) member C21 [Source:MGI Symbol;Acc:MGI:1925371]	14.0268	14.1029	14.0077	13.7584	14.398	13.9659	15.3752	13.3074	13.5663	14.0576	13.183	13.3199	13.8227	13.5393	0.852844413	0.966018836	4	4	24	0.642490463	0.878809617	24	22.46428882	0.01838142
ENSMUSG00000044229	Nxpe4	neurexophilin and PC-esterase domain family, member 4 [Source:MGI Symbol;Acc:MGI:1924792]	3.98297	6.27693	6.27631	5.64745	5.79666	5.7866	4.2011	5.81931	5.79852	5.41164	5.20762	5.48234	4.27306	5.94041	0.002268551	0.104113924	8	20	24	0.088854534	0.707896147	24	10.04204581	0.14837392
ENSMUSG00000044231	Nhlrc1	NHL repeat containing 1 [Source:MGI Symbol;Acc:MGI:2145264]	4.71982	4.23739	4.28732	4.24292	5.20337	4.97579	4.70167	4.35836	3.9561	4.74924	4.60653	5.09653	4.45417	4.3338	7.90E-04	0.071344007	24	12	24	6.94E-04	0.314096774	24	18.84824716	0.112468776
ENSMUSG00000044243	Bhlha9	basic helix-loop-helix family, member a9 [Source:MGI Symbol;Acc:MGI:2444198]	5.74053	3.51827	2.81204	3.88839	4.24849	3.97905	4.22367	4.87406	4.05376	4.49529	4.55543	4.71957	5.15998	4.35867	0.105280763	0.518002936	4	8	24	0.146761499	0.755260456	24	21.64495987	0.141171494
ENSMUSG00000044244	Il20rb	interleukin 20 receptor beta [Source:MGI Symbol;Acc:MGI:2143266]	0.847863	0.613113	0.560589	0.566095	0.835971	0.427866	0.346646	0.864317	0.585705	0.693367	0.673419	0.644364	0.768793	0.605182	0.903465533	0.966018836	20	16	24	0.989474373	0.997867764	24	10.49557632	0.008227841
ENSMUSG00000044250	Pced1b	PC-esterase domain containing 1B [Source:MGI Symbol;Acc:MGI:2446270]	2.92565	2.92091	3.02986	2.36856	2.35025	3.18629	3.58499	3.41786	2.30039	3.6492	2.81313	3.30915	3.10762	2.43353	0.46694635	0.966018836	24	20	24	0.413891213	0.799546792	24	22.82413425	0.089539286
ENSMUSG00000044252	Osbpl1a	oxysterol binding protein-like 1A [Source:MGI Symbol;Acc:MGI:1927551]	54.5859	55.3339	55.5439	56.1422	53.6009	56.7497	58.0307	55.6286	51.8816	59.5821	50.3005	55.9678	56.8443	57.1918	0.796193867	0.966018836	4	16	24	0.657674851	0.884531138	24	2.161694857	0.022356559
ENSMUSG00000044254	Pcsk9	proprotein convertase subtilisin/kexin type 9 [Source:MGI Symbol;Acc:MGI:2140260]	1.11935	0.701964	0.833668	0.721033	0.861427	1.14513	0.907728	0.688814	0.597201	0.840333	1.02616	0.881877	1.19618	1.02745	0.004320961	0.140598674	4	8	24	0.025198384	0.586541342	24	20.95805115	0.13474113
ENSMUSG00000044258	Ctla2a	cytotoxic T lymphocyte-associated protein 2 alpha [Source:MGI Symbol;Acc:MGI:88554]	12.7716	16.4737	17.3252	18.1039	19.339	9.95309	10.5954	14.6122	23.1645	9.95646	15.4831	15.3333	13.0338	13.5966	0.037219247	0.334069441	12	16	24	0.21516603	0.768040178	24	9.920389095	0.230024382
ENSMUSG00000044268	Gm4895	predicted gene 4895 [Source:MGI Symbol;Acc:MGI:3648582]	37.5827	29.76	31.1244	31.6517	32.3597	31.147	31.5463	31.1633	25.4328	33.4176	32.9712	32.6848	32.0929	32.77	0.251986462	0.75129457	20	16	24	0.212091821	0.768040178	24	20.13666705	0.080950628
ENSMUSG00000044285	Ubb-ps	ubiquitin B, pseudogene [Source:MGI Symbol;Acc:MGI:3037679]	282.651	248.882	250.201	218.108	216.116	17.8368	246.954	249.754	15.3162	246.764	236.752	255.955	269.108	286.503	0.112015648	0.530777648	4	16	24	0.908333236	0.974606327	24	1.50650035	0.246208624
ENSMUSG00000044287	Nrn1l	neuritin 1-like [Source:MGI Symbol;Acc:MGI:2443642]	23.4786	17.8331	14.9341	11.6747	14.5532	14.0507	14.1446	19.2759	14.2372	16.2507	17.6546	17.7273	17.5232	15.9877	0.45229395	0.966018836	8	8	24	0.168942471	0.767020009	24	0.307669509	0.164755628
ENSMUSG00000044288	Cnr1	cannabinoid receptor 1 (brain) [Source:MGI Symbol;Acc:MGI:104615]	8.37959	7.87697	7.99695	7.50215	7.42614	8.16988	7.70722	7.3661	6.46774	8.34818	7.45979	7.50454	6.97236	7.91796	0.801280095	0.966018836	24	20	24	0.757457593	0.921562129	24	20.52134455	0.02610597
ENSMUSG00000044296	Zfp879	zinc finger protein 879 [Source:MGI Symbol;Acc:MGI:3053099]	0.652547	0.691276	0.863196	0.707148	0.730303	0.695135	0.73618	0.618179	0.712666	0.658668	0.864795	0.760816	0.679147	0.69189	0.17953325	0.655480352	20	12	24	0.353904002	0.781118317	24	14.45046913	0.032629152
ENSMUSG00000044303	Cdkn2a	cyclin dependent kinase inhibitor 2A [Source:MGI Symbol;Acc:MGI:104738]	0.962876	1.34754	1.69405	0.498012	0.87768	1.07025	0.74792	1.214	1.90389	1.03043	1.07898	1.1367	1.51344	1.07825	0.087978327	0.472021905	12	4	24	0.228081867	0.768040178	24	5.633623998	0.163141562
ENSMUSG00000044308	Ubr3	ubiquitin protein ligase E3 component n-recognin 3 [Source:MGI Symbol;Acc:MGI:1861100]	51.282	45.6858	43.0867	49.5339	46.1785	45.5816	42.3043	47.2031	45.363	56.3778	46.9773	50.0647	49.8474	52.8077	0.883051071	0.966018836	16	20	24	0.870209957	0.963299648	24	12.49386697	0.021880944
ENSMUSG00000044312	Neurog3	neurogenin 3 [Source:MGI Symbol;Acc:MGI:893591]	0.471081	0.49568	0.465905	0.291129	0.422303	0.397127	0.726807	0.627741	0.55673	0.830229	0.382378	0.525326	0.48753	0.545384	0.335484691	0.85758369	8	12	24	0.403943098	0.799546792	24	4.698954639	0.066242621
ENSMUSG00000044317	Gpr4	G protein-coupled receptor 4 [Source:MGI Symbol;Acc:MGI:2441992]	1.09174	0.852271	0.950635	0.974332	0.975945	0.848381	1.3526	1.20406	1.14632	0.932458	1.16667	0.748162	0.879198	0.904297	0.879201926	0.966018836	20	4	24	0.84518817	0.953278734	24	5.505956986	0.028237835
ENSMUSG00000044320	1700001O22Rik	RIKEN cDNA 1700001O22 gene [Source:MGI Symbol;Acc:MGI:1923631]	0.31245	1.04836	0.985	0.835187	0.760631	1.10453	1.35074	1.74602	1.05803	0.708263	0.734035	0.902902	0.980035	0.710596	0.496745696	0.966018836	8	8	24	0.541147009	0.830298514	24	4.402223181	0.108387925
ENSMUSG00000044328	Trp53i13	transformation related protein 53 inducible protein 13 [Source:MGI Symbol;Acc:MGI:1915125]	7.0233	6.14418	4.52638	5.75324	5.38588	5.14452	7.06978	6.30583	6.66838	7.7089	5.8861	6.2513	5.90504	6.17833	0.834424969	0.966018836	4	16	24	0.798932829	0.935664328	24	2.805408401	0.044231631
ENSMUSG00000044330	Gm9790	predicted gene 9790 [Source:MGI Symbol;Acc:MGI:3704221]	103.291	102.164	106.829	120.02	106.553	99.3381	106.744	108.896	101.185	94.1282	131.774	109.584	121.526	115.322	0.850906022	0.966018836	20	16	24	0.877600263	0.965881478	24	17.62185561	0.025175267
ENSMUSG00000044337	Ackr3	atypical chemokine receptor 3 [Source:MGI Symbol;Acc:MGI:109562]	5.2503	5.58241	5.44758	5.79006	5.83514	4.6122	4.96891	5.48124	7.82905	3.8094	5.79535	4.94465	5.07728	5.07942	0.069101108	0.433403132	20	4	24	0.529145587	0.824292689	24	8.266747427	0.088520143
ENSMUSG00000044338	Aplnr	apelin receptor [Source:MGI Symbol;Acc:MGI:1346086]	0.783213	0.820433	1.25447	0.658921	0.754231	0.902014	1.03988	1.64038	1.04995	0.864466	0.611008	0.844451	0.890837	0.917625	0.039226672	0.338446795	12	8	24	0.063195975	0.663285417	24	4.780297365	0.156402629
ENSMUSG00000044339	Alkbh2	alkB homolog 2, alpha-ketoglutarate-dependent dioxygenase [Source:MGI Symbol;Acc:MGI:2141032]	3.7512	2.61165	2.85728	3.50794	3.00053	3.01558	3.13113	4.0013	3.49537	3.52113	3.98307	4.34995	4.72642	3.34899	0.941955432	0.966018836	4	8	24	0.591804493	0.856165204	24	20.8067586	0.062891356
ENSMUSG00000044340	Phlpp1	PH domain and leucine rich repeat protein phosphatase 1 [Source:MGI Symbol;Acc:MGI:2138327]	3.81524	3.93632	5.13333	3.67061	3.31632	4.28458	4.79459	4.16132	3.84762	4.58637	3.32021	4.1129	3.50329	4.47065	0.351315142	0.870418653	12	8	24	0.329781004	0.768040178	24	5.257766987	0.090757335
ENSMUSG00000044345	Marveld1	MARVEL (membrane-associating) domain containing 1 [Source:MGI Symbol;Acc:MGI:2147570]	10.0884	9.23254	8.69097	11.0047	11.2534	9.05684	10.0351	9.68326	10.3571	12.2666	10.4816	10.109	9.58201	9.28817	0.01518376	0.253245931	16	16	24	0.040146429	0.616921492	24	14.08494919	0.107954532
ENSMUSG00000044348	Slc25a53	solute carrier family 25, member 53 [Source:MGI Symbol;Acc:MGI:1914312]	15.6804	13.6382	12.1989	12.5356	13.5367	14.1626	13.3962	13.5537	11.9244	13.3626	13.9899	14.5866	13.0306	13.2346	0.004712708	0.140598674	24	12	24	0.007543706	0.478114494	24	20.92780486	0.101962692
ENSMUSG00000044349	Snhg11	small nucleolar RNA host gene 11 [Source:MGI Symbol;Acc:MGI:2441845]	432.474	472.314	454.065	451.304	480.782	432.503	439.319	384.266	475.301	383.083	490.471	439.98	441.702	464.344	0.581070934	0.966018836	20	8	24	0.859380773	0.95855262	24	15.21051132	0.023618054
ENSMUSG00000044350	Lacc1	laccase domain containing 1 [Source:MGI Symbol;Acc:MGI:2445077]	4.2117	5.05517	4.96994	4.48531	5.16796	4.64539	4.45253	4.82434	5.44781	4.43741	4.87274	4.86325	4.3427	4.33731	0.112800839	0.530777648	12	16	24	0.388843884	0.799546792	24	11.1453083	0.049036249
ENSMUSG00000044352	Sowaha	sosondowah ankyrin repeat domain family member A [Source:MGI Symbol;Acc:MGI:2687280]	0.860803	1.21822	1.20473	1.08436	0.910099	0.818156	1.20305	0.854689	1.35535	1.05567	0.950509	1.25432	1.20903	1.23898	0.588682825	0.966018836	12	8	24	0.187338978	0.768040178	24	6.498760987	0.084605377
ENSMUSG00000044362	Ccdc89	coiled-coil domain containing 89 [Source:MGI Symbol;Acc:MGI:1917304]	0.677223	0.637136	0.446667	0.496816	0.710342	0.749609	0.652123	0.424068	0.421394	0.460818	0.595026	0.52131	0.463395	0.501692	0.010049466	0.194720062	24	12	24	0.003097397	0.414576087	24	19.93960319	0.107091764
ENSMUSG00000044364	Tmem74b	transmembrane protein 74B [Source:MGI Symbol;Acc:MGI:1918629]	1.10386	1.30895	1.23649	1.30316	0.895246	1.16337	1.23101	0.979129	1.14148	0.892388	1.04039	1.16056	0.87394	1.21338	0.951846915	0.966018836	8	12	24	0.595771568	0.857839743	24	4.747538615	0.03986474
ENSMUSG00000044365	Cxxc4	CXXC finger 4 [Source:MGI Symbol;Acc:MGI:2442112]	5.1001	5.46156	5.43143	4.87646	4.59973	5.15956	5.22042	4.6885	4.76816	5.23627	5.07132	4.97479	4.71642	5.52437	0.865233232	0.966018836	8	12	24	0.584196716	0.852087247	24	4.730992146	0.029715665
ENSMUSG00000044367	Slc16a13	solute carrier family 16 (monocarboxylic acid transporters), member 13 [Source:MGI Symbol;Acc:MGI:1916559]	1.38239	1.26405	2.00338	1.04146	1.70225	1.27954	1.08321	1.32995	1.20579	1.2833	1.49972	1.29462	1.0211	1.1877	0.787022682	0.966018836	20	8	24	0.504745147	0.811111334	24	13.09416499	0.066354017
ENSMUSG00000044377	Gm13080	predicted gene 13080 [Source:MGI Symbol;Acc:MGI:3650235]	56.1505	61.0941	55.8069	51.0858	57.9916	54.7631	55.0009	58.1033	52.3563	46.6652	58.7307	46.1538	56.0336	46.0831	0.935593607	0.966018836	8	8	24	0.647776009	0.881026758	24	23.24093877	0.04184186
ENSMUSG00000044378	Slc15a5	solute carrier family 15, member 5 [Source:MGI Symbol;Acc:MGI:3607714]	0.672533	0.44911	0.957083	0.498614	0.437428	1.09703	0.739591	1.07571	0.479157	0.510269	0.965117	0.649374	1.0709	0.756081	0.697196105	0.966018836	24	20	24	0.362174199	0.787064638	24	23.5217131	0.111180627
ENSMUSG00000044390	Tigd3	tigger transposable element derived 3 [Source:MGI Symbol;Acc:MGI:2681860]	0.385504	0.557649	0.596184	0.444432	0.50385	0.474806	0.297576	0.58335	0.569402	0.549404	0.583518	0.530254	0.435735	0.515406	0.004861081	0.141782417	12	16	24	0.083178772	0.699403344	24	10.11392403	0.070778158
ENSMUSG00000044405	Adig	adipogenin [Source:MGI Symbol;Acc:MGI:2675492]	2.81114	2.03047	6.19626	14.5379	3.67066	4.84724	4.87338	6.75561	4.63521	9.91642	12.6945	7.90401	13.195	11.9351	0.90767227	0.966018836	16	20	24	0.233425164	0.768040178	24	12.32561156	0.354976087
ENSMUSG00000044408	Sptssa	serine palmitoyltransferase, small subunit A [Source:MGI Symbol;Acc:MGI:1913399]	53.7183	59.2621	52.1656	55.4066	56.2063	49.9832	54.9957	49.3908	55.9907	45.8879	56.0603	48.1847	50.7635	49.895	0.882617464	0.966018836	20	4	24	0.989614604	0.997887893	24	9.378843688	0.005390553
ENSMUSG00000044424	Gm9493	predicted gene 9493 [Source:MGI Symbol;Acc:MGI:3779903]	88.463	109.047	105.759	98.4775	102.386	89.3144	98.4342	95.668	133.04	78.8833	113.071	95.3582	111.893	98.9719	0.601647512	0.966018836	12	12	24	0.568900818	0.84466645	24	7.172291754	0.079151747
ENSMUSG00000044433	Camsap3	calmodulin regulated spectrin-associated protein family, member 3 [Source:MGI Symbol;Acc:MGI:1916947]	7.63495	6.30649	6.45125	7.22536	6.68157	8.75925	5.87355	7.71811	5.91177	9.4109	6.12217	7.56804	6.53137	7.14396	0.960338592	0.967043964	24	12	24	0.711374145	0.904655191	24	16.74485434	0.060556699
ENSMUSG00000044434	Gm9791	predicted pseudogene 9791 [Source:MGI Symbol;Acc:MGI:3642317]	19.65	15.2989	19.0175	19.9489	18.3136	17.9872	15.0963	17.4075	13.9992	19.0749	20.0632	18.2608	16.9204	17.5498	0.073826349	0.448301997	16	16	24	0.129292573	0.740162193	24	15.77464903	0.112522915
ENSMUSG00000044442	N6amt1	N-6 adenine-specific DNA methyltransferase 1 (putative) [Source:MGI Symbol;Acc:MGI:1915018]	15.8809	17.9305	17.1681	17.0742	15.4153	16.2753	15.1392	15.8915	17.0349	16.2099	18.604	18.8687	18.3229	18.2487	0.816361374	0.966018836	24	4	24	0.936510307	0.984336379	24	7.853209183	0.013248878
ENSMUSG00000044447	Dock5	dedicator of cytokinesis 5 [Source:MGI Symbol;Acc:MGI:2652871]	9.89377	8.37994	8.29724	8.71649	8.88596	8.93375	8.59706	8.51325	7.44178	9.1891	8.50244	8.79359	8.46105	8.71302	0.080164531	0.458678242	24	12	24	0.1418642	0.751766185	24	19.88575558	0.060367295
ENSMUSG00000044452	Zfp507	zinc finger protein 507 [Source:MGI Symbol;Acc:MGI:1916378]	4.73128	4.42273	4.50553	4.10773	3.89702	4.80227	4.54288	4.55744	4.0158	4.66296	4.04891	4.46472	4.26549	4.46995	0.522551072	0.966018836	24	20	24	0.191857659	0.768040178	24	0.720340626	0.05169623
ENSMUSG00000044456	Rin3	Ras and Rab interactor 3 [Source:MGI Symbol;Acc:MGI:2385708]	1.14213	1.56629	1.24149	2.17766	1.23537	1.49457	1.80515	1.16012	1.40381	1.56285	1.18299	1.40721	1.44047	1.18356	0.914502381	0.966018836	16	16	24	0.771441014	0.926312181	24	13.48564675	0.046021051
ENSMUSG00000044461	Shisa2	shisa family member 2 [Source:MGI Symbol;Acc:MGI:2444716]	2.18218	2.45599	2.31515	2.47249	3.11275	2.74959	2.78279	3.72768	2.16236	2.98731	2.91831	2.95545	2.83278	2.40513	0.179603275	0.655480352	20	16	24	0.541562939	0.830485817	24	18.30890415	0.068954327
ENSMUSG00000044465	Fam160a2	family with sequence similarity 160, member A2 [Source:MGI Symbol;Acc:MGI:1921599]	9.92804	9.13208	8.2216	9.42615	9.15933	8.72067	10.4159	7.7804	9.13705	8.43523	9.00499	10.102	9.48459	8.22571	0.054328576	0.395890765	4	4	24	0.096139128	0.71175955	24	20.43354243	0.090357781
ENSMUSG00000044468	Tent5c	terminal nucleotidyltransferase 5C [Source:MGI Symbol;Acc:MGI:1921895]	2.2867	3.26245	3.25486	3.03695	3.04067	2.63064	3.10872	2.6147	3.11664	2.16928	3.11802	2.91076	3.62971	2.28601	0.854443826	0.966018836	4	12	24	0.993641845	0.998731933	24	19.49462054	0.007865185
ENSMUSG00000044469	Tnfaip8l1	tumor necrosis factor, alpha-induced protein 8-like 1 [Source:MGI Symbol;Acc:MGI:1913693]	4.19247	4.03257	3.62651	4.16988	4.21551	3.33281	4.30655	3.69754	4.80896	4.34534	3.69419	3.99534	4.99545	3.72124	0.928780444	0.966018836	4	20	24	0.898833567	0.971427194	24	6.306093588	0.024604871
ENSMUSG00000044471	Lncpint	long non-protein coding RNA, Trp53 induced transcript [Source:MGI Symbol;Acc:MGI:2673128]	0.822567	0.948987	1.11892	0.817747	0.834511	1.75823	0.563371	1.19108	1.19028	0.837344	1.30657	0.756741	1.20669	1.27078	0.896753532	0.966018836	4	20	24	0.961298879	0.99001568	24	6.572447214	0.024278469
ENSMUSG00000044475	Aacs1	activating signal cointegrator 1 complex subunit 1 [Source:MGI Symbol;Acc:MGI:1916340]	14.4846	14.2373	13.1441	14.2256	13.765	15.6751	14.3327	13.6783	12.3186	15.5136	14.6167	14.8486	15.4931	14.9713	0.010886922	0.200176658	24	12	24	0.108345347	0.72082227	24	20.3374391	0.068240321
ENSMUSG00000044477	Zfand3	zinc finger, AN1-type domain 3 [Source:MGI Symbol;Acc:MGI:1096572]	72.4636	58.456	58.3388	55.175	58.757	56.1716	63.1787	64.6857	55.2704	73.674	57.3589	65.8685	66.8073	61.7808	0.15509859	0.607609206	4	8	24	0.490760625	0.805393068	24	23.81427436	0.062918466

ENSMUSG00000044496	2510039O18Rik	RIKEN cDNA 2510039O18 gene [Source:MGI Symbol;Acc:MGI:1924284]	9.34092	7.94144	8.03613	8.51935	8.50997	8.77957	7.93366	8.86855	8.22198	10.2201	8.39119	9.23408	8.28757	8.6544	0.069141379	0.970883022	24	12	24	0.584108911	0.852073789	24	15.94803334	0.037096437
ENSMUSG00000044499	Hs3st5	heparan sulfate (glucosamine) 3-O-sulfotransferase 5 [Source:MGI Symbol;Acc:MGI:2441996]	6.40515	6.37935	6.90059	6.28214	6.13665	6.48822	5.5724	6.74768	5.53545	7.56812	6.41521	7.01602	6.55106	7.21652	0.835668938	0.966018836	8	12	24	0.873046667	0.964303715	24	10.20841721	0.02255511
ENSMUSG00000044501	Zfp758	zinc finger protein 758 [Source:MGI Symbol;Acc:MGI:2385044]	9.78764	10.3793	9.9314	9.50075	10.1745	8.82718	9.96313	8.47587	10.4501	7.79789	10.6405	8.96353	9.01451	9.96579	0.942429894	0.966018836	12	12	24	0.959882725	0.989375125	24	5.393748917	0.013496535
ENSMUSG00000044502	Bod1	biorientation of chromosomes in cell division 1 [Source:MGI Symbol;Acc:MGI:1916806]	35.8541	35.497	36.9646	37.7967	34.9314	39.517	37.3428	39.7467	37.5319	38.5586	35.8306	37.4344	37.0348	37.4597	0.924553956	0.966018836	16	4	24	0.808601393	0.939135535	24	6.287421663	0.013915156
ENSMUSG00000044505	Lingo4	leucine rich repeat and Ig domain containing 4 [Source:MGI Symbol;Acc:MGI:2444651]	1.20443	1.24396	1.65737	1.20706	1.10484	1.2603	1.57013	1.18549	1.26081	1.49317	1.04461	1.42691	0.972539	1.44406	0.85447369	0.966018836	12	8	24	0.56773402	0.844120153	24	7.016068147	0.055050326
ENSMUSG00000044528	Tram1l1	translocation associated membrane protein 1-like 1 [Source:MGI Symbol;Acc:MGI:2443503]	6.35988	5.99064	5.62859	5.14988	4.9306	5.9438	6.15463	5.4438	4.39282	5.57399	5.09394	5.64256	5.58604	6.13365	0.013969059	0.241049421	4	16	24	0.027187071	0.589075452	24	0.01232756	0.113620851
ENSMUSG00000044533	Rps2	ribosomal protein S2 [Source:MGI Symbol;Acc:MGI:105110]	12.3477	12.6378	12.2509	14.7202	13.8856	12.9945	14.2966	16.4977	15.1032	16.3217	21.4505	22.4996	25.6127	19.6122	0.865490576	0.966018836	20	16	24	0.509070636	0.813832486	24	20.13113495	0.070891515
ENSMUSG00000044548	Dact1	dishevelled-binding antagonist of beta-catenin 1 [Source:MGI Symbol;Acc:MGI:1891740]	3.28918	3.26654	3.40218	2.85147	3.46766	2.70387	2.98345	2.9653	4.45964	3.12917	3.49839	3.06109	2.96758	3.14933	0.581070934	0.966018836	12	12	24	0.274616842	0.768040178	24	9.341203824	0.086949248
ENSMUSG00000044550	Tceal3	transcription elongation factor A (SII)-like 3 [Source:MGI Symbol;Acc:MGI:1913354]	38.8045	40.7038	42.8514	34.9584	38.2908	42.047	38.6929	36.5297	31.9657	32.8304	30.0875	37.4359	36.026	37.8911	0.569060064	0.966018836	24	20	24	0.107220641	0.71991576	24	0.345606016	0.096622552
ENSMUSG00000044551	9930012K11Rik	RIKEN cDNA 9930012K11 gene [Source:MGI Symbol;Acc:MGI:2145726]	4.37836	4.34915	3.54409	2.97095	5.35534	5.11201	4.43372	4.65212	4.60212	3.34623	3.97093	4.44606	4.66704	3.84048	0.03782572	0.334069441	24	16	24	0.068195164	0.672582187	24	22.08513265	0.162667152
ENSMUSG00000044562	Rasip1	Ras interacting protein 1 [Source:MGI Symbol;Acc:MGI:1917153]	1.41596	1.16654	1.39868	1.44241	1.2785	1.33313	1.62129	1.60682	1.70209	2.08881	1.4722	1.39773	1.50223	1.68227	0.539040394	0.966018836	16	4	24	0.205893527	0.768040178	24	8.876706437	0.075442584
ENSMUSG00000044566	Cage1	cancer antigen 1 [Source:MGI Symbol;Acc:MGI:1918463]	1.74485	1.95596	2.03244	1.3625	1.63295	1.69615	1.49668	1.3059	1.86557	1.7695	1.80527	1.9756	2.2388	2.00958	0.911371589	0.966018836	12	4	24	0.791294939	0.933267004	24	2.548414164	0.036079307
ENSMUSG00000044573	Acp1	acid phosphatase 1, soluble [Source:MGI Symbol;Acc:MGI:87881]	19.0048	21.4567	19.6312	20.5759	20.0296	19.7239	18.5765	19.2619	21.1748	18.5494	21.7977	19.722	19.1513	20.8599	0.310940564	0.820837787	20	8	24	0.515855795	0.817421075	24	10.57288766	0.033531774
ENSMUSG00000044583	Tlr7	toll-like receptor 7 [Source:MGI Symbol;Acc:MGI:2176882]	0.493155	1.16509	0.873589	0.822044	0.891481	0.591549	0.755586	0.789443	1.51143	0.554012	0.74181	0.799845	0.569068	0.891297	0.109040811	0.524035616	12	16	24	0.129833667	0.740383911	24	8.018636286	0.155442375
ENSMUSG00000044595	Dnd1	DND microRNA-mediated repression inhibitor 1 [Source:MGI Symbol;Acc:MGI:2447763]	0.468811	0.492895	0.404307	0.532292	0.400205	0.438346	0.541199	0.637735	0.582764	0.422562	0.533952	0.475359	0.593683	0.552511	0.232647885	0.733359426	8	12	24	0.105256453	0.717252017	24	3.8880826	0.05012534
ENSMUSG00000044600	Smim7	small integral membrane protein 7 [Source:MGI Symbol;Acc:MGI:1914068]	49.8453	49.9292	49.4971	53.0085	50.5274	49.4061	50.8961	51.3688	49.7693	44.2255	50.6523	45.3001	50.135	50.1632	0.92655264	0.966018836	8	16	24	0.74684917	0.917587548	24	3.695395634	0.019355983
ENSMUSG00000044609	Gm9294	predicted pseudogene 9294 [Source:MGI Symbol;Acc:MGI:3648888]	22.5757	28.6441	25.9925	27.2095	29.574	23.8673	24.1085	21.202	29.0659	17.1034	22.8665	21.2107	23.5454	23.3688	0.92721845	0.966018836	12	12	24	0.885004951	0.967256182	24	8.096693447	0.038689572
ENSMUSG00000044617	Zbtb39	zinc finger and BTB domain containing 39 [Source:MGI Symbol;Acc:MGI:2443316]	1.6985	1.67808	1.80453	1.82095	1.80111	1.68236	1.96916	1.78019	1.69662	2.03141	1.47205	1.74673	1.5019	1.74671	0.496745696	0.966018836	16	8	24	0.616488531	0.867682235	24	9.776543586	0.031318308
ENSMUSG00000044627	Swi5	SWI5 recombination repair homolog (yeast) [Source:MGI Symbol;Acc:MGI:1920181]	198.386	207.61	190.38	191.23	210.248	190.25	186.429	185.617	191.445	164.187	211.685	189.242	204.667	196.886	0.891022863	0.966018836	20	20	24	0.568290929	0.844336151	24	21.47811097	0.040756108
ENSMUSG00000044628	Rnf208	ring finger protein 208 [Source:MGI Symbol;Acc:MGI:1916096]	14.208	12.3393	13.9946	12.839	12.0794	14.3263	12.9337	13.841	11.7319	18.8445	10.7804	14.7347	12.5011	15.2658	0.911135828	0.966018836	24	20	24	0.922020733	0.979411448	24	7.225536151	0.031153425
ENSMUSG00000044629	Cnrip1	cannabinoid receptor interacting protein 1 [Source:MGI Symbol;Acc:MGI:1917505]	23.7994	22.6013	23.3758	22.3815	20.8033	24.6945	21.8119	21.75	20.6709	25.6718	21.2725	22.3721	21.6703	23.6	0.88067614	0.966018836	24	20	24	0.999274629	0.999783065	24	3.412752464	0.001353583
ENSMUSG00000044636	Csrnp2	cysteine-serine-rich nuclear protein 2 [Source:MGI Symbol;Acc:MGI:2386852]	3.1366	2.75471	3.2509	3.04818	3.29278	3.12606	3.33078	3.37097	3.00439	3.45413	3.31154	3.16186	2.90927	3.2595	0.912686312	0.966018836	20	8	24	0.530134768	0.824795587	24	14.24785079	0.02906012
ENSMUSG00000044641	Pard6b	par-6 family cell polarity regulator beta [Source:MGI Symbol;Acc:MGI:2135605]	1.16162	1.07711	0.891753	0.847016	1.21641	1.25118	1.33072	1.25362	1.22271	1.38077	1.20006	1.36509	1.40368	1.37363	0.451149628	0.966018836	4	8	24	0.06495353	0.663876076	24	22.51439228	0.071297803
ENSMUSG00000044645	Gm7334	predicted gene 7334 [Source:MGI Symbol;Acc:MGI:3647393]	6.78324	7.91079	8.02999	6.75709	6.31258	6.30676	6.71167	7.08055	8.33611	6.30228	6.67919	8.15756	6.23004	7.61312	0.081336319	0.462794877	12	8	24	0.145263631	0.754184974	24	6.23398168	0.102196363
ENSMUSG00000044646	Zbtb7c	zinc finger and BTB domain containing 7C [Source:MGI Symbol;Acc:MGI:2443302]	0.831918	1.0782	1.16213	0.799058	0.980931	0.887733	0.976507	1.2258	1.24105	1.02114	0.964279	1.104	1.23677	0.98637	0.308023687	0.814993005	12	4	24	0.146977846	0.755442533	24	5.634963937	0.071918999
ENSMUSG00000044647	Csrnp3	cysteine-serine-rich nuclear protein 3 [Source:MGI Symbol;Acc:MGI:1925021]	9.68794	9.45548	9.44343	9.41579	8.96307	9.13322	8.49066	8.14153	9.61926	7.78335	9.94053	10.56	10.4305	10.6328	0.963760335	0.966018836	24	16	24	0.662564067	0.886140924	24	23.17624702	0.042587523
ENSMUSG00000044667	Plppr4	phospholipid phosphatase related 4 [Source:MGI Symbol;Acc:MGI:106530]	1.58819	1.84518	2.27112	1.57113	1.93422	1.97277	2.13761	1.62088	1.74562	1.66952	1.6496	1.85221	1.4188	1.84576	0.880247609	0.966018836	12	4	24	0.984490972	0.996767829	24	4.913009178	0.008597363
ENSMUSG00000044674	Fzd1	frizzled class receptor 1 [Source:MGI Symbol;Acc:MGI:1196625]	4.40284	4.02487	4.69675	4.80222	4.27345	4.07707	4.92893	4.13721	4.39316	4.93417	4.10848	4.21578	3.72097	4.51921	0.337071598	0.85758369	16	8	24	0.262903935	0.768040178	24	10.42638001	0.061696742
ENSMUSG00000044676	Zfp612	zinc finger protein 612 [Source:MGI Symbol;Acc:MGI:2443465]	7.88303	8.3314	9.02081	7.39254	7.38173	8.20936	8.40097	6.96249	8.35669	8.21414	7.62655	8.95193	8.2499	8.81959	0.581070934	0.966018836	12	8	24	0.655373731	0.883697438	24	3.187145885	0.035041828
ENSMUSG00000044689	Gm13749	predicted gene 13749 [Source:MGI Symbol;Acc:MGI:3649721]	0.531269	0.635864	0.544001	1.51023	1.00915	0.729788	0.419867	0.588866	1.0045	1.05276	0.430836	0.655929	0.768787	0.673423	0.097542159	0.501112642	16	12	24	0.069763548	0.674183933	24	12.04968329	0.192184812
ENSMUSG00000044700	Tmem201	transmembrane protein 201 [Source:MGI Symbol;Acc:MGI:1196277]	7.83572	7.19663	7.62682	8.27986	7.77414	7.22889	8.34214	8.74402	8.12728	10.4217	8.11399	7.78178	7.61001	8.40486	0.826609618	0.966018836	16	8	24	0.189936915	0.768040178	24	10.36328744	0.079373323
ENSMUSG00000044702	Palb2	partner and localizer of BRCA2 [Source:MGI Symbol;Acc:MGI:3040695]	0.449818	0.910509	0.640237	0.853418	0.849005	0.66989	0.530364	0.627135	0.589925	0.5301	0.84553	1.08392	0.547843	0.898366	0.671726933	0.966018836	24	4	24	0.619111791	0.869347961	24	15.19872843	0.059492332
ENSMUSG00000044707	Ccnjl	cyclin J-like [Source:MGI Symbol;Acc:MGI:2685723]	0.631088	0.515647	0.463643	0.436885	0.487182	0.693987	0.597938	0.737513	0.56175	0.635573	0.697538	0.660847	0.52365	0.455216	0.459019924	0.966018836	24	12	24	0.319766188	0.768040178	24	20.30098949	0.055075359
ENSMUSG00000044708	Kcnj10	potassium inwardly-rectifying channel, subfamily J, member 10 [Source:MGI Symbol;Acc:MGI:1194504]	14.1776	18.8124	28.2003	14.4911	14.312	24.3095	24.7577	15.2887	17.2007	17.7327	12.4809	16.4972	11.6139	21.235	0.70641939	0.966018836	12	8	24	0.618326376	0.868968106	24	5.148337676	0.141349308
ENSMUSG00000044709	Gemin7	gem nuclear organelle associated protein 7 [Source:MGI Symbol;Acc:MGI:1916981]	37.4533	32.0516	30.6931	35.3443	31.9773	36.4902	32.7014	37.0968	36.6508	36.2259	36.7656	36.5904	37.9015	36.2225	0.63897419	0.966018836	4	8	24	0.717212926	0.906810602	24	21.6160887	0.027770295
ENSMUSG00000044712	Slc38a6	solute carrier family 38, member 6 [Source:MGI Symbol;Acc:MGI:3648156]	6.85678	7.32638	8.10797	6.36514	8.35539	6.51339	7.59501	7.60348	8.92318	7.16679	8.96363	6.93685	7.6858	6.63667	0.745118544	0.966018836	12	12	24	0.627578321	0.872795898	24	10.97881425	0.054810201
ENSMUSG00000044715	Gskip	GSK3B interacting protein [Source:MGI Symbol;Acc:MGI:1914037]	27.5621	25.5446	23.6875	24.3862	25.7435	25.509	24.7564	25.2654	24.2508	22.581	27.0147	25.8026	26.0059	25.3054	0.025422665	0.29519482	20	20	24	0.024117196	0.586541342	24	21.62449996	0.069563333
ENSMUSG00000044716	Dok7	docking protein 7 [Source:MGI Symbol;Acc:MGI:3584043]	2.58992	2.58648	2.89931	3.28541	2.08157	2.30832	2.60378	2.91186	3.15163	2.88892	2.49885	2.98143	2.30606	2.90296	0.184044621	0.662239322	16	4	24	0.050284263	0.644435324	24	8.255976621	0.124171073
ENSMUSG00000044730	9930104L06Rik	RIKEN cDNA 9930104L06 gene [Source:MGI Symbol;Acc:MGI:3041172]	5.37934	7.11424	6.68591	5.05326	6.5459	6.48287	6.81877	5.84926	6.77967	8.10122	5.73729	7.12867	6.16927	6.82104	0.858266942	0.966018836	8	20	24	0.845617722	0.953396028	24	7.938800927	0.036071123
ENSMUSG00000044734	Serpinb1a	serine (or cysteine) peptidase inhibitor, clade B, member 1a [Source:MGI Symbol;Acc:MGI:1913472]	36.2685	40.4262	48.1149	36.3123	43.8798	39.3066	36.0886	34.3625	38.4084	33.59	54.067	34.9016	35.6294	36.1647	0.745199643	0.966018836	20	8	24	0.366758828	0.790399468	24	14.26791737	0.103771081
ENSMUSG00000044749	Abca6	ATP-binding cassette, sub-family A (ABC1), member 6 [Source:MGI Symbol;Acc:MGI:1923434]	16.6893	20.4545	16.2796	21.5432	19.6288	16.1347	17.4497	15.985	22.4282	15.397	19.9071	20.9289	22.5327	20.6382	0.731683656	0.966018836	4	16	24	0.974688494	0.993631841	24	9.434631156	0.016836945
ENSMUSG00000044751	Atp5pb-ps	ATP synthase peripheral stalk-membrane subunit b, pseudogene [Source:MGI Symbol;Acc:MGI:3651344]	114.194	121.991	121.165	131.747	117.77	114.998	117.015	121.462	125.991	106.273	124.389	113.364	124.865	117.544	0.390686537	0.915479136	12	12	24	0.586259006	0.853300537	24	8.098990578	0.032985192
ENSMUSG00000044763	Trmt10c	tRNA methyltransferase 10C, mitochondrial RNase P subunit [Source:MGI Symbol;Acc:MGI:1196261]	6.80284	6.89168	6.57006	7.51373	7.36234	6.4925	6.93375	7.25741	7.11212	5.66163	7.57117	6.88121	7.09618	6.99903	0.122551072	0.613778672	20	4	24	0.94568672	0.986808796	24	13.67471968	0.027778672
ENSMUSG00000044768	Macir	macrophage immunometabolism regulator [Source:MGI Symbol;Acc:MGI:1277184]	15.4329	14.8488	17.4935	14.7067	14.9289	15.1953	14.6044	13.3236	12.4857	14.0263	16.0668	13.9223	12.3582	13.7195	0.745199643	0.966018836	20	12	24	0.36677852	0.790399468	24	15.85850548	0.056566454
ENSMUSG00000044770	Scml4	Scm polycomb group protein like 4 [Source:MGI Symbol;Acc:MGI:2446140]	2.84815	2.59821	2.36635	2.32944	2.22576	2.67324	2.68624	2.22436	2.11155	2.97708	2.0675	2.57372	2.50857	2.66768	0.297978647	0.80820842	4	16	24	0.392340305	0.799546792	24	23.57143857	0.061569351
ENSMUSG00000044783	Hjurp	Holliday junction recognition protein [Source:MGI Symbol;Acc:MGI:2685821]	23.5261	23.1739	23.6589	25.1872	25.9442	23.4599	24.44	24.8334	26.748	23.6736	27.2679	24.6455	24.3368	24.2198	0.371875382	0.894537464	20	12	24	0.182408383	0.768040178	24	13.55462652	0.04499924
ENSMUSG00000044786	Zfp36	zinc finger protein 36 [Source:MGI Symbol;Acc:MGI:99180]	2.67893	3.22122	3.01392	3.74935	2.80302	2.57892	2.9745	3.04309	3.41481	3.87781	3.00974	3.1241	2.70962	2.68166	0.016024075	0.253395174	16	12	24	0.015173746	0.537924552	24	10.77422072	0.136518989
ENSMUSG00000044788	Fads6	fatty acid desaturase domain family, member 6 [Source:MGI Symbol;Acc:MGI:3039592]	10.1075	8.53381	9.0012	8.29495	8.95456	9.40854	10.1722	8.75769	7.88941	10.5171	8.90507	9.16732	9.28854	9.2204	0.097760727	0.501112642	4	8	24	0.300492298	0.768040178	24	21.17593145	0.064012558
ENSMUSG00000044791	Setd2	SET domain containing 2 [Source:MGI Symbol;Acc:MGI:1918177]	16.2443	15.392	16.779	15.5552	14.4521	15.7566	15.9092	16.4661	16.2218	15.4442	16.7646	16.6188	15.905	17.4473	0.91695334	0.966018836	12	4	24	0.247563543	0.768040178	24	4.21891956	0.037058916
ENSMUSG00000044792	Iscu	iron-sulfur cluster assembly 1 [Source:MGI Symbol;Acc:MGI:1916296]	33.1688	36.6449	38.5991	39.9081	32.4648	36.3775	37.0812	40.2793	37.0874	41.2571	37.9366	38.1067	39.3874	43.0556	0.360033827	0.883787544	16	4	24	0.038857485	0.611732283	24	7.545046331	0.081455671
ENSMUSG00000044795	Cyb5d1	cytochrome b5 domain containing 1 [Source:MGI Symbol;Acc:MGI:2685586]	20.6117	15.2023	15.9259	16.8987	15.3882	17.0338	16.2681	16.2777	12.9714	17.4462	16.2498	18.1762	17.1164	18.5633	0.300823756	0.809362476	4	8	24	0.233014828	0.768040178	24	21.67408036	0.098186209
ENSMUSG00000044807	Zfp354c	zinc finger protein 354C [Source:MGI Symbol;Acc:MGI:1353621]	8.53355	9.10515	8.03996	8.24931	7.80706	7.6931	8.24384	7.21696	8.6786	7.81775	9.59794	8.36759	8.09975	8.8863	0.879201926	0.966018836	8	16	24	0.990047849	0.998140337	24	7.788671741	0.005720439
ENSMUSG00000044811	Cd300c2	CD300C molecule 2 [Source:MGI Symbol;Acc:MGI:2153249]	1.1816	1.6786	1.94571	1.09193	1.69775	1.40053	1.49397	1.02294	2.28053	1.14582	1.19694	1.0703	1.72536	1.34613	0.649212555	0.966018836	12	4	24	0.517576798	0.8183265	24	6.73944189	0.099368087
ENSMUSG00000044813	Shb	src homology 2 domain-containing transforming protein B [Source:MGI Symbol;Acc:MGI:98294]	0.761164	1.03373	1.90335	1.89763	1.13953	0.920968	1.13936	0.98456	1.50298	2.30306	1.12171	1.12322	1.0807	1.52709	0.028632001	0.316608413	16	12	24	8.55E-04	0.319044294	24	10.38104049	0.305193564
ENSMUSG00000044816	D630023F18Rik	RIKEN cDNA D630023F18 gene [Source:MGI Symbol;Acc:MGI:2138198]	0.49797	0.619157	1.02006	0.503026	0.481233	0.756334	0.761236	0.678842	0.669985	0.615643	0.503685	0.578628	0.615647	0.860906	0.05475651	0.398010585	8	12	24	0.130693368	0.740728512	24	5.231945021	0.099259176
ENSMUSG00000044827	Tlr1	toll-like receptor 1 [Source:MGI Symbol;Acc:MGI:1341295]	0.470534	0.745955	0.460415	0.331226	0.61436	0.424022	0.553766	0.698801	0.768327	0.369145	0.711645	0.537758	0.78292	0.480391	0.657586167	0.966018836	8	8	24	0.480510805	0.805393068	24	2.314956878	0.064859836
ENSMUSG00000044835	Ankrd45	ankyrin repeat domain 45 [Source:MGI Symbol;Acc:MGI:1921094]	7.2865	6.09887	7.08267	5.95672	5.83688	7.37215	6.81585	5.92252	5.07492	7.18984	6.79474	6.96151	6.11335	6.49491	0.431555283	0.966018836	24	12	24	0.268017525	0.768040178	24	20.0103629	0.087415639
ENSMUSG00000044847	Lsm11	U7 snRNP-specific Sm-like protein LSM11 [Source:MGI Symbol;Acc:MGI:1919540]	3.58944	2.81443	3.2439	3.10228	2.74137	3.48246	3.42959	3.13423	2.5414	3.47431	2.80035	3.27896	2.98482	3.24212	0.428730128	0.966018836	24	20	24	0.410930233	0.799546792	24	22.32662903	0.060240403
ENSMUSG00000044857	Lemd2	LEM domain containing 2 [Source:MGI Symbol;Acc:MGI:2385045]	13.0524	13.1533	14.3211	13.4293	15.1191	13.2016	14.7849	14.9636	16.379	13.3283	13.779	13.3856	15.6559	14.729	0.896753532	0.966018836	12	12	24	0.354728685	0.781734364	24	6.147055985	0.052564534
ENSMUSG00000044864	Ankrd50	ankyrin repeat domain 50 [Source:MGI Symbol;Acc:MGI:2139777]	31.1141	24.2434	25.5068	24.6147	30.1183	26.9171	31.6145	27.3366	23.7054	29.0031	28.3685	28.0955	27.9825	25.7523	0.046934151	0.375011045	4	8	24	0.044772947	0.628432994	24	20.15811785	0.117668465
ENSMUSG00000044876	Zfp444	zinc finger protein 444 [Source:MGI Symbol;Acc:MGI:1923365]	6.01003	5.52356	4.47248	4.58788	3.90499	5.8626	5.40709	6.21509	5.63106	7.11939	7.0608	8.7255	6.57139	5.54515	0.885955579	0.966018836	24	12	24	0.438863083	0.805393068	24	21.43128705	0.108926507
ENSMUSG00000044881	Coa4	cytochrome c oxidase assembly factor 4 [Source:MGI Symbol;Acc:MGI:1915435]	6.23151	5.71045	5.00254	5.33584	5.71482	6.02944	6.12942	5.21865	4.81031	5.44947	6.3127	5.89897	6.5302	5.72443	1.13E-04	0.029372629	4	8	24	0.001023298	0.336500758	24	20.96962463	0.12924658
ENSMUSG00000044894	Uqcrq	ubiquinol-cytochrome c reductase, complex III subunit VII [Source:MGI Symbol;Acc:MGI:107807]	259.866	260.082	275.529	269.347	219.517	199.736	258.393	299.503	337.756	254.012	338.008	280.19	327.708	295.509	0.777867261	0.966018836	12	12	24	0.161047923	0.765941695	24	6.640339217	0.139655352
ENSMUSG00000044906	4930503L19Rik	RIKEN cDNA 4930503L19 gene [Source:MGI Symbol;Acc:MGI:1922045]	13.3733	14.4653	14.1161	14.1087	16.2687	13.7466	13.8759	10.9308	13.8683	10.9446	14.6595	10.8135	12.0641	13.1938	0.664568268	0.966018836	20	4	24	0.650989377	0.882698308	24	14.84910417	0.052904222
ENSMUSG00000044912	Syt16	synaptotagmin XVI [Source:MGI Symbol;Acc:MGI:2673872]	2.3493	2.97018	4.11105	3.26627	2.50755	3.65632	2.8265	2.8166	4.31664	3.25077	3.10051	2.50532	2.08389	2.93106	0.002004172	0.100255953	12	16	24	0.043114276	0.625007356	24	9.946598071	0.201967363
ENSMUSG00000044916	1700029I15Rik	RIKEN cDNA 1700029I15 gene [Source:MGI Symbol;Acc:MGI:1916742]	6.20955	6.87695	7.97491	9.48798	6.11513	6.07987	7.26444	6.87626	7.58761	5.38136	6.42812	5.57321	4.3988	5.75975	0.236893212	0.7385769	12	12	24	0.111374522	0.72334045	24	9.673038481	0.154795489
ENSMUSG00000044921	Rassf9	Ras association (RalGDS/AF-6) domain family (N-terminal) member 9 [Source:MGI Symbol;Acc:MGI:2384307]	1.98204	2.16944	2.31076	2.77697	2.43673	2.69862	2.70072	2.03879	1.92181	1.91866	2.43457	1.97172	2.27524	2.0522	0.893609703	0.966018836	20	16	24	0.292140619	0.768040178	24	17.74911738	0.079509378
ENSMUSG00000044927	H1f10	H1.10 linker histone [Source:MGI Symbol;Acc:MGI:2685307]	2.83588	2.43729	2.82958	2.79095	1.72966	1.614	2.83427	2.67429	2.77979	4.27662	3.59763	3.55798	4.08348	4.03653	0.854540463	0.966018836	4	20	24	0.431996928	0.805393068	24	6.880989652	0.122808601
ENSMUSG00000044934	Zfp367	zinc finger protein 367 [Source:MGI Symbol;Acc:MGI:2442266]	12.9752	12.0353	12.9482	12.1978	12.4727	11.7367	10.6828	11.1062	10.2069	10.2545	12.896	11.1376	11.2121	10.6442	0.881355613	0.966018836	20	12	24	0.29558949	0.768040178	24	17.73590549	0.055837089
ENSMUSG00000044937	Ttc41	tetratricopeptide repeat domain 41 [Source:MGI Symbol;Acc:MGI:2387653]	1.48918	1.71864	1.2755	1.65459	1.55917	1.61173	1.65227	1.56093	1.59591	1.4117	1.94273	1.3644	1.68237	1.75627	0.939335547	0.966018836	8	4	24	0.911298216	0.97486689	24	22.20394949	0.016566399
ENSMUSG00000044938	Klhl31	kelch-like 31 [Source:MGI Symbol;Acc:MGI:3045305]	0.56834	1.63509	1.60464	2.11495	1.14506	1.67277	1.61965	3.68164	2.91866	3.0064	1.16214	1.59124	2.11227	1.65431	0.248056532	0.746745468	16	4	24	0.072368685	0.679262305	24	8.142552952	0.329806186
ENSMUSG00000044948	Cfap43	cilia and flagella associated protein 43 [Source:MGI Symbol;Acc:MGI:1289258]	0.442772	0.915366	0.978886	0.802948	0.791398	0.550822	0.830724	0.545587	0.81236	0.647715	0.749148	0.733994	0.483379	0.849529	0.105280783	0.518002936	12	16	24	0.181548842	0.768040178	24	9.636856523	0.10133494
ENSMUSG00000044949	Ubtd2	ubiquitin domain containing 2 [Source:MGI Symbol;Acc:MGI:2444752]	11.6993	9.94208	9.41517	11.3837	9.70648	10.4441	9.65042	9.68177	8.98229	11.4084	9.75291	11.395	11.1257	11.3862	0.581070934	0.966018836	24	12	24	0.629307132	0.873280786	24	20.47628341	0.046522964
ENSMUSG00000044950	Pwwp2a	PWWP domain containing 2A [Source:MGI Symbol;Acc:MGI:1918052]	9.93242	9.10056	9.39803	10.5188	9.95492	8.76963	8.685	8.95887	9.58941	9.87803	8.81107	8.8154	8.57346	8.59979	0.054935173	0.398010585	16	12	24	0.013108968	0.529134128	24	12.17957854	0.070699063
ENSMUSG00000044951	Mylk4	myosin light chain kinase family, member 4 [Source:MGI Symbol;Acc:MGI:3643758]	1.13655	4.38117	7.49812	14.951	7.43359	8.23744	3.4949	11.6441	14.6032	24.1822	6.32897	6.71416	5.0396	3.71788	7.90E-04	0.071344007	16	12	24	0.004481116	0.427431763	24	11.54395611	0.95261108
ENSMUSG00000044952	Kctd21	potassium channel tetramerisation domain containing 21 [Source:MGI Symbol;Acc:MGI:3643121]	4.53239	2.95458	3.61159	4.02507	3.90392	4.30856	4.88559	3.50392	2.9781	3.15163	4.0098	3.60142	3.51415	3.39332	0.116913252	0.538362324	4	4	24	0.028461977	0.593964798	24	20.04730221	0.161186109
ENSMUSG00000044968	Napepld	N-acyl phosphatidylethanolamine phospholipase D [Source:MGI Symbol;Acc:MGI:2140885]	4.52231	4.21148	4.40213	4.49434	4.32858	4.43543	4.23636	4.18458	4.11186	4.16266	4.15053	4.38029	3.75238	4.21007	0.919571723	0.966018836	24	12	24	0.41903903	0.803497501	24	16.45206603	0.022377317
ENSMUSG00000044976	Wdr72	WD repeat domain 72 [Source:MGI Symbol;Acc:MGI:3583957]	2.08158	1.80279	1.71751	1.70044	1.62499	1.56269	1.4047	1.79931	1.61121	1.76959	2.01078	1.5609	1.88198	1.73341	0.864782464	0.966018836	4	20	24	0.952392588	0.988407044	24	5.707407785	0.012371813
ENSMUSG00000044982	Sft2d3	SFT2 domain containing 3 [Source:MGI Symbol;Acc:MGI:1914408]	2.26941	2.6408	2.58768	2.387	2.1279	2.34604	2.18941	2.52593	2.98103	2.27355	2.76232	2.68443	2.78734	2.56956	0.91724565	0.966018836	12	4	24	0.328375352	0.768040178	24	6.513844631	0.055845748
ENSMUSG00000044986	Tst	thiosulfate sulfurtransferase, mitochondrial [Source:MGI Symbol;Acc:MGI:98852]	7.55824	9.21821	11.6873	10.3887	7.8068	8.646	9.51277	10.4665	11.0998	9.53374	8.25504	8.61224	8.82977	9.82645	4.29E-04	0.054040373	12	8	24	0.001777355	0.382759815	24	7.753476219	0.196158536
ENSMUSG00000044991	Shld1	shieldin complex subunit 1 [Source:MGI Symbol;Acc:MGI:1920997]	10.4338	13.1939	11.1368	11.8021	14.0787	11.9229	13.7273	10.8323	11.7641	9.7531	12.2395	11.6976	12.4613	11.4328	0.840483986	0.966018836	20	20	24	0.497166212	0.807576732	24	20.43797414	0.065705225
ENSMUSG00000045005	Fzd5	frizzled class receptor 5 [Source:MGI Symbol;Acc:MGI:108571]	0.537819	0.429305	0.414227	0.575309	0.362314	0.522701	0.450172	0.615879	0.493339	0.618268	0.492244	0.545204	0.481202	0.488172	0.934818505	0.966018836	16	20	24	0.941358535	0.985472945	24	9.806309176	0.010129591
ENSMUSG00000045007	Tubg2	tubulin, gamma 2 [Source:MGI Symbol;Acc:MGI:2144208]	8.21949	7.75651	8.04959	8.34915	5.97389	8.47965	8.00403	8.09656	7.41347	10.4956	7.0284	9.93981	7.57964	9.23729	0.93243477	0.966018836	24	20	24	0.861667554	0.95949572	24	5.270218142	0.038851094
ENSMUSG00000045008	9030612E09Rik	RIKEN cDNA 9030612E09 gene [Source:MGI Symbol;Acc:MGI:1921780]	0.373714	0.570568	0.330411	0.565201	0.614864	0.359734	0.485231	0.608399	0.607416	0.52453	0.499688	0.563926	0.605198	0.588818	0.951945551	0.966018836	8	16	24	0.666989198	0.887459929	24	8.271204331	0.03185872
ENSMUSG00000045009	Prrt3	proline-rich transmembrane protein 3 [Source:MGI Symbol;Acc:MGI:2444810]	5.51273	4.06367	4.82076	4.14464	3.86762	5.03349	5.08908	5.48946	3.77814	7.27669	4.48255	6.01467	5.26965	5.37292	0.46694635	0.966018836	4	8	24	0.757374432	0.921562129	24	23.02308973	0.062210074
ENSMUSG00000045031	Cetn4	centrin 4 [Source:MGI Symbol;Acc:MGI:2677454]	3.61377	3.63057	4.22116	3.2807	2.72511	2.81088	3.75609	4.25873	3.41323	2.63589	3.10027	3.39205	3.56899	3.10133	0.023460887	0.278499043	8	8	24	0.024008894	0.586541342	24	3.687265696	0.15029105
ENSMUSG00000045034	Ankrd34b	ankyrin repeat domain 34B [Source:MGI Symbol;Acc:MGI:2443245]	1.0638	1.53124	2.99047	1.44776	0.782403	1.86266	2.02313	1.40162	1.6259	1.37742	0.85954	1.20695	0.785506	1.83452	0.167216842	0.634643192	12	8	24	0.183426038	0.768040178	24	6.433134334	0.225761102
ENSMUSG00000045038	Prkce	protein kinase C, epsilon [Source:MGI Symbol;Acc:MGI:97599]	47.961	35.1746	36.8564	40.7914	36.1111	42.8421	37.0209	41.573	29.6213	53.3785	36.2461	41.5468	39.3274	41.4587	0.883024237	0.966018836	24	12	24	0.742629168	0.916215545	24	19.44808841	0.064972492
ENSMUSG00000045039	Megf8	multiple EGF-like-domains 8 [Source:MGI Symbol;Acc:MGI:2446294]	17.7074	14.986	15.3898	15.933	15.1906	16.2045	15.9745	17.777	14.5891	21.8803	14.9585	17.3995	15.7117	16.8518	0.760769894	0.966018836	24	12	24	0.980471712	0.995264516	24	12.63813239	0.01132707
ENSMUSG00000045045	Lrfn4	leucine rich repeat and fibronectin type III domain containing 4 [Source:MGI Symbol;Acc:MGI:2385612]	6.6778	5.82016	5.6951	5.2073	5.36183	5.33506	6.28958	6.34455	6.24238	6.60323	6.85164	6.59361	6.73628	6.44529	0.762156552	0.966018836	4	12	24	0.378173516	0.797706855	24	1.439445617	0.051393747
ENSMUSG00000045048	Gm9795	predicted pseudogene 9795 [Source:MGI Symbol;Acc:MGI:3704361]	2.63254	3.43543	3.51472	2.49028	2.80123	3.14383	4.30619	3.08828	4.04985	2.18725	2.52497	2.88833	3.41618	2.65998	0.035050838	0.328311403	12	4	24	0.171675298	0.768040178	24	2.629449811	0.146393725
ENSMUSG00000045053	Kcng3	potassium voltage-gated channel, subfamily G, member 3 [Source:MGI Symbol;Acc:MGI:2663923]	0.588536	0.693056	0.422537	0.621615	0.820987	0.60125	0.658399	0.484219	0.866197	0.468558	0.995244	0.979802	0.944239	0.721928	0.602156008	0.966018836	20	20	24	0.266091351	0.768040178	24	19.16194036	0.09032732
ENSMUSG00000045055	Rpsa-ps2	ribosomal protein SA, pseudogene 2 [Source:MGI Symbol;Acc:MGI:3643356]	16.9181	17.8941	19.6612	17.7303	16.7531	15.6927	16.5741	15.1862	16.3571	12.7177	18.2911	16.8865	19.2999	16.7668	0.801251296	0.966018836	4	12	24	0.792283023	0.933537459	24	0.648939915	0.039632417
ENSMUSG00000045062	Pcdhb7	protocadherin beta 7 [Source:MGI Symbol;Acc:MGI:2136741]	1.13793	1.08039	1.23865	0.780094	0.96486	1.00292	1.23205	0.970118	0.958003	1.05672	1.14701	1.02252	0.802247	1.16503	0.912686312	0.966018836	8	8	24	0.749368712	0.918511706	24	2.329235663	0.030350848
ENSMUSG00000045064	Zc2hc1c	zinc finger, C2HC-type containing 1C [Source:MGI Symbol;Acc:MGI:1919600]	0.586304	0.594956	0.96603	0.642033	0.517165	0.593016	0.655221	0.488182	0.420504	0.681623	0.571762	0.574858	0.700687	0.548431	0.886952527	0.966018836	4	16	24	0.947528027	0.987232058	24	9.547949378	0.014702114
ENSMUSG00000045071	E130308A19Rik	RIKEN cDNA E130308A19 gene [Source:MGI Symbol;Acc:MGI:2442164]	1.54114	1.99846	2.12603	1.74191	2.22109	1.53978	1.90807	1.88871	2.04522	1.77266	1.68787	1.64561	2.14779	1.85023	0.148073804	0.590732968	12	12	24	0.292037862	0.768040178	24	7.383578358	0.070882913
ENSMUSG00000045078	Rnf216	ring finger protein 216 [Source:MGI Symbol;Acc:MGI:1344349]	6.952	6.97458	6.91624	6.83858	7.0543	6.80246	7.27191	7.76024	6.96544	8.7864	6.53701	7.59088	7.10208	7.027	0.840483986	0.966018836	8	12	24	0.817264903	0.941826666	24	9.074801407	0.023962893
ENSMUSG00000045083	Lingo2	leucine rich repeat and Ig domain containing 2 [Source:MGI Symbol;Acc:MGI:2442298]	5.08473	4.52829	5.23726	3.73329	3.80641	3.74281	3.88299	4.68987	3.46448	4.57605	3.77239	5.04078	5.88004	5.4824	0.23195324	0.731832877	4	16	24	0.12705419	0.739868076	24	2.49772771	0.145723122
ENSMUSG00000045087	S1pr5	sphingosine-1-phosphate receptor 5 [Source:MGI Symbol;Acc:MGI:2150641]	0.423518	1.14697	1.84378	1.01618	0.408192	1.06429	0.786954	1.03542	1.03227	0.660079	0.479737	1.16133	0.292415	1.25371	0.150109238	0.595846665	8	12	24	0.189936236	0.768040178	24	7.142383488	0.228567592
ENSMUSG00000045092	S1pr1	sphingosine-1-phosphate receptor 1 [Source:MGI Symbol;Acc:MGI:1096355]	7.13812	7.2407	9.98091	7.93785	6.27789	8.29979	9.60054	6.88744	7.88718	9.01075	6.12768	6.87161	5.51169	7.35851	0.212359178	0.701530532	16	4	24	0.444013492	0.805393068	24	8.567533988	0.106587911
ENSMUSG00000045094	Arhgef37	Rho guanine nucleotide exchange factor (GEF) 37 [Source:MGI Symbol;Acc:MGI:3045339]	3.82812	3.47299	3.00597	4.07591	3.79515	3.41748	3.87352	3.51981	4.18046	3.68464	3.3511	3.82943	4.0108	3.06822	0.935316621	0.966018836	4	4	24	0.749388834	0.918511706	24	18.18091214	0.035803596
ENSMUSG00000045095	Magi1	membrane associated guanylate kinase, WW and PDZ domain containing 1 [Source:MGI Symbol;Acc:MGI:1203522]	43.4088	35.0672	34.412	34.5608	35.1369	38.3688	37.8887	36.1929	31.6448	40.0685	36.7949	39.0776	39.9695	40.014	0.017495149	0.255326033	4	8	24	0.06866302	0.673061483	24	22.03561041	0.097885923
ENSMUSG00000045098	Kmt5b	lysine methyltransferase 5B [Source:MGI Symbol;Acc:MGI:2444557]	11.4198	13.0042	12.1004	12.8615	12.1641	11.1988	11.8422	11.8723	13.4683	11.8767	11.8521	12.6804	12.511	12.4926	0.336988702	0.85758369	16	16	24	0.166549375	0.767020009	24	8.099825082	0.05018714
ENSMUSG00000045100	Slc25a26	solute carrier family 25 (mitochondrial carrier, phosphate carrier), member 26 [Source:MGI Symbol;Acc:MGI:1914832]	7.43497	7.34564	6.31298	7.24547	6.85007	7.18796	7.87324	8.26636	6.00584	8.33427	7.97201	7.37107	7.54785	6.95304	0.643325306	0.966018836	4	8	24	0.517292445	0.818279867	24	20.59500884	0.055107784
ENSMUSG00000045102	Poln	DNA polymerase N [Source:MGI Symbol;Acc:MGI:2675617]	1.18045	1.37588	1.46646	4.62099	1.65985	1.31628	1.35837	1.3818	2.21086	2.57449	3.41243	1.55084	2.95385	2.14033	0.028632001	0.316608413	16	12	24	0.025895873	0.586541342	24	11.37216661	0.406071614
ENSMUSG00000045103	Dmd	dystrophin, muscular dystrophy [Source:MGI Symbol;Acc:MGI:94909]	37.3632	39.7536	35.6112	38.4046	45.972	34.9539	40.7575	34.5277	41.6321	35.1253	40.0162	35.8519	33.9566	36.9633	0.939920159	0.966018836	20	4	24	0.546833862	0.833502152	24	14.4431901	0.050608074
ENSMUSG00000045104	Ldhb-ps	lactate dehydrogenase B, pseudogene [Source:MGI Symbol;Acc:MGI:3645435]	81.6451	91.2465	92.6522	94.9228	89.4599	88.848	95.3462	86.3932	89.6017	83.476	80.9717	81.9656	86.0089	84.7018	0.734765071	0.966018836	12	12	24	0.558070192	0.839541809	24	7.659009023	0.030962238
ENSMUSG00000045106	Ccdc73	coiled-coil domain containing 73 [Source:MGI Symbol;Acc:MGI:3606488]	4.48181	6.31759	7.03631	5.33947	6.13963	5.3	6.13967	4.95605	6.69358	6.5927	6.48114	6.30781	7.87843	6.27749	0.648024224	0.966018836	12	12	24	0.579231217	0.849727189	24	9.574622685	0.067801886

ENSMUSG00000045438	Cox19	cytochrome c oxidase assembly protein 19 [Source:MGI Symbol;Acc:MGI:1915283]	35.3376	38.2942	37.4151	38.4316	37.5063	36.6791	36.5909	37.4543	42.5169	32.0666	40.8037	34.3568	37.0718	34.1151	0.46694635	0.966018836	20	4	24	0.767006658	0.924684154	24	10.11888455	0.029850247
ENSMUSG00000045440	Insm2	insulinoma-associated 2 [Source:MGI Symbol;Acc:MGI:1930787]	3.5194	2.31627	2.07055	2.8168	2.45955	2.10101	2.32573	2.40844	2.18182	3.51772	3.154	3.26987	3.33596	3.48341	0.918193129	0.966018836	4	8	24	0.795406389	0.934545847	24	19.40208155	0.054799033
ENSMUSG00000045455	Gm9797	predicted pseudogene 9797 [Source:MGI Symbol;Acc:MGI:3704349]	10.732	14.7764	14.629	12.8138	16.0042	12.2999	13.4828	12.2108	10.9474	13.2991	16.4528	11.3202	15.9237	10.8975	0.636884864	0.966018836	20	12	24	0.509767963	0.813985618	24	15.48206377	0.09209976
ENSMUSG00000045464	2810002D19Rik	RIKEN cDNA 2810002D19 gene [Source:MGI Symbol;Acc:MGI:1913707]	3.23248	2.99643	2.31907	2.57846	2.75827	2.60134	2.64035	2.74381	2.57327	2.06272	2.96666	2.82564	3.13312	2.83006	0.015486342	0.253395174	4	8	24	0.034893263	0.608551791	24	23.07254477	0.113871617
ENSMUSG00000045466	Zfp956	zinc finger protein 956 [Source:MGI Symbol;Acc:MGI:2141515]	4.22136	4.26565	4.45156	4.21169	4.29719	4.59634	4.37486	4.13125	5.24504	4.53735	3.94253	4.22071	3.91631	4.34261	0.760769894	0.966018836	12	16	24	0.346910175	0.778844143	24	9.005271388	0.049563695
ENSMUSG00000045467	Ttll13	tubulin tyrosine ligase-like family, member 13 [Source:MGI Symbol;Acc:MGI:1920845]	1.08862	0.702015	0.914288	0.722803	0.684945	0.415406	1.35749	0.584504	0.508327	0.445224	0.677228	0.362719	0.821548	0.533185	0.05137861	0.38924256	4	20	24	0.347057474	0.778844143	24	0.934442207	0.110937666
ENSMUSG00000045482	Trrap	transformation/transcription domain-associated protein [Source:MGI Symbol;Acc:MGI:2153272]	20.6331	20.8501	22.4526	18.1334	22.5744	21.3546	23.859	20.6832	23.1667	21.9937	20.4906	23.4253	22.1015	20.2089	0.94086481	0.966018836	24	8	24	0.734302585	0.913333287	24	22.16124227	0.031849284
ENSMUSG00000045498	Pcdhb3	protocadherin beta 3 [Source:MGI Symbol;Acc:MGI:2136737]	0.720508	0.628169	0.903131	0.882455	0.732414	0.661668	0.894459	0.697172	0.57578	0.958787	0.815025	0.699274	0.608408	0.621027	0.143673396	0.585944911	16	16	24	0.179705597	0.768040178	24	13.52116075	0.069205514
ENSMUSG00000045503	Sys1	SYS1 Golgi-localized integral membrane protein homolog (S. cerevisiae) [Source:MGI Symbol;Acc:MGI:1913710]	9.65235	10.9459	12.0054	9.99562	9.77067	9.55512	9.96066	10.3152	12.192	11.2577	9.28722	9.82452	10.7344	10.9261	1.58E-04	0.031913438	12	8	24	0.00117671	0.336500758	24	7.383675807	0.133101367
ENSMUSG00000045519	Zfp560	zinc finger protein 560 [Source:MGI Symbol;Acc:MGI:1915280]	13.0467	17.4387	15.3542	13.6905	16.7312	13.1853	13.4585	16.9232	17.6521	12.2353	17.3583	17.3654	17.0244	16.3545	0.92655264	0.966018836	12	4	24	0.741563047	0.915904517	24	5.259994886	0.0533543
ENSMUSG00000045532	C1ql1	complement component 1, q subcomponent-like 1 [Source:MGI Symbol;Acc:MGI:1344400]	0.817277	1.77078	1.38319	0.905697	0.773011	0.827704	0.702488	1.22236	1.86324	0.699778	0.918194	1.05873	0.78481	1.30418	0.059717207	0.406067671	12	4	24	0.025100826	0.586541342	24	6.540050557	0.244747498
ENSMUSG00000045534	Kcna5	potassium voltage-gated channel, shaker-related subfamily, member 5 [Source:MGI Symbol;Acc:MGI:96662]	0.615914	0.440495	0.606536	0.829237	0.651764	0.728976	0.663386	0.58012	0.642685	0.561692	0.526865	0.604843	0.4964	0.585667	0.671726933	0.966018836	16	16	24	0.379172314	0.798545485	24	15.17988007	0.044665861
ENSMUSG00000045538	Ddx28	DEAD box helicase 28 [Source:MGI Symbol;Acc:MGI:1919236]	3.82978	3.33219	3.51098	3.95945	3.76314	4.04986	3.36677	4.49261	3.3449	3.9995	3.72575	4.00074	4.2217	4.54007	0.934818505	0.966018836	8	4	24	0.867847413	0.962222482	24	23.42944099	0.021861066
ENSMUSG00000045551	Fpr1	formyl peptide receptor 1 [Source:MGI Symbol;Acc:MGI:107443]	0.3417	0.955828	1.82147	0.982398	0.743752	0.914215	0.95973	0.674292	0.957894	0.58685	0.847593	0.60878	0.795396	0.431995	0.801995186	0.966018836	12	20	24	0.419559726	0.803639585	24	10.12183456	0.129063796
ENSMUSG00000045573	Penk	preproenkephalin [Source:MGI Symbol;Acc:MGI:104629]	8.01208	15.9525	20.4023	12.5172	10.4166	13.4771	12.9782	8.8327	15.2911	14.342	7.99291	13.1737	7.35844	13.7261	0.060394272	0.407012001	12	16	24	0.198367897	0.768040178	24	8.784829124	0.278098651
ENSMUSG00000045576	St7l	suppression of tumorigenicity 7-like [Source:MGI Symbol;Acc:MGI:2386964]	20.6346	18.5222	15.9698	16.4256	16.3722	17.5202	17.7015	15.7354	18.4333	15.3355	18.3757	16.1645	16.1747	16.1484	0.93447422	0.966018836	4	12	24	0.350787084	0.780345856	24	22.6803883	0.058534731
ENSMUSG00000045589	Frrs1l	ferric-chelate reductase 1 like [Source:MGI Symbol;Acc:MGI:2442704]	7.72475	8.04176	9.09553	8.04241	6.81943	8.20289	8.67176	6.70102	7.58571	8.63598	7.07423	7.85183	6.25341	8.21979	0.941955432	0.966018836	16	4	24	0.682286903	0.894159472	24	8.936561471	0.048004731
ENSMUSG00000045594	Glb1	galactosidase, beta 1 [Source:MGI Symbol;Acc:MGI:88151]	11.1442	10.0721	9.44062	9.16414	9.93073	11.4401	10.2198	11.6318	8.52862	11.4669	9.66625	10.6585	8.91879	9.96264	0.390686537	0.915479136	24	12	24	0.433322648	0.805393068	24	21.24961132	0.067302118
ENSMUSG00000045598	Zfp553	zinc finger protein 553 [Source:MGI Symbol;Acc:MGI:2384725]	3.12308	2.62121	2.96327	2.83336	2.77555	2.89026	3.03225	2.86622	2.79198	3.31048	2.85777	3.12355	2.71009	3.08351	0.874054778	0.966018836	24	12	24	0.931636949	0.982352286	24	16.35846168	0.010348332
ENSMUSG00000045608	Dbx2	developing brain homeobox 2 [Source:MGI Symbol;Acc:MGI:107445]	1.03437	2.56772	5.07138	2.03391	1.79447	2.78242	2.15483	2.1423	2.25745	1.50632	1.73421	2.03751	0.749585	2.24984	0.287488775	0.793714531	12	4	24	0.364450931	0.788837108	24	8.756278127	0.229067166
ENSMUSG00000045613	Chrm2	cholinergic receptor, muscarinic 2, cardiac [Source:MGI Symbol;Acc:MGI:88397]	3.42868	4.02963	4.83328	3.75412	3.15461	4.23936	5.29944	3.32263	3.7696	3.97392	3.84984	4.52399	3.59646	5.54791	0.910436409	0.966018836	24	20	24	0.598952053	0.859281363	24	3.608826473	0.07653369
ENSMUSG00000045620	Odf3l1	outer dense fiber of sperm tails 3-like 1 [Source:MGI Symbol;Acc:MGI:2681875]	0.378347	0.508041	0.618347	0.498908	0.551211	0.615794	0.646692	1.08611	0.770187	0.649854	0.715274	0.690652	0.691502	0.704184	0.90332508	0.966018836	12	4	24	0.388629013	0.799546792	24	6.402463777	0.06135158
ENSMUSG00000045624	Esf1	ESF1 nucleolar pre-rRNA processing protein homolog [Source:MGI Symbol;Acc:MGI:1913830]	10.8855	12.0361	11.5258	10.6232	10.5154	9.66518	10.5059	9.97171	13.2112	11.2336	13.0208	12.7581	13.3238	13.0616	0.896753532	0.966018836	12	12	24	0.587995462	0.853828785	24	6.858779641	0.051417998
ENSMUSG00000045625	Pigz	phosphatidylinositol glycan anchor biosynthesis, class Z [Source:MGI Symbol;Acc:MGI:2443822]	12.2639	9.09574	9.00403	9.49907	8.15963	10.5521	8.96963	10.4497	8.91149	11.3854	8.55811	11.1136	9.91977	9.99337	0.503972924	0.966018836	24	20	24	0.509504487	0.813985618	24	23.41007497	0.071635937
ENSMUSG00000045629	Sh3tc2	SH3 domain and tetratricopeptide repeats 2 [Source:MGI Symbol;Acc:MGI:2444417]	6.50794	6.67448	6.7628	7.37085	7.9042	6.15669	7.79802	6.41166	7.77089	7.02745	6.2519	6.29549	7.15871	5.32613	0.669104912	0.966018836	16	8	24	0.591072703	0.855623646	24	13.28020771	0.053073059
ENSMUSG00000045636	Mtus1	mitochondrial tumor suppressor 1 [Source:MGI Symbol;Acc:MGI:2142572]	41.2559	34.7201	31.3258	36.3191	33.2214	37.2699	37.294	37.9698	27.6169	38.7384	31.2887	37.2703	36.8626	35.5648	0.101520755	0.511659635	4	8	24	0.139102858	0.748793588	24	22.2887407	0.111601971
ENSMUSG00000045639	Zfp629	zinc finger protein 629 [Source:MGI Symbol;Acc:MGI:2444524]	2.82465	3.0058	3.5347	3.2996	2.98217	3.04323	3.30916	3.73837	3.60037	3.64205	3.10772	3.50151	2.91253	3.21705	0.158469451	0.614860056	12	16	24	0.071344026	0.677164282	24	9.06663315	0.084662795
ENSMUSG00000045648	Vwc2l	von Willebrand factor C domain-containing protein 2-like [Source:MGI Symbol;Acc:MGI:2444069]	4.22967	4.47611	3.97738	3.48447	3.96541	3.9609	4.07923	3.49738	3.45755	3.71975	4.53463	3.53096	3.62351	4.56312	0.960269811	0.967043964	8	4	24	0.716366832	0.906378296	24	23.55172426	0.038055017
ENSMUSG00000045655	Fam216b	family with sequence similarity 216, member B [Source:MGI Symbol;Acc:MGI:2145738]	0.312873	0.901477	0.944024	0.819521	0.688967	0.284252	1.02491	0.119872	0.66064	0.541186	0.421077	1.06039	0.0424094	1.13168	0.891690964	0.966018836	12	16	24	0.671408351	0.889179947	24	9.725955208	0.123777385
ENSMUSG00000045657	Pcdhb10	protocadherin beta 10 [Source:MGI Symbol;Acc:MGI:2136745]	0.971839	1.01204	1.34626	0.985559	0.942826	0.992783	1.2602	0.979649	0.736064	1.24468	1.08856	0.8783	0.899145	0.816775	0.860560779	0.966018836	16	16	24	0.811953879	0.939785122	24	13.57399428	0.029464969
ENSMUSG00000045658	Pid1	phosphotyrosine interaction domain containing 1 [Source:MGI Symbol;Acc:MGI:2138391]	7.96174	8.28188	8.84572	6.3916	6.89063	7.78348	8.01282	7.70932	7.82774	6.93028	7.30057	7.66	7.1774	8.54035	0.00789564	0.192760771	12	4	24	0.031127234	0.60008277	24	3.199410113	0.103676314
ENSMUSG00000045659	Plekha7	pleckstrin homology domain containing, family A member 7 [Source:MGI Symbol;Acc:MGI:2445094]	11.7005	17.4384	14.4095	12.7086	17.8166	12.3491	15.719	16.8519	18.7147	16.6003	14.0755	14.9184	15.2063	16.2961	0.268052211	0.774280302	12	12	24	0.202955682	0.768040178	24	7.568393483	0.130017703
ENSMUSG00000045664	Cdc42ep2	CDC42 effector protein (Rho GTPase binding) 2 [Source:MGI Symbol;Acc:MGI:1929744]	6.38353	5.01827	4.99921	5.42902	5.83006	6.45563	6.2864	6.0039	4.71421	5.89683	5.22875	5.07693	5.09272	5.48313	0.210566453	0.69936851	24	12	24	0.20288895	0.768040178	24	20.80039391	0.085946052
ENSMUSG00000045665	Mfsd5	major facilitator superfamily domain containing 5 [Source:MGI Symbol;Acc:MGI:2145901]	16.0654	13.3584	13.0907	13.5423	12.7343	14.4594	14.669	15.4774	13.1056	17.7504	13.146	14.419	15.1262	14.6873	0.104170975	0.518002936	4	8	24	0.659522485	0.884879972	24	0.81861265	0.044837977
ENSMUSG00000045667	Smtnl2	smoothelin-like 2 [Source:MGI Symbol;Acc:MGI:2442764]	2.27398	4.20754	3.99942	5.70736	3.31788	5.50944	4.33907	11.0454	5.78229	8.4557	3.88996	5.16628	5.96804	4.17685	0.911685024	0.966018836	16	4	24	0.416848886	0.801604539	24	8.525933579	0.23237087
ENSMUSG00000045671	Spred2	sprouty-related EVH1 domain containing 2 [Source:MGI Symbol;Acc:MGI:2150019]	13.6945	10.5602	10.074	10.8931	11.1207	11.9771	10.8746	11.2808	9.0553	16.3345	11.33	12.6422	11.6949	12.8658	0.08302755	0.462922111	24	12	24	0.62838859	0.872795898	24	18.916283	0.073976076
ENSMUSG00000045672	Col27a1	collagen, type XXVII, alpha 1 [Source:MGI Symbol;Acc:MGI:2672118]	4.65708	5.39939	6.1854	5.39859	5.84453	5.41968	5.3066	5.60129	5.87448	5.80731	5.97596	5.18104	6.0005	4.83226	0.27817759	0.789849983	12	20	24	0.150690445	0.758865539	24	11.85001024	0.073475589
ENSMUSG00000045679	Pqlc3	PQ loop repeat containing [Source:MGI Symbol;Acc:MGI:2444067]	35.692	35.0765	30.657	33.1826	33.2176	34.0436	33.1755	33.5538	31.7733	33.4252	35.4204	37.164	35.6966	37.1621	0.071575474	0.440262583	24	12	24	0.061671708	0.661743448	24	22.11655129	0.061012628
ENSMUSG00000045689	Pcdhb4	protocadherin beta 4 [Source:MGI Symbol;Acc:MGI:2136738]	0.979347	0.765044	0.775308	0.655402	0.922787	0.916298	0.884613	0.940932	0.724173	1.00682	0.923156	0.649362	1.00421	0.74825	0.94086481	0.966018836	4	20	24	0.552282476	0.836643755	24	20.9994923	0.044886183
ENSMUSG00000045690	Wdr89	WD repeat domain 89 [Source:MGI Symbol;Acc:MGI:1919588]	12.6145	11.0166	9.9539	8.76257	6.6515	5.97909	10.4353	12.2833	12.972	12.4319	12.1966	12.3852	14.3429	12.3524	0.595952838	0.966018836	4	16	24	0.109171662	0.72082227	24	4.782147963	0.235103406
ENSMUSG00000045691	Thtpa	thiamine triphosphatase [Source:MGI Symbol;Acc:MGI:2446078]	7.23471	6.23445	5.63213	6.9428	6.17772	7.12935	6.11293	7.10268	6.15189	7.48515	5.10499	6.72128	5.91832	6.94905	0.916556573	0.966018836	24	12	24	0.909450533	0.974606327	24	1.812148299	0.023722031
ENSMUSG00000045725	Prr15	proline rich 15 [Source:MGI Symbol;Acc:MGI:1925254]	3.09082	3.7619	3.62065	4.60467	4.74646	4.31533	4.4325	3.38998	4.19103	4.74353	4.48173	4.18241	3.64129	3.75069	0.013969059	0.241049421	16	16	24	0.003724419	0.426426863	24	14.63304532	0.160466521
ENSMUSG00000045730	Adrb2	adrenergic receptor, beta 2 [Source:MGI Symbol;Acc:MGI:87938]	2.0792	2.23863	2.65439	2.90424	3.06922	2.00843	2.95666	2.22742	3.39377	3.18207	2.60871	2.8184	2.40311	2.20643	0.092596422	0.487919468	12	20	24	0.045328044	0.629099373	24	12.25578392	0.15378532
ENSMUSG00000045731	Pnoc	prepronociceptin [Source:MGI Symbol;Acc:MGI:105308]	0.194004	0.559894	1.23683	0.334207	0.291036	0.605751	1.06391	0.406678	0.742018	0.426403	0.335674	0.715099	0.205736	0.632396	0.496745696	0.966018836	12	8	24	0.559655063	0.840636989	24	6.136203395	0.120673542
ENSMUSG00000045733	Sprn	shadow of prion protein [Source:MGI Symbol;Acc:MGI:3582583]	16.2964	11.9297	13.0103	12.7856	12.9752	15.4854	12.5918	13.4988	10.0898	14.6907	12.9184	14.7606	13.1599	13.1261	0.148073804	0.590732968	24	12	24	0.096940054	0.71175955	24	20.12085713	0.13061032
ENSMUSG00000045751	Mms22l	MMS22-like, DNA repair protein [Source:MGI Symbol;Acc:MGI:2684980]	0.512065	0.708878	0.811444	1.24951	0.923705	0.949865	0.456461	0.97707	0.577291	0.669116	0.709466	0.785207	0.499751	0.56796	0.037716301	0.334069441	24	4	24	0.126824797	0.739868076	24	13.63078141	0.128790061
ENSMUSG00000045752	Tssc4	tumor-suppressing subchromosomal transferable fragment 4 [Source:MGI Symbol;Acc:MGI:1861712]	15.4141	15.7946	14.7359	13.2239	14.5577	15.1474	12.6344	15.4347	13.5499	14.0302	13.6503	14.6517	13.6942	13.8398	0.515451644	0.966018836	8	8	24	0.611236985	0.865079333	24	0.43808379	0.031447913
ENSMUSG00000045757	Zfp764	zinc finger protein 764 [Source:MGI Symbol;Acc:MGI:2443580]	2.48179	2.32972	2.67106	2.55133	2.79678	2.38414	2.35346	2.26375	2.56611	2.79078	2.26225	2.44397	2.55964	2.18648	0.111887343	0.530777648	12	20	24	0.122675756	0.735333681	24	13.28127622	0.05805945
ENSMUSG00000045761	Togaram2	TOG array regulator of axonemal microtubules 2 [Source:MGI Symbol;Acc:MGI:2443498]	2.00988	1.86307	1.54848	1.65744	2.52086	2.06605	1.78194	1.99048	1.86183	1.75599	2.18645	1.91667	1.90053	1.80426	0.00448422	0.140598674	20	20	24	0.079441973	0.693027736	24	18.79543078	0.097734942
ENSMUSG00000045763	Basp1	brain abundant, membrane attached signal protein 1 [Source:MGI Symbol;Acc:MGI:1917600]	69.3834	52.8489	60.1621	50.1576	48.6412	58.8571	55.7982	59.0975	56.357	81.613	82.3467	80.5895	78.5132	80.5916	0.759203569	0.966018836	24	8	24	0.796937164	0.935133209	24	21.44554996	0.056215199
ENSMUSG00000045767	B230219D22Rik	RIKEN cDNA B230219D22 gene [Source:MGI Symbol;Acc:MGI:1925771]	33.5697	33.2546	34.1365	34.0983	34.2062	32.1503	33.2235	31.9547	31.9861	34.3797	35.2702	32.7299	31.2858	33.1724	0.061928188	0.409814092	20	4	24	0.047841663	0.63658289	24	13.67718647	0.039866086
ENSMUSG00000045775	Slc16a5	solute carrier family 16 (monocarboxylic acid transporters), member 5 [Source:MGI Symbol;Acc:MGI:2443515]	0.936028	0.805677	0.70225	0.959123	0.90598	0.972356	0.960094	0.710024	0.556187	0.980448	0.929087	1.04269	0.950338	0.911797	8.24E-04	0.071344007	24	12	24	0.015284446	0.538738573	24	19.17454523	0.110579754
ENSMUSG00000045777	Ifitm10	interferon induced transmembrane protein 10 [Source:MGI Symbol;Acc:MGI:2444776]	8.67014	8.77006	7.50485	6.30921	6.01772	8.18831	9.20973	7.93377	7.25148	9.7651	7.85555	9.30544	7.74438	10.0552	0.347075276	0.870418653	8	12	24	0.187705276	0.768040178	24	1.574377319	0.124145301
ENSMUSG00000045790	Ccdc149	coiled-coil domain containing 149 [Source:MGI Symbol;Acc:MGI:2685293]	11.9568	10.4076	10.5211	11.0759	9.80409	12.2089	11.0623	11.077	10.0018	12.6926	9.59602	11.5739	11.1157	12.0121	0.503972924	0.966018836	24	20	24	0.636961085	0.876483853	24	23.923122	0.042467594

ENSMUSG00000045795	Whamm	WAS protein homolog associated with actin, golgi membranes and microtubules [Source:MGI Symbol;Acc:MGI:2142282]	4.08398	5.4465	5.92831	5.09274	4.95247	4.89187	5.15477	4.90478	5.7421	5.40315	4.35962	5.44544	5.33904	5.53174	0.035023815	0.328311403	12	8	24	0.089161618	0.708638675	24	7.678319194	0.099750636
ENSMUSG00000045799	Gm9800	predicted gene 9800 [Source:MGI Symbol;Acc:MGI:3710580]	131.156	129.95	127.642	110.596	140.142	123.696	123.336	97.3957	84.2609	84.8236	80.7863	104.943	93.6667	100.109	0.881996584	0.966018836	24	16	24	0.105184528	0.717252017	24	22.11406997	0.14280796
ENSMUSG00000045817	Zfp36l2	zinc finger protein 36, C3H type-like 2 [Source:MGI Symbol;Acc:MGI:107945]	5.45253	6.19307	5.78168	6.40413	5.57103	4.85046	6.46643	6.22503	6.92189	6.63482	7.00961	6.05969	6.89275	6.79073	0.956719163	0.96629412	12	12	24	0.082542901	0.699403344	24	8.023775151	0.089378782
ENSMUSG00000045822	Zswim3	zinc finger SWIM-type containing 3 [Source:MGI Symbol;Acc:MGI:1914788]	3.07962	2.74764	2.40388	3.66104	2.90522	3.27874	3.09949	3.03417	2.4495	3.49233	2.85815	3.29679	3.06633	3.09513	0.043989163	0.363663264	24	12	24	0.320023596	0.768040178	24	18.30217444	0.076091605
ENSMUSG00000045826	Ptprcap	protein tyrosine phosphatase, receptor type, C polypeptide-associated protein [Source:MGI Symbol;Acc:MGI:97811]	0.749273	0.663433	0.606433	0.78904	0.578666	0.597085	0.435656	1.04608	1.39319	0.784619	1.47432	0.843236	1.23907	1.08593	0.831272156	0.966018836	8	20	24	0.481342412	0.805393068	24	8.493103031	0.093692435
ENSMUSG00000045827	Serpinb9	serine (or cysteine) peptidase inhibitor, clade B, member 9 [Source:MGI Symbol;Acc:MGI:106603]	6.59473	6.71585	5.42042	6.1164	6.5284	5.5953	6.05929	6.81114	6.8715	4.68621	6.60439	5.79747	5.51264	6.08572	0.850398351	0.966018836	8	16	24	0.702881926	0.901733679	24	2.314328913	0.043416825
ENSMUSG00000045838	Ccdc9b	coiled-coil domain containing 9B [Source:MGI Symbol;Acc:MGI:2685199]	3.95403	4.33363	4.0383	3.94737	4.59742	4.31547	3.8483	4.65136	4.3089	4.97317	4.05562	4.67901	4.18917	4.35237	0.921442568	0.966018836	24	4	24	0.672843191	0.889786694	24	12.23745054	0.029333581
ENSMUSG00000045854	Lyrm2	LYR motif containing 2 [Source:MGI Symbol;Acc:MGI:1917573]	35.7016	43.2669	34.9121	37.4864	36.6741	32.2618	32.0694	30.3328	38.0187	33.027	34.7796	36.7453	41.3511	33.2331	0.771160198	0.966018836	12	20	24	0.984395672	0.996767829	24	5.616559975	0.010157297
ENSMUSG00000045867	Cradd	CASP2 and RIPK1 domain containing adaptor with death domain [Source:MGI Symbol;Acc:MGI:1336168]	5.60823	6.08642	5.47161	6.07096	5.36498	6.42999	5.95703	6.13296	6.59846	5.91528	5.95558	6.14205	7.30271	6.1933	0.7207001	0.966018836	24	20	24	0.38450294	0.799546792	24	1.981835684	0.042545788
ENSMUSG00000045875	Adra1a	adrenergic receptor, alpha 1a [Source:MGI Symbol;Acc:MGI:104773]	0.345274	0.378891	0.661703	0.989411	0.248306	0.519604	0.625728	0.446642	0.440538	0.493414	0.557898	0.613014	0.722662	0.682439	0.795556128	0.966018836	16	4	24	0.825118846	0.945127573	24	9.310991016	0.042112949
ENSMUSG00000045876	Pcdhb8	protocadherin beta 8 [Source:MGI Symbol;Acc:MGI:2136742]	0.820487	0.792901	1.04051	0.914559	0.761562	0.808929	1.02098	0.628436	1.38133	1.08291	0.944005	1.15212	0.641292	0.525725	0.05137861	0.38924256	12	20	24	0.289294175	0.768040178	24	12.92579918	0.104578305
ENSMUSG00000045886	Gm9803	predicted gene 9803 [Source:MGI Symbol;Acc:MGI:3704359]	36.5808	40.4236	38.6417	40.7876	35.6464	34.7267	36.1052	45.2768	50.9256	36.9367	49.9523	43.9711	44.2394	44.0019	0.607427393	0.966018836	12	12	24	0.301389577	0.768040178	24	7.706283838	0.091555057
ENSMUSG00000045896	Paip2b	poly(A) binding protein interacting protein 2B [Source:MGI Symbol;Acc:MGI:2386865]	41.2221	40.0774	37.4153	39.1896	40.7444	39.7772	39.6458	36.3634	38.0962	40.4686	39.2605	39.8406	43.1292	41.8939	0.182849367	0.659303873	4	8	24	0.289632215	0.768040178	24	21.21639942	0.037785492
ENSMUSG00000045912	C2cd4c	C2 calcium-dependent domain containing 4C [Source:MGI Symbol;Acc:MGI:2685084]	2.45184	1.62095	1.34317	1.73948	1.59484	1.32253	1.86264	1.86178	1.45014	2.30401	1.99978	1.7304	1.44641	1.66832	0.896799496	0.966018836	16	20	24	0.909566483	0.974606327	24	18.31480959	0.031226746
ENSMUSG00000045917	Tmem268	transmembrane protein 268 [Source:MGI Symbol;Acc:MGI:1913920]	2.91269	3.2497	3.34149	3.34268	3.25245	3.13394	3.8585	3.64809	3.07717	3.22336	3.50832	3.59375	3.1985	3.61365	0.858891143	0.966018836	8	4	24	0.882249978	0.966683041	24	2.327189527	0.015592702
ENSMUSG00000045930	Clec14a	C-type lectin domain family 14, member a [Source:MGI Symbol;Acc:MGI:1914114]	1.30109	2.24372	1.78754	1.79324	1.56308	1.71428	1.38278	2.38958	2.4343	1.28578	1.58216	1.79209	1.47613	1.55702	0.286642595	0.793714531	12	16	24	0.235845496	0.768040178	24	6.947723834	0.12909993
ENSMUSG00000045932	Ifit2	interferon-induced protein with tetratricopeptide repeats 2 [Source:MGI Symbol;Acc:MGI:99449]	15.7213	15.6108	15.8911	14.5825	15.8754	16.7102	14.8165	14.7813	15.7582	13.7785	16.9376	16.6168	16.298	17.1717	0.212359178	0.701530532	20	20	24	0.373802722	0.794450623	24	21.62792407	0.046053233
ENSMUSG00000045934	Mtmr11	myotubularin related protein 11 [Source:MGI Symbol;Acc:MGI:2652817]	6.94912	8.56955	8.80439	9.43182	9.12219	7.91707	7.92489	7.80999	8.33231	9.09767	7.89662	7.5487	7.29616	7.1522	0.008961721	0.192760771	16	12	24	8.51E-04	0.319044294	24	11.99867117	0.131505303
ENSMUSG00000045942	BC049762	cDNA sequence BC049762 [Source:MGI Symbol;Acc:MGI:3039622]	0.331421	0.803384	0.435047	0.696433	0.547427	0.594276	0.202122	0.774841	0.682435	0.881264	1.00661	0.810343	0.842252	0.801134	0.910436409	0.966018836	16	20	24	0.310134378	0.768040178	24	11.49990759	0.101618933
ENSMUSG00000045948	Mrps12	mitochondrial ribosomal protein S12 [Source:MGI Symbol;Acc:MGI:1346333]	41.7319	38.6247	40.8077	40.2003	44.6176	40.5076	39.3253	41.3102	37.1831	41.8672	42.1872	40.6231	40.5178	41.5134	0.560714538	0.966018836	20	16	24	0.198594309	0.768040178	24	16.98232059	0.041902628
ENSMUSG00000045954	Cavin2	caveolae associated 2 [Source:MGI Symbol;Acc:MGI:99513]	6.56849	8.33989	9.14549	12.4305	8.0833	7.88297	7.94765	9.52382	8.84662	8.9194	9.06836	7.27557	7.57422	7.4569	0.016024075	0.253395174	16	12	24	0.012694449	0.526249719	24	10.93374359	0.198954636
ENSMUSG00000045962	Wnk1	WNK lysine deficient protein kinase 1 [Source:MGI Symbol;Acc:MGI:2442092]	164.764	151.323	148.109	142.95	148.899	160.752	158.114	136.32	130.27	154.525	135.664	133.099	131.022	144.44	0.885059765	0.966018836	4	8	24	0.47911177	0.805393068	24	21.15102242	0.043809969
ENSMUSG00000045967	Gpr158	G protein-coupled receptor 158 [Source:MGI Symbol;Acc:MGI:2441697]	26.9014	23.9828	23.774	23.4306	23.7499	24.6997	22.7545	22.3882	19.9126	24.75	24.6274	24.5662	23.2799	25.6456	0.945451136	0.966018836	24	12	24	0.315722206	0.768040178	24	20.19386796	0.059352646
ENSMUSG00000045969	Ing1	inhibitor of growth family, member 1 [Source:MGI Symbol;Acc:MGI:1349481]	13.7539	12.196	11.5951	13.4179	12.7913	11.9615	14.407	11.8708	13.5938	11.7845	12.988	12.398	13.2533	12.0919	0.432054949	0.966018836	4	12	24	0.590690991	0.855420653	24	21.35106777	0.038092767
ENSMUSG00000045973	Slc25a51	solute carrier family 25, member 51 [Source:MGI Symbol;Acc:MGI:2684984]	38.5656	30.4067	34.5332	37.8236	33.8625	32.8741	36.9217	35.2123	32.128	40.1265	38.148	35.4509	36.2836	36.9872	0.967855455	0.970142211	16	20	24	0.69148	0.897724829	24	15.7209522	0.035670942
ENSMUSG00000045975	C2cd2	C2 calcium-dependent domain containing 2 [Source:MGI Symbol;Acc:MGI:1891883]	9.2226	9.03417	9.63034	10.5922	9.61453	10.7464	8.24577	13.5251	9.11475	12.7055	8.98586	9.99823	10.3409	9.26274	0.92769718	0.966018836	16	12	24	0.6966249	0.89922881	24	10.75714449	0.061758639
ENSMUSG00000045980	Tmem104	transmembrane protein 104 [Source:MGI Symbol;Acc:MGI:2444222]	5.57212	3.81289	4.10945	4.76942	4.48697	5.33571	4.86973	5.15864	3.23239	6.27933	4.34225	4.68391	4.31026	4.32675	0.338172159	0.85758369	24	12	24	0.387902196	0.799546792	24	18.89144284	0.109381774
ENSMUSG00000045983	Eif4g1	eukaryotic translation initiation factor 4, gamma 1 [Source:MGI Symbol;Acc:MGI:2384784]	70.4259	58.6185	62.5471	62.7559	62.107	65.8762	64.9458	71.8158	58.2927	81.125	62.8087	67.0873	68.6659	69.7037	0.45229395	0.966018836	4	8	24	0.947754329	0.987252246	24	22.88341478	0.01585682
ENSMUSG00000045991	Onecut2	one cut domain, family member 2 [Source:MGI Symbol;Acc:MGI:1891408]	4.26186	3.92018	3.52905	4.10134	4.29847	3.63238	3.82743	3.4277	3.77917	3.90036	4.65849	3.89762	4.1554	4.15259	0.185796491	0.662239322	20	16	24	0.283480032	0.768040178	24	17.25742026	0.058772105
ENSMUSG00000045994	B3gat1	beta-1,3-glucuronyltransferase 1 (glucuronosyltransferase P) [Source:MGI Symbol;Acc:MGI:1924148]	2.71888	4.29927	6.53509	3.97926	3.24269	3.80327	4.86097	3.00971	5.85582	4.22744	2.89291	4.42616	2.40507	5.04671	0.089801637	0.477824925	12	16	24	0.145310432	0.754184974	24	8.265555226	0.259851386
ENSMUSG00000045996	Polr2k	polymerase (RNA) II (DNA directed) polypeptide K [Source:MGI Symbol;Acc:MGI:102725]	4.74976	5.46116	4.36232	4.46626	5.06363	3.68186	4.63671	4.30798	4.97229	3.82124	4.82267	5.40925	3.96533	4.39743	0.813775138	0.966018836	20	20	24	0.984148369	0.996767829	24	22.49954905	0.010375283
ENSMUSG00000046008	Pnlip	pancreatic lipase [Source:MGI Symbol;Acc:MGI:97722]	1.31843	1.47779	1.14164	1.1374	1.16978	1.67876	1.17087	1.23632	1.22901	1.41687	1.28028	1.52571	1.35382	1.60165	0.217933169	0.704989686	24	12	24	0.38005839	0.799474855	24	22.62007243	0.055676797
ENSMUSG00000046010	Zfp830	zinc finger protein 830 [Source:MGI Symbol;Acc:MGI:1914233]	6.07944	5.50811	5.67847	5.20961	5.73663	5.83824	5.79912	5.23301	4.99436	5.53732	5.13078	5.45307	5.77491	5.33721	0.080148772	0.458678242	4	8	24	0.026754592	0.586541342	24	21.79319609	0.062085423
ENSMUSG00000046020	Pofut1	protein O-fucosyltransferase 1 [Source:MGI Symbol;Acc:MGI:2153207]	8.36818	7.71246	8.12114	7.80493	7.98484	8.37226	7.47688	7.97693	7.57265	8.47879	7.35272	8.51313	8.09289	8.3935	0.85387321	0.966018836	24	12	24	0.830407092	0.947343852	24	21.85872548	0.015713694
ENSMUSG00000046027	Stard5	StAR-related lipid transfer (START) domain containing 5 [Source:MGI Symbol;Acc:MGI:2156765]	10.2155	14.0448	13.5116	14.6784	16.4186	10.5872	13.6978	13.0294	18.0627	13.5695	13.0986	13.3446	14.2922	12.0172	0.4776989	0.966018836	16	8	24	0.140929054	0.751014756	24	10.48397231	0.150114997
ENSMUSG00000046032	Snx12	sorting nexin 12 [Source:MGI Symbol;Acc:MGI:1919331]	34.0867	24.7496	29.3092	26.0922	25.0833	29.1931	26.7153	25.0114	22.9127	31.4747	25.6999	27.8557	27.9924	26.4518	0.530814489	0.966018836	4	4	24	0.507105256	0.812385664	24	20.59571383	0.068810245
ENSMUSG00000046034	Otulin	OTU deubiquitinase with linear linkage specificity [Source:MGI Symbol;Acc:MGI:3577015]	9.04654	8.61213	10.4477	8.70227	8.63243	8.72644	8.69324	9.16405	9.26449	9.20999	8.54293	8.9005	10.1844	8.80442	0.117335378	0.538362324	12	8	24	0.378446144	0.797729687	24	6.085996712	0.044885579
ENSMUSG00000046056	Sbsn	suprabasin [Source:MGI Symbol;Acc:MGI:2446326]	3.55466	3.87689	4.40621	2.53365	3.91617	2.94068	3.24189	3.96033	4.91736	3.27316	3.4548	4.16095	2.73202	3.48264	0.310940564	0.820837787	12	4	24	0.473071566	0.805393068	24	7.181686806	0.103515651
ENSMUSG00000046057	Gm15428	predicted pseudogene 15428 [Source:MGI Symbol;Acc:MGI:3642670]	0.474121	0.596632	1.79676	0.264052	0.859634	1.30366	0.286506	0.624438	1.27953	0.470835	0.318508	0.774179	0.643156	0.185713	0.823657258	0.966018836	12	20	24	0.904807764	0.973704275	24	10.91841557	0.065028983
ENSMUSG00000046058	Eid2	EP300 interacting inhibitor of differentiation 2 [Source:MGI Symbol;Acc:MGI:2681174]	13.167	11.8189	12.8854	10.6551	10.9281	12.313	11.5484	13.5504	12.3586	13.7708	13.0563	14.473	14.163	14.5122	0.935593607	0.966018836	8	8	24	0.276422026	0.768040178	24	2.314742637	0.061538932
ENSMUSG00000046062	Ppp1r15b	protein phosphatase 1, regulatory subunit 15B [Source:MGI Symbol;Acc:MGI:2444211]	15.1149	13.771	15.0128	15.3582	14.1957	14.5827	15.3224	13.4627	14.3143	17.5361	15.0498	15.292	15.8759	14.9221	0.249881062	0.749643185	4	4	24	0.48776933	0.805393068	24	14.72660148	0.03860947
ENSMUSG00000046079	Lrrc8d	leucine rich repeat containing 8D [Source:MGI Symbol;Acc:MGI:1922368]	22.3797	15.9528	17.1237	16.4035	16.9784	19.8025	16.9659	20.1002	12.8472	23.851	15.9015	17.8154	17.8636	17.8441	0.725061903	0.966018836	4	8	24	0.63265663	0.875112738	24	21.11109683	0.083434633
ENSMUSG00000046093	Hpcal4	hippocalcin-like 4 [Source:MGI Symbol;Acc:MGI:2157521]	1.15298	1.82075	3.57953	1.82083	1.30124	1.7605	2.63877	1.40311	2.85851	1.75824	1.00383	2.04037	0.825287	3.09248	0.10560184	0.518002936	12	8	24	0.158974436	0.765350752	24	7.289648284	0.299252109
ENSMUSG00000046096	Mosmo	modulator of smoothened [Source:MGI Symbol;Acc:MGI:2446240]	27.2319	25.4168	23.9554	25.1937	23.8986	23.3599	25.8045	25.1403	23.1953	23.9491	26.3447	25.0894	23.0835	28.4729	0.912923872	0.966018836	8	4	24	0.56102518	0.841531815	24	1.674773723	0.036530583
ENSMUSG00000046101	Mcmdc2	minichromosome maintenance domain containing 2 [Source:MGI Symbol;Acc:MGI:3045334]	0.850848	0.567778	0.441039	0.35934	0.26332	0.611642	0.467556	0.377832	1.05486	0.366118	0.69219	0.63976	0.663055	0.827391	0.530814489	0.966018836	4	12	24	0.380666894	0.799546792	24	2.223578483	0.103031403
ENSMUSG00000046110	Serinc4	serine incorporator 4 [Source:MGI Symbol;Acc:MGI:2441842]	0.542664	0.415323	0.202454	0.615048	0.634402	0.727977	0.683853	0.754059	0.580669	0.867175	0.185669	0.389805	0.434207	0.65613	0.853736882	0.966018836	16	4	24	0.967521509	0.992187127	24	3.914933217	0.020464645
ENSMUSG00000046111	Cep295	centrosomal protein 295 [Source:MGI Symbol;Acc:MGI:2442521]	5.49146	7.01919	7.32595	7.71961	7.49316	6.88422	6.33975	6.15552	7.82851	5.59837	7.23487	7.17079	6.42278	6.68605	0.017495149	0.255326033	12	16	24	0.302763396	0.768040178	24	12.49941437	0.08269053
ENSMUSG00000046138	9930021J03Rik	RIKEN cDNA 9930021J03 gene [Source:MGI Symbol;Acc:MGI:2444398]	8.50401	8.52364	9.06622	8.74638	8.39876	8.32628	8.80728	7.7697	7.74428	7.81927	8.81716	8.33672	8.62782	7.76899	0.916746631	0.966018836	4	4	24	0.379145433	0.79853649	24	18.92913846	0.035234457
ENSMUSG00000046139	Patl1	protein associated with topoisomerase II homolog 1 (yeast) [Source:MGI Symbol;Acc:MGI:2147679]	5.26847	4.31926	4.52614	5.33659	4.77299	4.95528	5.41965	4.83962	4.88742	5.45814	4.84716	4.56738	4.65643	4.7239	0.743750765	0.966018836	16	16	24	0.655862778	0.883738698	24	15.47685729	0.031214657
ENSMUSG00000046152	Fut10	fucosyltransferase 10 [Source:MGI Symbol;Acc:MGI:2384748]	2.49584	2.87956	3.2421	2.73785	2.6517	3.0254	3.07759	3.21217	3.08602	2.87761	2.38893	3.085	2.88044	2.56759	0.424718357	0.966018836	12	8	24	0.523360145	0.821170929	24	5.12169064	0.046676104
ENSMUSG00000046157	Tmem229b	transmembrane protein 229B [Source:MGI Symbol;Acc:MGI:2444389]	30.3475	22.5617	21.5832	24.074	25.7924	27.2893	26.6357	26.8029	18.2508	33.6432	24.122	25.4466	24.6455	25.352	0.143321297	0.585944911	24	12	24	0.34590963	0.778673045	24	19.73951219	0.119798207
ENSMUSG00000046159	Chrm3	cholinergic receptor, muscarinic 3, cardiac [Source:MGI Symbol;Acc:MGI:88398]	0.938408	1.00387	1.13163	0.711025	0.720501	0.930684	0.927425	0.664129	0.732816	0.632193	0.731045	0.927777	0.635092	0.955087	0.25849765	0.761738864	12	4	24	0.356480468	0.783133725	24	1.302485466	0.063269356
ENSMUSG00000046160	Olig1	oligodendrocyte transcription factor 1 [Source:MGI Symbol;Acc:MGI:1355334]	1.48589	4.64866	8.99096	3.22076	1.77428	4.27478	5.88423	4.0231	5.84489	3.72906	2.06545	4.8561	1.71038	6.6179	0.229694208	0.729069536	12	8	24	0.134768907	0.746461599	24	6.688803021	0.476566086
ENSMUSG00000046167	Gldn	gliomedin [Source:MGI Symbol;Acc:MGI:2388361]	16.4808	19.2288	18.4104	19.1204	22.9263	16.7256	22.288	19.4512	22.0994	24.642	16.8662	18.6443	19.0977	16.5672	0.724067924	0.966018836	16	8	24	0.383224785	0.799546792	24	11.23606335	0.097553635
ENSMUSG00000046168	Kcnrg	potassium channel regulator [Source:MGI Symbol;Acc:MGI:2685591]	0.573556	0.566299	0.546123	0.633323	0.581036	0.470796	0.584045	0.475111	0.756221	0.501582	0.626594	0.592181	0.714473	0.354417	0.644063931	0.966018836	20	12	24	0.781623111	0.929899462	24	15.2316542	0.026448974
ENSMUSG00000046169	Adamts6	a disintegrin-like and metallopeptidase (reprolysin type) with thrombospondin type 1 motif, 6 [Source:MGI Symbol;Acc:MGI:1347348]	0.662859	1.19855	1.34677	0.914569	1.05961	0.867499	1.03792	1.02953	1.42439	0.972693	0.90065	0.9615	0.783832	1.05805	0.010478767	0.196621011	12	16	24	0.032451763	0.606585237	24	8.327773862	0.138978446
ENSMUSG00000046178	Nxph1	neurexophilin 1 [Source:MGI Symbol;Acc:MGI:107492]	2.03711	2.84417	3.76639	2.6186	1.94181	2.42909	3.06432	2.10338	2.73896	2.3997	2.29896	2.34074	1.57483	2.97193	0.268242676	0.774280302	12	8	24	0.184378404	0.768040178	24	7.807321534	0.158047973
ENSMUSG00000046182	Gsg1l	GSG1-like [Source:MGI Symbol;Acc:MGI:2685483]	1.01005	1.21086	1.27129	1.08142	1.07081	1.12507	1.23523	0.946416	1.06665	1.2784	0.923749	1.22771	1.07497	1.1503	0.853736882	0.966018836	16	4	24	0.888406613	0.96864266	24	7.222015035	0.016181444
ENSMUSG00000046185	Zfp84	zinc finger protein 84 [Source:MGI Symbol;Acc:MGI:107780]	3.46508	3.51893	3.53213	3.26181	3.31472	3.03544	3.35587	3.29727	3.38897	3.20078	3.45499	3.37213	3.38836	3.25466	0.892685629	0.966018836	12	4	24	0.547859924	0.833842569	24	4.811990784	0.019088376
ENSMUSG00000046186	Cd109	CD109 antigen [Source:MGI Symbol;Acc:MGI:2445221]	1.65893	2.0434	2.24885	1.58489	1.89862	1.77542	1.51129	1.85516	2.47246	1.87002	1.92381	2.03702	1.61009	2.07455	0.033248781	0.324908923	12	16	24	0.129212588	0.740162193	24	8.756389583	0.102564842
ENSMUSG00000046191	Pcdhb20	protocadherin beta 20 [Source:MGI Symbol;Acc:MGI:2136758]	2.10877	2.05265	2.37153	1.92368	1.7475	1.95162	2.1513	1.81279	2.21842	2.03882	2.19848	2.10606	2.34233	2.01458	0.72778087	0.966018836	12	4	24	0.692824737	0.898196933	24	4.177470854	0.028888729
ENSMUSG00000046201	Scaf8	SR-related CTD-associated factor 8 [Source:MGI Symbol;Acc:MGI:1925212]	10.7957	9.69287	10.5748	9.55611	9.8041	10.2744	9.93372	10.396	9.43917	12.2154	8.43273	10.4946	10.1085	10.289	0.904690479	0.966018836	24	20	24	0.858483189	0.958368659	24	4.39125958	0.024408615
ENSMUSG00000046204	Pnma2	paraneoplastic antigen MA2 [Source:MGI Symbol;Acc:MGI:2444129]	21.3688	17.3732	17.3586	18.24	17.5238	20.4911	18.5833	18.7173	15.4781	22.1024	19.6945	20.5918	19.7241	20.0054	0.047644929	0.377556215	24	12	24	0.198302444	0.768040178	24	20.0887443	0.090651155
ENSMUSG00000046207	Pik3r6	phosphoinositide-3-kinase regulatory subunit 5 [Source:MGI Symbol;Acc:MGI:2144613]	0.399596	0.671396	0.801886	0.374293	0.678178	1.19625	0.7769	0.897805	0.900371	0.921126	0.750309	0.657465	0.749304	0.687108	0.883907406	0.966018836	12	16	24	0.99223854	0.998516607	24	8.253882831	0.008804436
ENSMUSG00000046223	Plaur	plasminogen activator, urokinase receptor [Source:MGI Symbol;Acc:MGI:97612]	5.52501	3.28069	4.78214	3.244	3.70049	4.87274	3.71449	3.48299	2.90625	3.45753	5.62846	3.88184	3.79738	2.91096	0.057005497	0.401309455	24	8	24	0.092546865	0.71175955	24	19.23813888	0.201672987
ENSMUSG00000046229	Scand1	SCAN domain-containing 1 [Source:MGI Symbol;Acc:MGI:1343132]	1.87645	1.21614	0.923748	1.03701	0.897677	0.570257	0.998285	1.53066	2.44374	2.79837	8.81415	6.18381	8.34431	5.33538	0.787484004	0.966018836	4	20	24	0.87835345	0.965980735	24	21.00060524	0.117876229
ENSMUSG00000046230	Vps13a	vacuolar protein sorting 13A [Source:MGI Symbol;Acc:MGI:2444304]	27.9261	28.7398	26.5972	27.1473	28.4977	26.3923	26.1776	24.3646	26.0922	24.3421	30.3845	25.7988	26.9019	25.2419	0.784278077	0.966018836	20	12	24	0.284284373	0.768040178	24	17.01230611	0.051189129
ENSMUSG00000046240	Hepacam	hepatocyte cell adhesion molecule [Source:MGI Symbol;Acc:MGI:1920177]	6.54961	8.31184	11.6487	8.98281	7.2396	8.7908	10.5764	8.02903	10.1098	11.5738	6.81142	9.88314	7.65498	8.93887	0.191123261	0.669841558	16	4	24	0.260879936	0.768040178	24	8.87767543	0.15590983
ENSMUSG00000046245	Pilra	paired immunoglobin-like type 2 receptor alpha [Source:MGI Symbol;Acc:MGI:2450529]	0.565433	1.00551	1.28489	1.32384	1.09813	0.675169	1.07635	1.28516	1.0027	0.511996	1.01408	0.843405	1.20791	0.99075	0.924553956	0.966018836	12	12	24	0.498521276	0.808235473	24	7.159787985	0.096418931
ENSMUSG00000046269	Usp27x	ubiquitin specific peptidase 27, X chromosome [Source:MGI Symbol;Acc:MGI:1859645]	0.964342	0.870996	0.951808	0.74182	0.842703	0.801252	0.88033	0.92518	0.704585	0.755748	0.755146	0.946161	0.673995	0.940997	0.68887295	0.966018836	8	8	24	0.296023679	0.768040178	24	1.249043855	0.045206574
ENSMUSG00000046275	Trarg1	trafficking regulator of GLUT4 (SLC2A4) 1 [Source:MGI Symbol;Acc:MGI:3029307]	171.744	124.451	121.765	140.013	136.321	157.118	131.515	145.549	100.53	173.277	132.295	148.946	149.762	140.269	0.08302755	0.462922111	24	12	24	0.226325012	0.768040178	24	20.03058093	0.139487966
ENSMUSG00000046280	She	src homology 2 domain-containing transforming protein E [Source:MGI Symbol;Acc:MGI:1099462]	0.619162	0.726803	0.749702	0.969674	0.74979	0.683267	0.870197	1.0248	0.825072	0.793267	0.603976	0.702017	0.593266	0.775443	0.180336821	0.657605589	16	8	24	0.178923432	0.768040178	24	8.213672286	0.07512429
ENSMUSG00000046287	Pnma3	paraneoplastic antigen MA3 [Source:MGI Symbol;Acc:MGI:2180565]	0.890956	1.38584	2.01933	1.28226	0.919818	1.3884	1.52228	0.889081	1.62168	1.25731	0.944772	1.50965	0.939447	1.65973	0.745199643	0.966018836	12	8	24	0.353004925	0.780629136	24	7.458693582	0.127964028
ENSMUSG00000046311	Zfp62	zinc finger protein 62 [Source:MGI Symbol;Acc:MGI:99662]	15.1607	15.4158	14.7583	14.3852	15.6099	12.9869	15.2661	12.7301	15.9409	12.2324	16.3089	13.7589	14.5906	15.001	0.939103211	0.966018836	12	12	24	0.985934638	0.997339197	24	2.670576364	0.007899617
ENSMUSG00000046312	Myorg	myogenesis regulating glycosidase (putative) [Source:MGI Symbol;Acc:MGI:2140300]	5.00387	4.56521	5.13987	4.60307	4.92174	5.61905	4.99942	6.12879	4.45898	7.27633	4.82487	5.58223	4.56131	5.02723	0.886572916	0.966018836	24	4	24	0.821689805	0.943784856	24	13.96588423	0.040498695
ENSMUSG00000046314	Stxbp6	syntaxin binding protein 6 (amisyn) [Source:MGI Symbol;Acc:MGI:2384963]	20.8775	20.58	18.6711	19.1687	21.1275	16.5342	20.9278	17.4685	23.346	18.5904	19.5074	18.9547	18.849	18.0184	0.887862353	0.966018836	20	12	24	0.894992042	0.970212381	24	8.94572807	0.02356326
ENSMUSG00000046321	Hs3st2	heparan sulfate (glucosamine) 3-O-sulfotransferase 2 [Source:MGI Symbol;Acc:MGI:1333802]	10.0067	6.808	5.51043	7.36105	7.33072	7.93877	7.0762	8.4316	5.87575	11.445	7.26546	8.56102	8.97342	8.28714	0.217827365	0.704989686	12	12	24	0.5136404	0.816307374	24	20.51780967	0.113504761
ENSMUSG00000046324	Ermp1	endoplasmic reticulum metallopeptidase 1 [Source:MGI Symbol;Acc:MGI:106250]	43.0113	37.5272	37.72	37.8667	38.9719	39.222	39.637	39.9234	32.6863	40.9122	38.1959	38.2242	40.8273	39.0266	0.009848456	0.194720062	4	8	24	0.16853774	0.767020009	24	21.65543518	0.063868906
ENSMUSG00000046329	Slc25a23	solute carrier family 25 (mitochondrial carrier; phosphate carrier), member 23 [Source:MGI Symbol;Acc:MGI:1914222]	16.5191	19.2112	21.1848	21.5319	20.841	21.4954	23.0817	19.8011	21.448	23.9932	17.4357	20.1594	20.2462	19.8755	0.223774686	0.717349224	16	16	24	0.441497097	0.805393068	24	10.94622381	0.068336395
ENSMUSG00000046330	Rpl37a	ribosomal protein L37a [Source:MGI Symbol;Acc:MGI:98068]	9.05587	8.71927	8.79003	8.97245	11.3336	8.29543	9.689	9.28598	10.918	8.47871	12.413	8.96293	13.1425	9.47383	0.944053021	0.966018836	4	12	24	0.84747182	0.954020745	24	17.93731365	0.041227826
ENSMUSG00000046334	Gm6195	predicted pseudogene 6195 [Source:MGI Symbol;Acc:MGI:3647661]	4.77745	4.6065	3.60045	3.48355	8.16895	5.04676	5.78013	4.75347	6.17487	4.12439	4.75859	5.27425	4.35649	3.23443	0.238201401	0.741459408	20	20	24	0.270044255	0.768040178	24	18.79841615	0.186918988
ENSMUSG00000046337	Fam178b	family with sequence similarity 178, member B [Source:MGI Symbol;Acc:MGI:3026913]	116.17	121.736	107.838	110.592	158.02	115.35	133.889	121.616	134.366	107.703	117.422	115.475	122.802	97.4066	0.953141285	0.966018836	4	12	24	0.568786931	0.844618938	24	18.78856166	0.071985026
ENSMUSG00000046341	Gm11223	predicted gene 11223 [Source:MGI Symbol;Acc:MGI:3651631]	295.826	280.198	269.354	270.236	284.842	270.378	311.824	263.099	264.24	293.266	282.809	296.339	322.364	278.359	0.004861081	0.141782417	4	8	24	0.090632431	0.71175955	24	21.62299369	0.070376281
ENSMUSG00000046345	Smco1	single-pass membrane protein with coiled-coil domains 1 [Source:MGI Symbol;Acc:MGI:1916826]	0.0636091	0.905948	1.34947	2.48819	1.21797	1.28602	0.738043	2.65384	2.65349	2.18483	0.701492	1.11266	1.14195	1.46546	0.055920623	0.401074772	12	16	24	0.016485571	0.544099157	24	9.66075466	0.482502016
ENSMUSG00000046351	Zfp322a	zinc finger protein 322A [Source:MGI Symbol;Acc:MGI:2442566]	11.7607	13.0942	13.2915	12.2185	11.8294	10.987	12.1645	10.4471	11.9016	9.98303	13.0964	11.0719	12.6623	11.2475	0.890648739	0.966018836	12	12	24	0.972018091	0.993193546	24	4.156775512	0.010654283
ENSMUSG00000046352	Gjb2	gap junction protein, beta 2 [Source:MGI Symbol;Acc:MGI:95720]	3.40687	4.8163	3.266	2.58572	3.05669	2.70152	3.3446	4.83879	4.7373	1.67871	2.85271	3.36172	2.66042	3.31883	0.01457641	0.247599677	8	8	24	0.064294143	0.663876076	24	3.661936122	0.252762899
ENSMUSG00000046364	Rpl27a	ribosomal protein L27A [Source:MGI Symbol;Acc:MGI:1347076]	35.031	34.3905	32.9932	33.4406	36.719	34.4102	32.1916	31.1026	35.9237	26.8089	37.7311	33.5217	36.6634	33.6367	0.369262716	0.893596186	20	20	24	0.509747716	0.813985618	24	20.52267479	0.057319615
ENSMUSG00000046378	Asphd1	aspartate beta-hydroxylase domain containing 1 [Source:MGI Symbol;Acc:MGI:2685014]	5.44772	5.7743	5.88748	4.76092	5.4458	5.79313	4.84901	5.73579	4.23924	5.66149	5.67019	5.04256	4.96668	3.90232	0.846563228	0.966018836	24	4	24	0.424906798	0.805393068	24	17.68319282	0.069191156
ENSMUSG00000046380	Jrk	jerky [Source:MGI Symbol;Acc:MGI:106214]	1.22293	1.14314	1.15	1.0601	1.09022	1.33221	1.24963	1.26286	0.985694	1.36753	1.05727	1.4132	1.21217	1.19498	0.062786747	0.41072308	24	12	24	0.21583268	0.768040178	24	21.93491875	0.054868071
ENSMUSG00000046387	Pcdhb17	protocadherin beta 17 [Source:MGI Symbol;Acc:MGI:2136754]	2.65617	2.63826	2.72874	2.15403	2.27504	2.53566	2.68929	2.69046	2.20534	2.60191	2.49353	2.5428	2.2403	2.2859	0.612141595	0.966018836	8	8	24	0.475774647	0.805393068	24	0.360300579	0.038761791
ENSMUSG00000046402	Rbp1	retinol binding protein 1, cellular [Source:MGI Symbol;Acc:MGI:97876]	7.39546	9.86166	8.20502	6.25724	8.85165	7.82232	7.31074	9.03565	10.9878	4.16974	8.23994	8.14555	7.7175	8.11895	0.297978647	0.80820842	8	20	24	0.43835881	0.805393068	24	2.563762779	0.14096923
ENSMUSG00000046404	Yod1	YOD1 deubiquitinase [Source:MGI Symbol;Acc:MGI:2442596]	3.71225	3.99089	4.24347	3.60066	3.8809	3.52037	3.51923	3.51977	3.42937	3.57186	4.18634	3.54195	3.77195	3.99644	0.920872048	0.966018836	20	4	24	0.859271955	0.958530654	24	9.628208695	0.018405102
ENSMUSG00000046410	Kcnk6	potassium inwardly-rectifying channel, subfamily K, member 6 [Source:MGI Symbol;Acc:MGI:1891291]	0.575063	0.67817	0.625994	0.617566	0.494418	0.588497	0.629255	0.517307	0.746391	0.492999	0.551026	0.415846	0.481902	0.499277	0.435739761	0.966018836	12	12	24	0.196648254	0.768040178	24	7.191356008	0.04886501
ENSMUSG00000046417	Lrrc75a	leucine rich repeat containing 75A [Source:MGI Symbol;Acc:MGI:2682293]	5.11207	3.89538	3.57643	4.73863	6.16967	4.19546	6.11452	3.47836	4.25366	4.89917	4.99148	4.2419	4.20467	3.33938	0.004545736	0.140598674	20	12	24	0.045390777	0.629400324	24	17.55040937	0.199786453
ENSMUSG00000046432	Bex3	brain expressed X-linked 3 [Source:MGI Symbol;Acc:MGI:1338016]	146.022	143.137	139.817	137.758	139.243	156.573	133.118	128.584	116.296	126.098	126.233	139.059	132.826	127.522	0.308023687	0.814993005	24	12	24	0.016051153	0.544099157	24	20.64004944	0.089809153
ENSMUSG00000046434	Hnrnpa1	heterogeneous nuclear ribonucleoprotein A1 [Source:MGI Symbol;Acc:MGI:104820]	16.3513	18.2749	19.1015	17.9693	17.3508	15.5943	17.2198	16.3477	19.227	15.5873	16.5427	16.4638	16.1238	16.813	0.047644929	0.377556215	12	12	24	0.063110434	0.663164648	24	8.184980468	0.075980061
ENSMUSG00000046441	Cmtr2	cap methyltransferase 2 [Source:MGI Symbol;Acc:MGI:2384580]	2.9837	2.93315	2.96021	2.35844	2.89825	2.84159	2.50867	2.81227	2.60796	2.68526	2.85961	2.94638	3.11022	2.87882	0.47028201	0.966018836	4	12	24	0.291639192	0.768040178	24	23.27625676	0.048303374
ENSMUSG00000046442	Ppm1e	protein phosphatase 1E (PP2C domain containing) [Source:MGI Symbol;Acc:MGI:2444096]	17.5964	15.5171	15.7726	14.7497	13.5593	16.1648	16.4141	14.8131	12.687	17.0222	13.9689	15.8338	15.4437	16.9372	0.432054949	0.966018836	4	16	24	0.259487103	0.768040178	24	0.109302073	0.078795389
ENSMUSG00000046447	Camk2n1	calcium/calmodulin-dependent protein kinase II inhibitor 1 [Source:MGI Symbol;Acc:MGI:1913509]	15.199	18.1862	20.4781	16.7457	15.8922	16.0603	20.0127	14.9623	21.9892	18.2488	16.1823	19.6606	16.8366	20.189	0.285027487	0.793714531	12	8	24	0.231553468	0.768040178	24	7.294713366	0.111253484
ENSMUSG00000046449	Nexmif	neurite extension and migration factor [Source:MGI Symbol;Acc:MGI:2148050]	0.435095	0.642232	0.732352	0.374868	0.510065	0.471596	0.603016	0.402044	0.576514	0.367241	0.520403	0.469937	0.428897	0.620167	0.676344318	0.966018836	12	4	24	0.394824976	0.799546792	24	5.104912327	0.055763185
ENSMUSG00000046456	Tmem150b	transmembrane protein 150B [Source:MGI Symbol;Acc:MGI:2679718]	0.988849	0.722239	0.693891	0.82958	0.510511	0.771824	0.689993	0.475975	0.640808	0.711842	0.831698	0.82119	0.727662	0.692431	0.29905572	0.809362476	24	12	24	0.464238453	0.805393068	24	19.73255285	0.055867601
ENSMUSG00000046470	Sox18	SRY (sex determining region Y)-box 18 [Source:MGI Symbol;Acc:MGI:103559]	1.06102	1.10734	0.888058	1.40966	1.47212	1.0235	1.263	1.39212	1.4517	1.31175	1.33223	1.48726	1.91656	1.43365	0.771670154	0.966018836	20	16	24	0.965017033	0.99132528	24	18.4792544	0.012618737
ENSMUSG00000046480	Scn4b	sodium channel, type IV, beta [Source:MGI Symbol;Acc:MGI:2687406]	55.1755	49.3002	48.263	52.8712	44.2819	55.2283	52.4488	55.8998	42.5868	75.3289	43.582	55.4318	52.8626	58.6785	0.671726933	0.966018836	24	12	24	0.945708061	0.986808796	24	3.046518859	0.027025656
ENSMUSG00000046491	C1qtnf2	C1q and tumor necrosis factor related protein 2 [Source:MGI Symbol;Acc:MGI:1916433]	3.10542	5.70014	4.49027	3.6995	4.60158	3.70548	3.86029	5.67036	8.48272	2.35703	3.25077	3.97809	3.8299	4.62022	0.064405315	0.414867207	12	4	24	0.137643206	0.747972781	24	5.85291984	0.277215249
ENSMUSG00000046500	Tafa4	TAFA chemokine like family member 4 [Source:MGI Symbol;Acc:MGI:2444563]	28.5963	24.1127	21.5934	22.482	25.7193	23.514	20.8951	22.4474	21.0726	20.3942	25.6108	25.0253	27.7358	24.947	0.158091814	0.614628746	20	20	24	0.089233923	0.708638675	24	21.44117904	0.12226826
ENSMUSG00000046516	Cox17	cytochrome c oxidase assembly protein 17, copper chaperone [Source:MGI Symbol;Acc:MGI:1333806]	133.998	143.197	135.002	148.468	114.195	114.027	130.06	138.376	152.635	128.891	163.502	142.521	174.511	145.665	0.833406028	0.966018836	12	12	24	0.463006747	0.805393068	24	5.903166371	0.076304892
ENSMUSG00000046519	Golph3l	golgi phosphoprotein 3-like [Source:MGI Symbol;Acc:MGI:1917129]	12.0593	12.6291	11.4477	11.9276	12.0922	11.4344	11.4181	11.7688	12.9045	10.3152	12.808	12.0083	12.2952	12.4632	0.916556573	0.966018836	8	8	24	0.766397643	0.92437333	24	2.409505938	0.022406629
ENSMUSG00000046523	Kctd4	potassium channel tetramerisation domain containing 4 [Source:MGI Symbol;Acc:MGI:1914766]	1.05217	1.63892	1.94259	1.34621	1.4465	1.19747	1.61474	1.28051	1.80892	1.09669	1.36923	1.34754	1.12234	1.37916	0.210566453	0.69936851	12	12	24	0.225846771	0.768040178	24	7.99219231	0.104826374

ENSMUSG00000046532	Ar	androgen receptor [Source:MGI Symbol;Acc:MGI:88064]	3.76702	3.98251	3.48741	4.20753	3.63722	3.5397	3.39535	3.49196	4.08906	3.74159	3.85455	4.08429	4.11216	4.03379	0.813775138	0.966018836	16	12	24	0.792053172	0.93349015	24	9.966297816	0.021980592
ENSMUSG00000046541	Zfp526	zinc finger protein 526 [Source:MGI Symbol;Acc:MGI:2445181]	1.40218	1.37783	1.12463	1.05082	1.28847	1.23747	1.24835	1.39376	1.37314	1.5189	1.26665	1.17812	1.32221	1.14543	0.874473208	0.966018836	4	20	24	0.871177234	0.963464461	24	2.875505819	0.01713915
ENSMUSG00000046546	Fam43a	family with sequence similarity 43, member A [Source:MGI Symbol;Acc:MGI:2676309]	2.37829	2.47715	2.21888	2.47844	1.78169	1.94945	2.04388	2.41959	2.56874	2.26163	2.51682	2.47458	2.24579	2.8436	0.40165019	0.933249185	8	16	24	0.10147689	0.713208839	24	6.410528392	0.093108452
ENSMUSG00000046550	Spin2c	spindlin family, member 2C [Source:MGI Symbol;Acc:MGI:3605548]	1.64196	1.70336	2.23474	1.77607	1.24711	1.82141	1.11272	1.91302	1.67871	1.14936	1.55039	1.5923	1.13873	1.34485	0.619261897	0.966018836	12	16	24	0.567979912	0.844165911	24	8.041584251	0.072341109
ENSMUSG00000046556	Zfp319	zinc finger protein 319 [Source:MGI Symbol;Acc:MGI:1890618]	2.18597	1.85768	1.87545	2.06743	1.91297	1.95599	2.38746	2.03878	1.7707	2.29344	1.8998	2.03452	2.06781	1.90804	0.035806379	0.331814682	4	8	24	0.514162048	0.816624526	24	21.30586884	0.038771553
ENSMUSG00000046562	Unc119b	unc-119 lipid binding chaperone B [Source:MGI Symbol;Acc:MGI:2147162]	13.2745	9.99331	10.8048	13.4741	12.1904	11.6866	12.4882	11.2607	11.0883	11.7099	12.0004	12.3327	11.7851	11.1772	0.059717207	0.406067671	4	4	24	0.095932859	0.71175955	24	18.09537942	0.086793873
ENSMUSG00000046567	4930430F08Rik	RIKEN cDNA 4930430F08 gene [Source:MGI Symbol;Acc:MGI:1921197]	13.1924	15.7257	13.4133	15.2177	14.7813	12.3854	12.1733	12.9496	11.5178	11.4384	17.2909	12.2374	11.3248	12.388	0.886952527	0.966018836	20	8	24	0.386350368	0.799546792	24	14.7400006	0.085228294
ENSMUSG00000046572	Zfp518b	zinc finger protein 518B [Source:MGI Symbol;Acc:MGI:2140750]	4.54134	4.10091	4.14832	4.3353	4.20927	4.46714	3.80177	4.10419	3.64804	4.42871	3.91815	4.01112	3.97945	4.21539	0.874672901	0.966018836	16	12	24	0.684377878	0.895095838	24	17.59023908	0.023884436
ENSMUSG00000046573	Lyrm4	LYR motif containing 4 [Source:MGI Symbol;Acc:MGI:2683538]	14.0058	13.9842	13.3144	12.252	13.8245	13.8711	13.2662	12.1577	12.5725	13.3581	14.8303	13.826	15.3753	13.4123	0.143370478	0.585944911	4	8	24	0.098969565	0.71175955	24	20.48312033	0.071209636
ENSMUSG00000046574	Prr12	proline rich 12 [Source:MGI Symbol;Acc:MGI:2679002]	3.21312	3.29376	3.41596	2.99103	3.12409	2.88949	4.08381	3.80914	3.728	5.29376	3.65533	3.34154	3.48444	3.26341	0.92769718	0.966018836	12	12	24	0.475854523	0.805393068	24	9.195325893	0.082537522
ENSMUSG00000046580	Gm7862	predicted gene 7862 [Source:MGI Symbol;Acc:MGI:3648002]	6.84501	4.99562	6.39297	5.15592	4.69303	5.42474	6.7539	5.97738	5.44243	4.53381	6.85234	5.93433	4.88144	4.20023	0.93447422	0.966018836	4	12	24	0.509379185	0.813985618	24	21.38551692	0.088848007
ENSMUSG00000046598	Bdh1	3-hydroxybutyrate dehydrogenase, type 1 [Source:MGI Symbol;Acc:MGI:1919161]	12.5199	11.407	14.749	13.6618	10.9197	13.1511	13.7598	15.0142	13.1363	14.6458	11.2963	12.9922	11.5465	13.1327	0.308023687	0.814993005	16	4	24	0.279505354	0.768040178	24	7.454106383	0.088945059
ENSMUSG00000046603	Tcaim	T cell activation inhibitor, mitochondrial [Source:MGI Symbol;Acc:MGI:1196217]	10.0329	9.44172	9.84029	9.60256	9.96535	9.66773	9.7543	9.98844	8.92218	9.67328	9.59322	10.0377	10.1705	9.42822	0.202982082	0.689605535	4	8	24	0.114148417	0.727799063	24	20.90515762	0.035779827
ENSMUSG00000046605	B3gntl1	UDP-GlcNAc:betaGal beta-1,3-N-acetylglucosaminyltransferase-like 1 [Source:MGI Symbol;Acc:MGI:2441705]	3.14018	3.30544	3.3852	3.18192	3.54048	3.00629	2.95245	3.43404	3.55595	3.30434	3.10267	2.9834	3.01096	2.99358	0.015345591	0.253245931	12	12	24	0.032691086	0.606585237	24	9.781684977	0.064133006
ENSMUSG00000046613	Vwa5b2	von Willebrand factor A domain containing 5B2 [Source:MGI Symbol;Acc:MGI:2681859]	13.4639	13.868	15.2812	16.6929	12.6739	12.6355	13.2511	13.1422	16.0914	18.1004	12.7559	14.891	14.4156	16.0997	0.022000259	0.267229408	16	4	24	0.021661302	0.574400012	24	9.254944664	0.150165351
ENSMUSG00000046618	Olfml2a	olfactomedin-like 2A [Source:MGI Symbol;Acc:MGI:2444741]	6.65901	6.60087	6.70879	5.77948	6.36708	6.04532	7.2074	7.16833	6.00834	7.06817	5.73173	6.12788	6.37281	6.43973	0.143609806	0.585944911	8	12	24	0.167365159	0.767020009	24	3.427354066	0.065606326
ENSMUSG00000046658	Zfp316	zinc finger protein 316 [Source:MGI Symbol;Acc:MGI:1860402]	2.11676	2.10132	2.01868	1.94798	1.88053	1.98095	1.9448	2.1419	2.23353	2.58013	2.20264	2.56457	2.32197	2.3661	0.804027134	0.966018836	8	12	24	0.822827922	0.944355004	24	7.105804648	0.01873399
ENSMUSG00000046667	Rbm12b1	RNA binding motif protein 12 B1 [Source:MGI Symbol;Acc:MGI:1919647]	3.30492	2.2854	1.89282	2.95193	2.76283	2.49819	3.2617	3.16438	2.53101	2.14833	3.46436	2.05693	2.10444	2.26448	0.92721845	0.966018836	20	16	24	0.631410392	0.87436603	24	19.55594886	0.08109614
ENSMUSG00000046668	Cxxc5	CXXC finger 5 [Source:MGI Symbol;Acc:MGI:1914643]	11.5738	12.7062	14.0488	11.2539	10.5492	10.4755	14.1077	12.1831	12.5342	15.4375	11.5467	13.922	12.7117	14.0168	0.582224696	0.966018836	12	8	24	0.198167012	0.768040178	24	6.194420318	0.103025882
ENSMUSG00000046671	Mtfr1l	mitochondrial fission regulator 1-like [Source:MGI Symbol;Acc:MGI:1924074]	60.4262	54.6872	53.5351	57.5104	55.4582	59.2093	53.5541	60.5363	53.3114	66.0777	54.4641	58.0796	57.4558	57.1945	0.601647512	0.966018836	24	12	24	0.906891129	0.974587616	24	17.12085999	0.015529091
ENSMUSG00000046675	Tmem251	transmembrane protein 251 [Source:MGI Symbol;Acc:MGI:2443862]	2.44527	2.59707	2.9171	2.4666	2.61532	2.52026	2.1595	2.45453	2.52906	2.16119	2.75696	2.48335	2.36835	2.19066	0.133766544	0.568189321	20	8	24	0.526611621	0.823075573	24	13.57857506	0.036109024
ENSMUSG00000046679	C87436	expressed sequence C87436 [Source:MGI Symbol;Acc:MGI:2141787]	9.3469	8.84114	9.18616	8.76795	9.98351	9.53019	9.51454	8.81204	9.76114	10.146	9.70974	9.66416	9.40838	9.39113	0.017417473	0.255326033	20	12	24	0.114600808	0.727799063	24	16.19525608	0.041470606
ENSMUSG00000046687	Gm5424	predicted gene 5424 [Source:MGI Symbol;Acc:MGI:3643173]	29.0721	24.0963	22.486	25.0312	23.4403	28.1343	24.4289	26.1677	22.0901	34.2222	21.8514	27.8871	23.4096	26.6002	0.601647512	0.966018836	24	12	24	0.863815061	0.960525796	24	19.67382415	0.039830774
ENSMUSG00000046688	Tifa	TRAF-interacting protein with forkhead-associated domain [Source:MGI Symbol;Acc:MGI:2182965]	10.7304	9.8245	12.439	11.391	13.366	10.8273	9.01837	8.82307	8.92656	8.75132	12.5913	9.4377	10.0936	8.87704	0.03484427	0.328311403	20	12	24	0.048699164	0.638992719	24	15.97500099	0.154443267
ENSMUSG00000046691	Chtf8	CTF8, chromosome transmission fidelity factor 8 [Source:MGI Symbol;Acc:MGI:2443370]	18.3343	10.6339	13.3797	13.0778	9.53442	14.7138	15.4305	14.1759	10.6438	13.2579	15.3755	14.6711	14.395	15.2891	0.080148772	0.458678242	4	8	24	0.188733426	0.768040178	24	22.85664138	0.162442582
ENSMUSG00000046699	Slitrk4	SLIT and NTRK-like family, member 4 [Source:MGI Symbol;Acc:MGI:2442509]	6.93264	7.62672	7.17589	7.38292	6.76168	7.28948	6.93804	6.64126	8.47047	6.37041	8.15922	7.82058	7.81239	7.31497	0.816300547	0.966018836	24	16	24	0.999034974	0.999770694	24	7.525071587	0.001835829
ENSMUSG00000046707	Csnk2a2	casein kinase 2, alpha prime polypeptide [Source:MGI Symbol;Acc:MGI:88547]	21.1757	15.475	17.7466	15.7844	17.3258	18.4789	20.5527	16.3454	16.0521	20.5416	14.2201	18.8047	16.5653	19.6442	0.454881285	0.966018836	4	16	24	0.491081151	0.805393068	24	23.2211207	0.079210245
ENSMUSG00000046709	Mapk10	mitogen-activated protein kinase 10 [Source:MGI Symbol;Acc:MGI:1346863]	105.122	90.6812	107.3	95.6058	92.5167	109.594	102.533	98.6231	76.9969	108.644	95.7027	99.4523	105.401	108.596	0.94086481	0.966018836	4	8	24	0.490193274	0.805393068	24	21.99349664	0.066382672
ENSMUSG00000046711	Hmga1	high mobility group AT-hook 1 [Source:MGI Symbol;Acc:MGI:96160]	6.47662	5.89156	7.0819	6.75043	6.02413	7.68123	7.38291	7.698	6.4629	8.0352	5.9002	6.95212	6.79995	7.71882	0.858932988	0.966018836	8	12	24	0.700758649	0.900900643	24	4.636857215	0.042366514
ENSMUSG00000046714	Foxc2	forkhead box C2 [Source:MGI Symbol;Acc:MGI:1347481]	2.00853	2.13946	1.91705	1.78273	1.76882	1.97716	2.00223	1.80564	2.64482	1.12236	2.41224	2.35317	2.02035	2.21727	0.892555977	0.966018836	12	4	24	0.533731427	0.825979252	24	0.651172811	0.07998844
ENSMUSG00000046718	Bst2	bone marrow stromal cell antigen 2 [Source:MGI Symbol;Acc:MGI:1916800]	21.6967	22.7541	19.0569	18.1741	24.4256	18.9888	19.8271	27.7196	24.5152	17.1048	24.1474	21.1483	18.3866	28.0002	0.223774686	0.717349224	8	8	24	0.509862336	0.813985618	24	4.390472358	0.099186182
ENSMUSG00000046719	Nxph3	neurexophilin 3 [Source:MGI Symbol;Acc:MGI:1336188]	3.47789	2.86342	2.58263	2.66507	2.7556	3.07089	3.02547	2.84899	2.49252	3.62104	2.90942	3.53386	3.38312	2.94012	0.001971398	0.100255953	24	12	24	0.111332645	0.723334499	24	21.01780249	0.099529231
ENSMUSG00000046721	Rpl14-ps1	ribosomal protein L14, pseudogene 1 [Source:MGI Symbol;Acc:MGI:3710579]	248.234	264.884	265.912	252.546	254.648	267.002	254.678	253.577	263.725	238.44	257.023	250.645	257.649	260.694	0.503972924	0.966018836	12	4	24	0.575609413	0.848060327	24	3.707251457	0.018351897
ENSMUSG00000046722	Cdc42se1	CDC42 small effector 1 [Source:MGI Symbol;Acc:MGI:1889510]	28.4069	21.9193	23.3594	26.0239	28.3659	25.7373	27.35	23.8496	23.7659	27.6395	25.5983	24.7313	24.246	22.5254	0.038707602	0.336039716	20	12	24	0.02951775	0.596648309	24	17.49254904	0.10794337
ENSMUSG00000046727	Cystm1	cysteine-rich transmembrane module containing 1 [Source:MGI Symbol;Acc:MGI:1913310]	190.063	157.742	161.982	152.495	151.156	177.033	157.76	166.357	145.339	149.981	185.542	157.633	161.14	156.292	0.242077645	0.741580974	4	12	24	0.128337647	0.740162193	24	21.2637359	0.087528802
ENSMUSG00000046731	Kctd11	potassium channel tetramerisation domain containing 11 [Source:MGI Symbol;Acc:MGI:2448712]	8.124	6.31808	6.46754	6.71345	8.61302	7.01042	8.9863	6.88608	6.81311	7.99145	6.26447	6.68754	7.15938	5.7465	0.369262716	0.893596186	4	4	24	0.325900283	0.768040178	24	19.39295358	0.095198114
ENSMUSG00000046743	Fat4	FAT atypical cadherin 4 [Source:MGI Symbol;Acc:MGI:3045256]	0.709985	0.799187	0.87101	0.78465	0.731118	0.816818	0.933552	0.733412	0.906032	0.814627	0.694239	0.854209	0.689049	0.936169	0.616047374	0.966018836	12	8	24	0.397719004	0.799546792	24	6.421736205	0.03746183
ENSMUSG00000046750	Selenov	selenoprotein V [Source:MGI Symbol;Acc:MGI:3608324]	0.287534	0.47523	0.351887	0.247923	0.727802	0.491425	0.438863	0.580176	0.76267	0.308647	0.297107	0.987281	0.525983	0.61686	0.952127548	0.966018836	8	8	24	0.731327968	0.912429036	24	23.51102385	0.057365152
ENSMUSG00000046753	Ccdc66	coiled-coil domain containing 66 [Source:MGI Symbol;Acc:MGI:2443639]	6.07165	6.6402	5.9327	6.07851	6.78117	5.99175	7.34098	5.89949	6.66237	5.5788	6.0968	7.74102	6.66593	6.90555	0.628286596	0.966018836	24	16	24	0.257229902	0.768040178	24	22.88216175	0.070656396
ENSMUSG00000046756	Mrps7	mitchondrial ribosomal protein S7 [Source:MGI Symbol;Acc:MGI:1354367]	22.4053	18.2613	20.4562	22.4213	19.8564	23.5977	19.7984	20.8715	18.4724	19.9242	21.9517	21.1779	20.9585	20.1681	0.289496388	0.794869797	24	12	24	0.104053127	0.715480277	24	18.80498839	0.081586248
ENSMUSG00000046761	Fam83h	family with sequence similarity 83, member H [Source:MGI Symbol;Acc:MGI:2145900]	2.08151	1.75018	1.84576	1.40052	1.76664	2.00222	1.70654	2.32915	1.72361	1.98716	1.97257	2.24335	2.08689	1.94445	0.417963425	0.963884303	24	16	24	0.203982838	0.768040178	24	23.20440656	0.076238401
ENSMUSG00000046764	A530053G22Rik	RIKEN cDNA A530053G22 gene [Source:MGI Symbol;Acc:MGI:2443347]	0.245782	0.244748	0.831592	1.85759	0.506375	0.279691	0.774188	1.08083	0.664621	1.01841	1.76029	1.22053	1.08732	1.4005	0.865233232	0.966018836	16	12	24	0.172815378	0.768040178	24	11.58297588	0.226016535
ENSMUSG00000046768	Rhoj	ras homolog family member J [Source:MGI Symbol;Acc:MGI:1931551]	11.4159	10.8686	8.33365	7.20064	10.4051	9.01961	11.7213	11.2144	9.64036	6.78843	10.4227	10.5406	10.8815	9.00223	1.06E-04	0.029372629	4	12	24	0.008511036	0.501317957	24	23.44126539	0.229342297
ENSMUSG00000046785	Epm2aip1	EPM2A (laforin) interacting protein 1 [Source:MGI Symbol;Acc:MGI:1925031]	34.4713	35.3606	34.1832	34.6974	34.8632	33.2574	34.1449	31.2621	35.382	32.2959	37.96	35.4051	34.6866	37.2525	0.887862353	0.966018836	20	8	24	0.912428944	0.975295827	24	16.57807591	0.011858679
ENSMUSG00000046791	Riox1	ribosomal oxygenase 1 [Source:MGI Symbol;Acc:MGI:1919202]	2.05152	1.37696	1.87709	1.73787	1.94075	1.7934	1.95291	1.6479	1.55359	1.67859	1.8054	1.68276	2.14569	2.0295	0.118984653	0.542554768	4	8	24	0.373539506	0.794388449	24	21.44639423	0.061289836
ENSMUSG00000046792	Zfp787	zinc finger protein 787 [Source:MGI Symbol;Acc:MGI:1914359]	4.65232	4.34885	4.96443	3.8239	3.48641	3.73058	4.48899	4.78578	4.94732	5.41865	4.18519	5.18332	5.31978	4.69091	0.745118544	0.966018836	12	8	24	0.137560972	0.747972781	24	5.011491627	0.108283875
ENSMUSG00000046793	Gpr61	G protein-coupled receptor 61 [Source:MGI Symbol;Acc:MGI:2441719]	3.16311	2.8697	2.81265	2.67107	2.51961	3.08539	3.17863	2.85214	2.18049	3.10051	2.61218	2.84778	2.76647	3.09405	0.128810631	0.560209134	4	16	24	0.176612402	0.768040178	24	23.4293067	0.077635582
ENSMUSG00000046794	Ppp1r3b	protein phosphatase 1, regulatory subunit 3B [Source:MGI Symbol;Acc:MGI:2177268]	3.03233	2.06893	3.49558	9.73297	3.57669	3.28254	4.16694	3.04996	3.1957	4.18156	9.89705	4.39814	5.9886	3.95768	0.108029159	0.524035616	20	12	24	0.10850449	0.72082227	24	14.93831959	0.376059666
ENSMUSG00000046798	Cldn12	claudin 12 [Source:MGI Symbol;Acc:MGI:1929288]	9.98072	10.8392	11.9145	10.3877	11.6703	10.6884	11.5889	10.733	11.7063	12.0274	11.5151	11.5141	10.6385	11.1834	0.155518775	0.609118119	12	16	24	0.165421861	0.767020009	24	11.70241956	0.050784904
ENSMUSG00000046805	Mpeg1	macrophage expressed gene 1 [Source:MGI Symbol;Acc:MGI:1333743]	3.99253	5.99343	7.03032	5.56472	5.54903	5.28298	5.60203	5.41757	6.39269	3.58832	5.41611	5.15333	4.94478	4.58032	0.143321297	0.585944911	12	12	24	0.680812788	0.893366006	24	8.099436498	0.074196606
ENSMUSG00000046806	Cyren	cell cycle regulator of NHEJ [Source:MGI Symbol;Acc:MGI:1925662]	2.78954	1.77196	2.459	2.68183	3.15697	2.68654	2.22489	2.52466	2.70475	1.97956	3.64232	2.48545	2.74963	2.12609	0.064091035	0.414867207	20	12	24	0.100662297	0.711862888	24	16.9282507	0.152440244
ENSMUSG00000046807	Lrrc75b	leucine rich repeat containing 75B [Source:MGI Symbol;Acc:MGI:2143657]	26.8082	19.5849	21.0144	23.0928	22.6829	25.0474	23.2611	22.5956	16.8272	29.5257	20.2997	24.2717	22.6955	23.7563	0.133266423	0.567728672	24	12	24	0.402009122	0.799546792	24	19.45197574	0.104229417
ENSMUSG00000046808	Atp10d	ATPase, class V, type 10D [Source:MGI Symbol;Acc:MGI:2450125]	2.00468	2.15082	2.12891	1.53295	2.0587	1.87701	1.50119	1.90815	2.00681	1.89499	1.94607	1.78093	1.96513	1.79494	0.843639218	0.966018836	12	12	24	0.83197868	0.948003732	24	7.114820005	0.024892414
ENSMUSG00000046814	Gchfr	GTP cyclohydrolase I feedback regulator [Source:MGI Symbol;Acc:MGI:2443977]	0.910574	1.42778	0.899398	1.32134	1.29302	1.30731	2.01577	1.32879	1.69159	1.14901	1.35966	1.85629	1.05134	1.62296	0.873350015	0.966018836	8	4	24	0.888084524	0.968515338	24	23.22709198	0.036580107
ENSMUSG00000046818	Ddit4l	DNA-damage-inducible transcript 4-like [Source:MGI Symbol;Acc:MGI:1920534]	2.35646	4.60441	4.2012	3.03419	4.79453	5.79585	5.106	6.38622	5.81348	5.42472	3.65708	3.41611	5.46043	4.85776	0.910436409	0.966018836	8	12	24	0.514199099	0.816624526	24	6.016692992	0.142646393
ENSMUSG00000046822	Slc39a3	solute carrier family 39 (zinc transporter), member 3 [Source:MGI Symbol;Acc:MGI:2147269]	14.6402	11.55	11.3826	12.4864	12.2013	13.998	13.6184	14.0547	12.1588	14.5314	13.4311	14.7483	14.2328	13.6326	0.047644929	0.377556215	24	12	24	0.071215067	0.676901434	24	21.23061889	0.090480927
ENSMUSG00000046836	Brox	BRO1 domain and CAAX motif containing [Source:MGI Symbol;Acc:MGI:1918928]	28.9664	28.4912	27.7812	28.0671	26.6705	28.4129	27.0617	26.5532	26.2596	29.2306	29.5897	27.1112	27.941	27.8409	0.946423879	0.966018836	16	20	24	0.662713537	0.886154038	24	16.81843292	0.01881249
ENSMUSG00000046841	Ckap4	cytoskeleton-associated protein 4 [Source:MGI Symbol;Acc:MGI:2444926]	9.28922	8.07178	8.6293	8.28302	8.56771	8.77951	9.15381	9.16764	8.55876	9.70082	7.82289	9.03597	8.63497	8.86588	0.628286596	0.966018836	4	16	24	0.700941953	0.900900643	24	1.112618558	0.024774177
ENSMUSG00000046844	Vat1l	vesicle amine transport protein 1 like [Source:MGI Symbol;Acc:MGI:2142534]	119.669	105.798	99.2243	105.779	119.927	113	129.605	115.047	99.8375	130.482	102.659	117.497	113.569	109.424	0.16456443	0.628002127	4	8	24	0.262155727	0.768040178	24	20.68547001	0.081608852
ENSMUSG00000046854	Pip5kl1	phosphatidylinositol-4-phosphate 5-kinase-like 1 [Source:MGI Symbol;Acc:MGI:2448520]	3.34494	3.42053	4.27365	2.82512	4.1657	3.59828	3.1249	3.05124	3.36928	3.25582	3.4275	2.7012	2.57581	2.90977	0.708020493	0.966018836	20	8	24	0.364269546	0.788789286	24	12.91645679	0.073380963
ENSMUSG00000046861	Hectd3	HECT domain E3 ubiquitin protein ligase 3 [Source:MGI Symbol;Acc:MGI:1923858]	13.6164	12.4022	11.7685	13.4849	13.3743	13.2706	12.192	13.3351	12.0971	14.1461	11.7718	13.3163	12.6091	13.2192	0.369262716	0.893596186	16	20	24	0.754062987	0.920300959	24	17.72356192	0.024263063
ENSMUSG00000046862	Pramel12	PRAME like 12 [Source:MGI Symbol;Acc:MGI:2140473]	6.94122	6.27681	6.16672	6.40136	6.80777	6.0514	6.50428	6.51542	6.17601	5.6867	6.58145	6.13157	6.82267	5.78157	0.305564675	0.811201458	4	12	24	0.230375575	0.768040178	24	21.28111278	0.04791348
ENSMUSG00000046865	Fbl	fibrillarin [Source:MGI Symbol;Acc:MGI:95486]	1.4025	3.1332	6.33212	1.85482	2.94966	2.79395	2.28997	3.77282	2.38942	2.83745	1.69333	1.6684	1.87294	2.31416	0.108524038	0.524035616	12	16	24	0.166318302	0.767020009	24	7.928896676	0.294680592
ENSMUSG00000046873	Mbtps2	membrane-bound transcription factor peptidase, site 2 [Source:MGI Symbol;Acc:MGI:2444506]	7.5156	6.87504	6.61692	6.54945	7.17289	6.78662	7.60808	7.62747	6.98771	7.29093	7.34309	6.74617	6.62481	6.99026	0.920620634	0.966018836	8	4	24	0.769234622	0.925619883	24	23.66578133	0.019689721
ENSMUSG00000046876	Atxn1	ataxin 1 [Source:MGI Symbol;Acc:MGI:104783]	4.73228	5.07469	4.97585	6.23093	5.36947	5.47107	5.26629	5.1913	5.24571	6.23194	4.98198	5.70422	4.69848	5.23563	0.109247218	0.524035616	16	12	24	0.066544962	0.669010807	24	13.18698165	0.087536137
ENSMUSG00000046879	Irgm1	immunity-related GTPase family M member 1 [Source:MGI Symbol;Acc:MGI:107567]	7.71201	8.73289	7.99947	6.27876	9.14362	7.42409	6.93984	10.7002	8.78748	6.35705	8.13657	7.13079	6.37106	10.3614	0.200145651	0.684570416	8	8	24	0.31425684	0.768040178	24	5.214976838	0.132894015
ENSMUSG00000046897	Zfp740	zinc finger protein 740 [Source:MGI Symbol;Acc:MGI:1915994]	25.7716	30.0693	30.3883	32.2113	30.2587	27.1352	28.1364	26.8273	32.6859	30.2622	26.0309	30.0144	28.8881	29.085	0.008024668	0.192760771	12	16	24	0.061416052	0.661066697	24	10.03711005	0.09026968
ENSMUSG00000046908	Ltb4r1	leukotriene B4 receptor 1 [Source:MGI Symbol;Acc:MGI:1309472]	0.322195	1.19635	0.666003	0.741314	0.725744	0.458697	0.629603	0.346882	0.705056	0.319233	0.66619	0.758329	0.792689	0.455005	0.782779623	0.966018836	24	4	24	0.996298154	0.999034519	24	11.27959556	0.007229476
ENSMUSG00000046909	Tefm	transcription elongation factor, mitochondrial [Source:MGI Symbol;Acc:MGI:1915800]	5.82646	6.54881	6.28041	6.55007	6.61393	5.83518	5.94165	7.08507	6.30214	5.44783	6.14704	6.37332	6.10886	6.71607	0.265774856	0.773510818	8	20	24	0.455495523	0.805393068	24	5.959598891	0.04317504
ENSMUSG00000046916	Myct1	myc target 1 [Source:MGI Symbol;Acc:MGI:1915882]	0.723129	0.871554	1.04408	0.868549	0.755943	0.843208	0.938241	1.37988	0.94993	1.04281	1.02982	0.917719	0.901162	0.949591	0.250898337	0.75129457	12	12	24	0.173672582	0.768040178	24	7.360393497	0.077910344
ENSMUSG00000046934	Csl	citrate synthase like [Source:MGI Symbol;Acc:MGI:1919082]	3.03197	2.5229	2.84187	3.29024	3.01332	3.61139	3.21846	3.26028	2.57483	4.58961	2.70364	3.12086	3.83683	3.87097	0.920858634	0.966018836	4	8	24	0.969675911	0.992868519	24	17.93431645	0.017253909
ENSMUSG00000046947	Adck2	aarF domain containing kinase 2 [Source:MGI Symbol;Acc:MGI:1889336]	9.13692	7.46381	8.2019	10.3954	9.55719	9.34595	9.16567	9.81663	8.57685	9.19066	8.10422	9.23724	9.17226	8.79061	0.393470605	0.921010321	16	20	24	0.585782845	0.853141382	24	17.38569485	0.045885245
ENSMUSG00000046949	Nqo2	N-ribosyldihydronicotinamide quinone reductase 2 [Source:MGI Symbol;Acc:MGI:104513]	28.6388	27.7432	24.7228	26.3102	28.4839	26.8048	24.3233	25.0389	26.0713	22.4505	28.2163	25.7331	26.5909	26.1355	0.435739761	0.966018836	20	20	24	0.32132842	0.768040178	24	20.37426257	0.055103596
ENSMUSG00000046952	Gm5815	predicted pseudogene 5815 [Source:MGI Symbol;Acc:MGI:3648184]	1.93955	1.63115	1.54179	1.45759	1.56681	1.50661	2.04928	1.57185	1.37681	1.5769	1.18169	1.66205	1.83628	1.44368	0.013006247	0.228055093	4	8	24	0.020837425	0.568066329	24	23.8372494	0.116181067
ENSMUSG00000046961	Gpr156	G protein-coupled receptor 156 [Source:MGI Symbol;Acc:MGI:2653880]	1.61933	1.81795	1.62646	1.73629	1.40331	1.5813	1.54285	1.87545	1.68551	1.94957	1.39192	1.71306	1.90501	1.82989	0.113148904	0.530777648	8	12	24	0.062716746	0.661961741	24	5.509939251	0.079671918
ENSMUSG00000046962	Zbtb21	zinc finger and BTB domain containing 21 [Source:MGI Symbol;Acc:MGI:1927240]	5.48512	4.04186	5.27583	4.53688	5.50681	4.97304	5.84057	4.69408	5.02222	5.14738	5.05097	4.46067	4.35158	3.62709	0.305564675	0.811201458	20	12	24	0.284104036	0.768040178	24	16.93471002	0.087479847
ENSMUSG00000046982	Tshz1	teashirt zinc finger family member 1 [Source:MGI Symbol;Acc:MGI:1346031]	3.83856	3.78465	4.20414	3.5766	3.61815	4.10189	4.36873	4.11239	4.08459	5.23653	3.43631	4.17463	3.8879	4.15431	0.892685629	0.966018836	12	8	24	0.653795061	0.883343578	24	6.79416179	0.044714909
ENSMUSG00000046985	Tapt1	transmembrane anterior posterior transformation 1 [Source:MGI Symbol;Acc:MGI:2683537]	17.1323	19.238	19.7057	18.5167	18.837	18.0739	18.5248	18.3319	20.2512	18.4091	16.7606	19.3178	18.0828	20.06	0.173809281	0.649460502	12	8	24	0.094842596	0.71175955	24	7.156953394	0.061567344
ENSMUSG00000046991	Wdr27	WD repeat domain 27 [Source:MGI Symbol;Acc:MGI:1918932]	1.96295	2.1958	2.22831	2.29558	1.91724	2.20537	2.59588	1.59332	2.58591	2.84307	2.3257	2.08323	2.11851	3.32588	0.772122688	0.966018836	16	8	24	0.408341717	0.799546792	24	8.765440514	0.090191241
ENSMUSG00000046993	Gm5637	predicted pseudogene 5637 [Source:MGI Symbol;Acc:MGI:3648120]	36.3833	29.1816	29.9508	30.3381	36.3605	36.2176	34.5244	34.8022	26.2247	29.2486	34.6492	29.0212	27.1464	23.7712	0.483374602	0.966018836	20	16	24	0.019679878	0.565108098	24	18.95632524	0.163549919
ENSMUSG00000046994	Mars2	methionine-tRNA synthetase 2 (mitochondrial) [Source:MGI Symbol;Acc:MGI:2444136]	2.12357	2.05503	2.01434	2.2502	2.29731	2.01099	2.01158	1.9086	2.03711	1.7328	2.31729	1.93708	1.80338	1.91648	0.496745696	0.966018836	20	12	24	0.170550201	0.768040178	24	15.93763837	0.051619277
ENSMUSG00000046997	Spsb4	splA/ryanodine receptor domain and SOCS box containing 4 [Source:MGI Symbol;Acc:MGI:2183445]	3.09019	2.23654	3.26092	2.19721	2.76622	3.68473	3.18665	3.07658	2.07459	2.85308	2.46099	3.27628	2.86805	3.03859	0.054126506	0.395890765	24	16	24	0.128504748	0.740162193	24	21.97155687	0.139638772
ENSMUSG00000046999	1110032F04Rik	RIKEN cDNA 1110032F04 gene [Source:MGI Symbol;Acc:MGI:1915975]	17.2873	13.6793	11.6469	13.2495	13.1877	13.9652	13.0755	13.6065	10.2057	16.5163	12.7941	13.515	13.6272	13.9495	0.43280837	0.966018836	8	4	24	0.396465473	0.799546792	24	21.14622795	0.093662709
ENSMUSG00000047003	Zfp41	zinc finger protein 41 [Source:MGI Symbol;Acc:MGI:99186]	1.67927	1.90528	1.86244	1.80527	2.08626	1.88656	1.59529	2.12633	1.62089	1.87723	1.81425	1.81706	1.87441	1.96588	0.970223078	0.971116366	8	20	24	0.921754676	0.979395417	24	11.10446466	0.01278238
ENSMUSG00000047013	Fbxo41	F-box protein 41 [Source:MGI Symbol;Acc:MGI:1261912]	3.76634	2.97187	3.73896	3.1282	2.78866	3.77387	3.49058	3.28606	2.97886	4.6543	2.96454	3.72286	3.34442	3.96114	0.915475528	0.966018836	24	20	24	0.938604089	0.984866431	24	2.96025174	0.022321203
ENSMUSG00000047021	Cfap65	cilia and flagella associated protein 65 [Source:MGI Symbol;Acc:MGI:2444274]	0.301911	1.17722	1.32607	0.912382	0.66587	0.479429	1.02018	0.213927	0.574068	0.782943	0.401996	1.082	0.0464489	1.18501	0.840483986	0.966018836	12	16	24	0.532519325	0.825678486	24	9.4359464	0.161880819
ENSMUSG00000047022	Mipol1	mirror-image polydactyly 1 [Source:MGI Symbol;Acc:MGI:1920740]	5.00098	5.57727	5.53303	5.19432	4.75465	5.08211	4.86961	4.73098	5.30997	5.16764	5.13875	5.32748	5.53239	5.81273	0.539001179	0.966018836	8	12	24	0.265929335	0.768040178	24	6.115311333	0.046076748
ENSMUSG00000047030	Spata2	spermatogenesis associated 2 [Source:MGI Symbol;Acc:MGI:2146885]	7.14717	5.42323	6.89001	6.13844	6.09248	6.31904	7.02177	6.59544	5.63812	8.68012	5.98002	6.97244	6.13517	6.76541	0.91695334	0.966018836	4	16	24	0.961941842	0.9903359	24	15.30552068	0.016486839
ENSMUSG00000047033	Pcdhb15	protocadherin beta 15 [Source:MGI Symbol;Acc:MGI:2136750]	0.675201	0.618748	0.555257	0.83108	0.757241	0.771547	0.80715	0.863322	0.667829	0.663884	0.663442	0.685544	0.577633	0.66588	0.880631923	0.966018836	24	12	24	0.728103549	0.910948356	24	18.21576559	0.025721698
ENSMUSG00000047036	Zfp445	zinc finger protein 445 [Source:MGI Symbol;Acc:MGI:2143340]	47.0367	57.4581	53.4972	51.0381	55.297	49.7233	51.6954	47.7611	63.1	45.1995	56.6451	55.6741	55.0658	57.1566	0.93717134	0.966018836	12	4	24	0.802224169	0.936565832	24	7.443080519	0.034901763
ENSMUSG00000047037	Nipa1	non imprinted in Prader-Willi/Angelman syndrome 1 homolog (human) [Source:MGI Symbol;Acc:MGI:2442058]	22.8003	23.233	22.464	22.1968	22.5338	22.8759	23.3805	20.7168	20.3692	22.0937	21.9201	23.974	22.8252	23.5116	0.094391858	0.492161865	24	16	24	0.112492534	0.726529814	24	21.51273539	0.050468989
ENSMUSG00000047040	Prr15l	proline rich 15-like [Source:MGI Symbol;Acc:MGI:2387599]	0.537943	0.408476	0.687558	0.887751	0.651839	0.548453	0.63416	0.683034	0.42937	0.706828	0.804286	0.778744	0.587389	0.403419	0.01457641	0.247599677	16	16	24	0.047652467	0.635670298	24	15.34569995	0.11683496
ENSMUSG00000047044	D030056L22Rik	RIKEN cDNA D030056L22 gene [Source:MGI Symbol;Acc:MGI:3583960]	11.2804	10.672	10.8219	10.0764	10.6032	10.7283	12.1932	10.3649	10.5297	9.88425	12.5322	11.9039	12.9969	11.5645	0.008961721	0.192760771	4	12	24	0.03149826	0.602663372	24	22.31164568	0.092518505
ENSMUSG00000047045	Tmem164	transmembrane protein 164 [Source:MGI Symbol;Acc:MGI:2148020]	18.3816	13.6352	14.4362	15.4161	15.9394	16.7182	14.6358	14.9303	11.6245	16.2001	15.3111	16.3149	16.0771	15.8042	0.010886922	0.200176658	24	12	24	0.058952609	0.658304792	24	19.87975326	0.129513648
ENSMUSG00000047067	Dusp28	dual specificity phosphatase 28 [Source:MGI Symbol;Acc:MGI:1914696]	8.58256	9.43115	9.97417	9.67134	8.27806	8.96632	9.21785	10.0786	10.2043	9.85861	11.4943	10.6374	11.7603	10.6386	0.881996584	0.966018836	8	12	24	0.488960308	0.805393068	24	7.11695787	0.042609394
ENSMUSG00000047084	Ngrn	neugrin, neurite outgrowth associated [Source:MGI Symbol;Acc:MGI:1933212]	21.2254	21.0729	19.9325	20.1056	20.2964	22.1558	20.4578	23.0581	20.6538	22.5026	22.1662	21.887	22.5903	21.3847	0.834424969	0.966018836	8	4	24	0.513364701	0.816307374	24	22.39836492	0.025681211
ENSMUSG00000047085	Lrrc4b	leucine rich repeat containing 4B [Source:MGI Symbol;Acc:MGI:3027390]	2.1142	2.73405	4.60225	2.27923	2.06562	2.77737	3.73564	2.37234	3.26517	3.67906	2.58	3.48643	2.12025	3.96128	0.708020493	0.966018836	12	8	24	0.360546542	0.786181564	24	7.55364998	0.161442812
ENSMUSG00000047090	Tmem198b	transmembrane protein 198b [Source:MGI Symbol;Acc:MGI:1921077]	5.67016	5.97682	6.58585	5.9364	6.24893	6.39672	5.90755	5.59858	7.07216	6.36117	5.44679	5.49507	6.22648	5.86408	0.441501717	0.966018836	12	20	24	0.268732984	0.768040178	24	9.330298562	0.059131008
ENSMUSG00000047098	Rnf31	ring finger protein 31 [Source:MGI Symbol;Acc:MGI:1934704]	5.28793	5.37213	4.95752	5.62308	6.1103	6.66949	6.91341	6.69263	5.49545	6.47106	6.73495	5.8767	6.81882	5.42531	0.313786068	0.824470524	4	8	24	0.171786768	0.768040178	24	19.39615649	0.092634323
ENSMUSG00000047109	Cldn14	claudin 14 [Source:MGI Symbol;Acc:MGI:1860425]	1.06116	1.0034	1.58417	0.987404	1.26589	1.30924	1.44038	1.26498	1.93042	1.13355	1.14181	1.71508	1.17225	1.27696	0.953629935	0.966018836	12	4	24	0.89843487	0.971196215	24	7.071783738	0.030997876
ENSMUSG00000047115	Fam221a	family with sequence similarity 221, member A [Source:MGI Symbol;Acc:MGI:2442161]	1.57139	1.50363	1.27832	1.29404	1.97258	1.4729	1.19035	2.08149	1.59791	1.36161	1.53551	1.68654	1.67118	1.86711	0.886952527	0.966018836	8	8	24	0.787028197	0.931456435	24	1.011670363	0.037943561
ENSMUSG00000047123	Ticam1	toll-like receptor adaptor molecule 1 [Source:MGI Symbol;Acc:MGI:2147032]	2.43419	1.83764	2.16224	1.6792	1.99201	2.24642	2.10389	2.32706	1.82024	2.12485	1.93331	2.1569	1.83837	1.84132	0.625825704	0.966018836	24	16	24	0.289828111	0.768040178	24	22.01595107	0.063379116
ENSMUSG00000047126	Cltc	clathrin, heavy polypeptide (Hc) [Source:MGI Symbol;Acc:MGI:2388633]	285.314	268.481	261.488	266.964	259.039	264.981	252.441	247.4	231.634	252.723	253.887	256.416	258.342	260.831	0.82502117	0.966018836	24	12	24	0.203402387	0.768040178	24	21.4103912	0.040311107
ENSMUSG00000047139	Cd24a	CD24a antigen [Source:MGI Symbol;Acc:MGI:88323]	815.088	766.129	701.296	728.186	739.465	771.172	678.768	688.101	716.186	644.648	823.673	771.863	823.335	751.413	0.177969241	0.655480352	20	20	24	0.13480186	0.746461599	24	21.07365891	0.083765394
ENSMUSG00000047141	Zfp654	zinc finger protein 654 [Source:MGI Symbol;Acc:MGI:1919270]	5.09028	5.6791	6.04863	5.90041	5.40391	4.99866	5.37013	5.16279	5.78596	4.95364	5.86851	4.51209	4.72575	5.04471	0.023472439	0.278499043	12	12	24	0.035711441	0.609176161	24	10.045367	0.088428052
ENSMUSG00000047146	Tet1	tet methylcytosine dioxygenase 1 [Source:MGI Symbol;Acc:MGI:1098693]	0.714779	1.18513	1.26954	1.12256	1.23305	1.02	1.22947	0.993661	1.31466	0.830707	1.12094	1.0978	1.09555	1.35618	0.321662653	0.83607295	12	12	24	0.513664847	0.816307374	24	7.914674289	0.059306503
ENSMUSG00000047153	Khnyn	KH and NYN domain containing [Source:MGI Symbol;Acc:MGI:2451333]	6.46942	5.99836	6.39099	6.03565	6.88176	6.14122	6.54618	6.80868	6.23444	6.68125	6.15385	6.5998	6.47236	6.82562	0.804027134	0.966018836	8	4	24	0.867902002	0.962222593	24	1.352280588	0.011418074
ENSMUSG00000047155	Cyp4x1	cytochrome P450, family 4, subfamily x, polypeptide 1 [Source:MGI Symbol;Acc:MGI:1932403]	9.42386	7.68771	7.74579	8.10539	7.2778	8.23954	7.272	7.87725	6.3416	9.09389	7.67849	7.90183	8.61164	8.35231	0.956794505	0.96629412	4	8	24	0.653026141	0.883278597	24	21.75057617	0.048295468

ENSMUSG00000047161	Chst9	carbohydrate (N-acetylgalactosamine 4-0) sulfotransferase 9 [Source:MGI Symbol;Acc:MGI:1918617]	1.43174	1.42044	1.334	1.69588	1.33596	1.15883	1.4287	1.26587	1.22795	1.18055	1.17618	1.25382	1.52191	0.930925	0.675497492	0.966018836	4	20	24	0.896735826	0.970634157	24	19.78980098	0.019554451
ENSMUSG00000047180	Neurl3	neuralized E3 ubiquitin protein ligase 3 [Source:MGI Symbol;Acc:MGI:2429944]	1.28868	1.88433	1.68895	1.65874	1.32676	1.31921	1.64467	1.62613	2.01143	1.04801	1.74332	1.54834	2.13416	1.62569	0.865233232	0.966018836	12	12	24	0.250985906	0.768040178	24	4.545483822	0.104189535
ENSMUSG00000047181	Samd14	sterile alpha motif domain containing 14 [Source:MGI Symbol;Acc:MGI:2384945]	36.3401	26.7755	26.3846	27.7064	29.5572	31.6959	27.0376	31.0035	26.5063	41.367	33.4159	36.8029	36.6879	34.4465	0.0444686	0.366018836	24	12	24	0.399713698	0.799546792	24	19.43410826	0.098092931
ENSMUSG00000047182	Irs3	insulin receptor substrate 3 [Source:MGI Symbol;Acc:MGI:1194882]	1.65744	0.973472	0.753256	1.13241	0.822674	0.790385	1.00186	0.81842	0.598502	1.0104	1.50934	1.2835	1.37987	1.10569	0.105280783	0.518002936	4	8	24	0.222625786	0.768040178	24	20.73233709	0.14391193
ENSMUSG00000047187	Rab2a	RAB2A, member RAS oncogene family [Source:MGI Symbol;Acc:MGI:1928750]	152.088	162.881	157.31	149.87	157.978	148.454	147.529	151.461	154.082	148.323	154.232	140.932	143.259	151.165	0.26148192	0.768150062	12	12	24	0.173334069	0.768040178	24	8.674009246	0.032666657
ENSMUSG00000047193	Dync2h1	dynein cytoplasmic 2 heavy chain 1 [Source:MGI Symbol;Acc:MGI:107736]	2.2313	2.6694	2.59314	3.11395	3.00273	2.10094	2.4551	2.05027	2.87012	2.38091	2.66078	2.97036	2.86175	2.8991	0.801995186	0.966018836	20	8	24	0.451769765	0.805393068	24	12.22147578	0.066929157
ENSMUSG00000047205	Dusp18	dual specificity phosphatase 18 [Source:MGI Symbol;Acc:MGI:1922469]	3.25695	3.10102	3.26612	3.42775	3.60411	3.5691	4.35149	3.73469	3.00739	4.21956	3.27414	3.24335	3.21717	3.2413	0.671726933	0.966018836	16	20	24	0.7908303	0.933002007	24	17.3715454	0.034930029
ENSMUSG00000047213	Ythdf3	YTH N6-methyladenosine RNA binding protein 3 [Source:MGI Symbol;Acc:MGI:1918850]	23.8086	24.2819	24.0769	24.4925	23.3613	22.1153	23.3435	21.713	22.0021	21.8438	23.5111	20.2361	19.785	21.6793	0.787022682	0.966018836	16	8	24	0.247726279	0.768040178	24	11.44419427	0.036049663
ENSMUSG00000047215	Rpl9	ribosomal protein L9 [Source:MGI Symbol;Acc:MGI:1298373]	2.68769	3.27125	3.72466	2.97878	3.5863	2.46642	4.72378	2.87446	3.6596	2.89084	3.33297	2.91933	3.72562	2.81867	0.919571723	0.966018836	12	12	24	0.892920856	0.969717376	24	5.407471986	0.037495701
ENSMUSG00000047216	Cdh19	cadherin 19, type 2 [Source:MGI Symbol;Acc:MGI:3588198]	51.7794	54.9177	49.9279	66.3662	68.5877	54.2943	53.7847	44.6647	55.8946	46.0872	71.3625	56.3371	48.1988	44.0634	0.004196064	0.140598674	20	12	24	0.010104087	0.503967859	24	15.96470904	0.21421069
ENSMUSG00000047221	Fam185a	family with sequence similarity 185, member A [Source:MGI Symbol;Acc:MGI:2140983]	5.07222	4.96933	4.66283	4.88495	4.7674	5.10913	5.09164	5.27636	3.81622	5.15197	5.17834	4.66131	4.95226	5.4117	0.675497492	0.966018836	8	4	24	0.496856975	0.807576732	24	22.29648923	0.047939245
ENSMUSG00000047227	Gm527	predicted gene 527 [Source:MGI Symbol;Acc:MGI:2685373]	15.7065	13.9146	11.9323	14.0766	15.5789	13.2241	15.2332	11.3025	12.551	14.8589	16.1645	14.3992	15.8114	14.3628	0.047000349	0.375011045	20	16	24	0.091941393	0.71175955	24	18.93428179	0.121353019
ENSMUSG00000047228	A2ml1	alpha-2-macroglobulin like 1 [Source:MGI Symbol;Acc:MGI:3039594]	91.0059	78.4031	68.9166	79.2636	82.513	79.5117	78.4884	75.7221	75.1662	75.0145	84.9413	84.3587	89.2888	86.8388	0.017495149	0.255326033	4	8	24	0.040834389	0.616921492	24	21.34254294	0.097449655
ENSMUSG00000047238	Mageh1	MAGE family member H1 [Source:MGI Symbol;Acc:MGI:1922875]	13.153	14.6569	14.6019	12.6605	14.7828	13.1138	15.3824	13.2977	12.6367	11.1132	12.2542	13.3115	12.075	13.78	0.885025617	0.966018836	8	8	24	0.339678462	0.774150091	24	1.043778079	0.061560677
ENSMUSG00000047242	Taf9b	TATA-box binding protein associated factor 9B [Source:MGI Symbol;Acc:MGI:3039562]	22.7695	23.6777	22.3237	21.3473	23.9475	21.7255	21.6951	20.1178	23.5629	18.788	24.5044	22.0481	22.4004	20.7644	0.722296281	0.966018836	20	20	24	0.635863079	0.876315013	24	19.22815632	0.038260625
ENSMUSG00000047246	H2bc6	H2B clustered histone 6 [Source:MGI Symbol;Acc:MGI:2448380]	5.77205	4.86516	4.76677	4.19625	5.03796	4.57283	5.22678	5.02058	4.09927	4.93624	5.79897	4.8045	5.28777	4.52995	0.062631097	0.410322149	4	8	24	0.154955386	0.760294912	24	21.15808404	0.091853082
ENSMUSG00000047248	C2cd3	C2 calcium-dependent domain containing 3 [Source:MGI Symbol;Acc:MGI:2142166]	5.05807	4.64281	4.24698	5.74877	5.42021	4.72348	4.86823	4.02999	5.16292	4.95385	4.2896	4.58623	4.95821	4.73116	0.916746631	0.966018836	16	16	24	0.517170047	0.818196189	24	14.94003209	0.050650577
ENSMUSG00000047250	Ptgs1	prostaglandin-endoperoxide synthase 1 [Source:MGI Symbol;Acc:MGI:97797]	1.85638	3.14591	3.08405	2.59274	2.14161	2.08394	2.50783	2.65319	3.92514	1.90868	2.35876	2.1339	2.03893	2.53104	0.015345591	0.253245931	12	12	24	0.020311479	0.565108098	24	7.369573818	0.220524131
ENSMUSG00000047260	Emc6	ER membrane protein complex subunit 6 [Source:MGI Symbol;Acc:MGI:1913298]	26.2838	23.1648	23.775	25.4861	23.3574	24.7278	25.7201	27.0915	25.8807	26.5838	29.9722	26.8749	29.6701	26.8976	0.874054778	0.966018836	4	8	24	0.737366408	0.91440753	24	21.37605323	0.024231867
ENSMUSG00000047261	Gap43	growth associated protein 43 [Source:MGI Symbol;Acc:MGI:95639]	131.124	102.698	124.124	109.436	116.329	133.098	114.063	112.685	85.8808	112.394	137.087	109.308	107.788	115.645	0.481530854	0.966018836	20	16	24	0.153995109	0.760223561	24	19.10159819	0.126678842
ENSMUSG00000047264	Zfp358	zinc finger protein 358 [Source:MGI Symbol;Acc:MGI:2153740]	7.77391	7.05703	6.67756	7.13996	6.98021	7.13735	7.8702	7.91326	6.88216	7.6977	7.59953	7.95674	7.63947	7.43242	0.097760727	0.501112642	4	8	24	0.117935546	0.728323355	24	22.28986187	0.052192291
ENSMUSG00000047281	Sfn	stratifin [Source:MGI Symbol;Acc:MGI:1891831]	3.33517	2.0863	2.26063	2.04598	2.4448	2.66456	2.67181	2.38048	1.73924	2.41954	2.65847	2.67618	2.45275	2.15083	0.001028555	0.076130598	4	8	24	0.004614934	0.433484671	24	20.63441039	0.175971522
ENSMUSG00000047284	Neurl4	neuralized E3 ubiquitin protein ligase 4 [Source:MGI Symbol;Acc:MGI:1921092]	14.0012	15.026	11.9554	17.2062	14.7143	13.2485	16.7127	12.7941	12.9842	17.0655	13.7888	12.7679	13.989	12.4124	0.369262716	0.893596186	16	20	24	0.530076181	0.824795587	24	15.06544656	0.069465157
ENSMUSG00000047307	Pcdhb13	protocadherin beta 13 [Source:MGI Symbol;Acc:MGI:2136748]	0.814248	0.522541	0.728493	0.579599	0.87442	0.829079	0.845689	0.732371	0.527165	0.824953	0.678508	0.622534	0.577245	0.733462	0.532553009	0.966018836	20	16	24	0.412379653	0.799546792	24	19.00758421	0.05652319
ENSMUSG00000047330	Kcne4	potassium voltage-gated channel, Isk-related subfamily, gene 4 [Source:MGI Symbol;Acc:MGI:1891125]	1.06159	1.25902	1.36107	0.917258	1.21094	1.20605	1.29052	1.20578	1.59489	0.901872	1.34478	1.02763	1.0881	1.26581	0.195343269	0.673387182	12	4	24	0.498491236	0.808231807	24	5.491854185	0.058160352
ENSMUSG00000047342	Zfp286	zinc finger protein 286 [Source:MGI Symbol;Acc:MGI:2384758]	3.71959	4.71612	3.91754	3.49891	4.14768	4.19081	4.77394	3.98979	4.41515	3.65225	4.96966	4.53696	3.88321	4.84191	0.718720526	0.966018836	8	8	24	0.626383076	0.872596805	24	23.89968261	0.047927947
ENSMUSG00000047343	Mettl21c	methyltransferase like 21C [Source:MGI Symbol;Acc:MGI:3611450]	1.13174	3.71381	3.79612	5.22634	1.92586	5.71667	5.33952	12.0558	8.48669	5.96157	4.26406	4.3874	5.32044	2.94821	0.003207257	0.966018836	16	4	24	0.423464615	0.805346055	24	7.940834061	0.32787708
ENSMUSG00000047344	Lancl3	LanC lantibiotic synthetase component C-like 3 (bacterial) [Source:MGI Symbol;Acc:MGI:2443335]	3.69868	4.36757	3.36279	4.38018	3.9345	3.85309	3.8294	3.55059	3.50346	4.08089	4.49385	4.01728	4.27783	5.0077	0.602156008	0.966018836	16	20	24	0.945205247	0.986693398	24	17.53293784	0.015874236
ENSMUSG00000047361	Gm973	predicted gene 973 [Source:MGI Symbol;Acc:MGI:2685819]	0.918628	2.2527	3.22954	1.51768	1.44533	1.11765	1.53906	1.33388	3.32037	1.50653	1.1625	3.08887	0.890309	2.60209	0.018894761	0.256345714	12	16	24	0.162671404	0.766365035	24	7.986128541	0.314299008
ENSMUSG00000047363	Cstad	CSA-conditional, T cell activation-dependent protein [Source:MGI Symbol;Acc:MGI:1925867]	0.980816	0.682753	0.923298	0.981955	0.951474	0.691996	0.739617	1.03061	0.78647	0.928781	0.499745	0.975981	0.937958	0.727304	0.813775138	0.966018836	16	4	24	0.938267034	0.984659033	24	6.551229585	0.018840849
ENSMUSG00000047368	Abhd17b	abhydrolase domain containing 17B [Source:MGI Symbol;Acc:MGI:1917816]	26.8894	23.2054	23.6368	24.1595	21.8414	23.6337	22.4801	21.9116	18.6513	24.5736	23.6848	19.8309	18.1522	20.893	0.700065774	0.966018836	16	16	24	0.553096854	0.837286821	24	15.77698516	0.049016573
ENSMUSG00000047369	Dnah14	dynein, axonemal, heavy chain 14 [Source:MGI Symbol;Acc:MGI:2444525]	0.675661	0.488378	0.545827	0.647785	0.475192	0.546046	0.784941	0.505546	0.63329	0.393484	0.422508	0.610712	0.543366	0.386648	0.601395222	0.966018836	4	4	24	0.369917336	0.792235171	24	22.95887611	0.054677499
ENSMUSG00000047370	Gm7367	predicted pseudogene 7367 [Source:MGI Symbol;Acc:MGI:3646404]	7.30824	6.35112	5.28827	4.41428	5.57759	6.34335	7.99009	6.70433	6.70092	9.9073	7.71164	10.2788	9.23722	8.35704	0.481530854	0.966018836	4	4	24	0.190716209	0.768040178	24	22.80815669	0.152013265
ENSMUSG00000047371	Zfp768	zinc finger protein 768 [Source:MGI Symbol;Acc:MGI:2384582]	3.50277	3.29948	3.59604	3.37344	2.96318	3.2579	3.39887	3.8457	3.57224	4.30453	3.34812	4.15584	4.41677	3.88799	0.769899911	0.966018836	4	16	24	0.42435578	0.805393068	24	4.876665611	0.053642658
ENSMUSG00000047379	B4gat1	beta-1,4-glucuronyltransferase 1 [Source:MGI Symbol;Acc:MGI:1919680]	29.6183	24.3334	23.4866	24.4245	24.7447	25.9253	26.9734	27.2125	22.556	32.4794	23.3736	26.959	26.5064	27.4668	0.182849367	0.659303873	4	8	24	0.652330856	0.88299052	24	22.91948511	0.050943485
ENSMUSG00000047388	Atmin	ATM interactor [Source:MGI Symbol;Acc:MGI:2682328]	15.8067	13.2204	14.577	13.5896	12.7058	15.6131	13.9765	15.4752	12.7221	18.5486	12.7873	15.3401	14.8309	15.6339	0.633249925	0.966018836	24	20	24	0.859905463	0.958681059	24	0.674486233	0.032712176
ENSMUSG00000047394	Odf3b	outer dense fiber of sperm tails 3B [Source:MGI Symbol;Acc:MGI:1917363]	1.03955	1.39585	1.66291	1.70668	1.36659	0.9243	1.3763	1.06446	1.17032	1.47511	0.866206	1.59129	0.456882	2.3097	0.291637091	0.798132248	16	4	24	0.318315416	0.768040178	24	9.062814668	0.174791314
ENSMUSG00000047407	Tgif1	TGFB-induced factor homeobox 1 [Source:MGI Symbol;Acc:MGI:1194497]	4.90257	4.93565	4.31068	4.87581	5.85549	4.66777	5.92517	4.78507	5.93997	4.11338	5.68876	4.56798	4.47151	4.57515	0.835668938	0.966018836	20	20	24	0.805045851	0.937541099	24	17.77536645	0.039917053
ENSMUSG00000047409	Ctdspl	CTD (carboxy-terminal domain, RNA polymerase II, polypeptide A) small phosphatase-like [Source:MGI Symbol;Acc:MGI:1916524]	6.84772	6.37072	6.96001	7.08455	7.38284	7.02108	6.60969	6.20029	7.04977	7.15227	6.61078	7.02552	6.62784	6.09025	3.83E-04	0.052429523	16	16	24	0.006385651	0.470534175	24	15.22890388	0.071425908
ENSMUSG00000047412	Zbtb44	zinc finger and BTB domain containing 44 [Source:MGI Symbol;Acc:MGI:1925123]	4.31168	4.28408	4.55241	5.08238	4.19736	4.02358	4.37297	4.2993	4.52617	4.36877	4.39733	4.42675	4.06894	4.16282	0.216776163	0.704989686	12	20	24	0.066845991	0.669146244	24	11.00133567	0.05543286
ENSMUSG00000047414	Flrt2	fibronectin leucine rich transmembrane protein 2 [Source:MGI Symbol;Acc:MGI:3603594]	3.13904	3.69565	4.22638	3.29171	3.43668	3.01851	3.24189	3.09445	4.39024	2.55295	3.58734	3.18363	2.83577	3.39693	0.091424683	0.483797062	12	12	24	0.280202276	0.768040178	24	8.169582353	0.099164164
ENSMUSG00000047415	Gpr68	G protein-coupled receptor 68 [Source:MGI Symbol;Acc:MGI:2441763]	1.56669	1.24281	1.86576	1.92914	1.25549	1.69458	1.72621	1.21126	1.38318	1.56614	1.3235	1.41386	1.24867	1.57853	0.932092277	0.966018836	16	16	24	0.764282043	0.92403577	24	11.54933079	0.038654985
ENSMUSG00000047417	Rexo1	REX1, RNA exonuclease 1 [Source:MGI Symbol;Acc:MGI:1914182]	7.24379	6.33593	7.18018	6.30766	7.10702	5.95709	6.09283	7.32682	6.72781	8.93043	6.80741	6.08569	6.74841	6.55299	0.387380622	0.915479136	12	12	24	0.25177766	0.768040178	24	10.11357027	0.060431183
ENSMUSG00000047419	Cmya5	cardiomyopathy associated 5 [Source:MGI Symbol;Acc:MGI:1923719]	1.87707	4.97753	4.95634	6.78149	3.84233	5.46328	4.19999	11.7588	9.02313	10.0516	4.14262	4.94529	6.37455	5.0342	0.230338208	0.730447182	16	4	24	0.064732058	0.663876076	24	8.862920662	0.44639416
ENSMUSG00000047420	Fam180a	family with sequence similarity 180, member A [Source:MGI Symbol;Acc:MGI:3039626]	5.87869	5.00351	3.97535	3.79549	4.12881	3.5856	4.53059	4.19221	6.89679	1.97772	3.846	3.99896	3.46887	3.97613	0.446653834	0.966018836	8	8	24	0.28679806	0.768040178	24	2.53303655	0.1806446
ENSMUSG00000047423	AI837181	expressed sequence AI837181 [Source:MGI Symbol;Acc:MGI:2147598]	21.5787	18.4249	18.9906	19.7575	16.9745	19.1647	17.9503	19.2365	17.3428	22.5974	17.5227	19.5917	19.4206	18.0089	0.873337044	0.966018836	16	4	24	0.994247507	0.998755922	24	20.31142434	0.005008016
ENSMUSG00000047434	Xylt1	xyloside xylosyltransferase 1 [Source:MGI Symbol;Acc:MGI:2146443]	5.10712	4.14276	5.29201	4.26854	4.39153	4.60323	4.25754	4.77769	3.85156	5.3174	4.0687	4.4093	4.35855	4.83663	0.795269577	0.966018836	16	4	24	0.885039592	0.967256182	24	6.010292658	0.023998655
ENSMUSG00000047446	Arl4a	ADP-ribosylation factor-like 4A [Source:MGI Symbol;Acc:MGI:99437]	18.5699	20.6541	20.838	23.8923	19.9387	18.0683	19.319	21.6021	23.4915	20.1889	23.7634	24.112	24.7456	21.7289	0.854443826	0.966018836	16	8	24	0.438688469	0.805393068	24	10.10974356	0.06075814
ENSMUSG00000047454	Gphn	gephyrin [Source:MGI Symbol;Acc:MGI:109602]	18.6779	16.6638	17.8728	16.8849	16.2844	16.8054	19.8427	17.3286	15.3081	17.67	15.4305	17.3372	15.5262	17.0231	0.953350544	0.966018836	4	16	24	0.423716875	0.805393068	24	0.409212731	0.047203705
ENSMUSG00000047459	Dynlrb1	dynein light chain roadblock-type 1 [Source:MGI Symbol;Acc:MGI:1914318]	284.498	297.046	272.615	279.156	292.81	284.33	272.458	251.052	307.745	221.873	305.099	292.659	297.716	285.38	0.874607729	0.966018836	20	20	24	0.599677001	0.859762618	24	21.53818743	0.015499834
ENSMUSG00000047466	8030462N17Rik	RIKEN cDNA 8030462N17 gene [Source:MGI Symbol;Acc:MGI:2444951]	7.24099	7.25537	7.10128	7.49414	6.82127	7.27834	7.31781	7.224	6.70388	6.63117	7.04106	6.476	6.83887	6.92254	0.891105762	0.966018836	4	16	24	0.60254952	0.861114219	24	1.6627423	0.016777788
ENSMUSG00000047473	Zfp30	zinc finger protein 30 [Source:MGI Symbol;Acc:MGI:99178]	2.01889	2.01773	2.4607	2.17955	1.86418	2.39794	2.33662	2.0443	2.33157	2.22851	2.11352	2.23416	2.11913	2.08795	0.675497492	0.966018836	12	20	24	0.872403105	0.96394484	24	9.251166772	0.01676078
ENSMUSG00000047485	Klhl34	kelch-like 34 [Source:MGI Symbol;Acc:MGI:2685234]	2.5263	2.50306	2.50587	2.44763	2.72436	2.7679	2.89602	3.05241	2.75401	2.96787	2.40707	2.6841	2.86616	3.47981	0.636884864	0.966018836	8	12	24	0.144430575	0.752803875	24	3.858326479	0.063432992
ENSMUSG00000047496	Rnf152	ring finger protein 152 [Source:MGI Symbol;Acc:MGI:2443787]	2.03641	2.19985	2.22492	2.14015	2.16447	1.78645	2.41188	1.9227	2.61224	1.95155	2.08561	2.28627	1.98037	2.23859	0.94397916	0.966018836	12	4	24	0.425170692	0.805393068	24	7.073920703	0.052494485
ENSMUSG00000047497	Adamts12	a disintegrin-like and metallopeptidase (reprolysin type) with thrombospondin type 1 motif, 12 [Source:MGI Symbol;Acc:MGI:2146046]	0.662328	1.06861	1.07533	0.695103	0.965594	0.858752	1.0783	1.10588	1.09446	0.527286	0.858007	0.944686	0.975165	1.01851	0.012922042	0.227495952	12	4	24	0.174379487	0.768040178	24	3.326906396	0.100040364
ENSMUSG00000047502	Mroh7	maestro heat-like repeat family member 7 [Source:MGI Symbol;Acc:MGI:2685873]	0.616825	1.1246	1.04682	1.40032	1.47661	0.938103	0.870568	0.54391	0.888441	0.680886	0.940227	0.696496	0.727038	0.967001	0.496745696	0.966018836	20	8	24	0.190070207	0.768040178	24	13.21767607	0.128961996
ENSMUSG00000047507	Baiap3	BAI1-associated protein 3 [Source:MGI Symbol;Acc:MGI:2685783]	1.76931	3.43257	6.19781	2.57003	2.68417	2.76144	4.32606	3.55796	6.12154	2.38253	1.18602	3.38294	1.76973	3.65511	0.024253829	0.286402646	12	8	24	0.091261964	0.71175955	24	6.540740483	0.405096444
ENSMUSG00000047509	Gm6776	predicted pseudogene 6776 [Source:MGI Symbol;Acc:MGI:3647201]	9.3341	11.4583	10.3251	10.4494	12.3058	9.69964	9.54213	9.12517	10.9028	8.27054	11.7153	10.3626	11.1468	11.0159	0.920992137	0.966018836	20	20	24	0.896880052	0.970634157	24	15.6480138	0.027007275
ENSMUSG00000047514	Tspyl1	testis-specific protein, Y-encoded-like 1 [Source:MGI Symbol;Acc:MGI:1298395]	53.2353	43.0766	44.0334	42.6627	40.9053	46.2628	46.0706	45.2636	36.6706	52.8584	42.9525	46.8996	45.1416	48.0281	0.215777641	0.704989686	24	20	24	0.451298823	0.805393068	24	22.52859722	0.068707707
ENSMUSG00000047515	BC049715	cDNA sequence BC049715 [Source:MGI Symbol;Acc:MGI:3605234]	1.58798	1.66678	1.56841	1.91616	1.91518	1.41065	2.30183	1.63651	1.93079	1.20692	1.95005	1.68617	1.82549	1.21787	0.93447422	0.966018836	20	12	24	0.731660008	0.912544394	24	19.27667436	0.053367374
ENSMUSG00000047528	Flacc1	flagellum associated containing coiled-coil domains 1 [Source:MGI Symbol;Acc:MGI:1918359]	1.12747	0.980886	0.826527	0.770205	1.12881	0.909906	0.814741	0.919792	0.786998	0.644107	0.848558	0.99119	1.37428	0.874529	0.028632001	0.316608413	4	12	24	0.046388386	0.633507364	24	22.54134836	0.121170971
ENSMUSG00000047534	Mis18bp1	MIS18 binding protein 1 [Source:MGI Symbol;Acc:MGI:2145099]	0.464993	0.609075	0.524747	0.854951	0.689844	0.534042	0.512665	0.610344	0.687863	0.43114	0.673508	0.599812	0.716614	0.675581	0.927004797	0.966018836	20	8	24	0.663505106	0.886493109	24	11.39775147	0.036083701
ENSMUSG00000047539	Fbxo28	F-box protein 28 [Source:MGI Symbol;Acc:MGI:1261890]	18.1281	14.1324	14.4689	15.8343	14.4819	15.7902	16.0354	14.5886	12.8051	15.9019	15.978	15.2029	15.6116	15.16	0.101520755	0.511659635	4	8	24	0.115579677	0.727799063	24	20.33026292	0.086665373
ENSMUSG00000047547	Cltb	clathrin, light polypeptide (Lcb) [Source:MGI Symbol;Acc:MGI:1921575]	158.406	123.613	129.3	122.304	134.027	140.209	140.431	141.151	121.175	156.853	147.092	156.013	148.782	139.717	0.101520755	0.511659635	4	8	24	0.092888508	0.71175955	24	20.60074635	0.101500898
ENSMUSG00000047554	Tmem41b	transmembrane protein 41B [Source:MGI Symbol;Acc:MGI:1289225]	21.2474	22.3318	21.6654	19.4756	22.6952	18.2321	19.6552	20.8558	18.7121	19.474	23.1621	21.0325	21.3343	20.6342	0.932092277	0.966018836	20	20	24	0.927584118	0.981244452	24	20.0603989	0.016264263
ENSMUSG00000047557	Lxn	latexin [Source:MGI Symbol;Acc:MGI:107633]	208.659	188.501	177.268	170.077	172.842	190.552	167.936	179.726	172.108	194.298	189.529	198.102	200.321	194.254	0.379902653	0.908319827	4	8	24	0.607962147	0.768040178	24	22.47478622	0.060506521
ENSMUSG00000047565	Acot10	acyl-CoA thioesterase 10 [Source:MGI Symbol;Acc:MGI:1928940]	7.41567	7.70587	8.13127	8.03796	8.5198	7.15338	6.06266	6.41548	7.30276	5.78795	7.62041	6.7072	7.25003	6.94212	0.633249925	0.966018836	20	4	24	0.594010201	0.857184401	24	13.88780519	0.05029952
ENSMUSG00000047583	Tyw3	tRNA-yW synthesizing protein 3 homolog (S. cerevisiae) [Source:MGI Symbol;Acc:MGI:2445040]	2.46739	2.77226	2.61344	3.41303	2.15602	3.07437	4.31224	2.84115	3.17408	2.64775	3.38764	2.59704	2.37008	2.78027	0.880247609	0.966018836	16	12	24	0.991684696	0.998516607	24	6.755922133	0.010487614
ENSMUSG00000047591	Mafa	v-maf musculoaponeurotic fibrosarcoma oncogene family, protein A (avian) [Source:MGI Symbol;Acc:MGI:2673307]	1.73637	1.369	1.45505	1.71986	1.42357	1.20268	1.7659	2.08592	1.88339	2.90224	1.4225	2.31107	2.06034	2.04601	0.883870616	0.966018836	8	12	24	0.573361672	0.847005958	24	7.450408169	0.080746481
ENSMUSG00000047592	Nxpe5	neurexophilin and PC-esterase domain family, member 5 [Source:MGI Symbol;Acc:MGI:3584036]	0.719575	0.654315	0.491264	0.764491	0.715411	0.795376	0.717547	0.624532	0.539057	0.49811	0.623668	0.614371	0.499106	0.654848	0.601647512	0.966018836	24	12	24	0.133386234	0.743843563	24	20.02149127	0.064200829
ENSMUSG00000047603	Zfp235	zinc finger protein 235 [Source:MGI Symbol;Acc:MGI:1929117]	4.57365	5.56999	6.13109	5.28415	5.04071	6.18018	5.52694	5.10992	5.78826	4.46201	5.75327	5.44145	4.40005	5.43377	0.956178643	0.96629412	12	16	24	0.906876809	0.974587616	24	10.86229977	0.023830809
ENSMUSG00000047604	Frat2	frequently rearranged in advanced T cell lymphomas 2 [Source:MGI Symbol;Acc:MGI:2673967]	2.23987	1.80591	1.76889	1.85115	1.76025	2.08188	2.30157	1.97694	1.70957	1.96365	1.94332	2.02649	2.56686	2.04069	1.23E-04	0.029372629	4	8	24	0.005792509	0.455634368	24	22.90399442	0.112970124
ENSMUSG00000047606	Ankrd34c	ankyrin repeat domain 34C [Source:MGI Symbol;Acc:MGI:2685617]	4.04336	3.59725	2.86571	3.40632	3.38687	3.70789	3.38603	2.9729	2.51562	3.86387	3.29201	3.62164	3.47979	3.69872	0.245061943	0.741580974	24	12	24	0.138764795	0.748341961	24	20.52173965	0.107695041
ENSMUSG00000047613	A430005L14Rik	RIKEN cDNA A430005L14 gene [Source:MGI Symbol;Acc:MGI:2140680]	10.9786	11.1531	10.2386	11.3777	11.1717	9.77268	11.2415	12.155	12.9827	9.94586	12.2651	10.2617	10.4218	10.9223	0.791413662	0.966018836	12	12	24	0.507669863	0.812880742	24	7.898174579	0.052559333
ENSMUSG00000047617	Paxx	non-homologous end joining factor [Source:MGI Symbol;Acc:MGI:2442831]	32.788	33.3251	31.837	32.9673	30.3898	31.7631	28.0894	32.6705	33.6214	32.8604	30.8507	34.1967	31.5976	31.4977	0.671726933	0.966018836	16	4	24	0.580764467	0.850320846	24	8.865929555	0.030241888
ENSMUSG00000047632	Fgfbp3	fibroblast growth factor binding protein 3 [Source:MGI Symbol;Acc:MGI:1919764]	1.34329	1.54427	2.05722	1.54737	0.86034	1.16323	1.00303	1.25092	1.44677	1.31439	1.14989	1.02748	1.05781	1.73439	0.018923071	0.256345714	12	8	24	0.005673315	0.451284058	24	7.39476557	0.208043634
ENSMUSG00000047635	Mtrfr	mitochondrial translation release factor in rescue [Source:MGI Symbol;Acc:MGI:1919900]	8.1504	7.90354	8.01692	7.70067	9.20083	7.65922	7.2653	7.15099	7.38969	7.5273	8.89191	8.42957	9.19382	8.64987	0.446653834	0.966018836	20	16	24	0.371128588	0.792854157	24	18.54552768	0.059352088
ENSMUSG00000047643	Gm5454	predicted gene 5454 [Source:MGI Symbol;Acc:MGI:3648286]	14.3866	11.9782	10.1673	13.0175	11.0358	11.9772	12.6159	11.8615	9.60797	14.6601	11.9519	13.0402	13.1413	13.2693	0.182849367	0.659303873	4	8	24	0.448698098	0.805393068	24	21.31400448	0.080818633
ENSMUSG00000047648	Fbxo30	F-box protein 30 [Source:MGI Symbol;Acc:MGI:1919115]	8.80016	8.3251	7.38812	9.48744	9.20308	8.05396	8.93602	7.81185	8.33049	7.51133	9.86553	8.19161	8.79564	7.84781	0.235141005	0.73629392	20	16	24	0.177858605	0.768040178	24	17.95558664	0.080867959
ENSMUSG00000047649	Cd3eap	CD3E antigen, epsilon polypeptide associated protein [Source:MGI Symbol;Acc:MGI:1917583]	4.83851	4.46844	4.86543	5.30502	4.35036	4.48618	4.83421	4.81664	4.83591	4.51736	4.03044	5.43083	4.3236	4.86411	0.745118544	0.966018836	12	8	24	0.753965019	0.920255919	24	5.4567784	0.029420516
ENSMUSG00000047656	Trpt1	tRNA phosphotransferase 1 [Source:MGI Symbol;Acc:MGI:1333115]	6.38615	6.46776	7.87208	7.90644	6.66219	6.95251	7.81895	8.3553	7.34925	8.76564	6.59721	7.29423	7.11382	7.64939	0.125573493	0.555618842	16	4	24	0.128832973	0.740162193	24	8.688986916	0.093853622
ENSMUSG00000047657	Crry-ps	complement receptor related protein, pseudogene [Source:MGI Symbol;Acc:MGI:88514]	4.08506	4.84183	3.7661	4.14016	4.23499	3.92136	3.42425	3.88135	3.95281	4.36778	4.24569	4.07542	4.9965	4.24854	0.890648739	0.966018836	16	20	24	0.995957625	0.998898684	24	6.709798099	0.004166202
ENSMUSG00000047658	Gal3st3	galactose-3-O-sulfotransferase 3 [Source:MGI Symbol;Acc:MGI:3617843]	0.56111	0.526888	0.629769	0.465173	0.475264	0.690927	0.514431	0.535823	0.540207	0.949571	0.460184	0.65422	0.523877	0.791709	0.945728192	0.966018836	12	8	24	0.789662181	0.932573819	24	8.521558238	0.032727238
ENSMUSG00000047669	Msl3l2	MSL3 like 2 [Source:MGI Symbol;Acc:MGI:1920640]	3.28539	2.80396	2.52897	2.67915	3.0905	2.57278	2.93816	2.9879	2.74245	3.26791	3.48532	3.29482	3.08649	2.87866	0.064422003	0.414867207	20	16	24	0.279972138	0.768040178	24	18.84244519	0.062989014
ENSMUSG00000047675	Rps8	ribosomal protein S8 [Source:MGI Symbol;Acc:MGI:98166]	56.4411	78.994	73.6387	81.721	64.9786	62.4624	72.3958	81.2397	80.7924	57.3995	81.7105	73.3492	82.9061	77.4428	0.834424969	0.966018836	8	16	24	0.336843689	0.771960538	24	6.466578594	0.10695659
ENSMUSG00000047676	Rpsa-ps10	ribosomal protein SA, pseudogene 10 [Source:MGI Symbol;Acc:MGI:3704228]	75.1326	84.5951	84.8648	80.277	91.2595	76.0485	71.7475	82.157	97.2357	69.0775	91.8827	83.7338	92.6654	83.8587	0.823677655	0.966018836	12	12	24	0.798822568	0.935664328	24	9.555517287	0.038848658
ENSMUSG00000047694	Yipf6	Yip1 domain family, member 6 [Source:MGI Symbol;Acc:MGI:1925179]	22.5228	21.2989	19.9354	20.9609	21.5216	20.0856	20.7498	19.8602	20.527	19.1632	21.9827	20.9859	20.3156	21.5013	0.814077113	0.966018836	20	20	24	0.43040768	0.805393068	24	20.99031337	0.031472081
ENSMUSG00000047710	Champ1	chromosome alignment maintaining phosphoprotein 1 [Source:MGI Symbol;Acc:MGI:1196398]	7.87074	6.70553	6.58308	6.41617	6.73804	7.76946	7.02441	7.59461	5.26938	8.14019	5.76425	6.77705	6.82446	6.93094	0.23195324	0.731832877	4	8	24	0.388674728	0.799546792	24	22.56936013	0.080244952
ENSMUSG00000047712	Ust	uronyl-2-sulfotransferase [Source:MGI Symbol;Acc:MGI:2442406]	14.4065	12.2937	10.9362	12.8239	12.2905	12.7921	12.9641	12.6422	10.2503	14.2069	11.3547	13.1223	12.7655	12.3408	0.042880436	0.356048751	24	12	24	0.231736276	0.768040178	24	21.07996283	0.082779914
ENSMUSG00000047714	Ppp1r2	protein phosphatase 1, regulatory inhibitor subunit 2 [Source:MGI Symbol;Acc:MGI:1914099]	88.4041	72.7959	75.2544	73.5526	75.7997	80.9895	73.1934	72.6759	62.2043	80.1308	83.0236	75.9193	77.8924	75.0644	0.059082035	0.406067671	24	8	24	0.089448252	0.708706636	24	19.5460754	0.096252611
ENSMUSG00000047719	Ubiad1	UbiA prenyltransferase domain containing 1 [Source:MGI Symbol;Acc:MGI:1918957]	2.32228	1.76641	1.97952	2.69641	2.19226	2.32637	2.06968	2.38995	2.20345	2.42049	2.22331	2.17465	2.11691	1.99649	0.128457847	0.560209134	16	16	24	0.188208742	0.768040178	24	14.74395492	0.069669723
ENSMUSG00000047721	Bola2	bolA-like 2 (E. coli) [Source:MGI Symbol;Acc:MGI:1913412]	48.3027	44.0935	36.7836	46.1924	51.1022	48.7386	35.9801	42.2868	46.5523	34.5488	56.4157	37.7857	44.9922	38.9194	0.280810068	0.792139038	20	20	24	0.335629224	0.771468587	24	17.56853801	0.118948321
ENSMUSG00000047731	Wbp1l	WW domain binding protein 1 like [Source:MGI Symbol;Acc:MGI:107577]	8.66158	7.84395	7.56032	8.18401	8.56375	8.12923	8.26838	9.03703	8.06721	9.37839	7.45189	9.39735	8.78938	7.79935	0.835401638	0.966018836	24	12	24	0.669519356	0.888703971	24	20.31758697	0.03517079
ENSMUSG00000047735	Samd9l	sterile alpha motif domain containing 9-like [Source:MGI Symbol;Acc:MGI:1343184]	6.69702	7.5935	6.89067	6.24358	8.82136	6.10001	7.40509	7.43801	8.37982	4.83161	8.64396	7.17252	7.44189	6.83011	0.93717134	0.966018836	20	20	24	0.879331379	0.966161692	24	22.8942501	0.040926092
ENSMUSG00000047746	Fbxo40	F-box protein 40 [Source:MGI Symbol;Acc:MGI:2443753]	1.96637	2.97633	3.37571	3.43578	2.18891	4.14402	2.5585	7.54809	4.43458	4.91847	2.58615	2.97386	4.24001	3.03442	0.332336445	0.853485917	16	4	24	0.254114576	0.768040178	24	7.340581597	0.25397487
ENSMUSG00000047747	Rnf150	ring finger protein 150 [Source:MGI Symbol;Acc:MGI:2443860]	4.83096	4.29709	4.59369	4.63573	4.18885	4.92043	4.18303	4.55634	3.9205	5.21721	4.16044	4.80587	4.59886	4.79474	0.8972237	0.966018836	24	12	24	0.913846386	0.975875087	24	20.09407558	0.016823005
ENSMUSG00000047749	Zc3hav1l	zinc finger CCCH-type, antiviral 1-like [Source:MGI Symbol;Acc:MGI:2443387]	1.10437	1.17241	0.965891	1.31224	1.34348	1.05812	1.39647	1.16143	1.13086	1.04878	1.12444	1.07348	1.11911	1.08033	0.896311169	0.966018836	4	8	24	0.76934061	0.925619883	24	18.8876508	0.023847506
ENSMUSG00000047757	Fancb	Fanconi anemia, complementation group B [Source:MGI Symbol;Acc:MGI:2448558]	1.46259	1.36832	1.20433	1.24425	1.50686	1.54825	1.40487	1.27545	1.45713	1.03047	1.63136	1.3861	1.80765	1.53154	0.079180494	0.458678242	20	20	24	0.085089513	0.70291653	24	21.92584854	0.092578816
ENSMUSG00000047766	Lrrc49	leucine rich repeat containing 49 [Source:MGI Symbol;Acc:MGI:2442689]	110.697	110.071	95.0834	100.874	104.619	102.326	99.1203	97.5399	97.6872	100.483	105.414	101.982	107.335	107.417	0.239699179	0.741580974	4	8	24	0.244852159	0.768040178	24	22.15883135	0.04427862
ENSMUSG00000047767	Atg16l2	autophagy related 16-like 2 (S. cerevisiae) [Source:MGI Symbol;Acc:MGI:1920933]	13.1625	12.2005	12.3681	13.834	14.9832	11.5974	16.7541	13.6395	15.0094	13.2493	15.3796	13.1366	13.486	13.3364	0.941830768	0.966018836	20	4	24	0.85434604	0.956491441	24	14.93467662	0.029161257
ENSMUSG00000047773	Ankfn1	ankyrin-repeat and fibronectin type III domain containing 1 [Source:MGI Symbol;Acc:MGI:2686021]	6.92938	8.79163	7.11681	8.74428	12.6675	8.16204	9.78968	8.07172	9.92201	7.63248	9.408	9.29587	9.00557	7.69893	0.542718076	0.966018836	20	12	24	0.312783619	0.768040178	24	16.69112388	0.120151902
ENSMUSG00000047777	Phf13	PHD finger protein 13 [Source:MGI Symbol;Acc:MGI:2446217]	3.29116	4.01382	4.36115	4.35479	3.48965	3.89602	3.86521	3.74334	3.90217	4.39834	3.82071	3.85392	3.54803	3.96195	0.029756552	0.323426482	16	4	24	0.027808118	0.590220615	24	10.12699175	0.092831886
ENSMUSG00000047786	Lix1	limb and CNS expressed 1 [Source:MGI Symbol;Acc:MGI:1913893]	82.9909	62.7346	67.5029	63.2721	68.5562	74.1384	65.0786	62.9405	51.742	69.0977	74.4409	66.4431	67.1196	65.5958	0.079622943	0.458678242	24	12	24	0.051555584	0.64767123	24	19.70554443	0.135534894
ENSMUSG00000047787	Flrt1	fibronectin leucine rich transmembrane protein 1 [Source:MGI Symbol;Acc:MGI:3026647]	1.05976	0.977216	0.957654	0.944514	0.903979	1.07411	1.00823	1.04718	1.00614	1.31723	0.91505	1.25125	1.11166	1.30345	0.187699199	0.662239322	24	20	24	0.662849099	0.886190881	24	2.518107653	0.027141037
ENSMUSG00000047789	Slc38a9	solute carrier family 38, member 9 [Source:MGI Symbol;Acc:MGI:1918839]	8.87353	8.56435	8.12347	8.98809	8.42607	8.20165	7.62678	8.15256	8.69521	8.55822	9.0584	9.03249	8.46485	8.88203	0.92655264	0.966018836	20	8	24	0.5015276	0.80908345	24	13.72994098	0.028550647
ENSMUSG00000047793	Sned1	sushi, nidogen and EGF-like domains 1 [Source:MGI Symbol;Acc:MGI:3045960]	11.2432	13.095	12.7682	12.9324	15.945	13.0461	14.0066	11.3697	15.57	9.43479	14.5326	12.6202	12.9233	11.2282	0.702630284	0.966018836	20	12	24	0.636082822	0.876347693	24	17.05947355	0.071488228
ENSMUSG00000047797	Gjb1	gap junction protein, beta 1 [Source:MGI Symbol;Acc:MGI:95719]	17.1447	16.8495	16.3492	16.9143	20.9274	16.1109	22.3069	16.1657	19.1465	19.8862	18.0719	18.9276	18.2135	15.2935	0.185945151	0.662239322	20	12	24	0.398413793	0.799546792	24	17.09019617	0.080327539
ENSMUSG00000047798	Cd300lf	CD300 molecule like family member F [Source:MGI Symbol;Acc:MGI:2442359]	0.681524	0.905048	1.57874	1.15809	0.986327	1.31337	1.07451	0.8565	1.06636	0.617304	0.826401	0.996272	1.19569	0.718867	0.890648739	0.966018836	24	8	24	0.984292668	0.996767829	24	15.97054116	0.01316088

ENSMUSG00000047804	Akap10	A kinase (PRKA) anchor protein 10 [Source:MGI Symbol;Acc:MGI:1890218]	9.54746	8.66195	8.55696	8.23591	8.69917	8.74047	8.92194	8.15791	8.48088	7.76197	9.63278	9.29572	9.33399	8.72055	0.008961721	0.192760771	4	12	24	0.025151437	0.586541342	24	21.35897509	0.077941489
ENSMUSG00000047810	Ccdc88b	coiled-coil domain containing 88B [Source:MGI Symbol;Acc:MGI:1925567]	1.20105	0.848506	1.47063	0.657503	0.616072	0.596087	0.864605	0.734788	1.0142	0.653434	1.37507	0.69977	0.82023	0.603664	0.830364743	0.966018836	12	12	24	0.869477419	0.962941471	24	7.037651125	0.045473698
ENSMUSG00000047821	Trim16	tripartite motif-containing 16 [Source:MGI Symbol;Acc:MGI:2137356]	5.02805	4.44188	4.4677	4.10115	3.82121	4.85203	4.37663	5.19837	3.9827	6.41043	4.37274	4.35343	4.96989	5.18956	0.185945151	0.662239322	8	12	24	0.700924494	0.900900643	24	4.18688233	0.052341441
ENSMUSG00000047822	Angptl8	angiopoietin-like 8 [Source:MGI Symbol;Acc:MGI:3643534]	0.339975	0.14392	0.369636	2.53121	1.05877	0.738703	0.829194	0.506889	0.502769	0.71138	2.88007	0.940397	1.24651	1.14505	0.217933169	0.704989686	20	16	24	0.05422381	0.654887447	24	15.42418961	0.391208519
ENSMUSG00000047824	Pygo2	pygopus 2 [Source:MGI Symbol;Acc:MGI:1916161]	13.8451	10.7556	11.8292	13.999	12.1812	11.2475	13.0387	12.7017	11.6487	13.4144	13.0774	12.4351	11.8601	11.6296	0.251986462	0.75129457	16	16	24	0.472442882	0.805393068	24	15.42390898	0.050505352
ENSMUSG00000047832	Cdca4	cell division cycle associated 4 [Source:MGI Symbol;Acc:MGI:1919213]	3.61905	3.43694	3.84844	3.66755	3.50027	3.70789	3.76599	3.8521	3.38792	3.56779	3.29338	3.573	4.25007	3.65312	0.231491055	0.731037655	4	16	24	0.244058678	0.768040178	24	1.524347638	0.045605709
ENSMUSG00000047842	Diras2	DIRAS family, GTP-binding RAS-like 2 [Source:MGI Symbol;Acc:MGI:1915453]	11.9261	9.20647	10.2726	9.05349	8.71031	11.1975	9.99297	9.39859	7.39101	10.3835	7.95118	10.2188	9.18751	10.0305	0.893307667	0.966018836	24	20	24	0.200525723	0.768040178	24	22.71551825	0.11072719
ENSMUSG00000047843	Bri3	brain protein I3 [Source:MGI Symbol;Acc:MGI:1933174]	20.1433	19.6833	18.9209	22.4561	20.9538	18.442	17.1683	19.5344	20.7959	21.7469	20.1348	21.2245	22.1769	17.7252	0.223774686	0.717349224	16	16	24	0.205133524	0.768040178	24	13.71427072	0.07480028
ENSMUSG00000047844	Bex4	brain expressed X-linked 4 [Source:MGI Symbol;Acc:MGI:3606746]	5.54909	3.71807	4.68507	3.71743	4.05749	4.81624	3.52701	3.69558	4.06879	3.3546	4.29667	4.4857	3.66075	4.46555	0.959856266	0.967043964	24	16	24	0.478474466	0.805393068	24	21.25274011	0.079323293
ENSMUSG00000047866	Lonp2	lon peptidase 2, peroxisomal [Source:MGI Symbol;Acc:MGI:1914137]	33.7818	33.7321	34.4254	37.4726	34.961	32.8687	32.3524	33.2792	33.1381	33.7943	34.3988	33.5576	37.3206	34.9102	0.760769894	0.966018836	20	4	24	0.679501848	0.892680535	24	12.10376419	0.021131495
ENSMUSG00000047867	Gimap6	GTPase, IMAP family member 6 [Source:MGI Symbol;Acc:MGI:1918876]	4.87428	5.70301	5.64311	7.32886	4.98386	5.05621	6.47826	5.71896	6.91626	5.61794	5.62996	4.57562	5.22862	5.55159	0.068712455	0.432567661	12	12	24	0.065711395	0.667854689	24	8.799394335	0.14265392
ENSMUSG00000047875	Gpr157	G protein-coupled receptor 157 [Source:MGI Symbol;Acc:MGI:2442046]	3.67364	3.19761	3.34477	3.84874	3.49478	3.31954	3.61501	3.8763	3.495	4.26175	3.81642	3.75815	3.66614	3.20524	0.338172159	0.85758369	16	16	24	0.316714633	0.768040178	24	14.46898238	0.05201132
ENSMUSG00000047878	A4galt	alpha 1,4-galactosyltransferase [Source:MGI Symbol;Acc:MGI:3512453]	15.7864	14.4543	14.1459	15.4409	16.1933	17.1623	14.4359	14.782	14.2423	16.4111	15.6957	16.7147	14.3982	14.613	0.01130565	0.204837764	24	12	24	0.002286118	0.39629122	24	17.45695291	0.100334699
ENSMUSG00000047879	Usp14	ubiquitin specific peptidase 14 [Source:MGI Symbol;Acc:MGI:1928898]	110.103	104.052	99.8745	106.792	109.813	106.409	103.656	96.8685	98.8439	102.742	106.328	106.982	110.986	108.142	0.094000699	0.490854203	4	8	24	0.054687887	0.655263328	24	19.71818248	0.053642512
ENSMUSG00000047881	Rell1	RELT-like 1 [Source:MGI Symbol;Acc:MGI:2140767]	11.1114	12.2368	10.8834	10.1484	11.1028	10.8462	13.1963	12.123	12.6327	11.4681	10.1311	11.828	12.4743	11.7107	0.07098829	0.440262583	4	16	24	0.023081695	0.581965446	24	2.703514458	0.09653649
ENSMUSG00000047888	Tnrc6b	trinucleotide repeat containing 6b [Source:MGI Symbol;Acc:MGI:2443730]	5.34346	7.30018	7.86071	5.4965	6.0407	5.75445	5.76625	5.21541	6.52815	6.75054	5.4427	8.14985	6.43322	7.86334	0.920858634	0.966018836	12	16	24	0.546564962	0.833378191	24	6.698371333	0.084276459
ENSMUSG00000047897	Ripply2	ripply transcriptional repressor 2 [Source:MGI Symbol;Acc:MGI:2685968]	0.752975	0.656451	0.865241	0.286174	0.248859	0.37741	0.712953	0.372977	0.740731	0.718829	0.686944	0.522056	0.391476	0.537986	0.44861185	0.966018836	12	8	24	0.482245265	0.805393068	24	5.990733602	0.091069994
ENSMUSG00000047904	Sstr2	somatostatin receptor 2 [Source:MGI Symbol;Acc:MGI:98328]	2.10026	1.98525	2.20211	1.56816	1.5956	2.40308	1.90691	2.17463	1.4512	2.09903	1.32873	2.23584	1.87299	1.91031	0.503972924	0.966018836	24	20	24	0.227983415	0.768040178	24	0.428161569	0.106375229
ENSMUSG00000047905	Gm8566	predicted pseudogene 8566 [Source:MGI Symbol;Acc:MGI:3647296]	238.045	234.487	239.711	244.176	230.897	245.887	224.328	228.27	225.29	224.556	252.035	244.585	243.113	244.576	0.369326376	0.893596186	24	12	24	0.434720036	0.805393068	24	18.78252127	0.028839746
ENSMUSG00000047907	Tshz2	teashirt zinc finger family member 2 [Source:MGI Symbol;Acc:MGI:2153084]	42.1359	30.5055	30.9149	34.335	32.5453	37.8606	34.4543	35.4981	27.8358	49.9982	30.8647	39.2219	37.8608	38.9311	0.321662653	0.83607295	24	12	24	0.666749866	0.887459929	24	20.49682671	0.078464956
ENSMUSG00000047909	Ankrd16	ankyrin repeat domain 16 [Source:MGI Symbol;Acc:MGI:2444796]	15.885	20.8664	23.2248	21.1361	21.1942	19.0382	16.1944	20.1345	25.6437	18.8114	18.1847	19.9867	19.3159	21.165	0.008389425	0.192760771	12	16	24	0.026154379	0.586541342	24	9.179091253	0.170148408
ENSMUSG00000047910	Pcdhb16	protocadherin beta 16 [Source:MGI Symbol;Acc:MGI:2136752]	1.0469	1.15789	1.15669	1.04713	1.09607	0.961022	1.14672	1.02637	1.16155	0.928606	1.02118	0.916244	1.04154	1.04382	0.0444686	0.365196805	12	12	24	0.121696275	0.734072261	24	5.807964177	0.04088064
ENSMUSG00000047921	Trappc9	trafficking protein particle complex 9 [Source:MGI Symbol;Acc:MGI:1923760]	18.6892	13.8498	14.4313	15.0663	13.8435	17.4978	14.7959	17.6145	11.9979	22.1542	13.4348	16.3923	14.9985	16.6784	0.759899911	0.966018836	24	12	24	0.877451529	0.965881478	24	21.41093173	0.045857792
ENSMUSG00000047945	Marcksl1	MARCKS-like 1 [Source:MGI Symbol;Acc:MGI:97143]	9.18153	10.1119	12.6223	8.10265	9.68876	8.13195	10.3436	10.4138	10.3806	10.531	9.74489	9.97817	9.59173	9.1413	0.593560247	0.966018836	12	12	24	0.279826342	0.768040178	24	7.210156026	0.093480464
ENSMUSG00000047963	Stbd1	starch binding domain 1 [Source:MGI Symbol;Acc:MGI:1261768]	43.1544	34.158	31.2578	33.1281	36.2799	36.2743	34.8106	36.0625	27.2551	31.875	39.7516	36.1216	39.3315	35.444	0.009821544	0.194720062	20	16	24	0.010267703	0.503967859	24	21.22361	0.167270678
ENSMUSG00000047965	Rpl9-ps7	ribosomal protein L9, pseudogene 7 [Source:MGI Symbol;Acc:MGI:3642824]	52.6413	60.0427	55.5056	56.0555	57.4497	53.8299	50.9535	56.6222	60.3334	40.325	54.335	50.9325	54.0118	50.0246	0.648082424	0.966018836	12	12	24	0.78709587	0.931456435	24	3.64162943	0.036291556
ENSMUSG00000047976	Kcna1	potassium voltage-gated channel, shaker-related subfamily, member 1 [Source:MGI Symbol;Acc:MGI:96654]	47.8437	40.9946	39.582	35.2977	33.4453	38.8516	45.1316	42.9876	32.8704	46.7994	33.572	41.658	44.4512	44.3355	0.008157655	0.192760771	4	16	24	0.056449757	0.65534544	24	1.386921028	0.154861851
ENSMUSG00000047986	Palm3	paralemmin 3 [Source:MGI Symbol;Acc:MGI:1921587]	15.5177	10.2487	10.8304	10.1105	10.7379	12.072	10.5725	11.6936	8.7182	14.6357	12.3716	14.9909	14.5548	12.3924	0.321662653	0.83607295	24	12	24	0.177246041	0.768040178	24	20.90036633	0.160123394
ENSMUSG00000047988	4933428G20Rik	RIKEN cDNA 4933428G20 gene [Source:MGI Symbol;Acc:MGI:1918475]	1.07303	1.43593	1.23688	1.15266	1.21551	1.53088	1.47334	1.0785	1.75154	1.05853	1.15508	1.36038	1.24125	1.32768	0.708020493	0.966018836	24	16	24	0.771863026	0.926423608	24	2.355241541	0.035186572
ENSMUSG00000047989	Ino80c	INO80 complex subunit C [Source:MGI Symbol;Acc:MGI:2443014]	12.1182	14.1295	14.099	14.7277	13.7538	13.3152	12.8093	14.4497	13.7909	14.0752	12.4756	13.2268	13.2468	13.7488	0.018311724	0.256345714	8	20	24	0.024419932	0.586541342	24	9.034566521	0.073645858
ENSMUSG00000047996	Prrg1	proline rich Gla (G-carboxyglutamic acid) 1 [Source:MGI Symbol;Acc:MGI:1917364]	6.12462	6.91953	5.83193	5.67942	6.34039	4.72004	6.0263	5.33084	6.1227	4.19235	5.34515	4.04913	4.12546	4.03902	0.896753532	0.966018836	12	12	24	0.729501246	0.911734411	24	7.730187359	0.045121348
ENSMUSG00000048000	Gigyf2	GRB10 interacting GYF protein 2 [Source:MGI Symbol;Acc:MGI:2138584]	20.7395	18.0012	19.2471	17.3712	19.8367	20.1684	17.6752	17.0353	16.002	17.5392	17.6502	18.4587	18.1184	17.7121	0.138350222	0.580158868	24	16	24	0.025252425	0.586541342	24	20.1431339	0.084872996
ENSMUSG00000048001	Hes5	hes family bHLH transcription factor 5 [Source:MGI Symbol;Acc:MGI:104876]	1.0724	0.636267	0.695476	0.821951	0.88513	1.41681	0.829322	1.15057	0.786551	0.68478	1.49552	1.16183	1.09174	1.29412	0.088675218	0.474297892	24	12	24	0.085044479	0.70291653	24	20.5256775	0.148742269
ENSMUSG00000048004	Tmem196	transmembrane protein 196 [Source:MGI Symbol;Acc:MGI:2685374]	4.71321	5.97938	5.39843	5.57391	5.15259	4.51095	5.35062	4.85361	4.21888	4.87614	4.53886	4.17276	4.63821	4.8837	0.203305541	0.689605535	16	8	24	0.435393883	0.805393068	24	7.118816677	0.053440532
ENSMUSG00000048007	Timm8a1	translocase of inner mitochondrial membrane 8A1 [Source:MGI Symbol;Acc:MGI:1353433]	2.93186	3.23925	3.08684	2.93801	3.40897	2.56756	2.38726	3.30951	3.42796	2.89447	3.47681	3.2083	3.79394	3.18609	0.888482515	0.966018836	20	4	24	0.697178112	0.899338675	24	8.741493982	0.043959115
ENSMUSG00000048022	Tmem229a	transmembrane protein 229A [Source:MGI Symbol;Acc:MGI:2442812]	23.401	22.6384	21.7249	21.072	22.9483	20.7051	24.1379	19.624	19.9176	19.8031	22.9252	20.9142	20.7841	19.852	0.435596482	0.966018836	4	4	24	0.122964602	0.735704523	24	20.27451113	0.065194768
ENSMUSG00000048027	Rgmb	repulsive guidance molecule family member B [Source:MGI Symbol;Acc:MGI:1916049]	52.8238	39.331	39.1332	41.5486	43.3778	46.4849	42.316	44.7849	32.8351	52.4022	44.0677	44.4242	43.4297	42.1684	0.021795836	0.26698364	24	12	24	0.196759596	0.768040178	24	19.59134503	0.119166103
ENSMUSG00000048029	Eno4	enolase 4 [Source:MGI Symbol;Acc:MGI:2441717]	0.475934	0.687149	0.895578	0.40395	0.368964	0.432912	0.73525	0.355479	0.565538	0.540141	0.369784	0.749086	0.334634	0.610443	0.881718085	0.966018836	12	8	24	0.509687169	0.813985618	24	5.294054225	0.074978553
ENSMUSG00000048039	Isg20l2	interferon stimulated exonuclease gene 20-like 2 [Source:MGI Symbol;Acc:MGI:2140076]	5.74114	5.0561	5.2074	5.5213	5.44985	5.31003	5.3354	5.24	5.40075	5.4094	5.54944	5.39996	4.96268	5.51101	0.365996436	0.893596186	20	12	24	0.396264951	0.799546792	24	15.52121566	0.024402544
ENSMUSG00000048040	Arxes2	adipocyte-related X-chromosome expressed sequence 2 [Source:MGI Symbol;Acc:MGI:1924226]	25.9486	26.6229	28.926	26.2686	27.5453	27.8063	25.1884	24.3035	23.2562	24.7334	27.6175	21.7467	21.3103	24.6237	0.756435587	0.966018836	20	8	24	0.275743635	0.768040178	24	13.77247501	0.058907447
ENSMUSG00000048047	Zbtb33	zinc finger and BTB domain containing 33 [Source:MGI Symbol;Acc:MGI:1927290]	6.58051	6.83013	6.68815	6.41647	6.46026	5.8548	6.78369	5.4338	6.14159	5.40774	7.08986	7.01944	6.63039	6.18693	0.823657258	0.966018836	20	20	24	0.471342727	0.805393068	24	20.53438841	0.053692861
ENSMUSG00000048058	Ldlrad3	low density lipoprotein receptor class A domain containing 3 [Source:MGI Symbol;Acc:MGI:2138856]	8.403	8.3259	7.84592	7.02931	9.00378	7.79016	10.2264	9.12722	7.70669	8.41295	7.3746	7.65191	8.36145	7.62431	0.428730128	0.966018836	4	12	24	0.25897972	0.768040178	24	0.293987646	0.078466474
ENSMUSG00000048065	Cyb5r2	cytochrome b5 reductase 2 [Source:MGI Symbol;Acc:MGI:2444415]	3.20953	2.41566	1.95531	2.56645	3.74699	2.93691	3.0408	2.53548	2.74745	2.57435	2.40703	2.7647	2.64759	2.30117	0.121015203	0.548303839	4	4	24	0.054319055	0.654981352	24	19.45976858	0.147767931
ENSMUSG00000048070	Pirt	phosphoinositide-interacting regulator of transient receptor potential channels [Source:MGI Symbol;Acc:MGI:2443635]	255.106	195.92	199.666	211.842	212.479	246.354	197.813	213.556	157.828	243.717	211.457	220.548	217.872	219.156	0.085890569	0.466404237	24	12	24	0.164590741	0.767020009	24	19.76955345	0.126558494
ENSMUSG00000048076	Arf1	ADP-ribosylation factor 1 [Source:MGI Symbol;Acc:MGI:99431]	132.338	118.954	120.053	118.879	120.329	129.444	122.13	126.964	112.117	132.356	119.759	120.054	123.269	124.335	0.607427393	0.966018836	4	8	24	0.469973288	0.805393068	24	21.8412661	0.032988254
ENSMUSG00000048078	Tenm4	teneurin transmembrane protein 4 [Source:MGI Symbol;Acc:MGI:2447063]	2.13986	2.41466	3.64142	2.28853	2.19585	2.62239	2.79987	2.1513	2.27652	3.03877	2.0423	2.80663	1.87072	3.30577	0.725061903	0.966018836	12	16	24	0.554692972	0.837923284	24	8.015488776	0.091354979
ENSMUSG00000048087	Gm4737	predicted gene 4737 [Source:MGI Symbol;Acc:MGI:3643647]	9.6453	9.47348	9.17901	8.56415	10.3	9.95574	9.63136	9.97481	9.93086	9.5823	9.80377	10.2828	9.39057	9.76018	0.356953959	0.881188925	20	20	24	0.339790502	0.774150091	24	20.63622256	0.034943028
ENSMUSG00000048096	Lmod1	leiomodin 1 (smooth muscle) [Source:MGI Symbol;Acc:MGI:2135671]	0.480782	0.553527	0.716773	0.458683	0.354579	0.436302	0.409022	0.752132	0.443886	0.717794	0.419084	0.397688	0.540862	0.563616	0.015345591	0.253245931	8	12	24	0.036242764	0.609176161	24	6.727941687	0.11358958
ENSMUSG00000048100	Taf13	TATA-box binding protein associated factor 13 [Source:MGI Symbol;Acc:MGI:1913500]	33.5732	34.1128	32.3291	31.1724	34.887	34.37	32.3321	31.1329	31.8032	28.9472	34.725	29.1413	31.8577	30.9373	0.265774856	0.773510818	20	20	24	0.412305262	0.799546792	24	20.04958062	0.03993276
ENSMUSG00000048106	4632415L05Rik	RIKEN cDNA 4632415L05 gene [Source:MGI Symbol;Acc:MGI:1918058]	5.14673	4.98371	5.12835	4.87137	5.0642	5.13059	5.26118	4.89337	4.3867	5.5129	4.91269	5.14648	4.96424	4.80696	0.215777641	0.704989686	4	4	24	0.309792428	0.768040178	24	19.09441973	0.03807857
ENSMUSG00000048108	Tmem72	transmembrane protein 72 [Source:MGI Symbol;Acc:MGI:2442707]	5.92443	3.50466	3.01267	3.83677	4.19899	5.14064	4.08282	4.88336	3.47949	5.61645	3.96727	4.78435	5.16292	4.44814	0.038363978	0.335328154	4	8	24	0.15526439	0.760445083	24	21.08196666	0.167924347
ENSMUSG00000048109	Rbm15	RNA binding motif protein 15 [Source:MGI Symbol;Acc:MGI:2443205]	2.10373	2.08317	2.37429	2.4796	2.08593	2.1137	2.12355	2.0886	2.04299	2.1083	2.45218	1.75012	1.96022	2.08	0.505728924	0.966018836	16	16	24	0.120883451	0.732894732	24	11.5291446	0.062587145
ENSMUSG00000048118	Arid4a	AT rich interactive domain 4A (RBP1-like) [Source:MGI Symbol;Acc:MGI:2444354]	8.07671	7.34081	6.92361	6.89741	7.52196	7.50996	7.29879	6.68388	7.76402	7.98725	7.75827	8.25486	7.72653	8.53726	0.854443826	0.966018836	24	16	24	0.597049571	0.858276814	24	20.62220231	0.032524276
ENSMUSG00000048120	Entpd1	ectonucleoside triphosphate diphosphohydrolase 1 [Source:MGI Symbol;Acc:MGI:102805]	5.71714	5.20695	6.68064	4.92883	5.75512	7.48343	5.10881	5.91013	5.93308	6.56629	5.86324	6.10549	5.17199	4.18821	0.398261528	0.929472831	24	4	24	0.313099185	0.768040178	24	16.10650843	0.102315968
ENSMUSG00000048126	Col6a3	collagen, type VI, alpha 3 [Source:MGI Symbol;Acc:MGI:88461]	8.63028	10.2339	9.0409	9.66297	11.1401	10.0354	11.0142	11.9235	7.99817	7.66729	10.2138	7.7434	10.0974	7.26279	0.917370979	0.966018836	20	20	24	0.581009423	0.850503204	24	21.02828712	0.082136483
ENSMUSG00000048142	Nat8l	N-acetyltransferase 8-like [Source:MGI Symbol;Acc:MGI:2447776]	35.0805	30.1176	34.026	32.7989	28.2233	33.9149	35.5341	35.5309	28.0639	46.9696	29.0012	37.2998	31.9051	38.2152	0.951846915	0.966018836	8	12	24	0.932909401	0.98297963	24	4.993578959	0.028852856
ENSMUSG00000048148	Nwd1	NACHT and WD repeat domain containing 1 [Source:MGI Symbol;Acc:MGI:2442268]	2.50001	2.51918	3.18602	2.49296	2.31077	2.70081	3.18428	2.4921	2.54757	2.28145	2.29435	2.76686	1.71654	2.69157	0.823657258	0.966018836	12	4	24	0.846070506	0.953396028	24	4.885104301	0.034633285
ENSMUSG00000048154	Kmt2d	lysine (K)-specific methyltransferase 2D [Source:MGI Symbol;Acc:MGI:2682319]	9.49897	9.40618	10.5674	9.58508	11.2279	8.68397	9.20507	9.01553	11.9431	10.6709	10.8802	10.2779	11.1099	8.99579	0.118123238	0.541691431	20	12	24	0.175012314	0.768040178	24	12.51851725	0.088967723
ENSMUSG00000048163	Selplg	selectin, platelet (p-selectin) ligand [Source:MGI Symbol;Acc:MGI:106689]	1.9848	3.73785	4.83082	4.19526	2.81098	3.67535	3.52358	3.18072	3.95397	2.7648	2.89338	3.39474	2.6835	2.43677	0.43565473	0.966018836	12	16	24	0.335756375	0.771468587	24	10.00410831	0.146085847
ENSMUSG00000048164	Gm7204	predicted pseudogene 7204 [Source:MGI Symbol;Acc:MGI:3645884]	30.7394	41.952	41.088	34.467	41.9607	32.8536	36.734	34.1059	44.6037	32.6705	42.2099	35.7804	36.2699	35.6961	0.823677655	0.966018836	12	12	24	0.440816597	0.805393068	24	10.13225887	0.08393753
ENSMUSG00000048170	Mcmbp	minichromosome maintenance complex binding protein [Source:MGI Symbol;Acc:MGI:1920977]	6.90978	7.451	8.18112	7.96089	8.17452	6.34879	8.19206	6.14642	6.90407	7.54518	8.07144	6.43414	6.17999	6.2536	0.278111325	0.789849983	20	12	24	0.069872959	0.674363692	24	13.26721513	0.107878539
ENSMUSG00000048174	Tmem81	transmembrane protein 81 [Source:MGI Symbol;Acc:MGI:1921876]	1.55065	2.64735	2.25772	1.78427	2.10511	2.03862	1.95288	1.79681	2.13949	2.22855	2.03639	2.30425	1.85889	2.09486	0.643325306	0.966018836	12	16	24	0.628335945	0.872795898	24	9.992095525	0.05010149
ENSMUSG00000048175	Asb8	ankyrin repeat and SOCS box-containing 8 [Source:MGI Symbol;Acc:MGI:1925791]	25.2803	21.8591	25.4397	23.5061	20.8936	26.0356	24.738	28.075	21.1272	31.1333	21.4239	25.1308	22.8825	24.7605	0.85387321	0.966018836	24	20	24	0.91496011	0.976279302	24	5.054379243	0.027240653
ENSMUSG00000048186	Bend7	BEN domain containing 7 [Source:MGI Symbol;Acc:MGI:2443100]	1.72092	1.95088	2.0307	2.47616	1.88246	1.97347	2.44885	1.79096	1.56744	2.20599	1.94005	2.3255	1.74018	2.04776	0.951846915	0.966018836	16	12	24	0.593721869	0.856999712	24	15.87195819	0.052968093
ENSMUSG00000048188	Gm8181	predicted gene 8181 [Source:MGI Symbol;Acc:MGI:3643329]	18.3955	19.5121	16.8007	18.0014	18.8851	17.2741	19.9882	15.8458	19.3408	17.0845	17.1642	19.5998	17.3253	18.8751	0.910436409	0.966018836	4	12	24	0.730381718	0.912198956	24	21.67063226	0.030777177
ENSMUSG00000048200	Cracr2b	calcium release activated channel regulator 2B [Source:MGI Symbol;Acc:MGI:2446129]	3.90664	4.3398	3.18187	6.45007	3.48601	3.33673	3.52095	7.02834	6.30073	5.94596	4.55026	3.31397	4.8045	4.51002	0.179603275	0.655480352	16	8	24	0.077071303	0.687041208	24	8.583788288	0.273748381
ENSMUSG00000048216	Gpr85	G protein-coupled receptor 85 [Source:MGI Symbol;Acc:MGI:1927851]	5.6047	5.85173	7.07626	5.5149	6.43755	6.79535	6.24957	5.11525	5.41626	6.08089	6.11342	6.02339	5.79878	6.29969	0.796183667	0.966018836	4	8	24	0.432166014	0.805393068	24	19.16496927	0.056288901
ENSMUSG00000048218	Amigo2	adhesion molecule with Ig like domain 2 [Source:MGI Symbol;Acc:MGI:2145995]	13.5249	9.39361	9.71658	10.8803	9.92705	12.5495	10.6943	10.4123	7.44431	11.6817	10.7905	9.93481	10.2205	9.62299	0.160291962	0.620016786	16	20	24	0.096602193	0.71175955	24	19.78389485	0.148399726
ENSMUSG00000048222	Mfap1b	microfibrillar-associated protein 1B [Source:MGI Symbol;Acc:MGI:3694697]	7.16175	7.4444	7.84943	7.14004	7.56923	7.25441	6.67014	7.03027	7.67528	6.118	7.18956	7.42249	7.3296	7.62451	0.503972924	0.966018836	12	4	24	0.81144983	0.939535552	24	4.321377666	0.020932945
ENSMUSG00000048232	Fbxo10	F-box protein 10 [Source:MGI Symbol;Acc:MGI:2686937]	3.21936	3.27913	3.58162	3.11627	3.18816	3.26324	3.47237	3.44552	3.55068	3.58597	2.81954	3.4206	3.0742	3.35665	0.113424199	0.530777648	12	8	24	0.193964356	0.768040178	24	6.360599996	0.053065819
ENSMUSG00000048234	Rnf149	ring finger protein 149 [Source:MGI Symbol;Acc:MGI:2677438]	12.2284	12.8483	12.6506	13.7187	12.2961	11.5069	11.8946	11.8425	12.0035	13.051	12.1844	13.149	10.835	12.1981	0.117050591	0.538362324	16	12	24	0.0991967	0.71175955	24	11.9460854	0.060300737
ENSMUSG00000048240	Gng7	guanine nucleotide binding protein (G protein), gamma 7 [Source:MGI Symbol;Acc:MGI:95787]	3.94575	4.32848	5.82219	3.74137	4.37286	4.29183	5.09712	4.73936	4.88241	5.87293	3.71872	4.34677	4.15315	4.79959	0.413946331	0.958746003	12	8	24	0.181491711	0.768040178	24	7.562898833	0.123497301
ENSMUSG00000048249	Crebrf	CREB3 regulatory factor [Source:MGI Symbol;Acc:MGI:1924378]	12.1658	15.6243	14.5705	16.6491	14.8623	12.9752	14.6421	12.7435	17.6748	12.6048	14.5504	14.844	14.0157	14.5376	0.669104912	0.966018836	12	16	24	0.330660514	0.768040178	24	9.984838705	0.084732972
ENSMUSG00000048251	Bcl11b	B cell leukemia/lymphoma 11B [Source:MGI Symbol;Acc:MGI:1929913]	1.03959	0.82599	0.991306	0.988492	0.936887	0.888382	0.940352	1.04321	0.861226	1.31355	1.01883	0.964499	0.936677	1.18415	0.91241026	0.966018836	8	8	24	0.52048478	0.820018348	24	10.41822769	0.038603508
ENSMUSG00000048261	Gm4879	predicted pseudogene 4879 [Source:MGI Symbol;Acc:MGI:3648111]	7.13777	7.65129	7.33633	6.46655	6.97637	7.41404	6.64542	6.70392	7.10544	6.90686	7.0085	6.95333	7.4732	7.49006	0.395544145	0.870418653	8	8	24	0.396472077	0.799546792	24	2.20561881	0.03307285
ENSMUSG00000048264	Dip2c	disco interacting protein 2 homolog C [Source:MGI Symbol;Acc:MGI:1920179]	21.439	18.0551	17.0167	18.5559	18.0801	19.6579	18.3653	17.4912	14.5977	19.5476	16.409	18.3641	18.2565	18.7402	0.217827365	0.704989686	24	12	24	0.15822187	0.764753215	24	21.32255114	0.089032396
ENSMUSG00000048271	Rbm33	RNA binding motif protein 33 [Source:MGI Symbol;Acc:MGI:1919670]	9.66151	10.2279	10.0249	9.46226	9.52389	9.21466	9.29911	10.1211	10.995	9.98872	10.2096	9.9537	10.6596	10.2067	0.27074138	0.780327352	8	16	24	0.056023111	0.65534544	24	6.794350462	0.052786095
ENSMUSG00000048277	Syngr2	synaptogyrin 2 [Source:MGI Symbol;Acc:MGI:1328324]	12.1837	12.8033	13.7195	14.2079	14.7407	14.1939	13.3838	18.8989	15.4011	17.2701	12.73	13.6431	14.0122	11.914	0.313239838	0.824470524	16	12	24	0.422451778	0.804917634	24	10.37040471	0.090481765
ENSMUSG00000048279	Sacs	sacsin [Source:MGI Symbol;Acc:MGI:1354724]	12.0797	13.4421	12.0105	12.9763	12.6563	11.5175	11.8121	10.7299	13.2236	10.7454	13.4701	12.7624	13.5864	13.5644	0.79625133	0.966018836	4	12	24	0.992725463	0.998516607	24	2.859183314	0.005223582
ENSMUSG00000048280	Zfp738	zinc finger protein 738 [Source:MGI Symbol;Acc:MGI:3040706]	5.81172	5.72286	4.85589	5.3576	5.97933	4.51279	6.4704	5.84187	6.08436	4.61828	6.08653	4.44855	6.23754	4.78006	0.510431574	0.966018836	4	20	24	0.735835174	0.913952055	24	1.613748415	0.049465263
ENSMUSG00000048281	Dleu7	deleted in lymphocytic leukemia, 7 [Source:MGI Symbol;Acc:MGI:2447771]	1.17191	2.06305	1.86516	1.14946	1.43569	1.31453	1.59944	1.61963	1.20909	1.05482	1.21118	1.36815	1.4782	1.29749	0.05211941	0.391073722	8	8	24	0.152571159	0.759088284	24	3.018009337	0.11215292
ENSMUSG00000048285	Frmd6	FERM domain containing 6 [Source:MGI Symbol;Acc:MGI:2442579]	8.44858	8.97373	8.3614	7.6335	8.72587	8.39088	9.6852	9.7142	9.26673	8.60151	8.54097	8.54643	9.54582	8.64884	0.20866337	0.698797644	8	8	24	0.064514565	0.663876076	24	2.257584551	0.068944032
ENSMUSG00000048304	Slitrk3	SLIT and NTRK-like family, member 3 [Source:MGI Symbol;Acc:MGI:2679447]	16.6445	15.6344	14.2318	15.0813	13.7345	16.3007	15.0252	14.8335	12.2938	18.088	13.6268	16.1404	15.1607	16.6985	0.492524295	0.966018836	24	12	24	0.607302921	0.863811892	24	22.69370107	0.054595025
ENSMUSG00000048307	Ankrd46	ankyrin repeat domain 46 [Source:MGI Symbol;Acc:MGI:1916089]	142.142	126.709	124.835	127.659	126.059	135.398	127.079	128.432	110.544	131.35	132.464	131.367	134.09	128.107	0.009630739	0.194720062	24	12	24	0.043534837	0.626787045	24	20.72984477	0.074370495
ENSMUSG00000048310	Pskh1	protein serine kinase H1 [Source:MGI Symbol;Acc:MGI:3528383]	4.68402	5.2456	5.32139	5.58452	5.04382	5.72035	5.58663	5.48969	5.29402	6.63432	5.14754	5.52066	5.19752	5.73282	0.907068194	0.966018836	16	4	24	0.35757158	0.784480156	24	10.79687431	0.050854559
ENSMUSG00000048327	Ckap2l	cytoskeleton associated protein 2-like [Source:MGI Symbol;Acc:MGI:1917716]	0.665323	0.596204	1.11525	0.75262	0.711868	0.752894	0.764699	0.741855	0.674353	0.623897	0.708046	0.759767	0.838912	0.54106	0.46238066	0.966018836	4	4	24	0.95875205	0.989144435	24	14.83470795	0.01226444
ENSMUSG00000048330	Ric3	RIC3 acetylcholine receptor chaperone [Source:MGI Symbol;Acc:MGI:2443887]	20.6353	20.1058	20.0411	19.7308	19.2714	20.775	18.7698	18.719	16.7186	20.4535	19.6013	20.4428	19.3473	20.9857	0.879206539	0.966018836	24	12	24	0.512739696	0.815901806	24	20.0823558	0.038503804
ENSMUSG00000048332	Lhfp	lipoma HMGIC fusion partner [Source:MGI Symbol;Acc:MGI:1920048]	23.1137	22.0751	21.8083	19.2748	23.9158	21.3057	24.6203	22.8666	20.7099	20.9282	19.2051	22.837	21.9201	20.7799	0.057224139	0.401309455	4	12	24	0.06290039	0.662016894	24	23.40497979	0.084164214
ENSMUSG00000048334	Gm8258	predicted gene 8258 [Source:MGI Symbol;Acc:MGI:3644429]	2.06605	1.99213	1.37552	2.05804	2.54749	1.98484	2.48584	1.96461	2.36202	1.75168	2.86973	2.21613	2.15265	2.10779	0.151535314	0.599319068	20	20	24	0.215469626	0.768040178	24	18.8657975	0.111431579
ENSMUSG00000048347	Pcdhb18	protocadherin beta 18 [Source:MGI Symbol;Acc:MGI:2136756]	1.01346	1.13552	1.26214	0.908627	1.09458	1.01117	1.15032	0.984835	0.972077	0.931549	1.09334	0.87378	0.919574	1.10127	0.759899911	0.966018836	8	16	24	0.657365269	0.884466305	24	5.179113499	0.026985794
ENSMUSG00000048349	Pou4f1	POU domain, class 4, transcription factor 1 [Source:MGI Symbol;Acc:MGI:102525]	63.7011	47.6628	43.8585	51.3892	49.4531	54.9414	46.8502	49.8966	38.9606	64.4729	45.987	52.5414	52.6833	53.6788	0.390686537	0.915479136	24	12	24	0.4727419	0.805393068	24	20.23172377	0.095633579
ENSMUSG00000048351	Coa7	cytochrome c oxidase assembly factor 7 [Source:MGI Symbol;Acc:MGI:1917143]	4.76785	7.31783	5.84881	6.51794	7.67404	7.4559	7.08586	6.74651	8.81694	8.05694	7.90966	8.16241	8.73384	6.72087	0.851656735	0.966018836	20	12	24	0.455680879	0.805393068	24	14.42894386	0.077385912
ENSMUSG00000048355	Arxes1	adipocyte-related X-chromosome expressed sequence 1 [Source:MGI Symbol;Acc:MGI:1923469]	7.77739	6.8177	8.97971	7.2147	8.59804	7.98813	8.30493	7.13196	6.7747	7.50221	8.00669	6.31384	6.89849	7.28983	0.801995186	0.966018836	20	12	24	0.592455957	0.856568887	24	15.65129291	0.047583291
ENSMUSG00000048368	Omd	osteomodulin [Source:MGI Symbol;Acc:MGI:1350918]	3.87946	6.32819	5.46311	5.34853	5.59955	4.0646	4.63902	5.84575	7.12126	3.53067	5.17451	4.57115	3.58844	4.96739	0.058395306	0.404936103	12	16	24	0.1632832	0.767020009	24	8.302082286	0.183873104
ENSMUSG00000048371	Pdp2	pyruvate dehyrogenase phosphatase catalytic subunit 2 [Source:MGI Symbol;Acc:MGI:1918878]	2.9181	2.90692	2.9977	3.19023	3.18865	3.15531	2.98324	3.08935	2.9403	3.43818	3.00765	3.09188	3.16861	2.90314	0.007831348	0.192760771	16	16	24	0.075692453	0.68634302	24	14.76219652	0.042235824
ENSMUSG00000048373	Fgfbp1	fibroblast growth factor binding protein 1 [Source:MGI Symbol;Acc:MGI:1096350]	3.32097	5.08479	3.12034	2.87902	3.44974	3.06166	2.6997	3.9423	4.99452	1.44966	3.39847	3.41787	2.394	3.73652	0.249881062	0.749643185	8	20	24	0.422337055	0.804917634	24	4.029845021	0.165299838
ENSMUSG00000048376	F2r	coagulation factor II (thrombin) receptor [Source:MGI Symbol;Acc:MGI:101802]	6.33511	4.95919	5.23969	6.42107	4.7554	5.6101	5.58115	5.80937	4.44135	6.43174	5.07651	5.52166	5.41204	5.70556	0.93717134	0.966018836	16	4	24	0.943935781	0.986212398	24	21.18528505	0.019373852
ENSMUSG00000048379	Socs4	suppressor of cytokine signaling 4 [Source:MGI Symbol;Acc:MGI:1914546]	7.956	7.30697	7.3601	7.80721	8.26484	7.01061	7.62172	6.52983	6.75522	6.43947	8.70572	6.78507	7.04487	7.15124	0.834424969	0.966018836	20	12	24	0.237287783	0.768040178	24	17.49697639	0.070779533
ENSMUSG00000048385	Scrt1	scratch family zinc finger 1 [Source:MGI Symbol;Acc:MGI:2176606]	6.83933	5.59807	6.21464	5.21152	4.45383	5.83504	6.6962	6.02277	6.18867	11.1678	6.85504	8.13956	7.55538	8.09123	0.872380105	0.966018836	4	16	24	0.910003543	0.97464686	24	5.574818916	0.041034151
ENSMUSG00000048387	Osr1	odd-skipped related transcription factor 1 [Source:MGI Symbol;Acc:MGI:1344424]	1.77813	2.43138	1.39882	1.20995	1.43496	1.36919	1.41382	1.9053	2.66855	1.00457	1.97832	1.79611	1.49407	1.93669	0.148627651	0.59172107	8	8	24	0.268183057	0.768040178	24	3.959981692	0.150132109
ENSMUSG00000048388	Fam171b	family with sequence similarity 171, member B [Source:MGI Symbol;Acc:MGI:2444579]	30.6508	31.4779	33.7671	31.1942	29.5201	31.4106	27.9434	27.2358	28.3874	31.8495	31.5973	31.9724	32.1275	33.3097	0.782779623	0.966018836	24	4	24	0.864764644	0.960819377	24	13.33942594	0.019194255
ENSMUSG00000048399	Tprg	transformation related protein 63 regulated [Source:MGI Symbol;Acc:MGI:1918588]	0.781972	0.684341	0.771171	0.54704	0.368644	0.663855	0.573686	0.313056	0.40731	0.508165	0.594013	0.321163	0.715124	0.657294	0.664568268	0.966018836	4	16	24	0.558160216	0.839605711	24	1.760000845	0.061312642
ENSMUSG00000048406	B330016D10Rik	RIKEN cDNA B330016D10 gene [Source:MGI Symbol;Acc:MGI:2444063]	0.797263	0.785157	0.923488	1.15521	0.870007	0.762516	1.13121	1.45479	1.28451	1.05316	1.29514	1.02972	0.854475	1.01125	0.669104912	0.966018836	16	8	24	0.251433947	0.768040178	24	9.558185715	0.09175315
ENSMUSG00000048410	Zfp407	zinc finger protein 407 [Source:MGI Symbol;Acc:MGI:2685179]	3.65948	3.30292	3.40391	2.89269	2.99895	3.19425	3.24607	2.58256	2.58734	2.98372	3.22875	3.44433	2.68881	3.38859	0.746385265	0.966018836	4	12	24	0.386350343	0.799546792	24	21.16437495	0.064336508
ENSMUSG00000048416	Mlf1	myeloid leukemia factor 1 [Source:MGI Symbol;Acc:MGI:1341819]	7.14394	18.0538	16.5881	24.0441	15.0805	15.4727	12.3942	30.4472	29.7622	23.1699	14.1778	14.2379	17.3553	14.4432	0.085562135	0.466404237	12	16	24	0.024381385	0.586541342	24	9.291698863	0.490023454
ENSMUSG00000048424	Ranbp3l	RAN binding protein 3-like [Source:MGI Symbol;Acc:MGI:2444654]	2.05107	6.13757	2.05003	1.65291	1.95731	1.8066	1.98242	3.46839	4.11	1.21278	2.03394	3.10203	1.91828	3.13517	0.008483961	0.192760771	8	8	24	0.100303399	0.71175955	24	4.21786647	0.353497472
ENSMUSG00000048429	Timm29	translocase of inner mitochondrial membrane 29 [Source:MGI Symbol;Acc:MGI:1917023]	18.5822	16.1083	16.86	16.4824	15.9951	18.2549	16.3972	18.4474	16.0428	19.806	17.0159	18.3342	19.2311	18.592	0.823657258	0.966018836	4	8	24	0.61527819	0.866892532	24	23.39515449	0.035356345
ENSMUSG00000048439	Nupl2	nucleoporin like 2 [Source:MGI Symbol;Acc:MGI:2387631]	3.3795	4.69604	4.17264	3.06377	4.50149	4.0873	3.57629	4.8469	4.20251	4.98342	4.02578	4.38811	3.92218	4.79148	0.187699199	0.662239322	8	20	24	0.54743601	0.833664411	24	7.412924271	0.069255168

ENSMUSG00000048440	Cyp4f16	cytochrome P450, family 4, subfamily f, polypeptide 16 [Source:MGI Symbol;Acc:MGI:1917351]	4.534	4.66762	4.99502	4.20003	4.38215	4.66899	3.81212	4.64031	4.45721	4.87501	4.50401	5.92022	3.76327	4.52666	0.924553956	0.966018836	24	4	24	0.777685845	0.928689964	24	13.94528568	0.037171852
ENSMUSG00000048442	Smim5	small integral membrane protein 5 [Source:MGI Symbol;Acc:MGI:1913778]	10.5313	11.6868	10.6061	11.3296	12.9169	11.2199	9.8121	10.0334	10.9805	9.60187	10.8595	12.8515	11.6364	10.31	0.696669634	0.966018836	24	4	24	0.24242389	0.768040178	24	18.12883512	0.079322919
ENSMUSG00000048445	Ccdc57	coiled-coil domain containing 57 [Source:MGI Symbol;Acc:MGI:1918526]	0.867343	1.37304	1.71379	1.43133	1.31707	1.1143	1.00994	1.30812	1.56884	1.51232	1.41618	1.20659	1.06307	1.58188	1.96E-06	0.006828104	12	16	24	1.10E-04	0.117264499	24	9.805377533	0.191760008
ENSMUSG00000048458	Inka2	inka box actin regulator 2 [Source:MGI Symbol;Acc:MGI:1923497]	12.5462	9.532	8.03338	10.6062	12.4714	10.4225	13.0664	10.3382	8.82846	11.0239	9.79829	9.53377	10.095	8.84964	0.127415507	0.560209134	4	8	24	0.100883833	0.712175739	24	19.83219484	0.147823628
ENSMUSG00000048478	Spata33	spermatogenesis associated 33 [Source:MGI Symbol;Acc:MGI:2444920]	3.54488	2.69523	2.4816	2.89727	2.8295	3.16675	2.57303	2.93763	3.39921	3.00161	3.29941	2.93046	3.24854	3.96156	0.807813307	0.966018836	4	12	24	0.842953959	0.952259721	24	1.652446598	0.028774764
ENSMUSG00000048481	Mypop	Myb-related transcription factor, partner of profilin [Source:MGI Symbol;Acc:MGI:2446472]	10.0473	7.75787	7.26929	7.3746	7.58526	8.49441	7.95229	8.58994	8.0707	11.6321	9.45716	10.9849	9.75593	10.7417	0.801995186	0.966018836	24	12	24	0.659556603	0.884879972	24	21.36331291	0.060163213
ENSMUSG00000048482	Bdnf	brain derived neurotrophic factor [Source:MGI Symbol;Acc:MGI:88145]	10.9035	5.96902	8.28807	5.52685	11.1464	8.43235	6.54828	5.49293	4.70193	6.16458	10.0797	5.71024	5.86287	5.62848	0.123511911	0.552144108	20	12	24	0.049293995	0.641475801	24	18.2641583	0.297355377
ENSMUSG00000048483	Zdhhc22	zinc finger, DHHC-type containing 22 [Source:MGI Symbol;Acc:MGI:2685108]	7.13921	6.10714	5.90099	5.69134	5.78996	6.93265	6.30648	6.58133	5.13171	7.86443	5.70743	6.55057	5.80911	6.70138	0.384378736	0.915479136	24	12	24	0.655889652	0.883738698	24	22.14465248	0.052523008
ENSMUSG00000048486	Fitm2	fat storage-inducing transmembrane protein 2 [Source:MGI Symbol;Acc:MGI:2444508]	15.6513	12.2436	13.3242	16.1179	12.9909	15.1586	14.3332	17.0068	10.9275	17.731	13.665	14.5802	14.8734	15.4327	0.921572192	0.966018836	16	20	24	0.896270021	0.970549275	24	20.16756293	0.034350301
ENSMUSG00000048489	Depp1	DEPP1 autophagy regulator [Source:MGI Symbol;Acc:MGI:1918730]	1.96062	1.72263	1.95297	3.15783	1.39891	1.7815	2.43041	2.28223	1.89127	2.7035	2.42151	2.13449	1.72036	1.81624	0.70641939	0.966018836	16	16	24	0.414819003	0.800009695	24	11.88735889	0.108216088
ENSMUSG00000048490	Nrip1	nuclear receptor interacting protein 1 [Source:MGI Symbol;Acc:MGI:1315213]	7.87054	7.30207	7.525	7.74181	8.0341	7.04802	6.77042	7.09858	7.29875	8.14693	7.77959	7.52293	7.63825	7.57975	0.193549995	0.672246127	16	16	24	0.117630054	0.728323355	24	13.63101516	0.050356148
ENSMUSG00000048495	Tyw5	tRNA-yW synthesizing protein 5 [Source:MGI Symbol;Acc:MGI:1915986]	19.6627	18.3303	17.2123	16.4556	18.8949	17.5321	15.3504	17.5553	17.6427	16.2112	20.9061	16.3946	18.0047	16.9416	0.890311217	0.966018836	20	20	24	0.720613977	0.908366535	24	18.31662575	0.036863537
ENSMUSG00000048497	Mmgt2	membrane magnesium transporter 2 [Source:MGI Symbol;Acc:MGI:2448491]	12.3613	11.7875	10.5962	9.39889	13.9761	10.2246	12.964	10.7217	11.6152	10.3723	12.664	13.8857	11.3667	9.66711	0.082113105	0.462922111	20	20	24	0.133603956	0.743843563	24	19.90689889	0.132005829
ENSMUSG00000048502	Duxbl1	double homeobox B-like 1 [Source:MGI Symbol;Acc:MGI:1916048]	5.53945	8.46643	7.44795	6.91645	8.31599	7.84937	7.26428	7.94691	9.17998	6.47838	6.72013	6.59721	7.65252	6.20368	0.94086481	0.966018836	12	16	24	0.718169503	0.907213187	24	8.234020441	0.058762558
ENSMUSG00000048503	Tlcd5	TLC domain containing 5 [Source:MGI Symbol;Acc:MGI:2685030]	2.25079	2.50933	2.86266	2.59876	2.84714	2.60058	2.3902	2.93006	2.64977	2.77711	2.45641	2.64491	2.2638	2.81889	0.037085178	0.334054683	12	16	24	0.095356506	0.71175955	24	10.05639394	0.072482447
ENSMUSG00000048534	Jaml	junction adhesion molecule like [Source:MGI Symbol;Acc:MGI:2685484]	0.999756	0.841577	0.934138	1.79706	1.07469	0.828768	1.0821	1.10294	0.991292	0.685078	0.989912	0.84259	0.656921	0.795256	0.858266942	0.966018836	20	4	24	0.454085322	0.805393068	24	12.61458238	0.080880855
ENSMUSG00000048537	Phldb1	pleckstrin homology like domain, family B, member 1 [Source:MGI Symbol;Acc:MGI:2143230]	31.4581	35.7854	39.4101	33.2575	33.4092	30.277	43.3551	31.7428	39.5097	34.9836	30.2135	33.2376	30.79	29.3356	0.494171275	0.966018836	12	12	24	0.513187068	0.816282691	24	7.64840356	0.074263276
ENSMUSG00000048538	Gm9826	predicted gene 9826 [Source:MGI Symbol;Acc:MGI:3642725]	5.44832	5.54175	5.927	5.98368	6.89315	5.82436	5.62948	6.76601	5.15768	6.13667	5.60644	5.73404	5.52865	4.62419	0.223125229	0.717349224	16	16	24	0.263631516	0.768040178	24	15.4620669	0.073000666
ENSMUSG00000048540	Nhlh2	nescient helix loop helix 2 [Source:MGI Symbol;Acc:MGI:97324]	0.887376	0.940831	0.924402	0.688449	0.535197	0.796848	0.801586	0.607874	0.744081	0.932309	0.75966	0.945697	0.618756	1.05688	0.920872048	0.966018836	8	12	24	0.632418949	0.875020466	24	4.606767552	0.047840871
ENSMUSG00000048546	Tob2	transducer of ERBB2, 2 [Source:MGI Symbol;Acc:MGI:1888525]	3.55436	3.56879	4.36935	6.18741	3.98487	3.94031	4.53438	4.81821	5.7192	7.52261	4.76658	4.42976	5.11018	4.81274	0.179603275	0.655480352	16	8	24	0.011035384	0.513501556	24	10.75058381	0.230302273
ENSMUSG00000048550	Thnsl1	threonine synthase-like 1 (bacterial) [Source:MGI Symbol;Acc:MGI:2139347]	6.91178	6.89334	7.10881	6.77356	6.69	6.35452	7.15242	5.82058	6.70947	5.6848	6.63589	7.26479	7.39992	7.03001	0.142596848	0.585944911	4	16	24	0.370309145	0.792393371	24	23.56246621	0.053521385
ENSMUSG00000048572	Tmem252	transmembrane protein 252 [Source:MGI Symbol;Acc:MGI:3583948]	0.738855	1.29842	1.20208	0.900552	0.661366	0.915064	0.893776	1.38075	1.19042	0.873913	0.729938	0.844149	0.872428	0.941027	7.90E-04	0.071344007	8	12	24	0.004017104	0.427431763	24	5.928944918	0.173231413
ENSMUSG00000048578	Mlec	malectin [Source:MGI Symbol;Acc:MGI:1924015]	73.025	55.6327	53.1718	59.6661	69.248	63.0437	67.9862	60.8073	51.1912	60.0297	63.2791	61.3354	63.8771	55.4624	8.30E-04	0.071344007	4	8	24	0.003301411	0.416356043	24	20.078385	0.160764502
ENSMUSG00000048581	E130311K13Rik	RIKEN cDNA E130311K13 gene [Source:MGI Symbol;Acc:MGI:3607716]	5.12936	5.61988	4.74262	4.81372	5.74836	5.39852	5.11464	5.20891	5.16736	5.10481	5.72073	5.67245	6.00548	5.04392	0.054328576	0.395890765	20	20	24	0.083005235	0.699403344	24	20.16546975	0.068966607
ENSMUSG00000048583	Igf2	insulin-like growth factor 2 [Source:MGI Symbol;Acc:MGI:96434]	64.2344	81.2163	59.0807	47.9986	53.8585	42.7316	58.6957	69.2353	81.8745	24.2358	45.5213	58.4376	45.0205	54.3892	0.032547207	0.324908923	12	4	24	0.097030799	0.71175955	24	3.193719214	0.322787447
ENSMUSG00000048592	Gm5946	predicted gene 5946 [Source:MGI Symbol;Acc:MGI:3644989]	0.639519	0.725303	0.53425	0.903728	0.624348	0.614978	0.721001	0.749591	0.806496	0.650731	0.778809	0.533842	0.7873	0.587646	0.918193129	0.966018836	16	8	24	0.732563516	0.912739737	24	9.456876581	0.030595869
ENSMUSG00000048600	Gm5763	predicted pseudogene 5763 [Source:MGI Symbol;Acc:MGI:3647841]	22.0423	16.009	16.7247	17.7606	17.8456	21.3024	18.4938	19.4192	14.4423	23.4479	16.0388	18.7728	17.1581	18.0267	0.245061943	0.741580974	24	12	24	0.458644463	0.805393068	24	19.91335688	0.092932396
ENSMUSG00000048602	Morc2b	microrchidia 2B [Source:MGI Symbol;Acc:MGI:3045293]	1.32619	1.1579	1.15862	0.794036	1.10412	0.986454	1.56545	1.20206	0.687711	0.862958	1.20978	1.00218	0.97054	1.20504	0.243857167	0.741580974	4	12	24	0.076083796	0.686372559	24	23.62768316	0.128413204
ENSMUSG00000048603	Gm9828	predicted gene 9828 [Source:MGI Symbol;Acc:MGI:3642913]	1.37312	1.00594	1.10726	0.75207	1.32423	1.1725	1.34181	1.03549	1.33911	1.1949	0.746755	1.25257	1.32187	1.24807	0.241264473	0.741580974	4	12	24	0.241428311	0.768040178	24	0.36881251	0.09363253
ENSMUSG00000048612	Myof	myoferlin [Source:MGI Symbol;Acc:MGI:1919192]	3.54366	4.80323	4.48684	3.54206	4.76602	3.42784	3.96465	3.64667	4.5138	2.7639	2.82215	3.28792	2.48552	3.59352	0.25851124	0.761738864	12	4	24	0.391701744	0.799546792	24	6.798570284	0.102709849
ENSMUSG00000048616	Nog	noggin [Source:MGI Symbol;Acc:MGI:104327]	1.78501	1.82965	1.74525	1.75787	1.61409	1.59297	1.83323	1.6027	1.32746	2.47755	2.28426	1.9072	2.01128	1.93854	0.90767227	0.966018836	16	20	24	0.671952902	0.889365603	24	15.96907781	0.048022556
ENSMUSG00000048617	Rtbdn	retbindin [Source:MGI Symbol;Acc:MGI:2443686]	1.73056	1.89031	1.49129	1.86667	1.23772	1.63573	1.96632	1.59244	2.20393	1.75297	1.72857	1.913	1.92096	2.01995	0.622293809	0.966018836	4	16	24	0.249417287	0.768040178	24	4.273179127	0.076375198
ENSMUSG00000048636	A730049H05Rik	RIKEN cDNA A730049H05 gene [Source:MGI Symbol;Acc:MGI:1921766]	4.91977	6.90837	5.96554	6.52918	8.06761	5.65661	6.20754	6.40095	9.05424	4.70382	6.40193	6.15569	6.67676	5.46625	0.914502381	0.966018836	12	12	24	0.778281268	0.928832041	24	10.33111152	0.063135734
ENSMUSG00000048644	Ctxn1	cortexin 1 [Source:MGI Symbol;Acc:MGI:88566]	20.0524	14.3329	14.1164	17.2189	14.5578	16.1419	16.1211	17.5376	14.8445	22.6937	17.8744	21.2924	18.1493	18.1953	0.791413662	0.966018836	24	12	24	0.637032881	0.876483853	24	18.98471778	0.070908565
ENSMUSG00000048647	Exd1	exonuclease 3'-5' domain containing 1 [Source:MGI Symbol;Acc:MGI:3045306]	0.786708	0.94087	1.03598	1.25023	1.00638	0.900182	1.20754	0.84665	1.08329	1.02879	1.21952	1.09033	1.14066	0.9336	0.515451644	0.966018836	16	16	24	0.24021734	0.768040178	24	13.88486743	0.061405616
ENSMUSG00000048661	Lemd3	LEM domain containing 3 [Source:MGI Symbol;Acc:MGI:3580376]	6.27814	6.32431	7.16347	6.33505	5.59434	6.13046	6.85482	6.33808	5.73286	6.83155	6.64041	6.4222	6.23226	6.74575	0.90332508	0.966018836	16	4	24	0.694002611	0.898390361	24	6.021502462	0.029511025
ENSMUSG00000048668	Rhno1	RAD9-HUS1-RAD1 interacting nuclear orphan 1 [Source:MGI Symbol;Acc:MGI:1915315]	11.5038	9.05541	9.5179	10.1282	9.56643	11.1317	10.0505	9.68363	8.51514	8.74523	9.69886	9.43791	9.95488	9.62416	0.062631097	0.410322149	4	8	24	0.0175	0.550995814	24	21.33036152	0.101471752
ENSMUSG00000048677	Tpcn2	two pore segment channel 2 [Source:MGI Symbol;Acc:MGI:2385297]	4.20164	5.1733	4.88717	5.30747	5.64424	4.42861	4.2292	4.48107	5.47841	5.03044	4.68787	4.50108	4.78378	4.38315	0.024691579	0.28960575	16	12	24	0.016956674	0.547196997	24	11.52497559	0.111363445
ENSMUSG00000048696	Mex3d	mex3 RNA binding family member D [Source:MGI Symbol;Acc:MGI:2681847]	3.37075	3.49192	3.45975	3.27978	3.40657	3.26288	4.04731	3.9243	3.22808	3.92959	3.81226	4.34872	3.55319	4.18581	0.79867683	0.966018836	8	4	24	0.644133849	0.879140522	24	0.36294674	0.032887525
ENSMUSG00000048701	Ccdc6	coiled-coil domain containing 6 [Source:MGI Symbol;Acc:MGI:1923801]	10.457	9.39625	9.38608	9.78287	8.93147	10.086	10.185	9.54907	9.23626	12.1169	8.99657	10.4052	9.82938	9.92193	0.108029159	0.524035616	24	20	24	0.960113187	0.989397471	24	20.28660399	0.012260428
ENSMUSG00000048706	Lurap1l	leucine rich adaptor protein 1-like [Source:MGI Symbol;Acc:MGI:106510]	4.43054	3.86608	3.65025	3.54036	3.51278	3.389	3.45037	3.35871	3.51963	3.58303	4.10722	3.96127	3.76367	3.57817	0.77722623	0.966018836	4	4	24	0.528292335	0.82388535	24	20.95255225	0.041815022
ENSMUSG00000048707	Tprn	taperin [Source:MGI Symbol;Acc:MGI:2139535]	3.30128	4.51437	4.21416	2.83725	3.79971	4.12885	2.97238	2.82038	3.2761	3.93403	2.65809	3.30453	2.79304	3.41501	0.928148816	0.966018836	12	16	24	0.889635171	0.968953708	24	9.142224525	0.035363902
ENSMUSG00000048709	Gm8666	predicted gene 8666 [Source:MGI Symbol;Acc:MGI:3648747]	0.933411	0.936679	0.336471	1.37491	0.670433	1.5257	1.12565	2.17359	1.88392	1.03041	1.45353	1.22779	0.848947	1.23536	0.873350015	0.966018836	24	4	24	0.968435065	0.992338543	24	4.837801433	0.03133922
ENSMUSG00000048720	Tbc1d12	TBC1D12: TBC1 domain family, member 12 [Source:MGI Symbol;Acc:MGI:2384803]	5.06679	4.94627	4.79003	4.44747	4.88304	4.79545	4.91575	4.56256	4.5453	4.33409	4.82886	4.61702	4.33406	4.85305	0.463629316	0.966018836	4	12	24	0.23724993	0.768040178	24	22.766072	0.034641135
ENSMUSG00000048728	Zfp454	zinc finger protein 454 [Source:MGI Symbol;Acc:MGI:2679253]	2.29791	2.49894	2.40146	1.94435	2.37243	2.32606	2.13128	2.59648	2.46858	2.18806	2.58782	2.30698	2.62006	2.26564	0.935593607	0.966018836	8	8	24	0.615162996	0.866892532	24	2.185307306	0.031810455
ENSMUSG00000048731	Ggnbp1	gametogenetin binding protein 1 [Source:MGI Symbol;Acc:MGI:3055306]	4.49316	7.98465	5.71784	10.089	8.91864	6.77464	6.20454	9.79738	9.31369	8.37802	8.55228	7.60007	9.12566	6.63088	0.588208558	0.966018836	16	12	24	0.189964017	0.768040178	24	11.71160174	0.189946428
ENSMUSG00000048732	Klhl11	kelch-like 11 [Source:MGI Symbol;Acc:MGI:2388648]	4.75842	5.4138	5.21746	5.65209	5.30683	5.05935	5.25237	4.64359	6.08988	3.7171	5.84903	6.12035	5.84709	6.29548	0.756529255	0.966018836	20	20	24	0.872791313	0.964262367	24	0.582897033	0.03303006
ENSMUSG00000048747	E130114P18Rik	RIKEN cDNA E130114P18 gene [Source:MGI Symbol;Acc:MGI:2442873]	2.4017	3.57753	2.59734	2.58697	2.89569	2.80944	3.40143	2.26113	2.74471	2.62694	2.71171	2.96309	2.59695	2.47868	0.800065124	0.966018836	24	8	24	0.688182869	0.896839325	24	20.80708222	0.047370889
ENSMUSG00000048755	Mcat	malonyl CoA:ACP acyltransferase (mitochondrial) [Source:MGI Symbol;Acc:MGI:2388651]	6.1782	6.61201	6.28082	6.51684	6.43622	5.91299	6.39455	6.89123	6.97348	5.87544	7.85074	7.35324	6.58218	7.17585	0.92721845	0.966018836	8	8	24	0.979802011	0.995083382	24	8.094508961	0.007523972
ENSMUSG00000048756	Foxo3	forkhead box O3 [Source:MGI Symbol;Acc:MGI:1890081]	12.9683	10.6839	11.4926	13.0155	11.5244	12.1638	12.344	11.0322	9.73363	14.7217	10.5687	11.7445	11.294	11.6117	0.483881213	0.966018836	16	20	24	0.604708429	0.862089625	24	16.72373462	0.054617026
ENSMUSG00000048758	Rpl29	ribosomal protein L29 [Source:MGI Symbol;Acc:MGI:99687]	99.7608	104.295	101.929	95.9007	104.337	97.4196	92.7225	103.095	126.76	88.9731	117.988	108.004	112.28	104.854	0.907000607	0.966018836	20	20	24	0.901570295	0.972353488	24	7.045802115	0.024039546
ENSMUSG00000048763	Hoxb3	homeobox B3 [Source:MGI Symbol;Acc:MGI:96184]	4.69986	5.7567	4.87131	3.5537	5.45296	4.44699	5.24463	5.20959	6.13208	5.35764	4.72024	6.14889	5.39654	5.06086	0.786317982	0.966018836	12	4	24	0.578184835	0.849184181	24	2.763829696	0.065203863
ENSMUSG00000048772	Tmem53	transmembrane protein 53 [Source:MGI Symbol;Acc:MGI:1916027]	8.08937	7.6759	7.53199	6.71364	7.43367	7.865	6.92944	8.03588	7.37359	7.56941	8.28597	7.20345	8.75143	7.45564	0.787283372	0.966018836	4	12	24	0.492736601	0.805558817	24	23.46302662	0.041663528
ENSMUSG00000048776	Pthlh	parathyroid hormone-like peptide [Source:MGI Symbol;Acc:MGI:97800]	2.05893	1.84113	1.77491	1.91838	2.02319	1.63562	2.42819	2.00781	1.91654	1.30912	1.61258	1.60496	1.67872	1.55328	0.959160181	0.967043964	4	12	24	0.320944941	0.768040178	24	0.671326137	0.070240765
ENSMUSG00000048779	P2ry6	pyrimidinergic receptor P2Y, G-protein coupled, 6 [Source:MGI Symbol;Acc:MGI:2673874]	1.3331	0.915887	1.06385	1.00605	1.21301	1.10011	1.22185	1.33989	1.51698	1.23263	1.21145	1.07262	0.959303	0.838321	0.84680398	0.966018836	12	20	24	0.82189259	0.943925679	24	13.72530315	0.030791828
ENSMUSG00000048782	Insc	INSC spindle orientation adaptor protein [Source:MGI Symbol;Acc:MGI:1917942]	10.1044	9.03172	9.27239	7.49333	10.3488	8.83632	11.3891	7.39105	9.39861	8.88368	8.71703	8.2024	9.80505	7.32127	0.510431574	0.966018836	4	4	24	0.392232291	0.799546792	24	20.93652131	0.0901771
ENSMUSG00000048787	Dcun1d3	DCN1, defective in cullin neddylation 1, domain containing 3 (S. cerevisiae) [Source:MGI Symbol;Acc:MGI:2679003]	7.35841	5.79787	5.96361	6.76602	6.23044	6.56442	6.58784	5.7969	5.54659	7.0396	6.33977	6.77909	6.4394	6.35845	0.078417851	0.458179412	24	12	24	0.128700843	0.740162193	24	18.99424132	0.077870091
ENSMUSG00000048794	Cfap100	cilia and flagella associated protein 100 [Source:MGI Symbol;Acc:MGI:2141635]	46.2691	57.2275	48.6885	53.9422	70.4295	44.7751	55.4004	50.8951	68.2022	47.0983	55.6852	50.7582	52.3119	43.2584	0.941955432	0.966018836	20	4	24	0.553858912	0.837374348	24	12.9665304	0.08619532
ENSMUSG00000048796	Cyb561d1	cytochrome b-561 domain containing 1 [Source:MGI Symbol;Acc:MGI:1919273]	10.1118	9.56443	9.02304	8.89129	9.52184	10.947	8.36156	10.0628	8.09754	10.1654	9.59316	9.47667	10.2757	9.63737	0.601647512	0.966018836	24	12	24	0.330447065	0.768040178	24	20.68807594	0.064092449
ENSMUSG00000048799	Cep120	centrosomal protein 120 [Source:MGI Symbol;Acc:MGI:2147298]	31.3364	26.2028	25.4007	26.3683	26.1379	28.266	26.6877	26.8751	23.1101	29.7009	26.255	28.4303	28.9681	28.6291	0.060394272	0.407012001	4	8	24	0.161561501	0.76636332	24	21.7028121	0.077812395
ENSMUSG00000048807	Slc35e4	solute carrier family 35, member E4 [Source:MGI Symbol;Acc:MGI:2144150]	6.2451	5.12782	5.78611	5.0158	5.15835	5.45869	5.31307	6.38959	5.28753	6.73218	6.01519	6.14681	5.64105	6.10273	0.816361374	0.966018836	8	4	24	0.948644371	0.987444112	24	3.761031334	0.013862448
ENSMUSG00000048814	Lonrf2	LON peptidase N-terminal domain and ring finger 2 [Source:MGI Symbol;Acc:MGI:1920209]	21.2305	16.6254	18.8299	17.8532	15.8828	20.1619	18.8622	16.8035	13.2698	21.6717	14.997	19.1693	17.2764	18.9492	0.44861185	0.966018836	24	20	24	0.553272419	0.837286821	24	21.74651522	0.08175378
ENSMUSG00000048826	Dact2	dishevelled-binding antagonist of beta-catenin 2 [Source:MGI Symbol;Acc:MGI:1920347]	0.82039	1.59653	1.80626	1.34069	1.23916	1.35066	0.909103	1.21579	1.27297	1.54086	1.08805	1.36251	0.850646	1.64531	0.064867922	0.416505219	12	16	24	0.074879971	0.68394125	24	9.554241166	0.165080897
ENSMUSG00000048832	Vps37c	vacuolar protein sorting 37C [Source:MGI Symbol;Acc:MGI:2147661]	15.3631	11.0842	11.7552	11.4187	11.3342	13.1265	12.2417	15.2613	11.4898	16.8953	12.138	14.0841	13.479	12.4291	0.625825704	0.966018836	4	8	24	0.779633661	0.929226662	24	22.40869948	0.050261752
ENSMUSG00000048833	Slc39a9	solute carrier family 39 (zinc transporter), member 9 [Source:MGI Symbol;Acc:MGI:1914820]	12.5472	12.3217	13.4727	12.2563	12.3306	12.2874	12.6559	12.5184	11.4282	13.3356	10.8352	12.3339	11.8597	12.285	0.874607729	0.966018836	4	16	24	0.722911726	0.909188168	24	5.862914038	0.022661435
ENSMUSG00000048834	Vstm2a	V-set and transmembrane domain containing 2A [Source:MGI Symbol;Acc:MGI:2384826]	12.9164	13.045	13.6797	12.2597	10.8266	13.7508	13.878	11.7878	11.4902	11.3054	12.587	12.5343	10.6615	12.3959	0.916746631	0.966018836	24	20	24	0.531582448	0.82545228	24	23.62857408	0.046621095
ENSMUSG00000048856	Slc25a47	solute carrier family 25, member 47 [Source:MGI Symbol;Acc:MGI:2144766]	3.1191	2.22182	2.7015	2.97698	2.74431	2.56985	2.40931	2.71321	3.08288	2.90064	2.49803	2.07206	2.55996	2.20943	0.388056094	0.915479136	12	20	24	0.282396517	0.768040178	24	11.2036152	0.074578459
ENSMUSG00000048865	Arhgap30	Rho GTPase activating protein 30 [Source:MGI Symbol;Acc:MGI:2684948]	1.37704	1.75044	2.12755	1.71113	1.54888	1.55067	2.0268	1.70163	2.0275	1.15261	1.53927	1.42832	1.81777	1.52219	0.217933169	0.704989686	12	12	24	0.276963303	0.768040178	24	5.194160718	0.091965321
ENSMUSG00000048874	Phf3	PHD finger protein 3 [Source:MGI Symbol;Acc:MGI:2446126]	18.1366	15.3537	16.8682	17.1758	16.8988	15.2121	17.6455	16.1005	14.2224	14.1888	15.7126	16.0004	15.1256	16.966	0.915835339	0.966018836	4	20	24	0.463490178	0.805393068	24	22.77997634	0.047167927
ENSMUSG00000048878	Hexim1	hexamethylene bis-acetamide inducible 1 [Source:MGI Symbol;Acc:MGI:2385923]	17.5984	14.1247	12.4734	14.588	14.2304	15.8921	14.7242	16.2227	13.1922	16.1852	15.1185	16.9847	16.783	16.5329	0.031969981	0.324385159	4	8	24	0.061113831	0.660703626	24	21.92649299	0.110448877
ENSMUSG00000048895	Cdk5r1	cyclin-dependent kinase 5, regulatory subunit 1 (p35) [Source:MGI Symbol;Acc:MGI:101764]	9.1796	6.97676	8.08673	7.17493	6.65269	7.97655	7.97228	7.67055	6.51135	10.4292	7.40663	8.50554	7.7172	8.13885	0.823677655	0.966018836	24	12	24	0.892913846	0.969717376	24	21.48648415	0.031809481
ENSMUSG00000048897	Zfp710	zinc finger protein 710 [Source:MGI Symbol;Acc:MGI:1921747]	3.73549	3.02257	3.45988	3.77886	3.65286	3.08241	3.45343	3.67229	3.7878	3.94448	3.46225	3.50556	4.24016	3.51562	0.891105762	0.966018836	16	8	24	0.734464576	0.913383989	24	10.16030119	0.030204531
ENSMUSG00000048899	Rimkla	ribosomal modification protein rimK-like family member A [Source:MGI Symbol;Acc:MGI:3040686]	36.9857	28.7311	27.5414	30.1785	28.3864	33.9744	32.0374	31.1627	22.6974	35.3829	27.8259	31.2724	30.8491	31.5461	0.069101108	0.433403132	4	8	24	0.189965506	0.768040178	24	21.51278779	0.120561592
ENSMUSG00000048911	Rnf24	ring finger protein 24 [Source:MGI Symbol;Acc:MGI:1261771]	6.74078	6.34517	6.58565	6.6772	6.50042	7.06589	6.90773	6.70351	5.51703	7.67815	6.23693	6.77978	6.53338	6.4215	0.22872448	0.72665454	24	12	24	0.50554359	0.811334839	24	18.81890573	0.043704776
ENSMUSG00000048915	Efna5	ephrin A5 [Source:MGI Symbol;Acc:MGI:107444]	5.5706	4.22253	4.93672	4.33379	4.20118	4.7829	4.56406	4.13889	3.75412	6.23192	3.89488	5.04544	4.73524	5.35469	0.95032543	0.966018836	24	20	24	0.908835903	0.974606327	24	23.0605396	0.030559912
ENSMUSG00000048916	Gm5540	predicted pseudogene 5540 [Source:MGI Symbol;Acc:MGI:3644824]	1.45788	2.04617	2.32848	2.60092	2.03365	1.90461	2.24121	2.2808	2.2688	2.18976	2.02827	1.80766	2.21823	2.28388	0.010049466	0.194720062	12	12	24	0.026817179	0.586541342	24	9.006941125	0.126572647
ENSMUSG00000048920	Fkrp	fukutin related protein [Source:MGI Symbol;Acc:MGI:2447586]	8.14908	7.29943	7.78766	7.73865	6.81774	7.40509	7.37604	7.35928	6.86909	9.17022	7.01649	8.57788	7.9855	7.92043	0.718086778	0.966018836	24	20	24	0.995319734	0.998759942	24	0.172787122	0.004132586
ENSMUSG00000048921	Zfp689	zinc finger protein 689 [Source:MGI Symbol;Acc:MGI:1918381]	1.0069	1.03802	0.943976	0.797767	1.07047	0.83037	0.782698	1.07619	0.932871	0.952257	1.00031	0.897674	0.843445	0.923347	0.801995186	0.966018836	20	4	24	0.807818943	0.938844629	24	8.40356371	0.019548783
ENSMUSG00000048924	Ccdc125	coiled-coil domain containing 125 [Source:MGI Symbol;Acc:MGI:1923291]	0.651094	1.03013	1.07251	0.87276	0.940654	0.753673	0.946479	0.917301	0.939816	0.813497	1.27608	0.799477	1.15665	0.924933	0.944053021	0.966018836	12	12	24	0.676869049	0.891927029	24	9.135662711	0.042331431
ENSMUSG00000048930	Tada3	transcriptional adaptor 3 [Source:MGI Symbol;Acc:MGI:1915724]	13.858	10.5906	10.5521	11.8438	11.6367	12.8917	10.8841	12.3496	10.4493	13.6587	11.0671	12.5638	12.4346	12.2793	0.041292272	0.346472636	24	12	24	0.412283462	0.799546792	24	20.21336601	0.067314432
ENSMUSG00000048939	Atp13a5	ATPase type 13A5 [Source:MGI Symbol;Acc:MGI:2444068]	1.25748	2.35812	1.8132	1.52477	1.36521	1.33145	1.75953	1.84073	2.25451	0.877481	1.47459	1.50402	0.96577	1.75824	0.062704268	0.410492299	8	16	24	0.129950704	0.740483001	24	5.739518764	0.179545881
ENSMUSG00000048949	Gm6206	predicted pseudogene 6206 [Source:MGI Symbol;Acc:MGI:3644890]	20.6764	19.0285	18.84	19.3775	20.6561	19.4981	19.8041	18.7712	18.7603	18.4983	19.2586	18.8967	20.912	19.4757	0.009118941	0.192760771	4	8	24	0.082427853	0.699403344	24	21.38132469	0.045902636
ENSMUSG00000048960	Prex2	phosphatidylinositol-3,4,5-trisphosphate-dependent Rac exchange factor 2 [Source:MGI Symbol;Acc:MGI:1923385]	5.60336	6.18733	6.47319	8.07588	7.71769	5.76236	6.5374	5.17608	7.1372	5.522	7.22998	5.70054	5.2334	5.34531	0.06180207	0.409814092	20	12	24	0.027913393	0.590904845	24	13.51625971	0.16013541
ENSMUSG00000048965	Mrgpre	MAS-related GPR, member E [Source:MGI Symbol;Acc:MGI:2441884]	6.25656	4.75932	5.43405	5.11711	5.69438	6.36256	6.02411	6.48803	4.15751	6.86173	5.1535	5.82295	5.77796	5.37797	0.46238066	0.966018836	24	12	24	0.313607778	0.768040178	24	20.58382445	0.100018373
ENSMUSG00000048967	Yjefn3	YjeF N-terminal domain containing 3 [Source:MGI Symbol;Acc:MGI:2681845]	2.1179	3.28132	4.24979	3.06982	1.78988	1.66655	2.88672	3.27257	3.71123	2.87605	4.61146	4.17165	4.69708	3.26441	0.957256673	0.966536401	12	12	24	0.332737506	0.769110818	24	7.15209876	0.182805944
ENSMUSG00000048970	C1galt1c1	C1GALT1-specific chaperone 1 [Source:MGI Symbol;Acc:MGI:1913493]	28.5238	28.0466	25.2291	25.7255	29.0646	24.5733	28.0599	24.3778	26.8891	20.1035	30.1003	26.0052	26.2624	23.8959	0.454881285	0.966018836	20	20	24	0.337602785	0.772537663	24	20.84835641	0.080919701
ENSMUSG00000048978	Nrsn1	neurensin 1 [Source:MGI Symbol;Acc:MGI:894662]	30.111	29.1893	31.2744	26.0808	27.2847	33.9499	30.8926	28.4014	24.2806	31.9705	26.2694	29.9564	28.1669	31.2528	0.779660587	0.966018836	24	20	24	0.397901988	0.799546792	24	22.98041898	0.068939929
ENSMUSG00000048988	Elfn1	leucine rich repeat and fibronectin type III, extracellular 1 [Source:MGI Symbol;Acc:MGI:2442479]	6.5837	4.30362	4.52735	4.18481	4.02808	4.52577	5.18418	5.60876	4.00133	6.6338	4.40245	5.47688	5.34919	5.12288	0.289496388	0.794869797	4	16	24	0.442436505	0.805393068	24	0.238219002	0.099910093
ENSMUSG00000048997	Atxn7l2	ataxin 7-like 2 [Source:MGI Symbol;Acc:MGI:1919772]	3.37113	4.26268	4.54379	3.50493	3.75706	3.81988	3.78184	4.08128	4.68597	4.33965	4.11108	4.06546	4.05937	4.26391	0.030691182	0.324150946	12	16	24	0.029935466	0.596648309	24	7.968608493	0.095813223
ENSMUSG00000049001	Ndnf	neuron-derived neurotrophic factor [Source:MGI Symbol;Acc:MGI:1915419]	2.15854	1.79402	2.31633	2.02502	1.90375	2.45219	2.68559	2.32911	2.31768	2.57033	2.44495	1.98141	1.8849	3.10503	0.722624967	0.966018836	8	12	24	0.672966479	0.889847627	24	6.64878593	0.052979136
ENSMUSG00000049036	Tmem121	transmembrane protein 121 [Source:MGI Symbol;Acc:MGI:1916445]	1.9105	1.82961	1.43677	1.97306	1.41446	1.79335	1.51018	1.94189	1.74893	2.28254	2.24752	2.23271	2.0728	2.10666	0.847312766	0.966018836	16	20	24	0.938257928	0.984659033	24	15.98907367	0.017481912
ENSMUSG00000049037	Clec4a1	C-type lectin domain family 4, member a1 [Source:MGI Symbol;Acc:MGI:3036291]	1.25043	1.98076	2.00987	2.02836	1.58012	1.44138	1.56224	1.81111	1.80789	1.14502	1.86174	1.87907	1.30337	1.4137	0.510431574	0.966018836	12	16	24	0.531857807	0.82547989	24	9.953491785	0.074486929
ENSMUSG00000049038	Mterf2	mitochondrial transcription termination factor 2 [Source:MGI Symbol;Acc:MGI:1921488]	11.7842	13.6466	14.3615	12.9256	14.6309	12.3282	11.4996	14.2842	17.5644	11.0737	15.5644	14.2537	13.9084	15.3821	0.403954478	0.937351147	12	16	24	0.415645052	0.800637996	24	8.554512091	0.087999382
ENSMUSG00000049044	Rapgef4	Rap guanine nucleotide exchange factor (GEF) 4 [Source:MGI Symbol;Acc:MGI:1917723]	60.5296	53.68	60.2415	55.2558	56.4544	62.4838	60.6237	56.1551	46.0563	62.8893	51.2358	53.8049	57.7164	58.9385	0.873337044	0.966018836	4	8	24	0.567537506	0.843934556	24	21.98493135	0.051895286
ENSMUSG00000049047	Armcx3	armadillo repeat containing, X-linked 3 [Source:MGI Symbol;Acc:MGI:1918953]	25.2066	25.0643	22.1786	21.9702	23.7163	22.9254	23.0109	23.6621	23.8878	23.4857	25.4899	25.025	25.5783	23.15	0.628286596	0.966018836	4	12	24	0.2474871	0.768040178	24	22.02626693	0.044774044
ENSMUSG00000049076	Acap2	ArfGAP with coiled-coil, ankyrin repeat and PH domains 2 [Source:MGI Symbol;Acc:MGI:1925868]	16.3377	17.3023	19.1479	16.3052	16.9789	16.8131	17.1957	15.9298	18.7376	16.9695	16.2198	16.2813	15.9747	16.8405	0.390686537	0.915479136	12	12	24	0.12547097	0.739416874	24	8.485560596	0.056913012
ENSMUSG00000049086	Bmyc	brain expressed myelocytomatosis oncogene [Source:MGI Symbol;Acc:MGI:88184]	14.4155	12.0102	11.7948	9.91541	7.98085	8.74192	11.3526	13.9081	12.9051	18.4941	15.8344	17.4963	19.1411	16.5423	0.91241026	0.966018836	4	16	24	0.444671593	0.805393068	24	4.017142441	0.13896718
ENSMUSG00000049090	Zadh2	zinc binding alcohol dehydrogenase, domain containing 2 [Source:MGI Symbol;Acc:MGI:2444835]	21.0127	18.8893	19.3421	19.0699	18.7989	19.4148	20.7401	20.2914	18.0789	20.8392	19.0267	20.5527	20.8087	21.7567	0.128989168	0.560209134	4	16	24	0.118862178	0.729829751	24	0.534969524	0.052454601
ENSMUSG00000049091	Sephs2	selenophosphate synthetase 2 [Source:MGI Symbol;Acc:MGI:108388]	7.8932	7.78633	8.3572	9.7292	7.40984	8.26334	7.34946	8.36912	7.71849	9.85389	9.21855	9.04079	9.17461	9.55575	0.702630284	0.966018836	16	12	24	0.313609296	0.768040178	24	11.86855999	0.06512509
ENSMUSG00000049092	Gpr137c	G protein-coupled receptor 137C [Source:MGI Symbol;Acc:MGI:1917963]	9.77556	9.51468	9.91983	10.0034	9.69121	10.3587	10.3788	8.91488	8.77839	9.9983	10.7058	8.70763	8.22099	9.76365	0.668329321	0.966018836	20	12	24	0.333935443	0.770000654	24	15.34315673	0.053658369
ENSMUSG00000049097	Ankrd34a	ankyrin repeat domain 34A [Source:MGI Symbol;Acc:MGI:3617846]	1.01423	0.899392	1.37094	0.981362	0.628269	1.0652	1.33989	1.08814	1.28805	1.3288	0.802281	1.22515	1.06424	1.56145	0.285027487	0.793714531	12	8	24	0.084919551	0.70291653	24	5.379451127	0.135320235
ENSMUSG00000049100	Pcdh10	protocadherin 10 [Source:MGI Symbol;Acc:MGI:1338042]	5.29643	5.68201	6.0713	5.70925	6.2481	5.99279	6.33417	5.59701	4.88746	6.64065	5.63651	6.74987	5.49072	6.84469	0.892685629	0.966018836	24	4	24	0.747300203	0.91776718	24	17.41177033	0.03560506
ENSMUSG00000049103	Ccr2	chemokine (C-C motif) receptor 2 [Source:MGI Symbol;Acc:MGI:106185]	3.41227	4.80117	4.63618	3.45371	4.5335	3.5473	4.03269	3.34357	4.28124	2.58556	4.33326	3.49939	4.05701	3.58186	0.919571723	0.966018836	12	4	24	0.921953254	0.979411448	24	3.085198202	0.029721048
ENSMUSG00000049106	Dcaf5	DDB1 and CUL4 associated factor 5 [Source:MGI Symbol;Acc:MGI:2444785]	16.6736	12.4195	12.5126	12.0305	14.1168	14.7139	16.5799	14.0334	11.398	16.0977	11.3482	13.9761	14.5431	12.9561	0.058157448	0.40386543	4	8	24	0.113727939	0.727440321	24	22.05059256	0.138219738
ENSMUSG00000049107	Ntf3	neurotrophin 3 [Source:MGI Symbol;Acc:MGI:97380]	0.336176	0.444553	0.402292	0.541297	0.585692	0.606112	0.656862	0.547185	0.551676	0.310885	0.463303	0.488289	0.552473	0.54006	0.843639218	0.966018836	24	16	24	0.638312673	0.877195603	24	22.15539133	0.037316277
ENSMUSG00000049112	Oxtr	oxytocin receptor [Source:MGI Symbol;Acc:MGI:109147]	0.84503	0.769978	0.544635	0.603416	0.687251	0.680157	1.11467	0.66999	0.883297	0.638734	0.653648	0.656148	0.745156	0.583115	0.066380001	0.42386701	4	12	24	0.205669467	0.768040178	24	23.86355198	0.079197407
ENSMUSG00000049115	Agtr1a	angiotensin II receptor, type 1a [Source:MGI Symbol;Acc:MGI:87964]	8.19667	6.56345	6.09653	8.18608	5.72398	7.78286	7.17986	7.9068	6.27117	10.4168	6.26324	8.40437	7.4999	7.81562	0.92655264	0.966018836	24	12	24	0.971351894	0.993017774	24	21.91955138	0.020125474
ENSMUSG00000049119	Fam110b	family with sequence similarity 110, member B [Source:MGI Symbol;Acc:MGI:1916593]	10.2802	7.42548	8.32003	8.74507	6.28006	8.53916	8.50486	8.57384	7.80847	10.4812	7.71566	10.0575	10.0358	9.06598	0.81705122	0.966018836	4	16	24	0.611286529	0.865079333	24	0.699237199	0.068731568

ENSMUSG00000049122	Frmd3	FERM domain containing 3 [Source:MGI Symbol;Acc:MGI:2442466]	34.3231	34.2466	34.0261	34.2749	35.3354	32.4155	30.7592	31.0442	34.4533	36.1024	34.3772	33.7492	35.1136	35.2178	0.622293809	0.966018836	20	4	24	0.238011647	0.768040178	24	12.07647753	0.041861745
ENSMUSG00000049124	Gm8186	predicted gene 8186 [Source:MGI Symbol;Acc:MGI:3643110]	55.5942	64.1852	58.7397	71.1887	68.275	52.7798	62.1303	53.2415	65.8473	41.9912	59.2232	44.1769	48.8167	55.0254	0.401437339	0.933249185	20	4	24	0.639604148	0.877957092	24	9.815963126	0.074055538
ENSMUSG00000049130	C5ar1	complement component 5a receptor 1 [Source:MGI Symbol;Acc:MGI:88232]	1.32499	1.38243	1.68183	1.28062	1.1703	1.17265	1.71796	1.28404	1.49678	0.92493	1.27598	1.27366	1.24731	0.796351	0.43280837	0.966018836	12	8	24	0.804711204	0.937484836	24	1.714175542	0.036535745
ENSMUSG00000049134	Nrap	nebulin-related anchoring protein [Source:MGI Symbol;Acc:MGI:1098765]	1.29834	3.5952	5.80309	6.01648	3.94724	5.78126	2.98909	10.971	6.53306	12.8248	2.82215	3.27639	6.08555	4.56559	0.144748194	0.589637022	16	4	24	0.074530681	0.683293901	24	9.385424561	0.554550559
ENSMUSG00000049148	Plcxd3	phosphatidylinositol-specific phospholipase C, X domain containing 3 [Source:MGI Symbol;Acc:MGI:2442605]	8.52043	10.1121	9.093	11.1425	10.4503	9.31871	9.49075	8.41131	12.0627	6.17478	11.1924	12.3373	11.4144	12.0602	0.831272156	0.966018836	20	20	24	0.907109248	0.974606327	24	22.34691353	0.042445602
ENSMUSG00000049154	Fam183b	family with sequence similarity 183, member B [Source:MGI Symbol;Acc:MGI:1922679]	1.03182	3.54262	2.07273	3.08286	2.53223	1.69413	3.95216	1.25153	3.00666	1.66027	2.29371	3.48414	1.5472	3.27357	0.710938687	0.966018836	8	20	24	0.901558285	0.972353488	24	10.00805583	0.077504733
ENSMUSG00000049164	Zfp518a	zinc finger protein 518A [Source:MGI Symbol;Acc:MGI:1919922]	3.85707	4.70242	4.56264	4.41948	5.19163	3.68217	4.41571	4.7917	5.24802	3.6694	5.43347	4.16496	4.22067	4.17627	0.195343269	0.673387182	20	4	24	0.524370674	0.822002818	24	10.47718532	0.068031638
ENSMUSG00000049173	Myoz3	myozenin 3 [Source:MGI Symbol;Acc:MGI:2179296]	0.314317	1.21266	1.26327	1.91217	1.12978	1.783	1.09393	3.3813	2.46743	3.73616	1.06952	1.40329	1.5482	1.09	0.432054949	0.966018836	16	12	24	0.126265067	0.739868076	24	9.9505636	0.354958428
ENSMUSG00000049176	Frmpd4	FERM and PDZ domain containing 4 [Source:MGI Symbol;Acc:MGI:3042378]	6.4162	6.35356	5.49689	6.24366	5.74559	5.76796	6.41977	5.52458	5.36593	6.37394	5.73403	6.05593	6.37728	6.81443	0.035806379	0.331814682	4	8	24	0.435333527	0.805393068	24	0.261687219	0.045172394
ENSMUSG00000049184	Purg	purine-rich element binding protein G [Source:MGI Symbol;Acc:MGI:1922279]	14.0407	14.1881	12.0807	13.1539	15.0229	12.8904	14.3163	12.0562	14.2561	12.0293	14.6883	14.2486	14.82	13.5498	0.23919737	0.741580974	20	20	24	0.234999577	0.768040178	24	20.53569824	0.070983064
ENSMUSG00000049191	Rtl5	retrotransposon Gag like 5 [Source:MGI Symbol;Acc:MGI:3045324]	5.70373	5.14909	4.99092	3.19545	4.59224	5.45144	4.93709	4.53108	6.40037	5.3376	4.608	4.77939	4.3896	5.8305	0.814077113	0.966018836	12	4	24	0.450020454	0.805393068	24	3.336804987	0.095962362
ENSMUSG00000049225	Pdp1	pyruvate dehyrdogenase phosphatase catalytic subunit 1 [Source:MGI Symbol;Acc:MGI:2685870]	20.6704	23.3158	21.4138	20.4878	24.9737	22.9443	21.2485	21.8354	21.632	21.1278	23.4018	21.2314	21.77	21.59	0.676344318	0.966018836	20	20	24	0.470920242	0.805393068	24	17.31360078	0.037542711
ENSMUSG00000049230	Gm9833	predicted gene 9833 [Source:MGI Symbol;Acc:MGI:3641855]	12.8827	12.434	11.1218	10.0503	11.9402	11.7949	12.6052	11.1642	11.4666	10.6279	12.4978	13.1988	12.9012	12.5485	7.56E-04	0.071344007	4	12	24	0.002781668	0.414500244	24	22.7081033	0.116033871
ENSMUSG00000049232	Tigd2	tigger transposable element derived 2 [Source:MGI Symbol;Acc:MGI:1915390]	4.88441	5.32085	5.05898	4.78621	4.67594	5.00993	4.83212	5.2677	5.51768	5.42191	5.84629	5.49172	5.09802	5.67988	0.858269825	0.966018836	8	20	24	0.414871368	0.800066997	24	8.024448011	0.034462845
ENSMUSG00000049233	Apoo-ps	apolipoprotein O, pseudogene [Source:MGI Symbol;Acc:MGI:3649039]	61.4894	60.7671	61.5556	64.6981	57.5567	63.311	60.9085	63.4242	69.1662	61.3787	68.7979	61.4651	69.0292	66.5305	0.880631923	0.966018836	12	8	24	0.557570155	0.839231952	24	6.997252356	0.02922924
ENSMUSG00000049235	Gm7324	predicted gene 7324 [Source:MGI Symbol;Acc:MGI:3646999]	1.12351	0.693211	1.04175	0.642304	0.775243	0.767611	0.678118	0.85904	1.03776	0.795242	0.932111	0.612404	1.1196	1.08427	0.495929335	0.966018836	4	20	24	0.333291294	0.769685083	24	4.89421645	0.082124018
ENSMUSG00000049241	Hcar1	hydrocarboxylic acid receptor 1 [Source:MGI Symbol;Acc:MGI:2441671]	0.86188	0.658268	0.67602	0.673073	0.639945	0.717236	0.402957	1.03724	0.694453	0.652861	0.928519	0.812075	0.967247	0.871291	0.801995186	0.966018836	8	8	24	0.809098545	0.939184036	24	1.8367013	0.033278542
ENSMUSG00000049252	Lrp1b	low density lipoprotein-related protein 1B [Source:MGI Symbol;Acc:MGI:2151136]	2.48861	2.90341	3.40144	2.63871	2.4451	2.96855	2.62489	2.45779	2.45828	2.50499	2.34313	2.31787	1.89338	2.7333	0.446069941	0.966018836	12	8	24	0.418362039	0.802974112	24	8.149208995	0.063165562
ENSMUSG00000049265	Kcnk3	potassium channel, subfamily K, member 3 [Source:MGI Symbol;Acc:MGI:1100509]	3.57116	2.78881	3.87207	5.25605	3.61347	3.72061	3.5237	4.20429	3.69204	5.37257	4.19242	3.97842	4.61939	5.02615	0.47028201	0.966018836	16	12	24	0.173663554	0.768040178	24	11.47657811	0.123561035
ENSMUSG00000049281	Scn3b	sodium channel, voltage-gated, type III, beta [Source:MGI Symbol;Acc:MGI:1918882]	30.3283	27.4874	27.2768	28.2082	28.4935	32.0494	27.6519	27.4062	24.9776	31.3936	25.6322	30.4082	30.674	31.824	0.042880436	0.356048751	24	12	24	0.348396266	0.779121086	24	21.45476432	0.063325217
ENSMUSG00000049285	Mblac1	metallo-beta-lactamase domain containing 1 [Source:MGI Symbol;Acc:MGI:2679717]	2.04432	1.85032	1.50776	1.39454	1.50824	1.34113	1.26935	1.61605	1.42141	1.96837	1.83822	2.42536	1.91279	2.08749	0.832506215	0.966018836	8	4	24	0.752062079	0.91939047	24	23.31693232	0.049837113
ENSMUSG00000049287	Iba57	IBA57 homolog, iron-sulfur cluster assembly [Source:MGI Symbol;Acc:MGI:3041174]	1.73906	1.48858	1.73597	2.26134	1.89753	1.79395	1.68685	2.01548	1.65824	2.08287	1.82535	1.80578	1.99565	2.15132	0.351315142	0.870418653	16	20	24	0.427773002	0.805393068	24	12.15606006	0.049470253
ENSMUSG00000049288	Lix1l	Lix1-like [Source:MGI Symbol;Acc:MGI:3036267]	9.2177	10.2958	10.6786	9.37337	10.501	9.32374	10.0268	10.4638	10.4522	10.5958	8.99846	9.77777	9.51773	9.61266	0.118459043	0.541945448	12	12	24	0.129586573	0.740162193	24	8.104237967	0.059938214
ENSMUSG00000049295	Zfp219	zinc finger protein 219 [Source:MGI Symbol;Acc:MGI:1917140]	13.8389	13.5396	12.9404	12.5227	13.0312	12.144	14.756	15.0641	16.8066	17.3342	17.4394	17.3007	16.4339	14.9803	0.74401906	0.966018836	20	12	24	0.915766015	0.976747021	24	9.511670924	0.020481416
ENSMUSG00000049299	Trappc1	trafficking protein particle complex 1 [Source:MGI Symbol;Acc:MGI:1098727]	98.9624	105.706	107.917	97.0274	111.275	110.807	91.2104	94.889	102.541	87.3596	110.66	94.5585	103.5	104.325	0.951523604	0.966018836	20	8	24	0.856197978	0.957451799	24	17.49769792	0.024987166
ENSMUSG00000049300	Prmt6	protein arginine N-methyltransferase 6 [Source:MGI Symbol;Acc:MGI:2139971]	5.40435	5.73741	5.27199	4.97697	5.20587	5.69846	5.40547	5.85751	5.86246	6.37664	5.57669	6.74988	6.47316	6.48786	0.774195442	0.966018836	8	8	24	0.285967135	0.768040178	24	1.866767272	0.043062655
ENSMUSG00000049303	Syt12	synaptotagmin XII [Source:MGI Symbol;Acc:MGI:2159601]	3.3549	3.19191	3.74729	3.26821	2.95331	3.77713	3.79599	3.40231	2.90216	3.3274	3.25622	3.56152	2.79175	3.46807	0.9143748	0.966018836	24	20	24	0.76545978	0.924119887	24	22.52368783	0.031599096
ENSMUSG00000049305	Ccdc71	coiled-coil domain containing 71 [Source:MGI Symbol;Acc:MGI:1919704]	7.40474	7.05184	7.25231	6.51888	6.94381	8.05719	6.78987	8.94265	6.93319	7.65347	6.46096	7.33193	7.17212	6.90268	0.91241026	0.966018836	24	20	24	0.573538782	0.847005958	24	1.704220287	0.044938703
ENSMUSG00000049307	Fut4	fucosyltransferase 4 [Source:MGI Symbol;Acc:MGI:95594]	0.637223	0.545984	0.627596	0.514006	0.640573	0.566864	0.507166	0.600519	0.561225	0.601129	0.671313	0.609333	0.56366	0.493087	0.059513104	0.406067671	20	12	24	0.241476158	0.768040178	24	16.32908438	0.031912072
ENSMUSG00000049313	Sorl1	sortilin-related receptor, LDLR class A repeats-containing [Source:MGI Symbol;Acc:MGI:1202296]	37.1242	29.2208	35.2348	32.4412	30.4956	36.7205	29.6496	29.4039	25.1255	39.1617	25.9694	34.0471	29.8583	34.7756	0.893609703	0.966018836	24	12	24	0.8755174	0.965124369	24	19.39615095	0.040210943
ENSMUSG00000049321	Zfp2	zinc finger protein 2 [Source:MGI Symbol;Acc:MGI:99167]	9.06895	10.6095	9.91	8.57177	10.4344	9.58021	9.48551	9.81302	9.71197	8.42715	10.8079	9.68723	10.1498	10.667	0.777666236	0.966018836	8	8	24	0.653787362	0.883343578	24	1.112906758	0.035488786
ENSMUSG00000049323	Smcr8	Smith-Magenis syndrome chromosome region, candidate 8 homolog (human) [Source:MGI Symbol;Acc:MGI:2444720]	5.62878	4.87089	5.10493	5.7485	5.40672	5.6618	5.33952	5.38468	4.71738	5.84087	5.10811	5.31934	5.51827	5.34863	0.187699199	0.662239322	16	20	24	0.3534097	0.780711577	24	18.25347516	0.043080471
ENSMUSG00000049327	Kmt5a	lysine methyltransferase 5A [Source:MGI Symbol;Acc:MGI:1915206]	40.6073	29.7432	29.8425	35.5448	36.9138	36.1661	40.6661	33.0023	30.4836	41.0381	36.0521	35.953	35.4799	32.7426	0.040741957	0.345521986	20	16	24	0.059928844	0.658304792	24	18.86252449	0.135926196
ENSMUSG00000049336	Tenm2	teneurin transmembrane protein 2 [Source:MGI Symbol;Acc:MGI:1345184]	1.96737	2.14905	2.77275	2.14717	2.04285	2.69383	2.19269	3.01805	2.53138	3.50727	1.70519	2.96398	1.8523	2.82547	0.417963425	0.963884303	8	12	24	0.419207339	0.803638254	24	8.243769858	0.114177647
ENSMUSG00000049339	Retreg2	reticulophagy regulator family member 2 [Source:MGI Symbol;Acc:MGI:2388278]	34.0901	28.777	32.124	33.47	31.7155	33.392	33.3225	32.199	29.3279	37.3641	29.0506	32.2508	31.1441	31.7491	0.850398351	0.966018836	16	20	24	0.780896249	0.929765633	24	16.57635262	0.027705905
ENSMUSG00000049354	Dcaf7	DDB1 and CUL4 associated factor 7 [Source:MGI Symbol;Acc:MGI:1919083]	22.7195	21.197	23.2403	22.9245	21.7846	23.1868	23.0544	22.4136	19.432	26.3871	19.9787	22.8992	21.8124	22.7826	0.915082018	0.966018836	16	16	24	0.958538942	0.989144435	24	15.97366761	0.011958836
ENSMUSG00000049396	Gemin4	gem nuclear organelle associated protein 4 [Source:MGI Symbol;Acc:MGI:2449313]	4.99571	3.07116	3.77574	3.41581	4.85641	4.17881	3.43765	4.00613	4.16957	4.10124	4.25923	3.56629	4.00085	3.24166	0.117050591	0.538362324	20	12	24	0.28294267	0.768040178	24	17.38918593	0.101064427
ENSMUSG00000049401	Ogfr	opioid growth factor receptor [Source:MGI Symbol;Acc:MGI:1919325]	12.7125	11.3535	12.8933	12.8557	12.2514	12.296	11.9374	13.0699	11.9993	12.6871	11.6621	11.4465	12.5134	12.184	0.832506215	0.966018836	16	4	24	0.572814562	0.846790202	24	8.489712698	0.025595484
ENSMUSG00000049404	Rarres1	retinoic acid receptor responder (tazarotene induced) 1 [Source:MGI Symbol;Acc:MGI:1924461]	24.7519	20.7843	19.0532	20.8482	20.7047	23.8269	20.3454	22.2937	19.5615	26.0923	23.3828	24.4169	27.1335	23.5359	0.143321297	0.585944911	24	12	24	0.2041388	0.768040178	24	20.76065009	0.092574578
ENSMUSG00000049409	Prokr1	prokineticin receptor 1 [Source:MGI Symbol;Acc:MGI:1929676]	7.75131	5.3813	5.40529	6.19215	6.30622	6.68282	6.49516	6.72479	4.17522	6.84779	6.26775	5.73854	6.11109	5.765	0.393470605	0.921010321	4	8	24	0.139541636	0.74956518	24	20.16315041	0.136041214
ENSMUSG00000049411	Tmem241	transmembrane protein 241 [Source:MGI Symbol;Acc:MGI:2442435]	3.82736	4.88842	4.98114	4.45739	5.20033	4.77653	4.19747	5.09766	5.18199	5.00011	4.49932	4.76833	5.52625	5.26103	0.892578749	0.966018836	8	20	24	0.268492102	0.768040178	24	8.254612054	0.065832204
ENSMUSG00000049414	Gm5417	predicted gene 5417 [Source:MGI Symbol;Acc:MGI:3648626]	11.9767	8.56559	11.0902	9.88549	7.19724	9.77284	9.74588	9.31102	9.50709	12.7523	11.3987	11.5152	10.8079	8.62848	0.310940564	0.820837787	16	16	24	0.891181808	0.969291345	24	16.71560848	0.039225853
ENSMUSG00000049420	Tmem200a	transmembrane protein 200A [Source:MGI Symbol;Acc:MGI:1924470]	14.5768	11.7105	12.5332	12.9344	10.7789	14.0836	11.9632	13.5217	9.87621	13.7849	11.867	13.5352	13.4803	12.9503	0.338172159	0.85758369	24	12	24	0.375182629	0.795216953	24	22.24075329	0.081082233
ENSMUSG00000049421	Zfp260	zinc finger protein 260 [Source:MGI Symbol;Acc:MGI:1347071]	28.3291	31.5602	32.584	33.9737	33.137	30.2281	31.402	28.6445	32.2379	26.952	35.039	32.355	33.5705	32.8365	0.784278077	0.966018836	20	8	24	0.763409867	0.92366044	24	14.77495415	0.030127649
ENSMUSG00000049422	Chchd10	coiled-coil-helix-coiled-coil-helix domain containing 10 [Source:MGI Symbol;Acc:MGI:2143558]	62.2279	66.8892	78.9006	96.2132	69.1154	85.6153	76.7417	105.698	74.5406	113.853	89.2125	77.5304	87.041	96.067	0.47028201	0.966018836	16	12	24	0.208568035	0.768040178	24	10.3892242	0.145024889
ENSMUSG00000049439	Cyp20a1	cytochrome P450, family 20, subfamily a, polypeptide 1 [Source:MGI Symbol;Acc:MGI:1925201]	11.8674	12.8268	13.3891	12.1937	12.778	12.7331	12.9105	13.3054	13.904	13.0649	13.0306	12.4713	12.0944	12.5253	0.016795343	0.253395174	12	16	24	0.111543842	0.724173789	24	9.206948769	0.045910957
ENSMUSG00000049470	Aff4	AF4/FMR2 family, member 4 [Source:MGI Symbol;Acc:MGI:2136171]	27.0424	25.6789	25.4035	26.8113	26.2864	24.7044	24.7703	22.8422	26.1466	22.0724	28.3207	25.9591	26.3925	25.8103	0.895493752	0.966018836	20	12	24	0.614764359	0.866687196	24	18.75231117	0.036790906
ENSMUSG00000049477	Gm8508	predicted gene 8508 [Source:MGI Symbol;Acc:MGI:3643536]	4.63332	5.07837	5.67484	3.83207	4.40562	3.7857	4.38817	4.61274	4.90143	3.3332	5.1616	3.53884	3.67797	5.16881	0.077301512	0.453790923	12	12	24	0.191227723	0.768040178	24	5.988986161	0.131614393
ENSMUSG00000049482	Ctu2	cytosolic thiouridylase subunit 2 [Source:MGI Symbol;Acc:MGI:1914215]	5.90801	5.59498	4.44504	6.01004	5.8572	6.35094	6.09684	6.22305	6.38019	5.68347	7.4182	7.80401	9.97366	7.62169	0.588682825	0.966018836	24	16	24	0.045266825	0.628927515	24	21.93529956	0.138794346
ENSMUSG00000049488	Tmem67	transmembrane protein 67 [Source:MGI Symbol;Acc:MGI:1923928]	2.47212	3.02641	2.83875	2.65134	2.62337	2.7415	2.922	2.68378	2.44407	2.29792	2.83994	2.8105	2.51791	2.76815	0.948582958	0.966018836	8	8	24	0.569775143	0.844900526	24	23.81088105	0.033436355
ENSMUSG00000049489	Ccnq	cyclin Q [Source:MGI Symbol;Acc:MGI:1916359]	8.3787	8.74951	8.82943	8.4263	8.64176	8.59701	7.67234	8.65287	9.71608	8.10455	9.8409	9.83409	10.7256	8.26928	0.860560779	0.966018836	20	20	24	0.904462335	0.973594617	24	18.44264456	0.020125792
ENSMUSG00000049493	Pls1	plastin 1 (I-isoform) [Source:MGI Symbol;Acc:MGI:104809]	5.64234	5.56992	5.25404	5.56067	4.57319	5.02754	5.60194	4.99459	4.40814	5.79922	5.03563	5.08079	4.50718	5.47318	0.890176313	0.966018836	16	4	24	0.910184815	0.974679582	24	4.932281235	0.018264731
ENSMUSG00000049502	Dtx3l	deltex 3-like, E3 ubiquitin ligase [Source:MGI Symbol;Acc:MGI:2656973]	3.6995	3.12011	3.41741	3.29169	3.66548	3.72213	3.52622	4.07082	3.09985	3.27089	3.46812	3.57362	3.51839	3.43853	0.027439001	0.308438477	24	12	24	0.145012847	0.753947259	24	21.60083841	0.062421097
ENSMUSG00000049504	Proser1	proline and serine rich 1 [Source:MGI Symbol;Acc:MGI:1919933]	4.92472	5.32981	5.15999	5.35993	5.04097	4.83801	4.46109	4.91942	5.45386	6.40352	4.86522	4.89227	5.12786	5.50201	0.018923071	0.256345714	16	8	24	0.020039028	0.565108098	24	9.795363956	0.100929324
ENSMUSG00000049511	Htr1b	5-hydroxytryptamine (serotonin) receptor 1B [Source:MGI Symbol;Acc:MGI:96274]	7.43711	5.78777	5.81114	5.86745	6.83148	6.61218	6.77972	6.13182	4.35397	6.25455	6.18633	5.8878	6.04226	6.11262	0.094000699	0.490854203	4	8	24	0.037961223	0.61140397	24	20.55208048	0.136065294
ENSMUSG00000049516	Spty2d1	SPT2 chromatin protein domain containing 1 [Source:MGI Symbol;Acc:MGI:2142062]	9.20439	7.68951	7.52492	8.08297	9.09807	8.30975	8.75356	8.12593	6.89046	7.87223	8.66127	7.63199	7.87536	7.66866	0.029825002	0.323426482	4	8	24	0.024457478	0.586541342	24	19.55706893	0.096020284
ENSMUSG00000049517	Rps23	ribosomal protein S23 [Source:MGI Symbol;Acc:MGI:1913725]	2.37043	2.34871	2.32905	2.2734	2.04577	2.63122	2.76341	2.28618	3.27013	1.9969	2.61049	2.14814	2.24535	2.14368	0.951523604	0.966018836	4	12	24	0.869999583	0.963168206	24	3.967064211	0.028964077
ENSMUSG00000049521	Cdc42ep1	CDC42 effector protein (Rho GTPase binding) 1 [Source:MGI Symbol;Acc:MGI:1929763]	8.25792	8.67674	9.05968	8.82166	10.5964	9.49948	11.4682	9.33791	9.98496	12.7177	8.7158	10.3464	10.2734	9.19035	0.92721845	0.966018836	16	16	24	0.699725651	0.900387881	24	14.89794314	0.048330318
ENSMUSG00000049526	Tmem202	transmembrane protein 202 [Source:MGI Symbol;Acc:MGI:1921143]	2.27225	2.70433	2.42503	1.8999	2.44148	2.56003	2.69344	2.14943	2.36867	2.7876	3.12779	3.28165	2.50107	3.1459	0.920620634	0.966018836	24	12	24	0.561021277	0.841531815	24	20.28691179	0.05665167
ENSMUSG00000049530	Clrn2	clarin 2 [Source:MGI Symbol;Acc:MGI:3646230]	2.08675	2.87076	1.99241	2.04252	2.43724	2.05053	2.17313	2.14139	2.05051	2.45477	1.88931	2.30479	2.84833	2.20579	0.691437565	0.966018836	8	4	24	0.600748361	0.860170376	24	1.292913136	0.051049568
ENSMUSG00000049532	Sall2	spalt like transcription factor 2 [Source:MGI Symbol;Acc:MGI:1354373]	5.38113	5.81538	7.10604	5.02675	5.47497	6.21662	6.45779	5.87614	5.18125	6.45846	4.5756	5.38262	4.80437	6.48139	0.745475226	0.966018836	8	12	24	0.436555517	0.805393068	24	5.483646946	0.080277349
ENSMUSG00000049536	Tceal1	transcription elongation factor A (SII)-like 1 [Source:MGI Symbol;Acc:MGI:2385317]	11.6345	11.648	11.3741	9.51559	9.82533	10.6048	9.5868	10.4599	11.3958	9.04969	10.7313	9.39036	10.0909	11.5886	0.124028266	0.552144108	8	8	24	0.105274122	0.717252017	24	4.262995992	0.088776163
ENSMUSG00000049550	Clip1	CAP-GLY domain containing linker protein 1 [Source:MGI Symbol;Acc:MGI:1928401]	16.5849	15.8669	15.1206	19.5189	18.5449	18.8603	16.1902	18.1939	17.1456	22.2904	17.0879	16.6486	17.6026	16.859	0.111887343	0.530777648	16	20	24	0.122668006	0.735333681	24	13.93991615	0.09932474
ENSMUSG00000049551	Fzd9	frizzled class receptor 9 [Source:MGI Symbol;Acc:MGI:1313278]	0.278861	0.586701	0.728378	0.578852	0.419823	0.551946	0.636274	1.08592	0.623601	0.736663	0.359452	0.46032	0.629556	0.729525	0.055738947	0.401074772	8	12	24	0.026527357	0.586541342	24	6.459260779	0.165287438
ENSMUSG00000049553	Polr1a	polymerase (RNA) I polypeptide A [Source:MGI Symbol;Acc:MGI:1096397]	6.68537	5.98282	5.68299	5.84393	5.68219	6.80005	6.53361	6.96961	5.25434	6.89926	5.22702	6.27947	5.84824	6.20042	0.341022016	0.86129475	24	20	24	0.317973065	0.768040178	24	23.68254543	0.069649635
ENSMUSG00000049555	Tmie	transmembrane inner ear [Source:MGI Symbol;Acc:MGI:2159400]	0.524952	1.04526	1.61954	0.709612	0.924061	0.745531	0.825181	0.728152	1.02704	0.752777	0.632101	1.1941	0.724223	0.820938	0.873337044	0.966018836	12	16	24	0.497968882	0.807927978	24	8.355393344	0.096115695
ENSMUSG00000049556	Lingo1	leucine rich repeat and Ig domain containing 1 [Source:MGI Symbol;Acc:MGI:1915522]	14.7304	10.5342	13.2744	11.7849	9.88267	12.4579	11.971	12.9856	10.2253	19.1082	9.92651	13.9566	12.9022	14.5523	0.784278077	0.966018836	4	16	24	0.869621131	0.963010037	24	4.517755522	0.051569755
ENSMUSG00000049562	Ap5b1	adaptor-related protein complex 5, beta 1 subunit [Source:MGI Symbol;Acc:MGI:2685808]	2.87187	2.06916	2.27123	1.79893	1.8762	2.04823	2.47524	2.85333	2.13961	3.12818	2.52393	2.72222	2.36676	2.3624	0.952127548	0.966018836	4	16	24	0.703553384	0.902058318	24	1.171489337	0.053056505
ENSMUSG00000049571	Cfap46	cilia and flagella associated protein 46 [Source:MGI Symbol;Acc:MGI:2444387]	0.377969	0.720757	1.39528	0.639013	0.34599	0.259336	0.834175	0.352577	0.573467	0.440773	0.514935	0.992471	0.216337	0.933774	0.745199643	0.966018836	12	16	24	0.439944231	0.805393068	24	7.40240771	0.148242514
ENSMUSG00000049577	Zfpm1	zinc finger protein, multitype 1 [Source:MGI Symbol;Acc:MGI:1095400]	1.88466	1.42596	2.50638	1.91351	1.49424	1.61494	1.97041	2.77954	1.94046	2.0398	1.63018	1.28538	2.12484	1.39593	0.390686537	0.915479136	12	12	24	0.282570644	0.768040178	24	6.812657452	0.13168711
ENSMUSG00000049580	Tsku	tsukushi, small leucine rich proteoglycan [Source:MGI Symbol;Acc:MGI:2443855]	1.1807	1.10064	1.22887	1.63844	2.04399	1.3056	1.22165	1.21894	1.99457	1.57579	1.2498	1.78828	1.06162	1.14489	0.010275722	0.19555351	16	16	24	0.043108908	0.625007356	24	13.80171264	0.168270176
ENSMUSG00000049583	Grm5	glutamate receptor, metabotropic 5 [Source:MGI Symbol;Acc:MGI:1351342]	0.706811	1.81903	2.95631	0.953549	1.00657	1.25465	1.80621	1.08203	1.99957	1.06124	0.927831	1.43942	0.506826	1.59652	0.10560184	0.518002936	12	8	24	0.215322897	0.768040178	24	7.222400885	0.249317641
ENSMUSG00000049588	Ccdc69	coiled-coil domain containing 69 [Source:MGI Symbol;Acc:MGI:1196234]	0.834675	0.689777	0.979487	1.14163	0.672997	1.05951	1.18052	1.44821	0.877622	0.915155	1.0149	1.35091	1.70781	1.18615	0.612331511	0.966018836	24	20	24	0.288192755	0.768040178	24	0.652080367	0.094127871
ENSMUSG00000049600	Zbtb45	zinc finger and BTB domain containing 45 [Source:MGI Symbol;Acc:MGI:2685003]	3.16586	2.30763	3.23391	3.03837	2.63476	2.83447	3.17096	2.90693	2.95811	4.32156	2.96769	3.38925	2.94537	3.50637	0.890235289	0.966018836	16	4	24	0.593054022	0.856634016	24	11.21867746	0.058430055
ENSMUSG00000049606	Zfp644	zinc finger protein 644 [Source:MGI Symbol;Acc:MGI:1277212]	16.2449	17.2895	15.9489	16.4404	16.9544	15.3182	16.3965	15.1554	15.5272	14.5123	18.3484	15.2989	14.9766	15.8996	0.823144114	0.966018836	20	4	24	0.69652787	0.89922881	24	16.59677575	0.027747981
ENSMUSG00000049612	Omg	oligodendrocyte myelin glycoprotein [Source:MGI Symbol;Acc:MGI:106586]	9.49274	12.6309	17.3227	10.0835	8.28512	11.3312	13.5484	9.52951	12.1692	11.0693	8.96449	11.1171	7.56427	13.0651	0.185796491	0.662239322	12	8	24	0.214960246	0.768040178	24	6.616069719	0.195924451
ENSMUSG00000049624	Slc17a5	solute carrier family 17 (anion/sugar transporter), member 5 [Source:MGI Symbol;Acc:MGI:1924105]	12.0153	9.90345	9.31465	10.5514	9.63198	10.7243	9.31539	10.5654	9.28056	11.5454	10.5984	12.013	10.6471	11.9382	0.085890569	0.466404237	24	12	24	0.580168191	0.85017573	24	20.92092811	0.050687455
ENSMUSG00000049625	Tifab	TRAF-interacting protein with forkhead-associated domain, family member B [Source:MGI Symbol;Acc:MGI:2385852]	1.66918	1.77514	1.84514	1.84948	1.72173	1.80682	1.56611	1.75374	1.79919	1.52357	1.30459	1.64721	1.65431	1.43559	0.669104912	0.966018836	12	8	24	0.720523617	0.908336488	24	6.430651905	0.02409575
ENSMUSG00000049630	C1ql3	C1q-like 3 [Source:MGI Symbol;Acc:MGI:2387350]	4.95379	4.63894	5.03652	4.50156	5.24937	4.2783	5.74264	4.58817	4.92757	4.77677	4.75902	5.01801	5.11161	4.19842	0.798309651	0.966018836	4	4	24	0.696674397	0.89922881	24	20.63455485	0.034879606
ENSMUSG00000049641	Vgll2	vestigial like family member 2 [Source:MGI Symbol;Acc:MGI:2447460]	0.293206	0.393298	0.493055	1.01292	0.861475	0.92691	0.390784	1.29224	0.893549	1.32041	0.69865	0.660753	0.974258	0.850564	0.192357053	0.669841558	16	12	24	0.157533618	0.76387796	24	11.5790338	0.16659322
ENSMUSG00000049643	2310022A10Rik	RIKEN cDNA 2310022A10 gene [Source:MGI Symbol;Acc:MGI:1913617]	3.75629	3.92675	4.57324	4.02224	3.98105	3.98891	3.8727	4.85385	4.66539	5.42061	3.80404	4.41868	3.69641	4.10794	0.018194955	0.256345714	12	16	24	0.06267387	0.661961741	24	9.542321741	0.114877885
ENSMUSG00000049649	Gpr3	G-protein coupled receptor 3 [Source:MGI Symbol;Acc:MGI:101908]	1.11134	1.06389	0.941639	1.22149	0.991005	0.972215	1.22341	1.72927	0.702098	1.10447	1.3805	0.941359	1.11181	0.751788	0.893609703	0.966018836	4	8	24	0.862265333	0.959779249	24	20.41819449	0.039364468
ENSMUSG00000049657	Zbtb5	zinc finger and BTB domain containing 5 [Source:MGI Symbol;Acc:MGI:1924601]	3.04682	2.60043	2.47188	2.9217	3.17178	2.99369	3.01959	3.06845	2.77001	3.06755	2.66172	2.99107	2.88454	2.77811	0.455507782	0.966018836	24	12	24	0.22320323	0.768040178	24	19.38253871	0.053175831
ENSMUSG00000049658	Bdp1	B double prime 1, subunit of RNA polymerase III transcription initiation factor IIIB [Source:MGI Symbol;Acc:MGI:1347077]	6.55973	6.70226	6.53366	7.02441	6.93876	6.33845	7.08024	5.80271	6.38921	5.30959	7.43914	6.40976	6.48046	6.16399	0.883024237	0.966018836	20	12	24	0.400730868	0.799546792	24	18.75847739	0.056323203
ENSMUSG00000049659	Aftph	aftiphilin [Source:MGI Symbol;Acc:MGI:1923012]	26.2042	28.5295	26.6035	27.5568	25.6353	26.4188	24.5358	23.5324	24.1743	24.753	26.945	26.0882	26.4525	25.4896	0.847846021	0.966018836	20	12	24	0.681084023	0.893456358	24	17.79063809	0.024610083
ENSMUSG00000049670	Morn4	MORN repeat containing 4 [Source:MGI Symbol;Acc:MGI:2449568]	27.7132	22.0274	21.2267	23.041	21.7641	24.8714	22.7662	23.6318	19.6103	26.0678	21.8826	25.7833	24.7127	24.5169	0.0444686	0.365196805	24	12	24	0.175348415	0.768040178	24	21.62734773	0.093583608
ENSMUSG00000049672	Zbtb14	zinc finger and BTB domain containing 14 [Source:MGI Symbol;Acc:MGI:1195345]	4.64849	3.93889	3.87303	4.65082	4.34463	4.56831	4.21584	3.50888	3.77052	4.86118	4.35205	4.33808	3.77718	4.222	0.011964854	0.213452992	16	20	24	0.042529299	0.623698598	24	16.59754413	0.099242673
ENSMUSG00000049676	Catsperg1	cation channel sperm associated auxiliary subunit gamma 1 [Source:MGI Symbol;Acc:MGI:2443617]	0.68668	0.970398	1.49634	0.694142	0.794348	0.560034	0.88321	0.942726	0.971298	0.488409	0.520572	0.625033	0.575834	0.684897	0.013764091	0.238696502	12	4	24	0.039682004	0.616581176	24	6.283447011	0.163240627
ENSMUSG00000049680	Urgcp	upregulator of cell proliferation [Source:MGI Symbol;Acc:MGI:1919296]	11.186	9.11894	9.76921	9.31088	9.03342	11.4225	11.1236	11.1662	9.09297	13.1485	9.70091	11.519	11.0435	11.3415	0.300823756	0.809362476	24	20	24	0.480438375	0.805393068	24	22.80920538	0.06763472
ENSMUSG00000049686	Orai1	ORAI calcium release-activated calcium modulator 1 [Source:MGI Symbol;Acc:MGI:1925542]	5.68035	4.34057	4.98342	4.66758	4.80435	5.15058	4.25072	6.04074	4.52958	5.73545	5.42863	5.10581	4.86519	3.97327	0.867888967	0.966018836	24	8	24	0.5890202	0.853984101	24	16.68951721	0.060133272
ENSMUSG00000049687	Pheta2	PH domain containing endocytic trafficking adaptor 2 [Source:MGI Symbol;Acc:MGI:2443609]	0.582535	0.679009	0.717489	0.512991	0.611986	0.621839	0.543501	0.840681	0.77701	0.445441	0.603668	0.583624	0.824195	0.532855	0.676344318	0.966018836	12	4	24	0.273598912	0.768040178	24	3.641912505	0.064166985
ENSMUSG00000049717	Lig4	ligase IV, DNA, ATP-dependent [Source:MGI Symbol;Acc:MGI:1335098]	4.44591	4.20635	3.71894	3.64833	4.65285	4.47507	3.86035	4.07421	4.06139	3.84257	4.45805	4.76557	4.62859	3.99029	0.003383846	0.140598674	24	16	24	0.010347036	0.503967859	24	20.30082235	0.106275893
ENSMUSG00000049721	Gal3st1	galactose-3-O-sulfotransferase 1 [Source:MGI Symbol;Acc:MGI:1858277]	15.2234	12.4454	12.4814	12.3767	14.6865	11.7937	15.764	13.6907	14.5475	16.0522	13.5123	12.9592	14.6189	12.0408	0.949152198	0.966018836	4	4	24	0.951587094	0.988339231	24	19.27824578	0.018572622
ENSMUSG00000049728	Zfp668	zinc finger protein 668 [Source:MGI Symbol;Acc:MGI:2442943]	1.88345	1.7441	1.78804	2.49553	2.05929	2.00143	1.77827	1.8705	1.62759	2.48522	1.72046	2.10092	1.70889	1.94084	0.181442559	0.659020016	16	20	24	0.128813866	0.740162193	24	14.18873762	0.094537912
ENSMUSG00000049734	Trex1	three prime repair exonuclease 1 [Source:MGI Symbol;Acc:MGI:1328317]	7.7198	6.0239	7.44854	6.5927	7.27459	7.81206	6.81489	5.88069	6.61239	7.08622	5.56447	6.34383	6.27287	5.96733	0.481811994	0.966018836	24	8	24	0.515819634	0.817421075	24	17.98350214	0.052579409
ENSMUSG00000049739	Zfp646	zinc finger protein 646 [Source:MGI Symbol;Acc:MGI:3665412]	4.56974	4.19716	4.41757	4.7072	4.78254	4.48666	4.88485	5.05495	4.49548	4.93706	4.57299	4.70232	4.57174	4.58885	0.937921962	0.966018836	16	20	24	0.804363625	0.937432708	24	15.68073019	0.014603187
ENSMUSG00000049744	Arhgap15	Rho GTPase activating protein 15 [Source:MGI Symbol;Acc:MGI:1923367]	5.17155	6.84106	6.05509	5.82626	6.3913	5.48238	4.81376	5.48172	6.40375	4.25371	6.11434	4.97978	5.61487	5.335	0.46694635	0.966018836	12	16	24	0.732644893	0.912745963	24	8.948111477	0.048517342
ENSMUSG00000049751	Rpl36al	ribosomal protein L36A-like [Source:MGI Symbol;Acc:MGI:1913733]	67.2703	71.0262	64.3535	65.4996	65.8777	62.8451	69.5978	67.2543	84.6787	56.9642	83.6724	68.4639	79.3894	78.1143	0.722296281	0.966018836	8	8	24	0.586458456	0.853300537	24	3.605176308	0.059408658
ENSMUSG00000049755	Zfp672	zinc finger protein 672 [Source:MGI Symbol;Acc:MGI:2442105]	12.7791	15.8932	18.1513	14.7925	13.5456	13.766	16.0142	13.4779	16.7683	13.9538	15.8173	13.6045	14.8122	14.7859	0.245061943	0.741580974	12	12	24	0.15895971	0.765350752	24	8.053161933	0.100711051
ENSMUSG00000049760	Micos13	mitochondrial contact site and cristae organizing system subunit 13 [Source:MGI Symbol;Acc:MGI:2442174]	64.2733	71.5971	69.9657	70.1709	77.2439	63.6381	66.8717	68.9124	78.8665	59.5513	80.7808	69.9182	71.8703	68.6172	0.718086778	0.966018836	20	4	24	0.73044327	0.912198956	24	12.25239485	0.03746739
ENSMUSG00000049764	Zfp280b	zinc finger protein 280B [Source:MGI Symbol;Acc:MGI:1927865]	3.3585	2.99231	3.28093	3.26914	3.32509	3.45763	3.57845	3.56817	3.00664	3.87304	3.1892	3.62811	3.20052	3.5798	0.912686312	0.966018836	24	12	24	0.81023179	0.939224719	24	19.09535918	0.021140131
ENSMUSG00000049775	Tmsb4x	thymosin, beta 4, X chromosome [Source:MGI Symbol;Acc:MGI:99510]	2494.5	2730.55	2504.11	2331.4	2605.88	2349.75	2374.64	2340.95	2950.57	1948.49	3000.21	2651.76	2940.47	2708.03	0.829428469	0.966018836	12	4	24	0.764580025	0.92403577	24	0.859074	0.047743671
ENSMUSG00000049791	Fzd4	frizzled class receptor 4 [Source:MGI Symbol;Acc:MGI:108520]	2.23113	2.55235	4.14898	4.41173	3.19397	2.91277	2.88419	3.57103	3.6775	5.0847	3.0701	2.51083	2.97508	3.04749	3.11E-04	0.049770315	16	12	24	6.42E-04	0.296483534	24	10.65525474	0.301536298
ENSMUSG00000049792	Bag5	BCL2-associated athanogene 5 [Source:MGI Symbol;Acc:MGI:1917619]	23.9482	23.33	20.8926	19.8006	22.2577	22.479	22.1848	22.1544	20.519	21.2987	21.719	22.8068	23.6247	21.2485	7.94E-04	0.071344007	4	8	24	0.001132813	0.336500758	24	22.60255786	0.087335465
ENSMUSG00000049799	Lrrc19	leucine rich repeat containing 19 [Source:MGI Symbol;Acc:MGI:2140219]	0.695739	0.616008	0.428012	1.02972	0.581017	0.527111	0.610216	0.516703	0.486762	0.479435	0.637405	0.268736	0.34133	0.469871	0.90332508	0.966018836	16	20	24	0.515864948	0.817421075	24	12.94217633	0.059629014
ENSMUSG00000049800	Sertad2	SERTA domain containing 2 [Source:MGI Symbol;Acc:MGI:1931026]	8.65249	8.20293	8.03732	8.8971	8.06584	8.38009	8.10687	8.96058	7.3675	10.1968	8.08215	8.81461	8.9286	9.15368	0.722296281	0.966018836	8	4	24	0.992768641	0.998516607	24	11.42369396	0.004760982
ENSMUSG00000049804	Armcx4	armadillo repeat containing, X-linked 4 [Source:MGI Symbol;Acc:MGI:2147887]	6.0112	5.47349	6.48401	5.37946	5.3477	6.1337	6.0201	5.77418	4.93913	6.30212	4.71755	6.01919	5.59892	6.49612	0.881355613	0.966018836	24	20	24	0.477797293	0.805393068	24	2.148601694	0.056379106
ENSMUSG00000049807	Arhgap23	Rho GTPase activating protein 23 [Source:MGI Symbol;Acc:MGI:3697726]	8.96278	8.55185	8.99544	7.81845	8.10442	8.23614	9.73488	8.28619	9.71813	10.4806	8.784	10.2698	9.16245	9.83055	0.880247609	0.966018836	4	16	24	0.735274032	0.913704946	24	4.697924504	0.031452272

ENSMUSG00000049811	Fam161a	family with sequence similarity 161, member A [Source:MGI Symbol;Acc:MGI:1921123]	3.58615	5.02374	4.79968	3.25971	4.6896	4.46387	4.54579	4.55592	3.99892	4.10166	4.41832	3.64106	4.21913	3.94983	0.928780444	0.966018836	8	8	24	0.831003244	0.947661285	24	3.230792246	0.03441124
ENSMUSG00000049823	Zbtb12	zinc finger and BTB domain containing 12 [Source:MGI Symbol;Acc:MGI:88133]	1.79865	1.92617	2.13246	1.85841	1.79313	1.73296	2.05659	1.97384	1.73525	1.66616	1.67447	1.78208	1.83186	1.51775	0.455156031	0.966018836	12	8	24	0.562247791	0.842208433	24	3.086174629	0.028956944
ENSMUSG00000049832	Rbx1-ps	ring-box 1, pseudogene [Source:MGI Symbol;Acc:MGI:3710517]	172.97	179.258	174.225	166.218	187.588	170.858	181.38	176.992	180.12	152.425	185.696	170.737	189.277	169.865	0.47028201	0.966018836	4	12	24	0.448883115	0.805393068	24	22.9111207	0.040889235
ENSMUSG00000049858	Suox	sulfite oxidase [Source:MGI Symbol;Acc:MGI:2446117]	7.15819	6.86474	6.62769	8.00291	7.63789	7.89552	8.08702	8.355	6.95645	8.24482	7.53334	8.09671	8.17565	7.26838	0.745118544	0.966018836	24	12	24	0.269139636	0.768040178	24	18.87420325	0.056864501
ENSMUSG00000049866	Arl4c	ADP-ribosylation factor-like 4C [Source:MGI Symbol;Acc:MGI:2445172]	11.1053	9.31064	10.7911	8.96959	9.10911	10.4306	10.8358	9.74557	8.19879	11.1129	9.33564	9.90639	9.74772	10.8188	0.481530854	0.966018836	4	8	24	0.458289969	0.805393068	24	23.76430547	0.060136952
ENSMUSG00000049871	Nlrc3	NLR family, CARD domain containing 3 [Source:MGI Symbol;Acc:MGI:2444070]	0.734153	0.486641	0.55321	0.48279	0.617284	0.520532	0.473371	0.561644	0.687095	0.379171	0.710725	0.527412	0.659917	0.526692	0.919571723	0.966018836	20	20	24	0.741058088	0.915481114	24	21.89550227	0.030391185
ENSMUSG00000049872	Calhm5	calcium homeostasis modulator family member 5 [Source:MGI Symbol;Acc:MGI:2143897]	4.04666	4.98854	4.05491	4.25069	5.48036	4.14381	4.85593	4.3999	5.13554	3.59826	4.79693	4.58146	4.94598	4.24846	0.853736882	0.966018836	20	20	24	0.814206247	0.940708369	24	21.43944674	0.036316921
ENSMUSG00000049878	Rlf	rearranged L-myc fusion sequence [Source:MGI Symbol;Acc:MGI:1924705]	6.19466	7.32501	6.91862	6.65062	6.62942	6.45731	6.49549	6.26355	6.85958	5.92725	6.51498	6.95588	7.71387	6.4939	0.829428469	0.966018836	12	16	24	0.844338265	0.952833645	24	4.445052699	0.015699186
ENSMUSG00000049881	2810025M15Rik	RIKEN cDNA 2810025M15 gene [Source:MGI Symbol;Acc:MGI:1917203]	12.486	18.9954	12.4926	14.5988	13.6826	14.2289	14.32	7.47693	10.1119	13.8011	12.2525	12.2207	14.467	10.8621	0.886002469	0.966018836	4	4	24	0.515415292	0.81731462	24	18.49160628	0.126618263
ENSMUSG00000049882	Vcpkmt	valosin containing protein lysine (K) methyltransferase [Source:MGI Symbol;Acc:MGI:2684917]	8.58438	7.32449	7.53173	8.42312	7.34656	8.02458	7.6942	7.76253	8.86949	7.21171	8.42066	8.99448	7.82844	9.01131	0.872380105	0.966018836	24	20	24	0.913176717	0.975605762	24	23.41697568	0.016394606
ENSMUSG00000049891	Gm7984	predicted gene 7984 [Source:MGI Symbol;Acc:MGI:3645839]	29.02	31.784	24.9072	26.1661	29.5507	29.1757	28.0189	28.7487	31.0481	23.7721	33.2599	26.9545	28.9547	29.483	0.907000607	0.966018836	20	20	24	0.571504957	0.845931407	24	23.19152735	0.05405878
ENSMUSG00000049892	Rasd1	RAS, dexamethasone-induced 1 [Source:MGI Symbol;Acc:MGI:1270848]	1.09501	1.0446	1.84676	1.7336	1.62526	1.35752	1.30431	2.08887	1.40455	3.8694	1.41603	1.61838	2.11048	2.23511	0.696393581	0.966018836	16	12	24	0.140630679	0.75049778	24	10.31973208	0.209230135
ENSMUSG00000049904	Tmem17	transmembrane protein 17 [Source:MGI Symbol;Acc:MGI:2144205]	4.40169	5.23957	4.69716	3.89797	4.51588	4.08439	4.51544	4.60952	5.40434	4.19327	5.15635	5.59681	4.81981	4.90187	0.718086778	0.966018836	12	4	24	0.625583487	0.872249537	24	3.389708573	0.045008588
ENSMUSG00000049907	Rasl11b	RAS-like, family 11, member B [Source:MGI Symbol;Acc:MGI:1916189]	6.39193	7.01671	7.98557	7.46071	5.95485	6.58898	7.11196	6.6427	8.50377	9.50624	6.44271	7.25983	7.06518	6.80734	0.079180494	0.458678242	16	4	24	0.051324723	0.64767123	24	9.322943379	0.140601192
ENSMUSG00000049922	Slc35c1	solute carrier family 35, member C1 [Source:MGI Symbol;Acc:MGI:2443301]	8.71668	5.56179	5.55439	6.18222	7.30516	7.31828	6.43972	6.56607	4.83751	7.80147	6.97562	6.84852	6.32159	5.77807	0.01813461	0.256345714	20	16	24	0.02809711	0.590935816	24	18.97951485	0.185666726
ENSMUSG00000049929	Lpar4	lysophosphatidic acid receptor 4 [Source:MGI Symbol;Acc:MGI:1925384]	0.883348	1.27045	1.19406	0.784846	1.03932	0.960443	1.12782	1.16143	1.18366	0.884588	1.0537	1.1141	0.734114	1.00368	0.265774856	0.773510818	12	4	24	0.549243748	0.834838463	24	5.681211331	0.051333002
ENSMUSG00000049932	H2ax	H2A.X variant histone [Source:MGI Symbol;Acc:MGI:102688]	5.68477	4.52727	5.61917	5.45583	4.4876	4.95741	5.31619	5.12927	4.75373	7.03798	7.11941	7.68725	7.58187	6.68854	0.895493752	0.966018836	24	12	24	0.703378577	0.901975795	24	18.64180683	0.057914642
ENSMUSG00000049939	Lrrc4	leucine rich repeat containing 4 [Source:MGI Symbol;Acc:MGI:2182081]	3.63326	3.6593	4.30423	3.79822	3.35195	3.63027	4.49906	3.47399	3.49344	4.299	2.66986	3.72921	3.0343	3.54004	0.907068194	0.966018836	16	4	24	0.66561558	0.887035358	24	6.603093606	0.053892478
ENSMUSG00000049940	Pgrmc2	progesterone receptor membrane component 2 [Source:MGI Symbol;Acc:MGI:1918054]	22.4975	18.6159	18.7404	21.6571	19.2999	20.8407	20.8291	18.8986	18.1111	23.2627	18.9735	21.5636	21.1002	20.71	0.064165315	0.414867207	16	20	24	0.442941712	0.805393068	24	19.38470072	0.055844415
ENSMUSG00000049946	BC030500	cDNA sequence BC030500 [Source:MGI Symbol;Acc:MGI:2447770]	0.357502	0.43868	0.866383	0.284861	0.328729	0.915642	0.638081	0.461332	0.538564	0.527467	0.366693	0.69703	0.660378	0.619474	0.265774856	0.773510818	24	20	24	0.581447629	0.850792036	24	0.970174575	0.070849414
ENSMUSG00000049950	Rpp38	ribonuclease P/MRP 38 subunit [Source:MGI Symbol;Acc:MGI:2443607]	6.05564	5.62782	5.79953	5.29062	5.82206	5.49367	4.46708	4.53399	5.68053	5.70242	5.19209	5.18397	6.018	5.05698	0.916963526	0.966018836	12	20	24	0.750961964	0.91921305	24	14.11296371	0.033377758
ENSMUSG00000049957	Ccdc137	coiled-coil domain containing 137 [Source:MGI Symbol;Acc:MGI:1914541]	14.2723	10.7025	10.9632	10.2881	10.9518	12.4541	11.6563	11.7404	11.7236	12.0592	11.5495	12.2796	12.7513	12.3276	0.080006922	0.458678242	4	12	24	0.068790796	0.673096105	24	23.00349287	0.091285621
ENSMUSG00000049960	Mrps16	mitochondrial ribosomal protein S16 [Source:MGI Symbol;Acc:MGI:1913492]	27.8503	29.0028	27.7734	28.6168	31.9567	29.6622	26.5279	30.2314	30.6084	23.9587	29.9225	25.9774	29.6871	25.7516	0.896799496	0.966018836	20	20	24	0.905152247	0.97389694	24	18.00897213	0.019974712
ENSMUSG00000049969	Plekhf2	pleckstrin homology domain containing, family F (with FYVE domain) member 2 [Source:MGI Symbol;Acc:MGI:1919051]	4.73967	4.11736	4.94459	5.29169	5.11779	4.93469	4.59134	5.04056	5.00037	4.0495	5.22097	4.27514	4.61436	4.61758	0.44931827	0.966018836	20	8	24	0.572328578	0.846321055	24	13.86826985	0.041509532
ENSMUSG00000049971	Glt1d1	glycosyltransferase 1 domain containing 1 [Source:MGI Symbol;Acc:MGI:2442755]	3.0279	2.79081	2.54064	2.27359	2.31949	2.62809	2.66543	2.69716	2.56363	2.45062	2.16682	2.66306	2.23843	3.03327	7.90E-04	0.071344007	8	12	24	0.023546194	0.584402298	24	2.420549127	0.100415694
ENSMUSG00000049985	Ankrd55	ankyrin repeat domain 55 [Source:MGI Symbol;Acc:MGI:1924568]	6.31702	5.84342	5.93709	5.12638	5.34315	5.802	5.90821	4.95141	5.05469	5.78729	5.59453	6.11073	5.81059	5.8357	0.268242676	0.774280302	4	12	24	0.14441205	0.752803875	24	22.22111089	0.067620393
ENSMUSG00000049988	Lrrc25	leucine rich repeat containing 25 [Source:MGI Symbol;Acc:MGI:2445284]	0.728399	0.936937	0.742085	0.60243	0.803639	0.440884	0.777731	0.816631	1.05773	0.39031	0.925504	0.678646	0.989711	0.548942	0.665691261	0.966018836	12	4	24	0.616210894	0.867514503	24	3.436496628	0.066080604
ENSMUSG00000049999	Ppp1r3d	protein phosphatase 1, regulatory subunit 3D [Source:MGI Symbol;Acc:MGI:1917664]	1.82301	1.41505	1.88719	2.04564	1.75804	1.72697	1.80824	1.93747	1.44935	2.35722	2.17617	1.90047	2.08956	1.95063	0.94791825	0.966018836	16	20	24	0.364110158	0.788741571	24	14.81527034	0.063827469
ENSMUSG00000050002	Idnk	idnK gluconokinase homolog (E. coli) [Source:MGI Symbol;Acc:MGI:1922981]	26.1307	22.2423	24.9419	24.1487	25.101	27.1932	25.3508	26.7404	24.846	22.2685	24.6647	24.4546	24.2447	22.056	0.622132989	0.966018836	24	16	24	0.245138299	0.768040178	24	20.53700167	0.057599629
ENSMUSG00000050010	Shisa3	shisa family member 3 [Source:MGI Symbol;Acc:MGI:3041225]	5.33492	6.78759	5.60525	5.37588	6.5142	5.10662	5.59157	6.16715	6.56937	3.76348	6.02295	5.78911	5.42703	5.91844	0.27817759	0.789849983	8	20	24	0.6284983	0.872844283	24	2.953431667	0.066217196
ENSMUSG00000050017	Pitpnb	phosphatidylinositol transfer protein, beta [Source:MGI Symbol;Acc:MGI:1927542]	22.93	22.9703	23.3981	22.8273	24.6422	23.1862	24.0796	21.9285	23.4666	24.3207	21.5762	24.9677	25.1144	23.3322	0.904690479	0.966018836	4	4	24	0.707058684	0.903240808	24	20.09284654	0.021025131
ENSMUSG00000050022	Amz1	archaelysin family metallopeptidase 1 [Source:MGI Symbol;Acc:MGI:2442258]	1.68231	1.06636	1.24096	1.22774	1.44511	1.21915	0.979461	1.39613	1.14002	0.937072	1.58307	1.37783	1.30995	1.2654	0.503972924	0.966018836	20	24	24	0.39526008	0.799546792	24	19.83954535	0.074764876
ENSMUSG00000050029	Rap2c	RAP2C, member of RAS oncogene family [Source:MGI Symbol;Acc:MGI:1919315]	25.8547	23.7092	23.9409	24.0441	23.411	23.5161	23.791	21.9593	20.5293	23.2848	23.9842	20.0423	20.8114	23.3072	0.844448841	0.966018836	20	4	24	0.899783848	0.971554188	24	16.8508201	0.014407302
ENSMUSG00000050043	Tmx2	thioredoxin-related transmembrane protein 2 [Source:MGI Symbol;Acc:MGI:1914208]	72.0145	73.8257	69.4439	70.2318	71.0223	73.8106	67.7308	69.2213	71.059	64.3633	73.7375	70.5073	74.3655	73.8902	0.664568268	0.966018836	8	8	24	0.368715612	0.791761501	24	23.05075001	0.032290581
ENSMUSG00000050052	Tdrp	testis development related protein [Source:MGI Symbol;Acc:MGI:1919398]	15.2858	13.6351	13.3124	13.4123	11.6137	15.2752	12.0773	13.9346	10.6172	16.3612	12.125	15.7381	12.3927	15.2876	0.389563591	0.966018836	24	20	24	0.700812077	0.900900643	24	22.54075341	0.068592964
ENSMUSG00000050063	Klk6	kallikrein related-peptidase 6 [Source:MGI Symbol;Acc:MGI:1343166]	1.39634	5.41429	8.34539	2.39176	3.29146	4.28289	5.30995	4.38678	6.28749	2.19706	2.34414	4.88901	1.25076	3.86245	0.187699199	0.662239322	12	4	24	0.436801267	0.805393068	24	6.832791989	0.315160251
ENSMUSG00000050064	Zfp697	zinc finger protein 697 [Source:MGI Symbol;Acc:MGI:2139736]	1.50435	1.46911	2.10475	2.0441	1.54068	1.57323	1.78289	1.37797	1.24003	1.35538	2.09446	1.57584	1.60236	1.39981	0.477618579	0.966018836	20	12	24	0.406945058	0.799546792	24	15.75533505	0.077240146
ENSMUSG00000050069	Grem2	gremlin 2, DAN family BMP antagonist [Source:MGI Symbol;Acc:MGI:1344367]	0.94986	1.58603	1.91162	1.31368	1.10733	1.40197	1.62081	1.51997	1.74004	1.45871	0.923832	1.29589	1.01127	1.81158	0.010153564	0.194720062	12	8	24	0.023662295	0.584818005	24	6.747741534	0.193165422
ENSMUSG00000050071	Bex1	brain expressed X-linked 1 [Source:MGI Symbol;Acc:MGI:1328321]	11.4653	10.9247	14.1879	11.5962	8.22267	11.5908	11.6499	9.53609	10.4815	10.7408	8.04985	9.11702	8.98427	11.9463	0.328944792	0.847277612	12	8	24	0.154748282	0.760257003	24	5.795329122	0.141853094
ENSMUSG00000050079	Rspry1	ring finger and SPRY domain containing 1 [Source:MGI Symbol;Acc:MGI:1914860]	14.323	14.0903	14.2152	13.6364	13.7384	13.8858	12.6181	13.5865	12.9362	13.1198	14.3836	14.0402	13.8997	14.0765	0.860560779	0.966018836	20	20	24	0.757277045	0.921562129	24	20.51064515	0.016465962
ENSMUSG00000050088	1600012H06Rik	RIKEN cDNA 1600012H06 gene [Source:MGI Symbol;Acc:MGI:1915162]	16.2414	16.8599	16.2538	15.4263	16.2529	15.4065	15.2628	16.5254	15.6697	14.9954	15.5408	16.5662	15.5095	15.8663	0.953350544	0.966018836	8	8	24	0.821054848	0.943551411	24	0.836268514	0.01220105
ENSMUSG00000050103	Agmo	alkylglycerol monooxygenase [Source:MGI Symbol;Acc:MGI:2442495]	6.83113	7.18004	7.86068	8.17187	6.80712	4.96182	7.16272	5.45022	8.38224	6.72281	8.2434	6.17874	6.08071	6.68875	0.1321346	0.567728672	12	12	24	0.093019523	0.71175955	24	10.41216362	0.156332418
ENSMUSG00000050105	Fam110d	family with sequence similarity 110, member D [Source:MGI Symbol;Acc:MGI:1919940]	2.14012	1.21816	1.13713	1.15869	0.5601	0.955879	1.95492	1.50344	1.78913	1.19367	1.00287	1.5709	1.47676	1.52374	0.088810267	0.192760771	4	16	24	0.031718401	0.604316769	24	2.553845709	0.239981225
ENSMUSG00000050121	Opalin	oligodendrocytic myelin paranodal and inner loop protein [Source:MGI Symbol;Acc:MGI:2657025]	0.687067	2.79403	4.41649	1.35877	0.82027	1.68798	2.26813	1.56798	3.1203	1.37199	0.75354	1.83637	0.683739	3.1945	0.059650003	0.406067671	12	8	24	0.048993946	0.639929063	24	6.554503634	0.492977623
ENSMUSG00000050122	Vwa3b	von Willebrand factor A domain containing 3B [Source:MGI Symbol;Acc:MGI:1918103]	0.540551	0.40211	0.226227	0.680601	0.524212	0.506206	0.899958	0.688959	0.615001	0.509258	0.721347	0.493443	0.717384	0.608663	0.953609935	0.966018836	4	8	24	0.615118741	0.866892532	24	21.82555163	0.056290332
ENSMUSG00000050132	Sarm1	sterile alpha and HEAT/Armadillo motif containing 1 [Source:MGI Symbol;Acc:MGI:2136419]	7.68612	6.34189	7.08851	6.78697	7.15368	8.33235	7.85343	7.06297	5.00952	9.52113	6.629	7.20265	6.2001	7.09995	0.601647512	0.966018836	24	12	24	0.437789789	0.805393068	24	18.44678146	0.097596337
ENSMUSG00000050138	Kcnk12	potassium channel, subfamily K, member 12 [Source:MGI Symbol;Acc:MGI:2684043]	1.48907	0.757826	0.929378	0.807623	0.708364	0.937111	1.12398	0.924535	0.959158	1.82229	1.64748	1.40485	1.49817	1.00617	0.649148181	0.966018836	4	4	24	0.589450669	0.854256	24	18.92461774	0.088490165
ENSMUSG00000050141	Fam205c	family with sequence similarity 205, member C [Source:MGI Symbol;Acc:MGI:2679716]	3.073	3.35651	2.08714	3.06682	2.82093	2.89583	1.84132	2.90532	2.50978	4.30266	1.79576	3.52079	2.97218	3.05827	0.933543173	0.966018836	8	12	24	0.98033207	0.995255072	24	9.788139821	0.021301685
ENSMUSG00000050144	Slc25a44	solute carrier family 25, member 44 [Source:MGI Symbol;Acc:MGI:2444391]	9.57742	7.97964	9.53656	10.3666	8.30259	9.81253	8.94931	9.4972	7.86466	10.5978	8.67846	9.2671	8.98068	9.21368	0.928892845	0.966018836	16	16	24	0.768039049	0.925197875	24	14.68246503	0.03336024
ENSMUSG00000050147	F2rl3	coagulation factor II (thrombin) receptor-like 3 [Source:MGI Symbol;Acc:MGI:1298207]	1.62637	0.879989	0.841783	1.37729	1.10441	1.15897	1.46907	1.27954	1.15265	1.05649	1.23196	0.962408	1.26961	0.89559	0.286642595	0.793714531	4	8	24	0.381636977	0.799546792	24	20.90496603	0.084616069
ENSMUSG00000050148	Ubqln2	ubiquilin 2 [Source:MGI Symbol;Acc:MGI:1860283]	22.9007	19.2294	20.8924	19.2291	18.8801	22.4267	21.9034	20.8969	17.6355	27.9786	18.5011	20.1924	17.8048	21.4314	0.916746631	0.966018836	4	16	24	0.994635691	0.998755922	24	16.51249959	0.007126097
ENSMUSG00000050164	Mchr1	melanin-concentrating hormone receptor 1 [Source:MGI Symbol;Acc:MGI:2180756]	1.09465	1.3531	1.68058	2.11859	1.70653	1.69608	1.19459	1.35244	1.87325	2.20754	1.28215	1.48945	1.06441	1.20157	9.36E-06	0.009754434	16	12	24	9.99E-04	0.336500758	24	12.20349034	0.231708289
ENSMUSG00000050174	Nudt6	nudix (nucleoside diphosphate linked moiety X)-type motif 6 [Source:MGI Symbol;Acc:MGI:2387618]	2.54719	2.95547	2.36053	3.79755	3.96404	2.895	2.62504	2.656	3.17063	3.5159	2.70504	2.8028	3.22855	3.49051	0.487714334	0.966018836	16	12	24	0.231543287	0.768040178	24	12.81170027	0.108613198
ENSMUSG00000050188	Lsm10	U7 snRNP-specific Sm-like protein LSM10 [Source:MGI Symbol;Acc:MGI:2151045]	8.64882	8.28132	5.76542	8.88433	9.81288	8.84345	7.02513	9.11016	7.32607	7.07247	9.63243	6.7623	7.54301	7.85144	0.616047374	0.966018836	20	16	24	0.423635569	0.805393068	24	18.01786231	0.101750501
ENSMUSG00000050192	Eif5a2	eukaryotic translation initiation factor 5A2 [Source:MGI Symbol;Acc:MGI:1933735]	73.0569	70.543	64.6607	69.6959	68.1267	65.4165	67.3584	62.4134	62.7152	63.7319	74.2852	67.0004	68.5951	72.1736	0.962348911	0.967745676	4	8	24	0.492611589	0.805558817	24	20.64853736	0.039043013
ENSMUSG00000050199	Lgr4	leucine-rich repeat-containing G protein-coupled receptor 4 [Source:MGI Symbol;Acc:MGI:1891468]	15.2952	14.216	13.2798	13.9639	14.8873	12.909	14.2308	14.2891	13.7211	13.9248	15.9368	13.3124	13.7325	13.0553	0.881718085	0.966018836	20	16	24	0.578365689	0.849227023	24	17.26595753	0.033255958
ENSMUSG00000050211	Pla2g4e	phospholipase A2, group IVE [Source:MGI Symbol;Acc:MGI:1919144]	0.32166	0.708418	0.919828	0.790376	0.656707	0.883792	0.550716	1.01503	0.858811	1.23871	0.52266	0.692402	0.798689	0.606799	0.226403091	0.722427815	12	16	24	0.123658835	0.736492734	24	9.903313328	0.146085086
ENSMUSG00000050212	Eva1b	eva-1 homolog B (C. elegans) [Source:MGI Symbol;Acc:MGI:1922063]	2.81323	2.85221	2.69819	2.73646	2.43759	1.90108	3.03689	3.49344	5.61437	5.28233	5.06094	4.94566	5.74511	4.592	0.873350015	0.966018836	4	20	24	0.325444288	0.768040178	24	8.184402743	0.148602608
ENSMUSG00000050213	Snip1	Smad nuclear interacting protein 1 [Source:MGI Symbol;Acc:MGI:2156003]	5.60593	5.4071	5.595	5.83056	5.88322	5.44202	5.74898	5.82493	5.51232	6.24089	5.81771	5.71447	6.21389	5.95206	0.960132359	0.967043964	16	20	24	0.685336747	0.895376881	24	12.8389087	0.014964529
ENSMUSG00000050222	Il17d	interleukin 17D [Source:MGI Symbol;Acc:MGI:2446510]	2.03572	1.6528	2.02027	2.05303	1.92946	1.95115	1.61354	1.88971	2.51487	2.12326	2.90316	1.95476	2.39941	2.1523	0.663757733	0.966018836	12	20	24	0.260928634	0.768040178	24	12.74533567	0.082380542
ENSMUSG00000050229	Pigm	phosphatidylinositol glycan anchor biosynthesis, class M [Source:MGI Symbol;Acc:MGI:1914806]	4.83034	4.41678	4.31536	4.66225	4.61787	4.40298	4.06372	4.35731	4.43912	4.46462	4.8035	4.54915	4.56145	4.48638	0.351315142	0.870418653	20	16	24	0.304775081	0.768040178	24	16.22950733	0.031397787
ENSMUSG00000050234	Gja4	gap junction protein, alpha 4 [Source:MGI Symbol;Acc:MGI:95715]	0.755166	1.66945	1.70008	1.4995	1.35063	1.2768	1.20898	2.01069	1.54998	1.65112	0.934074	1.22471	1.15937	1.27518	0.004320961	0.140598674	12	16	24	0.036235274	0.609176161	24	8.294132299	0.193869142
ENSMUSG00000050240	Hic2	hypermethylated in cancer 2 [Source:MGI Symbol;Acc:MGI:1929869]	1.09746	0.724865	0.688363	0.765622	0.766646	0.762694	0.836135	0.848739	0.90421	0.899672	0.798675	0.909898	0.876749	0.893156	0.920858634	0.966018836	4	8	24	0.443495932	0.805393068	24	23.88377525	0.03868018
ENSMUSG00000050243	Gm5446	predicted gene 5446 [Source:MGI Symbol;Acc:MGI:3644854]	8.20602	7.65026	8.29298	7.48029	7.79968	7.69293	8.9086	5.90183	6.16624	6.56645	7.30223	6.71844	6.88606	7.30898	0.897570341	0.966018836	4	4	24	0.330818066	0.768040178	24	20.86137022	0.074940275
ENSMUSG00000050244	Heatr1	HEAT repeat containing 1 [Source:MGI Symbol;Acc:MGI:2442524]	6.58884	6.19129	6.16021	7.0779	6.22418	6.67695	6.25108	6.58505	6.34852	5.24929	7.15779	5.94624	5.99307	6.15283	0.880247609	0.966018836	20	12	24	0.793220232	0.933800612	24	18.50663164	0.026208057
ENSMUSG00000050248	Evc2	EvC ciliary complex subunit 2 [Source:MGI Symbol;Acc:MGI:1915775]	7.98683	7.21224	6.48781	10.0539	11.5099	8.08434	9.50788	7.44196	9.38595	8.00727	9.63349	7.99082	8.17485	6.23275	0.004720572	0.140598674	20	12	24	0.03835232	0.61140397	24	16.20207807	0.198597723
ENSMUSG00000050270	Tmem220	transmembrane protein 220 [Source:MGI Symbol;Acc:MGI:2443691]	1.18867	2.15182	2.10545	2.00513	1.45414	1.63864	1.21577	2.15889	2.26925	2.31884	1.25829	1.81003	1.73082	1.7647	0.0312832	0.324385159	12	16	24	0.012537831	0.525280095	24	8.43729301	0.220456389
ENSMUSG00000050271	Prag1	PEAK1 related kinase activating pseudokinase 1 [Source:MGI Symbol;Acc:MGI:1196223]	1.2638	1.23901	1.5125	1.17769	1.30546	1.23591	1.27825	1.28397	1.15029	1.74793	1.15552	1.35813	1.13114	1.38264	0.860993221	0.966018836	16	12	24	0.504354964	0.810907526	24	10.3592357	0.046752556
ENSMUSG00000050272	Dscam	DS cell adhesion molecule [Source:MGI Symbol;Acc:MGI:1196281]	3.91715	2.80938	3.6457	2.93565	2.83938	2.99671	3.63236	3.50358	2.98282	4.13552	2.23439	3.58583	3.14966	3.85798	0.708665292	0.966018836	4	16	24	0.350561608	0.780012278	24	3.689388873	0.103033423
ENSMUSG00000050288	Fzd2	frizzled class receptor 2 [Source:MGI Symbol;Acc:MGI:1888513]	3.1086	3.66088	3.01467	2.91078	3.51903	2.79202	3.11027	3.56869	3.84813	2.70969	3.79751	3.29794	3.15758	3.26575	0.83638114	0.966018836	8	8	24	0.808603944	0.939135535	24	5.574417761	0.032836889
ENSMUSG00000050295	Foxc1	forkhead box C1 [Source:MGI Symbol;Acc:MGI:1347466]	1.45478	1.85771	1.34884	1.23874	1.42304	1.21881	1.45595	1.62236	1.91728	0.721618	1.65617	1.40302	1.46799	1.55132	0.050258003	0.385784509	8	8	24	0.251904578	0.768040178	24	2.83679893	0.115878175
ENSMUSG00000050299	Gm9843	predicted gene 9843 [Source:MGI Symbol;Acc:MGI:3708621]	365.432	367.392	350.401	351.984	336.478	343.946	345.767	349.303	445.189	322.856	403.98	361.082	401.603	376.754	0.888991156	0.966018836	12	4	24	0.618659899	0.869022404	24	5.203415991	0.045489209
ENSMUSG00000050310	Rictor	RPTOR independent companion of MTOR, complex 2 [Source:MGI Symbol;Acc:MGI:1926007]	6.6268	7.77373	7.32816	7.61256	7.23946	6.85592	6.89566	6.68545	7.98781	6.07141	7.41605	7.54686	7.42264	7.39552	0.907000607	0.966018836	12	16	24	0.837630524	0.950322752	24	7.610401503	0.023330463
ENSMUSG00000050312	Nsun3	NOL1/NOP2/Sun domain family member 3 [Source:MGI Symbol;Acc:MGI:2146565]	6.18478	5.32623	5.26647	4.95525	4.90333	4.5408	4.25121	4.61668	4.90742	5.90253	4.77361	4.14546	4.46784	4.49401	0.110277874	0.526825967	16	8	24	0.370935903	0.792839127	24	9.964090626	0.064803186
ENSMUSG00000050315	Synpo2	synaptopodin 2 [Source:MGI Symbol;Acc:MGI:2153070]	1.72315	2.97586	3.4673	3.67992	2.96738	3.4764	2.64002	5.35383	4.0459	5.97149	2.56537	3.02	3.39164	2.82198	0.140377635	0.585715181	16	12	24	0.085124136	0.70291653	24	9.756790866	0.277060167
ENSMUSG00000050321	Neto1	neuropilin (NRP) and tolloid (TLL)-like 1 [Source:MGI Symbol;Acc:MGI:2180216]	1.64716	1.79607	2.26942	1.64639	1.74857	1.83031	1.75641	1.42533	1.9441	1.66138	1.69677	1.45918	1.35402	1.76603	0.608967886	0.966018836	12	16	24	0.240722828	0.768040178	24	9.605073588	0.0717758
ENSMUSG00000050323	Ndufaf6	NADH:ubiquinone oxidoreductase complex assembly factor 6 [Source:MGI Symbol;Acc:MGI:1924197]	6.34766	6.55523	5.99336	8.22825	5.98548	6.75008	5.85898	7.4305	8.19231	6.76701	6.69719	6.8852	7.2612	8.49416	0.68887295	0.966018836	16	4	24	0.176781246	0.768040178	24	7.729189797	0.09923022
ENSMUSG00000050332	Amer1	APC membrane recruitment 1 [Source:MGI Symbol;Acc:MGI:1919595]	4.7252	4.15416	4.07007	4.2999	3.99935	4.40786	4.12075	4.39	3.67711	4.32925	4.18505	4.45812	4.26231	4.62206	0.313239838	0.824470524	24	12	24	0.25829866	0.768040178	24	22.61920443	0.047195856
ENSMUSG00000050334	C130071C03Rik	RIKEN cDNA C130071C03 gene [Source:MGI Symbol;Acc:MGI:2443574]	2.76522	3.9116	4.73299	3.34073	3.5907	3.61602	3.72416	2.87316	4.1662	3.38124	3.26542	3.23347	2.86301	3.39711	0.182849367	0.659303873	12	16	24	0.219053054	0.768040178	24	9.55361429	0.110297001
ENSMUSG00000050335	Lgals3	lectin, galactose binding, soluble 3 [Source:MGI Symbol;Acc:MGI:96778]	14.9869	15.8282	22.1371	14.5441	18.3436	16.2011	16.0701	15.3643	15.4621	11.8906	17.9825	14.0591	15.7294	13.3533	0.92655264	0.966018836	20	20	24	0.941621927	0.985544192	24	16.1783741	0.026121095
ENSMUSG00000050357	Carmil2	capping protein regulator and myosin 1 linker 2 [Source:MGI Symbol;Acc:MGI:2685431]	10.6441	7.8606	8.24438	7.72771	7.56398	8.20081	8.7123	9.51259	8.74968	12.1396	10.2021	11.4307	10.0389	11.2076	0.872380105	0.966018836	4	16	24	0.85411592	0.956445086	24	0.133932985	0.036290915
ENSMUSG00000050359	Sprr1a	small proline-rich protein 1A [Source:MGI Symbol;Acc:MGI:106660]	1.7951	0.188616	7.13283	0.425017	5.87	4.10101	1.28286	1.43881	0.126696	0.327223	11.5563	0.869918	0.206434	0.139196	0.113148904	0.530777648	20	12	24	0.136597381	0.747972781	24	16.85557259	0.862371952
ENSMUSG00000050368	Hoxd10	homeobox D10 [Source:MGI Symbol;Acc:MGI:96202]	2.06148	3.67834	4.59081	4.19773	2.14506	4.67581	4.12996	1.76524	3.85461	4.9237	1.50515	4.11219	1.57661	3.53722	0.387503547	0.915479136	16	12	24	0.592885981	0.856634016	24	10.0462625	0.190181677
ENSMUSG00000050370	Ch25h	cholesterol 25-hydroxylase [Source:MGI Symbol;Acc:MGI:1333869]	0.550876	0.869895	0.614698	0.447327	0.645221	0.571136	0.672786	0.83759	0.642616	0.663538	0.446665	0.453499	0.237022	0.457644	0.723119329	0.966018836	8	16	24	0.419526329	0.803639585	24	6.803953802	0.071215551
ENSMUSG00000050373	Snx21	sorting nexin family member 21 [Source:MGI Symbol;Acc:MGI:1917729]	32.4197	29.9426	27.7044	26.5415	26.0836	31.9993	29.9495	29.4748	24.322	29.7507	30.072	31.8788	31.8508	27.6774	0.004319982	0.140598674	24	12	24	0.009960139	0.503967859	24	21.87134575	0.126978569
ENSMUSG00000050377	Il31ra	interleukin 31 receptor A [Source:MGI Symbol;Acc:MGI:2180511]	11.2037	8.46172	9.50883	9.65452	11.0899	10.2219	9.7701	9.07595	8.2018	10.8607	9.2231	9.09144	9.75423	9.37115	0.170973042	0.644539467	4	4	24	0.164021164	0.767020009	24	18.1728282	0.087790981
ENSMUSG00000050379	Septin6	septin 6 [Source:MGI Symbol;Acc:MGI:1888939]	38.4641	35.7314	35.0905	34.4696	37.698	38.2873	32.568	33.4027	32.3502	31.2619	35.869	34.8279	35.3104	35.3132	0.341022016	0.86129475	20	20	24	0.069341553	0.673520298	24	20.3969793	0.071222957
ENSMUSG00000050382	Kif7	kinesin family member 7 [Source:MGI Symbol;Acc:MGI:1098239]	0.612491	0.823914	0.777939	0.842021	0.676129	0.711167	0.913097	0.785463	0.592941	0.796676	0.755878	0.767566	0.705239	0.789842	0.93717134	0.966018836	16	4	24	0.931561452	0.982350631	24	6.493064958	0.011480153
ENSMUSG00000050390	C77080	expressed sequence C77080 [Source:MGI Symbol;Acc:MGI:2140651]	18.2059	12.753	11.6231	15.4176	13.4364	16.2536	13.9821	17.157	12.0952	24.9806	14.7953	16.6592	16.0944	15.8488	0.245061943	0.741580974	24	12	24	0.751241029	0.91921305	24	17.96954116	0.079064031
ENSMUSG00000050394	Armcx6	armadillo repeat containing, X-linked 6 [Source:MGI Symbol;Acc:MGI:2147993]	6.28491	6.02732	5.93898	5.82476	5.35957	6.82334	5.77033	5.51764	5.62382	5.64498	6.11132	7.09672	6.99479	5.45313	0.121741977	0.549378164	24	12	24	0.052509021	0.650526959	24	20.93073499	0.102682541
ENSMUSG00000050410	Tcf19	transcription factor 19 [Source:MGI Symbol;Acc:MGI:103180]	3.4888	3.07662	3.78268	3.29528	3.82795	3.77835	2.87664	3.04038	3.37797	2.62422	3.68816	3.37067	3.37419	2.60379	0.06180207	0.409814092	20	12	24	0.133587149	0.743843563	24	17.48202877	0.100088111
ENSMUSG00000050428	Fbxo46	F-box protein 46 [Source:MGI Symbol;Acc:MGI:2444918]	1.25863	1.0512	1.12062	1.21267	1.05789	1.26933	1.26629	1.23896	1.28096	1.30112	1.15441	1.03418	1.30154	1.4298	0.94499634	0.966018836	4	20	24	0.343672875	0.777219607	24	4.691096963	0.039616997
ENSMUSG00000050447	Lypd6	LY6/PLAUR domain containing 6 [Source:MGI Symbol;Acc:MGI:2443848]	3.06443	2.35815	4.36657	2.45526	2.14553	3.10448	2.74844	2.36024	1.91124	2.70537	2.25804	2.14663	2.69148	2.72041	0.92655264	0.966018836	4	16	24	0.77206674	0.926501074	24	3.27417173	0.059108661
ENSMUSG00000050471	Fam118b	family with sequence similarity 118, member B [Source:MGI Symbol;Acc:MGI:1924483]	7.79848	8.19275	7.7353	8.29556	8.26427	7.66382	8.3116	7.71871	9.12813	7.01899	8.47806	6.75663	8.01025	7.52683	0.911135828	0.966018836	20	4	24	0.760813961	0.92268731	24	8.6167445	0.029640054
ENSMUSG00000050473	Slc35d3	solute carrier family 35, member D3 [Source:MGI Symbol;Acc:MGI:1923407]	0.770223	0.857733	1.01296	0.629447	0.737159	0.687456	0.843532	0.418308	0.71415	0.731193	0.520973	0.692513	0.543249	0.997265	0.697196105	0.966018836	12	8	24	0.602089886	0.860902594	24	5.706392152	0.056646511
ENSMUSG00000050490	Psma5-ps	proteasome subunit alpha 5, pseudogene [Source:MGI Symbol;Acc:MGI:3647964]	61.1074	66.175	63.9842	62.5046	65.9698	58.7503	59.3685	57.4117	63.1263	56.5491	67.5688	60.0157	68.2294	63.1932	0.882263435	0.966018836	20	4	24	0.988800788	0.997581486	24	12.9419196	0.005147541
ENSMUSG00000050493	Fam167b	family with sequence similarity 167, member B [Source:MGI Symbol;Acc:MGI:2668032]	0.848277	0.702621	0.80701	0.972212	0.872304	0.661538	0.517315	1.04378	0.978492	0.630479	0.801499	0.556216	0.741863	0.672724	0.364640768	0.892062098	12	12	24	0.240104863	0.768040178	24	9.381481369	0.083713667
ENSMUSG00000050511	Oprd1	opioid receptor, delta 1 [Source:MGI Symbol;Acc:MGI:97438]	2.40964	1.82234	1.54034	1.94214	1.74711	1.96435	2.33243	2.27827	1.63658	2.73552	1.50284	2.32785	2.10201	2.49945	0.232647885	0.733359426	4	8	24	0.410127755	0.799546792	24	0.577818994	0.090691587
ENSMUSG00000050530	Fam171a1	family with sequence similarity 171, member A1 [Source:MGI Symbol;Acc:MGI:2442917]	14.5644	11.1237	11.4652	11.8446	11.7109	12.7451	12.645	11.9639	9.39614	14.1112	11.2114	11.8742	11.7407	11.5626	0.128989168	0.560209134	24	12	24	0.29698328	0.768040178	24	20.43724066	0.086114519
ENSMUSG00000050533	Gm9845	predicted pseudogene 9845 [Source:MGI Symbol;Acc:MGI:3704215]	4.86647	4.37638	4.38852	4.59903	4.77287	6.10074	5.81022	4.49883	5.12509	4.79509	5.96235	6.00827	5.8977	7.14996	0.140377635	0.585715181	24	12	24	0.067164707	0.670273692	24	21.57850523	0.111653861
ENSMUSG00000050534	Htr5b	5-hydroxytryptamine (serotonin) receptor 5B [Source:MGI Symbol;Acc:MGI:96284]	2.3698	2.70785	2.40855	2.24151	2.25348	2.62759	2.15835	2.57045	2.11141	2.96787	2.49965	3.05334	2.70281	2.80042	0.938993891	0.966018836	24	12	24	0.845476257	0.953396028	24	22.42120319	0.025263831
ENSMUSG00000050538	B230217C12Rik	RIKEN cDNA B230217C12 gene [Source:MGI Symbol;Acc:MGI:1915377]	12.0274	12.309	14.3736	12.2227	10.8178	12.5175	12.359	10.6605	12.1582	12.1278	11.5006	11.961	10.0605	13.9327	0.723119329	0.966018836	12	8	24	0.434515995	0.805393068	24	7.752743699	0.065491062
ENSMUSG00000050541	Adra1b	adrenergic receptor, alpha 1b [Source:MGI Symbol;Acc:MGI:104774]	0.543615	0.842054	0.967059	0.596277	0.780005	0.746585	0.937554	0.800087	1.11667	0.543768	0.852507	0.879081	0.464744	0.64148	0.745118544	0.966018836	12	16	24	0.808748075	0.939182735	24	8.777049524	0.041954525
ENSMUSG00000050545	Fam228b	family with sequence similarity 228, member B [Source:MGI Symbol;Acc:MGI:2442121]	3.13665	2.86226	2.0835	3.80856	3.59108	3.92508	2.31349	1.18921	2.85002	3.03151	2.98691	1.8945	3.75413	2.8143	0.302438464	0.811201458	16	20	24	0.312803612	0.768040178	24	16.53614815	0.192921679
ENSMUSG00000050549	Fam241a	family with sequence similarity 241, member A [Source:MGI Symbol;Acc:MGI:1917867]	8.32034	7.31803	6.88192	8.2958	7.88129	7.9376	7.35783	6.72722	6.29538	7.51013	7.61119	6.14737	6.83908	7.16045	0.478349072	0.966018836	16	20	24	0.248499299	0.768040178	24	17.19378763	0.066130086
ENSMUSG00000050550	Gm11868	predicted gene 11868 [Source:MGI Symbol;Acc:MGI:3650507]	9.52867	8.28328	8.36589	8.95319	8.40291	9.05302	8.43458	8.52402	7.85768	8.04661	8.91838	8.11139	7.3169	7.97415	0.823657258	0.966018836	24	12	24	0.214012497	0.768040178	24	18.19844852	0.046498335
ENSMUSG00000050552	Lamtor4	late endosomal/lysosomal adaptor, MAPK and MTOR activator 4 [Source:MGI Symbol;Acc:MGI:1913346]	54.1038	54.329	54.7756	54.8485	60.967	52.6754	48.7475	51.1458	51.0942	44.7681	58.8059	49.9861	56.9652	53.8254	0.833301348	0.966018836	20	4	24	0.72161907	0.90866625	24	18.10133206	0.03674805
ENSMUSG00000050555	Hyls1	HYLS1, centriolar and ciliogenesis associated [Source:MGI Symbol;Acc:MGI:1924082]	4.36271	5.16432	5.18422	5.43587	5.76879	5.06164	4.82776	4.64439	4.97283	3.70093	6.47526	5.06991	5.4543	4.8106	0.192357053	0.669841558	20	12	24	0.473132991	0.805393068	24	17.03357058	0.076454143

ENSMUSG00000050556	Kcnb1	potassium voltage gated channel, Shab-related subfamily, member 1 [Source:MGI Symbol;Acc:MGI:96666]	26.6546	18.4024	19.1952	23.9698	19.9223	23.6851	20.2482	22.7405	15.7045	29.1198	21.534	23.5903	22.2729	22.1371	0.195343269	0.673387182	16	20	24	0.449723387	0.805393068	24	18.54172428	0.11013807
ENSMUSG00000050558	Prokr2	prokineticin receptor 2 [Source:MGI Symbol;Acc:MGI:2181363]	3.48011	2.38949	2.15079	2.37063	2.29208	2.65027	2.421	2.70395	1.89038	2.58607	2.83745	2.46948	2.97659	2.36182	0.016795343	0.253395174	4	8	24	0.077452811	0.687420473	24	21.57394501	0.130683846
ENSMUSG00000050565	Tor1aip2	torsin A interacting protein 2 [Source:MGI Symbol;Acc:MGI:3582695]	39.3344	39.9119	38.6701	39.1312	40.5152	36.463	39.3263	39.2632	40.4945	37.9127	40.3072	37.784	40.1407	38.9942	0.891690964	0.966018836	20	4	24	0.6684688	0.887968245	24	7.11229393	0.015817957
ENSMUSG00000050567	Maml1	mastermind like transcriptional coactivator 1 [Source:MGI Symbol;Acc:MGI:1890504]	2.15152	2.14496	2.15228	2.4855	2.31791	2.02446	2.46898	2.52634	2.34472	2.69325	2.05646	2.18025	2.12118	2.12807	0.275526571	0.789849983	16	8	24	0.282317495	0.768040178	24	9.996332675	0.055668374
ENSMUSG00000050578	Mmp13	matrix metallopeptidase 13 [Source:MGI Symbol;Acc:MGI:1340026]	1.90828	2.76993	3.46735	2.29273	2.23302	3.24333	1.50389	3.998	2.81843	2.02052	2.08089	3.10274	3.37096	2.46185	0.665691261	0.966018836	12	4	24	0.58717411	0.853591039	24	4.991109644	0.116479738
ENSMUSG00000050587	Lrrc4c	leucine rich repeat containing 4C [Source:MGI Symbol;Acc:MGI:2442636]	7.68773	8.78622	9.54719	7.90617	8.09282	8.21424	8.90269	8.29624	8.36068	9.00059	9.09434	8.95014	8.34272	10.1572	0.915475528	0.966018836	8	20	24	0.362856374	0.787522977	24	7.072991048	0.049993921
ENSMUSG00000050592	Fam78a	family with sequence similarity 78, member A [Source:MGI Symbol;Acc:MGI:2443569]	0.270258	0.428001	0.708501	0.767464	0.631548	0.527928	0.706411	0.662246	0.736982	0.855105	0.586227	0.596788	0.575276	0.495027	0.023858628	0.282503052	16	12	24	0.022430795	0.578704263	24	11.40242715	0.129773974
ENSMUSG00000050605	Zfp61	zinc finger protein 61 [Source:MGI Symbol;Acc:MGI:99663]	6.05484	6.69225	7.84188	7.92696	6.39948	7.5672	6.37678	6.48915	7.33128	6.10586	6.70723	6.52717	6.44895	7.10765	0.112800839	0.530777648	12	16	24	0.311248601	0.768040178	24	9.993957653	0.067549732
ENSMUSG00000050608	Micos10	mitochondrial contact site and cristae organizing system subunit 10 [Source:MGI Symbol;Acc:MGI:1913628]	164.308	163.902	161.931	187.983	171.806	149.197	159.61	174.772	208.276	151.859	179.697	166.935	171.565	170.331	0.7207001	0.966018836	12	12	24	0.301651878	0.768040178	24	9.269360058	0.075065352
ENSMUSG00000050619	Zscan29	zinc finger SCAN domains 29 [Source:MGI Symbol;Acc:MGI:2139317]	7.43292	6.11041	6.03047	6.87199	6.27871	6.76101	6.82847	6.58242	5.60431	6.03045	7.72319	6.23429	6.15269	5.71856	0.097779482	0.501112642	20	16	24	0.074345535	0.683293901	24	19.13350798	0.096283666
ENSMUSG00000050621	Rps27rt	ribosomal protein S27, retrogene [Source:MGI Symbol;Acc:MGI:3704345]	355.038	407.201	373.997	360.851	345.646	297.463	331.856	316.173	470.114	260.978	373.94	370.015	414.068	436.037	0.593560247	0.966018836	12	4	24	0.315483849	0.768040178	24	4.977702813	0.130929104
ENSMUSG00000050623	Catsperz	cation channel sperm associated auxiliary subunit zeta [Source:MGI Symbol;Acc:MGI:1914327]	7.35041	6.43888	7.67553	9.26484	10.4128	9.03338	6.09872	5.68678	7.76631	8.76528	9.34325	8.82334	5.78033	5.49731	9.36E-06	0.009754434	20	12	24	3.16E-07	0.003664625	24	15.25973671	0.344494402
ENSMUSG00000050627	Gpd1l	glycerol-3-phosphate dehydrogenase 1-like [Source:MGI Symbol;Acc:MGI:1289257]	41.9163	34.9784	34.3292	36.6344	34.7023	38.4902	35.077	37.0479	30.7906	39.1681	35.993	36.9879	39.5222	36.2241	0.112800839	0.530777648	4	8	24	0.185102339	0.768040178	24	21.15317074	0.075816763
ENSMUSG00000050628	Ubald2	UBA-like domain containing 2 [Source:MGI Symbol;Acc:MGI:1914635]	5.4112	5.1594	4.90615	3.97868	2.95809	3.92195	5.58149	6.4288	5.83938	5.48045	5.36797	5.33372	5.68757	5.15836	0.241264473	0.741580974	4	20	24	0.041443839	0.618736234	24	4.054820451	0.198437342
ENSMUSG00000050640	Tmem150c	transmembrane protein 150C [Source:MGI Symbol;Acc:MGI:3041258]	30.5457	22.1062	22.6186	19.622	22.1953	25.1577	22.5226	25.3362	17.4039	26.0657	23.3646	23.9477	24.7993	23.9387	0.44861185	0.966018836	4	8	24	0.133163177	0.743835554	24	22.16551909	0.140230113
ENSMUSG00000050663	Trhde	TRH-degrading enzyme [Source:MGI Symbol;Acc:MGI:2384311]	1.28427	1.06602	0.890581	1.03575	1.08932	1.11122	1.07896	0.960207	0.935769	0.976939	1.16221	1.0099	1.16988	1.14443	0.004320961	0.140598674	4	8	24	0.040965981	0.61771321	24	21.41273777	0.069059654
ENSMUSG00000050666	Vstm4	V-set and transmembrane domain containing 4 [Source:MGI Symbol;Acc:MGI:2444633]	8.03309	7.32079	6.39066	6.93422	8.77564	7.86495	8.67779	7.64899	6.79173	6.37816	7.33956	7.53968	7.29612	5.87421	0.059380727	0.406067671	4	8	24	0.005753833	0.454617194	24	20.37128532	0.150938385
ENSMUSG00000050668	Gpatch11	G patch domain containing 11 [Source:MGI Symbol;Acc:MGI:1858435]	20.9209	23.044	25.1078	21.7121	21.2796	19.986	18.2568	18.51	25.4702	16.5153	19.6796	21.4335	22.6807	23.0362	0.125887884	0.555618842	12	8	24	0.432960352	0.805393068	24	6.227643378	0.087532658
ENSMUSG00000050677	Ccdc96	coiled-coil domain containing 96 [Source:MGI Symbol;Acc:MGI:1913967]	0.612787	0.571078	0.553763	0.614107	0.47931	0.502375	0.494855	0.478352	0.395964	0.481012	0.391601	0.616001	0.421937	0.563876	0.896799496	0.966018836	24	20	24	0.770263345	0.926036042	24	0.292492174	0.019605661
ENSMUSG00000050697	Prkaa1	protein kinase, AMP-activated, alpha 1 catalytic subunit [Source:MGI Symbol;Acc:MGI:2145955]	30.0347	26.9293	26.6654	28.2556	25.8497	27.4434	25.767	26.1755	22.9847	28.8727	26.4957	24.7332	24.9695	26.9558	0.883870616	0.966018836	16	12	24	0.878267822	0.965980735	24	17.33889539	0.017593068
ENSMUSG00000050705	2310061I04Rik	RIKEN cDNA 2310061I04 gene [Source:MGI Symbol;Acc:MGI:1916912]	22.9249	19.7558	23.9114	24.2372	25.2072	23.8615	22.6929	23.5002	21.8394	28.922	20.8663	23.7508	22.643	23.6062	0.10031279	0.511533473	16	12	24	0.287236522	0.768040178	24	13.54081449	0.073119339
ENSMUSG00000050708	Ftl1	ferritin light polypeptide 1 [Source:MGI Symbol;Acc:MGI:95589]	317.862	276.2	275.637	284.675	284.425	287.019	299.562	297.237	301.49	283.323	313.108	288.506	300.838	259.768	0.210566453	0.69936851	4	4	24	0.369675856	0.792235171	24	20.13564894	0.04413068
ENSMUSG00000050711	Scg2	secretogranin II [Source:MGI Symbol;Acc:MGI:103033]	167.533	160.971	163.759	158.541	165.71	169.605	154.583	151.395	140.624	150.144	172.212	158.839	157.018	161.603	0.287488775	0.793714531	20	20	24	0.085647073	0.703577714	24	19.24484869	0.063396621
ENSMUSG00000050714	Zbtb26	zinc finger and BTB domain containing 26 [Source:MGI Symbol;Acc:MGI:2444402]	3.10803	2.82147	2.54002	2.86591	3.07704	2.50343	3.26953	2.49275	2.82842	2.142	3.62252	2.54675	2.9587	2.77246	0.323424237	0.839647936	4	4	24	0.510451367	0.814224951	24	20.50441293	0.067573141
ENSMUSG00000050721	Plekho2	pleckstrin homology domain containing, family O member 2 [Source:MGI Symbol;Acc:MGI:2143132]	4.64745	5.07238	5.88387	4.78467	5.92196	5.26363	5.20714	5.72945	5.78224	5.25371	5.12412	4.99912	4.94725	4.95778	0.286642595	0.793714531	12	16	24	0.358736046	0.785239521	24	10.07867643	0.054121578
ENSMUSG00000050730	Arhgap42	Rho GTPase activating protein 42 [Source:MGI Symbol;Acc:MGI:1918794]	8.40009	7.64509	7.32375	8.23566	9.24084	7.43196	8.11287	6.41183	7.89964	7.68203	8.58126	7.81653	8.09837	8.13732	0.308023687	0.814993005	20	12	24	0.185454545	0.768040178	24	17.03391854	0.07663789
ENSMUSG00000050732	Vamp8	vesicle-associated membrane protein 8 [Source:MGI Symbol;Acc:MGI:1336882]	35.5771	43.8765	41.7134	41.4006	43.9693	34.8917	39.0829	37.4144	51.2099	31.5604	41.0558	35.3706	43.2461	36.72	0.148073804	0.590732968	12	12	24	0.545203046	0.832813511	24	8.129149365	0.07944032
ENSMUSG00000050737	Ptges	prostaglandin E synthase [Source:MGI Symbol;Acc:MGI:1927593]	4.35389	3.60432	3.31723	3.25916	4.10892	3.49359	2.89596	4.75694	3.6495	2.92217	2.7011	3.40558	4.03978	3.9938	0.306258944	0.812796472	8	8	24	0.170364937	0.767957732	24	2.321045587	0.128254376
ENSMUSG00000050751	Pgbd5	piggyBac transposable element derived 5 [Source:MGI Symbol;Acc:MGI:2429955]	12.286	9.80741	9.86015	10.2673	10.118	11.8175	10.6238	11.4424	8.44654	13.6048	9.49378	11.6579	10.1617	11.1048	0.390686537	0.915479136	24	8	24	0.580348086	0.850271896	24	20.6264211	0.069403299
ENSMUSG00000050761	Gp1bb	glycoprotein Ib, beta polypeptide [Source:MGI Symbol;Acc:MGI:107852]	0.882486	0.990359	1.50917	1.05805	0.939726	1.2847	1.05065	1.00383	1.1486	1.13217	0.729474	1.36364	0.389079	1.27841	0.43565473	0.966018836	12	16	24	0.583309719	0.851793019	24	9.709533522	0.088754204
ENSMUSG00000050777	Tmem37	transmembrane protein 37 [Source:MGI Symbol;Acc:MGI:2157899]	1.03676	1.24789	1.76697	2.70232	1.7118	1.08191	1.43483	1.73581	1.72486	1.41533	1.92866	0.901613	2.16058	1.31086	0.193864198	0.67239758	16	8	24	0.1623507	0.76636332	24	10.67476222	0.192500347
ENSMUSG00000050783	Htr1f	5-hydroxytryptamine (serotonin) receptor 1F [Source:MGI Symbol;Acc:MGI:99842]	2.21207	1.47645	1.26621	1.47448	1.80155	1.76892	1.62255	1.44343	1.48091	1.67728	1.91281	1.62239	1.92534	1.7338	0.070873295	0.440262583	4	8	24	0.042461858	0.623698598	24	20.33624886	0.12040951
ENSMUSG00000050786	Ccdc126	coiled-coil domain containing 126 [Source:MGI Symbol;Acc:MGI:1889376]	10.0946	9.09029	8.52814	8.32537	8.09078	8.41302	9.26872	8.11215	6.85018	8.135	9.53175	8.20137	8.28892	7.83729	0.628286596	0.966018836	4	8	24	0.102058677	0.713240751	24	21.22486613	0.090079189
ENSMUSG00000050796	B3galt6	UDP-Gal:betaGal beta 1,3-galactosyltransferase, polypeptide 6 [Source:MGI Symbol;Acc:MGI:2152819]	2.42334	2.34101	2.42388	2.253	2.438	2.40326	2.14626	2.42653	2.30555	2.21329	2.51656	2.86697	2.58178	2.78887	0.602156008	0.966018836	20	20	24	0.504261497	0.810849916	24	22.34568972	0.032819308
ENSMUSG00000050812	Ecpas	Ecm29 proteasome adaptor and scaffold [Source:MGI Symbol;Acc:MGI:2140220]	39.5607	39.9778	39.4239	42.9897	41.4748	37.3372	37.711	38.4416	39.9736	36.2689	44.6789	40.1612	41.1187	40.0612	0.650398351	0.966018836	20	16	24	0.530511209	0.824858059	24	14.75275923	0.03443818
ENSMUSG00000050821	Fam131a	family with sequence similarity 131, member A [Source:MGI Symbol;Acc:MGI:1925658]	19.3613	15.9823	15.1353	15.0111	15.0197	17.1852	16.4973	17.3202	15.4267	20.3278	17.7926	19.954	18.2325	18.9391	0.413946331	0.958746003	24	12	24	0.347919589	0.778844143	24	22.08578834	0.072807196
ENSMUSG00000050822	Slc29a4	solute carrier family 29 (nucleoside transporters), member 4 [Source:MGI Symbol;Acc:MGI:2385330]	6.37588	5.75611	5.29908	5.3919	5.27944	6.81186	5.98917	7.00589	4.43815	9.51055	5.70665	6.552	5.776	7.92094	0.44861185	0.966018836	8	4	24	0.947706996	0.987232058	24	23.75250938	0.029931241
ENSMUSG00000050830	Vwc2	von Willebrand factor C domain containing 2 [Source:MGI Symbol;Acc:MGI:2442987]	2.92975	2.65441	2.42464	1.94909	2.3747	2.55755	2.35689	2.68496	1.89086	2.4022	2.3916	2.53917	2.41492	2.44423	0.105467177	0.518002936	8	8	24	0.041349103	0.618736234	24	23.1728857	0.102657886
ENSMUSG00000050840	Cdh20	cadherin 20 [Source:MGI Symbol;Acc:MGI:1346069]	1.56222	1.7638	2.65155	1.22261	1.28627	1.99245	2.12421	1.75661	1.62632	1.58814	1.47083	1.7011	1.35008	1.93592	0.160842982	0.620479606	8	12	24	0.305207666	0.768040178	24	3.906159606	0.113097146
ENSMUSG00000050846	Zfp623	zinc finger protein 623 [Source:MGI Symbol;Acc:MGI:1926084]	3.9199	3.75663	4.17354	4.48238	4.67114	4.34117	4.27102	4.02878	3.97895	4.11946	4.44958	4.05679	4.46933	4.22542	0.109247218	0.524035616	20	12	24	0.067358346	0.670376655	24	16.22114595	0.057799755
ENSMUSG00000050854	Tmem125	transmembrane protein 125 [Source:MGI Symbol;Acc:MGI:1923409]	0.276484	1.30385	2.10376	0.870782	0.655289	0.783921	1.48007	0.782696	1.4186	0.876872	0.582396	1.09505	0.400143	1.61469	0.154830409	0.607427992	12	8	24	0.11548952	0.727799063	24	7.323817295	0.290989296
ENSMUSG00000050855	Zfp940	zinc finger protein 940 [Source:MGI Symbol;Acc:MGI:2446235]	2.59335	3.03671	2.88868	2.58475	3.18073	3.06563	3.40757	2.90452	3.04714	2.23617	2.45209	3.07713	2.68989	2.9167	0.708020493	0.966018836	24	16	24	0.295020753	0.768040178	24	0.199211056	0.071948698
ENSMUSG00000050856	Atp5k	ATP synthase, H+ transporting, mitochondrial F1F0 complex, subunit E [Source:MGI Symbol;Acc:MGI:106636]	264.847	286.125	282.405	285.97	289.246	271.628	275.623	275.028	295.508	265.332	309.541	293.26	330.821	343.81	0.826055781	0.966018836	8	8	24	0.655482103	0.883697438	24	4.703840068	0.03046066
ENSMUSG00000050860	Phospho1	phosphatase, orphan 1 [Source:MGI Symbol;Acc:MGI:2447348]	15.3381	12.763	14.4644	16.9037	10.9409	12.3702	15.9634	19.7427	20.1138	29.5578	19.6707	22.614	25.2606	21.7919	0.835668938	0.966018836	16	4	24	0.381460208	0.799546792	24	8.006887404	0.143775434
ENSMUSG00000050875	Minar2	membrane integral NOTCH2 associated receptor 2 [Source:MGI Symbol;Acc:MGI:2442934]	14.8523	17.0203	19.3995	15.2829	14.4305	18.0782	16.672	16.4033	16.3828	16.7102	15.1007	18.6141	16.2489	20.4088	0.47028201	0.966018836	8	12	24	0.29786711	0.768040178	24	4.593655892	0.079915429
ENSMUSG00000050890	Pdik1l	PDLIM1 interacting kinase 1 like [Source:MGI Symbol;Acc:MGI:2385213]	3.00486	3.41761	3.67322	5.18655	3.59446	3.47328	3.08635	2.74898	3.9208	2.85816	3.61711	3.18047	3.06936	3.92803	0.170659409	0.643496587	12	16	24	0.177184525	0.768040178	24	11.5942934	0.129665745
ENSMUSG00000050891	Tatdn1	TatD DNase domain containing 1 [Source:MGI Symbol;Acc:MGI:1916944]	13.8235	16.7481	15.5903	16.5096	17.4792	13.1712	14.0292	16.3593	17.9484	13.9955	16.7285	13.7534	15.7329	14.9535	0.039590247	0.338535904	12	12	24	0.144937181	0.753868423	24	9.539412595	0.103050492
ENSMUSG00000050896	Rtn4rl2	reticulon 4 receptor-like 2 [Source:MGI Symbol;Acc:MGI:2669796]	3.65011	2.24142	2.86141	3.34886	3.46999	2.57476	2.73578	3.09544	2.32251	5.53635	3.43563	3.6336	3.4567	3.24467	0.27909437	0.790764844	16	20	24	0.283406617	0.768040178	24	14.96204264	0.145427116
ENSMUSG00000050900	Gm7327	predicted gene 7327 [Source:MGI Symbol;Acc:MGI:3646274]	41.7705	37.5813	35.1025	35.8191	39.0276	35.5776	34.7686	33.9813	35.9023	28.5792	34.6227	34.8062	36.6968	36.0995	0.886952527	0.966018836	4	12	24	0.159139239	0.765350752	24	23.12925303	0.076609174
ENSMUSG00000050908	Tvp23a	trans-golgi network vesicle protein 23A [Source:MGI Symbol;Acc:MGI:3665441]	26.9355	21.1623	21.6354	22.6305	21.9237	26.8864	21.6058	24.6576	17.3206	28.767	22.1511	21.8247	23.2689	23.3356	0.90767227	0.966018836	16	20	24	0.566082476	0.843471947	24	19.8907963	0.077047357
ENSMUSG00000050910	Cdr2l	cerebellar degeneration-related protein 2-like [Source:MGI Symbol;Acc:MGI:2684867]	18.1878	12.8327	14.0266	13.8593	15.16	16.1677	15.5664	17.0431	11.845	19.0129	15.6807	15.515	15.5184	14.7109	0.300823756	0.809362476	4	8	24	0.325488981	0.768040178	24	19.81659721	0.103489279
ENSMUSG00000050912	Tmem123	transmembrane protein 123 [Source:MGI Symbol;Acc:MGI:1919179]	14.9193	16.0141	15.3027	14.6774	14.9689	13.3434	17.0179	16.2011	14.9358	12.6751	14.6256	12.694	13.6245	12.8628	0.437217485	0.966018836	8	16	24	0.395282793	0.799546792	24	3.932598819	0.054893366
ENSMUSG00000050914	Ankrd37	ankyrin repeat domain 37 [Source:MGI Symbol;Acc:MGI:3603344]	2.89927	3.55827	4.11862	3.19346	3.7983	2.95223	2.77837	2.92817	3.66608	3.69295	3.36506	3.23488	2.77668	3.63819	8.66E-04	0.071344007	12	16	24	0.014339906	0.533974075	24	10.47577541	0.143344324
ENSMUSG00000050917	Fgf4	fibroblast growth factor 4 [Source:MGI Symbol;Acc:MGI:95518]	2.87121	3.64525	3.01968	3.69607	3.86519	3.00553	3.45997	3.50458	3.59221	3.42755	3.23355	3.73472	3.49043	3.18936	0.935732159	0.966018836	20	8	24	0.653458955	0.883312134	24	12.67695162	0.036229762
ENSMUSG00000050919	Zfp366	zinc finger protein 366 [Source:MGI Symbol;Acc:MGI:2178429]	0.546793	0.498718	0.567591	0.830346	0.594826	0.687702	0.715529	0.732423	0.548938	0.776693	0.483781	0.447298	0.484886	0.506661	0.326899754	0.845515883	16	8	24	0.494325175	0.806389073	24	11.80402202	0.04943444
ENSMUSG00000050930	Map10	microtubule-associated protein 10 [Source:MGI Symbol;Acc:MGI:1921643]	1.21777	1.13881	1.13438	1.00047	1.22675	0.890335	1.40324	1.06366	1.01855	0.966553	0.906477	1.20317	1.07996	1.03834	0.159799452	0.619467724	4	12	24	0.210443366	0.768040178	24	0.087604864	0.061556346
ENSMUSG00000050931	Sgms2	sphingomyelin synthase 2 [Source:MGI Symbol;Acc:MGI:1921692]	8.86783	7.42919	8.46552	8.43616	7.32019	7.41078	7.64149	7.83875	7.21689	9.5075	7.22435	8.54843	8.75919	9.52994	0.722296281	0.966018836	4	16	24	0.701011281	0.900900643	24	5.624768968	0.042714495
ENSMUSG00000050944	Efcab5	EF-hand calcium binding domain 5 [Source:MGI Symbol;Acc:MGI:2442440]	0.633119	0.510447	0.301631	0.552849	0.66679	0.393171	0.451762	0.645766	0.314645	0.270624	0.535251	0.718439	0.654031	0.624513	0.200364953	0.684570416	20	20	24	0.144830106	0.753662838	24	21.98016533	0.109271453
ENSMUSG00000050945	Zfp438	zinc finger protein 438 [Source:MGI Symbol;Acc:MGI:2444919]	1.05984	1.52047	1.6023	1.42377	1.49418	1.49896	1.23869	1.46979	1.3078	1.51753	1.54646	1.57452	1.20199	1.43466	0.108029159	0.524035616	24	4	24	0.147724691	0.756090042	24	12.51804745	0.073137478
ENSMUSG00000050947	Amigo1	adhesion molecule with Ig like domain 1 [Source:MGI Symbol;Acc:MGI:2653612]	30.9516	25.7665	24.8766	27.701	26.3638	30.1967	27.2072	27.7777	21.5629	33.3104	26.0926	28.6068	27.473	28.0517	0.148073804	0.590732968	24	12	24	0.353860747	0.781118317	24	19.78569472	0.084471027
ENSMUSG00000050953	Gja1	gap junction protein, alpha 1 [Source:MGI Symbol;Acc:MGI:95713]	14.7929	21.0204	33.841	17.3679	14.9146	19.9732	24.2369	17.9878	22.2294	16.0197	16.0924	18.3706	13.2697	22.5912	0.315627348	0.828057631	12	8	24	0.200208481	0.768040178	24	6.540327043	0.232947166
ENSMUSG00000050954	Zfp169	zinc finger protein 169 [Source:MGI Symbol;Acc:MGI:1915161]	3.1771	2.97259	2.57909	2.59281	2.96717	3.00184	2.16701	2.57279	2.65239	2.0074	3.08445	3.0242	2.57177	2.41177	0.510031987	0.966018836	24	8	24	0.184384641	0.768040178	24	19.75229934	0.097837831
ENSMUSG00000050957	Insl6	insulin-like 6 [Source:MGI Symbol;Acc:MGI:1351595]	1.75506	1.78156	2.06629	2.42204	1.96165	1.7768	2.04904	1.24049	1.89425	1.59485	2.20502	2.01191	2.14863	1.92469	0.891105762	0.966018836	20	12	24	0.438066555	0.805393068	24	15.79495457	0.074520291
ENSMUSG00000050963	Kcns2	K+ voltage-gated channel, subfamily S, 2 [Source:MGI Symbol;Acc:MGI:1197011]	0.658637	0.789714	0.789747	0.645417	0.786055	0.825086	0.724027	0.787605	0.753997	0.732204	0.588387	0.803769	0.702749	0.677145	0.93717134	0.966018836	24	20	24	0.859478861	0.958575172	24	1.569458376	0.012849048
ENSMUSG00000050965	Prkca	protein kinase C, alpha [Source:MGI Symbol;Acc:MGI:97595]	61.2327	50.0311	48.9924	54.6149	56.7036	57.99	51.6067	51.3198	43.8882	58.8991	52.246	56.6668	56.4699	53.0634	0.020119233	0.259417302	24	12	24	0.043029211	0.625007356	24	19.32205743	0.115059905
ENSMUSG00000050967	Creg2	cellular repressor of E1A-stimulated genes 2 [Source:MGI Symbol;Acc:MGI:1928333]	5.02566	4.72431	5.59062	4.45381	4.28134	4.96425	5.46417	4.45839	4.22615	5.21968	4.29742	4.54694	4.08612	5.60862	0.681718085	0.966018836	8	12	24	0.572329466	0.846321055	24	4.286279737	0.054342444
ENSMUSG00000050973	Gdpgp1	GDP-D-glucose phosphorylase 1 [Source:MGI Symbol;Acc:MGI:2443429]	1.88648	1.78627	1.67922	1.8271	1.4446	2.07911	1.64149	2.27844	2.13628	1.6847	1.98748	1.64257	2.08569	2.00904	0.756219163	0.96629412	8	4	24	0.898552184	0.971263574	24	22.67729591	0.032791958
ENSMUSG00000050974	Gm9847	predicted pseudogene 9847 [Source:MGI Symbol;Acc:MGI:3642840]	1.46572	1.25415	1.39312	1.14713	0.680284	1.13483	1.22625	2.12184	1.68365	1.75614	2.56733	1.82175	1.53227	1.45429	0.715742619	0.966018836	8	20	24	0.797405827	0.935271704	24	7.013070448	0.065220859
ENSMUSG00000050989	Selenon	selenoprotein N [Source:MGI Symbol;Acc:MGI:2151208]	9.23784	8.45481	8.21956	8.71825	9.57092	8.83263	8.99278	9.31475	8.87307	9.6421	9.1796	8.90666	8.49618	8.09725	0.388056094	0.915479136	20	12	24	0.153188587	0.759484836	24	16.50654103	0.050309891
ENSMUSG00000050994	Adgb	androglobin [Source:MGI Symbol;Acc:MGI:3605549]	2.41403	2.47651	2.35262	2.36363	2.47057	2.36579	2.76929	1.92928	2.5096	1.82022	2.39239	2.32781	2.16032	2.28	0.873171835	0.966018836	20	12	24	0.539591004	0.829390353	24	21.43076834	0.045381221
ENSMUSG00000051000	Fam160a1	family with sequence similarity 160, member A1 [Source:MGI Symbol;Acc:MGI:2444746]	0.720299	0.969681	0.805577	1.15082	0.799943	0.845238	0.79538	1.62748	0.977191	1.03425	0.797752	0.949543	0.906514	0.894356	0.166168222	0.633011401	16	12	24	0.311247802	0.768040178	24	7.417859289	0.093544908
ENSMUSG00000051007	Gatd1	glutamine amidotransferase like class 1 domain containing 1 [Source:MGI Symbol;Acc:MGI:2387178]	17.4759	17.9517	16.5415	18.6722	16.7888	18.8545	18.3173	16.7526	15.6983	19.4654	15.6708	19.2252	17.306	18.0551	0.148073804	0.590732968	24	12	24	0.64287587	0.878809617	24	20.26407539	0.037644694
ENSMUSG00000051022	Hs3st1	heparan sulfate (glucosamine) 3-O-sulfotransferase 1 [Source:MGI Symbol;Acc:MGI:1201606]	5.8108	4.90026	5.33043	4.36102	4.25044	5.15754	5.31666	6.23553	4.79563	5.61647	4.90077	5.18758	4.18471	4.67877	0.890311217	0.966018836	8	12	24	0.62179377	0.871137783	24	2.720619102	0.053106664
ENSMUSG00000051029	Serpinb1b	serine (or cysteine) peptidase inhibitor, clade B, member 1b [Source:MGI Symbol;Acc:MGI:2445361]	10.8323	14.9046	17.0634	13.3793	14.1802	12.198	12.7159	10.9795	18.0919	11.4184	13.3922	13.1773	11.7481	13.1811	0.033248781	0.324908923	12	16	24	0.167843471	0.767020009	24	9.567088793	0.149201693
ENSMUSG00000051034	Zfp11	zinc finger protein 11 [Source:MGI Symbol;Acc:MGI:99156]	1.50215	1.51919	1.39505	1.23741	1.63929	1.59783	1.33603	1.89927	1.27362	1.55383	1.54681	1.63877	1.49141	1.51092	0.481272608	0.966018836	20	20	24	0.547315294	0.833663107	24	21.93442076	0.04235421
ENSMUSG00000051037	Zfp455	zinc finger protein 455 [Source:MGI Symbol;Acc:MGI:3040708]	1.48979	1.38098	1.36832	1.47772	1.4698	1.31553	1.51785	1.18948	1.65341	0.951098	1.474	1.24939	1.26278	1.37115	0.874672901	0.966018836	12	12	24	0.958130471	0.989075848	24	23.86839295	0.012318176
ENSMUSG00000051041	Olfml1	olfactomedin-like 1 [Source:MGI Symbol;Acc:MGI:2679264]	8.54119	10.7144	10.4272	7.85856	10.0532	8.38666	10.4915	8.93428	12.5808	7.67853	8.84849	9.29111	8.72087	9.9508	0.085045716	0.466404237	12	4	24	0.290588191	0.768040178	24	5.359043207	0.109767469
ENSMUSG00000051043	Gprc5c	G protein-coupled receptor, family C, group 5, member C [Source:MGI Symbol;Acc:MGI:1917605]	8.0777	6.09567	5.67573	7.08521	7.01817	7.27262	6.43056	9.24815	5.79738	9.3671	6.95018	7.29112	7.78232	7.28118	0.649212555	0.966018836	4	8	24	0.822034754	0.943977421	24	19.99347405	0.048755658
ENSMUSG00000051048	P4ha3	procollagen-proline, 2-oxoglutarate 4-dioxygenase (proline 4-hydroxylase), alpha polypeptide III [Source:MGI Symbol;Acc:MGI:2444049]	6.4708	5.73007	6.62157	5.43931	6.29351	5.85966	5.88853	4.72585	4.53894	6.40602	5.40506	5.82472	4.94497	5.01794	0.843639218	0.966018836	16	16	24	0.197506456	0.768040178	24	17.38898506	0.086926187
ENSMUSG00000051062	Fbll1	fibrillarin-like 1 [Source:MGI Symbol;Acc:MGI:3034689]	2.36716	1.77622	2.92962	1.77091	1.88935	2.17476	2.45778	1.83233	2.12505	2.34532	2.38694	2.25784	1.71128	2.68112	0.765626279	0.966018836	12	4	24	0.935654952	0.983894024	24	8.002635454	0.025425988
ENSMUSG00000051065	Mb21d2	Mab-21 domain containing 2 [Source:MGI Symbol;Acc:MGI:1917028]	6.15796	5.22549	5.64518	5.32283	4.93865	5.49775	4.75186	5.91855	4.73256	6.00049	4.77531	5.69544	5.19479	5.50295	0.887862353	0.966018836	8	12	24	0.82565623	0.945421318	24	2.865510403	0.026265299
ENSMUSG00000051067	Lingo3	leucine rich repeat and Ig domain containing 3 [Source:MGI Symbol;Acc:MGI:3609246]	0.555589	0.584249	0.854558	0.613566	0.418728	0.676644	0.708925	0.890716	0.827239	0.749737	0.592704	0.781441	0.785411	0.955101	0.048889741	0.379129806	8	12	24	0.00762176	0.479831204	24	5.508438603	0.113398266
ENSMUSG00000051074	4930579K19Rik	RIKEN cDNA 4930579K19 gene [Source:MGI Symbol;Acc:MGI:1923131]	0.815563	0.664939	0.803698	0.370316	0.854672	0.791396	0.314807	0.446251	0.998054	0.806021	0.498541	0.88761	0.60025	0.492817	0.671726933	0.966018836	12	20	24	0.763936839	0.924028949	24	14.6929042	0.055024797
ENSMUSG00000051098	Mblac2	metallo-beta-lactamase domain containing 2 [Source:MGI Symbol;Acc:MGI:1920102]	15.4632	14.4106	15.5613	13.4629	14.2124	13.5005	13.5549	13.3281	12.6993	13.8476	14.9936	12.299	12.4324	14.7009	0.879201926	0.966018836	20	4	24	0.85204199	0.955622342	24	9.022608937	0.021277473
ENSMUSG00000051111	Sv2c	synaptic vesicle glycoprotein 2c [Source:MGI Symbol;Acc:MGI:1922459]	73.7455	54.7574	58.566	60.1915	58.3436	68.5657	61.2999	57.8331	42.1691	83.4744	55.9565	63.6987	62.6612	64.0922	0.439800617	0.966018836	24	12	24	0.458021504	0.805393068	24	19.6318631	0.113562511
ENSMUSG00000051113	Fam71e1	family with sequence similarity 71, member E1 [Source:MGI Symbol;Acc:MGI:1922788]	3.42176	3.77315	4.12117	4.3811	2.69983	3.50175	3.63253	4.2655	5.09334	5.75767	6.07024	4.339	3.78499	4.29521	0.105345531	0.518002936	16	12	24	0.06374135	0.663876076	24	10.44938068	0.189428635
ENSMUSG00000051116	Gm8121	predicted pseudogene 8121 [Source:MGI Symbol;Acc:MGI:3645325]	19.793	21.5517	17.4726	18.2378	21.8498	17.1676	18.6875	19.2709	23.2462	16.2423	22.005	17.7368	21.6001	20.5285	0.814576414	0.966018836	12	12	24	0.792609882	0.933721061	24	3.952314596	0.04144994
ENSMUSG00000051124	Gimap9	GTPase, IMAP family member 9 [Source:MGI Symbol;Acc:MGI:3511744]	0.833847	0.712158	0.520343	0.706024	0.816429	0.898998	1.0318	0.921341	0.967389	0.490096	0.710463	0.637925	0.979683	0.507073	0.057224139	0.401309455	4	12	24	0.201314504	0.768040178	24	22.77000816	0.104593008
ENSMUSG00000051146	Camk2n2	calcium/calmodulin-dependent protein kinase II inhibitor 2 [Source:MGI Symbol;Acc:MGI:1920297]	19.8029	17.6737	21.2365	17.9972	15.2828	20.2392	19.4137	17.7291	19.1901	27.3703	17.302	22.2721	18.0381	23.2872	0.912686312	0.966018836	24	20	24	0.732610738	0.912739737	24	7.903218436	0.063091481
ENSMUSG00000051147	Nat2	N-acetyltransferase 2 (arylamine N-acetyltransferase) [Source:MGI Symbol;Acc:MGI:109201]	3.60271	3.35415	3.3246	2.88644	3.16395	3.64532	2.67147	3.29003	3.1456	2.33723	3.19253	3.1102	2.9219	2.91681	0.916860713	0.966018836	24	16	24	0.357578342	0.784480156	24	22.73591542	0.063586543
ENSMUSG00000051149	Adnp	activity-dependent neuroprotective protein [Source:MGI Symbol;Acc:MGI:1338758]	1.84451	1.41432	2.23941	2.12881	1.51409	1.84833	1.82767	2.41149	1.82787	1.9961	2.40068	2.21273	2.75291	2.10392	0.658515291	0.966018836	24	12	24	0.965423695	0.991608289	24	5.45960911	0.015552746
ENSMUSG00000051154	Commd3	COMM domain containing 3 [Source:MGI Symbol;Acc:MGI:88218]	65.7634	73.2233	68.4488	67.889	67.343	63.6819	66.1919	69.1089	75.7309	61.0207	74.3532	66.4131	73.4898	69.6225	0.321662653	0.83607295	12	12	24	0.438049226	0.805393068	24	5.813070071	0.044692083
ENSMUSG00000051159	Cited1	Cbp/p300-interacting transactivator with Glu/Asp-rich carboxy-terminal domain 1 [Source:MGI Symbol;Acc:MGI:108023]	1.74247	1.94584	1.95558	1.56474	1.73258	2.08502	1.67569	1.63149	1.28749	1.58028	1.6564	1.38521	1.04513	2.02071	0.890648739	0.966018836	8	8	24	0.989272199	0.997861299	24	4.411283301	0.008770293
ENSMUSG00000051166	Eml5	echinoderm microtubule associated protein like 5 [Source:MGI Symbol;Acc:MGI:2442513]	8.65042	11.3026	11.2548	10.5096	10.8854	9.6363	10.3176	10.7301	12.5634	9.52671	12.5957	13.3086	11.3188	13.9037	0.914502381	0.966018836	8	8	24	0.549645955	0.835049505	24	7.890825406	0.062184429
ENSMUSG00000051169	Rpusd3	RNA pseudouridylate synthase domain containing 3 [Source:MGI Symbol;Acc:MGI:2141440]	2.8713	2.7752	2.6581	2.04687	2.96971	2.97081	4.02895	3.05619	3.23005	4.00299	2.69259	4.7176	3.55298	2.72651	0.336988702	0.85758369	24	12	24	0.359288883	0.785405943	24	21.65376151	0.118970186
ENSMUSG00000051177	Plcb1	phospholipase C, beta 1 [Source:MGI Symbol;Acc:MGI:97613]	8.53924	8.48035	8.77693	8.98835	9.11664	8.98462	9.63583	7.25079	8.26256	8.74923	8.06971	8.44796	8.54909	8.50783	0.671726933	0.966018836	16	16	24	0.441420554	0.805393068	24	17.8915649	0.042433623
ENSMUSG00000051184	Zfp524	zinc finger protein 524 [Source:MGI Symbol;Acc:MGI:1916740]	5.96129	5.10189	5.21637	5.21736	5.35407	5.53366	5.79183	5.01592	5.71569	5.20711	5.83441	5.56152	6.13252	5.54638	0.036302654	0.33234124	4	4	24	0.11991688	0.731708105	24	21.59691183	0.056318814
ENSMUSG00000051185	Fam174a	family with sequence similarity 174, member A [Source:MGI Symbol;Acc:MGI:1914948]	24.2586	24.4088	22.5737	22.8127	21.0672	21.5101	22.199	22.2982	19.9443	22.1388	25.5839	22.0739	19.89	21.227	0.825856869	0.966018836	20	4	24	0.82206332	0.943977421	24	17.59347025	0.023274345
ENSMUSG00000051207	Mrgprd	MAS-related GPR, member D [Source:MGI Symbol;Acc:MGI:3033142]	48.0561	34.5121	34.2313	37.0983	34.5896	46.5927	36.035	41.3628	26.0107	48.6459	33.7138	43.3563	40.2522	39.041	0.14313801	0.585944911	24	12	24	0.258124057	0.768040178	24	20.93626312	0.157102524
ENSMUSG00000051209	Gpr119	G-protein coupled receptor 119 [Source:MGI Symbol;Acc:MGI:2668412]	0.67691	0.545541	0.491129	0.477026	0.452908	0.3972	0.321766	0.591392	0.592098	0.422211	0.636207	0.429777	0.624275	0.62652	0.46238066	0.966018836	4	20	24	0.403340216	0.799546792	24	5.004414372	0.054885168
ENSMUSG00000051223	Bzw1	basic leucine zipper and W2 domains 1 [Source:MGI Symbol;Acc:MGI:1914132]	60.4016	60.2791	58.0721	57.942	60.3622	56.5875	55.7942	53.6319	59.2261	53.4938	64.1255	57.5427	56.2049	58.4825	0.929527443	0.966018836	20	20	24	0.65847551	0.884596426	24	16.50999229	0.025076388
ENSMUSG00000051224	Tceanc	transcription elongation factor A (SII) N-terminal and central domain containing [Source:MGI Symbol;Acc:MGI:2685236]	0.641162	1.0729	1.35432	1.43833	1.08188	1.1645	0.983259	1.05282	1.26184	1.23247	1.16798	1.07132	1.14105	1.27043	0.004122392	0.140598674	12	16	24	0.009601342	0.502965379	24	10.6555238	0.13490273
ENSMUSG00000051232	Tmem199	transmembrane protein 199 [Source:MGI Symbol;Acc:MGI:2144113]	29.6911	28.5436	27.9962	28.5331	26.4958	29.2767	27.2645	29.6173	25.2024	28.0432	28.4152	28.365	27.452	30.4357	0.3576519	0.882035609	8	4	24	0.491499515	0.805393068	24	0.411240979	0.031953808
ENSMUSG00000051234	Rnf7	ring finger protein 7 [Source:MGI Symbol;Acc:MGI:1337096]	135.364	136.264	127.468	127.841	135.243	128.961	131.45	129.513	135.69	120.844	138.86	131.182	139.037	129.514	0.192357053	0.669841558	4	12	24	0.356045399	0.782989503	24	22.51142013	0.031819429
ENSMUSG00000051235	Gen1	GEN1, Holliday junction 5' flap endonuclease [Source:MGI Symbol;Acc:MGI:2443149]	0.723385	0.832611	0.784613	0.886153	0.573794	0.860973	0.644343	0.659761	0.75491	0.724307	0.710343	0.716444	0.877561	0.714776	0.897570341	0.966018836	16	4	24	0.895599833	0.971474297	24	6.136212956	0.014655963
ENSMUSG00000051236	Msrb3	methionine sulfoxide reductase B3 [Source:MGI Symbol;Acc:MGI:2443538]	5.91271	6.58564	6.05009	7.14133	6.19522	6.33769	6.86266	8.50952	7.59211	8.20652	6.17574	6.78878	6.93988	6.81953	0.676344318	0.966018836	16	4	24	0.173929429	0.768040178	24	7.82449532	0.094865023
ENSMUSG00000051238	Swsap1	SWIM type zinc finger 7 associated protein 1 [Source:MGI Symbol;Acc:MGI:1914212]	6.57607	5.23629	5.58576	5.94147	5.55027	5.8963	5.72324	6.4796	4.9694	6.68675	6.20274	6.22204	6.49035	5.7178	0.44861185	0.966018836	24	8	24	0.447883877	0.805393068	24	19.86723612	0.055325916
ENSMUSG00000051242	Pcdhb9	protocadherin beta 9 [Source:MGI Symbol;Acc:MGI:2136744]	3.25688	2.67545	3.03857	3.06418	2.25596	3.01869	2.86115	2.73164	2.38971	2.90988	2.78954	2.94323	2.30678	2.64078	0.94086481	0.966018836	24	8	24	0.535774779	0.827176676	24	17.86053943	0.040456722

ENSMUSG00000051243	Islr2	immunoglobulin superfamily containing leucine-rich repeat 2 [Source:MGI Symbol;Acc:MGI:2444277]	1.61095	1.8886	1.55686	1.58548	1.32219	1.70399	1.31876	2.02762	1.7168	2.43322	1.22944	2.44918	1.5582	2.69371	0.192357053	0.669841558	8	12	24	0.391459637	0.799546792	24	5.813096994	0.113000355
ENSMUSG00000051246	Msantd1	Myb/SANT-like DNA-binding domain containing 1 [Source:MGI Symbol;Acc:MGI:2684990]	2.09336	2.43056	2.91342	2.4083	2.06826	2.11714	2.2071	1.61878	2.07305	2.07971	2.10824	2.02439	2.19385	2.14317	0.835401638	0.966018836	12	8	24	0.626142919	0.872592654	24	9.677895235	0.046161244
ENSMUSG00000051255	Gm6563	predicted pseudogene 6563 [Source:MGI Symbol;Acc:MGI:3646907]	61.9025	65.684	58.5814	62.6159	65.9136	55.5824	56.1322	53.5244	66.3898	47.4836	65.4165	55.6728	61.4854	60.0339	0.885059765	0.966018836	20	4	24	0.999353884	0.999806823	24	10.32340566	0.002004061
ENSMUSG00000051256	Jagn1	jagunal homolog 1 [Source:MGI Symbol;Acc:MGI:1915017]	26.088	24.4472	25.1869	24.2892	24.6055	26.3764	24.0195	25.9991	23.041	24.8818	26.6323	26.1201	27.8032	26.8928	0.403750665	0.937128244	24	16	24	0.155226891	0.760374942	24	22.0976727	0.047844263
ENSMUSG00000051278	Zgrf1	zinc finger, GRF-type containing 1 [Source:MGI Symbol;Acc:MGI:1918893]	1.18289	1.62066	1.43437	1.61534	1.36773	1.34105	1.00216	1.55253	1.88237	1.30232	1.291	1.28915	1.61949	1.41351	0.085992778	0.466404237	12	12	24	0.088918495	0.708099981	24	8.168665233	0.111128981
ENSMUSG00000051285	Pcmtd1	protein-L-isoaspartate (D-aspartate) O-methyltransferase domain containing 1 [Source:MGI Symbol;Acc:MGI:2441773]	30.2065	36.1603	32.3185	33.4152	31.2871	29.4955	32.2125	29.475	34.8205	31.1795	34.5073	30.8103	29.042	32.852	0.235177833	0.73629392	12	16	24	0.188747601	0.768040178	24	9.42343661	0.06688973
ENSMUSG00000051306	Usp42	ubiquitin specific peptidase 42 [Source:MGI Symbol;Acc:MGI:1924050]	3.95376	3.37347	3.63485	3.16547	3.55824	3.19366	3.64341	3.41086	3.15894	3.6092	3.47398	3.62923	3.99074	3.56863	0.085329704	0.466404237	4	12	24	0.230200277	0.768040178	24	23.2552409	0.053585901
ENSMUSG00000051316	Taf7	TATA-box binding protein associated factor 7 [Source:MGI Symbol;Acc:MGI:1346348]	6.07608	5.76393	6.02375	5.41365	5.90884	5.3826	5.67248	5.30547	5.36148	5.2778	6.77808	5.7736	5.62139	5.69313	0.893307667	0.966018836	20	20	24	0.521305973	0.82025681	24	18.5053031	0.039048163
ENSMUSG00000051319	Mtln	mitoregulin [Source:MGI Symbol;Acc:MGI:1915135]	43.0537	43.665	40.4174	38.6105	36.81	31.8774	41.9728	45.5261	55.335	40.0113	54.122	43.9522	46.2245	45.0198	0.417963425	0.963884303	8	16	24	0.332221577	0.768996731	24	6.323504726	0.108443541
ENSMUSG00000051323	Pcdh19	protocadherin 19 [Source:MGI Symbol;Acc:MGI:2685563]	1.98743	1.74098	1.86927	2.07816	2.10726	1.50606	2.65728	1.36185	1.60236	1.38671	1.73521	1.45415	1.28549	1.51439	0.885059765	0.966018836	20	12	24	0.768191809	0.925214784	24	18.24571455	0.04727949
ENSMUSG00000051329	Nup160	nucleoporin 160 [Source:MGI Symbol;Acc:MGI:1926227]	5.33213	5.05498	5.28956	4.20168	5.25343	5.42526	6.36386	5.60517	6.4332	4.25513	6.71965	4.81049	5.66471	4.98799	0.47028201	0.966018836	4	12	24	0.589400661	0.854253633	24	23.8173541	0.068729235
ENSMUSG00000051331	Cacna1c	calcium channel, voltage-dependent, L type, alpha 1C subunit [Source:MGI Symbol;Acc:MGI:103013]	8.28961	7.23171	7.548	7.88529	7.39349	8.25717	7.49887	7.86341	7.33744	9.4279	7.57791	7.80766	7.33473	8.15189	0.935194469	0.966018836	16	20	24	0.730801124	0.912268461	24	13.751894	0.028301834
ENSMUSG00000051335	Gfod1	glucose-fructose oxidoreductase domain containing 1 [Source:MGI Symbol;Acc:MGI:2145304]	13.528	8.93681	9.21345	10.4235	9.30947	11.4976	10.2668	11.101	7.02467	13.2498	9.21359	11.2111	11.0323	10.5854	0.12833063	0.560209134	24	12	24	0.332032541	0.768814351	24	21.08920695	0.132476955
ENSMUSG00000051339	2900026A02Rik	RIKEN cDNA 2900026A02 gene [Source:MGI Symbol;Acc:MGI:1920194]	6.82359	5.47585	5.07863	5.61267	5.7306	6.07695	5.83753	5.81978	5.0216	8.36703	5.58267	5.87095	6.47562	5.69408	0.009640466	0.194720062	4	8	24	0.684679005	0.895095838	24	18.77424626	0.057513038
ENSMUSG00000051341	Zfp52	zinc finger protein 52 [Source:MGI Symbol;Acc:MGI:99199]	7.87536	6.79454	5.80443	6.03635	8.32754	6.49547	7.34086	5.87459	6.1468	5.00714	8.17243	6.36725	7.27079	6.58165	0.021214536	0.264116537	20	20	24	0.069158703	0.673520298	24	20.65703168	0.157969019
ENSMUSG00000051343	Rab11fip5	RAB11 family interacting protein 5 (class I) [Source:MGI Symbol;Acc:MGI:1098586]	19.2712	16.5979	18.0887	17.1533	17.1074	19.1311	18.0775	19.493	15.0997	25.7657	15.4631	19.0779	17.5145	19.7101	0.94791825	0.966018836	8	12	24	0.99895239	0.999770694	24	8.786889313	0.003426756
ENSMUSG00000051344	Plekhm3	pleckstrin homology domain containing, family M, member 3 [Source:MGI Symbol;Acc:MGI:2443627]	15.4867	11.9339	11.7132	12.9118	11.5572	15.3064	13.3568	13.7576	8.85734	16.5795	11.7069	14.3446	13.6127	13.8356	0.243586913	0.741580974	24	12	24	0.298952097	0.768040178	24	21.19847568	0.131397394
ENSMUSG00000051346	Spryd4	SPRY domain containing 4 [Source:MGI Symbol;Acc:MGI:1913951]	7.64924	7.68387	7.10781	7.39233	7.1744	8.22586	7.98042	9.0966	7.71522	7.81821	7.02716	7.80895	8.20121	7.52295	0.25849765	0.761738864	24	20	24	0.128200121	0.740162193	24	1.427146788	0.06368046
ENSMUSG00000051351	Zfp46	zinc finger protein 46 [Source:MGI Symbol;Acc:MGI:99192]	6.01944	6.55304	7.23045	6.37944	5.64405	6.27099	6.12522	6.88325	6.4317	8.38908	5.57983	6.84127	6.21659	6.09471	0.191123261	0.669841558	16	4	24	0.25746103	0.768040178	24	8.7355633	0.086560359
ENSMUSG00000051355	Commd1	COMM domain containing 1 [Source:MGI Symbol;Acc:MGI:109474]	60.8173	73.8972	64.2961	67.9836	68.7698	62.7494	68.6716	67.0715	79.1168	54.9197	69.8593	64.8253	77.8566	70.019	0.94086481	0.966018836	8	16	24	0.480727317	0.805393068	24	4.170217865	0.063870228
ENSMUSG00000051359	Ncald	neurocalcin delta [Source:MGI Symbol;Acc:MGI:1196326]	63.0928	51.9302	49.2155	51.2854	46.1416	57.1847	52.7209	49.1809	43.985	62.2057	49.6275	61.8929	57.5953	62.4465	0.208438456	0.698704663	4	8	24	0.284492058	0.768040178	24	22.47293625	0.102512125
ENSMUSG00000051367	Six1	sine oculis-related homeobox 1 [Source:MGI Symbol;Acc:MGI:102780]	15.4545	12.4728	11.8968	12.906	12.6517	14.4259	12.6307	15.3555	11.7962	19.0117	13.4664	12.7549	14.4147	13.4865	0.628286596	0.966018836	24	12	24	0.946370322	0.986887534	24	18.02814425	0.023724523
ENSMUSG00000051373	Plpp7	phospholipid phosphatase 7 (inactive) [Source:MGI Symbol;Acc:MGI:2445183]	7.07794	6.95942	7.87266	8.33309	6.00869	7.25432	6.97925	10.2356	8.41413	10.1378	7.12484	7.04287	8.69214	7.22519	0.590017078	0.966018836	16	4	24	0.138992968	0.748397521	24	7.923479147	0.145579275
ENSMUSG00000051375	Pcdh1	protocadherin 1 [Source:MGI Symbol;Acc:MGI:104692]	17.3022	12.6533	13.1198	13.8868	14.1937	15.5144	14.5828	15.8372	11.8415	20.7355	14.122	15.852	15.5174	15.2636	0.091424683	0.483797062	24	12	24	0.617599795	0.868431324	24	19.4253687	0.074531995
ENSMUSG00000051379	Flrt3	fibronectin leucine rich transmembrane protein 3 [Source:MGI Symbol;Acc:MGI:1918686]	7.16039	6.12336	8.10822	5.65648	7.81577	7.62337	6.511	5.96473	5.84393	5.59951	8.42193	6.56377	6.52816	6.65423	0.022000259	0.267229408	20	20	24	0.241065115	0.768040178	24	18.74689463	0.112898839
ENSMUSG00000051390	Zbtb22	zinc finger and BTB domain containing 22 [Source:MGI Symbol;Acc:MGI:1931870]	8.04991	8.03972	8.4096	7.27314	8.26567	8.27311	7.54883	9.27041	7.18296	9.74632	7.11272	8.80238	8.39127	8.31222	0.838692453	0.966018836	8	12	24	0.912828415	0.975351666	24	3.020356	0.020095023
ENSMUSG00000051391	Ywhag	tyrosine 3-monooxygenase/tryptophan 5-monooxygenase activation protein, gamma polypeptide [Source:MGI Symbol;Acc:MGI:108109]	422.263	290.355	308.614	325.251	291.95	375.083	357.762	366.817	237.162	479.401	310.34	362.552	338.192	352.423	0.390686537	0.915479136	24	12	24	0.557413598	0.839161699	24	20.97863765	0.110464827
ENSMUSG00000051396	Gm45902	predicted gene 45902 [Source:MGI Symbol;Acc:MGI:5805017]	1.22763	1.17704	1.06662	1.27065	1.12647	1.12094	1.1217	1.26588	1.30282	1.06982	1.41795	1.24718	1.13529	1.2301	0.8635847	0.966018836	20	8	24	0.942180037	0.985597432	24	12.93034989	0.008729954
ENSMUSG00000051401	Kctd16	potassium channel tetramerisation domain containing 16 [Source:MGI Symbol;Acc:MGI:1914659]	9.18301	7.06819	6.3938	6.69591	9.87651	8.15479	8.36747	5.86482	6.3225	6.85982	9.78387	7.86085	10.2483	6.66593	0.009067305	0.192760771	4	8	24	0.005003943	0.442351542	24	19.59210445	0.263995105
ENSMUSG00000051403	Ppp1r37	protein phosphatase 1, regulatory subunit 37 [Source:MGI Symbol;Acc:MGI:2687042]	20.1335	17.812	17.4737	19.0021	18.6275	19.4836	18.751	19.3624	17.6442	20.4214	18.2375	17.5242	18.1408	20.0408	0.941885246	0.966018836	16	20	24	0.972249265	0.993238187	24	22.0230629	0.007295497
ENSMUSG00000051413	Plagl2	pleiomorphic adenoma gene-like 2 [Source:MGI Symbol;Acc:MGI:1933165]	1.59758	1.44927	1.71493	1.76853	1.71515	1.68349	1.86322	1.7786	1.83268	1.67316	1.65995	1.74736	1.63124	1.57934	0.858932988	0.966018836	12	20	24	0.67405002	0.890479233	24	13.7064444	0.020445043
ENSMUSG00000051427	Ccdc157	coiled-coil domain containing 157 [Source:MGI Symbol;Acc:MGI:3041210]	8.6517	8.63505	8.17358	9.1513	8.29601	8.17834	8.73638	9.00326	8.35447	9.53721	8.43287	9.06068	8.14428	8.54632	0.927004797	0.966018836	16	20	24	0.693634926	0.898364349	24	11.25981066	0.021601382
ENSMUSG00000051435	Fhad1	forkhead-associated (FHA) phosphopeptide binding domain 1 [Source:MGI Symbol;Acc:MGI:1920323]	0.478719	0.766802	0.421209	0.502282	0.609187	0.860689	0.498132	0.215592	0.459535	0.478474	0.423615	0.691826	0.317197	0.531002	0.390686537	0.915479136	24	12	24	0.304034566	0.768040178	24	18.68760267	0.096325335
ENSMUSG00000051439	Cd14	CD14 antigen [Source:MGI Symbol;Acc:MGI:88318]	2.00888	2.6971	2.24688	2.68882	2.47937	2.14175	2.55525	2.32123	2.97269	1.66619	2.9363	2.0039	2.28761	2.02932	0.942429894	0.966018836	12	20	24	0.833277683	0.948610791	24	10.37618144	0.04069391
ENSMUSG00000051444	Bbs12	Bardet-Biedl syndrome 12 (human) [Source:MGI Symbol;Acc:MGI:2686651]	1.91861	2.07555	1.97325	2.07816	2.33759	1.73365	1.7942	1.85717	1.69937	2.42806	2.40618	2.21182	[illegible]	2.14892	0.935194469	0.966018836	24	20	24	0.582414057	0.851359615	24	17.3784532	0.044916682
ENSMUSG00000051451	Crebzf	CREB/ATF bZIP transcription factor [Source:MGI Symbol;Acc:MGI:2675296]	36.1761	34.8031	31.7539	34.3067	36.81	31.715	39.6968	29.372	32.2223	30.4166	38.9527	32.5141	32.2781	33.6115	0.570349842	0.966018836	20	16	24	0.330299121	0.768040178	24	19.40286564	0.074069884
ENSMUSG00000051456	Hspb3	heat shock protein 3 [Source:MGI Symbol;Acc:MGI:1928479]	0.128846	1.07953	1.12047	0.810599	1.0578	0.984494	0.785264	2.01521	1.66991	1.87315	0.972457	0.864535	1.11751	0.676549	0.498426201	0.966018836	12	16	24	0.115991351	0.727799063	24	9.664625096	0.268154502
ENSMUSG00000051457	Spn	sialophorin [Source:MGI Symbol;Acc:MGI:98384]	0.289589	0.767087	1.05865	0.727396	0.549801	0.746374	0.89186	0.564888	0.798247	0.578038	0.589699	0.688881	0.569999	0.48989	0.791413662	0.966018836	12	20	24	0.580824286	0.850337927	24	9.46991349	0.070048206
ENSMUSG00000051469	Zfp24	zinc finger protein 24 [Source:MGI Symbol;Acc:MGI:1929704]	19.5238	20.4825	19.8808	20.7416	20.023	19.7727	18.2031	17.9508	19.8967	17.527	19.3435	19.0179	18.8121	19.176	0.725736046	0.966018836	12	16	24	0.751865333	0.919370646	24	13.40237669	0.017464961
ENSMUSG00000051483	Cbr1	carbonyl reductase 1 [Source:MGI Symbol;Acc:MGI:88284]	32.7555	30.5404	30.3645	30.1545	28.3649	31.9172	30.5078	33.0903	31.4114	35.6062	32.5074	34.2266	33.6852	35.1621	0.933543173	0.966018836	24	20	24	0.593762194	0.857022893	24	2.774528982	0.026961155
ENSMUSG00000051486	Pcdhb11	protocadherin beta 11 [Source:MGI Symbol;Acc:MGI:2136746]	0.799274	0.657889	0.667733	0.526041	0.763885	0.611321	0.659265	0.677258	0.611217	0.570752	0.777561	0.589291	0.542536	0.672589	0.664568268	0.966018836	20	20	24	0.716552752	0.906516105	24	21.28249946	0.023856117
ENSMUSG00000051495	Irf2bp2	interferon regulatory factor 2 binding protein 2 [Source:MGI Symbol;Acc:MGI:2443921]	5.51767	4.61255	4.80709	4.79997	4.86534	4.73961	5.6863	4.27719	5.0379	5.41744	5.32876	5.21627	5.1577	4.86286	0.265774856	0.773510818	4	4	24	0.40891283	0.799546792	24	19.08513063	0.049990924
ENSMUSG00000051497	Kcnj16	potassium inwardly-rectifying channel, subfamily J, member 16 [Source:MGI Symbol;Acc:MGI:1314842]	2.16335	2.85431	5.20347	1.84928	1.99084	2.91463	3.5721	1.94365	3.12866	2.38325	1.89445	2.36766	1.60887	2.75559	0.446653834	0.966018836	12	12	24	0.411741695	0.799546792	24	6.257456256	0.116257948
ENSMUSG00000051499	Zfp786	zinc finger protein 786 [Source:MGI Symbol;Acc:MGI:3026883]	0.590411	0.583962	0.851434	0.704085	0.641803	0.958744	0.543314	0.690821	0.520687	0.775972	0.702104	0.704444	0.565282	0.604568	0.10338739	0.517709628	24	4	24	0.263497609	0.768040178	24	15.17006259	0.064159384
ENSMUSG00000051502	Ufsp1	UFM1-specific peptidase 1 [Source:MGI Symbol;Acc:MGI:1917490]	5.51048	5.71492	6.12943	7.69548	5.64877	5.75672	5.84156	6.18087	5.40614	7.86718	5.31962	5.21398	6.80141	7.61854	0.265774856	0.773510818	16	4	24	0.238858701	0.768040178	24	8.760885301	0.116673098
ENSMUSG00000051504	Siglech	sialic acid binding Ig-like lectin H [Source:MGI Symbol;Acc:MGI:2443256]	0.588853	1.43511	2.14965	0.688897	1.20355	1.27591	1.83006	1.38188	2.06587	0.86471	0.931672	1.55435	1.51814	1.41387	0.087978327	0.472021905	12	4	24	0.290296358	0.768040178	24	4.671817961	0.179380779
ENSMUSG00000051506	Wdfy4	WD repeat and FYVE domain containing 4 [Source:MGI Symbol;Acc:MGI:3584510]	0.380033	0.710248	0.821867	0.498435	1.0335	0.521556	0.44518	0.917408	0.811605	0.809916	0.394076	0.422086	0.644723	0.552973	0.196044629	0.673646834	12	12	24	0.156199546	0.761842768	24	8.951179495	0.138099905
ENSMUSG00000051510	Mafg	v-maf musculoaponeurotic fibrosarcoma oncogene family, protein G (avian) [Source:MGI Symbol;Acc:MGI:96911]	38.3377	30.6164	29.935	34.3699	36.555	33.7277	37.4665	34.6776	31.8901	36.0752	38.515	34.9091	36.1675	32.7516	0.019711532	0.258568924	20	16	24	0.044442489	0.628432994	24	19.06670982	0.100881133
ENSMUSG00000051515	Fam181b	family with sequence similarity 181, member B [Source:MGI Symbol;Acc:MGI:1930951]	3.57564	3.62194	4.00137	3.15132	3.04123	3.53592	4.34254	3.36424	3.16678	3.90559	4.47755	4.56661	3.7654	4.2353	0.888991156	0.966018836	16	16	24	0.565542157	0.843471947	24	22.30669494	0.058299344
ENSMUSG00000051517	Arhgef39	Rho guanine nucleotide exchange factor (GEF) 39 [Source:MGI Symbol;Acc:MGI:3036286]	0.577418	1.33758	1.58075	1.0822	0.77541	1.29957	1.40762	1.05157	1.42941	0.934608	1.33893	1.04581	1.1856	1.34982	0.198504154	0.680216953	12	4	24	0.364772797	0.789112192	24	6.856640023	0.108798705
ENSMUSG00000051518	Rps19bp1	ribosomal protein S19 binding protein 1 [Source:MGI Symbol;Acc:MGI:1913788]	13.6253	12.1021	11.5991	11.8886	14.886	12.763	11.7488	11.516	11.3525	11.2435	16.0739	14.2404	14.3695	12.8262	0.005076782	0.144054728	20	20	24	0.019911686	0.565108098	24	18.99390212	0.149280566
ENSMUSG00000051527	Usp29	ubiquitin specific peptidase 29 [Source:MGI Symbol;Acc:MGI:1888998]	1.06909	1.61794	2.55329	1.46738	1.1331	1.51347	2.50405	1.22636	1.9214	1.14889	1.11599	1.51119	1.002	1.91618	0.173410058	0.648600527	12	8	24	0.318864943	0.768040178	24	5.90195408	0.16260833
ENSMUSG00000051537	Gm5124	predicted pseudogene 5124 [Source:MGI Symbol;Acc:MGI:3643622]	10.2982	8.17739	9.1739	7.96309	8.27883	9.82406	8.61504	8.26837	7.20646	9.54884	7.87641	9.01932	8.24465	8.97468	0.708665292	0.966018836	24	12	24	0.339274773	0.773727252	24	21.51314762	0.072337772
ENSMUSG00000051548	Gm6365	predicted pseudogene 6365 [Source:MGI Symbol;Acc:MGI:3643502]	0.822711	1.32262	0.745172	0.86201	0.971466	1.00811	1.16966	1.16363	1.12203	1.49514	0.987281	0.964419	1.00673	1.02977	0.823677655	0.966018836	8	12	24	0.752507326	0.919493971	24	6.928537377	0.041489925
ENSMUSG00000051550	Zfp579	zinc finger protein 579 [Source:MGI Symbol;Acc:MGI:1915740]	7.92049	7.91274	9.18051	9.36631	8.89536	8.97435	8.74139	8.75382	9.67547	7.85504	10.236	7.18601	7.46799	7.84074	0.100794585	0.511659635	20	8	24	0.150133619	0.75876965	24	12.37764952	0.095287249
ENSMUSG00000051557	Pus1l	pseudouridylate synthase-like 1 [Source:MGI Symbol;Acc:MGI:3047787]	13.6347	10.6647	10.2435	11.1908	10.2822	11.0504	10.7319	10.9922	11.4017	12.663	13.0248	13.0307	11.4511	10.7238	0.387380622	0.915479136	24	12	24	0.403502851	0.799546792	24	18.44277293	0.066955774
ENSMUSG00000051579	Tceal8	transcription elongation factor A (SII)-like 8 [Source:MGI Symbol;Acc:MGI:1913934]	55.5873	61.4136	55.3684	53.8953	56.3283	50.9856	50.8201	48.6986	50.4774	44.7275	54.6013	43.4557	44.5081	47.6584	0.890235289	0.966018836	20	4	24	0.863744493	0.960525796	24	10.78319117	0.019253943
ENSMUSG00000051586	Mical3	microtubule associated monooxygenase, calponin and LIM domain containing 3 [Source:MGI Symbol;Acc:MGI:2442733]	22.0555	18.2469	18.5189	19.3616	23.4832	16.8955	22.93	18.8496	20.5403	20.7233	21.9778	19.1649	20.5107	18.4248	0.117050591	0.538362324	20	12	24	0.545268761	0.832868977	24	17.31085566	0.059200801
ENSMUSG00000051596	Otop1	otopetrin 1 [Source:MGI Symbol;Acc:MGI:2388363]	1.69256	1.42093	1.93004	1.37861	1.51822	1.5124	1.95456	2.52453	1.93152	3.02761	2.57126	2.02332	2.31888	2.57399	0.892044196	0.966018836	16	8	24	0.326822514	0.768040178	24	9.977019098	0.136119716
ENSMUSG00000051615	Rap2a	RAS related protein 2a [Source:MGI Symbol;Acc:MGI:97855]	27.9492	23.245	23.0698	23.3322	26.6044	25.0942	27.1075	24.1446	22.1869	26.6272	25.2783	24.5042	24.5286	23.551	0.026841899	0.305155548	20	16	24	0.05027336	0.644435324	24	19.70447285	0.086768856
ENSMUSG00000051627	H1f4	H1.4 linker histone, cluster member [Source:MGI Symbol;Acc:MGI:1931527]	1.64855	1.04852	1.17351	0.990515	1.20115	1.28819	1.2084	1.27432	1.24802	1.28294	1.21835	1.71694	1.23335	1.20421	0.215777641	0.704989686	24	12	24	0.152841985	0.759115906	24	21.26716804	0.089001936
ENSMUSG00000051648	Kctd19	potassium channel tetramerisation domain containing 19 [Source:MGI Symbol;Acc:MGI:3045294]	0.59976	0.503455	0.321243	0.676832	0.476474	0.506238	0.919144	0.435882	0.45235	0.46406	0.427291	0.419112	0.373106	0.540407	0.903465533	0.966018836	4	8	24	0.631484955	0.8744133	24	22.55225581	0.050714337
ENSMUSG00000051650	B3gnt2	UDP-GlcNAc:betaGal beta-1,3-N-acetylglucosaminyltransferase 2 [Source:MGI Symbol;Acc:MGI:1889505]	7.53326	6.59463	5.71724	7.57686	7.38789	7.38594	6.48683	6.47958	5.95689	8.57219	8.15773	8.94823	8.74272	6.10636	0.012705314	0.224589673	16	20	24	0.014302629	0.533411025	24	18.02280327	0.18107661
ENSMUSG00000051652	Lrrc3	leucine rich repeat containing 3 [Source:MGI Symbol;Acc:MGI:2447899]	5.77115	4.05513	3.85485	4.10134	4.63301	5.40268	4.16701	4.3739	2.79958	5.42887	4.4336	4.53328	4.61575	4.81343	0.133266423	0.567728672	24	12	24	0.12945495	0.740162193	24	20.16274784	0.16176832
ENSMUSG00000051669	AU021092	expressed sequence AU021092 [Source:MGI Symbol;Acc:MGI:2146559]	1.67879	2.17656	1.11015	1.66601	1.72986	1.36823	1.28643	2.30338	2.36345	1.5003	1.13426	1.40307	1.64173	1.19909	0.907000607	0.966018836	8	16	24	0.440710024	0.805393068	24	5.725152316	0.111883995
ENSMUSG00000051671	Coa6	cytochrome c oxidase assembly factor 6 [Source:MGI Symbol;Acc:MGI:1915142]	30.1633	28.8901	25.2972	28.1869	24.244	24.0298	28.7045	26.6782	31.299	24.906	36.1999	31.2875	33.8386	31.2205	0.923635729	0.966018836	4	20	24	0.649520493	0.88199334	24	0.853745461	0.056629922
ENSMUSG00000051674	Dcun1d4	DCN1, defective in cullin neddylation 1, domain containing 4 (S. cerevisiae) [Source:MGI Symbol;Acc:MGI:2140972]	17.3974	16.5574	18.4805	16.164	16.265	18.5416	17.3879	16.0794	15.6817	19.0503	16.2122	17.3214	16.1486	19.1293	0.828910443	0.966018836	24	12	24	0.983290628	0.996355202	24	2.407668025	0.006919868
ENSMUSG00000051675	Trim32	tripartite motif-containing 32 [Source:MGI Symbol;Acc:MGI:1917057]	21.1433	16.99	19.2221	17.3099	16.9458	20.705	20.0259	19.7092	15.5713	21.3615	16.4167	19.6691	18.2275	19.9679	0.417653118	0.963884303	4	16	24	0.377936755	0.797706855	24	23.31128596	0.076514544
ENSMUSG00000051678	Pcdhb6	protocadherin beta 6 [Source:MGI Symbol;Acc:MGI:2136740]	0.747093	0.660104	0.64007	0.474022	0.552245	0.576465	0.583399	0.843175	0.738276	0.77326	0.41414	0.829454	0.471107	0.832559	0.06180207	0.409814092	8	12	24	0.214194046	0.768040178	24	4.672571156	0.085901955
ENSMUSG00000051695	Pcbp1	poly(rC) binding protein 1 [Source:MGI Symbol;Acc:MGI:1345635]	53.5748	44.6921	44.2802	42.7207	42.4521	46.6644	48.0972	47.9657	42.0118	55.8237	54.2391	49.4126	49.773	50.2467	0.579092016	0.966018836	4	8	24	0.560554793	0.841237111	24	21.16178108	0.053579738
ENSMUSG00000051703	Tmem198	transmembrane protein 198 [Source:MGI Symbol;Acc:MGI:2443133]	2.85005	2.40011	2.66889	2.31984	2.39994	2.32858	2.18821	3.10297	2.6815	3.14946	2.76159	2.71866	2.78211	2.58211	0.77722623	0.966018836	16	8	24	0.631123988	0.874290528	24	7.989790211	0.04325272
ENSMUSG00000051705	Senp8	SUMO/sentrin specific peptidase 8 [Source:MGI Symbol;Acc:MGI:1918849]	9.86124	9.25266	6.39969	7.48248	9.02584	7.9121	9.78546	9.26581	7.72023	6.9778	9.29786	8.92061	8.88805	8.75086	0.014841078	0.250870466	4	8	24	0.015937201	0.544099157	24	22.7065969	0.168177106
ENSMUSG00000051721	Wdcp	WD repeat and coiled coil containing [Source:MGI Symbol;Acc:MGI:3040699]	4.29579	3.53578	4.44401	3.75243	4.52338	4.07984	4.27468	4.13575	3.82359	3.8876	4.65685	4.05949	4.24115	4.21096	0.483881213	0.966018836	20	20	24	0.406862743	0.799546792	24	19.0026112	0.047462081
ENSMUSG00000051726	Kcnf1	potassium voltage-gated channel, subfamily F, member 1 [Source:MGI Symbol;Acc:MGI:2687399]	0.991769	0.962285	0.977269	1.03712	0.825331	1.09555	0.867935	0.900018	0.884471	1.41707	0.780414	1.18708	0.935735	0.833228	0.70641939	0.966018836	24	8	24	0.680675404	0.893322401	24	14.57280537	0.040657409
ENSMUSG00000051727	Kctd14	potassium channel tetramerisation domain containing 14 [Source:MGI Symbol;Acc:MGI:1289222]	0.707133	0.88097	1.01876	0.693892	0.555203	0.767599	0.913257	1.04347	0.873563	0.613985	0.662366	0.829655	0.830053	0.933481	7.90E-04	0.071344007	8	12	24	0.002323714	0.39629122	24	4.045732149	0.131428545
ENSMUSG00000051730	Mettl5	methyltransferase like 5 [Source:MGI Symbol;Acc:MGI:1922672]	10.5208	12.2048	11.2631	11.1942	10.473	9.74857	10.666	12.3607	11.7403	11.3074	11.9496	11.3661	12.553	12.3698	0.142596848	0.585944911	8	16	24	0.022297237	0.578379975	24	6.172703997	0.085208215
ENSMUSG00000051735	Rinl	Ras and Rab interactor-like [Source:MGI Symbol;Acc:MGI:2444024]	2.46346	2.06104	2.20943	2.13978	2.39834	2.38696	3.08201	2.4513	2.41691	1.85698	1.83962	1.73934	2.75327	1.94191	0.037716301	0.334069441	4	20	24	0.189156984	0.768040178	24	0.61526051	0.10844199
ENSMUSG00000051736	Fam229b	family with sequence similarity 229, member B [Source:MGI Symbol;Acc:MGI:1913587]	16.8295	18.1383	15.8609	15.9346	14.3029	13.4939	16.107	14.4611	18.8801	11.9936	17.1112	15.5122	17.8994	16.4815	0.386774257	0.915479136	12	4	24	0.2910774	0.768040178	24	3.433960027	0.100697878
ENSMUSG00000051747	Ttn	titin [Source:MGI Symbol;Acc:MGI:98864]	1.71424	4.0458	4.74147	6.94868	3.51319	5.36292	3.52167	10.0163	8.16678	8.54093	3.67704	3.95632	5.27707	4.30302	0.192997592	0.670595308	16	4	24	0.051996696	0.649509116	24	9.290940469	0.462342685
ENSMUSG00000051748	Wfdc21	WAP four-disulfide core domain 21 [Source:MGI Symbol;Acc:MGI:1913357]	3.70585	10.3412	15.7575	14.0417	7.95569	9.83258	9.3506	6.69232	10.8538	7.25599	7.94526	7.76179	11.7697	6.37261	0.834424969	0.966018836	12	20	24	0.38369018	0.799546792	24	10.36914531	0.256674266
ENSMUSG00000051768	Xrcc1	X-ray repair complementing defective repair in Chinese hamster cells 1 [Source:MGI Symbol;Acc:MGI:99137]	8.34115	9.14586	9.56495	7.65946	7.45701	8.76519	7.69353	8.10165	9.11282	8.91855	6.63987	7.88916	8.10557	8.15257	0.021288455	0.264116537	12	8	24	0.09461528	0.71175955	24	6.023704375	0.097386541
ENSMUSG00000051786	Tubgcp6	tubulin, gamma complex associated protein 6 [Source:MGI Symbol;Acc:MGI:2146071]	6.28576	6.61947	6.95194	8.84464	6.87683	7.32107	7.75926	9.4049	7.78747	7.39387	6.39847	6.8621	6.68172	6.53058	0.628286596	0.966018836	16	12	24	0.488216256	0.805393068	24	8.937079205	0.075007132
ENSMUSG00000051790	Nlgn2	neuroligin 2 [Source:MGI Symbol;Acc:MGI:2681835]	17.0211	15.3039	15.0531	15.1616	17.2441	16.3379	15.3669	15.7154	15.099	20.7164	16.4407	16.2414	15.5313	17.7441	0.179603275	0.655480352	20	16	24	0.516192282	0.817725344	24	14.71327055	0.051300836
ENSMUSG00000051811	Cox6b2	cytochrome c oxidase subunit 6B2 [Source:MGI Symbol;Acc:MGI:3044182]	5.56039	6.22542	4.78092	5.89215	5.7966	5.42903	5.25444	5.52801	7.43497	4.59598	3.47759	4.86666	6.34921	6.44082	0.309932729	0.819919376	8	12	24	0.145700961	0.754301054	24	4.430875183	0.163186745
ENSMUSG00000051817	Sox12	SRY (sex determining region Y)-box 12 [Source:MGI Symbol;Acc:MGI:98360]	3.1625	3.24927	2.991	2.90759	3.14257	3.01653	3.06721	3.02766	3.56874	3.82353	3.11109	3.49007	3.12538	3.24088	0.710703678	0.966018836	8	12	24	0.618103188	0.868871403	24	10.52647102	0.031165317
ENSMUSG00000051839	Gypa	glycophorin A [Source:MGI Symbol;Acc:MGI:95880]	1.50491	4.30772	4.96402	5.09347	2.77637	3.66168	2.78715	2.89324	3.58825	1.4909	2.66109	2.85341	4.49895	2.49309	0.888379597	0.966018836	12	8	24	0.752362432	0.919493971	24	7.680963973	0.127157371
ENSMUSG00000051844	B230319C09Rik	RIKEN cDNA B230319C09 gene [Source:MGI Symbol;Acc:MGI:2444713]	0.389212	0.504	0.489995	0.403004	0.694941	0.779384	0.459474	0.606241	0.561458	0.314308	0.48856	0.427988	0.651725	0.798325	0.691437565	0.966018836	8	8	24	0.608253455	0.864339564	24	0.660192272	0.051888638
ENSMUSG00000051851	Rtl8c	retrotransposon Gag like 8C [Source:MGI Symbol;Acc:MGI:1920115]	67.1086	58.9004	62.8624	53.5862	54.2472	65.8244	69.2399	64.5079	50.7035	75.6052	55.3694	60.0395	56.1866	68.0921	0.504585278	0.966018836	4	8	24	0.579646282	0.849916859	24	1.180946565	0.067806841
ENSMUSG00000051853	Arf3	ADP-ribosylation factor 3 [Source:MGI Symbol;Acc:MGI:99432]	62.7715	49.1664	53.6188	55.1657	53.1531	60.6947	56.8046	56.9517	43.6048	70.2362	47.8881	56.185	54.1688	56.5011	0.46694635	0.966018836	4	8	24	0.693759721	0.898364349	24	20.19732114	0.05941409
ENSMUSG00000051855	Mest	mesoderm specific transcript [Source:MGI Symbol;Acc:MGI:96968]	26.2301	19.1115	18.1329	20.3007	21.3724	21.7815	21.9267	20.9937	14.5965	24.3928	20.4038	20.4643	21.0296	20.1484	0.009483698	0.194720062	4	8	24	0.13361299	0.743843563	24	20.27850779	0.143976686
ENSMUSG00000051864	Tbc1d22a	TBC1 domain family, member 22a [Source:MGI Symbol;Acc:MGI:1289265]	15.8493	12.8212	12.1661	13.2453	13.3826	14.0679	14.1877	14.566	11.4115	16.6457	12.6212	14.656	13.918	13.2139	0.056400419	0.401074772	24	12	24	0.353417993	0.780711577	24	20.76332488	0.079111466
ENSMUSG00000051890	Klhdc1	kelch domain containing 1 [Source:MGI Symbol;Acc:MGI:2672853]	5.54244	7.9557	8.57057	4.51015	5.98749	6.90448	7.19814	6.04385	6.48835	6.10574	6.35556	6.24483	6.92559	7.23542	0.163587268	0.626886304	12	4	24	0.262828739	0.768040178	24	3.417043132	0.122360358
ENSMUSG00000051910	Sox6	SRY (sex determining region Y)-box 6 [Source:MGI Symbol;Acc:MGI:98368]	1.87954	2.87734	2.6376	2.51972	2.55901	3.20873	3.02546	4.22277	2.97669	3.83144	2.85201	2.68906	3.2063	2.9377	0.868508291	0.966018836	8	12	24	0.534282181	0.826328258	24	7.363194915	0.083651173
ENSMUSG00000051920	Rspo2	R-spondin 2 [Source:MGI Symbol;Acc:MGI:1922667]	22.3018	18.4307	18.1631	18.0328	18.3979	19.6644	17.6117	18.5245	15.889	21.1567	19.1872	20.3333	19.513	19.1821	0.08302755	0.462922111	24	12	24	0.239096321	0.768040178	24	20.28799411	0.076668024
ENSMUSG00000051934	Spats2	spermatogenesis associated, serine-rich 2 [Source:MGI Symbol;Acc:MGI:1919822]	7.95671	7.24245	7.16791	7.07578	7.009	8.06273	6.48785	7.88088	5.79013	8.84404	6.53334	7.36587	6.78276	7.63106	0.923635729	0.966018836	24	12	24	0.937947588	0.984659033	24	20.40140408	0.020513715
ENSMUSG00000051950	B3glct	beta-3-glucosyltransferase [Source:MGI Symbol;Acc:MGI:2685903]	16.0647	14.3925	13.4095	14.183	13.0571	14.4601	13.762	14.136	12.9171	13.8109	14.3035	15.7172	16.3843	15.7245	0.031969981	0.324385159	4	8	24	0.037873493	0.61140397	24	23.30164024	0.08905158
ENSMUSG00000051951	Xkr4	X-linked Kx blood group related 4 [Source:MGI Symbol;Acc:MGI:3528744]	1.12603	1.39804	1.4543	1.37274	1.18908	1.01935	1.26399	1.17543	1.92029	0.947802	1.47828	1.59186	1.32917	1.58067	0.834434272	0.966018836	12	4	24	0.386234205	0.799546792	24	7.206922665	0.083419241
ENSMUSG00000051977	Prdm9	PR domain containing 9 [Source:MGI Symbol;Acc:MGI:2384854]	2.01986	2.07795	2.53743	2.34249	2.9689	2.35209	2.18031	2.81119	2.41153	2.33787	2.69081	2.63171	2.88153	2.89417	0.874672901	0.966018836	20	8	24	0.770038239	0.928523363	24	13.05668408	0.028303961
ENSMUSG00000051978	Erich1	glutamate rich 1 [Source:MGI Symbol;Acc:MGI:3588201]	5.631	5.67164	4.64184	4.48524	5.49165	5.42931	5.2807	5.69249	5.07741	5.57896	5.33429	4.683	5.42517	5.44776	0.24468385	0.741580974	8	4	24	0.371919882	0.793398709	24	0.506248592	0.051929058
ENSMUSG00000051984	Sec31b	Sec31 homolog B (S. cerevisiae) [Source:MGI Symbol;Acc:MGI:2685187]	0.536949	0.688216	0.832504	0.859046	0.664777	0.646988	0.785319	0.997007	0.955514	1.00313	0.630183	0.684299	0.869435	0.820354	0.035050838	0.328311403	16	4	24	0.00689755	0.472731997	24	8.082044765	0.12044007
ENSMUSG00000051985	Igfn1	immunoglobulin-like and fibronectin type III domain containing 1 [Source:MGI Symbol;Acc:MGI:3045352]	1.79385	5.46163	4.18201	4.79378	2.55473	5.50408	4.82539	11.7746	13.2189	12.9137	5.09293	5.77377	8.62767	5.0293	0.881996584	0.966018836	4	8	24	0.128927915	0.740162193	24	8.044705458	0.461000275
ENSMUSG00000051989	Smim11	small integral membrane protein 11 [Source:MGI Symbol;Acc:MGI:1916186]	23.3421	27.3833	22.6953	29.262	28.3838	24.9414	28.9419	27.3794	31.2478	22.4027	30.9606	28.5652	31.7838	29.1261	0.756529255	0.966018836	4	12	24	0.948756051	0.987480217	24	22.87051991	0.018904498
ENSMUSG00000052013	Btla	B and T lymphocyte associated [Source:MGI Symbol;Acc:MGI:2658978]	0.738906	0.576768	1.03855	0.777929	0.702002	0.461443	0.658102	0.553242	0.710879	0.477072	0.460514	0.682719	0.646283	0.520982	0.938993891	0.966018836	12	20	24	0.663246154	0.88640646	24	7.836629367	0.044259482
ENSMUSG00000052026	Slc6a7	solute carrier family 6 (neurotransmitter transporter, L-proline), member 7 [Source:MGI Symbol;Acc:MGI:2147363]	0.86476	1.4845	1.99016	1.35491	1.1079	1.46885	1.51061	1.111	1.53145	1.55818	0.734281	1.44189	0.959115	1.63438	0.059650003	0.406067671	12	8	24	0.227632424	0.768040178	24	7.688127735	0.148652065
ENSMUSG00000052031	Tagap1	T cell activation GTPase activating protein 1 [Source:MGI Symbol;Acc:MGI:1919786]	12.315	11.9605	11.8316	11.2691	11.3928	12.3561	10.9714	11.2853	9.78842	11.442	11.9811	11.2407	10.9311	11.9406	0.886572916	0.966018836	20	16	24	0.451439866	0.805393068	24	20.45447815	0.039241682
ENSMUSG00000052033	Pfdn4	prefoldin 4 [Source:MGI Symbol;Acc:MGI:1923512]	42.2775	48.0631	48.6645	37.6162	45.9933	39.2734	40.3755	40.0784	52.2624	33.1062	51.3936	47.3695	47.986	46.6603	0.503972924	0.966018836	12	4	24	0.828967101	0.946784793	24	3.286669709	0.045078569
ENSMUSG00000052040	Klf13	Kruppel-like factor 13 [Source:MGI Symbol;Acc:MGI:1354948]	12.931	10.61	11.7431	13.3369	11.7641	12.3245	12.3245	11.9144	10.5133	16.8154	10.9936	12.5277	11.8736	12.2178	0.300823756	0.809362476	16	20	24	0.58160013	0.85090942	24	14.43394021	0.062877565
ENSMUSG00000052056	Zfp217	zinc finger protein 217 [Source:MGI Symbol;Acc:MGI:2685408]	1.48242	1.44464	1.53218	1.52678	1.63048	1.38725	1.5283	1.61546	1.56458	1.62638	1.754	1.56578	1.73352	1.4377	0.47028201	0.966018836	20	8	24	0.409203189	0.799546792	24	14.29644622	0.027837357

ENSMUSG00000052062	Pard3b	par-3 family cell polarity regulator beta [Source:MGI Symbol;Acc:MGI:1919301]	1.30797	1.44428	1.31218	1.5472	1.45357	1.31786	1.44837	1.35894	1.56352	1.4667	1.32503	1.40076	1.39904	1.3021	0.328944792	0.847277612	16	16	24	0.284325244	0.768040178	24	11.17698396	0.032263078
ENSMUSG00000052085	Dock8	dedicator of cytokinesis 8 [Source:MGI Symbol;Acc:MGI:1921396]	1.5572	1.40716	1.50946	1.519	1.79908	1.41849	1.73987	1.54132	1.24792	1.68069	1.65352	1.80309	2.14156	1.93127	0.286642595	0.793714531	4	8	24	0.304008396	0.768040178	24	21.36685714	0.063216152
ENSMUSG00000052087	Rgs14	regulator of G-protein signaling 14 [Source:MGI Symbol;Acc:MGI:1859709]	2.18517	1.75515	2.47921	3.12019	2.3786	2.31508	1.65534	2.21918	2.15604	2.22292	2.19412	2.60713	2.13364	2.23182	0.049705476	0.793714531	16	12	24	0.103827285	0.715480277	24	13.58987105	0.118265112
ENSMUSG00000052102	Gnpda1	glucosamine-6-phosphate deaminase 1 [Source:MGI Symbol;Acc:MGI:1347054]	7.76797	7.08592	8.7383	6.81711	8.89163	8.78525	8.51611	8.69713	7.67604	8.16967	8.79038	8.29858	7.97123	7.89965	0.147689593	0.590732968	20	20	24	0.416577514	0.801257196	24	18.78040603	0.055673196
ENSMUSG00000052105	Mtcl1	microtubule crosslinking factor 1 [Source:MGI Symbol;Acc:MGI:1915867]	24.7301	18.5985	19.008	21.0149	22.0253	21.8106	19.8784	21.5369	17.3655	27.7088	18.6549	20.8228	22.0403	19.8341	0.287703522	0.793714531	16	20	24	0.576579443	0.848661503	24	18.21964705	0.072099886
ENSMUSG00000052125	F730043M19Rik	RIKEN cDNA F730043M19 gene [Source:MGI Symbol;Acc:MGI:2443237]	5.74398	4.82015	5.81554	4.47282	5.12013	5.43008	5.05458	5.32634	4.87075	4.85811	5.46069	5.2157	4.3862	4.88254	0.928780444	0.966018836	20	20	24	0.720170717	0.908295161	24	20.26581503	0.03215785
ENSMUSG00000052133	Sema5b	sema domain, seven thrombospondin repeats (type 1 and type 1-like), transmembrane domain (TM) and short cytoplasmic domain, (semaphorin) 5B [Source:MGI Symbol;Acc:MGI:107555]	1.24278	1.40067	1.62672	1.50386	0.644438	1.37196	1.7601	1.4947	1.10234	1.42689	1.05347	1.49464	1.1333	1.25334	0.718720526	0.966018836	16	4	24	0.410249127	0.799546792	24	3.976231343	0.096972028
ENSMUSG00000052137	Rbm12b2	RNA binding motif protein 12 B2 [Source:MGI Symbol;Acc:MGI:1924854]	3.81439	3.33039	3.82524	3.86937	3.64738	3.32416	4.05678	3.07039	3.61456	2.94017	3.95866	3.26847	3.07892	3.1983	0.94663657	0.966018836	20	12	24	0.636762041	0.876483853	24	16.31890109	0.039912592
ENSMUSG00000052139	Babam2	BRISC and BRCA1 A complex member 2 [Source:MGI Symbol;Acc:MGI:1333875]	33.3958	31.0611	28.9198	34.8978	35.159	40.1515	30.8719	33.9447	27.7842	37.668	30.3024	31.854	33.0923	32.3534	0.300823756	0.809362476	16	20	24	0.262604998	0.768040178	24	18.08489804	0.089828963
ENSMUSG00000052142	Rasal3	RAS protein activator like 3 [Source:MGI Symbol;Acc:MGI:2444128]	0.445791	0.847092	1.07674	1.04922	1.47369	1.58493	0.785944	0.640777	1.42125	0.708579	0.997668	0.794853	0.950855	0.384516	0.10338739	0.517709628	12	20	24	0.205031117	0.768040178	24	13.95801584	0.193918404
ENSMUSG00000052143	Gm9869	predicted gene 9869 [Source:MGI Symbol;Acc:MGI:3642719]	0.565117	1.04576	0.61227	1.03189	0.658062	0.411637	0.92293	1.82524	0.877707	1.62134	0.476332	1.09928	1.37594	0.437635	0.921572192	0.966018836	16	4	24	0.626424769	0.872596805	24	6.664749707	0.128640005
ENSMUSG00000052144	Ppp4r2	protein phosphatase 4, regulatory subunit 2 [Source:MGI Symbol;Acc:MGI:3027896]	14.4372	14.8362	12.829	16.4044	14.9106	12.7102	13.0552	14.0236	17.4675	14.2102	15.456	14.9359	15.3223	14.1684	0.892578749	0.966018836	16	16	24	0.403665723	0.799546792	24	11.18979767	0.06343941
ENSMUSG00000052146	Rps10	ribosomal protein S10 [Source:MGI Symbol;Acc:MGI:1914347]	4.12982	4.95953	5.28773	4.80254	5.85847	3.92881	3.76551	4.69757	6.09364	3.38934	5.72582	4.17182	5.37865	4.66787	0.347075276	0.870418653	12	12	24	0.560009205	0.840850554	24	9.812866666	0.09283636
ENSMUSG00000052151	Plpp2	phospholipid phosphatase 2 [Source:MGI Symbol;Acc:MGI:1354945]	9.36286	8.2268	8.7131	8.20677	8.30742	9.59761	8.05219	8.27516	7.7392	10.4171	7.01298	8.79502	7.85701	7.82252	0.918193129	0.966018836	24	12	24	0.835114778	0.949440189	24	17.65486513	0.03078654
ENSMUSG00000052155	Acvr2a	activin receptor IIA [Source:MGI Symbol;Acc:MGI:102806]	9.24239	9.17296	8.09534	8.53868	9.23421	8.30401	9.22769	8.47129	8.35901	7.85176	9.3885	7.92791	8.94154	8.26886	0.291042071	0.797585788	4	12	24	0.322231682	0.768040178	24	21.61120618	0.046379924
ENSMUSG00000052160	Pld4	phospholipase D family, member 4 [Source:MGI Symbol;Acc:MGI:2144765]	4.42478	6.16952	7.33627	6.55762	6.6055	4.96939	5.26678	6.01676	7.79258	4.49482	5.63914	5.05717	5.52234	5.15221	0.019280932	0.257669847	12	12	24	0.092049319	0.71175955	24	9.247764566	0.171746976
ENSMUSG00000052180	Serpinb6c	serine (or cysteine) peptidase inhibitor, clade B, member 6c [Source:MGI Symbol;Acc:MGI:2145481]	0.853512	1.71864	1.0028	1.29565	0.819987	1.29989	1.04229	1.40867	1.82076	0.789308	1.15268	1.25995	1.27099	1.31934	0.157744865	0.614233844	8	12	24	0.242310614	0.768040178	24	5.088154768	0.128567553
ENSMUSG00000052188	Gm14964	predicted gene 14964 [Source:MGI Symbol;Acc:MGI:3641621]	0.834808	1.77459	1.92012	1.23747	1.41044	1.27054	1.62193	1.44603	2.01444	0.943067	1.99496	1.63421	1.27999	1.28709	0.608967886	0.966018836	12	4	24	0.720075228	0.908238012	24	8.905142995	0.074181254
ENSMUSG00000052192	Gm5963	predicted pseudogene 5963 [Source:MGI Symbol;Acc:MGI:3648418]	3.14389	5.21967	7.3463	6.83522	7.37203	6.16147	8.18757	8.14689	8.41913	4.16601	10.007	6.98711	5.78838	8.97467	0.920858634	0.966018836	12	16	24	0.68911186	0.897070579	24	10.30034768	0.122347988
ENSMUSG00000052212	Cd177	CD177 antigen [Source:MGI Symbol;Acc:MGI:1916141]	1.1017	2.75766	4.30666	3.68825	2.75416	2.65938	2.21955	2.4272	2.5181	1.84452	2.29401	2.32397	2.884	1.56553	0.764889554	0.966018836	12	16	24	0.371987004	0.793398709	24	11.35834785	0.180320714
ENSMUSG00000052214	Opa3	optic atrophy 3 [Source:MGI Symbol;Acc:MGI:2686271]	17.5002	13.516	14.1579	15.6056	15.7311	17.2955	16.0374	17.0029	12.8453	19.8322	15.4734	17.5911	16.9468	16.7311	0.041292272	0.346472636	24	12	24	0.282306255	0.768040178	24	19.59443358	0.094222917
ENSMUSG00000052229	Gpr17	G protein-coupled receptor 17 [Source:MGI Symbol;Acc:MGI:3584514]	1.0464	1.66714	2.20023	2.15721	1.6318	1.63222	2.04867	1.36549	1.86012	1.30302	1.49128	1.36101	1.12113	1.84485	0.414321087	0.959486219	12	16	24	0.238992049	0.768040178	24	9.542587922	0.133074975
ENSMUSG00000052248	Zeb2os	zinc finger E-box binding homeobox 2, opposite strand [Source:MGI Symbol;Acc:MGI:3652108]	2.15487	3.45855	3.31641	2.42586	3.17067	2.22196	2.88537	3.03278	3.86097	2.23664	3.24085	2.56461	2.93461	2.87139	0.080164531	0.458678242	12	12	24	0.245006831	0.768040178	24	7.26455204	0.127858695
ENSMUSG00000052253	Zfp622	zinc finger protein 622 [Source:MGI Symbol;Acc:MGI:1289282]	16.5097	14.7899	14.1238	15.5329	16.5626	16.8323	14.5036	16.6541	16.4106	15.0073	16.9451	15.6776	[illegible]	16.8747	0.074944929	0.437756215	24	12	24	0.288427054	0.768040178	24	19.9756096	0.05790291
ENSMUSG00000052270	Fpr2	formyl peptide receptor 2 [Source:MGI Symbol;Acc:MGI:1278319]	0.866103	2.50319	4.30465	3.2858	2.22174	2.40904	2.10866	2.24728	2.74701	1.22049	1.46331	1.84165	1.9471	1.72529	0.642806037	0.966018836	12	16	24	0.292199961	0.768040178	24	8.908219255	0.230742846
ENSMUSG00000052281	Tspan32os	tetraspanin 32, opposite strand [Source:MGI Symbol;Acc:MGI:3705185]	2.23518	3.07832	2.63133	3.00938	3.15235	1.38802	1.65891	2.50044	3.88303	2.87273	2.70327	1.87018	2.61112	1.82105	0.031018002	0.324235048	20	4	24	0.027487417	0.590220615	24	10.11131983	0.271283478
ENSMUSG00000052291	5330438D12Rik	RIKEN cDNA 5330438D12 gene [Source:MGI Symbol;Acc:MGI:3026931]	3.56417	3.94003	2.95527	3.11123	2.69075	2.95247	2.54698	3.56963	3.7965	4.5083	3.6172	3.78307	3.60659	4.67376	0.664568268	0.966018836	8	20	24	0.23553948	0.768040178	24	6.822054843	0.117820157
ENSMUSG00000052293	Taf9	TATA-box binding protein associated factor 9 [Source:MGI Symbol;Acc:MGI:1888697]	7.9614	10.8257	13.156	8.39128	7.1207	6.23869	8.37293	13.7517	12.2177	11.9371	13.6983	14.822	11.9431	13.1262	0.566932234	0.966018836	8	20	24	0.098084991	0.71175955	24	7.176938249	0.249448116
ENSMUSG00000052296	Ppp6r1	protein phosphatase 6, regulatory subunit 1 [Source:MGI Symbol;Acc:MGI:2442163]	35.8273	27.6302	30.4732	31.2053	30.088	32.9615	31.8373	32.9462	26.1263	36.8263	29.7637	32.179	30.7058	31.3265	0.591351901	0.966018836	24	12	24	0.54725897	0.833663107	24	19.89088099	0.057075023
ENSMUSG00000052298	Cdc42se2	CDC42 small effector 2 [Source:MGI Symbol;Acc:MGI:1919079]	23.89	23.5817	24.0471	22.2672	22.9863	22.9868	24.4586	23.2064	21.3589	26.1595	22.188	24.0948	22.6137	24.3346	0.85447369	0.966018836	4	16	24	0.903340688	0.973174993	24	1.804696466	0.01269595
ENSMUSG00000052299	Ltn1	listerin E3 ubiquitin protein ligase 1 [Source:MGI Symbol;Acc:MGI:1926163]	15.891	15.0145	14.5819	15.3559	15.6479	14.5006	15.2807	13.7858	14.5572	13.3157	16.3749	15.174	15.5949	15.5176	0.326890754	0.845515883	20	16	24	0.285338995	0.768040178	24	20.35714076	0.048203191
ENSMUSG00000052301	Doc2a	double C2, alpha [Source:MGI Symbol;Acc:MGI:109446]	0.255478	0.907848	1.37315	0.909742	0.36891	0.804581	0.676977	0.584092	1.58828	0.689321	0.500604	0.856375	0.401804	1.04051	0.018194955	0.256345714	12	16	24	0.050632339	0.644469829	24	8.25833451	0.270011563
ENSMUSG00000052302	Tbc1d30	TBC1 domain family, member 30 [Source:MGI Symbol;Acc:MGI:1921944]	8.27457	6.6523	6.91043	7.02018	7.14954	7.75515	7.9389	7.01802	5.55732	8.01963	6.99096	7.363	6.72728	7.49131	0.217827365	0.704989686	24	12	24	0.182501912	0.768040178	24	20.41767141	0.061099163
ENSMUSG00000052305	Hbb-bs	hemoglobin, beta adult s chain [Source:MGI Symbol;Acc:MGI:5474852]	512.959	1376.51	937.164	1089.94	1026.71	896.789	738.335	1205.35	1579.1	597.333	983.676	1288.06	1408.25	961.901	0.874473208	0.966018836	8	20	24	0.687120744	0.896182667	24	6.910938411	0.16119913
ENSMUSG00000052310	Slc39a1	solute carrier family 39 (zinc transporter), member 1 [Source:MGI Symbol;Acc:MGI:1353474]	13.125	13.7268	13.9794	11.9924	14.1933	13.3849	14.5356	16.0383	13.7936	12.95	12.523	13.2821	13.1747	12.7714	0.886952527	0.966018836	12	4	24	0.308063778	0.768040178	24	2.789543009	0.055518905
ENSMUSG00000052331	Ankrd44	ankyrin repeat domain 44 [Source:MGI Symbol;Acc:MGI:3045243]	7.57342	9.64456	9.75805	10.5495	9.23751	9.10356	9.35613	8.57144	9.85451	9.10714	7.667	8.49259	8.542	8.8455	0.052964544	0.392528876	12	12	24	0.113681167	0.727440321	24	9.418037495	0.092556367
ENSMUSG00000052336	Cx3cr1	chemokine (C-X3-C motif) receptor 1 [Source:MGI Symbol;Acc:MGI:1333815]	1.79857	3.06363	3.39741	1.22098	1.89314	2.01711	2.9435	2.26046	2.39996	1.4234	1.86432	2.24882	2.24729	2.51275	0.00448422	0.140598674	12	4	24	0.054785987	0.655263328	24	3.182456621	0.235251676
ENSMUSG00000052337	Immt	inner membrane protein, mitochondrial [Source:MGI Symbol;Acc:MGI:1923864]	75.703	73.0747	73.3188	78.7868	73.912	76.5994	73.604	76.5245	70.294	81.369	75.7634	77.5386	84.0269	80.076	0.649215555	0.966018836	16	20	24	0.862354993	0.959779249	24	19.85588574	0.013207706
ENSMUSG00000052353	Cemip	cell migration inducing protein, hyaluronan binding [Source:MGI Symbol;Acc:MGI:2443629]	2.02635	2.2913	2.51264	1.60281	2.57227	2.02738	2.61966	2.49131	2.42289	1.81182	2.47967	1.9674	2.53381	1.69209	0.945282292	0.966018836	4	12	24	0.761220087	0.92277295	24	0.205773218	0.048387403
ENSMUSG00000052364	B630019K06Rik	RIKEN cDNA B630019K06 gene [Source:MGI Symbol;Acc:MGI:2147918]	8.11385	7.86135	9.38295	7.28473	7.14502	8.93276	8.81928	7.27875	6.84348	9.23678	7.03973	8.2542	7.42795	8.31565	0.8635847	0.966018836	24	20	24	0.878488236	0.965987302	24	0.229720115	0.027888751
ENSMUSG00000052369	Tmem106c	transmembrane protein 106C [Source:MGI Symbol;Acc:MGI:1196384]	16.9438	16.4899	16.603	15.904	16.5308	16.9062	19.0095	17.6694	15.4006	16.7074	15.3852	17.5606	16.8516	17.3234	0.057224304	0.401309455	4	16	24	0.040794051	0.616921492	24	6.048512625	0.067595354
ENSMUSG00000052371	Hoxd3os1	homeobox D3, opposite strand 1 [Source:MGI Symbol;Acc:MGI:1923875]	7.71877	6.59626	7.23825	6.23036	5.59083	5.24009	5.23476	6.82251	6.32735	6.68856	7.66802	7.36016	6.52501	5.89026	0.710938687	0.966018836	12	12	24	0.904626488	0.97359657	24	9.227041469	0.030251016
ENSMUSG00000052372	Il1rapl1	interleukin 1 receptor accessory protein-like 1 [Source:MGI Symbol;Acc:MGI:2687319]	0.577329	0.736342	0.801286	0.728611	0.681931	0.524798	0.722476	0.638714	0.791312	0.587821	0.649505	0.4925	0.561025	0.679249	2.98E-04	0.049770315	12	12	24	0.011147594	0.516680757	24	8.202874424	0.083658971
ENSMUSG00000052373	Mpp3	membrane protein, palmitoylated 3 (MAGUK p55 subfamily member 3) [Source:MGI Symbol;Acc:MGI:1328354]	1.75636	2.07635	3.02671	1.92972	1.9572	2.5676	2.23386	1.8008	2.26225	2.4296	1.56179	2.26917	2.11718	1.98862	0.916746631	0.966018836	12	20	24	0.802114794	0.936565832	24	8.512033076	0.046253189
ENSMUSG00000052374	Actn2	actinin alpha 2 [Source:MGI Symbol;Acc:MGI:109192]	5.31369	8.32284	17.6407	15.0776	12.42	10.9679	7.41687	18.3987	11.3	17.1499	8.6483	8.62259	10.5615	12.5698	0.026323104	0.301028315	12	16	24	0.016554275	0.54430656	24	9.723537285	0.501876315
ENSMUSG00000052384	Nrros	negative regulator of reactive oxygen species [Source:MGI Symbol;Acc:MGI:2445095]	2.15707	2.33559	2.91964	2.71101	2.80277	2.3767	2.38344	2.73435	2.66272	1.8094	2.78464	2.21292	2.58423	2.16759	0.570349842	0.966018836	20	4	24	0.777617621	0.928689964	24	11.37592961	0.038475177
ENSMUSG00000052387	Trpm3	transient receptor potential cation channel, subfamily M, member 3 [Source:MGI Symbol;Acc:MGI:2443101]	8.64567	11.003	11.0961	10.0125	10.0373	9.86575	9.64388	8.98543	10.9591	9.16351	9.65945	9.99695	8.94158	10.451	0.019594567	0.258568924	12	16	24	0.247634798	0.768040178	24	8.995378682	0.069089552
ENSMUSG00000052395	Rft1	RFT1 homolog [Source:MGI Symbol;Acc:MGI:3607791]	8.07304	9.61586	9.22535	8.80543	9.58101	9.13148	9.13669	10.1481	9.77807	10.1613	9.21934	9.71752	9.9518	9.76957	0.694910419	0.966018836	8	20	24	0.262083023	0.768040178	24	8.063552762	0.041039609
ENSMUSG00000052397	Ezr	ezrin [Source:MGI Symbol;Acc:MGI:98931]	14.3733	14.3456	13.7293	13.1209	14.4624	13.5586	15.5667	14.591	12.749	14.4206	13.4943	14.6376	14.1058	13.6403	0.267533088	0.774280302	4	8	24	0.131918401	0.742323615	24	23.01974493	0.052416631
ENSMUSG00000052406	Rexo4	REX4, 3'-5' exonuclease [Source:MGI Symbol;Acc:MGI:2684957]	23.5001	22.9061	23.9341	25.9135	26.751	23.305	23.4808	22.0754	21.3834	21.3584	22.4586	22.1501	22.1825	22.2733	0.890648739	0.966018836	20	12	24	0.194427617	0.768040178	24	16.4135136	0.051093015
ENSMUSG00000052407	Ccdc171	coiled-coil domain containing 171 [Source:MGI Symbol;Acc:MGI:1922152]	1.86849	1.80269	1.64388	1.73681	2.13847	1.57603	2.0165	1.59931	2.07522	1.41927	2.0299	1.89271	2.12097	1.44273	0.446653834	0.966018836	20	12	24	0.43517857	0.805393068	24	19.73255254	0.068089607
ENSMUSG00000052419	2610001J05Rik	RIKEN cDNA 2610001J05 gene [Source:MGI Symbol;Acc:MGI:1913770]	55.3825	59.422	58.0197	56.9685	64.2219	52.3691	54.4158	56.4808	55.9592	49.1281	58.3566	53.9988	57.2017	56.4742	0.503972924	0.966018836	20	4	24	0.991236738	0.998516607	24	10.11031832	0.004714764
ENSMUSG00000052423	B4galt3	UDP-Gal:betaGlcNAc beta 1,4-galactosyltransferase, polypeptide 3 [Source:MGI Symbol;Acc:MGI:1928767]	13.044	13.3174	11.7063	11.8157	11.35	13.0533	13.3841	13.5036	12.3363	14.4664	10.9853	11.0623	11.0942	12.732	0.192357053	0.669841558	8	12	24	0.349696264	0.779170188	24	4.805407541	0.065385828
ENSMUSG00000052428	Tmco1	transmembrane and coiled-coil domains 1 [Source:MGI Symbol;Acc:MGI:1921173]	54.2861	58.6643	56.052	59.1023	58.9603	53.5982	56.5262	60.513	56.2142	46.4921	57.5087	52.8401	57.1352	56.2892	0.643253306	0.966018836	8	16	24	0.659801825	0.88506517	24	3.258033057	0.032859952
ENSMUSG00000052430	Bmpr1b	bone morphogenetic protein receptor, type 1B [Source:MGI Symbol;Acc:MGI:107191]	1.33853	1.87727	2.0837	1.54629	1.3057	1.7397	1.85615	1.38096	1.69804	1.69477	1.30318	1.81769	1.34069	1.99741	0.054623609	0.397541027	12	8	24	0.353779063	0.781118317	24	6.068636361	0.086074822
ENSMUSG00000052435	Cebpe	CCAAT/enhancer binding protein (C/EBP), epsilon [Source:MGI Symbol;Acc:MGI:103572]	0.331312	0.82368	1.20134	0.677038	0.635175	0.586461	0.674205	0.726206	0.855604	0.510667	0.836734	0.679535	0.783671	0.493148	0.46238066	0.966018836	12	12	24	0.383157093	0.799546792	24	9.031638645	0.096848279
ENSMUSG00000052446	Zfp961	zinc finger protein 961 [Source:MGI Symbol;Acc:MGI:3583954]	4.73665	6.17039	6.1265	5.77097	6.32844	5.1346	5.50224	5.56936	6.33444	5.19425	6.27347	5.28643	4.83201	5.75802	0.032082657	0.324385159	12	16	24	0.08758249	0.707747429	24	10.44019733	0.09999914
ENSMUSG00000052456	Get3	guided entry of tail-anchored proteins factor 3, ATPase [Source:MGI Symbol;Acc:MGI:1928379]	49.0204	47.2081	48.7492	46.6024	46.4134	49.126	47.2991	47.252	44.3418	44.0212	46.3431	46.5527	44.124	46.1098	0.81872541	0.966018836	24	16	24	0.163316999	0.767020009	24	22.22710832	0.028204743
ENSMUSG00000052459	Atp6v1a	ATPase, H+ transporting, lysosomal V1 subunit A [Source:MGI Symbol;Acc:MGI:1201780]	141.956	129.342	131.64	126.064	125.593	134.462	125.314	122.246	116.969	130.841	133.405	132.858	134.487	139.7	0.560714538	0.966018836	4	8	24	0.284558765	0.768040178	24	21.73723826	0.04675771
ENSMUSG00000052468	Pmp2	peripheral myelin protein 2 [Source:MGI Symbol;Acc:MGI:102667]	127.105	196.097	173.183	209.563	228.46	148.62	155.472	177.207	234.384	186.263	185.996	147.393	150.155	163.759	0.00574313	0.157813586	16	8	24	0.006579619	0.470633744	24	10.92760212	0.270254019
ENSMUSG00000052488	Cherp	calcium homeostasis endoplasmic reticulum protein [Source:MGI Symbol;Acc:MGI:106417]	7.14861	8.04528	7.35876	9.80218	7.11205	6.87275	7.68123	7.99816	7.91525	9.07539	6.89903	8.01411	7.23733	7.18544	0.085045716	0.466404237	16	4	24	0.160950461	0.765941695	24	10.18976665	0.097421348
ENSMUSG00000052504	Epha3	Eph receptor A3 [Source:MGI Symbol;Acc:MGI:99612]	0.517676	0.745746	0.668202	0.697912	0.603738	0.600877	0.649073	0.692298	0.904098	0.484271	0.671337	0.669322	0.504874	0.680034	0.156415999	0.611943293	8	20	24	0.257439843	0.768040178	24	7.774693928	0.061554382
ENSMUSG00000052512	Nav2	neuron navigator 2 [Source:MGI Symbol;Acc:MGI:2183691]	2.80698	3.33302	3.95864	2.74614	3.50818	2.73821	2.90391	3.14605	3.60754	3.66093	3.34602	3.02955	3.66353	3.88937	0.041292272	0.346472636	12	16	24	0.059809901	0.658304792	24	8.181723939	0.117176916
ENSMUSG00000052516	Robo2	roundabout guidance receptor 2 [Source:MGI Symbol;Acc:MGI:1890110]	34.1204	29.3218	29.7262	30.0639	26.7547	32.3971	29.173	28.7153	23.8092	36.1159	28.0119	30.9258	29.1757	31.5086	0.928780444	0.966018836	24	12	24	0.730475902	0.912198956	24	20.81731819	0.047182206
ENSMUSG00000052525	Spdya	speedy/RINGO cell cycle regulator family, member A [Source:MGI Symbol;Acc:MGI:1918141]	0.67087	1.01396	1.1208	0.805555	0.84906	0.618676	0.962115	0.928091	0.757239	0.855344	0.636301	0.91488	0.759164	0.943416	0.188763171	0.664348865	8	12	24	0.128892071	0.740162193	24	6.19287463	0.089483775
ENSMUSG00000052533	Nup188	nucleoporin 188 [Source:MGI Symbol;Acc:MGI:2446190]	3.51039	2.87318	3.54398	3.57816	3.12289	4.26484	3.85665	3.44392	3.62714	3.48595	2.76619	2.70783	2.90439	2.98184	0.92655264	0.966018836	12	20	24	0.984309569	0.996767829	24	8.723373126	0.010283531
ENSMUSG00000052534	Pbx1	pre B cell leukemia homeobox 1 [Source:MGI Symbol;Acc:MGI:97495]	7.48473	8.27307	8.07659	7.94894	7.84647	7.63264	8.12634	7.97846	8.35143	8.29192	8.51104	7.98929	8.13516	8.67372	0.649215555	0.966018836	8	16	24	0.029812081	0.596648309	24	8.239054629	0.039370629
ENSMUSG00000052539	Magi3	membrane associated guanylate kinase, WW and PDZ domain containing 3 [Source:MGI Symbol;Acc:MGI:1923484]	50.8297	41.9105	39.8709	41.0089	40.7599	44.6681	40.6526	41.8518	35.4463	44.0955	43.0203	45.142	46.9528	44.7129	0.035787737	0.331814682	4	8	24	0.062515161	0.661961741	24	21.70820884	0.105104928
ENSMUSG00000052544	St6galnac3	ST6 (alpha-N-acetyl-neuraminyl-2,3-beta-galactosyl-1,3)-N-acetylgalactosaminide alpha-2,6-sialyltransferase 3 [Source:MGI Symbol;Acc:MGI:1341828]	2.79904	3.91547	4.40794	2.66927	2.84973	3.01358	3.3773	3.3364	4.6013	3.48475	3.1331	2.93922	4.4468	4.52312	0.073518583	0.448301997	12	8	24	0.0044756	0.427431763	24	5.858299347	0.227317923
ENSMUSG00000052557	Gan	giant axonal neuropathy [Source:MGI Symbol;Acc:MGI:1890619]	7.49599	6.18079	6.76018	7.30403	7.03912	7.07214	6.14576	6.59101	6.08706	8.70385	6.78586	7.36212	7.29828	7.08346	0.560714538	0.966018836	16	20	24	0.315962874	0.768040178	24	15.5777271	0.068592091
ENSMUSG00000052560	Cpne8	copine VIII [Source:MGI Symbol;Acc:MGI:1914121]	41.8354	34.0297	33.9411	30.204	33.3772	35.3731	33.4172	33.547	32.9925	32.2758	37.4324	35.8157	38.5404	35.4247	0.016720774	0.253395174	4	12	24	0.03724392	0.609176161	24	22.56561496	0.105076232
ENSMUSG00000052565	D930048N14Rik	RIKEN cDNA D930048N14 gene [Source:MGI Symbol;Acc:MGI:2144709]	0.862487	0.909685	0.899935	0.900424	0.987971	1.02911	0.923878	0.920571	1.06636	0.748384	0.913553	1.00942	0.872009	0.743839	0.542718076	0.966018836	24	8	24	0.485085919	0.805393068	24	18.55413163	0.034094271
ENSMUSG00000052566	Hook2	hook microtubule tethering protein 2 [Source:MGI Symbol;Acc:MGI:2181664]	2.89352	3.28904	3.08027	2.67148	2.451	2.86188	2.46835	2.82761	3.62656	3.8182	2.73842	3.21435	2.46079	3.23248	0.652082538	0.801049287	12	8	24	0.138052295	0.747972781	24	8.526672327	0.116982442
ENSMUSG00000052572	Dlg2	discs large MAGUK scaffold protein 2 [Source:MGI Symbol;Acc:MGI:1344351]	13.303	11.4265	13.1574	11.5108	9.29172	11.7049	12.692	11.3991	11.0787	14.0335	9.70824	12.6561	10.6312	15.468	0.700065774	0.966018836	8	12	24	0.284091424	0.768040178	24	4.602462355	0.113777116
ENSMUSG00000052581	Lrrtm4	leucine rich repeat transmembrane neuronal 4 [Source:MGI Symbol;Acc:MGI:2389180]	2.5029	3.31973	3.30004	2.38785	2.62645	2.28598	3.36722	2.11156	2.48091	2.26364	2.31126	2.32138	2.35606	2.5459	0.520414931	0.966018836	4	20	24	0.418439661	0.80300217	24	4.009859039	0.073960362
ENSMUSG00000052584	Serp2	stress-associated endoplasmic reticulum protein family member 2 [Source:MGI Symbol;Acc:MGI:1919911]	38.1348	36.8463	35.5539	33.3245	32.5636	35.3443	33.1762	35.0348	37.8883	35.3001	39.8442	39.6654	41.8116	39.2332	0.951846915	0.966018836	12	20	24	0.404123208	0.799546792	24	1.024286884	0.046674016
ENSMUSG00000052593	Adam17	a disintegrin and metallopeptidase domain 17 [Source:MGI Symbol;Acc:MGI:1096335]	11.4516	11.4611	10.4353	10.5961	12.281	10.5302	12.7858	11.4843	10.8149	10.2767	11.9657	11.0268	11.3687	10.1907	0.432054949	0.966018836	20	20	24	0.200493331	0.768040178	24	21.07444538	0.063751074
ENSMUSG00000052605	Isoc2b	isochorismatase domain containing 2b [Source:MGI Symbol;Acc:MGI:1914691]	5.27359	6.27926	9.36936	5.91429	6.15889	6.11109	4.8849	6.20727	6.21818	5.59374	5.94422	5.62325	6.7871	6.45964	0.304354943	0.811201458	8	20	24	0.247829353	0.768040178	24	8.143369175	0.124369567
ENSMUSG00000052609	Plekhg3	pleckstrin homology domain containing, family G (with RhoGef domain) member 3 [Source:MGI Symbol;Acc:MGI:2388284]	17.0451	18.0774	16.0898	16.2785	18.9439	17.3161	20.8316	18.2932	19.6435	17.3823	18.3661	17.3045	18.6561	17.2218	0.904831057	0.966018836	20	20	24	0.635717656	0.876220581	24	23.52309598	0.036078784
ENSMUSG00000052613	Pcdh15	protocadherin 15 [Source:MGI Symbol;Acc:MGI:1891428]	0.515262	0.629155	1.10037	0.799912	0.643954	0.628014	0.678686	0.498723	0.875535	0.662691	0.547298	0.799217	0.613169	0.709521	0.101520755	0.511659635	12	16	24	0.188126425	0.768040178	24	9.703565103	0.093136737
ENSMUSG00000052629	Gm9885	predicted gene 9885 [Source:MGI Symbol;Acc:MGI:3641726]	1.14415	0.779745	0.886382	0.715495	0.656752	0.763826	1.18514	1.20235	1.00885	1.19108	1.29881	1.15747	1.12035	0.948085	0.860709018	0.966018836	4	12	24	0.627905093	0.872795898	24	0.786880402	0.015928015
ENSMUSG00000052632	Asap2	ArfGAP with SH3 domain, ankyrin repeat and PH domain 2 [Source:MGI Symbol;Acc:MGI:2685438]	4.67467	4.61652	5.13509	4.42845	4.47771	4.8293	4.79392	4.96647	4.65601	5.43508	4.30754	5.0938	4.53307	5.10416	0.966749204	0.969592092	12	8	24	0.538675895	0.828721316	24	6.23712253	0.035408899
ENSMUSG00000052656	Rnf103	ring finger protein 103 [Source:MGI Symbol;Acc:MGI:109483]	24.8721	23.0023	22.4321	23.231	22.7498	23.4318	23.8263	22.4599	19.997	23.1832	23.8166	23.4037	23.576	23.3033	3.15E-04	0.049770315	4	8	24	0.047362863	0.63386192	24	20.82059654	0.061736733
ENSMUSG00000052658	5830454E08Rik	RIKEN cDNA 5830454E08 gene [Source:MGI Symbol;Acc:MGI:1923350]	1.05963	1.11163	0.921318	0.96858	1.4219	0.798705	0.946812	1.00942	1.63632	1.30718	0.846262	0.813277	1.51396	1.16786	0.446967971	0.966018836	12	8	24	0.400420567	0.799546792	24	7.723296084	0.096372898
ENSMUSG00000052675	Zfp112	zinc finger protein 112 [Source:MGI Symbol;Acc:MGI:1929115]	3.24826	4.24882	3.84375	3.30812	3.45121	3.65119	2.82114	3.20135	4.20488	3.25871	3.68101	3.98793	3.52197	3.8673	0.969174623	0.970883022	12	16	24	0.654194319	0.883406766	24	8.205050311	0.0497508
ENSMUSG00000052676	Zmat1	zinc finger, matrin type 1 [Source:MGI Symbol;Acc:MGI:2442284]	5.69784	7.05694	6.41594	6.91885	6.68472	6.29456	6.79649	6.05963	7.88842	6.48421	7.148	6.51693	6.2597	6.60882	0.360140629	0.883787544	20	8	24	0.175336442	0.768040178	24	10.76168968	0.070233802
ENSMUSG00000052681	Rap1b	RAS related protein 1b [Source:MGI Symbol;Acc:MGI:894315]	61.686	60.6363	60.3047	60.9119	65.8462	59.3861	62.1938	55.935	56.2448	52.7716	62.694	51.246	52.9492	51.1888	0.47028201	0.966018836	20	12	24	0.197290363	0.768040178	24	16.92951733	0.05487893
ENSMUSG00000052684	Jun	jun proto-oncogene [Source:MGI Symbol;Acc:MGI:96646]	16.4104	10.2394	13.4232	12.7988	12.9065	11.8155	14.1463	14.7155	11.9934	18.7759	18.9476	14.9043	15.3997	14.7366	0.945728192	0.966018836	20	16	24	0.627189111	0.872795898	24	16.44455626	0.079829201
ENSMUSG00000052688	Rab7b	RAB7B, member RAS oncogene family [Source:MGI Symbol;Acc:MGI:2442295]	2.37505	3.28769	3.1021	3.00343	3.08727	2.3202	2.33952	2.68396	3.02195	2.17549	2.57545	2.47814	1.76706	2.88182	0.05005312	0.385784509	12	16	24	0.10278257	0.713699364	24	9.253876625	0.129942691
ENSMUSG00000052698	Tln2	talin 2 [Source:MGI Symbol;Acc:MGI:1917799]	28.0635	21.8511	22.3562	24.9986	21.917	27.4596	23.614	25.7683	18.3887	29.4044	20.1105	24.1745	24.6437	25.2998	0.823657258	0.966018836	24	12	24	0.611973151	0.865342826	24	22.00503277	0.072992381
ENSMUSG00000052707	Tnrc6a	trinucleotide repeat containing 6a [Source:MGI Symbol;Acc:MGI:2385292]	20.0742	21.6098	22.3464	22.3489	20.5402	20.0919	23.3488	18.5455	24.7265	20.1037	20.5891	21.2536	21.8068	21.2807	0.888991156	0.966018836	12	12	24	0.594238241	0.857257137	24	7.804491511	0.042339893
ENSMUSG00000052712	BC004004	cDNA sequence BC004004 [Source:MGI Symbol;Acc:MGI:2136782]	66.0119	65.9103	69.3738	63.1459	62.8509	64.3657	59.2893	68.9459	72.5192	70.2829	70.5415	65.433	73.4448	71.8609	0.702220766	0.966018836	12	12	24	0.097271186	0.71175955	24	7.46789817	0.063081485
ENSMUSG00000052713	Zfp608	zinc finger protein 608 [Source:MGI Symbol;Acc:MGI:2442338]	4.7723	4.05024	4.396	3.54957	4.54438	3.89233	4.63745	4.41186	4.33827	4.46022	3.66878	4.46516	4.76331	4.34257	0.052640391	0.391647859	4	12	24	0.183381511	0.768040178	24	1.293669223	0.073030858
ENSMUSG00000052726	Kcnt2	potassium channel, subfamily T, member 2 [Source:MGI Symbol;Acc:MGI:3036273]	3.51109	2.83633	3.1292	2.89139	3.39034	3.00229	2.9688	2.98334	2.99239	2.29256	3.25969	2.87361	3.05229	2.92211	0.722296281	0.966018836	20	20	24	0.323410991	0.768040178	24	21.40577819	0.060568268
ENSMUSG00000052727	Map1b	microtubule-associated protein 1B [Source:MGI Symbol;Acc:MGI:1306778]	339.673	250.629	254.785	280.087	253.795	301.995	277.261	283.028	185.515	372.335	244.275	296.581	274.493	299.323	0.850398351	0.966018836	24	12	24	0.551305559	0.83632237	24	20.65850081	0.106466401
ENSMUSG00000052738	Suclg1	succinate-CoA ligase, GDP-forming, alpha subunit [Source:MGI Symbol;Acc:MGI:1927234]	61.4888	67.7236	76.1114	84.4058	64.579	66.8844	66.299	81.2246	73.824	78.3792	79.98	71.0284	82.1296	80.5039	0.664568268	0.966018836	16	8	24	0.035949422	0.609176161	24	9.240829642	0.116650464
ENSMUSG00000052748	Swt1	SWT1 RNA endoribonuclease homolog (S. cerevisiae) [Source:MGI Symbol;Acc:MGI:1914125]	9.29539	8.71334	8.35069	8.28684	8.74517	7.7746	14.3405	5.53017	9.45033	5.2594	8.06558	6.85704	6.8201	7.12346	0.896773941	0.966018836	4	20	24	0.602862851	0.861114219	24	22.57988752	0.118122607
ENSMUSG00000052751	Repin1	replication initiator 1 [Source:MGI Symbol;Acc:MGI:1889817]	6.28913	6.49429	6.6924	7.49779	7.07578	6.16717	7.27846	7.04412	8.27297	7.59523	7.79462	8.01889	7.46649	7.22881	0.687668716	0.966018836	16	16	24	0.106571398	0.718721872	24	11.7180446	0.065514887
ENSMUSG00000052752	Traf7	TNF receptor-associated factor 7 [Source:MGI Symbol;Acc:MGI:3042141]	25.4715	31.9147	31.5163	30.6495	30.6032	28.1514	28.7019	29.5758	32.6311	28.7272	28.7925	27.526	30.471	28.0782	0.020119233	0.259417302	12	12	24	0.071139292	0.676901434	24	9.02894638	0.080282487
ENSMUSG00000052760	A630001G21Rik	RIKEN cDNA A630001G21 gene [Source:MGI Symbol;Acc:MGI:2443131]	1.18799	1.14564	1.20519	0.730605	0.927779	0.771898	1.50207	0.948052	1.49997	0.890977	0.832958	1.00064	1.2233	1.23522	0.040074386	0.34094463	12	4	24	0.030758915	0.5982067	24	3.571702605	0.156277451
ENSMUSG00000052763	Zfp212	Zinc finger protein 212 [Source:MGI Symbol;Acc:MGI:2682609]	4.33625	4.72844	4.34407	4.44974	4.32868	4.92543	4.31091	5.20089	4.48987	4.5481	3.91844	4.41788	4.57601	4.62786	0.038715855	0.336039716	8	12	24	0.229642476	0.768040178	24	3.659548769	0.051717931
ENSMUSG00000052776	Oas1a	2'-5' oligoadenylate synthetase 1A [Source:MGI Symbol;Acc:MGI:2180860]	1.2194	1.10059	1.00147	1.08697	1.57471	0.945003	1.34953	1.32254	0.865961	0.85095	1.11144	0.9793	0.947608	1.00631	0.622773557	0.966018836	4	12	24	0.501780148	0.809142348	24	20.88808347	0.06098403
ENSMUSG00000052783	Grk4	G protein-coupled receptor kinase 4 [Source:MGI Symbol;Acc:MGI:95801]	3.8701	3.51463	3.85707	3.05527	3.47635	3.7212	2.25274	3.82628	3.60256	2.1862	3.47175	3.41031	2.5657	2.7003	0.920872048	0.966018836	12	4	24	0.98454556	0.996773483	24	19.70910758	0.014108174
ENSMUSG00000052794	1700030K09Rik	RIKEN cDNA 1700030K09 gene [Source:MGI Symbol;Acc:MGI:1919504]	4.35165	3.77294	4.70122	4.94526	4.28092	4.71896	4.92198	3.87214	4.62488	4.12125	3.99976	4.14634	4.16799	3.84495	0.280850951	0.792139038	12	20	24	0.637232608	0.876564021	24	15.50811669	0.03789568
ENSMUSG00000052798	Nup107	nucleoporin 107 [Source:MGI Symbol;Acc:MGI:2143854]	11.7817	12.3636	13.5958	9.62274	9.42514	12.5202	9.56471	10.1817	10.8464	10.6547	12.8071	9.65264	11.6801	12.6956	0.777867261	0.966018836	12	4	24	0.636435566	0.876483853	24	4.734850458	0.064589589
ENSMUSG00000052812	Atad2b	ATPase family, AAA domain containing 2B [Source:MGI Symbol;Acc:MGI:2444798]	4.45096	3.79025	3.51399	4.11841	4.13102	3.55592	4.71346	3.54863	3.90031	2.97273	4.16713	3.55094	3.43478	3.64856	0.912923872	0.966018836	4	8	24	0.4622465	0.805393068	24	21.23849137	0.063729918
ENSMUSG00000052825	Gm9892	predicted gene 9892 [Source:MGI Symbol;Acc:MGI:3701610]	56.3207	60.5115	59.7886	55.6482	58.7422	51.2173	55.1509	51.9375	69.0775	54.8801	66.5205	63.1953	67.0729	63.6634	0.842838536	0.966018836	12	4	24	0.78319906	0.930229965	24	8.122302096	0.03502133

ENSMUSG00000052833	Sae1	SUMO1 activating enzyme subunit 1 [Source:MGI Symbol;Acc:MGI:1929264]	45.7466	37.2446	35.4262	37.4913	36.363	42.5761	39.3057	41.547	31.9469	45.1391	34.3488	39.0612	41.4175	39.5961	0.170659409	0.643496587	4	8	24	0.254413521	0.768040178	24	22.63643281	0.093231463
ENSMUSG00000052837	Junb	jun B proto-oncogene [Source:MGI Symbol;Acc:MGI:96647]	5.05531	3.44221	3.30061	2.32965	2.37139	2.07323	3.91223	4.90748	4.57933	5.69198	4.91669	5.07811	5.72567	4.802	0.776666236	0.966018836	4	16	24	0.29467238	0.768040178	24	3.689233631	0.191496436
ENSMUSG00000052852	Reep1	receptor accessory protein 1 [Source:MGI Symbol;Acc:MGI:1098827]	142.607	106.156	113.727	113.209	115.316	134.71	112.58	122.238	82.586	136.046	115.03	120.465	115.682	118.282	0.390686537	0.915479136	24	12	24	0.217618754	0.768040178	24	20.1069634	0.128597459
ENSMUSG00000052889	Prkcb	protein kinase C, beta [Source:MGI Symbol;Acc:MGI:97596]	34.6705	37.425	39.0084	34.8216	35.6374	38.5048	35.271	33.3427	34.7846	36.3736	32.9912	35.2776	31.7252	36.2589	0.946423879	0.966018836	12	16	24	0.801571874	0.936422183	24	10.01907827	0.019861393
ENSMUSG00000052906	Ubxn8	UBX domain protein 8 [Source:MGI Symbol;Acc:MGI:1337129]	9.5773	8.50998	8.2742	8.16795	8.2963	8.83206	8.23307	8.67764	7.53161	9.24997	8.57088	8.54639	7.41974	8.89902	0.891742979	0.966018836	24	12	24	0.798875345	0.935664328	24	19.98701872	0.024572282
ENSMUSG00000052911	Lamb2	laminin, beta 2 [Source:MGI Symbol;Acc:MGI:99916]	22.7448	21.6736	21.9229	23.0669	26.2647	18.6504	25.0537	25.656	25.2611	21.5276	21.6337	21.4898	20.9021	20.2223	0.935316621	0.966018836	12	12	24	0.743505457	0.916215545	24	8.921411794	0.042663879
ENSMUSG00000052912	Smarca5-ps	SWI/SNF related, matrix associated, actin depenent regulator of chromatin, subfamily a, member 5, pseudogene [Source:MGI Symbol;Acc:MGI:2443330]	1.12186	1.39621	1.11015	1.17288	1.28713	0.917119	0.895916	1.28847	1.12174	0.980151	1.37659	1.18567	1.34941	1.30258	0.849179819	0.966018836	8	4	24	0.823721635	0.944521304	24	6.139749988	0.028398762
ENSMUSG00000052914	Cyp2j6	cytochrome P450, family 2, subfamily j, polypeptide 6 [Source:MGI Symbol;Acc:MGI:1270148]	22.978	24.6126	22.297	20.546	25.0913	22.2916	24.4021	23.6959	24.6039	21.3146	24.6944	23.8621	25.1077	24.2071	0.446653834	0.966018836	4	12	24	0.25421499	0.768040178	24	0.15501874	0.052208786
ENSMUSG00000052915	Msi1	male specific lethal 1 [Source:MGI Symbol;Acc:MGI:1921276]	35.4451	31.7262	30.9864	34.4509	34.0038	34.3143	42.0424	33.1648	29.917	36.5322	34.369	34.4877	33.0169	34.1635	0.175185919	0.651010555	16	20	24	0.228937309	0.768040178	24	20.28937309	0.077092505
ENSMUSG00000052917	Senp7	SUMO1/sentrin specific peptidase 7 [Source:MGI Symbol;Acc:MGI:1913565]	8.76065	9.97246	10.254	9.81546	9.18056	9.36172	9.6061	9.01776	9.95358	7.73863	10.5241	8.98016	9.31011	9.41275	0.68887295	0.966018836	12	12	24	0.896447295	0.970591958	24	7.351461539	0.019483895
ENSMUSG00000052920	Prkg1	protein kinase, cGMP-dependent, type I [Source:MGI Symbol;Acc:MGI:108174]	20.9499	18.3518	17.8067	18.6376	18.2074	19.2194	17.7669	16.4882	16.5863	16.8035	21.142	18.9402	19.3775	18.498	0.140905066	0.585944911	24	12	24	0.056605348	0.655587475	24	19.90198514	0.093710123
ENSMUSG00000052921	Arhgef15	Rho guanine nucleotide exchange factor (GEF) 15 [Source:MGI Symbol;Acc:MGI:3045246]	1.93937	2.14753	2.88221	2.35104	2.55699	2.62466	2.44691	2.39501	2.39496	2.53132	2.10323	2.1783	1.82195	2.63313	0.865233232	0.966018836	12	16	24	0.244309252	0.768040178	24	10.29913911	0.083438294
ENSMUSG00000052926	Rnaseh2a	ribonuclease H2, large subunit [Source:MGI Symbol;Acc:MGI:1916974]	23.6791	24.7318	27.4378	21.3905	21.3242	23.5302	22.1805	24.5078	22.0099	21.321	25.1079	19.877	24.476	22.9264	0.361555248	0.885260695	8	8	24	0.344686801	0.778067777	24	4.485261132	0.065068501
ENSMUSG00000052928	Ctif	CBP80/20-dependent translation initiation factor [Source:MGI Symbol;Acc:MGI:2685518]	19.8926	14.937	15.4262	16.0346	16.184	18.3246	17.3183	18.1687	12.5583	25.9105	14.2115	18.3054	17.5169	18.3432	0.46238066	0.966018836	24	12	24	0.792650344	0.933721061	24	20.59973988	0.066345039
ENSMUSG00000052934	Fbxo31	F-box protein 31 [Source:MGI Symbol;Acc:MGI:1354708]	25.8764	20.6648	19.8107	22.7793	19.964	22.3953	23.064	27.512	19.6498	33.1566	21.1229	24.8415	23.1471	23.9192	0.45229395	0.966018836	4	8	24	0.980526619	0.995264516	24	0.816659569	0.01557447
ENSMUSG00000052949	Rnf157	ring finger protein 157 [Source:MGI Symbol;Acc:MGI:2442484]	55.9277	49.9895	43.8191	45.8655	46.2176	52.7749	49.0516	49.5609	34.1159	68.4653	41.1696	49.9691	47.093	48.1893	0.123394836	0.552144108	24	12	24	0.58782934	0.853689229	24	19.56883142	0.097530458
ENSMUSG00000052951	C130021I20Rik	Riken cDNA C130021I20 gene [Source:MGI Symbol;Acc:MGI:3639863]	0.841431	0.920358	1.3445	0.809418	0.777244	1.10908	0.905755	0.989125	1.47393	0.92348	0.807524	0.808367	0.754255	1.17339	0.005378037	0.149433822	12	8	24	0.061295671	0.661066697	24	7.181516286	0.137547529
ENSMUSG00000052957	Gas1	growth arrest specific 1 [Source:MGI Symbol;Acc:MGI:95655]	3.86121	4.03904	3.67921	3.68912	2.86596	2.86908	3.75154	3.99106	4.9867	2.62316	3.75931	4.30461	3.59435	3.72985	0.446653834	0.966018836	8	8	24	0.198716486	0.768040178	24	4.350350936	0.130399596
ENSMUSG00000052962	Mrpl35	mitochondrial ribosomal protein L35 [Source:MGI Symbol;Acc:MGI:1913473]	24.4293	24.9274	24.8105	24.0713	27.5144	24.5204	22.9846	25.2259	26.3005	21.7429	27.9816	25.6351	26.7212	23.3369	0.676344318	0.966018836	20	20	24	0.594049066	0.857184401	24	17.82410068	0.040265318
ENSMUSG00000052974	Cyp2f2	cytochrome P450, family 2, subfamily f, polypeptide 2 [Source:MGI Symbol;Acc:MGI:88608]	0.263184	0.426212	0.851547	0.857822	0.437433	0.517578	0.298446	0.376115	0.55722	0.860471	0.818793	0.591219	0.510028	0.551565	3.11E-04	0.049770315	16	12	24	0.0014865	0.358147394	24	12.05022518	0.20855285
ENSMUSG00000052981	Ube2ql1	ubiquitin-conjugating enzyme E2Q family-like 1 [Source:MGI Symbol;Acc:MGI:1924230]	16.8681	14.8794	16.4548	15.1879	13.8796	17.1468	15.5725	17.006	13.3093	20.0098	14.299	18.3489	16.3981	17.1444	0.893307667	0.966018836	24	20	24	0.829667794	0.947045266	24	23.44252159	0.036535282
ENSMUSG00000052997	Uba2	ubiquitin-like modifier activating enzyme 2 [Source:MGI Symbol;Acc:MGI:1858313]	46.4513	46.8474	46.6222	46.9617	46.0496	46.7133	46.6251	44.5548	45.6759	44.9482	46.7977	44.8711	44.8956	46.9117	0.844448841	0.966018836	16	12	24	0.973197099	0.993305115	24	14.87655454	0.002314451
ENSMUSG00000053004	Hrh1	histamine receptor H1 [Source:MGI Symbol;Acc:MGI:107619]	1.60213	1.18491	1.33242	1.16845	1.24083	1.74332	1.45972	1.27718	1.22108	1.23268	1.30834	1.1221	1.52485	1.2298	0.185945151	0.662239322	4	12	24	0.068655498	0.673061483	24	22.08495834	0.092620769
ENSMUSG00000053007	Creb5	cAMP responsive element binding protein 5 [Source:MGI Symbol;Acc:MGI:2443973]	1.1158	1.44513	1.26327	1.21543	1.39933	1.34269	1.5554	1.17369	1.54708	1.58372	1.27096	1.45462	1.36216	1.83158	0.759203569	0.966018836	8	12	24	0.60196697	0.860847507	24	6.71238455	0.039247988
ENSMUSG00000053012	Krcc1	lysine-rich coiled-coil 1 [Source:MGI Symbol;Acc:MGI:1889377]	23.6128	25.8899	24.5969	25.7637	25.2402	21.6234	26.3798	22.8567	28.4716	24.2844	27.1527	23.9201	24.2493	25.4712	0.581070934	0.966018836	12	12	24	0.276258838	0.768040178	24	9.617488571	0.062447954
ENSMUSG00000053024	Cntn2	contactin 2 [Source:MGI Symbol;Acc:MGI:104518]	34.94	26.5411	27.7268	29.1677	28.788	31.3685	30.4358	29.6602	23.0137	35.4435	26.3252	29.6941	28.0549	30.0418	0.377663653	0.903463289	24	12	24	0.47852811	0.805393068	24	20.44595322	0.076654319
ENSMUSG00000053025	Sv2b	synaptic vesicle glycoprotein 2 b [Source:MGI Symbol;Acc:MGI:1927338]	50.9696	46.6365	43.4885	47.8136	43.0765	44.9264	46.0788	44.3962	38.8222	51.0109	45.1635	46.6081	47.2695	48.7081	0.745199643	0.966018836	4	8	24	0.667690404	0.887623355	24	21.99635352	0.03712907
ENSMUSG00000053030	Spink2	serine peptidase inhibitor, Kazal type 2 [Source:MGI Symbol;Acc:MGI:1917232]	3.43396	4.45543	2.40894	3.30447	2.62538	3.79816	4.15272	2.8131	3.48473	2.87486	3.50065	3.22488	4.56164	4.24734	0.133875138	0.568511754	4	8	24	0.076720444	0.687041208	24	0.546680731	0.175140668
ENSMUSG00000053038	Gm6180	predicted pseudogene 6180 [Source:MGI Symbol;Acc:MGI:3643972]	278.896	254.796	263.009	248.091	267.214	299.614	264.3	264.968	220.892	249.591	262.566	241.992	237.766	249.935	0.708020493	0.966018836	20	20	24	0.095594894	0.71175955	24	20.27062938	0.077316153
ENSMUSG00000053040	Aph1c	aph1 homolog C, gamma secretase subunit [Source:MGI Symbol;Acc:MGI:1915568]	1.35695	1.52601	1.70777	1.42285	1.50863	1.46743	1.28558	1.4045	1.66594	1.31557	1.38867	1.60144	1.54511	1.47063	0.648082424	0.966018836	12	4	24	0.633313537	0.875353057	24	7.989727307	0.029092136
ENSMUSG00000053046	Brsk2	BR serine/threonine kinase 2 [Source:MGI Symbol;Acc:MGI:1923020]	24.7588	23.1712	25.6994	24.4752	24.7509	25.7168	28.1593	24.2277	21.7304	30.7052	22.2923	24.8084	26.2348	26.0976	0.590017078	0.966018836	4	8	24	0.955101807	0.988639288	24	20.97530629	0.015617067
ENSMUSG00000053062	Jam2	junction adhesion molecule 2 [Source:MGI Symbol;Acc:MGI:1933820]	14.5512	16.0204	16.095	15.1351	17.1353	14.3016	14.4803	14.837	16.7408	16.4748	16.397	14.8978	14.7118	15.2945	0.00424198	0.140598674	20	4	24	0.011980511	0.525280095	24	11.37403585	0.083109427
ENSMUSG00000053063	Clec12a	C-type lectin domain family 12, member a [Source:MGI Symbol;Acc:MGI:3040968]	1.72857	3.41157	4.0187	2.69362	3.01441	2.89288	2.26008	2.68555	3.37114	1.64653	2.13481	2.55133	3.06221	1.9029	0.700065774	0.966018836	12	4	24	0.722994791	0.909188168	24	7.533515124	0.088807737
ENSMUSG00000053070	Cfap300	cilia and flagella associated protein 300 [Source:MGI Symbol;Acc:MGI:3045346]	8.82148	8.46808	8.18744	6.54738	8.4009	7.68402	8.91286	8.04324	7.53374	7.2924	8.4943	8.34137	8.29674	8.36051	0.017417473	0.255326033	4	12	24	0.030853858	0.599063533	24	23.49523961	0.098444174
ENSMUSG00000053080	Zfta	zinc finger translocation associated [Source:MGI Symbol;Acc:MGI:1919667]	11.3179	9.97711	10.1865	10.1179	9.89872	9.86669	9.92901	10.4869	10.5631	12.3336	11.935	12.227	12.2712	12.0478	0.768363194	0.966018836	16	4	24	0.96750672	0.992187127	24	19.51467533	0.009209981
ENSMUSG00000053081	Lins1	lines homolog 1 [Source:MGI Symbol;Acc:MGI:1919885]	5.74451	5.10856	5.61513	4.95858	5.00841	6.27378	5.1666	5.43819	5.08187	6.32276	5.42094	5.58709	5.28509	5.5339	0.852844413	0.966018836	24	20	24	0.802726054	0.936748018	24	18.88490491	0.025742362
ENSMUSG00000053093	Myh7	myosin, heavy polypeptide 7, cardiac muscle, beta [Source:MGI Symbol;Acc:MGI:2155600]	3.17791	2.76539	9.79999	12.0259	5.72685	5.4584	5.22985	9.7509	4.79239	13.8626	2.63005	0.769539	5.12714	5.90974	0.092972576	0.489012695	16	8	24	0.081186614	0.697074851	24	9.390153409	0.690111176
ENSMUSG00000053094	Tmem248	transmembrane protein 248 [Source:MGI Symbol;Acc:MGI:1918917]	15.519	13.3736	14.2257	14.1381	13.4109	14.7944	14.6151	14.2841	12.3241	16.9985	12.7702	14.5088	13.6672	14.6827	0.593560247	0.966018836	24	20	24	0.912753572	0.97533472	24	21.48308455	0.019489554
ENSMUSG00000053110	Yap1	yes-associated protein 1 [Source:MGI Symbol;Acc:MGI:103262]	13.733	12.4638	10.6337	11.5321	13.1244	10.9022	15.0958	12.4428	13.3244	12.9132	12.1511	12.3297	13.4599	11.8751	0.154289127	0.606139807	4	12	24	0.437461948	0.805393068	24	23.63814801	0.062765928
ENSMUSG00000053111	Fank1	fibronectin type 3 and ankyrin repeat domains 1 [Source:MGI Symbol;Acc:MGI:1914180]	1.16189	0.865063	0.702396	0.768808	0.75942	0.957857	1.02553	0.599203	0.736321	0.79579	0.876261	0.792652	0.743165	0.850961	0.158469451	0.614860056	4	8	24	0.075854122	0.686372559	24	21.37972524	0.090577144
ENSMUSG00000053113	Socs3	suppressor of cytokine signaling 3 [Source:MGI Symbol;Acc:MGI:1201791]	2.96014	3.06223	3.05483	2.92986	3.49403	2.47273	3.34259	3.56659	2.87051	2.84537	3.41306	2.78726	3.46532	3.02081	0.595952838	0.966018836	8	16	24	0.853563167	0.956231172	24	3.355367006	0.025532697
ENSMUSG00000053119	Chmp3	charged multivesicular body protein 3 [Source:MGI Symbol;Acc:MGI:1913950]	109.143	105.7	96.6456	101.749	99.7257	100.161	97.4233	96.508	96.252	92.5514	105.302	101.04	102.119	100.982	0.360033827	0.883787544	4	8	24	0.166637268	0.767020009	24	21.78081764	0.044338139
ENSMUSG00000053128	Rnf26	ring finger protein 26 [Source:MGI Symbol;Acc:MGI:2388131]	5.71152	5.30817	4.9432	5.58257	5.22947	5.75158	4.66973	5.99595	4.65723	7.50003	4.3644	5.77855	5.30268	5.52994	0.945282252	0.966018836	24	12	24	0.950021731	0.988090608	24	11.96215169	0.021230756
ENSMUSG00000053134	Supt7l	SPT7-like, STAGA complex gamma subunit [Source:MGI Symbol;Acc:MGI:1919445]	5.89156	5.06141	5.42776	5.02252	5.29978	5.99799	5.12721	6.09648	5.14209	6.20535	4.98869	5.5123	5.28452	5.28319	0.829428469	0.966018836	24	20	24	0.899479185	0.971453495	24	22.12157349	0.017767459
ENSMUSG00000053137	Mapk11	mitogen-activated protein kinase 11 [Source:MGI Symbol;Acc:MGI:1338024]	36.7085	27.5565	27.3831	28.7701	33.4481	33.7036	27.2917	30.1913	24.2115	35.0523	29.3196	32.939	30.725	29.9167	0.074438493	0.448301997	24	12	24	0.089720375	0.709708691	24	19.18259794	0.134394491
ENSMUSG00000053141	Ptprt	protein tyrosine phosphatase, receptor type, T [Source:MGI Symbol;Acc:MGI:1321152]	22.6259	18.0975	19.5938	19.6702	20.4217	21.9317	18.0771	17.6213	15.7306	20.1257	19.5852	20.8789	20.8524	20.4277	0.042880436	0.356048751	12	12	24	0.066948921	0.66926181	24	19.50663077	0.114766042
ENSMUSG00000053158	Fes	feline sarcoma oncogene [Source:MGI Symbol;Acc:MGI:95514]	2.10682	2.71557	4.12832	3.0598	3.13008	2.57024	2.68538	2.76346	2.88909	2.48919	2.16898	2.0945	2.83048	2.02979	0.364640768	0.892062098	12	12	24	0.208915029	0.768040178	24	9.514405427	0.127598203
ENSMUSG00000053164	Gpr21	G protein-coupled receptor 21 [Source:MGI Symbol;Acc:MGI:2441890]	0.469373	0.577118	0.672926	0.647726	0.45734	0.42544	0.489984	0.638399	0.682197	0.740876	0.466883	1.16019	0.554502	0.710573	0.080511813	0.4593055	16	4	24	0.643124643	0.878809617	24	8.683252207	0.055382979
ENSMUSG00000053173	Rpl18-ps2	ribosomal protein L18, pseudogene 2 [Source:MGI Symbol;Acc:MGI:3646997]	62.2375	70.8642	73.5027	67.7228	67.5832	64.0415	67.279	57.0706	72.7683	48.9951	73.4451	53.7546	61.0835	62.138	0.46238066	0.966018836	12	4	24	0.854675238	0.956688008	24	8.872791124	0.037435572
ENSMUSG00000053175	Bcl3	B cell leukemia/lymphoma 3 [Source:MGI Symbol;Acc:MGI:88140]	2.82149	2.86757	2.38685	2.7538	4.74934	2.35991	2.1319	3.96984	3.52286	3.21364	3.7643	2.27027	3.02477	2.50426	0.265774856	0.773510818	20	4	24	0.387616628	0.799546792	24	12.53580238	0.137043528
ENSMUSG00000053178	Mterf1b	mitochondrial transcription termination factor 1b [Source:MGI Symbol;Acc:MGI:3704243]	1.41545	1.47336	1.61151	1.13592	1.13676	1.08708	1.76884	1.26981	1.5081	0.934967	1.65602	1.25648	1.31338	1.37039	0.265774856	0.773510818	12	4	24	0.335410945	0.771377467	24	2.876465756	0.08287498
ENSMUSG00000053181	A830005F24Rik	RIKEN cDNA A830005F24 gene [Source:MGI Symbol;Acc:MGI:3045249]	2.36438	2.4986	2.22284	1.82754	2.1464	2.49583	2.33681	2.51308	2.39753	2.0269	2.67475	2.73577	3.36787	2.20464	0.097779482	0.501112642	24	16	24	0.043151952	0.625119217	24	23.1309163	0.123194215
ENSMUSG00000053182	Gm609	predicted gene 609 [Source:MGI Symbol;Acc:MGI:2685455]	0.219209	0.482323	0.727716	0.673721	0.683029	0.578055	0.274194	0.576237	0.696957	0.525506	0.57768	0.365953	0.564287	0.429194	0.008754183	0.192760771	12	16	24	0.020820706	0.568066329	24	11.23653326	0.150120139
ENSMUSG00000053192	Mllt11	myeloid/lymphoid or mixed-lineage leukemia; translocated to, 11 [Source:MGI Symbol;Acc:MGI:1929671]	167.99	155.296	155.517	150.105	152.711	167.003	148.592	151.447	134.663	158.833	163.342	153.134	157.142	162.93	0.724067924	0.966018836	24	12	24	0.266202033	0.768040178	24	20.56402543	0.052842203
ENSMUSG00000053198	Prx	periaxin [Source:MGI Symbol;Acc:MGI:108176]	196.213	197.707	181.529	204.967	257.387	194.058	251.318	215.798	241.449	284.202	193.479	233.666	232.622	185.834	0.924770496	0.966018836	16	16	24	0.630877157	0.874133182	24	15.0844277	0.072898611
ENSMUSG00000053199	Arhgap20	Rho GTPase activating protein 20 [Source:MGI Symbol;Acc:MGI:2445175]	4.64408	4.7692	4.3425	3.95748	3.84806	4.69802	4.61684	4.45391	4.33496	4.34404	4.21425	5.1516	4.63434	4.88041	0.006767691	0.181382464	24	20	24	0.008906832	0.502965379	24	0.664827719	0.090555088
ENSMUSG00000053205	Styx	serine/threonine/tyrosine interaction protein [Source:MGI Symbol;Acc:MGI:1891150]	1.56527	1.91969	1.72353	1.91506	1.92505	1.66309	1.67854	1.53494	2.36814	1.58417	2.04904	2.05651	1.57195	1.46587	0.151986936	0.600422612	20	12	24	0.252347072	0.768040178	24	13.53422993	0.081828175
ENSMUSG00000053208	Kat2b-ps	K(lysine) acetyltransferase 2B, pseudogene [Source:MGI Symbol;Acc:MGI:3648817]	0.969779	1.27315	1.11103	1.07942	1.17859	0.855084	0.977525	1.05516	1.02506	0.966294	0.987097	0.95035	0.835847	0.856212	0.195388358	0.673387182	12	12	24	0.201925368	0.768040178	24	10.02055679	0.046771018
ENSMUSG00000053214	Gm9899	predicted gene 9899 [Source:MGI Symbol;Acc:MGI:3708711]	0.825982	0.656812	0.846587	1.11113	0.642253	0.724863	0.832698	0.805426	0.529498	0.914465	0.759221	0.687699	0.842375	0.85523	0.602156008	0.966018836	16	4	24	0.887045322	0.968043979	24	10.52504523	0.022809452
ENSMUSG00000053226	Dand5	DAN domain family member 5, BMP antagonist [Source:MGI Symbol;Acc:MGI:1344365]	4.52446	4.49336	3.82863	3.44276	4.1348	4.29188	4.42172	4.19918	3.27311	3.64122	3.97049	4.39442	4.6291	3.62911	1.18E-04	0.029372629	4	8	24	2.53E-04	0.182644645	24	22.30812017	0.153795301
ENSMUSG00000053253	Ndfip2	Nedd4 family interacting protein 2 [Source:MGI Symbol;Acc:MGI:1923523]	152.648	139.317	127.987	131.104	134.048	138.501	140.01	135.518	120.251	140.218	136.686	133.862	143.504	139.511	0.002171187	0.102623968	4	8	24	0.042742679	0.624436905	24	22.42709525	0.07259754
ENSMUSG00000053263	Gm12592	predicted gene 12592 [Source:MGI Symbol;Acc:MGI:3649892]	0.728214	0.833045	0.765353	1.15866	0.860896	0.917248	0.659904	0.918426	0.931992	0.972265	0.839957	0.702202	0.895174	0.984089	0.101443845	0.511659635	16	12	24	0.088627005	0.707747429	24	10.50091412	0.081694157
ENSMUSG00000053279	Aldh1a1	aldehyde dehydrogenase family 1, subfamily A1 [Source:MGI Symbol;Acc:MGI:1353450]	37.235	46.9604	44.2268	43.2924	44.3886	43.4074	50.1404	44.4109	51.4294	31.2276	40.4423	47.6188	37.1212	37.5818	0.911685024	0.966018836	12	4	24	0.886409114	0.967763983	24	1.364940161	0.036611881
ENSMUSG00000053286	Trmt1l	tRNA methyltransferase 1 like [Source:MGI Symbol;Acc:MGI:1916185]	15.5586	12.5283	12.4543	13.1482	12.5267	13.4171	14.009	13.3313	12.4299	13.4619	14.0926	12.6735	12.7375	12.3067	0.235187365	0.73629392	4	8	24	0.247136897	0.768040178	24	20.57950248	0.05618918
ENSMUSG00000053289	Ddx10	DEAD box helicase 10 [Source:MGI Symbol;Acc:MGI:1924841]	11.7453	11.838	11.8837	11.6163	11.2233	11.1293	10.3466	11.1856	10.5299	10.3388	11.4369	11.5328	11.7717	12.6501	0.722296281	0.966018836	8	8	24	0.608851417	0.864393298	24	2.555136238	0.029800444
ENSMUSG00000053291	Rab4b	RAB4B, member RAS oncogene family [Source:MGI Symbol;Acc:MGI:105071]	63.6152	50.5199	56.7963	49.0916	46.8815	56.7751	53.3328	58.5044	49.8936	67.4468	56.7582	61.817	60.4883	59.3205	0.581070934	0.966018836	4	16	24	0.655379877	0.883697438	24	23.77108869	0.051607974
ENSMUSG00000053293	Pom121	nuclear pore membrane protein 121 [Source:MGI Symbol;Acc:MGI:2137624]	4.25582	4.09407	4.13962	4.15427	4.09565	4.30283	4.38529	4.44989	4.33785	6.198	3.91064	4.3816	4.01358	4.52629	0.890311217	0.966018836	8	12	24	0.511274572	0.814808034	24	10.30350199	0.060485663
ENSMUSG00000053297	AI854703	expressed sequence AI854703 [Source:MGI Symbol;Acc:MGI:2141510]	5.3128	4.66672	5.92343	4.97908	4.85425	5.89918	5.64171	4.97153	4.32913	4.75455	4.74513	4.99532	5.32836	5.14518	0.208071297	0.698704663	24	20	24	0.256245884	0.768040178	24	22.54872493	0.069236237
ENSMUSG00000053310	Nrgn	neurogranin [Source:MGI Symbol;Acc:MGI:1927184]	1.31155	2.4449	2.65885	1.8176	2.63963	1.59037	1.39448	1.83272	3.6973	2.915	2.57503	2.69795	3.52265	2.02391	0.608967886	0.966018836	12	16	24	0.188754846	0.768040178	24	10.59411831	0.203084027
ENSMUSG00000053317	Sec61b	Sec61 beta subunit [Source:MGI Symbol;Acc:MGI:1913462]	23.9124	21.9369	21.3758	20.3524	24.5223	19.5065	25.032	24.4393	23.3018	17.7389	25.9266	21.5857	24.8812	21.0625	0.117050591	0.538362324	4	12	24	0.399761613	0.799546792	24	23.29409531	0.081125098
ENSMUSG00000053329	Gatd3a	glutamine amidotransferase like class 1 domain containing 3A [Source:MGI Symbol;Acc:MGI:1351861]	42.5119	40.6398	43.4115	48.4672	40.2228	41.978	40.2372	48.2172	43.5273	51.7262	46.3746	48.8412	50.9062	48.7256	0.865490576	0.966018836	16	4	24	0.452781873	0.805393068	24	9.976365745	0.04781127
ENSMUSG00000053332	Gas5	growth arrest specific 5 [Source:MGI Symbol;Acc:MGI:95659]	198.312	238.264	224.306	209.316	226.81	192.511	228.193	200.642	256.895	175.474	226.921	216.734	231.454	229.036	0.522551072	0.966018836	12	4	24	0.570205169	0.845047627	24	4.977339965	0.060258623
ENSMUSG00000053333	Dis3l2	DIS3 like 3'-5' exoribonuclease 2 [Source:MGI Symbol;Acc:MGI:2442555]	6.85524	5.93234	6.55075	6.14325	5.49509	6.55509	6.12582	6.95745	5.76486	7.30468	5.4404	6.79298	5.93115	6.19839	0.892685629	0.966018836	8	12	24	0.810565984	0.939224719	24	3.11235726	0.028867636
ENSMUSG00000053334	Ficd	FIC domain containing [Source:MGI Symbol;Acc:MGI:1098550]	3.99732	3.18178	3.27774	3.39904	3.25289	3.73907	3.56798	3.42672	2.98393	4.61954	3.13065	3.55037	3.48398	3.37881	0.119434414	0.542554768	24	12	24	0.736114381	0.914041192	24	19.18810911	0.0411285
ENSMUSG00000053347	Zfp943	zinc finger prtoein 943 [Source:MGI Symbol;Acc:MGI:1921920]	14.4033	17.3443	14.7787	15.0988	16.815	14.5838	14.5729	15.2657	17.0644	12.6624	16.8689	15.1274	14.7204	14.7824	0.432054949	0.966018836	8	20	24	0.975415345	0.993762379	24	10.66809117	0.010561529
ENSMUSG00000053390	Zfp952	zinc finger protein 952 [Source:MGI Symbol;Acc:MGI:2441928]	6.69884	7.16858	6.57532	6.14107	6.69521	6.09665	6.65188	6.19621	7.70789	5.96932	7.16047	6.78474	7.32605	6.75314	0.849179819	0.966018836	4	12	24	0.650570412	0.882536809	24	3.090766038	0.033829975
ENSMUSG00000053395	Cacng8	calcium channel, voltage-dependent, gamma subunit 8 [Source:MGI Symbol;Acc:MGI:1932376]	0.681142	0.705296	1.38816	0.505436	0.691081	0.914308	0.521114	0.49639	0.576114	0.643907	0.500001	0.821596	0.60573	0.744522	0.905762334	0.966018836	24	20	24	0.980622449	0.995276271	24	6.939430656	0.014669016
ENSMUSG00000053398	Phgdh	3-phosphoglycerate dehydrogenase [Source:MGI Symbol;Acc:MGI:1355330]	7.52351	7.21256	7.38032	7.33368	9.90001	7.1807	7.63993	7.5997	7.58579	7.25792	8.0771	6.66209	7.05014	5.92531	0.960829223	0.967043964	20	4	24	0.129268652	0.740162193	24	15.74235222	0.09706736
ENSMUSG00000053411	Cbx7	chromobox 7 [Source:MGI Symbol;Acc:MGI:1196439]	12.597	15.0264	13.5333	13.2905	12.6051	12.9625	13.9913	14.1986	16.9341	16.0839	11.8104	15.2908	15.2693	14.59	0.510431574	0.966018836	12	8	24	0.102814074	0.713699364	24	6.291528407	0.103905194
ENSMUSG00000053414	Hunk	hormonally upregulated Neu-associated kinase [Source:MGI Symbol;Acc:MGI:1347352]	2.4849	2.47478	2.52713	2.1196	2.69911	2.37778	2.97814	2.4974	2.39988	2.91113	2.6211	2.21347	2.5276	2.86994	0.896753532	0.966018836	4	20	24	0.841674667	0.952050674	24	4.164106849	0.023343037
ENSMUSG00000053436	Mapk14	mitogen-activated protein kinase 14 [Source:MGI Symbol;Acc:MGI:1346865]	25.7777	21.7822	22.6337	24.1802	24.6593	25.1588	24.7808	24.6236	22.9039	24.867	24.3928	22.7093	22.7969	22.0104	0.275983441	0.789849983	4	4	24	0.134066979	0.744355886	24	17.93937369	0.055359482
ENSMUSG00000053453	Thoc7	THO complex 7 [Source:MGI Symbol;Acc:MGI:1913481]	58.6694	62.1139	64.176	58.8398	63.8248	59.2302	56.097	62.8303	63.5541	53.9166	60.804	56.9115	61.4116	63.2841	0.071575474	0.440262583	12	12	24	0.358194046	0.784922891	24	6.372549006	0.044100823
ENSMUSG00000053460	Ggcx	gamma-glutamyl carboxylase [Source:MGI Symbol;Acc:MGI:1927655]	7.6062	10.0098	9.48062	8.01634	8.48385	7.27037	8.2376	9.91645	7.45708	7.62742	8.47517	8.16177	8.59686	8.22906	0.522551072	0.966018836	8	16	24	0.292446105	0.768040178	24	5.011930211	0.078595353
ENSMUSG00000053461	Hhipl2	hedgehog interacting protein-like 2 [Source:MGI Symbol;Acc:MGI:1926022]	1.87272	0.375515	0.895448	0.510693	0.94231	0.762061	0.495965	0.627325	0.533836	1.33735	0.446878	0.634076	1.25682	0.648476	0.860993221	0.966018836	4	4	24	0.77554389	0.927742146	24	21.02671067	0.090575295
ENSMUSG00000053465	Hs6st3	heparan sulfate 6-O-sulfotransferase 3 [Source:MGI Symbol;Acc:MGI:1354960]	2.48818	2.27105	2.3446	2.59989	2.08262	2.64728	2.94606	2.53353	2.07095	3.20209	2.10579	2.85287	2.45448	2.91164	0.918193129	0.966018836	24	12	24	0.817207964	0.941826666	24	0.207800677	0.034673937
ENSMUSG00000053470	Kdm3a	lysine (K)-specific demethylase 3A [Source:MGI Symbol;Acc:MGI:98847]	9.14814	13.225	13.0639	11.3968	12.1562	10.9719	11.6295	11.6	13.4502	10.3845	11.2977	11.9612	11.7891	11.6398	0.43280837	0.966018836	12	4	24	0.299128264	0.768040178	24	7.866384511	0.082650108
ENSMUSG00000053472	Gm13534	predicted gene 13534 [Source:MGI Symbol;Acc:MGI:3651249]	0.800853	1.1588	1.36622	1.94596	1.60758	1.28303	1.66845	1.52479	1.01069	1.43734	1.16943	1.10438	2.225	1.04449	0.911685024	0.966018836	16	20	24	0.880259877	0.966381821	24	14.55854196	0.045905461
ENSMUSG00000053475	Tnfaip6	tumor necrosis factor alpha induced protein 6 [Source:MGI Symbol;Acc:MGI:1195266]	2.63867	3.4152	5.21275	3.39319	2.85028	3.43265	3.44305	3.24333	3.90867	2.5042	2.96904	2.63751	2.05976	2.67977	0.360140629	0.883787544	12	12	24	0.179658765	0.768040178	24	8.650607355	0.155528895
ENSMUSG00000053477	Tcf4	transcription factor 4 [Source:MGI Symbol;Acc:MGI:98506]	8.98096	11.0083	12.0039	10.3212	10.5889	9.4017	12.3842	9.37829	13.8536	10.2873	10.8897	10.5008	10.3774	11.9423	0.628286596	0.966018836	12	12	24	0.179370298	0.768040178	24	7.986018211	0.111826553
ENSMUSG00000053483	Usp21	ubiquitin specific peptidase 21 [Source:MGI Symbol;Acc:MGI:1353665]	12.9114	14.3011	12.5187	15.3428	14.9647	12.5516	14.5848	11.7404	13.3239	11.5346	12.7716	9.95165	11.0048	12.4734	0.917839756	0.966018836	20	4	24	0.635654062	0.876201318	24	11.32246922	0.050065061
ENSMUSG00000053510	Nrd1	nardilysin, N-arginine dibasic convertase, NRD convertase 1 [Source:MGI Symbol;Acc:MGI:1201386]	53.691	52.5672	58.2895	49.1659	52.2049	47.4591	50.4369	51.9655	51.0403	53.1235	51.7909	50.2444	49.424	49.9229	0.601647512	0.966018836	12	12	24	0.274988896	0.768040178	24	8.386820469	0.040722279
ENSMUSG00000053519	Kcnip1	Kv channel-interacting protein 1 [Source:MGI Symbol;Acc:MGI:1917607]	16.4426	19.3607	19.9886	17.4487	16.3743	15.4404	17.4623	15.4762	17.6359	17.4664	17.4072	17.5715	16.9136	18.8126	0.118761453	0.542330458	12	12	24	0.155387251	0.76052757	24	7.645211599	0.077233169
ENSMUSG00000053522	Lgals7	lectin, galactose binding, soluble 7 [Source:MGI Symbol;Acc:MGI:1316742]	0.673968	0.650067	0.641978	1.6382	0.603223	0.870164	0.389978	0.421453	0.321707	0.489872	0.415972	0.430824	0.629641	0.503921	0.806004771	0.966018836	16	20	24	0.569442135	0.844856622	24	14.68313328	0.092300974
ENSMUSG00000053536	Cstf2t	cleavage stimulation factor, 3' pre-RNA subunit 2, tau [Source:MGI Symbol;Acc:MGI:1932622]	10.4188	10.4681	11.5215	10.5299	10.2887	10.5	11.0425	9.93241	9.83375	10.2045	9.83767	10.144	10.69	10.1928	0.917370979	0.966018836	4	16	24	0.73326922	0.91292127	24	1.681163361	0.016372232
ENSMUSG00000053550	Shisa7	shisa family member 7 [Source:MGI Symbol;Acc:MGI:3605641]	1.34692	2.08713	2.75297	1.76541	2.00004	2.22023	2.13084	2.2893	2.35737	1.97265	1.72341	1.95602	1.67843	2.2594	0.079732457	0.458678242	12	16	24	0.177536323	0.768040178	24	7.67117429	0.122618625
ENSMUSG00000053552	Ebf4	early B cell factor 4 [Source:MGI Symbol;Acc:MGI:2385972]	4.15716	4.54836	4.65588	4.01847	5.22891	4.21757	4.60614	4.50668	4.81897	5.06415	5.19692	4.65825	5.12323	4.59112	0.192357053	0.669841558	20	12	24	0.522516615	0.820883629	24	13.5986921	0.037622608
ENSMUSG00000053553	3110082I17Rik	RIKEN cDNA 3110082I17 gene [Source:MGI Symbol;Acc:MGI:1920462]	3.63551	3.4221	3.57404	3.07919	2.92482	3.47457	2.85266	3.95375	3.63188	3.03696	3.47703	3.41487	3.28938	3.66316	0.152177471	0.600454138	8	12	24	0.194447204	0.768040178	24	4.351282656	0.072389725
ENSMUSG00000053559	Smagp	small cell adhesion glycoprotein [Source:MGI Symbol;Acc:MGI:2448476]	2.65008	1.68987	2.33794	2.00718	2.74625	2.43347	1.84681	2.52355	2.40356	1.71623	2.98149	1.92067	2.04164	1.84787	0.446653834	0.966018836	20	12	24	0.447056535	0.805393068	24	16.94606361	0.091005247
ENSMUSG00000053560	Ier2	immediate early response 2 [Source:MGI Symbol;Acc:MGI:104815]	2.79661	2.59349	2.72582	2.31367	1.99157	2.07488	2.59772	3.58197	2.94904	2.59761	2.59117	3.22297	3.73732	2.8556	0.118902121	0.542554768	4	16	24	0.030433454	0.597890721	24	3.4117903	0.153708846
ENSMUSG00000053565	Eif3k	eukaryotic translation initiation factor 3, subunit K [Source:MGI Symbol;Acc:MGI:1921080]	123.745	125.611	122.16	116.249	124.392	130.677	113.308	124.473	123.607	110.146	127.235	125.768	127.3	122.429	0.039226672	0.338446795	24	16	24	0.356163337	0.783018041	24	22.02354549	0.039050618
ENSMUSG00000053574	4930563E22Rik	RIKEN cDNA 4930563E22 gene [Source:MGI Symbol;Acc:MGI:1922554]	1.5216	1.58066	1.71216	1.92601	1.79775	1.48875	1.67441	1.27831	1.71364	1.88987	1.73328	1.69026	1.21055	1.85845	0.001684975	0.100255953	16	12	24	0.034263014	0.608494889	24	12.41033238	0.110059508
ENSMUSG00000053580	Tanc2	tetratricopeptide repeat, ankyrin repeat and coiled-coil containing 2 [Source:MGI Symbol;Acc:MGI:2444121]	13.5726	12.5219	12.0548	12.2713	12.9559	12.3887	13.0358	11.285	12.4602	14.7979	11.7335	13.4922	13.3814	13.6634	0.930944473	0.966018836	4	8	24	0.880226547	0.966381821	24	20.31921333	0.019527026
ENSMUSG00000053581	Zfand2a	zinc finger, AN1-type domain 2A [Source:MGI Symbol;Acc:MGI:2140729]	23.9603	20.0417	20.6583	20.8839	21.0362	22.1357	20.7423	20.4987	18.3362	23.7298	20.3358	22.3642	20.5993	21.1889	0.22872448	0.72665454	24	12	24	0.2748208	0.768040178	24	19.37584722	0.060964846
ENSMUSG00000053588	A730061H03Rik	RIKEN cDNA A730061H03 gene [Source:MGI Symbol;Acc:MGI:2445099]	0.984826	1.30837	0.711126	0.650099	1.603	0.727262	0.869119	1.06103	1.1967	0.983283	0.733767	1.26466	0.813772	0.902274	0.908406451	0.966018836	8	8	24	0.976607929	0.994103202	24	22.45401715	0.017448932
ENSMUSG00000053600	Zfp472	zinc finger protein 472 [Source:MGI Symbol;Acc:MGI:2385049]	2.6838	2.8871	2.76511	1.9604	3.09807	2.44181	2.64638	2.68535	2.79275	2.04419	3.03964	2.35118	2.95797	2.59595	0.72778087	0.966018836	20	20	24	0.533579338	0.825955545	24	0.485791214	0.062290355
ENSMUSG00000053604	Rpia	ribose 5-phosphate isomerase A [Source:MGI Symbol;Acc:MGI:103254]	5.74718	6.43181	6.29687	7.17482	6.97991	6.70194	6.6684	6.84534	7.20667	6.48737	7.35537	6.97407	7.73337	6.47456	0.612128753	0.966018836	20	12	24	0.423272592	0.805346055	24	14.82749761	0.039619919
ENSMUSG00000053617	Sh3pxd2a	SH3 and PX domains 2A [Source:MGI Symbol;Acc:MGI:1298393]	23.0635	19.1805	17.3432	20.0929	22.0869	20.0618	24.2131	21.0483	18.8632	21.9515	20.8023	20.3114	22.6857	20.3426	0.004320961	0.140598674	4	8	24	0.108421744	0.72082227	24	21.13222385	0.096946355

ENSMUSG00000053626	Tll1	tolloid-like [Source:MGI Symbol;Acc:MGI:106923]	2.63474	3.08785	2.54292	2.56232	3.55102	2.52985	3.17724	2.82197	3.42764	3.17786	2.34219	2.98633	2.77273	2.71767	0.814221431	0.966018836	12	12	24	0.914352361	0.976006964	24	8.451783632	0.023924624
ENSMUSG00000053641	Dennd4a	DENN/MADD domain containing 4A [Source:MGI Symbol;Acc:MGI:2142979]	13.6071	12.3274	13.1272	14.2341	12.8841	12.325	13.4685	12.4799	11.8378	12.6806	12.8217	12.1181	11.5702	12.7397	0.928127947	0.966018836	20	4	24	0.637945981	0.876998799	24	13.8347054	0.023525504
ENSMUSG00000053644	Aldh7a1	aldehyde dehydrogenase family 7, member A1 [Source:MGI Symbol;Acc:MGI:108186]	30.6917	29.1918	26.9137	27.5638	31.2531	31.152	33.1379	30.083	27.1807	28.9927	27.6766	27.3019	27.3582	29.2996	0.268178777	0.774280302	4	8	24	0.160244035	0.76578501	24	22.11522303	0.064146067
ENSMUSG00000053646	Plxnb1	plexin B1 [Source:MGI Symbol;Acc:MGI:2154238]	11.1585	11.0418	10.8322	12.6839	13.7929	10.7316	11.9708	9.40182	13.5583	12.2952	10.6169	10.6932	10.8524	9.6157	0.595952838	0.966018836	16	16	24	0.113291088	0.727393008	24	13.64810814	0.111114568
ENSMUSG00000053647	Gper1	G protein-coupled estrogen receptor 1 [Source:MGI Symbol;Acc:MGI:1924104]	1.15066	1.24792	0.929766	1.09655	0.97948	0.990571	0.795063	1.52893	1.01617	0.777384	0.909969	1.11139	0.85245	1.24638	0.06935957	0.433815306	8	12	24	0.263990255	0.768040178	24	3.89189344	0.087137596
ENSMUSG00000053684	BC048403	cDNA sequence BC048403 [Source:MGI Symbol;Acc:MGI:2670984]	4.02422	3.64056	3.55232	3.71917	3.69447	4.13858	4.0732	4.40833	3.28022	4.99091	3.39471	4.08141	3.72833	3.99692	0.245061943	0.741580974	24	12	24	0.883402816	0.966818623	24	22.13218815	0.025862278
ENSMUSG00000053693	Mast1	microtubule associated serine/threonine kinase 1 [Source:MGI Symbol;Acc:MGI:1861901]	26.3397	18.9325	19.6162	22.0438	20.9358	22.0193	21.6478	23.0601	20.8613	33.2877	24.9994	24.7468	24.4043	25.3076	0.892555977	0.966018836	16	20	24	0.716289197	0.90635549	24	16.08746794	0.057519828
ENSMUSG00000053702	Nebl	nebulette [Source:MGI Symbol;Acc:MGI:1921353]	29.3359	28.9564	25.8256	27.8918	28.5959	27.5244	27.3823	24.12	26.4301	24.6175	30.213	26.5975	27.7034	28.0784	0.745118544	0.966018836	20	16	24	0.281585222	0.768040178	24	19.83242618	0.056317015
ENSMUSG00000053714	4732471J01Rik	RIKEN cDNA 4732471J01 gene [Source:MGI Symbol;Acc:MGI:3603586]	2.06344	2.19139	2.78504	3.71201	3.13231	1.91399	2.13324	2.44375	2.23174	2.4162	2.31029	2.81053	2.91635	1.78884	0.360033827	0.883787544	16	16	24	0.189182734	0.768040178	24	13.68399339	0.141865518
ENSMUSG00000053716	Dusp7	dual specificity phosphatase 7 [Source:MGI Symbol;Acc:MGI:2387100]	5.10444	4.60314	5.44496	6.02656	4.9223	5.26824	5.76194	6.06626	4.98702	6.37273	5.95511	5.10262	4.96752	5.30106	0.814077113	0.966018836	16	12	24	0.309329798	0.768040178	24	12.09421029	0.068810939
ENSMUSG00000053730	Tmem39b	transmembrane protein 39b [Source:MGI Symbol;Acc:MGI:2682939]	2.46596	2.44429	2.4853	2.69481	2.55235	2.79748	2.75254	3.15469	2.15024	2.57224	2.96394	2.60048	2.24506	2.28563	0.40303616	0.935844475	20	16	24	0.451610314	0.805393068	24	17.77409212	0.055769439
ENSMUSG00000053740	Gm6457	predicted pseudogene 6457 [Source:MGI Symbol;Acc:MGI:3648519]	29.7202	25.9594	29.7465	27.4513	23.7497	25.8508	25.7459	24.979	23.9909	26.8222	24.7933	27.3056	25.668	28.5068	0.852921792	0.966018836	16	4	24	0.679506866	0.892680535	24	3.761368576	0.034050714
ENSMUSG00000053746	Ptrh1	peptidyl-tRNA hydrolase 1 homolog [Source:MGI Symbol;Acc:MGI:1913779]	9.96238	7.07057	7.15997	5.98385	6.6722	7.06276	6.63516	10.5335	7.58038	9.27476	9.42205	10.1542	9.79053	8.47349	0.867888967	0.966018836	8	8	24	0.503843897	0.810473618	24	0.132058474	0.098264383
ENSMUSG00000053754	Chd8	chromodomain helicase DNA binding protein 8 [Source:MGI Symbol;Acc:MGI:1915022]	17.977	22.6779	20.9195	19.4683	21.4118	20.4916	22.9918	21.4408	21.1258	20.4475	17.4271	21.3619	20.0527	20.0116	0.798309651	0.966018836	8	8	24	0.516705752	0.817817397	24	3.973771749	0.048140335
ENSMUSG00000053768	Chchd3	coiled-coil-helix-coiled-coil-helix domain containing 3 [Source:MGI Symbol;Acc:MGI:1913325]	49.5991	58.3181	61.7351	59.2667	56.9891	54.1064	57.8992	65.447	61.6619	55.4854	58.4698	56.3194	64.0795	59.3643	0.047000349	0.375011045	12	12	24	0.0824175	0.699403344	24	7.087512323	0.079519702
ENSMUSG00000053769	Lysmd1	LysM, putative peptidoglycan-binding, domain containing 1 [Source:MGI Symbol;Acc:MGI:1919409]	1.9877	1.7255	1.54522	1.57615	1.49647	1.76339	1.99587	1.40362	1.7739	1.89874	1.37274	1.99413	1.76748	2.04938	0.350544145	0.870418653	4	16	24	0.261768319	0.768040178	24	0.521033166	0.07518762
ENSMUSG00000053774	Ubxn7	UBX domain protein 7 [Source:MGI Symbol;Acc:MGI:2146388]	8.0944	8.37403	8.49718	8.31249	8.2327	8.0713	7.94722	8.08577	8.75311	8.0282	8.35425	8.08783	7.93924	8.51422	0.020294373	0.261094911	12	16	24	0.036862359	0.609176161	24	8.912008867	0.035386406
ENSMUSG00000053799	Exoc6	exocyst complex component 6 [Source:MGI Symbol;Acc:MGI:1351611]	13.2488	14.1293	13.5107	13.4503	13.957	12.914	12.5397	13.7496	13.7771	12.2432	13.6771	13.1871	14.7838	14.6882	0.371875382	0.894537464	8	16	24	0.378780915	0.798197156	24	4.349322243	0.038205071
ENSMUSG00000053801	Grwd1	glutamate-rich WD repeat containing 1 [Source:MGI Symbol;Acc:MGI:2141989]	5.50507	4.82364	4.76827	3.97323	5.57452	5.60653	4.54843	5.12909	3.86239	5.80459	4.98383	5.43853	4.63982	5.07549	0.435739761	0.966018836	24	12	24	0.225490128	0.768040178	24	19.57162539	0.101701609
ENSMUSG00000053819	Camk2d	calcium/calmodulin-dependent protein kinase II, delta [Source:MGI Symbol;Acc:MGI:1341265]	92.4171	75.9727	82.1626	79.163	81.3569	90.2688	79.5004	75.6816	65.9075	76.5967	81.5103	82.9486	77.7726	77.9434	0.031969981	0.324385159	24	8	24	0.009944173	0.503967859	24	20.01442306	0.115304082
ENSMUSG00000053825	Ppfia2	protein tyrosine phosphatase, receptor type, f polypeptide (PTPRF), interacting protein (liprin), alpha 2 [Source:MGI Symbol;Acc:MGI:2443834]	14.4093	12.6462	12.8699	11.6977	12.5595	13.031	12.8193	11.9249	11.2896	10.969	13.6379	12.3895	12.5741	13.3683	0.295379186	0.805301811	4	12	24	0.059646565	0.658304792	24	22.30110526	0.084362257
ENSMUSG00000053830	Gm9923	predicted pseudogene 9923 [Source:MGI Symbol;Acc:MGI:3704365]	2.5578	2.55499	2.70602	2.75598	2.42224	2.93141	2.74929	3.04161	2.13924	2.70606	2.30429	2.55802	2.45451	2.65163	0.919571723	0.966018836	16	4	24	0.727227481	0.910400545	24	1.055118669	0.029828797
ENSMUSG00000053835	H2-T24	histocompatibility 2, T region locus 24 [Source:MGI Symbol;Acc:MGI:95958]	2.93923	2.78001	2.87599	3.03058	3.26751	2.72656	2.77357	2.62384	3.19437	2.09782	2.88467	2.69035	2.78754	2.78925	0.697196105	0.966018836	20	4	24	0.968894132	0.992577968	24	18.32884127	0.011000978
ENSMUSG00000053838	Nudcd3	NudC domain containing 3 [Source:MGI Symbol;Acc:MGI:2144158]	28.8831	27.5239	30.1542	29.0571	28.7088	31.165	29.9002	29.064	28.1526	33.3228	26.5867	30.5666	27.6082	29.099	0.921572192	0.966018836	24	20	24	0.796645083	0.935045591	24	14.29585315	0.021477994
ENSMUSG00000053841	Txlna	taxilin alpha [Source:MGI Symbol;Acc:MGI:105968]	16.7691	13.9214	13.9237	16.1207	16.2191	17.1163	16.7646	16.2527	12.8083	16.9609	15.5147	15.5773	14.8664	14.2988	0.077301512	0.453790923	24	12	24	0.049162452	0.641092103	24	18.89088947	0.109410701
ENSMUSG00000053861	Gm9925	predicted gene 9925 [Source:MGI Symbol;Acc:MGI:3641738]	0.464766	0.683813	0.389204	1.13437	0.462335	0.490756	1.02029	0.611994	0.394431	0.828449	0.156393	0.801272	0.205666	0.573545	0.93717134	0.966018836	16	4	24	0.957788789	0.988896555	24	9.228341775	0.031146413
ENSMUSG00000053870	Fpgt	fucose-1-phosphate guanylyltransferase [Source:MGI Symbol;Acc:MGI:1922790]	16.8062	16.4922	15.7948	15.0397	16.5449	14.7662	15.1966	12.8118	15.4884	12.8041	17.9438	15.5607	15.3828	13.8811	0.722296281	0.966018836	20	20	24	0.32382238	0.768040178	24	18.78929006	0.074756917
ENSMUSG00000053877	Srcap	Snf2-related CREBBP activator protein [Source:MGI Symbol;Acc:MGI:2444036]	5.77619	6.25318	6.51908	6.32889	7.57578	5.99024	8.24522	7.27427	7.3434	10.1435	6.95805	8.02477	7.44098	8.09384	0.718363231	0.966018836	16	20	24	0.531796744	0.82547989	24	10.91253817	0.065720699
ENSMUSG00000053886	Sh2d4a	SH2 domain containing 4A [Source:MGI Symbol;Acc:MGI:1919531]	1.58025	1.77405	1.75373	1.9128	1.50603	1.8144	1.62894	1.99165	1.88832	1.87232	1.63754	2.00574	2.30695	2.09732	0.888991156	0.966018836	8	12	24	0.164678531	0.767020009	24	4.863512997	0.061313805
ENSMUSG00000053889	Kirrel3os	kirre like nephrin family adhesion molecule 3, opposite strand [Source:MGI Symbol;Acc:MGI:3045250]	2.03469	1.363	1.81214	1.85962	1.501	1.63624	1.58342	1.60505	1.60984	1.42985	1.84279	1.75273	1.57374	1.42282	0.493417188	0.966018836	24	8	24	0.524351895	0.822002818	24	18.01538437	0.046071937
ENSMUSG00000053897	Slc39a8	solute carrier family 39 (metal ion transporter), member 8 [Source:MGI Symbol;Acc:MGI:1914797]	1.14779	1.63058	1.47225	1.48212	1.57984	1.46205	1.71507	1.93268	1.46294	1.10522	1.51591	1.37026	1.28488	1.64008	0.387380622	0.915479136	8	16	24	0.549444047	0.834995331	24	4.351588138	0.055795142
ENSMUSG00000053898	Ech1	enoyl coenzyme A hydratase 1, peroxisomal [Source:MGI Symbol;Acc:MGI:1858208]	70.0016	81.7874	86.549	109.926	84.711	75.9655	82.8193	95.4605	95.6911	81.8017	97.6746	87.1947	99.4176	91.2644	0.869721097	0.966018836	20	4	24	0.163954291	0.767020009	24	10.06183324	0.111871081
ENSMUSG00000053907	Mat2a	methionine adenosyltransferase II, alpha [Source:MGI Symbol;Acc:MGI:2443731]	99.9452	93.7668	94.6652	121.576	96.8985	92.5228	97.3857	94.0766	110.784	100.138	101.892	86.0104	89.145	93.0067	0.033622862	0.324908923	16	8	24	0.047720061	0.636231449	24	11.27932897	0.106751483
ENSMUSG00000053914	Kdm4d	lysine (K)-specific demethylase 4D [Source:MGI Symbol;Acc:MGI:3606484]	0.613366	0.499396	0.68063	0.563268	0.576959	0.63057	0.687368	0.744872	0.599469	0.821158	0.496277	0.734192	0.529255	0.556417	0.858266942	0.966018836	24	20	24	0.955169654	0.988639288	24	10.66906553	0.010805478
ENSMUSG00000053925	Gm9929	predicted gene 9929 [Source:MGI Symbol;Acc:MGI:3708526]	1.42694	2.18542	1.77169	1.22024	1.97876	1.42357	1.46804	1.68626	2.54586	1.52921	2.02369	1.98417	1.93084	1.59173	0.94086481	0.966018836	12	4	24	0.803151104	0.936962113	24	7.748945832	0.05019321
ENSMUSG00000053929	Cyhr1	cysteine and histidine rich 1 [Source:MGI Symbol;Acc:MGI:1859320]	57.4172	51.7553	50.5373	52.9767	53.0566	52.4874	54.8602	52.0746	51.9064	54.1399	52.3507	54.9657	54.2632	52.2505	0.008389425	0.192760771	4	8	24	0.072938646	0.680039457	24	21.07264755	0.039942963
ENSMUSG00000053930	Shisa6	shisa family member 6 [Source:MGI Symbol;Acc:MGI:2685725]	1.88444	1.54144	2.83546	2.24337	0.917441	2.28962	1.4305	1.70887	2.09048	1.04426	1.21279	3.18728	0.61761	1.23443	0.891105762	0.966018836	24	20	24	0.979790115	0.995083382	24	10.7131488	0.030839242
ENSMUSG00000053931	Cnn3	calponin 3, acidic [Source:MGI Symbol;Acc:MGI:1919244]	135.69	128.503	119.905	118.929	137.411	132.197	137.214	132.66	115.376	117.952	133.333	125.087	131.552	120.02	0.019837701	0.259417302	20	20	24	0.003535434	0.423269403	24	21.25392553	0.097315277
ENSMUSG00000053950	Adnp2	ADNP homeobox 2 [Source:MGI Symbol;Acc:MGI:2448562]	4.45326	4.14142	4.21751	4.42894	4.32453	4.45142	4.39938	4.30782	4.2245	4.5456	4.46154	4.47261	4.42525	4.32314	0.010478767	0.196621011	16	20	24	0.027688038	0.590220615	24	17.97750384	0.028453035
ENSMUSG00000053963	Stum	mechanosensory transduction mediator [Source:MGI Symbol;Acc:MGI:2138735]	0.960872	0.984452	1.73733	0.98703	1.00279	1.30842	1.18194	1.13293	1.4525	1.22598	0.883067	1.29369	0.649016	1.16824	0.43565473	0.966018836	12	16	24	0.396697984	0.799546792	24	9.029544062	0.097690501
ENSMUSG00000053964	Lgals4	lectin, galactose binding, soluble 4 [Source:MGI Symbol;Acc:MGI:107536]	8.77572	9.29141	7.09612	11.1703	9.23218	9.19907	9.70815	10.0818	10.8245	9.00642	9.14193	8.16375	10.6436	9.451	0.826609618	0.966018836	8	16	24	0.893784115	0.969947957	24	6.439947882	0.029559205
ENSMUSG00000053965	Pde5a	phosphodiesterase 5A, cGMP-specific [Source:MGI Symbol;Acc:MGI:2651499]	2.28161	2.82315	2.51561	2.31431	2.82033	2.45194	2.80552	2.51489	3.4625	1.87312	2.52927	2.58099	2.23343	2.30114	0.921572192	0.966018836	12	4	24	0.894829145	0.970208625	24	4.92191842	0.028388869
ENSMUSG00000053985	Zfp14	zinc finger protein 14 [Source:MGI Symbol;Acc:MGI:99160]	8.40078	8.82819	7.93499	8.99737	8.64376	7.55614	9.43857	8.54033	9.03779	6.66362	8.29284	7.50688	7.8117	7.60091	0.903465533	0.966018836	12	12	24	0.955157756	0.988639288	24	19.0637319	0.016599787
ENSMUSG00000054000	Tusc1	tumor suppressor candidate 1 [Source:MGI Symbol;Acc:MGI:2684283]	1.01537	0.982916	1.03337	0.67818	1.22951	1.13766	1.20004	0.999063	1.16466	1.44956	1.46723	1.66694	1.35039	1.82613	0.669104912	0.966018836	20	12	24	0.569382554	0.844856622	24	20.72016508	0.0509142
ENSMUSG00000054008	Ndst1	N-deacetylase/N-sulfotransferase (heparan glucosaminyl) 1 [Source:MGI Symbol;Acc:MGI:104719]	21.7963	19.9836	20.7445	21.5123	21.7476	22.0862	21.336	21.5067	19.1899	26.5374	19.7156	22.5218	21.2073	21.1584	0.023472439	0.278499043	24	12	24	0.57274378	0.846721006	24	16.09236081	0.043831829
ENSMUSG00000054013	Tmem179	transmembrane protein 179 [Source:MGI Symbol;Acc:MGI:2144891]	22.0745	18.5095	20.1955	19.6098	19.6076	23.0279	21.4843	21.9678	17.9122	27.2813	18.501	22.6302	20.5142	23.652	0.649215555	0.966018836	24	20	24	0.888186648	0.96858941	24	21.66540887	0.030894619
ENSMUSG00000054021	Sirt5	sirtuin 5 [Source:MGI Symbol;Acc:MGI:1915596]	6.55372	7.32965	8.41766	8.05986	7.46659	7.21875	6.33069	7.33656	7.19282	7.95262	6.23227	8.31501	7.77722	8.39275	0.714044782	0.966018836	16	12	24	0.340449016	0.774843691	24	8.985215618	0.073727315
ENSMUSG00000054027	Nt5dc3	5'-nucleotidase domain containing 3 [Source:MGI Symbol;Acc:MGI:3513266]	13.0808	10.4694	11.2363	11.8249	10.6	13.6374	11.5806	12.2573	9.78759	14.5959	11.4555	12.1358	11.9032	12.1028	0.390686537	0.915479136	24	12	24	0.589060871	0.854006479	24	19.4859741	0.059386793
ENSMUSG00000054034	Tceal5	transcription elongation factor A (SII)-like 5 [Source:MGI Symbol;Acc:MGI:3036236]	7.0914	8.61687	11.2294	8.12292	6.91732	10.0659	10.1657	7.67413	7.93996	8.30966	5.79073	9.24319	7.64901	8.61867	0.850398351	0.966018836	12	8	24	0.595008558	0.857661186	24	3.718686064	0.088569324
ENSMUSG00000054051	Ercc6	excision repair cross-complementing rodent repair deficiency, complementation group 6 [Source:MGI Symbol;Acc:MGI:1100494]	3.71097	3.79048	3.67939	3.86274	4.01731	3.55136	3.91016	3.23051	3.85463	3.62469	4.06353	3.86406	3.52603	3.57468	0.057224139	0.401309455	20	12	24	0.113844095	0.727440321	24	15.75443796	0.052500983
ENSMUSG00000054057	A930004D18Rik	RIKEN cDNA A930004D18 gene [Source:MGI Symbol;Acc:MGI:1925190]	3.92599	3.05991	2.93653	3.26249	2.76542	2.8798	3.62742	3.38592	3.4326	4.11059	4.00811	3.31665	5.01209	2.90322	0.849179819	0.966018836	4	20	24	0.815121354	0.940891459	24	22.85674946	0.045727637
ENSMUSG00000054065	Pkp3	plakophilin 3 [Source:MGI Symbol;Acc:MGI:1891830]	2.89111	2.02793	2.02242	1.97697	2.17106	2.22343	2.37968	2.28394	1.65665	2.3182	2.23911	2.02255	2.37865	2.15432	0.004320961	0.140598674	4	8	24	0.081003446	0.697074851	24	22.01010525	0.103431916
ENSMUSG00000054072	Iigp1	interferon inducible GTPase 1 [Source:MGI Symbol;Acc:MGI:1926259]	3.3075	4.62365	3.99044	2.64335	4.32251	2.89332	2.70814	8.61896	4.90092	3.15053	4.61733	2.71199	2.7756	6.0132	0.038123942	0.334069441	8	16	24	0.127745872	0.740162193	24	6.427241975	0.32215103
ENSMUSG00000054074	Skida1	SKI/DACH domain containing 1 [Source:MGI Symbol;Acc:MGI:1919918]	2.85604	2.72623	2.46927	2.53288	2.69347	2.34436	2.31988	2.87236	2.99595	2.58269	2.38148	2.64041	2.9545	2.31207	0.81829516	0.966018836	20	12	24	0.911747377	0.975053334	24	13.92282926	0.018960329
ENSMUSG00000054079	Utp18	UTP18 small subunit processome component [Source:MGI Symbol;Acc:MGI:1923402]	6.63122	5.66937	5.41398	5.67972	5.75902	5.29637	5.88253	4.70594	5.65106	5.27518	6.5533	6.00831	5.94728	5.58717	0.114657038	0.534533508	4	4	24	0.242677022	0.768040178	24	19.54955965	0.072088951
ENSMUSG00000054091	1810037I17Rik	RIKEN cDNA 1810037I17 gene [Source:MGI Symbol;Acc:MGI:1914954]	38.2645	41.6437	38.3728	38.4254	39.3772	34.8457	40.5868	35.1914	44.6015	30.2753	40.216	38.1097	40.2225	35.7982	0.939103211	0.966018836	12	12	24	0.810484199	0.939224719	24	1.810126767	0.033657363
ENSMUSG00000054099	Slc25a40	solute carrier family 25, member 40 [Source:MGI Symbol;Acc:MGI:2442486]	7.27013	7.84366	7.36932	7.50059	7.90044	7.43486	7.48808	7.91941	7.27909	7.39886	7.72833	7.35686	7.71274	7.31562	0.893609703	0.966018836	16	20	24	0.935407477	0.98380253	24	19.26006637	0.006110809
ENSMUSG00000054115	Skp2	S-phase kinase-associated protein 2 [Source:MGI Symbol;Acc:MGI:1351663]	0.966654	1.07738	1.25706	1.34229	1.27861	1.25784	1.16163	1.15628	1.39728	0.920418	1.43856	1.01355	0.968328	1.15906	0.128810631	0.560209134	20	8	24	0.25342144	0.768040178	24	12.35767283	0.069819861
ENSMUSG00000054135	A430110L20Rik	RIKEN cDNA A430110L20 gene [Source:MGI Symbol;Acc:MGI:3036249]	0.650511	0.711928	0.765391	0.777359	0.735813	0.673134	0.885576	0.569214	0.899734	0.545716	1.00874	0.630911	0.770264	0.689721	0.939103211	0.966018836	20	12	24	0.851706258	0.955514806	24	14.1603772	0.023916757
ENSMUSG00000054150	Syne3	spectrin repeat containing, nuclear envelope family member 3 [Source:MGI Symbol;Acc:MGI:2442408]	3.37172	3.29958	3.1883	4.37072	3.83271	3.19488	4.16996	3.15266	3.88017	4.00187	3.28155	3.6411	3.56574	3.03808	0.053980818	0.395677346	16	16	24	0.321948983	0.768040178	24	14.08676068	0.07504363
ENSMUSG00000054162	Spock3	sparc/osteonectin, cwcv and kazal-like domains proteoglycan 3 [Source:MGI Symbol;Acc:MGI:1920152]	44.3864	43.6646	44.5245	41.5953	38.6747	41.7201	41.1087	35.7847	35.4483	37.0804	41.7934	36.111	32.2277	39.6778	0.700614329	0.966018836	20	12	24	0.947128049	0.987072308	24	16.81844568	0.013187029
ENSMUSG00000054169	Ceacam10	carcinoembryonic antigen-related cell adhesion molecule 10 [Source:MGI Symbol;Acc:MGI:1347248]	9.60322	8.32411	7.35311	6.95418	7.12943	7.93592	5.72715	7.73519	7.70144	6.27514	7.34702	7.53958	7.99897	8.66049	0.0518376	0.253245931	8	8	24	0.286458148	0.768040178	24	1.618695393	0.097724965
ENSMUSG00000054191	Klf1	Kruppel-like factor 1 (erythroid) [Source:MGI Symbol;Acc:MGI:1342771]	0.260943	0.634374	1.41994	1.3353	0.653605	0.925396	0.495922	0.676726	0.73394	0.421366	0.639869	0.792878	1.2916	0.624499	0.943890799	0.966018836	12	16	24	0.700362667	0.900629492	24	10.71506593	0.094202659
ENSMUSG00000054196	Cthrc1	collagen triple helix repeat containing 1 [Source:MGI Symbol;Acc:MGI:1915838]	4.97823	5.22146	5.16556	5.63991	5.348	4.89924	4.68203	5.82021	5.60675	5.35438	5.06387	5.23432	4.55105	5.45142	0.01065666	0.198248077	16	12	24	0.032860123	0.606585237	24	9.608829352	0.080539943
ENSMUSG00000054199	Gon4l	gon-4-like (C.elegans) [Source:MGI Symbol;Acc:MGI:1917579]	3.85109	3.87918	4.19984	4.12509	4.08144	4.22335	3.93182	3.83384	4.3361	4.60275	3.54904	4.31455	3.9381	4.37388	0.591351901	0.966018836	16	12	24	0.388369258	0.799546792	24	10.21321768	0.042312072
ENSMUSG00000054204	Alkal2	ALK and LTK ligand 2 [Source:MGI Symbol;Acc:MGI:3697448]	4.2404	2.85266	3.1942	3.0389	5.28375	3.45497	3.12891	2.54307	1.87683	2.76239	5.16152	3.54577	2.75958	1.92924	0.082447763	0.192760771	20	12	24	9.03E-04	0.319044294	24	17.89954096	0.374731164
ENSMUSG00000054206	Gzmm	granzyme M (lymphocyte met-ase 1) [Source:MGI Symbol;Acc:MGI:99549]	0.741291	0.433184	0.422406	1.03161	0.317866	0.642882	0.387934	0.405594	0.779872	0.629576	0.510692	0.695559	0.710947	0.552408	0.896753532	0.966018836	16	4	24	0.831191789	0.947661285	24	13.29715739	0.041886495
ENSMUSG00000054226	Tprkb	Tp53rk binding protein [Source:MGI Symbol;Acc:MGI:1917036]	27.4816	29.8598	35.4213	27.0242	28.7863	27.9665	30.3245	28.615	30.8003	26.9067	27.9882	31.547	28.1646	32.0621	0.087978327	0.472021905	12	4	24	0.30832082	0.768040178	24	5.537621602	0.065636073
ENSMUSG00000054237	Fra10ac1	FRA10AC1 homolog (human) [Source:MGI Symbol;Acc:MGI:1917817]	14.2288	14.5423	12.4565	13.0427	14.3367	13.4986	14.1488	14.1281	14.1918	12.4051	15.2615	13.5196	15.4865	14.8979	0.27817759	0.789849983	4	12	24	0.168680986	0.767020009	24	23.80441185	0.061170732
ENSMUSG00000054252	Fgfr3	fibroblast growth factor receptor 3 [Source:MGI Symbol;Acc:MGI:95524]	2.47936	5.10669	10.0246	6.90148	3.07314	6.94884	6.49501	2.88181	6.50678	4.05432	2.0784	4.91656	2.16934	5.07002	0.46238066	0.966018836	12	8	24	0.445781064	0.805393068	24	8.555068069	0.286542721
ENSMUSG00000054256	Msi1	musashi RNA-binding protein 1 [Source:MGI Symbol;Acc:MGI:107376]	2.01981	3.08228	3.80138	2.72636	2.21456	2.96755	3.01108	2.93997	3.58981	3.68785	2.76103	3.08314	2.34666	3.2397	0.080511813	0.4593055	12	16	24	0.049069861	0.640446914	24	8.610221373	0.177540283
ENSMUSG00000054263	Lifr	LIF receptor alpha [Source:MGI Symbol;Acc:MGI:96788]	63.4389	56.4689	55.2914	58.5641	58.3151	59.308	52.2584	54.9164	49.9395	59.4056	62.3802	61.9818	64.0502	60.6302	0.290796297	0.79743194	4	8	24	0.154032784	0.760223561	24	19.39703875	0.076514479
ENSMUSG00000054277	Arfgap3	ADP-ribosylation factor GTPase activating protein 3 [Source:MGI Symbol;Acc:MGI:1913501]	32.0403	32.616	26.5205	30.1677	28.7987	31.6475	28.5883	34.9915	32.0556	32.4444	27.9771	32.9651	32.6507	33.4518	0.066380001	0.42386701	8	12	24	0.23022887	0.768040178	24	2.600780283	0.06766151
ENSMUSG00000054280	Prr14l	proline rich 14-like [Source:MGI Symbol;Acc:MGI:2443658]	5.68842	5.1035	5.0868	4.84977	5.27774	5.43573	5.58753	4.83989	4.80151	5.60729	5.18506	5.67925	5.43914	5.70249	0.060394272	0.407012001	4	8	24	0.076700185	0.687041208	24	21.38399454	0.062835414
ENSMUSG00000054302	Eapp	E2F-associated phosphoprotein [Source:MGI Symbol;Acc:MGI:1913516]	24.0973	26.9618	24.9046	24.8102	25.031	22.728	27.0634	23.6203	25.1808	24.4696	26.132	26.0932	25.2958	24.2088	0.835668938	0.966018836	4	12	24	0.985423784	0.997026304	24	23.6531387	0.004772383
ENSMUSG00000054309	Cpsf3	cleavage and polyadenylation specificity factor 3 [Source:MGI Symbol;Acc:MGI:1859328]	15.3496	18.2565	18.0367	17.1668	16.8314	17.8334	18.2553	18.2832	20.2041	16.6389	17.0768	17.9742	18.8074	19.872	0.017462757	0.255326033	8	12	24	0.042417761	0.623698598	24	5.476527193	0.076637871
ENSMUSG00000054312	Mrps21	mitochondrial ribosomal protein S21 [Source:MGI Symbol;Acc:MGI:1913542]	78.8128	80.8081	80	80.7828	79.5963	80.5978	79.6733	76.925	83.2428	70.9286	87.357	72.5087	86.6623	80.5936	0.776152743	0.966018836	8	12	24	0.930492624	0.982155977	24	1.658568929	0.012316753
ENSMUSG00000054320	Lrrc36	leucine rich repeat containing 36 [Source:MGI Symbol;Acc:MGI:2448585]	0.402222	0.609336	0.533411	1.25404	0.585821	0.593041	0.791693	0.781528	0.492574	0.597213	0.636585	1.06604	0.706007	1.5783	0.917370979	0.966018836	8	4	24	0.851884306	0.955545499	24	6.33792749	0.050411128
ENSMUSG00000054321	Taf4b	TATA-box binding protein associated factor 4b [Source:MGI Symbol;Acc:MGI:2152345]	3.03911	2.69855	2.67622	2.79441	3.03032	3.04503	2.70521	2.77038	2.47465	2.70028	2.90504	2.94035	2.70174	2.9423	0.005105434	0.144396606	24	12	24	0.023630686	0.584818005	24	19.45647178	0.064237353
ENSMUSG00000054342	Kcnn4	potassium intermediate/small conductance calcium-activated channel, subfamily N, member 4 [Source:MGI Symbol;Acc:MGI:1277957]	4.32399	3.44183	3.50257	3.96711	4.42546	3.89583	3.06667	3.7439	2.75575	3.99223	4.36161	3.401	4.08696	3.01824	0.033404002	0.324908923	20	12	24	0.060430772	0.65893609	24	17.33854771	0.141393827
ENSMUSG00000054364	Rhob	ras homolog family member B [Source:MGI Symbol;Acc:MGI:107949]	30.177	29.8919	35.9575	34.1975	27.2994	30.232	36.9317	30.8967	32.6004	42.6102	32.1477	33.1065	28.7439	35.6557	0.545835653	0.966018836	16	4	24	0.230525464	0.768040178	24	9.568635963	0.107998339
ENSMUSG00000054381	Zfp747	zinc finger protein 747 [Source:MGI Symbol;Acc:MGI:2443581]	2.65929	2.59972	2.98115	2.28028	2.11685	2.32322	2.60459	2.61136	2.28959	2.45329	2.8189	2.32184	2.4291	2.32851	0.566932234	0.966018836	4	20	24	0.591447617	0.855955866	24	4.397186055	0.03841645
ENSMUSG00000054383	Pnma1	paraneoplastic antigen MA1 [Source:MGI Symbol;Acc:MGI:2180564]	2.01729	1.33436	1.39326	1.39329	1.44819	1.76051	1.21222	1.3368	1.13095	1.65938	1.60419	1.4721	1.60328	1.56902	0.233309673	0.735312275	24	12	24	0.227851504	0.768040178	24	19.78650231	0.089694234
ENSMUSG00000054387	Mdm4	transformed mouse 3T3 cell double minute 4 [Source:MGI Symbol;Acc:MGI:107934]	20.2329	25.3505	23.6349	23.7825	25.139	20.016	24.2827	19.69	30.3304	18.6457	24.8595	22.3548	24.26	23.3614	0.908406451	0.966018836	12	12	24	0.682906354	0.894308158	24	8.617079646	0.064496768
ENSMUSG00000054400	Cklf	chemokine-like factor [Source:MGI Symbol;Acc:MGI:1922708]	11.4605	12.6986	13.6015	13.6686	12.6703	12.123	11.6819	10.9472	12.5157	11.4393	13.0215	12.5801	13.8182	12.2446	0.92655264	0.966018836	20	12	24	0.588862891	0.853936396	24	13.07267155	0.038543807
ENSMUSG00000054404	Slfn5	schlafen 5 [Source:MGI Symbol;Acc:MGI:1329004]	3.28434	3.63109	4.00436	3.78717	4.10539	2.89787	3.12737	4.32331	4.44545	3.34625	3.13216	2.89292	3.8826	3.75942	0.002057111	0.100255953	12	12	24	0.020781567	0.568066329	24	7.62687291	0.160836687
ENSMUSG00000054405	Dnajc8	DnaJ heat shock protein family (Hsp40) member C8 [Source:MGI Symbol;Acc:MGI:1915848]	45.7296	51.8952	50.2811	47.832	48.1923	47.2852	48.3894	44.8462	54.8732	44.6311	51.8375	49.5038	52.9933	49.5413	0.922029406	0.966018836	12	4	24	0.810234944	0.939224719	24	6.494752647	0.023044378
ENSMUSG00000054408	Spcs3	signal peptidase complex subunit 3 homolog (S. cerevisiae) [Source:MGI Symbol;Acc:MGI:1923937]	22.0417	21.6051	20.3285	19.5705	21.3884	19.5309	20.5811	20.0389	20.9139	17.9413	21.9661	19.1783	21.3185	20.1668	0.702630284	0.966018836	4	12	24	0.449355758	0.805393068	24	23.99265134	0.038325226
ENSMUSG00000054409	Tmem74	transmembrane protein 74 [Source:MGI Symbol;Acc:MGI:2443417]	14.3684	12.7408	11.5063	13.8476	13.4706	12.5232	11.4452	11.1673	12.1769	11.0413	13.5155	12.9022	13.0135	13.6938	0.945282252	0.966018836	20	16	24	0.447695283	0.805393068	24	19.35240118	0.059236112
ENSMUSG00000054414	Slc30a7	solute carrier family 30 (zinc transporter), member 7 [Source:MGI Symbol;Acc:MGI:1913750]	3.00153	4.52221	3.50691	4.09081	3.67577	3.83374	3.47133	3.66749	4.32923	3.26873	3.63259	3.58481	3.50337	3.43061	0.636884864	0.966018836	12	16	24	0.532061726	0.825565809	24	9.277430323	0.055868932
ENSMUSG00000054418	2900041M22Rik	RIKEN cDNA 2900041M22 gene [Source:MGI Symbol;Acc:MGI:1925653]	2.22452	2.64953	2.15462	2.56265	2.33438	2.64886	2.21035	2.74403	3.1279	2.06661	2.51311	2.79593	2.93971	2.68314	0.767183862	0.966018836	8	8	24	0.614415135	0.866471559	24	3.147270048	0.044903738
ENSMUSG00000054423	Cadps	Ca2+-dependent secretion activator [Source:MGI Symbol;Acc:MGI:1350922]	142.897	125.988	123.927	122.631	126.283	135.082	120.008	126.856	106.688	131.6	130.033	133.715	128.232	132.848	0.041292272	0.346472636	24	12	24	0.13109324	0.741427049	24	20.71055432	0.076057533
ENSMUSG00000054426	A930005H10Rik	RIKEN cDNA A930005H10 gene [Source:MGI Symbol;Acc:MGI:1915411]	15.8444	15.0436	17.7052	17.217	14.5521	12.6223	16.2477	14.0956	13.6953	17.1488	12.9792	13.4504	15.0155	16.8502	0.084464639	0.466404237	16	8	24	0.189043565	0.768040178	24	7.522634818	0.106391223
ENSMUSG00000054428	Atpif1	ATPase inhibitory factor 1 [Source:MGI Symbol;Acc:MGI:1196457]	400.314	431.36	356.774	382.531	377.439	356.068	407	351.425	435.849	307.961	411.489	417.737	450.154	434.425	0.832506215	0.966018836	4	12	24	0.239053357	0.768040178	24	0.993732639	0.095597129
ENSMUSG00000054434	Tmem120b	transmembrane protein 120B [Source:MGI Symbol;Acc:MGI:3603158]	8.72407	6.55911	7.88121	8.28948	7.20516	7.72785	6.95139	9.73005	6.81411	9.16699	8.6246	8.65989	8.64674	7.38556	0.888482515	0.966018836	16	20	24	0.814057228	0.940708369	24	16.78646766	0.039104007
ENSMUSG00000054435	Gimap4	GTPase, IMAP family member 4 [Source:MGI Symbol;Acc:MGI:1349656]	2.48548	2.47148	2.83774	3.1078	2.57514	1.76279	2.92307	2.49388	3.76866	2.49513	2.52772	2.02023	3.14799	2.29214	0.236893212	0.7385769	12	12	24	0.158056549	0.764630328	24	8.169434324	0.14829476
ENSMUSG00000054452	Tle5	TLE family member 5, transcriptional modulator [Source:MGI Symbol;Acc:MGI:95806]	190.174	161.04	165.322	149.684	158.049	175.699	170.323	199.882	166.039	215.313	181.192	187.539	180.816	183.209	0.858269825	0.966018836	8	4	24	0.797364619	0.935271704	24	0.408792402	0.033829151
ENSMUSG00000054455	Vapb	vesicle-associated membrane protein, associated protein B and C [Source:MGI Symbol;Acc:MGI:1928744]	82.0051	67.5809	63.7597	70.143	64.2034	74.4654	76.0579	75.5292	62.2463	81.3875	68.9153	69.0765	74.3398	74.7446	0.062631097	0.410322149	4	8	24	0.334260319	0.770433279	24	23.22412145	0.071223252
ENSMUSG00000054457	9430021M05Rik	RIKEN cDNA 9430021M05 gene [Source:MGI Symbol;Acc:MGI:1924538]	2.63839	2.52762	2.5372	2.98804	2.92513	3.15374	2.42751	2.36541	2.48293	2.80378	2.92284	3.06737	3.61269	2.87539	0.031969981	0.324385159	24	8	24	0.0907162	0.71175955	24	18.28166606	0.09466391
ENSMUSG00000054459	Vsnl1	visinin-like 1 [Source:MGI Symbol;Acc:MGI:1349453]	80.0555	87.5037	104.421	75.6179	65.9765	84.9519	92.1983	72.0698	81.251	85.3403	63.6872	77.0597	67.6807	90.7063	0.185796491	0.662239322	12	8	24	0.181481882	0.768040178	24	5.446186663	0.137021092
ENSMUSG00000054469	Lclat1	lysocardiolipin acyltransferase 1 [Source:MGI Symbol;Acc:MGI:2684937]	26.484	18.5733	20.3773	18.8206	19.6858	23.0157	20.4701	21.657	17.0642	23.5621	21.7674	22.5731	21.4578	21.9821	0.245061943	0.741580974	12	4	24	0.174230384	0.768040178	24	21.09349138	0.111986393
ENSMUSG00000054474	Thnsl2	threonine synthase-like 2 (bacterial) [Source:MGI Symbol;Acc:MGI:3041254]	5.3135	4.61488	4.69441	4.07408	4.27641	4.70798	4.19281	4.5008	4.39387	4.69187	4.40883	4.70729	4.21433	4.58984	0.915475528	0.966018836	24	20	24	0.585172752	0.852642873	24	0.094511128	0.031574634
ENSMUSG00000054477	Kcnn2	potassium intermediate/small conductance calcium-activated channel, subfamily N, member 2 [Source:MGI Symbol;Acc:MGI:2153182]	4.7939	4.37686	5.18579	4.96548	4.26474	4.95734	5.00599	4.17352	4.11682	5.04197	3.97573	5.26098	4.4493	4.95391	0.95032543	0.966018836	20	20	24	0.92499185	0.980084153	24	21.29012175	0.018645314
ENSMUSG00000054484	Tmem62	transmembrane protein 62 [Source:MGI Symbol;Acc:MGI:2139461]	20.6128	21.6154	20.2203	22.8325	23.0988	21.3847	20.3938	19.9977	20.6247	20.0908	23.5635	22.767	22.5192	19.1149	0.008603252	0.192760771	20	20	24	0.01708763	0.547196097	24	17.14445616	0.089353491
ENSMUSG00000054493	Gm9947	predicted gene 9947 [Source:MGI Symbol;Acc:MGI:3642739]	1.89208	2.4842	2.13297	1.87741	1.47949	1.75653	1.39792	1.33484	1.37107	1.52505	1.58845	2.27682	2.18629	2.03508	0.890235289	0.966018836	24	20	24	0.623284799	0.87183699	24	0.929627696	0.073083492
ENSMUSG00000054499	Dedd2	death effector domain-containing DNA binding protein 2 [Source:MGI Symbol;Acc:MGI:1914629]	6.72402	5.18119	5.13628	6.9264	5.79599	5.63956	5.61846	6.15228	5.80141	6.7398	7.06803	6.8066	6.2527	6.0061	0.175185919	0.651010555	16	20	24	0.217439862	0.768040178	24	16.29505515	0.081322292
ENSMUSG00000054509	Parp4	poly (ADP-ribose) polymerase family, member 4 [Source:MGI Symbol;Acc:MGI:2685589]	8.57907	9.1459	8.45354	9.11714	8.29305	8.43748	9.02946	8.73685	8.41366	11.0295	9.58779	8.96316	8.44841	8.74472	0.921572192	0.966018836	12	8	24	0.318964547	0.768040178	24	12.44568109	0.055290438
ENSMUSG00000054514	Atad3aos	ATPase family, AAA domain containing 3A, opposite strand [Source:MGI Symbol;Acc:MGI:1917698]	1.00591	3.26287	2.5071	2.91525	2.98735	3.30172	5.53611	1.74926	3.47581	2.03961	1.63202	2.84491	1.70944	1.85655	0.873350015	0.966018836	24	4	24	0.967021193	0.992134979	24	15.4193897	0.044506015

ENSMUSG00000054517	Trim65	tripartite motif-containing 65 [Source:MGI Symbol;Acc:MGI:2442815]	1.71957	1.50061	1.69426	1.94106	1.86433	1.51006	1.55629	1.42945	1.86784	1.60148	2.04629	1.69158	1.6564	1.64924	0.036134798	0.331852526	20	12	24	0.04414216	0.628306416	24	13.94944417	0.083231354
ENSMUSG00000054519	Zfp867	zinc finger protein 867 [Source:MGI Symbol;Acc:MGI:2681848]	3.09335	3.96253	3.57924	2.60119	3.68971	3.27208	3.41029	3.29675	3.67846	2.79068	3.73728	3.3604	3.81432	3.7083	0.743750765	0.966018836	8	8	24	0.412750056	0.799546792	24	1.928967954	0.06708013
ENSMUSG00000054520	Sh3bp2	SH3-domain binding protein 2 [Source:MGI Symbol;Acc:MGI:1346349]	1.29311	1.67861	1.60442	1.58475	1.55898	1.14143	1.27826	1.26385	1.87165	1.3192	1.29285	1.43492	1.29666	1.29029	0.125887884	0.555618842	12	16	24	0.073227156	0.680409547	24	9.418720197	0.097915385
ENSMUSG00000054545	Ugt1a6a	UDP glucuronosyltransferase 1 family, polypeptide A6A [Source:MGI Symbol;Acc:MGI:2137698]	8.849	8.23659	8.12359	8.26615	8.26576	8.52684	7.66478	8.5604	7.72663	9.96345	7.69464	8.28029	8.50552	7.52654	0.93447422	0.966018836	24	12	24	0.774650921	0.927647959	24	15.33146396	0.026170599
ENSMUSG00000054555	Adam12	a disintegrin and metallopeptidase domain 12 (meltrin alpha) [Source:MGI Symbol;Acc:MGI:105378]	0.573557	0.707211	0.59352	1.12689	0.634832	0.706892	0.666311	0.986552	0.97527	0.619912	0.861581	0.622725	0.636121	0.647773	0.893609703	0.966018836	8	20	24	0.299394521	0.768040178	24	10.21239349	0.085201399
ENSMUSG00000054556	Gm4876	predicted gene 4876 [Source:MGI Symbol;Acc:MGI:3647654]	0.644516	0.50871	1.49649	1.30258	0.722878	0.5105	0.768182	0.752864	0.820026	0.41389	0.789083	1.23732	0.426622	0.599784	0.648082424	0.966018836	12	20	24	0.501274526	0.80908345	24	11.81908088	0.116162848
ENSMUSG00000054580	Pla2r1	phospholipase A2 receptor 1 [Source:MGI Symbol;Acc:MGI:102468]	2.67384	2.30567	2.25773	2.59645	2.68751	1.84666	2.29232	2.76205	2.6032	2.14318	2.39241	2.45777	2.99502	2.5116	0.948454836	0.966018836	4	20	24	0.665479761	0.886999446	24	3.920664711	0.043736235
ENSMUSG00000054582	Pabpc1l	poly(A) binding protein, cytoplasmic 1-like [Source:MGI Symbol;Acc:MGI:1922908]	1.30549	0.897666	2.04517	1.10317	1.14251	1.00929	0.813746	0.737693	1.0336	0.584282	0.852049	0.908751	1.24573	0.768124	0.917370979	0.966018836	12	20	24	0.988494765	0.997559852	24	6.56080235	0.012953673
ENSMUSG00000054589	Gm9949	predicted gene 9949 [Source:MGI Symbol;Acc:MGI:3647947]	0.248714	0.581398	0.540922	0.399328	0.521176	0.621114	0.485494	0.445351	0.679397	0.427238	0.376199	0.475815	0.695861	0.520196	0.904387595	0.966018836	12	4	24	0.687388213	0.89619155	24	5.246026164	0.040148551
ENSMUSG00000054594	Oscar	osteoclast associated receptor [Source:MGI Symbol;Acc:MGI:2179720]	0.727942	0.667426	0.607289	0.876848	0.93054	0.91337	0.711583	0.675509	0.878834	0.583326	0.853864	0.832564	0.553061	0.647801	0.19766851	0.679092646	20	12	24	0.057049911	0.65610184	24	16.54878726	0.092465645
ENSMUSG00000054604	Cggbp1	CGG triplet repeat binding protein 1 [Source:MGI Symbol;Acc:MGI:2146370]	18.7904	17.2292	17.6906	18.9737	19.1061	17.1746	18.6433	15.5618	17.1252	17.4857	19.1737	16.3739	16.2238	16.5295	0.033553741	0.324908923	20	12	24	0.05053806	0.644469829	24	15.67328412	0.070754988
ENSMUSG00000054611	Kdm2a	lysine (K)-specific demethylase 2A [Source:MGI Symbol;Acc:MGI:1354736]	20.5681	18.2106	18.2293	19.666	20.6221	19.7817	20.8951	20.2533	18.1956	21.3971	18.7477	20.1074	19.2519	19.3144	0.297978647	0.80820842	16	20	24	0.303061937	0.768040178	24	19.14472501	0.045679
ENSMUSG00000054612	Mgmt	O-6-methylguanine-DNA methyltransferase [Source:MGI Symbol;Acc:MGI:96977]	0.768102	0.90755	0.757162	0.936604	0.971661	0.770129	0.850517	0.886044	0.75424	0.689087	0.961281	0.827625	0.846465	0.868147	0.70641939	0.966018836	20	16	24	0.781998532	0.930013946	24	18.13685939	0.019719274
ENSMUSG00000054619	Mettl7a1	methyltransferase like 7A1 [Source:MGI Symbol;Acc:MGI:1916523]	25.7383	21.5823	24.3262	32.0954	25.5957	24.2678	25.583	23.5107	22.059	27.8965	25.9366	23.6041	26.355	24.0015	0.031431762	0.324385159	16	16	24	0.174093721	0.768040178	24	14.82584437	0.096272008
ENSMUSG00000054640	Slc8a1	solute carrier family 8 (sodium/calcium exchanger), member 1 [Source:MGI Symbol;Acc:MGI:107956]	2.06154	1.73037	2.32396	1.88546	1.44325	2.09704	2.26482	1.47865	2.06448	2.02021	1.40241	1.71002	1.6068	2.17558	0.285027487	0.793714531	12	8	24	0.469697428	0.805393068	24	5.315791882	0.077341911
ENSMUSG00000054648	Zfp869	zinc finger protein 869 [Source:MGI Symbol;Acc:MGI:1914119]	15.6684	17.9214	17.5717	16.116	17.426	15.891	17.0359	15.7053	19.765	14.448	15.5654	18.5415	18.6061	17.6482	0.851656735	0.966018836	12	4	24	0.485305819	0.805393068	24	3.666101434	0.055088454
ENSMUSG00000054659	Pm20d2	peptidase M20 domain containing 2 [Source:MGI Symbol;Acc:MGI:2685270]	0.711461	0.959839	0.706741	0.889847	0.720165	0.8986	0.75564	1.19355	0.785993	1.00438	0.818668	0.649224	1.13378	0.850374	0.918497267	0.966018836	8	16	24	0.474839664	0.805393068	24	5.317049159	0.059013664
ENSMUSG00000054675	Tmem119	transmembrane protein 119 [Source:MGI Symbol;Acc:MGI:2385228]	2.66669	3.07342	3.37898	2.17446	2.97909	2.5011	2.70073	2.73862	3.3771	1.45924	2.73569	2.65556	2.44113	2.55298	0.122530972	0.550724475	12	4	24	0.528871267	0.824228903	24	2.988154845	0.088018682
ENSMUSG00000054676	1600014C10Rik	RIKEN cDNA 1600014C10 gene [Source:MGI Symbol;Acc:MGI:1919494]	14.1763	12.2749	13.0433	14.0079	12.605	14.2868	12.8295	14.1804	10.7517	14.7051	13.4997	13.932	14.4625	13.1596	0.791413662	0.966018836	24	12	24	0.369779005	0.792235171	24	19.60922108	0.063875524
ENSMUSG00000054679	Srsf12	serine and arginine-rich splicing factor 12 [Source:MGI Symbol;Acc:MGI:2661424]	5.57711	5.51184	5.42914	5.38192	4.18439	5.73456	5.31074	5.14787	4.26857	5.58451	4.94696	5.84603	5.02241	5.01691	0.300823756	0.809362476	24	20	24	0.538641605	0.828704661	24	22.53269847	0.053592325
ENSMUSG00000054690	Emcn	endomucin [Source:MGI Symbol;Acc:MGI:1891716]	7.09555	8.69895	9.13379	9.69515	9.23096	8.44693	6.71378	8.96141	10.1471	7.54086	9.10513	8.2719	8.58923	7.29285	0.017495149	0.255326033	12	16	24	0.12186024	0.734196514	24	11.553098	0.12033358
ENSMUSG00000054693	Adam10	a disintegrin and metallopeptidase domain 10 [Source:MGI Symbol;Acc:MGI:109548]	66.9291	65.7198	59.1096	59.3989	78.7572	60.2194	72.3404	62.7243	64.5077	62.5739	67.4736	61.6545	64.5137	58.9224	0.337071598	0.85758369	20	20	24	0.362007637	0.787054939	24	19.241321	0.0664966
ENSMUSG00000054708	Ankrd24	ankyrin repeat domain 24 [Source:MGI Symbol;Acc:MGI:1890394]	19.3523	17.078	20.1301	16.9843	19.8308	18.2293	19.682	21.7318	17.7479	19.194	19.6399	22.0439	21.6783	21.6856	0.774195442	0.966018836	8	8	24	0.272402382	0.768040178	24	23.88873224	0.059882409
ENSMUSG00000054715	Zscan22	zinc finger and SCAN domain containing 22 [Source:MGI Symbol;Acc:MGI:2443312]	4.05486	2.70259	2.79174	2.75871	3.01822	3.30998	3.73981	2.90217	2.75198	3.31365	3.23376	3.04953	2.76391	2.7827	0.178701474	0.655480352	4	4	24	0.086487422	0.705702571	24	20.44922254	0.113558562
ENSMUSG00000054716	Zfp771	zinc finger protein 771 [Source:MGI Symbol;Acc:MGI:2442050]	3.99573	3.20648	9.50403	3.0937	2.81139	4.09284	3.90251	6.56512	6.70786	5.71918	7.51963	7.74348	6.25439	6.66613	0.895137379	0.966018836	12	4	24	0.483859206	0.805393068	24	7.463557543	0.193996615
ENSMUSG00000054717	Hmgb2	high mobility group box 2 [Source:MGI Symbol;Acc:MGI:96157]	1.04378	2.19229	2.51678	1.94436	1.282	1.73301	1.70296	1.67342	1.72605	1.21962	1.10779	1.62258	1.79251	1.4163	0.616047374	0.966018836	12	8	24	0.276353795	0.768040178	24	6.02406728	0.136785762
ENSMUSG00000054720	Lrrc8c	leucine rich repeat containing 8 family, member C [Source:MGI Symbol;Acc:MGI:2140839]	13.5972	10.4553	10.6269	11.4636	11.1049	13.0569	12.0285	11.4858	8.1483	14.4314	10.6869	11.0593	10.9985	11.5939	0.496745696	0.966018836	24	12	24	0.390105145	0.799546792	24	19.89136112	0.100066622
ENSMUSG00000054723	Vmac	vimentin-type intermediate filament associated coiled-coil protein [Source:MGI Symbol;Acc:MGI:2146912]	5.14962	6.9322	5.62609	5.59928	6.28759	5.56921	5.91127	6.16388	7.54102	6.6945	6.44656	6.52929	6.43381	6.37372	0.92655264	0.966018836	12	16	24	0.294750805	0.768040178	24	8.759269384	0.067898523
ENSMUSG00000054728	Phactr1	phosphatase and actin regulator 1 [Source:MGI Symbol;Acc:MGI:2659021]	36.1505	25.511	31.4173	24.4642	24.9668	34.744	28.055	30.119	24.4132	40.8116	24.5786	32.7027	32.0575	33.6901	0.541384199	0.966018836	24	12	24	0.651902976	0.882906761	24	23.75293609	0.085560015
ENSMUSG00000054733	Msra	methionine sulfoxide reductase A [Source:MGI Symbol;Acc:MGI:106916]	9.41466	10.6332	12.9533	13.1788	10.7401	11.5265	11.7394	11.3179	11.019	11.4	11.8566	12.0772	12.7274	11.9412	0.877082091	0.966018836	16	16	24	0.505987124	0.811776745	24	11.19534432	0.050016829
ENSMUSG00000054737	Zfp182	zinc finger protein 182 [Source:MGI Symbol;Acc:MGI:2442220]	5.3829	7.40803	5.70877	6.94545	6.75252	5.99281	6.59009	5.36901	6.9399	4.57358	7.20294	6.21567	6.29434	6.2026	0.881996584	0.966018836	20	12	24	0.943640561	0.986195696	24	17.87434797	0.023459054
ENSMUSG00000054752	Fsd1l	fibronectin type III and SPRY domain containing 1-like [Source:MGI Symbol;Acc:MGI:2442443]	26.0927	25.2207	22.2646	24.0858	25.3556	22.7122	24.2207	20.8813	22.9931	19.1549	26.1634	24.0463	23.6785	22.9364	0.338172159	0.85758369	20	16	24	0.187218831	0.768040178	24	20.51900234	0.082622881
ENSMUSG00000054763	Defb42	defensin beta 42 [Source:MGI Symbol;Acc:MGI:3033850]	6.23151	6.50433	7.51829	5.78302	5.8832	6.96376	6.97421	5.91992	5.22193	6.70944	6.53758	5.31527	6.46676	7.49398	0.860560779	0.966018836	8	8	24	0.714200866	0.905831242	24	3.223768378	0.046233653
ENSMUSG00000054766	Set	SET nuclear oncogene [Source:MGI Symbol;Acc:MGI:1860267]	24.4989	21.2275	22.06	21.6657	21.515	21.3733	21.8995	20.4482	21.1384	23.7421	22.2278	21.988	21.9504	23.1438	0.890176313	0.966018836	4	4	24	0.932815696	0.98297963	24	19.41792321	0.010469567
ENSMUSG00000054770	Kctd18	potassium channel tetramerisation domain containing 18 [Source:MGI Symbol;Acc:MGI:3603813]	12.3901	11.9041	11.1788	11.5893	12.8378	11.7213	11.9577	11.5275	12.6919	10.3911	12.1464	12.2778	11.9663	10.8783	0.246839642	0.745922296	20	20	24	0.257897997	0.768040178	24	20.13586811	0.050108516
ENSMUSG00000054792	Klhl18	kelch-like 18 [Source:MGI Symbol;Acc:MGI:2143315]	5.21127	4.65783	4.91452	5.51	5.19343	5.12623	5.52234	4.98569	4.87606	4.85998	5.11387	5.37162	4.81887	5.05431	0.369326376	0.893596186	24	12	24	0.173932276	0.768040178	24	18.48981748	0.044632657
ENSMUSG00000054793	Cadm4	cell adhesion molecule 4 [Source:MGI Symbol;Acc:MGI:2449088]	117.266	116.55	112.238	108.646	130.077	104.772	135.163	113.487	132.26	136.993	109.524	126.841	125.824	109.83	0.885059765	0.966018836	4	20	24	0.98906412	0.997679907	24	14.28455844	0.007571764
ENSMUSG00000054808	Actn4	actinin alpha 4 [Source:MGI Symbol;Acc:MGI:1890773]	45.6082	38.6853	41.5055	40.8416	47.4849	47.0767	45.5367	47.6814	37.2623	47.7495	38.2933	41.523	41.8659	37.94	0.85447369	0.966018836	16	20	24	0.353577528	0.780917462	24	19.50663464	0.074123067
ENSMUSG00000054814	Usp46	ubiquitin specific peptidase 46 [Source:MGI Symbol;Acc:MGI:1916977]	28.6413	25.9911	25.8798	25.6026	26.1268	27.1709	25.9967	25.3586	23.4784	26.4011	25.6325	25.4758	25.6692	25.232	0.443191651	0.966018836	4	4	24	0.044284065	0.628432994	24	20.61868302	0.04836762
ENSMUSG00000054823	Nsd3	nuclear receptor binding SET domain protein 3 [Source:MGI Symbol;Acc:MGI:2142581]	12.9151	12.0574	12.4558	11.4133	13.1956	12.5471	12.6443	11.3632	11.8062	11.9882	12.4072	12.5666	12.3093	11.8142	0.054939831	0.398010585	4	4	24	0.019602872	0.564709807	24	19.67686595	0.057707628
ENSMUSG00000054836	Elp6	elongator acetyltransferase complex subunit 6 [Source:MGI Symbol;Acc:MGI:1919349]	7.90894	7.48688	7.7872	7.10632	7.06153	7.40322	7.12597	7.58058	6.8873	7.37383	7.86252	7.79208	7.63039	6.47564	0.603018435	0.966018836	4	4	24	0.403131112	0.799546792	24	19.97107044	0.038941488
ENSMUSG00000054843	Atrnl1	attractin like 1 [Source:MGI Symbol;Acc:MGI:2147749]	50.3436	37.5197	39.7488	40.2703	40.1998	46.1705	40.3327	41.2754	29.1903	46.0755	40.8461	42.905	41.566	43.1303	0.085890569	0.466404237	24	12	24	0.161366488	0.76636332	24	20.44319639	0.130622015
ENSMUSG00000054850	Smim10l2a	small integral membrane protein 10 like 2A [Source:MGI Symbol;Acc:MGI:2443645]	4.33229	3.91348	4.25105	3.37897	3.71612	3.8224	3.7765	3.49466	3.76819	4.04514	3.34756	4.54162	3.74856	4.8888	0.787283372	0.966018836	24	20	24	0.432947887	0.805393068	24	2.375088278	0.062695527
ENSMUSG00000054855	Rnd1	Rho family GTPase 1 [Source:MGI Symbol;Acc:MGI:2444878]	1.01302	1.6123	2.92348	1.17575	0.951545	1.77376	2.08804	1.18616	1.55357	1.44108	0.916194	1.35371	0.995929	1.96211	0.152782337	0.601788038	12	8	24	0.274918884	0.768040178	24	5.912328705	0.187998511
ENSMUSG00000054863	Tafa5	TAFA chemokine like family member 5 [Source:MGI Symbol;Acc:MGI:2146182]	11.5995	10.6051	12.2348	9.69904	9.90396	10.5537	12.1083	10.4346	11.5354	13.0395	10.2806	11.0928	11.617	11.8848	0.455507782	0.966018836	12	8	24	0.352822164	0.780629136	24	4.925511039	0.063613641
ENSMUSG00000054871	Tmem158	transmembrane protein 158 [Source:MGI Symbol;Acc:MGI:1919559]	18.0374	16.2598	16.0913	18.5059	14.2907	15.7261	13.5668	16.2416	15.9296	24.0971	16.5204	20.5686	18.5648	18.7529	0.859936714	0.966018836	16	20	24	0.664522519	0.886705913	24	11.41096696	0.067969084
ENSMUSG00000054874	Pcnx3	pecanex homolog 3 [Source:MGI Symbol;Acc:MGI:1861733]	9.76021	10.9935	10.8053	8.45257	10.2216	9.57531	10.7101	11.7466	10.7424	12.1904	10.2069	9.03506	11.0419	11.028	0.030691182	0.324150946	8	16	24	0.107614178	0.720228013	24	5.887341148	0.101284368
ENSMUSG00000054893	Zfp667	zinc finger protein 667 [Source:MGI Symbol;Acc:MGI:2442757]	3.82013	3.53451	3.78096	3.60241	3.82102	3.37436	3.58635	3.43533	4.07243	3.34597	4.05336	3.88749	3.35152	3.63742	0.851656735	0.966018836	20	12	24	0.642113474	0.878809617	24	13.71769643	0.028262804
ENSMUSG00000054894	Dmac2l	distal membrane arm assembly complex 2 like [Source:MGI Symbol;Acc:MGI:1915305]	26.0995	24.6665	25.9731	23.946	26.3522	22.9505	21.0398	24.7793	24.9025	23.7738	27.2626	25.0125	23.8644	24.183	0.850398351	0.966018836	20	8	24	0.490097207	0.805393068	24	12.97180537	0.041628642
ENSMUSG00000054901	Arhgef33	Rho guanine nucleotide exchange factor (GEF) 33 [Source:MGI Symbol;Acc:MGI:2685787]	1.18289	1.20248	1.26421	1.22448	0.890247	1.1445	1.43735	0.838221	1.97904	1.56817	1.08945	1.10837	0.731095	2.33893	0.166168222	0.633011401	12	8	24	0.180212844	0.768040178	24	7.695190967	0.183731122
ENSMUSG00000054920	Klhl5	kelch-like 5 [Source:MGI Symbol;Acc:MGI:1919028]	50.2686	43.713	39.7487	40.6547	42.117	46.2227	42.6794	42.6324	37.4914	41.2508	44.1348	44.533	46.5295	45.4354	3.15E-04	0.049770315	4	8	24	0.00328083	0.416356043	24	22.14805218	0.116363633
ENSMUSG00000054931	Zkscan4	zinc finger with KRAB and SCAN domains 4 [Source:MGI Symbol;Acc:MGI:3649412]	1.05867	1.00034	1.24906	1.32303	1.09131	1.13124	1.01689	1.30025	1.08688	1.03819	0.970911	1.42404	1.22152	0.930223	0.92769718	0.966018836	24	8	24	0.919096572	0.978264112	24	16.65658352	0.016702017
ENSMUSG00000054934	Kcnmb4	potassium large conductance calcium-activated channel, subfamily M, beta member 4 [Source:MGI Symbol;Acc:MGI:1913272]	15.2252	14.6764	12.4181	12.5524	13.2329	12.2678	15.4733	15.4634	14.1073	16.3563	14.4124	15.3667	15.8605	16.3679	0.246839642	0.745922296	8	4	24	0.129507453	0.740162193	24	2.332320154	0.084221257
ENSMUSG00000054939	Zfp174	zinc finger protein 174 [Source:MGI Symbol;Acc:MGI:2686600]	1.17237	1.26098	1.32886	1.23527	1.26942	1.08127	1.31595	1.06123	1.12934	1.30694	1.22111	1.26327	1.11596	1.24177	0.916963526	0.966018836	12	20	24	0.535956433	0.82731247	24	12.40399455	0.025314347
ENSMUSG00000054942	Miga1	mitoguardin 1 [Source:MGI Symbol;Acc:MGI:1924567]	38.3418	37.6472	35.2968	37.6235	36.194	36.5072	35.2385	33.7889	35.2445	36.3363	37.8693	36.8849	38.4571	40.3679	0.911135828	0.966018836	4	8	24	0.795556274	0.934624211	24	21.89151467	0.017573047
ENSMUSG00000054945	Gm9958	predicted gene 9958 [Source:MGI Symbol;Acc:MGI:3641866]	0.88192	1.51911	1.23332	0.655849	1.54387	1.00756	0.681145	1.1111	1.54858	1.05249	1.09929	0.797285	1.46768	1.2893	0.331157065	0.850959667	12	12	24	0.462019816	0.805393068	24	7.03155585	0.108899693
ENSMUSG00000054951	9130008F23Rik	RIKEN cDNA 9130008F23 gene [Source:MGI Symbol;Acc:MGI:1918833]	0.39692	0.727152	0.923734	0.510302	0.716751	0.566295	0.708316	0.802318	0.842061	0.711385	0.794616	0.861789	0.658831	0.73488	0.176754388	0.653256853	12	16	24	0.316050323	0.768040178	24	8.755226182	0.069821276
ENSMUSG00000054958	Nt5c1a	5'-nucleotidase, cytosolic IA [Source:MGI Symbol;Acc:MGI:2155700]	0.413018	0.725528	0.857705	0.345229	0.478781	0.598081	0.916838	0.740633	0.667304	0.868104	0.749909	0.860485	0.449646	1.64903	0.881996584	0.966018836	8	12	24	0.250343598	0.768040178	24	5.48268386	0.138097393
ENSMUSG00000054966	Lmntd1	lamin tail domain containing 1 [Source:MGI Symbol;Acc:MGI:1921321]	0.65106	1.10977	0.821539	0.906455	1.10927	0.852169	0.912259	1.09107	1.49021	0.572989	0.979694	1.20584	0.680552	1.03767	0.918193129	0.966018836	8	20	24	0.801492003	0.936422183	24	7.906211657	0.049640842
ENSMUSG00000054967	Zfp647	zinc finger protein 647 [Source:MGI Symbol;Acc:MGI:3052806]	1.23703	1.12855	1.03066	1.02514	1.30402	1.25035	1.12719	1.1291	1.21716	1.02114	1.22709	1.30208	1.1551	0.983052	0.020428812	0.261278501	20	20	24	0.021907798	0.574400012	24	19.52618447	0.072331998
ENSMUSG00000054976	Nyap2	neuronal tyrosine-phophorylated phosphoinositide 3-kinase adaptor 2 [Source:MGI Symbol;Acc:MGI:2443135]	2.31033	1.89237	1.82286	2.00953	1.81831	2.07257	2.05471	2.03435	1.73203	2.42764	1.65402	2.14678	1.62526	2.00305	0.745118544	0.966018836	24	12	24	0.909040982	0.974606327	24	21.04515195	0.020402904
ENSMUSG00000054988	Agtr1b	angiotensin II receptor, type 1b [Source:MGI Symbol;Acc:MGI:87965]	2.95438	2.79405	2.52004	2.8078	2.52378	2.90555	2.8058	2.51103	2.66445	2.3335	2.59099	2.7127	2.50551	3.07542	0.640622311	0.966018836	8	4	24	0.226411244	0.768040178	24	0.113158576	0.052766919
ENSMUSG00000055003	Lrtm2	leucine-rich repeats and transmembrane domains 2 [Source:MGI Symbol;Acc:MGI:2141485]	1.15041	1.70893	2.54887	1.6647	1.81093	1.93405	2.2871	1.46003	1.99837	2.08692	1.16387	1.72706	1.19288	1.66607	0.773177465	0.966018836	12	16	24	0.425183337	0.805393068	24	9.714805167	0.114951641
ENSMUSG00000055013	Agap1	ArfGAP with GTPase domain, ankyrin repeat and PH domain 1 [Source:MGI Symbol;Acc:MGI:2653690]	7.22706	6.38541	8.05431	5.56298	6.9456	7.15927	7.24898	6.5985	6.03174	9.21003	6.23273	7.5139	5.63671	7.78339	0.733896783	0.966018836	24	20	24	0.973388168	0.993305115	24	11.82187164	0.016885757
ENSMUSG00000055022	Cntn1	contactin 1 [Source:MGI Symbol;Acc:MGI:105980]	87.5344	81.3613	80.0467	81.8752	74.935	79.5215	79.0783	74.0985	70.8661	86.0902	82.4936	79.8408	80.0326	88.159	0.830949782	0.966018836	16	8	24	0.906047534	0.974325939	24	21.95918248	0.016337486
ENSMUSG00000055024	Ep300	E1A binding protein p300 [Source:MGI Symbol;Acc:MGI:1276116]	7.24884	5.76361	6.50258	7.22748	8.69869	7.03298	8.04236	6.61908	6.99961	6.99844	7.17044	7.95782	6.24845	6.33644	0.37467485	0.897795059	20	12	24	0.110013879	0.721134035	24	17.50297833	0.110281736
ENSMUSG00000055026	Gabrg3	gamma-aminobutyric acid (GABA) A receptor, subunit gamma 3 [Source:MGI Symbol;Acc:MGI:95624]	0.591551	0.728999	0.987497	0.640491	0.482043	0.564326	0.681522	0.43987	0.579344	0.571712	0.383774	0.629554	0.535644	0.661351	0.021288455	0.264116537	12	8	24	0.14242704	0.751766186	24	6.480399911	0.094218654
ENSMUSG00000055027	Smyd1	SET and MYND domain containing 1 [Source:MGI Symbol;Acc:MGI:104790]	2.22716	4.89892	5.87345	7.79085	4.44501	6.69334	4.84255	11.5409	8.74341	10.2366	4.08438	4.90792	6.5772	5.60203	0.248056532	0.746745468	16	4	24	0.080338032	0.695795041	24	9.018673406	0.410196438
ENSMUSG00000055041	Commd5	COMM domain containing 5 [Source:MGI Symbol;Acc:MGI:1913648]	12.4165	12.9422	13.2576	11.4707	13.3743	13.108	12.1691	13.5837	15.0802	13.966	14.0377	15.1012	13.8259	14.2361	0.879201959	0.966018836	24	4	24	0.818805366	0.942446688	24	9.178718674	0.019881429
ENSMUSG00000055044	Pdlim1	PDZ and LIM domain 1 (elfin) [Source:MGI Symbol;Acc:MGI:1860611]	12.0131	9.06969	10.4178	10.5206	9.02953	10.9238	7.90085	12.6574	8.57688	10.181	9.06012	7.89689	11.8799	9.53345	0.935543173	0.966018836	8	12	24	0.721743942	0.90866625	24	3.420621476	0.062541062
ENSMUSG00000055053	Nfic	nuclear factor I/C [Source:MGI Symbol;Acc:MGI:109591]	25.8512	19.9332	19.9615	20.3534	19.1405	23.083	23.5502	26.4163	23.7807	43.6446	22.3859	27.9936	25.3229	25.6924	0.879901313	0.966018836	4	16	24	0.96363667	0.990884307	24	9.765461061	0.029111177
ENSMUSG00000055065	Ddx17	DEAD box helicase 17 [Source:MGI Symbol;Acc:MGI:1914290]	151.652	175.724	171.06	172.474	165.49	145.063	160.705	152.711	192.143	150.567	169.775	156.04	160.287	165.583	0.020957534	0.262822894	12	12	24	0.09575906	0.71175955	24	8.827085111	0.088583622
ENSMUSG00000055067	Smyd3	SET and MYND domain containing 3 [Source:MGI Symbol;Acc:MGI:1916976]	12.0219	10.2564	10.5408	10.9204	10.3477	11.6686	10.8233	9.83213	9.62354	10.4022	11.8458	11.4777	10.3685	12.4697	0.632729413	0.966018836	24	12	24	0.253071635	0.768040178	24	20.27990631	0.068240129
ENSMUSG00000055069	Rab39	RAB39, member RAS oncogene family [Source:MGI Symbol;Acc:MGI:2442855]	18.2805	15.4139	12.7024	14.6727	16.4798	16.3982	15.6302	14.3456	13.1652	13.8033	18.5817	14.532	16.0053	14.9352	0.002466754	0.109177422	20	16	24	0.016404439	0.544099157	24	20.17106734	0.153248063
ENSMUSG00000055078	Gabra5	gamma-aminobutyric acid (GABA) A receptor, subunit alpha 5 [Source:MGI Symbol;Acc:MGI:95617]	3.2513	4.16509	6.27114	3.52061	3.41537	4.26466	4.45917	3.04034	3.81383	3.81875	3.75243	3.74808	2.65506	4.27621	0.937920971	0.966018836	12	16	24	0.576830768	0.848735805	24	8.886632554	0.098068747
ENSMUSG00000055093	Rps27a-ps3	ribosomal protein S27A, pseudogene 3 [Source:MGI Symbol;Acc:MGI:3645406]	96.8818	126.313	117.004	118.994	114.375	100.624	102.931	108.451	150.881	87.2386	124.592	127.06	131.373	126.16	0.810949269	0.966018836	4	12	24	0.58863125	0.853936396	24	6.916406686	0.08287078
ENSMUSG00000055116	Arntl	aryl hydrocarbon receptor nuclear translocator-like [Source:MGI Symbol;Acc:MGI:1096381]	5.65458	3.37883	1.88996	0.844316	1.63464	3.4169	6.68639	3.71998	1.39589	1.08172	1.98313	3.81845	5.00334	3.66283	2.51E-07	0.001551842	4	12	24	1.93E-08	6.81E-04	24	23.87993776	0.85100119
ENSMUSG00000055128	Cgrrf1	cell growth regulator with ring finger domain 1 [Source:MGI Symbol;Acc:MGI:1916368]	20.6331	21.8279	22.2064	22.8213	26.8966	22.2908	22.2307	22.1324	25.39	19.9934	26.7677	25.5999	23.0689	21.3499	0.018114172	0.256345714	20	12	24	0.164128806	0.767020009	24	16.13265315	0.092672839
ENSMUSG00000055137	Sugct	succinyl-CoA glutarate-CoA transferase [Source:MGI Symbol;Acc:MGI:1923221]	2.47428	2.46707	2.48168	3.27325	2.67108	2.94059	2.61306	2.03367	2.36875	2.54981	3.32298	2.82028	2.97917	2.51362	0.008754183	0.192760771	24	8	24	0.064771457	0.663876076	24	18.15771356	0.227506503
ENSMUSG00000055148	Klf2	Kruppel-like factor 2 (lung) [Source:MGI Symbol;Acc:MGI:1342772]	3.78803	3.49612	4.04527	3.65971	2.74263	2.68414	3.76633	3.97264	6.06984	4.84832	4.51356	3.73706	4.23592	4.01227	0.148073804	0.590732968	12	4	24	0.046371404	0.633507364	24	8.035687127	0.201486996
ENSMUSG00000055150	Zfp78	zinc finger protein 78 [Source:MGI Symbol;Acc:MGI:107783]	1.96829	2.59204	2.75845	3.299	2.75158	2.90755	1.98667	3.26779	2.36278	2.90171	3.26587	2.25613	2.78665	2.4122	0.192357053	0.669841558	12	12	24	0.118477067	0.729286647	24	12.36003759	0.13462086
ENSMUSG00000055172	C1ra	complement component 1, r subcomponent A [Source:MGI Symbol;Acc:MGI:1355313]	2.96335	2.79583	2.78216	3.12837	3.02611	2.58423	3.41715	3.29585	3.69318	2.64327	3.21212	3.4396	3.04798	2.66897	0.804027134	0.966018836	4	12	24	0.994777486	0.998755922	24	22.85505727	0.004967354
ENSMUSG00000055177	Cstl1	cystatin-like 1 [Source:MGI Symbol;Acc:MGI:2652834]	0.342594	0.561002	1.00918	0.778953	0.529076	0.370334	0.729853	0.305417	0.466838	0.848722	0.468745	0.439182	0.45815	0.503434	0.154126887	0.606139807	16	8	24	0.10155522	0.713208839	24	10.5926756	0.145165587
ENSMUSG00000055184	Fam72a	family with sequence similarity 72, member A [Source:MGI Symbol;Acc:MGI:1919669]	0.742061	0.55661	0.446296	0.78806	0.661365	0.468193	0.739916	0.438669	0.668358	0.631944	0.593251	0.52079	0.64839	0.636344	0.92655264	0.966018836	4	20	24	0.922788473	0.979425536	24	15.82385913	0.016392078
ENSMUSG00000055188	Rbm3os	RNA binding motif protein 3, opposite strand [Source:MGI Symbol;Acc:MGI:1917476]	0.765147	0.597919	0.31994	0.336955	0.839181	0.606627	0.823196	0.579022	0.441825	0.429035	0.525393	0.33824	0.667166	0.396718	0.015486342	0.253395174	4	8	24	0.055928868	0.65534544	24	21.91389406	0.142637368
ENSMUSG00000055200	Sertad3	SERTA domain containing 3 [Source:MGI Symbol;Acc:MGI:2180697]	4.8993	5.15659	4.51558	4.49969	4.69879	4.7452	4.52875	6.0426	4.25712	4.85437	4.85277	4.97906	4.61599	4.45297	0.773177465	0.966018836	24	12	24	0.678126252	0.892430843	24	0.317385204	0.035757773
ENSMUSG00000055202	Zfp811	zinc finger protein 811 [Source:MGI Symbol;Acc:MGI:2682944]	2.12942	2.2728	2.59108	2.20553	2.33143	2.2742	1.80738	2.30003	2.3721	1.69645	2.34037	2.35799	2.22603	2.40978	0.277087842	0.789849983	12	16	24	0.880934518	0.966661503	24	6.839557154	0.023067648
ENSMUSG00000055204	Ankrd17	ankyrin repeat domain 17 [Source:MGI Symbol;Acc:MGI:1932101]	24.8392	22.7116	23.4403	22.8525	23.309	25.0692	23.3976	22.6785	19.2027	23.0595	22.1673	23.2577	22.2552	23.5555	0.888991156	0.966018836	24	16	24	0.104941415	0.717030134	24	20.85861296	0.066675741
ENSMUSG00000055210	Foxd2	forkhead box D2 [Source:MGI Symbol;Acc:MGI:1347471]	1.02676	1.21963	0.88692	1.01533	0.957326	0.884385	1.17553	1.09875	1.08242	0.72113	1.33969	1.25163	1.39568	1.32651	0.362530951	0.887524753	4	4	24	0.12752447	0.740162193	24	0.641276281	0.091625574
ENSMUSG00000055214	Pld5	phospholipase D family, member 5 [Source:MGI Symbol;Acc:MGI:2442056]	14.86	12.0016	12.2082	13.016	11.2457	13.618	11.5567	11.5393	9.41254	15.0024	10.3396	12.798	13.851	12.1622	0.70641939	0.966018836	4	24	24	0.568532833	0.844419134	24	21.22710062	0.075918814
ENSMUSG00000055228	Gm49359	predicted gene, 49359 [Source:MGI Symbol;Acc:MGI:6121568]	0.98014	0.626559	0.203995	1.30879	0.945053	0.540745	0.04377	1.39586	0.0365436	1.65664	0.8204	0.48744	0.293132	0.104511	0.03970002	0.966018836	16	20	24	0.569837775	0.844900526	24	17.71977259	0.200567731
ENSMUSG00000055235	Wdr86	WD repeat domain 86 [Source:MGI Symbol;Acc:MGI:1915466]	0.357507	0.526221	0.8343	0.654998	0.69705	0.429512	0.400982	0.841732	0.87214	0.486594	0.617089	0.667504	0.510439	0.45001	0.018894761	0.256345714	12	8	24	0.095916752	0.71175955	24	10.04580451	0.125723837
ENSMUSG00000055239	Kcmf1	potassium channel modulatory factor 1 [Source:MGI Symbol;Acc:MGI:1921537]	37.7488	35.4135	35.8201	36.7046	35.8582	37.0287	35.5467	39.3953	35.6796	40.6687	36.4296	37.7792	37.6417	37.705	0.896753532	0.966018836	24	12	24	0.983880759	0.996654399	24	9.410044643	0.004019507
ENSMUSG00000055240	Zfp101	zinc finger protein 101 [Source:MGI Symbol;Acc:MGI:107547]	5.07758	6.39911	5.83739	5.45774	5.84569	5.4346	4.80649	5.69696	6.50504	4.46298	5.98473	5.42176	5.25612	6.07558	0.058456626	0.404936103	12	16	24	0.46616059	0.805393068	24	7.37301587	0.065442955
ENSMUSG00000055254	Ntrk2	neurotrophic tyrosine kinase, receptor, type 2 [Source:MGI Symbol;Acc:MGI:97384]	30.8404	26.6247	31.4163	27.0306	24.9482	30.558	28.7086	28.3756	24.8887	32.7712	25.7203	28.94	26.595	28.7856	0.890235289	0.966018836	24	20	24	0.910589073	0.9747563	24	1.343921908	0.020818794
ENSMUSG00000055296	Tmem245	transmembrane protein 245 [Source:MGI Symbol;Acc:MGI:2445107]	10.5793	12.3537	10.9821	11.8007	12.8129	10.0483	12.1379	11.0604	13.5545	10.7773	11.7855	11.8774	12.2316	11.3906	0.849180783	0.966018836	12	12	24	0.770370663	0.926095918	24	9.193724793	0.03186041
ENSMUSG00000055301	Adh7	alcohol dehydrogenase 7 (class IV), mu or sigma polypeptide [Source:MGI Symbol;Acc:MGI:87926]	0.69673	0.760793	0.74505	0.447183	0.92924	0.779351	0.841552	0.945257	0.91074	0.414146	0.952518	0.445304	0.592434	0.908056	0.72778087	0.966018836	8	8	24	0.65125216	0.882725346	24	3.189652288	0.061843166
ENSMUSG00000055302	Mrfap1	Morf4 family associated protein 1 [Source:MGI Symbol;Acc:MGI:1914818]	81.6214	80.8571	78.3273	76.6555	82.873	81.2974	83.0884	85.8582	82.9382	90.9492	95.3836	94.7516	101.176	94.4619	0.745469888	0.966018836	20	20	24	0.239396993	0.768040178	24	21.67481493	0.038076089
ENSMUSG00000055305	Zfp93	zinc finger protein 93 [Source:MGI Symbol;Acc:MGI:107611]	3.33478	3.05908	2.71233	2.99961	3.26867	3.09679	2.92624	3.2112	2.87761	2.85143	3.34212	3.07178	2.95947	3.23536	0.093849599	0.490854203	20	16	24	0.260992125	0.768040178	24	20.50002798	0.045080197
ENSMUSG00000055313	Pgbd1	piggyBac transposable element derived 1 [Source:MGI Symbol;Acc:MGI:2441675]	0.410393	0.785666	0.73949	0.483053	0.457485	0.681642	0.634702	0.55015	0.582637	0.649978	0.588791	0.683106	0.55509	0.52332	0.866861748	0.966018836	24	20	24	0.886100354	0.967667196	24	6.245614448	0.020175743
ENSMUSG00000055319	Sec23ip	Sec23 interacting protein [Source:MGI Symbol;Acc:MGI:2450915]	8.12336	6.96022	7.2477	7.40561	7.17574	7.68422	8.09727	7.3731	6.67353	7.80014	7.06991	7.22919	7.11403	7.66027	0.860398351	0.966018836	4	8	24	0.383458166	0.799546792	24	22.14095483	0.039772145
ENSMUSG00000055320	Tead1	TEA domain family member 1 [Source:MGI Symbol;Acc:MGI:101876]	8.80418	9.32572	9.0332	10.3401	11.2719	9.16748	10.5228	10.4267	11.1026	12.1931	9.11416	9.18444	9.27372	8.97125	0.784278077	0.966018836	24	12	24	0.149465813	0.758126113	24	11.61092486	0.100070937
ENSMUSG00000055322	Tns1	tensin 1 [Source:MGI Symbol;Acc:MGI:104552]	48.7729	35.62	33.6598	44.3799	42.7912	42.1635	47.3237	46.9576	37.2845	66.4382	43.439	45.0767	48.4082	43.6476	0.112800839	0.530777648	4	8	24	0.645519751	0.879875982	24	17.78673201	0.082917425
ENSMUSG00000055323	Gm9967	predicted gene 9967 [Source:MGI Symbol;Acc:MGI:3704300]	1.5618	1.56035	1.54603	1.637	1.41655	1.64526	1.15326	2.1451	2.01308	1.41729	1.1558	1.38218	1.50131	1.91188	0.109247218	0.524035616	8	12	24	0.055047463	0.655263328	24	6.101852845	0.142609462
ENSMUSG00000055334	Snupn	snurportin 1 [Source:MGI Symbol;Acc:MGI:1913319]	12.2411	10.9085	10.9984	11.5927	11.9797	10.8884	11.7156	11.2432	9.40901	10.4112	11.3974	11.1359	11.0176	10.7311	0.310940564	0.820837787	20	16	24	0.050507511	0.658304792	24	20.20549454	0.070206621
ENSMUSG00000055341	Zfp457	zinc finger protein 457 [Source:MGI Symbol;Acc:MGI:2664334]	0.756498	0.620444	0.870984	0.789314	0.819417	0.486481	0.750663	0.892666	1.06368	0.57507	0.695175	0.664884	0.662338	0.650649	0.390686537	0.915479136	12	12	24	0.212326349	0.768040178	24	8.040676571	0.083312735
ENSMUSG00000055368	Slc6a2	solute carrier family 6 (neurotransmitter transporter, noradrenalin), member 2 [Source:MGI Symbol;Acc:MGI:1270850]	0.128559	0.227456	1.69414	3.29984	0.474732	5.35886	7.13433	0.0974259	0.0880878	5.85736	6.31825	5.71244	0.143594	21.5402	0.800065124	0.966018836	20	16	24	0.667804797	0.88767519	24	15.98904168	0.461600187
ENSMUSG00000055371	Stam2	signal transducing adaptor molecule (SH3 domain and ITAM motif) 2 [Source:MGI Symbol;Acc:MGI:1929100]	11.6105	10.5805	11.2323	10.873	10.7574	10.9518	10.0679	11.087	10.0259	10.695	10.5558	11.5582	11.4337	10.6166	0.586972936	0.966018836	24	20	24	0.435138759	0.805393068	24	21.33372799	0.031571423

ENSMUSG00000055373	Fut9	fucosyltransferase 9 [Source:MGI Symbol;Acc:MGI:1330859]	0.201413	0.745144	1.28698	0.538356	0.461128	0.588012	0.961727	0.419143	0.794611	0.438217	0.370161	0.447644	0.172928	0.730254	0.310940564	0.820837787	12	8	24	0.224316505	0.768040178	24	7.638914605	0.176857193
ENSMUSG00000055401	Fbxo6	F-box protein 6 [Source:MGI Symbol;Acc:MGI:1354743]	13.7419	13.3733	14.448	14.8423	14.0035	14.0098	13.1931	14.768	14.9549	11.7319	15.1937	12.9288	15.5817	12.938	0.862909389	0.966018836	12	12	24	0.995537371	0.998760424	24	7.759852137	0.004223424
ENSMUSG00000055407	Map6	microtubule-associated protein 6 [Source:MGI Symbol;Acc:MGI:1201690]	50.424	35.2807	35.9831	39.8127	40.3597	44.0907	46.807	42.6028	37.3531	58.6544	41.3289	45.8768	42.7231	43.5278	0.034356228	0.328311403	4	8	24	0.495384996	0.807162937	24	19.42326454	0.089603526
ENSMUSG00000055409	Nell1	NEL-like 1 [Source:MGI Symbol;Acc:MGI:2443902]	1.72868	2.42642	3.23567	1.91811	1.4895	2.24402	2.78425	1.94279	2.46976	2.28204	1.60371	1.96113	1.76802	2.83379	0.019711532	0.258568924	12	8	24	0.067695212	0.671778447	24	5.970494449	0.196681379
ENSMUSG00000055413	H2-Q5	histocompatibility 2, Q region locus 5 [Source:MGI Symbol;Acc:MGI:95934]	0.472549	0.612875	0.381827	0.587186	0.818155	0.627812	0.571688	1.11627	1.74858	0.650305	0.358907	0.528026	0.818277	0.621268	0.910436409	0.966018836	8	12	24	0.371964539	0.793398709	24	6.901083986	0.146983354
ENSMUSG00000055421	Pcdh9	protocadherin 9 [Source:MGI Symbol;Acc:MGI:1306801]	17.4861	16.8546	18.4022	16.196	15.2468	17.4533	17.7726	15.7177	15.6132	17.9367	15.0313	17.5975	15.8346	17.7004	0.714044782	0.966018836	4	16	24	0.633468321	0.875436572	24	2.337224053	0.034460456
ENSMUSG00000055430	Nap1l5	nucleosome assembly protein 1-like 5 [Source:MGI Symbol;Acc:MGI:1923555]	169.299	155.771	178.971	149.775	137.937	164.393	173.46	145.933	141.554	155.738	157.607	152.442	148.846	174.821	0.881718085	0.966018836	4	16	24	0.458524074	0.805393068	24	1.761427351	0.055912431
ENSMUSG00000055435	Maf	avian musculoaponeurotic fibrosarcoma oncogene homolog [Source:MGI Symbol;Acc:MGI:96909]	23.6451	21.6659	19.7455	22.4467	26.2122	20.6082	26.4687	21.3643	22.5884	27.9476	25.0134	21.9342	23.1656	19.8612	0.203345222	0.689605535	20	12	24	0.244556099	0.768040178	24	16.29356104	0.096383021
ENSMUSG00000055436	Srsf11	serine and arginine-rich splicing factor 11 [Source:MGI Symbol;Acc:MGI:1916457]	65.7929	74.509	76.9079	72.2195	75.4833	67.6576	71.0829	64.9389	79.8897	65.0996	75.7756	70.9624	68.9498	68.7747	0.300218476	0.809362476	12	20	24	0.358630923	0.785204076	24	11.35500985	0.052260244
ENSMUSG00000055447	Cd47	CD47 antigen (Rh-related antigen, integrin-associated signal transducer) [Source:MGI Symbol;Acc:MGI:96617]	55.8251	46.5374	46.5552	48.9332	45.2983	51.5967	47.5005	47.5508	44.2351	56.0074	51.7811	53.2771	57.0681	55.267	0.43565473	0.966018836	4	8	24	0.384321293	0.799546792	24	21.35601001	0.061521825
ENSMUSG00000055452	Gm7353	predicted pseudogene 7353 [Source:MGI Symbol;Acc:MGI:3643014]	0.541786	0.559122	0.525948	0.642131	0.694923	0.568932	0.769821	0.645086	0.752031	0.715578	0.54632	0.603944	0.466507	0.508988	0.916746631	0.966018836	16	16	24	0.496069356	0.807537659	24	12.38242654	0.039232705
ENSMUSG00000055480	Zfp458	zinc finger protein 458 [Source:MGI Symbol;Acc:MGI:3040691]	1.33816	1.95232	1.46214	1.43193	1.8576	1.86	1.69078	1.48793	2.01236	1.37676	1.8368	1.78662	1.6054	1.61696	0.924770496	0.966018836	20	20	24	0.806251166	0.938114514	24	19.02883453	0.033022675
ENSMUSG00000055485	Soga1	suppressor of glucose, autophagy associated 1 [Source:MGI Symbol;Acc:MGI:2444575]	2.41286	2.34355	2.22448	2.10082	2.39036	2.08646	2.75706	2.49066	2.15306	2.62714	2.2518	2.51797	2.53943	2.2682	0.227302972	0.723724673	4	8	24	0.385941393	0.799546792	24	23.50456244	0.04544361
ENSMUSG00000055489	Ano5	anoctamin 5 [Source:MGI Symbol;Acc:MGI:3576659]	0.638193	1.30744	1.26495	1.98701	1.2345	1.5036	1.04635	3.13648	2.74822	2.43914	1.122	1.1444	1.60702	1.3807	0.212679822	0.702364312	8	20	24	0.052823827	0.650526959	24	8.924815694	0.322780421
ENSMUSG00000055491	Pprc1	peroxisome proliferative activated receptor, gamma, coactivator-related 1 [Source:MGI Symbol;Acc:MGI:2385096]	5.7123	4.47768	6.14097	5.6342	6.96722	5.12683	7.40311	4.82389	4.70898	6.21593	5.881	5.31397	5.36406	5.28202	0.17953325	0.655480352	20	12	24	0.296813176	0.768040178	24	16.78946135	0.106644733
ENSMUSG00000055493	Epm2a	epilepsy, progressive myoclonic epilepsy, type 2 gene alpha [Source:MGI Symbol;Acc:MGI:1341085]	12.5966	12.5313	13.7599	12.7948	11.6644	12.6051	11.5852	13.0995	12.2579	14.743	11.3627	13.4911	12.3126	13.0984	0.570349842	0.966018836	16	4	24	0.361172108	0.786651663	24	8.452534815	0.054773929
ENSMUSG00000055494	Gm14168	predicted gene 14168 [Source:MGI Symbol;Acc:MGI:3651463]	0.681456	0.515326	0.508545	0.516342	0.527534	0.522387	0.594945	0.717296	0.559763	0.540065	0.443626	0.593424	0.504246	0.475839	0.912686312	0.966018836	4	16	24	0.280236655	0.768040178	24	1.064641226	0.040125916
ENSMUSG00000055497	Gm9974	predicted gene 9974 [Source:MGI Symbol;Acc:MGI:3708610]	0.41907	0.3459	0.653899	0.51523	0.560271	0.769753	0.824589	0.60423	0.666431	0.347972	0.596294	0.722083	0.653093	0.433117	0.435739761	0.966018836	24	8	24	0.405611479	0.799546792	24	20.4478634	0.075282644
ENSMUSG00000055531	Cpsf6	cleavage and polyadenylation specific factor 6 [Source:MGI Symbol;Acc:MGI:1913948]	18.8029	20.4377	20.8022	21.1464	21.6826	18.9631	20.857	16.8321	21.2111	17.6107	21.421	17.9806	17.7238	18.6219	0.185945151	0.662239322	20	12	24	0.286625844	0.768040178	24	13.2463324	0.065113448
ENSMUSG00000055538	Zcchc24	zinc finger, CCHC domain containing 24 [Source:MGI Symbol;Acc:MGI:1919168]	19.1705	18.8278	19.5163	19.8921	19.4776	17.865	23.2507	16.9116	18.4192	18.3323	18.1816	16.9926	17.2538	16.1273	0.522551072	0.966018836	12	20	24	0.823785851	0.944564271	24	16.98671632	0.025850219
ENSMUSG00000055540	Epha6	Eph receptor A6 [Source:MGI Symbol;Acc:MGI:108034]	1.62156	1.48359	1.77948	1.70506	1.46771	1.45678	1.99224	1.24409	1.49084	1.75371	1.64696	1.36928	1.61802	1.82779	0.718720526	0.966018836	4	20	24	0.871973972	0.963802358	24	8.367388012	0.024556322
ENSMUSG00000055541	Lair1	leukocyte-associated Ig-like receptor 1 [Source:MGI Symbol;Acc:MGI:105492]	1.95548	2.62017	2.71627	1.98653	2.18652	2.47233	2.48019	1.59742	2.9433	1.93108	1.74268	2.2845	2.18168	1.86597	0.924770496	0.966018836	12	4	24	0.899160761	0.971453495	24	5.209086965	0.032122688
ENSMUSG00000055553	Kxd1	KxDL motif containing 1 [Source:MGI Symbol;Acc:MGI:1922870]	20.2222	22.4785	22.938	22.384	22.5754	23.1777	21.8099	25.7614	26.7647	25.3498	24.0668	23.6501	23.7472	22.1519	0.759899911	0.966018836	12	16	24	0.120124718	0.731805836	24	10.07016953	0.070055965
ENSMUSG00000055555	Ct55	cancer/testis antigen 55 [Source:MGI Symbol;Acc:MGI:1922263]	0.878506	1.32465	1.22886	0.938595	1.17806	1.19729	1.18795	0.6475	0.879738	1.30563	1.05972	0.838148	0.834431	0.97921	0.891105762	0.966018836	16	12	24	0.527351018	0.8233276	24	14.09944449	0.062270362
ENSMUSG00000055567	Unc80	unc-80, NALCN activator [Source:MGI Symbol;Acc:MGI:2652882]	20.6054	20.8656	21.1542	19.9493	17.6649	19.4418	21.0418	17.0958	16.0645	19.3258	18.6349	17.5107	16.5813	19.747	0.873350015	0.966018836	4	20	24	0.912585239	0.975327891	24	2.003609245	0.018122356
ENSMUSG00000055612	Cdca7	cell division cycle associated 7 [Source:MGI Symbol;Acc:MGI:1914203]	0.739741	1.3455	1.52132	1.22927	1.56101	1.50648	1.43116	1.66598	1.16136	1.2635	1.08846	1.36506	1.35404	1.03618	0.816300547	0.966018836	24	16	24	0.956070064	0.988639288	24	13.53126043	0.019357649
ENSMUSG00000055629	B4galnt4	beta-1,4-N-acetyl-galactosaminyl transferase 4 [Source:MGI Symbol;Acc:MGI:2652891]	4.74583	5.64057	5.84596	4.59474	5.51173	5.17889	4.9713	5.02998	5.82497	6.38132	4.72345	6.08192	4.98151	5.45919	0.510431574	0.966018836	12	16	24	0.456746021	0.805393068	24	9.645380288	0.062027858
ENSMUSG00000055632	Hmcn2	hemicentin 2 [Source:MGI Symbol;Acc:MGI:2677838]	0.515986	0.775072	0.737896	0.951273	0.804404	1.13424	0.873724	1.40951	1.92614	1.06111	1.10873	0.755171	1.40287	2.00191	0.910436409	0.966018836	8	12	24	0.054059048	0.654752212	24	6.971634785	0.190438374
ENSMUSG00000055633	Zfp580	zinc finger protein 580 [Source:MGI Symbol;Acc:MGI:1916242]	3.78808	4.21776	4.57822	3.76333	4.38435	3.61652	3.99255	5.66221	5.31605	7.53645	5.94403	6.51136	6.82727	5.66399	0.768363194	0.966018836	20	4	24	0.428177875	0.805393068	24	9.725914824	0.093129744
ENSMUSG00000055639	Dach1	dachshund family transcription factor 1 [Source:MGI Symbol;Acc:MGI:1277991]	2.52422	2.11226	2.43066	2.46138	2.11769	2.41179	2.26493	2.22145	1.7568	2.55451	1.73273	1.96756	1.92993	2.18348	0.891022863	0.966018836	16	4	24	0.988484791	0.997559852	24	6.509105622	0.006835743
ENSMUSG00000055652	Klhl25	kelch-like 25 [Source:MGI Symbol;Acc:MGI:2668031]	4.31589	3.41501	3.72018	4.24824	4.17721	4.02554	4.43347	3.61625	3.1091	3.70192	3.71224	3.63236	3.93535	3.50993	0.112583132	0.530777648	4	4	24	0.052655345	0.650526959	24	19.58199696	0.096799712
ENSMUSG00000055653	Gpc3	glypican 3 [Source:MGI Symbol;Acc:MGI:104903]	42.6498	55.51	49.3743	47.6064	57.4656	38.3229	51.0901	47.1584	60.808	38.0722	50.7583	46.5534	51.7688	45.4341	0.914502381	0.966018836	12	12	24	0.713936649	0.905765245	24	7.205287614	0.066601703
ENSMUSG00000055660	Mettl4	methyltransferase like 4 [Source:MGI Symbol;Acc:MGI:1924031]	8.51071	7.63035	7.02797	7.2914	8.78258	7.86702	8.15144	6.63191	7.16923	6.71925	9.15134	7.9019	8.63108	7.04512	0.016110955	0.253395174	20	20	24	0.008866322	0.502965379	24	19.61418766	0.14212622
ENSMUSG00000055670	Zzef1	zinc finger, ZZ-type with EF hand domain 1 [Source:MGI Symbol;Acc:MGI:2444286]	13.1567	13.3183	13.2658	12.8381	13.1911	13.9132	12.6211	13.2248	12.458	13.9886	12.6891	12.6424	13.7391	13.1131	0.768363194	0.966018836	16	20	24	0.966656791	0.992039848	24	21.64870212	0.005348334
ENSMUSG00000055675	Kbtbd11	kelch repeat and BTB (POZ) domain containing 11 [Source:MGI Symbol;Acc:MGI:1922151]	12.017	9.92193	9.13919	10.1447	9.3622	11.0909	10.5234	10.0062	8.07325	12.0485	8.86214	10.5674	10.2223	11.3856	0.210566453	0.69936851	24	12	24	0.373415693	0.794388449	24	22.49804298	0.084220223
ENSMUSG00000055676	Gm5069	predicted pseudogene 5069 [Source:MGI Symbol;Acc:MGI:3644516]	5.00102	3.89661	4.18917	4.19082	5.05703	4.72983	4.85814	4.19771	3.88898	4.1222	4.26358	4.39829	4.48339	4.44989	0.004501001	0.140598674	4	8	24	0.011605772	0.520458883	24	20.50217319	0.104218134
ENSMUSG00000055681	Cope	coatomer protein complex, subunit epsilon [Source:MGI Symbol;Acc:MGI:1891702]	55.3841	53.01	56.1387	51.7399	51.4198	57.3451	54.3252	55.232	48.0859	54.9484	53.1738	50.6238	48.5129	49.0367	0.740423399	0.966018836	24	20	24	0.705690924	0.902918222	24	19.160889	0.02308191
ENSMUSG00000055692	Tmem191c	transmembrane protein 191C [Source:MGI Symbol;Acc:MGI:107238]	4.98256	6.35298	10.0804	4.88885	4.22362	5.01271	7.92059	5.5572	8.39949	7.42976	7.20409	6.78362	6.52082	7.5574	0.308023687	0.814993005	12	12	24	0.136048852	0.747972781	24	7.024943712	0.224229867
ENSMUSG00000055707	Klhl26	kelch-like 26 [Source:MGI Symbol;Acc:MGI:2443079]	10.0881	8.43329	8.14534	9.09811	8.62353	9.4555	9.3195	9.52597	9.00343	11.1591	9.70639	10.9523	10.159	9.37429	0.22872448	0.72665454	24	12	24	0.313883807	0.768040178	24	19.43155933	0.061233328
ENSMUSG00000055717	Slain1	SLAIN motif family, member 1 [Source:MGI Symbol;Acc:MGI:2145578]	2.7273	4.62395	6.73438	2.59574	2.7457	3.83883	4.55885	2.66102	3.93996	3.43627	2.3958	3.42646	1.70521	3.6978	0.581070934	0.966018836	12	8	24	0.394510058	0.799546792	24	6.847789244	0.196233249
ENSMUSG00000055720	Ubl7	ubiquitin-like 7 (bone marrow stromal cell-derived) [Source:MGI Symbol;Acc:MGI:1916709]	23.2615	20.7782	22.2198	22.3621	19.1367	23.591	24.3785	23.0613	22.0221	27.5738	19.7028	23.9025	21.7531	22.9636	0.644063931	0.966018836	24	20	24	0.86879527	0.962729397	24	4.146802154	0.026725777
ENSMUSG00000055723	Rras2	related RAS viral (r-ras) oncogene 2 [Source:MGI Symbol;Acc:MGI:1914172]	7.43544	8.31138	7.6461	7.78658	9.52426	7.70314	8.98709	8.23971	8.09526	6.6935	9.3146	8.15313	8.21524	7.24007	0.503972924	0.966018836	20	20	24	0.40296232	0.799546792	24	19.11398993	0.068129026
ENSMUSG00000055725	Paqr3	progestin and adipoQ receptor family member III [Source:MGI Symbol;Acc:MGI:2679683]	4.62667	4.41038	4.64199	4.28402	3.4663	5.32598	4.17579	4.34805	3.4203	4.40843	4.57389	5.31857	4.8873	4.63822	0.193864198	0.67239758	24	16	24	0.21667621	0.768040178	24	21.91778369	0.101331549
ENSMUSG00000055733	Nap1l3	nucleosome assembly protein 1-like 3 [Source:MGI Symbol;Acc:MGI:1859565]	23.5647	22.7735	23.3931	21.6591	20.583	22.9615	22.8567	20.9997	18.4879	22.6293	21.4557	19.3676	19.7279	22.3552	0.879206539	0.966018836	4	16	24	0.849369856	0.954760051	24	1.022041009	0.020389987
ENSMUSG00000055737	Ghr	growth hormone receptor [Source:MGI Symbol;Acc:MGI:95708]	20.8952	19.9506	19.9913	24.3384	20.623	19.7695	17.6175	22.3187	21.4685	21.0957	24.1027	20.1415	22.3187	22.0325	0.772442842	0.966018836	16	8	24	0.247991704	0.768040178	24	11.45454137	0.071953868
ENSMUSG00000055745	Rtl6	retrotransposon Gag like 6 [Source:MGI Symbol;Acc:MGI:2675858]	6.91252	4.94731	4.94926	4.99455	5.30342	5.91734	5.70802	5.94341	3.87739	6.80712	4.58712	6.24201	5.68108	5.17999	0.114657038	0.534533508	24	12	24	0.186848784	0.768040178	24	21.35845874	0.136291917
ENSMUSG00000055760	Gemin6	gem nuclear organelle associated protein 6 [Source:MGI Symbol;Acc:MGI:1914492]	3.23021	3.83627	3.29109	3.64178	3.90121	3.47173	3.34096	3.03959	3.37207	2.99841	3.7911	3.28129	3.81668	3.6804	0.847312766	0.966018836	20	20	24	0.775053103	0.927711986	24	18.28403088	0.028943748
ENSMUSG00000055762	Eef1d	eukaryotic translation elongation factor 1 delta (guanine nucleotide exchange protein) [Source:MGI Symbol;Acc:MGI:1913906]	57.0349	62.9965	59.5714	62.0903	66.2944	65.0729	64.143	68.6281	66.1761	61.7333	57.5936	58.2024	58.3678	56.8285	0.858891143	0.966018836	12	16	24	0.83034942	0.947325117	24	9.783775257	0.021929608
ENSMUSG00000055763	Rybp-ps	RING1 and YY1 binding protein, pseudogene [Source:MGI Symbol;Acc:MGI:3648043]	4.07961	3.28106	3.51308	4.30474	4.29868	3.8682	4.21888	3.3659	2.98995	3.66289	3.11059	3.89285	2.46825	3.70864	0.723119329	0.966018836	16	20	24	0.364301827	0.788789286	24	17.29841463	0.089876889
ENSMUSG00000055771	Gm7936	predicted pseudogene 7936 [Source:MGI Symbol;Acc:MGI:3646364]	24.9082	27.3822	29.0996	30.0773	32.3771	24.7633	28.7382	24.5221	37.4966	21.5069	26.3932	26.061	27.2219	29.6096	0.601647512	0.966018836	12	12	24	0.558363787	0.839674578	24	8.652480473	0.084679368
ENSMUSG00000055782	Abcd2	ATP-binding cassette, sub-family D (ALD), member 2 [Source:MGI Symbol;Acc:MGI:1349467]	6.34172	8.12162	8.31258	8.20463	6.74587	7.2951	8.31579	7.83907	8.0363	8.02016	7.44155	7.17806	7.5464	8.16169	0.077415205	0.453845658	12	12	24	0.021328429	0.572080066	24	7.633608034	0.104422327
ENSMUSG00000055799	Tcf7l1	transcription factor 7 like 1 (T cell specific, HMG box) [Source:MGI Symbol;Acc:MGI:1202876]	1.64615	1.82996	2.27523	1.44843	1.63324	1.1935	1.76547	1.51375	2.10813	2.21622	1.80185	1.76888	1.64916	1.57831	0.591351901	0.966018836	12	12	24	0.134781631	0.746461599	24	9.077577598	0.121368496
ENSMUSG00000055805	Fmnl1	formin-like 1 [Source:MGI Symbol;Acc:MGI:1888994]	23.1229	19.3274	17.3706	19.6381	22.8145	22.6654	20.7538	22.7421	17.6572	22.9087	19.6435	22.5444	22.8414	21.7218	0.105280783	0.518002936	4	8	24	0.064748558	0.663876076	24	20.85468156	0.118025034
ENSMUSG00000055809	Dnaaf3	dynein, axonemal assembly factor 3 [Source:MGI Symbol;Acc:MGI:3588207]	0.941512	1.16219	1.27395	0.952703	0.94003	0.899476	0.962913	0.719964	1.13115	0.899182	0.99504	1.06142	0.650723	1.23792	0.454881285	0.966018836	12	16	24	0.411017628	0.799546792	24	8.680414882	0.07005629
ENSMUSG00000055817	Mta3	metastasis associated 3 [Source:MGI Symbol;Acc:MGI:2151172]	22.0542	18.0139	17.5932	19.9947	17.4704	21.8328	19.781	19.9505	17.3677	24.8303	16.739	21.5464	19.1753	20.191	0.446653834	0.966018836	24	12	24	0.750148734	0.918827661	24	21.01523645	0.048825474
ENSMUSG00000055818	A230083G16Rik	RIKEN cDNA A230083G16 gene [Source:MGI Symbol;Acc:MGI:3045289]	1.5357	0.975601	1.14816	1.2112	1.32931	0.844659	0.995441	1.33224	1.31111	1.10972	1.38614	0.979073	1.05479	1.00324	0.503972924	0.966018836	20	4	24	0.646035665	0.880103669	24	11.5237926	0.047824435
ENSMUSG00000055835	Zfp1	zinc finger protein 1 [Source:MGI Symbol;Acc:MGI:99154]	7.95684	8.84037	9.72339	8.46614	9.03583	9.21411	7.55359	7.66836	9.34008	8.7634	8.16003	7.94845	8.79462	8.67163	0.182849367	0.659303873	12	16	24	0.242377378	0.768040178	24	10.47655961	0.064375705
ENSMUSG00000055839	Elob	elongin B [Source:MGI Symbol;Acc:MGI:1914923]	161.581	148.185	148.143	138.6	149.063	155.299	140.659	156.425	148.031	136.49	165.545	146.531	157.259	149.74	0.172322601	0.648362388	4	12	24	0.304288384	0.768040178	24	22.37929973	0.049007841
ENSMUSG00000055850	Rnf181	ring finger protein 181 [Source:MGI Symbol;Acc:MGI:1913760]	83.8484	77.4528	74.048	74.6215	82.3715	79.2941	78.2838	73.6457	74.2204	73.5598	78.5631	72.9495	77.7545	76.38	0.135859247	0.575390196	20	20	24	0.050291988	0.644435324	24	20.72135427	0.052207029
ENSMUSG00000055862	Izumo4	IZUMO family member 4 [Source:MGI Symbol;Acc:MGI:1918814]	32.2509	38.8858	37.1513	33.376	37.014	32.953	32.981	32.4783	42.0564	35.8033	33.214	36.237	35.5635	34.5722	0.105280783	0.518002936	12	16	24	0.241049906	0.768040178	24	8.867096533	0.072945764
ENSMUSG00000055865	Tafa3	TAFA chemokine like family member 3 [Source:MGI Symbol;Acc:MGI:3046463]	3.39845	3.22736	2.9699	3.80222	3.47133	3.79566	3.17784	4.12802	3.42751	5.01054	3.14813	3.89262	3.9591	3.68737	0.903207257	0.966018836	16	20	24	0.797949635	0.935424343	24	12.96033097	0.037767078
ENSMUSG00000055866	Per2	period circadian clock 2 [Source:MGI Symbol;Acc:MGI:1195265]	2.72691	2.03182	3.67476	7.27763	4.55623	2.72969	2.25215	1.53764	4.89329	5.471	4.09057	1.89644	1.71598	1.72933	1.45E-04	0.031913438	16	16	24	3.20E-05	0.062734842	24	12.80213084	0.645154924
ENSMUSG00000055884	Fancm	Fanconi anemia, complementation group M [Source:MGI Symbol;Acc:MGI:2442306]	1.61691	2.61622	2.18059	3.52561	2.6889	2.11795	3.71714	3.8346	3.34788	3.27782	3.34823	4.74633	3.32767	2.68715	0.788254138	0.966018836	16	20	24	0.792197103	0.933498508	24	12.83111535	0.065591012
ENSMUSG00000055897	Ppp4r1l-ps	protein phosphatase 4, regulatory subunit 1-like, pseudogene [Source:MGI Symbol;Acc:MGI:1924560]	7.09954	7.911	8.04295	8.06488	7.70241	8.01117	6.30059	8.72028	9.93248	8.84454	8.87916	9.29294	8.7918	8.49045	0.759899911	0.966018836	12	16	24	0.130886331	0.741164574	24	10.43820925	0.092421986
ENSMUSG00000055900	Tmem69	transmembrane protein 69 [Source:MGI Symbol;Acc:MGI:3045357]	7.12945	8.06568	9.17119	8.46761	8.01728	8.6268	7.95541	7.66287	7.24091	7.8752	8.48196	7.91268	8.81447	8.06085	0.801251296	0.966018836	24	4	24	0.74955198	0.918524199	24	14.87094022	0.027246159
ENSMUSG00000055912	Tmem150a	transmembrane protein 150A [Source:MGI Symbol;Acc:MGI:2385244]	3.69446	4.4043	4.99039	4.64812	5.01833	4.4506	4.7975	5.48047	5.03938	5.31156	4.07417	3.73632	4.22991	4.23576	0.094056402	0.490854203	12	16	24	0.082077842	0.699186014	24	9.224258996	0.119192179
ENSMUSG00000055917	Zfp277	zinc finger protein 277 [Source:MGI Symbol;Acc:MGI:1890393]	7.51888	9.21622	7.6357	8.88495	8.54872	7.77725	8.32384	7.84555	8.09075	7.52898	8.60931	7.92637	8.47067	8.59025	0.903207257	0.966018836	8	4	24	0.976845152	0.99411866	24	8.318000483	0.007606667
ENSMUSG00000055923	Aasdh	aminoadipate-semialdehyde dehydrogenase [Source:MGI Symbol;Acc:MGI:2442517]	6.96868	7.05544	6.72757	7.89521	6.47314	6.58489	7.82247	5.88974	8.19933	5.91905	7.32136	5.84749	6.26656	5.61643	0.953760335	0.966018836	12	20	24	0.788323443	0.932106039	24	12.10626454	0.037911081
ENSMUSG00000055926	Gm14137	predicted gene 14137 [Source:MGI Symbol;Acc:MGI:3651144]	1.25375	1.31138	1.39433	1.25836	1.43749	0.957939	1.31635	1.29891	1.46523	0.881949	1.31838	1.03753	1.30236	1.06804	0.245061943	0.741580974	12	12	24	0.677476014	0.892060593	24	6.328341187	0.041107468
ENSMUSG00000055932	Fto	fat mass and obesity associated [Source:MGI Symbol;Acc:MGI:1347093]	23.8515	22.1073	24.6166	22.4984	20.8106	26.0638	25.2681	24.7108	19.7165	31.0391	19.0493	25.0629	22.7095	25.523	0.893307667	0.966018836	24	20	24	0.883689224	0.966856754	24	2.281857178	0.034504345
ENSMUSG00000055937	Krt28	keratin 28 [Source:MGI Symbol;Acc:MGI:1918093]	0.651143	0.455525	0.472331	0.28116	0.795217	0.525815	0.729207	0.779401	0.612499	0.501756	0.954339	0.655349	0.757299	0.704775	0.187699199	0.662239322	20	20	24	0.276067347	0.768040178	24	21.68190015	0.087370722
ENSMUSG00000055943	Emc7	ER membrane protein complex subunit 7 [Source:MGI Symbol;Acc:MGI:1920274]	19.8452	20.3789	20.4871	18.737	20.1227	19.503	20.8923	19.1846	20.1954	19.8269	20.4492	20.7671	21.2008	20.7571	0.192357053	0.669841558	4	12	24	0.314756513	0.768040178	24	0.353422376	0.024902039
ENSMUSG00000055945	Prr18	proline rich 18 [Source:MGI Symbol;Acc:MGI:2443403]	1.03792	3.636	5.72076	2.12272	1.56487	3.18806	3.62482	2.49547	3.32629	2.72055	1.31049	2.3896	1.22527	3.12079	0.161023625	0.620479606	12	8	24	0.162486293	0.76636332	24	7.053543101	0.358527636
ENSMUSG00000055963	Triqk	triple QxxK/R motif containing [Source:MGI Symbol;Acc:MGI:3650048]	23.9545	24.9622	22.7581	24.2438	22.5557	21.6125	23.1016	21.2104	22.9524	20.6446	24.7604	22.121	24.6181	23.4497	0.93357733	0.966018836	4	20	24	0.791165281	0.933169725	24	1.139618386	0.022195868
ENSMUSG00000055980	Irs1	insulin receptor substrate 1 [Source:MGI Symbol;Acc:MGI:99454]	1.77445	1.89766	1.65976	1.8801	2.25124	1.80082	2.45483	2.021	2.24281	2.08236	1.88674	2.11958	2.2885	1.99563	0.869755137	0.966018836	4	8	24	0.827425729	0.946154971	24	22.50944338	0.024070364
ENSMUSG00000055991	Zkscan5	zinc finger with KRAB and SCAN domains 5 [Source:MGI Symbol;Acc:MGI:107533]	4.28409	3.99093	4.6498	4.67818	4.0175	4.24631	4.13646	4.27644	3.73807	3.9141	3.77968	4.20159	4.40874	3.51689	0.93717134	0.966018836	4	4	24	0.844243697	0.952833645	24	19.83573384	0.020188126
ENSMUSG00000055994	Nod2	nucleotide-binding oligomerization domain containing 2 [Source:MGI Symbol;Acc:MGI:2429397]	0.519705	0.448596	0.683385	0.498616	0.657641	0.428305	0.47403	0.539534	0.482769	0.482779	0.655843	0.464792	0.477316	0.435758	0.187699199	0.662239322	20	4	24	0.249308938	0.768040178	24	13.35929903	0.04858031
ENSMUSG00000056004	Elapor2	endosome-lysosome associated apoptosis and autophagy regulator family member 2 [Source:MGI Symbol;Acc:MGI:2443264]	31.0339	26.524	26.4513	26.279	25.8165	28.4403	26.3208	24.9839	22.5025	26.622	27.4181	27.6188	26.8837	28.1094	0.133266423	0.567728672	24	12	24	0.078461211	0.688981542	24	21.10705598	0.084381294
ENSMUSG00000056014	A430033K04Rik	RIKEN cDNA A430033K04 gene [Source:MGI Symbol;Acc:MGI:3583896]	2.13588	2.80472	2.30486	2.59679	2.66712	2.43521	2.33551	2.34096	2.79121	2.23379	2.56972	2.64599	2.59547	2.33583	0.915082018	0.966018836	20	8	24	0.769897078	0.92585938	24	13.67600614	0.024928757
ENSMUSG00000056019	Zfp709	zinc finger protein 709 [Source:MGI Symbol;Acc:MGI:2384299]	2.06419	2.38878	1.80521	2.12293	2.48519	1.7939	2.21225	2.15452	2.45876	1.53629	2.16619	2.10531	2.3377	2.30901	0.892578749	0.966018836	8	8	24	0.585103041	0.852625163	24	1.897060163	0.052710164
ENSMUSG00000056050	Mia3	melanoma inhibitory activity 3 [Source:MGI Symbol;Acc:MGI:2443183]	27.2154	23.3342	23.3994	24.7643	22.6646	26.1746	25.4211	24.8433	20.9764	31.4083	23.7676	25.7749	24.8996	25.3503	0.074438493	0.448301997	24	12	24	0.739399388	0.914968207	24	19.60153115	0.042256202
ENSMUSG00000056054	S100a8	S100 calcium binding protein A8 (calgranulin A) [Source:MGI Symbol;Acc:MGI:88244]	159.918	393.464	580.96	419.574	313.489	373.02	295.589	287.169	336.766	206.283	306.852	310.872	411.124	229.658	0.885059765	0.966018836	12	20	24	0.735930893	0.913953743	24	10.30465177	0.148799583
ENSMUSG00000056069	Otulinl	OTU deubiquitinase with linear linkage specificity like [Source:MGI Symbol;Acc:MGI:2687281]	5.00335	5.24375	6.27234	5.26396	4.69694	6.23469	4.91569	5.97717	4.56033	4.30394	4.68649	5.07321	6.17549	6.39137	0.351315142	0.870418653	8	8	24	0.180684797	0.768040178	24	2.438969256	0.112404857
ENSMUSG00000056071	S100a9	S100 calcium binding protein A9 (calgranulin B) [Source:MGI Symbol;Acc:MGI:1338947]	211.35	499.235	779.996	549.358	390.386	492.153	377.459	355.583	410.907	239.974	357.826	396.238	537.011	291.878	0.869755137	0.966018836	12	20	24	0.849552952	0.954767108	24	9.605735019	0.115693577
ENSMUSG00000056073	Grik2	glutamate receptor, ionotropic, kainate 2 (beta 2) [Source:MGI Symbol;Acc:MGI:95815]	5.11681	6.25338	5.80342	5.93376	5.61279	4.91873	5.26617	4.78488	5.32365	4.73691	5.16929	5.4962	5.31355	5.71416	0.892578749	0.966018836	12	12	24	0.698889672	0.899901033	24	7.351851134	0.033753166
ENSMUSG00000056076	Eif3b	eukaryotic translation initiation factor 3, subunit B [Source:MGI Symbol;Acc:MGI:106478]	44.4	37.7914	40.1885	44.1151	44.0163	49.1619	40.502	40.9332	36.7124	47.1131	43.073	41.6055	38.9296	42.2772	0.05238827	0.391426791	16	20	24	0.057891898	0.656248849	24	16.98668444	0.099395455
ENSMUSG00000056091	St3gal5	ST3 beta-galactoside alpha-2,3-sialyltransferase 5 [Source:MGI Symbol;Acc:MGI:1339963]	16.0156	15.2096	16.8801	18.2641	14.0946	16.0667	14.2132	18.8323	15.1854	18.2924	13.0042	13.888	15.5834	16.168	0.082113105	0.462922111	16	4	24	0.142366137	0.751766185	24	7.585173544	0.114205381
ENSMUSG00000056116	H2-T22	histocompatibility 2, T region locus 22 [Source:MGI Symbol;Acc:MGI:95956]	13.1297	12.0279	12.5024	13.0702	14.1805	13.2327	11.2683	13.9418	13.5892	12.4196	13.937	13.1362	12.7352	12.9744	0.373702442	0.896700272	20	8	24	0.36921379	0.792120801	24	14.66327476	0.044819931
ENSMUSG00000056121	Fez2	fasciculation and elongation protein zeta 2 (zygin II) [Source:MGI Symbol;Acc:MGI:2675856]	37.8653	35.3783	32.2799	33.654	35.9022	37.8108	36.7253	38.0876	32.7981	39.0233	35.4464	37.8809	38.4751	35.1643	0.042945285	0.356048751	4	8	24	0.050993998	0.645887282	24	21.31384108	0.074627077
ENSMUSG00000056124	B4galt6	UDP-Gal:betaGlcNAc beta 1,4-galactosyltransferase, polypeptide 6 [Source:MGI Symbol;Acc:MGI:1928380]	30.2901	23.655	25.0213	24.7871	25.7198	26.7723	26.34	24.2132	20.9078	26.5743	26.2171	24.7542	25.1663	25.1298	0.133476931	0.568189321	4	4	24	0.077377089	0.687324958	24	20.10243371	0.093946415
ENSMUSG00000056131	Pgm3	phosphoglucomutase 3 [Source:MGI Symbol;Acc:MGI:97566]	6.32244	6.30891	6.20161	5.82371	6.26016	6.02081	4.21945	5.10294	4.80432	5.68907	5.68768	5.54231	5.79908	5.22867	0.816300547	0.966018836	20	16	24	0.485787397	0.805393068	24	16.20937203	0.060432838
ENSMUSG00000056144	Trim34a	tripartite motif-containing 34A [Source:MGI Symbol;Acc:MGI:2137359]	1.75331	1.93708	1.60967	1.59349	2.11244	1.79349	2.15385	2.19862	2.40872	1.5926	1.90091	1.74879	1.55316	1.77287	0.92655264	0.966018836	8	8	24	0.839869477	0.95142285	24	4.12948671	0.031175654
ENSMUSG00000056145	AI504432	expressed sequence AI504432 [Source:MGI Symbol;Acc:MGI:2139742]	1.15578	1.62812	2.08644	1.54686	1.32433	1.57377	1.54794	1.19803	1.81436	1.32351	1.45708	1.66464	1.2614	1.58188	0.43565473	0.966018836	12	16	24	0.404861032	0.799546792	24	9.119311293	0.077969501
ENSMUSG00000056153	Socs6	suppressor of cytokine signaling 6 [Source:MGI Symbol;Acc:MGI:1924885]	4.0854	4.12377	4.33059	4.63894	4.48428	4.08441	4.07164	4.37369	4.12234	4.65042	4.61431	3.93926	4.34774	4.26115	0.009821544	0.194720062	16	8	24	0.018319749	0.559812298	24	12.13475646	0.061714545
ENSMUSG00000056158	Car10	carbonic anhydrase 10 [Source:MGI Symbol;Acc:MGI:1919855]	26.4915	20.1011	19.7464	18.3771	18.6049	22.4769	20.3299	22.29	17.9152	26.3493	16.7074	21.4208	20.927	24.0327	0.224031297	0.717774981	24	20	24	0.385321879	0.799546792	24	1.163196131	0.101816062
ENSMUSG00000056162	Cndp1	carnosine dipeptidase 1 (metallopeptidase M20 family) [Source:MGI Symbol;Acc:MGI:2451097]	1.03691	1.10005	1.31411	1.43212	1.91853	1.04333	1.15098	1.36536	1.44034	1.07854	1.20129	1.51542	1.04497	1.06289	0.216058318	0.704989686	20	8	24	0.212583605	0.768040178	24	13.76706589	0.100486644
ENSMUSG00000056167	Cnot10	CCR4-NOT transcription complex, subunit 10 [Source:MGI Symbol;Acc:MGI:1926143]	15.7526	16.9716	17.908	18.3758	16.7546	16.2008	17.5624	16.5305	17.7465	16.8705	16.6508	16.5048	17.662	16.8954	0.038115943	0.334069441	12	12	24	0.119989417	0.731805836	24	9.053389415	0.046251757
ENSMUSG00000056174	Col8a2	collagen, type VIII, alpha 2 [Source:MGI Symbol;Acc:MGI:88464]	4.11779	4.827	4.32763	3.52894	4.98006	3.56528	4.32828	5.01045	5.4598	3.52739	4.27067	4.94524	4.61986	4.47742	0.338172159	0.85758369	8	8	24	0.397555769	0.799546792	24	3.683629146	0.084588276
ENSMUSG00000056185	Snx32	sorting nexin 32 [Source:MGI Symbol;Acc:MGI:2444704]	38.244	36.2138	37.1533	37.7089	35.9391	41.3204	38.169	37.382	33.5728	40.9079	34.5984	35.6484	36.4449	37.2766	0.956794505	0.96629412	4	8	24	0.803590244	0.937199097	24	20.43925021	0.02172008
ENSMUSG00000056201	Cfl1	cofilin 1, non-muscle [Source:MGI Symbol;Acc:MGI:101757]	481.594	432.862	433.39	418.839	445.668	495.091	448.845	456.176	411.291	455.703	471.215	454.447	446.626	447.366	0.039704107	0.338535904	24	12	24	0.022088351	0.5765827	24	20.47399985	0.071986278
ENSMUSG00000056204	Pgpep1	pyroglutamyl-peptidase I [Source:MGI Symbol;Acc:MGI:1913772]	9.4892	6.96232	6.36672	6.32984	6.7615	8.42758	8.50099	8.24891	5.90112	6.81541	8.41925	7.32225	7.85365	6.21837	0.001837158	0.100255953	4	8	24	0.005228141	0.4424469	24	21.59542114	0.201683988
ENSMUSG00000056209	Npm3	nucleoplasmin 3 [Source:MGI Symbol;Acc:MGI:894653]	2.39821	1.5329	1.35119	1.06846	1.92758	1.79673	1.99041	1.44272	2.47781	1.54605	2.32343	1.9776	2.33319	1.89167	0.113148904	0.530777648	4	12	24	0.219492368	0.768040178	24	21.8556926	0.143145988
ENSMUSG00000056211	R3hdm1	R3H domain containing 1 [Source:MGI Symbol;Acc:MGI:2448514]	22.7949	21.5934	22.1317	22.8792	22.1186	22.1596	23.1029	21.3098	20.9226	23.9842	21.2209	21.3659	21.0622	21.8449	0.919571723	0.966018836	16	20	24	0.655919219	0.883738698	24	14.50354893	0.018770777
ENSMUSG00000056214	Pard6g	par-6 family cell polarity regulator gamma [Source:MGI Symbol;Acc:MGI:2135606]	4.09789	3.88259	3.32008	3.35249	3.74728	3.90346	3.82689	3.66107	3.47853	4.03734	3.63835	4.0637	3.37957	3.80407	0.080164531	0.458678242	24	12	24	0.216985709	0.768040178	24	21.06174595	0.056703535
ENSMUSG00000056215	Lrguk	leucine-rich repeats and guanylate kinase domain containing [Source:MGI Symbol;Acc:MGI:1921604]	1.81572	2.26999	1.75761	2.56684	1.91904	2.03999	1.66431	1.68091	1.97522	2.40342	1.54885	2.57911	2.04351	2.51029	0.893609703	0.966018836	16	12	24	0.808555131	0.939135535	24	10.80638816	0.043473582
ENSMUSG00000056216	Cebpg	CCAAT/enhancer binding protein (C/EBP), gamma [Source:MGI Symbol;Acc:MGI:104982]	14.9698	14.5915	13.9152	15.7069	15.3544	14.0861	14.618	14.2476	14.5221	14.5991	16.8562	13.4236	14.82	13.7814	0.216933149	0.704989686	20	12	24	0.195421713	0.768040178	24	15.02740892	0.052776855
ENSMUSG00000056220	Pla2g4a	phospholipase A2, group IVA (cytosolic, calcium-dependent) [Source:MGI Symbol;Acc:MGI:1195256]	1.6461	2.35061	2.27159	2.26868	2.66822	1.89788	1.67115	2.41815	3.55271	1.13339	2.12231	1.72567	1.18407	1.91312	0.03452758	0.328311403	12	16	24	0.332728733	0.769110818	24	9.366141004	0.164115584
ENSMUSG00000056222	Spock1	sparc/osteonectin, cwcv and kazal-like domains proteoglycan 1 [Source:MGI Symbol;Acc:MGI:105371]	40.3928	30.2633	33.0083	33.2124	29.8283	35.4544	33.5549	34.5847	26.4219	48.1238	27.3228	37.4523	32.2885	36.6963	0.745118544	0.966018836	24	12	24	0.92486428	0.98002538	24	22.24906258	0.035865694

ENSMUSG00000056228	Cars2	cysteinyl-tRNA synthetase 2 (mitochondrial)(putative) [Source:MGI Symbol;Acc:MGI:1919191]	9.16101	9.02494	8.91607	8.79851	9.2882	9.84142	9.64103	9.14151	8.4233	8.23676	10.0092	9.42881	9.59149	9.77347	0.006221724	0.169094792	24	16	24	0.016652223	0.544809764	24	21.45303568	0.072991648
ENSMUSG00000056234	Ncoa4	nuclear receptor coactivator 4 [Source:MGI Symbol;Acc:MGI:1350932]	6.90058	7.00925	7.42333	6.20825	6.78029	6.4014	6.25654	6.25227	6.70844	5.46451	7.5272	6.73589	7.22938	6.546	0.915835339	0.966018836	20	20	24	0.766885864	0.924587797	24	23.11574541	0.031110378
ENSMUSG00000056258	Kcnq3	potassium voltage-gated channel, subfamily Q, member 3 [Source:MGI Symbol;Acc:MGI:1336181]	4.8845	4.28929	4.66112	4.76233	4.25534	4.9184	4.94755	4.08314	4.20962	4.80758	4.69654	5.04652	4.58427	5.8826	0.454881285	0.966018836	24	12	24	0.735422045	0.91372816	24	22.16841075	0.033562838
ENSMUSG00000056260	Lrif1	ligand dependent nuclear receptor interacting factor 1 [Source:MGI Symbol;Acc:MGI:2445214]	16.3685	17.9104	14.4186	16.5831	16.64	14.3889	14.7966	15.0111	18.3577	16.1649	17.9628	14.6943	14.8188	17.3239	0.256915694	0.76168256	20	4	24	0.326462111	0.768040178	24	9.891315417	0.071362282
ENSMUSG00000056267	Cep70	centrosomal protein 70 [Source:MGI Symbol;Acc:MGI:1915371]	7.17289	9.15286	8.10272	8.9816	8.16438	7.11113	7.2321	7.14071	9.67673	8.20611	8.43927	8.39296	8.7428	9.0691	0.430715935	0.966018836	12	12	24	0.105600655	0.717832234	24	9.406026878	0.102078249
ENSMUSG00000056268	Dennd1b	DENN/MADD domain containing 1B [Source:MGI Symbol;Acc:MGI:2447812]	5.7404	5.51577	5.36917	5.77827	5.4876	5.37539	4.98774	5.08911	5.31523	5.04217	6.01276	5.14016	5.74774	5.81444	0.845386467	0.966018836	20	16	24	0.935244823	0.983765461	24	16.3517698	0.010854998
ENSMUSG00000056290	Ms4a4b	membrane-spanning 4-domains, subfamily A, member 4B [Source:MGI Symbol;Acc:MGI:1913083]	0.567615	0.989787	1.55429	0.647982	0.680516	0.66809	0.437467	1.24758	1.12855	0.36764	0.628806	0.500797	0.706969	0.514654	0.235187365	0.73629392	12	4	24	0.095855931	0.71175955	24	6.884069512	0.203614204
ENSMUSG00000056296	Synpr	synaptoporin [Source:MGI Symbol;Acc:MGI:1919253]	164.466	166.168	146.503	154.025	151.967	151.631	146.018	138.146	150.438	133.052	168.501	145.549	161.942	155.349	0.90332508	0.966018836	20	20	24	0.541062802	0.830298514	24	22.07588706	0.043936378
ENSMUSG00000056305	Usp39	ubiquitin specific peptidase 39 [Source:MGI Symbol;Acc:MGI:107622]	11.0181	10.6791	11.2352	10.0939	10.284	11.2333	10.3041	11.0978	9.19667	11.1851	10.6038	11.3333	10.6143	11.809	0.885025617	0.966018836	8	8	24	0.584896655	0.852600138	24	23.50013936	0.033820276
ENSMUSG00000056306	Sertm1	serine rich and transmembrane domain containing 1 [Source:MGI Symbol;Acc:MGI:3607715]	3.71351	3.05729	2.96111	2.64374	3.05233	4.28278	2.41961	3.2915	2.33411	3.26192	3.00716	3.39642	2.97428	2.82556	0.085890569	0.466404237	24	12	24	0.142786052	0.752449685	24	20.13127578	0.131999432
ENSMUSG00000056310	Tyw1	tRNA-yW synthesizing protein 1 homolog (S. cerevisiae) [Source:MGI Symbol;Acc:MGI:2141161]	4.15946	3.64783	4.2383	4.01555	4.0524	3.86815	3.30146	4.0544	3.67397	4.57943	3.96328	3.771	4.50832	3.49939	0.886952527	0.966018836	16	16	24	0.494797878	0.806761911	24	13.45345271	0.048806116
ENSMUSG00000056313	Tcim	transcriptional and immune response regulator [Source:MGI Symbol;Acc:MGI:1916318]	8.9386	10.6031	11.2777	14.2139	11.8967	9.16012	7.90252	9.99799	13.0328	13.9424	9.22842	9.51577	10.4824	9.24852	7.90E-04	0.071344007	16	12	24	0.001415016	0.354367925	24	10.99210671	0.267005691
ENSMUSG00000056316	Gm9996	predicted gene 9996 [Source:MGI Symbol;Acc:MGI:3642549]	0.552188	0.580653	0.356924	0.504568	0.649574	0.526941	0.200049	0.490844	0.488867	0.612305	0.491802	0.657344	0.325682	0.654289	0.843639218	0.966018836	24	4	24	0.553789717	0.837374348	24	13.94273521	0.057284759
ENSMUSG00000056328	Myh1	myosin, heavy polypeptide 1, skeletal muscle, adult [Source:MGI Symbol;Acc:MGI:1339711]	7.01505	12.7557	21.7072	33.516	23.5535	25.1271	10.3228	68.7657	21.5894	54.1852	12.3999	11.8242	23.2609	17.8576	0.06180207	0.409814092	16	12	24	0.141388268	0.751766185	24	10.10126627	0.648426589
ENSMUSG00000056342	Usp34	ubiquitin specific peptidase 34 [Source:MGI Symbol;Acc:MGI:109473]	37.9228	39.884	37.8076	41.6923	39.3027	34.9085	34.7103	35.1878	39.8471	37.2027	38.7601	37.0002	37.7779	37.947	0.176949612	0.653256853	20	4	24	0.122260903	0.735058232	24	11.04740072	0.056725091
ENSMUSG00000056366	Fabp3-ps1	fatty acid binding protein 3, muscle and heart, pseudogene 1 [Source:MGI Symbol;Acc:MGI:101929]	86.3507	109.741	119.538	134.927	101.758	111.75	83.4329	176.659	113.178	119.275	101.849	86.2889	122.444	117.143	0.121531327	0.548996929	16	4	24	0.098711919	0.71175955	24	8.072918494	0.231815333
ENSMUSG00000056367	Actr3b	ARP3 actin-related protein 3B [Source:MGI Symbol;Acc:MGI:2661120]	33.9053	27.7608	28.3429	29.5637	25.9847	29.8933	26.6604	27.3987	25.8761	31.4617	26.8001	30.0079	29.249	30.0833	0.953350544	0.966018836	24	12	24	0.719460553	0.907983128	24	22.35863846	0.035891053
ENSMUSG00000056383	AI987944	expressed sequence AI987944 [Source:MGI Symbol;Acc:MGI:2142079]	6.49351	7.7819	7.43662	6.8508	7.52092	7.09802	6.38045	6.85371	8.88585	5.51139	7.39116	7.71763	7.06549	6.90858	0.883024237	0.966018836	12	16	24	0.934743077	0.983765461	24	7.565928558	0.021806462
ENSMUSG00000056394	Lig1	ligase I, DNA, ATP-dependent [Source:MGI Symbol;Acc:MGI:101789]	4.89942	5.46746	6.76245	5.39509	4.93711	5.75524	4.61638	5.43323	5.64258	6.29855	5.40081	5.83022	6.0715	5.74881	0.943890799	0.966018836	12	16	24	0.245320745	0.768040178	24	9.271459765	0.079162387
ENSMUSG00000056399	Prss34	protease, serine 34 [Source:MGI Symbol;Acc:MGI:2681414]	0.788489	1.48924	1.59288	1.98507	1.12654	1.068	1.23773	1.75059	1.12928	0.91771	0.982639	0.932192	1.49882	0.83849	0.793403332	0.966018836	24	12	24	0.423067677	0.805102873	24	7.641632806	0.117441936
ENSMUSG00000056412	Psenen-ps	presenilin enhancer gamma secretase subunit, pseudogene [Source:MGI Symbol;Acc:MGI:3649201]	69.7737	70.389	71.535	66.0982	77.281	72.611	75.7216	71.3977	75.9792	56.0821	72.28	67.177	70.2778	69.3466	0.93717134	0.966018836	20	20	24	0.502779246	0.809737567	24	23.40676464	0.051297631
ENSMUSG00000056413	Adap1	ArfGAP with dual PH domains 1 [Source:MGI Symbol;Acc:MGI:2442201]	54.8928	45.623	47.354	47.969	52.3108	53.9012	52.137	49.7705	44.1419	59.3181	47.1566	51.4066	51.6984	49.9652	0.109879661	0.525500915	24	12	24	0.255731522	0.768040178	24	19.46818504	0.073361238
ENSMUSG00000056418	BC043934	cDNA sequence BC043934 [Source:MGI Symbol;Acc:MGI:2679715]	0.983291	1.45125	1.12011	1.04934	1.03306	1.08254	1.23519	1.31437	1.46062	1.40979	1.43343	1.42357	1.31297	1.50655	0.481530854	0.966018836	8	20	24	0.149487237	0.758126113	24	6.446828654	0.065387479
ENSMUSG00000056423	Uts2b	urotensin 2B [Source:MGI Symbol;Acc:MGI:2677064]	1.42104	6.16518	5.3428	7.72807	3.62718	5.19951	3.97071	2.01214	5.25268	3.48203	3.13044	3.02471	2.76371	2.66089	0.226403091	0.722427815	12	16	24	0.185903936	0.768040178	24	11.40184285	0.338313195
ENSMUSG00000056427	Slit3	slit guidance ligand 3 [Source:MGI Symbol;Acc:MGI:1315202]	1.03847	0.904045	1.46879	1.13672	1.30743	1.30851	1.46848	1.19378	1.20889	1.84064	1.06918	1.13348	1.23254	1.34812	0.890235289	0.966018836	12	20	24	0.497401604	0.807576732	24	11.31173139	0.064597259
ENSMUSG00000056429	Tgoln1	trans-golgi network protein [Source:MGI Symbol;Acc:MGI:105080]	76.3504	70.6886	70.8928	74.1554	74.7073	73.5686	72.2806	69.9894	65.2874	69.0207	76.9245	74.5351	74.2942	75.0674	0.064422003	0.414867207	20	16	24	0.039555322	0.615919442	24	19.79719912	0.060032558
ENSMUSG00000056445	Hoxaas2	Hoxa cluster antisense RNA 2 [Source:MGI Symbol;Acc:MGI:1913890]	2.23779	2.1446	1.91845	1.82797	2.26809	2.17688	2.43551	2.04901	2.05995	1.86112	2.38625	2.1278	1.77808	2.11894	0.054328576	0.395890765	20	20	24	0.204658977	0.768040178	24	20.75928224	0.065700197
ENSMUSG00000056458	Mok	MOK protein kinase [Source:MGI Symbol;Acc:MGI:1336881]	2.36071	2.1863	1.92179	2.34683	1.82847	1.67299	1.60884	1.53339	1.74841	1.3639	2.02284	2.05325	1.85487	1.58495	0.874054778	0.966018836	20	12	24	0.679136561	0.892648327	24	19.32831776	0.04865473
ENSMUSG00000056459	Zbtb25	zinc finger and BTB domain containing 25 [Source:MGI Symbol;Acc:MGI:99197]	5.43175	6.58966	6.68642	5.55371	6.1384	5.87826	5.70155	6.06048	7.33257	6.66626	5.67693	6.6063	6.62251	6.29559	0.310940564	0.820837787	12	8	24	0.157052	0.763141258	24	7.740700914	0.07748309
ENSMUSG00000056476	Med12l	mediator complex subunit 12-like [Source:MGI Symbol;Acc:MGI:2139916]	4.37966	4.3701	4.901	4.63568	4.43466	4.66491	4.51409	4.2783	3.56875	4.91466	4.43874	4.80505	4.41285	4.87208	0.633249925	0.966018836	16	16	24	0.721735596	0.90866625	24	17.54837968	0.031523788
ENSMUSG00000056481	Cd248	CD248 antigen, endosialin [Source:MGI Symbol;Acc:MGI:1917695]	1.91289	2.51704	2.09961	1.77674	2.36838	1.61121	2.02144	2.43354	2.88586	1.28469	2.13881	2.01659	1.91553	2.03907	0.119598685	0.54287566	12	12	24	0.398159531	0.799546792	24	5.065721855	0.101800397
ENSMUSG00000056486	Chn1	chimerin 1 [Source:MGI Symbol;Acc:MGI:1915674]	94.0625	85.3339	86.9932	83.3073	89.63	97.1592	94.8448	85.4458	72.0026	89.6643	81.938	87.3619	86.4948	85.056	0.133576635	0.568189321	24	12	24	0.028025275	0.590904845	24	20.96834216	0.09783703
ENSMUSG00000056492	Adgrf5	adhesion G protein-coupled receptor F5 [Source:MGI Symbol;Acc:MGI:2182928]	11.3917	11.2088	11.8712	14.0387	11.3969	12.4741	10.5299	14.0619	10.9096	15.2267	9.4727	11.2621	11.8712	10.5079	0.952551093	0.966018836	16	16	24	0.507284595	0.812455891	24	10.51687578	0.080094126
ENSMUSG00000056493	Foxk1	forkhead box K1 [Source:MGI Symbol;Acc:MGI:1347488]	3.44504	3.21896	3.29509	3.36037	3.43612	3.49281	3.71583	3.5605	3.4071	5.14519	3.26297	3.68797	3.57071	3.81591	0.759899911	0.966018836	4	8	24	0.74532381	0.916764484	24	11.62130051	0.038121351
ENSMUSG00000056498	Tmem154	transmembrane protein 154 [Source:MGI Symbol;Acc:MGI:2444725]	1.90322	2.21261	2.42256	2.0345	2.08607	2.2976	2.24717	2.15565	2.21292	1.73211	1.92593	2.24247	2.14469	2.31707	0.265774856	0.773510818	12	4	24	0.278264923	0.768040178	24	2.780402119	0.053437853
ENSMUSG00000056501	Cebpb	CCAAT/enhancer binding protein (C/EBP), beta [Source:MGI Symbol;Acc:MGI:88373]	1.73766	1.32527	2.19357	1.97081	1.49874	1.25797	1.27883	2.2614	2.0292	3.04106	3.00812	1.88877	3.0785	2.26192	0.85447369	0.966018836	16	8	24	0.19428403	0.768040178	24	10.23580811	0.168125569
ENSMUSG00000056508	1700001K19Rik	RIKEN cDNA 1700001K19 gene [Source:MGI Symbol;Acc:MGI:1913573]	1.15426	1.02009	1.2006	0.690594	0.788649	1.13924	0.979594	0.763787	0.753955	1.39259	0.897049	1.18419	1.20357	1.04977	0.61767505	0.966018836	24	20	24	0.628157279	0.872795898	24	22.60398978	0.059346175
ENSMUSG00000056515	Rab31	RAB31, member RAS oncogene family [Source:MGI Symbol;Acc:MGI:1914603]	11.4667	13.7801	14.6383	12.0488	12.505	12.1503	14.3251	12.988	13.4191	12.0594	12.5539	12.6975	12.3458	12.4737	0.265774856	0.773510818	12	4	24	0.289256949	0.768040178	24	5.85184903	0.057909948
ENSMUSG00000056529	Ptafr	platelet-activating factor receptor [Source:MGI Symbol;Acc:MGI:106066]	0.933875	0.887279	1.06614	0.986284	1.20181	0.97019	0.988813	0.952627	1.1779	0.774628	0.850205	0.895683	1.1637	0.77419	0.924770496	0.966018836	12	20	24	0.98502391	0.996964449	24	17.8099217	0.007134515
ENSMUSG00000056536	Pign	phosphatidylinositol glycan anchor biosynthesis, class N [Source:MGI Symbol;Acc:MGI:1351629]	3.6167	5.48398	4.9112	4.50112	4.61181	3.96997	4.44365	4.90389	5.12393	3.37545	4.16432	4.57083	4.06778	5.52957	0.045120335	0.368634624	8	20	24	0.117908906	0.728323355	24	5.867285894	0.134982946
ENSMUSG00000056537	Rlim	ring finger protein, LIM domain interacting [Source:MGI Symbol;Acc:MGI:1342291]	11.5011	12.8285	12.7664	12.3994	12.5178	11.3609	11.8604	10.8024	12.2913	11.1419	12.7294	11.9005	12.07	12.3876	0.777666236	0.966018836	20	4	24	0.657076029	0.884431998	24	11.00030784	0.026556688
ENSMUSG00000056553	Ptprn2	protein tyrosine phosphatase, receptor type, N polypeptide 2 [Source:MGI Symbol;Acc:MGI:107418]	13.1441	10.3917	13.1334	11.386	9.5514	13.5862	13.2738	11.8496	8.22886	16.6639	8.77804	11.4247	10.1986	13.1059	0.893609703	0.966018836	24	20	24	0.902924921	0.972952843	24	1.597415419	0.046098868
ENSMUSG00000056569	Mpz	myelin protein zero [Source:MGI Symbol;Acc:MGI:103177]	3400.71	3210.38	2947.36	3226.58	4180.19	3342.08	4427.25	3699.75	3562.57	4767.33	3132.68	3717.39	3751.15	3061.83	0.772442842	0.966018836	4	8	24	0.693931644	0.898364349	24	17.86049278	0.071441194
ENSMUSG00000056579	Tug1	taurine upregulated gene 1 [Source:MGI Symbol;Acc:MGI:2144114]	23.8318	25.3652	24.3784	24.6971	25.393	23.135	23.8057	22.5119	26.1619	21.7237	26.4239	25.1453	25.1403	24.9049	0.935194469	0.966018836	20	8	24	0.949493247	0.987927248	24	14.9877237	0.009971221
ENSMUSG00000056592	Zfp658	zinc finger protein 658 [Source:MGI Symbol;Acc:MGI:2652821]	2.15398	2.24342	1.97771	2.1941	2.56436	2.216	2.38119	2.19278	2.39119	1.92122	2.64243	2.31988	2.08018	2.00675	0.280810068	0.792139038	20	20	24	0.189322351	0.768040178	24	17.79499987	0.066185042
ENSMUSG00000056596	Trnp1	TMF1-regulated nuclear protein 1 [Source:MGI Symbol;Acc:MGI:1916789]	363.134	289.589	269.887	307.206	317.001	346.504	288.994	315.571	245.55	317.588	292.26	308.553	310.701	300.467	0.236893212	0.7385769	24	12	24	0.052695627	0.650526959	24	20.3675688	0.124388551
ENSMUSG00000056598	Drc3	dynein regulatory complex subunit 3 [Source:MGI Symbol;Acc:MGI:1921915]	2.94388	3.52148	3.60946	2.99248	2.83568	2.46659	2.9029	2.52781	2.86571	2.79258	3.08535	3.17277	2.42315	3.87603	0.839634479	0.966018836	8	20	24	0.389221253	0.799546792	24	7.328886393	0.084660443
ENSMUSG00000056602	Fry	FRY microtubule binding protein [Source:MGI Symbol;Acc:MGI:2443895]	17.0004	15.5083	17.1642	19.0039	13.8681	17.2426	16.531	18.2808	15.4304	22.3166	15.0328	16.9239	16.81	17.9878	0.503972924	0.966018836	16	4	24	0.568529612	0.844419134	24	8.54457513	0.068051792
ENSMUSG00000056608	Chd9	chromodomain helicase DNA binding protein 9 [Source:MGI Symbol;Acc:MGI:1924001]	8.67432	8.9151	8.70497	9.62113	9.27957	8.38036	8.40392	7.84891	9.37138	7.60027	9.64963	9.38599	8.74579	9.1779	0.725061903	0.966018836	20	8	24	0.633353305	0.875353057	24	15.18615383	0.035539049
ENSMUSG00000056612	Ppp1r14b	protein phosphatase 1, regulatory inhibitor subunit 14B [Source:MGI Symbol;Acc:MGI:107682]	24.7173	23.8009	21.6671	26.266	24.1105	22.6839	24.0306	25.1956	24.8644	23.3037	28.0426	21.6086	26.1731	22.4029	0.880247609	0.966018836	20	4	24	0.84503057	0.953147907	24	14.81319614	0.024325418
ENSMUSG00000056629	Fkbp2	FK506 binding protein 2 [Source:MGI Symbol;Acc:MGI:95542]	90.309	78.9698	74.2833	70.6558	75.4637	76.7202	80.3706	70.6337	67.7883	67.591	78.2291	82.5464	80.8473	74.5518	7.56E-04	0.071344007	4	12	24	7.74E-04	0.319044294	24	22.03110177	0.13395997
ENSMUSG00000056648	Hoxb8	homeobox B8 [Source:MGI Symbol;Acc:MGI:96189]	4.7428	6.04599	8.06687	4.14333	3.50813	4.80414	6.56508	5.18709	8.02876	6.57273	4.26532	8.79145	4.23002	7.25382	0.185796491	0.662239322	12	8	24	0.2312806	0.768040178	24	5.998931863	0.234563648
ENSMUSG00000056656	Apol8	apolipoprotein L 8 [Source:MGI Symbol;Acc:MGI:2444921]	1.0053	1.6097	1.98244	1.24366	0.960743	1.73731	1.65116	1.2364	1.08165	1.20981	1.04946	1.30257	1.06853	1.54975	0.784278077	0.966018836	8	12	24	0.505516122	0.811334839	24	4.147108942	0.086400527
ENSMUSG00000056665	Them6	thioesterase superfamily member 6 [Source:MGI Symbol;Acc:MGI:1925301]	41.4347	29.8537	29.4995	32.4694	30.1077	34.0726	32.2888	35.4309	27.2975	45.0455	34.4245	37.6828	35.3414	35.2068	0.321662653	0.83607295	24	12	24	0.584970106	0.852625163	24	20.03092482	0.079072489
ENSMUSG00000056666	Retsat	retinol saturase (all trans retinol 13,14 reductase) [Source:MGI Symbol;Acc:MGI:1914692]	4.29239	4.77742	8.13646	8.98369	5.5798	6.20806	6.34714	8.16189	6.63039	7.41553	6.28397	5.87132	6.61609	6.97235	0.056291566	0.401074772	16	8	24	0.05419897	0.654887447	24	9.859805848	0.21664629
ENSMUSG00000056671	Prelid2	PRELI domain containing 2 [Source:MGI Symbol;Acc:MGI:1924869]	2.31321	2.09226	1.5949	1.42181	1.7712	1.40718	1.72011	1.46372	1.73293	1.23021	2.90658	1.80065	2.63271	1.73133	0.305564675	0.811201458	4	12	24	0.380837876	0.799546792	24	22.09240935	0.128320218
ENSMUSG00000056673	Kdm5d	lysine (K)-specific demethylase 5D [Source:MGI Symbol;Acc:MGI:99780]	4.83688	6.21484	5.17821	5.44228	5.44849	5.41955	5.38888	5.66489	6.43241	4.84232	5.149	5.57	5.43051	5.86178	0.325142889	0.842977406	8	20	24	0.23860506	0.768040178	24	5.464620075	0.065162428
ENSMUSG00000056679	Gpr173	G-protein coupled receptor 173 [Source:MGI Symbol;Acc:MGI:1918021]	6.78775	6.50264	5.93067	6.33082	6.74729	7.5062	6.55373	6.80218	5.92207	7.69705	6.31594	7.22223	7.51299	7.3394	0.148073804	0.590732968	24	12	24	0.218646321	0.768040178	24	21.12450114	0.066104583
ENSMUSG00000056687	Gm11696	predicted gene 11696 [Source:MGI Symbol;Acc:MGI:3701776]	2.03881	1.53244	1.96589	1.40286	1.88009	2.13271	1.50839	1.79355	2.11539	1.9937	1.42602	2.17663	1.76656	1.67778	0.892555977	0.966018836	24	8	24	0.919679388	0.978586305	24	20.31449241	0.024420553
ENSMUSG00000056692	Ilrun	inflammation and lipid regulator with UBA-like and NBR1-like domains [Source:MGI Symbol;Acc:MGI:106281]	46.3603	40.02	39.4383	43.2727	43.8903	44.8698	44.6731	45.6845	40.5231	55.0052	38.6628	46.4121	45.1741	43.7458	0.245061943	0.741580974	24	12	24	0.815924243	0.941295238	24	19.16036151	0.03294168
ENSMUSG00000056698	Elmod3	ELMO/CED-12 domain containing 3 [Source:MGI Symbol;Acc:MGI:2445168]	4.84832	5.08504	5.59186	5.43495	4.7325	5.53844	6.88753	5.74939	5.07199	5.58768	4.90563	5.25949	4.96379	5.72103	0.305564675	0.811201458	8	12	24	0.5512206	0.83632237	24	3.750782132	0.051157725
ENSMUSG00000056708	Ier5	immediate early response 5 [Source:MGI Symbol;Acc:MGI:1337072]	2.2245	2.17469	2.11695	2.17179	1.84875	1.75831	2.72006	2.11476	2.21127	1.91389	2.30165	2.28202	2.71614	2.23624	0.217660303	0.704989686	4	12	24	0.15669364	0.762682854	24	1.600958884	0.082532911
ENSMUSG00000056718	Gm13199	predicted gene 13199 [Source:MGI Symbol;Acc:MGI:3649231]	0.870252	1.39843	1.38272	1.1019	1.51884	1.39437	1.33625	0.681582	1.11459	0.49738	1.64152	0.877987	0.733835	1.14525	0.892555977	0.966018836	20	20	24	0.689046673	0.897070579	24	17.01415852	0.081983485
ENSMUSG00000056724	Nbeal2	neurobeachin-like 2 [Source:MGI Symbol;Acc:MGI:2448554]	4.55506	3.28304	3.11443	3.25633	2.97128	3.94385	4.00282	2.74889	3.98422	3.02497	4.96577	3.70624	3.57947	2.87153	0.352925135	0.87272957	4	4	24	0.190096185	0.768040178	24	19.76820071	0.145347209
ENSMUSG00000056735	A930024E05Rik	RIKEN cDNA A930024E05 gene [Source:MGI Symbol;Acc:MGI:1924414]	1.41739	1.80327	1.81588	1.47524	1.39279	1.73457	1.73209	1.76589	1.89301	1.2985	1.7828	1.5208	1.5794	1.36632	0.369262716	0.893596186	12	4	24	0.643586416	0.878958277	24	4.387094481	0.041220163
ENSMUSG00000056737	Capg	capping protein (actin filament), gelsolin-like [Source:MGI Symbol;Acc:MGI:1098259]	41.8732	42.7809	41.1395	39.4683	47.9101	38.6297	48.4426	43.6302	49.5657	43.5069	36.9544	43.9633	44.9401	38.7389	0.890311217	0.966018836	4	16	24	0.838357855	0.95056415	24	3.396232527	0.030115453
ENSMUSG00000056749	Nfil3	nuclear factor, interleukin 3, regulated [Source:MGI Symbol;Acc:MGI:109495]	3.67829	1.77465	1.96674	2.67133	2.75145	2.98477	3.61189	2.4067	2.13915	1.85358	2.94999	2.18766	2.81023	2.07955	0.009428683	0.194720062	4	8	24	0.026418493	0.586541342	24	20.6216067	0.23419099
ENSMUSG00000056752	Dnah9	dynein, axonemal, heavy chain 9 [Source:MGI Symbol;Acc:MGI:1289279]	0.755713	0.572159	0.824099	0.672625	0.316999	0.423039	0.650322	0.194021	0.371898	0.448019	0.500458	0.681772	0.450889	0.534855	0.859936714	0.966018836	4	16	24	0.92779186	0.98127006	24	22.53177145	0.025868144
ENSMUSG00000056753	C330011M18Rik	RIKEN cDNA C330011M18 gene [Source:MGI Symbol;Acc:MGI:3045248]	0.634507	0.502269	0.716031	0.51446	0.406489	0.38874	0.315887	0.654287	0.5146	0.713971	0.551363	0.537497	0.48398	0.609832	0.23734581	0.739722813	12	12	24	0.128341653	0.740162193	24	8.319630815	0.089162168
ENSMUSG00000056755	Grm7	glutamate receptor, metabotropic 7 [Source:MGI Symbol;Acc:MGI:1351344]	19.4699	18.698	17.8768	16.1482	15.1006	19.0449	16.1171	16.4697	13.0184	19.3234	15.5779	17.6949	17.5959	19.3372	0.644063931	0.966018836	8	12	24	0.436977187	0.805393068	24	23.88370738	0.08017762
ENSMUSG00000056763	Cspp1	centrosome and spindle pole associated protein 1 [Source:MGI Symbol;Acc:MGI:2681832]	11.312	12.935	12.8739	12.6606	14.1673	11.5708	15.8162	11.489	14.4102	12.0612	13.9115	13.2497	11.9836	11.8434	0.849179819	0.966018836	12	20	24	0.71568784	0.906323093	24	14.6209065	0.043308735
ENSMUSG00000056770	Setd3	SET domain containing 3 [Source:MGI Symbol;Acc:MGI:1289184]	46.0109	43.1125	42.2013	44.1448	40.532	43.274	43.4936	43.1306	39.6161	45.8034	40.3444	44.2859	42.6269	43.1385	0.454881285	0.966018836	24	12	24	0.601055403	0.860341846	24	23.42797608	0.024709683
ENSMUSG00000056771	Gm10010	predicted gene 10010 [Source:MGI Symbol;Acc:MGI:3641978]	0.996611	0.904004	1.19682	1.08358	0.867105	0.894632	1.13152	0.538151	0.909297	1.05211	1.11168	1.32051	1.03997	1.16061	0.813775138	0.966018836	24	8	24	0.707919937	0.903565455	24	16.62820298	0.046818365
ENSMUSG00000056812	St8sia3	ST8 alpha-N-acetyl-neuraminide alpha-2,8-sialyltransferase 3 [Source:MGI Symbol;Acc:MGI:106019]	43.4299	38.1064	39.5916	34.9304	40.7409	43.3003	38.072	35.2605	32.8494	36.1389	40.4788	41.7086	36.9427	38.8631	0.087130946	0.470523339	24	16	24	0.010166382	0.503967859	24	20.17922967	0.121693553
ENSMUSG00000056820	Tsnax	translin-associated factor X [Source:MGI Symbol;Acc:MGI:1855672]	55.5931	53.6269	53.3339	53.8953	54.4897	55.2214	55.9134	53.7905	49.4034	55.4622	51.9063	53.2169	55.1156	53.6004	0.062631097	0.410322149	4	8	24	0.113124617	0.727393008	24	20.75281617	0.037165205
ENSMUSG00000056824	Zfp663	zinc finger protein 663 [Source:MGI Symbol;Acc:MGI:2685854]	0.449392	1.35712	0.957053	1.14086	0.918578	1.08993	0.597843	1.07763	1.22771	1.26161	0.810403	1.10689	0.667764	1.11392	0.036134798	0.331852526	16	12	24	0.084318814	0.702277994	24	9.947667411	0.170810925
ENSMUSG00000056832	Ttc26	tetratricopeptide repeat domain 26 [Source:MGI Symbol;Acc:MGI:2444853]	1.98079	2.57145	2.62722	2.06235	2.42627	2.45122	2.3277	2.50069	2.2716	2.15808	2.42154	2.59418	2.64242	2.5714	0.718720526	0.966018836	8	8	24	0.555899739	0.838702246	24	1.965821886	0.036223999
ENSMUSG00000056836	Gm6851	predicted pseudogene 6851 [Source:MGI Symbol;Acc:MGI:3643618]	53.5981	61.1267	56.225	47.9357	52.4502	48.432	48.9679	46.7137	61.408	44.875	56.2	55.68	63.0604	59.6283	0.676344318	0.966018836	12	4	24	0.426067883	0.805393068	24	3.477986879	0.078558249
ENSMUSG00000056849	Gm16415	predicted pseudogene 16415 [Source:MGI Symbol;Acc:MGI:3647855]	1.96703	1.05795	0.80061	1.25285	1.18016	0.825956	0.597337	0.654769	1.16888	0.975586	1.7573	0.871283	0.742648	1.03125	0.465479825	0.966018836	20	8	24	0.475818656	0.805393068	24	15.19698133	0.118790191
ENSMUSG00000056851	Pcbp2	poly(rC) binding protein 2 [Source:MGI Symbol;Acc:MGI:108202]	124.515	121.683	121.907	145.024	140.656	130.41	141.095	122.215	133.738	142.21	141.711	131.341	127.417	131.126	0.002034622	0.100255953	16	16	24	0.012793511	0.526249719	24	14.79065828	0.086811786
ENSMUSG00000056856	Jakmip3	janus kinase and microtubule interacting protein 3 [Source:MGI Symbol;Acc:MGI:1921254]	5.70894	5.71736	4.82964	4.73522	4.99583	5.40329	5.53664	5.27509	4.17602	5.48848	4.2772	5.23847	5.04105	5.10525	0.097373374	0.501112642	8	4	24	0.06873931	0.673096105	24	23.62826628	0.093848852
ENSMUSG00000056870	Gulp1	GULP, engulfment adaptor PTB domain containing 1 [Source:MGI Symbol;Acc:MGI:1920407]	22.7151	28.2621	22.7907	22.7018	25.2046	20.6091	26.993	21.8817	26.9425	17.4025	25.0437	19.7354	20.6814	20.3068	0.934152439	0.966018836	24	4	24	0.849541567	0.954767108	24	3.970735846	0.03961831
ENSMUSG00000056880	Gad1l	glutamate decarboxylase-like 1 [Source:MGI Symbol;Acc:MGI:1920998]	17.7682	19.0078	18.138	16.8848	18.7492	15.5098	16.3628	17.7855	22.3675	16.1578	21.0723	18.2961	20.5538	19.2042	0.888991156	0.966018836	12	12	24	0.601669426	0.860619632	24	7.236088977	0.056141404
ENSMUSG00000056888	Glipr1	GLI pathogenesis-related 1 (glioma) [Source:MGI Symbol;Acc:MGI:1920940]	0.628279	2.11259	2.24345	2.4685	1.35867	2.6068	1.58234	1.73489	1.62372	0.946541	1.48781	1.55929	1.67264	0.892706	0.928127947	0.966018836	12	8	24	0.87416756	0.964836189	24	12.76922505	0.06727929
ENSMUSG00000056895	H2bu2	H2B.U histone 2 [Source:MGI Symbol;Acc:MGI:1925553]	10.0923	8.79968	9.42488	8.57095	8.81445	8.8784	10.8829	9.23076	10.7894	12.8157	14.1057	13.5673	14.9807	13.9751	0.935316621	0.966018836	4	12	24	0.742872929	0.916215545	24	22.09894125	0.044701869
ENSMUSG00000056899	Immp2l	IMP2 inner mitochondrial membrane peptidase-like (S. cerevisiae) [Source:MGI Symbol;Acc:MGI:2135611]	7.70352	7.32481	8.12602	7.20906	9.31785	6.58477	3.92034	13.1659	8.43411	7.40925	9.45915	6.18698	10.7397	7.61532	0.917867465	0.966018836	12	12	24	0.622191101	0.871331547	24	8.607556735	0.142152022
ENSMUSG00000056900	Usp13	ubiquitin specific peptidase 13 (isopeptidase T-3) [Source:MGI Symbol;Acc:MGI:1919857]	10.9274	9.28588	9.7611	11.0138	9.64509	12.3661	10.0548	13.3306	9.45403	13.7125	9.34838	11.1497	10.7026	10.4236	0.935316621	0.966018836	12	8	24	0.978464512	0.994576186	24	18.6736083	0.014701412
ENSMUSG00000056904	Gm5620	predicted gene 5620 [Source:MGI Symbol;Acc:MGI:3647798]	1.50638	1.14074	1.2382	1.27055	1.10565	1.3722	1.2673	1.50144	0.846909	1.21616	0.945777	0.660236	1.04631	0.771585	0.853786882	0.966018836	4	16	24	0.826978364	0.946041107	24	0.365340267	0.032891557
ENSMUSG00000056917	Sipa1	signal-induced proliferation associated gene 1 [Source:MGI Symbol;Acc:MGI:107576]	6.84246	5.31251	5.93251	7.34422	7.1323	6.30501	6.50831	6.35071	5.93502	6.15531	7.10181	4.4111	6.8642	4.86812	0.208438456	0.698704663	20	12	24	0.417944194	0.802574655	24	15.70771674	0.089660784
ENSMUSG00000056919	Cep162	centrosomal protein 162 [Source:MGI Symbol;Acc:MGI:1925343]	3.00802	2.99707	3.17135	2.6973	2.62599	2.95836	3.85485	2.40288	2.82357	3.81798	3.07074	2.6215	2.78276	2.67379	0.896773941	0.966018836	12	20	24	0.952240769	0.988398268	24	14.44169512	0.018413316
ENSMUSG00000056938	Acbd4	acyl-Coenzyme A binding domain containing 4 [Source:MGI Symbol;Acc:MGI:1914381]	46.2696	42.6151	44.4819	43.239	46.7563	51.3342	49.5995	51.3488	37.4653	44.2544	41.1476	45.3654	44.2383	41.0351	0.505728924	0.966018836	8	4	24	0.09566359	0.71175955	24	21.38916216	0.099602865
ENSMUSG00000056941	Commd7	COMM domain containing 7 [Source:MGI Symbol;Acc:MGI:1914197]	23.1916	21.1819	17.7399	20.5196	22.8201	22.3593	21.4373	20.5569	20.2256	19.039	22.2401	21.662	22.9371	20.6436	0.003285255	0.140598674	4	8	24	0.002974164	0.414576087	24	20.78409778	0.117670459
ENSMUSG00000056943	Gm10015	predicted gene 10015 [Source:MGI Symbol;Acc:MGI:3809114]	0.664189	1.00811	1.41702	1.9522	1.46881	1.85603	0.645085	1.17786	1.35228	1.40567	1.08363	1.60849	1.05108	1.42195	0.019287464	0.257669847	24	4	24	0.076192863	0.686372559	24	12.97376836	0.203743524
ENSMUSG00000056947	Mab21l1	mab-21-like 1 [Source:MGI Symbol;Acc:MGI:1333773]	3.47236	2.71027	3.3422	2.68906	2.59663	3.28761	2.57723	2.1179	2.54569	2.84749	2.63441	3.05532	2.35069	3.47116	0.801273005	0.966018836	24	20	24	0.914468021	0.976044534	24	20.63344602	0.028157532
ENSMUSG00000056952	Tatdn2	TatD DNase domain containing 2 [Source:MGI Symbol;Acc:MGI:3576210]	14.1964	13.3695	14.5363	14.5144	12.5963	14.2661	12.7973	12.9325	13.3422	12.8971	12.6479	13.8509	13.5883	12.8953	0.880631923	0.966018836	24	4	24	0.756017452	0.921194485	24	20.91935418	0.021309337
ENSMUSG00000056962	Jmjd6	jumonji domain containing 6 [Source:MGI Symbol;Acc:MGI:1858910]	13.6914	12.053	11.882	12.6645	12.6663	12.4715	13.9069	11.9659	11.9951	11.8408	13.9198	11.1631	11.4532	10.9099	0.711168999	0.966018836	20	12	24	0.241034588	0.768040178	24	18.43668353	0.060401188
ENSMUSG00000056966	Gjc3	gap junction protein, gamma 3 [Source:MGI Symbol;Acc:MGI:2153041]	35.1764	39.1218	36.6321	32.4317	44.4573	34.0561	42.6945	35.1973	37.2757	33.7834	38.8995	32.0812	33.4832	30.8374	0.90767227	0.966018836	20	4	24	0.79191202	0.93349015	24	17.59950194	0.039044401
ENSMUSG00000056973	Ces1d	carboxylesterase 1D [Source:MGI Symbol;Acc:MGI:2148202]	4.78303	3.15375	7.189	27.7945	8.1262	6.23721	6.99466	9.39392	8.71481	15.2496	18.1439	11.2639	18.1322	11.9388	0.483374602	0.966018836	16	12	24	0.061341188	0.661066697	24	13.11141741	0.520905144
ENSMUSG00000056999	Ide	insulin degrading enzyme [Source:MGI Symbol;Acc:MGI:96412]	29.8006	25.1948	27.2059	22.5915	20.353	20.008	24.9889	19.4151	19.5837	21.5931	21.4645	19.3862	19.4177	26.7163	0.591351901	0.966018836	12	12	24	0.314118935	0.768040178	24	3.837506242	0.104892841
ENSMUSG00000057003	Myh4	myosin, heavy polypeptide 4, skeletal muscle [Source:MGI Symbol;Acc:MGI:1339713]	23.281	58.5868	62.164	99.2803	47.9151	95.8009	65.5668	197.814	143.573	213.339	57.9264	92.1003	89.094	62.5725	0.745118544	0.966018836	16	12	24	0.194579224	0.768040178	24	9.684541763	0.530113976
ENSMUSG00000057036	Gm7536	predicted gene 7536 [Source:MGI Symbol;Acc:MGI:3645137]	307.081	320.316	306.162	279.171	274.446	246.743	296.149	313.379	392.396	263.89	350.031	323.544	366.143	343.867	0.591033264	0.966018836	8	8	24	0.164049681	0.767020009	24	4.840860273	0.120847147
ENSMUSG00000057054	Inca1	inhibitor of CDK, cyclin A1 interacting protein 1 [Source:MGI Symbol;Acc:MGI:2144284]	0.952793	1.52981	1.69559	1.99949	1.50027	1.33847	1.23192	1.60767	1.51392	2.17279	1.35977	1.86378	1.29818	1.80501	8.93E-04	0.071344007	16	12	24	0.016226446	0.544099157	24	10.70328798	0.177329538
ENSMUSG00000057058	Skap1	src family associated phosphoprotein 1 [Source:MGI Symbol;Acc:MGI:1925723]	0.48547	0.48661	0.68951	0.581372	0.690159	0.359592	0.330346	0.35014	0.70406	0.507444	0.540729	0.36993	0.455035	0.506866	0.013234702	0.231593979	12	16	24	0.015967518	0.544099157	24	10.86243649	0.118970934
ENSMUSG00000057060	Slc35f3	solute carrier family 35, member F3 [Source:MGI Symbol;Acc:MGI:2444426]	5.9102	4.7034	5.22525	4.63828	4.03405	5.5409	5.20587	5.27763	3.92755	5.69972	4.47973	4.98346	4.79468	5.47262	0.724067924	0.966018836	4	16	24	0.364070217	0.788727838	24	0.615712555	0.080813708
ENSMUSG00000057069	Ero1b	endoplasmic reticulum oxidoreductase 1 beta [Source:MGI Symbol;Acc:MGI:1914725]	8.58712	7.50709	7.53938	7.01626	7.35335	7.84008	8.38115	6.86983	6.88169	7.1274	8.06526	7.60569	7.76614	7.10553	0.015345591	0.253245931	4	4	24	0.007315318	0.47735824	24	21.23723478	0.094480723
ENSMUSG00000057092	Fxyd3	FXYD domain-containing ion transport regulator 3 [Source:MGI Symbol;Acc:MGI:107497]	38.4259	40.6566	34.9324	32.93	37.5827	34.452	41.9443	36.5008	39.1486	33.4135	35.0526	38.0323	40.4744	34.414	0.004196064	0.140598674	12	4	24	0.049239927	0.641244748	24	0.424660671	0.095488429
ENSMUSG00000057093	Zfp607b	zinc finger protein 607B [Source:MGI Symbol;Acc:MGI:2148237]	2.94285	3.0784	3.12805	3.1146	3.20403	2.73081	3.14667	2.61551	3.12159	2.91488	3.48754	2.90109	3.54995	3.10735	0.94397916	0.966018836	20	4	24	0.86276558	0.959967665	24	14.26323071	0.01883667
ENSMUSG00000057098	Ebf1	early B cell factor 1 [Source:MGI Symbol;Acc:MGI:95275]	23.9486	20.2712	20.8662	21.4178	18.3895	22.3916	19.6786	20.1743	17.5317	25.4397	19.9961	22.2084	20.943	21.6355	0.935932447	0.966018836	24	12	24	0.826957053	0.946041107	24	19.81850029	0.034802556

ENSMUSG00000057101	Zfp180	zinc finger protein 180 [Source:MGI Symbol;Acc:MGI:1923701]	19.1652	16.3214	16.0532	15.5808	18.7362	17.5943	18.6611	16.7656	15.7	15.0858	18.9263	16.7543	18.108	16.8501	0.00218545	0.1030182	20	20	24	0.007553879	0.478114494	24	21.07557329	0.116981323
ENSMUSG00000057103	Nat8f1	N-acetyltransferase 8 (GCN5-related) family member 1 [Source:MGI Symbol;Acc:MGI:1913366]	4.95162	5.00904	5.30282	5.10438	5.47145	5.73526	4.7542	5.42266	6.17912	5.48185	5.58606	5.13755	5.38233	5.37058	0.315232163	0.827645017	20	8	24	0.221907972	0.768040178	24	11.49508608	0.050241339
ENSMUSG00000057110	Cntrl	centriolin [Source:MGI Symbol;Acc:MGI:1889576]	6.11847	7.84028	7.94144	7.42364	8.37814	6.80268	7.62227	8.52788	9.32002	6.10614	8.21105	8.17131	8.26975	7.43299	0.923635729	0.966018836	12	4	24	0.734339811	0.913347424	24	7.512554123	0.049147187
ENSMUSG00000057113	Npm1	nucleophosmin 1 [Source:MGI Symbol;Acc:MGI:106184]	51.4418	58.749	57.9668	58.0805	56.2535	51.2769	53.9953	52.2407	62.0658	45.0163	56.2079	47.357	52.6628	54.3997	0.010468194	0.196621011	12	12	24	0.31339086	0.768040178	24	7.865583449	0.071296148
ENSMUSG00000057130	Txnl4a	thioredoxin-like 4A [Source:MGI Symbol;Acc:MGI:1351613]	17.0532	19.1603	13.3445	16.6559	18.8405	18.5674	15.3776	18.2348	16.9058	14.2499	19.191	15.3467	17.545	15.7032	0.814077113	0.966018836	20	20	24	0.467754948	0.805393068	24	20.34582527	0.07793396
ENSMUSG00000057132	Rpgrip1	retinitis pigmentosa GTPase regulator interacting protein 1 [Source:MGI Symbol;Acc:MGI:1932134]	1.33148	1.29677	1.77978	0.937697	0.942857	1.03836	2.02473	0.772857	1.68347	0.86562	0.957001	1.08023	1.96695	1.38755	0.032263213	0.324385159	4	12	24	0.149392035	0.75807872	24	2.752073106	0.18665484
ENSMUSG00000057133	Chd6	chromodomain helicase DNA binding protein 6 [Source:MGI Symbol;Acc:MGI:1918639]	11.6786	11.7738	11.2213	13.3899	11.1476	10.9539	11.0737	12.451	12.2833	13.4131	9.87092	12.3826	12.5492	13.3064	0.125573493	0.555618842	16	4	24	0.204324662	0.768040178	24	7.112067344	0.080689971
ENSMUSG00000057134	Ado	2-aminoethanethiol (cysteamine) dioxygenase [Source:MGI Symbol;Acc:MGI:2685083]	11.8795	13.3051	13.461	12.1843	13.8582	12.2465	13.0763	13.7321	13.0213	13.9477	11.4937	13.004	13.0977	12.7324	0.691437565	0.966018836	12	12	24	0.433519319	0.805393068	24	7.308391237	0.041530344
ENSMUSG00000057137	Tmem140	transmembrane protein 140 [Source:MGI Symbol;Acc:MGI:1915737]	4.95345	4.92561	5.25148	5.75763	4.63178	5.22066	6.00365	6.88859	5.2427	4.56137	4.92522	4.89937	5.69131	5.73798	0.184505544	0.662239322	8	12	24	0.102704878	0.713699364	24	3.642545685	0.106778584
ENSMUSG00000057143	Trim12c	tripartite motif-containing 12C [Source:MGI Symbol;Acc:MGI:4821183]	3.48401	4.29166	3.96103	3.81884	4.66894	3.80072	3.44905	3.90513	4.40164	2.92882	4.25862	3.78791	3.54442	4.43724	0.138543921	0.580158868	8	20	24	0.855693182	0.957160062	24	9.046878096	0.033342087
ENSMUSG00000057147	Dph6	diphthamine biosynthesis 6 [Source:MGI Symbol;Acc:MGI:1913882]	12.9443	13.5535	13.0147	13.304	13.0118	13.4833	12.9372	12.603	13.8671	12.8852	14.0236	12.337	13.0284	13.1291	0.920858634	0.966018836	12	16	24	0.558631806	0.839921589	24	11.47731994	0.020084007
ENSMUSG00000057156	Homez	homeodomain leucine zipper-encoding gene [Source:MGI Symbol;Acc:MGI:2678023]	4.4373	3.56609	3.19292	2.88737	3.58382	3.92995	4.35556	3.89222	2.84688	3.29355	4.75515	3.40476	3.205	3.09091	0.053096754	0.393341118	4	8	24	0.059019731	0.658304792	24	20.81314724	0.15979586
ENSMUSG00000057157	Gm6054	predicted gene 6054 [Source:MGI Symbol;Acc:MGI:3645581]	21.727	20.4118	18.4724	25.6195	21.7569	24.4842	21.9995	26.2615	17.7361	22.5585	19.1001	18.6147	18.4682	19.1617	0.905762334	0.966018836	16	20	24	0.832765215	0.948359912	24	17.4507398	0.041224019
ENSMUSG00000057160	Gm16372	predicted pseudogene 16372 [Source:MGI Symbol;Acc:MGI:3648102]	0.839222	0.778131	0.606996	0.473821	0.411918	0.85884	1.52772	1.11535	0.664158	0.740105	1.42497	1.26448	0.846348	0.428575	0.17432128	0.650395613	24	16	24	0.184342429	0.768040178	24	21.61606479	0.190127871
ENSMUSG00000057176	Ccdc189	coiled-coil domain containing 189 [Source:MGI Symbol;Acc:MGI:2685012]	2.16582	2.9079	2.28862	2.15685	2.19953	2.02792	3.58675	1.94529	2.07225	2.48928	1.95803	2.07739	2.29477	1.78088	0.916963526	0.966018836	4	20	24	0.689089536	0.897070579	24	0.563391287	0.06016298
ENSMUSG00000057177	Gsk3a	glycogen synthase kinase 3 alpha [Source:MGI Symbol;Acc:MGI:2152453]	29.9809	26.7745	27.9515	29.1861	28.4492	30.6455	31.6967	29.5567	25.4208	35.9065	26.8474	30.3702	29.5883	30.228	0.14313801	0.585944911	24	12	24	0.749858018	0.918674671	24	19.52766158	0.036536399
ENSMUSG00000057181	5730455P16Rik	RIKEN cDNA 5730455P16 gene [Source:MGI Symbol;Acc:MGI:1917841]	28.5547	29.887	29.1188	28.7667	26.0414	28.2204	28.4789	28.5545	29.628	29.3386	30.3705	30.017	31.5305	31.2523	0.886572916	0.966018836	8	12	24	0.16752095	0.767020009	24	4.521333204	0.037980904
ENSMUSG00000057182	Scn3a	sodium channel, voltage-gated, type III, alpha [Source:MGI Symbol;Acc:MGI:98249]	1.28593	1.57995	1.29946	1.22971	1.58002	1.38793	1.30353	1.08809	1.65095	0.988314	1.98954	2.19269	1.0021	2.11174	0.865233232	0.966018836	20	20	24	0.838522733	0.950702194	24	18.05039034	0.05254367
ENSMUSG00000057191	AB124611	cDNA sequence AB124611 [Source:MGI Symbol;Acc:MGI:3043001]	1.76403	2.69343	3.57467	3.02288	2.54502	2.28085	2.2386	2.93261	3.06256	2.0335	2.59678	2.27622	3.10765	2.08381	0.300823756	0.809362476	12	16	24	0.230360725	0.768040178	24	8.83927877	0.13884397
ENSMUSG00000057193	Slc44a2	solute carrier family 44, member 2 [Source:MGI Symbol;Acc:MGI:1915932]	73.1481	59.1933	65.4062	63.0725	68.3172	73.8948	70.4795	75.8588	54.1604	81.682	60.1857	66.9137	66.508	62.3326	0.787283372	0.966018836	24	12	24	0.528767406	0.824139773	24	20.11911954	0.07272928
ENSMUSG00000057219	Armc7	armadillo repeat containing 7 [Source:MGI Symbol;Acc:MGI:2679719]	2.97325	2.40307	3.29711	2.36132	2.65512	3.0566	2.94898	2.96514	2.47538	3.1674	2.69952	2.54295	2.89773	2.92896	0.826055781	0.966018836	4	16	24	0.806246016	0.938114514	24	1.718003967	0.029074424
ENSMUSG00000057229	Dmac2	distal membrane arm assembly complex 2 [Source:MGI Symbol;Acc:MGI:1913599]	14.6314	15.9007	19.2882	17.8516	14.4889	16.9286	15.8034	18.987	17.9189	20.8737	14.9076	15.6794	16.0599	16.8296	0.012443448	0.220221977	16	4	24	0.019837874	0.565108098	24	8.759383631	0.150817474
ENSMUSG00000057230	Aak1	AP2 associated kinase 1 [Source:MGI Symbol;Acc:MGI:1098687]	41.5026	33.878	36.3182	32.5815	29.5425	33.9106	33.1896	36.8439	27.0518	42.411	29.9486	36.5404	33.2408	39.1081	0.836635326	0.966018836	8	12	24	0.594692595	0.857513003	24	2.231976276	0.071913205
ENSMUSG00000057234	Mettl15	methyltransferase like 15 [Source:MGI Symbol;Acc:MGI:1924144]	3.06993	3.41259	2.68495	2.67689	2.90777	2.58651	3.40841	3.12866	3.97743	3.20809	3.64687	2.90926	3.3672	3.57087	0.462767035	0.966018836	8	16	24	0.268069409	0.768040178	24	5.393315692	0.079424294
ENSMUSG00000057236	Rbbp4	retinoblastoma binding protein 4, chromatin remodeling factor [Source:MGI Symbol;Acc:MGI:1194912]	53.9137	53.4239	58.3077	55.327	55.9795	52.395	53.5226	51.6425	54.1132	51.7753	58.8951	51.7667	54.2949	50.3736	0.109247218	0.524035616	20	12	24	0.231021393	0.768040178	24	13.98032094	0.039116659
ENSMUSG00000057246	BC051142	cDNA sequence BC051142 [Source:MGI Symbol;Acc:MGI:3039565]	1.79357	1.70687	1.59752	1.52705	1.61286	1.22151	2.04349	0.98586	1.65028	2.66908	2.64945	2.08111	1.60476	1.7894	0.934818505	0.966018836	20	16	24	0.374543659	0.794636296	24	14.95318276	0.123291932
ENSMUSG00000057265	Bbof1	basal body orientation factor 1 [Source:MGI Symbol;Acc:MGI:1920123]	2.57608	1.96389	2.92467	2.06673	1.91847	1.73169	2.01916	2.25441	2.69536	1.69537	2.32155	2.36927	2.51797	2.2154	0.503972924	0.966018836	12	4	24	0.403690851	0.799546792	24	5.033324675	0.08620873
ENSMUSG00000057278	Snrpg	small nuclear ribonucleoprotein polypeptide G [Source:MGI Symbol;Acc:MGI:1915261]	4.2679	3.87177	4.31094	3.15209	3.57716	4.25327	3.90941	3.34969	4.89449	2.09119	3.85031	3.02574	3.70153	3.52569	0.503972924	0.966018836	12	4	24	0.408074546	0.799546792	24	1.484377512	0.11052173
ENSMUSG00000057280	Musk	muscle, skeletal, receptor tyrosine kinase [Source:MGI Symbol;Acc:MGI:103581]	0.84594	1.05544	0.899591	1.30105	0.916388	0.886578	0.834477	1.27211	1.22222	1.14568	1.21197	1.02322	1.07539	1.15725	0.128665061	0.560209134	16	12	24	0.038329461	0.61140397	24	9.537377594	0.096890891
ENSMUSG00000057286	St6galnac2	ST6 (alpha-N-acetyl-neuraminyl-2,3-beta-galactosyl-1,3)-N-acetylgalactosaminide alpha-2,6-sialyltransferase 2 [Source:MGI Symbol;Acc:MGI:107553]	3.1877	2.50085	1.98936	2.50045	2.49062	1.89553	1.67432	2.73446	2.18646	2.19683	2.05933	2.89393	2.1847	2.10071	0.794347041	0.966018836	8	4	24	0.902754163	0.97295148	24	22.20208286	0.032156738
ENSMUSG00000057315	Arhgap24	Rho GTPase activating protein 24 [Source:MGI Symbol;Acc:MGI:1922647]	17.073	17.2894	15.6974	16.3096	17.1704	17.6062	17.3691	17.6992	15.1183	18.7389	14.4791	16.8503	16.8297	16.0941	0.964321074	0.968944646	24	12	24	0.588389843	0.853936396	24	22.58342426	0.038007945
ENSMUSG00000057322	Rpl38	ribosomal protein L38 [Source:MGI Symbol;Acc:MGI:1914921]	18.7703	20.6095	16.1256	17.4256	17.524	13.4997	17.6828	20.909	22.9553	15.9725	27.351	21.6343	23.9897	25.4834	0.850398351	0.966018836	8	8	24	0.497896787	0.807927978	24	4.288372559	0.104101581
ENSMUSG00000057329	Bcl2	B cell leukemia/lymphoma 2 [Source:MGI Symbol;Acc:MGI:88138]	10.0408	9.06007	8.38326	8.02636	9.3159	9.03269	9.30621	9.21558	8.03708	9.56628	9.00845	9.90962	9.55835	9.25069	0.030691182	0.324150946	4	8	24	0.022653073	0.581862709	24	21.82652211	0.085784434
ENSMUSG00000057335	Cep170	centrosomal protein 170 [Source:MGI Symbol;Acc:MGI:1918348]	30.3029	24.4248	24.0921	24.2498	24.3283	26.6324	25.3346	21.9587	20.5351	22.9883	27.6641	27.3094	26.148	26.957	0.019280932	0.257669847	24	12	24	0.019557667	0.564709807	24	20.92996365	0.141246807
ENSMUSG00000057337	Chst3	carbohydrate sulfotransferase 3 [Source:MGI Symbol;Acc:MGI:1858224]	2.70234	2.40255	2.3279	2.77459	2.45126	2.43946	3.3629	2.32323	2.26597	2.63307	2.3055	2.42787	2.52852	1.91351	0.256915694	0.76168256	4	4	24	0.395609882	0.799546792	24	19.93405061	0.067995615
ENSMUSG00000057342	Sphk2	sphingosine kinase 2 [Source:MGI Symbol;Acc:MGI:1861380]	11.3062	8.58285	8.49269	8.90684	9.25485	10.1941	10.9115	10.5311	7.86269	12.15	9.8533	10.3193	10.4547	9.30811	0.004861081	0.141782417	4	8	24	0.194017337	0.768040178	24	20.7179296	0.112043982
ENSMUSG00000057359	Gm17494	predicted gene, 17494 [Source:MGI Symbol;Acc:MGI:4937128]	0.654627	0.720344	0.826563	0.38652	0.670559	0.587983	0.35365	0.506111	0.783347	0.433427	0.508199	0.524716	0.61847	0.579268	0.369262716	0.893596186	12	4	24	0.55926406	0.840399223	24	5.783929524	0.054905845
ENSMUSG00000057363	Uxs1	UDP-glucuronate decarboxylase 1 [Source:MGI Symbol;Acc:MGI:1915133]	22.1514	20.4446	19.7055	19.8467	20.6077	20.5717	18.3004	20.3094	17.8735	21.5383	21.3849	20.5566	19.9736	20.2378	0.113424199	0.530777648	20	16	24	0.372635052	0.793962643	24	18.19333284	0.044415757
ENSMUSG00000057367	Birc2	baculoviral IAP repeat-containing 2 [Source:MGI Symbol;Acc:MGI:1197009]	16.0723	16.5017	15.299	15.9724	16.1077	13.933	16.5158	16.4873	17.7469	13.3649	17.3081	17.1037	16.9378	16.0057	0.907068194	0.966018836	20	20	24	0.638867233	0.877547951	24	2.006051013	0.039220374
ENSMUSG00000057375	Yipf1	Yip1 domain family, member 1 [Source:MGI Symbol;Acc:MGI:1915532]	43.4005	41.3162	40.0235	38.9068	42.3391	39.3948	40.0875	37.3487	38.2853	33.6919	43.4596	37.5504	39.496	39.4587	0.510431574	0.966018836	20	20	24	0.316420406	0.768040178	24	21.28033261	0.052489899
ENSMUSG00000057378	Ryr3	ryanodine receptor 3 [Source:MGI Symbol;Acc:MGI:99684]	1.68406	2.19519	2.35443	2.33953	2.46222	2.89586	2.40912	2.10204	2.44858	2.2943	2.35331	2.15503	1.72579	2.39645	0.708020493	0.966018836	20	8	24	0.28206811	0.768040178	24	13.35354982	0.083465628
ENSMUSG00000057388	Mrpl18	mitochondrial ribosomal protein L18 [Source:MGI Symbol;Acc:MGI:1914931]	34.6318	38.9418	37.0053	35.8204	36.4801	33.8509	37.4909	34.7201	39.9156	35.3322	40.9599	34.0335	40.0983	39.8772	0.601647512	0.966018836	12	12	24	0.398836945	0.799546792	24	6.91994462	0.048218578
ENSMUSG00000057396	Zfp759	zinc finger protein 759 [Source:MGI Symbol;Acc:MGI:2446280]	2.52732	2.44631	2.50447	2.28485	2.47985	1.96958	2.2304	2.01567	2.81698	1.74768	2.5461	2.26093	2.09623	2.50916	0.353166621	0.966018836	12	12	24	0.77820823	0.928832041	24	6.996937507	0.036968998
ENSMUSG00000057406	Nsd2	nuclear receptor binding SET domain protein 2 [Source:MGI Symbol;Acc:MGI:1276574]	23.426	23.0835	21.0975	19.9508	23.8591	22.2369	20.3946	20.3008	21.1157	18.5143	22.5401	22.265	21.5193	22.8399	0.129903614	0.560263689	20	20	24	0.213495214	0.768040178	24	21.64323244	0.067099351
ENSMUSG00000057409	Zfp53	zinc finger protein 53 [Source:MGI Symbol;Acc:MGI:99200]	2.7611	3.24316	3.00241	2.38448	3.05315	2.82615	3.29397	3.08097	3.63482	2.637	2.94518	2.61934	3.23694	2.7302	0.349109869	0.870418653	12	4	24	0.32739195	0.768040178	24	3.683093841	0.069224413
ENSMUSG00000057411	Antkmt	adenine nucleotide translocase lysine methyltransferase [Source:MGI Symbol;Acc:MGI:2384888]	43.2743	43.821	44.1164	42.2617	44.5216	45.1697	37.0156	41.8876	46.4351	42.1162	43.0998	44.11	48.0856	42.1618	0.941830768	0.966018836	24	8	24	0.934594857	0.983690057	24	13.90328314	0.012434721
ENSMUSG00000057421	Las1l	LAS1-like (S. cerevisiae) [Source:MGI Symbol;Acc:MGI:1923380]	28.6622	32.8413	31.7616	28.2846	31.2474	29.4091	29.5124	29.4761	33.4116	28.5881	26.0959	28.7495	29.3735	30.483	0.033799022	0.325496079	12	4	24	0.092865458	0.71175955	24	5.910628829	0.071812757
ENSMUSG00000057440	Mpp7	membrane protein, palmitoylated 7 (MAGUK p55 subfamily member 7) [Source:MGI Symbol;Acc:MGI:1922989]	3.24056	4.01075	4.18126	4.36343	4.00419	4.15324	3.71734	4.00108	3.80821	3.74703	3.87338	3.86101	3.47965	4.18953	0.135537027	0.574447137	16	12	24	0.21616882	0.768040178	24	10.89376612	0.060803613
ENSMUSG00000057455	Rit2	Ras-like without CAAX 2 [Source:MGI Symbol;Acc:MGI:108054]	47.4559	53.5539	54.6524	51.2583	46.8969	52.6744	51.7528	45.7045	45.8668	49.3515	48.0064	52.9978	46.1508	58.0659	0.847846021	0.966018836	24	20	24	0.857647614	0.958007588	24	4.34859619	0.023983425
ENSMUSG00000057469	E2f6	E2F transcription factor 6 [Source:MGI Symbol;Acc:MGI:1354159]	24.6693	22.1965	23.1943	25.213	23.0663	25.6953	25.2868	26.5725	21.6645	26.031	25.1334	24.2283	25.607	25.0595	0.940625808	0.966018836	16	20	24	0.571762588	0.846185279	24	20.60259494	0.035122332
ENSMUSG00000057497	Fam136a	family with sequence similarity 136, member A [Source:MGI Symbol;Acc:MGI:1913738]	15.736	15.165	14.1272	14.4526	16.0784	15.6787	15.8589	15.1942	14.3634	11.8844	15.4944	15.9081	16.1589	15.2526	0.001649265	0.100255953	4	12	24	0.009695638	0.502965379	24	22.15870068	0.113302347
ENSMUSG00000057506	Bloc1s2	biogenesis of lysosomal organelles complex-1, subunit 2 [Source:MGI Symbol;Acc:MGI:1920939]	16.5938	18.7767	17.7719	17.9804	20.4089	19.3002	17.1848	19.2885	20.4972	15.5118	23.0408	18.5146	20.7009	20.1989	0.897570341	0.966018836	20	20	24	0.922673359	0.979411448	24	17.39989463	0.021271192
ENSMUSG00000057522	Spop	speckle-type BTB/POZ protein [Source:MGI Symbol;Acc:MGI:1343085]	76.5181	76.4997	77.8079	78.4707	77.6484	73.1925	81.1166	78.9593	79.5093	81.8684	76.8742	82.9689	80.8576	82.8838	0.826055781	0.966018836	4	16	24	0.406951827	0.799546792	24	6.333647039	0.020573706
ENSMUSG00000057530	Ece1	endothelin converting enzyme 1 [Source:MGI Symbol;Acc:MGI:1101357]	30.426	24.0651	27.9638	21.4137	17.2002	26.9268	34.6625	32.9977	30.0382	39.6164	21.0385	33.0546	29.1463	33.0774	0.350544145	0.870418653	4	16	24	0.146276484	0.754849922	24	3.545876299	0.215924083
ENSMUSG00000057531	Dtnbp1	dystrobrevin binding protein 1 [Source:MGI Symbol;Acc:MGI:2137586]	32.4252	34.2026	34.056	34.5174	35.4352	36.0891	33.1	33.8984	36.067	33.0408	35.987	36.1029	36.6538	35.301	0.648082424	0.966018836	24	4	24	0.542812048	0.831118582	24	16.98419507	0.020408054
ENSMUSG00000057541	Pus7	pseudouridylate synthase 7 [Source:MGI Symbol;Acc:MGI:1925947]	7.5548	7.10654	7.68466	8.28224	6.82773	7.20159	7.36086	7.10598	8.46741	6.72081	6.77691	7.24031	6.88076	6.63666	0.498748888	0.966018836	12	8	24	0.503333458	0.810121468	24	8.809613151	0.041147009
ENSMUSG00000057551	Zfp317	zinc finger protein 317 [Source:MGI Symbol;Acc:MGI:107775]	10.4881	12.4087	11.1389	11.1851	12.4857	11.1484	10.7869	10.2105	11.9883	9.81518	11.0745	10.8636	10.2217	10.7048	0.612141595	0.966018836	12	16	24	0.755268268	0.921118175	24	13.2759419	0.02566443
ENSMUSG00000057554	Lgals8	lectin, galactose binding, soluble 8 [Source:MGI Symbol;Acc:MGI:1928481]	85.1504	76.8691	75.7767	78.5795	80.1281	78.3215	72.6355	72.4149	71.4626	75.1686	83.9722	74.1034	75.9739	76.9597	0.285027487	0.793714531	20	16	24	0.169380373	0.767229759	24	17.96771268	0.051410978
ENSMUSG00000057561	Eif1a	eukaryotic translation initiation factor 1A [Source:MGI Symbol;Acc:MGI:95298]	51.7384	46.87	46.7793	46.1053	49.6464	46.0179	42.2002	42.3162	45.2522	38.6763	53.5346	43.1491	43.4957	45.4341	0.882617464	0.966018836	20	8	24	0.543480562	0.831703329	24	18.09966641	0.050430607
ENSMUSG00000057572	Zbtb8os	zinc finger and BTB domain containing 8 opposite strand [Source:MGI Symbol;Acc:MGI:1914356]	6.25042	7.78785	5.76739	5.54381	6.94151	6.03772	8.3038	5.78904	5.7464	4.92793	7.05449	6.1927	7.00241	7.09946	0.032263213	0.324385159	4	12	24	0.099972028	0.71175955	24	23.42019031	0.142900864
ENSMUSG00000057594	Arl16	ADP-ribosylation factor-like 16 [Source:MGI Symbol;Acc:MGI:1917567]	39.2034	33.7722	34.9081	35.4405	35.5224	38.6375	35.895	36.6038	33.361	35.4205	36.0234	35.1396	37.1964	35.2876	0.008754183	0.192760771	4	8	24	0.036806246	0.609176161	24	21.10614462	0.058822725
ENSMUSG00000057596	Trim30d	tripartite motif-containing 30D [Source:MGI Symbol;Acc:MGI:3035181]	1.07162	1.16134	1.16195	1.19843	1.27901	1.12828	0.872134	1.19938	1.55993	0.974995	1.16563	0.976556	0.72552	1.13904	0.033248781	0.324908923	12	16	24	0.108393285	0.72082227	24	10.26757697	0.102678865
ENSMUSG00000057604	Lmcd1	LIM and cysteine-rich domains 1 [Source:MGI Symbol;Acc:MGI:1353635]	2.27611	3.15278	4.01778	3.96168	3.1562	4.00902	3.49174	6.99429	4.01447	5.04449	2.49498	2.60363	4.08345	2.77654	0.622132989	0.966018836	16	4	24	0.298138513	0.768040178	24	7.937096771	0.214443198
ENSMUSG00000057606	Colq	collagen-like tail subunit (single strand of homotrimer) of asymmetric acetylcholinesterase [Source:MGI Symbol;Acc:MGI:1338761]	1.25816	0.964126	0.932835	1.24485	0.880854	1.31033	1.27886	1.39492	1.00824	1.73267	0.797686	1.10074	1.26409	1.34656	0.658895034	0.966018836	8	12	24	0.724752407	0.909665611	24	3.371601467	0.050957986
ENSMUSG00000057614	Gnai1	guanine nucleotide binding protein (G protein), alpha inhibiting 1 [Source:MGI Symbol;Acc:MGI:95771]	52.3163	51.1861	47.901	47.9541	47.978	46.3151	53.565	44.6012	44.2181	51.8447	46.7349	43.336	43.4408	44.5544	0.903465533	0.966018836	16	8	24	0.958966607	0.989178508	24	19.117587	0.009781215
ENSMUSG00000057637	Prdm2	PR domain containing 2, with ZNF domain [Source:MGI Symbol;Acc:MGI:107628]	9.45652	6.95167	7.19705	8.46688	7.88781	8.55637	8.75748	8.27722	6.59041	8.99003	7.99754	8.09934	8.00531	8.12358	0.286642595	0.793714531	4	8	24	0.199298243	0.768040178	24	19.9631907	0.090058903
ENSMUSG00000057649	Brd9	bromodomain containing 9 [Source:MGI Symbol;Acc:MGI:2145317]	29.8208	30.6319	29.0606	27.7136	32.9224	29.5556	29.3001	27.6361	31.0886	27.4881	30.7437	28.8088	30.2702	29.515	0.883051071	0.966018836	20	20	24	0.627097677	0.872795898	24	17.22254528	0.029452028
ENSMUSG00000057657	Rps18-ps3	ribosomal protein S18, pseudogene 3 [Source:MGI Symbol;Acc:MGI:3642474]	1.3697	2.56348	2.82365	1.54738	2.42148	1.9124	1.16377	1.57815	1.95154	0.724737	1.76819	1.41462	1.56506	1.36295	0.643325306	0.966018836	12	16	24	0.915893826	0.976753216	24	8.540016487	0.0478818
ENSMUSG00000057666	Gapdh	glyceraldehyde-3-phosphate dehydrogenase [Source:MGI Symbol;Acc:MGI:95640]	4.76238	5.04509	5.53352	4.40305	4.62777	5.6081	4.20206	4.88285	5.69872	4.81307	5.60086	4.52241	5.36854	4.86622	0.942429894	0.966018836	12	16	24	0.775294053	0.927711986	24	8.853736193	0.035171133
ENSMUSG00000057667	Bloc1s3	biogenesis of lysosomal organelles complex-1, subunit 3 [Source:MGI Symbol;Acc:MGI:2678952]	4.12076	4.17628	4.27178	3.98662	3.68667	4.65063	4.52678	4.22316	3.44928	3.93933	4.68294	4.52298	4.49693	4.49689	0.10560184	0.518002936	24	16	24	0.083291548	0.699490063	24	22.13588853	0.083383491
ENSMUSG00000057672	Pkn1	protein kinase N1 [Source:MGI Symbol;Acc:MGI:108022]	11.4333	12.2632	14.1089	13.6993	13.6383	13.1156	12.3859	12.9421	12.5509	16.0259	12.3606	11.5754	13.0903	12.4362	0.057224139	0.401309455	16	12	24	0.035898879	0.609176161	24	11.46532901	0.106386793
ENSMUSG00000057691	Zfp746	zinc finger protein 746 [Source:MGI Symbol;Acc:MGI:1916478]	5.17484	5.56841	5.54173	5.68846	4.9975	5.71785	5.67012	5.71307	5.70734	6.94854	5.80946	5.85015	5.48276	6.78544	0.960453992	0.967043964	16	12	24	0.212867585	0.768040178	24	8.804821687	0.060858237
ENSMUSG00000057706	Mex3b	mex3 RNA binding family member B [Source:MGI Symbol;Acc:MGI:1918252]	2.91314	2.25968	2.77965	2.47587	2.23277	2.35258	2.60721	2.39687	1.84237	2.85697	2.84443	2.1939	2.04825	2.40051	0.890235289	0.966018836	16	8	24	0.797942797	0.935424343	24	15.53503868	0.0353837
ENSMUSG00000057715	A830018L16Rik	RIKEN cDNA A830018L16 gene [Source:MGI Symbol;Acc:MGI:2444149]	20.0665	18.5768	18.0737	16.9364	18.1957	18.7358	18.628	17.1594	15.3517	19.5818	19.8585	20.6709	18.6663	22.5119	0.071575474	0.440262583	24	12	24	0.315347242	0.768040178	24	21.55335391	0.072895943
ENSMUSG00000057716	Tmem178b	transmembrane protein 178B [Source:MGI Symbol;Acc:MGI:3647581]	21.9598	19.3335	21.1174	19.6719	19.0199	21.7393	20.865	18.4167	17.598	24.0732	19.374	21.8039	19.6441	23.3678	0.934474422	0.966018836	24	12	24	0.864724546	0.960819377	24	20.29494345	0.028041331
ENSMUSG00000057722	Lepr	leptin receptor [Source:MGI Symbol;Acc:MGI:104993]	2.65267	4.40909	2.91696	2.83503	3.67444	2.45453	2.84243	3.14315	4.59561	2.17504	2.91982	2.96627	3.08494	2.72379	0.849179819	0.966018836	4	4	24	0.52669312	0.823075573	24	6.242406891	0.103321256
ENSMUSG00000057729	Prtn3	proteinase 3 [Source:MGI Symbol;Acc:MGI:893580]	2.17478	3.66711	6.48734	4.29912	3.56387	4.61901	2.97247	3.32291	4.07461	3.35806	3.5853	3.84246	6.39015	2.60342	0.887010723	0.966018836	12	20	24	0.753952086	0.920255919	24	11.61672563	0.104413607
ENSMUSG00000057738	Sptan1	spectrin alpha, non-erythrocytic 1 [Source:MGI Symbol;Acc:MGI:98386]	244.285	217.911	213.716	233.335	240.362	241.926	212.141	227.552	195.512	239.817	218.524	223.303	221.689	223.777	0.542718076	0.966018836	24	12	24	0.170068465	0.767957732	24	18.37520503	0.063525578
ENSMUSG00000057751	Megf6	multiple EGF-like-domains 6 [Source:MGI Symbol;Acc:MGI:1919351]	1.49846	2.21763	1.64885	1.41687	1.65141	1.46247	1.03894	1.4119	1.66946	1.29519	1.24623	1.03169	1.57657	1.12352	0.601647512	0.966018836	12	4	24	0.44752905	0.805393068	24	7.106501076	0.075367249
ENSMUSG00000057762	Gm6169	predicted gene 6169 [Source:MGI Symbol;Acc:MGI:3646298]	7.92693	7.54584	8.17244	8.20324	8.71633	8.19804	8.66675	7.14138	7.79385	8.26408	7.84856	7.06719	7.47514	7.93173	0.420039465	0.966018836	16	16	24	0.343491699	0.777127004	24	14.53670235	0.042894273
ENSMUSG00000057766	Ankrd29	ankyrin repeat domain 29 [Source:MGI Symbol;Acc:MGI:2687055]	19.0416	15.9443	15.5079	14.4333	16.0395	17.2665	15.6452	14.597	13.2269	16.9781	17.4264	16.4388	16.2648	15.9256	0.028632001	0.316608413	24	12	24	0.048230766	0.638872072	24	20.13739318	0.11164769
ENSMUSG00000057777	Mab21l2	mab-21-like 2 [Source:MGI Symbol;Acc:MGI:1346022]	3.94157	3.57117	4.23333	3.05236	3.2068	3.74458	4.14076	3.76181	2.91753	4.92159	2.85269	3.85635	3.08315	4.07364	0.588208558	0.966018836	4	16	24	0.42545651	0.805393068	24	3.862255819	0.113552223
ENSMUSG00000057778	Cyb5d2	cytochrome b5 domain containing 2 [Source:MGI Symbol;Acc:MGI:2684848]	13.7196	11.2155	10.7413	12.2418	11.7893	13.2631	12.78	11.6378	12.5319	12.3992	12.224	12.7473	11.8304	0.067104747	0.425837275	4	8	24	0.211656792	0.768040178	24	21.77490602	0.059457699	
ENSMUSG00000057788	Ddx49	DEAD box helicase 49 [Source:MGI Symbol;Acc:MGI:2136689]	11.8233	14.1123	14.5375	15.0568	14.9649	14.009	12.5901	11.9712	13.7157	13.6915	13.0923	15.2749	12.8948	12.3341	0.097373374	0.501112642	24	4	24	0.049571612	0.642484473	24	14.18596931	0.098151308
ENSMUSG00000057789	Bak1	BCL2-antagonist/killer 1 [Source:MGI Symbol;Acc:MGI:1097161]	6.51778	5.56856	5.36132	5.33156	6.21538	5.90037	5.84264	5.93266	5.63844	5.70746	5.10761	5.61748	5.52535	5.47814	0.699015352	0.966018836	4	12	24	0.183578819	0.768040178	24	22.29319586	0.051088752
ENSMUSG00000057816	Cfap299	cilia and flagella associated protein 299 [Source:MGI Symbol;Acc:MGI:1916571]	0.721518	1.05454	1.11529	0.718006	1.44955	0.844813	1.11195	0.5575	1.12567	1.19238	0.569797	1.20114	0.625809	1.08649	0.550682606	0.966018836	24	4	24	0.753438084	0.920152554	24	11.9939106	0.063310869
ENSMUSG00000057818	1600029O15Rik	RIKEN cDNA 1600029O15 gene [Source:MGI Symbol;Acc:MGI:3045362]	0.361099	0.269462	0.498209	1.28586	0.416355	0.43388	0.470676	0.995696	0.690094	0.476982	0.766143	0.709344	0.511819	0.660497	0.775247401	0.966018836	24	12	24	0.33642109	0.771892574	24	10.97326524	0.126667047
ENSMUSG00000057835	Zfp119a	zinc finger protein 119a [Source:MGI Symbol;Acc:MGI:1345189]	2.09267	2.58652	2.64914	2.87974	2.37772	2.42491	2.5836	2.0526	2.61606	2.49623	2.80057	2.30564	2.21254	2.68986	0.127318615	0.560209134	12	16	24	0.095947494	0.71175955	24	11.3299171	0.085056011
ENSMUSG00000057836	Xlr3a	X-linked lymphocyte-regulated 3A [Source:MGI Symbol;Acc:MGI:109506]	0.882538	0.719182	0.75102	0.965457	0.777032	0.602036	0.400094	0.321173	0.299623	0.508891	0.648408	0.575403	0.47147	0.429401	0.496745696	0.966018836	20	12	24	0.089768938	0.709751366	24	16.31893083	0.114678348
ENSMUSG00000057841	Rpl32	ribosomal protein L32 [Source:MGI Symbol;Acc:MGI:98038]	117.248	147.901	138.868	140.249	145.629	133.983	126.077	133.036	162.033	105.344	148.509	130.695	143.884	130.908	0.625825704	0.966018836	20	8	24	0.856553705	0.957486395	24	9.205235359	0.036155319
ENSMUSG00000057842	Zfp595	zinc finger protein 595 [Source:MGI Symbol;Acc:MGI:3040707]	1.63772	1.93182	1.82333	1.83161	1.99435	1.74284	1.98119	1.58975	2.05147	1.33215	2.1067	1.65321	1.89768	1.73546	0.953350544	0.966018836	20	12	24	0.962914402	0.990726108	24	19.09406525	0.012919319
ENSMUSG00000057858	Fam204a	family with sequence similarity 204, member A [Source:MGI Symbol;Acc:MGI:1289174]	15.7631	23.0739	23.8532	19.5062	21.0791	17.2908	17.2775	18.097	25.1694	20.5042	21.1197	21.4262	19.7126	23.4016	0.033248781	0.324908923	12	16	24	0.034086335	0.607550038	24	9.235077208	0.173433758
ENSMUSG00000057863	Rpl36	ribosomal protein L36 [Source:MGI Symbol;Acc:MGI:1860603]	2.56213	2.67824	2.57867	2.87432	2.67579	4.26033	2.65971	3.39039	5.32396	1.69543	3.70958	1.90106	2.3474	2.83393	0.772442842	0.966018836	12	16	24	0.887973078	0.968493323	24	8.018252316	0.066384762
ENSMUSG00000057880	Abat	4-aminobutyrate aminotransferase [Source:MGI Symbol;Acc:MGI:2443582]	22.2388	25.6399	36.3186	22.0858	19.886	29.1547	29.8725	22.5435	24.2529	24.794	18.1157	24.5128	18.4577	27.7974	0.510431574	0.966018836	12	8	24	0.423664924	0.805393068	24	5.343855855	0.135643025
ENSMUSG00000057886	Cbx3-ps6	chromobox 3, pseudogene 6 [Source:MGI Symbol;Acc:MGI:3646710]	20.8694	22.6366	23.3826	23.7722	21.3691	12.5351	16.9004	17.7011	25.051	20.6803	23.1577	23.992	21.2765	25.2268	0.579092016	0.966018836	12	16	24	0.16604289	0.767020009	24	9.163397915	0.189148799
ENSMUSG00000057894	Zfp329	zinc finger protein 329 [Source:MGI Symbol;Acc:MGI:1921283]	7.53783	7.91222	5.49211	7.72809	7.46911	6.72768	6.64129	6.79339	7.08842	5.98706	8.05453	7.86627	6.91684	6.51767	0.885025617	0.966018836	20	16	24	0.302417735	0.768040178	24	19.17730572	0.087155607
ENSMUSG00000057895	Zfp105	zinc finger protein 105 [Source:MGI Symbol;Acc:MGI:1277119]	2.98106	2.92519	2.73168	2.71916	2.53322	2.79327	2.54239	2.59721	3.11461	3.12272	2.78309	3.06488	2.99934	3.16173	0.879844076	0.966018836	12	8	24	0.623229307	0.87183478	24	6.077134153	0.029798933
ENSMUSG00000057897	Camk2b	calcium/calmodulin-dependent protein kinase II, beta [Source:MGI Symbol;Acc:MGI:88257]	16.0166	18.9932	24.1442	18.3712	15.8046	19.9206	20.9977	17.0992	20.8076	23.8106	14.4902	19.828	16.7321	21.239	0.117335378	0.538362324	12	8	24	0.2358982	0.768040178	24	7.684000426	0.143015394
ENSMUSG00000057914	Cacnb2	calcium channel, voltage-dependent, beta 2 subunit [Source:MGI Symbol;Acc:MGI:894644]	2.42835	2.79595	3.42029	2.01501	1.48388	2.55061	2.42771	1.52415	2.58295	2.23099	1.6337	2.32491	2.12399	2.8046	0.069194004	0.433403132	12	8	24	0.240889572	0.768040178	24	4.718338345	0.152920565
ENSMUSG00000057948	Unc13d	unc-13 homolog D [Source:MGI Symbol;Acc:MGI:1917700]	0.994242	1.08052	1.23985	1.28032	1.07387	1.10632	1.00147	1.14545	1.18429	1.34274	1.16614	1.02957	1.33025	1.10346	0.030972684	0.324150946	16	12	24	0.043465503	0.626787045	24	10.44377315	0.067560383
ENSMUSG00000057963	Itpk1	inositol 1,3,4-triphosphate 5/6 kinase [Source:MGI Symbol;Acc:MGI:2446159]	23.4351	19.7261	21.2155	21.425	24.5014	23.7916	23.2492	22.1779	19.549	25.2317	21.775	23.375	24.1628	19.9305	0.046934151	0.375011045	24	12	24	0.034496815	0.608551791	24	18.68516012	0.109368936
ENSMUSG00000057967	Fgf18	fibroblast growth factor 18 [Source:MGI Symbol;Acc:MGI:1277980]	4.47556	3.96856	3.77816	4.32685	3.83497	4.84716	5.50401	4.58464	3.18	5.48601	4.34694	4.6552	4.38376	4.80609	0.143321297	0.585944911	24	12	24	0.370887521	0.792839127	24	21.18921734	0.094175786
ENSMUSG00000057969	Sema3b	sema domain, immunoglobulin domain (Ig), short basic domain, secreted, (semaphorin) 3B [Source:MGI Symbol;Acc:MGI:107561]	94.4298	85.4251	79.8716	83.3398	98.5137	84.8262	109.106	102.322	92.4781	123.439	85.3128	98.9486	101.614	83.5103	0.85447369	0.966018836	4	8	24	0.885304652	0.967345379	24	20.06783915	0.035523817
ENSMUSG00000057982	Zfp809	zinc finger protein 809 [Source:MGI Symbol;Acc:MGI:2143362]	10.5776	10.9775	10.1537	11.4911	12.4949	10.3137	11.3281	8.25127	11.6976	9.09612	11.3292	10.5472	10.136	9.73976	0.47028201	0.966018836	20	12	24	0.272184521	0.768040178	24	16.56174165	0.085056676
ENSMUSG00000057990	Gm53055	predicted gene 53055 [Source:MGI Symbol;Acc:MGI:6435211]	0.484236	0.551579	0.657002	0.822716	0.715381	0.406799	0.588262	0.537408	0.328902	0.560376	0.659373	0.389882	0.487434	0.369077	0.23919737	0.741580974	20	4	24	0.132907642	0.74343239	24	13.71569314	0.087503323
ENSMUSG00000058006	Mdn1	midasin AAA ATPase 1 [Source:MGI Symbol;Acc:MGI:1926159]	8.27572	8.25286	8.09966	6.86176	9.3635	8.50612	10.8174	8.64215	7.75429	6.91544	9.7799	8.3053	8.85344	8.32909	0.007613653	0.192760771	20	20	24	0.082404864	0.699403344	24	21.96690819	0.130299298
ENSMUSG00000058013	Septin11	septin 11 [Source:MGI Symbol;Acc:MGI:1277214]	26.3823	25.7052	23.7839	24.1752	27.4163	25.3433	28.5318	25.1996	24.8526	26.6651	24.1795	25.553	25.716	24.4458	0.060394272	0.407012001	4	8	24	0.228725227	0.768040178	24	21.26649449	0.04827966
ENSMUSG00000058019	Ces5a	carboxylesterase 5A [Source:MGI Symbol;Acc:MGI:1915185]	1.31631	0.498982	0.721591	0.48123	0.626096	0.600319	0.516283	0.584286	0.557282	0.681951	0.762267	0.564912	0.440397	0.805463	0.859936714	0.966018836	20	8	24	0.885797631	0.967604872	24	22.44903017	0.034087981

ENSMUSG00000058022	Adtrp	androgen dependent TFPI regulating protein [Source:MGI Symbol;Acc:MGI:1924596]	2.31817	2.0422	3.10354	7.06715	2.10176	1.75119	2.40471	2.47651	2.60202	3.06084	3.94811	2.39409	3.6611	4.24406	0.285027487	0.793714531	16	8	24	0.208951321	0.768040178	24	10.54303833	0.270165956
ENSMUSG00000058056	Palld	palladin, cytoskeletal associated protein [Source:MGI Symbol;Acc:MGI:1919583]	5.77545	5.04153	4.07224	5.35219	5.59601	5.86375	5.08205	5.67786	4.73385	6.13065	4.9738	5.00955	5.49647	5.38127	0.628286596	0.966018836	24	12	24	0.463656785	0.805393068	24	19.79153069	0.063683602
ENSMUSG00000058057	Mettl7a3	methyltransferase like 7A3 [Source:MGI Symbol;Acc:MGI:3710670]	12.0319	10.8756	9.61611	10.3609	10.724	11.6543	10.2768	8.3644	6.81098	10.2677	10.5575	8.64585	8.7034	10.1929	0.432054949	0.966018836	20	16	24	0.17369408	0.768040178	24	19.28071133	0.127638347
ENSMUSG00000058064	Gm10036	predicted gene 10036 [Source:MGI Symbol;Acc:MGI:3642334]	3.01995	3.2238	2.69348	3.06092	3.91303	2.60364	2.62247	2.49346	4.61964	2.13933	3.53094	3.53665	3.75865	3.03058	0.885059765	0.966018836	20	12	24	0.94062787	0.985398871	24	16.27339939	0.031636234
ENSMUSG00000058070	Eml1	echinoderm microtubule associated protein like 1 [Source:MGI Symbol;Acc:MGI:1915769]	200.298	151.496	144.219	164.97	156.638	182.158	163.518	169.261	126.855	195.497	147.952	172.224	173.728	162.734	0.042880436	0.356048751	24	12	24	0.213746489	0.768040178	24	20.90456063	0.120858434
ENSMUSG00000058073	Gm11189	predicted gene 11189 [Source:MGI Symbol;Acc:MGI:3650984]	23.1767	21.0836	23.0583	17.6716	25.9426	23.1403	27.1409	23.4473	22.9969	19.1093	26.4208	25.3049	23.9976	23.5721	0.012922042	0.227495952	20	20	24	0.103787977	0.715480277	24	21.76458612	0.127987284
ENSMUSG00000058076	Sdhc	succinate dehydrogenase complex, subunit C, integral membrane protein [Source:MGI Symbol;Acc:MGI:1913302]	60.8913	60.5816	64.097	77.966	64.414	64.226	61.1012	73.264	66.0237	75.98	61.7721	62.3248	62.616	67.7601	0.032263213	0.324385159	16	12	24	0.048501845	0.638992719	24	10.41464831	0.106793712
ENSMUSG00000058093	Zfp729b	zinc finger protein 729b [Source:MGI Symbol;Acc:MGI:2145180]	3.99566	4.85456	4.13781	3.96094	4.62871	3.82575	4.02772	4.0893	5.20235	3.62234	5.06957	4.41165	4.53705	4.29997	0.888991156	0.966018836	12	12	24	0.937433175	0.984507582	24	8.252238748	0.018944458
ENSMUSG00000058099	Nfam1	Nfat activating molecule with ITAM motif 1 [Source:MGI Symbol;Acc:MGI:1921289]	0.986715	1.50325	1.92064	1.70604	1.47568	1.21313	1.466	1.267	1.6011	1.01423	1.54491	1.30086	1.27685	0.894156	0.119434414	0.542554768	12	20	24	0.327771114	0.768040178	24	11.66030521	0.096590336
ENSMUSG00000058126	Tpm3-rs7	tropomyosin 3, related sequence 7 [Source:MGI Symbol;Acc:MGI:99705]	7.82512	8.73777	10.8839	9.2059	8.30744	9.17242	9.5397	9.86475	8.49891	8.75391	7.30439	7.54233	8.32519	8.17647	0.243586913	0.741580974	12	8	24	0.193523172	0.768040178	24	6.868176535	0.091027854
ENSMUSG00000058135	Gstm1	glutathione S-transferase, mu 1 [Source:MGI Symbol;Acc:MGI:95860]	27.295	34.957	42.6909	32.8046	31.4379	38.3505	34.5939	37.5521	37.2815	37.2323	29.2649	31.4287	29.8157	31.7381	0.101520755	0.511659635	12	16	24	0.167198216	0.767020009	24	8.448687014	0.127065615
ENSMUSG00000058145	Adamts17	a disintegrin-like and metallopeptidase (reprolysin type) with thrombospondin type 1 motif, 17 [Source:MGI Symbol;Acc:MGI:3588195]	1.04767	0.750092	0.82536	0.461436	0.665127	0.638167	0.945701	0.933774	0.603447	0.635125	0.722242	0.468225	0.554214	1.77061	0.128810631	0.560209134	8	8	24	0.099432411	0.71175955	24	3.548544448	0.187150874
ENSMUSG00000058153	Sez6l	seizure related 6 homolog like [Source:MGI Symbol;Acc:MGI:1935121]	1.77474	2.08212	3.58016	1.76401	1.84215	2.38904	2.591	1.62346	2.12403	2.30399	1.69295	1.89212	1.18386	2.3735	0.923635729	0.966018836	12	8	24	0.514506721	0.816799944	24	8.490951831	0.115177479
ENSMUSG00000058159	T2	brachyury 2 [Source:MGI Symbol;Acc:MGI:104658]	1.08583	1.0684	1.13125	0.933863	1.16791	1.11145	1.077	0.694816	1.20083	1.10614	1.26889	1.26044	0.69919	1.19154	0.210154775	0.69936851	20	12	24	0.360709746	0.786233489	24	15.07202618	0.073344796
ENSMUSG00000058173	Smco4	single-pass membrane protein with coiled-coil domains 4 [Source:MGI Symbol;Acc:MGI:3039636]	3.48949	3.74874	2.62464	3.46322	4.7935	3.76664	3.32041	4.59971	4.04864	3.76036	4.2363	3.19676	4.19618	3.49451	0.895137379	0.966018836	20	4	24	0.816672657	0.941697244	24	17.0868928	0.045121595
ENSMUSG00000058174	Gm5148	predicted gene 5148 [Source:MGI Symbol;Acc:MGI:3646006]	29.4276	31.5508	33.4444	26.4349	27.5871	23.0224	26.6246	27.9852	34.4306	22.2668	27.7929	29.9999	35.5093	31.728	0.036302654	0.33234124	12	4	24	0.117763267	0.728323355	24	4.385405876	0.148614007
ENSMUSG00000058192	Zfp846	zinc finger protein 846 [Source:MGI Symbol;Acc:MGI:1924012]	5.39877	4.60051	4.77811	5.03241	4.68311	4.60386	4.87509	4.64547	5.29676	3.94788	4.7386	5.00877	5.39598	5.20677	0.515451644	0.966018836	4	12	24	0.270426084	0.768040178	24	1.151667124	0.060621504
ENSMUSG00000058230	Arhgap35	Rho GTPase activating protein 35 [Source:MGI Symbol;Acc:MGI:1929494]	30.7319	23.2304	23.1031	24.6391	24.7756	27.4094	26.9511	26.1944	19.1788	29.5668	23.3649	26.4136	26.1927	27.3115	0.074036902	0.448301997	4	8	24	0.1936417	0.768040178	24	21.52559441	0.116556116
ENSMUSG00000058239	Usf2	upstream transcription factor 2 [Source:MGI Symbol;Acc:MGI:99961]	24.4099	21.8083	20.8816	23.1524	22.9985	22.8609	22.294	25.4617	21.782	25.2124	22.973	20.7116	24.1812	21.4263	0.92769718	0.966018836	16	20	24	0.965561572	0.991637933	24	18.37124002	0.010131721
ENSMUSG00000058240	Cryzl1	crystallin, zeta (quinone reductase)-like 1 [Source:MGI Symbol;Acc:MGI:1913859]	44.7684	45.6186	41.7928	39.4191	44.3459	44.264	44.0585	43.2579	48.2592	38.3611	47.2763	43.719	46.5753	44.5136	0.918193129	0.966018836	4	12	24	0.283002067	0.768040178	24	23.93824155	0.053986293
ENSMUSG00000058246	Gm10037	predicted gene 10037 [Source:MGI Symbol;Acc:MGI:3645096]	2.94154	2.97644	3.65469	2.09016	3.57121	2.91394	3.82792	2.36857	3.57388	2.06185	3.01404	3.23967	3.65812	4.23088	0.47028201	0.966018836	4	12	24	0.319136247	0.768040178	24	1.069160494	0.133352329
ENSMUSG00000058248	Kcnh1	potassium voltage-gated channel, subfamily H (eag-related), member 1 [Source:MGI Symbol;Acc:MGI:1341721]	7.27659	5.43599	5.23317	6.23819	5.52156	6.77976	6.45946	6.22531	4.13998	7.59173	5.0869	6.31284	6.2203	6.53153	0.321662653	0.83607295	24	12	24	0.361374023	0.786774635	24	21.50184718	0.111959591
ENSMUSG00000058254	Tspan7	tetraspanin 7 [Source:MGI Symbol;Acc:MGI:1298407]	99.8989	90.042	104.065	90.6544	83.4321	110.49	103.403	94.8722	78.1293	111.034	82.6923	98.7515	85.5163	101.15	0.890235289	0.966018836	24	20	24	0.785541723	0.931158434	24	23.33327388	0.044749886
ENSMUSG00000058258	Idi1	isopentenyl-diphosphate delta isomerase [Source:MGI Symbol;Acc:MGI:2442264]	13.2833	12.349	13.1371	12.6829	14.5146	12.7302	13.5533	11.3662	12.9489	12.0443	14.7645	10.9602	11.6324	11.6583	0.305564675	0.811201458	20	12	24	0.191247281	0.768040178	24	15.57864653	0.073572869
ENSMUSG00000058267	Mrps14	mitochondrial ribosomal protein S14 [Source:MGI Symbol;Acc:MGI:1928141]	36.7561	43.7033	38.7756	38.3516	42.7793	38.0526	37.7039	42.9558	45.6485	35.632	42.8354	39.2244	43.4197	44.1302	0.215777641	0.704989686	8	8	24	0.404046115	0.799546792	24	5.756975275	0.057108102
ENSMUSG00000058290	Espl1	extra spindle pole bodies 1, separase [Source:MGI Symbol;Acc:MGI:2146156]	1.04945	1.37979	0.785882	0.78143	0.416331	0.971885	0.419514	1.1964	2.23738	0.797239	0.89548	0.831554	0.544616	1.23934	0.138610659	0.580158868	8	12	24	0.126801767	0.739868076	24	6.630904398	0.246344736
ENSMUSG00000058291	Zfp68	zinc finger protein 68 [Source:MGI Symbol;Acc:MGI:1344427]	15.4001	17.4884	17.3957	16.8878	18.4929	16.1646	16.3402	15.8951	17.96	14.1805	19.1853	16.7693	17.3082	17.2356	0.581070934	0.966018836	20	8	24	0.847810065	0.954054905	24	13.87125546	0.023938901
ENSMUSG00000058297	Spock2	sparc/osteonectin, cwcv and kazal-like domains proteoglycan 2 [Source:MGI Symbol;Acc:MGI:1891351]	233.757	170.383	169.43	194.749	179.743	220.036	194.842	209.872	140.789	287.02	175.045	214.331	198.159	207.918	0.148073804	0.590732968	24	12	24	0.602226839	0.861006659	24	19.88728816	0.10151694
ENSMUSG00000058298	Mcm9	minichromosome maintenance 9 homologous recombination repair factor [Source:MGI Symbol;Acc:MGI:1918817]	2.66345	3.01794	3.11328	2.86712	2.90117	3.07968	2.90759	3.02199	2.97767	2.97633	3.25025	2.88701	3.2255	3.35682	0.912923872	0.966018836	8	16	24	0.507867202	0.813057152	24	7.014805336	0.024294232
ENSMUSG00000058301	Upf1	UPF1 regulator of nonsense transcripts homolog (yeast) [Source:MGI Symbol;Acc:MGI:107995]	7.94164	6.64936	6.2658	6.98818	7.47197	7.35062	8.15143	8.10422	7.2833	10.04	7.86319	8.4583	8.29755	7.70774	0.195343269	0.673387182	4	8	24	0.574523138	0.847329862	24	18.92831971	0.053101546
ENSMUSG00000058317	Ube2e2	ubiquitin-conjugating enzyme E2E 2 [Source:MGI Symbol;Acc:MGI:2384997]	40.4142	42.1276	40.345	37.3969	35.8316	43.7134	37.9779	38.2196	34.9543	44.1364	36.5005	38.3296	39.2723	40.4963	0.954584434	0.966018836	8	12	24	0.758094895	0.921733004	24	1.179810469	0.029183692
ENSMUSG00000058318	Phf21a	PHD finger protein 21A [Source:MGI Symbol;Acc:MGI:2384756]	10.7615	9.27991	11.342	7.7368	8.94776	10.5293	12.2754	9.11988	10.55	8.70159	8.52896	10.4555	10.4331	9.19298	0.04166704	0.34886238	4	12	24	0.086262666	0.705563884	24	0.337514397	0.133645436
ENSMUSG00000058325	Dock1	dedicator of cytokinesis 1 [Source:MGI Symbol;Acc:MGI:2429765]	4.77584	4.45856	4.25209	4.33887	4.48519	4.60852	5.22824	5.51427	4.60451	4.83831	4.03009	4.53526	4.25076	4.26934	0.915082018	0.966018836	4	16	24	0.4581312	0.805393068	24	1.808882252	0.04837673
ENSMUSG00000058331	Zfp85	zinc finger protein 85 [Source:MGI Symbol;Acc:MGI:107767]	2.12383	2.85914	2.22366	2.32798	2.44044	1.93285	2.17689	2.35992	3.34312	1.80803	2.54931	2.64704	2.78332	2.08651	0.852921792	0.966018836	12	4	24	0.791869331	0.93349015	24	5.967936551	0.047484209
ENSMUSG00000058351	Smim4	small integral membrane protein 4 [Source:MGI Symbol;Acc:MGI:1913737]	14.1981	23.5263	21.9678	23.1241	18.8777	19.0377	22.7121	24.2957	25.7974	16.5317	24.3484	22.0842	21.9749	22.9473	0.40165019	0.933249185	8	16	24	0.338516323	0.773151665	24	7.221790933	0.127294975
ENSMUSG00000058355	Abce1	ATP-binding cassette, sub-family E (OABP), member 1 [Source:MGI Symbol;Acc:MGI:1195458]	46.5479	45.4708	45.6677	44.8886	49.1811	45.8771	44.7856	41.5049	45.1605	40.5406	51.2493	43.5474	46.7406	43.2614	0.235141005	0.73629392	20	20	24	0.21360736	0.768040178	24	18.090041	0.058015618
ENSMUSG00000058385	H2bc8	H2B clustered histone 8 [Source:MGI Symbol;Acc:MGI:2448386]	0.701941	0.622992	1.04852	0.999528	0.652046	0.741644	1.06799	0.547413	0.896162	0.927981	1.20016	0.900101	0.528133	0.993865	0.658942124	0.966018836	16	16	24	0.258792214	0.768040178	24	12.49483635	0.103822746
ENSMUSG00000058388	Phtf1	putative homeodomain transcription factor 1 [Source:MGI Symbol;Acc:MGI:1332671]	30.6304	23.8335	21.9236	24.4222	24.9765	25.5788	25.4046	25.3831	19.8454	24.6915	24.6679	24.8352	26.5213	25.9395	1.23E-04	0.029372629	4	8	24	0.030051394	0.596794384	24	21.79190456	0.12744281
ENSMUSG00000058392	Rrp1b	ribosomal RNA processing 1B [Source:MGI Symbol;Acc:MGI:1919712]	3.21113	4.19865	4.94386	4.40539	4.79572	4.27412	4.52805	6.028	5.77226	4.7058	4.01291	4.38607	4.06373	5.13548	0.087592959	0.472021905	12	16	24	0.041952216	0.622498612	24	8.187535057	0.166525119
ENSMUSG00000058402	Zfp420	zinc finger protein 420 [Source:MGI Symbol;Acc:MGI:2444666]	6.49245	7.04904	6.58286	6.65334	7.14733	6.41552	7.54934	6.83283	6.52549	5.00592	7.53608	6.3237	6.23285	6.06247	0.823144114	0.966018836	20	4	24	0.584904262	0.852600138	24	21.53303123	0.051698949
ENSMUSG00000058407	Txndc9	thioredoxin domain containing 9 [Source:MGI Symbol;Acc:MGI:2138153]	21.9835	25.1933	21.7076	21.7149	24.3615	21.6702	19.1478	21.0211	20.8272	16.5734	23.9112	20.3061	19.7804	21.2134	0.919571723	0.966018836	20	8	24	0.892249481	0.969493574	24	19.61273447	0.026399061
ENSMUSG00000058420	Syt17	synaptotagmin XVII [Source:MGI Symbol;Acc:MGI:104966]	0.448175	0.570009	0.632314	0.76596	0.394067	0.853209	0.831976	0.484347	0.306938	0.898917	0.588759	0.673303	0.462795	1.51099	0.887862353	0.966018836	16	12	24	0.942916842	0.985884155	24	5.452640369	0.031923603
ENSMUSG00000058440	Nrf1	nuclear respiratory factor 1 [Source:MGI Symbol;Acc:MGI:1332235]	6.56376	6.4739	6.8384	6.89962	6.46101	6.39977	7.54532	6.30838	6.32645	6.18462	6.01229	6.26795	6.33788	5.6258	0.914502381	0.966018836	4	16	24	0.814472444	0.940820791	24	21.65822462	0.018342367
ENSMUSG00000058441	Panx2	pannexin 2 [Source:MGI Symbol;Acc:MGI:1890615]	10.6045	9.98548	12.5028	9.73845	8.4084	11.2777	10.4107	10.125	10.0715	15.5285	9.53094	11.8614	9.79079	11.6837	0.927004797	0.966018836	24	20	24	0.678539427	0.892463655	24	8.803300494	0.070838935
ENSMUSG00000058443	Rpl10-ps3	ribosomal protein L10, pseudogene 3 [Source:MGI Symbol;Acc:MGI:3704336]	1.38218	1.53727	1.78858	1.05375	1.52243	1.63867	1.92528	0.823404	2.64862	1.5223	2.1221	2.27168	1.93241	2.76591	0.865490576	0.966018836	24	16	24	0.960007792	0.989397471	24	2.777628695	0.027668605
ENSMUSG00000058444	Map2k5	mitogen-activated protein kinase kinase 5 [Source:MGI Symbol;Acc:MGI:1346345]	9.60024	8.55974	9.33019	9.14631	7.93559	9.20358	8.11582	9.3313	7.99207	11.7296	8.24744	9.39051	8.9782	9.43832	0.911135828	0.966018836	8	12	24	0.828542263	0.946606788	24	9.551348817	0.033572255
ENSMUSG00000058446	Znrf2	zinc and ring finger 2 [Source:MGI Symbol;Acc:MGI:1196246]	6.27387	6.36627	6.8	6.85095	6.38535	6.10867	6.29661	6.19309	6.49314	7.27267	7.11308	6.84091	6.65363	6.94602	0.16079538	0.620479606	16	12	24	0.026759105	0.586541342	24	11.81669642	0.056038167
ENSMUSG00000058454	Dhcr7	7-dehydrocholesterol reductase [Source:MGI Symbol;Acc:MGI:1298378]	36.7696	24.0676	25.8794	26.1379	27.7725	30.6605	28.1607	30.036	21.9846	31.6409	25.7372	28.2897	29.5667	27.3001	0.098024777	0.501188411	4	8	24	0.149039254	0.757594757	24	21.3168959	0.132553635
ENSMUSG00000058486	Wdr91	WD repeat domain 91 [Source:MGI Symbol;Acc:MGI:2141558]	7.39056	5.88597	6.99805	6.93804	5.90354	7.19376	6.87243	7.35921	6.76256	7.0255	7.47621	7.60333	6.66247	6.62973	0.43565473	0.966018836	24	8	24	0.743969024	0.916218012	24	19.0729408	0.030833034
ENSMUSG00000058488	Kl	klotho [Source:MGI Symbol;Acc:MGI:1101771]	0.658038	0.712539	0.625599	0.561028	0.467839	0.539592	0.661227	0.740325	0.701513	0.379472	0.594541	0.537843	0.542252	0.629873	0.004122392	0.140598674	8	8	24	0.012269112	0.525280095	24	3.566788538	0.089312311
ENSMUSG00000058492	Scp2-ps2	sterol carrier protein 2, pseudogene 2 [Source:MGI Symbol;Acc:MGI:107679]	112.671	123.982	128.964	147.974	127.511	119.845	117.897	118.031	126.995	109.836	135.545	120.146	130.074	118.126	0.327540787	0.846670301	20	8	24	0.305222363	0.768040178	24	12.81980005	0.065223804
ENSMUSG00000058498	Rnf207	ring finger protein 207 [Source:MGI Symbol;Acc:MGI:2684989]	4.44199	5.74021	4.83129	6.38502	4.90531	4.89025	5.80196	6.10445	5.90922	4.60163	5.25146	5.06066	4.93996	4.74426	0.816300547	0.966018836	16	8	24	0.566322166	0.843471947	24	7.785742499	0.061085467
ENSMUSG00000058503	Fam133b	family with sequence similarity 133, member B [Source:MGI Symbol;Acc:MGI:1915402]	13.6086	14.376	15.4229	15.778	13.1984	12.9551	15.195	13.3865	15.9934	14.8745	17.4678	15.739	16.7652	15.8202	0.880247609	0.966018836	12	12	24	0.526490225	0.823075573	24	9.893944771	0.047641398
ENSMUSG00000058546	Rpl23a	ribosomal protein L23A [Source:MGI Symbol;Acc:MGI:3040672]	0.306284	1.03085	0.664958	0.786109	0.520135	0.756691	0.6961	0.4282	1.16513	0.566587	0.529806	0.734849	0.7126	0.603324	0.935732159	0.966018836	12	16	24	0.575850763	0.848260645	24	8.178355289	0.08229207
ENSMUSG00000058558	Rpl5	ribosomal protein L5 [Source:MGI Symbol;Acc:MGI:102854]	11.1763	12.3011	10.1333	10.165	11.7498	10.5472	8.83468	9.64143	12.0015	8.94198	11.6533	9.456	10.5603	10.5388	0.858266942	0.966018836	20	20	24	0.975151425	0.993747214	24	10.61129391	0.012593782
ENSMUSG00000058569	Tmed9	transmembrane p24 trafficking protein 9 [Source:MGI Symbol;Acc:MGI:1914761]	58.1452	55.4652	56.7135	51.7798	59.6348	57.366	55.0765	57.225	52.445	49.421	54.1516	51.4795	54.5365	52.7666	0.636884864	0.966018836	4	12	24	0.163480148	0.767020009	24	22.52279944	0.046515324
ENSMUSG00000058571	Gpc6	glypican 6 [Source:MGI Symbol;Acc:MGI:1346322]	2.38241	3.48783	3.16517	2.87999	2.89021	2.13502	2.32023	2.6908	3.49177	2.70319	2.35593	2.96865	2.4915	3.08325	0.032924718	0.324980923	12	16	24	0.025544345	0.586541342	24	8.014941068	0.159464061
ENSMUSG00000058581	Gm5801	predicted pseudogene 5801 [Source:MGI Symbol;Acc:MGI:3646055]	12.6476	11.0422	12.5557	13.3596	11.2212	12.746	13.0566	12.5146	11.5763	14.2848	10.1388	12.4121	12.0115	11.9561	0.646840613	0.966018836	16	16	24	0.95603241	0.986639288	24	7.596734022	0.014614088
ENSMUSG00000058586	Serhl	serine hydrolase-like [Source:MGI Symbol;Acc:MGI:1890404]	37.3636	42.8611	44.4982	46.6485	57.7465	41.4704	42.9342	41.5804	49.8452	41.0982	42.6902	40.9645	44.7078	37.6007	0.455156031	0.966018836	20	4	24	0.193602631	0.768040178	24	13.34751575	0.105386838
ENSMUSG00000058587	Tmod3	tropomodulin 3 [Source:MGI Symbol;Acc:MGI:1355315]	18.678	19.7822	18.8645	18.9609	19.9743	17.6674	18.4476	16.889	20.3774	14.8747	20.0027	16.3471	16.8054	17.9178	0.44861185	0.966018836	20	4	24	0.909373111	0.974606327	24	10.46841082	0.020032229
ENSMUSG00000058589	Anks1b	ankyrin repeat and sterile alpha motif domain containing 1B [Source:MGI Symbol;Acc:MGI:1924781]	16.1417	14.2332	15.2362	12.299	12.59	15.0998	13.3331	14.333	13.0123	15.8084	14.2733	16.1089	13.6754	16.203	0.935593607	0.966018836	8	8	24	0.596161367	0.857839743	24	0.651667927	0.050318249
ENSMUSG00000058594	Fbh1	F-box DNA helicase 1 [Source:MGI Symbol;Acc:MGI:1354699]	31.6246	33.5252	31.069	33.9268	34.7178	33.9337	31.8375	31.8209	31.5388	31.7983	35.1084	32.5344	32.9202	31.7343	0.002569536	0.112580623	20	16	24	0.026492805	0.586541342	24	16.97169561	0.052682533
ENSMUSG00000058600	Rpl30	ribosomal protein L30 [Source:MGI Symbol;Acc:MGI:98037]	32.312	44.8937	42.0245	42.3849	49.9922	37.5907	41.2398	41.5248	46.4897	25.8141	39.5889	29.1497	41.4206	27.9395	0.601647512	0.966018836	12	12	24	0.951111275	0.988300568	24	9.705315232	0.034264867
ENSMUSG00000058603	Rpl28-ps1	ribosomal protein L28, pseudogene 1 [Source:MGI Symbol;Acc:MGI:3705349]	163.394	147.051	145.398	153.447	146.335	162.9	163.719	157.778	155.729	149.746	143.146	149.552	162.3	153.445	0.004516736	0.140598674	4	16	24	0.049336646	0.641729576	24	0.129879894	0.059285941
ENSMUSG00000058624	Gda	guanine deaminase [Source:MGI Symbol;Acc:MGI:95678]	13.8174	12.9447	12.7939	14.301	11.6125	14.0416	12.4305	12.1164	12.2374	14.3041	13.2919	14.1257	13.3259	14.6553	0.916556753	0.966018836	16	20	24	0.887459234	0.968341734	24	20.27617343	0.019680283
ENSMUSG00000058638	Zfp110	zinc finger protein 110 [Source:MGI Symbol;Acc:MGI:1890378]	10.7565	12.7265	11.189	9.80023	11.9278	10.7816	10.8013	11.5655	9.33239	11.6855	12.1077	12.1378	[illegible]	12.0647	0.148621761	0.59172107	8	8	24	0.251489722	0.768040178	24	1.05390056	0.070395398
ENSMUSG00000058655	Eif4b	eukaryotic translation initiation factor 4B [Source:MGI Symbol;Acc:MGI:95304]	47.6513	46.8368	48.9796	49.837	42.0759	47.4669	44.284	48.2148	46.4955	55.1837	42.1494	49.6364	47.7651	48.8631	0.187699199	0.662239322	16	4	24	0.426384423	0.805393068	24	7.838251818	0.052453886
ENSMUSG00000058656	Samd12	sterile alpha motif domain containing 12 [Source:MGI Symbol;Acc:MGI:2444518]	9.89284	8.50157	8.15551	8.32519	9.41697	9.3753	8.27893	7.6668	9.03191	7.79504	10.4052	10.1859	10.0402	10.3239	0.356953959	0.881188925	20	20	24	0.1227692	0.735412761	24	20.35195649	0.101732572
ENSMUSG00000058672	Tubb2a	tubulin, beta 2A class IIA [Source:MGI Symbol;Acc:MGI:107861]	365.385	298.989	311.752	291.919	324.267	368.829	316.832	325.941	260.716	319.085	338.968	318.73	316.81	324.765	0.133012827	0.567728672	24	12	24	0.031982717	0.604838544	24	20.58811751	0.119670214
ENSMUSG00000058690	Ccser2	coiled-coil serine rich 2 [Source:MGI Symbol;Acc:MGI:101859]	106.416	98.3946	92.3181	102.382	100.928	98.6445	94.6428	86.5247	92.6668	98.111	104.236	102.759	107.243	102.27	0.104994359	0.518002936	4	8	24	0.147921126	0.756155497	24	19.04168965	0.065142211
ENSMUSG00000058704	Memo1	mediator of cell motility 1 [Source:MGI Symbol;Acc:MGI:1924140]	34.4711	32.9181	33.5752	32.2597	33.975	31.7651	30.3462	32.5237	34.5737	32.1348	35.1961	30.8792	35.4707	33.2237	0.914502381	0.966018836	12	12	24	0.819883438	0.942844597	24	8.959372631	0.017500751
ENSMUSG00000058706	0610030E20Rik	RIKEN cDNA 0610030E20 gene [Source:MGI Symbol;Acc:MGI:1915614]	6.3951	6.67275	7.1664	7.11571	7.47177	6.09553	6.74425	6.99131	7.82052	6.83019	7.50451	6.08316	7.1696	7.00897	0.040082266	0.34094463	20	4	24	0.051833486	0.648938347	24	9.815768249	0.083430491
ENSMUSG00000058709	Egln2	egl-9 family hypoxia-inducible factor 2 [Source:MGI Symbol;Acc:MGI:1932287]	35.4972	26.7046	26.8374	25.9562	27.1548	27.8425	29.8199	30.461	28.1338	34.5727	32.9261	33.6922	32.1823	32.0564	0.662399683	0.966018836	4	8	24	0.421270173	0.804499836	24	21.91756358	0.068610707
ENSMUSG00000058715	Fcer1g	Fc receptor, IgE, high affinity I, gamma polypeptide [Source:MGI Symbol;Acc:MGI:95496]	11.7822	18.4095	23.9227	14.6826	15.9766	14.1915	15.0168	18.112	20.0001	11.0919	16.0801	14.3001	15.2215	11.7713	0.236893212	0.7385769	12	12	24	0.386479018	0.799546792	24	7.759431679	0.155792574
ENSMUSG00000058729	Lin9	lin-9 homolog (C. elegans) [Source:MGI Symbol;Acc:MGI:1919818]	1.21943	1.57939	1.56307	1.56806	1.5711	1.64712	1.25226	1.48554	1.41543	1.47276	1.7362	1.53679	1.3049	1.1734	0.024928639	0.291208026	20	8	24	0.031957405	0.604838544	24	14.52681139	0.090306176
ENSMUSG00000058740	Kcnt1	potassium channel, subfamily T, member 1 [Source:MGI Symbol;Acc:MGI:1924627]	70.7493	63.509	62.2251	61.6205	69.1817	67.7788	57.4833	65.9143	66.7796	71.4024	66.4006	68.8511	67.597	69.1835	0.916556573	0.966018836	24	12	24	0.732884328	0.91282299	24	17.08044794	0.026188971
ENSMUSG00000058745	Gm9174	predicted pseudogene 9174 [Source:MGI Symbol;Acc:MGI:3643688]	15.8278	14.3176	16.1644	17.312	14.564	17.5072	14.4122	12.6154	17.2139	12.1865	13.5924	11.916	13.8151	15.1951	0.888482515	0.966018836	12	20	24	0.852322089	0.955628938	24	8.636915435	0.036433704
ENSMUSG00000058748	Zfp958	zinc finger protein 958 [Source:MGI Symbol;Acc:MGI:2385298]	6.62583	8.36187	7.1778	6.0163	6.38819	6.42175	7.35794	6.06487	8.38972	5.17207	8.04569	7.14587	7.09253	6.7319	0.503972924	0.966018836	12	4	24	0.615073697	0.866892532	24	2.223268274	0.066024722
ENSMUSG00000058756	Thra	thyroid hormone receptor alpha [Source:MGI Symbol;Acc:MGI:98742]	74.1471	66.5226	64.7192	66.7686	60.3945	70.5913	70.3586	70.6748	66.1202	84.2685	55.1287	71.2136	72.7567	74.1624	0.302438464	0.811201458	4	16	24	0.444748275	0.805393068	24	3.000577308	0.069893851
ENSMUSG00000058761	Rnf169	ring finger protein 169 [Source:MGI Symbol;Acc:MGI:1920257]	2.91314	2.90203	3.4393	2.97733	3.38929	2.90348	3.22423	2.79731	3.30366	3.2403	3.13635	2.79479	3.06572	2.90765	0.071311157	0.440262583	12	20	24	0.171418844	0.768040178	24	11.76288616	0.054100692
ENSMUSG00000058793	Cds2	CDP-diacylglycerol synthase (phosphatidate cytidylyltransferase) 2 [Source:MGI Symbol;Acc:MGI:1332236]	118.982	90.045	94.2826	102.785	92.6802	111.614	96.554	100.721	79.3942	128.531	91.4764	110.214	104.624	104.512	0.220555749	0.710961211	24	12	24	0.523244879	0.821146401	24	19.95100006	0.083253049
ENSMUSG00000058794	Nfe2	nuclear factor, erythroid derived 2 [Source:MGI Symbol;Acc:MGI:97308]	0.974034	2.19317	2.3334	2.86537	1.81123	2.33771	2.04401	2.29665	1.80209	1.48728	1.77949	1.81811	2.23603	1.43011	0.846563228	0.966018836	24	4	24	0.814466527	0.940820791	24	10.87075367	0.063581212
ENSMUSG00000058799	Nap1l1	nucleosome assembly protein 1-like 1 [Source:MGI Symbol;Acc:MGI:1855693]	47.7474	49.0234	46.369	46.5958	47.5365	45.8424	42.5229	44.0731	43.9843	45.1684	47.2921	43.6911	45.3994	45.7322	0.868022237	0.966018836	20	4	24	0.762150984	0.92297519	24	14.38300362	0.014113892
ENSMUSG00000058806	Col13a1	collagen, type XIII, alpha 1 [Source:MGI Symbol;Acc:MGI:1277201]	0.450215	0.570894	0.492321	1.19496	0.341376	0.362511	0.61453	0.926617	0.7132	0.534857	0.333006	0.868261	0.304064	0.747241	0.393470605	0.921010321	8	12	24	0.344826366	0.778238779	24	7.684203342	0.133862372
ENSMUSG00000058809	Hspd1-ps3	heat shock protein 1 (chaperonin), pseudogene 3 [Source:MGI Symbol;Acc:MGI:3651246]	67.5672	54.1055	52.3339	59.2612	60.5808	60.0953	59.9442	59.1631	46.4731	55.7751	59.8885	58.9487	60.4024	56.0262	0.001920665	0.100255953	4	8	24	0.009448384	0.502965379	24	20.46789564	0.130350589
ENSMUSG00000058816	Ppp1r2-ps3	protein phosphatase 1, regulatory (inhibitor) subunit 2, pseudogene 3 [Source:MGI Symbol;Acc:MGI:3645534]	5.26456	5.38443	4.00344	5.19755	5.02076	5.53761	4.37877	6.02155	5.49646	7.48185	6.78091	6.30071	6.51364	6.02945	0.869755137	0.966018836	16	20	24	0.658623269	0.884596426	24	15.4640784	0.055377036
ENSMUSG00000058818	Pirb	paired Ig-like receptor B [Source:MGI Symbol;Acc:MGI:894311]	1.17625	1.57846	1.7341	1.80007	1.55911	1.80201	1.39476	1.67949	1.74845	1.03023	1.40119	1.39772	1.63252	1.21986	0.870491976	0.966018836	12	16	24	0.935717381	0.983894024	24	9.108688399	0.021784291
ENSMUSG00000058833	Rex1bd	required for excision 1-B domain containing [Source:MGI Symbol;Acc:MGI:1913712]	55.9079	44.7558	45.9308	37.8211	42.0867	36.1337	45.3293	54.0625	57.3706	53.8985	66.2983	60.59	69.5163	64.9725	0.922029406	0.966018836	4	12	24	0.413451564	0.799546792	24	3.278849832	0.108411483
ENSMUSG00000058835	Abi1	abl interactor 1 [Source:MGI Symbol;Acc:MGI:104913]	39.1132	37.0078	38.3657	40.5299	35.6696	38.188	37.8664	37.7058	33.1312	39.2364	37.5922	37.7953	37.6922	40.0195	0.859936714	0.966018836	16	4	24	0.944427181	0.986443184	24	21.4674802	0.009972012
ENSMUSG00000058881	Zfp516	zinc finger protein 516 [Source:MGI Symbol;Acc:MGI:2443957]	5.52309	4.07124	3.6763	4.23678	3.81512	4.58428	4.68256	4.22802	4.06485	5.42219	4.13199	4.56381	4.52516	4.06777	0.030691182	0.324150946	4	8	24	0.406446831	0.799546792	24	21.19079534	0.076088919
ENSMUSG00000058883	Zfp708	zinc finger protein 708 [Source:MGI Symbol;Acc:MGI:3040674]	2.05845	2.15134	2.28404	1.89759	2.25939	1.60753	1.95603	1.37675	2.14818	1.29302	2.15151	1.94955	1.52724	1.56499	0.723119329	0.966018836	20	12	24	0.761297589	0.922782206	24	15.70312772	0.04493603
ENSMUSG00000058886	Deaf1	DEAF1, transcription factor [Source:MGI Symbol;Acc:MGI:1858496]	15.9639	14.5998	14.8992	16.4684	16.3284	16.7822	15.4038	15.4565	14.4539	19.7344	14.9969	16.52	15.7191	15.2406	0.010607268	0.197540491	24	12	24	0.186782309	0.768040178	24	15.75440263	0.073717428
ENSMUSG00000058897	Col25a1	collagen, type XXV, alpha 1 [Source:MGI Symbol;Acc:MGI:1924268]	2.29923	2.84227	2.39187	2.31591	1.9022	2.37346	2.20033	2.01236	2.47632	2.14582	2.03905	2.34177	1.79731	2.4559	0.302438464	0.811201458	12	8	24	0.359101575	0.785405943	24	5.849457767	0.063454803
ENSMUSG00000058900	Rsl1	regulator of sex limited protein 1 [Source:MGI Symbol;Acc:MGI:3044162]	1.63046	1.89782	1.70359	1.09099	1.87764	1.47148	1.67432	1.59751	2.00294	1.2275	2.02319	1.9289	1.69932	1.81248	0.922029406	0.966018836	20	20	24	0.598047335	0.858819856	24	0.418804964	0.061291948
ENSMUSG00000058914	C1qtnf3	C1q and tumor necrosis factor related protein 3 [Source:MGI Symbol;Acc:MGI:1932136]	1.26622	1.63381	1.27038	1.23879	1.15122	0.917931	0.973709	1.47897	1.16259	0.709373	0.857941	1.31912	0.81284	1.25346	0.157744865	0.614233844	8	12	24	0.170853172	0.768040178	24	4.403633506	0.114717973
ENSMUSG00000058922	Gm10052	predicted pseudogene 10052 [Source:MGI Symbol;Acc:MGI:3704348]	57.6978	65.5593	68.2196	67.5981	62.9587	61.0779	60.2311	59.0678	66.8165	60.5905	58.5599	60.9955	63.4841	63.0303	0.184814212	0.662239322	12	16	24	0.073812895	0.682440791	24	8.900372746	0.065361106
ENSMUSG00000058925	Ccdc192	coiled-coil domain containing 192 [Source:MGI Symbol;Acc:MGI:1922694]	3.51445	4.11638	4.29235	4.36445	4.58357	4.3875	3.16198	2.89238	3.37449	2.63761	5.07201	4.65415	3.9333	4.55202	0.126473451	0.558021618	24	4	24	0.368768057	0.791761501	24	17.51870414	0.125804838
ENSMUSG00000058927	Gm10053	predicted gene 10053 [Source:MGI Symbol;Acc:MGI:3704493]	46.2981	50.4836	52.001	56.6374	46.4555	45.9475	50.2256	55.7611	57.7873	50.3443	60.6528	56.4345	69.739	62.2081	0.895493752	0.966018836	8	16	24	0.286785206	0.768040178	24	6.2783757	0.074983632
ENSMUSG00000058966	Tlcd3b	TLC domain containing 3B [Source:MGI Symbol;Acc:MGI:1916202]	37.8314	27.7866	26.7112	29.14	26.0067	29.9572	30.6129	34.2513	22.8428	42.6271	30.3806	32.4953	34.3535	33.6518	0.105280783	0.518002936	4	8	24	0.662273246	0.885924039	24	22.58110919	0.080213334
ENSMUSG00000058975	Kcnc1	potassium voltage gated channel, Shaw-related subfamily, member 1 [Source:MGI Symbol;Acc:MGI:96667]	9.96879	9.80689	13.3666	9.20293	8.13234	11.3099	12.3563	9.97058	10.1811	13.7632	8.26833	11.2907	8.78519	13.3951	0.664568268	0.966018836	12	8	24	0.442029867	0.805393068	24	5.921732147	0.12006487
ENSMUSG00000058979	Hdhd5	haloacid dehalogenase like hydrolase domain containing 5 [Source:MGI Symbol;Acc:MGI:2136976]	10.226	10.7963	11.088	11.0194	11.4706	10.618	11.2438	11.0518	10.9208	11.1272	10.4549	10.3186	10.247	10.427	0.241950772	0.741580974	16	12	24	0.150470245	0.758865539	24	10.95938957	0.034376394
ENSMUSG00000058997	Vwa8	von Willebrand factor A domain containing 8 [Source:MGI Symbol;Acc:MGI:1919008]	12.1448	11.4939	12.6758	14.7882	11.169	12.6805	12.145	13.695	11.8128	14.679	11.4057	12.6098	12.6751	13.3416	0.369262716	0.893596186	16	4	24	0.434688905	0.805393068	24	9.193875793	0.060594534
ENSMUSG00000059005	Hnrnpa3	heterogeneous nuclear ribonucleoprotein A3 [Source:MGI Symbol;Acc:MGI:1917171]	14.8889	16.3934	15.6164	15.5987	16.7681	14.4805	15.6435	13.9043	18.4279	14.1094	16.5386	15.8171	16.2056	15.9844	0.90332508	0.966018836	20	20	24	0.809255574	0.939184036	24	10.17598496	0.026944512
ENSMUSG00000059013	Sh2d3c	SH2 domain containing 3C [Source:MGI Symbol;Acc:MGI:1351631]	5.68343	5.41672	5.81337	5.69299	5.40461	5.74042	6.40091	6.25413	5.6946	8.09526	6.31849	6.11364	5.67767	6.50689	0.794858476	0.966018836	16	12	24	0.553362101	0.837286821	24	10.87331018	0.050282187
ENSMUSG00000059022	Kcp	kielin/chordin-like protein [Source:MGI Symbol;Acc:MGI:2141640]	1.29515	1.01553	0.928208	0.973099	1.92812	1.29076	1.10568	1.13388	1.47042	1.45282	1.44333	1.64951	2.14446	1.50407	0.714062999	0.966018836	20	20	24	0.226650991	0.768040178	24	19.04894475	0.112722606
ENSMUSG00000059027	9630013D21Rik	RIKEN cDNA 9630013D21 gene [Source:MGI Symbol;Acc:MGI:2442649]	0.242635	0.16476	0.430031	1.11727	0.432803	0.23986	0.282499	0.760657	0.687912	0.868691	0.467113	0.5266	0.64684	0.451867	0.235141005	0.73629392	20	8	24	0.034216824	0.608494889	24	10.93480741	0.216092939
ENSMUSG00000059031	Olfr482	olfactory receptor 482 [Source:MGI Symbol;Acc:MGI:3030316]	1.29316	1.51413	1.40746	1.49193	1.48512	1.28344	0.948544	1.38039	1.64909	1.35573	1.5409	1.34395	1.69542	1.50696	0.459019924	0.966018836	12	12	24	0.30164676	0.768040178	24	9.776557656	0.068347919
ENSMUSG00000059040	Eno1b	enolase 1B, retrotransposed [Source:MGI Symbol;Acc:MGI:3648653]	18.4883	15.0979	16.8783	18.9621	16.6276	18.689	16.9654	18.3315	13.9682	19.118	18.2959	18.0285	18.1856	19.6519	0.880059765	0.966018836	16	20	24	0.556806437	0.838702296	24	18.92372047	0.055511855

ENSMUSG00000059049	Frem1	Fras1 related extracellular matrix protein 1 [Source:MGI Symbol;Acc:MGI:2670972]	1.64935	2.2619	2.03427	1.7469	2.0114	1.68935	2.42098	1.60116	2.23835	2.06984	1.73962	1.90879	2.25462	2.16442	0.849179819	0.966018836	4	20	24	0.430684858	0.805393068	24	5.223655416	0.065130657
ENSMUSG00000059058	Tma7-ps	translational machinery associated 7 homolog (S. cerevisiae), pseudogene [Source:MGI Symbol;Acc:MGI:3705453]	206.363	228.447	199.633	207.711	188.375	206.81	217.253	178.78	190.405	146.074	190.878	186.179	184.057	190.406	0.826055781	0.966018836	4	16	24	0.17694489	0.768040178	24	23.98112902	0.089397489
ENSMUSG00000059070	Rpl18	ribosomal protein L18 [Source:MGI Symbol;Acc:MGI:98003]	6.646	7.36486	6.01602	6.23372	7.56383	7.64778	5.98179	7.59754	9.47831	5.80165	10.0578	7.27058	7.99318	6.80054	0.911685024	0.966018836	20	20	24	0.861027181	0.959154979	24	17.48853854	0.044830387
ENSMUSG00000059089	Fcgr4	Fc receptor, IgG, low affinity IV [Source:MGI Symbol;Acc:MGI:2179523]	0.907626	0.941586	1.61923	1.00165	0.898862	0.681726	0.753591	0.781353	0.94312	0.599553	1.10035	0.762552	0.748678	0.567327	0.323424237	0.839647936	12	12	24	0.372553017	0.793835745	24	10.69879644	0.088693127
ENSMUSG00000059108	Ifitm6	interferon induced transmembrane protein 6 [Source:MGI Symbol;Acc:MGI:2686976]	3.40029	10.0142	13.9295	11.6779	8.61537	10.8485	6.06142	7.03183	8.73126	5.56871	7.84511	9.00715	10.8762	6.04787	0.897570341	0.966018836	12	20	24	0.591316604	0.855808161	24	11.93873232	0.188481796
ENSMUSG00000059119	Nap1l4	nucleosome assembly protein 1-like 4 [Source:MGI Symbol;Acc:MGI:1316687]	78.4996	75.7034	75.1928	77.435	73.7675	74.5378	73.1181	75.5995	74.7535	75.5643	74.7325	76.2329	80.2327	79.1733	0.510431574	0.966018836	4	16	24	0.393991656	0.799546792	24	2.875295878	0.019636528
ENSMUSG00000059142	Zfp945	zinc finger protein 945 [Source:MGI Symbol;Acc:MGI:2445132]	7.7976	11.2038	9.73329	9.53634	9.26069	8.67558	8.7374	8.70037	10.3276	7.77679	9.12165	10.0968	9.94624	9.98095	0.459028788	0.966018836	8	20	24	0.520954284	0.820180873	24	6.057353405	0.062625473
ENSMUSG00000059146	Ntrk3	neurotrophic tyrosine kinase, receptor, type 3 [Source:MGI Symbol;Acc:MGI:97385]	24.121	25.2621	24.1891	22.9565	23.4033	22.451	23.7115	25.4618	23.2128	24.8219	25.1248	25.9741	26.4853	26.4029	0.315232163	0.827645017	8	8	24	0.17035662	0.767957732	24	2.89022145	0.041570505
ENSMUSG00000059149	Mfsd4a	major facilitator superfamily domain containing 4A [Source:MGI Symbol;Acc:MGI:2442786]	8.76608	9.07746	8.88396	9.72064	9.39939	9.33216	10.4202	10.0581	10.0014	10.7645	9.22648	9.12434	9.71916	9.46418	0.893609703	0.966018836	16	8	24	0.543296759	0.831565139	24	9.406900259	0.03296526
ENSMUSG00000059173	Pde1a	phosphodiesterase 1A, calmodulin-dependent [Source:MGI Symbol;Acc:MGI:1201792]	5.38062	6.93544	6.81616	7.16095	6.06242	5.28373	5.55135	6.03621	9.14536	4.84858	6.56642	6.63116	5.40047	6.17354	0.122530972	0.550724475	12	16	24	0.185991215	0.768040178	24	8.97487665	0.145873189
ENSMUSG00000059182	Skap2	src family associated phosphoprotein 2 [Source:MGI Symbol;Acc:MGI:1889206]	19.7309	19.0868	17.6576	18.6181	19.0889	17.1049	17.7198	17.0449	18.2919	15.4547	18.0546	17.8217	18.9637	17.0205	0.910436409	0.966018836	4	4	24	0.435037177	0.805393068	24	22.27644491	0.040323276
ENSMUSG00000059183	Mtfmt	mitochondrial methionyl-tRNA formyltransferase [Source:MGI Symbol;Acc:MGI:1916856]	5.40469	7.46142	6.66539	7.05336	7.00899	6.90169	6.25292	6.83536	7.94264	7.21714	6.85516	7.31075	7.71051	7.12396	0.907000607	0.966018836	16	12	24	0.240162526	0.768040178	24	9.929111136	0.067486714
ENSMUSG00000059187	Tafa1	TAFA chemokine like family member 1 [Source:MGI Symbol;Acc:MGI:2443695]	10.5436	9.5263	9.12957	8.47515	9.47334	9.16072	10.0342	9.14848	9.1475	7.8585	10.0739	9.78507	10.239	9.67187	1.02E-04	0.029372629	4	12	24	0.008561736	0.501317957	24	23.00360463	0.105044746
ENSMUSG00000059195	Gm12715	predicted gene 12715 [Source:MGI Symbol;Acc:MGI:3650759]	170.748	120.488	126.961	124.571	110.627	138.026	142.984	154.135	112.888	182.544	132.122	155.059	148.31	141.367	0.34708369	0.870418653	4	8	24	0.524880885	0.822296127	24	23.09433845	0.093337896
ENSMUSG00000059208	Hnrnpm	heterogeneous nuclear ribonucleoprotein M [Source:MGI Symbol;Acc:MGI:1926465]	61.7784	60.7506	63.1024	63.5924	64.5324	59.2489	63.6827	54.4745	58.1853	58.6058	61.9871	54.9186	59.6539	54.6762	0.305564675	0.811201458	20	12	24	0.214436297	0.768040178	24	15.82911616	0.043527728
ENSMUSG00000059213	Ddn	dendrin [Source:MGI Symbol;Acc:MGI:108101]	21.3259	24.0891	20.1114	23.4343	26.834	20.1006	25.7955	24.7903	30.9389	28.4678	27.4444	29.8307	32.5586	24.9498	0.807813307	0.966018836	20	12	24	0.880142133	0.966366217	24	13.05931944	0.030418378
ENSMUSG00000059237	Ppp1r2-ps4	protein phosphatase 1, regulatory (inhibitor) subunit 2, pseudogene 4 [Source:MGI Symbol;Acc:MGI:3645199]	85.4237	76.1935	79.7998	77.8102	82.1394	84.6422	73.9125	84.2612	68.0091	90.9823	87.0865	87.6181	87.3172	88.2889	0.916746631	0.966018836	24	12	24	0.394469187	0.799546792	24	19.01045643	0.059096609
ENSMUSG00000059248	Septin9	septin 9 [Source:MGI Symbol;Acc:MGI:1858222]	21.632	19.5739	17.6344	20.1705	23.2824	20.3093	23.1755	23.08	20.8169	23.1675	21.124	20.3359	22.1954	19.5778	0.542718076	0.966018836	4	8	24	0.543871032	0.831894202	24	19.53580686	0.05048713
ENSMUSG00000059263	Usp47	ubiquitin specific peptidase 47 [Source:MGI Symbol;Acc:MGI:1922246]	22.8524	24.516	24.937	25.7231	24.2399	23.7381	22.0179	24.9263	25.699	22.8054	23.9608	25.0148	25.1183	23.9902	0.724067924	0.966018836	12	16	24	0.368495994	0.791588198	24	9.340093116	0.036613874
ENSMUSG00000059273	Zc3h4	zinc finger CCCH-type containing 4 [Source:MGI Symbol;Acc:MGI:2682314]	6.87913	6.43907	6.88162	7.52204	7.27582	6.47792	7.84333	7.17695	6.98878	10.0459	7.18661	8.1089	7.36658	7.31218	0.718086778	0.966018836	16	20	24	0.33724551	0.772183698	24	13.174414	0.072354117
ENSMUSG00000059277	R74862	expressed sequence R74862 [Source:MGI Symbol;Acc:MGI:2142382]	1.02569	1.21021	1.62917	1.44579	1.36657	1.12044	1.77503	1.0368	1.44789	1.06964	1.26401	1.38187	1.27784	1.43534	0.932671476	0.966018836	12	20	24	0.850806253	0.955422325	24	8.626137182	0.032705561
ENSMUSG00000059278	Naa38	N(alpha)-acetyltransferase 38, NatC auxiliary subunit [Source:MGI Symbol;Acc:MGI:1925554]	91.5991	103.476	86.5881	89.7692	94.8164	90.7164	81.8941	87.1855	103.81	72.3644	108.362	91.3696	96.5472	92.5242	0.885059765	0.966018836	20	20	24	0.946171179	0.986887534	24	22.48220209	0.020271105
ENSMUSG00000059288	Cdyl	chromodomain protein, Y chromosome-like [Source:MGI Symbol;Acc:MGI:1339956]	3.42189	3.08729	3.23055	3.57022	3.24685	3.51124	2.7558	3.4214	2.95081	3.60347	3.86155	3.4933	3.42532	3.11217	0.030043575	0.324150946	16	20	24	0.094827528	0.71175955	24	15.88222082	0.077403646
ENSMUSG00000059291	Rpl11	ribosomal protein L11 [Source:MGI Symbol;Acc:MGI:1914275]	5.80802	5.45774	5.65673	5.24891	5.03453	4.93789	4.45522	3.17965	6.44106	3.64227	6.38916	4.35751	5.68807	5.47784	0.912686312	0.966018836	12	12	24	0.985968899	0.997339197	24	12.45615572	0.016256559
ENSMUSG00000059301	Gm5434	predicted gene 5434 [Source:MGI Symbol;Acc:MGI:3646221]	0.369461	0.626465	0.770433	0.673139	0.601202	0.859348	0.752114	0.828828	0.779098	0.793126	0.594279	0.744081	0.573143	0.823489	0.664568268	0.966018836	8	20	24	0.390511931	0.799546792	24	8.754030231	0.061903426
ENSMUSG00000059316	Slc27a4	solute carrier family 27 (fatty acid transporter), member 4 [Source:MGI Symbol;Acc:MGI:1347347]	27.1832	20.1122	20.3956	22.5313	24.13	25.261	23.4868	23.4901	17.7988	25.98	21.5501	22.6158	21.1792	21.4256	0.261443857	0.768150062	24	12	24	0.1109694	0.723072658	24	19.38146434	0.122590747
ENSMUSG00000059323	Tonsl	tonsoku-like, DNA repair protein [Source:MGI Symbol;Acc:MGI:1919999]	2.347	2.353	3.2333	3.07487	3.89361	3.01397	1.86508	3.56044	2.40016	2.66248	3.12549	2.40942	2.58433	2.56622	0.050258003	0.385784509	20	8	24	0.177468403	0.768040178	24	13.35057303	0.142473415
ENSMUSG00000059325	Hopx	HOP homeobox [Source:MGI Symbol;Acc:MGI:1916782]	147.849	137.28	126.373	130.583	113.105	124.321	122.728	126.268	123.148	133.839	143.867	133.837	137.941	144.454	0.934110497	0.966018836	8	4	24	0.607174123	0.863796174	24	1.808442334	0.041361992
ENSMUSG00000059326	Csf2ra	colony stimulating factor 2 receptor, alpha, low-affinity (granulocyte-macrophage) [Source:MGI Symbol;Acc:MGI:1339754]	9.3788	11.4132	10.7808	9.33654	11.0654	8.38752	11.3834	13.4989	11.2842	11.0441	11.5342	11.9344	12.0212	11.9931	0.138719141	0.580158868	8	8	24	0.18971728	0.768040178	24	5.266714963	0.095638869
ENSMUSG00000059327	Eda	ectodysplasin-A [Source:MGI Symbol;Acc:MGI:1195272]	1.6456	1.73178	1.75564	1.71202	1.74371	1.93191	2.10318	2.12645	1.62401	1.97093	1.70438	1.66734	2.01073	1.75609	0.914360142	0.966018836	8	4	24	0.546910392	0.833582824	24	0.939723481	0.036577804
ENSMUSG00000059336	Slc14a1	solute carrier family 14 (urea transporter), member 1 [Source:MGI Symbol;Acc:MGI:1351654]	0.962545	1.43672	2.80388	1.41758	1.20553	1.83405	1.56117	1.46388	1.8185	1.25335	1.03113	1.45456	1.1592	1.45247	0.46238066	0.966018836	12	8	24	0.32065121	0.768040178	24	7.623319325	0.142761321
ENSMUSG00000059343	Aldoart1	aldolase 1 A, retrogene 1 [Source:MGI Symbol;Acc:MGI:2447811]	1.82601	1.75186	1.82728	2.39526	2.57418	2.37169	2.07779	3.22452	1.27846	1.95453	1.40534	1.27962	1.14299	1.04179	0.912923872	0.966018836	16	16	24	0.73504104	0.913625254	24	15.49990011	0.079699832
ENSMUSG00000059355	Wdr83os	WD repeat domain 83 opposite strand [Source:MGI Symbol;Acc:MGI:3041257]	16.4472	14.4538	14.282	14.8798	15.5718	17.9762	16.4333	20.4684	14.9406	18.4971	14.73	14.9556	16.3282	15.3449	0.633249925	0.966018836	4	8	24	0.772959682	0.92697438	24	23.78120616	0.041386304
ENSMUSG00000059361	Nrsn2	neurensin 2 [Source:MGI Symbol;Acc:MGI:2684969]	3.27928	5.33807	8.91358	4.87111	3.17589	6.30654	6.89596	3.44239	5.41376	5.97782	2.41192	5.91127	2.81382	7.15739	0.424718357	0.966018836	12	8	24	0.468594435	0.805393068	24	6.893078947	0.235199569
ENSMUSG00000059363	Fxn	frataxin [Source:MGI Symbol;Acc:MGI:1096879]	2.53013	2.69125	2.64794	3.48687	2.78051	2.34605	2.52518	2.83191	3.14595	3.00977	2.5215	2.68178	3.65246	2.92388	0.243586913	0.741580974	16	8	24	0.263534725	0.768040178	24	8.674711677	0.084502418
ENSMUSG00000059401	Mamld1	mastermind-like domain containing 1 [Source:MGI Symbol;Acc:MGI:3045303]	5.83545	5.05941	5.10731	5.15803	5.51106	5.38135	5.18155	5.41974	5.13324	6.78894	5.37764	5.19907	5.37488	4.93988	0.200364953	0.684570416	20	12	24	0.466077782	0.805393068	24	15.0967904	0.047069334
ENSMUSG00000059409	Ppp2r5d	protein phosphatase 2, regulatory subunit B', delta [Source:MGI Symbol;Acc:MGI:2388481]	18.2484	16.2387	16.8554	17.1646	17.1793	19.6265	18.9279	19.2729	14.5324	21.1693	16.2248	17.5447	17.283	16.7727	0.377663653	0.903463289	24	12	24	0.561308796	0.841538771	24	20.01173219	0.057626756
ENSMUSG00000059412	Fxyd2	FXYD domain-containing ion transport regulator 2 [Source:MGI Symbol;Acc:MGI:1195260]	801.462	701.904	590.246	679.187	648.037	729.672	622.265	642.373	669.21	622.954	695.612	734.299	782.504	698.546	0.073139747	0.447768907	24	12	24	0.064641951	0.663876076	24	21.98547486	0.109863451
ENSMUSG00000059422	Gm8116	predicted gene 8116 [Source:MGI Symbol;Acc:MGI:3648797]	8.94757	9.93547	9.23373	10.6778	10.4961	9.89622	9.38325	11.21	11.709	10.4826	11.2437	9.03171	11.9829	9.67952	0.838532766	0.966018836	20	4	24	0.41365919	0.799546792	24	10.48768626	0.062397314
ENSMUSG00000059423	Zfp933	zinc finger protein 933 [Source:MGI Symbol;Acc:MGI:1922865]	5.71749	4.77035	4.62832	5.15756	5.54793	4.78556	5.61536	4.6632	5.09371	4.08962	5.60408	4.4687	4.8297	4.99692	0.628286596	0.966018836	4	8	24	0.491189503	0.805393068	24	20.55037519	0.055793057
ENSMUSG00000059430	Actg2	actin, gamma 2, smooth muscle, enteric [Source:MGI Symbol;Acc:MGI:104589]	1.6081	0.984842	0.777725	0.741888	0.788946	0.810719	0.872966	1.12667	0.904212	0.91827	0.818102	0.818724	1.01478	0.806828	0.080148772	0.458678242	4	16	24	0.103482505	0.715292223	24	1.014823264	0.111788361
ENSMUSG00000059436	Max	Max protein [Source:MGI Symbol;Acc:MGI:96921]	26.722	26.3563	25.4295	24.612	24.5334	25.3553	29.946	23.1	28.2073	26.3198	23.4146	26.5415	23.762	23.1519	0.93447422	0.966018836	4	16	24	0.875492813	0.965124369	24	23.68497829	0.021247055
ENSMUSG00000059439	Bcas3	BCAS3 microtubule associated cell migration factor [Source:MGI Symbol;Acc:MGI:2385848]	17.5005	14.7823	16.9427	17.1208	14.8619	19.8569	18.1664	18.2075	13.5713	22.6591	15.3765	18.5459	18.0581	19.1607	0.745118544	0.966018836	24	12	24	0.821064462	0.943551411	24	21.40798463	0.04602615
ENSMUSG00000059447	Hadhb	hydroxyacyl-CoA dehydrogenase trifunctional multienzyme complex subunit beta [Source:MGI Symbol;Acc:MGI:2136381]	14.4957	15.9292	17.839	26.6148	16.9154	16.2539	17.7326	19.3757	18.0484	20.2549	24.8918	16.5759	22.1099	21.021	0.496745696	0.966018836	16	8	24	0.130438032	0.740728512	24	11.48612705	0.162102552
ENSMUSG00000059456	Ptk2b	PTK2 protein tyrosine kinase 2 beta [Source:MGI Symbol;Acc:MGI:104908]	4.16155	4.53692	4.36348	4.66779	3.68965	3.89985	4.05581	4.25464	3.90683	4.78105	3.76872	4.55258	3.99628	4.04192	0.129903614	0.560263689	16	4	24	0.506921767	0.812342247	24	8.407063478	0.04501306
ENSMUSG00000059461	Gm7331	predicted gene 7331 [Source:MGI Symbol;Acc:MGI:3644702]	1.4377	1.36056	1.75319	0.80682	1.44592	1.39524	3.26522	0.317176	0.972842	1.30808	1.09324	1.1663	0.606424	0.834215	0.921572192	0.966018836	4	4	24	0.566784895	0.843547308	24	19.71786989	0.162106175
ENSMUSG00000059474	Mbtd1	mbt domain containing 1 [Source:MGI Symbol;Acc:MGI:2143977]	5.06995	6.6041	6.0024	6.40283	5.76173	5.67713	5.87312	5.26527	6.98135	5.74346	6.06059	5.26777	5.49952	5.55739	0.097760727	0.501112642	12	16	24	0.078330642	0.68893025	24	9.989439316	0.09282383
ENSMUSG00000059475	Zfp426	zinc finger protein 426 [Source:MGI Symbol;Acc:MGI:1920248]	19.384	20.4081	19.9017	20.4564	21.1842	19.6751	19.2724	19.1828	21.254	18.2153	22.1162	21.3801	22.9943	21.5111	0.911685024	0.966018836	20	20	24	0.877601694	0.965881478	24	19.63028232	0.016728711
ENSMUSG00000059479	B3gnt8	UDP-GlcNAc:betaGal beta-1,3-N-acetylglucosaminyltransferase 8 [Source:MGI Symbol;Acc:MGI:2385269]	6.87286	6.10584	5.54142	6.82745	5.52259	6.28673	5.67796	6.07331	5.21272	8.51045	6.51147	7.34507	6.11352	5.64754	0.071569005	0.440262583	16	20	24	0.370166761	0.792263516	24	16.35731871	0.090478493
ENSMUSG00000059482	2610301B20Rik	RIKEN cDNA 2610301B20 gene [Source:MGI Symbol;Acc:MGI:1914407]	13.922	15.672	15.4283	14.914	15.1704	15.1457	15.1103	14.4363	15.2897	13.5785	16.6604	16.0225	15.6974	16.3055	0.8635847	0.966018836	20	20	24	0.923305375	0.979439431	24	21.46324085	0.011692146
ENSMUSG00000059486	Kbtbd2	kelch repeat and BTB (POZ) domain containing 2 [Source:MGI Symbol;Acc:MGI:2384811]	25.4934	22.9863	21.3313	23.4661	20.9592	24.5875	22.6984	22.7125	19.8736	24.1039	23.8012	23.3848	23.4492	24.3582	0.601647512	0.966018836	24	12	24	0.248822189	0.768040178	24	21.48632828	0.061299515
ENSMUSG00000059493	Nhs	NHS actin remodeling regulator [Source:MGI Symbol;Acc:MGI:2684894]	2.32036	2.26465	2.21194	2.23492	2.25043	2.16357	2.07903	2.04652	2.0257	1.86828	2.14072	2.23498	1.82612	2.29067	0.873350015	0.966018836	8	4	24	0.844541838	0.952844769	24	20.62962647	0.01547127
ENSMUSG00000059495	Arhgef12	Rho guanine nucleotide exchange factor (GEF) 12 [Source:MGI Symbol;Acc:MGI:1916882]	26.6725	23.2271	22.1355	24.5039	24.1934	23.7714	26.9296	23.2879	21.3443	24.9621	24.3556	24.6764	25.6425	24.1158	0.009483698	0.194720062	4	8	24	0.049814513	0.643621507	24	21.00761267	0.080602251
ENSMUSG00000059498	Fcgr3	Fc receptor, IgG, low affinity III [Source:MGI Symbol;Acc:MGI:95500]	5.558	7.68088	9.89204	8.11841	8.9814	6.3832	8.1786	7.27125	9.66752	4.30671	8.08482	6.31201	7.33486	6.57654	0.148073804	0.590732968	12	12	24	0.688891229	0.897070579	24	8.695617493	0.099408511
ENSMUSG00000059518	Znhit1	zinc finger, HIT domain containing 1 [Source:MGI Symbol;Acc:MGI:1917353]	50.9659	48.8204	47.5616	45.1835	42.5349	46.1607	49.2929	47.0756	45.4595	44.2747	51.8275	51.0567	47.8777	48.056	0.077415205	0.453845658	4	12	24	0.13167957	0.741998858	24	23.52353266	0.060749306
ENSMUSG00000059534	Uqcr10	ubiquinol-cytochrome c reductase, complex III subunit X [Source:MGI Symbol;Acc:MGI:1913402]	299.695	345.859	339.412	363.835	333.873	314.813	314.647	346.043	415.683	291.802	390.285	346.124	401.862	379.313	0.891022863	0.966018836	12	16	24	0.458214947	0.805393068	24	7.694416505	0.069804412
ENSMUSG00000059540	Tcea2	transcription elongation factor A (SII), 2 [Source:MGI Symbol;Acc:MGI:107368]	27.8658	34.2813	34.0765	36.2616	34.4152	34.0041	31.6818	29.5623	35.3004	30.7934	32.1078	34.4906	32.2056	30.672	0.207447292	0.698704663	12	20	24	0.231884742	0.768040178	24	13.17775586	0.067273195
ENSMUSG00000059552	Trp53	transformation related protein 53 [Source:MGI Symbol;Acc:MGI:98834]	9.71392	10.1302	9.56226	9.21817	10.1502	9.55975	9.8701	10.6459	11.5796	9.87297	8.76554	9.689	9.54445	9.99737	0.17395618	0.649730255	8	16	24	0.140545711	0.750453905	24	5.832331446	0.065212722
ENSMUSG00000059554	Ccdc28a	coiled-coil domain containing 28A [Source:MGI Symbol;Acc:MGI:2443508]	38.4848	39.7379	39.9779	37.5057	38.1569	36.9056	38.0425	37.195	37.4165	35.8625	42.979	38.6117	39.1127	39.6125	0.904831057	0.966018836	8	8	24	0.950968112	0.988261248	24	21.9969216	0.00820027
ENSMUSG00000059555	Tor4a	torsin family 4, member A [Source:MGI Symbol;Acc:MGI:2442720]	1.03663	1.30325	1.19801	1.13759	1.2923	1.32035	1.23626	1.30049	1.22709	0.988809	1.26477	1.19344	1.20596	1.16359	0.849179819	0.966018836	20	20	24	0.748375244	0.918047827	24	22.47775733	0.020659589
ENSMUSG00000059586	Nsmce2	NSE2/MMS21 homolog, SMC5-SMC6 complex SUMO ligase [Source:MGI Symbol;Acc:MGI:1915751]	11.0946	14.5997	15.5428	13.4997	13.6089	12.1519	13.4203	12.4951	14.8547	12.2555	12.0813	13.9037	14.0694	13.3027	0.374467935	0.89742288	12	8	24	0.266778479	0.768040178	24	7.577551791	0.081260089
ENSMUSG00000059588	Calcrl	calcitonin receptor-like [Source:MGI Symbol;Acc:MGI:1926944]	3.68784	4.60961	4.3083	4.75904	4.08935	4.37951	4.64279	4.79983	5.13184	3.81149	4.60533	3.61443	3.20615	3.44237	0.651661131	0.966018836	12	16	24	0.352952292	0.780629136	24	10.24819461	0.086636134
ENSMUSG00000059602	Syn3	synapsin III [Source:MGI Symbol;Acc:MGI:1351334]	2.42407	1.96694	2.41029	1.73621	1.87677	1.96712	2.42494	1.89261	2.04317	2.48877	1.98014	2.01471	1.71127	2.17199	0.850906022	0.966018836	12	8	24	0.824800068	0.945021534	24	5.303419406	0.030639437
ENSMUSG00000059657	Stfa2l1	stefin A2 like 1 [Source:MGI Symbol;Acc:MGI:3524944]	0.157026	0.793449	2.04352	0.530512	0.691661	0.786589	0.761609	0.277758	0.425597	0.727161	0.744962	0.609735	1.26899	1.08734	0.820042705	0.966018836	12	4	24	0.800911384	0.936344695	24	8.374066852	0.095428944
ENSMUSG00000059658	Mif-ps6	macrophage migration inhibitory factor, pseudogene 6 [Source:MGI Symbol;Acc:MGI:103158]	11.328	9.43855	11.0899	6.32416	6.02012	6.84689	11.8559	9.19585	9.12088	10.6709	12.1705	11.7856	11.5065	10.1991	0.397360907	0.927993503	4	12	24	0.317981857	0.768040178	24	2.034522651	0.167471058
ENSMUSG00000059659	Gm10069	predicted gene 10069 [Source:MGI Symbol;Acc:MGI:3641875]	1.33292	1.0449	1.0348	1.23541	1.43322	1.16648	1.28091	1.02157	0.883713	0.747697	1.54765	1.12825	0.981095	1.01199	0.069194004	0.433403132	20	16	24	0.038272471	0.61140397	24	16.90588574	0.132383438
ENSMUSG00000059668	Taf1b	TATA-box binding protein associated factor, RNA polymerase I, B [Source:MGI Symbol;Acc:MGI:109577]	8.96434	11.7076	11.3612	11.5026	9.38109	10.6894	9.79491	10.6689	12.0344	9.14482	10.5221	11.2159	[illegible]	12.1545	0.135268864	0.573729971	12	8	24	0.106652523	0.718749374	24	6.539392908	0.124317533
ENSMUSG00000059674	Cdh24	cadherin-like 24 [Source:MGI Symbol;Acc:MGI:1928330]	0.91772	0.76324	0.932342	0.906523	0.753152	0.850104	0.989202	0.862282	1.23508	0.887709	1.07371	0.882843	0.797254	0.914018	0.941885246	0.966018836	12	12	24	0.485586482	0.805393068	24	9.527098335	0.044214949
ENSMUSG00000059689	Zfp637	zinc finger protein 637 [Source:MGI Symbol;Acc:MGI:2448537]	24.1173	23.2268	23.8357	19.0414	24.5938	24.3485	23.7027	22.024	22.8014	22.2432	25.2939	23.8669	24.4899	22.3105	0.004003768	0.140598674	20	20	24	0.121125195	0.733270421	24	20.95284418	0.077166965
ENSMUSG00000059708	Akap17b	A kinase (PRKA) anchor protein 17B [Source:MGI Symbol;Acc:MGI:2443758]	6.62341	6.17277	6.07398	6.20514	6.28814	5.9941	6.19526	5.25251	5.90604	5.70058	6.33194	6.43324	6.56504	6.37065	0.097760727	0.501112642	4	8	24	0.238702068	0.768040178	24	20.53940889	0.050497822
ENSMUSG00000059713	Rcan3	regulator of calcineurin 3 [Source:MGI Symbol;Acc:MGI:1858220]	19.3906	14.6256	15.7933	15.9433	15.7063	18.674	16.0228	16.3954	12.3991	21.9166	14.3809	17.6309	16.2265	17.3947	0.44861185	0.966018836	24	20	24	0.602795636	0.861114219	24	19.83785103	0.079052027
ENSMUSG00000059714	Flot1	flotillin 1 [Source:MGI Symbol;Acc:MGI:1100500]	125.031	110.277	114.033	118.609	113.395	133.151	114.935	114.349	97.622	145.534	106.657	116.808	113.168	117.755	0.745118544	0.966018836	24	12	24	0.624418641	0.871960321	24	17.92783075	0.054077665
ENSMUSG00000059734	Ndufs8	NADH:ubiquinone oxidoreductase core subunit S8 [Source:MGI Symbol;Acc:MGI:2385079]	115.853	126.141	118.556	130.343	118.077	116.546	113.604	122.749	133.297	99.6729	141.58	119.906	132.437	126.844	0.916556573	0.966018836	8	20	24	0.923403047	0.979439431	24	6.158050296	0.020167544
ENSMUSG00000059741	Myl3	myosin, light polypeptide 3 [Source:MGI Symbol;Acc:MGI:97268]	4.42507	9.08462	16.0561	16.8289	8.15182	14.3015	11.1631	19.214	8.3941	15.7563	5.68458	1.19502	10.1823	14.8067	0.19937048	0.683051041	16	4	24	0.136533149	0.747972781	24	7.860330888	0.664776103
ENSMUSG00000059742	Kcnh7	potassium voltage-gated channel, subfamily H (eag-related), member 7 [Source:MGI Symbol;Acc:MGI:2159566]	11.3253	12.7543	10.6049	12.1298	11.7325	10.1565	10.7137	10.4043	11.7332	10.4199	12.2046	10.5964	11.6518	12.2764	0.954584434	0.966018836	8	16	24	0.75544534	0.921118175	24	8.08671069	0.031105406
ENSMUSG00000059743	Fdps	farnesyl diphosphate synthetase [Source:MGI Symbol;Acc:MGI:104888]	31.0706	27.9599	27.662	28.9766	33.4479	33.7317	31.7773	32.1924	27.3452	31.8777	29.4465	29.9408	30.6389	29.9459	0.08302755	0.462922111	24	12	24	0.081377777	0.697102286	24	19.77408144	0.080229826
ENSMUSG00000059751	Rps3a3	ribosomal protein S3A3 [Source:MGI Symbol;Acc:MGI:3643406]	9.82571	13.5298	11.9136	12.6113	13.072	11.1255	11.5939	11.9924	12.6329	8.07445	10.5036	8.61164	11.0501	10.9567	0.559102416	0.966018836	12	12	24	0.478717657	0.805393068	24	6.250244866	0.088727647
ENSMUSG00000059772	Slx1b	SLX1 structure-specific endonuclease subunit homolog B (S. cerevisiae) [Source:MGI Symbol;Acc:MGI:1915220]	6.80621	6.69443	7.03816	7.85723	6.76894	7.60357	6.40263	7.05302	7.7355	8.369	6.54457	7.88031	6.53055	7.71667	0.018810871	0.256345714	16	12	24	0.18260992	0.768040178	24	11.25423096	0.074851974
ENSMUSG00000059775	Rps26-ps1	ribosomal protein S26, pseudogene 1 [Source:MGI Symbol;Acc:MGI:3704322]	191.178	198.979	197.817	178.298	177.634	166.172	182.437	171.368	231.652	159.408	205.338	189.177	208.949	205.933	0.570592509	0.966018836	12	4	24	0.367704265	0.791015017	24	5.12798323	0.079714658
ENSMUSG00000059791	Nrm	nurim (nuclear envelope membrane protein) [Source:MGI Symbol;Acc:MGI:2146855]	2.64379	2.7859	2.71505	2.27207	2.42246	2.51376	2.56487	2.94549	2.82387	3.21624	2.36335	2.22408	3.09345	2.68854	0.096263602	0.501112642	12	8	24	0.084052827	0.701763292	24	5.62788918	0.095720093
ENSMUSG00000059796	Eif4a1	eukaryotic translation initiation factor 4A1 [Source:MGI Symbol;Acc:MGI:95303]	42.6057	43.7289	45.1096	42.1267	49.402	42.6638	36.36	42.4678	47.7148	38.9764	46.3193	41.299	43.4487	43.895	0.313239838	0.824470524	12	12	24	0.531751178	0.82547989	24	11.78363198	0.048941683
ENSMUSG00000059810	Rgs3	regulator of G-protein signaling 3 [Source:MGI Symbol;Acc:MGI:1354734]	18.6286	14.4751	14.1585	17.644	19.1471	18.8756	17.4011	18.3654	12.9988	18.9897	16.6953	16.1041	17.38	16.7746	0.137763286	0.580158868	16	20	24	0.133865349	0.744007455	24	19.05176724	0.127248766
ENSMUSG00000059811	Atl2	atlastin GTPase 2 [Source:MGI Symbol;Acc:MGI:1929492]	21.7971	23.1825	24.3106	24.979	21.7743	23.4873	20.3672	24.5745	25.9554	25.7739	22.7393	23.3274	23.8731	25.6348	0.048507657	0.379129806	16	4	24	0.005206937	0.4424469	24	8.875557671	0.101854055
ENSMUSG00000059820	Nkapd1	NKAP domain containing 1 [Source:MGI Symbol;Acc:MGI:2143205]	4.7006	5.30262	4.96558	5.13958	4.5355	4.34787	5.78186	5.28868	5.5494	4.56205	5.01288	5.08383	5.25852	5.12872	0.137758149	0.580158868	8	12	24	0.088978294	0.708139275	24	4.565523532	0.076819874
ENSMUSG00000059824	Dbp	D site albumin promoter binding protein [Source:MGI Symbol;Acc:MGI:94866]	3.96887	20.5005	37.4301	28.2509	14.7942	6.34612	5.13994	18.9772	45.1724	34.0429	6.49509	5.61993	5.19503	17.673	2.68E-07	0.001551842	12	16	24	2.14E-06	0.010811858	24	9.243948202	1.502907885
ENSMUSG00000059830	Gm16380	predicted pseudogene 16380 [Source:MGI Symbol;Acc:MGI:3643564]	1.0183	1.80372	1.51954	2.30441	2.16017	1.78023	1.70037	1.46814	2.41971	1.26268	2.33798	1.31292	1.62397	1.55488	0.708020493	0.966018836	20	8	24	0.220832698	0.768040178	24	12.66069039	0.150266708
ENSMUSG00000059834	Sclt1	sodium channel and clathrin linker 1 [Source:MGI Symbol;Acc:MGI:1914411]	6.16655	6.14557	6.66061	5.67303	6.11629	5.55996	6.24552	5.067	6.44421	5.1892	7.30714	6.12665	6.15921	6.35851	0.893307667	0.966018836	12	4	24	0.973125117	0.993305115	24	18.42073642	0.011638917
ENSMUSG00000059835	Rpl13-ps3	ribosomal protein L13, pseudogene 3 [Source:MGI Symbol;Acc:MGI:3646682]	0.952479	0.922258	1.2229	0.789269	0.514888	0.829658	0.737816	0.771842	1.17928	0.744061	0.592285	0.555076	0.984351	0.980476	0.005378037	0.149433822	12	8	24	0.009206529	0.502965379	24	5.623573818	0.173339901
ENSMUSG00000059839	Zfp874b	zinc finger protein 874b [Source:MGI Symbol;Acc:MGI:3040702]	7.80885	7.91464	7.34428	8.87901	7.83801	7.99904	8.37634	8.30695	8.08265	6.00423	8.97293	8.0606	8.65416	8.01776	0.866861748	0.966018836	4	8	24	0.513656658	0.816307374	24	22.29303287	0.058026592
ENSMUSG00000059842	Zfp341	zinc finger protein 341 [Source:MGI Symbol;Acc:MGI:2682937]	1.17484	0.799374	0.82408	0.73544	1.07457	1.06024	0.922713	1.10038	0.963591	1.06019	1.02993	1.12262	0.946243	0.910826	0.251797405	0.75129457	24	12	24	0.158037238	0.764630328	24	19.99513063	0.071572693
ENSMUSG00000059851	Kmt5c	lysine methyltransferase 5C [Source:MGI Symbol;Acc:MGI:2385262]	17.1422	13.5267	13.3774	13.7194	16.019	14.7181	18.9119	14.4649	16.5861	14.5512	17.9202	14.9824	15.1549	14.6065	0.104994359	0.518002936	4	4	24	0.301961077	0.768040178	24	20.29673832	0.089080687
ENSMUSG00000059852	Kcng2	potassium voltage-gated channel, subfamily G, member 2 [Source:MGI Symbol;Acc:MGI:3694646]	2.45067	1.30032	1.18676	1.37887	1.33792	1.28007	1.46089	1.952	1.64307	2.35493	2.30601	3.16475	3.02451	2.58644	0.763955526	0.966018836	4	8	24	0.387522162	0.799546792	24	22.66799037	0.128135048
ENSMUSG00000059857	Ntng1	netrin G1 [Source:MGI Symbol;Acc:MGI:1934028]	10.2405	8.43718	7.67136	8.73629	8.11472	9.51693	9.51041	10.0782	7.57112	14.2411	8.57558	10.3196	10.4982	10.6611	0.397301892	0.927993503	4	8	24	0.846475163	0.953644767	24	22.27530952	0.047165573
ENSMUSG00000059866	Tnip2	TNFAIP3 interacting protein 2 [Source:MGI Symbol;Acc:MGI:2386643]	4.00889	4.99416	5.64884	6.28138	4.42414	3.84192	5.5485	4.26952	6.37167	4.25702	4.72343	4.90798	4.96187	5.53031	0.133012827	0.567728672	12	12	24	0.117648547	0.728323355	24	8.158006406	0.152909025
ENSMUSG00000059878	Zfp422	zinc finger protein 422 [Source:MGI Symbol;Acc:MGI:1914505]	10.1215	9.94071	9.54515	11.1502	9.64152	10.571	9.8954	10.3279	9.34729	10.0593	9.6029	9.24221	8.53315	9.49229	0.890311217	0.966018836	16	20	24	0.587387317	0.853591039	24	13.38521257	0.028420235
ENSMUSG00000059883	Irak4	interleukin-1 receptor-associated kinase 4 [Source:MGI Symbol;Acc:MGI:2182474]	1.51368	1.74512	1.69717	1.78564	2.00743	1.78673	1.86453	2.14512	2.12582	1.44698	1.80125	1.89853	1.72431	1.54378	0.920872048	0.966018836	20	20	24	0.991431426	0.998516607	24	14.68397905	0.005742879
ENSMUSG00000059890	Ube4a	ubiquitination factor E4A [Source:MGI Symbol;Acc:MGI:2154580]	40.3172	36.5408	35.6299	37.5094	36.3206	40.5324	37.0915	34.7937	31.9862	37.4889	37.455	39.0761	37.9828	38.4496	0.010468194	0.196621011	24	12	24	0.025485148	0.586541342	24	20.37780103	0.084110067
ENSMUSG00000059895	Ptp4a3	protein tyrosine phosphatase 4a3 [Source:MGI Symbol;Acc:MGI:1277098]	12.8532	13.7251	15.9387	18.1171	14.0846	14.2633	13.6967	23.8264	17.9643	22.1034	14.263	14.2469	15.5938	13.7534	0.093640472	0.490854203	16	12	24	0.078023729	0.688222295	24	9.384305608	0.217717323
ENSMUSG00000059897	Zfp930	zinc finger protein 930 [Source:MGI Symbol;Acc:MGI:2675306]	6.7946	8.60948	7.70686	7.292	9.20201	7.14045	7.31797	6.73465	9.79661	5.75053	8.58833	7.38526	8.15739	8.61665	0.939103211	0.966018836	12	12	24	0.904920388	0.973766133	24	7.672133001	0.034059127
ENSMUSG00000059900	Tmem40	transmembrane protein 40 [Source:MGI Symbol;Acc:MGI:2137870]	9.89859	10.9396	10.359	10.0727	13.1128	10.3303	11.5933	11.029	13.2314	12.0959	11.0888	11.5671	10.9416	10.1604	0.929640773	0.966018836	12	20	24	0.463975986	0.805393068	24	13.11010569	0.057530167
ENSMUSG00000059901	Adamts14	a disintegrin-like and metallopeptidase (reprolysin type) with thrombospondin type 1 motif, 14 [Source:MGI Symbol;Acc:MGI:2179942]	0.448603	0.71348	0.779666	0.836592	0.694534	0.59436	0.673845	0.899372	0.868141	0.696391	0.560614	0.658572	0.642063	0.673906	0.008047769	0.192760771	12	12	24	0.019857415	0.565108098	24	8.51967226	0.105430384
ENSMUSG00000059920	4930453N24Rik	RIKEN cDNA 4930453N24 gene [Source:MGI Symbol;Acc:MGI:1914859]	9.79601	11.0074	10.8299	10.6105	11.4389	10.831	10.361	10.1608	11.211	9.20288	10.9968	11.7202	12.1661	10.2511	0.759899911	0.966018836	20	20	24	0.622319781	0.871390795	24	20.0126243	0.03825191
ENSMUSG00000059921	Unc5c	unc-5 netrin receptor C [Source:MGI Symbol;Acc:MGI:1095412]	2.32552	2.44725	2.44458	2.55466	2.51775	2.07972	2.47018	2.09156	2.24317	2.23388	2.54508	2.51769	2.23059	2.63762	0.85447369	0.966018836	20	8	24	0.787742882	0.931762722	24	13.50316144	0.021300217
ENSMUSG00000059923	Grb2	growth factor receptor bound protein 2 [Source:MGI Symbol;Acc:MGI:95805]	35.1488	28.8248	30.9619	31.2463	28.5319	32.8503	32.35	32.127	27.4943	38.9487	27.3767	33.0551	30.6361	32.2631	0.300823756	0.809362476	24	20	24	0.89151	0.969321764	24	22.64594352	0.026635675
ENSMUSG00000059939	9430015G10Rik	RIKEN cDNA 9430015G10 gene [Source:MGI Symbol;Acc:MGI:2444364]	6.63051	5.80658	7.402	7.70109	7.07809	6.52028	6.90816	5.97648	7.19621	6.34199	6.92927	6.83096	6.29728	5.80303	0.003689808	0.140598674	12	20	24	0.059527957	0.658304792	24	14.26711253	0.089635327
ENSMUSG00000059970	Hspa2	heat shock protein 2 [Source:MGI Symbol;Acc:MGI:96243]	6.63577	5.61257	6.21899	6.99642	5.71497	5.98137	6.30884	5.49748	5.05889	7.65716	6.12229	7.17924	5.62935	6.3109	0.371875382	0.894537464	16	20	24	0.405119625	0.799546792	24	15.86623783	0.074453307
ENSMUSG00000059974	Ntm	neurotrimin [Source:MGI Symbol;Acc:MGI:2446259]	7.09271	9.65164	12.0959	8.77841	7.16178	9.19397	9.70076	7.14018	11.5414	9.87371	5.59862	8.352	5.5273	11.9109	0.073139747	0.447768907	12	8	24	0.107307336	0.71991576	24	7.551984616	0.264975847
ENSMUSG00000059975	Zfp74	zinc finger protein 74 [Source:MGI Symbol;Acc:MGI:107784]	5.16485	4.92575	4.53769	4.36453	4.79008	4.60294	5.30074	4.3975	4.77999	5.07676	5.02929	4.86967	4.84613	4.98888	0.643325306	0.966018836	4	8	24	0.423537271	0.805393068	24	22.27949752	0.036061765

ENSMUSG00000059981	Taok2	TAO kinase 2 [Source:MGI Symbol;Acc:MGI:1915919]	22.7369	21.0123	21.7499	22.9546	23.2823	21.3856	24.7014	22.1024	25.4278	24.1364	24.1189	22.6111	22.5087	21.5666	0.159799452	0.619467724	16	16	24	0.383419433	0.799546792	24	13.45117972	0.041242558
ENSMUSG00000059991	Nptx2	neuronal pentraxin 2 [Source:MGI Symbol;Acc:MGI:1858209]	25.8256	16.1279	18.4482	18.0333	22.839	21.878	17.2132	17.905	12.7246	25.2305	21.1571	18.9652	18.2745	17.358	0.076929733	0.453790923	20	12	24	0.071876019	0.678310325	24	17.94139526	0.216182949
ENSMUSG00000059995	Atxn7l3	ataxin 7-like 3 [Source:MGI Symbol;Acc:MGI:3036270]	36.5134	28.8831	29.6545	32.4207	29.3252	33.1942	31.8422	32.7002	26.5274	39.8299	28.1937	34.6587	31.4097	33.3447	0.850398351	0.966018836	24	12	24	0.748998802	0.918384241	24	20.78041166	0.047875486
ENSMUSG00000060002	Chpt1	choline phosphotransferase 1 [Source:MGI Symbol;Acc:MGI:2384841]	27.6785	30.3319	38.4235	55.1806	35.2116	31.0357	37.2662	40.4513	37.1325	39.3563	50.9266	35.5936	43.8918	39.6827	0.729086532	0.966018836	16	8	24	0.098660207	0.71175955	24	11.73107154	0.189989834
ENSMUSG00000060012	Kif13b	kinesin family member 13B [Source:MGI Symbol;Acc:MGI:1098265]	21.0369	20.7407	20.1956	20.9237	24.579	21.4196	24.0282	20.4404	22.2312	23.1723	22.0975	23.6766	21.8239	19.1694	0.017417473	0.255326033	20	12	24	0.055717957	0.65534544	24	17.43361105	0.087148747
ENSMUSG00000060019	Gm10073	predicted pseudogene 10073 [Source:MGI Symbol;Acc:MGI:3641908]	213.223	245.274	236.526	230.569	271.523	216.917	216.697	230.934	255.805	163.21	232.306	188.442	225.184	203.056	0.432054949	0.966018836	12	12	24	0.935065599	0.983765461	24	7.421583897	0.026146645
ENSMUSG00000060032	H2aj	H2A.J histone [Source:MGI Symbol;Acc:MGI:3606192]	7.39123	5.67835	5.47577	5.72483	5.03761	5.29583	5.79657	6.46522	8.52014	7.14306	11.816	10.589	10.3873	8.17012	0.807813307	0.966018836	4	12	24	0.83537711	0.949499418	24	20.57158321	0.062815187
ENSMUSG00000060036	Rpl3	ribosomal protein L3 [Source:MGI Symbol;Acc:MGI:1351605]	59.0785	70.1062	65.4004	64.5171	69.7108	64.9517	61.3544	63.5298	69.5461	56.0864	65.5973	60.1266	63.2906	62.6282	0.446967971	0.966018836	20	8	24	0.78294274	0.930229965	24	9.366587516	0.025842685
ENSMUSG00000060038	Dhps	deoxyhypusine synthase [Source:MGI Symbol;Acc:MGI:2683592]	31.3254	28.9804	26.5182	27.9441	29.9205	31.6016	28.7831	29.3592	29.3141	30.3341	33.4794	31.2765	33.9575	27.0905	0.069704757	0.435817592	24	12	24	0.026495751	0.586541342	24	19.50088051	0.094382114
ENSMUSG00000060063	Alox5ap	arachidonate 5-lipoxygenase activating protein [Source:MGI Symbol;Acc:MGI:107505]	9.34592	9.99735	15.2449	12.0258	9.82769	9.44272	8.95001	7.998	11.5145	6.05881	9.63528	7.57098	9.48502	6.56269	0.297978647	0.80820842	12	20	24	0.63649077	0.876483853	24	10.36518143	0.098641456
ENSMUSG00000060073	Psma3	proteasome subunit alpha 3 [Source:MGI Symbol;Acc:MGI:104883]	30.5032	34.7269	32.1195	35.4112	37.1648	29.4297	31.9709	33.8878	35.1543	25.258	36.8845	29.5212	29.1633	27.9718	0.175272072	0.651010555	20	4	24	0.696201639	0.89922881	24	12.94136354	0.049818574
ENSMUSG00000060090	Rp2	retinitis pigmentosa 2 homolog [Source:MGI Symbol;Acc:MGI:1277953]	5.33622	5.59425	5.42811	5.53481	5.14251	4.79955	5.33714	5.23571	6.36638	3.8164	5.41397	5.36753	6.4715	5.29203	0.939103211	0.966018836	4	20	24	0.399323384	0.799546792	24	2.758262521	0.077661473
ENSMUSG00000060096	Amd-ps3	S-adenosylmethionine decarboxylase, pseudogene 3 [Source:MGI Symbol;Acc:MGI:3704487]	9.10734	10.1689	9.85301	9.04167	9.43359	11.013	9.38539	11.664	13.3762	11.3791	10.1585	10.8592	11.062	9.5102	0.906657897	0.966018836	12	8	24	0.595470962	0.857839743	24	7.936636288	0.05730013
ENSMUSG00000060098	Prmt7	protein arginine N-methyltransferase 7 [Source:MGI Symbol;Acc:MGI:2384879]	11.1737	9.10923	9.21011	8.93844	9.23694	10.835	10.1539	10.2554	8.97909	10.693	8.84996	9.73239	9.2279	10.1687	0.320283544	0.83607295	24	20	24	0.289125016	0.768040178	24	23.08013167	0.063891954
ENSMUSG00000060121	Gemin2	gem nuclear organelle associated protein 2 [Source:MGI Symbol;Acc:MGI:1913853]	4.90959	4.78664	4.57977	5.27667	5.42763	4.8299	3.88784	5.20349	5.08947	4.52435	5.12538	4.73202	5.57226	4.79718	0.837133694	0.966018836	20	16	24	0.760889981	0.92268731	24	13.75393618	0.031468851
ENSMUSG00000060126	Tpt1	tumor protein, translationally-controlled 1 [Source:MGI Symbol;Acc:MGI:104890]	4.59217	4.8792	4.39459	4.35615	4.5654	3.76991	4.79008	5.37184	5.81011	4.68067	22.214	18.6004	26.7538	17.5267	0.801995186	0.966018836	8	8	24	0.462776395	0.805393068	24	21.96842622	0.283627441
ENSMUSG00000060143	Gm10076	predicted gene 10076 [Source:MGI Symbol;Acc:MGI:3704451]	14.4576	13.6108	14.4801	8.78904	11.3219	11.5125	14.8499	11.9469	14.5999	9.52335	13.5811	12.4176	15.4964	16.4996	0.025720395	0.297892358	4	12	24	0.020512247	0.56691777	24	2.132606552	0.226436033
ENSMUSG00000060147	Serpinb6a	serine (or cysteine) peptidase inhibitor, clade B, member 6a [Source:MGI Symbol;Acc:MGI:103123]	363.984	349.563	334.925	341.04	355.503	344.475	319.934	345.385	330.965	315.265	385.498	377.645	386.332	352.408	0.121789249	0.549378164	20	20	24	0.174839102	0.768040178	24	20.62721029	0.064031873
ENSMUSG00000060149	BC002059	cDNA sequence BC002059 [Source:MGI Symbol;Acc:MGI:2384864]	4.04819	6.61122	4.1649	4.80702	5.18505	5.00014	6.42656	4.99093	6.30921	4.02772	6.84372	5.19764	6.06193	4.61176	0.956178643	0.96629412	20	20	24	0.806534264	0.938308753	24	22.46742069	0.058897796
ENSMUSG00000060152	Pop5	processing of precursor 5, ribonuclease P/MRP family (S. cerevisiae) [Source:MGI Symbol;Acc:MGI:2151221]	112.975	101.179	98.7716	99.839	117.876	113.261	92.2565	95.9681	85.7764	79.0426	104.759	96.8298	102.886	94.5379	0.087592959	0.472021905	20	16	24	0.031736222	0.604316769	24	20.12482057	0.135712097
ENSMUSG00000060166	Zdhhc8	zinc finger, DHHC domain containing 8 [Source:MGI Symbol;Acc:MGI:1338012]	18.3652	15.3025	14.2473	15.9464	17.7195	14.6475	18.0382	18.9473	17.9872	27.4486	17.147	21.0186	18.9986	18.3774	0.909604019	0.966018836	4	8	24	0.87058946	0.963313607	24	13.40411128	0.040560092
ENSMUSG00000060181	Slc35e3	solute carrier family 35, member E3 [Source:MGI Symbol;Acc:MGI:2448489]	10.7741	8.81531	9.29667	9.67572	9.45292	10.0242	9.57732	8.92305	8.45016	10.3585	9.34045	9.46226	9.20822	9.26608	0.100274585	0.511533473	24	8	24	0.163954905	0.767020009	24	18.64944341	0.059435114
ENSMUSG00000060183	Cxcl11	chemokine (C-X-C motif) ligand 11 [Source:MGI Symbol;Acc:MGI:1860203]	1.62106	2.25096	1.67212	1.60167	1.57398	0.974904	1.74008	2.00319	2.52577	1.53481	1.56142	1.56391	1.801	1.40923	0.090761402	0.481752771	12	12	24	0.077621396	0.687420473	24	6.316060352	0.167270315
ENSMUSG00000060206	Zfp462	zinc finger protein 462 [Source:MGI Symbol;Acc:MGI:107690]	7.66088	7.64697	7.00726	7.19691	7.59078	7.24514	6.81762	6.63423	6.94	7.6969	6.41506	7.23043	7.13958	7.41192	0.816406072	0.966018836	16	20	24	0.981693056	0.995786699	24	20.88143504	0.005514027
ENSMUSG00000060212	Pcnx2	pecanex homolog 2 [Source:MGI Symbol;Acc:MGI:2445010]	7.44283	6.01003	5.58967	6.93969	5.66823	7.04968	6.07318	7.05931	5.4083	9.05714	5.66815	6.76894	6.43694	6.77941	0.774195442	0.966018836	24	12	24	0.963495211	0.990884307	24	19.12709998	0.020251419
ENSMUSG00000060216	Arrb2	arrestin, beta 2 [Source:MGI Symbol;Acc:MGI:99474]	27.8353	24.0838	24.0888	26.1726	30.9446	25.9238	25.7056	26.2017	25.2542	26.0833	25.6773	23.9097	23.9763	22.7105	0.801995186	0.966018836	16	16	24	0.080777908	0.696683497	24	16.62321568	0.077778234
ENSMUSG00000060224	Pyroxd2	pyridine nucleotide-disulphide oxidoreductase domain 2 [Source:MGI Symbol;Acc:MGI:1921830]	0.776465	1.47914	1.26616	1.09641	1.20784	0.876573	1.25416	1.58945	1.48685	1.01499	0.883541	1.28301	1.06613	1.08695	0.160477553	0.620479606	8	20	24	0.127465456	0.740162193	24	6.379569375	0.127171941
ENSMUSG00000060227	Golm2	golgi membrane protein 2 [Source:MGI Symbol;Acc:MGI:2443129]	22.5625	29.0362	28.1766	27.2046	25.6001	27.6594	27.6	25.2772	24.2697	24.3911	25.237	24.3415	22.7984	26.2606	0.924770496	0.966018836	8	20	24	0.762005385	0.922884575	24	8.644082111	0.030842007
ENSMUSG00000060240	Ccnd1	cell cycle exit and neuronal differentiation 1 [Source:MGI Symbol;Acc:MGI:1929898]	195.669	154.74	161.372	156.823	149.986	188.061	164.928	171.642	130.189	200.837	154.191	174.376	162.053	174.754	0.478724519	0.966018836	24	20	24	0.456247242	0.805393068	24	21.50599202	0.083680576
ENSMUSG00000060244	Alyref2	Aly/REF export factor 2 [Source:MGI Symbol;Acc:MGI:1913144]	1.69428	1.78084	1.63707	1.78873	1.59459	1.6777	1.28629	1.35227	1.56733	1.25536	2.03001	2.07053	1.83498	1.7296	0.79867683	0.966018836	24	12	24	0.505223387	0.811334839	24	18.85671493	0.064153454
ENSMUSG00000060257	Scrt2	scratch family zinc finger 2 [Source:MGI Symbol;Acc:MGI:2139287]	7.92103	4.43222	4.46429	5.76683	6.34791	6.08865	5.95825	6.0006	4.59502	9.83098	7.59002	6.55435	6.12535	6.33381	0.154830409	0.607427992	20	16	24	0.20536073	0.768040178	24	17.44870955	0.181133962
ENSMUSG00000060260	Pwwp2b	PWWP domain containing 2B [Source:MGI Symbol;Acc:MGI:2142008]	2.98125	3.0274	2.71973	2.20334	2.79709	3.52648	2.76423	3.50687	2.63336	3.88235	2.76547	3.22175	2.88316	3.23271	0.649212555	0.966018836	8	4	24	0.730026129	0.911943391	24	23.70389192	0.04749021
ENSMUSG00000060261	Gtf2i	general transcription factor II I [Source:MGI Symbol;Acc:MGI:1202722]	55.3824	48.699	45.4531	44.4035	47.7719	51.1224	51.0767	50.4724	42.9595	58.3422	45.3675	48.9943	49.59	49.0745	0.058157448	0.40386543	4	8	24	0.39730076	0.799546792	24	22.42901839	0.063791526
ENSMUSG00000060268	Armh1	armadillo-like helical domain containing 1 [Source:MGI Symbol;Acc:MGI:2686507]	0.537171	0.652872	0.845365	0.694777	0.548161	0.482069	0.70368	0.750437	0.673509	0.642002	0.61259	0.47527	0.587724	0.593754	8.58E-04	0.071344007	12	12	24	0.006663355	0.470810671	24	7.703337925	0.096036095
ENSMUSG00000060275	Nrg2	neuregulin 2 [Source:MGI Symbol;Acc:MGI:1098246]	9.71817	6.94983	5.99468	7.32591	7.9376	7.27477	8.14025	6.65247	5.3547	6.93709	7.3487	7.07912	7.60134	6.82731	0.001920665	0.100255953	4	8	24	0.010305086	0.503967859	24	20.7276155	0.179964519
ENSMUSG00000060279	Ap2a1	adaptor-related protein complex 2, alpha 1 subunit [Source:MGI Symbol;Acc:MGI:101921]	30.7291	25.3195	26.766	27.3079	25.9852	29.3226	27.6645	29.9087	25.2341	42.1008	27.5073	33.3984	30.6994	31.1687	0.46238066	0.966018836	24	12	24	0.90187634	0.972475685	24	17.03396096	0.031763716
ENSMUSG00000060288	Ppih	peptidyl prolyl isomerase H [Source:MGI Symbol;Acc:MGI:106499]	3.16898	2.87022	3.8595	3.8221	3.72655	2.62106	3.1895	2.66239	3.31699	2.60306	3.43266	2.7489	2.97521	2.64643	0.498426201	0.966018836	12	20	24	0.199894115	0.768040178	24	12.83110323	0.0954759
ENSMUSG00000060301	2610008E11Rik	RIKEN cDNA 2610008E11 gene [Source:MGI Symbol;Acc:MGI:1919378]	9.16105	11.004	10.2844	10.1255	10.6404	8.79951	9.58159	10.3588	12.524	9.18558	12.071	10.2971	10.5306	10.8743	0.459019924	0.966018836	12	12	24	0.29577612	0.768040178	24	8.844751743	0.078372266
ENSMUSG00000060314	Zfp941	zinc finger protein 941 [Source:MGI Symbol;Acc:MGI:3039601]	6.16183	7.90526	7.0929	5.4789	6.78901	7.34322	6.21964	7.17053	6.49235	6.0723	6.9197	7.93681	7.34427	8.02895	0.050258003	0.385784509	8	8	24	0.246116288	0.768040178	24	1.007903044	0.084603496
ENSMUSG00000060336	Zfp937	zinc finger protein 937 [Source:MGI Symbol;Acc:MGI:3644476]	1.41255	1.6984	1.58652	1.5433	1.64128	1.38228	1.52489	1.28344	1.66697	0.952684	1.76494	1.4113	1.61584	1.82285	0.843639218	0.966018836	8	16	24	0.823234597	0.94441254	24	2.976061913	0.033935896
ENSMUSG00000060371	Calm1	calneuron 1 [Source:MGI Symbol;Acc:MGI:2155987]	5.47759	5.43792	8.27118	5.27888	4.29611	5.77456	6.85148	5.23055	6.23929	5.86702	3.65912	5.18944	4.58601	7.40448	0.039226672	0.338446795	12	8	24	0.077912773	0.687802873	24	5.658590108	0.217951349
ENSMUSG00000060373	Hnrnpc	heterogeneous nuclear ribonucleoprotein C [Source:MGI Symbol;Acc:MGI:107795]	86.9358	84.9534	80.0342	74.1737	85.7936	81.887	85.7648	80.8889	75.8294	66.3206	88.3731	79.6407	88.6793	80.4319	0.001845448	0.100255953	4	12	24	0.025762539	0.586541342	24	22.54767753	0.108548192
ENSMUSG00000060376	Bckdha	branched chain ketoacid dehydrogenase E1, alpha polypeptide [Source:MGI Symbol;Acc:MGI:107701]	21.321	21.3423	24.294	30.2441	22.0148	23.6795	22.5495	27.1805	23.3401	29.6543	25.8958	24.2325	27.8893	26.1074	0.704290284	0.966018836	16	12	24	0.150391976	0.758865539	24	10.90475873	0.104026312
ENSMUSG00000060377	Rpl36a-ps1	ribosomal protein L36A, pseudogene 1 [Source:MGI Symbol;Acc:MGI:3642585]	26.9502	26.9044	28.6295	25.3579	30.8973	22.0936	21.1225	25.2451	29.8015	16.7114	29.4565	24.5649	26.9916	26.3466	0.745118544	0.966018836	12	12	24	0.953219763	0.988600916	24	5.999392408	0.028762374
ENSMUSG00000060380	C030014I23Rik	RIKEN cDNA C030014I23 gene [Source:MGI Symbol;Acc:MGI:1924631]	0.8698	1.13656	1.16718	1.0108	0.863375	0.820465	0.659026	0.67361	1.31181	0.64468	0.851577	1.30946	1.02964	0.930871	0.625825704	0.966018836	12	4	24	0.81509789	0.940891459	24	7.770506633	0.044399603
ENSMUSG00000060397	Zfp128	zinc finger protein 128 [Source:MGI Symbol;Acc:MGI:2389445]	2.19796	2.29013	2.18709	1.99026	2.26333	2.52525	2.37928	2.34089	2.56573	2.04248	2.60797	2.1346	2.34769	2.17602	0.718720526	0.966018836	20	20	24	0.696638025	0.89922881	24	22.17583054	0.0283035
ENSMUSG00000060402	Chst8	carbohydrate sulfotransferase 8 [Source:MGI Symbol;Acc:MGI:1916197]	6.82006	5.48501	7.36112	4.7423	5.16379	4.6305	7.74023	5.00768	5.22528	7.53867	4.94185	5.73495	9.26208	5.92545	0.122341925	0.550724475	4	16	24	0.442843054	0.805393068	24	2.161016148	0.131716119
ENSMUSG00000060419	Rps16-ps2	ribosomal protein S16, pseudogene 2 [Source:MGI Symbol;Acc:MGI:3704259]	259.01	291.579	269.066	275.958	274.502	257.429	257.822	259.567	354.715	215.563	306.523	278.704	283.14	266.668	0.953350544	0.966018836	12	12	24	0.874787915	0.964976615	24	7.636668451	0.034495081
ENSMUSG00000060424	Pantr1	POU domain, class 3, transcription factor 3 adjacent noncoding transcript 1 [Source:MGI Symbol;Acc:MGI:1913547]	0.596952	1.60103	3.08366	1.49475	0.84132	2.50343	2.15359	1.33638	2.43866	1.04841	0.750231	1.4691	1.1455	1.94866	0.285027487	0.793714531	12	8	24	0.34434726	0.7777007	24	6.210481997	0.237834986
ENSMUSG00000060427	Zfp868	zinc finger protein 868 [Source:MGI Symbol;Acc:MGI:2142546]	7.95259	7.69421	7.57904	8.94615	7.97092	8.34961	7.76269	6.71666	8.18841	7.16111	8.43027	7.753	7.58763	7.90416	0.365877735	0.893596186	20	12	24	0.240581695	0.768040178	24	16.03208933	0.057305754
ENSMUSG00000060429	Sntb1	syntrophin, basic 1 [Source:MGI Symbol;Acc:MGI:101781]	10.6941	9.52485	9.88985	10.1556	11.4225	10.0521	10.7102	9.74563	7.93629	9.8073	10.9492	9.14688	9.69129	8.72611	0.06180207	0.409814092	20	12	24	0.034673306	0.608551791	24	18.2960283	0.106758449
ENSMUSG00000060438	Rps10-ps1	ribosomal protein S10, pseudogene 1 [Source:MGI Symbol;Acc:MGI:3644167]	48.3966	49.6138	50.4353	46.7547	51.7995	46.3719	51.4636	53.3648	62.5484	45.0124	58.2616	47.729	55.072	46.4492	0.928064146	0.966018836	12	12	24	0.851633747	0.955514806	24	7.363434744	0.031514149
ENSMUSG00000060441	Trim5	tripartite motif-containing 5 [Source:MGI Symbol;Acc:MGI:3646853]	0.498517	0.807701	0.776978	0.614887	0.546571	0.576524	0.59577	0.652192	0.892453	0.568619	0.579546	0.75452	0.646498	0.87064	0.033622862	0.324908923	12	8	24	0.024337051	0.586541342	24	6.35123977	0.101551034
ENSMUSG00000060450	Rnf14	ring finger protein 14 [Source:MGI Symbol;Acc:MGI:1929668]	133.23	120.505	121.865	117.569	116.609	121.32	118.002	114.883	110.675	127.306	123.226	125.672	127.898	129.431	0.787022682	0.966018836	4	8	24	0.41696003	0.801686744	24	22.15105436	0.038392145
ENSMUSG00000060459	Kng2	kininogen 2 [Source:MGI Symbol;Acc:MGI:3027157]	0.756953	0.692892	1.57775	1.33097	0.753563	0.687057	1.07452	1.70968	1.50761	1.5321	1.78385	0.901012	2.4651	1.70468	0.91695334	0.966018836	8	16	24	0.089941382	0.7097999	24	7.823235516	0.215888829
ENSMUSG00000060467	Gm10080	predicted gene 10080 [Source:MGI Symbol;Acc:MGI:3710529]	31.9853	34.41	30.102	32.7869	39.2073	26.7124	29.5909	33.7524	28.6203	24.0554	29.141	27.6554	33.8975	28.6869	0.759899911	0.966018836	8	16	24	0.80000735	0.935884746	24	0.496908766	0.042945962
ENSMUSG00000060470	Adgrg3	adhesion G protein-coupled receptor G3 [Source:MGI Symbol;Acc:MGI:1859670]	1.77958	1.05004	0.97138	1.71905	1.10395	0.993001	1.60093	0.933708	0.881282	0.632388	1.25159	0.788782	1.32109	0.947695	0.339604637	0.858712466	4	8	24	0.373053138	0.794374214	24	21.85386365	0.11746346
ENSMUSG00000060475	Wtap	Wilms tumour 1-associating protein [Source:MGI Symbol;Acc:MGI:1926395]	28.1729	26.0148	24.8672	28.2674	25.7466	25.0146	23.7932	26.3923	26.5597	24.4058	26.1627	27.1722	27.5888	27.0721	0.860560779	0.966018836	4	16	24	0.91206078	0.975239559	24	1.381284718	0.012548898
ENSMUSG00000060477	Irak2	interleukin-1 receptor-associated kinase 2 [Source:MGI Symbol;Acc:MGI:2429603]	3.28592	3.66592	3.36314	3.20652	4.07087	3.84417	3.25079	4.23304	3.80125	4.90862	4.16751	3.10704	3.51201	3.74237	0.496745696	0.966018836	20	8	24	0.289729591	0.768040178	24	11.61749125	0.087470961
ENSMUSG00000060487	Samd5	sterile alpha motif domain containing 5 [Source:MGI Symbol;Acc:MGI:2444815]	2.82208	3.34393	3.48631	3.02146	3.29041	2.96171	3.24143	2.97838	3.89203	3.21299	2.91804	3.33606	3.30549	3.35317	0.121546923	0.548996929	12	8	24	0.160223922	0.76578501	24	7.655606506	0.068090384
ENSMUSG00000060510	Zfp266	zinc finger protein 266 [Source:MGI Symbol;Acc:MGI:1924769]	8.4849	10.7446	9.72759	9.55318	10.036	9.31891	9.0856	9.72515	11.3102	8.75699	9.96043	10.6672	9.60182	9.9476	0.644063931	0.966018836	12	16	24	0.604958679	0.862224803	24	8.46610932	0.04171824
ENSMUSG00000060512	0610040J01Rik	RIKEN cDNA 0610040J01 gene [Source:MGI Symbol;Acc:MGI:1923511]	2.37994	2.42573	1.98597	2.15294	2.06753	2.22975	2.09909	2.62305	2.16422	2.51401	2.23777	2.05912	2.48096	2.48184	0.159799452	0.619467724	8	12	24	0.229900474	0.768040178	24	3.82291499	0.056031969
ENSMUSG00000060519	Tor3a	torsin family 3, member A [Source:MGI Symbol;Acc:MGI:1353652]	3.14333	3.77186	3.118	2.89714	3.74516	3.34716	3.84282	3.94523	3.71786	3.43304	3.56372	3.47594	3.19612	4.12847	0.297978647	0.80820842	8	8	24	0.415459821	0.800637996	24	3.662866645	0.055199736
ENSMUSG00000060530	A930017M01Rik	RIKEN cDNA A930017M01 gene [Source:MGI Symbol;Acc:MGI:2685151]	5.99649	6.11647	6.078	5.81249	4.89377	5.18521	6.31835	5.19355	5.3986	4.67018	5.15351	4.87771	5.66886	6.81305	0.035046129	0.328311403	8	12	24	0.02698029	0.587116368	24	3.612785496	0.125802106
ENSMUSG00000060538	Tmem219	transmembrane protein 219 [Source:MGI Symbol;Acc:MGI:1915992]	21.5846	22.169	23.0726	24.5692	22.0088	18.8521	18.4731	21.1862	24.9888	20.4288	21.4468	20.781	23.3166	21.5602	0.148073804	0.590732968	12	12	24	0.089854636	0.7097999	24	9.065197435	0.099470671
ENSMUSG00000060548	Tnfrsf19	tumor necrosis factor receptor superfamily, member 19 [Source:MGI Symbol;Acc:MGI:1352474]	2.46375	3.20158	4.1333	2.25516	2.675	2.92036	3.81217	3.83054	2.99334	2.36731	2.46589	2.70506	2.44838	2.67563	0.056268883	0.401074772	12	4	24	0.172699358	0.768040178	24	4.158491611	0.14428495
ENSMUSG00000060568	Fam78b	family with sequence similarity 78, member B [Source:MGI Symbol;Acc:MGI:2443050]	10.7108	7.07867	7.27782	7.75836	8.79929	9.15039	8.95002	8.69104	6.11811	10.0133	7.97527	8.13329	7.99534	8.50628	0.106193509	0.519440352	4	8	24	0.193412614	0.768040178	24	20.30671871	0.131397654
ENSMUSG00000060572	Mfap2	microfibrillar-associated protein 2 [Source:MGI Symbol;Acc:MGI:99559]	1.44025	2.8398	2.72613	1.90422	1.96197	2.30636	2.34375	2.42441	2.6837	1.5858	1.62174	1.52942	2.00481	2.08284	0.071804903	0.441489742	12	4	24	0.0670204	0.669597304	24	5.73870776	0.181725727
ENSMUSG00000060579	Fhit	fragile histidine triad gene [Source:MGI Symbol;Acc:MGI:1277947]	10.3741	11.6202	9.98746	8.12046	11.0441	9.75679	13.27	8.69221	9.99119	7.79847	10.3716	8.20309	9.13481	12.0867	0.25849765	0.761738864	4	12	24	0.293372909	0.768040178	24	1.210931032	0.122020593
ENSMUSG00000060586	H2-Eb1	histocompatibility 2, class II antigen E beta [Source:MGI Symbol;Acc:MGI:95901]	38.603	44.4909	45.5344	32.0151	40.0978	34.5768	43.9185	48.8488	57.5885	30.5441	47.4423	43.1253	43.6907	42.2453	0.056268883	0.401074772	12	4	24	0.257811343	0.768040178	24	4.264840645	0.148725558
ENSMUSG00000060591	Ifitm2	interferon induced transmembrane protein 2 [Source:MGI Symbol;Acc:MGI:1933382]	50.9998	50.9256	49.1587	40.1306	51.7011	45.1059	47.7855	52.0714	56.2306	39.9787	46.5116	45.496	41.1292	42.5333	0.676344318	0.966018836	12	4	24	0.627947363	0.872795898	24	3.554302428	0.052564264
ENSMUSG00000060594	Layn	layilin [Source:MGI Symbol;Acc:MGI:2685357]	1.75221	2.15975	3.24521	2.47512	1.76123	2.23676	1.89631	2.92517	2.53664	2.71721	2.499	2.68496	1.46846	2.97055	0.016795343	0.253395174	12	16	24	0.058626947	0.658304792	24	9.194163837	0.202667162
ENSMUSG00000060600	Eno3	enolase 3, beta muscle [Source:MGI Symbol;Acc:MGI:95395]	34.8967	85.9405	105.159	131.941	92.1302	124.348	72.7597	223.146	172.713	182.041	81.6335	92.6046	114.26	84.732	0.217827365	0.704989686	16	12	24	0.088664342	0.707747429	24	9.848804521	0.517636982
ENSMUSG00000060601	Nr1h2	nuclear receptor subfamily 1, group H, member 2 [Source:MGI Symbol;Acc:MGI:1352463]	25.7564	23.8577	24.0849	21.0486	22.4183	23.3998	23.8805	30.0121	27.1439	30.1031	27.6673	27.2001	28.173	26.0311	0.807813307	0.966018836	8	12	24	0.579276333	0.849727189	24	4.866991035	0.050238264
ENSMUSG00000060636	Rpl35a	ribosomal protein L35A [Source:MGI Symbol;Acc:MGI:1928894]	20.7683	24.7044	21.3364	23.8613	21.5402	22.351	21.4375	21.5188	25.5151	15.9369	24.4292	18.3306	22.2408	19.333	0.866861748	0.966018836	12	16	24	0.939299877	0.985099549	24	5.428117537	0.024115041
ENSMUSG00000060639	H4c9	H4 clustered histone 9 [Source:MGI Symbol;Acc:MGI:2448432]	2.54315	2.97635	3.1479	3.79368	2.47519	2.39617	3.70124	3.72229	4.28665	3.08125	3.82584	3.70501	3.82008	3.71596	0.86340968	0.966018836	12	12	24	0.152836145	0.759115906	24	7.24057102	0.12523978
ENSMUSG00000060647	Gm7099	predicted gene 7099 [Source:MGI Symbol;Acc:MGI:3646387]	14.4208	15.0823	15.9934	13.0159	16.0701	17.3003	16.7913	16.2889	14.3299	17.1747	16.335	14.3242	13.4266	12.3303	0.858269825	0.966018836	20	12	24	0.469511672	0.805393068	24	16.9186104	0.068989824
ENSMUSG00000060657	Marf1	meiosis regulator and mRNA stability 1 [Source:MGI Symbol;Acc:MGI:2444505]	19.3347	18.6045	17.4261	19.1308	19.0348	18.5412	19.7597	17.9735	18.0611	18.2827	19.0674	20.1157	19.8964	19.3231	0.008389425	0.192760771	4	8	24	0.017504312	0.550995814	24	20.94817262	0.054313843
ENSMUSG00000060671	Atp8b2	ATPase, class I, type 8B, member 2 [Source:MGI Symbol;Acc:MGI:1859660]	24.6902	19.7542	22.5307	23.6834	20.962	23.801	23.6413	22.2245	19.1172	23.6977	20.6824	19.7096	20.7535	19.8297	0.92655264	0.966018836	4	4	24	0.655391711	0.883697438	24	18.65357057	0.039932146
ENSMUSG00000060675	Plaat3	phospholipase A and acyltransferase 3 [Source:MGI Symbol;Acc:MGI:2179715]	64.7803	73.6758	66.0429	70.9459	86.4122	64.1236	74.267	74.7373	85.9749	65.9607	79.0373	69.9418	79.8758	65.0162	0.882617464	0.966018836	20	4	24	0.834662404	0.949290995	24	13.21322892	0.034804427
ENSMUSG00000060679	Mrps9	mitochondrial ribosomal protein S9 [Source:MGI Symbol;Acc:MGI:1916777]	26.9005	29.9259	29.0847	29.8603	27.4606	27.9772	27.747	27.8085	31.1096	27.7179	28.6908	30.1288	32.2093	30.8036	0.834424969	0.966018836	12	8	24	0.319201923	0.768040178	24	6.130931291	0.039742472
ENSMUSG00000060680	Gm8894	predicted gene 8894 [Source:MGI Symbol;Acc:MGI:3643084]	34.0951	44.0531	41.614	40.7067	50.6946	42.4245	35.1483	36.0067	37.493	24.7359	34.9093	30.2121	36.0281	31.4187	0.860560779	0.966018836	20	8	24	0.912778414	0.97533472	24	18.06891637	0.036593876
ENSMUSG00000060681	Slc9a6	solute carrier family 9 (sodium/hydrogen exchanger), member 6 [Source:MGI Symbol;Acc:MGI:2443511]	72.4776	70.5625	69.6306	64.401	62.2675	65.5852	64.2179	64.4119	66.1165	68.6713	69.3112	68.5041	70.7424	71.2081	0.483881213	0.966018836	4	16	24	0.407507142	0.799546792	24	2.884395948	0.034506718
ENSMUSG00000060703	Cd302	CD302 antigen [Source:MGI Symbol;Acc:MGI:1913455]	6.66129	9.58943	8.60629	10.1111	9.9965	8.39509	9.37261	8.62365	11.9098	6.29204	11.184	9.51952	8.97494	9.15801	0.783267549	0.966018836	20	8	24	0.793878159	0.933877786	24	11.77507853	0.060039923
ENSMUSG00000060708	Bloc1s4	biogenesis of lysosomal organelles complex-1, subunit 4, cappuccino [Source:MGI Symbol;Acc:MGI:1929230]	4.18236	4.34479	4.00531	3.69878	4.64022	4.06856	4.93229	4.58426	5.25423	5.43102	6.25479	5.6421	5.49273	5.31917	0.915475528	0.966018836	20	20	24	0.770302737	0.926051878	24	17.70228225	0.030489592
ENSMUSG00000060716	Plekhh1	pleckstrin homology domain containing, family H (with MyTH4 domain) member 1 [Source:MGI Symbol;Acc:MGI:2144989]	4.41154	4.99886	6.44184	5.04298	4.54754	5.45019	6.05483	4.56325	4.49987	5.30759	4.79498	4.77016	3.70982	5.16686	0.907068194	0.966018836	16	12	24	0.723954595	0.909494838	24	10.05648749	0.052728085
ENSMUSG00000060727	Gm12571	predicted gene 12571 [Source:MGI Symbol;Acc:MGI:3649675]	31.0005	28.501	26.1303	21.6401	28.3724	24.9259	18.0555	28.4962	22.0664	26.9572	24.2914	24.8166	28.3735	29.5129	0.172865085	0.648600527	8	8	24	0.754675205	0.920677682	24	2.609780311	0.060733804
ENSMUSG00000060733	Ipmk	inositol polyphosphate multikinase [Source:MGI Symbol;Acc:MGI:1916968]	8.46539	6.77008	7.72495	7.86391	7.07638	7.51952	7.30614	7.35196	6.31014	8.9477	6.69102	7.69761	7.1102	7.54159	0.919571723	0.966018836	16	4	24	0.903351519	0.973174993	24	16.60618467	0.021292628
ENSMUSG00000060739	Nsa2	NSA2 ribosome biogenesis homolog [Source:MGI Symbol;Acc:MGI:1913883]	8.61118	9.05532	8.55297	8.06249	8.61711	8.14066	7.29201	8.24072	8.72936	6.63789	8.80714	7.91726	8.44825	8.40878	0.945544484	0.966018836	12	12	24	0.765207841	0.924110464	24	2.821301559	0.029599401
ENSMUSG00000060743	H3f3a	H3.3 histone A [Source:MGI Symbol;Acc:MGI:1097686]	26.6256	28.8605	32.3274	29.9246	28.1443	27.6107	28.1147	28.1433	31.951	23.9957	30.5196	24.1496	24.2191	26.2144	0.038115943	0.334069441	12	12	24	0.185886199	0.768040178	24	9.588752286	0.086367885
ENSMUSG00000060771	Tsga10	testis specific 10 [Source:MGI Symbol;Acc:MGI:2685063]	10.0759	9.87025	8.20451	9.52166	10.2065	8.63915	9.86532	8.47546	9.56039	7.69245	10.2205	9.19209	9.96376	9.3235	0.328944792	0.847277612	20	20	24	0.350144509	0.77951311	24	21.3597013	0.067438115
ENSMUSG00000060780	Lrrtm1	leucine rich repeat transmembrane neuronal 1 [Source:MGI Symbol;Acc:MGI:2389173]	5.12455	4.00623	3.56701	3.28924	3.62549	4.35378	5.07422	4.11262	3.29927	4.51203	4.41506	4.56722	4.26307	4.54257	0.033248781	0.324908923	4	8	24	0.01887591	0.561252866	24	22.47896811	0.160073291
ENSMUSG00000060791	Gmfg	glia maturation factor, gamma [Source:MGI Symbol;Acc:MGI:1927135]	0.339188	0.542935	1.15402	0.685012	0.850155	0.564221	0.851952	0.669038	1.09699	0.322188	0.903509	0.835792	0.962851	0.54007	0.94397916	0.966018836	12	20	24	0.869461422	0.962941471	24	10.79293892	0.046965602
ENSMUSG00000060798	Intu	inturned planar cell polarity protein [Source:MGI Symbol;Acc:MGI:2443752]	1.16957	1.23932	1.17577	1.19676	1.18823	1.25777	1.0802	1.17079	1.26836	1.13524	1.09894	1.12377	1.15971	1.32944	0.274035549	0.788124063	8	20	24	0.302900109	0.768040178	24	6.275023026	0.027980478
ENSMUSG00000060802	B2m	beta-2 microglobulin [Source:MGI Symbol;Acc:MGI:88127]	399.674	445.046	424.485	395.274	449.44	373.446	410.413	425.212	460.226	331.812	467.366	355.071	364.228	398.241	0.390686537	0.915479136	12	12	24	0.697975685	0.899609709	24	8.05966335	0.049899165
ENSMUSG00000060803	Gstp1	glutathione S-transferase, pi 1 [Source:MGI Symbol;Acc:MGI:95865]	26.9512	25.8599	26.2118	25.0094	22.437	27.7412	33.2983	29.1152	22.982	29.6698	30.4573	25.258	27.8699	25.697	0.918193129	0.966018836	4	8	24	0.551603801	0.83632237	24	22.98822426	0.064873608
ENSMUSG00000060843	Ctnna3	catenin (cadherin associated protein), alpha 3 [Source:MGI Symbol;Acc:MGI:2661445]	2.84291	3.81088	3.56521	3.40024	4.32721	2.72899	3.95948	3.13254	4.6054	3.02332	3.58797	4.33802	3.70842	3.69781	0.831272156	0.966018836	12	4	24	0.874866957	0.964976615	24	9.523137863	0.037285029
ENSMUSG00000060860	Ube2s	ubiquitin-conjugating enzyme E2S [Source:MGI Symbol;Acc:MGI:1925141]	5.80612	3.50143	4.01359	3.64829	4.41164	4.29269	3.94941	4.83572	4.0416	5.94991	4.0547	4.27849	3.91756	3.30346	0.711168999	0.966018836	24	8	24	0.690245756	0.897408416	24	17.35366762	0.069284755
ENSMUSG00000060862	Zbtb40	zinc finger and BTB domain containing 40 [Source:MGI Symbol;Acc:MGI:2682254]	2.60221	2.09142	1.76963	1.93552	2.31058	1.86646	3.04697	1.65931	1.28663	1.90715	1.89331	1.42674	1.98342	1.50875	0.286642595	0.793714531	4	8	24	0.148525458	0.756728257	24	21.16721148	0.144976401
ENSMUSG00000060882	Kcnd2	potassium voltage-gated channel, Shal-related family, member 2 [Source:MGI Symbol;Acc:MGI:102663]	0.927467	1.52204	2.38178	1.04412	0.938703	1.49208	1.83252	0.855932	1.64562	1.08877	0.772979	1.48245	0.736086	1.68526	0.424718357	0.966018836	12	8	24	0.436424183	0.805393068	24	5.91306304	0.14984215
ENSMUSG00000060904	Arl1	ADP-ribosylation factor-like 1 [Source:MGI Symbol;Acc:MGI:99436]	54.1607	55.8185	54.9588	52.9703	53.7297	50.3523	54.6524	52.0701	52.8335	49.6973	58.6646	50.3488	51.09	52.2644	0.942429894	0.966018836	12	12	24	0.969172005	0.992631251	24	9.787581508	0.00634989
ENSMUSG00000060913	Trim55	tripartite motif-containing 55 [Source:MGI Symbol;Acc:MGI:3036269]	0.106132	0.455761	0.58534	0.769952	0.57614	0.688039	0.578025	1.49246	1.04773	1.21473	0.404783	0.537222	0.940104	0.614253	0.557587426	0.966018836	16	4	24	0.165753254	0.767020009	24	8.698734251	0.201186667
ENSMUSG00000060923	Acyp2	acylphosphatase 2, muscle type [Source:MGI Symbol;Acc:MGI:1922822]	63.5877	63.0446	65.5514	62.8746	59.8422	60.4884	63.7537	53.5273	56.2387	49.0368	63.5813	47.9905	50.7622	59.8443	0.862909389	0.966018836	4	20	24	0.976460025	0.994103202	24	5.209912329	0.010591451
ENSMUSG00000060924	Csmd1	CUB and Sushi multiple domains 1 [Source:MGI Symbol;Acc:MGI:2137383]	8.9325	7.65496	7.85635	8.66152	8.26329	7.84031	7.98452	7.43709	7.03386	8.53841	7.62259	6.99898	7.50872	8.18617	0.932092277	0.966018836	16	20	24	0.793858133	0.933877786	24	14.8237538	0.022811043
ENSMUSG00000060935	Tmem263	transmembrane protein 263 [Source:MGI Symbol;Acc:MGI:2143652]	10.4612	9.88365	9.16723	9.75078	9.13747	9.97048	10.4808	9.23381	8.7036	8.11125	9.90017	8.62629	8.75052	8.86445	0.46238066	0.966018836	4	12	24	0.087672242	0.707747429	24	22.02410659	0.067163926
ENSMUSG00000060938	Rpl26	ribosomal protein L26 [Source:MGI Symbol;Acc:MGI:106022]	65.6331	74.7728	68.4138	68.1862	75.9819	70.3723	69.693	63.584	78.9553	41.6222	73.9929	59.6952	64.9564	51.3992	0.948582958	0.966018836	4	12	24	0.786817121	0.931447579	24	20.60173354	0.063122791
ENSMUSG00000060950	Trmt61a	tRNA methyltransferase 61A [Source:MGI Symbol;Acc:MGI:2443487]	4.07439	3.78637	3.8594	4.04625	4.15996	4.18706	4.15555	3.3019	3.07325	3.83987	3.86773	3.62534	3.56751	3.81808	0.177969241	0.655480352	20	12	24	0.07281092	0.67982958	24	18.53389319	0.080668929
ENSMUSG00000060961	Slc4a4	solute carrier family 4 (anion exchanger), member 4 [Source:MGI Symbol;Acc:MGI:1927555]	13.8182	17.533	22.785	18.6756	14.0564	16.827	17.7964	14.7865	19.3599	18.3635	15.0005	17.4088	13.9457	20.382	0.030691182	0.324150946	12	16	24	0.067789681	0.671868099	24	8.479996628	0.184224493

ENSMUSG00000060962	Dmkn	dermokine [Source:MGI Symbol;Acc:MGI:1920962]	1.75612	2.19424	1.26906	2.30799	1.54364	1.33907	1.79492	1.22903	1.90664	1.18514	1.93972	2.703	0.480734	3.92528	0.816361374	0.966018836	24	4	24	0.92841297	0.981515518	24	8.022369858	0.066664685
ENSMUSG00000060981	H4c8	H4 clustered histone 8 [Source:MGI Symbol;Acc:MGI:2448427]	1.62194	2.11542	1.98484	2.80716	1.15611	1.82613	2.37945	3.82376	3.01507	4.23378	5.95218	4.97139	5.96751	4.99174	0.809772024	0.966018836	4	8	24	0.810009906	0.939224719	24	4.323340513	0.082339649
ENSMUSG00000060988	Galnt13	polypeptide N-acetylgalactosaminyltransferase 13 [Source:MGI Symbol;Acc:MGI:2139447]	9.96745	10.6634	9.96089	10.7591	10.922	9.69497	10.0105	8.36591	10.1862	9.16807	11.2585	11.2361	11.1777	10.2759	0.628286596	0.966018836	20	16	24	0.352787777	0.780629136	24	17.83689733	0.062718261
ENSMUSG00000060989	Gm11847	predicted gene 11847 [Source:MGI Symbol;Acc:MGI:3651787]	2.74121	2.5972	2.43944	2.19427	1.36974	1.32215	2.83901	2.08441	2.41244	2.16758	4.17391	3.62434	4.75476	3.99283	0.348367381	0.870418653	4	12	24	0.349423678	0.779170188	24	2.0120363	0.175716339
ENSMUSG00000060992	Copz1	coatomer protein complex, subunit zeta 1 [Source:MGI Symbol;Acc:MGI:1929063]	51.8269	47.7968	51.242	51.2257	51.0977	52.0952	51.5328	51.6525	46.2953	47.8264	48.5405	47.9777	51.8095	48.6437	0.203788871	0.689899499	4	8	24	0.232734336	0.768040178	24	21.71407093	0.0358348
ENSMUSG00000061013	Mkx	mohawk homeobox [Source:MGI Symbol;Acc:MGI:2687286]	3.74425	2.70535	3.14942	2.6596	2.8482	3.49066	3.95765	3.17153	2.94416	3.16928	4.44209	3.30794	3.5622	3.02862	0.046820236	0.375011045	4	8	24	0.116304404	0.727799063	24	20.70726546	0.126457863
ENSMUSG00000061024	Rrs1	ribosome biogenesis regulator 1 [Source:MGI Symbol;Acc:MGI:1929721]	5.73656	5.18685	5.85409	4.50528	4.71148	5.87599	5.98371	5.51234	4.83238	6.18955	5.55555	5.76529	5.94533	5.74488	0.360033827	0.883787544	4	16	24	0.254037375	0.768040178	24	23.74125848	0.071633784
ENSMUSG00000061028	Clasrp	CLK4-associating serine/arginine rich protein [Source:MGI Symbol;Acc:MGI:1855695]	22.3103	25.396	23.7699	24.0257	23.4549	20.3579	23.7453	21.9249	26.1641	22.4534	24.7764	21.9122	23.3528	23.808	0.041292272	0.346472636	12	12	24	0.140675489	0.750510107	24	8.365867612	0.0700862
ENSMUSG00000061032	Rrp1	ribosomal RNA processing 1 [Source:MGI Symbol;Acc:MGI:1203500]	90.8967	82.8624	91.1012	80.0092	84.0742	96.1692	88.1239	88.4099	67.8695	92.2436	74.0036	80.3907	79.1614	82.5527	0.801273005	0.966018836	24	20	24	0.553539137	0.837374348	24	22.16739512	0.054616002
ENSMUSG00000061039	Olfr920	olfactory receptor 920 [Source:MGI Symbol;Acc:MGI:3030754]	0.976493	1.17621	1.51311	1.49081	1.3012	1.1746	0.863134	1.16737	1.42116	1.29902	1.38036	1.07076	0.945433	1.16779	3.56E-05	0.023823328	12	16	24	4.53E-05	0.07998307	24	11.30229619	0.164657438
ENSMUSG00000061046	Haghl	hydroxyacylglutathione hydrolase-like [Source:MGI Symbol;Acc:MGI:1919877]	37.0847	37.014	36.0731	33.6072	35.6592	38.3934	36.2652	35.8224	34.5726	41.132	34.8591	37.4225	34.3921	38.9872	0.904831057	0.966018836	24	20	24	0.959042145	0.989178508	24	23.77795456	0.00897613
ENSMUSG00000061048	Cdh3	cadherin 3 [Source:MGI Symbol;Acc:MGI:88356]	0.899614	0.663346	0.703878	0.671119	1.01046	0.927245	0.85957	0.800901	0.731841	1.31187	0.615541	0.9804	0.839213	0.906556	0.454881285	0.966018836	24	12	24	0.643968085	0.879114352	24	18.13997149	0.051078865
ENSMUSG00000061062	Hdac1-ps	histone deacetylase 1, pseudogene [Source:MGI Symbol;Acc:MGI:3704479]	16.1288	16.3615	18.7601	17.5971	17.1812	16.3814	16.5236	15.8616	16.8952	16.1336	16.3679	15.574	16.7538	16.3079	0.495929335	0.966018836	12	20	24	0.156155968	0.761842768	24	10.3239621	0.043534874
ENSMUSG00000061068	Mcpt4	mast cell protease 4 [Source:MGI Symbol;Acc:MGI:96940]	2.18501	2.07347	2.28513	1.73719	2.3304	1.26585	1.84637	2.56053	1.98117	1.11035	1.87986	1.84688	1.79	1.87911	0.313786068	0.824470524	12	4	24	0.369201754	0.792120801	24	3.829702494	0.106569997
ENSMUSG00000061079	Zfp143	zinc finger protein 143 [Source:MGI Symbol;Acc:MGI:1277969]	4.75413	4.70646	4.76201	4.78304	4.58547	4.60575	5.4803	4.262	4.16387	4.47073	4.56189	4.64759	5.59272	4.24834	0.128457847	0.560209134	4	8	24	0.190029066	0.768040178	24	21.92938122	0.071709794
ENSMUSG00000061080	Lsamp	limbic system-associated membrane protein [Source:MGI Symbol;Acc:MGI:1261760]	25.7057	23.9968	28.3006	23.0671	19.5404	25.9368	24.6795	22.4312	20.7058	27.8912	19.6513	24.5748	21.2988	26.7164	0.836635326	0.966018836	8	12	24	0.61121521	0.865079333	24	4.323651652	0.066661907
ENSMUSG00000061086	Myl4	myosin, light polypeptide 4 [Source:MGI Symbol;Acc:MGI:97267]	2.44852	3.31433	2.51553	2.75276	2.32312	2.48456	2.09531	2.36955	2.5537	2.38424	2.64864	1.74138	2.61297	2.40719	0.742579234	0.966018836	12	12	24	0.320958446	0.768040178	24	7.708222042	0.081159795
ENSMUSG00000061100	Retnla	resistin like alpha [Source:MGI Symbol;Acc:MGI:1888504]	4.68252	8.51967	6.88169	6.27109	4.22708	5.06641	4.58084	7.27963	9.43737	3.27736	6.65244	7.59838	8.53911	6.59433	0.481811994	0.966018836	12	4	24	0.234864189	0.768040178	24	4.853863503	0.242664331
ENSMUSG00000061104	Sap18b	Sin3-associated polypeptide 18B [Source:MGI Symbol;Acc:MGI:3704317]	66.1374	60.9696	60.8335	57.1835	51.79	60.6582	65.1161	56.8417	58.0451	63.1606	57.6464	62.0879	67.4118	60.9019	0.008810267	0.192760771	4	16	24	0.088844537	0.707896147	24	0.736340736	0.078486301
ENSMUSG00000061111	Mcrip1	MAPK regulated corepressor interacting protein 1 [Source:MGI Symbol;Acc:MGI:2384752]	42.9501	36.2486	35.4152	37.2692	40.7388	41.2823	40.8953	40.7979	37.6951	40.4543	35.9399	38.5454	37.8397	37.0154	0.102109143	0.512431169	4	8	24	0.177048374	0.768040178	24	20.99684975	0.061196544
ENSMUSG00000061118	Dnajc30	DnaJ heat shock protein family (Hsp40) member C30 [Source:MGI Symbol;Acc:MGI:1913364]	8.0318	6.99771	7.06064	5.88199	6.71044	6.73984	7.27869	6.81588	7.28209	8.35486	7.35699	8.55278	7.60626	7.10363	0.835401638	0.966018836	4	4	24	0.598106543	0.858819856	24	22.12270421	0.04732808
ENSMUSG00000061119	Prcp	prolylcarboxypeptidase (angiotensinase C) [Source:MGI Symbol;Acc:MGI:1919711]	10.9897	10.8378	9.84878	9.71243	10.4624	10.5466	10.3755	10.6692	11.2659	9.46378	10.2666	11.053	11.5739	10.5402	0.057224139	0.401309455	4	12	24	0.031003672	0.59941366	24	0.209645436	0.067518885
ENSMUSG00000061126	Cyp4f39	cytochrome P450, family 4, subfamily f, polypeptide 39 [Source:MGI Symbol;Acc:MGI:2445210]	3.9834	2.81061	2.58765	3.31105	3.62939	4.37053	3.5321	3.68466	2.94531	5.56186	2.78275	3.69125	4.06433	3.53489	0.074438493	0.448301997	24	12	24	0.532947412	0.825678486	24	19.34504173	0.103215285
ENSMUSG00000061130	Ppm1b	protein phosphatase 1B, magnesium dependent, beta isoform [Source:MGI Symbol;Acc:MGI:101841]	27.5883	24.4746	24.648	26.9365	24.8869	26.99	25.1103	28.6921	23.618	29.5999	25.78	26.2124	27.6507	27.9165	0.959160181	0.967043964	8	4	24	0.935955322	0.983958032	24	22.78725344	0.013186599
ENSMUSG00000061132	Blnk	B cell linker [Source:MGI Symbol;Acc:MGI:96878]	0.921641	0.866519	1.2329	0.949035	0.928907	0.986135	1.25904	0.762332	1.37069	0.715469	0.799409	1.11417	1.21109	0.707814	0.671726933	0.966018836	4	4	24	0.868895951	0.962810755	24	0.220321934	0.032245253
ENSMUSG00000061136	Prpf40a	pre-mRNA processing factor 40A [Source:MGI Symbol;Acc:MGI:1860512]	15.3117	19.136	15.7959	17.7416	18.4519	16.3504	14.6712	14.3139	26.5187	16.8685	19.1924	21.5818	20.9121	24.2169	0.891742979	0.966018836	20	8	24	0.613278404	0.866078878	24	8.944257425	0.086759416
ENSMUSG00000061143	Maml3	mastermind like transcriptional coactivator 3 [Source:MGI Symbol;Acc:MGI:2389461]	0.860069	1.09474	1.0649	1.02856	0.920637	1.00358	1.281	1.27069	1.18906	1.36944	0.771673	1.38736	1.0662	1.12323	0.850398351	0.966018836	8	12	24	0.441377975	0.805393068	24	5.621665855	0.061758486
ENSMUSG00000061171	Slc38a11	solute carrier family 38, member 11 [Source:MGI Symbol;Acc:MGI:2443383]	2.3004	2.55437	2.05088	2.84783	2.37515	1.95171	2.05986	2.38306	2.0961	2.87079	2.68771	2.43239	2.36896	3.34968	0.443191651	0.966018836	16	12	24	0.449675501	0.805393068	24	9.902803146	0.073630376
ENSMUSG00000061175	Fnip2	folliculin interacting protein 2 [Source:MGI Symbol;Acc:MGI:2683054]	2.451	2.60946	2.34703	2.77057	2.71751	2.64852	2.61146	2.3227	2.30495	2.64216	2.51431	2.88157	2.48526	2.6359	0.038123942	0.334069441	24	12	24	0.093310823	0.71175955	24	17.73985271	0.05677216
ENSMUSG00000061184	Tmprss11c	transmembrane protease, serine 11c [Source:MGI Symbol;Acc:MGI:3521861]	1.85638	1.72253	1.60402	1.60311	1.25487	1.54052	1.43412	1.17384	1.79289	1.3163	1.7892	1.61426	1.74179	1.6818	0.90767227	0.966018836	4	12	24	0.802103281	0.936565832	24	1.452128033	0.032477513
ENSMUSG00000061186	Sfmbt2	Scm-like with four mbt domains 2 [Source:MGI Symbol;Acc:MGI:2447794]	0.442791	0.549836	0.627059	0.45441	0.300685	0.591636	0.432355	0.417948	0.699124	0.607441	0.375076	0.488914	0.517098	0.664823	0.064422003	0.414867207	12	8	24	0.090079206	0.709927873	24	6.625650002	0.091414533
ENSMUSG00000061207	Stk19	serine/threonine kinase 19 [Source:MGI Symbol;Acc:MGI:1860085]	16.8506	16.6699	15.0916	13.6511	14.2421	16.12	15.6028	15.9202	16.5056	16.5724	17.3986	18.1316	16.2855	16.573	0.800065124	0.966018836	16	24	24	0.338960559	0.773546719	24	23.59135303	0.052152277
ENSMUSG00000061232	H2-K1	histocompatibility 2, K1, K region [Source:MGI Symbol;Acc:MGI:95904]	91.9059	103.439	110.443	99.1118	101.47	97.6967	99.125	122.519	120.363	113.473	118.554	105.821	111.449	118.698	0.215777641	0.704989686	8	20	24	0.005482407	0.445165967	24	8.166342485	0.10824971
ENSMUSG00000061244	Exoc5	exocyst complex component 5 [Source:MGI Symbol;Acc:MGI:2145645]	24.5026	24.9581	24.895	25.5719	25.1203	23.7465	22.2145	22.4021	22.9728	21.9119	26.4182	23.4917	25.873	25.283	0.806523736	0.966018836	20	4	24	0.847768986	0.954054905	24	16.75163981	0.019688121
ENSMUSG00000061273	Mmgt1	membrane magnesium transporter 1 [Source:MGI Symbol;Acc:MGI:2384305]	25.788	25.6912	24.2115	25.8244	26.7412	25.0238	25.4663	24.5277	24.2622	24.3758	27.235	25.3599	24.9587	26.2292	0.117335378	0.538362324	20	16	24	0.228555158	0.768040178	24	18.33449636	0.034098617
ENSMUSG00000061286	Exosc5	exosome component 5 [Source:MGI Symbol;Acc:MGI:107889]	10.7085	8.44045	9.26945	8.53766	9.28397	8.57647	8.6625	9.86197	10.6752	12.4653	10.2202	9.90691	10.2853	11.4083	0.776152743	0.966018836	16	8	24	0.523890362	0.821614556	24	8.716379186	0.060499088
ENSMUSG00000061288	Taok3	TAO kinase 3 [Source:MGI Symbol;Acc:MGI:3041177]	11.4604	8.84541	8.89686	9.68903	9.44125	10.1677	11.6044	9.93625	8.56285	11.3945	9.41036	10.4354	10.07	10.2159	0.03452758	0.328311403	4	8	24	0.205606789	0.768040178	24	21.46550525	0.087922874
ENSMUSG00000061298	Agbl4	ATP/GTP binding protein-like 4 [Source:MGI Symbol;Acc:MGI:1918244]	1.45895	1.84782	1.76293	1.69073	1.6663	1.50289	1.1555	1.40819	2.05865	1.76368	1.7447	1.38076	1.60767	2.47575	0.018194955	0.256345714	12	16	24	0.044233367	0.628432994	24	8.690982959	0.150916353
ENSMUSG00000061306	Slc38a10	solute carrier family 38, member 10 [Source:MGI Symbol;Acc:MGI:1919305]	29.084	25.386	25.1652	28.0324	29.1294	28.0565	27.7079	29.965	26.4155	32.7219	27.9992	28.8409	29.1636	26.4026	0.560714538	0.966018836	16	20	24	0.321630739	0.768040178	24	17.20326666	0.055355602
ENSMUSG00000061313	Ddhd2	DDHD domain containing 2 [Source:MGI Symbol;Acc:MGI:1919358]	28.6929	30.9745	30.2499	33.2776	28.904	30.3533	25.7754	30.9467	29.778	30.1516	29.6426	31.7556	33.7548	33.8567	0.702630284	0.966018836	8	12	24	0.606251956	0.863126574	24	7.784886307	0.038577064
ENSMUSG00000061315	Naca	nascent polypeptide-associated complex alpha polypeptide [Source:MGI Symbol;Acc:MGI:106095]	11.6078	15.6613	14.9271	13.3322	16.636	15.1937	12.6656	14.6188	17.7236	13.2274	16.2258	16.2568	16.5444	14.7824	0.941579937	0.966018836	24	4	24	0.769102433	0.925585646	24	12.38573899	0.042884869
ENSMUSG00000061322	Dnai1	dynein axonemal intermediate chain 1 [Source:MGI Symbol;Acc:MGI:1916172]	0.507595	1.03508	1.06891	0.668486	0.711128	0.79501	0.415521	0.509028	0.683958	0.727277	0.33387	0.943225	0.139782	0.972507	0.448396961	0.966018836	12	16	24	0.483374757	0.805393068	24	9.143401572	0.118481444
ENSMUSG00000061353	Cxcl12	chemokine (C-X-C motif) ligand 12 [Source:MGI Symbol;Acc:MGI:103556]	14.1466	17.3335	22.3223	20.5539	16.427	17.7323	16.2758	20.75	20.5938	17.8811	15.4962	16.2521	18.9255	14.9187	0.121092416	0.512431169	12	16	24	0.085039135	0.70291653	24	8.879936582	0.15776146
ENSMUSG00000061360	Phf5a	PHD finger protein 5A [Source:MGI Symbol;Acc:MGI:2156864]	28.6525	26.7455	26.3772	27.2036	25.6522	28.582	28.5867	30.4122	25.6501	25.2006	26.5122	26.5767	28.603	28.1677	0.004194713	0.140598674	4	12	24	0.011009645	0.513501556	24	0.66513124	0.076396882
ENSMUSG00000061371	Zfp873	zinc finger protein 873 [Source:MGI Symbol;Acc:MGI:3040689]	1.88145	2.00486	2.03252	2.40129	2.23158	1.65431	2.12419	2.09891	2.81054	1.46793	2.58743	2.19549	1.83944	2.23142	0.591351901	0.966018836	20	8	24	0.634269895	0.875590992	24	10.05858323	0.063818248
ENSMUSG00000061374	Fiz1	Flt3 interacting zinc finger protein 1 [Source:MGI Symbol;Acc:MGI:1344336]	5.08928	5.60976	5.71624	5.16766	5.4702	5.04169	5.53578	6.21637	5.58255	5.70653	5.31567	6.11547	5.39397	5.50973	0.305259556	0.811201458	12	8	24	0.424381573	0.805393068	24	6.331569292	0.037827304
ENSMUSG00000061393	Acvr2b	activin receptor IIB [Source:MGI Symbol;Acc:MGI:87912]	2.18832	1.54017	2.42393	1.82376	1.90876	1.45127	1.42248	2.17763	2.25535	1.98671	1.76783	1.83287	1.94474	1.88187	0.077301512	0.453790923	12	12	24	0.162369616	0.76636332	24	8.388715648	0.112512215
ENSMUSG00000061410	Zcchc14	zinc finger, CCHC domain containing 14 [Source:MGI Symbol;Acc:MGI:2159407]	8.59087	6.82956	6.30992	8.90708	9.31682	7.57217	6.80752	8.81779	9.25259	8.05002	9.50094	8.19382	7.07059	8.08452	0.208341949	0.698704663	20	8	24	0.221499902	0.768040178	24	14.35859171	0.108689123
ENSMUSG00000061411	Nol4l	nucleolar protein 4-like [Source:MGI Symbol;Acc:MGI:1918765]	15.6015	11.9292	11.5714	11.9338	13.3055	13.4619	13.3397	11.8778	11.062	16.8635	12.7279	14.0237	12.8859	12.0391	0.001971398	0.100255953	24	12	24	0.182714093	0.768040178	24	18.77497323	0.113925599
ENSMUSG00000061414	Cracr2a	calcium release activated channel regulator 2A [Source:MGI Symbol;Acc:MGI:2685919]	0.74176	0.864677	0.849986	1.03984	0.796244	0.820472	0.942819	0.946601	1.01125	0.965488	0.833749	1.02865	0.92095	0.95173	0.503972924	0.966018836	16	12	24	0.239343591	0.768040178	24	8.663598764	0.040719183
ENSMUSG00000061436	Hipk2	homeodomain interacting protein kinase 2 [Source:MGI Symbol;Acc:MGI:1314872]	2.54607	3.00436	3.48026	3.10182	3.08612	2.90824	4.04252	3.0309	3.72033	3.48904	2.7633	3.49693	2.83312	3.0197	0.57851058	0.849238815	12	16	24	0.106919972	0.735544234	24	9.106919972	0.059442534
ENSMUSG00000061451	Tmem151a	transmembrane protein 151A [Source:MGI Symbol;Acc:MGI:2147713]	18.3925	14.4926	15.8938	15.1137	13.8066	16.2729	15.4517	17.3746	14.0648	25.0716	14.5461	18.835	17.0843	18.6878	0.896545154	0.966018836	24	20	24	0.976629378	0.994103202	24	4.916883092	0.018282435
ENSMUSG00000061455	Stx17	syntaxin 17 [Source:MGI Symbol;Acc:MGI:1914977]	5.99382	6.51738	7.12349	7.12001	5.7698	5.95684	7.19873	6.39362	6.87623	7.21936	5.66488	6.38617	5.971	6.60855	0.002443055	0.109177422	16	4	24	0.05700368	0.65610184	24	7.953296832	0.096405455
ENSMUSG00000061458	Nol10	nucleolar protein 10 [Source:MGI Symbol;Acc:MGI:2684913]	8.3223	7.3605	8.13907	7.79945	7.20911	8.23779	7.92375	8.41691	7.24498	9.30004	7.22423	8.6803	7.42566	7.86351	0.893307667	0.966018836	24	20	24	0.992939315	0.998516607	24	20.62470476	0.0050202
ENSMUSG00000061461	Smim20	small integral membrane protein 20 [Source:MGI Symbol;Acc:MGI:1913528]	33.9891	38.1619	37.892	37.4451	40.6468	35.7885	37.0586	42.1409	40.6362	34.827	43.7966	36.8352	40.468	38.6627	0.625825704	0.966018836	20	4	24	0.726369984	0.910133575	24	9.561941642	0.031574603
ENSMUSG00000061462	Obscn	obscurin, cytoskeletal calmodulin and titin-interacting RhoGEF [Source:MGI Symbol;Acc:MGI:2681862]	2.72525	5.41558	6.27437	8.81296	5.9258	6.73779	4.6502	14.1282	11.0286	17.1307	5.16836	5.31243	7.79724	6.83163	0.13938715	0.582812519	16	12	24	0.031702479	0.604316769	24	9.580031943	0.53145861
ENSMUSG00000061474	Mrps36	mitochondrial ribosomal protein S36 [Source:MGI Symbol;Acc:MGI:1913378]	4.88149	4.1221	3.54623	3.40829	3.60846	3.7958	3.77098	4.17018	3.81654	3.8694	4.22469	4.02714	5.01097	5.16743	0.104170975	0.518002936	8	4	24	0.026605949	0.586541342	24	1.253530674	0.12714799
ENSMUSG00000061477	Rps7	ribosomal protein S7 [Source:MGI Symbol;Acc:MGI:1333818]	3.15807	5.25565	4.01182	3.3185	4.51703	3.90652	4.33844	3.5772	6.97237	4.79311	4.95084	5.17121	4.15543	3.93218	0.920858634	0.966018836	12	16	24	0.600679507	0.860170376	24	10.84692453	0.095488563
ENSMUSG00000061479	Snrpa	small nuclear ribonucleoprotein polypeptide A [Source:MGI Symbol;Acc:MGI:1855690]	18.0697	15.2189	15.321	15.1741	14.1849	16.225	14.9049	14.6814	14.692	16.0515	13.4512	14.0115	15.566	11.9862	0.945282252	0.966018836	4	16	24	0.606149243	0.863057202	24	21.28035843	0.041787617
ENSMUSG00000061518	Cox5b	cytochrome c oxidase subunit 5B [Source:MGI Symbol;Acc:MGI:88475]	47.8811	45.8275	43.9956	44.9477	41.0137	42.6114	49.2168	45.275	46.7253	43.6568	47.1449	46.2901	47.7155	43.5848	0.108029159	0.524035616	4	12	24	0.234169499	0.768040178	24	1.00923792	0.045820741
ENSMUSG00000061524	Zic2	zinc finger protein of the cerebellum 2 [Source:MGI Symbol;Acc:MGI:106679]	2.59934	3.41792	1.77581	2.0984	2.01635	1.98581	2.29508	2.53853	3.67491	1.21562	1.98086	2.90626	1.85595	2.869	0.4776989	0.966018836	8	8	24	0.184787162	0.768040178	24	3.075502067	0.197612406
ENSMUSG00000061533	Cep128	centrosomal protein 128 [Source:MGI Symbol;Acc:MGI:1922466]	2.16848	2.31427	2.50564	3.607	1.90011	2.36105	2.1555	2.32324	2.40373	2.22379	2.53708	3.18933	2.52868	2.85556	0.886572916	0.966018836	16	12	24	0.765542401	0.924119887	24	11.06562851	0.049783932
ENSMUSG00000061535	C1qtnf7	C1q and tumor necrosis factor related protein 7 [Source:MGI Symbol;Acc:MGI:1925911]	4.45596	5.50227	6.16122	5.51428	5.65773	4.56375	4.74322	4.88488	7.80526	4.7916	5.04199	4.9764	4.74745	5.82104	8.30E-04	0.071344007	12	16	24	0.023815297	0.584955728	24	8.885421739	0.181204975
ENSMUSG00000061536	Sec22c	SEC22 homolog C, vesicle trafficking protein [Source:MGI Symbol;Acc:MGI:2447871]	10.3211	7.45749	7.74686	8.88701	8.70971	8.93512	8.82646	8.99788	7.5286	11.3848	7.55653	9.54326	9.05175	8.7792	0.289496388	0.794869797	24	12	24	0.630768173	0.874071894	24	19.45852632	0.062548865
ENSMUSG00000061544	Zfp229	zinc finger protein 229 [Source:MGI Symbol;Acc:MGI:2679295]	4.236	4.62536	3.73317	4.49927	4.08706	3.70492	3.68164	3.91355	3.65553	3.62563	4.5682	3.95803	4.20905	3.65297	0.891742979	0.966018836	20	16	24	0.706939129	0.903220087	24	18.15320115	0.033933817
ENSMUSG00000061559	Wdr61	WD repeat domain 61 [Source:MGI Symbol;Acc:MGI:1917493]	29.1745	29.1583	29.5994	28.1975	30.4286	27.6836	27.195	26.0119	30.9875	26.6483	30.4477	28.2535	29.0308	30.2487	0.823657258	0.966018836	12	12	24	0.832400269	0.948131477	24	11.35150392	0.018401623
ENSMUSG00000061576	Dpp6	dipeptidylpeptidase 6 [Source:MGI Symbol;Acc:MGI:94921]	82.7006	60.9453	64.1293	63.0168	57.6428	77.6266	68.0206	71.4272	47.0455	86.506	57.2469	67.719	63.5767	70.8253	0.612141595	0.966018836	4	16	24	0.534359564	0.826328258	24	22.21991537	0.102460058
ENSMUSG00000061578	Ksr2	kinase suppressor of ras 2 [Source:MGI Symbol;Acc:MGI:3610315]	2.35407	1.99769	2.19417	2.47192	2.06725	2.44329	2.41012	2.31435	1.91955	2.99196	1.97942	2.349	2.11246	2.40969	0.913451828	0.966018836	16	20	24	0.905870657	0.97423153	24	14.79285314	0.021017047
ENSMUSG00000061589	Dot1l	DOT1-like, histone H3 methyltransferase (S. cerevisiae) [Source:MGI Symbol;Acc:MGI:2143886]	9.31849	10.2373	11.9902	12.0851	11.2998	9.92519	10.1621	10.8811	12.6718	11.2825	11.8541	9.84837	11.0228	10.2489	0.00630769	0.171163753	12	12	24	0.001187362	0.336500758	24	10.81526261	0.138929048
ENSMUSG00000061601	Pclo	piccolo (presynaptic cytomatrix protein) [Source:MGI Symbol;Acc:MGI:1349390]	12.222	12.3765	11.8084	11.7604	11.5513	11.7662	12.071	10.6249	10.7923	10.6517	11.2242	12.0356	11.8177	12.8849	0.073123909	0.447768907	8	8	24	0.160024632	0.76564405	24	23.80165846	0.054637373
ENSMUSG00000061603	Akap6	A kinase (PRKA) anchor protein 6 [Source:MGI Symbol;Acc:MGI:3050566]	35.7795	31.4639	30.9613	31.4985	31.8457	32.3259	30.4493	29.3622	26.4102	32.7437	32.9921	32.5546	31.5005	33.9277	0.212359178	0.701530532	24	12	24	0.233249503	0.768040178	24	19.92825405	0.066994264
ENSMUSG00000061607	Mdc1	mediator of DNA damage checkpoint 1 [Source:MGI Symbol;Acc:MGI:3525201]	5.03688	4.84481	4.51286	5.67203	4.83121	5.15315	5.59877	4.61235	4.78101	4.73895	5.33092	4.73779	4.54972	4.19377	0.335107163	0.85758369	16	20	24	0.133099409	0.743835554	24	17.39777201	0.071484948
ENSMUSG00000061613	U2af1	U2 small nuclear ribonucleoprotein auxiliary factor (U2AF) 1 [Source:MGI Symbol;Acc:MGI:98884]	16.2753	18.3801	15.9041	17.2431	17.8697	17.6201	17.2471	16.6125	21.1775	15.3088	20.9795	18.4385	19.4133	18.1533	0.897570341	0.966018836	20	20	24	0.940564934	0.985398871	24	18.47495113	0.017305456
ENSMUSG00000061619	Vma21-ps	VMA21 vacuolar H+-ATPase homolog (S. cerevisiae), pseudogene [Source:MGI Symbol;Acc:MGI:3643145]	3.93884	5.5124	3.95726	2.18249	4.8681	5.23045	4.75382	3.69299	3.78949	5.21694	5.64688	6.45933	2.6809	4.22303	0.270970204	0.780857476	24	4	24	0.435660428	0.805393068	24	19.00431498	0.176527002
ENSMUSG00000061650	Med9	mediator complex subunit 9 [Source:MGI Symbol;Acc:MGI:2183151]	21.7363	17.9351	17.7157	19.3842	18.3681	20.7882	18.8197	20.8405	14.9679	22.7251	17.4134	18.9644	18.8723	19.2704	0.869721097	0.966018836	24	12	24	0.587526942	0.853591039	24	20.87487992	0.060877909
ENSMUSG00000061665	Cd2ap	CD2-associated protein [Source:MGI Symbol;Acc:MGI:1330281]	24.8658	26.1036	24.1923	26.0179	23.9485	24.0279	24.7065	22.5858	24.6319	24.5528	25.6227	25.3434	24.6867	26.4186	0.833301348	0.966018836	8	16	24	0.974061342	0.993499248	24	10.74106534	0.005407518
ENSMUSG00000061666	Gdpd1	glycerophosphodiester phosphodiesterase domain containing 1 [Source:MGI Symbol;Acc:MGI:1913819]	19.0603	18.1427	19.1177	16.3498	16.4199	18.697	17.9528	16.4251	15.7054	18.5206	18.5609	17.5956	17.9186	19.318	0.935593607	0.966018836	8	8	24	0.553736763	0.837374348	24	23.30358838	0.040006993
ENSMUSG00000061669	Rpl34-ps2	ribosomal protein L34, pseudogene 2 [Source:MGI Symbol;Acc:MGI:3648994]	1.89886	2.44553	1.37132	2.59365	2.91817	2.21259	2.55232	2.78483	1.71031	1.67582	1.27904	1.51162	1.85625	0.821942	0.825856869	0.966018836	4	8	24	0.662828582	0.886190881	24	19.74650804	0.101399714
ENSMUSG00000061684	Rpl21-ps8	ribosomal protein L21, pseudogene 8 [Source:MGI Symbol;Acc:MGI:3648345]	10.3977	13.5055	13.4601	10.2106	9.5241	10.8667	11.4113	14.897	17.4379	10.9355	12.0194	12.3546	11.6062	10.3238	0.435739761	0.966018836	12	4	24	0.128037052	0.740162193	24	6.362634685	0.173242942
ENSMUSG00000061689	Dlgap4	DLG associated protein 4 [Source:MGI Symbol;Acc:MGI:2138865]	17.4988	14.328	14.272	15.5513	15.1562	14.8384	16.9295	16.2873	17.606	21.2572	16.1692	16.7299	15.6173	15.4372	0.882263435	0.966018836	4	4	24	0.734796299	0.91349698	24	12.51751421	0.042368361
ENSMUSG00000061702	Tmem91	transmembrane protein 91 [Source:MGI Symbol;Acc:MGI:2443589]	0.928631	2.74831	2.76928	1.38228	1.39684	1.85521	2.7917	1.42374	2.80517	1.7886	1.68847	1.91463	1.35782	1.54596	0.628286596	0.966018836	12	8	24	0.485970071	0.805393068	24	7.332793584	0.154361468
ENSMUSG00000061718	Ppp1r1b	protein phosphatase 1, regulatory inhibitor subunit 1B [Source:MGI Symbol;Acc:MGI:94860]	5.1705	6.16038	8.65924	4.26379	3.93384	6.19545	7.49205	4.22007	5.27832	4.81998	4.2485	6.60219	4.26138	6.09764	0.676344318	0.966018836	24	20	24	0.462224173	0.805393068	24	2.732543636	0.141855389
ENSMUSG00000061723	Tnnt3	troponin T3, skeletal, fast [Source:MGI Symbol;Acc:MGI:109550]	146.843	423.586	470.289	588.312	352.611	513.912	361.097	999.328	906.791	903.573	378.69	452.342	562.962	385.222	0.291042071	0.797585788	16	4	24	0.071573341	0.67819049	24	9.427022055	0.567002566
ENSMUSG00000061724	Gm2423	predicted gene 2423 [Source:MGI Symbol;Acc:MGI:3805957]	12.441	12.8439	15.1929	12.7793	13.3406	13.0804	14.5633	10.6748	11.2945	10.9469	11.2131	12.4709	11.431	11.8148	0.865514257	0.966018836	24	16	24	0.738852293	0.914739906	24	20.7508835	0.037979026
ENSMUSG00000061731	Ext1	exostosin glycosyltransferase 1 [Source:MGI Symbol;Acc:MGI:894663]	9.53025	8.54795	8.91205	7.94146	8.99849	8.88883	8.69705	9.61319	7.391	9.3221	8.53764	9.20181	8.27697	8.92695	0.891742979	0.966018836	8	4	24	0.452598941	0.805393068	24	21.87994905	0.047048535
ENSMUSG00000061740	Cyp2d22	cytochrome P450, family 2, subfamily d, polypeptide 22 [Source:MGI Symbol;Acc:MGI:1929474]	11.0929	12.9588	12.8709	12.7823	14.1544	12.6522	13.9889	12.6892	13.9178	10.4449	9.50008	11.8365	12.2859	12.1614	0.94397916	0.966018836	12	12	24	0.557287289	0.839072789	24	4.077161313	0.061734718
ENSMUSG00000061751	Kalrn	kalirin, RhoGEF kinase [Source:MGI Symbol;Acc:MGI:2685385]	6.61475	6.72175	6.96152	6.30005	6.62711	6.60046	6.02127	6.52002	6.52382	6.6501	7.02776	7.41762	7.23713	7.39979	0.838692453	0.966018836	20	20	24	0.936352438	0.984199739	24	23.84864554	0.00915718
ENSMUSG00000061755	Bod1l	biorientation of chromosomes in cell division 1-like [Source:MGI Symbol;Acc:MGI:2444804]	8.41613	8.53368	8.22708	8.86238	8.84487	8.37714	8.3864	7.65624	9.0243	7.27164	9.09537	9.53322	9.44327	9.92451	0.8635847	0.966018836	20	20	24	0.705938829	0.902918222	24	21.92375817	0.033802925
ENSMUSG00000061758	Akr1b10	aldo-keto reductase family 1, member B10 (aldose reductase) [Source:MGI Symbol;Acc:MGI:1915111]	5.14338	7.37101	8.11024	7.55525	8.03283	7.69804	6.90064	7.96544	10.1176	7.69125	6.84104	7.98929	7.8082	7.96909	0.182849367	0.659303873	12	16	24	0.098304783	0.71175955	24	9.468771998	0.137814981
ENSMUSG00000061759	Armt1	acidic residue methyltransferase 1 [Source:MGI Symbol;Acc:MGI:1920669]	8.47568	7.32853	7.40734	8.20868	8.18188	8.82495	9.13976	8.26698	8.15819	7.76889	8.17138	8.67054	8.48914	7.2374	0.007992495	0.192760771	4	4	24	0.019763713	0.565108098	24	20.13009199	0.090539587
ENSMUSG00000061762	Tac1	tachykinin 1 [Source:MGI Symbol;Acc:MGI:98474]	314.895	303.091	307.235	299.151	324.687	325.669	277.372	295.086	270.604	260.219	348.002	273.668	291.95	274.489	0.814077113	0.966018836	20	20	24	0.173494345	0.768040178	24	18.19478946	0.079630354
ENSMUSG00000061778	Mospd2	motile sperm domain containing 2 [Source:MGI Symbol;Acc:MGI:1924013]	17.6199	18.1118	17.7053	18.786	18.3038	16.9797	18.9065	16.5621	18.6599	14.5865	18.8012	15.8284	17.0025	16.7343	0.904831057	0.966018836	20	4	24	0.998452848	0.999730606	24	17.42922239	0.0021642
ENSMUSG00000061780	Cfd	complement factor D (adipsin) [Source:MGI Symbol;Acc:MGI:87931]	27.4292	28.2266	46.1706	85.6924	28.6306	48.8565	36.4123	66.3444	41.964	81.8514	69.5034	46.3372	83.5699	72.9966	0.892555977	0.966018836	16	12	24	0.246479181	0.768040178	24	10.63226864	0.300367259
ENSMUSG00000061787	Rps17	ribosomal protein S17 [Source:MGI Symbol;Acc:MGI:1309526]	52.8208	55.6539	68.129	62.8464	76.6891	57.0583	57.4309	56.7057	66.7693	35.879	56.2395	50.2022	47.3638	46.795	0.708020493	0.966018836	12	20	24	0.809706515	0.939184036	24	13.5821142	0.059469841
ENSMUSG00000061808	Ttr	transthyretin [Source:MGI Symbol;Acc:MGI:98865]	0.698413	0.651057	0.590876	0.668769	0.814719	0.595835	0.42632	0.486976	0.636478	0.538262	0.801529	0.494096	0.903622	0.520077	0.702630284	0.966018836	20	12	24	0.529813009	0.824750346	24	16.45309381	0.054192134
ENSMUSG00000061816	Myl1	myosin, light polypeptide 1 [Source:MGI Symbol;Acc:MGI:97269]	44.1123	153.613	154.764	200.422	127.492	192.033	138.141	330.535	336.731	260.365	136.452	152.809	167.866	103.433	0.326899754	0.845515883	12	16	24	0.144387557	0.752803875	24	9.866621284	0.530768442
ENSMUSG00000061838	Suclg2	succinate-Coenzyme A ligase, GDP-forming, beta subunit [Source:MGI Symbol;Acc:MGI:1306824]	3.12583	3.7989	4.13477	4.2854	3.46784	3.69576	4.11068	3.86794	4.41899	4.83988	3.77375	3.68077	3.98341	4.08173	0.010249997	0.195277595	16	4	24	0.008256519	0.49701405	24	9.351449132	0.123391203
ENSMUSG00000061848	Gm5805	predicted gene 5805 [Source:MGI Symbol;Acc:MGI:3644633]	60.8149	73.7866	75.4954	74.5835	75.2676	70.7588	65.8359	61.9121	84.474	54.0835	74.3572	60.1211	68.2184	53.26	0.196044629	0.673646834	12	20	24	0.540440221	0.830021415	24	12.76094265	0.077882907
ENSMUSG00000061859	Patj	PATJ, crumbs cell polarity complex component [Source:MGI Symbol;Acc:MGI:1277960]	26.4946	22.7433	21.9465	21.9286	19.7515	25.5515	23.6308	23.2326	17.9613	28.2026	17.8255	23.1488	22.2892	23.6244	0.160013844	0.619746337	4	16	24	0.482739181	0.805393068	24	23.97678758	0.085551123
ENSMUSG00000061878	Sphk1	sphingosine kinase 1 [Source:MGI Symbol;Acc:MGI:1316649]	2.27659	2.34651	2.26893	1.98597	1.8989	2.45491	1.54714	1.90505	2.45085	2.84152	2.48542	1.87814	2.01642	2.30636	0.729086532	0.966018836	16	12	24	0.291324005	0.768040178	24	10.78375253	0.094369607
ENSMUSG00000061882	Ccdc62	coiled-coil domain containing 62 [Source:MGI Symbol;Acc:MGI:2684996]	3.1065	3.52118	2.49297	3.99605	3.2601	2.4416	4.14775	2.90514	4.49918	1.95592	2.39202	3.69971	2.49183	3.32323	0.928127947	0.966018836	12	8	24	0.71465601	0.905979936	24	3.674825256	0.085923844
ENSMUSG00000061887	Ssbp3	single-stranded DNA binding protein 3 [Source:MGI Symbol;Acc:MGI:1919725]	77.1723	59.9912	59.3546	59.1115	56.1876	63.8451	63.0138	62.3251	53.2946	80.037	56.9418	66.6358	69.3813	65.4652	0.08333408	0.462922111	4	16	24	0.528441703	0.823973486	24	22.94560644	0.074163459
ENSMUSG00000061894	Zscan20	zinc finger and SCAN domains 20 [Source:MGI Symbol;Acc:MGI:2679268]	1.79639	1.22522	1.18851	1.39192	1.43609	1.33576	1.76288	1.18964	1.23251	1.31708	1.19378	1.23014	1.39998	1.22293	0.056400419	0.401074772	4	4	24	0.117078106	0.728077475	24	21.64461935	0.086326041
ENSMUSG00000061897	Gm14292	predicted gene 14292 [Source:MGI Symbol;Acc:MGI:3649964]	29.9965	29.8611	30.3171	35.1254	32.6899	31.7458	31.9204	30.4705	28.8085	32.1602	30.0593	30.5911	30.3807	31.2431	0.129903614	0.560263689	16	20	24	0.222055731	0.768040178	24	15.04795024	0.044692477
ENSMUSG00000061898	Rbak	RB-associated KRAB zinc finger [Source:MGI Symbol;Acc:MGI:1927369]	3.66941	4.89097	4.67666	4.22698	4.53576	4.20233	4.03735	4.77233	5.5839	4.62642	4.45054	4.45146	4.75883	4.61576	0.039617363	0.338535904	8	20	24	0.031914421	0.604838544	24	8.27481113	0.104852724
ENSMUSG00000061904	Slc25a3	solute carrier family 25 (mitochondrial carrier, phosphate carrier), member 3 [Source:MGI Symbol;Acc:MGI:1353498]	193.516	187.538	197.435	204.767	189.722	206.886	203.447	219.119	188.275	236.895	181.945	200.601	200.305	204.303	0.905275015	0.966018836	16	4	24	0.827392511	0.946154971	24	8.46996386	0.024744186
ENSMUSG00000061911	Myt1l	myelin transcription factor 1-like [Source:MGI Symbol;Acc:MGI:1100511]	20.6091	16.4073	18.9495	19.9623	16.7862	21.6588	20.1788	18.6783	14.6616	22.0715	15.5978	20.6712	18.1033	19.7427	0.236893212	0.7385769	24	12	24	0.598616239	0.859078762	24	21.02022768	0.07187848
ENSMUSG00000061950	Ppp4r1	protein phosphatase 4, regulatory subunit 1 [Source:MGI Symbol;Acc:MGI:1917601]	15.6849	14.5841	13.5452	11.8787	17.4265	14.2636	15.3434	13.0389	12.575	13.7889	13.8763	12.3023	13.0837	12.8024	0.496745696	0.966018836	4	8	24	0.219146018	0.768040178	24	20.10220235	0.086668759

ENSMUSG00000061979	Rcc1l	reculator of chromosome condensation 1-like [Source:MGI Symbol;Acc:MGI:2137600]	5.89204	7.00462	7.54203	8.08119	7.57923	6.77005	6.49956	7.04709	9.01427	7.54376	6.47036	7.56707	7.72342	6.69943	0.315409647	0.827946344	16	12	24	0.067357598	0.670376655	24	10.46117294	0.115608425
ENSMUSG00000061981	Flot2	flotillin 2 [Source:MGI Symbol;Acc:MGI:103309]	99.4624	74.8246	77.1189	81.0545	75.1416	88.6001	79.3592	87.8737	67.9192	105.628	76.0849	90.0599	84.7085	85.1476	0.601647512	0.966018836	24	12	24	0.639595664	0.877957092	24	20.97529078	0.066129434
ENSMUSG00000061983	Rps12	ribosomal protein S12 [Source:MGI Symbol;Acc:MGI:98105]	5.42777	6.34199	5.85853	3.75242	6.8229	5.5965	5.31805	6.19936	7.34672	4.51374	6.02806	5.02774	7.40391	7.78269	0.223774686	0.717349224	8	8	24	0.216149278	0.768040178	24	3.254027324	0.153356039
ENSMUSG00000062006	Rpl34	ribosomal protein L34 [Source:MGI Symbol;Acc:MGI:1915686]	11.3207	14.5462	11.415	10.7249	13.2045	10.0646	9.43939	12.9451	16.9387	13.0744	16.1143	13.9859	14.4665	13.9058	0.896311169	0.966018836	12	16	24	0.456012401	0.805393068	24	9.150670067	0.103675646
ENSMUSG00000062012	Zfp13	zinc finger protein 13 [Source:MGI Symbol;Acc:MGI:99159]	3.00453	2.65809	2.32814	2.59022	3.6561	3.3413	2.75012	3.07306	3.65385	4.29472	2.19687	3.30737	3.28398	2.98547	0.86340968	0.966018836	16	16	24	0.901728128	0.972406468	24	15.38894594	0.035183974
ENSMUSG00000062014	Gmfb	glia maturation factor, beta [Source:MGI Symbol;Acc:MGI:1927133]	183.232	202.405	177.908	185.797	211.995	173.04	188.11	170.216	216.595	159.367	212.588	183.119	196.966	186.868	0.897425765	0.966018836	20	4	24	0.954648162	0.988601783	24	16.04758281	0.016068705
ENSMUSG00000062031	Pgghg	protein glucosylgalactosylhydroxylysine glucosidase [Source:MGI Symbol;Acc:MGI:2444047]	3.46331	4.14014	3.93254	3.40986	4.19724	3.62121	3.55001	4.14994	4.5634	3.47137	3.73014	3.65692	3.80671	3.76432	0.360675801	0.883978426	12	4	24	0.323614345	0.768040178	24	6.961195656	0.060145383
ENSMUSG00000062040	Zfp27	zinc finger protein 27 [Source:MGI Symbol;Acc:MGI:99174]	3.89805	3.93924	4.99316	3.81672	4.09281	4.00671	4.46439	3.68416	3.9221	3.74483	4.19514	3.99971	4.1573	4.21041	0.923635729	0.966018836	20	20	24	0.944870634	0.986547066	24	3.305080008	0.01235647
ENSMUSG00000062044	Lmtk3	lemur tyrosine kinase 3 [Source:MGI Symbol;Acc:MGI:3039582]	6.78418	7.2928	7.40787	7.21426	7.80257	6.96735	8.46458	7.07101	8.96662	8.42733	7.64722	8.63679	7.70682	9.43454	0.799229803	0.966018836	12	16	24	0.447012594	0.805393068	24	8.282696001	0.049105653
ENSMUSG00000062054	Iah1	isoamyl acetate-hydrolyzing esterase 1 homolog [Source:MGI Symbol;Acc:MGI:1914982]	19.8638	19.2159	17.6166	19.2104	20.7039	20.5744	20.1621	20.3475	20.9666	18.3657	20.5338	18.85	20.2662	17.0343	0.745118544	0.966018836	20	12	24	0.308791832	0.768040178	24	19.36000486	0.053716379
ENSMUSG00000062070	Pgk1	phosphoglycerate kinase 1 [Source:MGI Symbol;Acc:MGI:97555]	16.1365	16.19	16.3926	17.17	16.6919	17.0207	16.3684	16.221	15.6123	15.1321	16.7445	14.3138	15.2032	13.9648	0.57656008	0.966018836	20	12	24	0.222316482	0.768040178	24	15.84313546	0.04041102
ENSMUSG00000062075	Lmnb2	lamin B2 [Source:MGI Symbol;Acc:MGI:96796]	4.2251	3.92045	4.07973	4.14018	3.99706	4.02932	4.21435	4.37071	3.90376	4.32464	4.18547	4.21822	4.15528	4.41478	0.973308424	0.973980366	8	4	24	0.771676078	0.926392944	24	2.244887846	0.011158948
ENSMUSG00000062077	Trim54	tripartite motif-containing 54 [Source:MGI Symbol;Acc:MGI:1889623]	0.748843	2.40891	2.3423	4.34221	3.0227	3.88499	2.24012	8.64813	5.11546	7.48006	2.75302	3.2137	4.25084	2.75896	0.800065124	0.966018836	16	12	24	0.185999544	0.768040178	24	10.2420534	0.398253869
ENSMUSG00000062078	Qk	quaking, KH domain containing RNA binding [Source:MGI Symbol;Acc:MGI:97837]	88.9074	100.774	99.2056	101.338	106.736	81.2941	106.473	82.0137	105.601	89.6288	96.3376	82.2934	84.5624	80.842	0.466710516	0.966018836	20	4	24	0.273073963	0.768040178	24	11.59803889	0.085470028
ENSMUSG00000062081	Gm6055	predicted gene 6055 [Source:MGI Symbol;Acc:MGI:3648620]	177	140.237	150.012	149.517	127.394	155.827	144.017	159.716	118.072	160.017	151.916	151.351	150.655	145.31	0.951523604	0.966018836	24	12	24	0.461202141	0.805393068	24	22.01530795	0.070713681
ENSMUSG00000062083	Rpl13a-ps1	ribosomal protein 13A, pseudogene 1 [Source:MGI Symbol;Acc:MGI:3648883]	17.8744	20.4742	17.6786	18.1294	18.0373	17.635	16.0186	18.6007	21.9174	14.1667	20.006	16.5374	16.5952	16.8609	0.446967971	0.966018836	12	12	24	0.714375906	0.905903854	24	7.379961472	0.049529463
ENSMUSG00000062093	Gm10110	predicted gene 10110 [Source:MGI Symbol;Acc:MGI:3641718]	5.31772	4.46641	5.08581	5.09902	5.22465	5.64779	4.81698	7.61734	5.41234	7.25065	4.50882	5.57597	6.2439	5.48836	0.862909389	0.966018836	24	20	24	0.800018739	0.935884746	24	6.522584128	0.050141143
ENSMUSG00000062098	Btbd3	BTB (POZ) domain containing 3 [Source:MGI Symbol;Acc:MGI:2385155]	28.3497	24.4871	26.1957	25.3557	24.6977	27.1699	25.4223	24.3363	22.5685	28.9579	22.9876	25.3678	23.6755	24.3165	0.94086481	0.966018836	4	8	24	0.694828679	0.898603365	24	18.63589536	0.032915253
ENSMUSG00000062101	Zfp119b	zinc finger protein 119b [Source:MGI Symbol;Acc:MGI:2385323]	1.53792	2.16103	2.29803	1.90442	1.95089	1.74159	1.93545	1.62873	2.16868	1.66762	1.86923	2.07069	2.08903	2.16106	0.198504154	0.680216953	12	4	24	0.450354861	0.805393068	24	7.22668533	0.058550875
ENSMUSG00000062110	Scfd2	Sec1 family domain containing 2 [Source:MGI Symbol;Acc:MGI:2443446]	7.48115	6.82523	7.41736	7.77485	5.85492	9.4172	7.76032	8.05053	6.02089	8.95316	7.30425	7.35426	8.23597	8.92401	0.851656735	0.966018836	4	16	24	0.736582995	0.914180155	24	0.121583927	0.052258679
ENSMUSG00000062115	Rai1	retinoic acid induced 1 [Source:MGI Symbol;Acc:MGI:103291]	9.77325	9.11157	8.79878	8.00764	8.30455	8.1609	9.30339	9.22855	9.59226	10.354	9.34466	9.4443	9.65122	9.14813	0.810949269	0.966018836	4	16	24	0.509734673	0.813985618	24	3.899861803	0.039803286
ENSMUSG00000062116	Zfp954	zinc finger protein 954 [Source:MGI Symbol;Acc:MGI:1917764]	10.5028	9.79479	10.0022	10.4785	11.1411	10.5546	9.38546	8.91105	9.20544	8.31234	10.227	9.79418	8.86173	9.42605	0.296502765	0.807732044	20	12	24	0.054250054	0.654887447	24	17.89256063	0.079870948
ENSMUSG00000062127	Cttnbp2nl	CTTNBP2 N-terminal like [Source:MGI Symbol;Acc:MGI:1933137]	13.1528	11.8739	11.9795	11.9208	13.7028	12.8457	13.5197	12.513	11.4555	14.3471	11.5515	12.6078	12.3679	11.6029	0.351840494	0.870418653	24	12	24	0.311961246	0.768040178	24	18.79213936	0.056638961
ENSMUSG00000062148	Ear6	eosinophil-associated, ribonuclease A family, member 6 [Source:MGI Symbol;Acc:MGI:1890463]	0.507196	1.06653	1.96864	1.65525	1.07995	0.879447	1.19171	1.09982	0.839703	1.19713	0.893864	0.859851	0.822485	1.144	0.097542159	0.501112642	16	12	24	0.06667095	0.669134584	24	9.832927627	0.205537834
ENSMUSG00000062151	Unc13c	unc-13 homolog C [Source:MGI Symbol;Acc:MGI:2149021]	9.2546	9.59168	10.6091	8.74573	8.72999	8.9761	9.25473	7.86072	9.63426	8.06715	8.06977	9.12832	8.37989	10.3238	0.05475651	0.398010585	12	4	24	0.301017361	0.768040178	24	4.884197841	0.069006985
ENSMUSG00000062168	Ppef1	protein phosphatase with EF hand calcium-binding domain 1 [Source:MGI Symbol;Acc:MGI:1097157]	95.2862	79.7423	80.7354	74.8865	80.5744	82.9307	68.8261	70.0713	70.1463	69.1834	84.1789	81.0469	81.2975	83.5469	0.632729413	0.966018836	20	20	24	0.226882468	0.768040178	24	21.02975734	0.089875161
ENSMUSG00000062169	Cnih4	cornichon family AMPA receptor auxiliary protein 4 [Source:MGI Symbol;Acc:MGI:1925828]	18.8312	18.8623	18.9474	19.1477	18.7661	18.3322	19.6577	19.5283	20.4074	16.952	19.7751	18.3645	20.3151	17.473	0.941685246	0.966018836	20	4	24	0.806207996	0.938114514	24	1.531284682	0.018760224
ENSMUSG00000062175	Tgif2	TGFB-induced factor homeobox 2 [Source:MGI Symbol;Acc:MGI:1915299]	1.40944	1.3307	1.78717	1.67161	1.43201	1.42317	2.03567	1.72651	1.55874	2.25485	0.998433	2.02091	1.23785	1.36064	0.774195442	0.966018836	16	16	24	0.808302382	0.939135535	24	9.629762902	0.052427986
ENSMUSG00000062184	Hs6st2	heparan sulfate 6-O-sulfotransferase 2 [Source:MGI Symbol;Acc:MGI:1354959]	29.0423	26.0492	25.8989	24.5195	24.409	27.9906	23.925	25.5253	23.3557	27.6356	27.9137	26.1754	26.0518	28.0909	0.767183862	0.966018836	24	12	24	0.610562276	0.864861374	24	21.17663295	0.038157614
ENSMUSG00000062190	Lancl2	LanC (bacterial lantibiotic synthetase component C)-like 2 [Source:MGI Symbol;Acc:MGI:1919085]	19.9555	19.263	20.1317	20.0192	18.3135	20.2118	19.3578	19.6358	16.7598	21.7369	18.3035	19.4947	18.1717	20.2731	0.890311217	0.966018836	16	4	24	0.995961043	0.998898684	24	11.9748204	0.003156543
ENSMUSG00000062198	2700097O09Rik	RIKEN cDNA 2700097O09 gene [Source:MGI Symbol;Acc:MGI:1919908]	7.22143	8.20286	8.67553	7.79477	9.14266	7.77215	8.53856	8.12747	8.98549	6.93092	9.96993	8.18626	8.26855	9.03679	0.95032543	0.966018836	20	4	24	0.91352305	0.975739644	24	11.4713711	0.020500601
ENSMUSG00000062202	Btbd9	BTB (POZ) domain containing 9 [Source:MGI Symbol;Acc:MGI:1916625]	8.3488	8.24391	8.73815	8.1006	7.35639	7.82666	7.77237	9.2825	6.10049	9.65116	7.11599	8.72115	8.56742	8.56042	0.956178643	0.96629412	8	12	24	0.73707857	0.91430672	24	2.793938406	0.046275673
ENSMUSG00000062203	Gspt1	G1 to S phase transition 1 [Source:MGI Symbol;Acc:MGI:1316728]	30.297	31.1221	28.8313	30.7168	34.489	29.7732	32.9661	28.6878	30.9375	29.7741	32.9583	31.42	30.4607	29.9209	0.351315142	0.870418653	20	16	24	0.117895339	0.728323355	24	17.69423898	0.057476435
ENSMUSG00000062209	Erbb4	erb-b2 receptor tyrosine kinase 4 [Source:MGI Symbol;Acc:MGI:104771]	0.513688	0.764256	1.11479	0.727748	0.690753	0.656416	0.936482	0.59103	0.996823	0.550278	0.546164	0.568924	0.408213	0.683709	0.118446606	0.541945448	12	16	24	0.171584426	0.768040178	24	8.115908907	0.113101617
ENSMUSG00000062210	Tnfaip8	tumor necrosis factor, alpha-induced protein 8 [Source:MGI Symbol;Acc:MGI:2147191]	7.38325	7.14063	7.28337	6.37366	7.37451	7.0857	6.2121	7.2407	7.55409	5.42885	8.21543	7.20865	7.58178	6.45624	0.896799496	0.966018836	20	20	24	0.675374936	0.89119716	24	21.40274226	0.04814858
ENSMUSG00000062232	Rapgef2	Rap guanine nucleotide exchange factor (GEF) 2 [Source:MGI Symbol;Acc:MGI:2659071]	5.41039	5.24296	5.83487	5.50038	5.02518	5.55332	6.2206	5.23407	5.14574	6.10232	4.84259	5.66412	4.70649	5.55688	0.879206539	0.966018836	16	4	24	0.940414921	0.985398871	24	7.68317373	0.014498
ENSMUSG00000062234	Gak	cyclin G associated kinase [Source:MGI Symbol;Acc:MGI:2442153]	24.1396	21.4455	22.648	23.2319	22.2823	24.8904	20.9536	23.7745	19.4497	26.7677	20.2913	22.6263	22.6885	22.2961	0.919571723	0.966018836	24	12	24	0.845793927	0.953396028	24	17.59952603	0.027187745
ENSMUSG00000062248	Cks2	CDC28 protein kinase regulatory subunit 2 [Source:MGI Symbol;Acc:MGI:1913447]	1.2811	1.64449	2.29301	1.72066	1.1226	2.25589	2.38667	1.4798	2.00308	1.54396	1.91021	1.01286	1.76171	2.0414	0.917839756	0.966018836	12	8	24	0.508847792	0.813704895	24	6.151387227	0.110820501
ENSMUSG00000062252	Lhfpl5	lipoma HMGIC fusion partner-like 5 [Source:MGI Symbol;Acc:MGI:1915382]	10.4662	8.76307	8.75544	9.52105	8.24676	10.8986	10.8883	9.56907	8.39404	9.80885	10.626	9.48223	9.82674	10.1001	0.094000699	0.490854203	4	8	24	0.1322126	0.742420495	24	21.47491899	0.091637582
ENSMUSG00000062257	Opcml	opioid binding protein/cell adhesion molecule-like [Source:MGI Symbol;Acc:MGI:97397]	15.6422	15.091	14.1446	13.9485	14.249	14.6893	14.9923	13.3586	12.8879	15.3981	14.9365	14.4257	14.8665	16.0476	0.247964668	0.746745468	4	8	24	0.408324435	0.799546792	24	22.21130205	0.043181484
ENSMUSG00000062270	Morf4l1	mortality factor 4 like 1 [Source:MGI Symbol;Acc:MGI:1096551]	28.8848	29.9905	27.0869	30.5764	29.1919	30.3061	29.0694	30.7722	32.5222	32.4202	33.3507	29.6581	31.895	29.4764	0.833406028	0.966018836	16	12	24	0.435590891	0.805393068	24	13.13777717	0.033680405
ENSMUSG00000062284	Gm6030	predicted gene 6030 [Source:MGI Symbol;Acc:MGI:3645112]	3.36899	2.41663	2.88316	3.38442	3.50307	2.36083	3.222	1.72159	2.87999	2.34655	2.51756	1.84065	2.39281	3.42758	0.880631923	0.966018836	20	4	24	0.87845844	0.965987302	24	11.2429684	0.027581563
ENSMUSG00000062296	Trank1	tetratricopeptide repeat and ankyrin repeat containing 1 [Source:MGI Symbol;Acc:MGI:1341834]	1.77541	4.15618	6.04259	2.65186	2.35093	3.80726	4.57042	2.50212	4.94295	3.06883	1.64526	3.74632	1.34404	4.22568	0.064412003	0.414867207	12	8	24	0.303554415	0.768040178	24	7.05983456	0.293316162
ENSMUSG00000062300	Nectin2	nectin cell adhesion molecule 2 [Source:MGI Symbol;Acc:MGI:97822]	13.2615	10.0187	10.0167	10.1524	11.0624	11.955	10.0347	12.0068	8.67774	12.862	11.4968	11.6971	11.3805	12.5325	0.390685537	0.915479136	24	12	24	0.366326198	0.790197806	24	20.68904164	0.088246663
ENSMUSG00000062309	Rpp25	ribonuclease P/MRP 25 subunit [Source:MGI Symbol;Acc:MGI:2143151]	1.29048	1.55301	1.40399	1.44857	1.08725	1.34449	0.999515	0.988454	1.42066	1.53849	1.21495	1.8218	1.61142	1.66065	0.853736882	0.966018836	24	20	24	0.895860687	0.970467191	24	8.023709901	0.028624798
ENSMUSG00000062312	Erbb2	erb-b2 receptor tyrosine kinase 2 [Source:MGI Symbol;Acc:MGI:95410]	3.0069	3.93626	3.38096	3.26701	3.54099	3.42686	3.74447	4.07776	3.32613	3.99688	3.49165	3.40428	3.58448	3.29499	0.860004771	0.966018836	8	4	24	0.807555785	0.938693233	24	6.667285746	0.02627512
ENSMUSG00000062328	Rpl17	ribosomal protein L17 [Source:MGI Symbol;Acc:MGI:2448270]	1.04095	0.825878	0.630296	0.818515	0.72827	0.736371	0.87229	0.988413	0.887541	0.472918	0.714165	0.873156	0.868174	0.829791	0.026323104	0.301028315	4	16	24	0.02754831	0.590220615	24	0.813627806	0.110143921
ENSMUSG00000062329	Cytl1	cytokine-like 1 [Source:MGI Symbol;Acc:MGI:2684993]	2.64616	4.2086	3.78346	7.47529	3.70447	5.61889	2.57082	6.88856	3.55054	8.02901	4.61615	4.15566	4.99401	4.0622	0.47028201	0.966018836	16	12	24	0.210725777	0.768040178	24	11.84722767	0.270559014
ENSMUSG00000062352	Itgb1bp1	integrin beta 1 binding protein 1 [Source:MGI Symbol;Acc:MGI:1306802]	27.4255	26.2129	23.5735	24.9343	28.4262	24.209	27.7368	26.9782	23.7645	21.0822	27.3589	25.1926	26.8763	23.5324	0.091669402	0.484208195	4	8	24	0.08271075	0.699403344	24	21.69315298	0.096477951
ENSMUSG00000062353	Gm15772	predicted gene 15772 [Source:MGI Symbol;Acc:MGI:3805541]	207.685	262.21	243.51	228.19	253.182	212.396	215.642	213.119	304.227	180.271	270.992	237.667	276.289	279.249	0.869721097	0.966018836	12	4	24	0.67029404	0.888925819	24	6.378763794	0.07318956
ENSMUSG00000062372	Otof	otoferlin [Source:MGI Symbol;Acc:MGI:1891247]	0.353947	0.612688	1.13384	0.361137	0.371306	0.63978	0.603098	0.466476	1.01202	0.400519	0.263524	0.626586	0.252935	0.694556	0.138543921	0.580158868	12	8	24	0.195452632	0.768040178	24	6.636561464	0.163715662
ENSMUSG00000062373	Tmem65	transmembrane protein 65 [Source:MGI Symbol;Acc:MGI:1922118]	48.079	43.0518	44.4835	45.9299	44.321	45.0105	42.6868	43.8179	38.2153	49.1324	44.8951	39.6064	39.841	41.9392	0.581070934	0.966018836	16	16	24	0.379922967	0.79941134	24	14.82766444	0.047141576
ENSMUSG00000062376	Borcs7	BLOC-1 related complex subunit 7 [Source:MGI Symbol;Acc:MGI:1913689]	9.9746	10.0737	10.3628	9.20953	8.43963	9.80413	9.42616	8.43218	9.49412	10.7843	8.34021	10.1315	8.43989	10.4911	0.89563591	0.966018836	12	8	24	0.643624147	0.878958277	24	6.454927261	0.045140047
ENSMUSG00000062380	Tubb3	tubulin, beta 3 class III [Source:MGI Symbol;Acc:MGI:107813]	879.658	637.515	686.567	661.49	699.055	837.27	707.657	749.199	506.109	773.993	746.136	708.854	675.438	663.132	0.204218286	0.690546825	24	12	24	0.078357757	0.68893025	24	20.02616803	0.159735708
ENSMUSG00000062382	Ftl1-ps1	ferritin light polypeptide 1, pseudogene 1 [Source:MGI Symbol;Acc:MGI:3779109]	106.073	90.8414	90.4675	99.1504	92.869	101.338	98.8238	97.6926	97.4498	95.8983	105.046	98.3201	101.969	91.4753	0.051961445	0.391073722	4	8	24	0.115389627	0.727799063	24	19.81006669	0.058586075
ENSMUSG00000062391	4932435O22Rik	RIKEN cDNA 4932435O22 gene [Source:MGI Symbol;Acc:MGI:2442791]	0.69539	0.642155	0.345309	0.53669	0.803612	0.390914	0.550248	0.541727	0.550942	0.485557	0.637909	0.534365	0.630112	0.467835	0.700065774	0.966018836	20	20	24	0.518564936	0.818715145	24	19.7189071	0.052165109
ENSMUSG00000062393	Dgkk	diacylglycerol kinase kappa [Source:MGI Symbol;Acc:MGI:3580254]	1.44026	1.06114	1.03041	0.880024	1.04418	1.2337	1.32144	1.09729	0.736712	1.22952	1.18148	1.23211	1.22445	1.17616	7.94E-04	0.071344007	4	8	24	0.014275223	0.533194527	24	21.79304453	0.127818571
ENSMUSG00000062397	Zfp706	zinc finger protein 706 [Source:MGI Symbol;Acc:MGI:1915286]	44.1247	41.5297	41.9873	43.9798	47.3135	44.8185	49.3696	49.6659	37.2039	47.3372	40.2605	36.979	40.0599	35.7226	0.832742389	0.966018836	16	20	24	0.764991744	0.92403577	24	18.11587928	0.040647338
ENSMUSG00000062421	Arf2	ADP-ribosylation factor 2 [Source:MGI Symbol;Acc:MGI:99595]	16.7366	17.9651	17.9357	15.2261	17.7948	16.9144	17.6989	16.2948	18.6484	15.2869	16.7416	16.3325	16.2838	16.0148	0.919119843	0.966018836	12	4	24	0.752694429	0.919658958	24	2.872092326	0.023558808
ENSMUSG00000062444	Ap3b2	adaptor-related protein complex 3, beta 2 subunit [Source:MGI Symbol;Acc:MGI:1100869]	53.21	42.9312	45.0901	43.3706	45.8803	54.1938	45.8767	48.5121	36.9906	51.7688	44.4607	46.6142	45.2049	48.6002	0.245061943	0.741580974	24	12	24	0.239109178	0.768040178	24	20.86026329	0.093069675
ENSMUSG00000062456	Rpl9-ps6	ribosomal protein L9, pseudogene 6 [Source:MGI Symbol;Acc:MGI:3642682]	94.0214	105.32	96.5227	96.4919	96.4809	91.393	93.842	96.0922	122.226	87.0394	106.623	103.828	114.404	108.151	0.888991156	0.966018836	12	12	24	0.441196806	0.805393068	24	5.274489299	0.061323734
ENSMUSG00000062470	Fbxl12os	F-box and leucine-rich repeat protein 12, opposite strand [Source:MGI Symbol;Acc:MGI:1913912]	1.49894	1.70695	1.71019	3.0429	2.20664	2.08702	1.76471	1.32669	1.11399	1.52979	1.76182	1.77254	2.55964	1.8575	0.374467935	0.89742288	20	16	24	0.36159624	0.786932131	24	17.50322709	0.136420079
ENSMUSG00000062488	Ifit3b	interferon-induced protein with tetratricopeptide repeats 3B [Source:MGI Symbol;Acc:MGI:3698419]	4.50678	4.80678	5.67884	5.29391	6.22957	5.46327	3.82119	5.67574	5.79998	4.48609	6.98069	5.68394	4.36839	5.65661	0.008810267	0.192760771	20	8	24	0.183554296	0.768040178	24	13.23249262	0.132053006
ENSMUSG00000062515	Fabp4	fatty acid binding protein 4, adipocyte [Source:MGI Symbol;Acc:MGI:88038]	77.2095	64.7923	191.733	406.128	126.595	118.583	149.66	227.581	161.458	271.494	357.264	238.123	355.711	285.944	0.787022682	0.966018836	16	16	24	0.115228973	0.727799063	24	11.97180687	0.460627411
ENSMUSG00000062519	Zfp398	zinc finger protein 398 [Source:MGI Symbol;Acc:MGI:1917856]	4.50495	4.10939	4.28062	4.04136	4.29079	4.03663	4.89358	3.73832	4.43904	3.90121	4.56399	4.37378	4.24598	4.30811	0.784278077	0.966018836	4	12	24	0.488124038	0.805393068	24	21.42700824	0.039361784
ENSMUSG00000062526	Mppe1	metallophosphoesterase 1 [Source:MGI Symbol;Acc:MGI:2661311]	8.02146	8.16477	8.01405	8.06186	7.25524	8.18295	8.52825	7.91322	7.7883	8.6967	7.50099	9.09102	8.3524	8.60009	0.478724519	0.966018836	24	20	24	0.437457937	0.805393068	24	1.2690468	0.035152951
ENSMUSG00000062542	Syt9	synaptotagmin IX [Source:MGI Symbol;Acc:MGI:1926373]	46.9522	31.8186	34.1078	35.9061	34.2121	41.7397	34.3288	36.2398	25.4659	38.7267	34.1051	38.2895	36.4613	36.0746	0.08302755	0.462922111	24	12	24	0.10082958	0.712175739	24	20.67757077	0.157664607
ENSMUSG00000062554	Gm12751	predicted gene 12751 [Source:MGI Symbol;Acc:MGI:3651049]	13.4833	11.65	13.2124	14.0868	10.2594	11.9747	12.7496	12.0557	12.3697	10.1447	10.3561	11.6342	12.9698	11.159	0.483374602	0.966018836	4	16	24	0.28229353	0.768040178	24	2.78364187	0.076637393
ENSMUSG00000062563	Cys1	cystin 1 [Source:MGI Symbol;Acc:MGI:2177632]	6.28604	4.29141	5.09845	8.9301	8.75335	7.19198	4.99179	3.4388	7.63007	6.80187	9.00148	6.40644	4.94272	4.22094	9.36E-06	0.009754434	20	12	24	1.73E-04	0.14183874	24	15.27532312	0.448722179
ENSMUSG00000062580	Timm17a	translocase of inner mitochondrial membrane 17a [Source:MGI Symbol;Acc:MGI:1343131]	28.4434	35.6878	33.495	35.6343	38.9864	34.3942	29.4876	39.1585	32.725	28.3334	33.8663	27.3478	34.4828	32.2384	0.921572192	0.966018836	20	4	24	0.755399711	0.921118175	24	9.416486153	0.048767031
ENSMUSG00000062588	Gm6104	predicted gene 6104 [Source:MGI Symbol;Acc:MGI:3648587]	10.4381	12.703	15.2825	13.4345	12.1345	13.3417	11.2668	12.5345	12.4812	12.6599	11.6033	14.0891	13.8447	16.4639	0.93717134	0.966018836	8	20	24	0.356964691	0.783888514	24	7.524526563	0.085253568
ENSMUSG00000062590	Armc9	armadillo repeat containing 9 [Source:MGI Symbol;Acc:MGI:1926045]	5.57576	7.73743	6.61639	6.41002	6.28601	8.47083	7.33573	5.30665	7.41218	6.59906	6.12108	6.96923	6.55241	7.07625	0.94791825	0.966018836	24	20	24	0.954025026	0.988601783	24	22.06562007	0.020032724
ENSMUSG00000062591	Tubb4a	tubulin, beta 4A class IVA [Source:MGI Symbol;Acc:MGI:107848]	136.35	111.902	134.199	116.027	101.587	155.68	143.618	139.462	98.9417	174.539	97.4176	126.867	105.976	127.101	0.960338592	0.967043964	24	20	24	0.881592247	0.966661503	24	0.570793754	0.051136824
ENSMUSG00000062604	Srpk2	serine/arginine-rich protein specific kinase 2 [Source:MGI Symbol;Acc:MGI:1201408]	25.1936	24.1142	26.5336	23.8488	24.5855	24.835	24.4831	22.5584	22.4902	26.7401	23.9081	25.5203	24.9729	27.6132	0.891742979	0.966018836	24	12	24	0.994605543	0.998755922	24	21.05239153	0.003540897
ENSMUSG00000062611	Rps3a2	ribosomal protein S3A2 [Source:MGI Symbol;Acc:MGI:3642853]	65.0601	83.4638	80.149	75.448	70.9617	63.7022	71.0534	66.9231	100.554	63.6514	82.7787	88.0706	94.4014	88.1296	0.967855455	0.970142211	12	4	24	0.440335909	0.805393068	24	6.37942022	0.099359297
ENSMUSG00000062619	2310039H08Rik	RIKEN cDNA 2310039H08 gene [Source:MGI Symbol;Acc:MGI:1914351]	4.32138	4.05062	3.83	3.04076	3.02562	2.74581	4.45647	5.70275	6.48401	5.79926	5.24431	6.41704	6.52162	5.23943	0.908406451	0.966018836	4	16	24	0.205059359	0.768040178	24	4.772869656	0.171076649
ENSMUSG00000062627	Mysm1	myb-like, SWIRM and MPN domains 1 [Source:MGI Symbol;Acc:MGI:2444584]	6.13823	6.84538	6.79732	6.67927	7.07713	6.28069	6.43214	6.43497	7.7112	5.45421	7.7892	5.9666	6.14195	7.22111	0.160013844	0.619746337	12	12	24	0.558835005	0.839991153	24	9.406230109	0.055188735
ENSMUSG00000062646	Ganc	glucosidase, alpha; neutral C [Source:MGI Symbol;Acc:MGI:1923301]	7.39904	7.31371	8.41947	9.01298	7.84903	7.64042	7.89169	7.95393	7.54256	7.88889	8.06037	9.38375	9.01748	7.93953	0.890176313	0.966018836	16	16	24	0.652379938	0.88299052	24	16.10136185	0.033321287
ENSMUSG00000062647	Rpl7a	ribosomal protein L7A [Source:MGI Symbol;Acc:MGI:1353472]	12.5296	14.4702	14.6271	14.7401	16.2965	14.7259	13.7888	14.202	13.8591	12.5292	14.0658	13.2033	13.4222	13.1738	0.664568268	0.966018836	20	8	24	0.444552461	0.805393068	24	14.44067102	0.04372138
ENSMUSG00000062661	Ncs1	neuronal calcium sensor 1 [Source:MGI Symbol;Acc:MGI:109166]	23.194	19.7348	23.4462	19.7116	20.4275	24.4638	23.1963	21.264	17.984	27.1242	18.0911	21.9659	19.4521	23.4542	0.862909389	0.966018836	24	20	24	0.919528134	0.978484322	24	22.83132359	0.027964805
ENSMUSG00000062683	Atp5g2	ATP synthase, H+ transporting, mitochondrial F0 complex, subunit C2 (subunit 9) [Source:MGI Symbol;Acc:MGI:1915192]	0.845508	1.10036	0.789768	0.542808	1.20211	0.880264	0.699872	0.850574	1.06126	0.748946	1.12772	1.14963	1.33278	1.18993	0.503972924	0.966018836	20	20	24	0.565957101	0.843471947	24	22.85657101	0.064563749
ENSMUSG00000062691	Cebpzos	CCAAT/enhancer binding protein (C/EBP), zeta, opposite strand [Source:MGI Symbol;Acc:MGI:1915804]	38.7707	42.0705	40.5418	37.0428	41.1675	40.1473	41.1329	40.59	43.4742	34.3726	45.7247	41.4877	41.8805	44.7039	0.969487593	0.970883022	8	8	24	0.545489625	0.832999074	24	1.238465508	0.041463685
ENSMUSG00000062694	Cav3	caveolin 3 [Source:MGI Symbol;Acc:MGI:107570]	0.439319	1.56357	1.91142	2.47764	1.55707	1.95305	1.21885	3.04289	2.89326	2.92854	1.78727	1.50055	1.80729	1.13049	0.076187236	0.453790923	16	12	24	0.035128055	0.608551791	24	10.57824687	0.365662239
ENSMUSG00000062729	Ppox	protoporphyrinogen oxidase [Source:MGI Symbol;Acc:MGI:104968]	12.8398	15.7655	16.1961	12.3345	13.7347	12.7814	13.9854	14.7874	15.1301	13.7849	12.7186	13.3014	14.6164	13.5939	0.013764091	0.238696502	12	8	24	0.028035817	0.590904845	24	5.787734572	0.107428224
ENSMUSG00000062743	Zfp677	zinc finger protein 677 [Source:MGI Symbol;Acc:MGI:3053207]	2.66009	3.33041	2.52006	2.74154	2.97881	2.44868	3.07492	2.44492	2.69801	2.25053	3.0029	2.50912	3.02945	2.96674	0.836635326	0.966018836	4	12	24	0.583778343	0.851924181	24	0.701718748	0.048557525
ENSMUSG00000062753	AI413582	expressed sequence AI413582 [Source:MGI Symbol;Acc:MGI:2146839]	78.3618	69.7023	66.5168	65.5491	71.3349	69.7072	63.8917	66.8439	63.8695	56.2514	76.4452	68.1084	71.1406	65.7332	0.033799022	0.325496079	20	20	24	0.093661423	0.71175955	24	21.13921605	0.093487118
ENSMUSG00000062760	Shisal1	shisa like 1 [Source:MGI Symbol;Acc:MGI:1919551]	12.6856	9.34144	9.58007	10.2241	10.1529	12.3741	10.3482	11.1584	7.63872	14.5039	8.27332	10.9672	10.7832	11.256	0.628286596	0.966018836	24	12	24	0.614280321	0.866356765	24	21.20880892	0.089658006
ENSMUSG00000062761	Zfp512	zinc finger protein 512 [Source:MGI Symbol;Acc:MGI:1917345]	16.4725	15.3506	15.1821	15.2707	15.9315	15.89	16.1298	16.147	15.3535	16.294	16.2195	16.1848	16.2457	17.2277	0.643325306	0.966018836	4	8	24	0.180089328	0.768040178	24	22.8659762	0.0272911
ENSMUSG00000062762	Ei24	etoposide induced 2.4 mRNA [Source:MGI Symbol;Acc:MGI:108090]	46.6486	43.8406	44.3542	45.9606	45.6391	47.4868	45.5076	45.5849	42.1367	46.0632	43.5405	45.1202	47.1397	43.4424	0.210418232	0.69936851	24	12	24	0.089839906	0.7097999	24	20.20691991	0.040013541
ENSMUSG00000062778	Chia1	chitinase, acidic 1 [Source:MGI Symbol;Acc:MGI:1932052]	5.19965	5.9968	4.90445	5.33864	4.00685	4.63807	4.6845	4.20376	5.59633	4.81936	4.76437	5.26524	5.61646	5.04128	0.708665292	0.966018836	4	16	24	0.454857497	0.805393068	24	4.656724584	0.06643287
ENSMUSG00000062785	Kcnc3	potassium voltage gated channel, Shaw-related subfamily, member 3 [Source:MGI Symbol;Acc:MGI:96669]	11.1209	12.0628	15.7367	12.6521	9.46607	12.3765	14.1422	11.0771	12.1161	18.8267	11.4266	13.4487	11.2862	15.6733	0.893307667	0.966018836	16	4	24	0.329863031	0.768040178	24	8.558187341	0.139987807
ENSMUSG00000062797	Hikeshi	heat shock protein nuclear import factor [Source:MGI Symbol;Acc:MGI:96738]	36.3065	33.9755	34.2527	34.5773	35.3337	33.5537	32.8105	38.5748	34.9467	38.8359	38.166	35.496	37.8646	35.6712	0.858514388	0.966018836	20	4	24	0.78652181	0.931349942	24	11.47995606	0.018956717
ENSMUSG00000062822	4833420G17Rik	RIKEN cDNA 4833420G17 gene [Source:MGI Symbol;Acc:MGI:1914642]	14.3726	16.2676	16.4439	14.9704	14.1913	14.5459	13.3656	15.1112	16.8274	15.277	13.6792	15.5943	14.7487	15.8842	0.004947794	0.141782417	12	8	24	0.020618338	0.567777991	24	7.374963422	0.093192231
ENSMUSG00000062825	Actg1	actin, gamma, cytoplasmic 1 [Source:MGI Symbol;Acc:MGI:87906]	194.467	140.645	146.855	154.299	184.362	204.368	161.233	183.067	109.542	150.352	120.885	122.625	117.846	109.264	0.723119329	0.966018836	24	12	24	0.186970108	0.768040178	24	19.72567084	0.165221438
ENSMUSG00000062846	Gm14176	predicted gene 14176 [Source:MGI Symbol;Acc:MGI:3650021]	10.5989	7.94884	8.73078	9.96914	9.13899	10.8579	8.99431	8.38365	9.62309	10.4719	8.86037	8.47962	9.60416	9.75043	0.883024237	0.966018836	16	16	24	0.68938907	0.897245516	24	16.59886058	0.042923947
ENSMUSG00000062861	Zfp28	zinc finger protein 28 [Source:MGI Symbol;Acc:MGI:99175]	2.87847	5.39988	5.54884	3.98915	4.22246	3.56286	3.54905	5.58673	6.1236	5.13264	3.87036	4.79075	4.31225	3.82699	0.016795343	0.253395174	12	16	24	0.035755301	0.609176161	24	8.483654744	0.240074878
ENSMUSG00000062866	Phactr2	phosphatase and actin regulator 2 [Source:MGI Symbol;Acc:MGI:2446138]	20.5591	17.6979	17.5345	18.9694	19.4586	20.0891	19.6198	18.7402	15.8467	21.825	19.4047	19.7434	18.6178	19.8956	0.0444686	0.365196805	24	12	24	0.160109991	0.76564405	24	18.88372567	0.079083528
ENSMUSG00000062867	Impdh2	inosine monophosphate dehydrogenase 2 [Source:MGI Symbol;Acc:MGI:109367]	1.79169	3.28033	2.59366	3.62151	3.2539	2.23243	1.2149	3.6613	3.28759	2.05581	2.84879	2.0968	1.87703	3.14822	0.019643089	0.258568924	8	20	24	0.083026878	0.699403344	24	9.715834078	0.271790948
ENSMUSG00000062896	Rpl31-ps11	ribosomal protein L31, pseudogene 11 [Source:MGI Symbol;Acc:MGI:3646595]	0.376368	0.759521	1.01973	1.68767	1.09969	0.312663	0.683577	0.331862	0.509107	0.87173	0.508296	0.489002	0.762603	0.583513	0.161023625	0.620479606	16	8	24	0.091829338	0.71175955	24	11.74614986	0.224681864
ENSMUSG00000062901	Klhl24	kelch-like 24 [Source:MGI Symbol;Acc:MGI:1923035]	12.2271	13.7706	13.8748	15.4841	13.6565	12.1949	13.653	13.8532	16.361	13.1287	15.3955	13.5865	14.6266	14.9269	0.039704107	0.338535904	12	12	24	0.064802206	0.663876076	24	9.269559297	0.091997022
ENSMUSG00000062906	Hdac10	histone deacetylase 10 [Source:MGI Symbol;Acc:MGI:2158340]	8.34973	8.90831	7.31237	8.02835	8.3785	8.48924	7.38524	8.47918	8.79268	8.30157	7.83468	7.75986	8.41609	7.12082	0.735210701	0.966018836	8	8	24	0.986016991	0.997339197	24	17.38390726	0.005968592
ENSMUSG00000062908	Acadm	acyl-Coenzyme A dehydrogenase, medium chain [Source:MGI Symbol;Acc:MGI:87867]	47.5327	49.6327	65.4505	89.489	54.3432	53.3848	60.0272	65.6904	56.8187	66.4796	74.3633	58.2257	76.7929	67.4044	0.965734317	0.969272194	16	8	24	0.216758661	0.768040178	24	11.14853636	0.152464003
ENSMUSG00000062929	Cfl2	cofilin 2, muscle [Source:MGI Symbol;Acc:MGI:101763]	171.308	172.093	164.359	178.63	174.39	172.701	171.644	169.675	171.339	179.364	183.523	163.052	162.054	168.909	0.06620568	0.42386701	20	8	24	0.05984032	0.658304792	24	13.79598428	0.042680492
ENSMUSG00000062931	Zfp938	zinc finger protein 938 [Source:MGI Symbol;Acc:MGI:3621440]	14.4523	16.6928	14.9774	13.9825	15.0548	14.6318	15.1393	13.7764	17.0054	12.8622	15.8119	14.2672	15.5154	15.8007	0.966095896	0.969272194	12	4	24	0.531161926	0.825104543	24	3.78058063	0.045400999
ENSMUSG00000062933	Gm10123	predicted pseudogene 10123 [Source:MGI Symbol;Acc:MGI:3704342]	572.838	612.962	584.104	576.783	594.173	557.616	537.885	521.889	683.195	490.171	670.663	615.322	673.609	638.732	0.822716578	0.966018836	12	4	24	0.973815822	0.993334889	24	3.033394011	0.012506685
ENSMUSG00000062937	Mtap	methylthioadenosine phosphorylase [Source:MGI Symbol;Acc:MGI:1914152]	8.30579	8.51759	8.64092	8.19387	7.75181	8.20781	8.15287	8.22114	7.79687	9.04854	8.01112	8.38083	8.17627	8.48129	0.401437339	0.933249185	8	12	24	0.604602465	0.861995392	24	6.83591283	0.021481225
ENSMUSG00000062939	Stat4	signal transducer and activator of transcription 4 [Source:MGI Symbol;Acc:MGI:103062]	0.707769	0.942258	0.671692	0.614495	0.585374	0.804424	1.19614	0.999994	0.820632	0.88539	0.66543	0.271432	0.628233	0.482808	0.636884864	0.966018836	4	20	24	0.478485012	0.805393068	24	4.718465598	0.088837085
ENSMUSG00000062944	9130023H24Rik	RIKEN cDNA 9130023H24 gene [Source:MGI Symbol;Acc:MGI:2442738]	1.14162	1.32883	1.41325	1.06025	1.05633	1.35512	1.02189	1.32883	1.18709	1.22817	1.0653	1.18268	1.04053	1.2752	0.210566453	0.69936851	8	20	24	0.264004073	0.768040178	24	6.073319726	0.055115714

ENSMUSG00000062949	Atp11c	ATPase, class VI, type 11C [Source:MGI Symbol;Acc:MGI:1859661]	7.77171	7.87009	7.51688	8.28307	7.65658	6.99963	8.01564	6.5796	7.59646	6.66528	8.11439	7.07647	6.50781	7.3717	0.93717134	0.966018836	20	4	24	0.701395623	0.901104245	24	13.91359428	0.029642098
ENSMUSG00000062960	Kdr	kinase insert domain protein receptor [Source:MGI Symbol;Acc:MGI:96683]	2.57491	2.83622	2.63031	2.33205	2.83246	3.06495	3.71105	4.20644	3.01747	3.63804	2.74411	2.82484	3.70317	2.86778	0.671726933	0.966018836	8	8	24	0.377216736	0.797219305	24	2.262617685	0.090329244
ENSMUSG00000062963	Ufc1	ubiquitin-fold modifier conjugating enzyme 1 [Source:MGI Symbol;Acc:MGI:1913405]	90.29	83.0828	85.8175	79.4993	78.2585	83.6866	79.3591	77.9548	80.9772	81.7435	89.5467	83.7175	87.0489	86.7411	0.851656735	0.966018836	4	12	24	0.638122326	0.8771388	24	22.77099287	0.026287238
ENSMUSG00000062980	Cped1	cadherin-like and PC-esterase domain containing 1 [Source:MGI Symbol;Acc:MGI:2444814]	3.11233	4.06595	3.57421	3.84385	3.87525	3.14548	3.48662	4.217	4.85341	3.03712	4.34354	3.95586	3.73037	4.04532	0.47793208	0.966018836	8	20	24	0.373434538	0.794388449	24	8.004871117	0.0835327
ENSMUSG00000062981	Mrpl42	mitochondrial ribosomal protein L42 [Source:MGI Symbol;Acc:MGI:1333774]	82.6351	95.3033	84.1847	92.7171	97.9831	78.3311	82.8433	89.8123	104.071	80.5337	97.1208	82.5715	96.674	90.2799	0.300823756	0.809362476	20	4	24	0.461230629	0.805393068	24	9.072037706	0.06321145
ENSMUSG00000062991	Nrg1	neuregulin 1 [Source:MGI Symbol;Acc:MGI:96083]	29.5268	24.5221	23.5252	29.6591	24.9416	28.3939	25.7733	27.1365	20.321	30.8999	27.3564	27.6031	26.5557	29.5102	0.265774856	0.773510818	16	20	24	0.496476874	0.807537659	24	19.00696685	0.074546076
ENSMUSG00000062995	Ica1	islet cell autoantigen 1 [Source:MGI Symbol;Acc:MGI:96391]	22.1158	21.7752	21.8852	20.8741	20.6109	22.0747	19.1129	19.5738	17.7758	20.7499	19.1876	20.6536	21.7867	22.5838	0.92655264	0.966018836	4	16	24	0.599291629	0.859493187	24	23.54967332	0.03814261
ENSMUSG00000062997	Rpl35	ribosomal protein L35 [Source:MGI Symbol;Acc:MGI:1913739]	3.34838	4.45053	3.57174	3.38381	4.92105	2.67578	4.35969	3.21315	4.9532	3.47006	5.67645	4.3721	5.34157	3.45127	0.93447422	0.966018836	20	12	24	0.865491681	0.961058769	24	16.37497299	0.053299537
ENSMUSG00000063011	Msln	mesothelin [Source:MGI Symbol;Acc:MGI:1888992]	4.51988	5.56429	4.31587	2.08113	4.86855	5.15878	3.70356	4.91691	3.65953	2.00097	3.61367	4.58076	3.39322	3.76529	0.212359178	0.701530532	8	8	24	0.085094651	0.70291653	24	23.5981325	0.267044807
ENSMUSG00000063015	Ccni	cyclin I [Source:MGI Symbol;Acc:MGI:1341077]	72.3686	63.0674	62.2416	70.4731	64.8043	66.3668	65.6267	62.0078	56.6511	69.9444	68.0562	65.6105	66.2729	65.951	0.079180494	0.458678242	16	20	24	0.197341793	0.768040178	24	18.49699435	0.061971696
ENSMUSG00000063018	2010204K13Rik	RIKEN cDNA 2010204K13 gene [Source:MGI Symbol;Acc:MGI:1922859]	2.09472	1.58844	1.72363	1.55848	1.14865	1.88025	1.58057	1.63754	2.51405	1.62564	0.830105	1.49479	1.25143	1.58788	0.039226672	0.338446795	12	8	24	0.202051577	0.768040178	24	5.0949889	0.147267667
ENSMUSG00000063019	Manbal	mannosidase, beta A, lysosomal-like [Source:MGI Symbol;Acc:MGI:1916411]	26.9631	25.298	27.5422	25.0227	28.9401	29.8386	25.8767	26.7633	23.7993	26.2045	23.4797	24.839	24.7508	25.2073	0.755349378	0.966018836	24	16	24	0.498308189	0.808120517	24	19.68214657	0.040929242
ENSMUSG00000063047	Zfp780b	zinc finger protein 780B [Source:MGI Symbol;Acc:MGI:2444764]	6.73625	6.40445	6.47428	6.38208	6.99759	6.21829	6.63525	5.39345	7.28245	5.34431	7.35812	6.19946	7.19703	6.80923	0.933543173	0.966018836	20	20	24	0.859229224	0.958530654	24	20.33839799	0.028416119
ENSMUSG00000063049	Ing2	inhibitor of growth family, member 2 [Source:MGI Symbol;Acc:MGI:1916510]	5.0323	6.13536	5.51521	6.22213	6.25928	6.33284	5.82947	6.62923	5.86481	4.96475	5.75668	5.81724	6.84169	5.76356	0.916963526	0.966018836	8	4	24	0.737032443	0.914284314	24	23.58260623	0.035230518
ENSMUSG00000063052	Lrrc40	leucine rich repeat containing 40 [Source:MGI Symbol;Acc:MGI:1914394]	11.252	12.5909	12.3323	11.7678	10.9079	11.2493	11.818	11.4795	12.5486	11.4471	11.9565	11.0754	10.0494	12.7577	0.030691182	0.324150946	8	16	24	0.053327098	0.652202934	24	7.719519001	0.077869272
ENSMUSG00000063060	Sox7	SRY (sex determining region Y)-box 7 [Source:MGI Symbol;Acc:MGI:98369]	0.365923	0.379061	0.439509	0.786604	0.503967	0.425537	0.478302	0.669491	0.440736	0.774931	0.383703	0.328178	0.636363	0.447681	0.285027487	0.793714531	16	8	24	0.28533055	0.768040178	24	10.37077455	0.08714909
ENSMUSG00000063063	Ctnna2	catenin (cadherin associated protein), alpha 2 [Source:MGI Symbol;Acc:MGI:88275]	83.4464	70.7119	71.1565	69.7354	68.7534	76.6431	68.2395	69.9221	61.9796	73.5964	71.3462	74.5022	75.5109	76.6024	0.478349072	0.966018836	24	12	24	0.144696908	0.753524831	24	21.95214601	0.075335273
ENSMUSG00000063065	Mapk3	mitogen-activated protein kinase 3 [Source:MGI Symbol;Acc:MGI:1346859]	412.845	328.634	304.388	352.746	354.579	378.456	356.886	360.849	291.038	408.835	330.134	374.302	359.646	336.662	0.020957534	0.262822894	24	12	24	0.121859668	0.734196514	24	20.06572895	0.113013372
ENSMUSG00000063077	Kif1b	kinesin family member 1B [Source:MGI Symbol;Acc:MGI:108426]	171.973	139.892	148.442	155.579	141.613	165.242	151.021	153.206	119.505	194.244	139.579	157.222	151.281	158.766	0.883024237	0.966018836	24	12	24	0.734902646	0.913580967	24	19.52460419	0.052990656
ENSMUSG00000063087	Gm10125	predicted gene 10125 [Source:MGI Symbol;Acc:MGI:3642044]	3.39797	3.23736	3.09711	2.77216	2.43929	2.2373	3.95674	3.45941	4.32925	2.12523	3.09332	2.78108	3.18343	2.78562	0.051961445	0.391073722	4	20	24	0.098140294	0.71175955	24	3.812742151	0.171219691
ENSMUSG00000063108	Zfp26	zinc finger protein 26 [Source:MGI Symbol;Acc:MGI:99173]	3.60998	5.97523	4.75216	4.79919	4.52709	4.13838	3.9863	4.05867	5.66803	4.31318	5.63743	5.44272	5.49314	5.19594	0.953760335	0.966018836	12	16	24	0.570304433	0.845131203	24	9.30952992	0.075120918
ENSMUSG00000063142	Kcnma1	potassium large conductance calcium-activated channel, subfamily M, alpha member 1 [Source:MGI Symbol;Acc:MGI:99923]	7.41492	7.57531	8.21297	7.52117	7.50968	7.44889	7.02092	6.97936	7.30742	7.02035	7.58507	8.01435	7.39496	8.25689	0.896311169	0.966018836	12	16	24	0.966359132	0.991934487	24	8.648136459	0.007530007
ENSMUSG00000063145	Bbs5	Bardet-Biedl syndrome 5 (human) [Source:MGI Symbol;Acc:MGI:1919819]	10.2343	12.9684	12.4571	10.9979	11.8968	11.5131	11.1302	11.0898	13.3459	10.8478	11.5966	12.053	11.7759	11.7037	0.887027852	0.966018836	12	4	24	0.478098429	0.805393068	24	7.875867693	0.047271053
ENSMUSG00000063146	Clip2	CAP-GLY domain containing linker protein 2 [Source:MGI Symbol;Acc:MGI:1313136]	25.1341	20.1068	20.1788	20.5706	21.7757	21.2451	22.5301	22.6716	19.2204	29.2208	20.6981	24.1856	23.4582	21.8926	0.064867922	0.416505219	4	8	24	0.698038916	0.899652089	24	19.87228928	0.052252593
ENSMUSG00000063160	Numbl	numb-like [Source:MGI Symbol;Acc:MGI:894702]	16.1594	12.5674	12.3511	14.6888	12.1575	13.2417	14.1068	14.4878	12.8642	17.94	14.2712	16.9172	14.9996	15.2679	0.850398351	0.966018836	24	12	24	0.848035783	0.954124678	24	20.16780635	0.034982395
ENSMUSG00000063171	Rps4l	ribosomal protein S4-like [Source:MGI Symbol;Acc:MGI:1913434]	20.7415	17.8164	17.6777	20.276	19.4465	18.6808	18.0913	19.6165	19.3831	22.2225	20.6339	23.6304	20.9148	22.1629	0.942429894	0.966018836	24	12	24	0.563245426	0.84250706	24	16.87230656	0.039242914
ENSMUSG00000063172	Hspb11	heat shock protein family B (small), member 11 [Source:MGI Symbol;Acc:MGI:1920188]	19.3805	19.2677	17.5285	14.3945	19.2863	18.5026	17.7529	18.7268	18.9811	14.6056	16.4459	18.5939	20.601	17.361	0.036134798	0.331852526	4	12	24	0.024107122	0.586541342	24	0.106532999	0.135187328
ENSMUSG00000063179	Pstk	phosphoseryl-tRNA kinase [Source:MGI Symbol;Acc:MGI:2685945]	17.5468	21.1157	22.3835	19.5853	19.376	20.3906	20.3977	20.434	22.9931	19.1108	23.3272	24.498	25.3689	24.9488	0.745118544	0.966018836	8	8	24	0.533126831	0.825678486	24	4.363876596	0.046717373
ENSMUSG00000063193	Cd300lb	CD300 molecule like family member B [Source:MGI Symbol;Acc:MGI:2685099]	0.431855	0.810833	0.832052	1.08218	0.790424	0.627675	0.585959	0.732204	0.5492	0.390122	0.606617	0.567973	0.906796	0.555482	0.715742619	0.966018836	8	20	24	0.878731645	0.966077307	24	9.832849823	0.033535121
ENSMUSG00000063200	Nol7	nucleolar protein 7 [Source:MGI Symbol;Acc:MGI:1917328]	38.5387	43.8813	46.9091	44.6734	39.5183	37.4486	41.1727	41.5433	47.6064	41.2522	44.1383	46.1014	47.3943	49.6672	0.277221318	0.789849983	12	12	24	0.045824222	0.631948403	24	7.218251274	0.096625806
ENSMUSG00000063229	Ldha	lactate dehydrogenase A [Source:MGI Symbol;Acc:MGI:96759]	175.887	161.53	169.571	194.366	180.002	192.895	164.176	214.697	180.449	196.289	183.922	175.312	193.011	172.616	0.664568268	0.966018836	16	16	24	0.661612728	0.885525866	24	13.2974455	0.039158665
ENSMUSG00000063232	Serpina11	serine (or cysteine) peptidase inhibitor, clade A (alpha-1 antiproteinase, antitrypsin), member 11 [Source:MGI Symbol;Acc:MGI:2685741]	2.00748	1.86953	1.8614	1.59921	1.44565	1.8309	1.62207	1.78207	1.75314	1.26479	1.83095	1.98388	1.74791	1.93033	0.303804925	0.811201458	8	8	24	0.096296433	0.71175955	24	0.89279638	0.089768546
ENSMUSG00000063235	Ptpmt1	protein tyrosine phosphatase, mitochondrial 1 [Source:MGI Symbol;Acc:MGI:1913711]	22.681	24.7343	23.7242	23.3293	24.388	23.547	24.6793	22.1691	26.0105	22.9934	23.8092	24.7333	24.0943	23.0136	0.866861748	0.966018836	24	8	24	0.959755189	0.989375125	24	15.35820016	0.006753104
ENSMUSG00000063236	1110038F14Rik	RIKEN cDNA 1110038F14 gene [Source:MGI Symbol;Acc:MGI:2152337]	11.0993	10.734	10.7806	10.4625	10.7575	10.6429	11.2646	11.7444	10.7676	11.6995	11.0103	10.9266	11.1902	10.4018	0.81829516	0.966018836	4	4	24	0.906636846	0.974544241	24	1.04910695	0.008559905
ENSMUSG00000063239	Grm4	glutamate receptor, metabotropic 4 [Source:MGI Symbol;Acc:MGI:1351341]	3.36028	2.44	3.31845	2.46672	1.89577	2.80961	2.9811	2.67871	2.46453	3.92436	2.12263	3.0335	2.741	3.31053	0.9678483	0.970142211	4	16	24	0.506370643	0.81209692	24	4.119780469	0.0946288
ENSMUSG00000063253	Scoc	short coiled-coil protein [Source:MGI Symbol;Acc:MGI:1927654]	135.157	126.341	123.911	120.832	126.405	126.272	123.572	116.57	122.378	118.843	142.879	124.184	135.086	139.241	0.369262716	0.893596186	20	20	24	0.46714648	0.805393068	24	21.60183023	0.041824516
ENSMUSG00000063268	Parp10	poly (ADP-ribose) polymerase family, member 10 [Source:MGI Symbol;Acc:MGI:3712326]	4.65536	5.03174	4.41275	3.61768	6.29841	4.10749	4.94711	5.17182	4.88694	4.14256	5.28616	4.89499	4.10579	4.63914	0.867888967	0.966018836	20	20	24	0.852438778	0.955729429	24	20.34120509	0.038985853
ENSMUSG00000063273	Naa15	N(alpha)-acetyltransferase 15, NatA auxiliary subunit [Source:MGI Symbol;Acc:MGI:1922088]	18.3107	18.9388	18.4271	17.848	19.093	17.9495	18.1475	17.3446	18.4835	16.7345	20.3892	18.0242	18.9552	18.8818	0.924553956	0.966018836	20	20	24	0.814910417	0.940889579	24	19.80393042	0.017362682
ENSMUSG00000063275	Hacd1	3-hydroxyacyl-CoA dehydratase 1 [Source:MGI Symbol;Acc:MGI:1353592]	6.72985	8.07834	7.02712	7.18132	7.87639	7.26298	6.57991	8.27537	9.25335	7.61966	7.91065	7.74662	8.17509	7.41639	0.921572192	0.966018836	8	20	24	0.416982431	0.801686744	24	8.986958214	0.057535903
ENSMUSG00000063281	Zfp35	zinc finger protein 35 [Source:MGI Symbol;Acc:MGI:99179]	11.775	12.7467	11.5439	11.876	13.1209	11.3672	12.1098	11.1814	12.4819	9.76539	13.8493	11.7647	12.8906	12.195	0.894678274	0.966018836	20	20	24	0.746727155	0.917587548	24	20.80355896	0.035765115
ENSMUSG00000063286	Gvin-ps7	GTPase, very large interferon inducible, pseudogene 7 [Source:MGI Symbol;Acc:MGI:3644223]	1.70521	1.75436	2.01886	1.61677	1.81617	1.28597	1.85818	1.7388	2.05561	1.14225	1.76568	1.4789	1.74145	1.44572	0.245061943	0.741580974	12	12	24	0.536294156	0.827399772	24	5.309273634	0.062237454
ENSMUSG00000063296	Tmem117	transmembrane protein 117 [Source:MGI Symbol;Acc:MGI:2444580]	22.2716	20.6571	20.5131	21.2988	19.7163	21.799	19.7449	20.7185	18.2011	22.3848	20.0988	22.7409	21.4	21.0743	0.148073804	0.590732968	24	12	24	0.419400412	0.803639585	24	20.43863878	0.044352889
ENSMUSG00000063297	Luzp2	leucine zipper protein 2 [Source:MGI Symbol;Acc:MGI:1889615]	41.1846	43.452	41.9589	40.2991	38.973	39.1264	38.7948	37.1379	39.0773	37.4606	41.3036	38.0288	38.4111	41.892	0.939103211	0.966018836	12	12	24	0.655348869	0.883697438	24	5.720839752	0.023066257
ENSMUSG00000063316	Rpl27	ribosomal protein L27 [Source:MGI Symbol;Acc:MGI:98036]	3.74284	4.42225	4.50955	2.83128	3.98171	3.60725	3.59436	6.0649	5.15031	4.77526	6.85451	6.9431	5.30753	6.0765	0.858514388	0.966018836	8	8	24	0.783551506	0.93037654	24	4.747368824	0.062581812
ENSMUSG00000063317	Usp31	ubiquitin specific peptidase 31 [Source:MGI Symbol;Acc:MGI:1923429]	20.4556	17.7203	16.6359	18.3822	18.7595	19.1177	18.106	16.969	15.4508	18.0227	18.8418	19.0431	18.7176	18.5857	8.68E-05	0.029372629	24	12	24	0.006898361	0.472731997	24	20.02020304	0.102982198
ENSMUSG00000063320	1190007I07Rik	RIKEN cDNA 1190007I07 gene [Source:MGI Symbol;Acc:MGI:3698433]	7.85495	8.6632	8.88482	9.31998	8.11083	8.28085	8.46364	10.5493	11.8258	9.52523	11.1006	7.74392	8.93976	8.53879	0.034356228	0.328311403	16	8	24	0.061646991	0.661743448	24	9.464260799	0.1435498
ENSMUSG00000063334	Krr1	KRR1, small subunit (SSU) processome component, homolog (yeast) [Source:MGI Symbol;Acc:MGI:1289274]	10.2051	11.2408	11.5667	11.6697	11.185	10.8702	10.0676	10.4757	10.7207	8.52194	11.6551	10.6866	9.69785	10.8062	0.089506974	0.477279066	20	8	24	0.855284172	0.956952522	24	13.41474027	0.02352727
ENSMUSG00000063358	Mapk1	mitogen-activated protein kinase 1 [Source:MGI Symbol;Acc:MGI:1346858]	121.537	102.566	105.72	108.266	106.297	112.984	102.471	108.211	94.1768	104.419	99.2449	107.386	104.677	106.461	0.885955579	0.966018836	24	12	24	0.1443583	0.752803875	24	21.6199316	0.05864135
ENSMUSG00000063362	Alg11	asparagine-linked glycosylation 11 (alpha-1,2-mannosyltransferase) [Source:MGI Symbol;Acc:MGI:2142632]	10.2246	9.86335	9.56733	9.32708	9.28998	9.53395	8.6693	8.71426	8.88692	8.88806	9.41319	9.50387	9.19121	10.1461	0.883907406	0.966018836	8	8	24	0.696269056	0.89922881	24	23.36857385	0.022657923
ENSMUSG00000063364	3300002I08Rik	RIKEN cDNA 3300002I08 gene [Source:MGI Symbol;Acc:MGI:1916527]	1.70514	1.86137	1.25212	1.4053	2.05406	1.81618	1.82286	1.91576	2.04038	1.92922	1.63294	2.15956	2.38444	1.84332	0.896753532	0.966018836	8	4	24	0.243932197	0.768040178	24	22.54485594	0.083457199
ENSMUSG00000063382	Bcl9l	B cell CLL/lymphoma 9-like [Source:MGI Symbol;Acc:MGI:1933114]	3.21764	3.16016	3.79525	3.54461	2.77461	2.70456	3.94496	3.41758	3.61807	4.21689	4.75732	3.49788	4.00461	3.74621	0.920872048	0.966018836	16	8	24	0.442311051	0.805393068	24	9.461276302	0.076697556
ENSMUSG00000063383	Zfp947	zinc finger protein 947 [Source:MGI Symbol;Acc:MGI:3646759]	0.452922	0.580198	0.636099	0.826178	0.583324	0.49254	0.535894	0.472891	0.550267	0.405238	0.706624	0.556248	0.746692	0.594298	0.915082018	0.966018836	20	8	24	0.743083394	0.916215545	24	12.70793013	0.032087371
ENSMUSG00000063388	BC023105	cDNA sequence BC023105 [Source:MGI Symbol;Acc:MGI:2384767]	0.489974	1.49491	1.84495	2.12254	1.21562	2.48715	2.16277	3.64965	4.09635	2.43997	2.56975	1.48763	1.27888	1.15036	0.633085074	0.966018836	12	16	24	0.226601858	0.768040178	24	10.17781351	0.309912509
ENSMUSG00000063406	Tmed5	transmembrane p24 trafficking protein 5 [Source:MGI Symbol;Acc:MGI:1921586]	14.2677	14.3807	15.4819	16.8215	13.9499	13.4022	14.821	12.7331	13.0998	12.9489	15.9394	11.3363	11.8165	13.0051	0.7207001	0.966018836	20	4	24	0.243882747	0.768040178	24	12.23888406	0.077923433
ENSMUSG00000063410	Stk24	serine/threonine kinase 24 [Source:MGI Symbol;Acc:MGI:2385007]	32.6114	25.0224	27.6917	32.7503	23.2441	30.9523	27.0797	28.9155	20.518	36.5546	23.3774	27.4379	27.6943	29.0291	0.93447422	0.966018836	16	4	24	0.970352009	0.993003194	24	20.61830931	0.022236577
ENSMUSG00000063412	Gm10131	predicted pseudogene 10131 [Source:MGI Symbol;Acc:MGI:3704308]	31.3555	33.7056	31.5859	29.2391	30.5339	28.1474	28.55	27.9445	31.9042	24.9025	32.8915	31.1223	31.5118	30.0309	0.896799496	0.966018836	12	4	24	0.775418553	0.927742146	24	1.080251827	0.031012764
ENSMUSG00000063415	Cyp26b1	cytochrome P450, family 26, subfamily b, polypeptide 1 [Source:MGI Symbol;Acc:MGI:2176159]	1.14867	1.91343	1.01694	1.07154	1.58316	1.34032	1.12783	1.54809	1.9902	1.4129	1.22718	1.78052	1.795	1.77281	0.924553956	0.966018836	8	8	24	0.495361395	0.807162937	24	3.911388288	0.080780399
ENSMUSG00000063428	Ddo	D-aspartate oxidase [Source:MGI Symbol;Acc:MGI:1925528]	1.06829	1.7164	2.40453	2.80125	1.34997	1.84929	2.01513	2.43862	2.50277	2.35734	2.12506	2.14571	1.77527	2.4986	0.058157448	0.40386543	12	16	24	0.015366795	0.53936769	24	9.243114896	0.227360131
ENSMUSG00000063430	Wscd2	WSC domain containing 2 [Source:MGI Symbol;Acc:MGI:2445030]	1.74546	1.32524	1.56988	1.58476	1.69573	1.54139	2.32417	1.01292	1.64702	1.47741	1.34487	1.28974	1.31725	1.38241	0.934119097	0.966018836	12	20	24	0.760894386	0.92268731	24	19.01001432	0.048365791
ENSMUSG00000063434	Sorcs3	sortilin-related VPS10 domain containing receptor 3 [Source:MGI Symbol;Acc:MGI:1913923]	2.98193	2.45803	2.87118	2.63101	2.45165	2.89581	2.91262	2.45644	2.17028	3.15493	2.27964	2.68438	2.36186	2.80458	0.93447422	0.966018836	4	16	24	0.872124673	0.963835087	24	21.3629322	0.025189663
ENSMUSG00000063439	B9d2	B9 protein domain 2 [Source:MGI Symbol;Acc:MGI:2387643]	8.49479	10.1587	8.95138	8.5519	9.21905	8.27885	8.49775	10.2091	10.0996	8.44597	10.3701	8.65114	8.10592	9.33529	0.116913252	0.538362324	8	20	24	0.318843659	0.768040178	24	8.011619701	0.069182653
ENSMUSG00000063445	Nmral1	NmrA-like family domain containing 1 [Source:MGI Symbol;Acc:MGI:1915074]	2.47367	3.88121	5.12641	3.06487	3.27551	3.12674	3.57312	3.06511	4.14199	2.42972	2.67475	3.45113	2.88994	3.62622	0.054328576	0.395890765	12	4	24	0.220421533	0.768040178	24	6.513949592	0.154076188
ENSMUSG00000063450	Syne2	spectrin repeat containing, nuclear envelope 2 [Source:MGI Symbol;Acc:MGI:2449316]	16.5863	17.5844	14.7336	15.6504	18.9167	14.0356	18.3977	19.6643	18.3757	16.6494	16.0197	16.1503	17.5789	17.2605	0.4776989	0.966018836	8	16	24	0.404495765	0.799546792	24	4.205048421	0.067647435
ENSMUSG00000063455	D630045J12Rik	RIKEN cDNA D630045J12 gene [Source:MGI Symbol;Acc:MGI:2669829]	4.07682	3.62588	3.64805	3.31605	3.41074	3.60458	4.08223	3.53945	3.27355	4.34342	3.31358	3.35764	3.94303	3.85823	0.138719141	0.580158868	4	16	24	0.405039359	0.799546792	24	1.633572303	0.053686089
ENSMUSG00000063457	Rps15	ribosomal protein S15 [Source:MGI Symbol;Acc:MGI:98117]	188.443	181.675	200.079	187.442	214.237	191.447	180.358	178.383	181.191	134.044	169.29	159.534	172.881	151.754	0.82502117	0.966018836	20	12	24	0.68743402	0.89619155	24	20.20177258	0.045382244
ENSMUSG00000063458	Lrmda	leucine rich melanocyte differentiation associated [Source:MGI Symbol;Acc:MGI:1923883]	2.4206	3.27287	2.91467	2.93176	2.97831	3.50898	3.28705	3.69592	2.74357	2.75639	3.24708	2.94338	[illegible]	2.87541	0.181151038	0.659020016	4	12	24	0.034926983	0.608551791	24	0.193498013	0.112140723
ENSMUSG00000063480	Snu13	SNU13 homolog, small nuclear ribonucleoprotein (U4/U6.U5) [Source:MGI Symbol;Acc:MGI:893586]	62.6705	69.8577	64.2292	60.1942	66.8942	62.4264	63.5844	57.7115	68.9436	53.1024	68.682	67.4628	67.7941	64.2184	0.81265116	0.966018836	12	4	24	0.651800905	0.88284964	24	22.56784783	0.040168336
ENSMUSG00000063488	Zkscan7	zinc finger with KRAB and SCAN domains 7 [Source:MGI Symbol;Acc:MGI:3040678]	2.65933	3.57594	3.09294	3.80866	3.14673	2.63194	3.09638	3.33786	4.32844	2.64017	3.4218	3.60717	3.45293	3.53441	0.801995186	0.966018836	8	16	24	0.297743848	0.768040178	24	7.869054388	0.096333139
ENSMUSG00000063506	Arhgap22	Rho GTPase activating protein 22 [Source:MGI Symbol;Acc:MGI:2443418]	14.1696	9.9753	9.84973	12.441	11.673	11.3441	9.44788	12.5767	9.22405	11.9331	11.0651	11.7659	13.5426	12.7283	0.793403332	0.966018836	4	8	24	0.585722072	0.853141382	24	21.1846757	0.074286245
ENSMUSG00000063511	Snrnp70	small nuclear ribonucleoprotein 70 (U1) [Source:MGI Symbol;Acc:MGI:98341]	105.941	105.214	103.408	114.103	116.28	103.532	109	105.815	127.965	107.363	114.909	109.453	114.074	105.948	0.275008207	0.789849983	20	12	24	0.367349251	0.790714337	24	12.52275013	0.044651374
ENSMUSG00000063524	Eno1	enolase 1, alpha non-neuron [Source:MGI Symbol;Acc:MGI:95393]	73.1164	60.2521	63.8919	69.422	75.141	77.4138	61.5624	67.2093	44.857	57.4627	63.786	55.9211	54.6936	49.7466	0.622132989	0.966018836	20	16	24	0.020095298	0.565108098	24	18.52630896	0.173034972
ENSMUSG00000063531	Sema3e	sema domain, immunoglobulin domain (Ig), short basic domain, secreted, (semaphorin) 3E [Source:MGI Symbol;Acc:MGI:1340034]	5.07329	4.59448	4.18523	4.47581	4.50621	4.6672	4.79477	4.37247	3.75547	4.28139	4.32153	4.26669	4.51643	4.62939	0.004140921	0.140598674	4	8	24	0.01663483	0.544809764	24	22.3486623	0.078965449
ENSMUSG00000063543	Gm5616	predicted gene 5616 [Source:MGI Symbol;Acc:MGI:3646416]	13.6631	19.2145	16.1915	16.3517	20.2458	14.5421	14.1209	16.7152	16.023	11.6932	14.8951	13.8367	14.019	14.8073	0.266108356	0.773510818	8	20	24	0.804619389	0.937447587	24	8.67222411	0.047982362
ENSMUSG00000063550	Nup98	nucleoporin 98 [Source:MGI Symbol;Acc:MGI:109404]	7.15008	7.07987	7.39572	6.75551	6.67408	7.57543	6.81675	7.39259	6.28255	7.49278	6.52999	7.24252	7.02061	7.23397	0.911685024	0.966018836	24	20	24	0.625213976	0.872089543	24	0.779529642	0.02687565
ENSMUSG00000063556	Gm10132	predicted gene 10132 [Source:MGI Symbol;Acc:MGI:3704449]	1.97272	2.66171	2.42611	2.8254	2.13329	2.05853	2.23547	3.03238	2.41008	1.91352	2.05662	2.46008	3.00383	2.31199	0.459019924	0.966018836	8	12	24	0.234414714	0.768040178	24	4.818009626	0.101428605
ENSMUSG00000063558	Aox1	aldehyde oxidase 1 [Source:MGI Symbol;Acc:MGI:88035]	19.3555	18.1819	16.7123	18.4829	18.4681	18.8471	16.0189	17.4866	16.1731	18.0127	18.5789	18.8627	18.8663	18.6747	0.027960311	0.313364468	24	12	24	0.191915056	0.768040178	24	19.30279816	0.058738704
ENSMUSG00000063564	Col23a1	collagen, type XXIII, alpha 1 [Source:MGI Symbol;Acc:MGI:2653243]	16.8128	16.0323	14.6369	15.5515	18.3845	15.793	18.9199	17.3824	16.8418	19.5335	16.2313	18.3304	17.0102	14.4943	0.796193867	0.966018836	4	4	24	0.441890406	0.805393068	24	18.44036567	0.063820985
ENSMUSG00000063568	Jazf1	JAZF zinc finger 1 [Source:MGI Symbol;Acc:MGI:2141450]	4.2711	3.7419	4.21053	4.25288	3.91603	4.2444	4.98282	3.6789	3.48159	4.21898	3.80666	3.66097	4.07362	3.99672	0.648082424	0.966018836	4	4	24	0.607725182	0.864036051	24	21.28080266	0.042071181
ENSMUSG00000063576	Klhdc3	kelch domain containing 3 [Source:MGI Symbol;Acc:MGI:2651568]	28.7831	25.6618	26.205	27.758	26.0042	28.5179	28.103	29.5583	26.0655	32.7044	25.4865	27.9479	28.3292	27.8678	0.591351901	0.966018836	4	16	24	0.974276028	0.993545331	24	0.198289552	0.008476858
ENSMUSG00000063594	Gng8	guanine nucleotide binding protein (G protein), gamma 8 [Source:MGI Symbol;Acc:MGI:109163]	13.3244	11.7499	8.93686	11.9176	14.9917	13.8254	14.3716	13.5706	9.8061	11.1224	13.9877	10.7629	9.55473	10.3828	0.039226672	0.338446795	20	16	24	0.080256156	0.695750349	24	19.14674921	0.183029643
ENSMUSG00000063600	Egfem1	EGF-like and EMI domain containing 1 [Source:MGI Symbol;Acc:MGI:1922990]	4.30855	3.49806	3.2745	1.97311	4.43841	4.19663	3.69194	2.54889	3.17816	4.44403	4.04617	3.64531	2.76603	3.73839	0.480187506	0.966018836	20	16	24	0.325650437	0.768040178	24	18.99854814	0.154386256
ENSMUSG00000063605	Ccdc102a	coiled-coil domain containing 102A [Source:MGI Symbol;Acc:MGI:2686927]	3.64351	3.85984	3.17219	3.32731	3.51814	3.91721	4.64096	4.50049	4.09557	4.61567	3.85803	4.13402	4.60085	3.56028	0.5562083	0.966018836	4	8	24	0.434326226	0.805393068	24	23.8983548	0.064108549
ENSMUSG00000063611	Gm10134	predicted gene 10134 [Source:MGI Symbol;Acc:MGI:3642322]	2.56887	2.89421	2.82373	2.38485	2.6994	2.4509	3.29869	2.89478	3.46123	2.31999	2.69417	2.88224	2.21224	2.72301	0.265774856	0.773510818	12	4	24	0.481529488	0.805393068	24	4.961062367	0.064543481
ENSMUSG00000063623	C230062I16Rik	RIKEN cDNA C230062I16 gene [Source:MGI Symbol;Acc:MGI:2442417]	11.284	9.03691	8.88025	7.87825	8.46267	8.7635	9.49842	10.4008	8.5696	9.3232	11.0975	10.2647	9.53736	9.23845	0.114657038	0.534533508	4	12	24	0.216593422	0.768040178	24	22.65846876	0.090445655
ENSMUSG00000063626	Unc5d	unc-5 netrin receptor D [Source:MGI Symbol;Acc:MGI:2389364]	0.624864	0.670797	1.03086	0.756938	0.722905	0.935337	0.907768	0.487204	0.756655	0.619638	0.682527	0.739251	0.620327	0.895596	0.896753532	0.966018836	12	20	24	0.981441329	0.995695365	24	13.45434227	0.009503386
ENSMUSG00000063632	Sox11	SRY (sex determining region Y)-box 11 [Source:MGI Symbol;Acc:MGI:98359]	1.50896	1.1789	1.6798	1.16107	1.68072	1.33321	1.17527	1.05254	1.11721	1.20782	2.05779	1.18983	1.22894	1.20238	0.066380001	0.42386701	20	12	24	0.159812172	0.76564405	24	16.2721337	0.117785816
ENSMUSG00000063646	Jakmip1	janus kinase and microtubule interacting protein 1 [Source:MGI Symbol;Acc:MGI:1923321]	31.2357	23.9022	22.9411	26.1162	23.9644	29.5719	26.3054	29.0565	20.0166	33.6367	28.7909	31.6031	32.0138	33.6585	0.217827365	0.704989686	24	12	24	0.26281127	0.768040178	24	21.42525186	0.125335854
ENSMUSG00000063652	Slc22a21	solute carrier family 22 (organic cation transporter), member 21 [Source:MGI Symbol;Acc:MGI:1929481]	1.3237	2.47319	3.07602	1.98128	1.75502	2.46336	1.58861	2.68783	2.82898	2.40882	1.79479	2.99633	2.41721	2.53264	0.083608421	0.463738799	12	16	24	0.206877155	0.768040178	24	7.564812672	0.163135739
ENSMUSG00000063659	Zbtb18	zinc finger and BTB domain containing 18 [Source:MGI Symbol;Acc:MGI:1353609]	22.28	19.8153	18.4859	19.8321	20.1292	21.216	20.0911	20.2776	16.8783	20.6648	19.2805	18.113	19.4524	19.5345	0.949874888	0.966018836	4	8	24	0.228382066	0.768040178	24	21.12025912	0.058938447
ENSMUSG00000063663	Brwd3	bromodomain and WD repeat domain containing 3 [Source:MGI Symbol;Acc:MGI:3029414]	0.993699	1.25464	1.14576	1.20091	1.27105	1.10363	1.05957	1.08099	1.0825	0.897625	1.26784	0.939943	0.958435	0.943734	0.338172159	0.85758369	20	8	24	0.369910217	0.792235171	24	13.86425932	0.041213985
ENSMUSG00000063683	Glyat	glycine-N-acyltransferase [Source:MGI Symbol;Acc:MGI:2147502]	0.560011	0.697775	0.862948	0.760441	0.878924	0.732276	0.785295	0.817168	0.573243	0.47957	1.11584	0.641761	0.608712	0.806242	0.867888967	0.966018836	20	8	24	0.751134276	0.91921305	24	15.55891887	0.04043333
ENSMUSG00000063684	Hadhb-ps	hydroxyacyl-CoA dehydrogenase trifunctional multienzyme complex subunit beta, pseudogene [Source:MGI Symbol;Acc:MGI:3651143]	84.3951	88.3143	99.2881	140.534	93.0877	89.6186	98.675	118.117	100.296	101.903	122.874	102.97	127.483	112.263	0.922029406	0.966018836	16	12	24	0.435037628	0.805393068	24	10.67253738	0.09638254
ENSMUSG00000063687	Pcdhb5	protocadherin beta 5 [Source:MGI Symbol;Acc:MGI:2136739]	1.13817	0.991813	1.04733	0.73159	1.01705	1.36847	1.13353	1.06587	0.739382	1.06929	1.04624	0.945195	0.973206	0.979807	0.800065124	0.966018836	4	8	24	0.092802263	0.71175955	24	21.45073819	0.093909121
ENSMUSG00000063694	Cycs	cytochrome c, somatic [Source:MGI Symbol;Acc:MGI:88578]	5.37088	5.94263	5.51944	6.53906	6.24129	5.70923	4.83523	5.41789	5.77056	5.45926	6.52035	4.9094	5.3555	6.30002	0.050258003	0.385784509	20	8	24	0.13997658	0.749574609	24	11.90502992	0.087099935
ENSMUSG00000063696	Gm8730	predicted pseudogene 8730 [Source:MGI Symbol;Acc:MGI:3644565]	71.7053	76.2976	73.1108	64.652	66.2548	60.3418	65.7773	79.5343	87.7755	62.5802	82.7709	73.2251	78.5662	74.6676	0.313786068	0.824470524	12	4	24	0.220264635	0.768040178	24	5.459865901	0.101682599
ENSMUSG00000063698	Sfxn4	sideroflexin 4 [Source:MGI Symbol;Acc:MGI:2137680]	12.2312	15.2268	13.3656	13.8448	13.9334	13.2431	12.4012	13.117	15.2984	12.1829	15.5437	14.3108	14.7427	13.2942	0.948582958	0.966018836	20	8	24	0.829496132	0.947008621	24	12.12895525	0.026679116
ENSMUSG00000063704	Mapk15	mitogen-activated protein kinase 15 [Source:MGI Symbol;Acc:MGI:2652894]	0.185049	1.46457	0.777458	0.306694	0.185411	0.343976	1.02582	0.491327	0.823089	0.433449	0.397444	0.592836	0.125396	0.691567	0.187223811	0.662239322	12	8	24	0.149952543	0.758419028	24	5.508002678	0.239373653
ENSMUSG00000063714	Sp3os	trans-acting transcription factor 3, opposite strand [Source:MGI Symbol;Acc:MGI:1914936]	6.56862	7.29436	10.1873	6.2547	5.052	6.9774	6.88179	7.98402	8.11545	6.82708	7.27258	7.90433	7.53819	7.00387	0.087978327	0.472021905	12	4	24	0.180165709	0.768040178	24	5.715939241	0.140814934
ENSMUSG00000063727	Tnfrsf11b	tumor necrosis factor receptor superfamily, member 11b (osteoprotegerin) [Source:MGI Symbol;Acc:MGI:109587]	1.43117	1.37377	1.41169	0.946187	1.28019	0.996576	1.58573	1.30009	1.87836	0.718696	1.19479	1.23927	0.603606	1.47755	0.129903614	0.560263689	12	4	24	0.431699915	0.805393068	24	4.71081525	0.113506026
ENSMUSG00000063754	Gm10136	predicted pseudogene 10136 [Source:MGI Symbol;Acc:MGI:3704242]	5.46425	6.19673	6.24759	6.49565	7.70965	6.25685	6.27042	5.88336	4.70599	3.80985	6.61356	3.34114	4.4715	4.42092	0.644063931	0.966018836	20	12	24	0.716002196	0.906323093	24	16.12126723	0.073552582
ENSMUSG00000063760	Rnf217	ring finger protein 217 [Source:MGI Symbol;Acc:MGI:3610311]	2.25874	2.1992	2.11412	2.26634	2.12579	1.93581	2.01466	1.95803	2.01248	1.84024	2.37478	2.04961	1.95881	1.99304	0.93243477	0.966018836	20	12	24	0.636949292	0.876483853	24	16.20258108	0.02394415
ENSMUSG00000063765	Chadl	chondroadherin-like [Source:MGI Symbol;Acc:MGI:3036284]	3.50699	3.43378	3.52515	3.4502	3.26845	3.19904	4.04823	4.351	4.73195	7.56447	5.40217	5.00907	5.13114	4.89981	0.77722623	0.966018836	16	8	24	0.34951739	0.779170188	24	10.52416392	0.111540308
ENSMUSG00000063767	S100a7a	S100 calcium binding protein A7A [Source:MGI Symbol;Acc:MGI:2687194]	7.77925	4.11879	2.62766	5.02339	7.03677	5.05391	6.54954	3.37683	3.93416	2.88613	6.80293	4.20481	5.45804	3.46191	0.024898193	0.291208026	20	16	24	0.01407716	0.530670295	24	19.84492772	0.412649252
ENSMUSG00000063785	Utp14a	UTP14A small subunit processome component [Source:MGI Symbol;Acc:MGI:1919804]	9.43508	9.27982	8.77276	9.06586	9.166	8.42668	8.57177	8.09414	9.6569	8.564	9.79613	8.79022	9.26066	9.21821	0.858266942	0.966018836	20	4	24	0.888211937	0.96858941	24	12.5708161	0.013899491
ENSMUSG00000063787	Chchd1	coiled-coil-helix-coiled-coil-helix domain containing 1 [Source:MGI Symbol;Acc:MGI:1913371]	74.8252	84.538	75.1218	75.5995	77.9443	72.6288	71.4098	81.0285	92.6099	62.1539	85.7486	70.6957	84.9334	85.4957	0.431555283	0.966018836	12	12	24	0.355359359	0.782134973	24	5.136275719	0.083136234
ENSMUSG00000063796	Slc22a8	solute carrier family 22 (organic anion transporter), member 8 [Source:MGI Symbol;Acc:MGI:1336187]	2.53252	3.93945	2.96391	2.69544	2.52406	2.86175	2.44173	3.22884	3.79431	1.43773	2.52837	2.9082	1.60194	2.7735	0.150959199	0.598641364	8	20	24	0.465567507	0.805393068	24	5.208528057	0.129223556
ENSMUSG00000063800	Prpf38a	PRP38 pre-mRNA processing factor 38 (yeast) domain containing A [Source:MGI Symbol;Acc:MGI:1916962]	15.2793	18.2379	18.0214	16.552	17.4448	15.9875	16.6417	17.4601	21.3526	15.1181	16.9528	16.8223	18.0932	18.7417	0.107707354	0.524035616	12	4	24	0.091798615	0.71175955	24	6.586473423	0.097659548
ENSMUSG00000063801	Ap3s2	adaptor-related protein complex 3, sigma 2 subunit [Source:MGI Symbol;Acc:MGI:1337060]	24.7691	21.2421	21.2045	22.8483	20.5755	24.4498	21.8992	22.7558	17.5804	27.6876	20.9173	23.3614	22.9299	23.1706	0.236893212	0.7385769	24	12	24	0.624127611	0.871917209	24	20.18181031	0.058662958

ENSMUSG00000063802	Hspbp1	HSPA (heat shock 70kDa) binding protein, cytoplasmic cochaperone 1 [Source:MGI Symbol;Acc:MGI:1913495]	33.9584	27.9384	28.3948	25.9024	26.4472	28.0127	30.621	33.5594	27.9512	35.521	33.8841	33.7961	33.8011	32.8507	0.833406028	0.966018836	4	8	24	0.447343911	0.805393068	24	23.97778581	0.063176174
ENSMUSG00000063804	Lin28b	lin-28 homolog B (C. elegans) [Source:MGI Symbol;Acc:MGI:3584032]	2.37314	2.19288	2.28387	2.43111	2.32411	1.95741	1.87279	1.99745	2.46472	1.97429	2.51674	2.04994	1.94358	2.17782	0.125855811	0.555618842	20	8	24	0.16725798	0.767020009	24	11.74118738	0.067584846
ENSMUSG00000063808	Gpatch1	G patch domain containing 1 [Source:MGI Symbol;Acc:MGI:1914721]	6.45899	5.45073	6.03872	5.74894	5.59491	6.53078	5.7278	6.07511	5.59838	6.6771	5.56732	6.82779	5.73927	6.17698	0.893307667	0.966018836	24	20	24	0.680510656	0.893265165	24	20.13546367	0.033488752
ENSMUSG00000063810	Alms1	ALMS1, centrosome and basal body associated [Source:MGI Symbol;Acc:MGI:1934606]	0.621898	0.738278	0.751896	0.740186	0.824802	0.719617	0.858695	0.577702	0.900659	0.647062	0.80413	0.833477	0.789996	0.792022	0.924553956	0.966018836	20	12	24	0.830977031	0.947661285	24	15.901725	0.017848935
ENSMUSG00000063821	Dupd1	dual specificity phosphatase and pro isomerase domain containing 1 [Source:MGI Symbol;Acc:MGI:3647127]	0.447922	1.57342	1.06299	1.82419	0.579986	1.31962	1.34207	2.55547	2.17056	2.17891	1.20537	0.64835	1.97833	1.06187	0.470374729	0.966018836	16	4	24	0.162361643	0.76636332	24	7.000462259	0.390641356
ENSMUSG00000063838	Cdc42ep5	CDC42 effector protein (Rho GTPase binding) 5 [Source:MGI Symbol;Acc:MGI:1929745]	2.25828	2.24213	1.70415	1.21645	2.59811	1.8395	2.34802	2.90281	2.37209	2.38715	2.67273	2.46747	3.15418	2.68246	0.912923872	0.966018836	8	4	24	0.214460771	0.768040178	24	0.460377755	0.12399756
ENSMUSG00000063849	Ppcdc	phosphopantothenoylcysteine decarboxylase [Source:MGI Symbol;Acc:MGI:1914062]	13.4319	13.2224	11.2246	17.2137	13.9712	13.149	13.3853	11.8146	13.0728	11.2164	11.763	11.282	12.2783	12.0272	0.935732159	0.966018836	4	8	24	0.777510349	0.928654033	24	15.77053512	0.038376299
ENSMUSG00000063856	Gpx1	glutathione peroxidase 1 [Source:MGI Symbol;Acc:MGI:104887]	176.276	184.841	192.285	189.128	191.234	192.419	183.399	187.329	192.812	161.344	214.313	187.999	217.727	179.085	0.798829513	0.966018836	20	12	24	0.703680946	0.902090877	24	19.61697026	0.03635375
ENSMUSG00000063870	Chd4	chromodomain helicase DNA binding protein 4 [Source:MGI Symbol;Acc:MGI:1344380]	42.9318	36.0699	38.0768	40.9519	38.056	39.8548	44.3643	40.2116	35.983	47.669	37.5764	38.0549	41.3458	38.6135	0.371875382	0.894537464	4	8	24	0.875064702	0.964979482	24	19.84352543	0.024547893
ENSMUSG00000063873	Slc24a3	solute carrier family 24 (sodium/potassium/calcium exchanger), member 3 [Source:MGI Symbol;Acc:MGI:2137513]	6.76367	6.06704	7.91861	6.86695	6.33806	7.15051	7.52179	6.83469	7.36916	8.13916	6.94267	7.38947	6.35538	8.08105	0.93717134	0.966018836	16	4	24	0.418114946	0.80272238	24	9.339384209	0.058196448
ENSMUSG00000063882	Uqcrh	ubiquinol-cytochrome c reductase hinge protein [Source:MGI Symbol;Acc:MGI:1913826]	428.991	546.691	534.728	531.826	528.272	463.102	478.812	489.636	583.249	413.967	556.766	489.267	581.807	548.236	0.580049522	0.966018836	12	12	24	0.530096788	0.824795587	24	7.61441418	0.069537165
ENSMUSG00000063884	Ptcd3	pentatricopeptide repeat domain 3 [Source:MGI Symbol;Acc:MGI:1917206]	21.2312	23.1537	24.4375	22.9897	23.3042	20.6198	21.7615	22.0101	26.4893	21.0781	24.5292	21.8592	23.1429	23.0636	0.046056756	0.374351104	12	12	24	0.117196508	0.728210112	24	9.147784946	0.074877919
ENSMUSG00000063887	Nlgn1	neuroligin 1 [Source:MGI Symbol;Acc:MGI:2179435]	2.28908	2.81762	3.60858	2.73159	2.21474	2.48676	2.87302	2.31233	3.03457	2.95414	2.14936	2.7032	2.27462	3.2835	0.010153564	0.194720062	12	8	24	0.050373052	0.644469829	24	7.506061901	0.152747919
ENSMUSG00000063888	Rpl7l1	ribosomal protein L7-like 1 [Source:MGI Symbol;Acc:MGI:1913479]	20.6136	20.0967	19.585	20.7264	17.6913	19.9649	18.0552	20.9583	20.654	16.8157	21.3775	18.4257	19.1695	16.9389	0.768363194	0.966018836	12	4	24	0.988398206	0.997559852	24	3.654772758	0.006322275
ENSMUSG00000063889	Crem	cAMP responsive element modulator [Source:MGI Symbol;Acc:MGI:88495]	11.1147	10.608	11.789	11.3935	11.4994	9.00291	9.9076	10.1209	10.2762	7.17066	12.9002	9.53694	7.80917	9.09131	0.503972924	0.966018836	20	4	24	0.706940168	0.903220087	24	13.97215087	0.059784183
ENSMUSG00000063894	Zkscan8	zinc finger with KRAB and SCAN domains 8 [Source:MGI Symbol;Acc:MGI:1913815]	4.63849	5.43727	4.82083	5.14407	5.57917	4.44214	4.80119	4.26499	5.81537	4.13207	5.86901	5.01519	5.31727	5.30365	0.93243477	0.966018836	20	4	24	0.861338301	0.959281853	24	12.52945639	0.028886295
ENSMUSG00000063902	Gm7964	predicted gene 7964 [Source:MGI Symbol;Acc:MGI:3646150]	96.043	88.9344	95.1823	93.1686	85.8071	90.5281	92.2179	87.8038	80.3423	95.5056	84.2571	87.0577	88.5695	86.0756	0.935316621	0.966018836	4	16	24	0.939082633	0.984988133	24	21.96759026	0.009569932
ENSMUSG00000063904	Dpp3	dipeptidylpeptidase 3 [Source:MGI Symbol;Acc:MGI:1922471]	28.3154	23.2616	21.239	24.6061	25.0733	25.4434	24.4198	25.0753	21.577	30.4086	24.7664	25.4672	24.5696	25.4952	0.148073804	0.590732968	24	12	24	0.417408176	0.802199595	24	18.76833624	0.067838135
ENSMUSG00000063919	Srrm4	serine/arginine repetitive matrix 4 [Source:MGI Symbol;Acc:MGI:1916205]	2.39119	1.87247	2.1669	2.00598	2.10914	2.20786	2.15192	1.84277	1.93431	2.61082	2.36553	1.97615	2.23397	2.2677	0.787283372	0.966018836	4	4	24	0.534572705	0.826549286	24	16.55698962	0.043223602
ENSMUSG00000063931	Pepd	peptidase D [Source:MGI Symbol;Acc:MGI:97542]	20.2375	18.3153	19.7725	20.945	20.6942	21.7487	19.6823	22.2346	17.2548	21.2441	19.3501	19.5142	20.5873	19.8304	0.852844413	0.966018836	16	20	24	0.588596165	0.853936396	24	18.70430606	0.038949477
ENSMUSG00000063952	Brpf3	bromodomain and PHD finger containing, 3 [Source:MGI Symbol;Acc:MGI:2146836]	6.82015	6.50693	5.59326	7.95604	6.72158	5.63946	7.31257	5.53948	8.20475	7.75212	7.62408	6.36275	6.68219	6.99822	0.406690444	0.943196477	16	16	24	0.30605073	0.768040178	24	12.03523219	0.094525035
ENSMUSG00000063953	Amd2	S-adenosylmethionine decarboxylase 2 [Source:MGI Symbol;Acc:MGI:1333111]	1.72276	2.04453	1.8893	1.91394	1.84477	1.78425	1.86894	2.17821	2.87425	2.32666	1.92461	1.98858	2.09738	1.7427	0.519468828	0.966018836	16	8	24	0.127793782	0.740162193	24	8.801139779	0.10196516
ENSMUSG00000063972	Nr6a1	nuclear receptor subfamily 6, group A, member 1 [Source:MGI Symbol;Acc:MGI:1352459]	3.52526	3.2783	2.92342	2.69459	2.88897	2.74787	3.09038	2.71935	2.90965	2.87332	3.16911	3.13213	3.44567	3.56722	0.105345531	0.518002936	4	12	24	0.089754099	0.709751366	24	0.807779051	0.080636728
ENSMUSG00000063975	Slco1a5	solute carrier organic anion transporter family, member 1a5 [Source:MGI Symbol;Acc:MGI:1351865]	0.631519	0.67371	0.752661	0.476893	0.731892	0.517224	0.483565	0.841172	0.890277	0.593895	0.646241	0.721451	0.49092	0.612343	0.277087842	0.789849983	12	16	24	0.362342884	0.787151161	24	8.098670161	0.06604519
ENSMUSG00000064023	Klk8	kallikrein related-peptidase 8 [Source:MGI Symbol;Acc:MGI:1343327]	1.63197	2.61194	2.38582	2.25259	2.42866	2.30495	2.18941	3.06501	2.42494	2.30475	2.37063	1.54895	2.26699	2.15249	0.441501717	0.966018836	8	20	24	0.201007329	0.768040178	24	8.221278357	0.118766138
ENSMUSG00000064030	Pym1	PYM homolog 1, exon junction complex associated factor [Source:MGI Symbol;Acc:MGI:1925678]	9.99741	9.90933	10.7286	10.259	9.48175	10.4923	9.57285	11.8516	11.289	10.9895	9.84699	10.8081	11.2919	11.1867	0.308023687	0.814993005	8	12	24	0.06014764	0.658304792	24	6.228779169	0.068957269
ENSMUSG00000064036	Mro	maestro [Source:MGI Symbol;Acc:MGI:2152817]	1.53419	2.14083	2.1769	2.1493	1.41517	1.7447	2.15502	1.72552	2.5744	1.37134	1.12594	1.59145	1.11593	2.23809	0.035023815	0.328311403	12	8	24	0.057648689	0.656248849	24	6.419815838	0.20345009
ENSMUSG00000064037	Gpn1	GPN-loop GTPase 1 [Source:MGI Symbol;Acc:MGI:1921504]	15.2554	12.6386	11.8948	12.4408	12.8403	13.2875	11.0061	12.73	12.4571	9.79888	12.267	10.695	13.2326	12.4944	0.305564675	0.811201458	4	12	24	0.292183392	0.768040178	24	23.9957287	0.07883715
ENSMUSG00000064043	Trerf1	transcriptional regulating factor 1 [Source:MGI Symbol;Acc:MGI:2442086]	1.63863	1.8714	2.08562	2.05779	1.76565	1.96116	1.89855	1.98359	2.28177	2.35047	1.74927	2.04118	1.85328	2.18919	0.033814615	0.325496079	12	16	24	0.013239635	0.529134128	24	9.009766479	0.094921625
ENSMUSG00000064061	Dzip3	DAZ interacting protein 3, zinc finger [Source:MGI Symbol;Acc:MGI:1917433]	20.0697	21.2627	21.4761	20.7345	21.0086	19.169	19.8101	18.7341	21.4851	16.1253	20.5026	20.2734	19.0082	20.2537	0.112800839	0.530777648	12	16	24	0.941937441	0.985597432	24	4.922817044	0.013852217
ENSMUSG00000064065	Ipcef1	interaction protein for cytohesin exchange factors 1 [Source:MGI Symbol;Acc:MGI:2444159]	0.501882	0.827325	0.960654	0.692604	0.63644	0.6784	0.55614	0.475542	0.814563	0.480393	0.519578	0.712126	0.654346	0.57703	0.941955432	0.966018836	12	16	24	0.581050769	0.850505598	24	8.295074697	0.051559864
ENSMUSG00000064068	Mtx1	metaxin 1 [Source:MGI Symbol;Acc:MGI:103025]	36.1975	34.1198	28.8718	32.5496	30.8273	34.5717	34.5036	34.4681	35.0858	40.8141	33.2038	34.7956	39.1807	36.7545	0.671726933	0.966018836	4	16	24	0.55791563	0.839532165	24	1.204542994	0.044646165
ENSMUSG00000064080	Fbln2	fibulin 2 [Source:MGI Symbol;Acc:MGI:95488]	25.6026	22.8713	20.5408	23.207	24.1442	25.4165	24.0148	25.6693	20.8597	24.306	23.03	20.6807	22.5221	20.372	0.926137994	0.966018836	4	8	24	0.28935215	0.768040178	24	19.97509284	0.066808712
ENSMUSG00000064090	Vrk2	vaccinia related kinase 2 [Source:MGI Symbol;Acc:MGI:1917172]	2.94533	2.53766	2.85339	2.75991	3.02201	2.23272	2.95167	2.80392	3.60562	2.15049	3.36859	2.71674	3.84467	2.28334	0.850398351	0.966018836	4	12	24	0.966837675	0.992082154	24	23.35406485	0.019408639
ENSMUSG00000064105	Cnnm2	cyclin M2 [Source:MGI Symbol;Acc:MGI:2151054]	4.49807	3.3953	4.03691	4.0273	3.77387	3.89022	3.79709	4.21457	3.26204	4.90458	3.13047	4.24822	3.51027	3.62958	0.888482515	0.966018836	16	20	24	0.941142776	0.985472945	24	16.65902211	0.020101422
ENSMUSG00000064109	Hcst	hematopoietic cell signal transducer [Source:MGI Symbol;Acc:MGI:1344360]	0.863591	1.38711	1.87368	2.2739	0.635404	0.883162	1.40776	1.14729	1.86492	1.21609	1.27535	0.936317	0.968077	1.68402	0.013719501	0.238398597	12	12	24	0.010373878	0.503967859	24	8.299671146	0.302795966
ENSMUSG00000064115	Cadm2	cell adhesion molecule 2 [Source:MGI Symbol;Acc:MGI:2442722]	5.92596	6.68028	7.96516	6.43682	6.80169	6.74142	8.78765	6.99818	8.04033	6.16085	6.96421	8.63899	6.36557	7.36332	0.885059765	0.966018836	12	4	24	0.874464882	0.964864591	24	3.5312205	0.031589586
ENSMUSG00000064120	Mocs1	molybdenum cofactor synthesis 1 [Source:MGI Symbol;Acc:MGI:1928904]	4.19728	5.63311	6.00293	5.85144	5.28574	5.20627	5.42138	6.07669	6.19384	6.01612	4.98869	5.00471	6.68743	6.48434	0.061112935	0.409814092	8	16	24	0.00896756	0.502965379	24	7.407959745	0.1444139
ENSMUSG00000064125	Prr36	proline rich 36 [Source:MGI Symbol;Acc:MGI:3605626]	0.959508	0.975124	0.939113	1.02866	0.926696	0.797832	0.75126	1.48408	1.03654	1.08442	0.878722	1.23605	0.806884	1.24571	0.156415999	0.611943293	8	20	24	0.281681352	0.768040178	24	7.175098857	0.086228995
ENSMUSG00000064127	Med14	mediator complex subunit 14 [Source:MGI Symbol;Acc:MGI:1349442]	13.9879	11.8099	12.1539	11.9513	11.8557	13.1003	12.1032	12.494	10.1026	13.4874	11.432	12.2684	12.2734	11.9138	0.441501717	0.966018836	24	20	24	0.297465928	0.768040178	24	21.18434547	0.062302811
ENSMUSG00000064128	Cenpj	centromere protein J [Source:MGI Symbol;Acc:MGI:2684927]	4.52788	3.76149	3.97714	4.0086	3.9326	3.80949	3.22857	3.41225	4.09542	3.01691	4.40798	4.30679	4.38774	4.20851	0.921572192	0.966018836	20	20	24	0.715263522	0.90631377	24	20.86137657	0.047126528
ENSMUSG00000064138	Fam172a	family with sequence similarity 172, member A [Source:MGI Symbol;Acc:MGI:1915925]	13.073	13.3983	13.2091	12.6004	12.3042	11.4379	12.3506	12.8129	13.0121	14.7741	12.3024	14.4508	12.8467	13.3657	0.428730128	0.966018836	8	12	24	0.451008799	0.805393068	24	7.956770439	0.044119961
ENSMUSG00000064141	Zfp869	zinc finger protein 869 [Source:MGI Symbol;Acc:MGI:107794]	0.589491	0.533877	0.503036	0.773557	0.484827	0.523781	0.332652	0.895986	0.693849	0.428096	0.812481	0.656188	0.796562	0.713529	0.828428469	0.966018836	8	8	24	0.803109181	0.936962113	24	6.909500044	0.028014296
ENSMUSG00000064145	Arih2	ariadne RBR E3 ubiquitin protein ligase 2 [Source:MGI Symbol;Acc:MGI:1344361]	9.88003	10.1579	10.73	10.187	9.92091	10.2269	10.3796	11.0485	10.663	12.2186	12.0901	10.6841	10.0955	10.8091	0.628286596	0.966018836	16	12	24	0.110492595	0.722224739	24	11.39420689	0.059807749
ENSMUSG00000064147	Rab44	RAB44, member RAS oncogene family [Source:MGI Symbol;Acc:MGI:3045302]	1.3477	1.44485	1.82209	1.7483	1.11046	1.44794	2.78165	1.25584	0.978384	1.05648	1.00153	1.23881	1.86622	0.901547	0.360140629	0.883787544	4	16	24	0.377285742	0.797219305	24	23.69630201	0.14032052
ENSMUSG00000064177	Ghrl	ghrelin [Source:MGI Symbol;Acc:MGI:1930008]	0.456772	0.855365	1.22636	1.0711	2.04404	1.18798	1.37047	0.328343	0.877278	0.577393	0.946687	0.557742	1.61324	0.963727	0.912686312	0.966018836	20	12	24	0.634994415	0.875919107	24	17.00313313	0.128335811
ENSMUSG00000064179	Tnnt1	troponin T1, skeletal, slow [Source:MGI Symbol;Acc:MGI:1333868]	5.12071	15.8849	24.0806	23.9477	14.1083	18.9077	14.5124	23.7665	10.4901	21.7128	7.12094	2.19066	14.6052	21.1577	0.105687008	0.518128471	16	4	24	0.131400552	0.741500219	24	8.054811158	0.682934149
ENSMUSG00000064181	Rab3ip	RAB3A interacting protein [Source:MGI Symbol;Acc:MGI:105933]	13.234	11.745	11.0894	11.9064	11.9568	13.118	14.1163	12.2874	9.91471	14.7902	12.4143	13.1429	12.3801	11.6585	0.008747574	0.192760771	24	12	24	0.129703375	0.740162193	24	19.85130538	0.104986762
ENSMUSG00000064193	Gm4735	predicted gene 4735 [Source:MGI Symbol;Acc:MGI:3645521]	52.8761	43.3489	49.9433	47.8237	46.034	51.092	47.1288	49.0806	43.6532	51.3564	55.3889	51.5579	53.3095	53.9947	0.916746631	0.966018836	4	8	24	0.43337136	0.805393068	24	19.86920771	0.050577772
ENSMUSG00000064202	Spata6l	spermatogenesis associated 6 like [Source:MGI Symbol;Acc:MGI:1918036]	0.525356	1.24824	1.50366	1.35838	1.23038	1.94374	1.54276	1.57906	1.48774	1.12615	1.51887	1.72723	1.59241	1.82216	0.285027487	0.793714531	24	16	24	0.988743461	0.997581486	24	7.99543648	0.012095459
ENSMUSG00000064208	Gm10145	predicted gene 10145 [Source:MGI Symbol;Acc:MGI:3704353]	12.7478	11.88	12.3763	12.4363	8.80143	11.3217	13.1948	13.681	10.9272	15.0873	12.7686	11.3427	11.3611	14.5744	0.481611551	0.966018836	8	16	24	0.28914891	0.768040178	24	5.913031925	0.109056744
ENSMUSG00000064210	Ano6	anoctamin 6 [Source:MGI Symbol;Acc:MGI:2145890]	24.0872	19.0959	19.7793	19.8951	20.3673	22.3698	22.366	21.7723	15.9058	23.067	18.3828	20.3591	21.355	20.4585	0.101520755	0.511659635	4	8	24	0.170798356	0.768040178	24	21.85125666	0.104630719
ENSMUSG00000064215	Ifi27	interferon, alpha-inducible protein 27 [Source:MGI Symbol;Acc:MGI:1277180]	220.986	202.102	188.039	234.289	227.995	201.079	214.84	215.007	213.542	189.691	265.014	219.787	248.504	229.597	0.791413662	0.966018836	20	16	24	0.465257789	0.805393068	24	19.12984658	0.06380071
ENSMUSG00000064225	Paqr9	progestin and adipoQ receptor family member IX [Source:MGI Symbol;Acc:MGI:1922802]	9.55089	8.1248	8.75611	8.89653	8.28444	9.36807	9.06677	8.44961	6.9481	10.7452	8.76509	9.19708	8.61088	9.14097	0.377663653	0.903463289	24	12	24	0.496685595	0.807576732	24	18.57747757	0.060764818
ENSMUSG00000064246	Chil1	chitinase-like 1 [Source:MGI Symbol;Acc:MGI:1340899]	2.42161	5.32356	7.27429	5.53796	4.84498	6.13553	6.05701	4.70901	5.96713	3.53136	5.46915	4.47885	4.39485	3.27424	0.648082424	0.966018836	12	20	24	0.634744889	0.875768419	24	11.89961218	0.12756376
ENSMUSG00000064247	Plcxd1	phosphatidylinositol-specific phospholipase C, X domain containing 1 [Source:MGI Symbol;Acc:MGI:2685422]	0.602535	0.778684	1.23508	0.814228	0.692612	0.858752	1.3019	0.620094	0.717743	0.732458	0.731158	0.74036	0.585538	0.76073	0.881996584	0.966018836	16	12	24	0.946368067	0.986887534	24	7.739497441	0.021780984
ENSMUSG00000064254	Ethe1	ethylmalonic encephalopathy 1 [Source:MGI Symbol;Acc:MGI:1913321]	7.20398	7.55846	6.43362	7.66898	8.20699	8.66998	7.40626	7.59637	6.87756	7.07807	7.7397	7.73068	8.60045	7.22401	0.009067305	0.192760771	24	12	24	0.021822557	0.574400012	24	19.82156073	0.102560017
ENSMUSG00000064262	Gimap8	GTPase, IMAP family member 8 [Source:MGI Symbol;Acc:MGI:2685303]	1.0242	1.34821	1.49062	1.4282	1.26305	1.25554	1.53624	1.65539	1.71181	1.59444	1.41998	1.39978	1.37276	1.37329	0.073139747	0.447768907	12	12	24	0.029928051	0.596648309	24	8.742463882	0.096426043
ENSMUSG00000064264	Zfp428	zinc finger protein 428 [Source:MGI Symbol;Acc:MGI:1916463]	5.89686	4.42397	3.47748	2.78073	4.16297	3.62056	5.27342	4.54504	3.73135	4.55617	5.85104	5.7195	5.75865	5.40531	0.058525295	0.405089131	4	8	24	0.033608709	0.606793499	24	22.88046941	0.223990288
ENSMUSG00000064267	Hvcn1	hydrogen voltage-gated channel 1 [Source:MGI Symbol;Acc:MGI:1921346]	0.75008	1.21816	2.15616	1.69369	1.31445	1.39327	1.57813	1.59531	1.76122	0.850011	1.48618	1.48135	1.51777	1.20066	0.591351901	0.966018836	12	12	24	0.637857427	0.876945331	24	9.064337625	0.08721821
ENSMUSG00000064281	Rpl19-ps1	ribosomal protein L19, pseudogene 1 [Source:MGI Symbol;Acc:MGI:3642668]	3.6402	2.98136	3.14291	3.1867	2.70238	3.25755	2.61217	2.6603	3.11283	2.09787	2.55144	2.755	3.06054	2.32964	0.919571723	0.966018836	24	8	24	0.42179248	0.804844498	24	23.10214178	0.063984652
ENSMUSG00000064284	Cdpf1	cysteine rich, DPF motif domain containing 1 [Source:MGI Symbol;Acc:MGI:1919605]	16.4973	16.5887	14.9516	14.2817	16.469	15.9767	15.5331	16.1714	17.5078	12.7585	15.9308	15.3781	15.6408	13.7649	0.894678274	0.966018836	8	8	24	0.397389044	0.799546792	24	23.02559709	0.057598409
ENSMUSG00000064289	Tank	TRAF family member-associated Nf-kappa B activator [Source:MGI Symbol;Acc:MGI:107676]	16.0722	15.3658	15.3909	14.6552	14.7886	15.1178	15.4745	14.8075	14.6222	13.988	15.4584	14.175	15.2936	14.7984	0.036134798	0.331852526	4	12	24	0.080832651	0.696814151	24	23.95309021	0.036565225
ENSMUSG00000064293	Cntn4	contactin 4 [Source:MGI Symbol;Acc:MGI:1095737]	5.54454	8.25497	7.98692	6.72081	6.56441	6.04279	7.13786	5.6841	6.69152	7.62351	6.03842	6.96884	6.23619	7.73957	0.29905572	0.809362476	12	8	24	0.227390262	0.768040178	24	8.143974262	0.10882101
ENSMUSG00000064294	Aox3	aldehyde oxidase 3 [Source:MGI Symbol;Acc:MGI:1918974]	0.939447	0.843882	0.51554	0.662989	0.556443	0.64708	0.359303	0.739543	1.01289	0.286759	0.610722	0.569101	0.432259	0.650238	0.643501552	0.966018836	8	8	24	0.551342548	0.83632237	24	4.387170456	0.074516273
ENSMUSG00000064302	Clasp1	CLIP associating protein 1 [Source:MGI Symbol;Acc:MGI:1923957]	27.2115	21.6952	23.4201	23.8098	21.8757	27.5237	24.2553	25.8657	18.7991	32.1136	21.0568	25.1579	23.7472	25.344	0.745118544	0.966018836	24	12	24	0.793256894	0.933812645	24	20.89683395	0.052165642
ENSMUSG00000064307	Lrrc51	leucine rich repeat containing 51 [Source:MGI Symbol;Acc:MGI:1916608]	5.71241	7.89205	7.17369	7.08791	7.61656	7.27094	5.90505	5.58919	8.79373	5.40356	7.68689	8.33879	7.07419	7.66394	0.948454836	0.966018836	24	4	24	0.82367665	0.944500387	24	13.29058668	0.047449463
ENSMUSG00000064317	Gm10146	predicted gene 10146 [Source:MGI Symbol;Acc:MGI:3704367]	57.8884	63.4423	55.1817	56.2436	61.4551	50.5292	46.9177	61.5373	66.6605	51.4783	63.8426	58.9171	68.2648	60.4786	0.920858634	0.966018836	8	16	24	0.705013718	0.902587251	24	6.455503613	0.051591735
ENSMUSG00000064325	Hhip	Hedgehog-interacting protein [Source:MGI Symbol;Acc:MGI:1341847]	0.430909	0.809618	0.729316	0.441086	0.481292	0.465966	0.852021	0.502599	0.760069	0.395453	0.54279	0.447393	0.364699	0.516905	0.530814489	0.966018836	12	4	24	0.271094968	0.768040178	24	5.570273689	0.088608577
ENSMUSG00000064326	Siva1	SIVA1, apoptosis-inducing factor [Source:MGI Symbol;Acc:MGI:1353606]	12.2425	12.6279	12.4143	11.5573	11.4993	10.9051	11.8405	13.2863	14.9524	9.4106	13.5228	12.7163	12.9912	10.4763	0.93243477	0.966018836	12	4	24	0.737441203	0.91440753	24	3.279642684	0.04823282
ENSMUSG00000064329	Scn1a	sodium channel, voltage-gated, type I, alpha [Source:MGI Symbol;Acc:MGI:98246]	18.3748	21.0844	21.6991	19.3183	17.6884	18.5221	20.3104	17.0593	19.1904	18.7337	18.4844	19.7	17.7107	21.9612	0.632729413	0.966018836	12	8	24	0.332001706	0.768793356	24	6.171970343	0.063169889
ENSMUSG00000064337	mt-Rnr1	mitochondrially encoded 12S rRNA [Source:MGI Symbol;Acc:MGI:102493]	1146.73	1036.95	1122.94	1048.26	984.835	1168.52	1253.94	913.714	1048.82	617.945	1314.11	958.134	1004.74	1038.53	0.90332508	0.966018836	4	4	24	0.358687948	0.785236952	24	22.23233347	0.143889078
ENSMUSG00000064339	mt-Rnr2	mitochondrially encoded 16S rRNA [Source:MGI Symbol;Acc:MGI:102492]	999.183	924.918	971.174	867.183	896.171	1025.13	1083.82	787.222	910.79	546.333	1130.36	818.586	841.034	853.914	0.94073613	0.966018836	20	20	24	0.314295111	0.768040178	24	21.82008643	0.147128216
ENSMUSG00000064341	mt-Nd1	mitochondrially encoded NADH dehydrogenase 1 [Source:MGI Symbol;Acc:MGI:101787]	4854.77	7104.82	7090.14	7166.27	6095.64	5764.36	5818.01	6941.47	8269.98	6248.44	6541.1	6799.38	7484.06	7279.31	0.140905066	0.585944911	12	12	24	0.019167681	0.562219749	24	8.114163265	0.177759391
ENSMUSG00000064345	mt-Nd2	mitochondrially encoded NADH dehydrogenase 2 [Source:MGI Symbol;Acc:MGI:102500]	3308.04	4301.11	4360.43	4793.12	4105.78	3801.71	4047.44	4495.71	4861.76	4022.4	4466.35	4066.98	4534.86	4573.82	0.192658608	0.670488962	12	12	24	0.022729992	0.581965446	24	8.858102963	0.132489257
ENSMUSG00000064354	mt-Co2	mitochondrially encoded cytochrome c oxidase II [Source:MGI Symbol;Acc:MGI:102503]	36.1971	22.0967	58.2117	82.1074	45.0771	81.3288	32.2509	51.8369	82.9742	4.24852	55.4066	71.2763	8.07706	4.43546	0.388849454	0.915479136	12	20	24	0.44582617	0.805393068	24	16.71451985	0.64565037
ENSMUSG00000064360	mt-Nd3	mitochondrially encoded NADH dehydrogenase 3 [Source:MGI Symbol;Acc:MGI:102499]	2.39631	1.75601	1.88765	1.45942	2.53479	0.480483	1.84425	1.9158	2.50089	2.59563	1.46632	1.56195	2.21279	2.37978	0.851656735	0.966018836	12	12	24	0.258588835	0.768040178	24	7.130786798	0.218746294
ENSMUSG00000064363	mt-Nd4	mitochondrially encoded NADH dehydrogenase 4 [Source:MGI Symbol;Acc:MGI:102498]	2129.92	2801.04	2781.84	2993.38	2716.96	2367.52	2487.05	2666.21	3191.95	2397.99	2764.15	2529.76	2816.12	2718.8	0.12833063	0.560209134	12	12	24	0.065082941	0.663876076	24	9.178058057	0.128407586
ENSMUSG00000064367	mt-Nd5	mitochondrially encoded NADH dehydrogenase 5 [Source:MGI Symbol;Acc:MGI:102496]	1528.84	2053.69	2099.43	2139.62	1920.37	1754.85	1757.58	1969.09	2323.1	2191.6	2037.19	1915.88	1993.67	2122.66	0.001079996	0.077947897	16	8	24	5.59E-05	0.089877732	24	9.633206757	0.183173534
ENSMUSG00000064368	mt-Nd6	mitochondrially encoded NADH dehydrogenase 6 [Source:MGI Symbol;Acc:MGI:102495]	1963.93	2691.29	2796.94	2723.91	2436.29	2305.37	2236.51	2456.34	3298.09	2978.75	2699.52	2920.05	2915.8	2893.84	0.158469451	0.614860056	12	16	24	0.006758466	0.472731997	24	9.611558693	0.1756903
ENSMUSG00000064370	mt-Cytb	mitochondrially encoded cytochrome b [Source:MGI Symbol;Acc:MGI:102501]	6288.38	8159.94	8171.54	9094.46	7899.32	6928.26	7128.01	8115.93	9657.78	7651.06	8233.54	7800.87	9124.61	8700.93	0.212387017	0.701530532	12	12	24	0.02535916	0.586541342	24	8.941683212	0.148252603
ENSMUSG00000064373	Selenop	selenoprotein P [Source:MGI Symbol;Acc:MGI:894288]	344.148	391.073	371.943	367.805	401.162	339.714	402.517	368.913	421.779	322.375	380.78	357.715	357.103	355.259	0.953350544	0.966018836	12	12	24	0.79934208	0.935782011	24	7.413071832	0.029488516
ENSMUSG00000064966	Snord15b	small nucleolar RNA, C/D box 14B [Source:MGI Symbol;Acc:MGI:3779515]	70.675	23.0684	41.8634	17.9708	27.3553	82.5851	25.437	44.3052	13.2822	1.96088	26.2052	21.571	9.06583	46.2466	0.671726933	0.966018836	24	16	24	0.184214643	0.768040178	24	23.29225868	0.802380625
ENSMUSG00000065353	Snora73b	small nucleolar RNA, H/ACA box 73b [Source:MGI Symbol;Acc:MGI:4360049]	9.25991	5.64531	3.36184	3.9045	4.57157	8.81823	2.06381	4.46866	5.78152	3.33279	4.24126	5.2802	4.00703	4.67629	0.723119329	0.966018836	24	16	24	0.507528132	0.81273537	24	21.55266126	0.20907801
ENSMUSG00000065954	Tacc1	transforming, acidic coiled-coil containing protein 1 [Source:MGI Symbol;Acc:MGI:2443510]	22.9237	20.9992	22.2469	22.5282	22.8463	20.1274	24.7736	20.9225	22.8593	26.0624	23.5549	24.5915	26.013	24.3995	0.85447369	0.966018836	4	4	24	0.941778274	0.985597432	24	15.83944726	0.012149609
ENSMUSG00000065979	Cpped1	calcineurin-like phosphoesterase domain containing 1 [Source:MGI Symbol;Acc:MGI:2443300]	19.2882	17.5039	17.6969	18.5226	16.2637	18.9544	18.2184	18.2888	15.3053	19.8065	17.3528	17.9653	17.7316	19.1534	0.699015352	0.966018836	4	8	24	0.721044697	0.908651132	24	22.97854236	0.030809842
ENSMUSG00000065990	Aurkaip1	aurora kinase A interacting protein 1 [Source:MGI Symbol;Acc:MGI:1913327]	72.532	72.0428	71.2645	71.9011	74.3717	73.0741	70.928	71.0359	77.0336	61.9051	82.2065	69.9846	77.5656	73.4045	0.890648739	0.966018836	20	20	24	0.811339381	0.939504402	24	21.13202589	0.024274175
ENSMUSG00000066000	Zfp979	zinc finger protein 979 [Source:MGI Symbol;Acc:MGI:2148252]	3.02492	3.58467	3.50816	3.16492	3.03717	2.95512	3.01088	2.95674	3.32732	2.57623	3.00283	2.87432	2.16174	3.35104	0.074438493	0.448301997	12	12	24	0.248592988	0.768040178	24	7.71914562	0.07954193
ENSMUSG00000066026	Dhrs3	dehydrogenase/reductase (SDR family) member 3 [Source:MGI Symbol;Acc:MGI:1315215]	16.8234	17.659	12.6739	10.3525	18.8276	16.2016	18.1479	18.6312	15.2997	11.0066	15.7527	16.7167	22.9004	14.9298	0.0043709	0.140598674	4	12	24	0.008158147	0.496066371	24	23.26536528	0.298045381
ENSMUSG00000066036	Ubr4	ubiquitin protein ligase E3 component n-recognin 4 [Source:MGI Symbol;Acc:MGI:1916366]	41.5215	32.4496	35.4672	36.9413	34.2749	47.7006	46.7189	41.7986	26.814	47.1621	31.9703	33.081	33.3707	31.7622	0.851656735	0.966018836	24	12	24	0.490811433	0.805393068	24	20.44714916	0.11717991
ENSMUSG00000066037	Hnrnpr	heterogeneous nuclear ribonucleoprotein R [Source:MGI Symbol;Acc:MGI:1891692]	19.4078	19.6632	19.8998	18.7741	17.4615	18.5718	19.194	19.1888	19.3113	20.2846	18.1401	18.6819	17.9228	19.5316	0.019711532	0.258568924	12	8	24	0.037250857	0.609176161	24	6.518500626	0.05307427
ENSMUSG00000066042	Med18	mediator complex subunit 18 [Source:MGI Symbol;Acc:MGI:1914469]	2.38698	1.79548	1.98469	2.36477	2.107	2.29358	2.27385	2.24629	2.27705	2.26233	2.56489	2.2681	2.44815	2.52057	0.909604019	0.966018836	4	4	24	0.563694591	0.842726277	24	18.78368007	0.032400397
ENSMUSG00000066043	Phactr4	phosphatase and actin regulator 4 [Source:MGI Symbol;Acc:MGI:2140327]	2.65791	2.99619	3.21103	3.33167	3.12288	2.95533	3.20481	2.88932	3.12625	3.75665	2.63717	3.24442	2.57198	2.54335	0.048771079	0.379129806	16	16	24	0.105299731	0.717252017	24	12.39830102	0.092601445
ENSMUSG00000066057	Gm1976	predicted gene 1976 [Source:MGI Symbol;Acc:MGI:3780145]	12.8291	12.9853	13.5594	12.5686	11.9353	12.1389	10.9498	11.2287	11.7164	10.2073	13.1169	12.5063	12.7146	12.8019	0.830949782	0.966018836	12	4	24	0.892318765	0.969510679	24	23.95101076	0.020013259
ENSMUSG00000066058	Cldn19	claudin 19 [Source:MGI Symbol;Acc:MGI:3033992]	83.1294	77.4105	69.2023	77.324	95.3702	71.5389	98.3764	81.47	91.2615	106.105	73.3567	78.8155	86.3198	67.0322	0.921572192	0.966018836	4	4	24	0.848322575	0.954264952	24	15.12345136	0.045112842
ENSMUSG00000066068	Gm13611	predicted gene 13611 [Source:MGI Symbol;Acc:MGI:3651300]	57.6866	59.5837	66.3743	63.5041	58.6153	50.2914	61.8216	57.8487	69.0067	47.1447	65.8411	53.9243	66.7431	55.6166	0.245061943	0.741580974	12	12	24	0.623447624	0.87183699	24	6.786990183	0.06350933
ENSMUSG00000066090	Insl5	insulin-like 5 [Source:MGI Symbol;Acc:MGI:1346085]	0.83152	0.646464	0.169239	0.462508	0.669946	0.87305	1.02694	0.247893	0.10564	0.474415	0.410702	0.358273	0.350321	0.507384	0.138543921	0.580158868	24	12	24	0.028197014	0.591016114	24	20.90141469	0.237009839
ENSMUSG00000066113	Adamtsl1	ADAMTS-like 1 [Source:MGI Symbol;Acc:MGI:1924989]	2.81169	3.30884	3.54935	2.95738	3.18465	2.86345	3.06542	3.17691	3.8734	3.76555	2.54484	2.89162	2.61655	2.53311	0.097760727	0.501112642	12	16	24	0.057024622	0.65610184	24	9.384366582	0.125017242
ENSMUSG00000066129	Kndc1	kinase non-catalytic C-lobe domain (KIND) containing 1 [Source:MGI Symbol;Acc:MGI:1923734]	3.39535	5.14178	8.44628	4.65283	3.22668	5.39728	5.79568	4.02464	5.17884	5.3862	2.77375	4.67194	2.7154	6.55921	0.285027487	0.793714531	12	8	24	0.191370961	0.768040178	24	7.151068693	0.273954678
ENSMUSG00000066148	Prpf4	pre-mRNA processing factor 4 [Source:MGI Symbol;Acc:MGI:1917302]	4.67334	4.88879	4.76036	4.88794	5.04107	5.29005	4.73101	5.38491	4.9895	5.27486	4.96382	5.68319	4.92824	5.14171	0.3576519	0.882035609	24	4	24	0.670533583	0.888925819	24	16.85304146	0.020320239
ENSMUSG00000066149	Cdc26	cell division cycle 26 [Source:MGI Symbol;Acc:MGI:1913690]	12.1269	14.7103	12.4285	13.1675	14.1334	11.019	11.4934	13.0446	14.6995	11.4655	13.9123	13.3798	14.1054	13.9358	0.843639218	0.966018836	8	16	24	0.573642587	0.847005958	24	7.581817829	0.054713863
ENSMUSG00000066150	Slc31a1	solute carrier family 31, member 1 [Source:MGI Symbol;Acc:MGI:1333843]	15.802	14.9262	15.1425	16.4787	15.4536	17.4016	16.3637	14.7827	13.6712	16.684	17.9173	18.7532	15.4604	23.2753	0.0444686	0.365196805	24	12	24	0.729463377	0.911734411	24	20.00344483	0.050227453
ENSMUSG00000066151	Fkbp15	FK506 binding protein 15 [Source:MGI Symbol;Acc:MGI:2444782]	17.3448	15.8859	16.5935	16.492	15.7185	17.4641	16.2467	16.7221	13.6407	19.645	14.8139	16.8588	16.3799	16.3861	0.928780444	0.966018836	24	12	24	0.839679523	0.95137802	24	19.77475385	0.027922454
ENSMUSG00000066152	Slc31a2	solute carrier family 31, member 2 [Source:MGI Symbol;Acc:MGI:1333844]	18.341	15.4322	15.3264	16.6953	17.263	15.9834	18.4084	15.1831	16.9357	14.7985	18.4638	15.1934	15.6326	13.6711	0.185945151	0.662239322	20	12	24	0.19517349	0.768040178	24	18.45953031	0.081105482
ENSMUSG00000066175	2510046G10Rik	RIKEN cDNA 2510046G10 gene [Source:MGI Symbol;Acc:MGI:3698051]	1.54798	1.72361	2.44451	1.96614	2.66886	1.24327	1.79523	1.15314	2.75223	1.95292	1.7315	1.10618	2.64885	2.43834	0.080164531	0.458678242	12	12	24	0.228719636	0.768040178	24	9.140019172	0.195059084
ENSMUSG00000066189	Cacng3	calcium channel, voltage-dependent, gamma subunit 3 [Source:MGI Symbol;Acc:MGI:1859165]	0.831418	0.872753	1.02077	0.670303	0.465748	0.704447	0.736514	0.553463	0.723972	0.994942	0.314904	0.995224	0.57956	0.714123	0.869721097	0.966018836	12	8	24	0.566427794	0.843471947	24	5.314284648	0.074842959
ENSMUSG00000066191	Anks6	ankyrin repeat and sterile alpha motif domain containing 6 [Source:MGI Symbol;Acc:MGI:1922941]	3.63138	3.2386	3.03125	3.98017	4.01423	3.6219	3.78919	3.99953	3.93431	3.14591	3.68815	3.5323	3.54068	2.95168	0.922029406	0.966018836	20	12	24	0.449969289	0.805393068	24	17.8747493	0.058981247
ENSMUSG00000066196	Spag8	sperm associated antigen 8 [Source:MGI Symbol;Acc:MGI:3056295]	0.373719	0.714299	0.760244	0.263423	0.663626	0.31342	0.629039	0.480268	0.489393	0.491237	0.872197	0.581959	0.282028	0.625884	0.839634479	0.966018836	20	8	24	0.826591934	0.945877848	24	10.2502084	0.045152802

ENSMUSG00000066197	Gpr139	G protein-coupled receptor 139 [Source:MGI Symbol;Acc:MGI:2685341]	3.98886	3.64976	3.56034	3.83702	3.63292	3.63551	3.30763	3.8931	2.99333	4.25233	3.96224	3.69151	3.8286	3.73731	0.92721845	0.966018836	16	20	24	0.689143382	0.897070579	24	16.84683736	0.033605122
ENSMUSG00000066232	Ipo7	importin 7 [Source:MGI Symbol;Acc:MGI:2152414]	38.9536	38.0448	37.7049	39.0484	37.0678	36.279	39.3193	35.0079	33.5424	30.3605	38.0665	33.5824	35.5249	36.3198	0.907000607	0.966018836	4	20	24	0.417668553	0.802361287	24	22.73139526	0.045620173
ENSMUSG00000066233	Tmem42	transmembrane protein 42 [Source:MGI Symbol;Acc:MGI:1277176]	11.6764	11.1673	10.2316	11.1547	10.9604	11.0942	10.5819	9.94554	12.0551	12.2382	11.9505	12.002	13.8271	12.5101	0.742519053	0.966018836	16	20	24	0.895517983	0.970467191	24	20.26400544	0.017870809
ENSMUSG00000066235	Pomgnt2	protein O-linked mannose beta 1,4-N-acetylglucosaminyltransferase 2 [Source:MGI Symbol;Acc:MGI:2143424]	13.1382	11.3206	11.1397	11.01	10.9927	12.3475	11.3735	13.0099	11.0067	14.0616	11.7115	13.1284	13.4352	13.0862	0.241264473	0.741580974	4	8	24	0.425390952	0.805393068	24	23.64717873	0.052916346
ENSMUSG00000066245	Gm10156	predicted gene 10156 [Source:MGI Symbol;Acc:MGI:3642344]	6.70938	10.9975	14.1235	8.79639	7.61472	6.59807	5.70144	6.66207	11.4735	10.0286	9.66767	12.2951	9.3774	10.9822	0.062631097	0.410322149	12	16	24	0.125129293	0.73913972	24	9.326219809	0.269839871
ENSMUSG00000066258	Trim12a	tripartite motif-containing 12A [Source:MGI Symbol;Acc:MGI:1923931]	5.61282	6.05792	6.41531	6.46065	5.83344	5.9808	5.68056	6.8385	8.19855	4.956	5.74741	6.86989	5.42424	6.23514	0.267533088	0.774280302	12	16	24	0.403283732	0.799546792	24	7.314039975	0.08415672
ENSMUSG00000066270	Gm10157	predicted gene 10157 [Source:MGI Symbol;Acc:MGI:3642264]	41.6464	46.4619	34.4339	43.2171	42.6244	37.9936	38.4439	37.9839	47.1613	51.7289	41.57	45.7107	49.7577	51.0812	0.880992536	0.966018836	16	8	24	0.777082125	0.928544389	24	8.458422307	0.043652189
ENSMUSG00000066278	Vps37b	vacuolar protein sorting 37B [Source:MGI Symbol;Acc:MGI:1916724]	2.33278	2.21369	2.66126	2.28567	2.29337	2.56542	2.33401	2.9353	2.42875	2.69227	2.30945	2.20927	2.45093	2.49538	0.822170541	0.966018836	12	8	24	0.303983885	0.768040178	24	6.972803675	0.052509628
ENSMUSG00000066306	Numa1	nuclear mitotic apparatus protein 1 [Source:MGI Symbol;Acc:MGI:2443665]	24.574	21.0523	22.9302	22.6874	23.3762	23.7882	26.8852	23.6898	21.7643	26.1009	22.2069	25.2974	24.3296	23.5686	0.094000699	0.490854203	4	8	24	0.307965405	0.768040178	24	21.03949745	0.056218386
ENSMUSG00000066315	Gm12918	predicted gene 12918 [Source:MGI Symbol;Acc:MGI:3652005]	42.5226	44.3375	45.3739	46.1607	38.2699	38.6767	43.1405	44.0826	59.0143	42.6062	52.4753	47.9046	48.1146	47.7375	0.941885246	0.966018836	12	12	24	0.220036009	0.768040178	24	7.94879595	0.098508803
ENSMUSG00000066324	Bpnt2	3'(2'), 5'-bisphosphate nucleotidase 2 [Source:MGI Symbol;Acc:MGI:1915720]	21.871	24.2022	25.0114	23.623	21.8182	20.5257	22.8041	20.9943	22.9788	20.2446	22.8028	20.0871	19.7684	22.0172	0.039704107	0.338535904	12	12	24	0.09832179	0.71175955	24	8.393427227	0.068640442
ENSMUSG00000066357	Wdr6	WD repeat domain 6 [Source:MGI Symbol;Acc:MGI:1930140]	15.7077	16.3312	18.7107	16.3259	13.3342	17.6643	16.0179	17.6716	16.6675	21.7242	12.3813	18.0067	14.5359	19.6781	0.210789085	0.699573621	12	8	24	0.254038524	0.768040178	24	6.829996114	0.132415495
ENSMUSG00000066361	Serpina3c	serine (or cysteine) peptidase inhibitor, clade A, member 3C [Source:MGI Symbol;Acc:MGI:102848]	1.58407	1.62239	1.80804	1.96017	1.33763	1.48878	1.55838	1.45811	1.71907	1.582	1.52057	1.37379	1.47522	1.65949	0.011674605	0.209564579	12	12	24	0.025706417	0.586541342	24	8.490202837	0.085837991
ENSMUSG00000066392	Nrxn3	neurexin III [Source:MGI Symbol;Acc:MGI:1096389]	13.7591	11.9472	14.4098	12.6519	11.9781	14.2562	13.1683	11.7862	11.5767	14.3405	12.0683	12.7876	11.418	13.6466	0.866861748	0.966018836	24	8	24	0.950845919	0.988257443	24	15.95883815	0.014209781
ENSMUSG00000066406	Akap13	A kinase (PRKA) anchor protein 13 [Source:MGI Symbol;Acc:MGI:2676556]	6.11773	5.67939	6.37464	6.4493	6.33851	5.49793	5.93556	5.93652	5.58204	7.01315	5.3279	6.42367	6.0391	5.82538	0.743750765	0.966018836	16	16	24	0.52311217	0.82106433	24	12.35568012	0.041347245
ENSMUSG00000066407	Gm10263	predicted gene 10263 [Source:MGI Symbol;Acc:MGI:3642825]	10.2696	8.39797	7.61768	6.17014	6.97012	5.7673	10.6204	5.13084	7.70326	6.9958	6.27535	7.32187	10.5942	8.59499	0.006734756	0.181382464	4	16	24	0.123653173	0.736492734	24	1.122163153	0.216924502
ENSMUSG00000066415	Msl2	MSL complex subunit 2 [Source:MGI Symbol;Acc:MGI:1925103]	9.04336	9.72722	9.5255	9.83424	9.31521	9.07696	8.8719	8.9666	9.02263	9.31842	8.95352	8.84273	8.47897	9.06763	0.032263213	0.324385159	16	12	24	0.009059411	0.502965379	24	10.67310526	0.041039503
ENSMUSG00000066438	Plekhd1	pleckstrin homology domain containing, family D (with coiled-coil domains) member 1 [Source:MGI Symbol;Acc:MGI:3036228]	17.3331	14.917	14.0142	14.0911	15.2849	17.0131	14.2227	16.6827	13.1815	19.1773	14.7632	15.9699	14.8841	16.2598	0.390686537	0.915479136	24	12	24	0.70227192	0.901574817	24	20.18413798	0.048246132
ENSMUSG00000066440	Zfyve26	zinc finger, FYVE domain containing 26 [Source:MGI Symbol;Acc:MGI:1924767]	5.96656	6.93404	6.92444	5.18226	6.35257	6.77396	7.27268	7.61052	7.4415	5.75111	6.61769	5.90768	5.56776	6.48179	0.175272072	0.651010555	12	4	24	0.330309603	0.768040178	24	4.282138949	0.082831561
ENSMUSG00000066441	Rdh11	retinol dehydrogenase 11 [Source:MGI Symbol;Acc:MGI:102581]	29.8945	25.8159	27.5415	24.8137	29.2255	28.5369	24.6606	24.4451	24.029	24.5425	28.3249	26.5686	28.8695	24.2545	0.073522892	0.448301997	4	4	24	0.029884	0.596648309	24	19.51302374	0.103915199
ENSMUSG00000066442	Mthfs	5, 10-methenyltetrahydrofolate synthetase [Source:MGI Symbol;Acc:MGI:1340032]	8.23763	8.71865	7.1158	6.09995	7.1361	7.62266	8.35738	7.95678	8.25064	5.19451	6.74269	9.94946	9.41002	6.78891	0.00932019	0.194720062	4	12	24	0.010914726	0.51149922	24	23.81785877	0.222718447
ENSMUSG00000066456	Hmgn3	high mobility group nucleosomal binding domain 3 [Source:MGI Symbol;Acc:MGI:2138069]	33.8668	42.3748	36.2715	30.6264	35.7283	35.4905	36.8308	37.8199	38.8644	33.181	35.5212	33.2341	38.7268	35.4639	0.10031279	0.511533473	12	4	24	0.095542101	0.71175955	24	3.376362373	0.08953663
ENSMUSG00000066477	Gm16551	predicted gene 16551 [Source:MGI Symbol;Acc:MGI:4414971]	5.00826	4.26161	2.56772	4.86689	3.63671	3.53041	5.31869	4.51112	3.39665	5.77087	4.74364	4.46001	4.89851	4.5026	0.176754388	0.653256853	4	8	24	0.680046843	0.892958114	24	21.38013505	0.085182861
ENSMUSG00000066487	Gm5786	predicted pseudogene 5786 [Source:MGI Symbol;Acc:MGI:3645003]	3.20821	2.7539	2.94694	2.72505	2.85338	2.94795	3.30556	3.90971	3.24719	2.74109	3.04005	3.21244	3.28132	3.43487	0.01579111	0.253395174	8	8	24	0.025637868	0.586541342	24	2.186850554	0.094689474
ENSMUSG00000066491	Cox6c2	cytochrome c oxidase subunit 6C2 [Source:MGI Symbol;Acc:MGI:3649160]	192.912	213.858	218.176	223.822	193.511	188.287	191.067	232.152	229.535	183.435	220.442	191.557	232.167	209.187	0.390686537	0.915479136	12	12	24	0.12456052	0.738497304	24	6.902553515	0.096829318
ENSMUSG00000066551	Hmgb1	high mobility group box 1 [Source:MGI Symbol;Acc:MGI:96113]	12.0737	14.9068	15.0939	15.2726	14.4996	12.9759	15.3083	14.4421	15.6547	13.3148	15.4397	13.2091	12.6039	13.0682	0.128916799	0.560209134	12	16	24	0.134795563	0.746461599	24	10.37738059	0.087839036
ENSMUSG00000066553	Gm6969	predicted pseudogene 6969 [Source:MGI Symbol;Acc:MGI:3645320]	57.1897	58.1718	57.0589	54.7538	56.5946	55.3145	50.9075	51.2527	53.0458	51.1241	60.7267	53.6098	59.9576	48.3885	0.92655264	0.966018836	20	12	24	0.386455134	0.799546792	24	18.19950156	0.050202887
ENSMUSG00000066554	Gm10167	predicted pseudogene 10167 [Source:MGI Symbol;Acc:MGI:3704263]	11.0882	12.3388	12.4901	11.9979	12.5156	12.6852	11.1149	11.9516	11.5406	12.2	10.5111	10.6919	9.99545	10.9001	0.593560247	0.966018836	16	12	24	0.168668279	0.767020009	24	11.1631326	0.055467449
ENSMUSG00000066568	Lsm14a	LSM14A mRNA processing body assembly factor [Source:MGI Symbol;Acc:MGI:1914320]	30.7589	33.777	33.2174	29.8813	33.3364	30.3297	33.8746	32.1436	34.467	32.83	30.92	29.7286	31.6346	30.8041	0.085890569	0.466404237	12	12	24	0.244126341	0.768040178	24	7.07318356	0.045846385
ENSMUSG00000066571	Garre1	granule associated Rac and RHOG effector 1 [Source:MGI Symbol;Acc:MGI:1924311]	10.1747	11.3595	9.84573	9.97829	10.2738	10.4439	11.4877	10.0111	11.2916	12.2377	9.20219	10.8744	10.6681	9.88361	0.93447422	0.966018836	4	16	24	0.905422918	0.974083049	24	4.651339268	0.018420864
ENSMUSG00000066595	Flvcr1	feline leukemia virus subgroup C cellular receptor 1 [Source:MGI Symbol;Acc:MGI:2444881]	14.4372	14.6556	15.5737	15.6307	14.0588	14.4146	14.3162	15.2652	13.6931	14.1898	14.5961	13.0562	12.3283	14.3238	0.467283192	0.966018836	16	8	24	0.159191255	0.765350752	24	9.582450632	0.051814284
ENSMUSG00000066607	Insyn1	inhibitory synaptic factor 1 [Source:MGI Symbol;Acc:MGI:2442108]	29.1647	20.3649	20.3675	23.1769	21.0123	22.9313	22.8217	25.9769	18.3723	34.4654	22.6939	27.776	25.2784	25.8835	0.649212555	0.966018836	4	8	24	0.772696464	0.92687884	24	20.45127781	0.066284931
ENSMUSG00000066613	Zfp932	zinc finger protein 932 [Source:MGI Symbol;Acc:MGI:1916754]	13.4702	18.5735	15.0699	16.4717	17.0994	14.2372	15.3252	15.7838	19.1319	12.6996	17.58	16.0534	16.3216	16.1685	0.873337044	0.966018836	20	4	24	0.742833578	0.916215545	24	7.962825056	0.050052743
ENSMUSG00000066621	Tecpr1	tectonin beta-propeller repeat containing 1 [Source:MGI Symbol;Acc:MGI:1917631]	16.7058	16.1012	14.9173	17.317	16.7623	16.4907	17.133	16.8263	16.331	18.6195	15.4576	16.0005	15.6987	16.582	0.897425765	0.966018836	16	20	24	0.795847058	0.934774232	24	12.80910043	0.019835446
ENSMUSG00000066629	Rpl36-ps3	ribosomal protein L36, pseudogene 3 [Source:MGI Symbol;Acc:MGI:3642480]	1.85445	1.73143	1.72028	1.17648	0.957037	1.3064	3.5188	2.08897	1.07116	1.22798	1.42	1.73513	0.900436	0.625989	0.787022682	0.966018836	4	12	24	0.343574338	0.777219607	24	23.5400471	0.191931643
ENSMUSG00000066637	Ttc32	tetratricopeptide repeat domain 32 [Source:MGI Symbol;Acc:MGI:1922766]	2.81549	3.41953	3.14939	3.25086	3.07487	2.73857	3.41223	3.23572	3.23987	2.82548	3.39295	3.13292	3.68537	2.72691	0.849180783	0.966018836	4	20	24	0.908307268	0.974606327	24	3.316477006	0.017804316
ENSMUSG00000066640	Fbxl18	F-box and leucine-rich repeat protein 18 [Source:MGI Symbol;Acc:MGI:2444450]	4.21994	3.58198	3.87404	3.88519	4.03801	4.0533	4.41409	4.13038	4.03378	4.48412	4.38267	4.41087	4.23687	3.88993	0.121015203	0.548303839	4	4	24	0.141008652	0.751039598	24	18.53679605	0.050102629
ENSMUSG00000066643	Wdr35	WD repeat domain 35 [Source:MGI Symbol;Acc:MGI:1921932]	10.3044	10.3037	10.1504	9.89374	9.6791	10.1816	9.37538	9.63286	9.56477	9.7071	9.85175	10.1071	10.37	10.4005	0.483881213	0.966018836	8	8	24	0.456927966	0.805393068	24	0.552109508	0.021769053
ENSMUSG00000066647	Gm5113	predicted gene 5113 [Source:MGI Symbol;Acc:MGI:3644651]	4.63021	4.88708	5.05795	5.35894	4.68451	4.96	4.70643	5.12878	4.70376	5.81447	4.53262	5.10132	4.72841	5.33911	0.235141005	0.73629392	16	4	24	0.216585756	0.768040178	24	9.678858875	0.056915829
ENSMUSG00000066682	Pilrb2	paired immunoglobin-like type 2 receptor beta 2 [Source:MGI Symbol;Acc:MGI:2450535]	0.667917	1.1091	1.52414	0.993309	0.920089	1.18159	0.71835	1.59953	0.988872	1.15498	0.665527	1.31743	1.68855	0.834836	0.924553956	0.966018836	8	12	24	0.677347942	0.892060593	24	5.692566476	0.082301979
ENSMUSG00000066684	Pilrb1	paired immunoglobin-like type 2 receptor beta 1 [Source:MGI Symbol;Acc:MGI:2450532]	0.842092	1.24644	0.52931	1.12212	1.18372	0.686339	0.519478	0.761754	1.44621	0.222898	0.694351	0.946582	1.09855	0.751228	0.801280095	0.966018836	8	20	24	0.931800835	0.982456396	24	2.138232799	0.040085652
ENSMUSG00000066687	Zbtb16	zinc finger and BTB domain containing 16 [Source:MGI Symbol;Acc:MGI:103222]	1.42186	0.856075	1.71395	4.19402	1.02416	1.58488	1.16509	1.29224	1.2233	1.95861	0.983306	1.55597	0.970348	0.774563	0.053980818	0.395677346	16	16	24	0.151118472	0.758865539	24	12.99487385	0.266963858
ENSMUSG00000066705	Fxyd6	FXYD domain-containing ion transport regulator 6 [Source:MGI Symbol;Acc:MGI:1890226]	83.2879	90.4749	80.3209	85.8855	89.3062	77.8158	88.6588	85.9698	98.0181	80.3899	82.4146	93.893	99.5459	94.4852	0.628899575	0.966018836	8	8	24	0.288669158	0.768040178	24	3.318837927	0.058531804
ENSMUSG00000066720	Cldn9	claudin 9 [Source:MGI Symbol;Acc:MGI:1913100]	2.13773	1.46565	1.4157	1.78247	1.18663	1.46471	1.66501	1.77922	1.41963	1.84178	1.70855	1.69458	1.46247	1.6574	0.896753532	0.966018836	16	20	24	0.852888233	0.956013379	24	0.65928703	0.029475237
ENSMUSG00000066735	Vkorc1l1	vitamin K epoxide reductase complex, subunit 1-like 1 [Source:MGI Symbol;Acc:MGI:1916818]	23.275	21.8981	22.5683	22.7385	22.1222	22.884	21.8655	20.7082	20.6575	24.0696	21.1481	22.4725	22.8067	22.2595	0.492524295	0.966018836	4	4	24	0.60891948	0.864393298	24	17.89505466	0.02412757
ENSMUSG00000066798	Zbtb6	zinc finger and BTB domain containing 6 [Source:MGI Symbol;Acc:MGI:2442998]	5.81698	5.91193	5.52549	5.82091	6.48108	5.65595	5.99721	5.73813	5.82624	5.06063	6.46353	5.23718	5.08378	5.36434	0.807012301	0.966018836	20	4	24	0.50159706	0.80908345	24	16.43692116	0.039720967
ENSMUSG00000066800	Rnasel	ribonuclease L (2', 5'-oligoisoadenylate synthetase-dependent) [Source:MGI Symbol;Acc:MGI:1098272]	10.4713	10.5736	10.8254	10.3011	11.1449	11.1576	10.0287	9.72415	10.5644	10.5986	10.5365	11.5101	11.4525	10.8681	0.4776989	0.966018836	24	8	24	0.304932385	0.768040178	24	18.589492	0.036940795
ENSMUSG00000066807	Gm10179	predicted gene 10179 [Source:MGI Symbol;Acc:MGI:3708748]	16.9342	18.5748	15.5966	19.2442	19.6248	17.9266	18.592	15.6562	20.7015	16.4618	18.6232	19.4426	19.5797	17.9454	0.841143063	0.966018836	20	12	24	0.561375317	0.841538771	24	17.78677631	0.050342346
ENSMUSG00000066829	Zfp810	zinc finger protein 810 [Source:MGI Symbol;Acc:MGI:2384563]	4.49328	6.41035	6.99492	5.4171	6.57651	5.9495	5.33522	6.77075	7.12138	5.58	6.1076	6.42825	6.48764	6.32148	0.326899754	0.845515883	12	16	24	0.324929805	0.768040178	24	8.329189397	0.088017228
ENSMUSG00000066838	Zfp772	zinc finger protein 772 [Source:MGI Symbol;Acc:MGI:2385265]	4.27013	4.35561	4.61475	3.65186	4.414	4.26504	3.98027	3.82532	4.20969	3.55996	4.10086	4.05502	3.99021	4.15539	0.911685024	0.966018836	12	4	24	0.689907134	0.897375425	24	0.129202856	0.026686869
ENSMUSG00000066839	Ecsit	ECSIT signalling integrator [Source:MGI Symbol;Acc:MGI:1349469]	14.0343	12.0497	13.5977	14.4182	12.9454	14.9371	12.7559	14.4459	12.8986	16.6021	13.6528	13.895	15.4521	13.926	0.928780444	0.966018836	16	20	24	0.721765582	0.90866625	24	14.9886292	0.033459495
ENSMUSG00000066842	Hmcn1	hemicentin 1 [Source:MGI Symbol;Acc:MGI:2685047]	2.05679	2.17082	1.78984	1.54838	1.96496	1.50318	0.763353	1.66834	2.18866	1.46234	1.18929	1.17472	1.55624	2.4735	0.055920623	0.401074772	8	16	24	0.171141003	0.768040178	24	6.747921038	0.189183679
ENSMUSG00000066877	Nck2	non-catalytic region of tyrosine kinase adaptor protein 2 [Source:MGI Symbol;Acc:MGI:1306821]	2.51845	2.70334	3.17047	2.24083	2.6169	2.60959	2.85645	2.70059	2.30702	2.68511	2.57843	2.36837	2.25573	2.2454	0.814221431	0.966018836	12	12	24	0.971746539	0.993121308	24	7.487054405	0.009410788
ENSMUSG00000066878	Gm10184	predicted pseudogene 10184 [Source:MGI Symbol;Acc:MGI:3704480]	2.93027	2.3198	2.38571	2.42667	2.66169	2.43993	2.2017	2.39832	2.54026	2.62339	2.71175	2.17832	2.42209	2.81489	0.904831057	0.966018836	20	4	24	0.845211902	0.953278734	24	12.04311197	0.021208002
ENSMUSG00000066880	Zfp617	zinc finger protein 617 [Source:MGI Symbol;Acc:MGI:2684459]	7.86369	8.23114	8.40338	8.06234	7.41887	7.80462	7.82813	7.65396	8.91369	6.50343	8.30215	8.19572	8.62668	8.02058	0.834434272	0.966018836	12	4	24	0.535748897	0.827176676	24	3.08544005	0.042280595
ENSMUSG00000066892	Fbxl12	F-box and leucine-rich repeat protein 12 [Source:MGI Symbol;Acc:MGI:1354738]	6.16325	6.41321	6.29968	6.1014	5.7969	5.57753	6.30187	6.87663	8.31674	6.12342	7.25931	7.16588	6.78308	6.18609	0.891105762	0.966018836	12	4	24	0.518446867	0.818673182	24	7.202433969	0.056134776
ENSMUSG00000066894	Vsig10	V-set and immunoglobulin domain containing 10 [Source:MGI Symbol;Acc:MGI:2448533]	4.74521	6.06449	4.54111	5.90467	6.44445	4.97617	4.84164	6.4477	6.55351	5.53295	5.23868	6.50478	5.84737	5.88295	0.93357733	0.966018836	8	20	24	0.736289497	0.914122853	24	9.509356396	0.047108396
ENSMUSG00000066900	Suds3	suppressor of defective silencing 3 homolog (S. cerevisiae) [Source:MGI Symbol;Acc:MGI:1919204]	39.647	37.7412	38.2066	37.0381	38.2983	41.2042	41.6978	41.6951	36.4088	43.719	38.8689	39.8122	40.5269	40.1933	0.277087842	0.789849983	4	8	24	0.46757111	0.805393068	24	22.30867032	0.0336125
ENSMUSG00000066952	Myo1h	myosin 1H [Source:MGI Symbol;Acc:MGI:1914674]	0.298661	0.718279	0.887261	0.901405	1.02637	0.756477	0.452503	0.547586	1.1525	0.601236	0.610313	0.784967	0.465698	0.546916	0.00819923	0.192760771	12	16	24	0.044463841	0.628432994	24	11.99957816	0.178945097
ENSMUSG00000066979	Bub3	BUB3 mitotic checkpoint protein [Source:MGI Symbol;Acc:MGI:1343463]	31.8124	32.1303	32.4449	28.8632	32.9685	33.274	34.2452	32.6984	31.347	31.9743	31.9847	30.5247	30.9606	30.9616	0.881996584	0.966018836	4	12	24	0.505306858	0.811334839	24	22.71119839	0.026999097
ENSMUSG00000067006	Serpinb5	serine (or cysteine) peptidase inhibitor, clade B, member 5 [Source:MGI Symbol;Acc:MGI:109579]	0.769115	0.65757	0.637718	1.01854	0.791284	0.62691	0.572255	0.751365	0.633154	0.639446	0.838684	0.651055	0.743116	0.735513	0.784278077	0.966018836	20	16	24	0.432636523	0.805393068	24	13.8179512	0.046779702
ENSMUSG00000067017	Capza1-ps1	capping protein (actin filament) muscle Z-line, alpha 1, pseudogene 1 [Source:MGI Symbol;Acc:MGI:106236]	12.3614	11.6973	10.5943	10.8429	12.1319	11.0894	11.3619	10.8234	10.9579	9.99568	12.2227	10.9739	12.0341	11.3139	0.032586909	0.324908923	4	12	24	0.107524932	0.719994886	24	21.55984528	0.065632882
ENSMUSG00000067028	Cntnap5b	contactin associated protein-like 5B [Source:MGI Symbol;Acc:MGI:3664583]	0.811187	0.962171	0.816966	0.96037	0.820879	0.769476	0.916907	0.876853	1.12547	0.461461	0.873317	0.866152	0.933607	1.33505	0.203788871	0.689899499	8	16	24	0.188397477	0.768040178	24	3.977630573	0.100144307
ENSMUSG00000067058	Rps15a-ps5	ribosomal protein S15A, pseudogene 5 [Source:MGI Symbol;Acc:MGI:3642245]	23.4473	34.0095	27.6945	29.0862	33.4295	32.3371	23.5313	24.4082	31.9776	21.0719	27.8092	21.1099	22.3094	21.3651	0.93447422	0.966018836	20	8	24	0.466100838	0.805393068	24	13.15532708	0.099885262
ENSMUSG00000067071	Hes6	hairy and enhancer of split 6 [Source:MGI Symbol;Acc:MGI:1859852]	10.6608	9.8972	8.68822	10.1592	9.32428	10.0585	8.45172	10.1072	9.63781	12.1789	12.8613	11.5129	12.758	11.6242	0.879206539	0.966018836	16	20	24	0.664747564	0.886815534	24	17.39540544	0.049230857
ENSMUSG00000067081	Asb18	ankyrin repeat and SOCS box-containing 18 [Source:MGI Symbol;Acc:MGI:2655109]	0.465692	0.551433	0.47691	0.531227	0.421086	0.569908	0.504255	1.34532	0.589501	0.895342	0.310532	0.516447	0.76025	0.675671	0.117050591	0.538362324	8	12	24	0.178318437	0.768040178	24	5.292143141	0.14164452
ENSMUSG00000067106	Gm5529	predicted pseudogene 5529 [Source:MGI Symbol;Acc:MGI:3646377]	4.80706	5.41171	4.2764	4.63184	4.69705	4.7082	4.33055	4.9367	3.4307	3.64537	4.39118	3.69504	3.89559	3.75922	0.675497492	0.966018836	8	4	24	0.368507538	0.791588198	24	22.21022853	0.061325433
ENSMUSG00000067121	Gm7027	predicted gene 7027 [Source:MGI Symbol;Acc:MGI:3644555]	14.2268	14.5697	15.7772	15.7737	16.0771	16.7725	17.6268	15.9335	19.8865	14.2509	18.4489	19.0352	17.1476	15.9733	0.745118544	0.966018836	24	16	24	0.73856722	0.914690529	24	17.92075359	0.038760885
ENSMUSG00000067144	Slc22a7	solute carrier family 22 (organic anion transporter), member 7 [Source:MGI Symbol;Acc:MGI:1859559]	0.674286	0.565623	0.48546	0.80432	0.408775	0.827523	0.543821	0.514078	0.414274	0.653445	0.506028	0.649233	0.466711	0.543615	0.390686537	0.915479136	24	12	24	0.490365367	0.805393068	24	18.57434025	0.055644853
ENSMUSG00000067148	Polr1c	polymerase (RNA) I polypeptide C [Source:MGI Symbol;Acc:MGI:103288]	14.4527	15.3305	15.0128	14.4239	13.8542	14.6519	13.834	13.8133	12.3713	12.8834	15.7921	15.2178	13.758	14.0861	0.759899911	0.966018836	20	20	24	0.371091481	0.792854157	24	19.96902798	0.050394767
ENSMUSG00000067149	Jchain	immunoglobulin joining chain [Source:MGI Symbol;Acc:MGI:96493]	0.751063	1.60256	1.65703	1.16664	0.834186	1.07556	0.392671	1.07786	2.43347	0.797708	0.799734	0.874555	1.06085	0.685328	0.182849367	0.659303873	12	16	24	0.104023361	0.715480277	24	8.380834663	0.259575735
ENSMUSG00000067150	Xpo5	exportin 5 [Source:MGI Symbol;Acc:MGI:1913789]	10.3912	9.7816	10.9718	10.3195	9.91334	11.1799	11.0719	10.4925	9.12402	11.9127	9.66379	10.6473	10.4126	10.4429	0.928780444	0.966018836	24	20	24	0.866215734	0.961290808	24	20.03293546	0.020223025
ENSMUSG00000067158	Col4a4	collagen, type IV, alpha 4 [Source:MGI Symbol;Acc:MGI:104687]	0.715467	0.969878	0.921767	0.808856	0.804063	0.826798	0.831557	0.901715	0.972511	0.816239	0.684849	0.825579	0.809033	0.811849	0.010153564	0.194720062	12	8	24	0.042348966	0.623698598	24	6.426459675	0.059340918
ENSMUSG00000067161	Gm5560	predicted pseudogene 5560 [Source:MGI Symbol;Acc:MGI:3647980]	22.2019	21.9219	21.7084	21.1273	20.9651	21.9931	22.4214	19.9149	20.2208	20.2201	24.1746	18.3103	20.1054	19.5042	0.919571723	0.966018836	20	12	24	0.638226219	0.877145054	24	17.66176874	0.03267313
ENSMUSG00000067189	Gm7335	predicted gene 7335 [Source:MGI Symbol;Acc:MGI:3644473]	89.7606	86.2002	88.9576	93.4812	86.1136	91.1938	78.947	79.1199	82.8744	75.522	91.0108	81.8947	86.5609	73.2103	0.644063931	0.966018836	20	12	24	0.164369213	0.767020009	24	17.24600185	0.064853982
ENSMUSG00000067194	Eif1ax	eukaryotic translation initiation factor 1A, X-linked [Source:MGI Symbol;Acc:MGI:1913485]	47.7039	47.8353	46.388	45.7855	45.134	44.5521	46.0838	43.7269	43.8865	44.1589	52.728	42.9492	52.9407	47.9091	0.869721097	0.966018836	4	20	24	0.777472962	0.928654033	24	23.22970736	0.025961842
ENSMUSG00000067199	Frat1	frequently rearranged in advanced T cell lymphomas [Source:MGI Symbol;Acc:MGI:109450]	0.532992	1.18401	1.19537	0.857159	0.596005	1.02407	0.98726	1.14697	0.818343	1.27996	0.903706	1.11733	0.964956	1.29062	0.529387702	0.966018836	8	12	24	0.240899277	0.768040178	24	6.496003187	0.108893588
ENSMUSG00000067203	H2-K2	histocompatibility 2, K region locus 2 [Source:MGI Symbol;Acc:MGI:95906]	3.72483	3.08546	3.39493	3.10105	3.93357	3.4112	3.82447	4.17399	4.12341	3.54114	4.49183	4.04079	3.64152	3.64198	0.897425765	0.966018836	20	20	24	0.71570155	0.906323093	24	18.66701803	0.037197446
ENSMUSG00000067212	H2-T23	histocompatibility 2, T region locus 23 [Source:MGI Symbol;Acc:MGI:95957]	10.4536	12.1156	11.6395	9.88797	10.0763	9.61831	10.0047	12.3154	15.3875	9.60517	11.461	9.06706	10.7218	13.7142	0.004501001	0.140598674	8	16	24	0.007478406	0.478114494	24	6.420322726	0.21221556
ENSMUSG00000067219	Nipal1	NIPA-like domain containing 1 [Source:MGI Symbol;Acc:MGI:1917951]	2.23076	2.83297	2.29413	2.06804	3.27933	1.99763	2.47692	2.28844	3.00419	2.40543	2.22057	2.0902	2.44049	2.03055	0.8972237	0.966018836	12	12	24	0.786068713	0.931220622	24	9.721896296	0.043859963
ENSMUSG00000067235	H2-Q10	histocompatibility 2, Q region locus 10 [Source:MGI Symbol;Acc:MGI:95929]	1.16781	0.863507	2.74498	4.35134	1.64529	1.72265	2.21121	2.22522	2.09508	3.52123	5.04403	2.96028	5.05037	3.3818	0.896545154	0.966018836	16	16	24	0.181859843	0.768040178	24	12.82557907	0.267923813
ENSMUSG00000067242	Lgi1	leucine-rich repeat LGI family, member 1 [Source:MGI Symbol;Acc:MGI:1861691]	20.1273	18.3637	19.1124	18.1601	18.323	20.7919	18.9103	18.7126	17.089	18.9882	18.3359	19.1585	18.7473	19.464	0.127415507	0.560209134	24	20	24	0.093435158	0.71175955	24	21.78956767	0.053016853
ENSMUSG00000067261	Foxd3	forkhead box D3 [Source:MGI Symbol;Acc:MGI:1347473]	2.80349	2.44367	1.9942	2.09204	3.14494	2.20754	3.27007	2.61823	3.12342	4.47743	3.83401	3.30376	3.46743	2.68601	0.371724951	0.894537464	20	16	24	0.474892785	0.805393068	24	16.71613979	0.099784563
ENSMUSG00000067274	Rplp0	ribosomal protein, large, P0 [Source:MGI Symbol;Acc:MGI:1927636]	114.26	124.76	119.26	122.41	149.459	124.86	103.768	126.962	128.933	96.3198	128.928	96.0289	112.594	98.2573	0.745118544	0.966018836	20	8	24	0.577635275	0.848840315	24	13.99662111	0.067810622
ENSMUSG00000067276	Capn6	calpain 6 [Source:MGI Symbol;Acc:MGI:1100850]	1.24324	1.5119	1.54344	1.27416	1.63039	1.38978	1.52033	1.79648	1.61538	1.15754	1.21349	1.20951	1.53997	1.85656	0.085992778	0.466404237	8	8	24	0.049632711	0.642568944	24	4.628686938	0.114300295
ENSMUSG00000067279	Ppp1r3c	protein phosphatase 1, regulatory subunit 3C [Source:MGI Symbol;Acc:MGI:1858229]	5.09857	7.65329	7.41596	11.9265	6.69124	10.5125	10.5212	13.1673	7.57476	6.62191	10.4388	10.9913	9.94794	6.58324	0.891742979	0.966018836	24	12	24	0.813636819	0.940453305	24	19.13146896	0.093949327
ENSMUSG00000067288	Rps28	ribosomal protein S28 [Source:MGI Symbol;Acc:MGI:1859516]	7.54609	8.85417	8.43083	5.05703	11.5692	8.07228	5.29898	6.67122	8.37712	5.94461	8.59177	6.4368	7.87805	6.5632	0.94499634	0.966018836	20	20	24	0.845327857	0.953294887	24	17.1443438	0.066155972
ENSMUSG00000067297	Ifit1bl2	interferon induced protein with tetratricopeptide repeats 1B like 2 [Source:MGI Symbol;Acc:MGI:2148249]	0.740991	0.515641	0.793127	0.598828	0.92141	0.698896	0.724867	0.499792	0.727421	0.530104	0.87062	0.689384	0.70477	0.556793	0.038715855	0.336039716	20	12	24	0.114275175	0.727799063	24	17.46999826	0.087458649
ENSMUSG00000067336	Bmpr2	bone morphogenetic protein receptor, type II (serine/threonine kinase) [Source:MGI Symbol;Acc:MGI:1095407]	28.5138	28.5479	26.9706	28.5384	28.4615	25.6073	26.533	23.9957	24.9293	23.4036	30.4433	23.7538	24.1163	25.3944	0.852921792	0.966018836	20	4	24	0.493510301	0.806076716	24	16.21257882	0.04568014
ENSMUSG00000067344	Rps25-ps1	ribosomal protein S25, pseudogene 1 [Source:MGI Symbol;Acc:MGI:3642902]	104.514	120.392	110.225	121.588	112.753	102.501	100.699	102.734	153.037	84.6565	140.405	103.537	121.941	102.341	0.869721097	0.966018836	12	12	24	0.758213578	0.921778498	24	10.38007015	0.06581758
ENSMUSG00000067365	Tmem128	transmembrane protein 128 [Source:MGI Symbol;Acc:MGI:1913559]	29.3021	36.03	34.4591	32.9504	36.5601	30.8831	32.0425	31.692	39.0918	29.1733	35.2717	30.5263	34.5135	35.1209	0.549437303	0.966018836	12	12	24	0.363371785	0.788241076	24	8.495786301	0.071020639
ENSMUSG00000067367	Lyar	Ly1 antibody reactive clone [Source:MGI Symbol;Acc:MGI:107470]	9.47497	9.30511	8.43799	8.87964	9.76918	8.70173	9.3533	9.83063	9.34121	9.73577	9.85962	10.7868	11.9884	10.4021	0.451149628	0.966018836	4	8	24	0.17971649	0.768040178	24	23.05356469	0.057011127
ENSMUSG00000067369	Trmt2b	TRM2 tRNA methyltransferase 2B [Source:MGI Symbol;Acc:MGI:2442530]	12.0159	13.0268	12.5312	13.6693	12.2715	12.7722	12.6788	13.4617	12.1459	11.4454	12.4714	12.0903	12.5886	13.6662	0.373702442	0.896700272	8	12	24	0.391931227	0.799546792	24	4.435828212	0.037003952
ENSMUSG00000067370	B3galt4	UDP-Gal:betaGlcNAc beta 1,3-galactosyltransferase, polypeptide 4 [Source:MGI Symbol;Acc:MGI:1859517]	2.35	2.03535	1.70462	1.28834	2.14425	1.80171	1.26454	1.4063	1.72238	2.08805	1.85215	1.68962	1.65489	2.13918	0.879201926	0.966018836	20	8	24	0.955660344	0.988639288	24	18.24817839	0.021998043
ENSMUSG00000067377	Tspan6	tetraspanin 6 [Source:MGI Symbol;Acc:MGI:1926264]	7.67861	9.68024	9.74208	7.76459	9.06327	8.37427	9.03423	7.26579	8.99663	6.4043	8.12256	7.80324	7.87245	8.843	0.922035832	0.966018836	12	4	24	0.663462474	0.886493109	24	3.822764751	0.051674795
ENSMUSG00000067389	Gm17080	predicted gene 17080 [Source:MGI Symbol;Acc:MGI:4937907]	0.514122	0.387875	0.694945	0.807754	0.939438	0.618443	0.615244	0.630801	0.694775	0.546413	0.395097	0.694462	0.688902	0.334939	0.223125229	0.717349224	12	20	24	0.368078866	0.79131734	24	15.29768732	0.080712836
ENSMUSG00000067424	Zfp563	zinc finger protein 563 [Source:MGI Symbol;Acc:MGI:2677168]	4.69186	5.34573	3.93908	4.2054	5.66509	3.88245	5.20924	4.47058	4.3912	3.36334	4.61571	4.48775	5.23965	5.14326	0.105345531	0.518002936	4	12	24	0.208426542	0.768040178	24	23.91972699	0.115081458
ENSMUSG00000067430	Zfp763	zinc finger protein 763 [Source:MGI Symbol;Acc:MGI:1920701]	5.14728	6.50261	5.82855	5.36529	6.05728	5.27127	5.11915	5.77744	6.40292	4.72969	6.00476	5.79626	5.84524	6.03081	0.560714538	0.966018836	8	20	24	0.565337336	0.843471947	24	6.142641551	0.048655244
ENSMUSG00000067438	Hmx1	H6 homeobox 1 [Source:MGI Symbol;Acc:MGI:107178]	0.669034	0.488339	0.506383	0.602682	0.240386	0.464092	0.35533	0.731008	0.696383	0.691676	0.722056	0.811439	0.780302	0.864049	0.880631923	0.966018836	8	12	24	0.389632606	0.799546792	24	5.530071392	0.073549898
ENSMUSG00000067547	Gm7666	predicted pseudogene 7666 [Source:MGI Symbol;Acc:MGI:3648907]	67.0342	65.0993	65.0266	65.6183	68.3696	68.2034	64.6407	74.6859	63.7847	64.0075	74.0005	62.4128	67.7172	64.4554	0.745118544	0.966018836	20	16	24	0.83083692	0.94763783	24	19.06716998	0.019381912
ENSMUSG00000067567	Hdac8	histone deacetylase 8 [Source:MGI Symbol;Acc:MGI:1917565]	5.33613	5.04478	5.8113	5.14483	5.30897	4.75275	5.2376	4.77292	5.13079	5.37143	5.24321	5.33711	5.60995	6.29615	0.767868806	0.966018836	4	20	24	0.571571065	0.845961394	24	6.464253859	0.036044093
ENSMUSG00000067586	S1pr3	sphingosine-1-phosphate receptor 3 [Source:MGI Symbol;Acc:MGI:1339365]	35.0192	24.8937	25.7749	21.3471	30.5133	32.2524	32.6483	28.5096	17.6006	31.5921	28.7298	26.5374	30.6318	26.111	0.008389425	0.192760771	4	8	24	0.036016527	0.609176161	24	20.96299249	0.241601035
ENSMUSG00000067608	Pcna-ps2	proliferating cell nuclear antigen pseudogene 2 [Source:MGI Symbol;Acc:MGI:97505]	32.2116	34.4141	33.7541	34.4136	34.6671	31.0663	29.7615	27.8675	35.9471	28.3101	34.92	32.1525	33.6972	30.6863	0.373702442	0.896700272	20	8	24	0.607306642	0.863811892	24	13.66645816	0.04332901
ENSMUSG00000067629	Syngap1	synaptic Ras GTPase activating protein 1 homolog (rat) [Source:MGI Symbol;Acc:MGI:3039785]	9.44988	8.09103	8.86414	8.81222	10.0706	7.62888	10.7218	7.1098	7.96028	8.33778	8.30265	7.01404	7.4172	7.70994	0.767183862	0.966018836	20	4	24	0.702365925	0.901574817	24	16.508867	0.046942108
ENSMUSG00000067653	Ankrd23	ankyrin repeat domain 23 [Source:MGI Symbol;Acc:MGI:1925571]	10.2744	26.8151	30.1849	29.6855	18.0419	27.8602	24.8347	64.3042	50.2451	49.9976	19.838	24.4243	40.0763	27.7562	0.078207999	0.457568093	12	8	24	0.053085235	0.650879532	24	7.856057271	0.541270927
ENSMUSG00000067713	Prkag1	protein kinase, AMP-activated, gamma 1 non-catalytic subunit [Source:MGI Symbol;Acc:MGI:108411]	36.0906	35.9409	36.06	35.7587	34.3472	36.0445	35.6636	37.4636	34.5546	35.88	34.0407	37.6797	37.8059	34.862	0.074313825	0.448301997	24	20	24	0.214529855	0.768040178	24	0.501817944	0.030576989
ENSMUSG00000067714	Lpar5	lysophosphatidic acid receptor 5 [Source:MGI Symbol;Acc:MGI:2685918]	5.06266	2.50954	2.63113	4.06488	3.2789	4.10618	4.43936	3.65812	2.21222	4.60071	2.84472	3.98889	3.00649	3.42733	0.210566453	0.69936851	24	12	24	0.2449934	0.768040178	24	20.06813764	0.184833163
ENSMUSG00000067719	Gm10221	predicted gene 10221 [Source:MGI Symbol;Acc:MGI:3708709]	349.536	413.622	384.482	399.819	376.288	340.431	382.662	400.837	483.521	363.634	468.117	400.131	475.228	446.628	0.570349842	0.966018836	8	16	24	0.259739083	0.768040178	24	7.077466789	0.086016297

ENSMUSG00000067722	BC003965	cDNA sequence BC003965 [Source:MGI Symbol;Acc:MGI:2670966]	8.06276	8.11433	8.30844	8.18878	8.40397	8.27507	8.16965	8.38838	8.12184	7.88579	8.70731	8.60237	9.05297	8.42631	0.893307667	0.966018836	20	20	24	0.332757527	0.769110818	24	21.10066486	0.022593169
ENSMUSG00000067724	Gbx1	gastrulation brain homeobox 1 [Source:MGI Symbol;Acc:MGI:95667]	1.03935	0.699555	0.689211	0.744834	0.900393	0.881012	0.64278	0.687637	0.883702	1.05714	0.693607	0.909319	0.871976	0.891698	0.917610614	0.966018836	24	8	24	0.728412553	0.911205843	24	17.49915727	0.034120836
ENSMUSG00000067736	Gm10222	predicted gene 10222 [Source:MGI Symbol;Acc:MGI:3642643]	0.919558	3.53799	2.65202	3.04906	2.64343	3.34193	2.21815	0.267752	3.50622	1.82717	3.06961	3.42575	2.29918	3.40501	0.920992137	0.966018836	24	4	24	0.534332389	0.826328258	24	14.33674198	0.229904445
ENSMUSG00000067768	Xlr4b	X-linked lymphocyte-regulated 4B [Source:MGI Symbol;Acc:MGI:1350975]	0.652055	0.847007	0.404396	0.74774	0.320841	0.924694	0.530571	0.392863	0.266104	0.114823	0.387902	0.614634	0.71055	0.507402	0.14745801	0.590732968	24	20	24	0.057705806	0.656248849	24	22.73351226	0.187618811
ENSMUSG00000067786	Nnat	neuronatin [Source:MGI Symbol;Acc:MGI:104716]	36.2357	44.1472	39.5204	29.6105	33.9313	31.6874	37.5104	29.7079	36.336	26.5398	34.7149	41.2497	25.1397	41.5856	0.446653834	0.966018836	8	8	24	0.619737531	0.869740528	24	2.717652556	0.090420183
ENSMUSG00000067787	Blcap	bladder cancer associated protein [Source:MGI Symbol;Acc:MGI:1858907]	22.0497	20.4078	23.1408	21.1674	19.2654	23.3175	21.5921	21.922	19.7115	24.4724	20.2598	20.9904	21.153	21.7645	0.847312766	0.966018836	16	4	24	0.937627973	0.984533266	24	5.539105513	0.013610517
ENSMUSG00000067795	4930444P10Rik	RIKEN cDNA 4930444P10 gene [Source:MGI Symbol;Acc:MGI:1923049]	0.456033	0.500205	0.735542	0.615032	1.03009	0.702571	0.770124	0.657076	0.77082	0.593906	0.871145	0.791599	0.724844	0.675709	0.105345531	0.518002936	20	12	24	0.15483077	0.760257003	24	15.89269764	0.082613256
ENSMUSG00000067818	Myl9	myosin, light polypeptide 9, regulatory [Source:MGI Symbol;Acc:MGI:2138915]	36.3303	49.1194	42.2128	41.9657	54.1383	42.2528	45.445	53.0156	55.047	40.7583	42.1312	40.2999	47.704	39.594	0.928780444	0.966018836	12	12	24	0.686245894	0.895774045	24	7.578666173	0.06574871
ENSMUSG00000067825	Pex26	peroxisomal biogenesis factor 26 [Source:MGI Symbol;Acc:MGI:1921293]	3.9793	3.26454	3.69344	3.90259	3.6713	4.0205	3.88517	4.10254	3.45828	4.47941	4.28324	4.47068	4.36357	3.94974	0.377663653	0.903463289	24	12	24	0.157125955	0.763141258	24	18.46993298	0.064369256
ENSMUSG00000067847	Romo1	reactive oxygen species modulator 1 [Source:MGI Symbol;Acc:MGI:1914317]	75.5121	74.5081	70.8936	71.2279	61.7803	58.5835	69.6308	80.3159	85.0686	70.9885	95.9732	86.3344	88.1436	84.8716	0.813775138	0.966018836	8	16	24	0.491119107	0.805393068	24	5.125706867	0.075734801
ENSMUSG00000067851	Arfgef1	ADP-ribosylation factor guanine nucleotide-exchange factor 1(brefeldin A-inhibited) [Source:MGI Symbol;Acc:MGI:2442988]	41.1681	41.4859	38.2337	37.7417	39.6521	38.1821	33.6758	40.2684	37.2224	38.9968	40.1456	37.9863	41.8285	34.7059	0.908406451	0.966018836	8	4	24	0.88152255	0.966661503	24	18.68805145	0.018222368
ENSMUSG00000067869	Tcea1-ps1	transcription elongation factor A (SII) 1, pseudogene 1 [Source:MGI Symbol;Acc:MGI:1861432]	26.455	30.8353	27.8316	30.2569	31.2211	26.2643	29.1913	24.8747	30.5899	27.3004	31.4533	29.3084	29.181	28.5715	0.628286596	0.966018836	20	8	24	0.422465182	0.804917634	24	13.84678869	0.050600855
ENSMUSG00000067870	Rpl31-ps8	ribosomal protein L31, pseudogene 8 [Source:MGI Symbol;Acc:MGI:3647726]	93.2763	119.352	112.458	94.4982	108.1	95.343	95.7092	100.091	128.333	80.4422	106.379	104.388	121.055	110.47	0.369262716	0.893596186	12	4	24	0.404727267	0.799546792	24	4.841590595	0.093729812
ENSMUSG00000067873	Htatsf1	HIV TAT specific factor 1 [Source:MGI Symbol;Acc:MGI:1919709]	23.9695	26.2618	27.366	25.6312	26.347	24.7694	25.2658	27.5781	26.5011	26.9755	25.3091	26.0215	27.5987	28.6583	0.101520755	0.511659635	8	16	24	0.009492414	0.502965379	24	7.098420979	0.060993008
ENSMUSG00000067879	Vxn	vexin [Source:MGI Symbol;Acc:MGI:1924232]	0.294256	1.33095	2.29535	0.678106	0.653607	1.16722	1.48046	0.629629	1.39905	0.76976	0.571049	0.968662	0.277467	1.21592	0.275526571	0.789849983	12	8	24	0.352287001	0.780629136	24	7.41401641	0.222082091
ENSMUSG00000067889	Sptbn2	spectrin beta, non-erythrocytic 2 [Source:MGI Symbol;Acc:MGI:1313261]	13.4195	10.6571	11.8617	10.9334	11.4975	12.7031	13.0608	11.9321	9.67623	15.0484	11.2539	12.1994	10.925	12.6648	0.648082424	0.966018836	24	12	24	0.692888949	0.89819974	24	20.20381816	0.053149661
ENSMUSG00000067916	Zfp991	zinc finger protein 991 [Source:MGI Symbol;Acc:MGI:3701604]	1.49362	1.68813	1.60945	1.72358	1.50762	1.263	1.27828	1.24883	1.91259	1.44997	1.51694	1.16579	1.497	1.55769	0.010049466	0.194720062	12	12	24	0.031664837	0.604316769	24	9.017264686	0.111443495
ENSMUSG00000067924	Rtl8b	retrotransposon Gag like 8B [Source:MGI Symbol;Acc:MGI:3576504]	22.0144	13.9131	21.2011	17.3668	19.1317	17.5515	20.8501	21.2706	18.6267	23.8365	16.4408	14.5097	18.1033	20.5576	0.745469888	0.966018836	4	20	24	0.60735785	0.86383153	24	7.292110585	0.088777705
ENSMUSG00000067925	Rtl8a	retrotransposon Gag like 8A [Source:MGI Symbol;Acc:MGI:1913408]	12.1536	14.3545	16.5252	15.2036	9.63391	10.0192	16.9038	12.8268	9.38491	14.3549	9.29618	9.30514	9.82682	15.1491	0.141084603	0.585944911	16	4	24	0.117590858	0.728323355	24	6.221124457	0.23270495
ENSMUSG00000067928	Zfp760	zinc finger protein 760 [Source:MGI Symbol;Acc:MGI:2679257]	7.64544	8.24493	6.76879	7.34006	8.52893	6.94459	8.44159	7.03614	7.28011	5.52639	9.03755	7.66874	7.26415	7.14134	0.212359178	0.701530532	20	20	24	0.298353056	0.768040178	24	20.64637109	0.095638894
ENSMUSG00000067929	Gm10226	predicted gene 10226 [Source:MGI Symbol;Acc:MGI:3704470]	5.13335	7.26566	7.38942	6.55049	7.88881	7.37144	5.14722	4.45248	5.45107	4.25112	9.91111	7.944	4.40908	5.18013	0.043210264	0.357642487	24	4	24	0.092492091	0.71175955	24	16.35067402	0.259822329
ENSMUSG00000067931	Zfp948	zinc finger protein 948 [Source:MGI Symbol;Acc:MGI:3040683]	11.9441	12.6955	11.7017	12.2759	13.1757	10.9113	10.7522	10.5082	12.3272	8.81213	13.0521	11.4361	11.5287	12.8138	0.919571723	0.966018836	20	4	24	0.993920081	0.998755922	24	19.86448273	0.00663985
ENSMUSG00000067942	Zfp160	zinc finger protein 160 [Source:MGI Symbol;Acc:MGI:108187]	6.94882	9.08357	9.35278	8.88436	9.19896	7.12215	9.01433	7.71258	10.4246	7.26454	9.31441	8.23928	8.60774	9.82119	0.085890569	0.466404237	12	12	24	0.247247063	0.768040178	24	8.519009994	0.10496394
ENSMUSG00000067995	Gtf2f2	general transcription factor IIF, polypeptide 2 [Source:MGI Symbol;Acc:MGI:1915955]	11.4035	12.5918	11.4143	11.4082	12.3564	10.3938	10.5116	11.3248	12.3956	10.284	12.2011	12.8991	13.2823	12.3119	0.852844413	0.966018836	8	8	24	0.867671071	0.962111884	24	2.388811493	0.021906509
ENSMUSG00000068014	Gm14648	predicted gene 14648 [Source:MGI Symbol;Acc:MGI:3705508]	1.12746	1.64856	2.03941	1.84332	1.57194	1.12172	0.608601	1.00054	1.36101	0.994138	1.70488	1.29179	1.59796	1.0515	0.57057742	0.966018836	12	16	24	0.253168839	0.768040178	24	12.07973405	0.154474905
ENSMUSG00000068015	Lrch1	leucine-rich repeats and calponin homology (CH) domain containing 1 [Source:MGI Symbol;Acc:MGI:2443390]	6.14562	6.35654	6.00188	22.2385	5.74858	6.68643	6.5192	18.8581	5.85015	6.77293	5.37951	6.0454	5.39667	6.01486	0.908406451	0.966018836	16	4	24	0.607621008	0.864031555	24	9.153800253	0.238937739
ENSMUSG00000068036	Afdn	afadin, adherens junction formation factor [Source:MGI Symbol;Acc:MGI:1314653]	16.683	16.545	16.2762	14.6915	15.8629	15.3415	17.4017	17.1306	16.1438	17.1315	16.5144	14.9087	15.3211	16.7202	0.337748746	0.85758369	8	16	24	0.287161404	0.768040178	24	4.896275074	0.044697088
ENSMUSG00000068039	Tcp1	t-complex protein 1 [Source:MGI Symbol;Acc:MGI:98535]	153.547	147.103	144.713	142.694	151.954	149.36	142.411	140.608	144.533	139.43	159.363	140.928	155.933	151.961	0.510431574	0.966018836	20	20	24	0.457542991	0.805393068	24	20.5117856	0.030982047
ENSMUSG00000068040	Tm9sf4	transmembrane 9 superfamily member 4 [Source:MGI Symbol;Acc:MGI:2139220]	22.6353	18.8486	21.1015	16.6853	21.2523	22.0746	20.8813	20.6871	19.351	22.7391	19.0171	19.2432	21.2633	18.7187	0.741628485	0.966018836	4	4	24	0.496094233	0.807537659	24	21.72021831	0.057403936
ENSMUSG00000068099	1500009C09Rik	RIKEN cDNA 1500009C09 gene [Source:MGI Symbol;Acc:MGI:1923755]	2.39754	4.71921	8.04323	3.81137	3.17751	4.58676	5.19514	2.76683	5.09571	3.37455	2.55865	3.48416	2.0705	4.70468	0.266025655	0.773510818	12	8	24	0.273833466	0.768040178	24	7.75663837	0.262587245
ENSMUSG00000068101	Cenpm	centromere protein M [Source:MGI Symbol;Acc:MGI:1913820]	2.04039	1.51507	1.16704	2.46514	3.30333	1.8734	1.77239	2.11988	2.2252	1.45341	2.69408	1.87957	1.77191	1.56759	0.173121348	0.648600527	20	12	24	0.115916131	0.727799063	24	16.57024201	0.199943894
ENSMUSG00000068105	Tnfrsf13c	tumor necrosis factor receptor superfamily, member 13c [Source:MGI Symbol;Acc:MGI:1919299]	0.292451	0.598123	0.465136	0.677521	0.355355	0.487718	0.687295	0.733238	0.734737	0.534888	0.263941	0.314393	0.597753	0.689059	0.030972684	0.324150946	8	12	24	0.019823418	0.565108098	24	6.01970651	0.161741719
ENSMUSG00000068114	Ccdc134	coiled-coil domain containing 134 [Source:MGI Symbol;Acc:MGI:1923707]	6.81163	4.57579	5.29399	7.30285	5.96042	5.39525	6.65961	5.89158	5.83353	5.97744	6.11851	5.90895	5.64681	5.23308	0.146309959	0.590732968	16	16	24	0.405260634	0.799546792	24	16.56589153	0.07704582
ENSMUSG00000068115	Ninl	ninein-like [Source:MGI Symbol;Acc:MGI:1925427]	1.0824	1.87172	1.64409	2.00393	1.57737	1.86151	1.53769	2.61921	2.38881	1.77944	1.7054	2.09536	1.95265	1.66876	0.93717134	0.966018836	8	20	24	0.376869273	0.796956096	24	8.429776563	0.101683387
ENSMUSG00000068130	Zfp442	zinc finger protein 442 [Source:MGI Symbol;Acc:MGI:3651999]	0.659883	0.693838	0.724376	0.738196	0.943852	0.689449	0.754192	0.617535	0.955935	0.54164	0.743381	0.773683	0.609395	0.891458	0.95261238	0.966018836	20	8	24	0.84048152	0.951645964	24	11.81426149	0.023998874
ENSMUSG00000068134	Zfp120	zinc finger protein 120 [Source:MGI Symbol;Acc:MGI:1345179]	4.70561	5.89404	5.00668	5.27157	5.534	4.38531	4.96516	5.05214	6.15189	4.62264	6.3207	5.3786	5.08232	5.54898	0.669104912	0.966018836	20	4	24	0.523919883	0.821624369	24	9.790028203	0.058709267
ENSMUSG00000068141	Gm10232	predicted pseudogene 10232 [Source:MGI Symbol;Acc:MGI:3641637]	27.4708	31.4829	31.0997	24.0277	30.2337	29.4774	27.1072	26.0641	27.0683	24.3008	29.2745	27.2739	30.1389	27.2085	0.814077113	0.966018836	20	20	24	0.515941709	0.817497949	24	22.80711098	0.053637671
ENSMUSG00000068154	Insm1	insulinoma-associated 1 [Source:MGI Symbol;Acc:MGI:1859980]	1.60345	0.981048	0.938651	1.36866	0.964487	0.939486	1.23565	1.13892	1.01505	1.63726	1.58291	1.27374	1.3771	1.30503	0.954584434	0.966018836	16	20	24	0.810181771	0.939224719	24	17.3825338	0.040976703
ENSMUSG00000068184	Ndufaf2	NADH:ubiquinone oxidoreductase complex assembly factor 2 [Source:MGI Symbol;Acc:MGI:1922847]	44.2887	44.3058	46.7325	39.1355	44.2082	39.9169	45.9966	42.2495	47.296	37.604	41.3581	40.4469	46.0706	46.2793	0.004003768	0.140598674	12	4	24	0.0623691	0.661961741	24	3.565539918	0.088259099
ENSMUSG00000068196	Col8a1	collagen, type VIII, alpha 1 [Source:MGI Symbol;Acc:MGI:88463]	5.38944	6.45551	5.71144	4.48968	7.36979	4.87236	5.22367	6.303	6.36574	3.62812	5.98926	4.937	4.77752	5.43671	0.289105414	0.794869797	12	4	24	0.835840671	0.949722382	24	3.584683816	0.050676978
ENSMUSG00000068200	Gm5931	predicted pseudogene 5931 [Source:MGI Symbol;Acc:MGI:3647036]	9.19425	9.82296	8.45361	10.2581	8.47947	10.215	11.7857	10.083	7.10895	11.3164	10.0765	9.63219	7.97488	10.0429	0.914502381	0.966018836	16	20	24	0.77322564	0.927135738	24	19.49602014	0.053553372
ENSMUSG00000068205	Macrod2	mono-ADP ribosylhydrolase 2 [Source:MGI Symbol;Acc:MGI:1920149]	20.8727	17.9357	17.9503	18.834	18.427	18.7878	19.1211	16.8339	17.6827	19.4169	20.1424	19.3236	18.7781	20.5965	0.351840494	0.870418653	24	12	24	0.365328925	0.789589804	24	19.5084927	0.046261265
ENSMUSG00000068206	Pick1	protein interacting with C kinase 1 [Source:MGI Symbol;Acc:MGI:894645]	19.3153	21.8051	17.9823	18.5125	21.204	20.9635	19.919	20.1429	17.9762	21.1655	19.6512	19.0186	18.4005	20.0053	0.886952527	0.966018836	8	4	24	0.702305417	0.901574817	24	19.65700563	0.030454361
ENSMUSG00000068220	Lgals1	lectin, galactose binding, soluble 1 [Source:MGI Symbol;Acc:MGI:96777]	295.636	328.388	326.959	304.776	381.754	297.718	277.625	294.058	336.936	221.094	355.762	286.665	322.452	288.552	0.581070934	0.966018836	20	8	24	0.898633947	0.971322226	24	16.78947597	0.034704711
ENSMUSG00000068240	Gm11808	predicted gene 11808 [Source:MGI Symbol;Acc:MGI:3649356]	154.936	159.997	167.295	146.841	159.495	146.932	134.307	138.45	155.221	123.967	148.424	141.72	166.187	161.98	0.977455189	0.977820713	12	4	24	0.63366333	0.875436572	24	3.676889808	0.046670793
ENSMUSG00000068245	Phf11d	PHD finger protein 11D [Source:MGI Symbol;Acc:MGI:1277133]	1.61873	1.3519	1.62611	1.01233	1.70839	1.74219	1.81609	1.93433	2.19343	1.10605	1.89561	1.30045	1.77603	1.50281	0.941885246	0.966018836	12	4	24	0.542373295	0.83094351	24	1.09265467	0.08094529
ENSMUSG00000068250	Amn1	antagonist of mitotic exit network 1 [Source:MGI Symbol;Acc:MGI:2442933]	22.3873	19.9778	17.5328	22.2723	19.174	17.6245	19.5016	19.3813	19.515	16.2908	22.0315	19.8733	22.961	18.6017	0.70641939	0.966018836	4	8	24	0.522109758	0.820717128	24	21.93238502	0.064897621
ENSMUSG00000068263	Efcc1	EF hand and coiled-coil domain containing 1 [Source:MGI Symbol;Acc:MGI:3611451]	4.46523	4.02017	3.60437	4.32061	4.35306	3.32695	4.51866	2.98702	4.30191	4.21212	4.07572	3.95852	3.95614	3.82226	0.784278077	0.966018836	20	12	24	0.65370256	0.883325058	24	15.70382604	0.048804033
ENSMUSG00000068264	Ap5s1	adaptor-related protein 5 complex, sigma 1 subunit [Source:MGI Symbol;Acc:MGI:1916846]	6.83598	7.62505	6.43435	8.30796	8.67405	8.83649	7.33796	8.67058	7.36433	9.71554	8.11717	8.3677	7.8154	7.69634	0.15498249	0.607427992	16	20	24	0.133713698	0.744007455	24	15.5373939	0.097306932
ENSMUSG00000068267	Cenpb	centromere protein B [Source:MGI Symbol;Acc:MGI:88376]	21.2364	22.3687	21.7326	22.3385	22.4707	20.2589	21.8261	24.7432	24.6064	26.2823	21.3739	23.8476	24.0451	23.5791	0.61767505	0.966018836	16	4	24	0.091159662	0.71175955	24	8.113456288	0.070475427
ENSMUSG00000068270	Shroom4	shroom family member 4 [Source:MGI Symbol;Acc:MGI:2685570]	1.12358	1.09375	1.37387	1.20951	1.39176	1.20504	1.11915	1.43747	1.3142	1.71364	1.31838	1.20176	1.2751	1.32139	0.188783717	0.664348865	16	12	24	0.032434563	0.606585237	24	11.07804798	0.085777453
ENSMUSG00000068284	Usf3	upstream transcription factor family member 3 [Source:MGI Symbol;Acc:MGI:2685454]	2.86232	3.42727	3.17274	3.6908	3.52304	3.28287	3.47489	3.18061	3.81258	3.12785	3.38342	3.43999	3.4583	3.40235	0.776152743	0.966018836	12	20	24	0.556827805	0.838702296	24	11.50669051	0.033264669
ENSMUSG00000068290	Ddrgk1	DDRGK domain containing 1 [Source:MGI Symbol;Acc:MGI:1924256]	38.5098	40.0761	41.107	40.9548	35.8332	38.3568	41.5059	43.9061	44.0943	41.9917	40.963	40.511	42.1916	39.6146	0.308023687	0.814993005	12	12	24	0.068852551	0.673424097	24	6.753775394	0.062822825
ENSMUSG00000068299	Nat8f4	N-acetyltransferase 8 (GCN5-related) family member 4 [Source:MGI Symbol;Acc:MGI:1922791]	1.63514	1.63801	2.19258	1.31148	1.7436	2.10263	1.72738	1.47679	1.88931	1.88837	1.47766	1.69943	1.32431	2.00777	0.934152439	0.966018836	12	16	24	0.844310915	0.952833645	24	8.277021722	0.035545438
ENSMUSG00000068327	Tlx2	T cell leukemia, homeobox 2 [Source:MGI Symbol;Acc:MGI:1350935]	15.196	9.32662	7.59953	9.49195	8.94989	9.30516	9.21322	13.7097	10.7981	16.5191	17.8466	18.5573	20.2764	18.1058	0.92655264	0.966018836	4	8	24	0.553365097	0.837286821	24	22.46363589	0.135601452
ENSMUSG00000068328	Aup1	ancient ubiquitous protein 1 [Source:MGI Symbol;Acc:MGI:107789]	44.4449	44.5228	41.5904	40.96	47.812	41.0839	44.7196	47.4128	49.8385	44.1808	43.3014	45.0287	41.675	45.2334	0.851656735	0.966018836	8	8	24	0.677494232	0.892060593	24	6.453634122	0.030207574
ENSMUSG00000068329	Htra2	HtrA serine peptidase 2 [Source:MGI Symbol;Acc:MGI:1928676]	20.2319	19.0838	17.3989	21.661	20.6202	17.4888	20.1309	20.2853	21.5639	21.4525	18.053	20.294	21.0594	18.4237	0.843639218	0.966018836	16	16	24	0.851980244	0.955609701	24	10.87682845	0.024393145
ENSMUSG00000068335	Dok1	docking protein 1 [Source:MGI Symbol;Acc:MGI:893587]	7.0713	5.47431	6.08754	5.40821	6.22056	6.06765	4.50625	6.2483	5.98274	6.84914	6.01506	6.62651	6.73616	5.40328	0.9143748	0.966018836	4	4	24	0.736052846	0.913997413	24	16.93925279	0.046372935
ENSMUSG00000068373	D430041D05Rik	RIKEN cDNA D430041D05 gene [Source:MGI Symbol;Acc:MGI:2181743]	7.55323	7.26775	6.72602	6.50955	9.00656	6.42685	9.15406	7.31231	7.07278	8.27158	7.14652	6.96902	8.16251	6.59018	0.481530854	0.966018836	4	20	24	0.691886728	0.897856241	24	20.44745528	0.052843444
ENSMUSG00000068391	Chrac1	chromatin accessibility complex 1 [Source:MGI Symbol;Acc:MGI:2135796]	17.9005	22.0245	20.542	21.2314	20.7749	19.7776	20.0463	21.5587	23.2963	20.0696	19.2704	19.1743	21.2239	21.2952	0.008754183	0.192760771	8	16	24	0.025593971	0.586541342	24	7.523022196	0.088932342
ENSMUSG00000068394	Cep152	centrosomal protein 152 [Source:MGI Symbol;Acc:MGI:2139083]	0.79723	0.92349	0.91399	1.14139	0.968644	0.783183	0.854353	0.939633	0.971642	0.866513	0.975944	0.849672	0.814149	0.947614	0.019643089	0.258568924	20	4	24	0.021439696	0.573684034	24	10.65313476	0.068011514
ENSMUSG00000068396	Rpl34-ps1	ribosomal protein L34, pseudogene 1 [Source:MGI Symbol;Acc:MGI:3704270]	163.513	190.22	176.9	171.181	171.404	148.593	168.198	175.816	247.425	148.869	215.021	182.493	210.613	199.732	0.838532766	0.966018836	12	12	24	0.376165576	0.796215348	24	6.603228871	0.104910974
ENSMUSG00000068457	Uty	ubiquitously transcribed tetratricopeptide repeat containing, Y-linked [Source:MGI Symbol;Acc:MGI:894810]	8.44429	8.9305	8.27214	8.59851	8.12978	7.11546	7.89972	8.00256	9.28983	7.1745	8.49264	7.80634	7.16771	9.30305	0.047000349	0.375011045	12	12	24	0.168238526	0.767020009	24	7.192569901	0.083854969
ENSMUSG00000068479	Mfap1a	microfibrillar-associated protein 1A [Source:MGI Symbol;Acc:MGI:1914782]	6.96045	7.12416	6.74457	7.23551	6.90382	6.43648	6.5343	7.16997	6.86344	6.55137	7.00167	6.93752	7.35554	7.56635	0.349109869	0.870418653	8	16	24	0.336243912	0.771737966	24	4.930543108	0.032517875
ENSMUSG00000068522	Aard	alanine and arginine rich domain containing protein [Source:MGI Symbol;Acc:MGI:2181621]	3.58185	3.74571	4.07878	2.54111	3.01541	3.33522	3.72014	3.18306	2.79251	3.84749	2.72199	3.89752	2.98309	3.26057	0.920872048	0.966018836	24	20	24	0.496227968	0.807537659	24	1.233409478	0.074253172
ENSMUSG00000068523	Gng5	guanine nucleotide binding protein (G protein), gamma 5 [Source:MGI Symbol;Acc:MGI:109164]	7.47013	7.13053	8.01246	7.24012	4.59041	6.57596	6.86236	7.4375	9.83967	6.80076	8.58619	6.32298	11.1878	7.39293	0.236893212	0.7385769	12	12	24	0.22417376	0.768040178	24	5.203270973	0.167619501
ENSMUSG00000068551	Zfp467	zinc finger protein 467 [Source:MGI Symbol;Acc:MGI:1916160]	5.23246	7.915	8.10428	8.1343	6.27379	6.90372	7.34499	6.5247	9.21648	8.01673	8.7981	7.74136	6.89241	7.02606	0.046820236	0.375011045	12	16	24	0.04525942	0.628927515	24	10.73783259	0.162331419
ENSMUSG00000068566	Myadm	myeloid-associated differentiation marker [Source:MGI Symbol;Acc:MGI:1355332]	43.2019	29.8773	32.6913	31.1222	33.7273	38.3123	37.0935	37.3932	25.8261	41.8378	33.8556	34.9009	35.3319	34.5449	0.058157448	0.40386543	4	8	24	0.210204846	0.768040178	24	20.98483191	0.130345509
ENSMUSG00000068580	Zfyve19	zinc finger, FYVE domain containing 19 [Source:MGI Symbol;Acc:MGI:1919258]	32.8743	36.041	28.3873	29.754	32.9664	28.0166	33.9665	29.6411	31.4581	29.1345	29.0527	29.9125	31.5097	30.312	0.325142889	0.842977406	4	20	24	0.237682249	0.768040178	24	1.204893248	0.062147839
ENSMUSG00000068614	Actc1	actin, alpha, cardiac muscle 1 [Source:MGI Symbol;Acc:MGI:87905]	2.4821	6.4518	1.71251	6.40955	4.16062	6.10432	7.24672	20.1283	13.9122	8.59728	2.90066	3.09919	3.09899	2.45096	0.877082091	0.966018836	8	20	24	0.563903406	0.842860066	24	7.706584426	0.380629204
ENSMUSG00000068615	Gjd2	gap junction protein, delta 2 [Source:MGI Symbol;Acc:MGI:1334209]	0.888932	0.801279	1.28478	0.622243	0.6916	1.10744	1.17592	0.733713	0.84822	1.15483	0.614187	0.822967	0.628695	1.08852	0.959836008	0.967043964	12	8	24	0.682612473	0.89423365	24	4.885121558	0.061379582
ENSMUSG00000068617	Efcab1	EF-hand calcium binding domain 1 [Source:MGI Symbol;Acc:MGI:1914043]	4.81102	4.99798	4.03329	3.91003	3.78283	3.9689	4.28619	4.05379	4.13291	3.56879	4.43369	4.54281	4.04777	4.91341	0.016398461	0.253395174	8	8	24	0.077046269	0.687041208	24	1.396484965	0.096317219
ENSMUSG00000068631	Gm7676	predicted gene 7676 [Source:MGI Symbol;Acc:MGI:3644053]	0.5164	1.42831	0.558939	2.00814	2.42733	1.69913	1.24736	1.81067	1.56639	0.341933	0.865518	0.676005	1.4034	0.609334	0.8635847	0.966018836	20	8	24	0.901039799	0.972137578	24	17.58080079	0.078868843
ENSMUSG00000068663	Clec16a	C-type lectin domain family 16, member A [Source:MGI Symbol;Acc:MGI:1921624]	11.4023	10.2808	12.5785	12.5402	11.0506	12.7591	12.2066	12.3455	10.098	12.8072	13.2008	11.4124	13.1152	10.9424	0.428730128	0.966018836	16	16	24	0.4461701	0.805393068	24	17.00296056	0.058957786
ENSMUSG00000068686	Cd59b	CD59b antigen [Source:MGI Symbol;Acc:MGI:1888996]	2.28476	3.17939	2.70828	2.31486	2.23805	3.27607	3.39361	3.39028	2.74515	1.99462	3.19454	1.62185	2.86243	2.47629	0.531498969	0.966018836	8	8	24	0.391318894	0.799546792	24	2.686580457	0.11896299
ENSMUSG00000068697	Myoz1	myozenin 1 [Source:MGI Symbol;Acc:MGI:1929471]	13.0456	43.07	46.725	63.1157	34.5897	47.5313	33.976	99.0906	86.2415	81.8653	36.1632	42.4325	47.0049	34.2338	0.203788871	0.689899499	12	16	24	0.056902624	0.65610184	24	9.582983122	0.607006005
ENSMUSG00000068699	Flnc	filamin C, gamma [Source:MGI Symbol;Acc:MGI:95557]	1.79342	2.20024	2.94294	3.09863	2.95572	2.94663	2.62272	5.37094	3.28349	5.34084	2.74679	2.46241	3.07121	2.56018	0.26715327	0.774280302	16	12	24	0.167303678	0.767020009	24	10.19213691	0.227967191
ENSMUSG00000068706	Gm10250	predicted pseudogene 10250 [Source:MGI Symbol;Acc:MGI:3642408]	334.16	387.805	372.484	375.926	376.118	357.955	353.491	365.895	413	323.042	405.917	355.337	417.648	394.551	0.914360142	0.966018836	12	12	24	0.646198736	0.880190017	24	6.851309971	0.040028896
ENSMUSG00000068732	Tmem167b	transmembrane protein 167B [Source:MGI Symbol;Acc:MGI:1914745]	28.3915	27.2239	25.9816	25.625	26.6641	26.1284	27.2188	26.3046	23.9808	26.1979	27.6499	25.8085	26.667	26.967	0.075936367	0.453790923	4	8	24	0.128067572	0.740162193	24	22.15903336	0.042552235
ENSMUSG00000068735	Trp53i11	transformation related protein 53 inducible protein 11 [Source:MGI Symbol;Acc:MGI:2670995]	5.98574	8.40502	6.02552	4.71041	5.4431	6.4988	6.13829	6.9683	8.52372	4.29882	6.1409	7.7037	5.29514	7.90617	0.092593422	0.487919468	8	8	24	0.160901566	0.765941695	24	3.01780154	0.183466488
ENSMUSG00000068739	Sars	seryl-aminoacyl-tRNA synthetase [Source:MGI Symbol;Acc:MGI:102809]	94.3053	78.7093	85.5422	78.1831	77.0576	86.2924	80.7709	82.5806	73.9879	84.483	82.7189	82.7277	82.9567	83.6768	0.723119329	0.966018836	24	20	24	0.275508404	0.768040178	24	22.5001498	0.051965035
ENSMUSG00000068740	Celsr2	cadherin, EGF LAG seven-pass G-type receptor 2 [Source:MGI Symbol;Acc:MGI:1858235]	7.65995	6.61894	7.9567	7.45709	7.63672	7.30295	7.51208	7.68775	8.97087	8.37286	6.67075	7.67649	7.32538	6.98365	0.500660563	0.966018836	12	20	24	0.373110228	0.794388449	24	9.951921085	0.058241459
ENSMUSG00000068742	Cry2	cryptochrome 2 (photolyase-like) [Source:MGI Symbol;Acc:MGI:1270859]	18.062	16.6914	18.712	21.3887	17.7881	18.4278	17.0074	16.9094	19.5569	25.7543	16.5935	18.502	16.7585	17.7972	0.057707116	0.403601759	16	16	24	0.04284977	0.624864512	24	11.84904997	0.146739657
ENSMUSG00000068744	Psrc1	proline/serine-rich coiled-coil 1 [Source:MGI Symbol;Acc:MGI:1913099]	1.18776	0.789605	0.678072	0.496457	0.949728	0.588444	0.711528	1.08271	0.710337	1.0621	0.694517	0.597578	1.23633	0.78429	0.10338739	0.517709628	4	20	24	0.469809165	0.805393068	24	1.853773457	0.08722464
ENSMUSG00000068747	Sort1	sortilin 1 [Source:MGI Symbol;Acc:MGI:1338015]	16.8711	16.5529	18.1467	16.3315	16.7769	17.3806	20.1762	18.2479	16.1025	18.3651	13.6087	17.3295	16.6629	16.8677	0.951945551	0.966018836	24	20	24	0.415484193	0.800637996	24	2.445090869	0.060765587
ENSMUSG00000068748	Ptprz1	protein tyrosine phosphatase, receptor type Z, polypeptide 1 [Source:MGI Symbol;Acc:MGI:97816]	13.8188	14.2364	16.5973	13.2432	13.7699	14.898	15.1864	13.1236	12.7287	14.2668	14.0209	12.7832	11.9097	13.6897	0.710703678	0.966018836	12	20	24	0.908161195	0.974606327	24	11.52815438	0.017455491
ENSMUSG00000068749	Psma5	proteasome subunit alpha 5 [Source:MGI Symbol;Acc:MGI:1347009]	12.9453	16.9732	15.1652	14.353	17.9009	14.8985	15.2204	13.7574	16.2867	12.5956	16.5625	14.2031	14.6232	15.1517	0.935593607	0.966018836	20	8	24	0.883618333	0.966818623	24	13.98454474	0.026748809
ENSMUSG00000068758	Il3ra	interleukin 3 receptor, alpha chain [Source:MGI Symbol;Acc:MGI:96553]	2.28688	2.30131	2.21386	2.19065	2.18798	2.20891	1.29191	2.54393	2.70452	1.95527	2.56108	2.77572	2.71528	2.23794	0.945728192	0.966018836	24	16	24	0.947981909	0.987280175	24	10.3573437	0.023708112
ENSMUSG00000068762	Gstm6	glutathione S-transferase, mu 6 [Source:MGI Symbol;Acc:MGI:1309467]	3.16839	3.44381	3.97066	3.06841	2.52579	2.30125	3.03663	2.66103	2.69981	1.96058	2.39982	3.18798	2.5129	3.10736	0.465479825	0.966018836	12	4	24	0.182533988	0.768040178	24	3.973325182	0.123138278
ENSMUSG00000068794	Col28a1	collagen, type XXVIII, alpha 1 [Source:MGI Symbol;Acc:MGI:2685312]	26.4562	27.3754	25.0502	25.9508	28.1865	25.5533	26.849	25.8116	25.397	28.6769	27.5281	25.7658	26.5829	26.9615	0.628286596	0.966018836	16	20	24	0.592271589	0.856446446	24	15.61025795	0.02257324
ENSMUSG00000068798	Rap1a	RAS-related protein 1a [Source:MGI Symbol;Acc:MGI:97852]	28.1507	29.947	27.4355	27.3396	27.4631	26.7058	32.3059	26.5107	26.0971	21.3365	27.8635	23.2246	26.378	22.4417	0.95261238	0.966018836	4	12	24	0.332563619	0.769110818	24	23.08919868	0.070657797
ENSMUSG00000068823	Csde1	cold shock domain containing E1, RNA binding [Source:MGI Symbol;Acc:MGI:92356]	89.5347	94.4096	90.7674	96.2391	95.6919	88.3739	89.0469	94.4323	99.9489	90.7483	99.736	95.1473	103.712	93.0423	0.93717134	0.966018836	20	4	24	0.751046315	0.91921305	24	10.91176905	0.018762783
ENSMUSG00000068854	H2bc21	H2B clustered histone 21 [Source:MGI Symbol;Acc:MGI:2448415]	4.02794	3.8058	2.94604	3.55039	3.36103	3.23958	3.50605	3.69319	3.48601	3.68151	3.77294	4.24364	3.79689	3.54049	0.608967886	0.966018836	4	8	24	0.353950526	0.781118317	24	22.02872219	0.05673697
ENSMUSG00000068856	Sf3b4	splicing factor 3b, subunit 4 [Source:MGI Symbol;Acc:MGI:109580]	3.00747	3.16789	3.61726	3.43969	3.11259	2.98005	3.78575	3.51281	3.06403	4.53788	3.23853	3.35759	3.30461	3.16791	0.868508291	0.966018836	16	8	24	0.407793816	0.799546792	24	10.41939544	0.06694911
ENSMUSG00000068874	Selenbp1	selenium binding protein 1 [Source:MGI Symbol;Acc:MGI:96825]	1.80906	2.9825	3.58938	4.28683	3.30671	2.6797	2.82882	3.63738	4.31965	3.41003	2.81349	2.89212	3.25282	2.78419	0.018263889	0.256345714	12	16	24	0.010102717	0.503967859	24	10.02111144	0.24364413
ENSMUSG00000068876	Cgn	cingulin [Source:MGI Symbol;Acc:MGI:1927237]	0.50211	0.582819	0.710935	0.491107	0.431849	0.65831	0.557943	0.581063	0.736298	0.628272	0.423855	0.590782	0.47078	0.591436	0.03782572	0.334069441	12	8	24	0.176955506	0.768040178	24	6.743641647	0.064771501
ENSMUSG00000068882	Ssb	Sjogren syndrome antigen B [Source:MGI Symbol;Acc:MGI:98423]	81.3775	85.7733	79.546	78.5864	81.7095	74.7892	78.6188	71.847	81.7221	70.3176	87.8761	82.4467	89.1443	81.1548	0.924770496	0.966018836	20	20	24	0.685000778	0.895269122	24	22.20353193	0.034695842
ENSMUSG00000068917	Clk2	CDC-like kinase 2 [Source:MGI Symbol;Acc:MGI:1098669]	14.5438	13.7693	14.5345	15.3329	15.2539	13.8441	15.1306	14.5514	15.8479	14.0126	14.9708	14.4267	15.1915	13.6995	0.664568268	0.966018836	12	20	24	0.661152004	0.885520687	24	13.49586462	0.021995582
ENSMUSG00000068921	Dap3	death associated protein 3 [Source:MGI Symbol;Acc:MGI:1929538]	44.6777	47.8907	47.592	48.0862	45.7832	48.8073	46.8944	47.04	45.6824	43.5809	47.087	46.6985	49.8592	49.5458	0.91695334	0.966018836	8	8	24	0.432534823	0.805393068	24	1.493019625	0.025946015
ENSMUSG00000068922	Msto1	misato 1, mitochondrial distribution and morphology regulator [Source:MGI Symbol;Acc:MGI:2385175]	10.4544	10.6587	12.0208	10.6745	10.1538	11.5018	10.2617	11.2148	9.97058	12.2597	10.13	11.0816	11.2889	10.4764	0.880247609	0.966018836	16	4	24	0.890206492	0.969000219	24	9.570521459	0.017345237
ENSMUSG00000068923	Syt11	synaptotagmin XI [Source:MGI Symbol;Acc:MGI:1859547]	272.889	214.426	226.709	227.854	218.235	265.191	240.124	247.661	171.118	287.696	216.865	237.245	227.095	239.111	0.636884864	0.966018836	4	8	24	0.455078337	0.805393068	24	20.78429191	0.092765721
ENSMUSG00000068959	Zfp619	zinc finger protein 619 [Source:MGI Symbol;Acc:MGI:1917477]	0.660637	0.808251	0.808183	0.558819	0.801536	0.713213	0.766447	0.815167	0.849329	0.706068	0.81497	0.86769	0.794954	0.728759	0.94663657	0.966018836	12	4	24	0.801940292	0.936565832	24	1.986152042	0.017386717
ENSMUSG00000068966	Zbtb34	zinc finger and BTB domain containing 34 [Source:MGI Symbol;Acc:MGI:2685195]	1.9063	1.7801	1.95919	2.00881	2.2614	1.67568	2.04993	1.65396	2.22509	1.97029	1.69787	1.86755	2.00942	1.88366	0.699015352	0.966018836	12	20	24	0.596210409	0.857839743	24	11.31209588	0.037401403
ENSMUSG00000069011	Gm10254	predicted gene 10254 [Source:MGI Symbol;Acc:MGI:3708673]	27.2897	26.3197	27.7048	30.2885	27.4079	24.1161	23.358	23.8569	27.7278	25.274	30.2325	25.8585	27.8716	27.5282	0.279886222	0.792139038	20	4	24	0.236520028	0.768040178	24	12.15479681	0.072190216
ENSMUSG00000069020	Urm1	ubiquitin related modifier 1 [Source:MGI Symbol;Acc:MGI:1915455]	38.4142	32.776	33.0894	31.934	35.0451	35.9651	32.493	33.9059	30.6827	30.3582	32.9454	33.9747	31.1941	30.8212	0.166168222	0.633011401	24	16	24	0.005285044	0.4424469	24	20.66015093	0.084521554
ENSMUSG00000069045	Ddx3y	DEAD box helicase 3, Y-linked [Source:MGI Symbol;Acc:MGI:1349406]	22.3302	24.479	22.9005	23.9453	21.3923	19.5636	22.7829	19.656	25.7339	18.5865	23.0717	18.8652	17.5958	21.8268	0.245061943	0.741580974	12	12	24	0.262018722	0.768040178	24	8.526662212	0.090882646
ENSMUSG00000069049	Eif2s3y	eukaryotic translation initiation factor 2, subunit 3, structural gene Y-linked [Source:MGI Symbol;Acc:MGI:1349430]	36.4462	43.2553	36.6139	41.3129	30.09	34.3844	38.6433	33.9969	42.9569	35.804	39.3781	35.0533	38.0178	30.2903	0.888991156	0.966018836	12	12	24	0.704144406	0.902338136	24	7.853967823	0.053516816
ENSMUSG00000069072	Slc7a14	solute carrier family 7 (cationic amino acid transporter, y+ system), member 14 [Source:MGI Symbol;Acc:MGI:3040688]	19.8159	17.2205	18.3707	17.9643	17.7611	19.5653	16.915	16.525	14.2625	20.3755	18.1221	19.3245	17.544	20.3442	0.92655264	0.966018836	16	20	24	0.467848256	0.805393068	24	18.82841953	0.065991232

ENSMUSG00000069089	Cdk7	cyclin-dependent kinase 7 [Source:MGI Symbol;Acc:MGI:102956]	19.3166	21.1388	20.1642	22.1338	20.2513	19.2028	19.6525	18.2662	20.5346	19.5295	19.5685	19.9768	20.1428	19.5457	0.70641939	0.966018836	12	20	24	0.3583729	0.784979786	24	11.52618872	0.033926828
ENSMUSG00000069094	Pde7a	phosphodiesterase 7A [Source:MGI Symbol;Acc:MGI:1202402]	7.98852	8.23407	9.0797	10.3624	8.40957	7.77029	9.23824	7.45977	8.72421	6.96622	10.1445	8.2204	8.18307	7.85801	0.644063931	0.966018836	20	12	24	0.521261688	0.82025681	24	14.05607975	0.062956846
ENSMUSG00000069114	Zbtb10	zinc finger and BTB domain containing 10 [Source:MGI Symbol;Acc:MGI:2139883]	5.12541	4.67706	4.42246	4.90351	4.61648	4.22268	4.62246	4.05373	4.45759	4.63729	4.84343	4.60946	4.44354	4.65766	0.9143748	0.966018836	16	20	24	0.667289521	0.887459929	24	16.56740988	0.024843697
ENSMUSG00000069117	Rps18-ps6	ribosomal protein S18, pseudogene 6 [Source:MGI Symbol;Acc:MGI:3642298]	8.23735	10.3692	10.4731	9.24874	10.8739	9.62394	8.33273	9.67398	10.5386	6.78886	8.58804	8.09634	7.34807	9.68732	0.102109143	0.512431169	12	16	24	0.51604273	0.817621333	24	7.877475487	0.080339432
ENSMUSG00000069135	Cep43	centrosomal protein 43 [Source:MGI Symbol;Acc:MGI:1922546]	15.1028	13.2734	13.1192	13.946	14.9715	13.431	13.8029	12.346	12.843	11.112	16.1118	12.2042	13.6472	11.8111	0.280850951	0.792139038	20	12	24	0.143703243	0.752803875	24	18.75194505	0.095486773
ENSMUSG00000069136	A130006I12Rik	RIKEN cDNA A130006I12 gene [Source:MGI Symbol;Acc:MGI:2441774]	0.635747	0.675014	0.18314	0.357814	0.699748	0.530525	0.578006	0.296254	0.323442	0.755822	1.23609	0.319149	0.345934	0.51289	0.285027487	0.793714531	20	16	24	0.261277381	0.768040178	24	17.18727734	0.152298342
ENSMUSG00000069171	Nr2f1	nuclear receptor subfamily 2, group F, member 1 [Source:MGI Symbol;Acc:MGI:1352451]	7.36968	5.72179	5.62518	6.21433	7.63376	5.74783	7.79294	6.09638	5.22309	5.62822	8.39122	6.50027	6.9842	5.11809	0.018923071	0.256345714	20	16	24	0.03401983	0.607131058	24	19.28163599	0.18775639
ENSMUSG00000069184	Zfp72	zinc finger protein 72 [Source:MGI Symbol;Acc:MGI:3033356]	2.9541	3.75997	3.46893	3.46038	2.9526	3.03507	3.21101	3.01344	3.48755	2.61845	3.49954	2.95003	3.78112	2.99198	0.869721097	0.966018836	12	4	24	0.654532865	0.883569804	24	5.162054713	0.044312407
ENSMUSG00000069206	Zfp874a	zinc finger protein 874a [Source:MGI Symbol;Acc:MGI:3040703]	8.51292	9.4465	8.35564	8.76512	9.2072	8.37169	8.78543	8.00244	9.85456	6.74059	9.75426	8.39064	9.40445	9.40775	0.896753532	0.966018836	12	12	24	0.818842826	0.942446688	24	1.246107573	0.032105059
ENSMUSG00000069208	Zfp825	zinc finger protein 825 [Source:MGI Symbol;Acc:MGI:2385315]	8.34303	9.64061	8.22727	8.96321	8.48952	8.43131	8.66068	7.12815	9.08068	7.03963	8.29165	9.46079	8.99635	9.05036	0.935593607	0.966018836	8	8	24	0.652252868	0.88299052	24	23.4253712	0.044212999
ENSMUSG00000069227	Gprin1	G protein-regulated inducer of neurite outgrowth 1 [Source:MGI Symbol;Acc:MGI:1349455]	2.32692	1.93683	2.13231	1.7228	1.7994	1.99044	2.03096	2.07006	1.69836	2.40208	1.7119	2.12255	2.22357	2.38474	0.622773557	0.966018836	4	16	24	0.230961402	0.768040178	24	1.895387401	0.073645341
ENSMUSG00000069237	Fam8a1	family with sequence similarity 8, member A1 [Source:MGI Symbol;Acc:MGI:2145496]	37.5863	33.044	31.7864	33.1271	31.8323	32.6915	32.8824	32.2203	29.2675	32.3605	33.1303	31.4072	32.5537	33.6377	0.595952838	0.966018836	4	8	24	0.21135745	0.768040178	24	22.58633194	0.047702224
ENSMUSG00000069255	Dusp22	dual specificity phosphatase 22 [Source:MGI Symbol;Acc:MGI:1915926]	18.9252	15.6282	14.0938	17.6468	16.8214	16.8633	17.161	17.6614	14.9953	19.5694	16.6325	17.6873	18.8138	19.1427	0.43565473	0.966018836	4	8	24	0.487941936	0.805393068	24	21.34543157	0.057808445
ENSMUSG00000069300	H2bc11	H2B clustered histone 11 [Source:MGI Symbol;Acc:MGI:2448388]	1.73855	1.75911	1.45114	1.05813	1.1828	0.971502	1.62352	1.1857	1.36133	1.50363	1.45443	1.81204	1.43774	1.63645	0.630352198	0.966018836	4	16	24	0.315334489	0.768040178	24	1.956336916	0.092191527
ENSMUSG00000069372	Ctxn3	cortexin 3 [Source:MGI Symbol;Acc:MGI:3642816]	98.8573	99.8046	87.078	95.1997	94.5543	96.4941	87.7034	87.1594	92.9275	87.825	101.659	99.9675	103.375	97.7058	0.278774299	0.790764844	24	12	24	0.242577723	0.768040178	24	20.59795222	0.056581041
ENSMUSG00000069378	Prdm6	PR domain containing 6 [Source:MGI Symbol;Acc:MGI:2684938]	1.41033	1.58448	1.05218	0.868115	1.04433	0.930727	1.38918	1.39724	2.41805	0.685152	1.67023	1.09875	1.07202	1.07513	0.338172159	0.85758369	8	8	24	0.496614055	0.8075609	24	4.234371252	0.120451144
ENSMUSG00000069379	Gm4950	predicted pseudogene 4950 [Source:MGI Symbol;Acc:MGI:3649015]	38.6007	41.3471	40.782	38.2328	43.4503	40.6115	34.2962	36.1329	46.3696	32.8726	48.9889	37.5512	39.9371	40.0946	0.724067924	0.966018836	20	8	24	0.723165532	0.909188168	24	13.72285612	0.048426066
ENSMUSG00000069495	Epc2	enhancer of polycomb homolog 2 [Source:MGI Symbol;Acc:MGI:1278321]	9.52188	9.647	8.59153	8.62121	9.88255	9.07557	7.55924	7.95531	9.05229	8.94227	9.33067	8.97634	8.97686	8.37732	0.483881213	0.966018836	20	12	24	0.292528554	0.768040178	24	16.36111782	0.056033898
ENSMUSG00000069515	Lyz1	lysozyme 1 [Source:MGI Symbol;Acc:MGI:96902]	0.757534	1.22909	1.09875	0.787937	0.676634	0.903198	0.880924	1.13222	1.43033	0.953152	1.04365	1.56122	1.46375	1.23469	0.575202741	0.966018836	8	12	24	0.156046894	0.761695095	24	4.370279815	0.111164119
ENSMUSG00000069516	Lyz2	lysozyme 2 [Source:MGI Symbol;Acc:MGI:96897]	99.1985	135.429	152.946	128.873	135.68	114.128	128.49	120.857	144.753	66.7763	135.607	118.536	132.982	99.1109	0.515451644	0.966018836	12	12	24	0.964378413	0.991159965	24	2.823135222	0.031900132
ENSMUSG00000069520	Tmem19	transmembrane protein 19 [Source:MGI Symbol;Acc:MGI:1914476]	19.5662	17.5905	17.7695	17.8033	18.2844	18.9859	17.4012	19.1201	16.623	21.3213	15.715	18.428	17.6197	18.9754	0.875755063	0.966018836	24	12	24	0.972574515	0.993250534	24	1.62428342	0.009609205
ENSMUSG00000069539	Scyl2	SCY1-like 2 (S. cerevisiae) [Source:MGI Symbol;Acc:MGI:1289172]	15.0233	13.6067	14.2843	13.3599	14.7881	14.3521	13.496	12.9089	11.2388	13.6809	14.668	13.7526	14.7651	13.1188	0.079174302	0.458678242	20	12	24	0.041199813	0.61831089	24	19.44637748	0.092548977
ENSMUSG00000069565	Dazap1	DAZ associated protein 1 [Source:MGI Symbol;Acc:MGI:1917498]	26.4472	23.3199	24.8287	28.8764	26.7123	26.0457	29.7633	28.3656	25.6742	28.2583	30.282	28.0622	27.9362	26.7236	0.602156008	0.966018836	16	20	24	0.258993118	0.768040178	24	17.22237793	0.05619497
ENSMUSG00000069581	Tspear	thrombospondin type laminin G domain and EAR repeats [Source:MGI Symbol;Acc:MGI:2671932]	0.627132	0.562009	0.885616	0.771757	0.466564	0.490965	0.560959	0.663689	0.390087	0.455059	0.563363	0.393589	0.486989	0.808764	0.409451058	0.94909271	8	16	24	0.247585629	0.768040178	24	6.518385574	0.087439738
ENSMUSG00000069601	Ank3	ankyrin 3, epithelial [Source:MGI Symbol;Acc:MGI:88026]	98.4773	79.4613	91.712	87.0604	80.6059	97.3439	89.5083	90.5189	66.3467	111.799	75.0982	89.6132	83.1618	87.5691	0.924770496	0.966018836	24	20	24	0.813850166	0.940622376	24	20.43028214	0.048475245
ENSMUSG00000069631	Strada	STE20-related kinase adaptor alpha [Source:MGI Symbol;Acc:MGI:1919399]	10.7116	10.1765	10.3994	10.8441	10.0085	11.6316	10.5062	11.1102	9.67001	11.3699	9.6061	10.6668	10.5981	11.0034	0.883051071	0.966018836	24	20	24	0.776647952	0.928308257	24	23.65095602	0.021375167
ENSMUSG00000069633	Pex11g	peroxisomal biogenesis factor 11 gamma [Source:MGI Symbol;Acc:MGI:1920905]	1.37472	2.10939	1.66858	2.12017	1.77989	1.64518	2.22309	2.69661	1.72314	1.54683	2.01874	1.84639	1.67964	2.63607	0.37215823	0.894599261	8	16	24	0.335699391	0.771466587	24	5.11342364	0.104303729
ENSMUSG00000069662	Marcks	myristoylated alanine rich protein kinase C substrate [Source:MGI Symbol;Acc:MGI:96907]	32.4718	33.7251	31.7341	24.5316	31.2756	29.511	35.6397	32.6359	30.523	29.4039	32.4457	30.2836	32.1279	33.0257	0.001953237	0.100255953	8	8	24	0.042420832	0.623698598	24	1.306876454	0.107205753
ENSMUSG00000069670	Nkain2	Na+/K+ transporting ATPase interacting 2 [Source:MGI Symbol;Acc:MGI:1923447]	18.761	17.8538	17.3447	16.284	18.8501	14.7589	17.9156	14.5491	19.105	16.0087	17.1095	17.0237	16.0075	15.4917	0.90767227	0.966018836	12	20	24	0.931666207	0.982352286	24	14.98625784	0.016699394
ENSMUSG00000069678	Pcgf1	polycomb group ring finger 1 [Source:MGI Symbol;Acc:MGI:1917087]	26.3163	25.5235	20.8716	28.5644	27.927	25.3458	26.5587	25.9998	28.1627	28.7501	33.9328	28.4163	30.903	28.4748	0.539040394	0.966018836	16	20	24	0.222440855	0.768040178	24	16.7952139	0.079963242
ENSMUSG00000069682	Gm10275	predicted pseudogene 10275 [Source:MGI Symbol;Acc:MGI:3704354]	278.157	334.037	311.225	300.045	319.061	282.221	283.293	288.108	396.57	245.057	351.933	304.478	335.525	317.518	0.95261238	0.966018836	12	12	24	0.725905567	0.909996439	24	7.676892919	0.055942524
ENSMUSG00000069727	Zfp975	zinc finger protein 975 [Source:MGI Symbol;Acc:MGI:3648690]	2.25265	2.61643	2.63167	2.0881	2.54528	2.25718	2.36669	2.30621	2.78239	1.8998	2.72925	2.79566	2.70914	2.36204	0.897570341	0.966018836	12	4	24	0.806717986	0.938416757	24	23.8983802	0.030596269
ENSMUSG00000069729	Arid1b	AT rich interactive domain 1B (SWI-like) [Source:MGI Symbol;Acc:MGI:1926129]	5.72696	4.59585	4.66705	4.94263	4.89174	5.5774	5.38717	5.38683	4.38011	6.62729	4.60437	5.4048	5.30266	5.27287	0.08302755	0.462922111	24	16	24	0.609777208	0.864522156	24	20.76487246	0.053869194
ENSMUSG00000069743	Zfp820	zinc finger protein 820 [Source:MGI Symbol;Acc:MGI:1922674]	2.36588	2.66564	2.29818	2.00999	2.59586	2.22437	2.23794	2.23941	2.36149	1.67599	2.65261	2.41327	2.37767	2.64619	0.718086778	0.966018836	20	20	24	0.433283953	0.805393068	24	23.8384111	0.062321272
ENSMUSG00000069744	Psmb3	proteasome (prosome, macropain) subunit, beta type 3 [Source:MGI Symbol;Acc:MGI:1347014]	25.6345	31.8149	29.5337	30.7868	30.5366	27.9775	25.7497	28.2276	33.3858	22.0407	34.4095	30.3146	31.619	28.1087	0.36381114	0.966018836	20	8	24	0.932983874	0.982999447	24	12.59216626	0.024651101
ENSMUSG00000069755	Zfp125	zinc finger protein 125 [Source:MGI Symbol;Acc:MGI:1336211]	3.0651	3.30768	3.31659	3.00365	2.92906	2.92149	4.03681	2.80646	3.55283	2.82463	3.30672	3.01247	3.36202	3.59533	0.29491602	0.805301811	12	4	24	0.274724663	0.768040178	24	3.318829397	0.070128722
ENSMUSG00000069763	Tmem100	transmembrane protein 100 [Source:MGI Symbol;Acc:MGI:1915138]	28.3614	25.8966	24.0064	22.9949	30.7795	26.7407	25.568	23.0058	20.64	21.5222	30.6131	21.0989	21.5687	21.365	0.251986462	0.75129457	20	16	24	0.056202103	0.65534544	24	18.56028098	0.148286361
ENSMUSG00000069769	Msi2	musashi RNA-binding protein 2 [Source:MGI Symbol;Acc:MGI:1923876]	12.4566	13.4734	16.693	13.279	11.9385	13.0988	13.2154	12.9077	13.1973	16.0641	12.6111	12.9067	13.0758	14.7005	0.029825002	0.323426482	16	4	24	0.071906503	0.678310325	24	8.33776104	0.110786061
ENSMUSG00000069792	Wfdc17	WAP four-disulfide core domain 17 [Source:MGI Symbol;Acc:MGI:3649773]	4.54231	4.93104	2.93568	3.9285	4.24381	3.70344	3.287	2.98101	6.17623	1.57314	3.24542	2.99016	3.04423	4.02478	0.860560779	0.966018836	8	8	24	0.668360696	0.887912848	24	2.928313536	0.116887606
ENSMUSG00000069805	Fbp1	fructose bisphosphatase 1 [Source:MGI Symbol;Acc:MGI:95492]	0.934062	1.05423	0.786778	1.45654	1.24238	1.10649	1.14099	0.703363	0.968582	1.35346	0.852149	1.07303	1.24806	1.47931	0.874607729	0.966018836	16	20	24	0.774333474	0.927520217	24	14.84886404	0.04549229
ENSMUSG00000069806	Cacng7	calcium channel, voltage-dependent, gamma subunit 7 [Source:MGI Symbol;Acc:MGI:1932374]	23.3891	19.5972	22.9925	20.3067	18.6867	23.2731	22.2123	21.3577	19.422	26.6406	20.7063	21.2157	19.5616	22.2769	0.860560779	0.966018836	24	20	24	0.994453404	0.998755922	24	10.00097768	0.005835802
ENSMUSG00000069808	Tlcd3a	TLC domain containing 3A [Source:MGI Symbol;Acc:MGI:2151840]	19.8399	14.8095	18.4748	24.4933	24.2851	19.7152	17.7856	14.2813	17.9679	22.8576	20.2831	18.7927	15.6657	13.4434	1.62E-05	0.014856385	16	16	24	1.77E-05	0.047905911	24	15.2093883	0.306068333
ENSMUSG00000069814	Ccdc92b	coiled-coil domain containing 92B [Source:MGI Symbol;Acc:MGI:3588240]	5.34808	4.66499	4.74486	5.1769	4.6535	5.49202	4.53673	5.1816	4.2564	6.56648	4.93691	5.65247	5.0931	5.24447	0.390686537	0.915479136	24	12	24	0.621235748	0.870701383	24	16.80771026	0.049922582
ENSMUSG00000069833	Ahnak	AHNAK nucleoprotein (desmoyokin) [Source:MGI Symbol;Acc:MGI:1316648]	80.9529	79.7581	72.4977	83.3172	88.9352	75.778	79.8673	72.5149	88.4613	74.4669	85.8015	85.0386	87.4631	78.2548	0.305564675	0.811201458	20	12	24	0.399840839	0.799546792	24	17.59753492	0.053959463
ENSMUSG00000069835	Sat2	spermidine/spermine N1-acetyl transferase 2 [Source:MGI Symbol;Acc:MGI:1916465]	7.49153	9.45762	9.66543	8.31718	9.131	8.26025	7.26294	8.17845	10.2201	8.7801	7.4344	9.49082	8.44711	10.0629	0.105280783	0.518002936	12	16	24	0.137050841	0.747972781	24	8.21547263	0.111193697
ENSMUSG00000069844	Sco1	SCO1 cytochrome c oxidase assembly protein [Source:MGI Symbol;Acc:MGI:106362]	4.32563	3.86319	4.04745	4.49624	4.0025	4.36727	4.67972	4.07072	3.98938	4.85484	4.93443	4.6237	4.56984	4.5634	0.198504154	0.680216953	16	20	24	0.197208914	0.768040178	24	17.66008373	0.052862865
ENSMUSG00000069855	Slc47a2	solute carrier family 47, member 2 [Source:MGI Symbol;Acc:MGI:3588190]	2.16395	1.76607	1.5571	1.62949	1.7503	2.1037	1.42097	2.04123	1.56715	1.65351	1.69205	1.9414	1.69799	1.76074	0.080164531	0.458678242	24	12	24	0.207753076	0.768040178	24	21.73312015	0.077770764
ENSMUSG00000069874	Irgm2	immunity-related GTPase family M member 2 [Source:MGI Symbol;Acc:MGI:1926262]	6.63402	5.66662	6.0109	5.1364	6.23387	5.78897	5.50476	7.93527	4.87217	5.37651	5.47327	4.7322	4.67257	7.01756	0.591351901	0.966018836	8	8	24	0.43448813	0.805393068	24	3.805332785	0.092769972
ENSMUSG00000069895	Atxn1l	ataxin 1-like [Source:MGI Symbol;Acc:MGI:3694797]	6.06638	5.74639	5.90094	6.18661	5.93101	6.03387	6.67449	6.53633	5.5963	6.9286	5.90707	6.38237	6.06746	6.17901	0.949874888	0.966018836	4	8	24	0.930866473	0.982245205	24	20.28137426	0.011303888
ENSMUSG00000069899	Gm12166	predicted gene 12166 [Source:MGI Symbol;Acc:MGI:3650635]	14.1367	15.3449	14.8859	15.063	14.5538	14.3222	14.8701	13.5947	15.2859	11.7516	16.8901	14.2012	13.8139	13.2584	0.916746631	0.966018836	12	20	24	0.810480364	0.939224719	24	17.33313152	0.028885816
ENSMUSG00000069910	Spdl1	spindle apparatus coiled-coil protein 1 [Source:MGI Symbol;Acc:MGI:1917635]	0.895938	0.727625	0.551951	0.667426	0.744507	0.617901	0.557957	0.774908	0.735536	0.789433	0.618824	0.84802	0.718247	0.615247	0.806004771	0.966018836	16	20	24	0.849205189	0.954760051	24	20.93161526	0.021240989
ENSMUSG00000069911	Insyn2b	inhibitory synaptic factor family member 2B [Source:MGI Symbol;Acc:MGI:3643491]	2.43039	2.37277	2.09873	1.69953	2.01999	2.20416	2.22043	2.09247	1.68206	2.24398	1.80531	2.22061	2.24382	2.36654	0.082807861	0.462922111	8	4	24	0.028958416	0.595626873	24	0.25019852	0.10708412
ENSMUSG00000069913	Anp32-ps	acidic (leucine-rich) nuclear phosphoprotein 32 family, pseudogene [Source:MGI Symbol;Acc:MGI:3525200]	15.2072	14.719	14.1568	13.0535	14.2653	15.4792	16.0367	10.7364	15.1332	12.075	16.3265	13.351	13.522	15.2224	0.718720526	0.966018836	20	20	24	0.555278804	0.838355251	24	20.68509639	0.069527099
ENSMUSG00000069917	Hba-a2	hemoglobin alpha, adult chain 2 [Source:MGI Symbol;Acc:MGI:96016]	62.8991	149.579	157.49	146.545	133.034	124.55	46.3937	196.558	204.792	108.052	113.787	163.414	231.677	135.711	0.68441981	0.966018836	12	8	24	0.34657225	0.778844143	24	8.767869817	0.348644281
ENSMUSG00000069919	Hba-a1	hemoglobin alpha, adult chain 1 [Source:MGI Symbol;Acc:MGI:96015]	116.833	283.33	298.616	304.079	210.246	33.2897	243.797	282.081	327.803	144.207	212.064	286.359	314.387	169.843	0.602156008	0.966018836	12	12	24	0.242974028	0.768040178	24	7.554308798	0.580875987
ENSMUSG00000069920	B3gnt9	UDP-GlcNAc:betaGal beta-1,3-N-acetylglucosaminyltransferase 9 [Source:MGI Symbol;Acc:MGI:2142841]	1.50262	2.31124	1.89923	1.23162	1.63833	1.30488	1.88848	2.24199	2.55644	1.45095	1.91678	2.06042	1.85375	2.04399	0.040123816	0.34094463	12	4	24	0.070498245	0.67524425	24	4.825874435	0.161692974
ENSMUSG00000069939	Gm12070	predicted gene 12070 [Source:MGI Symbol;Acc:MGI:3650751]	0.805086	0.529524	0.721029	0.760687	0.815528	0.672276	0.404499	0.721003	0.452861	0.531894	0.390853	0.452849	0.536176	0.388865	0.874672901	0.966018836	16	16	24	0.730651122	0.912261334	24	15.64799857	0.032810625
ENSMUSG00000069972	Rps13-ps2	ribosomal protein S13, pseudogene 2 [Source:MGI Symbol;Acc:MGI:3704295]	27.4043	33.6718	31.2343	32.5151	33.8396	30.0612	27.213	32.3794	36.1496	33.7792	31.4029	31.7216	32.9362	30.9464	0.16079538	0.620479606	16	12	24	0.041496604	0.618736234	24	10.41079874	0.097970535
ENSMUSG00000070000	Fcho1	FCH domain only 1 [Source:MGI Symbol;Acc:MGI:1921265]	6.39189	6.02839	7.12139	8.73233	7.18358	6.91986	7.41706	7.57968	7.66268	8.12116	7.02419	6.89841	7.06388	7.2237	0.036424767	0.332409444	16	8	24	0.044080414	0.628008091	24	11.39123423	0.101321974
ENSMUSG00000070002	Ell	elongation factor RNA polymerase II [Source:MGI Symbol;Acc:MGI:109377]	4.84312	4.57846	5.15311	5.04051	4.74581	4.31499	4.79996	5.17446	5.59929	5.28258	4.50299	4.79892	5.23657	4.65707	0.1321346	0.567728672	16	4	24	0.070313384	0.675025526	24	8.122594169	0.074554161
ENSMUSG00000070003	Ssbp4	single stranded DNA binding protein 4 [Source:MGI Symbol;Acc:MGI:1924150]	12.8466	14.5032	17.3563	17.3148	11.5309	11.5899	13.1442	19.8912	19.2834	22.0521	19.8149	27.3677	18.7909	21.3529	0.832506215	0.966018836	16	4	24	0.179546835	0.768040178	24	8.89540472	0.187020952
ENSMUSG00000070031	Sp140	Sp140 nuclear body protein [Source:MGI Symbol;Acc:MGI:3702467]	0.67699	1.04134	0.675657	1.32183	0.828955	0.982777	1.25163	1.21162	1.94005	0.649702	0.876402	0.702627	1.01368	0.948833	0.852772142	0.966018836	8	12	24	0.420443276	0.804069238	24	6.604455741	0.12338139
ENSMUSG00000070034	Sp110	Sp110 nuclear body protein [Source:MGI Symbol;Acc:MGI:1923364]	0.760721	1.22241	1.75917	0.735635	1.10171	1.29612	1.08378	1.44694	1.48941	0.72694	1.21619	1.41317	0.923197	1.18037	0.129903614	0.560263689	12	4	24	0.680097978	0.892992059	24	5.527173155	0.074402986
ENSMUSG00000070044	Fam149a	family with sequence similarity 149, member A [Source:MGI Symbol;Acc:MGI:2387177]	10.8033	8.78458	9.29085	8.98224	9.08957	10.5104	9.05898	10.2015	7.43257	11.0916	8.54895	9.99978	9.47631	10.3323	0.916746631	0.966018836	24	12	24	0.515286448	0.817300608	24	21.9449215	0.065753296
ENSMUSG00000070047	Fat1	FAT atypical cadherin 1 [Source:MGI Symbol;Acc:MGI:109168]	6.06496	6.92002	6.92485	6.79811	8.0096	7.26159	6.59378	7.38396	6.8763	7.66646	5.5604	6.45886	5.84029	5.93231	0.644063931	0.966018836	16	12	24	0.389802814	0.799546792	24	12.0512365	0.066619776
ENSMUSG00000070056	Mfhas1	malignant fibrous histiocytoma amplified sequence 1 [Source:MGI Symbol;Acc:MGI:1098644]	13.1209	7.98381	8.0204	8.22481	8.14793	9.7557	9.79756	10.2154	7.13952	12.629	9.06853	10.1541	10.22	9.62635	0.018894761	0.256345714	4	8	24	0.317227022	0.768040178	24	21.85665249	0.135537975
ENSMUSG00000070271	Gm13268	predicted gene 13268 [Source:MGI Symbol;Acc:MGI:3651947]	0.29759	0.827364	0.645568	0.33555	0.984424	0.497572	1.08296	0.658532	0.706096	0.393691	0.504719	0.480954	0.300367	0.571197	0.928892845	0.966018836	8	20	24	0.889297064	0.968820559	24	4.509836615	0.0419198
ENSMUSG00000070282	3000002C10Rik	RIKEN cDNA 3000002C10 gene [Source:MGI Symbol;Acc:MGI:3036226]	0.987646	1.36369	1.4834	1.29545	1.40481	1.22094	0.917494	1.65155	1.45286	1.0253	0.747841	1.45947	1.34918	1.02452	0.588208558	0.966018836	12	16	24	0.578259558	0.849207394	24	6.555433497	0.073757867
ENSMUSG00000070283	Ndufaf3	NADH:ubiquinone oxidoreductase complex assembly factor 3 [Source:MGI Symbol;Acc:MGI:1913956]	21.4637	20.5919	21.2557	19.4881	22.2694	23.3166	17.6588	20.798	21.6798	21.464	23.1107	18.6351	21.3732	16.92	0.105345531	0.518002936	20	12	24	0.267961608	0.768040178	24	15.44142326	0.078218173
ENSMUSG00000070284	Gmppb	GDP-mannose pyrophosphorylase B [Source:MGI Symbol;Acc:MGI:2660880]	6.99542	5.54836	6.83283	5.05001	5.78242	6.11897	6.53539	5.37976	4.85086	5.2323	4.99795	4.95303	5.44419	5.5512	0.522551072	0.966018836	4	12	24	0.1283702	0.740162193	24	23.92113782	0.091229379
ENSMUSG00000070287	Slc35g2	solute carrier family 35, member G2 [Source:MGI Symbol;Acc:MGI:2685365]	15.6399	16.3065	16.4429	14.0406	14.5499	14.8037	14.7964	14.3884	14.3475	13.5778	15.6139	15.4877	17.0237	16.456	0.115745527	0.537734429	4	12	24	0.133584021	0.743843563	24	1.318109123	0.068129431
ENSMUSG00000070304	Scn2b	sodium channel, voltage-gated, type II, beta [Source:MGI Symbol;Acc:MGI:106921]	10.9648	9.26627	10.7469	8.87412	8.37197	10.4567	10.34	8.83366	7.99266	11.0506	8.1519	10.7826	8.7721	11.6157	0.93717134	0.966018836	24	20	24	0.589528357	0.854298478	24	0.715859926	0.068141976
ENSMUSG00000070305	Mpzl3	myelin protein zero-like 3 [Source:MGI Symbol;Acc:MGI:2442647]	3.62084	3.27358	3.78022	3.07522	3.54539	3.63322	4.10333	3.06078	2.73169	2.78273	3.6549	3.40775	3.81315	3.11631	0.06180207	0.409814092	4	12	24	0.027365952	0.590220615	24	21.33568902	0.129172419
ENSMUSG00000070306	Ccdc153	coiled-coil domain containing 153 [Source:MGI Symbol;Acc:MGI:2448587]	0.702262	4.0482	5.08	2.31868	3.22509	2.0665	4.17465	0.844274	2.0621	2.18462	1.12724	3.87847	0.147713	3.88153	0.887862353	0.966018836	12	16	24	0.704246381	0.902338136	24	10.14036594	0.243338851
ENSMUSG00000070315	4930581F22Rik	RIKEN cDNA 4930581F22 gene [Source:MGI Symbol;Acc:MGI:1926184]	3.12869	5.92661	6.8278	5.97014	5.72474	5.42002	6.13029	5.03966	6.78424	4.83621	5.51533	6.71227	5.71739	6.10818	0.625825704	0.966018836	12	16	24	0.471701365	0.805393068	24	9.763873092	0.110272856
ENSMUSG00000070319	Eif3g	eukaryotic translation initiation factor 3, subunit G [Source:MGI Symbol;Acc:MGI:1858258]	60.9624	53.7296	56.1728	52.5667	52.9302	55.5904	54.5995	58.7989	53.6279	55.9505	53.0516	54.6604	57.9755	53.9163	0.048396595	0.379129806	4	16	24	0.123495281	0.736432529	24	0.972198562	0.046213154
ENSMUSG00000070327	Rnf213	ring finger protein 213 [Source:MGI Symbol;Acc:MGI:1289196]	11.3201	13.893	12.3279	13.3071	13.8047	12.5317	12.6821	14.491	14.9032	11.8615	13.4307	12.5	12.3379	12.6641	0.72778087	0.966018836	8	20	24	0.411673586	0.799546792	24	8.934339106	0.056179679
ENSMUSG00000070336	Fbxo47	F-box protein 47 [Source:MGI Symbol;Acc:MGI:1920223]	0.45569	0.614737	0.931406	0.778807	0.67885	0.675478	0.560287	0.511592	0.89519	0.458187	0.66473	0.80374	0.722363	0.575919	0.321662653	0.83607295	12	20	24	0.517928387	0.818483315	24	11.7123688	0.05883072
ENSMUSG00000070337	Gpr179	G protein-coupled receptor 179 [Source:MGI Symbol;Acc:MGI:2443409]	0.628591	0.514815	0.513274	0.519694	0.434897	0.66086	0.544721	0.527337	0.445747	0.573589	0.456075	0.756465	0.544116	0.630808	0.021214536	0.264116537	24	20	24	0.159512338	0.765591735	24	22.32925915	0.059298756
ENSMUSG00000070343	Gm10288	predicted gene 10288 [Source:MGI Symbol;Acc:MGI:3704227]	238.137	282.977	274.835	269.158	283.853	249.282	238.931	257.146	327.972	217.5	300.137	256.668	294.64	270.554	0.636884864	0.966018836	12	12	24	0.65398484	0.883343578	24	8.862662923	0.058423461
ENSMUSG00000070348	Ccnd1	cyclin D1 [Source:MGI Symbol;Acc:MGI:88313]	14.8052	13.0974	12.8908	10.8162	16.2654	13.6864	15.3757	12.9166	12.9812	11.2763	15.2004	12.5608	12.1221	12.5283	0.129903614	0.560263689	20	20	24	0.163910398	0.767020009	24	20.52117792	0.114483824
ENSMUSG00000070366	Plpp4	phospholipid phosphatase 4 [Source:MGI Symbol;Acc:MGI:2685936]	3.04963	2.98819	3.47202	3.05668	2.66018	3.842	3.10612	2.96525	1.89463	2.91359	3.13787	2.93029	2.75145	2.62054	0.834424969	0.966018836	24	8	24	0.270702452	0.768040178	24	19.74050942	0.101840547
ENSMUSG00000070371	Prss36	protease, serine 36 [Source:MGI Symbol;Acc:MGI:1924863]	7.63075	7.17419	6.62384	7.32413	7.96246	7.69472	6.46314	7.08494	7.37115	7.34525	7.82082	7.04366	6.96427	7.78042	0.231491055	0.731037655	20	8	24	0.326821803	0.768040178	24	15.72419573	0.045440485
ENSMUSG00000070372	Capza1	capping protein (actin filament) muscle Z-line, alpha 1 [Source:MGI Symbol;Acc:MGI:106227]	12.5769	12.4883	12.9815	12.8686	13.3553	12.6759	13.2145	11.0279	12.2926	11.3211	13.971	11.9539	12.4278	11.6679	0.068668966	0.432567661	20	12	24	0.162538595	0.76636332	24	17.35765257	0.060021835
ENSMUSG00000070385	Ampd1	adenosine monophosphate deaminase 1 [Source:MGI Symbol;Acc:MGI:88015]	4.94894	13.4899	16.7951	18.3418	11.1493	17.0288	10.5691	30.6321	37.6285	36.2159	9.9637	16.2942	21.0438	15.5765	0.203345222	0.689605535	16	4	24	0.04134753	0.618736234	24	8.984820163	0.622695319
ENSMUSG00000070394	Tmem256	transmembrane protein 256 [Source:MGI Symbol;Acc:MGI:1916436]	121.123	125.77	121.989	124.555	125.309	108.717	117.451	120.362	140.452	96.2348	142.464	114.491	136.269	116.952	0.913451828	0.966018836	20	4	24	0.937020303	0.984474875	24	5.471356212	0.02187
ENSMUSG00000070407	Hs3st3b1	heparan sulfate (glucosamine) 3-O-sulfotransferase 3B1 [Source:MGI Symbol;Acc:MGI:1333853]	0.882962	0.716947	0.562215	0.615913	0.728748	0.671901	0.590917	0.644554	0.530739	0.58305	0.754604	0.686515	0.546988	0.701054	0.275526571	0.789849983	20	16	24	0.187039209	0.768040178	24	20.52104095	0.057064429
ENSMUSG00000070420	Zscan25	zinc finger and SCAN domain containing 25 [Source:MGI Symbol;Acc:MGI:3647079]	3.76109	4.29644	3.6702	2.93521	3.35652	4.05395	3.82482	4.22778	3.98836	4.79156	3.71316	4.26689	4.04317	4.23078	0.397301892	0.927993503	8	12	24	0.3345116	0.770673394	24	3.040959952	0.06995423
ENSMUSG00000070423	Olfr558	olfactory receptor 558 [Source:MGI Symbol;Acc:MGI:3030392]	0.508788	0.518691	0.553797	0.269816	0.445499	0.550089	0.823854	0.581133	0.461434	0.581179	0.522612	0.514743	0.49281	0.416863	0.801280095	0.966018836	24	20	24	0.304306582	0.768040178	24	23.70065945	0.067264246
ENSMUSG00000070424	Art5	ADP-ribosyltransferase 5 [Source:MGI Symbol;Acc:MGI:107948]	0.449959	1.1167	1.11523	1.27723	0.956535	0.8603	0.562328	1.81431	1.85884	1.95412	0.918114	0.914838	1.06956	1.07811	0.002437897	0.109215643	16	12	24	0.003324435	0.416356043	24	9.326473983	0.32629186
ENSMUSG00000070425	Xntrpc	Xndc1-transient receptor potential cation channel, subfamily C, member 2 readthrough [Source:MGI Symbol;Acc:MGI:5546370]	0.595755	1.2411	0.940163	1.02175	0.886845	0.659094	0.604706	0.556532	0.609464	1.00451	0.61416	0.732955	0.600581	0.43585	0.029756552	0.323426482	16	16	24	0.135507242	0.747573586	24	12.19519439	0.114684265
ENSMUSG00000070426	Rnf121	ring finger protein 121 [Source:MGI Symbol;Acc:MGI:1922462]	12.5386	13.4527	12.5071	12.3214	14.2447	12.5277	13.8797	12.681	12.6819	11.6869	14.6506	13.5898	12.7364	12.6491	0.058209189	0.40386543	20	20	24	0.255504938	0.768040178	24	19.3464199	0.055375848
ENSMUSG00000070427	Il18bp	interleukin 18 binding protein [Source:MGI Symbol;Acc:MGI:1333800]	2.64256	2.83908	3.12333	2.60759	3.29927	2.78227	3.45756	3.03382	3.13401	2.22713	2.64178	2.44183	2.40994	2.77157	0.676344318	0.966018836	12	4	24	0.705236189	0.902806616	24	4.205833934	0.042160376
ENSMUSG00000070436	Serpinh1	serine (or cysteine) peptidase inhibitor, clade H, member 1 [Source:MGI Symbol;Acc:MGI:88283]	99.0161	62.307	50.7041	46.1631	88.7617	62.0075	83.8516	78.5828	56.7448	44.5559	87.7159	61.6197	73.5336	53.478	0.016899511	0.254502248	4	12	24	0.037670461	0.611100708	24	21.63208706	0.340362854
ENSMUSG00000070461	9230112E08Rik	RIKEN cDNA 9230112E08 gene [Source:MGI Symbol;Acc:MGI:2444774]	3.87172	2.92818	2.73568	3.26723	3.18562	3.65411	3.50203	3.31474	2.5916	3.4242	3.70513	3.58732	3.27467	3.43749	0.009630739	0.194720062	24	12	24	0.015612273	0.542177229	24	20.11828296	0.127733066
ENSMUSG00000070462	Tlnrd1	talin rod domain containing 1 [Source:MGI Symbol;Acc:MGI:1891420]	5.93779	4.25516	4.15459	4.28014	4.3238	4.28635	4.35047	5.3503	4.46121	6.30659	5.91826	5.99094	6.18792	5.78028	0.833406028	0.966018836	4	8	24	0.719086819	0.907835864	24	21.85785477	0.053974796
ENSMUSG00000070469	Adamtsl3	ADAMTS-like 3 [Source:MGI Symbol;Acc:MGI:3028499]	2.08097	2.1242	1.71504	2.02792	2.85121	1.50739	2.00773	1.58693	2.42792	1.62306	1.70483	1.89117	1.47255	1.96438	0.911685024	0.966018836	12	12	24	0.801269964	0.936384198	24	12.39985845	0.045289454
ENSMUSG00000070471	Erich6	glutamate rich 6 [Source:MGI Symbol;Acc:MGI:3588212]	1.07192	1.05151	0.912042	0.988756	0.762742	1.02355	0.976671	0.788051	0.840472	0.84951	0.888125	1.05189	0.905951	0.995923	0.319330027	0.83607295	24	20	24	0.187800404	0.768040178	24	23.1129608	0.052288249
ENSMUSG00000070476	Fam217b	family with sequence similarity 217, member B [Source:MGI Symbol;Acc:MGI:1918782]	9.20023	7.53914	7.43813	7.52138	7.36055	7.98412	9.12289	8.00231	6.65371	9.20934	7.07849	8.18715	8.25676	8.1442	0.015984991	0.253395174	4	16	24	0.215340395	0.768040178	24	23.04444143	0.085516094
ENSMUSG00000070493	Chchd2	coiled-coil-helix-coiled-coil-helix domain containing 2 [Source:MGI Symbol;Acc:MGI:1261428]	84.753	91.1731	84.9103	94.5914	95.6204	90.9279	83.1923	86.8774	91.5468	79.9989	105.375	92.7961	99.1137	88.58	0.481611551	0.966018836	20	12	24	0.326335711	0.768040178	24	17.10279471	0.05939614
ENSMUSG00000070498	Tmem132b	transmembrane protein 132B [Source:MGI Symbol;Acc:MGI:3609245]	1.41445	1.4939	2.03888	1.40047	1.02473	1.41876	1.45118	1.23586	1.41136	1.48324	1.16295	1.39144	1.0282	1.70149	0.142596848	0.585944911	12	8	24	0.169723316	0.767809411	24	6.836220882	0.109501796
ENSMUSG00000070509	Rgma	repulsive guidance molecule family member A [Source:MGI Symbol;Acc:MGI:2679262]	2.10919	2.48291	3.1165	2.85576	2.4678	2.21321	2.7804	2.99459	3.72215	3.6087	2.79046	2.93771	2.7866	3.25762	0.031969981	0.324385159	12	16	24	1.39E-04	0.132802361	24	8.95414041	0.18534258
ENSMUSG00000070520	Nsmce3	NSE3 homolog, SMC5-SMC6 complex component [Source:MGI Symbol;Acc:MGI:1913897]	1.80518	1.79707	1.93545	1.84906	1.81063	1.93294	1.75646	2.07371	1.63045	2.11309	1.83506	1.90699	1.90914	1.96288	0.908406451	0.966018836	16	12	24	0.950723475	0.988257443	24	9.761471943	0.008060048
ENSMUSG00000070522	Gm6505	predicted pseudogene 6505 [Source:MGI Symbol;Acc:MGI:3648080]	5.59385	4.88268	5.72027	5.44042	5.62984	4.40825	5.50891	4.40191	5.37948	3.58859	4.67313	4.84857	4.17887	4.89978	0.887862353	0.966018836	12	12	24	0.972282742	0.993238187	24	3.292931528	0.013121422
ENSMUSG00000070527	Mkrn3	makorin, ring finger protein, 3 [Source:MGI Symbol;Acc:MGI:2181178]	0.714062	0.710102	0.650654	0.708811	0.650251	0.55012	0.649621	0.65677	0.500267	0.701871	0.550574	0.537545	0.683499	0.663568	0.321662653	0.83607295	4	20	24	0.549524379	0.834997353	24	4.624025979	0.026364709
ENSMUSG00000070532	Ccdc190	coiled-coil domain containing 190 [Source:MGI Symbol;Acc:MGI:1925715]	0.898117	1.33668	2.07311	0.98979	0.802136	0.970404	1.62586	0.709651	1.4975	0.823986	0.601558	0.951993	0.573072	1.46359	0.185796491	0.662239322	12	8	24	0.144751353	0.753586127	24	6.198806235	0.210244949
ENSMUSG00000070544	Top1	topoisomerase (DNA) I [Source:MGI Symbol;Acc:MGI:98788]	21.5645	18.8537	20.7195	19.0906	20.2314	19.7896	21.0971	18.1245	18.2777	19.0258	21.7937	20.6627	20.496	21.458	0.46238066	0.966018836	4	4	24	0.194886455	0.768040178	24	20.62982337	0.06015959
ENSMUSG00000070550	Mrgprb4	MAS-related GPR, member B4 [Source:MGI Symbol;Acc:MGI:3033119]	15.3714	15.4417	8.54432	14.2542	12.6574	14.3675	12.1137	11.1537	15.6195	10.6009	14.7276	15.1207	16.3027	16.2264	0.593560247	0.966018836	4	12	24	0.35975335	0.785405943	24	22.32056565	0.139038701

ENSMUSG00000070551	Mrgprb5	MAS-related GPR, member B5 [Source:MGI Symbol;Acc:MGI:3033121]	5.95411	6.19147	5.58569	6.15544	5.92335	5.73153	4.71456	5.74062	6.92402	4.14378	7.75447	7.61821	7.05149	5.93418	0.880247609	0.966018836	20	20	24	0.76325694	0.923617783	24	19.72595117	0.062258418
ENSMUSG00000070552	Mrgprx1	MAS-related GPR, member X1 [Source:MGI Symbol;Acc:MGI:3033139]	12.2166	11.0938	10.0619	11.1221	11.0927	12.342	10.2445	10.2168	8.50984	10.2103	11.3413	10.6615	12.3624	9.91162	0.025723486	0.297892358	24	12	24	0.013549353	0.529134128	24	20.22034256	0.135980135
ENSMUSG00000070564	Ntn5	netrin 5 [Source:MGI Symbol;Acc:MGI:2685330]	0.732936	0.855597	0.728132	0.828944	0.831392	0.664158	0.758887	0.863683	0.777623	1.23193	0.950853	0.932757	0.980908	0.97102	0.858266942	0.966018836	16	20	24	0.364909656	0.789214894	24	11.18406464	0.047185912
ENSMUSG00000070565	Rasal2	RAS protein activator like 2 [Source:MGI Symbol;Acc:MGI:2443881]	22.7593	28.0667	24.8897	22.1993	30.4562	20.7627	25.1187	25.5666	31.6591	23.46	26.296	28.0798	27.6563	27.364	0.890176313	0.966018836	12	4	24	0.695630825	0.898982381	24	6.773296303	0.055880425
ENSMUSG00000070570	Slc17a7	solute carrier family 17 (sodium-dependent inorganic phosphate cotransporter), member 7 [Source:MGI Symbol;Acc:MGI:1920211]	24.21	18.3999	14.3175	20.154	18.7948	19.7843	19.4386	22.644	13.9737	29.2914	18.2379	22.0909	22.6809	22.7417	0.43565473	0.966018836	4	8	24	0.595624521	0.857839743	24	21.20802208	0.108763289
ENSMUSG00000070572	Trmt112-ps2	tRNA methyltransferase 11-2, pseudogene 2 [Source:MGI Symbol;Acc:MGI:3651720]	28.4699	21.9238	20.5023	21.8396	20.1149	19.7118	22.2263	24.9402	26.3507	29.4996	26.9426	23.4813	28.9672	28.4523	0.928780444	0.966018836	4	20	24	0.523712066	0.821553822	24	5.092666858	0.077455641
ENSMUSG00000070583	Fv1	Friend virus susceptibility 1 [Source:MGI Symbol;Acc:MGI:95595]	3.74803	3.19942	2.90749	3.37421	3.62143	3.28485	3.82116	2.98241	2.91957	2.86324	4.42494	3.13071	3.72763	3.12525	0.029825002	0.323426482	4	8	24	0.068537631	0.672999758	24	19.63932004	0.126066014
ENSMUSG00000070604	Vsig10l	V-set and immunoglobulin domain containing 10 like [Source:MGI Symbol;Acc:MGI:1922940]	1.98729	1.72141	1.69846	1.09836	1.49285	1.75545	1.36386	1.98685	1.60419	1.64883	1.55841	1.53123	1.66609	1.27889	0.665690018	0.966018836	4	16	24	0.417327229	0.802114794	24	0.754535584	0.069545381
ENSMUSG00000070610	Gm13127	predicted gene 13127 [Source:MGI Symbol;Acc:MGI:3651260]	13.9111	13.625	13.9729	13.919	14.2096	14.6643	13.9079	15.7604	12.4774	15.2791	13.9578	13.2739	13.5311	13.3218	0.881355613	0.966018836	16	20	24	0.857962219	0.958106625	24	16.05853125	0.017456274
ENSMUSG00000070637	Srarp	steroid receptor associated and regulated protein [Source:MGI Symbol;Acc:MGI:2685540]	0.911003	0.707899	0.990559	1.10547	0.841598	0.759821	0.980445	1.00708	0.768287	0.46029	0.736482	0.615455	0.464259	0.561026	0.874607729	0.966018836	12	12	24	0.948337039	0.987393283	24	9.412893829	0.017014527
ENSMUSG00000070639	Lrrc8b	leucine rich repeat containing 8 family, member B [Source:MGI Symbol;Acc:MGI:2141353]	25.5441	23.3491	23.2904	23.5564	22.9647	23.7016	21.6816	22.285	22.2512	25.5514	23.1881	23.3213	24.4221	23.7131	0.919119843	0.966018836	4	8	24	0.868490015	0.96247509	24	17.34905103	0.014283506
ENSMUSG00000070643	Sox13	SRY (sex determining region Y)-box 13 [Source:MGI Symbol;Acc:MGI:98361]	5.02845	4.38916	5.23524	5.52554	5.56707	4.68393	6.72024	5.5454	5.84042	5.23595	5.70116	5.25835	5.3528	4.24954	0.945544484	0.966018836	20	12	24	0.702790254	0.901733679	24	15.59235102	0.047817411
ENSMUSG00000070644	Etnk2	ethanolamine kinase 2 [Source:MGI Symbol;Acc:MGI:2443760]	1.08473	1.08138	1.357	1.00523	1.06942	1.00773	0.883367	1.43391	1.46312	0.748892	1.25687	1.05382	1.07919	1.43845	0.050447964	0.385880335	12	4	24	0.161008154	0.765941695	24	6.067028533	0.108055065
ENSMUSG00000070687	Htr1d	5-hydroxytryptamine (serotonin) receptor 1D [Source:MGI Symbol;Acc:MGI:96276]	9.72089	6.73906	5.31913	6.42862	6.44222	9.19243	7.57844	7.74989	5.53822	10.7989	6.62316	7.29401	7.85483	7.96993	0.062631097	0.410322149	4	8	24	0.351298379	0.780345856	24	21.57192154	0.148061948
ENSMUSG00000070690	Albfm1	albumin superfamily member 1 [Source:MGI Symbol;Acc:MGI:1923342]	0.561647	0.689069	0.847205	0.404496	0.646902	0.590252	0.298453	0.613819	0.454904	0.651097	0.647939	0.701433	0.720339	0.848705	0.90332508	0.966018836	8	20	24	0.859425474	0.958572205	24	6.48356374	0.031674513
ENSMUSG00000070691	Runx3	runt related transcription factor 3 [Source:MGI Symbol;Acc:MGI:102672]	1.10706	0.868688	0.532368	0.736685	0.697302	0.937776	0.871128	0.698125	0.633591	1.34578	0.887105	1.04267	1.08953	0.887845	0.033552374	0.324908923	24	12	24	0.276261549	0.768040178	24	20.51407207	0.100601042
ENSMUSG00000070695	Cntnap5a	contactin associated protein-like 5A [Source:MGI Symbol;Acc:MGI:3643623]	2.37931	2.72106	2.77807	2.46715	2.47958	2.52949	2.72259	2.26742	2.66337	2.18833	2.46079	2.73381	2.52777	3.10846	0.65208848	0.966018836	12	4	24	0.384546102	0.799546792	24	3.433379225	0.049680372
ENSMUSG00000070697	Utp3	UTP3 small subunit processome component [Source:MGI Symbol;Acc:MGI:1919230]	22.3876	23.7577	23.271	21.42	21.7026	21.7778	22.1648	22.7683	23.6764	23.5554	25.7353	25.2818	27.0075	27.4316	0.949874888	0.966018836	8	8	24	0.299176264	0.768040178	24	3.63663668	0.042263892
ENSMUSG00000070699	Sars2	seryl-aminoacyl-tRNA synthetase 2 [Source:MGI Symbol;Acc:MGI:1919234]	4.88375	4.73958	4.34563	5.00337	4.52782	5.20039	4.99791	5.90272	4.78079	6.28698	3.87427	5.74248	4.82554	5.20939	0.929527443	0.966018836	24	20	24	0.848847708	0.954659791	24	3.658591038	0.033649357
ENSMUSG00000070705	Eid2b	EP300 interacting inhibitor of differentiation 2B [Source:MGI Symbol;Acc:MGI:1924095]	5.44013	5.68871	5.45802	5.28915	4.80314	5.10877	5.48841	6.40882	5.41267	6.44446	5.50938	6.12112	5.23956	5.77507	0.648082424	0.966018836	8	12	24	0.389568035	0.799546792	24	6.254308351	0.053240688
ENSMUSG00000070709	Zfp974	zinc finger protein 974 [Source:MGI Symbol;Acc:MGI:1920680]	2.23831	3.00353	3.5783	3.58104	2.83827	2.54995	2.8345	3.06414	3.3972	2.58487	3.06386	2.85585	3.29399	2.44775	0.278774299	0.790764844	12	12	24	0.199116965	0.768040178	24	9.778574539	0.107206565
ENSMUSG00000070713	Gm10282	predicted pseudogene 10282 [Source:MGI Symbol;Acc:MGI:3704312]	1.60727	1.85911	2.36013	2.07721	1.26525	1.25661	1.96203	1.76479	2.66371	2.25627	2.57269	2.04713	2.03228	2.27493	0.102109143	0.512431169	12	16	24	0.033525353	0.606793499	24	8.58896369	0.18500608
ENSMUSG00000070720	Tmem200b	transmembrane protein 200B [Source:MGI Symbol;Acc:MGI:3646343]	0.591528	0.401158	0.643534	0.793827	0.616785	0.50522	0.521744	0.589632	0.921339	0.738045	0.792665	0.564473	0.874126	0.635184	0.143268475	0.585944911	16	8	24	0.111663649	0.724377021	24	11.2395832	0.090962011
ENSMUSG00000070729	Gm12966	predicted gene 12966 [Source:MGI Symbol;Acc:MGI:3652043]	79.9072	107.968	104.589	89.8302	89.2535	97.7489	88.9431	105.484	105.22	95.9292	85.7568	106.053	97.4418	98.0185	0.101443845	0.511659635	8	12	24	0.182763942	0.768040178	24	6.299904135	0.093160361
ENSMUSG00000070730	Rmdn3	regulator of microtubule dynamics 3 [Source:MGI Symbol;Acc:MGI:1915059]	13.9884	14.6351	14.1893	14.5854	13.35	14.3081	15.1402	13.2812	13.6142	14.5349	16.1035	15.7951	15.8848	15.2344	0.801561896	0.966018836	24	12	24	0.415526795	0.800637996	24	20.42404805	0.038007784
ENSMUSG00000070733	Fryl	FRY like transcription coactivator [Source:MGI Symbol;Acc:MGI:1919563]	54.3817	48.1499	45.5963	48.6755	53.9725	49.8813	52.2809	53.3448	44.5585	55.2425	42.8009	50.1517	50.5959	45.4111	0.643325306	0.966018836	4	8	24	0.428178857	0.805393068	24	21.06964332	0.05921042
ENSMUSG00000070737	Tmem35b	transmembrane protein 35B [Source:MGI Symbol;Acc:MGI:3758095]	3.27525	3.96484	3.69125	4.07051	3.84305	3.75389	4.16863	4.04777	3.82003	3.48074	4.34561	3.79859	3.5021	3.34361	0.941830768	0.966018836	20	8	24	0.592998793	0.856634016	24	14.83134068	0.038187176
ENSMUSG00000070738	Dgkd	diacylglycerol kinase, delta [Source:MGI Symbol;Acc:MGI:2138334]	9.38474	8.42782	9.41563	9.65508	9.89445	9.22787	9.00415	9.20716	8.97814	10.8801	9.12995	9.64089	10.171	9.25391	0.350544145	0.870418653	16	16	24	0.231914848	0.768040178	24	14.61317713	0.049245117
ENSMUSG00000070802	Pnmal2	PNMA-like 2 [Source:MGI Symbol;Acc:MGI:3645856]	6.68229	9.28347	14.959	7.55744	5.29277	9.38642	10.9652	6.93127	10.7671	9.14654	4.94948	9.32467	5.2235	12.3424	0.113424199	0.530777648	12	8	24	0.177899376	0.768040178	24	6.314595035	0.312399661
ENSMUSG00000070803	Cited4	Cbp/p300-interacting transactivator, with Glu/Asp-rich carboxy-terminal domain, 4 [Source:MGI Symbol;Acc:MGI:1861694]	0.328861	0.520289	0.397488	0.378034	0.411201	0.270285	0.490767	1.17958	0.574521	0.893086	0.352365	0.372128	0.704927	0.435077	0.094000699	0.490854203	8	16	24	0.186779545	0.768040178	24	6.402810678	0.149185733
ENSMUSG00000070808	Bicra	BRD4 interacting chromatin remodeling complex associated protein [Source:MGI Symbol;Acc:MGI:2154263]	2.21227	1.96709	1.99361	2.49505	2.09151	1.73334	2.32205	2.7125	2.34982	2.73254	2.64866	2.27745	2.24416	2.25638	0.784278077	0.966018836	16	8	24	0.334959751	0.770984245	24	10.43540869	0.069293998
ENSMUSG00000070814	Zswim9	zinc finger SWIM-type containing 9 [Source:MGI Symbol;Acc:MGI:2447816]	6.4733	6.08478	5.44006	5.73461	6.52893	6.54848	6.14625	6.22841	5.71641	6.33251	5.87303	6.48123	6.57716	6.57362	0.020957534	0.262822894	24	12	24	0.025952005	0.586541342	24	21.66379692	0.070480115
ENSMUSG00000070822	Zscan18	zinc finger and SCAN domain containing 18 [Source:MGI Symbol;Acc:MGI:3643810]	5.35814	6.47574	6.70639	4.90918	5.02301	6.23989	6.27701	5.95488	6.0594	5.15383	4.92754	6.16268	6.1474	6.29878	0.040123816	0.34094463	12	4	24	0.017950801	0.557105788	24	2.883627989	0.129492604
ENSMUSG00000070858	Gm1673	predicted gene 1673 [Source:MGI Symbol;Acc:MGI:2686519]	13.5628	12.4652	9.31024	7.37742	6.76007	7.54705	10.8746	12.3729	12.8256	15.1179	19.3186	17.4247	17.6298	14.0761	0.869755137	0.966018836	4	12	24	0.546092499	0.833198402	24	1.927252664	0.150877223
ENSMUSG00000070866	Zfp804a	zinc finger protein 804A [Source:MGI Symbol;Acc:MGI:2442949]	4.77435	4.16692	3.3039	3.86487	3.9685	4.55165	4.3198	3.67271	3.34378	4.24852	4.27704	4.20297	4.60919	4.41641	3.29E-04	0.049770315	4	8	24	0.012217728	0.525280095	24	21.28693517	0.132869951
ENSMUSG00000070867	Trabd2b	TraB domain containing 2B [Source:MGI Symbol;Acc:MGI:3650152]	1.02311	1.17641	0.813699	0.637266	0.989192	0.802921	1.05668	1.02555	1.27619	0.687709	0.953527	0.896915	1.05629	0.867853	0.083354723	0.462922111	8	8	24	0.152424507	0.759088284	24	1.970233942	0.095350402
ENSMUSG00000070871	Ccnyl1	cyclin Y-like 1 [Source:MGI Symbol;Acc:MGI:2138614]	3.25507	3.54091	3.49624	3.69053	3.66219	2.96617	3.71694	3.19749	3.69714	3.39969	3.81464	3.50305	3.21227	3.4783	0.628899575	0.966018836	20	4	24	0.243629689	0.768040178	24	11.69300082	0.051603866
ENSMUSG00000070873	Lilra5	leukocyte immunoglobulin-like receptor, subfamily A (with TM domain), member 5 [Source:MGI Symbol;Acc:MGI:3647196]	0.753053	0.828709	0.863826	0.516199	0.980188	0.557591	0.945258	0.694873	0.738337	0.322601	1.16622	0.723801	0.675836	0.527686	0.893307667	0.966018836	20	20	24	0.630357215	0.873885049	24	20.53557404	0.071719743
ENSMUSG00000070880	Gad1	glutamate decarboxylase 1 [Source:MGI Symbol;Acc:MGI:95632]	1.88621	4.9368	7.86626	3.33707	2.2165	4.6557	5.53553	2.25238	5.63981	3.47926	1.6087	4.08786	1.47433	5.70396	0.062036003	0.409814092	12	8	24	0.293849492	0.768040178	24	6.943252135	0.363541165
ENSMUSG00000070883	Ccdc173	coiled-coil domain containing 173 [Source:MGI Symbol;Acc:MGI:1923100]	2.04548	2.25724	2.14322	1.91903	2.18284	1.89002	2.3132	1.88669	2.11036	1.5202	2.30045	2.14047	2.44419	2.38473	0.47028201	0.966018836	4	12	24	0.249256099	0.768040178	24	0.508645949	0.073810758
ENSMUSG00000070923	Klhl9	kelch-like 9 [Source:MGI Symbol;Acc:MGI:2180122]	34.7878	33.9998	33.1621	32.5812	32.9613	31.1429	32.7451	31.7763	32.1901	29.9131	37.0503	33.1601	33.3563	33.5869	0.767183862	0.966018836	4	12	24	0.69602693	0.899204829	24	21.6370509	0.024857308
ENSMUSG00000070934	Rraga	Ras-related GTP binding A [Source:MGI Symbol;Acc:MGI:1915691]	67.8901	62.3043	63.8504	61.7022	60.03	66.8825	63.2038	61.8266	54.6256	66.3259	61.63	59.261	64.7461	63.4111	0.593560247	0.966018836	4	16	24	0.415056124	0.800292198	24	22.228004	0.041115136
ENSMUSG00000070939	Tgfbrap1	transforming growth factor, beta receptor associated protein 1 [Source:MGI Symbol;Acc:MGI:2447427]	11.2413	9.40995	9.60615	10.5306	10.4523	10.3487	10.3699	9.70589	9.42596	11.9754	9.29274	10.1684	10.3949	9.59747	0.371875382	0.894537464	16	20	24	0.463447706	0.805393068	24	17.67111883	0.047518783
ENSMUSG00000070942	Il1rl2	interleukin 1 receptor-like 2 [Source:MGI Symbol;Acc:MGI:1913107]	0.662681	0.856225	0.79671	0.886992	0.81228	0.693983	0.981177	0.840416	0.981758	0.839843	0.652019	0.715773	0.866012	0.741935	0.402483494	0.934810776	16	4	24	0.214344823	0.768040178	24	7.149454077	0.058781682
ENSMUSG00000070953	Rabepk	Rab9 effector protein with kelch motifs [Source:MGI Symbol;Acc:MGI:2139530]	12.9651	12.9206	13.2859	16.7809	13.1811	14.3519	13.0237	14.9706	13.6481	10.8201	12.5953	11.8771	13.2765	12.711	0.862909389	0.966018836	12	8	24	0.916936029	0.977109697	24	6.986358295	0.022670314
ENSMUSG00000070960	Gm19680	predicted gene, 19680 [Source:MGI Symbol;Acc:MGI:5011865]	1.7328	1.79291	2.17291	2.4339	2.09602	1.65667	1.79379	2.13507	2.18965	2.25028	1.92376	1.99822	2.20612	2.35566	0.057264003	0.401309455	16	8	24	0.0060249	0.466639081	24	9.539677223	0.115167407
ENSMUSG00000070972	Dnajc25	DnaJ heat shock protein family (Hsp40) member C25 [Source:MGI Symbol;Acc:MGI:1919679]	2.85753	2.22379	2.31762	2.55096	1.94862	2.51606	2.53572	2.09909	2.56911	1.93907	2.70285	2.64927	2.39567	2.04465	0.632729413	0.966018836	24	8	24	0.335126357	0.771074441	24	20.74706587	0.074822281
ENSMUSG00000071001	Hrct1	histidine rich carboxyl terminus 1 [Source:MGI Symbol;Acc:MGI:1917945]	0.707583	0.938967	0.63595	0.637217	0.877999	0.736014	0.6388	0.828185	0.778493	0.711225	0.551403	0.782631	0.77723	0.959982	0.226403091	0.722427815	8	8	24	0.280310871	0.768040178	24	3.420534917	0.059450057
ENSMUSG00000071014	Ndufb6	NADH:ubiquinone oxidoreductase subunit B6 [Source:MGI Symbol;Acc:MGI:2684983]	151.745	139.849	140.046	142.547	130.717	139.24	142.299	149.116	138.768	139.003	158.887	140.246	158.764	151.332	0.325142889	0.842977406	4	8	24	0.359152421	0.785405943	24	1.170408915	0.040763285
ENSMUSG00000071035	Gm5499	predicted pseudogene 5499 [Source:MGI Symbol;Acc:MGI:3643804]	128.067	122.345	126.728	120.163	124.329	122.677	119.646	112.023	115.284	109.621	124.314	114.563	117.374	122.512	0.868022237	0.966018836	20	20	24	0.59771623	0.858729277	24	20.86964745	0.024119086
ENSMUSG00000071037	Camkmt	calmodulin-lysine N-methyltransferase [Source:MGI Symbol;Acc:MGI:1920832]	5.79611	5.301	6.1655	5.06875	4.84167	5.79529	5.18343	5.44276	5.06004	6.00471	5.75787	5.58031	5.7831	5.47623	0.832742389	0.966018836	24	20	24	0.956455342	0.988639288	24	0.02314118	0.010480639
ENSMUSG00000071041	Impdh2-ps	inosine monophosphate dehydrogenase 2, pseudogene [Source:MGI Symbol;Acc:MGI:3705743]	19.3065	18.0532	18.79	16.7756	19.0101	20.189	17.4517	19.4916	16.7971	19.5734	15.6472	18.677	17.9813	17.9779	0.789670753	0.966018836	24	20	24	0.606820741	0.8634501	24	22.98841893	0.03825693
ENSMUSG00000071042	Rasgrp3	RAS, guanyl releasing protein 3 [Source:MGI Symbol;Acc:MGI:3028579]	1.44649	2.65534	2.4895	1.80394	2.04126	2.98978	3.47297	3.3421	2.60615	2.8039	2.42593	2.61294	2.74388	2.45238	0.657586167	0.966018836	24	20	24	0.579912875	0.850060717	24	3.073886338	0.087014271
ENSMUSG00000071052	Rpl7a-ps5	ribosomal protein L7A, pseudogene 5 [Source:MGI Symbol;Acc:MGI:3643553]	2.72138	3.34241	3.30227	3.47553	1.98428	2.75876	2.85448	2.60225	3.45396	2.34197	3.127	2.74968	2.44869	2.89494	0.401437339	0.933249185	12	12	24	0.273835604	0.768040178	24	7.351337352	0.107049786
ENSMUSG00000071054	Safb	scaffold attachment factor B [Source:MGI Symbol;Acc:MGI:2146974]	30.9602	28.8254	32.6206	30.1312	29.823	26.266	33.9416	28.5149	32.1487	32.0954	32.4439	26.6115	27.641	27.9997	0.08302755	0.462922111	12	12	24	0.303992407	0.768040178	24	10.24245762	0.067339195
ENSMUSG00000071064	Zfp827	zinc finger protein 827 [Source:MGI Symbol;Acc:MGI:2444807]	4.27131	4.05669	4.19732	3.57316	4.13292	4.07999	4.00895	4.32943	3.47855	4.70414	3.82969	4.5402	4.0258	4.46087	0.88067614	0.966018836	8	4	24	0.703908708	0.90225186	24	23.34303151	0.032496775
ENSMUSG00000071068	Treml2	triggering receptor expressed on myeloid cells-like 2 [Source:MGI Symbol;Acc:MGI:2147038]	0.514716	0.68719	0.859711	0.881632	0.667219	0.655162	0.598001	0.608007	0.634621	0.29701	0.553972	0.431556	0.720027	0.352106	0.880247609	0.966018836	12	12	24	0.935011063	0.983765461	24	9.800054769	0.020051878
ENSMUSG00000071072	Ptges3	prostaglandin E synthase 3 [Source:MGI Symbol;Acc:MGI:1929282]	59.0031	49.6528	45.3271	49.967	53.5785	50.1442	52.1259	48.8693	43.3126	48.0331	57.7502	52.7449	49.6757	49.0435	4.29E-04	0.054040373	20	16	24	0.01134371	0.518288666	24	19.78633968	0.122678473
ENSMUSG00000071073	Lrrc73	leucine rich repeat containing 73 [Source:MGI Symbol;Acc:MGI:2684934]	3.51765	2.78958	2.87328	2.73352	2.25802	3.29377	3.26284	2.67713	1.80979	3.84482	3.12274	3.3584	2.37045	3.8435	0.919571723	0.966018836	24	12	24	0.677404818	0.892060593	24	21.2207282	0.081900585
ENSMUSG00000071074	Yipf3	Yip1 domain family, member 3 [Source:MGI Symbol;Acc:MGI:106280]	30.0873	26.6669	27.6934	25.9069	29.0012	31.7079	28.5379	31.9203	23.9514	31.3678	29.6258	31.8936	29.2117	27.5338	0.042880436	0.356048751	24	12	24	0.087383062	0.707460769	24	20.07665134	0.09913906
ENSMUSG00000071076	Jund	jun D proto-oncogene [Source:MGI Symbol;Acc:MGI:96648]	16.7755	13.9181	13.4533	11.5738	11.1719	11.7335	13.3931	19.3792	19.6731	24.9405	25.5308	25.6816	28.5374	22.9472	0.860560779	0.966018836	8	8	24	0.759129343	0.92214435	24	4.076590906	0.082789653
ENSMUSG00000071078	Nr2c2ap	nuclear receptor 2C2-associated protein [Source:MGI Symbol;Acc:MGI:1922942]	25.0396	22.4961	22.408	20.4519	23.7928	25.1065	20.88	23.3331	18.6173	19.3603	23.6986	21.9487	23.2023	20.6901	0.076247883	0.453790923	20	20	24	0.020085146	0.565108098	24	20.979327	0.120223821
ENSMUSG00000071103	1700029J07Rik	RIKEN cDNA 1700029J07 gene [Source:MGI Symbol;Acc:MGI:1916729]	3.55132	4.52098	4.02091	3.88602	5.15687	3.06877	3.97322	3.25941	5.16396	3.36241	4.19069	3.69133	3.43683	3.56413	0.561701903	0.966018836	12	12	24	0.427739295	0.805393068	24	11.25940193	0.09299278
ENSMUSG00000071112	Spx	spexin hormone [Source:MGI Symbol;Acc:MGI:2442262]	1.75006	1.98617	1.224	2.54243	1.67372	2.30473	1.85983	2.4283	2.61386	1.03191	1.8512	1.27748	1.54422	2.1241	0.756435587	0.966018836	8	12	24	0.720831825	0.908547187	24	4.666994856	0.083984791
ENSMUSG00000071151	Gm4799	predicted gene 4799 [Source:MGI Symbol;Acc:MGI:3648850]	18.4109	19.5007	19.0393	17.6831	20.1285	20.503	17.1092	19.8079	14.4682	15.1041	20.3979	18.1025	17.5927	15.7616	0.421215102	0.966018836	20	20	24	0.127853276	0.740162193	24	19.69511942	0.110589175
ENSMUSG00000071172	Srsf3	serine and arginine-rich splicing factor 3 [Source:MGI Symbol;Acc:MGI:98285]	42.1683	47.7468	44.2985	45.7653	47.2724	40.9189	43.9169	41.05	48.4052	38.7635	48.3946	39.7101	41.7821	43.0143	0.195343269	0.673387182	20	4	24	0.531225705	0.825167324	24	10.59488051	0.045916019
ENSMUSG00000071176	Arhgef10	Rho guanine nucleotide exchange factor (GEF) 10 [Source:MGI Symbol;Acc:MGI:2444453]	23.6038	20.3794	21.2623	27.0148	28.6693	21.8652	25.5655	20.2587	21.845	24.8028	24.786	22.5198	21.3152	16.4187	0.001017311	0.075944664	20	12	24	0.003814381	0.427278365	24	16.03615538	0.196760056
ENSMUSG00000071180	Smim15	small integral membrane protein 15 [Source:MGI Symbol;Acc:MGI:1922866]	32.978	35.3961	34.9545	32.1538	34.9449	30.7361	36.0711	31.2778	34.5334	30.1726	36.1149	32.2257	32.8813	32.5872	0.93447422	0.966018836	4	12	24	0.979771461	0.995083382	24	3.105342285	0.006627386
ENSMUSG00000071181	3830408C21Rik	RIKEN cDNA 3830408C21 gene [Source:MGI Symbol;Acc:MGI:1917940]	2.02328	2.35126	2.12712	2.2687	2.44702	2.21626	2.70256	2.07251	2.63725	2.03487	2.34534	2.61962	2.25122	2.10287	0.910436409	0.966018836	20	12	24	0.687046717	0.896182667	24	18.78327278	0.03533466
ENSMUSG00000071203	Naip5	NLR family, apoptosis inhibitory protein 5 [Source:MGI Symbol;Acc:MGI:1298220]	0.54132	0.71521	0.674703	0.679841	0.609488	0.650824	0.527768	0.621209	0.811634	0.497322	0.558835	0.616691	0.520343	0.573313	0.105280783	0.518002936	12	16	24	0.278349321	0.768040178	24	8.650576293	0.046501328
ENSMUSG00000071226	Cecr2	CECR2, histone acetyl-lysine reader [Source:MGI Symbol;Acc:MGI:1923799]	0.846721	0.828254	1.01918	1.06755	0.776572	0.93765	1.02919	1.35389	0.928656	0.733299	0.910279	1.33644	0.846413	1.36158	0.887010723	0.966018836	8	12	24	0.433194043	0.805393068	24	3.460326881	0.070291596
ENSMUSG00000071234	Syndig1l	synapse differentiation inducing 1 like [Source:MGI Symbol;Acc:MGI:2685107]	2.59078	2.2894	2.73473	2.72113	2.47628	2.91697	3.172	2.99002	2.10561	3.18703	2.75216	2.71664	1.96688	2.15574	0.928780444	0.966018836	16	20	24	0.44461142	0.805393068	24	16.41682353	0.077026874
ENSMUSG00000071252	2210408I21Rik	RIKEN cDNA 2210408I21 gene [Source:MGI Symbol;Acc:MGI:1919621]	4.08025	3.96132	4.47834	3.22845	4.0091	4.19642	4.39491	3.92924	4.6414	3.13486	5.19363	5.1944	4.30482	4.22677	0.671726933	0.966018836	24	16	24	0.39633782	0.799546792	24	21.92335485	0.086788769
ENSMUSG00000071253	Slc25a16	solute carrier family 25 (mitochondrial carrier, Graves disease autoantigen), member 16 [Source:MGI Symbol;Acc:MGI:1920382]	13.6132	13.4061	15.0848	15.3778	13.2021	13.0451	11.4687	14.2423	12.7127	16.7035	13.5215	13.3942	13.4968	13.9374	0.069194004	0.433403132	16	8	24	0.057800642	0.656248849	24	10.72754863	0.106495349
ENSMUSG00000071256	Zfp213	zinc finger protein 213 [Source:MGI Symbol;Acc:MGI:3053094]	2.74566	2.1486	2.1858	2.0456	2.42241	2.03457	2.58946	2.60929	2.47087	2.23622	2.95751	2.5042	2.62939	2.21066	0.305564675	0.811201458	4	12	24	0.452079843	0.805393068	24	21.65061699	0.057793807
ENSMUSG00000071265	1700086L19Rik	RIKEN cDNA 1700086L19 gene [Source:MGI Symbol;Acc:MGI:1921534]	6.73943	5.46886	5.8116	6.39741	6.28835	6.02632	5.71842	5.79044	4.48619	5.45871	6.52578	6.44836	4.9341	5.82751	0.319330027	0.83607295	20	16	24	0.079861067	0.694370548	24	18.32693362	0.111804084
ENSMUSG00000071266	Zfp946	zinc finger protein 946 [Source:MGI Symbol;Acc:MGI:1921399]	12.6907	14.4697	13.3466	12.6977	12.5848	12.2624	13.4797	11.3189	12.9391	10.1505	13.6285	13.1915	13.3935	14.7342	0.633249925	0.966018836	8	8	24	0.401666426	0.799546792	24	1.464771546	0.066099912
ENSMUSG00000071267	Zfp942	zinc finger protein 942 [Source:MGI Symbol;Acc:MGI:1920483]	7.69951	8.43457	8.45768	8.5748	8.06637	7.50113	8.18836	7.51979	8.8747	6.16945	8.25874	7.76511	7.68764	7.9412	0.138202217	0.580158868	12	12	24	0.805651673	0.937961689	24	5.996312809	0.029479744
ENSMUSG00000071273	Gm5145	predicted pseudogene 5145 [Source:MGI Symbol;Acc:MGI:3645482]	2.32133	1.36352	2.0754	1.42815	1.60033	1.92032	1.52749	2.02722	1.50623	1.80636	2.04435	1.71435	1.55148	1.90967	0.816406072	0.966018836	20	20	24	0.886999006	0.968043979	24	21.87099832	0.030441158
ENSMUSG00000071281	Zfp65	zinc finger protein 65 [Source:MGI Symbol;Acc:MGI:107769]	3.21876	3.64745	3.40539	3.24678	3.55984	3.20671	3.18894	3.5741	4.17798	3.09122	3.75237	3.78066	2.92995	4.19766	0.503972924	0.966018836	8	20	24	0.439877627	0.805393068	24	7.852796037	0.061888375
ENSMUSG00000071291	Zfp58	zinc finger protein 58 [Source:MGI Symbol;Acc:MGI:99205]	3.39025	3.6604	3.7101	3.88874	3.75144	3.07822	3.35287	3.17999	3.72871	2.74173	3.85261	3.80103	3.2107	3.56677	0.327174535	0.845975006	20	8	24	0.79283713	0.93379199	24	12.65060346	0.03073303
ENSMUSG00000071302	2610044O15Rik8	RIKEN cDNA 2610044O15 gene [Source:MGI Symbol;Acc:MGI:1919389]	12.9319	14.8048	14.8655	14.3856	13.7228	12.9309	11.7453	13.6856	16.0456	11.4578	14.3795	13.7099	13.5853	13.9892	0.008389425	0.192760771	12	16	24	0.313206212	0.768040178	24	8.334690302	0.074228217
ENSMUSG00000071317	Bves	blood vessel epicardial substance [Source:MGI Symbol;Acc:MGI:1346013]	7.02121	6.43693	6.51534	7.4652	6.50068	6.81445	5.76379	6.96983	6.25364	7.72922	5.62915	6.39666	6.84265	6.87106	0.893307667	0.966018836	16	4	24	0.661002409	0.885520687	24	9.242747962	0.041153556
ENSMUSG00000071324	Armc2	armadillo repeat containing 2 [Source:MGI Symbol;Acc:MGI:1916449]	4.47	3.14089	3.36844	3.85389	3.68248	3.87662	3.79388	3.39818	3.03141	2.40836	2.95048	3.53227	3.39703	2.86087	0.200364953	0.684570416	24	12	24	0.013338081	0.529134128	24	21.46021047	0.147738711
ENSMUSG00000071337	Tia1	cytotoxic granule-associated RNA binding protein 1 [Source:MGI Symbol;Acc:MGI:107914]	24.365	33.4399	32.3273	26.4313	33.3633	26.1435	30.4652	25.3624	33.48	20.7575	30.5117	25.9597	27.479	28.0004	0.601647512	0.966018836	12	12	24	0.860389616	0.958920776	24	6.750265319	0.044604654
ENSMUSG00000071340	Trappc3l	trafficking protein particle complex 3 like [Source:MGI Symbol;Acc:MGI:3642034]	59.9403	63.261	52.1295	60.6583	58.3786	50.1785	56.468	46.6958	63.1739	48.6599	66.6025	64.5307	68.0989	66.4357	0.891742979	0.966018836	4	12	24	0.84599158	0.953396028	24	22.40525489	0.040208755
ENSMUSG00000071342	Lsmem1	leucine-rich single-pass membrane protein 1 [Source:MGI Symbol;Acc:MGI:2685735]	0.529468	0.99567	0.968139	1.17029	0.585332	0.981754	0.646074	1.80723	2.1699	1.57149	0.649724	0.685047	1.0138	1.24875	0.094056402	0.490854203	16	4	24	0.005953517	0.46443032	24	7.87391115	0.340547598
ENSMUSG00000071343	Gm10327	predicted pseudogene 10327 [Source:MGI Symbol;Acc:MGI:3704356]	0.663012	0.457316	0.824033	0.923691	0.567324	1.10253	0.866783	0.763415	0.22643	0.531894	0.325711	0.452849	0.378477	0.318162	0.890648739	0.966018836	24	8	24	0.579769649	0.850007285	24	19.58070668	0.083836992
ENSMUSG00000071347	C1qtnf9	C1q and tumor necrosis factor related protein 9 [Source:MGI Symbol;Acc:MGI:3045252]	0.865217	0.753161	1.23886	2.38763	1.23306	1.09112	1.27991	1.67699	1.1063	1.8647	1.25708	1.22947	1.48226	1.06332	0.421215102	0.966018836	16	16	24	0.150527396	0.758865539	24	12.29579511	0.175105626
ENSMUSG00000071350	Setdb2	SET domain, bifurcated 2 [Source:MGI Symbol;Acc:MGI:2685139]	2.56982	3.15295	3.25044	3.32159	2.96728	3.10045	3.16234	3.26525	3.02063	4.74258	3.4477	3.36009	3.46547	3.89013	0.856752753	0.966018836	16	12	24	0.123346225	0.736234617	24	10.53140349	0.097115482
ENSMUSG00000071359	Tbpl1	TATA box binding protein-like 1 [Source:MGI Symbol;Acc:MGI:1339946]	26.8519	22.7732	20.9394	21.2941	21.9233	25.47	24.4883	22.929	19.4807	22.2433	23.8338	22.4424	23.4742	22.7466	3.15E-04	0.049770315	4	8	24	0.002418029	0.39630666	24	21.79611892	0.126905468
ENSMUSG00000071369	Map3k5	mitogen-activated protein kinase kinase kinase 5 [Source:MGI Symbol;Acc:MGI:1346876]	7.36842	5.87697	5.76923	6.37631	5.76066	7.01436	6.01892	6.57279	5.65916	7.06299	5.66922	6.70293	6.51368	6.66389	0.243586913	0.741580974	24	12	24	0.416465282	0.801257196	24	22.34266321	0.059149335
ENSMUSG00000071379	Hpcal1	hippocalcin-like 1 [Source:MGI Symbol;Acc:MGI:1855689]	53.8167	38.849	41.112	40.2157	39.9064	50.997	46.3889	46.3948	32.1598	51.3762	41.1705	45.2787	44.3691	44.6435	0.27074138	0.780327352	4	8	24	0.157516613	0.76387796	24	21.48130729	0.141249338
ENSMUSG00000071414	Gm6736	predicted gene 6736 [Source:MGI Symbol;Acc:MGI:3643048]	7.69663	6.6643	6.05676	6.32867	8.60075	7.3272	6.54785	7.25269	7.22658	5.85455	8.42385	5.94213	6.98293	5.97764	0.369262716	0.893596186	20	20	24	0.30748694	0.768040178	24	18.62221439	0.096128093
ENSMUSG00000071415	Rpl23	ribosomal protein L23 [Source:MGI Symbol;Acc:MGI:1929455]	16.7408	17.054	17.7202	14.0078	16.6184	15.6995	16.9297	19.0155	19.2826	13.3592	17.7917	16.0028	19.158	16.6605	0.079180494	0.458678242	12	4	24	0.153147243	0.759484836	24	2.753735957	0.104232011
ENSMUSG00000071419	Rps15-ps2	ribosomal protein S15, pseudogene 2 [Source:MGI Symbol;Acc:MGI:3647234]	72.7337	82.6284	69.2347	72.9879	92.9159	81.6847	69.1046	80.6955	83.3851	59.6071	82.6654	66.3542	74.8693	63.9853	0.935194469	0.966018836	20	8	24	0.747433235	0.917814248	24	18.07462818	0.05247355
ENSMUSG00000071424	Grid2	glutamate receptor, ionotropic, delta 2 [Source:MGI Symbol;Acc:MGI:95813]	1.78469	1.79758	2.83388	1.8458	1.89916	2.13608	1.9651	1.54475	1.68193	2.12732	1.43879	1.74243	1.49728	1.95944	0.882263435	0.966018836	16	4	24	0.614143293	0.866356765	24	9.848548433	0.061791413
ENSMUSG00000071451	Psmg4	proteasome (prosome, macropain) assembly chaperone 4 [Source:MGI Symbol;Acc:MGI:1916916]	11.9919	13.434	11.9353	11.6307	12.5796	11.37	13.1177	11.5222	12.4901	13.408	14.277	12.2116	11.9192	14.4018	0.888379597	0.966018836	20	4	24	0.793543351	0.933825946	24	9.247122225	0.027243795
ENSMUSG00000071454	Dtnb	dystrobrevin, beta [Source:MGI Symbol;Acc:MGI:1203728]	20.0689	17.5815	16.0868	16.2818	18.2889	17.999	17.8585	16.0684	16.4637	18.572	15.9973	18.2457	17.3533	17.5742	0.499268446	0.966018836	24	12	24	0.118932376	0.729851534	24	21.22225536	0.071934773
ENSMUSG00000071456	1110002L01Rik	RIKEN cDNA 1110002L01 gene [Source:MGI Symbol;Acc:MGI:1915821]	5.44508	5.23311	5.10339	4.89961	4.9466	5.80781	6.16058	5.27051	5.16429	5.32696	5.43178	5.55	5.70468	4.91665	0.009528986	0.194720062	4	8	24	0.020775723	0.568066329	24	21.86918328	0.079945648
ENSMUSG00000071477	Zfp777	zinc finger protein 777 [Source:MGI Symbol;Acc:MGI:1919556]	4.91849	5.05038	5.24253	3.56384	4.46284	4.43416	4.33992	5.86882	4.64665	5.4319	5.11795	4.88903	4.50731	4.70717	0.668329321	0.966018836	8	16	24	0.576210857	0.848480086	24	5.209426464	0.059913214
ENSMUSG00000071489	Ptgdr	prostaglandin D receptor [Source:MGI Symbol;Acc:MGI:102966]	19.3777	15.3922	15.7549	17.4692	15.8795	17.343	15.1347	15.6817	13.5315	17.3597	16.1418	16.0303	17.4031	16.8191	0.63897419	0.966018836	4	8	24	0.389263153	0.799546792	24	20.00812881	0.064673224
ENSMUSG00000071497	Nutf2-ps1	nuclear transport factor 2, pseudogene 1 [Source:MGI Symbol;Acc:MGI:108008]	14.1706	13.2495	13.4161	12.4111	12.0475	13.0173	13.4065	13.2594	11.7708	13.7061	12.8842	13.4769	12.8551	12.1289	0.94086481	0.966018836	4	8	24	0.342171101	0.77630879	24	22.63846553	0.039604633
ENSMUSG00000071528	Atp5md	ATP synthase membrane subunit DAPIT [Source:MGI Symbol;Acc:MGI:1891435]	44.1657	49.2684	50.4523	40.824	46.4668	45.0277	46.4039	48.5873	58.8969	38.519	55.7044	51.3253	56.5455	55.791	0.503972924	0.966018836	12	4	24	0.358968643	0.785405943	24	3.555869449	0.085581808
ENSMUSG00000071531	Gprin2	G protein regulated inducer of neurite outgrowth 2 [Source:MGI Symbol;Acc:MGI:2444560]	2.6376	1.92214	1.73989	1.95989	2.03484	2.29637	2.17818	1.7599	1.47007	1.98747	2.4032	2.17491	2.55325	1.90476	1.18E-04	0.029372629	4	8	24	0.002569791	0.396968951	24	20.4469176	0.176334152
ENSMUSG00000071532	Gm10335	predicted gene 10335 [Source:MGI Symbol;Acc:MGI:3641693]	2.89332	2.329	1.56984	1.93317	2.23026	1.69094	2.23547	2.79912	1.96377	1.73956	2.50372	2.46008	1.41357	2.11094	0.61767505	0.966018836	4	8	24	0.613999581	0.866266017	24	22.72092622	0.081628532
ENSMUSG00000071533	Pcnp	PEST proteolytic signal containing nuclear protein [Source:MGI Symbol;Acc:MGI:1923552]	36.5581	39.7027	40.8627	39.2268	39.3143	38.0207	39.2698	36.4363	37.6734	38.1388	41.1125	37.0085	37.6744	39.322	0.718086778	0.966018836	20	4	24	0.343490641	0.777127004	24	13.96783112	0.030304446
ENSMUSG00000071537	Klrg2	killer cell lectin-like receptor subfamily G, member 2 [Source:MGI Symbol;Acc:MGI:1921503]	0.777911	1.03284	0.409569	1.0081	0.72053	0.839479	0.91636	1.02712	0.544832	1.11406	1.20818	0.873433	0.825468	0.748353	0.932092277	0.966018836	16	20	24	0.56281293	0.842470381	24	18.13910062	0.075654978

ENSMUSG00000071540	3425401B19Rik	RIKEN cDNA 3425401B19 gene [Source:MGI Symbol;Acc:MGI:3588196]	0.705119	2.05466	2.6467	2.46981	1.85251	2.50167	1.28474	4.50766	3.11405	6.56253	1.36144	1.98732	2.22135	2.26072	0.055569815	0.401074772	12	16	24	0.05146337	0.64767123	24	9.516292671	0.459231802
ENSMUSG00000071547	Nt5dc2	5'-nucleotidase domain containing 2 [Source:MGI Symbol;Acc:MGI:1917271]	2.60687	2.70421	3.13959	2.90783	2.21948	2.42488	2.10464	2.34517	2.67447	2.69579	1.79659	1.99841	2.16446	2.98469	0.016811431	0.253395174	16	4	24	0.009364322	0.502965379	24	7.539869651	0.165020891
ENSMUSG00000071550	Cfap44	cilia and flagella associated protein 44 [Source:MGI Symbol;Acc:MGI:1277238]	0.802132	1.51595	0.855036	1.00662	0.688562	0.591505	0.736757	0.567791	1.02136	1.1922	0.518302	1.48523	0.536761	1.09661	0.61767505	0.966018836	16	4	24	0.581899811	0.850995343	24	7.981886663	0.105922755
ENSMUSG00000071568	Gm5874	predicted gene 5874 [Source:MGI Symbol;Acc:MGI:3648027]	16.4843	12.8739	10.3335	13.1597	11.8439	13.0797	14.1064	12.5812	11.5948	14.7381	14.7104	12.5534	13.3924	11.6634	0.105280783	0.518002936	4	8	24	0.238687394	0.768040178	24	20.1752404	0.107569428
ENSMUSG00000071573	Rnls	renalase, FAD-dependent amine oxidase [Source:MGI Symbol;Acc:MGI:1915045]	1.10876	1.49569	1.2416	1.6241	1.19998	1.19734	1.48698	1.33945	1.39214	1.6554	1.65815	1.22052	1.72102	1.44419	0.924770496	0.966018836	16	8	24	0.431390429	0.805393068	24	9.743561538	0.056993569
ENSMUSG00000071604	Fam189a2	family with sequence similarity 189, member A2 [Source:MGI Symbol;Acc:MGI:2685813]	7.94626	6.45544	6.92717	6.59737	6.4099	7.27316	6.00627	7.74945	6.34105	9.47603	6.48067	6.51984	6.75812	7.16684	0.853736882	0.966018836	8	12	24	0.831330769	0.947661285	24	9.343834726	0.038517415
ENSMUSG00000071632	2510002D24Rik	RIKEN cDNA 2510002D24 gene [Source:MGI Symbol;Acc:MGI:1919557]	6.06532	7.73737	7.16423	7.4107	5.94056	6.7932	6.9761	9.34888	9.15987	6.58838	7.7239	6.73356	8.48533	8.09555	0.083354723	0.462922111	8	16	24	0.018609241	0.559830643	24	5.955922894	0.161816451
ENSMUSG00000071637	Cebpd	CCAAT/enhancer binding protein (C/EBP), delta [Source:MGI Symbol;Acc:MGI:103573]	1.98073	1.3085	1.70744	1.65452	1.73514	1.41918	1.76375	1.74617	1.98793	1.69446	3.36669	2.34732	2.50697	1.63745	0.622132989	0.966018836	20	12	24	0.370177602	0.792263516	24	17.66539467	0.114523481
ENSMUSG00000071640	Stxbp3-ps	syntaxin-binding protein 3, pseudogene [Source:MGI Symbol;Acc:MGI:3586869]	7.36266	7.95205	6.62052	5.81613	6.64098	6.36139	7.9942	6.93715	8.81014	6.41361	6.42723	7.14554	6.92346	6.28049	0.295379186	0.805301811	4	12	24	0.218360929	0.768040178	24	2.690298487	0.09300655
ENSMUSG00000071644	Eef1g	eukaryotic translation elongation factor 1 gamma [Source:MGI Symbol;Acc:MGI:1914410]	25.7378	25.4954	27.3337	26.6264	27.8286	29.1468	23.772	27.4652	24.0183	25.9919	25.7414	24.0408	23.9688	23.0959	0.376911625	0.902906046	20	8	24	0.17775151	0.768040178	24	15.26492985	0.055922183
ENSMUSG00000071645	Tut1	terminal uridylyl transferase 1, U6 snRNA-specific [Source:MGI Symbol;Acc:MGI:1917294]	5.95338	5.91525	5.47121	5.79784	5.17732	6.16983	5.64798	6.3584	5.13994	5.86123	5.57636	6.37274	5.84486	5.20394	0.148073804	0.590732968	24	12	24	0.270424414	0.768040178	24	21.82127239	0.055838122
ENSMUSG00000071646	Mta2	metastasis-associated gene family, member 2 [Source:MGI Symbol;Acc:MGI:1346340]	17.2646	18.2596	17.6172	19.3453	19.1483	17.9054	19.4935	18.5308	20.4308	18.2774	17.3578	18.9638	18.4335	18.3359	0.782779623	0.966018836	12	20	24	0.702620753	0.901733679	24	9.270280461	0.022341775
ENSMUSG00000071647	Eml3	echinoderm microtubule associated protein like 3 [Source:MGI Symbol;Acc:MGI:2387612]	11.6325	12.9599	14.3699	13.2663	14.0958	13.1857	15.2075	13.017	14.4378	14.3819	13.3954	13.6388	13.397	12.6452	0.175612094	0.651716826	12	20	24	0.390102423	0.799546792	24	12.09653012	0.047769936
ENSMUSG00000071648	Rom1	rod outer segment membrane protein 1 [Source:MGI Symbol;Acc:MGI:97998]	8.22837	6.97167	7.55079	7.75203	7.32543	7.57484	7.01083	7.75288	7.05023	7.50448	7.18776	7.22429	7.45514	7.02412	0.860993221	0.966018836	16	16	24	0.84089917	0.951840198	24	18.96687907	0.013647008
ENSMUSG00000071649	B3gat3	beta-1,3-glucuronyltransferase 3 (glucuronosyltransferase I) [Source:MGI Symbol;Acc:MGI:1919977]	55.5837	48.5903	47.755	49.9926	49.986	51.1996	48.4124	52.4269	43.9909	54.6513	49.9599	51.7312	52.1916	52.6637	0.595952838	0.966018836	4	8	24	0.415022541	0.800271135	24	21.10775258	0.045187183
ENSMUSG00000071650	Ganab	alpha glucosidase 2 alpha neutral subunit [Source:MGI Symbol;Acc:MGI:1097667]	41.2454	39.5482	39.0016	39.9316	42.2185	42.4391	41.7566	41.0328	36.8161	43.1558	36.3138	38.309	41.0029	40.3194	0.628286596	0.966018836	24	12	24	0.624897505	0.872055388	24	21.86414374	0.028613491
ENSMUSG00000071652	Ints5	integrator complex subunit 5 [Source:MGI Symbol;Acc:MGI:1923578]	3.83944	3.59443	3.39206	3.7884	4.15995	4.57308	3.26617	4.65547	3.32774	4.72901	3.5426	3.89726	3.7481	3.83602	0.745118544	0.966018836	24	12	24	0.69075455	0.897666745	24	16.81147264	0.048520763
ENSMUSG00000071653	1810009A15Rik	RIKEN cDNA 1810009A15 gene [Source:MGI Symbol;Acc:MGI:1913526]	15.0026	15.7671	15.5161	17.4195	18.0566	14.1952	17.2196	16.5398	22.169	13.684	15.2996	16.3479	18.5655	17.9008	0.916556573	0.966018836	12	12	24	0.472502361	0.805393068	24	6.331662304	0.081153747
ENSMUSG00000071654	Uqcc3	ubiquinol-cytochrome c reductase complex assembly factor 3 [Source:MGI Symbol;Acc:MGI:2147553]	23.9496	26.2626	23.9324	23.468	22.2694	22.3006	22.7107	24.6594	27.7727	23.8979	25.3681	25.9953	26.3778	25.0592	0.459019924	0.966018836	8	12	24	0.198423235	0.768040178	24	5.744572276	0.059340984
ENSMUSG00000071655	Ubxn1	UBX domain protein 1 [Source:MGI Symbol;Acc:MGI:1289301]	72.2861	73.2673	72.3152	77.1854	70.5246	76.1909	71.8444	72.63	75.9477	76.6757	72.603	79.0857	77.0723	74.1003	0.952551093	0.966018836	16	12	24	0.841321966	0.951908032	24	14.61540183	0.010295575
ENSMUSG00000071656	Lrrn4cl	LRRN4 C-terminal like [Source:MGI Symbol;Acc:MGI:1916102]	2.0843	2.4449	2.52437	2.59629	1.89715	1.70477	1.69496	2.31181	4.08085	2.10736	1.62809	2.41687	1.68339	2.58471	0.016110955	0.253395174	12	16	24	0.020389706	0.565578297	24	7.912890357	0.254538027
ENSMUSG00000071657	Bscl2	Berardinelli-Seip congenital lipodystrophy 2 (seipin) [Source:MGI Symbol;Acc:MGI:1298392]	37.6517	30.0733	33.2916	36.757	32.2564	38.4433	36.9584	39.0131	27.9299	42.3028	32.9861	33.7812	34.1465	34.7949	0.891105762	0.966018836	4	8	24	0.765363003	0.924119887	24	19.52938423	0.047165618
ENSMUSG00000071658	Gng3	guanine nucleotide binding protein (G protein), gamma 3 [Source:MGI Symbol;Acc:MGI:102704]	149.592	115.014	116.646	115.458	117.242	147.029	128.473	132.606	93.7475	140.425	122.24	116.467	118.176	124.632	0.43565473	0.966018836	4	8	24	0.230030487	0.768040178	24	21.14501576	0.115471889
ENSMUSG00000071659	Hnrnpul2	heterogeneous nuclear ribonucleoprotein U-like 2 [Source:MGI Symbol;Acc:MGI:1915943]	39.6078	33.6312	36.0884	34.8942	34.9905	35.7772	38.3984	34.1197	32.3409	38.1757	32.593	36.2646	36.594	36.2695	0.062631097	0.410322149	4	8	24	0.325028464	0.768040178	24	22.52957291	0.049590598
ENSMUSG00000071660	Ttc9c	tetratricopeptide repeat domain 9C [Source:MGI Symbol;Acc:MGI:1917637]	24.4011	23.5224	23.1013	22.2435	23.2428	23.0389	23.596	21.2092	21.8954	21.6152	25.3075	22.3775	24.239	23.6457	0.113148904	0.530777648	4	12	24	0.242131306	0.768040178	24	21.23678185	0.045980729
ENSMUSG00000071661	Zbtb3	zinc finger and BTB domain containing 3 [Source:MGI Symbol;Acc:MGI:1922541]	1.76436	1.53307	1.52199	1.19041	1.12058	1.82942	1.8407	1.44968	1.44316	1.25053	1.10897	1.62629	1.38626	1.73791	0.004644371	0.140598674	20	20	24	0.105591628	0.717832234	24	19.86455799	0.12817165
ENSMUSG00000071662	Polr2g	polymerase (RNA) II (DNA directed) polypeptide G [Source:MGI Symbol;Acc:MGI:1914960]	71.7123	77.1464	76.8721	72.2606	81.458	73.999	69.917	71.8167	76.5545	59.8123	76.9008	68.349	72.7451	72.5282	0.81705122	0.966018836	20	8	24	0.992823278	0.998516607	24	22.90741367	0.004901488
ENSMUSG00000071669	Snx29	sorting nexin 29 [Source:MGI Symbol;Acc:MGI:1921728]	6.49643	5.70867	5.59661	6.75244	6.68189	5.39735	6.53259	5.783	4.79675	5.1628	5.73906	6.60897	6.3658	6.35828	0.271166436	0.780905461	20	16	24	0.218234791	0.768040178	24	21.07002128	0.091202966
ENSMUSG00000071679	Rtl4	retrotransposon Gag like 4 [Source:MGI Symbol;Acc:MGI:3588192]	0.724838	0.713723	0.594412	0.826936	0.712913	0.895604	0.627074	0.755558	0.710553	0.776923	0.677405	0.77355	0.846809	0.690712	0.390686537	0.915479136	24	12	24	0.45921467	0.805393068	24	18.7610829	0.034414994
ENSMUSG00000071691	Gm960	predicted gene 960 [Source:MGI Symbol;Acc:MGI:2685806]	0.884071	0.809189	1.44653	0.559376	0.616977	1.20661	1.2447	1.60489	1.19699	1.21635	1.19004	1.68327	0.982793	1.47441	0.894563866	0.966018836	24	20	24	0.424597118	0.805393068	24	3.404846178	0.105012178
ENSMUSG00000071708	Sms	spermine synthase [Source:MGI Symbol;Acc:MGI:109490]	22.5615	23.2918	22.7979	22.9275	20.9676	22.5076	22.4218	21.1974	21.7177	24.0382	21.8951	22.6101	21.7561	24.3294	0.891690964	0.966018836	16	4	24	0.609584957	0.864491738	24	7.142308472	0.02443041
ENSMUSG00000071711	Mpst	mercaptopyruvate sulfurtransferase [Source:MGI Symbol;Acc:MGI:2179733]	11.2875	12.1796	11.8494	12.2332	9.72891	10.0682	10.3771	13.0595	12.4206	14.6529	11.0437	12.1289	12.7121	12.6597	0.083354723	0.462922111	4	8	24	0.02913348	0.596648309	24	7.519567193	0.1330632
ENSMUSG00000071713	Csf2rb	colony stimulating factor 2 receptor, beta, low-affinity (granulocyte-macrophage) [Source:MGI Symbol;Acc:MGI:1339759]	1.14322	1.19404	1.14229	0.875124	1.11307	1.2383	1.10569	1.14225	1.10977	1.0293	0.91035	0.79005	1.15801	0.796699	0.699015352	0.966018836	4	12	24	0.488951949	0.805393068	24	1.345821048	0.042448881
ENSMUSG00000071714	Csf2rb2	colony stimulating factor 2 receptor, beta 2, low-affinity (granulocyte-macrophage) [Source:MGI Symbol;Acc:MGI:1339760]	0.554732	0.513477	0.672282	0.429269	0.651688	0.519276	0.500717	0.470266	0.62858	0.533944	0.472065	0.410959	0.372577	0.376564	0.608967886	0.966018836	12	20	24	0.298899344	0.768040178	24	11.62065546	0.043793602
ENSMUSG00000071715	Ncf4	neutrophil cytosolic factor 4 [Source:MGI Symbol;Acc:MGI:109186]	2.18583	3.48875	5.12173	3.95346	3.29773	3.79087	3.43507	2.4907	2.67636	2.15952	1.96105	2.30963	2.79823	1.71294	0.833786882	0.966018836	12	20	24	0.858326541	0.958262235	24	11.35468109	0.05786062
ENSMUSG00000071719	Tmem28	transmembrane protein 28 [Source:MGI Symbol;Acc:MGI:3648377]	3.30551	2.5706	2.58658	2.86611	2.66711	2.9651	3.02213	2.48888	1.99659	3.0894	2.56258	2.89324	2.70451	2.64471	0.076241493	0.453790923	4	8	24	0.096813289	0.71175955	24	20.09568995	0.103045445
ENSMUSG00000071723	Gspt2	G1 to S phase transition 2 [Source:MGI Symbol;Acc:MGI:1316727]	8.09859	7.19626	7.73995	7.05799	7.07337	8.40708	7.23444	7.50697	6.41915	7.76706	7.51595	7.78113	7.30657	7.53385	0.471214947	0.966018836	24	12	24	0.219670751	0.768040178	24	20.71435286	0.05812244
ENSMUSG00000071724	Smpd5	sphingomyelin phosphodiesterase 5 [Source:MGI Symbol;Acc:MGI:3709877]	1.73352	1.24713	1.36053	1.23401	1.71314	1.99396	1.10406	1.52693	1.58052	2.2302	1.66148	2.15746	1.39027	1.97383	0.608967886	0.966018836	24	4	24	0.398888634	0.799546792	24	16.55745895	0.093594398
ENSMUSG00000071748	Gm14698	predicted gene 14698 [Source:MGI Symbol;Acc:MGI:3709613]	0.749658	1.04117	1.05982	1.23697	1.04239	0.846348	0.857295	1.11231	1.02042	0.932207	1.07028	0.981581	0.891294	0.985089	0.039104	0.338446795	12	16	24	0.029799743	0.596648309	24	10.5559698	0.086533468
ENSMUSG00000071753	Cdr1os	cerebellar degeneration related antigen 1, opposite strand [Source:MGI Symbol;Acc:MGI:5637821]	6.3496	6.72762	7.6935	5.24714	6.29426	5.57232	5.94331	5.90163	6.19104	5.62871	5.8163	5.63889	6.11211	5.76823	0.23919737	0.741580974	12	4	24	0.293785081	0.768040178	24	5.223888438	0.06442458
ENSMUSG00000071757	Zhx2	zinc fingers and homeoboxes 2 [Source:MGI Symbol;Acc:MGI:2683087]	3.59591	2.88793	2.87196	2.74379	3.6389	3.00181	3.80078	2.99008	3.10658	3.6207	3.14197	3.19088	3.3757	2.90512	0.174261892	0.650395613	4	4	24	0.282545137	0.768040178	24	20.00958646	0.073928056
ENSMUSG00000071793	2610005L07Rik	RIKEN cDNA 2610005L07 gene [Source:MGI Symbol;Acc:MGI:1914283]	2.2174	0.727435	1.09197	0.713635	0.690165	0.835245	0.593215	0.666183	0.669357	0.834973	0.97051	1.42943	1.32804	1.17781	0.694784292	0.966018836	24	12	24	0.32024156	0.768040178	24	22.62207157	0.168278705
ENSMUSG00000071796	6820431F20Rik	RIKEN cDNA 6820431F20 gene [Source:MGI Symbol;Acc:MGI:3694236]	1.53335	1.45159	1.36946	1.33132	1.20743	1.67347	1.35571	1.2743	1.25961	1.50456	1.56945	1.54498	1.67964	1.41692	0.123394836	0.552144108	24	12	24	0.137117836	0.747972781	24	20.91400975	0.066120147
ENSMUSG00000071847	Apcdd1	adenomatosis polyposis coli down-regulated 1 [Source:MGI Symbol;Acc:MGI:3513977]	2.35153	3.02895	2.93344	2.24156	2.72367	2.46809	2.50698	3.45183	2.82349	3.10437	1.82074	2.06107	2.04679	2.52293	0.070873295	0.440262583	8	16	24	0.051548108	0.64767123	24	6.961022666	0.160002053
ENSMUSG00000071855	Ccdc112	coiled-coil domain containing 112 [Source:MGI Symbol;Acc:MGI:1918800]	3.73205	4.26963	3.78741	3.60916	3.82084	3.76448	3.08302	3.93423	4.38127	3.97725	3.92466	4.35404	3.19952	3.84472	0.243586913	0.741580974	12	16	24	0.431544465	0.805393068	24	10.52205001	0.059742462
ENSMUSG00000071856	Mcc	mutated in colorectal cancers [Source:MGI Symbol;Acc:MGI:96930]	1.81312	2.01946	2.20623	1.65587	1.58377	1.99378	1.50896	1.50099	1.62802	2.17085	1.40436	2.24713	1.70126	1.6797	0.860993221	0.966018836	24	20	24	0.988017052	0.997530201	24	12.33550453	0.008971186
ENSMUSG00000071862	Lrrtm2	leucine rich repeat transmembrane neuronal 2 [Source:MGI Symbol;Acc:MGI:2389174]	8.69836	8.09579	8.29752	8.15462	8.43133	8.20607	8.33326	7.23049	7.96859	6.74807	8.61198	8.30777	8.51239	7.78146	0.048507657	0.379129806	4	4	24	0.123727733	0.736651392	24	20.6234267	0.068513777
ENSMUSG00000071866	Ppia	peptidylprolyl isomerase A [Source:MGI Symbol;Acc:MGI:97749]	7.28582	7.96763	6.33183	7.90206	6.43572	6.01245	5.69416	8.30116	8.73789	7.05847	7.56029	7.22324	6.97947	9.72856	0.084464639	0.466404237	8	16	24	0.067939239	0.671988997	24	7.029057291	0.156027165
ENSMUSG00000071867	Mrpl27-ps	mitochondrial ribosomal protein L27, pseudogene [Source:MGI Symbol;Acc:MGI:3647929]	24.1737	28.8106	26.0723	27.7689	32.465	26.823	21.6152	25.2972	24.4507	22.3475	27.5509	22.7346	23.9547	26.5414	0.350544145	0.870418653	20	8	24	0.480789643	0.805393068	24	13.6507412	0.067771665
ENSMUSG00000071984	Fndc1	fibronectin type III domain containing 1 [Source:MGI Symbol;Acc:MGI:1915905]	3.18284	5.38948	4.06268	5.11468	4.75276	4.45277	5.70878	4.77237	5.60301	5.46736	4.62514	4.3182	5.61309	3.67853	0.914502381	0.966018836	4	20	24	0.695765333	0.899065372	24	10.71974454	0.069992286
ENSMUSG00000072066	6720489N17Rik	RIKEN cDNA 6720489N17 gene [Source:MGI Symbol;Acc:MGI:2443901]	4.32043	4.33201	3.20434	3.59129	3.47885	3.65694	4.18108	3.47661	4.19073	2.80141	3.78719	4.05956	4.09014	3.65298	0.036134798	0.331852526	4	24	24	0.044642843	0.628432994	24	23.79569526	0.125186333
ENSMUSG00000072082	Ccnf	cyclin F [Source:MGI Symbol;Acc:MGI:102551]	0.565871	0.629378	0.835194	0.888342	0.769784	0.74331	0.658042	0.709678	0.715547	0.521601	0.688329	0.669756	0.927061	0.822851	0.883907406	0.966018836	12	16	24	0.901729549	0.972406468	24	8.030004792	0.018503159
ENSMUSG00000072115	Ang	angiogenin, ribonuclease, RNase A family, 5 [Source:MGI Symbol;Acc:MGI:88022]	3.87397	3.92584	4.02797	3.93101	4.61048	2.85522	3.09356	3.87354	4.83415	2.22265	3.95192	3.52619	2.95246	3.70698	0.080164531	0.458678242	12	12	24	0.676191442	0.891741508	24	8.456472227	0.079973043
ENSMUSG00000072214	Septin5	septin 5 [Source:MGI Symbol;Acc:MGI:1195461]	107.84	76.048	75.9071	88.7018	86.0475	93.2328	84.0806	85.3998	64.1289	104.86	77.0359	87.8976	76.373	82.1384	0.069101108	0.433403132	16	20	24	0.297917334	0.768040178	24	19.0586923	0.117883326
ENSMUSG00000072235	Tuba1a	tubulin, alpha 1A [Source:MGI Symbol;Acc:MGI:98869]	886.354	760.702	768.865	782.81	880.075	901.285	784.515	823.676	683.563	822.462	825.107	841.311	792.041	789.089	0.001971398	0.100255953	24	12	24	0.008076787	0.494192776	24	19.32067335	0.111001449
ENSMUSG00000072244	Trim6	tripartite motif-containing 6 [Source:MGI Symbol;Acc:MGI:2137352]	0.698773	0.742154	0.648983	1.07457	0.667118	0.535407	0.697679	0.724881	0.800739	0.597211	0.846817	0.577925	0.741046	0.644266	0.496745696	0.966018836	12	12	24	0.409520241	0.799546792	24	10.22397113	0.057313717
ENSMUSG00000072258	Taf1a	TATA-box binding protein associated factor, RNA polymerase I, A [Source:MGI Symbol;Acc:MGI:109578]	8.52149	8.42136	8.09079	8.03954	8.01259	8.18473	8.47711	8.39554	8.01851	7.85291	8.76599	8.45398	8.61694	8.08952	0.003846392	0.140598674	4	12	24	0.037181218	0.609176161	24	22.54882984	0.039118552
ENSMUSG00000072294	Klf12	Kruppel-like factor 12 [Source:MGI Symbol;Acc:MGI:1333796]	1.45394	2.31272	1.75143	1.8517	1.87796	1.63739	1.98974	1.70676	2.04256	1.74283	1.96484	1.89038	1.56911	2.02284	0.745199643	0.966018836	8	20	24	0.37193861	0.793398709	24	7.33349137	0.076023284
ENSMUSG00000072381	Gm10355	predicted gene 10355 [Source:MGI Symbol;Acc:MGI:3641762]	2.26198	1.94756	2.94684	2.43723	2.42618	1.90299	3.02406	2.41212	2.45962	2.6174	3.46876	1.4554	2.35093	2.06435	0.1321346	0.567728672	20	4	24	0.424234445	0.805393068	24	11.05553215	0.112631609
ENSMUSG00000072407	Gm6419	predicted gene 6419 [Source:MGI Symbol;Acc:MGI:3647564]	51.3585	56.0415	59.8015	53.6346	58.0546	47.1289	53.3155	55.7444	65.2233	47.863	57.2699	52.749	62.5262	55.7018	0.047644929	0.377556215	12	12	24	0.303483187	0.768040178	24	6.897656461	0.079396031
ENSMUSG00000072437	Nanos1	nanos C2HC-type zinc finger 1 [Source:MGI Symbol;Acc:MGI:2669254]	1.25038	1.47143	1.5098	1.5047	1.26742	1.29182	1.33639	1.12474	1.29555	1.39465	1.5109	1.51926	1.3733	1.4903	0.943890799	0.966018836	24	4	24	0.56168655	0.841693408	24	12.33493929	0.03191672
ENSMUSG00000072494	Ppp1r3e	protein phosphatase 1, regulatory subunit 3E [Source:MGI Symbol;Acc:MGI:2145790]	12.94	9.36225	9.70094	11.1312	10.0276	10.1287	9.73617	11.3185	10.0505	14.7576	10.8011	12.4631	11.8491	10.0625	0.63897419	0.966018836	16	20	24	0.696333131	0.89922881	24	16.53063812	0.058071057
ENSMUSG00000072501	Phf20l1	PHD finger protein 20-like 1 [Source:MGI Symbol;Acc:MGI:2444412]	11.4889	14.343	13.9526	13.6391	13.5424	12.3609	14.2252	12.3931	14.5397	11.0519	14.0659	12.2595	12.2676	12.5606	0.245061943	0.741580974	12	12	24	0.654579182	0.883569804	24	9.152251612	0.043750534
ENSMUSG00000072553	Gm525	predicted gene 525 [Source:MGI Symbol;Acc:MGI:2685371]	7.54124	6.36974	3.50683	4.63455	4.90893	5.2571	3.90552	4.8704	5.3956	4.30119	4.9877	6.5335	6.09725	6.02369	0.134026105	0.56887508	4	12	24	0.188396157	0.768040178	24	23.70040079	0.168779634
ENSMUSG00000072568	Lratd2	LRAT domain containing 2 [Source:MGI Symbol;Acc:MGI:3026924]	0.577536	0.697163	0.804737	0.608904	0.603759	0.627445	0.700023	0.899439	0.63633	0.660555	0.680295	0.563733	0.674567	0.768076	0.017495149	0.255326033	8	16	24	0.035840034	0.609176161	24	5.747812096	0.072504305
ENSMUSG00000072571	Tmem253	transmembrane protein 253 [Source:MGI Symbol;Acc:MGI:3588246]	1.09184	0.326498	1.37486	0.571803	0.985593	0.936168	0.703602	1.13186	0.902683	0.706414	0.621765	1.18074	1.19471	0.423674	0.94086481	0.966018836	4	4	24	0.744315188	0.916430276	24	20.57738232	0.08146189
ENSMUSG00000072572	Slc39a2	solute carrier family 39 (zinc transporter), member 2 [Source:MGI Symbol;Acc:MGI:2684326]	0.897796	0.841507	0.875694	1.23312	1.06785	0.937091	1.57293	0.969233	1.00727	0.752721	0.962241	0.843601	0.718847	0.90368	0.890176313	0.966018836	20	8	24	0.928637727	0.981515518	24	18.49184078	0.023152849
ENSMUSG00000072582	Ptrh2	peptidyl-tRNA hydrolase 2 [Source:MGI Symbol;Acc:MGI:2444848]	5.27215	5.71898	5.38969	5.41783	4.92634	5.19982	5.57103	5.76788	5.83523	5.03609	5.69766	5.65063	6.13003	6.13738	0.083354723	0.462922111	8	8	24	0.007250588	0.47735824	24	3.696728837	0.070362994
ENSMUSG00000072591	Fzd10os	frizzled class receptor 10, opposite strand [Source:MGI Symbol;Acc:MGI:2442398]	0.50061	1.4241	1.45122	0.54125	1.75032	1.95118	1.25129	2.22987	1.62272	1.52818	1.6384	1.51124	0.97309	0.668811	0.17882595	0.655480352	20	8	24	0.785415333	0.931158434	24	14.75656048	0.094892489
ENSMUSG00000072594	Gm16439	predicted pseudogene 16439 [Source:MGI Symbol;Acc:MGI:3647611]	62.9056	70.0312	61.1708	63.1238	58.7684	61.0378	60.8711	63.6183	62.4618	62.6135	65.4817	61.7214	71.162	67.379	0.4776989	0.966018836	8	16	24	0.170210788	0.767957732	24	3.60780368	0.052426311
ENSMUSG00000072596	Ear2	eosinophil-associated, ribonuclease A family, member 2 [Source:MGI Symbol;Acc:MGI:108020]	1.93504	2.91025	3.50505	3.60242	1.76278	3.28113	2.53225	2.81589	2.93158	2.22609	2.16146	3.34672	3.15816	1.93937	0.759899911	0.966018836	12	16	24	0.904021883	0.97351114	24	6.898856151	0.04733481
ENSMUSG00000072601	Ear1	eosinophil-associated, ribonuclease A family, member 1 [Source:MGI Symbol;Acc:MGI:108021]	1.66655	1.76528	2.51985	2.6484	1.61992	2.37494	2.11861	1.61854	1.28424	0.81405	1.44011	1.23457	2.41907	1.03505	0.865490576	0.966018836	4	12	24	0.670254117	0.888925819	24	22.12602745	0.100244847
ENSMUSG00000072620	Slfn2	schlafen 2 [Source:MGI Symbol;Acc:MGI:1313258]	2.61416	3.50661	5.2005	3.1017	3.41382	2.90538	2.58089	2.91599	4.49829	2.65098	2.93248	2.68533	3.42178	2.72358	0.074036902	0.448301997	12	16	24	0.124259113	0.738073221	24	8.668932954	0.180703924
ENSMUSG00000072623	Zfp9	zinc finger protein 9 [Source:MGI Symbol;Acc:MGI:99210]	7.96527	7.73722	7.20324	7.78816	7.37048	7.40025	7.42392	7.81746	7.72099	7.70225	7.60265	8.13334	7.84608	8.19602	0.34708369	0.870418653	8	12	24	0.418908539	0.803307367	24	2.362617446	0.021764858
ENSMUSG00000072640	Lyrm9	LYR motif containing 9 [Source:MGI Symbol;Acc:MGI:1913524]	10.074	8.65317	12.6877	8.47322	10.3733	9.63324	8.03239	10.1453	10.0438	9.88003	8.85861	11.4108	11.1937	11.5672	0.897570341	0.966018836	12	4	24	0.755825305	0.921118175	24	5.746370578	0.050786626
ENSMUSG00000072647	Adam1a	a disintegrin and metallopeptidase domain 1a [Source:MGI Symbol;Acc:MGI:2429504]	3.58877	3.67988	2.56552	3.62372	3.09859	3.17186	3.48966	3.18707	4.01988	2.76708	3.69984	3.28621	3.36363	3.01288	0.814723813	0.966018836	4	12	24	0.816103781	0.94140005	24	22.54481329	0.035160432
ENSMUSG00000072653	Zfp783	zinc finger protein 783 [Source:MGI Symbol;Acc:MGI:3040704]	2.36541	3.62323	3.70081	2.45694	3.45807	2.95052	2.70385	3.25454	4.41968	3.63103	3.14772	3.36909	2.78277	3.89584	0.005041121	0.143276577	12	16	24	0.069167422	0.673520298	24	8.621313159	0.16407562
ENSMUSG00000072676	Tmem254a	transmembrane protein 254a [Source:MGI Symbol;Acc:MGI:1196450]	62.4464	51.5707	50.7602	44.9473	48.6412	60.7408	50.4147	52.0118	45.9482	56.2731	50.2564	56.53	52.6188	48.7177	0.081336319	0.462794877	24	12	24	0.064723511	0.663876076	24	21.42784541	0.11804209
ENSMUSG00000072692	Rpl37rt	ribosomal protein L37, retrotransposed [Source:MGI Symbol;Acc:MGI:3651519]	518.819	635.614	554.708	608.135	589.689	528.006	539.001	510.758	673.549	423.426	598.25	533.719	599.103	543.141	0.78898997	0.966018836	12	12	24	0.908314212	0.974606327	24	7.038908956	0.030234614
ENSMUSG00000072693	Gm10401	predicted gene 10401 [Source:MGI Symbol;Acc:MGI:3704254]	12.4744	10.3595	10.6158	12.0013	9.44281	11.3609	8.14893	8.53748	6.36559	7.029	9.39487	8.23884	10.2694	8.71878	0.822716578	0.966018836	4	8	24	0.291959807	0.768040178	24	20.93419804	0.13206718
ENSMUSG00000072694	1500011B03Rik	RIKEN cDNA 1500011B03 gene [Source:MGI Symbol;Acc:MGI:1913486]	53.8378	52.6016	55.2445	51.5008	50.2542	54.8895	50.3376	52.4923	51.3682	56.4548	57.8638	60.743	55.4987	59.6763	0.868022237	0.966018836	24	12	24	0.877970022	0.965978948	24	19.59801019	0.014698943
ENSMUSG00000072704	Smim10l1	small integral membrane protein 10 like 1 [Source:MGI Symbol;Acc:MGI:1914379]	48.1367	41.4253	45.143	44.5512	42.0269	42.2849	45.8835	40.7503	34.4315	42.0233	41.5153	36.9904	33.8717	39.9283	0.882263435	0.966018836	4	4	24	0.790418804	0.932883394	24	17.89131773	0.029490511
ENSMUSG00000072720	Myo18b	myosin XVIIIb [Source:MGI Symbol;Acc:MGI:1921626]	0.489817	1.02394	1.22946	1.6817	1.06634	1.72992	1.17613	3.37231	2.0356	2.95593	1.00773	1.25448	1.36346	1.08546	0.868638024	0.966018836	16	4	24	0.227967641	0.768040178	24	9.547209363	0.267008633
ENSMUSG00000072723	Gm10044	predicted gene 10044 [Source:MGI Symbol;Acc:MGI:3641749]	0.93587	1.14044	1.42886	0.476445	1.15793	0.978461	1.40715	0.742157	0.763263	0.876519	0.977809	1.29359	1.06469	1.01756	0.696393581	0.966018836	4	12	24	0.335655722	0.771468587	24	22.65045259	0.113519593
ENSMUSG00000072761	Gm6712	predicted gene 6712 [Source:MGI Symbol;Acc:MGI:3645888]	2.09496	2.9722	1.79569	1.87675	2.1217	1.88058	1.82638	2.25434	2.02365	0.947752	2.48087	1.94464	1.92172	2.34614	0.337748746	0.85758369	8	8	24	0.368139899	0.79131734	24	1.279277061	0.122306923
ENSMUSG00000072762	4930522L14Rik	RIKEN cDNA 4930522L14 gene [Source:MGI Symbol;Acc:MGI:1925270]	1.70809	2.12173	1.56434	1.10775	1.81527	1.27353	1.40023	1.08567	2.10195	1.0965	1.66439	1.09746	1.7724	1.35253	0.933543173	0.966018836	12	4	24	0.778550212	0.928832041	24	3.808961051	0.057251652
ENSMUSG00000072763	5430403G16Rik	RIKEN cDNA 5430403G16 gene [Source:MGI Symbol;Acc:MGI:1924450]	0.498041	0.710057	0.636988	0.747467	0.910176	0.683257	0.700352	0.727828	1.02331	0.633116	0.981004	0.742431	0.66924	0.754631	0.350544145	0.870418653	20	8	24	0.207399992	0.768040178	24	12.60525671	0.072156167
ENSMUSG00000072769	Gm10419	predicted gene 10419 [Source:MGI Symbol;Acc:MGI:3642823]	18.1296	18.1484	19.8766	15.4131	16.912	17.4699	17.882	17.9991	15.962	19.1239	17.8076	17.3672	19.4928	18.5647	0.275983441	0.789849983	8	12	24	0.394123681	0.799546792	24	2.469347232	0.04998758
ENSMUSG00000072770	Acrbp	proacrosin binding protein [Source:MGI Symbol;Acc:MGI:1859515]	5.65433	5.79917	6.07265	6.61221	6.41499	4.99955	6.89253	5.5449	5.43801	4.00447	5.99152	4.93606	4.79803	4.08704	0.885059765	0.966018836	20	12	24	0.754290746	0.920457316	24	17.3893253	0.04857695
ENSMUSG00000072772	Grcc10	gene rich cluster, C10 gene [Source:MGI Symbol;Acc:MGI:1315201]	111.409	115.392	113.052	108.941	114.891	108.878	108.555	118.1	119.537	94.6318	120.302	115.444	118.831	113.553	0.904690479	0.966018836	8	8	24	0.587087518	0.853586751	24	1.357951506	0.035266271
ENSMUSG00000072774	Zfp951	zinc finger protein 951 [Source:MGI Symbol;Acc:MGI:2441896]	1.21335	2.06938	1.71905	1.4018	1.61777	1.2048	1.3832	1.7119	1.6782	1.22004	1.58888	1.73092	1.61518	1.68394	0.183755367	0.662021716	8	20	24	0.295677776	0.768040178	24	5.810651344	0.086064142
ENSMUSG00000072825	Cep170b	centrosomal protein 170B [Source:MGI Symbol;Acc:MGI:2145043]	19.6617	16.1889	17.5311	19.853	16.0365	18.9029	17.4436	19.2821	14.7369	20.9763	17.58	18.2771	17.0291	18.1838	0.934818505	0.966018836	16	20	24	0.843200707	0.952379736	24	17.2329386	0.031280465
ENSMUSG00000072844	G530011O06Rik	RIKEN cDNA G530011O06 gene [Source:MGI Symbol;Acc:MGI:3603513]	0.339333	0.600015	0.485936	0.700351	0.524379	0.611901	0.436628	0.573867	0.412228	0.483586	0.690705	0.317457	0.379453	0.500727	0.463629316	0.966018836	20	8	24	0.266439195	0.768040178	24	12.35934926	0.071046361
ENSMUSG00000072847	A530017D24Rik	RIKEN cDNA A530017D24 gene [Source:MGI Symbol;Acc:MGI:2144320]	10.7468	10.8734	10.5271	8.84059	10.2626	10.041	9.6064	10.0319	10.0759	10.9644	10.8578	9.63232	10.3474	10.3624	0.916556573	0.966018836	20	4	24	0.896472557	0.970591958	24	4.929499678	0.014908712
ENSMUSG00000072849	Serpina1e	serine (or cysteine) peptidase inhibitor, clade A, member 1E [Source:MGI Symbol;Acc:MGI:891967]	1.3197	1.40056	1.03911	1.38742	1.31469	1.31636	1.15804	0.882061	1.57486	1.57552	1.25869	1.92129	1.12104	1.09772	0.590017078	0.966018836	16	16	24	0.249397003	0.768040178	24	15.89302201	0.102042023
ENSMUSG00000072872	Rybp	RING1 and YY1 binding protein [Source:MGI Symbol;Acc:MGI:1929059]	3.21716	2.92049	3.14135	3.04844	3.05141	3.16139	3.48389	2.56339	2.78463	3.20353	3.11937	3.11555	3.06306	2.69866	0.056268883	0.401074772	4	4	24	0.076034564	0.686372559	24	18.55642428	0.070762447
ENSMUSG00000072875	Gpr27	G protein-coupled receptor 27 [Source:MGI Symbol;Acc:MGI:1202299]	6.53087	4.6832	4.97743	4.74117	4.84983	5.17232	5.78625	5.74157	5.27514	5.82913	5.41974	5.39987	5.38768	5.50619	0.664568268	0.966018836	4	8	24	0.337676499	0.772537663	24	23.92161468	0.062503999
ENSMUSG00000072889	Nfxl1	nuclear transcription factor, X-box binding-like 1 [Source:MGI Symbol;Acc:MGI:1923646]	4.33519	5.73009	5.68218	5.22464	5.90329	4.80164	5.69689	4.76955	6.13387	4.23407	5.10932	5.59231	4.72703	4.56003	0.943890799	0.966018836	12	16	24	0.839078246	0.950923036	24	11.64452532	0.034136743
ENSMUSG00000072893	4933439C10Rik	RIKEN cDNA 4933439C10 gene [Source:MGI Symbol;Acc:MGI:1921726]	7.63111	8.84155	9.51798	9.5275	11.0245	9.39321	7.58525	7.56896	9.1577	6.98766	8.26926	9.63568	8.38359	7.7307	0.215777641	0.704989686	24	4	24	0.268140203	0.768040178	24	15.6460561	0.098822805
ENSMUSG00000072915	Gm12258	predicted gene 12258 [Source:MGI Symbol;Acc:MGI:3651534]	6.51201	7.73714	7.63485	7.67025	7.55352	7.53473	6.62889	6.8941	7.72625	6.73748	7.27164	7.45192	7.35667	7.30953	0.112800839	0.530777648	12	16	24	0.455472281	0.805393068	24	11.4078152	0.037332237
ENSMUSG00000072919	Noxred1	NADP+ dependent oxidoreductase domain containing 1 [Source:MGI Symbol;Acc:MGI:1918525]	0.886071	0.473484	0.540814	0.623953	0.764207	0.627127	1.24203	0.51087	0.376074	0.420946	0.967365	0.516697	0.767611	0.536119	0.036657636	0.332441566	4	8	24	0.071224315	0.676901434	24	20.62694752	0.17195839
ENSMUSG00000072940	Gm10443	predicted pseudogene 10443 [Source:MGI Symbol;Acc:MGI:3704272]	205.752	221.842	215.969	219.941	211.816	195.897	225.82	211.392	282.204	185.671	226.348	221.221	259.746	230.523	0.91724565	0.966018836	12	4	24	0.390416581	0.799546792	24	5.125105077	0.076955973
ENSMUSG00000072941	Sod3	superoxide dismutase 3, extracellular [Source:MGI Symbol;Acc:MGI:103181]	4.10569	4.06302	4.01144	4.62606	4.17937	3.79652	5.11556	5.91262	4.67699	5.87553	4.60525	4.58314	5.11769	4.43974	0.916963526	0.966018836	20	4	24	0.587943662	0.85378867	24	7.719117899	0.060891714
ENSMUSG00000072946	Ptgr2	prostaglandin reductase 2 [Source:MGI Symbol;Acc:MGI:1916372]	20.5563	23.531	24.9801	26.6323	23.2821	21.5094	22.9479	23.4965	24.6628	24.2202	22.8911	23.7119	25.4119	24.3078	0.094391858	0.492161865	16	4	24	0.030903666	0.599371601	24	9.377565332	0.083512679
ENSMUSG00000072949	Acot1	acyl-CoA thioesterase 1 [Source:MGI Symbol;Acc:MGI:1349396]	10.5747	9.64217	7.74317	8.32734	9.20175	10.3234	11.144	9.86921	7.91347	9.86733	7.34061	8.63826	8.88098	10.7628	0.064303071	0.414867207	8	4	24	0.076907134	0.687041208	24	0.299616538	0.140172662
ENSMUSG00000072955	Tmsb15l	thymosin beta 15b like [Source:MGI Symbol;Acc:MGI:3026988]	13.1096	16.9079	14.7604	12.8268	13.5094	12.1682	14.0434	13.7528	18.1065	11.906	16.2896	14.5331	17.4763	19.0631	0.175220702	0.651010555	12	4	24	0.098646338	0.71175955	24	4.856090278	0.146911907
ENSMUSG00000072964	Bhlhb9	basic helix-loop-helix domain containing, class B9 [Source:MGI Symbol;Acc:MGI:1917487]	17.7237	18.1825	18.6366	16.5548	16.645	16.6281	16.9841	17.5875	16.6033	16.2908	16.9126	17.2891	17.4601	18.3039	9.70E-04	0.073964894	8	8	24	0.023630733	0.584818005	24	3.500828043	0.054023536
ENSMUSG00000072966	Gprasp2	G protein-coupled receptor associated sorting protein 2 [Source:MGI Symbol;Acc:MGI:2442071]	25.0247	25.9477	29.325	23.3228	22.4964	25.4367	27.6661	23.9399	25.3134	23.363	22.474	26.9158	23.823	29.3372	0.074677099	0.449147115	8	12	24	0.109085017	0.72082227	24	3.479882717	0.096097234

ENSMUSG00000072969	Armcx5	armadillo repeat containing, X-linked 5 [Source:MGI Symbol;Acc:MGI:2148026]	7.76322	10.1336	10.9644	9.61139	8.32111	9.10143	7.49663	8.57266	11.1901	11.4624	7.93345	9.22052	9.49732	10.6406	0.024253829	0.286402646	12	16	24	0.011298114	0.517565995	24	8.678982661	0.189127443
ENSMUSG00000072978	Gm5830	predicted pseudogene 5830 [Source:MGI Symbol;Acc:MGI:3647375]	3.58529	2.39305	2.64482	3.23695	2.63807	2.40013	2.78419	2.54035	1.26437	2.46633	2.43399	3.24272	3.08649	3.29873	0.708665292	0.966018836	4	8	24	0.279327724	0.768040178	24	21.69269252	0.157537794
ENSMUSG00000072980	Oip5	Opa interacting protein 5 [Source:MGI Symbol;Acc:MGI:1917895]	0.690939	0.912425	1.21811	1.03292	0.783355	0.629934	0.869581	1.00345	1.00393	0.828019	0.886409	1.16602	1.18933	1.15199	0.505728924	0.966018836	12	8	24	0.119130353	0.730142553	24	6.750048543	0.099475531
ENSMUSG00000073000	Gm10451	predicted gene 10451 [Source:MGI Symbol;Acc:MGI:3641898]	0.913176	0.501287	0.410026	1.15251	0.409447	0.453924	0.509261	0.682246	0.660654	0.638788	0.597524	0.659744	0.726015	0.581875	0.9143748	0.966018836	4	16	24	0.961292809	0.99001568	24	9.765808256	0.019890933
ENSMUSG00000073002	Vamp5	vesicle-associated membrane protein 5 [Source:MGI Symbol;Acc:MGI:1858622]	19.1523	20.3327	19.347	19.872	20.2039	16.232	20.0437	22.8088	23.4369	17.0412	19.7976	17.7357	23.7724	17.1974	0.658895034	0.966018836	12	12	24	0.420150525	0.803839449	24	5.477242773	0.082984318
ENSMUSG00000073016	Uprt	uracil phosphoribosyltransferase [Source:MGI Symbol;Acc:MGI:2685620]	4.66074	5.01779	4.40816	5.33223	4.94241	4.44571	5.06401	4.23828	4.558	3.63992	5.15974	4.52073	4.70345	4.66944	0.912686312	0.966018836	20	16	24	0.693192914	0.898256039	24	20.29750966	0.039774407
ENSMUSG00000073062	Zxdb	zinc finger, X-linked, duplicated B [Source:MGI Symbol;Acc:MGI:3694898]	1.33363	1.26988	1.36505	1.1558	1.13724	1.26075	1.23232	1.0566	1.1571	1.20608	1.05773	1.00156	0.783541	1.00031	0.924553956	0.966018836	12	12	24	0.628262395	0.872795898	24	10.83324054	0.024813554
ENSMUSG00000073063	Hbq1b	hemoglobin, theta 1B [Source:MGI Symbol;Acc:MGI:3613460]	0.871087	0.645795	0.867915	0.964817	1.14399	1.06448	0.457938	0.580537	0.76757	0.250463	0.836017	0.677263	1.16734	0.754956	0.784278077	0.966018836	20	12	24	0.537507269	0.827958985	24	19.22907774	0.099853203
ENSMUSG00000073073	Gm8098	predicted gene 8098 [Source:MGI Symbol;Acc:MGI:3645749]	1.06842	1.69096	1.38848	1.26384	1.18158	1.3189	1.12382	1.22521	1.25855	1.45063	1.10496	1.2833	1.28239	1.30054	0.455507782	0.966018836	16	12	24	0.322275268	0.768040178	24	7.497522845	0.058754261
ENSMUSG00000073079	Srp54a	signal recognition particle 54A [Source:MGI Symbol;Acc:MGI:1346087]	5.56847	3.93999	4.04482	4.1298	4.09157	3.5938	4.06171	3.35564	4.88727	4.34767	3.85759	4.34507	5.03782	4.01859	0.864782464	0.966018836	4	4	24	0.87641163	0.965584799	24	0.039641715	0.032428935
ENSMUSG00000073096	Lrrc61	leucine rich repeat containing 61 [Source:MGI Symbol;Acc:MGI:2652848]	10.5432	6.98278	7.4283	7.27552	6.65074	8.82654	6.98131	8.54346	5.65007	9.8259	7.06353	7.88104	7.36801	8.08703	0.93717134	0.966018836	24	20	24	0.611888923	0.865342826	24	21.97183346	0.083938831
ENSMUSG00000073102	Drc1	dynein regulatory complex subunit 1 [Source:MGI Symbol;Acc:MGI:2685906]	1.2955	1.83378	1.40024	1.00915	1.2294	1.56747	0.98558	1.43388	1.3687	1.04224	1.58101	1.78798	1.29109	1.40938	0.632729413	0.966018836	8	8	24	0.65761612	0.884531138	24	23.59314419	0.058780943
ENSMUSG00000073125	Xlr3b	X-linked lymphocyte-regulated 3B [Source:MGI Symbol;Acc:MGI:109505]	5.10853	4.52259	2.48017	5.52773	3.47538	5.06644	3.09032	3.39583	1.95825	2.70283	3.65463	3.01803	4.01383	3.17794	0.622773557	0.966018836	4	8	24	0.157587978	0.763993442	24	20.57728502	0.227942708
ENSMUSG00000073131	Vma21	VMA21 vacuolar H+-ATPase homolog (S. cerevisiae) [Source:MGI Symbol;Acc:MGI:1914298]	20.0574	20.5046	21.0683	19.6568	18.7995	18.1387	19.6211	18.1716	17.238	18.8461	19.7932	16.201	15.2604	18.0366	0.917610614	0.966018836	12	12	24	0.398038772	0.799546792	24	10.37710095	0.046348782
ENSMUSG00000073139	Tmem185a	transmembrane protein 185A [Source:MGI Symbol;Acc:MGI:2448555]	19.0705	17.3925	20.0537	18.9026	17.5398	18.5535	20.2681	15.0881	16.6604	20.7179	17.6205	17.9559	17.6962	18.7242	0.93717134	0.966018836	4	4	24	0.781149926	0.929846332	24	15.52977463	0.031901177
ENSMUSG00000073147	5031425E22Rik	RIKEN cDNA 5031425E22 gene [Source:MGI Symbol;Acc:MGI:1923227]	7.13748	8.69214	8.38197	9.14052	7.75513	6.74399	8.48944	8.54734	10.3197	8.2837	10.1527	9.17763	8.88331	9.34573	0.916746631	0.966018836	12	16	24	0.098592249	0.71175955	24	8.911679911	0.106882202
ENSMUSG00000073154	9330158H04Rik	RIKEN cDNA 9330158H04 gene [Source:MGI Symbol;Acc:MGI:2442094]	1.01734	1.0358	0.9834	0.637822	0.792953	1.10392	1.92026	0.826086	1.30108	0.880593	0.896783	0.915218	0.614594	1.25737	0.549118437	0.966018836	12	4	24	0.410970357	0.799546792	24	2.062801425	0.112098405
ENSMUSG00000073155	1810058I24Rik	RIKEN cDNA 1810058I24 gene [Source:MGI Symbol;Acc:MGI:1914955]	50.7256	50.0022	44.1774	50.8035	50.143	55.6829	49.1793	60.0309	50.3309	58.9313	51.3431	50.6414	52.5765	50.6759	0.880631923	0.966018836	16	20	24	0.953957028	0.988601783	24	18.49665452	0.013419419
ENSMUSG00000073164	2410018L13Rik	RIKEN cDNA 2410018L13 gene [Source:MGI Symbol;Acc:MGI:1916982]	0.528507	0.44517	1.00835	0.744755	0.666361	0.687232	0.749757	0.874563	0.591367	0.658896	0.596819	0.599825	0.498953	0.730715	0.890648739	0.966018836	16	12	24	0.439700038	0.805393068	24	9.281691478	0.065785534
ENSMUSG00000073176	Zfp449	zinc finger protein 449 [Source:MGI Symbol;Acc:MGI:1925869]	3.58556	3.20082	3.3007	3.73152	3.22837	2.96761	3.43744	2.5482	3.32771	2.58699	3.51896	3.24259	3.26005	3.4504	0.844511766	0.966018836	4	4	24	0.889357846	0.968827007	24	19.7765502	0.024517487
ENSMUSG00000073179	Gm10478	predicted gene 10478 [Source:MGI Symbol;Acc:MGI:3714531]	1.01856	0.942964	0.736203	0.763248	1.74112	1.50864	0.617156	0.749752	1.03444	1.01088	1.14878	1.4305	1.03016	1.17922	0.097587539	0.501112642	20	16	24	0.066111585	0.668186543	24	17.83788148	0.183580551
ENSMUSG00000073198	Bnip3l-ps	BCL2/adenovirus E1B interacting protein 3-like, pseudogene [Source:MGI Symbol;Acc:MGI:3642435]	31.0347	33.7955	34.2577	33.641	35.1188	32.2718	30.2598	33.2453	35.7684	28.3738	31.2072	31.2156	31.9698	30.0487	0.43565473	0.966018836	12	16	24	0.608819131	0.864393298	24	9.337653776	0.034104105
ENSMUSG00000073207	Ccdc160	coiled-coil domain containing 160 [Source:MGI Symbol;Acc:MGI:3588225]	4.15812	4.42175	3.92723	3.62304	5.01142	4.57429	4.40922	4.25044	3.31827	3.45217	4.18134	4.49813	3.95397	4.67437	0.084464639	0.466404237	24	16	24	0.065265811	0.665048441	24	21.52113067	0.119764688
ENSMUSG00000073233	Nfyc-ps	nuclear transcription factor-Y gamma, pseudogene [Source:MGI Symbol;Acc:MGI:3643264]	5.9245	6.42851	7.67021	7.0135	5.94995	6.59509	8.08975	6.73773	6.80882	5.312	6.50035	6.65399	6.01218	5.89605	0.852921792	0.966018836	12	8	24	0.855140657	0.9569057	24	3.431568371	0.029944706
ENSMUSG00000073234	Gm8773	predicted gene 8773 [Source:MGI Symbol;Acc:MGI:3646213]	1.72364	1.60701	2.26566	1.48619	1.88602	1.67564	1.44693	1.57794	1.49749	1.42323	1.78103	1.50839	1.69594	1.54964	0.676344318	0.966018836	20	20	24	0.931674279	0.982352286	24	14.51483418	0.016139208
ENSMUSG00000073236	2500004C02Rik	RIKEN cDNA 2500004C02 gene [Source:MGI Symbol;Acc:MGI:1919576]	3.23148	2.75352	3.28298	2.64322	4.05032	3.19494	2.5672	3.24134	3.80969	2.48354	2.4111	2.64172	2.72804	3.63822	0.280810068	0.792139038	12	4	24	0.69690212	0.899309039	24	6.257895412	0.063514371
ENSMUSG00000073293	Nudt10	nudix (nucleoside diphosphate linked moiety X)-type motif 10 [Source:MGI Symbol;Acc:MGI:2147931]	7.22714	6.41694	6.41978	6.51314	6.50615	7.17776	6.75822	5.76153	5.53385	6.85152	5.5122	6.20122	5.34078	6.39927	0.896311169	0.966018836	24	8	24	0.550007534	0.835275459	24	19.64256551	0.045451478
ENSMUSG00000073295	Nudt11	nudix (nucleoside diphosphate linked moiety X)-type motif 11 [Source:MGI Symbol;Acc:MGI:1930957]	6.19876	6.24196	6.67213	6.12116	6.17518	6.61254	6.36651	5.87099	6.04683	7.08637	5.42559	5.75946	5.21096	5.94815	0.93717134	0.966018836	16	4	24	0.48238007	0.805393068	24	10.8088664	0.041892875
ENSMUSG00000073374	C030034I22Rik	RIKEN cDNA C030034I22 gene [Source:MGI Symbol;Acc:MGI:1924783]	1.53387	1.46345	1.337	1.16425	1.43501	1.54774	1.48952	1.44407	1.9481	1.4119	1.26516	1.59916	1.50503	1.60489	0.133012827	0.567728672	24	20	24	0.305969236	0.768040178	24	2.504121732	0.058704505
ENSMUSG00000073375	Lrrc30	leucine rich repeat containing 30 [Source:MGI Symbol;Acc:MGI:2685172]	0.545901	1.23738	0.648022	1.94369	1.45522	2.39143	1.15212	2.69614	1.4941	1.76999	1.43807	1.79754	2.50601	0.957033	0.891690964	0.966018836	24	4	24	0.714598504	0.905979936	24	17.05274676	0.116770925
ENSMUSG00000073400	Trim10	tripartite motif-containing 10 [Source:MGI Symbol;Acc:MGI:1338757]	0.608649	1.26826	2.0286	1.97394	1.17394	1.44421	1.30192	1.00841	1.1588	0.640636	1.10321	1.3405	2.00818	0.943654	0.874607729	0.966018836	24	8	24	0.978981557	0.994840185	24	12.62192477	0.023910172
ENSMUSG00000073411	H2-D1	histocompatibility 2, D region locus 1 [Source:MGI Symbol;Acc:MGI:95896]	85.1665	77.4936	80.6506	78.3369	81.389	76.2387	75.9175	84.005	81.7383	80.3153	92.0698	86.1839	86.8042	83.1504	0.886002469	0.966018836	20	20	24	0.846173303	0.953426343	24	18.26707185	0.01637909
ENSMUSG00000073412	Lst1	leukocyte specific transcript 1 [Source:MGI Symbol;Acc:MGI:1096324]	3.6696	6.20686	10.3811	5.38823	9.01509	7.10122	6.38339	4.09112	9.40389	4.01095	8.10742	9.29585	6.77079	7.36998	0.537381546	0.966018836	12	16	24	0.73809729	0.91451866	24	14.14420656	0.125639263
ENSMUSG00000073413	Ly6g6d	lymphocyte antigen 6 complex, locus G6D [Source:MGI Symbol;Acc:MGI:2148931]	2.0282	1.67245	2.04083	2.25217	1.52454	1.25204	1.98755	1.55835	2.90111	1.06809	2.23419	1.41024	1.99326	2.00869	0.245061943	0.741580974	12	12	24	0.430769196	0.805393068	24	6.709249197	0.127444831
ENSMUSG00000073415	Gm10501	predicted gene 10501 [Source:MGI Symbol;Acc:MGI:3704476]	1.12876	0.929144	1.21733	1.05152	1.02563	1.00501	1.04187	1.26155	1.12829	0.899638	0.914424	0.969848	0.720314	0.725486	0.954106009	0.966018836	12	12	24	0.75115389	0.92103958	24	9.590109016	0.028248677
ENSMUSG00000073418	C4b	complement component 4B (Chido blood group) [Source:MGI Symbol;Acc:MGI:88228]	19.0656	12.9496	16.938	15.2042	17.5951	12.3549	16.3269	17.6258	16.7234	15.3029	16.3735	12.6512	14.5321	16.1058	0.777666236	0.966018836	12	12	24	0.608802995	0.864393298	24	7.795803307	0.072611804
ENSMUSG00000073421	H2-Ab1	histocompatibility 2, class II antigen A, beta 1 [Source:MGI Symbol;Acc:MGI:103070]	28.9181	35.6347	33.8784	26.2194	35.0093	27.0068	32.4476	37.5465	41.8729	22.0994	35.4591	33.0464	33.7878	33.3298	0.239653024	0.741580974	12	4	24	0.391945288	0.799546792	24	3.99577127	0.122115286
ENSMUSG00000073422	H2-Ke6	H2-K region expressed gene 6 [Source:MGI Symbol;Acc:MGI:95911]	22.3137	28.4869	27.5316	32.9311	28.0076	25.4098	25.6723	28.5138	26.6153	23.7463	28.733	27.02	28.4193	24.7608	0.952127548	0.966018836	20	8	24	0.485460758	0.805393068	24	11.94718101	0.063504195
ENSMUSG00000073423	Zfp414	zinc finger protein 414 [Source:MGI Symbol;Acc:MGI:1915641]	17.5532	16.4082	15.8128	15.1776	16.8087	17.1653	16.1742	16.0656	16.3782	17.7174	16.3009	15.8382	17.4645	17.7188	0.893609703	0.966018836	4	8	24	0.592840133	0.856634016	24	23.69641399	0.025934399
ENSMUSG00000073424	Cyp4f15	cytochrome P450, family 4, subfamily f, polypeptide 15 [Source:MGI Symbol;Acc:MGI:2146921]	2.21618	1.73908	1.62665	2.68013	1.67165	2.47112	1.93829	1.99283	2.11073	2.59854	2.71803	2.42851	2.3009	1.69886	0.040123816	0.34094463	16	20	24	0.114674242	0.727799063	24	16.45390851	0.134350102
ENSMUSG00000073427	Gm4924	predicted gene 4924 [Source:MGI Symbol;Acc:MGI:3643133]	2.77348	2.72413	2.43101	2.39689	2.70448	2.68499	2.26919	2.40492	2.96795	1.84261	2.6425	3.04182	2.97885	2.81506	0.70641939	0.966018836	24	16	24	0.320594055	0.768040178	24	23.13638581	0.079155942
ENSMUSG00000073433	Arhgdig	Rho GDP dissociation inhibitor (GDI) gamma [Source:MGI Symbol;Acc:MGI:108430]	46.1534	36.9616	34.8511	38.8922	38.0388	44.4251	38.2622	37.0786	30.2675	40.4684	38.4013	40.6765	38.1493	38.4115	0.01130565	0.204837764	24	12	24	0.021489341	0.574100263	24	20.19100118	0.139354836
ENSMUSG00000073434	Wdr90	WD repeat domain 90 [Source:MGI Symbol;Acc:MGI:1921267]	2.79898	2.98723	3.85694	3.19468	3.46058	3.05365	3.8058	3.76926	3.54054	3.17712	3.21182	3.34432	2.97807	3.04601	0.783267549	0.966018836	12	16	24	0.557579075	0.839231952	24	9.069200511	0.050066006
ENSMUSG00000073435	Nme3	NME/NM23 nucleoside diphosphate kinase 3 [Source:MGI Symbol;Acc:MGI:1930182]	20.5381	21.4899	20.3759	18.3582	21.1252	17.2543	21.3824	23.9875	25.462	18.0698	24.0457	20.9562	25.0811	20.3905	0.798829513	0.966018836	4	12	24	0.434195501	0.805393068	24	3.879336429	0.078983651
ENSMUSG00000073436	Eme2	essential meiotic structure-specific endonuclease subunit 2 [Source:MGI Symbol;Acc:MGI:1919889]	12.1491	12.1444	13.112	10.6009	11.4068	12.2815	12.2582	12.5916	11.9635	12.4595	11.9711	12.7839	11.8214	13.198	0.835401638	0.966018836	8	12	24	0.217185999	0.768040178	24	3.229727383	0.047282517
ENSMUSG00000073437	D330041H03Rik	RIKEN cDNA D330041H03 gene [Source:MGI Symbol;Acc:MGI:3603827]	1.10908	2.00018	1.3948	2.26252	3.36493	2.21999	1.22422	1.47172	2.7849	1.43326	3.08121	1.5117	1.55267	2.24083	0.030347909	0.324150946	20	8	24	0.133603244	0.743843563	24	13.65218029	0.244407387
ENSMUSG00000073448	Gm10509	predicted gene 10509 [Source:MGI Symbol;Acc:MGI:3796835]	5.33216	4.61324	4.71408	2.81793	4.81576	3.6521	4.00109	3.30153	4.47862	2.59407	3.90294	4.33992	4.41482	4.08085	0.581070934	0.966018836	4	12	24	0.212814109	0.768040178	24	23.93279653	0.151848556
ENSMUSG00000073460	Pnldc1	poly(A)-specific ribonuclease (PARN)-like domain containing 1 [Source:MGI Symbol;Acc:MGI:2685159]	1.06022	0.727005	0.603315	0.774701	0.915859	0.513538	1.24771	0.910474	0.58916	1.16396	0.46501	2.03308	0.472283	0.578739	0.888482515	0.966018836	24	12	24	0.586698414	0.853342401	24	19.95507002	0.126371105
ENSMUSG00000073468	Sft2d1	SFT2 domain containing 1 [Source:MGI Symbol;Acc:MGI:1918689]	14.1085	16.0023	14.2967	15.3253	14.4923	14.273	15.1761	14.4007	16.3552	13.9505	16.6263	16.0277	15.4991	15.7581	0.826055781	0.966018836	20	8	24	0.955296995	0.988639288	24	6.632461244	0.009257837
ENSMUSG00000073471	Rsph3a	radial spoke 3A homolog (Chlamydomonas) [Source:MGI Symbol;Acc:MGI:1914082]	14.5831	14.6715	10.3931	9.66147	13.0243	11.8065	13.9113	14.3098	12.5683	18.0231	15.8179	13.3031	17.5144	12.232	0.917867465	0.966018836	4	8	24	0.624913925	0.872055388	24	22.43499917	0.087410733
ENSMUSG00000073478	D730003I15Rik	RIKEN cDNA D730003I15 gene [Source:MGI Symbol;Acc:MGI:1925971]	5.18024	5.54204	4.40868	4.18605	5.07227	3.9345	4.30507	3.53291	6.75363	4.1694	5.16902	5.20893	4.87825	5.71813	0.888379597	0.966018836	12	4	24	0.845680659	0.953396028	24	6.247331024	0.048662676
ENSMUSG00000073481	Mtarc2	mitochondrial amidoxime reducing component 2 [Source:MGI Symbol;Acc:MGI:1914497]	70.5127	73.0508	73.2858	76.9547	78.2658	74.7381	73.7118	80.2287	86.0968	71.642	77.2658	73.041	82.4241	73.0232	0.881996584	0.966018836	20	12	24	0.699240365	0.900024462	24	9.104080267	0.02754221
ENSMUSG00000073489	Ifi204	interferon activated gene 204 [Source:MGI Symbol;Acc:MGI:96429]	1.37204	1.17372	1.64002	1.39938	1.49206	1.08881	1.29167	1.19842	1.73028	0.912383	1.44021	1.02311	1.08936	1.39885	0.046056765	0.374351104	12	12	24	0.360136383	0.785733735	24	8.954235312	0.082455392
ENSMUSG00000073490	Ifi207	interferon activated gene 207 [Source:MGI Symbol;Acc:MGI:2138302]	0.83774	0.834584	0.991178	1.06532	1.14378	0.694055	0.920475	0.797102	1.1725	0.761687	0.864323	0.670011	0.588113	0.628111	0.187873422	0.662719589	12	20	24	0.049968858	0.643727973	24	11.56122749	0.106666861
ENSMUSG00000073491	Ifi213	interferon activated gene 213 [Source:MGI Symbol;Acc:MGI:3695276]	0.698924	0.911782	0.371711	0.511287	0.462181	0.498093	0.628323	0.899811	0.718922	0.643615	0.818231	0.523982	0.780366	0.490339	0.671726933	0.966018836	8	8	24	0.460045288	0.805393068	24	2.663517252	0.072489897
ENSMUSG00000073514	Dok6	docking protein 6 [Source:MGI Symbol;Acc:MGI:3639495]	4.06992	4.43022	4.52205	3.99275	4.12906	3.97938	4.43899	3.49552	4.05444	3.26155	4.25483	3.37769	3.29973	3.83998	0.879206539	0.966018836	12	12	24	0.95927925	0.98927861	24	7.539973622	0.012293948
ENSMUSG00000073530	Pappa2	pappalysin 2 [Source:MGI Symbol;Acc:MGI:3051647]	0.566234	0.623636	0.683605	1.07955	0.527554	0.649186	0.496397	0.539453	0.399642	0.82589	0.441499	0.677884	0.367015	0.659822	0.117050591	0.538362324	16	12	24	0.28013392	0.768040178	24	12.09328505	0.093210628
ENSMUSG00000073542	Cep76	centrosomal protein 76 [Source:MGI Symbol;Acc:MGI:1923401]	2.26055	2.3851	2.54902	2.54329	2.21953	2.82399	2.09875	2.52325	2.2868	2.22024	2.56415	2.34553	2.34603	2.2101	0.888379597	0.966018836	24	4	24	0.704798181	0.902490129	24	15.52039973	0.026800549
ENSMUSG00000073555	Gm4951	predicted gene 4951 [Source:MGI Symbol;Acc:MGI:3644953]	0.510373	0.625198	0.503412	0.496837	0.576558	0.48287	0.484462	0.860234	0.67707	0.421313	0.683089	0.415011	0.508192	0.802399	0.03452758	0.328311403	8	16	24	0.168983081	0.767020009	24	5.990129934	0.088920154
ENSMUSG00000073557	Ppp1r12b	protein phosphatase 1, regulatory subunit 12B [Source:MGI Symbol;Acc:MGI:1916417]	12.317	11.6879	11.6915	12.7912	11.6121	13.3702	13.5632	16.3159	12.2997	16.1081	11.0845	11.8629	13.6419	11.7338	0.743750765	0.966018836	4	16	24	0.825067058	0.945127573	24	5.347072615	0.040507593
ENSMUSG00000073563	Csnk1g3	casein kinase 1, gamma 3 [Source:MGI Symbol;Acc:MGI:1917675]	26.0911	24.3378	25.7716	23.4806	26.3182	26.2421	27.0122	24.9021	21.7335	27.1693	26.5654	25.109	26.784	25.8991	0.246839642	0.745922296	20	16	24	0.184319041	0.768040178	24	20.2290802	0.061338095
ENSMUSG00000073565	Prr16	proline rich 16 [Source:MGI Symbol;Acc:MGI:1918623]	1.30705	1.36456	1.55706	1.12903	0.994095	1.39706	1.41437	0.926909	1.18724	1.18397	1.34979	1.26509	1.11004	1.48882	0.93717134	0.966018836	12	4	24	0.798664256	0.935594023	24	1.98046964	0.031618435
ENSMUSG00000073574	Grxcr2	glutaredoxin, cysteine rich 2 [Source:MGI Symbol;Acc:MGI:2685697]	1.25205	1.41724	1.29205	0.821076	1.60781	1.13326	1.76758	1.43503	0.945556	1.45423	1.68375	1.20659	1.51986	1.72764	0.664568268	0.966018836	4	8	24	0.503495964	0.810250042	24	23.56792511	0.078986818
ENSMUSG00000073590	3222401L13Rik	RIKEN cDNA 3222401L13 gene [Source:MGI Symbol;Acc:MGI:2444186]	0.741971	0.793084	0.742798	0.582042	0.70425	0.694981	0.815382	0.973813	0.996301	0.597677	0.820111	0.894774	0.748129	0.89948	0.092593422	0.487919468	8	8	24	0.107848847	0.72073763	24	3.300799564	0.072883178
ENSMUSG00000073591	Pcdhb22	protocadherin beta 22 [Source:MGI Symbol;Acc:MGI:2136760]	2.08363	2.46946	2.40976	1.95828	2.23739	2.06358	2.09536	2.15704	2.87874	2.2713	2.55997	2.15538	2.15989	2.16475	0.038115943	0.334069441	12	12	24	0.225514064	0.768040178	24	9.145770401	0.070623866
ENSMUSG00000073599	Ecscr	endothelial cell surface expressed chemotaxis and apoptosis regulator [Source:MGI Symbol;Acc:MGI:1915795]	29.39	40.5357	33.185	34.5002	44.7894	31.1886	37.4321	35.8952	47.7374	33.4769	35.9846	32.7894	35.2515	32.6678	0.913451828	0.966018836	20	4	24	0.502828088	0.809737567	24	9.866815627	0.090637946
ENSMUSG00000073600	Prob1	proline rich basic protein 1 [Source:MGI Symbol;Acc:MGI:2686460]	1.28295	1.55291	2.11168	1.98542	1.8698	2.62659	2.26344	2.58245	2.79184	2.26968	1.60791	2.38784	1.61268	1.38442707	0.90703203	0.966018836	16	16	24	0.524849669	0.822296127	24	12.38442707	0.082280174
ENSMUSG00000073609	D2hgdh	D-2-hydroxyglutarate dehydrogenase [Source:MGI Symbol;Acc:MGI:2138209]	4.68049	4.52666	4.07956	4.97269	4.47857	4.30371	5.95104	5.68829	4.29354	4.33842	5.54942	4.15365	5.94355	4.49669	0.105280783	0.518002936	4	8	24	0.467567499	0.805393068	24	23.75932455	0.077315977
ENSMUSG00000073616	Cops9	COP9 signalosome subunit 9 [Source:MGI Symbol;Acc:MGI:1914165]	235.809	270.386	247.967	231.766	253.978	218.055	259.547	229.509	286.958	189.543	283.954	252.084	270.766	252.723	0.933543173	0.966018836	4	12	24	0.72715018	0.91035631	24	2.061106545	0.04997818
ENSMUSG00000073627	C130036L24Rik	RIKEN cDNA C130036L24 gene [Source:MGI Symbol;Acc:MGI:2441877]	1.06129	0.671115	0.509114	0.736252	0.598584	0.634922	0.918096	0.515324	0.808154	0.62232	0.772604	0.840595	0.739401	0.863544	0.147689593	0.590732968	4	8	24	0.277150058	0.768040178	24	22.72742508	0.080655424
ENSMUSG00000073633	Fbxo36	F-box protein 36 [Source:MGI Symbol;Acc:MGI:1289192]	10.1587	10.5229	10.416	8.18414	10.5737	10.8543	8.82549	9.07211	10.355	10.6209	10.6725	10.804	9.47322	10.5586	0.068670003	0.432567661	24	4	24	0.792588635	0.933721061	24	17.74173913	0.032524672
ENSMUSG00000073639	Rab18	RAB18, member RAS oncogene family [Source:MGI Symbol;Acc:MGI:102790]	96.5891	88.7001	89.2567	92.6812	90.95	86.6053	89.4926	87.7858	81.7403	89.3147	86.7092	87.1948	90.0818	90.0859	0.922029406	0.966018836	4	20	24	0.598712898	0.859134895	24	21.78683475	0.020387326
ENSMUSG00000073647	Wdfy1	WD repeat and FYVE domain containing 1 [Source:MGI Symbol;Acc:MGI:1916618]	5.52382	5.92748	5.33803	5.74461	6.10005	5.27454	5.43509	5.19831	6.05791	5.49586	5.86979	5.38367	5.65645	5.72513	0.369262716	0.893596186	20	4	24	0.481670539	0.805393068	24	11.82134809	0.030196952
ENSMUSG00000073648	Gm10557	predicted gene 10557 [Source:MGI Symbol;Acc:MGI:3708638]	49.027	52.0383	51.8346	52.5602	50.4234	53.134	51.5769	46.3501	48.3459	45.4281	50.7156	50.9101	51.9926	51.9603	0.912686312	0.966018836	24	12	24	0.495128812	0.806978832	24	21.21807184	0.03244405
ENSMUSG00000073650	Catip	ciliogenesis associated TTC17 interacting protein [Source:MGI Symbol;Acc:MGI:2685062]	0.569231	0.978715	1.37097	1.0518	0.729519	0.64287	1.04976	0.774286	0.965461	0.691453	0.541575	0.764841	0.362727	1.10759	0.121531327	0.548996929	12	8	24	0.066662222	0.669134584	24	7.758321647	0.180435917
ENSMUSG00000073664	Nbeal1	neurobeachin like 1 [Source:MGI Symbol;Acc:MGI:2444343]	6.33099	6.96426	6.42669	7.22889	6.80636	5.67979	6.67355	5.52108	6.89473	4.89092	7.17107	5.5069	5.56462	6.34166	0.718720526	0.966018836	16	20	24	0.805594838	0.937926438	24	10.7771059	0.037046773
ENSMUSG00000073676	Hspe1	heat shock protein 1 (chaperonin 10) [Source:MGI Symbol;Acc:MGI:104680]	47.7487	39.9468	36.3615	39.848	45.285	38.4377	38.5169	44.7681	43.7463	33.6115	50.6996	40.1269	45.0638	42.0071	0.676344318	0.966018836	20	4	24	0.604434619	0.861894646	24	21.57903421	0.064425563
ENSMUSG00000073678	Pgap1	post-GPI attachment to proteins 1 [Source:MGI Symbol;Acc:MGI:2443342]	4.62723	4.42606	4.19275	4.20064	4.67517	4.10901	4.67543	3.88191	4.29866	3.2933	4.96591	4.11566	4.19105	4.23209	0.936399519	0.966018836	20	20	24	0.389082217	0.799546792	24	20.99067713	0.062237583
ENSMUSG00000073680	Tmem88b	transmembrane protein 88B [Source:MGI Symbol;Acc:MGI:2444329]	2.85869	5.90774	10.163	4.15678	4.21893	5.22408	7.10838	4.46429	7.06998	4.0915	3.93519	5.29562	2.89688	6.29367	0.212359178	0.701530532	12	4	24	0.269983468	0.768040178	24	7.231265798	0.273334009
ENSMUSG00000073684	Faap20	Fanconi anemia core complex associated protein 20 [Source:MGI Symbol;Acc:MGI:1914763]	23.8086	21.2697	22.1234	24.6107	24.5833	29.9846	23.0321	24.9894	19.6018	29.3839	22.7921	24.4626	23.2981	22.7252	0.069101108	0.433403132	16	20	24	0.178403488	0.768040178	24	17.54650515	0.111726699
ENSMUSG00000073700	Klhl21	kelch-like 21 [Source:MGI Symbol;Acc:MGI:1919288]	6.90157	6.32308	6.34133	7.34968	6.48286	6.78708	6.97264	7.90248	6.67541	9.82487	6.64421	7.36709	7.17211	7.03774	0.90332508	0.966018836	16	4	24	0.659458238	0.884879972	24	11.42423739	0.049600301
ENSMUSG00000073702	Rpl31	ribosomal protein L31 [Source:MGI Symbol;Acc:MGI:2149632]	8.78257	9.56346	8.8173	6.89183	9.58638	8.17054	8.54608	10.7417	11.0352	7.87101	12.9634	9.96335	12.0551	9.05451	0.916860713	0.966018836	8	8	24	0.780366414	0.929485273	24	0.332009175	0.075891224
ENSMUSG00000073705	Cenps	centromere protein S [Source:MGI Symbol;Acc:MGI:1917178]	3.92188	4.30806	4.32012	3.96617	3.38825	4.48203	4.05332	3.47338	4.21482	4.3031	4.76615	4.76475	5.18071	4.24814	0.911371589	0.966018836	24	8	24	0.774939466	0.927711986	24	20.91251989	0.035105286
ENSMUSG00000073725	Lmbrd1	LMBR1 domain containing 1 [Source:MGI Symbol;Acc:MGI:1915671]	40.7618	42.7412	47.3874	41.1903	41.4738	40.5503	44.625	39.331	43.6783	42.3152	41.9239	40.4719	43.5631	42.7195	0.148073804	0.590732968	12	12	24	0.336957193	0.771960538	24	7.217232482	0.040036346
ENSMUSG00000073733	Cplane2	ciliogenesis and planar polarity effector 2 [Source:MGI Symbol;Acc:MGI:1923416]	1.18618	1.21075	0.920844	1.16016	1.11488	1.12619	1.35549	1.29006	1.48769	1.2563	1.60623	1.30816	0.910179	1.51675	0.832293447	0.966018836	8	20	24	0.899847583	0.971563601	24	10.10367214	0.023196679
ENSMUSG00000073755	5730409E04Rik	RIKEN cDNA 5730409E04Rik gene [Source:MGI Symbol;Acc:MGI:3609248]	35.5529	30.881	32.332	30.5965	28.8304	33.7111	32.261	31.7561	27.6696	36.0138	28.9519	33.7082	32.9468	33.71	0.43280837	0.966018836	24	20	24	0.396804601	0.799546792	24	23.58075476	0.058487138
ENSMUSG00000073758	Sh3d21	SH3 domain containing 21 [Source:MGI Symbol;Acc:MGI:1914188]	0.912739	1.24394	1.21151	1.28294	1.19565	1.26164	1.38773	1.32467	1.41057	1.46045	1.03125	1.05266	1.49873	1.24236	0.870491976	0.966018836	16	4	24	0.286015872	0.768040178	24	7.451800913	0.066414991
ENSMUSG00000073771	Btbd19	BTB (POZ) domain containing 19 [Source:MGI Symbol;Acc:MGI:1925861]	2.16714	2.2351	2.34902	2.14274	3.23029	2.59043	2.35058	2.90463	2.58541	3.10158	2.92781	2.2096	2.00729	3.23879	0.5169953	0.966018836	20	8	24	0.3410116	0.775221917	24	11.84926946	0.09280725
ENSMUSG00000073775	Kti12	KTI12 homolog, chromatin associated [Source:MGI Symbol;Acc:MGI:1923547]	4.48833	3.87937	3.41269	4.85685	4.24704	4.09972	3.99694	4.83037	4.10638	4.62536	5.42678	5.01855	5.22899	4.55314	0.602156008	0.966018836	16	20	24	0.331414484	0.76821181	24	18.02249323	0.071482226
ENSMUSG00000073787	Gm10575	predicted gene 10575 [Source:MGI Symbol;Acc:MGI:3708765]	1.72384	1.51986	1.90365	1.28069	1.63551	1.56842	1.76637	1.64998	1.75649	2.23244	1.73029	1.99377	1.80453	1.82105	0.782779623	0.966018836	12	20	24	0.971620817	0.993121308	24	7.727274375	0.010551324
ENSMUSG00000073791	Efcab7	EF-hand calcium binding domain 7 [Source:MGI Symbol;Acc:MGI:2385199]	2.91606	3.53649	3.41891	2.43278	3.33699	2.89742	2.88422	2.73528	2.72933	2.44279	2.52387	3.02212	3.00705	3.03162	0.588268558	0.966018836	8	8	24	0.291095526	0.768040178	24	1.275420928	0.043778479
ENSMUSG00000073792	Alg6	asparagine-linked glycosylation 6 (alpha-1,3,-glucosyltransferase) [Source:MGI Symbol;Acc:MGI:2444031]	9.13048	10.1893	8.68364	8.88298	9.46022	8.73945	8.95906	8.7905	10.2416	8.39949	9.23123	9.17181	9.29749	9.36097	0.912686312	0.966018836	8	8	24	0.63965696	0.877957092	24	4.153836417	0.027833939
ENSMUSG00000073802	Cdkn2b	cyclin dependent kinase inhibitor 2B [Source:MGI Symbol;Acc:MGI:104737]	3.29715	2.18677	1.96937	2.19399	2.28139	1.4726	2.56612	2.26208	2.16628	2.07113	2.67344	2.69372	2.83726	2.59489	0.015486342	0.253395174	4	8	24	0.364583882	0.788976047	24	23.72452619	0.104650896
ENSMUSG00000073838	Tufm	Tu translation elongation factor, mitochondrial [Source:MGI Symbol;Acc:MGI:1923686]	2.31172	2.47145	2.62008	2.30957	3.02741	2.91666	2.38829	3.12594	2.50861	4.02949	2.83737	2.63032	2.58798	2.85858	0.658895034	0.966018836	20	8	24	0.311344407	0.768040178	24	12.65683477	0.087454058
ENSMUSG00000073856	Iqck	IQ motif containing K [Source:MGI Symbol;Acc:MGI:3612188]	2.09391	2.86759	2.25442	2.26765	2.43852	2.12089	2.18784	1.96054	2.72907	2.07395	2.15931	2.64249	2.40641	2.62869	0.860993221	0.966018836	12	4	24	0.697197012	0.899338675	24	5.596106944	0.040849988
ENSMUSG00000073888	Ccl27a	chemokine (C-C motif) ligand 27A [Source:MGI Symbol;Acc:MGI:1343459]	17.5901	14.331	14.244	15.0083	15.6755	14.0661	12.2587	12.3938	16.317	13.0876	17.3772	13.413	15.6849	12.8813	0.914360142	0.966018836	20	12	24	0.512660731	0.815866858	24	16.20356612	0.071378721
ENSMUSG00000073889	Il11ra1	interleukin 11 receptor, alpha chain 1 [Source:MGI Symbol;Acc:MGI:107426]	21.8589	31.5072	24.9809	29.2479	30.901	24.909	28.8255	26.9225	32.1578	22.0219	28.29	29.1181	30.1602	25.1996	0.814221431	0.966018836	24	12	24	0.992763939	0.998516607	24	11.2058916	0.008608545
ENSMUSG00000073910	Mob3b	MOB kinase activator 3B [Source:MGI Symbol;Acc:MGI:2664539]	7.33967	7.72382	6.90448	7.20472	8.1202	6.75332	8.57407	7.11466	7.73995	9.26138	6.44233	7.19816	7.72373	7.21659	0.911371589	0.966018836	4	20	24	0.905452869	0.974083049	24	8.490910053	0.023217048
ENSMUSG00000073940	Hbb-bt	hemoglobin, beta adult t chain [Source:MGI Symbol;Acc:MGI:5474850]	61.9269	183.223	136.764	187.626	144.465	126.231	105.922	200.909	256.087	73.2686	149.405	157.701	213.14	94.1545	0.887862353	0.966018836	24	16	24	0.729506278	0.911734411	24	8.559640839	0.193436657
ENSMUSG00000073968	Trim68	tripartite motif-containing 68 [Source:MGI Symbol;Acc:MGI:2142077]	8.14217	9.59223	8.95845	8.96997	10.1764	9.21921	11.3984	10.21	10.2806	8.9847	10.7076	9.67583	8.49921	9.6858	0.869755137	0.966018836	20	8	24	0.973346451	0.993305115	24	13.89285749	0.010931769
ENSMUSG00000073982	Rhog	ras homolog family member G [Source:MGI Symbol;Acc:MGI:1928370]	14.3147	13.7306	16.3526	13.9824	13.7667	16.2699	16.2085	14.1243	14.7406	15.428	14.3836	13.5553	13.2927	13.2933	0.675497492	0.966018836	12	20	24	0.877180471	0.965814767	24	13.60854147	0.019438694
ENSMUSG00000073985	Gm10602	predicted gene 10602 [Source:MGI Symbol;Acc:MGI:3708759]	0.758497	1.06791	0.710307	0.5507	1.05977	0.646659	0.969464	0.737145	0.890988	1.01835	0.935456	1.0091	0.91757	0.873359	0.872380105	0.966018836	20	16	24	0.907316695	0.974606327	24	20.99150411	0.022636526
ENSMUSG00000073987	Ggh	gamma-glutamyl hydrolase [Source:MGI Symbol;Acc:MGI:1329035]	12.7944	15.07	14.5212	14.619	13.9245	12.7816	13.1641	14.2181	16.4142	12.2831	15.2642	14.1453	14.792	14.5164	0.390685537	0.915479136	12	12	24	0.282657329	0.768040178	24	7.862480397	0.067214575
ENSMUSG00000074001	Klhl40	kelch-like 40 [Source:MGI Symbol;Acc:MGI:1919580]	0.535594	1.33015	1.38632	1.69555	1.22668	1.07461	0.98613	3.64184	2.19798	2.43999	1.0527	1.2618	2.27432	1.43082	0.135252616	0.573729971	16	4	24	0.072475289	0.679262305	24	8.037181329	0.325140561
ENSMUSG00000074006	Omp	olfactory marker protein [Source:MGI Symbol;Acc:MGI:97436]	1.91926	1.37371	1.4795	1.86701	2.22669	2.52195	2.54126	1.8502	1.39583	2.24461	2.12562	2.65336	1.56667	3.02088	0.071575474	0.440262583	24	12	24	0.177318008	0.768040178	24	19.30595829	0.159526876
ENSMUSG00000074024	4632427E13Rik	RIKEN cDNA 4632427E13 gene [Source:MGI Symbol;Acc:MGI:1915436]	0.304528	0.849669	0.511446	0.805165	0.841648	0.866621	1.08724	0.82265	1.23322	0.385862	0.62509	0.983603	0.93194	0.728987	0.729086532	0.966018836	24	16	24	0.839294554	0.951051187	24	0.734128995	0.049409445
ENSMUSG00000074030	Exoc8	exocyst complex component 8 [Source:MGI Symbol;Acc:MGI:2142527]	6.17148	4.5237	4.81541	5.08942	5.18529	5.56557	5.69378	5.56046	4.41126	5.67123	5.38978	5.38303	5.43074	5.31422	0.010213213	0.195026763	4	8	24	0.084571081	0.702664096	24	20.76698029	0.093036066
ENSMUSG00000074039	4930520O04Rik	RIKEN cDNA 4930520O04 gene [Source:MGI Symbol;Acc:MGI:1922366]	0.888953	0.844711	0.908027	0.676871	1.055	0.632945	1.02037	0.710166	1.12988	0.688619	0.804572	0.925837	0.817086	0.950572	0.932671476	0.966018836	12	4	24	0.79065942	0.932939823	24	3.856720948	0.032197446

ENSMUSG00000074063	Osgin1	oxidative stress induced growth inhibitor 1 [Source:MGI Symbol;Acc:MGI:1919089]	1.08799	1.49941	1.59137	1.67058	1.87675	1.34773	1.16003	1.5648	1.62112	1.97419	1.9297	1.14357	1.76589	1.14735	0.0140901	0.241938013	20	4	24	0.020119192	0.565108098	24	12.29274963	0.170804106
ENSMUSG00000074064	Mlycd	malonyl-CoA decarboxylase [Source:MGI Symbol;Acc:MGI:1928485]	7.71609	7.57024	7.9426	8.92727	6.71781	8.0554	7.51305	10.6867	8.37992	9.16263	8.24763	8.12741	8.76666	8.69978	0.718720526	0.966018836	16	12	24	0.226556115	0.768040178	24	7.051013164	0.088502804
ENSMUSG00000074071	Fam169b	family with sequence similarity 169, member B [Source:MGI Symbol;Acc:MGI:3644026]	0.859458	0.539832	0.643007	0.46502	0.795091	1.02297	0.462992	0.599205	0.633733	0.581512	0.701173	0.595983	0.573114	0.571438	0.492524295	0.966018836	24	8	24	0.177393512	0.768040178	24	19.0041277	0.092230161
ENSMUSG00000074088	Snrnp40	small nuclear ribonucleoprotein 40 (U5) [Source:MGI Symbol;Acc:MGI:1913835]	9.02306	10.5359	10.819	9.97857	10.3181	10.5222	9.68818	10.884	10.5184	10.4539	10.9094	10.8225	10.6795	11.3369	0.966095896	0.969272194	8	20	24	0.308946119	0.768040178	24	8.89113386	0.036167094
ENSMUSG00000074093	Svip	small VCP/p97-interacting protein [Source:MGI Symbol;Acc:MGI:1922994]	57.6057	58.2221	53.1048	54.2865	58.7925	52.0377	59.5748	52.2634	56.7262	49.3887	59.0802	53.5537	56.3452	56.8605	0.895493752	0.966018836	4	12	24	0.537011343	0.827674738	24	23.07986398	0.03606847
ENSMUSG00000074102	Rbm15b	RNA binding motif protein 15B [Source:MGI Symbol;Acc:MGI:1923598]	9.08706	5.77547	6.2486	7.01107	7.99201	7.50813	6.94971	6.58469	5.55418	6.89063	7.15638	6.8798	6.23835	5.76579	0.015486342	0.253395174	20	16	24	0.004430895	0.427431763	24	18.72724482	0.172925776
ENSMUSG00000074111	Mrgpra9	MAS-related GPR, member A9 [Source:MGI Symbol;Acc:MGI:3033148]	1.11384	0.413972	0.65231	0.514594	0.642032	0.637278	0.547116	0.691049	0.446735	0.444267	0.854977	0.627714	0.598859	0.479083	0.503972924	0.966018836	20	20	24	0.162524291	0.76636332	24	20.39915688	0.112741995
ENSMUSG00000074129	Rpl13a	ribosomal protein L13A [Source:MGI Symbol;Acc:MGI:1351455]	267.32	304.541	287.095	279.384	298.674	243.161	251.827	270.287	366.611	223.005	318.41	295.523	309.54	291.585	0.912686312	0.966018836	12	12	24	0.716688109	0.906575756	24	7.519776348	0.059985354
ENSMUSG00000074151	Nlrc5	NLR family, CARD domain containing 5 [Source:MGI Symbol;Acc:MGI:3612191]	2.19014	2.04799	2.38927	1.67061	2.9057	1.61698	1.6446	3.04449	1.83649	2.39323	2.02708	1.4662	1.96018	2.95927	0.060394272	0.407012001	8	16	24	0.349276606	0.779170188	24	7.235877615	0.137277641
ENSMUSG00000074155	Klk5	kallikrein related-peptidase 5 [Source:MGI Symbol;Acc:MGI:1915918]	2.02545	1.18156	1.20358	0.975047	1.07903	1.46887	0.900614	1.32283	1.21016	1.19378	1.11772	1.45553	1.1041	1.20775	0.869721097	0.966018836	24	20	24	0.358173191	0.784922891	24	22.81407437	0.090232313
ENSMUSG00000074158	Zfp976	zinc finger protein 976 [Source:MGI Symbol;Acc:MGI:3036263]	1.08372	1.26696	1.15516	1.23205	1.1932	1.0011	1.43409	1.22063	1.3239	0.946038	1.59877	1.30382	1.15855	1.31077	0.853736882	0.966018836	8	8	24	0.98735251	0.997463271	24	2.545756688	0.006671507
ENSMUSG00000074165	Zfp788	zinc finger protein 788 [Source:MGI Symbol;Acc:MGI:1914857]	14.3695	17.2446	16.7644	15.852	16.7413	14.4789	15.5888	15.1005	19.0592	12.7717	16.6184	16.3744	16.6807	15.1432	0.887027852	0.966018836	12	12	24	0.816289468	0.941409072	24	7.304333124	0.035313017
ENSMUSG00000074166	AW146154	expressed sequence AW146154 [Source:MGI Symbol;Acc:MGI:2142212]	2.09918	3.19101	3.08825	2.90757	3.18469	3.11005	3.10973	2.83362	4.40219	2.83923	3.54782	2.77361	2.9403	2.68897	0.622773557	0.966018836	20	8	24	0.282208994	0.768040178	24	10.97485796	0.107537977
ENSMUSG00000074170	Plekhf1	pleckstrin homology domain containing, family F (with FYVE domain) member 1 [Source:MGI Symbol;Acc:MGI:1919537]	1.69045	1.61923	1.67145	1.79195	1.75468	1.73025	1.87798	1.59785	1.98701	1.64878	1.73756	1.96135	1.70238	1.42523	0.052640391	0.391647859	20	12	24	0.348376632	0.779121086	24	17.05902354	0.044698501
ENSMUSG00000074171	Gm6658	predicted gene 6658 [Source:MGI Symbol;Acc:MGI:3645470]	1.15038	0.971797	1.00147	1.00744	0.847923	0.826877	0.506075	1.0539	0.865961	1.07888	0.825641	1.03777	0.978176	0.83575	0.891022863	0.966018836	16	4	24	0.751351211	0.91921305	24	9.476609515	0.039558666
ENSMUSG00000074178	Gm10638	predicted gene 10638 [Source:MGI Symbol;Acc:MGI:3704316]	0.546313	0.641885	0.697303	0.607055	0.350539	0.70039	0.591782	0.61832	1.16653	1.26722	0.91715	0.61247	0.789484	0.479243	0.78898997	0.966018836	12	20	24	0.234881437	0.768040178	24	10.47001404	0.128350957
ENSMUSG00000074182	Znhit6	zinc finger, HIT type 6 [Source:MGI Symbol;Acc:MGI:1916996]	9.4006	10.0491	8.88334	10.9041	10.8282	9.43401	8.34799	10.5175	11.1198	10.1748	9.30654	11.0897	9.50557	9.74655	0.494171275	0.966018836	16	12	24	0.341859025	0.776129793	24	11.93745212	0.064984193
ENSMUSG00000074194	Zfp791	zinc finger protein 791 [Source:MGI Symbol;Acc:MGI:3648473]	1.08081	1.38992	1.3394	1.27981	1.21547	1.31557	1.22324	1.22346	1.35573	1.05155	1.20157	1.22718	1.0855	1.51257	0.160291962	0.620016786	8	20	24	0.317788216	0.768040178	24	6.48686591	0.049805038
ENSMUSG00000074199	Krtdap	keratinocyte differentiation associated protein [Source:MGI Symbol;Acc:MGI:1928282]	3.64935	3.80654	3.96832	4.43236	8.45429	2.69397	4.21606	5.01123	8.42432	1.03703	4.02482	7.44678	3.11105	7.3478	0.935593607	0.966018836	20	8	24	0.937723528	0.984586008	24	3.876483556	0.086737832
ENSMUSG00000074207	Adh1	alcohol dehydrogenase 1 (class I) [Source:MGI Symbol;Acc:MGI:87921]	2.8834	5.49109	6.26328	8.13063	7.24039	5.53181	5.6224	6.74742	7.28398	6.70135	7.13967	6.16804	6.09866	5.48015	0.02233689	0.270751126	16	12	24	0.012802553	0.526249719	24	11.90277049	0.277212873
ENSMUSG00000074211	Sdhaf1	succinate dehydrogenase complex assembly factor 1 [Source:MGI Symbol;Acc:MGI:1915582]	2.62011	2.61559	3.33309	3.23826	2.66464	2.88591	3.16136	3.47133	3.41298	3.14607	3.02005	3.21823	3.43375	3.13525	0.724067924	0.966018836	12	8	24	0.173335219	0.768040178	24	7.678855442	0.065981741
ENSMUSG00000074212	Dnajb14	DnaJ heat shock protein family (Hsp40) member B14 [Source:MGI Symbol;Acc:MGI:1917854]	17.3721	17.3542	17.0296	17.3815	17.7118	17.02	17.4636	15.4759	16.7895	16.9243	18.5596	17.6874	17.0803	18.5973	0.424718357	0.966018836	20	16	24	0.460991172	0.805393068	24	17.48640291	0.029614203
ENSMUSG00000074215	Gm10643	predicted gene 10643 [Source:MGI Symbol;Acc:MGI:3642507]	0.700894	0.884334	0.854125	0.903747	0.716265	1.23032	1.06173	0.981467	0.997267	0.96292	0.831368	1.17635	0.65219	1.01985	0.921741413	0.966018836	24	20	24	0.99405069	0.998755922	24	21.09543209	0.005773534
ENSMUSG00000074217	Misp3	MISP family member 3 [Source:MGI Symbol;Acc:MGI:1917384]	1.19459	1.04937	1.8535	1.07668	1.71762	1.31763	0.910647	1.61493	1.20917	1.8033	2.49473	1.27467	1.72179	1.68411	0.652290823	0.966018836	20	8	24	0.309169354	0.768040178	24	13.03504502	0.12648409
ENSMUSG00000074218	Cox7a1	cytochrome c oxidase subunit 7A1 [Source:MGI Symbol;Acc:MGI:1316714]	29.58	45.4487	59.9643	106.021	56.6995	55.8136	44.9352	95.53	69.0465	76.7262	71.0882	52.319	92.3077	72.338	0.160297544	0.620016786	16	12	24	0.067832946	0.671868099	24	10.26210736	0.365575651
ENSMUSG00000074220	Zfp382	zinc finger protein 382 [Source:MGI Symbol;Acc:MGI:3588204]	8.51925	6.06558	5.75478	5.53787	8.66619	7.45601	6.48077	7.3353	4.78646	6.11333	7.10208	6.11618	7.25894	7.09886	0.051054571	0.387791798	4	8	24	0.057177787	0.656248849	24	20.96964005	0.185416551
ENSMUSG00000074221	Zfp568	zinc finger protein 568 [Source:MGI Symbol;Acc:MGI:2142347]	1.35681	1.51117	1.20936	1.46561	2.28085	1.48802	2.00245	2.09065	1.60507	1.55877	1.73748	1.6754	1.48685	1.52915	0.942051135	0.966018836	20	8	24	0.663174536	0.88640646	24	18.5452089	0.057600114
ENSMUSG00000074227	Spint2	serine protease inhibitor, Kunitz type 2 [Source:MGI Symbol;Acc:MGI:1338031]	1.64126	2.24878	3.09778	2.95959	1.48899	2.50673	2.49359	2.40246	2.67621	2.0791	1.48184	3.28813	2.47244	2.7477	0.960933467	0.967043964	12	8	24	0.384004685	0.799546792	24	5.727376386	0.137669426
ENSMUSG00000074235	Gm10649	predicted gene 10649 [Source:MGI Symbol;Acc:MGI:3642042]	0.621	0.470031	0.535893	0.415253	0.524475	0.487889	0.595013	0.70065	0.4908	0.589808	0.690746	0.735524	0.622507	0.515267	0.569060064	0.966018836	4	12	24	0.298942088	0.768040178	24	21.47632521	0.049111875
ENSMUSG00000074236	Gm9387	predicted pseudogene 9387 [Source:MGI Symbol;Acc:MGI:3643269]	2.84596	3.48778	3.31986	3.11509	3.60291	3.29862	3.4335	3.41387	3.25874	2.62735	3.68559	2.94543	3.28389	3.03456	0.890235289	0.966018836	20	20	24	0.936695693	0.984438075	24	20.56595178	0.01571391
ENSMUSG00000074238	Ap1ar	adaptor-related protein complex 1 associated regulatory protein [Source:MGI Symbol;Acc:MGI:2384822]	83.4264	67.2204	66.0581	72.8266	69.6464	73.8343	67.1007	65.522	61.2335	77.7413	68.7163	73.3885	76.4459	71.6345	0.152177471	0.600454138	24	12	24	0.223008423	0.768040178	24	19.94308052	0.079103422
ENSMUSG00000074243	Gm10651	predicted pseudogene 10651 [Source:MGI Symbol;Acc:MGI:3704290]	8.23693	6.58336	6.80814	9.68612	8.24754	7.86774	7.08275	7.41952	6.33923	8.40397	6.85627	7.46434	6.22847	6.08789	0.023283676	0.278156767	16	16	24	0.03635976	0.609176161	24	15.68860308	0.138160915
ENSMUSG00000074247	Dda1	DET1 and DDB1 associated 1 [Source:MGI Symbol;Acc:MGI:1913748]	27.6179	23.1277	24.4386	23.3593	23.9112	23.7246	27.6147	27.7776	23.2024	29.7079	26.4463	28.0797	27.1253	25.8233	0.725736046	0.966018836	4	8	24	0.563517978	0.842665559	24	22.37457876	0.046300653
ENSMUSG00000074252	Gm10654	predicted gene 10654 [Source:MGI Symbol;Acc:MGI:3643366]	0.647144	1.04265	1.17358	0.68308	0.770091	0.937609	0.889448	1.13774	1.38351	1.33573	0.743921	1.04552	0.949753	1.04061	0.064422003	0.414867207	12	8	24	0.052927983	0.650874134	24	7.370055636	0.14186083
ENSMUSG00000074259	Gramd2	GRAM domain containing 2 [Source:MGI Symbol;Acc:MGI:3528937]	3.5717	3.02451	3.95455	3.57621	4.15345	3.5268	3.81225	2.36784	3.27358	3.85437	3.67049	3.44465	2.92234	3.32611	0.038580265	0.335882166	20	12	24	0.050605515	0.644469829	24	15.38202694	0.134465863
ENSMUSG00000074264	Amy1	amylase 1, salivary [Source:MGI Symbol;Acc:MGI:88019]	22.3705	28.819	27.415	34.0526	28.8597	21.9924	25.8475	21.4161	29.8662	22.2717	31.4258	22.963	27.0986	25.904	0.226438798	0.722427815	20	4	24	0.235834994	0.768040178	24	11.45259018	0.133359607
ENSMUSG00000074269	Rec114	REC114 meiotic recombination protein [Source:MGI Symbol;Acc:MGI:1920923]	0.922452	1.4715	1.63747	1.35706	1.55233	1.06313	1.1741	1.22503	2.05535	0.888023	0.833758	1.486	1.84767	1.09133	0.608967886	0.966018836	12	4	24	0.570028019	0.845005229	24	6.532235329	0.098190227
ENSMUSG00000074272	Ceacam1	carcinoembryonic antigen-related cell adhesion molecule 1 [Source:MGI Symbol;Acc:MGI:1347245]	4.21287	3.74076	3.54946	3.53912	3.90562	3.68321	3.89905	3.56017	3.64633	2.90346	4.37425	2.81857	3.9697	2.86985	0.235187365	0.73629392	4	12	24	0.494080968	0.806249091	24	20.71376688	0.067794104
ENSMUSG00000074274	D930028M14Rik	RIKEN cDNA D930028M14 gene [Source:MGI Symbol;Acc:MGI:3687343]	26.2049	27.8075	23.1965	25.1814	27.7659	25.5589	24.5295	27.1937	25.1654	23.2511	29.549	24.0455	26.9532	26.1636	0.675497492	0.966018836	8	4	24	0.710253836	0.90416311	24	21.24755772	0.034000548
ENSMUSG00000074280	Gm6166	predicted gene 6166 [Source:MGI Symbol;Acc:MGI:3645893]	73.4041	88.1134	88.9899	76.8823	84.762	87.5332	69.5579	70.3694	75.4073	61.7592	79.2705	73.1682	80.8613	81.7649	0.896753532	0.966018836	12	4	24	0.945687845	0.986808796	24	23.13240361	0.019807281
ENSMUSG00000074282	Zfp94	zinc finger protein 94 [Source:MGI Symbol;Acc:MGI:107610]	5.00235	5.76027	5.80912	5.07548	5.51177	6.41049	5.08886	5.65715	4.8795	5.15431	5.93596	5.29902	5.55963	5.54759	0.916963526	0.966018836	24	4	24	0.720859315	0.908547187	24	20.01591117	0.031782764
ENSMUSG00000074283	Zfp109	zinc finger protein 109 [Source:MGI Symbol;Acc:MGI:1929099]	2.21066	1.91091	1.85036	2.16505	2.06779	2.43581	2.56853	2.10833	1.53074	1.77529	1.99251	2.51608	2.21417	2.10436	7.55E-04	0.071344007	24	12	24	0.00210462	0.395705644	24	21.2577672	0.156206152
ENSMUSG00000074305	Peak1	pseudopodium-enriched atypical kinase 1 [Source:MGI Symbol;Acc:MGI:2442366]	6.56291	6.71954	6.28758	6.57842	6.70429	6.58275	6.80158	6.51453	5.90798	7.00376	6.20715	7.025	6.67048	6.59577	0.245061943	0.741580974	24	12	24	0.326768075	0.768040178	24	20.583213	0.034639919
ENSMUSG00000074340	Ovgp1	oviductal glycoprotein 1 [Source:MGI Symbol;Acc:MGI:106661]	2.07117	2.5337	2.47669	2.0163	2.31038	2.26068	2.23666	1.98763	2.91379	2.23104	2.52809	2.05279	2.33331	2.18703	0.93447422	0.966018836	12	12	24	0.472206898	0.805393068	24	9.210739281	0.053991089
ENSMUSG00000074345	Tnfaip8l3	tumor necrosis factor, alpha-induced protein 8-like 3 [Source:MGI Symbol;Acc:MGI:2685363]	1.08216	0.878912	0.974916	0.894409	0.956695	1.05649	0.935583	0.864454	0.792469	0.991809	0.935301	0.905014	0.860198	0.902478	0.47094626	0.966018836	24	8	24	0.116475136	0.727799063	24	19.01961022	0.04221817
ENSMUSG00000074346	Kcnd3os	potassium voltage-gated channel, Shal-related family, member 3, opposite strand [Source:MGI Symbol;Acc:MGI:1925885]	0.858864	0.807217	0.674719	0.491464	0.668922	0.326901	0.483848	0.609252	0.586318	0.500054	1.24845	0.678932	0.43981	0.568402	0.858266942	0.966018836	20	4	24	0.891718019	0.969342045	24	14.8206064	0.03558371
ENSMUSG00000074358	Ccdc61	coiled-coil domain containing 61 [Source:MGI Symbol;Acc:MGI:2685005]	4.4848	5.70778	4.91911	5.3625	5.32503	6.23318	5.41809	5.88167	6.34032	5.02873	4.99808	5.20843	5.62551	5.49347	0.759899911	0.966018836	8	12	24	0.704308155	0.902338136	24	5.018271342	0.037955528
ENSMUSG00000074364	Ehd2	EH-domain containing 2 [Source:MGI Symbol;Acc:MGI:2154274]	13.0152	11.1073	10.7981	12.2625	12.8321	11.8198	13.6841	13.5922	11.3555	14.7678	10.8882	11.448	12.2317	10.6268	0.893609703	0.966018836	4	8	24	0.872255228	0.963902069	24	18.724076	0.02954431
ENSMUSG00000074384	AI429214	expressed sequence AI429214 [Source:MGI Symbol;Acc:MGI:2142538]	1.50214	1.77993	1.63465	1.26603	1.63586	1.48379	1.53431	1.08748	1.24282	1.3919	1.51641	1.43844	1.35525	1.56208	0.860560779	0.966018836	20	20	24	0.715980165	0.906323093	24	20.48562301	0.035279716
ENSMUSG00000074385	Gm10684	predicted gene 10684 [Source:MGI Symbol;Acc:MGI:3641851]	1.38214	1.20385	1.42789	0.995781	0.565697	0.982342	1.00326	1.43614	1.27724	1.38768	0.867404	0.828235	0.833632	1.62754	0.004021228	0.140598674	8	12	24	0.00525165	0.4424469	24	5.946212595	0.230703111
ENSMUSG00000074405	Zfp865	zinc finger protein 865 [Source:MGI Symbol;Acc:MGI:2442656]	4.95568	4.65912	4.86245	5.12814	5.3853	4.15721	4.79077	5.56264	6.19445	6.10221	6.09273	6.17317	6.59788	6.30593	0.733896783	0.966018836	20	4	24	0.305841588	0.768040178	24	9.581683731	0.061653886
ENSMUSG00000074406	Zfp628	zinc finger protein 628 [Source:MGI Symbol;Acc:MGI:2665174]	1.21289	1.12727	1.08953	1.12849	1.10055	0.888633	1.15198	1.30267	1.29854	1.57857	1.24531	1.47141	1.21891	1.3227	0.823246279	0.966018836	8	16	24	0.489787012	0.805393068	24	8.846098639	0.044973395
ENSMUSG00000074415	Mir100hg	Mir100 Mirlet7a-2 Mir125b-1 cluster host gene [Source:MGI Symbol;Acc:MGI:1920394]	28.9943	36.3256	33.5584	29.6964	36.4569	28.8394	34.4525	28.555	42.0209	26.9563	36.5354	31.4014	32.7882	33.5807	0.945282252	0.966018836	12	12	24	0.789036374	0.932379688	24	7.950048774	0.050151079
ENSMUSG00000074457	S100a16	S100 calcium binding protein A16 [Source:MGI Symbol;Acc:MGI:1915110]	62.8392	62.1225	61.0369	51.0005	61.6787	59.2934	60.402	61.7192	60.6332	43.0824	61.9617	55.0455	61.1743	53.0671	0.47028201	0.966018836	4	12	24	0.159271792	0.765350752	24	23.78787774	0.103887288
ENSMUSG00000074461	Gm10699	predicted gene 10699 [Source:MGI Symbol;Acc:MGI:3642530]	1.24837	1.22977	1.18327	0.974963	1.00004	1.11479	1.03359	0.850273	1.35064	0.718083	0.986965	1.04848	1.05637	1.19316	0.085045716	0.466404237	12	4	24	0.300163339	0.768040178	24	2.546433521	0.067998985
ENSMUSG00000074466	Gm15417	predicted gene 15417 [Source:MGI Symbol;Acc:MGI:3642531]	6.89948	5.96248	7.786	5.819	4.65853	5.89391	8.11722	8.21029	8.58029	6.13302	7.61556	4.75557	7.8576	6.63899	0.088482991	0.473415302	4	20	24	0.09536822	0.71175955	24	5.072028062	0.198876769
ENSMUSG00000074476	Spc24	SPC24, NDC80 kinetochore complex component, homolog (S. cerevisiae) [Source:MGI Symbol;Acc:MGI:1914879]	0.766672	0.895554	1.07884	1.13321	0.685766	1.28637	0.500263	1.15566	1.11611	0.846905	0.694165	0.873328	1.0026	0.730801	0.843639218	0.966018836	12	8	24	0.610835998	0.864861374	24	8.733017773	0.070414757
ENSMUSG00000074479	Rtraf-ps	RNA transcription, translation and transport factor, pseudogene [Source:MGI Symbol;Acc:MGI:3642446]	79.868	87.8759	85.0631	78.6279	82.243	78.9162	76.8017	78.5705	91.6838	71.6536	90.2731	85.464	89.9688	88.4992	0.890235289	0.966018836	12	4	24	0.721171429	0.90866625	24	4.390856957	0.032622878
ENSMUSG00000074482	Gm16589	predicted gene 16589 [Source:MGI Symbol;Acc:MGI:4418561]	31.7539	29.111	27.8284	29.5165	27.8871	27.8403	31.282	29.2679	28.0799	30.7571	29.2783	32.52	31.8618	28.739	0.094000699	0.490854203	4	8	24	0.18994921	0.768040178	24	22.21158727	0.052502769
ENSMUSG00000074483	Bglap	bone gamma carboxyglutamate protein [Source:MGI Symbol;Acc:MGI:88156]	5.43379	10.5135	16.0126	12.3326	4.14337	14.3375	8.08252	12.5661	9.03962	13.9937	8.60741	8.14883	15.1593	3.83702	0.921572192	0.966018836	16	4	24	0.771896948	0.926423608	24	9.50669642	0.178637645
ENSMUSG00000074486	Bglap2	bone gamma-carboxyglutamate protein 2 [Source:MGI Symbol;Acc:MGI:88157]	3.51587	6.06197	7.20677	7.3635	3.16909	7.42667	4.42715	9.23967	5.21499	6.54505	4.87032	3.69508	8.22173	2.58688	0.907000607	0.966018836	16	4	24	0.701080487	0.900900643	24	8.271504024	0.164106601
ENSMUSG00000074500	Zfp558	zinc finger protein 558 [Source:MGI Symbol;Acc:MGI:1921681]	1.23972	0.980281	0.915389	1.35888	0.977299	0.914621	0.960227	1.01353	1.1327	1.05141	1.09483	0.966929	0.942215	1.38921	0.483374602	0.966018836	16	8	24	0.528293822	0.82388535	24	8.627841716	0.049805668
ENSMUSG00000074505	Fat3	FAT atypical cadherin 3 [Source:MGI Symbol;Acc:MGI:2444314]	7.23574	6.80956	6.94199	6.48338	6.54244	6.42744	6.29152	5.93566	6.2573	6.60627	6.22321	7.08951	6.85292	6.88818	0.907000607	0.966018836	4	16	24	0.650041252	0.882327323	24	23.29595168	0.026277164
ENSMUSG00000074506	Gm10705	predicted gene 10705 [Source:MGI Symbol;Acc:MGI:3708678]	18.7155	17.5054	18.116	17.1948	18.5543	19.8599	18.4696	16.4722	17.3596	18.7897	16.7461	19.4622	18.8194	20.1876	0.280810068	0.792139038	24	12	24	0.304965702	0.768040178	24	21.54582132	0.050504183
ENSMUSG00000074513	Arfip1	ADP-ribosylation factor interacting protein 1 [Source:MGI Symbol;Acc:MGI:1277120]	28.8908	32.5107	29.9427	30.9034	29.5572	26.8516	30.4365	28.3015	32.0032	27.3945	31.2311	27.6663	30.5869	27.9514	0.894563866	0.966018836	12	12	24	0.563150209	0.842499957	24	7.748790198	0.036320463
ENSMUSG00000074519	Zfp971	zinc finger protein 971 [Source:MGI Symbol;Acc:MGI:1261426]	13.2099	17.977	14.4828	16.4994	17.6216	14.1743	15.5691	13.0349	18.1822	11.3023	17.4101	16.1068	15.5881	17.9728	0.94086481	0.966018836	12	16	24	0.988229219	0.997559852	24	9.03699046	0.012654376
ENSMUSG00000074521	Gm14327	predicted gene 14327 [Source:MGI Symbol;Acc:MGI:3652188]	1.78573	2.19263	2.3448	2.16113	2.17358	1.70116	1.84268	2.01682	2.12673	1.99298	2.33467	1.82104	1.88319	2.37897	0.047773766	0.378060456	12	12	24	0.020836293	0.568066329	24	9.419907406	0.105552924
ENSMUSG00000074553	Eif1-ps1	eukaryotic translation initiation factor 1, pseudogene 1 [Source:MGI Symbol;Acc:MGI:3642383]	18.0978	17.973	13.0628	16.9243	16.6156	13.5276	17.204	11.649	13.8357	12.4613	12.4768	10.5643	12.443	13.2439	0.85387321	0.966018836	4	20	24	0.830258802	0.947325117	24	0.017174729	0.039760608
ENSMUSG00000074575	Kcng1	potassium voltage-gated channel, subfamily G, member 1 [Source:MGI Symbol;Acc:MGI:3616086]	0.604558	0.364372	0.492645	0.497519	0.717773	0.531884	0.884194	0.379077	0.447674	0.500877	0.571583	0.41595	0.421514	0.487608	0.036134798	0.331852526	20	12	24	0.290986955	0.768040178	24	18.72296898	0.077980736
ENSMUSG00000074576	Mocs3	molybdenum cofactor synthesis 3 [Source:MGI Symbol;Acc:MGI:1916622]	1.59768	1.8601	1.85773	1.53868	1.81109	1.92314	1.71178	2.06431	1.79743	1.92246	2.06954	2.23818	1.90438	2.20586	0.868638024	0.966018836	24	12	24	0.894435316	0.970031575	24	0.798229604	0.01352193
ENSMUSG00000074577	Ripor3	RIPOR family member 3 [Source:MGI Symbol;Acc:MGI:1916803]	0.78356	1.08391	0.783717	0.935296	0.769556	1.03869	0.753284	1.50872	0.799254	1.08535	1.13317	0.975362	0.985006	0.741034	0.890311217	0.966018836	16	12	24	0.996572816	0.999147209	24	10.88729965	0.005074333
ENSMUSG00000074578	Zfas1	zinc finger, NFX1-type containing 1, antisense RNA 1 [Source:MGI Symbol;Acc:MGI:1916199]	27.6144	34.3079	31.8974	33.5178	28.1207	30.0718	30.4856	29.3056	35.186	27.2338	33.7894	31.8166	37.0172	34.5936	0.937921962	0.966018836	12	4	24	0.448727797	0.805393068	24	5.661221612	0.06407117
ENSMUSG00000074579	Lekr1	leucine, glutamate and lysine rich 1 [Source:MGI Symbol;Acc:MGI:3645902]	2.26582	2.79154	2.41651	2.60426	2.51875	2.54852	2.2741	2.2417	2.51083	1.86404	2.82947	2.41647	2.52885	2.12669	0.697196105	0.966018836	20	12	24	0.663097699	0.886390077	24	18.09407953	0.04018052
ENSMUSG00000074580	4931440P22Rik	RIKEN cDNA 4931440P22 gene [Source:MGI Symbol;Acc:MGI:1918254]	0.634911	0.602433	0.653064	0.678737	0.667964	0.580887	0.562609	0.480414	0.852331	0.902417	0.849569	1.036	0.634021	0.791724	0.140728644	0.585944911	16	12	24	0.120966867	0.732894732	24	13.6138891	0.082090342
ENSMUSG00000074582	Arfgef2	ADP-ribosylation factor guanine nucleotide-exchange factor 2 (brefeldin A-inhibited) [Source:MGI Symbol;Acc:MGI:2139354]	26.4672	20.5836	21.4894	22.4322	20.4072	25.634	21.0582	22.7204	16.6279	27.0564	20.1132	22.7878	22.8597	22.983	0.46694635	0.966018836	24	12	24	0.494611962	0.806559646	24	20.80202846	0.084317798
ENSMUSG00000074591	Ankub1	ankrin repeat and ubiquitin domain containing 1 [Source:MGI Symbol;Acc:MGI:2685256]	0.943945	1.13427	1.25104	0.72124	1.12274	0.932823	0.790096	1.15626	1.55231	0.81993	0.854487	1.14309	0.853872	1.14675	0.021214536	0.264116537	12	4	24	0.251841459	0.768040178	24	6.450147934	0.103119403
ENSMUSG00000074604	Mgst2	microsomal glutathione S-transferase 2 [Source:MGI Symbol;Acc:MGI:2448481]	0.758851	1.39516	2.9353	2.56405	1.75294	1.89362	1.00135	1.48487	2.42814	1.53165	2.26967	1.13645	2.07035	1.6254	0.055920623	0.401074772	12	16	24	0.033464171	0.606585237	24	10.96569784	0.308553186
ENSMUSG00000074607	Tox2	TOX high mobility group box family member 2 [Source:MGI Symbol;Acc:MGI:3611233]	4.52457	3.30177	4.46211	3.24858	3.13378	4.00252	3.92586	5.18681	3.55456	7.60196	4.25151	4.38833	4.2458	5.46823	0.896753532	0.966018836	8	12	24	0.708052594	0.903565455	24	7.203249038	0.087364859
ENSMUSG00000074622	Mafb	v-maf musculoaponeurotic fibrosarcoma oncogene family, protein B (avian) [Source:MGI Symbol;Acc:MGI:104555]	1.14997	1.30331	1.65554	1.37046	1.11826	1.42074	1.64811	1.67918	1.69379	1.67396	1.30756	1.19225	1.36207	1.28863	0.187179226	0.662239322	12	8	24	0.104885985	0.717030134	24	7.657293151	0.102766201
ENSMUSG00000074629	4930518I15Rik	RIKEN cDNA 4930518I15 gene [Source:MGI Symbol;Acc:MGI:1921954]	0.746618	1.1405	0.992606	1.05103	1.40075	1.19743	1.20297	1.09443	1.50759	0.949441	1.45173	1.2087	1.10238	0.841338	0.304354943	0.811201458	20	12	24	0.326626341	0.768040178	24	15.33204074	0.084664172
ENSMUSG00000074634	Tmem267	transmembrane protein 267 [Source:MGI Symbol;Acc:MGI:3648543]	6.61902	5.06559	7.37709	8.29836	5.73738	6.89307	8.34771	6.6129	4.89305	9.50199	7.99082	6.29487	6.40375	5.15258	0.338172159	0.85758369	16	16	24	0.282466804	0.768040178	24	15.31914537	0.154441762
ENSMUSG00000074635	3110070M22Rik	RIKEN cDNA 3110070M22 gene [Source:MGI Symbol;Acc:MGI:1914554]	0.372206	0.669872	0.629769	0.712516	0.899003	0.393999	0.959183	0.699511	0.763074	1.20217	0.992913	0.786325	0.73152	0.61639	0.224031297	0.717774981	20	12	24	0.124600834	0.738549711	24	12.61882245	0.127479418
ENSMUSG00000074637	Sox2	SRY (sex determining region Y)-box 2 [Source:MGI Symbol;Acc:MGI:98364]	4.22442	4.61935	5.75795	4.36337	3.83943	4.5114	6.15971	3.9643	3.8647	4.93081	4.28763	4.68849	3.4585	5.67087	0.873567285	0.966018836	8	12	24	0.776747141	0.928317108	24	5.373478865	0.058045122
ENSMUSG00000074643	Cpne1	copine I [Source:MGI Symbol;Acc:MGI:2386621]	20.0866	17.8459	18.4204	17.4347	17.8856	18.7585	17.7193	18.2348	16.2221	21.473	18.4843	19.0408	18.5063	18.6282	0.601647512	0.966018836	24	12	24	0.678686611	0.892520664	24	18.96479814	0.032655623
ENSMUSG00000074646	6430550D23Rik	RIKEN cDNA 6430550D23 gene [Source:MGI Symbol;Acc:MGI:2443361]	1.05755	0.860969	1.22404	1.43705	1.2915	0.602813	1.29975	1.1299	0.964304	0.447122	1.12104	1.01048	0.93856	1.10349	0.838244962	0.966018836	20	4	24	0.968405209	0.992338543	24	4.705213063	0.020829263
ENSMUSG00000074649	BC029722	cDNA sequence BC029722 [Source:MGI Symbol;Acc:MGI:3584273]	18.5635	18.5053	19.1394	18.3731	18.3713	19.0967	20.3007	19.4282	19.06	19.8807	16.8468	20.1235	20.0406	20.4829	0.223774686	0.717349224	4	16	24	0.129434358	0.740162193	24	2.765202148	0.049080103
ENSMUSG00000074652	Myh7b	myosin, heavy chain 7B, cardiac muscle, beta [Source:MGI Symbol;Acc:MGI:3710243]	0.282338	0.62135	0.496227	0.375818	0.403512	0.583032	0.343147	0.81013	0.75736	1.03465	0.323876	0.359253	0.332106	0.574366	0.073315272	0.447768907	8	20	24	0.072758117	0.679705968	24	8.409640663	0.173604186
ENSMUSG00000074653	Lrrc31	leucine rich repeat containing 31 [Source:MGI Symbol;Acc:MGI:2443864]	2.84619	2.85939	2.5846	2.89296	2.40591	2.81961	2.33955	2.89239	2.47011	2.55874	2.21295	2.90107	2.97115	2.31714	0.939335547	0.966018836	24	20	24	0.517755641	0.818405353	24	0.086802835	0.046116425
ENSMUSG00000074656	Eif2s2	eukaryotic translation initiation factor 2, subunit 2 (beta) [Source:MGI Symbol;Acc:MGI:1914454]	15.9068	18.1781	16.8095	17.9093	18.1244	15.9683	15.9332	16.1457	18.8526	15.4429	18.7801	17.0021	18.4964	18.0202	0.935593607	0.966018836	20	8	24	0.670783438	0.888957954	24	10.27343572	0.034168614
ENSMUSG00000074657	Kif5a	kinesin family member 5A [Source:MGI Symbol;Acc:MGI:109564]	199.528	161.608	175.877	172.151	154.231	201.963	181.941	184.11	138.333	233.973	146.683	180.069	167.24	184.24	0.849179819	0.966018836	24	20	24	0.841461659	0.951974594	24	22.46240983	0.047435713
ENSMUSG00000074671	Tspyl3	TSPY-like 3 [Source:MGI Symbol;Acc:MGI:2139328]	4.65266	4.76548	4.70887	3.87852	4.07955	4.70372	4.7407	4.50166	4.04341	4.80643	3.94834	4.84208	4.46011	4.73753	0.63897419	0.966018836	24	20	24	0.111092737	0.723334499	24	1.159298022	0.072195869
ENSMUSG00000074673	Ttll9	tubulin tyrosine ligase-like family, member 9 [Source:MGI Symbol;Acc:MGI:1913589]	2.3503	3.17528	2.78417	2.856	3.34922	2.3462	3.56291	3.326	2.11866	2.5604	2.45422	3.55333	3.02935	2.65841	0.864782464	0.966018836	8	4	24	0.569967417	0.845005229	24	22.72453127	0.076533932
ENSMUSG00000074676	Foxs1	forkhead box S1 [Source:MGI Symbol;Acc:MGI:95546]	4.56173	6.08127	3.49635	4.33166	6.5619	4.58337	4.6843	5.6414	5.90705	3.65247	5.95266	6.17935	5.93011	4.16392	0.265774856	0.773510818	20	20	24	0.482345143	0.805393068	24	20.60253407	0.121410427
ENSMUSG00000074682	Zcchc3	zinc finger, CCHC domain containing 3 [Source:MGI Symbol;Acc:MGI:1915167]	1.82234	1.88338	2.38323	1.97784	1.92636	1.97411	2.12842	2.2256	1.98376	2.24522	1.9265	2.15704	2.34662	2.22215	0.841143063	0.966018836	12	8	24	0.30754227	0.768040178	24	6.171917227	0.044571793
ENSMUSG00000074698	Csnk2a1	casein kinase 2, alpha 1 polypeptide [Source:MGI Symbol;Acc:MGI:88543]	31.0315	29.1353	28.6999	29.6741	29.3804	29.9124	28.2847	29.5888	28.2016	30.4319	30.7978	30.2388	30.9338	31.7048	0.830364743	0.966018836	4	8	24	0.586351768	0.853300537	24	20.65728186	0.019191763
ENSMUSG00000074715	Ccl28	chemokine (C-C motif) ligand 28 [Source:MGI Symbol;Acc:MGI:1861731]	0.841266	0.92786	0.950582	1.14175	0.961707	0.772573	1.08692	0.75882	0.766837	1.19851	0.868659	1.04419	0.884183	0.734885	0.086420527	0.467848941	16	16	24	0.245632335	0.768040178	24	14.52628628	0.06931259
ENSMUSG00000074733	Zfp950	zinc finger protein 950 [Source:MGI Symbol;Acc:MGI:2652824]	19.7901	26.8575	21.0999	28.3331	24.0323	18.7498	22.0012	19.2607	26.9044	16.4802	24.9309	22.4081	23.3443	22.2717	0.917867465	0.966018836	20	4	24	0.81464274	0.940847489	24	10.12157213	0.056290938
ENSMUSG00000074736	Syndig1	synapse differentiation inducing 1 [Source:MGI Symbol;Acc:MGI:3702158]	3.6248	3.88868	5.46684	3.04873	2.98499	3.8887	4.10462	3.80197	3.72101	3.67309	2.72181	3.352	2.98344	5.65906	0.018114172	0.256345714	8	12	24	0.04860088	0.638992719	24	5.170625051	0.217886983
ENSMUSG00000074738	Fndc10	fibronectin type III domain containing 10 [Source:MGI Symbol;Acc:MGI:2444790]	8.15338	7.47383	7.5433	7.80649	7.10062	7.88987	7.17402	8.02774	7.39091	9.43327	7.3579	8.57415	7.87586	8.48759	0.888379597	0.966018836	24	20	24	0.944360874	0.986443184	24	8.611996839	0.01339594
ENSMUSG00000074743	Thbd	thrombomodulin [Source:MGI Symbol;Acc:MGI:98736]	15.3918	14.1702	9.73821	8.98309	13.5459	11.3041	13.5631	14.454	11.9283	8.43952	13.3439	12.4824	10.4998	11.249	0.157744865	0.614233844	4	12	24	0.0706577	0.675829204	24	23.22175023	0.201559654
ENSMUSG00000074746	Pdzd8	PDZ domain containing 8 [Source:MGI Symbol;Acc:MGI:2677270]	25.5578	24.0134	23.2592	23.63	23.6079	22.9002	23.5488	22.1438	21.251	24.6257	23.6942	24.8913	25.3095	24.6972	0.43565473	0.966018836	4	8	24	0.320607741	0.768040178	24	21.2747242	0.042485859
ENSMUSG00000074748	Atxn7l3b	ataxin 7-like 3B [Source:MGI Symbol;Acc:MGI:1914971]	43.5775	37.643	40.1565	38.7554	37.2174	40.9316	41.982	39.0122	35.5148	45.9027	37.0391	41.3388	40.0592	40.8326	0.297978647	0.80820842	4	16	24	0.66154525	0.885525866	24	22.20371454	0.036059005
ENSMUSG00000074749	Kiz	kizuna centrosomal protein [Source:MGI Symbol;Acc:MGI:2684960]	14.3926	16.3323	15.0451	14.4048	15.574	14.5446	13.0433	15.3249	16.8942	15.9908	15.9732	17.4713	17.4046	18.565	0.879201926	0.966018836	8	20	24	0.48204624	0.805393068	24	7.066769716	0.047356394
ENSMUSG00000074754	Smim26	small integral membrane protein 26 [Source:MGI Symbol;Acc:MGI:2685407]	37.2422	46.519	40.4145	42.7083	41.5823	39.3177	41.2673	44.4659	48.8795	33.7587	50.0315	43.7531	42.858	45.5688	0.714062999	0.966018836	8	20	24	0.756142141	0.921194485	24	5.943240156	0.043836362
ENSMUSG00000074766	Ism1	isthmin 1, angiogenesis inhibitor [Source:MGI Symbol;Acc:MGI:2442963]	1.45536	1.18103	1.17724	1.16613	0.943472	1.58577	0.869998	1.42032	1.40303	1.46498	1.12277	1.33581	1.4348	1.32602	0.882617464	0.966018836	24	20	24	0.802451401	0.93658224	24	3.453490425	0.035352091
ENSMUSG00000074771	Ankef1	ankyrin repeat and EF-hand domain containing 1 [Source:MGI Symbol;Acc:MGI:2441685]	0.602241	0.702035	1.04595	1.59926	0.607264	0.943774	1.52781	1.16317	0.836039	1.08289	2.38653	1.38491	2.07836	1.57357	0.831272156	0.966018836	24	20	24	0.906769924	0.974587616	24	17.04622222	0.045340692
ENSMUSG00000074776	Gm10754	predicted gene 10754 [Source:MGI Symbol;Acc:MGI:3642925]	1.76694	1.56765	1.32811	1.83723	1.81698	1.34092	1.14066	1.41701	2.7438	1.29705	1.40263	2.48307	1.88853	2.48528	0.742519053	0.966018836	8	20	24	0.82846809	0.946583288	24	6.525032358	0.05998025
ENSMUSG00000074780	Anapc15-ps	anaphase promoting complex C subunit 15, pseudogene [Source:MGI Symbol;Acc:MGI:3646302]	6.64897	8.09222	6.83892	5.17341	6.36904	6.24905	7.43731	7.22788	7.38718	4.51204	7.38299	5.85749	6.8976	5.13269	0.328944792	0.847277612	8	8	24	0.272469101	0.768040178	24	1.122763578	0.122694614

ENSMUSG00000074781	Ube2n	ubiquitin-conjugating enzyme E2N [Source:MGI Symbol;Acc:MGI:1934835]	57.6867	56.9126	54.9136	51.0698	57.7011	55.2795	55.8129	52.7652	48.14	57.6501	57.7473	53.5585	58.1448	56.1336	0.297756231	0.80820842	4	8	24	0.277007256	0.768040178	24	20.70750967	0.050335746
ENSMUSG00000074782	4833422C13Rik	RIKEN cDNA 4833422C13 gene [Source:MGI Symbol;Acc:MGI:3603826]	2.1684	2.42985	2.64735	2.81493	2.14385	2.46743	2.75476	2.50533	3.10125	2.4805	1.96932	2.51859	2.45947	2.87145	0.117335378	0.538362324	12	8	24	0.062605004	0.661961741	24	6.616244368	0.109288426
ENSMUSG00000074785	Plxnc1	plexin C1 [Source:MGI Symbol;Acc:MGI:1890127]	54.8288	44.1321	41.963	46.4652	45.3111	49.8746	42.3847	43.1708	38.1569	50.3685	45.9329	50.4484	50.7793	48.6731	0.085329704	0.466404237	24	12	24	0.121299104	0.733729587	24	20.25252738	0.108360409
ENSMUSG00000074793	Hspa12b	heat shock protein 12B [Source:MGI Symbol;Acc:MGI:1919880]	4.4438	4.39076	4.74516	3.68033	5.28628	4.6099	4.58594	5.47162	4.40655	6.22872	4.75092	4.74266	4.71405	4.56514	0.588682825	0.966018836	20	12	24	0.860035181	0.958767795	24	13.34688722	0.030428221
ENSMUSG00000074794	Arrdc3	arrestin domain containing 3 [Source:MGI Symbol;Acc:MGI:2145242]	14.6025	16.0218	17.0357	14.0386	16.1565	15.3151	17.6222	14.7114	14.908	15.4742	15.0101	15.7089	14.5747	14.2738	0.881996584	0.966018836	20	12	24	0.897275716	0.970715669	24	20.5749863	0.016893833
ENSMUSG00000074796	Slc4a11	solute carrier family 4, sodium bicarbonate transporter-like, member 11 [Source:MGI Symbol;Acc:MGI:2138987]	6.17905	4.49521	4.04693	4.97322	5.57819	5.56133	4.68738	5.50793	4.35786	5.86166	5.13088	5.68753	4.80099	4.69716	0.038115943	0.334069441	24	12	24	0.075918375	0.686372559	24	18.77637384	0.128801778
ENSMUSG00000074797	Itpa	inosine triphosphatase (nucleoside triphosphate pyrophosphatase) [Source:MGI Symbol;Acc:MGI:96622]	19.9541	21.3103	20.4283	19.8058	21.8946	20.2528	19.2394	20.9524	20.9706	19.4607	21.562	20.0768	21.8091	22.6016	0.951523604	0.966018836	8	8	24	0.751254593	0.91921305	24	5.184619682	0.01924018
ENSMUSG00000074800	Gm4149	predicted pseudogene 4149 [Source:MGI Symbol;Acc:MGI:3782325]	317.682	369.052	333.012	345.276	351.315	305.284	329.096	322.515	480.094	284.419	398.919	360.278	410.233	370.892	0.862909389	0.966018836	12	4	24	0.704304967	0.902338136	24	6.933366237	0.062938688
ENSMUSG00000074802	Gas2l3	growth arrest-specific 2 like 3 [Source:MGI Symbol;Acc:MGI:1918780]	31.4933	28.6075	28.3151	34.2819	36.8766	25.5256	35.3223	24.8181	33.1324	29.9901	30.1696	26.3268	29.2469	25.7264	0.295379186	0.805301811	20	12	24	0.381442766	0.799546792	24	14.45870241	0.089207451
ENSMUSG00000074807	Gm10762	predicted gene 10762 [Source:MGI Symbol;Acc:MGI:3704209]	2.28272	2.16173	1.94797	2.25489	1.47594	2.20019	1.59348	2.9344	2.91514	2.98748	2.24405	2.31603	2.85795	2.3675	0.92655264	0.966018836	16	4	24	0.272580088	0.768040178	24	6.931096081	0.122323663
ENSMUSG00000074811	Hps6	HPS6, biogenesis of lysosomal organelles complex 2 subunit 3 [Source:MGI Symbol;Acc:MGI:2181763]	2.97324	2.25436	2.7369	2.18966	2.47748	2.56577	2.76485	2.5164	2.33902	2.96619	2.61111	2.75014	2.3411	2.26987	0.631736745	0.966018836	4	4	24	0.49707492	0.807576732	24	19.14233905	0.050482529
ENSMUSG00000074813	Morrbid	myeloid RNA regulator of BCL2L11 induced cell death [Source:MGI Symbol;Acc:MGI:3652191]	2.19771	1.73495	2.31027	1.46969	1.69537	1.57756	1.26365	1.94501	1.75517	1.91394	1.42057	2.04594	1.56243	1.56392	0.951523604	0.966018836	12	8	24	0.73929516	0.914957371	24	6.78178721	0.048796232
ENSMUSG00000074818	Pdzd7	PDZ domain containing 7 [Source:MGI Symbol;Acc:MGI:3608325]	2.44623	2.21881	2.22776	2.14887	2.36196	2.58751	2.79418	2.60674	2.52308	2.62236	2.57495	2.73772	2.45494	2.55328	0.798309651	0.966018836	24	12	24	0.179736878	0.768040178	24	21.62515014	0.046844704
ENSMUSG00000074825	Itpripl1	inositol 1,4,5-triphosphate receptor interacting protein-like 1 [Source:MGI Symbol;Acc:MGI:1920588]	3.6073	2.22459	2.41835	2.7895	3.01381	3.18425	2.83648	3.20743	1.79816	2.94699	2.71356	2.63791	2.51188	2.47109	0.280850951	0.792139038	24	12	24	0.102877522	0.713714981	24	19.76880031	0.143106947
ENSMUSG00000074826	Gm10767	predicted gene 10767 [Source:MGI Symbol;Acc:MGI:3642136]	1.70322	1.93398	2.23138	2.39023	2.01668	2.06416	2.10417	1.70302	1.99067	1.67765	1.95639	1.49591	1.47875	1.9503	0.521828922	0.966018836	12	16	24	0.218901789	0.768040178	24	11.10301441	0.078600863
ENSMUSG00000074829	2010315B03Rik	RIKEN cDNA 2010315B03 gene [Source:MGI Symbol;Acc:MGI:1919321]	11.8724	13.0886	12.003	12.0691	12.1601	11.0116	11.3376	12.4887	12.7391	10.6531	12.4594	12.2738	11.8776	13.2086	0.160842982	0.620479606	8	20	24	0.27453191	0.768040178	24	5.517998549	0.051308866
ENSMUSG00000074846	Gm10774	predicted pseudogene 10774 [Source:MGI Symbol;Acc:MGI:3642325]	125.906	119.495	106.204	102.956	118.23	111.561	88.1991	114.822	122.714	118.004	125.349	124.641	138.505	144.379	0.864782464	0.966018836	8	8	24	0.847501876	0.954020745	24	3.068449157	0.035100864
ENSMUSG00000074862	BC025920	cDNA sequence BC025920 [Source:MGI Symbol;Acc:MGI:2670983]	1.22886	1.67826	1.25794	1.35529	1.42446	1.47308	1.27885	1.57671	1.55692	1.20394	1.71424	1.19727	1.3356	1.42986	0.714062999	0.966018836	8	20	24	0.836299331	0.949953371	24	7.770251786	0.025870629
ENSMUSG00000074863	Platr25	pluripotency associated transcript 25 [Source:MGI Symbol;Acc:MGI:3645613]	7.63774	8.93437	7.67447	7.99023	7.55974	6.8817	7.94433	7.23177	9.03045	6.63442	7.97802	7.04789	8.39223	8.53499	0.196044629	0.673646834	12	12	24	0.17220538	0.768040178	24	5.297092845	0.086489575
ENSMUSG00000074865	Zfp934	zinc finger protein 934 [Source:MGI Symbol;Acc:MGI:1924367]	4.8532	6.03935	5.20637	5.81112	6.06252	4.71716	5.36453	5.06142	5.73702	4.02662	5.64297	5.62411	5.25401	5.49312	0.909604019	0.966018836	20	8	24	0.997259846	0.999495538	24	7.317088178	0.00416354
ENSMUSG00000074867	Zfp808	zinc finger protein 808 [Source:MGI Symbol;Acc:MGI:3704127]	1.18752	1.75621	1.58852	1.35613	1.59221	1.5214	1.60479	1.57001	1.8258	0.986469	1.57792	1.55689	1.27976	1.45801	0.834434272	0.966018836	12	4	24	0.830279678	0.947325117	24	4.217716811	0.033148982
ENSMUSG00000074868	Gm6478	predicted pseudogene 6478 [Source:MGI Symbol;Acc:MGI:3647766]	2.54553	1.78125	1.27121	1.65132	1.847	1.59517	1.5929	1.59784	1.87655	1.90954	1.46244	2.02361	1.83683	2.0152	0.832293447	0.966018836	4	8	24	0.503153954	0.809997329	24	23.19816072	0.07234532
ENSMUSG00000074872	Ctxn2	cortexin 2 [Source:MGI Symbol;Acc:MGI:2139444]	3.16989	4.87853	6.29484	3.3163	4.39863	4.57088	5.01974	3.52422	4.4779	3.10013	3.36303	3.93425	2.73708	4.24045	0.893307667	0.966018836	12	4	24	0.705818375	0.902918222	24	6.17488639	0.085609244
ENSMUSG00000074874	Ctla2b	cytotoxic T lymphocyte-associated protein 2 beta [Source:MGI Symbol;Acc:MGI:88555]	1.52854	1.81224	1.12666	1.23235	1.34515	0.886342	1.10908	1.71938	2.1044	1.02722	1.13482	0.761309	1.25686	1.01157	0.245061943	0.741580974	12	12	24	0.10957157	0.72082227	24	6.137567728	0.171272381
ENSMUSG00000074884	Serf2	small EDRK-rich factor 2 [Source:MGI Symbol;Acc:MGI:1337041]	99.4834	100.368	92.519	94.2286	94.3236	96.9937	95.3702	93.813	88.0198	83.7458	86.5093	92.2987	95.2281	91.4537	0.073139747	0.447768907	4	12	24	8.11E-04	0.319044294	24	23.72816791	0.068213756
ENSMUSG00000074886	Grk6	G protein-coupled receptor kinase 6 [Source:MGI Symbol;Acc:MGI:1347078]	19.8215	17.6339	18.4617	18.1116	19.0449	18.6548	16.8798	18.5859	17.7078	21.1918	17.1907	18.6225	18.5277	18.0274	0.92655264	0.966018836	16	20	24	0.723759549	0.909411547	24	14.36189892	0.025116319
ENSMUSG00000074890	Lcmt2	leucine carboxyl methyltransferase 2 [Source:MGI Symbol;Acc:MGI:1353659]	2.6825	2.8025	2.68548	2.36041	2.79208	2.98731	2.05489	3.13522	2.43348	3.29923	2.63811	3.6144	2.96126	2.74186	0.94397916	0.966018836	24	12	24	0.799317594	0.935782011	24	18.60630584	0.039766829
ENSMUSG00000074892	B3galt5	UDP-Gal:betaGlcNAc beta 1,3-galactosyltransferase, polypeptide 5 [Source:MGI Symbol;Acc:MGI:2136878]	5.12187	6.20071	9.22994	6.05457	4.56111	7.10728	6.76886	5.77776	6.32276	6.69005	4.25874	6.1426	4.38415	6.9348	0.424718357	0.966018836	12	8	24	0.268880411	0.768040178	24	7.03471005	0.16634473
ENSMUSG00000074896	Ifit3	interferon-induced protein with tetratricopeptide repeats 3 [Source:MGI Symbol;Acc:MGI:1101055]	15.2302	18.7242	17.9627	17.6011	25.5944	18.6697	13.4977	20.649	23.0526	14.1623	24.0912	17.351	13.9311	20.0115	0.089506974	0.477279066	20	8	24	0.383345724	0.799546792	24	12.3917105	0.149684725
ENSMUSG00000074899	Sptbn5	spectrin beta, non-erythrocytic 5 [Source:MGI Symbol;Acc:MGI:2685200]	15.5185	17.3235	14.1958	17.3028	21.8247	14.5768	19.4162	17.617	21.0609	17.6883	18.74	16.6712	17.9426	14.3561	0.956178643	0.96629412	20	12	24	0.576320997	0.848564012	24	14.28066147	0.074678836
ENSMUSG00000074903	Gm2058	predicted gene 2058 [Source:MGI Symbol;Acc:MGI:3805010]	15.006	11.0249	12.5777	14.194	11.4237	14.2667	13.2289	12.1889	12.7423	16.6455	12.0322	13.9081	13.6913	13.9684	0.569060064	0.966018836	16	16	24	0.903813672	0.973435328	24	17.10621878	0.026988602
ENSMUSG00000074909	Ranbp6	RAN binding protein 6 [Source:MGI Symbol;Acc:MGI:2683212]	19.902	20.7175	19.3095	19.8052	20.7578	19.0833	20.5366	18.6315	19.7629	17.3111	21.938	20.4396	21.3448	21.1985	0.593560247	0.966018836	20	20	24	0.432927312	0.805393068	24	21.98134905	0.043268714
ENSMUSG00000074916	Chst14	carbohydrate sulfotransferase 14 [Source:MGI Symbol;Acc:MGI:1919386]	3.65862	4.17402	3.42247	3.82268	3.53856	3.60141	3.63015	4.33962	4.19238	5.2941	3.71761	4.58165	3.99963	4.59846	0.702630284	0.966018836	8	12	24	0.358297561	0.784922891	24	7.817340698	0.070333002
ENSMUSG00000074918	Inafm2	InaF motif containing 2 [Source:MGI Symbol;Acc:MGI:1915354]	3.08112	3.20719	3.81574	3.52466	2.85793	2.99729	3.53287	2.93054	3.40418	3.70769	3.17943	3.64031	3.18992	3.74623	0.435739761	0.966018836	16	4	24	0.251622354	0.768040178	24	8.557496147	0.068595683
ENSMUSG00000074922	Fam122a	family with sequence similarity 122, member A [Source:MGI Symbol;Acc:MGI:1915284]	5.46127	4.02982	3.81257	4.477	3.69463	4.33313	4.55659	4.31937	4.11966	5.46985	5.25109	4.73427	5.28896	6.32642	0.793403332	0.966018836	4	8	24	0.685900537	0.89571585	24	0.43256596	0.05309025
ENSMUSG00000074923	Pak6	p21 (RAC1) activated kinase 6 [Source:MGI Symbol;Acc:MGI:2679420]	0.112589	0.577189	0.879303	0.770866	0.545228	0.31853	0.792834	0.496517	0.589023	0.443719	0.140064	0.589519	0.203566	0.580373	0.588208558	0.966018836	12	16	24	0.222896546	0.768040178	24	8.417257516	0.161841074
ENSMUSG00000074925	Ptar1	protein prenyltransferase alpha subunit repeat containing 1 [Source:MGI Symbol;Acc:MGI:1921875]	4.4099	5.08134	4.844	5.05187	5.08508	4.46672	4.79222	4.38118	5.10989	3.97019	5.33124	4.5408	4.8285	5.07578	0.718086778	0.966018836	20	4	24	0.8678159	0.962217749	24	9.941420372	0.021532158
ENSMUSG00000074968	Ano3	anoctamin 3 [Source:MGI Symbol;Acc:MGI:3613666]	58.1404	61.4701	51.7523	58.8174	54.5395	55.3639	54.5523	52.429	57.321	52.4346	60.4334	59.1457	62.6954	62.3854	0.801995186	0.966018836	4	8	24	0.562358567	0.842285613	24	23.72869257	0.037948992
ENSMUSG00000074971	Fibin	fin bud initiation factor homolog (zebrafish) [Source:MGI Symbol;Acc:MGI:1914856]	4.27236	5.09656	4.6292	5.49101	4.49088	4.25515	4.34798	4.65107	5.96526	4.22614	4.26274	4.28993	3.48678	4.19474	0.321662653	0.83607295	12	12	24	0.048397558	0.638992719	24	9.441466261	0.128399655
ENSMUSG00000074978	Actg-ps1	actin, gamma, pseudogene 1 [Source:MGI Symbol;Acc:MGI:87907]	0.702377	0.546138	0.674082	0.427085	0.371629	0.493236	0.302593	0.814616	0.564675	0.928002	0.483477	0.631581	0.577475	0.76971	0.438929876	0.966018836	8	12	24	0.240314437	0.768040178	24	6.612516175	0.103042361
ENSMUSG00000074994	Qser1	glutamine and serine rich 1 [Source:MGI Symbol;Acc:MGI:2138986]	4.76986	5.20232	4.83156	5.49376	5.53038	5.08379	5.34134	4.94804	5.56292	5.20579	5.60397	5.13197	5.52779	4.85117	0.305564675	0.811201458	20	12	24	0.182231927	0.768040178	24	14.81528965	0.046247497
ENSMUSG00000075000	Nrbf2	nuclear receptor binding factor 2 [Source:MGI Symbol;Acc:MGI:1354950]	7.58854	6.51332	6.35	6.41869	6.19404	7.46419	6.07417	7.3973	6.26077	7.31179	7.71609	6.72887	6.66898	7.22988	0.930944473	0.966018836	24	12	24	0.778701879	0.928873919	24	20.42672246	0.030704795
ENSMUSG00000075010	AW112010	expressed sequence AW112010 [Source:MGI Symbol;Acc:MGI:2147706]	5.3389	6.99309	8.37948	7.82683	8.1271	5.67701	6.39239	7.86917	9.54378	5.63402	8.98262	6.76419	8.60829	7.36472	0.374467935	0.89742288	12	12	24	0.246781636	0.768040178	24	9.443286389	0.148033748
ENSMUSG00000075012	Fjx1	four jointed box 1 [Source:MGI Symbol;Acc:MGI:1341907]	1.02563	1.12579	1.63832	0.891755	0.833023	0.883069	1.20521	1.14984	1.29534	1.04674	1.10456	1.17461	1.17567	1.52635	0.029825002	0.323426482	12	4	24	0.016395473	0.544099157	24	5.564454086	0.143244541
ENSMUSG00000075020	Mir670hg	MIR670 host gene (non-protein coding) [Source:MGI Symbol;Acc:MGI:3041234]	1.16925	1.55131	1.88847	1.79846	1.28685	1.54795	2.54537	1.00556	1.38269	1.27783	1.50742	1.62422	1.22989	1.42715	0.852844413	0.966018836	12	20	24	0.951989373	0.988339231	24	18.36329344	0.026618782
ENSMUSG00000075028	Prdm11	PR domain containing 11 [Source:MGI Symbol;Acc:MGI:2685553]	0.805459	1.32217	1.48788	1.34611	1.18588	1.35017	1.38593	1.152	1.41157	1.52858	1.35468	1.28629	1.32423	1.4215	0.23195324	0.731832877	12	16	24	0.102335185	0.713699364	24	10.33275229	0.087208628
ENSMUSG00000075033	Nxpe3	neurexophilin and PC-esterase domain family, member 3 [Source:MGI Symbol;Acc:MGI:2686598]	8.47792	7.64108	7.19161	7.17569	6.94334	8.2211	7.7722	7.36896	5.87835	8.39925	6.74953	7.49456	7.08102	7.83198	0.38940266	0.915479136	4	16	24	0.313505379	0.768040178	24	22.38257377	0.072583719
ENSMUSG00000075040	Zfp408	zinc finger protein 408 [Source:MGI Symbol;Acc:MGI:2685857]	3.7206	2.81875	3.26089	2.38384	3.40539	2.70467	2.69187	3.2476	3.5464	3.49655	2.58306	2.93971	3.4092	2.98301	0.94397916	0.966018836	12	8	24	0.655757301	0.883738698	24	5.047796994	0.054912308
ENSMUSG00000075053	Vdac3-ps1	voltage-dependent anion channel 3, pseudogene 1 [Source:MGI Symbol;Acc:MGI:1270159]	103.259	100.98	102.737	97.721	98.2757	102.302	95.7557	101.747	94.9091	90.858	99.3856	102.841	102.784	99.4933	0.080148772	0.458678242	24	16	24	0.08902748	0.708329025	24	23.2581241	0.043244731
ENSMUSG00000075054	Yae1d1	Yae1 domain containing 1 [Source:MGI Symbol;Acc:MGI:1914258]	4.1002	4.43275	4.50287	4.56522	4.93066	3.92445	3.78512	3.68345	4.3774	3.37794	4.4369	4.05195	4.05935	4.28254	0.25849765	0.761738864	20	4	24	0.637072052	0.876483853	24	13.06145266	0.041210035
ENSMUSG00000075122	Cd80	CD80 antigen [Source:MGI Symbol;Acc:MGI:101775]	0.562917	0.534177	0.516618	0.458403	0.488527	0.503188	0.482512	0.612371	0.700994	0.403041	0.463747	0.568205	0.472238	0.569762	0.017525419	0.25552149	8	8	24	0.158584158	0.764943091	24	4.199876527	0.050791156
ENSMUSG00000075184	F930017D23Rik	RIKEN cDNA F930017D23 gene [Source:MGI Symbol;Acc:MGI:3822538]	0.60353	0.61455	0.766124	0.500844	0.437207	1.35628	1.30919	0.465315	0.899377	0.467168	0.669558	0.903594	1.03558	0.820397	0.133012827	0.567728672	24	20	24	0.137774645	0.747972781	24	22.92349273	0.17158173
ENSMUSG00000075224	Lrrc55	leucine rich repeat containing 55 [Source:MGI Symbol;Acc:MGI:2685197]	1.92651	2.28781	3.25577	2.26514	1.94052	2.42809	2.42296	1.9554	2.24733	2.13298	2.20468	2.41784	1.91156	2.62318	0.94086481	0.966018836	12	16	24	0.570148537	0.845047627	24	8.042880559	0.064879909
ENSMUSG00000075225	Ccdc162	coiled-coil domain containing 162 [Source:MGI Symbol;Acc:MGI:1923223]	1.90264	2.07602	2.90881	2.76479	2.08645	1.62611	1.66072	2.32331	2.27994	1.93673	2.74618	2.22741	1.86608	2.39706	0.031969981	0.324385159	12	16	24	0.045565483	0.629609444	24	10.19394387	0.163508097
ENSMUSG00000075227	Znhit2	zinc finger, HIT domain containing 2 [Source:MGI Symbol;Acc:MGI:1352481]	9.47578	7.97751	7.56703	7.83439	7.77615	7.99825	7.93041	9.29389	8.73474	11.6279	10.5131	10.5703	11.0479	9.61063	0.890648739	0.966018836	24	12	24	0.896037789	0.970467191	24	19.24425246	0.026979908
ENSMUSG00000075229	Ccdc58	coiled-coil domain containing 58 [Source:MGI Symbol;Acc:MGI:2146423]	11.4867	12.7351	15.0616	13.4816	12.8325	10.2879	13.0733	13.577	14.6482	8.17933	15.4622	10.8082	13.2174	10.384	0.245061943	0.741580974	12	12	24	0.815046587	0.940891459	24	7.439026319	0.061512328
ENSMUSG00000075232	Amd1	S-adenosylmethionine decarboxylase 1 [Source:MGI Symbol;Acc:MGI:88004]	14.4773	17.0192	14.0255	16.1242	13.9135	16.0912	16.7375	20.8523	22.7485	17.5088	17.2953	17.5005	17.9959	14.4173	0.844448841	0.966018836	8	12	24	0.581853355	0.85096264	24	6.726021968	0.078442307
ENSMUSG00000075254	Hegl	heart development protein with EGF-like domains 1 [Source:MGI Symbol;Acc:MGI:1924696]	5.42563	6.00333	6.15042	5.9869	6.1154	5.5069	5.7183	6.46202	5.92798	6.52993	4.84016	5.04291	5.55972	5.65258	0.033622862	0.324908923	16	8	24	0.042357384	0.623698598	24	8.110191357	0.093648249
ENSMUSG00000075256	Cerkl	ceramide kinase-like [Source:MGI Symbol;Acc:MGI:3037816]	2.09014	1.50108	1.80724	0.882913	0.994202	0.989391	1.3907	0.89325	1.12487	1.44064	1.15709	1.50526	0.968375	1.48131	0.860560779	0.966018836	12	4	24	0.570205185	0.845047627	24	2.487393438	0.088424604
ENSMUSG00000075266	Cenpw	centromere protein W [Source:MGI Symbol;Acc:MGI:1913561]	0.82493	1.15986	1.03339	0.818524	0.816572	1.00094	0.724134	1.02765	0.921993	0.714658	0.939885	0.862309	0.953388	1.02723	0.050258003	0.385784509	8	8	24	0.226051671	0.768040178	24	4.341513782	0.064201345
ENSMUSG00000075268	Gm10819	predicted gene 10819 [Source:MGI Symbol;Acc:MGI:3642135]	70.6202	59.16	62.9475	64.0561	63.2324	65.5646	70.8647	69.3738	61.6376	69.7333	68.6516	64.5867	65.5899	64.7872	0.067104747	0.425837275	4	8	24	0.502782863	0.809737567	24	20.74592043	0.037042616
ENSMUSG00000075270	Pde11a	phosphodiesterase 11A [Source:MGI Symbol;Acc:MGI:3036251]	16.796	15.5444	15.0752	15.6946	14.4066	16.0919	14.1368	13.1141	13.9769	15.1719	15.23	16.8724	17.9231	16.3004	0.217827365	0.704989686	24	12	24	0.307820124	0.768040178	24	21.20655541	0.06914847
ENSMUSG00000075271	Ttc30a1	tetratricopeptide repeat domain 30A1 [Source:MGI Symbol;Acc:MGI:1926052]	1.06747	1.19472	1.19829	1.00154	1.11479	1.15711	1.25101	1.10016	0.944439	0.95457	0.99924	1.09309	0.936958	1.18304	0.954584434	0.966018836	8	8	24	0.262865936	0.768040178	24	0.749214673	0.042408921
ENSMUSG00000075273	Ttc30b	tetratricopeptide repeat domain 30B [Source:MGI Symbol;Acc:MGI:1919671]	4.2751	4.72869	4.58613	4.66674	4.33195	4.54209	5.00102	4.64513	3.92766	4.25519	4.33455	4.44033	4.47094	4.72089	0.165587374	0.633636735	8	12	24	0.322509876	0.768040178	24	1.097744043	0.040148294
ENSMUSG00000075277	Hasgr	Hoxd antisense growth associated long non-coding RNA [Source:MGI Symbol;Acc:MGI:3026978]	6.08234	5.74148	5.24979	6.25442	5.29033	5.62304	5.43003	5.90812	5.77623	6.12588	7.08448	5.51469	5.21173	6.2671	0.823677655	0.966018836	16	8	24	0.530064757	0.824795587	24	12.1950629	0.046466158
ENSMUSG00000075279	Mrpl23-ps1	mitochondrial ribosomal protein L23, pseudogene 1 [Source:MGI Symbol;Acc:MGI:3650679]	30.4733	32.6936	33.0979	31.2857	28.6564	32.2965	30.3119	33.3629	39.9081	32.5618	33.9763	33.5846	37.5233	33.4359	0.904831057	0.966018836	12	4	24	0.199627938	0.768040178	24	6.532205484	0.070472856
ENSMUSG00000075284	Wipf1	WAS/WASL interacting protein family, member 1 [Source:MGI Symbol;Acc:MGI:2178801]	10.9698	10.2539	10.5211	10.9042	10.5928	10.327	10.9939	10.8661	10.5959	13.153	10.3471	10.6161	10.1385	10.6847	0.903332508	0.966018836	12	16	24	0.387811446	0.799546792	24	10.9888756	0.045594895
ENSMUSG00000075289	Carns1	carnosine synthase 1 [Source:MGI Symbol;Acc:MGI:2147595]	26.5042	31.285	28.7967	29.9689	34.8078	27.7369	33.1523	24.5878	35.4639	30.2541	27.5147	24.9046	28.4305	22.7431	0.714044782	0.966018836	12	20	24	0.423865957	0.805393068	24	12.81506152	0.086589719
ENSMUSG00000075297	H60b	histocompatibility 60b [Source:MGI Symbol;Acc:MGI:3649078]	0.688005	0.638458	0.648472	0.397802	0.722027	0.364685	0.951558	0.465639	0.858137	0.743827	0.625867	0.303306	0.490121	0.322915	0.850398351	0.966018836	12	8	24	0.812362564	0.94001603	24	9.170893951	0.04692116
ENSMUSG00000075307	Klhl41	kelch-like 41 [Source:MGI Symbol;Acc:MGI:2683854]	2.00792	6.48218	8.54117	8.95643	5.00214	7.39703	5.80929	16.093	9.57743	15.1611	5.00441	5.08729	8.87557	6.82271	0.048507657	0.379129806	16	4	24	0.054201972	0.654887447	24	8.789655679	0.544832803
ENSMUSG00000075312	Gm13597	predicted gene 13597 [Source:MGI Symbol;Acc:MGI:3651139]	44.0738	42.0713	38.2932	41.9435	47.307	42.9147	42.0914	44.8849	36.8721	42.6188	38.0921	41.9691	44.0171	37.5681	0.286642595	0.793714531	4	8	24	0.154001045	0.760223561	24	20.30088202	0.075202399
ENSMUSG00000075316	Scn9a	sodium channel, voltage-gated, type IX, alpha [Source:MGI Symbol;Acc:MGI:107636]	51.5141	51.5335	46.7084	51.7258	51.3244	48.1843	44.8801	43.7831	49.5648	42.8925	56.7609	52.7646	54.1745	54.0232	0.93717134	0.966018836	20	20	24	0.612577442	0.865589526	24	19.33351401	0.048210515
ENSMUSG00000075318	Scn2a	sodium channel, voltage-gated, type II, alpha [Source:MGI Symbol;Acc:MGI:98248]	4.33024	5.60937	5.6486	4.34432	5.04192	4.96782	4.91818	4.12865	6.05136	3.72932	4.85825	5.29024	4.65541	4.92213	0.887027852	0.966018836	12	4	24	0.865416691	0.961005713	24	4.678555104	0.034381456
ENSMUSG00000075324	Fign	fidgetin [Source:MGI Symbol;Acc:MGI:1890647]	2.21979	2.15005	1.65479	2.23588	2.51729	2.32228	2.30416	1.90736	2.16869	2.30964	2.61065	1.8684	2.40688	2.23935	0.033622862	0.324908923	20	16	24	0.232232505	0.768040178	24	17.68919367	0.077700739
ENSMUSG00000075327	Zbtb2	zinc finger and BTB domain containing 2 [Source:MGI Symbol;Acc:MGI:2685949]	2.40981	2.09712	2.18507	2.95426	2.26499	2.03273	2.66557	2.39852	2.30224	2.5038	2.30585	2.32264	2.14851	2.44533	0.917839756	0.966018836	16	8	24	0.619469662	0.869468249	24	10.99541689	0.039669221
ENSMUSG00000075334	Rprm	reprimo, TP53 dependent G2 arrest mediator candidate [Source:MGI Symbol;Acc:MGI:1915124]	24.6427	21.3539	20.6569	23.1152	19.5415	22.0839	21.7344	23.4303	20.6595	29.0604	23.7749	25.4524	26.0381	24.8037	0.850398351	0.966018836	24	12	24	0.953849305	0.988601783	24	21.15478393	0.016495997
ENSMUSG00000075376	Rc3h2	ring finger and CCCH-type zinc finger domains 2 [Source:MGI Symbol;Acc:MGI:2442789]	12.0635	12.913	12.9258	12.6637	12.1926	11.9963	11.8784	11.4104	13.2413	11.4705	12.719	11.7976	11.5185	12.8706	0.122530972	0.550724475	12	16	24	0.218977851	0.768040178	24	9.035877669	0.045169717
ENSMUSG00000075389	2810410L24Rik	RIKEN cDNA 2810410L24 gene [Source:MGI Symbol;Acc:MGI:1923627]	1.1701	2.35301	2.92119	1.82493	1.59529	1.54177	1.58369	2.39824	4.098	3.29097	2.94637	3.18664	3.44879	3.15866	0.688644847	0.966018836	12	16	24	0.014890703	0.537924552	24	8.489495242	0.284153163
ENSMUSG00000075391	Glo1-ps	glyoxalase 1, pseudogene [Source:MGI Symbol;Acc:MGI:3649615]	105.581	102.927	102.249	102.124	107.291	104.857	97.0157	102.295	100.469	95.7006	112.383	105.447	102.965	100.391	0.012922042	0.227495952	20	20	24	0.136629263	0.747972781	24	18.29239795	0.044297901
ENSMUSG00000075394	Hoxc4	homeobox C4 [Source:MGI Symbol;Acc:MGI:96195]	2.10871	3.02758	2.79804	2.58875	2.39628	2.49999	2.53423	2.53357	3.22925	3.108	2.82823	3.00204	2.48812	3.47352	0.153859465	0.605619266	12	16	24	0.034595975	0.608551791	24	8.533950621	0.116812009
ENSMUSG00000075408	Smim41	small integral membrane protein 41 [Source:MGI Symbol;Acc:MGI:1924967]	0.96529	1.21985	0.86771	1.42375	1.22822	0.977403	1.02458	0.823988	1.09171	0.898098	1.29696	1.14451	1.25657	0.982904	0.602156008	0.966018836	20	12	24	0.327889471	0.768040178	24	16.33011493	0.07208682
ENSMUSG00000075410	Prcd	photoreceptor disc component [Source:MGI Symbol;Acc:MGI:3649529]	1.27577	1.52315	1.08272	1.3356	1.18527	1.52518	1.39025	1.53747	1.83738	1.22729	1.56203	1.57792	1.09911	1.58086	0.890176313	0.966018836	8	20	24	0.869554824	0.962966803	24	4.857429576	0.027324648
ENSMUSG00000075415	Fnbp1	formin binding protein 1 [Source:MGI Symbol;Acc:MGI:109606]	53.6201	50.0515	46.9417	45.8096	42.2674	49.5156	54.3828	47.9082	43.2621	57.0444	46.5153	48.6187	54.7059	49.7321	0.01579111	0.253395174	4	16	24	0.244079601	0.768040178	24	0.309583242	0.079481736
ENSMUSG00000075419	Dolk	dolichol kinase [Source:MGI Symbol;Acc:MGI:2677836]	11.3862	9.55473	10.9256	10.5387	9.41251	11.1223	10.4995	11.3116	9.59519	11.8997	9.1284	10.3538	11.2615	10.6265	0.743750765	0.966018836	4	16	24	0.627504293	0.872795898	24	2.348891698	0.040855526
ENSMUSG00000075420	Smim6	small integral membrane protein 6 [Source:MGI Symbol;Acc:MGI:1915778]	0.682649	0.718629	0.424204	0.727092	0.620473	0.525923	0.713904	0.567591	0.732636	0.386586	0.637055	0.963799	0.876923	0.655433	0.92655264	0.966018836	4	12	24	0.219487126	0.768040178	24	22.63921259	0.088860336
ENSMUSG00000075463	4930594M22Rik	RIKEN cDNA 4930594M22 gene [Source:MGI Symbol;Acc:MGI:1925478]	0.672689	0.788121	0.606263	0.418799	0.79248	0.3896	0.518788	0.818784	0.894056	0.536214	0.749984	0.687669	0.597356	0.519009	0.798829513	0.966018836	12	12	24	0.768082639	0.925197875	24	6.514201475	0.041653382
ENSMUSG00000075467	Dnlz	DNL-type zinc finger [Source:MGI Symbol;Acc:MGI:106559]	13.7179	10.8903	11.2139	12.1134	11.9345	12.0968	11.313	12.0336	10.2975	11.0837	12.7175	12.2951	12.2702	11.8778	0.068712455	0.432567661	24	12	24	0.057731746	0.656248849	24	20.28953591	0.085591564
ENSMUSG00000075470	Alg10b	asparagine-linked glycosylation 10B (alpha-1,2-glucosyltransferase) [Source:MGI Symbol;Acc:MGI:2146159]	9.46194	10.5494	10.7003	10.1836	10.1044	9.4505	9.71562	9.7017	10.225	9.93444	10.158	9.69664	9.45366	9.7877	0.001885685	0.100255953	12	12	24	0.009186732	0.502965379	24	9.955228898	0.051525852
ENSMUSG00000075478	Slitrk1	SLIT and NTRK-like family, member 1 [Source:MGI Symbol;Acc:MGI:2679446]	3.94249	3.43443	4.13451	3.33303	3.20918	4.12383	4.5523	3.62292	2.75856	4.25681	3.18984	3.54613	3.07925	4.21894	0.907068194	0.966018836	4	16	24	0.660893182	0.885520687	24	0.529773036	0.060009336
ENSMUSG00000075486	Commd6	COMM domain containing 6 [Source:MGI Symbol;Acc:MGI:1913450]	36.1622	39.6265	41.7692	37.2099	40.152	34.0366	36.7473	35.4734	40.9064	30.935	46.8072	37.0568	36.5387	39.9777	0.243857167	0.741580974	12	12	24	0.822801127	0.94435494	24	10.49789421	0.036366815
ENSMUSG00000075511	1700001L05Rik	RIKEN cDNA 1700001L05 gene [Source:MGI Symbol;Acc:MGI:1916541]	19.2785	15.8497	15.7274	21.2118	16.75	20.5703	16.0079	16.8745	14.5322	23.2249	17.0213	18.8815	17.5953	18.0682	0.058209189	0.40386543	16	20	24	0.30377237	0.768040178	24	16.40398334	0.107197815
ENSMUSG00000075514	Gm13375	predicted gene 13375 [Source:MGI Symbol;Acc:MGI:3649913]	2.07254	1.60842	2.62885	1.78018	1.80694	1.63133	1.61467	2.52326	3.14333	3.68988	2.88632	3.38133	2.26454	3.10321	0.694910419	0.966018836	16	12	24	0.126416069	0.739868076	24	9.870079007	0.178953196
ENSMUSG00000075528	Aarsd1	alanyl-tRNA synthetase domain containing 1 [Source:MGI Symbol;Acc:MGI:1916934]	19.4705	19.0019	17.6231	18.8261	16.3669	19.3565	17.0071	17.5824	16.9807	19.426	17.4818	18.011	19.2396	18.7963	0.83638114	0.966018836	4	16	24	0.740367789	0.915274254	24	0.703104281	0.024738835
ENSMUSG00000075555	Gm10863	predicted gene 10863 [Source:MGI Symbol;Acc:MGI:3641623]	9.41106	12.0341	7.62916	6.9362	11.8337	9.16293	11.3076	10.7173	9.92285	8.42826	9.876	8.1424	11.9705	9.37065	0.241264473	0.741580974	4	12	24	0.200350051	0.768040178	24	0.239999538	0.152954429
ENSMUSG00000075569	Rsph10b	radial spoke head 10 homolog B (Chlamydomonas) [Source:MGI Symbol;Acc:MGI:1922386]	1.36705	1.1906	1.97721	2.31921	1.44387	1.20076	1.45579	0.890337	1.44479	1.35372	0.999987	1.99989	1.18016	2.2206	0.912686312	0.966018836	12	20	24	0.641185291	0.878540546	24	10.28410923	0.097125933
ENSMUSG00000075576	Gm12359	predicted gene 12359 [Source:MGI Symbol;Acc:MGI:3702436]	0.885395	0.887424	1.19667	1.09747	1.56989	0.707629	0.991205	0.904541	1.33083	1.07232	1.45248	1.17029	1.06946	1.10812	0.04467378	0.365845448	20	8	24	0.068479367	0.672999758	24	12.88851458	0.127190097
ENSMUSG00000075585	6330403L08Rik	RIKEN cDNA 6330403L08 gene [Source:MGI Symbol;Acc:MGI:1917994]	2.20208	1.95557	1.87438	1.95253	2.01562	2.20029	2.2977	2.17995	2.1644	2.9402	2.03032	2.81344	2.6548	2.69238	0.289496388	0.794869797	24	12	24	0.622443713	0.871391591	24	23.00630369	0.04008343
ENSMUSG00000075588	Hoxb2	homeobox B2 [Source:MGI Symbol;Acc:MGI:96183]	3.14749	2.46809	2.63469	2.42036	1.80928	2.36977	2.64941	3.1192	2.9231	2.45541	3.9405	2.75997	2.70587	2.99083	0.46694635	0.966018836	4	12	24	0.661395045	0.885520687	24	3.403493183	0.063635685
ENSMUSG00000075589	Gm11536	predicted gene 11536 [Source:MGI Symbol;Acc:MGI:3650116]	0.430247	0.326958	0.467161	0.61055	0.557513	0.36233	0.523077	0.573869	0.731297	0.426701	0.660276	0.345773	0.648747	0.816781	0.957256673	0.966536401	12	12	24	0.264810037	0.768040178	24	8.143619682	0.07948043
ENSMUSG00000075590	Nrbp2	nuclear receptor binding protein 2 [Source:MGI Symbol;Acc:MGI:2385017]	125.037	150.453	144.597	145.014	163.562	135.632	139.579	145.331	165.523	151.083	134.96	141.174	144.388	137.843	0.146381309	0.590732968	16	8	24	0.113020619	0.727393008	24	10.31646219	0.083949396
ENSMUSG00000075591	Gm10874	predicted gene 10874 [Source:MGI Symbol;Acc:MGI:3704261]	0.929248	0.967327	0.622962	1.18143	0.751564	1.27168	1.06364	0.907457	0.90427	1.41942	0.99549	0.959811	1.34931	1.30423	0.791413662	0.966018836	16	20	24	0.890955782	0.969222457	24	21.38103095	0.028847311
ENSMUSG00000075592	Nynrin	NYN domain and retroviral integrase containing [Source:MGI Symbol;Acc:MGI:2652872]	1.78574	1.81981	2.17327	1.53001	1.7865	1.89408	2.08654	2.00495	1.98367	2.12829	1.64528	1.91627	1.63405	1.79794	0.883024237	0.966018836	12	8	24	0.574048623	0.847226836	24	6.137064523	0.042049338
ENSMUSG00000075595	Zfp652	zinc finger protein 652 [Source:MGI Symbol;Acc:MGI:2442221]	3.8947	4.85809	5.04545	4.65387	4.5952	4.64489	5.04901	5.1784	5.03805	5.59844	4.4167	4.7872	4.89969	5.01068	0.10560184	0.518002936	12	8	24	0.070721257	0.675973954	24	8.065346361	0.080385144
ENSMUSG00000075600	Zc3h3	zinc finger CCCH type containing 3 [Source:MGI Symbol;Acc:MGI:2663721]	0.581351	0.428274	0.812429	0.771136	0.522322	0.403397	0.453751	0.545068	0.618427	0.838047	0.590478	0.643388	0.402184	0.566821	0.016720774	0.253395174	16	12	24	0.009078916	0.502965379	24	11.09003245	0.134152428
ENSMUSG00000075602	Ly6a	lymphocyte antigen 6 complex, locus A [Source:MGI Symbol;Acc:MGI:107527]	29.8763	34.5035	36.0004	31.1069	32.9636	27.8719	26.4571	40.3981	42.2705	25.6489	29.7078	31.7281	27.1834	31.8526	0.070795672	0.440262583	12	16	24	0.1050781	0.717252017	24	7.245227384	0.16908586
ENSMUSG00000075605	Slurp2	secreted Ly6/Plaur domain containing 2 [Source:MGI Symbol;Acc:MGI:1916712]	1.25656	1.0081	0.909644	1.54614	1.24121	1.17639	0.763962	1.58342	1.56657	0.900003	1.35752	1.21134	1.26276	1.19178	0.832293447	0.966018836	8	16	24	0.835319964	0.949499418	24	11.49003759	0.04069399
ENSMUSG00000075609	Gm7935	predicted pseudogene 7935 [Source:MGI Symbol;Acc:MGI:3646370]	3.06037	3.18513	2.59367	2.82304	2.32308	2.73399	2.86786	3.06375	3.0654	4.34874	2.62309	3.05741	3.07718	3.41526	0.018114172	0.256345714	8	12	24	0.359189768	0.785405943	24	6.427703012	0.086936671
ENSMUSG00000075700	Selenot	selenoprotein T [Source:MGI Symbol;Acc:MGI:1916477]	133.114	124.537	122.595	117.484	123.99	122.059	121.798	111.01	104.123	107.549	126.973	98.9245	104.102	110.558	0.865490576	0.966018836	20	20	24	0.508560689	0.813517676	24	19.45103613	0.04209634

ENSMUSG00000075701	Selenos	selenoprotein S [Source:MGI Symbol;Acc:MGI:95994]	37.9258	41.0111	36.6316	34.4664	40.9894	36.2144	36.7841	38.2759	41.5138	35.0388	38.4	38.2083	41.8471	38.3452	0.767183862	0.966018836	8	8	24	0.493327612	0.805944647	24	1.821879681	0.039593963
ENSMUSG00000075702	Selenom	selenoprotein M [Source:MGI Symbol;Acc:MGI:2149786]	102.137	103.759	98.3486	84.6857	85.3993	83.2945	94.8673	107.375	114.575	94.9977	104.541	105.151	110.144	105.703	0.270034343	0.778934887	8	8	24	0.036700913	0.609176161	24	4.307039585	0.11877374
ENSMUSG00000075703	Selenoi	selenoprotein I [Source:MGI Symbol;Acc:MGI:107898]	24.4633	20.7177	20.1524	19.9974	20.4375	22.0345	20.4799	20.1808	18.4508	20.4823	21.8291	22.3088	23.2664	21.9263	0.004140921	0.140598674	4	8	24	0.013245683	0.529134128	24	21.71375573	0.101539254
ENSMUSG00000075704	Txnrd2	thioredoxin reductase 2 [Source:MGI Symbol;Acc:MGI:1347023]	8.33759	8.06406	9.79253	8.7979	7.16911	8.58815	7.33794	8.80847	8.47275	9.33507	9.169	9.79039	9.20828	7.81987	0.838532766	0.966018836	12	20	24	0.670877474	0.888971832	24	11.83534919	0.043845874
ENSMUSG00000075705	Msrb1	methionine sulfoxide reductase B1 [Source:MGI Symbol;Acc:MGI:1351642]	51.1769	54.9965	60.266	62.0028	62.2201	61.6782	53.4529	65.9413	57.0312	56.8583	61.7405	54.262	57.6498	53.397	0.133764886	0.568189321	20	8	24	0.222746001	0.768040178	24	13.00432846	0.069139987
ENSMUSG00000075706	Gpx4	glutathione peroxidase 4 [Source:MGI Symbol;Acc:MGI:104767]	44.7094	52.3357	42.5239	51.2991	55.7931	48.7124	49.8713	50.4499	62.8656	50.3085	66.5489	63.0349	74.1897	58.7181	0.869755137	0.966018836	20	16	24	0.748495667	0.918086059	24	18.53717801	0.046009868
ENSMUSG00000076431	Sox4	SRY (sex determining region Y)-box 4 [Source:MGI Symbol;Acc:MGI:98366]	4.84995	4.06158	4.7034	3.78798	3.88949	4.26418	4.4176	4.22538	3.59765	4.849	4.58785	4.23871	4.29879	4.19855	0.767566073	0.966018836	4	8	24	0.729059389	0.911453005	24	21.62309336	0.034395345
ENSMUSG00000076432	Ywhaq	tyrosine 3-monooxygenase/tryptophan 5-monooxygenase activation protein theta [Source:MGI Symbol;Acc:MGI:891963]	67.1369	65.332	64.2858	62.618	64.602	63.2075	63.049	62.4992	63.1271	60.3327	70.3705	61.8535	63.9816	64.4015	0.85447369	0.966018836	20	20	24	0.738098047	0.91451866	24	20.44267265	0.017145509
ENSMUSG00000076434	Wfdc3	WAP four-disulfide core domain 3 [Source:MGI Symbol;Acc:MGI:1923897]	0.908821	0.931468	0.8056	0.749116	1.20076	0.748439	0.530923	0.662094	0.327614	0.514376	1.65677	0.718885	0.512591	0.620646	0.628286596	0.966018836	20	16	24	0.138534966	0.747972781	24	17.12922682	0.18236738
ENSMUSG00000076435	Acsf2	acyl-CoA synthetase family member 2 [Source:MGI Symbol;Acc:MGI:2388287]	1.29008	1.26796	1.45656	1.70904	1.50358	1.48103	1.58541	1.47528	1.3501	1.66881	1.4974	1.35989	1.6328	1.81102	0.895733497	0.966018836	16	20	24	0.577881679	0.849052597	24	11.02005469	0.033717283
ENSMUSG00000076437	Selenoh	selenoprotein H [Source:MGI Symbol;Acc:MGI:1919907]	24.5035	26.0518	23.0882	23.5804	19.0907	22.4809	20.6054	26.0082	27.2189	24.0568	25.3954	24.8007	27.869	25.9767	0.408386288	0.946876978	8	12	24	0.109027346	0.72082227	24	5.188072042	0.105069148
ENSMUSG00000076439	Mog	myelin oligodendrocyte glycoprotein [Source:MGI Symbol;Acc:MGI:97435]	3.3043	12.1961	20.8205	7.73101	5.47138	9.63409	11.2178	7.11299	12.4403	6.08113	4.55959	8.69539	2.52924	10.0744	0.064422003	0.414867207	12	8	24	0.2301289	0.768040178	24	7.632550883	0.468041961
ENSMUSG00000076441	Ass1	argininosuccinate synthetase 1 [Source:MGI Symbol;Acc:MGI:88090]	1.38005	1.81844	1.59264	1.8814	1.33026	1.43107	1.70826	1.60746	1.89238	1.89874	1.54765	1.52476	1.64807	1.95454	0.073315272	0.447768907	16	4	24	0.007119839	0.47735824	24	7.6524421	0.116501592
ENSMUSG00000076498	Trbc2	T cell receptor beta, constant 2 [Source:MGI Symbol;Acc:MGI:4835227]	0.301436	0.650148	0.655146	0.860332	0.505171	0.510768	0.826154	0.269054	0.719056	0.597662	0.464646	0.530548	0.503345	0.340233	0.479398357	0.966018836	12	20	24	0.392951539	0.799546792	24	11.94547301	0.088154911
ENSMUSG00000076609	Igkc	immunoglobulin kappa constant [Source:MGI Symbol;Acc:MGI:96495]	9.00109	14.339	17.6623	13.2944	8.26959	10.2879	8.83317	12.2456	27.7949	5.87963	10.2803	13.1097	16.6443	9.30786	0.251986462	0.75129457	12	8	24	0.320557878	0.768040178	24	6.333776916	0.311474652
ENSMUSG00000076612	Ighg2c	immunoglobulin heavy constant gamma 2C [Source:MGI Symbol;Acc:MGI:2686979]	4.53407	3.41782	2.30056	3.12649	3.41484	3.64274	3.91806	4.42922	3.01516	2.71445	3.70434	3.93663	4.78502	3.08026	1.13E-04	0.029372629	4	8	24	0.003588333	0.425687943	24	22.4153933	0.250154867
ENSMUSG00000076617	Ighm	immunoglobulin heavy constant mu [Source:MGI Symbol;Acc:MGI:96448]	22.7378	30.8657	30.7464	30.618	26.9507	30.3259	29.3952	36.7796	39.8228	28.3807	24.8558	29.0105	34.2974	27.4218	0.097779482	0.501112642	12	8	24	0.163290208	0.767020009	24	6.611983442	0.149177221
ENSMUSG00000076928	Trac	T cell receptor alpha constant [Source:MGI Symbol;Acc:MGI:4439838]	0.308959	0.834249	0.914832	0.897807	0.500815	0.467557	0.251267	0.701999	0.949371	0.507512	0.778758	0.755443	0.658757	0.962706	0.004681041	0.140598674	12	16	24	0.035127234	0.608551791	24	8.79782042	0.195510416
ENSMUSG00000076934	Iglv1	immunoglobulin lambda variable 1 [Source:MGI Symbol;Acc:MGI:96530]	0.70612	3.11892	2.48771	1.78402	1.29185	1.37122	1.92471	0.934963	2.50895	1.6353	2.02919	1.71781	1.31012	2.18353	0.277087842	0.789849983	12	16	24	0.206225192	0.768040178	24	8.671574876	0.243962878
ENSMUSG00000076937	Iglc2	immunoglobulin lambda constant 2 [Source:MGI Symbol;Acc:MGI:99547]	1.10177	2.45894	1.77482	2.2735	1.48601	1.13984	1.97473	1.20782	3.09564	0.694605	1.11804	1.89154	1.93006	1.50354	0.850398351	0.966018836	12	8	24	0.470814596	0.805393068	24	5.695616529	0.168527741
ENSMUSG00000077167	Gm24119	predicted gene, 24119 [Source:MGI Symbol;Acc:MGI:5453896]	2.81408	4.21164	2.02444	1.02309	3.31424	4.78238	2.25616	2.03016	3.1429	1.55367	1.54869	2.80626	1.61484	4.56481	0.435739761	0.966018836	8	8	24	0.342414345	0.776561345	24	0.417674388	0.262311155
ENSMUSG00000077450	Rab11b	RAB11B, member RAS oncogene family [Source:MGI Symbol;Acc:MGI:99425]	25.1752	21.568	24.7341	24.8783	21.3507	28.2326	26.9242	27.4857	22.706	29.2962	20.6564	25.6223	23.3022	23.3756	0.951082476	0.966018836	24	20	24	0.920102752	0.978771393	24	23.57998796	0.025019673
ENSMUSG00000078117	Gm16485	predicted gene 16485 [Source:MGI Symbol;Acc:MGI:3642771]	0.869896	1.06526	1.14985	1.05249	0.99154	0.91199	0.873868	0.968813	0.971855	0.88901	0.809011	0.880009	0.790102	1.05566	0.015647695	0.253395174	12	16	24	0.023161047	0.581965446	24	8.082460151	0.069784799
ENSMUSG00000078122	F630028O10Rik	RIKEN cDNA F630028O10 gene [Source:MGI Symbol;Acc:MGI:3641813]	1.28976	2.49541	3.78959	2.85446	2.128	2.56029	1.91068	1.82819	2.20416	1.45552	1.88012	1.95336	1.69736	1.27158	0.337748746	0.85758369	12	16	24	0.376033593	0.796208299	24	11.5533217	0.141959394
ENSMUSG00000078126	Rpl23a-ps3	ribosomal protein L23A, pseudogene 3 [Source:MGI Symbol;Acc:MGI:3781353]	139.011	165.558	160.266	141.419	153.985	146.229	153.289	143.921	200.036	125.336	175.975	151.677	181.908	156.813	0.924770496	0.966018836	12	4	24	0.725190748	0.909811519	24	5.134201462	0.054310189
ENSMUSG00000078129	Actl10	actin-like 10 [Source:MGI Symbol;Acc:MGI:1917612]	1.5706	1.74876	1.41865	1.02324	1.15757	0.928619	1.01356	1.2766	1.54047	1.01784	1.43021	1.48865	1.55626	1.43064	0.327540787	0.846670301	8	8	24	0.231813432	0.768040178	24	3.642865141	0.106029209
ENSMUSG00000078134	Srsf3-ps	serine and arginine rich splicing factor 3, pseudogene [Source:MGI Symbol;Acc:MGI:3649769]	10.9551	12.4789	10.9026	12.6771	11.827	10.1503	10.7612	10.3219	13.2554	8.94855	11.0005	9.30201	10.4581	11.2323	0.236893212	0.7385769	12	12	24	0.371667838	0.793385596	24	7.970242052	0.07776817
ENSMUSG00000078139	AK157302	cDNA sequence AK157302 [Source:MGI Symbol;Acc:MGI:3574096]	9.13363	9.25056	9.91485	9.02198	8.44403	9.40794	9.64963	11.6797	10.038	11.7973	11.4118	12.2044	12.2091	13.3733	0.911685024	0.966018836	8	12	24	0.146719832	0.755260456	24	4.696213853	0.067954448
ENSMUSG00000078151	Gm12226	predicted pseudogene 12226 [Source:MGI Symbol;Acc:MGI:3649742]	2.84491	2.97553	3.03252	3.00353	3.336	3.51027	3.4015	3.19401	2.51305	2.46946	3.21889	2.67022	2.90027	2.71311	0.793403332	0.966018836	20	20	24	0.184705241	0.768040178	24	20.15520289	0.080499109
ENSMUSG00000078153	Psme2b	protease (prosome, macropain) activator subunit 2B [Source:MGI Symbol;Acc:MGI:1341073]	56.6716	60.9584	58.4029	54.2069	63.6409	54.3165	52.2574	55.9088	60.1456	47.9006	59.7886	55.11	61.288	56.8142	0.93717134	0.966018836	12	4	24	0.926730569	0.980741025	24	1.579500856	0.016412834
ENSMUSG00000078154	Gm12184	predicted gene 12184 [Source:MGI Symbol;Acc:MGI:3652174]	13.3359	11.7252	12.9585	11.5876	12.8424	15.29	14.2203	14.6697	11.8003	14.0766	11.8573	13.8456	13.4863	12.0742	0.217933169	0.704989686	24	12	24	0.189257213	0.768040178	24	21.60568681	0.08725324
ENSMUSG00000078161	Erich3	glutamate rich 3 [Source:MGI Symbol;Acc:MGI:1919095]	5.56578	5.39079	5.99049	5.69007	4.86275	5.76257	5.76852	5.51924	5.389	6.66926	5.05969	5.32905	5.07359	5.90439	0.265774856	0.773510818	16	4	24	0.375125585	0.795192031	24	8.101941488	0.05636475
ENSMUSG00000078180	Gm20900	predicted gene, 20900 [Source:MGI Symbol;Acc:MGI:5434256]	0.519163	0.591089	0.422543	1.02758	1.05299	0.943505	0.630392	0.460491	0.792923	0.0858364	0.706128	0.418495	0.435874	0.793263	0.911371589	0.966018836	20	8	24	0.7247646	0.909665611	24	17.31705655	0.080248001
ENSMUSG00000078184	Rbm8a2	RNA binding motif protein 8A2 [Source:MGI Symbol;Acc:MGI:3612447]	7.0561	6.14727	5.54602	5.66899	6.03134	6.63097	6.0607	5.54209	5.72496	5.61986	6.04862	6.14351	6.64602	5.77405	0.001920665	0.100255953	4	8	24	0.001269254	0.346832852	24	21.52155601	0.106758661
ENSMUSG00000078185	Chml	choroideremia-like [Source:MGI Symbol;Acc:MGI:101913]	8.26645	8.89836	8.24799	8.03677	8.47875	7.52918	8.12415	7.35008	8.37596	7.62259	8.50845	8.21566	8.26385	8.17711	0.886572916	0.966018836	20	20	24	0.996707083	0.999212239	24	1.828297092	0.002120419
ENSMUSG00000078193	Gm2000	predicted gene 2000 [Source:MGI Symbol;Acc:MGI:3780170]	291.598	346.895	321.903	308.888	300.519	269.871	296.649	284.021	404.423	245.801	367.938	307.093	367.396	348.48	0.93447422	0.966018836	12	12	24	0.504749204	0.811111334	24	5.984721682	0.089550081
ENSMUSG00000078201	Tmem203	transmembrane protein 203 [Source:MGI Symbol;Acc:MGI:2443597]	5.18139	5.07205	6.25916	5.73613	4.9906	5.28553	5.93172	7.10634	6.49192	6.73309	5.33772	5.44995	6.15336	5.04272	0.714044782	0.966018836	16	4	24	0.187216989	0.768040178	24	7.902548557	0.102307011
ENSMUSG00000078202	Nrarp	Notch-regulated ankyrin repeat protein [Source:MGI Symbol;Acc:MGI:1914372]	1.93868	2.3162	3.48704	2.9289	1.63166	2.68959	2.36384	4.95987	4.73237	6.52713	6.00162	5.39035	5.33199	5.42437	0.90767227	0.966018836	16	12	24	0.090476645	0.711365572	24	9.130813009	0.245948778
ENSMUSG00000078234	Klhdc7a	kelch domain containing 7A [Source:MGI Symbol;Acc:MGI:2444612]	0.672367	0.80616	1.21626	1.27348	0.874283	0.950345	1.17053	1.17315	1.06684	1.22262	1.53506	1.22906	1.63586	1.41153	0.847846021	0.966018836	16	12	24	0.476856814	0.805393068	24	10.42051424	0.05720261
ENSMUSG00000078235	Fam43b	family with sequence similarity 43, member B [Source:MGI Symbol;Acc:MGI:3651622]	7.34689	5.35994	4.99202	5.75627	5.58907	6.44215	6.46761	6.44991	5.03611	9.2169	6.82794	7.85455	7.61472	7.58048	0.143321297	0.585944911	24	12	24	0.382492901	0.799546792	24	20.49849302	0.106286806
ENSMUSG00000078238	Gm12854	predicted gene 12854 [Source:MGI Symbol;Acc:MGI:3650410]	64.7638	70.5904	76.5364	61.0183	81.7348	78.8454	59.6786	70.4866	61.3451	55.0074	71.5018	61.4653	59.8766	59.6472	0.658895034	0.966018836	20	20	24	0.548341215	0.834310879	24	17.76947616	0.068391876
ENSMUSG00000078247	Airn	antisense Igf2r RNA [Source:MGI Symbol;Acc:MGI:1353471]	6.00506	6.57466	6.02998	5.06273	5.57553	5.14568	5.8353	6.56481	6.74469	5.42382	6.07931	6.74128	5.73502	6.94503	0.048396595	0.379129806	8	8	24	0.062241618	0.661961741	24	4.221938966	0.10263259
ENSMUSG00000078249	Hmga1b	high mobility group AT-hook 1B [Source:MGI Symbol;Acc:MGI:96161]	4.25372	3.80463	4.12723	3.30693	3.09508	3.84556	4.20375	4.49975	3.63289	5.24331	3.60658	4.65002	4.30346	4.42389	0.371875382	0.894537464	8	12	24	0.339508678	0.773997385	24	2.898620967	0.084169538
ENSMUSG00000078302	Foxd1	forkhead box D1 [Source:MGI Symbol;Acc:MGI:1347463]	0.936066	0.867164	0.658852	0.522108	0.645148	0.595539	0.799472	0.907605	0.768482	0.314386	0.675245	0.767351	0.685099	0.637399	0.016720774	0.253395174	4	12	24	0.014078885	0.530670295	24	1.015268121	0.137550016
ENSMUSG00000078307	AI593442	expressed sequence AI593442 [Source:MGI Symbol;Acc:MGI:2143099]	18.341	17.4949	16.2058	16.8795	15.0695	16.2789	16.9713	15.2971	14.8672	18.7103	16.0277	15.2886	15.385	17.4317	0.838532766	0.966018836	8	12	24	0.894512017	0.970039184	24	4.885537026	0.01876484
ENSMUSG00000078317	F8a	factor 8-associated gene A [Source:MGI Symbol;Acc:MGI:95474]	3.83732	3.3733	3.64288	3.31504	2.81307	3.51431	3.78171	4.04168	3.4374	4.42088	3.91143	4.21687	4.05136	3.82811	0.935316621	0.966018836	4	16	24	0.621728881	0.871115984	24	1.893191479	0.042342263
ENSMUSG00000078348	Sf3b5	splicing factor 3b, subunit 5 [Source:MGI Symbol;Acc:MGI:1913375]	5.14148	4.85291	4.16828	4.44691	3.85159	4.26843	5.16953	5.52452	6.00478	4.78027	6.61781	5.48448	6.0088	5.67369	0.840483986	0.966018836	4	12	24	0.368710574	0.791761501	24	2.749809322	0.078445098
ENSMUSG00000078349	AW011738	expressed sequence AW011738 [Source:MGI Symbol;Acc:MGI:2140540]	2.47452	2.75792	2.56309	2.26192	2.26851	2.74076	2.63247	2.76358	2.85429	2.81008	2.93352	2.71207	2.39497	3.34088	0.714062999	0.966018836	8	20	24	0.308858118	0.768040178	24	5.515945167	0.05699211
ENSMUSG00000078350	Smim1	small integral membrane protein 1 [Source:MGI Symbol;Acc:MGI:1916109]	9.53598	9.60872	8.66528	11.5933	14.6192	11.4844	14.7374	9.61565	13.5177	9.92606	11.2528	11.822	9.56958	11.4063	0.800065124	0.966018836	24	4	24	0.42399836	0.805393068	24	17.13661433	0.114405552
ENSMUSG00000078427	Sarnp	SAP domain containing ribonucleoprotein [Source:MGI Symbol;Acc:MGI:1913368]	6.23522	6.36639	5.85519	6.86433	5.41634	6.10518	5.58139	5.18073	6.07572	4.94514	6.98772	4.93594	5.88721	5.59193	0.770162999	0.966018836	20	4	24	0.908591211	0.974606327	24	13.76305362	0.021952061
ENSMUSG00000078429	Ctdsp2	CTD (carboxy-terminal domain, RNA polymerase II, polypeptide A) small phosphatase 2 [Source:MGI Symbol;Acc:MGI:1098748]	19.2631	21.0571	21.7691	24.9693	23.8261	20.8231	22.6926	23.9814	26.0824	26.6628	21.5373	23.7901	22.2371	21.0839	0.097542159	0.501112642	16	12	24	0.032299229	0.606585237	24	11.33725271	0.114783433
ENSMUSG00000078435	AU041133	expressed sequence AU041133 [Source:MGI Symbol;Acc:MGI:2143755]	2.70743	3.62926	2.91208	2.77678	3.0295	2.44367	3.08168	3.1494	4.04819	2.46513	3.13184	3.47795	3.60056	3.30695	0.338172159	0.85758369	8	8	24	0.247090115	0.768040178	24	4.469252837	0.097013587
ENSMUSG00000078439	Smim24	small integral membrane protein 24 [Source:MGI Symbol;Acc:MGI:1919523]	1.04955	1.43447	1.49504	0.987945	1.09738	0.975748	1.05183	0.950707	1.4021	0.744353	1.34097	1.08466	0.909652	0.984317	0.978555823	0.978780908	12	16	24	0.680668313	0.893322401	24	7.865137202	0.051840817
ENSMUSG00000078440	Dohh	deoxyhypusine hydroxylase/monooxygenase [Source:MGI Symbol;Acc:MGI:1915964]	35.1477	26.4781	31.172	32.5028	28.5623	29.7679	24.5745	30.858	26.7546	29.8085	31.3301	29.6001	30.9302	27.8836	0.940625808	0.966018836	16	16	24	0.68571807	0.895588997	24	16.33984747	0.044106704
ENSMUSG00000078444	Gm10941	predicted gene 10941 [Source:MGI Symbol;Acc:MGI:3779151]	0.146255	0.371158	0.476838	0.584629	0.708375	0.247929	0.979977	0.718179	0.797581	0.724934	0.551254	0.514674	0.488327	0.714888	0.723119329	0.966018836	16	8	24	0.248379237	0.768040178	24	9.346737419	0.12579563
ENSMUSG00000078451	Ppil6	peptidylprolyl isomerase (cyclophilin)-like 6 [Source:MGI Symbol;Acc:MGI:1920325]	1.33901	1.6977	1.82379	1.10842	1.30341	1.31066	1.64007	1.26258	1.49954	1.28612	1.30878	1.65888	1.8269	2.01893	0.053432515	0.394331071	8	8	24	0.050385066	0.644469829	24	3.124226391	0.125954046
ENSMUSG00000078453	Abracl	ABRA C-terminal like [Source:MGI Symbol;Acc:MGI:1920362]	14.4855	15.1925	17.1256	17.7888	16.4665	16.2805	15.219	13.0002	16.8102	12.7624	16.161	12.8136	15.5872	14.2162	0.253121224	0.753386477	12	20	24	0.508239238	0.813214197	24	12.87109078	0.062309116
ENSMUSG00000078480	Mrpl48-ps	mitochondrial ribosomal protein L48 pseudogene [Source:MGI Symbol;Acc:MGI:3050089]	28.264	30.5569	26.6744	30.4853	31.7452	26.6408	29.7875	24.7494	33.0411	24.5376	30.9425	31.4137	29.3877	29.47	0.933543173	0.966018836	20	12	24	0.823124546	0.94441254	24	17.64915994	0.033021197
ENSMUSG00000078485	Plekhn1	pleckstrin homology domain containing, family N member 1 [Source:MGI Symbol;Acc:MGI:2387630]	2.22051	2.74766	3.01393	2.72247	2.6721	2.64037	2.81281	2.84505	3.15018	2.37971	2.73256	2.6137	2.73089	2.75021	0.020957534	0.262822894	12	12	24	0.192066328	0.768040178	24	7.568740867	0.064293425
ENSMUSG00000078486	Perm1	PPARGC1 and ESRR induced regulator, muscle 1 [Source:MGI Symbol;Acc:MGI:1921433]	1.68275	2.18808	2.66124	3.55668	2.92015	3.17116	2.51089	5.54056	3.0738	4.39593	2.35588	2.08063	3.02628	2.68948	0.47028201	0.966018836	16	12	24	0.247830379	0.768040178	24	9.630528783	0.21404967
ENSMUSG00000078490	Cfap74	cilia and flagella associated protein 74 [Source:MGI Symbol;Acc:MGI:1917130]	1.46684	1.97396	2.22262	1.9837	2.00975	2.14961	1.93237	1.5671	1.66986	1.84834	2.09678	2.07772	1.25441	2.37189	0.253121224	0.753386477	24	4	24	0.456867504	0.805393068	24	13.32485955	0.078688433
ENSMUSG00000078495	Zfp984	zinc finger protein 984 [Source:MGI Symbol;Acc:MGI:3651978]	2.8264	3.69746	3.37639	2.85316	3.32305	3.12777	3.60215	3.13417	3.75942	2.4246	3.24962	2.94663	2.75355	3.36931	0.332336445	0.853485917	12	4	24	0.477300989	0.805393068	24	4.606993081	0.064405774
ENSMUSG00000078496	Zfp982	zinc finger protein 982 [Source:MGI Symbol;Acc:MGI:3701121]	4.32508	3.88099	3.43059	4.38963	3.79718	3.69249	4.79139	3.32391	4.26277	3.82915	4.0183	4.2742	4.17163	4.36374	0.922035832	0.966018836	4	8	24	0.647673859	0.881026758	24	22.0769875	0.043888379
ENSMUSG00000078515	Ddi2	DNA-damage inducible protein 2 [Source:MGI Symbol;Acc:MGI:1917244]	10.1666	8.53992	8.82918	9.72302	9.32412	9.25817	9.91598	9.16687	7.78692	10.2699	9.22077	8.98548	8.59213	9.03696	0.129903614	0.560263689	16	20	24	0.360086037	0.785733735	24	18.11068829	0.053948052
ENSMUSG00000078517	Emc1	ER membrane protein complex subunit 1 [Source:MGI Symbol;Acc:MGI:2443696]	11.6108	9.90782	11.3615	11.0316	10.7487	12.0296	11.9314	11.4521	10.0977	13.2115	9.33659	10.9618	9.89143	12.4635	0.858891143	0.966018836	8	12	24	0.951450835	0.988339231	24	5.206420628	0.016976046
ENSMUSG00000078532	Nkain1	Na+/K+ transporting ATPase interacting 1 [Source:MGI Symbol;Acc:MGI:1914399]	4.58456	4.00417	7.66086	5.11542	4.77186	7.22316	5.37331	5.87562	4.51991	5.25573	5.2539	4.077	3.689	4.93769	0.922029406	0.966018836	16	12	24	0.697362782	0.899338675	24	11.80399327	0.082360479
ENSMUSG00000078546	Zfp995	zinc finger protein 995 [Source:MGI Symbol;Acc:MGI:1917331]	3.77373	4.44301	3.4551	3.88922	4.26777	4.08303	4.3815	4.03384	4.29264	2.91327	4.87633	4.29525	4.30797	3.3128	0.718720526	0.966018836	20	20	24	0.273069858	0.768040178	24	20.56192404	0.10024399
ENSMUSG00000078552	Dcdc2b	doublecortin domain containing 2b [Source:MGI Symbol;Acc:MGI:2686212]	2.84176	2.27946	2.25293	2.54168	3.36692	2.55525	1.34522	2.30953	2.38863	1.62722	2.59037	2.00128	2.02801	1.39486	0.371875382	0.894537464	20	12	24	0.168880357	0.767020009	24	16.18751952	0.152916847
ENSMUSG00000078566	Bnip3	BCL2/adenovirus E1B interacting protein 3 [Source:MGI Symbol;Acc:MGI:109326]	62.9117	62.9105	59.2026	65.9239	53.9344	56.849	56.6778	57.6645	61.8331	60.645	62.7829	56.3206	56.4801	61.0934	0.40198845	0.933910392	16	8	24	0.213123185	0.768040178	24	8.865496027	0.055249798
ENSMUSG00000078570	1110065P20Rik	RIKEN cDNA 1110065P20 gene [Source:MGI Symbol;Acc:MGI:1916170]	22.5187	22.4238	24.122	23.129	23.8785	23.4421	19.721	25.8276	26.8235	23.6989	27.3592	27.3239	30.827	25.1971	0.860560779	0.966018836	20	8	24	0.938962826	0.984940091	24	11.4362313	0.016709679
ENSMUSG00000078572	Ndufaf8	NADH:ubiquinone oxidoreductase complex assembly factor 8 [Source:MGI Symbol;Acc:MGI:1913676]	21.1902	23.4367	16.5798	16.4878	17.6457	14.7239	18.1265	26.5048	26.0013	17.6593	28.7207	21.6255	27.5717	23.0206	0.65208848	0.966018836	8	8	24	0.319837962	0.768040178	24	3.687495964	0.153810873
ENSMUSG00000078578	Ube2d3	ubiquitin-conjugating enzyme E2D 3 [Source:MGI Symbol;Acc:MGI:1913355]	116.093	108.266	106.202	108.878	110.662	108.493	103.747	108.384	96.1993	106.932	112.286	96.8724	96.133	105.535	0.580049522	0.966018836	20	8	24	0.624575112	0.871960321	24	16.4567582	0.026169729
ENSMUSG00000078580	E430018J23Rik	RIKEN cDNA E430018J23 gene [Source:MGI Symbol;Acc:MGI:2141981]	1.64572	1.68992	1.39364	1.57944	1.83202	1.7217	1.70592	1.8165	1.69254	1.86148	1.24995	1.8471	1.7488	1.61607	0.894563866	0.966018836	24	12	24	0.684629837	0.895095838	24	23.37087838	0.034259037
ENSMUSG00000078584	AU022252	expressed sequence AU022252 [Source:MGI Symbol;Acc:MGI:2140466]	8.96044	7.37545	7.11955	7.69727	7.70228	8.00996	7.14532	7.8947	6.53589	9.47061	7.71751	8.83134	7.81591	8.10173	0.148073804	0.590732968	24	12	24	0.423630107	0.805393068	24	18.895246	0.066206228
ENSMUSG00000078591	Hs3st4	heparan sulfate (glucosamine) 3-O-sulfotransferase 4 [Source:MGI Symbol;Acc:MGI:1333792]	1.49083	1.73122	1.63697	1.72093	1.24162	1.47246	1.48711	1.21126	1.83602	1.74447	1.46406	1.69244	1.62287	1.68215	0.121243373	0.548623108	16	4	24	0.349719854	0.779170188	24	8.338539068	0.06272941
ENSMUSG00000078607	1810010H24Rik	RIKEN cDNA 1810010H24 gene [Source:MGI Symbol;Acc:MGI:1916316]	1.27921	1.68083	1.42888	1.70333	1.14905	1.2557	2.01736	1.56326	1.64971	2.09932	1.40722	1.75497	1.4999	1.83178	0.676344318	0.966018836	16	4	24	0.291206048	0.768040178	24	6.919164517	0.090777463
ENSMUSG00000078612	Fyb2	FYN binding protein 2 [Source:MGI Symbol;Acc:MGI:2685466]	0.776941	0.735729	1.02314	0.739029	0.73902	0.887536	0.767038	0.734972	0.597081	0.602752	1.01668	0.7433	0.804377	0.634898	0.211043643	0.699706585	24	8	24	0.446483201	0.805393068	24	18.85694073	0.052518294
ENSMUSG00000078619	Smarcd2	SWI/SNF related, matrix associated, actin dependent regulator of chromatin, subfamily d, member 2 [Source:MGI Symbol;Acc:MGI:1933621]	12.8574	11.5502	11.997	13.8803	12.2206	13.3423	12.3174	12.5848	11.2225	12.3759	13.3019	12.3395	12.8267	12.5015	0.243586913	0.741580974	24	12	24	0.18937309	0.768040178	24	17.99788539	0.053420783
ENSMUSG00000078622	Ccdc47	coiled-coil domain containing 47 [Source:MGI Symbol;Acc:MGI:1914413]	66.636	66.6236	67.396	64.8643	62.1176	63.3145	58.8281	60.5796	67.985	64.3384	66.9531	66.5358	69.4277	66.6289	0.934818505	0.966018836	12	12	24	0.768451781	0.925370112	24	8.151681568	0.020020893
ENSMUSG00000078627	Marchf10	membrane associated ring-CH-type finger 10 [Source:MGI Symbol;Acc:MGI:2443469]	0.358079	0.673977	0.788644	0.712421	0.725551	0.567269	1.12014	0.9138	0.734857	0.44804	0.693859	0.393557	0.259662	0.475245	0.44861185	0.966018836	12	16	24	0.632599989	0.875112738	24	8.864441108	0.077506312
ENSMUSG00000078640	Gm11627	predicted gene 11627 [Source:MGI Symbol;Acc:MGI:3650659]	0.628451	0.920482	1.25998	0.752626	1.13058	1.1718	0.865421	0.591622	0.97916	0.633508	1.07704	0.823384	1.14875	1.00174	0.910436409	0.966018836	20	12	24	0.870613114	0.963313607	24	16.89739101	0.035899666
ENSMUSG00000078651	Aoc2	amine oxidase, copper containing 2 (retina-specific) [Source:MGI Symbol;Acc:MGI:2668431]	1.04794	0.889123	0.977629	1.13217	1.1148	1.1306	1.15909	0.912052	0.945272	0.900787	1.02257	1.01356	0.949681	0.800101	0.050721923	0.386400877	24	8	24	0.006334343	0.470033096	24	18.14440071	0.077002654
ENSMUSG00000078652	Psme3	proteaseome (prosome, macropain) activator subunit 3 (PA28 gamma, Ki) [Source:MGI Symbol;Acc:MGI:1096366]	31.9835	30.9156	32.9801	29.1174	31.0286	30.4184	27.4958	32.271	25.852	35.3198	27.0451	29.4908	32.4079	31.9721	0.910436409	0.966018836	16	4	24	0.831902544	0.947968019	24	5.062455121	0.02972361
ENSMUSG00000078656	Vps25	vacuolar protein sorting 25 [Source:MGI Symbol;Acc:MGI:106354]	64.9966	60.8767	57.341	61.7474	57.6898	60.7823	59.6353	59.2286	54.9592	66.4231	59.8468	62.2497	60.3918	60.4119	0.390686537	0.915479136	24	12	24	0.587834333	0.853689229	24	19.7079563	0.029368448
ENSMUSG00000078670	Fam174b	family with sequence similarity 174, member B [Source:MGI Symbol;Acc:MGI:3698178]	5.90525	4.47757	4.69821	4.45834	4.59022	5.27601	5.26096	4.87066	4.04188	4.9365	4.92758	5.131	4.88977	5.78096	0.055920623	0.401074772	4	8	24	0.063991727	0.663876076	24	22.62502181	0.104583728
ENSMUSG00000078671	Chd2	chromodomain helicase DNA binding protein 2 [Source:MGI Symbol;Acc:MGI:2448567]	9.28139	10.588	9.04677	8.77689	9.21094	9.04957	9.06354	9.77314	9.09469	9.38121	9.17812	10.0107	9.66653	9.0461	0.917867465	0.966018836	8	4	24	0.379433532	0.798762204	24	1.169013999	0.035327359
ENSMUSG00000078676	Casc3	cancer susceptibility candidate 3 [Source:MGI Symbol;Acc:MGI:2179723]	13.7158	13.3699	12.775	13.3649	12.8084	13.6128	13.9409	13.637	13.3359	15.348	12.4784	13.6219	13.3611	14.263	0.291478323	0.798044066	8	12	24	0.731802537	0.912550805	24	4.692486963	0.021160031
ENSMUSG00000078681	Tm2d3	TM2 domain containing 3 [Source:MGI Symbol;Acc:MGI:1915884]	28.8854	29.8326	30.0722	29.4856	29.5094	29.6964	29.9454	26.7157	31.3281	22.4304	30.8004	26.1631	27.1168	28.9718	0.920620634	0.966018836	12	12	24	0.935014396	0.983765461	24	1.35207122	0.016704195
ENSMUSG00000078684	5830417I10Rik	RIKEN cDNA 5830417I10 gene [Source:MGI Symbol;Acc:MGI:1923272]	2.4375	1.52876	1.50458	1.91133	2.21725	2.10768	2.51658	1.7718	1.54094	1.24636	2.47018	1.55496	1.57854	1.36175	0.087130946	0.470523339	20	16	24	0.033059511	0.606585237	24	19.63009173	0.195420325
ENSMUSG00000078695	Cisd3	CDGSH iron sulfur domain 3 [Source:MGI Symbol;Acc:MGI:101788]	54.3933	47.0802	46.4168	48.0596	51.7575	56.9309	48.7502	46.6104	49.6169	45.8641	54.8093	47.2807	50.8727	46.7316	0.058087297	0.40386543	20	20	24	0.028769437	0.594683	24	19.29978718	0.095731351
ENSMUSG00000078698	Mrgpra3	MAS-related GPR, member A3 [Source:MGI Symbol;Acc:MGI:2684085]	13.4939	11.8358	9.95149	10.8408	11.3099	12.747	11.3844	10.8764	11.9094	8.79779	13.3774	11.9551	14.85	12.4623	0.029682156	0.323426482	4	12	24	0.037966429	0.61140397	24	22.47192446	0.158803068
ENSMUSG00000078700	D030028A08Rik	RIKEN cDNA D030028A08 gene [Source:MGI Symbol;Acc:MGI:2441931]	1.31513	0.961713	0.965553	0.639933	1.04517	1.11915	1.0281	1.0932	1.14648	1.19929	1.01126	0.894608	0.85492	0.837452	0.73379652	0.966018836	20	12	24	0.833531037	0.948715732	24	22.30332697	0.030493184
ENSMUSG00000078706	Gm53	predicted gene 53 [Source:MGI Symbol;Acc:MGI:2684899]	3.826	3.27252	5.98327	2.60248	2.41668	3.89304	3.75993	3.11668	3.02423	3.93835	2.50382	3.35418	2.29908	3.15024	0.911685024	0.966018836	24	20	24	0.650123892	0.882337763	24	5.546332227	0.099190247
ENSMUSG00000078713	Tomm5	translocase of outer mitochondrial membrane 5 [Source:MGI Symbol;Acc:MGI:1915762]	58.4318	60.6909	58.0405	56.0443	65.7889	54.4566	57.004	56.2089	71.3955	46.9348	67.2059	60.0082	62.3848	62.1189	0.919119843	0.966018836	20	20	24	0.980400847	0.995255072	24	2.697422158	0.011502282
ENSMUSG00000078716	Tmem8b	transmembrane protein 8B [Source:MGI Symbol;Acc:MGI:2441680]	8.52042	8.1206	7.41239	8.84667	8.1923	8.8707	7.36524	8.57255	7.88529	11.2747	9.14909	9.29822	8.70349	9.41295	0.44861185	0.966018836	16	20	24	0.341692137	0.77606964	24	14.42479936	0.069121677
ENSMUSG00000078762	Haus5	HAUS augmin-like complex, subunit 5 [Source:MGI Symbol;Acc:MGI:1919159]	1.88515	2.8941	2.55225	2.46995	2.00818	1.66284	2.07155	2.73764	2.58915	2.00183	2.12666	2.86383	1.94329	1.86628	0.83638114	0.966018836	8	20	24	0.337399146	0.772378988	24	7.646784342	0.105979709
ENSMUSG00000078763	Slfn1	schlafen 1 [Source:MGI Symbol;Acc:MGI:1313259]	0.628894	0.995666	1.59683	0.644122	0.980999	0.943363	1.13851	1.00415	0.92448	0.584581	0.851903	0.562322	1.16053	0.656209	0.481811994	0.966018836	12	4	24	0.603749867	0.861540092	24	4.417703559	0.081316667
ENSMUSG00000078765	U2af1l4	U2 small nuclear RNA auxiliary factor 1-like 4 [Source:MGI Symbol;Acc:MGI:2678374]	25.3643	24.1685	25.1363	24.0237	25.5803	27.1314	23.4433	23.173	23.7965	26.6604	22.5732	26.1719	26.2501	23.1338	0.176754388	0.653256853	24	8	24	0.310702916	0.768040178	24	18.57884465	0.050322512
ENSMUSG00000078768	Zfp566	zinc finger protein 566 [Source:MGI Symbol;Acc:MGI:1919806]	3.14496	2.8625	3.65945	2.59821	2.51476	2.67455	1.87925	2.62773	3.24473	2.92396	2.37179	2.46873	2.75258	2.51867	0.069194004	0.433403132	12	8	24	0.166650642	0.767020009	24	8.23386848	0.11618425
ENSMUSG00000078771	Evi2a	ecotropic viral integration site 2a [Source:MGI Symbol;Acc:MGI:95458]	2.76132	6.29429	8.71864	4.77962	4.38102	4.6537	6.3119	4.74064	6.25158	2.97822	3.50431	4.73387	2.73941	6.14679	0.20866337	0.698797644	12	8	24	0.205603084	0.768040178	24	6.75606362	0.282438276
ENSMUSG00000078773	Rad54b	RAD54 homolog B (S. cerevisiae) [Source:MGI Symbol;Acc:MGI:3605986]	0.651217	1.18658	0.898004	0.495809	0.758642	0.834493	1.49273	0.417364	1.0001	0.573247	0.689565	0.778962	0.871072	0.591173	0.47028201	0.966018836	4	12	24	0.466974386	0.805393068	24	0.118130596	0.104177054
ENSMUSG00000078779	Zfp59	zinc finger protein 59 [Source:MGI Symbol;Acc:MGI:99206]	2.34457	2.26458	2.13112	1.89763	2.5139	2.07953	2.37931	2.01077	2.40046	1.84357	2.30544	2.32276	2.34164	2.35796	0.305564675	0.811201458	4	12	24	0.281870593	0.768040178	24	22.86003674	0.057146593
ENSMUSG00000078784	Rbis	ribosomal biogenesis factor [Source:MGI Symbol;Acc:MGI:1916376]	15.9267	21.2455	20.4785	14.4629	17.9886	16.2892	16.5351	14.9759	18.8357	12.8549	21.2399	15.8542	17.8526	16.6939	0.96317972	0.967965568	12	4	24	0.91447176	0.976044534	24	3.890889495	0.03411675
ENSMUSG00000078786	BC024978	cDNA sequence BC024978 [Source:MGI Symbol;Acc:MGI:3041247]	3.2743	3.68635	3.42822	3.30365	3.67222	3.79175	3.63876	3.2625	3.66094	4.72257	3.15773	3.74548	3.10824	3.2446	0.714062999	0.966018836	24	4	24	0.441352642	0.805393068	24	13.47112991	0.06119098
ENSMUSG00000078789	Dph1	diphthamide biosynthesis 1 [Source:MGI Symbol;Acc:MGI:2151233]	6.76123	6.6828	7.19033	7.09393	7.28832	7.38286	7.60172	8.2839	7.17215	6.65925	7.8666	7.16636	6.74981	7.14532	0.91241026	0.966018836	20	20	24	0.962642259	0.99069291	24	18.51095	0.009176502
ENSMUSG00000078794	Dact3	dishevelled-binding antagonist of beta-catenin 3 [Source:MGI Symbol;Acc:MGI:3654828]	4.66349	4.9127	5.62476	4.9783	5.11718	5.12183	5.34237	4.98645	5.22677	5.88193	5.1771	5.45574	4.9633	5.83017	0.836635326	0.966018836	16	16	24	0.214283094	0.768040178	24	10.16710421	0.048284822
ENSMUSG00000078812	Eif5a	eukaryotic translation initiation factor 5A [Source:MGI Symbol;Acc:MGI:106248]	172.803	150.283	160.969	160.539	162.823	174.972	155.931	165.252	147.881	163.797	165.909	161.333	167.754	154.825	0.116174595	0.538362324	24	12	24	0.066080878	0.668186543	24	19.29237163	0.06047863
ENSMUSG00000078813	Leng1	leukocyte receptor cluster (LRC) member 1 [Source:MGI Symbol;Acc:MGI:1917007]	5.28593	4.00655	4.01439	4.31859	4.6062	4.55934	5.33478	4.4191	3.96993	4.83344	4.6541	4.38853	4.49137	3.97146	0.052138666	0.391073722	4	8	24	0.065081322	0.663876076	24	20.00709669	0.100201495

ENSMUSG00000078815	Cacng6	calcium channel, voltage-dependent, gamma subunit 6 [Source:MGI Symbol;Acc:MGI:1859168]	0.677667	1.43367	1.63974	2.39103	1.44568	1.68216	1.60126	4.35123	2.97416	3.74989	1.40177	1.88751	2.63936	1.69927	0.723119329	0.966018836	16	4	24	0.127691608	0.740162193	24	8.856799113	0.313483249
ENSMUSG00000078816	Prkcg	protein kinase C, gamma [Source:MGI Symbol;Acc:MGI:97597]	0.293731	0.768166	1.06088	0.843007	0.61289	0.60368	0.670406	0.636306	1.17158	0.722992	0.507936	0.728457	0.549353	0.938614	0.008389425	0.192760771	12	16	24	0.010031645	0.503967859	24	8.595844653	0.201083961
ENSMUSG00000078851	H2aw	H2A.W histone [Source:MGI Symbol;Acc:MGI:2448458]	15.1205	11.4172	11.187	10.377	12.7444	12.7366	13.8339	12.5594	10.444	11.1877	14.3394	11.7067	12.3528	11.7575	0.01118458	0.204559658	20	20	24	0.017875686	0.555944581	24	21.30823048	0.151834623
ENSMUSG00000078853	Igtp	interferon gamma induced GTPase [Source:MGI Symbol;Acc:MGI:107729]	14.525	13.7706	13.7381	13.2407	16.8497	14.9631	12.99	19.3643	12.7959	11.5772	16.1748	12.9144	12.8548	16.06	0.892555977	0.966018836	20	20	24	0.881043341	0.966661503	24	23.72941313	0.038150367
ENSMUSG00000078861	Zfp931	zinc finger protein 931 [Source:MGI Symbol;Acc:MGI:2441662]	16.653	17.7835	14.4001	14.4716	16.7114	14.6415	17.3474	13.081	15.6694	9.605	16.6895	11.9467	13.7652	13.9353	0.865514257	0.966018836	20	20	24	0.485751356	0.805393068	24	23.24913966	0.094069789
ENSMUSG00000078862	Gm14326	predicted gene 14326 [Source:MGI Symbol;Acc:MGI:3709298]	15.7461	19.1849	16.3875	21.5011	16.9551	15.9346	18.8621	15.9586	21.555	14.8392	20.2879	18.2274	18.8152	19.0608	0.867888967	0.966018836	12	12	24	0.777948607	0.928772941	24	9.13270155	0.047492751
ENSMUSG00000078864	Gm14322	predicted gene 14322 [Source:MGI Symbol;Acc:MGI:3652185]	1.00259	1.32958	1.65943	1.25118	1.30644	1.28806	1.38328	1.36699	1.90621	0.984369	1.01987	1.3054	1.10276	1.13767	0.265774856	0.773510818	12	4	24	0.312426741	0.768040178	24	7.345481099	0.092997182
ENSMUSG00000078866	Zfp970	zinc finger protein 970 [Source:MGI Symbol;Acc:MGI:3652255]	15.6483	18.0312	15.3179	17.0839	17.1617	15.6064	14.4663	13.8242	18.7475	13.1051	17.4115	16.4266	15.0919	16.1564	0.772122688	0.966018836	20	8	24	0.815847053	0.941291601	24	13.22811646	0.034756603
ENSMUSG00000078867	Gm14418	predicted gene 14418 [Source:MGI Symbol;Acc:MGI:3702408]	1.44006	1.78042	2.27252	1.87812	1.69775	1.30375	1.96181	1.7703	2.1535	1.71956	1.9598	1.45531	1.60497	1.96931	8.24E-04	0.071344007	12	12	24	0.008267675	0.49701405	24	8.45848325	0.154926979
ENSMUSG00000078868	Gm14412	predicted gene 14412 [Source:MGI Symbol;Acc:MGI:3652251]	1.92899	2.21881	2.19011	1.96333	1.89066	1.94384	1.3661	1.58098	2.40963	1.40336	1.77373	2.4171	1.95102	2.28293	0.885025617	0.966018836	12	8	24	0.831992856	0.948003732	24	5.962549402	0.042869792
ENSMUSG00000078870	Gm14410	predicted gene 14410 [Source:MGI Symbol;Acc:MGI:3709158]	4.23686	3.71453	3.36412	4.18902	4.3941	3.45769	3.78903	3.40181	4.68665	2.9694	4.3174	4.10532	4.5122	3.78634	0.846840613	0.966018836	20	20	24	0.718675639	0.907511396	24	19.59062701	0.051159773
ENSMUSG00000078877	Gm14295	predicted gene 14295 [Source:MGI Symbol;Acc:MGI:3709624]	23.3797	19.1106	23.747	17.0125	25.0646	20.3897	31.8639	19.3582	32.1931	19.0487	28.6243	22.4656	33.5071	21.9273	0.305564675	0.811201458	4	12	24	0.662981067	0.886333762	24	22.98520576	0.104668458
ENSMUSG00000078878	Gm14305	predicted gene 14305 [Source:MGI Symbol;Acc:MGI:3709632]	12.0599	14.6511	13.6049	12.8437	14.0856	11.9821	13.4584	11.8	14.5575	10.1239	14.0887	13.6447	12.788	14.4863	0.93717134	0.966018836	8	20	24	0.866038589	0.961251055	24	4.209167658	0.030336738
ENSMUSG00000078902	Gm14443	predicted gene 14443 [Source:MGI Symbol;Acc:MGI:3652324]	0.55658	1.08099	0.772498	0.898687	1.00379	0.795608	0.653108	0.761153	1.10433	0.554027	0.788067	0.815169	0.825978	0.842343	0.868638024	0.966018836	12	16	24	0.660614737	0.885496082	24	9.16894484	0.050474375
ENSMUSG00000078905	Gm14393	predicted gene 14393 [Source:MGI Symbol;Acc:MGI:3649576]	0.671276	0.736521	0.63266	1.08867	0.640973	0.672195	0.727568	0.698132	0.678212	0.461367	0.590123	0.914554	0.44662	0.669527	0.910436409	0.966018836	24	20	24	0.947584173	0.987232058	24	15.66198616	0.017037465
ENSMUSG00000078908	Mon1b	MON1 homolog B, secretory trafficking associated [Source:MGI Symbol;Acc:MGI:1923231]	7.48822	5.63328	5.78094	5.7027	5.6597	6.43712	6.64451	5.93123	5.09759	7.44789	5.74828	6.37039	6.21168	6.5252	0.16456443	0.628002127	4	8	24	0.354849181	0.78177053	24	21.88466238	0.078016656
ENSMUSG00000078915	Hsp25-ps1	heat shock protein 25, pseudogene 1 [Source:MGI Symbol;Acc:MGI:96241]	76.9187	54.3986	50.3199	59.6072	68.8761	73.8091	58.6794	71.9927	38.3562	56.0369	72.3845	56.0385	55.8306	48.6225	0.028632001	0.316608413	24	12	24	0.026166806	0.586541342	24	19.51767354	0.258579174
ENSMUSG00000078919	Dpm1	dolichol-phosphate (beta-D) mannosyltransferase 1 [Source:MGI Symbol;Acc:MGI:1330239]	17.4959	16.6044	22.7294	20.3992	21.9693	18.5356	15.9122	17.0883	23.89	16.3934	19.1354	16.6947	20.2587	21.3007	0.277087842	0.789849983	12	16	24	0.236012647	0.768040178	24	10.1279124	0.122930053
ENSMUSG00000078920	Ifi47	interferon gamma inducible protein 47 [Source:MGI Symbol;Acc:MGI:99448]	4.48619	4.92613	4.45744	3.37235	5.18614	4.25883	5.29567	7.53959	5.02543	2.6156	4.7549	4.00401	4.02363	5.08165	0.08333408	0.462922111	8	8	24	0.183227104	0.768040178	24	2.383375805	0.194199105
ENSMUSG00000078921	Tgtp2	T cell specific GTPase 2 [Source:MGI Symbol;Acc:MGI:3710083]	3.00259	2.40743	3.29045	2.60363	2.05694	1.87215	2.24375	2.31448	2.55748	1.18839	1.23469	0.954793	1.35734	2.75476	0.048889741	0.379129806	12	12	24	0.016061988	0.544099157	24	5.643940493	0.28236883
ENSMUSG00000078923	Ube2v1	ubiquitin-conjugating enzyme E2 variant 1 [Source:MGI Symbol;Acc:MGI:1913839]	4.41532	4.9886	4.8835	5.72513	5.76997	5.09254	4.86318	4.66028	5.00729	7.32934	5.47961	5.62811	5.2961	5.90836	0.06180207	0.409814092	16	12	24	0.019314674	0.563108074	24	13.12036212	0.130702783
ENSMUSG00000078931	Pdf	peptide deformylase (mitochondrial) [Source:MGI Symbol;Acc:MGI:1915273]	7.61205	6.40714	7.2185	10.1172	7.59635	7.31572	7.42106	8.05796	6.75001	7.23369	6.80077	7.70105	7.84061	7.96522	0.915475528	0.966018836	8	4	24	0.899447821	0.971453495	24	14.0680716	0.025428478
ENSMUSG00000078937	Cpt1b	carnitine palmitoyltransferase 1b, muscle [Source:MGI Symbol;Acc:MGI:1098297]	2.75089	3.43852	6.33738	12.5565	5.55724	4.4566	5.00014	11.1771	7.08624	12.3577	7.57295	5.27104	10.0891	8.24105	0.096178527	0.501112642	16	8	24	0.027604012	0.590220615	24	10.31787599	0.474339056
ENSMUSG00000078941	Ak6	adenylate kinase 6 [Source:MGI Symbol;Acc:MGI:5510732]	38.385	39.7486	36.0999	37.8541	38.4681	40.4367	39.611	30.3089	39.7624	29.7548	36.0318	31.9872	41.5651	37.3432	0.708665292	0.966018836	4	12	24	0.521285076	0.82025681	24	23.22764858	0.066536548
ENSMUSG00000078958	Atp6ap1l	ATPase, H+ transporting, lysosomal accessory protein 1-like [Source:MGI Symbol;Acc:MGI:3648665]	3.45667	4.94941	4.21188	5.03724	6.16483	3.6307	3.97042	3.72801	3.9293	3.36253	5.43638	4.08744	4.53995	5.00228	0.581070934	0.966018836	20	8	24	0.576823484	0.848735805	24	14.21310662	0.088082154
ENSMUSG00000078963	Hsbp1l1	heat shock factor binding protein 1-like 1 [Source:MGI Symbol;Acc:MGI:1913505]	26.3236	26.1081	24.1416	22.917	25.1678	24.1449	23.7925	23.3582	24.5102	23.3651	27.6462	24.8133	25.4494	26.519	0.758699911	0.966018836	20	20	24	0.577225639	0.848840315	24	22.36934218	0.032415996
ENSMUSG00000078970	Wdr92	WD repeat domain 92 [Source:MGI Symbol;Acc:MGI:2144224]	6.16044	5.52507	6.51651	5.1392	5.65563	5.49421	5.71238	5.34062	5.16405	6.5568	5.303	6.23782	5.77809	5.77809	0.834434272	0.966018836	12	4	24	0.869448011	0.962941471	24	3.938840535	0.022371406
ENSMUSG00000078974	Sec61g	SEC61, gamma subunit [Source:MGI Symbol;Acc:MGI:1202066]	2.92592	3.22551	2.71804	3.3219	3.27193	2.85351	2.50014	2.92565	3.11157	2.29434	3.49629	2.6132	3.10142	2.72707	0.91695334	0.966018836	20	4	24	0.802011645	0.936565832	24	14.87624774	0.03306038
ENSMUSG00000078994	Zfp429	zinc finger protein 429 [Source:MGI Symbol;Acc:MGI:1920057]	1.63207	1.70388	1.63949	1.44718	1.76328	1.58002	1.84121	1.44745	2.10522	1.09171	1.63218	1.43519	1.61204	1.50373	0.89563591	0.966018836	12	4	24	0.676710456	0.891857599	24	1.63419353	0.044280265
ENSMUSG00000079003	Samd1	sterile alpha motif domain containing 1 [Source:MGI Symbol;Acc:MGI:2142433]	5.18077	4.32469	4.83864	5.17036	5.37416	4.91509	5.24123	4.41098	4.46457	4.82737	5.88881	4.70071	4.60053	4.08636	3.52E-04	0.049770315	20	12	24	0.004044435	0.427431763	24	16.83480163	0.123641682
ENSMUSG00000079014	Serpina3i	serine (or cysteine) peptidase inhibitor, clade A, member 3I [Source:MGI Symbol;Acc:MGI:2182841]	2.39049	2.17109	2.5335	1.32423	2.34115	2.12001	1.28319	2.32319	1.78654	1.57482	2.30523	1.66635	1.32976	1.52276	0.915082018	0.966018836	20	8	24	0.955424817	0.988639288	24	15.70336894	0.024371796
ENSMUSG00000079017	Ifi27l2a	interferon, alpha-inducible protein 27 like 2A [Source:MGI Symbol;Acc:MGI:1924183]	39.1842	37.0089	37.8746	46.5576	44.9463	42.5966	27.4375	46.1237	40.2045	32.2838	58.2681	44.9559	47.8948	50.73	0.886572916	0.966018836	20	16	24	0.72043132	0.908336488	24	15.60653584	0.080218345
ENSMUSG00000079018	Ly6c1	lymphocyte antigen 6 complex, locus C1 [Source:MGI Symbol;Acc:MGI:96882]	27.9625	30.3755	33.7197	41.8443	40.2787	31.7155	27.2242	35.8317	34.6673	31.2516	41.4749	31.3397	28.3156	26.7175	0.007831348	0.192760771	20	8	24	0.005139734	0.4424469	24	13.42181591	0.221457803
ENSMUSG00000079019	Insl3	insulin-like 3 [Source:MGI Symbol;Acc:MGI:108427]	0.954458	1.27632	0.614386	0.00989813	0.645002	0.635619	0.63366	0.610156	1.00457	0.48558	1.07907	0.051184	0.769456	0.415054	0.922029406	0.966018836	4	12	24	0.571167815	0.845721742	24	2.68115791	0.136090539
ENSMUSG00000079020	Slc45a4	solute carrier family 45, member 4 [Source:MGI Symbol;Acc:MGI:2146236]	13.1369	9.80671	11.2475	11.026	9.73777	11.7769	9.93107	12.3456	8.86188	15.3565	10.3403	11.0206	10.5201	12.2657	0.890235289	0.966018836	8	8	24	0.988795592	0.997581486	24	10.10050326	0.031846272
ENSMUSG00000079022	Col22a1	collagen, type XXII, alpha 1 [Source:MGI Symbol;Acc:MGI:1916950]	0.370267	0.492631	0.858555	0.546435	0.412227	0.689835	0.660248	1.1343	0.856835	0.528424	0.692538	0.680706	0.695513	0.391271	0.829428469	0.966018836	12	16	24	0.66154247	0.885525866	24	6.725635508	0.06717329
ENSMUSG00000079036	Alkbh1	alkB homolog 1, histone H2A dioxygenase [Source:MGI Symbol;Acc:MGI:2384034]	4.58812	5.27236	5.11031	5.39124	5.20686	4.43682	4.45002	5.22351	5.43976	4.98419	5.13053	5.04402	4.73295	5.2029	0.036657636	0.332441566	12	16	24	0.008729503	0.502965379	24	9.697573429	0.086419618
ENSMUSG00000079037	Prnp	prion protein [Source:MGI Symbol;Acc:MGI:97769]	285.431	245.978	251.582	240.399	256.465	269.538	273.425	257.969	218.922	267.762	239.543	261.04	253.882	245.79	0.094000699	0.490854203	4	8	24	0.039491601	0.615919442	24	21.46393638	0.086394301
ENSMUSG00000079038	D130040H23Rik	RIKEN cDNA D130040H23 gene [Source:MGI Symbol;Acc:MGI:2444324]	1.37701	2.07615	2.54114	2.13694	2.54913	1.95946	1.81595	1.81597	2.3627	1.81896	2.34381	1.69492	1.72441	1.80895	0.007831348	0.192760771	20	8	24	0.032920245	0.606585237	24	11.7954199	0.152302758
ENSMUSG00000079043	Fastkd5	FAST kinase domains 5 [Source:MGI Symbol;Acc:MGI:2139469]	4.09467	3.46909	3.84773	3.44548	3.61515	3.85476	4.24166	4.1518	3.06001	3.24299	3.60611	3.77332	3.72153	3.92979	0.008244783	0.192760771	4	12	24	0.017941899	0.557105788	24	23.49228817	0.103266069
ENSMUSG00000079051	Vinac1	vinculin/alpha-catenin family member 1 [Source:MGI Symbol;Acc:MGI:3649276]	1.23159	1.23463	1.17178	1.55695	1.19917	1.05379	1.29276	1.05561	1.18914	1.20391	1.10631	1.40816	1.17085	1.29634	0.823246279	0.966018836	16	4	24	0.874026169	0.964832663	24	12.56397159	0.017720174
ENSMUSG00000079055	Slc8a3	solute carrier family 8 (sodium/calcium exchanger), member 3 [Source:MGI Symbol;Acc:MGI:107976]	4.16899	4.12843	5.26911	4.08619	3.44313	5.06997	4.5436	4.02103	3.86194	6.07757	3.38835	4.02671	3.74309	4.62587	0.911135828	0.966018836	16	4	24	0.671437808	0.889179947	24	7.919597415	0.071646495
ENSMUSG00000079056	Kcnip3	Kv channel interacting protein 3, calsenilin [Source:MGI Symbol;Acc:MGI:1929258]	73.5514	55.1326	54.9843	63.0197	57.1988	66.8664	58.2695	62.9084	47.0983	77.0738	57.479	66.6739	63.7551	62.2532	0.046505526	0.374351104	24	12	24	0.406681209	0.799546792	24	19.88741682	0.098206215
ENSMUSG00000079057	Cyp4v3	cytochrome P450, family 4, subfamily v, polypeptide 3 [Source:MGI Symbol;Acc:MGI:2142763]	6.09833	5.85167	5.89557	5.76561	5.68646	5.42414	5.66598	6.01386	6.34562	5.44479	5.28214	6.2902	6.34418	5.82151	0.277917941	0.789849983	4	16	24	0.158489471	0.764909967	24	3.24292104	0.051310343
ENSMUSG00000079067	Hmgn2-ps1	high mobility group nucleosomal binding domain 2, pseudogene 1 [Source:MGI Symbol;Acc:MGI:3783162]	0.344662	0.518098	1.11769	0.379551	0.49316	0.935446	0.415344	0.900952	1.3901	0.456925	0.229469	0.681877	0.471295	0.550521	0.140377635	0.585715181	12	4	24	0.326602226	0.768040178	24	6.897183659	0.165370398
ENSMUSG00000079076	Gm3086	predicted gene 3086 [Source:MGI Symbol;Acc:MGI:3781262]	1.52499	1.72393	1.43341	0.497588	1.56894	0.443051	1.62954	2.16477	1.24661	1.44538	0.597585	1.04291	0.972145	0.932558	0.515451644	0.966018836	8	16	24	0.176934404	0.768040178	24	4.306780267	0.233661396
ENSMUSG00000079083	Jrkl	Jrk-like [Source:MGI Symbol;Acc:MGI:1924782]	6.84098	6.86761	6.53135	6.24001	6.93828	5.92056	5.40791	5.46569	6.16422	5.22641	7.00664	5.48014	5.77552	5.04743	0.702630284	0.966018836	20	12	24	0.385484722	0.799546792	24	15.80151003	0.058297669
ENSMUSG00000079084	Ccdc82	coiled-coil domain containing 82 [Source:MGI Symbol;Acc:MGI:1913646]	12.2475	14.0209	12.8187	14.2663	13.4166	12.5868	11.772	11.2097	13.7208	11.3428	13.46	11.5871	11.828	12.7626	0.323424237	0.839647936	12	16	24	0.2955848	0.768040178	24	11.57332555	0.060478214
ENSMUSG00000079104	Prps1l3	phosphoribosyl pyrophosphate synthetase 1-like 3 [Source:MGI Symbol;Acc:MGI:3779453]	12.7053	11.609	12.7795	11.7821	12.2848	12.2928	13.3871	11.9737	11.8416	10.8226	13.3462	12.5319	12.5086	12.1767	0.537381546	0.966018836	4	4	24	0.303612806	0.768040178	24	20.53602284	0.046116873
ENSMUSG00000079108	Srp54c	signal recognition particle 54C [Source:MGI Symbol;Acc:MGI:3714359]	2.33355	3.39657	2.23893	3.49651	2.28262	3.09627	2.38188	2.16417	2.98081	2.41461	2.72801	3.24373	3.85666	2.85812	0.880631923	0.966018836	24	12	24	0.908762578	0.974606327	24	21.67188936	0.055455883
ENSMUSG00000079109	Pms2	PMS1 homolog2, mismatch repair system component [Source:MGI Symbol;Acc:MGI:104288]	3.73647	4.62442	5.41054	4.08536	5.68391	4.98822	4.11272	4.31574	4.86611	4.80622	5.17731	4.96446	4.37212	4.79971	0.016398461	0.253395174	20	8	24	0.158773139	0.765350752	24	13.53196732	0.096431155
ENSMUSG00000079110	Capn3	calpain 3 [Source:MGI Symbol;Acc:MGI:107437]	1.07973	2.43187	3.28653	3.2864	2.18178	2.12581	2.49958	6.24078	4.10974	4.83948	2.16384	2.47502	2.7898	2.43635	0.088482991	0.473415302	16	4	24	0.029716022	0.596648309	24	8.710954848	0.413013527
ENSMUSG00000079111	Kdelr2	KDEL (Lys-Asp-Glu-Leu) endoplasmic reticulum protein retention receptor 2 [Source:MGI Symbol;Acc:MGI:1914163]	23.6166	19.6667	19.7959	20.6881	20.9794	21.0671	22.7262	21.7438	20.0664	20.8601	19.5949	19.236	20.2246	20.4465	0.767183862	0.966018836	4	8	24	0.28803646	0.768040178	24	23.56535124	0.044465011
ENSMUSG00000079139	Gm4204	predicted gene 4204 [Source:MGI Symbol;Acc:MGI:3782381]	154.58	146.933	141.514	143.122	141.963	141.262	139.101	134.506	138.485	144.087	145.572	150.675	156.088	153.914	0.763955526	0.966018836	4	8	24	0.385620004	0.799546792	24	22.71221006	0.034133981
ENSMUSG00000079140	Gm6374	predicted gene 6374 [Source:MGI Symbol;Acc:MGI:3647016]	1.84719	1.48787	1.31557	1.31163	1.85429	1.65859	1.08701	1.19559	0.912483	1.09436	1.35495	1.50588	1.48725	1.19424	0.122696414	0.550756498	24	12	24	0.011959431	0.525280095	24	19.89543002	0.16387613
ENSMUSG00000079144	A130010J15Rik	RIKEN cDNA A130010J15 gene [Source:MGI Symbol;Acc:MGI:2441776]	3.5708	3.72118	3.41053	4.19765	3.84462	3.63653	3.93304	3.36371	3.58944	3.77111	3.3419	3.95799	4.31003	3.71242	0.725061903	0.966018836	4	8	24	0.711013785	0.904572112	24	19.8950646	0.029883084
ENSMUSG00000079157	Fam155a	family with sequence similarity 155, member A [Source:MGI Symbol;Acc:MGI:2142765]	4.66093	4.91824	5.20788	4.20556	3.55892	4.55654	4.68874	4.19866	4.36282	5.87128	4.53906	5.72211	4.62116	6.82779	0.868638024	0.966018836	8	12	24	0.451783859	0.805393068	24	5.025367106	0.087991233
ENSMUSG00000079164	Tlr5	toll-like receptor 5 [Source:MGI Symbol;Acc:MGI:1858171]	1.42494	1.04214	1.38225	1.21631	1.42195	1.45373	1.13332	1.37749	1.09644	1.69315	1.37467	1.22754	1.58521	1.43059	0.896753532	0.966018836	24	12	24	0.691597905	0.897724829	24	16.46637175	0.037601312
ENSMUSG00000079165	Sap25	sin3 associated polypeptide [Source:MGI Symbol;Acc:MGI:3802945]	3.60803	2.82149	3.30676	4.37442	6.64482	4.51808	7.58607	3.79846	3.19272	2.96145	3.21559	3.09247	4.38493	2.52305	0.398261528	0.929472831	4	4	24	0.228082719	0.768040178	24	19.93050638	0.247119296
ENSMUSG00000079179	Rab10os	RAB10, member RAS oncogene family, opposite strand [Source:MGI Symbol;Acc:MGI:1921423]	8.61732	9.37418	10.4207	8.72538	7.54575	9.05955	9.21878	8.24181	9.78448	8.93729	9.83001	9.29338	8.75854	8.40878	0.911685024	0.966018836	12	20	24	0.717129026	0.906810602	24	8.571290605	0.036177827
ENSMUSG00000079184	Mphosph8	M-phase phosphoprotein 8 [Source:MGI Symbol;Acc:MGI:1922589]	31.768	31.6037	31.2946	31.7947	33.2479	31.5294	28.7207	28.9564	33.0944	32.0507	38.7388	35.4922	37.1918	34.9824	0.696393581	0.966018836	12	20	24	0.345085394	0.778298718	24	16.53043677	0.055015759
ENSMUSG00000079197	Psme2	proteasome (prosome, macropain) activator subunit 2 (PA28 beta) [Source:MGI Symbol;Acc:MGI:1096365]	8.4086	7.49334	7.06424	6.92104	8.78295	7.40172	7.32222	7.61628	9.39172	5.32232	9.93228	6.34043	6.74335	6.32711	0.91241026	0.966018836	20	20	24	0.844590118	0.95285845	24	17.82173897	0.049309545
ENSMUSG00000079215	Zfp664	zinc finger protein 664 [Source:MGI Symbol;Acc:MGI:2442505]	56.7873	49.4244	49.8991	56.1333	51.5699	50.3465	51.1662	47.8931	46.496	52.0574	49.6192	50.1521	50.0812	48.145	0.064405315	0.414867207	16	20	24	0.218293712	0.768040178	24	17.85455396	0.045662959
ENSMUSG00000079225	Gm9531	predicted gene 9531 [Source:MGI Symbol;Acc:MGI:3779940]	81.0766	72.2921	75.3278	75.0464	78.3454	77.7315	78.2232	69.4388	66.7538	76.5335	72.9125	75.0903	75.1266	76.3288	0.251986462	0.75129457	4	8	24	0.087706712	0.707747429	24	19.90322505	0.059329706
ENSMUSG00000079227	Ccr5	chemokine (C-C motif) receptor 5 [Source:MGI Symbol;Acc:MGI:107182]	1.57628	1.77548	1.04884	1.3691	1.79484	1.09989	1.12414	1.17496	1.20538	0.545579	1.7582	0.359547	0.658626	1.00312	0.904831057	0.966018836	20	4	24	0.96012287	0.989397471	24	12.31381005	0.029530235
ENSMUSG00000079235	Ccdc13	coiled-coil domain containing 13 [Source:MGI Symbol;Acc:MGI:1920144]	6.43723	8.08385	6.12198	6.56011	9.76715	8.04316	7.86543	8.70288	7.86362	6.56053	8.68138	8.27411	7.11368	6.89512	0.187699199	0.662239322	20	20	24	0.36980068	0.792235171	24	18.91684387	0.099622962
ENSMUSG00000079242	C730034F03Rik	RIKEN cDNA C730034F03 gene [Source:MGI Symbol;Acc:MGI:2441921]	1.58993	1.41544	1.30048	1.51508	1.2543	1.36403	1.13439	1.13347	1.5895	1.55745	1.33119	1.59694	1.51908	1.40215	0.767183862	0.966018836	12	16	24	0.224825295	0.768040178	24	10.91644235	0.062009073
ENSMUSG00000079243	Xirp1	xin actin-binding repeat containing 1 [Source:MGI Symbol;Acc:MGI:1333878]	0.280556	0.451802	0.32614	0.491104	0.516927	0.622133	0.588084	1.245	0.477838	0.760242	0.432604	0.395229	0.776885	0.452449	0.81544087	0.966018836	8	4	24	0.815950904	0.941295249	24	4.593240658	0.050926813
ENSMUSG00000079260	Tmppe	transmembrane protein with metallophosphoesterase domain [Source:MGI Symbol;Acc:MGI:5317335]	2.38724	1.82148	2.15152	1.77208	1.9296	2.08533	1.84497	2.22755	1.74273	2.48364	1.91695	2.26459	2.04716	2.15126	0.896753532	0.966018836	24	12	24	0.850593551	0.95526784	24	22.59537821	0.025267818
ENSMUSG00000079277	Hoxd3	homeobox D3 [Source:MGI Symbol;Acc:MGI:96207]	1.83596	1.66766	2.06283	1.89314	1.85755	1.7889	1.653	1.72377	1.93513	1.89048	2.05679	2.39741	1.4031	2.11016	0.170659409	0.643496587	12	16	24	0.335822433	0.771482605	24	13.28243155	0.067259212
ENSMUSG00000079278	Tmem233	transmembrane protein 233 [Source:MGI Symbol;Acc:MGI:3651514]	192.593	145.643	146.771	156.911	154.784	176.566	147.724	166.787	131.185	193.424	160.173	175.748	174.338	169.373	0.041292272	0.346472636	24	12	24	0.305908537	0.768040178	24	20.1554438	0.097193009
ENSMUSG00000079283	2310009B15Rik	RIKEN cDNA 2310009B15 gene [Source:MGI Symbol;Acc:MGI:1916799]	5.63178	5.85991	5.99816	4.72275	5.17464	4.51384	5.28484	6.77057	7.37367	8.27312	8.45819	8.4268	8.69967	6.32358	0.795556128	0.966018836	12	4	24	0.820431744	0.943168034	24	8.916342826	0.05333749
ENSMUSG00000079293	Clec7a	C-type lectin domain family 7, member a [Source:MGI Symbol;Acc:MGI:1861431]	1.5706	2.7718	3.1292	2.12583	2.66972	2.13226	2.05552	2.05362	3.3319	1.36062	2.705	2.54977	2.56703	2.35813	0.643325306	0.966018836	12	16	24	0.748176453	0.918016105	24	8.279524824	0.077598096
ENSMUSG00000079297	Gm2223	predicted pseudogene 2223 [Source:MGI Symbol;Acc:MGI:3780393]	38.7321	37.7337	39.5555	37.8988	36.7261	36.3784	35.7102	35.1645	37.7724	38.1007	37.1686	37.9309	38.2032	39.0351	0.86340968	0.966018836	12	12	24	0.545976784	0.833198211	24	8.475389476	0.021296549
ENSMUSG00000079311	Gm3222	predicted pseudogene 3222 [Source:MGI Symbol;Acc:MGI:3781401]	0.912932	0.732588	0.888295	0.992176	0.971205	1.03664	0.703516	1.03266	0.590714	0.571405	0.264346	0.275701	0.352025	0.573989	0.801273005	0.966018836	8	12	24	0.775032525	0.927711986	24	6.67491245	0.049229993
ENSMUSG00000079316	Rab9	RAB9, member RAS oncogene family [Source:MGI Symbol;Acc:MGI:1890695]	31.1009	29.7748	29.4351	27.9735	28.354	28.5346	30.782	26.2448	29.5925	26.3091	32.34	30.0531	30.1266	30.0604	0.009678659	0.194720062	4	12	24	0.337063352	0.771960538	24	22.16135464	0.048624786
ENSMUSG00000079317	Trappc2	trafficking protein particle complex 2 [Source:MGI Symbol;Acc:MGI:1913476]	55.229	55.7846	50.804	54.2952	49.1487	50.791	52.5334	47.7723	50.3614	44.5986	55.4318	45.4739	50.2027	48.6339	0.801273005	0.966018836	4	12	24	0.789851242	0.932694713	24	23.42965177	0.023264476
ENSMUSG00000079330	Lemd1	LEM domain containing 1 [Source:MGI Symbol;Acc:MGI:1922403]	1.22309	0.465478	0.622417	0.497453	0.699365	0.700101	0.771051	0.568615	0.870927	0.74231	0.636243	0.707598	0.50326	0.52795	0.648082424	0.966018836	4	4	24	0.597857275	0.858787489	24	20.25986136	0.063876342
ENSMUSG00000079334	Naa80	N(alpha)-acetyltransferase 80, NatH catalytic subunit [Source:MGI Symbol;Acc:MGI:1888902]	5.68331	5.55551	5.38172	6.18853	6.03072	6.57482	5.8569	6.86823	6.83028	7.11996	5.88155	6.84573	5.78902	6.03378	0.823677655	0.966018836	16	12	24	0.53608683	0.827369107	24	13.21747967	0.044026845
ENSMUSG00000079339	Ifit1bl1	interferon induced protein with tetratricpeptide repeats 1B like 1 [Source:MGI Symbol;Acc:MGI:3650685]	2.04514	2.32353	2.39088	2.51927	2.80432	4.22034	2.2414	2.31385	2.18253	1.81044	3.47668	2.46913	2.29299	2.9084	0.253121224	0.753386477	24	4	24	0.229904651	0.768040178	24	18.22937374	0.154863052
ENSMUSG00000079363	Gbp4	guanylate binding protein 4 [Source:MGI Symbol;Acc:MGI:97072]	1.66875	2.20815	1.6492	1.39606	1.80122	1.36422	1.64589	2.78773	1.88098	1.62235	1.80502	1.4189	1.631	1.84359	0.004681041	0.140598674	8	16	24	0.103719746	0.715474111	24	5.255480538	0.142017984
ENSMUSG00000079414	Gm11110	predicted gene 11110 [Source:MGI Symbol;Acc:MGI:3779362]	0.524927	0.480831	0.668561	0.372095	0.681338	0.728127	0.397187	0.435543	0.385615	0.533681	0.400556	0.561873	0.625859	0.484604	0.588208558	0.966018836	24	16	24	0.420893656	0.80434587	24	19.5548617	0.059910927
ENSMUSG00000079415	Cntf	ciliary neurotrophic factor [Source:MGI Symbol;Acc:MGI:88439]	173.981	182.191	147.909	214.957	232.115	174.217	195.645	148.631	204.617	150.51	228.725	205.164	187.292	146.255	0.004021228	0.140598674	20	12	24	0.062535513	0.661961741	24	17.06672321	0.201995475
ENSMUSG00000079418	Atg4a	autophagy related 4A, cysteine peptidase [Source:MGI Symbol;Acc:MGI:2147903]	6.10532	6.97242	6.6658	6.28396	6.85961	5.65506	5.75469	5.78147	7.31263	5.98752	7.03344	6.12096	7.3826	6.37508	0.95269569	0.966018836	12	12	24	0.596230153	0.857839743	24	9.425099802	0.047115267
ENSMUSG00000079419	Ms4a6c	membrane-spanning 4-domains, subfamily A, member 6C [Source:MGI Symbol;Acc:MGI:2385644]	2.34272	3.13785	3.76143	2.99365	3.61918	2.48051	3.19157	3.33748	3.53837	1.66379	2.49794	2.88481	2.64612	1.88782	0.784278077	0.966018836	12	12	24	0.919410221	0.97844729	24	6.948645853	0.039375516
ENSMUSG00000079426	Arpc4	actin related protein 2/3 complex, subunit 4 [Source:MGI Symbol;Acc:MGI:1915339]	72.5621	62.2316	64.1086	64.8284	67.1875	69.4975	63.3998	66.5669	59.1164	76.1998	63.818	66.8122	67.6824	68.2321	0.791413662	0.966018836	24	12	24	0.578903594	0.849624393	24	18.98336112	0.039331293
ENSMUSG00000079427	Mthfsl	5, 10-methenyltetrahydrofolate synthetase-like [Source:MGI Symbol;Acc:MGI:3780550]	9.16668	10.3246	10.3974	8.63964	10.2856	10.1248	9.67193	8.57424	9.1381	8.31842	11.2963	10.2113	10.2898	8.935	0.503972924	0.966018836	20	20	24	0.239034098	0.768040178	24	19.45862425	0.080628937
ENSMUSG00000079428	Tceal7	transcription elongation factor A (SII)-like 7 [Source:MGI Symbol;Acc:MGI:1915746]	0.541046	0.799864	0.843519	0.846199	0.441764	0.558384	0.720109	1.05694	1.72573	1.26735	0.57493	0.627268	0.589736	0.587758	0.012443448	0.220221977	16	4	24	0.014152245	0.530991098	24	8.467809644	0.260990681
ENSMUSG00000079429	Mroh2a	maestro heat-like repeat family member 2A [Source:MGI Symbol;Acc:MGI:3705228]	0.554987	0.453437	0.534506	0.517621	0.687495	0.447779	0.468598	0.539018	0.554569	0.463124	0.574797	0.564128	0.457916	0.465628	0.702630284	0.966018836	20	12	24	0.235320148	0.768040178	24	15.18341955	0.039783722
ENSMUSG00000079434	Neu2	neuraminidase 2 [Source:MGI Symbol;Acc:MGI:1344417]	0.270999	0.568254	0.706338	1.03791	0.762079	0.546048	0.584645	0.969215	0.924874	1.13533	0.476185	0.448021	0.732205	0.501987	0.03782572	0.334069441	16	8	24	0.019778949	0.565108098	24	10.19976472	0.211584446
ENSMUSG00000079435	Rpl36a	ribosomal protein L36A [Source:MGI Symbol;Acc:MGI:1201789]	5.96748	7.19982	5.86984	6.41668	8.57851	6.3162	5.64363	5.80593	7.47019	5.39103	6.87701	7.44607	6.28882	6.67036	0.798829513	0.966018836	20	8	24	0.569099092	0.844691814	24	16.15963826	0.068504047
ENSMUSG00000079436	Kcnj13	potassium inwardly-rectifying channel, subfamily J, member 13 [Source:MGI Symbol;Acc:MGI:3781032]	2.14921	3.13839	2.17338	1.64299	2.64823	1.94987	1.78266	1.87114	3.15232	1.20211	2.02623	2.13527	1.57242	1.88967	0.956178643	0.96629412	12	4	24	0.838691449	0.9507575	24	4.585301305	0.056671536
ENSMUSG00000079442	St6galnac4	ST6 (alpha-N-acetyl-neuraminyl-2,3-beta-galactosyl-1,3)-N-acetylgalactosaminide alpha-2,6-sialyltransferase 4 [Source:MGI Symbol;Acc:MGI:1341894]	3.62146	3.3295	3.47261	3.00915	3.61631	3.95131	4.4456	3.73879	3.10148	3.96328	4.00417	3.30056	4.0933	3.1513	0.277087842	0.789849983	4	8	24	0.198049799	0.768040178	24	20.78189703	0.093615951
ENSMUSG00000079444	Gm21981	predicted gene 21981 [Source:MGI Symbol;Acc:MGI:5439450]	2.04673	1.49923	1.23135	1.20946	1.77074	1.27599	1.33512	2.17921	1.44361	1.68925	1.18251	2.12872	1.58588	1.68603	0.665910519	0.966018836	8	4	24	0.425396608	0.805393068	24	0.373113816	0.09483743
ENSMUSG00000079450	Cldn34c1	claudin 34C1 [Source:MGI Symbol;Acc:MGI:1920311]	1.73289	1.96292	1.4056	1.54014	1.98613	2.05053	1.92187	1.36614	1.59298	1.78508	2.12768	2.05196	1.37868	2.20908	0.288043872	0.793763484	24	12	24	0.254394331	0.768040178	24	18.81514048	0.099723302
ENSMUSG00000079465	Col4a3	collagen, type IV, alpha 3 [Source:MGI Symbol;Acc:MGI:104688]	0.477309	0.561605	0.512936	0.533773	0.588018	0.399171	0.432281	0.597387	0.690754	0.494277	0.510845	0.528408	0.459206	0.517147	0.081336319	0.462794877	12	16	24	0.081048522	0.697074851	24	8.939679735	0.0589263
ENSMUSG00000079466	Prdm12	PR domain containing 12 [Source:MGI Symbol;Acc:MGI:2685844]	33.8951	23.4346	20.9801	28.8082	25.3659	26.9007	27.3179	29.4134	21.1173	43.3996	28.2193	32.7055	32.4409	30.4754	0.44861185	0.966018836	4	8	24	0.59191733	0.856249693	24	19.18526498	0.106225621
ENSMUSG00000079467	Gm14966	predicted gene 14966 [Source:MGI Symbol;Acc:MGI:3705223]	0.574642	1.08283	0.960958	1.12331	1.25524	0.833548	0.685425	0.951824	1.01161	0.373592	0.56189	0.736276	0.823048	0.990304	0.330576288	0.850598217	8	20	24	0.633331978	0.875353057	24	6.963575844	0.076522374
ENSMUSG00000079469	Pigb	phosphatidylinositol glycan anchor biosynthesis, class B [Source:MGI Symbol;Acc:MGI:1891825]	14.3172	15.2004	13.0888	14.558	14.3203	14.6073	15.3912	13.7372	11.7382	13.7023	13.4688	12.6597	13.9081	13.9431	0.643325306	0.966018836	4	8	24	0.268447379	0.768040178	24	22.13093719	0.056528028
ENSMUSG00000079470	Utp14b	UTP14B small subunit processome component [Source:MGI Symbol;Acc:MGI:2445092]	2.20696	2.38975	2.64398	2.17783	2.50299	2.32182	2.42567	2.17231	2.47834	2.40004	2.187	2.49462	2.32527	2.43612	0.920858634	0.966018836	12	16	24	0.787847022	0.931832121	24	8.452581564	0.017915907
ENSMUSG00000079477	Rab7	RAB7, member RAS oncogene family [Source:MGI Symbol;Acc:MGI:105068]	130.362	118.419	118.401	120.425	121.533	133.909	122.963	124.375	103.219	128.689	122.092	118.917	118.136	117.393	0.331157065	0.850959667	24	12	24	0.113881024	0.727446192	24	19.71279496	0.068288238
ENSMUSG00000079478	Znrd2	zinc ribbon domain containing 2 [Source:MGI Symbol;Acc:MGI:1913482]	16.2465	14.7369	14.9544	11.177	10.2493	11.3348	12.1637	16.1243	15.711	18.1081	15.5937	17.429	17.477	18.3695	0.729086532	0.966018836	8	12	24	0.136673362	0.747972781	24	4.849372824	0.165674358
ENSMUSG00000079481	Nhsl2	NHS-like 2 [Source:MGI Symbol;Acc:MGI:3645090]	0.970646	1.47903	1.63696	1.36906	1.28299	1.14416	1.21802	1.30612	1.48154	1.20083	1.05016	1.23957	1.15584	1.15102	0.030691182	0.324150946	12	16	24	0.033291896	0.606585237	24	8.442188493	0.103967589
ENSMUSG00000079484	Phyhd1	phytanoyl-CoA dioxygenase domain containing 1 [Source:MGI Symbol;Acc:MGI:3612860]	7.38844	6.54032	8.62174	8.84378	7.61441	8.11307	7.24614	8.39405	9.26251	6.64861	6.87307	7.27814	7.36485	7.15407	0.953226423	0.966018836	12	20	24	0.460645561	0.805393068	24	9.253564623	0.068773888
ENSMUSG00000079487	Med12	mediator complex subunit 12 [Source:MGI Symbol;Acc:MGI:1926212]	9.90458	10.8127	10.9541	11.2277	12.1488	10.1916	10.8342	12.5375	14.0014	12.7241	10.8784	10.9648	10.6895	10.8443	0.008047769	0.192760771	16	12	24	0.029965149	0.596794384	24	9.895656768	0.117588641
ENSMUSG00000079491	H2-T10	histocompatibility 2, T region locus 10 [Source:MGI Symbol;Acc:MGI:95942]	0.572572	0.824616	0.603323	0.453017	0.462908	0.601026	0.61384	0.659821	0.679406	0.680491	0.5038	0.514304	0.480792	0.640249	0.048889741	0.379129806	8	12	24	0.04335555	0.626520598	24	5.258085135	0.083268884
ENSMUSG00000079499	6530402F18Rik	RIKEN cDNA 6530402F18 gene [Source:MGI Symbol;Acc:MGI:1923470]	1.74172	1.4334	1.2828	1.43251	1.52145	1.57802	1.78697	1.91442	1.66118	2.2338	2.07776	1.71761	1.89114	1.81625	0.890311217	0.966018836	4	8	24	0.872965829	0.964303715	24	19.56786664	0.023120431
ENSMUSG00000079507	H2-Q1	histocompatibility 2, Q region locus 1 [Source:MGI Symbol;Acc:MGI:95928]	0.763627	1.5031	0.627125	0.763742	0.598667	0.614394	0.917457	0.607183	1.24155	0.309722	0.633463	0.822632	0.487179	1.00817	0.496745696	0.966018836	8	8	24	0.228307494	0.768040178	24	3.981765795	0.154502063

ENSMUSG00000079508	Apoo	apolipoprotein O [Source:MGI Symbol;Acc:MGI:1915566]	1.73478	2.37665	2.85582	2.75848	2.30021	2.45212	2.33368	2.1252	2.43168	2.2923	2.62455	2.1811	2.2624	1.92138	0.128989168	0.560209134	12	20	24	0.070060506	0.67501971	24	12.37259119	0.108452925
ENSMUSG00000079509	Zfx	zinc finger protein X-linked [Source:MGI Symbol;Acc:MGI:99211]	5.17066	5.61731	6.11377	5.67224	6.662	5.81036	5.65104	4.79393	6.53866	5.10152	6.22538	5.40673	5.29594	5.46892	0.10031279	0.511533473	20	12	24	0.212480074	0.768040178	24	13.81663142	0.076347188
ENSMUSG00000079511	Gm42688	predicted gene 42688 [Source:MGI Symbol;Acc:MGI:5662825]	1.34896	1.79864	1.6474	1.4021	1.65864	1.41771	1.56308	1.5568	1.85543	1.65099	1.59561	1.69722	1.67213	1.71545	0.869721097	0.966018836	12	4	24	0.20668145	0.768040178	24	7.446122965	0.052268086
ENSMUSG00000079523	Tmsb10	thymosin, beta 10 [Source:MGI Symbol;Acc:MGI:109146]	141.653	117.498	120.501	126.33	132.226	133.667	118.319	114.773	129.136	116.634	142.808	134.653	127.65	125.008	0.028612069	0.316608413	24	8	24	0.048468826	0.638992719	24	18.42772679	0.092299385
ENSMUSG00000079547	H2-DMb1	histocompatibility 2, class II, locus Mb1 [Source:MGI Symbol;Acc:MGI:95922]	14.3815	12.9398	12.2736	12.2436	20.1488	14.3965	17.7988	17.1183	19.2721	16.6347	16.3198	12.6816	16.7451	13.3725	0.874672901	0.966018836	20	20	24	0.904013703	0.97351114	24	16.37054198	0.04093699
ENSMUSG00000079550	Mpp4	membrane protein, palmitoylated 4 (MAGUK p55 subfamily member 4) [Source:MGI Symbol;Acc:MGI:2386681]	0.726103	0.650191	0.752745	0.602913	0.691707	0.630434	0.454583	0.586733	0.485488	0.420031	0.679194	0.554167	0.575146	0.536031	0.847312766	0.966018836	20	20	24	0.685467454	0.895481396	24	19.25188933	0.027558859
ENSMUSG00000079553	Kifc1	kinesin family member C1 [Source:MGI Symbol;Acc:MGI:109596]	0.804789	0.70905	1.13649	1.1794	0.889319	0.813909	0.652915	1.1638	0.728779	0.58504	0.719037	0.890188	0.856966	0.495112	0.873350015	0.966018836	12	20	24	0.849265528	0.954760051	24	12.91968703	0.034396632
ENSMUSG00000079555	Haus3	HAUS augmin-like complex, subunit 3 [Source:MGI Symbol;Acc:MGI:2387633]	9.61921	9.93678	9.21493	9.07944	10.8904	8.56676	9.77018	8.64046	10.6784	7.06616	9.55365	8.72587	9.29799	9.56437	0.836635326	0.966018836	12	12	24	0.81830345	0.942408493	24	1.390462871	0.033850311
ENSMUSG00000079557	Marchf2	membrane associated ring-CH-type finger 2 [Source:MGI Symbol;Acc:MGI:1925915]	84.7643	81.9814	76.4758	81.0271	80.3393	84.8336	79.6048	83.2652	79.5084	80.0871	83.4239	82.3112	84.6578	79.8739	0.010049466	0.194720062	24	12	24	0.039512483	0.615919442	24	21.2112307	0.040245331
ENSMUSG00000079559	Colca2	COLCA2 homolog [Source:MGI Symbol;Acc:MGI:2685530]	0.495783	0.569935	0.827531	0.759249	0.627891	0.563689	0.403449	1.03521	0.791468	0.545872	0.656128	0.657634	0.797872	0.492228	0.865233232	0.966018836	12	16	24	0.365252069	0.789568647	24	8.840793804	0.082561058
ENSMUSG00000079560	Hoxa3	homeobox A3 [Source:MGI Symbol;Acc:MGI:96175]	1.51203	1.61642	1.70195	1.67898	1.22242	1.22465	1.83624	1.41355	2.01723	1.78875	1.88168	1.90851	2.16402	1.55009	0.880247609	0.966018836	4	4	24	0.71144453	0.904655191	24	7.076220812	0.046761312
ENSMUSG00000079562	Maea	macrophage erythroblast attacher [Source:MGI Symbol;Acc:MGI:1891748]	51.3746	41.1055	43.3866	42.0254	40.6686	49.4355	45.3645	48.2197	37.2329	49.4436	41.0566	43.463	45.418	44.7656	0.210566453	0.69976851	4	16	24	0.281397795	0.768040178	24	22.82091158	0.07979225
ENSMUSG00000079575	Rbpj-ps3	recombination signal binding protein for immunoglobulin kappa J region, pseudogene 3 [Source:MGI Symbol;Acc:MGI:96525]	5.77168	5.54033	5.30032	4.96301	6.15217	5.24937	5.49557	4.81697	5.05076	5.53401	4.83419	5.22992	5.11641	5.30286	0.84680398	0.966018836	4	12	24	0.596440657	0.857839743	24	19.98562009	0.031983257
ENSMUSG00000079588	Tmem182	transmembrane protein 182 [Source:MGI Symbol;Acc:MGI:1923725]	1.33341	4.12037	5.26519	6.51199	3.83036	5.11087	3.35514	9.35275	8.08984	7.48492	3.71681	3.70952	5.48558	4.57644	0.085562135	0.466404237	12	16	24	0.03059546	0.597890721	24	9.234493026	0.492505663
ENSMUSG00000079592	C1qtnf5	C1q and tumor necrosis factor related protein 5 [Source:MGI Symbol;Acc:MGI:2385958]	5.04965	5.53918	5.95215	5.11898	4.87709	5.61512	6.7817	5.92721	7.53109	5.39856	6.94759	6.14165	5.72239	5.58951	0.880247609	0.966018836	12	4	24	0.793328936	0.933814107	24	6.314532368	0.040529283
ENSMUSG00000079598	Clec2l	C-type lectin domain family 2, member L [Source:MGI Symbol;Acc:MGI:2141402]	56.8589	43.7278	37.5012	46.5301	44.0963	44.3383	45.2066	47.0986	35.4852	55.2619	46.8762	45.8322	45.4784	47.0904	0.927004797	0.966018836	16	20	24	0.467005862	0.805393068	24	20.21771738	0.087545676
ENSMUSG00000079605	Zbtb9	zinc finger and BTB domain containing 9 [Source:MGI Symbol;Acc:MGI:1918022]	3.23397	2.8766	2.63875	3.35988	3.00245	3.71395	2.75053	2.7235	2.29425	3.36154	2.94377	3.41338	3.04991	3.13757	0.005498159	0.152089577	24	12	24	0.07472198	0.683760931	24	18.64983026	0.115550046
ENSMUSG00000079608	Stard6	StAR-related lipid transfer (START) domain containing 6 [Source:MGI Symbol;Acc:MGI:2156774]	1.11306	0.928025	1.09001	0.9291	1.01471	0.905139	0.883849	0.91973	1.21983	1.20333	1.02842	1.24883	0.942848	1.35013	0.895493752	0.966018836	12	16	24	0.483510851	0.805393068	24	9.178369134	0.0463981
ENSMUSG00000079610	Ankrd39	ankyrin repeat domain 39 [Source:MGI Symbol;Acc:MGI:1914816]	7.51058	6.26361	7.58362	5.4475	5.17554	6.22511	6.38269	6.38252	6.67523	7.52715	6.11363	7.01736	6.39205	5.91523	0.892578749	0.966018836	12	8	24	0.672578954	0.889601659	24	4.106504251	0.051080369
ENSMUSG00000079614	Seh1l	SEH1-like (S. cerevisiae [Source:MGI Symbol;Acc:MGI:1919374]	16.7658	18.4286	18.8691	18.0648	17.3395	16.9979	16.1116	16.8969	16.7531	17.0418	18.1335	15.8836	16.228	17.7761	0.093936711	0.490854203	12	16	24	0.07051081	0.67524425	24	9.907314136	0.056555502
ENSMUSG00000079641	Rpl39	ribosomal protein L39 [Source:MGI Symbol;Acc:MGI:1914498]	43.3534	55.9443	48.4794	49.6514	53.8934	49.2302	51.2129	50.5032	68.8881	39.7221	65.204	48.5997	57.7397	52.9404	0.888482515	0.966018836	12	12	24	0.899262582	0.971453495	24	7.644090109	0.038274883
ENSMUSG00000079645	Gm17193	predicted gene 17193 [Source:MGI Symbol;Acc:MGI:4938020]	0.981538	1.49435	0.902544	1.37913	1.46221	1.1514	1.28757	0.673909	1.33793	0.698606	0.775874	1.25645	1.05906	0.852423	0.822716578	0.966018836	20	12	24	0.711219833	0.904630031	24	19.45626334	0.064193282
ENSMUSG00000079654	Prrt4	proline-rich transmembrane protein 4 [Source:MGI Symbol;Acc:MGI:2141677]	0.377651	0.452823	0.609984	0.436009	0.312161	0.466588	0.476694	0.591626	0.567374	0.699897	0.555713	0.649105	0.646694	0.891347	0.919571723	0.966018836	8	12	24	0.018742225	0.56081134	24	6.402093066	0.087436909
ENSMUSG00000079657	Rab26	RAB26, member RAS oncogene family [Source:MGI Symbol;Acc:MGI:2443284]	0.390819	0.537617	1.39674	0.441658	0.525919	0.370852	0.585762	0.793309	0.691249	0.48606	0.589327	0.706678	0.402214	0.752472	0.200364953	0.684570416	8	20	24	0.149799554	0.758419028	24	7.533367409	0.159583393
ENSMUSG00000079658	Eloc	elongin C [Source:MGI Symbol;Acc:MGI:1915173]	143.348	152.226	140.514	138.953	135.947	132.53	137.144	125.252	137.96	123.744	149.487	132.823	136.387	133.83	0.891742979	0.966018836	12	12	24	0.929721842	0.981769638	24	22.53713022	0.011900397
ENSMUSG00000079659	Tmem243	transmembrane protein 243, mitochondrial [Source:MGI Symbol;Acc:MGI:3606159]	10.7908	11.0278	11.3989	13.4802	10.4213	9.61825	11.3039	11.5502	10.9748	12.1313	9.85673	10.6162	11.0926	11.191	0.007331665	0.192760771	16	4	24	0.091075266	0.71175955	24	8.444445004	0.091762548
ENSMUSG00000079669	Gm17396	predicted gene, 17396 [Source:MGI Symbol;Acc:MGI:4937030]	5.71168	4.52805	4.10462	4.3135	4.45276	4.99766	4.77127	4.86553	4.19831	5.42179	4.32445	5.47084	5.10204	5.71727	0.050732383	0.386400877	4	8	24	0.130361953	0.740714253	24	23.19328428	0.098052112
ENSMUSG00000079671	2610203C22Rik	RIKEN cDNA 2610203C22 gene [Source:MGI Symbol;Acc:MGI:1919731]	1.18876	1.41624	1.6189	1.65866	0.892745	1.28908	1.40908	0.94119	1.74505	0.99162	1.23962	1.47113	1.39304	1.73422	0.285027487	0.793714531	12	8	24	0.398259441	0.799546792	24	6.04827928	0.096427538
ENSMUSG00000079677	Fdx2	ferredoxin 2 [Source:MGI Symbol;Acc:MGI:1915415]	19.6083	22.0977	21.5012	21.4996	23.9438	21.813	20.6855	21.5972	20.4081	23.6201	26.5909	26.2539	26.4099	25.3934	0.616047374	0.966018836	20	16	24	0.226446924	0.768040178	24	17.1336106	0.063073717
ENSMUSG00000079737	3110001I22Rik	RIKEN cDNA 3110001I22 gene [Source:MGI Symbol;Acc:MGI:1913848]	1.20456	1.28337	0.900378	1.29232	1.1046	1.052	1.50371	1.4255	1.07037	1.13887	1.26527	1.47402	1.53246	1.18256	0.101520755	0.511659635	4	8	24	0.123354155	0.736234617	24	23.07540713	0.087344767
ENSMUSG00000079884	Tmed2b	transmembrane p24 trafficking protein 2B [Source:MGI Symbol;Acc:MGI:5434895]	57.323	57.7676	58.5307	58.2565	58.5179	58.8057	58.9608	58.496	54.065	61.1189	55.1612	57.155	61.0665	56.8037	0.921572192	0.966018836	4	4	24	0.842915365	0.952259721	24	21.44321089	0.011556795
ENSMUSG00000079941	Cox5b-ps	cytochrome c oxidase subunit 5B, pseudogene [Source:MGI Symbol;Acc:MGI:3649411]	144.512	138.285	143.581	128.409	126.889	136.83	134.465	147.9	150.96	142.084	221.905	208.799	236.529	208.26	0.909604019	0.966018836	8	8	24	0.441884502	0.805393068	24	23.11642878	0.097936829
ENSMUSG00000080002	Gm5566	predicted pseudogene 5566 [Source:MGI Symbol;Acc:MGI:3648428]	14.641	15.6356	17.5041	15.4716	16.9802	17.342	15.9622	17.1841	15.2696	14.7044	17.5957	14.8733	16.3989	15.4555	0.903207257	0.966018836	20	20	24	0.859699022	0.958681059	24	16.78026169	0.020446354
ENSMUSG00000080021	Gm5915	predicted gene 5915 [Source:MGI Symbol;Acc:MGI:3642938]	7.17204	10.6038	8.67935	8.56849	11.8849	8.70277	8.70485	8.43379	5.05286	4.83485	5.80935	5.60952	6.21171	5.27994	0.882263435	0.966018836	20	20	24	0.592549076	0.856602197	24	20.96546559	0.106524062
ENSMUSG00000080059	Rps19-ps3	ribosomal protein S19, pseudogene 3 [Source:MGI Symbol;Acc:MGI:3701125]	3.05597	2.54843	2.32014	3.27157	2.02053	2.04253	3.33565	2.29837	3.21042	2.62804	3.10834	2.66849	2.46863	1.99643	0.952127548	0.966018836	16	16	24	0.933223107	0.983163516	24	12.91246428	0.028435417
ENSMUSG00000080242	Atp6v0c-ps2	ATPase, H+ transporting, lysosomal V0 subunit C, pseudogene 2 [Source:MGI Symbol;Acc:MGI:2151816]	219.448	188.853	210.017	180.503	177.236	213.37	216.587	216.02	172.371	222.272	196.408	210.648	197.507	204.558	0.505132703	0.966018836	4	8	24	0.327595884	0.768040178	24	23.47620279	0.070495155
ENSMUSG00000080268	Brms1	breast cancer metastasis-suppressor 1 [Source:MGI Symbol;Acc:MGI:2388804]	19.5788	17.5731	16.8653	15.9196	19.43	17.8073	18.4749	17.3959	16.4336	17.2448	17.8558	17.6144	19.2963	17.8397	0.002250501	0.104113924	4	12	24	0.014438951	0.534237819	24	21.76847471	0.085182846
ENSMUSG00000080316	Spaca6	sperm acrosome associated 6 [Source:MGI Symbol;Acc:MGI:1922452]	21.3663	26.2623	27.6892	26.6618	28.0219	20.2121	23.1572	23.8599	30.9065	24.7463	23.6379	20.1939	22.4242	22.6398	2.74E-04	0.049770315	12	12	24	0.002804959	0.414500244	24	9.661986618	0.19106866
ENSMUSG00000080465	Snord94	small nucleolar RNA, C/D box 94 [Source:MGI Symbol;Acc:MGI:5452263]	31.2807	25.1558	11.1754	21.8004	16.9773	34.0113	38.1232	21.8598	10.3817	3.43827	6.53391	18.2087	7.55424	13.9504	0.107220884	0.523582274	4	12	24	0.021914514	0.574400012	24	23.18929826	0.749151854
ENSMUSG00000080717	B230307C23Rik	RIKEN cDNA B230307C23 gene [Source:MGI Symbol;Acc:MGI:3643396]	5.98089	3.77632	4.18347	3.59359	4.04734	2.87934	3.65852	3.61059	3.78822	2.96679	3.85681	3.93718	3.82062	3.17684	0.93717134	0.966018836	20	20	24	0.521814978	0.820509389	24	23.22740622	0.08120448
ENSMUSG00000080727	C920021L13Rik	RIKEN cDNA C920021L13 gene [Source:MGI Symbol;Acc:MGI:1923991]	0.978295	1.84958	2.29826	2.1273	1.6869	1.47861	1.05553	1.28505	2.2795	1.40659	1.68516	2.52616	1.38654	1.99218	0.018894761	0.256345714	12	16	24	0.164657086	0.767020009	24	10.78757506	0.185636258
ENSMUSG00000080772	Gm12543	predicted gene 12543 [Source:MGI Symbol;Acc:MGI:3649591]	35.2657	35.417	34.8644	35.0299	37.6646	33.8206	32.6402	34.2694	34.7899	24.3601	33.4698	37.8897	34.4897	34.8309	0.7866493	0.966018836	24	4	24	0.455365777	0.805393068	24	22.88021969	0.071841527
ENSMUSG00000080775	Gm6368	predicted gene 6368 [Source:MGI Symbol;Acc:MGI:3644537]	5.76903	4.82609	3.99728	4.74104	6.18927	6.62009	5.17242	5.72155	3.04704	5.07568	3.84514	3.96764	3.63233	3.62249	0.723119329	0.966018836	24	12	24	0.140187686	0.749574609	24	19.64352163	0.177701588
ENSMUSG00000080776	Gm12174	predicted gene 12174 [Source:MGI Symbol;Acc:MGI:3651722]	50.6972	64.3377	60.9709	60.4757	62.8259	54.8568	53.1486	52.1154	64.9202	38.4359	51.1497	46.7353	53.6996	47.0337	0.9678483	0.970142211	12	12	24	0.814843507	0.940847489	24	7.313755459	0.047332009
ENSMUSG00000080783	Gm8250	predicted gene 8250 [Source:MGI Symbol;Acc:MGI:3647615]	1.62917	1.38976	1.62924	1.49064	1.41028	1.40138	0.794294	1.1065	1.51129	1.11965	1.47537	1.16615	1.52183	1.32012	0.913451828	0.966018836	12	20	24	0.726319939	0.910103158	24	11.49822504	0.047300846
ENSMUSG00000080791	Akirin1-ps	akirin 1, pseudogene [Source:MGI Symbol;Acc:MGI:3650065]	0.680151	0.756921	0.737981	0.768493	0.801748	0.607866	0.330238	1.08518	1.19988	0.629785	0.647045	0.351182	1.00564	0.416388	0.390686537	0.915479136	12	12	24	0.315263131	0.768040178	24	8.289440869	0.131756478
ENSMUSG00000080797	Gm15760	predicted gene 15760 [Source:MGI Symbol;Acc:MGI:3642834]	1.51637	1.04104	1.81724	1.1561	1.46354	1.71068	1.06757	1.97109	0.835371	1.34813	1.24967	1.95831	1.75833	1.29162	0.834424969	0.966018836	24	12	24	0.445414033	0.805393068	24	21.17192045	0.11141502
ENSMUSG00000080816	Gm12447	predicted gene 12447 [Source:MGI Symbol;Acc:MGI:3650247]	0.783882	1.18992	0.850644	0.886472	1.25245	0.803496	1.11041	0.8111	0.975464	0.777632	0.533031	0.758348	0.877575	1.19802	0.46694635	0.966018836	8	16	24	0.407831702	0.799546792	24	4.346893116	0.080398027
ENSMUSG00000080818	Gm9434	predicted gene 9434 [Source:MGI Symbol;Acc:MGI:3645161]	2.44914	2.42549	2.12999	2.38259	1.96617	1.59508	2.09011	1.47806	1.96348	1.41606	2.35364	2.07278	2.00097	1.56014	0.879844076	0.966018836	4	12	24	0.757976841	0.921733004	24	19.56796634	0.048637779
ENSMUSG00000080824	Gm9001	predicted gene 9001 [Source:MGI Symbol;Acc:MGI:3647168]	30.8581	34.6072	34.1946	39.6629	37.3401	35.222	32.2284	33.7302	40.2833	29.4412	41.068	31.94	32.9936	32.2439	0.128810631	0.560209134	20	8	24	0.210200764	0.768040178	24	12.92758791	0.095140163
ENSMUSG00000080829	Gm8545	predicted gene 8545 [Source:MGI Symbol;Acc:MGI:3648875]	0.998218	0.887133	1.08514	0.928061	1.07137	1.37767	0.608447	1.05968	0.851279	0.412261	0.813557	0.47828	0.49938	0.374172	0.816361374	0.966018836	24	4	24	0.700941391	0.900900643	24	16.86497903	0.057629707
ENSMUSG00000080832	M6pr-ps	mannose-6-phosphate receptor, pseudogene [Source:MGI Symbol;Acc:MGI:96905]	12.7666	12.8474	13.0361	12.908	11.5421	11.5581	11.929	12.0566	10.4622	11.3528	12.165	11.8481	11.8947	10.9934	0.816300547	0.966018836	12	8	24	0.84525807	0.953278734	24	22.07515701	0.015802283
ENSMUSG00000080836	Gm6444	predicted gene 6444 [Source:MGI Symbol;Acc:MGI:3648226]	43.1216	45.7593	46.4928	52.7186	44.8979	39.0554	38.803	48.3506	50.0258	37.6071	47.7889	41.3517	44.5226	42.7839	0.123394836	0.552144108	12	12	24	0.216963397	0.768040178	24	9.157724208	0.095551541
ENSMUSG00000080840	Gm11349	predicted gene 11349 [Source:MGI Symbol;Acc:MGI:3652298]	2.16657	2.28767	2.0846	2.24901	2.90423	2.3684	1.98341	1.97896	2.04992	1.98177	2.29427	2.33545	1.94123	1.98728	0.044528129	0.365513105	24	4	24	0.01386842	0.530091843	24	16.94334148	0.104393177
ENSMUSG00000080845	Gm9115	predicted gene 9115 [Source:MGI Symbol;Acc:MGI:3648181]	1.00426	1.08966	1.6388	1.34756	1.53283	1.35315	1.2673	1.59153	1.44204	1.34883	1.08418	1.66124	1.4025	1.19471	0.94086481	0.966018836	24	4	24	0.692402938	0.898097138	24	11.64795538	0.044166284
ENSMUSG00000080848	Gm9385	predicted pseudogene 9385 [Source:MGI Symbol;Acc:MGI:3646182]	199.477	228.048	232.21	211.457	221.609	205.675	202.77	212.55	249.592	168.508	235.029	203.885	230.631	209.795	0.321662653	0.83607295	12	12	24	0.761374666	0.922782206	24	6.278900445	0.042286558
ENSMUSG00000080850	Gm12439	predicted gene 12439 [Source:MGI Symbol;Acc:MGI:3651708]	0.398886	0.502829	1.51269	1.78586	0.436307	0.771879	0.241379	1.40542	0.808857	1.31998	0.672543	1.1676	1.61809	0.775009	0.339604637	0.858712466	16	4	24	0.264246879	0.768040178	24	9.903300928	0.233780753
ENSMUSG00000080875	Gm7332	predicted gene 7332 [Source:MGI Symbol;Acc:MGI:3643344]	35.9981	33.8345	28.2827	37.5456	33.7247	30.9603	29.2952	34.9574	37.8029	30.5599	38.5104	28.7599	34.8597	30.7292	0.852844413	0.966018836	20	4	24	0.799512303	0.935842908	24	11.78066624	0.040010649
ENSMUSG00000080877	Rpl22-ps1	ribosomal protein L22, pseudogene 1 [Source:MGI Symbol;Acc:MGI:3644276]	21.8436	25.4199	23.4813	21.475	25.2168	22.2219	18.8046	22.1418	22.9642	18.5157	20.7415	16.1693	20.957	16.3439	0.917610614	0.966018836	12	12	24	0.748178706	0.918016105	24	10.19430226	0.043309698
ENSMUSG00000080893	Ndufa12-ps	NADH:ubiquinone oxidoreductase subunit A12, pseudogene [Source:MGI Symbol;Acc:MGI:3801934]	91.2319	94.3555	106.58	101.432	87.4499	90.522	93.6326	97.103	99.6148	89.4287	98.4316	94.4706	104.741	97.273	0.212359178	0.701530532	12	12	24	0.200019561	0.768040178	24	6.84657708	0.058471082
ENSMUSG00000080896	Gm14567	predicted gene 14567 [Source:MGI Symbol;Acc:MGI:3705732]	71.2307	89.1316	76.463	58.5654	85.4538	63.107	108.36	106.152	118.993	80.8469	81.2945	60.5869	100.724	93.0854	0.157813674	0.614233844	8	8	24	0.071894202	0.678310325	24	4.931749783	0.258250445
ENSMUSG00000080902	Ywhaq-ps3	tyrosine 3-monooxygenase/tryptophan 5-monooxygenase activation protein theta, pseudogene 3 [Source:MGI Symbol;Acc:MGI:3650241]	19.0521	18.7811	16.3401	15.6774	16.8931	17.3941	20.2613	16.0277	15.2587	15.9787	18.4788	13.8668	16.0471	14.8837	0.351315142	0.870418653	4	8	24	0.285353807	0.768040178	24	22.05558391	0.076739235
ENSMUSG00000080921	Rpl38-ps2	ribosomal protein L38, pseudogene 2 [Source:MGI Symbol;Acc:MGI:3646625]	421.232	486.013	436.908	434.292	444.29	413.114	425.364	414.524	489.73	333.32	427.593	433.277	476.397	466.477	0.745475226	0.966018836	12	12	24	0.341087127	0.775335259	24	2.999611369	0.077346987
ENSMUSG00000080935	Got2-ps1	glutamatic-oxaloacetic transaminase 2, mitochondrial, pseudogene 1 [Source:MGI Symbol;Acc:MGI:104721]	35.0958	27.799	32.9369	31.4613	31.7166	40.2013	35.2355	40.4999	27.0268	46.4565	28.2583	32.8749	31.2358	30.5186	0.92721845	0.966018836	24	12	24	0.869298046	0.962936155	24	18.79065945	0.048364714
ENSMUSG00000080950	Gm7278	predicted gene 7278 [Source:MGI Symbol;Acc:MGI:3645261]	0.544623	0.635524	0.766788	0.884556	0.938144	0.87573	0.704291	0.603611	0.788697	0.592519	0.728669	0.652113	0.511249	0.452399	0.052733588	0.391647859	20	12	24	0.014730099	0.537860185	24	14.81628204	0.111219327
ENSMUSG00000080966	Gm6263	predicted gene 6263 [Source:MGI Symbol;Acc:MGI:3646097]	5.98903	5.95524	6.0762	5.495	5.93516	6.07218	6.49208	5.82507	5.24539	6.51194	6.09767	5.56589	4.83322	5.55404	0.910436409	0.966018836	20	12	24	0.671504633	0.889179947	24	16.1760253	0.032764087
ENSMUSG00000080968	Atf1-ps	activating transcription factor 1, pseudogene [Source:MGI Symbol;Acc:MGI:3037720]	2.95269	2.40554	2.94239	2.112	3.49113	3.34422	2.5498	2.80484	3.90325	2.84536	3.08235	3.26135	2.95403	3.36564	0.759899911	0.966018836	20	20	24	0.912743208	0.97533472	24	17.44620624	0.027609868
ENSMUSG00000080981	Gm12161	predicted gene 12161 [Source:MGI Symbol;Acc:MGI:3649484]	5.08736	3.59061	5.07083	4.3252	3.11429	4.27275	3.69364	3.942	3.98804	4.3099	4.11541	4.76747	3.57836	5.85684	0.895137379	0.966018836	16	4	24	0.691992373	0.89792735	24	5.687173773	0.068413579
ENSMUSG00000080989	Gm14048	predicted gene 14048 [Source:MGI Symbol;Acc:MGI:3650660]	9.39778	7.7102	8.34117	8.57271	7.65714	8.38675	7.61377	9.08902	6.06933	9.50966	8.21703	7.57888	7.83562	6.8116	0.858932988	0.966018836	16	20	24	0.647523173	0.880947922	24	17.71266216	0.055292268
ENSMUSG00000081010	Gm13880	predicted gene 13880 [Source:MGI Symbol;Acc:MGI:3649539]	15.7313	13.1809	17.6316	14.8779	16.2648	16.6587	13.9355	14.6411	12.955	11.3418	12.5412	12.2223	13.5096	10.8339	0.847846021	0.966018836	20	12	24	0.53918569	0.829144433	24	19.29543531	0.061556039
ENSMUSG00000081021	Gm11964	predicted gene 11964 [Source:MGI Symbol;Acc:MGI:3652241]	33.0087	32.8048	29.9186	25.6431	28.7065	33.6431	31.7303	28.4775	25.5847	28.733	30.2688	26.4004	26.812	26.7294	0.68887295	0.966018836	4	8	24	0.104263722	0.716417536	24	21.94561137	0.088679972
ENSMUSG00000081049	Rps24-ps3	ribosomal protein S24, pseudogene 3 [Source:MGI Symbol;Acc:MGI:3644407]	213.267	269.891	260.063	252.255	245.575	220.507	216.64	212.199	300.782	188.435	259.636	254.067	288.578	266.105	0.93717134	0.966018836	12	4	24	0.683687821	0.894811431	24	6.862368359	0.067632882
ENSMUSG00000081051	Gm15427	predicted pseudogene 15427 [Source:MGI Symbol;Acc:MGI:3642341]	262.946	313.18	305.805	311.78	319.036	271.991	280.129	278.353	375.996	222.1	323.112	286.281	315.445	290.27	0.777666236	0.966018836	20	4	24	0.772602322	0.92687884	24	8.673180241	0.052969107
ENSMUSG00000081052	Gm6305	predicted gene 6305 [Source:MGI Symbol;Acc:MGI:3644967]	0.748753	0.832471	0.610714	0.698055	1.19141	0.930139	0.685237	1.04781	0.895033	0.83476	0.772508	0.924729	0.903767	1.04781	0.891105762	0.966018836	20	20	24	0.881418295	0.966661503	24	20.81806794	0.023988273
ENSMUSG00000081058	H3c15	H3 clustered histone 15 [Source:MGI Symbol;Acc:MGI:2448357]	2.17882	1.39017	1.05873	1.86188	1.94406	2.3432	1.69706	1.82693	1.01333	0.888174	2.0727	1.47741	1.91397	1.45299	0.018263889	0.256345714	20	16	24	0.012501788	0.525280095	24	20.53061775	0.26623435
ENSMUSG00000081059	Gm11945	predicted gene 11945 [Source:MGI Symbol;Acc:MGI:3650072]	2.17789	3.56578	4.17092	2.17407	3.11898	2.36476	2.48916	2.15943	2.5222	1.94865	2.61428	2.97351	1.37652	3.61973	0.909604019	0.966018836	12	16	24	0.678757976	0.892520664	24	8.146244601	0.105620884
ENSMUSG00000081068	Gm12804	predicted gene 12804 [Source:MGI Symbol;Acc:MGI:3651639]	4.03996	4.05819	4.70358	3.9261	4.23457	4.10206	3.5376	3.87334	4.41503	3.20289	3.92254	3.66625	4.05173	3.54806	0.834434272	0.966018836	12	16	24	0.898268638	0.971075964	24	8.104213135	0.019407102
ENSMUSG00000081070	Gm13181	predicted gene 13181 [Source:MGI Symbol;Acc:MGI:3650464]	1.27129	1.62674	2.09785	1.4595	1.57649	1.89307	1.23054	1.21755	1.13813	1.50597	1.08593	1.42453	1.22575	1.04611	0.840483986	0.966018836	16	16	24	0.566228555	0.843471947	24	14.74153811	0.060824595
ENSMUSG00000081076	Rpsa-ps4	ribosomal protein S4, pseudogene 4 [Source:MGI Symbol;Acc:MGI:3651545]	0.603385	0.836028	0.83498	0.439954	0.779234	0.373232	0.602104	0.539885	0.524228	0.434918	0.527612	0.560083	0.402159	0.20517	0.847846021	0.966018836	12	4	24	0.964579332	0.99117464	24	14.08088759	0.013501627
ENSMUSG00000081087	Rps15a-ps7	ribosomal protein S15A, pseudogene 7 [Source:MGI Symbol;Acc:MGI:3650538]	162.57	199.448	193.57	191.846	195.134	168.62	165.014	168.495	223.274	120.943	183.383	175.454	178.564	175.374	0.182849367	0.659303873	12	16	24	0.81753702	0.941925115	24	7.003746949	0.052690546
ENSMUSG00000081093	Gm12565	predicted gene 12565 [Source:MGI Symbol;Acc:MGI:3652206]	0.820915	0.624689	1.07033	0.468041	0.793893	0.463078	0.599729	0.585714	0.503285	0.542595	0.505684	0.368137	0.384089	0.309453	0.92769718	0.966018836	12	12	24	0.765709382	0.924199664	24	9.826402952	0.034954782
ENSMUSG00000081094	Rpl19-ps11	ribosomal protein L19, pseudogene 11 [Source:MGI Symbol;Acc:MGI:3642193]	232.226	256.208	242.105	238.896	257.974	235.016	229.643	231.114	275.073	191.585	262.096	242.979	257.21	255.413	0.935593607	0.966018836	20	8	24	0.902686584	0.97295148	24	2.716311481	0.022648182
ENSMUSG00000081104	Gm15989	predicted gene 15989 [Source:MGI Symbol;Acc:MGI:3801755]	1.31555	1.45321	0.908717	1.35495	0.79528	1.27296	1.1624	1.2744	0.97488	0.949014	0.81441	0.770184	0.882696	0.911174	0.285193697	0.793714531	8	12	24	0.216207411	0.768040178	24	2.751602947	0.082564624
ENSMUSG00000081111	Gm5913	predicted gene 5913 [Source:MGI Symbol;Acc:MGI:3648292]	10.3646	9.36878	8.588	8.12153	10.3362	10.6719	9.95445	10.1053	6.8245	10.1104	8.44853	8.24636	8.51336	8.34733	0.688644847	0.966018836	4	8	24	0.173559206	0.768040178	24	20.84258295	0.113233379
ENSMUSG00000081113	Gm7308	predicted pseudogene 7308 [Source:MGI Symbol;Acc:MGI:3644558]	289.519	305.835	280.507	281.519	288.751	289.452	283.398	268.375	302.421	241.708	311.987	282.859	292.78	269.097	0.951846915	0.966018836	20	20	24	0.587505342	0.853591039	24	21.18366983	0.038153159
ENSMUSG00000081121	Gm12791	predicted gene 12791 [Source:MGI Symbol;Acc:MGI:3649397]	14.9189	13.0399	14.4481	13.316	13.2722	16.2857	16.488	15.9107	13.0358	18.7568	13.5983	15.8405	13.1136	13.6889	0.887010723	0.966018836	24	12	24	0.801032037	0.936384198	24	19.28518939	0.043045556
ENSMUSG00000081126	Gm15784	predicted gene 15784 [Source:MGI Symbol;Acc:MGI:3783226]	0.641062	0.807494	0.963167	0.843094	0.779543	0.886866	0.969454	0.739629	0.626345	1.27031	0.610295	1.02894	0.811796	0.81643	0.916556573	0.966018836	24	20	24	0.833039495	0.948410241	24	13.69702376	0.032365567
ENSMUSG00000081128	Gm13328	predicted gene 13328 [Source:MGI Symbol;Acc:MGI:3651185]	12.5167	10.4616	10.4865	11.7033	11.1437	11.4265	10.8046	11.1692	10.8934	11.9751	12.2767	10.8442	12.0086	12.3158	0.928127947	0.966018836	16	20	24	0.764287046	0.92403577	24	18.25717756	0.02342054
ENSMUSG00000081137	BC022960	cDNA sequence BC022960 [Source:MGI Symbol;Acc:MGI:2670977]	2.08671	3.2222	4.0977	3.0673	3.26462	2.73604	2.78428	2.69254	4.03749	2.74057	3.16108	2.88029	2.67709	2.86352	0.004681041	0.140598674	12	16	24	0.045158704	0.628927515	24	10.06062811	0.16960973
ENSMUSG00000081138	Gm12834	predicted gene 12834 [Source:MGI Symbol;Acc:MGI:3649428]	0.772015	0.521539	0.533258	0.582505	0.469705	0.729507	0.580847	0.787516	0.416819	0.70592	0.533385	0.423941	0.541982	0.538056	0.892578749	0.966018836	4	8	24	0.707343997	0.903441895	24	0.001519635	0.035153903
ENSMUSG00000081151	Gm11448	predicted gene 11448 [Source:MGI Symbol;Acc:MGI:3651347]	3.7765	4.24435	3.18673	3.00086	2.98641	3.98189	3.05543	6.32032	4.26959	2.22031	3.13494	4.15471	4.51385	4.28575	0.00384759	0.140598674	8	8	24	0.028055319	0.590904845	24	2.291047961	0.262812627
ENSMUSG00000081152	Gm12430	predicted gene 12430 [Source:MGI Symbol;Acc:MGI:3651061]	4.38134	4.52627	4.31952	4.60554	4.1876	4.26719	3.55296	4.26765	3.93181	3.54982	3.65565	3.52836	4.14325	3.94567	0.801995186	0.966018836	8	16	24	0.516847446	0.817924119	24	5.20739273	0.037114701
ENSMUSG00000081157	Gm13552	predicted gene 13552 [Source:MGI Symbol;Acc:MGI:3650819]	6.80592	7.83723	6.69417	5.52457	9.75836	8.19114	5.94912	6.15857	6.4343	6.9108	5.57723	6.45424	6.07643	6.57807	0.786317982	0.966018836	24	4	24	0.652495122	0.883020861	24	18.40976498	0.070365293
ENSMUSG00000081160	Gm14897	predicted gene 14897 [Source:MGI Symbol;Acc:MGI:3705528]	5.40345	4.96978	4.15459	3.32049	5.34553	3.66321	8.34217	3.30976	3.60758	2.55715	4.53189	2.59891	4.3032	3.84838	0.066380001	0.42386701	4	12	24	0.213177993	0.768040178	24	23.36812825	0.228607187
ENSMUSG00000081176	H3f3a-ps2	H3.3 histone A, pseudogene 2 [Source:MGI Symbol;Acc:MGI:1101758]	19.9445	20.2324	24.6163	22.8082	23.074	21.6419	20.0498	23.0772	24.2738	15.9841	23.0638	19.2162	24.3635	19.3622	0.8972237	0.966018836	12	12	24	0.941330857	0.985472945	24	8.295080714	0.024493574
ENSMUSG00000081179	Gm13136	predicted gene 13136 [Source:MGI Symbol;Acc:MGI:3649290]	1.06506	1.22911	0.973781	0.917801	0.920061	0.965653	1.11122	0.94485	1.31283	0.844018	1.33446	1.01817	1.0556	1.02913	0.305564675	0.811201458	4	12	24	0.764786569	0.92403577	24	2.588153152	0.02979392
ENSMUSG00000081185	Gm4852	predicted pseudogene 4852 [Source:MGI Symbol;Acc:MGI:3643039]	1.12626	1.17917	1.14757	0.965192	1.10205	1.15407	0.943469	1.25734	0.911886	0.651267	1.20644	1.21555	0.798868	1.3223	0.350544145	0.870418653	8	8	24	0.587325575	0.853591039	24	23.95670702	0.056480438
ENSMUSG00000081194	Gm8424	predicted gene 8424 [Source:MGI Symbol;Acc:MGI:3646584]	0.0963325	0.538538	0.471419	0.442033	0.324517	0.464879	0.293844	0.992028	0.657944	0.891021	0.496848	0.368504	0.577453	0.395593	0.293953646	0.803303962	8	20	24	0.097190647	0.71175955	24	8.851885031	0.170098504
ENSMUSG00000081208	Gm11400	predicted gene 11400 [Source:MGI Symbol;Acc:MGI:3705645]	0.490962	0.748893	0.862686	0.651038	0.821435	1.03112	0.737706	0.643307	0.98092	0.548852	0.442109	0.673142	0.527607	0.590365	0.892555977	0.966018836	12	16	24	0.92332497	0.979439431	24	9.58779557	0.023901832
ENSMUSG00000081219	Bambi-ps1	BMP and activin membrane-bound inhibitor, pseudogene (Xenopus laevis) [Source:MGI Symbol;Acc:MGI:1932402]	0.279355	0.660971	0.505729	0.74184	0.898708	0.681452	0.339918	0.580585	0.380271	0.984903	0.954564	0.597483	0.373868	1.26764	0.013006247	0.228055093	16	12	24	0.157196122	0.763141258	24	12.67928988	0.163820521
ENSMUSG00000081225	Cyp2j12	cytochrome P450, family 2, subfamily j, polypeptide 12 [Source:MGI Symbol;Acc:MGI:3717097]	5.49073	6.43939	6.90895	6.46717	6.64118	7.18332	6.74227	5.80628	6.88118	5.73489	7.66279	8.07971	7.29328	8.14017	0.896545154	0.966018836	24	16	24	0.731900229	0.912550805	24	19.27587209	0.039636835
ENSMUSG00000081226	Gm8802	predicted gene 8802 [Source:MGI Symbol;Acc:MGI:3647981]	0.690384	0.665913	0.52526	0.315622	0.669273	0.585612	0.757155	0.555016	0.517841	0.593114	0.568066	0.706346	0.829485	0.311133	0.004140921	0.140598674	4	8	24	0.05839488	0.658304792	24	21.59295825	0.123382362
ENSMUSG00000081227	Gm13082	predicted gene 13082 [Source:MGI Symbol;Acc:MGI:3650233]	16.5752	14.2045	15.055	12.6742	14.2254	18.1842	12.8289	15.7343	10.4552	13.5482	13.7852	12.5818	12.378	12.4093	0.820042705	0.966018836	20	16	24	0.247513876	0.768040178	24	20.76605236	0.107890937
ENSMUSG00000081228	Gm16089	predicted gene 16089 [Source:MGI Symbol;Acc:MGI:3801884]	55.9102	55.3578	60.794	64.6441	59.6748	53.3242	52.9389	50.9486	64.2961	49.8146	61.3726	47.7569	65.5509	53.7928	0.892555977	0.966018836	12	12	24	0.54664069	0.833423522	24	10.72856014	0.064180682
ENSMUSG00000081232	Gm14373	predicted gene 14373 [Source:MGI Symbol;Acc:MGI:3651800]	5.00263	4.51653	3.84558	5.28713	4.48193	4.31733	4.41596	4.24521	4.74237	4.30031	4.8793	4.42468	4.52186	4.45516	0.880247609	0.966018836	20	12	24	0.689540484	0.897245516	24	16.62652011	0.032014792
ENSMUSG00000081249	Gm11517	predicted gene 11517 [Source:MGI Symbol;Acc:MGI:3650064]	0.359426	1.08845	0.779196	0.806823	1.18303	0.498318	0.870724	0.951529	0.729633	0.832418	0.8503	0.816409	0.363854	1.39036	0.580049522	0.966018836	8	20	24	0.358280903	0.784922891	24	9.24567117	0.133846802
ENSMUSG00000081254	Gm12112	predicted gene 12112 [Source:MGI Symbol;Acc:MGI:3650115]	51.7026	52.0121	47.9508	39.8174	53.0594	40.6226	52.7774	47.006	55.0588	40.1495	50.8149	42.692	49.201	43.2017	0.843639218	0.966018836	4	12	24	0.578228878	0.849207394	24	2.053525693	0.064713529
ENSMUSG00000081262	Gm12261	predicted gene 12261 [Source:MGI Symbol;Acc:MGI:3650914]	1.15891	1.63774	1.25916	1.60448	1.41617	1.30731	1.41104	1.6979	1.29688	1.42258	1.35966	1.37896	1.27267	1.31085	0.745118544	0.966018836	16	12	24	0.662499113	0.886140924	24	9.070325521	0.034339719

ENSMUSG00000081272	Ap2m1-ps	adaptor-related protein complex 2, mu 1 subunit, pseudogene [Source:MGI Symbol;Acc:MGI:3649640]	189.602	171.34	182.854	168.779	187.724	198.113	177.921	186.84	154.46	184.203	175.777	176.61	178.65	182.668	0.772122688	0.966018836	4	8	24	0.204221486	0.768040178	24	20.70838711	0.06045048
ENSMUSG00000081274	Gm15727	predicted gene 15727 [Source:MGI Symbol;Acc:MGI:3783170]	0.586365	0.815232	0.954114	1.65353	0.646826	0.817338	0.355682	0.648951	1.68971	0.387862	0.696394	0.75794	1.08484	1.01239	0.843639218	0.966018836	12	8	24	0.303856728	0.768040178	24	8.880068396	0.181537325
ENSMUSG00000081277	Gm15285	predicted gene 15285 [Source:MGI Symbol;Acc:MGI:3705624]	24.0622	24.7452	28.6394	38.6306	24.2366	27.636	26.3906	26.5077	24.9401	29.0034	26.3068	24.4423	29.9758	28.8562	0.676344318	0.966018836	16	4	24	0.362528618	0.78719863	24	10.72477995	0.098217917
ENSMUSG00000081281	Rpl7-ps9	ribosomal protein L7, pseudogene 9 [Source:MGI Symbol;Acc:MGI:3645367]	45.4565	55.6058	52.1443	58.9072	57.4204	49.481	45.999	47.5375	63.5216	39.2065	56.7045	44.8455	52.3096	44.2497	0.4776989	0.966018836	12	16	24	0.527519861	0.823409555	24	11.68771876	0.083212038
ENSMUSG00000081295	Gm6275	predicted gene 6275 [Source:MGI Symbol;Acc:MGI:3645368]	0.786655	0.399936	0.63046	0.618184	0.341368	0.800233	0.808602	0.816315	0.565855	0.847894	0.398987	0.58017	0.33068	0.431952	0.853079521	0.966018836	16	4	24	0.966308676	0.991934487	24	1.94038293	0.017741984
ENSMUSG00000081309	Gm12397	predicted gene 12397 [Source:MGI Symbol;Acc:MGI:3649202]	2.38494	2.23982	2.4949	1.81103	3.08484	1.99076	2.68779	2.36163	1.50355	2.91941	2.47663	1.9257	2.06775	2.26825	0.880247609	0.966018836	20	4	24	0.777898711	0.928772941	24	17.29590038	0.054844103
ENSMUSG00000081325	Gm12599	predicted gene 12599 [Source:MGI Symbol;Acc:MGI:3651469]	1.13918	0.964168	0.481582	0.868883	1.3701	1.07484	1.15722	1.3577	1.16571	0.543672	1.04233	0.930038	1.18213	0.949161	0.305564675	0.811201458	4	12	24	0.17478174	0.768040178	24	22.38535833	0.142992356
ENSMUSG00000081339	Gm16044	predicted gene 16044 [Source:MGI Symbol;Acc:MGI:3801992]	0.979093	0.707392	0.758744	0.473821	0.308939	0.156153	0.169746	0.619798	0.664158	0.832618	0.380033	0.632238	0.564232	1.17873	0.125343232	0.555618842	8	16	24	0.072924434	0.680039457	24	6.253741269	0.228026678
ENSMUSG00000081344	Gm14303	predicted gene 14303 [Source:MGI Symbol;Acc:MGI:3651379]	993.614	1172.22	1212.38	1116.61	1163.06	965.882	1112.23	1168.05	1419.91	817.256	1197.93	1089.95	1192.29	1081.23	0.148073804	0.590732968	12	12	24	0.539514909	0.829375145	24	6.283292536	0.084106545
ENSMUSG00000081352	Gm12389	predicted gene 12389 [Source:MGI Symbol;Acc:MGI:3650084]	2.99534	4.06251	5.12901	5.14814	3.24022	3.50946	5.35225	4.04321	3.7386	2.19015	2.8846	2.14271	2.44202	2.79022	0.801995186	0.966018836	12	12	24	0.362430855	0.787179935	24	6.772299543	0.155958712
ENSMUSG00000081359	Cox20b	cytochrome c oxidase assembly protein 20B [Source:MGI Symbol;Acc:MGI:3783125]	10.1809	12.7194	11.9473	11.8499	11.578	9.71502	10.2628	9.89074	10.3155	8.84466	10.428	11.882	12.6338	9.58719	0.757932695	0.966018836	4	12	24	0.939300425	0.985099549	24	22.36894303	0.021371532
ENSMUSG00000081370	Gm9105	predicted gene 9105 [Source:MGI Symbol;Acc:MGI:3648833]	2.31227	2.25425	1.67707	2.32759	1.66382	2.12148	2.03957	1.93049	1.64712	1.73947	2.08064	2.02242	2.02865	1.71858	0.300823756	0.809362476	4	8	24	0.145263472	0.754184974	24	21.4880561	0.084784191
ENSMUSG00000081382	Rpl18-ps1	ribosomal protein L18, pseudogene 1 [Source:MGI Symbol;Acc:MGI:98018]	7.73986	8.7932	7.08276	7.77852	7.77361	6.45245	7.25773	7.56797	8.11705	3.94784	7.99083	6.54285	6.81917	6.13435	0.928148816	0.966018836	12	12	24	0.680807747	0.893366006	24	0.941199601	0.074854128
ENSMUSG00000081390	Gm14538	predicted gene 14538 [Source:MGI Symbol;Acc:MGI:3705591]	0.374898	0.589349	0.593229	0.509533	0.588356	0.582041	0.541055	0.825407	0.559271	0.697895	0.469672	0.54214	0.566664	0.405521	0.832506215	0.966018836	12	16	24	0.650413201	0.882429682	24	9.163483274	0.039315833
ENSMUSG00000081400	Gm13680	predicted gene 13680 [Source:MGI Symbol;Acc:MGI:3652285]	21.0412	27.9801	26.5134	25.3746	29.5757	24.1099	24.1089	24.1173	30.562	19.7914	25.3666	24.0612	27.2295	25.0644	0.245061943	0.741580974	12	12	24	0.769634775	0.925855377	24	8.303561266	0.049906581
ENSMUSG00000081402	Gm15455	predicted gene 15455 [Source:MGI Symbol;Acc:MGI:3705563]	1.96785	1.78467	1.81781	1.56432	1.54413	1.76626	1.94071	1.99181	1.53982	1.55076	1.50776	1.87876	1.44909	1.59076	0.241264473	0.741580974	8	12	24	0.062685936	0.661961741	24	0.529931535	0.076968444
ENSMUSG00000081406	Rps6-ps4	ribosomal protein S6, pseudogene 4 [Source:MGI Symbol;Acc:MGI:3650907]	189.377	210.962	205.691	205.242	193.48	192.163	194.403	182.824	232.379	165.325	206.795	208.1	218.457	208.019	0.890235289	0.966018836	12	4	24	0.727087586	0.91035631	24	4.718782913	0.038144502
ENSMUSG00000081418	Gm14830	predicted gene 14830 [Source:MGI Symbol;Acc:MGI:3705386]	2.44947	3.05139	3.14593	2.54272	2.9308	3.18693	2.35096	3.11429	2.78265	2.01129	2.59383	1.94746	2.37946	1.95423	0.895493752	0.966018836	12	16	24	0.918282212	0.977931782	24	8.060902203	0.025161945
ENSMUSG00000081451	Gm12734	predicted gene 12734 [Source:MGI Symbol;Acc:MGI:3650213]	2.22795	2.18709	1.70693	1.85798	2.46275	2.38495	2.47089	1.81317	1.31392	1.37163	1.94932	1.71159	1.41682	1.62682	0.140905066	0.585944911	20	16	24	0.0137206	0.529134128	24	20.43889622	0.170335062
ENSMUSG00000081453	Gm6767	predicted gene 6767 [Source:MGI Symbol;Acc:MGI:3645053]	0.911813	0.612855	0.708561	0.936513	0.660126	0.74173	0.676107	0.701168	0.378564	0.430207	0.56108	0.538616	0.34647	0.532545	0.748433002	0.966018836	24	12	24	0.552973877	0.837224306	24	19.56788257	0.047898552
ENSMUSG00000081473	Gm4853	predicted pseudogene 4853 [Source:MGI Symbol;Acc:MGI:3644655]	0.69686	1.23135	0.377725	0.978454	0.510151	0.290087	0.422148	0.615273	0.471692	0.230545	0.942643	1.01688	0.70509	0.673033	0.832506215	0.966018836	8	4	24	0.860662511	0.959073596	24	23.53780295	0.057511964
ENSMUSG00000081476	Itpa-ps1	inosine triphosphatase (nucleoside triphosphate pyrophosphatase) pseudogene 1 [Source:MGI Symbol;Acc:MGI:3613363]	40.2189	43.4453	41.188	38.1337	44.626	41.4556	39.4218	38.1232	40.845	37.7201	43.4051	43.8693	47.5389	42.4456	0.265774856	0.773510818	20	20	24	0.341889814	0.776129793	24	21.19866893	0.05340953
ENSMUSG00000081485	Gm12338	predicted gene 12338 [Source:MGI Symbol;Acc:MGI:3650622]	755.357	751.693	828.173	827.433	712.895	668.15	684.804	770.732	940.145	665.852	913.749	722.812	891.561	853.988	0.390686537	0.915479136	12	12	24	0.259577298	0.768040178	24	7.724968346	0.105398671
ENSMUSG00000081512	Gm15821	predicted gene 15821 [Source:MGI Symbol;Acc:MGI:3801940]	0.635533	0.590583	0.339487	0.327696	0.621965	0.293097	0.736944	0.576053	0.859421	0.284728	0.725952	0.566819	0.467027	0.616319	0.850398351	0.966018836	4	12	24	0.571991608	0.846185279	24	1.625283447	0.069742358
ENSMUSG00000081516	Gm12470	predicted gene 12470 [Source:MGI Symbol;Acc:MGI:3652113]	0.806983	0.843181	0.877656	1.18358	1.17539	0.840483	0.820684	0.843407	0.704447	0.444275	0.586054	0.571393	0.865647	0.769166	0.814221431	0.966018836	12	12	24	0.983800866	0.996611948	24	23.37504949	0.010365528
ENSMUSG00000081519	Gm13331	predicted gene 13331 [Source:MGI Symbol;Acc:MGI:3649454]	6.3925	5.92877	3.89236	7.02118	4.48602	4.81841	5.26702	3.65959	6.39279	3.2953	6.44346	4.63395	6.74985	5.11714	0.807012301	0.966018836	4	8	24	0.790440484	0.932883394	24	22.36517763	0.082043526
ENSMUSG00000081534	Slc48a1	solute carrier family 48 (heme transporter), member 1 [Source:MGI Symbol;Acc:MGI:1914989]	83.8614	74.0394	79.1139	78.4146	70.667	81.4681	85.2153	83.8523	64.6231	92.1058	67.0387	81.9069	74.0675	76.3599	0.784278077	0.966018836	4	16	24	0.745024967	0.916569459	24	23.31201461	0.043061511
ENSMUSG00000081540	Gm12538	predicted gene 12538 [Source:MGI Symbol;Acc:MGI:3650715]	5.3361	3.39194	4.17527	6.60293	4.4892	4.93185	3.31604	4.18533	3.70788	3.9391	2.53366	3.99825	3.582	3.40782	0.920858634	0.966018836	24	8	24	0.746972071	0.917587548	24	15.35761063	0.06979424
ENSMUSG00000081544	Gm13077	predicted gene 13077 [Source:MGI Symbol;Acc:MGI:3649703]	3.4966	2.33867	4.20153	3.81661	3.13418	3.42467	2.12343	2.65982	1.44155	2.39491	2.20947	1.851	2.76654	1.61974	0.858366225	0.966018836	16	16	24	0.511982537	0.81526923	24	16.75354517	0.1143849
ENSMUSG00000081553	Ccdc50-ps	Ccdc50 retrotransposed pseudogene [Source:MGI Symbol;Acc:MGI:3651098]	2.24812	2.07702	2.33349	2.9291	2.79066	2.4353	2.43085	2.14964	2.96554	1.82291	2.42342	2.47674	3.23389	2.29083	0.4776989	0.966018836	20	12	24	0.825701488	0.945421318	24	17.71912394	0.040013004
ENSMUSG00000081559	Gm12411	predicted gene 12411 [Source:MGI Symbol;Acc:MGI:3649918]	17.0372	18.6414	17.6749	14.0376	15.2571	14.9329	17.6494	15.3417	23.6475	13.0895	18.2422	18.8925	16.4001	21.9859	0.187699199	0.662239322	12	4	24	0.233421942	0.768040178	24	4.233240647	0.140493093
ENSMUSG00000081564	Gm13717	predicted gene 13717 [Source:MGI Symbol;Acc:MGI:3650430]	0.997215	0.70396	0.432197	0.446465	0.436307	1.10268	0.241379	0.702712	0.539238	0.395994	0.807051	0.259467	0.269682	1.39502	0.88067614	0.966018836	8	20	24	0.757089614	0.92154584	24	2.705220437	0.087113285
ENSMUSG00000081600	Gm12286	predicted gene 12286 [Source:MGI Symbol;Acc:MGI:3649467]	0.331506	0.505454	0.51502	0.760687	0.673697	0.618494	0.404499	0.636179	0.517555	0.907348	0.91199	0.513228	0.788494	0.424216	0.042812373	0.356048751	20	8	24	0.020061707	0.565108098	24	13.81111152	0.145952303
ENSMUSG00000081603	Gm14681	predicted gene 14681 [Source:MGI Symbol;Acc:MGI:3705734]	157.286	181.191	192.886	174.802	184.235	159.609	163.544	163.404	191.555	141.283	173.748	170.531	178.957	178.632	0.148073804	0.590732968	12	12	24	0.567271994	0.843774904	24	7.469052242	0.053572745
ENSMUSG00000081611	Gm13651	predicted gene 13651 [Source:MGI Symbol;Acc:MGI:3649524]	11.9082	12.0226	10.8542	9.53443	11.4075	11.2495	11.5666	10.709	11.031	9.91657	10.4184	11.5918	10.6055	11.7505	0.04467378	0.365845448	8	8	24	0.023383596	0.581965446	24	0.148953308	0.085594914
ENSMUSG00000081629	Eif3s6-ps1	eukaryotic translation initiation factor 3, subunit 6, pseudogene 1 [Source:MGI Symbol;Acc:MGI:103203]	4.05006	6.14793	4.96209	5.31883	6.06958	4.74793	5.55206	4.40311	6.11882	4.57939	4.70844	3.96026	5.26545	4.5924	0.93447422	0.966018836	12	12	24	0.58273083	0.851636365	24	9.208372238	0.07281948
ENSMUSG00000081643	Gm11605	predicted gene 11605 [Source:MGI Symbol;Acc:MGI:3651339]	26.5993	24.2636	25.3999	27.5967	28.8795	30.9382	24.4406	25.1948	19.9193	22.4407	21.2573	19.7409	21.0377	20.119	0.881996584	0.966018836	20	12	24	0.221041675	0.768040178	24	18.17390912	0.091528587
ENSMUSG00000081651	Gm15530	predicted gene 15530 [Source:MGI Symbol;Acc:MGI:3782978]	2.14223	2.32633	1.10779	1.04327	1.89026	1.77724	0.744366	1.72458	1.45707	1.07908	1.67113	1.1141	1.43481	1.16015	0.714044782	0.966018836	8	8	24	0.684025938	0.895068737	24	22.30690613	0.08869432
ENSMUSG00000081669	Npm3-ps1	nucleoplasmin 3, pseudogene 1 [Source:MGI Symbol;Acc:MGI:894683]	7.78513	5.88772	5.83349	5.59961	6.57398	5.10757	5.73153	6.23859	6.03756	5.65493	6.87858	5.50035	6.5413	6.16508	0.510431574	0.966018836	4	20	24	0.774842602	0.927711986	24	0.022601232	0.037989128
ENSMUSG00000081700	Atp5k-ps2	ATP synthase, H+ transporting, mitochondrial F1F0 complex, subunit E, pseudogene 2 [Source:MGI Symbol;Acc:MGI:107356]	15.4867	16.4463	20.6441	25.4084	15.349	22.5885	14.1691	18.3544	11.3276	13.1924	9.63183	11.4083	15.4	18.344	0.833406028	0.966018836	8	12	24	0.741852469	0.916071144	24	5.446463678	0.105933852
ENSMUSG00000081718	Gm8261	predicted gene 8261 [Source:MGI Symbol;Acc:MGI:3647364]	1.55719	1.62226	1.61124	2.13876	1.56674	1.36109	1.6693	1.56638	1.47976	1.45517	1.8832	1.57167	1.18767	1.44205	0.891105762	0.966018836	20	4	24	0.208244827	0.768040178	24	12.97754999	0.076273648
ENSMUSG00000081729	Hspa9-ps1	heat shock protein 9, pseudogene 1 [Source:MGI Symbol;Acc:MGI:96246]	0.649076	0.626473	0.560197	0.902775	0.656604	0.689563	0.629093	0.64334	0.336982	0.473032	0.432525	0.511838	0.401421	0.431325	0.924770496	0.966018836	16	20	24	0.478684749	0.805393068	24	18.33770125	0.048054715
ENSMUSG00000081731	Calr-ps	calreticulin, pseudogene [Source:MGI Symbol;Acc:MGI:3651104]	2.24075	1.76429	2.43197	1.69109	3.49217	2.57804	1.9656	1.8782	1.35253	0.905553	1.5249	1.20331	1.83848	1.56982	0.835401638	0.966018836	20	20	24	0.13691109	0.747972781	24	19.29991073	0.124629745
ENSMUSG00000081752	Sms-ps	spermine synthase, pseudogene [Source:MGI Symbol;Acc:MGI:3705601]	41.0644	41.946	43.2363	43.3156	41.4344	44.947	41.5958	40.5535	38.8782	45.9221	40.5581	43.9583	44.6363	45.9144	0.912686312	0.966018836	24	12	24	0.941991891	0.985597432	24	18.58310348	0.009877401
ENSMUSG00000081758	Gm11460	predicted gene 11460 [Source:MGI Symbol;Acc:MGI:3649774]	3.25591	3.38543	1.76377	3.64228	3.11429	3.71055	1.96994	5.913	2.61286	3.09774	2.88079	1.72159	2.3397	2.2161	0.847846021	0.966018836	8	4	24	0.964803018	0.99124136	24	9.60536807	0.038770968
ENSMUSG00000081769	Gm12216	predicted gene 12216 [Source:MGI Symbol;Acc:MGI:3650088]	1.23742	1.52818	2.16565	1.41063	2.03937	2.10026	1.15389	2.58827	2.80322	1.53735	2.09816	1.62623	1.801	2.16514	0.286642595	0.793714531	12	16	24	0.318908179	0.768040178	24	8.832610585	0.14198153
ENSMUSG00000081772	Gm13131	predicted gene 13131 [Source:MGI Symbol;Acc:MGI:3651062]	2.29465	1.79529	2.75978	4.25077	2.95129	2.51778	4.61938	3.35323	2.23449	2.70109	3.93262	3.00291	2.94406	2.40527	0.542718076	0.966018836	20	12	24	0.442085264	0.805393068	24	16.51752179	0.140965996
ENSMUSG00000081787	Gm13991	predicted gene 13991 [Source:MGI Symbol;Acc:MGI:3651689]	53.5528	43.3181	45.8516	44.3675	42.4074	48.166	47.6771	46.1467	40.2901	51.7083	40.538	51.7005	46.4074	48.0054	0.160842982	0.620479606	24	20	24	0.309032228	0.768040178	24	22.78315419	0.074930646
ENSMUSG00000081788	Gm5898	predicted gene 5898 [Source:MGI Symbol;Acc:MGI:3644921]	8.26213	8.4418	8.42658	8.47134	7.90374	8.41171	8.93067	9.73547	7.31741	9.63825	6.59208	9.37371	8.25851	8.84324	0.702630284	0.966018836	8	12	24	0.523344511	0.821170929	24	2.937362911	0.059651996
ENSMUSG00000081792	Anp32b-ps1	acidic (leucine-rich) nuclear phosphoprotein 32 family, member B, pseudogene 1 [Source:MGI Symbol;Acc:MGI:3651262]	17.8712	14.001	15.3509	18.2902	16.8771	15.7142	19.1698	18.0007	14.6988	19.1524	15.4254	15.8742	17.4203	15.2742	0.496745696	0.966018836	4	4	24	0.567271994	0.835424243	24	18.59366985	0.038619055
ENSMUSG00000081809	Gm15539	predicted gene 15539 [Source:MGI Symbol;Acc:MGI:3782987]	0.574425	0.502768	0.512383	0.338321	0.735619	0.613547	0.844594	0.353162	0.674834	0.327945	1.08375	0.445968	0.956281	0.751573	0.184631358	0.662239322	4	12	24	0.410545708	0.799546792	24	21.27411953	0.103518842
ENSMUSG00000081810	Gm11878	predicted gene 11878 [Source:MGI Symbol;Acc:MGI:3651784]	5.60208	6.515	6.25773	8.01409	6.34264	5.46883	5.96583	4.4985	6.20664	3.48191	7.00753	6.05209	6.06651	6.28914	0.838532766	0.966018836	20	4	24	0.931054363	0.982245205	24	17.19111156	0.036939933
ENSMUSG00000081819	Gm12722	predicted gene 12722 [Source:MGI Symbol;Acc:MGI:3652209]	7.28582	7.3768	7.24913	6.60152	9.0588	8.50041	6.93315	8.40895	5.19597	4.57708	6.20029	5.6318	6.6008	6.757	0.863237861	0.966018836	8	8	24	0.256040609	0.768040178	24	22.58167921	0.13563449
ENSMUSG00000081824	Ndufs5-ps	NADH:ubiquinone oxidoreductase core subunit S5, pseudogene [Source:MGI Symbol;Acc:MGI:3612445]	85.9806	83.1016	91.3865	94.0957	88.6911	90.4788	89.1861	78.085	70.6626	70.2673	86.2056	62.0235	72.6262	74.7454	0.860560779	0.966018836	16	16	24	0.723297986	0.909188168	24	16.47790949	0.042005666
ENSMUSG00000081865	Gm15484	predicted gene 15484 [Source:MGI Symbol;Acc:MGI:3705726]	2.75253	3.25193	3.44778	2.74004	2.81595	2.67785	2.55351	3.37103	2.49077	2.49096	2.50603	2.36921	2.47397	3.04819	0.229314076	0.728394638	8	16	24	0.04885236	0.638992719	24	5.786040617	0.10621969
ENSMUSG00000081871	Gm11488	predicted gene 11488 [Source:MGI Symbol;Acc:MGI:3650705]	2.95492	4.58979	4.32479	3.64491	3.99231	3.6074	3.60347	4.21915	4.57666	3.86737	3.74696	3.7725	3.14423	3.61533	0.002004172	0.100255953	12	16	24	0.064077209	0.663876076	24	9.046251212	0.126291949
ENSMUSG00000081883	Gm11821	predicted gene 11821 [Source:MGI Symbol;Acc:MGI:3649777]	0.389604	0.717746	0.688483	0.446894	0.911346	0.663512	0.713026	0.697717	0.498911	0.546982	0.73674	0.776292	0.713645	0.400042	0.369262716	0.893596186	20	20	24	0.340356174	0.774739261	24	18.62587353	0.082127147
ENSMUSG00000081887	Gm12097	predicted gene 12097 [Source:MGI Symbol;Acc:MGI:3651582]	10.5569	10.643	10.1403	10.5046	10.2364	8.90123	10.0672	10.3823	10.5366	9.9677	9.87691	10.4051	11.0454	12.5103	0.082282215	0.462922111	8	12	24	0.058420605	0.658304792	24	5.025810425	0.07849129
ENSMUSG00000081888	Spcs2-ps	signal peptidase complex subunit 2, pseudogene [Source:MGI Symbol;Acc:MGI:3650451]	3.53391	4.09338	4.42456	4.03343	3.37515	3.34023	2.99428	2.94806	3.97889	3.35112	3.52557	3.24586	3.69511	3.70127	0.068712455	0.432567661	12	12	24	0.119346294	0.730262024	24	8.773449441	0.101833803
ENSMUSG00000081895	Rpl17-ps10	ribosomal protein L17, pseudogene 10 [Source:MGI Symbol;Acc:MGI:3642871]	52.7397	63.4312	61.9255	59.93	59.8836	57.2158	59.4252	58.2725	82.845	53.1445	73.4599	64.0937	73.7902	69.6862	0.914502381	0.966018836	12	12	24	0.550133588	0.83543097	24	7.386138356	0.07068103
ENSMUSG00000081907	Gm13731	predicted gene 13731 [Source:MGI Symbol;Acc:MGI:3650010]	1.85144	3.65765	2.98685	1.98197	2.54559	2.42588	6.66961	1.58276	4.93921	2.45309	4.20893	3.75669	4.53478	0.775554	0.933543173	0.966018836	4	4	24	0.763207134	0.923599528	24	19.56512182	0.171050212
ENSMUSG00000081911	Mrps36-ps2	mitochondrial ribosomal protein S36, pseudogene 2 [Source:MGI Symbol;Acc:MGI:3704298]	18.1935	16.2069	17.0019	18.0347	15.9107	20.2117	18.6342	14.4794	19.4968	16.4292	21.2194	17.3498	20.2544	19.7969	0.843639218	0.966018836	4	4	24	0.638765457	0.877476407	24	19.84050492	0.054756981
ENSMUSG00000081920	Selenot-ps	selenoprotein T, pseudogene [Source:MGI Symbol;Acc:MGI:3705375]	4.36628	4.52177	5.44907	5.6038	5.31082	5.28213	3.8434	5.95785	4.5526	4.17599	5.0157	5.24533	3.60548	3.79374	0.200145651	0.684570416	16	12	24	0.236895682	0.768040178	24	13.95250536	0.112430823
ENSMUSG00000081921	Gm12858	predicted gene 12858 [Source:MGI Symbol;Acc:MGI:3651259]	1.52026	0.376538	4.09144	4.08885	2.10686	2.00508	1.82552	1.30403	2.53773	2.01449	0.992071	1.02329	2.64746	3.78691	0.435596482	0.966018836	12	20	24	0.343404339	0.777109916	24	9.578630823	0.320793203
ENSMUSG00000081929	Rps11-ps2	ribosomal protein S11, pseudogene 2 [Source:MGI Symbol;Acc:MGI:3651796]	3.97253	3.39488	3.56708	3.71406	4.64116	2.90738	4.15647	4.12784	3.25306	3.08037	2.79218	3.09471	3.31449	1.8874	0.904831057	0.966018836	4	20	24	0.75211024	0.91939047	24	19.03350809	0.059438343
ENSMUSG00000081944	Gm12276	predicted gene 12276 [Source:MGI Symbol;Acc:MGI:3649700]	3.20498	3.95014	4.09717	4.19266	3.77592	4.45503	3.51522	4.84046	6.08013	4.8237	5.06346	4.20817	3.37353	4.8419	0.089766625	0.477784783	12	16	24	0.029815039	0.596648309	24	10.10215171	0.179958617
ENSMUSG00000081952	Gm12178	predicted gene 12178 [Source:MGI Symbol;Acc:MGI:3651496]	3.37826	2.91455	4.44134	2.6864	4.19763	3.58084	2.04115	4.0242	2.27882	2.07688	2.26852	1.89211	2.61958	3.21637	0.94086481	0.966018836	8	16	24	0.7913136	0.933267004	24	4.924881004	0.07493099
ENSMUSG00000081957	Ak3l2-ps	adenylate kinase 3-like 2, pseudogene [Source:MGI Symbol;Acc:MGI:3574349]	1.56695	0.954107	1.27702	0.89398	1.28312	1.10568	1.62211	1.04859	1.60157	1.19099	0.751134	0.953151	1.09929	1.2391	0.714044782	0.966018836	4	20	24	0.432182668	0.805393068	24	3.213630226	0.087294564
ENSMUSG00000081967	Gm14017	predicted gene 14017 [Source:MGI Symbol;Acc:MGI:3702155]	21.8436	24.2355	25.8294	25.1217	26.6691	24.7243	20.1166	22.4604	20.1547	18.3962	24.1578	18.1612	22.2972	24.7255	0.708020493	0.966018836	20	4	24	0.902864693	0.97295148	24	11.6797663	0.029227755
ENSMUSG00000081975	Gm12482	predicted gene 12482 [Source:MGI Symbol;Acc:MGI:3651627]	0.341861	0.780295	0.835663	0.38959	1.65201	0.481802	0.624249	0.533349	0.407425	0.508427	0.52622	0.657343	0.400014	0.386977	0.94499634	0.966018836	20	8	24	0.475030042	0.805393068	24	15.77946724	0.11712523
ENSMUSG00000081984	Dnajb3	DnaJ heat shock protein family (Hsp40) member B3 [Source:MGI Symbol;Acc:MGI:1306822]	1.38666	1.43244	0.827022	1.07824	1.30678	1.19436	1.14636	1.32237	1.43627	1.27071	1.41567	1.39672	1.87627	1.06769	0.864782464	0.966018836	4	12	24	0.370974412	0.792839127	24	21.90435649	0.080938632
ENSMUSG00000081992	Gm13408	predicted gene 13408 [Source:MGI Symbol;Acc:MGI:3649613]	0.626856	1.08234	0.95401	0.430352	0.561604	0.461383	0.916998	0.72847	0.898077	0.372802	0.55927	0.680818	0.794472	0.469982	0.032547207	0.324908923	12	4	24	0.086682639	0.70594329	24	3.371489142	0.146575705
ENSMUSG00000081999	Gm13461	predicted gene 13461 [Source:MGI Symbol;Acc:MGI:3650103]	67.1433	61.0763	62.7948	61.8208	63.7098	64.4597	70.6681	62.251	59.9049	60.0689	61.0194	65.0653	62.7625	61.611	0.027467587	0.308438477	4	8	24	0.00741014	0.47735824	24	21.95171274	0.066789118
ENSMUSG00000082016	Pgam1-ps2	phosphoglycerate mutase 1, pseudogene 2 [Source:MGI Symbol;Acc:MGI:3645709]	74.6159	57.8691	62.8422	60.589	57.3002	72.5714	69.4956	71.0474	51.2971	83.754	60.9396	72.3859	70.5805	72.1326	0.431765373	0.966018836	24	12	24	0.41593932	0.800710663	24	22.3481275	0.095928267
ENSMUSG00000082028	Gm1826	predicted gene 1826 [Source:MGI Symbol;Acc:MGI:3037684]	3.09951	2.72757	3.06884	3.97182	3.6349	2.86977	3.88076	2.92081	2.63433	2.80881	2.84533	2.8341	3.25255	2.84145	0.702220766	0.966018836	4	8	24	0.618456556	0.868968106	24	18.01382805	0.055911779
ENSMUSG00000082029	H3f3c	H3 histone, family 3C [Source:MGI Symbol;Acc:MGI:3650546]	16.0529	18.3484	18.2864	17.6992	16.4135	15.1494	15.1357	15.767	21.5279	13.195	16.1447	19.9512	20.7581	18.3629	0.93717134	0.966018836	12	4	24	0.51847177	0.818673182	24	4.820557374	0.083335202
ENSMUSG00000082044	Snrpert	small nuclear ribonucleoprotein E, pseudogene [Source:MGI Symbol;Acc:MGI:3650419]	76.1713	80.8238	86.0551	78.5651	79.6458	80.8227	76.4199	77.1823	78.9899	61.1889	80.9879	70.6881	81.0633	75.3928	0.874054778	0.966018836	12	4	24	0.771489688	0.926312181	24	1.692816575	0.030995617
ENSMUSG00000082045	Gm11585	predicted gene 11585 [Source:MGI Symbol;Acc:MGI:3651395]	4.62733	6.33769	6.20172	5.70922	6.42587	5.09384	6.5868	5.60885	6.49733	4.36088	5.64771	6.57476	6.98728	5.59287	0.887862353	0.966018836	4	12	24	0.781616519	0.929899462	24	1.878334076	0.047063313
ENSMUSG00000082051	Gm16072	predicted gene 16072 [Source:MGI Symbol;Acc:MGI:3801776]	0.572286	0.755589	0.622843	0.639427	0.686657	0.643367	0.721418	0.546478	0.967233	0.602358	0.654588	0.715255	0.688694	0.688409	0.862909389	0.966018836	12	4	24	0.774534767	0.927605007	24	7.548382527	0.024741348
ENSMUSG00000082070	Gm1866	predicted gene 1866 [Source:MGI Symbol;Acc:MGI:3037724]	8.81116	10.4692	11.9182	9.5357	10.1754	8.71489	9.30446	9.06341	10.1433	8.19169	9.72903	8.97358	11.4049	8.87289	0.8972237	0.966018836	12	12	24	0.674005758	0.890479233	24	6.852329515	0.050852679
ENSMUSG00000082087	Gm12138	predicted gene 12138 [Source:MGI Symbol;Acc:MGI:3651749]	5.14502	6.61311	4.66232	6.36249	7.96413	5.63915	6.21517	5.82522	7.57791	5.26556	6.55392	6.75562	6.72081	5.80366	0.933543173	0.966018836	20	20	24	0.667637124	0.887585935	24	16.76628467	0.064639197
ENSMUSG00000082088	Gm15753	predicted gene 15753 [Source:MGI Symbol;Acc:MGI:3783195]	0.353445	0.628829	0.816862	0.862205	0.794362	0.527143	0.377434	0.435397	1.02627	0.525375	0.607987	0.563035	0.558866	0.691945	0.004320961	0.140598674	12	16	24	0.018715778	0.560647889	24	10.63806173	0.167299162
ENSMUSG00000082100	Glns-ps1	glutamine synthetase pseudogene 1 [Source:MGI Symbol;Acc:MGI:95740]	11.309	10.5688	15.7826	15.6147	11.7485	14.7007	12.2307	11.29	11.0236	15.0792	10.6174	11.1494	10.5988	10.6279	0.17882595	0.655480352	16	16	24	0.138807116	0.748341961	24	12.70953132	0.14329091
ENSMUSG00000082120	Gm15720	predicted gene 15720 [Source:MGI Symbol;Acc:MGI:3783164]	494.762	501.713	469.751	444.426	459.316	471.096	446.718	393.211	420.329	347.12	431.029	436.456	427.948	436.05	0.881718085	0.966018836	24	16	24	0.121367242	0.733729587	24	22.72132002	0.083930998
ENSMUSG00000082134	Gm15236	predicted gene 15236 [Source:MGI Symbol;Acc:MGI:3645927]	2.79641	2.44562	3.03074	3.34467	3.95026	4.3376	4.51498	3.61701	2.64812	2.59074	3.14782	3.8747	3.27325	1.87795	0.094000699	0.490854203	24	8	24	0.038312576	0.61140397	24	19.15455987	0.24490973
ENSMUSG00000082144	Gm12788	predicted gene 12788 [Source:MGI Symbol;Acc:MGI:3649859]	1.94271	1.25504	1.80177	1.23073	1.4013	0.75911	0.66998	0.971709	1.49545	0.918941	1.30017	1.45588	0.944203	1.0955	0.956794505	0.96629412	12	16	24	0.72980071	0.911863904	24	10.4875228	0.071924496
ENSMUSG00000082145	Gm12312	predicted gene 12312 [Source:MGI Symbol;Acc:MGI:3649321]	5.06015	3.99771	3.60513	5.20171	5.98489	4.11719	3.93896	5.17826	4.57681	3.61876	5.05254	5.79331	5.10148	4.12868	0.729086532	0.966018836	20	16	24	0.249002451	0.768040178	24	18.56611504	0.13056761
ENSMUSG00000082154	Gm16464	predicted gene 16464 [Source:MGI Symbol;Acc:MGI:3643716]	4.38867	3.37908	2.8269	4.06092	3.67194	3.68083	3.48435	3.85619	3.34809	4.36298	3.40158	3.80014	3.88568	3.27158	0.289105414	0.794869797	16	20	24	0.457302565	0.805393068	24	18.8384202	0.068650552
ENSMUSG00000082160	Gm11578	predicted gene 11578 [Source:MGI Symbol;Acc:MGI:3650505]	80.1188	62.6111	75.1645	63.5715	63.526	78.8343	66.4815	62.6079	48.1085	66.5284	77.5686	59.9306	64.2292	60.7847	0.671726933	0.966018836	4	4	24	0.187211699	0.768040178	24	19.48021237	0.129726682
ENSMUSG00000082163	Gm14276	predicted gene 14276 [Source:MGI Symbol;Acc:MGI:3701955]	0.429231	0.944124	0.932991	0.980721	0.853155	1.17263	0.958777	0.51106	1.02421	1.13467	1.02976	1.14404	0.764219	1.18298	0.3576519	0.882035609	24	4	24	0.248611778	0.768040178	24	13.32732626	0.107063206
ENSMUSG00000082183	Gm15994	predicted gene 15994 [Source:MGI Symbol;Acc:MGI:3801929]	0.673298	0.85005	1.6425	0.756954	0.616713	0.467572	0.407985	1.04092	0.79783	0.33437	0.569627	0.545441	0.907834	0.259741	0.588208558	0.966018836	12	12	24	0.359536674	0.785405943	24	6.857880537	0.143980956
ENSMUSG00000082185	Gm12428	predicted gene 12428 [Source:MGI Symbol;Acc:MGI:3650848]	5.72209	4.69762	3.99246	4.52013	3.8971	5.91054	4.47592	4.54246	2.79355	3.92159	4.31012	3.52428	3.0655	2.22958	0.887862353	0.966018836	24	12	24	0.046163431	0.633283523	24	19.48387216	0.194087758
ENSMUSG00000082189	Gm4784	predicted gene 4784 [Source:MGI Symbol;Acc:MGI:3648818]	0.834921	0.886152	0.363416	0.632128	1.00458	0.938829	0.670432	0.921217	0.506029	0.614459	0.493816	0.575948	0.412055	0.495279	0.921741413	0.966018836	24	12	24	0.316653987	0.768040178	24	20.55047506	0.096891529
ENSMUSG00000082192	Gm14719	predicted gene 14719 [Source:MGI Symbol;Acc:MGI:3705771]	13.662	9.28772	9.61683	7.61761	4.29927	5.89287	9.35963	14.6059	13.6786	13.5663	13.0143	14.1147	13.8889	12.9969	0.745199643	0.966018836	4	16	24	0.153907364	0.760223561	24	4.596038961	0.309647877
ENSMUSG00000082194	Gm12444	predicted gene 12444 [Source:MGI Symbol;Acc:MGI:3650375]	20.4433	20.4315	21.5006	20.0381	22.0285	20.4638	21.2635	19.8848	21.2169	18.6237	21.4292	20.2293	22.1296	20.2567	0.481530854	0.966018836	20	20	24	0.693795924	0.898364349	24	21.36794126	0.02255228
ENSMUSG00000082195	Gm13034	predicted gene 13034 [Source:MGI Symbol;Acc:MGI:3650252]	0.682738	0.695246	0.771729	0.636548	0.643069	0.690382	0.770597	0.637557	0.827253	0.815023	0.925487	0.874591	0.575065	0.931795	0.920858634	0.966018836	12	16	24	0.659588599	0.884879972	24	11.05555906	0.030684759
ENSMUSG00000082197	Gm8577	predicted gene 8577 [Source:MGI Symbol;Acc:MGI:3779807]	1.25538	1.05516	1.47022	1.3847	1.15663	1.31578	0.589121	1.55651	0.791455	1.1484	1.09572	1.44686	0.996945	0.757093	0.941830768	0.966018836	24	4	24	0.548830699	0.834692497	24	15.20753647	0.082872533
ENSMUSG00000082223	Gm15079	predicted gene 15079 [Source:MGI Symbol;Acc:MGI:3705703]	1.35205	1.34183	2.91262	2.91823	1.41196	1.07423	4.05949	1.16219	3.16209	3.13534	2.21199	1.81477	0.469591	0.558236	0.066882443	0.425513244	12	20	24	0.15691691	0.763141258	24	12.00951623	0.385640122
ENSMUSG00000082226	Gm715	predicted gene 715 [Source:MGI Symbol;Acc:MGI:2685561]	0.947683	0.790301	0.907161	0.697038	0.625501	0.776285	0.971895	0.951627	0.621865	1.05172	1.12255	0.941183	1.00884	1.07239	0.969174623	0.970883022	4	8	24	0.530660174	0.824858059	24	0.608877144	0.049359424
ENSMUSG00000082229	Nap1l2	nucleosome assembly protein 1-like 2 [Source:MGI Symbol;Acc:MGI:106654]	55.6038	51.7687	53.9843	47.9502	45.1816	52.058	54.2642	46.7458	42.7314	51.771	50.9479	46.3	45.9932	53.2074	0.893609703	0.966018836	4	16	24	0.609610282	0.864491738	24	0.438095034	0.042913284
ENSMUSG00000082241	Gng5-ps	G protein subunit gamma 5, pseudogene [Source:MGI Symbol;Acc:MGI:3783217]	8.76123	10.6452	12.8582	8.55834	11.0172	8.39055	7.65098	14.3353	15.9498	6.3323	14.0177	10.7346	16.6626	8.61581	0.924770496	0.966018836	12	4	24	0.757135048	0.92154584	24	5.59484356	0.117002547
ENSMUSG00000082252	Gm11204	predicted gene 11204 [Source:MGI Symbol;Acc:MGI:3650271]	13.3084	19.3578	21.3877	6.97427	13.1394	10.1007	16.1161	11.1968	13.249	13.1811	10.5055	4.6143	11.9555	8.77799	0.22872448	0.72665454	12	12	24	0.25678881	0.768040178	24	5.715231754	0.290876071
ENSMUSG00000082253	Gm14639	predicted gene 14639 [Source:MGI Symbol;Acc:MGI:3641909]	0.226325	1.06349	0.861247	1.16659	0.930398	0.865945	1.03501	0.759244	0.695149	0.44863	1.08821	0.830689	0.452622	0.508171	0.711168999	0.966018836	20	8	24	0.450122024	0.805393068	24	14.29155255	0.116743003
ENSMUSG00000082264	Gm12799	predicted gene 12799 [Source:MGI Symbol;Acc:MGI:3652118]	3.66652	3.43034	3.37941	3.54391	3.33314	3.74508	3.87656	3.83017	3.36978	3.38121	3.94721	2.92467	3.20154	3.11641	0.823246279	0.966018836	20	16	24	0.884510853	0.967066659	24	19.53437987	0.01893505
ENSMUSG00000082272	Gm11675	predicted gene 11675 [Source:MGI Symbol;Acc:MGI:3650970]	4.99609	4.471	5.69019	6.7412	6.66472	5.62707	5.29393	5.74436	5.03277	4.834	3.92037	4.29186	4.08508	3.6087	0.864782464	0.966018836	12	20	24	0.258376412	0.768040178	24	13.71705704	0.108546698
ENSMUSG00000082274	Gm14026	predicted gene 14026 [Source:MGI Symbol;Acc:MGI:3649712]	10.3572	10.0446	9.91783	10.6122	9.5654	11.7721	10.3626	10.439	9.13803	11.2186	10.156	10.22	9.43951	10.0124	0.581070934	0.966018836	12	4	24	0.49806836	0.807927978	24	18.07947447	0.040959329
ENSMUSG00000082283	Gm14472	predicted gene 14472 [Source:MGI Symbol;Acc:MGI:3650138]	1.00903	0.842932	1.45434	0.370663	1.20429	0.730948	0.485371	0.586443	0.45221	0.668395	0.89611	1.32998	1.37899	0.80452	0.933431033	0.966018836	20	20	24	0.599200497	0.859466291	24	20.54040726	0.118533057
ENSMUSG00000082286	Ptsd-ps1	phosphatidylserine decarboxylase, pseudogene 1 [Source:MGI Symbol;Acc:MGI:3842428]	4.40687	4.30354	5.58407	5.58801	4.93356	4.67622	4.81215	5.3664	4.95343	4.45276	4.82058	4.08456	4.23549	3.70602	0.149537283	0.594118663	12	20	24	0.143498822	0.752803875	24	11.56853234	0.091099091

ENSMUSG00000082292	Gm12250	predicted gene 12250 [Source:MGI Symbol;Acc:MGI:3649299]	0.392858	0.871807	0.660473	0.451667	0.884335	0.528437	0.436092	2.14594	0.634823	0.353876	0.738091	0.227297	0.617669	1.03629	0.109040811	0.524035616	8	16	24	0.24417312	0.768040178	24	5.507495138	0.218934754
ENSMUSG00000082308	Gm15770	predicted gene 15770 [Source:MGI Symbol;Acc:MGI:3783212]	2.18287	2.16516	2.16477	1.61969	2.39484	1.76249	2.42488	1.69033	1.61468	2.50962	2.33439	1.5837	2.54081	1.93897	0.714044782	0.966018836	4	8	24	0.864794674	0.960819377	24	19.99839308	0.038828169
ENSMUSG00000082319	Actr3-ps	actin related protein 3, pseudogene [Source:MGI Symbol;Acc:MGI:3648640]	28.4279	23.8192	23.4609	23.3559	25.2771	24.9386	23.4692	24.0491	22.3162	26.3655	25.4537	26.749	26.5072	24.884	0.076438025	0.453790923	24	12	24	0.088087693	0.707747429	24	20.22608818	0.076596288
ENSMUSG00000082321	Gm14253	predicted gene 14253 [Source:MGI Symbol;Acc:MGI:3650994]	66.899	82.4572	73.048	72.1517	77.8057	67.4611	70.1186	70.1923	83.5935	63.2251	82.6062	72.8982	82.441	77.2102	0.836635326	0.966018836	12	12	24	0.805904267	0.938090863	24	6.662216823	0.034128087
ENSMUSG00000082361	Btc	betacellulin, epidermal growth factor family member [Source:MGI Symbol;Acc:MGI:99439]	0.539786	0.700979	0.726156	0.663532	0.484657	0.627844	0.543835	0.500587	0.531625	0.569943	0.665599	0.654764	0.668123	0.809204	0.860993221	0.966018836	8	12	24	0.727101724	0.91035631	24	5.951283892	0.026818769
ENSMUSG00000082381	Gm12928	predicted gene 12928 [Source:MGI Symbol;Acc:MGI:3652022]	3.02196	3.06362	4.02321	3.43665	2.60329	2.30292	1.77878	2.86385	2.52042	4.38225	1.99741	3.44227	3.002	2.58355	0.92655264	0.966018836	16	4	24	0.291615471	0.768040178	24	9.769335556	0.164232384
ENSMUSG00000082389	Mkrn1-ps1	makorin, ring finger protein 1, pseudogene 1 [Source:MGI Symbol;Acc:MGI:2663191]	0.937424	0.937188	1.37153	1.22715	1.11643	1.25171	0.868532	0.986036	0.862091	1.13165	1.0471	0.945887	0.752303	0.864963	0.096454606	0.501112642	16	12	24	0.019224952	0.562219749	24	13.50184549	0.104872397
ENSMUSG00000082394	Gm4596	predicted gene 4596 [Source:MGI Symbol;Acc:MGI:3782779]	27.491	28.165	25.1386	28.2613	27.639	25.3708	27.7677	26.6415	25.9639	28.1803	31.0707	30.4083	28.7519	27.041	0.137763286	0.580158868	16	20	24	0.223502562	0.768040178	24	17.98315749	0.053044597
ENSMUSG00000082396	Zfp389	zinc finger protein 389 [Source:MGI Symbol;Acc:MGI:3641940]	0.629436	1.03364	1.10081	1.15421	0.631383	1.09537	0.747005	0.551128	1.37605	1.27452	0.631897	0.759365	0.980464	0.773279	0.088274239	0.473025915	16	4	24	0.165730784	0.767020009	24	9.805047926	0.148157771
ENSMUSG00000082420	Gm6517	predicted gene 6517 [Source:MGI Symbol;Acc:MGI:3643656]	9.0911	7.80883	8.37012	7.69809	10.7216	9.80659	9.50946	11.2464	7.97597	6.9242	7.29206	6.29603	6.56024	7.88074	0.866861748	0.966018836	8	16	24	0.782984448	0.930229965	24	0.144432431	0.056309963
ENSMUSG00000082431	Prdx2-ps1	peroxiredoxin 2, pseudogene 1 [Source:MGI Symbol;Acc:MGI:1321403]	49.0542	57.8181	51.385	53.6386	61.8477	57.7787	54.317	46.1578	48.6757	41.6746	53.7216	44.6369	47.6005	43.9765	0.92655264	0.966018836	20	12	24	0.24460691	0.768040178	24	18.14952901	0.088041546
ENSMUSG00000082454	Gm12183	predicted gene 12183 [Source:MGI Symbol;Acc:MGI:3652175]	176.474	173.505	175.174	181.956	181.861	167.337	181.28	160.986	178.576	177.371	182.106	182.11	180.271	181.717	0.275008207	0.789849983	20	12	24	0.463333916	0.805393068	24	15.30446826	0.024511763
ENSMUSG00000082456	Gm11598	predicted gene 11598 [Source:MGI Symbol;Acc:MGI:3649374]	2.28481	2.40794	1.57514	3.71563	4.23575	2.52338	3.01562	4.02216	3.36809	2.19968	3.63892	2.83998	2.10798	2.42582	0.632729413	0.966018836	20	8	24	0.464867645	0.805393068	24	15.35381622	0.144919374
ENSMUSG00000082474	Gm8213	predicted pseudogene 8213 [Source:MGI Symbol;Acc:MGI:3643597]	0.433368	0.584326	0.626828	0.977996	0.42509	0.241718	0.52581	0.127928	2.05717	1.14661	1.96112	1.40005	0.485826	1.21867	0.431789666	0.966018836	12	16	24	0.103986558	0.715480277	24	11.67186879	0.312123962
ENSMUSG00000082491	Gm5909	predicted gene 5909 [Source:MGI Symbol;Acc:MGI:3646686]	14.5153	12.0385	12.9335	13.3837	13.2354	12.8861	12.654	11.7447	12.2286	12.7297	14.4494	13.4613	12.6059	14.5216	0.575387897	0.966018836	20	12	24	0.419795761	0.803639585	24	17.90111393	0.047894594
ENSMUSG00000082530	Gm12168	predicted gene 12168 [Source:MGI Symbol;Acc:MGI:3650839]	12.9694	12.0901	11.4982	7.26112	9.03155	8.58672	14.0334	11.4271	11.8232	12.4849	11.4011	10.6058	12.8836	11.1412	0.031384475	0.324385159	4	20	24	0.097366763	0.71175955	24	2.716377607	0.18240872
ENSMUSG00000082536	Gm13456	predicted gene 13456 [Source:MGI Symbol;Acc:MGI:3651389]	175.065	182.13	179.972	188.576	199.033	191.728	171.966	180.793	183.817	165.73	179.91	172.068	177.686	168.395	0.893609703	0.966018836	20	8	24	0.321949291	0.768040178	24	15.80780298	0.037558758
ENSMUSG00000082575	Eef2-ps2	eukaryotic translation elongation factor 2, pseudogene 2 [Source:MGI Symbol;Acc:MGI:3650664]	20.3041	18.1787	18.8029	19.8014	19.4231	22.7751	18.0742	20.9231	14.3193	19.2865	14.7545	16.5268	15.891	14.1239	0.888482515	0.966018836	24	12	24	0.457093732	0.805393068	24	19.09618339	0.07813514
ENSMUSG00000082588	Gm15443	predicted gene 15443 [Source:MGI Symbol;Acc:MGI:3642525]	3.02857	3.81466	3.3583	3.46661	4.07157	2.84754	3.61859	3.03295	3.7197	2.8017	3.20182	3.36566	3.23162	2.72314	0.854443826	0.966018836	20	4	24	0.865403065	0.961005713	24	13.99329397	0.026803575
ENSMUSG00000082665	Gm11470	predicted gene 11470 [Source:MGI Symbol;Acc:MGI:3649221]	1.72272	1.53746	1.61125	1.14779	1.31666	1.5668	1.29208	1.2205	1.44697	2.03623	1.39603	1.47994	1.38638	1.59403	0.794858476	0.966018836	12	8	24	0.950403999	0.988257443	24	8.648417057	0.017354279
ENSMUSG00000082674	Gm11914	predicted gene 11914 [Source:MGI Symbol;Acc:MGI:3649889]	3.02713	2.82992	2.40138	3.29941	2.75433	2.54516	2.26362	2.89587	3.24336	1.92468	3.17269	2.09849	2.96761	1.9498	0.841589986	0.966018836	20	4	24	0.953366714	0.988601783	24	13.42666518	0.022293814
ENSMUSG00000082675	Gm6382	predicted gene 6382 [Source:MGI Symbol;Acc:MGI:3646438]	38.1687	35.2898	36.8992	36.6839	35.8503	35.2088	33.3034	36.4057	37.6313	39.1756	35.2524	39.3484	38.7222	39.6558	0.882263435	0.966018836	16	4	24	0.654650759	0.883584166	24	7.673755002	0.024829375
ENSMUSG00000082691	Dynlt1-ps1	dynein light chain Tctex-type 1, pseudogene 1 [Source:MGI Symbol;Acc:MGI:3642625]	54.9382	70.5319	68.5195	54.0003	72.7879	63.9961	61.2523	67.8752	66.5732	48.8884	62.544	63.4505	63.9243	64.4007	0.25849765	0.761738864	12	4	24	0.654398344	0.883555345	24	2.275801622	0.056845541
ENSMUSG00000082693	Gm15190	predicted gene 15190 [Source:MGI Symbol;Acc:MGI:3705630]	20.849	22.6564	16.0396	19.0524	17.6794	16.6728	15.7524	15.3872	16.1456	12.4068	15.7499	15.1575	17.8888	14.4064	0.79625133	0.966018836	4	12	24	0.276951163	0.768040178	24	23.6034957	0.095807857
ENSMUSG00000082706	Gm11663	predicted gene 11663 [Source:MGI Symbol;Acc:MGI:3651529]	2.52711	0.792331	1.02475	1.39526	1.36056	0.172017	0.380902	1.65456	0.849972	0.627704	1.68591	1.87057	1.07776	0.753133	0.844448841	0.966018836	20	12	24	0.922885304	0.979430367	24	17.82805611	0.071896307
ENSMUSG00000082709	Gm12362	predicted gene 12362 [Source:MGI Symbol;Acc:MGI:3652292]	4.72829	3.96884	1.70689	3.5163	4.41634	3.84473	4.35572	2.9683	4.55981	2.6829	2.12013	2.4969	2.44102	2.63937	0.845386467	0.966018836	4	12	24	0.604733384	0.862089625	24	22.33112019	0.125909263
ENSMUSG00000082715	Gm11633	predicted gene 11633 [Source:MGI Symbol;Acc:MGI:3650676]	3.71526	3.3961	2.17029	4.03194	2.94427	3.42814	2.42749	3.67048	2.22897	3.11906	3.39554	1.84622	2.21034	1.64325	0.854540463	0.966018836	16	20	24	0.450873863	0.805393068	24	15.90592973	0.119307071
ENSMUSG00000082718	Gm14928	predicted gene 14928 [Source:MGI Symbol;Acc:MGI:3648477]	3.57554	2.97274	2.91026	2.45616	2.63806	2.71447	2.63626	2.26585	3.2077	2.19571	2.86659	2.55212	2.74405	1.75508	0.890648739	0.966018836	4	12	24	0.603953293	0.861730796	24	20.77166173	0.06141335
ENSMUSG00000082729	Gm14845	predicted gene 14845 [Source:MGI Symbol;Acc:MGI:3704515]	11.8165	12.1555	17.351	13.4067	11.7646	9.69172	17.3175	13.0486	12.5053	12.6697	15.4348	14.9487	16.6598	14.6695	0.854443826	0.966018836	4	20	24	0.688525353	0.896969506	24	5.037626741	0.074271936
ENSMUSG00000082740	Gm14127	predicted gene 14127 [Source:MGI Symbol;Acc:MGI:3652004]	2.86477	3.02525	3.36542	2.91592	3.51652	2.75402	2.73474	3.93824	3.48228	2.71216	3.81805	3.22202	3.82777	2.50672	0.860560779	0.966018836	20	20	24	0.942748664	0.985847022	24	12.83680911	0.02212919
ENSMUSG00000082743	Gm12988	predicted gene 12988 [Source:MGI Symbol;Acc:MGI:3650876]	36.0223	44.708	42.6852	37.0146	51.7501	45.5738	39.8815	40.4302	36.3821	31.9037	40.2651	33.1511	35.8983	31.292	0.892685629	0.966018836	20	8	24	0.501531614	0.80908345	24	18.03698325	0.079242048
ENSMUSG00000082755	Gm8692	predicted gene 8692 [Source:MGI Symbol;Acc:MGI:3646818]	2.92103	3.17518	2.85177	2.96565	3.43735	3.17617	1.59472	1.68116	2.27774	1.06269	2.18062	2.17091	1.76857	1.90499	0.772442842	0.966018836	20	8	24	0.526375993	0.823075573	24	18.09917075	0.11237378
ENSMUSG00000082762	Gm12366	predicted gene 12366 [Source:MGI Symbol;Acc:MGI:3649557]	2.18477	1.30909	1.58361	1.54017	1.54941	1.87367	1.29805	1.95457	1.18537	1.99879	1.2699	2.03807	1.8671	1.38338	0.850398351	0.966018836	24	12	24	0.421244047	0.804493443	24	20.77889565	0.097836596
ENSMUSG00000082779	Gm13162	predicted pseudogene 13162 [Source:MGI Symbol;Acc:MGI:3701119]	0.689619	0.697619	0.448932	0.778953	0.711063	0.385078	0.837005	0.855601	0.935714	0.273671	0.374797	0.35616	0.278136	0.422395	0.588208558	0.966018836	12	12	24	0.453673697	0.805393068	24	5.635174448	0.093651792
ENSMUSG00000082791	Gm4875	predicted gene 4875 [Source:MGI Symbol;Acc:MGI:3648108]	3.0034	2.07676	2.29231	2.04684	2.00331	2.35114	2.02524	2.79866	1.35133	2.70253	2.10472	2.32745	2.00367	1.58159	0.887862353	0.966018836	24	12	24	0.475898814	0.805393068	24	20.29473905	0.09573692
ENSMUSG00000082809	Gm14150	predicted gene 14150 [Source:MGI Symbol;Acc:MGI:3651006]	47.4942	37.5559	39.6034	36.7374	41.9535	45.1879	42.6674	42.486	35.6413	41.3382	44.6409	39.2433	39.9123	38.4318	0.097587539	0.501112642	4	8	24	0.044030571	0.628008091	24	20.28188202	0.106588069
ENSMUSG00000082820	Gm13803	predicted gene 13803 [Source:MGI Symbol;Acc:MGI:3651056]	16.105	18.6696	17.2131	16.7047	18.0655	16.3126	16.1281	18.5518	20.1215	21.0564	18.1588	22.891	24.5074	24.0938	0.880631923	0.966018836	8	20	24	0.36981161	0.792235171	24	5.510132103	0.066266107
ENSMUSG00000082836	Gm13612	predicted gene 13612 [Source:MGI Symbol;Acc:MGI:3651734]	7.44724	6.53385	8.26505	6.96792	7.26562	5.90208	7.51801	7.3559	5.27999	2.93319	4.55558	4.83172	5.14454	3.976	0.951846915	0.966018836	4	12	24	0.342196781	0.776317183	24	0.491813647	0.135316247
ENSMUSG00000082840	Gm16421	predicted gene 16421 [Source:MGI Symbol;Acc:MGI:3645976]	0.739424	0.766829	0.649809	0.720258	0.769258	0.713104	1.18121	0.842387	1.0505	0.713806	0.823677	0.669	0.793928	0.844614	0.622773557	0.966018836	4	20	24	0.484326003	0.805393068	24	3.352163078	0.051505006
ENSMUSG00000082860	Gm13204	predicted gene 13204 [Source:MGI Symbol;Acc:MGI:3651444]	1.20959	1.44367	1.24926	0.899954	1.62617	0.753629	2.06463	1.46012	1.65071	1.27882	1.53621	1.19742	1.20999	1.2235	0.581070934	0.966018836	12	12	24	0.775470742	0.927742146	24	4.828870422	0.058525458
ENSMUSG00000082872	Gm15773	predicted gene 15773 [Source:MGI Symbol;Acc:MGI:3783214]	1.85164	1.821	1.7795	1.52697	1.92369	1.91606	1.99776	1.79999	1.96285	1.61035	1.74445	1.8057	1.62088	1.53623	0.510431574	0.966018836	24	16	24	0.379479993	0.798771123	24	21.54652733	0.042176983
ENSMUSG00000082876	Gm11889	predicted gene 11889 [Source:MGI Symbol;Acc:MGI:3651112]	9.84192	14.3922	11.0343	13.6395	13.1243	11.7029	11.5357	12.3922	13.3305	12.0013	11.5166	11.5466	11.8338	11.8642	0.329755693	0.849240527	16	12	24	0.292586829	0.768040178	24	9.85668626	0.08017336
ENSMUSG00000082896	Gm5844	predicted gene 5844 [Source:MGI Symbol;Acc:MGI:3645252]	66.3363	60.7898	55.2982	61.6488	72.4856	63.3844	58.525	54.781	58.7219	45.8353	70.8935	60.6968	63.7756	57.6759	0.061981717	0.409814092	20	16	24	0.082935582	0.699403344	24	19.48238205	0.136686206
ENSMUSG00000082908	Gm13736	predicted gene 13736 [Source:MGI Symbol;Acc:MGI:3650137]	89.8907	102.859	101.748	92.0587	98.3431	95.8013	84.5228	93.8256	106.507	81.2361	109.709	97.5806	103.792	98.0305	0.94086481	0.966018836	12	16	24	0.949816782	0.988075055	24	8.310943829	0.016367865
ENSMUSG00000082925	Gm13135	predicted gene 13135 [Source:MGI Symbol;Acc:MGI:3650404]	1.52515	1.70293	1.77659	1.46705	1.51207	1.59803	1.76669	1.64813	1.7683	1.54611	1.58092	1.723	1.81123	1.68146	0.079180494	0.458678242	12	4	24	0.050795825	0.644862314	24	3.278808091	0.050374059
ENSMUSG00000082935	Gm7658	predicted gene 7658 [Source:MGI Symbol;Acc:MGI:3648029]	40.3247	48.3542	41.0291	47.0268	43.6044	40.2809	39.1859	41.6565	51.3007	35.0457	46.101	42.658	46.143	42.8267	0.892578749	0.966018836	12	12	24	0.776165732	0.928029314	24	7.642670555	0.041463397
ENSMUSG00000082938	Gm2810	predicted pseudogene 2810 [Source:MGI Symbol;Acc:MGI:3780980]	5.0932	6.73981	5.9568	6.17354	6.61858	5.91974	6.15577	6.97043	6.9573	6.0315	6.14604	6.9538	6.84849	6.09863	0.860560779	0.966018836	8	20	24	0.776771973	0.928317108	24	7.559253337	0.029612705
ENSMUSG00000082953	Gm13217	predicted gene 13217 [Source:MGI Symbol;Acc:MGI:3649713]	4.47785	5.6759	4.97873	4.78867	5.19563	4.96821	5.21731	5.13483	5.32543	5.3851	5.70721	5.19811	5.93269	5.3752	0.832293447	0.966018836	8	16	24	0.877013778	0.965814767	24	6.495554393	0.015100498
ENSMUSG00000082978	Rpsa-ps11	ribosomal protein SA, pseudogene 11 [Source:MGI Symbol;Acc:MGI:3649246]	1.09554	1.36362	0.833824	0.627643	1.31059	1.36664	0.601272	1.02927	0.860043	0.687665	0.903233	0.873907	0.401598	0.696594	0.954744729	0.966018836	20	20	24	0.624949122	0.872055388	24	19.04019107	0.076900586
ENSMUSG00000082985	Gm14042	predicted gene 14042 [Source:MGI Symbol;Acc:MGI:3650966]	0.950205	0.874761	0.839335	1.08787	0.621115	0.605609	0.434466	0.5842	1.09421	1.02028	0.652421	1.06186	0.831621	1.02313	0.463629316	0.966018836	12	16	24	0.289038226	0.768040178	24	9.179951235	0.111263646
ENSMUSG00000082988	Gm5903	predicted gene 5903 [Source:MGI Symbol;Acc:MGI:3644470]	3.38827	3.30028	3.44838	3.46402	3.30897	3.49282	2.70887	3.48936	3.13452	3.61657	3.51608	3.88363	3.12532	3.1729	0.046240749	0.375011045	24	4	24	0.242316644	0.768040178	24	14.79620589	0.059540663
ENSMUSG00000082998	Gm11221	predicted gene 11221 [Source:MGI Symbol;Acc:MGI:3651633]	12.4246	11.293	10.7969	14.2478	11.5495	11.7112	10.4154	10.9041	12.7797	8.33492	11.6376	11.3954	12.0695	11.8291	0.732668271	0.966018836	4	4	24	0.947928597	0.987280175	24	22.29876203	0.021311429
ENSMUSG00000083012	Fam220a	family with sequence similarity 220, member A [Source:MGI Symbol;Acc:MGI:1914488]	2.846	5.36736	5.18925	4.942	5.26592	4.25087	6.93609	5.29918	5.23986	3.46	3.52419	5.00753	5.63621	2.14709	0.849179819	0.966018836	4	12	24	0.969247447	0.992631251	24	22.21740952	0.03641401
ENSMUSG00000083013	Gm12978	predicted gene 12978 [Source:MGI Symbol;Acc:MGI:3705382]	4.97692	3.62299	4.51671	3.15542	3.62278	4.30148	5.44944	3.80889	3.07192	4.0629	3.58598	3.54314	3.09201	4.58567	0.673956234	0.966018836	4	16	24	0.323618793	0.768040178	24	0.172456518	0.115895805
ENSMUSG00000083022	Rps15a-ps6	ribosomal protein S15A, pseudogene 6 [Source:MGI Symbol;Acc:MGI:3650298]	12.0736	13.9572	15.7442	14.15	12.9363	11.4588	12.2956	8.80548	14.2123	11.4622	11.9521	12.5978	14.5188	14.7392	0.390686537	0.915479136	12	12	24	0.638349644	0.877212277	24	8.360530432	0.077441176
ENSMUSG00000083027	Gm13140	predicted gene 13140 [Source:MGI Symbol;Acc:MGI:3649297]	0.503272	0.478773	0.562216	0.794959	0.608742	0.650968	0.421042	0.54783	0.56235	0.620968	0.503378	0.50413	0.650397	0.469973	0.080247632	0.458851846	16	16	24	0.140030042	0.749574609	24	13.422729	0.063713394
ENSMUSG00000083041	Gmfg-ps	glia maturation factor, gamma, pseudogene [Source:MGI Symbol;Acc:MGI:3704264]	3.34868	7.46237	7.42912	9.26334	5.03246	6.52864	5.35366	6.8691	7.73766	4.83313	4.74819	4.8239	5.94266	4.67879	0.208071297	0.698704663	12	8	24	0.163248647	0.767020009	24	8.794777464	0.241217941
ENSMUSG00000083049	Gm14822	predicted gene 14822 [Source:MGI Symbol;Acc:MGI:3801968]	4.07545	1.41553	1.35604	2.42376	2.02596	2.04913	1.89019	1.36858	1.68716	2.07647	2.09182	2.47469	2.99435	2.49043	0.277087842	0.789849983	4	8	24	0.197759551	0.768040178	24	20.63614717	0.21564227
ENSMUSG00000083055	Gm14140	predicted gene 14140 [Source:MGI Symbol;Acc:MGI:3649719]	2.48345	2.08743	1.71393	2.13683	1.93077	2.22355	2.21438	1.75352	1.72885	1.76695	2.10207	2.00951	2.44921	2.21058	3.44E-04	0.049770315	4	8	24	0.017472948	0.550995814	24	22.07043372	0.120712419
ENSMUSG00000083061	Gm12191	predicted gene 12191 [Source:MGI Symbol;Acc:MGI:3651293]	1.96062	4.71927	3.06743	4.6215	3.32691	2.16217	3.6885	4.02318	2.50089	1.44202	3.2259	2.55592	2.65535	2.88973	0.7207001	0.966018836	8	16	24	0.731620539	0.912544394	24	5.648057294	0.109337832
ENSMUSG00000083064	Gm7785	predicted gene 7785 [Source:MGI Symbol;Acc:MGI:3645449]	6.60058	6.95625	7.53269	7.50082	5.79191	7.26918	6.91935	7.32167	7.70846	6.84815	6.82136	6.30068	6.3261	6.97744	0.004947794	0.141782417	12	8	24	0.057622413	0.656248849	24	7.620293638	0.088259027
ENSMUSG00000083083	Gm15382	predicted gene 15382 [Source:MGI Symbol;Acc:MGI:3705661]	0.748858	0.541998	1.04544	0.97169	1.02989	1.02115	1.04053	0.570802	1.09106	0.91951	0.8027	0.5502	0.50182	1.29957	0.515451644	0.966018836	12	16	24	0.367754661	0.791056648	24	11.07739535	0.110172036
ENSMUSG00000083097	Gm14494	predicted gene 14494 [Source:MGI Symbol;Acc:MGI:3704210]	53.6087	56.0693	54.9468	65.1231	61.1343	56.5586	60.9127	60.0102	60.5771	54.3148	56.5159	62.8135	59.7193	57.9254	0.866861748	0.966018836	24	8	24	0.857667833	0.958007588	24	15.70721919	0.017882498
ENSMUSG00000083116	Gm13410	predicted gene 13410 [Source:MGI Symbol;Acc:MGI:3650681]	3.29442	3.35148	2.55027	2.80628	3.49663	2.70613	3.1725	2.5864	2.64288	3.07579	3.20962	2.92157	3.1172	3.08385	0.217827365	0.704989686	20	16	24	0.343750269	0.777295089	24	20.05548065	0.065179505
ENSMUSG00000083139	Gm12418	predicted gene 12418 [Source:MGI Symbol;Acc:MGI:3650582]	7.24593	8.42703	7.21338	7.31338	7.29224	8.02043	6.84743	7.81101	7.90954	6.7765	7.85725	7.388	7.98922	8.31246	0.5169953	0.966018836	8	8	24	0.387810156	0.799546792	24	3.195988483	0.045263617
ENSMUSG00000083161	Gm11427	predicted gene 11427 [Source:MGI Symbol;Acc:MGI:3651774]	0.0573787	0.615333	0.871211	0.667727	0.540082	0.69134	0.551268	0.845449	0.822759	0.433117	0.465921	0.574821	0.805748	0.408874	0.581070934	0.966018836	12	8	24	0.454386693	0.805393068	24	8.320831889	0.110108288
ENSMUSG00000083169	Gm6580	predicted gene 6580 [Source:MGI Symbol;Acc:MGI:3646421]	1.62787	1.68973	1.69869	1.83418	1.84552	1.91042	1.50116	1.51993	1.52202	1.40446	1.78573	1.14134	1.52441	1.53138	0.885025617	0.966018836	20	8	24	0.53232664	0.825678486	24	14.52859257	0.043384508
ENSMUSG00000083179	Gm12693	predicted gene 12693 [Source:MGI Symbol;Acc:MGI:3650224]	54.1182	59.7285	65.3972	66.5014	67.5009	57.418	60.2515	56.364	66.179	57.0801	60.8643	60.5528	61.227	63.759	0.256685902	0.76168256	20	8	24	0.154583326	0.760257003	24	11.10983097	0.070955669
ENSMUSG00000083186	Gm11805	predicted gene 11805 [Source:MGI Symbol;Acc:MGI:3649544]	1.18814	1.34395	0.992789	1.35262	1.76472	1.28665	1.00099	1.05849	1.11985	1.02708	1.43692	1.17257	1.34525	1.03632	0.113424199	0.530777648	20	16	24	0.070686706	0.675829204	24	17.17368993	0.113293809
ENSMUSG00000083226	Gm7831	predicted gene 7831 [Source:MGI Symbol;Acc:MGI:3643841]	2.39571	1.3195	1.24419	1.18318	1.77501	1.17031	0.886621	1.11189	1.34588	1.10185	1.35047	1.47261	1.7445	1.5014	0.961536114	0.967151979	20	20	24	0.525311046	0.822381894	24	21.68993413	0.097505084
ENSMUSG00000083240	Gm13453	predicted gene 13453 [Source:MGI Symbol;Acc:MGI:3651618]	107.178	111.184	104.184	94.0518	107.255	103.458	104.542	112.803	109.884	91.699	121.064	98.8583	109.474	96.8712	0.70641939	0.966018836	8	8	24	0.6572029	0.884447211	24	23.62285365	0.039688381
ENSMUSG00000083258	Gm5939	predicted pseudogene 5939 [Source:MGI Symbol;Acc:MGI:3648845]	0.423388	0.594636	0.492613	0.454906	0.582894	0.592281	0.821097	0.538173	0.639838	0.645973	0.585057	0.537308	0.455435	0.615451	0.831272156	0.966018836	8	20	24	0.990623923	0.998255413	24	6.256503888	0.005131391
ENSMUSG00000083261	Gm7816	predicted pseudogene 7816 [Source:MGI Symbol;Acc:MGI:3646286]	84.8502	65.1278	60.847	66.2706	75.5001	68.7134	72.4278	60.3996	57.8377	54.4491	76.3237	67.896	74.7467	67.8512	0.011207621	0.204559658	20	20	24	0.009269123	0.502965379	24	20.9092059	0.181944639
ENSMUSG00000083270	Gm13498	predicted gene 13498 [Source:MGI Symbol;Acc:MGI:3650381]	1.60155	0.915796	0.989824	1.29941	1.54754	1.51237	1.35333	1.51009	0.717309	1.1883	0.900978	0.828651	0.695909	0.439329	0.800065124	0.966018836	20	12	24	0.135114518	0.746923165	24	18.35368804	0.155456233
ENSMUSG00000083280	Gm11838	predicted gene 11838 [Source:MGI Symbol;Acc:MGI:3650938]	14.4545	13.003	13.7859	18.8758	12.0944	13.6397	8.01682	14.3896	5.20826	12.5237	8.04265	8.90963	6.9189	12.2784	0.882263435	0.966018836	8	20	24	0.992240315	0.998516607	24	9.717339529	0.018672326
ENSMUSG00000083282	Ctsf	cathepsin F [Source:MGI Symbol;Acc:MGI:1861434]	31.0147	29.9531	33.5492	33.7198	29.9393	30.0289	32.5676	30.2295	34.6772	32.253	27.817	28.8062	29.0787	31.9629	0.011038458	0.202748617	16	4	24	0.029442256	0.596648309	24	8.20184477	0.085972904
ENSMUSG00000083283	Gm15361	predicted pseudogene 15361 [Source:MGI Symbol;Acc:MGI:3642247]	26.4355	29.1919	24.493	25.9657	27.4446	28.3017	28.4359	32.7808	27.6199	32.3247	30.6568	29.6313	30.656	28.5917	0.890235289	0.966018836	8	4	24	0.873901331	0.96480494	24	22.96541121	0.01962389
ENSMUSG00000083284	Gm13688	predicted gene 13688 [Source:MGI Symbol;Acc:MGI:3652062]	0.717881	0.773587	0.974796	1.22624	0.799932	1.01094	1.52896	0.239877	0.610916	0.474539	0.736247	0.805701	0.600264	0.542406	0.643325306	0.966018836	24	8	24	0.372497448	0.793806676	24	18.48682469	0.133918256
ENSMUSG00000083287	Idi1-ps1	isopentenyl-diphosphate delta isomerase, pseudogene 1 [Source:MGI Symbol;Acc:MGI:3649894]	84.6384	75.6878	72.3489	69.6786	82.8598	79.9891	84.8217	72.9404	81.879	67.774	86.8054	76.224	83.5311	78.5135	0.018810871	0.256345714	4	12	24	0.087426815	0.707548182	24	21.58534589	0.092869188
ENSMUSG00000083307	AA414768	expressed sequence AA414768 [Source:MGI Symbol;Acc:MGI:3035137]	2.86547	3.1173	2.91983	2.62693	2.80298	2.93081	3.32162	3.02498	3.57066	3.23461	3.07316	3.55529	3.40214	3.77867	0.798309651	0.966018836	8	8	24	0.044607607	0.628432994	24	3.497727133	0.065538726
ENSMUSG00000083325	Gm14121	predicted gene 14121 [Source:MGI Symbol;Acc:MGI:3651769]	18.0557	17.804	17.0549	18.2957	17.0305	16.2015	17.5932	18.567	19.1221	16.6627	17.8121	16.7109	19.245	18.8242	0.109685864	0.524718348	8	16	24	0.059000762	0.658304792	24	5.090607232	0.062465065
ENSMUSG00000083327	Vcp-rs	valosin containing protein, related sequence [Source:MGI Symbol;Acc:MGI:894298]	169.072	141.29	145.491	148.18	147.784	169.702	150.593	153.445	119.303	174.738	138.942	153.518	152.347	154.195	0.390686537	0.915479136	24	12	24	0.32553547	0.768040178	24	20.56234659	0.082650197
ENSMUSG00000083328	Gm11826	predicted gene 11826 [Source:MGI Symbol;Acc:MGI:3651142]	23.2222	25.7706	25.8244	30.3155	28.7878	20.1972	18.2507	17.0702	19.0142	10.0472	19.2291	13.7805	16.9979	12.2143	0.840483986	0.966018836	20	4	24	0.721420719	0.90866625	24	15.85270312	0.08931835
ENSMUSG00000083337	Gm11539	predicted gene 11539 [Source:MGI Symbol;Acc:MGI:3651911]	8.7279	8.33202	7.71483	7.15071	10.6826	11.1802	6.54167	9.03066	8.76692	6.77665	9.2832	7.95369	9.03998	7.3088	0.265774856	0.773510818	20	20	24	0.29757943	0.768040178	24	19.19577128	0.127542816
ENSMUSG00000083364	Llph-ps2	LLP homolog, pseudogene 2 [Source:MGI Symbol;Acc:MGI:3642864]	27.1219	26.8543	26.3667	23.9764	27.4096	24.18	23.7433	23.3268	28.6614	21.6508	26.6259	27.352	32.2248	25.9627	0.951523604	0.966018836	20	20	24	0.611565319	0.865117073	24	23.83049963	0.054336653
ENSMUSG00000083367	Gm8806	predicted gene 8806 [Source:MGI Symbol;Acc:MGI:3643511]	23.9112	24.1305	24.7654	22.631	24.6813	22.7865	22.4674	19.4226	24.0917	20.3639	23.227	24.0311	24.7175	25.2817	0.915475528	0.966018836	12	4	24	0.865382923	0.961005713	24	22.85606527	0.023254424
ENSMUSG00000083372	Gm11235	predicted gene 11235 [Source:MGI Symbol;Acc:MGI:3651442]	5.45239	4.41507	3.8502	3.43267	4.29817	4.04136	5.73012	3.33034	2.55905	1.88334	3.02583	4.49024	2.57262	3.15712	0.178701474	0.655480352	4	12	24	0.008184385	0.49666	24	22.20954321	0.300685765
ENSMUSG00000083378	Gm13196	predicted gene 13196 [Source:MGI Symbol;Acc:MGI:3651918]	3.3557	4.04467	5.45534	4.18083	2.99675	3.20156	6.09522	2.79496	2.39592	1.85053	3.14782	1.93735	2.89556	2.7447	0.823144114	0.966018836	4	12	24	0.628254333	0.872795898	24	2.427438191	0.117686473
ENSMUSG00000083392	Gm6335	predicted gene 6335 [Source:MGI Symbol;Acc:MGI:3644202]	1.03945	0.74387	0.921289	0.79631	1.21262	0.853991	0.799009	0.828338	0.55259	0.559496	0.821825	0.80887	0.53793	0.601975	0.718720526	0.966018836	20	20	24	0.063265436	0.663620042	24	18.58994652	0.103828297
ENSMUSG00000083396	Gm15542	predicted gene 15542 [Source:MGI Symbol;Acc:MGI:3782990]	97.1312	77.9106	74.2894	73.4817	82.516	80.0342	83.6609	74.5504	73.5735	69.6064	94.5629	84.2695	89.0146	84.9358	0.001765212	0.100255953	4	12	24	0.0200808	0.565108098	24	21.45551289	0.140712222
ENSMUSG00000083405	Gm15725	predicted gene 15725 [Source:MGI Symbol;Acc:MGI:3833682]	13.6384	11.0331	9.56382	12.2152	14.3307	15.3366	13.8422	13.0386	6.54868	10.7902	7.82472	8.80998	7.9951	5.98303	0.63897419	0.966018836	24	12	24	0.082626372	0.699403344	24	19.55119095	0.250666347
ENSMUSG00000083443	Gm15519	predicted pseudogene 15519 [Source:MGI Symbol;Acc:MGI:3782965]	6.9228	7.389	8.23946	7.8721	7.94057	8.1643	8.35342	8.1162	7.55754	8.23057	6.95637	7.32941	7.56875	7.82493	0.880247609	0.966018836	16	4	24	0.650191939	0.882388586	24	8.792379094	0.030504944
ENSMUSG00000083449	Gm13487	predicted gene 13487 [Source:MGI Symbol;Acc:MGI:3650141]	1.4186	1.78717	1.97421	1.94985	1.65403	1.67248	1.79916	1.26902	1.47173	1.16229	1.71491	1.23153	1.05917	1.82626	0.729086532	0.966018836	12	12	24	0.690373474	0.897541425	24	10.36227621	0.054671281
ENSMUSG00000083458	Gm5510	predicted pseudogene 5510 [Source:MGI Symbol;Acc:MGI:3648472]	28.6661	23.8064	25.1863	26.5522	26.9352	28.3408	24.1167	26.2134	24.4383	24.8656	27.0257	25.109	24.3243	21.8668	0.048771079	0.379129806	24	8	24	0.015765995	0.544099157	24	17.71203291	0.089509996
ENSMUSG00000083465	Gm11652	predicted gene 11652 [Source:MGI Symbol;Acc:MGI:3651204]	7.11647	8.38964	7.89791	8.09881	8.71051	7.91534	7.10957	7.44559	7.22396	7.52623	8.09616	7.09132	7.77021	7.46116	0.223774686	0.717349224	20	8	24	0.254606888	0.768040178	24	13.97546847	0.049683927
ENSMUSG00000083474	Gm15267	predicted gene 15267 [Source:MGI Symbol;Acc:MGI:3705447]	3.73201	5.73175	6.07433	3.91732	6.09105	5.36294	6.95788	5.43616	3.88946	6.61132	5.07377	4.29968	4.65677	4.68844	0.79625133	0.966018836	20	4	24	0.972609398	0.993250534	24	13.53679447	0.023000865
ENSMUSG00000083477	Gm5555	predicted pseudogene 5555 [Source:MGI Symbol;Acc:MGI:3648970]	24.9639	26.8306	25.1201	25.8313	26.872	28.4036	24.8981	25.1114	24.0708	25.2909	26.206	25.8803	25.8442	25.4737	0.061697418	0.409814092	20	16	24	0.088547553	0.707747429	24	18.62359378	0.047352576
ENSMUSG00000083482	Gm14706	predicted gene 14706 [Source:MGI Symbol;Acc:MGI:3646357]	4.70557	8.33414	7.85126	6.0826	9.40589	3.91608	6.17666	6.67872	5.73797	3.6843	5.09714	8.22771	7.34847	5.18013	0.849180783	0.966018836	8	8	24	0.910653161	0.9747563	24	2.458254139	0.060394069
ENSMUSG00000083537	Gm14942	predicted gene 14942 [Source:MGI Symbol;Acc:MGI:3646389]	0.465918	0.938268	2.01806	1.03846	1.3524	1.53819	0.281589	0.613839	1.57203	1.2338	1.2525	0.608367	1.42159	1.09409	0.239653024	0.741580974	12	16	24	0.115141078	0.727799063	24	11.37532535	0.271130386
ENSMUSG00000083557	Gm6877	predicted pseudogene 6877 [Source:MGI Symbol;Acc:MGI:3644703]	3.13275	4.0461	4.00592	3.3544	3.55334	3.39268	3.35867	3.94533	5.12012	3.13232	3.70034	3.15529	3.52243	3.56586	0.056291566	0.401074772	12	12	24	0.067998609	0.671988997	24	7.394425382	0.133489558
ENSMUSG00000083563	Gm13340	predicted gene 13340 [Source:MGI Symbol;Acc:MGI:3650227]	4.79076	6.31935	6.30496	7.19897	5.8841	5.57444	4.87107	6.1024	5.84759	4.62334	5.83555	3.53842	4.51917	4.66488	0.195388358	0.673387182	12	16	24	0.069324228	0.673520298	24	10.01529095	0.158041
ENSMUSG00000083567	Gm11451	predicted gene 11451 [Source:MGI Symbol;Acc:MGI:3705412]	14.7	13.7419	14.4848	12.5643	14.741	13.4423	13.9416	17.1433	17.6357	14.0665	15.4358	15.5571	16.8577	15.1566	0.896799496	0.966018836	12	4	24	0.370328019	0.792393371	24	4.153107554	0.061509275
ENSMUSG00000083595	Gm9200	predicted gene 9200 [Source:MGI Symbol;Acc:MGI:3779839]	0.466284	0.914759	0.797141	0.842782	0.750063	0.87228	0.528767	0.866451	0.888185	0.550003	0.7115	0.593313	0.598021	0.643832	0.132887428	0.567728672	12	16	24	0.301564385	0.768040178	24	9.497358113	0.077430516
ENSMUSG00000083610	Oaz2-ps	ornithine decarboxylase antizyme 2, pseudogene [Source:MGI Symbol;Acc:MGI:3644634]	27.1729	26.3411	28.4592	32.0658	26.381	30.1541	26.0132	28.1595	24.5852	32.4658	27.0729	31.9804	28.0303	29.8902	0.636884864	0.966018836	12	16	24	0.517700167	0.818405353	24	14.75695925	0.052510239
ENSMUSG00000083621	Gm14586	predicted gene 14586 [Source:MGI Symbol;Acc:MGI:3705507]	189.439	239.22	227.306	223.265	233.4	200.866	197.788	206.307	251.05	149.117	207.426	187.891	209.196	190.676	0.245061943	0.741580974	12	12	24	0.732936027	0.91285519	24	7.198130827	0.057733625
ENSMUSG00000083626	Gm4459	predicted gene 4459 [Source:MGI Symbol;Acc:MGI:3782643]	76.8045	81.3818	81.4888	91.9	79.1256	82.2678	83.8301	81.7576	84.442	90.2834	93.6211	86.1028	107.873	96.2992	0.846643334	0.966018836	16	8	24	0.890106576	0.968985114	24	9.291612827	0.017781382
ENSMUSG00000083627	Gm12502	predicted gene 12502 [Source:MGI Symbol;Acc:MGI:3650307]	3.85816	2.89029	2.63532	4.19322	2.8391	3.98615	3.64118	4.42984	2.61662	4.52123	3.88061	3.9693	4.99722	2.73867	0.195343269	0.673387182	16	20	24	0.350896572	0.780345856	24	19.71949527	0.014070878
ENSMUSG00000083649	Rasl2-9	RAS-like, family 2, locus 9 [Source:MGI Symbol;Acc:MGI:104605]	0.5609	0.475136	0.304996	0.375435	0.595006	0.583973	0.456286	0.795399	0.86205	0.463207	0.35365	0.595889	0.622546	0.209311	0.880992536	0.966018836	24	16	24	0.814591399	0.940847489	24	22.85011559	0.045008868

ENSMUSG00000083669	Mif-ps4	macrophage migration inhibitory factor, pseudogene 4 [Source:MGI Symbol;Acc:MGI:103182]	52.007	36.7034	41.6037	43.0208	33.4326	40.9844	47.6671	49.7447	35.7538	46.6484	41.5822	40.6256	45.2949	40.3126	0.45229395	0.966018836	4	16	24	0.426202431	0.805393068	24	0.671548848	0.090560625
ENSMUSG00000083670	Gm6829	predicted pseudogene 6829 [Source:MGI Symbol;Acc:MGI:3648590]	43.1563	43.4081	46.5341	39.8063	42.0273	48.4758	51.2302	44.8398	44.5045	43.0486	43.7031	43.2643	44.4084	42.8078	0.530814489	0.966018836	12	4	24	0.261977802	0.768040178	24	23.927823	0.055090888
ENSMUSG00000083672	Kpna2-ps	Kpna2 retrotransposed pseudogene [Source:MGI Symbol;Acc:MGI:3647335]	0.466572	0.71684	1.13322	1.22872	0.729865	0.637564	0.47954	0.242081	0.453041	0.486335	1.06377	0.517095	0.0640818	0.648258	0.046535108	0.375011045	20	8	24	0.046374004	0.633507364	24	12.94361521	0.235337553
ENSMUSG00000083674	Zfp133-ps	zinc finger protein 133, pseudogene [Source:MGI Symbol;Acc:MGI:2176612]	3.26626	3.37803	2.92915	2.30554	3.34519	3.18611	3.09551	3.00284	2.82008	2.82713	3.75488	2.85627	3.42086	2.76793	0.121243373	0.548623108	20	20	24	0.190187042	0.768040178	24	21.23389208	0.09297383
ENSMUSG00000083678	Gm12989	predicted gene 12989 [Source:MGI Symbol;Acc:MGI:3652245]	41.9446	43.2969	39.5653	39.6746	44.1888	37.5148	35.2978	38.2764	36.5379	32.1232	37.8451	35.5283	38.7236	36.6269	0.822716578	0.966018836	20	4	24	0.762782481	0.923472507	24	22.74402643	0.027904509
ENSMUSG00000083688	Gm13743	predicted gene 13743 [Source:MGI Symbol;Acc:MGI:3650802]	3.12506	3.53313	3.62799	2.59669	3.33546	2.52936	2.42305	3.17135	4.01911	2.53463	3.11596	3.04376	3.27182	2.88237	0.14313801	0.585944911	12	12	24	0.36930428	0.792167634	24	6.795247731	0.092895724
ENSMUSG00000083692	Gm9575	predicted gene 9575 [Source:MGI Symbol;Acc:MGI:3779984]	15.4495	15.3722	15.0459	14.7812	16.6101	16.0544	13.8412	16.3094	14.4386	15.3746	16.3174	15.0451	14.3126	15.3507	0.435739761	0.966018836	20	8	24	0.418277659	0.80286578	24	15.98304944	0.037163919
ENSMUSG00000083708	Gm13123	predicted gene 13123 [Source:MGI Symbol;Acc:MGI:3651473]	7.79546	6.47444	7.53579	8.00088	7.10693	8.66242	8.28602	8.43447	7.28001	9.42402	7.62872	8.63107	7.68406	7.7999	0.601647512	0.966018836	24	12	24	0.628327799	0.872795898	24	17.41051487	0.04438693
ENSMUSG00000083716	Gm13436	predicted gene 13436 [Source:MGI Symbol;Acc:MGI:3651005]	61.3469	63.8429	66.8402	61.7605	73.6737	59.7573	60.6595	63.4118	69.7391	52.3966	71.8911	54.8348	56.1809	53.8929	0.348367381	0.870418653	20	4	24	0.498072384	0.807927978	24	13.10411751	0.062957594
ENSMUSG00000083718	Ccnb2-ps	cyclin B2, pseudogene [Source:MGI Symbol;Acc:MGI:3779896]	2.1769	1.76674	1.39408	2.85811	2.7066	1.53919	1.61113	1.51002	2.00921	1.7473	1.74411	2.15234	2.23049	1.3118	0.481530854	0.966018836	16	16	24	0.197470601	0.768040178	24	16.27100448	0.151176184
ENSMUSG00000083734	Gm16209	predicted gene 16209 [Source:MGI Symbol;Acc:MGI:3802095]	0.688188	0.610849	0.747654	0.882217	0.703432	0.533391	0.523621	0.690222	0.820141	0.398031	0.765004	0.771975	0.460173	0.519422	0.268178777	0.774280302	12	16	24	0.460857124	0.805393068	24	12.51719622	0.059386989
ENSMUSG00000083736	Gm6039	predicted gene 6039 [Source:MGI Symbol;Acc:MGI:3648055]	1.82532	1.37378	1.27079	1.51075	1.31463	1.24633	1.91669	1.14576	1.67206	1.47102	1.63448	1.30124	1.72426	1.33118	0.675497492	0.966018836	4	20	24	0.763611284	0.923793359	24	22.43517803	0.0412746
ENSMUSG00000083744	Gm14824	predicted gene 14824 [Source:MGI Symbol;Acc:MGI:3802065]	0.971631	1.19789	0.905182	1.11043	1.96651	0.51017	0.845698	0.743888	0.473232	0.366919	1.3324	0.976395	0.880215	0.729878	0.581070934	0.966018836	20	16	24	0.386205216	0.799546792	24	17.9518174	0.152814979
ENSMUSG00000083757	Rps15a-ps4	ribosomal protein S15A, pseudogene 4 [Source:MGI Symbol;Acc:MGI:3652187]	4.72667	6.76097	6.41352	3.74943	5.93851	3.90665	4.30726	5.0375	5.61944	3.59437	5.3119	5.63018	6.18346	6.02959	0.184631358	0.662239322	8	8	24	0.380717801	0.799546792	24	3.903226811	0.134557554
ENSMUSG00000083767	Gm11405	predicted gene 11405 [Source:MGI Symbol;Acc:MGI:3651226]	0.910715	0.78186	0.712207	1.44155	1.32578	0.924455	0.938694	0.894082	1.05125	0.80831	0.765392	1.06756	0.813418	0.760705	0.313530643	0.824470524	24	8	24	0.212012197	0.768040178	24	15.59031859	0.09900315
ENSMUSG00000083774	Gm7180	predicted pseudogene 7180 [Source:MGI Symbol;Acc:MGI:3648560]	24.9409	28.7382	30.0568	31.3948	30.7979	30.0871	29.3369	26.1247	27.2885	25.4938	27.3646	26.6524	27.3905	25.6772	0.801995186	0.966018836	20	12	24	0.353979125	0.781120571	24	15.03437274	0.052497727
ENSMUSG00000083798	Gm14584	predicted gene 14584 [Source:MGI Symbol;Acc:MGI:3705780]	20.7109	20.4135	18.4769	18.4255	20.1591	19.4639	20.807	18.5417	17.8904	21.2577	19.7592	19.4766	20.7692	20.6411	0.060394272	0.407012001	4	8	24	0.269183349	0.768040178	24	21.88272055	0.048136512
ENSMUSG00000083811	Gm13071	predicted gene 13071 [Source:MGI Symbol;Acc:MGI:3651971]	2.26889	1.9077	2.45889	2.33124	2.62377	1.9893	1.37357	1.16692	1.02298	1.75173	1.78728	1.84263	2.04339	1.17279	0.696669634	0.966018836	20	12	24	0.057370891	0.656248849	24	17.0583137	0.198438545
ENSMUSG00000083813	Gm15502	predicted gene 15502 [Source:MGI Symbol;Acc:MGI:3782949]	45.6079	44.8081	42.551	61.3328	54.2517	57.7463	60.2423	59.9855	43.1414	40.2898	44.1471	50.653	39.7119	49.861	0.911371589	0.966018836	24	12	24	0.647442336	0.880898698	24	20.20604607	0.08423986
ENSMUSG00000083819	Gm11977	predicted gene 11977 [Source:MGI Symbol;Acc:MGI:3650690]	0.488806	0.730414	0.631528	0.808587	0.711306	0.783131	0.895571	0.603125	0.605934	0.80717	0.652886	0.79659	0.751493	0.771674	0.823677655	0.966018836	16	20	24	0.706300827	0.902918222	24	17.33122134	0.02966871
ENSMUSG00000083820	Ndufs6b	NADH:ubiquinone oxidoreductase subunit S6B [Source:MGI Symbol;Acc:MGI:3648526]	23.9631	22.3891	25.7192	26.0263	21.0704	23.0632	20.8137	25.2886	20.4484	19.3231	20.3818	20.1634	20.2101	17.5084	0.854443826	0.966018836	12	8	24	0.931500869	0.982348071	24	8.794923636	0.016895305
ENSMUSG00000083829	Gm2199	predicted gene 2199 [Source:MGI Symbol;Acc:MGI:3780369]	12.1391	13.3566	12.8942	17.1745	13.7995	13.8267	12.0071	13.0122	12.2188	11.9018	13.4113	11.3183	13.3494	13.3534	0.93447422	0.966018836	20	8	24	0.480133087	0.805393068	24	12.53711657	0.061883562
ENSMUSG00000083836	Ldha-ps2	lactate dehydrogenase A, pseudogene 2 [Source:MGI Symbol;Acc:MGI:96761]	11.3739	8.19805	8.64938	11.3222	8.52044	10.9045	9.75986	11.6622	10.9273	11.4472	11.9526	10.3618	11.0925	9.60984	0.886572916	0.966018836	16	16	24	0.770872553	0.926171026	24	16.68531824	0.045394748
ENSMUSG00000083844	Ube2d-ps	ubiquitin-conjugating enzyme E2D, pseudogene [Source:MGI Symbol;Acc:MGI:1913774]	12.79	13.6225	13.9538	14.4668	15.5556	14.6312	13.5172	14.156	14.6011	13.8083	13.2665	14.2188	14.3469	15.2603	0.806523736	0.966018836	24	4	24	0.668449958	0.887968245	24	11.532644	0.024329596
ENSMUSG00000083853	Gm15696	predicted gene 15696 [Source:MGI Symbol;Acc:MGI:3783137]	0.427152	0.504003	0.463396	0.803564	0.419137	0.635551	0.172777	0.504488	0.579416	0.565064	0.773396	1.28761	0.86176	1.41881	0.833301348	0.966018836	24	4	24	0.969576703	0.992868519	24	13.035576	0.020841954
ENSMUSG00000083854	Dnajc19-ps	DnaJ heat shock protein family (Hsp40) member C19, pseudogene [Source:MGI Symbol;Acc:MGI:3709029]	37.299	47.9576	48.9936	49.5671	40.0841	40.6809	42.5227	41.863	53.8593	38.5944	45.4669	39.8063	45.5666	46.2981	0.038115943	0.334069441	12	12	24	0.050025464	0.643877315	24	8.010856953	0.139682111
ENSMUSG00000083856	Gm13886	predicted gene 13886 [Source:MGI Symbol;Acc:MGI:3649359]	1.36204	1.42468	1.05825	1.29675	1.60504	1.39436	1.23522	0.976816	1.48949	0.874048	1.23262	1.04069	1.13935	1.1302	0.832742389	0.966018836	20	20	24	0.634070104	0.875589926	24	19.02534286	0.048090395
ENSMUSG00000083860	Gm13313	predicted gene 13313 [Source:MGI Symbol;Acc:MGI:3651091]	0.991758	0.745287	0.937966	0.495096	0.646202	0.833147	0.789419	0.579541	0.648167	0.569727	0.830868	0.796679	0.631501	0.482234	0.360140629	0.883787544	24	16	24	0.156512165	0.762206981	24	21.0655414	0.089323613
ENSMUSG00000083863	Gm13341	predicted gene 13341 [Source:MGI Symbol;Acc:MGI:3650228]	2.27208	3.41889	3.40538	2.14556	2.04136	2.78631	3.24421	3.77494	2.93621	1.60526	3.32645	2.55846	2.77439	1.65214	0.446653834	0.966018836	8	8	24	0.515239157	0.817300608	24	1.941372412	0.122622432
ENSMUSG00000083864	Gm13719	predicted gene 13719 [Source:MGI Symbol;Acc:MGI:3649492]	0.752278	0.672634	0.774431	0.726626	0.673188	0.742692	0.646091	0.767229	0.459247	0.523688	0.477239	0.271145	0.594122	0.376529	0.832742389	0.966018836	12	8	24	0.855121388	0.9569057	24	2.844267107	0.021335355
ENSMUSG00000083879	Gm14038	predicted gene 14038 [Source:MGI Symbol;Acc:MGI:3650657]	7.10735	9.51024	8.46541	9.33591	8.16837	8.04936	7.38379	8.951	10.1621	8.16778	7.93261	8.25118	9.72837	9.21864	0.216688884	0.704989686	8	20	24	0.052138123	0.64978825	24	7.482636242	0.118885129
ENSMUSG00000083889	E530001F21Rik	RIKEN cDNA E530001F21 gene [Source:MGI Symbol;Acc:MGI:3036288]	0.772928	1.35542	1.52738	1.36495	1.36204	1.03287	1.11303	0.878583	0.574909	1.20734	1.15614	1.25831	1.12579	1.00313	0.360675801	0.883978426	16	16	24	0.430390189	0.805393068	24	14.9947502	0.091461154
ENSMUSG00000083892	Gm1848	predicted gene 1848 [Source:MGI Symbol;Acc:MGI:3037706]	65.247	48.6336	43.3439	49.1808	54.8281	59.3622	52.8681	50.5323	44.2496	50.4276	55.2026	50.9544	55.0664	48.216	9.86E-04	0.074544544	4	8	24	0.001829637	0.38463758	24	20.41493124	0.177629045
ENSMUSG00000083898	Gm7340	predicted gene 7340 [Source:MGI Symbol;Acc:MGI:3648496]	0.500821	0.404897	0.504258	0.627002	0.608297	0.922042	0.722919	0.429007	0.942565	0.523267	0.58348	0.70644	0.879194	0.611151	0.286642595	0.793714531	24	8	24	0.548155342	0.834148407	24	19.29056355	0.062441897
ENSMUSG00000083899	Gm12346	predicted gene 12346 [Source:MGI Symbol;Acc:MGI:3649810]	318.975	283.433	261.487	270.055	303.881	272.766	274.848	261.511	288.469	242.713	351.522	309.165	329.419	312.064	0.172322601	0.648362388	4	12	24	0.231432071	0.768040178	24	20.86695638	0.095751134
ENSMUSG00000083902	Tent2-ps1	terminal nucleotidyltransferase 2, pseudogene 1 [Source:MGI Symbol;Acc:MGI:3801944]	4.38547	5.8942	4.87561	6.55518	5.23976	5.25015	5.12153	5.42811	5.34129	4.60944	4.36849	5.37555	5.08705	5.87638	0.446653834	0.966018836	8	12	24	0.574293468	0.847244567	24	6.791895248	0.059738576
ENSMUSG00000083906	Gm15186	predicted gene 15186 [Source:MGI Symbol;Acc:MGI:3705337]	0.89953	0.911812	0.759811	0.795307	1.33396	0.561928	0.850966	1.06743	0.747906	1.1233	0.887135	1.0913	0.602365	1.13604	0.916963526	0.966018836	8	4	24	0.782710618	0.930229965	24	12.33247182	0.045981136
ENSMUSG00000083907	Plk-ps1	polo like kinase, pseudogene 1 [Source:MGI Symbol;Acc:MGI:103247]	0.634399	0.34765	0.345163	0.674618	0.532064	0.654036	0.447214	0.613528	0.584353	0.53181	0.454554	0.682178	0.37286	0.597151	0.390686537	0.915479136	24	12	24	0.636647341	0.876483853	24	17.27400562	0.044530837
ENSMUSG00000083912	Gm5391	predicted gene 5391 [Source:MGI Symbol;Acc:MGI:3647141]	0.443639	0.764943	0.674714	1.41481	0.329624	0.649854	0.647951	0.553495	0.785335	0.410555	0.424715	0.455167	0.415424	0.469142	0.185796491	0.662239322	12	8	24	0.336266934	0.771737966	24	8.981891554	0.119701417
ENSMUSG00000083992	Gm11478	predicted gene 11478 [Source:MGI Symbol;Acc:MGI:3650888]	262.73	301.924	290.465	273.748	287.557	256.988	265.762	251.928	332.614	204.257	290.547	249.815	284.205	259.557	0.601647512	0.966018836	12	12	24	0.796528511	0.935045591	24	5.924294312	0.044497571
ENSMUSG00000084013	Gm14270	predicted gene 14270 [Source:MGI Symbol;Acc:MGI:3649849]	35.4158	34.5586	33.8809	31.8169	33.7313	30.5317	29.8318	33.4992	30.411	29.0207	35.761	31.017	34.2546	31.9276	0.93717134	0.966018836	20	20	24	0.781755457	0.929907172	24	23.83192389	0.025562238
ENSMUSG00000084020	Wsb2-ps	WD repeat and SOCS box-containing 2, pseudogene [Source:MGI Symbol;Acc:MGI:3649891]	6.747	4.52443	4.48007	4.98049	5.92264	6.48623	5.89307	5.82816	4.32685	6.01278	4.8877	4.50567	5.03616	4.47496	0.16079538	0.620479606	4	8	24	0.149884957	0.758419028	24	19.99455915	0.135063335
ENSMUSG00000084038	Gm11295	predicted gene 11295 [Source:MGI Symbol;Acc:MGI:3651595]	4.04249	3.7269	3.98888	4.12769	3.57559	3.73279	3.63923	2.66872	3.30959	4.15072	4.22583	3.57571	4.18979	3.35676	0.360140629	0.883787544	16	16	24	0.264748117	0.768040178	24	16.1848542	0.08695827
ENSMUSG00000084064	Gm12079	predicted gene 12079 [Source:MGI Symbol;Acc:MGI:3650745]	3.83329	2.38967	4.13601	3.65591	1.6915	3.08337	2.30471	2.48781	3.21029	3.8095	2.21386	4.14348	4.29158	3.89051	0.883024237	0.966018836	12	8	24	0.688808131	0.897070579	24	5.45587501	0.107721157
ENSMUSG00000084067	Gm14269	predicted gene 14269 [Source:MGI Symbol;Acc:MGI:3650060]	106.349	115.902	102.889	92.2881	119.877	95.225	92.6995	94.2943	108.379	81.9805	117.831	106.487	104.555	109.132	0.676344318	0.966018836	20	20	24	0.862194912	0.959779249	24	21.56891747	0.035339143
ENSMUSG00000084071	Gm13378	predicted gene 13378 [Source:MGI Symbol;Acc:MGI:3650302]	3.02847	1.17284	2.77483	2.59615	1.85954	2.17911	1.97112	1.22768	2.35805	1.38802	1.72219	3.34602	1.42159	2.73523	0.92769718	0.966018836	24	8	24	0.874411823	0.964836189	24	18.35013575	0.071140543
ENSMUSG00000084088	Gm12941	predicted gene 12941 [Source:MGI Symbol;Acc:MGI:3650501]	2.55965	1.58898	1.3787	3.45776	1.78578	2.71642	1.53714	3.85499	0.8556	2.1197	1.67728	1.71544	0.887725	2.10916	0.928148816	0.966018836	16	20	24	0.921161905	0.979223729	24	16.91931578	0.065158239
ENSMUSG00000084093	Gm16418	predicted pseudogene 16418 [Source:MGI Symbol;Acc:MGI:3644548]	67.2841	71.2992	41.9801	71.2377	41.1235	50.8359	58.3402	75.6575	71.8281	58.0055	82.3388	54.6048	59.8363	68.3302	0.339604637	0.858712466	8	16	24	0.537411964	0.827949555	24	5.339692045	0.132171942
ENSMUSG00000084094	Gm13961	predicted gene 13961 [Source:MGI Symbol;Acc:MGI:3650627]	1.43308	1.49027	1.35454	1.52671	1.63086	1.3254	1.52187	1.32798	1.41097	1.11064	1.77427	1.26764	1.44579	1.33683	0.909604019	0.966018836	20	4	24	0.601689367	0.860619632	24	17.98470666	0.03910189
ENSMUSG00000084098	Gm13422	predicted gene 13422 [Source:MGI Symbol;Acc:MGI:3650167]	18.5595	19.2116	17.5965	20.2356	17.3647	18.4323	22.6938	21.1796	19.2331	19.2631	20.3232	19.2842	18.2025	16.1891	0.801280095	0.966018836	16	20	24	0.934201285	0.983548589	24	20.38806511	0.018308635
ENSMUSG00000084099	Gm12929	predicted gene 12929 [Source:MGI Symbol;Acc:MGI:3652021]	6.61624	7.16453	9.27813	10.2437	9.79834	10.2283	11.5424	10.8034	8.68342	10.8178	6.82808	8.09663	7.75889	7.25133	0.965971228	0.969272194	16	12	24	0.652437779	0.883020861	24	12.52512323	0.089670575
ENSMUSG00000084106	Gm6136	predicted pseudogene 6136 [Source:MGI Symbol;Acc:MGI:3646897]	88.4389	87.7807	93.0116	82.4968	83.6484	87.0398	84.3459	82.4086	100.795	78.1769	95.7803	89.4479	91.2734	90.0986	0.893307667	0.966018836	12	4	24	0.82465869	0.944988393	24	4.837723557	0.023711243
ENSMUSG00000084113	Gm14277	predicted gene 14277 [Source:MGI Symbol;Acc:MGI:3650350]	23.1122	22.6836	20.3864	21.2758	24.8885	22.4864	19.5516	18.5482	20.1791	17.057	23.1613	20.2344	16.0479	19.733	0.34708369	0.870418653	20	12	24	0.18534426	0.768040178	24	17.36525385	0.098015464
ENSMUSG00000084145	Gm12263	predicted gene 12263 [Source:MGI Symbol;Acc:MGI:3650086]	4.0584	3.73144	4.35012	3.7019	4.08713	4.81253	4.66348	4.01977	3.59821	4.24933	4.41459	3.40287	3.65727	3.52823	0.81872541	0.966018836	20	12	24	0.448025764	0.805393068	24	18.07851783	0.060537795
ENSMUSG00000084159	Gm12696	predicted gene 12696 [Source:MGI Symbol;Acc:MGI:3649288]	27.598	24.8309	24.123	24.1976	25.6086	24.7094	25.8184	24.6494	24.073	18.4824	25.4086	23.2847	26.7485	24.5249	0.001845448	0.100255953	4	12	24	0.060110679	0.658304792	24	23.29332458	0.10700423
ENSMUSG00000084166	Gm6451	predicted gene 6451 [Source:MGI Symbol;Acc:MGI:3648305]	0.647439	0.584177	0.234519	0.821675	0.428893	0.650455	1.05163	0.642284	0.588446	0.76059	0.887503	0.782265	0.432247	0.757083	0.649212555	0.966018836	16	20	24	0.667441832	0.887459929	24	19.39738486	0.067268223
ENSMUSG00000084183	Gm12009	predicted gene 12009 [Source:MGI Symbol;Acc:MGI:3651311]	1.0595	1.07724	1.24455	1.07066	0.9528	1.44523	0.931088	1.2912	0.752912	1.17588	0.641687	0.72893	0.987343	0.60022	0.880631923	0.966018836	16	4	24	0.984296342	0.996767829	24	0.61225035	0.010794845
ENSMUSG00000084215	Gm11814	predicted gene 11814 [Source:MGI Symbol;Acc:MGI:3650512]	1.81647	2.10967	1.74945	2.73686	2.60127	2.47331	1.64603	3.06594	2.90578	2.26314	1.8906	2.25121	2.38604	1.74561	0.851656735	0.966018836	16	12	24	0.586380597	0.853300537	24	11.90191676	0.083161682
ENSMUSG00000084230	Gm14388	predicted gene 14388 [Source:MGI Symbol;Acc:MGI:3650926]	38.1819	41.2306	38.2491	40.7434	38.9114	34.5738	35.8222	33.6479	41.8735	35.3847	42.7382	38.1995	42.0182	42.2194	0.916556573	0.966018836	20	4	24	0.749607084	0.918524199	24	9.221352944	0.035023894
ENSMUSG00000084235	Gm15421	predicted gene 15421 [Source:MGI Symbol;Acc:MGI:3705475]	171.51	223.742	208.865	190.458	209.804	165.695	197.915	178.137	258.206	157.077	211.223	202.139	221.413	220.738	0.777666236	0.966018836	12	4	24	0.459277391	0.805393068	24	6.429801255	0.096260882
ENSMUSG00000084241	Gm13416	predicted gene 13416 [Source:MGI Symbol;Acc:MGI:3652158]	4.0761	4.48169	4.04814	3.8743	4.50236	4.28806	4.17759	4.56778	4.5289	3.88479	3.66028	3.56492	3.56351	4.12192	0.446653834	0.966018836	8	16	24	0.330619201	0.768040178	24	5.74084593	0.052979857
ENSMUSG00000084260	Gm12844	predicted gene 12844 [Source:MGI Symbol;Acc:MGI:3651053]	23.4599	24.1245	23.202	20.8305	23.2127	23.7722	22.1607	22.9284	21.7404	22.5066	19.4156	22.0966	22.7872	18.9085	0.910436409	0.966018836	4	12	24	0.428889635	0.805393068	24	22.94483628	0.041557905
ENSMUSG00000084274	Ptma-ps2	prothymosin alpha, pseudogene 2 [Source:MGI Symbol;Acc:MGI:3651567]	0.555331	0.624142	0.793381	0.48656	1.51305	0.824071	0.686052	0.96071	1.86465	0.493223	0.680359	0.608176	0.496311	1.83427	0.669104912	0.966018836	12	4	24	0.481673138	0.805393068	24	7.54390053	0.161838045
ENSMUSG00000084289	Fth1-ps	ferritin heavy polypeptide 1, pseudogene [Source:MGI Symbol;Acc:MGI:3647597]	15.1456	15.4564	15.6947	15.0915	17.7337	16.5338	17.1509	14.7818	14.5022	12.0632	11.4931	12.2578	10.3881	9.16667	0.807813307	0.966018836	20	20	24	0.558369298	0.839674578	24	17.84989745	0.068117317
ENSMUSG00000084304	Gm6142	predicted pseudogene 6142 [Source:MGI Symbol;Acc:MGI:3644670]	47.5039	49.5501	48.1943	47.859	44.4815	32.7745	41.1645	33.5989	59.2034	36.2281	58.7458	47.3278	57.0717	56.0485	0.881718085	0.966018836	12	12	24	0.759258729	0.922160315	24	7.467210894	0.083708987
ENSMUSG00000084319	Tpt1-ps3	tumor protein, translationally-controlled, pseudogene 3 [Source:MGI Symbol;Acc:MGI:2664997]	856.914	893.434	846.443	879.778	905.415	834.668	897.992	873.381	983.879	756.36	891.614	919.134	980.429	865.023	0.887862353	0.966018836	4	12	24	0.625164799	0.872055388	24	0.746537866	0.035417738
ENSMUSG00000084329	Gm6733	predicted gene 6733 [Source:MGI Symbol;Acc:MGI:3645402]	9.56035	7.80294	7.77822	9.848	8.85343	8.91476	9.23076	10.2886	8.00862	10.067	7.84305	9.1528	8.43262	7.52097	0.934152439	0.966018836	16	20	24	0.666260086	0.88724859	24	18.00476745	0.04993241
ENSMUSG00000084342	Gm11224	predicted gene 11224 [Source:MGI Symbol;Acc:MGI:3651640]	2.17806	2.93859	2.68789	2.16464	2.60558	2.26181	1.89164	2.36475	2.118	2.43501	2.56582	2.24118	2.73218	2.52864	0.882263435	0.966018836	8	20	24	0.790622431	0.932939823	24	8.10394241	0.035020415
ENSMUSG00000084347	Akt2-ps	thymoma viral proto-oncogene 2, pseudogene [Source:MGI Symbol;Acc:MGI:108506]	13.6297	13.2547	14.3256	15.7191	15.4008	15.1761	14.3878	15.7722	13.9823	16.0299	13.1795	13.2968	14.6585	14.113	0.850398351	0.966018836	16	16	24	0.482738141	0.805393068	24	11.90234666	0.043803573
ENSMUSG00000084349	Rpl3-ps1	ribosomal protein L3, pseudogene 1 [Source:MGI Symbol;Acc:MGI:3644217]	178.506	212.745	200.276	204.276	200.359	185.769	184.105	188.184	230.634	168.977	196.759	199.555	211.15	200	0.648082424	0.966018836	12	12	24	0.52429741	0.821960934	24	7.330149965	0.052986281
ENSMUSG00000084350	Znf41-ps	ZNF41, pseudogene [Source:MGI Symbol;Acc:MGI:1917255]	1.09102	1.29886	1.28318	1.46137	1.28512	1.03504	1.14008	0.980563	1.43363	0.871604	1.15455	1.01653	1.06318	1.27648	0.236893212	0.7385769	12	12	24	0.31006246	0.768040178	24	9.019934294	0.06795929
ENSMUSG00000084353	Gm15452	predicted gene 15452 [Source:MGI Symbol;Acc:MGI:3768636]	3.00407	3.49354	3.24056	4.90505	3.17575	1.87638	1.80577	3.03008	2.68256	2.3221	2.97091	3.00081	1.03593	0.814845	0.595037318	0.966018836	16	12	24	0.056445736	0.65534544	24	13.12902842	0.295117741
ENSMUSG00000084375	Gm11637	predicted gene 11637 [Source:MGI Symbol;Acc:MGI:3651875]	0.931184	0.472122	0.404583	0.847817	0.829577	0.83871	0.906448	0.580585	0.570407	0.554008	0.954564	0.597483	0.373868	0.422547	0.258307762	0.761738864	20	16	24	0.021457518	0.573684034	24	17.94437072	0.171759445
ENSMUSG00000084381	AA413626	expressed sequence AA413626 [Source:MGI Symbol;Acc:MGI:3035311]	0.242118	0.796725	1.1826	0.548477	0.35769	0.474598	0.588262	0.859853	0.575579	0.160107	0.247265	0.62381	0.731152	0.645898	0.025129001	0.292995596	8	12	24	0.070188955	0.675025526	24	4.676119824	0.211172345
ENSMUSG00000084384	Gm12251	predicted gene 12251 [Source:MGI Symbol;Acc:MGI:3652216]	45.8205	49.0113	48.0894	52.4226	46.9613	49.3351	47.6775	50.5742	50.2984	53.4471	47.7737	50.5051	51.5914	51.3496	0.676344318	0.966018836	16	4	24	0.15691255	0.763141258	24	9.224919461	0.03981136
ENSMUSG00000084416	Rpl10a-ps1	ribosomal protein L10A, pseudogene 1 [Source:MGI Symbol;Acc:MGI:3705885]	247.561	277.243	253.007	258.798	262.596	235.658	232.491	240.451	318.922	205.299	286.144	264.696	282.745	252.858	0.912686312	0.966018836	20	8	24	0.917396264	0.977511653	24	7.050270674	0.026839039
ENSMUSG00000084755	Gm16276	predicted gene 16276 [Source:MGI Symbol;Acc:MGI:3826598]	1.2011	1.20025	1.29605	0.876871	1.70695	1.29622	0.482264	1.04234	1.0738	1.32521	1.06118	1.05936	1.92114	3.21828	0.653906707	0.966018836	20	20	24	0.740325879	0.915274254	24	5.456777704	0.10015083
ENSMUSG00000084781	D930015M05Rik	RIKEN cDNA D930015M05 gene [Source:MGI Symbol;Acc:MGI:3612068]	0.587032	0.775243	0.848153	0.946302	0.561547	0.846952	0.47445	0.844804	1.63061	0.828067	1.05559	0.728656	0.652742	0.845104	0.008389425	0.192760771	12	16	24	0.044960247	0.628432994	24	9.772165094	0.181601124
ENSMUSG00000084786	Ubl5	ubiquitin-like 5 [Source:MGI Symbol;Acc:MGI:1913427]	211.641	216.05	217.237	213.101	209.231	207.019	209.152	198.671	204.944	173.59	208.878	186.995	208.155	213.101	0.800065124	0.966018836	12	12	24	0.49900496	0.808512079	24	2.431477675	0.036646485
ENSMUSG00000084790	Gm15879	predicted gene 15879 [Source:MGI Symbol;Acc:MGI:3802012]	3.89009	2.51717	2.25829	2.50566	2.817	3.18719	3.49539	3.43227	2.72138	2.56081	2.52641	3.29	2.74386	2.36016	0.018894761	0.256345714	4	8	24	0.014686966	0.537860185	24	22.1139449	0.178845482
ENSMUSG00000084792	1700056N10Rik	RIKEN cDNA 1700056N10 gene [Source:MGI Symbol;Acc:MGI:1920642]	1.35419	0.963669	0.883665	0.777151	0.879081	0.846195	1.10173	1.13221	0.894282	1.13322	1.14904	0.920535	1.14211	0.976551	0.154289127	0.606139807	4	12	24	0.292519064	0.768040178	24	0.216760077	0.068010985
ENSMUSG00000084796	Mir142hg	Mir142 host gene (non-protein coding) [Source:MGI Symbol;Acc:MGI:1925841]	3.3783	3.5694	4.46089	2.88569	2.67642	4.26541	2.71227	3.59124	3.78094	2.98403	3.24113	5.95438	9.00003	3.01001	0.7207001	0.966018836	24	20	24	0.400333003	0.799546792	24	23.49195983	0.197503283
ENSMUSG00000084799	Ino80dos	INO80 complex subunit D, opposite strand [Source:MGI Symbol;Acc:MGI:3651861]	3.73624	5.0236	5.51131	5.53136	4.81857	3.95804	4.46642	5.00481	6.38165	4.15704	4.5799	5.19069	5.04911	4.55669	0.286642595	0.793714531	12	16	24	0.106830229	0.72082227	24	8.775814651	0.137488836
ENSMUSG00000084800	Gm12205	predicted gene 12205 [Source:MGI Symbol;Acc:MGI:3650261]	0.87304	0.885096	0.526778	0.330974	0.647684	0.654806	0.826046	0.518177	0.72604	0.513025	0.463805	0.560477	0.649373	0.587554	0.016024075	0.253395174	4	12	24	0.021594037	0.574198497	24	0.541309634	0.129403459
ENSMUSG00000084803	5830444B04Rik	RIKEN cDNA 5830444B04 gene [Source:MGI Symbol;Acc:MGI:3603170]	1.0336	0.604377	1.02346	0.166184	0.768965	0.624494	0.416387	0.479525	0.715516	0.792444	0.370848	0.545496	0.577853	0.805861	0.914502381	0.966018836	12	4	24	0.671487646	0.889179947	24	4.164439922	0.077430635
ENSMUSG00000084808	9430091E24Rik	RIKEN cDNA 9430091E24 gene [Source:MGI Symbol;Acc:MGI:1924618]	2.94363	2.77565	2.10247	2.82452	2.87832	2.73966	2.84186	2.16494	2.88164	2.52518	2.7744	2.93115	2.26844	2.36878	0.68887295	0.966018836	24	8	24	0.11605143	0.727799063	24	18.05677211	0.094089057
ENSMUSG00000084817	Gm5526	predicted pseudogene 5526 [Source:MGI Symbol;Acc:MGI:3643066]	114.7	131.554	119.536	102.226	128.515	105.01	115.476	110.906	137.444	96.4318	137.495	125.312	133.429	131.58	0.897425765	0.966018836	12	8	24	0.710630921	0.904369518	24	2.281849637	0.051562329
ENSMUSG00000084826	AI847159	expressed sequence AI847159 [Source:MGI Symbol;Acc:MGI:3528181]	1.01919	1.02559	0.919831	0.545402	0.96048	1.15354	0.838306	1.041	0.35571	0.943181	0.8288	0.876998	0.891692	0.766906	0.836635326	0.966018836	8	4	24	0.125509302	0.739416874	24	21.6439575	0.132715162
ENSMUSG00000084828	Gm12367	predicted gene 12367 [Source:MGI Symbol;Acc:MGI:3649554]	0.996227	0.758215	0.530388	0.692037	1.01929	0.754736	0.680507	0.993478	0.867096	0.640236	0.806369	0.707256	0.821535	0.746405	0.918497267	0.966018836	20	8	24	0.572295845	0.846321055	24	22.11405178	0.049628548
ENSMUSG00000084830	Gm14539	predicted gene 14539 [Source:MGI Symbol;Acc:MGI:3705371]	428.838	501.276	449.415	459.276	453.789	395.921	520.827	419.672	534.985	338.787	458.8	421.129	451.203	492.732	0.867888967	0.966018836	16	12	24	0.359759911	0.785405943	24	4.128156903	0.094606773
ENSMUSG00000084838	Gm10241	predicted gene 10241 [Source:MGI Symbol;Acc:MGI:3704461]	12.381	12.9078	13.7382	12.2204	11.4468	9.74734	12.5689	11.0853	13.5841	11.0067	14.9862	13.9456	14.6837	15.0424	0.927172192	0.966018836	12	4	24	0.579978527	0.850086388	24	5.209046595	0.06573336
ENSMUSG00000084843	B230312C02Rik	RIKEN cDNA B230312C02 gene [Source:MGI Symbol;Acc:MGI:2444130]	2.18747	2.94625	3.19231	3.14183	2.80517	2.67054	2.60976	2.3569	3.43345	3.28051	2.2724	2.84584	2.6164	2.44387	0.016795343	0.253395174	12	16	24	0.039837618	0.616921492	24	10.55587134	0.136418792
ENSMUSG00000084844	Hoxb3os	homeobox B3 and homeobox B2, opposite strand [Source:MGI Symbol;Acc:MGI:103211]	2.56546	4.01068	3.67307	3.43771	2.91723	2.8549	3.08228	2.83788	3.81835	3.45902	3.2442	2.73392	2.40541	3.1971	7.94E-04	0.071344007	12	16	24	0.0061604	0.469371824	24	9.30564709	0.174228259
ENSMUSG00000084845	Tmem240	transmembrane protein 240 [Source:MGI Symbol;Acc:MGI:3648074]	1.22415	1.36663	1.67865	0.826499	0.923286	1.35135	1.42316	1.46474	2.09092	1.61892	1.45433	1.65306	1.53573	2.29874	0.446653834	0.966018836	12	8	24	0.029903232	0.596648309	24	5.288675034	0.171086903
ENSMUSG00000084846	A730011C13Rik	RIKEN cDNA A730011C13 gene [Source:MGI Symbol;Acc:MGI:2442973]	1.18857	1.64441	1.69053	1.23742	1.23963	0.935173	1.17093	1.13821	1.5289	1.14363	1.1938	1.11242	1.04735	1.24626	0.004712708	0.140598674	12	12	24	0.013160939	0.529134128	24	7.784905127	0.128881341
ENSMUSG00000084866	A930006K02Rik	RIKEN cDNA A930006K02 gene [Source:MGI Symbol;Acc:MGI:1925683]	2.22241	2.51577	2.23869	2.44704	2.18855	2.35468	2.90541	2.53759	2.98248	2.26146	3.2465	2.95475	2.88399	2.64817	0.835668938	0.966018836	8	4	24	0.806102982	0.938114514	24	23.37220879	0.027923017
ENSMUSG00000084874	Gm12906	predicted gene 12906 [Source:MGI Symbol;Acc:MGI:3650884]	20.3683	20.5245	19.4317	17.6039	23.4862	20.0626	19.2797	19.8289	19.984	18.8235	20.0034	18.854	21.8622	19.9419	0.446653834	0.966018836	4	12	24	0.551707339	0.83632237	24	21.77936042	0.041929912
ENSMUSG00000084880	Tomm6os	translocase of outer mitochondrial membrane 6, opposite strand [Source:MGI Symbol;Acc:MGI:3705171]	0.766957	0.743593	0.978317	0.664065	0.740178	0.733303	0.658076	0.855728	0.761398	0.72756	0.856123	0.763632	0.721429	0.83104	0.669104912	0.966018836	12	4	24	0.455221174	0.805393068	24	7.488250626	0.034795334
ENSMUSG00000084883	Ccdc85c	coiled-coil domain containing 85C [Source:MGI Symbol;Acc:MGI:3644008]	2.05671	2.59656	3.249	2.0773	1.82171	2.78484	3.1979	2.53873	2.61474	3.30644	1.99093	2.71483	2.33531	3.41508	0.496745696	0.966018836	12	8	24	0.19715191	0.768040178	24	5.445064309	0.142214856
ENSMUSG00000084885	3010001F23Rik	RIKEN cDNA 3010001F23 gene [Source:MGI Symbol;Acc:MGI:1922943]	4.15177	3.1256	1.9649	2.28373	2.28759	2.91457	2.21253	2.96824	2.74308	1.94009	2.93501	2.40413	[illegible]	1.49518	0.10031279	0.511533473	8	12	24	0.115832109	0.733729587	24	2.371336623	0.203707241
ENSMUSG00000084896	Gm11632	predicted gene 11632 [Source:MGI Symbol;Acc:MGI:3705198]	0.212554	1.51199	1.12499	2.94866	0.85538	0.382042	2.90125	0.244528	1.78782	0.914807	1.23352	1.84631	1.29278	0.964397	0.860560779	0.966018836	16	16	24	0.721212309	0.90866625	24	11.42380559	0.172920703
ENSMUSG00000084898	Gm12371	predicted gene 12371 [Source:MGI Symbol;Acc:MGI:3652322]	3.42344	3.06737	2.46679	3.4396	2.91811	2.60225	2.71596	2.68413	2.5146	2.74221	3.00392	2.99884	3.24741	3.38164	0.894563866	0.966018836	8	4	24	0.704374216	0.902363261	24	22.11689555	0.042324334
ENSMUSG00000084899	Gm15344	predicted gene 15344 [Source:MGI Symbol;Acc:MGI:3708098]	2.33709	1.57458	0.844838	2.28186	1.4872	1.90817	2.07813	1.65369	1.37247	1.13371	3.06256	1.41134	1.36379	2.39671	0.873337044	0.966018836	8	4	24	0.508090809	0.813175565	24	20.32435952	0.146390752
ENSMUSG00000084904	Gm14827	predicted gene 14827 [Source:MGI Symbol;Acc:MGI:3705192]	1.20373	1.36349	1.57521	1.01474	1.52859	1.22128	1.42221	1.3013	1.36158	1.00035	1.34585	1.2099	1.53489	1.22996	0.920620634	0.966018836	12	4	24	0.589969105	0.854726756	24	1.26762966	0.045274516
ENSMUSG00000084910	C630043F03Rik	RIKEN cDNA C630043F03 gene [Source:MGI Symbol;Acc:MGI:1915535]	0.802234	0.850129	0.980057	0.849957	0.887701	0.935161	1.18088	1.07432	1.1546	0.616295	0.961271	0.841693	0.934699	1.02972	0.662339683	0.966018836	4	4	24	0.298954018	0.768040178	24	3.319910663	0.065961613
ENSMUSG00000084911	Gm16185	predicted gene 16185 [Source:MGI Symbol;Acc:MGI:3802169]	1.729	2.61455	2.05179	2.29602	2.31184	1.85757	2.49564	1.77692	2.16348	1.28773	2.37178	1.79786	1.79441	2.27758	0.865514257	0.966018836	20	8	24	0.940497925	0.985398871	24	4.265008794	0.025019821
ENSMUSG00000084915	C230037L18Rik	RIKEN cDNA C230037L18 gene [Source:MGI Symbol;Acc:MGI:3697701]	3.53026	3.32561	2.18544	3.30554	1.95686	2.85973	2.3193	2.89173	2.18286	2.30742	2.11147	2.68971	3.02154	3.2853	0.059513104	0.406067671	8	12	24	0.121327199	0.733729587	24	1.664277232	0.155180607
ENSMUSG00000084925	1810062O18Rik	RIKEN cDNA 1810062O18 gene [Source:MGI Symbol;Acc:MGI:1922852]	1.8846	2.27693	3.25355	2.7	2.73692	2.00136	3.32234	1.96428	2.95149	2.76847	2.53236	3.36842	2.59539	1.96215	0.253121224	0.753386477	12	20	24	0.450146223	0.805393068	24	13.37523826	0.102879094

ENSMUSG00000084950	Gm5577	predicted gene 5577 [Source:MGI Symbol;Acc:MGI:3648213]	2.04958	2.35626	2.96696	2.97209	3.32305	2.68583	2.65088	2.61109	3.43169	2.33607	2.52514	2.9836	2.45702	2.44324	0.25851124	0.761738864	12	20	24	0.23524125	0.768040178	24	12.62464912	0.097572396
ENSMUSG00000084957	Bbip1	BBSome interacting protein 1 [Source:MGI Symbol;Acc:MGI:1913610]	47.529	53.6224	49.3327	46.9653	53.6133	48.4391	50.794	44.2043	50.3417	38.9996	53.1637	46.2035	50.8404	48.5853	0.885059765	0.966018836	20	20	24	0.676475051	0.891831571	24	22.12951443	0.043163194
ENSMUSG00000084970	1700060J05Rik	RIKEN cDNA 1700060J05 gene [Source:MGI Symbol;Acc:MGI:1920641]	1.01843	1.32379	1.10765	1.41915	0.708926	0.933574	0.67419	0.833069	1.00178	0.949929	1.36069	0.828388	1.17958	1.35181	0.708665292	0.966018836	16	8	24	0.430689567	0.805393068	24	8.46637607	0.093269697
ENSMUSG00000084973	Gm13848	predicted gene 13848 [Source:MGI Symbol;Acc:MGI:3651996]	0.879447	0.756821	0.612091	0.666408	0.644312	0.746308	0.927883	1.09076	0.536916	1.03454	0.579697	0.794062	0.833138	1.00346	0.046240749	0.375011045	8	4	24	0.175485933	0.768040178	24	1.837743341	0.09520702
ENSMUSG00000084974	Gm15567	predicted gene 15567 [Source:MGI Symbol;Acc:MGI:3783016]	0.449052	0.687071	0.718572	0.568365	0.744427	0.675483	0.455687	0.478629	0.847871	0.618184	0.53398	0.600258	0.508128	0.731436	0.018894761	0.256345714	12	16	24	0.177767154	0.768040178	24	10.18977846	0.078813334
ENSMUSG00000084980	Slc36a1os	solute carrier family 36 (proton/amino acid symporter), member 1, opposite strand [Source:MGI Symbol;Acc:MGI:1921295]	1.21774	1.15256	1.64743	0.869777	1.53831	1.06953	1.24989	1.33415	1.54984	1.39945	0.907433	1.29992	1.2945	1.46301	0.287843582	0.793714531	12	12	24	0.315581853	0.768040178	24	6.260199028	0.090504629
ENSMUSG00000084990	Gm14549	predicted gene 14549 [Source:MGI Symbol;Acc:MGI:3705175]	0.865565	1.27356	1.18907	1.41167	1.09184	1.29384	1.00067	1.26502	1.02699	0.963094	1.59832	1.01618	1.48637	1.25759	0.845386467	0.966018836	20	16	24	0.908724603	0.974606327	24	12.74011929	0.023741593
ENSMUSG00000084998	Gm16279	predicted gene 16279 [Source:MGI Symbol;Acc:MGI:3826576]	0.49416	0.56728	0.576566	0.546725	0.658066	0.592974	0.645351	0.762354	0.729558	0.506793	0.771815	0.67086	0.688087	0.633337	0.883870616	0.966018836	20	20	24	0.978485064	0.994576186	24	0.400397972	0.005526472
ENSMUSG00000085007	Smim43	small integral membrane protein 43 [Source:MGI Symbol;Acc:MGI:3650339]	4.22134	3.6957	3.35703	4.06554	3.64366	4.00103	3.74126	4.30909	3.09579	4.35299	3.81628	3.97299	4.63424	4.14272	0.625825704	0.966018836	4	8	24	0.394772762	0.799546792	24	22.15442448	0.063837002
ENSMUSG00000085015	Gm14424	predicted gene 14424 [Source:MGI Symbol;Acc:MGI:3650516]	0.543253	0.690004	0.918395	0.996024	0.568702	0.43129	0.662587	0.663572	1.42133	1.09617	0.89584	0.756634	0.877757	0.73139	0.016462359	0.253395174	16	8	24	0.00444046	0.427431763	24	9.719683914	0.208155153
ENSMUSG00000085022	Gm5860	predicted gene 5860 [Source:MGI Symbol;Acc:MGI:3646172]	0.311715	0.550972	1.04813	1.41375	0.743059	0.737189	0.850782	0.854183	0.958819	0.504292	0.336362	1.39784	0.544053	0.304835	0.664568268	0.966018836	12	16	24	0.666120214	0.887215207	24	12.78913187	0.104016948
ENSMUSG00000085024	C230035I16Rik	RIKEN cDNA C230035I16 gene [Source:MGI Symbol;Acc:MGI:2444850]	0.169907	0.823919	0.565654	0.682576	0.503813	0.384346	0.416688	0.748479	1.16739	0.505479	0.698843	0.825646	0.457736	0.520375	0.109040811	0.524035616	12	16	24	0.136347269	0.747972781	24	9.3655139	0.162645131
ENSMUSG00000085025	Gm13715	predicted gene 13715 [Source:MGI Symbol;Acc:MGI:3650277]	0.648602	0.245738	0.703322	0.547398	0.951505	0.18035	0.982836	0.716683	0.549182	0.429105	0.329483	0.31502	0.327759	0.374753	0.8972237	0.966018836	12	12	24	0.908208084	0.974606327	24	8.246868912	0.040972678
ENSMUSG00000085028	Slc2a4rg-ps	Slc2a4 regulator, pseudogene [Source:MGI Symbol;Acc:MGI:3651388]	2.40585	2.21159	2.00611	2.76045	2.81949	2.27527	2.72192	2.35926	2.4694	2.07192	2.17762	2.2674	2.36243	2.1377	0.879901313	0.966018836	4	8	24	0.583417506	0.851822355	24	18.69203214	0.044761457
ENSMUSG00000085030	2810455O05Rik	RIKEN cDNA 2810455O05 gene [Source:MGI Symbol;Acc:MGI:1920072]	2.10095	1.43853	1.04426	1.49362	1.46233	1.31616	1.67958	1.63018	1.22446	1.49043	1.59243	1.17041	1.46504	1.57803	0.337748746	0.85758369	4	8	24	0.389253311	0.799546792	24	23.17476619	0.078987995
ENSMUSG00000085037	4933421O10Rik	RIKEN cDNA 4933421O10 gene [Source:MGI Symbol;Acc:MGI:1918323]	2.137	2.64124	2.23271	2.46786	2.22624	2.24127	2.2588	2.33758	2.73637	2.31178	2.22709	2.12483	2.21578	2.38794	0.029283281	0.322165609	8	16	24	0.02919185	0.596648309	24	7.764599975	0.073498668
ENSMUSG00000085042	Abhd11os	abhydrolase domain containing 11, opposite strand [Source:MGI Symbol;Acc:MGI:1917062]	3.59239	3.56836	3.76918	2.63201	4.03596	3.15383	2.61263	4.10733	5.85969	4.16778	3.38154	4.88792	4.34025	5.14947	0.926137994	0.966018836	12	4	24	0.324264671	0.768040178	24	6.735627188	0.136224845
ENSMUSG00000085049	Parvaos	parvin, alpha, opposite strand [Source:MGI Symbol;Acc:MGI:2445046]	1.62669	1.56462	1.26949	1.70768	1.72484	1.81132	1.03634	1.36374	1.08795	1.26141	1.51045	1.91291	1.88174	1.57844	0.01130565	0.204837764	24	12	24	0.129491185	0.740162193	24	19.54530309	0.130184616
ENSMUSG00000085054	Gm15834	predicted gene 15834 [Source:MGI Symbol;Acc:MGI:3802168]	0.518712	0.880731	0.694853	0.751719	0.735074	0.772493	0.68235	0.915292	0.908114	0.712191	0.702352	0.869797	0.85629	0.82876	0.644063931	0.966018836	8	12	24	0.37304235	0.794374214	24	6.261243367	0.046106542
ENSMUSG00000085069	Prdm16os	Prdm16 opposite strand transcript [Source:MGI Symbol;Acc:MGI:3651882]	6.59237	5.17534	4.56302	4.79957	5.74543	4.77969	7.04483	5.37095	4.96445	4.63946	6.15919	5.3474	6.26219	4.6206	0.008360387	0.192760771	4	8	24	0.043851522	0.627786	24	21.76944944	0.16110731
ENSMUSG00000085072	Ict1os	immature colon carcinoma transcript 1, opposite strand [Source:MGI Symbol;Acc:MGI:2441927]	0.804247	1.02202	0.631716	0.923168	0.803279	0.964577	0.98149	1.16077	0.518918	1.0036	0.952998	0.854165	1.07093	0.799652	0.46238066	0.966018836	8	4	24	0.411616089	0.799546792	24	21.76039606	0.072826997
ENSMUSG00000085084	4930570G19Rik	RIKEN cDNA 4930570G19 gene [Source:MGI Symbol;Acc:MGI:1923158]	1.28455	1.34134	1.85923	1.25136	1.07471	1.00316	1.3535	1.03467	2.15068	1.00945	0.894929	1.57478	1.0607	1.08556	0.424718357	0.966018836	12	8	24	0.341166411	0.775424114	24	6.957230885	0.121886094
ENSMUSG00000085095	Gm15635	predicted gene 15635 [Source:MGI Symbol;Acc:MGI:3783079]	0.458076	0.265383	0.539959	0.439033	0.418014	0.370991	0.548486	0.932551	0.686397	0.358881	0.788023	0.339869	0.436476	0.449231	0.8972237	0.966018836	12	12	24	0.653468296	0.883312134	24	7.590091439	0.064171236
ENSMUSG00000085105	Gm12758	predicted gene 12758 [Source:MGI Symbol;Acc:MGI:3702543]	0.733407	0.629088	0.750918	0.601569	0.564033	0.592274	0.728832	0.574081	0.858787	0.637059	0.516758	0.687725	0.453841	0.525259	0.424718357	0.966018836	12	8	24	0.645581261	0.879878902	24	7.382319751	0.033956878
ENSMUSG00000085112	A530072M11Rik	RIKEN cDNA gene A530072M11 [Source:MGI Symbol;Acc:MGI:4440477]	0.891416	0.620751	0.382905	0.810865	0.6704	0.682433	0.716634	0.863808	0.54579	0.898615	0.809389	0.655764	0.935051	0.765271	0.643325306	0.966018836	4	8	24	0.5633449	0.842513738	24	20.94240926	0.05594902
ENSMUSG00000085133	B930095G15Rik	RIKEN cDNA B930095G15 gene [Source:MGI Symbol;Acc:MGI:2443701]	3.46686	3.43385	4.00105	3.55538	3.54499	4.0508	3.63448	3.61169	3.30374	3.70648	3.35865	3.36036	3.05465	3.48993	0.849180783	0.966018836	16	12	24	0.753750207	0.920255919	24	12.92758451	0.02219664
ENSMUSG00000085148	Mir22hg	Mir22 host gene (non-protein coding) [Source:MGI Symbol;Acc:MGI:1914348]	17.7338	24.9537	27.0501	24.68	21.3564	21.867	15.069	23.1775	25.7324	26.1021	18.3407	21.8795	19.8531	24.9694	9.02E-04	0.071344007	12	16	24	0.009012636	0.502965379	24	9.189813464	0.250361781
ENSMUSG00000085151	1110018N20Rik	RIKEN cDNA 1110018N20 gene [Source:MGI Symbol;Acc:MGI:1915753]	9.02387	7.58582	7.2735	8.03046	8.73646	8.09639	6.33747	7.70462	6.87465	8.7305	9.03214	7.13185	8.34018	8.40194	0.424718357	0.966018836	20	16	24	0.438605376	0.805393068	24	16.1008875	0.069474879
ENSMUSG00000085154	C130046K22Rik	RIKEN cDNA C130046K22 gene [Source:MGI Symbol;Acc:MGI:3026935]	0.963131	1.47053	1.21866	1.37076	0.83603	1.13221	1.27051	1.28868	1.03887	1.07289	0.769215	0.87427	1.88044	1.58188	0.0043709	0.140598674	8	12	24	0.033341301	0.606585237	24	4.069079528	0.181367351
ENSMUSG00000085156	Snhg15	small nucleolar RNA host gene 15 [Source:MGI Symbol;Acc:MGI:3650059]	3.24395	3.82405	4.52111	2.63161	2.98742	3.26519	2.14972	2.24008	3.83443	3.08215	3.29131	2.97283	3.04755	2.686	0.773177465	0.966018836	12	16	24	0.54052383	0.830021415	24	10.24695775	0.09423712
ENSMUSG00000085169	Gm10785	predicted gene 10785 [Source:MGI Symbol;Acc:MGI:3642149]	0.499099	0.662037	0.536592	0.628788	0.619767	0.410327	0.540955	0.489691	0.68779	0.725483	0.533096	0.660703	0.749311	0.565785	0.945728192	0.966018836	16	8	24	0.456060464	0.805393068	24	9.904526211	0.042953193
ENSMUSG00000085171	D830026I12Rik	RIKEN cDNA D830026I12 gene [Source:MGI Symbol;Acc:MGI:2442531]	0.46673	0.65643	0.537644	0.504832	0.536378	0.675415	0.959649	0.690004	0.907528	0.308237	0.748917	0.418813	0.419623	0.570737	0.952127548	0.966018836	12	4	24	0.718588378	0.907466097	24	3.287074075	0.053332371
ENSMUSG00000085172	Gm6542	predicted gene 6542 [Source:MGI Symbol;Acc:MGI:3644649]	33.3451	35.7548	35.3931	32.8265	33.616	31.0053	33.1516	31.6097	37.996	29.8123	38.2612	32.9701	36.3838	31.5444	0.93447422	0.966018836	12	12	24	0.936092689	0.98404386	24	7.994105761	0.016363098
ENSMUSG00000085175	Gm11423	predicted gene 11423 [Source:MGI Symbol;Acc:MGI:3651335]	0.874257	1.71506	1.61128	1.50291	1.37973	1.77466	1.33331	2.00313	2.07839	1.75772	1.13839	2.15311	1.87317	2.04476	0.708665292	0.966018836	8	12	24	0.246403362	0.768040178	24	6.641775561	0.12191485
ENSMUSG00000085178	Kdm6bos	KDM1 lysine (K)-specific demethylase 6B, opposite strand [Source:MGI Symbol;Acc:MGI:1921523]	0.536882	0.773709	0.77102	0.488503	0.770742	0.682244	0.655494	0.5256	0.820016	0.430172	0.366796	0.598642	0.446922	0.577934	0.893307667	0.966018836	12	4	24	0.664798461	0.886849936	24	5.009202946	0.043543758
ENSMUSG00000085180	AI838599	expressed sequence AI838599 [Source:MGI Symbol;Acc:MGI:3510989]	2.654	3.27553	4.44741	2.43924	3.22149	3.38657	4.48459	4.18521	3.47396	3.71602	2.96221	3.36178	2.71032	2.98972	0.932092277	0.966018836	12	8	24	0.64404	0.879114352	24	5.804409777	0.080093191
ENSMUSG00000085203	Gm12927	predicted gene 12927 [Source:MGI Symbol;Acc:MGI:3651776]	1.15078	0.773963	0.623535	0.429799	0.560088	0.424651	0.232197	0.507179	0.64847	0.380783	0.388382	0.498598	0.777493	0.446404	0.450844752	0.966018836	4	12	24	0.371030214	0.792839127	24	2.06897207	0.099155163
ENSMUSG00000085207	Gm11767	predicted gene 11767 [Source:MGI Symbol;Acc:MGI:3650674]	0.728582	0.910555	0.790814	1.10025	0.717528	0.884043	0.442541	0.83807	0.907233	0.642397	0.413353	0.782195	0.733422	0.740493	0.351840494	0.870418653	12	8	24	0.48573554	0.805393068	24	7.73177161	0.071366578
ENSMUSG00000085208	Brip1os	BRCA1 interacting protein C-terminal helicase 1, opposite strand [Source:MGI Symbol;Acc:MGI:1921288]	8.89626	7.54923	7.54931	7.75112	7.79216	8.52186	8.29437	8.40702	7.19893	8.95394	7.50991	8.23944	7.67813	7.63022	0.245061943	0.741580974	24	12	24	0.325965309	0.768040178	24	20.65549801	0.052330279
ENSMUSG00000085218	BB218582	expressed sequence BB218582 [Source:MGI Symbol;Acc:MGI:2139448]	2.22733	2.29943	2.54739	2.92124	2.59651	2.22437	1.85347	2.89538	2.97393	2.23747	3.50681	2.04693	1.88171	2.78799	0.02438554	0.287293295	12	16	24	0.066475901	0.668887716	24	10.83229706	0.165445756
ENSMUSG00000085224	Gm13425	predicted gene 13425 [Source:MGI Symbol;Acc:MGI:3649915]	11.6648	8.20341	7.53698	9.07348	8.68486	9.18885	8.42222	10.103	7.35908	14.5955	9.85726	11.2319	11.0096	10.8235	0.245061943	0.741580974	12	16	24	0.703363572	0.901975795	24	19.27754063	0.078766714
ENSMUSG00000085227	6330418K02Rik	RIKEN cDNA 6330418K02 gene [Source:MGI Symbol;Acc:MGI:3697416]	4.66051	4.56124	3.80382	3.57238	5.41013	4.2133	4.47964	4.24337	3.97911	3.25922	3.45051	4.34442	4.66064	5.67385	0.016024075	0.253395174	4	12	24	0.084767462	0.70291653	24	0.360977508	0.150122865
ENSMUSG00000085241	Snhg3	small nucleolar RNA host gene 3 [Source:MGI Symbol;Acc:MGI:2684817]	16.1444	22.6147	23.957	20.0483	15.5114	17.0811	16.2356	19.7755	24.3322	16.6925	21.8028	21.8807	18.7596	20.041	0.101520755	0.511659635	12	16	24	0.139904119	0.74956518	24	7.691363239	0.161652826
ENSMUSG00000085255	Taco1os	translational activator of mitochondrially encoded cytochrome c oxidase I, opposite strand [Source:MGI Symbol;Acc:MGI:1915441]	1.42829	0.810928	2.09399	0.661732	0.999554	1.1458	1.32228	0.359197	0.737607	0.511133	0.852107	0.922379	1.04191	0.659027	0.060621686	0.408386372	4	12	24	0.475181719	0.805393068	24	21.52760068	0.136207091
ENSMUSG00000085260	Med9os	mediator complex subunit 9, opposite strand [Source:MGI Symbol;Acc:MGI:1916628]	1.97966	1.63287	0.835667	0.873427	1.36269	1.07457	0.668388	1.36844	0.747433	1.13456	1.3334	1.29712	1.54036	1.08616	0.321662653	0.83607295	8	4	24	0.29195188	0.768040178	24	22.09279322	0.147557018
ENSMUSG00000085269	Gm15777	predicted gene 15777 [Source:MGI Symbol;Acc:MGI:3783219]	0.278913	0.595106	0.667154	0.639049	0.417	0.442737	0.544241	0.598877	0.761531	0.294861	0.498162	0.427236	0.334646	0.626103	0.019088001	0.257576951	12	12	24	0.059730835	0.658304792	24	7.627681799	0.127838974
ENSMUSG00000085272	Sbk3	SH3 domain binding kinase family, member 3 [Source:MGI Symbol;Acc:MGI:2685924]	0.807614	0.694325	0.662904	0.672433	0.62849	0.711285	0.647539	0.627021	0.556159	0.515843	0.6313	0.587954	0.738041	0.831964	0.181151038	0.659020016	4	12	24	0.07621731	0.686372559	24	0.681171275	0.061757076
ENSMUSG00000085282	Gm15663	predicted gene 15663 [Source:MGI Symbol;Acc:MGI:3783105]	2.606	2.4449	2.11326	2.19602	2.29753	2.50423	2.60596	2.37695	2.11766	2.10815	1.92701	2.78583	1.96466	2.50529	0.077415205	0.453845658	24	20	24	0.075644033	0.68634302	24	23.12537437	0.093902306
ENSMUSG00000085286	Ube4bos3	ubiquitination factor E4B, opposite strand 3 [Source:MGI Symbol;Acc:MGI:1921560]	1.13801	1.41834	1.41119	1.45897	0.991406	1.21184	0.982264	1.49816	1.93769	1.32623	1.31961	2.1022	2.15978	1.81514	0.696393581	0.966018836	8	12	24	0.300464433	0.768040178	24	5.616021661	0.104470372
ENSMUSG00000085287	4833418N02Rik	RIKEN cDNA 4833418N02 gene [Source:MGI Symbol;Acc:MGI:1921847]	0.638668	1.0149	0.932167	1.04035	0.963223	0.80773	0.728011	1.05669	1.13878	1.25042	0.859849	1.02822	1.11406	1.12947	0.160291962	0.620016786	16	12	24	0.010647728	0.509561578	24	9.22256825	0.11450938
ENSMUSG00000085311	Gm15445	predicted gene 15445 [Source:MGI Symbol;Acc:MGI:3707341]	1.63378	1.79498	1.97193	1.55013	1.88759	1.27794	1.78734	1.37475	2.47153	2.01999	1.79944	2.387	2.00383	2.81258	0.102974476	0.966018836	12	4	24	0.524728021	0.822234538	24	3.885495528	0.102754694
ENSMUSG00000085315	A430018G15Rik	RIKEN cDNA A430018G15 gene [Source:MGI Symbol;Acc:MGI:3040796]	2.54027	2.02772	3.26958	3.03732	2.57789	2.25894	1.19481	2.93584	1.93799	3.31018	2.28551	2.20622	2.24066	2.5663	0.054935173	0.398010585	16	12	24	0.126535217	0.739868076	24	11.00064881	0.193614457
ENSMUSG00000085316	D330050G23Rik	RIKEN cDNA D330050G23 gene [Source:MGI Symbol;Acc:MGI:2445155]	1.61424	1.05157	1.3613	1.12605	1.35801	1.27901	1.0268	1.10066	0.952058	0.816553	1.27134	1.26488	1.22262	0.913017	0.303922122	0.811201458	20	20	24	0.052366307	0.65045829	24	20.04059692	0.11392735
ENSMUSG00000085323	9130410C08Rik	RIKEN cDNA 9130410C08 gene [Source:MGI Symbol;Acc:MGI:1918862]	0.51519	0.632929	0.653726	0.0985921	0.579074	0.585568	0.523998	0.653973	0.498687	0.397074	0.325002	0.51968	0.80022	0.51212	0.14606006	0.590732968	4	12	24	0.053909165	0.654390023	24	1.273527542	0.146464604
ENSMUSG00000085331	Gm11274	predicted gene 11274 [Source:MGI Symbol;Acc:MGI:3649225]	0.504286	0.510457	0.273648	0.570713	0.744343	0.775984	0.918677	0.894957	1.0272	0.416866	0.686225	0.731341	0.761809	0.384881	0.72778087	0.966018836	4	12	24	0.508754532	0.813629441	24	20.94877892	0.091532083
ENSMUSG00000085334	Gm12940	predicted gene 12940 [Source:MGI Symbol;Acc:MGI:3702626]	28.0449	33.2209	35.9613	34.5904	30.3389	24.0858	32.2413	22.4943	39.3693	27.1945	35.7226	24.2992	27.6966	29.5634	0.023472439	0.278499043	12	12	24	0.152222177	0.759088284	24	9.930389732	0.1722385
ENSMUSG00000085340	Gm16335	predicted gene 16335 [Source:MGI Symbol;Acc:MGI:3840152]	1.10264	0.831122	1.4914	0.611344	1.78995	1.81	0.33275	1.20661	2.41389	1.46038	1.84516	1.26509	1.68787	2.39304	0.891105762	0.966018836	12	16	24	0.642897908	0.878809617	24	11.01494237	0.129758529
ENSMUSG00000085342	Gm12254	predicted gene 12254 [Source:MGI Symbol;Acc:MGI:3650324]	125.53	136.658	134.809	132.396	138.287	128.292	119.691	124.967	149.644	106.529	144.073	114.631	127.349	124.071	0.441501717	0.966018836	12	16	24	0.703618359	0.902090877	24	10.1935103	0.042783257
ENSMUSG00000085347	Hk1os	hexokinase 1, opposite strand [Source:MGI Symbol;Acc:MGI:1917412]	1.02288	1.13818	1.01152	0.615065	0.662776	1.46797	0.526826	0.396182	0.61429	0.89217	1.60957	1.48182	1.04426	1.17091	0.388056094	0.915479136	24	12	24	0.316792606	0.768040178	24	19.21113302	0.167114059
ENSMUSG00000085348	Myhas	myosin heavy chain gene antisense RNA [Source:MGI Symbol;Acc:MGI:3650429]	1.18204	2.68346	3.50077	4.20634	2.61109	3.15217	2.66528	4.46923	7.63896	6.12565	1.99109	2.84653	2.50645	1.66765	0.046820236	0.375011045	12	16	24	0.042453607	0.623698598	24	10.11844799	0.48704274
ENSMUSG00000085376	Gm14508	predicted gene 14508 [Source:MGI Symbol;Acc:MGI:3705279]	0.700449	0.711749	0.820198	1.11145	0.765558	1.0695	0.78862	0.916102	0.662111	0.890006	0.777493	0.721978	0.789979	0.564152	0.853771773	0.966018836	16	16	24	0.283701677	0.768040178	24	15.31942255	0.068231942
ENSMUSG00000085379	2310058D17Rik	RIKEN cDNA 2310058D17 gene [Source:MGI Symbol;Acc:MGI:1922944]	1.60987	1.57184	1.53996	1.36831	1.53752	1.42943	1.48123	1.14791	1.26676	1.3763	1.68333	1.6279	1.51272	1.42511	0.300956784	0.809595158	20	20	24	0.129303883	0.740162193	24	19.36116849	0.068519108
ENSMUSG00000085382	Gm13861	predicted gene 13861 [Source:MGI Symbol;Acc:MGI:3649466]	0.84483	1.40532	0.655004	1.23054	1.42649	0.946364	1.17301	1.28599	1.14768	1.35676	1.80783	1.32149	0.890871	0.919786	0.080247632	0.458851846	20	8	24	0.211051193	0.768040178	24	15.12498178	0.130820777
ENSMUSG00000085385	Snhg17	small nucleolar RNA host gene 17 [Source:MGI Symbol;Acc:MGI:1915358]	6.65997	5.58352	7.1109	7.58818	6.23865	6.016	5.52324	5.33849	6.88278	6.26374	5.65673	5.51536	6.2514	7.23887	0.191123261	0.669841558	12	12	24	0.181168488	0.768040178	24	9.559179705	0.101900694
ENSMUSG00000085389	1700003M07Rik	RIKEN cDNA 1700003M07 gene [Source:MGI Symbol;Acc:MGI:1919475]	0.20553	0.680313	1.02098	0.587219	0.23517	0.820355	0.624984	0.558339	0.487178	0.410144	0.418408	0.466913	0.0709127	0.481966	0.708020493	0.966018836	12	8	24	0.522167031	0.820770626	24	8.381422373	0.106312145
ENSMUSG00000085396	Firre	functional intergenic repeating RNA element [Source:MGI Symbol;Acc:MGI:2147989]	44.7758	53.2554	49.5101	50.5884	52.4295	43.3868	46.3095	46.2243	57.5163	46.1289	52.9072	50.0653	51.3465	50.6029	0.638532766	0.966018836	12	12	24	0.278312768	0.768040178	24	9.728224037	0.068858031
ENSMUSG00000085399	Foxd2os	forkhead box D2, opposite strand [Source:MGI Symbol;Acc:MGI:2444065]	2.84933	3.06676	2.49243	2.30938	2.36627	2.17224	2.70741	2.92161	3.48983	2.06977	2.29854	2.47153	4.01076	2.28363	0.387380665	0.915479136	8	8	24	0.074402347	0.683293901	24	2.23367698	0.146556573
ENSMUSG00000085400	Pard3bos3	par-3 family cell polarity regulator beta, opposite strand 3 [Source:MGI Symbol;Acc:MGI:3651159]	0.853611	0.549253	0.556844	1.0429	0.595447	0.290293	0.346985	0.509059	0.815659	0.864832	0.70356	0.726533	0.493183	0.553742	0.046934151	0.375011045	16	8	24	0.100218912	0.71175955	24	11.66020649	0.146848559
ENSMUSG00000085401	Slc39a1-ps	solute carrier family 39 (zinc transporter), member 1, pseudogene [Source:MGI Symbol;Acc:MGI:3615375]	1.67648	1.65382	1.76947	1.58348	2.28809	2.15509	1.68443	2.20742	2.28985	2.01979	1.66126	1.76075	1.70796	1.43628	0.914360142	0.966018836	12	20	24	0.633818386	0.875522631	24	13.87074626	0.052966917
ENSMUSG00000085408	C530005A16Rik	RIKEN cDNA C530005A16 gene [Source:MGI Symbol;Acc:MGI:3612454]	1.34636	1.23849	1.41959	1.50949	1.39566	1.41463	0.873203	1.63217	1.18722	1.56118	1.18461	1.44773	1.10487	1.66121	0.251986462	0.75129457	16	12	24	0.439038467	0.805393068	24	10.38112817	0.071555329
ENSMUSG00000085412	Halr1	Hoxa adjacent long noncoding RNA 1 [Source:MGI Symbol;Acc:MGI:3705267]	0.918503	0.792751	0.596192	0.708768	0.968664	1.06844	1.11241	0.568498	0.761678	0.421168	0.580413	0.354669	0.840543	0.301654	0.48023977	0.966018836	4	12	24	0.166550292	0.767020009	24	21.11502021	0.136123851
ENSMUSG00000085415	Selenok-ps1	selenoprotein K, pseudogene 1 [Source:MGI Symbol;Acc:MGI:3649370]	0.640057	0.812544	0.487034	0.951843	1.14658	0.398523	0.702176	0.686487	0.392955	0.253773	0.614862	0.408992	0.469709	0.432054	0.896773941	0.966018836	8	4	24	0.784044269	0.930629322	24	17.75370969	0.052188453
ENSMUSG00000085419	Gm11734	predicted gene 11734 [Source:MGI Symbol;Acc:MGI:3652086]	0.423102	0.859102	0.459847	0.564021	0.315425	0.637852	1.03107	0.566803	1.05774	0.652431	0.435085	0.631423	0.659074	0.317931	0.965163262	0.969272194	4	16	24	0.649206896	0.881804828	24	3.867779047	0.075348148
ENSMUSG00000085433	Gm16001	predicted gene 16001 [Source:MGI Symbol;Acc:MGI:3802119]	1.55639	2.28237	2.3308	2.67172	2.46204	2.07126	2.52909	2.10107	2.51318	2.84547	2.57462	2.61804	2.3581	2.50095	0.077161185	0.453790923	16	16	24	0.019516383	0.564709807	24	12.02643048	0.120250185
ENSMUSG00000085436	Zfp335os	zinc finger protein 335, opposite strand [Source:MGI Symbol;Acc:MGI:3649775]	1.34132	1.24573	1.47241	1.14673	1.117	1.63027	1.03831	0.987613	1.34467	1.41281	1.00888	1.45835	0.968109	1.57366	0.954106009	0.966018836	12	16	24	0.951661818	0.988339231	24	10.24053692	0.018437665
ENSMUSG00000085438	Oip5os1	Opa interacting protein 5, opposite strand 1 [Source:MGI Symbol;Acc:MGI:1913852]	49.6735	60.2827	56.6804	59.5537	55.2464	50.6285	57.0356	53.9482	65.6479	52.5528	59.125	53.8504	57.4392	59.3429	0.22872448	0.72665454	12	12	24	0.095416621	0.71175955	24	8.246362901	0.086542038
ENSMUSG00000085442	Gm3362	predicted pseudogene 3362 [Source:MGI Symbol;Acc:MGI:3781540]	2.91266	3.14827	3.59158	3.14324	2.83043	2.07197	2.89325	2.70016	4.40282	2.71438	3.78025	4.09955	4.00258	2.93529	0.785563354	0.966018836	12	4	24	0.709363454	0.903951515	24	8.625710988	0.072871652
ENSMUSG00000085457	1110046J04Rik	RIKEN cDNA 1110046J04 gene [Source:MGI Symbol;Acc:MGI:1916058]	17.3208	14.5408	13.2543	13.5494	16.333	13.6473	16.2358	11.9935	14.8576	12.9864	16.1095	15.4952	15.8157	15.7706	0.079732457	0.458678242	4	12	24	0.156712993	0.762862854	24	21.10380555	0.108166343
ENSMUSG00000085479	9430073C21Rik	Riken cDNA 9430073C21 gene [Source:MGI Symbol;Acc:MGI:3699860]	0.326028	0.760777	0.949254	1.30696	0.640808	0.645064	0.345882	1.27747	0.899175	1.09174	0.499099	0.415713	0.731902	0.513821	0.016720774	0.253395174	12	12	24	0.014680743	0.537860185	24	9.560877182	0.268770747
ENSMUSG00000085492	Trmt61b	tRNA methyltransferase 61B [Source:MGI Symbol;Acc:MGI:1916039]	5.25275	6.13709	6.04706	5.84619	6.01819	7.12766	6.99974	5.89456	5.85691	6.53425	6.36356	6.16782	6.0644	6.72492	0.881718085	0.966018836	24	12	24	0.79363836	0.933825946	24	19.27569368	0.026048847
ENSMUSG00000085500	Gm16976	predicted gene, 16976 [Source:MGI Symbol;Acc:MGI:4439900]	3.17586	2.6372	1.44939	2.32489	1.83761	4.23364	6.6496	2.05127	4.0338	4.72508	4.26081	3.62322	2.29465	2.08482	0.476029854	0.966018836	24	8	24	0.460061163	0.805393068	24	19.29375576	0.234171943
ENSMUSG00000085517	Gm12963	predicted gene 12963 [Source:MGI Symbol;Acc:MGI:3651569]	0.228397	0.650869	2.0127	0.847691	1.07295	0.754102	0.899594	1.59355	1.73233	1.14127	1.35649	0.969717	0.969205	1.18052	0.018194955	0.256345714	12	16	24	0.028240835	0.591583525	24	9.46524743	0.293671975
ENSMUSG00000085521	4930526A20Rik	RIKEN cDNA 4930526A20 gene [Source:MGI Symbol;Acc:MGI:3612449]	6.91781	5.41257	5.9252	6.2533	5.25183	5.4717	5.54533	6.55332	5.27482	5.77012	5.97021	5.73061	5.85202	5.46949	0.856752753	0.966018836	4	16	24	0.861916714	0.959653684	24	0.8153529	0.021217226
ENSMUSG00000085526	Gm16083	predicted gene 16083 [Source:MGI Symbol;Acc:MGI:3802163]	0.548282	0.727311	0.669104	0.823936	0.787982	0.588183	0.588793	0.492577	0.648208	0.394855	0.940741	0.506094	0.716813	0.639427	0.676344318	0.966018836	20	4	24	0.60419225	0.861793218	24	14.4625484	0.049742973
ENSMUSG00000085527	Gm15535	predicted gene 15535 [Source:MGI Symbol;Acc:MGI:3782983]	1.71026	2.01445	2.07921	2.16455	1.57349	1.32041	1.7619	1.66717	1.36545	1.91318	2.23585	1.10885	2.07993	1.80063	0.581070934	0.966018836	16	8	24	0.456030647	0.805393068	24	8.791415901	0.093736926
ENSMUSG00000085543	Gm13568	predicted gene 13568 [Source:MGI Symbol;Acc:MGI:3702049]	1.00802	2.29679	1.79101	4.45889	2.18717	1.21646	1.94544	2.07744	4.37003	1.75581	2.95016	3.95728	2.6337	2.32377	0.920858634	0.966018836	12	16	24	0.272558616	0.768040178	24	11.13390556	0.252531082
ENSMUSG00000085553	Gm14808	predicted gene 14808 [Source:MGI Symbol;Acc:MGI:3705252]	0.813977	1.23107	0.881883	0.910569	1.6313	1.23685	1.23121	1.61264	1.23767	0.404054	0.960263	0.794579	1.10103	1.10805	0.93447422	0.966018836	8	8	24	0.532523709	0.825678486	24	1.010778944	0.102602105
ENSMUSG00000085558	4930412C18Rik	RIKEN cDNA 4930412C18 gene [Source:MGI Symbol;Acc:MGI:2443490]	3.20351	3.09813	3.69384	2.85506	2.91955	3.04285	3.07171	3.22259	3.02104	2.72992	2.8372	2.77925	2.51349	2.95049	0.203345222	0.689605535	12	4	24	0.19780028	0.768040178	24	5.65399296	0.053233766
ENSMUSG00000085566	A730017L22Rik	RIKEN cDNA A730017L22 gene [Source:MGI Symbol;Acc:MGI:3584452]	4.19934	4.10633	3.96582	4.13138	4.2639	4.00908	4.64058	3.87918	3.98528	4.26592	4.115	3.75087	4.02913	3.62785	0.328944792	0.847277612	16	16	24	0.523374982	0.821170929	24	17.78358219	0.028964698
ENSMUSG00000085573	Gm15418	predicted gene 15418 [Source:MGI Symbol;Acc:MGI:3705275]	0.543049	0.393658	0.252901	0.619727	1.09743	0.613189	0.755885	0.55479	0.740106	0.513781	0.425051	0.447011	0.363553	0.467302	0.881996584	0.966018836	20	12	24	0.462149322	0.805393068	24	16.73365339	0.090255816
ENSMUSG00000085584	Rtl9	retrotransposon Gag like 9 [Source:MGI Symbol;Acc:MGI:2685231]	1.48556	1.52324	1.49694	1.51869	1.30924	1.66469	1.15391	1.3454	1.32018	1.95535	1.31853	1.77586	1.43835	1.58658	0.93447422	0.966018836	16	12	24	0.744650427	0.916522362	24	13.59301856	0.035842765
ENSMUSG00000085586	Gm11613	predicted gene 11613 [Source:MGI Symbol;Acc:MGI:3651819]	4.42588	4.64983	4.07723	5.02518	4.1866	4.68814	4.65907	4.17255	4.34859	5.6725	3.91681	4.80143	4.80264	4.98497	0.95032543	0.966018836	16	20	24	0.967350804	0.992187127	24	9.385751406	0.012257125
ENSMUSG00000085611	Ap3s1-ps1	adaptor-related protein complex 3, sigma 1 subunit, pseudogene 1 [Source:MGI Symbol;Acc:MGI:1929216]	22.094	19.0403	18.3628	19.7779	18.6034	19.5107	22.0876	15.9945	16.6031	17.5906	20.7366	18.4985	19.8686	21.1587	0.037085178	0.334054683	4	8	24	0.161882152	0.76636332	24	21.51472149	0.099149018
ENSMUSG00000085614	1700123M08Rik	RIKEN cDNA 1700123M08 gene [Source:MGI Symbol;Acc:MGI:1923915]	2.95791	3.37688	3.12891	3.32284	3.63639	2.86341	2.71033	2.43191	3.94822	3.70612	2.24783	2.63521	3.77186	2.8453	0.628286596	0.966018836	16	8	24	0.425305232	0.805393068	24	9.584431328	0.098573421
ENSMUSG00000085615	A330035P11Rik	RIKEN cDNA A330035P11 gene [Source:MGI Symbol;Acc:MGI:2442610]	0.439164	0.561512	0.325547	0.455014	0.451417	0.535383	0.507177	0.48422	0.655446	0.493796	0.42855	0.558576	0.508638	0.656661	0.644063931	0.966018836	8	12	24	0.410594659	0.799546792	24	3.232705949	0.038292998
ENSMUSG00000085622	3110056K07Rik	RIKEN cDNA 3110056K07 gene [Source:MGI Symbol;Acc:MGI:1920454]	5.51435	7.61652	6.91914	6.29814	8.37184	6.74294	8.01529	6.55632	7.5737	5.6279	9.00092	6.60051	7.01913	7.03736	0.887862353	0.966018836	20	20	24	0.871267936	0.963486859	24	16.69361808	0.037247392
ENSMUSG00000085642	3110053B16Rik	RIKEN cDNA 3110053B16 gene [Source:MGI Symbol;Acc:MGI:1920435]	3.69436	3.09999	3.48334	3.01693	3.68289	4.37323	2.94251	3.6747	2.25568	3.17136	3.1439	3.87681	2.73837	4.33181	0.601647512	0.966018836	24	12	24	0.425713119	0.805393068	24	20.8527348	0.106318608
ENSMUSG00000085645	Hoxb5os	homeobox B5 and homeobox B6, opposite strand [Source:MGI Symbol;Acc:MGI:1922645]	0.83024	1.16934	0.801629	0.525018	0.410981	0.727137	1.01028	0.821747	1.25582	0.853734	1.13492	0.769381	0.864135	1.32524	0.149354198	0.593798152	8	16	24	0.058009139	0.656248849	24	4.612762825	0.164578302
ENSMUSG00000085653	Gm15179	predicted gene 15179 [Source:MGI Symbol;Acc:MGI:3705125]	0.099434	0.842605	0.778522	1.03552	0.597044	1.12841	0.293114	2.71706	1.91694	1.16582	1.02462	1.11396	1.37315	1.51633	0.708665292	0.966018836	8	12	24	0.119831056	0.731610562	24	8.00915997	0.296867306
ENSMUSG00000085655	Gm15952	predicted gene 15952 [Source:MGI Symbol;Acc:MGI:3802149]	0.727022	1.28503	1.03951	1.54548	0.70895	0.880649	0.96673	1.75317	1.73205	0.822623	1.29249	1.33481	0.888315	1.14961	0.38940266	0.915479136	8	20	24	0.196807309	0.768040178	24	7.781970163	0.162763423
ENSMUSG00000085667	Gm12992	predicted gene 12992 [Source:MGI Symbol;Acc:MGI:3702644]	0.572592	0.669464	0.397052	0.799516	0.47563	0.476583	0.538779	0.438421	0.701393	0.530158	0.436985	0.481582	0.645047	0.694068	0.401437339	0.933249185	16	4	24	0.469707458	0.805393068	24	6.364267856	0.055641562
ENSMUSG00000085669	Gm14817	predicted gene 14817 [Source:MGI Symbol;Acc:MGI:3705159]	1.06444	0.655327	0.713429	0.493212	0.731692	0.834433	0.542499	0.763653	0.540457	0.833959	0.837969	0.276101	0.170686	0.575802	0.858932988	0.966018836	20	8	24	0.778495934	0.928832041	24	13.25199965	0.052209899
ENSMUSG00000085682	Gm14267	predicted gene 14267 [Source:MGI Symbol;Acc:MGI:3649851]	0.527553	0.852	0.847692	0.804095	0.829667	0.541639	0.369645	0.855749	0.307195	0.394813	0.808106	0.707736	0.597962	0.585815	0.95261238	0.966018836	8	20	24	0.883595143	0.966818623	24	14.48275708	0.034279368
ENSMUSG00000085683	Tmem238l	transmembrane protein 238 like [Source:MGI Symbol;Acc:MGI:1918826]	5.7653	5.85881	4.5698	5.95209	4.5414	4.9986	4.9119	4.91193	4.13022	5.4017	5.40094	6.55457	5.46314	4.74629	0.601647512	0.966018836	24	12	24	0.351278998	0.780345856	24	19.95317182	0.089667242
ENSMUSG00000085684	4930469K13Rik	RIKEN cDNA 4930469K13 gene [Source:MGI Symbol;Acc:MGI:1924124]	3.30869	4.08534	2.68151	4.97771	4.21564	4.32627	6.42086	2.15502	3.59404	1.89703	3.39224	2.11152	2.87656	4.21551	0.79256873	0.966018836	4	8	24	0.795097689	0.934298757	24	21.84092326	0.104628208
ENSMUSG00000085687	Gm16153	predicted gene 16153 [Source:MGI Symbol;Acc:MGI:3802057]	0.580116	0.753831	0.751262	0.67002	0.739026	0.572002	0.648307	0.611833	0.829043	0.640666	0.857468	0.653276	0.643635	0.879121	0.279136262	0.790764844	12	16	24	0.154524139	0.760257003	24	9.287686991	0.060603112
ENSMUSG00000085696	Hoxaas3	Hoxa cluster antisense RNA 3 [Source:MGI Symbol;Acc:MGI:1919878]	1.15675	1.07913	1.06634	1.37276	0.543248	0.859513	0.406019	0.467254	1.09867	0.931954	0.881863	0.610206	0.946186	1.54299	0.366334701	0.893596186	12	12	24	0.292545102	0.768040178	24	8.192041563	0.16093738
ENSMUSG00000085704	4921531C22Rik	RIKEN cDNA 4921531C22 gene [Source:MGI Symbol;Acc:MGI:1913980]	1.11439	1.02582	1.49985	0.920169	1.53031	1.01271	1.19506	0.927721	1.55192	0.881526	1.52909	1.25694	1.32652	1.46311	0.93717134	0.966018836	20	20	24	0.962177742	0.990445744	24	12.03052106	0.017842409

ENSMUSG00000085705	Gm16046	predicted gene 16046 [Source:MGI Symbol;Acc:MGI:3802093]	0.720508	1.05599	0.515638	0.380796	1.0461	0.775015	1.31738	1.27269	1.06873	0.594179	0.674906	0.664992	0.845933	0.543835	0.371875382	0.894537464	4	12	24	0.274430331	0.768040178	24	0.848067085	0.139571921
ENSMUSG00000085711	Gm15163	predicted gene 15163 [Source:MGI Symbol;Acc:MGI:3642430]	5.25975	6.05857	6.40395	8.13083	5.59414	6.50297	5.35941	6.90439	7.07311	7.73107	5.90367	5.69664	6.85771	6.84002	0.038123942	0.334069441	16	12	24	0.021332733	0.572080066	24	9.581550872	0.159711945
ENSMUSG00000085735	Mettl5os	methyltransferase like 5, opposite strand [Source:MGI Symbol;Acc:MGI:1922537]	0.237396	0.521961	0.859851	0.722246	0.471195	0.93793	0.963689	0.211799	0.647004	0.679919	0.433471	0.557412	0.793133	0.715309	0.867488717	0.966018836	24	20	24	0.957231697	0.988639288	24	12.37970875	0.025577054
ENSMUSG00000085741	5430405H02Rik	RIKEN cDNA 5430405H02 gene [Source:MGI Symbol;Acc:MGI:1921737]	3.38253	5.07254	4.0578	3.94971	4.6736	4.11542	4.1814	5.02969	6.14354	4.63767	5.30061	4.97234	5.65053	4.71725	0.891742979	0.966018836	8	8	24	0.516491199	0.817817397	24	8.108427899	0.069524943
ENSMUSG00000085749	Gm12843	predicted gene 12843 [Source:MGI Symbol;Acc:MGI:3651051]	0.851215	1.06427	1.86271	1.68075	0.825275	1.24198	1.9406	0.812369	0.973165	1.98947	0.878706	1.28683	1.80404	1.05774	0.93717134	0.966018836	16	4	24	0.89422245	0.970031575	24	10.33071304	0.054500414
ENSMUSG00000085761	4930455G09Rik	RIKEN cDNA 4930455G09 gene [Source:MGI Symbol;Acc:MGI:1926167]	0.512791	0.563546	0.835663	0.194795	1.08016	0.578163	0.936374	1.14289	0.931258	0.677902	0.760096	0.602564	1.1429	0.644962	0.881996584	0.966018836	4	20	24	0.675900721	0.891524562	24	2.442983687	0.077012485
ENSMUSG00000085763	Smc2os	structural maintenance of chromosomes 2, opposite strand [Source:MGI Symbol;Acc:MGI:1919898]	0.258633	0.34981	0.74928	0.491173	0.70493	0.631721	0.314834	0.461105	0.821915	0.455883	0.412812	0.828937	0.518833	0.260291	0.214902712	0.704989686	12	20	24	0.303240942	0.768040178	24	13.84554071	0.110176518
ENSMUSG00000085766	2810430I11Rik	RIKEN cDNA 2810430I11 gene [Source:MGI Symbol;Acc:MGI:3590515]	1.10688	1.58554	1.48289	1.16559	1.50341	1.23769	1.21965	1.46705	1.73283	1.18154	1.05087	1.39651	1.33175	1.35743	0.200364953	0.684570416	12	4	24	0.186477696	0.768040178	24	6.317528975	0.085893774
ENSMUSG00000085767	Gm13563	predicted gene 13563 [Source:MGI Symbol;Acc:MGI:3652052]	4.56458	3.53133	3.28425	5.12396	5.41179	4.29011	5.16241	2.58401	3.55749	2.68352	4.76619	2.91957	4.25473	5.63414	0.945728192	0.966018836	20	16	24	0.590167969	0.854944722	24	19.58464345	0.128870981
ENSMUSG00000085779	Atcayos	ataxia, cerebellar, Cayman type, opposite strand [Source:MGI Symbol;Acc:MGI:1916928]	0.691789	2.16698	2.6555	2.98025	2.12273	2.35644	2.17897	3.81485	4.22183	4.87698	1.40455	2.74116	2.8626	1.94335	0.344645202	0.86981408	16	4	24	0.085930362	0.704313881	24	9.536673536	0.36294907
ENSMUSG00000085783	Gm9816	predicted pseudogene 9816 [Source:MGI Symbol;Acc:MGI:3704465]	45.2077	43.1849	42.2511	42.8208	43.3737	46.3839	40.23	43.2359	40.3253	41.3576	46.1724	40.8946	44.1818	40.8529	0.387380622	0.915479136	20	16	24	0.181320103	0.768040178	24	19.17277708	0.047098683
ENSMUSG00000085787	Gm13092	predicted gene 13092 [Source:MGI Symbol;Acc:MGI:3649661]	54.2116	55.4228	59.4233	57.5978	57.3549	46.6161	47.0974	53.4388	57.1601	48.0667	52.9506	54.5385	54.5537	57.771	0.118459043	0.541945448	12	12	24	0.254369798	0.768040178	24	8.205190241	0.073294184
ENSMUSG00000085793	Lin52	lin-52 homolog (C. elegans) [Source:MGI Symbol;Acc:MGI:3045391]	5.56989	7.57014	7.03316	7.72118	6.51463	7.10753	7.68772	6.53732	7.69282	7.61535	6.81496	7.09631	7.01311	6.81448	0.636884864	0.966018836	16	4	24	0.31506677	0.768040178	24	10.17580786	0.066779465
ENSMUSG00000085795	Zfp703	zinc finger protein 703 [Source:MGI Symbol;Acc:MGI:2662729]	5.94005	4.26533	5.28433	5.08853	5.21857	4.15765	5.00521	6.28079	5.6645	8.39768	5.28828	5.44277	7.56448	5.75591	0.896753532	0.966018836	4	20	24	0.638066502	0.87713034	24	8.389047631	0.081175292
ENSMUSG00000085829	Gm4285	predicted gene 4285 [Source:MGI Symbol;Acc:MGI:3782463]	0.753303	0.683445	0.526553	1.10995	0.241429	0.884957	0.525033	0.866712	0.918348	1.031	0.776442	0.995648	0.753039	1.12671	0.702630284	0.966018836	16	12	24	0.636723221	0.876483853	24	7.632964858	0.076321766
ENSMUSG00000085830	Grin1os	glutamate receptor, ionotropic, NMDA1 (zeta 1), opposite strand [Source:MGI Symbol;Acc:MGI:2443867]	0.632521	0.775763	0.729835	0.6487	0.648942	0.63842	0.777479	0.561964	0.99261	0.689133	0.452571	0.877701	0.746505	0.901636	0.668329321	0.966018836	12	8	24	0.250129098	0.768040178	24	4.969697715	0.074672261
ENSMUSG00000085837	Kcnmb4os2	potassium large conductance calcium-activated channel, subfamily M, beta member 4, opposite strand 2 [Source:MGI Symbol;Acc:MGI:1918263]	1.44889	1.87428	1.4913	1.06487	1.18511	1.28611	1.15922	1.64761	1.94765	2.2144	1.10848	1.81869	2.06655	1.92699	0.455507782	0.966018836	12	8	24	0.097927054	0.71175955	24	5.39240373	0.159857068
ENSMUSG00000085838	Chn1os1	chimerin 1, opposite strand 1 [Source:MGI Symbol;Acc:MGI:3702041]	1.46854	3.84984	3.16798	2.84806	5.52908	1.47849	3.60415	3.9726	2.65428	0.507874	2.7532	2.50938	3.58779	1.84362	0.940625808	0.966018836	8	16	24	0.865707007	0.961207208	24	1.229789876	0.113725158
ENSMUSG00000085843	Kank4os	KN motif and ankyrin repeat domains 4, opposite strand [Source:MGI Symbol;Acc:MGI:3026921]	0.891379	1.07154	0.660493	0.721701	0.899339	0.736274	0.811358	0.66466	1.16845	0.588689	1.07385	0.879043	1.14602	0.85666	0.943920575	0.966018836	20	20	24	0.693048998	0.898227246	24	23.67074634	0.048694827
ENSMUSG00000085862	Gm13483	predicted gene 13483 [Source:MGI Symbol;Acc:MGI:3651423]	1.403	1.09962	0.821254	0.686815	1.00963	1.11217	0.872799	0.795265	0.542628	0.964198	1.14404	1.08839	0.371987	1.34737	0.518444157	0.966018836	24	12	24	0.564601859	0.843293894	24	20.20827105	0.098009068
ENSMUSG00000085873	Ttc39aos1	Ttc39a opposite strand RNA 1 [Source:MGI Symbol;Acc:MGI:3651956]	4.87569	7.13651	6.25165	7.11494	5.53568	5.69646	4.57595	6.79983	5.47937	5.39615	6.94546	4.81097	5.61319	4.48249	0.823677655	0.966018836	16	12	24	0.296305357	0.768040178	24	11.0232839	0.114438272
ENSMUSG00000085875	Gm12905	predicted gene 12905 [Source:MGI Symbol;Acc:MGI:3702581]	0.457259	0.592255	0.52683	0.77553	0.623385	0.655425	0.610759	0.565862	0.741896	0.660465	0.876154	0.825159	0.649808	0.53757	0.044869393	0.367274458	16	16	24	0.022751429	0.581965446	24	15.06088189	0.088067547
ENSMUSG00000085881	Gm15912	predicted gene 15912 [Source:MGI Symbol;Acc:MGI:3801895]	0.683643	1.25607	1.27369	1.00391	0.77289	1.29166	0.732093	0.715086	1.58958	1.19465	0.889736	1.2621	0.83838	1.14004	0.064867922	0.416505219	12	16	24	0.280395316	0.768040178	24	9.705533568	0.123752003
ENSMUSG00000085882	2610507I01Rik	RIKEN cDNA 2610507I01 gene [Source:MGI Symbol;Acc:MGI:1919453]	4.53995	4.87106	4.9751	4.84079	5.07492	4.77355	4.61359	4.1619	4.73587	4.20276	4.72719	4.596	4.49556	4.60711	0.519468828	0.966018836	20	8	24	0.519859039	0.819573919	24	15.19674819	0.027253767
ENSMUSG00000085886	D030047H15Rik	RIKEN cDNA D030047H15 gene [Source:MGI Symbol;Acc:MGI:3698136]	0.600597	0.824654	1.1984	0.791519	0.868363	0.878891	0.795399	0.602899	0.925218	0.794257	0.901564	0.837744	0.776623	0.802467	0.105280783	0.518002936	12	16	24	0.262534387	0.768040178	24	11.85524977	0.069723274
ENSMUSG00000085888	Gm12224	predicted gene 12224 [Source:MGI Symbol;Acc:MGI:3651369]	1.49862	3.60608	2.78303	3.85145	2.78169	4.70979	1.72929	4.13483	6.70909	4.63945	2.95257	3.05198	3.54826	2.70314	0.46238066	0.966018836	16	12	24	0.14592492	0.754579257	24	10.31140868	0.309222341
ENSMUSG00000085890	Tnfsf13os	tumor necrosis factor (ligand) superfamily, member 13, opposite strand [Source:MGI Symbol;Acc:MGI:1919587]	1.00099	1.21951	0.882196	1.36939	1.47962	1.53379	1.20797	1.19021	2.26043	1.1903	0.949798	1.00642	0.94843	0.753432	0.823657258	0.966018836	24	4	24	0.566384141	0.843471947	24	12.3727687	0.092728198
ENSMUSG00000085891	Gm14634	predicted gene 14634 [Source:MGI Symbol;Acc:MGI:3705147]	0.802046	0.791895	0.755039	0.855104	0.725049	0.856408	1.01214	0.91934	0.936262	0.745797	0.681291	1.05627	0.746045	0.93552	0.265774856	0.773510818	24	20	24	0.342669002	0.776697379	24	1.689315868	0.048276435
ENSMUSG00000085894	Gm15832	predicted gene 15832 [Source:MGI Symbol;Acc:MGI:3834078]	1.57604	1.42673	1.6619	1.73192	1.89292	1.29997	2.01473	1.61024	1.16712	1.07605	1.92385	1.0782	1.04298	1.1302	0.894678274	0.966018836	20	4	24	0.627835252	0.872795898	24	16.64410974	0.066309602
ENSMUSG00000085912	Trp53cor1	tumor protein p53 pathway corepressor 1 [Source:MGI Symbol;Acc:MGI:3801771]	0.573107	0.639924	0.494206	0.67287	0.788608	0.472091	0.653101	0.810794	0.612108	0.462409	0.668307	0.558765	0.748648	0.886002	0.478724519	0.966018836	8	16	24	0.516859123	0.817924119	24	3.568218777	0.048409877
ENSMUSG00000085913	Gm15601	predicted gene 15601 [Source:MGI Symbol;Acc:MGI:3783048]	1.77114	1.70216	1.41122	1.73926	1.7594	1.38249	1.55471	1.36317	1.97622	1.22824	2.119	1.30467	1.90183	1.60384	0.91241026	0.966018836	20	4	24	0.988183879	0.997559852	24	14.92255835	0.009178494
ENSMUSG00000085917	Gm15899	predicted gene 15899 [Source:MGI Symbol;Acc:MGI:3801945]	51.2095	48.7328	51.3681	55.1489	48.6777	55.884	44.9381	40.7136	43.2057	48.518	52.6399	54.5961	51.2391	59.2402	0.830364743	0.966018836	24	12	24	0.487534247	0.805393068	24	17.89691982	0.070541376
ENSMUSG00000085925	Rtl1	retrotransposon Gag like 1 [Source:MGI Symbol;Acc:MGI:2656842]	1.07868	1.14434	1.18127	1.15943	1.31465	1.04103	1.05403	1.17756	1.23334	0.973576	1.14938	0.954131	0.781192	1.09732	0.010049466	0.194720062	12	12	24	0.171336994	0.768040178	24	10.61506659	0.059532848
ENSMUSG00000085929	Gm13421	predicted gene 13421 [Source:MGI Symbol;Acc:MGI:3649922]	0.449039	0.473759	1.14948	0.569343	0.602336	0.634548	0.758174	0.454982	0.721702	0.452081	0.639254	0.623851	0.705227	0.698536	0.891022863	0.966018836	12	4	24	0.811107113	0.93944887	24	8.07057311	0.040931971
ENSMUSG00000085936	2610307P16Rik	RIKEN cDNA 2610307P16 gene [Source:MGI Symbol;Acc:MGI:1919768]	3.4768	5.6516	3.49091	7.0669	4.41946	2.96851	4.98346	4.01748	5.478	3.9569	5.9503	4.54579	6.22046	5.7652	0.852844413	0.966018836	20	4	24	0.652615332	0.883085974	24	8.770358584	0.109350149
ENSMUSG00000085939	Cd63-ps	CD63 antigen, pseudogene [Source:MGI Symbol;Acc:MGI:105972]	80.7483	83.5684	80.4189	74.4614	82.8158	72.5874	78.9345	86.0009	91.4362	61.07	84.4182	76.9251	81.9104	84.6764	0.06935957	0.433815306	8	16	24	0.296511143	0.768040178	24	3.440637427	0.082612101
ENSMUSG00000085945	2310014F06Rik	RIKEN cDNA 2310014F06 gene [Source:MGI Symbol;Acc:MGI:1916875]	2.77459	3.24026	2.86458	3.29162	3.91415	2.79148	3.1128	3.4566	3.48488	2.97325	4.02143	2.93315	3.80051	3.04714	0.676344318	0.966018836	20	4	24	0.640906505	0.878468414	24	13.73427877	0.04917876
ENSMUSG00000085946	AA387200	expressed sequence AA387200 [Source:MGI Symbol;Acc:MGI:3034170]	1.05696	0.736469	0.766465	0.764649	0.998059	1.22448	0.955805	1.05278	1.04347	1.16371	0.862547	0.822569	1.04196	0.87461	0.896311169	0.966018836	4	8	24	0.665800924	0.887206076	24	20.26186309	0.042116175
ENSMUSG00000085953	Gm15496	predicted gene 15496 [Source:MGI Symbol;Acc:MGI:3782942]	1.26045	0.949262	0.995984	1.01574	0.975037	1.36219	0.80207	0.915618	1.0621	1.29994	0.965952	1.6086	0.905605	1.1757	0.495929335	0.966018836	24	4	24	0.490957068	0.805393068	24	17.90252537	0.070025112
ENSMUSG00000085956	4930481B07Rik	RIKEN cDNA 4930481B07 gene [Source:MGI Symbol;Acc:MGI:1922187]	0.472507	0.740285	0.746238	1.5595	0.735364	0.61713	0.60127	0.422702	1.12539	0.567399	0.587818	0.908336	0.687874	0.95424	0.662399683	0.966018836	12	16	24	0.257401596	0.768040178	24	10.79098393	0.143666166
ENSMUSG00000085957	Syna	syncytin a [Source:MGI Symbol;Acc:MGI:2684898]	1.84978	2.54294	2.05929	2.37724	2.75145	2.48731	2.41286	2.75051	2.80855	3.39823	1.98134	2.6603	2.52779	1.92201	0.916746631	0.966018836	16	12	24	0.552359995	0.836653527	24	12.3555044	0.07377979
ENSMUSG00000085962	Gm16984	predicted gene, 16984 [Source:MGI Symbol;Acc:MGI:4439908]	0.497913	0.728241	0.549575	0.520587	0.677802	0.631073	0.672565	0.715992	0.829547	0.59576	0.618949	0.546504	0.669481	0.594504	0.965163262	0.969272194	8	8	24	0.525262363	0.822381894	24	5.65048878	0.036284662
ENSMUSG00000085971	Gm15411	predicted gene 15411 [Source:MGI Symbol;Acc:MGI:3705213]	0.531557	0.903614	0.633577	0.69548	0.415424	0.532491	0.483091	0.789263	0.634009	0.72274	0.36662	0.594368	0.537343	0.671263	0.026194135	0.300938431	8	12	24	0.02084529	0.568066329	24	6.324278517	0.127417698
ENSMUSG00000085987	Gm13403	predicted gene 13403 [Source:MGI Symbol;Acc:MGI:3649380]	1.33847	1.53128	1.21948	2.27773	1.93759	1.13144	2.10289	1.87518	2.09171	1.21259	1.62028	2.01192	2.10416	1.18782	0.835668938	0.966018836	4	4	24	0.951659324	0.988339231	24	17.74516874	0.029441558
ENSMUSG00000085998	AW822252	expressed sequence AW822252 [Source:MGI Symbol;Acc:MGI:2148030]	0.366729	0.427	0.959771	0.983257	0.95773	0.772504	0.458882	0.818584	1.40421	0.290193	0.692294	0.677611	0.585137	0.875485	0.03452758	0.328311403	12	16	24	0.306799017	0.768040178	24	10.19608305	0.150449275
ENSMUSG00000086003	B230206L02Rik	RIKEN cDNA B230206L02 gene [Source:MGI Symbol;Acc:MGI:1924598]	6.70165	6.7263	5.86325	4.57988	5.62906	6.65237	7.40356	6.07285	8.22982	5.44602	6.83246	6.31543	6.1269	6.47308	0.305564675	0.811201458	4	12	24	0.237817803	0.768040178	24	1.249577521	0.111016107
ENSMUSG00000086013	Gm15706	predicted gene 15706 [Source:MGI Symbol;Acc:MGI:3783146]	4.59239	5.65462	4.99337	5.38654	5.37342	5.05554	5.66718	6.38179	6.71289	5.60453	5.16023	5.20567	5.97715	5.85904	0.267533088	0.774280302	8	16	24	0.043501448	0.626787045	24	6.551448043	0.101198519
ENSMUSG00000086040	Wipf3	WAS/WASL interacting protein family, member 3 [Source:MGI Symbol;Acc:MGI:3044681]	19.5225	15.5655	13.9135	16.8406	16.4728	17.7108	17.2218	18.6598	14.0307	23.4489	16.3756	17.6192	19.4302	18.9454	0.109040811	0.524035616	4	8	24	0.674066463	0.890479233	24	21.02663418	0.064990134
ENSMUSG00000086043	Gm12473	predicted gene 12473 [Source:MGI Symbol;Acc:MGI:3652337]	3.44715	2.26409	2.8928	1.7916	2.21147	1.65434	2.37678	4.2766	4.66494	6.6196	5.18173	5.70004	4.85925	4.90235	0.658515291	0.966018836	12	12	24	0.600241001	0.860027394	24	7.835545117	0.145635623
ENSMUSG00000086055	Gm13837	predicted gene 13837 [Source:MGI Symbol;Acc:MGI:3650972]	0.694868	1.74926	1.00326	2.06534	1.84922	1.52965	0.559974	1.42423	1.09418	1.68672	1.24548	1.05839	1.41327	0.725011	0.068668966	0.432567661	16	12	24	0.118351508	0.728974286	24	13.38591143	0.224046626
ENSMUSG00000086095	Gm15328	predicted gene 15328 [Source:MGI Symbol;Acc:MGI:3705182]	0.63217	0.707538	0.653032	0.745547	0.57701	0.63778	0.774473	0.389431	0.622017	0.391363	0.625464	0.596641	0.684661	0.665531	0.763955526	0.966018836	4	16	24	0.618590699	0.868995186	24	23.04874502	0.040284003
ENSMUSG00000086096	Gm12688	predicted gene 12688 [Source:MGI Symbol;Acc:MGI:3650434]	8.96454	13.4612	11.755	12.7747	11.5911	11.4266	13.1769	11.7065	16.6648	13.4651	13.1481	12.0893	12.2212	12.4831	0.433658121	0.966018836	16	8	24	0.079603817	0.69332863	24	9.531402174	0.140696601
ENSMUSG00000086098	Gm14291	predicted gene 14291 [Source:MGI Symbol;Acc:MGI:3649962]	0.683039	0.725293	0.877527	1.1369	0.880919	0.879007	0.454192	0.777164	0.720192	0.492379	0.852373	0.634674	0.911186	0.465471	0.783267549	0.966018836	20	8	24	0.380540801	0.799546792	24	14.669247	0.081753257
ENSMUSG00000086108	Gm5602	predicted gene 5602 [Source:MGI Symbol;Acc:MGI:3645136]	0.767784	0.794031	0.970233	0.627677	0.651803	0.649743	0.696031	0.55601	0.694815	0.736115	0.51268	0.676774	0.674693	0.734791	0.237522906	0.74000957	12	8	24	0.261339662	0.768040178	24	5.629650397	0.054225774
ENSMUSG00000086119	Gm2415	predicted gene 2415 [Source:MGI Symbol;Acc:MGI:3780582]	2.98675	2.88678	3.12372	2.90692	2.92059	3.17644	3.36085	2.98079	3.00169	3.15036	2.98552	3.24211	3.14145	2.67287	0.067104747	0.425837275	24	8	24	0.329990719	0.768040178	24	19.85977744	0.037702017
ENSMUSG00000086122	Gm14507	predicted gene 14507 [Source:MGI Symbol;Acc:MGI:3705240]	3.06646	2.31718	2.37236	1.71167	3.02593	2.05283	0.794663	2.88906	2.21855	2.31982	2.21118	1.1423	1.63171	1.88048	0.798829513	0.966018836	12	12	24	0.541586127	0.830485817	24	10.05564501	0.138352527
ENSMUSG00000086124	A530076I17Rik	RIKEN cDNA A530076I17 gene [Source:MGI Symbol;Acc:MGI:2443897]	0.527516	0.554351	0.584305	0.492012	0.609346	0.450412	0.342865	0.738956	0.692927	0.48714	0.463274	0.467759	0.676842	0.525066	0.124211791	0.552144108	12	12	24	0.218613432	0.768040178	24	6.710147136	0.069536928
ENSMUSG00000086129	Gm13558	predicted gene 13558 [Source:MGI Symbol;Acc:MGI:3702047]	1.2273	1.34756	0.999585	1.51078	0.909694	1.55185	1.61694	0.727994	1.25213	1.01426	0.698079	1.18273	0.890538	1.2408	0.894678274	0.966018836	24	20	24	0.73862108	0.914690529	24	23.82292513	0.059373388
ENSMUSG00000086155	9430041J12Rik	RIKEN cDNA 9430041J12 gene [Source:MGI Symbol;Acc:MGI:1924573]	0.991094	1.10445	1.13286	1.10467	0.816491	1.01417	1.22107	0.906728	1.01313	1.15605	1.13739	0.877764	1.17686	1.20673	0.920872048	0.966018836	4	20	24	0.443701124	0.805393068	24	6.009578647	0.04597215
ENSMUSG00000086158	Ccpg1os	cell cycle progression 1, opposite strand [Source:MGI Symbol;Acc:MGI:3648770]	10.581	10.5943	7.10301	8.69067	10.6697	9.79551	10.676	9.55044	9.02196	8.17348	10.4289	9.18584	8.7564	8.51591	0.196044629	0.673646834	20	20	24	0.060338468	0.658743124	24	20.91017394	0.137493265
ENSMUSG00000086165	Gm15690	predicted gene 15690 [Source:MGI Symbol;Acc:MGI:3783131]	0.298733	0.754957	1.94396	0.505211	0.658718	1.08203	0.905811	0.396583	0.708756	0.493966	0.506579	0.772477	1.10558	0.45878	0.893609703	0.966018836	24	8	24	0.984490629	0.996767829	24	3.547633025	0.021818125
ENSMUSG00000086171	Pcsk2os1	proprotein convertase subtilisin/kexin type 2, opposite strand 1 [Source:MGI Symbol;Acc:MGI:3605632]	2.8967	3.35596	3.88581	2.31135	2.80385	2.4493	3.14168	3.56641	2.46618	2.34823	1.76264	3.26033	3.31705	3.00196	0.01457641	0.247599677	8	8	24	0.025262735	0.586541342	24	2.843084707	0.204483002
ENSMUSG00000086179	Gm14317	predicted gene 14317 [Source:MGI Symbol;Acc:MGI:3651690]	0.91439	0.480649	0.431099	0.77165	0.457851	0.520886	0.819044	0.683789	0.583689	0.563711	0.483283	0.562132	0.628168	0.747229	0.086420527	0.467848941	4	16	24	0.267544506	0.768040178	24	1.389828591	0.080279877
ENSMUSG00000086191	Zfp652os	zinc finger protein 652, opposite strand [Source:MGI Symbol;Acc:MGI:3044900]	1.28376	1.89699	1.97472	1.21461	1.18833	1.08122	1.69086	1.71252	1.81843	1.90976	1.38648	1.71938	1.3621	1.21835	0.184044621	0.662239322	12	12	24	0.171134905	0.768040178	24	7.137588734	0.132716534
ENSMUSG00000086212	Mkln1os	muskelin 1, intracellular mediator containing kelch motifs, opposite strand [Source:MGI Symbol;Acc:MGI:3651306]	3.14952	3.58093	3.70071	3.441	2.95472	3.11407	3.78119	3.06855	3.21957	3.11319	3.59882	3.32164	3.41095	3.40927	0.873350015	0.966018836	4	20	24	0.68396426	0.895068737	24	4.442614863	0.029682773
ENSMUSG00000086213	A330040F15Rik	RIKEN cDNA A330040F15 gene [Source:MGI Symbol;Acc:MGI:3698434]	1.06507	0.76078	0.867131	0.669613	0.641294	0.678618	0.627531	0.754768	1.07598	1.18498	1.0816	0.985643	1.2435	1.08958	0.816300547	0.966018836	12	8	24	0.735382864	0.91372816	24	6.785941995	0.044731217
ENSMUSG00000086216	Gm13610	predicted gene 13610 [Source:MGI Symbol;Acc:MGI:3651297]	6.13844	4.82995	2.90844	7.57028	4.4808	6.3482	6.41701	6.27732	4.49829	4.69112	4.455	4.70699	5.90222	3.80422	0.418381424	0.963884303	4	8	24	0.330140285	0.768040178	24	21.19307939	0.177512333
ENSMUSG00000086224	2700069I18Rik	RIKEN cDNA 2700069I18 gene [Source:MGI Symbol;Acc:MGI:1919858]	0.370862	0.753989	0.608099	0.31918	0.347154	0.658204	0.452549	0.373691	0.569997	0.398147	0.637753	0.531866	0.715501	0.939954	0.350544145	0.870418653	8	8	24	0.293344959	0.768040178	24	3.186672193	0.091006115
ENSMUSG00000086228	Ubap1l	ubiquitin-associated protein 1-like [Source:MGI Symbol;Acc:MGI:2685360]	2.58659	2.96553	4.68429	2.67758	2.43566	2.87353	2.90723	3.28811	3.62531	2.74878	3.02951	2.5991	2.17137	4.65179	0.039104	0.338446795	12	12	24	0.034507538	0.608551791	24	7.100010142	0.235230265
ENSMUSG00000086229	Gm4887	predicted gene 4887 [Source:MGI Symbol;Acc:MGI:3645730]	5.5341	5.08165	4.65971	5.3791	4.3796	4.9696	4.35726	4.11812	4.72598	4.21627	4.36031	4.4366	4.69487	4.69374	0.859936714	0.966018836	4	16	24	0.779836861	0.929226662	24	0.535832453	0.024965508
ENSMUSG00000086231	Rapgef4os3	Rap guanine nucleotide exchange factor (GEF) 4, opposite strand 3 [Source:MGI Symbol;Acc:MGI:2442080]	0.524065	0.873326	0.505627	0.522568	0.700194	0.510681	0.524326	0.674855	0.962619	0.550572	0.872632	0.702594	0.766605	0.660149	0.860560779	0.966018836	8	8	24	0.809802041	0.939224719	24	7.41723483	0.033686402
ENSMUSG00000086235	Gm16284	predicted gene 16284 [Source:MGI Symbol;Acc:MGI:3826541]	2.71616	2.31454	2.02456	1.90812	3.6066	3.67732	4.11437	3.44885	2.06888	3.59425	2.98682	3.52122	3.43387	3.14457	0.042880436	0.356048751	24	12	24	0.028615649	0.594253518	24	20.79511791	0.234564817
ENSMUSG00000086240	Gm7846	predicted gene 7846 [Source:MGI Symbol;Acc:MGI:3645212]	14.3764	15.2987	21.3611	15.8102	14.3755	11.6876	14.7206	16.583	21.4891	11.4284	16.9679	14.7113	19.4313	16.3907	0.046056765	0.374351104	12	12	24	0.143089178	0.752803875	24	6.576275708	0.194483868
ENSMUSG00000086247	Gm15787	predicted gene 15787 [Source:MGI Symbol;Acc:MGI:3783229]	2.91783	2.62607	3.42279	3.0463	2.97725	2.14832	2.02913	2.79623	2.82379	2.42919	2.09932	1.87529	1.64953	1.51314	0.180494254	0.657903823	12	16	24	0.006296226	0.46954479	24	10.6755218	0.160198952
ENSMUSG00000086283	2810433D01Rik	RIKEN cDNA 2810433D01 gene [Source:MGI Symbol;Acc:MGI:1924382]	2.01395	1.89399	2.15319	1.18894	2.19636	1.65327	1.13501	2.52211	2.04628	1.16809	1.11545	1.83135	1.87013	1.96197	0.201875332	0.688108864	12	4	24	0.231119798	0.768040178	24	3.565799484	0.157985099
ENSMUSG00000086287	Gm15972	predicted gene 15972 [Source:MGI Symbol;Acc:MGI:3801965]	0.630597	0.5576	0.854834	0.710088	0.810055	0.701892	0.191152	0.975948	0.74772	1.04293	0.74786	1.32644	0.637048	1.09182	0.347721987	0.870418653	16	12	24	0.405195077	0.799546792	24	11.62787145	0.11023903
ENSMUSG00000086288	Gm15265	predicted gene 15265 [Source:MGI Symbol;Acc:MGI:3705193]	2.08705	2.80557	2.56842	2.9484	2.94691	2.29139	1.93307	2.52179	3.65422	2.95362	2.49231	2.31153	1.95762	2.54659	3.50E-05	0.023823328	16	12	24	0.001496658	0.358147394	24	10.50961433	0.21161497
ENSMUSG00000086290	Snhg12	small nucleolar RNA host gene 12 [Source:MGI Symbol;Acc:MGI:1916721]	16.1345	17.5636	19.0504	18.6482	16.7751	14.5097	14.1416	17.8164	19.2207	16.6686	14.0772	17.0439	20.5263	17.2353	0.059650003	0.406067671	12	8	24	0.096131754	0.71175955	24	7.162557423	0.130365069
ENSMUSG00000086291	Gm15513	predicted gene 15513 [Source:MGI Symbol;Acc:MGI:3782961]	1.3816	1.53489	1.59528	1.91514	1.92795	1.22123	1.48111	1.24014	1.98661	1.26639	1.82774	1.50575	2.23014	1.69558	0.888991156	0.966018836	12	12	24	0.724982282	0.909665611	24	11.47475201	0.053252141
ENSMUSG00000086298	Gm11716	predicted gene 11716 [Source:MGI Symbol;Acc:MGI:3649215]	0.522588	1.52046	3.2608	2.21618	2.98662	2.33744	2.22082	4.28724	3.90186	4.40658	1.06606	0.92687	1.57963	1.79703	0.194398468	0.673387182	12	16	24	0.054044009	0.654752212	24	9.462149818	0.503687727
ENSMUSG00000086316	Nbdy	negative regulator of P-body association [Source:MGI Symbol;Acc:MGI:1917373]	60.0759	56.6837	54.265	53.6252	55.61	59.5566	55.4896	58.1388	53.0704	55.436	59.1981	57.1513	58.2424	59.6085	0.030777129	0.324150946	4	8	24	0.032150109	0.606234683	24	22.40365836	0.053868396
ENSMUSG00000086324	Gm15564	predicted gene 15564 [Source:MGI Symbol;Acc:MGI:3783013]	1.00995	0.79649	0.547532	0.118944	1.11009	0.807065	0.816099	0.743517	0.755064	0.699878	0.761918	0.872707	0.567531	0.129875	0.077415205	0.453845658	20	20	24	0.176397839	0.768040178	24	20.08929109	0.190785331
ENSMUSG00000086331	Gm16310	predicted gene 16310 [Source:MGI Symbol;Acc:MGI:3826522]	0.947503	0.734912	0.813429	1.54413	1.4928	0.764189	1.33847	1.02688	1.15788	0.626004	1.26857	0.890446	0.863729	0.931299	0.922029406	0.966018836	20	4	24	0.523405925	0.821182997	24	15.20891368	0.089270891
ENSMUSG00000086350	B230369F24Rik	RIKEN cDNA B230369F24 gene [Source:MGI Symbol;Acc:MGI:2443392]	0.957919	1.02574	0.916754	1.04793	1.23127	1.04711	1.09028	1.05404	1.06295	0.850756	1.01532	1.04827	1.0087	1.36878	0.890648739	0.966018836	8	8	24	0.793215081	0.933800612	24	0.850523711	0.022427786
ENSMUSG00000086358	Gm13270	predicted gene 13270 [Source:MGI Symbol;Acc:MGI:3651704]	0.592507	0.633554	0.742986	0.63607	0.400298	0.528292	0.669502	0.383253	0.692524	0.455769	0.653173	0.866098	0.716407	0.675096	0.890648739	0.966018836	12	4	24	0.794288085	0.933999221	24	1.503786222	0.032298297
ENSMUSG00000086363	A330102I10Rik	RIKEN cDNA A330102I10 gene [Source:MGI Symbol;Acc:MGI:1925170]	8.83545	9.81474	8.50092	6.86654	7.97825	7.93907	11.3456	7.63171	9.29181	7.54355	11.311	9.82664	8.41176	10.2805	0.47028201	0.966018836	4	12	24	0.406638492	0.799546792	24	23.99592743	0.1003196
ENSMUSG00000086368	Gm13830	predicted gene 13830 [Source:MGI Symbol;Acc:MGI:3651141]	1.13745	0.813768	0.815547	1.16994	1.40382	0.952652	0.962508	0.898316	0.790452	0.862601	1.26951	1.26231	1.35712	0.881239	0.001051376	0.07619846	20	16	24	0.012402787	0.525280095	24	18.48097919	0.155189968
ENSMUSG00000086370	Ftx	Ftx transcript, Xist regulator (non-protein coding) [Source:MGI Symbol;Acc:MGI:1926128]	5.26875	5.18772	5.412	4.81342	5.30796	4.41245	5.56138	4.07301	5.86531	3.8323	5.63247	4.44821	4.54084	4.87824	0.93447422	0.966018836	12	12	24	0.979424064	0.994983268	24	6.812046389	0.012585009
ENSMUSG00000086382	Chrna1os	cholinergic receptor, nicotinic, alpha polypeptide 1 (muscle), opposite strand [Source:MGI Symbol;Acc:MGI:3651120]	1.26056	2.972	2.29489	2.82624	2.6236	2.08159	2.21271	2.70996	3.19429	2.49538	2.16474	2.38916	2.21834	3.08567	0.030043575	0.324150946	8	20	24	0.037134373	0.609176161	24	8.808776601	0.202694174
ENSMUSG00000086392	Mccc1os	methylcrotonoyl-Coenzyme A carboxylase 1 (alpha), opposite strand [Source:MGI Symbol;Acc:MGI:3590669]	0.945225	0.854066	0.77828	0.69463	0.651908	0.746893	0.650943	0.662681	0.56791	0.747146	0.769654	0.67254	0.523492	0.737801	0.785563354	0.966018836	8	20	24	0.93985006	0.985383376	24	22.09236855	0.011011967
ENSMUSG00000086399	Gm16144	predicted gene 16144 [Source:MGI Symbol;Acc:MGI:3801782]	1.91771	1.5219	1.23787	2.19533	1.52201	1.42581	2.0459	1.87562	1.85714	1.06918	2.46866	1.60741	0.83924	1.34687	0.877082091	0.966018836	20	16	24	0.77865758	0.928855622	24	16.16814035	0.069479029
ENSMUSG00000086401	Gm15559	predicted gene 15559 [Source:MGI Symbol;Acc:MGI:3783008]	0.664618	0.695726	0.660072	0.635457	0.712373	0.797747	0.491017	0.72489	0.545477	0.685547	0.696042	0.810289	0.680849	0.872512	0.377663653	0.903463289	24	12	24	0.797588883	0.935389147	24	20.03990091	0.022787214
ENSMUSG00000086411	Gm14236	predicted gene 14236 [Source:MGI Symbol;Acc:MGI:3651467]	1.06589	1.02596	1.52486	0.963962	1.04836	1.01766	1.43672	0.953571	0.804128	0.852544	1.15608	0.823556	0.822697	1.09151	0.807813307	0.966018836	20	20	24	0.910688088	0.9747563	24	2.463173723	0.023368786
ENSMUSG00000086425	F730016J06Rik	RIKEN cDNA F730016J06 gene [Source:MGI Symbol;Acc:MGI:2443559]	0.904064	1.17498	1.13435	1.46697	0.915374	1.0395	0.82997	0.841285	1.22504	1.05924	0.852534	1.30896	1.10483	1.30251	0.714044782	0.966018836	12	16	24	0.399986958	0.799546792	24	9.219553288	0.074751933
ENSMUSG00000086427	Hoxa11os	homeobox A11, opposite strand [Source:MGI Symbol;Acc:MGI:107208]	0.410969	0.974036	1.2804	0.729589	0.577871	0.56599	0.990567	0.418039	0.75748	0.663332	0.677444	0.819882	0.511304	0.969294	0.834434272	0.966018836	12	16	24	0.391963468	0.799546792	24	7.800137889	0.11253269
ENSMUSG00000086429	Gt(ROSA)26Sor	gene trap ROSA 26, Philippe Soriano [Source:MGI Symbol;Acc:MGI:104735]	18.6839	19.2136	18.8419	18.6356	19.5803	16.3112	20.0365	17.8454	15.216	12.5317	19.3744	12.3114	12.3431	15.4482	0.874607729	0.966018836	20	4	24	0.99891572	0.999770694	24	8.590576879	0.003549358
ENSMUSG00000086454	Platr14	pluripotency associated transcript 14 [Source:MGI Symbol;Acc:MGI:1922209]	1.61939	1.28713	2.05765	1.33844	2.18819	1.29935	1.37854	2.04332	1.17787	1.57298	1.55177	2.50421	1.86594	1.27123	0.834424969	0.966018836	20	12	24	0.594271988	0.857257137	24	18.68242886	0.090674384
ENSMUSG00000086468	Etaa1os	Ewing tumor-associated antigen 1, opposite strand [Source:MGI Symbol;Acc:MGI:1920221]	1.64572	1.08898	0.612304	0.117407	0.775525	0.52994	1.43881	1.05129	0.976252	1.10672	1.08821	1.27795	0.969175	1.24061	0.223125229	0.717349224	4	12	24	0.102332311	0.713699364	24	0.606963155	0.238209156
ENSMUSG00000086472	Gm16172	predicted gene 16172 [Source:MGI Symbol;Acc:MGI:3801735]	2.61046	2.60592	2.74773	3.77549	3.60016	3.21848	3.24857	2.35513	2.29135	2.84663	3.29144	2.64048	2.79328	3.11544	0.033404002	0.324908923	16	20	24	0.075029869	0.68394125	24	16.27163155	0.134942105
ENSMUSG00000086477	Gm15506	predicted gene 15506 [Source:MGI Symbol;Acc:MGI:3782954]	1.41207	1.95243	1.24496	1.86271	1.3663	1.77005	2.01573	1.96976	2.06544	1.37495	1.47967	1.16106	1.38131	1.41524	0.631736745	0.966018836	8	16	24	0.485705791	0.805393068	24	5.201742441	0.081370877
ENSMUSG00000086495	Gm13778	predicted gene 13778 [Source:MGI Symbol;Acc:MGI:3649233]	1.28376	1.77978	1.84022	1.21461	1.58444	1.38156	1.82092	2.26692	1.81843	1.55609	1.31351	1.23795	1.63895	0.974676	0.433658121	0.966018836	12	4	24	0.410390849	0.799546792	24	5.589938848	0.099308386
ENSMUSG00000086496	Gm14204	predicted gene 14204 [Source:MGI Symbol;Acc:MGI:3651576]	0.265653	0.51176	1.60529	0.622797	0.267476	0.510744	0.733764	0.349156	0.612308	0.54161	0.181274	0.360211	0.214169	0.81601	0.10951302	0.524035616	12	8	24	0.099579999	0.71175955	24	7.603988372	0.240456664
ENSMUSG00000086502	B130055M24Rik	RIKEN cDNA B130055M24 gene [Source:MGI Symbol;Acc:MGI:3590645]	4.15245	4.25234	3.5893	3.89725	4.36797	3.65563	4.42699	3.46207	4.77686	4.02548	4.50297	5.48472	4.86597	4.7973	0.796193867	0.966018836	24	16	24	0.565396263	0.843471947	24	21.17893485	0.054328357
ENSMUSG00000086533	Mypopos	Myb-related transcription factor, partner of profilin, opposite strand [Source:MGI Symbol;Acc:MGI:3826523]	1.74019	2.07747	2.3451	1.64491	1.8385	1.53437	1.71581	1.24003	2.29114	1.11857	1.71976	1.51206	1.62294	1.62116	0.390686537	0.915479136	12	12	24	0.643362811	0.87886261	24	6.829475512	0.065487272
ENSMUSG00000086536	Gm12264	predicted gene 12264 [Source:MGI Symbol;Acc:MGI:3702404]	3.00537	4.06506	4.12087	5.05578	4.56696	4.90105	2.97953	4.92951	5.14374	3.75124	4.93957	3.19526	4.64015	3.12934	0.143609806	0.585944911	12	16	24	0.23682281	0.768040178	24	12.16017534	0.154976177
ENSMUSG00000086539	Gm16759	predicted gene, 16759 [Source:MGI Symbol;Acc:MGI:4439683]	0.541448	1.18568	1.16092	0.63262	0.564688	0.475006	1.24242	0.778203	0.682531	0.742563	0.511034	0.67202	0.784612	0.68792	0.031811505	0.324385159	8	16	24	0.093008901	0.71175955	24	4.798326908	0.156898091
ENSMUSG00000086541	Has2os	hyaluronan synthase 2, opposite strand [Source:MGI Symbol;Acc:MGI:3643465]	1.77721	1.97517	1.47416	2.10356	1.45039	1.49201	2.14602	2.26937	2.41934	1.66869	1.92583	1.97556	2.10377	1.37146	0.81705122	0.966018836	4	16	24	0.733401158	0.912983754	24	4.273114994	0.053874351
ENSMUSG00000086544	Chn1os3	chimerin 1, opposite strand 3 [Source:MGI Symbol;Acc:MGI:3705094]	0.481447	1.1533	0.855709	1.20771	1.19673	0.751644	0.419416	0.805979	1.05834	1.17395	0.84617	1.0113	1.04923	0.861965	0.03484427	0.328311403	16	12	24	0.053789191	0.654390023	24	11.60751266	0.172521437

ENSMUSG00000086555	Gm13446	predicted gene 13446 [Source:MGI Symbol;Acc:MGI:3649869]	1.18623	1.8231	1.95806	1.3673	1.86503	1.8718	1.81483	1.39148	1.6635	2.11759	1.24024	2.09652	1.48033	1.80564	0.955654267	0.96629412	24	4	24	0.892275571	0.969493574	24	11.80601242	0.032451022
ENSMUSG00000086560	Gm13372	predicted gene 13372 [Source:MGI Symbol;Acc:MGI:3650806]	0.61629	1.27434	0.825232	0.520119	0.961453	0.774351	0.924976	0.677761	0.879815	0.652452	1.14444	1.1245	1.0682	0.516355	0.893307667	0.966018836	20	20	24	0.532994079	0.825678486	24	20.30308892	0.087266814
ENSMUSG00000086583	Gm15500	predicted pseudogene 15500 [Source:MGI Symbol;Acc:MGI:3782947]	157.662	199.994	181.053	179.908	182.92	159.761	171.717	163.826	215.806	141.692	186.282	177.824	195.826	187.831	0.798829513	0.966018836	12	12	24	0.577115213	0.848822643	24	6.239873008	0.066258009
ENSMUSG00000086587	Gm11837	predicted gene 11837 [Source:MGI Symbol;Acc:MGI:3702175]	2.15793	2.17455	2.06051	2.08284	2.06518	1.67763	2.16375	1.38237	1.68981	1.87634	2.46549	2.02483	1.14958	2.3071	0.860993221	0.966018836	20	4	24	0.622228126	0.871331547	24	14.49487693	0.070281572
ENSMUSG00000086596	Susd5	sushi domain containing 5 [Source:MGI Symbol;Acc:MGI:2685972]	0.43393	0.611536	0.966694	0.664344	0.65432	0.906055	0.888587	0.780697	0.663628	0.89962	0.516283	0.69752	0.702953	0.872095	0.908406451	0.966018836	12	8	24	0.634651551	0.875714377	24	7.396619461	0.051971527
ENSMUSG00000086604	Gm15510	predicted gene 15510 [Source:MGI Symbol;Acc:MGI:3782958]	1.33763	2.19991	1.73481	1.94367	1.9175	1.83461	1.89911	2.04546	2.33757	2.51899	1.86776	2.07538	1.96093	2.23633	0.291694073	0.798132248	16	12	24	0.066407367	0.668887716	24	9.244811976	0.10497048
ENSMUSG00000086606	Gm13205	predicted gene 13205 [Source:MGI Symbol;Acc:MGI:3700976]	0.801607	1.28066	2.06755	1.26235	1.50397	1.05378	1.98538	1.08465	1.84039	1.50873	1.51939	1.71532	1.49953	1.36678	0.9045879	0.966018836	12	20	24	0.4860223	0.805393068	24	10.40377487	0.099618105
ENSMUSG00000086629	2810403D21Rik	RIKEN cDNA 2810403D21 gene [Source:MGI Symbol;Acc:MGI:1917214]	1.0243	2.07284	2.31481	2.96895	1.81665	1.34386	1.43181	2.04682	3.36553	1.92618	1.71029	2.1214	1.76497	3.04787	0.004320961	0.140598674	12	16	24	0.002872471	0.414576087	24	8.854456212	0.338587717
ENSMUSG00000086638	4930405A21Rik	RIKEN cDNA 4930405A21 gene [Source:MGI Symbol;Acc:MGI:1922096]	1.49687	1.53617	1.27919	2.019	1.11074	1.75857	2.12559	1.63091	1.94688	1.52054	2.02474	1.42194	2.0503	1.81959	0.888379597	0.966018836	4	8	24	0.789248863	0.932388659	24	2.717136149	0.043811211
ENSMUSG00000086644	Gm13470	predicted gene 13470 [Source:MGI Symbol;Acc:MGI:3649387]	0.62318	0.787276	0.844815	1.0529	0.800773	0.520387	0.755681	0.137807	0.633438	0.103064	0.739231	0.705669	0.314722	0.239662	0.832506215	0.966018836	12	20	24	0.365790463	0.789934347	24	16.43660537	0.129757233
ENSMUSG00000086652	Gm14029	predicted gene 14029 [Source:MGI Symbol;Acc:MGI:3650450]	1.13816	0.646985	1.0197	0.577525	0.753852	0.533635	0.776733	0.54052	0.846909	0.884481	0.599761	0.809322	0.667857	0.821364	0.918193129	0.966018836	12	12	24	0.799363651	0.935782011	24	5.561323592	0.03925821
ENSMUSG00000086657	Stamos	signal transducing adaptor molecule (SH3 domain and ITAM motif) 1, opposite strand [Source:MGI Symbol;Acc:MGI:1918207]	0.686275	0.708422	0.54257	0.523569	0.324854	0.718747	0.304224	0.305651	0.629785	0.535939	0.570459	0.647666	0.374535	0.441026	0.882617464	0.966018836	24	4	24	0.882360606	0.966683041	24	16.09999023	0.028231013
ENSMUSG00000086670	Gm13194	predicted gene 13194 [Source:MGI Symbol;Acc:MGI:3651272]	0.543977	0.956884	1.76595	0.301725	0.19633	1.19115	0.985063	0.714521	0.549712	0.72045	1.27483	0.35652	0.369983	1.71607	0.723119329	0.966018836	12	4	24	0.470327948	0.805393068	24	5.433480944	0.191369719
ENSMUSG00000086677	Tvp23bos	trans-golgi network vesicle protein 23B, opposite strand [Source:MGI Symbol;Acc:MGI:3649886]	0.37482	0.904894	0.968108	0.645116	0.526239	0.425706	0.400194	0.839237	0.832084	0.959566	0.386709	0.836236	0.717635	1.04907	0.045862815	0.374349041	8	20	24	0.007103108	0.47735824	24	7.400212117	0.218565539
ENSMUSG00000086679	Gm15551	predicted gene 15551 [Source:MGI Symbol;Acc:MGI:3783000]	0.645371	0.708415	0.817547	0.610564	0.637177	0.66428	0.915428	0.573877	0.731311	1.13788	1.32056	1.03756	1.00917	0.653426	0.887027852	0.966018836	20	12	24	0.338525671	0.773151665	24	16.29270161	0.08643318
ENSMUSG00000086681	Gm16178	predicted gene 16178 [Source:MGI Symbol;Acc:MGI:3801783]	0.620627	0.826681	0.590102	0.796792	1.15519	0.788386	0.566914	0.623037	1.21589	0.615332	1.06263	1.19203	0.932921	0.64254	0.714044782	0.966018836	24	4	24	0.230875419	0.768040178	24	16.12164079	0.116508802
ENSMUSG00000086682	Gm16023	predicted gene 16023 [Source:MGI Symbol;Acc:MGI:3802146]	0.837688	0.61495	0.793181	0.645157	0.7032	0.64024	0.603419	0.584723	1.22899	0.450173	0.98431	0.822937	0.837886	0.669122	0.948582958	0.966018836	20	12	24	0.989452256	0.997867764	24	12.21408561	0.009099716
ENSMUSG00000086688	Gm11560	predicted gene 11560 [Source:MGI Symbol;Acc:MGI:3652066]	5.26607	6.9215	6.77256	7.43183	9.851	7.44213	7.65975	8.65789	8.091	7.15927	7.03146	6.81407	7.86	6.3938	0.81544087	0.966018836	20	4	24	0.594734842	0.857515621	24	13.35171564	0.073499919
ENSMUSG00000086693	A730081D07Rik	RIKEN cDNA A730081D07 gene [Source:MGI Symbol;Acc:MGI:2441825]	1.55951	1.48308	1.77925	1.42479	1.93305	1.40397	1.48342	1.45749	1.32674	1.406	1.45405	1.29185	1.87456	1.28364	0.929527443	0.966018836	4	20	24	0.858408937	0.958316059	24	19.13279276	0.025522202
ENSMUSG00000086712	Mexis	macrophage expressed LXRa(NR1H3)-dependent amplifier of Abca1 transcription lncRNA [Source:MGI Symbol;Acc:MGI:2140270]	0.70848	1.0867	1.23372	0.823829	0.894565	0.666145	1.3843	0.827724	0.986263	0.637934	0.725519	0.725198	0.634162	0.499302	0.960829223	0.967043964	12	12	24	0.699210942	0.900024462	24	6.145800342	0.055595812
ENSMUSG00000086714	0610009E02Rik	RIKEN cDNA 0610009E02 gene [Source:MGI Symbol;Acc:MGI:3698435]	2.77514	2.63319	3.26158	2.84756	2.27444	2.52198	2.28148	1.74264	2.6035	1.92058	2.61676	2.71515	3.27614	2.48172	0.880631923	0.966018836	4	4	24	0.934860902	0.983765461	24	22.4769456	0.027074868
ENSMUSG00000086717	Gm15655	predicted gene 15655 [Source:MGI Symbol;Acc:MGI:3805551]	9.45042	6.50823	8.35914	5.27113	7.32032	6.53547	7.22983	8.83875	8.09319	9.63526	7.28062	7.18654	5.22195	7.16365	0.91695334	0.966018836	12	12	24	0.751792114	0.919351611	24	7.900288308	0.074215327
ENSMUSG00000086725	A630052C17Rik	RIKEN cDNA A630052C17 gene [Source:MGI Symbol;Acc:MGI:2444673]	2.07719	2.3319	2.4296	2.21599	2.43998	2.20532	2.20668	1.67459	2.36541	2.41031	2.62715	2.12015	2.29463	2.04148	0.041285459	0.346472636	20	12	24	0.09224009	0.71175955	24	14.16729437	0.083671133
ENSMUSG00000086728	Man2c1os	mannosidase, alpha, class 2C, member 1, opposite strand [Source:MGI Symbol;Acc:MGI:1924044]	0.877154	1.13349	0.768433	0.749509	1.00854	0.820587	0.593443	1.09566	1.08097	1.32907	1.61182	1.43645	1.00817	1.0174	0.955654267	0.96629412	20	8	24	0.459147506	0.805393068	24	14.21413714	0.081874767
ENSMUSG00000086741	Gm15816	predicted gene 15816 [Source:MGI Symbol;Acc:MGI:3802114]	0.554049	0.691171	0.863189	0.552483	0.549963	0.517824	0.793911	0.949374	1.23306	0.686871	0.956152	1.02351	0.656382	0.601043	0.777666236	0.966018836	12	12	24	0.521608189	0.820407334	24	7.954425521	0.077185473
ENSMUSG00000086742	Gm16201	predicted gene 16201 [Source:MGI Symbol;Acc:MGI:3802153]	1.92099	1.60788	1.56372	1.13683	1.3942	1.53363	1.56228	1.74515	1.14513	1.64693	1.32459	1.5967	1.65792	1.26369	0.125855811	0.555618842	4	12	24	0.072722612	0.679705968	24	23.45141575	0.108257254
ENSMUSG00000086753	Gm15751	predicted gene 15751 [Source:MGI Symbol;Acc:MGI:3783193]	4.73577	5.47556	5.47742	5.35114	4.96618	4.90749	4.64433	4.93302	6.28632	5.1458	5.62258	5.37593	5.81106	5.83815	0.300823736	0.809362476	12	16	24	0.086004829	0.704553215	24	8.485533584	0.079027146
ENSMUSG00000086770	Gm15351	predicted gene 15351 [Source:MGI Symbol;Acc:MGI:3705299]	2.8013	1.97872	2.0545	3.76784	1.78073	2.60321	2.3793	1.3448	2.13537	2.45165	2.9693	2.13435	1.85006	0.757631	0.050258003	0.385784509	16	16	24	0.046704973	0.633507364	24	16.08656139	0.27672642
ENSMUSG00000086779	Gm13562	predicted gene 13562 [Source:MGI Symbol;Acc:MGI:3651238]	2.16541	1.42874	1.32503	1.07396	1.74202	0.976562	0.994987	1.31593	0.94233	1.38229	1.42192	1.24892	1.25694	1.36515	0.911685024	0.966018836	20	4	24	0.799550952	0.935842908	24	21.01837211	0.050591958
ENSMUSG00000086782	E130102H24Rik	RIKEN cDNA E130102H24 gene [Source:MGI Symbol;Acc:MGI:1925116]	6.75396	7.55723	6.8914	8.0345	7.09236	5.92398	5.79477	6.15617	8.90477	6.78614	7.46661	7.84426	8.00427	8.17859	0.745199643	0.966018836	12	16	24	0.312318523	0.768040178	24	9.387558918	0.093424236
ENSMUSG00000086784	Isoc2a	isochorismatase domain containing 2a [Source:MGI Symbol;Acc:MGI:3609243]	8.35393	8.55275	8.73961	9.43557	10.3127	8.36522	7.79201	9.14844	8.98825	8.75957	8.86931	7.86563	8.82002	8.06017	0.06935957	0.433815306	20	4	24	0.078376388	0.68893025	24	12.48066215	0.07639116
ENSMUSG00000086794	Gm11642	predicted gene 11642 [Source:MGI Symbol;Acc:MGI:3651889]	1.59482	1.90595	1.73006	0.817907	1.70642	1.85978	1.58315	1.1555	1.86799	1.43851	1.77134	1.31158	0.877729	1.77934	0.95032543	0.966018836	12	4	24	0.992434578	0.998516607	24	4.503882133	0.011083886
ENSMUSG00000086796	4932702P03Rik	RIKEN cDNA 4932702P03 gene [Source:MGI Symbol;Acc:MGI:1921673]	0.561557	0.496369	0.482413	0.419491	0.482804	0.805764	0.631907	0.411988	0.534527	0.524124	0.534553	0.503185	0.7179	0.365212	0.031295391	0.324385159	4	4	24	0.08116865	0.697074851	24	20.65364194	0.092605845
ENSMUSG00000086804	Gm43154	predicted gene 43154 [Source:MGI Symbol;Acc:MGI:5663291]	2.35313	1.78285	1.79531	2.20446	2.46005	2.56527	1.94519	2.31734	1.99122	1.83279	2.02553	2.44274	2.35109	1.49452	0.019199841	0.192760771	24	8	24	0.021964638	0.574400012	24	19.21112055	0.149094835
ENSMUSG00000086806	Gm13054	predicted gene 13054 [Source:MGI Symbol;Acc:MGI:3702728]	1.79765	1.11827	1.04058	2.14159	1.81923	1.528	1.20638	1.30303	1.5023	1.6025	1.12785	1.0123	1.72051	1.03728	0.616109934	0.966018836	16	16	24	0.400239693	0.799546792	24	16.2327911	0.105619509
ENSMUSG00000086824	4930448D08Rik	RIKEN cDNA 4930448D08 gene [Source:MGI Symbol;Acc:MGI:1921921]	0.549117	0.688797	0.438046	0.547055	0.631668	0.499906	0.357581	0.59088	0.650658	0.532009	0.630384	0.652437	0.657533	0.572107	0.858514388	0.966018836	8	8	24	0.961413902	0.99009168	24	12.55795008	0.009653045
ENSMUSG00000086825	Gm15675	predicted gene 15675 [Source:MGI Symbol;Acc:MGI:3783117]	10.04	15.7942	14.0546	14.6692	10.9093	10.7403	10.2723	12.9375	17.8625	12.4986	11.8076	13.3005	12.3979	14.9722	0.008389425	0.192760771	12	16	24	0.005707913	0.453015886	24	7.982275127	0.249792888
ENSMUSG00000086826	Gm11739	predicted gene 11739 [Source:MGI Symbol;Acc:MGI:3650571]	2.49032	1.76973	1.62393	2.11356	1.85633	1.24682	2.42741	2.60515	2.27997	1.73982	1.65831	1.22882	1.90015	2.19249	0.030302256	0.324150946	4	20	24	0.116608591	0.727799063	24	4.954160436	0.168456609
ENSMUSG00000086841	2410006H16Rik	RIKEN cDNA 2410006H16 gene [Source:MGI Symbol;Acc:MGI:1916471]	29.2529	38.2195	37.2745	26.5408	27.3932	25.0427	34.3715	31.7638	44.346	30.7958	42.2276	39.3963	35.4358	37.5651	0.723119329	0.966018836	12	4	24	0.294111979	0.768040178	24	6.060774842	0.139722754
ENSMUSG00000086844	B230206H07Rik	RIKEN cDNA B230206H07 gene [Source:MGI Symbol;Acc:MGI:2444929]	38.6753	44.942	36.6894	38.4964	50.0316	33.4664	48.4415	42.2613	51.0292	38.1019	42.4406	36.9772	47.2823	36.8607	0.91241026	0.966018836	4	20	24	0.903004703	0.97297944	24	5.068530663	0.035104467
ENSMUSG00000086847	Tbx3os2	T-box 3, opposite strand 2 [Source:MGI Symbol;Acc:MGI:3801954]	3.49029	3.14074	2.33667	3.78272	2.82151	3.13349	2.21317	3.25135	2.91435	3.67995	2.93703	3.37369	3.6826	3.39372	0.921572192	0.966018836	16	20	24	0.970677365	0.99300601	24	15.00145661	0.016522989
ENSMUSG00000086855	Gm13844	predicted gene 13844 [Source:MGI Symbol;Acc:MGI:3651305]	0.669182	0.533082	0.622994	0.652501	0.354694	0.753051	0.930452	0.340492	0.390316	0.442386	0.391852	0.490908	0.767828	0.578792	0.128989168	0.560209134	4	16	24	0.151035746	0.758865539	24	23.38476203	0.117140459
ENSMUSG00000086859	Snhg20	small nucleolar RNA host gene 20 [Source:MGI Symbol;Acc:MGI:1924222]	16.6047	19.4106	17.2475	20.962	17.858	16.2948	18.2865	18.3488	25.5949	20.3855	29.1753	24.0307	25.4534	23.6904	0.882263435	0.966018836	20	4	24	0.498120837	0.807927978	24	11.33290467	0.079944097
ENSMUSG00000086863	Gm15543	predicted gene 15543 [Source:MGI Symbol;Acc:MGI:3782992]	0.406989	1.43625	1.54331	1.5935	1.6313	1.12443	1.72372	1.97102	1.65023	1.07749	0.960264	0.662176	3.16548	1.42464	0.834434272	0.966018836	4	20	24	0.389542392	0.799546792	24	6.050454362	0.20282074
ENSMUSG00000086873	Gm15672	predicted gene 15672 [Source:MGI Symbol;Acc:MGI:3783114]	1.23592	1.16886	0.900268	1.23356	1.31778	0.815392	0.952159	0.947949	1.07783	0.684583	0.986919	0.78923	0.830503	0.770383	0.860709018	0.966018836	20	4	24	0.87972205	0.966348076	24	15.17079555	0.021270024
ENSMUSG00000086877	A230072C01Rik	RIKEN cDNA A230072C01 gene [Source:MGI Symbol;Acc:MGI:2444644]	4.54288	4.91485	5.13088	5.25783	5.5078	4.5282	6.3852	4.86539	5.36271	4.5309	5.23654	5.21006	6.1341	5.01012	0.879206539	0.966018836	4	4	24	0.831220516	0.947661285	24	23.25027935	0.029989901
ENSMUSG00000086889	Phf2os1	PHD finger protein 2, opposite strand 1 [Source:MGI Symbol;Acc:MGI:3645592]	1.19595	1.87706	0.7789	1.76143	1.94402	1.00497	0.871797	1.4868	1.70604	1.18627	1.95458	1.61739	0.882696	1.09341	0.086420527	0.467848941	20	8	24	0.290275472	0.768040178	24	13.77639884	0.157088275
ENSMUSG00000086890	1700021J08Rik	RIKEN cDNA 1700021J08 gene [Source:MGI Symbol;Acc:MGI:1919485]	1.03356	1.00698	0.87609	1.27022	1.48732	1.18882	0.882764	1.00678	1.63198	0.935285	0.897568	1.15938	1.00511	0.783007	0.640622311	0.966018836	16	16	24	0.396883858	0.799546792	24	14.55266932	0.082867458
ENSMUSG00000086903	Hotair	HOX transcript antisense RNA (non-protein coding) [Source:MGI Symbol;Acc:MGI:3826586]	0.386877	1.17043	1.66067	1.31298	1.18042	0.833998	1.73363	0.463394	1.25036	1.05044	0.682445	1.02386	0.528202	0.732625	0.23143369	0.731037655	12	16	24	0.344612038	0.778015201	24	11.22268056	0.169838505
ENSMUSG00000086905	Gm13716	predicted gene 13716 [Source:MGI Symbol;Acc:MGI:3650432]	1.11658	0.528792	0.930818	0.885701	0.809457	0.932949	0.827503	0.598325	0.535284	0.978364	0.750788	0.536894	0.542622	1.06588	0.786317982	0.966018836	16	20	24	0.847341248	0.953981454	24	14.95528621	0.037963071
ENSMUSG00000086914	Gm16124	predicted gene 16124 [Source:MGI Symbol;Acc:MGI:3802001]	2.06797	2.69989	3.48592	3.73044	3.27197	2.87981	1.24064	3.32605	3.3916	3.2249	2.74292	2.84633	2.28169	2.67441	3.15E-04	0.049770315	12	16	24	0.011281727	0.517565995	24	11.2138593	0.285089045
ENSMUSG00000086915	Gm16364	predicted gene 16364 [Source:MGI Symbol;Acc:MGI:3840142]	8.70946	7.35511	7.05668	8.28874	5.81752	9.14398	7.51181	9.08429	5.65186	8.83408	6.6149	8.51956	9.32788	8.66183	0.140384419	0.585715181	4	8	24	0.287802791	0.768040178	24	23.94657918	0.126717544
ENSMUSG00000086918	4930429F24Rik	RIKEN cDNA 4930429F24 gene [Source:MGI Symbol;Acc:MGI:1921883]	7.47968	9.51279	8.32878	9.21464	8.40675	7.49154	8.13739	9.44023	11.3313	9.88084	7.99786	8.89879	10.0993	7.76784	0.435596482	0.966018836	12	12	24	0.143147088	0.752803875	24	8.477577179	0.120418779
ENSMUSG00000086920	Gm12207	predicted gene 12207 [Source:MGI Symbol;Acc:MGI:3702399]	1.08953	1.16444	0.394201	1.23407	1.16249	0.610198	1.17652	1.1823	0.493354	0.640429	1.31875	0.701787	1.05611	0.830423	0.791413662	0.966018836	8	4	24	0.518663477	0.818725157	24	21.10224678	0.114104113
ENSMUSG00000086922	Gm13835	predicted gene 13835 [Source:MGI Symbol;Acc:MGI:3650921]	176.817	189.379	183.594	184.041	194.412	172.311	172.759	178.457	202.899	154.72	198.601	180.507	184.39	192.13	0.44861185	0.966018836	20	4	24	0.854786418	0.956691175	24	8.134040962	0.023387398
ENSMUSG00000086935	Gm15956	predicted gene 15956 [Source:MGI Symbol;Acc:MGI:3801856]	0.827186	0.837642	1.02623	0.80347	0.611336	0.666161	0.574303	0.52474	0.321119	0.390703	0.685322	0.608675	0.792752	0.719952	0.732668271	0.966018836	20	20	24	0.772400626	0.926838763	24	22.17724664	0.048194784
ENSMUSG00000086938	4930481A15Rik	RIKEN cDNA 4930481A15 gene [Source:MGI Symbol;Acc:MGI:1922181]	2.21024	2.26233	3.21422	3.0929	2.92058	3.00836	2.95938	2.6255	2.37717	2.35852	2.69542	3.09079	2.82894	3.00476	0.939921059	0.966018836	20	4	24	0.66748724	0.887486893	24	17.0652519	0.047510404
ENSMUSG00000086939	Gm13530	predicted gene 13530 [Source:MGI Symbol;Acc:MGI:3651890]	0.35463	0.358019	1.53768	0.398167	0.648716	0.295104	1.5036	0.469525	0.839998	0.821293	1.19884	1.03537	1.19641	0.959723	0.846643334	0.966018836	12	12	24	0.925856629	0.980197974	24	8.114626627	0.049199372
ENSMUSG00000086965	Rtl10	retrotransposon Gag like 10 [Source:MGI Symbol;Acc:MGI:3833934]	1.07659	0.764497	0.924066	1.03844	0.860802	1.07988	0.906136	1.11514	0.398636	0.765431	1.01192	0.723382	1.09542	0.844484	0.833406028	0.966018836	4	8	24	0.248512794	0.768040178	24	21.32714238	0.105787152
ENSMUSG00000086968	4933431E20Rik	RIKEN cDNA 4933431E20 gene [Source:MGI Symbol;Acc:MGI:3584041]	15.6487	18.377	16.9904	16.8404	16.6204	16.8719	17.0006	15.2032	18.2341	18.0281	13.8518	17.5533	15.7184	18.6462	0.542718076	0.966018836	8	12	24	0.382245069	0.799546792	24	7.001037179	0.063641017
ENSMUSG00000087001	Gm15475	predicted gene 15475 [Source:MGI Symbol;Acc:MGI:3705288]	0.453378	0.478422	0.528611	0.538627	0.742873	0.385342	0.729738	0.327092	0.590656	0.458938	0.707682	0.476963	0.630631	0.367264	0.117050591	0.538362324	20	12	24	0.350827977	0.780345856	24	16.27917231	0.07146235
ENSMUSG00000087006	Gm13889	predicted gene 13889 [Source:MGI Symbol;Acc:MGI:3652053]	27.7435	17.1269	17.7974	15.7557	14.5391	14.738	19.2102	26.1352	21.9402	33.2781	33.9366	32.7432	35.3724	31.9225	0.84680398	0.966018836	4	16	24	0.713023445	0.905403147	24	1.934273183	0.106881668
ENSMUSG00000087013	2610027K06Rik	RIKEN cDNA 2610027K06 gene [Source:MGI Symbol;Acc:MGI:1917159]	1.6957	2.10712	1.75851	1.47106	1.91245	2.11659	2.33748	2.17002	1.84441	2.24246	2.14982	2.0845	1.68331	1.91101	0.854443826	0.966018836	20	16	24	0.759626053	0.92234382	24	21.38031659	0.03723907
ENSMUSG00000087014	Gm16364	predicted gene 16364 [Source:MGI Symbol;Acc:MGI:3840142]	8.69355	7.56202	6.45509	6.78375	7.22369	8.40463	7.66717	7.85947	6.29775	9.1608	6.77424	7.92466	8.28302	7.54342	0.020119233	0.259417302	24	12	24	0.167965047	0.767020009	24	22.0185576	0.104235404
ENSMUSG00000087022	9130024F11Rik	RIKEN cDNA 9130024F11 gene [Source:MGI Symbol;Acc:MGI:1926150]	0.996585	1.12981	0.690876	0.374343	0.674265	0.637807	0.471192	1.09134	1.35041	0.706123	0.740617	0.862659	0.492765	0.909029	0.260026661	0.765037845	12	4	24	0.257491123	0.768040178	24	5.242533348	0.142424023
ENSMUSG00000087026	A230103J11Rik	RIKEN cDNA A230103J11 gene [Source:MGI Symbol;Acc:MGI:2444082]	1.24982	1.29542	1.3145	1.37709	1.71765	1.44131	1.47048	1.57081	1.48654	1.35389	1.42712	1.52771	1.81551	1.33891	0.68887295	0.966018836	24	8	24	0.510268105	0.814059488	24	19.56095716	0.039713987
ENSMUSG00000087034	Cbfa2t2-ps1	CBFA2/RUNX1 translocation partner 2, pseudogene 1 [Source:MGI Symbol;Acc:MGI:1333788]	1.61612	1.28409	1.52738	1.52554	1.04772	1.2315	0.770563	1.69441	0.766545	1.43953	1.01162	1.30325	0.797436	1.63668	0.496745696	0.966018836	8	12	24	0.690174012	0.897381208	24	7.470514916	0.076501756
ENSMUSG00000087038	2900079G21Rik	RIKEN cDNA 2900079G21 gene [Source:MGI Symbol;Acc:MGI:1920268]	0.675424	0.716865	0.882085	0.867953	0.583903	0.62889	1.39407	0.932687	0.979218	1.31787	1.42252	1.94757	1.19417	1.39359	0.715742619	0.966018836	24	12	24	0.983179207	0.996355202	24	20.8395606	0.013137665
ENSMUSG00000087042	Gm11611	predicted gene 11611 [Source:MGI Symbol;Acc:MGI:3707011]	2.86247	3.04466	3.628	2.92607	3.07804	2.908	2.76712	2.69782	3.64196	2.21505	3.24974	2.5424	2.23127	3.08119	0.112800839	0.530777648	12	16	24	0.383528438	0.799546792	24	9.166237058	0.083666552
ENSMUSG00000087055	Gm11948	predicted gene 11948 [Source:MGI Symbol;Acc:MGI:3650068]	1.38325	2.26421	1.38644	1.69147	2.60053	2.03153	2.19929	2.08181	2.38751	2.04016	1.5243	1.77838	2.56675	2.1001	0.860560779	0.966018836	8	8	24	0.84235873	0.952050674	24	2.630970369	0.043834507
ENSMUSG00000087064	Sap30bpos	SAP30 binding protein, opposite strand [Source:MGI Symbol;Acc:MGI:3650575]	1.08531	1.74023	1.43711	1.31429	1.78388	1.42535	1.28243	1.67439	1.86988	1.46936	1.24917	2.07851	1.58534	1.37926	0.92769718	0.966018836	12	16	24	0.930651372	0.982155977	24	9.458444739	0.02392217
ENSMUSG00000087088	Gm16638	predicted gene, 16638 [Source:MGI Symbol;Acc:MGI:4439562]	2.23501	2.17912	2.18566	2.02593	2.08092	1.84484	1.91175	2.13118	2.13303	2.06662	2.14543	2.26832	1.87426	2.07792	0.890311217	0.966018836	12	4	24	0.666104726	0.887215207	24	8.81865211	0.022706455
ENSMUSG00000087090	Nctc1	non-coding transcript 1 [Source:MGI Symbol;Acc:MGI:1306816]	0.377057	1.12643	1.43585	1.87741	0.841231	1.55645	0.823302	2.01048	2.19829	2.80398	0.911685	1.23782	1.54998	1.2333	0.113148904	0.530777648	16	12	24	0.034859281	0.608551791	24	9.673260228	0.341190954
ENSMUSG00000087107	AI662270	expressed sequence AI662270 [Source:MGI Symbol;Acc:MGI:2144254]	1.33402	2.41267	3.96787	2.84518	1.71112	2.20506	1.60789	1.9207	2.64373	1.20704	1.72094	2.03805	2.12697	1.22833	0.851656735	0.966018836	12	16	24	0.462584292	0.805393068	24	9.226110879	0.154192145
ENSMUSG00000087114	Gm16099	predicted gene 16099 [Source:MGI Symbol;Acc:MGI:3802061]	0.826068	1.57186	0.203631	0.60898	0.555481	0.824522	1.56785	0.978646	0.710814	0.737945	0.990869	1.0312	1.25916	0.604677	0.053683798	0.394331071	4	8	24	0.074339508	0.683293901	24	23.22886217	0.244256156
ENSMUSG00000087119	Atg4a-ps	autophagy related 4A, pseudogene [Source:MGI Symbol;Acc:MGI:3615624]	4.687	5.07055	4.96826	4.81866	5.20653	4.40071	3.99258	4.79447	5.77742	4.5427	5.05678	5.34731	5.64601	5.25474	0.896753532	0.966018836	12	4	24	0.59889083	0.859228432	24	8.476978614	0.046066057
ENSMUSG00000087128	Gm12655	predicted gene 12655 [Source:MGI Symbol;Acc:MGI:3651648]	2.77632	2.40629	1.81541	2.66441	1.99043	2.01267	1.71834	2.09667	2.88334	2.00857	1.79293	2.18191	1.8426	2.06696	0.839634479	0.966018836	8	12	24	0.655285829	0.883697438	24	7.578654452	0.059537052
ENSMUSG00000087135	Gm16096	predicted gene 16096 [Source:MGI Symbol;Acc:MGI:3801985]	0.390445	0.881619	1.35939	0.741345	0.911835	0.545248	0.97252	1.01151	0.902163	0.497273	0.809278	0.348142	0.685567	0.643434	0.148073804	0.590732968	12	12	24	0.166892792	0.767020009	24	7.811179965	0.160337623
ENSMUSG00000087138	Gm15545	predicted gene 15545 [Source:MGI Symbol;Acc:MGI:3782994]	1.61821	2.31657	2.26703	2.32691	2.16634	1.42998	1.54305	2.01552	2.06968	1.91043	1.88008	2.03346	1.70759	1.79495	0.026323104	0.301028315	12	16	24	0.026601113	0.586541342	24	10.03891127	0.135510134
ENSMUSG00000087141	Plcxd2	phosphatidylinositol-specific phospholipase C, X domain containing 2 [Source:MGI Symbol;Acc:MGI:3647874]	19.5903	16.5423	16.4601	16.7029	16.0522	17.6413	16.5611	15.7143	14.1272	16.6364	16.7277	16.9436	16.8873	17.5747	0.020957534	0.262822894	24	12	24	0.098032414	0.71175955	24	21.52553659	0.077733269
ENSMUSG00000087142	Gm12454	predicted gene 12454 [Source:MGI Symbol;Acc:MGI:3649695]	1.09072	1.08835	1.35266	0.950813	0.87699	0.664323	0.416444	0.907523	1.09895	1.0631	1.27291	1.22958	1.29915	1.27445	0.937921962	0.966018836	12	12	24	0.43669765	0.805393068	24	8.848087256	0.102462599
ENSMUSG00000087143	A830082K12Rik	RIKEN cDNA A830082K12 gene [Source:MGI Symbol;Acc:MGI:2443527]	7.94042	10.0453	6.91477	6.96122	7.87871	8.44636	8.85218	7.16005	6.63904	6.53422	8.17449	6.39024	5.41655	7.25036	0.696669634	0.966018836	8	4	24	0.614857921	0.866749903	24	22.03886499	0.066989322
ENSMUSG00000087150	BC064078	cDNA sequence BC064078 [Source:MGI Symbol;Acc:MGI:3040692]	0.871304	1.0663	1.29125	0.684122	0.879155	0.827906	0.997047	1.06621	0.901045	0.587671	0.832099	0.706186	0.738565	0.988248	0.0140901	0.241938013	12	4	24	0.060457068	0.659019358	24	4.390497076	0.111635958
ENSMUSG00000087153	Gm6483	predicted gene 6483 [Source:MGI Symbol;Acc:MGI:3644574]	1.02433	1.0696	1.05182	0.977706	0.765574	0.87088	0.485779	1.17084	0.893341	0.864832	0.625386	0.726533	0.986365	0.468551	0.388056094	0.915479136	8	12	24	0.595191862	0.857704078	24	7.043484213	0.063384208
ENSMUSG00000087159	Gm15246	predicted gene 15246 [Source:MGI Symbol;Acc:MGI:3708120]	0.848947	1.50578	1.72841	1.80751	1.41474	1.31106	1.0322	0.849392	1.22665	0.771773	1.44787	0.68213	0.995861	1.28879	0.268242676	0.774280302	12	16	24	0.252992414	0.768040178	24	10.93514534	0.139338599
ENSMUSG00000087165	2010001A14Rik	RIKEN cDNA 2010001A14 gene [Source:MGI Symbol;Acc:MGI:1923766]	7.76525	6.47937	6.74992	7.48862	5.27293	5.10271	7.65902	5.65048	5.5334	5.4132	4.08769	5.25013	5.80494	5.65682	0.010310139	0.19599406	4	20	24	0.136462838	0.747972781	24	3.352823847	0.131902293
ENSMUSG00000087174	5530601H04Rik	RIKEN cDNA 5530601H04 gene [Source:MGI Symbol;Acc:MGI:1918695]	12.8026	13.4596	12.6664	12.1029	11.8214	9.91389	12.2724	11.8382	13.8491	11.1012	12.2119	11.8882	12.872	11.8518	0.259197958	0.763633007	4	20	24	0.171646684	0.768040178	24	5.626832583	0.078277343
ENSMUSG00000087177	E130307A14Rik	RIKEN cDNA E130307A14 gene [Source:MGI Symbol;Acc:MGI:3036287]	13.8471	15.6049	17.2052	18.6705	12.9764	14.3124	15.5225	13.9354	17.6582	16.5523	13.9196	15.9476	15.7241	16.2296	0.020428812	0.261278501	16	4	24	0.067399644	0.670457112	24	8.591988618	0.12457686
ENSMUSG00000087178	A230056P14Rik	RIKEN cDNA A230056P14 gene [Source:MGI Symbol;Acc:MGI:2444856]	3.99389	2.89028	2.62519	2.80324	3.68739	3.27875	3.16639	3.31533	3.12345	2.43477	3.93219	3.43241	3.23098	2.73976	0.006767691	0.181382464	20	20	24	0.030134423	0.596794384	24	20.22378681	0.156205944
ENSMUSG00000087179	5730471H19Rik	RIKEN cDNA 5730471H19 gene [Source:MGI Symbol;Acc:MGI:3642639]	0.699275	1.04717	0.608219	0.897176	1.6519	0.856241	1.4688	1.12434	1.49629	1.16324	0.957116	0.866512	0.926831	1.33217	0.93717134	0.966018836	20	4	24	0.85878935	0.958407503	24	9.336898429	0.04737922
ENSMUSG00000087187	Gm13431	predicted gene 13431 [Source:MGI Symbol;Acc:MGI:3650776]	0.128111	0.524121	0.623988	0.623803	0.902007	0.428437	0.480385	0.682354	0.701333	0.249722	0.362812	0.616015	0.499561	0.236216	0.596159714	0.966018836	12	16	24	0.662251284	0.885924039	24	12.17482963	0.074596971
ENSMUSG00000087196	Gm13373	predicted gene 13373 [Source:MGI Symbol;Acc:MGI:3650390]	1.45945	1.89223	1.32147	1.75697	1.38754	2.19582	1.28349	2.53125	1.2152	1.66131	1.8316	1.61002	2.60105	1.71111	0.9045879	0.966018836	8	4	24	0.578060166	0.849184181	24	23.68523339	0.088709106
ENSMUSG00000087200	Lrp8os3	low density lipoprotein receptor-related protein 8, apolipoprotein e receptor, opposite strand 3 [Source:MGI Symbol;Acc:MGI:3651133]	0.567165	0.677585	0.64398	0.766966	0.517384	0.758484	0.873786	0.3986	0.556812	0.602613	0.612494	0.735907	0.622593	0.566964	0.725061903	0.966018836	24	8	24	0.451040362	0.805393068	24	19.3797131	0.054816569
ENSMUSG00000087211	Lhx1os	LIM homeobox 1, opposite strand [Source:MGI Symbol;Acc:MGI:1925615]	0.183215	0.71723	1.70396	0.25161	0.136057	0.46425	0.796984	0.163219	1.07524	0.65574	0.151186	0.646537	0.074326	1.1383	0.062036003	0.409814092	12	8	24	0.141729785	0.751766185	24	7.080370918	0.317133252
ENSMUSG00000087231	E230016M11Rik	RIKEN cDNA E230016M11 gene [Source:MGI Symbol;Acc:MGI:2443524]	2.4255	4.25629	4.21135	3.1182	2.81292	3.25072	2.68002	3.55118	4.12659	2.82528	2.38438	4.12362	2.97216	3.41412	0.064422003	0.414867207	12	8	24	0.180614798	0.768040178	24	6.479710969	0.161522406
ENSMUSG00000087249	Gm16062	predicted gene 16062 [Source:MGI Symbol;Acc:MGI:3802162]	2.83721	2.22672	2.75923	3.40724	3.43308	2.93025	2.36671	3.17756	2.65422	2.34626	3.14237	2.52	2.89223	2.8231	0.658942124	0.966018836	20	16	24	0.405407907	0.799546792	24	15.25907326	0.075071385
ENSMUSG00000087259	2610035D17Rik	RIKEN cDNA 2610035D17 gene [Source:MGI Symbol;Acc:MGI:1919636]	7.9877	6.30683	7.05022	7.23013	6.48325	6.75564	7.31217	7.75127	6.94763	7.09762	6.46715	7.34182	7.39592	8.13404	0.200625581	0.685057195	4	16	24	0.142403687	0.751766185	24	2.794060217	0.069086551
ENSMUSG00000087260	Lamtor5	late endosomal/lysosomal adaptor, MAPK and MTOR activator 5 [Source:MGI Symbol;Acc:MGI:1915826]	62.085	71.0921	58.7465	62.9565	64.4814	61.056	62.6849	65.949	68.3771	53.6498	73.4672	67.8913	71.4196	70.2516	0.909604019	0.966018836	8	8	24	0.494927212	0.806761911	24	0.836201787	0.053251682
ENSMUSG00000087266	Gm15991	predicted gene 15991 [Source:MGI Symbol;Acc:MGI:3801813]	0.612332	0.682787	0.832313	0.954461	1.06902	0.90948	0.93374	0.642847	0.943754	0.687402	0.971693	0.783497	0.985904	0.762611	0.038715855	0.336039716	20	12	24	0.188081974	0.768040178	24	15.80893652	0.078411729
ENSMUSG00000087269	D330023K18Rik	RIKEN cDNA D330023K18 gene [Source:MGI Symbol;Acc:MGI:2445152]	2.62908	3.96005	3.8671	3.70275	3.73877	3.24408	3.46161	4.38974	4.7138	2.66352	3.96298	3.71157	4.30614	4.30761	0.188118386	0.663314657	8	16	24	0.23571676	0.768040178	24	6.322987651	0.120722801
ENSMUSG00000087298	Gm9392	predicted pseudogene 9392 [Source:MGI Symbol;Acc:MGI:3643262]	24.8529	26.2256	23.6568	24.5213	26.2277	20.3465	26.3686	25.8438	24.4861	19.8795	21.8499	21.4866	24.113	21.6364	0.714062999	0.966018836	4	20	24	0.30249179	0.768040178	24	3.189017129	0.069099726
ENSMUSG00000087299	Gm12953	predicted gene 12953 [Source:MGI Symbol;Acc:MGI:3649497]	1.24234	0.619687	1.01606	0.707373	1.7083	0.850391	0.885622	0.383159	0.887544	0.463416	0.642669	0.718758	0.610834	1.38409	0.846643334	0.966018836	12	4	24	0.853616656	0.956260763	24	19.70705754	0.062895415
ENSMUSG00000087301	Gm13629	predicted gene 13629 [Source:MGI Symbol;Acc:MGI:3650683]	2.65961	2.38352	2.50136	2.66453	2.08154	2.6926	3.1364	1.84539	1.91041	2.95826	1.99817	2.03796	1.79282	2.78376	0.858266942	0.966018836	16	4	24	0.999172555	0.999770694	24	3.24731563	0.002973532
ENSMUSG00000087305	A430035B10Rik	RIKEN cDNA A430035B10 gene [Source:MGI Symbol;Acc:MGI:2443789]	0.97226	1.42892	1.42626	1.04215	0.966137	1.13384	1.09828	1.15397	0.955026	1.22463	1.55308	1.17802	1.18288	1.40135	0.882263435	0.966018836	8	20	24	0.765084943	0.92403577	24	7.284346522	0.037035795
ENSMUSG00000087314	Prkag2os2	protein kinase, AMP-activated, gamma 2 non-catalytic subunit, opposite strand 2 [Source:MGI Symbol;Acc:MGI:3783001]	1.46096	1.46799	1.34181	1.38057	1.233	1.19043	1.38062	1.2811	1.79944	1.29078	1.34301	1.52872	1.10625	1.53522	0.723119329	0.966018836	12	8	24	0.351709598	0.780345856	24	7.015975911	0.060261804
ENSMUSG00000087328	Gm12120	predicted gene 12120 [Source:MGI Symbol;Acc:MGI:3651991]	0.372477	0.944244	0.606875	0.423913	0.829577	0.419367	0.566534	0.663528	0.760542	1.16957	0.445465	0.716981	0.685428	1.19722	0.648082424	0.966018836	8	20	24	0.179916604	0.768040178	24	7.508754337	0.144546869
ENSMUSG00000087331	1810021B22Rik	RIKEN cDNA 1810021B22 gene [Source:MGI Symbol;Acc:MGI:1916370]	1.67804	1.15396	1.62747	1.95406	0.99272	1.15154	1.35195	2.14057	0.907796	1.35541	1.55783	1.34537	1.25879	1.92492	0.834424969	0.966018836	8	16	24	0.616965487	0.867884935	24	5.631641031	0.086927394
ENSMUSG00000087336	Gm15860	predicted gene 15860 [Source:MGI Symbol;Acc:MGI:3801778]	2.09101	2.02094	1.43166	2.93438	1.42939	1.57284	1.9344	2.49869	3.60514	2.59284	1.73709	1.68174	1.93285	1.54904	0.356953959	0.881188925	16	4	24	0.210018628	0.768040178	24	8.605106169	0.193368714

ENSMUSG00000087352	Gm12999	predicted gene 12999 [Source:MGI Symbol;Acc:MGI:3651167]	0.856245	1.06834	0.492178	0.353601	0.35225	0.486919	1.11006	1.0522	0.965684	1.63185	1.51209	0.709132	0.957324	0.734399	0.85447369	0.966018836	4	20	24	0.796648707	0.935045591	24	4.98756901	0.077476962
ENSMUSG00000087354	4930404I05Rik	RIKEN cDNA 4930404I05 gene [Source:MGI Symbol;Acc:MGI:1914644]	0.873322	1.21166	0.712442	0.870824	0.84603	1.03062	0.72295	0.941257	1.18528	0.641087	1.0297	0.824027	1.15146	0.803679	0.911685024	0.966018836	8	8	24	0.724093329	0.909572065	24	1.336332561	0.043240631
ENSMUSG00000087365	C430049B03Rik	RIKEN cDNA C430049B03 gene [Source:MGI Symbol;Acc:MGI:1924903]	0.532922	0.347362	0.528134	0.373635	0.390398	0.355998	0.682397	0.750694	0.41326	0.395835	0.626462	0.678605	0.589816	0.634874	0.632729413	0.966018836	4	12	24	0.161790921	0.76636332	24	0.889621081	0.082758042
ENSMUSG00000087366	Junos	jun proto-oncogene, opposite strand [Source:MGI Symbol;Acc:MGI:2652837]	2.25032	1.41562	1.54429	1.63132	1.49279	1.67967	1.11031	1.66017	1.25711	1.39245	1.28083	1.21942	1.58975	1.11213	0.715742619	0.966018836	4	4	24	0.656166333	0.8838564	24	21.44889132	0.05017011
ENSMUSG00000087368	BC065397	cDNA sequence BC065397 [Source:MGI Symbol;Acc:MGI:3584520]	3.60764	3.43061	2.78806	3.506	3.31985	2.6238	5.23861	2.94525	2.84321	2.6126	4.95862	3.34887	2.87595	2.63051	0.714044782	0.966018836	4	8	24	0.389706477	0.799546792	24	19.62306051	0.13841669
ENSMUSG00000087370	Tmem170b	transmembrane protein 170B [Source:MGI Symbol;Acc:MGI:3647046]	5.22086	5.58747	6.42743	6.37392	5.44293	5.73057	6.11741	5.04581	5.92531	5.43384	5.71725	5.58772	5.40757	5.91943	0.648082424	0.966018836	12	20	24	0.395322514	0.799546792	24	10.43767006	0.047245632
ENSMUSG00000087371	Gm15541	predicted gene 15541 [Source:MGI Symbol;Acc:MGI:3782989]	2.14407	2.67478	2.02817	2.83012	3.45119	2.62614	2.63087	2.76733	3.51859	3.14994	2.56023	2.44641	2.63474	2.01862	0.496745696	0.966018836	16	8	24	0.237257628	0.768040178	24	13.2614128	0.115441769
ENSMUSG00000087373	Gm15892	predicted gene 15892 [Source:MGI Symbol;Acc:MGI:3801833]	0.709684	1.44639	0.950462	1.32045	0.435425	0.433038	0.387491	0.750237	0.717305	0.28333	0.607747	0.750839	0.8349	0.913959	0.27909437	0.790764844	8	12	24	0.219265883	0.768040178	24	5.825246244	0.177104132
ENSMUSG00000087378	Gm12414	predicted gene 12414 [Source:MGI Symbol;Acc:MGI:3650374]	1.69482	0.919298	0.262801	0.822716	0.0894226	0.542398	0.294131	1.07482	0.493354	0.560376	0.576952	0.467858	0.649913	0.830423	0.192997592	0.670595308	8	12	24	0.244619413	0.768040178	24	2.568654933	0.204989864
ENSMUSG00000087380	2210408F21Rik	RIKEN cDNA 2210408F21 gene [Source:MGI Symbol;Acc:MGI:1920902]	18.0263	19.4262	19.2734	17.8702	20.8816	21.1459	15.4126	21.4057	21.2117	22.0194	21.1606	20.2929	23.8035	17.8458	0.860560779	0.966018836	20	8	24	0.625646959	0.872249537	24	13.95555641	0.054520628
ENSMUSG00000087381	Gm16008	predicted gene 16008 [Source:MGI Symbol;Acc:MGI:3802048]	3.41146	5.17525	5.40133	5.4653	4.68536	4.53414	5.60408	4.87302	6.56745	4.97278	5.91755	4.78376	4.20999	4.86094	0.098024777	0.501188411	12	16	24	0.063635574	0.663836692	24	10.25447618	0.161156089
ENSMUSG00000087382	Ctcflos	CCCTC-binding factor (zinc finger protein)-like, opposite strand [Source:MGI Symbol;Acc:MGI:1921411]	0.812949	1.1824	1.09049	2.98513	1.75261	1.33385	1.36959	1.07375	1.35181	1.97076	1.61952	1.18997	1.61581	1.75295	0.106675896	0.521506614	16	16	24	0.049014364	0.639959076	24	12.81663673	0.235343342
ENSMUSG00000087383	Gm12446	predicted gene 12446 [Source:MGI Symbol;Acc:MGI:3651433]	0.724568	0.625206	0.629963	0.579337	0.43198	0.608412	0.882754	0.961773	0.486669	0.909821	0.54625	0.596309	0.628959	0.475331	0.510031987	0.966018836	4	16	24	0.595667322	0.857839743	24	2.378231193	0.055757583
ENSMUSG00000087385	Frg2f1	FSHD region gene 2 family member 1 [Source:MGI Symbol;Acc:MGI:3035485]	3.04656	2.67048	3.03584	2.80809	3.36446	2.24725	2.77895	3.02118	2.64866	3.55954	2.5009	3.12765	3.24395	3.2276	0.81544087	0.966018836	16	20	24	0.792938992	0.93379199	24	8.236251059	0.03724148
ENSMUSG00000087390	Gm7598	predicted gene 7598 [Source:MGI Symbol;Acc:MGI:3644312]	0.628366	0.776012	0.640302	0.648152	0.554053	0.481378	0.751972	0.54099	0.732481	0.591537	0.563572	0.610698	0.638823	0.563401	0.403750665	0.937128244	12	8	24	0.276405446	0.768040178	24	5.178261809	0.045264058
ENSMUSG00000087396	4933407K13Rik	RIKEN cDNA 4933407K13 gene [Source:MGI Symbol;Acc:MGI:1921646]	1.4959	1.76439	1.71156	1.53484	2.11651	1.32514	1.87213	1.33865	1.8859	1.7258	1.67125	1.73314	1.47883	1.72893	0.888482515	0.966018836	20	4	24	0.547671756	0.833771137	24	12.05173101	0.051568366
ENSMUSG00000087400	Gm15270	predicted gene 15270 [Source:MGI Symbol;Acc:MGI:3705149]	0.920602	0.269163	0.875028	0.508	0.67579	0.308604	0.592692	0.626699	0.374975	0.374949	0.97122	0.469033	0.949712	0.674307	0.929527443	0.966018836	4	12	24	0.851840638	0.955545499	24	22.26558389	0.050955126
ENSMUSG00000087403	Kantr	Kdm5c adjacent non-coding transcript [Source:MGI Symbol;Acc:MGI:1920247]	7.24141	6.94357	7.15696	6.57905	7.61824	6.83541	7.65396	6.4268	6.59571	5.94698	7.48753	5.97563	6.02567	6.80248	0.907000607	0.966018836	20	20	24	0.770687707	0.926171026	24	19.72451939	0.028888052
ENSMUSG00000087406	E130215H24Rik	RIKEN cDNA E130215H24 gene [Source:MGI Symbol;Acc:MGI:1926091]	0.922071	1.06723	0.835546	1.23338	1.11146	0.75572	0.562417	1.23808	1.78392	0.879974	0.915524	0.947539	1.12628	1.18671	0.478349072	0.966018836	8	16	24	0.131804209	0.742139265	24	8.313535626	0.151900383
ENSMUSG00000087408	Cers1	ceramide synthase 1 [Source:MGI Symbol;Acc:MGI:2136690]	4.1937	3.48989	4.27431	3.78219	2.91348	3.84286	4.68182	4.5185	4.38595	5.95436	4.22654	4.96967	4.29056	5.13428	0.920872048	0.966018836	8	12	24	0.484565796	0.805393068	24	6.114573807	0.080475917
ENSMUSG00000087410	2310065F04Rik	RIKEN cDNA 2310065F04 gene [Source:MGI Symbol;Acc:MGI:1921434]	0.543478	1.19769	0.731605	2.76827	1.42282	1.6026	1.4798	4.92354	2.42127	2.21019	1.13431	1.3501	1.88287	1.00165	0.910436409	0.966018836	16	4	24	0.560011516	0.840850554	24	9.198183564	0.215182912
ENSMUSG00000087412	Gm15501	predicted pseudogene 15501 [Source:MGI Symbol;Acc:MGI:3704296]	58.4571	60.919	61.0327	53.3947	54.7751	50.5769	53.5324	51.2999	65.4236	51.2714	61.3725	61.7956	69.3657	65.2629	0.94663657	0.966018836	12	4	24	0.426109306	0.805393068	24	4.07033649	0.069239852
ENSMUSG00000087413	Gm11266	predicted gene 11266 [Source:MGI Symbol;Acc:MGI:3649705]	0.644606	1.60265	1.05184	1.35866	0.967335	1.36542	1.09319	1.67001	2.10549	1.27742	1.11013	1.25355	1.1196	1.65196	0.142596848	0.585944911	8	20	24	0.065788255	0.668083862	24	7.478707902	0.188363052
ENSMUSG00000087424	5730405O15Rik	RIKEN cDNA 5730405O15 gene [Source:MGI Symbol;Acc:MGI:1917739]	0.574702	0.712594	0.583956	0.685313	0.69554	0.506864	0.99462	0.605791	0.547079	0.455513	0.792082	0.636537	0.514659	0.816278	0.935593607	0.966018836	8	4	24	0.795741904	0.93469372	24	23.73023807	0.033715442
ENSMUSG00000087435	Gm16323	predicted gene 16323 [Source:MGI Symbol;Acc:MGI:3826604]	0.508742	0.601684	0.964791	1.17657	0.6034	0.402347	1.36125	0.520532	0.781961	0.838231	0.376891	0.763871	0.613342	0.925681	0.356953959	0.881188925	16	4	24	0.44228501	0.805393068	24	7.861829146	0.120711538
ENSMUSG00000087445	Gm14286	predicted gene 14286 [Source:MGI Symbol;Acc:MGI:3650190]	3.33212	2.76471	2.60328	1.6573	2.06309	2.68147	3.55982	2.71806	2.89448	2.29192	2.35563	2.92374	2.41792	1.93652	0.059513104	0.406067671	4	12	24	0.060090589	0.658304792	24	0.00292941	0.177240055
ENSMUSG00000087466	A330041J22Rik	RIKEN cDNA A330041J22 gene [Source:MGI Symbol;Acc:MGI:2442269]	0.453041	0.715829	0.752437	0.686795	0.484142	0.707243	0.553573	0.471097	0.603677	0.503918	0.70742	0.590787	0.443548	0.760226	0.338172159	0.85758369	12	16	24	0.56740796	0.843848507	24	9.848235895	0.046458023
ENSMUSG00000087478	4930506C21Rik	RIKEN cDNA 4930506C21 gene [Source:MGI Symbol;Acc:MGI:1922310]	1.8566	4.25819	4.10809	4.31679	2.58557	2.7379	1.98361	3.66971	4.03442	2.80264	3.50272	3.62142	1.34732	3.10272	0.008754183	0.192760771	12	16	24	0.048630012	0.638992719	24	10.03567871	0.325485469
ENSMUSG00000087484	2900089D17Rik	RIKEN cDNA 2900089D17 gene [Source:MGI Symbol;Acc:MGI:1914527]	2.03159	2.51708	2.21539	3.20617	3.21689	2.38844	1.95958	2.83454	2.46071	2.62765	2.39059	2.53183	1.76397	1.99218	0.008603252	0.192760771	16	12	24	0.025253266	0.586541342	24	13.18557971	0.163494741
ENSMUSG00000087496	Gm13523	predicted gene 13523 [Source:MGI Symbol;Acc:MGI:3651426]	1.84983	1.62304	0.267699	1.53593	1.18375	0.828473	1.04857	0.985171	1.00499	1.30484	1.42694	0.715126	1.0761	1.22268	0.928127947	0.966018836	16	20	24	0.924690541	0.979951713	24	20.23090033	0.048697738
ENSMUSG00000087497	2810001G20Rik	RIKEN cDNA 2810001G20 gene [Source:MGI Symbol;Acc:MGI:1913706]	5.63315	7.76221	6.66737	6.66867	6.64031	6.65458	7.08426	8.04194	11.2115	6.90644	8.84861	8.45745	8.74917	8.46201	0.887862353	0.966018836	8	16	24	0.287289422	0.768040178	24	7.067730102	0.106097741
ENSMUSG00000087501	Gm12116	predicted gene 12116 [Source:MGI Symbol;Acc:MGI:3652289]	4.70676	4.51418	4.03223	4.60773	4.28861	4.38924	4.21372	3.99939	4.14822	4.69253	4.24823	4.44171	4.82736	4.0543	0.341022016	0.86129475	4	4	24	0.422122825	0.804917634	24	19.09279416	0.038803394
ENSMUSG00000087502	Gm16091	predicted gene 16091 [Source:MGI Symbol;Acc:MGI:3802007]	1.73881	1.91873	1.67475	3.41854	2.19365	1.52265	2.24259	1.82366	2.00171	1.24728	2.32269	1.83749	1.7871	1.59571	0.933543173	0.966018836	20	4	24	0.585962265	0.853257144	24	14.37681334	0.090578736
ENSMUSG00000087516	Tbx3os1	T-box 3, opposite strand 1 [Source:MGI Symbol;Acc:MGI:3780472]	2.46243	3.30988	2.32349	2.5464	3.17133	3.22283	1.71163	1.99591	1.96293	3.29159	1.78049	2.80373	2.10798	2.81522	0.94791825	0.966018836	16	12	24	0.719459223	0.907983128	24	15.5662387	0.075527212
ENSMUSG00000087519	AV039307	expressed sequence AV039307 [Source:MGI Symbol;Acc:MGI:2139243]	1.20428	1.72024	1.63191	0.765534	1.85013	1.8656	1.03642	1.17159	1.05204	1.74463	2.75064	1.18971	0.538043	1.68704	0.231491055	0.731037655	20	8	24	0.403431328	0.799546792	24	14.71114963	0.17255553
ENSMUSG00000087523	Gm12319	predicted gene 12319 [Source:MGI Symbol;Acc:MGI:3649749]	2.0771	4.81588	4.27537	4.87676	3.63064	3.67037	1.75911	6.03325	5.75382	7.42391	4.19876	3.78664	3.5133	2.91099	0.0043709	0.140598674	16	12	24	0.014109602	0.530991098	24	10.5002045	0.443446314
ENSMUSG00000087528	9830144P21Rik	RIKEN cDNA 9830144P21 gene [Source:MGI Symbol;Acc:MGI:3041171]	0.959405	1.13261	1.16765	1.14164	1.37342	0.940039	0.910921	1.01382	1.25479	0.884858	1.04078	0.765268	0.941351	1.06434	0.004516345	0.140598674	12	12	24	0.121055974	0.733023294	24	9.907414385	0.082244078
ENSMUSG00000087536	Gm14246	predicted gene 14246 [Source:MGI Symbol;Acc:MGI:3651253]	0.38562	0.469634	0.754268	0.176042	0.28713	0.827383	0.56361	0.275298	0.630684	0.560702	0.633883	0.543112	0.309138	0.754821	0.858266942	0.966018836	12	4	24	0.973560861	0.993305115	24	5.170945404	0.018348879
ENSMUSG00000087543	Gm16576	predicted gene 16576 [Source:MGI Symbol;Acc:MGI:4414996]	0.565918	0.480379	0.361434	0.592878	0.557783	0.483532	0.544537	0.536395	0.641065	0.533133	0.464453	0.507014	0.625566	0.501244	0.891022863	0.966018836	4	20	24	0.959905092	0.989375125	24	23.07572833	0.007925594
ENSMUSG00000087556	Gm15764	predicted gene 15764 [Source:MGI Symbol;Acc:MGI:3783206]	1.01856	1.01963	0.595941	0.96806	1.43172	1.0425	0.763344	1.46018	0.533893	0.86555	0.745507	0.927858	0.703326	0.649055	0.601647512	0.966018836	24	12	24	0.409180985	0.799546792	24	19.27881066	0.104768638
ENSMUSG00000087574	C030037D09Rik	RIKEN cDNA C030037D09 gene [Source:MGI Symbol;Acc:MGI:1924865]	0.823969	0.965989	1.88336	1.34192	0.613269	1.24606	2.02162	1.33295	1.00662	1.35133	1.09898	1.12034	1.39417	1.51795	0.885025617	0.966018836	12	8	24	0.406805508	0.799546792	24	5.197862479	0.125758214
ENSMUSG00000087575	Gm12976	predicted gene 12976 [Source:MGI Symbol;Acc:MGI:3650499]	0.542271	0.48942	0.588384	0.545229	1.45213	0.538903	0.164558	1.14088	0.656361	0.89661	0.303335	0.962388	0.637736	0.933554	0.897425765	0.966018836	8	20	24	0.764318121	0.92403577	24	11.32626187	0.082711242
ENSMUSG00000087578	Ccdc142os	coiled-coil domain containing 142, opposite strand [Source:MGI Symbol;Acc:MGI:3783052]	1.3431	1.74903	1.03553	1.39954	0.855722	1.43552	2.07164	0.985697	1.30101	0.991371	1.63916	1.24378	0.932209	1.15434	0.814221431	0.966018836	24	20	24	0.605592581	0.862773266	24	22.55989887	0.08350621
ENSMUSG00000087579	Hectd2os	Hectd2, opposite strand [Source:MGI Symbol;Acc:MGI:1919243]	0.761702	0.871955	1.38241	1.80473	0.830957	1.12096	0.838057	2.86028	1.53491	0.932724	0.994399	0.16674	1.12217	0.461367	0.416857905	0.963884303	12	12	24	0.240719561	0.768040178	24	8.307667373	0.268111333
ENSMUSG00000087586	Gm6938	predicted gene 6938 [Source:MGI Symbol;Acc:MGI:3648987]	1.24433	0.831335	0.996632	1.04857	0.537563	1.00061	1.27716	0.819342	0.759386	0.692446	0.898571	1.33943	0.917667	1.17938	0.112583132	0.530777648	24	20	24	0.143643556	0.752803875	24	23.61362182	0.129068745
ENSMUSG00000087589	D430040D24Rik	RIKEN cDNA D430040D24 gene [Source:MGI Symbol;Acc:MGI:2442526]	16.328	20.481	17.9886	19.4107	23.7278	17.8079	20.1232	19.6766	24.6338	19.6932	21.4687	19.1387	22.5367	17.2518	0.961261938	0.967043964	20	4	24	0.526682564	0.823075573	24	12.54694773	0.070758871
ENSMUSG00000087590	Epb41l4aos	erythrocyte membrane protein band 4.1 like 4a, opposite strand [Source:MGI Symbol;Acc:MGI:1916999]	6.92502	5.65496	6.77694	4.74987	6.29241	5.12076	4.96638	5.32723	7.09192	6.59941	7.72095	8.07639	8.25546	6.93532	0.847846021	0.966018836	20	20	24	0.968896719	0.992577968	24	18.88726776	0.020497605
ENSMUSG00000087593	Gm16174	predicted gene 16174 [Source:MGI Symbol;Acc:MGI:3801857]	1.17827	0.85005	0.730002	0.189238	0.863398	0.561088	0.81597	0.148703	0.455904	0.668209	0.227851	0.872708	0.453924	0.519482	0.590547452	0.966018836	24	16	24	0.303567509	0.768040178	24	22.14797689	0.157669462
ENSMUSG00000087598	Zfp111	zinc finger protein 111 [Source:MGI Symbol;Acc:MGI:1929114]	1.9128	1.75843	1.94971	2.02119	2.26215	2.00431	1.94151	1.73433	1.78849	1.72299	2.02399	1.89196	1.72172	1.76611	0.016720774	0.253395174	20	12	24	0.005250446	0.4424469	24	16.7793652	0.075986428
ENSMUSG00000087604	Cdrt4os1	CMT1A duplicated region transcript 4, opposite strand 1 [Source:MGI Symbol;Acc:MGI:1922134]	0.719388	0.776984	0.783805	0.618953	0.992007	0.762902	0.602898	0.664792	0.986725	0.447962	0.697535	0.719069	0.725872	0.730879	0.676344318	0.966018836	12	4	24	0.992684027	0.998516607	24	0.906566962	0.005631881
ENSMUSG00000087610	Gm16253	predicted gene 16253 [Source:MGI Symbol;Acc:MGI:3826531]	0.413383	0.680795	0.336679	1.17407	0.84241	0.5226	0.754047	0.643595	0.773136	0.614946	0.91686	0.39843	0.969175	0.470302	0.912923872	0.966018836	20	4	24	0.501802051	0.809142348	24	13.72470823	0.094248071
ENSMUSG00000087616	Gm14257	predicted gene 14257 [Source:MGI Symbol;Acc:MGI:3652145]	0.661623	1.44421	1.43804	1.28591	1.23386	1.2351	1.12872	1.24127	1.94002	1.04918	0.862156	1.26846	1.32421	1.39003	0.035023815	0.328311403	12	8	24	0.124203436	0.738003002	24	7.546888866	0.149507543
ENSMUSG00000087620	5330434G04Rik	RIKEN cDNA 5330434G04 gene [Source:MGI Symbol;Acc:MGI:1925519]	7.47809	7.68642	7.20205	6.80158	8.13144	7.5851	7.02897	6.77637	7.36651	7.23691	8.66459	7.2022	7.97108	9.25208	0.90767227	0.966018836	20	20	24	0.925469582	0.980131499	24	20.55081265	0.017230439
ENSMUSG00000087623	Gm12404	predicted gene 12404 [Source:MGI Symbol;Acc:MGI:3649601]	1.27002	1.427	1.75617	1.18832	1.49156	1.34247	1.19606	1.35402	1.55674	1.13592	1.75206	1.34537	2.00445	1.47253	0.913451828	0.966018836	12	4	24	0.916441235	0.976967742	24	4.625389115	0.023228226
ENSMUSG00000087624	9230111E07Rik	RIKEN cDNA 9230111E07 gene [Source:MGI Symbol;Acc:MGI:1924998]	0.614188	0.748618	1.00153	0.702916	0.596249	0.347821	0.299512	0.713857	0.880015	0.446432	0.548065	0.627708	0.40964	0.552381	0.030691182	0.324150946	12	16	24	0.033030358	0.606585237	24	8.49143012	0.158594013
ENSMUSG00000087626	Gm15050	predicted gene 15050 [Source:MGI Symbol;Acc:MGI:3705180]	2.15894	2.18809	2.12118	2.56976	2.43807	1.61754	1.62531	1.82934	2.30741	2.71877	2.80665	2.77427	2.06589	2.57281	0.08333408	0.462922111	20	8	24	0.05594157	0.65534544	24	12.67950669	0.146694141
ENSMUSG00000087635	Gm13414	predicted gene 13414 [Source:MGI Symbol;Acc:MGI:3652154]	10.7378	12.1564	12.1309	12.0268	11.5333	10.1837	11.7062	8.76947	12.1857	11.1112	11.3322	11.5217	12.262	11.3602	0.888991156	0.966018836	12	12	24	0.740266566	0.915274254	24	11.21738366	0.037664331
ENSMUSG00000087644	Gm14703	predicted gene 14703 [Source:MGI Symbol;Acc:MGI:3705165]	0.881509	0.894968	0.886063	0.655463	1.2182	0.708269	0.947445	0.922667	1.23171	0.606796	0.659445	1.24497	1.12242	1.06106	0.455507782	0.966018836	4	12	24	0.409168382	0.799546792	24	1.507322922	0.083257062
ENSMUSG00000087646	Gm1667	predicted gene 1667 [Source:MGI Symbol;Acc:MGI:2686513]	3.23927	2.35986	2.74826	1.95574	3.1517	2.78802	2.33916	2.8825	2.75422	2.54309	2.34949	2.73105	3.11852	2.45543	0.891105762	0.966018836	4	12	24	0.380066335	0.799474855	24	22.65777094	0.080346332
ENSMUSG00000087651	1500009L16Rik	RIKEN cDNA 1500009L16 gene [Source:MGI Symbol;Acc:MGI:1917034]	4.10239	4.26747	5.3029	4.0832	3.75037	5.39477	4.75504	5.23024	4.49524	4.72179	4.19422	5.46806	4.12413	5.89684	0.714044782	0.966018836	8	12	24	0.447917882	0.805393068	24	3.897716928	0.076341569
ENSMUSG00000087658	Hotairm1	Hoxa transcript antisense RNA, myeloid-specific 1 [Source:MGI Symbol;Acc:MGI:3705155]	4.46933	4.4797	3.0654	3.54363	3.8814	3.12607	2.89909	4.79338	8.72118	6.45187	8.27053	6.73351	5.79015	6.66606	0.873567285	0.966018836	20	8	24	0.517770119	0.818405353	24	9.919142961	0.152403778
ENSMUSG00000087670	9530036M11Rik	RIKEN cDNA 9530036M11 gene [Source:MGI Symbol;Acc:MGI:1925866]	0.799596	0.912865	0.470413	0.473256	0.520262	0.530254	0.264106	0.640642	0.488126	0.585183	0.537	0.603958	0.889948	0.640571	0.07395231	0.448301997	8	4	24	0.407361682	0.799546792	24	1.756717058	0.081675587
ENSMUSG00000087672	Gm15122	predicted gene 15122 [Source:MGI Symbol;Acc:MGI:3705268]	4.294	3.32639	4.95052	4.09487	3.9557	3.89886	6.02724	4.1638	6.00994	2.75121	5.56347	3.63658	3.60669	4.00284	0.912686312	0.966018836	12	12	24	0.955395878	0.988639288	24	5.378693968	0.032823873
ENSMUSG00000087674	4930447M23Rik	RIKEN cDNA 4930447M23 gene [Source:MGI Symbol;Acc:MGI:1918902]	0.568666	0.964555	1.00543	0.836499	1.11972	0.839941	1.0739	0.642922	0.907226	0.79608	0.886248	0.588285	1.25926	1.3183	0.915835339	0.966018836	8	16	24	0.720493023	0.908336488	24	6.280055725	0.056007814
ENSMUSG00000087679	Tmem250-ps	transmembrane protein 250, pseudogene [Source:MGI Symbol;Acc:MGI:1924939]	23.8165	20.1125	19.8519	21.5312	21.2397	21.6179	20.9486	22.0259	18.4218	24.0785	21.1769	23.183	21.9941	21.8139	0.390686537	0.915479136	24	12	24	0.285292375	0.768040178	24	19.82558359	0.061796089
ENSMUSG00000087687	Pet100	PET100 homolog [Source:MGI Symbol;Acc:MGI:3615306]	46.6867	52.5587	39.8148	52.675	50.5692	44.4299	49.8252	43.4822	53.8713	35.8505	51.9721	39.969	46.3579	46.4867	0.838244962	0.966018836	12	12	24	0.985343024	0.996973154	24	0.804034794	0.011832192
ENSMUSG00000087691	Cd55os	CD55 molecule, opposite strand sequence [Source:MGI Symbol;Acc:MGI:3783116]	2.9628	3.59645	3.7108	5.26728	3.64377	3.16701	3.21393	3.06551	5.49427	2.41379	3.94384	2.91948	3.54102	4.26571	0.080164531	0.458678242	12	12	24	0.248558477	0.768040178	24	9.281729794	0.169875218
ENSMUSG00000087694	A530058N18Rik	RIKEN cDNA A530058N18 gene [Source:MGI Symbol;Acc:MGI:2444858]	0.874535	1.03055	1.62112	0.838589	0.863789	0.973762	1.05435	0.918438	0.880372	0.672268	0.868572	1.17082	0.680742	1.19203	0.132887428	0.567728672	8	8	24	0.610849057	0.864861374	24	4.637601895	0.065315977
ENSMUSG00000087701	Gm13493	predicted gene 13493 [Source:MGI Symbol;Acc:MGI:3650824]	0.242861	0.983573	0.790814	1.10025	0.269073	0.476023	0.442541	0.215614	0.494854	0.160599	0.248012	0.625756	0.651931	0.55537	0.862909389	0.966018836	12	8	24	0.668288664	0.887912848	24	6.227466481	0.097380872
ENSMUSG00000088088	Rmrp	RNA component of mitochondrial RNAase P [Source:MGI Symbol;Acc:MGI:97937]	21.3075	3.26686	6.35082	4.45764	0.00851684	7.57959	6.17746	12.1912	2.53996	0.244166	9.75529	11.4258	5.03031	7.36284	0.281810132	0.793714531	24	16	24	0.096461004	0.71175955	24	0.204717098	0.984758448
ENSMUSG00000088948	Gm23262	predicted gene, 23262 [Source:MGI Symbol;Acc:MGI:5453039]	14.3429	11.2804	6.4167	10.5023	7.43853	19.7268	8.53558	3.68146	5.02152	2.28309	6.94391	7.80475	6.52387	4.80794	0.241950772	0.741580974	24	12	24	0.013460986	0.529134128	24	20.81851288	0.597849987
ENSMUSG00000089417	Gm22009	predicted gene, 22009 [Source:MGI Symbol;Acc:MGI:5451786]	2.46296	3.04483	1.21246	2.24827	0.976131	3.20725	1.35366	1.57446	3.17432	0.889321	1.95801	2.33665	2.4272	2.97389	0.513519678	0.966018836	8	8	24	0.366018255	0.790003135	24	1.364057942	0.218884746
ENSMUSG00000089542	Gm25835	predicted gene, 25835 [Source:MGI Symbol;Acc:MGI:5455612]	15.054	21.8659	28.2257	11.8223	9.87737	39.8919	4.56499	3.1632	7.49256	4.96806	7.11376	15.6899	2.71072	13.2922	0.896753532	0.966018836	24	4	24	0.851703139	0.955514806	24	17.95934324	0.21491071
ENSMUSG00000089628	Gm9727	predicted gene 9727 [Source:MGI Symbol;Acc:MGI:3779431]	10.7112	8.47823	10.7456	10.5486	8.77092	10.9502	8.56027	10.3475	10.8934	11.735	11.0016	11.8619	12.0252	9.59055	0.286642595	0.793714531	24	8	24	0.643043037	0.878809617	24	14.79321284	0.052060215
ENSMUSG00000089636	1700058P15Rik	RIKEN cDNA 1700058P15 gene [Source:MGI Symbol;Acc:MGI:1920627]	1.12587	0.89381	0.872067	1.26718	1.41602	1.5209	2.14488	1.84855	1.52539	1.70251	0.871653	0.520699	1.40863	1.36253	0.852844413	0.966018836	4	20	24	0.765093664	0.92403577	24	4.905814978	0.083688768
ENSMUSG00000089648	Gm15790	predicted pseudogene 15790 [Source:MGI Symbol;Acc:MGI:3783232]	4.12141	2.9676	3.01534	3.68956	3.20148	3.73555	2.89465	3.33601	2.86732	3.42119	3.26595	3.15722	3.03285	3.09414	0.369262716	0.893596186	16	20	24	0.310819709	0.768040178	24	18.24842524	0.06709542
ENSMUSG00000089659	Gm3470	predicted gene 3470 [Source:MGI Symbol;Acc:MGI:3781646]	0.782041	0.158136	1.01777	0.88084	0.344516	0.609458	0.758634	0.691753	0.529974	1.24166	0.954112	0.607354	0.210688	0.481302	0.350544145	0.870418653	16	16	24	0.225484637	0.768040178	24	12.73301552	0.179380788
ENSMUSG00000089661	Mia	melanoma inhibitory activity [Source:MGI Symbol;Acc:MGI:109615]	2.02078	2.35598	3.08454	1.72791	2.67066	2.04684	2.62232	1.97213	2.31371	2.40585	2.14809	2.14126	1.2844	1.4277	0.636884864	0.966018836	12	20	24	0.4362448	0.805393068	24	12.98120195	0.097506019
ENSMUSG00000089662	Gm14057	predicted gene 14057 [Source:MGI Symbol;Acc:MGI:3705433]	1.28279	0.795795	0.930793	0.28447	1.82471	0.993132	0.846453	0.892156	0.731305	1.06263	1.05095	0.78559	0.81392	1.50273	0.865514257	0.966018836	20	20	24	0.743340014	0.916215545	24	20.79629321	0.085659405
ENSMUSG00000089665	Fcor	Foxo1 corepressor [Source:MGI Symbol;Acc:MGI:1915484]	4.92105	4.61357	4.57253	8.6844	4.50209	6.43663	4.25842	6.51637	5.35286	6.74489	8.31938	8.20836	8.77406	7.60725	0.91241026	0.966018836	16	20	24	0.542316249	0.83094351	24	15.41477127	0.10916254
ENSMUSG00000089669	Tnfsf13	tumor necrosis factor (ligand) superfamily, member 13 [Source:MGI Symbol;Acc:MGI:1916833]	3.93666	4.67525	3.54197	5.00653	7.40739	5.29352	4.70201	4.30304	5.29275	3.27842	5.85305	4.43626	4.66388	4.18407	0.217933169	0.704989686	20	12	24	0.189570609	0.768040178	24	17.32837594	0.173435147
ENSMUSG00000089670	Gm16581	predicted gene 16581 [Source:MGI Symbol;Acc:MGI:4415001]	2.35155	2.34144	2.29633	2.25724	2.55084	1.93454	2.45932	2.49257	2.16333	2.44137	2.47525	2.14172	2.23743	2.72309	0.643325306	0.966018836	8	16	24	0.574247405	0.847244567	24	7.034106538	0.037077824
ENSMUSG00000089682	Bcl2l2	BCL2-like 2 [Source:MGI Symbol;Acc:MGI:108052]	60.7192	52.9307	50.5264	55.4049	56.7614	54.807	57.8212	53.4426	51.2819	61.6167	53.0454	57.0996	58.7613	55.1739	0.019594567	0.258568924	4	8	24	0.253108596	0.768040178	24	20.16105492	0.055713353
ENSMUSG00000089686	Mnd1-ps	Mnd1 retrotransposed pseudogene [Source:MGI Symbol;Acc:MGI:3782005]	1.71889	1.74196	1.37638	1.64787	1.27434	1.22088	1.75948	1.04897	1.22879	1.37261	1.79435	1.21063	1.33038	1.76995	0.888379597	0.966018836	4	20	24	0.938101458	0.984659033	24	1.567688211	0.021754425
ENSMUSG00000089695	Gm15946	predicted gene 15946 [Source:MGI Symbol;Acc:MGI:3801999]	2.78248	2.26547	3.85601	3.13471	2.95175	2.66828	3.18684	1.36752	3.14278	2.35199	2.41035	2.10021	2.49789	2.61497	0.675497492	0.966018836	12	20	24	0.643821384	0.879013224	24	12.31639121	0.086771879
ENSMUSG00000089698	Gm2541	predicted pseudogene 2541 [Source:MGI Symbol;Acc:MGI:3780709]	9.44067	9.07115	8.99735	9.12772	8.34246	8.50022	7.25832	9.07967	9.80859	9.11936	8.66456	9.06521	10.4774	9.89627	0.289828769	0.795531262	8	12	24	0.2949818	0.768040178	24	6.368048724	0.066832042
ENSMUSG00000089699	Gm1992	predicted gene 1992 [Source:MGI Symbol;Acc:MGI:3780162]	21.5732	27.5807	29.1168	27.4858	37.5305	25.3682	29.7338	22.9597	36.1423	19.0656	33.8796	28.3494	27.8298	29.8388	0.928892845	0.966018836	20	8	24	0.787279063	0.931463668	24	13.96913788	0.071461328
ENSMUSG00000089704	Galnt2	polypeptide N-acetylgalactosaminyltransferase 2 [Source:MGI Symbol;Acc:MGI:894694]	12.3233	11.0259	10.9603	10.0837	10.89	10.156	12.836	13.3117	10.4847	11.4786	10.0797	9.99136	11.9353	9.37129	0.119293142	0.542554768	4	20	24	0.254355189	0.768040178	24	1.576240906	0.083056414
ENSMUSG00000089706	B230216N24Rik	RIKEN cDNA B230216N24 gene [Source:MGI Symbol;Acc:MGI:1925853]	1.91866	2.14751	2.08144	1.63089	1.61342	1.79205	2.02367	1.72233	1.47202	1.76729	1.85467	2.08125	1.7241	1.65664	0.72778087	0.966018836	24	20	24	0.320046013	0.768040178	24	22.95805792	0.059148171
ENSMUSG00000089715	Cbx6	chromobox 6 [Source:MGI Symbol;Acc:MGI:3512628]	33.7777	28.7342	27.9665	30.8981	29.3254	29.4914	34.0476	30.8111	28.4431	39.3237	28.0935	32.2773	31.1796	33.3229	0.112800839	0.530777648	4	8	24	0.944108904	0.986282661	24	23.50446735	0.017981704
ENSMUSG00000089726	Mir17hg	Mir17 host gene (non-protein coding) [Source:MGI Symbol;Acc:MGI:1923207]	0.715543	0.87803	0.715039	0.758461	0.86644	0.637084	0.67418	0.970601	0.980195	0.642935	0.843225	0.624977	0.856166	0.771476	0.112800839	0.530777648	8	16	24	0.285916311	0.768040178	24	6.834312089	0.06121865
ENSMUSG00000089736	Tgfbr3l	transforming growth factor, beta receptor III-like [Source:MGI Symbol;Acc:MGI:3833469]	4.46724	6.94663	5.50216	5.60947	5.09607	3.92265	4.38159	6.11675	7.37246	6.9126	6.27261	5.16746	5.32266	5.81135	0.024898193	0.291208026	12	12	24	0.004233606	0.427431763	24	8.893615087	0.245368372
ENSMUSG00000089762	Ier5l	immediate early response 5-like [Source:MGI Symbol;Acc:MGI:1919750]	0.691161	0.599767	0.530004	0.362483	0.34309	0.349968	0.537714	0.508358	0.645342	0.912882	1.22622	0.907174	0.844598	0.848328	0.706093972	0.966018836	4	20	24	0.999531775	0.999956469	24	1.657668739	0.002073607
ENSMUSG00000089764	Gm16580	predicted gene 16580 [Source:MGI Symbol;Acc:MGI:4415000]	13.9587	14.9663	16.0598	16.8464	17.3145	15.8852	16.7062	15.2789	20.5134	20.9343	18.2191	18.9061	17.6315	16.9411	0.235147982	0.73629392	16	16	24	0.008680694	0.502965379	24	12.56642743	0.123267081
ENSMUSG00000089768	Tmsb15b1	thymosin beta 15b1 [Source:MGI Symbol;Acc:MGI:3843059]	7.71786	9.48207	10.4656	8.23199	7.61079	6.03757	8.25543	5.76469	10.5305	8.21906	9.1601	9.36304	7.42254	9.63859	0.210566453	0.69936851	12	12	24	0.276357596	0.768040178	24	9.020045329	0.149756735
ENSMUSG00000089774	Slc5a3	solute carrier family 5 (inositol transporters), member 3 [Source:MGI Symbol;Acc:MGI:1858226]	9.54979	7.13007	5.7181	6.33929	8.25788	6.6994	9.14643	6.58196	6.11352	5.28799	9.03907	6.72886	7.59172	6.56513	0.002250501	0.104113924	4	12	24	0.026100739	0.586541342	24	21.27134355	0.221183331
ENSMUSG00000089782	Btf3-ps1	basic transcription factor 3, pseudogene 1 [Source:MGI Symbol;Acc:MGI:3781708]	127.734	141.267	136.771	131.271	144.705	135.939	130.219	124.624	139.932	116.176	133.974	138.772	137.142	126	0.93717134	0.966018836	20	20	24	0.68137259	0.893635751	24	19.295614	0.030259078
ENSMUSG00000089791	Gm3555	predicted pseudogene 3555 [Source:MGI Symbol;Acc:MGI:3781732]	5.21531	5.07626	4.97344	5.2961	5.70456	5.24324	5.74046	5.28565	4.0948	5.44381	5.16988	5.40635	5.00912	5.59914	0.43280837	0.966018836	16	20	24	0.299403521	0.768040178	24	19.7581332	0.06208091
ENSMUSG00000089797	Gm16118	predicted gene 16118 [Source:MGI Symbol;Acc:MGI:3801926]	0.231372	0.352226	0.502848	0.704168	0.344556	0.479011	0.56361	0.344123	0.630684	0.407783	0.369776	0.444365	0.875907	0.754835	0.904690479	0.966018836	12	8	24	0.519501691	0.819306539	24	6.448992417	0.069632326
ENSMUSG00000089809	Rasgef1b	RasGEF domain family, member 1B [Source:MGI Symbol;Acc:MGI:2443755]	3.88832	3.39435	4.00817	3.51049	2.53942	2.28864	3.6014	3.16844	3.41095	4.21821	3.04495	3.95622	2.94472	4.13469	0.676344318	0.966018836	16	4	24	0.475856055	0.805393068	24	6.224951	0.080088057
ENSMUSG00000089810	Gm16536	predicted gene 16536 [Source:MGI Symbol;Acc:MGI:4414956]	9.93504	9.98294	10.7615	10.7306	8.49836	9.7939	10.8253	10.1308	9.3213	10.9785	9.3984	10.2574	9.82407	10.4822	0.265774856	0.773510818	16	4	24	0.445309932	0.805393068	24	5.821392237	0.047247901
ENSMUSG00000089824	Rbm12	RNA binding motif protein 12 [Source:MGI Symbol;Acc:MGI:1922960]	3.3887	3.09174	2.74358	2.36526	2.3578	2.56326	2.57551	2.38929	3.18649	2.50779	3.21896	2.94533	2.46597	2.81059	0.917839756	0.966018836	12	4	24	0.802227751	0.936565832	24	1.795612868	0.035107411
ENSMUSG00000089832	Shkbp1	Sh3kbp1 binding protein 1 [Source:MGI Symbol;Acc:MGI:2385803]	10.5189	7.97411	10.4104	9.69308	10.595	9.69453	8.11207	9.43099	7.38742	9.35279	10.7369	9.98371	8.94889	9.34926	0.057224139	0.401309455	20	12	24	0.12349867	0.736432529	24	16.95738876	0.113973683
ENSMUSG00000089838	Gm2962	predicted pseudogene 2962 [Source:MGI Symbol;Acc:MGI:3781140]	74.547	90.0737	77.9475	97.6152	90.2725	77.5506	78.8449	91.6666	100.138	86.2117	92.2154	90.3426	101.722	89.7155	0.882263435	0.966018836	20	4	24	0.335722321	0.771468587	24	9.93520727	0.071621075
ENSMUSG00000089847	Timm10b	translocase of inner mitochondrial membrane 10B [Source:MGI Symbol;Acc:MGI:1315196]	34.0544	24.2718	27.1815	23.6383	22.6005	23.6513	29.4213	33.9871	33.3788	30.978	38.7668	33.7396	34.7571	33.6025	0.702630284	0.966018836	4	12	24	0.511158642	0.814670619	24	2.635036233	0.087596059
ENSMUSG00000089854	Gm16133	predicted gene 16133 [Source:MGI Symbol;Acc:MGI:3802155]	2.85734	1.49948	1.29123	2.04128	1.78995	1.3907	2.05508	1.66447	1.27531	0.921376	2.11565	1.22647	1.88264	1.51189	0.147689593	0.590732968	4	8	24	0.22320672	0.768040178	24	21.95840132	0.165317267
ENSMUSG00000089857	Zfp882	zinc finger protein 882 [Source:MGI Symbol;Acc:MGI:3642748]	2.69135	2.79981	1.94548	2.78143	3.63159	2.11	3.54091	2.44114	3.064	2.04353	3.80659	2.72762	2.96623	2.62624	0.481530854	0.966018836	20	20	24	0.535077604	0.826859366	24	19.06634363	0.099844185
ENSMUSG00000089862	Umad1	UMAP1-MVP12 associated (UMA) domain containing 1 [Source:MGI Symbol;Acc:MGI:3840148]	8.30073	9.49006	8.58357	7.88985	8.43956	8.34531	9.34736	8.97776	8.88798	8.74005	8.612	7.78147	6.91309	8.52765	0.176754388	0.653256853	8	16	24	0.435256812	0.805393068	24	7.015860038	0.051000085

ENSMUSG00000089872	Rps6kc1	ribosomal protein S6 kinase polypeptide 1 [Source:MGI Symbol;Acc:MGI:2443419]	13.041	10.5879	11.3327	10.7724	10.748	12.7172	11.7344	11.2831	8.62319	11.8218	9.91152	12.08	11.4131	11.3407	0.036657636	0.332441566	24	12	24	0.074242561	0.683293901	24	21.77711043	0.116164815
ENSMUSG00000089875	Etohd2	ethanol decreased 2 [Source:MGI Symbol;Acc:MGI:1261417]	1.05094	1.27585	1.01692	1.41941	1.54716	1.10181	1.28974	1.20197	1.6844	1.35197	1.70982	1.29731	1.44136	1.50262	0.581070934	0.966018836	20	8	24	0.202034163	0.768040178	24	12.48008036	0.074904316
ENSMUSG00000089886	Gm16184	predicted gene 16184 [Source:MGI Symbol;Acc:MGI:3802154]	1.36475	0.741199	0.741118	1.1086	1.01239	0.548314	0.711512	0.520701	0.795922	0.773337	1.39818	0.625867	0.848391	0.811974	0.722296281	0.966018836	20	4	24	0.447889498	0.805393068	24	15.47417471	0.098460269
ENSMUSG00000089889	0610040B10Rik	RIKEN cDNA 0610040B10 gene [Source:MGI Symbol;Acc:MGI:1914922]	1.5601	3.73159	2.72186	4.50224	2.89475	2.97167	1.97666	3.49939	4.62006	3.49614	2.89658	3.8678	2.68236	4.45644	0.085638565	0.466404237	16	12	24	0.045755727	0.631496972	24	9.682548286	0.281006329
ENSMUSG00000089911	Mfsd14a	major facilitator superfamily domain containing 14A [Source:MGI Symbol;Acc:MGI:1201609]	30.2561	30.2723	28.0625	30.9084	30.3931	28.4483	31.1642	27.8678	28.4438	27.018	30.8753	27.753	27.327	28.0729	0.881996584	0.966018836	20	16	24	0.485808641	0.805393068	24	18.26473015	0.029783452
ENSMUSG00000089917	Uckl1	uridine-cytidine kinase 1-like 1 [Source:MGI Symbol;Acc:MGI:1915806]	28.0943	27.6726	26.7566	25.6523	32.8545	28.5126	30.9917	29.5892	29.0441	25.54	28.4848	27.1535	29.1449	25.0833	0.446653834	0.966018836	4	12	24	0.303597539	0.768040178	24	20.71627318	0.064534112
ENSMUSG00000089929	Bcl2a1b	B cell leukemia/lymphoma 2 related protein A1b [Source:MGI Symbol;Acc:MGI:1278326]	0.851917	1.51946	1.2569	0.81895	1.68141	1.00158	1.70634	1.25138	1.6608	0.666016	0.994038	1.17805	1.58274	1.32148	0.348367381	0.878416653	4	12	24	0.327334742	0.768040178	24	2.436626383	0.130303884
ENSMUSG00000089940	Gm4117	predicted gene 4117 [Source:MGI Symbol;Acc:MGI:3782293]	1.0656	0.827564	0.840014	0.97745	0.285579	0.495673	0.764747	0.654973	0.831901	1.29017	0.862372	0.817867	0.343765	1.19925	0.245586913	0.741580974	16	8	24	0.291576633	0.768040178	24	8.232056573	0.133567232
ENSMUSG00000089941	Gm16168	predicted gene 16168 [Source:MGI Symbol;Acc:MGI:3802010]	4.46176	3.57085	3.29391	3.3113	5.80459	3.65808	4.69613	3.92907	4.23152	2.95799	3.548	4.2645	4.8031	2.76303	0.057224139	0.401309455	4	12	24	0.140189112	0.749574609	24	20.70916176	0.179240531
ENSMUSG00000089945	Pakap	paralemmin A kinase anchor protein [Source:MGI Symbol;Acc:MGI:5141924]	4.77578	4.01231	4.39167	4.11336	3.85278	5.2398	3.89719	4.76501	3.25646	5.23655	3.56368	4.73526	4.50148	5.49446	0.830364743	0.966018836	24	20	24	0.624282523	0.871960321	24	0.238808563	0.068426415
ENSMUSG00000089996	Tmsb15b2	thymosin beta 15b2 [Source:MGI Symbol;Acc:MGI:3843061]	35.3337	28.8039	26.0925	36.5705	29.1037	19.8574	29.476	32.4974	29.2077	16.643	23.593	30.3146	30.2242	24.1808	0.89563591	0.966018836	4	16	24	0.469596171	0.805393068	24	1.508207803	0.135368679
ENSMUSG00000089997	1810020O05Rik	Riken cDNA 1810020O05 gene [Source:MGI Symbol;Acc:MGI:3586838]	0.690537	0.758653	0.667798	0.712375	0.652142	0.79815	0.735365	0.624834	1.19904	0.584562	0.75041	0.926206	0.686362	0.826123	0.919571723	0.966018836	24	16	24	0.905021688	0.973815799	24	5.755233705	0.021350276
ENSMUSG00000089998	Phtf1os	putative homeodomain transcription factor 1, opposite strand [Source:MGI Symbol;Acc:MGI:3698050]	0.930668	0.91809	0.921019	0.624963	0.779945	0.688015	0.755997	0.854073	0.964489	0.976805	1.02347	1.00642	0.845463	0.937759	0.91241026	0.966018836	20	8	24	0.707311428	0.903432968	24	6.61549452	0.030979155
ENSMUSG00000089999	Gm6485	predicted gene 6485 [Source:MGI Symbol;Acc:MGI:3644007]	17.3164	21.1889	17.6795	18.551	19.48	18.3397	15.5361	21.2607	17.2677	15.6893	14.7156	17.9606	16.2494	14.4005	0.823144114	0.966018836	8	20	24	0.934642031	0.983690057	24	3.490920806	0.020009771
ENSMUSG00000090000	Ier3ip1	immediate early response 3 interacting protein 1 [Source:MGI Symbol;Acc:MGI:1913441]	49.5264	50.5351	42.4736	47.4754	43.8374	41.8591	41.6969	47.167	52.2581	45.2319	50.8596	49.4793	50.7105	50.9557	0.886952527	0.966018836	8	16	24	0.566799398	0.843547308	24	5.388456375	0.045780575
ENSMUSG00000090008	Gm16111	predicted gene 16111 [Source:MGI Symbol;Acc:MGI:3801919]	0.328392	1.97583	1.06509	1.81086	4.943	0.71429	1.58343	3.43725	1.32487	0.435049	1.09432	0.64886	0.672832	1.30753	0.648082424	0.966018836	8	20	24	0.790825452	0.933002007	24	9.677302986	0.170044511
ENSMUSG00000090015	Gm15446	predicted gene 15446 [Source:MGI Symbol;Acc:MGI:3709333]	8.48654	10.2884	11.3964	15.4231	11.6117	8.54016	10.0098	9.0332	11.7658	8.35438	10.7948	9.68707	10.076	10.2481	0.125855811	0.555618842	12	12	24	0.152049441	0.759065259	24	11.19181066	0.15733163
ENSMUSG00000090019	Gimap1	GTPase, IMAP family member 1 [Source:MGI Symbol;Acc:MGI:109368]	1.19205	1.23528	1.6507	1.2361	0.883643	1.14791	1.18681	1.12707	1.11081	1.25048	0.914672	1.02988	1.02993	1.02334	0.087978327	0.472021905	16	4	24	0.125694408	0.739632637	24	7.004785488	0.080971728
ENSMUSG00000090021	Gm6493	predicted gene 6493 [Source:MGI Symbol;Acc:MGI:3644797]	3.53333	3.46728	3.65425	3.72608	4.17812	3.43394	3.34231	2.83128	3.9847	2.41787	4.32935	3.86595	3.41742	3.50132	0.074313825	0.448301997	20	12	24	0.492888452	0.805636409	24	17.21054652	0.079155702
ENSMUSG00000090024	Gm16350	predicted gene 16350 [Source:MGI Symbol;Acc:MGI:3840133]	0.425617	0.860929	1.23131	1.2812	1.25301	1.02916	1.03295	0.25135	1.1547	0.656875	0.867001	0.733092	0.954033	0.54367	0.166587374	0.633636735	12	20	24	0.221352588	0.768040178	24	14.07924248	0.168509603
ENSMUSG00000090031	4732440D04Rik	RIKEN cDNA 4732440D04 gene [Source:MGI Symbol;Acc:MGI:3604103]	1.64145	1.4313	1.26742	1.26714	1.16714	1.33016	1.24948	1.69277	1.60565	1.4215	1.40615	1.61046	1.3087	1.7335	0.113148904	0.530777648	8	12	24	0.173669137	0.768040178	24	3.95420584	0.073139182
ENSMUSG00000090035	Galnt4	polypeptide N-acetylgalactosaminyltransferase 4 [Source:MGI Symbol;Acc:MGI:894692]	1.11597	1.12445	1.13621	1.06675	1.07787	1.05183	1.11947	1.17751	1.22663	1.38369	1.04937	1.11973	1.081	1.16755	0.009730499	0.194720062	12	8	24	0.095323395	0.71175955	24	8.290758617	0.046596271
ENSMUSG00000090036	Gm15900	predicted gene 15900 [Source:MGI Symbol;Acc:MGI:3801848]	11.2578	11.7931	9.50982	14.5675	12.7583	9.42579	11.4338	10.6859	14.1354	8.64489	11.9636	13.072	12.8154	12.1411	0.761979	0.966018836	12	20	24	0.997571047	0.999623846	24	15.72304734	0.005679286
ENSMUSG00000090053	Pakap	paralemmin A kinase anchor protein [Source:MGI Symbol;Acc:MGI:5141924]	10.6492	6.15082	10.8172	7.91436	8.11259	9.8803	6.9754	5.76523	6.22949	8.95534	7.64837	9.95944	8.50826	8.74228	0.643325306	0.966018836	24	8	24	0.387373856	0.799546792	24	18.40412008	0.145855857
ENSMUSG00000090057	Gm16333	predicted gene 16333 [Source:MGI Symbol;Acc:MGI:3840150]	0.781104	0.783996	0.843741	0.57731	0.563648	0.285416	1.25526	0.683253	1.92658	0.17207	0.174183	0.680433	0.882988	0.81851	0.018857365	0.256345714	12	4	24	0.066062834	0.668186543	24	4.240360656	0.296887322
ENSMUSG00000090061	Nwd2	NACHT and WD repeat domain containing 2 [Source:MGI Symbol;Acc:MGI:1920464]	14.0285	13.3558	12.5749	11.8839	11.9902	12.4039	11.1309	11.7069	10.4792	13.2139	13.4676	13.0183	12.7518	13.611	0.916963526	0.966018836	8	4	24	0.706779635	0.903131193	24	21.07470252	0.038342996
ENSMUSG00000090066	1110002E22Rik	RIKEN cDNA 1110002E22 gene [Source:MGI Symbol;Acc:MGI:1915066]	0.349117	0.868652	1.26141	0.88756	0.980159	1.13817	1.04963	2.47874	1.51492	2.18155	0.883917	0.872567	1.4208	0.846994	0.823657258	0.966018836	12	20	24	0.23277644	0.768040178	24	8.710234532	0.222499528
ENSMUSG00000090069	E430024P14Rik	RIKEN cDNA E430024P14 gene [Source:MGI Symbol;Acc:MGI:2445079]	0.588384	0.584572	0.509737	0.543919	0.733878	0.611868	0.713135	0.523998	0.625568	0.723863	0.458287	0.439389	0.464167	0.647397	0.801273005	0.966018836	16	8	24	0.891531528	0.969321764	24	10.33875126	0.017581269
ENSMUSG00000090071	Cdk5r2	cyclin-dependent kinase 5, regulatory subunit 2 (p39) [Source:MGI Symbol;Acc:MGI:1330828]	22.3734	14.0508	13.941	14.6935	13.6519	16.1973	16.247	19.885	13.3667	26.883	19.1817	22.4207	21.3189	20.9357	0.217827365	0.704989686	24	12	24	0.622164972	0.871331547	24	21.95037363	0.106634639
ENSMUSG00000090077	Lime1	Lck interacting transmembrane adaptor 1 [Source:MGI Symbol;Acc:MGI:1919949]	10.1568	8.11656	9.78346	11.6326	11.684	9.48137	10.0825	9.61301	10.8957	8.7236	11.108	8.62936	8.53581	8.41114	0.03484427	0.328311403	20	12	24	0.090879462	0.71175955	24	14.34985205	0.120786171
ENSMUSG00000090083	Rnf8	ring finger protein 8 [Source:MGI Symbol;Acc:MGI:1929069]	13.7732	16.5878	17.2698	14.5964	16.2722	18.212	12.776	16.704	16.1447	15.2888	15.326	12.5812	13.7254	15.9984	0.338172159	0.85758369	12	16	24	0.313488162	0.768040178	24	9.348359012	0.089965195
ENSMUSG00000090084	Srpx	sushi-repeat-containing protein [Source:MGI Symbol;Acc:MGI:1858306]	6.12352	6.75321	6.27335	5.87241	8.05957	6.70307	5.96598	6.45869	5.54898	4.87189	7.73879	6.44322	6.88696	5.87815	0.022785983	0.272961011	20	20	24	0.172379517	0.768040178	24	19.41632841	0.118104962
ENSMUSG00000090086	AI480526	expressed sequence AI480526 [Source:MGI Symbol;Acc:MGI:2140910]	22.1412	27.6743	26.6165	30.3454	29.4123	26.6346	24.0617	25.0216	33.7657	27.0061	27.3918	29.2501	23.0333	26.9953	0.007288205	0.192760771	16	12	24	0.027441651	0.590220615	24	11.77663714	0.145669905
ENSMUSG00000090093	Gm14399	predicted gene 14399 [Source:MGI Symbol;Acc:MGI:3650082]	4.09085	4.85939	5.15742	4.51834	5.05159	4.58744	5.68719	3.99272	4.66997	2.7407	4.32662	3.69188	4.39461	4.06282	0.88067614	0.966018836	12	12	24	0.670346936	0.888925819	24	0.396327701	0.065558234
ENSMUSG00000090100	Ttbk2	tau tubulin kinase 2 [Source:MGI Symbol;Acc:MGI:2155779]	25.2424	25.3946	24.9699	24.9561	25.1829	24.2533	24.5482	22.6444	23.4263	22.6251	26.8473	26.1887	25.1847	27.1798	0.870491976	0.966018836	20	20	24	0.547463	0.833669565	24	20.60254966	0.034188231
ENSMUSG00000090101	Snhg9	small nucleolar RNA host gene 9 [Source:MGI Symbol;Acc:MGI:1920724]	9.50674	7.01637	5.10691	3.79499	2.42707	2.47163	7.13831	7.08357	9.49877	4.72237	8.44124	12.936	8.36533	4.02551	0.359479536	0.883787544	4	12	24	0.416550754	0.801257196	24	1.646564673	0.30702705
ENSMUSG00000090110	Cmc4	C-x(9)-C motif containing 4 [Source:MGI Symbol;Acc:MGI:5637812]	10.4199	11.3304	11.1912	11.5744	11.7528	11.8491	10.6592	11.2235	10.2438	9.95703	10.72	8.53713	10.9829	8.85195	0.844511766	0.966018836	20	8	24	0.781372813	0.929846332	24	15.12758318	0.028738632
ENSMUSG00000090112	Shprh	SNF2 histone linker PHD RING helicase [Source:MGI Symbol;Acc:MGI:1917581]	5.7004	6.22884	5.42078	5.88087	5.97021	5.20574	6.11173	4.97924	6.3005	4.77321	5.94019	5.71552	5.49296	5.65527	0.807813307	0.966018836	20	20	24	0.965307407	0.991586499	24	22.99690965	0.010870687
ENSMUSG00000090115	Usp49	ubiquitin specific peptidase 49 [Source:MGI Symbol;Acc:MGI:2685391]	3.90915	4.01597	4.73621	4.30148	4.63225	4.36288	3.89772	4.09158	4.42612	4.26663	4.24344	4.40658	4.05892	3.964	0.015511729	0.253395174	12	16	24	0.02518955	0.586541342	24	13.28223515	0.066416448
ENSMUSG00000090119	Gm8185	predicted gene 8185 [Source:MGI Symbol;Acc:MGI:3643116]	2.44331	3.75919	3.08314	2.42799	4.04808	3.55902	2.39966	3.23475	2.14813	2.34416	2.75247	2.42253	2.21184	1.84035	0.945728192	0.966018836	20	16	24	0.484133158	0.805393068	24	17.67591242	0.098593008
ENSMUSG00000090122	Kcne1l	potassium voltage-gated channel, Isk-related family, member 1-like, pseudogene [Source:MGI Symbol;Acc:MGI:1913490]	0.603306	0.672315	1.31284	0.793693	0.88497	1.11323	0.772079	1.03143	1.00221	0.815953	0.772597	1.11422	0.725649	1.02394	0.43565473	0.966018836	12	16	24	0.668752836	0.888066351	24	9.810349372	0.054786402
ENSMUSG00000090124	Ugt1a7c	UDP glucuronosyltransferase 1 family, polypeptide A7C [Source:MGI Symbol;Acc:MGI:3032636]	0.769901	0.751075	1.12607	0.958262	0.749549	0.717495	0.89896	0.891158	1.03005	0.469421	1.12333	0.54469	0.668693	0.941524	0.047644929	0.377556215	12	12	24	0.381544381	0.799546792	24	7.875745084	0.089482201
ENSMUSG00000090125	Pou3f1	POU domain, class 3, transcription factor 1 [Source:MGI Symbol;Acc:MGI:101896]	1.37381	1.14774	0.969708	0.9172	0.946649	0.797827	1.27317	1.01369	1.3895	1.701	1.16488	1.1299	1.40992	1.30036	0.675497492	0.966018836	4	20	24	0.549490528	0.834995331	24	5.500447635	0.065242431
ENSMUSG00000090129	Olfr287	olfactory receptor 287 [Source:MGI Symbol;Acc:MGI:3030121]	0.388375	0.869967	1.59629	0.214001	0.258605	1.01713	0.967362	0.769726	0.735917	0.72382	0.341962	0.444608	0.0966274	1.18818	0.243857167	0.741580974	8	12	24	0.189112394	0.768040178	24	5.724794709	0.252619892
ENSMUSG00000090136	Gm10177	predicted gene 10177 [Source:MGI Symbol;Acc:MGI:3704330]	81.5929	102.172	89.911	82.4128	93.2697	78.7664	83.2269	87.8373	112.224	67.8629	97.8883	96.0939	104.749	106.918	0.522551072	0.966018836	12	4	24	0.404208404	0.799546792	24	4.300552285	0.098579403
ENSMUSG00000090137	Uba52	ubiquitin A-52 residue ribosomal protein fusion product 1 [Source:MGI Symbol;Acc:MGI:98887]	17.4566	21.7537	23.1586	20.5119	24.861	21.6332	19.4224	22.0097	26.0138	19.2158	24.1796	20.4759	23.9702	20.8192	0.588208558	0.966018836	12	16	24	0.482320072	0.805393068	24	11.11934442	0.07189759
ENSMUSG00000090150	Acad11	acyl-Coenzyme A dehydrogenase family, member 11 [Source:MGI Symbol;Acc:MGI:2143169]	10.3167	10.1312	11.8099	16.5749	12.0317	10.5476	12.2019	11.0392	12.5594	13.7188	12.6282	11.7137	13.5576	11.3963	0.01518376	0.253245931	16	16	24	0.055928225	0.65534544	24	12.42941939	0.145041382
ENSMUSG00000090157	Gm16534	predicted gene 16534 [Source:MGI Symbol;Acc:MGI:4413442]	0.454645	0.75673	0.760792	0.594894	0.64983	0.752465	0.634705	0.849845	1.19683	0.709448	0.442856	0.607432	0.598167	0.689602	0.059650003	0.406067671	12	8	24	0.07584902	0.686372559	24	7.360302212	0.129059711
ENSMUSG00000090164	BC035044	cDNA sequence BC035044 [Source:MGI Symbol;Acc:MGI:2448540]	0.479671	0.947429	1.10638	0.722895	1.07857	0.707533	0.827257	0.607848	0.850315	0.485754	0.895064	0.663725	0.844019	0.691289	0.759899911	0.966018836	12	12	24	0.827874388	0.946180429	24	11.46222158	0.038909989
ENSMUSG00000090194	Gm16161	predicted gene 16161 [Source:MGI Symbol;Acc:MGI:3801899]	0.631778	1.42623	1.70811	1.74406	1.81428	1.37614	2.28552	1.37901	1.48753	2.0925	1.89665	1.45489	1.94002	1.75731	0.700065774	0.966018836	20	4	24	0.32660797	0.768040178	24	12.04221456	0.124113382
ENSMUSG00000090210	Itga10	integrin, alpha 10 [Source:MGI Symbol;Acc:MGI:2153482]	1.71507	1.83495	1.63423	1.35036	2.23753	1.59823	2.21672	1.72817	2.1017	1.40541	1.45894	1.67658	1.45538	1.67817	0.481530854	0.966018836	8	8	24	0.658718984	0.884596426	24	1.079188973	0.052548183
ENSMUSG00000090213	Tmem189	transmembrane protein 189 [Source:MGI Symbol;Acc:MGI:2142624]	92.4876	75.4273	70.8546	77.2554	92.8544	85.159	90.1795	85.6602	74.0343	100.1	81.6082	83.5028	86.7763	69.5755	0.16456443	0.628002127	4	8	24	0.121424329	0.733843269	24	18.87411507	0.122977022
ENSMUSG00000090223	Pcp4	Purkinje cell protein 4 [Source:MGI Symbol;Acc:MGI:97509]	373.577	374.4	329.419	333.919	319.205	311.604	326.31	316.424	368.574	321.68	339.051	366.968	392.308	375.88	0.37215823	0.894599261	4	20	24	0.195980533	0.768040178	24	2.537068507	0.07408455
ENSMUSG00000090231	Cfb	complement factor B [Source:MGI Symbol;Acc:MGI:105975]	2.17093	2.18847	3.72281	1.87298	2.16455	2.47819	1.64122	2.4799	2.18492	2.32674	2.28654	2.66118	2.27859	2.06017	0.891742979	0.966018836	24	4	24	0.790877572	0.933002007	24	9.843391158	0.053974838
ENSMUSG00000090236	Car15	carbonic anhydrase 15 [Source:MGI Symbol;Acc:MGI:1931324]	2.88117	2.05822	2.02688	1.50773	2.67762	2.89428	2.55249	2.38391	2.17803	2.36876	2.35626	1.64151	1.93108	2.364	0.714044782	0.966018836	4	8	24	0.48552772	0.805393068	24	21.84540516	0.090922962
ENSMUSG00000090247	Bloc1s1	biogenesis of lysosomal organelles complex-1, subunit 1 [Source:MGI Symbol;Acc:MGI:1195276]	65.4161	81.181	70.3975	71.2965	75.019	67.3657	73.5173	70.2376	86.6483	57.5343	71.8494	66.3292	69.2622	66.804	0.850398351	0.966018836	12	12	24	0.647886385	0.881026758	24	6.184756101	0.051960075
ENSMUSG00000090257	Gm4524	predicted gene 4524 [Source:MGI Symbol;Acc:MGI:3782709]	1.62517	1.40717	0.958371	1.45335	1.52456	1.22613	1.93337	1.38833	1.36299	1.4655	1.17099	1.3343	1.3398	1.56454	0.658895034	0.966018836	4	8	24	0.452701116	0.805393068	24	23.77711551	0.06757516
ENSMUSG00000090258	Churc1	churchill domain containing 1 [Source:MGI Symbol;Acc:MGI:1923684]	51.3284	66.6476	62.0229	62.3935	62.8881	53.7964	58.9254	55.8753	73.6527	52.5297	66.6659	60.9852	61.5867	64.1742	0.335107163	0.85758369	12	16	24	0.353398191	0.780711577	24	9.020885221	0.081704919
ENSMUSG00000090264	Eif4ebp3	eukaryotic translation initiation factor 4E binding protein 3 [Source:MGI Symbol;Acc:MGI:1270847]	3.70935	4.3178	3.026	4.53735	4.1792	2.80443	2.35523	3.12	4.62525	3.40216	3.35197	3.29711	3.75898	1.70923	0.532553009	0.966018836	16	8	24	0.343936333	0.777417177	24	13.2064871	0.14929763
ENSMUSG00000090266	Mettl23	methyltransferase like 23 [Source:MGI Symbol;Acc:MGI:1921569]	8.56018	10.3476	11.2343	11.493	11.4818	10.2617	11.2383	9.63952	11.4755	7.62133	11.4208	10.1243	11.8218	9.66396	0.909604019	0.966018836	20	12	24	0.942332582	0.985645387	24	16.24496917	0.023559558
ENSMUSG00000090270	Gm17226	predicted gene 17226 [Source:MGI Symbol;Acc:MGI:4938053]	1.37015	0.982988	1.01029	1.55482	1.50615	1.46595	1.22319	0.865062	1.0564	1.01326	1.15113	1.96288	1.34467	1.21747	0.008389425	0.192760771	24	8	24	0.017424923	0.550995814	24	18.80943363	0.181975622
ENSMUSG00000090272	Mndal	myeloid nuclear differentiation antigen like [Source:MGI Symbol;Acc:MGI:3780953]	3.8743	3.27143	4.23922	4.00309	4.2791	3.78987	4.36076	4.77048	4.58324	3.1596	4.15581	3.77421	3.25676	4.82269	0.492524295	0.966018836	8	16	24	0.66105529	0.885520687	24	6.638870906	0.059539739
ENSMUSG00000090273	Prr22	proline rich 22 [Source:MGI Symbol;Acc:MGI:2685392]	0.804484	0.963537	1.1551	1.14171	0.783104	0.903848	1.09319	0.694414	0.94268	0.640163	0.996554	0.87627	0.975187	0.804404	0.813775138	0.966018836	12	20	24	0.995863452	0.998861337	24	10.32444501	0.004407383
ENSMUSG00000090290	Tarbp1	TAR RNA binding protein 1 [Source:MGI Symbol;Acc:MGI:4936930]	1.25234	1.45382	1.29418	1.54287	1.33379	1.42975	1.53192	1.33302	1.3547	1.28448	1.53026	1.3175	1.23099	1.28052	0.85447369	0.966018836	20	8	24	0.552353913	0.836653527	24	15.60051984	0.02739245
ENSMUSG00000090291	Lrrc10b	leucine rich repeat containing 10B [Source:MGI Symbol;Acc:MGI:2685551]	0.428733	0.561111	0.666541	0.470722	0.537696	0.419485	0.424188	0.641994	0.475116	0.59327	0.422829	0.816808	0.555907	0.80156	0.374467935	0.89742288	8	12	24	0.430272949	0.805393068	24	5.992547421	0.057221177
ENSMUSG00000090302	Smr2l	submaxillary gland androgen regulated protein 2 like [Source:MGI Symbol;Acc:MGI:3646267]	0.840165	0.69767	0.663993	0.52309	0.739425	0.690107	0.74425	0.681654	0.728728	0.605757	0.732459	0.880046	0.918379	0.747365	0.001845448	0.100255953	4	12	24	0.007807391	0.486810697	24	22.85080504	0.085033858
ENSMUSG00000090307	1700071M16Rik	RIKEN cDNA 1700071M16 gene [Source:MGI Symbol;Acc:MGI:1920754]	2.49715	2.56482	2.63914	1.906	2.62124	1.84117	2.27731	1.51178	3.20347	1.67763	2.49038	2.78371	3.19363	2.14773	0.90703203	0.966018836	12	4	24	0.878491029	0.965987302	24	23.27548618	0.047202792
ENSMUSG00000090322	Gm17090	predicted gene 17090 [Source:MGI Symbol;Acc:MGI:4937917]	0.755463	0.986028	1.12043	1.03401	1.51068	1.02971	1.22492	0.471014	2.06038	0.869967	1.38712	0.88672	1.01844	0.761482	0.084879179	0.466404237	20	12	24	0.315770687	0.768040178	24	13.52764864	0.148301399
ENSMUSG00000090327	Gm17111	predicted gene 17111 [Source:MGI Symbol;Acc:MGI:4937938]	2.10796	1.31089	0.175831	1.45989	1.30832	1.1724	1.1794	0.86002	1.42795	0.643657	1.31838	0.735081	1.2018	0.874489	0.832293447	0.966018836	4	8	24	0.390891658	0.799546792	24	20.91864937	0.181198341
ENSMUSG00000090330	9130221H12Rik	RIKEN cDNA 9130221H12 gene [Source:MGI Symbol;Acc:MGI:1924374]	3.08886	2.9531	3.26818	3.98692	3.31408	3.45333	2.68072	4.08659	3.98544	3.30036	3.01089	3.38027	3.29537	3.4766	0.850398351	0.966018836	16	12	24	0.227767037	0.768040178	24	9.433423687	0.089998399
ENSMUSG00000090334	Gm17149	predicted gene 17149 [Source:MGI Symbol;Acc:MGI:4937976]	12.5382	13.8003	10.088	10.4326	12.7632	11.1496	11.5004	11.8912	11.3057	10.4347	13.2105	11.4798	12.1916	11.3441	0.151986936	0.600422612	20	20	24	0.280494977	0.768040178	24	22.22095019	0.076774385
ENSMUSG00000090381	Gm6158	predicted gene 6158 [Source:MGI Symbol;Acc:MGI:3779562]	22.1084	21.5656	21.1217	21.0322	21.9912	22.3383	23.4971	20.4088	20.7718	20.1739	21.2762	20.7829	22.1718	19.3821	0.032514053	0.324908923	4	4	24	0.019584339	0.564709807	24	20.8883214	0.061630914
ENSMUSG00000090386	Mir99ahg	Mir99a and Mirlet7c-1 host gene (non-protein coding) [Source:MGI Symbol;Acc:MGI:1919929]	3.61318	7.02265	5.79154	4.07052	4.91133	4.47716	4.50528	5.53412	6.5401	4.93723	6.30398	4.40535	5.36897	5.24172	0.077301512	0.453790923	12	12	24	0.157836648	0.764449175	24	7.679788481	0.168538437
ENSMUSG00000090389	Cdv3-ps	Cdv3 retrotransposed pseudogene [Source:MGI Symbol;Acc:MGI:3646628]	10.7871	8.73565	9.12113	9.26152	8.59481	9.06402	10.0103	9.27089	9.24187	10.3354	9.46319	9.98887	10.6703	8.08119	0.187699199	0.662239322	4	4	24	0.461133855	0.805393068	24	20.90924182	0.055572283
ENSMUSG00000090394	4930523C07Rik	RIKEN cDNA 4930523C07 gene [Source:MGI Symbol;Acc:MGI:1914897]	1.32993	1.76509	1.78188	1.46171	1.58862	1.67831	1.46609	1.92139	3.20168	1.57698	2.72095	1.99059	2.93697	1.72014	0.890648729	0.966018836	12	8	24	0.784727223	0.930908103	24	8.361493524	0.055165676
ENSMUSG00000090397	Gm17096	predicted gene 17096 [Source:MGI Symbol;Acc:MGI:4937923]	0.489255	1.02895	0.98754	1.03843	1.04226	0.987946	1.19236	0.499423	0.905468	0.646682	0.623001	0.759879	0.746517	0.997698	0.710938687	0.966018836	20	8	24	0.944575628	0.986443184	24	10.33848977	0.02495292
ENSMUSG00000090475	Gm6245	predicted gene 6245 [Source:MGI Symbol;Acc:MGI:3646756]	4.14655	4.5784	3.00481	4.51608	4.32173	4.80429	3.52968	4.64635	4.21983	4.43642	4.10874	5.25853	4.47967	4.59338	0.658895034	0.966018836	24	12	24	0.569728345	0.844900526	24	19.80863365	0.064433041
ENSMUSG00000090516	Rps11-ps1	ribosomal protein S11, pseudogene 1 [Source:MGI Symbol;Acc:MGI:3644744]	10.4107	10.7318	11.9953	13.2313	14.3169	14.8087	9.20837	9.23543	11.6918	8.75494	10.0534	7.87266	7.59575	8.04905	0.459019924	0.966018836	20	8	24	0.070914532	0.676359558	24	14.34770253	0.176365606
ENSMUSG00000090523	Gypc	glycophorin C [Source:MGI Symbol;Acc:MGI:1098566]	8.75708	10.3622	9.04606	8.63828	9.8692	8.46279	10.3933	11.5206	10.7271	10.2315	8.46632	10.3892	10.8807	9.34704	0.268085731	0.774280302	8	12	24	0.225036411	0.768040178	24	4.198931109	0.084333455
ENSMUSG00000090553	Snrpe	small nuclear ribonucleoprotein E [Source:MGI Symbol;Acc:MGI:98346]	25.5156	28.3533	24.0024	26.4572	23.6939	21.4365	24.2908	25.1628	30.3363	21.0065	30.0768	27.6004	28.3187	25.6109	0.890176313	0.966018836	12	4	24	0.777513451	0.928654033	24	4.726752132	0.044244057
ENSMUSG00000090582	Gm17024	predicted gene 17024 [Source:MGI Symbol;Acc:MGI:4937851]	0.847321	1.16045	1.28879	1.06232	1.32307	0.429962	0.515768	0.831003	1.73124	0.448054	0.985545	1.08606	1.41626	1.02287	0.8972237	0.966018836	12	12	24	0.491239535	0.805393068	24	7.972040768	0.137487022
ENSMUSG00000090607	Gm17052	predicted gene 17052 [Source:MGI Symbol;Acc:MGI:4937879]	2.86485	3.35303	4.47454	2.81127	3.1981	3.9857	4.42188	2.50051	2.99749	3.16268	2.97245	1.25688	3.91043	3.54305	0.90767227	0.966018836	4	12	24	0.668156016	0.887908123	24	5.423289402	0.112561669
ENSMUSG00000090610	Gm3571	predicted gene 3571 [Source:MGI Symbol;Acc:MGI:3781748]	101.002	82.3807	88.0815	77.0086	86.5789	99.1723	86.0851	87.8685	67.4616	86.2963	80.2502	79.6234	81.1296	83.9153	0.910436409	0.966018836	4	12	24	0.109006191	0.72082227	24	21.68345149	0.105225202
ENSMUSG00000090622	A930033H14Rik	RIKEN cDNA A930033H14 gene [Source:MGI Symbol;Acc:MGI:2444562]	0.648806	0.768373	0.690842	0.683694	0.637566	0.733446	0.655314	0.748226	0.898269	1.02458	0.672459	0.83227	0.664632	0.88653	0.16079538	0.620479606	12	16	24	0.142023438	0.751766185	24	8.779541604	0.064116248
ENSMUSG00000090626	Tex9	testis expressed gene 9 [Source:MGI Symbol;Acc:MGI:1201610]	6.35277	9.21228	7.7797	6.50607	7.02708	7.79921	6.19545	6.75389	9.33284	7.43292	8.5653	8.1981	7.39111	7.90659	0.636884864	0.966018836	16	16	24	0.595443917	0.857839743	24	9.249586926	0.067571424
ENSMUSG00000090641	Zfp712	zinc finger protein 712 [Source:MGI Symbol;Acc:MGI:1925501]	1.91301	2.52542	2.16164	2.38943	2.26523	1.78955	2.05978	1.87045	2.52824	1.90862	2.2066	2.06367	2.22793	2.15041	0.539040394	0.966018836	12	8	24	0.316390379	0.768040178	24	9.059186945	0.064809181
ENSMUSG00000090659	Zfp493	zinc finger protein 493 [Source:MGI Symbol;Acc:MGI:1920208]	1.16003	1.4821	1.26603	0.919489	1.00706	0.836327	1.16558	1.38195	1.81798	0.788532	1.25983	1.51817	1.63649	0.885198	0.676344318	0.966018836	12	4	24	0.390482564	0.799546792	24	3.231480873	0.108255365
ENSMUSG00000090667	Mdfic2	MyoD family inhibitor domain containing 2 [Source:MGI Symbol;Acc:MGI:2685611]	94.3375	90.7783	84.9863	84.3329	84.5291	84.1885	82.6904	80.6713	82.9988	81.3724	87.996	87.2557	91.0601	90.4734	0.688644847	0.966018836	4	12	24	0.221864655	0.768040178	24	23.64612684	0.044986364
ENSMUSG00000090698	Apold1	apolipoprotein L domain containing 1 [Source:MGI Symbol;Acc:MGI:2685921]	0.499799	0.651041	0.721173	0.469724	0.438039	0.458514	0.540114	1.00358	0.788157	0.752204	0.506149	0.552918	0.825212	0.678528	0.057264003	0.401309455	12	8	24	0.004301728	0.427431763	24	5.882582721	0.150179366
ENSMUSG00000090704	Tp53-ps	transformation related protein 53, pseudogene [Source:MGI Symbol;Acc:MGI:98835]	0.913958	0.929394	0.778016	0.776103	0.834239	0.85867	0.824661	0.605533	0.733267	0.840017	0.629256	0.50609	0.52887	0.562371	0.757932695	0.966018836	16	8	24	0.731063551	0.912382349	24	14.0137596	0.021428866
ENSMUSG00000090733	Rps27	ribosomal protein S27 [Source:MGI Symbol;Acc:MGI:1888676]	2.9352	2.91574	3.61304	2.28571	2.13095	2.30566	2.01618	3.11813	4.08183	2.88437	3.34787	3.42201	3.21078	2.82284	0.818295516	0.966018836	12	4	24	0.284907768	0.768040178	24	7.261435417	0.135648548
ENSMUSG00000090761	Gm17201	predicted gene 17201 [Source:MGI Symbol;Acc:MGI:4938028]	4.94129	3.85781	2.58115	2.81639	3.10118	5.28738	3.47194	4.47423	4.23125	3.8078	4.12039	4.16203	3.64633	4.34801	0.904831057	0.966018836	20	8	24	0.979990776	0.993012781	24	17.95669545	0.033160056
ENSMUSG00000090778	Gm3235	predicted gene 3235 [Source:MGI Symbol;Acc:MGI:3781413]	0.374658	0.380277	0.895752	0.513318	1.0605	0.634894	0.365122	0.601958	0.868204	0.544751	0.616043	0.33568	0.400376	0.451011	0.046535188	0.375011045	20	8	24	0.072647411	0.679671854	24	11.9439145	0.161800264
ENSMUSG00000090790	Gm17377	predicted gene, 17377 [Source:MGI Symbol;Acc:MGI:4937011]	8.62436	13.1987	10.1267	11.7993	11.9372	10.743	14.1216	14.7874	7.32766	11.4279	15.3376	9.50652	8.19811	13.6905	0.869721097	0.966018836	20	16	24	0.981264632	0.995642502	24	12.90417908	0.023845351
ENSMUSG00000090799	Klhl33	kelch-like 33 [Source:MGI Symbol;Acc:MGI:3644593]	2.48013	3.63645	3.85116	4.35744	2.751	3.24985	3.10348	4.68201	4.77084	5.81937	2.53372	2.81727	3.30523	3.62301	0.001043432	0.07619846	16	4	24	0.005300954	0.4424469	24	8.604951351	0.318937948
ENSMUSG00000090812	Samd15	sterile alpha motif domain containing 15 [Source:MGI Symbol;Acc:MGI:2685109]	0.685526	0.742534	0.509404	1.63447	0.577988	1.02465	1.70916	0.559405	0.985292	0.848984	0.739659	0.774073	0.647851	0.970035	0.860560779	0.966018836	16	20	24	0.967388478	0.992187127	24	16.07258487	0.027065619
ENSMUSG00000090841	Myl6	myosin, light polypeptide 6, alkali, smooth muscle and non-muscle [Source:MGI Symbol;Acc:MGI:109318]	197.716	211.875	186.994	195.178	233.03	197.559	183.223	185.612	213.638	150.24	204.232	181.279	193.91	172.027	0.903207257	0.966018836	12	20	24	0.704494335	0.902419351	24	18.85669435	0.048134368
ENSMUSG00000090862	Rps13	ribosomal protein S13 [Source:MGI Symbol;Acc:MGI:1915302]	1.5578	1.73298	2.31923	1.37937	1.79615	1.73969	1.91533	1.62662	1.7529	1.28419	2.20232	1.54604	1.40927	2.00135	0.960333395	0.967043964	12	4	24	0.924427834	0.979866138	24	6.060092992	0.026051106
ENSMUSG00000090877	Hspa1b	heat shock protein 1B [Source:MGI Symbol;Acc:MGI:99517]	10.2702	2.11435	1.79506	2.94118	3.37675	2.57839	5.15084	3.78765	1.88904	2.89115	7.67556	4.69042	4.14981	3.0718	0.004501001	0.140598674	4	8	24	0.071234064	0.676901434	24	20.72164173	0.504128857
ENSMUSG00000090935	Synj2bp	synaptojanin 2 binding protein [Source:MGI Symbol;Acc:MGI:1344347]	19.9258	18.8365	17.0331	18.5381	18.0451	17.0046	18.5722	17.6896	18.9491	15.9339	19.9825	17.7728	19.3562	19.1236	0.45229395	0.966018836	12	12	24	0.445622648	0.805393068	24	0.333763673	0.044038649
ENSMUSG00000090946	Ccdc71l	coiled-coil domain containing 71 like [Source:MGI Symbol;Acc:MGI:1919373]	3.24058	3.25608	3.07347	3.55249	3.44564	3.06303	3.25127	3.27311	3.02415	3.22267	3.50167	3.24222	2.81272	3.14572	0.455007782	0.966018836	20	8	24	0.124137961	0.737882465	24	14.74079197	0.048578747
ENSMUSG00000090952	Gm17251	predicted gene, 17251 [Source:MGI Symbol;Acc:MGI:4936885]	4.21949	4.33435	3.61801	3.89941	3.88505	4.08097	5.60846	5.0198	5.64654	5.82183	5.7063	5.68712	5.62125	6.17513	0.748433002	0.966018836	8	4	24	0.409400081	0.799546792	24	3.26961529	0.059291233
ENSMUSG00000090955	Gm17097	predicted gene 17097 [Source:MGI Symbol;Acc:MGI:4937924]	0.506269	0.477229	0.393008	0.330454	0.8085	0.449608	0.440574	0.193743	0.739566	0.525775	0.644402	0.508963	0.482923	0.70687	0.833406028	0.966018836	20	12	24	0.586463677	0.853300537	24	15.12244975	0.062127808
ENSMUSG00000090957	Gm7535	predicted gene 7535 [Source:MGI Symbol;Acc:MGI:3644434]	2.2833	2.5486	2.96546	2.77173	2.8983	2.96837	2.42994	3.10869	2.5703	2.85395	2.3546	2.04369	2.36529	2.34892	0.437307241	0.966018836	20	12	24	0.215216197	0.768040178	24	10.44752504	0.081248854
ENSMUSG00000090958	Lrrc32	leucine rich repeat containing 32 [Source:MGI Symbol;Acc:MGI:93882]	1.26668	1.38936	1.80027	1.45852	1.50394	1.24436	1.37701	1.73349	1.81663	0.99372	1.41453	1.25655	1.28577	1.19976	0.042880436	0.356048751	12	20	24	0.307001963	0.768040178	24	7.713329419	0.083618226
ENSMUSG00000090963	Gm17655	predicted gene, 17655 [Source:MGI Symbol;Acc:MGI:4937289]	0.835272	0.962568	0.807895	0.820714	1.23762	0.840623	1.02061	0.992752	1.08438	0.68148	0.898355	0.993744	1.11178	0.819905	0.510431574	0.966018836	20	20	24	0.59566415	0.857839743	24	22.10242545	0.045661926
ENSMUSG00000091002	Tcergl1	transcription elongation regulator 1-like [Source:MGI Symbol;Acc:MGI:1917821]	5.78077	5.01014	5.19618	5.79635	4.52265	5.65885	4.84202	5.26898	4.15192	7.05499	4.04826	5.73744	5.72251	5.5246	0.823677655	0.966018836	24	20	24	0.967859513	0.99226729	24	21.18057525	0.018505158
ENSMUSG00000091020	Gm5828	predicted gene 5828 [Source:MGI Symbol;Acc:MGI:3644176]	1.07398	1.03366	1.16869	1.31818	0.877953	1.34512	1.19826	1.9904	0.894707	1.34155	0.982802	1.18664	0.884089	1.15975	0.720359655	0.966018836	16	4	24	0.886175647	0.967667196	24	14.42077455	0.021976101

ENSMUSG00000091021	Gm17300	predicted gene, 17300 [Source:MGI Symbol;Acc:MGI:4936934]	1.2532	1.54361	1.45132	1.31537	1.33938	1.36933	1.65982	1.38878	1.61487	1.14545	1.5441	1.16278	1.10417	1.37721	0.63897419	0.966018836	12	4	24	0.607558635	0.864022052	24	6.532458594	0.042121868
ENSMUSG00000091058	Gm17538	predicted gene, 17538 [Source:MGI Symbol;Acc:MGI:4937172]	70.6341	68.5693	57.0793	62.2443	69.1535	76.2007	66.4041	60.8708	68.26	51.7073	70.762	65.5433	68.3848	66.7895	0.116913252	0.538362324	20	20	24	0.055015726	0.655263328	24	21.45292369	0.125636482
ENSMUSG00000091060	Myocos	myocilin opposite strand [Source:MGI Symbol;Acc:MGI:3642786]	0.628273	0.424457	1.13705	0.713598	0.698173	0.352913	0.891284	0.403044	0.498451	0.207687	0.571582	0.472543	0.491068	0.476706	0.934152439	0.966018836	12	20	24	0.990355054	0.998212323	24	7.032324979	0.011190897
ENSMUSG00000091083	Gm4535	predicted gene 4535 [Source:MGI Symbol;Acc:MGI:3782719]	2.42945	3.43718	3.6277	3.91149	2.92658	2.56042	2.33916	2.70234	2.20337	2.54309	1.93487	2.66602	2.64396	2.30196	0.890648739	0.966018836	16	12	24	0.477879946	0.805393068	24	9.494146038	0.085148811
ENSMUSG00000091086	Rpl6l	ribosomal protein L6-like [Source:MGI Symbol;Acc:MGI:3647789]	62.3588	67.6094	63.7074	62.424	60.8063	59.7121	60.1535	59.5567	74.9665	55.9025	69.1154	63.0027	67.4453	67.9814	0.960333395	0.967043964	12	12	24	0.478758534	0.805393068	24	6.061617539	0.052257726
ENSMUSG00000091172	Gm17120	predicted gene 17120 [Source:MGI Symbol;Acc:MGI:4937947]	1.00058	1.74449	0.878277	1.96042	1.04563	1.43594	1.19718	0.933759	1.97031	0.662885	0.894834	1.67595	0.842662	1.02154	0.816361374	0.966018836	24	4	24	0.991797919	0.998516607	24	10.85643125	0.014228896
ENSMUSG00000091175	S100a7l2	S100 calcium binding protein A7 like 2 [Source:MGI Symbol;Acc:MGI:2684973]	4.17993	2.48929	2.6687	1.11364	3.26813	2.47787	1.79201	1.85518	2.42126	2.03247	3.17988	1.34653	2.22794	2.25	0.891690964	0.966018836	20	20	24	0.812680914	0.940101979	24	21.53175461	0.086741963
ENSMUSG00000091183	Gm5141	predicted gene 5141 [Source:MGI Symbol;Acc:MGI:3779466]	1.37155	1.85624	2.01814	2.11126	2.09225	1.65879	1.7604	1.95972	2.42592	1.59854	2.27239	1.84441	1.90577	2.00705	0.039617363	0.338535904	20	4	24	0.108489418	0.72082227	24	10.39258471	0.104783631
ENSMUSG00000091191	Gm17334	predicted gene, 17334 [Source:MGI Symbol;Acc:MGI:4936968]	0.322337	0.61856	0.817878	0.488573	0.3482	0.454146	0.382168	0.810886	0.612634	0.740577	0.580974	0.469713	0.59119	0.552453	0.045120335	0.368634624	12	12	24	0.01572062	0.544099157	24	8.005986717	0.147567327
ENSMUSG00000091204	Gm7271	predicted gene 7271 [Source:MGI Symbol;Acc:MGI:3647743]	76.3249	70.2068	60.6911	60.4772	67.9322	64.3258	57.1452	62.0278	60.4562	52.8572	62.8768	66.2549	73.4833	63.3345	0.082807861	0.462922111	4	12	24	0.0656507	0.667622064	24	22.79640604	0.116539709
ENSMUSG00000091228	Gm20390	predicted gene 20390 [Source:MGI Symbol;Acc:MGI:5141855]	34.71	31.5767	36.2776	33.3326	41.4385	36.1614	32.1276	35.6882	35.478	23.5715	35.4528	30.2846	28.3109	31.8028	0.870491976	0.966018836	20	8	24	0.855600174	0.957116652	24	18.17502714	0.039725898
ENSMUSG00000091230	H1f11-ps	H1.11 linker histone, pseudogene [Source:MGI Symbol;Acc:MGI:3645322]	1.82369	1.56901	1.55083	1.99011	1.53441	2.01382	1.93123	1.34406	1.51269	1.57219	1.79162	1.67563	2.01334	2.26979	0.43565473	0.966018836	4	8	24	0.438838639	0.805393068	24	21.81393422	0.067590029
ENSMUSG00000091243	Vgll3	vestigial like family member 3 [Source:MGI Symbol;Acc:MGI:1920819]	8.56999	8.67791	6.649	6.47091	9.33697	7.19421	10.2558	8.42342	8.20594	7.8449	7.93619	7.76124	8.11455	7.56974	0.285193697	0.793714531	4	12	24	0.258713872	0.768040178	24	23.19197329	0.100480872
ENSMUSG00000091264	Smim13	small integral membrane protein 13 [Source:MGI Symbol;Acc:MGI:2652854]	38.5925	36.7292	36.1409	34.9344	35.9164	36.4394	35.8789	34.811	33.7585	37.8243	36.4218	37.7695	36.5026	38.9777	0.800065124	0.966018836	4	8	24	0.432064141	0.805393068	24	22.14688593	0.028937684
ENSMUSG00000091269	Gm6682	predicted gene 6682 [Source:MGI Symbol;Acc:MGI:3647156]	24.646	20.227	22.143	20.0932	22.5278	25.3331	21.6216	23.1313	19.3973	21.1085	24.5624	24.4917	21.9876	20.2701	0.028632001	0.316608413	24	12	24	0.007916637	0.490448898	24	19.81695747	0.12990994
ENSMUSG00000091272	Gm17641	predicted gene, 17641 [Source:MGI Symbol;Acc:MGI:4937275]	0.840942	0.864778	0.832076	0.92758	0.691346	0.677542	0.808822	0.953973	0.864504	1.33235	0.826887	1.04683	0.983408	0.88458	0.961261938	0.967043964	16	4	24	0.434855008	0.805393068	24	9.04748052	0.056577821
ENSMUSG00000091277	Gm1818	predicted gene 1818 [Source:MGI Symbol;Acc:MGI:3037676]	8.74734	9.6269	10.0224	11.2126	9.30011	8.09264	8.5906	10.7693	10.2833	10.1701	9.55687	9.95099	9.59732	10.4222	0.030777129	0.324150946	16	8	24	0.005487012	0.445165967	24	8.97117076	0.1250955
ENSMUSG00000091329	1700112D23Rik	RIKEN cDNA 1700112D23 gene [Source:MGI Symbol;Acc:MGI:1920861]	4.2596	2.62662	3.29881	1.94334	3.6896	2.77171	3.03532	3.01794	1.73344	3.01637	3.50013	2.14961	2.08355	2.16949	0.95032543	0.966018836	20	20	24	0.410921246	0.799546792	24	19.29232123	0.134391744
ENSMUSG00000091337	Eid1	EP300 interacting inhibitor of differentiation 1 [Source:MGI Symbol;Acc:MGI:1889651]	72.3865	83.8966	84.9793	79.441	81.4161	71.981	81.1313	73.1772	88.5476	85.1746	78.6936	73.6788	68.6856	77.8705	0.004606005	0.140598674	12	16	24	0.010061414	0.503967859	24	9.978095888	0.11089078
ENSMUSG00000091345	Col6a5	collagen, type VI, alpha 5 [Source:MGI Symbol;Acc:MGI:3648134]	0.388573	1.05219	0.753555	1.09991	0.998292	0.775117	0.408356	0.783211	1.28291	0.969191	0.734545	1.028	0.513653	0.684553	0.016720774	0.253395174	16	12	24	0.030536152	0.597890721	24	11.47486503	0.203109442
ENSMUSG00000091363	Gm7701	predicted gene 7701 [Source:MGI Symbol;Acc:MGI:3646506]	0.903944	1.16491	1.1146	1.17898	1.40319	1.02992	1.36177	1.13567	0.721285	1.19413	0.955557	1.09221	0.981517	0.53717	0.851656735	0.966018836	16	20	24	0.254598809	0.768040178	24	17.14626414	0.09897264
ENSMUSG00000091387	Gcnt4	glucosaminyl (N-acetyl) transferase 4, core 2 (beta-1,6-N-acetylglucosaminyltransferase) [Source:MGI Symbol;Acc:MGI:2684919]	0.6559	0.556806	0.808753	0.52456	0.665389	0.569764	0.512668	0.517509	0.598274	0.767006	0.630557	0.524947	0.471917	0.647818	0.25824623	0.761738864	12	16	24	0.151355453	0.758865539	24	11.10870968	0.063038884
ENSMUSG00000091405	H4c14	H4 clustered histone 14 [Source:MGI Symbol;Acc:MGI:2140113]	1.2429	0.4949	0.440994	0.777812	0.812277	0.600678	0.82525	0.50939	0.591506	0.321233	0.950438	0.687	0.790096	0.38101	0.070382661	0.439424269	4	4	24	0.045471939	0.629609444	24	20.00695613	0.18736407
ENSMUSG00000091408	Gm6728	predicted gene 6728 [Source:MGI Symbol;Acc:MGI:3643418]	5.23992	4.34235	3.63966	4.49893	4.38749	4.70354	3.86923	4.73398	4.39224	4.82892	4.63564	4.46107	5.77509	5.12276	0.891105762	0.966018836	4	8	24	0.516993517	0.818056104	24	22.98222378	0.05793907
ENSMUSG00000091421	Gm4202	predicted gene 4202 [Source:MGI Symbol;Acc:MGI:3782379]	268.111	208.022	222.348	224.117	223.039	255.72	235.227	223.921	185.526	266.283	228.863	226.846	230.44	231.729	0.064867922	0.416505219	4	8	24	0.234248281	0.768040178	24	19.82397884	0.092537655
ENSMUSG00000091449	Gm10269	predicted gene 10269 [Source:MGI Symbol;Acc:MGI:3642381]	14.7892	17.8622	16.8693	17.2182	19.4547	15.0733	15.7824	15.0905	22.8926	15.4541	27.3123	19.2295	19.8575	17.6751	0.96317972	0.967965568	20	8	24	0.375760004	0.795959912	24	13.66210872	0.117986513
ENSMUSG00000091462	Gm17084	predicted gene 17084 [Source:MGI Symbol;Acc:MGI:4937911]	3.11337	3.22843	2.87098	2.73325	1.82452	2.68604	1.83214	2.88662	3.23745	1.54209	2.88775	2.30645	2.07118	1.94019	0.7207001	0.966018836	12	4	24	0.655278471	0.883697438	24	5.134421252	0.081268548
ENSMUSG00000091474	2610021A01Rik	RIKEN cDNA 2610021A01 gene [Source:MGI Symbol;Acc:MGI:1922662]	8.46118	7.46351	6.56373	7.00397	7.98319	6.98732	8.17651	6.43226	7.96818	5.4362	8.95658	7.41565	7.20456	6.34426	0.361555248	0.885260695	20	20	24	0.214525976	0.768040178	24	20.25764036	0.114703816
ENSMUSG00000091475	Cerox1	cytoplasmic endogenous regulator of oxidative phosphorylation 1 [Source:MGI Symbol;Acc:MGI:1920084]	8.64952	11.8905	13.0471	9.27256	12.9148	11.6135	10.3016	10.3475	11.4325	9.72681	10.705	10.8689	9.20385	9.61812	0.291478323	0.798044066	12	16	24	0.635328426	0.876170065	24	12.78246335	0.060803877
ENSMUSG00000091476	Catspere2	cation channel sperm associated auxiliary subunit epsilon 2 [Source:MGI Symbol;Acc:MGI:5589632]	0.427621	0.467404	0.586133	0.534928	0.518074	0.410332	0.476536	0.516229	0.580631	0.681262	0.503994	0.59178	0.379622	0.662978	0.025723486	0.297892358	16	12	24	0.015885329	0.544099157	24	10.00251853	0.081313175
ENSMUSG00000091478	Gm10039	predicted pseudogene 10039 [Source:MGI Symbol;Acc:MGI:3704382]	279.223	273.015	264.977	262.933	286.046	292.256	248.264	306.74	284.037	259.394	282.587	263.356	272.477	270.321	0.897570341	0.966018836	20	20	24	0.976927277	0.994118853	24	23.19932891	0.006974892
ENSMUSG00000091498	Mpc1-ps	mitochondrial pyruvate carrier 1, pseudogene [Source:MGI Symbol;Acc:MGI:3781628]	152.504	166.979	192.023	212.225	163.008	161.934	171.456	186.596	196.141	157.843	208.542	167.137	189.592	179.927	0.531498969	0.966018836	12	12	24	0.202476677	0.768040178	24	9.727151578	0.102291125
ENSMUSG00000091509	Gm17066	predicted gene 17066 [Source:MGI Symbol;Acc:MGI:4937893]	8.10462	9.1267	9.5993	10.4226	7.62125	7.54923	8.62924	8.81808	11.2063	6.77035	9.09371	7.42102	7.769	9.68307	0.001971398	0.100255953	12	12	24	0.05680807	0.65610184	24	7.474778956	0.164697739
ENSMUSG00000091510	Mtag2	metastasis associated gene 2 [Source:MGI Symbol;Acc:MGI:1860766]	1.19393	1.72152	1.16204	1.61529	1.36509	1.29395	1.16512	1.46999	1.4484	1.91268	1.43297	1.97793	1.43499	1.4588	0.702630284	0.966018836	16	12	24	0.50972049	0.813985618	24	12.41885512	0.061861294
ENSMUSG00000091512	Lamtor3	late endosomal/lysosomal adaptor, MAPK and MTOR activator 3 [Source:MGI Symbol;Acc:MGI:1929467]	26.0829	23.4345	24.338	26.1794	22.2971	23.0839	24.2421	23.7748	24.5873	25.0209	25.2438	24.3608	25.4313	25.1408	0.906657897	0.966018836	16	4	24	0.804818038	0.937541099	24	7.339040515	0.017304259
ENSMUSG00000091514	Gm17484	predicted gene, 17484 [Source:MGI Symbol;Acc:MGI:4937118]	2.1638	1.58816	1.69795	1.58707	1.87067	1.72652	1.49576	0.978507	1.64027	1.24819	1.87262	1.70562	2.42581	1.69997	0.221479046	0.71314449	20	20	24	0.289698897	0.768040178	24	20.47968206	0.121328999
ENSMUSG00000091519	Skor2	SKI family transcriptional corepressor 2 [Source:MGI Symbol;Acc:MGI:3645984]	1.36872	1.06225	0.838927	1.04305	0.851092	1.12403	1.02364	1.23078	0.846359	1.8604	1.1387	1.50062	1.45509	1.34591	0.245061943	0.741580974	24	12	24	0.727640586	0.910646611	24	21.87894749	0.054053691
ENSMUSG00000091534	Gm17195	predicted gene 17195 [Source:MGI Symbol;Acc:MGI:4938022]	1.21305	1.22624	0.98678	0.170904	2.33974	0.929192	0.919546	1.34182	0.719451	1.00305	0.925512	1.17676	1.22475	1.16506	0.371875382	0.894537464	8	4	24	0.457919923	0.805393068	24	22.23929937	0.161497213
ENSMUSG00000091537	Tma7	translational machinery associated 7 [Source:MGI Symbol;Acc:MGI:1913417]	28.031	29.685	30.0966	33.6107	32.1338	34.1378	32.8517	28.3792	29.2783	25.7839	31.1146	31.1266	27.3427	27.3938	0.247964668	0.746745468	24	8	24	0.11734223	0.728323355	24	17.76141771	0.087763828
ENSMUSG00000091549	Gm6548	predicted gene 6548 [Source:MGI Symbol;Acc:MGI:3644071]	27.257	28.9463	29.9498	28.2204	28.8141	30.1214	28.2929	34.9803	32.3836	30.4375	36.224	29.6176	31.5837	27.1477	0.896311169	0.966018836	12	16	24	0.707995272	0.903565455	24	12.04678718	0.037763028
ENSMUSG00000091561	Gm6665	predicted gene 6665 [Source:MGI Symbol;Acc:MGI:3643253]	9.02548	11.2024	8.93114	8.73187	9.29448	9.13961	9.61666	10.9262	12.5173	7.88285	8.38543	8.05189	8.77825	7.77325	0.892555977	0.966018836	12	4	24	0.25880835	0.768040178	24	5.231697589	0.108545712
ENSMUSG00000091570	Gm7707	predicted gene 7707 [Source:MGI Symbol;Acc:MGI:3646976]	0.639656	0.752085	0.652279	0.505212	0.449607	0.636516	0.488079	0.645026	0.573771	0.264137	0.880772	0.407365	0.505518	0.565824	0.431765373	0.966018836	8	8	24	0.690925927	0.897666745	24	2.182314768	0.046310368
ENSMUSG00000091586	Cyp4f17	cytochrome P450, family 4, subfamily f, polypeptide 17 [Source:MGI Symbol;Acc:MGI:3646233]	1.11981	1.57874	1.77031	1.03624	1.61102	1.68097	1.39486	1.63333	1.59306	1.50166	1.68047	2.00333	1.77227	1.80355	0.892578749	0.966018836	24	16	24	0.891458238	0.969321764	24	0.568311275	0.023600439
ENSMUSG00000091613	Gm17046	predicted gene 17046 [Source:MGI Symbol;Acc:MGI:4937873]	5.21173	4.7358	3.96636	4.77468	4.71664	5.11927	5.29599	3.93642	4.11018	3.59674	4.77222	3.80799	3.86084	3.46856	0.977065717	0.977627766	4	4	24	0.029874431	0.596648309	24	19.78211179	0.124529801
ENSMUSG00000091625	Lsm5	LSM5 homolog, U6 small nuclear RNA and mRNA degradation associated [Source:MGI Symbol;Acc:MGI:1913623]	1.91027	4.00002	2.25208	2.33449	3.26524	2.91303	1.81054	1.97461	3.64471	1.90826	2.81471	2.04095	2.80914	1.2479	0.85447369	0.966018836	20	8	24	0.711944691	0.904867662	24	14.63466899	0.099111631
ENSMUSG00000091636	Akain1	A kinase (PRKA) anchor inhibitor 1 [Source:MGI Symbol;Acc:MGI:2444600]	2.39482	2.28957	2.9094	2.25883	1.83065	2.31068	2.14827	1.98528	1.72837	2.18235	1.88447	2.36224	1.84902	2.73528	0.951856915	0.966018836	8	12	24	0.655675928	0.883738698	24	4.304222292	0.05421977
ENSMUSG00000091639	Gm3756	predicted gene 3756 [Source:MGI Symbol;Acc:MGI:3781931]	1.29784	1.23558	1.0861	0.918624	1.97351	0.947337	0.975363	1.22395	1.22876	0.923227	1.14662	1.58788	1.28355	1.23708	0.198504154	0.680216953	4	12	24	0.639786559	0.877957092	24	20.07726988	0.068323691
ENSMUSG00000091648	C2cd4d	C2 calcium-dependent domain containing 4D [Source:MGI Symbol;Acc:MGI:2685505]	3.22016	2.89745	1.27817	3.05061	2.58516	2.80401	2.96284	2.55839	4.12543	2.06016	3.46805	2.52727	3.09431	1.89476	0.94086481	0.966018836	4	4	24	0.620066951	0.869994203	24	20.10390247	0.117909619
ENSMUSG00000091649	Phf11b	PHD finger protein 11B [Source:MGI Symbol;Acc:MGI:3645789]	1.39958	0.869781	0.80348	0.759419	0.787157	1.10555	1.23401	1.39408	0.478295	0.436594	1.17788	1.3367	1.39691	1.04222	1.06E-04	0.029372629	4	12	24	0.001216178	0.340896499	24	22.75627835	0.28678409
ENSMUSG00000091655	Gm8741	predicted gene 8741 [Source:MGI Symbol;Acc:MGI:3644779]	2.73261	4.96451	4.73897	4.08792	4.52868	4.03946	4.85406	4.17879	4.19062	3.85756	3.5699	3.31134	2.8284	3.38453	0.44861185	0.966018836	12	16	24	0.350009942	0.779440054	24	9.665779141	0.113465961
ENSMUSG00000091694	Apol11b	apolipoprotein L 11b [Source:MGI Symbol;Acc:MGI:3036248]	0.453222	0.734471	1.07326	0.799382	0.697821	0.817259	0.711466	0.700386	0.597377	0.39912	0.726024	0.645799	0.966383	0.875181	0.887862353	0.966018836	8	8	24	0.781730369	0.929907172	24	4.054914376	0.042931296
ENSMUSG00000091697	Eif3s6-ps2	eukaryotic translation initiation factor 3, subunit 6, pseudogene 2 [Source:MGI Symbol;Acc:MGI:103204]	14.8768	17.5328	16.9283	16.3218	14.9648	13.8808	13.9072	14.111	18.1689	12.3571	17.0371	13.9706	16.1492	13.8868	0.390686537	0.915479136	12	12	24	0.550504532	0.835742738	24	8.331316058	0.066713311
ENSMUSG00000091712	Sec14l5	SEC14-like lipid binding 5 [Source:MGI Symbol;Acc:MGI:3616084]	14.0252	15.3123	16.2643	20.4188	20.9624	13.9855	17.7713	12.9274	19.9531	17.2441	15.8651	12.7957	13.8631	11.1931	0.073826349	0.448301997	16	16	24	0.025927092	0.586541342	24	12.88941088	0.221918726
ENSMUSG00000091722	Siah3	siah E3 ubiquitin protein ligase family member 3 [Source:MGI Symbol;Acc:MGI:2685758]	5.29651	3.63228	3.76722	4.22021	3.57865	4.86713	4.1539	4.63295	3.08765	5.07957	3.82729	4.09159	4.2746	4.5551	0.625825704	0.966018836	4	8	24	0.468577959	0.805393068	24	22.20263729	0.086375861
ENSMUSG00000091735	Gpr62	G protein-coupled receptor 62 [Source:MGI Symbol;Acc:MGI:3525078]	0.393078	0.777961	1.47339	0.906075	0.410706	0.922072	1.41444	0.725819	0.986403	0.504793	0.678277	0.818383	0.478668	0.938643	0.424718357	0.966018836	12	8	24	0.490823981	0.805393068	24	6.333064247	0.124956732
ENSMUSG00000091736	Yy2	Yy2 transcription factor [Source:MGI Symbol;Acc:MGI:3837947]	0.464499	0.701522	1.12573	0.769487	0.910813	0.792538	0.4932	0.543643	0.790627	0.812286	0.399185	0.763831	0.589049	0.584111	0.052672766	0.391647859	12	16	24	0.165166152	0.767020009	24	11.39676404	0.115133228
ENSMUSG00000091742	Gm5093	predicted gene 5093 [Source:MGI Symbol;Acc:MGI:3644404]	1.90633	1.41992	1.9479	0.763228	1.1615	1.5097	1.36296	0.896948	1.44808	0.889752	2.36957	2.30853	1.27813	1.44943	0.745118544	0.966018836	24	16	24	0.424128964	0.805393068	24	20.86680942	0.153910248
ENSMUSG00000091754	Gm3636	predicted gene 3636 [Source:MGI Symbol;Acc:MGI:3781812]	0.600811	0.585957	0.735487	0.7209	0.771678	0.642394	0.856453	0.809252	0.685147	0.380287	0.44905	0.493155	0.382571	0.761325	0.935593607	0.966018836	8	8	24	0.517445593	0.818298678	24	4.972966039	0.06074781
ENSMUSG00000091764	Zfp964	zinc finger protein 964 [Source:MGI Symbol;Acc:MGI:3646490]	1.52395	1.92303	1.68993	1.62162	1.73816	1.89582	1.85677	1.65831	2.0347	1.35579	1.91664	1.96977	1.71824	1.71947	0.839634479	0.966018836	24	16	24	0.711377769	0.904655191	24	22.10099897	0.033698187
ENSMUSG00000091780	Sco2	SCO2 cytochrome c oxidase assembly protein [Source:MGI Symbol;Acc:MGI:3818630]	4.79652	3.29525	3.87818	3.07163	3.30295	4.02018	3.67237	4.52026	2.94705	4.63931	4.27388	4.35838	3.81864	3.92598	0.78898997	0.966018836	24	12	24	0.470201745	0.805393068	24	21.67161404	0.086386995
ENSMUSG00000091784	Gm17022	predicted gene 17022 [Source:MGI Symbol;Acc:MGI:4937849]	1.30952	1.35736	1.08221	1.82214	1.29303	1.21833	1.66129	1.07843	0.965669	1.10749	1.18811	1.3699	1.57067	1.29217	0.037085178	0.334054683	4	8	24	0.410110979	0.799546792	24	20.99659143	0.07938758
ENSMUSG00000091803	Cox16	cytochrome c oxidase assembly protein 16 [Source:MGI Symbol;Acc:MGI:1913522]	78.215	83.8691	76.8107	72.598	69.8471	66.0016	78.078	71.6115	87.2317	64.2773	87.7631	75.7092	84.728	82.6151	0.602014439	0.966018836	12	4	24	0.331341768	0.768121337	24	3.788660776	0.079845636
ENSMUSG00000091811	Inafm1	InaF motif containing 1 [Source:MGI Symbol;Acc:MGI:1913550]	8.10944	10.3077	9.93033	10.3182	9.29625	9.29142	8.69805	10.241	10.1173	11.003	9.19508	10.7645	10.1764	10.5288	0.446653834	0.966018836	16	12	24	0.064292203	0.663876076	24	9.092908916	0.087567168
ENSMUSG00000091845	Rpl36-ps12	ribosomal protein L36, pseudogene 12 [Source:MGI Symbol;Acc:MGI:3782787]	84.0474	87.1777	88.9934	69.8782	62.4065	66.9886	69.3805	82.9495	93.7198	71.6872	89.6731	81.3339	98.3445	84.4556	0.198504154	0.680216953	12	12	24	0.106240335	0.71810271	24	4.753486241	0.150245934
ENSMUSG00000091864	Gm17102	predicted gene 17102 [Source:MGI Symbol;Acc:MGI:4937929]	0.452226	0.581804	0.791412	1.1302	0.759898	0.708699	0.811203	0.0782521	0.559019	0.538571	0.576702	0.464039	0.317583	0.304335	0.226403091	0.722427815	16	16	24	0.054344049	0.655059087	24	14.62792803	0.182531558
ENSMUSG00000091866	Gm6257	predicted gene 6257 [Source:MGI Symbol;Acc:MGI:3645370]	2.23555	2.35283	2.60443	1.64715	2.38853	2.4756	2.33727	2.7152	2.36209	2.18043	2.12152	2.41116	1.95087	2.02976	0.937921962	0.966018836	12	4	24	0.69589658	0.89909553	24	1.371063846	0.041516717
ENSMUSG00000091881	Gm17146	predicted gene 17146 [Source:MGI Symbol;Acc:MGI:4937973]	1.54176	0.583244	0.835371	1.72726	0.422031	0.319978	0.466614	0.50958	0.52125	0.637703	1.04057	0.751582	0.911523	1.04685	0.874672901	0.966018836	16	8	24	0.928805145	0.981515518	24	10.08101288	0.049739979
ENSMUSG00000091896	Ube2d2a	ubiquitin-conjugating enzyme E2D 2A [Source:MGI Symbol;Acc:MGI:1930715]	33.3597	32.129	29.5246	31.2124	31.6246	31.8875	32.1914	30.6981	29.8756	34.6854	33.9461	32.4286	33.2463	29.9945	0.051610136	0.389629649	4	8	24	0.107536469	0.719994886	24	18.68345738	0.055057035
ENSMUSG00000091898	Tnnc1	troponin C, cardiac/slow skeletal [Source:MGI Symbol;Acc:MGI:98779]	8.52154	17.3676	29.5062	28.945	18.7264	23.0909	15.2709	24.042	14.7054	19.3322	10.4562	2.84904	15.2862	21.803	0.180336821	0.657605589	12	16	24	0.125450942	0.739416874	24	8.444011734	0.593617435
ENSMUSG00000091900	Gm4353	predicted gene 4353 [Source:MGI Symbol;Acc:MGI:3782538]	8.24055	6.46766	6.57516	8.55731	8.54063	8.82997	8.1504	8.4702	7.79258	6.63632	8.21466	6.80877	8.11966	7.8652	0.466599742	0.966018836	20	16	24	0.430126657	0.805393068	24	19.82419053	0.074491745
ENSMUSG00000091905	Dnajb6-ps	DnaJ heat shock protein family (Hsp40) member B6, pseudogene [Source:MGI Symbol;Acc:MGI:3645295]	17.8187	18.2771	18.215	18.532	16.3646	17.6121	16.6465	13.9499	16.5697	16.7665	17.483	16.4304	16.8441	17.329	0.852844413	0.966018836	16	4	24	0.782106707	0.930017329	24	14.37408986	0.024963665
ENSMUSG00000091931	Gon7	GON7 subunit of KEOPS complex [Source:MGI Symbol;Acc:MGI:4845848]	3.67041	3.64248	3.25734	4.29145	4.23586	4.2149	4.17779	4.28227	3.511	4.18959	4.31051	3.99737	4.07248	4.26362	0.184631358	0.662239322	20	16	24	0.21609706	0.768040178	24	17.96433515	0.062686899
ENSMUSG00000091952	Gm17709	predicted gene, 17709 [Source:MGI Symbol;Acc:MGI:4937343]	3.83063	4.26259	3.56455	4.7897	4.49733	3.99138	4.36375	3.96649	4.42055	4.41451	4.36459	4.39254	4.39145	3.83913	0.048396595	0.379129806	16	16	24	0.181897392	0.768040178	24	15.18585448	0.063241238
ENSMUSG00000091955	Gm9844	predicted pseudogene 9844 [Source:MGI Symbol;Acc:MGI:3704288]	253.328	221.417	205.635	203.771	245.001	230.907	200.34	206.159	209.726	212.887	233.818	248.203	244.268	258.696	0.360033827	0.883787544	24	12	24	0.149644137	0.758377578	24	21.0097528	0.094054573
ENSMUSG00000091957	Rps2-ps10	ribosomal protein S2, pseudogene 10 [Source:MGI Symbol;Acc:MGI:3645604]	0.833205	0.56632	0.754449	0.341017	0.207627	0.196933	1.08955	0.700067	0.985829	1.57819	2.10134	1.73809	1.88163	1.88621	0.801251296	0.966018836	4	20	24	0.716265249	0.90635549	24	3.527696878	0.09896839
ENSMUSG00000091971	Hspa1a	heat shock protein 1A [Source:MGI Symbol;Acc:MGI:96244]	4.07911	1.46598	1.10614	2.21544	1.4887	1.33082	2.83022	1.8655	1.49818	2.12519	3.17939	2.14038	1.99532	1.30466	0.26093427	0.767318799	4	8	24	0.312769652	0.768040178	24	20.12005257	0.223106352
ENSMUSG00000091985	Gm17354	predicted gene, 17354 [Source:MGI Symbol;Acc:MGI:4936988]	1.76517	2.20652	1.88269	2.28623	2.01781	2.01212	2.03769	2.3972	1.87077	2.14656	1.79024	2.1409	2.02054	2.08972	0.590017078	0.966018836	8	12	24	0.739101333	0.914957371	24	6.608801011	0.027852197
ENSMUSG00000091989	Ndufab1-ps	NADH:ubiquinone oxidoreductase subunit AB1b [Source:MGI Symbol;Acc:MGI:4936891]	23.5411	21.0941	23.5476	20.224	18.3269	20.2178	20.1192	22.2764	24.3686	22.3535	29.3292	22.48	26.7695	25.9344	0.912686312	0.966018836	12	12	24	0.630622666	0.874000625	24	5.595981882	0.057547283
ENSMUSG00000091994	E130317F20Rik	RIKEN cDNA E130317F20 gene [Source:MGI Symbol;Acc:MGI:3041202]	0.535774	0.627532	0.630508	0.574332	0.495242	0.643694	0.586164	0.622428	0.800343	0.883532	0.661361	0.712621	0.715899	0.776349	0.928127947	0.966018836	12	8	24	0.102736765	0.713699364	24	8.397259926	0.05475839
ENSMUSG00000091996	BC049352	cDNA sequence BC049352 [Source:MGI Symbol;Acc:MGI:3040681]	0.939783	0.811543	0.925453	0.932097	0.786768	0.948906	1.38051	1.24054	1.04495	0.619661	0.609457	1.0774	0.683522	1.1427	0.173541845	0.648600527	8	12	24	0.150829022	0.758865539	24	2.448477968	0.12177409
ENSMUSG00000092021	Gbp11	guanylate binding protein 11 [Source:MGI Symbol;Acc:MGI:3646307]	0.64174	0.788873	0.562187	0.193603	0.660163	0.429198	0.497527	1.16788	0.750218	0.351458	0.508205	0.5364	0.488325	0.903187	0.008483961	0.192760771	8	8	24	0.052235062	0.650277726	24	3.731068827	0.18573346
ENSMUSG00000092029	Gm9027	predicted gene 9027 [Source:MGI Symbol;Acc:MGI:3646681]	0.320378	0.447009	0.69623	0.456817	0.655656	0.632777	0.390141	0.357251	0.654825	0.476444	0.383849	0.820755	0.53524	0.482806	0.591351901	0.966018836	24	4	24	0.575589813	0.848060327	24	14.75593418	0.05626464
ENSMUSG00000092035	Peg10	paternally expressed 10 [Source:MGI Symbol;Acc:MGI:2157785]	3.29079	2.29562	3.01135	2.38342	2.46899	2.85518	3.35341	2.23937	1.78965	2.80496	2.1056	2.44962	1.91568	2.54653	0.90332508	0.966018836	4	4	24	0.501455958	0.80908345	24	21.53473097	0.083193632
ENSMUSG00000092060	Bend4	BEN domain containing 4 [Source:MGI Symbol;Acc:MGI:3648414]	3.69815	2.56109	2.68574	3.09739	3.27138	3.00472	2.92469	2.56512	2.33628	2.90143	3.12917	2.90808	2.90407	2.84011	0.002363973	0.106802459	20	16	24	0.021859601	0.574400012	24	18.86749964	0.118570217
ENSMUSG00000092072	Gm4540	predicted gene 4540 [Source:MGI Symbol;Acc:MGI:3782724]	95.9196	120.176	101.581	100.969	107.007	92.4905	98.7528	89.699	121.846	90.0305	122.736	108.135	121.087	109.431	0.831272156	0.966018836	12	4	24	0.956681552	0.988639288	24	7.125656873	0.019568092
ENSMUSG00000092074	Dynlt1a	dynein light chain Tctex-type 1A [Source:MGI Symbol;Acc:MGI:3807506]	0.755308	0.798851	0.728185	0.657266	0.920064	0.705992	0.955138	0.638938	0.889354	0.533202	0.890322	0.797456	0.73559	0.688143	0.503972924	0.966018836	20	20	24	0.501189355	0.80908345	24	20.50958705	0.047660967
ENSMUSG00000092083	Kcnb2	potassium voltage gated channel, Shab-related subfamily, member 2 [Source:MGI Symbol;Acc:MGI:99632]	12.3279	12.2485	10.9574	12.7903	11.6455	11.2211	10.9058	10.5739	10.7809	10.587	12.5333	11.0507	11.416	12.0492	0.914502381	0.966018836	20	16	24	0.784824307	0.930960733	24	17.75553234	0.024178884
ENSMUSG00000092085	Gm17224	predicted gene 17224 [Source:MGI Symbol;Acc:MGI:4938051]	0.596833	0.96396	0.774234	1.21227	0.878447	0.599434	0.722161	1.26684	1.61512	1.57217	0.485756	0.612385	0.717815	1.35828	0.014875682	0.251211268	16	8	24	0.002927274	0.414576087	24	8.358685983	0.308174639
ENSMUSG00000092097	Gm5819	predicted gene 5819 [Source:MGI Symbol;Acc:MGI:3643122]	2.7648	1.39788	1.89423	1.97001	1.81956	2.43447	2.47165	1.67508	1.48009	2.40633	1.87648	1.69262	2.44721	2.4559	0.243586913	0.741580974	4	8	24	0.368411659	0.791553898	24	21.6455035	0.116453823
ENSMUSG00000092116	Gm10320	predicted pseudogene 10320 [Source:MGI Symbol;Acc:MGI:3642323]	16.3832	12.4764	11.6993	10.1068	10.8237	9.81104	14.0343	14.3686	15.5466	12.2865	16.1939	14.6383	15.699	12.3655	0.305564675	0.811201458	4	12	24	0.353380163	0.780711577	24	1.295323964	0.121939878
ENSMUSG00000092118	Fancf	Fanconi anemia, complementation group F [Source:MGI Symbol;Acc:MGI:3689889]	1.93429	2.32276	2.17486	2.19656	2.46782	2.21196	1.72705	1.99452	2.16379	2.73175	2.17298	2.46122	2.20241	2.31027	0.239653024	0.741580974	16	12	24	0.099026485	0.71175955	24	13.05354412	0.083480558
ENSMUSG00000092187	Gm20457	predicted gene 20457 [Source:MGI Symbol;Acc:MGI:5141922]	2.58415	2.59824	2.18049	2.85944	2.37827	2.42924	1.9277	2.44098	3.11016	2.61627	2.39415	2.57036	1.79485	2.39551	0.446653834	0.966018836	16	12	24	0.167968594	0.767020009	24	11.07311731	0.101171447
ENSMUSG00000092192	Dnaaf4	dynein axonemal assembly factor 4 [Source:MGI Symbol;Acc:MGI:1914935]	0.76356	1.10281	1.30849	0.723275	0.749421	0.794285	0.859496	1.29427	0.972691	0.95047	1.01695	1.18034	1.03974	0.989382	0.249881062	0.749643185	12	4	24	0.189949467	0.768040178	24	5.559455986	0.094750501
ENSMUSG00000092193	Cd9-ps	Cd9 antigen, pseudogene [Source:MGI Symbol;Acc:MGI:3645134]	5.40681	6.0262	6.31967	6.6326	6.88667	6.93198	7.28165	6.2835	4.74347	4.98151	6.16791	4.67079	5.47552	4.44667	0.865233232	0.966018836	20	12	24	0.384107101	0.799546792	24	18.35631076	0.092424252
ENSMUSG00000092203	1110038B12Rik	RIKEN cDNA 1110038B12 gene [Source:MGI Symbol;Acc:MGI:1916013]	24.125	26.1847	23.0459	24.7626	24.9778	22.7713	22.3889	24.6472	27.5301	21.3853	29.4086	26.2026	25.9176	25.9342	0.860993221	0.966018836	20	8	24	0.989378213	0.997867764	24	10.69647316	0.006825174
ENSMUSG00000092210	A930009A15Rik	RIKEN cDNA A930009A15 gene [Source:MGI Symbol;Acc:MGI:1925048]	1.09054	0.867531	0.67033	0.543994	0.922998	0.996154	0.867822	1.0191	0.712491	0.845788	0.619589	0.695208	1.16838	1.16801	0.016720774	0.253395174	4	12	24	0.02302037	0.581965446	24	0.789500319	0.146618603
ENSMUSG00000092212	Slc22a13b	solute carrier family 22 (organic cation transporter), member 13b [Source:MGI Symbol;Acc:MGI:1924762]	0.643055	0.716536	0.53305	0.916173	0.689954	0.593069	0.523629	0.796908	1.13091	0.667467	0.759989	0.428437	0.590384	0.65974	0.072609122	0.446119062	16	8	24	0.080406428	0.695795041	24	9.508556686	0.122966266
ENSMUSG00000092216	Gm19345	predicted gene, 19345 [Source:MGI Symbol;Acc:MGI:5011530]	1.96825	1.73952	1.85742	2.73434	1.53291	1.85557	2.20008	1.80127	1.60545	2.02634	1.87731	2.13481	2.24851	1.87884	0.483971878	0.966018836	4	8	24	0.895945062	0.970467191	24	18.84184749	0.027981752
ENSMUSG00000092223	Gm19807	predicted gene, 19807 [Source:MGI Symbol;Acc:MGI:5011992]	1.66177	1.44543	1.54773	1.62505	1.76698	1.25057	1.92738	2.40039	1.77926	0.889124	1.51715	1.21617	1.9564	1.19218	0.722296281	0.966018836	4	20	24	0.431411289	0.805393068	24	2.206851597	0.106462463
ENSMUSG00000092229	Gm5977	predicted gene 5977 [Source:MGI Symbol;Acc:MGI:3779541]	1.46512	1.99974	1.81401	1.43395	2.09797	1.2255	1.23261	1.99101	2.00805	1.26363	1.76587	1.65941	1.50842	2.09789	0.180494254	0.657903823	8	20	24	0.288656328	0.768040178	24	7.210082927	0.113209239
ENSMUSG00000092230	Gm18889	predicted gene, 18889 [Source:MGI Symbol;Acc:MGI:5011074]	0.934183	1.13395	1.24154	1.07094	1.78595	1.32499	1.3928	1.3007	1.52377	0.92583	1.17716	1.45667	1.24489	0.969783	0.814077113	0.966018836	20	20	24	0.594030892	0.857184401	24	18.01221228	0.064232484
ENSMUSG00000092241	Gm20522	predicted gene 20522 [Source:MGI Symbol;Acc:MGI:5141987]	0.393729	0.458826	0.526609	0.785802	0.314511	0.316474	0.811725	0.892256	0.936699	0.644912	0.69334	0.305698	0.70064	0.3757	0.208438456	0.698704663	12	12	24	0.211741466	0.768040178	24	7.933097931	0.143071024
ENSMUSG00000092260	Zfp963	zinc finger protein 963 [Source:MGI Symbol;Acc:MGI:4867078]	2.88337	3.60822	3.32129	3.44825	3.16536	3.33918	3.41341	3.26083	3.70691	3.0969	3.41172	3.65422	3.20986	3.60397	0.937921962	0.966018836	12	16	24	0.595505399	0.857839743	24	7.490039699	0.031198162
ENSMUSG00000092274	Neat1	nuclear paraspeckle assembly transcript 1 (non-protein coding) [Source:MGI Symbol;Acc:MGI:1914211]	262.619	349.146	306.675	316.401	366.946	280.972	324.09	325.534	380.63	319.255	328.845	314.515	339.204	290.666	0.926137994	0.966018836	20	4	24	0.395744715	0.799546792	24	10.4698546	0.076948183
ENSMUSG00000092300	Cdk3	cyclin-dependent kinase 3 [Source:MGI Symbol;Acc:MGI:1916931]	0.332632	0.671393	0.554945	0.372183	0.847505	0.62282	0.500105	0.567002	0.777496	0.71731	0.675933	0.605312	0.647813	0.680871	0.498426201	0.966018836	20	8	24	0.525444094	0.822480809	24	11.92175189	0.052324428
ENSMUSG00000092325	Gm18284	predicted gene, 18284 [Source:MGI Symbol;Acc:MGI:5010469]	23.3754	23.8435	25.1088	23.4377	27.6516	23.9276	22.3784	24.5016	26.5873	20.3703	25.6871	24.3044	24.474	24.9225	0.446967971	0.966018836	20	8	24	0.960768468	0.989831707	24	13.42966936	0.011637981
ENSMUSG00000092335	Zfp977	zinc finger protein 977 [Source:MGI Symbol;Acc:MGI:3645327]	0.597077	0.511519	0.991488	0.47004	0.873145	0.572743	0.383819	0.78951	0.838152	0.332116	0.844788	0.579646	0.501458	0.84067	0.588208558	0.966018836	12	16	24	0.644760139	0.879359107	24	8.902053724	0.07079537
ENSMUSG00000092341	Malat1	metastasis associated lung adenocarcinoma transcript 1 (non-coding RNA) [Source:MGI Symbol;Acc:MGI:1919539]	157.265	234.336	176.579	187.259	206.624	164.559	178.269	192.958	292.961	121.266	242.578	192.182	232.82	254.662	0.669104912	0.966018836	8	16	24	0.543748133	0.83189324	24	5.312088854	0.137971131

ENSMUSG00000092371	Gm20511	predicted gene 20511 [Source:MGI Symbol;Acc:MGI:5141976]	0.581115	1.10862	0.743065	1.08321	0.956023	0.765088	1.82881	0.607196	0.579817	0.271825	0.837787	1.3728	1.1363	0.838828	0.454881285	0.966018836	4	8	24	0.208234015	0.768040178	24	21.99817475	0.198323517
ENSMUSG00000092374	Gm6623	predicted gene 6623 [Source:MGI Symbol;Acc:MGI:3644901]	2.09982	2.6067	1.93811	3.10035	2.33332	2.65378	1.91495	2.24128	2.49884	2.7766	3.2236	2.42921	2.25094	2.3104	0.02233689	0.270751126	16	12	24	0.041155403	0.61831089	24	13.93544553	0.140122696
ENSMUSG00000092384	Gm4189	predicted gene 4189 [Source:MGI Symbol;Acc:MGI:3782365]	1.57315	1.3813	3.20089	1.78262	1.29953	1.31056	1.91249	2.95759	2.00241	1.93851	1.32006	1.40262	2.12508	1.38442	0.035023815	0.328311403	12	8	24	0.095678346	0.71175955	24	6.712555679	0.232718548
ENSMUSG00000092395	Gm20463	predicted gene 20463 [Source:MGI Symbol;Acc:MGI:5141928]	35.7402	34.4923	23.9006	35.3397	37.2839	36.0064	38.2399	37.537	32.9969	32.9812	35.9933	30.1795	33.2523	27.5966	0.446967071	0.966018836	20	16	24	0.273070431	0.768040178	24	19.59980966	0.11681008
ENSMUSG00000092416	Zfp141	zinc finger protein 141 [Source:MGI Symbol;Acc:MGI:3584269]	4.42564	4.86616	4.659	3.70169	5.21752	4.72273	3.67314	4.79954	5.08691	4.13737	4.86016	4.58352	4.63416	4.79113	0.588682825	0.966018836	20	8	24	0.975253063	0.993747214	24	6.084313104	0.011566457
ENSMUSG00000092417	Gpank1	G patch domain and ankyrin repeats 1 [Source:MGI Symbol;Acc:MGI:2148975]	7.92609	7.13115	6.96689	7.03829	7.58112	7.71343	6.39195	7.61261	7.65043	7.62107	7.43755	6.77271	7.20562	7.51204	0.654636128	0.966018836	16	12	24	0.881828479	0.966661503	24	11.59782654	0.01533603
ENSMUSG00000092448	Gm20387	predicted gene 20387 [Source:MGI Symbol;Acc:MGI:5141852]	1.56546	2.0801	1.78332	1.63435	1.7678	1.8972	1.8565	1.53967	1.79294	1.63169	1.60667	1.68	1.93571	1.91212	0.671726933	0.966018836	8	8	24	0.402863525	0.799546792	24	2.2456756	0.043401604
ENSMUSG00000092454	Gm2991	predicted pseudogene 2991 [Source:MGI Symbol;Acc:MGI:3781169]	8.47377	7.9372	7.13751	7.15623	5.88002	6.43955	9.06231	8.75332	9.01646	11.0405	9.12105	10.109	9.59588	8.30228	0.890176313	0.966018836	4	16	24	0.674722585	0.89097199	24	4.242597487	0.065843493
ENSMUSG00000092526	Gm17907	predicted gene, 17907 [Source:MGI Symbol;Acc:MGI:5010092]	0.947149	1.10939	1.26695	0.499286	0.488629	0.823415	0.976378	0.845665	0.844322	0.722257	0.798897	0.652598	0.729767	0.931329	0.018311724	0.256345714	8	8	24	0.028057858	0.590904845	24	3.918570777	0.153044528
ENSMUSG00000092536	Gm20501	predicted gene 20501 [Source:MGI Symbol;Acc:MGI:5141966]	2.00165	1.69706	2.2708	1.92671	2.19096	1.91211	1.59136	1.11268	1.88515	1.81473	1.33179	1.53531	1.78762	1.91416	0.943920575	0.966018836	12	20	24	0.72236417	0.908924547	24	12.18292132	0.051038273
ENSMUSG00000092558	Med20	mediator complex subunit 20 [Source:MGI Symbol;Acc:MGI:1929648]	13.7903	14.4755	12.9828	14.9986	15.4241	15.3889	15.0544	14.7989	13.9176	13.362	15.3882	13.4345	15.1372	14.5666	0.10560184	0.518002936	20	16	24	0.378317421	0.797706855	24	19.80435584	0.044661992
ENSMUSG00000092564	BC051226	cDNA sequence BC051226 [Source:MGI Symbol;Acc:MGI:3039585]	5.88883	6.55056	6.84522	6.68411	5.31794	5.22404	4.63482	4.55411	6.99218	5.35951	5.82667	5.53169	6.40991	5.20921	0.239699179	0.741580974	12	12	24	0.324454752	0.768040178	24	9.674781901	0.099649135
ENSMUSG00000092569	Gm20544	predicted gene 20544 [Source:MGI Symbol;Acc:MGI:5142009]	1.13471	1.11331	0.904268	0.690866	0.619762	0.811769	0.737158	0.742706	0.61868	1.04992	0.621875	0.968367	0.40423	0.683128	0.903207257	0.966018836	24	20	24	0.95003233	0.988090608	24	1.932905785	0.019064212
ENSMUSG00000092593	Gm20492	predicted gene 20492 [Source:MGI Symbol;Acc:MGI:5141957]	0.325895	1.14366	1.40905	0.359423	1.16804	0.531672	0.785558	2.27391	1.31459	1.07909	0.217192	3.21821	1.33488	1.03741	0.851656735	0.966018836	12	4	24	0.575078118	0.847865121	24	4.352340205	0.196896076
ENSMUSG00000092595	Gm20427	predicted gene 20427 [Source:MGI Symbol;Acc:MGI:5141892]	0.485044	0.615966	0.868035	0.523089	0.514713	0.265159	0.449718	0.61312	0.978777	0.58802	0.399932	0.807623	0.960582	0.990992	0.051054571	0.387791798	12	8	24	0.022955628	0.581965446	24	6.230379131	0.181295762
ENSMUSG00000092607	Scnm1	sodium channel modifier 1 [Source:MGI Symbol;Acc:MGI:1341284]	28.4364	32.9855	31.5095	30.7358	30.4782	30.6888	32.3649	31.1822	33.8902	25.4294	32.3859	31.2832	30.2659	30.5137	0.894563866	0.966018836	12	16	24	0.727039225	0.91035631	24	3.220624048	0.030995149
ENSMUSG00000092626	9130230N09Rik	RIKEN cDNA 9130230N09 gene [Source:MGI Symbol;Acc:MGI:1924921]	1.91457	1.78093	0.866811	1.91101	1.89978	1.57565	1.74858	1.70795	1.14134	1.52903	1.80235	1.53263	1.7058	1.68284	0.602156008	0.966018836	4	8	24	0.201009313	0.768040178	24	20.36472417	0.128450287
ENSMUSG00000093384	Gm20689	predicted gene 20689 [Source:MGI Symbol;Acc:MGI:5313136]	11.3739	12.4343	13.7839	12.9043	11.5893	9.54155	14.1209	13.214	9.72891	8.44763	12.1596	11.1373	10.1328	12.6572	0.643325306	0.966018836	8	16	24	0.523811839	0.821564372	24	3.596974713	0.086464961
ENSMUSG00000093394	Gm20621	predicted gene 20621 [Source:MGI Symbol;Acc:MGI:5313068]	0.905353	1.27217	1.36641	1.36238	1.18975	1.25287	0.815363	1.69726	1.79412	1.15892	0.94002	1.4727	1.3117	1.73217	0.125855811	0.555618842	12	8	24	0.071763021	0.678214502	24	7.2946903	0.156048998
ENSMUSG00000093402	Gm18588	predicted gene, 18588 [Source:MGI Symbol;Acc:MGI:5010773]	57.2479	53.707	50.3709	52.9714	49.6586	46.1086	49.7161	50.9312	52.201	46.3146	49.0892	46.137	51.1725	52.1851	0.047717257	0.377785147	8	16	24	0.084037631	0.701763292	24	4.335486482	0.06387681
ENSMUSG00000093424	6330562C20Rik	RIKEN cDNA 6330562C20 gene [Source:MGI Symbol;Acc:MGI:1923443]	1.58796	1.05279	1.23683	1.80828	2.05262	1.27378	2.00007	1.27881	2.02766	1.78645	2.49931	1.21437	1.53509	1.35044	0.192357053	0.669841558	20	12	24	0.132280323	0.74244083	24	14.60837592	0.165581679
ENSMUSG00000093445	Lrch4	leucine-rich repeats and calponin homology (CH) domain containing 4 [Source:MGI Symbol;Acc:MGI:1917193]	9.21046	7.15044	8.30328	7.66082	9.34407	9.22207	8.24713	6.04714	7.11167	8.53595	7.18604	6.55269	7.40208	6.4387	0.105345531	0.518002936	20	12	24	0.094137284	0.71175955	24	17.4575134	0.12259392
ENSMUSG00000093452	Zfhx2os	zinc finger homeobox 2, opposite strand [Source:MGI Symbol;Acc:MGI:3620246]	1.1109	0.970536	1.30789	0.875879	1.03576	0.640332	1.81315	0.940282	1.55088	1.02956	1.38609	1.67787	1.11928	0.749228	0.885025617	0.966018836	4	4	24	0.964100311	0.991038325	24	20.01725157	0.025977939
ENSMUSG00000093456	Gm20662	predicted gene 20662 [Source:MGI Symbol;Acc:MGI:5313109]	1.88301	1.37817	0.11017	1.71935	1.86652	1.04467	1.23256	1.46966	1.49176	0.630666	0.908255	1.61266	0.953031	1.39554	0.823246279	0.966018836	8	4	24	0.512473657	0.815772508	24	21.56442849	0.175325165
ENSMUSG00000093467	Gm20659	predicted gene 20659 [Source:MGI Symbol;Acc:MGI:5313106]	7.48354	7.57112	6.17164	8.41464	7.11566	5.30578	1.88779	7.66642	7.59893	6.28892	6.61208	1.46637	5.13716	9.23042	0.062631097	0.410322149	8	16	24	0.113781445	0.727440321	24	8.750962253	0.477330274
ENSMUSG00000093483	AA465934	expressed sequence AA465934 [Source:MGI Symbol;Acc:MGI:2671018]	2.88393	4.34216	3.09305	4.21973	3.21429	3.32272	3.13268	3.39897	3.69061	3.62202	2.5257	3.123	3.48796	3.09434	0.718742371	0.966018836	16	4	24	0.314854697	0.768040178	24	7.828232206	0.094087291
ENSMUSG00000093502	Gm20700	predicted gene 20700 [Source:MGI Symbol;Acc:MGI:5313147]	3.25655	2.51701	2.17287	2.548	2.7136	2.96699	1.99135	2.56044	2.03078	2.53549	2.70435	2.76936	2.69103	2.64211	0.004516345	0.140598674	24	12	24	0.111874367	0.72485395	24	19.87292755	0.113019279
ENSMUSG00000093505	Gm20691	predicted gene 20691 [Source:MGI Symbol;Acc:MGI:5313138]	6.6377	7.15958	5.90385	5.54509	5.50166	6.38795	8.02216	4.78736	4.47834	5.80697	5.73314	6.96761	7.23551	5.01425	0.033248781	0.324908923	4	8	24	0.025909955	0.586541342	24	21.75071229	0.200834189
ENSMUSG00000093507	Gm20627	predicted gene 20627 [Source:MGI Symbol;Acc:MGI:5313074]	1.79779	0.883739	0.378312	2.24157	0.910029	1.03817	1.31642	0.975799	0.813675	0.38504	1.5134	1.00578	0.954219	0.694748	0.121015203	0.548303839	24	12	24	0.283979184	0.768040178	24	19.73309572	0.212278112
ENSMUSG00000093536	Smim17	small integral membrane protein 17 [Source:MGI Symbol;Acc:MGI:4365374]	4.3543	3.75322	5.86051	4.5832	2.8129	4.9244	4.76071	3.31095	3.88757	4.13923	3.33781	4.10221	2.72846	4.5946	0.906657897	0.966018836	12	8	24	0.777765508	0.928715022	24	7.05611735	0.070089223
ENSMUSG00000093553	Gm20633	predicted gene 20633 [Source:MGI Symbol;Acc:MGI:5313080]	0.540717	0.725074	0.654508	0.523579	0.656306	0.747895	0.417171	0.700258	0.762742	0.965264	0.6646	0.658061	0.58568	0.548623	0.347075276	0.870418653	16	12	24	0.230934368	0.768040178	24	11.65643941	0.074587281
ENSMUSG00000093565	Rab26os	RAB26, member RAS oncogene family, opposite strand [Source:MGI Symbol;Acc:MGI:1922864]	16.4767	12.5918	16.1083	13.1719	14.0748	12.5831	14.9592	11.4478	18.1332	9.46664	13.1852	11.0588	12.1225	14.5946	0.745118544	0.966018836	12	12	24	0.596181493	0.857839743	24	4.836028758	0.089506766
ENSMUSG00000093577	Gm20632	predicted gene 20632 [Source:MGI Symbol;Acc:MGI:5313079]	0.559619	0.827903	0.87553	0.746341	0.724175	0.80894	0.47031	0.785887	1.02966	1.10874	0.724682	1.09903	0.859197	0.737819	0.351315142	0.870418653	12	16	24	0.198863124	0.768040178	24	10.98077487	0.094142017
ENSMUSG00000093579	Gm20036	predicted gene, 20036 [Source:MGI Symbol;Acc:MGI:5012221]	1.11002	0.771553	0.571642	0.869666	1.09129	0.893802	0.854851	0.470265	1.07649	0.77206	0.641896	0.857937	0.623248	0.568726	0.147689593	0.590732968	24	8	24	0.236023109	0.768040178	24	17.68196427	0.101129177
ENSMUSG00000093593	Gm20683	predicted gene 20683 [Source:MGI Symbol;Acc:MGI:5313130]	3.09125	3.59185	4.21831	3.85224	3.98671	3.94457	3.83187	4.19646	3.61517	4.4259	3.65546	4.44448	3.39314	3.16596	0.132411359	0.567728672	24	4	24	0.228488076	0.768040178	24	13.79134836	0.083858397
ENSMUSG00000093606	B130034C11Rik	RIKEN cDNA B130034C11 gene [Source:MGI Symbol;Acc:MGI:3041186]	0.552369	0.708202	0.910916	0.964015	0.737151	0.573994	0.433136	0.65478	0.634671	0.858382	0.788351	0.885043	0.620244	0.765574	0.004021228	0.140598674	16	12	24	0.018532395	0.559812298	24	11.81987437	0.125582151
ENSMUSG00000093629	Prox2os	prospero homeobox 2 opposite strand [Source:MGI Symbol;Acc:MGI:5011542]	1.72901	2.02648	2.09721	2.04914	1.57755	1.61009	1.88241	1.48622	1.93366	1.94901	2.10401	1.79247	1.80961	1.35124	0.449304156	0.966018836	12	20	24	0.314433275	0.768040178	24	12.31218602	0.070128768
ENSMUSG00000093651	Gm5873	predicted gene 5873 [Source:MGI Symbol;Acc:MGI:3648385]	16.362	13.9516	15.1643	14.2393	15.5535	17.8937	14.0641	14.3833	11.7197	13.6861	12.5296	12.1911	12.3572	11.3492	0.866861748	0.966018836	24	8	24	0.104707797	0.717030134	24	19.24370617	0.094690075
ENSMUSG00000093661	Eif4e3	eukaryotic translation initiation factor 4E member 3 [Source:MGI Symbol;Acc:MGI:1914142]	15.1156	12.0046	12.1279	14.2521	13.4221	13.6224	13.8688	13.0569	11.9855	15.6621	14.87	14.2632	15.6667	14.0261	0.156791576	0.61244839	4	8	24	0.161958244	0.76636332	24	18.8054441	0.083681769
ENSMUSG00000093662	Gm20649	predicted gene 20649 [Source:MGI Symbol;Acc:MGI:5313096]	0.657581	0.689375	0.385589	0.505719	0.648209	0.501049	0.662644	0.563288	0.311461	0.629179	0.682934	0.133917	0.472775	0.369795	0.943890799	0.966018836	20	16	24	0.907655697	0.974606327	24	18.81719804	0.028084407
ENSMUSG00000093674	Rpl41	ribosomal protein L41 [Source:MGI Symbol;Acc:MGI:1915195]	485.325	518.688	480.513	488.246	522.722	472.424	474.353	469.647	627.31	388.098	599.678	489.272	537.615	478.385	0.912686312	0.966018836	20	12	24	0.997732965	0.999623846	24	14.41419266	0.004484804
ENSMUSG00000093677	Gm20712	predicted gene 20712 [Source:MGI Symbol;Acc:MGI:5313159]	2.62749	2.56693	3.05362	2.26797	2.62409	2.63665	2.29768	2.78673	2.4506	2.93852	2.86137	2.22144	2.69943	2.68346	0.91695334	0.966018836	12	12	24	0.554613427	0.837869569	24	8.983939898	0.044797573
ENSMUSG00000093739	Gm20692	predicted gene 20692 [Source:MGI Symbol;Acc:MGI:5313139]	0.461843	0.507783	0.752802	0.614978	0.458526	0.434629	0.750023	0.343459	0.839284	0.610482	0.474496	0.640596	0.462796	0.579475	0.268242676	0.774280302	12	12	24	0.331757619	0.768621471	24	9.100771668	0.077870809
ENSMUSG00000093752	Gm20716	predicted gene 20716 [Source:MGI Symbol;Acc:MGI:5313163]	27.2361	26.4901	26.6839	27.5972	27.1055	26.1562	26.2457	25.9421	24.8081	27.6338	25.8709	26.5257	25.0389	26.4822	0.628286596	0.966018836	16	16	24	0.337970345	0.772742827	24	14.56480572	0.022555526
ENSMUSG00000093769	H3c14	H3 clustered histone 14 [Source:MGI Symbol;Acc:MGI:2448355]	3.85669	2.79057	2.33903	2.97853	3.56479	3.17418	3.62318	3.35009	2.10693	2.56439	3.89162	2.90732	2.95564	2.33349	0.033622862	0.324908923	20	16	24	0.011138873	0.516680757	24	19.89792689	0.214185607
ENSMUSG00000093798	Gm8355	predicted pseudogene 8355 [Source:MGI Symbol;Acc:MGI:3645191]	92.5774	72.967	75.0189	78.4196	77.9859	84.0157	89.0104	79.4953	64.15	75.2111	89.1609	80.745	85.1901	82.2072	8.30E-04	0.071344007	4	8	24	0.013856364	0.530091843	24	21.1276158	0.140070651
ENSMUSG00000093803	Ppp2r3d	protein phosphatase 2 (formerly 2A), regulatory subunit B'', delta [Source:MGI Symbol;Acc:MGI:1335093]	20.4406	16.3774	19.9433	18.9923	18.0405	17.6564	19.9411	17.6572	17.2701	19.9931	23.3648	20.3454	21.217	19.1391	0.541384199	0.966018836	4	4	24	0.377404937	0.797295584	24	17.90927135	0.066682097
ENSMUSG00000093826	Gm6900	predicted gene 6900 [Source:MGI Symbol;Acc:MGI:3645052]	2.47672	2.3813	1.70689	3.01397	1.47211	1.48828	1.90563	2.57253	3.03987	3.8753	6.20897	4.99379	3.8141	5.98258	0.77722623	0.966018836	16	8	24	0.798304887	0.935424343	24	10.08694516	0.09494892
ENSMUSG00000093862	Gm5117	predicted gene 5117 [Source:MGI Symbol;Acc:MGI:3648041]	0.480365	0.548103	0.468736	0.554161	0.623673	0.501435	0.425242	0.550897	0.578398	0.393809	0.52631	0.441568	0.538823	0.465775	0.952127548	0.966018836	20	4	24	0.874830435	0.964976615	24	11.9293301	0.012633374
ENSMUSG00000093904	Tomm20	translocase of outer mitochondrial membrane 20 [Source:MGI Symbol;Acc:MGI:1915202]	27.7009	28.9789	30.3083	29.0511	26.2218	27.3902	26.1825	25.8386	28.377	31.0761	25.3936	29.7245	28.1612	30.1042	0.181442559	0.659020016	16	4	24	0.288960332	0.768040178	24	8.363785616	0.056750462
ENSMUSG00000093908	Gm5784	predicted gene 5784 [Source:MGI Symbol;Acc:MGI:3647969]	2.82174	1.6972	2.14022	1.6755	1.91387	2.23234	1.90714	2.08927	1.19972	2.06126	1.50197	2.25384	1.47849	1.97342	0.924770496	0.966018836	24	20	24	0.285720404	0.768040178	24	21.76119317	0.118410779
ENSMUSG00000093930	Hmgcs1	3-hydroxy-3-methylglutaryl-Coenzyme A synthase 1 [Source:MGI Symbol;Acc:MGI:107592]	383.679	348.158	342.57	344.589	363.946	356.209	370.678	330.452	324.876	359.85	351.662	318.33	339.338	340.545	0.671726933	0.966018836	4	8	24	0.333970733	0.770000654	24	19.74302313	0.038282251
ENSMUSG00000093938	Evi2b	ecotropic viral integration site 2b [Source:MGI Symbol;Acc:MGI:1890682]	1.82919	2.90674	3.60449	3.01032	2.68018	2.56853	3.08697	2.65083	3.1983	2.06388	2.17806	2.30621	2.5809	1.83605	0.390686537	0.915479136	12	12	24	0.485930938	0.805393068	24	8.608863067	0.097403289
ENSMUSG00000093954	Gm16867	predicted gene, 16867 [Source:MGI Symbol;Acc:MGI:4439791]	0.584832	0.267019	0.619481	0.634752	0.895949	0.5467	1.26593	0.531782	0.374763	1.95527	0.893262	0.920125	1.65762	2.03572	0.94086481	0.966018836	4	4	24	0.966376404	0.991934487	24	18.3808506	0.031988265
ENSMUSG00000093973	Mrgpra2a	MAS-related GPR, member A2A [Source:MGI Symbol;Acc:MGI:3821888]	1.54756	1.78005	1.40287	1.52475	2.34956	2.26416	1.6196	2.08152	1.58686	1.46041	2.13184	1.85288	2.05906	2.22126	0.082113105	0.462922111	20	20	24	0.251600723	0.768040178	24	20.48914326	0.101628218
ENSMUSG00000093989	Rnasek	ribonuclease, RNase K [Source:MGI Symbol;Acc:MGI:106369]	141.959	111.064	114.328	107.968	110.352	118.039	109.675	123.961	100.98	151.85	115.202	125.107	123.952	120.051	0.81872541	0.966018836	24	12	24	0.81480483	0.940847489	24	21.25627251	0.041319755
ENSMUSG00000094002	Gm9866	predicted gene 9866 [Source:MGI Symbol;Acc:MGI:3643118]	2.96931	3.85362	6.20172	4.57854	3.48666	3.73709	4.06333	2.8615	4.30943	3.55419	3.17493	2.78463	2.86253	5.01444	0.123394836	0.552144108	12	12	24	0.066918414	0.669146244	24	8.560381248	0.233001337
ENSMUSG00000094082	Gm12452	predicted gene 12452 [Source:MGI Symbol;Acc:MGI:3650619]	0.71962	0.603635	0.259713	0.534186	0.869493	0.263726	1.15951	1.05284	0.809089	1.11167	0.966593	0.313437	0.488203	0.376045	0.90703203	0.966018836	20	4	24	0.884100764	0.966917127	24	11.50207158	0.05643706
ENSMUSG00000094090	Gm8494	predicted gene 8494 [Source:MGI Symbol;Acc:MGI:3645114]	5.60042	6.1296	6.05178	6.52247	6.78863	6.20376	5.97746	6.58472	5.93266	5.65954	5.9009	6.28003	5.4777	5.10693	0.705189716	0.966018836	24	4	24	0.24913257	0.768040178	24	15.49455153	0.054257338
ENSMUSG00000094127	G530012D18Rik	RIKEN cDNA G530012D1 gene [Source:MGI Symbol;Acc:MGI:3642025]	4.44E-04	1.61228	2.24245	1.27786	1.13658	1.1002	0.888303	2.83E-04	1.67574	1.04273	1.26887	1.15734	1.28531	0.435834	0.217827365	0.704989686	12	20	24	0.245169221	0.768040178	24	12.47995947	0.315982786
ENSMUSG00000094242	Gm5456	predicted gene 5456 [Source:MGI Symbol;Acc:MGI:3643775]	10.5159	12.5822	11.4963	10.9043	12.0478	10.9573	10.4007	10.607	11.6022	9.1875	13.1589	11.4143	12.8934	11.287	0.924770496	0.966018836	20	20	24	0.89734375	0.970734418	24	21.04033068	0.024650767
ENSMUSG00000094257	Ap3s1-ps2	adaptor-related protein complex 3, sigma 1 subunit, pseudogene 2 [Source:MGI Symbol;Acc:MGI:1929217]	13.2564	11.5897	10.4334	10.5992	12.4023	11.5659	9.583	11.6324	11.5045	10.9862	12.665	11.3457	11.3168	11.779	0.947524602	0.966018836	20	20	24	0.678341731	0.892463655	24	19.29412853	0.040047848
ENSMUSG00000094320	Chchd2-ps	coiled-coil-helix-coiled-coil-helix domain containing 2, pseudogene [Source:MGI Symbol;Acc:MGI:3649942]	2.54956	2.24022	1.31043	1.66828	1.18671	0.824759	2.44347	2.14138	1.82119	1.50873	4.28661	3.72316	3.51653	2.87383	0.268242676	0.774280302	4	12	24	0.361754989	0.787031962	24	23.86289027	0.21085995
ENSMUSG00000094344	Gm11942	predicted gene 11942 [Source:MGI Symbol;Acc:MGI:3650608]	5.75751	7.13881	6.7154	10.1327	10.6231	8.42087	5.89113	8.37139	8.22323	4.25102	9.38682	4.90947	6.90002	4.32304	0.729086532	0.966018836	20	8	24	0.359537642	0.785405943	24	14.83753865	0.193126452
ENSMUSG00000094392	Gm3788	predicted gene 3788 [Source:MGI Symbol;Acc:MGI:3781961]	1010.22	910.857	835.655	826.344	1003.73	938.735	1064.74	879.119	561.28	572.042	563.789	644.861	740.555	676.854	0.217827365	0.704989686	4	12	24	0.020461635	0.566351116	24	23.00869496	0.233636391
ENSMUSG00000094410	Zbed6	zinc finger, BED type containing 6 [Source:NCBI gene (formerly Entrezgene);Acc:667118]	3.00063	4.69117	3.85876	5.26755	4.64854	2.8355	4.37671	3.23754	6.60145	1.99755	5.82321	4.48426	4.51624	4.80231	0.777867261	0.966018836	12	12	24	0.862544613	0.959898873	24	9.218186882	0.0805005
ENSMUSG00000094437	Gm9830	predicted gene 9830 [Source:MGI Symbol;Acc:MGI:3704332]	51.9274	54.9639	52.2045	50.6254	54.3084	45.1914	47.4961	45.3408	50.2406	49.4927	49.4054	53.2834	56.6344	58.3815	0.920858634	0.966018836	8	16	24	0.775485692	0.927742146	24	4.676809467	0.030987804
ENSMUSG00000094441	Zfp955a	zinc finger protein 955A [Source:MGI Symbol;Acc:MGI:4834570]	3.06603	3.61702	2.94255	2.93209	3.8192	2.90359	3.09102	3.27329	4.20005	2.84385	3.72892	3.28076	3.39324	3.05263	0.885059765	0.966018836	20	20	24	0.943904675	0.986212398	24	11.00355986	0.018482164
ENSMUSG00000094446	Gm6344	predicted gene 6344 [Source:MGI Symbol;Acc:MGI:3779586]	3.30009	4.50978	4.8524	3.94015	2.17199	3.8937	4.36798	3.8131	3.53794	2.95742	2.21237	5.81229	4.36734	5.6437	0.17953325	0.655480352	8	12	24	0.067201969	0.670273692	24	3.027618906	0.274577386
ENSMUSG00000094463	Gm8546	predicted gene 8546 [Source:MGI Symbol;Acc:MGI:3647346]	15.0648	13.5627	14.3492	12.4713	14.4128	15.6276	15.2261	14.4793	12.5163	15.1651	13.3566	14.2905	15.5112	14.4272	0.083354723	0.462922111	4	8	24	0.067429177	0.670457112	24	22.36955633	0.083968594
ENSMUSG00000094483	Purb	purine rich element binding protein B [Source:MGI Symbol;Acc:MGI:1338779]	33.339	35.5349	37.7268	36.2886	36.6579	33.0783	36.4272	30.2451	40.6252	34.2977	39.7067	35.5522	33.74	37.9592	0.278111325	0.789849983	12	16	24	0.256270869	0.768040178	24	11.41208606	0.069075969
ENSMUSG00000094500	Smim18	small integral membrane protein 18 [Source:MGI Symbol;Acc:MGI:1919882]	6.2191	8.74233	9.51849	8.04149	7.97336	7.68985	7.33755	7.63661	9.55519	7.28808	7.59764	8.28439	7.88267	7.84152	0.182849367	0.659303873	12	16	24	0.140382784	0.749741291	24	8.905387294	0.10829983
ENSMUSG00000094530	Gm21399	predicted gene, 21399 [Source:MGI Symbol;Acc:MGI:5434754]	70.314	84.7906	76.4777	78.3626	79.8894	69.2308	78.1717	72.639	84.509	58.2228	79.3872	67.2506	75.9883	72.5055	0.44861185	0.966018836	20	4	24	0.741842043	0.916071144	24	5.635634624	0.043451555
ENSMUSG00000094568	Smarce1-ps1	SWI/SNF related, matrix associated, actin dependent regulator of chromatin, subfamily e, member 1, pseudogene 1 [Source:MGI Symbol;Acc:MGI:3037673]	9.21179	7.09042	8.27031	7.08406	8.52989	8.27162	6.7881	6.65736	7.09547	7.23385	8.10314	7.36407	7.98373	7.16029	0.217827365	0.704989686	20	12	24	0.160040674	0.76564405	24	18.7264169	0.087993528
ENSMUSG00000094594	Tpd52-ps	tumor protein D52, pseudogene [Source:MGI Symbol;Acc:MGI:107737]	7.22743	5.80103	5.31167	5.92915	6.91139	7.29899	6.62252	8.10414	5.74386	5.66102	7.35285	7.58153	7.00702	5.40923	0.010886922	0.200176658	24	12	24	0.015328385	0.539211046	24	20.53390696	0.178396626
ENSMUSG00000094683	Ap3m1-ps	adaptor-related protein complex 3, mu 1 subunit, pseudogene [Source:MGI Symbol;Acc:MGI:3645307]	26.9977	28.3617	26.1361	28.4533	27.9227	27.3018	26.0619	25.7376	28.0932	25.3026	30.5787	27.678	28.759	29.0688	0.845386467	0.966018836	20	20	24	0.777403281	0.928654033	24	17.11699333	0.020428546
ENSMUSG00000094685	Gm5900	predicted pseudogene 5900 [Source:MGI Symbol;Acc:MGI:3643907]	26.7862	33.0629	29.0287	31.2979	27.3299	28.2942	28.061	31.9889	26.5942	28.7897	29.3707	25.3049	30.7991	32.117	0.037085178	0.334054683	8	16	24	0.17692216	0.768040178	24	5.961703092	0.081270227
ENSMUSG00000094686	Ccl21a	chemokine (C-C motif) ligand 21A (serine) [Source:MGI Symbol;Acc:MGI:1349183]	2.86921	3.08661	3.91474	2.7067	2.69136	2.934	2.60715	3.8754	6.33607	2.15602	3.67127	2.33412	2.77518	3.62082	0.025223982	0.293279372	12	12	24	0.10245914	0.713699364	24	7.136750116	0.252274698
ENSMUSG00000094724	Rnaset2b	ribonuclease T2B [Source:MGI Symbol;Acc:MGI:3702087]	1.50107	2.01602	1.65665	1.45717	2.12282	2.1983	2.41943	2.1189	3.05231	1.4345	1.97216	2.40521	3.37673	1.55804	0.192357053	0.669841558	4	12	24	0.428299766	0.805393068	24	23.33099327	0.12544058
ENSMUSG00000094786	Gm14403	predicted gene 14403 [Source:MGI Symbol;Acc:MGI:3649813]	6.27911	6.53134	6.75908	7.89484	6.72916	4.46249	5.03299	5.15887	6.80744	4.11234	6.7792	5.70626	5.37286	6.47572	0.125855811	0.555618842	12	12	24	0.492573255	0.805558817	24	9.963949778	0.105403614
ENSMUSG00000094840	Muc3a	mucin 3A, cell surface associated [Source:MGI Symbol;Acc:MGI:3588263]	0.462222	0.563315	0.628764	0.612529	0.382387	0.461435	0.536604	0.498186	0.790836	0.549518	0.652502	0.528577	0.353095	0.633969	0.032924718	0.324908923	12	16	24	0.039914076	0.616921492	24	9.028408647	0.099932892
ENSMUSG00000094870	Zfp131	zinc finger protein 131 [Source:MGI Symbol;Acc:MGI:1919715]	15.9499	15.7699	16.0612	16.2244	17.35	15.0043	15.3716	15.0823	16.8058	14.0851	16.9883	14.2763	16.0745	15.3909	0.265774856	0.773510818	20	4	24	0.736139863	0.914041192	24	12.4948774	0.025099083
ENSMUSG00000094910	D430019H16Rik	RIKEN cDNA D430019H16 gene [Source:MGI Symbol;Acc:MGI:2443127]	9.18449	8.41015	9.06469	8.85001	9.12493	9.2796	9.17853	8.48658	7.5796	11.0893	7.66582	9.29413	8.30661	9.35749	0.945282252	0.966018836	24	12	24	0.847981077	0.954100589	24	16.39730128	0.0286349
ENSMUSG00000094935	Gm9726	predicted gene 9726 [Source:MGI Symbol;Acc:MGI:3648149]	5.11014	4.47894	4.87985	5.03659	6.11068	5.68291	4.43216	4.62916	4.64303	4.96377	4.2953	5.10566	4.91402	4.19729	0.03452758	0.328311403	24	8	24	0.045407781	0.629400324	24	17.36005568	0.10633386
ENSMUSG00000094936	Rbm4	RNA binding motif protein 4 [Source:MGI Symbol;Acc:MGI:1100865]	4.55283	3.90978	5.65648	4.71286	4.83985	4.18103	4.89097	4.54956	4.51142	4.45383	4.98496	4.06707	4.54365	4.86564	0.907068194	0.966018836	20	4	24	0.470824788	0.805393068	24	10.09882742	0.054766548
ENSMUSG00000094942	Gm3604	predicted gene 3604 [Source:MGI Symbol;Acc:MGI:3781781]	2.04322	2.53464	2.7633	2.35045	2.04891	2.14993	2.05152	2.49253	2.63621	2.14488	2.23906	2.26671	2.14534	2.22568	0.008754183	0.192760771	12	16	24	0.01357973	0.529134128	24	7.772846256	0.101504728
ENSMUSG00000094989	Rpl9-ps4	ribosomal protein L9, pseudogene 4 [Source:MGI Symbol;Acc:MGI:3704410]	13.2564	12.1254	12.8331	12.7846	10.6204	10.4849	12.7384	14.2839	16.6031	9.8431	12.8619	13.2573	13.6958	13.5241	0.359479536	0.883787544	12	4	24	0.106853676	0.71909525	24	4.653471908	0.138629277
ENSMUSG00000095041	-	\N	46.09	49.2472	56.836	57.0771	66.0244	52.5762	48.7876	46.682	48.8866	45.3768	56.8081	52.3008	51.9759	51.6874	0.140217658	0.585715181	20	12	24	0.102931978	0.713807763	24	15.32145859	0.113465931
ENSMUSG00000095079	Igha	immunoglobulin heavy constant alpha [Source:MGI Symbol;Acc:MGI:96444]	0.637665	3.15107	4.0563	2.28732	0.794	1.88528	0.672452	2.69632	2.00157	0.848184	1.10837	0.759115	2.34075	1.13726	0.087130946	0.470523339	8	20	24	0.106490295	0.718721872	24	6.930708683	0.425827824
ENSMUSG00000095098	Ccdc85b	coiled-coil domain containing 85B [Source:MGI Symbol;Acc:MGI:2147607]	4.85052	3.99126	4.84706	4.72304	4.17505	3.95991	4.08615	5.58998	6.3086	6.95168	8.15077	6.8485	7.80739	6.70488	0.7866493	0.966018836	20	4	24	0.528474076	0.823973486	24	10.65996783	0.083979378
ENSMUSG00000095115	Itpripl2	inositol 1,4,5-triphosphate receptor interacting protein-like 2 [Source:MGI Symbol;Acc:MGI:2442416]	6.87295	6.86631	6.42273	6.10284	7.02879	5.75359	7.4766	6.4725	7.57179	6.73538	6.94195	6.67759	7.52695	6.85505	0.595952838	0.966018836	4	12	24	0.577554634	0.848840315	24	3.22036152	0.038199184
ENSMUSG00000095123	Gm21781	predicted gene, 21781 [Source:MGI Symbol;Acc:MGI:5433945]	3.03715	3.76138	3.65625	3.82157	3.6687	2.88441	3.25943	3.47861	4.42815	3.06697	3.62717	3.38975	3.72047	3.91621	0.077301512	0.453790923	12	12	24	0.067991853	0.671988997	24	8.13795562	0.110465094
ENSMUSG00000095139	Pou3f2	POU domain, class 3, transcription factor 2 [Source:MGI Symbol;Acc:MGI:101895]	2.89444	2.87039	2.60336	2.03879	2.92586	2.48632	3.37989	2.58493	2.31787	2.46973	3.02552	2.56029	2.53375	2.73889	0.251986462	0.75129457	4	12	24	0.186419921	0.768040178	24	22.59541021	0.092038582
ENSMUSG00000095159	Tubb4b-ps1	tubulin, beta 4B class IVB, pseudogene 1 [Source:MGI Symbol;Acc:MGI:3642800]	36.4231	30.1181	32.1189	30.8046	31.9849	37.1388	32.0073	35.8573	24.3726	37.2842	32.4152	30.9856	32.9049	31.963	0.826609618	0.966018836	4	8	24	0.376591386	0.796625215	24	20.41145693	0.086331981
ENSMUSG00000095193	Gm20939	predicted gene, 20939 [Source:MGI Symbol;Acc:MGI:5434295]	2.76594	3.81881	2.75342	3.19034	3.91064	3.57877	3.35936	2.19367	3.89051	2.76039	3.80862	3.34094	3.41231	2.89623	0.702630284	0.966018836	20	12	24	0.317880897	0.768040178	24	17.22938511	0.110559516
ENSMUSG00000095253	Zfp799	zinc finger protein 799 [Source:MGI Symbol;Acc:MGI:2443934]	4.05569	4.66314	4.15879	4.31874	3.85622	4.50932	3.64694	3.5905	3.93546	3.99708	3.94035	3.79457	3.73675	4.96136	0.924770496	0.966018836	8	20	24	0.712543347	0.905210356	24	7.103346961	0.037778204
ENSMUSG00000095304	Plac9a	placenta specific 9a [Source:MGI Symbol;Acc:MGI:2663998]	48.88	57.5552	50.1609	41.5988	49.084	53.5486	52.1687	53.5383	70.7682	30.1945	50.4688	54.2888	49.0703	42.4368	0.745118544	0.966018836	24	16	24	0.443709005	0.805393068	24	1.033329485	0.132243748
ENSMUSG00000095315	Gm10130	predicted gene 10130 [Source:MGI Symbol;Acc:MGI:3642547]	1.23019	1.69664	1.55473	1.53273	1.65749	1.13259	1.11556	1.21231	1.41249	1.17937	1.411	1.24736	1.31766	1.1169	0.417963425	0.963884303	12	16	24	0.216052494	0.768040178	24	11.52764394	0.071983131
ENSMUSG00000095325	Zfp870	zinc finger protein 870 [Source:MGI Symbol;Acc:MGI:3029586]	2.55302	2.80715	2.67255	1.98934	2.36957	2.37204	2.72454	2.39687	2.34617	2.20561	2.21	3.15794	2.45372	2.46544	0.021406186	0.26465279	4	12	24	0.086019408	0.704553215	24	0.131575961	0.09633763
ENSMUSG00000095332	Gm9821	predicted gene 9821 [Source:MGI Symbol;Acc:MGI:3704208]	2.55916	2.57563	2.81779	2.83114	2.16426	2.81587	2.85369	3.09326	3.39598	2.87367	2.41214	2.74773	2.76548	2.96089	0.051961445	0.391073722	12	8	24	0.038641668	0.61140397	24	6.155293471	0.101953546
ENSMUSG00000095334	Gm21984	predicted gene 21984 [Source:MGI Symbol;Acc:MGI:5439453]	0.729663	1.07797	2.14635	0.939054	0.585069	1.31972	2.23551	0.609896	1.39266	0.690884	0.224861	1.04045	0.228798	0.876401	0.424718357	0.966018836	12	8	24	0.610691031	0.864861374	24	5.264618032	0.157106281
ENSMUSG00000095362	Gm14325	predicted gene 14325 [Source:MGI Symbol;Acc:MGI:3702875]	12.8974	17.3408	13.091	14.8703	16.7008	13.4479	12.929	17.7078	20.3902	10.506	17.657	15.7614	16.8462	15.3401	0.796193867	0.966018836	8	20	24	0.829850884	0.947121458	24	5.55867556	0.05830452
ENSMUSG00000095385	D630033O11Rik	RIKEN cDNA D630033O11 gene [Source:MGI Symbol;Acc:MGI:2685434]	0.317405	1.05751	0.826458	1.06118	1.2572	0.970652	0.382348	0.688226	1.27671	0.212895	0.983701	0.251875	1.06925	1.29352	0.858269825	0.966018836	12	12	24	0.597878328	0.858787489	24	8.831470801	0.140635132
ENSMUSG00000095403	Gm21092	predicted gene, 21092 [Source:MGI Symbol;Acc:MGI:5434447]	0.853147	0.918084	0.654871	0.805693	1.01398	1.02532	0.734696	0.988431	0.822497	0.828922	1.03506	1.21619	0.702081	0.674855	0.046934151	0.375011045	24	12	24	0.066896832	0.669146244	24	18.07842161	0.10793473
ENSMUSG00000095407	Tmem200c	transmembrane protein 200C [Source:MGI Symbol;Acc:MGI:3646281]	1.05058	1.0104	1.18589	1.10795	0.869841	0.978547	1.43025	1.03805	0.968222	1.49033	1.02183	1.25067	0.998428	1.29436	0.907000607	0.966018836	16	4	24	0.820959157	0.943551411	24	6.830990305	0.03080936
ENSMUSG00000095419	Gm14328	predicted gene 14328 [Source:MGI Symbol;Acc:MGI:3651951]	8.70039	10.4478	11.57	8.86416	8.18525	8.3048	7.56132	8.55986	7.56042	6.4078	7.1143	7.23067	7.85452	6.45287	0.928127947	0.966018836	12	8	24	0.406760036	0.799546792	24	5.082898586	0.070410033
ENSMUSG00000095432	Zfp748	zinc finger protein 748 [Source:MGI Symbol;Acc:MGI:1916455]	3.65335	4.29284	3.89292	3.63126	3.74132	3.67592	4.17988	3.32358	4.12893	3.11469	3.46117	3.62547	3.88488	3.24629	0.918497267	0.966018836	4	12	24	0.506668701	0.812342247	24	1.210033292	0.04456505

ENSMUSG00000095438	Mir133a-1hg	Mir133a-1, Mir1b and Mir1a-2 host gene [Source:MGI Symbol;Acc:MGI:5433911]	0.520371	0.61302	0.564133	0.584583	0.76199	0.674006	0.210164	1.5316	0.704492	0.91816	0.469313	0.449942	0.81897	0.267996	0.917610614	0.966018836	16	12	24	0.710523287	0.90429771	24	9.337281857	0.089744779
ENSMUSG00000095440	Fignl2	fidgetin-like 2 [Source:MGI Symbol;Acc:MGI:3646919]	1.29135	1.39003	1.38443	1.51412	1.37941	1.24768	1.22051	1.32893	1.46104	1.46646	1.25136	1.52606	1.44732	1.41622	0.305564675	0.811201458	16	12	24	0.213248262	0.768040178	24	9.94740243	0.040465439
ENSMUSG00000095478	Gm9824	predicted pseudogene 9824 [Source:MGI Symbol;Acc:MGI:3641964]	51.3328	46.2283	42.5468	44.9877	49.2304	49.0746	48.0169	48.1862	42.914	42.9026	45.2648	44.3268	45.1393	44.0796	0.078380335	0.458114095	4	8	24	0.005349907	0.4424469	24	21.50258315	0.081083571
ENSMUSG00000095567	Noc2l	NOC2 like nucleolar associated transcriptional repressor [Source:MGI Symbol;Acc:MGI:1931051]	18.2708	17.7281	17.4429	18.7978	19.2476	17.5002	20.4874	16.7113	16.7754	17.8299	19.3034	17.1921	17.5872	16.8578	0.128262369	0.560209134	20	12	24	0.150759128	0.758865539	24	17.81900218	0.059779251
ENSMUSG00000095588	Gm12350	predicted gene 12350 [Source:MGI Symbol;Acc:MGI:3649478]	88.2954	78.4078	80.9555	92.8171	89.3988	76.0278	79.3315	92.5552	95.4305	79.6963	104.703	96.8022	103.422	98.7367	0.773386789	0.966018836	20	4	24	0.975992787	0.993976564	24	13.01975289	0.010737441
ENSMUSG00000095597	Rps7-ps3	ribosomal protein S7, pseudogene 3 [Source:MGI Symbol;Acc:MGI:3648396]	155.419	192.328	180.846	174.037	177.864	152.993	166.231	156.695	223.464	139.835	184.134	174.91	200.482	180.77	0.745118544	0.966018836	12	12	24	0.568056031	0.844243515	24	6.66260162	0.0747299
ENSMUSG00000095600	Gm9457	predicted gene 9457 [Source:MGI Symbol;Acc:MGI:3779867]	1.34904	1.94415	1.04421	0.863629	1.26609	1.38657	1.16654	1.01916	1.69406	1.02032	2.73149	1.62843	1.95326	1.7946	0.481530854	0.966018836	20	20	24	0.653777766	0.883343578	24	21.94578672	0.095146168
ENSMUSG00000095677	Dynlt1f	dynein light chain Tctex-type 1F [Source:MGI Symbol;Acc:MGI:3780996]	2.54432	2.92576	3.7585	3.14176	3.75185	2.76598	2.07193	2.27384	4.23869	1.89592	2.84593	2.12452	2.88885	2.93873	0.085890569	0.466404237	12	12	24	0.339190695	0.773727252	24	9.870989576	0.144769562
ENSMUSG00000095687	Rnaset2a	ribonuclease T2A [Source:MGI Symbol;Acc:MGI:1915445]	0.842989	0.86234	0.867201	0.84826	1.13653	0.6615	0.849757	0.751767	1.1247	0.895514	0.797994	1.07746	1.2119	1.06421	0.809772024	0.966018836	12	12	24	0.911615739	0.975053334	24	7.280096102	0.019790744
ENSMUSG00000095690	Rab11b-ps2	RAB11B, member RAS oncogene family, pseudogene 2 [Source:MGI Symbol;Acc:MGI:3643959]	44.0361	37.6747	42.727	39.473	37.7209	45.0597	43.4313	43.1189	35.6449	51.3919	35.1493	40.5944	39.6397	38.336	0.608967886	0.966018836	24	20	24	0.940045084	0.985398871	24	21.63152519	0.021151583
ENSMUSG00000095742	-	\N	3.11684	4.8906	2.88367	0.972828	0.945914	1.4365	5.35631	10.4189	28.9558	48.3791	140.45	112.63	178.537	114.9	0.890176313	0.966018836	8	8	24	0.730036456	0.911943391	24	4.139014877	0.362712575
ENSMUSG00000095762	Gm16378	predicted gene 16378 [Source:MGI Symbol;Acc:MGI:3646886]	27.4691	26.7613	22.6773	26.1211	20.6096	21.208	30.2178	28.4957	27.6628	25.3385	25.5931	28.0534	32.3436	28.6812	0.007831348	0.192760771	4	16	24	0.025420329	0.586541342	24	2.75514488	0.149401743
ENSMUSG00000095789	Nupr1l	nuclear protein transcriptional regulator 1 like [Source:MGI Symbol;Acc:MGI:1923099]	1.24865	1.12187	1.90933	2.1005	0.818016	0.929852	0.924056	1.16037	1.08392	0.819369	1.12173	1.39091	2.23329	0.975238	0.90703203	0.966018836	4	16	24	0.881264694	0.966661503	24	6.190453344	0.057868745
ENSMUSG00000095930	Nim1k	NIM1 serine/threonine protein kinase [Source:MGI Symbol;Acc:MGI:2442399]	2.04384	2.10187	2.42389	1.91798	2.09182	2.5426	2.05258	2.33201	2.05828	2.20499	2.19018	2.48461	1.9676	2.48067	0.865233232	0.966018836	24	4	24	0.952764876	0.988554064	24	23.09981367	0.011505315
ENSMUSG00000095990	Zfp97	zinc finger protein 97 [Source:MGI Symbol;Acc:MGI:105921]	1.6016	1.59075	1.81155	1.35877	1.76207	1.51593	1.45893	1.42566	1.91376	1.29619	1.25012	1.82359	1.8136	1.74244	0.96317972	0.967965568	20	20	24	0.953302017	0.988601783	24	18.71337327	0.015191949
ENSMUSG00000095991	Gm17048	predicted gene 17048 [Source:MGI Symbol;Acc:MGI:4937875]	1.10511	1.00451	0.695592	0.666049	0.869218	0.758061	0.851088	0.468199	0.952673	0.49962	1.07845	1.00266	0.581698	1.13234	0.892044196	0.966018836	20	20	24	0.651176341	0.882725346	24	21.81122947	0.069393576
ENSMUSG00000096054	Syne1	spectrin repeat containing, nuclear envelope 1 [Source:MGI Symbol;Acc:MGI:1927152]	26.2498	27.1399	24.7293	26.2971	25.343	26.857	26.5973	24.7888	24.6731	26.2143	24.2883	28.0797	25.7859	28.798	0.290796297	0.79743194	24	20	24	0.359596308	0.785405943	24	23.59993898	0.03835258
ENSMUSG00000096056	Gm21986	predicted gene 21986 [Source:MGI Symbol;Acc:MGI:5439455]	0.838834	0.515895	0.719076	0.497375	0.647055	0.734226	0.267713	0.640377	0.54522	0.681829	0.558566	0.454631	0.563542	0.581011	0.710938687	0.966018836	12	12	24	0.899041146	0.971453495	24	12.28506676	0.023366896
ENSMUSG00000096140	Ankrd66	ankyrin repeat domain 66 [Source:MGI Symbol;Acc:MGI:1925106]	0.485518	0.670226	0.528179	0.518423	0.650671	0.65141	0.508185	0.528786	0.379375	0.526887	0.525665	0.507567	0.438252	0.386915	0.915082018	0.966018836	20	8	24	0.136891931	0.747972781	24	17.84465004	0.053425202
ENSMUSG00000096141	Dnah7a	dynein, axonemal, heavy chain 7A [Source:MGI Symbol;Acc:MGI:2685838]	2.3352	3.33011	3.59207	3.38428	2.51928	3.15387	2.56293	2.82779	3.11398	1.95444	2.84935	3.57192	2.94856	2.70554	0.956794505	0.96629412	12	16	24	0.949204477	0.987684983	24	5.649667362	0.024378749
ENSMUSG00000096145	Vkorc1	vitamin K epoxide reductase complex, subunit 1 [Source:MGI Symbol;Acc:MGI:106442]	36.0901	41.2585	40.5003	39.2088	42.9181	40.1489	41.062	41.14	45.1303	34.0157	40.8557	40.8329	41.4889	36.5042	0.868022237	0.966018836	12	4	24	0.988607588	0.997561007	24	21.87655867	0.006564697
ENSMUSG00000096146	Kcnj11	potassium inwardly rectifying channel, subfamily J, member 11 [Source:MGI Symbol;Acc:MGI:107501]	1.96605	2.65901	3.10258	2.02407	2.15332	2.86653	3.12877	3.66722	3.06924	4.28098	1.96452	2.61257	2.68881	2.85911	0.424718357	0.966018836	12	8	24	0.206083142	0.768040178	24	6.562345855	0.166184237
ENSMUSG00000096157	Gm5472	predicted gene 5472 [Source:MGI Symbol;Acc:MGI:3648309]	0.627862	0.625613	0.426989	0.632694	0.432623	0.626428	0.575326	0.469763	0.375864	0.518368	0.523332	0.665085	0.451997	0.524101	0.022634137	0.27189101	24	12	24	0.15775334	0.764314876	24	20.67662405	0.064682075
ENSMUSG00000096160	Gm3436	predicted pseudogene 3436 [Source:MGI Symbol;Acc:MGI:3781613]	19.2764	14.7166	14.2482	14.0638	15.6753	16.4495	14.0453	15.5052	12.9328	16.5428	13.1962	15.8732	15.8312	15.0084	0.245061943	0.741580974	24	12	24	0.149026997	0.757594757	24	21.68154831	0.109033901
ENSMUSG00000096173	Gm3150	predicted gene 3150 [Source:MGI Symbol;Acc:MGI:3781329]	11.3297	10.8135	10.451	19.1229	10.6276	11.929	14.5758	13.1747	23.7664	9.11475	16.8739	16.3669	17.5011	16.8472	0.756529255	0.966018836	4	12	24	0.879233969	0.966161692	24	6.936405281	0.070093584
ENSMUSG00000096188	Cmtm4	CKLF-like MARVEL transmembrane domain containing 4 [Source:MGI Symbol;Acc:MGI:2142888]	9.85905	9.05171	9.07353	9.79707	8.88226	9.52331	9.94643	8.85055	9.07148	11.0822	9.27747	9.99687	9.43207	10.0994	0.851656735	0.966018836	4	4	24	0.870887518	0.963364428	24	16.37261015	0.016692495
ENSMUSG00000096199	Ptrhd1	peptidyl-tRNA hydrolase domain containing 1 [Source:MGI Symbol;Acc:MGI:1916959]	3.90372	3.82122	3.64187	3.67203	3.73839	3.64312	3.97942	3.43624	3.95374	3.65496	4.10254	3.77922	3.98709	3.74957	0.46238066	0.966018836	4	12	24	0.602007679	0.860847507	24	20.77909664	0.022213285
ENSMUSG00000096210	H1f0	H1.0 linker histone [Source:MGI Symbol;Acc:MGI:95893]	50.2233	58.112	55.909	47.2755	49.5083	50.8247	50.4301	59.1325	57.3583	55.3057	46.7513	57.5049	58.5925	59.3129	7.90E-04	0.071344007	8	12	24	0.007257495	0.47735824	24	4.674297793	0.119964775
ENSMUSG00000096221	1500002C15Rik	RIKEN cDNA 1500002C15 gene [Source:MGI Symbol;Acc:MGI:1916196]	1.3293	1.61989	1.54075	1.3837	1.42461	1.3615	1.28909	1.4877	1.31024	0.961959	1.51414	1.41876	1.73343	1.19337	0.832742389	0.966018836	20	20	24	0.53383649	0.82605921	24	23.67849908	0.051367565
ENSMUSG00000096255	Dynlt1b	dynein light chain Tctex-type 1B [Source:MGI Symbol;Acc:MGI:98643]	0.65491	0.731574	0.996632	0.599185	1.07513	0.296477	0.878051	0.702293	1.16141	0.995391	0.988428	1.04643	0.699175	1.27766	0.649212555	0.966018836	12	16	24	0.233539866	0.768040178	24	9.197463738	0.131069505
ENSMUSG00000096257	Ccer2	coiled-coil glutamate-rich protein 2 [Source:MGI Symbol;Acc:MGI:3645242]	1.3273	1.63555	1.94825	2.00899	2.62306	1.66115	1.60716	1.49627	2.4898	2.17786	1.85172	1.53655	1.44047	1.38544	0.003752521	0.140598674	16	12	24	0.001863653	0.384740371	24	12.4760336	0.22051886
ENSMUSG00000096351	Samd11	sterile alpha motif domain containing 11 [Source:MGI Symbol;Acc:MGI:2446220]	1.00537	1.581	1.71598	1.658	1.25504	1.4283	1.3023	1.23162	1.71548	1.47741	1.32134	1.75698	1.7025	1.51938	0.658895034	0.966018836	12	16	24	0.333485088	0.769685083	24	9.269360628	0.077140659
ENSMUSG00000096370	Gm21992	predicted gene 21992 [Source:MGI Symbol;Acc:MGI:5439461]	5.89479	4.80506	3.80001	5.23623	5.01245	4.96361	6.68159	5.09889	5.63829	4.05932	6.08606	5.30255	2.69319	3.25577	0.91695334	0.966018836	20	12	24	0.427495881	0.805393068	24	17.94925458	0.142234194
ENSMUSG00000096375	Gm7094	predicted pseudogene 7094 [Source:MGI Symbol;Acc:MGI:3648114]	3.96909	4.00288	2.79541	1.99941	2.46052	2.96293	3.60289	2.79717	3.48786	3.02088	4.28341	2.7106	2.4149	3.39239	0.935593607	0.966018836	8	8	24	0.569690137	0.844900526	24	1.642526816	0.098489486
ENSMUSG00000096401	Gm21811	predicted gene, 21811 [Source:MGI Symbol;Acc:MGI:5433975]	0.362817	0.500391	1.63444	1.90143	0.891813	0.294059	0.569035	1.39179	1.0617	0.41094	1.53188	0.396748	1.72674	0.968492	0.245061943	0.741580974	12	12	24	0.239593599	0.768040178	24	9.30046583	0.277529684
ENSMUSG00000096403	Rnps1-ps	RNA binding protein with serine rich domain 1, pseudogene [Source:MGI Symbol;Acc:MGI:3708729]	18.8397	17.9799	17.4927	16.2129	13.4644	16.5098	17.0509	17.0066	17.2891	20.4126	18.6423	19.1269	17.3989	17.3065	0.757932695	0.966018836	4	16	24	0.819603094	0.942798491	24	4.5681501	0.032774189
ENSMUSG00000096433	Zfp994	zinc finger protein 994 [Source:MGI Symbol;Acc:MGI:3643318]	3.28538	3.56409	3.09295	2.62403	3.60923	2.90548	3.07526	3.00127	3.52556	2.60666	3.64551	3.5281	3.32993	3.39076	0.676344318	0.966018836	20	20	24	0.619254033	0.869407455	24	23.02257138	0.048007583
ENSMUSG00000096446	Gm8104	predicted gene 8104 [Source:MGI Symbol;Acc:MGI:3649000]	0.719145	0.653107	0.566664	0.485618	0.499594	0.563673	0.606001	0.512169	0.486678	0.491356	0.461946	0.515491	0.466107	0.625357	0.083354723	0.462922111	8	8	24	0.01520102	0.537924552	24	1.400394843	0.060732619
ENSMUSG00000096458	Moap1	modulator of apoptosis 1 [Source:MGI Symbol;Acc:MGI:1915555]	11.8052	12.0878	9.9935	9.20655	10.8621	11.3156	10.0032	11.7341	8.61928	11.2255	9.23139	11.9474	11.8967	12.9968	0.009517052	0.194720062	8	4	24	0.083081401	0.699403344	24	0.339075724	0.13028049
ENSMUSG00000096472	Cdkn2d	cyclin dependent kinase inhibitor 2D [Source:MGI Symbol;Acc:MGI:105387]	49.0618	45.7943	43.9256	42.9393	48.2801	47.1165	43.2543	47.8113	41.4738	46.3573	47.1265	46.0612	48.4973	48.5454	0.303973871	0.811201458	4	8	24	0.209734445	0.768040178	24	21.66277945	0.050914855
ENSMUSG00000096544	Gm4617	predicted pseudogene 4617 [Source:MGI Symbol;Acc:MGI:3704225]	166.712	181.694	159.983	164.529	173.029	161.4	147.258	147.819	135.603	144.359	147.889	162.203	164.362	159.067	0.920858634	0.966018836	4	8	24	0.369664976	0.792235171	24	21.5010952	0.057780685
ENSMUSG00000096549	Prickle4	prickle planar cell polarity protein 4 [Source:MGI Symbol;Acc:MGI:2685785]	0.954188	1.7399	2.25126	2.07094	1.52486	1.58966	1.65856	0.904767	1.04053	1.32066	1.48436	1.19132	1.53789	0.599371	0.915082018	0.966018836	16	16	24	0.304743585	0.768040178	24	14.75584002	0.145484137
ENSMUSG00000096606	Tpbgl	trophoblast glycoprotein-like [Source:MGI Symbol;Acc:MGI:3646425]	6.95442	5.58397	4.80324	6.4782	5.51549	6.38637	5.84243	6.9012	5.17188	9.21815	5.59895	7.18435	7.12859	6.94631	0.245061943	0.741580974	24	12	24	0.869768838	0.963087797	24	20.52473683	0.043189098
ENSMUSG00000096647	Tmem41b-ps	transmembrane protein 41B, pseudogene [Source:MGI Symbol;Acc:MGI:3646615]	32.64	27.1122	25.8372	27.4151	26.6238	27.6427	25.1439	26.6874	25.9219	25.687	29.0971	27.8259	28.6109	27.4532	0.244223134	0.741580974	24	12	24	0.201846001	0.768040178	24	21.34947059	0.064077608
ENSMUSG00000096687	Mfsd4b4	major facilitator superfamily domain containing 4B4 [Source:MGI Symbol;Acc:MGI:3035041]	3.83086	5.60389	5.48394	5.06695	4.54841	3.95335	4.34116	3.9624	5.34576	4.56305	4.69891	4.92333	5.32904	5.18914	0.203305541	0.689605535	12	12	24	0.156258406	0.761842768	24	8.358084635	0.11269836
ENSMUSG00000096696	Zfp960	zinc finger protein 960 [Source:MGI Symbol;Acc:MGI:3052731]	1.02757	1.13477	1.15282	1.13087	1.22634	1.25528	0.985671	0.980578	1.15822	0.835207	1.25589	1.28421	1.25928	1.15787	0.261443857	0.768150062	24	8	24	0.418833464	0.803273646	24	19.05543977	0.048423865
ENSMUSG00000096719	Mrgpra2b	MAS-related GPR, member A2B [Source:MGI Symbol;Acc:MGI:3033098]	7.18849	4.12653	5.64302	4.21648	5.64265	6.6196	4.70509	5.28278	3.67233	3.72052	6.30372	5.80236	6.70509	4.74355	0.053432515	0.394331071	4	8	24	0.017076933	0.547196997	24	20.89192746	0.272035636
ENSMUSG00000096727	Psmb9	proteasome (prosome, macropain) subunit, beta type 9 (large multifunctional peptidase 2) [Source:MGI Symbol;Acc:MGI:1346526]	4.2461	4.6119	5.09561	4.45594	4.48727	4.09016	3.70845	6.00803	4.86327	3.69905	4.30877	3.68288	4.37002	4.64018	0.004377091	0.140598674	12	12	24	0.064131453	0.663876076	24	6.556103764	0.136929109
ENSMUSG00000096740	Lbhd1	LBH domain containing 1 [Source:MGI Symbol;Acc:MGI:5516029]	7.4998	10.6518	10.5491	4.58736	7.67976	7.37441	1.5448	4.05196	4.9968	6.20321	10.8027	6.9047	3.18587	3.66917	0.231491055	0.731037655	20	8	24	0.29521946	0.768040178	24	14.2178393	0.350727393
ENSMUSG00000096764	Gm21985	predicted gene 21985 [Source:MGI Symbol;Acc:MGI:5439454]	2.22095	1.59335	1.05395	1.86116	1.34641	2.28253	1.28298	1.28685	0.279959	1.62901	0.952613	0.668279	1.58827	1.12161	0.834434272	0.966018836	16	20	24	0.345779428	0.778673045	24	20.92625102	0.192272021
ENSMUSG00000096780	Tmem181b-ps	transmembrane protein 181B, pseudogene [Source:MGI Symbol;Acc:MGI:3779544]	7.23949	6.23964	5.81878	6.64658	6.28777	6.34066	6.62378	6.16071	6.9403	5.73521	8.40816	6.79735	7.42947	6.20422	0.248787753	0.747781955	4	4	24	0.316480966	0.768040178	24	19.72503257	0.076689081
ENSMUSG00000096795	Zfp433	zinc finger protein 433 [Source:MGI Symbol;Acc:MGI:1920860]	0.556728	0.945491	0.948228	0.888571	0.598161	0.656975	0.828161	0.708985	1.13676	0.527574	0.701471	0.644526	0.706016	0.752578	0.01813461	0.256345714	12	8	24	0.067922811	0.671988997	24	7.094015263	0.119683688
ENSMUSG00000096847	Tmem151b	transmembrane protein 151B [Source:MGI Symbol;Acc:MGI:2685169]	8.90323	7.29253	7.41706	7.52318	7.38035	8.22627	7.85734	8.03091	7.08616	9.73949	7.83574	9.67561	8.8244	9.30381	0.390686537	0.915479136	24	12	24	0.440649888	0.805393068	24	21.67238089	0.061438193
ENSMUSG00000096910	Zfp955b	zinc finger protein 955B [Source:MGI Symbol;Acc:MGI:4834573]	7.64477	7.74488	7.91602	8.38234	7.89676	7.20665	7.16383	6.50637	7.38069	6.72026	7.91943	7.71362	6.74744	6.83103	0.203532277	0.689899499	20	12	24	0.094814138	0.71175955	24	15.0517193	0.065127378
ENSMUSG00000096916	Zfp850	zinc finger protein 850 [Source:MGI Symbol;Acc:MGI:3036281]	2.38744	2.3594	2.21995	1.99127	1.95801	2.27794	2.53832	1.91867	2.23367	1.73355	2.59456	2.36196	2.35152	2.41916	0.109247218	0.524035616	4	12	24	0.106380217	0.718519122	24	23.27293316	0.089720607
ENSMUSG00000096917	2500002B13Rik	RIKEN cDNA 2500002B13 gene [Source:MGI Symbol;Acc:MGI:1925603]	0.806245	1.9202	2.33163	3.06157	2.38412	1.72055	3.38014	1.25446	0.607237	0.811541	1.17418	1.54513	1.33907	1.11678	0.854443826	0.966018836	20	16	24	0.733612465	0.913085883	24	18.39414093	0.133835453
ENSMUSG00000096929	A330023F24Rik	RIKEN cDNA A330023F24 gene [Source:MGI Symbol;Acc:MGI:2443958]	11.6862	11.6568	12.567	13.158	12.0645	11.1807	13.62	9.77112	11.9865	8.96629	12.5417	11.0185	10.2766	11.3299	0.873337044	0.966018836	20	12	24	0.896192112	0.970549275	24	17.26358324	0.026964828
ENSMUSG00000096935	1700113A16Rik	RIKEN cDNA 1700113A16 gene [Source:MGI Symbol;Acc:MGI:1923892]	2.51042	1.8519	3.10331	1.58492	3.26846	1.62144	2.20571	1.92543	3.21786	2.29155	2.23697	3.18467	1.97093	2.34569	0.917867465	0.966018836	12	20	24	0.809426536	0.939184036	24	12.07749332	0.065719787
ENSMUSG00000096936	Gm3510	predicted gene 3510 [Source:MGI Symbol;Acc:MGI:3781687]	0.429699	0.593295	0.601076	0.847654	0.599344	0.437089	0.524847	0.330505	0.650734	0.466287	0.528965	0.527039	0.530546	0.889317	0.260713922	0.766800466	12	16	24	0.308174915	0.768040178	24	9.540217845	0.082984029
ENSMUSG00000096942	Rps19-ps6	ribosomal protein S19, pseudogene 6 [Source:MGI Symbol;Acc:MGI:5011818]	118.01	136.976	129.439	116.649	134.164	122.161	117.123	122.466	158.551	99.4096	140.551	126.668	142.271	132.117	0.948161972	0.966018836	12	4	24	0.788673869	0.932208289	24	4.613238485	0.04440201
ENSMUSG00000096948	Gm4221	predicted gene 4221 [Source:MGI Symbol;Acc:MGI:3782397]	2.0635	2.42666	1.73326	1.80924	2.03758	1.99182	1.80328	1.68684	3.43538	2.03561	1.99997	2.12455	2.88027	3.33972	0.837133694	0.966018836	12	4	24	0.278812721	0.768040178	24	5.112426163	0.12891702
ENSMUSG00000096956	Snhg18	small nucleolar RNA host gene 18 [Source:MGI Symbol;Acc:MGI:1914085]	10.8495	15.3684	11.0217	10.7618	13.6427	11.5938	13.3989	13.4215	16.5427	10.4525	11.468	12.6552	13.9669	13.0457	0.148627651	0.59172107	8	8	24	0.258308411	0.768040178	24	3.603822722	0.116166689
ENSMUSG00000096967	Gm26621	predicted gene, 26621 [Source:MGI Symbol;Acc:MGI:5477115]	0.465144	0.5999	0.523143	0.661934	0.470693	0.487172	0.758224	0.529664	0.594529	0.389093	0.610542	0.472299	0.566054	0.52761	0.832506215	0.966018836	4	20	24	0.73654444	0.914180155	24	3.527495341	0.027088929
ENSMUSG00000096971	4930556M19Rik	RIKEN cDNA 4930556M19 gene [Source:MGI Symbol;Acc:MGI:1922509]	0.926249	1.05306	1.00741	0.84924	0.936515	1.13658	0.752326	0.87999	1.21589	1.36901	0.922239	1.16075	0.806182	1.22051	0.588208558	0.966018836	12	16	24	0.39670729	0.799546792	24	10.40300995	0.06739548
ENSMUSG00000096974	Gm26881	predicted gene, 26881 [Source:MGI Symbol;Acc:MGI:5477375]	3.2984	1.77126	1.43665	1.11671	1.85791	1.79543	2.00555	2.01746	2.56056	3.40721	2.58499	2.11934	2.5956	1.87622	0.885955579	0.966018836	4	8	24	0.815853409	0.941291601	24	21.69556102	0.074493503
ENSMUSG00000096981	Gm16845	predicted gene, 16845 [Source:MGI Symbol;Acc:MGI:4439769]	0.658691	0.857978	0.565419	0.572587	0.483255	0.637024	0.620805	0.662108	1.02212	0.828741	0.684233	0.723071	0.835289	0.727295	0.801561896	0.966018836	8	12	24	0.240049497	0.768040178	24	5.701015989	0.071706557
ENSMUSG00000096982	Redrum	Redrum, erythroid developmental long intergenic non-protein coding transcript [Source:MGI Symbol;Acc:MGI:1924683]	2.32044	2.00104	1.85749	1.81327	1.81869	1.80642	1.66425	1.84387	1.78533	1.9251	1.88029	2.17959	2.76338	2.2722	0.793403332	0.966018836	4	8	24	0.207680631	0.768040178	24	0.424062772	0.083752827
ENSMUSG00000096995	2810029C07Rik	RIKEN cDNA 2810029C07 gene [Source:MGI Symbol;Acc:MGI:1919920]	0.755184	1.39284	1.59873	1.10379	1.09461	1.44985	1.2706	1.46502	1.43521	1.16968	1.14959	1.72615	1.40547	1.49338	0.360675801	0.883978426	12	4	24	0.515607107	0.817346854	24	5.338710553	0.067359115
ENSMUSG00000097000	Gm17435	predicted gene, 17435 [Source:MGI Symbol;Acc:MGI:4937069]	0.405731	0.73726	0.771296	0.470107	0.693316	0.416318	0.593737	0.410432	0.534071	0.537002	0.542215	0.446067	0.462137	0.583706	0.591351901	0.966018836	12	12	24	0.364466955	0.788837108	24	9.015298519	0.061747705
ENSMUSG00000097006	9530082P21Rik	RIKEN cDNA 9530082P21 gene [Source:MGI Symbol;Acc:MGI:3603340]	1.25936	1.90908	1.87639	1.79877	2.30418	1.75873	1.35899	2.05129	2.07937	1.82588	2.02157	1.4389	1.7314	1.64371	0.033248781	0.324908923	12	16	24	0.063753566	0.663876076	24	11.20727744	0.132855042
ENSMUSG00000097023	Mir9-3hg	Mir9-3 host gene [Source:MGI Symbol;Acc:MGI:2142071]	0.146142	0.834283	1.53326	0.37914	0.238538	0.788192	0.60485	0.395487	0.804839	0.562981	0.348596	0.634032	0.117432	1.15188	0.114881734	0.535150745	12	8	24	0.202133866	0.768040178	24	7.325928242	0.239960751
ENSMUSG00000097032	4930539J05Rik	RIKEN cDNA 4930539J05 gene [Source:MGI Symbol;Acc:MGI:1922477]	1.33291	1.32378	0.855329	0.917712	0.952913	1.17715	1.38623	0.715694	1.1729	0.980909	1.56947	0.887021	0.783249	1.02255	0.906657897	0.966018836	20	20	24	0.651284248	0.882725346	24	19.86772135	0.065791598
ENSMUSG00000097039	Pvt1	Pvt1 oncogene [Source:MGI Symbol;Acc:MGI:97824]	11.4653	11.6887	10.6005	9.36775	11.0002	10.5205	9.59003	9.70346	9.90604	10.0077	11.0774	10.1465	11.2532	13.23	0.595952838	0.966018836	8	8	24	0.487065183	0.805393068	24	1.589433371	0.057353801
ENSMUSG00000097040	2610316D01Rik	RIKEN cDNA 2610316D01 gene [Source:MGI Symbol;Acc:MGI:1919761]	2.9778	3.67904	3.02875	2.33998	3.09046	2.43472	2.45228	2.24824	3.46191	2.33391	2.19851	2.59073	2.47142	3.37394	0.401437339	0.933249185	12	4	24	0.226148947	0.768040178	24	5.370451075	0.120087805
ENSMUSG00000097041	Pafah1b1-ps1	platelet-activating factor acetylhydrolase, isoform 1b, beta1 subunit, pseudogene 1 [Source:MGI Symbol;Acc:MGI:103115]	1.61948	2.0351	1.81022	1.60067	1.8533	2.06999	1.97635	2.21643	1.07563	1.42654	1.64033	1.6054	1.49762	1.14096	0.70641939	0.966018836	8	4	24	0.247176357	0.768040178	24	21.17743313	0.106343205
ENSMUSG00000097042	Gm17491	predicted gene, 17491 [Source:MGI Symbol;Acc:MGI:4937125]	2.23058	2.10191	2.38748	2.42358	2.56724	2.18931	2.68148	1.98798	2.78899	1.65574	3.08067	2.11837	2.23999	1.99267	0.192357053	0.669841558	20	12	24	0.623012029	0.871634402	24	16.30899396	0.062140729
ENSMUSG00000097047	1110020A21Rik	RIKEN cDNA 1110020A21 gene [Source:MGI Symbol;Acc:MGI:1915781]	0.807174	2.22143	1.15971	1.80177	1.06362	0.99055	1.1976	1.56557	2.10447	2.11253	1.44728	1.16766	1.76532	1.36199	0.186921604	0.662239322	16	8	24	0.04649748	0.633507364	24	8.570788732	0.236810251
ENSMUSG00000097048	1600020E01Rik	RIKEN cDNA 1600020E01 gene [Source:MGI Symbol;Acc:MGI:1919262]	5.61204	9.01747	7.72839	10.5058	8.4379	6.35909	7.64254	7.84249	10.5365	6.05972	7.90268	8.82058	7.16394	7.71606	0.313530643	0.824470524	12	16	24	0.31047897	0.768040178	24	9.862443359	0.143956104
ENSMUSG00000097055	Gm4419	predicted gene 4419 [Source:MGI Symbol;Acc:MGI:3782604]	4.85919	6.43042	3.80988	4.1913	5.13953	5.4736	2.70617	5.97761	4.44975	4.31941	5.07797	5.68914	5.49978	3.85827	0.539040394	0.966018836	8	4	24	0.760864442	0.92268731	24	20.38336255	0.082132852
ENSMUSG00000097061	9330151L19Rik	RIKEN cDNA 9330151L19 gene [Source:MGI Symbol;Acc:MGI:3041168]	3.82888	4.0145	4.6509	4.62279	3.66946	4.27461	3.96565	4.3177	4.73151	4.6431	4.49978	4.06896	4.34131	4.43201	0.009118941	0.192760771	12	16	24	0.008923567	0.502965379	24	9.466258979	0.092761593
ENSMUSG00000097073	9430037G07Rik	RIKEN cDNA 9430037G07 gene [Source:MGI Symbol;Acc:MGI:2444544]	1.96601	1.98606	1.64576	1.75021	2.17085	1.84982	1.60835	1.44405	1.89984	1.69931	1.97408	1.74426	1.90767	1.80563	0.89563591	0.966018836	20	20	24	0.36814472	0.79131734	24	17.61554506	0.053988743
ENSMUSG00000097075	Cdiptos	CDIP transferase, opposite strand [Source:MGI Symbol;Acc:MGI:2443610]	0.367244	0.650691	1.01518	0.910153	1.17104	0.717171	1.12619	0.607792	0.843222	0.605978	0.500861	0.693608	0.753725	0.949815	0.833301348	0.966018836	12	12	24	0.76683496	0.92458474	24	9.959870306	0.057946901
ENSMUSG00000097078	Gm26566	predicted gene, 26566 [Source:MGI Symbol;Acc:MGI:5477060]	0.390812	0.582305	0.693278	0.297837	0.348967	0.320247	0.505143	0.501423	0.690139	0.623952	0.913463	0.796306	0.926402	0.947148	0.934152439	0.966018836	12	4	24	0.313081216	0.768040178	24	5.45561862	0.082897835
ENSMUSG00000097080	1700086O06Rik	RIKEN cDNA 1700086O06 gene [Source:MGI Symbol;Acc:MGI:1920766]	1.92933	2.20753	2.05246	1.83499	2.07798	1.69084	2.32042	2.82341	2.80564	2.94742	2.43561	2.69848	2.8956	3.14338	0.834424969	0.966018836	8	16	24	0.012957478	0.528286959	24	6.035963913	0.121461842
ENSMUSG00000097086	Gm7672	predicted gene 7672 [Source:MGI Symbol;Acc:MGI:3647232]	0.560993	0.826465	1.34188	0.642642	0.712883	0.73144	0.752566	0.752819	0.650961	0.815477	0.346824	0.465488	0.411349	0.377829	0.586972936	0.966018836	12	8	24	0.216311222	0.768040178	24	8.591429962	0.111469523
ENSMUSG00000097090	Gm26724	predicted gene, 26724 [Source:MGI Symbol;Acc:MGI:5477218]	1.75924	1.32908	1.68219	1.81099	2.08357	1.30679	1.66083	1.4119	1.96945	1.21477	1.72824	1.01654	1.23996	1.60223	0.44861185	0.966018836	20	4	24	0.449377558	0.805393068	24	10.96395759	0.083450379
ENSMUSG00000097093	C330013E15Rik	RIKEN cDNA C330013E15 gene [Source:MGI Symbol;Acc:MGI:1925695]	1.05244	0.675367	0.963614	0.634613	0.538338	0.690903	1.08088	1.13985	1.20994	1.31757	0.837178	1.2373	1.10762	0.787416	0.886572916	0.966018836	4	16	24	0.566238682	0.843471947	24	4.177834561	0.074626998
ENSMUSG00000097099	Gm9917	predicted gene 9917 [Source:MGI Symbol;Acc:MGI:3704343]	1.81661	1.34639	0.93923	2.06122	1.85754	1.06368	1.25165	1.7076	1.29545	1.01186	1.21613	1.25718	0.971291	0.831819	0.858266942	0.966018836	16	20	24	0.613867778	0.866162311	24	16.53085266	0.076432791
ENSMUSG00000097101	1810034E14Rik	RIKEN cDNA 1810034E14 gene [Source:MGI Symbol;Acc:MGI:1913753]	0.747147	0.951048	0.638119	0.714611	0.972378	0.64953	1.12682	0.700323	1.27255	0.755486	1.53342	1.10416	1.0214	0.733897	0.823657258	0.966018836	20	12	24	0.640004682	0.878006652	24	17.60462671	0.06884508
ENSMUSG00000097113	Gm19705	predicted gene, 19705 [Source:MGI Symbol;Acc:MGI:5011890]	4.14129	4.5381	3.89931	5.42106	4.88701	2.54177	4.18575	4.79204	6.79438	3.76072	5.3021	5.81438	3.37823	2.15797	0.696676764	0.966018836	20	8	24	0.436724428	0.805393068	24	12.84676767	0.175185509
ENSMUSG00000097119	B230354K17Rik	RIKEN cDNA B230354K17 gene [Source:MGI Symbol;Acc:MGI:2443272]	11.1383	11.5877	10.1619	10.5371	11.5971	10.1866	12.2411	9.44936	11.1231	8.30924	11.5929	11.1298	12.1337	10.9172	0.109247218	0.524035616	4	12	24	0.194849564	0.768040178	24	22.46464622	0.097398208
ENSMUSG00000097121	D130020L05Rik	RIKEN cDNA D130020L05 gene [Source:MGI Symbol;Acc:MGI:2450270]	2.50095	2.29645	3.119	1.69048	2.05752	1.61678	2.7773	3.55274	2.44208	2.60184	2.35582	2.12085	1.98011	2.79429	0.094000699	0.490854203	8	16	24	0.055232403	0.65534544	24	5.588562106	0.197121835
ENSMUSG00000097125	Gm26885	predicted gene, 26885 [Source:MGI Symbol;Acc:MGI:5477379]	0.996279	1.1934	0.689669	0.76254	1.2828	1.32843	1.22574	0.709821	1.30188	1.20821	1.26075	0.966273	0.837507	1.121	0.598711142	0.966018836	20	12	24	0.494905267	0.806761911	24	17.7236057	0.080255668
ENSMUSG00000097140	Gm26779	predicted gene, 26779 [Source:MGI Symbol;Acc:MGI:5477273]	0.8246	1.46251	1.34318	0.936851	1.37492	0.868756	1.50416	0.641918	0.84123	0.613319	1.33658	0.794745	1.10471	0.703558	0.897425765	0.966018836	20	20	24	0.784969342	0.931056644	24	19.48890298	0.061813807
ENSMUSG00000097141	Gm10524	predicted gene 10524 [Source:MGI Symbol;Acc:MGI:3642791]	2.40462	1.98123	2.51701	2.2431	2.30814	2.39081	2.21084	2.67401	2.82094	2.76791	1.88074	2.25504	2.23632	2.58045	0.417653118	0.963884303	12	8	24	0.177867802	0.768040178	24	7.617643321	0.086295302
ENSMUSG00000097145	9230114K14Rik	RIKEN cDNA 9230114K14 gene [Source:MGI Symbol;Acc:MGI:3041212]	1.89559	2.29131	1.7307	2.15043	2.24624	2.47484	2.1444	2.09096	2.77027	0.853142	2.42852	2.56437	2.04851	2.10415	0.669104912	0.966018836	24	4	24	0.520912799	0.820180873	24	22.09165653	0.11364675
ENSMUSG00000097156	Gm3764	predicted gene 3764 [Source:MGI Symbol;Acc:MGI:3781938]	2.95377	8.00545	16.4364	4.93532	3.64957	8.21178	8.55971	4.51149	9.82474	5.56069	2.06823	6.34712	1.491	9.77589	0.064422003	0.414867207	12	8	24	0.234827403	0.768040178	24	7.091791011	0.514543714
ENSMUSG00000097162	2310010J17Rik	RIKEN cDNA 2310010J17 gene [Source:MGI Symbol;Acc:MGI:1925579]	11.8025	15.135	10.7598	14.4767	11.658	11.3282	15.2027	10.7221	15.0397	13.7485	13.2011	10.8154	13.9766	15.888	0.595952838	0.966018836	8	16	24	0.41237383	0.799546792	24	6.63665161	0.102687722
ENSMUSG00000097164	Cep83os	centrosomal protein 83, opposite strand [Source:MGI Symbol;Acc:MGI:1914973]	4.90705	5.18792	5.4886	5.40776	4.81663	4.7268	4.50845	4.67227	5.1927	5.79159	5.0969	5.33649	5.25992	5.65647	0.208438456	0.698704663	16	12	24	0.063822889	0.663876076	24	9.818082897	0.074650158
ENSMUSG00000097167	Gm16740	predicted gene, 16740 [Source:MGI Symbol;Acc:MGI:4439664]	0.521896	0.548387	1.27417	0.244657	0.550604	0.771787	0.735333	0.453485	0.585612	0.636076	0.843888	0.592089	1.01443	0.584523	0.956794505	0.96629412	24	8	24	0.910744743	0.9747563	24	23.75982045	0.036609302
ENSMUSG00000097177	9330159M07Rik	RIKEN cDNA 9330159M07 gene [Source:MGI Symbol;Acc:MGI:2442519]	2.35879	1.97336	2.53481	2.84598	2.17596	2.4474	2.4928	2.16866	2.68734	2.47755	2.23862	2.13006	2.91826	2.86379	0.745475226	0.966018836	12	12	24	0.541646343	0.830485817	24	7.657754988	0.053756608
ENSMUSG00000097178	2310002F09Rik	RIKEN cDNA 2310002F09 gene [Source:MGI Symbol;Acc:MGI:1916779]	1.07883	1.03398	0.82661	1.31537	0.940052	1.14872	0.935015	1.11983	0.952299	1.00826	1.11208	1.34511	1.29803	1.27839	0.245061943	0.741580974	24	12	24	0.49973863	0.808791684	24	21.18208272	0.047149844
ENSMUSG00000097180	2700038G22Rik	RIKEN cDNA 2700038G22 gene [Source:MGI Symbol;Acc:MGI:1914444]	1.66513	1.21264	1.31735	0.903495	1.03193	1.17399	0.759397	1.83542	1.3836	0.886309	1.54374	1.4472	1.15587	0.851481	0.853736882	0.966018836	12	4	24	0.834074017	0.94899733	24	0.246092105	0.050467276
ENSMUSG00000097183	Gm17501	predicted gene, 17501 [Source:MGI Symbol;Acc:MGI:4937135]	0.924078	1.11309	1.64395	0.838355	1.48619	1.01346	0.926983	1.0727	1.37108	1.17035	0.937132	0.689113	1.16057	1.34279	0.039704107	0.338535904	12	12	24	0.130940609	0.741353066	24	8.167824901	0.139837364
ENSMUSG00000097184	4632428C04Rik	RIKEN cDNA 4632428C04 gene [Source:MGI Symbol;Acc:MGI:1921600]	4.33513	4.0843	3.61472	3.89136	4.00095	4.48215	3.9104	4.02196	3.17138	4.35218	3.92363	3.60633	3.99233	3.66158	0.759899911	0.966018836	24	12	24	0.193205308	0.768040178	24	19.97578285	0.0702966
ENSMUSG00000097187	Gm19426	predicted gene, 19426 [Source:MGI Symbol;Acc:MGI:5011611]	0.636927	1.25733	0.692237	0.485157	1.42443	0.880147	1.2072	0.821623	0.723757	0.982155	1.06731	1.17689	0.803192	0.531822	0.446653834	0.966018836	20	16	24	0.247230528	0.768040178	24	18.8688664	0.144844047
ENSMUSG00000097195	Snhg5	small nucleolar RNA host gene 5 [Source:MGI Symbol;Acc:MGI:1919905]	6.04542	10.2806	12.3158	7.94399	8.58263	7.3074	8.53943	9.87685	10.6818	8.4982	9.12827	9.728	8.54955	8.86216	0.073518583	0.448301997	12	16	24	0.087683592	0.707747429	24	8.15051603	0.18626705
ENSMUSG00000097204	Gm17690	predicted gene, 17690 [Source:MGI Symbol;Acc:MGI:4937324]	0.721457	0.821817	0.802233	1.01569	0.881376	1.15045	0.909434	0.907665	0.822515	1.1408	0.869079	1.62168	0.820677	0.953613	0.236246476	0.7385769	24	4	24	0.238654918	0.768040178	24	17.31461245	0.092757698

ENSMUSG00000097207	6030443J06Rik	RIKEN cDNA 6030443J06 gene [Source:MGI Symbol;Acc:MGI:2444595]	0.662278	0.607116	0.257338	0.710538	0.834965	0.557427	0.703984	0.512106	0.585987	0.517363	0.848348	0.663636	0.484634	0.874806	0.616047374	0.966018836	20	16	24	0.422537316	0.804917634	24	18.3719573	0.078595884
ENSMUSG00000097216	4932441J04Rik	RIKEN cDNA 4932441J04 gene [Source:MGI Symbol;Acc:MGI:2441691]	0.501603	0.422808	0.767776	0.640306	0.325309	0.268878	0.856199	0.770546	0.486156	0.615582	0.66232	0.452266	0.723921	0.752265	0.46238066	0.966018836	4	20	24	0.158616648	0.764990136	24	5.813638183	0.119148197
ENSMUSG00000097217	Gm26549	predicted gene, 26549 [Source:MGI Symbol;Acc:MGI:5477043]	1.02645	1.02455	0.82707	1.267	1.05499	0.865011	1.11394	1.19295	1.2733	0.878968	1.36139	1.01652	1.2647	1.04721	0.852844413	0.966018836	20	4	24	0.985183906	0.996973154	24	9.207856665	0.008041194
ENSMUSG00000097223	Gm2449	predicted gene 2449 [Source:MGI Symbol;Acc:MGI:3780616]	0.718992	0.932332	0.978652	0.869776	0.530766	0.688003	1.1543	0.553799	1.01038	0.538062	0.78452	0.916815	0.405929	0.965956	0.581070934	0.966018836	12	8	24	0.63996439	0.878006652	24	5.885276631	0.070209725
ENSMUSG00000097233	Gm17552	predicted gene, 17552 [Source:MGI Symbol;Acc:MGI:4937186]	4.33927	5.60693	4.9568	5.3644	5.57259	4.1963	5.30485	4.64593	7.3906	5.52903	5.11292	4.70197	5.22361	4.97002	0.289105414	0.794869797	16	8	24	0.082851824	0.699403344	24	9.472534176	0.140681256
ENSMUSG00000097239	Gm27029	predicted gene, 27029 [Source:MGI Symbol;Acc:MGI:5504144]	1.33136	1.31808	1.60926	0.961327	1.81367	1.30717	1.76431	1.35866	1.01319	1.02652	1.46351	1.00957	1.18193	1.44105	0.93717134	0.966018836	20	20	24	0.640438473	0.878305673	24	22.71836131	0.063296289
ENSMUSG00000097240	Gm26614	predicted gene, 26614 [Source:MGI Symbol;Acc:MGI:5477108]	3.35254	2.89139	2.83698	3.37561	2.98105	2.85725	2.81096	3.29152	2.65858	3.41941	2.81714	3.11583	3.41005	2.94662	0.890311217	0.966018836	16	20	24	0.959813546	0.989375125	24	20.10977409	0.011435131
ENSMUSG00000097245	Gm5421	predicted gene 5421 [Source:MGI Symbol;Acc:MGI:3645718]	13.301	12.6028	15.9315	20.405	14.19	17.5427	15.2868	17.6869	12.904	19.6213	15.6628	13.6184	16.2949	15.3586	0.628286596	0.966018836	16	12	24	0.360376729	0.785908323	24	12.61106323	0.111405286
ENSMUSG00000097247	1500012K07Rik	RIKEN cDNA 1500012K07 gene [Source:MGI Symbol;Acc:MGI:1916204]	1.44269	1.34273	1.03375	1.23276	1.27259	1.57004	1.11643	1.77242	1.66062	1.30914	1.82671	1.43369	1.2291	1.6288	0.882263435	0.966018836	8	20	24	0.996520085	0.999122704	24	0.387314911	0.004539073
ENSMUSG00000097248	Gm2694	predicted gene 2694 [Source:MGI Symbol;Acc:MGI:3780864]	3.67182	2.50014	4.19671	2.24177	3.20602	2.43634	2.08141	2.78723	3.74668	3.37268	4.01198	3.21481	3.6697	3.48328	0.934152439	0.966018836	12	12	24	0.6700868	0.888925819	24	9.970052595	0.085451118
ENSMUSG00000097251	5033417F24Rik	RIKEN cDNA 5033417F24 gene [Source:MGI Symbol;Acc:MGI:1923245]	0.609999	0.63322	0.345163	0.646509	0.715535	0.64012	0.566471	0.723087	0.734615	0.70908	0.707085	0.57365	0.48634	0.633342	0.108029159	0.524035616	16	12	24	0.604490891	0.861905906	24	14.13160398	0.038936536
ENSMUSG00000097267	1700109K24Rik	RIKEN cDNA 1700109K24 gene [Source:MGI Symbol;Acc:MGI:1921553]	0.91469	1.06558	1.4902	1.46746	1.3661	0.98581	0.950546	1.02188	1.90289	0.783737	1.50663	1.27844	1.45662	1.43136	0.267533088	0.774280302	12	16	24	0.356487935	0.783133725	24	10.13564887	0.109837922
ENSMUSG00000097277	2900076A07Rik	RIKEN cDNA 2900076A07 gene [Source:MGI Symbol;Acc:MGI:1920242]	8.13444	11.0899	10.8704	11.2906	10.6782	9.97034	11.0222	10.9224	14.4776	8.51029	10.9691	9.75017	11.9601	10.1719	0.801995186	0.966018836	12	12	24	0.413641698	0.799546792	24	7.738414855	0.098663765
ENSMUSG00000097287	D130017N08Rik	RIKEN cDNA D130017N08 gene [Source:MGI Symbol;Acc:MGI:2443273]	6.55766	5.53324	6.15231	5.91308	6.09191	6.73303	5.8938	6.50884	5.59959	8.74865	5.1297	5.90875	5.90316	6.2988	0.85387321	0.966018836	16	20	24	0.864790588	0.960819377	24	11.45094053	0.034269109
ENSMUSG00000097290	1300002E11Rik	RIKEN cDNA 1300002E11 gene [Source:MGI Symbol;Acc:MGI:1919001]	10.6183	13.8232	10.728	12.5671	14.6803	11.3564	12.6982	12.7101	15.6852	11.7839	14.6093	12.724	13.725	12.0478	0.93447422	0.966018836	20	8	24	0.729378383	0.911672396	24	12.58080781	0.047448026
ENSMUSG00000097292	A230107N01Rik	RIKEN cDNA A230107N01 gene [Source:MGI Symbol;Acc:MGI:2445042]	0.813555	1.03657	0.652129	0.409558	1.05098	0.922193	1.1711	0.972654	1.22477	0.624986	1.01346	1.02512	1.00387	0.895571	0.718720526	0.966018836	20	20	24	0.184553447	0.768040178	24	23.56123971	0.120147328
ENSMUSG00000097295	Hmgb1-ps8	high mobility group box 1, pseudogene 8 [Source:MGI Symbol;Acc:MGI:96114]	0.258656	0.699624	0.281002	0.687647	0.70493	0.340173	0.734632	0.845363	1.29158	0.398913	0.530758	0.773722	0.345892	0.455513	0.846643334	0.966018836	8	20	24	0.651266662	0.882725346	24	8.922491447	0.093343514
ENSMUSG00000097296	Gm26532	predicted gene, 26532 [Source:MGI Symbol;Acc:MGI:5477026]	6.95957	10.3269	9.20709	10.8811	9.69082	7.56227	7.09515	10.0603	12.2705	8.8493	8.49896	6.92382	9.80258	8.36832	0.019280932	0.257669847	12	12	24	0.017158706	0.548188101	24	9.021363853	0.232670938
ENSMUSG00000097303	3110083C13Rik	RIKEN cDNA 3110083C13 gene [Source:MGI Symbol;Acc:MGI:1920458]	1.30192	1.58713	1.12013	0.852204	1.17822	0.940751	1.46575	1.12946	1.67979	0.907665	0.864849	1.05195	1.32712	1.1819	0.027467587	0.308438477	8	8	24	0.032577167	0.606585237	24	3.203576524	0.153938045
ENSMUSG00000097305	Gm17276	predicted gene, 17276 [Source:MGI Symbol;Acc:MGI:4936910]	1.02657	1.03787	0.970304	0.928348	0.592115	0.855499	0.789794	0.708147	0.726076	0.943758	0.598384	0.944533	0.795261	0.903854	0.70641939	0.966018836	8	12	24	0.442274774	0.805393068	24	3.7115381	0.055417305
ENSMUSG00000097309	Gm17249	predicted gene, 17249 [Source:MGI Symbol;Acc:MGI:4936883]	0.452789	0.497618	0.623568	0.62854	0.535472	0.414953	0.593451	0.585302	0.595671	0.40702	0.493173	0.567732	0.505204	0.347382	0.838532766	0.966018836	12	12	24	0.772987204	0.926975725	24	10.29932698	0.023927137
ENSMUSG00000097311	Gm26871	predicted gene, 26871 [Source:MGI Symbol;Acc:MGI:5477365]	3.63124	3.42519	3.55286	2.67676	3.26203	4.47451	5.00497	2.3099	4.20992	3.36671	4.71047	3.16342	4.86937	2.64471	0.369262716	0.893596186	4	4	24	0.357434821	0.784480156	24	20.23733816	0.157233424
ENSMUSG00000097313	Gm26569	predicted gene, 26569 [Source:MGI Symbol;Acc:MGI:5477063]	0.729441	1.21059	0.68136	1.19454	0.916878	0.832991	1.07834	0.888092	1.3025	0.765636	1.36079	0.955975	1.32966	1.4835	0.845386467	0.966018836	8	4	24	0.716956513	0.906702121	24	5.660982689	0.052749888
ENSMUSG00000097316	Gm10516	predicted gene 10516 [Source:MGI Symbol;Acc:MGI:3641979]	7.6707	5.39564	5.15706	4.86146	5.02143	5.80329	5.13165	5.3931	5.46241	5.07667	5.40334	5.66936	6.10895	5.33547	0.048889741	0.379129806	4	12	24	0.050228275	0.644435324	24	23.11396393	0.122916793
ENSMUSG00000097318	1700007L15Rik	RIKEN cDNA 1700007L15 gene [Source:MGI Symbol;Acc:MGI:1916581]	1.043	1.51953	1.51036	1.56626	0.879761	1.16378	1.42061	1.36406	1.58602	1.49703	1.56779	1.24268	1.41477	1.63099	0.110090633	0.526220521	12	12	24	0.018656621	0.560301468	24	7.666261394	0.136758551
ENSMUSG00000097320	Tmem147os	transmembrane protein 147, opposite strand [Source:MGI Symbol;Acc:MGI:3642392]	6.86704	6.53836	6.65748	7.41673	7.5832	6.54971	6.17129	5.88932	6.68278	6.56091	6.51431	6.66659	6.35121	5.85062	0.061981717	0.409814092	16	16	24	0.003841627	0.427278365	24	15.11843335	0.083925958
ENSMUSG00000097323	4930426I24Rik	RIKEN cDNA 4930426I24 gene [Source:MGI Symbol;Acc:MGI:1921125]	0.760761	0.762846	0.84663	0.690441	0.675678	0.633774	0.181305	0.400246	1.08661	0.469286	0.23089	0.671526	0.375929	0.828875	0.049545731	0.38370367	12	16	24	0.340521779	0.774856529	24	8.049371374	0.133934506
ENSMUSG00000097328	Tnfsf12	tumor necrosis factor (ligand) superfamily, member 12 [Source:MGI Symbol;Acc:MGI:1196259]	12.2338	9.68904	10.6193	10.8798	13.3878	9.82708	12.9077	10.2879	14.0206	13.1427	12.3645	12.073	11.2424	9.37025	0.066380001	0.42386701	20	12	24	0.290351206	0.768040178	24	15.16467848	0.104446586
ENSMUSG00000097331	F420014N23Rik	RIKEN cDNA F420014N23 gene [Source:MGI Symbol;Acc:MGI:3642477]	0.373911	1.36113	1.25229	1.09169	1.12739	1.04868	1.44261	1.40232	1.26497	1.11113	1.11236	1.21526	1.27821	0.714029	0.550682606	0.966018836	8	8	24	0.769832546	0.925855377	24	9.789910906	0.064767163
ENSMUSG00000097333	Zfp87	zinc finger protein 87 [Source:MGI Symbol;Acc:MGI:107768]	6.07565	6.67991	6.46213	6.76545	7.18983	6.74387	7.85003	6.77315	8.68538	7.25785	6.81159	6.74157	8.59564	7.37931	0.830949782	0.966018836	4	20	24	0.656098029	0.8838564	24	6.212397874	0.040832212
ENSMUSG00000097336	Fendrr	Foxf1 adjacent non-coding developmental regulatory RNA [Source:MGI Symbol;Acc:MGI:1916040]	0.403873	0.939815	0.970183	0.694167	1.05693	0.62914	0.744636	1.16442	0.910572	0.801676	0.759845	0.46183	0.630103	0.659426	0.074438493	0.448301997	12	12	24	0.107811159	0.72073763	24	8.962456188	0.143288933
ENSMUSG00000097343	9030407P20Rik	RIKEN cDNA 9030407P20 gene [Source:MGI Symbol;Acc:MGI:1918818]	2.2951	2.46672	2.59786	2.51051	2.18338	2.40372	2.03047	2.50086	2.50474	2.60535	2.10244	2.73304	2.32187	3.00415	0.137504104	0.580158868	16	4	24	0.163624156	0.767020009	24	7.246939511	0.076084868
ENSMUSG00000097350	4732491K20Rik	RIKEN cDNA 4732491K20 gene [Source:MGI Symbol;Acc:MGI:3026975]	2.02164	2.54025	2.41764	2.12394	2.29596	2.33377	1.98335	1.83626	2.47523	2.31732	2.13296	2.70977	2.24942	2.44841	0.940625808	0.966018836	12	16	24	0.837535991	0.950322752	24	11.67397951	0.025135603
ENSMUSG00000097354	2310001H17Rik	RIKEN cDNA 2310001H17 gene [Source:MGI Symbol;Acc:MGI:1923682]	1.81906	2.73845	2.57717	2.46888	2.69764	3.06278	2.92485	4.0282	3.77567	2.37079	2.07262	3.02362	3.09661	1.69165	0.869721097	0.966018836	24	20	24	0.893919724	0.969958849	24	4.252235828	0.049544589
ENSMUSG00000097357	Gm16793	predicted gene, 16793 [Source:MGI Symbol;Acc:MGI:4439717]	1.09978	1.10515	1.05185	0.851242	1.4036	1.00418	0.86778	0.778024	0.947358	1.10596	1.08133	1.03638	1.04534	1.24775	0.853736882	0.966018836	20	4	24	0.666996338	0.887459929	24	16.61424488	0.040619423
ENSMUSG00000097360	9430065F17Rik	RIKEN cDNA 9430065F17 gene [Source:MGI Symbol;Acc:MGI:1924606]	3.09102	3.85028	3.09297	3.30646	2.92676	2.32907	4.23296	2.79766	2.90644	2.40588	2.88647	3.40049	2.8627	4.24677	0.851656735	0.966018836	8	12	24	0.223311177	0.768040178	24	2.962068585	0.130864334
ENSMUSG00000097366	Gm8177	predicted gene 8177 [Source:MGI Symbol;Acc:MGI:3805962]	0.676381	0.533216	0.478288	0.622316	0.736161	0.539081	0.495596	0.455041	0.554974	0.603159	0.605892	0.516426	0.427336	0.829882	0.481811994	0.966018836	20	8	24	0.671217956	0.889066748	24	13.58656614	0.035475962
ENSMUSG00000097375	6720427I07Rik	RIKEN cDNA 6720427I07 gene [Source:MGI Symbol;Acc:MGI:1924398]	1.44065	1.99372	1.82183	2.09352	1.88808	1.56538	1.89567	1.57139	2.01406	1.41734	1.9777	1.35135	1.57797	1.89561	0.387380622	0.915479136	12	12	24	0.287285131	0.768040178	24	9.382395596	0.083561854
ENSMUSG00000097378	B230208H11Rik	RIKEN cDNA B230208H11 gene [Source:MGI Symbol;Acc:MGI:2443719]	0.537379	1.26858	1.02277	0.851736	1.17407	1.14483	0.765106	0.704269	1.05826	1.1538	0.956865	1.37677	1.19264	0.894177	0.92655264	0.966018836	24	4	24	0.499593778	0.808791684	24	14.92000674	0.077160661
ENSMUSG00000097379	Gm26873	predicted gene, 26873 [Source:MGI Symbol;Acc:MGI:5477367]	0.564568	0.828507	0.635128	0.691006	0.734877	0.57589	0.652694	0.499175	0.898831	0.54369	0.627283	0.836585	0.704128	0.682287	0.789670753	0.966018836	12	16	24	0.943565434	0.986151003	24	8.684524947	0.014380676
ENSMUSG00000097383	1500026H17Rik	RIKEN cDNA 1500026H17 gene [Source:MGI Symbol;Acc:MGI:1916252]	1.14692	1.16789	0.857322	1.27592	0.864115	1.06607	1.09821	1.05612	1.1545	1.01292	1.04078	1.03454	0.96294	1.36913	0.47028201	0.966018836	8	12	24	0.446164878	0.805393068	24	4.658687391	0.048928264
ENSMUSG00000097388	Gm3200	predicted pseudogene 3200 [Source:MGI Symbol;Acc:MGI:3781379]	0.71037	0.457316	0.51502	0.706352	0.921901	0.672276	0.577856	0.678592	0.711638	0.87606	0.325711	0.664178	0.441558	0.459568	0.565721644	0.966018836	16	16	24	0.430689237	0.805393068	24	14.42542159	0.073450018
ENSMUSG00000097391	Mirg	miRNA containing gene [Source:MGI Symbol;Acc:MGI:3781106]	27.1882	23.4855	25.3033	27.9343	30.0441	25.0082	25.9997	18.6813	25.1563	21.9871	26.4203	21.2132	21.3273	22.986	0.305564675	0.811201458	20	12	24	0.116500417	0.727799063	24	15.68520173	0.11626862
ENSMUSG00000097392	Thoc2l	THO complex subunit 2-like [Source:MGI Symbol;Acc:MGI:3040669]	6.46265	7.0891	6.93525	7.54485	7.35908	7.15953	7.50646	6.4978	6.80682	6.27658	7.68644	5.77043	5.90989	6.09803	0.581070934	0.966018836	20	8	24	0.257064878	0.768040178	24	14.38405833	0.063207863
ENSMUSG00000097397	Gm16861	predicted gene, 16861 [Source:MGI Symbol;Acc:MGI:4439785]	0.751804	0.785191	0.787351	0.974274	0.881052	0.930623	0.677764	0.547703	0.977751	0.638314	1.16154	0.717857	0.717424	0.804679	0.200625581	0.685057195	20	8	24	0.141933003	0.751766185	24	14.57529725	0.092179403
ENSMUSG00000097402	Gm17399	predicted gene, 17399 [Source:MGI Symbol;Acc:MGI:4937033]	0.779844	0.51548	0.424135	0.806455	0.643272	0.554401	0.380816	0.279639	0.426406	0.386371	0.671219	0.496548	0.415131	0.522672	0.586972936	0.966018836	20	16	24	0.139477983	0.74956518	24	16.88730961	0.101142127
ENSMUSG00000097404	Gm10814	predicted gene 10814 [Source:MGI Symbol;Acc:MGI:3642580]	1.14984	0.844428	0.866175	0.916824	1.14822	0.882974	1.24463	1.03033	1.13273	1.03537	1.21858	0.855025	1.02646	1.17696	0.896311169	0.966018836	4	8	24	0.971127463	0.993012791	24	22.63642197	0.009859713
ENSMUSG00000097412	1810014B01Rik	RIKEN cDNA 1810014B01 gene [Source:MGI Symbol;Acc:MGI:1913513]	2.17032	3.67429	3.51762	4.44229	3.72935	2.74157	5.20462	4.97214	4.10968	5.55634	4.87786	2.79004	3.19697	3.32444	0.113424199	0.530777648	16	8	24	0.123279555	0.736234617	24	10.43891999	0.236194317
ENSMUSG00000097413	A830052D11Rik	RIKEN cDNA A830052D11 gene [Source:MGI Symbol;Acc:MGI:3028074]	1.11565	1.19486	1.37014	0.757773	1.34871	1.08707	0.716092	1.69584	1.35005	0.973087	0.801507	0.894389	0.907189	0.833838	0.413946331	0.958746003	12	4	24	0.435305639	0.805393068	24	6.663588055	0.095380104
ENSMUSG00000097414	B130046B21Rik	RIKEN cDNA B130046B21 gene [Source:MGI Symbol;Acc:MGI:3704458]	1.21809	2.28289	1.65769	2.66564	2.28713	2.26857	1.63903	2.46024	2.1631	1.53615	2.22663	2.06953	2.11342	2.39318	0.676344318	0.966018836	8	20	24	0.747888192	0.917930198	24	11.19657037	0.060575789
ENSMUSG00000097415	AU020206	expressed sequence AU020206 [Source:MGI Symbol;Acc:MGI:2142134]	1.90159	2.93114	3.63264	3.41861	3.13605	2.5191	2.77543	3.72451	3.50445	3.30699	3.29366	2.90716	2.88869	3.69826	5.90E-04	0.071238795	12	16	24	0.001325739	0.346832852	24	9.343509631	0.207520009
ENSMUSG00000097425	Vmn1r181	vomeronasal 1 receptor 181 [Source:MGI Symbol;Acc:MGI:3033489]	0.516082	0.813265	0.613696	0.696358	0.909226	0.470488	0.73742	0.621605	0.710403	0.629861	1.28183	0.523962	0.979852	0.612129	0.676344318	0.966018836	20	4	24	0.649019539	0.881754555	24	14.43582305	0.061288232
ENSMUSG00000097428	AW047730	expressed sequence AW047730 [Source:MGI Symbol;Acc:MGI:2139951]	0.607312	1.04779	1.7373	0.62119	0.590341	1.07506	1.1162	0.708532	1.22112	0.714172	0.505215	0.782266	0.440883	1.46245	0.064422003	0.414867207	12	8	24	0.147196835	0.75567997	24	6.051247614	0.209963199
ENSMUSG00000097431	Gm26782	predicted gene, 26782 [Source:MGI Symbol;Acc:MGI:5477276]	19.1168	19.8653	16.6061	17.6087	17.1154	18.6205	19.3226	15.8114	20.0188	17.3052	17.9782	19.0145	18.3678	19.7274	0.466710516	0.966018836	8	8	24	0.385577661	0.799546792	24	0.411721654	0.05241891
ENSMUSG00000097439	Gm16754	predicted gene, 16754 [Source:MGI Symbol;Acc:MGI:4439678]	0.91702	0.528925	1.04449	0.947079	1.03734	0.62402	0.760164	0.439344	0.845513	0.706048	0.820923	0.49984	0.698328	0.688586	0.107363828	0.523986091	12	20	24	0.20184972	0.768040178	24	13.0420651	0.100129546
ENSMUSG00000097440	Gm6277	predicted gene 6277 [Source:MGI Symbol;Acc:MGI:3779583]	0.917583	0.817551	0.734121	1.0444	0.882683	0.936806	0.923317	0.878388	1.03684	1.01375	1.0361	1.00271	0.918472	1.31539	0.745118544	0.966018836	16	16	24	0.836587947	0.949953371	24	11.15826786	0.018091615
ENSMUSG00000097445	Gm26631	predicted gene, 26631 [Source:MGI Symbol;Acc:MGI:5477125]	0.817604	0.564339	0.872178	1.04256	0.694297	0.563783	0.731703	0.766093	0.724399	0.606625	0.741699	0.583497	0.768031	0.654002	0.933421033	0.966018836	12	12	24	0.540502475	0.830021415	24	9.709544199	0.04681208
ENSMUSG00000097451	Rian	RNA imprinted and accumulated in nucleus [Source:MGI Symbol;Acc:MGI:1922995]	381.232	388.863	359.002	359.361	398.123	358.928	366.537	333.154	368.026	297.792	390.968	333.102	346.929	363.713	0.907000607	0.966018836	20	4	24	0.73171387	0.912550805	24	21.18825652	0.033246183
ENSMUSG00000097462	9530026P05Rik	RIKEN cDNA 9530026P05 gene [Source:MGI Symbol;Acc:MGI:1924659]	2.35894	3.09929	3.02699	3.6246	2.46679	2.24611	3.0149	2.79832	3.43408	3.09535	1.80203	2.45136	2.73346	3.1385	0.003683467	0.140598674	16	4	24	0.011517496	0.519508195	24	7.31307294	0.204208372
ENSMUSG00000097466	D430036J16Rik	RIKEN cDNA D430036J16 gene [Source:MGI Symbol;Acc:MGI:2441977]	2.29274	2.15273	1.78713	2.45009	2.08478	2.18294	2.61943	1.62172	1.459	1.96803	1.77118	2.00003	1.46217	1.89137	0.649212555	0.966018836	4	8	24	0.258686875	0.768040178	24	19.36842844	0.095281959
ENSMUSG00000097487	Ptges3l	prostaglandin E synthase 3 like [Source:MGI Symbol;Acc:MGI:1916146]	1.27024	2.82958	3.14127	4.17431	2.55411	3.2146	3.16077	4.54559	4.04449	3.67836	2.0093	2.80338	3.54133	2.69586	0.369262716	0.893596186	16	4	24	0.187982158	0.768040178	24	8.613250535	0.235206751
ENSMUSG00000097488	4732487G21Rik	RIKEN cDNA 4732487G21 gene [Source:MGI Symbol;Acc:MGI:2441820]	0.750416	0.745361	0.933045	0.90431	0.714654	0.838601	0.816529	0.748177	0.790078	0.954199	0.580058	0.764366	0.711515	0.805124	0.344645202	0.86981408	16	4	24	0.295881656	0.768040178	24	8.976109382	0.048690786
ENSMUSG00000097494	4933406C10Rik	RIKEN cDNA 4933406C10 gene [Source:MGI Symbol;Acc:MGI:1921326]	0.368905	0.830493	0.602344	0.870971	0.882892	0.595444	0.837534	0.597194	1.00803	0.884891	0.656403	0.637374	0.496345	0.461559	0.057224139	0.401309455	16	12	24	0.081495645	0.697102286	24	11.8926435	0.133600577
ENSMUSG00000097503	3110045C21Rik	RIKEN cDNA 3110045C21 gene [Source:MGI Symbol;Acc:MGI:1914553]	1.55217	2.0144	1.99936	1.45441	1.86513	1.46301	1.29636	1.35453	1.50734	1.26823	1.85581	1.63459	1.22331	1.43041	0.887027852	0.966018836	20	8	24	0.648335297	0.881219014	24	13.42497517	0.048272643
ENSMUSG00000097504	4930516B21Rik	RIKEN cDNA 4930516B21 gene [Source:MGI Symbol;Acc:MGI:1922364]	2.15381	2.62057	2.0918	2.6076	1.93255	2.1876	2.62947	2.20676	2.12935	2.54069	2.22473	2.32628	2.17521	2.20507	0.91241026	0.966018836	16	20	24	0.928288862	0.981449065	24	6.293634539	0.015583064
ENSMUSG00000097515	1700040D17Rik	RIKEN cDNA 1700040D17 gene [Source:MGI Symbol;Acc:MGI:1923852]	3.33103	4.42584	4.60574	4.31295	3.13518	4.10695	3.7741	3.36659	3.64027	4.40141	2.9127	3.73137	2.9474	4.47602	0.176965982	0.653256853	16	4	24	0.219589469	0.768040178	24	7.971298847	0.122926387
ENSMUSG00000097523	1700022N22Rik	RIKEN cDNA 1700022N22 gene [Source:MGI Symbol;Acc:MGI:1916681]	0.352695	0.953596	1.07407	0.667091	0.604732	0.802446	0.515247	0.539728	0.752737	0.839224	0.456808	1.0146	0.764252	0.600741	0.759899911	0.966018836	12	16	24	0.649111129	0.881796509	24	9.65157014	0.06982877
ENSMUSG00000097527	1700112J16Rik	RIKEN cDNA 1700112J16 gene [Source:MGI Symbol;Acc:MGI:1923878]	1.03506	1.08268	1.2705	0.984242	0.717834	0.877167	1.30486	0.826586	1.17639	1.24697	1.04176	1.05664	0.874245	0.74426	0.954584434	0.966018836	12	20	24	0.713671561	0.905681809	24	9.132568535	0.046028171
ENSMUSG00000097530	Kansl2-ps	KAT8 regulatory NSL complex subunit 2, pseudogene [Source:MGI Symbol;Acc:MGI:3648525]	17.5054	15.1833	17.1842	17.9295	14.8457	18.186	17.6502	16.723	14.3544	18.1815	16.617	17.253	17.1422	17.2492	0.390686537	0.915479136	24	12	24	0.65846042	0.884596426	24	20.31056894	0.038862745
ENSMUSG00000097535	Gm26592	predicted gene, 26592 [Source:MGI Symbol;Acc:MGI:5477086]	1.34563	0.969366	1.30013	1.10871	1.06262	1.19499	1.20738	1.09981	1.28916	1.32059	1.14814	1.2618	1.16258	1.11812	0.791413662	0.966018836	12	20	24	0.952665271	0.988490277	24	14.95078474	0.009120262
ENSMUSG00000097536	2610037D02Rik	RIKEN cDNA 2610037D02 gene [Source:MGI Symbol;Acc:MGI:1917290]	0.92846	1.32974	0.975899	1.1169	1.23484	0.875117	1.03041	0.859765	1.36238	0.710221	1.08475	1.03642	1.29349	1.35217	0.924770496	0.966018836	12	12	24	0.652689361	0.883085974	24	4.795756623	0.053211595
ENSMUSG00000097537	2610020C07Rik	RIKEN cDNA 2610020C07 gene [Source:MGI Symbol;Acc:MGI:1917168]	0.513866	0.700679	0.440898	0.907113	0.59961	0.492773	0.485056	0.871778	0.754012	0.37119	0.822091	0.799454	0.658085	0.552488	0.880631923	0.966018836	20	8	24	0.949863632	0.988075055	24	11.90314689	0.019587174
ENSMUSG00000097545	Mir124a-1hg	Mir124-1 host gene (non-protein coding) [Source:MGI Symbol;Acc:MGI:2442197]	27.4546	28.3798	26.5124	28.0862	25.6985	24.4324	26.3913	24.6515	29.5775	24.1708	28.1772	25.9385	26.6876	28.6271	0.496745696	0.966018836	12	12	24	0.451992037	0.805393068	24	6.596940397	0.045207165
ENSMUSG00000097547	B230110C06Rik	RIKEN cDNA B230110C06 gene [Source:MGI Symbol;Acc:MGI:1925231]	4.16445	3.23756	2.84885	2.85001	3.48736	2.73567	3.00303	2.48111	2.76738	2.80772	3.84314	2.98443	2.73843	3.59896	0.92721845	0.966018836	20	16	24	0.594174475	0.857257137	24	19.86750893	0.069116172
ENSMUSG00000097571	Jpx	Jpx transcript, Xist activator (non-protein coding) [Source:MGI Symbol;Acc:MGI:2180008]	2.24917	2.38747	2.79647	2.36233	2.47564	2.37147	2.05409	2.9115	2.78849	2.06666	2.11054	1.86437	2.41981	2.30429	0.220555749	0.710961211	12	12	24	0.070139244	0.675025526	24	6.965642276	0.10788798
ENSMUSG00000097572	Gm26797	predicted gene, 26797 [Source:MGI Symbol;Acc:MGI:5477291]	0.362268	0.536267	0.860222	0.442803	0.637508	0.438649	0.886987	0.442026	0.6238	0.368513	0.385234	0.491667	0.464096	0.61744	0.933421033	0.966018836	12	4	24	0.545951072	0.833198211	24	5.347240344	0.066827066
ENSMUSG00000097574	C920006O11Rik	RIKEN cDNA C920006O11 gene [Source:MGI Symbol;Acc:MGI:2443759]	2.1051	2.86602	2.29029	2.10168	2.02268	1.97493	2.19307	1.9445	3.0911	2.7196	2.09134	2.34298	2.07879	2.46113	0.277221318	0.789849983	12	12	24	0.102515393	0.713699364	24	7.83470665	0.121533442
ENSMUSG00000097576	D930030I03Rik	RIKEN cDNA D930030I03 gene [Source:MGI Symbol;Acc:MGI:2444245]	0.524504	0.479705	0.969533	0.721362	0.353097	0.357045	0.959688	0.375557	0.358115	0.346546	0.612814	0.467779	0.489297	0.352886	0.894678274	0.966018836	4	12	24	0.98863555	0.997561007	24	4.004788757	0.011124327
ENSMUSG00000097577	6230400D17Rik	RIKEN cDNA 6230400D17 gene [Source:MGI Symbol;Acc:MGI:1923383]	1.82118	2.70739	2.43278	2.67443	2.2358	2.09264	2.24429	2.46704	2.56253	2.91576	2.5579	2.09683	2.50034	2.61487	0.018923071	0.256345714	16	8	24	0.003629985	0.426426863	24	9.272932118	0.135440094
ENSMUSG00000097585	E230029C05Rik	RIKEN cDNA E230029C05 gene [Source:MGI Symbol;Acc:MGI:2442580]	1.06353	1.40203	1.21491	1.31446	1.41692	1.28883	0.953262	0.748854	1.49888	1.17113	1.31913	1.34434	1.2993	0.987897	0.316183628	0.829266891	20	8	24	0.169854557	0.767957732	24	14.73161497	0.099886403
ENSMUSG00000097587	4930578M01Rik	RIKEN cDNA 4930578M01 gene [Source:MGI Symbol;Acc:MGI:1923201]	0.699452	1.07622	0.741974	1.18983	0.656371	0.676492	0.879283	0.86699	0.914997	0.734935	0.959928	1.01321	0.827887	1.17805	0.822170541	0.966018836	8	12	24	0.444854377	0.805393068	24	6.773193199	0.067217652
ENSMUSG00000097589	Dleu2	deleted in lymphocytic leukemia, 2 [Source:MGI Symbol;Acc:MGI:1934030]	6.65239	6.73804	6.18288	7.09185	8.69832	5.40945	8.25879	6.78079	7.84482	5.22212	7.95655	5.7673	7.24372	5.39461	0.951846915	0.966018836	20	12	24	0.897923699	0.970959341	24	17.28489326	0.039351341
ENSMUSG00000097596	Gm26673	predicted gene, 26673 [Source:MGI Symbol;Acc:MGI:5477167]	1.36425	0.889012	1.12123	1.08894	0.86674	0.961354	0.848233	1.00066	0.804085	1.55723	1.14452	1.22783	1.19745	0.980586	0.904831057	0.966018836	16	20	24	0.724687705	0.909665611	24	15.83716715	0.046113532
ENSMUSG00000097601	Gm26660	predicted gene, 26660 [Source:MGI Symbol;Acc:MGI:5477154]	0.96983	1.23142	1.08548	0.969885	1.04021	0.893798	0.868309	1.11518	1.28586	1.21122	1.02032	1.13376	1.22588	0.995485	0.57656008	0.966018836	12	16	24	0.206804548	0.768040178	24	7.979150305	0.06262788
ENSMUSG00000097610	A930012L18Rik	RIKEN cDNA A930012L18 gene [Source:MGI Symbol;Acc:MGI:2442887]	1.39266	1.23786	1.82356	1.35619	1.30279	1.14779	0.997362	0.639632	1.07117	1.24017	0.978183	0.499464	0.733984	1.70156	0.602156008	0.966018836	12	12	24	0.25210247	0.768040178	24	9.195962888	0.153027632
ENSMUSG00000097613	Gm17597	predicted gene, 17597 [Source:MGI Symbol;Acc:MGI:4937231]	0.291962	0.678542	0.635114	0.766423	0.750264	0.498678	0.10182	0.37378	0.684027	0.523519	0.401941	0.531348	0.333134	0.466266	0.004021228	0.140598674	16	12	24	0.02546378	0.586541342	24	11.54664902	0.177182985
ENSMUSG00000097616	1110019D14Rik	RIKEN cDNA 1110019D14 gene [Source:MGI Symbol;Acc:MGI:1923561]	3.46508	3.16773	3.03933	3.59859	2.67479	3.48856	3.27772	3.31776	3.26926	3.03582	4.02204	2.61903	3.84122	3.16962	0.833406028	0.966018836	4	20	24	0.934639404	0.983690057	24	0.528914189	0.01964762
ENSMUSG00000097617	Gm10687	predicted gene 10687 [Source:MGI Symbol;Acc:MGI:3642689]	2.13969	2.38894	1.88006	2.72298	2.36027	2.0555	2.44296	2.29507	2.45412	2.57063	2.13791	2.10149	2.55756	2.63932	0.285027487	0.793714531	16	8	24	0.507591523	0.812800027	24	7.930632009	0.049468781
ENSMUSG00000097626	4921504A21Rik	RIKEN cDNA 4921504A21 gene [Source:MGI Symbol;Acc:MGI:1918082]	1.37131	1.55142	1.24072	2.34348	2.10094	1.68032	1.81055	1.13069	1.96123	2.1061	1.48218	0.99018	1.4577	1.6745	0.184814212	0.662239322	16	16	24	0.162403395	0.76636332	24	12.39690767	0.15502682
ENSMUSG00000097633	Gm16617	predicted gene, 16617 [Source:MGI Symbol;Acc:MGI:4439541]	1.33152	1.73277	0.942978	1.61042	2.31936	1.1033	2.18171	2.15441	1.7572	1.2516	1.71319	1.30136	1.70212	1.45051	0.920858634	0.966018836	8	16	24	0.930404654	0.982155977	24	0.214218233	0.035195751
ENSMUSG00000097636	Mirt1	myocardial infarction associated transcript 1 [Source:MGI Symbol;Acc:MGI:1922001]	0.263227	0.741993	0.96889	0.647424	0.628257	0.709263	0.407321	0.56047	0.443894	0.705459	0.647857	0.423286	0.798311	0.538091	0.909604019	0.966018836	16	12	24	0.373153726	0.794388449	24	10.80154948	0.092862051
ENSMUSG00000097640	Gm20033	predicted gene, 20033 [Source:MGI Symbol;Acc:MGI:5012218]	1.24209	0.818186	1.2702	1.46945	0.771769	1.07686	0.423218	0.854607	1.69292	1.20931	1.03448	1.11395	1.22195	1.43638	0.235187365	0.73629392	12	8	24	0.211471793	0.768040178	24	9.651689168	0.159748889
ENSMUSG00000097649	Gm10561	predicted gene 10561 [Source:MGI Symbol;Acc:MGI:3648163]	1.1081	1.37438	1.0561	0.910601	0.749878	1.09695	1.89987	1.33771	0.936608	0.684129	0.90141	1.08674	1.07105	1.22059	7.83E-04	0.071344007	8	8	24	0.003383805	0.417612737	24	1.428281661	0.210015732
ENSMUSG00000097656	Gm26712	predicted gene, 26712 [Source:MGI Symbol;Acc:MGI:5477206]	15.4769	13.1837	15.6809	6.40157	13.9621	15.4539	10.5047	15.8896	12.2852	6.27171	7.66206	4.8065	14.4976	6.31232	0.496745696	0.966018836	8	8	24	0.261302762	0.768040178	24	1.651883298	0.294239889
ENSMUSG00000097657	Gm7389	predicted gene 7389 [Source:MGI Symbol;Acc:MGI:3645153]	2.90917	2.34017	2.77065	2.4844	2.69558	2.90897	2.88105	2.52404	2.52006	2.68965	2.73364	2.42743	2.46187	2.57104	0.95032543	0.966018836	4	4	24	0.384227336	0.799546792	24	19.03587441	0.04014514
ENSMUSG00000097666	A330094K24Rik	RIKEN cDNA A330094K24 gene [Source:MGI Symbol;Acc:MGI:1924343]	0.789283	1.18592	1.05375	0.948655	0.995998	0.979976	1.18278	1.38347	1.03013	1.42193	0.988566	1.02751	1.67053	1.33467	0.227302972	0.723724673	8	16	24	0.145377627	0.754184974	24	4.837590186	0.093106232
ENSMUSG00000097675	1700101I11Rik	RIKEN cDNA 1700101I11 gene [Source:MGI Symbol;Acc:MGI:1915180]	1.73526	1.54733	1.07888	1.71657	1.34884	1.23333	1.28789	1.11427	1.99873	1.29478	1.21378	1.25684	1.38406	1.63593	0.800065124	0.966018836	16	4	24	0.625439985	0.872249537	24	6.684047132	0.062191963
ENSMUSG00000097680	Gm26642	predicted gene, 26642 [Source:MGI Symbol;Acc:MGI:5477136]	0.630595	0.515727	0.614373	0.49583	0.664471	0.438287	0.431684	0.534578	0.726533	0.430319	0.591301	0.556169	0.645803	0.582977	0.919571723	0.966018836	12	12	24	0.827645202	0.946180429	24	7.192166972	0.022062946
ENSMUSG00000097697	4833412C05Rik	RIKEN cDNA 4833412C05 gene [Source:MGI Symbol;Acc:MGI:1921154]	0.910666	0.891415	0.488336	0.528844	0.786982	0.640917	0.504775	0.875921	0.613868	0.618108	0.700093	0.557896	0.665244	0.60955	0.894563866	0.966018836	8	4	24	0.517503286	0.818310252	24	0.144318731	0.052635718
ENSMUSG00000097705	Gm26740	predicted gene, 26740 [Source:MGI Symbol;Acc:MGI:5477234]	0.37677	1.06516	1.29671	0.900726	1.15052	0.827231	1.12439	1.09251	1.9777	1.26994	0.479612	0.847673	1.18968	0.817813	0.22872448	0.72665454	12	12	24	0.115455122	0.727799063	24	8.249822668	0.219681618
ENSMUSG00000097709	2810429I04Rik	RIKEN cDNA 2810429I04 gene [Source:MGI Symbol;Acc:MGI:1924187]	0.956496	1.89742	2.66885	1.179	1.50159	1.40207	2.7483	1.58417	1.84024	1.37957	1.32399	1.33544	2.12238	1.861	0.207677584	0.698704663	12	4	24	0.188912752	0.768040178	24	4.70389534	0.188842591
ENSMUSG00000097715	Gpr137b-ps	G protein-coupled receptor 137B, pseudogene [Source:MGI Symbol;Acc:MGI:3710533]	16.8513	14.0144	13.4609	13.5369	16.873	15.8635	17.8386	13.4595	14.2887	13.5418	15.0493	15.1687	15.8157	13.2122	0.003683467	0.140598674	4	4	24	0.002459938	0.39630666	24	20.57469092	0.153699406
ENSMUSG00000097717	1700066J03Rik	RIKEN cDNA 1700066J03 gene [Source:MGI Symbol;Acc:MGI:1914589]	0.864384	0.505801	1.10789	0.491563	1.05655	0.0797231	0.528618	0.651187	0.683091	0.670913	0.877808	0.747713	0.485287	0.778004	0.777666236	0.966018836	20	4	24	0.520680165	0.82006084	24	10.14444092	0.112311196
ENSMUSG00000097729	2310015A10Rik	RIKEN cDNA 2310015A10 gene [Source:MGI Symbol;Acc:MGI:1916798]	9.96615	13.3493	13.064	11.5956	12.4234	12.4309	11.5697	13.7878	14.6002	14.7874	11.5871	13.4257	14.2122	13.9979	0.622132989	0.966018836	12	8	24	0.060950576	0.660449324	24	7.788784046	0.109201975
ENSMUSG00000097736	9530059O14Rik	RIKEN cDNA 9530059O14 gene [Source:MGI Symbol;Acc:MGI:2442421]	1.98777	1.21357	1.4218	1.09944	1.40083	1.3266	1.02686	1.25057	1.11741	0.884335	1.50852	1.29236	1.2827	1.33595	0.369262716	0.893596186	20	20	24	0.280085704	0.768040178	24	21.85697249	0.094839717
ENSMUSG00000097743	Gm16973	predicted gene, 16973 [Source:MGI Symbol;Acc:MGI:4439897]	3.47212	4.49481	4.09161	4.16651	3.77689	3.71752	3.29206	3.94644	4.95449	3.5207	4.13089	4.0069	3.79707	4.40967	0.054126506	0.395890765	12	16	24	0.088693673	0.707747429	24	8.259689061	0.105854042
ENSMUSG00000097745	AI115009	expressed sequence AI115009 [Source:MGI Symbol;Acc:MGI:2139623]	1.40666	1.88501	1.3011	2.13402	1.49869	1.52454	1.31782	2.18983	2.55086	1.64132	1.61782	2.05549	1.58864	1.63392	0.297978647	0.80820842	16	12	24	0.361651746	0.786932131	24	8.822045736	0.106617289
ENSMUSG00000097749	Gm16938	predicted gene, 16938 [Source:MGI Symbol;Acc:MGI:4439862]	0.385447	1.34511	0.417691	1.2584	0.619979	0.387039	0.233315	1.12958	0.651566	1.12896	1.17065	0.751589	1.04628	0.382312	0.47028201	0.966018836	16	12	24	0.40760492	0.799546792	24	11.3206675	0.168611959

ENSMUSG00000097750	Gm4673	predicted gene 4673 [Source:MGI Symbol;Acc:MGI:3782854]	1.21751	1.88766	1.75954	1.329	1.33902	1.2226	1.49674	1.3004	1.57978	1.31892	1.60757	1.66242	1.84925	1.53904	0.914502381	0.966018836	12	4	24	0.483412685	0.805393068	24	4.875270662	0.060564259
ENSMUSG00000097767	Miat	myocardial infarction associated transcript (non-protein coding) [Source:MGI Symbol;Acc:MGI:2444886]	113.914	116.399	115.203	124.095	128.346	107.768	105.349	106.955	122.693	107.502	130.506	113.74	118.94	115.655	0.175272072	0.651010555	20	4	24	0.205624208	0.768040178	24	13.54304389	0.065082181
ENSMUSG00000097768	2310043M15Rik	RIKEN cDNA 2310043M15 gene [Source:MGI Symbol;Acc:MGI:1919180]	2.25951	2.02435	2.1327	2.34108	2.11592	2.43034	1.75946	2.16976	2.59123	2.09242	2.62824	2.34239	1.90126	1.62888	0.162825822	0.624932115	24	4	24	0.111485001	0.72392485	24	14.87948756	0.109948475
ENSMUSG00000097772	5430416N02Rik	RIKEN cDNA 5430416N02 gene [Source:MGI Symbol;Acc:MGI:1918676]	3.93552	5.19453	4.56012	3.14073	3.59913	3.75612	3.65188	3.82399	4.05961	3.37917	3.7803	3.52778	5.04216	3.78956	0.018857365	0.256345714	12	4	24	0.098496377	0.71175955	24	3.115108308	0.129586115
ENSMUSG00000097777	Gm16794	predicted gene, 16794 [Source:MGI Symbol;Acc:MGI:4439718]	1.29701	1.60097	1.42266	1.28379	1.40584	1.34555	1.22409	1.40442	1.59837	1.20711	1.80596	1.39495	1.31873	1.41103	0.341022016	0.86129475	12	16	24	0.786801492	0.931447579	24	10.50404866	0.02699043
ENSMUSG00000097784	A430105J06Rik	RIKEN cDNA A430105J06 gene [Source:MGI Symbol;Acc:MGI:1924439]	0.499549	0.689558	0.73751	0.410238	0.776446	0.467045	0.435699	0.736003	0.829405	0.471409	0.516199	0.567728	0.545904	0.743326	0.175808942	0.651752074	12	4	24	0.156055622	0.761695095	24	6.770144483	0.096765563
ENSMUSG00000097785	B230217O12Rik	RIKEN cDNA B230217O12 gene [Source:MGI Symbol;Acc:MGI:2444948]	8.97521	8.2651	8.34075	7.83706	7.39935	8.70673	7.72342	8.66288	7.33735	7.7659	8.36568	7.39164	8.21844	8.98818	0.481530854	0.966018836	8	4	24	0.216508569	0.768040178	24	1.897162007	0.059409907
ENSMUSG00000097787	2700046G09Rik	RIKEN cDNA 2700046G09 gene [Source:MGI Symbol;Acc:MGI:1914438]	1.12178	1.27741	1.51123	0.799673	1.02381	0.97446	0.913677	1.41476	1.48009	1.1114	1.01326	1.23934	0.958491	1.38747	0.038093618	0.334069441	12	4	24	0.037165234	0.609176161	24	6.178299065	0.141792129
ENSMUSG00000097788	Gm16596	predicted gene, 16596 [Source:MGI Symbol;Acc:MGI:4439520]	0.529456	0.502911	0.513007	0.731531	1.09311	1.41568	0.916859	0.690904	0.381359	0.38311	0.521221	0.3807	0.6845	0.291195	0.369326376	0.893596186	20	16	24	0.089882041	0.7097999	24	19.30832431	0.201592393
ENSMUSG00000097789	Gm2115	predicted gene 2115 [Source:MGI Symbol;Acc:MGI:3780284]	13.1525	9.88176	9.56916	9.55007	9.70653	11.5603	12.1508	10.5412	7.24801	11.8305	9.61027	11.1691	11.3377	10.8899	0.002004172	0.100255953	4	8	24	0.040669555	0.616921492	24	22.02545984	0.177477188
ENSMUSG00000097792	Gm27042	predicted gene, 27042 [Source:MGI Symbol;Acc:MGI:5504157]	2.46332	2.20229	2.35586	2.34097	2.35525	2.75179	2.28087	1.63648	1.85729	1.57122	2.74761	1.97507	1.8376	1.8679	0.374467935	0.89742288	20	12	24	0.05447258	0.655263328	24	18.46423743	0.135868289
ENSMUSG00000097797	Gm26901	predicted gene, 26901 [Source:MGI Symbol;Acc:MGI:5477395]	0.817462	0.509459	0.813396	0.540013	0.693597	0.749466	0.517509	1.20943	0.479509	0.560042	0.970421	0.809663	1.03458	0.946894	0.636884864	0.966018836	8	4	24	0.4806316	0.805393068	24	23.86271056	0.080386845
ENSMUSG00000097820	E530011L22Rik	RIKEN cDNA E530011L22 gene [Source:MGI Symbol;Acc:MGI:2443770]	1.70146	2.22708	2.09276	2.49111	2.28446	2.00996	2.1202	2.69422	2.191	2.34161	2.63165	1.97322	2.00408	2.04448	0.034679785	0.328311403	20	4	24	0.099710127	0.71175955	24	11.33308232	0.096759368
ENSMUSG00000097823	Gm16701	predicted gene, 16701 [Source:MGI Symbol;Acc:MGI:4439625]	3.8983	3.84681	3.94378	3.89103	3.67301	3.46875	3.98711	3.33732	3.49149	4.07861	3.79495	4.12201	4.00444	4.10176	0.887862353	0.966018836	4	16	24	0.978635387	0.994660123	24	21.19761173	0.006511085
ENSMUSG00000097835	Gm26910	predicted gene, 26910 [Source:MGI Symbol;Acc:MGI:5477404]	0.968581	0.907512	0.809685	1.10594	1.11008	0.757606	0.544566	1.13066	1.26958	0.590893	0.66364	1.28736	1.04497	0.952854	0.768363194	0.966018836	8	20	24	0.893094164	0.96974576	24	5.563053338	0.035841292
ENSMUSG00000097840	Gm26756	predicted gene, 26756 [Source:MGI Symbol;Acc:MGI:5477250]	0.912085	1.39158	1.29751	1.41176	1.15831	0.838573	1.02808	1.10119	1.43903	1.32085	1.08757	0.803669	1.23666	1.0699	5.26E-04	0.064393065	12	12	24	0.002487216	0.39630666	24	9.011572085	0.161904076
ENSMUSG00000097842	9330104G04Rik	RIKEN cDNA 9330104G04 gene [Source:MGI Symbol;Acc:MGI:2445112]	1.81827	1.58317	1.67815	1.50411	1.80533	1.88551	2.12156	1.60678	1.75393	1.82266	1.33697	1.57609	1.73271	1.84156	0.962348911	0.967745676	4	16	24	0.383945674	0.799546792	24	0.594996373	0.055012716
ENSMUSG00000097863	1010001B22Rik	RIKEN cDNA 1010001B22 gene [Source:MGI Symbol;Acc:MGI:1922653]	0.468907	0.950933	0.713049	0.320158	0.417691	1.05572	1.02697	0.835247	0.702092	0.929928	0.640848	0.361075	1.06691	1.06409	0.170143858	0.643496587	4	16	24	0.123973605	0.737493648	24	3.236976837	0.169750083
ENSMUSG00000097876	Gm16892	predicted gene, 16892 [Source:MGI Symbol;Acc:MGI:4439816]	1.58386	2.43911	2.30263	2.3782	2.59534	1.62667	1.97648	2.01827	2.66387	1.47976	2.16469	2.32448	2.34687	1.86961	0.891105762	0.966018836	12	16	24	0.703324798	0.901975795	24	9.985652908	0.062392431
ENSMUSG00000097877	Gm26703	predicted gene, 26703 [Source:MGI Symbol;Acc:MGI:5477197]	0.25857	0.841243	1.62679	0.701668	0.355487	0.659691	0.609025	0.455427	1.18692	0.415014	0.411683	0.752491	0.303749	0.939459	0.031969981	0.324385159	12	16	24	0.105178329	0.717252017	24	7.742904777	0.246425106
ENSMUSG00000097885	5031434O11Rik	RIKEN cDNA 5031434O11 gene [Source:MGI Symbol;Acc:MGI:1923230]	1.80092	2.12705	2.49357	2.29672	2.02439	1.48113	2.26769	1.47243	2.46365	1.93628	1.73274	1.96783	1.99933	2.00677	0.077301512	0.453790923	12	12	24	0.215148506	0.768040178	24	8.702808897	0.106452136
ENSMUSG00000097906	Gm9625	predicted gene 9625 [Source:MGI Symbol;Acc:MGI:3780033]	36.4846	42.5379	39.3468	42.414	45.8919	39.3039	37.1634	43.3112	48.9728	35.6436	49.6137	36.0696	38.347	34.9425	0.446653834	0.966018836	20	8	24	0.307940727	0.768040178	24	12.34023018	0.096171648
ENSMUSG00000097908	4933404O12Rik	RIKEN cDNA 4933404O12 gene [Source:MGI Symbol;Acc:MGI:1914002]	1.39143	1.30005	1.356	1.28214	1.28212	1.45271	1.4076	1.51567	1.28207	1.57804	1.13321	1.37877	1.3469	1.39433	0.481530854	0.966018836	24	20	24	0.576106167	0.848424552	24	2.887527775	0.027901522
ENSMUSG00000097915	A330009N23Rik	RIKEN cDNA A330009N23 gene [Source:MGI Symbol;Acc:MGI:2443491]	1.01629	0.93038	1.16655	1.1061	0.855442	0.608899	1.08988	1.47621	0.971058	0.971328	0.984859	1.30981	0.915729	1.34311	0.708665292	0.966018836	8	12	24	0.188960001	0.768040178	24	5.73471282	0.107137106
ENSMUSG00000097923	Gm26577	predicted gene, 26577 [Source:MGI Symbol;Acc:MGI:5477071]	0.781683	0.665013	0.594604	0.70041	0.887412	0.750607	0.815043	0.619531	0.49615	0.670636	0.580624	0.66534	0.458179	0.718159	0.591351901	0.966018836	24	12	24	0.252277258	0.768040178	24	19.1910153	0.061980991
ENSMUSG00000097924	A730020E08Rik	RIKEN cDNA A730020E08 gene [Source:MGI Symbol;Acc:MGI:2444999]	1.64277	1.49745	1.23487	1.36143	1.36068	1.60945	1.0808	0.915094	1.17302	1.27679	1.64785	1.17358	1.00369	1.05256	0.173121348	0.648600527	20	12	24	0.054511201	0.655263328	24	16.96183258	0.114285995
ENSMUSG00000097929	Tunar	Tcl1 upstream neural differentiation associated RNA [Source:MGI Symbol;Acc:MGI:1917202]	0.277016	0.668311	1.42867	0.6736	0.482111	0.706827	0.981198	0.422149	1.04383	0.585318	0.393357	0.786206	0.308697	0.854285	0.321662653	0.83607295	12	8	24	0.302783579	0.768040178	24	8.143746184	0.164101742
ENSMUSG00000097934	6720483E21Rik	RIKEN cDNA 6720483E21 gene [Source:MGI Symbol;Acc:MGI:1924991]	0.497878	0.717174	0.541528	0.714776	0.684159	0.542462	0.405148	0.446193	0.708799	0.575587	0.742523	0.581396	0.441837	0.556547	0.008810267	0.192760771	20	8	24	0.030553319	0.597890721	24	12.69344034	0.094947942
ENSMUSG00000097944	A130014A01Rik	RIKEN cDNA A130014A01 gene [Source:MGI Symbol;Acc:MGI:3028043]	1.09992	1.35304	1.17712	1.16278	1.3524	1.32932	1.72543	1.43328	1.45576	1.25911	1.48114	1.44316	1.21721	1.2622	0.874054778	0.966018836	20	20	24	0.788168727	0.931998618	24	21.37728708	0.027282138
ENSMUSG00000097960	A330074K22Rik	RIKEN cDNA A330074K22 gene [Source:MGI Symbol;Acc:MGI:3045392]	2.24677	1.9842	2.51942	2.19904	1.61485	2.66356	2.5385	2.29482	2.051	3.04626	1.4225	2.26886	1.90972	2.64533	0.829428469	0.966018836	16	4	24	0.55568442	0.838702296	24	5.124081641	0.086756708
ENSMUSG00000097961	Gm27000	predicted gene, 27000 [Source:MGI Symbol;Acc:MGI:5504115]	62.1695	63.0674	61.0283	58.5727	62.999	54.9142	56.5154	55.3399	67.5792	56.5663	67.3961	60.6466	62.8233	62.8362	0.942429894	0.966018836	12	12	24	0.818118056	0.942317891	24	9.910169296	0.024802415
ENSMUSG00000097967	Gm27002	predicted gene, 27002 [Source:MGI Symbol;Acc:MGI:5504117]	2.09653	1.06331	0.759207	1.69181	0.843984	1.08303	0.744322	1.86866	0.654318	0.981626	1.01579	0.616067	1.46051	1.25321	0.665691261	0.966018836	8	4	24	0.57680648	0.848735805	24	1.848036145	0.119806318
ENSMUSG00000097970	Gm27028	predicted gene, 27028 [Source:MGI Symbol;Acc:MGI:5504143]	0.852434	0.688701	0.739344	0.191608	0.874184	0.378731	0.413185	0.752906	0.69255	0.564007	0.922876	0.552595	0.229921	0.526431	0.941579937	0.966018836	20	4	24	0.922169385	0.979411448	24	10.97812995	0.035279894
ENSMUSG00000097975	Gm10605	predicted gene 10605 [Source:MGI Symbol;Acc:MGI:3642596]	2.23548	2.16438	1.68562	2.16244	1.61755	2.29877	2.01747	2.23257	1.76243	2.39158	1.77459	2.03822	2.48187	2.19561	0.361555248	0.885260695	4	16	24	0.304170163	0.768040178	24	0.82916481	0.075216581
ENSMUSG00000097979	Gm4691	predicted gene 4691 [Source:MGI Symbol;Acc:MGI:3782871]	0.768332	0.386043	0.716026	0.653573	0.947185	0.53911	0.335894	0.540818	0.263203	0.436736	0.377219	0.489765	0.58459	0.655286	0.68642356	0.966018836	4	8	24	0.77947969	0.929129631	24	18.14426426	0.024339877
ENSMUSG00000097993	Ptprv	protein tyrosine phosphatase, receptor type, V [Source:MGI Symbol;Acc:MGI:108027]	0.174222	0.33421	0.60843	0.371836	0.455973	0.731276	0.198229	0.699808	0.553269	0.909357	0.653583	0.576682	0.743598	0.312925	0.945544484	0.966018836	24	4	24	0.268453285	0.768040178	24	13.03935349	0.119412936
ENSMUSG00000097994	Gm26982	predicted gene, 26982 [Source:MGI Symbol;Acc:MGI:5504097]	2.85291	2.86017	3.32167	3.46201	2.37988	2.6503	2.7959	2.41308	2.71863	2.38899	2.95997	2.63569	2.9682	3.20287	0.688644847	0.966018836	12	8	24	0.624265686	0.871960321	24	6.856275582	0.05038576
ENSMUSG00000098021	Gm9522	predicted gene 9522 [Source:MGI Symbol;Acc:MGI:3779931]	0.937879	0.873673	0.113313	0.477841	0.701586	0.147797	0.317793	0.512998	0.569216	0.378683	1.32503	0.598285	0.451333	0.467365	0.873171835	0.966018836	20	20	24	0.798195409	0.935424343	24	18.24341371	0.07991103
ENSMUSG00000098022	Zfp82	zinc finger protein 82 [Source:MGI Symbol;Acc:MGI:1890753]	2.77013	2.9231	2.36101	3.09304	2.3677	2.30913	3.28777	2.50491	3.03486	1.99439	2.48914	2.15144	2.03585	2.4766	0.896545154	0.966018836	4	20	24	0.545069921	0.832646172	24	4.967309357	0.063613136
ENSMUSG00000098024	Gm27003	predicted gene, 27003 [Source:MGI Symbol;Acc:MGI:5504118]	0.839706	1.21465	0.911463	1.22308	2.21567	0.873554	0.874422	0.745622	1.62968	0.555272	1.22526	0.849812	1.69032	1.37104	0.897425765	0.966018836	20	4	24	0.920879948	0.979223729	24	13.41325824	0.046275241
ENSMUSG00000098050	Gm5345	predicted gene 5345 [Source:MGI Symbol;Acc:MGI:3644320]	0.466865	0.616949	0.304636	1.07141	0.664228	0.556776	0.341811	0.543581	0.446463	0.370127	0.417459	0.654698	0.683988	0.522651	0.91724565	0.966018836	24	12	24	0.753215242	0.919977034	24	16.798268	0.05244979
ENSMUSG00000098051	Gm27032	predicted gene, 27032 [Source:MGI Symbol;Acc:MGI:5504147]	0.374984	0.826103	0.72508	0.909174	0.499652	0.521048	0.915418	0.560082	0.711779	0.688114	0.68828	0.690019	0.499172	0.962588	0.446069941	0.966018836	8	20	24	0.238276929	0.768040178	24	8.232768441	0.102130211
ENSMUSG00000098062	Gm26931	predicted gene, 26931 [Source:MGI Symbol;Acc:MGI:5504046]	2.14876	1.31667	0.875333	1.32547	1.33025	1.26086	1.52551	1.43604	1.76767	0.769377	1.71407	1.14836	1.01812	0.947005	0.895733497	0.966018836	20	20	24	0.518000426	0.818483315	24	21.60669833	0.101201925
ENSMUSG00000098066	Gm26944	predicted gene, 26944 [Source:MGI Symbol;Acc:MGI:5504059]	0.35306	0.904501	0.64578	0.591192	1.23008	0.765995	0.930368	0.302825	0.888509	0.561272	0.533104	0.502519	0.34064	0.576633	0.885059765	0.966018836	12	20	24	0.595424671	0.857839743	24	14.05595572	0.086261472
ENSMUSG00000098076	Gm6981	predicted pseudogene 6981 [Source:MGI Symbol;Acc:MGI:3646966]	1.26954	1.17099	0.97156	0.917151	0.844963	1.14813	0.68849	1.01066	0.867161	1.49109	1.35828	1.109	1.06466	1.5442	0.847846021	0.966018836	8	4	24	0.977365701	0.994257299	24	9.535810813	0.014438406
ENSMUSG00000098087	Gm17750	predicted gene, 17750 [Source:MGI Symbol;Acc:MGI:5009828]	0.32804	1.27401	0.712336	0.372294	0.485641	1.16603	1.19633	0.486059	0.520333	0.650686	0.745634	0.421917	0.219877	0.91382	0.897975397	0.966018836	8	20	24	0.855172216	0.956910697	24	2.356786658	0.063218758
ENSMUSG00000098090	2700099C18Rik	RIKEN cDNA 2700099C18 gene [Source:MGI Symbol;Acc:MGI:1924272]	1.09353	2.06467	1.75738	2.10511	2.02748	2.19991	1.39228	1.43309	2.12238	1.50491	2.17016	1.82763	2.04752	2.34196	0.773177465	0.966018836	8	8	24	0.502357103	0.809556861	24	12.4215231	0.085905929
ENSMUSG00000098098	Bvht	braveheart long non-coding RNA [Source:MGI Symbol;Acc:MGI:5434104]	6.35224	8.08529	7.84953	8.81028	8.94697	8.02218	8.01789	8.09568	7.5967	6.48903	8.14318	8.29078	8.3124	8.45683	0.94663657	0.966018836	20	16	24	0.909348816	0.974606327	24	16.60520983	0.021998861
ENSMUSG00000098104	Gm6085	predicted gene 6085 [Source:MGI Symbol;Acc:MGI:3646770]	1.48662	1.35815	1.35336	1.78063	1.2989	1.29625	1.33409	1.44298	1.57702	1.58314	1.06671	1.19624	1.37155	1.1269	0.056268883	0.401074772	16	4	24	0.260625202	0.768040178	24	9.259623634	0.067631224
ENSMUSG00000098112	Bin2	bridging integrator 2 [Source:MGI Symbol;Acc:MGI:3611448]	0.655601	1.21791	1.77725	1.35063	0.780957	1.35376	1.35478	1.10249	1.49952	0.97683	1.06481	0.913324	1.60394	1.05166	0.616047374	0.966018836	12	8	24	0.360052813	0.785733735	24	6.986546512	0.122060913
ENSMUSG00000098120	Gm5914	predicted gene 5914 [Source:MGI Symbol;Acc:MGI:3779534]	2.89375	2.5193	1.36346	2.71059	1.04703	1.2348	1.34865	2.49225	3.10748	3.20233	3.14153	2.70679	1.71395	2.21737	0.784278077	0.966018836	16	8	24	0.491764772	0.805393068	24	9.144374264	0.176604855
ENSMUSG00000098132	Rassf10	Ras association (RalGDS/AF-6) domain family (N-terminal) member 10 [Source:MGI Symbol;Acc:MGI:1925998]	0.807241	0.930889	0.839928	0.863502	0.912619	0.82796	0.856475	0.928867	0.723646	0.838806	1.00104	0.97134	0.83496	0.842189	0.772122688	0.966018836	20	16	24	0.390783824	0.799546792	24	18.20770109	0.030640601
ENSMUSG00000098134	Rnf113a2	ring finger protein 113A2 [Source:MGI Symbol;Acc:MGI:1913631]	5.29933	5.45704	5.44695	5.49771	5.85818	5.77603	5.20146	6.30017	4.64649	4.85336	6.2066	5.62545	5.49114	4.66413	0.097779482	0.501112642	20	16	24	0.214083061	0.768040178	24	18.80970717	0.077734817
ENSMUSG00000098146	Gm26935	predicted gene, 26935 [Source:MGI Symbol;Acc:MGI:5504050]	0.224912	1.42394	0.854645	1.40316	1.24795	0.883137	1.09345	1.39917	1.75788	0.892285	1.30309	1.0129	1.28183	1.19715	0.472013662	0.966018836	8	16	24	0.256773918	0.768040178	24	9.32135852	0.169743088
ENSMUSG00000098158	Gm4804	predicted gene 4804 [Source:MGI Symbol;Acc:MGI:3646648]	1.66615	3.34898	1.91001	2.6255	3.367	2.34759	3.00016	2.57428	3.39371	2.15613	2.28366	2.44445	3.29746	3.22907	0.912686312	0.966018836	8	8	24	0.697637625	0.899395227	24	4.92428295	0.077056098
ENSMUSG00000098176	Ccdc166	coiled-coil domain containing 166 [Source:MGI Symbol;Acc:MGI:1925902]	1.41496	1.34347	1.3894	1.21385	1.32489	1.37911	1.56056	1.47476	1.73604	1.19609	1.36307	1.54527	1.35222	1.34422	0.271126962	0.780905461	4	12	24	0.397831275	0.799546792	24	1.826215821	0.043237559
ENSMUSG00000098183	Gm27010	predicted gene, 27010 [Source:MGI Symbol;Acc:MGI:5504125]	0.62508	0.750536	0.864986	0.911227	1.06181	0.644182	0.415795	0.50835	1.20235	0.93869	1.1319	0.798028	0.947068	0.637999	0.028612069	0.316608413	20	8	24	0.015951782	0.544099157	24	12.79187182	0.181356672
ENSMUSG00000098188	Sowahc	sosondowah ankyrin repeat domain family member C [Source:MGI Symbol;Acc:MGI:3606051]	7.44555	7.05652	6.80914	7.82847	7.38935	6.54783	8.22135	7.26702	6.48725	7.15418	7.73185	6.788	6.70686	7.03318	0.961261938	0.967043964	16	20	24	0.778738664	0.928873919	24	17.48922019	0.025192496
ENSMUSG00000098198	Gm9169	predicted gene 9169 [Source:MGI Symbol;Acc:MGI:3648561]	13.516	12.4452	11.7639	11.8131	12.7093	13.8524	10.365	10.0158	12.1798	11.9861	12.219	12.7584	10.9984	9.04094	0.015994567	0.258568924	24	8	24	0.012631421	0.526249719	24	17.51599447	0.132836429
ENSMUSG00000098221	Gm27030	predicted gene, 27030 [Source:MGI Symbol;Acc:MGI:5504145]	0.266569	0.539456	0.289254	0.753317	0.294727	0.744853	0.97086	0.35453	0.72362	0.528904	0.543607	1.1179	0.716421	1.52883	0.911685024	0.966018836	24	12	24	0.661685131	0.885564505	24	1.074338044	0.080060975
ENSMUSG00000098222	Gm8318	predicted gene 8318 [Source:MGI Symbol;Acc:MGI:3646694]	0.392695	0.170993	0.304972	0.0642642	0.419343	0.19081	0.13683	0.451691	1.07217	1.07495	1.11754	0.644522	1.15926	0.79764	0.771575322	0.966018836	20	12	24	0.564062283	0.842960348	24	10.64499101	0.104772054
ENSMUSG00000098234	Snhg6	small nucleolar RNA host gene 6 [Source:MGI Symbol;Acc:MGI:1921074]	27.3836	30.5997	27.3687	26.5481	27.9399	25.6822	24.8315	29.4584	34.9931	29.0692	35.477	32.3646	35.4215	33.4584	0.849180783	0.966018836	8	20	24	0.605877176	0.862870954	24	7.205386061	0.052209911
ENSMUSG00000098274	Rpl24	ribosomal protein L24 [Source:MGI Symbol;Acc:MGI:1915443]	2.15421	3.64822	2.10353	1.20002	2.20698	2.09971	2.89741	2.87582	3.07378	2.00166	2.75665	1.48819	2.05984	1.88032	0.745118544	0.966018836	8	8	24	0.351249372	0.780345856	24	3.713454427	0.159525029
ENSMUSG00000098318	Lockd	lncRNA downstream of Cdkn1b [Source:MGI Symbol;Acc:MGI:1915081]	0.522591	1.09127	0.972757	0.921198	0.754851	0.894406	0.322371	1.00227	1.11995	0.417546	0.741682	0.537399	1.17667	0.634547	0.451149628	0.966018836	12	16	24	0.471311041	0.805393068	24	7.214007254	0.114337915
ENSMUSG00000098332	Pigbos1	Pigb opposite strand 1 [Source:MGI Symbol;Acc:MGI:1913614]	17.0122	17.9971	13.8615	17.9669	14.3533	13.4631	16.9045	14.0434	18.0662	15.6502	18.9373	17.8607	17.2119	15.0344	0.807813307	0.966018836	20	12	24	0.97114365	0.993012791	24	17.1147171	0.015680347
ENSMUSG00000098374	Gm28043	predicted gene, 28043 [Source:MGI Symbol;Acc:MGI:5547779]	2.29482	2.96605	2.71303	2.78592	2.9076	2.42318	3.35521	2.29841	2.65047	2.33717	2.78932	3.01098	2.84366	2.9262	0.914360142	0.966018836	24	12	24	0.848593858	0.954502769	24	22.16798993	0.028058112
ENSMUSG00000098404	Mrip-ps	Mom radiation induced polyposis, pseudogene [Source:MGI Symbol;Acc:MGI:3645947]	30.8494	25.9443	28.9838	27.9795	33.837	30.0158	34.1383	32.1946	28.8552	33.2699	30.5955	28.3891	29.0406	28.6731	0.943890799	0.966018836	4	4	24	0.548697757	0.834686108	24	17.30095291	0.050172593
ENSMUSG00000098424	Gm27202	predicted gene 27202 [Source:MGI Symbol;Acc:MGI:5521045]	5.62223	4.47423	4.35963	4.94378	5.2607	4.43903	5.71696	5.09718	3.58656	3.33824	5.16018	4.28285	4.15525	4.80159	0.521072381	0.966018836	8	8	24	0.179562768	0.768040178	24	21.88399594	0.125668715
ENSMUSG00000098449	Gm7467	predicted gene 7467 [Source:MGI Symbol;Acc:MGI:3648350]	0.489498	0.662225	0.531856	0.465184	0.485569	0.644355	0.596033	0.800416	0.500187	0.593153	0.446744	0.731661	0.436217	0.491987	0.787283372	0.966018836	8	12	24	0.613250574	0.866078878	24	1.058230988	0.039524368
ENSMUSG00000098488	Pla2g4b	phospholipase A2, group IVB (cytosolic) [Source:MGI Symbol;Acc:MGI:2384819]	5.47	5.78414	3.72761	5.35962	7.96189	5.40571	7.0441	4.95569	6.26058	4.33867	6.85565	5.46744	5.96055	4.8769	0.223535653	0.717349224	20	20	24	0.199145699	0.768040178	24	19.27143761	0.170456525
ENSMUSG00000098506	Phf20-ps	PHD finger protein 20, pseudogene [Source:MGI Symbol;Acc:MGI:5010233]	1.33723	1.19883	1.12326	1.35871	1.1935	1.24579	1.19067	1.08467	1.04511	1.19985	1.44616	1.38344	1.38921	1.26844	0.123511911	0.552144108	20	24	24	0.068008451	0.671988997	24	19.02460768	0.066300073
ENSMUSG00000098557	Kctd12	potassium channel tetramerisation domain containing 12 [Source:MGI Symbol;Acc:MGI:2145823]	4.93106	6.47877	7.72007	7.05543	6.34696	5.57064	7.11505	5.34325	7.55738	5.79893	6.55701	5.4557	4.1556	5.65863	0.033248781	0.324908923	12	16	24	0.057969234	0.656248849	24	10.64879275	0.176406346
ENSMUSG00000098620	Gm27209	predicted gene 27209 [Source:MGI Symbol;Acc:MGI:5521052]	1.51486	1.15182	1.64713	1.79798	1.61826	1.56476	1.5837	1.21032	1.73591	1.25113	1.82155	1.58802	1.6586	1.5787	0.113148904	0.530777648	20	12	24	0.576903897	0.848735805	24	15.78987239	0.042042574
ENSMUSG00000098650	Commd1b	COMM domain containing 1B [Source:MGI Symbol;Acc:MGI:5547784]	19.4443	19.2116	20.3702	19.7133	19.4252	19.44	18.3357	18.777	18.4366	19.0048	18.7111	18.2772	17.1146	20.2026	0.618239704	0.966018836	12	16	24	0.334316455	0.770442964	24	10.0632978	0.032178728
ENSMUSG00000098659	1110015O18Rik	RIKEN cDNA 1110015O18 gene [Source:MGI Symbol;Acc:MGI:1915766]	0.65904	1.9427	2.4887	1.16788	1.88902	1.13594	1.67406	1.66531	2.56284	1.38739	1.08106	1.4141	1.33062	1.53108	0.077301512	0.453790923	12	12	24	0.086501829	0.705702571	24	7.894317631	0.250888906
ENSMUSG00000098702	1500015A07Rik	RIKEN cDNA 1500015A07 gene [Source:MGI Symbol;Acc:MGI:1916232]	1.85623	2.46248	1.95053	1.79712	2.00023	1.50053	1.70677	2.15265	2.31971	2.03765	2.11133	1.71555	1.9176	2.24074	0.008389425	0.192760771	8	16	24	0.02576374	0.586541342	24	7.316980705	0.125014321
ENSMUSG00000098739	Gm27151	predicted gene 27151 [Source:MGI Symbol;Acc:MGI:5520994]	2.68061	2.3144	2.09636	0.882494	1.68708	2.45775	2.25698	1.34808	1.50479	1.35638	1.85049	2.28995	2.15751	2.01092	0.008012038	0.192760771	24	16	24	0.007038482	0.477130707	24	22.94016769	0.283069411
ENSMUSG00000098789	Jmjd7	jumonji domain containing 7 [Source:MGI Symbol;Acc:MGI:3845785]	2.93045	3.38815	2.6987	2.50213	4.5792	3.28059	2.96073	2.9359	3.17557	3.23283	2.67957	3.12652	3.11939	2.91289	0.935194469	0.966018836	4	12	24	0.577893141	0.849052597	24	18.434614	0.068135387
ENSMUSG00000098900	Gm18190	predicted gene, 18190 [Source:MGI Symbol;Acc:MGI:5010375]	0.345131	0.317178	0.754155	0.300733	0.428585	0.390318	1.46582	0.408195	0.893432	0.666045	1.05553	0.427414	1.03818	0.695714	0.881996584	0.966018836	24	12	24	0.902858809	0.97295148	24	3.995465907	0.046680216
ENSMUSG00000098905	Zfp953	zinc finger protein 953 [Source:MGI Symbol;Acc:MGI:3612873]	0.679796	0.717636	0.801608	0.557636	0.82067	0.627172	0.77821	0.614369	0.921035	0.62462	0.704337	0.725616	0.702089	0.670695	0.93717134	0.966018836	12	4	24	0.916737479	0.977104487	24	6.673422702	0.014039246
ENSMUSG00000098912	1500004A13Rik	RIKEN cDNA 1500004A13 gene [Source:MGI Symbol;Acc:MGI:2442808]	34.6782	30.8233	31.8899	33.8259	33.6622	35.1595	31.8485	31.743	30.2218	37.2344	33.7531	32.2596	33.5633	34.7916	0.175185919	0.651010555	16	20	24	0.292173034	0.768040178	24	16.60929767	0.047872952
ENSMUSG00000098923	Tmem185b	transmembrane protein 185B [Source:MGI Symbol;Acc:MGI:1917634]	3.97024	3.40738	3.59412	3.36427	3.61904	3.82484	4.03676	3.66315	3.24509	4.08606	3.82346	3.5788	4.19492	3.64383	0.035806379	0.331814682	4	8	24	0.172806451	0.768040178	24	21.33439257	0.061819746
ENSMUSG00000098950	Gm28036	predicted gene, 28036 [Source:MGI Symbol;Acc:MGI:5547772]	2.16434	1.20134	2.16416	2.60215	2.05125	2.2441	1.86854	2.66995	2.22367	1.91947	2.52176	1.91876	2.16606	1.54711	0.441501717	0.966018836	16	16	24	0.306562829	0.768040178	24	17.14105028	0.134471819
ENSMUSG00000098985	Gm27219	predicted gene 27219 [Source:MGI Symbol;Acc:MGI:5521062]	5.36624	7.02022	7.77673	6.36936	7.64453	6.88647	6.28774	6.207	6.47405	6.68338	7.26504	7.52253	6.52076	5.37382	0.088274239	0.473025915	20	8	24	0.126927581	0.739868076	24	14.99093774	0.107714454
ENSMUSG00000099034	2810039B14Rik	RIKEN cDNA 2810039B14 gene [Source:MGI Symbol;Acc:MGI:1919915]	2.0877	3.13971	3.23233	2.32068	2.69084	2.15198	2.91758	2.30966	2.66145	2.65878	2.35517	2.32688	1.98434	2.84552	0.287849332	0.793714531	12	16	24	0.135587344	0.747707672	24	7.918578978	0.119570875
ENSMUSG00000099041	Gm28035	predicted gene, 28035 [Source:MGI Symbol;Acc:MGI:5547771]	0.557171	0.538555	0.655923	0.626349	0.600915	0.591038	0.529755	0.474964	0.651523	0.488765	0.930783	0.483689	0.648417	0.34107	0.119293142	0.542554768	12	20	24	0.197041385	0.768040178	24	15.27111571	0.079907665
ENSMUSG00000099061	Gm28050	predicted gene, 28050 [Source:MGI Symbol;Acc:MGI:5547786]	2.44375	2.64583	3.65243	2.42433	2.48228	1.86041	1.84848	1.75972	2.92341	2.2236	2.6563	2.87999	2.47389	2.93206	0.920620634	0.966018836	24	16	24	0.36212326	0.787054939	24	9.306278238	0.119549325
ENSMUSG00000099083	Atf7	activating transcription factor 7 [Source:MGI Symbol;Acc:MGI:2443472]	15.7497	15.4461	11.8329	16.5064	10.6423	14.8548	17.8453	15.1666	13.4495	15.0914	14.2014	14.373	15.0756	12.895	0.217827365	0.704989686	4	16	24	0.429453503	0.805393068	24	23.96388062	0.091740939
ENSMUSG00000099137	Gm10603	predicted gene 10603 [Source:MGI Symbol;Acc:MGI:3642592]	1.99574	1.93083	2.09899	2.09894	2.79211	1.95426	2.15776	1.92464	2.20464	2.26843	2.17299	2.17763	2.11152	1.48486	0.017373137	0.255326033	16	16	24	0.141333663	0.751766185	24	14.2525291	0.119633297
ENSMUSG00000099338	2810030D12Rik	RIKEN cDNA 2810030D12 gene [Source:MGI Symbol;Acc:MGI:1925585]	0.54417	0.744754	0.542494	0.540322	0.455247	0.571843	0.279994	0.577876	0.663538	0.449377	1.0782	0.664859	0.845435	0.672741	0.767183862	0.966018836	12	8	24	0.994889747	0.998755922	24	13.5752941	0.006297663
ENSMUSG00000099342	Gm18180	predicted gene, 18180 [Source:MGI Symbol;Acc:MGI:5010365]	0.240133	0.519167	0.424554	0.518174	0.586214	0.581419	0.47964	0.484874	0.554311	0.594796	0.641526	0.744435	0.618594	0.646564	0.625825704	0.966018836	4	8	24	0.158329337	0.764753215	24	14.97748572	0.054368425
ENSMUSG00000099343	Gm28836	predicted gene 28836 [Source:MGI Symbol;Acc:MGI:5579542]	0.5944	0.473786	0.553525	0.677452	1.13644	0.574495	0.516873	0.605651	0.462649	0.673527	0.929542	0.598636	0.510961	0.961055	0.123511911	0.552144108	20	8	24	0.188933085	0.768040178	24	15.09057691	0.11102468
ENSMUSG00000099375	Gm28187	predicted gene 28187 [Source:MGI Symbol;Acc:MGI:5578893]	0.864042	0.705683	0.787245	0.648658	0.665344	0.596406	0.613983	0.613843	0.605178	0.570643	0.873982	0.778374	0.880737	0.759083	0.702630284	0.966018836	4	12	24	0.530239079	0.824795587	24	22.51262918	0.042043358
ENSMUSG00000099377	Gm6159	predicted gene 6159 [Source:MGI Symbol;Acc:MGI:3644323]	2.99574	3.39744	3.17689	2.80721	3.05379	3.09792	3.03989	2.57595	2.74451	2.96794	2.32942	3.40426	3.00273	3.40882	0.954584434	0.966018836	24	20	24	0.446805977	0.805393068	24	1.094141441	0.05696128
ENSMUSG00000099397	Gm7809	predicted gene 7809 [Source:MGI Symbol;Acc:MGI:3643362]	29.2391	28.5349	25.5735	24.3766	30.3729	25.834	27.092	25.3811	28.6641	24.9604	31.702	26.1749	28.9301	22.9421	0.256915694	0.76168256	20	20	24	0.360766506	0.786233489	24	18.97617618	0.073144471
ENSMUSG00000099478	Gm28370	predicted gene 28370 [Source:MGI Symbol;Acc:MGI:5579076]	3.35078	3.68831	3.52665	3.49871	2.98945	3.1196	3.41948	2.94697	3.6871	3.76656	3.55044	2.6725	2.93466	3.27777	0.101690661	0.511659635	16	8	24	0.059298001	0.658304792	24	9.284638517	0.096492842
ENSMUSG00000099481	Xndc1	Xrcc1 N-terminal domain containing 1 [Source:MGI Symbol;Acc:MGI:5546359]	7.315	8.99763	9.58386	9.56645	9.80146	9.02645	9.27326	8.88278	11.2795	8.70932	8.60351	9.27933	8.37955	9.0069	0.112800839	0.530777648	12	16	24	0.146352991	0.754936569	24	10.35206134	0.092801241
ENSMUSG00000099492	Gm5525	predicted gene 5525 [Source:MGI Symbol;Acc:MGI:3646042]	2.52057	2.14239	2.10349	2.1838	2.28042	2.4371	2.15037	1.82721	1.5581	1.68779	1.68909	1.51602	1.74532	1.51515	0.823657258	0.966018836	4	4	24	0.02188712	0.574400012	24	20.34192069	0.086332413
ENSMUSG00000099521	Gm28309	predicted gene 28309 [Source:MGI Symbol;Acc:MGI:5579015]	2.13636	3.24763	3.33514	4.50675	1.51367	3.85474	3.07873	1.12769	2.49545	3.55047	1.43416	4.3781	1.61525	2.94196	0.492524295	0.966018836	16	12	24	0.863846661	0.960530692	24	12.79982989	0.096371048
ENSMUSG00000099560	Mrgprx3-ps	MAS-related GPR, member X3, pseudogene [Source:MGI Symbol;Acc:MGI:2685506]	39.2316	31.7599	27.1985	29.5165	33.4409	31.3352	29.7404	29.5507	29.4102	25.8512	36.3717	30.8403	36.1779	29.3689	0.031295391	0.324385159	20	20	24	0.051255814	0.64758613	24	21.11002731	0.145939896
ENSMUSG00000099564	Gm28729	predicted gene 28729 [Source:MGI Symbol;Acc:MGI:5579435]	0.791844	0.761197	0.742025	0.583054	0.810785	0.727913	0.618251	0.671783	0.481711	0.552028	0.607263	0.824324	0.730514	0.619451	0.260025655	0.775510818	24	16	24	0.041773235	0.621198778	24	21.44944157	0.080409988
ENSMUSG00000099583	H3c4	H3 clustered histone 4 [Source:MGI Symbol;Acc:MGI:2448322]	0.767342	0.687525	0.278241	0.714065	0.548268	0.602943	0.446152	0.393195	0.374687	0.724124	0.855744	0.511676	0.729235	0.380692	0.056291566	0.401074772	16	20	24	0.162704256	0.766365035	24	17.92540379	0.122287755
ENSMUSG00000099631	Gm8641	predicted gene 8641 [Source:MGI Symbol;Acc:MGI:3647459]	0.720232	1.00597	0.978746	0.961898	0.806974	1.29195	0.658603	0.697707	0.737191	0.832845	0.535681	0.613283	0.640395	0.809368	0.388109084	0.915479136	16	4	24	0.830328117	0.947325117	24	10.31450623	0.033342714
ENSMUSG00000099632	2900093K20Rik	RIKEN cDNA 2900093K20 gene [Source:MGI Symbol;Acc:MGI:1915287]	3.16629	4.16262	3.65364	4.72485	4.18267	4.56247	4.45317	3.69902	4.17357	3.85942	4.12157	4.00302	4.77012	4.34741	0.911685024	0.966018836	16	4	24	0.839726167	0.95137802	24	16.65678516	0.028575455
ENSMUSG00000099634	Gm29462	predicted gene 29462 [Source:MGI Symbol;Acc:MGI:5580168]	14.1144	11.7448	13.1801	11.3294	12.4273	17.9821	13.9232	11.1975	13.8078	13.1737	9.62637	11.613	14.8897	11.4811	0.280850951	0.792139038	4	16	24	0.426168349	0.805393068	24	22.59322672	0.10578888
ENSMUSG00000099647	Gm5776	predicted gene 5776 [Source:MGI Symbol;Acc:MGI:3647650]	62.2104	70.914	68.7186	64.0449	67.1419	61.2661	72.0624	52.7575	59.1279	46.425	63.093	57.5557	64.2499	70.1538	0.823677655	0.966018836	4	20	24	0.49406715	0.806249091	24	0.647132376	0.077659803
ENSMUSG00000099662	Ppp1r2-ps2	protein phosphatase 1, regulatory (inhibitor) subunit 2, pseudogene 2 [Source:MGI Symbol;Acc:MGI:3647847]	46.8413	42.888	41.9802	43.3669	49.1641	54.6332	36.2328	40.4822	36.3361	45.1006	47.6692	44.8656	42.9614	37.3806	0.009067305	0.192760771	20	12	24	0.004712139	0.434006302	24	17.67862575	0.174935678
ENSMUSG00000099681	1700052K11Rik	RIKEN cDNA 1700052K11 gene [Source:MGI Symbol;Acc:MGI:1920681]	1.11754	1.17014	0.867333	0.973704	1.13999	1.33121	0.906182	1.27293	1.23627	1.36074	1.13544	1.36671	1.28088	1.23306	0.8972237	0.966018836	24	12	24	0.773873553	0.927334758	24	20.37188855	0.027929419

ENSMUSG00000099689	Zfp383	zinc finger protein 383 [Source:MGI Symbol;Acc:MGI:1920979]	4.01576	5.86435	6.09189	4.48078	4.97461	4.88996	5.46348	5.33272	5.56526	4.60932	4.73202	4.75621	4.65286	4.48285	0.503972024	0.966018836	12	4	24	0.299770712	0.768040178	24	6.801709618	0.084887435
ENSMUSG00000099696	2900052N01Rik	RIKEN cDNA 2900052N01 gene [Source:MGI Symbol;Acc:MGI:1920290]	12.4875	12.3571	10.7417	10.399	11.8854	12.0069	11.3128	10.7582	10.358	8.17778	13.1465	12.2574	13.2569	11.9845	0.016024075	0.253395174	4	12	24	0.029491927	0.596648309	24	22.15706998	0.15792213
ENSMUSG00000099757	BE692007	expressed sequence BE692007 [Source:MGI Symbol;Acc:MGI:3035348]	0.531112	0.519164	0.520714	0.520647	0.547138	0.485083	0.516862	0.785317	0.868132	0.451977	0.653241	0.550246	0.442698	0.573907	0.70641939	0.966018836	12	16	24	0.374032078	0.794557071	24	7.635069	0.060393908
ENSMUSG00000099759	1700030C10Rik	RIKEN cDNA 1700030C10 gene [Source:MGI Symbol;Acc:MGI:1916763]	2.34183	1.92256	2.19154	2.23589	1.95043	1.56371	1.50863	2.37537	1.44447	2.28525	1.9703	1.94146	1.94826	3.38774	0.833406028	0.966018836	8	16	24	0.468640165	0.805393068	24	6.643131109	0.105790728
ENSMUSG00000099764	Rps10-ps2	ribosomal protein S10, pseudogene 2 [Source:MGI Symbol;Acc:MGI:3645627]	168.331	159.783	168.74	124.308	133.336	115.248	152.317	179.913	222.469	148.232	194.662	176.145	190.948	179.437	0.767183862	0.966018836	12	4	24	0.146112594	0.754849922	24	5.158509961	0.173289125
ENSMUSG00000099779	Gm8228	predicted gene 8228 [Source:MGI Symbol;Acc:MGI:3646986]	5.36753	5.74218	5.65029	5.24364	5.25894	5.32586	5.39157	4.94575	5.79848	4.64056	5.10753	4.96984	5.0679	4.71239	0.935194469	0.966018836	12	4	24	0.768874638	0.925500646	24	4.62569046	0.018195316
ENSMUSG00000099835	Gm29668	predicted gene 29668 [Source:MGI Symbol;Acc:MGI:5580374]	0.529845	0.932311	0.573426	0.882358	1.72267	1.3064	1.59946	1.62476	1.07116	0.175426	0.887501	0.52054	0.900437	0.625996	0.912686312	0.966018836	8	8	24	0.648006368	0.881026758	24	22.17489299	0.13223446
ENSMUSG00000099843	Gm7160	predicted gene 7160 [Source:MGI Symbol;Acc:MGI:3646078]	1.54016	1.81674	1.47404	1.82478	2.21453	1.18471	1.32619	1.17824	2.17752	1.40266	2.9794	2.24647	1.8982	2.13059	0.722296281	0.966018836	20	8	24	0.397727065	0.799546792	24	14.4601817	0.122308488
ENSMUSG00000099858	Gm6652	predicted gene 6652 [Source:MGI Symbol;Acc:MGI:3647244]	15.3699	14.2238	15.1448	13.875	16.174	15.4513	13.895	16.9876	13.5228	17.9936	14.1015	14.871	14.1387	14.3677	0.844511766	0.966018836	24	12	24	0.781249551	0.929846332	24	14.17871114	0.031559
ENSMUSG00000099875	Rbm3-ps	RNA binding motif (RNP1, RRM) protein 3 [Source:MGI Symbol;Acc:MGI:3642874]	47.2902	72.9873	77.9816	76.7364	66.7819	59.5416	54.0259	61.0132	86.3656	68.6144	58.787	68.9876	64.2588	62.9722	0.004320961	0.140598674	12	16	24	0.013373525	0.529134128	24	9.919044171	0.228913595
ENSMUSG00000099876	Gm29650	predicted gene 29650 [Source:MGI Symbol;Acc:MGI:5580356]	8.32456	7.99816	7.2533	8.02068	6.66972	7.00319	8.29982	7.43611	7.92836	5.72861	9.24641	6.59871	9.00049	6.20056	0.591351901	0.966018836	4	12	24	0.605020242	0.862246789	24	23.42684063	0.070385523
ENSMUSG00000099881	2810013P06Rik	RIKEN cDNA 2810013P06 gene [Source:MGI Symbol;Acc:MGI:1914456]	7.58396	8.82294	9.62907	9.02116	8.15319	7.9362	8.72982	9.04944	11.0826	8.29485	9.55764	9.28799	9.20697	10.0164	0.182320384	0.659303873	12	16	24	0.026914446	0.586541342	24	7.811623184	0.110007834
ENSMUSG00000099891	Gm5575	predicted gene 5575 [Source:MGI Symbol;Acc:MGI:3647974]	0.17122	0.691636	0.928138	1.34688	0.877838	0.76063	0.829894	0.604871	0.927329	1.01959	0.926763	0.777029	1.03916	1.71856	0.274035549	0.788124063	16	8	24	0.08631637	0.705702571	24	10.27797297	0.189600063
ENSMUSG00000099902	Gm12115	predicted gene 12115 [Source:MGI Symbol;Acc:MGI:3652288]	7.63379	8.86479	7.20823	8.13917	9.15679	7.73133	7.54814	9.39251	6.99961	5.70376	6.43174	5.93475	7.41456	6.82492	0.916963526	0.966018836	8	4	24	0.509816556	0.813985618	24	2.075771543	0.071335085
ENSMUSG00000099913	Gm28551	predicted gene 28551 [Source:MGI Symbol;Acc:MGI:5579257]	0.380796	0.389327	0.780684	0.662318	0.613804	0.498783	0.603683	0.760192	0.617138	0.348119	0.584626	0.368543	0.515185	0.454007	0.245061943	0.741580974	12	12	24	0.467233026	0.805393068	24	9.250736272	0.066160989
ENSMUSG00000099966	2810402E24Rik	RIKEN cDNA 2810402E24 gene [Source:MGI Symbol;Acc:MGI:1913715]	0.75657	1.42175	1.76678	1.28604	1.43024	1.11628	1.27826	1.3482	1.92174	1.38986	1.21668	1.29167	1.55198	1.52567	0.041292272	0.346472636	12	12	24	0.020528068	0.56691777	24	8.450865765	0.16549139
ENSMUSG00000099997	Tubb4b-ps2	tubulin, beta 4B class IVB, pseudogene 2 [Source:MGI Symbol;Acc:MGI:3723458]	0.979914	0.996777	0.62492	0.738372	0.862352	1.28885	0.495173	1.09117	0.43898	0.848165	0.81493	0.666477	0.786522	0.804039	0.9045879	0.966018836	8	4	24	0.499691502	0.808791684	24	20.92864874	0.085556857
ENSMUSG00000099998	Gm6501	predicted gene 6501 [Source:MGI Symbol;Acc:MGI:3645570]	20.8035	18.3502	17.9702	19.0641	18.6406	18.3896	21.4469	17.2955	16.6909	17.0593	19.2683	18.7965	19.7033	19.2245	0.004320961	0.140598674	4	8	24	0.030725739	0.597890721	24	21.91063383	0.091865297
ENSMUSG00000100001	1810007D17Rik	RIKEN cDNA 1810007D17 gene [Source:MGI Symbol;Acc:MGI:1916305]	0.481586	0.523897	0.448658	0.393171	0.410445	0.855945	0.335346	0.553099	0.750614	0.591047	0.283053	0.527897	0.50513	0.671727	0.708665292	0.966018836	8	12	24	0.645900654	0.880062569	24	4.622071181	0.054270415
ENSMUSG00000100005	B130024G19Rik	RIKEN cDNA B130024G19 gene [Source:MGI Symbol;Acc:MGI:3604353]	2.11456	2.70639	2.13667	2.18646	3.29319	2.05189	2.24365	2.74196	3.41969	2.31908	2.2242	2.2535	2.23513	2.82451	0.579814065	0.966018836	8	16	24	0.349315507	0.779170188	24	8.061853321	0.102126209
ENSMUSG00000100009	Gm7967	predicted gene 7967 [Source:MGI Symbol;Acc:MGI:3644984]	1.39199	1.80587	1.84084	1.60503	0.982995	1.16697	1.28534	1.88093	1.70652	1.04647	1.10771	1.4471	1.94032	1.33563	0.048889741	0.379129806	8	12	24	0.021863607	0.574400012	24	4.74268133	0.18715822
ENSMUSG00000100017	2410022M11Rik	RIKEN cDNA 2410022M11 gene [Source:MGI Symbol;Acc:MGI:1917005]	0.543945	0.761243	0.764571	0.548473	0.511427	0.568926	0.443145	0.692151	0.961641	0.928068	0.782083	0.849466	1.06894	0.924006	0.945728192	0.966018836	12	8	24	0.112274334	0.726113335	24	7.362212405	0.097849083
ENSMUSG00000100039	Gm28959	predicted gene 28959 [Source:MGI Symbol;Acc:MGI:5579665]	7.03547	4.09823	5.71132	4.6617	1.61474	3.92548	3.56969	4.62806	3.73383	3.7614	3.57963	2.78266	3.34544	5.89937	0.173121348	0.648600527	8	12	24	0.08565192	0.703577714	24	4.817105194	0.316542708
ENSMUSG00000100078	Gm6170	predicted gene 6170 [Source:MGI Symbol;Acc:MGI:3643368]	1.35987	1.66335	1.23287	1.00567	1.45858	1.41969	1.50265	1.24908	1.59416	1.64679	1.42816	1.31818	1.57167	1.27552	0.801273005	0.966018836	4	20	24	0.852178952	0.955628938	24	23.71958618	0.025701529
ENSMUSG00000100104	Gm5644	predicted gene 5644 [Source:MGI Symbol;Acc:MGI:3646381]	51.541	62.9734	57.0808	61.4192	67.5652	55.7553	56.6062	52.4148	57.6071	39.5346	49.6664	46.0203	52.6566	49.3621	0.911685024	0.966018836	12	12	24	0.988124867	0.997530201	24	3.016282479	0.009829143
ENSMUSG00000100131	Gm28439	predicted gene 28439 [Source:MGI Symbol;Acc:MGI:5579145]	1.05987	1.13353	1.15236	1.21419	9.38249	1.29659	1.92045	0.69869	2.13228	1.58488	1.83936	1.53093	1.3014	1.54439	0.026012493	0.29944494	20	8	24	0.131100626	0.741427049	24	15.27770045	0.461674789
ENSMUSG00000100147	1700047M11Rik	RIKEN cDNA 1700047M11 gene [Source:MGI Symbol;Acc:MGI:1914580]	2.66502	5.53698	6.13828	4.93946	3.92647	3.76756	4.95006	3.98499	5.56707	3.95348	3.38213	3.7757	3.37	4.38883	0.008047769	0.192760771	12	12	24	0.03358504	0.606793499	24	8.094174139	0.250869017
ENSMUSG00000100153	Ppp1ccb	protein phosphatase 1 catalytic subunit gamma B [Source:MGI Symbol;Acc:MGI:3647492]	50.4826	43.9434	50.4815	47.1128	45.9902	48.5445	47.4018	45.7142	41.5377	46.7481	43.1287	45.8924	45.5303	47.0813	0.915082018	0.966018836	4	16	24	0.615981146	0.867388068	24	22.26249882	0.028292042
ENSMUSG00000100162	Gm20687	predicted gene 20687 [Source:MGI Symbol;Acc:MGI:5313134]	1.43144	2.34386	2.42008	2.11734	1.2759	1.29171	0.842975	1.33149	2.75875	1.9732	0.985649	1.92981	1.69524	2.03065	0.009730499	0.194720062	12	8	24	0.03224218	0.606585237	24	8.131275186	0.295833574
ENSMUSG00000100164	2610306M01Rik	RIKEN cDNA 2610306M01 gene [Source:MGI Symbol;Acc:MGI:1914420]	3.6605	4.43334	3.88274	3.94203	4.40039	4.08174	4.08103	3.8458	4.38389	3.88123	3.97956	4.11838	4.00197	4.51757	0.880247609	0.966018836	8	8	24	0.841879658	0.952050674	24	6.954752633	0.017308937
ENSMUSG00000100182	1810006J02Rik	RIKEN cDNA 1810006J02 gene [Source:MGI Symbol;Acc:MGI:1916280]	0.704132	0.666804	0.336579	0.239526	0.481517	0.531995	0.395733	0.835402	0.47518	0.639989	0.364643	0.524012	0.736954	0.492099	0.101491041	0.511659635	8	4	24	0.124976695	0.738693118	24	1.276432592	0.119432804
ENSMUSG00000100183	Gm28512	predicted gene 28512 [Source:MGI Symbol;Acc:MGI:5579218]	0.664828	0.877635	0.86717	0.684171	0.496003	0.60182	0.810319	0.950459	0.725484	0.834742	0.501629	0.934933	0.887296	0.74849	0.030972684	0.324150946	8	12	24	0.047141483	0.633507364	24	4.236169366	0.110806985
ENSMUSG00000100201	Gm29093	predicted gene 29093 [Source:MGI Symbol;Acc:MGI:5579799]	0.980901	1.32129	1.2409	1.2875	2.51768	1.27255	1.38719	0.577983	0.996572	1.29792	0.885746	0.952987	0.991487	1.00789	0.880992536	0.966018836	16	8	24	0.206405062	0.768040178	24	15.71114793	0.162370863
ENSMUSG00000100204	Gm4849	predicted gene 4849 [Source:MGI Symbol;Acc:MGI:3645366]	2.98475	2.18693	2.13107	2.76219	2.59001	3.22349	2.8582	2.69919	2.00415	3.08864	2.31037	2.49864	2.49954	1.91455	0.166457038	0.633636735	24	12	24	0.08989167	0.7097999	24	18.97155978	0.135234887
ENSMUSG00000100213	Gm28151	predicted gene 28151 [Source:MGI Symbol;Acc:MGI:5578857]	2.11854	3.8961	3.62016	2.8342	3.24604	3.03426	3.07682	4.19958	3.92673	3.46594	3.08624	3.79601	2.78913	2.97721	0.132887428	0.567728672	12	16	24	0.276034532	0.768040178	24	8.518450676	0.124132893
ENSMUSG00000100235	Gm28557	predicted gene 28557 [Source:MGI Symbol;Acc:MGI:5579263]	1.44891	1.82832	1.809	1.95754	1.70412	1.79243	1.67256	1.60754	1.9298	1.35913	2.63458	1.93677	2.04371	2.13962	0.801273005	0.966018836	20	8	24	0.855656581	0.957149435	24	14.78324267	0.030819123
ENSMUSG00000100252	Mir124-2hg	Mir124-2 host gene (non-protein coding) [Source:MGI Symbol;Acc:MGI:1917691]	1.76535	2.97445	4.84156	2.34507	2.39107	2.74502	3.01853	1.7947	3.28605	2.41401	1.76291	2.41783	2.17935	3.8147	0.185796491	0.662239322	12	8	24	0.20448579	0.768040178	24	7.091019319	0.21949222
ENSMUSG00000100261	Gm6473	predicted gene 6473 [Source:MGI Symbol;Acc:MGI:3645176]	1.09931	1.29147	0.783457	1.35051	1.05198	1.27225	0.786772	0.985614	0.932798	0.826355	0.887594	1.01358	0.958396	0.79018	0.937921962	0.966018836	24	12	24	0.500311564	0.808986978	24	18.82849712	0.056574007
ENSMUSG00000100281	4930422M22Rik	RIKEN cDNA 4930422M22 gene [Source:MGI Symbol;Acc:MGI:1921024]	0.607626	0.797646	0.770861	0.812857	0.644104	1.0057	0.555952	0.543961	1.03732	0.468363	0.765803	0.807355	0.539743	1.17283	0.924553956	0.966018836	12	16	24	0.827835306	0.946180429	24	8.124121892	0.043626676
ENSMUSG00000100301	6030407O03Rik	RIKEN cDNA 6030407O03 gene [Source:MGI Symbol;Acc:MGI:1924410]	1.41543	1.77946	1.29475	1.878	1.91477	1.15902	1.83233	1.01357	2.01531	1.0027	1.66191	1.7562	1.56064	1.21558	0.886572916	0.966018836	20	12	24	0.74930277	0.918501847	24	16.94059994	0.062634908
ENSMUSG00000100309	Gm6644	predicted gene 6644 [Source:MGI Symbol;Acc:MGI:3644951]	17.754	18.7819	16.9473	16.5804	22.3002	17.7504	19.6664	21.794	23.0313	17.3241	23.5005	18.037	20.3619	16.8478	0.91241026	0.966018836	20	20	24	0.925192511	0.980131499	24	16.73083838	0.026822807
ENSMUSG00000100319	Gm5256	predicted gene 5256 [Source:MGI Symbol;Acc:MGI:3644836]	3.95497	3.8769	2.90712	4.72236	3.41462	3.25273	3.19292	2.84068	2.81371	2.94445	3.09958	2.63087	2.63624	2.41746	0.86340968	0.966018836	16	20	24	0.325608608	0.768040178	24	15.85410458	0.073001171
ENSMUSG00000100337	Gm5066	predicted gene 5066 [Source:MGI Symbol;Acc:MGI:3645897]	2.53622	2.94429	2.69157	1.45157	2.34535	2.4969	2.94496	2.86176	2.38985	2.63353	3.1092	2.31399	2.77869	2.62418	0.325033326	0.842977406	8	8	24	0.265374558	0.768040178	24	1.353392473	0.100936283
ENSMUSG00000100347	Gm7895	predicted gene 7895 [Source:MGI Symbol;Acc:MGI:3648340]	13.3628	11.8303	11.4144	12.6891	11.8767	13.2095	12.8106	12.5072	11.4163	13.0075	11.5551	12.2127	13.155	13.2598	0.03452758	0.328311403	4	8	24	0.218823712	0.768040178	24	23.02461795	0.051942108
ENSMUSG00000100362	Gm1833	predicted gene 1833 [Source:MGI Symbol;Acc:MGI:3037691]	1.99743	1.79329	1.75313	2.01672	2.51706	1.86532	2.43286	2.74238	1.20414	1.47246	2.26268	1.67211	1.43699	1.9663	0.953350544	0.966018836	20	16	24	0.60781341	0.864036051	24	20.541923	0.086962134
ENSMUSG00000100397	Gm28071	predicted gene 28071 [Source:MGI Symbol;Acc:MGI:5578777]	16.0382	11.5309	13.814	12.6742	12.961	14.8827	11.9026	12.7615	10.7159	12.0375	12.5558	11.488	12.9063	14.1883	0.895493752	0.966018836	4	12	24	0.443265926	0.805393068	24	22.06720939	0.07214315
ENSMUSG00000100417	Gm28578	predicted gene 28578 [Source:MGI Symbol;Acc:MGI:5579284]	2.50211	1.40742	1.87766	2.33886	2.44439	2.03051	2.16152	1.62077	1.90464	1.7927	1.672	2.00869	2.10947	1.75061	0.056268883	0.401074772	4	4	24	0.185778918	0.768040178	24	18.596508	0.108770502
ENSMUSG00000100441	Gm7266	predicted gene 7266 [Source:MGI Symbol;Acc:MGI:3648608]	33.0769	41.3029	36.4049	35.8898	45.2022	38.4277	39.0826	32.3115	39.2841	24.7433	36.9086	29.1849	36.8596	32.6852	0.853736882	0.966018836	20	20	24	0.859868946	0.958681059	24	21.55827787	0.04529304
ENSMUSG00000100455	Gm29170	predicted gene 29170 [Source:MGI Symbol;Acc:MGI:5579876]	0.903553	1.44929	1.32778	0.965083	1.00458	1.17526	1.46403	1.06664	1.29193	1.0367	1.23354	1.1318	1.04163	1.2768	0.676344318	0.966018836	12	4	24	0.404126449	0.799546792	24	4.710277391	0.062227527
ENSMUSG00000100475	Gm29543	predicted gene 29543 [Source:MGI Symbol;Acc:MGI:5580249]	2.47158	3.24054	1.82765	2.32442	2.58973	2.2446	2.59325	2.12439	2.76817	2.38409	2.37912	2.86842	2.33788	2.03066	0.866861748	0.966018836	4	8	24	0.692454245	0.898111639	24	20.54010715	0.053162431
ENSMUSG00000100480	Gm29156	predicted gene 29156 [Source:MGI Symbol;Acc:MGI:5579862]	1.31159	0.724428	1.03541	0.945663	0.881038	0.868402	0.869187	1.16472	0.728976	1.57692	1.46159	0.537478	0.960005	0.817506	0.883024237	0.966018836	16	16	24	0.605706719	0.862773266	24	12.75094194	0.081897777
ENSMUSG00000100481	Gm7114	predicted gene 7114 [Source:MGI Symbol;Acc:MGI:3643085]	4.04539	3.81944	4.21641	3.90236	3.68323	4.21127	3.60308	4.44261	3.75193	4.84477	4.18685	3.82476	4.11258	3.79533	0.854540463	0.966018836	16	12	24	0.63142606	0.87436603	24	11.31878819	0.036227759
ENSMUSG00000100548	Gm29585	predicted gene 29585 [Source:MGI Symbol;Acc:MGI:5580291]	0.416747	0.729501	0.6562	0.674006	0.869411	0.661685	1.05117	1.19199	1.09655	1.13823	1.3632	0.708717	0.942541	0.978306	0.916860713	0.966018836	20	4	24	0.339800387	0.774150091	24	10.03486869	0.091704629
ENSMUSG00000100572	2410021H03Rik	RIKEN cDNA 2410021H03 gene [Source:MGI Symbol;Acc:MGI:1917000]	0.132525	0.773776	0.288096	1.28805	0.346054	0.337406	0.726882	0.414447	0.768263	0.175151	0.63634	1.05883	0.309523	1.04431	0.873567285	0.966018836	8	20	24	0.848980767	0.954680046	24	8.76926303	0.075182454
ENSMUSG00000100583	Gm29443	predicted gene 29443 [Source:MGI Symbol;Acc:MGI:5580149]	0.656661	0.578085	0.529643	1.09917	0.840944	0.908482	0.1998	0.868777	0.882252	0.650331	0.663635	1.16201	0.658244	0.376349	0.158050885	0.614628746	24	4	24	0.227495477	0.768040178	24	15.15767603	0.149064361
ENSMUSG00000100594	2810414N06Rik	RIKEN cDNA 2810414N06 gene [Source:MGI Symbol;Acc:MGI:1919065]	2.01901	2.13671	2.23197	2.50986	2.17597	2.0583	2.02832	2.07429	2.20458	1.8714	2.01989	2.17518	2.4686	2.32383	0.920858634	0.966018836	12	16	24	0.756235192	0.921259908	24	6.594940404	0.024780945
ENSMUSG00000100599	1700120C14Rik	RIKEN cDNA 1700120C14 gene [Source:MGI Symbol;Acc:MGI:1920850]	1.17667	1.74803	1.63785	3.13713	1.83974	2.22497	0.842532	2.49092	2.3678	3.60911	1.87396	2.12918	1.83354	1.75772	0.03484427	0.328311403	16	12	24	0.02381693	0.584955728	24	11.74501737	0.313766613
ENSMUSG00000100600	A230077H06Rik	RIKEN cDNA A230077H06 gene [Source:MGI Symbol;Acc:MGI:2443592]	0.597015	0.900907	0.907958	0.574089	0.818731	0.694261	1.04309	0.956462	0.886425	1.16743	0.601884	0.928281	0.436271	0.884955	0.90332508	0.966018836	8	20	24	0.464687233	0.805393068	24	7.873042336	0.084953111
ENSMUSG00000100629	Gm28192	predicted gene 28192 [Source:MGI Symbol;Acc:MGI:5578898]	2.20115	2.43262	2.04081	2.88791	2.65736	2.27167	1.89387	2.60362	2.73792	2.65243	3.28693	2.02726	2.63483	2.67118	0.06935957	0.433815306	20	4	24	0.114668777	0.727799063	24	11.81704009	0.11573701
ENSMUSG00000100642	Gm28230	predicted gene 28230 [Source:MGI Symbol;Acc:MGI:5578936]	2.9548	2.73248	2.71131	2.51355	2.68821	2.60718	2.91449	2.45355	2.93017	1.95929	3.3435	2.05343	2.95644	2.00821	0.47028201	0.966018836	4	12	24	0.745119188	0.916618581	24	21.20731137	0.049986849
ENSMUSG00000100665	Mrgprc1-ps	MAS-related GPR, member C1, pseudogene [Source:MGI Symbol;Acc:MGI:3033136]	1.7632	1.30558	1.09562	1.38642	1.01782	1.0292	0.614581	1.03729	1.13524	1.16493	1.38532	1.12449	1.00683	1.2044	0.914360142	0.966018836	16	12	24	0.817110672	0.941826666	24	11.88485536	0.042952845
ENSMUSG00000100680	1810044D09Rik	RIKEN cDNA 1810044D09 gene [Source:MGI Symbol;Acc:MGI:1917048]	2.23372	1.8216	1.88561	2.24781	2.47364	2.01442	1.80875	2.20281	2.35956	2.21574	2.44921	1.8392	2.74897	2.08256	0.446653834	0.966018836	20	12	24	0.613612008	0.866162311	24	14.17926213	0.047183833
ENSMUSG00000100691	2010320M18Rik	RIKEN cDNA 2010320M18 gene [Source:MGI Symbol;Acc:MGI:1919343]	6.99763	9.23749	8.73366	6.84358	8.12318	7.33406	6.87674	7.52893	9.62371	6.70191	8.85071	7.66388	7.85029	7.67667	0.591351901	0.966018836	12	4	24	0.531731035	0.82547989	24	7.989765563	0.069577463
ENSMUSG00000100701	Gm19587	predicted gene, 19587 [Source:MGI Symbol;Acc:MGI:5011772]	19.5813	17.9335	18.6477	17.959	18.6181	20.234	21.1363	18.8147	14.0992	17.8616	16.7904	14.4688	15.0128	14.4602	0.801995186	0.966018836	4	8	24	0.409165981	0.799546792	24	20.00068382	0.070820663
ENSMUSG00000100706	Gm19744	predicted gene, 19744 [Source:MGI Symbol;Acc:MGI:5011929]	2.29429	1.11976	1.04245	2.49222	1.56329	2.02962	1.6367	1.2446	1.39136	1.06201	1.37084	1.97736	1.5955	1.35218	0.065849436	0.422183965	24	12	24	0.086999983	0.706216588	24	19.6203069	0.205263409
ENSMUSG00000100755	Rps23-ps1	ribosomal protein S23, pseudogene 1 [Source:MGI Symbol;Acc:MGI:3705429]	208.805	255.814	246.47	248.15	238.401	220.292	242.565	232.42	295.142	197.613	255.654	263.214	267.299	273.673	0.904831057	0.966018836	12	4	24	0.488986405	0.805393068	24	5.93886667	0.06875561
ENSMUSG00000100774	Gm7329	predicted gene 7329 [Source:MGI Symbol;Acc:MGI:3646271]	36.1356	29.9688	36.5241	38.9066	39.3957	31.9354	30.1093	44.3037	29.5502	40.5544	38.5273	44.1451	43.6332	41.5798	0.852844413	0.966018836	24	12	24	0.844839773	0.953079218	24	15.55076969	0.041945579
ENSMUSG00000100783	2310047D07Rik	RIKEN cDNA 2310047D07 gene [Source:MGI Symbol;Acc:MGI:1916907]	1.1012	0.785193	0.779267	0.898807	0.870445	0.744881	0.714014	1.48197	1.51214	0.94689	0.959942	0.527421	0.526276	0.84867	0.250396044	0.751058594	12	12	24	0.182580139	0.768040178	24	8.298523276	0.152708435
ENSMUSG00000100801	Gm15459	predicted gene 15459 [Source:MGI Symbol;Acc:MGI:3705702]	414.047	326.645	314.745	335.325	347.576	372.725	365.708	336.354	277.841	330.482	366.808	341.21	361.007	352.163	7.57E-04	0.071344007	4	8	24	0.006232952	0.469371824	24	21.06186386	0.14449383
ENSMUSG00000100811	Gm29083	predicted gene 29083 [Source:MGI Symbol;Acc:MGI:5579789]	3.21509	3.97381	2.5828	3.03508	5.52395	2.88495	4.59896	2.55945	3.05	2.44805	3.83355	3.6653	4.10584	3.15063	0.142596848	0.585944911	20	20	24	0.182154748	0.768040178	24	20.13891336	0.194615531
ENSMUSG00000100812	A730098A19Rik	RIKEN cDNA A730098A19 gene [Source:MGI Symbol;Acc:MGI:2444037]	0.786673	0.892144	0.544645	0.671162	0.668422	0.569981	0.560667	0.593546	0.703384	0.939306	0.646591	0.910708	0.907953	1.05949	0.858514388	0.966018836	8	12	24	0.713713577	0.905681809	24	3.915775757	0.039907198
ENSMUSG00000100826	Snhg14	small nucleolar RNA host gene 14 [Source:MGI Symbol;Acc:MGI:1289201]	39.4412	42.9398	46.0888	41.8229	41.7222	35.1415	37.2651	32.0499	43.6809	33.9185	42.8788	35.56	32.7352	36.3705	0.210566453	0.69936851	12	12	24	0.190835215	0.768040178	24	11.25043846	0.098394878
ENSMUSG00000100837	1700063D05Rik	RIKEN cDNA 1700063D05 gene [Source:MGI Symbol;Acc:MGI:1925750]	0.318202	0.375136	0.959887	0.496943	0.423631	0.732139	0.966843	0.614719	0.754504	0.222195	0.63932	0.499933	0.223998	0.71737	0.895137379	0.966018836	12	4	24	0.809065187	0.939184036	24	6.157195597	0.061125732
ENSMUSG00000100839	Gm5920	predicted gene 5920 [Source:MGI Symbol;Acc:MGI:3645716]	0.576546	1.02503	0.814877	0.528221	0.904774	0.751497	0.632747	0.721978	0.590231	0.457147	1.02949	0.368076	0.615013	0.345313	0.905275015	0.966018836	20	20	24	0.834325073	0.949069766	24	16.45525648	0.040781934
ENSMUSG00000100862	Gm10925	predicted gene 10925 [Source:MGI Symbol;Acc:MGI:3809095]	1.4711	55.9081	3.87023	3.35773	58.8585	41.9661	56.7694	3.73096	3.42951	62.9938	2.59839	2.82546	4.96575	2.56501	0.820042705	0.966018836	16	16	24	0.725483978	0.90999235	24	17.62439467	0.610382142
ENSMUSG00000100863	Gm12669	predicted gene 12669 [Source:MGI Symbol;Acc:MGI:3651683]	127.883	136.032	127.481	123.777	132.468	119.585	116.618	122.482	131.749	107.417	138.446	122.951	136.019	127.173	0.890311217	0.966018836	20	4	24	0.906411923	0.97449058	24	1.524819661	0.017480946
ENSMUSG00000100865	Gm9320	predicted gene 9320 [Source:MGI Symbol;Acc:MGI:3645000]	0.810851	1.11993	0.842334	0.438985	1.14504	0.868148	0.628347	0.344256	0.360711	0.171111	0.70386	0.250263	0.695092	0.098823	0.943920575	0.966018836	20	20	24	0.204418869	0.768040178	24	20.11012308	0.163573937
ENSMUSG00000100872	1700065J18Rik	RIKEN cDNA 1700065J18 gene [Source:MGI Symbol;Acc:MGI:1920705]	0.435693	0.109744	0.23595	0.729703	0.633688	1.08108	0.790384	0.766308	0.441333	0.576802	0.733151	0.709977	0.885108	0.679937	0.077301512	0.453790923	24	12	24	0.068339889	0.672656585	24	19.16850034	0.179311846
ENSMUSG00000100891	2810049E08Rik	RIKEN cDNA 2810049E08 gene [Source:MGI Symbol;Acc:MGI:1919927]	0.126868	0.666464	0.87392	0.631318	0.443695	0.600901	0.412933	0.341059	0.924684	0.754305	0.5239	0.592664	0.450387	0.788053	0.002087679	0.100255953	12	16	24	0.021166136	0.571993737	24	9.761158308	0.198499375
ENSMUSG00000100937	Nscme3l	NSE3 homolog, SMC5-SMC6 complex component like [Source:MGI Symbol;Acc:MGI:1922805]	1.20389	1.60035	1.5107	1.48778	1.56038	1.18339	1.35606	1.20271	1.96103	1.19304	1.84733	1.35761	1.51087	1.17483	0.798829513	0.966018836	12	20	24	0.398741845	0.799546792	24	11.66684178	0.076845744
ENSMUSG00000100975	Gm28875	predicted gene 28875 [Source:MGI Symbol;Acc:MGI:5579581]	7.85341	7.65209	8.07966	9.8689	8.55862	7.50323	8.35972	7.14455	8.35575	8.2291	7.08613	7.95609	7.31062	6.10716	0.143673396	0.585944911	16	16	24	0.094778153	0.71175955	24	13.87981022	0.095974394
ENSMUSG00000101013	A630072M18Rik	RIKEN cDNA A630072M18 gene [Source:MGI Symbol;Acc:MGI:2444702]	0.755314	0.697674	0.801036	0.693692	0.392988	0.779978	0.387328	0.592147	0.503655	0.595187	0.627065	0.14812	0.391184	0.400652	0.885955579	0.966018836	12	16	24	0.439125997	0.805393068	24	9.168277319	0.073163714
ENSMUSG00000101037	Gm28424	predicted gene 28424 [Source:MGI Symbol;Acc:MGI:5579130]	0.549095	0.907609	0.970689	0.828777	0.592418	0.761877	0.670532	0.738772	1.00897	0.997446	0.969486	0.87348	0.616265	0.714641	0.001837158	0.100255953	12	16	24	0.031033556	0.59941366	24	10.56557105	0.1206051
ENSMUSG00000101054	Gm18406	predicted gene, 18406 [Source:MGI Symbol;Acc:MGI:5010591]	2.83739	3.19931	2.65493	3.5071	2.99206	1.77951	2.68762	2.17994	3.86663	2.60463	2.75247	2.77582	2.94912	2.7902	0.903207257	0.966018836	12	12	24	0.320487073	0.768040178	24	9.202420344	0.118790143
ENSMUSG00000101111	Gm28437	predicted gene 28437 [Source:MGI Symbol;Acc:MGI:5579143]	4.88024	12.7167	67.2193	8.15339	85.7904	10.0677	69.5056	112.201	14.4112	39.7174	11.5878	82.3539	105.302	117.041	0.885025617	0.966018836	8	8	24	0.597177482	0.858280068	24	3.742591175	0.539051284
ENSMUSG00000101122	Gm17971	predicted gene, 17971 [Source:MGI Symbol;Acc:MGI:5010156]	0.433299	0.943078	0.874781	0.637526	0.87819	0.560822	1.05647	1.21746	1.43589	0.736252	1.14713	0.67221	0.330296	0.881575	0.297978647	0.80820842	8	20	24	0.205517189	0.768040178	24	8.677024719	0.172055637
ENSMUSG00000101162	Gm26728	predicted gene, 26728 [Source:MGI Symbol;Acc:MGI:5477222]	0.31699	0.584518	0.529959	0.614956	0.394814	0.623897	0.439051	0.605177	0.549346	0.628772	0.538368	0.617801	0.551924	0.887707	0.850398351	0.966018836	16	12	24	0.212823699	0.768040178	24	7.710658455	0.068733531
ENSMUSG00000101174	Hoxd4	homeobox D4 [Source:MGI Symbol;Acc:MGI:96208]	3.5911	4.48487	5.30673	3.23756	3.43102	3.67963	4.36865	3.91107	4.77232	3.60903	3.9301	4.31538	3.94158	4.35539	0.013400636	0.233323991	12	4	24	0.108487723	0.72082227	24	5.23480713	0.125291103
ENSMUSG00000101188	Eif4a-ps4	eukaryotic translation initiation factor 4A, pseudogene 4 [Source:MGI Symbol;Acc:MGI:1195958]	178.011	177.04	183.08	185.436	184.764	171.952	165.941	163.72	180.437	157.65	181.252	166.128	172.318	170.065	0.335107163	0.85758369	20	12	24	0.502862297	0.809737567	24	13.11928182	0.029044823
ENSMUSG00000101225	1700008J07Rik	RIKEN cDNA 1700008J07 gene [Source:MGI Symbol;Acc:MGI:1916610]	0.327348	0.524457	0.52773	0.601393	0.552564	0.457701	0.688941	0.632598	0.691572	0.775026	0.564403	0.702354	0.600232	0.767537	0.92655264	0.966018836	16	12	24	0.073681605	0.682388921	24	9.059276163	0.068197676
ENSMUSG00000101249	Gm29216	predicted gene 29216 [Source:MGI Symbol;Acc:MGI:5579922]	27.4059	124.987	76.8121	64.0071	61.9832	57.9768	61.5533	7.13686	10.5891	43.6565	126.123	74.9333	12.0166	65.5563	0.834424969	0.966018836	20	8	24	0.339834157	0.774150091	24	16.28011696	0.705764447
ENSMUSG00000101304	Plet1os	placenta expressed transcript 1, opposite strand [Source:MGI Symbol;Acc:MGI:1916881]	0.242491	0.559635	0.545974	0.787376	0.664216	0.483974	0.220167	1.1654	0.799563	0.870191	0.795517	0.482294	1.14256	0.755776	0.305259556	0.811201458	16	8	24	0.129139276	0.740162193	24	9.411394479	0.167858144
ENSMUSG00000101309	Gm29397	predicted gene 29397 [Source:MGI Symbol;Acc:MGI:5580103]	33.4255	34.8433	24.6413	31.6862	39.3712	36.3722	45.2679	35.3848	31.1884	34.9988	28.3472	32.8252	32.801	25.257	0.46238066	0.966018836	24	12	24	0.19554172	0.768040178	24	20.57917845	0.157306048
ENSMUSG00000101316	Gm12663	predicted gene 12663 [Source:MGI Symbol;Acc:MGI:3652168]	52.0944	50.8624	47.5156	47.9304	48.4879	47.4413	44.8908	47.1761	46.1588	48.9518	53.5513	51.2035	53.0546	56.2081	0.888379597	0.966018836	20	16	24	0.75938045	0.922213009	24	23.23435635	0.024898568
ENSMUSG00000101330	Gm10193	predicted gene 10193 [Source:MGI Symbol;Acc:MGI:3708520]	38.36	27.9427	34.7643	32.5779	33.1621	36.1749	26.7227	30.2181	27.9014	34.9927	25.2247	37.1234	25.7537	35.9257	0.852844413	0.966018836	24	4	24	0.876181956	0.965492319	24	17.11917318	0.042074108
ENSMUSG00000101335	Gm28229	predicted gene 28229 [Source:MGI Symbol;Acc:MGI:5578935]	0.995085	1.27632	1.08294	1.17022	0.913613	1.0224	0.924334	1.01926	1.444	1.23551	1.09923	0.834092	0.938373	1.01711	0.012175926	0.216995965	12	16	24	0.011264017	0.517565995	24	9.058955146	0.115708948
ENSMUSG00000101344	Gm29183	predicted gene 29183 [Source:MGI Symbol;Acc:MGI:5579889]	0.761897	0.741554	0.897236	0.653733	1.13748	0.790719	0.851346	0.794515	0.909565	0.587376	0.609965	0.811352	0.635332	0.523755	0.90332508	0.966018836	12	4	24	0.768151574	0.925197875	24	18.11581634	0.034280025
ENSMUSG00000101356	Gm28876	predicted gene 28876 [Source:MGI Symbol;Acc:MGI:5579582]	0.738103	0.674301	0.721923	0.758596	0.274962	0.375314	0.539503	0.329467	0.704364	0.390246	0.859814	0.802912	0.690091	0.444125	0.79625133	0.966018836	20	12	24	0.999822609	0.999984043	24	6.924198764	0.001449884
ENSMUSG00000101389	Ms4a4a	membrane-spanning 4-domains, subfamily A, member 4A [Source:MGI Symbol;Acc:MGI:3643932]	0.768576	0.420409	0.707024	1.62455	0.662526	0.435443	0.50461	0.793086	1.12966	0.781308	0.568327	0.377518	0.433643	0.398526	0.097779482	0.501112642	16	8	24	0.030800409	0.598684007	24	10.77647316	0.233057739
ENSMUSG00000101431	Gm7901	predicted gene 7901 [Source:MGI Symbol;Acc:MGI:3645047]	17.8854	16.1175	16.1519	16.5502	20.7526	17.4731	20.2287	14.1941	16.263	15.5745	18.2449	16.9405	16.2781	13.5636	0.004196064	0.140598674	20	12	24	0.009501712	0.502965379	24	18.36224366	0.157746463
ENSMUSG00000101438	Gm19412	predicted gene, 19412 [Source:MGI Symbol;Acc:MGI:5011597]	1.17564	1.67393	1.45423	1.41969	1.26586	1.05746	0.742052	2.04153	1.28531	1.56008	1.08881	1.29506	1.37569	1.61317	0.009118941	0.192760771	8	16	24	0.071027704	0.676890567	24	7.317641558	0.168548893
ENSMUSG00000101439	Gm336	predicted pseudogene 336 [Source:MGI Symbol;Acc:MGI:2685182]	0.78281	0.750488	0.589634	0.727643	0.633221	0.909076	0.809076	0.702501	0.617774	0.457401	0.871803	1.07221	0.800626	0.738041	7.89E-05	0.029372629	24	12	24	0.007232217	0.47735824	24	21.27994088	0.1323512
ENSMUSG00000101450	Gm28941	predicted gene 28941 [Source:MGI Symbol;Acc:MGI:5579647]	0.511737	0.945808	0.910695	0.747364	0.870927	0.396305	0.567679	0.58334	0.794449	0.399554	0.735992	0.326186	0.712541	0.520781	0.022634137	0.27189101	12	12	24	0.254404803	0.768040178	24	8.654746959	0.108416439
ENSMUSG00000101464	Gm7436	predicted gene 7436 [Source:MGI Symbol;Acc:MGI:3646488]	1.58877	2.58425	4.01861	2.18047	3.48799	1.763	1.71085	2.18158	1.55316	1.28488	1.07427	2.17589	0.59551	1.36471	0.897425765	0.966018836	12	16	24	0.571107732	0.845721742	24	11.06842201	0.142154335
ENSMUSG00000101482	Gm28883	predicted gene 28883 [Source:MGI Symbol;Acc:MGI:5579589]	3.51983	2.85996	3.60218	2.84684	3.99499	3.7862	3.78721	3.96305	2.24745	2.58829	3.29843	2.92427	3.83265	4.25153	0.226403091	0.722427815	8	4	24	0.174856983	0.768040178	24	23.11507491	0.147909715
ENSMUSG00000101493	2810405F17Rik	RIKEN cDNA 2810405F17 gene [Source:MGI Symbol;Acc:MGI:1917225]	1.33187	1.92555	1.31282	1.19503	1.49739	1.77444	1.5749	1.67849	2.1331	1.70472	1.7762	1.45811	2.16079	2.10923	0.926552264	0.966018836	8	12	24	0.226112156	0.768040178	24	3.976813453	0.089586394
ENSMUSG00000101519	Ska2l-ps	spindle and kinetochore associated complex subunit 2-like, pseudogene [Source:MGI Symbol;Acc:MGI:3643639]	1.22719	2.37146	1.32955	1.83017	2.53356	1.35597	1.73242	1.26056	1.1057	0.812237	1.10292	0.798726	0.697719	0.636547	0.816406072	0.966018836	20	8	24	0.640518495	0.878323188	24	15.91603882	0.084583296
ENSMUSG00000101523	Csnk2a1-ps3	casein kinase 2, alpha 1 polypeptide, pseudogene 3 [Source:MGI Symbol;Acc:MGI:3704198]	29.922	26.5737	24.8047	25.7575	27.1694	28.6642	27.9203	26.9265	23.5493	26.3967	27.4341	28.2974	28.7465	28.4822	3.40E-05	0.023823328	4	8	24	7.42E-04	0.319044294	24	21.68757351	0.104881009

ENSMUSG00000101567	Txn-ps1	thioredoxin, pseudogene 1 [Source:MGI Symbol;Acc:MGI:98875]	111.091	105.691	88.8873	99.649	103.022	107.243	92.5044	104.734	85.2289	81.5986	104.99	80.243	105.398	78.9702	0.510431574	0.966018836	4	8	24	0.208466632	0.768040178	24	21.01176645	0.104445289
ENSMUSG00000101581	C430002N11Rik	RIKEN cDNA C430002N11 gene [Source:MGI Symbol;Acc:MGI:2442576]	1.20556	1.50651	0.983193	1.09204	1.56882	1.16141	1.14034	1.01039	1.26344	1.23883	1.30644	0.709811	1.08754	1.4561	0.928780444	0.966018836	20	4	24	0.818221508	0.942344852	24	9.372599185	0.039871165
ENSMUSG00000101586	Gm29017	predicted gene 29017 [Source:MGI Symbol;Acc:MGI:5579723]	0.851033	1.07125	1.01242	0.669275	0.911132	0.830334	0.91602	0.89317	1.31945	0.907245	1.01259	0.814467	1.01303	1.31933	0.124028266	0.552144108	12	12	24	0.055143778	0.65534544	24	5.919770507	0.106755179
ENSMUSG00000101589	Rbm6-ps1	RNA binding motif protein 6, pseudogene 1 [Source:MGI Symbol;Acc:MGI:1889580]	2.64644	2.5756	2.25982	2.48036	3.33731	2.41439	2.6457	2.32677	2.06252	1.99872	2.15175	2.31014	2.6008	2.38977	0.027467587	0.308438477	4	8	24	0.180823559	0.768040178	24	21.03685713	0.089718719
ENSMUSG00000101599	Gm20342	predicted gene, 20342 [Source:MGI Symbol;Acc:MGI:5012527]	0.649394	0.70796	0.734039	0.803428	0.783624	0.54705	0.688212	0.662784	0.833416	0.554434	0.898689	0.634217	0.737699	0.768627	0.118446606	0.541945448	20	4	24	0.458926997	0.805393068	24	10.18412431	0.042791065
ENSMUSG00000101655	2310040G24Rik	RIKEN cDNA 2310040G24 gene [Source:MGI Symbol;Acc:MGI:1916897]	2.60238	5.67814	5.18492	5.20873	4.49931	4.95479	4.02102	8.72164	10.1835	9.1701	5.28977	4.54073	7.32348	4.48122	0.282728968	0.793714531	12	16	24	0.05229686	0.65045829	24	8.992720266	0.372895799
ENSMUSG00000101678	Gm29609	predicted gene 29609 [Source:MGI Symbol;Acc:MGI:5580315]	0.75423	1.06837	0.494056	0.850026	0.557079	0.686666	0.725821	0.774311	0.761482	0.785537	0.797332	0.658064	0.826187	1.09824	0.227302972	0.723724673	8	16	24	0.246828015	0.768040178	24	4.40334547	0.082390412
ENSMUSG00000101693	Gm19461	predicted gene, 19461 [Source:MGI Symbol;Acc:MGI:5011646]	7.92383	6.21237	5.12015	4.95669	5.93864	5.97845	5.96875	6.34954	5.53323	9.22997	4.75638	6.57107	6.95857	6.56123	0.185192844	0.662239322	4	16	24	0.634906956	0.875864536	24	0.502514084	0.083600827
ENSMUSG00000101759	Gm5734	predicted gene 5734 [Source:MGI Symbol;Acc:MGI:3647104]	4.24106	3.44774	1.67166	3.09887	3.48922	3.45804	2.06924	2.75178	3.18507	1.85641	4.55397	3.85573	4.3452	3.96367	0.14745801	0.590732968	20	20	24	0.222524092	0.768040178	24	20.98017586	0.22439175
ENSMUSG00000101780	Gm7255	predicted gene 7255 [Source:MGI Symbol;Acc:MGI:3779706]	2.77531	1.65413	2.03113	1.1622	3.15331	2.11896	1.36708	1.48065	2.00406	1.09316	3.00743	2.38366	2.27627	2.19496	0.022785983	0.272961011	20	20	24	0.247372135	0.768040178	24	19.73057465	0.209213571
ENSMUSG00000101784	Gm7553	predicted gene 7553 [Source:MGI Symbol;Acc:MGI:3826364]	1.66421	1.64514	1.67394	1.6619	1.60343	1.78841	1.95592	1.91586	1.84677	1.94963	2.05619	1.95724	1.9416	1.91332	0.78898997	0.966018836	24	12	24	0.776637335	0.928308257	24	22.58193118	0.012213303
ENSMUSG00000101795	Gm5835	predicted gene 5835 [Source:MGI Symbol;Acc:MGI:3643957]	54.5545	60.8829	56.6557	57.9806	65.2463	57.2361	57.0723	58.9533	63.761	51.4036	58.2481	53.3816	55.0297	52.3245	0.879901313	0.966018836	20	8	24	0.761024558	0.92277295	24	12.20207963	0.026807256
ENSMUSG00000101797	Gm29266	predicted gene 29266 [Source:MGI Symbol;Acc:MGI:5579972]	0.731164	0.648802	0.595673	0.312143	1.01811	0.771991	0.667326	0.407113	0.31107	0.48333	0.749699	0.351772	0.489181	0.207263	0.078207999	0.457568093	20	16	24	0.033890822	0.606793499	24	18.84311207	0.172732103
ENSMUSG00000101800	Rnf170-ps	ring finger protein 170, pseudogene [Source:MGI Symbol;Acc:MGI:3645710]	7.31574	7.46151	6.95964	7.92071	8.79919	7.28849	7.94547	7.41317	8.85498	7.93059	7.06588	8.13628	7.92537	7.57266	0.807813307	0.966018836	20	12	24	0.717358889	0.906854196	24	13.55956314	0.029576929
ENSMUSG00000101834	Gm5829	predicted gene 5829 [Source:MGI Symbol;Acc:MGI:3644438]	30.2251	30.7836	24.3807	34.242	31.8178	33.1124	32.9291	30.1715	21.7174	25.5404	25.2594	21.23	25.6856	25.2383	0.700065774	0.966018836	16	20	24	0.480138531	0.805393068	24	20.74503271	0.089027265
ENSMUSG00000101840	Gm28294	predicted gene 28294 [Source:MGI Symbol;Acc:MGI:5579000]	2.39731	1.59432	1.00263	2.72879	1.60219	2.05338	1.79675	1.46297	1.12078	1.21787	1.87094	0.709526	1.85632	1.41984	0.280850951	0.792139038	4	8	24	0.586520877	0.853301382	24	20.11571217	0.120123928
ENSMUSG00000101841	Gm11993	predicted gene 11993 [Source:MGI Symbol;Acc:MGI:3651126]	6.784	7.5678	7.04584	7.00255	7.6455	6.51847	6.57254	6.76999	6.36482	6.04368	6.56997	6.38315	6.50363	6.98939	0.835401638	0.966018836	8	16	24	0.891538719	0.969321764	24	4.891285845	0.013712518
ENSMUSG00000101856	1700096K18Rik	RIKEN cDNA 1700096K18 gene [Source:MGI Symbol;Acc:MGI:1920821]	1.13652	2.01565	2.00514	1.97939	1.47645	1.64472	1.98846	1.92944	1.9117	2.11007	1.79987	1.48468	1.26613	1.98542	0.097587539	0.501112642	8	20	24	0.02469602	0.586541342	24	8.945405819	0.16875765
ENSMUSG00000101860	Mindy4b-ps	MINDY lysine 48 deubiquitinase 4B, pseudogene [Source:MGI Symbol;Acc:MGI:2140018]	13.7849	11.0075	10.4726	10.4088	12.4676	11.5464	9.08571	10.3327	9.75064	9.67127	12.72	11.8205	12.3968	10.2984	0.187699199	0.662239322	20	20	24	0.071576535	0.67819049	24	19.70414995	0.13877077
ENSMUSG00000101865	Gm9775	predicted gene 9775 [Source:MGI Symbol;Acc:MGI:3809039]	0.705395	1.16727	0.877985	0.442245	0.291846	0.628711	0.860621	0.0579759	0.685378	0.447569	0.51311	1.21379	0.260454	0.339466	0.891742979	0.966018836	24	20	24	0.812885679	0.940109891	24	21.69779303	0.077733375
ENSMUSG00000101872	Gm29237	predicted gene 29237 [Source:MGI Symbol;Acc:MGI:5579943]	15.4012	15.6377	14.9702	14.6856	17.9571	14.477	17.9606	12.9057	23.0597	11.1644	17.0202	15.931	14.1044	12.6288	0.942429894	0.966018836	20	12	24	0.909789948	0.974611343	24	17.29466754	0.042708055
ENSMUSG00000101878	Gm8203	predicted pseudogene 8203 [Source:MGI Symbol;Acc:MGI:3646499]	108.299	114.989	114.735	110.985	103.531	102.527	117.797	106.188	121.242	88.4901	114.374	107.004	114.472	110.519	0.280810068	0.792139038	12	4	24	0.340291799	0.774739261	24	3.326774347	0.062954481
ENSMUSG00000101892	9130401M01Rik	RIKEN cDNA 9130401M01 gene [Source:MGI Symbol;Acc:MGI:1923008]	16.14	15.0978	15.4197	12.6195	14.4921	14.0344	13.6333	14.7811	15.589	15.0202	16.8916	15.6886	16.8027	16.0256	0.911135828	0.966018836	4	12	24	0.658520075	0.884596426	24	1.767257894	0.035472317
ENSMUSG00000101900	Mrgprb12-ps	MAS-related GPR, member B12, pseudogene [Source:MGI Symbol;Acc:MGI:3033192]	1.16312	0.745232	0.714884	1.15975	0.605447	0.660047	0.55524	0.769617	0.656124	0.60141	0.834359	0.677304	0.842263	0.717896	0.897975397	0.966018836	4	20	24	0.986523441	0.997339197	24	23.92494959	0.009212495
ENSMUSG00000101932	Gm10074	predicted gene 10074 [Source:MGI Symbol;Acc:MGI:3708632]	0.908995	0.680527	1.47158	0.496189	0.964729	0.976789	0.546676	1.57488	0.304648	0.301114	1.20157	1.20764	0.31274	2.20825	0.850398351	0.966018836	8	8	24	0.810979574	0.93944887	24	1.76623041	0.103652598
ENSMUSG00000101939	Gm28438	predicted gene 28438 [Source:MGI Symbol;Acc:MGI:5579144]	2.43587	141.661	2.39836	2.96566	2.89721	76.6421	1.33889	2.3362	1.64465	0.586348	1.19198	1.01077	1.2	1.38337	0.860560779	0.966018836	8	8	24	0.64938214	0.881907191	24	23.39677292	0.640914276
ENSMUSG00000101970	Chaserr	CHD2 adjacent suppressive regulatory RNA [Source:MGI Symbol;Acc:MGI:1916420]	22.2151	22.4226	23.774	21.8786	24.1411	22.0458	23.8591	21.2138	27.5498	19.6284	21.3756	24.4553	22.5309	23.4413	0.907000607	0.966018836	12	4	24	0.824252392	0.944824178	24	4.261338788	0.028092575
ENSMUSG00000101995	Gm29480	predicted gene 29480 [Source:MGI Symbol;Acc:MGI:5580186]	0.717268	0.851538	0.953891	0.673024	0.778724	0.681499	0.649121	0.691657	0.690633	0.754491	0.659662	0.77672	0.776779	0.729402	0.866861748	0.966018836	8	20	24	0.60439704	0.861894646	24	7.068509984	0.027163881
ENSMUSG00000102014	2900009J06Rik	RIKEN cDNA 2900009J06 gene [Source:MGI Symbol;Acc:MGI:1920137]	1.7269	1.07532	0.720722	0.600677	1.03347	0.96513	1.77398	0.588921	0.963857	0.702835	1.26424	1.00966	1.05803	1.01568	0.01950757	0.258568924	4	12	24	0.036276629	0.609176161	24	22.17492178	0.227529817
ENSMUSG00000102018	Iqank1	IQ motif and ankyrin repeat containing 1 [Source:MGI Symbol;Acc:MGI:3588184]	0.74136	1.36018	1.65592	1.47751	1.72328	1.31696	1.22981	1.41431	1.30579	1.92427	1.71066	1.38447	1.69722	1.55726	0.118984653	0.542554768	20	8	24	0.030658616	0.597890721	24	11.96413537	0.14758574
ENSMUSG00000102038	Gm12345	predicted gene 12345 [Source:MGI Symbol;Acc:MGI:3649862]	4.65278	4.78275	3.82368	4.83586	4.49066	4.57149	6.13683	5.03492	4.46905	5.50497	3.82897	4.79858	4.83157	4.89607	0.339604637	0.858712466	8	4	24	0.492759859	0.805558817	24	1.095965428	0.067097218
ENSMUSG00000102051	Ly6a2	lymphocyte antigen 6 complex, locus A2 [Source:MGI Symbol;Acc:MGI:3612406]	1.68639	4.06627	6.64411	5.6204	2.98601	3.73423	2.99663	2.57299	3.97645	2.41469	3.13121	3.50529	4.6908	1.79507	0.923635729	0.966018836	12	20	24	0.532749561	0.825678486	24	11.27345172	0.189191745
ENSMUSG00000102055	Gm28913	predicted gene 28913 [Source:MGI Symbol;Acc:MGI:5579619]	1.80397	0.808513	1.7374	1.79455	1.60756	1.44045	1.45549	1.76541	0.812862	0.928619	1.622	1.17358	0.813165	0.623249	0.945282252	0.966018836	20	12	24	0.211714046	0.768040178	24	17.2115161	0.162139497
ENSMUSG00000102057	Gm29594	predicted gene 29594 [Source:MGI Symbol;Acc:MGI:5580300]	2.05464	1.72909	1.2994	1.15447	1.37946	1.99665	2.07474	1.20975	1.85467	1.47274	1.62184	2.22008	2.19379	1.18977	0.023460887	0.278499043	4	12	24	0.02190207	0.574400012	24	21.70547741	0.210651251
ENSMUSG00000102059	Gm20257	predicted gene, 20257 [Source:MGI Symbol;Acc:MGI:5012442]	1.54279	1.82919	1.39801	1.29711	1.34661	2.9822	2.00749	9.94125	1.86109	1.39307	1.7321	1.4761	4.53858	1.42201	0.052646208	0.391647859	4	12	24	0.262468613	0.768040178	24	1.890835606	0.383967751
ENSMUSG00000102070	Gm28661	predicted gene 28661 [Source:MGI Symbol;Acc:MGI:5579367]	10.3915	4.68723	21.3294	5.09541	5.1011	27.8163	3.1876	5.49534	24.3392	45.2774	7.17017	74.0134	7.7647	6.14244	0.43565473	0.966018836	24	8	24	0.577237438	0.848840315	24	14.72473569	0.517865135
ENSMUSG00000102101	Zbtb11os1	zinc finger and BTB domain containing 11, opposite strand 1 [Source:MGI Symbol;Acc:MGI:1913641]	0.623851	1.12403	1.25717	1.27372	0.786645	1.03143	0.829745	0.87985	1.77277	1.18591	1.3233	0.9808	1.18154	1.70505	0.086480643	0.468028719	12	16	24	0.009999734	0.503967859	24	8.913373984	0.201552589
ENSMUSG00000102128	Gm18349	predicted gene, 18349 [Source:MGI Symbol;Acc:MGI:5010534]	2.30743	1.74488	1.51703	2.09092	1.72559	2.13036	1.89809	1.72875	2.30798	1.70184	2.65491	2.15704	2.61056	2.04656	0.388109084	0.915479136	4	8	24	0.321752199	0.768040178	24	20.26069419	0.090028732
ENSMUSG00000102145	Gm38056	predicted gene, 38056 [Source:MGI Symbol;Acc:MGI:5611284]	1.5498	1.47527	1.15982	1.29964	1.56493	1.09268	1.57541	1.51713	1.68437	1.04789	1.61548	1.40371	1.48885	1.39824	0.881355613	0.966018836	4	12	24	0.569161448	0.844729091	24	0.807717686	0.051273935
ENSMUSG00000102205	9430092D12Rik	RIKEN cDNA 9430092D12 gene [Source:MGI Symbol;Acc:MGI:1924677]	0.607144	0.744668	0.699325	0.739092	0.482364	0.467464	0.566921	0.576521	0.952234	0.424652	0.639679	0.522784	0.617669	0.744004	0.038115943	0.334069441	12	12	24	0.044907237	0.628432994	24	6.756883868	0.11251343
ENSMUSG00000102206	Pcdha11	protocadherin alpha 11 [Source:MGI Symbol;Acc:MGI:1298372]	1.50628	1.25678	1.23728	1.12695	1.46766	1.96284	1.37961	1.34492	1.2478	1.51664	1.64497	1.65248	1.56346	1.46841	2.74E-04	0.049770315	24	12	24	0.004343098	0.427431763	24	19.76544541	0.129364005
ENSMUSG00000102207	Gm10344	predicted gene 10344 [Source:MGI Symbol;Acc:MGI:3642672]	1.32618	0.738828	0.927532	1.52583	0.924729	1.09699	0.848442	1.01993	0.431877	1.10541	0.783527	0.682858	0.923762	1.10285	0.852844413	0.966018836	16	20	24	0.901852521	0.972475685	24	17.73991843	0.036778864
ENSMUSG00000102215	Gm2464	predicted gene 2464 [Source:MGI Symbol;Acc:MGI:3780631]	3.78786	3.48072	2.60429	2.61644	2.83164	3.56853	2.98911	2.79084	2.92778	2.72362	4.05625	3.48413	3.91352	3.12603	0.02270465	0.272549814	24	16	24	0.027109535	0.588305652	24	21.53917367	0.160257394
ENSMUSG00000102222	Pcdhga10	protocadherin gamma subfamily A, 10 [Source:MGI Symbol;Acc:MGI:1935227]	0.989363	1.03745	1.05531	0.960076	1.00499	0.978656	1.20987	1.05623	0.817639	1.06914	0.640031	1.30319	0.911933	1.26609	0.91695334	0.966018836	8	12	24	0.386582218	0.799546792	24	1.673462752	0.066842828
ENSMUSG00000102234	Gm37885	predicted gene, 37885 [Source:MGI Symbol;Acc:MGI:5611113]	0.547451	1.10256	0.0885399	1.13635	0.37894	0.705796	0.217013	0.488614	0.21424	0.0435846	0.303985	0.952091	0.594382	0.659282	0.588208558	0.966018836	24	12	24	0.492653471	0.805558817	24	22.45600426	0.158997621
ENSMUSG00000102252	Snrpn	small nuclear ribonucleoprotein N [Source:MGI Symbol;Acc:MGI:98347]	214.731	182.49	182.602	184.684	183.23	216.656	198.427	198.058	154.727	218.325	172.906	196.487	189.756	195.809	0.236893212	0.7385769	24	12	24	0.256248986	0.768040178	24	21.54595319	0.087531197
ENSMUSG00000102254	Gm7496	predicted gene 7496 [Source:MGI Symbol;Acc:MGI:3643469]	0.527706	0.482066	0.722551	0.639069	0.940277	0.654964	0.528524	0.823549	0.745133	0.487987	0.833811	0.56455	0.577921	0.629596	0.231491055	0.731037655	20	8	24	0.375781063	0.795959912	24	13.27976288	0.065290237
ENSMUSG00000102270	Gm8388	predicted gene 8388 [Source:MGI Symbol;Acc:MGI:3645001]	1.47754	1.19344	1.095	1.91963	1.65329	1.0258	2.53734	1.80014	1.78631	1.2427	0.829141	1.07336	1.34461	1.00836	0.94663657	0.966018836	4	20	24	0.806576761	0.938327285	24	4.090245255	0.067175456
ENSMUSG00000102298	Gm33707	predicted gene, 33707 [Source:MGI Symbol;Acc:MGI:5592866]	1.18144	1.5969	2.0316	1.45529	1.31438	0.99662	0.83832	0.964021	1.33966	1.23667	1.41193	1.64369	1.91161	1.41686	0.883907406	0.966018836	12	16	24	0.644767071	0.879359107	24	9.431016176	0.079612608
ENSMUSG00000102302	Gm38190	predicted gene, 38190 [Source:MGI Symbol;Acc:MGI:5611418]	0.826858	0.991347	0.721974	1.00499	1.00682	0.620807	0.958641	0.588956	1.05034	0.591505	1.16737	0.830024	0.851265	0.928448	0.939335547	0.966018836	20	4	24	0.875126668	0.964979482	24	14.50941014	0.029559455
ENSMUSG00000102305	Gm38192	predicted gene, 38192 [Source:MGI Symbol;Acc:MGI:5611420]	0.541173	0.730382	0.716882	0.596411	0.619308	0.603931	1.15006	0.781864	0.69233	0.63712	0.746864	0.672679	0.687786	1.08157	0.138543921	0.580158868	8	8	24	0.156272722	0.761842768	24	3.512189819	0.090962959
ENSMUSG00000102312	Pcdha3	protocadherin alpha 3 [Source:MGI Symbol;Acc:MGI:2447313]	2.87156	1.61706	1.79619	1.96709	1.37152	2.19889	1.88506	1.74408	1.44128	2.42564	2.01794	2.13424	1.90627	2.10312	0.245061943	0.741580974	24	12	24	0.498025631	0.807927978	24	21.02984545	0.090328377
ENSMUSG00000102330	Gm37234	predicted gene, 37234 [Source:MGI Symbol;Acc:MGI:5610462]	0.65164	0.426179	0.776479	0.678397	1.11858	0.671636	0.720109	0.696094	0.360768	0.368934	1.00534	0.513124	0.557028	0.253615	0.305564675	0.811201458	20	12	24	0.059116867	0.658304792	24	17.40476342	0.173900905
ENSMUSG00000102349	Gm37376	predicted gene, 37376 [Source:MGI Symbol;Acc:MGI:5610604]	22.8509	15.3096	20.598	11.9989	25.5711	21.3528	17.4029	24.3465	25.4213	19.8185	14.6406	24.2549	33.8853	23.0724	0.94663657	0.966018836	4	12	24	0.351291903	0.780345856	24	0.689762927	0.190555309
ENSMUSG00000102374	Gm38387	predicted gene, 38387 [Source:MGI Symbol;Acc:MGI:5613622]	0.409983	0.651185	0.642556	0.62434	0.419892	0.487089	0.541299	0.530679	0.684927	0.314082	0.532416	0.572471	0.39192	0.521787	0.351840494	0.870418653	12	16	24	0.367548018	0.790901169	24	7.086017096	0.059031518
ENSMUSG00000102390	Gm37579	predicted gene, 37579 [Source:MGI Symbol;Acc:MGI:5610807]	36.9588	47.6718	39.5987	30.0828	16.1322	24.832	34.9098	17.2463	49.0515	40.6448	28.4165	57.1466	50.3599	76.3543	0.644063931	0.966018836	8	12	24	0.25153253	0.768040178	24	4.489398182	0.361287008
ENSMUSG00000102411	Gm36936	predicted gene, 36936 [Source:MGI Symbol;Acc:MGI:5610164]	1.30106	1.74554	1.59936	1.43063	1.51031	1.2505	1.66551	1.78889	2.19054	1.07149	1.96688	1.5833	1.42242	1.74367	0.173541845	0.648600527	12	4	24	0.389765984	0.799546792	24	6.273175227	0.089187383
ENSMUSG00000102422	Iqschfp	Iqcj and Schip1 fusion protein [Source:MGI Symbol;Acc:MGI:5439400]	0.886013	0.841291	1.01509	0.425289	0.774484	1.22824	1.52907	1.22515	0.198875	1.55915	1.11805	1.48397	0.586426	1.04634	0.833406028	0.966018836	24	12	24	0.424434969	0.805393068	24	20.76093647	0.168767197
ENSMUSG00000102428	Pcdhga12	protocadherin gamma subfamily A, 12 [Source:MGI Symbol;Acc:MGI:1935229]	0.784274	0.963272	0.863607	0.763611	1.06631	0.994063	0.986726	0.911537	0.961751	1.33969	0.904831	1.0206	1.00759	0.938595	0.580049522	0.966018836	24	12	24	0.6527005	0.883085974	24	14.64969268	0.032530948
ENSMUSG00000102440	Pcdhga9	protocadherin gamma subfamily A, 9 [Source:MGI Symbol;Acc:MGI:1935226]	0.701299	0.784576	0.569961	0.809104	0.863488	0.777566	0.794612	0.601274	0.603677	1.04216	0.484719	0.823368	0.674049	0.76016	0.265774856	0.773510818	16	20	24	0.708342258	0.903587413	24	16.77611181	0.039814504
ENSMUSG00000102463	1700003N22Rik	RIKEN cDNA 1700003N22 gene [Source:MGI Symbol;Acc:MGI:1919464]	4.38117	4.32465	3.75654	4.8726	5.66756	5.69574	4.58888	5.35124	3.45227	5.95316	3.60015	4.05997	4.07455	2.45131	0.892555977	0.966018836	16	20	24	0.207895037	0.768040178	24	17.06760567	0.173288462
ENSMUSG00000102493	Gm8010	predicted gene 8010 [Source:MGI Symbol;Acc:MGI:3648648]	1.45651	1.05297	1.03544	0.770087	1.29814	1.01623	1.36637	1.05246	0.573566	1.36954	0.577227	1.10842	0.858866	1.13186	0.723016199	0.966018836	4	8	24	0.376723347	0.796713484	24	22.92259429	0.108555106
ENSMUSG00000102496	Gm36989	predicted gene, 36989 [Source:MGI Symbol;Acc:MGI:5610217]	1.20974	0.999321	0.992804	1.05179	1.28354	0.984861	1.28468	1.10479	1.09956	0.419121	1.47876	0.554117	0.53997	0.866631	0.895137379	0.966018836	20	20	24	0.950590299	0.988257443	24	18.9795825	0.028163921
ENSMUSG00000102504	Gm21955	predicted gene, 21955 [Source:MGI Symbol;Acc:MGI:5439424]	1.42395	1.1947	1.02148	1.30281	1.24777	1.22899	1.19801	1.14791	1.35903	1.06529	1.73037	1.37758	1.59416	1.34896	0.291081671	0.797585788	20	20	24	0.312162657	0.768040178	24	19.9496676	0.066118978
ENSMUSG00000102525	Gm37784	predicted gene, 37784 [Source:MGI Symbol;Acc:MGI:5611012]	68.4643	50.8822	37.3627	57.3339	58.355	49.4785	56.9267	45.8275	47.0353	68.3766	50.474	57.1673	47.3175	63.0092	0.503972924	0.966018836	16	20	24	0.600210413	0.860027394	24	18.69356396	0.098835406
ENSMUSG00000102545	6430573P05Rik	RIKEN cDNA 6430573P05 gene [Source:MGI Symbol;Acc:MGI:2686494]	0.357443	0.673066	1.31944	0.430881	0.68058	0.516828	0.446334	0.566821	1.48767	0.659722	0.372107	0.89481	0.527546	0.590828	0.105280783	0.518002936	12	16	24	0.157466299	0.76387796	24	8.594751011	0.198143837
ENSMUSG00000102548	Gm16701	predicted gene, 16701 [Source:MGI Symbol;Acc:MGI:4439625]	1.0322	0.700808	0.287689	0.957145	0.86771	0.767784	1.22414	0.724482	0.721717	0.951404	0.949143	0.838442	0.737014	0.727486	0.019643089	0.258568924	16	20	24	0.20432036	0.768040178	24	19.31007662	0.127072915
ENSMUSG00000102550	Gm8276	predicted gene 8276 [Source:MGI Symbol;Acc:MGI:3648019]	1.29756	1.10048	1.22894	1.55028	1.33713	1.43903	1.40936	1.29477	1.29895	1.52356	1.29326	1.2672	1.26117	1.38984	0.338172159	0.85758369	16	16	24	0.256368266	0.768040178	24	14.28955462	0.045295105
ENSMUSG00000102553	D930036K23Rik	RIKEN cDNA D930036K23 gene [Source:MGI Symbol;Acc:MGI:2442341]	0.55161	0.741995	0.917682	0.769269	0.635559	0.737998	0.585493	0.571087	0.791354	0.749132	0.719517	0.666615	0.497151	0.691932	1.13E-04	0.029372629	12	16	24	0.01440762	0.534084587	24	10.82210343	0.094177517
ENSMUSG00000102555	6430511E19Rik	RIKEN cDNA 6430511E19 gene [Source:MGI Symbol;Acc:MGI:2443259]	0.641452	0.7721	0.677524	0.695858	0.668902	0.660832	0.89518	0.568434	0.826843	0.379262	0.942868	0.749282	0.639913	0.815225	0.816406072	0.966018836	20	20	24	0.779530458	0.929143691	24	23.27799355	0.035387056
ENSMUSG00000102562	Gm37694	predicted gene, 37694 [Source:MGI Symbol;Acc:MGI:5610922]	0.442293	0.49116	0.546976	0.672165	0.559777	0.459007	0.540581	0.343204	0.729335	0.398326	0.569071	0.472369	0.480647	0.536097	0.239362259	0.741580974	20	4	24	0.31014528	0.768040178	24	11.43591708	0.056805694
ENSMUSG00000102602	A930004J17Rik	RIKEN cDNA A930004J17 gene [Source:MGI Symbol;Acc:MGI:2442442]	0.800481	1.42682	1.06505	1.13021	1.12682	0.966642	1.18572	1.1363	1.67565	0.898602	1.43299	1.37595	1.39826	1.53019	0.952127548	0.966018836	8	8	24	0.439777099	0.805393068	24	6.214946767	0.072455738
ENSMUSG00000102644	Thap6	THAP domain containing 6 [Source:MGI Symbol;Acc:MGI:1922436]	1.90048	1.84269	2.14954	1.93247	2.20779	1.92582	1.8422	2.13543	1.88097	2.17174	1.96886	2.21653	1.8917	2.01931	0.122341925	0.550724475	20	8	24	0.319773114	0.768040178	24	13.45882421	0.037992314
ENSMUSG00000102697	Pcdhac2	protocadherin alpha subfamily C, 2 [Source:MGI Symbol;Acc:MGI:1891443]	21.9965	18.1859	17.4585	19.2612	16.7434	20.2139	17.9204	19.0018	14.0189	25.2649	17.582	19.9407	18.5899	20.073	0.601647512	0.966018836	24	12	24	0.775287922	0.927711986	24	19.94822069	0.055748393
ENSMUSG00000102704	Gm38309	predicted gene, 38309 [Source:MGI Symbol;Acc:MGI:5611537]	0.76078	1.32483	1.27345	0.875671	0.879283	0.833619	1.14378	0.945417	1.24089	1.33357	1.04912	0.855852	0.855829	0.977862	0.068712455	0.432567661	12	12	24	0.046058719	0.633009261	24	8.448928482	0.13023242
ENSMUSG00000102729	Gm32444	predicted gene, 32444 [Source:MGI Symbol;Acc:MGI:5591603]	9.8916	13.2809	10.8958	11.0934	12.577	10.4248	9.92814	9.86352	12.7001	9.95807	12.6611	9.69223	11.5193	11.8074	0.61767505	0.966018836	20	4	24	0.535955681	0.82731247	24	9.985610632	0.0675888
ENSMUSG00000102742	Pcdhga11	protocadherin gamma subfamily A, 11 [Source:MGI Symbol;Acc:MGI:1935228]	2.24856	2.12807	2.08847	2.02136	2.09481	2.47463	2.47826	2.58433	1.93189	2.7984	1.7307	2.32903	2.02058	2.60164	0.595952838	0.966018836	8	12	24	0.607868901	0.864036051	24	2.16284894	0.052065799
ENSMUSG00000102748	Pcdhgb2	protocadherin gamma subfamily B, 2 [Source:MGI Symbol;Acc:MGI:1935170]	1.52197	1.28857	1.13401	1.22161	1.26744	1.35817	1.36372	1.51969	1.35377	1.75725	1.05232	1.3815	1.20849	1.40005	0.839634479	0.966018836	8	12	24	0.792912956	0.93379199	24	3.664505417	0.029469021
ENSMUSG00000102752	Gm7694	predicted gene 7694 [Source:MGI Symbol;Acc:MGI:3649135]	2.24586	2.36816	2.17439	2.3317	2.58838	2.19038	2.28349	2.13667	2.17442	1.93939	2.44824	2.20412	2.56893	2.1765	0.251986462	0.75129457	20	16	24	0.372017184	0.793398709	24	19.78429861	0.044120082
ENSMUSG00000102753	Gm37056	predicted gene, 37056 [Source:MGI Symbol;Acc:MGI:5610284]	0.86131	1.24385	0.785279	0.911971	0.910394	0.969367	0.682707	0.982656	0.939866	1.0301	0.947788	0.635936	0.804156	0.862921	0.423605651	0.966018836	16	12	24	0.411778184	0.799546792	24	9.123107725	0.058714939
ENSMUSG00000102771	Gm33320	predicted gene, 33320 [Source:MGI Symbol;Acc:MGI:5592479]	3.92562	3.20392	3.0783	4.5792	2.98669	3.12142	3.57915	2.71452	2.66868	2.95605	2.15646	2.24392	3.58131	2.48516	0.515451644	0.966018836	4	16	24	0.874061025	0.964836189	24	0.869506426	0.038321721
ENSMUSG00000102788	Gm38255	predicted gene, 38255 [Source:MGI Symbol;Acc:MGI:5611483]	0.377185	0.88907	0.805893	0.66106	0.715689	0.478986	0.290371	0.414511	0.98537	0.405919	0.530911	0.638175	0.472848	0.619788	0.009848456	0.194720062	12	16	24	0.162686285	0.766365035	24	9.506094555	0.134455725
ENSMUSG00000102796	Gm37711	predicted gene, 37711 [Source:MGI Symbol;Acc:MGI:5610939]	2.27375	1.76725	1.82164	1.81324	1.54044	1.95204	2.13708	1.57057	1.57285	2.14537	1.44803	1.76245	1.61038	2.31668	0.767183862	0.966018836	4	16	24	0.532448124	0.825678486	24	1.75486552	0.062696627
ENSMUSG00000102803	Gm37477	predicted gene, 37477 [Source:MGI Symbol;Acc:MGI:5610705]	0.604962	0.549834	0.758134	0.481273	0.438949	0.17129	0.49424	0.67756	0.633035	0.317328	0.765363	0.59156	0.532036	0.581557	0.300823756	0.809362476	12	4	24	0.347328435	0.778844143	24	6.087358291	0.092241515
ENSMUSG00000102813	Gm37795	predicted gene, 37795 [Source:MGI Symbol;Acc:MGI:5611023]	6.10433	7.98795	7.0566	6.92191	6.24508	5.65269	7.06163	6.23047	7.98071	6.82143	6.51464	6.07497	6.92623	7.13426	0.001971398	0.100255953	12	12	24	0.014963151	0.537924552	24	7.124317794	0.130125645
ENSMUSG00000102824	Pdcd5-ps	programmed cell death 5, pseudogene [Source:MGI Symbol;Acc:MGI:3782009]	98.4467	111.187	106.099	102.751	111.098	102.348	94.6155	99.2152	116.7	86.2909	116.663	108.785	114.858	102.434	0.891105762	0.966018836	20	8	24	0.973809044	0.993334889	24	17.47657577	0.011720486
ENSMUSG00000102827	Gm8242	predicted gene 8242 [Source:MGI Symbol;Acc:MGI:3647216]	15.6888	14.9863	16.021	16.2142	17.6859	14.0794	14.3421	16.3976	19.6752	12.7107	15.771	15.9523	17.1876	12.6389	0.934110497	0.966018836	12	20	24	0.921130087	0.979223729	24	12.26980596	0.026574412
ENSMUSG00000102828	Gm38182	predicted gene, 38182 [Source:MGI Symbol;Acc:MGI:5611410]	1.38323	0.944772	0.823115	0.133845	1.02716	0.388789	0.937538	0.656865	0.464132	0.832401	0.46266	0.701387	0.529401	0.595682	0.918193129	0.966018836	4	12	24	0.344040238	0.777470047	24	0.141495811	0.143173022
ENSMUSG00000102854	C130023A14Rik	RIKEN cDNA C130023A14 gene [Source:MGI Symbol;Acc:MGI:2442654]	0.784993	0.999196	0.677659	0.765479	0.887191	0.781225	0.761238	0.547651	0.806385	0.482641	0.813676	0.609657	0.628748	0.7605	0.79625133	0.966018836	20	20	24	0.875832998	0.965318618	24	21.14575937	0.02208374
ENSMUSG00000102861	Gm37637	predicted gene, 37637 [Source:MGI Symbol;Acc:MGI:5610865]	1.19807	1.14467	1.57973	1.58594	1.17124	1.21884	1.10822	1.2697	1.56359	0.935303	1.00032	1.29403	1.30415	1.24272	0.628286596	0.966018836	12	8	24	0.367303	0.790704242	24	7.47467209	0.071958177
ENSMUSG00000102869	Norad	non-coding RNA activated by DNA damage [Source:MGI Symbol;Acc:MGI:1914767]	115.854	107.032	108.326	110.935	103.05	111.88	110.378	105.618	96.4789	122.074	102.35	116.403	112.463	119.287	0.791413662	0.966018836	24	12	24	0.696427829	0.89922881	24	22.60374997	0.031333012
ENSMUSG00000102882	Gm2065	predicted gene 2065 [Source:MGI Symbol;Acc:MGI:3780232]	0.610373	0.629256	0.481647	0.651481	0.655136	0.453329	0.335803	0.507575	0.752305	0.423239	0.600895	0.513223	0.45579	0.643387	0.450844752	0.966018836	12	16	24	0.426452628	0.805393068	24	10.00328878	0.056409869
ENSMUSG00000102899	Gm37850	predicted gene, 37850 [Source:MGI Symbol;Acc:MGI:5611078]	0.310846	0.630056	0.450095	0.588546	0.767773	0.349369	0.882001	0.46091	1.19794	0.411026	0.919229	0.199743	0.277641	0.628758	0.44861185	0.966018836	20	4	24	0.357429932	0.784480156	24	9.920573945	0.144891506
ENSMUSG00000102918	Pcdhgc3	protocadherin gamma subfamily C, 3 [Source:MGI Symbol;Acc:MGI:1935201]	7.98621	7.24081	7.88691	7.04046	7.39788	7.21614	8.71854	7.90392	7.37475	10.2265	7.26179	8.52589	8.00301	8.24104	0.777666236	0.966018836	4	16	24	0.943474542	0.986151003	24	4.789551496	0.016777668
ENSMUSG00000102923	Gm31728	predicted gene, 31728 [Source:MGI Symbol;Acc:MGI:5590887]	1.03379	0.896041	0.480724	0.832782	0.434366	0.411756	0.358318	1.30759	0.600906	0.488566	1.00206	1.43193	0.695679	0.793646	0.896773941	0.966018836	8	4	24	0.760061649	0.922505796	24	0.922040781	0.078096005
ENSMUSG00000102929	Gm37154	predicted gene, 37154 [Source:MGI Symbol;Acc:MGI:5610382]	0.528078	0.751976	0.612768	0.647663	0.581207	0.681248	0.644832	0.505188	0.938696	0.418595	0.800382	0.515294	0.796164	0.707102	0.816406072	0.966018836	12	4	24	0.787686073	0.931762722	24	5.558223892	0.034236811
ENSMUSG00000102953	Gm37019	predicted gene, 37019 [Source:MGI Symbol;Acc:MGI:5610247]	1.28602	1.24773	0.984061	1.94597	1.76921	0.997833	1.30164	1.19158	1.43042	0.889142	1.63698	1.32706	1.22769	1.05676	0.454881285	0.966018836	20	12	24	0.299257494	0.768040178	24	15.58436501	0.109360784
ENSMUSG00000102961	Gm19918	predicted gene, 19918 [Source:MGI Symbol;Acc:MGI:5012103]	4.09239	4.98961	8.10978	5.55239	5.34239	5.84245	0.00627979	3.44589	6.20907	3.61605	8.75633	4.05037	6.74596	3.03181	0.510431574	0.966018836	12	20	24	0.309356907	0.768040178	24	12.61239205	0.468066502
ENSMUSG00000102976	Zc3h11a	zinc finger CCCH type containing 11A [Source:MGI Symbol;Acc:MGI:1917829]	25.3984	32.1411	22.5528	20.7482	29.9579	26.8591	28.5786	22.4343	25.6703	23.715	30.6776	25.8751	21.5656	24.0354	0.369262716	0.893596186	20	20	24	0.401574645	0.799546792	24	19.49661925	0.102596787
ENSMUSG00000103009	Gm4430	predicted gene 4430 [Source:MGI Symbol;Acc:MGI:3782614]	3.38217	2.2227	2.38061	2.13631	2.25703	2.64978	2.84314	2.28918	2.02148	2.61838	2.72202	3.01807	3.18807	2.88871	0.004320961	0.140598674	4	8	24	0.012927587	0.527834117	24	22.11293674	0.160756215
ENSMUSG00000103013	Gm37503	predicted gene, 37503 [Source:MGI Symbol;Acc:MGI:5610731]	0.516836	1.16878	1.11801	0.860631	0.93359	0.141651	0.935666	0.226354	0.348433	0.854833	0.519311	0.338002	1.22798	1.01576	0.648082424	0.966018836	4	20	24	0.609305382	0.864393298	24	7.055314288	0.13414157
ENSMUSG00000103032	Gm37121	predicted gene, 37121 [Source:MGI Symbol;Acc:MGI:5610349]	0.430131	0.547415	0.486154	0.515297	0.535711	0.453597	0.586505	0.401502	0.702269	0.535345	0.559932	0.367511	0.757796	0.612089	0.905275015	0.966018836	4	20	24	0.51849147	0.818673182	24	7.040851517	0.044458719
ENSMUSG00000103034	Gm8797	predicted pseudogene 8797 [Source:MGI Symbol;Acc:MGI:3643769]	4.04443	2.91591	1.86232	2.22053	1.81012	2.33312	2.23312	2.71987	2.41382	3.72815	2.08565	4.02687	2.87902	4.67251	0.17953325	0.655480352	8	12	24	0.313990296	0.768040178	24	1.926395547	0.176600747
ENSMUSG00000103037	Pcdhgb1	protocadherin gamma subfamily B, 1 [Source:MGI Symbol;Acc:MGI:1935169]	0.757864	0.541806	0.743051	0.651217	0.437783	0.8404	0.628915	1.11777	0.617901	0.957477	0.434679	0.683488	0.613366	0.859734	0.983095273	0.983095273	8	12	24	0.349697116	0.779170188	24	4.934014688	0.090579171
ENSMUSG00000103039	Gm37123	predicted gene, 37123 [Source:MGI Symbol;Acc:MGI:5610351]	0.748236	0.881028	1.02806	0.995422	1.06304	0.627071	1.15381	0.741333	1.45342	0.598344	1.22017	1.35412	1.20545	0.909229	0.935732159	0.966018836	20	12	24	0.97269257	0.993250534	24	14.19798321	0.017185058
ENSMUSG00000103041	Gm37305	predicted gene, 37305 [Source:MGI Symbol;Acc:MGI:5610533]	1.6524	1.72876	1.75348	1.63554	1.5925	1.57884	1.65382	1.63483	1.61591	1.63248	1.64742	1.40608	1.74631	1.91739	0.047000349	0.375011045	4	20	24	0.053331504	0.652202934	24	5.57233559	0.05467568
ENSMUSG00000103042	Gm2719	predicted pseudogene 2719 [Source:MGI Symbol;Acc:MGI:3780888]	13.4321	13.4398	16.432	19.5146	14.8264	15.546	14.2179	11.7707	14.8275	18.5253	18.4329	14.031	15.0878	13.6933	3.51E-04	0.049770315	16	16	24	0.002874658	0.414576087	24	13.55773205	0.208680419
ENSMUSG00000103046	Gm37309	predicted gene, 37309 [Source:MGI Symbol;Acc:MGI:5610537]	1.66497	1.51662	1.57788	1.42246	1.43302	1.23997	1.542	1.27776	1.87392	1.20612	1.94486	1.21194	1.49911	1.49472	0.745118544	0.966018836	12	12	24	0.801217084	0.936384198	24	7.554828551	0.036132002

ENSMUSG00000103088	Pcdhgb6	protocadherin gamma subfamily B, 6 [Source:MGI Symbol;Acc:MGI:1935197]	0.999514	1.49318	1.58675	1.34234	1.07505	1.30443	1.19821	1.40191	1.55848	1.87023	1.04195	1.53419	1.24599	1.73639	0.052138666	0.391073722	12	16	24	0.030188824	0.596794384	24	7.956929989	0.145468612
ENSMUSG00000103092	Pcdha5	protocadherin alpha 5 [Source:MGI Symbol;Acc:MGI:1298371]	1.50834	1.12901	1.16529	1.16431	0.866733	1.02396	1.00192	1.17269	0.820728	1.23381	0.906091	0.969827	1.08869	0.999071	0.700065774	0.966018836	16	4	24	0.589297549	0.854191861	24	2.328166276	0.042015206
ENSMUSG00000103103	4833445I07Rik	RIKEN cDNA 4833445I07 gene [Source:MGI Symbol;Acc:MGI:2143268]	2.00337	1.74924	1.86415	1.26528	1.24585	1.9809	1.54937	1.90748	1.7811	2.02831	2.12134	1.87555	2.20282	1.60095	0.891742979	0.966018836	4	4	24	0.601185239	0.860439692	24	0.189960739	0.06047737
ENSMUSG00000103115	Gm37584	predicted gene, 37584 [Source:MGI Symbol;Acc:MGI:5610812]	0.281642	0.98473	0.459576	0.607184	0.766136	0.681248	0.687821	0.947227	0.601728	0.488361	0.557842	0.694527	0.585415	0.680913	0.886952527	0.966018836	8	4	24	0.798989197	0.935664328	24	3.358081717	0.043163596
ENSMUSG00000103133	Gm37303	predicted gene, 37303 [Source:MGI Symbol;Acc:MGI:5610531]	0.937155	1.08358	0.803369	1.34038	1.51498	0.971029	0.971366	0.968632	1.22382	0.900353	1.37083	1.0833	0.962665	1.13858	0.148627651	0.59172107	20	8	24	0.160870956	0.765941695	24	14.69794255	0.097393341
ENSMUSG00000103137	Gm37981	predicted gene, 37981 [Source:MGI Symbol;Acc:MGI:5611209]	0.968056	1.5258	0.467161	1.34321	1.27432	1.63048	1.56923	1.24338	1.60885	1.63569	1.8341	0.691547	1.58582	1.8786	0.807813307	0.966018836	16	20	24	0.996309493	0.999034519	24	9.335877189	0.009465007
ENSMUSG00000103144	Pcdhga1	protocadherin gamma subfamily A, 1 [Source:MGI Symbol;Acc:MGI:1935212]	1.06377	0.878633	1.23051	0.954028	1.06744	0.938006	0.997928	0.846443	0.774352	1.2638	0.872296	0.954596	0.986866	0.848454	0.643051552	0.966018836	16	16	24	0.550215795	0.835448041	24	14.78962678	0.041618648
ENSMUSG00000103149	AA914427	EST AA914427 [Source:MGI Symbol;Acc:MGI:1858439]	0.356203	0.603703	0.817952	0.773015	0.504488	0.495148	0.821365	0.638596	0.568041	0.601342	0.790248	0.831683	0.790114	0.442178	0.824031576	0.966018836	20	12	24	0.819359025	0.942753508	24	13.5272444	0.034968189
ENSMUSG00000103164	Gm38150	predicted gene, 38150 [Source:MGI Symbol;Acc:MGI:5611378]	0.138892	0.351236	1.05483	0.627433	0.715927	0.620339	0.337151	0.123091	1.03644	0.826779	0.566065	0.896754	0.840355	0.850926	0.17882595	0.655480352	12	16	24	0.121522287	0.733843269	24	11.84210274	0.197196622
ENSMUSG00000103174	Gm37168	predicted gene, 37168 [Source:MGI Symbol;Acc:MGI:5610396]	2.7688	1.65884	2.14215	1.84085	2.54158	2.36576	2.36732	2.18089	1.55699	2.64169	2.01474	2.1131	2.02489	2.05711	0.714062999	0.966018836	4	8	24	0.212093476	0.768040178	24	19.43992372	0.112092057
ENSMUSG00000103181	A330069K06Rik	RIKEN cDNA A330069K06 gene [Source:MGI Symbol;Acc:MGI:2685464]	9.33325	11.9493	6.99503	10.1541	12.0294	9.15276	12.285	9.95769	14.2566	8.01309	12.6111	9.75568	11.279	10.2497	0.872380105	0.966018836	20	4	24	0.916141176	0.976814265	24	22.41275686	0.042577955
ENSMUSG00000103183	Gm37090	predicted gene, 37090 [Source:MGI Symbol;Acc:MGI:5610318]	2.92302	3.3926	2.8957	2.61417	3.20411	3.11322	2.69046	2.92951	3.65069	2.94116	3.24963	3.21124	3.16997	3.34773	0.90332508	0.966018836	8	20	24	0.878792247	0.966097006	24	5.72577433	0.019611554
ENSMUSG00000103189	Gm37092	predicted gene, 37092 [Source:MGI Symbol;Acc:MGI:5610320]	10.2705	10.1223	8.27744	11.8216	10.8152	9.20315	9.90084	8.00978	9.22658	9.37365	11.3991	10.0842	9.70588	8.45307	0.082685511	0.462922111	20	16	24	0.035743147	0.609176161	24	16.83751079	0.135956964
ENSMUSG00000103206	Gm8515	predicted gene 8515 [Source:MGI Symbol;Acc:MGI:3648243]	6.77365	3.82607	3.57334	3.96213	4.09214	4.19157	3.53158	4.22151	3.62138	4.03017	3.17269	3.04392	3.82183	3.51642	0.79625133	0.966018836	4	8	24	0.470115	0.805393068	24	23.64272402	0.088118544
ENSMUSG00000103217	Gm38287	predicted gene, 38287 [Source:MGI Symbol;Acc:MGI:5611515]	2.88548	3.04292	1.53491	3.06813	3.51386	3.36764	2.27808	1.6744	0.756131	9.40E-04	0.00130483	0.0805617	0.00291208	0.902322	0.832742389	0.966018836	24	12	24	0.603920829	0.861719289	24	23.79882867	0.206896488
ENSMUSG00000103255	Pcdhac1	protocadherin alpha subfamily C, 1 [Source:MGI Symbol;Acc:MGI:1891442]	12.0448	9.18003	10.1236	9.27889	9.09277	10.7559	9.85486	9.1244	6.70669	12.3135	9.21093	9.61161	9.68174	9.40373	0.467283192	0.966018836	4	4	24	0.431429381	0.805393068	24	20.08037689	0.097593246
ENSMUSG00000103260	Gm8146	predicted gene 8146 [Source:MGI Symbol;Acc:MGI:3644273]	0.776429	0.658516	0.716882	0.759051	0.867026	1.01997	0.661263	0.824111	0.788918	0.590501	0.779319	0.911844	0.578336	0.697786	0.119293142	0.542554768	24	4	24	0.198238742	0.768040178	24	18.24025536	0.069382877
ENSMUSG00000103310	Pcdha12	protocadherin alpha 12 [Source:MGI Symbol;Acc:MGI:1298370]	3.19644	2.50108	2.78092	2.65265	2.62619	3.10218	3.02624	2.66921	2.02991	3.49278	2.61029	2.75323	2.50324	3.22908	0.888482515	0.966018836	24	12	24	0.70566597	0.902918222	24	20.5997069	0.050598682
ENSMUSG00000103332	Pcdhga2	protocadherin gamma subfamily A, 2 [Source:MGI Symbol;Acc:MGI:1935214]	1.03015	0.882403	1.17426	1.01883	0.949519	0.992693	1.09609	1.07461	0.85036	1.66139	0.802453	0.889646	0.940671	1.03456	0.870491976	0.966018836	16	4	24	0.532684951	0.825678486	24	9.725507859	0.062844565
ENSMUSG00000103355	Gm2147	predicted gene 2147 [Source:MGI Symbol;Acc:MGI:3780316]	2.012	2.21133	2.45922	1.43348	2.11971	1.70189	1.53097	2.76583	2.05558	1.82859	2.23725	1.66532	1.73737	2.3397	0.094000699	0.490854203	8	16	24	0.208426467	0.768040178	24	6.350667784	0.126186239
ENSMUSG00000103357	Gm7804	predicted gene 7804 [Source:MGI Symbol;Acc:MGI:3643373]	0.558197	1.62129	0.931546	0.758715	1.12984	0.48952	0.280812	1.72966	1.40746	1.32186	0.474748	1.43987	1.41904	1.10013	0.432054949	0.966018836	8	12	24	0.218454507	0.768040178	24	7.007491615	0.2351615
ENSMUSG00000103364	Gm38157	predicted gene, 38157 [Source:MGI Symbol;Acc:MGI:5611385]	0.464757	0.551762	0.505738	0.56499	0.562859	0.58885	0.347016	0.440473	0.635935	0.528657	0.552139	0.557907	0.56617	0.440578	0.278343887	0.790064226	24	8	24	0.167976551	0.767020009	24	13.81873138	0.051246344
ENSMUSG00000103367	Gm38158	predicted gene, 38158 [Source:MGI Symbol;Acc:MGI:5611386]	1.83195	2.76738	2.4798	3.57449	1.66265	2.01711	3.04517	2.01195	2.93656	2.88029	3.23313	2.68992	2.94907	3.22378	0.908406451	0.966018836	16	8	24	0.333615871	0.769755361	24	8.975075894	0.128698326
ENSMUSG00000103375	Gm37181	predicted gene, 37181 [Source:MGI Symbol;Acc:MGI:5610409]	0.304066	0.683339	0.661727	0.55453	0.476242	0.49125	0.743033	0.409541	0.7975	0.351451	0.541906	0.676316	0.67347	0.563903	0.892578749	0.966018836	12	4	24	0.692770202	0.898177673	24	5.03814311	0.047386274
ENSMUSG00000103388	Gm37581	predicted gene, 37581 [Source:MGI Symbol;Acc:MGI:5610809]	1.3005	1.32187	1.51902	1.54712	2.37979	1.72279	1.46922	1.46648	2.23701	0.859199	2.418	1.84252	1.8286	1.76219	0.173121348	0.648600527	20	12	24	0.525098678	0.822381894	24	17.08459063	0.103932457
ENSMUSG00000103409	Lsmem2	leucine-rich single-pass membrane protein 2 [Source:MGI Symbol;Acc:MGI:3612240]	1.07319	1.28554	1.23469	1.52353	1.50937	1.3794	1.13111	2.23523	1.70348	1.78816	1.24028	1.50267	1.60779	1.01838	0.772122688	0.966018836	16	12	24	0.537313386	0.827923483	24	10.60930756	0.082312886
ENSMUSG00000103419	Gm29808	predicted gene, 29808 [Source:MGI Symbol;Acc:MGI:5588967]	3.41938	2.66171	2.99696	1.93317	3.39387	3.97003	4.94997	3.14901	1.42819	1.82654	2.68255	2.62974	2.032	2.11094	0.157813674	0.614233844	4	12	24	0.018531752	0.559812298	24	21.50126772	0.322960958
ENSMUSG00000103436	Gm36995	predicted gene, 36995 [Source:MGI Symbol;Acc:MGI:5610223]	1.88222	1.80356	1.8247	1.81656	1.87509	1.79094	1.60919	1.72601	1.63257	1.47165	1.1788	1.47785	1.45351	1.71608	0.854443826	0.966018836	12	8	24	0.370015435	0.792235171	24	4.194945587	0.0451217
ENSMUSG00000103439	Gm7019	predicted pseudogene 7019 [Source:MGI Symbol;Acc:MGI:3645821]	0.977732	2.26824	2.11893	1.09494	2.71089	2.16354	0.946803	2.92921	1.98305	1.94121	0.92356	1.65285	1.45377	2.12577	0.181442559	0.659020016	8	20	24	0.498413188	0.808179467	24	7.444524165	0.169173259
ENSMUSG00000103442	Pcdha1	protocadherin alpha 1 [Source:MGI Symbol;Acc:MGI:2150982]	0.6083	0.601029	0.543314	0.598144	0.51758	0.62183	0.605411	0.637352	0.589271	0.719983	0.597887	0.517553	0.509982	0.918726	0.887027852	0.966018836	8	12	24	0.304679971	0.768040178	24	6.352888061	0.051739639
ENSMUSG00000103451	Gm33973	predicted gene, 33973 [Source:MGI Symbol;Acc:MGI:5593132]	0.754987	0.76497	0.58554	0.612184	1.11801	0.847697	1.04899	0.671317	0.806604	0.427867	1.02986	0.832154	1.15651	0.491446	0.044627045	0.365845448	20	20	24	0.05337655	0.652322075	24	20.31771089	0.167847542
ENSMUSG00000103472	Pcdhga7	protocadherin gamma subfamily A, 7 [Source:MGI Symbol;Acc:MGI:1935219]	1.64476	1.90333	1.63925	1.61015	1.7155	1.89892	1.91175	1.71128	1.60673	2.15745	1.50673	1.72475	1.37057	1.81545	0.914502381	0.966018836	24	12	24	0.971285156	0.993017774	24	9.818398454	0.01043109
ENSMUSG00000103477	5930409G06Rik	RIKEN cDNA 5930409G06 gene [Source:MGI Symbol;Acc:MGI:1924316]	1.8086	1.66496	1.74918	1.09054	1.57454	1.4396	1.29755	1.21106	1.11931	1.08083	1.60346	1.31418	1.22126	1.49215	0.893307667	0.966018836	20	20	24	0.472457358	0.805393068	24	22.49281565	0.065416984
ENSMUSG00000103520	1110025M09Rik	RIKEN cDNA 1110025M09 gene [Source:MGI Symbol;Acc:MGI:1915885]	1.26471	1.54768	1.56252	1.84345	1.50362	1.62899	1.47213	2.05683	2.07944	1.59536	1.77667	1.28449	1.3414	1.54833	0.008047769	0.192760771	16	12	24	0.054209285	0.654887447	24	9.543444276	0.124021294
ENSMUSG00000103532	Gm4430	predicted gene 4430 [Source:MGI Symbol;Acc:MGI:3782614]	1.35729	1.03486	1.52535	1.60976	1.11829	1.69623	1.43551	1.37796	0.989046	1.49449	1.52516	1.47048	1.50623	1.14758	0.300218476	0.809362476	16	16	24	0.268270872	0.768040178	24	17.91959879	0.088305735
ENSMUSG00000103548	Gm37670	predicted gene, 37670 [Source:MGI Symbol;Acc:MGI:5610898]	102.547	88.1402	76.7686	69.8105	83.4755	73.7723	70.5082	77.1332	84.2357	69.4534	86.8603	66.2359	91.8321	87.7157	0.142596848	0.585944911	4	20	24	0.383839587	0.799546792	24	2.026295402	0.0957658
ENSMUSG00000103567	Pcdhga5	protocadherin gamma subfamily A, 5 [Source:MGI Symbol;Acc:MGI:1935217]	1.10701	1.22808	1.38428	1.06757	1.24601	1.53298	1.55051	1.65171	1.00621	1.88586	1.00497	1.62018	1.48582	1.3375	0.927004797	0.966018836	24	20	24	0.764781199	0.92403577	24	23.56190563	0.046363743
ENSMUSG00000103585	Pcdhgb4	protocadherin gamma subfamily B, 4 [Source:MGI Symbol;Acc:MGI:1935173]	0.796321	0.657945	0.818041	0.55262	0.714716	0.64633	0.727828	0.745127	0.66822	1.07874	0.627762	0.745644	0.578791	0.974448	0.928127947	0.966018836	12	8	24	0.57552055	0.848060327	24	7.546659867	0.049924819
ENSMUSG00000103632	Gm38329	predicted gene, 38329 [Source:MGI Symbol;Acc:MGI:5611557]	2.73983	1.4617	1.9811	1.88699	1.11833	1.44139	1.29226	1.88564	0.825352	1.0069	1.4451	0.68913	1.0246	1.63758	0.631736745	0.966018836	8	16	24	0.492717465	0.805558817	24	3.52302825	0.116528135
ENSMUSG00000103649	Gm37768	predicted gene, 37768 [Source:MGI Symbol;Acc:MGI:5610996]	3.51548	2.90225	1.9709	2.89511	2.6284	3.11455	2.34476	2.8506	2.66227	2.35908	2.86813	2.76929	3.00211	2.46551	0.46238066	0.966018836	24	12	24	0.139676649	0.74956518	24	21.25398659	0.113533363
ENSMUSG00000103651	Gm37206	predicted gene, 37206 [Source:MGI Symbol;Acc:MGI:5610434]	0.777828	0.683929	0.770599	0.991972	1.44691	0.890999	1.40295	1.00861	1.65541	1.006	0.86784	1.32447	1.4755	1.3155	0.807813307	0.966018836	4	4	24	0.990861825	0.99839802	24	22.42151849	0.009630643
ENSMUSG00000103653	Gstp-ps	glutathione S-transferase, pi, pseudogene [Source:MGI Symbol;Acc:MGI:3782108]	53.8975	48.5339	53.5526	53.3575	54.2103	57.1646	55.1274	54.2093	48.3797	54.6223	47.6672	54.2216	52.5158	46.0128	0.217827365	0.704989686	24	12	24	0.201966578	0.768040178	24	19.08992086	0.061475869
ENSMUSG00000103658	Gm37201	predicted gene, 37201 [Source:MGI Symbol;Acc:MGI:5610429]	3.22455	3.09764	3.98375	3.65841	3.37342	3.93022	2.97337	4.1509	2.56962	3.16135	3.33531	3.21858	3.27988	2.6201	0.881996584	0.966018836	20	16	24	0.674754644	0.89097199	24	17.0282951	0.052536676
ENSMUSG00000103662	Gm34294	predicted gene, 34294 [Source:MGI Symbol;Acc:MGI:5593453]	1.03232	1.17426	0.955723	1.00294	1.08391	0.921484	0.92035	0.937369	0.97155	0.96838	1.00058	1.14399	1.23525	0.890662	0.928148816	0.966018836	20	12	24	0.580647447	0.850320846	24	20.84366727	0.031811714
ENSMUSG00000103677	Pcdhga4	protocadherin gamma subfamily A, 4 [Source:MGI Symbol;Acc:MGI:1935216]	1.81912	1.33592	1.34901	1.26317	1.27479	1.42911	2.0799	1.56583	1.43208	1.74189	1.09049	1.22216	1.19481	1.31933	0.834424969	0.966018836	4	16	24	0.450243394	0.805393068	24	1.877608695	0.072796044
ENSMUSG00000103685	Gm37074	predicted gene, 37074 [Source:MGI Symbol;Acc:MGI:5610302]	1.60373	0.984128	1.07789	1.52171	1.56081	1.00894	1.78679	0.762529	1.22654	1.13709	1.56235	0.929012	1.09677	1.01422	0.192357053	0.669841558	20	12	24	0.310678545	0.768040178	24	16.93373818	0.114615307
ENSMUSG00000103703	Gm42568	predicted gene 42568 [Source:MGI Symbol;Acc:MGI:5662705]	1.74733	1.45392	1.23335	1.30588	1.40285	1.50848	1.41382	1.44205	1.17953	1.59071	1.75973	1.65022	1.42751	1.52316	0.22872448	0.72665454	24	12	24	0.088493557	0.707747429	24	20.06498893	0.083608444
ENSMUSG00000103707	Pcdha6	protocadherin alpha 6 [Source:MGI Symbol;Acc:MGI:1298367]	2.12887	1.72418	2.15869	1.80315	1.45139	2.52192	2.04885	2.09262	1.39684	2.67641	1.67434	2.07639	2.2294	2.17665	0.92655264	0.966018836	24	20	24	0.650038889	0.882327323	24	23.60829443	0.065822087
ENSMUSG00000103711	Tstd1	thiosulfate sulfurtransferase (rhodanese)-like domain containing 1 [Source:MGI Symbol;Acc:MGI:3648482]	8.2602	8.36816	6.284	6.70327	7.58449	7.25002	6.64851	6.976	7.51972	7.1598	8.81212	8.50308	8.37048	8.53397	0.619356354	0.966018836	20	20	24	0.368982061	0.791960403	24	21.75933293	0.072372729
ENSMUSG00000103715	4933431K14Rik	RIKEN cDNA 4933431K14 gene [Source:MGI Symbol;Acc:MGI:1918545]	0.667272	0.833768	0.97996	0.729075	0.701233	0.664805	0.611199	0.697301	0.758369	0.872897	0.560415	0.890167	0.63236	0.724848	0.058157448	0.40386543	12	16	24	0.224375527	0.768040178	24	8.941480781	0.065504036
ENSMUSG00000103731	Gm17530	predicted gene, 17530 [Source:MGI Symbol;Acc:MGI:4937164]	3.24477	2.95544	4.08458	2.49562	2.58458	2.32247	2.36397	2.41758	3.70031	2.06007	3.8837	2.59467	2.4409	2.77646	0.581070934	0.966018836	12	12	24	0.588681308	0.853936396	24	8.543789306	0.094934655
ENSMUSG00000103735	Gm38317	predicted gene, 38317 [Source:MGI Symbol;Acc:MGI:5611545]	17.0168	16.803	22.5517	16.7371	23.1017	15.6662	21.6585	17.7127	18.7436	14.6738	14.4591	15.0422	17.5972	15.7441	0.44861185	0.966018836	12	4	24	0.825788688	0.94544881	24	5.408677256	0.048919332
ENSMUSG00000103749	Pcdhgb5	protocadherin gamma subfamily B, 5 [Source:MGI Symbol;Acc:MGI:1935196]	0.776171	0.513559	0.740786	0.799225	0.867892	0.714694	0.809451	0.754217	0.692114	0.96125	0.599277	0.864539	0.854193	0.80595	0.625825704	0.966018836	16	20	24	0.723860538	0.909441374	24	16.40804381	0.02941596
ENSMUSG00000103756	Gm37285	predicted gene, 37285 [Source:MGI Symbol;Acc:MGI:5610513]	0.366104	0.599787	0.486701	0.607458	0.609903	0.508805	0.496553	0.583343	0.750576	0.591212	0.448073	0.7254	0.270723	0.424304	0.150109238	0.595846665	16	12	24	0.244340123	0.768040178	24	11.79703983	0.08098128
ENSMUSG00000103770	Pcdha9	protocadherin alpha 9 [Source:MGI Symbol;Acc:MGI:2447322]	3.00998	1.8075	1.7866	2.33197	2.2146	2.44645	2.15512	2.40869	1.6543	2.85643	1.99163	2.64111	2.1326	2.63609	0.245061943	0.741580974	24	12	24	0.425786694	0.805393068	24	20.30529894	0.09511645
ENSMUSG00000103784	Gm37526	predicted gene, 37526 [Source:MGI Symbol;Acc:MGI:5610754]	1.13813	0.976828	0.760874	0.897613	1.17113	1.03604	0.532819	1.0144	0.834577	1.1574	1.07778	0.729692	0.702603	0.594965	0.246839642	0.745922296	20	8	24	0.141505881	0.751766185	24	15.01794273	0.110537276
ENSMUSG00000103793	Pcdhga6	protocadherin gamma subfamily A, 6 [Source:MGI Symbol;Acc:MGI:1935218]	1.70227	1.38989	2.23664	1.37128	1.64713	1.99588	1.70294	1.67772	1.50552	2.5323	1.29874	1.89989	1.63537	1.56002	0.892555977	0.966018836	24	20	24	0.916498534	0.976967742	24	13.67767885	0.029074335
ENSMUSG00000103839	Gm37607	predicted gene, 37607 [Source:MGI Symbol;Acc:MGI:5610835]	1.86668	1.94973	1.87405	1.63379	1.72089	1.64539	1.98696	1.75032	2.09346	1.65784	1.8008	2.07326	1.78396	2.1074	0.175272072	0.651010555	12	4	24	0.136182222	0.747972781	24	3.379059148	0.060124904
ENSMUSG00000103847	Gm20056	predicted gene, 20056 [Source:MGI Symbol;Acc:MGI:5012241]	54.8785	39.9271	41.2661	40.0537	40.1002	46.0481	43.4645	43.874	34.5462	51.009	44.3814	42.6865	44.1845	41.7291	0.156274615	0.611802982	4	8	24	0.287016663	0.768040178	24	20.82751247	0.099451533
ENSMUSG00000103897	Pcdhga8	protocadherin gamma subfamily A, 8 [Source:MGI Symbol;Acc:MGI:1935221]	0.836099	0.74943	0.96076	0.846355	0.728718	0.971033	1.18642	0.955728	0.756912	0.92329	0.700175	0.755453	0.722993	1.11485	0.849179819	0.966018836	8	12	24	0.401725579	0.799546792	24	3.662522829	0.060436815
ENSMUSG00000103898	Gm30238	predicted gene, 30238 [Source:MGI Symbol;Acc:MGI:5589397]	0.84366	1.06546	0.953548	0.898455	1.0752	0.898937	0.733869	0.962545	0.94423	0.867875	1.05347	1.1018	0.958552	1.17643	0.904387595	0.966018836	8	20	24	0.891517864	0.969321764	24	10.30554445	0.016076511
ENSMUSG00000103901	Gm37499	predicted gene, 37499 [Source:MGI Symbol;Acc:MGI:5610727]	2.06488	1.83543	1.48111	1.8604	2.00606	1.71267	1.66388	1.52431	1.70858	1.51333	2.02944	1.55958	1.79781	1.76413	0.7207001	0.966018836	20	16	24	0.375380815	0.795468467	24	18.96455323	0.056214125
ENSMUSG00000103906	Tigd5	tigger transposable element derived 5 [Source:MGI Symbol;Acc:MGI:2145902]	0.562821	0.456011	0.594126	0.50244	0.482342	0.595141	0.615369	0.613805	0.549173	0.709515	0.465857	0.605518	0.588267	0.547747	0.891742979	0.966018836	24	20	24	0.885519496	0.967390811	24	3.036839498	0.012190999
ENSMUSG00000103921	Gm5841	predicted gene 5841 [Source:MGI Symbol;Acc:MGI:3645248]	0.904543	1.35117	1.30541	1.4737	1.34671	0.920435	1.22575	1.06012	1.50754	1.0779	1.31694	1.53583	1.4103	1.22335	0.916963526	0.966018836	12	20	24	0.47817046	0.805393068	24	10.92788726	0.06271316
ENSMUSG00000103922	Gm6123	predicted gene 6123 [Source:MGI Symbol;Acc:MGI:3647047]	36.9112	36.39	35.4247	43.8349	36.292	35.2209	38.2	39.04	36.4801	38.315	42.9545	38.5737	42.5802	37.6923	0.911371589	0.966018836	16	20	24	0.699791866	0.900387881	24	14.90298662	0.030903634
ENSMUSG00000103928	Gm37893	predicted gene, 37893 [Source:MGI Symbol;Acc:MGI:5611121]	2.47237	2.26893	2.52029	2.93209	2.80533	2.0085	2.0858	2.39424	3.07538	1.72477	2.88599	2.21045	2.28879	2.4614	0.138202217	0.580158868	12	12	24	0.478820087	0.805393068	24	10.8710713	0.078632987
ENSMUSG00000103953	Gm29718	predicted gene, 29718 [Source:MGI Symbol;Acc:MGI:5588877]	5.36331	6.97947	9.56228	6.93505	8.00839	8.78632	8.21894	7.13743	7.54242	6.16488	5.82005	5.84518	7.97488	4.97272	0.819898687	0.966018836	12	20	24	0.984228544	0.996767829	24	12.33442748	0.016599412
ENSMUSG00000103955	Gm37270	predicted gene, 37270 [Source:MGI Symbol;Acc:MGI:5610498]	8.20945	2.03533	1.42092	9.19472	8.38593	14.1512	10.834	8.96465	7.00199	11.1088	7.40139	10.7863	4.41811	8.9704	0.148073804	0.590732968	24	12	24	0.181022463	0.768040178	24	18.29800715	0.515935041
ENSMUSG00000103957	Gm10766	predicted gene 10766 [Source:MGI Symbol;Acc:MGI:3642134]	0.540867	0.594264	1.41932	1.02646	0.334801	0.507736	0.548535	0.241	0.859157	0.604055	1.16042	0.637827	0.84718	0.885533	0.40165019	0.933249185	12	12	24	0.310612557	0.768040178	24	10.17240108	0.159742003
ENSMUSG00000103967	Gm38214	predicted gene, 38214 [Source:MGI Symbol;Acc:MGI:5611442]	0.337164	0.636454	0.523799	0.66305	0.757207	0.492224	0.705226	0.301921	0.394766	0.350044	0.695588	0.614174	0.384969	0.539444	0.478724519	0.966018836	20	12	24	0.285118726	0.768040178	24	16.67488572	0.090061301
ENSMUSG00000103983	Gm20045	predicted gene, 20045 [Source:MGI Symbol;Acc:MGI:5012230]	1.32207	1.43227	1.47902	1.67118	1.62542	1.34158	1.59032	1.43945	1.77164	1.15274	1.94496	1.58347	1.38064	1.5418	0.537028703	0.966018836	20	8	24	0.598480549	0.85898917	24	13.57280268	0.045201545
ENSMUSG00000104011	Gm32391	predicted gene, 32391 [Source:MGI Symbol;Acc:MGI:5591550]	1.06118	1.23661	1.38273	1.20501	1.17895	1.48984	1.16123	1.60441	1.29881	1.33306	1.52027	1.09141	1.77763	2.09429	0.92655264	0.966018836	8	16	24	0.221413623	0.768040178	24	5.26107568	0.088127458
ENSMUSG00000104018	4833412K13Rik	RIKEN cDNA 4833412K13 gene [Source:MGI Symbol;Acc:MGI:1921857]	0.542358	1.01163	0.787051	0.866899	0.860023	0.720946	0.625671	0.892322	0.824325	0.457749	0.747827	0.517352	0.761177	0.940047	0.018894761	0.256345714	8	16	24	0.271761476	0.768040178	24	6.577131202	0.089213436
ENSMUSG00000104033	Gm37773	predicted gene, 37773 [Source:MGI Symbol;Acc:MGI:5611001]	2.55	2.53394	2.33461	2.91649	3.09158	2.47197	2.79508	2.03654	2.82591	1.72421	3.80036	2.19769	1.66661	2.0688	0.305564675	0.811201458	20	12	24	0.225534863	0.768040178	24	15.72279724	0.143124109
ENSMUSG00000104063	Pcdhgb7	protocadherin gamma subfamily B, 7 [Source:MGI Symbol;Acc:MGI:1935199]	0.962147	0.857249	0.994791	0.597831	0.708201	0.960218	0.734247	0.989786	0.97496	1.14299	0.705045	1.08576	0.920731	1.04977	0.725061903	0.966018836	12	8	24	0.382608845	0.799546792	24	4.141844179	0.064645428
ENSMUSG00000104093	A330015K06Rik	RIKEN cDNA A330015K06 gene [Source:MGI Symbol;Acc:MGI:2443553]	3.56704	4.74453	3.94331	4.49464	4.87805	3.99275	4.44673	3.38584	4.79595	2.94188	4.39613	4.67072	4.30217	3.79985	0.945282252	0.966018836	20	12	24	0.731207912	0.912382349	24	18.47011751	0.056167997
ENSMUSG00000104097	Gm37313	predicted gene, 37313 [Source:MGI Symbol;Acc:MGI:5610541]	2.21122	2.69392	2.73267	2.09287	4.30843	2.75228	6.09443	1.3788	2.33739	1.67388	2.7396	2.90977	4.74214	2.76152	0.144748194	0.589637022	4	12	24	0.201496547	0.768040178	24	21.36782104	0.294686533
ENSMUSG00000104103	Gm9517	predicted gene 9517 [Source:MGI Symbol;Acc:MGI:3779430]	1.95792	2.92709	2.67616	2.94786	2.75991	2.28331	2.25133	1.95182	3.85657	1.88507	3.57378	2.85637	3.09497	2.80464	0.881996584	0.966018836	12	20	24	0.596330463	0.857839743	24	11.4255077	0.090342694
ENSMUSG00000104117	Gm20743	predicted gene, 20743 [Source:MGI Symbol;Acc:MGI:5434099]	0.521469	0.808013	0.703883	0.678289	0.654673	0.649026	0.616881	0.616585	0.778881	0.566799	0.670551	0.707981	0.72836	1.00108	0.521828922	0.966018836	8	20	24	0.191461991	0.768040178	24	6.011763325	0.062488386
ENSMUSG00000104126	Gm37486	predicted gene, 37486 [Source:MGI Symbol;Acc:MGI:5610714]	13.2972	16.2861	11.5342	10.6548	10.9655	10.7154	10.8786	13.9527	10.5808	10.4472	9.33969	13.4617	11.8757	11.536	0.013969059	0.241049421	8	8	24	0.033949373	0.606793499	24	2.126680675	0.167640488
ENSMUSG00000104148	Pcdha2	protocadherin alpha 2 [Source:MGI Symbol;Acc:MGI:2681880]	2.23245	1.34197	1.49853	1.96326	1.27996	1.79575	1.23678	1.67011	1.48255	2.16915	1.51595	1.6991	1.52197	1.63216	0.93717134	0.966018836	16	20	24	0.849455717	0.954767108	24	14.24907765	0.037147053
ENSMUSG00000104156	Gm38102	predicted gene, 38102 [Source:MGI Symbol;Acc:MGI:5611330]	8.33164	9.42973	9.19791	10.3609	10.6494	8.38963	9.64306	9.58562	12.5508	9.41011	10.1612	9.1258	8.8992	9.39633	0.168929279	0.640164678	20	4	24	0.062454327	0.661961741	24	10.55214038	0.118243615
ENSMUSG00000104168	Gm38250	predicted gene, 38250 [Source:MGI Symbol;Acc:MGI:5611478]	2.05141	2.11324	1.36386	1.8124	2.06946	2.76529	2.0141	1.88831	1.49027	1.74559	2.67515	2.36748	1.67655	1.93985	0.002399962	0.108147525	24	12	24	0.008627198	0.502965379	24	19.45553558	0.203656993
ENSMUSG00000104178	Gm9916	predicted gene 9916 [Source:MGI Symbol;Acc:MGI:3642633]	3.65185	2.80446	2.31051	2.74113	2.87911	2.9256	2.72981	2.60616	2.90386	2.09234	3.05412	3.43955	3.44597	3.07876	0.074318596	0.448301997	4	12	24	0.027294532	0.589956102	24	22.05141279	0.152033552
ENSMUSG00000104202	Gm8101	predicted gene 8101 [Source:MGI Symbol;Acc:MGI:3649004]	1.40016	2.19023	1.51954	1.29623	2.53585	1.28176	3.09159	1.24227	1.46911	1.34686	1.0391	0.984687	0.598306	1.36052	0.885955579	0.966018836	12	12	24	0.981119867	0.995579529	24	3.809020156	0.023741624
ENSMUSG00000104204	Gm37397	predicted gene, 37397 [Source:MGI Symbol;Acc:MGI:5610625]	0.850877	1.0584	0.87232	1.035	0.920905	0.863969	1.00974	1.0091	0.759053	0.663645	0.850419	0.778097	0.841293	0.879431	0.481811994	0.966018836	8	16	24	0.443743297	0.805393068	24	1.904506211	0.038140477
ENSMUSG00000104208	Gm37401	predicted gene, 37401 [Source:MGI Symbol;Acc:MGI:5610629]	60.1137	54.2173	53.9986	60.1578	60.3741	54.9187	63.1981	46.8012	73.0361	60.5201	53.779	66.3128	74.0713	62.2218	0.935593607	0.966018836	4	16	24	0.955858788	0.988639288	24	21.60584252	0.020330861
ENSMUSG00000104212	Gm37986	predicted gene, 37986 [Source:MGI Symbol;Acc:MGI:5611214]	1.46408	1.47765	0.595078	1.02654	1.3379	1.1158	0.886571	0.968682	0.495252	1.45323	1.11287	0.95053	0.864831	1.41697	0.9143748	0.966018836	8	4	24	0.625627535	0.872249537	24	19.86483871	0.092377195
ENSMUSG00000104213	Ighd	immunoglobulin heavy constant delta [Source:MGI Symbol;Acc:MGI:96447]	0.482358	1.0186	1.28592	1.55593	0.83422	0.696642	1.2377	0.952967	2.09607	0.876068	1.04837	1.19524	1.29785	0.625947	0.745469888	0.966018836	12	20	24	0.278340333	0.768040178	24	9.705458024	0.174091865
ENSMUSG00000104218	Gm9134	predicted gene 9134 [Source:MGI Symbol;Acc:MGI:3645181]	6.1845	5.06654	4.36533	3.7474	5.80522	3.88044	5.38992	3.30129	4.55666	3.9525	4.8539	4.70224	3.93553	3.90866	0.798829513	0.966018836	20	20	24	0.280202864	0.768040178	24	20.1465477	0.124878976
ENSMUSG00000104231	Gm37592	predicted gene, 37592 [Source:MGI Symbol;Acc:MGI:5610820]	0.412557	1.26757	0.41569	1.2894	0.931964	0.871122	1.23597	0.820696	1.07625	0.539877	1.34177	1.10257	0.285776	0.842832	0.851656735	0.966018836	20	8	24	0.677433496	0.892060593	24	14.875289	0.101110049
ENSMUSG00000104241	Gm9442	predicted gene 9442 [Source:MGI Symbol;Acc:MGI:3779852]	0.7746	0.389538	0.558939	1.43439	0.746872	0.849566	0.311839	0.905333	0.522132	0.5129	0.346207	1.18301	0.526276	0.203111	0.839634479	0.966018836	24	8	24	0.358162234	0.784922891	24	16.56099733	0.155465079
ENSMUSG00000104245	Gm38155	predicted gene, 38155 [Source:MGI Symbol;Acc:MGI:5611383]	0.593736	1.00758	0.977637	0.772948	0.81416	0.634934	0.818957	0.916999	0.969483	0.613285	1.0728	0.598982	0.838733	0.743238	0.245061943	0.741580974	12	12	24	0.288626407	0.768040178	24	7.8438779	0.082126688
ENSMUSG00000104252	Pcdha4	protocadherin alpha 4 [Source:MGI Symbol;Acc:MGI:1298406]	3.63209	2.91488	2.97667	3.21987	3.05645	3.39852	2.6553	2.82967	1.98771	3.45439	2.3473	2.49566	2.67061	3.02549	0.862909389	0.966018836	16	4	24	0.820726762	0.943353655	24	19.61555681	0.040588808
ENSMUSG00000104277	Gm38299	predicted gene, 38299 [Source:MGI Symbol;Acc:MGI:5611527]	0.338231	0.883393	0.570084	0.478784	0.41124	0.567713	0.600429	0.412184	0.702513	0.497141	0.61093	0.636603	0.868672	0.717412	0.783267549	0.966018836	8	8	24	0.390674428	0.799546792	24	3.838400316	0.071342983
ENSMUSG00000104279	Gm38300	predicted gene, 38300 [Source:MGI Symbol;Acc:MGI:5611528]	0.742728	0.614195	0.479887	0.454652	0.610973	0.662009	0.680704	0.604544	0.49238	0.827792	0.576618	0.57508	0.493408	0.567844	0.541963377	0.966018836	4	8	24	0.525950794	0.822909234	24	20.49147113	0.043527251
ENSMUSG00000104309	Gm5846	predicted gene 5846 [Source:MGI Symbol;Acc:MGI:3643624]	5.54793	4.77324	5.41505	4.8146	5.97519	5.1999	5.34335	4.43737	4.14093	4.81633	4.28291	4.77911	4.70064	5.04182	0.882263435	0.966018836	4	4	24	0.337865123	0.772644419	24	20.25608617	0.06455172
ENSMUSG00000104318	Pcdha7	protocadherin alpha 7 [Source:MGI Symbol;Acc:MGI:1298369]	1.6832	1.27464	1.68852	1.5583	1.22537	1.60876	1.34657	1.86182	1.07386	1.88938	1.47342	1.73275	1.48489	1.66781	0.858266942	0.966018836	16	4	24	0.986603075	0.997339197	24	23.40943802	0.009391552
ENSMUSG00000104330	Gm38285	predicted gene, 38285 [Source:MGI Symbol;Acc:MGI:5611513]	0.419045	0.452454	0.626524	0.240176	0.313454	0.356576	0.447262	0.703229	0.393403	0.865211	0.576004	0.868806	0.69806	0.783133	0.941885246	0.966018836	8	12	24	0.526051624	0.822948463	24	4.473627936	0.06517849
ENSMUSG00000104344	Gm38077	predicted gene, 38077 [Source:MGI Symbol;Acc:MGI:5611305]	0.526418	0.8433	0.654552	0.769473	0.880877	0.557746	0.832082	0.738954	1.28983	0.412467	0.835729	0.87152	0.988357	0.844695	0.790218349	0.966018836	20	20	24	0.740477826	0.915276515	24	5.446495524	0.051914306
ENSMUSG00000104346	Pcdhga3	protocadherin gamma subfamily A, 3 [Source:MGI Symbol;Acc:MGI:1935215]	2.80914	2.06302	2.26608	2.08644	1.86285	2.32085	2.49905	2.35546	1.84492	2.87595	1.85243	2.39001	1.88958	2.31681	0.591351901	0.966018836	4	16	24	0.721135835	0.90866625	24	0.487097601	0.047144946
ENSMUSG00000104348	Gm37691	predicted gene, 37691 [Source:MGI Symbol;Acc:MGI:5610919]	0.755629	0.869679	0.840287	0.928593	0.779314	0.638018	0.863506	0.885933	0.92225	0.65089	0.658106	0.813225	0.81786	0.887306	0.038471411	0.335882166	12	8	24	0.043742695	0.626975067	24	5.536564147	0.073259068
ENSMUSG00000104361	Gm38080	predicted gene, 38080 [Source:MGI Symbol;Acc:MGI:5611308]	1.51486	2.08467	2.04567	1.66321	3.23921	1.43402	2.28025	3.6226	2.03431	1.00356	1.76039	2.25334	0.778473	2.4306	0.496745696	0.966018836	8	8	24	0.850126448	0.955046946	24	5.595022095	0.086935952
ENSMUSG00000104362	Gm37928	predicted gene, 37928 [Source:MGI Symbol;Acc:MGI:5611156]	6.46003	7.13792	6.54385	7.36912	7.61411	5.92934	5.92536	5.41671	8.7413	5.20068	8.16062	6.08775	7.00383	7.66517	0.777666236	0.966018836	20	4	24	0.65310678	0.883297283	24	10.64455108	0.072722266
ENSMUSG00000104379	Gm37509	predicted gene, 37509 [Source:MGI Symbol;Acc:MGI:5610737]	1.78751	2.28657	1.76675	2.62318	3.47269	2.00948	2.79675	1.76366	2.7337	1.60509	3.09538	2.90486	2.55166	2.13758	0.136853693	0.578758359	20	12	24	0.229601825	0.768040178	24	17.34513323	0.159174602
ENSMUSG00000104394	Gm37254	predicted gene, 37254 [Source:MGI Symbol;Acc:MGI:5610482]	0.836086	0.565881	0.518425	0.80667	0.566477	0.720167	0.737737	0.637831	0.666956	0.496203	0.804805	0.570494	0.751303	0.453459	0.369262716	0.893596184	4	4	24	0.276689159	0.768040178	24	20.03506182	0.069445073
ENSMUSG00000104399	Gm37963	predicted gene, 37963 [Source:MGI Symbol;Acc:MGI:5611191]	1.77455	3.14977	1.92779	2.22327	2.50514	2.49946	2.6998	2.13491	2.65806	2.75658	2.42694	2.33273	2.61124	1.34041	0.786317982	0.966018836	16	16	24	0.665337184	0.886947643	24	16.41675596	0.057540598
ENSMUSG00000104420	E230020A03Rik	RIKEN cDNA E230020A03 gene [Source:MGI Symbol;Acc:MGI:2442139]	0.819321	0.689059	0.75601	0.719005	0.576603	0.640765	0.740262	0.624297	0.707237	0.682246	1.03326	0.689728	0.792326	0.777894	0.664568268	0.966018836	4	20	24	0.983251553	0.996355202	24	19.89076778	0.006701036

ENSMUSG00000104435	Gm37422	predicted gene, 37422 [Source:MGI Symbol;Acc:MGI:5610650]	1.07428	1.23097	1.14935	1.3417	1.30377	1.12772	1.13903	1.31313	1.57069	1.09471	1.42724	0.958983	1.18697	1.10494	0.182320384	0.659303873	20	4	24	0.194627588	0.768040178	24	10.31862548	0.072269115
ENSMUSG00000104453	Gm37829	predicted gene, 37829 [Source:MGI Symbol;Acc:MGI:5611057]	1.51468	2.05541	2.16081	2.61578	1.7883	2.30982	2.02903	3.45571	2.56596	3.23039	2.27003	1.83014	2.5293	2.07296	0.151535314	0.599319068	16	4	24	0.126201555	0.739868076	24	9.130451926	0.168802433
ENSMUSG00000104494	Gm37111	predicted gene, 37111 [Source:MGI Symbol;Acc:MGI:5610339]	0.338929	1.59024	3.22199	0.86917	0.882033	1.43321	2.26938	1.13318	1.78894	1.06413	0.811626	1.08606	0.509854	1.85396	0.218388267	0.706199204	12	8	24	0.229438867	0.768040178	24	7.00258884	0.301253285
ENSMUSG00000104496	Gm5837	predicted gene 5837 [Source:MGI Symbol;Acc:MGI:3648028]	14.2743	12.7139	13.6686	12.5604	12.1484	14.1579	13.2151	14.2219	10.5929	13.6	12.2398	12.8473	12.6417	12.1031	0.745118544	0.966018836	4	16	24	0.333703713	0.769855499	24	22.3014555	0.059709838
ENSMUSG00000104520	Gm37336	predicted gene, 37336 [Source:MGI Symbol;Acc:MGI:5610564]	0.526058	0.632753	0.778139	0.598662	0.466356	0.985804	0.322275	0.23326	0.979104	1.04611	0.675868	0.806115	0.873324	0.150213	0.20729371	0.698501896	12	20	24	0.303895989	0.768040178	24	14.67603736	0.154084831
ENSMUSG00000104523	Gm37335	predicted gene, 37335 [Source:MGI Symbol;Acc:MGI:5610563]	7.32821	6.24619	7.14731	10.0036	7.3801	9.55443	7.06117	7.38409	8.18939	8.42153	6.41275	8.64521	5.91356	6.58779	0.105163243	0.518002936	16	12	24	0.187069887	0.768040178	24	14.55400657	0.135173108
ENSMUSG00000104546	Gm43858	predicted gene 43858 [Source:MGI Symbol;Acc:MGI:5663995]	2.25734	2.6469	2.05597	2.77002	2.38395	2.07098	2.84816	2.84238	3.22349	2.23637	3.4197	2.69724	2.77137	2.42031	0.838244962	0.966018836	20	4	24	0.71354888	0.905668057	24	14.68912908	0.054120727
ENSMUSG00000104548	Gm43857	predicted gene 43857 [Source:MGI Symbol;Acc:MGI:5663994]	1.64853	1.61325	1.80643	1.69695	1.70649	1.31788	1.21523	1.76194	1.50404	1.21054	2.17634	1.43002	1.14876	1.92628	0.428730128	0.966018836	8	20	24	0.545886425	0.833198211	24	10.23570544	0.073843264
ENSMUSG00000104560	Gm8115	predicted gene 8115 [Source:MGI Symbol;Acc:MGI:3648794]	1.25144	0.635289	1.0579	1.43017	1.24417	1.21867	0.677744	0.931483	0.568872	0.551347	0.858038	0.711367	0.881775	0.365594	0.708665292	0.966018836	20	12	24	0.077526645	0.687420473	24	17.56755197	0.1651318
ENSMUSG00000104597	B230334C09Rik	RIKEN cDNA B230334C09 gene [Source:MGI Symbol;Acc:MGI:2442224]	0.510648	0.913998	1.09749	0.763749	0.756061	0.752943	0.842292	0.636681	1.26558	0.54122	0.637338	0.859379	0.541171	0.927876	0.170659409	0.643496587	12	16	24	0.25269055	0.768040178	24	7.722113225	0.113532903
ENSMUSG00000104610	Gm567	predicted gene 567 [Source:MGI Symbol;Acc:MGI:2685413]	1.70403	2.38767	3.69828	2.51981	2.60966	2.71141	3.02388	1.51124	3.11393	2.86077	1.87168	2.11362	3.08181	2.50442	0.675497492	0.966018836	12	20	24	0.56675676	0.843547308	24	9.615662775	0.111020709
ENSMUSG00000104623	Gm6394	predicted gene 6394 [Source:MGI Symbol;Acc:MGI:3648319]	16.0899	20.6799	18.5702	18.7824	23.931	21.6443	18.9191	17.6736	17.4256	16.0456	15.7228	14.4894	15.178	13.0656	0.7866493	0.966018836	20	8	24	0.40660088	0.799546792	24	16.08774609	0.086317242
ENSMUSG00000104642	Gm9207	predicted gene 9207 [Source:MGI Symbol;Acc:MGI:3779840]	3.04474	3.39271	3.76532	3.64051	4.18897	4.12533	3.66553	3.32664	3.64814	4.29837	3.7321	3.02868	3.72	2.73543	0.061928188	0.409814092	20	12	24	0.06680044	0.669146244	24	14.46483689	0.119413476
ENSMUSG00000104649	Gm43712	predicted gene 43712 [Source:MGI Symbol;Acc:MGI:5663849]	108.156	121.579	110.517	100.413	105.35	101.603	110.765	114.96	127.17	107.354	120.28	111.568	123.196	113.813	0.218388267	0.706199204	8	8	24	0.152498345	0.759088284	24	4.735427639	0.069516808
ENSMUSG00000104671	Gm43062	predicted gene 43062 [Source:MGI Symbol;Acc:MGI:5663199]	1.75024	1.39632	1.4187	0.947173	1.10756	1.26979	1.04771	1.10816	0.750863	1.08812	1.11136	1.02634	1.301	1.14853	0.515451644	0.966018836	4	12	24	0.117773007	0.728323355	24	23.61096748	0.107107403
ENSMUSG00000104677	Gm43376	predicted gene 43376 [Source:MGI Symbol;Acc:MGI:5663513]	3.08241	2.88018	2.12879	2.82499	2.63802	2.75598	2.7447	2.33776	2.24467	2.72857	2.83213	2.92927	2.84805	3.12377	0.122341925	0.550724475	4	8	24	0.139851129	0.74956518	24	21.01741941	0.089932351
ENSMUSG00000104693	Gm42941	predicted gene 42941 [Source:MGI Symbol;Acc:MGI:5663078]	0.753696	0.888614	1.00628	1.02434	1.41421	0.58505	0.87855	0.921871	1.49913	0.633637	1.29829	0.850159	0.865827	0.917215	0.011317069	0.204837764	20	4	24	0.316489245	0.768040178	24	11.16494719	0.113990968
ENSMUSG00000104699	Rps4x-ps	Rps4x retrotransposed pseudogene [Source:MGI Symbol;Acc:MGI:3644569]	6.25549	8.40688	8.72332	7.51841	8.6793	5.97382	6.77822	6.89066	7.80216	5.55328	7.54495	6.82273	6.40694	6.87021	0.071575474	0.440262583	12	12	24	0.406689524	0.799546792	24	9.645576686	0.088855478
ENSMUSG00000104713	Gbp6	guanylate binding protein 6 [Source:MGI Symbol;Acc:MGI:2140937]	2.26057	1.94757	1.77451	1.38403	2.28837	1.7687	1.99603	3.06874	1.80724	1.30862	1.96264	1.67445	1.92428	2.58158	0.027096347	0.306445942	8	8	24	0.106561555	0.718721872	24	1.89714246	0.165766812
ENSMUSG00000104737	Gm42937	predicted gene 42937 [Source:MGI Symbol;Acc:MGI:5663074]	4.2601	4.77199	3.01345	5.2567	4.64034	3.93968	3.79075	3.9872	4.7037	4.37926	4.31295	3.71789	4.64833	5.02622	0.46238066	0.966018836	16	8	24	0.822089932	0.943977421	24	10.12454154	0.042899769
ENSMUSG00000104777	Gm4865	predicted gene 4865 [Source:MGI Symbol;Acc:MGI:3647137]	1.83481	1.8487	1.46878	1.39332	1.22113	1.81289	1.64871	1.64569	1.34916	1.18913	1.57424	1.38784	1.28942	1.28744	0.373664134	0.896700272	4	12	24	0.041056091	0.61771321	24	23.64881642	0.098687942
ENSMUSG00000104814	Gm42979	predicted gene 42979 [Source:MGI Symbol;Acc:MGI:5663116]	4.85184	4.7244	4.80026	5.9484	5.35504	4.31351	5.32091	4.29086	5.77608	3.55891	5.54818	4.48095	5.00464	4.35978	0.515451644	0.966018836	20	12	24	0.837285855	0.950299676	24	14.56091434	0.038591339
ENSMUSG00000104864	Gm43655	predicted gene 43655 [Source:MGI Symbol;Acc:MGI:5663792]	14.3283	16.0939	16.1107	16.8457	16.9712	15.8842	14.2182	12.3351	7.08073	4.54034	9.39542	5.84101	9.06981	10.2483	0.759825336	0.966018836	20	4	24	0.561037095	0.841531815	24	23.49457157	0.166816526
ENSMUSG00000104868	Gm9954	predicted gene 9954 [Source:MGI Symbol;Acc:MGI:3642120]	1.8542	1.40897	1.39122	1.58982	1.32067	1.46088	1.45034	1.26985	1.08249	1.84566	1.4867	1.41958	1.66952	1.42017	0.300218476	0.809362476	16	20	24	0.568464267	0.844388316	24	19.41373257	0.053367197
ENSMUSG00000104872	1700008B11Rik	RIKEN cDNA 1700008B11 gene [Source:MGI Symbol;Acc:MGI:1913571]	0.338946	0.343836	0.368227	0.772598	0.441017	0.430007	0.722076	0.528838	0.346246	1.06415	0.231934	0.488729	0.623156	0.511437	0.917839756	0.966018836	16	4	24	0.790857661	0.933002007	24	11.16080718	0.053836474
ENSMUSG00000104882	Gm43096	predicted gene 43096 [Source:MGI Symbol;Acc:MGI:5663233]	31.0032	32.4358	27.372	29.3058	30.2006	28.3611	30.0383	28.6639	26.3932	26.1783	30.5197	29.4365	31.3672	28.9991	0.053683796	0.394331071	4	8	24	0.055259203	0.65534544	24	22.32893874	0.076326743
ENSMUSG00000104891	2510017J16Rik	RIKEN cDNA 2510017J16 gene [Source:MGI Symbol;Acc:MGI:1913824]	0.505027	0.660413	0.627862	0.953828	0.839031	0.615847	0.396418	0.646994	0.887793	0.691039	0.820019	0.551027	0.647896	0.778409	0.016169219	0.253395174	16	12	24	0.009418097	0.502965379	24	11.41322053	0.135009861
ENSMUSG00000104897	Gm43203	predicted gene 43203 [Source:MGI Symbol;Acc:MGI:5663340]	1.51847	1.53904	1.20923	1.38053	1.35073	1.36557	0.738681	1.17103	1.37711	1.07078	1.65874	2.01578	1.1525	1.38118	0.890235289	0.966018836	24	4	24	0.591024964	0.855623646	24	17.12365467	0.076594219
ENSMUSG00000104913	Gm6560	predicted gene 6560 [Source:MGI Symbol;Acc:MGI:3646204]	99.4086	92.3527	100.249	103.936	101.862	117.572	96.8215	110.566	82.0367	103.504	93.0432	91.2197	91.0332	87.9918	0.840483986	0.966018836	16	20	24	0.439024049	0.805393068	24	17.74105084	0.063706088
ENSMUSG00000104923	Gm5865	predicted gene 5865 [Source:MGI Symbol;Acc:MGI:3645504]	21.637	21.0541	23.8736	20.3448	20.2843	19.6538	21.3195	19.1422	19.0373	20.1447	18.6071	19.5845	17.561	19.2439	0.823246279	0.966018836	4	20	24	0.709256021	0.903951515	24	7.725035467	0.023624449
ENSMUSG00000104938	Gm42840	predicted gene 42840 [Source:MGI Symbol;Acc:MGI:5662977]	1.34655	1.63144	1.44483	1.57462	1.59883	1.66744	1.46044	1.27819	1.63583	1.69092	2.17388	1.734	1.87644	1.65714	0.341022016	0.86129475	24	8	24	0.105795488	0.717832234	24	15.94992824	0.077126279
ENSMUSG00000104950	4833413G10Rik	RIKEN cDNA 4833413G10 gene [Source:MGI Symbol;Acc:MGI:1921834]	2.67647	2.42929	2.65676	2.48721	2.66313	2.38328	2.78935	2.12082	2.94663	2.54828	2.67421	2.61093	2.49184	2.68227	0.877082091	0.966018836	12	8	24	0.841153908	0.951862945	24	12.814287	0.019107859
ENSMUSG00000104951	Gm43413	predicted gene 43413 [Source:MGI Symbol;Acc:MGI:5663550]	0.621639	0.646897	0.676374	0.748175	0.785564	0.708539	0.931993	0.583352	0.61852	0.522576	0.915824	0.610786	0.638558	0.902069	0.890311217	0.966018836	20	20	24	0.774038487	0.927465562	24	19.24885706	0.032381936
ENSMUSG00000104960	Snhg8	small nucleolar RNA host gene 8 [Source:MGI Symbol;Acc:MGI:1917145]	31.0643	39.0642	35.2828	34.0545	31.8532	28.097	34.7166	30.4324	45.2776	23.2629	36.6426	38.7482	37.7862	34.7235	0.9143748	0.966018836	12	4	24	0.609630465	0.864491738	24	4.171363758	0.089705132
ENSMUSG00000104965	Gm43437	predicted gene 43437 [Source:MGI Symbol;Acc:MGI:5663574]	0.897192	0.579068	0.885793	0.686755	0.920206	0.817302	0.918599	0.750744	0.671746	0.723356	0.959229	0.800519	0.809792	0.778352	0.068820236	0.375011045	20	20	24	0.133148002	0.743835554	24	19.18590515	0.069842893
ENSMUSG00000104986	1600023N17Rik	RIKEN cDNA 1600023N17 gene [Source:MGI Symbol;Acc:MGI:1917038]	0.56491	0.646008	0.614347	0.594152	0.599222	0.614843	0.631865	0.548089	0.578793	0.248811	0.615188	0.540159	0.595668	0.351379	0.90332508	0.966018836	20	20	24	0.370097063	0.792235171	24	21.51509997	0.057155435
ENSMUSG00000105044	Gm10416	predicted pseudogene 10416 [Source:MGI Symbol;Acc:MGI:3645037]	1.07613	0.713888	0.629579	0.849431	1.10831	0.606967	1.10867	0.812039	1.29688	0.656576	0.736483	0.795554	0.774671	0.873902	0.886002469	0.966018836	4	20	24	0.903749863	0.973407573	24	2.507971973	0.028422444
ENSMUSG00000105055	Gm43079	predicted gene 43079 [Source:MGI Symbol;Acc:MGI:5663216]	5.4975	6.38829	3.83994	7.37747	6.3898	5.04474	7.32941	6.09919	8.76054	6.73762	7.4422	7.33425	5.67185	7.61705	0.886952527	0.966018836	16	12	24	0.771688102	0.926392944	24	11.6040566	0.06651711
ENSMUSG00000105076	A930003O13Rik	RIKEN cDNA A930003O13 gene [Source:MGI Symbol;Acc:MGI:3612704]	1.05018	1.27416	1.12999	1.1703	0.958117	1.13598	1.14989	1.17435	1.13664	1.10724	0.996442	1.46629	1.18576	1.23639	0.018810871	0.256345714	8	12	24	0.342965805	0.776764544	24	2.448089343	0.04354656
ENSMUSG00000105081	Gm43110	predicted gene 43110 [Source:MGI Symbol;Acc:MGI:5663247]	75.7133	85.2873	84.7925	73.0447	72.0459	81.5299	78.1716	72.3814	84.2696	81.1259	70.294	80.7064	85.1925	84.9306	0.166709446	0.633685338	8	12	24	0.159945326	0.76564405	24	4.444601245	0.072398064
ENSMUSG00000105095	8430422M14Rik	RIKEN cDNA 8430422M14 gene [Source:MGI Symbol;Acc:MGI:1925753]	3.62318	5.55121	4.26712	5.5298	5.27704	4.92539	4.9328	4.69761	5.31535	3.95029	5.55511	4.97508	4.95333	5.29361	0.929640773	0.966018836	20	8	24	0.86301043	0.960084493	24	13.04960776	0.032517647
ENSMUSG00000105112	Gm42778	predicted gene 42778 [Source:MGI Symbol;Acc:MGI:5662915]	0.615241	0.623555	0.779466	0.815567	0.303982	0.576223	0.374102	0.912324	1.18561	1.08468	0.979811	1.11997	1.09892	0.622047	0.858269825	0.966018836	16	4	24	0.366250489	0.790179277	24	9.528683134	0.117466273
ENSMUSG00000105124	Gm5297	predicted gene 5297 [Source:MGI Symbol;Acc:MGI:3646414]	1.57523	1.5418	1.84194	1.62887	2.30326	1.50049	1.63456	1.90628	1.72257	1.743	1.68112	1.75534	1.38888	1.31985	0.426055651	0.966018836	20	8	24	0.165595483	0.767020009	24	13.92194464	0.085019938
ENSMUSG00000105135	Gm43667	predicted gene 43667 [Source:MGI Symbol;Acc:MGI:5663804]	2.85476	4.85201	5.14413	4.99059	5.23651	3.75348	4.31644	3.58895	5.09492	3.91263	4.80915	3.74587	4.28275	4.98679	0.048880363	0.379129806	12	12	24	0.097574801	0.71175955	24	10.33531765	0.16758758
ENSMUSG00000105139	Gm19391	predicted gene, 19391 [Source:MGI Symbol;Acc:MGI:5011576]	2.0717	2.94812	2.43186	2.3241	2.54394	2.09357	2.90273	2.79827	2.8874	2.42865	2.62685	2.21883	2.82971	2.56769	0.286642595	0.793714531	8	16	24	0.153007787	0.759413336	24	5.726417936	0.089721452
ENSMUSG00000105143	Gm8069	predicted pseudogene 8069 [Source:MGI Symbol;Acc:MGI:3645115]	54.0035	26.2709	38.583	10.8102	25.5746	37.9535	27.9441	28.1956	18.7856	18.696	29.384	21.3082	34.9486	20.1802	0.032263213	0.324385159	4	12	24	0.051031039	0.645587282	24	23.02592222	0.466291528
ENSMUSG00000105156	Gm9057	predicted gene 9057 [Source:MGI Symbol;Acc:MGI:3643991]	0.302068	0.454805	0.326672	1.66994	0.651542	0.494208	0.364319	1.0557	1.21955	0.200041	0.403434	0.198512	0.617664	0.718411	0.65208848	0.966018836	8	16	24	0.308003447	0.768040178	24	8.685370496	0.212509138
ENSMUSG00000105161	Gm42595	predicted gene 42595 [Source:MGI Symbol;Acc:MGI:5662732]	0.988251	1.49442	1.52332	1.48554	1.38219	1.42386	1.03964	1.28144	2.14084	1.66811	1.60928	1.39604	1.24063	1.54858	8.66E-04	0.071344007	12	16	24	0.004056825	0.427431763	24	10.49918871	0.172841138
ENSMUSG00000105176	Gm43668	predicted gene 43668 [Source:MGI Symbol;Acc:MGI:5663805]	3.61946	3.25904	2.93444	3.01752	3.17363	2.83542	3.11022	3.11261	2.95596	3.08587	3.77594	3.44646	3.05392	3.75375	0.675497492	0.966018836	8	4	24	0.731073601	0.912382349	24	22.1035076	0.03215616
ENSMUSG00000105189	Gm7774	predicted gene 7774 [Source:MGI Symbol;Acc:MGI:3646056]	11.8965	9.91687	10.451	9.33018	8.64777	8.68183	9.99249	9.61615	9.48493	9.00981	11.359	10.4308	10.8722	9.93875	0.111887343	0.530777648	4	12	24	0.3583247	0.784922891	24	0.407274126	0.069333188
ENSMUSG00000105195	Gm43584	predicted gene 43584 [Source:MGI Symbol;Acc:MGI:5663721]	6.61293	6.55132	5.64929	6.0075	4.80834	6.57619	6.28878	7.07683	6.20585	6.24946	5.30952	6.26373	6.79372	5.85689	0.01518376	0.253245931	4	16	24	0.044868672	0.628432994	24	2.071432822	0.110787312
ENSMUSG00000105230	Gm42433	predicted gene 42433 [Source:MGI Symbol;Acc:MGI:5662570]	10.9482	9.07271	8.01087	7.44947	8.26787	8.91194	8.0509	7.23576	6.98674	8.82213	7.98296	8.45147	8.55539	9.17538	0.326490454	0.845515883	4	8	24	0.152357912	0.759088284	24	22.35772416	0.107165771
ENSMUSG00000105245	Gm31305	predicted gene, 31305 [Source:MGI Symbol;Acc:MGI:5590464]	0.941526	0.711081	0.383133	0.977496	0.931166	0.593101	0.621331	0.948018	1.01326	0.902431	0.814837	0.83713	0.837821	0.773951	0.916746631	0.966018836	16	8	24	0.809459221	0.939184036	24	13.434155	0.038045155
ENSMUSG00000105265	Sox2ot	SOX2 overlapping transcript (non-protein coding) [Source:MGI Symbol;Acc:MGI:2444112]	13.2558	16.6787	16.8802	12.2406	15.916	14.702	15.9676	14.4623	16.6879	12.8365	14.7046	15.6357	14.6423	15.9059	0.265774856	0.773510818	12	4	24	0.490064492	0.805393068	24	3.549012026	0.063901512
ENSMUSG00000105272	Gm43071	predicted gene 43071 [Source:MGI Symbol;Acc:MGI:5663208]	1.33521	1.48852	1.38382	1.56348	1.3235	1.05223	1.29508	1.23758	1.65361	0.951818	1.36056	1.33261	1.15427	1.49182	0.220555749	0.710961211	12	12	24	0.359483309	0.785405943	24	7.247589846	0.068992862
ENSMUSG00000105340	Gm42878	predicted gene 42878 [Source:MGI Symbol;Acc:MGI:5663015]	3.07157	3.66425	3.76076	3.94956	3.46877	3.07036	3.88051	3.42827	3.90843	3.02962	3.91673	3.3304	3.68704	3.63129	0.628286596	0.966018836	12	12	24	0.422880785	0.805019467	24	8.186498652	0.056955191
ENSMUSG00000105345	BC030343	cDNA sequence BC030343 [Source:MGI Symbol;Acc:MGI:2679267]	1.44384	1.64707	1.36316	1.74567	1.84675	1.48021	1.53107	1.50095	1.92935	1.55092	1.56187	1.85514	1.62845	1.57081	0.924770496	0.966018836	20	8	24	0.51836848	0.818661985	24	13.97953633	0.03972911
ENSMUSG00000105349	Gm43604	predicted gene 43604 [Source:MGI Symbol;Acc:MGI:5663741]	0.441224	1.23164	1.0389	0.755808	0.821861	0.997166	0.89584	0.722204	0.852136	0.680394	1.05827	0.941053	0.933039	0.71896	0.928127947	0.966018836	20	20	24	0.969099194	0.992631251	24	15.44427796	0.015797921
ENSMUSG00000105355	Gm40117	predicted gene, 40117 [Source:MGI Symbol;Acc:MGI:5623002]	0.22501	0.970904	1.44996	1.53425	0.969956	0.495598	0.835723	0.589587	1.46266	0.439301	0.681283	0.557896	0.573582	1.07571	0.01844263	0.256345714	12	12	24	0.040794158	0.616921492	24	9.206197659	0.296611988
ENSMUSG00000105368	Gm43759	predicted gene 43759 [Source:MGI Symbol;Acc:MGI:5663896]	1.40943	1.10269	1.06145	1.24637	1.57264	1.52177	2.02982	1.13428	1.21058	1.09813	1.51854	1.10407	1.27412	1.23698	0.067920241	0.430600538	20	16	24	0.10622845	0.71810271	24	20.11646549	0.127345092
ENSMUSG00000105388	Rpl36a-ps2	ribosomal protein L36A, pseudogene 2 [Source:MGI Symbol;Acc:MGI:3780887]	245.017	306.482	292.537	293.586	298.818	262.549	265.768	246.351	345.142	218.282	306.403	293.784	302.892	300.459	0.888482515	0.966018836	12	4	24	0.842642243	0.952242624	24	8.698118472	0.041728992
ENSMUSG00000105389	BB187690	expressed sequence BB187690 [Source:MGI Symbol;Acc:MGI:2140033]	0.548598	0.637577	0.852472	0.315166	0.587672	0.534844	0.621888	0.772801	0.776681	0.336516	0.404717	0.449121	0.312898	0.437744	0.035050838	0.328311403	12	4	24	0.218562639	0.768040178	24	5.084405741	0.099666488
ENSMUSG00000105395	Gm42672	predicted gene 42672 [Source:MGI Symbol;Acc:MGI:5662809]	2.13225	1.61168	0.923881	2.14398	2.01718	1.52976	1.54752	0.75036	1.87236	0.564549	2.15386	1.1113	1.73199	1.32971	0.840483986	0.966018836	20	12	24	0.451810118	0.805393068	24	19.15856399	0.164633416
ENSMUSG00000105408	Gm7285	predicted gene 7285 [Source:MGI Symbol;Acc:MGI:3647212]	1.05411	1.36021	1.04082	1.41712	1.63548	1.34794	1.74876	1.36523	1.63023	1.07695	1.89848	1.04567	1.53942	1.01541	0.840483986	0.966018836	20	12	24	0.655347013	0.883697438	24	16.97658435	0.066227977
ENSMUSG00000105419	Gm43205	predicted gene 43205 [Source:MGI Symbol;Acc:MGI:5663342]	0.790085	0.8712	1.28969	0.708234	0.792481	0.81396	0.831445	0.768867	0.886185	0.927666	0.605918	0.892189	0.641421	0.975894	0.505728924	0.966018836	12	8	24	0.222927658	0.768040178	24	7.243344175	0.084210064
ENSMUSG00000105429	Gm43692	predicted gene 43692 [Source:MGI Symbol;Acc:MGI:5663829]	1.10452	0.988195	0.576608	0.862205	0.728207	0.727969	0.700948	0.752105	1.02628	0.642125	0.790398	1.15425	0.764764	0.889643	0.858266942	0.966018836	24	20	24	0.585935765	0.853257144	24	23.79679116	0.055889579
ENSMUSG00000105452	Gm5276	predicted gene 5276 [Source:MGI Symbol;Acc:MGI:3646382]	2.96567	2.78653	2.78556	2.99142	3.13022	4.08426	3.016	4.38759	2.08743	3.20606	1.82396	2.40534	2.45328	1.74299	0.849180783	0.966018836	16	20	24	0.769741687	0.925855377	24	20.84329342	0.07429574
ENSMUSG00000105461	Gm4742	predicted gene 4742 [Source:MGI Symbol;Acc:MGI:3645661]	1.4408	1.4299	1.56614	1.57286	1.66156	1.07473	1.19734	1.34606	0.804056	0.952113	0.988428	1.00457	0.873969	1.0811	0.801251296	0.966018836	20	4	24	0.954244473	0.988601783	24	11.99997842	0.014497798
ENSMUSG00000105504	Gbp5	guanylate binding protein 5 [Source:MGI Symbol;Acc:MGI:2429943]	2.31446	2.18675	2.07118	2.08719	2.85665	1.8675	1.76546	2.62888	2.29865	2.01369	2.15502	1.64177	1.46023	2.03569	0.459019924	0.966018836	20	4	24	0.365743524	0.789934347	24	10.48687511	0.086083032
ENSMUSG00000105509	C130013H08Rik	RIKEN cDNA C130013H08 gene [Source:MGI Symbol;Acc:MGI:3697343]	0.664603	0.558204	0.538855	0.607486	0.515315	0.490875	0.67653	0.477958	0.585028	0.706264	0.731377	0.654829	0.735289	0.687737	0.90703203	0.966018836	4	4	24	0.897125332	0.970698391	24	20.19165868	0.013470537
ENSMUSG00000105516	Gm36823	predicted gene, 36823 [Source:MGI Symbol;Acc:MGI:5595982]	0.70542	0.457983	0.513075	0.616504	0.859669	0.416191	0.891679	0.415045	0.682425	0.530173	0.805618	0.293667	0.775742	0.414906	0.192357053	0.669841558	20	12	24	0.728581268	0.911275824	24	17.07591739	0.055676885
ENSMUSG00000105547	Iglc3	immunoglobulin lambda constant 3 [Source:MGI Symbol;Acc:MGI:99886]	0.972232	3.09088	1.80827	2.03916	1.6364	1.62839	1.85422	1.84609	2.44975	0.275591	1.50951	1.34513	2.98786	1.48912	0.595037318	0.966018836	8	16	24	0.381266072	0.799546792	24	3.054808643	0.224624231
ENSMUSG00000105552	Gm19710	predicted gene, 19710 [Source:MGI Symbol;Acc:MGI:5011895]	0.752912	0.551685	0.727125	0.572214	0.435509	0.707644	0.712949	0.373007	0.854706	0.387069	0.744206	0.482533	0.503335	0.504785	0.91724565	0.966018836	12	4	24	0.972940319	0.993305115	24	22.86635344	0.012933833
ENSMUSG00000105556	Gm43080	predicted gene 43080 [Source:MGI Symbol;Acc:MGI:5663217]	0.415014	0.828854	0.590684	0.607734	0.661171	0.601794	0.702507	0.602649	1.09293	0.358405	0.860062	0.73952	0.890057	0.720384	0.823144114	0.966018836	8	8	24	0.810395177	0.939224719	24	4.488324056	0.040075314
ENSMUSG00000105572	Gm43300	predicted gene 43300 [Source:MGI Symbol;Acc:MGI:5663437]	0.758487	1.93015	1.71964	0.922879	1.3633	0.973917	1.21023	1.19247	1.87465	0.709661	1.06171	1.20431	1.0635	1.43617	0.076247883	0.453790923	12	4	24	0.173233539	0.768040178	24	6.189068505	0.175911478
ENSMUSG00000105576	Gm42164	predicted gene, 42164 [Source:MGI Symbol;Acc:MGI:5625049]	8.01639	8.37153	9.29428	7.94358	10.761	10.3276	6.77247	6.24756	7.94437	5.67495	9.19165	7.62831	6.2947	8.08965	0.885025617	0.966018836	20	20	24	0.30390783	0.768040178	24	16.73713652	0.134392482
ENSMUSG00000105594	Gm43398	predicted gene 43398 [Source:MGI Symbol;Acc:MGI:5663535]	1.58346	1.32519	1.05777	1.15851	1.95642	1.38954	1.5428	1.0896	1.23413	1.19658	1.60136	1.47496	1.13874	1.64176	0.142513744	0.585944911	20	20	24	0.133003641	0.743835554	24	19.29326403	0.120444142
ENSMUSG00000105632	Gm43272	predicted gene 43272 [Source:MGI Symbol;Acc:MGI:5663409]	0.836916	1.13545	1.15344	0.963539	1.00623	0.651944	0.954249	0.801348	1.52669	1.06872	0.961947	1.17053	0.852915	1.26329	0.109040811	0.524035616	12	16	24	0.060910052	0.660449324	24	8.554492686	0.134225128
ENSMUSG00000105677	Gm43328	predicted gene 43328 [Source:MGI Symbol;Acc:MGI:5663465]	1.7785	2.34899	1.96889	1.47196	2.15572	1.72386	2.17225	1.90381	2.51797	1.33963	1.67762	1.6273	1.90855	1.9475	0.022000259	0.267229408	12	4	24	0.175382319	0.768040178	24	3.692296098	0.113091373
ENSMUSG00000105679	Gm43338	predicted gene 43338 [Source:MGI Symbol;Acc:MGI:5663475]	0.499821	0.567525	0.460282	0.421167	0.477486	0.428006	0.516945	0.708243	0.847859	0.330095	0.517675	0.613147	0.678988	0.64062	0.029264055	0.322165609	8	8	24	0.069329827	0.673520298	24	3.702829184	0.096131656
ENSMUSG00000105687	Gm6157	predicted gene 6157 [Source:MGI Symbol;Acc:MGI:3779561]	2.35333	2.0464	2.11337	1.9483	1.92472	1.95515	2.34532	2.36	2.00137	2.18806	1.54525	2.2788	1.76061	2.06631	0.25849765	0.761738864	8	12	24	0.249066135	0.768040178	24	2.723183498	0.069486759
ENSMUSG00000105692	Gm43737	predicted gene 43737 [Source:MGI Symbol;Acc:MGI:5663874]	3.6083	2.68176	3.37254	3.45287	3.36877	3.67072	3.46656	3.21707	2.93869	4.37022	3.5973	3.22936	2.86295	3.24666	0.157813674	0.614233844	16	16	24	0.132671824	0.742819192	24	15.26271087	0.099382412
ENSMUSG00000105703	Gm43305	predicted gene 43305 [Source:MGI Symbol;Acc:MGI:5663442]	1.90891	0.976079	1.682	2.30854	2.5213	2.36957	2.22887	1.80226	1.04934	1.51497	1.76295	1.81239	2.15623	3.17146	0.542718076	0.966018836	24	12	24	0.424323529	0.805393068	24	20.04377691	0.150698475
ENSMUSG00000105706	Gm42855	predicted gene 42855 [Source:MGI Symbol;Acc:MGI:5662992]	1.74545	2.83297	2.35528	3.33892	2.7833	2.58288	2.62493	1.13499	3.51121	4.04735	3.75322	0.86867	1.49988	2.11577	0.034640964	0.328311403	16	12	24	0.036701976	0.609176161	24	12.0394077	0.385558632
ENSMUSG00000105710	Gm43859	predicted gene 43859 [Source:MGI Symbol;Acc:MGI:5663996]	1.04853	0.808432	0.898361	1.05945	1.09606	0.97733	1.16446	1.02698	1.54086	0.755806	1.5102	1.37193	1.26679	1.43956	0.897975397	0.966018836	20	20	24	0.883165949	0.966818623	24	21.03176163	0.02806047
ENSMUSG00000105741	C79130	expressed sequence C79130 [Source:MGI Symbol;Acc:MGI:2141307]	1.11978	0.916312	0.729303	0.84018	0.967018	0.990771	0.959818	0.843789	0.714652	0.865842	1.01245	1.01346	0.971406	0.869387	8.68E-05	0.029372629	24	12	24	1.63E-04	0.140635355	24	20.51675483	0.107819712
ENSMUSG00000105784	Gm35065	predicted gene, 35065 [Source:MGI Symbol;Acc:MGI:5594224]	0.909788	0.919681	0.98678	0.683614	0.445665	0.84472	0.183909	0.67091	1.04125	0.706497	0.94707	0.510009	0.519966	0.593205	0.138543921	0.580158868	12	12	24	0.32529402	0.768040178	24	9.487447986	0.125967792
ENSMUSG00000105787	Gm8099	predicted gene 8099 [Source:MGI Symbol;Acc:MGI:3645754]	15.7547	20.0129	16.5256	17.7713	22.164	21.243	16.3481	17.4533	14.2148	13.3213	14.6716	13.3294	14.0977	13.0212	0.823144114	0.966018836	20	8	24	0.450369264	0.805393068	24	18.56419707	0.088926965
ENSMUSG00000105802	Gm43012	predicted gene 43012 [Source:MGI Symbol;Acc:MGI:5663149]	1.07855	1.72243	1.1728	1.11879	1.26803	1.69018	1.75427	1.60907	2.10416	1.56093	1.58865	1.31037	1.43719	1.57366	0.965163262	0.969272194	8	8	24	0.549954041	0.835266054	24	5.268956201	0.06812854
ENSMUSG00000105807	Gm42622	predicted gene 42622 [Source:MGI Symbol;Acc:MGI:5662759]	7.76563	3.49065	4.09254	4.69176	5.04273	5.56137	3.80863	5.17167	4.11188	4.30653	4.80725	4.23722	4.40289	3.76027	0.209352197	0.69936851	24	8	24	0.214775979	0.768040178	24	19.60673935	0.157554682
ENSMUSG00000105814	Mir703	microRNA 703 [Source:MGI Symbol;Acc:MGI:3629657]	472.189	548.808	146.082	214.557	429.898	431.918	256.712	790.623	97.3349	382.027	532.68	209.379	108.623	205.404	0.977483417	0.977820713	8	4	24	0.585174998	0.852642873	24	19.49893186	0.375249735
ENSMUSG00000105843	Gm19439	predicted gene, 19439 [Source:MGI Symbol;Acc:MGI:5011624]	0.431648	0.515847	1.22549	1.16867	0.678076	0.8679	1.28462	0.337298	0.719447	0.570141	0.440613	0.560029	0.174464	0.361298	0.253121224	0.753386477	12	20	24	0.430100361	0.805393068	24	13.00871494	0.129524699
ENSMUSG00000105851	9130604C24Rik	RIKEN cDNA 9130604C24 gene [Source:MGI Symbol;Acc:MGI:1924957]	0.601458	0.865982	0.817551	0.517295	0.712646	0.768023	0.550325	1.30129	1.02682	0.662296	0.827002	0.799218	1.20203	0.524212	0.908406451	0.966018836	12	4	24	0.622611078	0.87142241	24	4.081512521	0.070960954
ENSMUSG00000105852	Gm42890	predicted gene 42890 [Source:MGI Symbol;Acc:MGI:5663027]	0.691269	0.923913	1.44628	1.36176	0.769312	0.787361	1.10347	0.949861	1.3809	1.00624	0.552095	1.24243	0.659233	1.17633	0.050760377	0.386400877	12	8	24	0.087895191	0.707747429	24	8.224149658	0.17802126
ENSMUSG00000105858	2310074N15Rik	RIKEN cDNA 2310074N15 gene [Source:MGI Symbol;Acc:MGI:1922838]	0.667558	0.725185	1.13939	0.108492	0.424623	0.751266	0.580123	0.849198	0.843659	0.819567	0.456072	0.91814	0.893616	0.866036	0.421215102	0.966018836	12	4	24	0.14094724	0.751014756	24	3.527948558	0.167928444
ENSMUSG00000105867	Gm42517	predicted gene 42517 [Source:MGI Symbol;Acc:MGI:5662654]	5.28439	3.54965	3.85915	3.70733	3.44428	4.2736	4.10401	4.68275	4.3715	5.89448	5.93175	6.01727	6.57945	5.93795	0.697196105	0.966018836	4	8	24	0.517079078	0.818088916	24	22.44962853	0.08044772
ENSMUSG00000105879	Gm6204	predicted gene 6204 [Source:MGI Symbol;Acc:MGI:3649038]	81.3532	118.691	106.486	120.135	124.186	103.532	95.7652	102.353	135.771	68.7028	108.979	97.7067	106.787	93.1859	0.505728924	0.966018836	12	16	24	0.821168592	0.943569442	24	10.54553358	0.064058941
ENSMUSG00000105881	4932422M17Rik	RIKEN cDNA 4932422M17 gene [Source:MGI Symbol;Acc:MGI:1921616]	5.81824	6.57276	7.1767	7.37402	6.35492	5.81935	6.14747	6.9749	7.64502	7.00943	6.06475	6.07407	6.36655	6.56612	2.86E-04	0.049770315	12	12	24	2.71E-05	0.056204232	24	8.881880956	0.143171588
ENSMUSG00000105891	A230001M10Rik	RIKEN cDNA A230001M10 gene [Source:MGI Symbol;Acc:MGI:2443043]	0.39242	0.517497	1.18463	0.172915	0.527225	0.293469	0.912551	0.422736	0.929873	0.416872	0.416201	0.389591	0.190171	0.748064	0.042880436	0.356048751	12	12	24	0.207635843	0.768040178	24	6.646470512	0.185698071
ENSMUSG00000105892	Gm35013	predicted gene, 35013 [Source:MGI Symbol;Acc:MGI:5594172]	1.72484	1.51047	1.81052	1.29377	1.42181	1.41519	1.59543	1.3829	1.42054	1.17902	1.51098	1.32934	1.15017	1.38111	0.858366225	0.966018836	12	4	24	0.630238886	0.873885049	24	1.930296088	0.032087692
ENSMUSG00000105895	Gm42829	predicted gene 42829 [Source:MGI Symbol;Acc:MGI:5662966]	2.1412	1.83058	1.95691	2.11196	1.8587	1.86724	1.89827	1.89532	1.56677	2.2776	1.77267	2.04368	2.20267	1.82624	0.718742371	0.966018836	20	4	24	0.751129091	0.91921305	24	19.23220396	0.029715353
ENSMUSG00000105906	Iglc1	immunoglobulin lambda constant 1 [Source:MGI Symbol;Acc:MGI:99546]	1.65509	1.95333	0.897864	1.40212	2.03161	2.23345	2.34361	1.7112	2.62	1.00346	1.96768	1.69176	1.48339	1.37278	0.823657588	0.966018836	20	4	24	0.391461568	0.799546792	24	21.2772875	0.148505488
ENSMUSG00000105928	Gm9256	predicted gene 9256 [Source:MGI Symbol;Acc:MGI:3648981]	5.26895	4.71831	4.1534	3.99305	4.43044	4.81576	5.24073	3.96325	4.87633	5.16837	5.06713	5.09726	4.86684	4.994	0.338172159	0.85758369	4	8	24	0.24956492	0.768040178	24	21.42021248	0.072623717
ENSMUSG00000105929	Gm43693	predicted gene 43693 [Source:MGI Symbol;Acc:MGI:5663830]	0.328911	0.557468	0.596261	0.798097	0.438614	0.457422	0.446165	0.327673	0.574552	0.41041	0.755078	0.465185	0.583407	0.516673	0.597914157	0.966018836	12	20	24	0.226428374	0.768040178	24	11.98886183	0.084994694
ENSMUSG00000105954	Gm42793	predicted gene 42793 [Source:MGI Symbol;Acc:MGI:5662930]	0.668002	0.742311	0.396395	0.676088	0.588078	0.420611	0.688029	0.664078	0.656308	0.410025	0.655434	0.49961	0.496585	0.40775	0.891690964	0.966018836	4	20	24	0.879461216	0.966193785	24	2.048357449	0.021927043
ENSMUSG00000105957	Gm43254	predicted gene 43254 [Source:MGI Symbol;Acc:MGI:5663391]	0.833311	1.05618	1.01801	1.05934	0.806732	0.932419	0.810789	0.886567	1.14105	0.999571	0.916197	0.852787	0.666131	0.994287	0.002087679	0.100255953	12	4	24	0.008454776	0.499340744	24	9.390219883	0.100380685
ENSMUSG00000105977	Gm43555	predicted gene 43555 [Source:MGI Symbol;Acc:MGI:5663692]	6.57149	6.88016	8.59241	10.0216	9.08955	9.44103	8.26345	7.19215	6.45585	7.4342	8.06563	5.92303	6.89154	8.42472	0.696676764	0.966018836	20	8	24	0.326544141	0.768040178	24	13.97869018	0.112032157
ENSMUSG00000105987	AI506816	expressed sequence AI506816 [Source:MGI Symbol;Acc:MGI:2140929]	2.88428	3.76378	3.47712	3.93648	4.27855	3.96058	4.18205	4.64754	4.33444	4.76702	3.77464	3.88947	3.31629	3.36233	0.438929876	0.966018836	16	12	24	0.245602317	0.768040178	24	12.08767926	0.100229428

ENSMUSG00000106019	Gm43672	predicted gene 43672 [Source:MGI Symbol;Acc:MGI:5663809]	1.9461	1.98048	2.39263	2.29981	2.47015	2.26264	1.9146	2.81409	2.55932	2.80441	2.40621	2.07192	2.26713	2.2873	0.016131606	0.253395174	12	16	24	0.021846142	0.574400012	24	10.71422877	0.115105282
ENSMUSG00000106025	Gm42940	predicted gene 42940 [Source:MGI Symbol;Acc:MGI:5663077]	0.614993	0.595951	0.662846	0.804975	0.560091	0.515651	0.638298	0.567965	0.58772	0.563455	0.5676	0.577131	0.542714	0.693607	0.269657278	0.777976231	12	8	24	0.303427757	0.768040178	24	8.45770999	0.039412301
ENSMUSG00000106037	Gm4332	predicted gene 4332 [Source:MGI Symbol;Acc:MGI:3782515]	411.612	479.149	465.37	442.484	446.688	414.927	402.95	441.108	519.275	372.623	491.729	422.721	474.045	437.222	0.46238066	0.966018836	12	12	24	0.538348834	0.82853828	24	7.744792627	0.058294794
ENSMUSG00000106067	Gm7902	predicted gene 7902 [Source:MGI Symbol;Acc:MGI:3645041]	2.07871	1.64886	2.26041	1.86032	2.04858	2.04271	1.60813	1.72471	1.1425	2.17734	1.4987	1.42278	1.41865	1.74259	0.852772142	0.966018836	16	12	24	0.672687086	0.889678044	24	14.63951312	0.052931156
ENSMUSG00000106087	Gm43609	predicted gene 43609 [Source:MGI Symbol;Acc:MGI:5663746]	0.559067	0.640394	0.692987	0.680899	0.748559	0.470218	0.721445	0.628229	0.680528	0.707622	0.665166	0.512464	0.536021	0.482502	0.050845331	0.38654007	20	4	24	0.071925888	0.678312022	24	11.33255802	0.066347397
ENSMUSG00000106093	Gm42722	predicted gene 42722 [Source:MGI Symbol;Acc:MGI:5662859]	0.450511	0.330823	0.719409	0.335662	0.680765	0.341085	0.263664	0.503759	0.524005	0.338013	0.590153	0.750519	0.84962	0.573462	0.882263435	0.966018836	20	20	24	0.978272107	0.994576186	24	19.2059292	0.014067681
ENSMUSG00000106099	Gm42664	predicted gene 42664 [Source:MGI Symbol;Acc:MGI:5662801]	0.540311	1.03493	0.616127	0.66828	0.930636	0.625009	0.597751	0.728825	1.38325	0.450256	0.853495	0.603913	0.79394	0.893898	0.607427393	0.966018836	8	20	24	0.460094896	0.805393068	24	7.293652957	0.095279932
ENSMUSG00000106133	Gm3724	predicted gene 3724 [Source:MGI Symbol;Acc:MGI:3804937]	164.021	159.272	142.692	150.259	157.755	156.895	161.427	152.454	150.717	141.129	165.989	151.38	150.524	148.574	0.079180494	0.458678242	20	20	24	0.044437528	0.628432994	24	20.98453015	0.061942417
ENSMUSG00000106139	Gm30648	predicted gene, 30648 [Source:MGI Symbol;Acc:MGI:5589807]	1.35069	1.4649	1.29401	0.585327	1.33254	1.50126	0.84572	0.857614	1.23747	1.2531	1.02649	1.08252	0.958879	1.07935	0.849180783	0.966018836	24	4	24	0.902893196	0.97295148	24	21.30746367	0.033851369
ENSMUSG00000106181	Gm9523	predicted gene 9523 [Source:MGI Symbol;Acc:MGI:3779932]	11.0365	9.88818	10.7504	13.4609	10.8168	10.3308	11.4286	10.1058	10.0782	9.95078	9.54787	8.50133	8.19556	8.99215	0.904690479	0.966018836	16	16	24	0.245895144	0.768040178	24	12.37509552	0.073386737
ENSMUSG00000106190	Gm20768	predicted gene, 20768 [Source:MGI Symbol;Acc:MGI:5434124]	0.947546	1.20581	1.09074	0.945683	1.06997	1.16531	1.1137	1.09719	1.2724	1.03494	1.3087	1.21468	1.30669	1.32204	0.850398351	0.966018836	8	8	24	0.53192586	0.82547989	24	2.413759466	0.027140624
ENSMUSG00000106239	Gm9260	predicted gene 9260 [Source:MGI Symbol;Acc:MGI:3645725]	2.02068	3.16626	3.22661	3.4492	2.91183	2.48683	3.50273	3.39176	3.63275	3.35838	2.76278	3.40024	3.29419	3.14948	0.760769894	0.966018836	16	4	24	0.098019715	0.71175955	24	8.405817734	0.128019214
ENSMUSG00000106251	Gm42658	predicted gene 42658 [Source:MGI Symbol;Acc:MGI:5662795]	0.729966	0.65705	0.54986	0.808058	0.864952	0.624028	0.685958	0.655238	0.845038	0.611953	0.697014	0.82105	0.783743	0.687823	0.826609618	0.966018836	20	12	24	0.5870381	0.853586751	24	17.52449763	0.033277602
ENSMUSG00000106288	Gm43593	predicted gene 43593 [Source:MGI Symbol;Acc:MGI:5663730]	1.20715	1.19384	0.839027	1.0413	0.95649	0.70635	0.778048	0.782072	0.8942	0.841683	1.08602	0.597359	0.490596	1.19676	0.791413662	0.966018836	20	4	24	0.520978087	0.820180873	24	8.748971578	0.074856333
ENSMUSG00000106291	1110003F10Rik	RIKEN cDNA 1110003F10 gene [Source:MGI Symbol;Acc:MGI:1915726]	0.725209	0.6026	0.716623	0.452362	0.688681	0.820686	0.803534	0.824767	1.12416	0.261402	1.08542	0.716364	0.703884	0.841086	0.932671476	0.966018836	12	4	24	0.609186463	0.864393298	24	0.484615104	0.07333107
ENSMUSG00000106294	Gm8539	predicted gene 8539 [Source:MGI Symbol;Acc:MGI:3779802]	21.9263	26.2242	21.7425	25.6516	22.5975	22.9773	22.8964	22.9463	30.3546	20.0635	28.5751	27.7024	26.3991	27.7136	0.826055781	0.966018836	8	8	24	0.903211437	0.973124041	24	5.95843168	0.028446053
ENSMUSG00000106317	Gm43848	predicted gene 43848 [Source:MGI Symbol;Acc:MGI:5663985]	2.3429	3.68599	2.90703	2.77736	2.93863	2.06211	2.32428	3.11853	3.72877	2.90187	2.39546	3.02917	3.11719	3.18746	0.070786393	0.440262583	8	16	24	0.029677691	0.596648309	24	7.139147741	0.174686122
ENSMUSG00000106321	Gm43674	predicted gene 43674 [Source:MGI Symbol;Acc:MGI:5663811]	0.824064	0.321899	0.689076	0.217565	0.283918	0.82535	0.463628	0.509946	0.389191	0.670209	0.782994	0.486023	0.676593	0.570427	0.718742371	0.966018836	24	16	24	0.517976351	0.818483315	24	21.87499644	0.087961841
ENSMUSG00000106379	Lhfpl3	lipoma HMGIC fusion partner-like 3 [Source:MGI Symbol;Acc:MGI:1925076]	23.8791	22.9696	22.3559	23.0917	19.9105	24.641	21.6563	20.3495	20.8649	22.4424	21.14	22.7023	22.4765	22.8065	0.913451828	0.966018836	24	20	24	0.525804426	0.822781291	24	22.58398457	0.037234377
ENSMUSG00000106383	E330034L11Rik	RIKEN cDNA E330034L11 gene [Source:MGI Symbol;Acc:MGI:3026962]	1.8823	1.91558	2.3213	2.02363	2.09446	1.8605	2.60654	2.29869	2.28389	1.74051	2.00853	1.89791	1.56754	2.2712	0.351840494	0.870418653	12	12	24	0.423350583	0.805346055	24	6.414812831	0.068569444
ENSMUSG00000106390	Gm5551	predicted gene 5551 [Source:MGI Symbol;Acc:MGI:3642937]	0.983417	0.844577	0.822062	0.777225	1.01416	0.811769	0.829303	0.810225	0.61868	0.299976	0.621875	0.677857	0.40423	0.455419	0.924770496	0.966018836	4	12	24	0.201058253	0.768040178	24	21.02207177	0.082702942
ENSMUSG00000106396	Gm42902	predicted gene 42902 [Source:MGI Symbol;Acc:MGI:5663039]	0.559528	1.1761	0.955384	0.82063	1.01127	0.631516	1.07073	0.57046	1.08918	1.37361	0.985066	1.12623	0.854638	0.60226	0.823657258	0.966018836	16	12	24	0.28621544	0.768040178	24	12.71589516	0.116612303
ENSMUSG00000106415	Gm42893	predicted gene 42893 [Source:MGI Symbol;Acc:MGI:5663030]	0.806323	0.957294	1.05353	0.689868	0.51139	0.767561	0.820214	0.660156	0.725465	0.573219	0.967878	1.20913	1.00129	0.482038	0.725061903	0.966018836	24	16	24	0.521537868	0.820365849	24	21.9111723	0.079984113
ENSMUSG00000106416	Gm5857	predicted gene 5857 [Source:MGI Symbol;Acc:MGI:3644956]	0.971443	1.29094	1.97704	2.33804	1.70413	1.08805	0.885082	1.61711	1.48456	1.92719	1.57074	1.01685	1.46684	1.2033	7.90E-04	0.071344007	16	12	24	7.75E-04	0.319044294	24	11.0707977	0.285981481
ENSMUSG00000106446	Gm42970	predicted gene 42970 [Source:MGI Symbol;Acc:MGI:5663107]	1.20104	1.25567	0.653308	1.38141	1.17707	1.15866	0.855628	1.25897	1.68811	1.25613	1.70127	1.33722	1.33119	1.31471	0.923635729	0.966018836	16	12	24	0.657402463	0.884466885	24	13.88967935	0.059131864
ENSMUSG00000106464	C130083M11Rik	RIKEN cDNA C130083M11 gene [Source:MGI Symbol;Acc:MGI:3041216]	1.65986	1.68959	1.33219	1.38723	1.73292	1.39727	1.67362	1.69629	1.63313	1.23327	2.10549	1.77888	1.91541	1.64474	0.05238827	0.391426791	20	20	24	0.232555718	0.768040178	24	22.08190472	0.08055766
ENSMUSG00000106485	3830422I06Rik	RIKEN cDNA 3830422I06 gene [Source:MGI Symbol;Acc:MGI:1917958]	0.89682	1.18957	0.678616	0.940864	1.75438	0.887041	1.42806	0.734051	1.62567	0.592436	1.36929	0.968268	1.14161	1.04534	0.914502381	0.966018836	20	20	24	0.778915228	0.928881414	24	18.92404999	0.070290759
ENSMUSG00000106496	Gm7733	predicted gene 7733 [Source:MGI Symbol;Acc:MGI:3643628]	1.41993	1.49637	2.00144	1.44675	1.73081	1.49161	1.66787	1.97686	1.79531	0.938161	1.59029	1.57685	1.71711	1.37604	0.907068194	0.966018836	12	4	24	0.641727672	0.878809617	24	2.767625575	0.058125784
ENSMUSG00000106498	Gm43032	predicted gene 43032 [Source:MGI Symbol;Acc:MGI:5663169]	1.20325	1.12318	1.2224	1.2963	1.18869	1.14614	0.915444	1.02187	1.07125	1.14776	1.40431	1.1281	1.13569	0.978538	0.021406186	0.26465279	20	12	24	0.023071628	0.581965446	24	14.81093648	0.076890711
ENSMUSG00000106535	Gm43031	predicted gene 43031 [Source:MGI Symbol;Acc:MGI:5663168]	1.74654	2.94709	2.70083	2.46272	1.98772	2.2549	2.21853	2.45625	2.57914	2.38292	2.09343	2.07311	1.95407	2.41914	0.004140921	0.140598674	12	16	24	0.00953396	0.502965379	24	7.831544678	0.148257609
ENSMUSG00000106558	Gm5552	predicted gene 5552 [Source:MGI Symbol;Acc:MGI:3643001]	2.27499	2.24674	2.09932	0.902991	2.69225	2.23266	2.62661	2.32474	2.86016	1.42906	3.48841	2.85462	3.20369	2.85532	0.510431574	0.966018836	20	20	24	0.165955887	0.767020009	24	23.12200102	0.205593583
ENSMUSG00000106565	Gm43582	predicted gene 43582 [Source:MGI Symbol;Acc:MGI:5663719]	0.671715	0.902173	0.242492	0.499616	0.162688	0.370068	1.08293	0.590423	0.75579	0.7411	0.451848	0.14604	0.6068	1.04961	0.030302252	0.324150946	4	20	24	0.050683516	0.644469829	24	4.316852345	0.243948207
ENSMUSG00000106568	Gm42814	predicted gene 42814 [Source:MGI Symbol;Acc:MGI:5662951]	0.405308	0.683451	0.293186	0.763367	0.497755	0.377387	0.656005	0.71859	0.641724	0.625484	0.275447	0.261559	0.363162	0.51681	0.097760727	0.501112642	8	16	24	0.189073593	0.768040178	24	7.299132524	0.119878706
ENSMUSG00000106617	Gm36266	predicted gene, 36266 [Source:MGI Symbol;Acc:MGI:5595425]	0.919205	0.853077	0.809541	1.02778	1.05405	0.794012	0.999197	0.958554	1.01785	0.5776	0.848213	0.76752	0.872951	0.665953	0.844511766	0.966018836	20	4	24	0.920988337	0.979223729	24	21.13592161	0.017186876
ENSMUSG00000106631	Gm42669	predicted gene 42669 [Source:MGI Symbol;Acc:MGI:5662806]	0.67409	0.68843	0.780575	0.485473	0.627311	0.533593	0.811314	0.158308	0.784031	0.513656	0.368866	0.631725	0.790712	0.62689	0.163587268	0.626886304	4	12	24	0.751200268	0.91921305	24	1.335321897	0.093074574
ENSMUSG00000106662	Gm43034	predicted gene 43034 [Source:MGI Symbol;Acc:MGI:5663171]	0.543071	0.457252	0.471925	0.726464	0.605046	0.759256	0.611878	0.663779	0.686889	0.955071	0.58191	0.916526	0.650941	0.6167	0.349109869	0.870418653	24	4	24	0.214065058	0.768040178	24	15.61189205	0.070890714
ENSMUSG00000106664	Gm17936	predicted gene, 17936 [Source:MGI Symbol;Acc:MGI:5010121]	10.5002	8.32286	7.01893	7.84611	8.9062	9.81719	9.75563	9.18706	7.40188	7.35094	7.81709	7.55434	8.27683	6.43738	0.053683798	0.394331071	4	8	24	0.004781304	0.435221931	24	21.49592218	0.174386782
ENSMUSG00000106666	Gm43587	predicted gene 43587 [Source:MGI Symbol;Acc:MGI:5663724]	46.9372	36.0451	40.933	37.3765	39.7136	40.8993	41.784	44.3779	30.7883	42.8928	36.9116	42.0084	41.2123	34.3683	0.481811994	0.966018836	4	8	24	0.172566121	0.768040178	24	21.11987589	0.111090729
ENSMUSG00000106688	Gm42851	predicted gene 42851 [Source:MGI Symbol;Acc:MGI:5662988]	0.558204	0.53323	0.869264	0.515664	0.492159	0.737718	0.681876	0.51453	0.653927	0.744879	1.06126	0.820136	0.607512	0.441087	0.142596848	0.585944911	24	8	24	0.279575193	0.768040178	24	15.89785031	0.088418056
ENSMUSG00000106691	2700029L08Rik	RIKEN cDNA 2700029L08 gene [Source:MGI Symbol;Acc:MGI:1924056]	1.37053	0.821123	0.828398	1.05144	1.098	0.867478	1.30202	0.901908	1.10399	0.583393	1.36475	1.08466	0.850408	1.13129	0.823657258	0.966018836	20	20	24	0.430457669	0.805393068	24	20.9919715	0.088099423
ENSMUSG00000106694	Gm42894	predicted gene 42894 [Source:MGI Symbol;Acc:MGI:5663031]	0.72344	0.643075	0.694039	0.707813	0.554454	0.672891	0.494792	0.507257	0.579467	0.688265	0.661024	0.598898	0.634641	0.724991	0.910436409	0.966018836	16	4	24	0.750083095	0.918827661	24	11.55877678	0.021029091
ENSMUSG00000106715	Tmem265	transmembrane protein 265 [Source:MGI Symbol;Acc:MGI:5613213]	1.20145	1.80788	1.62415	1.37806	1.88225	1.61094	1.8508	0.858827	1.71671	1.58961	1.66041	1.59016	1.57986	1.00096	0.702630284	0.966018836	20	12	24	0.267783771	0.768040178	24	16.33709301	0.117664008
ENSMUSG00000106734	Gm20559	predicted gene, 20559 [Source:MGI Symbol;Acc:MGI:5295666]	6.08074	6.56259	6.23499	5.35895	6.75565	5.91346	6.05881	5.74713	7.5045	4.28171	6.97498	5.513	5.76503	5.24241	0.916860713	0.966018836	20	20	24	0.962340649	0.990544581	24	22.60174579	0.018875978
ENSMUSG00000106738	Gm5	predicted gene 5 [Source:MGI Symbol;Acc:MGI:2684851]	1.61324	2.7638	2.82841	1.40488	3.20657	1.5282	1.50795	1.54198	3.11799	1.47716	3.63158	2.55888	3.33209	2.36654	0.941830768	0.966018836	20	20	24	0.916737445	0.977104487	24	12.11530961	0.05779759
ENSMUSG00000106743	Gm42847	predicted gene 42847 [Source:MGI Symbol;Acc:MGI:5662984]	0.861344	0.8601	0.793245	1.03189	0.748408	0.965026	0.769486	0.804012	0.839244	0.929828	0.938204	0.778001	0.79174	0.919293	0.916746631	0.966018836	16	12	24	0.400589809	0.799546792	24	12.55048821	0.034620437
ENSMUSG00000106746	2900064F13Rik	RIKEN cDNA 2900064F13 gene [Source:MGI Symbol;Acc:MGI:1920293]	1.41059	1.52946	1.68745	1.7264	1.37306	1.81565	2.00809	1.43177	1.1241	1.21151	1.26126	0.899187	1.06466	1.5442	0.847846021	0.966018836	8	4	24	0.826124006	0.945636513	24	3.252473995	0.038992982
ENSMUSG00000106755	Tpi-rs11	triosephosphate isomerase related sequence 11 [Source:MGI Symbol;Acc:MGI:98799]	359.241	337.953	338.067	317.048	323.864	349.468	309.053	380.465	369.459	350.859	366.62	383.202	387.603	397.906	0.800065124	0.966018836	8	8	24	0.285753966	0.768040178	24	2.915208952	0.05017493
ENSMUSG00000106791	4930553P18Rik	RIKEN cDNA 4930553P18 gene [Source:MGI Symbol;Acc:MGI:1922605]	1.16575	0.970579	0.720708	0.884799	1.03553	0.936449	0.971074	1.02282	0.634405	0.717465	0.840649	0.926187	0.897936	0.904374	0.047717257	0.377785147	4	8	24	0.006753882	0.472731997	24	22.15287812	0.11004073
ENSMUSG00000106803	Gm8930	predicted gene 8930 [Source:MGI Symbol;Acc:MGI:3646325]	10.9916	13.0668	11.8762	14.0751	13.7019	13.2583	13.7979	13.3509	15.0837	13.3963	14.1774	14.0929	13.9221	13.0393	0.4274437	0.966018836	20	12	24	0.327533252	0.768040178	24	13.4068467	0.050113855
ENSMUSG00000106825	2510016D11Rik	RIKEN cDNA 2510016D11 gene [Source:MGI Symbol;Acc:MGI:1917539]	1.20858	1.42498	1.26173	0.887284	1.5923	1.18015	1.35663	1.64498	1.74993	1.4692	1.52922	1.32535	1.48116	1.55216	0.588682825	0.966018836	8	8	24	0.516682905	0.817817397	24	5.712181029	0.057164829
ENSMUSG00000106831	Ube2n-ps1	ubiquitin-conjugating enzyme E2N, pseudogene 1 [Source:MGI Symbol;Acc:MGI:894296]	61.9531	53.7646	49.8645	58.3632	52.4389	58.0845	60.2283	54.3163	51.42	60.0728	59.7887	60.6305	66.2335	63.4817	0.009483698	0.194720062	4	8	24	0.094228595	0.71175955	24	21.76723215	0.084743727
ENSMUSG00000106832	Gm42632	predicted gene 42632 [Source:MGI Symbol;Acc:MGI:5662769]	0.457934	0.573331	0.36421	0.609444	0.570198	0.472721	0.667431	0.459057	0.601291	0.395486	0.681182	0.546325	0.746386	0.56059	0.906657897	0.966018836	20	16	24	0.600518091	0.860118739	24	21.16576986	0.039818568
ENSMUSG00000106836	Gm43578	predicted gene 43578 [Source:MGI Symbol;Acc:MGI:5663715]	0.813389	0.617196	0.882558	1.9914	1.69802	0.530106	1.15191	1.08152	0.6439	0.717288	0.829119	0.787409	1.27547	1.03728	0.887862353	0.966018836	4	20	24	0.760515081	0.922513876	24	13.93965345	0.081923644
ENSMUSG00000106841	Gm42656	predicted gene 42656 [Source:MGI Symbol;Acc:MGI:5662793]	0.591563	0.621049	0.48286	0.42638	0.640127	0.664963	0.768285	0.481775	0.65807	0.329637	0.727644	0.715935	0.761037	0.643286	0.048889741	0.379129806	4	12	24	0.026512981	0.586541342	24	22.13112808	0.112898368
ENSMUSG00000106847	Peg13	paternally expressed 13 [Source:MGI Symbol;Acc:MGI:2663476]	45.5945	38.3915	42.0652	40.3938	36.9872	46.0631	44.6527	41.5604	31.8423	53.7348	33.8007	42.6212	39.3529	43.733	0.644063931	0.966018836	24	20	24	0.800471121	0.93612712	24	22.62431785	0.0514009
ENSMUSG00000106864	Gtf3c2	general transcription factor IIIC, polypeptide 2, beta [Source:MGI Symbol;Acc:MGI:1919002]	23.3761	23.6017	24.2439	26.1417	24.9118	22.1472	27.4422	21.0851	23.3133	21.4195	25.8732	22.3244	20.8543	19.5173	0.658942124	0.966018836	20	12	24	0.240167495	0.768040178	24	15.83319762	0.074471642
ENSMUSG00000106870	Gm43681	predicted gene 43681 [Source:MGI Symbol;Acc:MGI:5663818]	3.09657	3.17486	2.52727	2.85991	2.96277	2.57192	3.25542	2.49502	3.05402	2.78201	3.12699	2.89286	2.63176	2.63298	0.896311169	0.966018836	4	8	24	0.712550401	0.905210356	24	18.8701059	0.032304994
ENSMUSG00000106874	Gm20186	predicted gene, 20186 [Source:MGI Symbol;Acc:MGI:5012371]	3.15712	2.94373	3.18118	3.40986	3.23404	3.32884	2.93922	2.78271	3.22154	3.74845	3.01187	3.02413	3.90568	3.53857	0.894563866	0.966018836	16	16	24	0.696354793	0.89922881	24	11.4913177	0.033697618
ENSMUSG00000106891	Gm43460	predicted gene 43460 [Source:MGI Symbol;Acc:MGI:5663597]	0.48872	0.790445	0.628167	0.61201	0.665659	0.656292	0.867543	0.676649	0.849899	0.498979	0.458538	0.537909	0.532442	0.662747	0.365996436	0.893596186	8	12	24	0.245007938	0.768040178	24	4.879829079	0.072111926
ENSMUSG00000106895	Gm4754	predicted gene 4754 [Source:MGI Symbol;Acc:MGI:3645925]	5.91459	7.97879	8.10417	7.33288	7.45309	5.47763	6.68485	5.50821	7.81861	3.82687	6.47025	5.19488	6.5748	6.00312	0.085890569	0.466404237	12	12	24	0.64163195	0.878809617	24	6.644378168	0.089911908
ENSMUSG00000106904	Gm43029	predicted gene 43029 [Source:MGI Symbol;Acc:MGI:5663166]	0.66463	0.749747	0.440171	0.631998	0.846699	0.461054	0.458981	0.674341	0.770874	0.515342	0.697397	0.587942	0.595482	0.622278	0.92655264	0.966018836	20	4	24	0.87642992	0.965584799	24	11.07074793	0.023200314
ENSMUSG00000106905	Gm42741	predicted gene 42741 [Source:MGI Symbol;Acc:MGI:5662878]	0.879974	0.962541	0.815058	0.604543	0.732848	0.919299	1.02984	0.8749	0.807464	0.828455	0.840358	0.891541	0.745929	0.804052	0.223902379	0.717349224	24	16	24	0.088194168	0.707747429	24	0.004040112	0.067010662
ENSMUSG00000106917	Gm7832	predicted gene 7832 [Source:MGI Symbol;Acc:MGI:3779767]	34.5573	36.859	36.7893	37.8119	37.4458	37.1719	37.2071	36.2284	31.3889	36.1741	34.2197	31.5073	36.5662	34.1562	0.846643334	0.966018836	16	20	24	0.961971255	0.9903359	24	17.64113264	0.008837417
ENSMUSG00000106918	Mrpl33	mitochondrial ribosomal protein L33 [Source:MGI Symbol;Acc:MGI:2137225]	73.7637	89.3079	90.2227	92.1236	103.308	84.9471	83.7462	74.6685	123.105	72.9022	92.1886	68.2915	109.75	98.202	0.942429894	0.966018836	12	12	24	0.532989921	0.825678486	24	8.727375563	0.109698866
ENSMUSG00000106926	Rpl7-ps7	ribosomal protein L7, pseudogene 7 [Source:MGI Symbol;Acc:MGI:3644615]	41.6199	56.0341	49.7342	54.7123	56.7266	47.1646	46.6931	48.1153	54.8244	38.5859	51.1573	39.8242	45.7972	44.2009	0.313786068	0.824470524	20	4	24	0.481645605	0.805393068	24	10.42775784	0.079676724
ENSMUSG00000106928	Gm43860	predicted gene 43860 [Source:MGI Symbol;Acc:MGI:5663997]	0.93432	0.66592	0.711846	0.898372	0.703704	1.08531	0.989961	0.895925	0.799692	0.637589	1.04349	0.928938	0.847599	0.760302	0.074318596	0.448301997	24	12	24	0.063346927	0.663715314	24	20.47405249	0.097647243
ENSMUSG00000106943	Dancr	differentiation antagonizing non-protein coding RNA [Source:MGI Symbol;Acc:MGI:1917286]	5.6006	6.40487	6.54627	3.95961	4.91801	3.65086	6.22128	5.40314	7.89857	4.9857	7.29788	6.45375	7.00841	6.16348	0.913451828	0.966018836	4	12	24	0.294811177	0.768040178	24	4.6014113	0.153281628
ENSMUSG00000106944	Gm43843	predicted gene 43843 [Source:MGI Symbol;Acc:MGI:5663980]	0.890824	0.849786	0.787532	1.12176	1.2133	0.349055	1.22431	0.599739	1.12356	0.404518	1.15191	0.690198	1.05463	0.909282	0.93717134	0.966018836	20	4	24	0.991582757	0.998516607	24	8.369293945	0.013010828
ENSMUSG00000106951	5930430L01Rik	RIKEN cDNA 5930430L01 gene [Source:MGI Symbol;Acc:MGI:2443110]	0.289128	0.68837	0.510146	0.87163	0.536687	0.372122	0.325724	0.881934	1.12303	0.859699	0.445535	0.507928	0.598046	0.813087	0.023467076	0.278499043	12	16	24	0.001419193	0.354367925	24	8.657749355	0.245914011
ENSMUSG00000106961	Gm43128	predicted gene 43128 [Source:MGI Symbol;Acc:MGI:5663265]	2.36527	1.77053	1.71573	2.12869	2.45185	1.85963	2.09311	1.29354	2.26905	1.32881	2.55014	1.7109	2.00558	1.33177	0.117050591	0.538362324	20	12	24	0.165316948	0.767020009	24	17.77027036	0.148148489
ENSMUSG00000106980	Gm43690	predicted gene 43690 [Source:MGI Symbol;Acc:MGI:5663827]	1.9279	0.964509	1.66604	0.423102	1.09852	1.2506	1.39278	1.00551	1.03541	0.510878	1.53554	1.78521	0.528608	0.30962	0.277917941	0.789849983	24	8	24	0.209028105	0.768040178	24	20.40219726	0.244211224
ENSMUSG00000106981	Gm45495	predicted gene 45495 [Source:MGI Symbol;Acc:MGI:5791331]	9.59213	8.5674	8.36113	7.24624	8.72267	8.40946	8.56919	9.09873	6.4922	8.02149	8.52034	8.16099	8.97833	8.39825	0.008747574	0.192760771	4	4	24	0.039871923	0.616921492	24	22.69907715	0.112331431
ENSMUSG00000106988	Tsg101-ps	tumor susceptibility gene 101, pseudogene [Source:MGI Symbol;Acc:MGI:1335078]	24.9924	21.1457	20.1958	22.4645	24.3559	23.2246	22.9067	22.7568	21.365	22.2389	22.4191	22.4908	22.6641	23.9668	0.031969981	0.324385159	4	8	24	0.113135635	0.727393008	24	20.71038727	0.062319439
ENSMUSG00000106990	Gm42547	predicted gene 42547 [Source:MGI Symbol;Acc:MGI:5662684]	3.73309	2.8653	2.99737	3.70767	3.15051	2.91244	3.63355	2.53551	2.60926	2.5586	3.41575	2.37443	2.56344	2.72582	0.909604019	0.966018836	20	12	24	0.495637205	0.807322367	24	18.02201593	0.065942831
ENSMUSG00000107000	Gm43481	predicted gene 43481 [Source:MGI Symbol;Acc:MGI:5663618]	2.01824	2.45155	2.08644	2.23934	2.37117	2.19824	1.87611	2.42497	2.21132	2.34592	2.16675	2.16198	2.41593	0.280850051	0.792139038	12	4	24	0.52134925	0.820288359	24	12.10464984	0.037540185
ENSMUSG00000107002	0610012G03Rik	RIKEN cDNA 0610012G03 gene [Source:MGI Symbol;Acc:MGI:1913301]	13.7961	11.7069	11.6107	11.9595	10.2449	12.4923	13.6015	14.1838	12.3759	12.6475	14.4787	13.0238	13.5735	14.0886	0.46238066	0.966018836	8	4	24	0.160504633	0.765941695	24	1.0668489	0.079203828
ENSMUSG00000107008	Gm2762	predicted gene 2762 [Source:MGI Symbol;Acc:MGI:3780931]	1.1425	0.53974	0.78098	1.05822	1.30006	0.956418	0.7919	0.883172	0.913926	0.488474	1.11539	1.08974	0.83581	1.06707	0.17847698	0.655480352	20	16	24	0.261660804	0.768040178	24	18.51885063	0.115842736
ENSMUSG00000107017	Gm43196	predicted gene 43196 [Source:MGI Symbol;Acc:MGI:5663333]	0.649616	0.619937	0.61859	0.39786	0.549945	0.53299	0.570062	0.674853	0.43075	0.42903	0.364387	0.464102	0.417939	0.631588	0.007831348	0.192760771	8	8	24	0.01656746	0.54430656	24	2.55026474	0.088085585
ENSMUSG00000107019	Gm43682	predicted gene 43682 [Source:MGI Symbol;Acc:MGI:5663819]	1.97318	2.36422	2.57234	2.25099	1.80955	2.28751	1.95942	2.56683	2.39162	2.6742	1.75948	2.59758	2.12949	2.31445	0.110739523	0.528348425	12	8	24	0.167040339	0.767020009	24	7.240066081	0.099181537
ENSMUSG00000107020	Gm6051	predicted gene 6051 [Source:MGI Symbol;Acc:MGI:3645584]	19.8299	17.0838	16.8608	25.4895	21.4131	21.8829	21.9232	18.1962	17.8588	18.9124	18.5232	20.698	16.8869	16.7876	0.085562135	0.466404237	24	8	24	0.042470562	0.623698598	24	17.18832409	0.150948358
ENSMUSG00000107021	Gm42853	predicted gene 42853 [Source:MGI Symbol;Acc:MGI:5662990]	0.388854	0.527433	0.599315	0.745545	0.851533	0.701197	0.554059	0.348866	0.753412	0.513707	0.736997	0.721475	0.646423	0.60216	0.029682156	0.323426482	20	12	24	0.041019752	0.61771321	24	15.14107628	0.11409739
ENSMUSG00000107023	Gm42715	predicted gene 42715 [Source:MGI Symbol;Acc:MGI:5662852]	3.15809	2.73746	4.46591	2.5396	2.10224	2.77564	2.45112	2.78915	3.17308	2.04882	3.28878	1.85471	3.72984	2.88433	0.44861185	0.966018836	12	4	24	0.307727558	0.768040178	24	5.221892453	0.151478162
ENSMUSG00000107028	Gm5043	predicted gene 5043 [Source:MGI Symbol;Acc:MGI:3646551]	1.81714	2.15344	1.44868	1.87197	2.1715	1.7497	2.14161	2.27472	1.48788	2.16131	1.82961	2.35983	1.85818	2.13312	0.9143748	0.966018836	8	4	24	0.545301253	0.832868977	24	21.58140864	0.061591972
ENSMUSG00000107035	Ybx1-ps2	Y box protein 1, pseudogene 2 [Source:MGI Symbol;Acc:MGI:2137671]	8.86026	8.72519	7.72969	7.57746	9.51495	7.699	10.0765	8.42532	9.50321	8.96273	8.77584	8.87469	9.57066	8.97494	0.702630284	0.966018836	4	12	24	0.539307742	0.829259908	24	23.95560444	0.045583876
ENSMUSG00000107060	Gm38901	predicted gene, 38901 [Source:MGI Symbol;Acc:MGI:5621786]	8.29084	6.90547	6.13223	7.73873	7.11625	7.4884	7.04153	7.29213	5.81106	7.35834	7.34374	8.07581	7.92131	7.03562	0.021795836	0.26698364	24	12	24	0.032782366	0.606585237	24	20.26487503	0.118198517
ENSMUSG00000107066	Gm4482	predicted gene 4482 [Source:MGI Symbol;Acc:MGI:3782666]	13.4844	12.5735	13.6093	16.5918	14.6221	14.3565	13.833	13.8889	13.2765	14.0942	15.4736	13.8573	15.7968	15.3464	0.297756231	0.80820842	16	20	24	0.32790642	0.768040178	24	15.28002683	0.053560075
ENSMUSG00000107068	Gm42742	predicted gene 42742 [Source:MGI Symbol;Acc:MGI:5662879]	24.4819	23.9288	25.7323	22.8146	24.9912	23.1344	23.2941	21.7696	27.89	25.3107	26.9459	26.104	29.0106	27.8202	0.846643334	0.966018836	12	20	24	0.8661151	0.961251055	24	8.732207881	0.021272946
ENSMUSG00000107086	Gm43808	predicted gene 43808 [Source:MGI Symbol;Acc:MGI:5663945]	2.74197	2.32305	1.98516	2.0624	2.9558	1.99208	3.04058	2.08001	3.28288	2.05256	3.72029	2.45166	2.84299	2.97392	0.951846915	0.966018836	20	20	24	0.820786213	0.943391287	24	20.75243444	0.053318173
ENSMUSG00000107092	Gm7993	predicted gene 7993 [Source:MGI Symbol;Acc:MGI:3647399]	7.40645	9.35194	10.1781	9.86533	9.92639	8.54598	10.2357	8.8305	10.1961	9.39828	8.61941	8.82381	9.33153	7.50952	0.377663653	0.903463289	12	12	24	0.288409658	0.768040178	24	11.27229024	0.08250745
ENSMUSG00000107096	Gm43597	predicted gene 43597 [Source:MGI Symbol;Acc:MGI:5663734]	5.47526	4.53471	4.91591	4.99342	4.89566	5.11138	4.65789	5.17789	5.80951	4.25655	5.40749	5.04181	4.77691	5.1393	0.804027134	0.966018836	12	4	24	0.987117112	0.997396641	24	1.391837105	0.006213464
ENSMUSG00000107108	Gm9936	predicted gene 9936 [Source:MGI Symbol;Acc:MGI:3641854]	0.546737	0.598563	0.42074	0.678103	0.575767	0.561297	0.596303	0.593255	0.639771	0.597612	0.636423	0.603716	0.59408	0.689241	0.965971228	0.966272194	16	12	24	0.938419269	0.984760273	24	11.73501133	0.007539488
ENSMUSG00000107120	Gm43059	predicted gene 43059 [Source:MGI Symbol;Acc:MGI:5663196]	0.615181	0.477595	0.598825	0.409716	0.7536	0.608439	0.672236	0.464774	0.819219	0.641607	0.825656	0.885654	0.602832	0.577595	0.030972684	0.324150946	20	12	24	0.19774103	0.768040178	24	17.39448162	0.07920257
ENSMUSG00000107121	1110060O24Rik	RIKEN cDNA 1110060O24 gene [Source:MGI Symbol;Acc:MGI:1913373]	0.39491	0.367609	0.786975	0.677412	0.589327	0.44691	0.882421	0.882219	0.763162	0.652415	0.85787	0.799296	0.878367	0.691476	0.81544087	0.966018836	4	20	24	0.708311018	0.903587413	24	9.132176881	0.04368684
ENSMUSG00000107134	Gm42528	predicted gene 42528 [Source:MGI Symbol;Acc:MGI:5662665]	1.3007	1.3246	1.5746	1.22101	1.20931	1.34116	1.23376	1.20257	1.52362	0.987378	1.28547	1.14891	1.12121	1.44643	0.256915694	0.76168256	12	4	24	0.299474231	0.768040178	24	6.061466574	0.05952657
ENSMUSG00000107145	Gm43442	predicted gene 43442 [Source:MGI Symbol;Acc:MGI:5663579]	19.0627	15.0531	17.373	16.4086	19.7464	18.9812	17.0904	18.6955	16.0612	22.8911	14.7595	16.179	18.1293	15.2951	0.93447422	0.966018836	16	16	24	0.722222389	0.908924547	24	16.36463768	0.053923382
ENSMUSG00000107176	Gm9794	predicted pseudogene 9794 [Source:MGI Symbol;Acc:MGI:3642653]	516.769	590.508	563.145	558.5	540.056	502.842	542.513	525.264	639.346	442.993	571.359	540.353	590.958	558.295	0.601647512	0.966018836	12	12	24	0.497980568	0.807927978	24	5.232912232	0.058935152
ENSMUSG00000107182	Gm43268	predicted gene 43268 [Source:MGI Symbol;Acc:MGI:5663405]	2.75447	2.98653	3.08693	3.63906	3.08822	2.74933	3.23188	3.03554	3.25151	2.74515	3.33079	2.96489	2.40727	3.07813	0.201875332	0.688108864	20	4	24	0.182968635	0.768040178	24	11.26284851	0.075815544
ENSMUSG00000107200	Gm43039	predicted gene 43039 [Source:MGI Symbol;Acc:MGI:5663176]	0.695524	1.21183	1.35061	1.48137	1.48722	0.93051	1.03038	1.06751	1.4928	0.984694	1.26382	1.10883	0.959737	1.15039	0.009118941	0.192760771	12	16	24	0.024489694	0.586541342	24	11.08775926	0.155253031
ENSMUSG00000107201	5930420M18Rik	RIKEN cDNA 5930420M18 gene [Source:MGI Symbol;Acc:MGI:2441703]	0.747852	0.704229	0.610428	0.711491	0.985133	0.693836	0.639883	0.672047	0.58875	0.691457	0.710065	0.890276	0.707629	1.01056	0.285027487	0.793741531	20	20	24	0.551891393	0.836481856	24	19.19505384	0.044783973
ENSMUSG00000107211	Gm5864	predicted gene 5864 [Source:MGI Symbol;Acc:MGI:3648579]	4.92469	4.83017	6.05155	5.76126	5.34456	5.92616	4.62642	4.93849	3.56504	5.40107	5.11305	4.67897	5.08464	4.92867	0.360675801	0.883978426	16	8	24	0.412472994	0.799546792	24	16.05567212	0.077870693
ENSMUSG00000107215	Gm43197	predicted gene 43197 [Source:MGI Symbol;Acc:MGI:5663334]	0.507116	0.541301	0.569743	0.547298	0.763778	0.53261	0.799935	0.764636	0.918437	0.540162	1.03957	0.643724	0.629855	0.67784	0.888482515	0.966018836	20	4	24	0.821298859	0.943612008	24	12.8480473	0.03047977
ENSMUSG00000107216	2210412B16Rik	RIKEN cDNA 2210412B16 gene [Source:MGI Symbol;Acc:MGI:1919625]	0.837168	1.63486	1.29817	1.76143	1.67893	1.47395	1.74359	1.2744	1.78728	0.474507	1.22161	1.46335	1.20368	0.546705	0.885059765	0.966018836	16	20	24	0.793195885	0.933800612	24	18.35164633	0.07968695
ENSMUSG00000107230	Gm19265	predicted gene, 19265 [Source:MGI Symbol;Acc:MGI:5011450]	1.632	1.65829	1.12384	1.18439	1.62708	1.60412	1.65874	1.3143	1.26265	1.22208	1.01839	1.42327	1.59527	1.5869	0.033814615	0.325496079	20	20	24	0.046993192	0.633507364	24	21.0088531	0.129900034
ENSMUSG00000107259	Gm5298	predicted gene 5298 [Source:MGI Symbol;Acc:MGI:3645526]	0.709153	1.27485	0.889688	1.50016	1.18286	1.4229	1.06452	1.17147	0.930974	0.756901	0.899521	0.800639	0.727138	0.571253	0.935593607	0.966018836	20	4	24	0.535409518	0.826974257	24	15.47832962	0.075274297
ENSMUSG00000107272	Gm34583	predicted gene, 34583 [Source:MGI Symbol;Acc:MGI:5593742]	1.93576	2.03794	1.79745	1.41176	2.8444	2.07906	2.01886	1.10136	1.97129	1.27746	2.06227	1.60422	1.29104	1.27337	0.243586913	0.741580974	20	12	24	0.148078992	0.756225271	24	17.89047739	0.16053082
ENSMUSG00000107283	Mpv17	MpV17 mitochondrial inner membrane protein [Source:MGI Symbol;Acc:MGI:97138]	39.7394	40.9296	38.7892	40.701	44.0701	37.5773	38.5935	39.1167	41.3496	37.2029	43.4421	39.1663	42.1261	39.3698	0.84100102	0.966018836	20	4	24	0.69165837	0.897724829	24	15.43698691	0.02441365
ENSMUSG00000107300	Gm43279	predicted gene 43279 [Source:MGI Symbol;Acc:MGI:5663416]	0.522474	0.84939	0.678708	0.816572	0.710692	0.606436	0.535689	0.681705	0.853903	0.623308	0.628622	0.79164	0.794422	1.03523	0.356953959	0.881188925	8	20	24	0.162590191	0.76636332	24	7.255226638	0.080262315
ENSMUSG00000107331	Gm42732	predicted gene 42732 [Source:MGI Symbol;Acc:MGI:5662869]	9.02073	9.29086	7.89167	7.98421	7.88802	9.32305	8.951	8.94767	8.57961	9.07798	8.43244	9.09144	8.93927	10.2034	0.202982082	0.689605535	24	20	24	0.033932492	0.606793499	24	1.399121625	0.076733865

ENSMUSG00000107336	Gm43461	predicted gene 43461 [Source:MGI Symbol;Acc:MGI:5663598]	0.381608	0.53335	0.570621	0.4378	0.535685	0.352093	0.349217	0.683479	0.52129	0.819381	0.426471	0.760252	0.571848	0.640996	0.885025617	0.966018836	8	20	24	0.285131649	0.768040178	24	8.73397208	0.07601862
ENSMUSG00000107352	Gm43660	predicted gene 43660 [Source:MGI Symbol;Acc:MGI:5663797]	0.600686	0.741236	0.815756	0.772981	0.658798	0.637934	0.50754	0.730101	0.920809	0.703325	0.642553	0.745708	0.772047	0.826996	0.029412383	0.322165609	12	16	24	0.015448392	0.539669932	24	8.221931319	0.088422828
ENSMUSG00000107355	AI839979	expressed sequence AI839979 [Source:MGI Symbol;Acc:MGI:2140975]	0.933096	1.51647	1.15992	1.33319	1.09189	1.54503	0.904131	1.00857	1.23571	1.24868	1.05573	1.44669	1.37599	1.46678	0.919571723	0.966018836	24	4	24	0.973874676	0.993366233	24	9.316145773	0.013286605
ENSMUSG00000107369	Gstm2-ps1	glutathione S-transferase mu 2 (muscle), pseudogene 1 [Source:MGI Symbol;Acc:MGI:3648223]	9.93217	12.5362	14.5646	12.065	10.3346	14.3521	14.651	15.7023	11.8276	13.1294	10.0607	12.62	10.1994	10.5888	0.951846915	0.966018836	8	12	24	0.711441686	0.904655191	24	5.833006208	0.070176735
ENSMUSG00000107374	Gm43172	predicted gene 43172 [Source:MGI Symbol;Acc:MGI:5663309]	0.722282	0.82715	0.786047	0.855854	0.877394	0.745321	0.61304	0.630937	0.769055	0.728781	0.834953	0.726222	0.706179	0.906532	0.08333408	0.462922111	20	8	24	0.22129909	0.768040178	24	12.13709281	0.047567634
ENSMUSG00000107383	Gm4366	predicted gene 4366 [Source:MGI Symbol;Acc:MGI:3782551]	120.79	114.827	117.388	116.556	122.517	125.715	115.584	125.254	115.698	129.172	114.418	119.453	126.648	116.26	0.916556573	0.966018836	24	12	24	0.737495529	0.91440753	24	19.09518149	0.019062084
ENSMUSG00000107388	Gm42788	predicted gene 42788 [Source:MGI Symbol;Acc:MGI:5662925]	1.73929	2.15879	2.26529	2.00299	2.67411	1.75779	2.02839	2.38965	2.47002	1.61873	2.19952	1.55771	2.48676	1.83814	0.601647512	0.966018836	12	12	24	0.655025958	0.883697438	24	7.883389783	0.063145542
ENSMUSG00000107451	Gm44421	predicted gene, 44421 [Source:MGI Symbol;Acc:MGI:5690813]	1.50046	0.991886	1.79539	2.88857	1.77263	1.51513	1.28236	0.773567	1.56412	1.58588	1.44673	1.26562	0.923392	1.22861	0.0014632	0.099807435	16	16	24	0.006846694	0.472731997	24	13.45992367	0.265655424
ENSMUSG00000107465	Gm5990	predicted gene 5990 [Source:MGI Symbol;Acc:MGI:3648301]	0.95651	0.797772	0.59408	0.859065	0.713373	1.00467	0.99911	1.22068	0.559087	0.812707	0.749638	0.698947	0.684015	0.820911	0.834434272	0.966018836	8	4	24	0.316721289	0.768040178	24	23.40188062	0.081122462
ENSMUSG00000107470	Gm3375	predicted gene 3375 [Source:MGI Symbol;Acc:MGI:3781553]	29.2252	29.9063	27.3876	29.2582	29.2003	27.5285	26.7964	31.6418	25.7815	27.1367	30.8192	28.0482	28.5286	27.0611	0.890648739	0.966018836	20	16	24	0.811246854	0.939484534	24	19.77116708	0.021422245
ENSMUSG00000107476	Zfp862-ps	zinc finger protein 862, pseudogene [Source:MGI Symbol;Acc:MGI:1889827]	2.76072	2.646	2.83831	2.82098	2.98853	3.24584	3.50753	2.77207	2.60468	2.93228	2.81458	2.79741	2.85015	2.41995	0.132411359	0.567728672	24	8	24	0.106019344	0.71810271	24	19.16389451	0.077533925
ENSMUSG00000107499	Ccdc142	coiled-coil domain containing 142 [Source:MGI Symbol;Acc:MGI:3045292]	1.17858	1.52633	1.04237	1.28453	0.636501	0.831213	1.49774	1.23847	1.13003	1.47427	1.3963	1.27574	1.14111	1.46389	0.830364743	0.966018836	8	16	24	0.300269955	0.768040178	24	5.242568246	0.105727101
ENSMUSG00000107504	Gm35549	predicted gene, 35549 [Source:MGI Symbol;Acc:MGI:5594708]	0.95789	0.359569	0.570297	0.797357	0.588499	0.30085	0.425985	0.443851	0.746833	0.560315	0.642879	0.478346	0.726986	0.382361	0.481530854	0.966018836	16	16	24	0.708231393	0.903587413	24	12.81466502	0.055526095
ENSMUSG00000107524	Gm44136	predicted gene, 44136 [Source:MGI Symbol;Acc:MGI:5690528]	0.673742	0.727857	0.686999	0.773719	0.978313	0.622294	0.82236	0.641491	1.06462	0.472914	0.869479	0.724241	0.756794	0.753267	0.915475528	0.966018836	20	4	24	0.933463997	0.983211997	24	13.33521634	0.01806883
ENSMUSG00000107560	Gm44199	predicted gene, 44199 [Source:MGI Symbol;Acc:MGI:5690591]	0.484115	0.912839	1.05235	1.02755	1.13457	1.05592	0.6742	0.988408	1.36759	0.548449	1.28036	0.796044	0.784784	0.727272	0.028180201	0.314814652	20	8	24	0.278869975	0.768040178	24	12.21038577	0.122708641
ENSMUSG00000107577	Gm44103	predicted gene, 44103 [Source:MGI Symbol;Acc:MGI:5690495]	1.4376	1.30228	1.41348	1.08784	1.43588	1.48882	1.30444	1.29928	1.4361	1.44128	1.37904	1.47929	1.39093	1.52104	0.920620634	0.966018836	24	8	24	0.680510908	0.893265165	24	21.91013165	0.023282796
ENSMUSG00000107585	3300002P13Rik	RIKEN cDNA 3300002P13 gene [Source:MGI Symbol;Acc:MGI:1917480]	0.25648	0.956159	1.44128	0.736265	0.48049	1.11752	1.09565	1.45553	1.72327	0.960148	0.470697	0.680812	1.22357	0.987886	0.066808003	0.425506559	12	8	24	0.045552668	0.629609444	24	6.310916769	0.283684186
ENSMUSG00000107596	Gm18913	predicted gene, 18913 [Source:MGI Symbol;Acc:MGI:5011098]	0.657986	0.79365	1.06073	0.954535	0.93455	0.87561	0.535996	1.18178	0.717035	0.659919	0.924806	0.805213	0.906711	0.921724	0.481811994	0.966018836	12	16	24	0.678879666	0.892553775	24	9.630634017	0.049083294
ENSMUSG00000107620	Gm44256	predicted gene, 44256 [Source:MGI Symbol;Acc:MGI:5690648]	1.64018	1.41548	1.26857	1.04431	2.54997	1.28903	3.39851	1.64622	1.26484	1.08585	2.04685	1.37696	1.74777	1.2842	0.009678659	0.194720062	4	12	24	0.207034005	0.768040178	24	21.27309077	0.215870985
ENSMUSG00000107639	Gm4374	predicted gene 4374 [Source:MGI Symbol;Acc:MGI:3782559]	16.1682	22.412	17.5355	19.778	20.5842	18.3502	15.8729	16.3476	24.3152	17.4187	23.0723	20.8194	20.9412	20.5987	0.95261238	0.966018836	20	8	24	0.544422949	0.832181275	24	12.04898993	0.078070854
ENSMUSG00000107655	Gm44220	predicted gene, 44220 [Source:MGI Symbol;Acc:MGI:5690612]	4.02042	5.14255	4.56111	5.06999	4.24583	3.95986	4.0401	4.30266	5.4235	5.00648	4.53003	4.80116	4.41085	4.4127	0.026323104	0.301028315	12	16	24	0.022297765	0.578379975	24	9.75765443	0.115656072
ENSMUSG00000107667	C530044C16Rik	RIKEN cDNA C530044C16 gene [Source:MGI Symbol;Acc:MGI:2443109]	2.2153	2.31737	2.04981	2.7039	1.92022	2.12669	1.5247	2.26085	2.2664	1.75847	1.98931	2.16231	1.91528	2.08655	0.652290823	0.966018836	8	12	24	0.622437575	0.871391591	24	8.901042241	0.050610643
ENSMUSG00000107689	Gm44386	predicted gene, 44386 [Source:MGI Symbol;Acc:MGI:5690778]	2.26582	4.19929	5.89823	8.17289	4.66213	4.20794	3.48108	6.14926	5.52914	7.99383	4.81518	5.47027	8.23516	7.19482	0.496745696	0.966018836	16	12	24	0.024792875	0.586541342	24	9.917766727	0.347634039
ENSMUSG00000107705	Gm45062	predicted gene 45062 [Source:MGI Symbol;Acc:MGI:5753638]	4.51275	4.65216	4.36011	4.86791	4.48455	4.70215	5.29572	4.47405	4.67902	4.63541	4.7247	4.6196	4.73457	4.58159	0.892578749	0.966018836	4	4	24	0.663671754	0.886493109	24	21.03071103	0.020644618
ENSMUSG00000107724	Gm16042	predicted gene 16042 [Source:MGI Symbol;Acc:MGI:3801935]	3.75975	5.76608	5.58623	4.07769	4.68735	5.62507	3.82103	5.28788	5.25886	4.9503	4.56155	4.62549	5.32222	4.85933	0.560714538	0.966018836	8	20	24	0.467728701	0.805393068	24	7.164354064	0.083361614
ENSMUSG00000107726	Gm44037	predicted gene, 44037 [Source:MGI Symbol;Acc:MGI:5690429]	0.687823	0.746732	0.923136	0.951107	0.79325	0.810931	0.784491	0.844313	0.972781	0.681418	0.822776	0.771964	0.868147	0.900807	0.321662653	0.83607295	12	12	24	0.199082364	0.768040178	24	8.450833346	0.050101046
ENSMUSG00000107728	Gm38910	predicted gene, 38910 [Source:MGI Symbol;Acc:MGI:5621795]	1.19557	1.00551	1.28235	1.31909	1.36983	1.38533	1.46149	1.38602	0.925287	1.59057	1.17277	1.22087	1.2654	1.16702	0.888991156	0.966018836	16	20	24	0.456016578	0.805393068	24	17.21270888	0.056572903
ENSMUSG00000107741	Gm2011	predicted gene 2011 [Source:MGI Symbol;Acc:MGI:3780180]	2.53858	2.7102	2.358	3.04322	2.42351	2.77073	2.52163	2.56194	3.0163	2.84812	2.87455	2.52297	2.73202	3.08584	0.895493752	0.966018836	16	12	24	0.327772715	0.768040178	24	9.654664649	0.049896029
ENSMUSG00000107742	9530085L11Rik	RIKEN cDNA 9530085L11 gene [Source:MGI Symbol;Acc:MGI:2443889]	0.689871	0.619619	0.552041	0.608181	0.564932	0.486401	0.371987	0.364611	0.70829	0.402009	0.532294	0.541174	0.687224	0.631278	0.919119843	0.966018836	4	20	24	0.898701856	0.9713659	24	5.134863649	0.020613782
ENSMUSG00000107747	Gm5881	predicted gene 5881 [Source:MGI Symbol;Acc:MGI:3779532]	98.5405	85.4424	85.8042	89.0993	98.6701	100.148	97.0411	87.51	61.8077	83.7747	76.8625	70.8906	67.9452	75.0152	0.888991156	0.966018836	20	16	24	0.199617862	0.768040178	24	19.56512947	0.11223986
ENSMUSG00000107761	2010008C14Rik	RIKEN cDNA 2010008C14 gene [Source:MGI Symbol;Acc:MGI:1917109]	6.68876	3.48553	3.02726	4.12355	3.44858	4.86374	4.18252	4.22014	2.97184	6.57104	4.44429	4.23202	4.91091	4.07476	0.040082266	0.34094463	4	8	24	0.370048442	0.792235171	24	20.27411079	0.163498947
ENSMUSG00000107770	Gm44126	predicted gene, 44126 [Source:MGI Symbol;Acc:MGI:5690518]	28.4053	24.3805	26.0074	25.0965	23.7081	25.2043	23.7073	22.4013	23.7995	25.1185	25.5766	25.0327	26.9281	22.1295	0.636884864	0.966018836	4	4	24	0.373254375	0.794388449	24	19.1567031	0.05038004
ENSMUSG00000107810	Gm18609	predicted gene, 18609 [Source:MGI Symbol;Acc:MGI:5010794]	2.00305	2.08667	2.1593	2.31479	2.25094	2.07661	1.68827	1.7491	2.00822	2.33435	2.01453	2.30889	2.14297	2.47088	0.192357053	0.669841558	16	12	24	0.285044532	0.768040178	24	12.73830294	0.060959525
ENSMUSG00000107838	Gm45769	predicted gene 45769 [Source:MGI Symbol;Acc:MGI:5804884]	1.49924	1.46529	1.57964	1.837	1.3243	1.28949	1.2504	1.62429	1.53072	1.50887	1.93768	1.31543	1.28061	1.44694	0.032593161	0.324908923	16	12	24	0.071295039	0.676901434	24	10.87575303	0.097889389
ENSMUSG00000107853	Gm18584	predicted gene, 18584 [Source:MGI Symbol;Acc:MGI:5010769]	3.17555	3.65615	4.37048	3.98334	4.64177	4.11168	3.93476	4.44239	4.50669	3.76992	3.54792	3.86017	3.73494	3.0391	0.648082424	0.966018836	12	20	24	0.428227776	0.805393068	24	12.38012049	0.071288636
ENSMUSG00000107868	Gm5112	predicted gene 5112 [Source:MGI Symbol;Acc:MGI:3779460]	0.502797	0.585003	0.527671	0.683447	0.62186	0.563958	0.526808	0.56484	0.700721	0.664111	0.582851	0.718184	0.560366	0.717825	0.109247218	0.524035616	16	12	24	0.123135451	0.736234617	24	11.3218564	0.044168471
ENSMUSG00000107875	Gm16630	predicted gene, 16630 [Source:NCBI gene (formerly Entrezgene);Acc:100503622]	0.595659	0.283969	0.526462	0.342321	0.251338	0.783667	0.45454	0.467333	0.458074	0.688591	0.512816	0.49767	0.693495	0.637333	0.843694678	0.966018836	24	20	24	0.652296951	0.88299052	24	23.18705262	0.050822121
ENSMUSG00000107877	-	novel protein	1.61208	1.97163	2.14345	0.774062	1.78556	2.21091	2.21107	1.70471	2.34955	1.97614	1.37807	0.488675	1.25846	2.22028	0.287843582	0.793714531	12	12	24	0.307595854	0.768040178	24	5.705802544	0.208692675
ENSMUSG00000107879	9330102E08Rik	RIKEN cDNA 9330102E08 gene [Source:MGI Symbol;Acc:MGI:3026973]	4.92367	4.46707	4.75113	4.7052	4.36006	4.99147	4.53567	4.38047	3.63773	5.18942	4.07257	4.78608	4.54337	4.59048	0.791413662	0.966018836	24	12	24	0.587655049	0.853689229	24	20.1405406	0.044207926
ENSMUSG00000107881	Gm44250	predicted gene, 44250 [Source:MGI Symbol;Acc:MGI:5690642]	2.01383	2.43257	2.66877	3.48344	2.31797	2.16439	2.20115	3.25571	3.05233	2.76243	3.4237	2.91172	3.46573	3.00338	0.652290823	0.966018836	16	8	24	0.116149208	0.727799063	24	9.773205545	0.126843159
ENSMUSG00000107892	Gm44524	predicted gene 44524 [Source:MGI Symbol;Acc:MGI:5753100]	0.368829	0.36933	1.19553	1.21586	0.263165	1.39795	1.77695	0.962732	1.4863	0.244399	0.980457	0.974661	0.505318	1.18196	0.869755137	0.966018836	12	4	24	0.872003183	0.963804482	24	5.013317255	0.082641996
ENSMUSG00000107906	Eif4a3l2	eukaryotic translation initiation factor 4A3 like 2 [Source:MGI Symbol;Acc:MGI:3646594]	3.96994	4.10571	4.03101	3.46282	4.02338	4.14401	4.40386	4.30335	3.55163	4.58907	3.41979	3.6201	3.85001	4.08282	0.622132989	0.966018836	8	12	24	0.486953129	0.805393068	24	2.656318506	0.04800646
ENSMUSG00000107928	Gm45140	predicted gene 45140 [Source:MGI Symbol;Acc:MGI:5753716]	4.92477	3.95599	1.78296	5.98022	3.55266	2.99805	3.22898	2.60463	2.82642	1.36135	2.53991	0.666815	2.98752	2.9292	0.834035723	0.966018836	4	20	24	0.842274713	0.952050674	24	2.438213093	0.103917821
ENSMUSG00000107937	Gm44200	predicted gene, 44200 [Source:MGI Symbol;Acc:MGI:5690592]	0.879258	0.486664	0.694825	1.18535	0.594863	0.405975	0.778711	0.285228	1.03472	0.211324	0.875613	0.716691	0.908052	0.843162	0.877082091	0.966018836	4	4	24	0.99977236	0.999970554	24	8.081122427	0.002262289
ENSMUSG00000107951	Gm6210	predicted gene 6210 [Source:MGI Symbol;Acc:MGI:3643375]	0.645784	0.657297	0.605823	0.436276	0.820797	0.775016	0.48984	0.580077	0.472434	0.250023	0.507185	0.325344	0.414172	0.280581	0.858932988	0.966018836	20	20	24	0.300260723	0.768040178	24	20.31431328	0.073478437
ENSMUSG00000107952	Gm6352	predicted gene 6352 [Source:MGI Symbol;Acc:MGI:3647816]	4.37517	3.36772	3.64689	4.03911	4.26049	4.25407	3.94302	4.3976	3.04573	3.99771	3.20878	3.724	4.599	3.89966	0.182849367	0.659303873	4	8	24	0.287474153	0.768040178	24	21.96706556	0.088336688
ENSMUSG00000107962	Gm43980	predicted gene, 43980 [Source:MGI Symbol;Acc:MGI:5690372]	7.90599	8.5876	8.09629	8.91403	9.95042	7.26632	9.69201	6.94991	9.63794	5.36643	10.8865	6.869	7.6179	7.655	0.676344318	0.966018836	20	4	24	0.875664468	0.965253361	24	16.23148633	0.048358953
ENSMUSG00000107968	Gm44419	predicted gene, 44419 [Source:MGI Symbol;Acc:MGI:5690811]	5.69705	3.68432	1.84245	4.94234	3.59464	5.16103	2.05364	4.42165	2.6681	3.39718	2.62078	1.52266	2.36478	1.86629	0.814723813	0.966018836	16	20	24	0.780386049	0.929485273	24	17.60706216	0.107432015
ENSMUSG00000107997	Gm44243	predicted gene, 44243 [Source:MGI Symbol;Acc:MGI:5690635]	1.6579	1.64449	1.88369	2.0648	1.85229	0.893845	1.00974	2.08517	1.48947	1.29493	1.85858	1.92927	1.91271	1.58775	0.748433002	0.966018836	8	20	24	0.656680069	0.884067495	24	9.524719881	0.082609266
ENSMUSG00000108030	9530062K07Rik	RIKEN cDNA 9530062K07 gene [Source:MGI Symbol;Acc:MGI:1925989]	0.382794	0.550752	1.17898	0.875819	0.428595	0.325024	0.466611	0.969406	1.17508	0.463747	0.612894	0.733786	0.808678	0.669875	0.039704107	0.338535904	12	12	24	0.029544172	0.596648309	24	7.822058349	0.224881208
ENSMUSG00000108037	Gm44597	predicted gene 44597 [Source:MGI Symbol;Acc:MGI:5753173]	1.21876	0.943617	1.2336	1.61208	1.96289	1.30882	1.81538	1.05608	1.42945	1.56983	1.92138	1.35275	1.33953	1.23128	1.06E-04	0.029372629	20	12	24	0.011660079	0.521279312	24	15.67610997	0.180280297
ENSMUSG00000108045	Gm5576	predicted pseudogene 5576 [Source:MGI Symbol;Acc:MGI:3644832]	1.36844	1.07202	0.764408	1.48791	1.13753	1.40984	0.812824	1.2602	0.78375	1.19713	0.993472	1.24775	0.984191	1.04601	0.338172159	0.85758369	24	12	24	0.324477404	0.768040178	24	18.35918103	0.094576886
ENSMUSG00000108049	Gm44168	predicted gene, 44168 [Source:MGI Symbol;Acc:MGI:5690560]	0.996181	1.37855	1.2758	1.48851	1.43985	1.21946	1.30417	1.0857	1.70178	1.27699	1.23136	1.0853	1.17423	1.37703	0.061112935	0.409814092	12	16	24	0.060717174	0.660111271	24	10.11846287	0.100513684
ENSMUSG00000108057	Gm44234	predicted gene, 44234 [Source:MGI Symbol;Acc:MGI:5690626]	0.75536	0.767543	0.879982	0.680289	0.484164	0.458973	0.59227	0.337969	0.47884	0.499084	1.14939	0.550835	0.719149	1.08948	0.890648739	0.966018836	12	12	24	0.936848304	0.984446525	24	6.422665154	0.030159243
ENSMUSG00000108064	Gm44423	predicted gene, 44423 [Source:MGI Symbol;Acc:MGI:5690815]	0.43606	0.59202	0.819777	0.854111	0.766617	0.558827	0.565461	0.594076	0.769161	0.479906	0.611774	0.754421	0.648873	0.77168	0.248423582	0.746945512	12	16	24	0.278230046	0.768040178	24	10.45182593	0.071246044
ENSMUSG00000108079	Gm44210	predicted gene, 44210 [Source:MGI Symbol;Acc:MGI:5690602]	1.62386	1.93852	1.67834	1.61863	1.72792	1.81574	1.11178	1.22612	1.92182	2.66272	1.30542	1.3564	1.43952	1.38165	0.579092016	0.966018836	16	12	24	0.154147224	0.760257003	24	11.46018466	0.144674004
ENSMUSG00000108126	Gm43909	predicted gene, 43909 [Source:MGI Symbol;Acc:MGI:5690301]	0.718499	0.917519	1.01722	1.57669	0.700664	0.740903	0.830751	0.861334	1.5373	0.889834	0.81204	0.973435	0.659021	0.828946	0.029756552	0.323426482	12	16	24	0.038867638	0.611732283	24	9.823346853	0.179375008
ENSMUSG00000108137	Gm44053	predicted gene, 44053 [Source:MGI Symbol;Acc:MGI:5690445]	2.75065	2.2679	2.42665	2.67736	2.91187	2.64997	3.41868	2.55495	2.58686	2.43459	2.56836	2.1519	2.69727	2.2111	0.187699199	0.662239322	4	4	24	0.498334282	0.808125725	24	20.12430838	0.058352781
ENSMUSG00000108154	Gm44033	predicted gene, 44033 [Source:MGI Symbol;Acc:MGI:5690425]	0.524511	0.380273	1.14	1.11215	0.446522	0.507904	0.730201	0.535075	0.970342	0.148565	0.718713	0.863178	0.450413	0.620136	0.948454836	0.966018836	12	20	24	0.753006826	0.919913355	24	9.726765218	0.078341436
ENSMUSG00000108158	Gm44002	predicted gene, 44002 [Source:MGI Symbol;Acc:MGI:5690394]	14.5224	14.6136	16.5012	12.4753	11.8918	18.6	16.2514	13.3689	15.5523	18.8787	11.1313	15.5494	12.8843	11.072	0.916963526	0.966018836	12	20	24	0.991334598	0.998516607	24	21.1679987	0.01272981
ENSMUSG00000108181	C030015A19Rik	RIKEN cDNA C030015A19 gene [Source:MGI Symbol;Acc:MGI:1924668]	1.45888	1.21528	1.28108	1.64475	1.49807	0.956364	1.49973	1.23683	1.7934	1.36854	1.45429	1.41	1.33168	1.43531	0.883024237	0.966018836	16	16	24	0.385803396	0.799546792	24	10.50771198	0.067042482
ENSMUSG00000108207	1810059H22Rik	RIKEN cDNA 1810059H22 gene [Source:MGI Symbol;Acc:MGI:1917070]	0.653963	1.25279	1.23431	1.03249	0.881384	0.683351	1.01239	0.97078	1.30131	1.15293	0.875875	1.06313	0.755938	1.48602	0.015345591	0.253245931	8	20	24	0.005110126	0.442351542	24	7.89079156	0.189287982
ENSMUSG00000108218	Olfr1372-ps1	olfactory receptor 1372, pseudogene 1 [Source:MGI Symbol;Acc:MGI:3031206]	1.5968	1.82926	1.12753	1.52591	1.3038	1.22818	0.937112	0.724804	1.40642	0.874771	1.2072	0.920741	1.20258	1.156	0.715742619	0.966018836	4	20	24	0.997687748	0.999623846	24	7.747469336	0.004808118
ENSMUSG00000108231	Gm4045	predicted gene 4045 [Source:MGI Symbol;Acc:MGI:3782220]	2.1905	2.20165	3.55548	3.03686	3.35918	3.29687	4.62753	3.35634	4.42687	2.53851	3.8495	2.87177	2.79387	2.80824	0.671726933	0.966018836	12	20	24	0.790487941	0.932883394	24	12.4933219	0.067394682
ENSMUSG00000108297	Gm44167	predicted gene, 44167 [Source:MGI Symbol;Acc:MGI:5690559]	1.01647	1.08158	0.703896	1.01155	0.886128	0.830247	1.15744	0.705469	0.980162	0.717114	0.844952	1.13009	1.50398	1.13034	0.076942821	0.453790923	4	8	24	0.109351182	0.72082227	24	23.80305253	0.116766822
ENSMUSG00000108312	Gm44521	predicted gene 44521 [Source:MGI Symbol;Acc:MGI:5753097]	3.33319	5.95557	10.7716	7.91682	5.57263	7.06559	4.80393	4.58541	5.79153	5.30031	5.23692	10.2865	13.4247	5.50125	0.916963526	0.966018836	12	20	24	0.954026271	0.988601783	24	14.55377166	0.056330143
ENSMUSG00000108313	Gm45052	predicted gene 45052 [Source:MGI Symbol;Acc:MGI:5753628]	0.997294	1.59503	0.540667	2.05619	2.1931	0.831369	1.20874	1.4687	1.57587	0.879763	1.80042	1.07964	1.00832	0.512709	0.322551072	0.768040178	12	20	24	0.326618579	0.768040178	24	14.92554927	0.189536834
ENSMUSG00000108314	Prkcz	protein kinase C, zeta 2 [Source:MGI Symbol;Acc:MGI:3029152]	16.3934	14.4302	16.6203	14.6428	14.0276	17.4346	15.3192	15.6814	11.0807	17.5145	12.9064	16.0448	14.6112	15.4436	0.88067614	0.966018836	12	20	24	0.594880282	0.857607093	24	21.83290494	0.068175884
ENSMUSG00000108322	5430431A17Rik	RIKEN cDNA 5430431A17 gene [Source:MGI Symbol;Acc:MGI:1918618]	0.496381	1.35553	0.794722	1.40992	1.39834	1.3887	1.25678	1.71317	2.18543	2.12203	1.09042	1.13922	1.71259	1.20478	0.826609618	0.966018836	16	12	24	0.281366257	0.768040178	24	9.938524936	0.164025876
ENSMUSG00000108325	Gm44898	predicted gene 44898 [Source:MGI Symbol;Acc:MGI:5753474]	1.61481	1.80042	1.6528	2.01716	1.69287	1.55838	1.50381	1.38194	1.71072	1.5743	1.81849	1.61121	1.39144	1.69408	0.185849533	0.662239322	16	12	24	0.08246799	0.699403344	24	12.41116952	0.070912745
ENSMUSG00000108348	Gm42372	predicted gene, 42372 [Source:MGI Symbol;Acc:MGI:5625257]	8.38718	7.78327	9.39386	7.92001	7.30518	8.58159	9.21296	7.48134	7.18285	8.68026	6.65909	8.21585	7.27472	9.07289	0.868022237	0.966018836	4	16	24	0.631951507	0.874893236	24	3.125958739	0.050368994
ENSMUSG00000108350	Gm44950	predicted gene 44950 [Source:MGI Symbol;Acc:MGI:5753526]	3.93568	4.01607	4.12166	4.77417	3.74515	4.19738	3.74419	3.95388	3.93968	4.79382	4.32018	3.92487	3.85829	3.77991	0.008961721	0.192760771	16	12	24	0.02626205	0.586541342	24	12.5958524	0.087045251
ENSMUSG00000108358	Gm44509	predicted gene 44509 [Source:MGI Symbol;Acc:MGI:5753085]	27.9511	28.727	27.4708	27.7429	26.9803	27.6599	26.33	25.6844	25.0221	27.7851	28.2766	28.8301	26.8389	28.1813	0.92655264	0.966018836	24	12	24	0.481161792	0.805393068	24	18.38854375	0.028299025
ENSMUSG00000108368	Gm45053	predicted gene 45053 [Source:MGI Symbol;Acc:MGI:5753629]	0.618296	0.482704	0.492387	0.598683	0.457239	0.407625	0.487391	0.469178	0.3761	0.660006	0.460494	0.646626	0.634923	0.698443	0.915082018	0.966018836	4	16	24	0.90576606	0.974171561	24	1.428021089	0.01632292
ENSMUSG00000108389	Gm45206	predicted gene 45206 [Source:MGI Symbol;Acc:MGI:5753782]	0.709116	0.465433	0.548346	0.408874	0.449497	0.575333	0.593055	0.368929	0.459884	0.455992	0.60564	0.521303	0.64433	0.442141	0.038123942	0.334069441	4	12	24	0.057918777	0.656248849	24	21.36515456	0.082094173
ENSMUSG00000108393	Gm32633	predicted gene, 32633 [Source:MGI Symbol;Acc:MGI:5591792]	2.48331	2.98885	2.24092	2.61496	1.79565	2.19146	2.20161	2.48287	2.82561	3.29127	2.41747	2.69284	2.67432	2.23019	0.893307667	0.966018836	16	4	24	0.457495814	0.805393068	24	7.849667371	0.07966715
ENSMUSG00000108402	9430064I24Rik	RIKEN cDNA 9430064I24 gene [Source:MGI Symbol;Acc:MGI:3704302]	0.54013	0.545906	0.652992	0.666364	0.53392	0.627304	0.862524	0.576465	0.820522	0.518287	0.569732	0.588685	0.434834	0.660706	0.951523604	0.966018836	12	8	24	0.675324215	0.89119716	24	7.057652265	0.037128106
ENSMUSG00000108414	Snhg1	small nucleolar RNA host gene 1 [Source:MGI Symbol;Acc:MGI:3763743]	14.048	21.1075	19.3348	19.7504	19.1126	16.9625	14.5434	15.992	22.9855	15.5613	20.0554	17.6816	18.9163	20.7483	0.101520755	0.511659635	12	16	24	0.180957144	0.768040178	24	9.610960544	0.142613736
ENSMUSG00000108421	Gm44718	predicted gene 44718 [Source:MGI Symbol;Acc:MGI:5753294]	1.23349	1.13994	0.890889	0.689736	1.49774	0.681353	0.497494	0.904929	0.555655	1.36068	0.969891	1.07055	1.25159	1.11993	0.924553956	0.966018836	20	16	24	0.87150965	0.96354675	24	17.81494298	0.04922898
ENSMUSG00000108425	Gm44706	predicted gene 44706 [Source:MGI Symbol;Acc:MGI:5753282]	0.732471	0.465006	0.729925	0.489035	0.729362	0.622254	0.744172	0.491177	0.791307	0.605006	0.377105	0.467846	0.569929	0.59465	0.767183862	0.966018836	12	8	24	0.824312488	0.944827078	24	4.542726723	0.028627492
ENSMUSG00000108438	Gm9278	predicted gene 9278 [Source:MGI Symbol;Acc:MGI:3646238]	0.512701	0.727481	1.55893	0.815567	1.6719	1.0372	0.997606	0.821092	0.278966	0.406756	0.839838	0.856444	0.480777	0.777559	0.40165019	0.933249185	20	20	24	0.523742522	0.821564372	24	17.30781826	0.13002011
ENSMUSG00000108456	4732496C06Rik	RIKEN cDNA 4732496C06 gene [Source:MGI Symbol;Acc:MGI:2442534]	0.914799	0.61229	0.645323	0.50809	0.625045	0.657923	0.60057	0.89838	0.719108	0.525826	0.807362	0.548404	0.742283	0.639393	0.628286596	0.966018836	4	12	24	0.32752152	0.768040178	24	1.143031566	0.061817537
ENSMUSG00000108461	AV356131	expressed sequence AV356131 [Source:MGI Symbol;Acc:MGI:2142184]	0.949939	1.30904	1.18001	1.08593	1.41707	0.997853	1.24034	1.09112	1.98971	0.94164	2.14054	1.21467	2.12842	1.57938	0.890235289	0.966018836	20	20	24	0.93060517	0.982155977	24	7.843613279	0.032881026
ENSMUSG00000108465	Gm45110	predicted gene 45110 [Source:MGI Symbol;Acc:MGI:5753686]	0.800267	1.04207	0.835819	0.552434	1.10564	0.528663	0.664627	0.660564	1.26965	0.486319	1.12522	0.794119	1.04296	0.904059	0.953350544	0.966018836	12	12	24	0.888644492	0.968707249	24	5.543316699	0.038777021
ENSMUSG00000108484	Gm18537	predicted gene, 18537 [Source:MGI Symbol;Acc:MGI:5010722]	4.18798	5.24384	4.81669	5.18944	5.22613	5.02687	5.3765	5.0866	5.68854	4.38681	5.19765	5.06591	4.10803	4.60624	0.945282252	0.966018836	20	8	24	0.629575224	0.873481213	24	12.19543993	0.043737325
ENSMUSG00000108503	Gm20949	predicted gene, 20949 [Source:MGI Symbol;Acc:MGI:5434304]	0.906789	0.71679	0.493031	0.635453	0.640849	0.628957	0.737498	0.90199	0.688026	1.19824	0.796557	0.674698	0.637689	1.09149	0.836635326	0.966018836	8	16	24	0.741573384	0.915904517	24	6.177454143	0.045934156
ENSMUSG00000108518	Gm5587	predicted gene 5587 [Source:MGI Symbol;Acc:MGI:3645641]	2.49722	2.93026	2.79836	3.2058	3.95058	3.65656	2.85129	3.06384	2.92468	2.26946	2.51181	2.898	3.28478	3.70231	0.890176313	0.966018836	8	8	24	0.837239561	0.950299676	24	21.91486297	0.042392505
ENSMUSG00000108540	Gm4768	predicted gene 4768 [Source:MGI Symbol;Acc:MGI:3643845]	2.82056	5.10641	5.21888	3.04555	3.06562	3.83142	5.19975	2.65337	5.40303	3.22447	3.0809	3.56211	2.95347	3.72946	0.791413662	0.966018836	12	8	24	0.392469631	0.799546792	24	5.991019678	0.157780776
ENSMUSG00000108551	Gm20274	predicted gene, 20274 [Source:MGI Symbol;Acc:MGI:5012459]	1.04398	1.01041	1.54627	0.971816	1.05655	0.534087	0.577353	0.507114	1.09799	0.627405	0.778132	0.487292	0.952802	1.22083	0.022634137	0.27189101	12	12	24	0.175003499	0.768040178	24	7.845752159	0.168206845
ENSMUSG00000108591	Particl	promoter of Mat2a antisense radiation induced circulating long non-coding RNA [Source:MGI Symbol;Acc:MGI:1925358]	3.19777	2.6618	3.41042	2.97926	2.94088	2.36133	2.45035	2.93635	3.45202	3.80991	3.14573	3.72069	3.30646	3.72438	0.635666629	0.966018836	12	12	24	0.203330996	0.768040178	24	9.532375983	0.099067835
ENSMUSG00000108594	Gm44770	predicted gene 44770 [Source:MGI Symbol;Acc:MGI:5753346]	1.15328	1.09968	0.862519	1.11869	1.16276	1.16899	0.836116	0.969595	1.25656	0.952282	1.66336	0.894587	1.51036	1.12629	0.855823117	0.966018836	20	12	24	0.708602993	0.903807891	24	17.11867612	0.050113522
ENSMUSG00000108607	Gm44646	predicted gene 44646 [Source:MGI Symbol;Acc:MGI:5753222]	1.71611	2.49244	1.73882	3.33371	2.92472	2.37361	1.59444	1.82975	3.25919	2.95033	1.93344	1.5862	1.84138	1.56674	0.057224139	0.401309455	16	12	24	0.020042932	0.565108098	24	12.18116317	0.262156343
ENSMUSG00000108608	Gm6916	predicted pseudogene 6916 [Source:MGI Symbol;Acc:MGI:3644024]	3.63657	2.59883	3.92495	3.84401	5.0407	2.77338	0.145915	8.64593	1.79394	2.45096	4.20202	2.46632	3.14703	2.27475	0.946423879	0.966018836	20	8	24	0.697942187	0.899599363	24	12.20315305	0.229453671
ENSMUSG00000108614	2610306O10Rik	RIKEN cDNA 2610306O10 gene [Source:MGI Symbol;Acc:MGI:1917710]	1.68962	0.992144	0.966041	2.32947	1.84998	0.951889	1.08234	1.3472	1.332	0.764177	1.70264	0.969433	1.18962	1.54502	0.880247609	0.966018836	20	4	24	0.707391463	0.903469848	24	14.08990888	0.084945221
ENSMUSG00000108617	Gm31749	predicted gene, 31749 [Source:MGI Symbol;Acc:MGI:5590908]	1.01002	0.791474	0.398957	1.25428	1.15913	0.930621	1.22909	1.39055	0.375007	0.728368	0.627616	0.883569	1.16725	0.763589	0.182849367	0.659303873	4	8	24	0.193146821	0.768040178	24	21.58127472	0.176058643
ENSMUSG00000108621	Gm33989	predicted gene, 33989 [Source:MGI Symbol;Acc:MGI:5593148]	2.98326	4.07969	4.26625	5.55461	4.39584	4.33651	4.67651	3.80423	4.17816	2.77894	4.40226	3.28194	4.47582	4.86043	0.795269577	0.966018836	20	4	24	0.909278506	0.974606327	24	8.947546543	0.039981903
ENSMUSG00000108638	Gm45150	predicted gene 45150 [Source:MGI Symbol;Acc:MGI:5753726]	0.659764	0.667629	1.14549	0.596761	0.389133	0.737582	0.320409	0.468051	0.537341	0.785416	0.986824	0.936297	0.620522	0.90781	0.853079521	0.966018836	24	4	24	0.732256049	0.912582601	24	11.53753181	0.063061944
ENSMUSG00000108655	Gm44949	predicted gene 44949 [Source:MGI Symbol;Acc:MGI:5753525]	1.04762	0.971777	0.804557	0.780029	1.18019	0.649399	0.790334	0.718876	1.0958	0.651278	0.807148	0.999417	1.18815	0.778351	0.93447422	0.966018836	4	12	24	0.740392391	0.915274254	24	22.60189613	0.044830471
ENSMUSG00000108659	Gm34121	predicted gene, 34121 [Source:MGI Symbol;Acc:MGI:5593280]	0.4386	0.496581	0.587947	0.380031	0.631058	0.465559	0.700178	0.483073	0.449241	0.454911	0.701507	0.636259	0.528337	0.788746	0.918497267	0.966018836	20	20	24	0.718629494	0.907485572	24	22.21235008	0.032848177
ENSMUSG00000108676	1700123J17Rik	RIKEN cDNA 1700123J17 gene [Source:MGI Symbol;Acc:MGI:1923978]	0.555029	0.699946	0.630223	1.02865	0.43551	0.646879	0.648714	0.888533	0.743067	1.00411	0.682686	0.689986	0.689404	0.518961	0.351315142	0.870418653	16	4	24	0.24931671	0.768040178	24	10.19630441	0.091991671
ENSMUSG00000108681	Gm17825	predicted gene, 17825 [Source:MGI Symbol;Acc:MGI:5010010]	0.820122	0.800909	0.617207	0.721783	1.03611	0.678584	0.692125	0.507522	0.81771	0.500247	0.562811	0.523986	0.589164	0.567652	0.809772024	0.966018836	4	12	24	0.793324305	0.933814107	24	17.73876286	0.028584528
ENSMUSG00000108701	Gm44616	predicted gene 44616 [Source:MGI Symbol;Acc:MGI:5753192]	1.04062	1.30421	1.02404	1.46625	1.14335	1.21512	1.48538	1.27983	1.44245	1.25943	1.19079	1.27485	1.13628	1.05481	0.795556128	0.966018836	16	4	24	0.84225958	0.952050674	24	11.51102645	0.023424621
ENSMUSG00000108702	Gm9333	predicted gene 9333 [Source:MGI Symbol;Acc:MGI:3643231]	9.61009	9.27084	9.5617	9.20316	9.3434	8.42969	7.68447	8.8347	9.28323	8.06488	8.1062	9.20492	9.05264	9.30872	0.961261938	0.967043964	12	4	24	0.556482643	0.838702296	24	5.837773239	0.038488817
ENSMUSG00000108736	Gm45151	predicted gene 45151 [Source:MGI Symbol;Acc:MGI:5753727]	0.653309	0.442129	0.473339	0.33165	0.432763	0.205108	0.265311	0.9722	0.841237	0.383787	0.596993	0.557293	0.436239	0.654943	0.192997592	0.670595308	12	4	24	0.205288897	0.768040178	24	5.992073551	0.134537395
ENSMUSG00000108738	Gm44777	predicted gene 44777 [Source:MGI Symbol;Acc:MGI:5753353]	1.91942	0.712717	1.23818	1.11552	1.36929	1.04406	0.766202	0.506762	0.88126	0.748854	1.00823	0.764509	0.613776	0.578706	0.064091035	0.414867207	20	12	24	0.102741172	0.713699364	24	16.94860146	0.154690785
ENSMUSG00000108753	Gm45094	predicted gene 45094 [Source:MGI Symbol;Acc:MGI:5753670]	0.615651	1.20415	0.9554	0.601091	0.784172	0.89192	1.07036	1.01886	1.2574	1.27882	1.02238	1.3532	1.41152	1.32818	0.886952527	0.966018836	8	8	24	0.182657754	0.768040178	24	4.514620268	0.091527895
ENSMUSG00000108772	Gm6063	predicted gene 6063 [Source:MGI Symbol;Acc:MGI:3645395]	1.7428	0.945148	2.02663	1.69105	1.74607	1.67223	1.9656	2.32012	1.18346	0.740907	1.10132	0.481325	0.417837	0.854724	0.860993221	0.966018836	12	12	24	0.945206346	0.986693398	24	8.156637509	0.042552321
ENSMUSG00000108799	Glud-ps	glutamate dehydrogenase, pseudogene [Source:MGI Symbol;Acc:MGI:95754]	23.3366	25.3592	27.9887	28.643	25.416	29.4332	24.3855	26.2184	21.9498	27.1661	21.1165	23.2229	21.3163	23.1251	0.914360142	0.966018836	16	12	24	0.510723528	0.814381398	24	12.25342118	0.058470163

ENSMUSG00000108801	Gm39090	predicted gene, 39090 [Source:MGI Symbol;Acc:MGI:5621975]	1.86672	2.32577	1.86972	2.55008	2.65934	2.4578	2.26663	1.53946	2.21939	1.88859	3.16729	2.16425	2.06651	1.98428	0.057224139	0.401309455	20	12	24	0.032873611	0.606585237	24	16.35758885	0.166048159
ENSMUSG00000108802	Gm44769	predicted gene 44769 [Source:MGI Symbol;Acc:MGI:5753345]	0.478684	0.898655	0.553576	0.535118	0.676074	0.444427	0.731552	0.457253	0.973105	0.592722	0.651099	0.569543	0.804245	0.796625	0.935316621	0.966018836	12	12	24	0.349593338	0.779170188	24	6.072641867	0.079523312
ENSMUSG00000108823	Gm18959	predicted gene, 18959 [Source:MGI Symbol;Acc:MGI:5011144]	0.972969	0.493625	0.616686	0.647305	0.723936	0.411717	0.888572	0.940179	0.497127	0.214363	0.444025	0.830977	1.02958	0.488888	0.105345531	0.518002936	4	12	24	0.063230473	0.663450344	24	23.69054358	0.185642591
ENSMUSG00000108825	Gm45838	predicted gene 45838 [Source:MGI Symbol;Acc:MGI:5804953]	1.21342	2.12662	1.6225	1.75955	2.40151	1.49483	2.22499	1.71381	3.08564	1.65996	2.02332	1.85962	1.72631	1.66791	0.890176313	0.966018836	20	4	24	0.539538601	0.829375145	24	10.51250244	0.096174138
ENSMUSG00000108852	Gm44911	predicted gene 44911 [Source:MGI Symbol;Acc:MGI:5753487]	5.69931	4.62638	4.90117	5.27467	5.26265	4.97021	4.20569	5.26434	4.67858	4.84513	5.53268	5.48959	4.58858	4.54707	0.215777641	0.704989686	24	4	24	0.155360897	0.760503971	24	16.64559502	0.076039883
ENSMUSG00000108880	Gm44560	predicted gene 44560 [Source:MGI Symbol;Acc:MGI:5753136]	0.208177	0.733308	1.28052	0.509429	0.45643	0.513886	0.391079	0.367286	0.820933	0.513184	0.716875	0.259767	0.169408	0.660376	0.002004172	0.100255953	12	16	24	0.025751391	0.586541342	24	9.450852883	0.246811533
ENSMUSG00000108883	Gm44623	predicted gene 44623 [Source:MGI Symbol;Acc:MGI:5753199]	2.37178	2.9229	3.69835	3.67767	4.14156	2.47902	2.51748	3.93666	3.71847	2.74555	3.01747	2.77913	3.79026	3.75532	0.418381424	0.963884303	8	20	24	0.128782125	0.740162193	24	8.82281803	0.16396642
ENSMUSG00000108911	Gm45113	predicted gene 45113 [Source:MGI Symbol;Acc:MGI:5753689]	9.16728	11.6502	15.7319	11.6158	14.7444	4.70628	13.2434	4.65638	7.31373	8.49218	17.586	6.29194	3.90803	14.2143	0.503972924	0.966018836	20	4	24	0.403805039	0.799546792	24	11.72389427	0.326302181
ENSMUSG00000108923	4933431C10Rik	RIKEN cDNA 4933431C10 gene [Source:MGI Symbol;Acc:MGI:1918572]	0.503643	0.750082	0.665282	0.537652	0.863741	0.327545	0.834597	0.70975	0.959151	0.617858	0.545189	0.641092	0.646115	0.481808	0.917610614	0.966018836	12	12	24	0.524413901	0.822024124	24	7.595169928	0.069592246
ENSMUSG00000108943	Gm44559	predicted gene 44559 [Source:MGI Symbol;Acc:MGI:5753135]	0.701971	0.499482	1.06881	0.268492	0.560667	0.611325	0.742356	0.293394	1.08683	0.742011	0.836884	0.41762	0.405148	0.873193	0.774195442	0.966018836	12	12	24	0.569918149	0.844984223	24	8.703134409	0.100897224
ENSMUSG00000109015	Gm31024	predicted gene, 31024 [Source:MGI Symbol;Acc:MGI:5590183]	0.560489	0.791488	0.767421	0.604715	0.530836	0.825914	0.640924	0.682451	0.915944	0.788075	0.74916	0.734604	0.696271	0.722777	0.43565473	0.966018836	12	16	24	0.387767786	0.799546792	24	7.979113513	0.047873167
ENSMUSG00000109061	Gm49320	predicted gene, 49320 [Source:MGI Symbol;Acc:MGI:6121499]	0.515518	0.70655	1.79113	0.792315	0.768599	0.754342	1.03586	1.31837	1.87175	1.82314	0.781308	1.27185	1.17619	0.74433	0.214902712	0.704989686	12	16	24	0.106027332	0.71810271	24	9.039119291	0.237001737
ENSMUSG00000109080	Gm38944	predicted gene, 38944 [Source:MGI Symbol;Acc:MGI:5621829]	0.917916	1.16214	0.779454	1.56119	1.1879	1.02934	1.02698	0.611662	0.935624	1.13804	1.25095	0.738423	1.07729	1.22222	0.360033827	0.883787544	20	4	24	0.32546401	0.768040178	24	13.94375453	0.098992098
ENSMUSG00000109097	Gm29683	predicted gene, 29683 [Source:MGI Symbol;Acc:MGI:5588842]	1.9669	1.94978	1.68651	1.46118	2.06341	1.52969	1.70694	1.64418	1.93497	1.91283	1.92334	1.39994	2.11186	1.65856	0.923635729	0.966018836	4	20	24	0.96102396	0.989941253	24	5.589021829	0.014029828
ENSMUSG00000109108	Gm36371	predicted gene, 36371 [Source:MGI Symbol;Acc:MGI:5595530]	0.887123	0.987891	1.05677	1.2613	1.17138	0.936411	1.13436	0.832814	0.865968	1.31165	0.814089	1.39933	1.09664	1.41696	0.882617464	0.966018836	16	20	24	0.852568591	0.95583907	24	13.55065379	0.03023802
ENSMUSG00000109118	Gm32031	predicted gene, 32031 [Source:MGI Symbol;Acc:MGI:5591190]	4.55072	4.06893	7.42494	3.51355	4.41193	4.14774	3.31376	5.6808	5.542	3.53496	5.29497	5.56322	6.25423	3.74705	0.896799496	0.966018836	12	4	24	0.875728881	0.965294237	24	5.54404927	0.061932664
ENSMUSG00000109122	A230103L15Rik	RIKEN cDNA A230103L15 gene [Source:MGI Symbol;Acc:MGI:1925005]	0.405316	0.510562	0.847582	0.578936	0.337717	0.47573	0.534804	0.383772	0.825604	0.683218	0.589888	0.500559	0.367981	0.606733	0.031969981	0.324385159	12	16	24	0.021302628	0.572080066	24	9.405437003	0.143468423
ENSMUSG00000109136	Gm45114	predicted gene 45114 [Source:MGI Symbol;Acc:MGI:5753690]	1.00341	1.34391	1.16438	0.846324	1.20515	1.18063	1.14369	1.20014	0.937675	0.729079	1.26766	1.31935	1.29009	1.01961	0.142513744	0.585944911	20	20	24	0.105756814	0.717832234	24	22.44770564	0.104826661
ENSMUSG00000109143	Gm10046	predicted gene 10046 [Source:MGI Symbol;Acc:MGI:3797981]	30.0494	31.2599	7.62714	6.7983	22.2137	11.6795	67.1373	18.8566	21.993	20.5374	19.6269	8.18097	8.25	29.4127	0.443191651	0.966018836	4	20	24	0.444662865	0.805393068	24	1.864223504	0.444830873
ENSMUSG00000109147	4930431P19Rik	RIKEN cDNA 4930431P19 gene [Source:MGI Symbol;Acc:MGI:1921136]	0.826593	1.11216	1.00215	1.06255	0.80339	0.804054	0.726315	1.05846	0.994888	0.938938	1.04363	1.03999	1.05008	1.24713	0.092972576	0.489012695	8	16	24	0.072268863	0.679008169	24	7.253377324	0.083530782
ENSMUSG00000109165	Gm45148	predicted gene 45148 [Source:MGI Symbol;Acc:MGI:5753724]	0.572038	0.463519	0.372605	0.389191	1.09993	0.641516	1.52941	0.711568	0.46647	0.680845	1.01351	0.515377	0.92069	0.609289	0.162391276	0.624504499	20	16	24	0.212032558	0.768040178	24	20.34652435	0.171245143
ENSMUSG00000109168	Gm44709	predicted gene 44709 [Source:MGI Symbol;Acc:MGI:5753285]	5.83359	4.34623	3.77685	9.19841	5.19101	5.37341	7.54224	5.97683	10.8452	1.52264	8.28287	5.52517	8.57916	3.16797	0.862909389	0.966018836	4	4	24	0.781966462	0.930007122	24	21.14730882	0.172500527
ENSMUSG00000109178	Gm3257	predicted gene 3257 [Source:MGI Symbol;Acc:MGI:3781435]	0.467305	0.750886	0.930951	0.79989	0.811775	0.307786	0.474282	0.83396	1.00837	0.720662	0.853418	1.09705	0.468314	1.23161	0.286642595	0.793714531	12	16	24	0.063972865	0.663876076	24	9.222693867	0.182818733
ENSMUSG00000109205	Gm44954	predicted gene 44954 [Source:MGI Symbol;Acc:MGI:5753530]	1.75224	0.871362	3.14784	1.89877	2.04533	3.10973	2.10526	2.51724	2.7349	2.32194	1.15082	1.56209	0.404313	3.35249	0.455507782	0.966018836	12	16	24	0.483426619	0.805393068	24	9.332720061	0.229879179
ENSMUSG00000109222	Gm10297	predicted pseudogene 10297 [Source:MGI Symbol;Acc:MGI:3642512]	29.1854	27.1657	27.6248	25.7305	26.4566	28.2249	29.2657	24.7908	26.7872	26.2609	30.4967	29.5372	28.8504	26.4387	0.054878003	0.398010585	24	16	24	0.042510294	0.623698598	24	20.45704818	0.077801362
ENSMUSG00000109224	Tmem147os	transmembrane protein 147, opposite strand [Source:MGI Symbol;Acc:MGI:3642392]	5.58175	6.40732	3.57462	4.7144	3.50178	6.02658	5.31868	6.37048	6.04852	5.06535	7.33	6.25904	8.22388	5.4895	0.513782337	0.966018836	8	8	24	0.181499808	0.768040178	24	23.93112251	0.175940603
ENSMUSG00000109231	Gm45737	predicted gene 45737 [Source:MGI Symbol;Acc:MGI:5804852]	0.465058	0.403631	0.56555	0.629967	0.555085	0.358638	0.735256	0.294696	0.786007	0.415582	0.698094	0.608646	0.745427	0.48733	0.91724565	0.966018836	12	20	24	0.829549933	0.947008621	24	14.76569671	0.034022744
ENSMUSG00000109237	9130214F15Rik	RIKEN cDNA 9130214F15 gene [Source:MGI Symbol;Acc:MGI:1924373]	0.186209	0.216554	0.49206	0.765825	0.379887	0.166812	0.584994	0.573174	0.254785	0.650085	0.75229	0.625598	0.972285	0.750132	0.921572192	0.966018836	16	20	24	0.733208863	0.91292127	24	11.19101123	0.058478684
ENSMUSG00000109241	Gm7434	predicted gene 7434 [Source:MGI Symbol;Acc:MGI:3644530]	6.89465	7.6285	8.18798	6.46343	6.13893	6.93587	7.36234	7.39919	5.89268	3.80124	6.89197	4.89133	5.08884	5.1876	0.522551072	0.966018836	12	4	24	0.482211236	0.805393068	24	1.765185074	0.091727913
ENSMUSG00000109243	Gm45867	predicted gene 45867 [Source:MGI Symbol;Acc:MGI:5804982]	1.4386	1.45282	1.58424	1.76861	1.4714	1.36943	1.5342	1.42576	1.63746	0.849842	1.88314	1.41734	1.49257	1.36334	0.671726933	0.966018836	20	4	24	0.975857591	0.993924858	24	18.77592757	0.013453994
ENSMUSG00000109255	Gm7407	predicted gene 7407 [Source:MGI Symbol;Acc:MGI:3647246]	7.10637	10.6057	9.75451	8.30429	10.8336	9.93513	9.48452	8.87331	4.63327	5.54629	5.32463	5.19707	4.75805	4.33626	0.816300547	0.966018836	20	16	24	0.707506105	0.903508876	24	19.93492188	0.092300782
ENSMUSG00000109274	Gm45133	predicted gene 45133 [Source:MGI Symbol;Acc:MGI:5753709]	2.2347	1.92055	2.16226	1.69573	1.62052	1.92962	2.2412	1.58087	1.43111	2.18786	1.6883	2.0386	2.02695	1.82135	0.277917941	0.789849983	4	16	24	0.33479489	0.770864198	24	22.67571202	0.077340367
ENSMUSG00000109284	B230311B06Rik	RIKEN cDNA B230311B06 gene [Source:MGI Symbol;Acc:MGI:2686541]	2.36229	3.07414	2.64578	2.84007	2.87181	2.05684	2.31369	2.11967	2.80568	1.64214	2.79988	1.94343	3.19146	2.56637	0.928064146	0.966018836	20	4	24	0.804511579	0.93744342	24	6.002326238	0.052619956
ENSMUSG00000109305	Smim38	small integral membrane protein 38 [Source:MGI Symbol;Acc:MGI:1916283]	1.00117	0.885297	1.12109	0.787234	1.06581	0.358431	0.334048	0.620765	1.20278	1.48593	1.50376	1.54312	1.50468	1.50422	0.847846021	0.966018836	20	12	24	0.419226207	0.803638254	24	11.09675884	0.143584322
ENSMUSG00000109321	A330076H08Rik	RIKEN cDNA A330076H08 gene [Source:MGI Symbol;Acc:MGI:2443193]	2.80419	3.19101	2.97541	2.64725	3.21151	2.71299	2.9716	2.49048	2.68752	2.40128	3.3689	2.46215	2.59084	2.82437	0.915475528	0.966018836	20	20	24	0.865784247	0.961251055	24	17.54380481	0.023462548
ENSMUSG00000109324	Prmt1	protein arginine N-methyltransferase 1 [Source:MGI Symbol;Acc:MGI:107846]	63.8334	69.5662	67.9344	63.0915	71.7017	68.1942	61.4235	62.7507	63.885	61.7004	68.3002	64.1737	65.2589	63.3318	0.664568268	0.966018836	20	20	24	0.447592541	0.805393068	24	16.95414854	0.032805238
ENSMUSG00000109326	Gm9165	predicted gene 9165 [Source:MGI Symbol;Acc:MGI:3647914]	2.40059	1.81123	2.06246	1.69914	1.99633	1.9468	1.69857	1.41885	1.64305	1.62718	1.87517	1.71189	2.02797	1.52901	0.481530854	0.966018836	4	4	24	0.15217991	0.759088284	24	19.9421609	0.084841124
ENSMUSG00000109336	Samd4b	sterile alpha motif domain containing 4B [Source:MGI Symbol;Acc:MGI:2448542]	8.82114	7.64301	8.15601	8.81683	8.8031	8.58992	9.10291	9.10541	9.87976	10.7422	8.3197	9.00302	8.10725	9.25201	0.911135828	0.966018836	16	4	24	0.380954249	0.799546792	24	10.97029047	0.057248155
ENSMUSG00000109337	Gm45047	predicted gene 45047 [Source:MGI Symbol;Acc:MGI:5753623]	0.551409	1.52381	2.0882	1.22273	0.596651	0.603396	0.33275	1.44798	0.557076	0.18262	0.55358	0.542217	0.750302	0.870229	0.149537283	0.594118663	8	16	24	0.122344589	0.735058232	24	6.297311706	0.279815309
ENSMUSG00000109355	Gm4880	predicted pseudogene 4880 [Source:MGI Symbol;Acc:MGI:3648786]	0.723878	0.749799	0.675141	0.863477	0.7385	0.825448	0.726494	0.673159	0.672141	0.665939	0.588527	0.525779	0.755884	0.824776	0.915082018	0.966018836	4	16	24	0.7738905	0.927334758	24	4.275358883	0.021671284
ENSMUSG00000109362	Gm44562	predicted gene 44562 [Source:MGI Symbol;Acc:MGI:5753138]	0.469061	0.834977	1.42154	0.809223	0.704191	0.801192	0.981978	0.540982	1.51198	0.995886	0.784965	0.703431	0.534748	0.946635	0.016795343	0.253395174	12	16	24	0.046806269	0.633507364	24	9.333534852	0.206440367
ENSMUSG00000109367	Gm18762	predicted gene, 18762 [Source:MGI Symbol;Acc:MGI:5010967]	0.577596	0.799774	0.768032	0.959817	0.746908	0.950239	0.901387	0.978856	0.833862	0.69151	0.575031	0.898118	0.746784	0.97783	0.305564675	0.811201458	8	12	24	0.587220726	0.853591039	24	4.504187412	0.045764327
ENSMUSG00000109378	Gm49396	predicted gene, 49396 [Source:MGI Symbol;Acc:MGI:6121629]	3.92451	3.97947	4.19144	4.44146	4.95551	3.59759	4.71587	3.78858	4.38975	3.6322	5.38483	3.88014	3.76376	3.55003	0.251986462	0.75129457	20	12	24	0.214632877	0.768040178	24	14.9982795	0.097724598
ENSMUSG00000109379	Gm44550	predicted gene 44550 [Source:MGI Symbol;Acc:MGI:5753126]	0.940638	0.989338	0.864381	0.698754	0.739179	0.88272	0.919638	0.882586	0.872706	1.11817	1.03888	0.796549	0.846814	0.72786	0.784548857	0.966018836	4	12	24	0.998928699	0.999770694	24	12.37492083	0.001702656
ENSMUSG00000109394	A230057D06Rik	RIKEN cDNA A230057D06 gene [Source:MGI Symbol;Acc:MGI:2442922]	4.91573	7.20077	6.99482	7.08409	6.32694	5.45608	4.96472	4.9155	8.13477	5.50184	6.57708	5.83365	5.51746	6.643	0.018194955	0.256345714	12	16	24	0.044371773	0.628432994	24	10.03686674	0.182448412
ENSMUSG00000109398	Gm3854	predicted gene 3854 [Source:MGI Symbol;Acc:MGI:3782026]	1.57629	1.67737	1.92812	1.63893	1.76503	1.67852	1.58955	1.41664	1.50592	1.37832	1.73503	1.64981	1.94188	1.6172	0.895493752	0.966018836	20	20	24	0.706359403	0.902918222	24	20.01440261	0.030834715
ENSMUSG00000109399	Gm44982	predicted gene 44982 [Source:MGI Symbol;Acc:MGI:5753558]	0.390683	0.725146	0.847996	0.883697	1.15261	0.728301	0.632462	0.115496	1.14911	0.430627	0.531557	0.756001	0.87467	0.199023	0.227302972	0.723724673	12	20	24	0.328990717	0.768040178	24	15.0492723	0.15926278
ENSMUSG00000109401	Cpeb1os1	cytoplasmic polyadenylation element binding protein 1, opposite strand 1 [Source:MGI Symbol;Acc:MGI:1922988]	0.528356	0.548173	0.597111	0.749681	0.397589	0.498741	0.372553	0.675546	0.556377	0.832101	0.37879	0.709776	0.634303	0.737615	0.121243373	0.548623108	16	4	24	0.224183725	0.768040178	24	7.990943188	0.087810831
ENSMUSG00000109408	A930037H05Rik	RIKEN cDNA A930037H05 gene [Source:MGI Symbol;Acc:MGI:1925689]	0.658501	0.90877	0.574838	0.626858	1.09524	0.706488	0.764939	0.596342	0.809326	0.67245	0.901253	0.860861	0.749476	0.854968	0.602156008	0.966018836	20	20	24	0.472168949	0.805393068	24	18.14604955	0.056461994
ENSMUSG00000109428	Gm45698	predicted gene 45698 [Source:MGI Symbol;Acc:MGI:5804813]	0.490158	0.560708	0.666424	0.844527	0.734869	0.348336	0.598321	0.585743	0.949066	0.917444	0.674838	0.494283	0.733989	0.973895	0.064422003	0.414867207	16	8	24	0.002532203	0.396968951	24	9.398111477	0.170484763
ENSMUSG00000109448	Gm45224	predicted gene 45224 [Source:MGI Symbol;Acc:MGI:5753800]	4.16496	3.80374	4.51654	3.24565	4.23138	3.65365	2.91314	4.24897	4.12383	3.7092	3.47283	4.3559	4.64024	3.33039	0.874054778	0.966018836	12	4	24	0.946600308	0.986959555	24	2.863606629	0.020942759
ENSMUSG00000109460	Gm45591	predicted gene 45591 [Source:MGI Symbol;Acc:MGI:5791427]	2.39893	2.28836	2.48078	2.81397	2.57924	2.32068	2.92507	1.88381	2.83498	2.31961	3.29455	1.90525	3.1989	2.62891	0.92655264	0.966018836	20	12	24	0.837722755	0.950325222	24	13.70046085	0.040340907
ENSMUSG00000109472	Gm18943	predicted gene, 18943 [Source:MGI Symbol;Acc:MGI:5011128]	5.35203	4.77222	5.61684	5.58792	5.96503	5.93914	4.96977	5.54206	5.04959	5.17871	6.25565	4.36692	4.46353	4.62948	0.050258003	0.385784509	20	8	24	0.061992981	0.661961741	24	14.61622837	0.103795892
ENSMUSG00000109489	Gm7451	predicted gene 7451 [Source:MGI Symbol;Acc:MGI:3645148]	1.58134	2.01906	2.1066	1.59641	1.74461	1.91128	1.57657	2.17154	1.39034	1.30034	1.42352	1.39597	1.86695	1.65511	0.33885934	0.858075318	8	8	24	0.199485854	0.768040178	24	2.924008882	0.093318316
ENSMUSG00000109509	Rps12-ps4	ribosomal protein S12, pseudogene 4 [Source:MGI Symbol;Acc:MGI:3780139]	0.687836	0.520947	0.372852	0.772883	0.881524	0.47739	0.208362	0.455579	1.0477	0.455109	0.349006	0.668961	0.927751	0.929541	0.807813307	0.966018836	8	12	24	0.864653833	0.960819377	24	8.07822706	0.051049732
ENSMUSG00000109511	Nup62	nucleoporin 62 [Source:MGI Symbol;Acc:MGI:1351500]	10.1497	8.72941	8.73012	9.16993	9.94486	10.7979	9.31036	11.0662	7.94734	10.0341	9.61268	9.40845	9.87959	8.47602	0.364640768	0.892062098	24	12	24	0.129786419	0.740277254	24	19.79763548	0.094944493
ENSMUSG00000109517	Gm44763	predicted gene 44763 [Source:MGI Symbol;Acc:MGI:5753339]	0.896427	1.08619	0.766452	0.959418	1.05977	0.718556	1.09548	0.843612	0.847239	0.859826	0.795523	0.935377	1.00645	0.822356	0.91724565	0.966018836	4	8	24	0.665034667	0.886947643	24	22.99259844	0.032883171
ENSMUSG00000109524	Gm44691	predicted gene 44691 [Source:MGI Symbol;Acc:MGI:5753267]	1.00098	1.88835	1.35448	2.22638	1.99276	1.6485	1.20924	1.09747	1.68739	0.828454	1.17528	0.818335	1.01948	2.35865	0.510031987	0.966018836	8	20	24	0.544435714	0.832181275	24	8.990756334	0.132748688
ENSMUSG00000109572	Cfap99	cilia and flagella associated protein 99 [Source:MGI Symbol;Acc:MGI:5434801]	0.484969	0.606801	0.417827	0.512517	0.760031	0.54208	0.592736	0.599132	0.497927	0.520473	0.585923	0.603114	0.523369	0.538855	0.21107051	0.699706585	20	16	24	0.286023325	0.768040178	24	18.76709668	0.045939492
ENSMUSG00000109584	Gm7997	predicted gene 7997 [Source:MGI Symbol;Acc:MGI:3646045]	1.07009	1.38429	1.58685	1.7296	1.60201	1.21496	1.24626	1.6115	1.72744	0.867666	1.37134	0.961409	1.52272	1.12598	0.236893212	0.7385769	12	12	24	0.421752817	0.804844498	24	8.75293671	0.092547078
ENSMUSG00000109587	Gm31105	predicted gene, 31105 [Source:MGI Symbol;Acc:MGI:5590264]	1.25525	0.863238	1.17818	2.2021	1.6597	1.20853	1.04535	1.33198	1.61949	1.10275	1.04311	0.765087	0.905041	1.30956	0.459019924	0.966018836	16	8	24	0.132496854	0.742700873	24	11.22052361	0.164803629
ENSMUSG00000109598	Gm36356	predicted gene, 36356 [Source:MGI Symbol;Acc:MGI:5595515]	6.72469	9.22951	7.4817	7.51716	10.025	7.52898	8.76926	8.43332	12.0023	8.36271	7.9031	8.11093	8.10961	7.71149	0.814221431	0.966018836	8	20	24	0.527192176	0.823319175	24	9.299240962	0.086361802
ENSMUSG00000109599	Gm31812	predicted gene, 31812 [Source:MGI Symbol;Acc:MGI:5590971]	3.86317	4.06879	3.25877	3.57801	5.29092	3.60927	4.18672	3.63975	4.79571	3.46345	5.12987	4.44295	4.11843	3.78749	0.125573493	0.555618842	20	20	24	0.335913301	0.771482605	24	17.70014675	0.098524029
ENSMUSG00000109609	Gm4972	predicted gene 4972 [Source:MGI Symbol;Acc:MGI:3643689]	3.92816	3.10739	3.7192	3.84668	3.16541	3.97365	4.13016	3.62038	3.25725	3.50784	3.45741	3.75036	3.41157	2.95018	0.321662653	0.83607295	4	4	24	0.272547539	0.768040178	24	19.97517519	0.069508433
ENSMUSG00000109618	Gm45527	predicted gene 45527 [Source:MGI Symbol;Acc:MGI:5791363]	0.391002	0.56252	0.637527	0.447191	0.826718	0.590056	0.714864	0.465897	0.577848	0.473699	0.581208	0.499777	0.521769	0.391146	0.759899911	0.966018836	20	12	24	0.458745966	0.805393068	24	16.69434406	0.053878372
ENSMUSG00000109644	0610005C13Rik	RIKEN cDNA 0610005C13 gene [Source:MGI Symbol;Acc:MGI:1918911]	0.629033	0.425496	0.461765	0.542545	0.549216	0.606283	0.67315	0.628539	0.829643	0.357074	0.566563	0.657584	0.553513	0.6112	0.254637229	0.757509828	4	12	24	0.530644357	0.824858059	24	23.87483686	0.046393868
ENSMUSG00000109645	Gm45246	predicted gene 45246 [Source:MGI Symbol;Acc:MGI:5791082]	5.33185	4.58083	6.63324	5.30616	5.9058	5.0238	4.30231	3.82747	3.60433	3.92081	3.33027	4.23803	4.53858	5.06238	0.68642356	0.966018836	4	16	24	0.987857406	0.997530201	24	21.92547767	0.012797043
ENSMUSG00000109695	Gm31166	predicted gene, 31166 [Source:MGI Symbol;Acc:MGI:5590325]	1.27578	2.10788	1.82953	1.59254	1.39757	1.82475	1.62274	3.60509	2.12922	2.40515	1.15458	1.71216	1.85692	2.02351	0.029825002	0.323426482	8	12	24	0.056610342	0.655587475	24	5.994189369	0.236811329
ENSMUSG00000109708	Gm45809	predicted gene 45809 [Source:MGI Symbol;Acc:MGI:5804924]	0.668004	1.0529	0.738699	0.783692	0.951227	0.533559	1.1022	0.686716	1.27105	0.399115	0.888521	0.759848	0.816029	0.736301	0.820042705	0.966018836	12	12	24	0.766838636	0.92458474	24	4.450329654	0.053629673
ENSMUSG00000109714	Gm35021	predicted gene, 35021 [Source:MGI Symbol;Acc:MGI:5594180]	2.93423	3.31222	2.85909	2.96955	3.22579	2.90579	2.72006	3.33503	2.84935	3.71171	3.21007	2.98949	3.47853	3.23127	0.91695334	0.966018836	8	4	24	0.764446437	0.92403577	24	10.11389354	0.027172533
ENSMUSG00000109715	Gm45606	predicted gene 45606 [Source:MGI Symbol;Acc:MGI:5791442]	0.813091	1.00322	1.15024	0.8636	0.821171	0.755186	0.960074	0.997539	1.04045	1.33769	0.835177	1.00132	1.1829	1.24055	0.134026105	0.56887508	12	8	24	0.016037552	0.544099157	24	6.905593578	0.111901605
ENSMUSG00000109727	Gm45464	predicted gene 45464 [Source:MGI Symbol;Acc:MGI:5791300]	0.616154	0.590523	0.727142	0.610899	0.516954	0.540262	0.473931	0.539814	0.688131	0.334912	0.620133	0.540154	0.640612	0.548219	0.718720526	0.966018836	12	12	24	0.665085421	0.886947643	24	5.074927896	0.032603011
ENSMUSG00000109780	Gm45447	predicted gene 45447 [Source:MGI Symbol;Acc:MGI:5791283]	1.15832	1.31393	1.10289	1.27886	0.907312	1.26623	1.38589	1.21361	1.10634	0.988171	1.11561	1.08864	1.34674	0.876864	0.297978647	0.80820842	4	16	24	0.267367209	0.768040178	24	23.90616257	0.064305287
ENSMUSG00000109783	Gm45338	predicted gene 45338 [Source:MGI Symbol;Acc:MGI:5791174]	2.39832	3.4711	2.67979	2.69104	2.41529	2.70863	3.02632	2.42522	3.25215	2.17186	2.20503	2.51497	3.03647	2.88983	0.15509859	0.607609206	8	12	24	0.080692209	0.696283271	24	3.757140798	0.121620249
ENSMUSG00000109793	Gm10043	predicted pseudogene 10043 [Source:MGI Symbol;Acc:MGI:3641648]	7.95165	8.97592	2.85838	5.1846	5.21831	5.09802	5.09719	4.57793	6.74248	3.16103	4.18804	4.59614	4.02718	5.19131	0.908406451	0.966018836	8	8	24	0.290296333	0.768040178	24	0.862039307	0.206025633
ENSMUSG00000109799	Gm45515	predicted gene 45515 [Source:MGI Symbol;Acc:MGI:5791351]	0.307246	0.474498	0.658798	0.400054	0.527087	0.351472	0.583461	0.455828	0.82007	0.57333	0.480107	0.580574	0.640792	0.486516	0.883024237	0.966018836	12	12	24	0.237035461	0.768040178	24	8.491469013	0.077736303
ENSMUSG00000109814	Gm45847	predicted gene 45847 [Source:MGI Symbol;Acc:MGI:5804962]	2.60753	3.62755	3.13059	3.00371	2.4575	3.79136	3.20135	2.91531	2.69859	3.16594	2.71667	3.1033	3.12756	2.93683	0.920872048	0.966018836	24	20	24	0.672137378	0.889365603	24	0.799117117	0.045730922
ENSMUSG00000109827	Gm6213	predicted gene 6213 [Source:MGI Symbol;Acc:MGI:3646754]	0.504069	0.724466	0.456378	0.383061	0.499799	0.473737	0.511404	0.374548	0.8583	0.499769	0.28743	0.37685	0.50548	0.570356	0.201269978	0.686853052	12	8	24	0.160367458	0.765941695	24	5.474814416	0.098319059
ENSMUSG00000109829	Gm45605	predicted gene 45605 [Source:MGI Symbol;Acc:MGI:5791441]	1.09825	1.36878	1.19553	1.29079	1.37289	1.12951	1.69662	1.42133	1.53769	1.20234	1.51867	1.50884	1.12479	1.46566	0.845386467	0.966018836	20	8	24	0.934232743	0.98355239	24	7.020194638	0.016619969
ENSMUSG00000109841	E330011O21Rik	RIKEN cDNA E330011O21 gene [Source:MGI Symbol;Acc:MGI:3759668]	0.818386	0.929765	0.885761	0.90844	0.67091	0.710401	0.986055	1.14684	0.626683	0.926956	1.3901	1.14271	1.38283	1.11267	0.729086532	0.966018836	4	4	24	0.605523758	0.862720716	24	0.13174535	0.055928648
ENSMUSG00000109844	Gm31816	predicted gene, 31816 [Source:MGI Symbol;Acc:MGI:5590975]	1.91804	1.24307	0.425459	1.02052	0.971821	1.76856	0.917739	1.13947	1.5308	1.28958	1.65408	2.11908	1.98947	1.24057	0.282728968	0.793714531	12	8	24	0.086394604	0.705702571	24	20.88124204	0.237904535
ENSMUSG00000109847	Gm45278	predicted gene 45278 [Source:MGI Symbol;Acc:MGI:5791114]	0.426965	0.574152	0.328616	0.722302	0.433953	0.401743	0.397995	0.513167	0.586429	0.496424	0.563473	0.529528	0.663972	0.609237	0.920858634	0.966018836	4	16	24	0.643233845	0.878809617	24	8.703301748	0.037480577
ENSMUSG00000109857	Gm53058	predicted gene 53058 [Source:MGI Symbol;Acc:MGI:6435685]	0.596541	0.848733	0.702562	1.49203	0.968181	0.418317	0.376871	1.03693	1.08308	2.01873	1.38886	0.902574	1.22155	0.933464	0.048889741	0.379129806	16	12	24	0.01118469	0.517565995	24	11.44724409	0.292118811
ENSMUSG00000109865	Hspa14	heat shock protein 14 [Source:MGI Symbol;Acc:MGI:1354164]	15.8351	17.1393	17.9461	17.5658	15.9619	15.301	16.437	16.6516	17.3844	16.2957	16.1358	15.9662	16.9039	15.2926	0.026341137	0.27189101	12	12	24	0.056092306	0.65534544	24	8.43605752	0.057360695
ENSMUSG00000109868	Gm39244	predicted gene, 39244 [Source:MGI Symbol;Acc:MGI:5622129]	5.46839	4.42037	4.8648	4.27281	4.54055	4.8832	4.43	4.86013	4.49948	4.64504	4.11812	4.85741	5.7456	5.21262	0.217933169	0.704989686	4	16	24	0.068354437	0.672656585	24	0.934714539	0.091658031
ENSMUSG00000109881	Gm45507	predicted gene 45507 [Source:MGI Symbol;Acc:MGI:5791343]	12.3189	23.9823	17.198	20.5456	22.4588	15.7098	16.4098	20.5273	25.1221	22.1292	16.9535	20.8029	22.5238	19.4199	0.632729413	0.966018836	12	16	24	0.169989432	0.767957732	24	9.366857172	0.185152226
ENSMUSG00000109894	Gm5904	predicted gene 5904 [Source:MGI Symbol;Acc:MGI:3644469]	14.7322	14.4208	14.0461	12.7859	13.6793	15.7376	14.2345	13.0776	12.2985	13.2536	12.6489	13.5996	13.3338	13.3589	0.210789085	0.699573621	24	16	24	0.017258853	0.549489822	24	22.12497241	0.077751549
ENSMUSG00000109901	Chmp1b	charged multivesicular body protein 1B [Source:MGI Symbol;Acc:MGI:1914314]	22.7972	22.7382	21.2305	22.8014	22.1216	20.7063	22.1439	20.3093	20.2152	19.9985	22.7923	19.4582	20.2914	21.0443	0.849180783	0.966018836	20	4	24	0.850524037	0.95526784	24	17.89031522	0.014636267
ENSMUSG00000109904	Gm45819	predicted gene 45819 [Source:MGI Symbol;Acc:MGI:5804934]	0.299457	0.279437	0.977582	0.948685	0.712966	0.384275	0.420712	0.870809	1.26911	0.67151	0.412362	0.315037	0.370283	[illegible]	9.24E-05	0.029372629	16	16	24	8.26E-05	0.108055894	24	12.0508586	0.350028862
ENSMUSG00000109933	Gm7600	predicted gene 7600 [Source:MGI Symbol;Acc:MGI:3647502]	19.0737	25.5139	22.6818	24.1622	22.4539	20.6507	20.9092	21.3383	31.6547	20.0904	26.3951	22.8626	25.0227	21.7947	0.846840413	0.966018836	16	16	24	0.359497155	0.785405943	24	9.57277431	0.102826521
ENSMUSG00000109946	Brd3os	bromodomain containing 3, opposite strand [Source:MGI Symbol;Acc:MGI:106557]	5.43465	4.99683	5.31763	4.81532	5.22123	5.18469	5.49716	5.23003	4.73488	5.12766	5.09009	5.29478	4.96023	5.6706	0.498269922	0.966018836	4	12	24	0.247659391	0.768040178	24	23.71126903	0.038677125
ENSMUSG00000109956	Gm45428	predicted gene 45428 [Source:MGI Symbol;Acc:MGI:5791264]	0.657988	0.508433	0.344738	0.421119	0.421496	0.514247	0.535998	0.503352	0.600324	0.515059	0.638959	0.588427	0.534314	0.487973	0.292304585	0.799299788	4	8	24	0.278637355	0.768040178	24	21.78685758	0.050474668
ENSMUSG00000109975	Gm19196	predicted gene, 19196 [Source:MGI Symbol;Acc:MGI:5011381]	7.25216	6.40187	9.17381	6.9879	7.6541	6.34662	6.58225	7.15405	8.57751	4.19174	6.8397	8.57686	7.00759	5.48207	0.927004797	0.966018836	20	8	24	0.958438767	0.989144435	24	22.62523057	0.028771414
ENSMUSG00000110003	Gm45403	predicted gene 45403 [Source:MGI Symbol;Acc:MGI:5791239]	2.47361	3.18606	3.43705	3.11059	1.51538	3.06626	1.70296	1.53871	4.0364	3.74543	1.88059	3.45043	2.17566	2.82477	0.158091814	0.614628746	12	16	24	0.300032974	0.768040178	24	9.343481802	0.211143167
ENSMUSG00000110016	Gm20751	predicted gene, 20751 [Source:MGI Symbol;Acc:MGI:5434107]	0.752723	0.419254	0.70186	0.576922	0.645471	0.571137	0.875342	0.546459	0.906671	0.805032	0.469145	0.615942	0.572233	0.693394	0.954744729	0.966018836	12	20	24	0.772959836	0.92697438	24	7.521582523	0.037824026
ENSMUSG00000110020	Gm45441	predicted gene 45441 [Source:MGI Symbol;Acc:MGI:5791277]	1.00971	1.22585	1.60291	1.05151	1.25395	1.20256	1.01369	1.36784	0.79385	0.684229	1.19672	0.961294	1.05344	0.726291	0.828910443	0.966018836	20	20	24	0.883576102	0.966818623	24	20.6996921	0.031486688
ENSMUSG00000110057	Gm2225	predicted gene 2225 [Source:MGI Symbol;Acc:MGI:3780395]	57.7725	70.4456	65.4666	70.4603	65.9816	57.2173	68.3577	68.3637	88.2028	53.5491	63.4099	67.849	74.608	71.6262	0.209185536	0.69936851	12	12	24	0.205348051	0.768040178	24	5.922140229	0.118607719
ENSMUSG00000110067	6330411D24Rik	RIKEN cDNA 6330411D24 gene [Source:MGI Symbol;Acc:MGI:1917975]	2.70883	3.13633	2.21742	3.77538	0.773475	2.804	2.29359	4.33746	1.85042	1.70459	1.8573	3.08904	3.47317	4.06053	0.018810871	0.256345714	8	12	24	0.072093997	0.678628941	24	2.880569512	0.349542216
ENSMUSG00000110070	Gm45619	predicted gene 45619 [Source:MGI Symbol;Acc:MGI:5791455]	0.978039	1.06017	1.17006	1.0667	2.08844	1.29052	0.907031	1.1206	0.713768	1.2297	1.32537	1.68858	1.31432	0.90421	0.028632001	0.316608413	20	12	24	0.020001426	0.565108098	24	17.39617084	0.210504783
ENSMUSG00000110077	Gm45632	predicted gene 45632 [Source:MGI Symbol;Acc:MGI:5791468]	0.482322	0.748032	0.774787	0.714569	0.34549	0.314454	0.645526	0.453834	0.754432	0.652269	0.570604	0.496089	0.640806	0.629969	0.009630739	0.194720062	12	12	24	0.020761599	0.568066329	24	7.516816436	0.139878401
ENSMUSG00000110080	Gm6145	predicted gene 6145 [Source:MGI Symbol;Acc:MGI:3779659]	0.577634	1.77606	2.56729	1.50892	1.05103	1.43517	2.0654	0.925647	2.27436	1.20796	1.10369	1.47259	0.496444	1.80643	0.138543921	0.580158868	12	8	24	0.23483723	0.768040178	24	8.570508802	0.250316342
ENSMUSG00000110105	Gm45844	predicted gene 45844 [Source:MGI Symbol;Acc:MGI:5804959]	1.9144	2.44172	2.27362	1.93325	3.27008	2.22663	2.89663	1.94571	2.4834	1.90649	2.45124	2.23315	2.66848	2.33276	0.897570341	0.966018836	20	20	24	0.680201658	0.893034613	24	20.08060427	0.0602172
ENSMUSG00000110126	Gm9347	predicted gene 9347 [Source:MGI Symbol;Acc:MGI:3648409]	1.23531	0.698329	0.746728	0.695359	0.969599	0.970104	0.871952	0.936434	0.751173	0.525816	1.13596	0.732922	0.565579	0.448275	0.503972924	0.966018836	20	20	24	0.089399177	0.708706636	24	19.43419715	0.132858475
ENSMUSG00000110129	Gm45453	predicted gene 45453 [Source:MGI Symbol;Acc:MGI:5791289]	1.09544	1.02503	1.19097	1.25453	1.20637	0.849518	0.913967	0.825117	1.14111	0.761912	0.989896	0.883383	0.999397	0.992775	0.628286596	0.966018836	12	12	24	0.509182418	0.813345173	24	10.63380024	0.045924697
ENSMUSG00000110144	Gm45564	predicted gene 45564 [Source:MGI Symbol;Acc:MGI:5791400]	16.466	20.0845	20.4826	20.6683	21.7948	15.4848	18.2935	18.7108	27.7988	18.0248	20.7467	18.6527	21.0494	20.544	0.44861185	0.966018836	20	4	24	0.078462937	0.688981542	24	0.064367318	0.160681654
ENSMUSG00000110148	5830408C22Rik	RIKEN cDNA 5830408C22 gene [Source:MGI Symbol;Acc:MGI:1921994]	0.733549	1.33306	0.562873	0.616313	0.590901	0.82365	1.34589	0.531246	0.601499	0.75752	0.626367	1.24572	0.510343	1.19236	0.895493752	0.966018836	24	12	24	0.318942971	0.768040178	24	0.035925786	0.139432157
ENSMUSG00000110150	Gm6523	predicted gene 6523 [Source:MGI Symbol;Acc:MGI:3779801]	1.64491	1.19825	1.67463	0.584036	1.52379	1.44423	1.6253	0.548808	1.25859	1.29141	0.982323	1.05714	0.964081	1.55928	0.858514388	0.966018836	12	12	24	0.775308539	0.927711986	24	22.85123236	0.074298247
ENSMUSG00000110156	Gm42067	predicted gene, 42067 [Source:MGI Symbol;Acc:MGI:5624952]	5.1594	4.90573	4.68545	3.25653	4.94775	3.94537	4.73406	6.31917	6.34351	5.08397	7.05122	4.5232	3.44416	4.76051	0.888991156	0.966018836	12	12	24	0.744776185	0.916569459	24	8.658828426	0.083087211

ENSMUSG00000110165	Btf3-ps18	basic transcription factor 3, pseudogene 18 [Source:MGI Symbol;Acc:MGI:3782206]	0.905744	0.62107	0.699826	0.638373	0.258314	0.435487	0.132199	0.676787	1.09859	0.677416	0.448075	1.22206	0.709228	0.707891	0.881718085	0.966018836	12	8	24	0.556030768	0.838702296	24	6.55084975	0.113931235
ENSMUSG00000110173	Gm6929	predicted gene 6929 [Source:MGI Symbol;Acc:MGI:3645961]	1.05212	0.998141	1.42712	1.33835	0.969676	1.02927	1.75644	0.816409	0.714097	0.260935	0.894184	0.933133	0.883479	0.502605	0.893609703	0.966018836	4	4	24	0.38048416	0.799546792	24	21.7650177	0.131732007
ENSMUSG00000110185	Igip	IgA inducing protein [Source:MGI Symbol;Acc:MGI:1924271]	54.4156	54.263	53.5609	53.5098	56.377	50.1312	54.2146	48.1382	51.6037	46.1428	54.1203	46.5148	47.9181	49.8073	0.882263435	0.966018836	20	4	24	0.86473021	0.960819377	24	15.99220493	0.015179348
ENSMUSG00000110195	Pde2a	phosphodiesterase 2A, cGMP-stimulated [Source:MGI Symbol;Acc:MGI:2446107]	23.6106	20.4372	22.283	23.3602	21.8987	25.2789	20.0908	21.9475	18.5438	26.99	21.5923	24.2547	23.7295	22.9366	0.390686537	0.915479136	24	12	24	0.381440273	0.799546792	24	17.52489692	0.07320198
ENSMUSG00000110206	Flt3l	FMS-like tyrosine kinase 3 ligand [Source:MGI Symbol;Acc:MGI:95560]	10.7799	11.5298	9.73193	10.0506	13.9332	10.274	11.5282	10.6924	12.0536	9.72551	11.1694	11.2244	10.8892	11.2528	0.911135828	0.966018836	8	8	24	0.745656996	0.91691176	24	20.20522397	0.039995563
ENSMUSG00000110218	Tincr	TINCR ubiquitin domain containing [Source:MGI Symbol;Acc:MGI:5012404]	1.14805	1.13119	1.01472	1.19613	1.02292	1.20455	1.44562	0.86869	1.25087	0.639871	1.13685	0.981839	1.00232	1.01435	0.838244962	0.966018836	4	4	24	0.460114793	0.805393068	24	22.59732034	0.064849047
ENSMUSG00000110221	Gm36210	predicted gene, 36210 [Source:MGI Symbol;Acc:MGI:5595369]	0.995503	0.650919	0.734858	0.817542	0.800348	1.31519	0.694614	0.988449	0.972268	1.22125	0.612322	0.882452	1.18246	1.05803	0.924770496	0.966018836	24	20	24	0.888532467	0.968671311	24	23.90644413	0.030527494
ENSMUSG00000110234	Gm45799	predicted gene 45799 [Source:MGI Symbol;Acc:MGI:5804914]	2.31844	1.15867	1.61469	1.42734	0.466435	0.947537	0.938939	1.19565	0.892366	1.44885	3.24025	1.21213	1.84692	1.92436	0.801273005	0.966018836	4	20	24	0.97566479	0.993814468	24	3.389473988	0.033633387
ENSMUSG00000110251	Gm2996	predicted gene 2996 [Source:MGI Symbol;Acc:MGI:3781174]	7.70254	7.80477	5.92416	8.01644	8.00143	8.22931	8.06409	7.03989	7.34645	9.90242	7.00539	6.60239	8.31588	6.98518	0.823677655	0.966018836	16	20	24	0.694401699	0.898522833	24	16.90759011	0.054486211
ENSMUSG00000110275	Gm5905	predicted gene 5905 [Source:MGI Symbol;Acc:MGI:3647422]	186.673	197.765	185.89	192.075	192.702	189.789	189.333	191.9	217.669	182.891	205.364	206.551	219.623	207.361	0.823144114	0.966018836	8	8	24	0.700983665	0.900900643	24	3.123399945	0.021739667
ENSMUSG00000110277	Gm45871	predicted gene 45871 [Source:MGI Symbol;Acc:MGI:5804986]	4.85839	6.38761	6.18715	6.74275	6.58863	5.2809	7.59024	5.62371	6.4223	5.08863	7.19444	6.11481	5.63443	6.39654	0.966095896	0.969272194	20	4	24	0.817356463	0.941870715	24	11.9314052	0.040815873
ENSMUSG00000110316	Gm45311	predicted gene 45311 [Source:MGI Symbol;Acc:MGI:5791147]	0.705357	0.749242	0.843328	0.579798	1.09204	0.742428	0.854246	1.01902	0.52614	0.894472	0.844671	0.638668	0.699896	0.865368	0.93717134	0.966018836	20	4	24	0.979877957	0.995092578	24	17.63472825	0.010740575
ENSMUSG00000110317	Gm45250	predicted gene 45250 [Source:MGI Symbol;Acc:MGI:5791086]	1.43076	1.66273	1.73325	1.55878	1.58285	1.4051	1.42509	1.42175	1.81772	1.39437	1.8264	1.57344	1.53884	1.90131	0.697196105	0.966018836	12	16	24	0.254632441	0.768040178	24	8.717542066	0.060334933
ENSMUSG00000110331	Nudc-ps1	nuclear distribution gene C homolog (Aspergillus), pseudogene 1 [Source:MGI Symbol;Acc:MGI:2142657]	47.1052	46.3824	48.0186	46.6083	44.6849	48.7082	42.618	45.3927	40.379	41.842	41.7857	42.9162	45.8455	45.8876	0.90332508	0.966018836	24	20	24	0.424235181	0.805393068	24	0.424235181	0.035880876
ENSMUSG00000110332	Gm19935	predicted gene, 19935 [Source:MGI Symbol;Acc:MGI:5012120]	3.60384	3.78673	3.33649	2.68137	3.17148	2.46052	4.0999	3.70662	3.82499	3.02449	3.51626	3.63305	2.33557	3.70948	0.313239838	0.824470524	8	8	24	0.387696585	0.799546792	24	4.558213466	0.102566245
ENSMUSG00000110344	Gm45716	predicted gene 45716 [Source:MGI Symbol;Acc:MGI:5804831]	0.435612	0.479563	1.10582	0.875502	0.708321	0.61951	0.576275	0.941202	0.893874	0.718324	0.674127	0.948008	0.580219	0.862016	0.005041121	0.143276577	12	16	24	0.07599966	0.686372559	24	9.876200272	0.134775916
ENSMUSG00000110350	Gm10252	predicted gene 10252 [Source:MGI Symbol;Acc:MGI:3704292]	0.726138	0.465397	0.593832	0.642192	0.877301	0.559349	0.66238	0.837437	0.518505	0.979862	0.536637	0.338611	0.703927	0.506627	0.928127947	0.966018836	16	8	24	0.765532154	0.924119887	24	12.16907527	0.044259373
ENSMUSG00000110355	Gm7514	predicted gene 7514 [Source:MGI Symbol;Acc:MGI:3643009]	1.64993	2.35794	1.73805	2.12804	2.04442	1.4627	2.07561	2.07685	1.83072	1.94177	2.16188	1.74309	1.88944	1.69552	0.676344318	0.966018836	20	4	24	0.638672776	0.877451476	24	9.901641737	0.046875866
ENSMUSG00000110357	A030001D20Rik	RIKEN cDNA A030001D20 gene [Source:MGI Symbol;Acc:MGI:3696869]	5.26758	4.51771	5.07614	4.758	4.6592	5.10826	4.70528	4.77763	3.98452	4.37907	4.89411	4.90924	4.88985	4.77001	0.237522906	0.74000957	24	16	24	0.081497688	0.697102286	24	21.19650267	0.069028382
ENSMUSG00000110360	Gm45380	predicted gene 45380 [Source:MGI Symbol;Acc:MGI:5791216]	4.50376	4.12909	4.87412	4.70591	5.16823	3.77405	6.07851	3.01741	3.74905	3.85936	3.7275	4.68492	2.34113	5.6339	0.640044239	0.966018836	16	16	24	0.945382641	0.986789931	24	14.96211303	0.037233336
ENSMUSG00000110390	Gm45869	predicted gene 45869 [Source:MGI Symbol;Acc:MGI:5804984]	11.2903	10.2725	8.50728	9.18243	8.12007	8.81293	10.6836	10.1142	8.39329	6.96443	7.68212	6.53789	9.33086	7.95646	0.021407754	0.26465279	4	16	24	0.006290649	0.46954479	24	1.755365806	0.172226563
ENSMUSG00000110393	Gm36445	predicted gene, 36445 [Source:MGI Symbol;Acc:MGI:5595604]	2.18392	2.30492	1.77246	1.81046	1.74189	1.70408	1.63281	2.09411	2.08561	1.7913	1.87629	2.16126	2.00612	1.94583	0.591351901	0.966018836	8	12	24	0.244678506	0.768040178	24	3.161253008	0.064200951
ENSMUSG00000110424	1700012D14Rik	RIKEN cDNA 1700012D14 gene [Source:MGI Symbol;Acc:MGI:1922729]	0.699427	1.13351	1.06626	1.5831	1.05619	1.17609	1.03933	1.15987	1.69967	1.35491	1.4244	1.71726	1.32691	1.30923	0.424589909	0.966018836	16	12	24	0.08480584	0.70291653	24	11.86997501	0.117751461
ENSMUSG00000110435	Gm3635	predicted gene 3635 [Source:MGI Symbol;Acc:MGI:3781811]	1.26591	0.744293	0.68566	0.471572	0.460764	0.349356	0.255232	0.556966	1.14021	0.558607	0.710579	0.274839	0.428366	0.493165	0.077301512	0.453790923	12	12	24	0.341105364	0.775335259	24	7.30455001	0.137744527
ENSMUSG00000110444	Gm10033	predicted gene 10033 [Source:MGI Symbol;Acc:MGI:3642803]	7.71139	7.83215	6.64613	7.89505	8.30175	7.62915	8.98646	8.04745	7.27986	7.35706	9.23029	7.80013	9.96679	6.88754	0.142513744	0.585944911	4	8	24	0.138328337	0.747972781	24	20.39279473	0.107441018
ENSMUSG00000110446	Gm18646	predicted gene, 18646 [Source:MGI Symbol;Acc:MGI:5010831]	4.369	4.82313	4.12775	4.45824	4.79395	5.81723	3.93171	4.78404	4.10051	4.05253	4.12972	3.99305	4.26024	2.86339	0.854443826	0.966018836	20	16	24	0.229060306	0.768040178	24	18.4687659	0.102495359
ENSMUSG00000110523	C230057M02Rik	RIKEN cDNA C230057M02 gene [Source:MGI Symbol;Acc:MGI:2442079]	0.569638	0.573068	0.817914	0.512176	0.556319	0.471601	0.589925	0.453275	0.584429	0.353487	0.659123	0.536923	0.40199	0.451017	0.91724565	0.966018836	12	12	24	0.909884142	0.974625908	24	10.97748124	0.016854293
ENSMUSG00000110528	Gm1943	predicted gene 1943 [Source:MGI Symbol;Acc:MGI:3037801]	37.7959	29.9482	32.7698	29.2827	35.3602	36.8124	26.2161	25.9209	21.2833	34.6487	21.4916	24.8307	28.7783	32.2696	0.829428469	0.966018836	4	8	24	0.804363079	0.937432708	24	20.69199661	0.065229547
ENSMUSG00000110529	Gm45694	predicted gene 45694 [Source:MGI Symbol;Acc:MGI:5804809]	0.512579	0.430632	0.530039	0.488169	0.531107	0.432145	0.642433	0.564661	0.456367	0.406192	0.628778	0.666592	0.476208	0.464148	0.472443528	0.966018836	20	20	24	0.359900893	0.785598254	24	20.06262024	0.044783492
ENSMUSG00000110540	Gm45743	predicted gene 45743 [Source:MGI Symbol;Acc:MGI:5804858]	0.616582	0.712033	0.654735	0.780912	0.634355	0.541302	0.661902	0.635447	0.710543	0.635606	0.581832	0.753903	0.688754	0.789764	0.455507782	0.966018836	8	12	24	0.254810794	0.768040178	24	6.670508875	0.04000783
ENSMUSG00000110545	Gm7730	predicted gene 7730 [Source:MGI Symbol;Acc:MGI:3647355]	63.9966	71.2269	67.6391	69.4827	81.569	79.2491	62.7753	75.2929	73.4536	60.7549	79.9254	62.0609	69.9049	65.788	0.949874888	0.966018836	20	8	24	0.583529082	0.851892977	24	15.62856885	0.056831888
ENSMUSG00000110547	Gm29773	predicted gene, 29773 [Source:MGI Symbol;Acc:MGI:5588932]	1.12104	1.52299	1.12655	1.90651	1.96014	1.62637	1.33025	1.78388	1.50753	1.18101	1.63231	1.25905	1.78906	1.02492	0.725061903	0.966018836	20	16	24	0.487996211	0.805393068	24	16.15290249	0.086439147
ENSMUSG00000110569	Gm18860	predicted gene, 18860 [Source:MGI Symbol;Acc:MGI:5011045]	4.10811	4.03709	4.24931	4.16719	4.10319	4.42373	4.36083	4.26283	4.02204	4.64135	3.54519	3.98119	4.50993	3.77939	0.903465533	0.966018836	16	16	24	0.941613748	0.985544192	24	1.268441878	0.011238909
ENSMUSG00000110605	Gm32856	predicted gene, 32856 [Source:MGI Symbol;Acc:MGI:5592015]	2.06448	2.40324	2.27815	1.92042	2.5339	1.89852	2.1844	1.58435	2.56096	1.28275	2.62237	1.87193	2.07051	1.86217	0.951846915	0.966018836	20	12	24	0.936065458	0.98404386	24	18.64007818	0.02776282
ENSMUSG00000110611	Gm20163	predicted gene, 20163 [Source:MGI Symbol;Acc:MGI:5012348]	1.34317	1.50248	2.13726	2.32106	1.33909	2.168	1.47975	2.15784	1.22935	2.54411	1.78636	2.2535	2.18903	1.81356	0.843639218	0.966018836	16	20	24	0.689067841	0.897070579	24	13.23607406	0.071600982
ENSMUSG00000110613	Lncbate1	brown adipose tissue enriched long non-coding RNA 1 [Source:MGI Symbol;Acc:MGI:1924437]	0.18397	0.249153	0.933313	1.75459	0.36636	0.347288	0.822385	0.274181	0.460248	0.688869	1.22073	0.509238	1.636	0.735345	0.81872541	0.966018836	16	16	24	0.497224654	0.807576732	24	12.43744773	0.170902512
ENSMUSG00000110617	Gm5910	predicted gene 5910 [Source:MGI Symbol;Acc:MGI:3646687]	3.705	3.57943	3.67744	3.63334	3.82075	3.69733	3.48699	3.91139	2.72346	3.24071	3.57513	3.24528	3.44314	2.8039	0.849180783	0.966018836	20	20	24	0.201505641	0.768040178	24	19.338331	0.068342196
ENSMUSG00000110630	K230015D01Rik	RIKEN cDNA K230015D01 gene [Source:MGI Symbol;Acc:MGI:3642019]	1.46965	0.972208	0.957788	0.675581	1.18965	1.03632	1.00587	0.924257	1.1474	1.13056	1.75439	1.21847	1.16004	1.33379	0.320027687	0.83607295	20	20	24	0.457956386	0.805393068	24	19.78249902	0.082256154
ENSMUSG00000110631	Gm42047	predicted gene, 42047 [Source:MGI Symbol;Acc:MGI:5624932]	1.03972	1.12907	1.34936	1.11594	1.60359	1.67971	1.13131	1.09418	1.27417	0.684892	0.91939	1.22017	1.4024	1.07537	0.912686312	0.966018836	24	16	24	0.508189832	0.813213314	24	23.86331655	0.093546237
ENSMUSG00000110644	Gm7390	predicted gene 7390 [Source:MGI Symbol;Acc:MGI:3645154]	7.87728	8.01119	9.14273	9.70306	9.47054	9.99148	8.05287	8.61722	8.14117	8.04195	8.69562	7.72608	8.14971	7.74386	0.345459565	0.870418653	20	8	24	0.095891676	0.71175955	24	14.8651864	0.081708755
ENSMUSG00000110656	Gm20735	predicted gene, 20735 [Source:MGI Symbol;Acc:MGI:5434091]	0.567152	0.854814	0.997003	0.978946	0.689227	0.911939	0.592504	0.744719	0.716593	0.873902	0.590676	0.692777	0.652006	0.921434	0.052672766	0.391647859	12	16	24	0.116073513	0.727799063	24	9.324455863	0.096207329
ENSMUSG00000110657	Gm7684	predicted gene 7684 [Source:MGI Symbol;Acc:MGI:3644097]	2.0314	1.76007	1.68881	1.57317	1.78523	1.38769	1.96688	1.49599	1.42695	1.69761	1.80492	1.52619	1.43431	1.25321	0.904387595	0.966018836	20	12	24	0.462879236	0.805393068	24	18.79304593	0.051044819
ENSMUSG00000110686	Gm45855	predicted gene 45855 [Source:MGI Symbol;Acc:MGI:5804970]	1.66408	2.11348	2.71396	1.33454	2.90278	2.29573	2.17478	2.58066	3.82422	2.89539	2.85799	2.93832	2.99481	3.3842	0.332293447	0.966018836	12	4	24	0.393004818	0.799546792	24	7.672233695	0.115000708
ENSMUSG00000110701	Gm6013	predicted gene 6013 [Source:MGI Symbol;Acc:MGI:3648988]	1.93352	1.62291	1.37388	1.69829	1.82822	1.8905	1.72123	2.05794	1.82478	1.327	1.42672	1.57045	1.63898	1.39856	0.596159714	0.966018836	4	12	24	0.278462684	0.768040178	24	22.89352223	0.071730506
ENSMUSG00000110702	Gm45767	predicted gene 45767 [Source:MGI Symbol;Acc:MGI:5804882]	1.5998	2.11956	1.9883	1.82089	1.66418	1.61414	2.33679	1.76824	2.59981	1.98512	1.83091	1.81009	1.71777	1.66989	0.217827365	0.704989686	12	12	24	0.235089014	0.768040178	24	7.789990591	0.093595291
ENSMUSG00000110710	C78859	expressed sequence C78859 [Source:MGI Symbol;Acc:MGI:2142856]	36.5534	31.6253	27.8179	34.4231	36.7939	28.7977	32.5449	27.0462	32.2322	25.7723	37.3071	28.043	31.8598	31.5285	0.602156008	0.966018836	20	12	24	0.547364341	0.833663107	24	18.57174914	0.073515892
ENSMUSG00000110715	Gm5131	predicted gene 5131 [Source:MGI Symbol;Acc:MGI:3644589]	0.849059	0.882301	0.682667	1.06082	0.638943	0.6301	0.631018	0.761334	0.88323	0.780341	1.09333	0.869436	0.933228	0.906665	0.891742979	0.966018836	16	8	24	0.704058138	0.902338136	24	10.23687017	0.038900637
ENSMUSG00000110723	Gm49353	predicted gene, 49353 [Source:MGI Symbol;Acc:MGI:6121556]	5.07189	4.56725	4.88308	3.98264	5.37015	6.06648	4.75129	4.92076	4.17067	6.61656	7.34892	5.23224	4.85898	5.63218	0.173410058	0.648600527	20	16	24	0.177720669	0.768040178	24	17.05533968	0.134668224
ENSMUSG00000110726	Gm18101	predicted gene, 18101 [Source:MGI Symbol;Acc:MGI:5010286]	11.8432	9.21233	8.23524	10.3851	9.69889	10.0971	11.117	9.61134	9.46722	9.06636	10.0668	9.27942	9.88242	8.35358	0.075079679	0.451256373	4	8	24	0.062682407	0.661961741	24	20.61402849	0.107483097
ENSMUSG00000110735	Gm47173	predicted gene, 47173 [Source:MGI Symbol;Acc:MGI:6095954]	0.646587	0.865198	0.806671	0.556646	0.445386	0.292562	0.511053	0.860057	0.681134	0.168115	0.523835	0.668098	0.324384	0.643068	0.069996239	0.437482784	8	16	24	0.101318619	0.713208839	24	4.819222657	0.15294594
ENSMUSG00000110744	Gm5171	predicted gene 5171 [Source:MGI Symbol;Acc:MGI:3642952]	7.53917	7.15415	6.4447	7.14327	7.87337	6.80141	6.6545	6.79285	6.62889	6.06791	6.81226	5.92376	5.93956	5.57919	0.858932988	0.966018836	20	16	24	0.187323872	0.768040178	24	17.14308085	0.052002222
ENSMUSG00000110768	Gm18541	predicted gene, 18541 [Source:MGI Symbol;Acc:MGI:5010726]	39.6891	44.703	37.4187	41.0663	46.2223	42.8949	40.4718	40.4189	48.0956	37.5275	44.8317	42.3179	45.1836	43.0906	0.883051071	0.966018836	20	20	24	0.893633882	0.969947957	24	19.62722448	0.020589261
ENSMUSG00000110773	Gm39326	predicted gene, 39326 [Source:MGI Symbol;Acc:MGI:5622211]	0.991172	0.832958	1.00423	0.621805	0.615309	0.884405	1.15471	0.486079	0.781335	1.05897	1.01691	0.505541	1.03434	0.96417	0.888379597	0.966018836	4	20	24	0.847735246	0.954054905	24	2.097481131	0.040485809
ENSMUSG00000110834	Gm39469	predicted gene, 39469 [Source:MGI Symbol;Acc:MGI:5622354]	3.50073	4.90425	2.92193	5.17364	4.16727	4.06225	4.57654	4.53583	5.02514	4.09633	4.1179	4.43112	3.70959	5.14991	0.706051896	0.966018836	8	20	24	0.770253371	0.926036042	24	7.678927457	0.054535289
ENSMUSG00000110841	Gpx4-ps2	glutathione peroxidase 4, pseudogene 2 [Source:MGI Symbol;Acc:MGI:3779731]	442.024	476.393	447.505	489.787	429.557	416.423	449.368	465.455	556.392	417.398	544.236	498.355	555.112	513.581	0.916556573	0.966018836	12	4	24	0.582135063	0.851114094	24	6.073954001	0.051849175
ENSMUSG00000110847	Gm18705	predicted gene, 18705 [Source:MGI Symbol;Acc:MGI:5010890]	0.795198	0.538709	0.749367	0.85067	0.436152	0.571328	0.7113	0.711668	0.832462	0.595453	0.692204	0.879376	0.67121	0.673823	0.897425765	0.966018836	12	8	24	0.791759508	0.933456119	24	5.7376018	0.030859274
ENSMUSG00000110867	Gm7368	predicted gene 7368 [Source:MGI Symbol;Acc:MGI:3643487]	4.73942	7.46874	5.56336	7.07488	7.63433	5.67907	7.88757	5.39326	6.67469	5.88122	7.45979	5.90755	4.80555	4.44876	0.371875382	0.894537464	20	12	24	0.226817128	0.768040178	24	14.64691034	0.147954347
ENSMUSG00000110905	Gm48007	predicted gene, 48007 [Source:MGI Symbol;Acc:MGI:6097311]	2.53243	2.71904	1.54481	2.67318	1.85874	1.84961	1.91907	2.93932	1.93029	1.25651	2.3596	1.12693	2.5585	1.70543	0.492524295	0.966018836	8	16	24	0.490382727	0.805393068	24	2.618195694	0.120794093
ENSMUSG00000110924	Gm40518	predicted gene, 40518 [Source:MGI Symbol;Acc:MGI:5623403]	5.05967	3.55375	2.88032	3.18712	3.97462	3.33824	3.51815	3.26409	2.76033	3.39088	3.4598	3.45186	4.53229	3.42619	0.002087679	0.100255953	4	8	24	0.039833469	0.616921492	24	21.80134554	0.16799134
ENSMUSG00000110935	Gm8834	predicted gene 8834 [Source:MGI Symbol;Acc:MGI:3646859]	1.69056	2.92335	4.64397	2.3741	2.21228	3.69037	3.02395	3.18636	2.90903	4.14184	2.51238	1.91592	1.93105	2.86304	0.388109084	0.915479136	12	16	24	0.166759539	0.767020009	24	8.930665602	0.231375995
ENSMUSG00000110945	Gm9856	predicted gene 9856 [Source:MGI Symbol;Acc:MGI:3704347]	0.95987	1.20503	0.804207	0.931202	0.978356	1.02482	0.903415	1.0848	1.23108	0.970052	1.15305	1.1234	0.945201	1.25003	0.909604019	0.966018836	8	20	24	0.739719413	0.915107718	24	5.120393859	0.027758576
ENSMUSG00000110949	Nudt8	nudix (nucleoside diphosphate linked moiety X)-type motif 8 [Source:MGI Symbol;Acc:MGI:1913637]	5.71321	5.90584	5.54734	5.67214	5.38452	5.48235	6.64548	4.49636	5.37053	5.55983	5.7965	5.09461	6.57734	4.72025	0.648082424	0.966018836	4	4	24	0.563594505	0.842665559	24	20.43177281	0.056941463
ENSMUSG00000110960	Supt4b	SPT4B, DSIF elongation factor subunit [Source:MGI Symbol;Acc:MGI:1335090]	59.9977	70.2958	61.9842	54.5646	71.5655	54.8304	53.6719	58.4171	64.9797	43.9488	68.8398	63.22	69.9148	66.5078	0.908406451	0.966018836	20	20	24	0.740424166	0.915274254	24	0.600779801	0.059746221
ENSMUSG00000110980	Gm47204	predicted gene, 47204 [Source:MGI Symbol;Acc:MGI:6096003]	0.473136	1.28084	1.70807	1.29953	0.978114	1.61906	1.35428	1.2657	1.55115	1.40078	1.15017	1.14434	1.13974	1.49444	0.101520755	0.511659635	12	16	24	0.177121143	0.768040178	24	8.954828014	0.152816997
ENSMUSG00000110984	Gm47131	predicted gene, 47131 [Source:MGI Symbol;Acc:MGI:6095884]	0.911839	0.605301	0.533637	0.721356	0.313767	0.872438	0.5128	0.626455	0.573894	0.463538	0.721317	0.762603	0.749186	0.316204	0.251760751	0.75129457	24	12	24	0.235149909	0.768040178	24	20.97220406	0.110821416
ENSMUSG00000110997	2610028D06Rik	RIKEN cDNA 2610028D06 gene [Source:MGI Symbol;Acc:MGI:1923609]	0.383526	0.572433	0.86247	0.441735	0.518981	0.583151	0.281161	0.367325	0.944661	0.455961	0.370412	0.568721	0.526681	0.588017	0.387380622	0.915479136	12	16	24	0.243001753	0.768040178	24	8.548980303	0.111725091
ENSMUSG00000111001	Gm8767	predicted gene 8767 [Source:MGI Symbol;Acc:MGI:3647013]	1.09472	1.11298	1.28588	1.80978	0.951413	1.49288	1.0154	1.92239	0.568436	0.867858	0.783418	0.753316	0.670496	0.718387	0.823246279	0.966018836	16	12	24	0.987813865	0.997530201	24	8.625544948	0.015052965
ENSMUSG00000111013	Gm32468	predicted gene, 32468 [Source:MGI Symbol;Acc:MGI:5591627]	0.0452949	0.124508	0.853124	2.05207	0.287176	0.212592	0.315196	0.350016	0.276889	0.254359	0.873947	0.768352	0.339563	0.337932	0.742579234	0.966018836	20	8	24	0.138903887	0.748341961	24	12.84755943	0.29486132
ENSMUSG00000111027	Gm2981	predicted gene 2981 [Source:MGI Symbol;Acc:MGI:3781159]	19.2936	18.9353	16.0718	16.5422	18.7331	18.5308	19.5698	15.5464	16.7316	17.2663	19.9046	18.6901	21.1598	18.0188	0.009483698	0.194720062	4	8	24	0.013302155	0.529134128	24	20.96806358	0.122975965
ENSMUSG00000111052	Gm20139	predicted gene, 20139 [Source:MGI Symbol;Acc:MGI:5012324]	0.868221	0.756885	0.427524	0.52606	0.804479	0.659899	0.801973	0.586468	0.63778	0.401941	0.714062	0.725036	0.836605	0.647823	7.56E-04	0.071344007	4	12	24	0.00535103	0.4424469	24	22.14610023	0.140666585
ENSMUSG00000111063	Zfp660	zinc finger protein 660 [Source:MGI Symbol;Acc:MGI:3645415]	0.576455	0.608334	0.550566	0.591293	0.559974	0.630979	0.727173	0.804189	0.82376	0.580592	0.720975	0.586501	0.654832	0.718061	0.858269825	0.966018836	8	8	24	0.23360805	0.768040178	24	4.789955217	0.046100236
ENSMUSG00000111068	Gm35501	predicted gene, 35501 [Source:MGI Symbol;Acc:MGI:5594660]	4.63766	6.87102	4.68422	6.25614	5.52779	5.04483	5.35456	5.70333	6.11489	4.93183	5.7919	4.15521	5.06603	5.75383	0.216933149	0.704989686	8	16	24	0.27631921	0.768040178	24	8.024545133	0.104639735
ENSMUSG00000111080	Gm20300	predicted gene, 20300 [Source:MGI Symbol;Acc:MGI:5012485]	38.7466	37.4666	34.6976	37.1512	36.1635	33.6286	34.0918	32.8921	33.3358	33.7518	37.7546	32.2744	33.8505	34.4266	0.872380105	0.966018836	20	4	24	0.76812957	0.925197875	24	16.1855275	0.019456159
ENSMUSG00000111085	Gm7607	predicted gene 7607 [Source:MGI Symbol;Acc:MGI:3644538]	0.33833	0.685426	0.367308	0.127916	0.250283	1.20186	0.274136	1.30289	0.689812	1.04397	0.230601	0.435357	0.831795	0.514571	0.633249925	0.966018836	8	12	24	0.522593085	0.820892304	24	3.669119331	0.14451042
ENSMUSG00000111088	9430081H08Rik	RIKEN cDNA 9430081H08 gene [Source:MGI Symbol;Acc:MGI:1924680]	0.303788	0.555439	0.462358	0.764974	0.771412	0.653829	0.222225	0.488927	0.912043	0.521918	0.667342	0.698504	0.769884	1.04714	0.941830768	0.966018836	20	8	24	0.327832873	0.768040178	24	11.19743876	0.101307753
ENSMUSG00000111092	Gm17875	predicted gene, 17875 [Source:MGI Symbol;Acc:MGI:5010060]	0.715312	0.700995	1.33342	1.05448	0.535194	0.492859	1.18426	0.411634	1.11644	0.337575	0.597075	0.48883	0.37445	0.839973	0.390686537	0.915479136	12	12	24	0.294586832	0.768040178	24	7.340712622	0.155534985
ENSMUSG00000111118	Gm6545	predicted gene 6545 [Source:MGI Symbol;Acc:MGI:3643874]	0.610376	0.807545	0.664186	0.843796	0.605825	0.710139	0.835316	0.723532	0.801893	0.693393	0.72437	0.713629	0.665077	0.77859	0.933543173	0.966018836	8	12	24	0.421582953	0.804749039	24	6.887158525	0.034498534
ENSMUSG00000111128	Gm49338	predicted gene, 49338 [Source:MGI Symbol;Acc:MGI:6121528]	6.56696	6.02824	6.98072	6.65684	6.93935	6.53796	6.60089	6.88324	6.28679	8.0452	5.10475	6.32339	6.08699	6.91871	0.664568268	0.966018836	16	8	24	0.539504559	0.829375145	24	8.751016166	0.057517218
ENSMUSG00000111133	Gm5831	predicted gene 5831 [Source:MGI Symbol;Acc:MGI:3779529]	23.1497	21.0016	20.6496	20.2475	21.5706	22.7761	22.0569	23.0418	18.8288	24.6421	18.9667	21.3013	21.5251	18.8717	0.607427393	0.966018836	4	8	24	0.490042015	0.805393068	24	20.98797735	0.053395105
ENSMUSG00000111151	Gm5120	predicted gene 5120 [Source:MGI Symbol;Acc:MGI:3646745]	0.844287	1.01176	0.689161	0.973812	1.00645	0.803561	0.774048	0.822042	0.830895	0.67374	0.947525	0.904423	0.747397	0.637955	0.725061903	0.966018836	20	16	24	0.227606774	0.768040178	24	17.22322267	0.059391706
ENSMUSG00000111186	Gm48135	predicted gene, 48135 [Source:MGI Symbol;Acc:MGI:6097497]	3.15873	3.11496	2.09704	3.89816	1.95614	2.32382	2.56318	2.65886	2.62874	3.76992	2.34031	3.65666	2.87539	3.11411	0.886952527	0.966018836	16	4	24	0.908795674	0.974606327	24	7.156497389	0.041654738
ENSMUSG00000111192	Gm47128	predicted gene, 47128 [Source:MGI Symbol;Acc:MGI:6095880]	5.56901	6.25202	4.00653	7.59496	5.57737	4.73396	7.25585	4.82222	5.28726	4.612	7.6641	4.62666	4.15341	6.39542	0.890648739	0.966018836	20	4	24	0.886753915	0.967901066	24	15.26579939	0.052174267
ENSMUSG00000111197	Gm10608	predicted gene 10608 [Source:MGI Symbol;Acc:MGI:3642009]	8.23949	6.42919	6.166	6.65281	6.59735	6.27543	7.38431	7.00548	3.6548	2.77422	3.83291	3.63297	3.37556	3.62887	0.910436409	0.966018836	4	12	24	0.112954464	0.727393008	24	23.29274978	0.190564448
ENSMUSG00000111221	Gm47133	predicted gene, 47133 [Source:MGI Symbol;Acc:MGI:6095888]	0.892596	0.612521	0.631002	0.614754	0.869234	0.557812	0.871452	0.999544	0.518334	0.678141	0.675542	0.881632	0.861717	0.865513	0.277087842	0.789849983	4	8	24	0.134448187	0.7455552	24	23.4660967	0.093028177
ENSMUSG00000111224	Gm18669	predicted gene, 18669 [Source:MGI Symbol;Acc:MGI:5010854]	2.20115	2.03805	1.26671	1.70312	1.64273	1.97855	1.49931	1.33074	1.14816	0.983965	1.33254	1.15843	1.46859	1.26274	0.510431574	0.966018836	4	8	24	0.016814547	0.547196997	24	22.03693528	0.150018006
ENSMUSG00000111244	Gm47130	predicted gene, 47130 [Source:MGI Symbol;Acc:MGI:6095883]	1.13211	0.798181	0.735618	0.757605	0.493373	0.498793	1.095	0.994888	0.458515	0.299778	0.456808	0.886328	0.153437	0.707973	0.296502765	0.807732044	4	16	24	0.245510374	0.768040178	24	1.361821949	0.14700248
ENSMUSG00000111256	Gm48094	predicted gene, 48094 [Source:MGI Symbol;Acc:MGI:6097440]	1.87541	1.4129	1.40437	1.55716	1.64235	1.39839	1.33495	1.3366	1.81019	1.67154	2.0806	2.15584	2.11536	2.37211	0.807813307	0.966018836	20	12	24	0.859781982	0.958681059	24	19.49076425	0.030923179
ENSMUSG00000111291	Gm48604	predicted gene, 48604 [Source:MGI Symbol;Acc:MGI:6098186]	2.85942	2.82182	2.80658	2.91368	2.95851	2.49444	2.9164	3.0114	2.60616	2.43291	2.95619	2.41649	3.03985	2.71379	0.722296281	0.966018836	4	20	24	0.734416237	0.913383989	24	2.376998586	0.025392492
ENSMUSG00000111293	Gm34006	predicted gene, 34006 [Source:MGI Symbol;Acc:MGI:5593165]	0.519196	0.575075	0.491303	0.425387	0.610268	0.633082	0.571608	0.680607	0.547194	0.634276	0.537476	0.762305	0.747429	0.562135	0.308023687	0.814993005	24	12	24	0.121515467	0.733843269	24	22.08871467	0.055451029
ENSMUSG00000111325	Gm47140	predicted gene, 47140 [Source:MGI Symbol;Acc:MGI:6095900]	0.929817	1.09268	1.60458	1.40168	1.01112	0.967732	0.978975	1.35203	1.184	1.05893	0.972343	1.08094	0.788817	0.97703	0.004501001	0.140598674	12	16	24	0.020048568	0.565108098	24	9.276348813	0.132739077
ENSMUSG00000111329	A830035O19Rik	RIKEN cDNA A830035O19 gene [Source:MGI Symbol;Acc:MGI:3704334]	0.601631	0.730448	0.683618	0.510274	0.679209	0.606626	0.312677	0.878943	0.901265	0.821586	0.705111	0.896167	0.728317	0.771526	0.648082424	0.966018836	8	20	24	0.415761691	0.800637996	24	8.718924788	0.070969476
ENSMUSG00000111334	Gm30015	predicted gene, 30015 [Source:MGI Symbol;Acc:MGI:5589174]	0.583721	0.475498	0.787183	0.38583	0.547229	0.381622	0.649144	0.516233	0.77364	0.350972	0.46014	0.62244	0.408224	0.582553	0.187699199	0.662239322	12	4	24	0.415774086	0.800637996	24	5.09744478	0.067641021
ENSMUSG00000111356	Rpl32l	ribosomal protein L32-like [Source:MGI Symbol;Acc:MGI:3644747]	15.1733	20.2306	17.3746	17.2447	18.6076	16.5695	16.9406	16.5639	16.6212	12.9722	17.4193	12.1571	12.5307	11.0551	0.718086778	0.966018836	20	4	24	0.605912805	0.862870954	24	13.09118676	0.064238187
ENSMUSG00000111357	Gm6980	predicted gene 6980 [Source:MGI Symbol;Acc:MGI:3779645]	1.18253	0.985661	0.874488	0.705513	1.16872	1.50411	1.26976	0.974315	0.969258	0.531257	1.07357	0.874452	1.07106	0.953328	0.004196064	0.140598674	4	12	24	0.013561858	0.529134128	24	22.01907726	0.176062612
ENSMUSG00000111374	Gm50490	predicted gene, 50490 [Source:MGI Symbol;Acc:MGI:6324767]	0.781265	1.91077	2.23855	1.37949	1.00593	1.3249	1.3846	1.58568	1.79165	0.891814	0.973738	1.18116	1.56478	2.45535	0.005288315	0.148362816	8	12	24	0.009170611	0.502965379	24	5.674681541	0.30032369
ENSMUSG00000111375	Btbd8	BTB (POZ) domain containing 8 [Source:MGI Symbol;Acc:MGI:3646208]	10.1716	11.5369	11.8625	10.8939	10.2981	10.4971	10.8327	9.93025	10.9778	10.4796	10.9058	10.4396	10.696	11.4659	0.351840494	0.870418653	12	12	24	0.195580804	0.768040178	24	7.491609265	0.047549536
ENSMUSG00000111380	D9Wsu149	DNA segment, Chr 9, Wayne State University 149 [Source:MGI Symbol;Acc:MGI:107281]	6.02431	5.07512	5.23497	4.74696	4.95451	5.26996	3.94721	5.6138	5.74058	4.41125	6.01757	3.92465	6.94582	4.76581	0.93447422	0.966018836	4	12	24	0.826767369	0.946041107	24	3.233053972	0.048328517
ENSMUSG00000111390	Gm48796	predicted gene, 48796 [Source:MGI Symbol;Acc:MGI:6098500]	2.88042	3.75724	3.49471	4.3943	4.48468	3.64161	3.26453	3.26433	5.06965	3.49488	4.27467	3.2813	3.29604	3.30759	0.007505042	0.192760771	20	8	24	0.025182534	0.586541342	24	12.33286769	0.170708583
ENSMUSG00000111394	Gm49759	predicted gene, 49759 [Source:MGI Symbol;Acc:MGI:6215263]	5.2004	6.93774	7.82491	7.75313	6.72606	6.16595	5.72702	6.53456	7.80466	6.24022	6.41555	6.43424	6.1815	6.86747	3.15E-04	0.049770315	12	16	24	0.00589342	0.461723519	24	9.679126249	0.159427765
ENSMUSG00000111446	Gm6581	predicted gene 6581 [Source:MGI Symbol;Acc:MGI:3648298]	4.89127	6.1128	5.72224	7.14392	3.18638	4.39774	3.92478	4.24679	2.46144	1.62023	2.70156	2.42993	2.51786	2.9542	0.909604019	0.966018836	8	12	24	0.483121597	0.805393068	24	3.461874147	0.138507689
ENSMUSG00000111447	Gm48249	predicted gene, 48249 [Source:MGI Symbol;Acc:MGI:6097661]	0.368409	1.00224	1.13531	0.958314	0.934104	1.0671	0.790848	0.499669	0.409239	0.737364	1.03051	0.765042	0.509709	0.597732	0.522551072	0.966018836	24	4	24	0.154307851	0.760257003	24	15.07670219	0.149890502
ENSMUSG00000111462	Gm49317	predicted gene, 49317 [Source:MGI Symbol;Acc:MGI:6121493]	0.953375	1.13852	0.664238	0.918281	1.31594	0.832195	1.13549	0.897089	1.48601	0.706249	0.773053	1.25592	0.704536	1.13006	0.7866493	0.966018836	20	20	24	0.935231116	0.983765461	24	1.55955427	0.026726533
ENSMUSG00000111474	Gm32926	predicted gene, 32926 [Source:MGI Symbol;Acc:MGI:5592085]	0.471877	0.639247	0.810861	0.858911	0.589207	0.59648	0.629888	1.05565	0.753854	0.673311	0.433791	0.851053	0.759694	0.852882	0.156791576	0.61244839	8	12	24	0.110365005	0.721828007	24	6.497568555	0.11023269
ENSMUSG00000111496	Gm47483	predicted gene, 47483 [Source:MGI Symbol;Acc:MGI:6096458]	1.53998	2.15532	1.90862	2.76114	2.23676	1.79417	2.14343	1.69086	2.86916	1.97763	2.17029	1.90186	1.83258	2.32974	0.128916799	0.560209134	12	16	24	0.074742263	0.683760931	24	10.61272675	0.143620817
ENSMUSG00000111497	Gm38431	predicted gene, 38431 [Source:MGI Symbol;Acc:MGI:5621316]	0.884707	0.458417	0.687483	0.54653	0.3432	0.231954	0.885592	0.442171	0.706672	0.456814	0.47505	0.662467	0.567441	0.633598	0.337748746	0.85758369	4	16	24	0.301649032	0.768040178	24	3.326636168	0.103373614
ENSMUSG00000111511	6720432D03Rik	RIKEN cDNA 6720432D03 gene [Source:MGI Symbol;Acc:MGI:1924990]	3.35581	3.96125	3.36835	3.78808	4.09414	2.89989	3.65279	3.74283	5.52585	2.96763	4.2584	2.50282	3.95584	3.35694	0.195343269	0.673387182	20	4	24	0.369475767	0.792235171	24	8.288258948	0.12502375
ENSMUSG00000111521	Gm48529	predicted gene, 48529 [Source:MGI Symbol;Acc:MGI:6098068]	0.83813	1.0933	1.22017	0.933917	0.981895	0.849484	0.715312	1.06698	0.886174	0.882452	0.927295	1.01045	1.00424	1.05781	0.287979583	0.793763484	8	20	24	0.284546888	0.768040178	24	7.409866564	0.059977308
ENSMUSG00000111547	Gm48069	predicted gene, 48069 [Source:MGI Symbol;Acc:MGI:6097398]	0.439194	0.578609	0.359238	0.329325	0.616732	0.509455	0.403311	0.431	0.589302	0.416357	0.698655	0.661783	0.723986	0.42363	0.187699199	0.662239322	20	20	24	0.260319354	0.768040178	24	19.56925482	0.073970909

ENSMUSG00000111548	Gm8162	predicted gene 8162 [Source:MGI Symbol;Acc:MGI:3644710]	1.58901	2.0559	1.89926	0.906387	1.53745	1.39005	1.93509	1.27586	1.78637	1.20778	2.99196	1.72992	2.44214	1.55621	0.305564675	0.811201458	4	12	24	0.60837335	0.864352842	24	22.78219499	0.105495495
ENSMUSG00000111554	Gm232	predicted gene 232 [Source:MGI Symbol;Acc:MGI:2685078]	1.02441	1.15234	0.809238	0.895876	0.846074	0.877676	0.706961	0.876258	0.882392	0.829289	0.99292	0.880916	1.00913	0.831391	0.896311169	0.966018836	4	8	24	0.798736671	0.935650162	24	23.97681384	0.021978845
ENSMUSG00000111577	Gm47136	predicted gene, 47136 [Source:MGI Symbol;Acc:MGI:6095893]	2.4913	2.31012	3.60395	4.06439	2.65009	3.1684	2.6877	2.82093	4.69476	1.46468	2.53856	2.14456	2.69817	2.54354	0.759899911	0.966018836	12	12	24	0.559261294	0.840399223	24	8.368269669	0.129151391
ENSMUSG00000111594	Gm3365	predicted gene 3365 [Source:MGI Symbol;Acc:MGI:3781543]	1.05378	0.918965	1.11375	1.52459	1.13075	0.78901	1.10329	0.918655	0.885476	0.85514	1.07937	1.26367	0.960751	0.984055	0.888991156	0.966018836	20	12	24	0.494209371	0.806249091	24	15.18847211	0.058969752
ENSMUSG00000111656	Gm47232	predicted gene, 47232 [Source:MGI Symbol;Acc:MGI:6096048]	1.30137	0.804802	1.29187	1.16659	0.972689	0.801801	0.414005	1.21479	1.04272	0.934647	1.08821	1.11962	0.867526	1.35512	0.879901313	0.966018836	8	20	24	0.429598951	0.805393068	24	9.294054348	0.102719442
ENSMUSG00000111662	Gm7435	predicted gene 7435 [Source:MGI Symbol;Acc:MGI:3646489]	0.832129	0.851818	1.40404	0.80343	0.72042	0.655703	0.537812	0.9126	1.29967	1.2541	0.868945	0.682033	0.771054	0.783216	2.86E-04	0.049770315	12	12	24	0.003405416	0.417612737	24	9.169819664	0.210814224
ENSMUSG00000111669	Gm31698	predicted gene, 31698 [Source:MGI Symbol;Acc:MGI:5590857]	0.989591	0.445584	0.542621	0.754857	0.826667	0.396391	0.534637	0.902149	0.677873	0.653721	0.340368	0.607307	0.592688	0.523583	0.842838536	0.966018836	16	4	24	0.909156585	0.974606327	24	5.804276031	0.028791007
ENSMUSG00000111681	Gm47640	predicted gene, 47640 [Source:MGI Symbol;Acc:MGI:6096715]	0.553149	1.00049	0.950635	0.890338	0.745666	0.756889	0.747489	0.772166	1.11642	0.922845	0.714084	0.729689	0.792244	0.839704	0.004815369	0.141782417	12	8	24	0.004454844	0.427431763	24	8.662713401	0.124767728
ENSMUSG00000111686	Rab7-ps1	RAB7, member RAS oncogene family, pseudogene 1 [Source:MGI Symbol;Acc:MGI:1276565]	77.6643	62.0179	62.4531	67.3787	72.6551	74.6004	69.3409	68.5281	58.2149	76.2861	66.1485	66.2369	66.3608	62.2456	0.152177471	0.600454138	24	12	24	0.065063195	0.663876076	24	18.86533025	0.103068951
ENSMUSG00000111722	Gm47270	predicted gene, 47270 [Source:MGI Symbol;Acc:MGI:6096107]	0.760675	0.643799	0.895799	0.362609	0.378558	0.681811	0.772714	0.283304	0.475678	0.460769	0.521996	0.526525	0.972602	0.475346	0.018810871	0.256345714	4	12	24	0.201023489	0.768040178	24	23.8255929	0.125368656
ENSMUSG00000111737	Gm47248	predicted gene, 47248 [Source:MGI Symbol;Acc:MGI:6096073]	2.95829	3.10732	2.87104	2.11456	2.83948	1.96341	2.00238	3.03549	3.01487	2.17352	2.82336	2.11128	1.977	3.34598	8.66E-04	0.071344007	8	16	24	0.145918649	0.754579257	24	6.588388639	0.154341885
ENSMUSG00000111761	9230112J17Rik	RIKEN cDNA 9230112J17 gene [Source:MGI Symbol;Acc:MGI:1924999]	1.04778	1.42403	1.26209	1.53418	1.10342	1.35994	1.01721	1.5201	0.928456	1.26459	1.04656	1.27939	1.20819	1.08547	0.92769718	0.966018836	16	12	24	0.939524997	0.985218048	24	9.501737571	0.017857039
ENSMUSG00000111793	Gm47950	predicted gene, 47950 [Source:MGI Symbol;Acc:MGI:6097220]	1.57735	2.31514	1.43834	2.08081	1.61998	1.64198	1.58796	1.33022	1.66866	1.62062	1.61492	1.73598	1.98248	1.25104	0.949382029	0.966018836	16	16	24	0.835824965	0.949722382	24	17.52365893	0.035600738
ENSMUSG00000111828	D830035M03Rik	RIKEN cDNA D830035M03 gene [Source:MGI Symbol;Acc:MGI:2686558]	0.226219	0.482938	0.492051	0.519312	0.508325	0.719621	0.552126	0.486352	0.339989	0.657579	0.591399	0.518995	0.481999	0.540041	0.248787753	0.747781955	24	4	24	0.304928784	0.768040178	24	15.4889611	0.066004241
ENSMUSG00000111837	Gm47135	predicted gene, 47135 [Source:MGI Symbol;Acc:MGI:6095892]	64.4678	61.9116	47.2274	56.5601	74.8351	55.7366	57.3728	53.9215	64.0512	52.1541	71.9986	53.451	64.8067	54.4298	0.519468828	0.966018836	20	20	24	0.388446551	0.799546792	24	18.43364876	0.101989827
ENSMUSG00000111847	Gm8899	predicted gene 8899 [Source:MGI Symbol;Acc:MGI:3646245]	0.441158	0.517785	0.406095	0.624441	0.585845	0.405691	0.538737	0.882757	0.672892	0.403488	0.444281	0.669353	0.40624	0.504703	0.873567285	0.966018836	8	16	24	0.74451903	0.916498249	24	6.465939373	0.040379574
ENSMUSG00000111864	Rpl26-ps6	ribosomal protein L26, pseudogene 6 [Source:MGI Symbol;Acc:MGI:3648682]	2.22351	1.19898	1.28633	1.50447	2.07066	1.2394	1.97801	0.65611	1.00498	1.07859	1.70974	1.43737	1.79537	1.25176	0.050447964	0.385880335	4	4	24	0.060224774	0.658304792	24	19.59709415	0.233755287
ENSMUSG00000111875	Gm5425	predicted gene 5425 [Source:MGI Symbol;Acc:MGI:3643901]	22.2326	24.7564	26.4767	20.9863	28.2671	27.717	21.0635	24.0337	25.2961	17.4839	25.6106	20.1227	20.9542	18.4636	0.843639218	0.966018836	20	8	24	0.727124318	0.91035631	24	16.97310069	0.059037563
ENSMUSG00000111877	Gm6477	predicted gene 6477 [Source:MGI Symbol;Acc:MGI:3647945]	13.252	13.9616	12.0277	10.7589	12.705	11.4342	10.8213	11.8412	12.3373	10.9466	12.09	9.69885	11.0537	11.9846	0.636884864	0.966018836	12	12	24	0.424731327	0.805393068	24	5.229973569	0.055510793
ENSMUSG00000111897	Gm19810	predicted gene, 19810 [Source:MGI Symbol;Acc:MGI:5011995]	54.3221	48.0168	51.4255	61.2882	56.7549	39.5039	42.8505	44.3767	50.7473	28.3195	43.2834	27.4924	43.4859	33.4976	0.496745696	0.966018836	12	12	24	0.802286679	0.936565832	24	9.072349868	0.069714576
ENSMUSG00000111905	Gm48045	predicted gene, 48045 [Source:MGI Symbol;Acc:MGI:6097362]	0.525827	0.622551	0.490887	0.544782	0.739998	0.53894	0.411788	0.706271	0.64957	0.798023	0.744746	0.840281	0.618645	0.668959	0.337748746	0.85758369	20	8	24	0.270449687	0.768040178	24	13.86670573	0.056881549
ENSMUSG00000111932	Gm18899	predicted gene, 18899 [Source:MGI Symbol;Acc:MGI:5011084]	8.77266	9.26073	9.5261	10.0832	8.27905	9.44545	10.2864	9.66151	12.6418	13.6024	11.687	15.1012	14.2031	15.134	0.759203569	0.966018836	16	4	24	0.552411632	0.836695859	24	7.221071736	0.057073473
ENSMUSG00000111938	2900045O20Rik	RIKEN cDNA 2900045O20 gene [Source:MGI Symbol;Acc:MGI:1920205]	0.491996	0.482555	0.611679	0.404074	0.553808	0.600039	0.472038	0.441256	0.504555	0.580347	0.508432	0.581465	0.350622	0.758116	0.9045879	0.966018836	24	4	24	0.880094629	0.966366217	24	10.86054662	0.0194112
ENSMUSG00000111942	Gm5182	predicted gene 5182 [Source:MGI Symbol;Acc:MGI:3649174]	15.1726	11.6242	14.18	21.3574	15.2958	15.0791	16.1122	15.467	11.8952	16.6246	18.6554	17.3525	20.5534	17.0477	0.503972924	0.966018836	16	20	24	0.307474092	0.768040178	24	17.32756736	0.128893617
ENSMUSG00000111966	Gm5781	predicted gene 5781 [Source:MGI Symbol;Acc:MGI:3646721]	1.2907	1.08775	1.4002	0.971085	1.89941	1.12006	1.39209	1.65123	1.06985	1.23317	1.36303	0.833843	0.964528	0.659745	0.879844076	0.966018836	20	4	24	0.625846201	0.872406749	24	15.741748	0.072260436
ENSMUSG00000111977	Gm47163	predicted gene, 47163 [Source:MGI Symbol;Acc:MGI:6095939]	9.07749	7.31735	8.32674	7.93143	7.08095	7.91303	7.02824	7.24958	6.48116	10.5794	7.33541	8.83886	7.97881	9.57507	0.822716578	0.966018836	16	4	24	0.985079533	0.996971113	24	12.65011843	0.011982354
ENSMUSG00000111990	Gm47033	predicted gene, 47033 [Source:MGI Symbol;Acc:MGI:6095730]	0.36206	0.66645	0.700486	0.463797	0.635745	0.619973	0.51627	0.307233	0.660865	0.876763	0.486038	0.485384	0.454176	0.715162	0.387380622	0.915479136	12	16	24	0.195103715	0.768040178	24	10.66680866	0.099219549
ENSMUSG00000111995	Gm48697	predicted gene, 48697 [Source:MGI Symbol;Acc:MGI:6098331]	4.91689	6.25925	6.05244	6.76511	6.93594	4.66329	6.63082	5.48657	7.90801	5.56073	6.3606	4.19694	4.49725	4.58392	0.112583132	0.530777648	20	4	24	0.069579917	0.673820536	24	10.72142698	0.187071328
ENSMUSG00000111997	Gm5176	predicted gene 5176 [Source:MGI Symbol;Acc:MGI:3644450]	0.896402	0.431213	0.324714	0.540559	1.04582	0.525845	0.637476	0.426695	0.879358	0.686934	0.60385	0.425774	0.7011	0.339756	0.665691261	0.966018836	20	12	24	0.408292017	0.799546792	24	17.15609818	0.101623814
ENSMUSG00000112000	Gm47956	predicted gene, 47956 [Source:MGI Symbol;Acc:MGI:6097228]	0.552165	0.565808	0.678149	0.831143	0.804795	0.548973	0.80154	0.329365	0.770231	0.53149	0.576585	0.770465	0.460073	0.455883	0.16942967	0.641781289	20	12	24	0.198911462	0.768040178	24	15.03761196	0.0920539
ENSMUSG00000112008	Gm47218	predicted gene, 47218 [Source:MGI Symbol;Acc:MGI:6096027]	1.09819	1.36141	1.56804	1.22343	1.08181	1.19166	1.13152	1.20051	1.31401	1.29215	1.27688	1.13552	1.25536	1.42928	0.025723486	0.297892358	12	12	24	0.013808812	0.529470441	24	7.702842468	0.08775897
ENSMUSG00000112023	Lilr4b	leukocyte immunoglobulin-like receptor, subfamily B, member 4B [Source:MGI Symbol;Acc:MGI:102702]	1.34847	2.38831	2.77789	1.38324	1.39628	1.74525	1.17936	1.85945	1.74673	0.886824	1.0164	1.2909	1.44107	1.11065	0.369262716	0.893596186	12	4	24	0.177077564	0.768040178	24	5.286001456	0.167221793
ENSMUSG00000112058	Gm47593	predicted gene, 47593 [Source:MGI Symbol;Acc:MGI:6096640]	2.69186	1.20783	2.22431	0.842448	1.89313	2.18385	1.79572	1.59544	2.22178	1.35845	1.81781	1.77201	2.24503	2.16574	0.305564675	0.811201458	4	12	24	0.2779598	0.768040178	24	23.63601885	0.162398962
ENSMUSG00000112060	Gm32220	predicted gene, 32220 [Source:MGI Symbol;Acc:MGI:5591379]	3.57198	3.58652	5.15159	2.63438	3.64069	3.24878	2.87276	2.60177	3.80663	2.76003	3.18196	2.34651	3.65125	0.471665	0.945451136	0.966018836	12	12	24	0.661025442	0.885520687	24	15.84150453	0.14027756
ENSMUSG00000112068	Gm9182	predicted gene 9182 [Source:MGI Symbol;Acc:MGI:3644174]	1.006	1.02061	1.82165	1.14678	1.49626	1.22914	1.42891	1.42029	1.42749	0.997415	1.43413	1.23556	1.12088	1.45441	0.846840613	0.966018836	12	16	24	0.669309926	0.888624758	24	9.210473265	0.054118236
ENSMUSG00000112071	5430401H09Rik	RIKEN cDNA 5430401H09 gene [Source:MGI Symbol;Acc:MGI:1918646]	0.347998	0.665878	0.821821	1.51686	0.317599	0.759957	0.643083	0.443204	0.516284	0.329935	0.359203	0.425953	0.754738	0.303841	0.874672901	0.966018836	12	8	24	0.854391301	0.956491441	24	10.1754357	0.057369207
ENSMUSG00000112080	Gm47595	predicted gene, 47595 [Source:MGI Symbol;Acc:MGI:6096644]	1.58177	0.709796	0.762089	0.592129	1.02905	1.07277	0.425842	1.24121	1.18952	0.697747	1.66389	0.34202	0.948552	0.543376	0.853187563	0.966018836	20	20	24	0.921377652	0.979266762	24	17.24935401	0.04981429
ENSMUSG00000112096	A430103D13Rik	RIKEN cDNA A430103D13 gene [Source:MGI Symbol;Acc:MGI:1925025]	2.63672	2.99906	3.92614	3.21677	2.57023	2.95625	3.63473	3.1789	3.87302	2.99207	2.99196	3.22012	3.17176	3.90477	0.036424767	0.332409444	12	8	24	0.029765589	0.596648309	24	6.505409131	0.136189407
ENSMUSG00000112099	Gm8960	predicted gene 8960 [Source:MGI Symbol;Acc:MGI:3642929]	3.12111	3.91376	4.08518	4.44498	4.23158	4.39886	3.0281	3.35903	3.43268	2.18872	2.40395	2.27918	2.93201	2.77064	0.801995186	0.966018836	12	12	24	0.891572643	0.969326231	24	9.074386789	0.038851743
ENSMUSG00000112105	Rps7-ps2	ribosomal protein S7, pseudogene 2 [Source:MGI Symbol;Acc:MGI:3646014]	1.27314	1.53363	1.05701	1.73403	2.01197	1.77392	1.43203	1.83331	1.60362	1.85898	1.43141	1.42818	1.73791	1.39502	0.745199643	0.966018836	16	20	24	0.499678246	0.808791684	24	15.39986349	0.068083808
ENSMUSG00000112110	Gm15608	predicted gene 15608 [Source:MGI Symbol;Acc:MGI:3801804]	2.82213	2.24284	2.88068	2.28952	2.68901	2.54648	2.73563	2.98144	2.21851	2.11855	2.54795	2.39344	2.83328	3.37121	0.077161185	0.453790923	8	8	24	0.146622256	0.755260456	24	1.594252993	0.098598629
ENSMUSG00000112112	Gm48508	predicted gene, 48508 [Source:MGI Symbol;Acc:MGI:6098039]	1.06035	1.08779	0.909275	1.0936	1.45191	0.770888	0.923123	1.06871	1.35304	0.753304	1.30957	0.948271	1.04517	0.891506	0.893307667	0.966018836	20	4	24	0.718737614	0.907557206	24	13.32698144	0.046261166
ENSMUSG00000112117	Rmst	rhabdomyosarcoma 2 associated transcript (non-coding RNA) [Source:MGI Symbol;Acc:MGI:1099806]	0.688097	1.77761	3.84893	1.86746	1.53643	2.11815	3.97433	1.88679	2.16095	1.12256	1.56088	0.937088	0.46577	1.33553	0.643325306	0.966018836	12	16	24	0.546058964	0.833198402	24	8.575277671	0.212889199
ENSMUSG00000112140	Gm5780	predicted gene 5780 [Source:MGI Symbol;Acc:MGI:3645288]	1.94436	2.24433	1.72877	1.91237	2.10416	2.31276	2.20207	2.51287	2.12558	1.73835	1.99618	1.76264	1.95336	1.89882	0.773177465	0.966018836	8	8	24	0.409980894	0.799546792	24	0.727235167	0.055399858
ENSMUSG00000112143	Gm35721	predicted gene, 35721 [Source:MGI Symbol;Acc:MGI:5594880]	1.0914	1.11091	0.978114	1.23331	1.07872	1.36772	0.941525	0.99968	1.01061	1.25789	1.15219	0.97863	1.40716	1.28443	0.874607729	0.966018836	16	20	24	0.846014058	0.953396028	24	17.49639106	0.023242858
ENSMUSG00000112146	Gm46210	predicted gene, 46210 [Source:MGI Symbol;Acc:MGI:5825847]	0.48265	0.63613	0.209684	0.658769	0.501337	0.434443	1.0569	0.343781	0.394102	0.255258	0.263753	0.61967	0.969255	0.365375	0.105280783	0.518002936	4	8	24	0.107376687	0.71991576	24	22.78498493	0.183685658
ENSMUSG00000112148	Lilrb4a	leukocyte immunoglobulin-like receptor, subfamily B, member 4A [Source:MGI Symbol;Acc:MGI:102701]	3.40044	6.69774	7.78763	5.30572	5.51808	5.58944	5.20928	6.3906	7.35629	3.68918	5.26143	4.65215	5.2332	5.11058	0.170659409	0.643496587	12	16	24	0.228796239	0.768040178	24	7.37671578	0.188999517
ENSMUSG00000112160	BC024063	cDNA sequence BC024063 [Source:MGI Symbol;Acc:MGI:2670978]	0.787653	1.02214	1.10944	0.855377	1.01202	0.92643	0.925198	1.05519	1.12096	0.652753	1.05628	1.09771	1.07642	0.902556	0.915475528	0.966018836	12	4	24	0.838640555	0.950732077	24	2.570689838	0.024438628
ENSMUSG00000112163	Gm8188	predicted gene 8188 [Source:MGI Symbol;Acc:MGI:3646272]	1.32369	1.43947	1.10142	1.37503	1.48279	1.4798	1.87095	1.2817	1.5449	1.2857	1.36072	1.5011	1.34527	0.982107	0.476029854	0.966018836	24	8	24	0.235862578	0.768040178	24	19.69485612	0.08457481
ENSMUSG00000112168	Gm34776	predicted gene, 34776 [Source:MGI Symbol;Acc:MGI:5593935]	0.0406364	0.743774	1.1934	0.513465	0.243706	0.670013	0.644857	0.473481	0.694265	0.348983	0.363668	0.698769	0.108178	0.726622	0.210418232	0.69936851	12	8	24	0.337038385	0.771960538	24	7.734361524	0.172274565
ENSMUSG00000112218	Gm18726	predicted gene, 18726 [Source:MGI Symbol;Acc:MGI:5010911]	2.93817	3.32904	3.22078	3.18883	2.87206	3.14521	3.43353	3.0248	3.11179	3.44191	3.31602	3.63699	3.40448	3.33729	0.886572916	0.966018836	24	12	24	0.946284178	0.986887534	24	23.78811863	0.008158268
ENSMUSG00000112233	Gm48698	predicted gene, 48698 [Source:MGI Symbol;Acc:MGI:6098333]	1.0875	1.27058	0.391706	1.59419	0.517651	0.785623	3.9299	0.946558	2.67857	0.24014	1.20493	1.19687	0.744387	0.870471	0.826055781	0.966018836	4	20	24	0.633252225	0.875353057	24	1.336412883	0.198711123
ENSMUSG00000112237	D630033A02Rik	RIKEN cDNA D630033A02 gene [Source:MGI Symbol;Acc:MGI:3028060]	3.012	2.54873	2.54636	2.21931	1.89742	1.81754	2.85507	2.27251	3.69752	2.56544	2.89225	1.96823	2.32271	2.61268	0.44861185	0.966018836	4	20	24	0.192136748	0.768040178	24	6.516472315	0.143190731
ENSMUSG00000112239	Gm17823	predicted gene, 17823 [Source:MGI Symbol;Acc:MGI:5010008]	5.84437	5.03049	6.30462	6.32716	5.29355	5.48663	5.52105	5.82508	4.53927	4.57736	5.31634	5.07763	4.83383	4.04575	0.79625133	0.966018836	4	4	24	0.588040792	0.853859503	24	17.58907283	0.046904032
ENSMUSG00000112269	Gm6906	predicted gene 6906 [Source:MGI Symbol;Acc:MGI:3644963]	3.8397	3.14389	2.99393	2.4628	3.65106	2.93449	3.83232	4.03136	3.14819	2.21298	3.56344	2.59197	3.03221	2.01343	0.47028201	0.966018836	4	12	24	0.324436053	0.768040178	24	22.3794666	0.123865775
ENSMUSG00000112278	Gm30025	predicted gene, 30025 [Source:MGI Symbol;Acc:MGI:5589184]	0.634653	0.393755	0.61313	0.483447	0.735991	0.558121	0.687379	0.881693	0.625029	1.02525	1.06354	0.902032	0.659074	0.847816	0.941830768	0.966018836	20	16	24	0.420483575	0.804069238	24	15.11295829	0.063028684
ENSMUSG00000112280	A830082N09Rik	RIKEN cDNA A830082N09 gene [Source:MGI Symbol;Acc:MGI:3041184]	5.46351	5.49144	4.85022	5.44974	4.90355	4.2346	5.75414	4.7092	5.94629	5.67474	4.63341	5.37948	5.37035	5.57563	0.399942636	0.932395417	16	4	24	0.32746328	0.768040178	24	6.036651082	0.069624205
ENSMUSG00000112288	Gm47879	predicted gene, 47879 [Source:MGI Symbol;Acc:MGI:6097104]	0.541895	0.586238	0.12789	0.491853	0.363201	0.171828	0.384343	0.714062	1.14507	0.627823	0.921049	0.211754	0.681782	0.496351	0.649212555	0.966018836	8	16	24	0.407295057	0.799546792	24	7.963451181	0.138780892
ENSMUSG00000112300	Gm47976	predicted gene, 47976 [Source:MGI Symbol;Acc:MGI:6097260]	2.12285	1.86808	2.02882	1.8823	1.93981	1.89629	2.20775	1.39042	2.37436	1.66743	2.01772	1.93336	1.77334	1.81057	0.965971228	0.969272194	12	20	24	0.877637439	0.965887029	24	17.82271954	0.024839508
ENSMUSG00000112302	Gm48226	predicted gene, 48226 [Source:MGI Symbol;Acc:MGI:6097627]	1.12547	1.24319	1.28635	1.46806	1.13999	1.23187	1.30526	1.4528	1.38481	1.40868	1.43492	1.25039	1.13202	1.27221	0.059513104	0.406067671	16	12	24	0.046965401	0.633507364	24	10.29134182	0.066958951
ENSMUSG00000112308	Gm48512	predicted gene, 48512 [Source:MGI Symbol;Acc:MGI:6098046]	4.5206	6.56288	2.07907	6.60901	8.93431	2.51956	3.15329	0.14864	4.59517	0.809556	7.32945	3.62761	6.52073	3.2648	0.784278077	0.966018836	20	12	24	0.456956248	0.805393068	24	17.91295746	0.44172481
ENSMUSG00000112329	Gm47908	predicted gene, 47908 [Source:MGI Symbol;Acc:MGI:6097152]	0.41328	2.39566	3.13411	3.00383	1.95678	2.1684	2.00015	5.82084	4.74714	4.23988	2.50657	1.61429	1.95713	1.60851	0.016795343	0.253395174	12	16	24	0.023938806	0.585506055	24	9.503379053	0.546829357
ENSMUSG00000112332	4930466K18Rik	RIKEN cDNA 4930466K18 gene [Source:MGI Symbol;Acc:MGI:1922219]	4.0672	4.89422	4.30601	4.17063	4.48251	3.33873	3.52484	4.07077	4.27468	3.88949	4.00843	3.2438	3.95866	3.84175	0.021795836	0.26698364	12	12	24	0.068564974	0.673034395	24	8.320809446	0.098548933
ENSMUSG00000112346	Gm48768	predicted gene, 48768 [Source:MGI Symbol;Acc:MGI:6098457]	1.00678	0.819755	0.83509	0.773499	0.642727	0.68394	0.901679	0.81059	0.973815	0.767033	0.867142	0.797227	0.713044	0.967494	0.230022832	0.72997941	12	4	24	0.150683054	0.758865539	24	4.699535534	0.063825844
ENSMUSG00000112375	Gm38560	predicted gene, 38560 [Source:MGI Symbol;Acc:MGI:5621445]	0.991277	1.04765	0.718017	1.50699	0.67591	0.792095	0.201158	0.810359	0.844036	0.709803	0.791497	1.0585	0.938689	0.62288	0.794347041	0.966018836	24	20	24	0.709820866	0.9039575	24	12.6936629	0.076100754
ENSMUSG00000112390	Gm20208	predicted gene, 20208 [Source:MGI Symbol;Acc:MGI:5012393]	0.493777	0.700676	0.252821	0.973797	0.879119	1.16605	0.738501	0.95513	0.947638	1.02236	1.03955	0.906137	0.847603	0.962822	0.226403091	0.722427815	24	4	24	0.313434787	0.768040178	24	16.19216543	0.101154993
ENSMUSG00000112400	Gm47725	predicted gene, 47725 [Source:MGI Symbol;Acc:MGI:6096856]	0.629607	0.348901	0.435606	0.282217	0.59886	0.616354	0.558382	[illegible]	0.46079	0.567292	0.432425	0.694238	0.64869	0.599849	0.016110955	0.253395174	24	12	24	0.019849338	0.565108098	24	21.40343963	0.115605204
ENSMUSG00000112404	Gm32442	predicted gene, 32442 [Source:MGI Symbol;Acc:MGI:5591601]	0.82198	1.13615	0.931765	1.0158	0.875669	0.909048	0.42759	0.677272	0.702173	1.06148	0.869744	1.12873	0.877133	1.23988	0.953350544	0.966018836	16	12	24	0.677102695	0.891969863	24	11.80088931	0.05889786
ENSMUSG00000112433	Gm30122	predicted gene, 30122 [Source:MGI Symbol;Acc:MGI:5589281]	2.13449	1.38997	1.84958	2.30549	2.15707	2.01967	2.03181	2.06916	2.08429	1.66822	1.95829	1.71895	1.74034	1.7854	0.907068194	0.966018836	20	12	24	0.547613198	0.833754491	24	15.78040069	0.052329778
ENSMUSG00000112466	Gm29674	predicted gene, 29674 [Source:MGI Symbol;Acc:MGI:5588833]	2.82652	2.64352	1.62532	1.93818	2.37294	2.47989	1.40992	2.58322	2.26136	2.06789	2.73373	2.19525	3.11782	2.37175	0.300218476	0.809362476	4	12	24	0.58211656	0.851114094	24	22.41004094	0.087929212
ENSMUSG00000112515	Gm4928	predicted gene 4928 [Source:MGI Symbol;Acc:MGI:3644549]	12.1607	13.9427	14.8581	15.1198	13.9335	13.7827	15.1112	13.0523	14.8994	11.5172	15.3109	12.3852	12.8566	13.2239	0.595037318	0.966018836	20	8	24	0.668186172	0.887912848	24	11.18672228	0.043441401
ENSMUSG00000112527	Gm35696	predicted gene, 35696 [Source:MGI Symbol;Acc:MGI:5594855]	0.528487	0.308233	0.167653	0.64186	0.632656	0.391986	1.04103	0.136752	0.516698	0.860661	0.701662	0.606902	0.406436	0.242834	0.01579111	0.253395174	16	16	24	0.100620172	0.711862888	24	16.83344194	0.196945481
ENSMUSG00000112546	Gm6992	predicted gene 6992 [Source:MGI Symbol;Acc:MGI:3647975]	0.76864	0.57263	0.65712	0.948217	0.701412	0.751048	0.402439	0.640718	0.507042	0.652737	0.473317	0.296659	0.346645	0.611284	0.833406028	0.966018836	16	12	24	0.236602789	0.768040178	24	11.38783781	0.083945531
ENSMUSG00000112550	Gm6627	predicted gene 6627 [Source:MGI Symbol;Acc:MGI:3644486]	4.27472	4.37068	4.38098	4.58111	4.62627	4.63791	4.76818	4.37008	5.37746	4.45816	4.44056	4.05922	4.37557	4.43004	0.537028703	0.966018836	20	4	24	0.459138031	0.805393068	24	9.700108045	0.038938798
ENSMUSG00000112596	Gm48804	predicted gene, 48804 [Source:MGI Symbol;Acc:MGI:6098514]	0.673845	0.735131	0.883081	0.74602	0.880702	0.628725	0.538075	0.680049	0.894618	0.644826	0.921733	0.721535	1.0495	0.894773	0.954584434	0.966018836	12	12	24	0.589440832	0.854256	24	9.661130397	0.045831188
ENSMUSG00000112627	4933412E12Rik	RIKEN cDNA 4933412E12 gene [Source:MGI Symbol;Acc:MGI:1918336]	0.852579	1.226	1.20106	0.712672	1.34976	1.20517	1.13564	1.08956	0.959761	1.07991	0.92951	1.28368	1.15183	1.17851	0.942429894	0.966018836	24	16	24	0.60122121	0.860439692	24	22.8868894	0.050391068
ENSMUSG00000112639	A730063M14Rik	RIKEN cDNA A730063M14 gene [Source:MGI Symbol;Acc:MGI:2443898]	2.39707	4.04259	3.15172	2.30894	2.30913	3.22595	1.68005	2.80602	4.50226	3.29184	3.33234	3.03252	2.633	2.34776	0.643325306	0.966018836	12	16	24	0.38364889	0.799546792	24	9.604746418	0.151588173
ENSMUSG00000112640	Gm32687	predicted gene, 32687 [Source:MGI Symbol;Acc:MGI:5591846]	0.768311	1.01867	0.855633	1.00978	0.874359	0.765221	0.69892	0.70656	0.978583	0.660806	0.900787	1.02086	0.972788	1.03167	0.854540463	0.966018836	8	20	24	0.878073168	0.965978948	24	8.117929225	0.02210875
ENSMUSG00000112673	Gm7413	predicted gene 7413 [Source:MGI Symbol;Acc:MGI:3644069]	0.402563	0.746741	0.43681	0.606647	1.18671	0.524846	1.14029	0.594828	0.364239	0.443743	0.729635	0.865852	0.901673	0.615822	0.187179226	0.662239322	20	16	24	0.182949576	0.768040178	24	20.23198056	0.155727699
ENSMUSG00000112693	Gm5512	predicted gene 5512 [Source:MGI Symbol;Acc:MGI:3645436]	8.89819	7.59901	7.61816	8.89894	9.2492	8.34499	9.19453	7.8815	7.74267	7.22625	9.07238	7.66127	9.36684	7.32296	0.152236561	0.600454138	4	4	24	0.087040721	0.706216588	24	19.48191942	0.102386388
ENSMUSG00000112714	Gm4798	predicted pseudogene 4798 [Source:MGI Symbol;Acc:MGI:3644096]	5.41845	2.90143	3.08834	3.17972	4.88787	5.00188	4.28739	4.2164	2.40085	4.95919	3.84466	3.70601	3.37765	3.5606	0.118761453	0.542330458	24	12	24	0.072149959	0.678728491	24	19.47312359	0.233575986
ENSMUSG00000112739	Gm20597	predicted gene, 20597 [Source:MGI Symbol;Acc:MGI:5295703]	0.38632	0.50703	0.420564	0.506489	0.532383	0.518595	0.572494	0.681892	0.503931	0.510196	0.375088	0.541573	0.615914	0.4935	0.742579234	0.966018836	4	8	24	0.504586034	0.811111334	24	2.114163492	0.03416004
ENSMUSG00000112742	Gm31793	predicted gene, 31793 [Source:MGI Symbol;Acc:MGI:5590952]	2.39322	3.14373	1.73018	1.97569	1.99034	2.0416	1.54749	2.68039	2.05372	1.26661	2.37818	1.44602	2.90177	2.33383	0.048396595	0.379129806	8	8	24	0.208748106	0.768040178	24	2.471852549	0.167444446
ENSMUSG00000112748	Gm6331	predicted gene 6331 [Source:MGI Symbol;Acc:MGI:3643973]	1.81832	1.48552	1.66924	1.89528	1.85363	2.10806	2.37645	1.98285	1.89759	1.75775	1.80496	2.34831	1.59866	1.07144	0.43565473	0.966018836	24	8	24	0.142910821	0.752803875	24	18.5653457	0.134274516
ENSMUSG00000112795	Gm2027	predicted gene 2027 [Source:MGI Symbol;Acc:MGI:3780196]	0.83513	0.67087	0.647234	0.716075	0.365774	0.633893	0.297293	0.542012	0.46243	0.885688	0.466218	0.756219	0.571459	0.622077	0.890648739	0.966018836	16	4	24	0.928196875	0.981410569	24	9.484249396	0.024959816
ENSMUSG00000112808	Gm4739	predicted gene 4739 [Source:MGI Symbol;Acc:MGI:3642984]	6.45348	11.2417	15.96	13.3555	8.77585	10.9633	9.09693	10.2961	11.971	8.75585	8.59827	11.3196	13.3417	8.52135	0.774195442	0.966018836	12	8	24	0.465976783	0.805393068	24	8.750295752	0.157287514
ENSMUSG00000112825	Gm9118	predicted gene 9118 [Source:MGI Symbol;Acc:MGI:3648185]	32.6841	37.6356	34.3535	35.2091	35.6907	32.0593	32.1119	34.4388	39.6619	31.5299	38.2694	35.543	39.4425	35.6177	0.912686312	0.966018836	12	12	24	0.712918302	0.905403147	24	7.696961785	0.034894536
ENSMUSG00000112829	Gm29696	predicted gene, 29696 [Source:MGI Symbol;Acc:MGI:5588855]	4.22405	4.90619	3.6045	5.00797	6.63932	6.46503	6.26543	5.79048	3.37059	5.61022	2.73996	4.41821	3.86147	4.73511	0.915835339	0.966018836	24	12	24	0.684228148	0.895093979	24	20.82078146	0.110364205
ENSMUSG00000112847	Gm47573	predicted gene, 47573 [Source:MGI Symbol;Acc:MGI:6096604]	0.683435	0.721282	0.562412	0.769183	0.589951	0.628368	0.978127	0.52441	0.821723	0.440336	0.771421	0.507851	0.6529	0.555083	0.891022863	0.966018836	4	12	24	0.836581986	0.949953371	24	23.55989745	0.029265694
ENSMUSG00000112850	Gm9176	predicted gene 9176 [Source:MGI Symbol;Acc:MGI:3644176]	0.12447	0.527334	1.17331	1.19129	0.404276	0.235853	0.202574	0.297462	1.38925	1.01413	0.114234	0.132135	0.138074	0.43287	0.002159991	0.102623968	12	16	24	5.85E-05	0.089877732	24	9.652829998	0.502059615
ENSMUSG00000112880	Gm20337	predicted gene, 20337 [Source:MGI Symbol;Acc:MGI:5012522]	0.415772	0.465028	0.814	0.429762	0.592098	0.354518	0.456748	0.707952	0.539817	0.576799	0.572367	0.52966	0.636483	0.712657	0.351617883	0.870418653	12	12	24	0.104061582	0.715480277	24	7.555461308	0.090693539
ENSMUSG00000112909	Gm10120	predicted gene 10120 [Source:MGI Symbol;Acc:MGI:3641745]	29.0794	32.4549	30.3776	32.5117	33.4322	36.589	29.6536	29.9834	29.0498	29.0082	30.9309	25.8303	27.4037	26.3606	0.896545154	0.966018836	20	8	24	0.203606334	0.768040178	24	15.83705114	0.070117456
ENSMUSG00000112922	Gm47572	predicted gene, 47572 [Source:MGI Symbol;Acc:MGI:6096602]	0.544414	0.711913	0.70034	0.614235	0.708671	0.495095	0.590061	0.734403	0.728786	0.52293	0.863701	0.674984	0.829252	0.695018	0.708665292	0.966018836	8	16	24	0.749557107	0.918524199	24	7.139341349	0.027554702
ENSMUSG00000112925	Gm31763	predicted gene, 31763 [Source:MGI Symbol;Acc:MGI:5590922]	0.359014	0.608548	0.551985	0.553451	0.624714	0.342392	0.861992	0.316394	0.728956	0.467561	0.696524	0.633992	0.567604	0.438793	0.903465533	0.966018836	20	12	24	0.811199442	0.939484534	24	14.17861256	0.037389912
ENSMUSG00000112941	Gm48623	predicted gene, 48623 [Source:MGI Symbol;Acc:MGI:6098220]	2.20403	1.73956	1.61278	0.920519	1.23583	1.39191	2.73487	2.37911	2.46663	3.5834	3.51439	3.3699	2.28709	3.99949	0.807813307	0.966018836	8	12	24	0.595482652	0.857839743	24	3.082356651	0.11725626
ENSMUSG00000112964	E430024I08Rik	RIKEN cDNA E430024I08 gene [Source:MGI Symbol;Acc:MGI:3649010]	6.49272	8.09485	7.64232	7.21109	7.4218	6.64789	6.56031	7.14421	8.77662	5.69891	7.08179	7.31382	6.93228	8.75433	0.084464639	0.466404237	12	16	24	0.198069644	0.768040178	24	6.399351448	0.104227431
ENSMUSG00000112969	Gm48496	predicted gene, 48496 [Source:MGI Symbol;Acc:MGI:6098022]	15.6732	11.0052	7.77499	9.26926	7.21372	5.47658	8.15561	11.0247	8.8078	8.98045	8.13392	10.3922	8.14804	5.81057	0.948582958	0.966018836	8	12	24	0.684787248	0.895095838	24	2.048559326	0.109223199
ENSMUSG00000113001	Gm40538	predicted gene, 40538 [Source:MGI Symbol;Acc:MGI:5623423]	1.44442	1.75732	1.46422	2.4097	3.35806	1.57109	2.22583	1.20033	2.35889	1.65513	2.23651	2.03986	2.90061	1.53074	0.173121348	0.648600527	20	12	24	0.241682529	0.768040178	24	16.33803037	0.180245182
ENSMUSG00000113029	Gm40578	predicted gene, 40578 [Source:MGI Symbol;Acc:MGI:5623463]	0.210082	0.912263	1.06117	0.180613	0.214306	0.508848	0.991544	0.491679	0.50202	0.635188	0.544121	0.431116	0.817153	1.06397	0.416857905	0.963884303	8	8	24	0.080587307	0.695888143	24	4.434961736	0.222754611
ENSMUSG00000113036	Gm47021	predicted gene, 47021 [Source:MGI Symbol;Acc:MGI:6095708]	15.1472	16.6199	17.9582	16.2749	14.7455	18.1583	16.8491	19.1987	15.4689	14.2262	13.1619	14.278	12.4789	14.6742	0.729086532	0.966018836	8	12	24	0.407197397	0.799546792	24	5.086197346	0.074136482
ENSMUSG00000113047	Gm47469	predicted gene, 47469 [Source:MGI Symbol;Acc:MGI:6096437]	3.28036	2.42282	2.86497	1.37079	3.09277	1.92312	1.79966	2.51455	2.67146	2.06449	3.19555	2.58736	2.13218	3.44332	0.921572192	0.966018836	12	4	24	0.884303494	0.967039336	24	3.274781759	0.052138738
ENSMUSG00000113061	Rps18-ps5	ribosomal protein S18, pseudogene 5 [Source:MGI Symbol;Acc:MGI:3649931]	65.8356	79.8143	76.4998	77.1192	75.4287	67.1751	72.8995	74.3691	107.091	64.7439	89.3477	81.9671	91.4498	80.7352	0.853736882	0.966018836	12	4	24	0.536614321	0.827423907	24	7.824697685	0.08110076
ENSMUSG00000113070	Gm48420	predicted gene, 48420 [Source:MGI Symbol;Acc:MGI:6097914]	2.793	3.65332	3.82963	3.22404	2.59724	2.98187	1.87015	2.81771	3.0275	3.29125	3.44061	3.2483	2.0709	3.00687	0.015486342	0.253395174	12	16	24	0.108317651	0.72082227	24	10.84208671	0.162570113
ENSMUSG00000113113	Gm2614	predicted gene 2614 [Source:MGI Symbol;Acc:MGI:3780782]	17.432	21.6704	21.263	22.8038	32.7975	22.0721	22.3454	24.2863	27.2401	16.7457	22.3507	18.1394	20.6779	20.3003	0.881718085	0.966018836	20	8	24	0.706377191	0.902918222	24	12.42185825	0.079313514
ENSMUSG00000113137	Gm7511	predicted gene 7511 [Source:MGI Symbol;Acc:MGI:3646392]	15.3857	16.4115	15.6301	15.3202	16.3738	14.1318	11.5427	13.756	18.5964	14.1545	15.2947	13.6383	14.576	13.0715	0.300218476	0.809362476	12	12	24	0.199462799	0.768040178	24	10.91268848	0.101210635
ENSMUSG00000113149	Gm49383	predicted gene, 49383 [Source:MGI Symbol;Acc:MGI:6121605]	2.68409	1.8605	3.17559	1.61995	2.39105	2.47327	1.58418	2.64968	1.18851	2.34695	2.25443	2.74464	2.11915	1.81744	0.956719163	0.96629412	24	12	24	0.543510755	0.831703329	24	19.40810361	0.114611107
ENSMUSG00000113165	Gm47863	predicted gene, 47863 [Source:MGI Symbol;Acc:MGI:6097080]	0.866941	0.852431	1.02926	0.840565	1.03801	0.708584	0.915126	1.02684	0.876652	1.19716	0.761805	1.16356	1.07292	0.715052	0.833301348	0.966018836	16	16	24	0.894045641	0.969984454	24	12.73790856	0.022175124
ENSMUSG00000113178	Myf-ps	myosin light chain, alkali, fast skeletal muscle, pseudogene [Source:MGI Symbol;Acc:MGI:97270]	58.7347	182.491	188.901	250.415	144.139	239.725	163.983	435.333	429.576	320.839	167.805	192.345	213.381	134.45	0.510431574	0.966018836	12	16	24	0.145438787	0.754184974	24	9.593839778	0.528600901

ENSMUSG00000113184	Gm49654	predicted gene, 49654 [Source:MGI Symbol;Acc:MGI:6215088]	11.6689	9.66746	8.89162	10.9725	10.0126	11.205	9.49283	10.4509	9.36946	14.867	8.76059	11.0344	11.5272	10.5204	0.46238066	0.966018836	24	12	24	0.874190162	0.964836189	24	17.30155235	0.038168323
ENSMUSG00000113188	Gm35638	predicted gene, 35638 [Source:MGI Symbol;Acc:MGI:5594797]	18.0299	15.6452	17.6399	15.4441	17.1164	17.1214	16.2916	18.1913	15.617	15.0445	18.3724	16.4101	17.3396	14.0831	0.718720526	0.966018836	20	20	24	0.284324706	0.768040178	24	20.18926946	0.066682984
ENSMUSG00000113192	Gm18032	predicted gene, 18032 [Source:MGI Symbol;Acc:MGI:5010217]	7.57511	6.10818	5.56068	7.14724	6.16297	8.20754	6.09671	8.729	5.58576	6.6308	7.27787	6.24139	7.50681	6.39881	0.46238066	0.966018836	24	12	24	0.402203681	0.799546792	24	21.25315027	0.095594437
ENSMUSG00000113204	Gm46430	predicted gene, 46430 [Source:MGI Symbol;Acc:MGI:5826067]	10.4196	10.5468	10.4745	10.2657	11.262	9.72559	9.5137	9.32742	12.081	8.23601	11.0655	10.9924	10.5172	10.9935	0.916746631	0.966018836	20	20	24	0.999880635	0.999984043	24	10.1357074	8.06E-04
ENSMUSG00000113216	Gm40841	predicted gene, 40841 [Source:MGI Symbol;Acc:MGI:5623726]	2.59494	2.77152	2.62184	2.63117	2.52224	2.46045	2.70606	2.2505	2.66777	1.6263	2.33736	2.08655	2.41631	2.37247	0.869721097	0.966018836	4	20	24	0.379349794	0.798714572	24	2.007283581	0.061972251
ENSMUSG00000113238	Gm47251	predicted gene, 47251 [Source:MGI Symbol;Acc:MGI:6096078]	2.14492	2.2105	1.71445	1.94881	2.49607	1.57146	1.76876	1.52257	2.02276	1.70918	2.29454	1.69286	1.93582	1.84485	0.915475528	0.966018836	20	4	24	0.626100298	0.872592654	24	15.5918354	0.052112347
ENSMUSG00000113255	Tes3-ps	testis derived transcript 3, pseudogene [Source:MGI Symbol;Acc:MGI:3582925]	4.27655	4.43923	4.79352	4.21695	4.64223	3.70301	4.21763	3.85728	4.94265	3.79909	4.49487	4.56996	4.789	4.6288	0.581070934	0.966018836	12	12	24	0.736324233	0.914122853	24	6.894532179	0.03431723
ENSMUSG00000113262	Gm48551	predicted gene, 48551 [Source:MGI Symbol;Acc:MGI:6098103]	2.8104	2.20828	2.13758	2.45566	2.99973	2.69215	3.27917	1.93495	2.90105	2.67132	2.62094	2.71532	3.00892	2.37909	0.013400636	0.233323991	4	4	24	0.131472442	0.741511289	24	19.20009712	0.11681793
ENSMUSG00000113263	Gm4811	predicted gene 4811 [Source:MGI Symbol;Acc:MGI:3648985]	1.35169	1.852	1.7971	1.98507	1.8588	1.9224	1.23773	1.41394	1.46294	1.41377	1.83598	1.14731	1.24902	1.17389	0.287843582	0.793714531	20	8	24	0.031970585	0.604838544	24	13.61124154	0.12918735
ENSMUSG00000113275	Tubb2a-ps2	tubulin, beta 2a, pseudogene 2 [Source:MGI Symbol;Acc:MGI:2145100]	28.2003	22.1491	25.1743	21.8808	25.9533	27.5576	22.4096	22.6032	19.8358	22.7276	25.9637	23.4798	21.6484	22.1984	0.201437924	0.687291339	24	8	24	0.028792908	0.594683	24	18.96986703	0.12564756
ENSMUSG00000113288	Gm47982	predicted gene, 47982 [Source:MGI Symbol;Acc:MGI:6097270]	0.945801	1.10175	0.969963	0.922971	1.12277	1.01778	1.35621	0.915368	1.07084	0.828829	1.25605	0.658182	0.724599	0.834847	0.88067614	0.966018836	20	4	24	0.916877389	0.977109697	24	15.67741784	0.021668925
ENSMUSG00000113294	Gm49326	predicted gene, 49326 [Source:MGI Symbol;Acc:MGI:6121511]	60.9382	82.8299	43.5932	53.0701	39.2288	20.2	37.5135	101.884	60.9042	67.03	126.375	136.483	164.079	148.008	0.823677655	0.966018836	8	4	24	0.414367879	0.799576286	24	4.265849752	0.348155168
ENSMUSG00000113302	Gm32219	predicted gene, 32219 [Source:MGI Symbol;Acc:MGI:5591378]	0.406761	0.523558	0.442626	0.4962	0.647751	0.39577	0.613985	0.709115	0.753152	0.537304	0.478796	0.41047	0.492788	0.514531	0.203305541	0.689605535	12	12	24	0.253939342	0.768040178	24	7.777416305	0.068394433
ENSMUSG00000113328	Gm47260	predicted gene, 47260 [Source:MGI Symbol;Acc:MGI:6096093]	1.80249	2.45187	2.2255	2.17371	2.65544	2.0526	2.61512	2.08044	2.49647	2.01645	2.16583	2.20177	2.3478	2.44648	0.916556573	0.966018836	12	4	24	0.852973213	0.956025132	24	6.53031511	0.025339248
ENSMUSG00000113361	B930059L03Rik	RIKEN cDNA B930059L03 gene [Source:MGI Symbol;Acc:MGI:2442724]	0.56687	0.497465	0.574376	0.49584	0.632594	0.513288	0.64508	0.737692	0.682398	0.542203	0.593836	0.732311	0.59711	0.738314	0.743750765	0.966018836	8	8	24	0.411667585	0.799546792	24	3.034619006	0.032298741
ENSMUSG00000113389	Gm9512	predicted gene 9512 [Source:MGI Symbol;Acc:MGI:3779922]	1.07962	0.364396	0.520836	1.22308	1.68391	1.20954	0.582948	0.958656	0.651874	0.793246	1.22526	1.31335	0.402457	0.914027	0.014841078	0.250870466	20	16	24	0.018835853	0.561252866	24	16.73164556	0.290148046
ENSMUSG00000113390	Gm32635	predicted gene, 32635 [Source:MGI Symbol;Acc:MGI:5591794]	3.02003	2.24267	1.77215	2.73919	2.23457	2.80198	2.571	2.49337	2.06401	3.17631	2.65382	3.54263	3.041	2.98944	0.021795836	0.26698364	24	12	24	0.113761686	0.727440321	24	20.34378759	0.135502321
ENSMUSG00000113425	Gm48653	predicted gene, 48653 [Source:MGI Symbol;Acc:MGI:6098263]	0.509136	0.739066	0.554101	0.521877	0.652913	0.516757	0.622324	0.813369	0.981046	0.523049	0.572919	0.442984	0.575981	0.628323	0.030691182	0.324150946	8	16	24	0.082409225	0.699403344	24	6.46538861	0.102826167
ENSMUSG00000113428	Gm7664	predicted gene 7664 [Source:MGI Symbol;Acc:MGI:3645656]	3.01138	2.60595	3.04992	2.73807	3.47089	2.82561	3.41951	3.11807	3.64033	2.39712	3.33223	2.58188	2.69515	2.77547	0.912686312	0.966018836	12	12	24	0.987652003	0.997530201	24	13.0693314	0.008591666
ENSMUSG00000113429	Gm20063	predicted gene, 20063 [Source:MGI Symbol;Acc:MGI:5012248]	0.428859	0.457831	0.373153	0.541672	0.257089	0.536193	0.889832	0.384275	0.673964	0.594965	0.531248	0.409825	0.884992	0.575502	0.891742979	0.966018836	4	16	24	0.539778495	0.829550362	24	2.158641698	0.065271635
ENSMUSG00000113432	8430406P12Rik	RIKEN cDNA 8430406P12 gene [Source:MGI Symbol;Acc:MGI:1918744]	0.744412	0.623266	0.857253	0.603261	0.49211	0.771322	0.961962	0.588486	0.867613	0.578572	0.482103	0.47431	0.553888	0.816349	0.101690661	0.511659635	12	8	24	0.142428795	0.751766185	24	4.357885524	0.099430774
ENSMUSG00000113440	Gm46404	predicted gene, 46404 [Source:MGI Symbol;Acc:MGI:5826041]	0.720873	0.984032	0.9809	0.878846	0.920943	0.893226	0.636719	0.900665	0.905327	0.879329	0.789116	0.920594	0.823626	0.724718	0.176754388	0.653256853	12	16	24	0.261486989	0.768040178	24	11.21177672	0.050408085
ENSMUSG00000113450	Zfp935	zinc finger protein 935 [Source:MGI Symbol;Acc:MGI:1918758]	4.72049	5.21459	4.99385	3.73119	5.34523	5.47669	5.01908	3.9307	6.27348	3.19531	6.19105	6.04118	6.13387	6.2621	0.879201926	0.966018836	20	20	24	0.400250267	0.799546792	24	22.75663843	0.125667942
ENSMUSG00000113473	Gm7045	predicted gene 7045 [Source:MGI Symbol;Acc:MGI:3779655]	0.813156	0.549789	0.58888	0.720138	0.738217	0.559765	0.32987	0.402508	0.492062	0.179174	0.679465	0.753348	0.90063	0.614499	0.439555938	0.966018836	4	4	24	0.392485488	0.799546792	24	20.52786456	0.104511779
ENSMUSG00000113491	Gm19221	predicted gene, 19221 [Source:MGI Symbol;Acc:MGI:5011406]	4.70762	4.52858	4.20255	4.16744	3.94398	4.41821	4.05463	3.57469	4.29304	4.20577	4.32739	4.31788	3.71141	3.78844	0.907000607	0.966018836	24	8	24	0.477449043	0.805393068	24	17.52225461	0.037679806
ENSMUSG00000113495	Gm19792	predicted gene, 19792 [Source:MGI Symbol;Acc:MGI:5011977]	0.724852	1.01992	0.533612	0.688058	0.882187	0.863773	0.521096	0.804392	0.80252	0.675847	0.470913	0.771814	0.707997	0.837239	0.725061903	0.966018836	8	8	24	0.701756544	0.901390662	24	2.384967004	0.042634958
ENSMUSG00000113512	Gm9063	predicted gene 9063 [Source:MGI Symbol;Acc:MGI:3647644]	1.23975	0.821877	1.0358	0.650952	0.636935	1.1269	1.62434	0.934118	0.908556	1.0087	0.651532	0.612093	1.02128	0.866036	0.089506974	0.477279066	4	16	24	0.111003119	0.723072658	24	1.405631757	0.149112591
ENSMUSG00000113521	Gm29787	predicted gene, 29787 [Source:MGI Symbol;Acc:MGI:5588946]	4.34949	4.03574	4.03522	4.58238	4.37681	5.65252	4.24299	4.25338	3.00798	4.67497	4.0623	3.25825	3.75054	3.30005	0.265774856	0.773510818	16	20	24	0.20006931	0.768040178	24	17.68933268	0.114934733
ENSMUSG00000113527	Gm48079	predicted gene, 48079 [Source:MGI Symbol;Acc:MGI:6097416]	0.826755	0.733155	0.785563	0.821849	0.765804	0.754852	0.125673	0.367757	1.05427	1.02487	0.775804	0.59764	0.346134	1.09737	0.053683798	0.394331071	12	16	24	0.091304872	0.71175955	24	11.14402214	0.206626196
ENSMUSG00000113528	Gm32699	predicted gene, 32699 [Source:MGI Symbol;Acc:MGI:5591858]	0.759699	0.574426	0.496038	0.625431	0.653174	0.631658	0.769269	0.721444	0.727912	0.745445	0.599281	0.703128	0.648775	0.756	0.622773557	0.966018836	4	16	24	0.549564343	0.834997353	24	0.450884177	0.028872163
ENSMUSG00000113529	Gm47484	predicted gene, 47484 [Source:MGI Symbol;Acc:MGI:6096460]	0.745116	0.731097	0.500374	0.78317	0.507392	0.559356	0.679788	0.335337	0.714488	0.574545	0.673662	0.618093	0.54208	0.646512	0.801280095	0.966018836	16	4	24	0.956844147	0.988639288	24	16.12149278	0.013293592
ENSMUSG00000113536	Gm49327	predicted gene, 49327 [Source:MGI Symbol;Acc:MGI:6121512]	8.57273	9.20131	12.1188	10.3941	9.77438	8.63428	6.52586	7.03555	8.65735	8.24005	9.20178	6.66199	8.11049	8.19272	0.053432515	0.394331071	12	16	24	0.044773712	0.628432994	24	11.34094117	0.170072578
ENSMUSG00000113585	Gm19605	predicted gene, 19605 [Source:MGI Symbol;Acc:MGI:5011790]	1.02894	2.34259	1.77015	2.17082	1.73706	1.47875	1.30256	2.9658	2.35494	1.91428	1.66187	2.02142	1.63393	1.85643	0.01148626	0.206824017	8	20	24	0.071546827	0.67819049	24	8.473306101	0.20907652
ENSMUSG00000113600	Gm7868	predicted gene 7868 [Source:MGI Symbol;Acc:MGI:3644628]	16.1961	18.5114	16.5101	17.4282	17.4575	14.0655	15.0296	14.9349	18.0796	12.4374	17.6431	15.4046	16.5389	15.5029	0.928780444	0.966018836	20	4	24	0.892143882	0.969410135	24	7.955438917	0.028684925
ENSMUSG00000113611	Gm47750	predicted gene, 47750 [Source:MGI Symbol;Acc:MGI:6096894]	26.8207	19.8803	17.3103	21.0731	14.7711	19.2331	19.3232	24.4714	19.084	14.8072	15.6915	17.521	18.7541	13.461	0.474557444	0.966018836	4	16	24	0.142283834	0.751766185	24	0.966875796	0.181573349
ENSMUSG00000113616	Gm45941	predicted gene, 45941 [Source:MGI Symbol;Acc:MGI:5825578]	0.790313	1.19737	0.36233	0.399899	0.839822	0.724678	0.647256	1.01267	1.74049	0.707116	0.602041	0.727427	1.06554	0.964191	0.138719141	0.580158868	8	8	24	0.244983151	0.768040178	24	3.944843577	0.162337408
ENSMUSG00000113622	Gm49749	predicted gene, 49749 [Source:MGI Symbol;Acc:MGI:6215244]	3.68722	5.52564	7.93788	5.07993	4.04364	5.30041	4.9834	5.61294	5.68431	6.39253	5.09251	4.86367	4.64385	6.27648	0.016095537	0.253395174	12	16	24	0.018876567	0.561252866	24	8.266849186	0.232265038
ENSMUSG00000113623	Gm48691	predicted gene, 48691 [Source:MGI Symbol;Acc:MGI:6098320]	0.816589	1.17101	1.39464	1.46528	0.660923	0.818756	0.501659	1.33667	1.23523	0.931539	1.47683	1.63622	0.701844	1.3228	0.21107051	0.699706585	16	12	24	0.232424211	0.768040178	24	9.729945555	0.169821911
ENSMUSG00000113626	Gm7240	predicted gene 7240 [Source:MGI Symbol;Acc:MGI:3643235]	0.477033	1.07929	2.08962	0.854917	1.62812	1.06389	1.58601	1.04305	1.18105	1.33588	0.712152	0.950348	0.906409	1.26858	0.702220766	0.966018836	12	16	24	0.42551901	0.805393068	24	9.020618808	0.144417764
ENSMUSG00000113632	Gm47446	predicted gene, 47446 [Source:MGI Symbol;Acc:MGI:6096401]	0.413378	0.785523	0.897786	0.117407	0.306322	0.406459	0.628364	0.827454	0.140569	0.5466	0.634749	0.531235	0.415361	0.548684	0.347721987	0.870418653	8	8	24	0.401891215	0.799546792	24	2.813914686	0.120822432
ENSMUSG00000113636	Gm8616	predicted gene 8616 [Source:MGI Symbol;Acc:MGI:3647863]	3.22704	3.48848	3.11415	3.02556	3.09784	3.07231	2.66234	3.70494	3.47753	2.49227	4.03358	4.17119	3.20797	3.86569	0.350544145	0.870418653	8	8	24	0.816903158	0.9417567	24	1.039535932	0.038806669
ENSMUSG00000113637	Gm7049	predicted gene 7049 [Source:MGI Symbol;Acc:MGI:3646400]	28.3869	23.0469	24.1851	31.4094	25.8492	27.1821	29.0401	32.492	21.8342	32.0585	28.9867	28.1297	30.0773	27.5572	0.915475528	0.966018836	16	20	24	0.6622689	0.885924039	24	18.68787234	0.060373489
ENSMUSG00000113642	Gm49329	predicted gene, 49329 [Source:MGI Symbol;Acc:MGI:6121514]	3.81802	2.90599	3.79607	2.88542	4.11398	3.32858	1.95618	2.2866	2.63305	2.60345	3.40914	3.38382	2.52222	2.54507	0.249881062	0.749643185	20	12	24	0.099375977	0.71175955	24	16.49535181	0.16781548
ENSMUSG00000113651	Gm4900	predicted gene 4900 [Source:MGI Symbol;Acc:MGI:3646967]	1.40695	1.43167	1.91359	1.33966	2.1758	1.95872	1.75192	2.04034	1.62887	1.69772	1.5489	1.16951	1.36659	1.34542	0.860560779	0.966018836	20	8	24	0.783709866	0.93037654	24	12.88901432	0.044330673
ENSMUSG00000113654	Gm30893	predicted gene, 30893 [Source:MGI Symbol;Acc:MGI:5590052]	1.20439	1.21676	1.26209	0.862129	1.25373	1.45028	2.24742	2.06394	0.756588	0.923106	1.46672	0.974778	0.928582	1.56951	0.371875382	0.894537464	8	4	24	0.267507764	0.768040178	24	0.344886206	0.1625494
ENSMUSG00000113660	Gm5626	predicted gene 5626 [Source:MGI Symbol;Acc:MGI:3779505]	1.81955	1.9175	2.12372	2.13965	2.3937	2.16839	2.70803	2.14695	1.66758	1.84708	1.86264	2.03609	1.97963	2.05218	0.911371589	0.966018836	24	12	24	0.496762308	0.807576732	24	20.87674069	0.057585919
ENSMUSG00000113662	Gm35558	predicted gene, 35558 [Source:MGI Symbol;Acc:MGI:5594717]	0.541976	0.701804	0.590539	0.580171	0.683353	0.651077	0.853759	0.748804	0.865775	0.680464	0.689185	0.711291	0.809069	0.450347	0.887862353	0.966018836	4	12	24	0.932969691	0.982999447	24	22.10961561	0.015576455
ENSMUSG00000113664	Gm47184	predicted gene, 47184 [Source:MGI Symbol;Acc:MGI:6095973]	1.19595	0.726604	0.7789	1.21945	1.14874	0.937968	1.3077	0.955798	1.05612	1.26535	1.14017	0.616147	0.882696	0.820057	0.5169953	0.966018836	16	16	24	0.447799477	0.805393068	24	14.52958857	0.077575431
ENSMUSG00000113668	Gm47451	predicted gene, 47451 [Source:MGI Symbol;Acc:MGI:6096409]	2.32578	2.16722	2.90893	2.61071	1.57049	2.77832	1.95041	1.421	2.05811	2.36765	4.47468	2.67019	3.62195	2.49094	0.851656735	0.966018836	24	8	24	0.566176639	0.843471947	24	16.95647331	0.124675683
ENSMUSG00000113679	Gm10432	predicted gene 10432 [Source:MGI Symbol;Acc:MGI:3642011]	1.04438	1.48342	1.41442	1.39902	1.50306	1.37019	0.792238	1.43277	1.70253	1.26552	1.55699	0.993664	1.28976	1.46343	0.093059154	0.48932011	12	16	24	0.069132341	0.673520298	24	10.04158487	0.137491431
ENSMUSG00000113683	Gm47123	predicted gene, 47123 [Source:MGI Symbol;Acc:MGI:6095872]	2.25545	1.97183	2.37281	1.77445	2.14308	1.68794	1.47949	2.01134	1.66745	1.73023	2.17885	1.31952	1.12655	1.89623	0.787283372	0.966018836	20	4	24	0.359501388	0.785405943	24	10.26686823	0.089906407
ENSMUSG00000113690	Gm36501	predicted gene, 36501 [Source:MGI Symbol;Acc:MGI:5595660]	2.80607	4.37795	5.28158	3.67683	4.13443	3.98683	3.33611	3.91288	4.76044	3.25647	4.13008	3.41333	4.9335	3.39224	0.913451828	0.966018836	12	16	24	0.495804924	0.807422206	24	8.704648859	0.099911831
ENSMUSG00000113722	Snhg10	small nucleolar RNA host gene 10 [Source:MGI Symbol;Acc:MGI:1916684]	3.18694	2.87262	2.62638	3.74326	2.84694	2.28307	4.41077	3.84183	3.73716	3.93703	4.04846	4.80871	4.57118	5.99243	0.942051135	0.966018836	4	8	24	0.376415497	0.796443956	24	3.966614119	0.099458457
ENSMUSG00000113743	Gm8712	predicted gene 8712 [Source:MGI Symbol;Acc:MGI:3644353]	15.0397	12.8596	16.1028	14.2129	14.4061	15.3192	13.1769	15.4102	13.5843	16.0601	13.1989	12.6582	13.9035	13.4305	0.72778087	0.966018836	16	8	24	0.587214876	0.853591039	24	10.70635746	0.043422489
ENSMUSG00000113750	Gm5628	predicted gene 5628 [Source:MGI Symbol;Acc:MGI:3779506]	1.30014	1.4129	1.02393	1.33796	1.00748	1.17158	0.930109	1.60649	1.40889	1.03664	1.14126	1.62847	1.22385	1.07014	0.939921059	0.966018836	8	12	24	0.762556046	0.923339085	24	2.694417075	0.041361364
ENSMUSG00000113769	5033406O09Rik	RIKEN cDNA 5033406O09 gene [Source:MGI Symbol;Acc:MGI:1923205]	0.447774	0.689487	1.24271	0.303265	0.711898	0.674005	0.866151	0.417089	0.380458	0.622184	0.902103	0.595679	0.920644	0.427563	0.843639218	0.966018836	20	20	24	0.796534912	0.935045591	24	19.56235068	0.062764751
ENSMUSG00000113786	Gm49384	predicted gene, 49384 [Source:MGI Symbol;Acc:MGI:6121607]	1.73275	1.42704	2.74756	2.25406	2.13429	2.47186	1.15443	2.85556	1.89827	1.3852	1.6285	2.23534	1.79213	2.09677	0.932671476	0.966018836	24	4	24	0.876389406	0.965584799	24	9.972037679	0.053147458
ENSMUSG00000113831	Gm49602	predicted gene, 49602 [Source:MGI Symbol;Acc:MGI:6215013]	2.12902	3.05086	3.1307	2.9128	2.76178	2.34458	2.2583	2.1171	3.26351	2.70951	2.64017	2.56748	2.72308	3.31548	0.018194955	0.256345714	12	16	24	0.035941561	0.609176161	24	9.224781267	0.145913434
ENSMUSG00000113835	Gm19554	predicted gene, 19554 [Source:MGI Symbol;Acc:MGI:5011739]	0.621142	0.453125	0.383048	0.33415	0.560853	0.508112	0.328963	0.576649	0.623542	0.642689	0.628898	0.631161	0.481283	0.720933	0.860560779	0.966018836	20	8	24	0.988014824	0.997530201	24	14.68830758	0.006405468
ENSMUSG00000113836	Gm3325	predicted gene 3325 [Source:MGI Symbol;Acc:MGI:3781503]	1.51525	1.94353	1.83986	1.62856	1.61413	1.29707	1.09377	1.51139	2.22036	1.18317	1.42086	1.83214	1.14708	1.70543	0.060394272	0.407012001	12	16	24	0.24297199	0.768040178	24	8.230599767	0.120650276
ENSMUSG00000113852	Gm48062	predicted gene, 48062 [Source:MGI Symbol;Acc:MGI:6097387]	1.08358	1.78348	1.81978	1.08257	1.44249	1.44274	1.42559	1.30154	1.6999	0.998544	1.38169	1.93346	1.01391	1.85213	0.887010723	0.966018836	12	4	24	0.72618269	0.910047737	24	4.93669101	0.063383572
ENSMUSG00000113853	Gm47583	predicted gene, 47583 [Source:MGI Symbol;Acc:MGI:6096621]	1.6618	2.01969	1.69005	1.70855	1.91064	1.89996	1.8096	1.76726	2.07698	1.8824	2.39776	2.16206	2.19735	2.1067	0.961261938	0.967043964	20	20	24	0.660739108	0.885520687	24	18.16800102	0.027394856
ENSMUSG00000113861	Gm6190	predicted gene 6190 [Source:MGI Symbol;Acc:MGI:3643523]	1.50667	1.31611	1.21154	1.95919	1.22951	1.58527	1.64005	1.41044	1.27665	1.73082	0.948059	1.20853	1.30683	1.31481	0.912923872	0.966018836	16	4	24	0.976651931	0.994103202	24	8.809318057	0.013477614
ENSMUSG00000113871	Gm48349	predicted gene, 48349 [Source:MGI Symbol;Acc:MGI:6097813]	0.527811	0.789208	0.859118	0.596761	1.26468	0.590066	1.28164	0.468051	1.16424	0.261805	0.986824	1.02141	0.797814	1.00868	0.911685024	0.966018836	20	20	24	0.881229177	0.966661503	24	21.44150159	0.052870993
ENSMUSG00000113880	A030005L19Rik	RIKEN cDNA A030005L19 gene [Source:MGI Symbol;Acc:MGI:1925172]	0.3095	0.565847	1.07665	0.708362	0.878189	0.595874	0.30185	0.608729	0.506786	0.736252	0.552321	0.355876	0.743164	0.37119	0.282213399	0.793714531	16	12	24	0.093575074	0.71175955	24	11.60659454	0.157239092
ENSMUSG00000113894	Gm48838	predicted gene, 48838 [Source:MGI Symbol;Acc:MGI:6098568]	0.444649	0.527594	0.403074	0.596271	0.444748	0.695692	0.633379	0.465151	0.785216	0.61193	0.612508	0.731253	0.345116	0.827366	0.883907406	0.966018836	24	4	24	0.85876074	0.958405859	24	11.76893838	0.027622892
ENSMUSG00000113899	-	NICE-3 protein pseudogene	1.8511	1.67067	1.26679	0.86201	1.58502	1.20197	1.33675	1.9598	0.841526	0.860838	1.08131	1.35895	1.55585	1.39019	0.00759188	0.192760771	8	4	24	0.009588556	0.502965379	24	0.29923063	0.221562395
ENSMUSG00000113902	Ndufb1	NADH:ubiquinone oxidoreductase subunit B1 [Source:MGI Symbol;Acc:MGI:3780865]	149.83	176.663	162.805	155.852	163.1	147.439	149.249	162.731	196.56	130.828	192.595	161.089	190.518	186.088	0.708665292	0.966018836	12	12	24	0.52775119	0.823537159	24	5.398348164	0.072419364
ENSMUSG00000113918	Gm6566	predicted gene 6566 [Source:MGI Symbol;Acc:MGI:3647745]	0.362502	0.901053	1.11373	0.758376	0.600864	0.647094	0.658786	0.43262	0.972283	0.633154	0.44755	0.635276	0.655134	0.93856	0.020499994	0.261611804	12	8	24	0.077800952	0.687802873	24	7.812700451	0.158872242
ENSMUSG00000113936	Gm8784	predicted gene 8784 [Source:MGI Symbol;Acc:MGI:3646031]	4.39598	4.02819	5.52891	4.84288	5.15094	4.86564	5.17882	4.64387	4.88272	4.96301	4.10479	4.97718	4.56031	4.95753	0.896753532	0.966018836	12	20	24	0.716850546	0.906600572	24	11.27523258	0.034654177
ENSMUSG00000113948	Rpl17-ps3	ribosomal protein L17, pseudogene 3 [Source:MGI Symbol;Acc:MGI:3642206]	34.0857	34.6509	34.2534	31.4502	30.5449	29.565	34.0295	32.0498	43.2545	27.7819	35.838	35.7543	39.7295	40.1891	0.575387897	0.966018836	12	4	24	0.136351814	0.747972781	24	4.044824728	0.117054366
ENSMUSG00000113949	Scamp4	secretory carrier membrane protein 4 [Source:MGI Symbol;Acc:MGI:1928947]	14.0965	10.9411	13.2598	11.9625	12.0926	12.654	15.2143	12.7006	10.4004	14.8026	12.0382	13.9326	12.451	12.4607	0.537381546	0.966018836	4	8	24	0.439498089	0.805393068	24	20.84571215	0.075151973
ENSMUSG00000113983	Gm47742	predicted gene, 47742 [Source:MGI Symbol;Acc:MGI:6096885]	0.804194	1.21886	0.697653	0.905128	0.708008	0.805181	0.584967	0.853189	0.980623	1.17048	1.41644	0.624878	0.758491	1.85804	0.34294592	0.866028069	8	20	24	0.290085323	0.768040178	24	8.336386696	0.142248193
ENSMUSG00000114003	Gm9616	predicted gene 9616 [Source:MGI Symbol;Acc:MGI:3780024]	76.0837	101.901	89.8475	100.146	104.098	88.2398	87.5569	89.8209	111.093	68.7532	97.113	83.6129	90.7762	87.0815	0.46694635	0.966018836	12	16	24	0.738155622	0.91451866	24	9.98302724	0.057543946
ENSMUSG00000114019	Gm47155	predicted gene, 47155 [Source:MGI Symbol;Acc:MGI:6095924]	2.81475	2.87534	3.51312	3.31345	3.65899	2.8427	2.71859	2.74138	3.79954	3.44491	3.27523	2.69976	2.71885	2.94481	0.002087679	0.100255953	12	16	24	3.24E-04	0.224563799	24	11.5712333	0.158891495
ENSMUSG00000114023	Gm6474	predicted gene 6474 [Source:MGI Symbol;Acc:MGI:3648182]	19.4095	19.8146	19.5576	18.5114	18.7808	17.6027	18.0276	17.5257	20.326	15.9732	19.2607	20.0352	18.0785	20.475	0.879831094	0.966018836	12	4	24	0.690122569	0.897381208	24	3.92594971	0.032793976
ENSMUSG00000114025	Gm49331	predicted gene, 49331 [Source:MGI Symbol;Acc:MGI:6121516]	1.43497	1.7858	1.39662	1.2152	2.23759	1.95483	1.42923	1.48972	1.91011	1.14748	1.58639	1.80301	1.93456	1.63048	0.142513744	0.585944911	20	20	24	0.394690873	0.799546792	24	21.02790244	0.093379322
ENSMUSG00000114036	Gm48228	predicted gene, 48228 [Source:MGI Symbol;Acc:MGI:6097631]	137.703	52.932	130.664	137.577	131.878	126.851	53.4605	118.669	127.158	33.241	120.333	113.021	140.191	125.862	0.885059765	0.966018836	4	4	24	0.894923018	0.970212381	24	20.31006064	0.129777728
ENSMUSG00000114041	Gm9269	predicted gene 9269 [Source:MGI Symbol;Acc:MGI:3646451]	2.86945	1.655	1.71936	2.36242	2.64169	3.22711	2.28677	2.46602	1.07705	2.8128	1.4189	2.22389	1.92128	1.64902	0.364640768	0.892062098	24	12	24	0.124980733	0.738693118	24	19.44362074	0.220373485
ENSMUSG00000114045	Gm34220	predicted gene, 34220 [Source:MGI Symbol;Acc:MGI:5593379]	0.637447	0.617382	0.456896	0.627873	0.50353	0.48851	0.551225	0.419305	0.777143	0.482573	0.644442	0.48347	0.485053	0.517568	0.81544087	0.966018836	20	4	24	0.791567069	0.93334777	24	10.33314244	0.024745436
ENSMUSG00000114102	Gm48048	predicted gene, 48048 [Source:MGI Symbol;Acc:MGI:6097366]	3.59877	4.71102	4.8487	5.76	5.0389	3.75099	3.91896	3.63463	5.14047	3.69291	4.56059	4.55801	5.58125	5.30105	0.834424969	0.966018836	12	16	24	0.468701982	0.805393068	24	9.948999466	0.095508607
ENSMUSG00000114110	Gm46379	predicted gene, 46379 [Source:MGI Symbol;Acc:MGI:5826016]	2.7238	5.64458	2.10154	4.69549	4.98747	4.41611	2.81118	3.38196	3.1343	2.83596	1.54928	2.31677	1.60063	2.50073	0.885059765	0.966018836	8	20	24	0.892870955	0.969717376	24	12.44538612	0.067225572
ENSMUSG00000114117	Gm48795	predicted gene, 48795 [Source:MGI Symbol;Acc:MGI:6098498]	2.64454	2.05584	1.95868	1.55917	1.76125	0.920712	1.16606	0.735174	1.05186	1.62565	2.18264	2.68437	1.7424	1.60429	0.933543173	0.966018836	20	12	24	0.621070596	0.870573556	24	18.39651711	0.131381918
ENSMUSG00000114133	Gm20075	predicted gene, 20075 [Source:MGI Symbol;Acc:MGI:5012260]	9.97038	11.0978	10.3114	11.5868	10.8449	10.9412	9.30319	9.27913	11.2208	9.90987	11.003	10.2835	10.4122	10.5999	0.432054949	0.966018836	16	12	24	0.23827513	0.768040178	24	13.14187731	0.056502303
ENSMUSG00000114147	Gm18939	predicted gene, 18939 [Source:MGI Symbol;Acc:MGI:5011124]	10.4202	11.9608	11.9616	13.4036	14.1054	10.4239	11.1639	10.8063	16.1474	6.82581	15.5608	15.3335	14.674	16.1808	0.890648739	0.966018836	20	8	24	0.986717388	0.997339197	24	0.74178925	0.019483797
ENSMUSG00000114152	Gm5374	predicted gene 5374 [Source:MGI Symbol;Acc:MGI:3779486]	0.322997	0.778391	0.965126	1.10519	0.781106	0.501184	1.67159	0.504201	1.04523	0.907314	1.0503	0.879263	0.917409	0.547699	0.417963425	0.963884303	12	20	24	0.581073905	0.850505598	24	12.40712368	0.104633698
ENSMUSG00000114172	Gm36298	predicted gene, 36298 [Source:MGI Symbol;Acc:MGI:5595457]	1.9196	2.68608	2.28116	2.61836	2.35464	2.03076	2.49973	2.51746	3.02429	1.38463	2.2827	2.56818	2.48385	2.64181	0.282728968	0.793714531	8	12	24	0.429106233	0.805393068	24	4.273514785	0.093737616
ENSMUSG00000114184	Gm7143	predicted gene 7143 [Source:MGI Symbol;Acc:MGI:3646528]	0.857314	0.319367	0.621397	0.392763	0.427146	0.615474	0.627275	0.818007	0.507115	0.717531	0.785099	0.875665	0.914469	0.641754	0.235141005	0.73629392	4	8	24	0.231493208	0.768040178	24	22.33294348	0.098084758
ENSMUSG00000114210	A330084C13Rik	RIKEN cDNA A330084C13 gene [Source:MGI Symbol;Acc:MGI:2685802]	2.58172	2.0283	2.3591	2.33446	2.40905	2.82796	2.08635	2.60094	1.94727	3.1026	2.38289	2.54703	2.28943	2.55786	0.885955579	0.966018836	24	12	24	0.488540767	0.805393068	24	16.70388305	0.060567957
ENSMUSG00000114217	Gm7054	predicted gene 7054 [Source:MGI Symbol;Acc:MGI:3779659]	4.48228	4.1223	3.75339	4.22926	5.06684	3.19062	3.54474	3.68287	4.25699	2.92178	5.07301	3.67515	3.79715	3.63085	0.893307667	0.966018836	20	4	24	0.673774813	0.890318765	24	15.82245128	0.062077606
ENSMUSG00000114230	Gm48239	predicted gene, 48239 [Source:MGI Symbol;Acc:MGI:6097649]	1.68166	2.04561	1.97035	1.82034	1.98048	1.79597	1.37095	1.586	2.54362	1.13364	2.31668	2.23696	2.07667	2.07113	0.90332508	0.966018836	20	20	24	0.988619653	0.997561007	24	10.37076627	0.012044257
ENSMUSG00000114251	Tmed10-ps	transmembrane p24 trafficking protein 10, pseudogene [Source:MGI Symbol;Acc:MGI:3782198]	77.5285	74.0685	70.2875	62.5265	71.2512	71.0581	76.2313	79.8273	68.4033	80.7889	65.2205	73.4029	74.7117	71.9666	0.103318612	0.517709628	4	16	24	0.169060093	0.767020009	24	1.375437478	0.07210404
ENSMUSG00000114252	Gm5630	predicted pseudogene 5630 [Source:MGI Symbol;Acc:MGI:3647174]	1.24095	1.26096	1.23197	1.36027	1.2911	1.31572	1.18454	1.20703	1.21552	1.06947	1.22343	0.895107	1.54617	0.887725	0.882263435	0.966018836	4	4	24	0.911113477	0.974779246	24	19.02792982	0.019107283
ENSMUSG00000114253	Gm47798	predicted gene, 47798 [Source:MGI Symbol;Acc:MGI:6096974]	0.769137	1.1474	1.07308	0.863184	1.25761	0.910551	1.00683	0.789268	1.43212	0.658129	1.04841	1.04427	1.11403	1.02441	0.939103211	0.966018836	12	4	24	0.951770751	0.988339231	24	7.286737712	0.019386614
ENSMUSG00000114254	Gm48067	predicted gene, 48067 [Source:MGI Symbol;Acc:MGI:6097396]	5.11855	10.4856	5.5148	6.91539	4.91091	7.47017	4.61111	9.35916	7.2709	5.08709	11.7396	8.57591	5.77021	10.5391	0.664568268	0.966018836	8	20	24	0.827118852	0.946112955	24	5.900505878	0.097794006
ENSMUSG00000114277	Gm48583	predicted gene, 48583 [Source:MGI Symbol;Acc:MGI:6098152]	23.6733	31.7746	35.1569	24.2039	27.6797	21.7314	20.75	16.5162	31.2221	24.5876	29.3491	29.1581	27.9634	28.9826	0.43565473	0.966018836	12	16	24	0.542580254	0.830991507	24	10.48500882	0.124045164
ENSMUSG00000114278	Gm49027	predicted gene, 49027 [Source:MGI Symbol;Acc:MGI:6118396]	27.7171	19.9175	20.9874	19.4941	21.9893	20.1247	18.9881	23.7864	17.067	28.2417	26.0254	25.8063	20.9958	25.885	0.9143748	0.966018836	20	16	24	0.757055295	0.92154584	24	18.48148347	0.065071785
ENSMUSG00000114293	Gm48078	predicted gene, 48078 [Source:MGI Symbol;Acc:MGI:6097415]	3.4155	6.42114	4.68512	6.85585	5.12605	6.39439	6.88208	5.20209	6.30138	6.18021	6.12438	4.3087	5.70986	6.05337	0.853736882	0.966018836	16	8	24	0.526580719	0.823075573	24	10.2721355	0.107071036
ENSMUSG00000114309	Gm48295	predicted gene, 48295 [Source:MGI Symbol;Acc:MGI:6097735]	0.851613	0.556688	0.396985	0.465835	0.729629	0.714762	0.396022	0.726944	0.360279	0.85727	0.502513	0.645673	0.458807	0.422983	0.289105414	0.794869797	20	16	24	0.312590478	0.768040178	24	18.65693125	0.093765747
ENSMUSG00000114321	Gm8971	predicted gene 8971 [Source:MGI Symbol;Acc:MGI:3648536]	37.9178	30.8938	36.6117	30.2454	30.1012	35.6092	35.9019	37.1606	35.7038	26.6768	39.2363	29.7959	29.342	30.9318	0.921572192	0.966018836	12	4	24	0.697282754	0.899338675	24	0.662664381	0.054420863
ENSMUSG00000114347	Gm47913	predicted gene, 47913 [Source:MGI Symbol;Acc:MGI:6097160]	1.89948	1.98042	2.3205	1.98559	1.673	1.309	2.13425	1.32138	1.5075	2.62534	1.43186	2.48086	3.09331	2.87562	0.916963526	0.966018836	4	20	24	0.65576098	0.883738698	24	4.127804206	0.092326355
ENSMUSG00000114362	4933433G19Rik	RIKEN cDNA 4933433G19 gene [Source:MGI Symbol;Acc:MGI:1918476]	0.57952	0.53872	0.80357	0.516364	0.82691	0.473488	0.745949	0.780775	0.950775	0.756686	0.688866	0.662556	0.548	0.682389	0.14313801	0.585944911	12	12	24	0.206480912	0.768040178	24	9.319283681	0.081608921
ENSMUSG00000114369	Gm41077	predicted gene, 41077 [Source:MGI Symbol;Acc:MGI:5623962]	1.17494	1.3589	1.0643	1.79168	1.60742	1.03428	0.716041	1.22498	1.11307	0.647021	1.11954	1.00122	0.871062	1.42029	0.696669634	0.966018836	8	20	24	0.725951347	0.909996439	24	10.92188224	0.065307364
ENSMUSG00000114375	Gm31544	predicted gene, 31544 [Source:MGI Symbol;Acc:MGI:5590703]	6.30212	6.93875	7.38314	9.01731	8.22081	7.81245	6.99623	9.90171	8.44074	7.16535	6.69775	7.80055	7.44326	5.45833	0.636884864	0.966018836	8	20	24	0.593475308	0.856923985	24	12.57857933	0.076153796
ENSMUSG00000114380	Gm47942	predicted gene, 47942 [Source:MGI Symbol;Acc:MGI:6097208]	5.40813	8.05082	7.28156	8.02513	7.05979	4.65498	8.93086	6.64872	8.58281	6.27067	7.52077	7.25907	11.7397	8.37352	0.934152439	0.966018836	4	20	24	0.778604041	0.928832041	24	6.12619391	0.088231806
ENSMUSG00000114419	5430414B19Rik	RIKEN cDNA 5430414B19 gene [Source:MGI Symbol;Acc:MGI:1921096]	0.882089	0.895663	0.590011	1.16351	0.961582	0.690824	0.909585	0.606183	0.508998	0.76228	0.465339	0.477193	0.633997	0.86611	0.916963526	0.966018836	4	20	24	0.998584813	0.999730606	24	4.747056124	0.003305858
ENSMUSG00000114429	Ncf2-rs	neutrophil cytosolic factor 2 related sequence [Source:MGI Symbol;Acc:MGI:97285]	2.14338	2.98908	3.9653	3.60691	3.98807	1.53721	3.80084	2.45652	3.24332	2.43697	3.01428	1.45788	2.8002	2.11599	0.128457847	0.560209134	12	12	24	0.224242257	0.768040178	24	9.862732298	0.214020422
ENSMUSG00000114432	Gm49391	predicted gene, 49391 [Source:MGI Symbol;Acc:MGI:6121619]	0.978427	0.45855	2.31968	1.18664	1.30061	1.30907	1.47001	0.629618	1.62551	1.16039	2.1766	1.88514	0.630657	1.66672	0.492524295	0.966018836	12	20	24	0.263382279	0.768040178	24	13.96080705	0.217110405

ENSMUSG00000114488	Gm5802	predicted gene 5802 [Source:MGI Symbol;Acc:MGI:3643080]	22.967	17.5597	19.8869	19.3735	20.864	22.351	20.2166	18.8494	16.6648	20.0752	18.0216	18.4896	17.5154	16.3284	0.446653834	0.966018836	24	8	24	0.028749565	0.594683	24	18.85575443	0.108231347
ENSMUSG00000114493	Gm47071	predicted gene, 47071 [Source:MGI Symbol;Acc:MGI:6095789]	1.07085	0.921987	1.02649	0.813174	1.10492	0.849086	1.12733	1.0247	1.03826	0.917619	1.09558	0.964126	1.02847	1.06008	0.305564675	0.811201458	4	12	24	0.604958485	0.862224803	24	0.994786649	0.027388766
ENSMUSG00000114500	Gm8983	predicted gene 8983 [Source:MGI Symbol;Acc:MGI:3644468]	12.027	9.91816	9.69781	10.6931	10.5621	10.7863	11.4392	11.5418	8.52576	11.1449	10.6848	11.4089	10.6611	10.9672	0.105280783	0.518002936	4	8	24	0.09911295	0.71175955	24	21.19070938	0.093452684
ENSMUSG00000114515	Aldoa	aldolase A, fructose-bisphosphate [Source:MGI Symbol;Acc:MGI:87994]	0.758137	0.969313	0.949753	0.644713	1.15208	0.635154	0.839451	1.05264	0.865141	1.47997	0.850642	1.09542	1.17136	1.48667	0.93243477	0.966018836	8	16	24	0.445714511	0.805393068	24	7.153284486	0.079806505
ENSMUSG00000114529	Gm4939	predicted gene 4939 [Source:MGI Symbol;Acc:MGI:3646780]	4.43826	4.53192	4.38261	4.24032	4.97117	4.02298	4.29422	4.89972	3.64153	4.08185	4.23432	4.06225	3.67835	3.60528	0.832742389	0.966018836	8	16	24	0.711433973	0.904655191	24	17.5151629	0.030499959
ENSMUSG00000114540	Gm6421	predicted pseudogene 6421 [Source:MGI Symbol;Acc:MGI:3647311]	2.12529	2.06451	2.12725	2.57501	2.99724	2.18151	1.38719	1.87844	1.32876	0.973437	1.54998	1.37654	2.20331	1.25986	0.928780444	0.966018836	20	12	24	0.529369756	0.824460072	24	18.93326113	0.10792341
ENSMUSG00000114547	Gm3226	predicted gene 3226 [Source:MGI Symbol;Acc:MGI:3804968]	3.49921	3.33699	4.0264	2.941	3.53584	3.79272	3.16387	3.13891	3.0138	4.17831	3.47777	3.8319	3.62492	4.00294	0.886572916	0.966018836	24	8	24	0.890355359	0.969016997	24	17.43762262	0.023901859
ENSMUSG00000114571	Gm35595	predicted gene, 35595 [Source:MGI Symbol;Acc:MGI:5594754]	356.402	300.842	287.733	335.944	309.289	358.06	296.797	304.681	218.56	306.636	245.321	277.995	264.961	272.995	0.928780444	0.966018836	24	12	24	0.250573502	0.768040178	24	20.27793574	0.104285646
ENSMUSG00000114575	Gm47814	predicted gene, 47814 [Source:MGI Symbol;Acc:MGI:6096999]	0.749207	0.850492	0.811974	1.25998	1.09469	0.829972	0.680397	0.495501	0.253373	0.61974	0.505964	0.729988	0.379483	1.01615	0.894678274	0.966018836	16	12	24	0.64074595	0.878446626	24	16.04194081	0.080580431
ENSMUSG00000114579	Gm4130	predicted gene 4130 [Source:MGI Symbol;Acc:MGI:3782306]	18.2706	17.5938	19.8684	16.0976	17.5818	18.3096	17.5444	14.8155	14.7983	16.3659	15.4407	16.2037	15.7132	15.9998	0.896311169	0.966018836	24	8	24	0.526936783	0.823139162	24	20.78619842	0.041333826
ENSMUSG00000114593	Gm19716	predicted gene, 19716 [Source:MGI Symbol;Acc:MGI:5011901]	5.33665	4.15108	2.23135	6.12855	4.49299	5.12159	3.46251	8.32378	0.922532	10.3717	1.52393	2.92302	3.0223	1.97892	0.883907406	0.966018836	16	20	24	0.877579926	0.965881478	24	17.95575335	0.144968996
ENSMUSG00000114607	Gm8990	predicted gene 8990 [Source:MGI Symbol;Acc:MGI:3647409]	2.03089	2.89588	3.26785	3.6879	2.51449	2.55914	3.59906	3.2863	2.71969	4.23208	2.60363	2.9098	2.47167	2.76102	0.251986462	0.75129457	16	12	24	0.153805183	0.760223561	24	10.0158096	0.155213285
ENSMUSG00000114621	Gm31107	predicted gene, 31107 [Source:MGI Symbol;Acc:MGI:5590266]	1.5058	1.23064	0.908899	1.90731	1.43093	1.46246	1.6296	1.49191	1.42448	1.43766	1.71736	1.44734	1.95736	1.57745	0.112800839	0.530777648	4	8	24	0.458935023	0.805393068	24	19.59802776	0.071521845
ENSMUSG00000114662	Gm31683	predicted gene, 31683 [Source:MGI Symbol;Acc:MGI:5590842]	0.833524	0.750145	0.321809	0.538963	0.710624	0.871028	1.01448	0.696455	0.523331	0.591552	0.861839	0.677571	0.714025	0.572473	0.002004172	0.100255953	4	8	24	0.00561224	0.448445896	24	21.1485173	0.168769911
ENSMUSG00000114687	Gm5452	predicted gene 5452 [Source:MGI Symbol;Acc:MGI:3645281]	13.8166	11.6227	12.4349	13.2276	14.3749	14.9055	13.6031	17.2371	12.4308	14.2904	14.4499	11.7848	11.9943	11.4883	0.47028201	0.966018836	20	12	24	0.613877026	0.866162311	24	16.68274127	0.061006202
ENSMUSG00000114705	Gm18760	predicted gene, 18760 [Source:MGI Symbol;Acc:MGI:5010945]	0.238351	0.564382	0.518052	1.08766	0.532206	0.403594	0.774059	0.708809	1.02853	0.472608	0.761591	0.864963	0.583989	0.658409	0.823246279	0.966018836	12	16	24	0.341460607	0.775806931	24	10.31103689	0.111907284
ENSMUSG00000114708	Gm30177	predicted gene, 30177 [Source:MGI Symbol;Acc:MGI:5589336]	1.44091	2.24005	1.87818	2.07659	2.20962	1.45925	1.05184	2.05277	1.43807	1.96864	1.90304	1.72653	2.31479	2.25407	0.935732159	0.966018836	8	20	24	0.483864069	0.805393068	24	9.675379432	0.098969419
ENSMUSG00000114733	Gm4815	predicted gene 4815 [Source:MGI Symbol;Acc:MGI:3643439]	7.17247	6.36704	6.032	5.1565	7.44598	6.51577	7.23458	6.16611	5.38877	5.68637	6.21514	5.36522	6.52856	4.78357	0.059513104	0.406067671	4	12	24	0.046709395	0.633507364	24	20.99199445	0.133830068
ENSMUSG00000114759	Gm10257	predicted gene 10257 [Source:MGI Symbol;Acc:MGI:3644245]	1.78366	1.47443	1.75831	1.27726	1.30832	1.0821	0.786268	1.43337	1.97705	0.965485	1.20811	2.73017	1.42029	1.74885	0.503972924	0.966018836	12	4	24	0.78446102	0.930811164	24	4.168391183	0.075288472
ENSMUSG00000114765	Gm8514	predicted gene 8514 [Source:MGI Symbol;Acc:MGI:3648245]	1.20638	0.823911	0.59995	1.22874	0.931285	0.882898	0.841813	0.711066	0.589164	0.774117	0.546219	0.636049	0.596038	0.512706	0.723119329	0.966018836	16	20	24	0.268333785	0.768040178	24	18.4485979	0.079271847
ENSMUSG00000114771	Gm49064	predicted gene, 49064 [Source:MGI Symbol;Acc:MGI:6118443]	1.12757	1.50529	1.26496	1.05339	1.18989	1.08294	1.41989	0.884864	1.30089	1.11725	1.40798	1.07636	1.26563	1.36305	0.671726933	0.966018836	8	8	24	0.804382238	0.937432708	24	4.175682747	0.030236305
ENSMUSG00000114780	AI197445	expressed sequence AI197445 [Source:MGI Symbol;Acc:MGI:2145191]	1.14291	2.72303	2.12549	2.17654	1.54458	1.06179	1.91697	2.77535	3.09316	1.22043	1.69644	1.45298	2.02112	2.60718	0.003960881	0.140598674	8	16	24	0.003561217	0.424915748	24	6.386995794	0.357502443
ENSMUSG00000114788	Gm48899	predicted gene, 48899 [Source:MGI Symbol;Acc:MGI:6098665]	1.97362	1.10163	0.844386	0.0822847	1.28578	0.738066	0.00158471	1.3274	0.0502451	1.04101	0.909203	1.37354	0.778214	2.05835	0.9045879	0.966018836	8	4	24	0.657675624	0.884531138	24	23.41500616	0.189429725
ENSMUSG00000114796	A930028N01Rik	RIKEN cDNA A930028N01 gene [Source:MGI Symbol;Acc:MGI:1925688]	0.775932	1.17638	0.673221	0.874072	0.683885	0.950412	0.564565	1.64733	1.36714	0.821287	1.4728	1.50612	1.04491	0.596723	0.822716578	0.966018836	24	4	24	0.99353779	0.998731933	24	14.62046539	0.011354325
ENSMUSG00000114797	Gm49336	predicted gene, 49336 [Source:MGI Symbol;Acc:MGI:6121524]	29.8239	28.9928	30.2841	29.1966	29.2629	27.4329	27.877	26.9001	26.5286	25.5968	32.0063	24.976	26.5591	28.4579	0.879844076	0.966018836	20	4	24	0.846056181	0.953396028	24	13.71872253	0.019722486
ENSMUSG00000114809	Atp5c1-ps	ATP synthase, H+ transporting, mitochondrial F1 complex, gamma polypeptide 1, pseudogene [Source:MGI Symbol;Acc:MGI:2145515]	1.4944	1.19314	1.62491	1.1622	1.19746	1.21084	1.30198	1.33736	0.947374	0.881584	0.880224	0.749151	1.42267	0.917894	0.159799452	0.619467724	4	16	24	0.168968074	0.767020009	24	1.999132952	0.096771395
ENSMUSG00000114827	Gm46432	predicted gene, 46432 [Source:MGI Symbol;Acc:MGI:5826069]	1.17619	1.15068	1.27795	1.14918	1.31201	0.947474	1.84105	1.27346	0.972783	0.77742	0.919848	0.969046	1.46034	0.88722	0.192357053	0.669841558	4	12	24	0.218412052	0.768040178	24	0.33639398	0.112777542
ENSMUSG00000114835	Gm48194	predicted gene, 48194 [Source:MGI Symbol;Acc:MGI:6097578]	2.14888	2.23397	1.59621	2.5655	1.67323	1.90299	2.33677	2.1106	2.38276	1.64522	2.15834	1.60094	1.59256	1.29022	0.859936714	0.966018836	16	8	24	0.971695894	0.993121308	24	11.93279398	0.016071592
ENSMUSG00000114842	Gm47797	predicted gene, 47797 [Source:MGI Symbol;Acc:MGI:6096972]	0.7371	1.35991	1.32997	1.91371	1.42382	2.02439	1.78115	1.72466	1.15994	1.13871	1.31934	1.44556	2.16793	1.54248	0.50155611	0.966018836	24	12	24	0.812757652	0.940101979	24	21.7699784	0.058460712
ENSMUSG00000114893	Gm48684	predicted gene, 48684 [Source:MGI Symbol;Acc:MGI:6098310]	0.2277	0.917306	1.7261	1.26962	0.826782	0.877662	1.10113	0.400161	0.768461	0.602953	0.765548	0.74288	0.462962	1.24628	0.724067924	0.966018836	12	16	24	0.363966111	0.788727838	24	10.13102185	0.171642
ENSMUSG00000114907	Gm47794	predicted gene, 47794 [Source:MGI Symbol;Acc:MGI:6096966]	0.326474	0.651712	0.683171	0.491765	0.339857	0.458259	0.490954	0.42846	0.652955	0.431257	0.433147	0.797948	0.48391	0.353938	0.970950683	0.971732806	12	8	24	0.826351101	0.945708264	24	7.585474564	0.034733964
ENSMUSG00000114934	Gm48342	predicted gene, 48342 [Source:MGI Symbol;Acc:MGI:6097804]	0.538131	0.882259	0.938853	1.0295	0.892765	0.837121	0.663944	0.879839	1.32906	0.609064	1.28451	0.81608	0.991174	0.868975	0.208438456	0.698704663	12	16	24	0.280548952	0.768040178	24	10.84478802	0.100780231
ENSMUSG00000114937	Gm30054	predicted gene, 30054 [Source:MGI Symbol;Acc:MGI:5589213]	0.241411	0.715731	0.565358	0.775443	0.741624	0.588346	0.391526	0.462578	0.883794	0.620167	0.764819	0.841482	0.839284	0.607284	0.570592509	0.966018836	20	8	24	0.153901638	0.760223561	24	12.72768324	0.111120831
ENSMUSG00000114995	Gm49284	predicted gene, 49284 [Source:MGI Symbol;Acc:MGI:6118771]	4.10464	3.71154	3.9454	3.45864	4.40537	3.82892	4.60912	3.98714	4.3068	4.49978	4.21431	4.44617	4.48356	3.73567	0.676344318	0.966018836	4	4	24	0.480673865	0.805393068	24	19.52964351	0.046671879
ENSMUSG00000114999	Gm7962	predicted gene 7962 [Source:MGI Symbol;Acc:MGI:3643213]	3.06443	2.83779	3.1141	2.78889	3.27954	2.98288	2.88832	2.91979	3.033	3.18202	2.44758	2.42607	2.32894	2.52851	0.470607371	0.966018836	12	16	24	0.32656909	0.768040178	24	10.93180083	0.051127625
ENSMUSG00000115007	5830448L01Rik	RIKEN cDNA 5830448L01 gene [Source:MGI Symbol;Acc:MGI:1923281]	0.838203	0.959149	1.06382	0.883496	0.760081	0.576473	1.02354	1.00237	1.02676	1.10737	0.986542	1.22237	0.975644	1.14306	0.714062999	0.966018836	12	8	24	0.133831966	0.744007455	24	6.864884205	0.084858473
ENSMUSG00000115017	Gm2573	predicted gene 2573 [Source:MGI Symbol;Acc:MGI:3780740]	39.2373	35.4105	32.6248	34.35	37.0103	38.0397	33.8921	39.1731	32.0515	32.8636	32.5722	39.4651	36.7264	38.2364	0.071575474	0.440262583	24	12	24	0.03654466	0.609176161	24	22.77147301	0.100366665
ENSMUSG00000115018	Ndor1	NADPH dependent diflavin oxidoreductase 1 [Source:MGI Symbol;Acc:MGI:1926047]	2.215	2.64541	2.41508	2.60131	2.82702	2.38037	1.92767	1.5204	2.977	2.86021	2.54321	2.82618	2.38091	1.8872	0.024198298	0.28633006	12	20	24	0.047786308	0.636393972	24	14.21306956	0.163205753
ENSMUSG00000115049	Gm49172	predicted gene, 49172 [Source:MGI Symbol;Acc:MGI:6118604]	10.2547	8.02942	8.4711	7.82615	7.75626	8.42355	8.8292	8.79598	7.12093	8.22528	8.16924	8.21	8.51221	7.09041	0.05688647	0.401309455	4	12	24	0.060016651	0.658304792	24	22.21843877	0.093881746
ENSMUSG00000115070	Gm4740	predicted gene 4740 [Source:MGI Symbol;Acc:MGI:3648965]	0.713325	0.463108	0.481783	0.623383	0.388392	0.519027	0.631159	0.574302	0.589073	0.796051	0.339423	0.484529	0.539314	0.456074	0.676344318	0.966018836	16	4	24	0.760482318	0.922513876	24	5.609829966	0.034366364
ENSMUSG00000115099	1700087I21Rik	RIKEN cDNA 1700087I21 gene [Source:MGI Symbol;Acc:MGI:1923869]	2.76501	2.55627	2.95936	3.12564	2.67803	3.18125	2.71521	2.55505	2.6757	3.17927	2.82596	2.63606	2.96009	2.90022	0.350544145	0.870418653	16	16	24	0.346641103	0.778844143	24	13.87988756	0.045498383
ENSMUSG00000115100	Gm34934	predicted gene, 34934 [Source:MGI Symbol;Acc:MGI:5594093]	0.730165	1.06742	1.03542	0.934395	0.899821	0.843473	1.32045	1.09916	1.33558	1.19646	0.853412	1.29788	1.31425	1.25872	0.622132989	0.966018836	4	16	24	0.097130602	0.71175955	24	5.33193419	0.092350752
ENSMUSG00000115116	Gm46496	predicted gene, 46496 [Source:MGI Symbol;Acc:MGI:5826133]	1.03197	1.01287	1.34597	1.41541	1.59047	1.43954	1.42522	1.6593	1.50123	1.59128	1.41527	1.27575	1.05618	1.18844	0.325142889	0.842977406	16	12	24	0.085920536	0.704313881	24	12.86121783	0.10675489
ENSMUSG00000115124	Gm49201	predicted gene, 49201 [Source:MGI Symbol;Acc:MGI:6118649]	1.92879	1.87587	1.82423	2.45584	1.38273	1.50152	1.48421	1.80362	1.63316	2.07749	1.41413	1.94977	1.98155	1.34381	0.917839756	0.966018836	16	4	24	0.746660117	0.917587548	24	9.558817243	0.050555388
ENSMUSG00000115138	Gm36899	predicted gene, 36899 [Source:MGI Symbol;Acc:MGI:5596058]	0.332551	0.657646	0.602916	0.552137	0.582097	0.525571	0.451147	0.496984	0.631467	0.463761	0.534449	0.540968	0.368725	0.495012	0.004861081	0.141782417	12	16	24	0.138765979	0.748341961	24	10.70957386	0.066598972
ENSMUSG00000115148	Gm49125	predicted gene, 49125 [Source:MGI Symbol;Acc:MGI:6118530]	0.771143	1.62207	1.29636	1.24932	1.63073	0.997422	1.19836	1.10022	1.7972	1.04184	1.15905	1.40518	1.44537	1.25144	0.953629935	0.966018836	12	12	24	0.544886019	0.832473263	24	9.07120709	0.079133169
ENSMUSG00000115149	9330188P03Rik	RIKEN cDNA 9330188P03 gene [Source:MGI Symbol;Acc:MGI:2686435]	2.04086	3.57724	3.32373	3.22965	2.47866	2.35747	2.27239	2.37424	3.42773	2.91076	2.26366	2.73136	2.785	3.12549	0.029412383	0.322165609	12	16	24	0.015163292	0.537924552	24	8.318272753	0.195391241
ENSMUSG00000115160	Gm6532	predicted pseudogene 6532 [Source:MGI Symbol;Acc:MGI:3646742]	7.47169	7.41978	8.00447	7.9181	7.18581	6.5269	7.36806	7.36775	8.51643	6.8755	9.23643	7.84763	7.50571	5.20408	0.675497492	0.966018836	12	20	24	0.466858363	0.805393068	24	14.30969624	0.078616814
ENSMUSG00000115186	Gm49417	predicted gene, 49417 [Source:MGI Symbol;Acc:MGI:6155048]	1.25788	1.39802	1.31843	1.33647	1.30752	1.29002	1.84485	0.893235	1.53222	1.18724	1.3534	1.43658	1.19735	1.15995	0.896799496	0.966018836	20	12	24	0.745758651	0.916958085	24	18.86790777	0.041474467
ENSMUSG00000115196	Gm6212	predicted gene 6212 [Source:MGI Symbol;Acc:MGI:3643635]	148.438	126.145	116.5	112.029	115.63	128.012	113.741	128.275	109.064	105.981	143.391	131.64	123.838	107.499	0.166168222	0.633011401	24	16	24	0.056965767	0.65610184	24	21.12513063	0.131121504
ENSMUSG00000115219	Eef1akmt4	EEF1A lysine methyltransferase 4 [Source:MGI Symbol;Acc:MGI:5903914]	1.51036	1.5806	1.29394	1.10859	1.41161	1.25877	1.24578	1.55747	1.26849	1.26505	2.0855	1.084	1.79057	1.27002	0.658895034	0.966018836	8	16	24	0.837836285	0.950338886	24	22.68834322	0.038305025
ENSMUSG00000115220	Gm49768	predicted gene, 49768 [Source:MGI Symbol;Acc:MGI:6215279]	1.56385	3.13441	2.71892	2.58748	3.37618	2.78087	3.71397	2.86054	4.10261	2.94575	2.1419	3.20419	3.03007	2.64985	0.759203569	0.966018836	12	4	24	0.749159141	0.918462643	24	8.217040429	0.075057015
ENSMUSG00000115222	Gm49747	predicted gene, 49747 [Source:MGI Symbol;Acc:MGI:6215240]	1.43882	1.55073	1.49939	1.76621	1.56795	1.26799	1.36609	1.11461	1.90552	1.30398	1.73424	1.33075	1.43257	1.32473	0.291478323	0.798044066	20	4	24	0.18398049	0.768040178	24	12.01632321	0.085588453
ENSMUSG00000115223	Gm48985	predicted gene, 48985 [Source:MGI Symbol;Acc:MGI:6118331]	3.72735	6.33331	4.79289	3.6346	3.88814	4.35821	5.0686	4.29687	4.8733	3.54717	3.28782	4.25857	5.68634	3.46462	0.202982082	0.689605535	4	16	24	0.123141562	0.736234617	24	2.498988898	0.17007103
ENSMUSG00000115232	Gm49378	predicted gene, 49378 [Source:MGI Symbol;Acc:MGI:6121598]	0.817816	0.566479	0.454201	0.940808	0.642631	0.699681	1.34431	0.966659	0.824233	1.02766	0.733855	0.855264	0.586787	1.53651	0.858514388	0.966018836	8	12	24	0.715824772	0.906323093	24	3.952995761	0.065917985
ENSMUSG00000115234	Gm49130	predicted gene, 49130 [Source:MGI Symbol;Acc:MGI:6118539]	1.24778	1.78815	1.75488	1.08972	1.85946	1.28564	1.87778	1.37612	1.35089	1.06719	1.55925	1.22107	1.41079	0.772796	0.922029406	0.966018836	4	12	24	0.771289502	0.926522557	24	20.24100839	0.054623037
ENSMUSG00000115247	Gm6201	predicted gene 6201 [Source:MGI Symbol;Acc:MGI:3779571]	8.87967	7.19695	7.09292	6.94222	5.93685	7.21026	7.06297	7.81934	5.45313	9.06523	6.85634	7.37734	7.99166	9.04851	0.281427347	0.793378811	8	12	24	0.425981615	0.805393068	24	1.594551635	0.0924154
ENSMUSG00000115280	Gm7107	predicted gene 7107 [Source:MGI Symbol;Acc:MGI:3648887]	4.73084	4.49273	4.67885	5.02299	4.56976	4.38888	3.87863	4.12528	3.95882	4.895	3.96695	5.26178	3.60801	4.30418	0.47028201	0.966018836	16	12	24	0.328291097	0.768040178	24	14.27894565	0.068476728
ENSMUSG00000115281	Gm49743	predicted gene, 49743 [Source:MGI Symbol;Acc:MGI:6215234]	2.50499	1.88991	1.75743	2.01992	1.60269	2.22436	1.56819	2.00785	1.75662	3.17899	2.21021	2.19289	2.23364	2.57838	0.879206539	0.966018836	4	8	24	0.96565109	0.991649758	24	13.9909669	0.018992771
ENSMUSG00000115289	Gm49164	predicted gene, 49164 [Source:MGI Symbol;Acc:MGI:6118590]	1.2001	0.698344	0.203237	0.0632754	0.0453364	8.22173	6.25959	2.93343	0.383724	1.88889	0.0763601	0.0583795	0.240402	0.0390498	0.942429894	0.966018836	4	16	24	0.355368931	0.782134973	24	22.8804245	0.602343871
ENSMUSG00000115312	Gm8518	predicted gene 8518 [Source:MGI Symbol;Acc:MGI:3645036]	13.2363	12.7183	14.3665	12.6293	13.0329	15.3711	13.101	13.4906	11.9026	14.4263	11.346	15.9496	11.8451	13.083	0.785563354	0.966018836	24	20	24	0.694354096	0.898522833	24	19.62513324	0.046611338
ENSMUSG00000115317	Gm32618	predicted gene, 32618 [Source:MGI Symbol;Acc:MGI:5591777]	1.45671	2.10205	2.13756	1.44156	1.72911	1.90928	1.90304	1.73211	2.20053	1.58881	2.1009	1.93993	1.82493	2.04051	0.265774856	0.773510818	12	4	24	0.623932705	0.871879816	24	5.449307484	0.048435235
ENSMUSG00000115332	Gm48952	predicted gene, 48952 [Source:MGI Symbol;Acc:MGI:6118279]	0.483735	1.2175	2.0919	0.815692	1.23343	0.922725	1.45805	1.06215	2.72134	1.58238	0.82217	0.77904	1.75645	2.22364	0.071575474	0.440262583	12	4	24	0.018059014	0.558889165	24	7.29156084	0.360400395
ENSMUSG00000115338	Pnp	purine-nucleoside phosphorylase [Source:MGI Symbol;Acc:MGI:97365]	8.022	7.67842	8.78062	9.03982	10.096	8.64658	8.80718	6.70873	8.35657	6.80051	9.22693	8.79005	7.74765	7.89349	0.001925685	0.100255953	20	12	24	0.075500724	0.685660727	24	16.90056426	0.120345563
ENSMUSG00000115379	Gm48990	predicted gene, 48990 [Source:MGI Symbol;Acc:MGI:6118337]	2.00957	2.10923	2.01183	2.61226	1.81646	1.80754	1.12477	1.64102	2.93326	1.53391	2.51515	2.50025	1.87341	2.37724	0.759899911	0.966018836	12	16	24	0.438767682	0.805393068	24	10.96323269	0.119263797
ENSMUSG00000115380	Gm46453	predicted gene, 46453 [Source:MGI Symbol;Acc:MGI:5826090]	1.57981	1.89208	1.49783	1.10545	2.73683	3.05796	2.15116	2.26169	2.1359	1.43763	2.531	1.66953	1.46836	1.53405	0.129903614	0.560263689	20	20	24	0.342787683	0.776697379	24	19.34430814	0.161406401
ENSMUSG00000115384	Gm49205	predicted gene, 49205 [Source:MGI Symbol;Acc:MGI:6118655]	1.32693	1.0268	0.824251	1.51814	1.88681	1.0016	1.46348	1.1861	1.07765	0.960343	1.17077	0.726682	1.68604	1.13452	0.595574256	0.966018836	4	8	24	0.788504222	0.932208289	24	19.99607095	0.060373836
ENSMUSG00000115417	Gm48949	predicted gene, 48949 [Source:MGI Symbol;Acc:MGI:6118274]	0.315896	0.641135	0.94395	1.35154	1.46958	0.846936	1.44266	1.0569	1.45287	1.04394	0.648921	0.758571	0.897073	0.713847	0.536543745	0.966018836	12	20	24	0.293731768	0.768040178	24	11.41400389	0.161975335
ENSMUSG00000115423	-	novel protein	27.4915	27.9168	27.6817	23.6804	26.8548	22.3456	24.6759	24.6648	31.0253	23.7294	30.4519	30.0932	31.3584	27.7232	0.93717134	0.966018836	4	12	24	0.837714347	0.950325222	24	3.32013047	0.034103507
ENSMUSG00000115424	D130079A08Rik	RIKEN cDNA D130079A08 gene [Source:MGI Symbol;Acc:MGI:2444500]	8.17618	7.72882	6.57923	6.7987	7.09142	6.05323	6.33099	5.96543	7.59337	6.47118	7.2543	7.24537	8.25612	7.89436	0.951082476	0.966018836	4	12	24	0.657182789	0.884447211	24	1.879899518	0.04877935
ENSMUSG00000115431	Gm3219	predicted pseudogene 3219 [Source:MGI Symbol;Acc:MGI:3781398]	24.4172	25.1045	27.3481	28.3142	29.5931	29.0372	28.7712	23.8705	27.0783	25.3024	22.4744	27.5259	28.1673	29.3058	0.865514257	0.966018836	24	8	24	0.931521155	0.982348071	24	20.52548141	0.019065649
ENSMUSG00000115432	D130009I18Rik	RIKEN cDNA D130009I18 gene [Source:MGI Symbol;Acc:MGI:2443663]	13.8529	14.5093	10.9399	13.9546	12.93	11.9555	12.4293	11.0149	12.3877	12.9823	13.4998	13.2355	13.5335	14.0501	0.92721845	0.966018836	8	4	24	0.835135311	0.949440189	24	19.89472454	0.029366516
ENSMUSG00000115463	Gm49356	predicted gene, 49356 [Source:MGI Symbol;Acc:MGI:6121562]	0.932263	0.809275	1.33455	1.0698	2.06338	1.04242	1.1713	1.411	1.33568	0.933017	0.978318	1.07938	2.33678	1.91306	0.880631923	0.966018836	4	12	24	0.807703811	0.938776658	24	3.379426384	0.063856698
ENSMUSG00000115505	Gm9247	predicted gene 9247 [Source:MGI Symbol;Acc:MGI:3648323]	16.445	15.967	16.6951	18.1582	18.3972	20.4023	15.6002	21.9631	14.6086	18.1183	17.3572	13.8166	15.6688	13.0569	0.505728924	0.966018836	20	8	24	0.524793176	0.822296127	24	15.38590344	0.080365834
ENSMUSG00000115518	Gm10791	predicted gene 10791 [Source:MGI Symbol;Acc:MGI:3641949]	0.37292	0.447245	0.492723	0.736178	0.620647	0.440367	0.520695	0.263058	0.80183	0.580587	0.606315	0.575652	0.672669	0.494178	0.122341026	0.554724475	16	16	24	0.101153155	0.713208839	24	13.07217606	0.101335558
ENSMUSG00000115539	Gm6997	predicted gene 6997 [Source:MGI Symbol;Acc:MGI:3644027]	11.7929	13.2911	12.1471	11.544	12.335	13.2423	12.8659	12.769	14.2756	11.8371	13.8611	13.0491	13.7286	13.4553	0.912923872	0.966018836	8	8	24	0.499837714	0.808791684	24	1.678484964	0.033445575
ENSMUSG00000115553	Gm49124	predicted gene, 49124 [Source:MGI Symbol;Acc:MGI:6118528]	0.145249	0.293223	0.629111	0.817907	0.533257	0.323439	0.703623	0.513556	0.393261	0.0959023	1.08249	0.65586	0.585152	1.11209	0.764371375	0.966018836	20	20	24	0.957198897	0.988639288	24	12.24263006	0.030512657
ENSMUSG00000115591	Gm31282	predicted gene, 31282 [Source:MGI Symbol;Acc:MGI:5590441]	1.64086	1.69583	1.41491	1.55546	1.8608	1.6036	1.65602	1.51849	2.16269	1.49543	1.47685	1.48079	1.88593	1.6939	0.586972936	0.966018836	8	8	24	0.758698562	0.92195554	24	4.109658566	0.031654665
ENSMUSG00000115593	Gm4824	predicted gene 4824 [Source:MGI Symbol;Acc:MGI:3643521]	0.730824	0.739936	0.528827	0.413839	0.719689	0.272832	0.591859	0.973209	0.910001	0.483283	0.663367	0.627714	0.326981	0.742848	0.101520755	0.511659635	8	16	24	0.316117282	0.768040178	24	5.898356832	0.111519766
ENSMUSG00000115602	Gm7908	predicted gene 7908 [Source:MGI Symbol;Acc:MGI:3645647]	7.58649	6.94669	9.12944	10.2215	9.24587	8.12257	7.5945	7.50384	6.60565	7.56495	8.50118	7.8384	8.40856	6.91677	0.00932019	0.194720062	20	12	24	0.057922383	0.656248849	24	15.46073582	0.130271488
ENSMUSG00000115624	Gm49204	predicted gene, 49204 [Source:MGI Symbol;Acc:MGI:6118653]	1.02481	1.13381	1.1637	1.09203	1.06598	0.823723	0.950862	0.786899	1.28312	0.641719	1.25844	0.901805	0.855597	0.821919	0.591351901	0.966018836	12	20	24	0.687451429	0.89619155	24	11.48421173	0.04500773
ENSMUSG00000115632	Gm17922	predicted gene, 17922 [Source:MGI Symbol;Acc:MGI:5010107]	1.86871	2.02455	1.76058	2.13834	1.34875	1.44606	1.97435	1.67056	2.20985	1.97573	1.71007	1.07442	1.95286	1.72777	0.076241493	0.453790923	16	4	24	0.07026454	0.675025526	24	7.091144734	0.148531365
ENSMUSG00000115635	Gm49271	predicted gene, 49271 [Source:MGI Symbol;Acc:MGI:6118753]	0.941852	0.580654	0.804364	0.461486	0.401473	0.456677	0.409942	0.240424	0.550564	0.577953	0.461411	0.559108	0.538767	0.252528	0.911371589	0.966018836	12	20	24	0.954512709	0.988601783	24	18.76934282	0.019295734
ENSMUSG00000115637	Gm30970	predicted gene, 30970 [Source:MGI Symbol;Acc:MGI:5590129]	46.1318	36.3611	42.3431	43.5053	40.238	43.3129	52.658	38.1438	27.3663	42.9316	27.438	32.8981	37.1884	38.8444	0.923635729	0.966018836	4	4	24	0.554060628	0.837433699	24	23.24888498	0.101499426
ENSMUSG00000115643	Gm49011	predicted gene, 49011 [Source:MGI Symbol;Acc:MGI:6118369]	7.82373	6.89992	8.39601	11.5968	12.6534	6.45535	9.40971	7.96395	12.4918	9.32231	10.0413	8.56546	5.5404	13.3981	0.218596088	0.706608504	20	4	24	0.105400588	0.717252017	24	11.21427005	0.270217544
ENSMUSG00000115650	Gm48992	predicted gene, 48992 [Source:MGI Symbol;Acc:MGI:6118339]	1.01603	0.753263	0.598585	0.826447	0.475893	0.697786	0.930093	0.720782	0.578545	0.531377	0.582731	0.889954	0.732666	0.536457	0.05211941	0.391073722	4	16	24	0.035928581	0.609176161	24	23.06888318	0.120677855
ENSMUSG00000115692	Gm49163	predicted gene, 49163 [Source:MGI Symbol;Acc:MGI:6118588]	2.2796	3.24704	2.87696	2.60334	3.06827	2.53446	1.88126	2.69166	3.15791	2.23555	3.27541	3.10546	2.30804	3.81602	0.369262716	0.893596186	8	20	24	0.514301747	0.816624526	24	9.399909765	0.092490584
ENSMUSG00000115700	Gm7517	predicted gene 7517 [Source:MGI Symbol;Acc:MGI:3643004]	2.25881	2.05805	2.37209	2.11735	2.01376	1.81187	2.24629	2.00443	1.94071	2.37666	1.50376	1.66937	1.60577	1.63709	0.602156008	0.966018836	16	4	24	0.292190277	0.768040178	24	8.411814609	0.06461928
ENSMUSG00000115702	Gm5206	predicted pseudogene 5206 [Source:MGI Symbol;Acc:MGI:3645516]	1.4829	1.74131	2.21479	1.79329	2.13318	1.56533	1.35333	2.14645	1.19858	1.22534	1.45688	1.44558	1.17014	1.26153	0.894678274	0.966018836	20	8	24	0.746608709	0.917587548	24	11.81884572	0.04867611
ENSMUSG00000115730	Gm7459	predicted gene 7459 [Source:MGI Symbol;Acc:MGI:3645341]	2.49713	2.61549	2.32748	2.45415	2.78352	2.6315	2.54894	2.50909	2.01834	2.22225	2.9427	2.08025	2.13901	2.1706	0.424718357	0.966018836	20	16	24	0.192518171	0.768040178	24	18.02174716	0.073088236
ENSMUSG00000115737	Rpl19-ps3	ribosomal protein L19, pseudogene 3 [Source:MGI Symbol;Acc:MGI:3648038]	1.01124	1.17201	1.22709	0.53956	0.875962	0.744912	1.06224	0.821285	1.26722	0.772963	1.54549	1.30507	0.728788	0.627005	0.910436409	0.966018836	20	12	24	0.946296393	0.986887534	24	18.16923886	0.029771175
ENSMUSG00000115738	Gm7543	predicted gene 7543 [Source:MGI Symbol;Acc:MGI:3645066]	3.3226	3.2693	2.97178	2.55597	2.60961	3.24291	2.73417	2.52287	1.32979	3.23022	2.53622	2.69797	3.0095	2.1461	0.492524295	0.966018836	4	8	24	0.199777	0.768040178	24	20.68702246	0.153826094
ENSMUSG00000115756	Gm49519	predicted gene, 49519 [Source:MGI Symbol;Acc:MGI:6155213]	0.557138	0.525808	0.708332	0.785759	0.612214	0.572142	0.717135	0.712087	0.62846	0.490561	0.709456	0.603987	0.621905	0.524809	0.84680398	0.966018836	12	20	24	0.805051501	0.937541099	24	12.77699961	0.020519791
ENSMUSG00000115808	Gm18949	predicted gene, 18949 [Source:MGI Symbol;Acc:MGI:5011134]	8.92662	9.99366	8.91823	10.6104	10.3151	9.45765	8.0918	10.1669	9.71523	7.97211	9.84073	8.19562	10.4768	8.87127	0.830949782	0.966018836	20	4	24	0.908127579	0.974606327	24	10.77426778	0.022831827
ENSMUSG00000115810	Gm49066	predicted gene, 49066 [Source:MGI Symbol;Acc:MGI:6118447]	0.625863	0.953501	0.884465	1.07419	0.794359	0.708709	0.762959	1.11896	1.87872	1.03392	1.11678	1.23951	1.46117	1.45459	0.867888967	0.966018836	8	20	24	0.027223681	0.58914582	24	7.764521559	0.162015928
ENSMUSG00000115843	Gm17873	predicted gene, 17873 [Source:MGI Symbol;Acc:MGI:5010058]	2.12886	2.2564	1.99687	2.20118	1.723	2.55849	1.64807	2.15383	3.02879	2.17501	1.71854	2.42271	2.33196	1.97799	0.864782464	0.966018836	24	20	24	0.745019204	0.916569459	24	6.372169213	0.051867831
ENSMUSG00000115852	Gm52969	predicted gene, 52969 [Source:MGI Symbol;Acc:MGI:6388851]	2.32186	2.70535	2.68467	2.54003	2.54354	2.64516	3.16296	3.27233	3.4109	3.4232	1.77204	2.75861	2.59575	2.72998	0.387380622	0.915479136	12	8	24	0.197266828	0.768040178	24	6.279523618	0.124796729
ENSMUSG00000115868	Gm2999	predicted gene 2999 [Source:MGI Symbol;Acc:MGI:3781177]	272.936	291.386	285.015	276.009	279.543	266.847	282.485	265.278	298.264	247.653	285.778	294.191	289.756	274.255	0.832742389	0.966018836	12	4	24	0.793777057	0.933877786	24	1.013072395	0.019190814
ENSMUSG00000115923	Gm49521	predicted gene, 49521 [Source:MGI Symbol;Acc:MGI:6155216]	3.12805	3.13923	1.68194	2.30433	2.9061	2.65377	1.86351	3.16691	1.48099	2.49902	1.89033	2.19621	3.5391	3.30114	0.031384475	0.324385159	8	4	24	0.250434302	0.768040178	24	0.124142947	0.178050689
ENSMUSG00000115936	4833415N18Rik	RIKEN cDNA 4833415N18 gene [Source:MGI Symbol;Acc:MGI:1921011]	2.72543	3.23341	3.17934	3.1313	3.05054	3.14469	3.18247	2.76174	3.42845	2.6093	3.2091	2.86731	3.46952	3.23608	0.823657258	0.966018836	12	4	24	0.804254436	0.937432307	24	4.87516122	0.024541634
ENSMUSG00000115970	8430426J06Rik	RIKEN cDNA 8430426J06 gene [Source:MGI Symbol;Acc:MGI:1925352]	2.01032	1.67472	1.90408	3.50706	2.56726	1.27992	2.19773	1.97769	2.73818	1.79886	2.36622	2.1463	1.90958	1.76808	0.249881062	0.749643185	20	12	24	0.200531527	0.768040178	24	12.34889576	0.160928792
ENSMUSG00000115987	Vps28	vacuolar protein sorting 28 [Source:MGI Symbol;Acc:MGI:1914164]	104.777	110.45	103.6	101.557	115.609	114.053	94.1709	108.551	109.668	96.4094	110.414	103.849	107.621	103.924	0.893307667	0.966018836	20	20	24	0.779077204	0.928962679	24	18.44073754	0.024531649
ENSMUSG00000116033	Gm49442	predicted gene, 49442 [Source:MGI Symbol;Acc:MGI:6155088]	2.58667	1.98514	2.69081	2.23962	2.29578	1.80162	2.44373	2.26313	2.79273	1.79925	2.71181	1.71016	2.2715	2.06508	0.601647512	0.966018836	12	12	24	0.635375066	0.876170065	24	7.643283661	0.061661189
ENSMUSG00000116048	Septin2	septin 2 [Source:MGI Symbol;Acc:MGI:97298]	40.7081	45.2669	43.5712	41.1645	47.085	38.1182	44.8505	40.8656	48.4788	36.9574	45.2564	39.6301	40.0027	40.3161	0.245061943	0.741580974	12	12	24	0.765085501	0.92403577	24	9.097390898	0.033913073
ENSMUSG00000116056	Gm4544	predicted gene 4544 [Source:MGI Symbol;Acc:MGI:3782728]	0.174472	0.581849	0.721833	0.322558	0.264596	0.3514	0.304317	0.624894	1.29207	0.672441	0.410316	0.444031	0.678145	0.51504	0.064422003	0.414867207	12	8	24	0.011583445	0.520458883	24	7.691453547	0.243260906
ENSMUSG00000116066	Gm49477	predicted gene, 49477 [Source:MGI Symbol;Acc:MGI:6155147]	0.293411	0.655751	0.76558	0.739115	0.87688	0.93099	0.286215	1.25958	1.68197	1.12448	0.604437	0.337222	0.430403	0.83498	0.018894761	0.256345714	12	16	24	0.024085754	0.586541342	24	9.578350486	0.306111578
ENSMUSG00000116089	Gm7859	predicted gene 7859 [Source:MGI Symbol;Acc:MGI:3646834]	5.98749	5.6016	4.89931	5.61474	5.57043	5.86068	4.59135	5.9505	4.63392	4.53471	5.12166	4.60189	4.9387	5.80813	0.705189716	0.966018836	8	4	24	0.64547783	0.879852798	24	0.253761302	0.044662807
ENSMUSG00000116090	Rpl19-ps6	ribosomal protein L19, pseudogene 6 [Source:MGI Symbol;Acc:MGI:3644218]	6.42286	7.70962	9.54112	7.30809	9.18384	7.86736	7.12514	8.57993	9.51357	6.0567	8.5836	5.03116	6.511	6.78883	0.071569005	0.440262583	12	16	24	0.284301821	0.768040178	24	9.606200843	0.14088881

ENSMUSG00000116093	Gm3888	predicted gene 3888 [Source:MGI Symbol;Acc:MGI:3782061]	9.91178	9.79967	9.82011	9.31789	7.96086	10.3564	9.67259	8.32608	8.21136	9.32228	8.67965	9.42859	9.43737	9.78998	0.25849765	0.761738864	24	20	24	0.30009166	0.768040178	24	23.9286579	0.061161284
ENSMUSG00000116097	Gm36738	predicted gene, 36738 [Source:MGI Symbol;Acc:MGI:5595897]	1.21951	1.23135	1.88862	1.36984	1.0203	1.54713	1.68859	2.15345	1.29715	0.806908	1.76746	0.903897	1.52769	1.61528	0.867888967	0.966018836	12	4	24	0.320700013	0.768040178	24	3.837823187	0.134870028
ENSMUSG00000116114	Gm35853	predicted gene, 35853 [Source:MGI Symbol;Acc:MGI:5595012]	0.233762	1.06963	0.773343	1.0496	0.647797	0.728006	0.819468	0.942188	0.678524	0.551246	1.0984	0.954497	1.1211	1.2348	0.89563591	0.966018836	8	8	24	0.74790077	0.917930198	24	6.368732173	0.062036826
ENSMUSG00000116121	Gm49486	predicted gene, 49486 [Source:MGI Symbol;Acc:MGI:6155161]	1.10506	0.532359	0.546562	0.330935	0.418072	0.516516	0.545984	0.735029	0.595505	0.569625	0.644516	0.433605	0.768159	0.387756	0.644063931	0.966018836	4	12	24	0.24682289	0.768040178	24	0.531049814	0.105324557
ENSMUSG00000116125	Gm41361	predicted gene, 41361 [Source:MGI Symbol;Acc:MGI:5624246]	1.02781	1.65084	1.30273	1.65929	0.827768	1.6899	1.35546	1.37441	1.69154	2.03812	0.996101	1.26267	1.20267	1.48671	0.05238827	0.391426791	16	4	24	0.21942639	0.768040178	24	8.050669203	0.139033542
ENSMUSG00000116138	C030006K11Rik	RIKEN cDNA C030006K11 gene [Source:MGI Symbol;Acc:MGI:1925941]	5.58625	5.1911	5.43579	6.9399	4.36483	5.27659	6.00197	5.54207	5.50398	8.08692	5.50913	5.704	5.8895	5.78597	0.920620634	0.966018836	16	4	24	0.457937445	0.805393068	24	10.16109554	0.085179437
ENSMUSG00000116145	5730521K06Rik	RIKEN cDNA 5730521K06 gene [Source:MGI Symbol;Acc:MGI:1917889]	0.370907	0.495126	0.608819	0.509176	0.728401	0.481517	0.499958	0.563846	0.440303	0.557587	0.582983	0.431787	0.528849	0.454032	0.265774856	0.773510818	20	4	24	0.190637263	0.768040178	24	13.27078896	0.056572044
ENSMUSG00000116164	1110013H19Rik	RIKEN cDNA 1110013H19 gene [Source:MGI Symbol;Acc:MGI:1915772]	0.314013	0.51783	0.682425	1.16445	0.409066	0.576077	0.28682	1.05066	0.641893	0.570739	0.752611	0.904799	0.419615	0.591247	0.109247218	0.524035616	16	12	24	0.22694884	0.768040178	24	11.01434074	0.147858959
ENSMUSG00000116165	Pdxp	pyridoxal (pyridoxine, vitamin B6) phosphatase [Source:MGI Symbol;Acc:MGI:1919282]	6.83191	6.83332	7.73965	5.29105	5.57763	7.29271	7.16414	6.0293	7.1981	7.10268	5.38515	8.29539	5.82611	8.09696	0.708020493	0.966018836	24	20	24	0.406067853	0.799546792	24	3.031184809	0.096650627
ENSMUSG00000116180	Gm49492	predicted gene, 49492 [Source:MGI Symbol;Acc:MGI:6155169]	1.83096	2.43518	2.42366	2.28354	1.98745	2.13872	2.05685	2.16475	2.04419	2.69069	1.72047	2.55457	2.00392	2.62425	0.327894132	0.846828588	16	4	24	0.326547793	0.768040178	24	7.866171254	0.080134326
ENSMUSG00000116207	Nnt	nicotinamide nucleotide transhydrogenase [Source:MGI Symbol;Acc:MGI:109279]	4.63727	3.5968	3.39871	3.09805	3.34663	4.34347	4.27132	3.53633	2.52225	4.4248	3.35245	3.12179	3.29315	2.71807	0.801561896	0.966018836	4	8	24	0.172231151	0.768040178	24	20.71563306	0.135563595
ENSMUSG00000116215	Gm5470	predicted gene 5470 [Source:MGI Symbol;Acc:MGI:3645123]	1.1322	1.47178	1.23179	1.77309	1.18086	1.50464	1.2292	0.903012	1.22596	1.12179	1.21399	1.0802	1.10827	0.958198	0.929640773	0.966018836	12	20	24	0.294387802	0.768040178	24	14.71303411	0.071730275
ENSMUSG00000116223	Gm41396	predicted gene, 41396 [Source:MGI Symbol;Acc:MGI:5624281]	0.597901	0.826453	1.31292	0.371119	0.826333	0.892544	0.327876	0.552916	1.0711	0.697804	0.654811	0.766325	0.555934	1.0048	0.182849367	0.659303873	12	16	24	0.463918957	0.805393068	24	8.653235372	0.113243485
ENSMUSG00000116226	Gm49502	predicted gene, 49502 [Source:MGI Symbol;Acc:MGI:6155188]	1.34259	1.35631	1.17332	1.85269	1.76037	1.26555	1.64092	1.49869	1.87971	1.08822	1.7695	1.49853	1.51309	1.29593	0.881996584	0.966018836	12	20	24	0.664235336	0.886686094	24	15.3859122	0.053166319
ENSMUSG00000116275	Zc3h11a	zinc finger CCCH type containing 11A [Source:MGI Symbol;Acc:MGI:1917829]	22.9609	15.2334	22.9178	27.0203	18.5019	19.8506	21.3319	22.5015	22.4907	20.6341	22.1481	20.0357	25.8536	20.7194	0.883024237	0.966018836	4	20	24	0.868186975	0.962417689	24	10.39640338	0.041764151
ENSMUSG00000116288	Gm41349	predicted gene, 41349 [Source:MGI Symbol;Acc:MGI:5624234]	1.39455	2.10615	2.23063	2.16713	2.5519	1.58563	2.20293	2.31589	2.18628	2.14056	1.63759	1.90702	1.52869	1.83372	0.248423582	0.746945512	12	16	24	0.117843287	0.728323355	24	9.917552353	0.132026552
ENSMUSG00000116295	Gm32885	predicted gene, 32885 [Source:MGI Symbol;Acc:MGI:5592044]	0.324323	1.00312	0.264294	0.388085	1.02052	0.640414	0.704602	1.54145	1.53897	0.696813	0.789133	0.974908	1.29603	0.932532	0.936399519	0.966018836	8	8	24	0.553947809	0.837374348	24	3.95228798	0.115560747
ENSMUSG00000116305	Lncppara	long noncoding RNA near Ppara [Source:MGI Symbol;Acc:MGI:5568843]	1.9852	2.58695	3.57126	2.31192	1.9694	2.15715	2.47655	2.17289	2.98353	2.22354	2.21852	2.44119	2.01277	2.20421	0.089177696	0.476253254	12	8	24	0.109027208	0.72082227	24	8.038276091	0.136654802
ENSMUSG00000116398	Gm5216	predicted gene 5216 [Source:MGI Symbol;Acc:MGI:3645017]	0.383786	0.418417	0.507071	0.410327	0.47383	0.640635	0.502352	0.492179	0.39382	0.579405	0.718448	0.313672	0.783422	0.406952	0.880247609	0.966018836	20	12	24	0.683686989	0.894811431	24	17.86781435	0.04356097
ENSMUSG00000116504	I730030J21Rik	RIKEN cDNA I730030J21 gene [Source:MGI Symbol;Acc:MGI:3588279]	3.75478	4.56099	4.66769	3.09744	4.47376	4.88449	3.44018	4.63197	4.93486	3.38404	3.81595	3.79025	3.92912	3.64954	0.503972924	0.966018836	12	4	24	0.784591741	0.930879688	24	4.236227768	0.047607301
ENSMUSG00000116508	Gm49463	predicted gene, 49463 [Source:MGI Symbol;Acc:MGI:6155122]	1.20441	1.67058	1.67731	1.89296	1.37084	1.46461	1.12337	1.89319	1.46798	1.55034	1.22042	1.56805	1.33276	1.30695	0.092972576	0.489012695	12	16	24	0.166221862	0.767020009	24	9.310085361	0.100703377
ENSMUSG00000116518	Gm49494	predicted gene, 49494 [Source:MGI Symbol;Acc:MGI:6155173]	0.808539	0.756237	0.282005	0.37678	0.489564	0.291409	0.591473	0.193601	0.554186	0.409476	0.740606	0.834588	0.407899	0.522616	0.68441981	0.966018836	24	16	24	0.473268588	0.805393068	24	20.84054258	0.098960398
ENSMUSG00000116536	Gm2805	predicted gene 2805 [Source:MGI Symbol;Acc:MGI:3780974]	6.16019	5.25741	5.44916	3.75154	8.35269	6.52504	4.991	5.57498	5.03897	4.60128	4.58668	4.25776	3.59551	3.29773	0.816406072	0.966018836	20	20	24	0.327519163	0.768040178	24	18.29925007	0.141420931
ENSMUSG00000116550	Gm3507	predicted gene 3507 [Source:MGI Symbol;Acc:MGI:3781684]	1.6773	1.35929	1.45681	2.15648	0.992812	1.2546	0.543651	0.795062	0.684009	0.739361	0.914698	0.79128	0.899629	1.10515	0.74401906	0.966018836	16	12	24	0.875364621	0.965073441	24	11.9459448	0.047411812
ENSMUSG00000116557	4933432I09Rik	RIKEN cDNA 4933432I09 gene [Source:MGI Symbol;Acc:MGI:1914029]	0.900318	0.621395	0.756433	0.826971	1.03937	0.504448	0.781962	1.00752	0.655144	0.595722	0.747812	0.484987	0.658663	0.567459	0.852921792	0.966018836	20	4	24	0.950759167	0.988257443	24	10.85372445	0.016821732
ENSMUSG00000116564	Riok2	RIO kinase 2 [Source:MGI Symbol;Acc:MGI:1914295]	11.3933	13.425	13.6403	12.3253	12.7681	11.9756	12.1398	12.1578	12.5497	10.3843	13.0253	12.6306	12.4534	12.0327	0.762156552	0.966018836	12	16	24	0.954440441	0.988601783	24	5.44954812	0.011330344
ENSMUSG00000116594	Gm49601	predicted gene, 49601 [Source:MGI Symbol;Acc:MGI:6215011]	15.5084	15.0704	12.747	13.6725	17.2418	14.48	12.8275	12.0768	14.4585	10.7215	15.9873	11.6332	12.8437	13.46	0.911135828	0.966018836	20	20	24	0.550975585	0.836129338	24	18.34672872	0.072315649
ENSMUSG00000116610	Gm49678	predicted gene, 49678 [Source:MGI Symbol;Acc:MGI:6215126]	16.3057	15.1371	15.7575	15.7133	14.8216	15.947	17.2626	15.353	13.1652	18.0419	14.9854	18.2067	19.2504	18.5488	0.210566453	0.69936851	4	16	24	0.28266369	0.768040178	24	23.02951205	0.077457259
ENSMUSG00000116614	Gm46610	predicted gene, 46610 [Source:MGI Symbol;Acc:MGI:5826247]	4.55761	4.74415	3.25011	3.53448	3.45719	4.22301	4.90149	3.34938	3.3588	1.89462	3.80771	3.27518	4.89805	3.58183	0.004194713	0.140598674	4	12	24	0.00741667	0.47735824	24	23.50337427	0.274405147
ENSMUSG00000116617	Gm49767	predicted gene, 49767 [Source:MGI Symbol;Acc:MGI:6215277]	1.75667	1.35074	1.01383	1.49738	1.51062	1.40868	1.34078	0.880087	1.39699	1.34689	1.63795	1.52551	1.60369	1.41141	0.133576635	0.568189321	20	16	24	0.104957214	0.717030134	24	18.77151613	0.118151882
ENSMUSG00000116620	Gm4828	predicted gene 4828 [Source:MGI Symbol;Acc:MGI:3643809]	0.367675	0.930611	0.532092	0.555133	0.905062	0.754833	1.191	1.19674	0.749118	0.891514	1.08454	0.947499	0.904836	0.0934502	0.798829513	0.966018836	20	16	24	0.575809706	0.848235546	24	18.20705332	0.120371654
ENSMUSG00000116622	Gm5221	predicted gene 5221 [Source:MGI Symbol;Acc:MGI:3648101]	0.790907	0.326566	0.483789	0.793704	0.554941	0.589207	0.964932	0.752388	0.303824	0.489881	0.373862	0.409775	0.329279	0.516896	0.956178643	0.96629412	4	8	24	0.668557949	0.888007884	24	22.1955003	0.063782775
ENSMUSG00000116632	Magef1	MAGE family member F1 [Source:MGI Symbol;Acc:MGI:1923472]	3.5515	3.7767	3.34267	4.00145	4.74763	3.60709	4.4464	3.328	4.47086	3.76957	3.25868	3.72028	4.67898	3.42937	0.890176313	0.966018836	16	16	24	0.884743338	0.967171141	24	18.7211329	0.03127343
ENSMUSG00000116635	Gm49699	predicted gene, 49699 [Source:MGI Symbol;Acc:MGI:6215157]	1.62234	0.914065	1.40512	1.18715	1.24565	1.421	2.29499	1.80411	0.911104	1.10936	0.906888	1.61076	1.44671	1.31159	0.017148991	0.255326033	4	12	24	0.061104742	0.660703626	24	23.40152468	0.19475384
ENSMUSG00000116637	Gm8130	predicted gene 8130 [Source:MGI Symbol;Acc:MGI:3647919]	1.17851	2.04097	1.49326	1.34006	1.5297	1.76745	1.79196	1.74846	2.13816	1.03881	1.3414	1.38731	1.64381	1.11572	0.718086778	0.966018836	12	4	24	0.539133543	0.829144433	24	2.761669046	0.080600261
ENSMUSG00000116640	Gm8670	predicted gene 8670 [Source:MGI Symbol;Acc:MGI:3643441]	0.851815	1.11664	1.02393	1.13212	0.705234	1.09517	0.875396	1.04422	1.43952	1.1255	0.987038	1.05708	0.955196	1.17047	0.083084111	0.462922111	12	16	24	0.05919187	0.658304792	24	7.980356401	0.105106399
ENSMUSG00000116673	A630089N07Rik	RIKEN cDNA A630089N07 gene [Source:MGI Symbol;Acc:MGI:2444323]	1.43306	1.15711	1.30898	1.15962	1.31986	1.3828	1.04828	0.99938	0.736511	1.20625	1.31824	1.03267	1.27135	1.12117	0.374467935	0.89742288	20	12	24	0.153925298	0.760223561	24	18.88180934	0.091179244
ENSMUSG00000116718	Gm49668	predicted gene, 49668 [Source:MGI Symbol;Acc:MGI:6215110]	2.23063	2.56038	2.2308	2.85451	1.95874	1.91366	2.32556	1.6959	2.73667	2.07241	2.25612	2.46073	2.76112	2.65435	0.906657897	0.966018836	16	4	24	0.659356773	0.884879972	24	6.595562193	0.057347533
ENSMUSG00000116733	6330408M09Rik	RIKEN cDNA 6330408M09 gene [Source:MGI Symbol;Acc:MGI:1917999]	0.205293	0.626239	0.491248	0.282961	0.523216	0.420158	0.300711	0.662369	0.81369	0.325487	0.56527	0.653771	0.546532	0.642478	0.387380622	0.915479136	8	8	24	0.406780673	0.799546792	24	6.600181819	0.082777519
ENSMUSG00000116753	Gm36001	predicted gene, 36001 [Source:MGI Symbol;Acc:MGI:5595160]	0.598029	1.28083	0.821848	0.832927	1.11089	0.752915	0.691972	1.07318	1.05474	0.727663	1.17156	0.96095	0.878954	1.21625	0.265774856	0.773510818	8	20	24	0.560397933	0.841060952	24	8.148089458	0.068071811
ENSMUSG00000116772	4930483P17Rik	RIKEN cDNA 4930483P17 gene [Source:MGI Symbol;Acc:MGI:1925419]	0.796889	0.455579	0.433283	0.571181	0.78274	0.537086	0.60763	0.312136	0.340125	0.329059	0.308185	0.50818	0.663565	0.372043	0.172322601	0.648362388	4	8	24	0.034421244	0.608551791	24	20.71514676	0.136960531
ENSMUSG00000116775	Gm49769	predicted gene, 49769 [Source:MGI Symbol;Acc:MGI:6215281]	3.48148	2.62736	3.13664	2.90337	2.8411	3.73422	3.41135	2.4226	1.96625	4.15342	1.6848	3.5918	2.66834	3.71608	0.745118544	0.966018836	24	12	24	0.712968103	0.905403147	24	21.98699151	0.09154381
ENSMUSG00000116802	Gm5165	predicted gene 5165 [Source:MGI Symbol;Acc:MGI:3646034]	2.34343	2.63006	2.07963	2.58552	2.87636	2.60123	2.75501	2.4354	2.92308	1.58396	2.96187	2.66199	2.49907	2.39718	0.401437339	0.933249185	20	20	24	0.482182486	0.805393068	24	20.51766203	0.079745089
ENSMUSG00000116808	Gm20164	predicted gene, 20164 [Source:MGI Symbol;Acc:MGI:5012349]	1.05284	1.32336	0.854605	2.3382	1.3315	0.721244	0.635682	0.461275	1.41908	0.52287	0.352762	1.03418	1.6101	0.828993	0.816300547	0.966018836	12	8	24	0.929015372	0.981515518	24	9.662654123	0.056421835
ENSMUSG00000116811	Gm16385	predicted gene 16385 [Source:MGI Symbol;Acc:MGI:3780125]	2.07217	2.12838	2.52977	2.03964	1.93324	1.56234	1.65173	1.81898	1.41592	2.09529	1.77544	2.02044	2.64602	1.95437	0.843694678	0.966018836	4	20	24	0.885693755	0.967521328	24	4.699536793	0.032552669
ENSMUSG00000116815	Gm49760	predicted gene, 49760 [Source:MGI Symbol;Acc:MGI:6215265]	0.635183	0.571845	0.770931	0.703592	0.918481	0.654841	0.358213	0.482652	0.534925	0.548604	0.876517	0.714071	0.68172	0.561659	0.016720774	0.253395174	20	12	24	0.042599863	0.623773613	24	15.62160967	0.118117846
ENSMUSG00000116838	Gm6475	predicted gene 6475 [Source:MGI Symbol;Acc:MGI:3648186]	7.70867	8.72164	8.29144	8.78075	7.47936	7.99306	8.57393	7.88765	8.60908	9.1642	8.55877	9.31095	9.81067	9.67168	0.874054778	0.966018836	16	4	24	0.473569865	0.805393068	24	5.87908783	0.037481006
ENSMUSG00000116840	Gm21691	predicted gene, 21691 [Source:MGI Symbol;Acc:MGI:5435046]	0.604362	0.598326	0.565017	0.557011	0.519131	0.56666	0.216808	0.654711	0.602718	0.548323	0.374193	0.678711	0.574971	0.707852	0.017417473	0.255326033	8	12	24	0.549314347	0.834904854	24	6.31704616	0.055831689
ENSMUSG00000116845	Gm46546	predicted gene, 46546 [Source:MGI Symbol;Acc:MGI:5826183]	9.99894	13.8374	11.7726	10.42	15.4357	12.2099	11.4802	12.2053	14.3184	12.9479	20.7271	9.3592	18.7644	18.7701	0.94663657	0.966018836	20	4	24	0.850546673	0.95526784	24	9.100202676	0.067121723
ENSMUSG00000116847	Gm30251	predicted gene, 30251 [Source:MGI Symbol;Acc:MGI:5589410]	0.989467	1.35409	0.42984	1.01247	0.513668	0.890239	0.361068	1.49722	1.279	0.98121	1.2162	0.762478	0.530009	0.599644	0.909604019	0.966018836	8	20	24	0.6833524	0.894571336	24	9.069953396	0.102505877
ENSMUSG00000116858	Gm49797	predicted gene, 49797 [Source:MGI Symbol;Acc:MGI:6215329]	4.79396	4.73276	4.41207	4.92155	5.00607	4.37435	4.20043	4.68518	4.45308	4.69302	4.08686	4.1132	5.04259	4.61769	0.911371589	0.966018836	4	20	24	0.767018149	0.924684154	24	6.868771434	0.024325241
ENSMUSG00000116875	Morf4l1-ps1	mortality factor 4 like 1, pseudogene 1 [Source:MGI Symbol;Acc:MGI:3612158]	63.1146	60.0137	58.2317	58.4756	54.4712	59.8666	57.5714	59.1587	63.0382	61.4406	60.4834	68.0718	67.6942	64.1906	0.961261938	0.967043964	24	20	24	0.297950484	0.768040178	24	0.966711839	0.042095728
ENSMUSG00000116883	Gm49700	predicted gene, 49700 [Source:MGI Symbol;Acc:MGI:6215158]	0.601993	0.379178	0.592611	0.593981	0.581523	0.653421	0.455237	0.643248	0.666692	0.492293	0.711621	0.583231	0.495413	0.681128	0.879206539	0.966018836	12	16	24	0.624022869	0.871879816	24	13.59138817	0.032495727
ENSMUSG00000116884	C130040N14Rik	RIKEN cDNA C130040N14 gene [Source:MGI Symbol;Acc:MGI:3588257]	0.415869	0.457314	0.767549	0.598212	0.275155	0.244285	0.392302	0.859987	0.655655	0.521734	0.53446	0.759432	0.593818	0.608716	0.162786057	0.624917386	12	8	24	0.070824051	0.676331884	24	7.08138555	0.140645728
ENSMUSG00000116895	Gm3435	predicted gene 3435 [Source:MGI Symbol;Acc:MGI:3810125]	0.749641	0.738203	0.203812	0.537597	0.14033	0.452312	0.9719	1.13297	1.05611	0.742889	0.90232	0.328521	0.405619	1.0141	0.235141005	0.73629392	8	16	24	0.18983171	0.768040178	24	4.946709278	0.201335463
ENSMUSG00000116898	Gm49785	predicted gene, 49785 [Source:MGI Symbol;Acc:MGI:6215307]	2.31623	1.86588	2.00652	1.29084	1.513	1.14724	2.23989	0.813844	2.34466	2.14674	2.17959	1.20958	1.5703	1.63128	0.8972237	0.966018836	12	12	24	0.822357945	0.944152607	24	8.905308306	0.071799904
ENSMUSG00000116912	Gm5487	predicted gene 5487 [Source:MGI Symbol;Acc:MGI:3647872]	1.41018	1.55681	1.51307	1.71746	1.79367	1.38314	1.52937	1.67752	1.49548	1.50849	1.24786	1.4378	1.53003	1.33869	0.9143748	0.966018836	16	8	24	0.690149375	0.897381208	24	10.10845161	0.027766339
ENSMUSG00000116936	Bod1-ps	biorientation of chromosomes in cell division 1, pseudogene [Source:MGI Symbol;Acc:MGI:3646992]	4.39829	4.68374	6.97886	5.24457	6.09023	5.62976	5.07151	4.61021	4.13887	3.87761	4.53516	4.42613	3.32718	3.68776	0.914360142	0.966018836	20	8	24	0.293940513	0.768040178	24	14.91015517	0.106275623
ENSMUSG00000116946	Gm41442	predicted gene, 41442 [Source:MGI Symbol;Acc:MGI:5624327]	2.21743	1.57455	2.52154	1.85347	2.13438	2.3408	1.37806	1.87334	1.46353	2.39856	2.28121	1.74292	1.63833	1.77048	0.40303616	0.935844475	16	16	24	0.162148728	0.76636332	24	14.78465733	0.126244781
ENSMUSG00000116957	Gm34680	predicted gene, 34680 [Source:MGI Symbol;Acc:MGI:5593839]	0.367614	0.65726	0.667124	0.612515	0.400215	0.562266	0.750641	0.443097	0.597722	0.401814	0.288021	0.338842	0.505899	0.457848	0.285027487	0.793714531	12	8	24	0.212353783	0.768040178	24	5.319018428	0.084544088
ENSMUSG00000116958	Gm6705	predicted gene 6705 [Source:MGI Symbol;Acc:MGI:3648191]	8.78354	6.12399	7.96792	8.63099	6.65483	8.92826	8.55888	7.77086	5.51751	6.71381	6.53981	6.40478	6.12253	5.74896	0.823657258	0.966018836	16	20	24	0.362221774	0.787064638	24	20.00041791	0.099317256
ENSMUSG00000116972	Gm6278	predicted gene 6278 [Source:MGI Symbol;Acc:MGI:3644862]	0.392296	0.630772	0.923915	0.972082	0.487894	0.554986	0.159382	0.35057	0.624347	0.475268	0.672823	0.459659	0.523508	0.588697	0.080148772	0.458678242	12	16	24	0.053606233	0.653526039	24	10.97969216	0.170097893
ENSMUSG00000116995	Gm21926	predicted gene, 21926 [Source:MGI Symbol;Acc:MGI:5439378]	11.6144	11.908	10.5498	12.2733	12.0876	12.5594	11.6288	10.7875	10.5356	11.6418	11.7681	11.3064	11.3786	11.3759	0.056712099	0.401309455	16	20	24	0.035806067	0.609176161	24	18.23063285	0.064463947
ENSMUSG00000117003	Gm49937	predicted gene, 49937 [Source:MGI Symbol;Acc:MGI:6270649]	0.965436	0.80357	0.917362	0.831078	0.842598	1.02432	0.861822	0.688832	0.755511	0.941172	0.836365	0.739087	0.844271	1.00888	0.891022863	0.966018836	4	4	24	0.873594227	0.96459704	24	19.75110756	0.016997498
ENSMUSG00000117004	Gm38593	predicted gene, 38593 [Source:MGI Symbol;Acc:MGI:5621478]	0.336047	1.06539	0.912755	1.05342	0.749698	0.947477	0.920527	0.674186	0.514982	0.721889	0.747318	0.592195	0.786302	0.633729	0.768363194	0.966018836	16	20	24	0.804626144	0.937447587	24	12.53357857	0.04532309
ENSMUSG00000117011	E130008D07Rik	RIKEN cDNA E130008D07 gene [Source:MGI Symbol;Acc:MGI:3584523]	0.498574	0.542631	0.635757	0.581412	0.663241	0.486295	0.379526	0.518838	0.50183	0.533739	0.533617	0.535374	0.620087	0.693107	0.582224696	0.966018836	8	20	24	0.463482975	0.805393068	24	10.04091133	0.037659767
ENSMUSG00000117013	Gm30531	predicted gene, 30531 [Source:MGI Symbol;Acc:MGI:5589690]	3.02015	2.89214	2.76325	2.95422	3.2902	2.88576	2.42437	2.72025	2.49816	2.97024	3.37938	2.88542	2.31536	3.33174	0.208341949	0.698704663	20	8	24	0.259868977	0.768040178	24	14.81941402	0.075964547
ENSMUSG00000117027	Gm49886	predicted gene, 49886 [Source:MGI Symbol;Acc:MGI:6270570]	0.785903	1.06411	0.640692	0.898786	0.733044	1.03767	1.357	0.877866	1.65278	0.649174	1.16814	1.33943	1.17986	0.933678	0.668329321	0.966018836	4	12	24	0.576398761	0.848607756	24	23.60927375	0.079520098
ENSMUSG00000117037	Gm50433	predicted gene, 50433 [Source:MGI Symbol;Acc:MGI:6303365]	1.67538	1.56598	1.05903	2.21757	1.63504	1.64675	2.04406	0.658068	1.33613	0.917628	1.05789	1.85882	1.62191	1.86302	0.470672946	0.966018836	4	8	24	0.360651952	0.786233489	24	21.00994775	0.158380095
ENSMUSG00000117042	2700054A10Rik	RIKEN cDNA 2700054A10 gene [Source:MGI Symbol;Acc:MGI:1919828]	0.564594	0.467973	0.608455	0.770709	0.609216	0.57779	0.55376	0.593552	0.694797	0.445859	0.422812	0.425541	0.411215	0.359258	0.46238066	0.966018836	12	20	24	0.310983167	0.768040178	24	11.36906412	0.051530036
ENSMUSG00000117049	Gm6540	predicted gene 6540 [Source:MGI Symbol;Acc:MGI:3647824]	0.493083	0.776795	0.536203	0.452423	0.627378	1.11952	0.782056	0.79467	0.808182	0.488658	0.542452	0.471629	0.459834	0.810209	0.5169953	0.966018836	8	8	24	0.535231819	0.82691856	24	2.011937489	0.072741536
ENSMUSG00000117050	1700023B13Rik	RIKEN cDNA 1700023B13 gene [Source:MGI Symbol;Acc:MGI:1920529]	1.4397	1.11	1.11951	1.52766	1.48243	1.35445	1.75681	1.20899	1.32152	1.17763	1.88796	1.46318	1.55376	1.04031	0.030972684	0.324150946	20	12	24	0.036207684	0.609176161	24	18.49467713	0.13900476
ENSMUSG00000117055	Gm9805	predicted gene 9805 [Source:MGI Symbol;Acc:MGI:3642566]	0.732009	0.642192	0.686659	0.814084	0.872942	0.734064	0.647066	0.770622	1.07078	0.666864	0.835876	0.786	0.754742	0.71152	0.24445174	0.741580974	20	12	24	0.374955407	0.794931845	24	12.76467692	0.04700725
ENSMUSG00000117059	Gm7912	predicted gene 7912 [Source:MGI Symbol;Acc:MGI:3644792]	0.525345	0.655355	1.03875	1.04102	0.786612	0.677913	0.874096	0.727069	0.554538	0.725713	0.492673	0.578495	0.858814	0.641782	0.872380105	0.966018836	12	8	24	0.658741315	0.884596426	24	9.352861247	0.052858905
ENSMUSG00000117098	Gm49909	predicted gene, 49909 [Source:MGI Symbol;Acc:MGI:6270609]	7.8811	9.87828	11.53	10.0277	6.03228	8.15604	10.1785	9.49456	7.13412	8.66019	8.02689	6.25109	6.2617	8.33623	0.23371652	0.736061043	16	4	24	0.133257563	0.743843563	24	6.970156704	0.078405559
ENSMUSG00000117102	-	Novel transcript	0.949483	1.05092	0.898674	0.952952	1.15081	0.943664	0.975852	1.07646	1.38527	0.935986	1.12202	0.934168	1.14156	1.27181	0.843639218	0.966018836	8	16	24	0.377636604	0.797541458	24	6.741559665	0.049800123
ENSMUSG00000117113	Gm49883	predicted gene, 49883 [Source:MGI Symbol;Acc:MGI:6270565]	2.59393	3.14412	3.17352	2.95695	2.95805	2.70006	3.0501	2.59237	3.16626	2.55474	2.94251	2.65047	2.66245	3.11224	0.22872448	0.72665454	12	8	24	0.274687913	0.768040178	24	8.075786082	0.056845538
ENSMUSG00000117115	Gm9586	predicted gene 9586 [Source:MGI Symbol;Acc:MGI:3779995]	3.05206	2.76055	2.05019	2.31832	2.11808	2.47329	2.51962	2.05059	2.51435	2.9652	2.2851	2.4598	3.1677	2.64496	0.138543921	0.580158868	4	16	24	0.397750571	0.799546792	24	0.368129454	0.075954015
ENSMUSG00000117171	C230013L11Rik	RIKEN cDNA C230013L11 gene [Source:MGI Symbol;Acc:MGI:2442581]	0.464403	0.528375	0.45138	0.53825	0.634354	0.518681	0.473243	0.484743	0.452592	0.642781	0.489025	0.603222	0.631876	0.426843	0.079180494	0.458678242	16	20	24	0.143768329	0.752803875	24	16.79921396	0.051602509
ENSMUSG00000117183	Gm20008	predicted gene, 20008 [Source:MGI Symbol;Acc:MGI:5012193]	13.3838	14.2922	12.8479	13.6773	16.0322	11.8017	14.3864	11.7864	16.7038	9.88152	15.4501	13.3483	14.318	12.9755	0.94499634	0.966018836	20	12	24	0.952081209	0.988339231	24	19.47593465	0.024304524
ENSMUSG00000117187	Gm4708	predicted gene 4708 [Source:MGI Symbol;Acc:MGI:3782888]	188.054	178.34	172.342	172.657	170.689	171.36	175.705	189.32	174.684	163.37	203.177	183.021	202.107	179.349	0.101443845	0.511659635	4	12	24	0.252823792	0.768040178	24	23.25784855	0.055268188
ENSMUSG00000117192	Gm18649	predicted gene, 18649 [Source:MGI Symbol;Acc:MGI:5010834]	3.23144	2.24778	2.48364	2.89115	2.57974	2.33203	2.45172	2.74537	1.91869	3.91823	3.0198	3.99669	2.26195	4.01693	0.942051135	0.966018836	16	20	24	0.797422783	0.935271704	24	16.00216369	0.063009059
ENSMUSG00000117202	Gm9349	predicted gene 9349 [Source:MGI Symbol;Acc:MGI:3648407]	33.4065	41.9877	38.4515	36.1772	43.6354	40.2319	31.5749	35.0981	38.2271	28.4685	35.3083	30.9982	36.0255	34.1463	0.92655264	0.966018836	12	16	24	0.979913558	0.995100119	24	11.54873923	0.01240586
ENSMUSG00000117217	Gm4766	predicted pseudogene 4766 [Source:MGI Symbol;Acc:MGI:3649016]	1.1782	1.11929	1.44828	0.998453	1.3031	1.42424	1.12483	1.05466	0.909449	1.18458	1.05637	1.04556	1.05802	1.56952	0.801273005	0.966018836	12	12	24	0.94546915	0.98679581	24	0.623264979	0.016695827
ENSMUSG00000117219	Gm49911	predicted gene, 49911 [Source:MGI Symbol;Acc:MGI:6270612]	2.26296	3.89099	1.74767	4.63728	3.47789	3.51789	2.67694	2.82073	3.9134	2.3555	4.52642	3.40533	2.42781	3.60119	0.832742389	0.966018836	20	8	24	0.717897677	0.907012192	24	16.91993476	0.106998741
ENSMUSG00000117220	Gm49932	predicted gene, 49932 [Source:MGI Symbol;Acc:MGI:6270641]	0.969298	0.707203	0.483597	0.879013	0.561904	0.663676	0.637014	0.425761	0.592041	0.839063	0.599179	0.750658	0.534277	0.857286	0.891742979	0.966018836	12	20	24	0.899059698	0.971453495	24	18.05894743	0.026582182
ENSMUSG00000117234	Gm7818	predicted gene 7818 [Source:MGI Symbol;Acc:MGI:3643608]	3.55729	4.25312	4.14154	3.81115	4.17087	4.15649	4.65048	3.46241	4.08505	3.95965	4.49665	4.55262	4.01644	4.05925	0.61767505	0.966018836	20	20	24	0.428698627	0.805393068	24	18.12789642	0.04955986
ENSMUSG00000117246	Gm17891	predicted gene, 17891 [Source:MGI Symbol;Acc:MGI:5010076]	0.969509	0.426656	0.843096	1.18287	0.723562	0.695031	0.630258	0.866466	0.793586	0.640665	0.886918	0.949998	0.819332	0.824261	0.7866493	0.966018836	16	16	24	0.703663487	0.902090877	24	14.51075804	0.048413253
ENSMUSG00000117252	Gm9514	predicted gene 9514 [Source:MGI Symbol;Acc:MGI:3779924]	7.55112	7.40805	7.74021	7.91283	6.70108	6.84018	5.73785	7.37575	6.7925	7.04955	6.84537	6.57656	6.18231	7.11185	0.184505544	0.662239322	8	20	24	0.097082081	0.71175955	24	9.290093537	0.078432584
ENSMUSG00000117256	Gm5497	predicted gene 5497 [Source:MGI Symbol;Acc:MGI:3646432]	1.98208	2.2188	1.43117	1.96659	2.40155	1.82092	1.33163	0.580915	4.01501	2.18675	2.66761	2.01022	2.23747	2.06318	0.784278077	0.966018836	20	8	24	0.410773897	0.799546792	24	13.55934852	0.188386756
ENSMUSG00000117257	Gm4948	predicted gene 4948 [Source:MGI Symbol;Acc:MGI:3645145]	1.15544	1.06742	0.742404	1.02343	1.17855	0.983165	1.15315	1.22215	0.50202	1.00631	0.577524	0.837628	0.279971	0.51041	0.829428469	0.966018836	8	4	24	0.62563445	0.872249537	24	18.69894105	0.075281774
ENSMUSG00000117284	Gm7072	predicted gene 7072 [Source:MGI Symbol;Acc:MGI:3648314]	7.0506	9.26126	7.5576	8.27843	9.01565	7.27567	8.05179	7.7275	9.4863	7.07866	9.18481	9.20206	9.01579	8.63708	0.826055781	0.966018836	20	8	24	0.974671047	0.993631841	24	11.0267255	0.011988638
ENSMUSG00000117285	Gm49863	predicted gene, 49863 [Source:MGI Symbol;Acc:MGI:6270537]	2.36333	2.03427	2.1806	2.14253	2.35794	1.98643	2.8715	2.20391	2.80972	3.0474	2.17305	2.20642	1.82333	1.88987	0.895493752	0.966018836	12	20	24	0.527270716	0.823319175	24	11.75445582	0.07026299
ENSMUSG00000117286	Gm1043	predicted gene 1043 [Source:MGI Symbol;Acc:MGI:2685889]	8.75821	7.38742	6.84902	7.59259	7.99809	8.09695	7.36757	6.97769	6.38399	7.93615	7.89138	7.73088	7.91219	8.54007	0.140377635	0.585715181	24	12	24	0.119439222	0.730450985	24	20.07913217	0.086739942
ENSMUSG00000117295	Gm5492	predicted gene 5492 [Source:MGI Symbol;Acc:MGI:3644513]	4.03931	5.49372	4.385	3.92531	6.22945	4.39969	3.6464	3.48604	2.90153	4.2735	4.40349	3.11976	5.26343	2.19568	0.814077113	0.966018836	20	12	24	0.268691164	0.768040178	24	17.45607619	0.174277465
ENSMUSG00000117300	Gm18736	predicted gene, 18736 [Source:MGI Symbol;Acc:MGI:5010921]	33.8821	36.9365	36.0515	34.5066	35.4017	33.2501	31.5385	30.2483	33.8097	30.8397	35.547	36.2593	40.8979	40.8353	0.890176313	0.966018836	8	8	24	0.777122334	0.928561019	24	1.67692445	0.034040569
ENSMUSG00000117310	Ptp4a1	protein tyrosine phosphatase 4a1 [Source:MGI Symbol;Acc:MGI:1277096]	2.68097	2.92868	4.23467	3.74111	3.51376	2.9454	3.09358	3.3852	3.35783	3.80246	4.47229	2.33436	2.14046	3.0475	0.005573591	0.153884205	20	4	24	0.004743895	0.434006302	24	11.49050254	0.239844601
ENSMUSG00000117327	Gm6467	predicted gene 6467 [Source:MGI Symbol;Acc:MGI:3645284]	49.1095	53.7412	54.4137	53.5953	54.1658	48.6038	45.994	49.7192	54.0668	37.4703	56.495	42.1238	49.899	47.1986	0.188831826	0.664348865	20	4	24	0.859199896	0.958530654	24	9.095208436	0.034352401
ENSMUSG00000117333	Gm16386	predicted gene 16386 [Source:MGI Symbol;Acc:MGI:3646848]	2.17045	2.09058	1.82623	2.36512	2.73948	1.45114	2.33317	2.07792	2.23682	1.35714	2.79366	2.04242	2.26989	1.97704	0.745199643	0.966018836	20	12	24	0.85655424	0.957486395	24	18.53263815	0.045214206
ENSMUSG00000117335	Gm49870	predicted gene, 49870 [Source:MGI Symbol;Acc:MGI:6270545]	0.498669	1.36783	0.470349	0.627835	0.833595	0.536837	0.760578	0.806648	1.00889	0.686766	0.739448	1.05367	0.535523	0.601816	0.895137379	0.966018836	20	8	24	0.943764928	0.986208939	24	6.822123956	0.028278375
ENSMUSG00000117345	Gm7926	predicted gene 7926 [Source:MGI Symbol;Acc:MGI:3646071]	0.949866	0.958344	0.411755	0.425822	0.554921	1.2622	0.690024	0.334962	0.770845	0.628596	0.384769	0.370316	0.641648	1.17861	0.783267549	0.966018836	4	16	24	0.333964415	0.770000654	24	0.96956627	0.14346386
ENSMUSG00000117381	Gm10350	predicted gene 10350 [Source:MGI Symbol;Acc:MGI:3642728]	5.25672	1.74272	3.14784	1.89877	2.04533	3.73168	7.01755	1.51034	1.5628	3.4829	0.383607	1.56209	3.23451	2.39464	0.297978647	0.80820842	4	16	24	0.238755129	0.768040178	24	0.122167415	0.394026194
ENSMUSG00000117406	Ntn3	netrin 3 [Source:MGI Symbol;Acc:MGI:1341188]	0.397518	0.653286	0.653256	0.664981	0.990476	0.55809	0.664539	0.782932	0.715825	0.942266	0.760393	0.864546	0.72089	0.808447	0.633249925	0.966018836	20	8	24	0.102998676	0.713946463	24	12.49100362	0.084041495
ENSMUSG00000117416	Gm5064	predicted gene 5064 [Source:MGI Symbol;Acc:MGI:3649060]	5.25853	4.95977	4.9404	5.61339	5.01372	5.20681	4.70115	6.1726	5.94958	5.62484	6.08732	5.54272	6.49414	5.74716	0.761468226	0.966018836	8	16	24	0.69786553	0.899566218	24	8.492201969	0.031209845
ENSMUSG00000117425	Gm7464	predicted gene 7464 [Source:MGI Symbol;Acc:MGI:3645335]	1.50351	1.88709	1.46175	1.62505	1.41358	1.87586	1.2528	1.34139	1.07834	1.04603	1.6797	1.1655	0.95176	1.13257	0.873350015	0.966018836	24	4	24	0.451567629	0.805393068	24	17.93584495	0.068686983
ENSMUSG00000117428	Gm4833	predicted gene 4833 [Source:MGI Symbol;Acc:MGI:3646586]	10.0951	13.219	13.3205	13.3652	13.5075	10.6747	12.3832	12.9392	11.911	7.7198	10.5873	9.08714	9.19113	7.28484	0.839634479	0.966018836	20	4	24	0.834704094	0.949290995	24	10.62065971	0.049262284
ENSMUSG00000117438	Rpsa-ps7	ribosomal protein SA, pseudogene 7 [Source:MGI Symbol;Acc:MGI:3643805]	7.49123	9.31861	7.5462	9.46663	9.47188	8.15139	8.59895	9.1176	11.0546	7.60653	10.3311	9.49124	9.83927	10.879	0.830949782	0.966018836	20	8	24	0.770453483	0.926138528	24	7.941853822	0.042039962
ENSMUSG00000117449	Gm6643	predicted gene 6643 [Source:MGI Symbol;Acc:MGI:3648552]	0.549884	0.612322	0.579771	0.753218	0.452848	0.579011	0.546162	0.679574	0.541179	0.624162	0.545948	0.610832	0.673097	0.686768	0.192357053	0.669841558	8	12	24	0.337794204	0.772605919	24	6.085361327	0.039650623

ENSMUSG00000117458	Gm6552	predicted gene 6552 [Source:MGI Symbol;Acc:MGI:3647048]	14.994	14.3443	13.2287	15.8924	12.492	13.9974	14.6284	12.7991	14.9882	12.5355	13.0558	12.2316	11.661	12.5351	0.921572192	0.966018836	16	4	24	0.84116589	0.951862945	24	8.052775711	0.023598956
ENSMUSG00000117465	Gm49980	predicted gene, 49980 [Source:MGI Symbol;Acc:MGI:6275257]	1.28421	1.86	1.18524	1.02845	1.54667	2.27966	1.23522	0.802384	0.797941	0.771219	0.937865	1.38758	1.11346	2.02856	0.167216842	0.634643192	24	16	24	0.231114753	0.768040178	24	22.28832589	0.188177012
ENSMUSG00000117468	Gm7059	predicted gene 7059 [Source:MGI Symbol;Acc:MGI:3779663]	6.09451	9.28578	7.75179	8.85893	8.37487	8.07697	6.68725	6.43001	9.06527	5.67828	6.52649	5.76113	6.38142	5.06943	0.881355613	0.966018836	12	16	24	0.430434391	0.805393068	24	11.63666805	0.099856314
ENSMUSG00000117484	Gm5500	predicted pseudogene 5500 [Source:MGI Symbol;Acc:MGI:3647082]	18.5642	11.2228	10.6934	11.8152	16.6001	15.9602	13.5354	14.0733	9.83828	11.8486	14.7825	13.6884	13.807	13.3279	0.009640466	0.194720062	20	16	24	0.004241823	0.427431763	24	20.40267215	0.264839141
ENSMUSG00000117486	Gm30938	predicted gene, 30938 [Source:MGI Symbol;Acc:MGI:5590097]	1.65784	1.05009	0.6751	0.588546	1.07488	1.10608	0.756001	1.19831	0.704672	0.753547	1.20207	0.865552	1.11056	1.17892	0.091669402	0.484208195	8	4	24	0.080414769	0.695795041	24	22.80152659	0.170673009
ENSMUSG00000117499	Gm50010	predicted gene, 50010 [Source:MGI Symbol;Acc:MGI:6275297]	10.3625	9.1317	8.85483	7.98396	10.2932	9.51993	10.942	8.19054	8.48819	6.61909	10.7454	9.53135	9.80327	10.3006	0.018114172	0.256345714	4	12	24	0.036897452	0.609176161	24	21.94647844	0.167627599
ENSMUSG00000117503	Gm7527	predicted gene 7527 [Source:MGI Symbol;Acc:MGI:3645979]	0.821657	0.584411	0.833824	0.627643	0.737209	0.90074	0.801696	0.686179	0.82265	0.832437	0.602156	0.664169	0.584143	0.73757	0.916746631	0.966018836	12	20	24	0.987682866	0.997530201	24	11.65489947	0.005617849
ENSMUSG00000117505	Gm9993	predicted gene 9993 [Source:MGI Symbol;Acc:MGI:3641793]	0.353641	0.677576	0.572865	0.746445	0.548959	0.219253	0.529826	0.517654	0.668582	0.419119	0.473748	0.425043	0.817388	0.467197	0.601647512	0.966018836	12	12	24	0.249064441	0.768040178	24	7.558145575	0.101443301
ENSMUSG00000117513	Gm3946	predicted gene 3946 [Source:MGI Symbol;Acc:MGI:3782120]	0.467375	0.593501	0.571642	1.00346	0.698426	0.728283	0.854849	0.731518	0.996749	0.965073	1.12332	0.447618	0.973824	0.743718	0.602156008	0.966018836	20	4	24	0.188004985	0.768040178	24	11.73816235	0.111373767
ENSMUSG00000117522	Gm50041	predicted gene, 50041 [Source:MGI Symbol;Acc:MGI:6275349]	4.66647	6.94817	13.644	2.87884	5.57263	7.06559	7.2059	8.02447	4.90052	5.742	6.10974	6.70858	5.55505	7.15163	0.5169953	0.966018836	8	8	24	0.515345444	0.817300608	24	3.682917951	0.192013312
ENSMUSG00000117545	Gm30794	predicted gene, 30794 [Source:MGI Symbol;Acc:MGI:5589953]	0.287328	0.692862	0.507087	0.944387	0.570352	0.604604	0.453933	1.52739	1.42236	1.29976	0.59071	0.560277	0.989576	0.429572	0.668329321	0.966018836	16	12	24	0.13393949	0.744019334	24	9.146373619	0.229046829
ENSMUSG00000117548	Gm41662	predicted gene, 41662 [Source:MGI Symbol;Acc:MGI:5624547]	1.31637	0.835475	1.14469	1.05375	1.32599	0.670365	0.882421	0.999848	0.897837	1.13085	1.08362	0.71516	0.922285	0.543303	0.344645202	0.86981408	20	4	24	0.267136018	0.768040178	24	13.78173792	0.091516585
ENSMUSG00000117552	Gm5686	predicted gene 5686 [Source:MGI Symbol;Acc:MGI:3644275]	1.21398	1.21221	0.943107	1.09514	1.33192	0.936231	1.37581	1.39849	0.770204	0.973853	0.716056	0.74583	0.606119	0.711205	0.81544087	0.966018836	8	16	24	0.865314356	0.960982718	24	0.423319424	0.030378962
ENSMUSG00000117621	Hspe1-rs1	heat shock protein 1 (chaperonin 10), related sequence 1 [Source:MGI Symbol;Acc:MGI:1935159]	85.8809	78.751	76.9339	74.0452	86.741	76.2293	79.6279	76.0088	81.6305	64.1573	94.032	80.222	85.6598	80.8143	0.129903614	0.560263689	20	20	24	0.334507445	0.770673394	24	21.20307078	0.075898198
ENSMUSG00000117627	Tpi-rs10	triosephosphate isomerase related sequence 10 [Source:MGI Symbol;Acc:MGI:98798]	15.2786	15.1546	16.1154	16.5129	19.3745	19.8954	12.8625	19.209	15.9418	14.5883	16.79	11.998	15.377	11.9475	0.515451644	0.966018836	20	8	24	0.456937558	0.805393068	24	14.79529452	0.09785762
ENSMUSG00000117648	Gm32282	predicted gene, 32282 [Source:MGI Symbol;Acc:MGI:5591441]	0.10965	0.777623	0.238102	0.622179	0.324641	0.553833	0.133291	0.682346	1.11807	0.579993	0.672904	0.423104	0.66148	0.583183	0.537381546	0.966018836	8	20	24	0.191895315	0.768040178	24	9.051144238	0.171487473
ENSMUSG00000117655	6720468P15Rik	RIKEN cDNA 6720468P15 gene [Source:MGI Symbol;Acc:MGI:1925323]	5.29043	4.93202	5.90391	4.68446	6.29779	4.88912	5.51834	4.72475	5.01745	4.46177	6.43516	5.76712	5.51195	4.64764	0.064951807	0.41673616	20	12	24	0.201936667	0.768040178	24	18.27239606	0.10001732
ENSMUSG00000117662	Gm4013	predicted gene 4013 [Source:MGI Symbol;Acc:MGI:3782187]	1.16727	1.70874	2.11693	2.03724	0.745418	1.04392	1.24555	1.39221	1.9414	1.6642	2.09294	1.66241	2.01446	2.47641	0.266108356	0.773510818	12	12	24	0.043825461	0.627667326	24	7.728572848	0.233823415
ENSMUSG00000117667	Gm18293	predicted gene, 18293 [Source:MGI Symbol;Acc:MGI:5010478]	6.35024	5.59842	1.51968	6.08465	7.84618	2.98547	7.62604	5.45427	4.24213	1.40247	5.53518	3.31831	4.41562	2.91913	0.588208558	0.966018836	4	8	24	0.357822548	0.78475603	24	21.49751689	0.332013944
ENSMUSG00000117669	Gm5509	predicted gene 5509 [Source:MGI Symbol;Acc:MGI:3645239]	0.929841	1.07807	0.768721	0.897752	0.837009	1.10034	0.817404	0.867091	0.762739	0.687943	1.12714	0.710285	0.890794	0.858197	0.630352198	0.966018836	20	20	24	0.467117079	0.805393068	24	21.05267688	0.05140317
ENSMUSG00000117673	Gm6974	predicted gene 6974 [Source:MGI Symbol;Acc:MGI:3647986]	0.728232	1.06734	1.58252	1.47657	0.602063	0.913081	1.18235	0.505223	1.10248	0.802213	1.1632	0.932913	1.13561	0.914744	0.927004797	0.966018836	12	20	24	0.628082117	0.872795898	24	9.894519081	0.086665518
ENSMUSG00000117679	Apbb3	amyloid beta (A4) precursor protein-binding, family B, member 3 [Source:MGI Symbol;Acc:MGI:108404]	36.8622	34.3711	33.9465	38.8818	37.5295	39.8188	28.191	32.9505	33.018	36.0916	32.5809	33.0487	32.2016	36.3279	0.7770058	0.966018836	16	12	24	0.356255064	0.783018041	24	14.30984211	0.066596284
ENSMUSG00000117694	Snhg4	small nucleolar RNA host gene 4 [Source:MGI Symbol;Acc:MGI:4937091]	1.15867	1.43939	1.30171	0.415895	1.12244	2.57474	0.805457	1.29961	1.54479	1.19239	1.31381	1.41302	5.4505	2.4616	0.708020493	0.966018836	8	8	24	0.191167235	0.768040178	24	0.551170371	0.30138485
ENSMUSG00000117698	BC037704	cDNA sequence BC037704 [Source:MGI Symbol;Acc:MGI:2652856]	0.582533	0.801567	0.798288	0.547086	1.0226	0.738682	0.843757	0.755886	0.738313	0.755215	0.804184	0.753706	1.00313	0.767419	0.96317972	0.967965568	20	20	24	0.814949885	0.940889579	24	21.1548943	0.026154794
ENSMUSG00000117714	Gm50439	predicted gene, 50439 [Source:MGI Symbol;Acc:MGI:6303373]	4.58544	3.44151	2.47675	4.19423	1.08905	3.30612	3.68096	2.99002	3.59173	3.29089	3.5516	3.03217	3.14253	2.14689	0.920872048	0.966018836	4	4	24	0.795752047	0.93469372	24	1.628483709	0.092613043
ENSMUSG00000117725	Gm50240	predicted gene, 50240 [Source:MGI Symbol;Acc:MGI:6303048]	3.18285	2.71351	3.05734	3.55546	3.12312	2.75611	3.4511	3.46226	3.07522	2.86133	2.79271	2.51696	2.74939	2.74548	0.918193129	0.966018836	16	8	24	0.655320516	0.883697438	24	8.1890014	0.039202401
ENSMUSG00000117730	Gm5503	predicted gene 5503 [Source:MGI Symbol;Acc:MGI:3646950]	5.87118	5.18142	5.27666	6.09732	6.52851	4.55186	5.66322	5.67492	4.68217	4.8185	5.32091	4.68179	5.15234	5.67818	0.934818505	0.966018836	20	4	24	0.997781373	0.999623846	24	13.89448677	0.003308514
ENSMUSG00000117733	Gm4221	predicted gene 4221 [Source:MGI Symbol;Acc:MGI:3782397]	0.531174	0.942823	0.961722	0.605008	0.657795	0.598544	1.07751	0.946745	0.843863	0.351115	0.787011	0.965035	1.12503	0.73553	0.450844752	0.966018836	4	12	24	0.144356903	0.752803875	24	2.021061737	0.133385275
ENSMUSG00000117739	Gm36787	predicted gene, 36787 [Source:MGI Symbol;Acc:MGI:5595946]	0.73541	0.742197	0.398539	0.412459	0.806329	0.509451	0.222655	0.648711	0.373132	0.486723	0.62105	0.835628	0.620642	0.427132	0.287488775	0.793714531	24	12	24	0.34934478	0.779170188	24	19.69855883	0.101455329
ENSMUSG00000117743	Gm50232	predicted gene, 50232 [Source:MGI Symbol;Acc:MGI:6303036]	0.735055	1.05664	0.799698	0.772121	1.03187	0.655214	0.936781	0.917566	1.07091	0.61219	1.07935	0.731841	0.892507	1.16386	0.267533088	0.774280302	8	16	24	0.443667654	0.805393068	24	5.506445037	0.067312247
ENSMUSG00000117748	Derpc	DERPC proline and glycine rich nuclear protein [Source:MGI Symbol;Acc:MGI:6303050]	2.2851	5.22311	3.05071	4.04122	7.81187	5.38956	4.45951	2.5245	3.88022	4.17488	1.84797	4.61722	3.81622	0.00554968	0.481811994	0.966018836	24	8	24	0.195493313	0.768040178	24	17.11646239	0.469220729
ENSMUSG00000117771	Gm25432	predicted gene, 25432 [Source:MGI Symbol;Acc:MGI:5455209]	11.3532	13.3877	14.8684	12.1667	11.6707	11.9199	12.5447	11.1471	13.943	12.07	12.2307	15.2443	11.7692	13.883	0.851656735	0.966018836	12	16	24	0.582392262	0.851359615	24	7.654786771	0.056990367
ENSMUSG00000117775	Gm50462	predicted gene, 50462 [Source:MGI Symbol;Acc:MGI:6324728]	1.58969	1.20993	1.33622	1.14409	1.61218	1.24081	1.75105	1.51396	1.5088	1.22653	1.80493	1.36847	1.68212	1.56032	0.03484427	0.328311403	4	12	24	0.376991001	0.796956096	24	22.74930385	0.065231568
ENSMUSG00000117789	Gm50388	predicted gene, 50388 [Source:MGI Symbol;Acc:MGI:6303291]	0.244937	0.250811	1.05262	0.886826	0.286846	0.874855	0.305969	0.783164	0.411001	0.474225	0.499597	0.892051	0.547427	0.504079	0.960269811	0.967043964	24	4	24	0.715087393	0.906238356	24	12.31260299	0.08062318
ENSMUSG00000117804	Gm6252	predicted gene 6252 [Source:MGI Symbol;Acc:MGI:3644748]	3.44981	4.95505	4.99591	3.27028	4.76422	3.98989	3.73069	4.94897	4.8907	2.72404	5.17173	3.56759	4.04097	4.85945	0.280768839	0.792139038	12	4	24	0.528819039	0.824183876	24	5.506788398	0.10504829
ENSMUSG00000117813	Gm5823	predicted gene 5823 [Source:MGI Symbol;Acc:MGI:3647390]	20.7082	20.1078	17.5662	17.9982	16.6903	19.9209	20.3242	19.0846	17.0853	22.463	20.8914	19.1395	16.4802	21.296	0.860993221	0.966018836	8	4	24	0.931045125	0.982245205	24	22.90227561	0.020796894
ENSMUSG00000117814	Gm50431	predicted gene, 50431 [Source:MGI Symbol;Acc:MGI:6303362]	2.24532	2.38029	2.70951	2.61325	2.10708	2.2197	2.55376	2.01689	2.00827	2.62816	2.11742	2.04761	2.17072	2.57037	0.602156008	0.966018836	16	4	24	0.429702376	0.805393068	24	8.466410172	0.057095632
ENSMUSG00000117819	Gm50253	predicted gene, 50253 [Source:MGI Symbol;Acc:MGI:6303071]	1.4008	1.51653	1.5415	1.37711	1.28452	0.949379	1.7362	1.45665	1.56523	1.29025	1.1833	1.11195	1.27754	1.15092	0.004909071	0.141782417	12	12	24	0.075040273	0.68394125	24	6.009950479	0.101194486
ENSMUSG00000117822	Eef1a1-ps1	eukaryotic translation elongation factor 1 alpha 1, pseudogene 1 [Source:MGI Symbol;Acc:MGI:2388571]	11.3092	10.9442	10.7877	11.5187	10.5833	10.7025	10.4876	9.09958	7.93298	8.1765	8.10697	8.28666	8.53279	8.33389	0.874672901	0.966018836	4	16	24	0.278745696	0.768040178	24	21.44632065	0.055104136
ENSMUSG00000117832	Gm50263	predicted gene, 50263 [Source:MGI Symbol;Acc:MGI:6303087]	29.143	29.9317	28.4501	22.5112	21.5554	21.2817	14.3279	27.0296	22.9302	30.702	28.1587	29.8095	21.4973	28.1701	0.870491976	0.966018836	8	20	24	0.578478502	0.849227023	24	9.199095171	0.125893742
ENSMUSG00000117864	Gm19500	predicted gene, 19500 [Source:MGI Symbol;Acc:MGI:5011685]	0.251431	0.808008	1.06043	0.507678	0.473415	0.49374	0.864526	0.579193	1.15132	0.456625	0.477655	0.757942	0.469409	0.534777	0.133012827	0.567728672	12	4	24	0.240247859	0.768040178	24	7.174747054	0.146938619
ENSMUSG00000117869	Snhg4	small nucleolar RNA host gene 4 [Source:MGI Symbol;Acc:MGI:4937091]	5.74742	5.74544	6.1683	6.16951	5.67273	4.77753	6.06559	5.75862	6.16337	4.40353	7.17333	5.30188	4.72455	5.64475	0.542718076	0.966018836	12	12	24	0.754429033	0.920525691	24	9.722436365	0.047432938
ENSMUSG00000117875	Gm6789	predicted gene 6789 [Source:MGI Symbol;Acc:MGI:3643581]	28.8549	28.1154	28.9203	29.7212	33.0934	30.3301	27.907	26.1204	30.7851	26.9711	28.5662	31.2605	29.3312	28.2519	0.089766625	0.477784783	24	8	24	0.13848446	0.747972781	24	17.05834939	0.064399731
ENSMUSG00000117877	Gm8184	predicted gene 8184 [Source:MGI Symbol;Acc:MGI:3643112]	0.916453	0.883305	0.763946	0.944276	0.983247	0.912742	0.714989	0.770918	0.640675	0.952952	0.753932	0.606686	0.673325	0.750923	0.530814489	0.966018836	16	20	24	0.257771105	0.768040178	24	14.69842807	0.050220808
ENSMUSG00000117884	Gm50156	predicted gene, 50156 [Source:MGI Symbol;Acc:MGI:6302916]	7.69874	7.40083	5.89638	8.49701	7.13419	7.68168	5.80132	8.95803	7.11965	8.92411	8.12894	6.1232	8.75862	7.65409	0.895137379	0.966018836	16	20	24	0.807047252	0.938616703	24	10.74299348	0.049091723
ENSMUSG00000117896	AA387883	expressed sequence AA387883 [Source:MGI Symbol;Acc:MGI:3035466]	1.45818	1.96092	1.6701	1.11963	1.69135	1.26914	1.82104	1.83801	1.87771	1.84171	1.18873	1.34734	1.9577	1.85913	0.03963404	0.338535904	8	12	24	0.023310826	0.581965446	24	4.76223538	0.161305722
ENSMUSG00000117901	-	novel transcript, antisense to Macrod1	1.25782	2.1732	2.46201	1.90393	2.22132	2.17002	2.1483	1.89178	2.0271	2.02772	1.9024	1.76479	1.54183	2.51845	0.446653834	0.966018836	12	16	24	0.28551583	0.768040178	24	9.420251702	0.10661454
ENSMUSG00000117905	Gm50230	predicted gene, 50230 [Source:MGI Symbol;Acc:MGI:6303033]	1.54329	1.51845	1.3857	1.64795	1.66531	1.25594	1.78309	1.31883	1.75427	1.49803	1.85938	1.53039	1.66777	1.42563	0.702630284	0.966018836	20	12	24	0.632005892	0.874894528	24	15.36024958	0.037950713
ENSMUSG00000117924	Tmem223	transmembrane protein 223 [Source:MGI Symbol;Acc:MGI:1914086]	23.8984	24.3349	25.5315	29.8092	24.3245	23.1057	23.8134	25.6074	25.9549	25.152	27.6886	24.1603	27.1747	22.3935	0.177811964	0.655480352	16	8	24	0.178366263	0.768040178	24	12.01578118	0.074437263
ENSMUSG00000117935	Gm8717	predicted gene 8717 [Source:MGI Symbol;Acc:MGI:3644350]	0.978139	0.434673	0.598141	0.699689	0.365239	0.553961	0.745318	0.54659	0.375407	0.403962	0.587508	0.507624	0.652361	0.229072	0.336755135	0.85758369	4	4	24	0.195459526	0.768040178	24	21.47437307	0.109480685
ENSMUSG00000117939	Gm9067	predicted gene 9067 [Source:MGI Symbol;Acc:MGI:3644658]	1.13251	0.925063	0.821352	0.670914	0.824234	0.800948	1.13152	0.98503	0.42236	0.5894	0.401982	0.633273	0.593441	0.459412	0.359479536	0.883787544	4	12	24	0.013641906	0.529134128	24	23.69377927	0.143886602
ENSMUSG00000117942	Maskbp3	multiple ankyrin repeats single KH domain binding protein 3 [Source:MGI Symbol;Acc:MGI:3845902]	8.51226	7.38316	7.2973	7.2235	7.29605	7.50272	7.17924	6.984	6.8487	7.1702	7.32272	7.47359	6.66961	7.79044	0.631736745	0.966018836	24	12	24	0.438014464	0.805393068	24	21.66844451	0.035960519
ENSMUSG00000117951	Gm50357	predicted gene, 50357 [Source:MGI Symbol;Acc:MGI:6303243]	3.38881	2.11639	2.74411	2.78078	2.104	2.05372	4.0778	4.4099	1.13649	2.52575	1.96336	1.9661	1.74601	2.39259	0.835401638	0.966018836	4	20	24	0.505081216	0.811334839	24	1.554564951	0.153131035
ENSMUSG00000117959	D330050I16Rik	RIKEN cDNA D330050I16 gene [Source:MGI Symbol;Acc:MGI:3041222]	0.590277	0.578522	0.472178	0.628206	0.379523	0.644281	0.365975	0.423076	0.843936	0.754619	0.706848	0.784807	0.508049	0.584977	0.373702442	0.896700272	24	4	24	0.415952366	0.800710663	24	12.73787776	0.066958767
ENSMUSG00000117969	Gm5826	predicted gene 5826 [Source:MGI Symbol;Acc:MGI:3646075]	0.624392	1.01132	0.677667	0.989491	0.460909	0.978477	0.606695	0.554128	1.78074	1.23875	1.95453	1.04622	1.25752	0.476395	0.89563591	0.966018836	16	16	24	0.359160106	0.785405943	24	13.34949838	0.168304236
ENSMUSG00000117975	Itprip	inositol 1,4,5-triphosphate receptor interacting protein [Source:MGI Symbol;Acc:MGI:3042776]	0.780913	0.834513	0.790191	1.01609	0.804236	1.03767	0.833926	0.791451	0.941738	0.864311	0.813536	1.0254	1.07272	0.836987	0.953629935	0.966018836	24	8	24	0.584808355	0.8525659	24	18.57096766	0.031972228
ENSMUSG00000117992	Gm41760	predicted gene, 41760 [Source:MGI Symbol;Acc:MGI:5624645]	0.797108	0.890307	0.522712	0.955756	0.600918	0.831925	0.851924	0.72392	0.893382	0.849283	0.864559	1.0141	0.762979	0.968913	0.838692453	0.966018836	16	12	24	0.923191108	0.979439431	24	22.13735316	0.017148976
ENSMUSG00000118012	Gm46620	predicted gene, 46620 [Source:MGI Symbol;Acc:MGI:5826257]	0.833122	0.68306	0.604399	0.77332	0.748435	0.871304	0.876672	0.748276	0.585961	0.993497	0.830461	0.808627	0.845555	0.686267	0.020646136	0.262705843	4	8	24	0.064848536	0.663876076	24	18.96720628	0.078973107
ENSMUSG00000118018	-	novel transcript	5.46357	4.6141	4.4635	4.82527	4.97745	4.58037	4.80947	3.78866	4.01784	4.08749	5.13603	4.28005	4.61097	4.79625	0.446967971	0.966018836	20	16	24	0.196399197	0.768040178	24	19.73399392	0.080534889
ENSMUSG00000118038	Gm9895	predicted gene 9895 [Source:MGI Symbol;Acc:MGI:3642802]	0.526456	1.06799	0.9532	1.19943	0.195614	0.642758	1.38839	0.782019	0.657186	1.10198	0.780105	0.562563	0.880222	0.662577	0.435739761	0.966018836	16	4	24	0.445661858	0.805393068	24	6.538092116	0.13233605
ENSMUSG00000118049	Gm50103	predicted gene, 50103 [Source:MGI Symbol;Acc:MGI:6302834]	1.26446	1.4822	1.29195	1.86871	1.7047	1.16254	1.94322	1.57998	1.68792	1.39887	1.69505	1.32553	1.48347	1.90501	0.922035832	0.966018836	20	4	24	0.575888883	0.848281418	24	8.589262616	0.060007798
ENSMUSG00000118052	Gm50432	predicted gene, 50432 [Source:MGI Symbol;Acc:MGI:6303364]	26.6913	20.5452	18.7802	24.4856	14.167	15.841	20.937	34.2113	30.1053	35.0679	39.4271	33.3422	31.0806	29.0141	0.733896783	0.966018836	16	20	24	0.655009325	0.883697438	24	7.468459032	0.131584228
ENSMUSG00000118053	Gm50244	predicted gene, 50244 [Source:MGI Symbol;Acc:MGI:6303055]	25.3885	19.159	16.8677	19.2338	19.3676	20.1151	18.1053	19.2425	14.2749	20.9688	17.7673	18.6696	17.8464	15.1927	0.44861185	0.966018836	16	20	24	0.090374237	0.711204167	24	19.82757849	0.136437572
ENSMUSG00000118057	B020010K11Rik	RIKEN cDNA B020010K11 gene [Source:MGI Symbol;Acc:MGI:3612155]	6.85675	5.11925	5.55998	4.03882	4.22533	4.71472	5.35383	4.69816	3.8952	5.19336	4.16307	5.95782	5.47947	7.49738	0.178785852	0.655480352	4	16	24	0.068943268	0.673520298	24	1.326899376	0.193711722
ENSMUSG00000118084	AA388235	expressed sequence AA388235 [Source:MGI Symbol;Acc:MGI:3035178]	1.79823	1.67946	1.48529	1.56118	1.92994	0.975677	2.45379	1.66994	1.32399	1.71131	2.36645	1.79455	1.48882	1.62215	0.266025655	0.773510818	20	16	24	0.807238877	0.938686319	24	18.96707386	0.055444295
ENSMUSG00000118087	4833438C02Rik	RIKEN cDNA 4833438C02 gene [Source:MGI Symbol;Acc:MGI:2147599]	0.737088	0.891509	0.690448	0.928023	0.803436	0.766834	0.386962	0.77309	1.00518	1.23528	1.02029	0.89913	0.639646	0.64499	0.001845448	0.100255953	16	12	24	0.021610313	0.574198497	24	12.51147937	0.158157608
ENSMUSG00000118088	Gm3815	predicted gene 3815 [Source:MGI Symbol;Acc:MGI:3781987]	6.0265	4.84636	6.02871	4.9546	5.58919	5.82687	6.46089	4.61771	3.93361	4.92272	3.8632	3.57593	3.96376	3.32977	0.860993221	0.966018836	4	4	24	0.519040656	0.819011931	24	19.8536019	0.073996491
ENSMUSG00000118099	Gm31356	predicted gene, 31356 [Source:MGI Symbol;Acc:MGI:5590515]	0.59594	0.721869	0.710164	0.588039	0.597029	0.517509	0.450671	0.509441	0.931769	0.543572	0.782771	0.595385	0.547004	0.442459	0.481811994	0.966018836	12	20	24	0.271638614	0.768040178	24	10.89211548	0.072713556
ENSMUSG00000118100	Gm50340	predicted gene, 50340 [Source:MGI Symbol;Acc:MGI:6303216]	0.983494	1.69824	1.66885	1.09949	0.920017	0.660834	1.00252	1.19622	1.94141	1.73034	1.29065	1.32952	0.988108	0.533246	0.201580999	0.687644618	12	16	24	0.129664855	0.740162193	24	9.64226769	0.211163291
ENSMUSG00000118107	Gm34455	predicted gene, 34455 [Source:MGI Symbol;Acc:MGI:5593614]	0.480357	0.855373	0.755926	1.01992	0.403398	0.525912	0.57673	1.25509	0.493555	0.823818	0.305588	0.678832	0.913981	0.794376	0.066380001	0.42386701	8	12	24	0.118955664	0.729851534	24	5.751943863	0.171899588
ENSMUSG00000118125	Gm50387	predicted gene, 50387 [Source:MGI Symbol;Acc:MGI:6303289]	4.47765	3.43562	2.47912	4.45853	3.97024	3.58692	3.90467	3.64441	3.4673	3.49668	3.91587	3.42075	3.43003	4.31317	0.928127947	0.966018836	16	20	24	0.631370218	0.87436603	24	19.89358789	0.066826609
ENSMUSG00000118132	4833408A19Rik	RIKEN cDNA 4833408A19 gene [Source:MGI Symbol;Acc:MGI:1921038]	0.859656	0.946623	0.727122	1.04102	0.607837	0.677913	0.75755	1.02645	0.652397	0.978135	0.656897	0.974307	1.01785	0.820054	0.702630284	0.966018836	8	12	24	0.563787937	0.842768475	24	3.895025338	0.052804627
ENSMUSG00000118133	Gm6978	predicted gene 6978 [Source:MGI Symbol;Acc:MGI:3647254]	0.737991	0.654825	1.10224	0.840023	0.342493	0.779068	0.785772	0.739571	0.690694	0.485326	0.630503	0.414275	0.308618	0.34875	0.864782464	0.966018836	12	8	24	0.54807386	0.834077197	24	7.936919973	0.069581341
ENSMUSG00000118140	Gm4949	predicted gene 4949 [Source:MGI Symbol;Acc:MGI:3646890]	1.44401	1.07955	1.61677	1.65818	1.55956	1.78625	1.0886	0.95144	0.928647	1.40287	0.964588	1.02812	1.01775	0.823141	0.310940564	0.820837787	16	16	24	0.040769173	0.616921492	24	15.76264191	0.139688367
ENSMUSG00000118163	Gm41757	predicted gene, 41757 [Source:MGI Symbol;Acc:MGI:5624642]	0.0639022	0.162247	1.12275	0.481908	0.346359	0.113559	0.737336	1.4964	1.20628	0.400862	0.875153	0.244994	1.362	1.14061	0.143321297	0.585944911	12	12	24	0.03794682	0.61140397	24	6.728492417	0.350702567
ENSMUSG00000118164	Gm20570	predicted gene, 20570 [Source:MGI Symbol;Acc:MGI:5295677]	144.084	125.474	80.921	157.69	128.891	106.28	83.9629	137.867	136.744	99.5144	154.192	134.829	159.889	148.106	0.844511766	0.966018836	4	8	24	0.970063642	0.993003194	24	20.12754382	0.032162441
ENSMUSG00000118171	Gm50390	predicted gene, 50390 [Source:MGI Symbol;Acc:MGI:6303294]	17.1743	17.6989	14.3965	17.4206	16.145	15.1844	17.9444	14.8811	14.9289	16.7829	15.5	17.5481	17.114	16.0957	0.286642595	0.793714531	4	8	24	0.374458686	0.794636296	24	21.56165261	0.056870105
ENSMUSG00000118181	Gm53015	predicted gene, 53015 [Source:MGI Symbol;Acc:MGI:6388904]	7.5958	7.40867	8.30686	8.8337	8.21536	8.60006	5.01713	6.97384	6.42381	8.55781	6.18095	4.60373	6.2249	7.24525	0.305564675	0.811201458	16	12	24	0.155985082	0.76165922	24	11.02448307	0.160890991
ENSMUSG00000118202	C030017B01Rik	RIKEN cDNA C030017B01 gene [Source:MGI Symbol;Acc:MGI:1924774]	19.3228	19.9455	17.5902	17.7647	21.2357	18.0033	17.4964	16.4862	18.2524	15.6553	21.5367	18.3747	19.6137	18.484	0.369262716	0.893596186	20	20	24	0.361619445	0.786932131	24	19.31547492	0.07037095
ENSMUSG00000118219	Gm29695	predicted gene, 29695 [Source:MGI Symbol;Acc:MGI:5588854]	0.625064	0.519476	0.419149	0.537108	0.678391	0.588648	0.537665	0.60684	0.425314	0.568058	0.614228	0.596029	0.575505	0.607643	0.039226672	0.338446795	20	16	24	0.052402106	0.650522874	24	19.66228816	0.060917763
ENSMUSG00000118220	Gm6192	predicted gene 6192 [Source:MGI Symbol;Acc:MGI:3779569]	0.560999	0.688169	0.753925	0.569373	0.941405	0.601264	0.523918	0.592084	0.315914	0.435958	0.341126	0.377894	0.548618	0.367997	0.701395851	0.966018836	8	4	24	0.855549275	0.957116652	24	19.50543989	0.02791452
ENSMUSG00000118239	Gm50107	predicted gene, 50107 [Source:MGI Symbol;Acc:MGI:6302841]	3.83625	4.23361	4.28711	4.83617	3.83854	3.97546	4.27538	3.74697	4.30942	3.68084	4.04106	4.01963	3.20374	3.69246	0.648082424	0.966018836	12	20	24	0.2734013	0.768040178	24	11.54456039	0.060128495
ENSMUSG00000118240	Gm1859	predicted gene 1859 [Source:MGI Symbol;Acc:MGI:3037717]	0.965517	0.855411	1.04801	1.23039	1.06987	1.48714	0.87972	1.07158	1.14768	1.11733	1.39696	1.47696	0.890871	1.01176	9.02E-04	0.071344007	24	4	24	0.025890862	0.586541342	24	15.93038419	0.128666163
ENSMUSG00000118245	Gm30082	predicted gene, 30082 [Source:MGI Symbol;Acc:MGI:5589241]	1.1842	1.23304	1.54491	1.15243	1.28278	1.50954	1.29951	1.21787	1.17175	1.44759	1.46358	1.24769	0.908076	1.59653	0.896773941	0.966018836	24	4	24	0.626587958	0.872696329	24	12.14931549	0.046082424
ENSMUSG00000118249	Gm36608	predicted gene, 36608 [Source:MGI Symbol;Acc:MGI:5595767]	0.979657	1.24142	0.851179	1.0026	1.08999	0.936671	0.834188	1.39534	1.93294	0.97147	0.735964	0.943517	1.18058	1.18707	0.080148772	0.458678242	8	16	24	0.095979833	0.71175955	24	6.203724676	0.155762865
ENSMUSG00000118256	Gm50303	predicted gene, 50303 [Source:MGI Symbol;Acc:MGI:6303153]	1.64204	1.9838	1.78429	1.78447	0.873151	1.58907	1.23799	1.74423	1.49186	2.17055	1.71352	1.15151	2.19419	1.53102	0.602156008	0.966018836	16	4	24	0.324810496	0.768040178	24	6.714653512	0.128621122
ENSMUSG00000118257	9630014M24Rik	RIKEN cDNA 9630014M24 gene [Source:MGI Symbol;Acc:MGI:3588234]	2.83929	2.82919	2.27867	2.35099	2.78266	2.98791	2.55754	2.26495	2.2769	1.8594	3.10081	2.7341	2.33815	3.09694	0.209352197	0.69936851	20	20	24	0.135485721	0.747573586	24	21.15825161	0.118840579
ENSMUSG00000118264	Rps15-ps3	ribosomal protein S15, pseudogene 3 [Source:MGI Symbol;Acc:MGI:3645421]	24.3656	26.251	24.9473	24.0272	32.501	26.0379	25.5797	23.8676	25.9681	15.6944	22.0476	21.2991	22.1842	14.7827	0.789670753	0.966018836	12	4	24	0.433799852	0.805393068	24	19.33082923	0.115031707
ENSMUSG00000118270	Gm17819	predicted gene, 17819 [Source:MGI Symbol;Acc:MGI:5010004]	153.689	145.997	116.889	124.409	138.99	164.34	138.866	149.72	49.3584	94.8278	90.1653	121.109	96.8925	48.5683	0.598711142	0.966018836	24	12	24	0.025134788	0.586541342	24	20.32304476	0.424031375
ENSMUSG00000118274	Gm50204	predicted gene, 50204 [Source:MGI Symbol;Acc:MGI:6302987]	0.536821	1.07936	0.871415	1.78725	1.74455	1.02901	0.648139	0.940412	0.904261	0.710958	0.539342	0.527569	0.730032	0.634643	0.566932234	0.966018836	16	12	24	0.216795093	0.768040178	24	12.48506202	0.165822655
ENSMUSG00000118280	Gm41804	predicted gene, 41804 [Source:MGI Symbol;Acc:MGI:5624689]	2.03537	1.56956	1.23844	0.83563	1.15088	1.69948	2.67665	0.798783	1.22018	2.31391	1.61615	1.66888	1.46917	1.71832	0.347794523	0.870418653	4	8	24	0.335717028	0.771468587	24	21.83038551	0.172133137
ENSMUSG00000118283	Gm7623	predicted gene 7623 [Source:MGI Symbol;Acc:MGI:3647114]	4.88392	5.62046	3.86732	5.08753	3.92182	4.23174	3.95979	4.52681	4.63688	3.76704	5.27847	3.20285	4.49767	5.014	0.671726933	0.966018836	8	16	24	0.49085836	0.805393068	24	5.633340741	0.085981923
ENSMUSG00000118310	Gm50268	predicted gene, 50268 [Source:MGI Symbol;Acc:MGI:6303093]	1.0228	1.11133	0.830751	1.00403	1.03483	1.08144	0.95181	0.946922	1.14983	1.07867	1.01242	1.16246	1.62121	1.0953	0.939103211	0.966018836	24	12	24	0.465390197	0.805393068	24	22.87040487	0.049279391
ENSMUSG00000118332	Fam220a	family with sequence similarity 220, member A [Source:MGI Symbol;Acc:MGI:1914488]	18.8041	17.0929	16.4203	18.2815	16.7829	17.9203	17.4602	18.726	17.0778	20.4874	18.4156	18.7205	20.5473	18.1063	0.43565473	0.966018836	4	8	24	0.766710278	0.924565474	24	20.59373707	0.02433136
ENSMUSG00000118344	Gm50152	predicted gene, 50152 [Source:MGI Symbol;Acc:MGI:6302910]	1.38896	1.81703	1.75912	1.04975	1.45486	1.9466	1.40654	1.33675	2.67401	1.22458	1.1829	1.04301	1.55265	1.58566	0.256915694	0.76168256	12	4	24	0.303745728	0.768040178	24	5.472709352	0.137332158
ENSMUSG00000118346	Tmem179b	transmembrane protein 179B [Source:MGI Symbol;Acc:MGI:1914956]	10.2391	10.5591	11.6123	10.5339	10.0139	9.58082	9.49078	11.2876	13.0294	9.58166	10.7274	10.1821	10.8566	8.64124	0.245061943	0.741580974	12	12	24	0.34889084	0.779170188	24	8.593157596	0.07663415
ENSMUSG00000118353	Nsa2-ps1	NSA2 ribosome biogenesis homolog, pseudogene 1 [Source:MGI Symbol;Acc:MGI:3648670]	15.2933	17.6938	16.2733	15.574	16.5188	15.0834	15.9668	13.5526	18.3989	12.2381	20.5875	17.3192	16.7472	17.6679	0.890235289	0.966018836	12	4	24	0.958585131	0.989144435	24	20.33320727	0.021185529
ENSMUSG00000118365	Gm41724	predicted gene, 41724 [Source:MGI Symbol;Acc:MGI:5624609]	0.725307	0.525047	0.787616	1.05938	0.844551	0.582148	0.504001	0.553065	1.19794	0.411026	0.707099	0.599229	0.416461	0.550163	0.160943355	0.620479606	12	16	24	0.149063665	0.757609805	24	11.6593781	0.131552819
ENSMUSG00000118401	Gpr52	G protein-coupled receptor 52 [Source:MGI Symbol;Acc:MGI:3643278]	0.618491	0.643046	0.497415	0.867021	0.525478	0.705134	0.65706	0.48283	0.551901	0.586121	0.574975	0.581158	0.607612	0.818234	0.956794505	0.96629412	4	8	24	0.984795261	0.996934195	24	22.34030701	0.007049086
ENSMUSG00000118426	Gm2102	predicted gene 2102 [Source:MGI Symbol;Acc:MGI:3780270]	0.273762	0.799149	0.371942	0.0782304	0.255212	0.232242	0.500416	0.366832	0.420038	0.859345	1.31412	1.2253	1.50746	0.976553	0.860709018	0.966018836	4	12	24	0.661611035	0.885525866	24	23.51516083	0.095997478
ENSMUSG00000118449	Gm3625	predicted gene 3625 [Source:MGI Symbol;Acc:MGI:3781801]	0.532054	0.691073	0.600129	0.595186	0.60221	0.554749	0.535875	0.577434	0.654279	0.749064	0.531438	0.601731	0.618999	0.566457	0.413946331	0.958746003	16	12	24	0.150338747	0.758865539	24	9.537378555	0.041913837
ENSMUSG00000118458	Gm10599	predicted pseudogene 10599 [Source:MGI Symbol;Acc:MGI:3710582]	0.354726	0.503665	0.650199	0.559776	0.632403	0.564944	0.75308	0.570432	0.63903	0.591837	0.502789	0.687498	0.557857	1.00911	0.906657897	0.966018836	12	8	24	0.489845449	0.805393068	24	6.584622177	0.052148858
ENSMUSG00000118502	Gm52964	predicted gene, 52964 [Source:MGI Symbol;Acc:MGI:6388844]	1.30594	0.91646	1.09096	1.14184	1.78758	1.58126	1.83276	1.34102	0.820055	1.59392	1.3031	1.16142	0.941825	0.456806	0.17432128	0.650395613	20	16	24	0.035839686	0.609176161	24	17.78424398	0.229903061
ENSMUSG00000118504	Gm53012	predicted gene, 53012 [Source:MGI Symbol;Acc:MGI:6388899]	2.63787	2.50982	2.65118	2.6342	2.42704	2.03899	2.34052	2.02831	2.83896	1.81699	2.23931	2.22462	2.24785	1.96722	0.601647512	0.966018836	12	12	24	0.802401631	0.936565832	24	8.084976638	0.029994523
ENSMUSG00000118506	1700094D03Rik	RIKEN cDNA 1700094D03 gene [Source:MGI Symbol;Acc:MGI:1920795]	2.38326	4.64768	4.1824	5.1789	4.68073	3.24841	4.81509	3.01045	3.09992	3.95201	2.74211	4.26683	2.22747	5.45005	0.928780444	0.966018836	16	12	24	0.579780155	0.850007285	24	10.26729902	0.139024557
ENSMUSG00000118537	Shld3	shieldin complex subunit 3 [Source:MGI Symbol;Acc:MGI:6194609]	0.766282	1.29328	1.20523	1.11494	1.28312	0.964767	1.16501	1.0586	1.28664	1.17089	1.04497	1.25668	1.18448	1.06426	0.865233232	0.966018836	12	16	24	0.374224366	0.794636296	24	10.13812952	0.056801581
ENSMUSG00000118550	Gm52965	predicted gene, 52965 [Source:MGI Symbol;Acc:MGI:6388845]	0.553495	0.577983	0.665607	0.771226	0.809758	0.464773	0.391438	0.49677	0.717334	0.538764	0.602597	0.555685	0.658388	0.778806	0.29214565	0.798990791	20	4	24	0.163792111	0.767020009	24	10.64855894	0.082093991
ENSMUSG00000118552	Rplp2-ps1	ribosomal protein, large P2, pseudogene 1 [Source:MGI Symbol;Acc:MGI:3826366]	253.016	262.272	266.704	263.374	234.596	237.782	262.339	268.59	282.039	274.493	273.771	279.036	309.101	273.915	0.93447422	0.966018836	12	8	24	0.160967317	0.765941695	24	5.768003552	0.056308189

ENSMUSG00000118559	A930007A09Rik	RIKEN cDNA A930007A09 gene [Source:MGI Symbol;Acc:MGI:3045309]	1.13206	1.27989	0.972281	0.971562	1.09619	0.997769	1.06691	1.13977	0.996006	1.11791	1.19907	1.23163	1.15758	1.03571	0.287979583	0.793763484	8	4	24	0.386724856	0.799546792	24	22.03155823	0.036829545
ENSMUSG00000118642	AY036118	cDNA sequence AY036118 [Source:MGI Symbol;Acc:MGI:2158419]	14.9515	19.645	17.9601	7.60059	5.33222	6.75271	11.1567	30.3442	40.7244	34.4183	28.6861	45.1667	50.3907	48.3408	0.708665292	0.966018836	8	12	24	0.044727253	0.628432994	24	5.351096825	0.661956091
ENSMUSG00000118653	Gm2296	predicted gene 2296 [Source:MGI Symbol;Acc:MGI:3780467]	21.0968	18.8978	21.6329	18.3444	19.6153	21.5758	19.9814	19.2367	18.554	21.5754	20.7351	20.5441	20.0505	19.3481	0.354332646	0.876085579	24	8	24	0.349548904	0.779170188	24	18.81094739	0.045658251
ENSMUSG00000118662	Tctn2	tectonic family member 2 [Source:MGI Symbol;Acc:MGI:1915228]	3.67406	2.97661	3.24962	2.59619	3.08712	3.54446	3.26066	3.02742	2.78047	2.98242	2.88081	3.39507	3.068	3.25097	0.010576629	0.197206032	24	16	24	0.016437124	0.544099157	24	22.47684058	0.102041912
ENSMUSG00000118663	Spata5l1	spermatogenesis associated 5-like 1 [Source:MGI Symbol;Acc:MGI:3036261]	2.5532	2.44994	2.37768	2.52419	2.06103	1.80999	2.0994	2.35255	2.36954	2.33979	2.65043	2.09057	2.54246	2.06162	0.924770496	0.966018836	4	20	24	0.499695823	0.808791684	24	8.432561319	0.052320352
ENSMUSG00000118664	Tusc3	tumor suppressor candidate 3 [Source:MGI Symbol;Acc:MGI:1933134]	62.0581	61.0761	60.1029	59.3043	57.8047	58.437	59.2156	58.0139	56.5971	53.979	63.1644	54.9436	56.9509	60.7506	0.94086481	0.966018836	8	16	24	0.709119175	0.903951515	24	1.279640698	0.019289898
ENSMUSG00000118665	Lin54	lin-54 homolog (C. elegans) [Source:MGI Symbol;Acc:MGI:2140902]	5.05412	5.15072	4.90039	5.51192	5.11083	5.1772	4.74184	4.52174	4.62548	5.20232	4.98656	4.81272	4.69471	4.81567	0.098459729	0.503116486	16	16	24	0.038490658	0.61140397	24	14.97759876	0.051281529
ENSMUSG00000118667	Ahnak2	AHNAK nucleoprotein 2 [Source:MGI Symbol;Acc:MGI:2144831]	25.3257	19.677	19.7635	21.9011	22.5134	25.1464	22.7016	26.0259	16.4129	32.6259	21.1884	23.9163	23.0287	23.5723	0.46238066	0.966018836	24	12	24	0.595628013	0.857839743	24	19.23482565	0.091348052
ENSMUSG00000118668	Rps6ka4	ribosomal protein S6 kinase, polypeptide 4 [Source:MGI Symbol;Acc:MGI:1930076]	7.54691	6.47062	7.59023	6.58803	6.83992	6.19968	6.864	7.20501	7.24962	9.08352	6.96039	8.24216	7.25145	8.30524	0.8635847	0.966018836	16	4	24	0.548608904	0.834655201	24	8.424430538	0.053183176
ENSMUSG00000118669	Arvcf	armadillo repeat gene deleted in velocardiofacial syndrome [Source:MGI Symbol;Acc:MGI:109620]	21.5445	21.6284	21.418	21.447	27.243	22.8779	25.977	22.1295	21.9605	22.4822	22.2508	21.2853	21.3979	22.0403	0.928127947	0.966018836	20	16	24	0.431174102	0.805393068	24	18.09716157	0.054101149
ENSMUSG00000119584	Rn18s-rs5	18s RNA, related sequence 5 [Source:MGI Symbol;Acc:MGI:107222]	3577.27	1508.85	1702.95	1144.54	952.457	3079.08	972.654	1623.47	732.839	195.813	1069.08	1204.77	819.633	1250.29	0.541384199	0.966018836	8	8	24	0.057831937	0.656248849	24	23.26008617	0.755906864
ENSMUSG00002074854	7SK	7SK RNA [Source:RFAM;Acc:RF00100]	1.33461	1.00353	1.08207	0.735585	0.956023	1.45063	1.60861	1.16371	1.5702	1.32609	1.11141	0.879377	0.91181	0.531835	0.853736882	0.966018836	4	4	24	0.878290002	0.965980735	24	20.77761185	0.045128916
ENSMUSG00002075137	7SK	7SK RNA [Source:RFAM;Acc:RF00100]	0.52644	1.72038	1.99408	1.16911	1.14129	1.29824	1.58923	1.1532	1.59649	1.39434	0.705533	0.861819	1.9679	1.03637	0.285027487	0.793714531	12	8	24	0.280792248	0.768040178	24	6.645859489	0.183497529
ENSMUSG00002075344	-	\N	1.39096	1.72483	1.49803	3.74995	3.86918	0.736047	3.34091	1.79253	1.39393	1.38239	2.72896	0.934178	2.90061	1.72523	0.941885246	0.966018836	20	4	24	0.79496612	0.93426902	24	14.07109518	0.1275395
ENSMUSG00002075580	-	\N	15.7163	19.0143	25.2582	28.7907	10.3011	17.8342	9.53745	13.2052	20.8588	7.77887	14.8419	19.1234	5.66427	17.37	0.286642595	0.793714531	12	16	24	0.485415619	0.805393068	24	9.596376447	0.274627261
ENSMUSG00002076083	Klhl17	kelch-like 17 [Source:NCBI gene (formerly Entrezgene);Acc:231003]	21.9351	24.1575	25.6736	21.8688	23.6052	23.8588	23.3441	23.7177	20.0592	23.137	22.2267	21.3906	20.5216	19.708	0.785563354	0.966018836	12	16	24	0.81147269	0.939535552	24	15.44078669	0.021181676
ENSMUSG00002076161	7SK	7SK RNA [Source:RFAM;Acc:RF00100]	33.5823	17.5054	16.3619	12.8205	14.9553	40.6138	10.7255	14.7398	9.70907	2.06454	8.37714	15.9853	9.27585	18.0502	0.387380622	0.915479136	24	16	24	0.065883828	0.668087926	24	23.01990196	0.686518318
ENSMUSG00002076304	7SK	7SK RNA [Source:RFAM;Acc:RF00100]	2.13891	5.1288	4.61839	4.21103	2.73077	3.22502	3.0885	4.45252	6.59872	1.70045	2.83175	4.25591	3.82122	2.76274	0.184631358	0.662239322	12	8	24	0.319763692	0.768040178	24	6.201479877	0.241305809
ENSMUSG00002076675	7SK	7SK RNA [Source:RFAM;Acc:RF00100]	2.4891	1.87336	1.00941	1.37491	1.78782	0.847613	1.50087	0.815097	1.88392	1.44257	2.28411	1.43242	1.69789	1.97657	0.881996584	0.966018836	4	20	24	0.95371831	0.988601783	24	20.93329472	0.037284327
ENSMUSG00002076957	7SK	7SK RNA [Source:RFAM;Acc:RF00100]	1.93096	1.33584	0.261256	1.88048	0.874506	0.795743	0.875189	1.059	1.95327	0.479288	0.648148	0.315352	1.47357	0.946182	0.518578174	0.966018836	4	20	24	0.549164748	0.834817817	24	3.719507435	0.170935116

ID	Description	GeneRatio	BgRatio	RichFactor	FoldEnrichm	zScore	pvalue	p.adjust	qvalue	geneID	Count
GO:0071375	cellular response to peptide hormone stimulus	5/22	292/14464	0.01712329	11.2577833	6.91124419	6.4361E-05	0.03520548	0.02770911	Hmgcs2/Rab8a/Rab13/Marcks/Fam114a1	5
GO:0032869	cellular response to insulin stimulus	4/22	212/14464	0.01886792	12.4048027	6.52896988	0.00026674	0.05755167	0.04529709	Hmgcs2/Rab8a/Rab13/Marcks	4
GO:0043434	response to peptide hormone	5/22	410/14464	0.01219512	8.01773836	5.62621066	0.00031564	0.05755167	0.04529709	Hmgcs2/Rab8a/Rab13/Marcks/Fam114a1	5
GO:0032868	response to insulin	4/22	265/14464	0.01509434	9.9238422	5.72232872	0.00062115	0.06881916	0.05416538	Hmgcs2/Rab8a/Rab13/Marcks	4
GO:0046942	carboxylic acid transport	4/22	281/14464	0.01423488	9.35878357	5.52254231	0.00077384	0.06881916	0.05416538	Fabp7/Slc6a13/Slc47a1/Crabp1	4
GO:0015849	organic acid transport	4/22	283/14464	0.01413428	9.29264375	5.49869538	0.00079464	0.06881916	0.05416538	Fabp7/Slc6a13/Slc47a1/Crabp1	4
GO:0015718	monocarboxylic acid transport	3/22	126/14464	0.02380952	15.6536797	6.44784429	0.00088068	0.06881916	0.05416538	Fabp7/Slc6a13/Crabp1	3
GO:0035725	sodium ion transmembrane transport	3/22	137/14464	0.02189781	14.3968149	6.14909315	0.00112204	0.07671952	0.0603835	Slc6a13/Slc9b2/Fxyd3	3
GO:0098876	vesicle-mediated transport to the plasma membrane	3/22	154/14464	0.01948052	12.8075561	5.74946486	0.0015711	0.08677652	0.06829904	Rab8a/Rab13/Arl3	3
GO:0015711	organic anion transport	4/22	341/14464	0.01173021	7.71207678	4.89550489	0.00158641	0.08677652	0.06829904	Fabp7/Slc6a13/Slc47a1/Crabp1	4
GO:0006814	sodium ion transport	3/22	185/14464	0.01621622	10.6614251	5.16184572	0.0026506	0.12748147	0.10033661	Slc6a13/Slc9b2/Fxyd3	3
GO:0089718	amino acid import across plasma membrane	2/22	52/14464	0.03846154	25.2867133	6.84754909	0.00279667	0.12748147	0.10033661	Slc6a13/Slc47a1	2
GO:0072348	sulfur compound transport	2/22	56/14464	0.03571429	23.4805195	6.57847713	0.00323609	0.13616489	0.10717106	Slc6a13/Slc47a1	2
GO:0006893	Golgi to plasma membrane transport	2/22	62/14464	0.03225806	21.2082111	6.22356422	0.00395182	0.148197	0.11664115	Rab13/Arl3	2
GO:0061512	protein localization to cilium	2/22	65/14464	0.03076923	20.2293706	6.06432469	0.00433483	0.148197	0.11664115	Rab8a/Arl3	2
GO:0140115	export across plasma membrane	2/22	65/14464	0.03076923	20.2293706	6.06432469	0.00433483	0.148197	0.11664115	Slc47a1/Fxyd3	2
GO:0007626	locomotory behavior	3/22	240/14464	0.0125	8.21818182	4.40098035	0.00550308	0.1770698	0.13936602	Npas2/Pbx3/Hexb	3
GO:0032456	endocytic recycling	2/22	86/14464	0.02325581	15.2896406	5.18738109	0.00747178	0.18634746	0.14666817	Rab8a/Rab13	2
GO:0015908	fatty acid transport	2/22	87/14464	0.02298851	15.1138976	5.15346481	0.00764057	0.18634746	0.14666817	Fabp7/Crabp1	2
GO:0048193	Golgi vesicle transport	3/22	284/14464	0.01056338	6.94494238	3.94907669	0.00874989	0.18634746	0.14666817	Rab8a/Rab13/Arl3	3
GO:0003333	amino acid transmembrane transport	2/22	96/14464	0.02083333	13.6969697	4.87151903	0.00923674	0.18634746	0.14666817	Slc6a13/Slc47a1	2
GO:0006892	post-Golgi vesicle-mediated transport	2/22	103/14464	0.01941748	12.7661077	4.67720013	0.01057243	0.18634746	0.14666817	Rab13/Arl3	2
GO:0033555	multicellular organismal response to stress	2/22	103/14464	0.01941748	12.7661077	4.67720013	0.01057243	0.18634746	0.14666817	Hmgcs2/Npas2	2
GO:0006855	xenobiotic transmembrane transport	1/22	10/14464	0.1	65.7454545	7.99359466	0.01511116	0.18634746	0.14666817	Slc47a1	1
GO:0009313	oligosaccharide catabolic process	1/22	10/14464	0.1	65.7454545	7.99359466	0.01511116	0.18634746	0.14666817	Hexb	1
GO:0046618	xenobiotic export from cell	1/22	10/14464	0.1	65.7454545	7.99359466	0.01511116	0.18634746	0.14666817	Slc47a1	1
GO:0051775	response to redox state	1/22	10/14464	0.1	65.7454545	7.99359466	0.01511116	0.18634746	0.14666817	Npas2	1
GO:0060179	male mating behavior	1/22	10/14464	0.1	65.7454545	7.99359466	0.01511116	0.18634746	0.14666817	Hexb	1
GO:0099639	neurotransmitter receptor transport, endosome to plasma membrane	1/22	10/14464	0.1	65.7454545	7.99359466	0.01511116	0.18634746	0.14666817	Rab8a	1
GO:1903278	positive regulation of sodium ion export across plasma membrane	1/22	10/14464	0.1	65.7454545	7.99359466	0.01511116	0.18634746	0.14666817	Fxyd3	1
GO:1903365	regulation of fear response	1/22	10/14464	0.1	65.7454545	7.99359466	0.01511116	0.18634746	0.14666817	Npas2	1
GO:0016114	terpenoid biosynthetic process	1/22	11/14464	0.09090909	59.768595	7.61008509	0.01661023	0.18634746	0.14666817	Hmgcs2	1
GO:0043652	engulfment of apoptotic cell	1/22	11/14464	0.09090909	59.768595	7.61008509	0.01661023	0.18634746	0.14666817	Megf10	1
GO:0045117	azole transmembrane transport	1/22	11/14464	0.09090909	59.768595	7.61008509	0.01661023	0.18634746	0.14666817	Slc47a1	1
GO:0050655	dermatan sulfate proteoglycan metabolic process	1/22	11/14464	0.09090909	59.768595	7.61008509	0.01661023	0.18634746	0.14666817	Hexb	1
GO:0072531	pyrimidine-containing compound transmembrane transport	1/22	11/14464	0.09090909	59.768595	7.61008509	0.01661023	0.18634746	0.14666817	Slc47a1	1
GO:1903276	regulation of sodium ion export across plasma membrane	1/22	11/14464	0.09090909	59.768595	7.61008509	0.01661023	0.18634746	0.14666817	Fxyd3	1
GO:1903441	protein localization to ciliary membrane	1/22	11/14464	0.09090909	59.768595	7.61008509	0.01661023	0.18634746	0.14666817	Arl3	1
GO:1905039	carboxylic acid transmembrane transport	2/22	133/14464	0.01503759	9.88653452	4.01834173	0.01718847	0.18634746	0.14666817	Slc6a13/Slc47a1	2
GO:1903825	organic acid transmembrane transport	2/22	136/14464	0.01470588	9.6684492	3.96410308	0.01792641	0.18634746	0.14666817	Slc6a13/Slc47a1	2
GO:0002087	regulation of respiratory gaseous exchange by nervous system process	1/22	12/14464	0.08333333	54.7878788	7.27508258	0.01810712	0.18634746	0.14666817	Pbx3	1
GO:0046479	glycosphingolipid catabolic process	1/22	12/14464	0.08333333	54.7878788	7.27508258	0.01810712	0.18634746	0.14666817	Hexb	1
GO:1902600	proton transmembrane transport	2/22	137/14464	0.01459854	9.59787658	3.94639655	0.01817536	0.18634746	0.14666817	Slc47a1/Slc9b2	2
GO:1990778	protein localization to cell periphery	3/22	378/14464	0.00793651	5.21789322	3.24320575	0.01886336	0.18634746	0.14666817	Rab8a/Rab13/Arl3	3
GO:0014816	skeletal muscle satellite cell differentiation	1/22	13/14464	0.07692308	50.5734266	6.97908564	0.01960184	0.18634746	0.14666817	Megf10	1
GO:0015846	polyamine transport	1/22	13/14464	0.07692308	50.5734266	6.97908564	0.01960184	0.18634746	0.14666817	Slc47a1	1
GO:0019377	glycolipid catabolic process	1/22	13/14464	0.07692308	50.5734266	6.97908564	0.01960184	0.18634746	0.14666817	Hexb	1
GO:0030214	hyaluronan catabolic process	1/22	13/14464	0.07692308	50.5734266	6.97908564	0.01960184	0.18634746	0.14666817	Hexb	1
GO:0051764	actin crosslink formation	1/22	13/14464	0.07692308	50.5734266	6.97908564	0.01960184	0.18634746	0.14666817	Marcks	1
GO:0097709	connective tissue replacement	1/22	13/14464	0.07692308	50.5734266	6.97908564	0.01960184	0.18634746	0.14666817	Fam114a1	1
GO:0010876	lipid localization	3/22	393/14464	0.00763359	5.01873699	3.15246476	0.0208963	0.18634746	0.14666817	Fabp7/Crabp1/Hexb	3
GO:0051451	myoblast migration	1/22	14/14464	0.07142857	46.961039	6.71501203	0.02109438	0.18634746	0.14666817	Megf10	1
GO:1903826	L-arginine transmembrane transport	1/22	14/14464	0.07142857	46.961039	6.71501203	0.02109438	0.18634746	0.14666817	Slc47a1	1
GO:1905274	regulation of modification of postsynaptic actin cytoskeleton	1/22	14/14464	0.07142857	46.961039	6.71501203	0.02109438	0.18634746	0.14666817	Marcks	1
GO:0002486	antigen processing and presentation of endogenous peptide antigen via MHC class I via ER pathway, T/	1/22	15/14464	0.06666667	43.830303	6.47746007	0.02258476	0.18634746	0.14666817	Mill2	1
GO:0006044	N-acetylglucosamine metabolic process	1/22	15/14464	0.06666667	43.830303	6.47746007	0.02258476	0.18634746	0.14666817	Hexb	1
GO:0006491	N-glycan processing	1/22	15/14464	0.06666667	43.830303	6.47746007	0.02258476	0.18634746	0.14666817	Hexb	1
GO:0044065	regulation of respiratory system process	1/22	15/14464	0.06666667	43.830303	6.47746007	0.02258476	0.18634746	0.14666817	Pbx3	1
GO:1990822	basic amino acid transmembrane transport	1/22	15/14464	0.06666667	43.830303	6.47746007	0.02258476	0.18634746	0.14666817	Slc47a1	1
GO:2000288	positive regulation of myoblast proliferation	1/22	15/14464	0.06666667	43.830303	6.47746007	0.02258476	0.18634746	0.14666817	Megf10	1
GO:0006865	amino acid transport	2/22	158/14464	0.01265823	8.32220944	3.61192267	0.02373829	0.18634746	0.14666817	Slc6a13/Slc47a1	2
GO:0002476	antigen processing and presentation of endogenous peptide antigen via MHC class Ib	1/22	16/14464	0.0625	41.0909091	6.26222824	0.02407297	0.18634746	0.14666817	Mill2	1
GO:0002484	antigen processing and presentation of endogenous peptide antigen via MHC class I via ER pathway	1/22	16/14464	0.0625	41.0909091	6.26222824	0.02407297	0.18634746	0.14666817	Mill2	1
GO:0035461	vitamin transmembrane transport	1/22	16/14464	0.0625	41.0909091	6.26222824	0.02407297	0.18634746	0.14666817	Slc47a1	1
GO:0036035	osteoclast development	1/22	16/14464	0.0625	41.0909091	6.26222824	0.02407297	0.18634746	0.14666817	Slc9b2	1
GO:0046514	ceramide catabolic process	1/22	16/14464	0.0625	41.0909091	6.26222824	0.02407297	0.18634746	0.14666817	Hexb	1
GO:0060134	prepulse inhibition	1/22	16/14464	0.0625	41.0909091	6.26222824	0.02407297	0.18634746	0.14666817	Fabp7	1
GO:0098751	bone cell development	1/22	16/14464	0.0625	41.0909091	6.26222824	0.02407297	0.18634746	0.14666817	Slc9b2	1
GO:0021915	neural tube development	2/22	162/14464	0.01234568	8.11672278	3.55521634	0.02486842	0.18634746	0.14666817	Inka1/Marcks	2
GO:0002428	antigen processing and presentation of peptide antigen via MHC class Ib	1/22	17/14464	0.05882353	38.6737968	6.06599263	0.02555901	0.18634746	0.14666817	Mill2	1
GO:0036376	sodium ion export across plasma membrane	1/22	17/14464	0.05882353	38.6737968	6.06599263	0.02555901	0.18634746	0.14666817	Fxyd3	1
GO:0098739	import across plasma membrane	2/22	167/14464	0.01197605	7.87370713	3.48701379	0.02631181	0.18634746	0.14666817	Slc6a13/Slc47a1	2
GO:0015802	basic amino acid transport	1/22	18/14464	0.05555556	36.5252525	5.88608439	0.0270429	0.18634746	0.14666817	Slc47a1	1
GO:0014841	skeletal muscle satellite cell proliferation	1/22	19/14464	0.05263158	34.6028708	5.72033249	0.02852463	0.18634746	0.14666817	Megf10	1
GO:0060009	Sertoli cell development	1/22	19/14464	0.05263158	34.6028708	5.72033249	0.02852463	0.18634746	0.14666817	Rab13	1
GO:0002475	antigen processing and presentation via MHC class Ib	1/22	20/14464	0.05	32.8727273	5.56695035	0.0300042	0.18634746	0.14666817	Mill2	1
GO:0002483	antigen processing and presentation of endogenous peptide antigen	1/22	20/14464	0.05	32.8727273	5.56695035	0.0300042	0.18634746	0.14666817	Mill2	1
GO:0006027	glycosaminoglycan catabolic process	1/22	20/14464	0.05	32.8727273	5.56695035	0.0300042	0.18634746	0.14666817	Hexb	1
GO:0019885	antigen processing and presentation of endogenous peptide antigen via MHC class I	1/22	20/14464	0.05	32.8727273	5.56695035	0.0300042	0.18634746	0.14666817	Mill2	1
GO:0033762	response to glucagon	1/22	20/14464	0.05	32.8727273	5.56695035	0.0300042	0.18634746	0.14666817	Hmgcs2	1
GO:0038166	angiotensin-activated signaling pathway	1/22	20/14464	0.05	32.8727273	5.56695035	0.0300042	0.18634746	0.14666817	Fam114a1	1
GO:0099638	endosome to plasma membrane protein transport	1/22	20/14464	0.05	32.8727273	5.56695035	0.0300042	0.18634746	0.14666817	Rab8a	1
GO:2000291	regulation of myoblast proliferation	1/22	20/14464	0.05	32.8727273	5.56695035	0.0300042	0.18634746	0.14666817	Megf10	1
GO:0043576	regulation of respiratory gaseous exchange	1/22	21/14464	0.04761905	31.3073593	5.4244524	0.03148163	0.18634746	0.14666817	Pbx3	1
GO:0045475	locomotor rhythm	1/22	21/14464	0.04761905	31.3073593	5.4244524	0.03148163	0.18634746	0.14666817	Npas2	1
GO:0072537	fibroblast activation	1/22	21/14464	0.04761905	31.3073593	5.4244524	0.03148163	0.18634746	0.14666817	Fam114a1	1
GO:0098969	neurotransmitter receptor transport to postsynaptic membrane	1/22	21/14464	0.04761905	31.3073593	5.4244524	0.03148163	0.18634746	0.14666817	Rab8a	1
GO:1901570	fatty acid derivative biosynthetic process	1/22	21/14464	0.04761905	31.3073593	5.4244524	0.03148163	0.18634746	0.14666817	Hmgcs2	1
GO:0050905	neuromuscular process	2/22	188/14464	0.0106383	6.99419729	3.22874355	0.03273501	0.18634746	0.14666817	Fabp7/Hexb	2
GO:0006910	phagocytosis, recognition	1/22	22/14464	0.04545455	29.8842975	5.2915919	0.0329569	0.18634746	0.14666817	Megf10	1
GO:0019883	antigen processing and presentation of endogenous antigen	1/22	22/14464	0.04545455	29.8842975	5.2915919	0.0329569	0.18634746	0.14666817	Mill2	1
GO:1903540	establishment of protein localization to postsynaptic membrane	1/22	22/14464	0.04545455	29.8842975	5.2915919	0.0329569	0.18634746	0.14666817	Rab8a	1
GO:0010737	protein kinase A signaling	1/22	23/14464	0.04347826	28.5849802	5.16731366	0.03443003	0.18634746	0.14666817	Rab13	1
GO:1901071	glucosamine-containing compound metabolic process	1/22	23/14464	0.04347826	28.5849802	5.16731366	0.03443003	0.18634746	0.14666817	Hexb	1
GO:1902307	positive regulation of sodium ion transmembrane transport	1/22	23/14464	0.04347826	28.5849802	5.16731366	0.03443003	0.18634746	0.14666817	Fxyd3	1
GO:0001573	ganglioside metabolic process	1/22	24/14464	0.04166667	27.3939394	5.05071779	0.03590102	0.18634746	0.14666817	Hexb	1
GO:0008299	isoprenoid biosynthetic process	1/22	24/14464	0.04166667	27.3939394	5.05071779	0.03590102	0.18634746	0.14666817	Hmgcs2	1
GO:0015812	gamma-aminobutyric acid transport	1/22	24/14464	0.04166667	27.3939394	5.05071779	0.03590102	0.18634746	0.14666817	Slc6a13	1
GO:0008654	phospholipid biosynthetic process	2/22	201/14464	0.00995025	6.54183627	3.08797049	0.03699128	0.18634746	0.14666817	Hmgcs2/Hexb	2
GO:0030204	chondroitin sulfate metabolic process	1/22	25/14464	0.04	26.2981818	4.94103146	0.03736987	0.18634746	0.14666817	Hexb	1
GO:0048641	regulation of skeletal muscle tissue development	1/22	25/14464	0.04	26.2981818	4.94103146	0.03736987	0.18634746	0.14666817	Megf10	1
GO:0055090	acylglycerol homeostasis	1/22	25/14464	0.04	26.2981818	4.94103146	0.03736987	0.18634746	0.14666817	Angptl4	1
GO:0060008	Sertoli cell differentiation	1/22	25/14464	0.04	26.2981818	4.94103146	0.03736987	0.18634746	0.14666817	Rab13	1
GO:0070328	triglyceride homeostasis	1/22	25/14464	0.04	26.2981818	4.94103146	0.03736987	0.18634746	0.14666817	Angptl4	1
GO:0098877	neurotransmitter receptor transport to plasma membrane	1/22	25/14464	0.04	26.2981818	4.94103146	0.03736987	0.18634746	0.14666817	Rab8a	1
GO:0099159	regulation of modification of postsynaptic structure	1/22	25/14464	0.04	26.2981818	4.94103146	0.03736987	0.18634746	0.14666817	Marcks	1
GO:0006026	aminoglycan catabolic process	1/22	26/14464	0.03846154	25.2867133	4.83758673	0.03883658	0.18634746	0.14666817	Hexb	1
GO:0014856	skeletal muscle cell proliferation	1/22	26/14464	0.03846154	25.2867133	4.83758673	0.03883658	0.18634746	0.14666817	Megf10	1
GO:0021846	cell proliferation in forebrain	1/22	26/14464	0.03846154	25.2867133	4.83758673	0.03883658	0.18634746	0.14666817	Fabp7	1
GO:0030149	sphingolipid catabolic process	1/22	26/14464	0.03846154	25.2867133	4.83758673	0.03883658	0.18634746	0.14666817	Hexb	1
GO:0034694	response to prostaglandin	1/22	26/14464	0.03846154	25.2867133	4.83758673	0.03883658	0.18634746	0.14666817	Hmgcs2	1
GO:0046628	positive regulation of insulin receptor signaling pathway	1/22	26/14464	0.03846154	25.2867133	4.83758673	0.03883658	0.18634746	0.14666817	Marcks	1
GO:0060416	response to growth hormone	1/22	26/14464	0.03846154	25.2867133	4.83758673	0.03883658	0.18634746	0.14666817	Hmgcs2	1
GO:0098885	modification of postsynaptic actin cytoskeleton	1/22	26/14464	0.03846154	25.2867133	4.83758673	0.03883658	0.18634746	0.14666817	Marcks	1
GO:0048520	positive regulation of behavior	1/22	27/14464	0.03703704	24.3501684	4.73980291	0.04030116	0.18889745	0.14867518	Npas2	1
GO:2000352	negative regulation of endothelial cell apoptotic process	1/22	27/14464	0.03703704	24.3501684	4.73980291	0.04030116	0.18889745	0.14867518	Angptl4	1
GO:0034284	response to monosaccharide	2/22	211/14464	0.00947867	6.23179664	2.98789824	0.04040403	0.18889745	0.14867518	Hmgcs2/Slc9b2	2
GO:0001916	positive regulation of T cell mediated cytotoxicity	1/22	28/14464	0.03571429	23.4805195	4.64717241	0.04176361	0.19037246	0.14983611	Mill2	1
GO:0035767	endothelial cell chemotaxis	1/22	28/14464	0.03571429	23.4805195	4.64717241	0.04176361	0.19037246	0.14983611	Rab13	1
GO:0055078	sodium ion homeostasis	1/22	28/14464	0.03571429	23.4805195	4.64717241	0.04176361	0.19037246	0.14983611	Slc9b2	1
GO:0042594	response to starvation	2/22	216/14464	0.00925926	6.08754209	2.94025367	0.04215421	0.19056492	0.14998759	Angptl4/Hmgcs2	2
GO:0046466	membrane lipid catabolic process	1/22	29/14464	0.03448276	22.6708464	4.55924932	0.04322393	0.19119392	0.15048266	Hexb	1
GO:0051450	myoblast proliferation	1/22	29/14464	0.03448276	22.6708464	4.55924932	0.04322393	0.19119392	0.15048266	Megf10	1
GO:0042745	circadian sleep/wake cycle	1/22	30/14464	0.03333333	21.9151515	4.47564009	0.04468213	0.19119392	0.15048266	Npas2	1
GO:0045672	positive regulation of osteoclast differentiation	1/22	30/14464	0.03333333	21.9151515	4.47564009	0.04468213	0.19119392	0.15048266	Slc9b2	1
GO:0060191	regulation of lipase activity	1/22	30/14464	0.03333333	21.9151515	4.47564009	0.04468213	0.19119392	0.15048266	Angptl4	1
GO:0030212	hyaluronan metabolic process	1/22	31/14464	0.03225806	21.2082111	4.39599586	0.0461382	0.19119392	0.15048266	Hexb	1
GO:0050654	chondroitin sulfate proteoglycan metabolic process	1/22	31/14464	0.03225806	21.2082111	4.39599586	0.0461382	0.19119392	0.15048266	Hexb	1
GO:0061003	positive regulation of dendritic spine morphogenesis	1/22	31/14464	0.03225806	21.2082111	4.39599586	0.0461382	0.19119392	0.15048266	Marcks	1
GO:0072337	modified amino acid transport	1/22	31/14464	0.03225806	21.2082111	4.39599586	0.0461382	0.19119392	0.15048266	Slc6a13	1
GO:1900078	positive regulation of cellular response to insulin stimulus	1/22	31/14464	0.03225806	21.2082111	4.39599586	0.0461382	0.19119392	0.15048266	Marcks	1
GO:1905244	regulation of modification of synaptic structure	1/22	31/14464	0.03225806	21.2082111	4.39599586	0.0461382	0.19119392	0.15048266	Marcks	1
GO:0001964	startle response	1/22	32/14464	0.03125	20.5454545	4.32000617	0.04759216	0.19427547	0.15290804	Fabp7	1
GO:0051180	vitamin transport	1/22	32/14464	0.03125	20.5454545	4.32000617	0.04759216	0.19427547	0.15290804	Slc47a1	1
GO:0009743	response to carbohydrate	2/22	234/14464	0.00854701	5.61926962	2.78038959	0.04868777	0.19439905	0.15300532	Hmgcs2/Slc9b2	2
GO:0002474	antigen processing and presentation of peptide antigen via MHC class I	1/22	33/14464	0.03030303	19.922865	4.24739363	0.049044	0.19439905	0.15300532	Mill2	1
GO:0043330	response to exogenous dsRNA	1/22	33/14464	0.03030303	19.922865	4.24739363	0.049044	0.19439905	0.15300532	Rftn2	1
GO:1904385	cellular response to angiotensin	1/22	33/14464	0.03030303	19.922865	4.24739363	0.049044	0.19439905	0.15300532	Fam114a1	1
GO:0001914	regulation of T cell mediated cytotoxicity	1/22	34/14464	0.02941176	19.3368984	4.17790956	0.05049373	0.19588703	0.15417645	Mill2	1
GO:0009311	oligosaccharide metabolic process	1/22	34/14464	0.02941176	19.3368984	4.17790956	0.05049373	0.19588703	0.15417645	Hexb	1
GO:0071398	cellular response to fatty acid	1/22	34/14464	0.02941176	19.3368984	4.17790956	0.05049373	0.19588703	0.15417645	Hmgcs2	1
GO:0007617	mating behavior	1/22	35/14464	0.02857143	18.7844156	4.11133028	0.05194135	0.19868474	0.15637845	Hexb	1
GO:0033574	response to testosterone	1/22	35/14464	0.02857143	18.7844156	4.11133028	0.05194135	0.19868474	0.15637845	Hmgcs2	1
GO:0042908	xenobiotic transport	1/22	36/14464	0.02777778	18.2626263	4.04745394	0.05338686	0.2012151	0.15837001	Slc47a1	1
GO:1902742	apoptotic process involved in development	1/22	36/14464	0.02777778	18.2626263	4.04745394	0.05338686	0.2012151	0.15837001	Megf10	1
GO:0006084	acetyl-CoA metabolic process	1/22	37/14464	0.02702703	17.7690418	3.98609785	0.05483027	0.2012151	0.15837001	Hmgcs2	1
GO:0043277	apoptotic cell clearance	1/22	37/14464	0.02702703	17.7690418	3.98609785	0.05483027	0.2012151	0.15837001	Megf10	1
GO:0048169	regulation of long-term neuronal synaptic plasticity	1/22	37/14464	0.02702703	17.7690418	3.98609785	0.05483027	0.2012151	0.15837001	Rab8a	1
GO:0099637	neurotransmitter receptor transport	1/22	37/14464	0.02702703	17.7690418	3.98609785	0.05483027	0.2012151	0.15837001	Rab8a	1
GO:0031503	protein-containing complex localization	2/22	251/14464	0.00796813	5.23868164	2.6439403	0.05517781	0.2012151	0.15837001	Rab8a/Arl3	2
GO:0044273	sulfur compound catabolic process	1/22	38/14464	0.02631579	17.3014354	3.92709624	0.05627158	0.20384472	0.1604397	Hexb	1
GO:0006904	vesicle docking involved in exocytosis	1/22	39/14464	0.02564103	16.8578089	3.87029826	0.05771079	0.20632549	0.16239223	Rab8a	1
GO:0042461	photoreceptor cell development	1/22	39/14464	0.02564103	16.8578089	3.87029826	0.05771079	0.20632549	0.16239223	Arl3	1
GO:0010765	positive regulation of sodium ion transport	1/22	41/14464	0.02439024	16.0354767	3.7627745	0.06058292	0.21107552	0.16613084	Fxyd3	1
GO:0030866	cortical actin cytoskeleton organization	1/22	41/14464	0.02439024	16.0354767	3.7627745	0.06058292	0.21107552	0.16613084	Rab13	1
GO:0043331	response to dsRNA	1/22	41/14464	0.02439024	16.0354767	3.7627745	0.06058292	0.21107552	0.16613084	Rftn2	1
GO:1990776	response to angiotensin	1/22	41/14464	0.02439024	16.0354767	3.7627745	0.06058292	0.21107552	0.16613084	Fam114a1	1
GO:0022604	regulation of cell morphogenesis	2/22	268/14464	0.00746269	4.9063772	2.51933223	0.06195953	0.21335013	0.16792111	Marcks/Hexb	2
GO:0006040	amino sugar metabolic process	1/22	42/14464	0.02380952	15.6536797	3.71180759	0.06201585	0.21335013	0.16792111	Hexb	1
GO:0016202	regulation of striated muscle tissue development	1/22	43/14464	0.02325581	15.2896406	3.66255966	0.0634467	0.21587661	0.16990962	Megf10	1
GO:0001662	behavioral fear response	1/22	44/14464	0.02272727	14.9421488	3.61493326	0.06487546	0.21587661	0.16990962	Npas2	1
GO:0001913	T cell mediated cytotoxicity	1/22	44/14464	0.02272727	14.9421488	3.61493326	0.06487546	0.21587661	0.16990962	Mill2	1
GO:1901861	regulation of muscle tissue development	1/22	44/14464	0.02272727	14.9421488	3.61493326	0.06487546	0.21587661	0.16990962	Megf10	1
GO:0016197	endosomal transport	2/22	276/14464	0.00724638	4.76416337	2.46427561	0.0652468	0.21587661	0.16990962	Rab8a/Rab13	2
GO:0002209	behavioral defense response	1/22	45/14464	0.02222222	14.610101	3.56883852	0.06630214	0.21587661	0.16990962	Npas2	1

GO:0003016	respiratory system process	1/22	45/14464	0.02222222	14.610101	3.56883852	0.06630214	0.21587661	0.16990962	Pbx3	1
GO:0048634	regulation of muscle organ development	1/22	45/14464	0.02222222	14.610101	3.56883852	0.06630214	0.21587661	0.16990962	Megf10	1
GO:0099010	modification of postsynaptic structure	1/22	45/14464	0.02222222	14.610101	3.56883852	0.06630214	0.21587661	0.16990962	Marcks	1
GO:0001912	positive regulation of leukocyte mediated cytotoxicity	1/22	46/14464	0.02173913	14.2924901	3.52419243	0.06772674	0.21664637	0.17051547	Mill2	1
GO:1901568	fatty acid derivative metabolic process	1/22	46/14464	0.02173913	14.2924901	3.52419243	0.06772674	0.21664637	0.17051547	Hmgcs2	1
GO:2000351	regulation of endothelial cell apoptotic process	1/22	46/14464	0.02173913	14.2924901	3.52419243	0.06772674	0.21664637	0.17051547	Angptl4	1
GO:0006790	sulfur compound metabolic process	2/22	285/14464	0.00701754	4.61371611	2.40480864	0.06901528	0.21738305	0.17109529	Hmgcs2/Hexb	2
GO:0019098	reproductive behavior	1/22	47/14464	0.0212766	13.9883946	3.48091819	0.06914927	0.21738305	0.17109529	Hexb	1
GO:0042073	intraciliary transport	1/22	47/14464	0.0212766	13.9883946	3.48091819	0.06914927	0.21738305	0.17109529	Arl3	1
GO:0006911	phagocytosis, engulfment	1/22	48/14464	0.02083333	13.6969697	3.43894459	0.07056973	0.2187886	0.17220155	Megf10	1
GO:0072583	clathrin-dependent endocytosis	1/22	48/14464	0.02083333	13.6969697	3.43894459	0.07056973	0.2187886	0.17220155	Slc9b2	1
GO:0003179	heart valve morphogenesis	1/22	49/14464	0.02040816	13.4174397	3.39820551	0.0719881	0.2187886	0.17220155	Fam114a1	1
GO:0006687	glycosphingolipid metabolic process	1/22	49/14464	0.02040816	13.4174397	3.39820551	0.0719881	0.2187886	0.17220155	Hexb	1
GO:0006721	terpenoid metabolic process	1/22	49/14464	0.02040816	13.4174397	3.39820551	0.0719881	0.2187886	0.17220155	Hmgcs2	1
GO:0072659	protein localization to plasma membrane	2/22	292/14464	0.00684932	4.50311333	2.36024401	0.07199625	0.2187886	0.17220155	Rab8a/Rab13	2
GO:0042596	fear response	1/22	50/14464	0.02	13.1490909	3.35863948	0.07340443	0.22061663	0.17364033	Npas2	1
GO:0072577	endothelial cell apoptotic process	1/22	50/14464	0.02	13.1490909	3.35863948	0.07340443	0.22061663	0.17364033	Angptl4	1
GO:0002711	positive regulation of T cell mediated immunity	1/22	51/14464	0.01960784	12.8912656	3.32018924	0.07481869	0.22122067	0.17411576	Mill2	1
GO:0006695	cholesterol biosynthetic process	1/22	51/14464	0.01960784	12.8912656	3.32018924	0.07481869	0.22122067	0.17411576	Hmgcs2	1
GO:1902653	secondary alcohol biosynthetic process	1/22	51/14464	0.01960784	12.8912656	3.32018924	0.07481869	0.22122067	0.17411576	Hmgcs2	1
GO:0031343	positive regulation of cell killing	1/22	52/14464	0.01923077	12.6433566	3.28280139	0.07623089	0.22298554	0.17550483	Mill2	1
GO:1902305	regulation of sodium ion transmembrane transport	1/22	52/14464	0.01923077	12.6433566	3.28280139	0.07623089	0.22298554	0.17550483	Fxyd3	1
GO:0001822	kidney development	2/22	303/14464	0.00660066	4.33963396	2.29297904	0.07676589	0.22335608	0.17579647	Hmgcs2/Arl3	2
GO:0048002	antigen processing and presentation of peptide antigen	1/22	53/14464	0.01886792	12.4048027	3.24642607	0.07764103	0.22352443	0.17592898	Mill2	1
GO:0099563	modification of synaptic structure	1/22	53/14464	0.01886792	12.4048027	3.24642607	0.07764103	0.22352443	0.17592898	Marcks	1
GO:0048512	circadian behavior	1/22	54/14464	0.01851852	12.1750842	3.21101664	0.07904911	0.22638672	0.17818179	Npas2	1
GO:0007622	rhythmic behavior	1/22	55/14464	0.01818182	11.953719	3.17652943	0.08045514	0.22685033	0.17854668	Npas2	1
GO:0030865	cortical cytoskeleton organization	1/22	55/14464	0.01818182	11.953719	3.17652943	0.08045514	0.22685033	0.17854668	Rab13	1
GO:0070542	response to fatty acid	1/22	55/14464	0.01818182	11.953719	3.17652943	0.08045514	0.22685033	0.17854668	Hmgcs2	1
GO:0016126	sterol biosynthetic process	1/22	56/14464	0.01785714	11.7402597	3.14292351	0.08185913	0.22791699	0.17938622	Hmgcs2	1
GO:0070830	bicellular tight junction assembly	1/22	56/14464	0.01785714	11.7402597	3.14292351	0.08185913	0.22791699	0.17938622	Rab13	1
GO:0072001	renal system development	2/22	315/14464	0.00634921	4.17431457	2.22315237	0.08208345	0.22791699	0.17938622	Hmgcs2/Arl3	2
GO:0048278	vesicle docking	1/22	57/14464	0.01754386	11.5342903	3.11016045	0.08326106	0.22886332	0.18013105	Rab8a	1
GO:0099024	plasma membrane invagination	1/22	57/14464	0.01754386	11.5342903	3.11016045	0.08326106	0.22886332	0.18013105	Megf10	1
GO:0006644	phospholipid metabolic process	2/22	319/14464	0.00626959	4.12197207	2.20064375	0.08388154	0.22941603	0.18056606	Hmgcs2/Hexb	2
GO:0046530	photoreceptor cell differentiation	1/22	58/14464	0.01724138	11.3354232	3.07820415	0.08466096	0.23039573	0.18133716	Arl3	1
GO:0002763	positive regulation of myeloid leukocyte differentiation	1/22	59/14464	0.01694915	11.1432974	3.04702064	0.08605881	0.23189245	0.18251518	Slc9b2	1
GO:0003170	heart valve development	1/22	59/14464	0.01694915	11.1432974	3.04702064	0.08605881	0.23189245	0.18251518	Fam114a1	1
GO:0071385	cellular response to glucocorticoid stimulus	1/22	60/14464	0.01666667	10.9575758	3.01657794	0.08745462	0.23449842	0.18456626	Hmgcs2	1
GO:0061001	regulation of dendritic spine morphogenesis	1/22	61/14464	0.01639344	10.7779434	2.98684591	0.0888484	0.2359227	0.18568726	Marcks	1
GO:0120192	tight junction assembly	1/22	61/14464	0.01639344	10.7779434	2.98684591	0.0888484	0.2359227	0.18568726	Rab13	1
GO:0007585	respiratory gaseous exchange by respiratory system	1/22	63/14464	0.01587302	10.4357864	2.92940161	0.09162987	0.2380989	0.18740008	Pbx3	1
GO:0050775	positive regulation of dendrite morphogenesis	1/22	63/14464	0.01587302	10.4357864	2.92940161	0.09162987	0.2380989	0.18740008	Marcks	1
GO:0010770	positive regulation of cell morphogenesis	1/22	64/14464	0.015625	10.2727273	2.90163707	0.09301756	0.2380989	0.18740008	Marcks	1
GO:0050885	neuromuscular process controlling balance	1/22	64/14464	0.015625	10.2727273	2.90163707	0.09301756	0.2380989	0.18740008	Hexb	1
GO:0060271	cilium assembly	2/22	339/14464	0.00589971	3.87878788	2.09335146	0.09305476	0.2380989	0.18740008	Rab8a/Arl3	2
GO:0001910	regulation of leukocyte mediated cytotoxicity	1/22	65/14464	0.01538462	10.1146853	2.87447844	0.09440323	0.2380989	0.18740008	Mill2	1
GO:0010324	membrane invagination	1/22	65/14464	0.01538462	10.1146853	2.87447844	0.09440323	0.2380989	0.18740008	Megf10	1
GO:0032922	circadian regulation of gene expression	1/22	65/14464	0.01538462	10.1146853	2.87447844	0.09440323	0.2380989	0.18740008	Npas2	1
GO:0043297	apical junction assembly	1/22	65/14464	0.01538462	10.1146853	2.87447844	0.09440323	0.2380989	0.18740008	Rab13	1
GO:0060999	positive regulation of dendritic spine development	1/22	65/14464	0.01538462	10.1146853	2.87447844	0.09440323	0.2380989	0.18740008	Marcks	1
GO:0006869	lipid transport	2/22	342/14464	0.00584795	3.84476342	2.07796375	0.09445605	0.2380989	0.18740008	Fabp7/Crabp1	2
GO:0071384	cellular response to corticosteroid stimulus	1/22	66/14464	0.01515152	9.96143251	2.84790294	0.09578687	0.23968866	0.18865132	Hmgcs2	1
GO:0060612	adipose tissue development	1/22	67/14464	0.01492537	9.81275441	2.82188902	0.0971685	0.23968866	0.18865132	Hmgcs2	1
GO:0061951	establishment of protein localization to plasma membrane	1/22	67/14464	0.01492537	9.81275441	2.82188902	0.0971685	0.23968866	0.18865132	Rab8a	1
GO:0120193	tight junction organization	1/22	67/14464	0.01492537	9.81275441	2.82188902	0.0971685	0.23968866	0.18865132	Rab13	1
GO:0009410	response to xenobiotic stimulus	2/22	348/14464	0.00574713	3.7784744	2.04770344	0.09727766	0.23968866	0.18865132	Hmgcs2/Npas2	2
GO:0006720	isoprenoid metabolic process	1/22	68/14464	0.01470588	9.6684492	2.7964162	0.09854811	0.24065097	0.18940873	Hmgcs2	1
GO:1902475	L-alpha-amino acid transmembrane transport	1/22	68/14464	0.01470588	9.6684492	2.7964162	0.09854811	0.24065097	0.18940873	Slc47a1	1
GO:0006887	exocytosis	2/22	352/14464	0.00568182	3.73553719	2.0279	0.09917257	0.24079016	0.18951828	Rab8a/Rab13	2
GO:0008347	glial cell migration	1/22	69/14464	0.01449275	9.52832675	2.77146504	0.09992571	0.24079016	0.18951828	Hexb	1
GO:0010761	fibroblast migration	1/22	69/14464	0.01449275	9.52832675	2.77146504	0.09992571	0.24079016	0.18951828	Fam114a1	1
GO:0045670	regulation of osteoclast differentiation	1/22	70/14464	0.01428571	9.39220779	2.7470171	0.10130131	0.24092093	0.18962121	Slc9b2	1
GO:0046626	regulation of insulin receptor signaling pathway	1/22	70/14464	0.01428571	9.39220779	2.7470171	0.10130131	0.24092093	0.18962121	Marcks	1
GO:1903539	protein localization to postsynaptic membrane	1/22	70/14464	0.01428571	9.39220779	2.7470171	0.10130131	0.24092093	0.18962121	Rab8a	1
GO:0007018	microtubule-based movement	2/22	359/14464	0.00557103	3.66269942	1.99392898	0.10251463	0.2427511	0.19106167	Slc9b2/Arl3	2
GO:0015800	acidic amino acid transport	1/22	72/14464	0.01388889	9.13131313	2.69956148	0.10404647	0.2442636	0.19225212	Slc6a13	1
GO:0051591	response to cAMP	1/22	72/14464	0.01388889	9.13131313	2.69956148	0.10404647	0.2442636	0.19225212	Hmgcs2	1
GO:0048168	regulation of neuronal synaptic plasticity	1/22	73/14464	0.01369863	9.00622665	2.6765212	0.10541605	0.24433296	0.19230671	Rab8a	1
GO:0062237	protein localization to postsynapse	1/22	73/14464	0.01369863	9.00622665	2.6765212	0.10541605	0.24433296	0.19230671	Rab8a	1
GO:1903510	mucopolysaccharide metabolic process	1/22	73/14464	0.01369863	9.00622665	2.6765212	0.10541605	0.24433296	0.19230671	Hexb	1
GO:0034109	homotypic cell-cell adhesion	1/22	74/14464	0.01351351	8.88452088	2.65391882	0.10678363	0.24645842	0.19397959	Megf10	1
GO:0044782	cilium organization	2/22	369/14464	0.00542005	3.56343927	1.94684125	0.10734466	0.24649008	0.19400451	Rab8a/Arl3	2
GO:0002709	regulation of T cell mediated immunity	1/22	75/14464	0.01333333	8.76606061	2.63173989	0.10814921	0.24649008	0.19400451	Mill2	1
GO:0070050	neuron cellular homeostasis	1/22	75/14464	0.01333333	8.76606061	2.63173989	0.10814921	0.24649008	0.19400451	Hexb	1
GO:0140056	organelle localization by membrane tethering	1/22	76/14464	0.01315789	8.6507177	2.60997064	0.1095128	0.24856226	0.19563545	Rab8a	1
GO:0043603	amide metabolic process	2/22	376/14464	0.00531915	3.49709865	1.91483701	0.11076327	0.24956101	0.19642154	Hmgcs2/Hexb	2
GO:0061178	regulation of insulin secretion involved in cellular response to glucose stimulus	1/22	77/14464	0.01298701	8.53837072	2.5885979	0.11087441	0.24956101	0.19642154	Slc9b2	1
GO:0031341	regulation of cell killing	1/22	78/14464	0.01282051	8.42890443	2.56760911	0.11223402	0.24956101	0.19642154	Mill2	1
GO:0050795	regulation of behavior	1/22	78/14464	0.01282051	8.42890443	2.56760911	0.11223402	0.24956101	0.19642154	Npas2	1
GO:0060997	dendritic spine morphogenesis	1/22	78/14464	0.01282051	8.42890443	2.56760911	0.11223402	0.24956101	0.19642154	Marcks	1
GO:0002028	regulation of sodium ion transport	1/22	79/14464	0.01265823	8.32220944	2.54699224	0.11359165	0.25054287	0.19719433	Fxyd3	1
GO:0035914	skeletal muscle cell differentiation	1/22	79/14464	0.01265823	8.32220944	2.54699224	0.11359165	0.25054287	0.19719433	Megf10	1
GO:0071230	cellular response to amino acid stimulus	1/22	80/14464	0.0125	8.21818182	2.52673582	0.1149473	0.25251474	0.19874633	Hmgcs2	1
GO:0021510	spinal cord development	1/22	81/14464	0.01234568	8.11672278	2.50682883	0.11630096	0.25446651	0.2002825	Pbx3	1
GO:0006029	proteoglycan metabolic process	1/22	82/14464	0.01219512	8.01773836	2.48726077	0.11765266	0.25527176	0.20091629	Hexb	1
GO:0014855	striated muscle cell proliferation	1/22	82/14464	0.01219512	8.01773836	2.48726077	0.11765266	0.25527176	0.20091629	Megf10	1
GO:0006637	acyl-CoA metabolic process	1/22	83/14464	0.01204819	7.9211391	2.46802153	0.11900237	0.25527176	0.20091629	Hmgcs2	1
GO:0035383	thioester metabolic process	1/22	83/14464	0.01204819	7.9211391	2.46802153	0.11900237	0.25527176	0.20091629	Hmgcs2	1
GO:1900076	regulation of cellular response to insulin stimulus	1/22	83/14464	0.01204819	7.9211391	2.46802153	0.11900237	0.25527176	0.20091629	Marcks	1
GO:0060998	regulation of dendritic spine development	1/22	84/14464	0.01190476	7.82683983	2.44910146	0.12035012	0.25577712	0.20131404	Marcks	1
GO:0006664	glycolipid metabolic process	1/22	85/14464	0.01176471	7.73475936	2.43049128	0.1216959	0.25577712	0.20131404	Hexb	1
GO:0019915	lipid storage	1/22	85/14464	0.01176471	7.73475936	2.43049128	0.1216959	0.25577712	0.20131404	Hexb	1
GO:0022406	membrane docking	1/22	85/14464	0.01176471	7.73475936	2.43049128	0.1216959	0.25577712	0.20131404	Rab8a	1
GO:0030203	glycosaminoglycan metabolic process	1/22	85/14464	0.01176471	7.73475936	2.43049128	0.1216959	0.25577712	0.20131404	Hexb	1
GO:0006672	ceramide metabolic process	1/22	86/14464	0.01162791	7.6448203	2.41218209	0.12303971	0.25577712	0.20131404	Hexb	1
GO:1903509	liposaccharide metabolic process	1/22	86/14464	0.01162791	7.6448203	2.41218209	0.12303971	0.25577712	0.20131404	Hexb	1
GO:0000281	mitotic cytokinesis	1/22	87/14464	0.01149425	7.5569488	2.39416537	0.12438156	0.25577712	0.20131404	Arl3	1
GO:0015807	L-amino acid transport	1/22	87/14464	0.01149425	7.5569488	2.39416537	0.12438156	0.25577712	0.20131404	Slc47a1	1
GO:0035773	insulin secretion involved in cellular response to glucose stimulus	1/22	87/14464	0.01149425	7.5569488	2.39416537	0.12438156	0.25577712	0.20131404	Slc9b2	1
GO:0048477	oogenesis	1/22	87/14464	0.01149425	7.5569488	2.39416537	0.12438156	0.25577712	0.20131404	Hexb	1
GO:0061515	myeloid cell development	1/22	91/14464	0.01098901	7.22477522	2.32486383	0.12972939	0.2647835	0.20840268	Slc9b2	1
GO:0071229	cellular response to acid chemical	1/22	91/14464	0.01098901	7.22477522	2.32486383	0.12972939	0.2647835	0.20840268	Hmgcs2	1
GO:0048565	digestive tract development	1/22	92/14464	0.01086957	7.14624506	2.3081926	0.13106147	0.2665079	0.20975989	Hmgcs2	1
GO:0002708	positive regulation of lymphocyte mediated immunity	1/22	95/14464	0.01052632	6.92057416	2.25963982	0.13504604	0.27158155	0.21375321	Mill2	1
GO:0019882	antigen processing and presentation	1/22	95/14464	0.01052632	6.92057416	2.25963982	0.13504604	0.27158155	0.21375321	Mill2	1
GO:0045639	positive regulation of myeloid cell differentiation	1/22	95/14464	0.01052632	6.92057416	2.25963982	0.13504604	0.27158155	0.21375321	Slc9b2	1
GO:0006022	aminoglycan metabolic process	1/22	96/14464	0.01041667	6.84848485	2.24392165	0.13637034	0.27224299	0.2142738	Hexb	1
GO:0048814	regulation of dendrite morphogenesis	1/22	96/14464	0.01041667	6.84848485	2.24392165	0.13637034	0.27224299	0.2142738	Marcks	1
GO:0008344	adult locomotory behavior	1/22	97/14464	0.01030928	6.77788191	2.22842666	0.13769272	0.27352589	0.21528353	Pbx3	1
GO:0007040	lysosome organization	1/22	98/14464	0.01020408	6.70871985	2.21314924	0.13901316	0.27352589	0.21528353	Hexb	1
GO:0080171	lytic vacuole organization	1/22	98/14464	0.01020408	6.70871985	2.21314924	0.13901316	0.27352589	0.21528353	Hexb	1
GO:0097120	receptor localization to synapse	1/22	98/14464	0.01020408	6.70871985	2.21314924	0.13901316	0.27352589	0.21528353	Rab8a	1
GO:0007338	single fertilization	1/22	101/14464	0.00990099	6.50945095	2.16856923	0.14296291	0.28028928	0.22060679	Hexb	1
GO:0001909	leukocyte mediated cytotoxicity	1/22	102/14464	0.00980392	6.4456328	2.15410982	0.14427564	0.28084974	0.2210479	Mill2	1
GO:0002824	positive regulation of adaptive immune response based on somatic recombination of immune receptors b	1/22	102/14464	0.00980392	6.4456328	2.15410982	0.14427564	0.28084974	0.2210479	Mill2	1
GO:0042752	regulation of circadian rhythm	1/22	103/14464	0.00970874	6.38305384	2.13984274	0.14558646	0.28139856	0.22147986	Npas2	1
GO:0140029	exocytic process	1/22	103/14464	0.00970874	6.38305384	2.13984274	0.14558646	0.28139856	0.22147986	Rab8a	1
GO:0055123	digestive system development	1/22	104/14464	0.00961538	6.32167832	2.12576343	0.14689536	0.281936	0.22190286	Hmgcs2	1
GO:0098754	detoxification	1/22	104/14464	0.00961538	6.32167832	2.12576343	0.14689536	0.281936	0.22190286	Slc47a1	1
GO:0035418	protein localization to synapse	1/22	105/14464	0.00952381	6.26147186	2.1118675	0.14820234	0.2824623	0.2223171	Rab8a	1
GO:0045445	myoblast differentiation	1/22	105/14464	0.00952381	6.26147186	2.1118675	0.14820234	0.2824623	0.2223171	Megf10	1
GO:0097061	dendritic spine organization	1/22	106/14464	0.00943396	6.20240137	2.09815069	0.14950741	0.28317074	0.22287469	Marcks	1
GO:0051223	regulation of protein transport	2/22	452/14464	0.00442478	2.90909091	1.60943198	0.1496094	0.28317074	0.22287469	Slc9b2/Rab8a	2
GO:0098771	inorganic ion homeostasis	2/22	454/14464	0.00440529	2.89627553	1.60227539	0.15066881	0.28323948	0.2229288	Slc9b2/Hexb	2
GO:0002456	T cell mediated immunity	1/22	107/14464	0.00934579	6.144435	2.08460889	0.15081058	0.28323948	0.2229288	Mill2	1
GO:0031667	response to nutrient levels	2/22	455/14464	0.0043956	2.88991009	1.59871164	0.15119914	0.28323948	0.2229288	Angptl4/Hmgcs2	2
GO:0030316	osteoclast differentiation	1/22	108/14464	0.00925926	6.08754209	2.07123812	0.15211184	0.28397671	0.22350904	Slc9b2	1
GO:0008037	cell recognition	1/22	109/14464	0.00917431	6.03169308	2.05803452	0.15341119	0.28446075	0.22389002	Megf10	1
GO:0046683	response to organophosphorus	1/22	109/14464	0.00917431	6.03169308	2.05803452	0.15341119	0.28446075	0.22389002	Hmgcs2	1
GO:0002821	positive regulation of adaptive immune response	1/22	110/14464	0.00909091	5.9768595	2.04499437	0.15470865	0.2858974	0.22502075	Mill2	1
GO:0001501	skeletal system development	2/22	466/14464	0.00429185	2.82169333	1.56013469	0.15705922	0.28873131	0.22725124	Slc9b2/Hexb	2
GO:0061640	cytoskeleton-dependent cytokinesis	1/22	112/14464	0.00892857	5.87012987	2.01939001	0.15729786	0.28873131	0.22725124	Arl3	1
GO:0010720	positive regulation of cell development	2/22	473/14464	0.00422833	2.77993465	1.53616234	0.16081277	0.29288578	0.23052109	Slc9b2/Marcks	2
GO:0002705	positive regulation of leukocyte mediated immunity	1/22	115/14464	0.00869565	5.71699605	1.98212244	0.1611675	0.29288578	0.23052109	Mill2	1
GO:0043200	response to amino acid	1/22	115/14464	0.00869565	5.71699605	1.98212244	0.1611675	0.29288578	0.23052109	Hmgcs2	1
GO:0008584	male gonad development	1/22	117/14464	0.00854701	5.61926962	1.95799941	0.16373782	0.29462036	0.23188632	Rab13	1
GO:0015695	organic cation transport	1/22	117/14464	0.00854701	5.61926962	1.95799941	0.16373782	0.29462036	0.23188632	Slc47a1	1
GO:0106027	neuron projection organization	1/22	117/14464	0.00854701	5.61926962	1.95799941	0.16373782	0.29462036	0.23188632	Marcks	1
GO:0008203	cholesterol metabolic process	1/22	118/14464	0.00847458	5.57164869	1.94614549	0.16502017	0.29498703	0.23217492	Hmgcs2	1
GO:0046546	development of primary male sexual characteristics	1/22	118/14464	0.00847458	5.57164869	1.94614549	0.16502017	0.29498703	0.23217492	Rab13	1
GO:0030317	flagellated sperm motility	1/22	119/14464	0.00840336	5.52482811	1.93442615	0.16630063	0.2960391	0.23300297	Slc9b2	1
GO:0002761	regulation of myeloid leukocyte differentiation	1/22	121/14464	0.00826446	5.43350864	1.91138031	0.16885594	0.2960391	0.23300297	Slc9b2	1
GO:0007043	cell-cell junction assembly	1/22	121/14464	0.00826446	5.43350864	1.91138031	0.16885594	0.2960391	0.23300297	Rab13	1
GO:0046165	alcohol biosynthetic process	1/22	121/14464	0.00826446	5.43350864	1.91138031	0.16885594	0.2960391	0.23300297	Hmgcs2	1
GO:0072329	monocarboxylic acid catabolic process	1/22	121/14464	0.00826446	5.43350864	1.91138031	0.16885594	0.2960391	0.23300297	Hexb	1
GO:0097722	sperm motility	1/22	121/14464	0.00826446	5.43350864	1.91138031	0.16885594	0.2960391	0.23300297	Slc9b2	1
GO:0045834	positive regulation of lipid metabolic process	1/22	122/14464	0.00819672	5.38897168	1.90004855	0.17013079	0.29732122	0.23401208	Angptl4	1
GO:0016125	sterol metabolic process	1/22	123/14464	0.00813008	5.34515891	1.88884084	0.17140377	0.29764401	0.23426614	Hmgcs2	1
GO:0051384	response to glucocorticoid	1/22	123/14464	0.00813008	5.34515891	1.88884084	0.17140377	0.29764401	0.23426614	Hmgcs2	1
GO:0008286	insulin receptor signaling pathway	1/22	124/14464	0.00806452	5.30205279	1.87775473	0.17267489	0.29795952	0.23451447	Marcks	1
GO:0045471	response to ethanol	1/22	124/14464	0.00806452	5.30205279	1.87775473	0.17267489	0.29795952	0.23451447	Hmgcs2	1
GO:0006694	steroid biosynthetic process	1/22	126/14464	0.00793651	5.21789322	1.85593785	0.17521155	0.30044112	0.23646766	Hmgcs2	1
GO:0014812	muscle cell migration	1/22	126/14464	0.00793651	5.21789322	1.85593785	0.17521155	0.30044112	0.23646766	Megf10	1
GO:1902652	secondary alcohol metabolic process	1/22	127/14464	0.00787402	5.17680744	1.8452025	0.17647709	0.30166553	0.23743135	Hmgcs2	1
GO:0014074	response to purine-containing compound	1/22	128/14464	0.0078125	5.13636364	1.83457961	0.17774078	0.30193853	0.23764622	Hmgcs2	1
GO:0060294	cilium movement involved in cell motility	1/22	128/14464	0.0078125	5.13636364	1.83457961	0.17774078	0.30193853	0.23764622	Slc9b2	1
GO:0006665	sphingolipid metabolic process	1/22	129/14464	0.00775194	5.09654686	1.82406705	0.17900262	0.30296681	0.23845554	Hexb	1
GO:0006906	vesicle fusion	1/22	130/14464	0.00769231	5.05734266	1.81366272	0.18026261	0.30296681	0.23845554	Rab8a	1
GO:0007030	Golgi organization	1/22	131/14464	0.00763359	5.01873699	1.80336462	0.18152075	0.30296681	0.23845554	Rab8a	1
GO:0009566	fertilization	1/22	131/14464	0.00763359	5.01873699	1.80336462	0.18152075	0.30296681	0.23845554	Hexb	1
GO:0001101	response to acid chemical	1/22	132/14464	0.00757576	4.98071625	1.79317077	0.18277705	0.30296681	0.23845554	Hmgcs2	1
GO:0001539	cilium or flagellum-dependent cell motility	1/22	132/14464	0.00757576	4.98071625	1.79317077	0.18277705	0.30296681	0.23845554	Slc9b2	1
GO:0060285	cilium-dependent cell motility	1/22	132/14464	0.00757576	4.98071625	1.79317077	0.18277705	0.30296681	0.23845554	Slc9b2	1
GO:0090174	organelle membrane fusion	1/22	132/14464	0.00757576	4.98071625	1.79317077	0.18277705	0.30296681	0.23845554	Rab8a	1
GO:0007292	female gamete generation	1/22	133/14464	0.0075188	4.94326726	1.78307926	0.18403151	0.30344437	0.23883142	Hexb	1

GO:0045739 positive regulation of DNA repair	1/22	134/14464	0.00746269	4.9063772	1.77308822	0.18528413	0.30344437	0.23883142	Npas2	1
GO:0046661 male sex differentiation	1/22	134/14464	0.00746269	4.9063772	1.77308822	0.18528413	0.30344437	0.23883142	Rab13	1
GO:0060996 dendritic spine development	1/22	134/14464	0.00746269	4.9063772	1.77308822	0.18528413	0.30344437	0.23883142	Marcks	1
GO:0050657 nucleic acid transport	1/22	136/14464	0.00735294	4.8342246	1.75340031	0.18778387	0.30389874	0.23918904	Rftn2	1
GO:0050658 RNA transport	1/22	136/14464	0.00735294	4.8342246	1.75340031	0.18778387	0.30389874	0.23918904	Rftn2	1
GO:0050773 regulation of dendrite development	1/22	136/14464	0.00735294	4.8342246	1.75340031	0.18778387	0.30389874	0.23918904	Marcks	1
GO:1904064 positive regulation of cation transmembrane transport	1/22	136/14464	0.00735294	4.8342246	1.75340031	0.18778387	0.30389874	0.23918904	Fxyd3	1
GO:1902414 protein localization to cell junction	1/22	137/14464	0.00729927	4.79893829	1.74369995	0.18903099	0.3050146	0.2400673	Rab8a	1
GO:0031960 response to corticosteroid	1/22	138/14464	0.00724638	4.76416337	1.73409305	0.19027628	0.30522324	0.24023151	Hmgcs2	1
GO:0048144 fibroblast proliferation	1/22	138/14464	0.00724638	4.76416337	1.73409305	0.19027628	0.30522324	0.24023151	Fam114a1	1
GO:0007584 response to nutrient	1/22	139/14464	0.00719424	4.72988882	1.72457797	0.19151975	0.30542654	0.24039152	Hmgcs2	1
GO:0051236 establishment of RNA localization	1/22	139/14464	0.00719424	4.72988882	1.72457797	0.19151975	0.30542654	0.24039152	Rftn2	1
GO:0002706 regulation of lymphocyte mediated immunity	1/22	140/14464	0.00714286	4.6961039	1.71515311	0.19276139	0.30562459	0.2405474	Mill2	1
GO:0008360 regulation of cell shape	1/22	140/14464	0.00714286	4.6961039	1.71515311	0.19276139	0.30562459	0.2405474	Hexb	1
GO:0007605 sensory perception of sound	1/22	141/14464	0.0070922	4.66279819	1.70581691	0.19400122	0.30581748	0.24069922	Hexb	1
GO:0055088 lipid homeostasis	1/22	141/14464	0.0070922	4.66279819	1.70581691	0.19400122	0.30581748	0.24069922	Angptl4	1
GO:0051147 regulation of muscle cell differentiation	1/22	144/14464	0.00694444	4.56565657	1.67832518	0.1977098	0.31076798	0.2445956	Megf10	1
GO:0001889 liver development	1/22	150/14464	0.00666667	4.3830303	1.6255408	0.20507814	0.3214262	0.25298435	Hmgcs2	1
GO:0007224 smoothened signaling pathway	1/22	151/14464	0.00662252	4.35400361	1.61701306	0.2062999	0.32241728	0.25376439	Arl3	1
GO:0034767 positive regulation of monoatomic ion transmembrane transport	1/22	153/14464	0.00653595	4.29708853	1.60017764	0.20873805	0.32345528	0.25458137	Fxyd3	1
GO:0044706 multi-multicellular organism process	1/22	153/14464	0.00653595	4.29708853	1.60017764	0.20873805	0.32345528	0.25458137	Hexb	1
GO:0061008 hepaticobiliary system development	1/22	153/14464	0.00653595	4.29708853	1.60017764	0.20873805	0.32345528	0.25458137	Hmgcs2	1
GO:0071333 cellular response to glucose stimulus	1/22	155/14464	0.00645161	4.24164223	1.58362793	0.21116905	0.32629793	0.25681873	Slc9b2	1
GO:0001906 cell killing	1/22	156/14464	0.00641026	4.21445221	1.57545742	0.21238187	0.32632832	0.25684265	Mill2	1
GO:0002822 regulation of adaptive immune response based on somatic recombination of immune receptors built from	1/22	156/14464	0.00641026	4.21445221	1.57545742	0.21238187	0.32632832	0.25684265	Mill2	1
GO:0071331 cellular response to hexose stimulus	1/22	158/14464	0.00632911	4.16110472	1.55931975	0.21480218	0.32730551	0.25761176	Slc9b2	1
GO:0003341 cilium movement	1/22	159/14464	0.00628931	4.13493425	1.5513505	0.21600967	0.32730551	0.25761176	Slc9b2	1
GO:0061025 membrane fusion	1/22	159/14464	0.00628931	4.13493425	1.5513505	0.21600967	0.32730551	0.25761176	Rab8a	1
GO:0071326 cellular response to monosaccharide stimulus	1/22	159/14464	0.00628931	4.13493425	1.5513505	0.21600967	0.32730551	0.25761176	Slc9b2	1
GO:0099072 regulation of postsynaptic membrane neurotransmitter receptor levels	1/22	159/14464	0.00628931	4.13493425	1.5513505	0.21600967	0.32730551	0.25761176	Rab8a	1
GO:0009266 response to temperature stimulus	1/22	161/14464	0.00621118	4.08356861	1.53560617	0.21841933	0.33004247	0.25976594	Hmgcs2	1
GO:0006403 RNA localization	1/22	163/14464	0.00613497	4.03346347	1.52011425	0.22082193	0.33183954	0.26118036	Rftn2	1
GO:0051017 actin filament bundle assembly	1/22	163/14464	0.00613497	4.03346347	1.52011425	0.22082193	0.33183954	0.26118036	Marcks	1
GO:0030534 adult behavior	1/22	164/14464	0.00609756	4.00886918	1.51246061	0.22202058	0.33272673	0.26187863	Pbx3	1
GO:0061572 actin filament bundle organization	1/22	166/14464	0.0060241	3.96056955	1.49733346	0.2244126	0.3353926	0.26397686	Marcks	1
GO:0000910 cytokinesis	1/22	168/14464	0.00595238	3.91341991	1.48244066	0.22679761	0.33691981	0.26517887	Arl3	1
GO:0016052 carbohydrate catabolic process	1/22	168/14464	0.00595238	3.91341991	1.48244066	0.22679761	0.33691981	0.26517887	Hexb	1
GO:0002819 regulation of adaptive immune response	1/22	170/14464	0.00588235	3.86737968	1.46777551	0.22917561	0.33691981	0.26517887	Mill2	1
GO:0001678 intracellular glucose homeostasis	1/22	171/14464	0.00584795	3.84476342	1.46052628	0.23036199	0.33691981	0.26517887	Slc9b2	1
GO:0042552 myelination	1/22	171/14464	0.00584795	3.84476342	1.46052628	0.23036199	0.33691981	0.26517887	Hexb	1
GO:0071322 cellular response to carbohydrate stimulus	1/22	171/14464	0.00584795	3.84476342	1.46052628	0.23036199	0.33691981	0.26517887	Slc9b2	1
GO:1902107 positive regulation of leukocyte differentiation	1/22	171/14464	0.00584795	3.84476342	1.46052628	0.23036199	0.33691981	0.26517887	Slc9b2	1
GO:1903708 positive regulation of hemopoiesis	1/22	171/14464	0.00584795	3.84476342	1.46052628	0.23036199	0.33691981	0.26517887	Slc9b2	1
GO:0006643 membrane lipid metabolic process	1/22	172/14464	0.00581395	3.82241015	1.45333157	0.23154663	0.33759669	0.26571162	Hexb	1
GO:0071222 cellular response to lipopolysaccharide	1/22	173/14464	0.00578035	3.80031529	1.44619061	0.23272953	0.33759669	0.26571162	Hmgcs2	1
GO:0007272 ensheathment of neurons	1/22	174/14464	0.00574713	3.7784744	1.43910265	0.23391068	0.33759669	0.26571162	Hexb	1
GO:0008366 axon ensheathment	1/22	174/14464	0.00574713	3.7784744	1.43910265	0.23391068	0.33759669	0.26571162	Hexb	1
GO:0048771 tissue remodeling	1/22	174/14464	0.00574713	3.7784744	1.43910265	0.23391068	0.33759669	0.26571162	Fam114a1	1
GO:0045216 cell-cell junction organization	1/22	176/14464	0.00568182	3.73553719	1.4250828	0.2362678	0.33832064	0.26628143	Rab13	1
GO:0048284 organelle fusion	1/22	176/14464	0.00568182	3.73553719	1.4250828	0.2362678	0.33832064	0.26628143	Rab8a	1
GO:0050954 sensory perception of mechanical stimulus	1/22	176/14464	0.00568182	3.73553719	1.4250828	0.2362678	0.33832064	0.26628143	Hexb	1
GO:0010970 transport along microtubule	1/22	177/14464	0.00564972	3.71443246	1.41814949	0.23744376	0.3391168	0.26690806	Arl3	1
GO:0050796 regulation of insulin secretion	1/22	179/14464	0.00558659	3.67293042	1.40443263	0.23979049	0.34068935	0.26814576	Slc9b2	1
GO:0051346 negative regulation of hydrolase activity	1/22	179/14464	0.00558659	3.67293042	1.40443263	0.23979049	0.34068935	0.26814576	Angptl4	1
GO:0071219 cellular response to molecule of bacterial origin	1/22	180/14464	0.00555556	3.65252525	1.39764773	0.24096128	0.34146585	0.26875692	Hmgcs2	1
GO:0009267 cellular response to starvation	1/22	183/14464	0.00546448	3.59264779	1.37757934	0.2444633	0.34464285	0.27125743	Angptl4	1
GO:0071383 cellular response to steroid hormone stimulus	1/22	183/14464	0.00546448	3.59264779	1.37757934	0.2444633	0.34464285	0.27125743	Hmgcs2	1
GO:0007519 skeletal muscle tissue development	1/22	184/14464	0.00543478	3.57312253	1.37098322	0.24562721	0.34539353	0.27184827	Megf10	1
GO:0044242 cellular lipid catabolic process	1/22	185/14464	0.00540541	3.55380835	1.36443275	0.24678941	0.34599131	0.27231877	Hexb	1
GO:0048813 dendrite morphogenesis	1/22	186/14464	0.00537634	3.53470186	1.35792733	0.2479499	0.34599131	0.27231877	Marcks	1
GO:0099175 regulation of postsynapse organization	1/22	186/14464	0.00537634	3.53470186	1.35792733	0.2479499	0.34599131	0.27231877	Marcks	1
GO:1901617 organic hydroxy compound biosynthetic process	1/22	187/14464	0.00534759	3.51579971	1.35146639	0.24910868	0.34672379	0.27289528	Hmgcs2	1
GO:0061351 neural precursor cell proliferation	1/22	189/14464	0.00529101	3.47859548	1.33867564	0.25142114	0.34905422	0.27472948	Fabp7	1
GO:0015931 nucleobase-containing compound transport	1/22	192/14464	0.00520833	3.42424242	1.31980906	0.25487708	0.35206506	0.27709922	Rftn2	1
GO:1901654 response to ketone	1/22	192/14464	0.00520833	3.42424242	1.31980906	0.25487708	0.35206506	0.27709922	Hmgcs2	1
GO:0006909 phagocytosis	1/22	194/14464	0.00515464	3.38894096	1.30743816	0.25717257	0.3525649	0.27749263	Megf10	1
GO:0030324 lung development	1/22	194/14464	0.00515464	3.38894096	1.30743816	0.25717257	0.3525649	0.27749263	Hmgcs2	1
GO:0045637 regulation of myeloid cell differentiation	1/22	194/14464	0.00515464	3.38894096	1.30743816	0.25717257	0.3525649	0.27749263	Slc9b2	1
GO:0002703 regulation of leukocyte mediated immunity	1/22	197/14464	0.00507614	3.33733272	1.28918185	0.26060316	0.3543901	0.27892918	Mill2	1
GO:0030323 respiratory tube development	1/22	197/14464	0.00507614	3.33733272	1.28918185	0.26060316	0.3543901	0.27892918	Hmgcs2	1
GO:0060538 skeletal muscle organ development	1/22	197/14464	0.00507614	3.33733272	1.28918185	0.26060316	0.3543901	0.27892918	Megf10	1
GO:0002064 epithelial cell development	1/22	198/14464	0.00505051	3.3204775	1.28317447	0.26174332	0.3543901	0.27892918	Rab13	1
GO:0030705 cytoskeleton-dependent intracellular transport	1/22	198/14464	0.00505051	3.3204775	1.28317447	0.26174332	0.3543901	0.27892918	Arl3	1
GO:0008406 gonad development	1/22	200/14464	0.005	3.28727273	1.27127395	0.26401862	0.35631318	0.28044278	Rab13	1
GO:0009636 response to toxic substance	1/22	201/14464	0.00497512	3.27091814	1.26537989	0.26515376	0.35631318	0.28044278	Slc47a1	1
GO:0009749 response to glucose	1/22	201/14464	0.00497512	3.27091814	1.26537989	0.26515376	0.35631318	0.28044278	Slc9b2	1
GO:0045137 development of primary sexual characteristics	1/22	203/14464	0.00492611	3.23869234	1.25370196	0.26741902	0.35631318	0.28044278	Rab13	1
GO:0007623 circadian rhythm	1/22	204/14464	0.00490196	3.2228164	1.24791722	0.26854915	0.35631318	0.28044278	Npas2	1
GO:0016054 organic acid catabolic process	1/22	204/14464	0.00490196	3.2228164	1.24791722	0.26854915	0.35631318	0.28044278	Hexb	1
GO:0046395 carboxylic acid catabolic process	1/22	204/14464	0.00490196	3.2228164	1.24791722	0.26854915	0.35631318	0.28044278	Hexb	1
GO:0009746 response to hexose	1/22	205/14464	0.00487805	3.20709534	1.24216805	0.26967762	0.35631318	0.28044278	Slc9b2	1
GO:0051651 maintenance of location in cell	1/22	205/14464	0.00487805	3.20709534	1.24216805	0.26967762	0.35631318	0.28044278	Hexb	1
GO:0071216 cellular response to biotic stimulus	1/22	205/14464	0.00487805	3.20709534	1.24216805	0.26967762	0.35631318	0.28044278	Hmgcs2	1
GO:0043270 positive regulation of monoatomic ion transport	1/22	206/14464	0.00485437	3.19152692	1.23645404	0.27080442	0.3569398	0.28093597	Fxyd3	1
GO:0060348 bone development	1/22	207/14464	0.00483092	3.17610892	1.23077478	0.27192956	0.35756123	0.28142508	Slc9b2	1
GO:0006282 regulation of DNA repair	1/22	208/14464	0.00480769	3.16083916	1.22512987	0.27305305	0.3581775	0.28191013	Npas2	1
GO:0043542 endothelial cell migration	1/22	210/14464	0.0047619	3.13073593	1.21394151	0.27529506	0.35939474	0.28286818	Rab13	1
GO:0090276 regulation of peptide hormone secretion	1/22	210/14464	0.0047619	3.13073593	1.21394151	0.27529506	0.35939474	0.28286818	Slc9b2	1
GO:0002699 positive regulation of immune effector process	1/22	212/14464	0.00471698	3.10120069	1.20288586	0.27753047	0.35973736	0.28313785	Mill2	1
GO:0006813 potassium ion transport	1/22	212/14464	0.00471698	3.10120069	1.20288586	0.27753047	0.35973736	0.28313785	Fxyd3	1
GO:0099111 microtubule-based transport	1/22	212/14464	0.00471698	3.10120069	1.20288586	0.27753047	0.35973736	0.28313785	Arl3	1
GO:0030073 insulin secretion	1/22	213/14464	0.00469484	3.08664106	1.19740686	0.27864571	0.36032908	0.28360357	Slc9b2	1
GO:0034764 positive regulation of transmembrane transport	1/22	214/14464	0.0046729	3.0722175	1.19195993	0.2797593	0.36064923	0.28385555	Fxyd3	1
GO:0002791 regulation of peptide secretion	1/22	215/14464	0.00465116	3.05792812	1.18654469	0.28087125	0.36064923	0.28385555	Slc9b2	1
GO:0090087 regulation of peptide transport	1/22	215/14464	0.00465116	3.05792812	1.18654469	0.28087125	0.36064923	0.28385555	Slc9b2	1
GO:0048863 stem cell differentiation	1/22	218/14464	0.00458716	3.01584654	1.17048569	0.28419728	0.36321474	0.28587478	Phf5a	1
GO:0055001 muscle cell development	1/22	218/14464	0.00458716	3.01584654	1.17048569	0.28419728	0.36321474	0.28587478	Megf10	1
GO:0006836 neurotransmitter transport	1/22	219/14464	0.00456621	3.00207555	1.16519377	0.28530268	0.36349034	0.28609169	Slc6a13	1
GO:0010976 positive regulation of neuron projection development	1/22	220/14464	0.00454545	2.98842975	1.15993185	0.28640646	0.36349034	0.28609169	Marcks	1
GO:0060541 respiratory system development	1/22	220/14464	0.00454545	2.98842975	1.15993185	0.28640646	0.36349034	0.28609169	Hmgcs2	1
GO:0007033 vacuole organization	1/22	223/14464	0.0044843	2.94822666	1.14432275	0.28970805	0.3668294	0.28871977	Hexb	1
GO:0008202 steroid metabolic process	1/22	225/14464	0.00444444	2.9220202	1.13406076	0.291901	0.36875253	0.2902334	Hmgcs2	1
GO:1901136 carbohydrate derivative catabolic process	1/22	229/14464	0.00436681	2.87098055	1.11387163	0.29626753	0.37340631	0.29389624	Hexb	1
GO:0001667 ameboidal-type cell migration	1/22	231/14464	0.004329	2.84612357	1.10393981	0.29844114	0.37528116	0.29537187	Fam114a1	1
GO:0002573 myeloid leukocyte differentiation	1/22	232/14464	0.00431034	2.8338558	1.09901361	0.29952554	0.3757809	0.2957652	Slc9b2	1
GO:0002449 lymphocyte mediated immunity	1/22	236/14464	0.00423729	2.78582435	1.07956719	0.30384717	0.38033044	0.299346	Mill2	1
GO:0007548 sex differentiation	1/22	237/14464	0.00421941	2.77406981	1.07476886	0.3049236	0.38080641	0.29972062	Rab13	1
GO:0048167 regulation of synaptic plasticity	1/22	239/14464	0.0041841	2.75085584	1.06524637	0.30707168	0.38261551	0.30114451	Rab8a	1
GO:0031669 cellular response to nutrient levels	1/22	241/14464	0.00414938	2.72802716	1.05582112	0.30921343	0.38440851	0.30255572	Angptl4	1
GO:0060326 cell chemotaxis	1/22	245/14464	0.00408163	2.68348794	1.03725464	0.31347798	0.38882642	0.30603292	Rab13	1
GO:0097305 response to alcohol	1/22	247/14464	0.00404858	2.66175929	1.02810974	0.31560082	0.39057386	0.30740827	Hmgcs2	1
GO:0033002 muscle cell proliferation	1/22	249/14464	0.00401606	2.6403797	1.01905469	0.31771739	0.39230567	0.30877133	Megf10	1
GO:0022409 positive regulation of cell-cell adhesion	1/22	252/14464	0.00396825	2.60894661	1.00563682	0.32088054	0.39531904	0.31114305	Megf10	1
GO:0006898 receptor-mediated endocytosis	1/22	254/14464	0.00393701	2.58840372	0.99679897	0.32298152	0.39691236	0.3123971	Slc9b2	1
GO:0030072 peptide hormone secretion	1/22	256/14464	0.00390625	2.56818182	0.98804514	0.3250763	0.39691236	0.3123971	Slc9b2	1
GO:0034976 response to endoplasmic reticulum stress	1/22	256/14464	0.00390625	2.56818182	0.98804514	0.3250763	0.39691236	0.3123971	Marcks	1
GO:0090150 establishment of protein localization to membrane	1/22	256/14464	0.00390625	2.56818182	0.98804514	0.3250763	0.39691236	0.3123971	Rab8a	1
GO:0016042 lipid catabolic process	1/22	259/14464	0.003861	2.53843454	0.97506855	0.32820687	0.39984222	0.31470311	Hexb	1
GO:0046883 regulation of hormone secretion	1/22	261/14464	0.00383142	2.51898293	0.96651805	0.33028621	0.40132582	0.3158708	Slc9b2	1
GO:0002460 adaptive immune response based on somatic recombination of immune receptors built from immunoglobu	1/22	263/14464	0.00380228	2.49982717	0.95804628	0.33235941	0.40132582	0.3158708	Mill2	1
GO:0002790 peptide secretion	1/22	263/14464	0.00380228	2.49982717	0.95804628	0.33235941	0.40132582	0.3158708	Slc9b2	1
GO:0050708 regulation of protein secretion	1/22	263/14464	0.00380228	2.49982717	0.95804628	0.33235941	0.40132582	0.3158708	Slc9b2	1
GO:0042593 glucose homeostasis	1/22	265/14464	0.00377358	2.48096055	0.94965183	0.33442648	0.40293234	0.31713524	Slc9b2	1
GO:0033500 carbohydrate homeostasis	1/22	266/14464	0.0037594	2.47163363	0.94548316	0.33545772	0.40328654	0.31741402	Slc9b2	1
GO:0048608 reproductive structure development	1/22	268/14464	0.00373134	2.4531886	0.93720209	0.33751563	0.40487073	0.31866088	Rab13	1
GO:0015833 peptide transport	1/22	272/14464	0.00367647	2.4171123	0.92086037	0.34161322	0.40567253	0.31929195	Slc9b2	1
GO:0061448 connective tissue development	1/22	272/14464	0.00367647	2.4171123	0.92086037	0.34161322	0.40567253	0.31929195	Hmgcs2	1
GO:0061458 reproductive system development	1/22	272/14464	0.00367647	2.4171123	0.92086037	0.34161322	0.40567253	0.31929195	Rab13	1
GO:0000375 RNA splicing, via transesterification reactions	1/22	273/14464	0.003663	2.40825841	0.91681992	0.34263383	0.40567253	0.31929195	Phf5a	1
GO:0000377 RNA splicing, via transesterification reactions with bulged adenosine as nucleophile	1/22	273/14464	0.003663	2.40825841	0.91681992	0.34263383	0.40567253	0.31929195	Phf5a	1
GO:0000398 mRNA splicing, via spliceosome	1/22	273/14464	0.003663	2.40825841	0.91681992	0.34263383	0.40567253	0.31929195	Phf5a	1
GO:0006066 alcohol metabolic process	1/22	275/14464	0.00363636	2.3907438	0.9087919	0.34467053	0.40720255	0.32049618	Hmgcs2	1
GO:0007281 germ cell development	1/22	276/14464	0.00362319	2.38208169	0.90480403	0.34568661	0.4075228	0.32074824	Hexb	1
GO:0006874 intracellular calcium ion homeostasis	1/22	278/14464	0.00359712	2.36494441	0.89687981	0.34771428	0.40903163	0.3219358	Hexb	1
GO:0032496 response to lipopolysaccharide	1/22	283/14464	0.00353357	2.32316094	0.87736302	0.35275723	0.4140734	0.32590401	Hmgcs2	1
GO:1902105 regulation of leukocyte differentiation	1/22	286/14464	0.0034965	2.29879212	0.86584876	0.35576511	0.41670988	0.3279791	Slc9b2	1
GO:0051054 positive regulation of DNA metabolic process	1/22	287/14464	0.00348432	2.29078239	0.86204249	0.35676477	0.41698788	0.32819791	Npas2	1
GO:0001666 response to hypoxia	1/22	289/14464	0.00346021	2.27492922	0.8544769	0.35875965	0.41807096	0.32905037	Angptl4	1
GO:0048545 response to steroid hormone	1/22	290/14464	0.00344828	2.26708464	0.85071732	0.35975487	0.41807096	0.32905037	Hmgcs2	1
GO:0048511 rhythmic process	1/22	291/14464	0.00343643	2.25929397	0.84697304	0.36074862	0.41807096	0.32905037	Npas2	1
GO:0050769 positive regulation of neurogenesis	1/22	291/14464	0.00343643	2.25929397	0.84697304	0.36074862	0.41807096	0.32905037	Marcks	1
GO:1904062 regulation of monoatomic cation transmembrane transport	1/22	294/14464	0.00340136	2.23623995	0.83583081	0.36372104	0.41910877	0.32986719	Fxyd3	1
GO:0010506 regulation of autophagy	1/22	295/14464	0.00338983	2.22865948	0.83214653	0.36470891	0.41910877	0.32986719	Rab8a	1
GO:0022412 cellular process involved in reproduction in multicellular organism	1/22	295/14464	0.00338983	2.22865948	0.83214653	0.36470891	0.41910877	0.32986719	Hexb	1
GO:0055074 calcium ion homeostasis	1/22	295/14464	0.00338983	2.22865948	0.83214653	0.36470891	0.41910877	0.32986719	Hexb	1
GO:0044282 small molecule catabolic process	1/22	296/14464	0.00337838	2.22113022	0.82847694	0.36569532	0.4193613	0.33006595	Hexb	1
GO:0002237 response to molecule of bacterial origin	1/22	297/14464	0.003367	2.21365167	0.82482193	0.36668026	0.41961109	0.33026256	Hmgcs2	1
GO:0019216 regulation of lipid metabolic process	1/22	298/14464	0.0033557	2.20622331	0.82118138	0.36766375	0.41985818	0.33045703	Angptl4	1
GO:0099003 vesicle-mediated transport in synapse	1/22	303/14464	0.00330033	2.16981698	0.80319156	0.37255934	0.42367975	0.33346487	Rab8a	1
GO:0099173 postsynapse organization	1/22	303/14464	0.00330033	2.16981698	0.80319156	0.37255934	0.42367975	0.33346487	Marcks	1
GO:0036293 response to decreased oxygen levels	1/22	304/14464	0.00328947	2.16267943	0.79963541	0.37353411	0.42390697	0.3336437	Angptl4	1
GO:0051235 maintenance of location	1/22	307/14464	0.00325733	2.14154575	0.78904861	0.37644975	0.42633129	0.33555181	Hexb	1
GO:0032102 negative regulation of response to external stimulus	1/22	308/14464	0.00324675	2.13459268	0.78554654	0.37741875	0.42654557	0.33572046	Fabp7	1
GO:0016358 dendrite development	1/22	309/14464	0.00323625	2.12768461	0.78205774	0.37838631	0.42675734	0.33588714	Marcks	1
GO:0010038 response to metal ion	1/22	311/14464	0.00321543	2.11400175	0.77511953	0.38031713	0.4280524	0.33690644	Hmgcs2	1
GO:0042886 amide transport	1/22	314/14464	0.00318471	2.09380428	0.76480917	0.38320262	0.42953245	0.33807134	Slc9b2	1
GO:0046879 hormone secretion	1/22	314/14464	0.00318471	2.09380428	0.76480917	0.38320262	0.42953245	0.33807134	Slc9b2	1
GO:0002697 regulation of immune effector process	1/22	320/14464	0.003125	2.05454545	0.744528	0.38893518	0.43469564	0.34213512	Mill2	1
GO:0009914 hormone transport	1/22	321/14464	0.00311526	2.048145	0.74119073	0.38988565	0.43469564	0.34213512	Slc9b2	1
GO:0007517 muscle organ development	1/22	323/14464	0.00309598	2.03546299	0.73455217	0.39178236	0.43469564	0.34213512	Megf10	1
GO:0009100 glycoprotein metabolic process	1/22	323/14464	0.00309598	2.03546299	0.73455217	0.39178236	0.43469564	0.34213512	Hexb	1
GO:0034765 regulation of monoatomic ion transmembrane transport	1/22	323/14464	0.00309598	2.03546299	0.73455217	0.39178236	0.43469564	0.34213512	Fxyd3	1
GO:0002443 leukocyte mediated immunity	1/22	327/14464	0.0030581	2.01056436	0.72141653	0.39555889	0.43711256	0.3440374	Mill2	1
GO:0070482 response to oxygen levels	1/22	327/14464	0.0030581	2.01056436	0.72141653	0.39555889	0.43711256	0.3440374	Angptl4	1
GO:0031589 cell-substrate adhesion	1/22	332/14464	0.00301205	1.98028478	0.70525528	0.4002481	0.44140264	0.34741399	Marcks	1
GO:0002250 adaptive immune response	1/22	337/14464	0.00296736	1.9509037	0.68937158	0.40490256	0.44563723	0.3507469	Mill2	1

GO:0001654 eye development	1/22	351/14464	0.002849	1.87308987	0.64629337	0.41775234	0.45702106	0.35970675	Pbx3	1
GO:0016050 vesicle organization	1/22	351/14464	0.002849	1.87308987	0.64629337	0.41775234	0.45702106	0.35970675	Rab8a	1
GO:0051962 positive regulation of nervous system development	1/22	351/14464	0.002849	1.87308987	0.64629337	0.41775234	0.45702106	0.35970675	Marcks	1
GO:0009306 protein secretion	1/22	352/14464	0.00284091	1.8677686	0.64329154	0.41865998	0.45709982	0.35976874	Slc9b2	1
GO:0035592 establishment of protein localization to extracellular region	1/22	354/14464	0.00282486	1.85721623	0.63731681	0.4204712	0.45725198	0.3598885	Slc9b2	1
GO:0150063 visual system development	1/22	354/14464	0.00282486	1.85721623	0.63731681	0.4204712	0.45725198	0.3598885	Pbx3	1
GO:0006935 chemotaxis	1/22	356/14464	0.00280899	1.84678243	0.63138022	0.42227703	0.45746705	0.36005778	Rab13	1
GO:0042330 taxis	1/22	357/14464	0.00280112	1.84160937	0.62842608	0.42317793	0.45746705	0.36005778	Rab13	1
GO:0071692 protein localization to extracellular region	1/22	357/14464	0.00280112	1.84160937	0.62842608	0.42317793	0.45746705	0.36005778	Slc9b2	1
GO:0048880 sensory system development	1/22	359/14464	0.00278552	1.83134971	0.62254578	0.42497571	0.45850437	0.36087421	Pbx3	1
GO:0007600 sensory perception	1/22	362/14464	0.00276243	1.81617278	0.61379437	0.42766235	0.45959	0.36172868	Hexb	1
GO:1903706 regulation of hemopoiesis	1/22	362/14464	0.00276243	1.81617278	0.61379437	0.42766235	0.45959	0.36172868	Slc9b2	1
GO:0042063 gliogenesis	1/22	366/14464	0.00273224	1.79632389	0.60225221	0.43122591	0.46251093	0.36402765	Hexb	1
GO:0050807 regulation of synapse organization	1/22	370/14464	0.0027027	1.77690418	0.5908511	0.43476829	0.46539776	0.36629978	Marcks	1
GO:0030900 forebrain development	1/22	373/14464	0.00268097	1.76261272	0.58239068	0.43741124	0.4673124	0.36780674	Fabp7	1
GO:0010959 regulation of metal ion transport	1/22	375/14464	0.00266667	1.75321212	0.57679264	0.43916665	0.46827321	0.36856296	Fxyd3	1
GO:0050803 regulation of synapse structure or activity	1/22	376/14464	0.00265957	1.74854932	0.57400614	0.44004239	0.46829414	0.36857943	Marcks	1
GO:0031329 regulation of cellular catabolic process	1/22	388/14464	0.00257732	1.69447048	0.54119888	0.45045	0.47843913	0.37656423	Rab8a	1
GO:0022407 regulation of cell-cell adhesion	1/22	398/14464	0.00251256	1.65189584	0.51470846	0.45898171	0.48561508	0.3822122	Megf10	1
GO:0043086 negative regulation of catalytic activity	1/22	398/14464	0.00251256	1.65189584	0.51470846	0.45898171	0.48561508	0.3822122	Angptl4	1
GO:0048732 gland development	1/22	408/14464	0.00245098	1.6114082	0.48894198	0.4673869	0.49355335	0.38846015	Hmgcs2	1
GO:0031349 positive regulation of defense response	1/22	410/14464	0.00243902	1.60354767	0.48387189	0.46905291	0.49435827	0.38909368	Npas2	1
GO:0014706 striated muscle tissue development	1/22	414/14464	0.00241546	1.58805446	0.4738124	0.47237002	0.49654588	0.39081548	Megf10	1
GO:0008380 RNA splicing	1/22	417/14464	0.00239808	1.57662961	0.46633717	0.47484487	0.49654588	0.39081548	Phf5a	1
GO:0050673 epithelial cell proliferation	1/22	417/14464	0.00239808	1.57662961	0.46633717	0.47484487	0.49654588	0.39081548	Fabp7	1
GO:1901615 organic hydroxy compound metabolic process	1/22	417/14464	0.00239808	1.57662961	0.46633717	0.47484487	0.49654588	0.39081548	Hmgcs2	1
GO:0050767 regulation of neurogenesis	1/22	418/14464	0.00239234	1.57285776	0.46385845	0.47566735	0.49654588	0.39081548	Marcks	1
GO:0007264 small GTPase-mediated signal transduction	1/22	420/14464	0.00238095	1.56536797	0.45892033	0.47730863	0.49702549	0.39119297	Arl3	1
GO:0048568 embryonic organ development	1/22	421/14464	0.0023753	1.56164975	0.45646085	0.47812743	0.49702549	0.39119297	Pbx3	1
GO:0045785 positive regulation of cell adhesion	1/22	422/14464	0.00236967	1.55794916	0.45400771	0.478945	0.49702549	0.39119297	Megf10	1
GO:0042692 muscle cell differentiation	1/22	423/14464	0.00236407	1.55426606	0.45156089	0.47976135	0.49702549	0.39119297	Megf10	1
GO:0031346 positive regulation of cell projection organization	1/22	426/14464	0.00234742	1.54332053	0.4442579	0.48220309	0.49861075	0.39244068	Marcks	1
GO:0030099 myeloid cell differentiation	1/22	428/14464	0.00233645	1.53610875	0.43942015	0.48382483	0.49923679	0.39293341	Slc9b2	1
GO:0007015 actin filament organization	1/22	429/14464	0.002331	1.53252808	0.43701044	0.48463388	0.49923679	0.39293341	Marcks	1
GO:0060537 muscle tissue development	1/22	435/14464	0.00229885	1.51138976	0.4226785	0.48946283	0.50326348	0.39610269	Megf10	1
GO:0043269 regulation of monoatomic ion transport	1/22	440/14464	0.00227273	1.49421488	0.41089709	0.49345394	0.5064152	0.39858331	Fxyd3	1
GO:0051052 regulation of DNA metabolic process	1/22	443/14464	0.00225734	1.48409604	0.40389712	0.49583428	0.50790516	0.39975601	Npas2	1
GO:0007409 axonogenesis	1/22	446/14464	0.00224215	1.47411333	0.39694783	0.49820395	0.50828368	0.40005393	Rab8a	1
GO:0030003 intracellular monoatomic cation homeostasis	1/22	447/14464	0.00223714	1.47081554	0.39464252	0.49899148	0.50828368	0.40005393	Hexb	1
GO:0034762 regulation of transmembrane transport	1/22	447/14464	0.00223714	1.47081554	0.39464252	0.49899148	0.50828368	0.40005393	Fxyd3	1
GO:0006091 generation of precursor metabolites and energy	1/22	449/14464	0.00222717	1.46426402	0.39004841	0.50056299	0.50893672	0.40056792	Hmgcs2	1
GO:0006873 intracellular monoatomic ion homeostasis	1/22	454/14464	0.00220264	1.44813777	0.37865811	0.5044712	0.51085284	0.40207604	Hexb	1
GO:0006397 mRNA processing	1/22	455/14464	0.0021978	1.44495504	0.37639611	0.50524934	0.51085284	0.40207604	Phf5a	1
GO:0010817 regulation of hormone levels	1/22	455/14464	0.0021978	1.44495504	0.37639611	0.50524934	0.51085284	0.40207604	Slc9b2	1
GO:0071407 cellular response to organic cyclic compound	1/22	462/14464	0.0021645	1.42306179	0.3607087	0.51066369	0.51537461	0.40563498	Hmgcs2	1
GO:0007005 mitochondrion organization	1/22	466/14464	0.00214592	1.41084666	0.35185732	0.51373217	0.51751657	0.40732085	Marcks	1
GO:0044283 small molecule biosynthetic process	1/22	470/14464	0.00212766	1.39883946	0.34308598	0.51678228	0.51963218	0.40898597	Hmgcs2	1
GO:0034329 cell junction assembly	1/22	485/14464	0.00206186	1.35557638	0.31087986	0.52805832	0.52999615	0.41714313	Rab13	1
GO:0005975 carbohydrate metabolic process	1/22	491/14464	0.00203666	1.33901129	0.2982888	0.53249804	0.53347331	0.41987989	Hexb	1
GO:0071396 cellular response to lipid	1/22	499/14464	0.00200401	1.31754418	0.28174799	0.53835567	0.53835567	0.42372264	Hmgcs2	1

ID	Description	GeneRatio	BgRatio	RichFactor	FoldEnrichm	zScore	pvalue	p.adjust	qvalue	geneID	Count
GO:0001503	ossification	7/28	359/14464	0.01949861	10.0724234	7.66599905	4.133E-06	0.00418854	0.00322131	Igfbp5/Ccn3/Bmp6/Comp/Col6a1/H3f3b/Kl	7
GO:0042246	tissue regeneration	4/28	67/14464	0.05970149	30.8400853	10.7816749	7.9178E-06	0.00418854	0.00322131	Ccn3/Fzd7/Col6a1/Cpq	4
GO:0007178	cell surface receptor protein serine/threonine kinase signaling pathway	6/28	313/14464	0.01916933	9.9023277	7.01240438	2.4673E-05	0.00870126	0.00669195	Fbn1/Cilp/Ccn3/Bmp6/Comp/Slc2a10	6
GO:0090092	regulation of transmembrane receptor protein serine/threonine kinase signaling pathway	5/28	223/14464	0.02242152	11.582319	7.01371009	6.1289E-05	0.01071373	0.00823969	Fbn1/Cilp/Ccn3/Bmp6/Slc2a10	5
GO:0030282	bone mineralization	4/28	115/14464	0.03478261	17.9677019	8.04536773	6.7003E-05	0.01071373	0.00823969	Bmp6/Comp/Col6a1/Kl	4
GO:0030278	regulation of ossification	4/28	119/14464	0.03361345	17.3637455	7.89388314	7.6552E-05	0.01071373	0.00823969	Ccn3/Bmp6/Comp/Kl	4
GO:0051224	negative regulation of protein transport	4/28	120/14464	0.03333333	17.2190476	7.85716016	7.9087E-05	0.01071373	0.00823969	Fbn1/Ccn3/Gbp4/Ndufaf2	4
GO:0048009	insulin-like growth factor receptor signaling pathway	3/28	45/14464	0.06666667	34.4380952	9.89384241	8.7308E-05	0.01071373	0.00823969	Cilp/Igfbp5/Col6a1	3
GO:0090101	negative regulation of transmembrane receptor protein serine/threonine kinase signaling pathway	4/28	126/14464	0.03174603	16.399093	7.64576416	9.5599E-05	0.01071373	0.00823969	Fbn1/Cilp/Ccn3/Slc2a10	4
GO:0060390	regulation of SMAD protein signal transduction	3/28	49/14464	0.06122449	31.6268222	9.45752342	0.00011277	0.01071373	0.00823969	Cilp/Ccn3/Bmp6	3
GO:1904950	negative regulation of establishment of protein localization	4/28	135/14464	0.02962963	15.3058201	7.35455626	0.00012489	0.01071373	0.00823969	Fbn1/Ccn3/Gbp4/Ndufaf2	4
GO:0046676	negative regulation of insulin secretion	3/28	51/14464	0.05882353	30.3865546	9.25851507	0.00012714	0.01071373	0.00823969	Fbn1/Ccn3/Ndufaf2	3
GO:0042692	muscle cell differentiation	6/28	423/14464	0.0141844	7.32725431	5.81662715	0.00013164	0.01071373	0.00823969	Igfbp5/Ccn3/Comp/Fzd7/Col6a1/H3f3b	6
GO:0048286	lung alveolus development	3/28	57/14464	0.05263158	27.1879699	8.72443387	0.00017725	0.01146817	0.00881992	Fbn1/Igfbp5/Col6a1	3
GO:0045778	positive regulation of ossification	3/28	58/14464	0.05172414	26.7192118	8.64340213	0.00018668	0.01146817	0.00881992	Ccn3/Bmp6/Kl	3
GO:0090278	negative regulation of peptide hormone secretion	3/28	58/14464	0.05172414	26.7192118	8.64340213	0.00018668	0.01146817	0.00881992	Fbn1/Ccn3/Ndufaf2	3
GO:0031214	biomineral tissue development	4/28	150/14464	0.02666667	13.7752381	6.92658069	0.00018744	0.01146817	0.00881992	Bmp6/Comp/Col6a1/Kl	4
GO:0002792	negative regulation of peptide secretion	3/28	60/14464	0.05	25.8285714	8.48731993	0.0002065	0.01146817	0.00881992	Fbn1/Ccn3/Ndufaf2	3
GO:0141091	transforming growth factor beta receptor superfamily signaling pathway	5/28	292/14464	0.01712329	8.84540117	5.96452389	0.00021751	0.01146817	0.00881992	Fbn1/Cilp/Bmp6/Comp/Slc2a10	5
GO:0001501	skeletal system development	6/28	466/14464	0.01287554	6.65113427	5.46110293	0.00022305	0.01146817	0.00881992	Fbn1/Ccn3/Bmp6/Comp/Col6a1/Slc2a10	6
GO:0030199	collagen fibril organization	3/28	62/14464	0.0483871	24.9953917	8.33867519	0.00022763	0.01146817	0.00881992	Loxl1/Comp/Col6a1	3
GO:0060395	SMAD protein signal transduction	3/28	68/14464	0.04411765	22.789916	7.93183853	0.0002993	0.01439367	0.01106985	Cilp/Ccn3/Bmp6	3
GO:0030500	regulation of bone mineralization	3/28	72/14464	0.04166667	21.5238095	7.68862042	0.00035434	0.01575341	0.0121156	Bmp6/Comp/Kl	3
GO:0090136	epithelial cell-cell adhesion	2/28	15/14464	0.13333333	68.8761905	11.583211	0.00037359	0.01575341	0.0121156	Ccn3/Kifc3	2
GO:0014823	response to activity	3/28	74/14464	0.04054054	20.9420849	7.57427025	0.00038414	0.01575341	0.0121156	Bmp6/Col6a1/Kl	3
GO:0050709	negative regulation of protein secretion	3/28	76/14464	0.03947368	20.3909774	7.46433384	0.00041552	0.01575341	0.0121156	Fbn1/Ccn3/Ndufaf2	3
GO:0051216	cartilage development	4/28	186/14464	0.02150538	11.109063	6.11108687	0.00042577	0.01575341	0.0121156	Ccn3/Bmp6/Comp/Col6a1	4
GO:0060392	negative regulation of SMAD protein signal transduction	2/28	16/14464	0.125	64.5714286	11.204768	0.00042645	0.01575341	0.0121156	Cilp/Ccn3	2
GO:0046888	negative regulation of hormone secretion	3/28	77/14464	0.03896104	20.1261596	7.4109313	0.0004318	0.01575341	0.0121156	Fbn1/Ccn3/Ndufaf2	3
GO:0031099	regeneration	4/28	191/14464	0.02094241	10.8182498	6.01558546	0.0004707	0.01660003	0.01276671	Ccn3/Fzd7/Col6a1/Cpq	4
GO:0043567	regulation of insulin-like growth factor receptor signaling pathway	2/28	18/14464	0.11111111	57.3968254	10.5439145	0.00054242	0.01851242	0.01423749	Cilp/Igfbp5	2
GO:0030512	negative regulation of transforming growth factor beta receptor signaling pathway	3/28	86/14464	0.03488372	18.0199336	6.97176305	0.00059712	0.01914395	0.01472318	Fbn1/Cilp/Slc2a10	3
GO:0070167	regulation of biomineral tissue development	3/28	86/14464	0.03488372	18.0199336	6.97176305	0.00059712	0.01914395	0.01472318	Bmp6/Comp/Kl	3
GO:0060348	bone development	4/28	207/14464	0.01932367	9.98205659	5.73233624	0.00063728	0.01983052	0.01525121	Fbn1/Bmp6/Comp/Col6a1	4
GO:1903828	negative regulation of protein localization	4/28	221/14464	0.01809955	9.34970911	5.50873039	0.00081422	0.02433584	0.01871616	Fbn1/Ccn3/Gbp4/Ndufaf2	4
GO:0043588	skin development	4/28	222/14464	0.01801802	9.30759331	5.49352362	0.00082806	0.02433584	0.01871616	Igfbp5/Comp/Col6a1/Slc2a10	4
GO:0035988	chondrocyte proliferation	2/28	23/14464	0.08695652	44.9192547	9.28335626	0.0008916	0.02549487	0.01960753	Ccn3/Comp	2
GO:0090025	regulation of monocyte chemotaxis	2/28	24/14464	0.08333333	43.047619	9.07921301	0.00097149	0.02704828	0.02080223	Cx3cr1/Ccn3	2
GO:0051051	negative regulation of transport	5/28	415/14464	0.01204819	6.22375215	4.75519879	0.00108129	0.0293334	0.02255966	Fbn1/Gstm7/Ccn3/Gbp4/Ndufaf2	5
GO:0002062	chondrocyte differentiation	3/28	107/14464	0.02803738	14.4833111	6.1651176	0.00112706	0.029448	0.0226478	Ccn3/Bmp6/Comp	3
GO:0071560	cellular response to transforming growth factor beta stimulus	4/28	242/14464	0.01652893	8.53837072	5.20822346	0.00114118	0.029448	0.0226478	Fbn1/Cx3cr1/Cilp/Slc2a10	4
GO:0071559	response to transforming growth factor beta	4/28	249/14464	0.01606426	8.2983362	5.11605116	0.00126811	0.03194432	0.02456766	Fbn1/Cx3cr1/Cilp/Slc2a10	4
GO:0046883	regulation of hormone secretion	4/28	261/14464	0.01532567	7.9168035	4.96615672	0.00150829	0.03655387	0.02811277	Fbn1/Ccn3/Bmp6/Ndufaf2	4
GO:0030261	chromosome condensation	2/28	30/14464	0.06666667	34.4380952	8.07408987	0.0015202	0.03655387	0.02811277	H3f3b/H1f0	2
GO:0030509	BMP signaling pathway	3/28	121/14464	0.02479339	12.8075561	5.74404026	0.00160591	0.0374276	0.02878473	Fbn1/Bmp6/Comp	3
GO:0010817	regulation of hormone levels	5/28	455/14464	0.01098901	5.67660911	4.46393615	0.00162729	0.0374276	0.02878473	Fbn1/Ccn3/Bmp6/Cpq/Ndufaf2	5
GO:0044342	type B pancreatic cell proliferation	2/28	32/14464	0.0625	32.2857143	7.80265808	0.00172924	0.03766485	0.0289672	Igfbp5/Ccn3	2
GO:0061448	connective tissue development	4/28	272/14464	0.01470588	7.59663866	4.83693352	0.00175493	0.03766485	0.0289672	Ccn3/Bmp6/Comp/Col6a1	4
GO:0002548	monocyte chemotaxis	2/28	33/14464	0.06060606	31.3073593	7.67611772	0.0018386	0.03766485	0.0289672	Cx3cr1/Ccn3	2
GO:0030198	extracellular matrix organization	4/28	276/14464	0.01449275	7.48654244	4.79172612	0.0018512	0.03766485	0.0289672	Loxl1/Comp/Col6a1/Slc2a10	4
GO:0043062	extracellular structure organization	4/28	276/14464	0.01449275	7.48654244	4.79172612	0.0018512	0.03766485	0.0289672	Loxl1/Comp/Col6a1/Slc2a10	4
GO:0045229	external encapsulating structure organization	4/28	276/14464	0.01449275	7.48654244	4.79172612	0.0018512	0.03766485	0.0289672	Loxl1/Comp/Col6a1/Slc2a10	4
GO:0017015	regulation of transforming growth factor beta receptor signaling pathway	3/28	129/14464	0.02325581	12.013289	5.53347326	0.00192895	0.03808013	0.02928658	Fbn1/Cilp/Slc2a10	3
GO:0071772	response to BMP	3/28	131/14464	0.02290076	11.82988	5.48372308	0.00201558	0.03808013	0.02928658	Fbn1/Bmp6/Comp	3
GO:0071773	cellular response to BMP stimulus	3/28	131/14464	0.02290076	11.82988	5.48372308	0.00201558	0.03808013	0.02928658	Fbn1/Bmp6/Comp	3
GO:1903844	regulation of cellular response to transforming growth factor beta stimulus	3/28	131/14464	0.02290076	11.82988	5.48372308	0.00201558	0.03808013	0.02928658	Fbn1/Cilp/Slc2a10	3
GO:0051154	negative regulation of striated muscle cell differentiation	2/28	37/14464	0.05405405	27.9227799	7.22133545	0.00230808	0.04284119	0.03294821	Ccn3/Fzd7	2
GO:0051146	striated muscle cell differentiation	4/28	311/14464	0.01286174	6.64400551	4.43126928	0.0028559	0.05209561	0.04006557	Igfbp5/Ccn3/Fzd7/Col6a1	4
GO:0046879	hormone secretion	4/28	314/14464	0.01273885	6.58052775	4.40297935	0.00295645	0.05301572	0.04077321	Fbn1/Ccn3/Bmp6/Ndufaf2	4
GO:0030501	positive regulation of bone mineralization	2/28	43/14464	0.04651163	24.0265781	6.65963941	0.00310707	0.05461502	0.04200319	Bmp6/Kl	2
GO:1903531	negative regulation of secretion by cell	3/28	154/14464	0.01948052	10.0630798	4.97967637	0.00318973	0.05461502	0.04200319	Fbn1/Ccn3/Ndufaf2	3
GO:0009914	hormone transport	4/28	321/14464	0.01246106	6.43702715	4.33838429	0.0032005	0.05461502	0.04200319	Fbn1/Ccn3/Bmp6/Ndufaf2	4
GO:0043403	skeletal muscle tissue regeneration	2/28	45/14464	0.04444444	22.9587302	6.49726649	0.00339829	0.05706977	0.04389109	Fzd7/Col6a1	2
GO:0070169	positive regulation of biomineral tissue development	2/28	48/14464	0.04166667	21.5238095	6.27250447	0.00385815	0.06378007	0.04905183	Bmp6/Kl	2
GO:0051048	negative regulation of secretion	3/28	170/14464	0.01764706	9.11596639	4.68783788	0.00421092	0.06854088	0.05271326	Fbn1/Ccn3/Ndufaf2	3
GO:0009306	protein secretion	4/28	352/14464	0.01136364	5.87012987	4.07382494	0.0044485	0.07131082	0.05484356	Fbn1/Ccn3/Comp/Ndufaf2	4
GO:0035592	establishment of protein localization to extracellular region	4/28	354/14464	0.01129944	5.83696529	4.05784886	0.00453884	0.07167307	0.05512216	Fbn1/Ccn3/Comp/Ndufaf2	4
GO:0071692	protein localization to extracellular region	4/28	357/14464	0.01120448	5.78791517	4.03411239	0.00467668	0.07276363	0.05596089	Fbn1/Ccn3/Comp/Ndufaf2	4
GO:0034332	adherens junction organization	2/28	54/14464	0.03703704	19.1322751	5.87897972	0.00485959	0.07350646	0.05653218	Bmp6/Kifc3	2
GO:0050796	regulation of insulin secretion	3/28	179/14464	0.01675978	8.65762171	4.54009639	0.00486338	0.07350646	0.05653218	Fbn1/Ccn3/Ndufaf2	3
GO:0050879	multicellular organismal movement	2/28	56/14464	0.03571429	18.4489796	5.76165129	0.00521729	0.07774494	0.0597919	Comp/Col6a1	2
GO:0001649	osteoblast differentiation	3/28	186/14464	0.01612903	8.33179724	4.43218624	0.00541096	0.07951111	0.06115022	Igfbp5/Bmp6/H3f3b	3
GO:0007179	transforming growth factor beta receptor signaling pathway	3/28	190/14464	0.01578947	8.15639098	4.37303331	0.00573992	0.08318959	0.06397926	Fbn1/Cilp/Slc2a10	3
GO:0030324	lung development	3/28	194/14464	0.01546392	7.98821797	4.31558915	0.00608071	0.08579415	0.06598237	Fbn1/Igfbp5/Col6a1	3
GO:0060350	endochondral bone morphogenesis	2/28	61/14464	0.03278689	16.9367681	5.49317537	0.00616291	0.08579415	0.06598237	Bmp6/Comp	2
GO:1903053	regulation of extracellular matrix organization	2/28	61/14464	0.03278689	16.9367681	5.49317537	0.00616291	0.08579415	0.06598237	Col6a1/Slc2a10	2
GO:0030323	respiratory tube development	3/28	197/14464	0.01522843	7.86656998	4.27357574	0.00634413	0.08627786	0.06635438	Fbn1/Igfbp5/Col6a1	3
GO:0051148	negative regulation of muscle cell differentiation	2/28	62/14464	0.03225806	16.6635945	5.44327962	0.00636075	0.08627786	0.06635438	Ccn3/Fzd7	2
GO:0009612	response to mechanical stimulus	3/28	202/14464	0.01485149	7.6718529	4.20549095	0.00679821	0.09104445	0.07002025	Bmp6/Col6a1/Kcnk2	3
GO:0090276	regulation of peptide hormone secretion	3/28	210/14464	0.01428571	7.37959184	4.10127543	0.00756434	0.09926615	0.07634338	Fbn1/Ccn3/Ndufaf2	3
GO:0043434	response to peptide hormone	4/28	410/14464	0.0097561	5.03972125	3.65450134	0.00759977	0.09926615	0.07634338	Fbn1/Igfbp5/Col6a1/Kl	4
GO:0030073	insulin secretion	3/28	213/14464	0.01408451	7.27565392	4.06359942	0.00786434	0.10042564	0.07723512	Fbn1/Ccn3/Ndufaf2	3
GO:0050673	epithelial cell proliferation	4/28	417/14464	0.00959233	4.95512162	3.60928229	0.00805903	0.10042564	0.07723512	Igfbp5/Ccn3/Bmp6/Fzd7	4
GO:0002791	regulation of peptide secretion	3/28	215/14464	0.01395349	7.20797342	4.03888658	0.00806822	0.10042564	0.07723512	Fbn1/Ccn3/Ndufaf2	3
GO:0090087	regulation of peptide transport	3/28	215/14464	0.01395349	7.20797342	4.03888658	0.00806822	0.10042564	0.07723512	Fbn1/Ccn3/Ndufaf2	3
GO:0045926	negative regulation of growth	3/28	216/14464	0.01388889	7.17460317	4.02664874	0.00817133	0.10052638	0.0773126	Igfbp5/Ccn3/Kcnk2	3
GO:0060541	respiratory system development	3/28	220/14464	0.01363636	7.04415584	3.97846722	0.00859159	0.10448166	0.08035452	Fbn1/Igfbp5/Col6a1	3
GO:0060537	muscle tissue development	4/28	435/14464	0.0091954	4.7500821	3.49749272	0.00932362	0.11209534	0.08621002	Igfbp5/Fzd7/Col6a1/Kcnk2	4
GO:0033627	cell adhesion mediated by integrin	2/28	80/14464	0.025	12.9142857	4.70606519	0.01040383	0.12367704	0.09511725	Fbn1/Ccn3	2
GO:0051223	regulation of protein transport	4/28	452/14464	0.00884956	4.57142857	3.39739383	0.01063199	0.12498494	0.09612313	Fbn1/Ccn3/Gbp4/Ndufaf2	4
GO:0001818	negative regulation of cytokine production	3/28	239/14464	0.0125523	6.48416019	3.76502372	0.01076107	0.12511217	0.09622098	Cx3cr1/Gbp4/Slc2a10	3
GO:0009611	response to wounding	4/28	465/14464	0.00860215	4.44362519	3.32413473	0.01170973	0.13466188	0.10356545	Comp/Fzd7/Col6a1/Kcnk2	4
GO:0033002	muscle cell proliferation	3/28	249/14464	0.01204819	6.22375215	3.6617912	0.01201973	0.13674064	0.10516418	Igfbp5/Ccn3/Kcnk2	3
GO:0051153	regulation of striated muscle cell differentiation	2/28	88/14464	0.02272727	11.7402597	4.45063379	0.01248377	0.13902982	0.10692473	Ccn3/Fzd7	2
GO:0120254	olefinic compound metabolic process	2/28	88/14464	0.02272727	11.7402597	4.45063379	0.01248377	0.13902982	0.10692473	Gstm7/Bmp6	2
GO:0030072	peptide hormone secretion	3/28	256/14464	0.01171875	6.05357143	3.59283017	0.01294949	0.14212087	0.10930199	Fbn1/Ccn3/Ndufaf2	3
GO:0001942	hair follicle development	2/28	90/14464	0.02222222	11.4793651	4.39189731	0.01302999	0.14212087	0.10930199	Igfbp5/Col6a1	2
GO:0090100	positive regulation of transmembrane receptor protein serine/threonine kinase signaling pathway	2/28	91/14464	0.02197802	11.3532182	4.36322012	0.01330697	0.14278557	0.1098132	Bmp6/Slc2a10	2
GO:0006112	energy reserve metabolic process	2/28	93/14464	0.02150538	11.109063	4.30718627	0.01386864	0.14278557	0.1098132	Col6a1/Kl	2
GO:0022404	molting cycle process	2/28	93/14464	0.02150538	11.109063	4.30718627	0.01386864	0.14278557	0.1098132	Igfbp5/Col6a1	2
GO:0022405	hair cycle process	2/28	93/14464	0.02150538	11.109063	4.30718627	0.01386864	0.14278557	0.1098132	Igfbp5/Col6a1	2
GO:0002790	peptide secretion	3/28	263/14464	0.01140684	5.89244976	3.52638363	0.01391968	0.14278557	0.1098132	Fbn1/Ccn3/Ndufaf2	3
GO:0050708	regulation of protein secretion	3/28	263/14464	0.01140684	5.89244976	3.52638363	0.01391968	0.14278557	0.1098132	Fbn1/Ccn3/Ndufaf2	3
GO:0060349	bone morphogenesis	2/28	94/14464	0.0212766	10.9908815	4.27980637	0.0141533	0.14278557	0.1098132	Bmp6/Comp	2
GO:0042593	glucose homeostasis	3/28	265/14464	0.01132075	5.84797844	3.50783784	0.01420434	0.14278557	0.1098132	Fbn1/Igfbp5/Ndufaf2	3
GO:0033500	carbohydrate homeostasis	3/28	266/14464	0.0112782	5.82599356	3.49863585	0.01434792	0.14278557	0.1098132	Fbn1/Igfbp5/Ndufaf2	3
GO:0019226	transmission of nerve impulse	2/28	95/14464	0.02105263	10.875188	4.25283637	0.01444051	0.14278557	0.1098132	Col6a1/Kcnk2	2
GO:0048608	reproductive structure development	3/28	268/14464	0.01119403	5.78251599	3.48037151	0.01463758	0.14297795	0.10996115	Bmp6/Col6a1/H3f3b	3
GO:0001704	formation of primary germ layer	2/28	96/14464	0.02083333	10.7619048	4.22626568	0.01473024	0.14297795	0.10996115	Fzd7/Col6a1	2
GO:0030336	negative regulation of cell migration	3/28	271/14464	0.01107011	5.7185029	3.45331776	0.01507833	0.14341296	0.11029571	Cx3cr1/Igfbp5/Ccn3	3
GO:0015833	peptide transport	3/28	272/14464	0.01102941	5.69747899	3.4443896	0.01522692	0.14341296	0.11029571	Fbn1/Ccn3/Ndufaf2	3
GO:0061458	reproductive system development	3/28	272/14464	0.01102941	5.69747899	3.4443896	0.01522692	0.14341296	0.11029571	Bmp6/Col6a1/H3f3b	3
GO:0018958	phenol-containing compound metabolic process	2/28	98/14464	0.02040816	10.5422741	4.1742818	0.01531726	0.14341296	0.11029571	Cpq/Kl	2
GO:0043491	phosphatidylinositol 3-kinase/protein kinase B signal transduction	3/28	278/14464	0.01079137	5.57451182	3.39173526	0.01613605	0.14664168	0.11277884	Cx3cr1/Igfbp5/Col6a1	3
GO:0090287	regulation of cellular response to growth factor stimulus	3/28	278/14464	0.01079137	5.57451182	3.39173526	0.01613605	0.14664168	0.11277884	Fbn1/Cilp/Slc2a10	3
GO:0002688	regulation of leukocyte chemotaxis	2/28	101/14464	0.01980198	10.2291372	4.09905722	0.01621652	0.14664168	0.11277884	Cx3cr1/Ccn3	2
GO:0007338	single fertilization	2/28	101/14464	0.01980198	10.2291372	4.09905722	0.01621652	0.14664168	0.11277884	Col6a1/H3f3b	2
GO:2000146	negative regulation of cell motility	3/28	282/14464	0.0106383	5.49544073	3.35747557	0.01675893	0.14791678	0.1137595	Cx3cr1/Igfbp5/Ccn3	3
GO:0042303	molting cycle	2/28	103/14464	0.01941748	10.0305132	4.05063839	0.01682839	0.14791678	0.1137595	Igfbp5/Col6a1	2
GO:0042633	hair cycle	2/28	103/14464	0.01941748	10.0305132	4.05063839	0.01682839	0.14791678	0.1137595	Igfbp5/Col6a1	2
GO:0032496	response to lipopolysaccharide	3/28	283/14464	0.01060071	5.47602221	3.3490127	0.01691676	0.14791678	0.1137595	Cx3cr1/Bmp6/Col6a1	3
GO:0060840	artery development	2/28	106/14464	0.01886792	9.74663073	3.98044403	0.01776454	0.15405642	0.11848136	Comp/Slc2a10	2
GO:0032341	aldosterone metabolic process	1/28	10/14464	0.1	51.6571429	7.05718396	0.01919656	0.15882451	0.12214839	Bmp6	1
GO:0048630	skeletal muscle tissue growth	1/28	10/14464	0.1	51.6571429	7.05718396	0.01919656	0.15882451	0.12214839	Col6a1	1
GO:0071281	cellular response to iron ion	1/28	10/14464	0.1	51.6571429	7.05718396	0.01919656	0.15882451	0.12214839	Bmp6	1
GO:1905208	negative regulation of cardiocyte differentiation	1/28	10/14464	0.1	51.6571429	7.05718396	0.01919656	0.15882451	0.12214839	Fzd7	1
GO:0002237	response to molecule of bacterial origin	3/28	297/14464	0.01010101	5.21789322	3.23459291	0.01921506	0.15882451	0.12214839	Cx3cr1/Bmp6/Col6a1	3
GO:0040013	negative regulation of locomotion	3/28	297/14464	0.01010101	5.21789322	3.23459291	0.01921506	0.15882451	0.12214839	Cx3cr1/Igfbp5/Ccn3	3
GO:0014909	smooth muscle cell migration	2/28	111/14464	0.01801802	9.30759331	3.86945809	0.01937307	0.15888919	0.12219813	Igfbp5/Ccn3	2
GO:0071675	regulation of mononuclear cell migration	2/28	114/14464	0.01754386	9.06265664	3.80618078	0.02036672	0.16411877	0.12622008	Cx3cr1/Ccn3	2
GO:0051053	negative regulation of DNA metabolic process	2/28	115/14464	0.0173913	8.98385093	3.78560498	0.02070263	0.16411877	0.12622008	H1f0/Kcnk2	2
GO:0007611	learning or memory	3/28	307/14464	0.00977199	5.04792927	3.15719393	0.0209585	0.16411877	0.12622008	Cx3cr1/Kcnk2/Bhlhb9	3
GO:0008212	mineralocorticoid metabolic process	1/28	11/14464	0.09090909	46.961039	6.71571026	0.02109655	0.16411877	0.12622008	Bmp6	1
GO:0034309	primary alcohol biosynthetic process	1/28	11/14464	0.09090909	46.961039	6.71571026	0.02109655	0.16411877	0.12622008	Bmp6	1
GO:0060231	mesenchymal to epithelial transition	1/28	11/14464	0.09090909	46.961039	6.71571026	0.02109655	0.16411877	0.12622008	Fzd7	1
GO:0071313	cellular response to caffeine	1/28	11/14464	0.09090909	46.961039	6.71571026	0.02109655	0.16411877	0.12622008	Gstm7	1
GO:0098773	skin epidermis development	2/28	118/14464	0.01694915	8.75544794	3.72535144	0.02172433	0.16762263	0.12891483	Igfbp5/Col6a1	2
GO:0042886	amide transport	3/28	314/14464	0.00955414	4.93539581	3.10498707	0.02222957	0.16762263	0.12891483	Fbn1/Ccn3/Ndufaf2	3
GO:0031507	heterochromatin formation	2/28	120/14464	0.01666667	8.60952381	3.68635993	0.022417	0.16762263	0.12891483	H3f3b/H1f0	2
GO:0002689	negative regulation of leukocyte chemotaxis	1/28	12/14464	0.08333333	43.047619	6.41730717	0.022993	0.16762263	0.12891483	Ccn3	1
GO:0010561	negative regulation of glycoprotein biosynthetic process	1/28	12/14464	0.08333333	43.047619	6.41730717	0.022993	0.16762263	0.12891483	Slc2a10	1
GO:0046886	positive regulation of hormone biosynthetic process	1/28	12/14464	0.08333333	43.047619	6.41730717	0.022993	0.16762263	0.12891483	Bmp6	1
GO:1990314	cellular response to insulin-like growth factor stimulus	1/28	12/14464	0.08333333	43.047619	6.41730717	0.022993	0.16762263	0.12891483	Fbn1	1
GO:2000833	positive regulation of steroid hormone secretion	1/28	12/14464	0.08333333	43.047619	6.41730717	0.022993	0.16762263	0.12891483	Bmp6	1
GO:0045667	regulation of osteoblast differentiation	2/28	124/14464	0.01612903	8.33179724	3.61103323	0.02382968	0.16762263	0.12891483	Igfbp5/Bmp6	2
GO:0014812	muscle cell migration	2/28	126/14464	0.01587302	8.19954649	3.57462836	0.02454954	0.16762263	0.12891483	Igfbp5/Ccn3	2
GO:0050728	negative regulation of inflammatory response	2/28	126/14464	0.01587302	8.19954649	3.57462836	0.02454954	0.16762263	0.12891483	Cx3cr1/Ccn3	2
GO:0001765	membrane raft assembly	1/28	13/14464	0.07692308	39.7362637	6.15354282	0.0248859	0.16762263	0.12891483	Col6a1	1
GO:0006103	2-oxoglutarate metabolic process	1/28	13/14464	0.07692308	39.7362637	6.15354282	0.0248859	0.16762263	0.12891483	Col6a1	1
GO:0014745	negative regulation of muscle adaptation	1/28	13/14464	0.07692308	39.7362637	6.15354282	0.0248859	0.16762263	0.12891483	Igfbp5	1
GO:0044857	plasma membrane raft organization	1/28	13/14464	0.07692308	39.7362637	6.15354282	0.0248859	0.16762263	0.12891483	Col6a1	1
GO:0051280	negative regulation of release of sequestered calcium ion into cytosol	1/28	13/14464	0.07692308	39.7362637	6.15354282	0.0248859	0.16762263	0.12891483	Gstm7	1
GO:0060623	regulation of chromosome condensation	1/28	13/14464	0.07692308	39.7362637	6.15354282	0.0248859	0.16762263	0.12891483	H3f3b	1
GO:0070341	fat cell proliferation	1/28	13/14464	0.07692308	39.7362637	6.15354282	0.0248859	0.16762263	0.12891483	Col6a1	1
GO:0071415	cellular response to purine-containing compound	1/28	13/14464	0.07692308	39.7362637	6.15354282	0.0248859	0.16762263	0.12891483	Gstm7	1
GO:0014074	response to purine-containing compound	2/28	128/14464	0.015625	8.07142857	3.53901921	0.02527831	0.16762263	0.12891483	Gstm7/Igfbp5	2
GO:0014902	myotube differentiation	2/28	128/14464	0.015625	8.07142857	3.53901921	0.02527831	0.16762263	0.12891483	Ccn3/Col6a1	2
GO:0009566	fertilization	2/28	131/14464	0.01526718	7.8865867	3.48703165	0.02638802	0.16762263	0.12891483	Col6a1/H3f3b	2
GO:0006590	thyroid hormone generation	1/28	14/14464	0.07142857	36.8979592	5.9181317	0.02677526	0.16762263	0.12891483	Cpq	1
GO:0006700	C21-steroid hormone biosynthetic process	1/28	14/14464	0.07142857	36.8979592	5.9181317	0.02677526	0.16762263	0.12891483	Bmp6	1
GO:0010832	negative regulation of myotube differentiation	1/28	14/14464	0.07142857	36.8979592	5.9181317	0.02677526	0.16762263	0.12891483	Ccn3	1
GO:0014733	regulation of skeletal muscle adaptation	1/28	14/14464	0.07142857	36.8979592	5.9181317	0.02677526	0.16762263	0.12891483	Igfbp5	1
GO:0030539	male genitalia development	1/28	14/14464	0.07142857	36.8979592	5.9181317	0.02677526	0.16762263	0.12891483	Bmp6	1
GO:0048103	somatic stem cell division	1/28	14/14464	0.07142857	36.8979592	5.9181317	0.02677526	0.16762263	0.12891483	Fzd7	1
GO:0060044	negative regulation of cardiac muscle cell proliferation	1/28	14/14464	0.07142857	36.8979592	5.9181317	0.02677526	0.16762263	0.12891483	Kcnk2	1

GO:0072498	embryonic skeletal joint development	1/28	14/14464	0.07142857	36.8979592	5.9181317	0.02677526	0.16762263	0.12891483 Slc2a10	1
GO:1900272	negative regulation of long-term synaptic potentiation	1/28	14/14464	0.07142857	36.8979592	5.9181317	0.02677526	0.16762263	0.12891483 Cx3cr1	1
GO:1903019	negative regulation of glycoprotein metabolic process	1/28	14/14464	0.07142857	36.8979592	5.9181317	0.02677526	0.16762263	0.12891483 Slc2a10	1
GO:1903977	positive regulation of glial cell migration	1/28	14/14464	0.07142857	36.8979592	5.9181317	0.02677526	0.16762263	0.12891483 Cx3cr1	1
GO:0051402	neuron apoptotic process	3/28	339/14464	0.00884956	4.57142857	2.93043681	0.02711046	0.1681504	0.12932072 Cx3cr1/Col6a1/Bhlhb9	3
GO:0046661	male sex differentiation	2/28	134/14464	0.01492537	7.71002132	3.43667114	0.02751738	0.1681504	0.12932072 Bmp6/H3f3b	2
GO:0050890	cognition	3/28	344/14464	0.00872093	4.50498339	2.89756114	0.02815067	0.1681504	0.12932072 Cx3cr1/Kcnk2/Bhlhb9	3
GO:0007565	female pregnancy	2/28	136/14464	0.01470588	7.59663866	3.40395755	0.02828107	0.1681504	0.12932072 Igfbp5/H3f3b	2
GO:0042178	xenobiotic catabolic process	1/28	15/14464	0.06666667	34.4380952	5.70627945	0.02866109	0.1681504	0.12932072 Gstm7	1
GO:0051284	positive regulation of sequestering of calcium ion	1/28	15/14464	0.06666667	34.4380952	5.70627945	0.02866109	0.1681504	0.12932072 Gstm7	1
GO:0097084	vascular associated smooth muscle cell development	1/28	15/14464	0.06666667	34.4380952	5.70627945	0.02866109	0.1681504	0.12932072 Comp	1
GO:0098528	skeletal muscle fiber differentiation	1/28	15/14464	0.06666667	34.4380952	5.70627945	0.02866109	0.1681504	0.12932072 Col6a1	1
GO:2000047	regulation of cell-cell adhesion mediated by cadherin	1/28	15/14464	0.06666667	34.4380952	5.70627945	0.02866109	0.1681504	0.12932072 Bmp6	1
GO:2000679	positive regulation of transcription regulatory region DNA binding	1/28	15/14464	0.06666667	34.4380952	5.70627945	0.02866109	0.1681504	0.12932072 H1f0	1
GO:2000725	regulation of cardiac muscle cell differentiation	1/28	15/14464	0.06666667	34.4380952	5.70627945	0.02866109	0.1681504	0.12932072 Fzd7	1
GO:0002544	chronic inflammatory response	1/28	16/14464	0.0625	32.2857143	5.5142566	0.0305434	0.1681504	0.12932072 Cx3cr1	1
GO:0007252	I-kappaB phosphorylation	1/28	16/14464	0.0625	32.2857143	5.5142566	0.0305434	0.1681504	0.12932072 Cx3cr1	1
GO:0007398	ectoderm development	1/28	16/14464	0.0625	32.2857143	5.5142566	0.0305434	0.1681504	0.12932072 Fzd7	1
GO:0036035	osteoclast development	1/28	16/14464	0.0625	32.2857143	5.5142566	0.0305434	0.1681504	0.12932072 Fbn1	1
GO:0042308	negative regulation of protein import into nucleus	1/28	16/14464	0.0625	32.2857143	5.5142566	0.0305434	0.1681504	0.12932072 Gbp4	1
GO:0043117	positive regulation of vascular permeability	1/28	16/14464	0.0625	32.2857143	5.5142566	0.0305434	0.1681504	0.12932072 Bmp6	1
GO:0098751	bone cell development	1/28	16/14464	0.0625	32.2857143	5.5142566	0.0305434	0.1681504	0.12932072 Fbn1	1
GO:1901862	negative regulation of muscle tissue development	1/28	16/14464	0.0625	32.2857143	5.5142566	0.0305434	0.1681504	0.12932072 Igfbp5	1
GO:0051147	regulation of muscle cell differentiation	2/28	144/14464	0.01388889	7.17460317	3.27946923	0.03142056	0.1681504	0.12932072 Ccn3/Fzd7	2
GO:0048872	homeostasis of number of cells	3/28	360/14464	0.00833333	4.3047619	2.79643706	0.03162257	0.1681504	0.12932072 Cx3cr1/Ccn3/Col6a1	3
GO:0007600	sensory perception	3/28	362/14464	0.00828729	4.28097869	2.78421079	0.03207188	0.1681504	0.12932072 Ccn3/Col6a1/Kcnk2	3
GO:0002281	macrophage activation involved in immune response	1/28	17/14464	0.05882353	30.3865546	5.33911244	0.03242219	0.1681504	0.12932072 Cx3cr1	1
GO:0003323	type B pancreatic cell development	1/28	17/14464	0.05882353	30.3865546	5.33911244	0.03242219	0.1681504	0.12932072 Bmp6	1
GO:0032095	regulation of response to food	1/28	17/14464	0.05882353	30.3865546	5.33911244	0.03242219	0.1681504	0.12932072 Fbn1	1
GO:0032352	positive regulation of hormone metabolic process	1/28	17/14464	0.05882353	30.3865546	5.33911244	0.03242219	0.1681504	0.12932072 Bmp6	1
GO:0042415	norepinephrine metabolic process	1/28	17/14464	0.05882353	30.3865546	5.33911244	0.03242219	0.1681504	0.12932072 Kl	1
GO:0042759	long-chain fatty acid biosynthetic process	1/28	17/14464	0.05882353	30.3865546	5.33911244	0.03242219	0.1681504	0.12932072 Gstm7	1
GO:0043171	peptide catabolic process	1/28	17/14464	0.05882353	30.3865546	5.33911244	0.03242219	0.1681504	0.12932072 Cpq	1
GO:0060314	regulation of ryanodine-sensitive calcium-release channel activity	1/28	17/14464	0.05882353	30.3865546	5.33911244	0.03242219	0.1681504	0.12932072 Gstm7	1
GO:0061179	negative regulation of insulin secretion involved in cellular response to glucose stimulus	1/28	17/14464	0.05882353	30.3865546	5.33911244	0.03242219	0.1681504	0.12932072 Ndufaf2	1
GO:0061760	antifungal innate immune response	1/28	17/14464	0.05882353	30.3865546	5.33911244	0.03242219	0.1681504	0.12932072 Cx3cr1	1
GO:0090030	regulation of steroid hormone biosynthetic process	1/28	17/14464	0.05882353	30.3865546	5.33911244	0.03242219	0.1681504	0.12932072 Bmp6	1
GO:0098883	synapse pruning	1/28	17/14464	0.05882353	30.3865546	5.33911244	0.03242219	0.1681504	0.12932072 Cx3cr1	1
GO:2000846	regulation of corticosteroid hormone secretion	1/28	17/14464	0.05882353	30.3865546	5.33911244	0.03242219	0.1681504	0.12932072 Bmp6	1
GO:0001911	negative regulation of leukocyte mediated cytotoxicity	1/28	18/14464	0.05555556	28.6984127	5.17847759	0.03429747	0.1716012	0.13197466 Cx3cr1	1
GO:0031000	response to caffeine	1/28	18/14464	0.05555556	28.6984127	5.17847759	0.03429747	0.1716012	0.13197466 Gstm7	1
GO:0031645	negative regulation of nervous system process	1/28	18/14464	0.05555556	28.6984127	5.17847759	0.03429747	0.1716012	0.13197466 Ccn3	1
GO:0032026	response to magnesium ion	1/28	18/14464	0.05555556	28.6984127	5.17847759	0.03429747	0.1716012	0.13197466 Bmp6	1
GO:0042659	regulation of cell fate specification	1/28	18/14464	0.05555556	28.6984127	5.17847759	0.03429747	0.1716012	0.13197466 Fzd7	1
GO:0060795	cell fate commitment involved in formation of primary germ layer	1/28	18/14464	0.05555556	28.6984127	5.17847759	0.03429747	0.1716012	0.13197466 Fzd7	1
GO:0090026	positive regulation of monocyte chemotaxis	1/28	18/14464	0.05555556	28.6984127	5.17847759	0.03429747	0.1716012	0.13197466 Cx3cr1	1
GO:0042445	hormone metabolic process	2/28	152/14464	0.01315789	6.79699248	3.16416512	0.03469219	0.1716012	0.13197466 Bmp6/Cpq	2
GO:0044703	multi-organism reproductive process	2/28	152/14464	0.01315789	6.79699248	3.16416512	0.03469219	0.1716012	0.13197466 Igfbp5/H3f3b	2
GO:0044706	multi-multicellular organism process	2/28	153/14464	0.0130719	6.75256769	3.1503386	0.03511021	0.1716012	0.13197466 Igfbp5/H3f3b	2
GO:0045814	negative regulation of gene expression, epigenetic	2/28	153/14464	0.0130719	6.75256769	3.1503386	0.03511021	0.1716012	0.13197466 H3f3b/H1f0	2
GO:0006575	cellular modified amino acid metabolic process	2/28	154/14464	0.01298701	6.70871985	3.13663545	0.03553022	0.1716012	0.13197466 Gstm7/Cpq	2
GO:0003417	growth plate cartilage development	1/28	19/14464	0.05263158	27.1879699	5.03042447	0.03616925	0.1716012	0.13197466 Comp	1
GO:0010566	regulation of ketone biosynthetic process	1/28	19/14464	0.05263158	27.1879699	5.03042447	0.03616925	0.1716012	0.13197466 Bmp6	1
GO:0032098	regulation of appetite	1/28	19/14464	0.05263158	27.1879699	5.03042447	0.03616925	0.1716012	0.13197466 Fbn1	1
GO:0032332	positive regulation of chondrocyte differentiation	1/28	19/14464	0.05263158	27.1879699	5.03042447	0.03616925	0.1716012	0.13197466 Bmp6	1
GO:0035930	corticosteroid hormone secretion	1/28	19/14464	0.05263158	27.1879699	5.03042447	0.03616925	0.1716012	0.13197466 Bmp6	1
GO:0045217	cell-cell junction maintenance	1/28	19/14464	0.05263158	27.1879699	5.03042447	0.03616925	0.1716012	0.13197466 Kifc3	1
GO:0051930	regulation of sensory perception of pain	1/28	19/14464	0.05263158	27.1879699	5.03042447	0.03616925	0.1716012	0.13197466 Ccn3	1
GO:0007613	memory	2/28	156/14464	0.01282051	6.62271062	3.10959148	0.03637615	0.17181237	0.13213706 Cx3cr1/Kcnk2	2
GO:0042403	thyroid hormone metabolic process	1/28	20/14464	0.05	25.8285714	4.8933667	0.03803753	0.17598593	0.13534686 Cpq	1
GO:0042537	benzene-containing compound metabolic process	1/28	20/14464	0.05	25.8285714	4.8933667	0.03803753	0.17598593	0.13534686 Gstm7	1
GO:0051931	regulation of sensory perception	1/28	20/14464	0.05	25.8285714	4.8933667	0.03803753	0.17598593	0.13534686 Ccn3	1
GO:2001044	regulation of integrin-mediated signaling pathway	1/28	20/14464	0.05	25.8285714	4.8933667	0.03803753	0.17598593	0.13534686 Slc2a10	1
GO:0065004	protein-DNA complex assembly	2/28	160/14464	0.0125	6.45714286	3.05690073	0.03809147	0.17598593	0.13534686 H3f3b/H1f0	2
GO:0051897	positive regulation of phosphatidylinositol 3-kinase/protein kinase B signal transduction	2/28	161/14464	0.01242236	6.41703638	3.04400873	0.03852514	0.17721564	0.13629259 Cx3cr1/Igfbp5	2
GO:0007369	gastrulation	2/28	162/14464	0.01234568	6.37742504	3.03122559	0.03896072	0.177698	0.13666357 Fzd7/Col6a1	2
GO:0071674	mononuclear cell migration	2/28	163/14464	0.01226994	6.33829974	3.01854967	0.03939821	0.177698	0.13666357 Cx3cr1/Ccn3	2
GO:0003309	type B pancreatic cell differentiation	1/28	21/14464	0.04761905	24.5986395	4.76598509	0.03990231	0.177698	0.13666357 Bmp6	1
GO:0009713	catechol-containing compound biosynthetic process	1/28	21/14464	0.04761905	24.5986395	4.76598509	0.03990231	0.177698	0.13666357 Kl	1
GO:0010560	positive regulation of glycoprotein biosynthetic process	1/28	21/14464	0.04761905	24.5986395	4.76598509	0.03990231	0.177698	0.13666357 Slc2a10	1
GO:0042423	catecholamine biosynthetic process	1/28	21/14464	0.04761905	24.5986395	4.76598509	0.03990231	0.177698	0.13666357 Kl	1
GO:0060065	uterus development	1/28	21/14464	0.04761905	24.5986395	4.76598509	0.03990231	0.177698	0.13666357 Col6a1	1
GO:0003012	muscle system process	3/28	395/14464	0.00759494	3.92332731	2.59435348	0.03997365	0.177698	0.13666357 Igfbp5/Comp/Col6a1	3
GO:0008207	C21-steroid hormone metabolic process	1/28	22/14464	0.04545455	23.4805195	4.64717241	0.04176361	0.18011366	0.1385214 Bmp6	1
GO:0031342	negative regulation of cell killing	1/28	22/14464	0.04545455	23.4805195	4.64717241	0.04176361	0.18011366	0.1385214 Cx3cr1	1
GO:0032691	negative regulation of interleukin-1 beta production	1/28	22/14464	0.04545455	23.4805195	4.64717241	0.04176361	0.18011366	0.1385214 Cx3cr1	1
GO:0071676	negative regulation of mononuclear cell migration	1/28	22/14464	0.04545455	23.4805195	4.64717241	0.04176361	0.18011366	0.1385214 Ccn3	1
GO:0120255	olefinic compound biosynthetic process	1/28	22/14464	0.04545455	23.4805195	4.64717241	0.04176361	0.18011366	0.1385214 Bmp6	1
GO:1904754	positive regulation of vascular associated smooth muscle cell migration	1/28	22/14464	0.04545455	23.4805195	4.64717241	0.04176361	0.18011366	0.1385214 Igfbp5	1
GO:1905207	regulation of cardiocyte differentiation	1/28	22/14464	0.04545455	23.4805195	4.64717241	0.04176361	0.18011366	0.1385214 Fzd7	1
GO:0048736	appendage development	2/28	172/14464	0.01162791	6.00664452	2.90903702	0.04342014	0.18011366	0.1385214 Comp/Col6a1	2
GO:0060173	limb development	2/28	172/14464	0.01162791	6.00664452	2.90903702	0.04342014	0.18011366	0.1385214 Comp/Col6a1	2
GO:0010737	protein kinase A signaling	1/28	23/14464	0.04347826	22.4596273	4.5359915	0.04362143	0.18011366	0.1385214 Fbn1	1
GO:0046885	regulation of hormone biosynthetic process	1/28	23/14464	0.04347826	22.4596273	4.5359915	0.04362143	0.18011366	0.1385214 Bmp6	1
GO:1901223	negative regulation of non-canonical NF-kappaB signal transduction	1/28	23/14464	0.04347826	22.4596273	4.5359915	0.04362143	0.18011366	0.1385214 Ccn3	1
GO:1903020	positive regulation of glycoprotein metabolic process	1/28	23/14464	0.04347826	22.4596273	4.5359915	0.04362143	0.18011366	0.1385214 Slc2a10	1
GO:2000831	regulation of steroid hormone secretion	1/28	23/14464	0.04347826	22.4596273	4.5359915	0.04362143	0.18011366	0.1385214 Bmp6	1
GO:0071222	cellular response to lipopolysaccharide	2/28	173/14464	0.01156069	5.97192403	2.89735025	0.04387626	0.18011366	0.1385214 Cx3cr1/Bmp6	2
GO:0048771	tissue remodeling	2/28	174/14464	0.01149425	5.93760263	2.88575474	0.04433419	0.18011366	0.1385214 Igfbp5/Col6a1	2
GO:0050920	regulation of chemotaxis	2/28	175/14464	0.01142857	5.90367347	2.87424922	0.04479394	0.18011366	0.1385214 Cx3cr1/Ccn3	2
GO:0014706	striated muscle tissue development	3/28	414/14464	0.00724638	3.74327122	2.49410957	0.04493683	0.18011366	0.1385214 Fzd7/Col6a1/Kcnk2	3
GO:0045216	cell-cell junction organization	2/28	176/14464	0.01136364	5.87012987	2.86283243	0.04525548	0.18011366	0.1385214 Bmp6/Kifc3	2
GO:0050954	sensory perception of mechanical stimulus	2/28	176/14464	0.01136364	5.87012987	2.86283243	0.04525548	0.18011366	0.1385214 Col6a1/Kcnk2	2
GO:0003085	negative regulation of systemic arterial blood pressure	1/28	24/14464	0.04166667	21.5238095	4.43164304	0.04547577	0.18011366	0.1385214 Kl	1
GO:0010880	regulation of release of sequestered calcium ion into cytosol by sarcoplasmic reticulum	1/28	24/14464	0.04166667	21.5238095	4.43164304	0.04547577	0.18011366	0.1385214 Gstm7	1
GO:0031664	regulation of lipopolysaccharide-mediated signaling pathway	1/28	24/14464	0.04166667	21.5238095	4.43164304	0.04547577	0.18011366	0.1385214 Bmp6	1
GO:0035883	enteroendocrine cell differentiation	1/28	24/14464	0.04166667	21.5238095	4.43164304	0.04547577	0.18011366	0.1385214 Bmp6	1
GO:0043501	skeletal muscle adaptation	1/28	24/14464	0.04166667	21.5238095	4.43164304	0.04547577	0.18011366	0.1385214 Igfbp5	1
GO:0046184	aldehyde biosynthetic process	1/28	24/14464	0.04166667	21.5238095	4.43164304	0.04547577	0.18011366	0.1385214 Bmp6	1
GO:0090335	regulation of brown fat cell differentiation	1/28	24/14464	0.04166667	21.5238095	4.43164304	0.04547577	0.18011366	0.1385214 Fbn1	1
GO:1901615	organic hydroxy compound metabolic process	3/28	417/14464	0.00719424	3.71634121	2.47882194	0.04574779	0.18011366	0.1385214 Bmp6/Cpq/Kl	3
GO:0030595	leukocyte chemotaxis	2/28	179/14464	0.01117318	5.77174781	2.82910227	0.04665084	0.18011366	0.1385214 Cx3cr1/Ccn3	2
GO:0035265	organ growth	2/28	180/14464	0.01111111	5.73968254	2.81802835	0.04711951	0.18011366	0.1385214 Comp/Kcnk2	2
GO:0048659	smooth muscle cell proliferation	2/28	180/14464	0.01111111	5.73968254	2.81802835	0.04711951	0.18011366	0.1385214 Igfbp5/Ccn3	2
GO:0071219	cellular response to molecule of bacterial origin	2/28	180/14464	0.01111111	5.73968254	2.81802835	0.04711951	0.18011366	0.1385214 Cx3cr1/Bmp6	2
GO:0071824	protein-DNA complex organization	2/28	180/14464	0.01111111	5.73968254	2.81802835	0.04711951	0.18011366	0.1385214 H3f3b/H1f0	2
GO:0003416	endochondral bone growth	1/28	25/14464	0.04	20.6628571	4.33344054	0.04732665	0.18011366	0.1385214 Comp	1
GO:0010039	response to iron ion	1/28	25/14464	0.04	20.6628571	4.33344054	0.04732665	0.18011366	0.1385214 Bmp6	1
GO:0014829	vascular associated smooth muscle contraction	1/28	25/14464	0.04	20.6628571	4.33344054	0.04732665	0.18011366	0.1385214 Comp	1
GO:0033280	response to vitamin D	1/28	25/14464	0.04	20.6628571	4.33344054	0.04732665	0.18011366	0.1385214 Kl	1
GO:0046823	negative regulation of nucleocytoplasmic transport	1/28	25/14464	0.04	20.6628571	4.33344054	0.04732665	0.18011366	0.1385214 Gbp4	1
GO:1900037	regulation of cellular response to hypoxia	1/28	25/14464	0.04	20.6628571	4.33344054	0.04732665	0.18011366	0.1385214 Kcnk2	1
GO:1903975	regulation of glial cell migration	1/28	25/14464	0.04	20.6628571	4.33344054	0.04732665	0.18011366	0.1385214 Cx3cr1	1
GO:0010893	positive regulation of steroid biosynthetic process	1/28	26/14464	0.03846154	19.8681319	4.24079062	0.04917407	0.18157254	0.13964339 Bmp6	1
GO:0030262	apoptotic nuclear changes	1/28	26/14464	0.03846154	19.8681319	4.24079062	0.04917407	0.18157254	0.13964339 Col6a1	1
GO:0060416	response to growth hormone	1/28	26/14464	0.03846154	19.8681319	4.24079062	0.04917407	0.18157254	0.13964339 Igfbp5	1
GO:0060479	lung cell differentiation	1/28	26/14464	0.03846154	19.8681319	4.24079062	0.04917407	0.18157254	0.13964339 Col6a1	1
GO:0060487	lung epithelial cell differentiation	1/28	26/14464	0.03846154	19.8681319	4.24079062	0.04917407	0.18157254	0.13964339 Col6a1	1
GO:1901020	negative regulation of calcium ion transmembrane transporter activity	1/28	26/14464	0.03846154	19.8681319	4.24079062	0.04917407	0.18157254	0.13964339 Gstm7	1
GO:1905523	positive regulation of macrophage migration	1/28	26/14464	0.03846154	19.8681319	4.24079062	0.04917407	0.18157254	0.13964339 Cx3cr1	1
GO:0002683	negative regulation of immune system process	3/28	433/14464	0.00692841	3.57901683	2.39959851	0.05019735	0.18157254	0.13964339 Fbn1/Cx3cr1/Ccn3	3
GO:0050679	positive regulation of epithelial cell proliferation	2/28	187/14464	0.01069519	5.52482811	2.74276868	0.05044903	0.18157254	0.13964339 Bmp6/Fzd7	2
GO:1901617	organic hydroxy compound biosynthetic process	2/28	187/14464	0.01069519	5.52482811	2.74276868	0.05044903	0.18157254	0.13964339 Bmp6/Kl	2
GO:0043524	negative regulation of neuron apoptotic process	2/28	188/14464	0.0106383	5.49544073	2.73232719	0.05093155	0.18157254	0.13964339 Cx3cr1/Bhlhb9	2
GO:0006921	cellular component disassembly involved in execution phase of apoptosis	1/28	27/14464	0.03703704	19.1322751	4.15317734	0.05101803	0.18157254	0.13964339 Col6a1	1
GO:0014808	release of sequestered calcium ion into cytosol by sarcoplasmic reticulum	1/28	27/14464	0.03703704	19.1322751	4.15317734	0.05101803	0.18157254	0.13964339 Gstm7	1
GO:0031069	hair follicle morphogenesis	1/28	27/14464	0.03703704	19.1322751	4.15317734	0.05101803	0.18157254	0.13964339 Igfbp5	1
GO:0031579	membrane raft organization	1/28	27/14464	0.03703704	19.1322751	4.15317734	0.05101803	0.18157254	0.13964339 Col6a1	1
GO:0032647	regulation of interferon-alpha production	1/28	27/14464	0.03703704	19.1322751	4.15317734	0.05101803	0.18157254	0.13964339 Gbp4	1
GO:0034508	centromere complex assembly	1/28	27/14464	0.03703704	19.1322751	4.15317734	0.05101803	0.18157254	0.13964339 H3f3b	1
GO:0035929	steroid hormone secretion	1/28	27/14464	0.03703704	19.1322751	4.15317734	0.05101803	0.18157254	0.13964339 Bmp6	1
GO:0002685	regulation of leukocyte migration	2/28	191/14464	0.0104712	5.40912491	2.70144559	0.05238929	0.18157254	0.13964339 Cx3cr1/Ccn3	2
GO:0002068	glandular epithelial cell development	1/28	28/14464	0.03571429	18.4489796	4.07014973	0.05285855	0.18157254	0.13964339 Bmp6	1
GO:0014850	response to muscle activity	1/28	28/14464	0.03571429	18.4489796	4.07014973	0.05285855	0.18157254	0.13964339 Col6a1	1
GO:0032692	negative regulation of interleukin-1 production	1/28	28/14464	0.03571429	18.4489796	4.07014973	0.05285855	0.18157254	0.13964339 Cx3cr1	1
GO:0035767	endothelial cell chemotaxis	1/28	28/14464	0.03571429	18.4489796	4.07014973	0.05285855	0.18157254	0.13964339 Ccn3	1
GO:0042832	defense response to protozoan	1/28	28/14464	0.03571429	18.4489796	4.07014973	0.05285855	0.18157254	0.13964339 Gbp4	1
GO:0050901	leukocyte tethering or rolling	1/28	28/14464	0.03571429	18.4489796	4.07014973	0.05285855	0.18157254	0.13964339 Cx3cr1	1
GO:0055022	negative regulation of cardiac muscle tissue growth	1/28	28/14464	0.03571429	18.4489796	4.07014973	0.05285855	0.18157254	0.13964339 Kcnk2	1
GO:0061036	positive regulation of cartilage development	1/28	28/14464	0.03571429	18.4489796	4.07014973	0.05285855	0.18157254	0.13964339 Bmp6	1
GO:0061117	negative regulation of heart growth	1/28	28/14464	0.03571429	18.4489796	4.07014973	0.05285855	0.18157254	0.13964339 Kcnk2	1
GO:1902932	positive regulation of alcohol biosynthetic process	1/28	28/14464	0.03571429	18.4489796	4.07014973	0.05285855	0.18157254	0.13964339 Bmp6	1
GO:1903514	release of sequestered calcium ion into cytosol by endoplasmic reticulum	1/28	28/14464	0.03571429	18.4489796	4.07014973	0.05285855	0.18157254	0.13964339 Gstm7	1
GO:0008015	blood circulation	3/28	443/14464	0.00677201	3.49822638	2.35195458	0.05308382	0.18175625	0.13978468 Bmp6/Comp/Kl	3
GO:0045671	negative regulation of osteoclast differentiation	1/28	29/14464	0.03448276	17.8128079	3.99131154	0.05469562	0.18218623	0.14011537 Fbn1	1
GO:0045940	positive regulation of steroid metabolic process	1/28	29/14464	0.03448276	17.8128079	3.99131154	0.05469562	0.18218623	0.14011537 Bmp6	1
GO:0051882	mitochondrial depolarization	1/28	29/14464	0.03448276	17.8128079	3.99131154	0.05469562	0.18218623	0.14011537 Col6a1	1
GO:0060586	multicellular organismal-level iron ion homeostasis	1/28	29/14464	0.03448276	17.8128079	3.99131154	0.05469562	0.18218623	0.14011537 Bmp6	1
GO:0061484	hematopoietic stem cell homeostasis	1/28	29/14464	0.03448276	17.8128079	3.99131154	0.05469562	0.18218623	0.14011537 Ccn3	1
GO:0098868	bone growth	1/28	29/14464	0.03448276	17.8128079	3.99131154	0.05469562	0.18218623	0.14011537 Comp	1
GO:0120178	steroid hormone biosynthetic process	1/28	29/14464	0.03448276	17.8128079	3.99131154	0.05469562	0.18218623	0.14011537 Bmp6	1
GO:2001258	negative regulation of cation channel activity	1/28	29/14464	0.03448276	17.8128079	3.99131154	0.05469562	0.18218623	0.14011537 Gstm7	1
GO:0097529	myeloid leukocyte migration	2/28	196/14464	0.01020408	5.27113703	2.65139701	0.0548522	0.18218623	0.14011537 Cx3cr1/Ccn3	2
GO:0098771	inorganic ion homeostasis	3/28	454/14464	0.00660793	3.41346759	2.30109793	0.05635154	0.18218623	0.14011537 Gstm7/Bmp6/Kl	3
GO:0001562	response to protozoan	1/28	30/14464	0.03333333	17.2190476	3.91631303	0.05652925	0.18218623	0.14011537 Gbp4	1
GO:0002063	chondrocyte development	1/28	30/14464	0.03333333	17.2190476	3.91631303	0.05652925	0.18218623	0.14011537 Comp	1
GO:0010453	regulation of cell fate commitment	1/28	30/14464	0.03333333	17.2190476	3.91631303	0.05652925	0.18218623	0.14011537 Fzd7	1
GO:0030511	positive regulation of transforming growth factor beta receptor signaling pathway	1/28	30/14464	0.03333333	17.2190476	3.91631303	0.05652925	0.18218623	0.14011537 Slc2a10	1
GO:0032107	regulation of response to nutrient levels	1/28	30/14464	0.03333333	17.2190476	3.91631303	0.05652925	0.18218623	0.14011537 Fbn1	1
GO:0032607	interferon-alpha production	1/28	30/14464	0.03333333	17.2190476	3.91631303	0.05652925	0.18218623	0.14011537 Gbp4	1
GO:0048048	embryonic eye morphogenesis	1/28	30/14464	0.03333333	17.2190476	3.91631303	0.05652925	0.18218623	0.14011537 Fbn1	1
GO:0150146	cell junction disassembly	1/28	30/14464	0.03333333	17.2190476	3.91631303	0.05652925	0.18218623	0.14011537 Cx3cr1	1
GO:1903846	positive regulation of cellular response to transforming growth factor beta stimulus	1/28	30/14464	0.03333333	17.2190476	3.91631303	0.05652925	0.18218623	0.14011537 Slc2a10	1
GO:0031667	response to nutrient levels	3/28	455/14464	0.00659341	3.40596546	2.29655204	0.05665337	0.18218623	0.14011537 Fbn1/Col6a1/Kl	3
GO:0009636	response to toxic substance	2/28	201/14464	0.00995025	5.14001421	2.60303011	0.05735599	0.18321752	0.14090851 Gstm7/Col6a1	2
GO:0035264	multicellular organism growth	2/28	202/14464	0.00990099	5.1145686	2.59354954	0.05786157	0.18321752	0.14090851 Comp/H3f3b	2

GO:0006099	tricarboxylic acid cycle	1/28	31/14464	0.03225806	16.6635945	3.84484418	0.05835946	0.18321752	0.14090851	Col6a1	1
GO:0045454	cell redox homeostasis	1/28	31/14464	0.03225806	16.6635945	3.84484418	0.05835946	0.18321752	0.14090851	Slc2a10	1
GO:0048730	epidermis morphogenesis	1/28	31/14464	0.03225806	16.6635945	3.84484418	0.05835946	0.18321752	0.14090851	Igfbp5	1
GO:0061003	positive regulation of dendritic spine morphogenesis	1/28	31/14464	0.03225806	16.6635945	3.84484418	0.05835946	0.18321752	0.14090851	Bhlhb9	1
GO:1901021	positive regulation of calcium ion transmembrane transporter activity	1/28	31/14464	0.03225806	16.6635945	3.84484418	0.05835946	0.18321752	0.14090851	Gstm7	1
GO:2000279	negative regulation of DNA biosynthetic process	1/28	31/14464	0.03225806	16.6635945	3.84484418	0.05835946	0.18321752	0.14090851	Kcnk2	1
GO:0071216	cellular response to biotic stimulus	2/28	205/14464	0.0097561	5.03972125	2.56547961	0.05938781	0.18377075	0.14133399	Cx3cr1/Bmp6	2
GO:0002021	response to dietary excess	1/28	32/14464	0.03125	16.1428571	3.77662902	0.06018624	0.18377075	0.14133399	Col6a1	1
GO:0009309	amine biosynthetic process	1/28	32/14464	0.03125	16.1428571	3.77662902	0.06018624	0.18377075	0.14133399	Kl	1
GO:0032094	response to food	1/28	32/14464	0.03125	16.1428571	3.77662902	0.06018624	0.18377075	0.14133399	Fbn1	1
GO:0042401	biogenic amine biosynthetic process	1/28	32/14464	0.03125	16.1428571	3.77662902	0.06018624	0.18377075	0.14133399	Kl	1
GO:0050922	negative regulation of chemotaxis	1/28	32/14464	0.03125	16.1428571	3.77662902	0.06018624	0.18377075	0.14133399	Ccn3	1
GO:0051180	vitamin transport	1/28	32/14464	0.03125	16.1428571	3.77662902	0.06018624	0.18377075	0.14133399	Slc2a10	1
GO:1900047	negative regulation of hemostasis	1/28	32/14464	0.03125	16.1428571	3.77662902	0.06018624	0.18377075	0.14133399	Comp	1
GO:2000677	regulation of transcription regulatory region DNA binding	1/28	32/14464	0.03125	16.1428571	3.77662902	0.06018624	0.18377075	0.14133399	H1f0	1
GO:0003018	vascular process in circulatory system	2/28	207/14464	0.00966184	4.9910283	2.5470688	0.06041314	0.18377075	0.14133399	Bmp6/Comp	2
GO:0003013	circulatory system process	3/28	470/14464	0.00638298	3.29726444	2.22984028	0.06127541	0.18377075	0.14133399	Bmp6/Comp/Kl	3
GO:0031348	negative regulation of defense response	2/28	210/14464	0.00952381	4.91972789	2.5198922	0.06196277	0.18377075	0.14133399	Cx3cr1/Ccn3	2
GO:0014047	glutamate secretion	1/28	33/14464	0.03030303	15.6536797	3.71142101	0.0620096	0.18377075	0.14133399	Kcnk2	1
GO:0022011	myelination in peripheral nervous system	1/28	33/14464	0.03030303	15.6536797	3.71142101	0.0620096	0.18377075	0.14133399	Col6a1	1
GO:0031018	endocrine pancreas development	1/28	33/14464	0.03030303	15.6536797	3.71142101	0.0620096	0.18377075	0.14133399	Bmp6	1
GO:0032292	peripheral nervous system axon ensheathment	1/28	33/14464	0.03030303	15.6536797	3.71142101	0.0620096	0.18377075	0.14133399	Col6a1	1
GO:0060351	cartilage development involved in endochondral bone morphogenesis	1/28	33/14464	0.03030303	15.6536797	3.71142101	0.0620096	0.18377075	0.14133399	Comp	1
GO:0060391	positive regulation of SMAD protein signal transduction	1/28	33/14464	0.03030303	15.6536797	3.71142101	0.0620096	0.18377075	0.14133399	Bmp6	1
GO:0070296	sarcoplasmic reticulum calcium ion transport	1/28	33/14464	0.03030303	15.6536797	3.71142101	0.0620096	0.18377075	0.14133399	Gstm7	1
GO:0090317	negative regulation of intracellular protein transport	1/28	33/14464	0.03030303	15.6536797	3.71142101	0.0620096	0.18377075	0.14133399	Gbp4	1
GO:0001958	endochondral ossification	1/28	34/14464	0.02941176	15.1932773	3.64899907	0.06382955	0.18552656	0.14268434	Bmp6	1
GO:0002052	positive regulation of neuroblast proliferation	1/28	34/14464	0.02941176	15.1932773	3.64899907	0.06382955	0.18552656	0.14268434	Cx3cr1	1
GO:0035987	endodermal cell differentiation	1/28	34/14464	0.02941176	15.1932773	3.64899907	0.06382955	0.18552656	0.14268434	Col6a1	1
GO:0036075	replacement ossification	1/28	34/14464	0.02941176	15.1932773	3.64899907	0.06382955	0.18552656	0.14268434	Bmp6	1
GO:0048806	genitalia development	1/28	34/14464	0.02941176	15.1932773	3.64899907	0.06382955	0.18552656	0.14268434	Bmp6	1
GO:0071398	cellular response to fatty acid	1/28	34/14464	0.02941176	15.1932773	3.64899907	0.06382955	0.18552656	0.14268434	Kcnk2	1
GO:1904752	regulation of vascular associated smooth muscle cell migration	1/28	34/14464	0.02941176	15.1932773	3.64899907	0.06382955	0.18552656	0.14268434	Igfbp5	1
GO:0048705	skeletal system morphogenesis	2/28	214/14464	0.00934579	4.82777036	2.48444909	0.06405031	0.18565815	0.14278555	Bmp6/Comp	2
GO:0014912	negative regulation of smooth muscle cell migration	1/28	35/14464	0.02857143	14.7591837	3.58916435	0.0656461	0.1892468	0.14554549	Igfbp5	1
GO:0051281	positive regulation of release of sequestered calcium ion into cytosol	1/28	35/14464	0.02857143	14.7591837	3.58916435	0.0656461	0.1892468	0.14554549	Gstm7	1
GO:0055001	muscle cell development	2/28	218/14464	0.00917431	4.73918742	2.44987419	0.06616185	0.19021531	0.14629036	Comp/Col6a1	2
GO:0014741	negative regulation of muscle hypertrophy	1/28	36/14464	0.02777778	14.3492063	3.53173738	0.06745925	0.19237705	0.1479529	Igfbp5	1
GO:0032350	regulation of hormone metabolic process	1/28	36/14464	0.02777778	14.3492063	3.53173738	0.06745925	0.19237705	0.1479529	Bmp6	1
GO:0045747	positive regulation of Notch signaling pathway	1/28	36/14464	0.02777778	14.3492063	3.53173738	0.06745925	0.19237705	0.1479529	Ccn3	1
GO:0005975	carbohydrate metabolic process	3/28	491/14464	0.00610998	3.15624091	2.14080924	0.06803941	0.19350994	0.14882418	Fbn1/Col6a1/Kl	3
GO:0002686	negative regulation of leukocyte migration	1/28	37/14464	0.02702703	13.96139	3.47655573	0.069269	0.19543095	0.15030159	Ccn3	1
GO:0045601	regulation of endothelial cell differentiation	1/28	37/14464	0.02702703	13.96139	3.47655573	0.069269	0.19543095	0.15030159	Bmp6	1
GO:0046621	negative regulation of organ growth	1/28	37/14464	0.02702703	13.96139	3.47655573	0.069269	0.19543095	0.15030159	Kcnk2	1
GO:0071396	cellular response to lipid	3/28	499/14464	0.00601202	3.10563985	2.1081362	0.07070441	0.1973694	0.15179241	Cx3cr1/Bmp6/Kcnk2	3
GO:0035886	vascular associated smooth muscle cell differentiation	1/28	38/14464	0.02631579	13.593985	3.42347197	0.07107537	0.1973694	0.15179241	Comp	1
GO:0042755	eating behavior	1/28	38/14464	0.02631579	13.593985	3.42347197	0.07107537	0.1973694	0.15179241	Col6a1	1
GO:0044331	cell-cell adhesion mediated by cadherin	1/28	38/14464	0.02631579	13.593985	3.42347197	0.07107537	0.1973694	0.15179241	Bmp6	1
GO:0050832	defense response to fungus	1/28	38/14464	0.02631579	13.593985	3.42347197	0.07107537	0.1973694	0.15179241	Cx3cr1	1
GO:0071312	cellular response to alkaloid	1/28	38/14464	0.02631579	13.593985	3.42347197	0.07107537	0.1973694	0.15179241	Gstm7	1
GO:0040029	epigenetic regulation of gene expression	2/28	230/14464	0.00869565	4.49192547	2.35098671	0.07263585	0.19821415	0.15244208	H3f3b/H1f0	2
GO:0010559	regulation of glycoprotein biosynthetic process	1/28	39/14464	0.02564103	13.2454212	3.37235196	0.07287836	0.19821415	0.15244208	Slc2a10	1
GO:0010830	regulation of myotube differentiation	1/28	39/14464	0.02564103	13.2454212	3.37235196	0.07287836	0.19821415	0.15244208	Ccn3	1
GO:0017145	stem cell division	1/28	39/14464	0.02564103	13.2454212	3.37235196	0.07287836	0.19821415	0.15244208	Fzd7	1
GO:0032228	regulation of synaptic transmission, GABAergic	1/28	39/14464	0.02564103	13.2454212	3.37235196	0.07287836	0.19821415	0.15244208	Kcnk2	1
GO:0046189	phenol-containing compound biosynthetic process	1/28	39/14464	0.02564103	13.2454212	3.37235196	0.07287836	0.19821415	0.15244208	Kl	1
GO:1904738	vascular associated smooth muscle cell migration	1/28	39/14464	0.02564103	13.2454212	3.37235196	0.07287836	0.19821415	0.15244208	Igfbp5	1
GO:1905521	regulation of macrophage migration	1/28	39/14464	0.02564103	13.2454212	3.37235196	0.07287836	0.19821415	0.15244208	Cx3cr1	1
GO:0043114	regulation of vascular permeability	1/28	40/14464	0.025	12.9142857	3.3230733	0.07467797	0.2005312	0.15422408	Bmp6	1
GO:0043388	positive regulation of DNA binding	1/28	40/14464	0.025	12.9142857	3.3230733	0.07467797	0.2005312	0.15422408	H1f0	1
GO:0060043	regulation of cardiac muscle cell proliferation	1/28	40/14464	0.025	12.9142857	3.3230733	0.07467797	0.2005312	0.15422408	Kcnk2	1
GO:0071711	basement membrane organization	1/28	40/14464	0.025	12.9142857	3.3230733	0.07467797	0.2005312	0.15422408	Col6a1	1
GO:1900181	negative regulation of protein localization to nucleus	1/28	40/14464	0.025	12.9142857	3.3230733	0.07467797	0.2005312	0.15422408	Gbp4	1
GO:0001706	endoderm formation	1/28	41/14464	0.02439024	12.5993031	3.27552408	0.07647421	0.20134977	0.15485362	Col6a1	1
GO:0032480	negative regulation of type I interferon production	1/28	41/14464	0.02439024	12.5993031	3.27552408	0.07647421	0.20134977	0.15485362	Gbp4	1
GO:0044060	regulation of endocrine process	1/28	41/14464	0.02439024	12.5993031	3.27552408	0.07647421	0.20134977	0.15485362	Bmp6	1
GO:0045910	negative regulation of DNA recombination	1/28	41/14464	0.02439024	12.5993031	3.27552408	0.07647421	0.20134977	0.15485362	H1f0	1
GO:0048741	skeletal muscle fiber development	1/28	41/14464	0.02439024	12.5993031	3.27552408	0.07647421	0.20134977	0.15485362	Col6a1	1
GO:1990776	response to angiotensin	1/28	41/14464	0.02439024	12.5993031	3.27552408	0.07647421	0.20134977	0.15485362	Kl	1
GO:0007548	sex differentiation	2/28	237/14464	0.00843882	4.35925256	2.29638624	0.0765053	0.20134977	0.15485362	Bmp6/H3f3b	2
GO:0048738	cardiac muscle tissue development	2/28	237/14464	0.00843882	4.35925256	2.29638624	0.0765053	0.20134977	0.15485362	Fzd7/Kcnk2	2
GO:0051896	regulation of phosphatidylinositol 3-kinase/protein kinase B signal transduction	2/28	240/14464	0.00833333	4.3047619	2.27362951	0.07818384	0.20345599	0.15647346	Cx3cr1/Igfbp5	2
GO:0042446	hormone biosynthetic process	1/28	42/14464	0.02380952	12.2993197	3.22960173	0.0782671	0.20345599	0.15647346	Bmp6	1
GO:0050974	detection of mechanical stimulus involved in sensory perception	1/28	42/14464	0.02380952	12.2993197	3.22960173	0.0782671	0.20345599	0.15647346	Kcnk2	1
GO:0060428	lung epithelium development	1/28	42/14464	0.02380952	12.2993197	3.22960173	0.0782671	0.20345599	0.15647346	Col6a1	1
GO:1904707	positive regulation of vascular associated smooth muscle cell proliferation	1/28	42/14464	0.02380952	12.2993197	3.22960173	0.0782671	0.20345599	0.15647346	Igfbp5	1
GO:0006458	'de novo' protein folding	1/28	43/14464	0.02325581	12.013289	3.18521204	0.08005662	0.20558229	0.15810876	Telo2	1
GO:0042181	ketone biosynthetic process	1/28	43/14464	0.02325581	12.013289	3.18521204	0.08005662	0.20558229	0.15810876	Bmp6	1
GO:0061756	leukocyte adhesion to vascular endothelial cell	1/28	43/14464	0.02325581	12.013289	3.18521204	0.08005662	0.20558229	0.15810876	Cx3cr1	1
GO:0140719	constitutive heterochromatin formation	1/28	43/14464	0.02325581	12.013289	3.18521204	0.08005662	0.20558229	0.15810876	H3f3b	1
GO:1903018	regulation of glycoprotein metabolic process	1/28	43/14464	0.02325581	12.013289	3.18521204	0.08005662	0.20558229	0.15810876	Slc2a10	1
GO:0051259	protein complex oligomerization	2/28	244/14464	0.00819672	4.23419204	2.24385966	0.08044029	0.20606738	0.15848183	Comp/Col6a1	2
GO:0060326	cell chemotaxis	2/28	245/14464	0.00816327	4.21690962	2.23651671	0.08100765	0.20616589	0.15855759	Cx3cr1/Ccn3	2
GO:0001774	microglial cell activation	1/28	44/14464	0.02272727	11.7402597	3.1422683	0.08184279	0.20616589	0.15855759	Cx3cr1	1
GO:0002066	columnar/cuboidal epithelial cell development	1/28	44/14464	0.02272727	11.7402597	3.1422683	0.08184279	0.20616589	0.15855759	Bmp6	1
GO:0030858	positive regulation of epithelial cell differentiation	1/28	44/14464	0.02272727	11.7402597	3.1422683	0.08184279	0.20616589	0.15855759	Bmp6	1
GO:0048265	response to pain	1/28	44/14464	0.02272727	11.7402597	3.1422683	0.08184279	0.20616589	0.15855759	Col6a1	1
GO:0071320	cellular response to cAMP	1/28	44/14464	0.02272727	11.7402597	3.1422683	0.08184279	0.20616589	0.15855759	Igfbp5	1
GO:1901861	regulation of muscle tissue development	1/28	44/14464	0.02272727	11.7402597	3.1422683	0.08184279	0.20616589	0.15855759	Igfbp5	1
GO:0003016	respiratory system process	1/28	45/14464	0.02222222	11.4793651	3.10069057	0.08362562	0.20965855	0.16124372	Col6a1	1
GO:2001259	positive regulation of cation channel activity	1/28	45/14464	0.02222222	11.4793651	3.10069057	0.08362562	0.20965855	0.16124372	Gstm7	1
GO:0008340	determination of adult lifespan	1/28	46/14464	0.02173913	11.2298137	3.06040498	0.08540511	0.21361374	0.16428556	Kl	1
GO:0002269	leukocyte activation involved in inflammatory response	1/28	47/14464	0.0212766	10.9908815	3.02134318	0.08718127	0.21400879	0.16458939	Cx3cr1	1
GO:0007566	embryo implantation	1/28	47/14464	0.0212766	10.9908815	3.02134318	0.08718127	0.21400879	0.16458939	H3f3b	1
GO:0014044	Schwann cell development	1/28	47/14464	0.0212766	10.9908815	3.02134318	0.08718127	0.21400879	0.16458939	Col6a1	1
GO:0030514	negative regulation of BMP signaling pathway	1/28	47/14464	0.0212766	10.9908815	3.02134318	0.08718127	0.21400879	0.16458939	Fbn1	1
GO:0032330	regulation of chondrocyte differentiation	1/28	47/14464	0.0212766	10.9908815	3.02134318	0.08718127	0.21400879	0.16458939	Bmp6	1
GO:0045776	negative regulation of blood pressure	1/28	47/14464	0.0212766	10.9908815	3.02134318	0.08718127	0.21400879	0.16458939	Kl	1
GO:0140236	translation at presynapse	1/28	47/14464	0.0212766	10.9908815	3.02134318	0.08718127	0.21400879	0.16458939	Rpl8	1
GO:1902930	regulation of alcohol biosynthetic process	1/28	47/14464	0.0212766	10.9908815	3.02134318	0.08718127	0.21400879	0.16458939	Bmp6	1
GO:0009620	response to fungus	1/28	48/14464	0.02083333	10.7619048	2.9834418	0.08895411	0.21469062	0.16511377	Cx3cr1	1
GO:0014904	myotube cell development	1/28	48/14464	0.02083333	10.7619048	2.9834418	0.08895411	0.21469062	0.16511377	Col6a1	1
GO:0085029	extracellular matrix assembly	1/28	48/14464	0.02083333	10.7619048	2.9834418	0.08895411	0.21469062	0.16511377	Col6a1	1
GO:0090102	cochlea development	1/28	48/14464	0.02083333	10.7619048	2.9834418	0.08895411	0.21469062	0.16511377	Kcnk2	1
GO:0140241	translation at synapse	1/28	48/14464	0.02083333	10.7619048	2.9834418	0.08895411	0.21469062	0.16511377	Rpl8	1
GO:0140242	translation at postsynapse	1/28	48/14464	0.02083333	10.7619048	2.9834418	0.08895411	0.21469062	0.16511377	Rpl8	1
GO:0140353	lipid export from cell	1/28	48/14464	0.02083333	10.7619048	2.9834418	0.08895411	0.21469062	0.16511377	Bmp6	1
GO:0008544	epidermis development	2/28	259/14464	0.00772201	3.98896856	2.13764401	0.0890824	0.21469062	0.16511377	Igfbp5/Col6a1	2
GO:0014888	striated muscle adaptation	1/28	49/14464	0.02040816	10.5422741	2.94664199	0.09072362	0.21618375	0.16626211	Igfbp5	1
GO:0045668	negative regulation of osteoblast differentiation	1/28	49/14464	0.02040816	10.5422741	2.94664199	0.09072362	0.21618375	0.16626211	Igfbp5	1
GO:0060074	synapse maturation	1/28	49/14464	0.02040816	10.5422741	2.94664199	0.09072362	0.21618375	0.16626211	Cx3cr1	1
GO:1900046	regulation of hemostasis	1/28	49/14464	0.02040816	10.5422741	2.94664199	0.09072362	0.21618375	0.16626211	Comp	1
GO:1903170	negative regulation of calcium ion transmembrane transport	1/28	49/14464	0.02040816	10.5422741	2.94664199	0.09072362	0.21618375	0.16626211	Gstm7	1
GO:0007162	negative regulation of cell adhesion	2/28	263/14464	0.00760456	3.92829984	2.11066217	0.09143317	0.21738492	0.1671859	Bmp6/Fzd7	2
GO:0002762	negative regulation of myeloid leukocyte differentiation	1/28	50/14464	0.02	10.3314286	2.91088904	0.09248982	0.21842461	0.1679855	Fbn1	1
GO:0032413	negative regulation of ion transmembrane transporter activity	1/28	50/14464	0.02	10.3314286	2.91088904	0.09248982	0.21842461	0.1679855	Gstm7	1
GO:0060443	mammary gland morphogenesis	1/28	50/14464	0.02	10.3314286	2.91088904	0.09248982	0.21842461	0.1679855	Igfbp5	1
GO:0060986	endocrine hormone secretion	1/28	51/14464	0.01960784	10.1288515	2.87613198	0.0942527	0.22110723	0.17004864	Bmp6	1
GO:0070098	chemokine-mediated signaling pathway	1/28	51/14464	0.01960784	10.1288515	2.87613198	0.0942527	0.22110723	0.17004864	Cx3cr1	1
GO:1902692	regulation of neuroblast proliferation	1/28	51/14464	0.01960784	10.1288515	2.87613198	0.0942527	0.22110723	0.17004864	Cx3cr1	1
GO:0060070	canonical Wnt signaling pathway	2/28	269/14464	0.00743494	3.84067977	2.07116878	0.09499418	0.22227791	0.17094898	Fzd7/Col6a1	2
GO:0002704	negative regulation of leukocyte mediated immunity	1/28	52/14464	0.01923077	9.93406593	2.84232327	0.09601229	0.22227791	0.17094898	Cx3cr1	1
GO:0006518	peptide metabolic process	1/28	52/14464	0.01923077	9.93406593	2.84232327	0.09601229	0.22227791	0.17094898	Cpq	1
GO:0006749	glutathione metabolic process	1/28	52/14464	0.01923077	9.93406593	2.84232327	0.09601229	0.22227791	0.17094898	Gstm7	1
GO:0032387	negative regulation of intracellular transport	1/28	52/14464	0.01923077	9.93406593	2.84232327	0.09601229	0.22227791	0.17094898	Gbp4	1
GO:0050881	musculoskeletal movement	1/28	52/14464	0.01923077	9.93406593	2.84232327	0.09601229	0.22227791	0.17094898	Comp	1
GO:0002067	glandular epithelial cell differentiation	1/28	53/14464	0.01886792	9.74663073	2.80941851	0.09776858	0.22389428	0.1721921	Bmp6	1
GO:0006334	nucleosome assembly	1/28	53/14464	0.01886792	9.74663073	2.80941851	0.09776858	0.22389428	0.1721921	H1f0	1
GO:0045599	negative regulation of fat cell differentiation	1/28	53/14464	0.01886792	9.74663073	2.80941851	0.09776858	0.22389428	0.1721921	Fbn1	1
GO:0050982	detection of mechanical stimulus	1/28	53/14464	0.01886792	9.74663073	2.80941851	0.09776858	0.22389428	0.1721921	Kcnk2	1
GO:0070527	platelet aggregation	1/28	53/14464	0.01886792	9.74663073	2.80941851	0.09776858	0.22389428	0.1721921	Comp	1
GO:0006584	catecholamine metabolic process	1/28	54/14464	0.01851852	9.56613757	2.77737618	0.09952158	0.22498682	0.17303235	Kl	1
GO:0009712	catechol-containing compound metabolic process	1/28	54/14464	0.01851852	9.56613757	2.77737618	0.09952158	0.22498682	0.17303235	Kl	1
GO:0014037	Schwann cell differentiation	1/28	54/14464	0.01851852	9.56613757	2.77737618	0.09952158	0.22498682	0.17303235	Col6a1	1
GO:0031663	lipopolysaccharide-mediated signaling pathway	1/28	54/14464	0.01851852	9.56613757	2.77737618	0.09952158	0.22498682	0.17303235	Bmp6	1
GO:0035567	non-canonical Wnt signaling pathway	1/28	54/14464	0.01851852	9.56613757	2.77737618	0.09952158	0.22498682	0.17303235	Fzd7	1
GO:0061900	glial cell activation	1/28	54/14464	0.01851852	9.56613757	2.77737618	0.09952158	0.22498682	0.17303235	Cx3cr1	1
GO:0010812	negative regulation of cell-substrate adhesion	1/28	55/14464	0.01818182	9.39220779	2.74615739	0.1012713	0.22652227	0.17421322	Fzd7	1
GO:0019228	neuronal action potential	1/28	55/14464	0.01818182	9.39220779	2.74615739	0.1012713	0.22652227	0.17421322	Kcnk2	1
GO:0060425	lung morphogenesis	1/28	55/14464	0.01818182	9.39220779	2.74615739	0.1012713	0.22652227	0.17421322	Col6a1	1
GO:0070542	response to fatty acid	1/28	55/14464	0.01818182	9.39220779	2.74615739	0.1012713	0.22652227	0.17421322	Kcnk2	1
GO:1905517	macrophage migration	1/28	55/14464	0.01818182	9.39220779	2.74615739	0.1012713	0.22652227	0.17421322	Cx3cr1	1
GO:0002886	regulation of myeloid leukocyte mediated immunity	1/28	56/14464	0.01785714	9.2244898	2.7157257	0.10301773	0.22897639	0.17610064	Cx3cr1	1
GO:0006497	protein lipidation	1/28	56/14464	0.01785714	9.2244898	2.7157257	0.10301773	0.22897639	0.17610064	Col6a1	1
GO:0050688	regulation of defense response to virus	1/28	56/14464	0.01785714	9.2244898	2.7157257	0.10301773	0.22897639	0.17610064	Gbp4	1
GO:0043523	regulation of neuron apoptotic process	2/28	283/14464	0.00706714	3.65068147	1.98327119	0.10345896	0.229475	0.17648411	Cx3cr1/Bhlhb9	2
GO:0032410	negative regulation of transporter activity	1/28	57/14464	0.01754386	9.06265664	2.68604687	0.1047609	0.23091048	0.1775881	Gstm7	1
GO:0060038	cardiac muscle cell proliferation	1/28	57/14464	0.01754386	9.06265664	2.68604687	0.1047609	0.23091048	0.1775881	Kcnk2	1
GO:1900271	regulation of long-term synaptic potentiation	1/28	57/14464	0.01754386	9.06265664	2.68604687	0.1047609	0.23091048	0.1775881	Cx3cr1	1
GO:0006805	xenobiotic metabolic process	1/28	58/14464	0.01724138	8.90640394	2.65708876	0.10650079	0.23377145	0.17978841	Gstm7	1
GO:0071709	membrane assembly	1/28	58/14464	0.01724138	8.90640394	2.65708876	0.10650079	0.23377145	0.17978841	Col6a1	1
GO:0030166	proteoglycan biosynthetic process	1/28	59/14464	0.01694915	8.75544794	2.62882111	0.10823743	0.23504872	0.18077073	Slc2a10	1
GO:0035176	social behavior	1/28	59/14464	0.01694915	8.75544794	2.62882111	0.10823743	0.23504872	0.18077073	Cx3cr1	1
GO:0042158	lipoprotein biosynthetic process	1/28	59/14464	0.01694915	8.75544794	2.62882111	0.10823743	0.23504872	0.18077073	Col6a1	1
GO:0050769	positive regulation of neurogenesis	2/28	291/14464	0.00687285	3.5503191	1.93550767	0.10838887	0.23504872	0.18077073	Cx3cr1/Bhlhb9	2
GO:0071375	cellular response to peptide hormone stimulus	2/28	292/14464	0.00684932	3.53816047	1.92965515	0.10900965	0.23504872	0.18077073	Fbn1/Col6a1	2
GO:0006879	intracellular iron ion homeostasis	1/28	60/14464	0.01666667	8.60952381	2.60121543	0.10997081	0.23504872	0.18077073	Bmp6	1
GO:0010257	NADH dehydrogenase complex assembly	1/28	60/14464	0.01666667	8.60952381	2.60121543	0.10997081	0.23504872	0.18077073	Ndufaf2	1
GO:0032147	activation of protein kinase activity	1/28	60/14464	0.01666667	8.60952381	2.60121543	0.10997081	0.23504872	0.18077073	Fbn1	1
GO:0032981	mitochondrial respiratory chain complex I assembly	1/28	60/14464	0.01666667	8.60952381	2.60121543	0.10997081	0.23504872	0.18077073	Ndufaf2	1
GO:0034331	cell junction maintenance	1/28	60/14464	0.01666667	8.60952381	2.60121543	0.10997081	0.23504872	0.18077073	Kifc3	1
GO:0048662	negative regulation of smooth muscle cell proliferation	1/28	60/14464	0.01666667	8.60952381	2.60121543	0.10997081	0.23504872	0.18077073	Igfbp5	1
GO:0050873	brown fat cell differentiation	1/28	60/14464	0.01666667	8.60952381	2.60121543	0.10997081	0.23504872	0.18077073	Fbn1	1
GO:0051703	biological process involved in intraspecies interaction between organisms	1/28	60/14464	0.01666667	8.60952381	2.60121543	0.10997081	0.23504872	0.18077073	Cx3cr1	1
GO:0050727	regulation of inflammatory response	2/28	294/14464	0.00680272	3.51409135	1.91802634	0.11025419	0.23517929	0.18087115	Cx3cr1/Ccn3	2
GO:0055074	calcium ion homeostasis	2/28	295/14464	0.00677966	3.50217918	1.91224963	0.11087792	0.23541752	0.18105437	Gstm7/Kl	2

GO:0002931	response to ischemia	1/28	61/14464	0.01639344	8.46838407	2.57424486	0.11170094	0.23541752	0.18105437	Cx3cr1	1
GO:0034308	primary alcohol metabolic process	1/28	61/14464	0.01639344	8.46838407	2.57424486	0.11170094	0.23541752	0.18105437	Bmp6	1
GO:0061001	regulation of dendritic spine morphogenesis	1/28	61/14464	0.01639344	8.46838407	2.57424486	0.11170094	0.23541752	0.18105437	Bhlhb9	1
GO:1990868	response to chemokine	1/28	61/14464	0.01639344	8.46838407	2.57424486	0.11170094	0.23541752	0.18105437	Cx3cr1	1
GO:1990869	cellular response to chemokine	1/28	61/14464	0.01639344	8.46838407	2.57424486	0.11170094	0.23541752	0.18105437	Cx3cr1	1
GO:0031016	pancreas development	1/28	62/14464	0.01612903	8.33179724	2.54788405	0.11342783	0.2385818	0.18348794	Bmp6	1
GO:0006081	cellular aldehyde metabolic process	1/28	63/14464	0.01587302	8.19954649	2.52210906	0.11515148	0.23888288	0.18371949	Bmp6	1
GO:0007585	respiratory gaseous exchange by respiratory system	1/28	63/14464	0.01587302	8.19954649	2.52210906	0.11515148	0.23888288	0.18371949	Col6a1	1
GO:0045669	positive regulation of osteoblast differentiation	1/28	63/14464	0.01587302	8.19954649	2.52210906	0.11515148	0.23888288	0.18371949	Bmp6	1
GO:0050775	positive regulation of dendrite morphogenesis	1/28	63/14464	0.01587302	8.19954649	2.52210906	0.11515148	0.23888288	0.18371949	Bhlhb9	1
GO:0051932	synaptic transmission, GABAergic	1/28	63/14464	0.01587302	8.19954649	2.52210906	0.11515148	0.23888288	0.18371949	Kcnk2	1
GO:0061035	regulation of cartilage development	1/28	63/14464	0.01587302	8.19954649	2.52210906	0.11515148	0.23888288	0.18371949	Bmp6	1
GO:1901019	regulation of calcium ion transmembrane transporter activity	1/28	63/14464	0.01587302	8.19954649	2.52210906	0.11515148	0.23888288	0.18371949	Gstm7	1
GO:0001822	kidney development	2/28	303/14464	0.00660066	3.4097124	1.86691411	0.11590238	0.2396327	0.18429616	Fbn1/Bmp6	2
GO:0036293	response to decreased oxygen levels	2/28	304/14464	0.00657895	3.39849624	1.86135401	0.11653467	0.2396327	0.18429616	Col6a1/Kcnk2	2
GO:0050900	leukocyte migration	2/28	304/14464	0.00657895	3.39849624	1.86135401	0.11653467	0.2396327	0.18429616	Cx3cr1/Ccn3	2
GO:0010770	positive regulation of cell morphogenesis	1/28	64/14464	0.015625	8.07142857	2.49689726	0.1168719	0.2396327	0.18429616	Bhlhb9	1
GO:0045428	regulation of nitric oxide biosynthetic process	1/28	64/14464	0.015625	8.07142857	2.49689726	0.1168719	0.2396327	0.18429616	Cx3cr1	1
GO:2000179	positive regulation of neural precursor cell proliferation	1/28	64/14464	0.015625	8.07142857	2.49689726	0.1168719	0.2396327	0.18429616	Cx3cr1	1
GO:0051235	maintenance of location	2/28	307/14464	0.00651466	3.36528618	1.84481167	0.11843705	0.2412832	0.18556553	Fbn1/Gstm7	2
GO:0001910	regulation of leukocyte mediated cytotoxicity	1/28	65/14464	0.01538462	7.94725275	2.47222722	0.11858909	0.2412832	0.18556553	Cx3cr1	1
GO:0051926	negative regulation of calcium ion transport	1/28	65/14464	0.01538462	7.94725275	2.47222722	0.11858909	0.2412832	0.18556553	Gstm7	1
GO:0060999	positive regulation of dendritic spine development	1/28	65/14464	0.01538462	7.94725275	2.47222722	0.11858909	0.2412832	0.18556553	Bhlhb9	1
GO:0032102	negative regulation of response to external stimulus	2/28	308/14464	0.00649351	3.35435993	1.83934294	0.11907299	0.24180274	0.18596509	Cx3cr1/Ccn3	2
GO:0014911	positive regulation of smooth muscle cell migration	1/28	66/14464	0.01515152	7.82683983	2.44807866	0.12030307	0.24243933	0.18645468	Igfbp5	1
GO:0044091	membrane biogenesis	1/28	66/14464	0.01515152	7.82683983	2.44807866	0.12030307	0.24243933	0.18645468	Col6a1	1
GO:0098869	cellular oxidant detoxification	1/28	66/14464	0.01515152	7.82683983	2.44807866	0.12030307	0.24243933	0.18645468	Gstm7	1
GO:0140962	multicellular organismal-level chemical homeostasis	1/28	66/14464	0.01515152	7.82683983	2.44807866	0.12030307	0.24243933	0.18645468	Bmp6	1
GO:0001708	cell fate specification	1/28	67/14464	0.01492537	7.71002132	2.42443236	0.12201383	0.24310853	0.18696935	Fzd7	1
GO:0033273	response to vitamin	1/28	67/14464	0.01492537	7.71002132	2.42443236	0.12201383	0.24310853	0.18696935	Kl	1
GO:0045123	cellular extravasation	1/28	67/14464	0.01492537	7.71002132	2.42443236	0.12201383	0.24310853	0.18696935	Cx3cr1	1
GO:0050810	regulation of steroid biosynthetic process	1/28	67/14464	0.01492537	7.71002132	2.42443236	0.12201383	0.24310853	0.18696935	Bmp6	1
GO:0060612	adipose tissue development	1/28	67/14464	0.01492537	7.71002132	2.42443236	0.12201383	0.24310853	0.18696935	Col6a1	1
GO:0080164	regulation of nitric oxide metabolic process	1/28	67/14464	0.01492537	7.71002132	2.42443236	0.12201383	0.24310853	0.18696935	Cx3cr1	1
GO:0072001	renal system development	2/28	315/14464	0.00634921	3.27981859	1.80167921	0.12354949	0.2446677	0.18816847	Fbn1/Bmp6	2
GO:0007492	endoderm development	1/28	68/14464	0.01470588	7.59663866	2.40127005	0.12372138	0.2446677	0.18816847	Col6a1	1
GO:0050830	defense response to Gram-positive bacterium	1/28	68/14464	0.01470588	7.59663866	2.40127005	0.12372138	0.2446677	0.18816847	Gbp4	1
GO:0055021	regulation of cardiac muscle tissue growth	1/28	68/14464	0.01470588	7.59663866	2.40127005	0.12372138	0.2446677	0.18816847	Kcnk2	1
GO:0008347	glial cell migration	1/28	69/14464	0.01449275	7.48654244	2.37857444	0.12542573	0.24574152	0.18899432	Cx3cr1	1
GO:0008543	fibroblast growth factor receptor signaling pathway	1/28	69/14464	0.01449275	7.48654244	2.37857444	0.12542573	0.24574152	0.18899432	Kl	1
GO:0010761	fibroblast migration	1/28	69/14464	0.01449275	7.48654244	2.37857444	0.12542573	0.24574152	0.18899432	Ccn3	1
GO:0048844	artery morphogenesis	1/28	69/14464	0.01449275	7.48654244	2.37857444	0.12542573	0.24574152	0.18899432	Comp	1
GO:0071260	cellular response to mechanical stimulus	1/28	69/14464	0.01449275	7.48654244	2.37857444	0.12542573	0.24574152	0.18899432	Bmp6	1
GO:0001676	long-chain fatty acid metabolic process	1/28	70/14464	0.01428571	7.37959184	2.35632905	0.12712688	0.24815542	0.1908508	Gstm7	1
GO:0045670	regulation of osteoclast differentiation	1/28	70/14464	0.01428571	7.37959184	2.35632905	0.12712688	0.24815542	0.1908508	Fbn1	1
GO:0042060	wound healing	2/28	321/14464	0.00623053	3.21851357	1.77022556	0.12742014	0.24826982	0.19093878	Comp/Fzd7	2
GO:0034728	nucleosome organization	1/28	71/14464	0.01408451	7.27565392	2.33451823	0.12882484	0.24917126	0.19163206	H1f0	1
GO:0042306	regulation of protein import into nucleus	1/28	71/14464	0.01408451	7.27565392	2.33451823	0.12882484	0.24917126	0.19163206	Gbp4	1
GO:0071496	cellular response to external stimulus	1/28	71/14464	0.01408451	7.27565392	2.33451823	0.12882484	0.24917126	0.19163206	Bmp6	1
GO:1904705	regulation of vascular associated smooth muscle cell proliferation	1/28	71/14464	0.01408451	7.27565392	2.33451823	0.12882484	0.24917126	0.19163206	Igfbp5	1
GO:0015800	acidic amino acid transport	1/28	72/14464	0.01388889	7.17460317	2.3131271	0.13051961	0.25107228	0.19309409	Kcnk2	1
GO:0051591	response to cAMP	1/28	72/14464	0.01388889	7.17460317	2.3131271	0.13051961	0.25107228	0.19309409	Igfbp5	1
GO:0097194	execution phase of apoptosis	1/28	72/14464	0.01388889	7.17460317	2.3131271	0.13051961	0.25107228	0.19309409	Col6a1	1
GO:0070482	response to oxygen levels	2/28	327/14464	0.00611621	3.15945828	1.73950162	0.13132065	0.25215471	0.19392656	Col6a1/Kcnk2	2
GO:0060420	regulation of heart growth	1/28	73/14464	0.01369863	7.07632094	2.29214148	0.13221121	0.25294658	0.19453558	Kcnk2	1
GO:1902476	chloride transmembrane transport	1/28	73/14464	0.01369863	7.07632094	2.29214148	0.13221121	0.25294658	0.19453558	Kcnk2	1
GO:0015980	energy derivation by oxidation of organic compounds	2/28	330/14464	0.00606061	3.13073593	1.72440323	0.13328173	0.25342721	0.19490522	Col6a1/Kl	2
GO:0032651	regulation of interleukin-1 beta production	1/28	74/14464	0.01351351	6.98069498	2.27154784	0.13389963	0.25342721	0.19490522	Cx3cr1	1
GO:0034109	homotypic cell-cell adhesion	1/28	74/14464	0.01351351	6.98069498	2.27154784	0.13389963	0.25342721	0.19490522	Comp	1
GO:0051145	smooth muscle cell differentiation	1/28	74/14464	0.01351351	6.98069498	2.27154784	0.13389963	0.25342721	0.19490522	Comp	1
GO:0051279	regulation of release of sequestered calcium ion into cytosol	1/28	74/14464	0.01351351	6.98069498	2.27154784	0.13389963	0.25342721	0.19490522	Gstm7	1
GO:0051851	modulation by host of symbiont process	1/28	74/14464	0.01351351	6.98069498	2.27154784	0.13389963	0.25342721	0.19490522	Cx3cr1	1
GO:0001656	metanephros development	1/28	75/14464	0.01333333	6.88761905	2.25133332	0.13558489	0.25479363	0.1959561	Fbn1	1
GO:0014743	regulation of muscle hypertrophy	1/28	75/14464	0.01333333	6.88761905	2.25133332	0.13558489	0.25479363	0.1959561	Igfbp5	1
GO:0150076	neuroinflammatory response	1/28	75/14464	0.01333333	6.88761905	2.25133332	0.13558489	0.25479363	0.1959561	Cx3cr1	1
GO:1990874	vascular associated smooth muscle cell proliferation	1/28	75/14464	0.01333333	6.88761905	2.25133332	0.13558489	0.25479363	0.1959561	Igfbp5	1
GO:0006809	nitric oxide biosynthetic process	1/28	76/14464	0.01315789	6.79699248	2.23148561	0.13726698	0.25704153	0.19768491	Cx3cr1	1
GO:0050886	endocrine process	1/28	76/14464	0.01315789	6.79699248	2.23148561	0.13726698	0.25704153	0.19768491	Bmp6	1
GO:0032370	positive regulation of lipid transport	1/28	77/14464	0.01298701	6.70871985	2.21199298	0.13894592	0.25835638	0.19869613	Bmp6	1
GO:0032414	positive regulation of ion transmembrane transporter activity	1/28	77/14464	0.01298701	6.70871985	2.21199298	0.13894592	0.25835638	0.19869613	Gstm7	1
GO:0033559	unsaturated fatty acid metabolic process	1/28	77/14464	0.01298701	6.70871985	2.21199298	0.13894592	0.25835638	0.19869613	Gstm7	1
GO:0061178	regulation of insulin secretion involved in cellular response to glucose stimulus	1/28	77/14464	0.01298701	6.70871985	2.21199298	0.13894592	0.25835638	0.19869613	Ndufaf2	1
GO:0015711	organic anion transport	2/28	341/14464	0.0058651	3.02974445	1.67047448	0.14053152	0.25867253	0.19893927	Kcnk2/Slc2a10	2
GO:0007422	peripheral nervous system development	1/28	78/14464	0.01282051	6.62271062	2.19284421	0.1406217	0.25867253	0.19893927	Col6a1	1
GO:0031341	regulation of cell killing	1/28	78/14464	0.01282051	6.62271062	2.19284421	0.1406217	0.25867253	0.19893927	Cx3cr1	1
GO:0050906	detection of stimulus involved in sensory perception	1/28	78/14464	0.01282051	6.62271062	2.19284421	0.1406217	0.25867253	0.19893927	Kcnk2	1
GO:0060997	dendritic spine morphogenesis	1/28	78/14464	0.01282051	6.62271062	2.19284421	0.1406217	0.25867253	0.19893927	Bhlhb9	1
GO:0007405	neuroblast proliferation	1/28	79/14464	0.01265823	6.53887884	2.17402859	0.14229434	0.25867253	0.19893927	Cx3cr1	1
GO:0031397	negative regulation of protein ubiquitination	1/28	79/14464	0.01265823	6.53887884	2.17402859	0.14229434	0.25867253	0.19893927	Gbp4	1
GO:0032611	interleukin-1 beta production	1/28	79/14464	0.01265823	6.53887884	2.17402859	0.14229434	0.25867253	0.19893927	Cx3cr1	1
GO:0035914	skeletal muscle cell differentiation	1/28	79/14464	0.01265823	6.53887884	2.17402859	0.14229434	0.25867253	0.19893927	Col6a1	1
GO:0051965	positive regulation of synapse assembly	1/28	79/14464	0.01265823	6.53887884	2.17402859	0.14229434	0.25867253	0.19893927	Bhlhb9	1
GO:0071677	positive regulation of mononuclear cell migration	1/28	79/14464	0.01265823	6.53887884	2.17402859	0.14229434	0.25867253	0.19893927	Cx3cr1	1
GO:0097009	energy homeostasis	1/28	79/14464	0.01265823	6.53887884	2.17402859	0.14229434	0.25867253	0.19893927	Col6a1	1
GO:1904063	negative regulation of cation transmembrane transport	1/28	79/14464	0.01265823	6.53887884	2.17402859	0.14229434	0.25867253	0.19893927	Gstm7	1
GO:0050678	regulation of epithelial cell proliferation	2/28	346/14464	0.00578035	2.98596201	1.64667226	0.14385631	0.26059609	0.20041865	Bmp6/Fzd7	2
GO:0071230	cellular response to amino acid stimulus	1/28	80/14464	0.0125	6.45714286	2.15553586	0.14396384	0.26059609	0.20041865	Col6a1	1
GO:0009410	response to xenobiotic stimulus	2/28	348/14464	0.00574713	2.96880131	1.63727059	0.14519116	0.26059609	0.20041865	Gstm7/Col6a1	2
GO:0002690	positive regulation of leukocyte chemotaxis	1/28	81/14464	0.01234568	6.37742504	2.13735621	0.14563021	0.26059609	0.20041865	Cx3cr1	1
GO:0033077	T cell differentiation in thymus	1/28	81/14464	0.01234568	6.37742504	2.13735621	0.14563021	0.26059609	0.20041865	Fzd7	1
GO:0045638	negative regulation of myeloid cell differentiation	1/28	81/14464	0.01234568	6.37742504	2.13735621	0.14563021	0.26059609	0.20041865	Fbn1	1
GO:0045666	positive regulation of neuron differentiation	1/28	81/14464	0.01234568	6.37742504	2.13735621	0.14563021	0.26059609	0.20041865	Bmp6	1
GO:0046209	nitric oxide metabolic process	1/28	81/14464	0.01234568	6.37742504	2.13735621	0.14563021	0.26059609	0.20041865	Cx3cr1	1
GO:1904427	positive regulation of calcium ion transmembrane transport	1/28	81/14464	0.01234568	6.37742504	2.13735621	0.14563021	0.26059609	0.20041865	Gstm7	1
GO:0001654	eye development	2/28	351/14464	0.00569801	2.94342694	1.62329286	0.14719862	0.26059609	0.20041865	Fbn1/Bmp6	2
GO:0051962	positive regulation of nervous system development	2/28	351/14464	0.00569801	2.94342694	1.62329286	0.14719862	0.26059609	0.20041865	Cx3cr1/Bhlhb9	2
GO:0006029	proteoglycan metabolic process	1/28	82/14464	0.01219512	6.29965157	2.11948025	0.14729344	0.26059609	0.20041865	Slc2a10	1
GO:0006576	biogenic amine metabolic process	1/28	82/14464	0.01219512	6.29965157	2.11948025	0.14729344	0.26059609	0.20041865	Kl	1
GO:0014855	striated muscle cell proliferation	1/28	82/14464	0.01219512	6.29965157	2.11948025	0.14729344	0.26059609	0.20041865	Kcnk2	1
GO:0032411	positive regulation of transporter activity	1/28	82/14464	0.01219512	6.29965157	2.11948025	0.14729344	0.26059609	0.20041865	Gstm7	1
GO:1901222	regulation of non-canonical NF-kappaB signal transduction	1/28	82/14464	0.01219512	6.29965157	2.11948025	0.14729344	0.26059609	0.20041865	Ccn3	1
GO:0030510	regulation of BMP signaling pathway	1/28	83/14464	0.01204819	6.22375215	2.10189897	0.14895356	0.26208219	0.20156157	Fbn1	1
GO:0043648	dicarboxylic acid metabolic process	1/28	83/14464	0.01204819	6.22375215	2.10189897	0.14895356	0.26208219	0.20156157	Col6a1	1
GO:2001057	reactive nitrogen species metabolic process	1/28	83/14464	0.01204819	6.22375215	2.10189897	0.14895356	0.26208219	0.20156157	Cx3cr1	1
GO:0150063	visual system development	2/28	354/14464	0.00564972	2.91848265	1.60946216	0.14921216	0.26208219	0.20156157	Fbn1/Bmp6	2
GO:0006935	chemotaxis	2/28	356/14464	0.00561798	2.90208668	1.6003219	0.15055783	0.26208219	0.20156157	Cx3cr1/Ccn3	2
GO:0008593	regulation of Notch signaling pathway	1/28	84/14464	0.01190476	6.14965986	2.08460376	0.15061056	0.26208219	0.20156157	Ccn3	1
GO:0032526	response to retinoic acid	1/28	84/14464	0.01190476	6.14965986	2.08460376	0.15061056	0.26208219	0.20156157	Bmp6	1
GO:0034766	negative regulation of monoatomic ion transmembrane transport	1/28	84/14464	0.01190476	6.14965986	2.08460376	0.15061056	0.26208219	0.20156157	Gstm7	1
GO:0042157	lipoprotein metabolic process	1/28	84/14464	0.01190476	6.14965986	2.08460376	0.15061056	0.26208219	0.20156157	Col6a1	1
GO:0060998	regulation of dendritic spine development	1/28	84/14464	0.01190476	6.14965986	2.08460376	0.15061056	0.26208219	0.20156157	Bhlhb9	1
GO:0042330	taxis	2/28	357/14464	0.00560224	2.89395758	1.59577551	0.15123165	0.26273085	0.20206044	Cx3cr1/Ccn3	2
GO:0003073	regulation of systemic arterial blood pressure	1/28	85/14464	0.01176471	6.07731092	2.06758633	0.15226445	0.26365922	0.20277443	Kl	1
GO:0051262	protein tetramerization	1/28	85/14464	0.01176471	6.07731092	2.06758633	0.15226445	0.26365922	0.20277443	Col6a1	1
GO:0048880	sensory system development	2/28	359/14464	0.00557103	2.87783526	1.58672966	0.15258123	0.26377604	0.20286427	Fbn1/Bmp6	2
GO:0001938	positive regulation of endothelial cell proliferation	1/28	86/14464	0.01162791	6.00664452	2.05083875	0.15391523	0.26478425	0.20363966	Bmp6	1
GO:0006821	chloride transport	1/28	86/14464	0.01162791	6.00664452	2.05083875	0.15391523	0.26478425	0.20363966	Kcnk2	1
GO:0030168	platelet activation	1/28	86/14464	0.01162791	6.00664452	2.05083875	0.15391523	0.26478425	0.20363966	Comp	1
GO:0035773	insulin secretion involved in cellular response to glucose stimulus	1/28	87/14464	0.01149425	5.93760263	2.03435339	0.15556291	0.26588943	0.20448963	Ndufaf2	1
GO:0048477	oogenesis	1/28	87/14464	0.01149425	5.93760263	2.03435339	0.15556291	0.26588943	0.20448963	H3f3b	1
GO:0051881	regulation of mitochondrial membrane potential	1/28	87/14464	0.01149425	5.93760263	2.03435339	0.15556291	0.26588943	0.20448963	Col6a1	1
GO:0090288	negative regulation of cellular response to growth factor stimulus	1/28	87/14464	0.01149425	5.93760263	2.03435339	0.15556291	0.26588943	0.20448963	Fbn1	1
GO:0006835	dicarboxylic acid transport	1/28	88/14464	0.01136364	5.87012987	2.01812294	0.1572075	0.26674585	0.20514828	Kcnk2	1
GO:0032652	regulation of interleukin-1 production	1/28	88/14464	0.01136364	5.87012987	2.01812294	0.1572075	0.26674585	0.20514828	Cx3cr1	1
GO:0043954	cellular component maintenance	1/28	88/14464	0.01136364	5.87012987	2.01812294	0.1572075	0.26674585	0.20514828	Kifc3	1
GO:1990748	cellular detoxification	1/28	88/14464	0.01136364	5.87012987	2.01812294	0.1572075	0.26674585	0.20514828	Gstm7	1
GO:0042063	gliogenesis	2/28	366/14464	0.00546448	2.82279469	1.55555122	0.15732458	0.26674585	0.20514828	Cx3cr1/Col6a1	2
GO:0002275	myeloid cell activation involved in immune response	1/28	89/14464	0.01123596	5.80417335	2.00214035	0.158849	0.26718957	0.20548954	Cx3cr1	1
GO:0019218	regulation of steroid metabolic process	1/28	89/14464	0.01123596	5.80417335	2.00214035	0.158849	0.26718957	0.20548954	Bmp6	1
GO:0098661	inorganic anion transmembrane transport	1/28	89/14464	0.01123596	5.80417335	2.00214035	0.158849	0.26718957	0.20548954	Kcnk2	1
GO:1903321	negative regulation of protein modification by small protein conjugation or removal	1/28	89/14464	0.01123596	5.80417335	2.00214035	0.158849	0.26718957	0.20548954	Gbp4	1
GO:2001251	negative regulation of chromosome organization	1/28	89/14464	0.01123596	5.80417335	2.00214035	0.158849	0.26718957	0.20548954	H3f3b	1
GO:0019233	sensory perception of pain	1/28	90/14464	0.01111111	5.73968254	1.98639887	0.16048741	0.26866406	0.20662353	Ccn3	1
GO:0051101	regulation of DNA binding	1/28	90/14464	0.01111111	5.73968254	1.98639887	0.16048741	0.26866406	0.20662353	H1f0	1
GO:0051702	biological process involved in interaction with symbiont	1/28	90/14464	0.01111111	5.73968254	1.98639887	0.16048741	0.26866406	0.20662353	Cx3cr1	1
GO:0042310	vasoconstriction	1/28	91/14464	0.01098901	5.67660911	1.970892	0.16212275	0.2690261	0.20690198	Comp	1
GO:0061515	myeloid cell development	1/28	91/14464	0.01098901	5.67660911	1.970892	0.16212275	0.2690261	0.20690198	Fbn1	1
GO:0071229	cellular response to acid chemical	1/28	91/14464	0.01098901	5.67660911	1.970892	0.16212275	0.2690261	0.20690198	Col6a1	1
GO:0090398	cellular senescence	1/28	91/14464	0.01098901	5.67660911	1.970892	0.16212275	0.2690261	0.20690198	Comp	1
GO:2001257	regulation of cation channel activity	1/28	91/14464	0.01098901	5.67660911	1.970892	0.16212275	0.2690261	0.20690198	Gstm7	1
GO:0007631	feeding behavior	1/28	92/14464	0.01086957	5.61490683	1.95561347	0.16375502	0.2690261	0.20690198	Col6a1	1
GO:0009581	detection of external stimulus	1/28	92/14464	0.01086957	5.61490683	1.95561347	0.16375502	0.2690261	0.20690198	Kcnk2	1
GO:0009582	detection of abiotic stimulus	1/28	92/14464	0.01086957	5.61490683	1.95561347	0.16375502	0.2690261	0.20690198	Kcnk2	1
GO:0044344	cellular response to fibroblast growth factor stimulus	1/28	92/14464	0.01086957	5.61490683	1.95561347	0.16375502	0.2690261	0.20690198	Kl	1
GO:0046889	positive regulation of lipid biosynthetic process	1/28	92/14464	0.01086957	5.61490683	1.95561347	0.16375502	0.2690261	0.20690198	Bmp6	1
GO:0048678	response to axon injury	1/28	92/14464	0.01086957	5.61490683	1.95561347	0.16375502	0.2690261	0.20690198	Kcnk2	1
GO:0051899	membrane depolarization	1/28	92/14464	0.01086957	5.61490683	1.95561347	0.16375502	0.2690261	0.20690198	Col6a1	1
GO:0032612	interleukin-1 production	1/28	93/14464	0.01075269	5.55453149	1.94055725	0.16538422	0.27002547	0.20767057	Cx3cr1	1
GO:0042116	macrophage activation	1/28	93/14464	0.01075269	5.55453149	1.94055725	0.16538422	0.27002547	0.20767057	Cx3cr1	1
GO:0045185	maintenance of protein location	1/28	93/14464	0.01075269	5.55453149	1.94055725	0.16538422	0.27002547	0.20767057	Fbn1	1
GO:1901874	negative regulation of post-translational protein modification	1/28	93/14464	0.01075269	5.55453149	1.94055725	0.16538422	0.27002547	0.20767057	Gbp4	1
GO:0007229	integrin-mediated signaling pathway	1/28	94/14464	0.0106383	5.49544073	1.92571756	0.16701036	0.2714239	0.20874607	Slc2a10	1
GO:0009308	amine metabolic process	1/28	94/14464	0.0106383	5.49544073	1.92571756	0.16701036	0.2714239	0.20874607	Kl	1
GO:0034446	substrate adhesion-dependent cell spreading	1/28	94/14464	0.0106383	5.49544073	1.92571756	0.16701036	0.2714239	0.20874607	Fzd7	1
GO:1990542	mitochondrial transmembrane transport	1/28	95/14464	0.01052632	5.43759398	1.9110888	0.16863344	0.27364138	0.21045148	Col6a1	1
GO:0048814	regulation of dendrite morphogenesis	1/28	96/14464	0.01041667	5.38095238	1.89666557	0.17025347	0.27515164	0.21161299	Bhlhb9	1
GO:1902106	negative regulation of leukocyte differentiation	1/28	96/14464	0.01041667	5.38095238	1.89666557	0.17025347	0.27515164	0.21161299	Fbn1	1
GO:0016055	Wnt signaling pathway	2/28	385/14464	0.00519481	2.68348794	1.47448121	0.17034435	0.27515164	0.21161299	Fzd7/Col6a1	2
GO:0071346	cellular response to type II interferon	1/28	97/14464	0.01030928	5.32547865	1.8824427	0.17187046	0.27677161	0.21285888	Gbp4	1
GO:0098656	monoatomic anion transmembrane transport	1/28	97/14464	0.01030928	5.32547865	1.8824427	0.17187046	0.27677161	0.21285888	Kcnk2	1
GO:0001558	regulation of cell growth	2/28	389/14464	0.00514139	2.65589423	1.45803505	0.17311027	0.27810077	0.2138811	Igfbp5/Ccn3	2
GO:0071774	response to fibroblast growth factor	1/28	98/14464	0.01020408	5.27113703	1.86841516	0.17348441	0.27810077	0.2138811	Kl	1
GO:1905954	positive regulation of lipid localization	1/28	98/14464	0.01020408	5.27113703	1.86841516	0.17348441	0.27810077	0.2138811	Bmp6	1
GO:0002444	myeloid leukocyte mediated immunity	1/28	99/14464	0.01010101	5.21789322	1.85457813	0.17509533	0.27820239	0.21395926	Cx3cr1	1
GO:0006096	glycolytic process	1/28	99/14464	0.01010101	5.21789322	1.85457813	0.17509533	0.27820239	0.21395926	Col6a1	1
GO:0033108	mitochondrial respiratory chain complex assembly	1/28	99/14464	0.01010101	5.21789322	1.85457813	0.17509533	0.27820239	0.21395926	Ndufaf2	1

GO:0046620 regulation of organ growth	1/28	99/14464	0.01010101	5.21789322	1.85457813	0.17509533	0.27820239	0.21395926	Kcnk2	1
GO:0048640 negative regulation of developmental growth	1/28	99/14464	0.01010101	5.21789322	1.85457813	0.17509533	0.27820239	0.21395926	Kcnk2	1
GO:0002065 columnar/cuboidal epithelial cell differentiation	1/28	100/14464	0.01	5.16571429	1.84092694	0.17670322	0.27820239	0.21395926	Bmp6	1
GO:0002698 negative regulation of immune effector process	1/28	100/14464	0.01	5.16571429	1.84092694	0.17670322	0.27820239	0.21395926	Cx3cr1	1
GO:0008645 hexose transmembrane transport	1/28	100/14464	0.01	5.16571429	1.84092694	0.17670322	0.27820239	0.21395926	Slc2a10	1
GO:0014910 regulation of smooth muscle cell migration	1/28	100/14464	0.01	5.16571429	1.84092694	0.17670322	0.27820239	0.21395926	Igfbp5	1
GO:0035270 endocrine system development	1/28	100/14464	0.01	5.16571429	1.84092694	0.17670322	0.27820239	0.21395926	Bmp6	1
GO:0055017 cardiac muscle tissue growth	1/28	100/14464	0.01	5.16571429	1.84092694	0.17670322	0.27820239	0.21395926	Kcnk2	1
GO:1904659 D-glucose transmembrane transport	1/28	100/14464	0.01	5.16571429	1.84092694	0.17670322	0.27820239	0.21395926	Slc2a10	1
GO:0007200 phospholipase C-activating G protein-coupled receptor signaling pathway	1/28	101/14464	0.00990099	5.1145686	1.82745708	0.17830809	0.27989608	0.21526183	Cx3cr1	1
GO:0097237 cellular response to toxic substance	1/28	101/14464	0.00990099	5.1145686	1.82745708	0.17830809	0.27989608	0.21526183	Gstm7	1
GO:0001909 leukocyte mediated cytotoxicity	1/28	102/14464	0.00980392	5.06442577	1.8141642	0.17990994	0.28074442	0.21591427	Cx3cr1	1
GO:0006939 smooth muscle contraction	1/28	102/14464	0.00980392	5.06442577	1.8141642	0.17990994	0.28074442	0.21591427	Comp	1
GO:0015749 monosaccharide transmembrane transport	1/28	102/14464	0.00980392	5.06442577	1.8141642	0.17990994	0.28074442	0.21591427	Slc2a10	1
GO:1903707 negative regulation of hemopoiesis	1/28	102/14464	0.00980392	5.06442577	1.8141642	0.17990994	0.28074442	0.21591427	Fbn1	1
GO:0022411 cellular component disassembly	2/28	400/14464	0.005	2.58285714	1.41384584	0.18075716	0.28116586	0.2162384	Cx3cr1/Col6a1	2
GO:0033555 multicellular organismal response to stress	1/28	103/14464	0.00970874	5.01525659	1.8010441	0.18150878	0.28116586	0.2162384	Col6a1	1
GO:0043502 regulation of muscle adaptation	1/28	103/14464	0.00970874	5.01525659	1.8010441	0.18150878	0.28116586	0.2162384	Igfbp5	1
GO:0046032 ADP catabolic process	1/28	103/14464	0.00970874	5.01525659	1.8010441	0.18150878	0.28116586	0.2162384	Col6a1	1
GO:2000278 regulation of DNA biosynthetic process	1/28	103/14464	0.00970874	5.01525659	1.8010441	0.18150878	0.28116586	0.2162384	Kcnk2	1
GO:0014897 striated muscle hypertrophy	1/28	104/14464	0.00961538	4.96703297	1.7880927	0.18310461	0.28239749	0.21718561	Igfbp5	1
GO:0043279 response to alkaloid	1/28	104/14464	0.00961538	4.96703297	1.7880927	0.18310461	0.28239749	0.21718561	Gstm7	1
GO:0098754 detoxification	1/28	104/14464	0.00961538	4.96703297	1.7880927	0.18310461	0.28239749	0.21718561	Gstm7	1
GO:0002377 immunoglobulin production	1/28	105/14464	0.00952381	4.91972789	1.77530609	0.18469744	0.2827929	0.21748971	Fbn1	1
GO:0009137 purine nucleoside diphosphate catabolic process	1/28	105/14464	0.00952381	4.91972789	1.77530609	0.18469744	0.2827929	0.21748971	Col6a1	1
GO:0009181 purine ribonucleoside diphosphate catabolic process	1/28	105/14464	0.00952381	4.91972789	1.77530609	0.18469744	0.2827929	0.21748971	Col6a1	1
GO:0019364 pyridine nucleotide catabolic process	1/28	105/14464	0.00952381	4.91972789	1.77530609	0.18469744	0.2827929	0.21748971	Col6a1	1
GO:0038061 non-canonical NF-kappaB signal transduction	1/28	105/14464	0.00952381	4.91972789	1.77530609	0.18469744	0.2827929	0.21748971	Ccn3	1
GO:0014896 muscle hypertrophy	1/28	106/14464	0.00943396	4.87331536	1.76268045	0.18628728	0.28440395	0.21872874	Igfbp5	1
GO:0097061 dendritic spine organization	1/28	106/14464	0.00943396	4.87331536	1.76268045	0.18628728	0.28440395	0.21872874	Bhlhb9	1
GO:0032963 collagen metabolic process	1/28	107/14464	0.00934579	4.82777036	1.75021212	0.18787413	0.28559027	0.21964111	Col6a1	1
GO:0060419 heart growth	1/28	107/14464	0.00934579	4.82777036	1.75021212	0.18787413	0.28559027	0.21964111	Kcnk2	1
GO:0097250 mitochondrial respirasome assembly	1/28	107/14464	0.00934579	4.82777036	1.75021212	0.18787413	0.28559027	0.21964111	Ndufaf2	1
GO:0030316 osteoclast differentiation	1/28	108/14464	0.00925926	4.78306878	1.73789752	0.18945799	0.28676188	0.22054217	Fbn1	1
GO:0046031 ADP metabolic process	1/28	108/14464	0.00925926	4.78306878	1.73789752	0.18945799	0.28676188	0.22054217	Col6a1	1
GO:0072526 pyridine-containing compound catabolic process	1/28	108/14464	0.00925926	4.78306878	1.73789752	0.18945799	0.28676188	0.22054217	Col6a1	1
GO:0009191 ribonucleoside diphosphate catabolic process	1/28	109/14464	0.00917431	4.73918742	1.72573321	0.19103887	0.28832971	0.22174795	Col6a1	1
GO:0046683 response to organophosphorus	1/28	109/14464	0.00917431	4.73918742	1.72573321	0.19103887	0.28832971	0.22174795	Igfbp5	1
GO:0006986 response to unfolded protein	1/28	110/14464	0.00909091	4.6961039	1.71371585	0.19261678	0.28856018	0.2219252	Comp	1
GO:0034219 carbohydrate transmembrane transport	1/28	110/14464	0.00909091	4.6961039	1.71371585	0.19261678	0.28856018	0.2219252	Slc2a10	1
GO:0045446 endothelial cell differentiation	1/28	110/14464	0.00909091	4.6961039	1.71371585	0.19261678	0.28856018	0.2219252	Bmp6	1
GO:0050767 regulation of neurogenesis	2/28	418/14464	0.00478469	2.47163363	1.34460984	0.19338664	0.28856018	0.2219252	Cx3cr1/Bhlhb9	2
GO:0043410 positive regulation of MAPK cascade	2/28	419/14464	0.00477327	2.46573474	1.34086883	0.19409213	0.28856018	0.2219252	Fzd7/Kl	2
GO:0009134 nucleoside diphosphate catabolic process	1/28	111/14464	0.00900901	4.65379665	1.70184222	0.19419173	0.28856018	0.2219252	Col6a1	1
GO:0048661 positive regulation of smooth muscle cell proliferation	1/28	111/14464	0.00900901	4.65379665	1.70184222	0.19419173	0.28856018	0.2219252	Igfbp5	1
GO:0051928 positive regulation of calcium ion transport	1/28	111/14464	0.00900901	4.65379665	1.70184222	0.19419173	0.28856018	0.2219252	Gstm7	1
GO:0060291 long-term synaptic potentiation	1/28	111/14464	0.00900901	4.65379665	1.70184222	0.19419173	0.28856018	0.2219252	Cx3cr1	1
GO:0099565 chemical synaptic transmission, postsynaptic	1/28	111/14464	0.00900901	4.65379665	1.70184222	0.19419173	0.28856018	0.2219252	Kcnk2	1
GO:2000177 regulation of neural precursor cell proliferation	1/28	111/14464	0.00900901	4.65379665	1.70184222	0.19419173	0.28856018	0.2219252	Cx3cr1	1
GO:0010657 muscle cell apoptotic process	1/28	112/14464	0.00892857	4.6122449	1.69010917	0.19576371	0.29008124	0.22309501	Col6a1	1
GO:0051209 release of sequestered calcium ion into cytosol	1/28	112/14464	0.00892857	4.6122449	1.69010917	0.19576371	0.29008124	0.22309501	Gstm7	1
GO:0009135 purine nucleoside diphosphate metabolic process	1/28	113/14464	0.00884956	4.57142857	1.67851366	0.19733273	0.29118275	0.22394216	Col6a1	1
GO:0009179 purine ribonucleoside diphosphate metabolic process	1/28	113/14464	0.00884956	4.57142857	1.67851366	0.19733273	0.29118275	0.22394216	Col6a1	1
GO:0051283 negative regulation of sequestering of calcium ion	1/28	113/14464	0.00884956	4.57142857	1.67851366	0.19733273	0.29118275	0.22394216	Gstm7	1
GO:0008643 carbohydrate transport	1/28	114/14464	0.00877193	4.53132832	1.66705276	0.1988988	0.29267723	0.22509154	Slc2a10	1
GO:0034341 response to type II interferon	1/28	114/14464	0.00877193	4.53132832	1.66705276	0.1988988	0.29267723	0.22509154	Gbp4	1
GO:0043200 response to amino acid	1/28	115/14464	0.00869565	4.49192547	1.6557236	0.20046193	0.29415911	0.22623121	Col6a1	1
GO:0051282 regulation of sequestering of calcium ion	1/28	115/14464	0.00869565	4.49192547	1.6557236	0.20046193	0.29415911	0.22623121	Gstm7	1
GO:0034763 negative regulation of transmembrane transport	1/28	116/14464	0.00862069	4.45320197	1.64452342	0.20202211	0.29545536	0.22722813	Gstm7	1
GO:0051208 sequestering of calcium ion	1/28	116/14464	0.00862069	4.45320197	1.64452342	0.20202211	0.29545536	0.22722813	Gstm7	1
GO:0000018 regulation of DNA recombination	1/28	117/14464	0.00854701	4.41514042	1.63344952	0.20357936	0.29545536	0.22722813	H1f0	1
GO:0006820 monoatomic anion transport	1/28	117/14464	0.00854701	4.41514042	1.63344952	0.20357936	0.29545536	0.22722813	Kcnk2	1
GO:0008584 male gonad development	1/28	117/14464	0.00854701	4.41514042	1.63344952	0.20357936	0.29545536	0.22722813	H3f3b	1
GO:0015698 inorganic anion transport	1/28	117/14464	0.00854701	4.41514042	1.63344952	0.20357936	0.29545536	0.22722813	Kcnk2	1
GO:0017148 negative regulation of translation	1/28	117/14464	0.00854701	4.41514042	1.63344952	0.20357936	0.29545536	0.22722813	Igfbp5	1
GO:0106027 neuron projection organization	1/28	117/14464	0.00854701	4.41514042	1.63344952	0.20357936	0.29545536	0.22722813	Bhlhb9	1
GO:0032479 regulation of type I interferon production	1/28	118/14464	0.00847458	4.37772397	1.6224993	0.20513367	0.29649102	0.22802463	Gbp4	1
GO:0043271 negative regulation of monoatomic ion transport	1/28	118/14464	0.00847458	4.37772397	1.6224993	0.20513367	0.29649102	0.22802463	Gstm7	1
GO:0046546 development of primary male sexual characteristics	1/28	118/14464	0.00847458	4.37772397	1.6224993	0.20513367	0.29649102	0.22802463	H3f3b	1
GO:0042391 regulation of membrane potential	2/28	437/14464	0.00457666	2.3641713	1.27529894	0.20685191	0.2985666	0.22962091	Col6a1/Kcnk2	2
GO:0048706 embryonic skeletal system development	1/28	120/14464	0.00833333	4.3047619	1.60095982	0.20823353	0.30015133	0.2308397	Slc2a10	1
GO:0002761 regulation of myeloid leukocyte differentiation	1/28	121/14464	0.00826446	4.26918536	1.59036571	0.20977909	0.30114827	0.23160642	Fbn1	1
GO:0046165 alcohol biosynthetic process	1/28	121/14464	0.00826446	4.26918536	1.59036571	0.20977909	0.30114827	0.23160642	Bmp6	1
GO:1901890 positive regulation of cell junction assembly	1/28	121/14464	0.00826446	4.26918536	1.59036571	0.20977909	0.30114827	0.23160642	Bhlhb9	1
GO:0051052 regulation of DNA metabolic process	2/28	443/14464	0.00451467	2.33215092	1.25415314	0.21112858	0.3013186	0.23173741	H1f0/Kcnk2	2
GO:0003231 cardiac ventricle development	1/28	122/14464	0.00819672	4.23419204	1.57988557	0.21132174	0.3013186	0.23173741	Kcnk2	1
GO:0030879 mammary gland development	1/28	122/14464	0.00819672	4.23419204	1.57988557	0.21132174	0.3013186	0.23173741	Igfbp5	1
GO:0032606 type I interferon production	1/28	122/14464	0.00819672	4.23419204	1.57988557	0.21132174	0.3013186	0.23173741	Gbp4	1
GO:0045834 positive regulation of lipid metabolic process	1/28	122/14464	0.00819672	4.23419204	1.57988557	0.21132174	0.3013186	0.23173741	Bmp6	1
GO:0006633 fatty acid biosynthetic process	1/28	123/14464	0.00813008	4.19976771	1.56951714	0.21286148	0.30229187	0.23248594	Gstm7	1
GO:0032368 regulation of lipid transport	1/28	123/14464	0.00813008	4.19976771	1.56951714	0.21286148	0.30229187	0.23248594	Bmp6	1
GO:0051384 response to glucocorticoid	1/28	123/14464	0.00813008	4.19976771	1.56951714	0.21286148	0.30229187	0.23248594	Bmp6	1
GO:0002831 regulation of response to biotic stimulus	2/28	446/14464	0.0044843	2.31646381	1.24370705	0.21327085	0.30244457	0.23260337	Bmp6/Gbp4	2
GO:0030003 intracellular monoatomic cation homeostasis	2/28	447/14464	0.00447427	2.31128156	1.24024347	0.2139855	0.30244457	0.23260337	Gstm7/Bmp6	2
GO:0008286 insulin receptor signaling pathway	1/28	124/14464	0.00806452	4.16589862	1.55925822	0.21439832	0.30244457	0.23260337	Col6a1	1
GO:0009185 ribonucleoside diphosphate metabolic process	1/28	124/14464	0.00806452	4.16589862	1.55925822	0.21439832	0.30244457	0.23260337	Col6a1	1
GO:0071466 cellular response to xenobiotic stimulus	1/28	124/14464	0.00806452	4.16589862	1.55925822	0.21439832	0.30244457	0.23260337	Gstm7	1
GO:0006091 generation of precursor metabolites and energy	2/28	449/14464	0.00445434	2.30098632	1.2333437	0.21541563	0.30347501	0.23339586	Col6a1/Kl	2
GO:0006694 steroid biosynthetic process	1/28	126/14464	0.00793651	4.09977324	1.53906046	0.21746334	0.3052665	0.23477366	Bmp6	1
GO:0043500 muscle adaptation	1/28	126/14464	0.00793651	4.09977324	1.53906046	0.21746334	0.3052665	0.23477366	Igfbp5	1
GO:0001936 regulation of endothelial cell proliferation	1/28	127/14464	0.00787402	4.06749156	1.52911753	0.21899151	0.3052665	0.23477366	Bmp6	1
GO:0010565 regulation of cellular ketone metabolic process	1/28	127/14464	0.00787402	4.06749156	1.52911753	0.21899151	0.3052665	0.23477366	Bmp6	1
GO:0035966 response to topologically incorrect protein	1/28	127/14464	0.00787402	4.06749156	1.52911753	0.21899151	0.3052665	0.23477366	Comp	1
GO:0046822 regulation of nucleocytoplasmic transport	1/28	127/14464	0.00787402	4.06749156	1.52911753	0.21899151	0.3052665	0.23477366	Gbp4	1
GO:0050921 positive regulation of chemotaxis	1/28	127/14464	0.00787402	4.06749156	1.52911753	0.21899151	0.3052665	0.23477366	Cx3cr1	1
GO:0006873 intracellular monoatomic ion homeostasis	2/28	454/14464	0.00440529	2.27564506	1.21625176	0.21899553	0.3052665	0.23477366	Gstm7/Bmp6	2
GO:0022612 gland morphogenesis	1/28	128/14464	0.0078125	4.03571429	1.51927592	0.22051682	0.30657923	0.23578325	Igfbp5	1
GO:0030856 regulation of epithelial cell differentiation	1/28	128/14464	0.0078125	4.03571429	1.51927592	0.22051682	0.30657923	0.23578325	Bmp6	1
GO:0006090 pyruvate metabolic process	1/28	129/14464	0.00775194	4.00442968	1.50953373	0.22203924	0.30788666	0.23678876	Col6a1	1
GO:0009132 nucleoside diphosphate metabolic process	1/28	129/14464	0.00775194	4.00442968	1.50953373	0.22203924	0.30788666	0.23678876	Col6a1	1
GO:0071407 cellular response to organic cyclic compound	2/28	462/14464	0.004329	2.23623995	1.18936046	0.22473615	0.31087452	0.23908666	Gstm7/Igfbp5	2
GO:0007030 Golgi organization	1/28	131/14464	0.00763359	3.94329335	1.49034021	0.2250755	0.31087452	0.23908666	Kifc3	1
GO:0009154 purine ribonucleotide catabolic process	1/28	131/14464	0.00763359	3.94329335	1.49034021	0.2250755	0.31087452	0.23908666	Col6a1	1
GO:0001101 response to acid chemical	1/28	132/14464	0.00757576	3.91341991	1.4808853	0.22658934	0.3125574	0.24038093	Col6a1	1
GO:0007005 mitochondrion organization	2/28	466/14464	0.00429185	2.21704476	1.17611905	0.22761189	0.31260382	0.24041663	Col6a1/Ndufaf2	2
GO:0002687 positive regulation of leukocyte migration	1/28	133/14464	0.0075188	3.8839957	1.47152266	0.22810033	0.31260382	0.24041663	Cx3cr1	1
GO:0007292 female gamete generation	1/28	133/14464	0.0075188	3.8839957	1.47152266	0.22810033	0.31260382	0.24041663	H3f3b	1
GO:0050731 positive regulation of peptidyl-tyrosine phosphorylation	1/28	133/14464	0.0075188	3.8839957	1.47152266	0.22810033	0.31260382	0.24041663	Bmp6	1
GO:0055007 cardiac muscle cell differentiation	1/28	133/14464	0.0075188	3.8839957	1.47152266	0.22810033	0.31260382	0.24041663	Fzd7	1
GO:0003158 endothelium development	1/28	134/14464	0.00746269	3.85501066	1.46225061	0.22960848	0.3134526	0.24106941	Bmp6	1
GO:0045598 regulation of fat cell differentiation	1/28	134/14464	0.00746269	3.85501066	1.46225061	0.22960848	0.3134526	0.24106941	Fbn1	1
GO:0060996 dendritic spine development	1/28	134/14464	0.00746269	3.85501066	1.46225061	0.22960848	0.3134526	0.24106941	Bhlhb9	1
GO:0044283 small molecule biosynthetic process	2/28	470/14464	0.00425532	2.19817629	1.16301011	0.23049095	0.31425184	0.24168408	Gstm7/Bmp6	2
GO:0050773 regulation of dendrite development	1/28	136/14464	0.00735294	3.79831933	1.4439718	0.23261624	0.31597707	0.24301092	Bhlhb9	1
GO:1904064 positive regulation of cation transmembrane transport	1/28	136/14464	0.00735294	3.79831933	1.4439718	0.23261624	0.31597707	0.24301092	Gstm7	1
GO:0010720 positive regulation of cell development	2/28	473/14464	0.00422833	2.18423437	1.15326371	0.2326523	0.31597707	0.24301092	Cx3cr1/Bhlhb9	2
GO:0016049 cell growth	2/28	476/14464	0.00420168	2.17046819	1.14358921	0.23481532	0.31836298	0.24484587	Igfbp5/Ccn3	2
GO:0031960 response to corticosteroid	1/28	138/14464	0.00724638	3.74327122	1.42603633	0.23561268	0.31836298	0.24484587	Bmp6	1
GO:0051099 positive regulation of binding	1/28	138/14464	0.00724638	3.74327122	1.42603633	0.23561268	0.31836298	0.24484587	H1f0	1
GO:0051606 detection of stimulus	1/28	138/14464	0.00724638	3.74327122	1.42603633	0.23561268	0.31836298	0.24484587	Kcnk2	1
GO:0006997 nucleus organization	1/28	139/14464	0.00719424	3.71634121	1.41719359	0.23710667	0.31875331	0.24514606	H3f3b	1
GO:0007584 response to nutrient	1/28	139/14464	0.00719424	3.71634121	1.41719359	0.23710667	0.31875331	0.24514606	Kl	1
GO:0035107 appendage morphogenesis	1/28	139/14464	0.00719424	3.71634121	1.41719359	0.23710667	0.31875331	0.24514606	Col6a1	1
GO:0035108 limb morphogenesis	1/28	139/14464	0.00719424	3.71634121	1.41719359	0.23710667	0.31875331	0.24514606	Col6a1	1
GO:0007204 positive regulation of cytosolic calcium ion concentration	1/28	140/14464	0.00714286	3.68979592	1.40843226	0.23859784	0.31913592	0.24544032	Cx3cr1	1
GO:0009261 ribonucleotide catabolic process	1/28	140/14464	0.00714286	3.68979592	1.40843226	0.23859784	0.31913592	0.24544032	Col6a1	1
GO:0021782 glial cell development	1/28	140/14464	0.00714286	3.68979592	1.40843226	0.23859784	0.31913592	0.24544032	Col6a1	1
GO:0071456 cellular response to hypoxia	1/28	140/14464	0.00714286	3.68979592	1.40843226	0.23859784	0.31913592	0.24544032	Kcnk2	1
GO:0006195 purine nucleotide catabolic process	1/28	141/14464	0.0070922	3.66362715	1.39975093	0.2400862	0.32031677	0.24634848	Col6a1	1
GO:0031644 regulation of nervous system process	1/28	141/14464	0.0070922	3.66362715	1.39975093	0.2400862	0.32031677	0.24634848	Ccn3	1
GO:0048592 eye morphogenesis	1/28	142/14464	0.00704225	3.63782696	1.39114825	0.24157175	0.32189284	0.2475606	Fbn1	1
GO:0007009 plasma membrane organization	1/28	143/14464	0.00699301	3.61238761	1.38262286	0.2430545	0.32305486	0.24845429	Col6a1	1
GO:0043123 positive regulation of canonical NF-kappaB signal transduction	1/28	143/14464	0.00699301	3.61238761	1.38262286	0.2430545	0.32305486	0.24845429	Cx3cr1	1
GO:0007596 blood coagulation	1/28	145/14464	0.00689655	3.56256158	1.36579884	0.24601163	0.32657504	0.25116158	Comp	1
GO:0007286 spermatid development	1/28	146/14464	0.00684932	3.53816047	1.35749768	0.24748601	0.32770989	0.25203437	H3f3b	1
GO:1900180 regulation of protein localization to nucleus	1/28	146/14464	0.00684932	3.53816047	1.35749768	0.24748601	0.32770989	0.25203437	Gbp4	1
GO:0001935 endothelial cell proliferation	1/28	148/14464	0.00675676	3.49034749	1.34111103	0.25042644	0.32992757	0.25373994	Bmp6	1
GO:0007599 hemostasis	1/28	148/14464	0.00675676	3.49034749	1.34111103	0.25042644	0.32992757	0.25373994	Comp	1
GO:0009411 response to UV	1/28	148/14464	0.00675676	3.49034749	1.34111103	0.25042644	0.32992757	0.25373994	Col6a1	1
GO:0045766 positive regulation of angiogenesis	1/28	148/14464	0.00675676	3.49034749	1.34111103	0.25042644	0.32992757	0.25373994	Cx3cr1	1
GO:0051604 protein maturation	2/28	498/14464	0.00401606	2.07458405	1.07474099	0.25072001	0.32992757	0.25373994	Comp/Telo2	2
GO:0032200 telomere organization	1/28	149/14464	0.00671141	3.46692234	1.3330232	0.2518925	0.33064797	0.25429398	H3f3b	1
GO:0036294 cellular response to decreased oxygen levels	1/28	149/14464	0.00671141	3.46692234	1.3330232	0.2518925	0.33064797	0.25429398	Kcnk2	1
GO:0050817 coagulation	1/28	150/14464	0.00666667	3.44380952	1.32500418	0.25335579	0.33215666	0.25545428	Comp	1
GO:1904018 positive regulation of vasculature development	1/28	151/14464	0.00662252	3.42100284	1.31705287	0.25481632	0.333658	0.25660893	Cx3cr1	1
GO:1905952 regulation of lipid localization	1/28	152/14464	0.00657895	3.39849624	1.30916819	0.25627409	0.33515203	0.25775795	Bmp6	1
GO:0034767 positive regulation of monoatomic ion transmembrane transport	1/28	153/14464	0.00653595	3.37628385	1.30134909	0.25772912	0.33604647	0.25844585	Gstm7	1
GO:0046887 positive regulation of hormone secretion	1/28	153/14464	0.00653595	3.37628385	1.30134909	0.25772912	0.33604647	0.25844585	Bmp6	1
GO:0035150 regulation of tube size	1/28	154/14464	0.00649351	3.35435993	1.29359454	0.2591814	0.33604647	0.25844585	Comp	1
GO:0035296 regulation of tube diameter	1/28	154/14464	0.00649351	3.35435993	1.29359454	0.2591814	0.33604647	0.25844585	Comp	1
GO:0048515 spermatid differentiation	1/28	154/14464	0.00649351	3.35435993	1.29359454	0.2591814	0.33604647	0.25844585	H3f3b	1
GO:0062013 positive regulation of small molecule metabolic process	1/28	154/14464	0.00649351	3.35435993	1.29359454	0.2591814	0.33604647	0.25844585	Bmp6	1
GO:0097746 blood vessel diameter maintenance	1/28	154/14464	0.00649351	3.35435993	1.29359454	0.2591814	0.33604647	0.25844585	Comp	1
GO:0001508 action potential	1/28	155/14464	0.00645161	3.33271889	1.28590352	0.26063094	0.33709967	0.25925584	Kcnk2	1
GO:0071333 cellular response to glucose stimulus	1/28	155/14464	0.00645161	3.33271889	1.28590352	0.26063094	0.33709967	0.25925584	Ndufaf2	1
GO:0001906 cell killing	1/28	156/14464	0.00641026	3.31135531	1.27827507	0.26207774	0.33814421	0.26005917	Cx3cr1	1
GO:0072330 monocarboxylic acid biosynthetic process	1/28	156/14464	0.00641026	3.31135531	1.27827507	0.26207774	0.33814421	0.26005917	Gstm7	1
GO:0072523 purine-containing compound catabolic process	1/28	157/14464	0.00636943	3.29026388	1.2707082	0.26352182	0.33959328	0.26117362	Col6a1	1
GO:0006865 amino acid transport	1/28	158/14464	0.00632911	3.26943942	1.26320199	0.26496317	0.34020756	0.26164605	Kcnk2	1
GO:0009166 nucleotide catabolic process	1/28	158/14464	0.00632911	3.26943942	1.26320199	0.26496317	0.34020756	0.26164605	Col6a1	1
GO:0071331 cellular response to hexose stimulus	1/28	158/14464	0.00632911	3.26943942	1.26320199	0.26496317	0.34020756	0.26164605	Ndufaf2	1
GO:0071326 cellular response to monosaccharide stimulus	1/28	159/14464	0.00628931	3.24887691	1.2557555	0.2664018	0.34081391	0.26211238	Ndufaf2	1
GO:0071897 DNA biosynthetic process	1/28	159/14464	0.00628931	3.24887691	1.2557555	0.2664018	0.34081391	0.26211238	Kcnk2	1
GO:0097553 calcium ion transmembrane import into cytosol	1/28	159/14464	0.00628931	3.24887691	1.2557555	0.2664018	0.34081391	0.26211238	Gstm7	1
GO:0002181 cytoplasmic translation	1/28	160/14464	0.00625	3.22857143	1.24836784	0.26783771	0.34182424	0.26288941	Rpl8	1
GO:0071356 cellular response to tumor necrosis factor	1/28	160/14464	0.00625	3.22857143	1.24836784	0.26783771	0.34182424	0.26288941	Cx3cr1	1

GO:0008217 regulation of blood pressure	1/28	161/14464	0.00621118	3.20851819	1.24103812	0.26927092	0.34241422	0.26334314	Kl	1
GO:0032680 regulation of tumor necrosis factor production	1/28	161/14464	0.00621118	3.20851819	1.24103812	0.26927092	0.34241422	0.26334314	Cx3cr1	1
GO:0033157 regulation of intracellular protein transport	1/28	161/14464	0.00621118	3.20851819	1.24103812	0.26927092	0.34241422	0.26334314	Gbp4	1
GO:0003205 cardiac chamber development	1/28	162/14464	0.00617284	3.18871252	1.23376548	0.27070141	0.34340779	0.26410727	Kcnk2	1
GO:0007219 Notch signaling pathway	1/28	162/14464	0.00617284	3.18871252	1.23376548	0.27070141	0.34340779	0.26410727	Ccn3	1
GO:0006606 protein import into nucleus	1/28	163/14464	0.00613497	3.16914987	1.22654908	0.27212921	0.34439319	0.26486512	Gbp4	1
GO:1903555 regulation of tumor necrosis factor superfamily cytokine production	1/28	163/14464	0.00613497	3.16914987	1.22654908	0.27212921	0.34439319	0.26486512	Cx3cr1	1
GO:0035051 cardiocyte differentiation	1/28	165/14464	0.00606061	3.13073593	1.21228168	0.27497672	0.34716632	0.26699787	Fzd7	1
GO:0071453 cellular response to oxygen levels	1/28	165/14464	0.00606061	3.13073593	1.21228168	0.27497672	0.34716632	0.26699787	Kcnk2	1
GO:0006457 protein folding	1/28	166/14464	0.0060241	3.11187608	1.20522908	0.27639645	0.34812791	0.26773741	Telo2	1
GO:0032640 tumor necrosis factor production	1/28	166/14464	0.0060241	3.11187608	1.20522908	0.27639645	0.34812791	0.26773741	Cx3cr1	1
GO:0071248 cellular response to metal ion	1/28	167/14464	0.00598802	3.09324209	1.1982295	0.27781349	0.34866747	0.26815238	Bmp6	1
GO:0098739 import across plasma membrane	1/28	167/14464	0.00598802	3.09324209	1.1982295	0.27781349	0.34866747	0.26815238	Slc2a10	1
GO:1903169 regulation of calcium ion transmembrane transport	1/28	167/14464	0.00598802	3.09324209	1.1982295	0.27781349	0.34866747	0.26815238	Gstm7	1
GO:0016052 carbohydrate catabolic process	1/28	168/14464	0.00595238	3.07482993	1.19128219	0.27922786	0.34961311	0.26887965	Col6a1	1
GO:0071706 tumor necrosis factor superfamily cytokine production	1/28	168/14464	0.00595238	3.07482993	1.19128219	0.27922786	0.34961311	0.26887965	Cx3cr1	1
GO:0019362 pyridine nucleotide metabolic process	1/28	169/14464	0.00591716	3.05663567	1.18438639	0.28063956	0.35013756	0.26928299	Col6a1	1
GO:0046496 nicotinamide nucleotide metabolic process	1/28	169/14464	0.00591716	3.05663567	1.18438639	0.28063956	0.35013756	0.26928299	Col6a1	1
GO:0051170 import into nucleus	1/28	169/14464	0.00591716	3.05663567	1.18438639	0.28063956	0.35013756	0.26928299	Gbp4	1
GO:0046890 regulation of lipid biosynthetic process	1/28	170/14464	0.00588235	3.03865546	1.17754138	0.28204859	0.35116551	0.27007356	Bmp6	1
GO:0001678 intracellular glucose homeostasis	1/28	171/14464	0.00584795	3.02088555	1.17074645	0.28345496	0.35116551	0.27007356	Ndufaf2	1
GO:0022408 negative regulation of cell-cell adhesion	1/28	171/14464	0.00584795	3.02088555	1.17074645	0.28345496	0.35116551	0.27007356	Bmp6	1
GO:0030308 negative regulation of cell growth	1/28	171/14464	0.00584795	3.02088555	1.17074645	0.28345496	0.35116551	0.27007356	Ccn3	1
GO:0042552 myelination	1/28	171/14464	0.00584795	3.02088555	1.17074645	0.28345496	0.35116551	0.27007356	Col6a1	1
GO:0071322 cellular response to carbohydrate stimulus	1/28	171/14464	0.00584795	3.02088555	1.17074645	0.28345496	0.35116551	0.27007356	Ndufaf2	1
GO:0048660 regulation of smooth muscle cell proliferation	1/28	172/14464	0.00581395	3.00332226	1.16400088	0.28485867	0.35249178	0.27109357	Igfbp5	1
GO:0007272 ensheathment of neurons	1/28	174/14464	0.00574713	2.96880131	1.15065511	0.28765814	0.35512522	0.27311889	Col6a1	1
GO:0008366 axon ensheathment	1/28	174/14464	0.00574713	2.96880131	1.15065511	0.28765814	0.35512522	0.27311889	Col6a1	1
GO:0006839 mitochondrial transport	1/28	175/14464	0.00571429	2.95183673	1.14405358	0.28905392	0.35643245	0.27412425	Col6a1	1
GO:0072524 pyridine-containing compound metabolic process	1/28	176/14464	0.00568182	2.93506494	1.13749875	0.29044705	0.35731742	0.27480486	Col6a1	1
GO:1901292 nucleoside phosphate catabolic process	1/28	176/14464	0.00568182	2.93506494	1.13749875	0.29044705	0.35731742	0.27480486	Col6a1	1
GO:0032412 regulation of monoatomic ion transmembrane transporter activity	1/28	177/14464	0.00564972	2.91848265	1.13098998	0.29183755	0.35861107	0.27579978	Gstm7	1
GO:0019827 stem cell population maintenance	1/28	179/14464	0.00558659	2.8858739	1.11810816	0.29461068	0.36159872	0.27809751	Fzd7	1
GO:0009060 aerobic respiration	1/28	180/14464	0.00555556	2.86984127	1.11173389	0.29599331	0.36245477	0.27875588	Col6a1	1
GO:0034612 response to tumor necrosis factor	1/28	180/14464	0.00555556	2.86984127	1.11173389	0.29599331	0.36245477	0.27875588	Cx3cr1	1
GO:0019722 calcium-mediated signaling	1/28	181/14464	0.00552486	2.85398579	1.10540327	0.29737333	0.36288464	0.27908648	Cx3cr1	1
GO:0048839 inner ear development	1/28	181/14464	0.00552486	2.85398579	1.10540327	0.29737333	0.36288464	0.27908648	Kcnk2	1
GO:0051260 protein homooligomerization	1/28	181/14464	0.00552486	2.85398579	1.10540327	0.29737333	0.36288464	0.27908648	Comp	1
GO:0042742 defense response to bacterium	1/28	182/14464	0.00549451	2.83830455	1.09911571	0.29875074	0.36372644	0.2797339	Gbp4	1
GO:0071805 potassium ion transmembrane transport	1/28	182/14464	0.00549451	2.83830455	1.09911571	0.29875074	0.36372644	0.2797339	Kcnk2	1
GO:0042180 cellular ketone metabolic process	1/28	183/14464	0.00546448	2.82279469	1.09287065	0.30012554	0.36498026	0.28069818	Bmp6	1
GO:0007519 skeletal muscle tissue development	1/28	184/14464	0.00543478	2.80745342	1.08666754	0.30149775	0.36622803	0.28165782	Col6a1	1
GO:0045664 regulation of neuron differentiation	1/28	185/14464	0.00540541	2.79227799	1.08050582	0.30286736	0.36704888	0.28228911	Bmp6	1
GO:0098727 maintenance of cell number	1/28	185/14464	0.00540541	2.79227799	1.08050582	0.30286736	0.36704888	0.28228911	Fzd7	1
GO:0022898 regulation of transmembrane transporter activity	1/28	186/14464	0.00537634	2.77726575	1.07438497	0.30423438	0.3674429	0.28259214	Gstm7	1
GO:0048813 dendrite morphogenesis	1/28	186/14464	0.00537634	2.77726575	1.07438497	0.30423438	0.3674429	0.28259214	Bhlhb9	1
GO:0099175 regulation of postsynapse organization	1/28	186/14464	0.00537634	2.77726575	1.07438497	0.30423438	0.3674429	0.28259214	Bhlhb9	1
GO:0050905 neuromuscular process	1/28	188/14464	0.00531915	2.74772036	1.06226378	0.30696068	0.36989111	0.28447501	Comp	1
GO:0055002 striated muscle cell development	1/28	188/14464	0.00531915	2.74772036	1.06226378	0.30696068	0.36989111	0.28447501	Col6a1	1
GO:0050806 positive regulation of synaptic transmission	1/28	189/14464	0.00529101	2.73318216	1.05626241	0.30831996	0.37068468	0.28508532	Cx3cr1	1
GO:0061351 neural precursor cell proliferation	1/28	189/14464	0.00529101	2.73318216	1.05626241	0.30831996	0.37068468	0.28508532	Cx3cr1	1
GO:0050730 regulation of peptidyl-tyrosine phosphorylation	1/28	191/14464	0.0052356	2.70456245	1.04437566	0.31103081	0.37351941	0.28726545	Bmp6	1
GO:0045637 regulation of myeloid cell differentiation	1/28	194/14464	0.00515464	2.66273932	1.02682827	0.3150779	0.37795058	0.29067337	Fbn1	1
GO:0008361 regulation of cell size	1/28	195/14464	0.00512821	2.64908425	1.02105269	0.31642182	0.37870394	0.29125276	Col6a1	1
GO:0045165 cell fate commitment	1/28	195/14464	0.00512821	2.64908425	1.02105269	0.31642182	0.37870394	0.29125276	Fzd7	1
GO:0032409 regulation of transporter activity	1/28	196/14464	0.00510204	2.63556851	1.01531312	0.3177632	0.37933704	0.29173966	Gstm7	1
GO:0002703 regulation of leukocyte mediated immunity	1/28	197/14464	0.00507614	2.62218999	1.00960914	0.31910205	0.37933704	0.29173966	Cx3cr1	1
GO:0006006 glucose metabolic process	1/28	197/14464	0.00507614	2.62218999	1.00960914	0.31910205	0.37933704	0.29173966	Fbn1	1
GO:0050821 protein stabilization	1/28	197/14464	0.00507614	2.62218999	1.00960914	0.31910205	0.37933704	0.29173966	Telo2	1
GO:0051963 regulation of synapse assembly	1/28	197/14464	0.00507614	2.62218999	1.00960914	0.31910205	0.37933704	0.29173966	Bhlhb9	1
GO:0060538 skeletal muscle organ development	1/28	197/14464	0.00507614	2.62218999	1.00960914	0.31910205	0.37933704	0.29173966	Col6a1	1
GO:0002064 epithelial cell development	1/28	198/14464	0.00505051	2.60894661	1.00394031	0.32043836	0.38049807	0.29263258	Bmp6	1
GO:0008406 gonad development	1/28	200/14464	0.005	2.58285714	0.99270641	0.3231034	0.3832325	0.29473557	H3f3b	1
GO:0000302 response to reactive oxygen species	1/28	201/14464	0.00497512	2.57000711	0.98714052	0.32443213	0.38394765	0.29528558	Col6a1	1
GO:0009749 response to glucose	1/28	201/14464	0.00497512	2.57000711	0.98714052	0.32443213	0.38394765	0.29528558	Ndufaf2	1
GO:0045137 development of primary sexual characteristics	1/28	203/14464	0.00492611	2.54468684	0.97610884	0.32708207	0.3866512	0.29736482	H3f3b	1
GO:0007623 circadian rhythm	1/28	204/14464	0.00490196	2.53221289	0.97064228	0.32840327	0.38734745	0.29790029	Col6a1	1
GO:0031396 regulation of protein ubiquitination	1/28	204/14464	0.00490196	2.53221289	0.97064228	0.32840327	0.38734745	0.29790029	Gbp4	1
GO:0009746 response to hexose	1/28	205/14464	0.00487805	2.51986063	0.96520804	0.32972197	0.38803765	0.29843111	Ndufaf2	1
GO:0051651 maintenance of location in cell	1/28	205/14464	0.00487805	2.51986063	0.96520804	0.32972197	0.38803765	0.29843111	Gstm7	1
GO:0010810 regulation of cell-substrate adhesion	1/28	206/14464	0.00485437	2.50762829	0.95980577	0.33103818	0.38872186	0.29895732	Fzd7	1
GO:0043270 positive regulation of monoatomic ion transport	1/28	206/14464	0.00485437	2.50762829	0.95980577	0.33103818	0.38872186	0.29895732	Gstm7	1
GO:0043583 ear development	1/28	207/14464	0.00483092	2.49551415	0.95443509	0.33235189	0.38983182	0.29981096	Kcnk2	1
GO:0043542 endothelial cell migration	1/28	210/14464	0.0047619	2.45986395	0.93850896	0.33627813	0.39400029	0.30301683	Ccn3	1
GO:0034284 response to monosaccharide	1/28	211/14464	0.00473934	2.44820582	0.93326106	0.33758192	0.39509034	0.30385517	Ndufaf2	1
GO:0006813 potassium ion transport	1/28	212/14464	0.00471698	2.43665768	0.92804299	0.33888324	0.39573782	0.30435313	Kcnk2	1
GO:0032869 cellular response to insulin stimulus	1/28	212/14464	0.00471698	2.43665768	0.92804299	0.33888324	0.39573782	0.30435313	Col6a1	1
GO:0002274 myeloid leukocyte activation	1/28	213/14464	0.00469484	2.42521797	0.92285442	0.34018209	0.39594351	0.30451133	Cx3cr1	1
GO:0043122 regulation of canonical NF-kappaB signal transduction	1/28	213/14464	0.00469484	2.42521797	0.92285442	0.34018209	0.39594351	0.30451133	Cx3cr1	1
GO:0072593 reactive oxygen species metabolic process	1/28	213/14464	0.00469484	2.42521797	0.92285442	0.34018209	0.39594351	0.30451133	Col6a1	1
GO:0034764 positive regulation of transmembrane transport	1/28	214/14464	0.0046729	2.41388518	0.91769501	0.34147848	0.39701564	0.30533588	Gstm7	1
GO:0044403 biological process involved in symbiotic interaction	1/28	216/14464	0.00462963	2.39153439	0.9074624	0.34406391	0.39958245	0.30730995	Cx3cr1	1
GO:0002440 production of molecular mediator of immune response	1/28	219/14464	0.00456621	2.35877365	0.8923243	0.34792369	0.403622	0.31041668	Fbn1	1
GO:0010976 positive regulation of neuron projection development	1/28	220/14464	0.00454545	2.34805195	0.88733326	0.34920542	0.40378069	0.31053873	Bhlhb9	1
GO:0016485 protein processing	1/28	220/14464	0.00454545	2.34805195	0.88733326	0.34920542	0.40378069	0.31053873	Comp	1
GO:0046034 ATP metabolic process	1/28	220/14464	0.00454545	2.34805195	0.88733326	0.34920542	0.40378069	0.31053873	Col6a1	1
GO:0046434 organophosphate catabolic process	1/28	222/14464	0.0045045	2.32689833	0.87743189	0.35176158	0.4062923	0.31247035	Col6a1	1
GO:0008202 steroid metabolic process	1/28	225/14464	0.00444444	2.29587302	0.86277734	0.35557767	0.40980521	0.31517205	Bmp6	1
GO:2001234 negative regulation of apoptotic signaling pathway	1/28	225/14464	0.00444444	2.29587302	0.86277734	0.35557767	0.40980521	0.31517205	Cx3cr1	1
GO:0060828 regulation of canonical Wnt signaling pathway	1/28	226/14464	0.00442478	2.28571429	0.85794405	0.35684489	0.41081816	0.31595109	Fzd7	1
GO:1901136 carbohydrate derivative catabolic process	1/28	229/14464	0.00436681	2.25577043	0.84359506	0.36063212	0.41472694	0.31895724	Col6a1	1
GO:0001894 tissue homeostasis	1/28	230/14464	0.00434783	2.24596273	0.8388615	0.36188975	0.41482053	0.31902922	Comp	1
GO:0019318 hexose metabolic process	1/28	230/14464	0.00434783	2.24596273	0.8388615	0.36188975	0.41482053	0.31902922	Fbn1	1
GO:0060249 anatomical structure homeostasis	1/28	230/14464	0.00434783	2.24596273	0.8388615	0.36188975	0.41482053	0.31902922	Comp	1
GO:0001667 ameboidal-type cell migration	1/28	231/14464	0.004329	2.23623995	0.83415222	0.36314499	0.41580887	0.31978933	Ccn3	1
GO:0002573 myeloid leukocyte differentiation	1/28	232/14464	0.00431034	2.22660099	0.82946699	0.36439784	0.41634225	0.32019954	Fbn1	1
GO:0046394 carboxylic acid biosynthetic process	1/28	232/14464	0.00431034	2.22660099	0.82946699	0.36439784	0.41634225	0.32019954	Gstm7	1
GO:0018108 peptidyl-tyrosine phosphorylation	1/28	233/14464	0.00429185	2.21704476	0.82480555	0.36564833	0.41732031	0.32095175	Bmp6	1
GO:0009743 response to carbohydrate	1/28	234/14464	0.0042735	2.20757021	0.82016766	0.36689644	0.4178433	0.32135397	Ndufaf2	1
GO:0045333 cellular respiration	1/28	234/14464	0.0042735	2.20757021	0.82016766	0.36689644	0.4178433	0.32135397	Col6a1	1
GO:0015850 organic hydroxy compound transport	1/28	235/14464	0.00425532	2.19817629	0.81555309	0.36814218	0.41791247	0.32140716	Bmp6	1
GO:0016053 organic acid biosynthetic process	1/28	235/14464	0.00425532	2.19817629	0.81555309	0.36814218	0.41791247	0.32140716	Gstm7	1
GO:0090257 regulation of muscle system process	1/28	235/14464	0.00425532	2.19817629	0.81555309	0.36814218	0.41791247	0.32140716	Igfbp5	1
GO:0007249 canonical NF-kappaB signal transduction	1/28	236/14464	0.00423729	2.18886199	0.81096159	0.36938555	0.41842604	0.32180213	Cx3cr1	1
GO:0033044 regulation of chromosome organization	1/28	236/14464	0.00423729	2.18886199	0.81096159	0.36938555	0.41842604	0.32180213	H3f3b	1
GO:0018212 peptidyl-tyrosine modification	1/28	237/14464	0.00421941	2.17962628	0.80639294	0.37062657	0.4193828	0.32253796	Bmp6	1
GO:0048167 regulation of synaptic plasticity	1/28	239/14464	0.0041841	2.16138673	0.79732328	0.37310155	0.42128222	0.32399876	Cx3cr1	1
GO:1903320 regulation of protein modification by small protein conjugation or removal	1/28	239/14464	0.0041841	2.16138673	0.79732328	0.37310155	0.42128222	0.32399876	Gbp4	1
GO:0051924 regulation of calcium ion transport	1/28	240/14464	0.00416667	2.15238095	0.79282182	0.37433551	0.42222492	0.32472377	Gstm7	1
GO:0002366 leukocyte activation involved in immune response	1/28	241/14464	0.00414938	2.14344991	0.78834231	0.37556714	0.4227128	0.32509899	Cx3cr1	1
GO:0045444 fat cell differentiation	1/28	241/14464	0.00414938	2.14344991	0.78834231	0.37556714	0.4227128	0.32509899	Fbn1	1
GO:0071695 anatomical structure maturation	1/28	242/14464	0.00413223	2.13459268	0.78388453	0.37679642	0.42319598	0.32547059	Cx3cr1	1
GO:0090596 sensory organ morphogenesis	1/28	242/14464	0.00413223	2.13459268	0.78388453	0.37679642	0.42319598	0.32547059	Fbn1	1
GO:0009205 purine ribonucleoside triphosphate metabolic process	1/28	243/14464	0.00411523	2.12580835	0.77944828	0.37802337	0.42412378	0.32618415	Col6a1	1
GO:1901873 regulation of post-translational protein modification	1/28	244/14464	0.00409836	2.11709602	0.77503335	0.37924799	0.42504701	0.32689418	Gbp4	1
GO:0002263 cell activation involved in immune response	1/28	245/14464	0.00408163	2.10845481	0.77063953	0.38047029	0.42596568	0.32760071	Cx3cr1	1
GO:0045860 positive regulation of protein kinase activity	1/28	246/14464	0.00406504	2.09988386	0.7662666	0.38169027	0.42687981	0.32830374	Fbn1	1
GO:0009199 ribonucleoside triphosphate metabolic process	1/28	248/14464	0.00403226	2.08294931	0.75758267	0.38412327	0.42869454	0.32969941	Col6a1	1
GO:0051607 defense response to virus	1/28	248/14464	0.00403226	2.08294931	0.75758267	0.38412327	0.42869454	0.32969941	Gbp4	1
GO:0005996 monosaccharide metabolic process	1/28	250/14464	0.004	2.06628571	0.74897998	0.38654704	0.43094496	0.33143016	Fbn1	1
GO:0009144 purine nucleoside triphosphate metabolic process	1/28	251/14464	0.00398406	2.0580535	0.74470862	0.38775548	0.43183715	0.33211633	Col6a1	1
GO:0010721 negative regulation of cell development	1/28	252/14464	0.00396825	2.04988662	0.740457	0.38896162	0.43272491	0.33279908	Fbn1	1
GO:0048562 embryonic organ morphogenesis	1/28	254/14464	0.00393701	2.03374578	0.73201224	0.39136702	0.4349436	0.33450543	Fbn1	1
GO:0050878 regulation of body fluid levels	1/28	256/14464	0.00390625	2.01785714	0.72364424	0.3937633	0.4371475	0.3362004	Comp	1
GO:0033674 positive regulation of kinase activity	1/28	258/14464	0.00387597	2.00221484	0.71535159	0.39615047	0.43933669	0.33788405	Fbn1	1
GO:0030217 T cell differentiation	1/28	263/14464	0.00380228	1.96414992	0.69494071	0.40207882	0.44544439	0.34258135	Fzd7	1
GO:0009141 nucleoside triphosphate metabolic process	1/28	265/14464	0.00377358	1.94932615	0.68690119	0.40443442	0.44711767	0.34386823	Col6a1	1
GO:0032868 response to insulin	1/28	265/14464	0.00377358	1.94932615	0.68690119	0.40443442	0.44711767	0.34386823	Col6a1	1
GO:0022604 regulation of cell morphogenesis	1/28	268/14464	0.00373134	1.92750533	0.67497174	0.40795103	0.45053465	0.34649615	Bhlhb9	1
GO:0045765 regulation of angiogenesis	1/28	270/14464	0.0037037	1.91322751	0.66710353	0.41028431	0.452639	0.34811456	Cx3cr1	1
GO:0009101 glycoprotein biosynthetic process	1/28	272/14464	0.00367647	1.89915966	0.65930177	0.41260872	0.45425601	0.34935817	Slc2a10	1
GO:0010001 glial cell differentiation	1/28	272/14464	0.00367647	1.89915966	0.65930177	0.41260872	0.45425601	0.34935817	Col6a1	1
GO:0006066 alcohol metabolic process	1/28	275/14464	0.00363636	1.87844156	0.64772118	0.41607878	0.45760015	0.35193007	Bmp6	1
GO:0007281 germ cell development	1/28	276/14464	0.00362319	1.87163561	0.64389298	0.41723106	0.45839093	0.35253824	H3f3b	1
GO:0032386 regulation of intracellular transport	1/28	277/14464	0.00361011	1.8648788	0.64008054	0.41838116	0.45870183	0.35277735	Gbp4	1
GO:1901342 regulation of vasculature development	1/28	277/14464	0.00361011	1.8648788	0.64008054	0.41838116	0.45870183	0.35277735	Cx3cr1	1
GO:0006874 intracellular calcium ion homeostasis	1/28	278/14464	0.00359712	1.85817061	0.63628374	0.41952906	0.45948421	0.35337906	Gstm7	1
GO:0006310 DNA recombination	1/28	279/14464	0.00358423	1.8515105	0.63250245	0.42067478	0.46026258	0.35397769	H1f0	1
GO:0001933 negative regulation of protein phosphorylation	1/28	281/14464	0.00355872	1.83833249	0.62498584	0.42295968	0.46133128	0.3547996	Gbp4	1
GO:0006936 muscle contraction	1/28	281/14464	0.00355872	1.83833249	0.62498584	0.42295968	0.46133128	0.3547996	Comp	1
GO:0046942 carboxylic acid transport	1/28	281/14464	0.00355872	1.83833249	0.62498584	0.42295968	0.46133128	0.3547996	Kcnk2	1
GO:0030111 regulation of Wnt signaling pathway	1/28	282/14464	0.0035461	1.83181358	0.62125027	0.42409887	0.46190921	0.35524408	Fzd7	1
GO:0007416 synapse assembly	1/28	283/14464	0.00353357	1.82534074	0.61752969	0.42523589	0.46190921	0.35524408	Bhlhb9	1
GO:0009416 response to light stimulus	1/28	283/14464	0.00353357	1.82534074	0.61752969	0.42523589	0.46190921	0.35524408	Col6a1	1
GO:0015849 organic acid transport	1/28	283/14464	0.00353357	1.82534074	0.61752969	0.42523589	0.46190921	0.35524408	Kcnk2	1
GO:0006790 sulfur compound metabolic process	1/28	285/14464	0.00350877	1.81253133	0.610133	0.42750344	0.46389605	0.35677211	Gstm7	1
GO:1902105 regulation of leukocyte differentiation	1/28	286/14464	0.0034965	1.80619381	0.60645665	0.42863398	0.46464626	0.35734908	Fbn1	1
GO:1901888 regulation of cell junction assembly	1/28	288/14464	0.00347222	1.79365079	0.59914732	0.43088861	0.46661223	0.35886106	Bhlhb9	1
GO:0001666 response to hypoxia	1/28	289/14464	0.00346021	1.78744439	0.59551411	0.4320127	0.46735116	0.35942936	Kcnk2	1
GO:0048545 response to steroid hormone	1/28	290/14464	0.00344828	1.78128079	0.59189504	0.43313465	0.46760863	0.35962737	Bmp6	1
GO:0070588 calcium ion transmembrane transport	1/28	290/14464	0.00344828	1.78128079	0.59189504	0.43313465	0.46760863	0.35962737	Gstm7	1
GO:0048511 rhythmic process	1/28	291/14464	0.00343643	1.77515955	0.58829001	0.43425446	0.46833967	0.3601896	Col6a1	1
GO:1904062 regulation of monoatomic cation transmembrane transport	1/28	294/14464	0.00340136	1.75704568	0.57755799	0.43760111	0.47146841	0.36259584	Gstm7	1
GO:0022412 cellular process involved in reproduction in multicellular organism	1/28	295/14464	0.00338983	1.75108959	0.57400797	0.43871242	0.47218488	0.36314686	H3f3b	1
GO:0019216 regulation of lipid metabolic process	1/28	298/14464	0.0033557	1.73346117	0.56343836	0.44203364	0.475276	0.36552418	Bmp6	1
GO:0042326 negative regulation of phosphorylation	1/28	301/14464	0.00332226	1.71618415	0.55298722	0.4453359	0.47737121	0.36713555	Gbp4	1
GO:0071214 cellular response to abiotic stimulus	1/28	301/14464	0.00332226	1.71618415	0.55298722	0.4453359	0.47737121	0.36713555	Bmp6	1
GO:0104004 cellular response to environmental stimulus	1/28	301/14464	0.00332226	1.71618415	0.55298722	0.4453359	0.47737121	0.36713555	Bmp6	1
GO:0099173 postsynapse organization	1/28	303/14464	0.00330033	1.7048562	0.54608425	0.44752693	0.47923431	0.36856842	Bhlhb9	1
GO:0034504 protein localization to nucleus	1/28	306/14464	0.00326797	1.68814192	0.53582448	0.45079784	0.48176173	0.37051221	Gbp4	1
GO:0051347 positive regulation of transferase activity	1/28	306/14464	0.00326797	1.68814192	0.53582448	0.45079784	0.48176173	0.37051221	Fbn1	1
GO:0051098 regulation of binding	1/28	308/14464	0.00324675	1.67717996	0.52904663	0.45296806	0.48359254	0.37192024	H1f0	1
GO:0016358 dendrite development	1/28	309/14464	0.00323625	1.6717522	0.52567602	0.45405007	0.48425905	0.37243283	Bhlhb9	1
GO:0009150 purine ribonucleotide metabolic process	1/28	311/14464	0.00321543	1.66100138	0.51897099	0.4562079	0.48509342	0.37307453	Col6a1	1
GO:0010038 response to metal ion	1/28	311/14464	0.00321543	1.66100138	0.51897099	0.4562079	0.48509342	0.37307453	Bmp6	1
GO:0043010 camera-type eye development	1/28	311/14464	0.00321543	1.66100138	0.51897099	0.4562079	0.48509342	0.37307453	Fbn1	1

GO:0009615	response to virus	1/28	312/14464	0.00320513	1.65567766	0.51563637	0.45728372	0.48574918	0.37357886	Gbp4	1
GO:1903532	positive regulation of secretion by cell	1/28	315/14464	0.0031746	1.6399093	0.50570308	0.4604989	0.48867386	0.37582817	Bmp6	1
GO:0062012	regulation of small molecule metabolic process	1/28	319/14464	0.0031348	1.61934617	0.49262006	0.46475725	0.49269857	0.37892348	Bmp6	1
GO:0002697	regulation of immune effector process	1/28	320/14464	0.003125	1.61428571	0.48937756	0.46581676	0.49332746	0.37940715	Cx3cr1	1
GO:0007517	muscle organ development	1/28	323/14464	0.00309598	1.59929235	0.47971655	0.46898318	0.49433065	0.38017868	Col6a1	1
GO:0009100	glycoprotein metabolic process	1/28	323/14464	0.00309598	1.59929235	0.47971655	0.46898318	0.49433065	0.38017868	Slc2a10	1
GO:0021700	developmental maturation	1/28	323/14464	0.00309598	1.59929235	0.47971655	0.46898318	0.49433065	0.38017868	Cx3cr1	1
GO:0034765	regulation of monoatomic ion transmembrane transport	1/28	323/14464	0.00309598	1.59929235	0.47971655	0.46898318	0.49433065	0.38017868	Gstm7	1
GO:0006913	nucleocytoplasmic transport	1/28	324/14464	0.00308642	1.59435626	0.47651811	0.47003462	0.49433065	0.38017868	Gbp4	1
GO:0031647	regulation of protein stability	1/28	324/14464	0.00308642	1.59435626	0.47651811	0.47003462	0.49433065	0.38017868	Telo2	1
GO:0051169	nuclear transport	1/28	324/14464	0.00308642	1.59435626	0.47651811	0.47003462	0.49433065	0.38017868	Gbp4	1
GO:0006417	regulation of translation	1/28	326/14464	0.00306748	1.58457493	0.47015361	0.47213149	0.49555071	0.381117	Igfbp5	1
GO:0007159	leukocyte cell-cell adhesion	1/28	326/14464	0.00306748	1.58457493	0.47015361	0.47213149	0.49555071	0.381117	Cx3cr1	1
GO:0002443	leukocyte mediated immunity	1/28	327/14464	0.0030581	1.57972914	0.46698739	0.47317692	0.49615578	0.38158235	Cx3cr1	1
GO:0002833	positive regulation of response to biotic stimulus	1/28	331/14464	0.00302115	1.56063876	0.45442761	0.47733872	0.49952954	0.38417703	Bmp6	1
GO:0048638	regulation of developmental growth	1/28	331/14464	0.00302115	1.56063876	0.45442761	0.47733872	0.49952954	0.38417703	Kcnk2	1
GO:0009259	ribonucleotide metabolic process	1/28	332/14464	0.00301205	1.55593804	0.45131357	0.47837421	0.49962479	0.38425029	Col6a1	1
GO:0031589	cell-substrate adhesion	1/28	332/14464	0.00301205	1.55593804	0.45131357	0.47837421	0.49962479	0.38425029	Fzd7	1
GO:0051047	positive regulation of secretion	1/28	334/14464	0.00299401	1.54662104	0.44511607	0.48043925	0.50128671	0.38552843	Bmp6	1
GO:0006631	fatty acid metabolic process	1/28	335/14464	0.00298507	1.54200426	0.44203246	0.48146881	0.50186601	0.38597396	Gstm7	1
GO:0002250	adaptive immune response	1/28	337/14464	0.00296736	1.5328529	0.4358952	0.48352203	0.50351015	0.38723843	Cx3cr1	1
GO:0019693	ribose phosphate metabolic process	1/28	339/14464	0.00294985	1.52380952	0.4297974	0.48556742	0.5051429	0.38849414	Col6a1	1
GO:0006869	lipid transport	1/28	342/14464	0.00292398	1.51044277	0.42072351	0.48862085	0.5078201	0.39055312	Bmp6	1
GO:0010563	negative regulation of phosphorus metabolic process	1/28	345/14464	0.00289855	1.49730849	0.41173541	0.49165681	0.50997343	0.3922092	Gbp4	1
GO:0045936	negative regulation of phosphate metabolic process	1/28	345/14464	0.00289855	1.49730849	0.41173541	0.49165681	0.50997343	0.3922092	Gbp4	1
GO:0019221	cytokine-mediated signaling pathway	1/28	353/14464	0.00283286	1.46337515	0.38817358	0.49966796	0.51777542	0.39820953	Cx3cr1	1
GO:0010639	negative regulation of organelle organization	1/28	355/14464	0.0028169	1.45513078	0.38237285	0.50165165	0.51932235	0.39939924	H3f3b	1
GO:0007018	microtubule-based movement	1/28	359/14464	0.00278552	1.43891763	0.37087578	0.50559629	0.52238367	0.40175363	Kifc3	1
GO:0030098	lymphocyte differentiation	1/28	359/14464	0.00278552	1.43891763	0.37087578	0.50559629	0.52238367	0.40175363	Fzd7	1
GO:1903706	regulation of hemopoiesis	1/28	362/14464	0.00276243	1.4269929	0.36234256	0.508535	0.52490734	0.40369453	Fbn1	1
GO:0032535	regulation of cellular component size	1/28	365/14464	0.00273973	1.41526419	0.35388449	0.51145686	0.52740873	0.40561829	Col6a1	1
GO:0050807	regulation of synapse organization	1/28	370/14464	0.0027027	1.396139	0.33995082	0.51628944	0.53187364	0.40905215	Bhlhb9	1
GO:1902903	regulation of supramolecular fiber organization	1/28	374/14464	0.0026738	1.38120703	0.32894703	0.52012228	0.53530095	0.41168802	Col6a1	1
GO:0010959	regulation of metal ion transport	1/28	375/14464	0.00266667	1.37752381	0.32621556	0.5210759	0.53569865	0.41199389	Gstm7	1
GO:0043603	amide metabolic process	1/28	376/14464	0.00265957	1.37386018	0.32349177	0.5220277	0.53569865	0.41199389	Gstm7	1
GO:0050803	regulation of synapse structure or activity	1/28	376/14464	0.00265957	1.37386018	0.32349177	0.5220277	0.53569865	0.41199389	Bhlhb9	1
GO:0006816	calcium ion transport	1/28	377/14464	0.00265252	1.37021599	0.32077563	0.52297768	0.53615347	0.41234368	Gstm7	1
GO:0031400	negative regulation of protein modification process	1/28	378/14464	0.0026455	1.36659108	0.31806708	0.52392583	0.53660554	0.41269136	Gbp4	1
GO:2001233	regulation of apoptotic signaling pathway	1/28	379/14464	0.00263852	1.3629853	0.31536609	0.52487216	0.53705488	0.41303694	Cx3cr1	1
GO:0006979	response to oxidative stress	1/28	388/14464	0.00257732	1.33136966	0.2913894	0.53330795	0.54515923	0.41926981	Col6a1	1
GO:0010876	lipid localization	1/28	393/14464	0.00254453	1.31443112	0.27831979	0.53793189	0.54935516	0.4224968	Bmp6	1
GO:0009314	response to radiation	1/28	394/14464	0.00253807	1.311095	0.27572672	0.53885137	0.5497635	0.42281085	Col6a1	1
GO:0006163	purine nucleotide metabolic process	1/28	395/14464	0.00253165	1.30777577	0.27314052	0.53976908	0.55016925	0.4231229	Col6a1	1
GO:0022407	regulation of cell-cell adhesion	1/28	398/14464	0.00251256	1.29791816	0.26542265	0.54251165	0.55243246	0.42486349	Bmp6	1
GO:0048732	gland development	1/28	408/14464	0.00245098	1.26610644	0.2401261	0.5515402	0.56108609	0.4315188	Igfbp5	1
GO:0043254	regulation of protein-containing complex assembly	1/28	413/14464	0.00242131	1.25077828	0.22771734	0.5559898	0.56506937	0.43458225	H3f3b	1
GO:0072594	establishment of protein localization to organelle	1/28	415/14464	0.00240964	1.24475043	0.22279712	0.5577577	0.56632211	0.43554571	Gbp4	1
GO:0048568	embryonic organ development	1/28	421/14464	0.0023753	1.22701052	0.20818137	0.56302075	0.57111788	0.43923403	Fbn1	1
GO:0007283	spermatogenesis	1/28	423/14464	0.00236407	1.22120905	0.20335686	0.56476164	0.57233507	0.44017014	H3f3b	1
GO:0031346	positive regulation of cell projection organization	1/28	426/14464	0.00234742	1.21260899	0.19616367	0.56736044	0.57386935	0.44135012	Bhlhb9	1
GO:0045859	regulation of protein kinase activity	1/28	426/14464	0.00234742	1.21260899	0.19616367	0.56736044	0.57386935	0.44135012	Fbn1	1
GO:0030099	myeloid cell differentiation	1/28	428/14464	0.00233645	1.20694259	0.19139688	0.56908465	0.57506357	0.44226857	Fbn1	1
GO:0048232	male gamete generation	1/28	437/14464	0.00228833	1.18208565	0.17022322	0.57676196	0.58226541	0.44780734	H3f3b	1
GO:0043269	regulation of monoatomic ion transport	1/28	440/14464	0.00227273	1.17402597	0.16326355	0.57929162	0.58426171	0.44934265	Gstm7	1
GO:0034762	regulation of transmembrane transport	1/28	447/14464	0.00223714	1.15564078	0.14720901	0.5851376	0.58903481	0.45301354	Gstm7	1
GO:1903131	mononuclear cell differentiation	1/28	447/14464	0.00223714	1.15564078	0.14720901	0.5851376	0.58903481	0.45301354	Fzd7	1
GO:0009117	nucleotide metabolic process	1/28	450/14464	0.00222222	1.14793651	0.14040589	0.58761897	0.59097041	0.45450216	Col6a1	1
GO:0043549	regulation of kinase activity	1/28	456/14464	0.00219298	1.13283208	0.12693521	0.59253884	0.59535242	0.45787227	Fbn1	1
GO:0042110	T cell activation	1/28	464/14464	0.00215517	1.11330049	0.10924751	0.5990107	0.60128399	0.46243411	Fzd7	1
GO:0010608	post-transcriptional regulation of gene expression	1/28	468/14464	0.00213675	1.1037851	0.10051729	0.60220935	0.60392179	0.46446278	Igfbp5	1
GO:0002009	morphogenesis of an epithelium	1/28	470/14464	0.00212766	1.09908815	0.09617993	0.60379943	0.60494299	0.46524816	Igfbp5	1
GO:0034655	nucleobase-containing compound catabolic process	1/28	471/14464	0.00212314	1.09675463	0.09401812	0.60459217	0.60516416	0.46541826	Col6a1	1
GO:0034329	cell junction assembly	1/28	485/14464	0.00206186	1.06509573	0.06421997	0.61553122	0.61553122	0.47339134	Bhlhb9	1

ID	Description	GeneRatio	BgRatio	RichFactor	FoldEnrichm	zScore	pvalue	p.adjust	qvalue	geneID	Count
GO:0061001	regulation of dendritic spine morphogenesis	5/67	61/14464	0.08196721	17.6951309	8.91371733	8.921E-06	0.01374724	0.01318428	Ngef/Slc30a1/Tiam1/Abi3bp/Il1rapl1	5
GO:0060997	dendritic spine morphogenesis	5/67	78/14464	0.06410256	13.8384998	7.75572041	2.9793E-05	0.02295572	0.02201567	Ngef/Slc30a1/Tiam1/Abi3bp/Il1rapl1	5
GO:0097061	dendritic spine organization	5/67	106/14464	0.04716981	10.183047	6.47326459	0.00012943	0.06648488	0.06376227	Ngef/Slc30a1/Tiam1/Abi3bp/Il1rapl1	5
GO:0106027	neuron projection organization	5/67	117/14464	0.04273504	9.22566654	6.0941631	0.00020577	0.06693338	0.0641924	Ngef/Slc30a1/Tiam1/Abi3bp/Il1rapl1	5
GO:0048813	dendrite morphogenesis	6/67	186/14464	0.03225806	6.96389023	5.58448356	0.00021718	0.06693338	0.0641924	Ngef/Slc30a1/Nfatc4/Tiam1/Abi3bp/Il1rapl1	6
GO:0060996	dendritic spine development	5/67	134/14464	0.03731343	8.05524616	5.597216	0.00038595	0.08567481	0.08216635	Ngef/Slc30a1/Tiam1/Abi3bp/Il1rapl1	5
GO:0061003	positive regulation of dendritic spine morphogenesis	3/67	31/14464	0.09677419	20.8916707	7.56317926	0.00038918	0.08567481	0.08216635	Slc30a1/Tiam1/Il1rapl1	3
GO:0060998	regulation of dendritic spine development	4/67	84/14464	0.04761905	10.2800284	5.81888935	0.00061421	0.1183119	0.11346692	Ngef/Slc30a1/Tiam1/Il1rapl1	4
GO:0048013	ephrin receptor signaling pathway	3/67	39/14464	0.07692308	16.6061998	6.6573616	0.0007706	0.13194354	0.12654034	Ngef/Epha8/Tiam1	3
GO:0048814	regulation of dendrite morphogenesis	4/67	96/14464	0.04166667	8.99502488	5.36151923	0.00101442	0.15632145	0.14991995	Slc30a1/Nfatc4/Tiam1/Il1rapl1	4
GO:0002269	leukocyte activation involved in inflammatory response	3/67	47/14464	0.06382979	13.7796126	5.98631768	0.0013316	0.16049506	0.15392265	Nr1d1/Ager/Enpp1	3
GO:0043031	negative regulation of macrophage activation	2/67	12/14464	0.16666667	35.9800995	8.26948149	0.00135395	0.16049506	0.15392265	Nr1d1/Cd200	2
GO:0150079	negative regulation of neuroinflammatory response	2/67	12/14464	0.16666667	35.9800995	8.26948149	0.00135395	0.16049506	0.15392265	Nr1d1/Cd200	2
GO:0099175	regulation of postsynapse organization	5/67	186/14464	0.02688172	5.80324186	4.49767252	0.00168894	0.18590385	0.17829093	Ngef/Slc30a1/Tiam1/Abi3bp/Il1rapl1	5
GO:0031648	protein destabilization	3/67	57/14464	0.05263158	11.3621367	5.34725085	0.00232494	0.23884874	0.22906768	Nr1d1/Ccdc88c/Dazap2	3
GO:0045472	response to ether	2/67	17/14464	0.11764706	25.3977173	6.86618358	0.00274857	0.24806575	0.23790725	Hnrnpd/Nfatc4	2
GO:0016358	dendrite development	6/67	309/14464	0.01941748	4.19185625	3.86899545	0.00302864	0.24806575	0.23790725	Ngef/Slc30a1/Nfatc4/Tiam1/Abi3bp/Il1rapl1	6
GO:0050775	positive regulation of dendrite morphogenesis	3/67	63/14464	0.04761905	10.2800284	5.03563042	0.00309364	0.24806575	0.23790725	Slc30a1/Tiam1/Il1rapl1	3
GO:0010770	positive regulation of cell morphogenesis	3/67	64/14464	0.046875	10.119403	4.98776221	0.00323512	0.24806575	0.23790725	Slc30a1/Tiam1/Il1rapl1	3
GO:0052548	regulation of endopeptidase activity	5/67	218/14464	0.02293578	4.95138984	4.01016505	0.00336191	0.24806575	0.23790725	Aim2/Ager/Psmb9/Tnfsf10/Fnip1	5
GO:0060999	positive regulation of dendritic spine development	3/67	65/14464	0.04615385	9.96371986	4.94093772	0.00338052	0.24806575	0.23790725	Slc30a1/Tiam1/Il1rapl1	3
GO:0050773	regulation of dendrite development	4/67	136/14464	0.02941176	6.34942932	4.27577372	0.00362609	0.25399113	0.24358998	Slc30a1/Nfatc4/Tiam1/Il1rapl1	4
GO:0048143	astrocyte activation	2/67	20/14464	0.1	21.5880597	6.28517348	0.00380568	0.25498038	0.24453871	Nr1d1/Ager	2
GO:1902074	response to salt	2/67	22/14464	0.09090909	19.6255088	5.96398315	0.00459938	0.29531873	0.28322518	Hnrnpd/Enpp1	2
GO:0150076	neuroinflammatory response	3/67	75/14464	0.04	8.63522388	4.52238668	0.00505701	0.3117138	0.29894886	Nr1d1/Ager/Cd200	3
GO:0032611	interleukin-1 beta production	3/67	79/14464	0.03797468	8.19799735	4.37623754	0.00584505	0.34173761	0.32774316	Aim2/Slamf9/Ager	3
GO:0150077	regulation of neuroinflammatory response	2/67	26/14464	0.07692308	16.6061998	5.43326527	0.00639432	0.34173761	0.32774316	Nr1d1/Cd200	2
GO:0071674	mononuclear cell migration	4/67	163/14464	0.02453988	5.29768336	3.76422527	0.00686918	0.34173761	0.32774316	Slamf9/Ager/Cd200/Enpp1	4
GO:2000116	regulation of cysteine-type endopeptidase activity	4/67	163/14464	0.02453988	5.29768336	3.76422527	0.00686918	0.34173761	0.32774316	Aim2/Psmb9/Tnfsf10/Fnip1	4
GO:1905874	regulation of postsynaptic density organization	2/67	27/14464	0.07407407	15.9911553	5.31874416	0.00688534	0.34173761	0.32774316	Slc30a1/Abi3bp	2
GO:0050807	regulation of synapse organization	6/67	370/14464	0.01621622	3.50076644	3.32420235	0.00722988	0.34173761	0.32774316	Ngef/Slc30a1/Nfatc4/Tiam1/Abi3bp/Il1rapl1	6
GO:0052547	regulation of peptidase activity	5/67	262/14464	0.01908397	4.11985872	3.47649342	0.00727254	0.34173761	0.32774316	Aim2/Ager/Psmb9/Tnfsf10/Fnip1	5
GO:0032640	tumor necrosis factor production	4/67	166/14464	0.02409639	5.2019421	3.71447169	0.0073182	0.34173761	0.32774316	Slamf9/H2-T23/Ager/Nfatc4	4
GO:0071706	tumor necrosis factor superfamily cytokine production	4/67	168/14464	0.02380952	5.14001421	3.68196618	0.00762808	0.34334168	0.32928155	Slamf9/H2-T23/Ager/Nfatc4	4
GO:0050803	regulation of synapse structure or activity	6/67	376/14464	0.01595745	3.44490314	3.27688728	0.00779816	0.34334168	0.32928155	Ngef/Slc30a1/Nfatc4/Tiam1/Abi3bp/Il1rapl1	6
GO:0001819	positive regulation of cytokine production	6/67	386/14464	0.01554404	3.35565695	3.20011665	0.00881621	0.37152315	0.35630896	Aim2/H2-T23/Ager/Rftn1/Cd200/Nfatc4	6
GO:0032612	interleukin-1 production	3/67	93/14464	0.03225806	6.96389023	3.9360283	0.00916151	0.37152315	0.35630896	Aim2/Slamf9/Ager	3
GO:0042116	macrophage activation	3/67	93/14464	0.03225806	6.96389023	3.9360283	0.00916151	0.37152315	0.35630896	Nr1d1/Ager/Cd200	3
GO:0010717	regulation of epithelial to mesenchymal transition	3/67	94/14464	0.03191489	6.88980629	3.9081133	0.00943262	0.37270943	0.35744666	Ager/Tiam1/Ldlrad4	3
GO:0019882	antigen processing and presentation	3/67	95/14464	0.03157895	6.81728201	3.88060323	0.0097084	0.37401593	0.35869966	H2-T23/Rftn1/Psmb9	3
GO:0001914	regulation of T cell mediated cytotoxicity	2/67	34/14464	0.05882353	12.6988586	4.65886771	0.0107781	0.39752612	0.38124709	H2-T23/Ager	2
GO:0032760	positive regulation of tumor necrosis factor production	3/67	99/14464	0.03030303	6.54183627	3.77440992	0.01085841	0.39752612	0.38124709	H2-T23/Ager/Nfatc4	3
GO:1903557	positive regulation of tumor necrosis factor superfamily cytokine production	3/67	100/14464	0.03	6.47641791	3.74877587	0.01115771	0.39752612	0.38124709	H2-T23/Ager/Nfatc4	3
GO:0097028	dendritic cell differentiation	2/67	35/14464	0.05714286	12.3360341	4.58044467	0.01139743	0.39752612	0.38124709	Slamf9/Ager	2
GO:0050727	regulation of inflammatory response	5/67	294/14464	0.0170068	3.67143872	3.15692707	0.01160849	0.39752612	0.38124709	Nr1d1/Aim2/H2-T23/Ager/Cd200	5
GO:0014002	astrocyte development	2/67	36/14464	0.05555556	11.9933665	4.50515236	0.0120321	0.40307531	0.38656904	Nr1d1/Ager	2
GO:0099173	postsynapse organization	5/67	303/14464	0.01650165	3.56238609	3.0750311	0.01309017	0.42098983	0.40374994	Ngef/Slc30a1/Tiam1/Abi3bp/Il1rapl1	5
GO:0050900	leukocyte migration	5/67	304/14464	0.01644737	3.55066771	3.06612349	0.01326235	0.42098983	0.40374994	Slamf9/Ager/Podxl2/Cd200/Enpp1	5
GO:0002456	T cell mediated immunity	3/67	107/14464	0.02803738	6.05272702	3.57862726	0.01338644	0.42098983	0.40374994	H2-T23/Ager/Rftn1	3
GO:0051098	regulation of binding	5/67	308/14464	0.01623377	3.50455515	3.03086276	0.01396645	0.43044596	0.41281883	Ager/Nfatc4/Tiam1/Gpsm1/Dazap2	5
GO:0060291	long-term synaptic potentiation	3/67	111/14464	0.02702703	5.83461073	3.48804638	0.01476595	0.4307297	0.41309095	Ager/Nfatc4/Enpp1	3
GO:0051345	positive regulation of hydrolase activity	5/67	317/14464	0.01577287	3.40505674	2.95361151	0.01564221	0.4307297	0.41309095	Aim2/Ager/Cd200/Tnfsf10/Tiam1	5
GO:0005978	glycogen biosynthetic process	2/67	43/14464	0.04651163	10.040958	4.05024769	0.01689185	0.4307297	0.41309095	Nr1d1/Enpp1	2
GO:0007616	long-term memory	2/67	43/14464	0.04651163	10.040958	4.05024769	0.01689185	0.4307297	0.41309095	Nfatc4/Ehmt2	2
GO:0009250	glucan biosynthetic process	2/67	43/14464	0.04651163	10.040958	4.05024769	0.01689185	0.4307297	0.41309095	Nr1d1/Enpp1	2
GO:0001774	microglial cell activation	2/67	44/14464	0.04545455	9.81275441	3.99379683	0.01764392	0.4307297	0.41309095	Nr1d1/Ager	2
GO:0001913	T cell mediated cytotoxicity	2/67	44/14464	0.04545455	9.81275441	3.99379683	0.01764392	0.4307297	0.41309095	H2-T23/Ager	2
GO:0034113	heterotypic cell-cell adhesion	2/67	44/14464	0.04545455	9.81275441	3.99379683	0.01764392	0.4307297	0.41309095	Ager/Cd200	2
GO:0051602	response to electrical stimulus	2/67	44/14464	0.04545455	9.81275441	3.99379683	0.01764392	0.4307297	0.41309095	Cdk2/Hnrnpd	2
GO:0002443	leukocyte mediated immunity	5/67	327/14464	0.01529052	3.30092656	2.87096962	0.01765688	0.4307297	0.41309095	Slamf9/H2-T23/Ager/Rftn1/Enpp1	5
GO:0032731	positive regulation of interleukin-1 beta production	2/67	46/14464	0.04347826	9.38611291	3.88613339	0.01918997	0.4307297	0.41309095	Aim2/Ager	2
GO:0010950	positive regulation of endopeptidase activity	3/67	125/14464	0.024	5.18113433	3.2027297	0.02020956	0.4307297	0.41309095	Aim2/Ager/Tnfsf10	3
GO:0043030	regulation of macrophage activation	2/67	49/14464	0.04081633	8.81145294	3.73639799	0.02161174	0.4307297	0.41309095	Nr1d1/Cd200	2
GO:0010952	positive regulation of peptidase activity	3/67	132/14464	0.02272727	4.9063772	3.07566007	0.02329322	0.4307297	0.41309095	Aim2/Ager/Tnfsf10	3
GO:0002449	lymphocyte mediated immunity	4/67	236/14464	0.01694915	3.65899317	2.80951844	0.02378725	0.4307297	0.41309095	H2-T23/Ager/Rftn1/Enpp1	4
GO:0006749	glutathione metabolic process	2/67	52/14464	0.03846154	8.30309989	3.59896297	0.02415341	0.4307297	0.41309095	Gstt2/Gstt3	2
GO:0048663	neuron fate commitment	2/67	52/14464	0.03846154	8.30309989	3.59896297	0.02415341	0.4307297	0.41309095	Ehmt2/Bhlhe22	2
GO:0032732	positive regulation of interleukin-1 production	2/67	53/14464	0.03773585	8.14643762	3.55558508	0.02502664	0.4307297	0.41309095	Aim2/Ager	2
GO:0035567	non-canonical Wnt signaling pathway	2/67	54/14464	0.03703704	7.99557767	3.51333097	0.02591265	0.4307297	0.41309095	Ccdc88c/Tiam1	2
GO:0061900	glial cell activation	2/67	54/14464	0.03703704	7.99557767	3.51333097	0.02591265	0.4307297	0.41309095	Nr1d1/Ager	2
GO:0051099	positive regulation of binding	3/67	138/14464	0.02173913	4.69305646	2.97367573	0.026129	0.4307297	0.41309095	Tiam1/Gpsm1/Dazap2	3
GO:1905517	macrophage migration	2/67	55/14464	0.03636364	7.85020353	3.4721501	0.0268113	0.4307297	0.41309095	Cd200/Enpp1	2
GO:0021782	glial cell development	3/67	140/14464	0.02142857	4.62601279	2.94097807	0.02711374	0.4307297	0.41309095	Nr1d1/Ager/Enpp1	3
GO:0002437	inflammatory response to antigenic stimulus	2/67	56/14464	0.03571429	7.71002132	3.43199507	0.02772247	0.4307297	0.41309095	H2-T23/Enpp1	2
GO:0000723	telomere maintenance	3/67	143/14464	0.02097902	4.52896357	2.89307115	0.02862782	0.4307297	0.41309095	Cdk2/Hnrnpd/Rpa2	3
GO:0030178	negative regulation of Wnt signaling pathway	3/67	144/14464	0.02083333	4.49751244	2.87739574	0.02914235	0.4307297	0.41309095	Ccdc88c/Ppp2r3a/Nfatc4	3
GO:0044088	regulation of vacuole organization	2/67	58/14464	0.03448276	7.44415852	3.3545873	0.02958181	0.4307297	0.41309095	Ehmt2/Fnip1	2
GO:0071709	membrane assembly	2/67	58/14464	0.03448276	7.44415852	3.3545873	0.02958181	0.4307297	0.41309095	Rftn1/Il1rapl1	2
GO:0097106	postsynaptic density organization	2/67	58/14464	0.03448276	7.44415852	3.3545873	0.02958181	0.4307297	0.41309095	Slc30a1/Abi3bp	2
GO:0001837	epithelial to mesenchymal transition	3/67	149/14464	0.02013423	4.3465892	2.80111188	0.03178866	0.4307297	0.41309095	Ager/Tiam1/Ldlrad4	3
GO:0032200	telomere organization	3/67	149/14464	0.02013423	4.3465892	2.80111188	0.03178866	0.4307297	0.41309095	Cdk2/Hnrnpd/Rpa2	3
GO:0016055	Wnt signaling pathway	5/67	385/14464	0.01298701	2.80364412	2.44695455	0.03275679	0.4307297	0.41309095	Ccdc88c/Ppp2r3a/Nfatc4/Tiam1/Enpp1	5
GO:0000271	polysaccharide biosynthetic process	2/67	62/14464	0.03225806	6.96389023	3.21029302	0.03344493	0.4307297	0.41309095	Nr1d1/Enpp1	2
GO:0002460	adaptive immune response based on somatic recombination of immune receptors built from immunoglobu	4/67	263/14464	0.01520913	3.28335509	2.54930887	0.03355808	0.4307297	0.41309095	H2-T23/Ager/Rftn1/Enpp1	4
GO:0032868	response to insulin	4/67	265/14464	0.01509434	3.25857505	2.53139067	0.03436257	0.4307297	0.41309095	Srsf5/Cdk2/Tnfsf10/Enpp1	4
GO:0042593	glucose homeostasis	4/67	265/14464	0.01509434	3.25857505	2.53139067	0.03436257	0.4307297	0.41309095	Nr1d1/Ager/Tiam1/Enpp1	4
GO:1903531	negative regulation of secretion by cell	3/67	154/14464	0.01948052	4.20546618	2.72811287	0.03455723	0.4307297	0.41309095	Cd200/Slc30a1/Il1rapl1	3
GO:0033500	carbohydrate homeostasis	4/67	266/14464	0.01503759	3.24632477	2.52249533	0.03476902	0.4307297	0.41309095	Nr1d1/Ager/Tiam1/Enpp1	4
GO:0071333	cellular response to glucose stimulus	3/67	155/14464	0.01935484	4.17833414	2.71388444	0.03512555	0.4307297	0.41309095	Nr1d1/Ager/Tiam1	3
GO:0001910	regulation of leukocyte mediated cytotoxicity	2/67	65/14464	0.03076923	6.64247991	3.11022012	0.03646463	0.4307297	0.41309095	H2-T23/Ager	2
GO:0071331	cellular response to hexose stimulus	3/67	158/14464	0.01898734	4.09899868	2.67190748	0.03685962	0.4307297	0.41309095	Nr1d1/Ager/Tiam1	3
GO:0071326	cellular response to monosaccharide stimulus	3/67	159/14464	0.01886792	4.07321881	2.65814524	0.03744731	0.4307297	0.41309095	Nr1d1/Ager/Tiam1	3
GO:0044091	membrane biogenesis	2/67	66/14464	0.03030303	6.54183627	3.07825906	0.03749385	0.4307297	0.41309095	Rftn1/Il1rapl1	2
GO:0032088	negative regulation of NF-kappaB transcription factor activity	2/67	67/14464	0.02985075	6.44419693	3.04695343	0.03853417	0.4307297	0.41309095	Aim2/Cd200	2
GO:0045123	cellular extravasation	2/67	67/14464	0.02985075	6.44419693	3.04695343	0.03853417	0.4307297	0.41309095	Ager/Podxl2	2
GO:0002695	negative regulation of leukocyte activation	3/67	161/14464	0.01863354	4.02261982	2.6309554	0.03863716	0.4307297	0.41309095	Nr1d1/H2-T23/Cd200	3
GO:0032680	regulation of tumor necrosis factor production	3/67	161/14464	0.01863354	4.02261982	2.6309554	0.03863716	0.4307297	0.41309095	H2-T23/Ager/Nfatc4	3
GO:1903555	regulation of tumor necrosis factor superfamily cytokine production	3/67	163/14464	0.01840491	3.97326252	2.60420068	0.03984625	0.4307297	0.41309095	H2-T23/Ager/Nfatc4	3
GO:0008347	glial cell migration	2/67	69/14464	0.02898551	6.25740861	2.98621474	0.04064769	0.4307297	0.41309095	Tiam1/Enpp1	2
GO:0030111	regulation of Wnt signaling pathway	4/67	282/14464	0.0141844	3.06213613	2.38563357	0.04165578	0.4307297	0.41309095	Ccdc88c/Ppp2r3a/Nfatc4/Tiam1	4
GO:0051051	negative regulation of transport	5/67	415/14464	0.01204819	2.60097105	2.25743582	0.04301483	0.4307297	0.41309095	Pkia/Cd200/Slc30a1/Enpp1/Il1rapl1	5
GO:0099084	postsynaptic specialization organization	2/67	72/14464	0.02777778	5.99668325	2.89946643	0.04389832	0.4307297	0.41309095	Slc30a1/Abi3bp	2
GO:0050767	regulation of neurogenesis	5/67	418/14464	0.01196172	2.58230379	2.23940307	0.04413646	0.4307297	0.41309095	Nr1d1/Slc30a1/Nfatc4/Tiam1/Il1rapl1	5
GO:0051048	negative regulation of secretion	3/67	170/14464	0.01764706	3.80965759	2.51380486	0.04422841	0.4307297	0.41309095	Cd200/Slc30a1/Il1rapl1	3
GO:0001678	intracellular glucose homeostasis	3/67	171/14464	0.01754386	3.78737889	2.50128373	0.0448734	0.4307297	0.41309095	Nr1d1/Ager/Tiam1	3
GO:0071322	cellular response to carbohydrate stimulus	3/67	171/14464	0.01754386	3.78737889	2.50128373	0.0448734	0.4307297	0.41309095	Nr1d1/Ager/Tiam1	3
GO:0001787	natural killer cell proliferation	1/67	10/14464	0.1	21.5880597	4.44275113	0.04538199	0.4307297	0.41309095	H2-T23	1
GO:0009313	oligosaccharide catabolic process	1/67	10/14464	0.1	21.5880597	4.44275113	0.04538199	0.4307297	0.41309095	Man2b1	1
GO:0021871	forebrain regionalization	1/67	10/14464	0.1	21.5880597	4.44275113	0.04538199	0.4307297	0.41309095	Bhlhe22	1
GO:0021957	corticospinal tract morphogenesis	1/67	10/14464	0.1	21.5880597	4.44275113	0.04538199	0.4307297	0.41309095	Bhlhe22	1
GO:0031953	negative regulation of protein autophosphorylation	1/67	10/14464	0.1	21.5880597	4.44275113	0.04538199	0.4307297	0.41309095	Enpp1	1
GO:0043252	sodium-independent organic anion transport	1/67	10/14464	0.1	21.5880597	4.44275113	0.04538199	0.4307297	0.41309095	Slco1a4	1
GO:0070874	negative regulation of glycogen metabolic process	1/67	10/14464	0.1	21.5880597	4.44275113	0.04538199	0.4307297	0.41309095	Enpp1	1
GO:0070934	CRD-mediated mRNA stabilization	1/67	10/14464	0.1	21.5880597	4.44275113	0.04538199	0.4307297	0.41309095	Hnrnpd	1
GO:0099545	trans-synaptic signaling by trans-synaptic complex	1/67	10/14464	0.1	21.5880597	4.44275113	0.04538199	0.4307297	0.41309095	Il1rapl1	1
GO:0140970	AIM2 inflammasome complex assembly	1/67	10/14464	0.1	21.5880597	4.44275113	0.04538199	0.4307297	0.41309095	Aim2	1
GO:2000402	negative regulation of lymphocyte migration	1/67	10/14464	0.1	21.5880597	4.44275113	0.04538199	0.4307297	0.41309095	Cd200	1
GO:0032651	regulation of interleukin-1 beta production	2/67	74/14464	0.02702703	5.83461073	2.84431418	0.0461176	0.4307297	0.41309095	Aim2/Ager	2
GO:0042102	positive regulation of T cell proliferation	2/67	74/14464	0.02702703	5.83461073	2.84431418	0.0461176	0.4307297	0.41309095	H2-T23/Ager	2
GO:0002709	regulation of T cell mediated immunity	2/67	75/14464	0.02666667	5.75681592	2.81748922	0.04724255	0.4307297	0.41309095	H2-T23/Ager	2
GO:0050866	negative regulation of cell activation	3/67	178/14464	0.01685393	3.63843703	2.41619512	0.04951961	0.4307297	0.41309095	Nr1d1/H2-T23/Cd200	3
GO:0061178	regulation of insulin secretion involved in cellular response to glucose stimulus	2/67	77/14464	0.02597403	5.60728823	2.76526155	0.04952249	0.4307297	0.41309095	Nr1d1/Tiam1	2
GO:0002407	dendritic cell chemotaxis	1/67	11/14464	0.09090909	19.6255088	4.21556781	0.04980702	0.4307297	0.41309095	Slamf9	1
GO:0002863	positive regulation of inflammatory response to antigenic stimulus	1/67	11/14464	0.09090909	19.6255088	4.21556781	0.04980702	0.4307297	0.41309095	H2-T23	1
GO:0032604	granulocyte macrophage colony-stimulating factor production	1/67	11/14464	0.09090909	19.6255088	4.21556781	0.04980702	0.4307297	0.41309095	Slamf9	1
GO:0032793	positive regulation of CREB transcription factor activity	1/67	11/14464	0.09090909	19.6255088	4.21556781	0.04980702	0.4307297	0.41309095	Cd200	1
GO:0035630	bone mineralization involved in bone maturation	1/67	11/14464	0.09090909	19.6255088	4.21556781	0.04980702	0.4307297	0.41309095	Enpp1	1
GO:0035701	hematopoietic stem cell migration	1/67	11/14464	0.09090909	19.6255088	4.21556781	0.04980702	0.4307297	0.41309095	Enpp1	1
GO:0050774	negative regulation of dendrite morphogenesis	1/67	11/14464	0.09090909	19.6255088	4.21556781	0.04980702	0.4307297	0.41309095	Nfatc4	1
GO:0097090	presynaptic membrane organization	1/67	11/14464	0.09090909	19.6255088	4.21556781	0.04980702	0.4307297	0.41309095	Il1rapl1	1
GO:0097501	stress response to metal ion	1/67	11/14464	0.09090909	19.6255088	4.21556781	0.04980702	0.4307297	0.41309095	Slc30a1	1
GO:1900147	regulation of Schwann cell migration	1/67	11/14464	0.09090909	19.6255088	4.21556781	0.04980702	0.4307297	0.41309095	Tiam1	1
GO:1900152	negative regulation of nuclear-transcribed mRNA catabolic process, deadenylation-dependent decay	1/67	11/14464	0.09090909	19.6255088	4.21556781	0.04980702	0.4307297	0.41309095	Hnrnpd	1
GO:1903044	protein localization to membrane raft	1/67	11/14464	0.09090909	19.6255088	4.21556781	0.04980702	0.4307297	0.41309095	Rftn1	1
GO:1903377	negative regulation of oxidative stress-induced neuron intrinsic apoptotic signaling pathway	1/67	11/14464	0.09090909	19.6255088	4.21556781	0.04980702	0.4307297	0.41309095	Pycr1	1
GO:1905456	regulation of lymphoid progenitor cell differentiation	1/67	11/14464	0.09090909	19.6255088	4.21556781	0.04980702	0.4307297	0.41309095	Fnip1	1
GO:1905806	regulation of synapse pruning	1/67	11/14464	0.09090909	19.6255088	4.21556781	0.04980702	0.4307297	0.41309095	Ngef	1
GO:0051091	positive regulation of DNA-binding transcription factor activity	3/67	179/14464	0.01675978	3.61811056	2.40439031	0.05020198	0.4307297	0.41309095	Aim2/Ager/Cd200	3
GO:0031341	regulation of cell killing	2/67	78/14464	0.02564103	5.53539992	2.73982897	0.05067725	0.4307297	0.41309095	H2-T23/Ager	2
GO:0050795	regulation of behavior	2/67	78/14464	0.02564103	5.53539992	2.73982897	0.05067725	0.4307297	0.41309095	Nr1d1/Aim2	2
GO:0042742	defense response to bacterium	3/67	182/14464	0.01648352	3.55847138	2.36947611	0.05227672	0.4307297	0.41309095	Slamf9/H2-T23/Slc30a1	3
GO:0002260	lymphocyte homeostasis	2/67	80/14464	0.025	5.39701493	2.69025649	0.05301579	0.4307297	0.41309095	Aim2/Enpp1	2
GO:0007611	learning or memory	4/67	307/14464	0.01302932	2.81277651	2.19007218	0.05386432	0.4307297	0.41309095	Ager/Nfatc4/Ehmt2/Man2b1	4
GO:0002524	hypersensitivity	1/67	12/14464	0.08333333	17.9900498	4.01653808	0.05421184	0.4307297	0.41309095	H2-T23	1
GO:0006013	mannose metabolic process	1/67	12/14464	0.08333333	17.9900498	4.01653808	0.05421184	0.4307297	0.41309095	Man2b1	1
GO:0007130	synaptonemal complex assembly	1/67	12/14464	0.08333333	17.9900498	4.01653808	0.05421184	0.4307297	0.41309095	Ehmt2	1
GO:0009143	nucleoside triphosphate catabolic process	1/67	12/14464	0.08333333	17.9900498	4.01653808	0.05421184	0.4307297	0.41309095	Enpp1	1
GO:0031453	positive regulation of heterochromatin formation	1/67	12/14464	0.08333333	17.9900498	4.01653808	0.05421184	0.4307297	0.41309095	Cdk2	1
GO:0032736	positive regulation of interleukin-13 production	1/67	12/14464	0.08333333	17.9900498	4.01653808	0.05421184	0.4307297	0.41309095	H2-T23	1
GO:0036135	Schwann cell migration	1/67	12/14464	0.08333333	17.9900498	4.01653808	0.05421184	0.4307297	0.41309095	Tiam1	1
GO:0051589	negative regulation of neurotransmitter transport	1/67	12/14464	0.08333333	17.9900498	4.01653808	0.05421184	0.4307297	0.41309095	Slc30a1	1
GO:0061430	bone trabecula morphogenesis	1/67	12/14464	0.08333333	17.9900498	4.01653808	0.05421184	0.4307297	0.41309095	Enpp1	1
GO:0071636	positive regulation of transforming growth factor beta production	1/67	12/14464	0.08333333	17.9900498	4.01653808	0.05421184	0.4307297	0.41309095	Cd200	1
GO:0090281	negative regulation of calcium ion import	1/67	12/14464	0.08333333	17.9900498	4.01653808	0.05421184	0.4307297	0.41309095	Slc30a1	1
GO:0097119	postsynaptic density protein 95 clustering	1/67	12/14464	0.08333333	17.9900498	4.01653808	0.05421184	0.4307297	0.41309095	Slc30a1	1
GO:0141161	regulation of cAMP/PKA signal transduction	1/67	12/14464	0.08333333	17.9900498	4.01653808	0.05421184	0.4307297	0.41309095	Pkia	1
GO:1904424	regulation of GTP binding	1/67	12/14464	0.08333333	17.9900498	4.01653808	0.05421184	0.4307297	0.41309095	Gpsm1	1
GO:2000105	positive regulation of DNA-templated DNA replication	1/67	12/14464	0.08333333	17.9900498	4.01653808	0.05421184	0.4307297	0.41309095	Cdk2	1
GO:2000489	regulation of hepatic stellate cell activation	1/67	12/14464	0.08333333	17.9900498	4.01653808	0.05421184	0.4307297	0.41309095	Nr1d1	1
GO:2000674	regulation of type B pancreatic cell apoptotic process	1/67	12/14464	0.08333333	17.9900498	4.01653808	0.05421184	0.4307297	0.41309095	Ager	1
GO:0032102	negative regulation of response to external stimulus	4/67	308/14464	0.01298701	2.80364412	2.18266218	0.05438929	0.4307297	0.41309095	Nr1d1/Ajap1/H2-T23/Cd200	4
GO:0051090	regulation of DNA-binding transcription factor activity	4/67	308/14464	0.01298701	2.80364412	2.18266218	0.05438929	0.4307297	0.41309095	Aim2/Ager/Cd200/Enpp1	4
GO:0046330	positive regulation of JNK cascade	2/67	82/14464	0.02439024	5.26538041	2.64232143	0.0553923	0.4307297	0.41309095	Ccdc88c/Ager	2
GO:0045089	positive regulation of innate immune response	4/67	310/14464	0.01290323	2.78555609	2.16793043	0.05544764	0.4307297	0.41309095	Nr1d1/Aim2/H2-T23/Rftn1	4
GO:0010038	response to metal ion	4/67	311/14464	0.01286174	2.77659932	2.16060822	0.05598102	0.4307297	0.41309095	Hnrnpd/Slc30a1/Nfatc4/Enpp1	4

GO:0050806 positive regulation of synaptic transmission	3/67	189/14464	0.01587302	3.42667614	2.29078988	0.05727752	0.4307297	0.41309095	Ager/Nfatc4/Enpp1	3
GO:0005977 glycogen metabolic process	2/67	84/14464	0.02380952	5.14001421	2.59592789	0.05780588	0.4307297	0.41309095	Nr1d1/Enpp1	2
GO:0001765 membrane raft assembly	1/67	13/14464	0.07692308	16.6061998	3.84017026	0.05859655	0.4307297	0.41309095	Rftn1	1
GO:0002328 pro-B cell differentiation	1/67	13/14464	0.07692308	16.6061998	3.84017026	0.05859655	0.4307297	0.41309095	Fnip1	1
GO:0009084 glutamine family amino acid biosynthetic process	1/67	13/14464	0.07692308	16.6061998	3.84017026	0.05859655	0.4307297	0.41309095	Pycr1	1
GO:0030502 negative regulation of bone mineralization	1/67	13/14464	0.07692308	16.6061998	3.84017026	0.05859655	0.4307297	0.41309095	Enpp1	1
GO:0045346 regulation of MHC class II biosynthetic process	1/67	13/14464	0.07692308	16.6061998	3.84017026	0.05859655	0.4307297	0.41309095	Nfx1	1
GO:0055062 phosphate ion homeostasis	1/67	13/14464	0.07692308	16.6061998	3.84017026	0.05859655	0.4307297	0.41309095	Enpp1	1
GO:0061687 detoxification of inorganic compound	1/67	13/14464	0.07692308	16.6061998	3.84017026	0.05859655	0.4307297	0.41309095	Slc30a1	1
GO:0071372 cellular response to follicle-stimulating hormone stimulus	1/67	13/14464	0.07692308	16.6061998	3.84017026	0.05859655	0.4307297	0.41309095	Epha8	1
GO:0097154 GABAergic neuron differentiation	1/67	13/14464	0.07692308	16.6061998	3.84017026	0.05859655	0.4307297	0.41309095	Bhlhe22	1
GO:1902902 negative regulation of autophagosome assembly	1/67	13/14464	0.07692308	16.6061998	3.84017026	0.05859655	0.4307297	0.41309095	Ehmt2	1
GO:1905269 positive regulation of chromatin organization	1/67	13/14464	0.07692308	16.6061998	3.84017026	0.05859655	0.4307297	0.41309095	Cdk2	1
GO:1905671 regulation of lysosome organization	1/67	13/14464	0.07692308	16.6061998	3.84017026	0.05859655	0.4307297	0.41309095	Fnip1	1
GO:2000767 positive regulation of cytoplasmic translation	1/67	13/14464	0.07692308	16.6061998	3.84017026	0.05859655	0.4307297	0.41309095	Hnrnpd	1
GO:0044042 glucan metabolic process	2/67	85/14464	0.02352941	5.07954346	2.57328138	0.0590263	0.4307297	0.41309095	Nr1d1/Enpp1	2
GO:0048708 astrocyte differentiation	2/67	85/14464	0.02352941	5.07954346	2.57328138	0.0590263	0.4307297	0.41309095	Nr1d1/Ager	2
GO:0006397 mRNA processing	5/67	455/14464	0.01098901	2.37231425	2.02901985	0.05942544	0.4307297	0.41309095	Srsf5/Arglu1/Luc7l2/Pcif1/Xrn2	5
GO:0035773 insulin secretion involved in cellular response to glucose stimulus	2/67	87/14464	0.02298851	4.96277235	2.52903713	0.06149388	0.4307297	0.41309095	Nr1d1/Tiam1	2
GO:0097529 myeloid leukocyte migration	3/67	196/14464	0.01530612	3.30429485	2.21572127	0.06249816	0.4307297	0.41309095	Ager/Cd200/Enpp1	3
GO:0032652 regulation of interleukin-1 production	2/67	88/14464	0.02272727	4.9063772	2.50741993	0.06274083	0.4307297	0.41309095	Aim2/Ager	2
GO:0015721 bile acid and bile salt transport	1/67	14/14464	0.07142857	15.4200426	3.68236837	0.06296124	0.4307297	0.41309095	Slco1a4	1
GO:0032693 negative regulation of interleukin-10 production	1/67	14/14464	0.07142857	15.4200426	3.68236837	0.06296124	0.4307297	0.41309095	Ager	1
GO:0045342 MHC class II biosynthetic process	1/67	14/14464	0.07142857	15.4200426	3.68236837	0.06296124	0.4307297	0.41309095	Nfx1	1
GO:0045953 negative regulation of natural killer cell mediated cytotoxicity	1/67	14/14464	0.07142857	15.4200426	3.68236837	0.06296124	0.4307297	0.41309095	H2-T23	1
GO:0050930 induction of positive chemotaxis	1/67	14/14464	0.07142857	15.4200426	3.68236837	0.06296124	0.4307297	0.41309095	Ager	1
GO:0071285 cellular response to lithium ion	1/67	14/14464	0.07142857	15.4200426	3.68236837	0.06296124	0.4307297	0.41309095	Nfatc4	1
GO:0071639 positive regulation of monocyte chemotactic protein-1 production	1/67	14/14464	0.07142857	15.4200426	3.68236837	0.06296124	0.4307297	0.41309095	Ager	1
GO:1900272 negative regulation of long-term synaptic potentiation	1/67	14/14464	0.07142857	15.4200426	3.68236837	0.06296124	0.4307297	0.41309095	Ager	1
GO:1903977 positive regulation of glial cell migration	1/67	14/14464	0.07142857	15.4200426	3.68236837	0.06296124	0.4307297	0.41309095	Tiam1	1
GO:1905274 regulation of modification of postsynaptic actin cytoskeleton	1/67	14/14464	0.07142857	15.4200426	3.68236837	0.06296124	0.4307297	0.41309095	Tiam1	1
GO:0009611 response to wounding	5/67	465/14464	0.01075269	2.32129674	1.97564507	0.06402163	0.4307297	0.41309095	2810403D21Rik/Ajap1/Srsf5/Fundc2/Enpp1	5
GO:0046634 regulation of alpha-beta T cell activation	2/67	90/14464	0.02222222	4.7973466	2.46514952	0.0652605	0.4307297	0.41309095	H2-T23/Ager	2
GO:0051101 regulation of DNA binding	2/67	90/14464	0.02222222	4.7973466	2.46514952	0.0652605	0.4307297	0.41309095	Ager/Dazap2	2
GO:0009749 response to glucose	3/67	201/14464	0.01492537	3.22209846	2.16414561	0.06635903	0.4307297	0.41309095	Nr1d1/Ager/Tiam1	3
GO:0032602 chemokine production	2/67	91/14464	0.02197802	4.74462851	2.44447902	0.06653303	0.4307297	0.41309095	Slamf9/Ager	2
GO:0043525 positive regulation of neuron apoptotic process	2/67	91/14464	0.02197802	4.74462851	2.44447902	0.06653303	0.4307297	0.41309095	Ager/Nfatc4	2
GO:0002833 positive regulation of response to biotic stimulus	4/67	331/14464	0.01208459	2.60882897	2.0199345	0.06723259	0.4307297	0.41309095	Nr1d1/Aim2/H2-T23/Rftn1	4
GO:0002486 antigen processing and presentation of endogenous peptide antigen via MHC class I via ER pathway, T/	1/67	15/14464	0.06666667	14.3920398	3.54000648	0.06730598	0.4307297	0.41309095	H2-T23	1
GO:0002716 negative regulation of natural killer cell mediated immunity	1/67	15/14464	0.06666667	14.3920398	3.54000648	0.06730598	0.4307297	0.41309095	H2-T23	1
GO:0002864 regulation of acute inflammatory response to antigenic stimulus	1/67	15/14464	0.06666667	14.3920398	3.54000648	0.06730598	0.4307297	0.41309095	H2-T23	1
GO:0006699 bile acid biosynthetic process	1/67	15/14464	0.06666667	14.3920398	3.54000648	0.06730598	0.4307297	0.41309095	Nr1d1	1
GO:0006895 Golgi to endosome transport	1/67	15/14464	0.06666667	14.3920398	3.54000648	0.06730598	0.4307297	0.41309095	Sys1	1
GO:0032966 negative regulation of collagen biosynthetic process	1/67	15/14464	0.06666667	14.3920398	3.54000648	0.06730598	0.4307297	0.41309095	Ager	1
GO:0035733 hepatic stellate cell activation	1/67	15/14464	0.06666667	14.3920398	3.54000648	0.06730598	0.4307297	0.41309095	Nr1d1	1
GO:0038065 collagen-activated signaling pathway	1/67	15/14464	0.06666667	14.3920398	3.54000648	0.06730598	0.4307297	0.41309095	Enpp1	1
GO:0061158 3'-UTR-mediated mRNA destabilization	1/67	15/14464	0.06666667	14.3920398	3.54000648	0.06730598	0.4307297	0.41309095	Hnrnpd	1
GO:0061469 regulation of type B pancreatic cell proliferation	1/67	15/14464	0.06666667	14.3920398	3.54000648	0.06730598	0.4307297	0.41309095	Nr1d1	1
GO:0070193 synaptonemal complex organization	1/67	15/14464	0.06666667	14.3920398	3.54000648	0.06730598	0.4307297	0.41309095	Ehmt2	1
GO:0097050 type B pancreatic cell apoptotic process	1/67	15/14464	0.06666667	14.3920398	3.54000648	0.06730598	0.4307297	0.41309095	Ager	1
GO:0097084 vascular associated smooth muscle cell development	1/67	15/14464	0.06666667	14.3920398	3.54000648	0.06730598	0.4307297	0.41309095	Nfatc4	1
GO:2000679 positive regulation of transcription regulatory region DNA binding	1/67	15/14464	0.06666667	14.3920398	3.54000648	0.06730598	0.4307297	0.41309095	Dazap2	1
GO:0006112 energy reserve metabolic process	2/67	93/14464	0.02150538	4.64259348	2.40402665	0.06910294	0.4307297	0.41309095	Nr1d1/Enpp1	2
GO:0009746 response to hexose	3/67	205/14464	0.01463415	3.15922825	2.12403438	0.06952539	0.4307297	0.41309095	Nr1d1/Ager/Tiam1	3
GO:0010389 regulation of G2/M transition of mitotic cell cycle	2/67	94/14464	0.0212766	4.59320419	2.38422936	0.07040012	0.4307297	0.41309095	Cdk2/Pkia	2
GO:0002250 adaptive immune response	4/67	337/14464	0.01186944	2.56238097	1.97973705	0.07082276	0.4307297	0.41309095	H2-T23/Ager/Rftn1/Enpp1	4
GO:0043087 regulation of GTPase activity	3/67	207/14464	0.01449275	3.1287043	2.10434542	0.0711341	0.4307297	0.41309095	Ngef/Tiam1/Gpsm1	3
GO:0002371 dendritic cell cytokine production	1/67	16/14464	0.0625	13.4925373	3.41065171	0.07163088	0.4307297	0.41309095	Slamf9	1
GO:0002476 antigen processing and presentation of endogenous peptide antigen via MHC class Ib	1/67	16/14464	0.0625	13.4925373	3.41065171	0.07163088	0.4307297	0.41309095	H2-T23	1
GO:0002484 antigen processing and presentation of endogenous peptide antigen via MHC class I via ER pathway	1/67	16/14464	0.0625	13.4925373	3.41065171	0.07163088	0.4307297	0.41309095	H2-T23	1
GO:0010713 negative regulation of collagen metabolic process	1/67	16/14464	0.0625	13.4925373	3.41065171	0.07163088	0.4307297	0.41309095	Ager	1
GO:0010960 magnesium ion homeostasis	1/67	16/14464	0.0625	13.4925373	3.41065171	0.07163088	0.4307297	0.41309095	Enpp1	1
GO:0030730 sequestering of triglyceride	1/67	16/14464	0.0625	13.4925373	3.41065171	0.07163088	0.4307297	0.41309095	Enpp1	1
GO:0032753 positive regulation of interleukin-4 production	1/67	16/14464	0.0625	13.4925373	3.41065171	0.07163088	0.4307297	0.41309095	H2-T23	1
GO:0034497 protein localization to phagophore assembly site	1/67	16/14464	0.0625	13.4925373	3.41065171	0.07163088	0.4307297	0.41309095	Stx17	1
GO:0042308 negative regulation of protein import into nucleus	1/67	16/14464	0.0625	13.4925373	3.41065171	0.07163088	0.4307297	0.41309095	Pkia	1
GO:0045954 positive regulation of natural killer cell mediated cytotoxicity	1/67	16/14464	0.0625	13.4925373	3.41065171	0.07163088	0.4307297	0.41309095	H2-T23	1
GO:0048712 negative regulation of astrocyte differentiation	1/67	16/14464	0.0625	13.4925373	3.41065171	0.07163088	0.4307297	0.41309095	Nr1d1	1
GO:0060100 positive regulation of phagocytosis, engulfment	1/67	16/14464	0.0625	13.4925373	3.41065171	0.07163088	0.4307297	0.41309095	Ager	1
GO:0060392 negative regulation of SMAD protein signal transduction	1/67	16/14464	0.0625	13.4925373	3.41065171	0.07163088	0.4307297	0.41309095	Ldlrad4	1
GO:0061000 negative regulation of dendritic spine development	1/67	16/14464	0.0625	13.4925373	3.41065171	0.07163088	0.4307297	0.41309095	Ngef	1
GO:1904355 positive regulation of telomere capping	1/67	16/14464	0.0625	13.4925373	3.41065171	0.07163088	0.4307297	0.41309095	Hnrnpd	1
GO:1905155 positive regulation of membrane invagination	1/67	16/14464	0.0625	13.4925373	3.41065171	0.07163088	0.4307297	0.41309095	Ager	1
GO:0051402 neuron apoptotic process	4/67	339/14464	0.01179941	2.54726368	1.96652986	0.07204123	0.4307297	0.41309095	Ager/Pycr1/Nfatc4/Enpp1	4
GO:0031348 negative regulation of defense response	3/67	210/14464	0.01428571	3.08400853	2.07525445	0.07357871	0.4307297	0.41309095	Nr1d1/H2-T23/Cd200	3
GO:0034284 response to monosaccharide	3/67	211/14464	0.01421801	3.06939237	2.06567276	0.07440193	0.4307297	0.41309095	Nr1d1/Ager/Tiam1	3
GO:0050890 cognition	4/67	344/14464	0.01162791	2.5102395	1.93391904	0.07513464	0.4307297	0.41309095	Ager/Nfatc4/Ehmt2/Man2b1	4
GO:0002327 immature B cell differentiation	1/67	17/14464	0.05882353	12.6988586	3.29237814	0.07593603	0.4307297	0.41309095	Fnip1	1
GO:0002428 antigen processing and presentation of peptide antigen via MHC class Ib	1/67	17/14464	0.05882353	12.6988586	3.29237814	0.07593603	0.4307297	0.41309095	H2-T23	1
GO:0010226 response to lithium ion	1/67	17/14464	0.05882353	12.6988586	3.29237814	0.07593603	0.4307297	0.41309095	Nfatc4	1
GO:0032656 regulation of interleukin-13 production	1/67	17/14464	0.05882353	12.6988586	3.29237814	0.07593603	0.4307297	0.41309095	H2-T23	1
GO:0036336 dendritic cell migration	1/67	17/14464	0.05882353	12.6988586	3.29237814	0.07593603	0.4307297	0.41309095	Slamf9	1
GO:0045056 transcytosis	1/67	17/14464	0.05882353	12.6988586	3.29237814	0.07593603	0.4307297	0.41309095	Ager	1
GO:0046325 negative regulation of D-glucose import	1/67	17/14464	0.05882353	12.6988586	3.29237814	0.07593603	0.4307297	0.41309095	Enpp1	1
GO:0070254 mucus secretion	1/67	17/14464	0.05882353	12.6988586	3.29237814	0.07593603	0.4307297	0.41309095	Enpp1	1
GO:0071732 cellular response to nitric oxide	1/67	17/14464	0.05882353	12.6988586	3.29237814	0.07593603	0.4307297	0.41309095	Hnrnpd	1
GO:0098814 spontaneous synaptic transmission	1/67	17/14464	0.05882353	12.6988586	3.29237814	0.07593603	0.4307297	0.41309095	Ager	1
GO:0098883 synapse pruning	1/67	17/14464	0.05882353	12.6988586	3.29237814	0.07593603	0.4307297	0.41309095	Ngef	1
GO:1903376 regulation of oxidative stress-induced neuron intrinsic apoptotic signaling pathway	1/67	17/14464	0.05882353	12.6988586	3.29237814	0.07593603	0.4307297	0.41309095	Pycr1	1
GO:2001185 regulation of CD8-positive, alpha-beta T cell activation	1/67	17/14464	0.05882353	12.6988586	3.29237814	0.07593603	0.4307297	0.41309095	H2-T23	1
GO:0002274 myeloid leukocyte activation	3/67	213/14464	0.01408451	3.04057179	2.04667838	0.07606079	0.4307297	0.41309095	Nr1d1/Ager/Cd200	3
GO:0001911 negative regulation of leukocyte mediated cytotoxicity	1/67	18/14464	0.05555556	11.9933665	3.18363849	0.08022151	0.4307297	0.41309095	H2-T23	1
GO:0002675 positive regulation of acute inflammatory response	1/67	18/14464	0.05555556	11.9933665	3.18363849	0.08022151	0.4307297	0.41309095	H2-T23	1
GO:0002717 positive regulation of natural killer cell mediated immunity	1/67	18/14464	0.05555556	11.9933665	3.18363849	0.08022151	0.4307297	0.41309095	H2-T23	1
GO:0002902 regulation of B cell apoptotic process	1/67	18/14464	0.05555556	11.9933665	3.18363849	0.08022151	0.4307297	0.41309095	Fnip1	1
GO:0014004 microglia differentiation	1/67	18/14464	0.05555556	11.9933665	3.18363849	0.08022151	0.4307297	0.41309095	Enpp1	1
GO:0032026 response to magnesium ion	1/67	18/14464	0.05555556	11.9933665	3.18363849	0.08022151	0.4307297	0.41309095	Enpp1	1
GO:0032354 response to follicle-stimulating hormone	1/67	18/14464	0.05555556	11.9933665	3.18363849	0.08022151	0.4307297	0.41309095	Epha8	1
GO:0032616 interleukin-13 production	1/67	18/14464	0.05555556	11.9933665	3.18363849	0.08022151	0.4307297	0.41309095	H2-T23	1
GO:0034114 regulation of heterotypic cell-cell adhesion	1/67	18/14464	0.05555556	11.9933665	3.18363849	0.08022151	0.4307297	0.41309095	Ager	1
GO:0042104 positive regulation of activated T cell proliferation	1/67	18/14464	0.05555556	11.9933665	3.18363849	0.08022151	0.4307297	0.41309095	Ager	1
GO:0044027 negative regulation of gene expression via chromosomal CpG island methylation	1/67	18/14464	0.05555556	11.9933665	3.18363849	0.08022151	0.4307297	0.41309095	Ehmt2	1
GO:0048148 behavioral response to cocaine	1/67	18/14464	0.05555556	11.9933665	3.18363849	0.08022151	0.4307297	0.41309095	Ehmt2	1
GO:0060099 regulation of phagocytosis, engulfment	1/67	18/14464	0.05555556	11.9933665	3.18363849	0.08022151	0.4307297	0.41309095	Ager	1
GO:0141156 cAMP/PKA signal transduction	1/67	18/14464	0.05555556	11.9933665	3.18363849	0.08022151	0.4307297	0.41309095	Pkia	1
GO:1903978 regulation of microglial cell activation	1/67	18/14464	0.05555556	11.9933665	3.18363849	0.08022151	0.4307297	0.41309095	Nr1d1	1
GO:1905809 negative regulation of synapse organization	1/67	18/14464	0.05555556	11.9933665	3.18363849	0.08022151	0.4307297	0.41309095	Nfatc4	1
GO:0001909 leukocyte mediated cytotoxicity	2/67	102/14464	0.01960784	4.23295288	2.23523193	0.08105793	0.4307297	0.41309095	H2-T23/Ager	2
GO:0002440 production of molecular mediator of immune response	3/67	219/14464	0.01369863	2.95726845	1.99100256	0.08113551	0.4307297	0.41309095	Slamf9/H2-T23/Enpp1	3
GO:0048762 mesenchymal cell differentiation	3/67	219/14464	0.01369863	2.95726845	1.99100256	0.08113551	0.4307297	0.41309095	Ager/Tiam1/Ldlrad4	3
GO:0010976 positive regulation of neuron projection development	3/67	220/14464	0.01363636	2.94382632	1.98190763	0.08199542	0.4307297	0.41309095	Slc30a1/Tiam1/Il1rapl1	3
GO:0042752 regulation of circadian rhythm	2/67	103/14464	0.01941748	4.19185625	2.21768668	0.08242375	0.4307297	0.41309095	Nr1d1/Hnrnpd	2
GO:0002507 tolerance induction	1/67	19/14464	0.05263158	11.3621367	3.0831733	0.0844874	0.4307297	0.41309095	H2-T23	1
GO:0015936 coenzyme A metabolic process	1/67	19/14464	0.05263158	11.3621367	3.0831733	0.0844874	0.4307297	0.41309095	Enpp1	1
GO:0016322 neuron remodeling	1/67	19/14464	0.05263158	11.3621367	3.0831733	0.0844874	0.4307297	0.41309095	Epha8	1
GO:0036480 neuron intrinsic apoptotic signaling pathway in response to oxidative stress	1/67	19/14464	0.05263158	11.3621367	3.0831733	0.0844874	0.4307297	0.41309095	Pycr1	1
GO:0045947 negative regulation of translational initiation	1/67	19/14464	0.05263158	11.3621367	3.0831733	0.0844874	0.4307297	0.41309095	Zfp598	1
GO:0046641 positive regulation of alpha-beta T cell proliferation	1/67	19/14464	0.05263158	11.3621367	3.0831733	0.0844874	0.4307297	0.41309095	H2-T23	1
GO:0070230 positive regulation of lymphocyte apoptotic process	1/67	19/14464	0.05263158	11.3621367	3.0831733	0.0844874	0.4307297	0.41309095	Fnip1	1
GO:0071371 cellular response to gonadotropin stimulus	1/67	19/14464	0.05263158	11.3621367	3.0831733	0.0844874	0.4307297	0.41309095	Epha8	1
GO:0150104 transport across blood-brain barrier	1/67	19/14464	0.05263158	11.3621367	3.0831733	0.0844874	0.4307297	0.41309095	Ager	1
GO:1900452 regulation of long-term synaptic depression	1/67	19/14464	0.05263158	11.3621367	3.0831733	0.0844874	0.4307297	0.41309095	Ager	1
GO:1902170 cellular response to reactive nitrogen species	1/67	19/14464	0.05263158	11.3621367	3.0831733	0.0844874	0.4307297	0.41309095	Hnrnpd	1
GO:1905153 regulation of membrane invagination	1/67	19/14464	0.05263158	11.3621367	3.0831733	0.0844874	0.4307297	0.41309095	Ager	1
GO:2000353 positive regulation of endothelial cell apoptotic process	1/67	19/14464	0.05263158	11.3621367	3.0831733	0.0844874	0.4307297	0.41309095	Ager	1
GO:0007033 vacuole organization	3/67	223/14464	0.01345291	2.90422328	1.95492747	0.08459903	0.4307297	0.41309095	Ehmt2/Fnip1/Stx17	3
GO:0002377 immunoglobulin production	2/67	105/14464	0.01904762	4.11201137	2.18325427	0.08517673	0.4307297	0.41309095	H2-T23/Enpp1	2
GO:0051092 positive regulation of NF-kappaB transcription factor activity	2/67	105/14464	0.01904762	4.11201137	2.18325427	0.08517673	0.4307297	0.41309095	Aim2/Ager	2
GO:0006275 regulation of DNA replication	2/67	106/14464	0.01886792	4.07321881	2.16635703	0.08656371	0.4307297	0.41309095	Cdk2/Ehmt2	2
GO:1902749 regulation of cell cycle G2/M phase transition	2/67	106/14464	0.01886792	4.07321881	2.16635703	0.08656371	0.4307297	0.41309095	Cdk2/Pkia	2
GO:0005976 polysaccharide metabolic process	2/67	107/14464	0.01869159	4.03515135	2.14966598	0.08795757	0.4307297	0.41309095	Nr1d1/Enpp1	2
GO:0016064 immunoglobulin mediated immune response	2/67	107/14464	0.01869159	4.03515135	2.14966598	0.08795757	0.4307297	0.41309095	H2-T23/Enpp1	2
GO:0002475 antigen processing and presentation via MHC class Ib	1/67	20/14464	0.05	10.7940299	2.98994551	0.08873381	0.4307297	0.41309095	H2-T23	1
GO:0002483 antigen processing and presentation of endogenous peptide antigen	1/67	20/14464	0.05	10.7940299	2.98994551	0.08873381	0.4307297	0.41309095	H2-T23	1
GO:0006775 fat-soluble vitamin metabolic process	1/67	20/14464	0.05	10.7940299	2.98994551	0.08873381	0.4307297	0.41309095	Enpp1	1
GO:0010232 vascular transport	1/67	20/14464	0.05	10.7940299	2.98994551	0.08873381	0.4307297	0.41309095	Ager	1
GO:0019885 antigen processing and presentation of endogenous peptide antigen via MHC class I	1/67	20/14464	0.05	10.7940299	2.98994551	0.08873381	0.4307297	0.41309095	H2-T23	1
GO:0032740 positive regulation of interleukin-17 production	1/67	20/14464	0.05	10.7940299	2.98994551	0.08873381	0.4307297	0.41309095	Rftn1	1
GO:0042749 regulation of circadian sleep/wake cycle	1/67	20/14464	0.05	10.7940299	2.98994551	0.08873381	0.4307297	0.41309095	Nr1d1	1
GO:0042753 positive regulation of circadian rhythm	1/67	20/14464	0.05	10.7940299	2.98994551	0.08873381	0.4307297	0.41309095	Nr1d1	1
GO:0044321 response to leptin	1/67	20/14464	0.05	10.7940299	2.98994551	0.08873381	0.4307297	0.41309095	Nr1d1	1
GO:0070168 negative regulation of biomineral tissue development	1/67	20/14464	0.05	10.7940299	2.98994551	0.08873381	0.4307297	0.41309095	Enpp1	1
GO:1905962 glutamatergic neuron differentiation	1/67	20/14464	0.05	10.7940299	2.98994551	0.08873381	0.4307297	0.41309095	Bhlhe22	1
GO:0050671 positive regulation of lymphocyte proliferation	2/67	108/14464	0.01851852	3.99778883	2.13317646	0.0893582	0.4307297	0.41309095	H2-T23/Ager	2
GO:2001056 positive regulation of cysteine-type endopeptidase activity	2/67	108/14464	0.01851852	3.99778883	2.13317646	0.0893582	0.4307297	0.41309095	Aim2/Tnfsf10	2
GO:0042063 gliogenesis	4/67	366/14464	0.01092896	2.35935079	1.79690007	0.08953349	0.4307297	0.41309095	Nr1d1/Ager/Tiam1/Enpp1	4
GO:0019724 B cell mediated immunity	2/67	110/14464	0.01818182	3.92510176	2.10078421	0.09217944	0.4307297	0.41309095	H2-T23/Enpp1	2
GO:0002221 pattern recognition receptor signaling pathway	3/67	232/14464	0.01293103	2.79155944	1.87660487	0.09262047	0.4307297	0.41309095	Nr1d1/Aim2/Rftn1	3
GO:0018022 peptidyl-lysine methylation	1/67	21/14464	0.04761905	10.2800284	2.90309228	0.09296081	0.4307297	0.41309095	Ehmt2	1
GO:0030859 polarized epithelial cell differentiation	1/67	21/14464	0.04761905	10.2800284	2.90309228	0.09296081	0.4307297	0.41309095	Ajap1	1
GO:0031445 regulation of heterochromatin formation	1/67	21/14464	0.04761905	10.2800284	2.90309228	0.09296081	0.4307297	0.41309095	Cdk2	1
GO:0032816 positive regulation of natural killer cell activation	1/67	21/14464	0.04761905	10.2800284	2.90309228	0.09296081	0.4307297	0.41309095	H2-T23	1
GO:0035561 regulation of chromatin binding	1/67	21/14464	0.04761905	10.2800284	2.90309228	0.09296081	0.4307297	0.41309095	Nfatc4	1
GO:0050927 positive regulation of positive chemotaxis	1/67	21/14464	0.04761905	10.2800284	2.90309228	0.09296081	0.4307297	0.41309095	Ager	1
GO:0060343 trabecula formation	1/67	21/14464	0.04761905	10.2800284	2.90309228	0.09296081	0.4307297	0.41309095	Enpp1	1
GO:0071605 monocyte chemotactic protein-1 production	1/67	21/14464	0.04761905	10.2800284	2.90309228	0.09296081	0.4307297	0.41309095	Ager	1
GO:0071637 regulation of monocyte chemotactic protein-1 production	1/67	21/14464	0.04761905	10.2800284	2.90309228	0.09296081	0.4307297	0.41309095	Ager	1
GO:0071731 response to nitric oxide	1/67	21/14464	0.04761905	10.2800284	2.90309228	0.09296081	0.4307297	0.41309095	Hnrnpd	1
GO:0072537 fibroblast activation	1/67	21/14464	0.04761905	10.2800284	2.90309228	0.09296081	0.4307297	0.41309095	Nr1d1	1
GO:2000001 regulation of DNA damage checkpoint	1/67	21/14464	0.04761905	10.2800284	2.90309228	0.09296081	0.4307297	0.41309095	Rpa2	1
GO:0014909 smooth muscle cell migration	2/67	111/14464	0.01801802	3.88974049	2.08487289	0.09359987	0.4307297	0.41309095	Ager/Enpp1	2
GO:0032946 positive regulation of mononuclear cell proliferation	2/67	111/14464	0.01801802	3.88974049	2.08487289	0.09359987	0.4307297	0.41309095	H2-T23/Ager	2
GO:0009743 response to carbohydrate	3/67	234/14464	0.01282051	2.76769996	1.85970742	0.09444496	0.4307297	0.41309095	Nr1d1/Ager/Tiam1	3
GO:0045088 regulation of innate immune response	4/67	374/14464	0.01069519	2.30888339	1.74949157	0.09508016	0.4307297	0.41309095	Nr1d1/Aim2/H2-T23/Rftn1	4
GO:0002758 innate immune response-activating signaling pathway	3/67	235/14464	0.01276596	2.75592252	1.85132507	0.09536282	0.4307297	0.41309095	Nr1d1/Aim2/Rftn1	3
GO:2001252 positive regulation of chromosome organization	2/67	113/14464	0.01769912	3.82089552	2.05359942	0.09645991	0.4307297	0.41309095	Cdk2/Hnrnpd	2

GO:0002693	positive regulation of cellular extravasation	1/67	22/14464	0.04545455	9.81275441	2.82188902	0.0971685	0.4307297	0.41309095	Ager	1
GO:0002888	positive regulation of myeloid leukocyte mediated immunity	1/67	22/14464	0.04545455	9.81275441	2.82188902	0.0971685	0.4307297	0.41309095	H2-T23	1
GO:0010829	negative regulation of D-glucose transmembrane transport	1/67	22/14464	0.04545455	9.81275441	2.82188902	0.0971685	0.4307297	0.41309095	Enpp1	1
GO:0019883	antigen processing and presentation of endogenous antigen	1/67	22/14464	0.04545455	9.81275441	2.82188902	0.0971685	0.4307297	0.41309095	H2-T23	1
GO:0031342	negative regulation of cell killing	1/67	22/14464	0.04545455	9.81275441	2.82188902	0.0971685	0.4307297	0.41309095	H2-T23	1
GO:0031571	mitotic G1 DNA damage checkpoint signaling	1/67	22/14464	0.04545455	9.81275441	2.82188902	0.0971685	0.4307297	0.41309095	Rpa2	1
GO:0032673	regulation of interleukin-4 production	1/67	22/14464	0.04545455	9.81275441	2.82188902	0.0971685	0.4307297	0.41309095	H2-T23	1
GO:0034260	negative regulation of GTPase activity	1/67	22/14464	0.04545455	9.81275441	2.82188902	0.0971685	0.4307297	0.41309095	Gpsm1	1
GO:0034349	glial cell apoptotic process	1/67	22/14464	0.04545455	9.81275441	2.82188902	0.0971685	0.4307297	0.41309095	Enpp1	1
GO:0042474	middle ear morphogenesis	1/67	22/14464	0.04545455	9.81275441	2.82188902	0.0971685	0.4307297	0.41309095	Enpp1	1
GO:0071676	negative regulation of mononuclear cell migration	1/67	22/14464	0.04545455	9.81275441	2.82188902	0.0971685	0.4307297	0.41309095	Cd200	1
GO:0099151	regulation of postsynaptic density assembly	1/67	22/14464	0.04545455	9.81275441	2.82188902	0.0971685	0.4307297	0.41309095	Abi3bp	1
GO:0071675	regulation of mononuclear cell migration	2/67	114/14464	0.01754386	3.78737889	2.03822946	0.09789934	0.4307297	0.41309095	Ager/Cd200	2
GO:0048167	regulation of synaptic plasticity	3/67	239/14464	0.0125523	2.70979829	1.81822776	0.09907122	0.4307297	0.41309095	Ager/Nfatc4/Enpp1	3
GO:0000956	nuclear-transcribed mRNA catabolic process	2/67	115/14464	0.0173913	3.75444517	2.02303234	0.09934494	0.4307297	0.41309095	Hnrnpd/Xrn2	2
GO:0050729	positive regulation of inflammatory response	2/67	115/14464	0.0173913	3.75444517	2.02303234	0.09934494	0.4307297	0.41309095	Aim2/H2-T23	2
GO:0034763	negative regulation of transmembrane transport	2/67	116/14464	0.01724138	3.72207926	2.00800444	0.10079661	0.4307297	0.41309095	Slc30a1/Enpp1	2
GO:0002320	lymphoid progenitor cell differentiation	1/67	23/14464	0.04347826	9.38611291	2.74572212	0.10135696	0.4307297	0.41309095	Fnip1	1
GO:0044819	mitotic G1/S transition checkpoint signaling	1/67	23/14464	0.04347826	9.38611291	2.74572212	0.10135696	0.4307297	0.41309095	Rpa2	1
GO:0048665	neuron fate specification	1/67	23/14464	0.04347826	9.38611291	2.74572212	0.10135696	0.4307297	0.41309095	Ehmt2	1
GO:0050926	regulation of positive chemotaxis	1/67	23/14464	0.04347826	9.38611291	2.74572212	0.10135696	0.4307297	0.41309095	Ager	1
GO:1900151	regulation of nuclear-transcribed mRNA catabolic process, deadenylation-dependent decay	1/67	23/14464	0.04347826	9.38611291	2.74572212	0.10135696	0.4307297	0.41309095	Hnrnpd	1
GO:0090596	sensory organ morphogenesis	3/67	242/14464	0.01239669	2.67620575	1.79384644	0.10189079	0.4307297	0.41309095	Ppp2r3a/Enpp1/Bhlhe22	3
GO:0001776	leukocyte homeostasis	2/67	117/14464	0.01709402	3.69026662	1.99314225	0.10225427	0.4307297	0.41309095	Aim2/Enpp1	2
GO:0006820	monoatomic anion transport	2/67	117/14464	0.01709402	3.69026662	1.99314225	0.10225427	0.4307297	0.41309095	Slco1a4/Gabrg1	2
GO:0015698	inorganic anion transport	2/67	117/14464	0.01709402	3.69026662	1.99314225	0.10225427	0.4307297	0.41309095	Gabrg1/Enpp1	2
GO:0017148	negative regulation of translation	2/67	117/14464	0.01709402	3.69026662	1.99314225	0.10225427	0.4307297	0.41309095	Pcif1/Zfp598	2
GO:0000045	autophagosome assembly	2/67	118/14464	0.01694915	3.65899317	1.97844237	0.10371783	0.4307297	0.41309095	Ehmt2/Stx17	2
GO:0002367	cytokine production involved in immune response	2/67	118/14464	0.01694915	3.65899317	1.97844237	0.10371783	0.4307297	0.41309095	Slamf9/H2-T23	2
GO:0006260	DNA replication	3/67	245/14464	0.0122449	2.64343588	1.76808313	0.10474259	0.4307297	0.41309095	Cdk2/Ehmt2/Rpa2	3
GO:0090090	negative regulation of canonical Wnt signaling pathway	2/67	119/14464	0.01680672	3.62824533	1.96390148	0.10518722	0.4307297	0.41309095	Ccdc88c/Ppp2r3a	2
GO:0001783	B cell apoptotic process	1/67	24/14464	0.04166667	8.99502488	2.67406793	0.10552627	0.4307297	0.41309095	Fnip1	1
GO:0006298	mismatch repair	1/67	24/14464	0.04166667	8.99502488	2.67406793	0.10552627	0.4307297	0.41309095	Rpa2	1
GO:0006882	intracellular zinc ion homeostasis	1/67	24/14464	0.04166667	8.99502488	2.67406793	0.10552627	0.4307297	0.41309095	Slc30a1	1
GO:0008206	bile acid metabolic process	1/67	24/14464	0.04166667	8.99502488	2.67406793	0.10552627	0.4307297	0.41309095	Nr1d1	1
GO:0010763	positive regulation of fibroblast migration	1/67	24/14464	0.04166667	8.99502488	2.67406793	0.10552627	0.4307297	0.41309095	Ager	1
GO:0034138	toll-like receptor 3 signaling pathway	1/67	24/14464	0.04166667	8.99502488	2.67406793	0.10552627	0.4307297	0.41309095	Rftn1	1
GO:0043931	ossification involved in bone maturation	1/67	24/14464	0.04166667	8.99502488	2.67406793	0.10552627	0.4307297	0.41309095	Enpp1	1
GO:0046716	muscle cell cellular homeostasis	1/67	24/14464	0.04166667	8.99502488	2.67406793	0.10552627	0.4307297	0.41309095	Enpp1	1
GO:0071542	dopaminergic neuron differentiation	1/67	24/14464	0.04166667	8.99502488	2.67406793	0.10552627	0.4307297	0.41309095	Tiam1	1
GO:0071634	regulation of transforming growth factor beta production	1/67	24/14464	0.04166667	8.99502488	2.67406793	0.10552627	0.4307297	0.41309095	Cd200	1
GO:1902176	negative regulation of oxidative stress-induced intrinsic apoptotic signaling pathway	1/67	24/14464	0.04166667	8.99502488	2.67406793	0.10552627	0.4307297	0.41309095	Pycr1	1
GO:1902993	positive regulation of amyloid precursor protein catabolic process	1/67	24/14464	0.04166667	8.99502488	2.67406793	0.10552627	0.4307297	0.41309095	Ager	1
GO:1903523	negative regulation of blood circulation	1/67	24/14464	0.04166667	8.99502488	2.67406793	0.10552627	0.4307297	0.41309095	Ager	1
GO:1904037	positive regulation of epithelial cell apoptotic process	1/67	24/14464	0.04166667	8.99502488	2.67406793	0.10552627	0.4307297	0.41309095	Ager	1
GO:0031507	heterochromatin formation	2/67	120/14464	0.01666667	3.59800995	1.94951637	0.10666234	0.4307297	0.41309095	Cdk2/Ehmt2	2
GO:0051224	negative regulation of protein transport	2/67	120/14464	0.01666667	3.59800995	1.94951637	0.10666234	0.4307297	0.41309095	Pkia/Cd200	2
GO:0002230	positive regulation of defense response to virus by host	1/67	25/14464	0.04	8.63522388	2.6064765	0.10967653	0.4307297	0.41309095	Aim2	1
GO:0002438	acute inflammatory response to antigenic stimulus	1/67	25/14464	0.04	8.63522388	2.6064765	0.10967653	0.4307297	0.41309095	H2-T23	1
GO:0006353	DNA-templated transcription termination	1/67	25/14464	0.04	8.63522388	2.6064765	0.10967653	0.4307297	0.41309095	Xrn2	1
GO:0030318	melanocyte differentiation	1/67	25/14464	0.04	8.63522388	2.6064765	0.10967653	0.4307297	0.41309095	Enpp1	1
GO:0034143	regulation of toll-like receptor 4 signaling pathway	1/67	25/14464	0.04	8.63522388	2.6064765	0.10967653	0.4307297	0.41309095	Nr1d1	1
GO:0035235	ionotropic glutamate receptor signaling pathway	1/67	25/14464	0.04	8.63522388	2.6064765	0.10967653	0.4307297	0.41309095	Tiam1	1
GO:0046823	negative regulation of nucleocytoplasmic transport	1/67	25/14464	0.04	8.63522388	2.6064765	0.10967653	0.4307297	0.41309095	Pkia	1
GO:0055090	acylglycerol homeostasis	1/67	25/14464	0.04	8.63522388	2.6064765	0.10967653	0.4307297	0.41309095	Fundc2	1
GO:0070328	triglyceride homeostasis	1/67	25/14464	0.04	8.63522388	2.6064765	0.10967653	0.4307297	0.41309095	Fundc2	1
GO:0071577	zinc ion transmembrane transport	1/67	25/14464	0.04	8.63522388	2.6064765	0.10967653	0.4307297	0.41309095	Slc30a1	1
GO:0099159	regulation of modification of postsynaptic structure	1/67	25/14464	0.04	8.63522388	2.6064765	0.10967653	0.4307297	0.41309095	Tiam1	1
GO:1903975	regulation of glial cell migration	1/67	25/14464	0.04	8.63522388	2.6064765	0.10967653	0.4307297	0.41309095	Tiam1	1
GO:1904353	regulation of telomere capping	1/67	25/14464	0.04	8.63522388	2.6064765	0.10967653	0.4307297	0.41309095	Hnrnpd	1
GO:1904862	inhibitory synapse assembly	1/67	25/14464	0.04	8.63522388	2.6064765	0.10967653	0.4307297	0.41309095	Gabrg1	1
GO:0070665	positive regulation of leukocyte proliferation	2/67	123/14464	0.01626016	3.51025361	1.90726496	0.11112131	0.4307297	0.41309095	H2-T23/Ager	2
GO:0071466	cellular response to xenobiotic stimulus	2/67	124/14464	0.01612903	3.48194511	1.89347263	0.11261857	0.4307297	0.41309095	Aim2/Ehmt2	2
GO:0002313	mature B cell differentiation involved in immune response	1/67	26/14464	0.03846154	8.30309989	2.5425587	0.11380782	0.4307297	0.41309095	Enpp1	1
GO:0002861	regulation of inflammatory response to antigenic stimulus	1/67	26/14464	0.03846154	8.30309989	2.5425587	0.11380782	0.4307297	0.41309095	H2-T23	1
GO:0010893	positive regulation of steroid biosynthetic process	1/67	26/14464	0.03846154	8.30309989	2.5425587	0.11380782	0.4307297	0.41309095	Nr1d1	1
GO:0036037	CD8-positive, alpha-beta T cell activation	1/67	26/14464	0.03846154	8.30309989	2.5425587	0.11380782	0.4307297	0.41309095	H2-T23	1
GO:0070269	pyroptotic inflammatory response	1/67	26/14464	0.03846154	8.30309989	2.5425587	0.11380782	0.4307297	0.41309095	Aim2	1
GO:0070977	bone maturation	1/67	26/14464	0.03846154	8.30309989	2.5425587	0.11380782	0.4307297	0.41309095	Enpp1	1
GO:0090200	positive regulation of release of cytochrome c from mitochondria	1/67	26/14464	0.03846154	8.30309989	2.5425587	0.11380782	0.4307297	0.41309095	Tnfsf10	1
GO:0098885	modification of postsynaptic actin cytoskeleton	1/67	26/14464	0.03846154	8.30309989	2.5425587	0.11380782	0.4307297	0.41309095	Tiam1	1
GO:1900745	positive regulation of p38MAPK cascade	1/67	26/14464	0.03846154	8.30309989	2.5425587	0.11380782	0.4307297	0.41309095	Ager	1
GO:0000086	G2/M transition of mitotic cell cycle	2/67	125/14464	0.016	3.45408955	1.87982133	0.11412115	0.4307297	0.41309095	Cdk2/Pkia	2
GO:0045727	positive regulation of translation	2/67	125/14464	0.016	3.45408955	1.87982133	0.11412115	0.4307297	0.41309095	Hnrnpd/Pcif1	2
GO:0022411	cellular component disassembly	4/67	400/14464	0.01	2.15880597	1.60330772	0.11419951	0.4307297	0.41309095	Ccdc88c/Ngef/Stx17/Zfp598	4
GO:0014812	muscle cell migration	2/67	126/14464	0.01587302	3.42667614	1.86630834	0.11562899	0.4307297	0.41309095	Ager/Enpp1	2
GO:0050728	negative regulation of inflammatory response	2/67	126/14464	0.01587302	3.42667614	1.86630834	0.11562899	0.4307297	0.41309095	Nr1d1/Cd200	2
GO:1905037	autophagosome organization	2/67	126/14464	0.01587302	3.42667614	1.86630834	0.11562899	0.4307297	0.41309095	Ehmt2/Stx17	2
GO:0050921	positive regulation of chemotaxis	2/67	127/14464	0.01574803	3.39969444	1.85293104	0.117142	0.4307297	0.41309095	Ager/Tiam1	2
GO:0002218	activation of innate immune response	3/67	258/14464	0.01162791	2.5102395	1.66974582	0.11746	0.4307297	0.41309095	Nr1d1/Aim2/Rftn1	3
GO:0006376	mRNA splice site recognition	1/67	27/14464	0.03703704	7.99557767	2.48197601	0.11792022	0.4307297	0.41309095	Luc7l2	1
GO:0006829	zinc ion transport	1/67	27/14464	0.03703704	7.99557767	2.48197601	0.11792022	0.4307297	0.41309095	Slc30a1	1
GO:0031579	membrane raft organization	1/67	27/14464	0.03703704	7.99557767	2.48197601	0.11792022	0.4307297	0.41309095	Rftn1	1
GO:0032633	interleukin-4 production	1/67	27/14464	0.03703704	7.99557767	2.48197601	0.11792022	0.4307297	0.41309095	H2-T23	1
GO:0071604	transforming growth factor beta production	1/67	27/14464	0.03703704	7.99557767	2.48197601	0.11792022	0.4307297	0.41309095	Cd200	1
GO:1904889	regulation of excitatory synapse assembly	1/67	27/14464	0.03703704	7.99557767	2.48197601	0.11792022	0.4307297	0.41309095	Abi3bp	1
GO:2000050	regulation of non-canonical Wnt signaling pathway	1/67	27/14464	0.03703704	7.99557767	2.48197601	0.11792022	0.4307297	0.41309095	Tiam1	1
GO:0046328	regulation of JNK cascade	2/67	128/14464	0.015625	3.37313433	1.83968684	0.1186601	0.4307297	0.41309095	Ccdc88c/Ager	2
GO:0030183	B cell differentiation	2/67	129/14464	0.01550388	3.346986	1.82657327	0.12018322	0.4307297	0.41309095	Enpp1/Fnip1	2
GO:0043433	negative regulation of DNA-binding transcription factor activity	2/67	130/14464	0.01538462	3.32123995	1.81358788	0.12171128	0.4307297	0.41309095	Aim2/Cd200	2
GO:0031349	positive regulation of defense response	4/67	410/14464	0.0097561	2.10615217	1.55002042	0.12197935	0.4307297	0.41309095	Nr1d1/Aim2/H2-T23/Rftn1	4
GO:0043434	response to peptide hormone	4/67	410/14464	0.0097561	2.10615217	1.55002042	0.12197935	0.4307297	0.41309095	Srsf5/Cdk2/Tnfsf10/Enpp1	4
GO:0001916	positive regulation of T cell mediated cytotoxicity	1/67	28/14464	0.03571429	7.71002132	2.42443236	0.12201383	0.4307297	0.41309095	H2-T23	1
GO:0002478	antigen processing and presentation of exogenous peptide antigen	1/67	28/14464	0.03571429	7.71002132	2.42443236	0.12201383	0.4307297	0.41309095	H2-T23	1
GO:0003401	axis elongation	1/67	28/14464	0.03571429	7.71002132	2.42443236	0.12201383	0.4307297	0.41309095	Ppp2r3a	1
GO:0021952	central nervous system projection neuron axonogenesis	1/67	28/14464	0.03571429	7.71002132	2.42443236	0.12201383	0.4307297	0.41309095	Bhlhe22	1
GO:0032735	positive regulation of interleukin-12 production	1/67	28/14464	0.03571429	7.71002132	2.42443236	0.12201383	0.4307297	0.41309095	Ager	1
GO:0034505	tooth mineralization	1/67	28/14464	0.03571429	7.71002132	2.42443236	0.12201383	0.4307297	0.41309095	Enpp1	1
GO:0034698	response to gonadotropin	1/67	28/14464	0.03571429	7.71002132	2.42443236	0.12201383	0.4307297	0.41309095	Epha8	1
GO:0042832	defense response to protozoan	1/67	28/14464	0.03571429	7.71002132	2.42443236	0.12201383	0.4307297	0.41309095	Enpp1	1
GO:0045686	negative regulation of glial cell differentiation	1/67	28/14464	0.03571429	7.71002132	2.42443236	0.12201383	0.4307297	0.41309095	Nr1d1	1
GO:0048799	animal organ maturation	1/67	28/14464	0.03571429	7.71002132	2.42443236	0.12201383	0.4307297	0.41309095	Enpp1	1
GO:0050901	leukocyte tethering or rolling	1/67	28/14464	0.03571429	7.71002132	2.42443236	0.12201383	0.4307297	0.41309095	Podxl2	1
GO:0099150	regulation of postsynaptic specialization assembly	1/67	28/14464	0.03571429	7.71002132	2.42443236	0.12201383	0.4307297	0.41309095	Abi3bp	1
GO:1902894	negative regulation of miRNA transcription	1/67	28/14464	0.03571429	7.71002132	2.42443236	0.12201383	0.4307297	0.41309095	Nfatc4	1
GO:0007162	negative regulation of cell adhesion	3/67	263/14464	0.01140684	2.46251632	1.6328629	0.1225007	0.4307297	0.41309095	Ajap1/H2-T23/Ager	3
GO:0050708	regulation of protein secretion	3/67	263/14464	0.01140684	2.46251632	1.6328629	0.1225007	0.4307297	0.41309095	Nr1d1/Cd200/Tiam1	3
GO:0006270	DNA replication initiation	1/67	29/14464	0.03448276	7.44415852	2.3696675	0.12608871	0.4307297	0.41309095	Cdk2	1
GO:0006929	substrate-dependent cell migration	1/67	29/14464	0.03448276	7.44415852	2.3696675	0.12608871	0.4307297	0.41309095	Epha8	1
GO:0007129	homologous chromosome pairing at meiosis	1/67	29/14464	0.03448276	7.44415852	2.3696675	0.12608871	0.4307297	0.41309095	Ehmt2	1
GO:0042269	regulation of natural killer cell mediated cytotoxicity	1/67	29/14464	0.03448276	7.44415852	2.3696675	0.12608871	0.4307297	0.41309095	H2-T23	1
GO:0045940	positive regulation of steroid metabolic process	1/67	29/14464	0.03448276	7.44415852	2.3696675	0.12608871	0.4307297	0.41309095	Nr1d1	1
GO:0046006	regulation of activated T cell proliferation	1/67	29/14464	0.03448276	7.44415852	2.3696675	0.12608871	0.4307297	0.41309095	Ager	1
GO:0071392	cellular response to estradiol stimulus	1/67	29/14464	0.03448276	7.44415852	2.3696675	0.12608871	0.4307297	0.41309095	Hnrnpd	1
GO:0098868	bone growth	1/67	29/14464	0.03448276	7.44415852	2.3696675	0.12608871	0.4307297	0.41309095	Enpp1	1
GO:0032675	regulation of interleukin-6 production	2/67	133/14464	0.01503759	3.24632477	1.77537749	0.12632433	0.4307297	0.41309095	Ager/Cd200	2
GO:0060485	mesenchyme development	3/67	267/14464	0.01123596	2.42562469	1.60395854	0.12659039	0.4307297	0.41309095	Ager/Tiam1/Ldlrad4	3
GO:0022604	regulation of cell morphogenesis	3/67	268/14464	0.01119403	2.41657385	1.59681357	0.12762058	0.4307297	0.41309095	Slc30a1/Tiam1/Il1rapl1	3
GO:0045598	regulation of fat cell differentiation	2/67	134/14464	0.01492537	3.22209846	1.7628818	0.12787138	0.4307297	0.41309095	Nr1d1/Enpp1	2
GO:1904950	negative regulation of establishment of protein localization	2/67	135/14464	0.01481481	3.19823107	1.75050307	0.129423	0.4307297	0.41309095	Pkia/Cd200	2
GO:0001562	response to protozoan	1/67	30/14464	0.03333333	7.1960199	2.3174516	0.13014497	0.4307297	0.41309095	Enpp1	1
GO:0005979	regulation of glycogen biosynthetic process	1/67	30/14464	0.03333333	7.1960199	2.3174516	0.13014497	0.4307297	0.41309095	Enpp1	1
GO:0007214	gamma-aminobutyric acid signaling pathway	1/67	30/14464	0.03333333	7.1960199	2.3174516	0.13014497	0.4307297	0.41309095	Gabrg1	1
GO:0010962	regulation of glucan biosynthetic process	1/67	30/14464	0.03333333	7.1960199	2.3174516	0.13014497	0.4307297	0.41309095	Enpp1	1
GO:0032814	regulation of natural killer cell activation	1/67	30/14464	0.03333333	7.1960199	2.3174516	0.13014497	0.4307297	0.41309095	H2-T23	1
GO:0036119	response to platelet-derived growth factor	1/67	30/14464	0.03333333	7.1960199	2.3174516	0.13014497	0.4307297	0.41309095	Enpp1	1
GO:0042745	circadian sleep/wake cycle	1/67	30/14464	0.03333333	7.1960199	2.3174516	0.13014497	0.4307297	0.41309095	Nr1d1	1
GO:0060071	Wnt signaling pathway, planar cell polarity pathway	1/67	30/14464	0.03333333	7.1960199	2.3174516	0.13014497	0.4307297	0.41309095	Ccdc88c	1
GO:0090128	regulation of synapse maturation	1/67	30/14464	0.03333333	7.1960199	2.3174516	0.13014497	0.4307297	0.41309095	Nfatc4	1
GO:0150146	cell junction disassembly	1/67	30/14464	0.03333333	7.1960199	2.3174516	0.13014497	0.4307297	0.41309095	Ngef	1
GO:2000108	positive regulation of leukocyte apoptotic process	1/67	30/14464	0.03333333	7.1960199	2.3174516	0.13014497	0.4307297	0.41309095	Fnip1	1
GO:0030336	negative regulation of cell migration	3/67	271/14464	0.01107011	2.38982211	1.57556873	0.13072946	0.4307297	0.41309095	Ager/Cd200/Ldlrad4	3
GO:0050657	nucleic acid transport	2/67	136/14464	0.01470588	3.17471466	1.73823921	0.1309791	0.4307297	0.41309095	Tst/Rftn1	2
GO:0050658	RNA transport	2/67	136/14464	0.01470588	3.17471466	1.73823921	0.1309791	0.4307297	0.41309095	Tst/Rftn1	2
GO:0010001	glial cell differentiation	3/67	272/14464	0.01102941	2.381036	1.56854955	0.1317718	0.4307297	0.41309095	Nr1d1/Ager/Enpp1	3
GO:0000375	RNA splicing, via transesterification reactions	3/67	273/14464	0.01098901	2.37231425	1.56156113	0.13281712	0.4307297	0.41309095	Srsf5/Arglu1/Luc7l2	3
GO:0000377	RNA splicing, via transesterification reactions with bulged adenosine as nucleophile	3/67	273/14464	0.01098901	2.37231425	1.56156113	0.13281712	0.4307297	0.41309095	Srsf5/Arglu1/Luc7l2	3
GO:0000398	mRNA splicing, via spliceosome	3/67	273/14464	0.01098901	2.37231425	1.56156113	0.13281712	0.4307297	0.41309095	Srsf5/Arglu1/Luc7l2	3
GO:0050768	negative regulation of neurogenesis	2/67	138/14464	0.01449275	3.1287043	1.71404808	0.13410449	0.4307297	0.41309095	Nr1d1/Nfatc4	2
GO:0022038	corpus callosum development	1/67	31/14464	0.03225806	6.96389023	2.26758082	0.13418268	0.4307297	0.41309095	Bhlhe22	1
GO:0032660	regulation of interleukin-17 production	1/67	31/14464	0.03225806	6.96389023	2.26758082	0.13418268	0.4307297	0.41309095	Rftn1	1
GO:0034067	protein localization to Golgi apparatus	1/67	31/14464	0.03225806	6.96389023	2.26758082	0.13418268	0.4307297	0.41309095	Sys1	1
GO:1905244	regulation of modification of synaptic structure	1/67	31/14464	0.03225806	6.96389023	2.26758082	0.13418268	0.4307297	0.41309095	Tiam1	1
GO:2000629	negative regulation of miRNA metabolic process	1/67	31/14464	0.03225806	6.96389023	2.26758082	0.13418268	0.4307297	0.41309095	Nfatc4	1
GO:0032635	interleukin-6 production	2/67	139/14464	0.01438849	3.10619564	1.70211691	0.13567362	0.4307297	0.41309095	Ager/Cd200	2
GO:0051236	establishment of RNA localization	2/67	139/14464	0.01438849	3.10619564	1.70211691	0.13567362	0.4307297	0.41309095	Tst/Rftn1	2
GO:0002706	regulation of lymphocyte mediated immunity	2/67	140/14464	0.01428571	3.08400853	1.69029282	0.13724695	0.4307297	0.41309095	H2-T23/Ager	2
GO:0002021	response to dietary excess	1/67	32/14464	0.03125	6.74626866	2.21987361	0.13820192	0.4307297	0.41309095	Enpp1	1
GO:0002715	regulation of natural killer cell mediated immunity	1/67	32/14464	0.03125	6.74626866	2.21987361	0.13820192	0.4307297	0.41309095	H2-T23	1
GO:0022010	central nervous system myelination	1/67	32/14464	0.03125	6.74626866	2.21987361	0.13820192	0.4307297	0.41309095	Enpp1	1
GO:0032291	axon ensheathment in central nervous system	1/67	32/14464	0.03125	6.74626866	2.21987361	0.13820192	0.4307297	0.41309095	Enpp1	1
GO:0032620	interleukin-17 production	1/67	32/14464	0.03125	6.74626866	2.21987361	0.13820192	0.4307297	0.41309095	Rftn1	1
GO:0044342	type B pancreatic cell proliferation	1/67	32/14464	0.03125	6.74626866	2.21987361	0.13820192	0.4307297	0.41309095	Nr1d1	1
GO:2000677	regulation of transcription regulatory region DNA binding	1/67	32/14464	0.03125	6.74626866	2.21987361	0.13820192	0.4307297	0.41309095	Dazap2	1
GO:2000765	regulation of cytoplasmic translation	1/67	32/14464	0.03125	6.74626866	2.21987361	0.13820192	0.4307297	0.41309095	Hnrnpd	1
GO:0044839	cell cycle G2/M phase transition	2/67	141/14464	0.0141844	3.06213613	1.67857397	0.13882441	0.4307297	0.41309095	Cdk2/Pkia	2
GO:0055088	lipid homeostasis	2/67	141/14464	0.0141844	3.06213613	1.67857397	0.13882441	0.4307297	0.41309095	Nr1d1/Fundc2	2
GO:0048592	eye morphogenesis	2/67	142/14464	0.01408451	3.04057179	1.66695858	0.14040593	0.4307297	0.41309095	Ppp2r3a/Bhlhe22	2
GO:0001933	negative regulation of protein phosphorylation	3/67	281/14464	0.01067616	2.30477506	1.50672969	0.14128473	0.4307297	0.41309095	Ager/Pkia/Enpp1	3
GO:0042129	regulation of T cell proliferation	2/67	143/14464	0.01398601	3.01930905	1.6554449	0.14199143	0.4307297	0.41309095	H2-T23/Ager	2
GO:0002474	antigen processing and presentation of peptide antigen via MHC class I	1/67	33/14464	0.03030303	6.54183627	2.1741677	0.14220279	0.4307297	0.41309095	H2-T23	1
GO:0006346	DNA methylation-dependent heterochromatin formation	1/67	33/14464	0.03030303	6.54183627	2.1741677	0.14220279	0.4307297	0.41309095	Ehmt2	1
GO:0010719	negative regulation of epithelial to mesenchymal transition	1/67	33/14464	0.03030303	6.54183627	2.1741677	0.14220279	0.4307297	0.41309095	Ldlrad4	1
GO:0019731	antibacterial humoral response	1/67	33/14464	0.03030303	6.54183627	2.1741677	0.14220279	0.4307297	0.41309095	H2-T23	1
GO:0030279	negative regulation of ossification	1/67	33/14464	0.03030303	6.54183627	2.1741677	0.14220279	0.4307297	0.41309095	Enpp1	1
GO:0043247	telomere maintenance in response to DNA damage	1/67	33/14464	0.03030303	6.54183627	2.1741677	0.14220279	0.4307297	0.41309095	Cdk2	1

GO:0043330 response to exogenous dsRNA	1/67	33/14464	0.03030303	6.54183627	2.1741677	0.14220279	0.4307297	0.41309095 Rftn1	1
GO:0048710 regulation of astrocyte differentiation	1/67	33/14464	0.03030303	6.54183627	2.1741677	0.14220279	0.4307297	0.41309095 Nr1d1	1
GO:0050691 regulation of defense response to virus by host	1/67	33/14464	0.03030303	6.54183627	2.1741677	0.14220279	0.4307297	0.41309095 Aim2	1
GO:0090317 negative regulation of intracellular protein transport	1/67	33/14464	0.03030303	6.54183627	2.1741677	0.14220279	0.4307297	0.41309095 Pkia	1
GO:2000146 negative regulation of cell motility	3/67	282/14464	0.0106383	2.2966021	1.50000644	0.142356	0.4307297	0.41309095 Ager/Cd200/Ldlrad4	3
GO:0007416 synapse assembly	3/67	283/14464	0.01060071	2.28848689	1.49331142	0.14343007	0.4307297	0.41309095 Gabrg1/Abi3bp/Il1rapl1	3
GO:0043523 regulation of neuron apoptotic process	3/67	283/14464	0.01060071	2.28848689	1.49331142	0.14343007	0.4307297	0.41309095 Ager/Pycr1/Nfatc4	3
GO:0016241 regulation of macroautophagy	2/67	144/14464	0.01388889	2.99834163	1.64403123	0.14358086	0.4307297	0.41309095 Ehmt2/Gpsm1	2
GO:0043281 regulation of cysteine-type endopeptidase activity involved in apoptotic process	2/67	144/14464	0.01388889	2.99834163	1.64403123	0.14358086	0.4307297	0.41309095 Tnfsf10/Fnip1	2
GO:0006790 sulfur compound metabolic process	3/67	285/14464	0.01052632	2.27242734	1.4800051	0.14558646	0.4307297	0.41309095 Gstt2/Gstt3/Enpp1	3
GO:0000132 establishment of mitotic spindle orientation	1/67	34/14464	0.02941176	6.34942932	2.13031752	0.14618535	0.4307297	0.41309095 Gpsm1	1
GO:0001953 negative regulation of cell-matrix adhesion	1/67	34/14464	0.02941176	6.34942932	2.13031752	0.14618535	0.4307297	0.41309095 Ajap1	1
GO:0001958 endochondral ossification	1/67	34/14464	0.02941176	6.34942932	2.13031752	0.14618535	0.4307297	0.41309095 Enpp1	1
GO:0002335 mature B cell differentiation	1/67	34/14464	0.02941176	6.34942932	2.13031752	0.14618535	0.4307297	0.41309095 Enpp1	1
GO:0009311 oligosaccharide metabolic process	1/67	34/14464	0.02941176	6.34942932	2.13031752	0.14618535	0.4307297	0.41309095 Man2b1	1
GO:0010043 response to zinc ion	1/67	34/14464	0.02941176	6.34942932	2.13031752	0.14618535	0.4307297	0.41309095 Slc30a1	1
GO:0019884 antigen processing and presentation of exogenous antigen	1/67	34/14464	0.02941176	6.34942932	2.13031752	0.14618535	0.4307297	0.41309095 H2-T23	1
GO:0034063 stress granule assembly	1/67	34/14464	0.02941176	6.34942932	2.13031752	0.14618535	0.4307297	0.41309095 Dazap2	1
GO:0035136 forelimb morphogenesis	1/67	34/14464	0.02941176	6.34942932	2.13031752	0.14618535	0.4307297	0.41309095 Enpp1	1
GO:0036075 replacement ossification	1/67	34/14464	0.02941176	6.34942932	2.13031752	0.14618535	0.4307297	0.41309095 Enpp1	1
GO:0045920 negative regulation of exocytosis	1/67	34/14464	0.02941176	6.34942932	2.13031752	0.14618535	0.4307297	0.41309095 Il1rapl1	1
GO:0071398 cellular response to fatty acid	1/67	34/14464	0.02941176	6.34942932	2.13031752	0.14618535	0.4307297	0.41309095 Nfatc4	1
GO:0097107 postsynaptic density assembly	1/67	34/14464	0.02941176	6.34942932	2.13031752	0.14618535	0.4307297	0.41309095 Abi3bp	1
GO:1902175 regulation of oxidative stress-induced intrinsic apoptotic signaling pathway	1/67	34/14464	0.02941176	6.34942932	2.13031752	0.14618535	0.4307297	0.41309095 Pycr1	1
GO:1902275 regulation of chromatin organization	1/67	34/14464	0.02941176	6.34942932	2.13031752	0.14618535	0.4307297	0.41309095 Cdk2	1
GO:1905606 regulation of presynapse assembly	1/67	34/14464	0.02941176	6.34942932	2.13031752	0.14618535	0.4307297	0.41309095 Il1rapl1	1
GO:2001235 positive regulation of apoptotic signaling pathway	2/67	146/14464	0.01369863	2.95726845	1.62149733	0.14677118	0.43163052	0.41395489 Nfatc4/Tnfsf10	2
GO:0051961 negative regulation of nervous system development	2/67	147/14464	0.01360544	2.93715098	1.61037388	0.14837195	0.43329718	0.41555329 Nr1d1/Nfatc4	2
GO:0060041 retina development in camera-type eye	2/67	147/14464	0.01360544	2.93715098	1.61037388	0.14837195	0.43329718	0.41555329 Bhlhe22/Xm2	2
GO:0051052 regulation of DNA metabolic process	4/67	443/14464	0.00902935	1.94926047	1.38429406	0.1492043	0.43329718	0.41555329 Cdk2/Hnmpd/Ehmt2/Rpa2	4
GO:0002673 regulation of acute inflammatory response	1/67	35/14464	0.02857143	6.16801706	2.08819204	0.15014971	0.43329718	0.41555329 H2-T23	1
GO:0003351 epithelial cilium movement involved in extracellular fluid movement	1/67	35/14464	0.02857143	6.16801706	2.08819204	0.15014971	0.43329718	0.41555329 Ccdc88c	1
GO:0016242 negative regulation of macroautophagy	1/67	35/14464	0.02857143	6.16801706	2.08819204	0.15014971	0.43329718	0.41555329 Ehmt2	1
GO:0032212 positive regulation of telomere maintenance via telomerase	1/67	35/14464	0.02857143	6.16801706	2.08819204	0.15014971	0.43329718	0.41555329 Hnmpd	1
GO:0046640 regulation of alpha-beta T cell proliferation	1/67	35/14464	0.02857143	6.16801706	2.08819204	0.15014971	0.43329718	0.41555329 H2-T23	1
GO:0072344 rescue of stalled ribosome	1/67	35/14464	0.02857143	6.16801706	2.08819204	0.15014971	0.43329718	0.41555329 Zfp598	1
GO:0090329 regulation of DNA-templated DNA replication	1/67	35/14464	0.02857143	6.16801706	2.08819204	0.15014971	0.43329718	0.41555329 Cdk2	1
GO:0046631 alpha-beta T cell activation	2/67	149/14464	0.01342282	2.89772613	1.58840626	0.15158437	0.43332448	0.41557948 H2-T23/Ager	2
GO:0002831 regulation of response to biotic stimulus	4/67	446/14464	0.00896861	1.93614885	1.36993481	0.15179035	0.43332448	0.41557948 Nr1d1/Aim2/H2-T23/Rftn1	4
GO:0050769 positive regulation of neurogenesis	3/67	291/14464	0.01030928	2.22557317	1.44073941	0.15212032	0.43332448	0.41557948 Slc30a1/Tiam1/Il1rapl1	3
GO:1903131 mononuclear cell differentiation	4/67	447/14464	0.00894855	1.93181742	1.36517285	0.15265626	0.43332448	0.41557948 Slamf9/Ager/Enpp1/Fnip1	4
GO:0002714 positive regulation of B cell mediated immunity	1/67	36/14464	0.02777778	5.99668325	2.04767296	0.15409592	0.43332448	0.41557948 H2-T23	1
GO:0002891 positive regulation of immunoglobulin mediated immune response	1/67	36/14464	0.02777778	5.99668325	2.04767296	0.15409592	0.43332448	0.41557948 H2-T23	1
GO:0007026 negative regulation of microtubule depolymerization	1/67	36/14464	0.02777778	5.99668325	2.04767296	0.15409592	0.43332448	0.41557948 Ccdc88c	1
GO:0031952 regulation of protein autophosphorylation	1/67	36/14464	0.02777778	5.99668325	2.04767296	0.15409592	0.43332448	0.41557948 Enpp1	1
GO:0045879 negative regulation of smoothened signaling pathway	1/67	36/14464	0.02777778	5.99668325	2.04767296	0.15409592	0.43332448	0.41557948 Enpp1	1
GO:0050798 activated T cell proliferation	1/67	36/14464	0.02777778	5.99668325	2.04767296	0.15409592	0.43332448	0.41557948 Ager	1
GO:0050931 pigment cell differentiation	1/67	36/14464	0.02777778	5.99668325	2.04767296	0.15409592	0.43332448	0.41557948 Enpp1	1
GO:0050951 sensory perception of temperature stimulus	1/67	36/14464	0.02777778	5.99668325	2.04767296	0.15409592	0.43332448	0.41557948 Enpp1	1
GO:0060292 long-term synaptic depression	1/67	36/14464	0.02777778	5.99668325	2.04767296	0.15409592	0.43332448	0.41557948 Ager	1
GO:1902742 apoptotic process involved in development	1/67	36/14464	0.02777778	5.99668325	2.04767296	0.15409592	0.43332448	0.41557948 Enpp1	1
GO:0010506 regulation of autophagy	3/67	295/14464	0.01016949	2.1953959	1.41508902	0.1565285	0.43805097	0.42011241 Ager/Ehmt2/Gpsm1	3
GO:0051223 regulation of protein transport	4/67	452/14464	0.00884956	1.91044776	1.34154356	0.15701462	0.43805097	0.42011241 Nr1d1/Pkia/Cd200/Tiam1	4
GO:0045862 positive regulation of proteolysis	3/67	296/14464	0.01013514	2.18797902	1.40874032	0.1576369	0.43805097	0.42011241 Aim2/Ager/Tnfsf10	3
GO:0002686 negative regulation of leukocyte migration	1/67	37/14464	0.02702703	5.83461073	2.00865315	0.15802409	0.43805097	0.42011241 Cd200	1
GO:0016233 telomere capping	1/67	37/14464	0.02702703	5.83461073	2.00865315	0.15802409	0.43805097	0.42011241 Hnmpd	1
GO:1904646 cellular response to amyloid-beta	1/67	37/14464	0.02702703	5.83461073	2.00865315	0.15802409	0.43805097	0.42011241 Ager	1
GO:1990806 ligand-gated ion channel signaling pathway	1/67	37/14464	0.02702703	5.83461073	2.00865315	0.15802409	0.43805097	0.42011241 Tiam1	1
GO:0045814 negative regulation of gene expression, epigenetic	2/67	153/14464	0.0130719	2.82196859	1.54554695	0.15805084	0.43805097	0.42011241 Cdk2/Ehmt2	2
GO:0040013 negative regulation of locomotion	3/67	297/14464	0.01010101	2.18061209	1.40241677	0.15874779	0.43831694	0.42036748 Ager/Cd200/Ldlrad4	3
GO:0006575 cellular modified amino acid metabolic process	2/67	154/14464	0.01298701	2.80364412	1.53504809	0.15967575	0.43831694	0.42036748 Gstt2/Gstt3	2
GO:0001569 branching involved in blood vessel morphogenesis	1/67	38/14464	0.02631579	5.68106834	1.97103533	0.16193428	0.43831694	0.42036748 Nfatc4	1
GO:0002691 regulation of cellular extravasation	1/67	38/14464	0.02631579	5.68106834	1.97103533	0.16193428	0.43831694	0.42036748 Ager	1
GO:0006858 extracellular transport	1/67	38/14464	0.02631579	5.68106834	1.97103533	0.16193428	0.43831694	0.42036748 Ccdc88c	1
GO:0021955 central nervous system neuron axonogenesis	1/67	38/14464	0.02631579	5.68106834	1.97103533	0.16193428	0.43831694	0.42036748 Bhlhe22	1
GO:0035886 vascular associated smooth muscle cell differentiation	1/67	38/14464	0.02631579	5.68106834	1.97103533	0.16193428	0.43831694	0.42036748 Nfatc4	1
GO:0040001 establishment of mitotic spindle localization	1/67	38/14464	0.02631579	5.68106834	1.97103533	0.16193428	0.43831694	0.42036748 Gpsm1	1
GO:0044273 sulfur compound catabolic process	1/67	38/14464	0.02631579	5.68106834	1.97103533	0.16193428	0.43831694	0.42036748 Enpp1	1
GO:0071312 cellular response to alkaloid	1/67	38/14464	0.02631579	5.68106834	1.97103533	0.16193428	0.43831694	0.42036748 Ehmt2	1
GO:2000404 regulation of T cell migration	1/67	38/14464	0.02631579	5.68106834	1.97103533	0.16193428	0.43831694	0.42036748 Cd200	1
GO:0001906 cell killing	2/67	156/14464	0.01282051	2.76769996	1.51430019	0.1629351	0.43831694	0.42036748 H2-T23/Ager	2
GO:0002822 regulation of adaptive immune response based on somatic recombination of immune receptors built from	2/67	156/14464	0.01282051	2.76769996	1.51430019	0.1629351	0.43831694	0.42036748 H2-T23/Ager	2
GO:0007254 JNK cascade	2/67	156/14464	0.01282051	2.76769996	1.51430019	0.1629351	0.43831694	0.42036748 Ccdc88c/Ager	2
GO:0007613 memory	2/67	156/14464	0.01282051	2.76769996	1.51430019	0.1629351	0.43831694	0.42036748 Nfatc4/Ehmt2	2
GO:0042326 negative regulation of phosphorylation	3/67	301/14464	0.00996678	2.15163386	1.37737005	0.16321596	0.43831694	0.42036748 Ager/Pkia/Enpp1	3
GO:0003382 epithelial cell morphogenesis	1/67	39/14464	0.02564103	5.53539992	1.93473092	0.16582659	0.43831694	0.42036748 Ccdc88c	1
GO:0007157 heterophilic cell-cell adhesion via plasma membrane cell adhesion molecules	1/67	39/14464	0.02564103	5.53539992	1.93473092	0.16582659	0.43831694	0.42036748 Il1rapl1	1
GO:0032715 negative regulation of interleukin-6 production	1/67	39/14464	0.02564103	5.53539992	1.93473092	0.16582659	0.43831694	0.42036748 Cd200	1
GO:0032885 regulation of polysaccharide biosynthetic process	1/67	39/14464	0.02564103	5.53539992	1.93473092	0.16582659	0.43831694	0.42036748 Enpp1	1
GO:0043001 Golgi to plasma membrane protein transport	1/67	39/14464	0.02564103	5.53539992	1.93473092	0.16582659	0.43831694	0.42036748 Sys1	1
GO:0090279 regulation of calcium ion import	1/67	39/14464	0.02564103	5.53539992	1.93473092	0.16582659	0.43831694	0.42036748 Slc30a1	1
GO:0099174 regulation of presynapse organization	1/67	39/14464	0.02564103	5.53539992	1.93473092	0.16582659	0.43831694	0.42036748 Il1rapl1	1
GO:1900744 regulation of p38MAPK cascade	1/67	39/14464	0.02564103	5.53539992	1.93473092	0.16582659	0.43831694	0.42036748 Ager	1
GO:1904358 positive regulation of telomere maintenance via telomere lengthening	1/67	39/14464	0.02564103	5.53539992	1.93473092	0.16582659	0.43831694	0.42036748 Hnmpd	1
GO:1904738 vascular associated smooth muscle cell migration	1/67	39/14464	0.02564103	5.53539992	1.93473092	0.16582659	0.43831694	0.42036748 Enpp1	1
GO:1905521 regulation of macrophage migration	1/67	39/14464	0.02564103	5.53539992	1.93473092	0.16582659	0.43831694	0.42036748 Cd200	1
GO:1901990 regulation of mitotic cell cycle phase transition	3/67	304/14464	0.00986842	2.13040063	1.35883945	0.16659233	0.43877412	0.42080594 Cdk2/Pkia/Rpa2	3
GO:0042110 T cell activation	4/67	464/14464	0.00862069	1.86103963	1.28602291	0.16766495	0.43877412	0.42080594 Slamf9/H2-T23/Ager/Enpp1	4
GO:0071356 cellular response to tumor necrosis factor	2/67	160/14464	0.0125	2.69850746	1.47376858	0.16949008	0.43877412	0.42080594 Nr1d1/Aim2	2
GO:0001782 B cell homeostasis	1/67	40/14464	0.025	5.39701493	1.89965909	0.16970109	0.43877412	0.42080594 Enpp1	1
GO:0007099 centriole replication	1/67	40/14464	0.025	5.39701493	1.89965909	0.16970109	0.43877412	0.42080594 Cdk2	1
GO:0032941 secretion by tissue	1/67	40/14464	0.025	5.39701493	1.89965909	0.16970109	0.43877412	0.42080594 Enpp1	1
GO:0043388 positive regulation of DNA binding	1/67	40/14464	0.025	5.39701493	1.89965909	0.16970109	0.43877412	0.42080594 Dazap2	1
GO:0045740 positive regulation of DNA replication	1/67	40/14464	0.025	5.39701493	1.89965909	0.16970109	0.43877412	0.42080594 Cdk2	1
GO:0046633 alpha-beta T cell proliferation	1/67	40/14464	0.025	5.39701493	1.89965909	0.16970109	0.43877412	0.42080594 H2-T23	1
GO:0070873 regulation of glycogen metabolic process	1/67	40/14464	0.025	5.39701493	1.89965909	0.16970109	0.43877412	0.42080594 Enpp1	1
GO:0140894 endolysosomal toll-like receptor signaling pathway	1/67	40/14464	0.025	5.39701493	1.89965909	0.16970109	0.43877412	0.42080594 Rftn1	1
GO:1900181 negative regulation of protein localization to nucleus	1/67	40/14464	0.025	5.39701493	1.89965909	0.16970109	0.43877412	0.42080594 Pkia	1
GO:1901532 regulation of hematopoietic progenitor cell differentiation	1/67	40/14464	0.025	5.39701493	1.89965909	0.16970109	0.43877412	0.42080594 Fnip1	1
GO:0002694 regulation of leukocyte activation	4/67	467/14464	0.00856531	1.84908434	1.27239626	0.17036812	0.43949958	0.4215017 Nr1d1/H2-T23/Ager/Cd200	4
GO:0016236 macroautophagy	3/67	309/14464	0.00970874	2.09592813	1.32842478	0.17226611	0.43949958	0.4215017 Ehmt2/Stx17/Gpsm1	3
GO:0001754 eye photoreceptor cell differentiation	1/67	41/14464	0.02439024	5.26538041	1.86574587	0.17355785	0.43949958	0.4215017 Ppp2r3a	1
GO:0002707 negative regulation of lymphocyte mediated immunity	1/67	41/14464	0.02439024	5.26538041	1.86574587	0.17355785	0.43949958	0.4215017 H2-T23	1
GO:0006284 base-excision repair	1/67	41/14464	0.02439024	5.26538041	1.86574587	0.17355785	0.43949958	0.4215017 Rpa2	1
GO:0010543 regulation of platelet activation	1/67	41/14464	0.02439024	5.26538041	1.86574587	0.17355785	0.43949958	0.4215017 Fundc2	1
GO:0043331 response to dsRNA	1/67	41/14464	0.02439024	5.26538041	1.86574587	0.17355785	0.43949958	0.4215017 Rftn1	1
GO:0043507 positive regulation of JUN kinase activity	1/67	41/14464	0.02439024	5.26538041	1.86574587	0.17355785	0.43949958	0.4215017 Ager	1
GO:0045143 homologous chromosome segregation	1/67	41/14464	0.02439024	5.26538041	1.86574587	0.17355785	0.43949958	0.4215017 Ehmt2	1
GO:0051294 establishment of spindle orientation	1/67	41/14464	0.02439024	5.26538041	1.86574587	0.17355785	0.43949958	0.4215017 Gpsm1	1
GO:1902991 regulation of amyloid precursor protein catabolic process	1/67	41/14464	0.02439024	5.26538041	1.86574587	0.17355785	0.43949958	0.4215017 Ager	1
GO:0006403 RNA localization	2/67	163/14464	0.01226994	2.64884168	1.44417608	0.17443614	0.43949958	0.4215017 Tst/Rftn1	2
GO:0010508 positive regulation of autophagy	2/67	163/14464	0.01226994	2.64884168	1.44417608	0.17443614	0.43949958	0.4215017 Ager/Gpsm1	2
GO:0010720 positive regulation of cell development	4/67	473/14464	0.00845666	1.82562873	1.24543647	0.17582137	0.43949958	0.4215017 Ager/Slc30a1/Tiam1/Il1rapl1	4
GO:0030534 adult behavior	2/67	164/14464	0.01219512	2.63269021	1.43445948	0.1760902	0.43949958	0.4215017 Ehmt2/Enpp1	2
GO:0001937 negative regulation of endothelial cell proliferation	1/67	42/14464	0.02380952	5.14001421	1.83292344	0.17739697	0.43949958	0.4215017 Ager	1
GO:0031114 regulation of microtubule depolymerization	1/67	42/14464	0.02380952	5.14001421	1.83292344	0.17739697	0.43949958	0.4215017 Ccdc88c	1
GO:0032655 regulation of interleukin-12 production	1/67	42/14464	0.02380952	5.14001421	1.83292344	0.17739697	0.43949958	0.4215017 Ager	1
GO:0032790 ribosome disassembly	1/67	42/14464	0.02380952	5.14001421	1.83292344	0.17739697	0.43949958	0.4215017 Zfp598	1
GO:0033628 regulation of cell adhesion mediated by integrin	1/67	42/14464	0.02380952	5.14001421	1.83292344	0.17739697	0.43949958	0.4215017 Epha8	1
GO:0046627 negative regulation of insulin receptor signaling pathway	1/67	42/14464	0.02380952	5.14001421	1.83292344	0.17739697	0.43949958	0.4215017 Enpp1	1
GO:0046677 response to antibiotic	1/67	42/14464	0.02380952	5.14001421	1.83292344	0.17739697	0.43949958	0.4215017 Ehmt2	1
GO:0070192 chromosome organization involved in meiotic cell cycle	1/67	42/14464	0.02380952	5.14001421	1.83292344	0.17739697	0.43949958	0.4215017 Ehmt2	1
GO:0090630 activation of GTPase activity	1/67	42/14464	0.02380952	5.14001421	1.83292344	0.17739697	0.43949958	0.4215017 Tiam1	1
GO:0098534 centriole assembly	1/67	42/14464	0.02380952	5.14001421	1.83292344	0.17739697	0.43949958	0.4215017 Cdk2	1
GO:1904645 response to amyloid-beta	1/67	42/14464	0.02380952	5.14001421	1.83292344	0.17739697	0.43949958	0.4215017 Ager	1
GO:0032653 regulation of interleukin-10 production	1/67	43/14464	0.02325581	5.020479	1.80112946	0.18121852	0.44609864	0.42783052 Ager	1
GO:0061756 leukocyte adhesion to vascular endothelial cell	1/67	43/14464	0.02325581	5.020479	1.80112946	0.18121852	0.44609864	0.42783052 Podxl2	1
GO:0099560 synaptic membrane adhesion	1/67	43/14464	0.02325581	5.020479	1.80112946	0.18121852	0.44609864	0.42783052 Il1rapl1	1
GO:0140719 constitutive heterochromatin formation	1/67	43/14464	0.02325581	5.020479	1.80112946	0.18121852	0.44609864	0.42783052 Ehmt2	1
GO:0016052 carbohydrate catabolic process	2/67	168/14464	0.01190476	2.57000711	1.39630271	0.18273165	0.44910602	0.43071475 Enpp1/Man2b1	2
GO:0032615 interleukin-12 production	1/67	44/14464	0.02272727	4.9063772	1.77030655	0.18502259	0.45113893	0.43266441 Ager	1
GO:0048265 response to pain	1/67	44/14464	0.02272727	4.9063772	1.77030655	0.18502259	0.45113893	0.43266441 Gja4	1
GO:1901976 regulation of cell cycle checkpoint	1/67	44/14464	0.02272727	4.9063772	1.77030655	0.18502259	0.45113893	0.43266441 Rpa2	1
GO:1902116 negative regulation of organelle assembly	1/67	44/14464	0.02272727	4.9063772	1.77030655	0.18502259	0.45113893	0.43266441 Ehmt2	1
GO:1904861 excitatory synapse assembly	1/67	44/14464	0.02272727	4.9063772	1.77030655	0.18502259	0.45113893	0.43266441 Abi3bp	1
GO:0002819 regulation of adaptive immune response	2/67	170/14464	0.01176471	2.53977173	1.37763607	0.18606673	0.45296813	0.4344187 H2-T23/Ager	2
GO:0021700 developmental maturation	3/67	323/14464	0.00928793	2.00508294	1.24621707	0.18844233	0.45350884	0.43493727 Epha8/Nfatc4/Enpp1	3
GO:0002639 positive regulation of immunoglobulin production	1/67	45/14464	0.02222222	4.7973466	1.74040177	0.18880923	0.45350884	0.43493727 H2-T23	1
GO:0003016 respiratory system process	1/67	45/14464	0.02222222	4.7973466	1.74040177	0.18880923	0.45350884	0.43493727 Ccdc88c	1
GO:0007628 adult walking behavior	1/67	45/14464	0.02222222	4.7973466	1.74040177	0.18880923	0.45350884	0.43493727 Enpp1	1
GO:0043029 T cell homeostasis	1/67	45/14464	0.02222222	4.7973466	1.74040177	0.18880923	0.45350884	0.43493727 Aim2	1
GO:0061383 trabecula morphogenesis	1/67	45/14464	0.02222222	4.7973466	1.74040177	0.18880923	0.45350884	0.43493727 Enpp1	1
GO:0098698 postsynaptic specialization assembly	1/67	45/14464	0.02222222	4.7973466	1.74040177	0.18880923	0.45350884	0.43493727 Abi3bp	1
GO:0099010 modification of postsynaptic structure	1/67	45/14464	0.02222222	4.7973466	1.74040177	0.18880923	0.45350884	0.43493727 Tiam1	1
GO:0031647 regulation of protein stability	3/67	324/14464	0.00925926	1.99889442	1.24050346	0.18961311	0.45350884	0.43493727 Nr1d1/Ccdc88c/Dazap2	3
GO:0043393 regulation of protein binding	2/67	173/14464	0.01156069	2.49572945	1.35012935	0.19108601	0.45350884	0.43493727 Nfatc4/Tiam1	2
GO:0006417 regulation of translation	3/67	326/14464	0.00920245	1.98663126	1.22913695	0.19196047	0.45350884	0.43493727 Hnmpd/Pcif1/Zfp598	3
GO:0007159 leukocyte cell-cell adhesion	3/67	326/14464	0.00920245	1.98663126	1.22913695	0.19196047	0.45350884	0.43493727 H2-T23/Ager/Podxl2	3
GO:0005975 carbohydrate metabolic process	4/67	491/14464	0.00814664	1.7587014	1.16680262	0.19253802	0.45350884	0.43493727 Nr1d1/Enpp1/Hexd/Man2b1	4
GO:0001912 positive regulation of leukocyte mediated cytotoxicity	1/67	46/14464	0.02173913	4.69305646	1.71136618	0.19257855	0.45350884	0.43493727 H2-T23	1
GO:0008340 determination of adult lifespan	1/67	46/14464	0.02173913	4.69305646	1.71136618	0.19257855	0.45350884	0.43493727 Enpp1	1
GO:0014014 negative regulation of gliogenesis	1/67	46/14464	0.02173913	4.69305646	1.71136618	0.19257855	0.45350884	0.43493727 Nr1d1	1
GO:0032613 interleukin-10 production	1/67	46/14464	0.02173913	4.69305646	1.71136618	0.19257855	0.45350884	0.43493727 Ager	1
GO:0032965 regulation of collagen biosynthetic process	1/67	46/14464	0.02173913	4.69305646	1.71136618	0.19257855	0.45350884	0.43493727 Ager	1
GO:0120163 negative regulation of cold-induced thermogenesis	1/67	46/14464	0.02173913	4.69305646	1.71136618	0.19257855	0.45350884	0.43493727 Nr1d1	1
GO:2000351 regulation of endothelial cell apoptotic process	1/67	46/14464	0.02173913	4.69305646	1.71136618	0.19257855	0.45350884	0.43493727 Ager	1
GO:2000785 regulation of autophagosome assembly	1/67	46/14464	0.02173913	4.69305646	1.71136618	0.19257855	0.45350884	0.43493727 Ehmt2	1
GO:0043484 regulation of RNA splicing	2/67	174/14464	0.01149425	2.48138617	1.34108835	0.19276333	0.45350884	0.43493727 Srsf5/Arglu1	2
GO:0021543 pallium development	2/67	175/14464	0.01142857	2.46720682	1.33210984	0.19444267	0.45359142	0.43501646 Bhlhe22/Xm2	2
GO:0046777 protein autophosphorylation	2/67	175/14464	0.01142857	2.46720682	1.33210984	0.19444267	0.45359142	0.43501646 Epha8/Enpp1	2
GO:0050920 regulation of chemotaxis	2/67	175/14464	0.01142857	2.46720682	1.33210984	0.19444267	0.45359142	0.43501646 Ager/Tiam1	2
GO:0033120 positive regulation of RNA splicing	1/67	47/14464	0.0212766	4.59320419	1.68315445	0.19633061	0.45359142	0.43501646 Srsf5	1
GO:0033173 calcineurin-NFAT signaling cascade	1/67	47/14464	0.0212766	4.59320419	1.68315445	0.19633061	0.45359142	0.43501646 Nfatc4	1
GO:0035458 cellular response to interferon-beta	1/67	47/14464	0.0212766	4.59320419	1.68315445	0.19633061	0.45359142	0.43501646 Aim2	1
GO:0042789 mRNA transcription by RNA polymerase II	1/67	47/14464	0.0212766	4.59320419	1.68315445	0.19633061	0.45359142	0.43501646 Enpp1	1
GO:0046686 response to cadmium ion	1/67	47/14464	0.0212766	4.59320419	1.68315445	0.19633061	0.45359142	0.43501646 Slc30a1	1

GO:0048066 developmental pigmentation	1/67	47/14464	0.0212766	4.59320419	1.68315445	0.19633061	0.45359142	0.43501646	Enpp1	1
GO:0090199 regulation of release of cytochrome c from mitochondria	1/67	47/14464	0.0212766	4.59320419	1.68315445	0.19633061	0.45359142	0.43501646	Tnfsf10	1
GO:0170038 proteinogenic amino acid biosynthetic process	1/67	47/14464	0.0212766	4.59320419	1.68315445	0.19633061	0.45359142	0.43501646	Pycr1	1
GO:1900077 negative regulation of cellular response to insulin stimulus	1/67	47/14464	0.0212766	4.59320419	1.68315445	0.19633061	0.45359142	0.43501646	Enpp1	1
GO:0015980 energy derivation by oxidation of organic compounds	3/67	330/14464	0.00909091	1.96255088	1.20664277	0.19667785	0.45371343	0.43513348	Nr1d1/Nfatc4/Enpp1	3
GO:0031589 cell-substrate adhesion	3/67	332/14464	0.00903614	1.95072829	1.19551281	0.19904756	0.45511601	0.43647862	Ajap1/Tiam1/Abi3bp	3
GO:0000288 nuclear-transcribed mRNA catabolic process, deadenylation-dependent decay	1/67	48/14464	0.02083333	4.49751244	1.65572456	0.2000655	0.45511601	0.43647862	Hnrnpd	1
GO:0006911 phagocytosis, engulfment	1/67	48/14464	0.02083333	4.49751244	1.65572456	0.2000655	0.45511601	0.43647862	Ager	1
GO:0010762 regulation of fibroblast migration	1/67	48/14464	0.02083333	4.49751244	1.65572456	0.2000655	0.45511601	0.43647862	Ager	1
GO:0050918 positive chemotaxis	1/67	48/14464	0.02083333	4.49751244	1.65572456	0.2000655	0.45511601	0.43647862	Ager	1
GO:0061045 negative regulation of wound healing	1/67	48/14464	0.02083333	4.49751244	1.65572456	0.2000655	0.45511601	0.43647862	Ajap1	1
GO:0097720 calcineurin-mediated signaling	1/67	48/14464	0.02083333	4.49751244	1.65572456	0.2000655	0.45511601	0.43647862	Nfatc4	1
GO:0170034 L-amino acid biosynthetic process	1/67	48/14464	0.02083333	4.49751244	1.65572456	0.2000655	0.45511601	0.43647862	Pycr1	1
GO:0050796 regulation of insulin secretion	2/67	179/14464	0.01117318	2.41207371	1.29680403	0.20117918	0.45511601	0.43647862	Nr1d1/Tiam1	2
GO:0051346 negative regulation of hydrolase activity	2/67	179/14464	0.01117318	2.41207371	1.29680403	0.20117918	0.45511601	0.43647862	Fnip1/Gpsm1	2
GO:0034612 response to tumor necrosis factor	2/67	180/14464	0.01111111	2.3986733	1.28812559	0.20286784	0.45511601	0.43647862	Nr1d1/Aim2	2
GO:0035265 organ growth	2/67	180/14464	0.01111111	2.3986733	1.28812559	0.20286784	0.45511601	0.43647862	Ehmt2/Enpp1	2
GO:0048659 smooth muscle cell proliferation	2/67	180/14464	0.01111111	2.3986733	1.28812559	0.20286784	0.45511601	0.43647862	Ager/Enpp1	2
GO:0007215 glutamate receptor signaling pathway	1/67	49/14464	0.02040816	4.40572647	1.62903747	0.20378329	0.45511601	0.43647862	Tiam1	1
GO:0010596 negative regulation of endothelial cell migration	1/67	49/14464	0.02040816	4.40572647	1.62903747	0.20378329	0.45511601	0.43647862	Ager	1
GO:0031111 negative regulation of microtubule polymerization or depolymerization	1/67	49/14464	0.02040816	4.40572647	1.62903747	0.20378329	0.45511601	0.43647862	Ccdc88c	1
GO:0032881 regulation of polysaccharide metabolic process	1/67	49/14464	0.02040816	4.40572647	1.62903747	0.20378329	0.45511601	0.43647862	Enpp1	1
GO:0042267 natural killer cell mediated cytotoxicity	1/67	49/14464	0.02040816	4.40572647	1.62903747	0.20378329	0.45511601	0.43647862	H2-T23	1
GO:0048599 oocyte development	1/67	49/14464	0.02040816	4.40572647	1.62903747	0.20378329	0.45511601	0.43647862	Ehmt2	1
GO:0060074 synapse maturation	1/67	49/14464	0.02040816	4.40572647	1.62903747	0.20378329	0.45511601	0.43647862	Nfatc4	1
GO:0060390 regulation of SMAD protein signal transduction	1/67	49/14464	0.02040816	4.40572647	1.62903747	0.20378329	0.45511601	0.43647862	Ldlrad4	1
GO:0099054 presynapse assembly	1/67	49/14464	0.02040816	4.40572647	1.62903747	0.20378329	0.45511601	0.43647862	Il1rapl1	1
GO:0000245 spliceosomal complex assembly	1/67	50/14464	0.02	4.31761194	1.60305684	0.20748405	0.45777351	0.43902729	Luc7l2	1
GO:0002712 regulation of B cell mediated immunity	1/67	50/14464	0.02	4.31761194	1.60305684	0.20748405	0.45777351	0.43902729	H2-T23	1
GO:0002889 regulation of immunoglobulin mediated immune response	1/67	50/14464	0.02	4.31761194	1.60305684	0.20748405	0.45777351	0.43902729	H2-T23	1
GO:0009994 oocyte differentiation	1/67	50/14464	0.02	4.31761194	1.60305684	0.20748405	0.45777351	0.43902729	Ehmt2	1
GO:0010718 positive regulation of epithelial to mesenchymal transition	1/67	50/14464	0.02	4.31761194	1.60305684	0.20748405	0.45777351	0.43902729	Ager	1
GO:0043154 negative regulation of cysteine-type endopeptidase activity involved in apoptotic process	1/67	50/14464	0.02	4.31761194	1.60305684	0.20748405	0.45777351	0.43902729	Fnip1	1
GO:0072577 endothelial cell apoptotic process	1/67	50/14464	0.02	4.31761194	1.60305684	0.20748405	0.45777351	0.43902729	Ager	1
GO:0072678 T cell migration	1/67	50/14464	0.02	4.31761194	1.60305684	0.20748405	0.45777351	0.43902729	Cd200	1
GO:0009267 cellular response to starvation	2/67	183/14464	0.01092896	2.35935079	1.26243309	0.20794384	0.45777351	0.43902729	Ehmt2/Fnip1	2
GO:0016051 carbohydrate biosynthetic process	2/67	183/14464	0.01092896	2.35935079	1.26243309	0.20794384	0.45777351	0.43902729	Nr1d1/Enpp1	2
GO:0001756 somitogenesis	1/67	51/14464	0.01960784	4.23295288	1.57774881	0.21116787	0.46136544	0.44247213	Ppp2r3a	1
GO:0002711 positive regulation of T cell mediated immunity	1/67	51/14464	0.01960784	4.23295288	1.57774881	0.21116787	0.46136544	0.44247213	H2-T23	1
GO:0009299 mRNA transcription	1/67	51/14464	0.01960784	4.23295288	1.57774881	0.21116787	0.46136544	0.44247213	Enpp1	1
GO:0050853 B cell receptor signaling pathway	1/67	51/14464	0.01960784	4.23295288	1.57774881	0.21116787	0.46136544	0.44247213	Rftn1	1
GO:0042098 T cell proliferation	2/67	185/14464	0.01081081	2.33384429	1.24558306	0.21133569	0.46136544	0.44247213	H2-T23/Ager	2
GO:0010563 negative regulation of phosphorus metabolic process	3/67	345/14464	0.00869565	1.87722258	1.12498822	0.21461769	0.46136544	0.44247213	Ager/Pkia/Enpp1	3
GO:0045936 negative regulation of phosphate metabolic process	3/67	345/14464	0.00869565	1.87722258	1.12498822	0.21461769	0.46136544	0.44247213	Ager/Pkia/Enpp1	3
GO:0002228 natural killer cell mediated immunity	1/67	52/14464	0.01923077	4.15154994	1.5530818	0.21483482	0.46136544	0.44247213	H2-T23	1
GO:0002704 negative regulation of leukocyte mediated immunity	1/67	52/14464	0.01923077	4.15154994	1.5530818	0.21483482	0.46136544	0.44247213	H2-T23	1
GO:0031343 positive regulation of cell killing	1/67	52/14464	0.01923077	4.15154994	1.5530818	0.21483482	0.46136544	0.44247213	H2-T23	1
GO:0032210 regulation of telomere maintenance via telomerase	1/67	52/14464	0.01923077	4.15154994	1.5530818	0.21483482	0.46136544	0.44247213	Hnrnpd	1
GO:0032387 negative regulation of intracellular transport	1/67	52/14464	0.01923077	4.15154994	1.5530818	0.21483482	0.46136544	0.44247213	Pkia	1
GO:0034142 toll-like receptor 4 signaling pathway	1/67	52/14464	0.01923077	4.15154994	1.5530818	0.21483482	0.46136544	0.44247213	Nr1d1	1
GO:0042220 response to cocaine	1/67	52/14464	0.01923077	4.15154994	1.5530818	0.21483482	0.46136544	0.44247213	Ehmt2	1
GO:0170036 import into the mitochondrion	1/67	52/14464	0.01923077	4.15154994	1.5530818	0.21483482	0.46136544	0.44247213	Tst	1
GO:2000401 regulation of lymphocyte migration	1/67	52/14464	0.01923077	4.15154994	1.5530818	0.21483482	0.46136544	0.44247213	Cd200	1
GO:0018105 peptidyl-serine phosphorylation	2/67	188/14464	0.0106383	2.2966021	1.2207113	0.21643428	0.46136544	0.44247213	Cdk2/Fnip1	2
GO:0043524 negative regulation of neuron apoptotic process	2/67	188/14464	0.0106383	2.2966021	1.2207113	0.21643428	0.46136544	0.44247213	Pycr1/Nfatc4	2
GO:0050870 positive regulation of T cell activation	2/67	188/14464	0.0106383	2.2966021	1.2207113	0.21643428	0.46136544	0.44247213	H2-T23/Ager	2
GO:0001738 morphogenesis of a polarized epithelium	1/67	53/14464	0.01886792	4.07321881	1.52902628	0.21848498	0.46136544	0.44247213	Ajap1	1
GO:0001941 postsynaptic membrane organization	1/67	53/14464	0.01886792	4.07321881	1.52902628	0.21848498	0.46136544	0.44247213	Slc30a1	1
GO:0006513 protein monoubiquitination	1/67	53/14464	0.01886792	4.07321881	1.52902628	0.21848498	0.46136544	0.44247213	Zfp598	1
GO:0008631 intrinsic apoptotic signaling pathway in response to oxidative stress	1/67	53/14464	0.01886792	4.07321881	1.52902628	0.21848498	0.46136544	0.44247213	Pycr1	1
GO:0014003 oligodendrocyte development	1/67	53/14464	0.01886792	4.07321881	1.52902628	0.21848498	0.46136544	0.44247213	Enpp1	1
GO:0045599 negative regulation of fat cell differentiation	1/67	53/14464	0.01886792	4.07321881	1.52902628	0.21848498	0.46136544	0.44247213	Enpp1	1
GO:0048002 antigen processing and presentation of peptide antigen	1/67	53/14464	0.01886792	4.07321881	1.52902628	0.21848498	0.46136544	0.44247213	H2-T23	1
GO:0051293 establishment of spindle localization	1/67	53/14464	0.01886792	4.07321881	1.52902628	0.21848498	0.46136544	0.44247213	Gpsm1	1
GO:0099563 modification of synaptic structure	1/67	53/14464	0.01886792	4.07321881	1.52902628	0.21848498	0.46136544	0.44247213	Tiam1	1
GO:1901607 alpha-amino acid biosynthetic process	1/67	53/14464	0.01886792	4.07321881	1.52902628	0.21848498	0.46136544	0.44247213	Pycr1	1
GO:0032259 methylation	2/67	190/14464	0.01052632	2.27242734	1.2043911	0.21983996	0.46136544	0.44247213	Pcif1/Ehmt2	2
GO:0002685 regulation of leukocyte migration	2/67	191/14464	0.0104712	2.26052981	1.19630733	0.22154465	0.46136544	0.44247213	Ager/Cd200	2
GO:0001654 eye development	3/67	351/14464	0.00854701	1.84513331	1.09345194	0.22189395	0.46136544	0.44247213	Ppp2r3a/Bhlhe22/Xrn2	3
GO:0051962 positive regulation of nervous system development	3/67	351/14464	0.00854701	1.84513331	1.09345194	0.22189395	0.46136544	0.44247213	Slc30a1/Tiam1/Il1rapl1	3
GO:0002381 immunoglobulin production involved in immunoglobulin-mediated immune response	1/67	54/14464	0.01851852	3.99778883	1.50555463	0.22211842	0.46136544	0.44247213	Enpp1	1
GO:0010712 regulation of collagen metabolic process	1/67	54/14464	0.01851852	3.99778883	1.50555463	0.22211842	0.46136544	0.44247213	Ager	1
GO:0043506 regulation of JUN kinase activity	1/67	54/14464	0.01851852	3.99778883	1.50555463	0.22211842	0.46136544	0.44247213	Ager	1
GO:0048512 circadian behavior	1/67	54/14464	0.01851852	3.99778883	1.50555463	0.22211842	0.46136544	0.44247213	Nr1d1	1
GO:0090659 walking behavior	1/67	54/14464	0.01851852	3.99778883	1.50555463	0.22211842	0.46136544	0.44247213	Enpp1	1
GO:1901224 positive regulation of non-canonical NF-kappaB signal transduction	1/67	54/14464	0.01851852	3.99778883	1.50555463	0.22211842	0.46136544	0.44247213	Ager	1
GO:0009306 protein secretion	3/67	352/14464	0.00852273	1.83989145	1.08825533	0.22311175	0.46136544	0.44247213	Nr1d1/Cd200/Tiam1	3
GO:0015931 nucleobase-containing compound transport	2/67	192/14464	0.01041667	2.24875622	1.18827361	0.22325051	0.46136544	0.44247213	Tst/Rftn1	2
GO:0050670 regulation of lymphocyte proliferation	2/67	192/14464	0.01041667	2.24875622	1.18827361	0.22325051	0.46136544	0.44247213	H2-T23/Ager	2
GO:0035592 establishment of protein localization to extracellular region	3/67	354/14464	0.00847458	1.82949658	1.07791203	0.22555154	0.46136544	0.44247213	Nr1d1/Cd200/Tiam1	3
GO:0150063 visual system development	3/67	354/14464	0.00847458	1.82949658	1.07791203	0.22555154	0.46136544	0.44247213	Ppp2r3a/Bhlhe22/Xrn2	3
GO:0006479 protein methylation	1/67	55/14464	0.01818182	3.92510176	1.48264097	0.22573522	0.46136544	0.44247213	Ehmt2	1
GO:0007622 rhythmic behavior	1/67	55/14464	0.01818182	3.92510176	1.48264097	0.22573522	0.46136544	0.44247213	Nr1d1	1
GO:0008213 protein alkylation	1/67	55/14464	0.01818182	3.92510176	1.48264097	0.22573522	0.46136544	0.44247213	Ehmt2	1
GO:0010812 negative regulation of cell-substrate adhesion	1/67	55/14464	0.01818182	3.92510176	1.48264097	0.22573522	0.46136544	0.44247213	Ajap1	1
GO:0035456 response to interferon-beta	1/67	55/14464	0.01818182	3.92510176	1.48264097	0.22573522	0.46136544	0.44247213	Aim2	1
GO:0070542 response to fatty acid	1/67	55/14464	0.01818182	3.92510176	1.48264097	0.22573522	0.46136544	0.44247213	Nfatc4	1
GO:1904263 positive regulation of TORC1 signaling	1/67	55/14464	0.01818182	3.92510176	1.48264097	0.22573522	0.46136544	0.44247213	Fnip1	1
GO:0010639 negative regulation of organelle organization	3/67	355/14464	0.0084507	1.82434307	1.07276512	0.2267735	0.46136544	0.44247213	Ccdc88c/Ehmt2/Fnip1	3
GO:0006935 chemotaxis	3/67	356/14464	0.00842697	1.81921851	1.06763456	0.2279968	0.46136544	0.44247213	Slamf9/Ager/Tiam1	3
GO:0045165 cell fate commitment	2/67	195/14464	0.01025641	2.21415997	1.16446661	0.22837462	0.46136544	0.44247213	Ehmt2/Bhlhe22	2
GO:0042330 taxis	3/67	357/14464	0.00840336	1.81412266	1.06252024	0.22922144	0.46136544	0.44247213	Slamf9/Ager/Tiam1	3
GO:0071692 protein localization to extracellular region	3/67	357/14464	0.00840336	1.81412266	1.06252024	0.22922144	0.46136544	0.44247213	Nr1d1/Cd200/Tiam1	3
GO:0002886 regulation of myeloid leukocyte mediated immunity	1/67	56/14464	0.01785714	3.85501066	1.46026101	0.22933545	0.46136544	0.44247213	H2-T23	1
GO:0007019 microtubule depolymerization	1/67	56/14464	0.01785714	3.85501066	1.46026101	0.22933545	0.46136544	0.44247213	Ccdc88c	1
GO:0032964 collagen biosynthetic process	1/67	56/14464	0.01785714	3.85501066	1.46026101	0.22933545	0.46136544	0.44247213	Ager	1
GO:0038066 p38MAPK cascade	1/67	56/14464	0.01785714	3.85501066	1.46026101	0.22933545	0.46136544	0.44247213	Ager	1
GO:0042987 amyloid precursor protein catabolic process	1/67	56/14464	0.01785714	3.85501066	1.46026101	0.22933545	0.46136544	0.44247213	Ager	1
GO:0043124 negative regulation of canonical NF-kappaB signal transduction	1/67	56/14464	0.01785714	3.85501066	1.46026101	0.22933545	0.46136544	0.44247213	Nr1d1	1
GO:0050688 regulation of defense response to virus	1/67	56/14464	0.01785714	3.85501066	1.46026101	0.22933545	0.46136544	0.44247213	Aim2	1
GO:0051653 spindle localization	1/67	56/14464	0.01785714	3.85501066	1.46026101	0.22933545	0.46136544	0.44247213	Gpsm1	1
GO:0060042 retina morphogenesis in camera-type eye	1/67	56/14464	0.01785714	3.85501066	1.46026101	0.22933545	0.46136544	0.44247213	Bhlhe22	1
GO:2001238 positive regulation of extrinsic apoptotic signaling pathway	1/67	56/14464	0.01785714	3.85501066	1.46026101	0.22933545	0.46136544	0.44247213	Tnfsf10	1
GO:0032944 regulation of mononuclear cell proliferation	2/67	196/14464	0.01020408	2.20286323	1.15662701	0.23008469	0.4619414	0.44302451	H2-T23/Ager	2
GO:0048880 sensory system development	3/67	359/14464	0.00835655	1.80401613	1.05233989	0.23167463	0.4619414	0.44302451	Ppp2r3a/Bhlhe22/Xrn2	3
GO:0002703 regulation of leukocyte mediated immunity	2/67	197/14464	0.01015228	2.19168119	1.14883447	0.23179569	0.4619414	0.44302451	H2-T23/Ager	2
GO:0051963 regulation of synapse assembly	2/67	197/14464	0.01015228	2.19168119	1.14883447	0.23179569	0.4619414	0.44302451	Abi3bp/Il1rapl1	2
GO:0009064 glutamine family amino acid metabolic process	1/67	57/14464	0.01754386	3.78737889	1.43839197	0.23291919	0.4619414	0.44302451	Pycr1	1
GO:0016601 Rac protein signal transduction	1/67	57/14464	0.01754386	3.78737889	1.43839197	0.23291919	0.4619414	0.44302451	Tiam1	1
GO:0030225 macrophage differentiation	1/67	57/14464	0.01754386	3.78737889	1.43839197	0.23291919	0.4619414	0.44302451	Enpp1	1
GO:0046635 positive regulation of alpha-beta T cell activation	1/67	57/14464	0.01754386	3.78737889	1.43839197	0.23291919	0.4619414	0.44302451	H2-T23	1
GO:0048278 vesicle docking	1/67	57/14464	0.01754386	3.78737889	1.43839197	0.23291919	0.4619414	0.44302451	Stx17	1
GO:0099024 plasma membrane invagination	1/67	57/14464	0.01754386	3.78737889	1.43839197	0.23291919	0.4619414	0.44302451	Ager	1
GO:1900271 regulation of long-term synaptic potentiation	1/67	57/14464	0.01754386	3.78737889	1.43839197	0.23291919	0.4619414	0.44302451	Ager	1
GO:0018209 peptidyl-serine modification	2/67	199/14464	0.01005025	2.16965424	1.13338834	0.23522034	0.46542237	0.44636292	Cdk2/Fnip1	2
GO:0032722 positive regulation of chemokine production	1/67	58/14464	0.01724138	3.72207926	1.4170124	0.23648651	0.46542237	0.44636292	Ager	1
GO:0045912 negative regulation of carbohydrate metabolic process	1/67	58/14464	0.01724138	3.72207926	1.4170124	0.23648651	0.46542237	0.44636292	Enpp1	1
GO:0046324 regulation of D-glucose import	1/67	58/14464	0.01724138	3.72207926	1.4170124	0.23648651	0.46542237	0.44636292	Enpp1	1
GO:0046530 photoreceptor cell differentiation	1/67	58/14464	0.01724138	3.72207926	1.4170124	0.23648651	0.46542237	0.44636292	Ppp2r3a	1
GO:2000117 negative regulation of cysteine-type endopeptidase activity	1/67	58/14464	0.01724138	3.72207926	1.4170124	0.23648651	0.46542237	0.44636292	Fnip1	1
GO:0009636 response to toxic substance	2/67	201/14464	0.00995025	2.14806564	1.11812382	0.23864822	0.46704264	0.44791685	Slc30a1/Ehmt2	2
GO:0031345 negative regulation of cell projection organization	2/67	201/14464	0.00995025	2.14806564	1.11812382	0.23864822	0.46704264	0.44791685	Ngef/Nfatc4	2
GO:0000381 regulation of alternative mRNA splicing, via spliceosome	1/67	59/14464	0.01694915	3.65899317	1.39610213	0.24003749	0.46704264	0.44791685	Arglu1	1
GO:0006278 RNA-templated DNA biosynthetic process	1/67	59/14464	0.01694915	3.65899317	1.39610213	0.24003749	0.46704264	0.44791685	Hnrnpd	1
GO:0007004 telomere maintenance via telomerase	1/67	59/14464	0.01694915	3.65899317	1.39610213	0.24003749	0.46704264	0.44791685	Hnrnpd	1
GO:0031103 axon regeneration	1/67	59/14464	0.01694915	3.65899317	1.39610213	0.24003749	0.46704264	0.44791685	Enpp1	1
GO:0048255 mRNA stabilization	1/67	59/14464	0.01694915	3.65899317	1.39610213	0.24003749	0.46704264	0.44791685	Hnrnpd	1
GO:1904035 regulation of epithelial cell apoptotic process	1/67	59/14464	0.01694915	3.65899317	1.39610213	0.24003749	0.46704264	0.44791685	Ager	1
GO:2000134 negative regulation of G1/S transition of mitotic cell cycle	1/67	59/14464	0.01694915	3.65899317	1.39610213	0.24003749	0.46704264	0.44791685	Rpa2	1
GO:0030101 natural killer cell activation	1/67	60/14464	0.01666667	3.59800995	1.37564214	0.2435722	0.47197061	0.45264301	H2-T23	1
GO:0070228 regulation of lymphocyte apoptotic process	1/67	60/14464	0.01666667	3.59800995	1.37564214	0.2435722	0.47197061	0.45264301	Fnip1	1
GO:0140895 cell surface toll-like receptor signaling pathway	1/67	60/14464	0.01666667	3.59800995	1.37564214	0.2435722	0.47197061	0.45264301	Nr1d1	1
GO:0007623 circadian rhythm	2/67	204/14464	0.00980392	2.11647644	1.09555826	0.24379533	0.47197061	0.45264301	Nr1d1/Hnrnpd	2
GO:0060350 endochondral bone morphogenesis	1/67	61/14464	0.01639344	3.53902618	1.35561451	0.24709071	0.47682237	0.45729609	Enpp1	1
GO:1904356 regulation of telomere maintenance via telomere lengthening	1/67	61/14464	0.01639344	3.53902618	1.35561451	0.24709071	0.47682237	0.45729609	Hnrnpd	1
GO:0010810 regulation of cell-substrate adhesion	2/67	206/14464	0.00970874	2.09592813	1.08072941	0.24722977	0.47682237	0.45729609	Ajap1/Abi3bp	2
GO:0000082 G1/S transition of mitotic cell cycle	2/67	207/14464	0.00966184	2.08580287	1.07337796	0.24894778	0.47792571	0.45835424	Cdk2/Rpa2	2
GO:0043583 ear development	2/67	207/14464	0.00966184	2.08580287	1.07337796	0.24894778	0.47792571	0.45835424	H2-T23/Enpp1	2
GO:1903039 positive regulation of leukocyte cell-cell adhesion	2/67	207/14464	0.00966184	2.08580287	1.07337796	0.24894778	0.47792571	0.45835424	H2-T23/Ager	2
GO:0006893 Golgi to plasma membrane transport	1/67	62/14464	0.01612903	3.48194511	1.3360023	0.2505931	0.47792571	0.45835424	Sys1	1
GO:0008652 amino acid biosynthetic process	1/67	62/14464	0.01612903	3.48194511	1.3360023	0.2505931	0.47792571	0.45835424	Pycr1	1
GO:0042130 negative regulation of T cell proliferation	1/67	62/14464	0.01612903	3.48194511	1.3360023	0.2505931	0.47792571	0.45835424	H2-T23	1
GO:0042551 neuron maturation	1/67	62/14464	0.01612903	3.48194511	1.3360023	0.2505931	0.47792571	0.45835424	Epha8	1
GO:0050805 negative regulation of synaptic transmission	1/67	62/14464	0.01612903	3.48194511	1.3360023	0.2505931	0.47792571	0.45835424	Ager	1
GO:2000514 regulation of CD4-positive, alpha-beta T cell activation	1/67	62/14464	0.01612903	3.48194511	1.3360023	0.2505931	0.47792571	0.45835424	Ager	1
GO:0043603 amide metabolic process	3/67	376/14464	0.00797872	1.72245157	0.96829699	0.25271822	0.47869799	0.4590949	Gstt2/Gstt3/Enpp1	3
GO:0001836 release of cytochrome c from mitochondria	1/67	63/14464	0.01587302	3.42667614	1.31678952	0.25407944	0.47869799	0.4590949	Tnfsf10	1
GO:0002752 cell surface pattern recognition receptor signaling pathway	1/67	63/14464	0.01587302	3.42667614	1.31678952	0.25407944	0.47869799	0.4590949	Nr1d1	1
GO:0007585 respiratory gaseous exchange by respiratory system	1/67	63/14464	0.01587302	3.42667614	1.31678952	0.25407944	0.47869799	0.4590949	Ccdc88c	1
GO:0051898 negative regulation of phosphatidylinositol 3-kinase/protein kinase B signal transduction	1/67	63/14464	0.01587302	3.42667614	1.31678952	0.25407944	0.47869799	0.4590949	Aim2	1
GO:0051932 synaptic transmission, GABAergic	1/67	63/14464	0.01587302	3.42667614	1.31678952	0.25407944	0.47869799	0.4590949	Gabrg1	1
GO:0097352 autophagosome maturation	1/67	63/14464	0.01587302	3.42667614	1.31678952	0.25407944	0.47869799	0.4590949	Stx17	1
GO:0099172 presynapse organization	1/67	63/14464	0.01587302	3.42667614	1.31678952	0.25407944	0.47869799	0.4590949	Il1rapl1	1
GO:0007160 cell-matrix adhesion	2/67	210/14464	0.00952381	2.05600569	1.05156983	0.25410445	0.47869799	0.4590949	Ajap1/Tiam1	2
GO:0090276 regulation of peptide hormone secretion	2/67	210/14464	0.00952381	2.05600569	1.05156983	0.25410445	0.47869799	0.4590949	Nr1d1/Tiam1	2
GO:0031400 negative regulation of protein modification process	3/67	378/14464	0.00793651	1.71333807	0.95868968	0.25521409	0.48020136	0.46053671	Ager/Pkia/Enpp1	3
GO:0051321 meiotic cell cycle	2/67	211/14464	0.00947867	2.04626158	1.04438099	0.25582409	0.4807621	0.46107449	Cdk2/Ehmt2	2
GO:2001233 regulation of apoptotic signaling pathway	3/67	379/14464	0.00791557	1.70881739	0.95390722	0.25646344	0.4807952	0.46110623	Pycr1/Nfatc4/Tnfsf10	3
GO:0032869 cellular response to insulin stimulus	2/67	212/14464	0.00943396	2.03660941	1.03723165	0.25754404	0.4807952	0.46110623	Cdk2/Enpp1	2
GO:0002637 regulation of immunoglobulin production	1/67	64/14464	0.015625	3.37313433	1.29796103	0.2575498	0.4807952	0.46110623	H2-T23	1
GO:0003407 neural retina development	1/67	64/14464	0.015625	3.37313433	1.29796103	0.2575498	0.4807952	0.46110623	Bhlhe22	1
GO:0045453 bone resorption	1/67	64/14464	0.015625	3.37313433	1.29796103	0.2575498	0.4807952	0.46110623	Enpp1	1
GO:0002757 immune response-activating signaling pathway	3/67	380/14464	0.00789474	1.7043205	0.94913877	0.25771372	0.4807952	0.46110623	Nr1d1/Aim2/Rftn1	3
GO:0030073 insulin secretion	2/67	213/14464	0.00938967	2.02704786	1.03012139	0.25926426	0.48165823	0.46193392	Nr1d1/Tiam1	2
GO:0043122 regulation of canonical NF-kappaB signal transduction	2/67	213/14464	0.00938967	2.02704786	1.03012139	0.25926426	0.48165823	0.46193392	Nr1d1/Tnfsf10	2
GO:0070663 regulation of leukocyte proliferation	2/67	213/14464	0.00938967	2.02704786	1.03012139	0.25926426	0.48165823	0.46193392	H2-T23/Ager	2

GO:0010324 membrane invagination	1/67	65/14464	0.01538462	3.32123995	1.27950251	0.26100426	0.48165823	0.46193392	Ager	1
GO:0032007 negative regulation of TOR signaling	1/67	65/14464	0.01538462	3.32123995	1.27950251	0.26100426	0.48165823	0.46193392	Fnip1	1
GO:0032922 circadian regulation of gene expression	1/67	65/14464	0.01538462	3.32123995	1.27950251	0.26100426	0.48165823	0.46193392	Nr1d1	1
GO:0050766 positive regulation of phagocytosis	1/67	65/14464	0.01538462	3.32123995	1.27950251	0.26100426	0.48165823	0.46193392	Ager	1
GO:0051926 negative regulation of calcium ion transport	1/67	65/14464	0.01538462	3.32123995	1.27950251	0.26100426	0.48165823	0.46193392	Slc30a1	1
GO:0140115 export across plasma membrane	1/67	65/14464	0.01538462	3.32123995	1.27950251	0.26100426	0.48165823	0.46193392	Slc30a1	1
GO:0002791 regulation of peptide secretion	2/67	215/14464	0.00930233	2.0081916	1.01601637	0.26270536	0.48165823	0.46193392	Nr1d1/Tiam1	2
GO:0090087 regulation of peptide transport	2/67	215/14464	0.00930233	2.0081916	1.01601637	0.26270536	0.48165823	0.46193392	Nr1d1/Tiam1	2
GO:0002429 immune response-activating cell surface receptor signaling pathway	2/67	216/14464	0.00925926	1.99889442	1.00902076	0.26442616	0.48165823	0.46193392	Nr1d1/Rftn1	2
GO:0042594 response to starvation	2/67	216/14464	0.00925926	1.99889442	1.00902076	0.26442616	0.48165823	0.46193392	Ehmt2/Fnip1	2
GO:0002720 positive regulation of cytokine production involved in immune response	1/67	66/14464	0.01515152	3.27091814	1.26140037	0.26444288	0.48165823	0.46193392	H2-T23	1
GO:0014911 positive regulation of smooth muscle cell migration	1/67	66/14464	0.01515152	3.27091814	1.26140037	0.26444288	0.48165823	0.46193392	Ager	1
GO:0031122 cytoplasmic microtubule organization	1/67	66/14464	0.01515152	3.27091814	1.26140037	0.26444288	0.48165823	0.46193392	Ccdc88c	1
GO:0061053 somite development	1/67	66/14464	0.01515152	3.27091814	1.26140037	0.26444288	0.48165823	0.46193392	Ppp2r3a	1
GO:1902373 negative regulation of mRNA catabolic process	1/67	66/14464	0.01515152	3.27091814	1.26140037	0.26444288	0.48165823	0.46193392	Hnmpd	1
GO:2000573 positive regulation of DNA biosynthetic process	1/67	66/14464	0.01515152	3.27091814	1.26140037	0.26444288	0.48165823	0.46193392	Hnmpd	1
GO:0031329 regulation of cellular catabolic process	3/67	388/14464	0.00773196	1.66917987	0.91148536	0.26774699	0.48165823	0.46193392	Ager/Ehmt2/Gpsm1	3
GO:0001708 cell fate specification	1/67	67/14464	0.01492537	3.22209846	1.24364174	0.26786574	0.48165823	0.46193392	Ehmt2	1
GO:0002220 innate immune response activating cell surface receptor signaling pathway	1/67	67/14464	0.01492537	3.22209846	1.24364174	0.26786574	0.48165823	0.46193392	Nr1d1	1
GO:0006766 vitamin metabolic process	1/67	67/14464	0.01492537	3.22209846	1.24364174	0.26786574	0.48165823	0.46193392	Enpp1	1
GO:0033273 response to vitamin	1/67	67/14464	0.01492537	3.22209846	1.24364174	0.26786574	0.48165823	0.46193392	Enpp1	1
GO:0035904 aorta development	1/67	67/14464	0.01492537	3.22209846	1.24364174	0.26786574	0.48165823	0.46193392	Enpp1	1
GO:0045132 meiotic chromosome segregation	1/67	67/14464	0.01492537	3.22209846	1.24364174	0.26786574	0.48165823	0.46193392	Ehmt2	1
GO:0050810 regulation of steroid biosynthetic process	1/67	67/14464	0.01492537	3.22209846	1.24364174	0.26786574	0.48165823	0.46193392	Nr1d1	1
GO:0060612 adipose tissue development	1/67	67/14464	0.01492537	3.22209846	1.24364174	0.26786574	0.48165823	0.46193392	Enpp1	1
GO:0061951 establishment of protein localization to plasma membrane	1/67	67/14464	0.01492537	3.22209846	1.24364174	0.26786574	0.48165823	0.46193392	Sys1	1
GO:0070534 protein K63-linked ubiquitination	1/67	67/14464	0.01492537	3.22209846	1.24364174	0.26786574	0.48165823	0.46193392	Zfp598	1
GO:1902807 negative regulation of cell cycle G1/S phase transition	1/67	67/14464	0.01492537	3.22209846	1.24364174	0.26786574	0.48165823	0.46193392	Rpa2	1
GO:0006414 translational elongation	1/67	68/14464	0.01470588	3.17471466	1.2262144	0.27127291	0.48327347	0.46348301	Zfp598	1
GO:0006446 regulation of translational initiation	1/67	68/14464	0.01470588	3.17471466	1.2262144	0.27127291	0.48327347	0.46348301	Zfp598	1
GO:0033138 positive regulation of peptidyl-serine phosphorylation	1/67	68/14464	0.01470588	3.17471466	1.2262144	0.27127291	0.48327347	0.46348301	Fnip1	1
GO:0048146 positive regulation of fibroblast proliferation	1/67	68/14464	0.01470588	3.17471466	1.2262144	0.27127291	0.48327347	0.46348301	Ager	1
GO:0050830 defense response to Gram-positive bacterium	1/67	68/14464	0.01470588	3.17471466	1.2262144	0.27127291	0.48327347	0.46348301	H2-T23	1
GO:0051865 protein autoubiquitination	1/67	68/14464	0.01470588	3.17471466	1.2262144	0.27127291	0.48327347	0.46348301	Nfx1	1
GO:0060395 SMAD protein signal transduction	1/67	68/14464	0.01470588	3.17471466	1.2262144	0.27127291	0.48327347	0.46348301	Ldlrad4	1
GO:1902893 regulation of miRNA transcription	1/67	68/14464	0.01470588	3.17471466	1.2262144	0.27127291	0.48327347	0.46348301	Nfatc4	1
GO:1903828 negative regulation of protein localization	2/67	221/14464	0.00904977	1.95367056	0.97459543	0.27303116	0.48584413	0.4659484	Pkia/Cd200	2
GO:0008543 fibroblast growth factor receptor signaling pathway	1/67	69/14464	0.01449275	3.1287043	1.20910674	0.27466446	0.48706321	0.46711755	Enpp1	1
GO:0010761 fibroblast migration	1/67	69/14464	0.01449275	3.1287043	1.20910674	0.27466446	0.48706321	0.46711755	Ager	1
GO:0070509 calcium ion import	1/67	69/14464	0.01449275	3.1287043	1.20910674	0.27466446	0.48706321	0.46711755	Slc30a1	1
GO:0032008 positive regulation of TOR signaling	1/67	70/14464	0.01428571	3.08400853	1.19230772	0.27804045	0.49022922	0.47015391	Fnip1	1
GO:0043489 RNA stabilization	1/67	70/14464	0.01428571	3.08400853	1.19230772	0.27804045	0.49022922	0.47015391	Hnmpd	1
GO:0046626 regulation of insulin receptor signaling pathway	1/67	70/14464	0.01428571	3.08400853	1.19230772	0.27804045	0.49022922	0.47015391	Enpp1	1
GO:0061614 miRNA transcription	1/67	70/14464	0.01428571	3.08400853	1.19230772	0.27804045	0.49022922	0.47015391	Nfatc4	1
GO:1903035 negative regulation of response to wounding	1/67	70/14464	0.01428571	3.08400853	1.19230772	0.27804045	0.49022922	0.47015391	Ajap1	1
GO:0002764 immune response-regulating signaling pathway	3/67	397/14464	0.00755668	1.63133952	0.87013789	0.27909279	0.49094884	0.47084406	Nr1d1/Aim2/Rftn1	3
GO:0008202 steroid metabolic process	2/67	225/14464	0.00888889	1.91893864	0.94769507	0.27991418	0.49094884	0.47084406	Nr1d1/Enpp1	2
GO:0043086 negative regulation of catalytic activity	3/67	398/14464	0.00753769	1.62724068	0.86560756	0.28035675	0.49094884	0.47084406	Pkia/Fnip1/Gpsm1	3
GO:0031102 neuron projection regeneration	1/67	71/14464	0.01408451	3.04057179	1.17580684	0.28140097	0.49094884	0.47084406	Enpp1	1
GO:0042306 regulation of protein import into nucleus	1/67	71/14464	0.01408451	3.04057179	1.17580684	0.28140097	0.49094884	0.47084406	Pkia	1
GO:0071347 cellular response to interleukin-1	1/67	71/14464	0.01408451	3.04057179	1.17580684	0.28140097	0.49094884	0.47084406	Nr1d1	1
GO:2000379 positive regulation of reactive oxygen species metabolic process	1/67	71/14464	0.01408451	3.04057179	1.17580684	0.28140097	0.49094884	0.47084406	Ager	1
GO:0044772 mitotic cell cycle phase transition	3/67	399/14464	0.0075188	1.62316238	0.86108971	0.28162132	0.49094884	0.47084406	Cdk2/Pkia/Rpa2	3
GO:0006402 mRNA catabolic process	2/67	226/14464	0.00884956	1.91044776	0.94105574	0.28163451	0.49094884	0.47084406	Hnmpd/Xrn2	2
GO:0060828 regulation of canonical Wnt signaling pathway	2/67	226/14464	0.00884956	1.91044776	0.94105574	0.28163451	0.49094884	0.47084406	Ccdc88c/Ppp2r3a	2
GO:0002768 immune response-regulating cell surface receptor signaling pathway	2/67	227/14464	0.00881057	1.90203169	0.93445001	0.28335458	0.49137033	0.4712483	Nr1d1/Rftn1	2
GO:0010833 telomere maintenance via telomere lengthening	1/67	72/14464	0.01388889	2.99834163	1.15959411	0.28474608	0.49137033	0.4712483	Hnmpd	1
GO:0019730 antimicrobial humoral response	1/67	72/14464	0.01388889	2.99834163	1.15959411	0.28474608	0.49137033	0.4712483	H2-T23	1
GO:0030500 regulation of bone mineralization	1/67	72/14464	0.01388889	2.99834163	1.15959411	0.28474608	0.49137033	0.4712483	Enpp1	1
GO:0032091 negative regulation of protein binding	1/67	72/14464	0.01388889	2.99834163	1.15959411	0.28474608	0.49137033	0.4712483	Nfatc4	1
GO:0042982 amyloid precursor protein metabolic process	1/67	72/14464	0.01388889	2.99834163	1.15959411	0.28474608	0.49137033	0.4712483	Ager	1
GO:0051591 response to cAMP	1/67	72/14464	0.01388889	2.99834163	1.15959411	0.28474608	0.49137033	0.4712483	Cdk2	1
GO:1903008 organelle disassembly	1/67	72/14464	0.01388889	2.99834163	1.15959411	0.28474608	0.49137033	0.4712483	Zfp598	1
GO:2000027 regulation of animal organ morphogenesis	1/67	72/14464	0.01388889	2.99834163	1.15959411	0.28474608	0.49137033	0.4712483	Ajap1	1
GO:1901987 regulation of cell cycle phase transition	3/67	402/14464	0.00746269	1.61104923	0.84761039	0.28541847	0.49197972	0.47183273	Cdk2/Pkia/Rpa2	3
GO:0006289 nucleotide-excision repair	1/67	73/14464	0.01369863	2.95726845	1.14366	0.28807585	0.49290308	0.47271828	Rpa2	1
GO:0032092 positive regulation of protein binding	1/67	73/14464	0.01369863	2.95726845	1.14366	0.28807585	0.49290308	0.47271828	Tiam1	1
GO:0032206 positive regulation of telomere maintenance	1/67	73/14464	0.01369863	2.95726845	1.14366	0.28807585	0.49290308	0.47271828	Hnmpd	1
GO:0072676 lymphocyte migration	1/67	73/14464	0.01369863	2.95726845	1.14366	0.28807585	0.49290308	0.47271828	Cd200	1
GO:1902476 chloride transmembrane transport	1/67	73/14464	0.01369863	2.95726845	1.14366	0.28807585	0.49290308	0.47271828	Gabrg1	1
GO:0031331 positive regulation of cellular catabolic process	2/67	230/14464	0.00869565	1.87722258	0.91483092	0.28851303	0.49290308	0.47271828	Ager/Gpsm1	2
GO:0040029 epigenetic regulation of gene expression	2/67	230/14464	0.00869565	1.87722258	0.91483092	0.28851303	0.49290308	0.47271828	Cdk2/Ehmt2	2
GO:1902115 regulation of organelle assembly	2/67	230/14464	0.00869565	1.87722258	0.91483092	0.28851303	0.49290308	0.47271828	Ehmt2/Abi3bp	2
GO:0002312 B cell activation involved in immune response	1/67	74/14464	0.01351351	2.91730537	1.12799544	0.29139034	0.49521999	0.47494031	Enpp1	1
GO:0051145 smooth muscle cell differentiation	1/67	74/14464	0.01351351	2.91730537	1.12799544	0.29139034	0.49521999	0.47494031	Nfatc4	1
GO:0051298 centrosome duplication	1/67	74/14464	0.01351351	2.91730537	1.12799544	0.29139034	0.49521999	0.47494031	Cdk2	1
GO:0046394 carboxylic acid biosynthetic process	2/67	232/14464	0.00862069	1.86103963	0.90191323	0.29195016	0.49521999	0.47494031	Nr1d1/Pycr1	2
GO:0044843 cell cycle G1/S phase transition	2/67	233/14464	0.00858369	1.85305233	0.89550192	0.29366807	0.49521999	0.47494031	Cdk2/Rpa2	2
GO:0002526 acute inflammatory response	1/67	75/14464	0.01333333	2.87840796	1.11259176	0.29468964	0.49521999	0.47494031	H2-T23	1
GO:0008625 extrinsic apoptotic signaling pathway via death domain receptors	1/67	75/14464	0.01333333	2.87840796	1.11259176	0.29468964	0.49521999	0.47494031	Tnfsf10	1
GO:0010569 regulation of double-strand break repair via homologous recombination	1/67	75/14464	0.01333333	2.87840796	1.11259176	0.29468964	0.49521999	0.47494031	Rpa2	1
GO:0010827 regulation of D-glucose transmembrane transport	1/67	75/14464	0.01333333	2.87840796	1.11259176	0.29468964	0.49521999	0.47494031	Enpp1	1
GO:0042632 cholesterol homeostasis	1/67	75/14464	0.01333333	2.87840796	1.11259176	0.29468964	0.49521999	0.47494031	Nr1d1	1
GO:0044773 mitotic DNA damage checkpoint signaling	1/67	75/14464	0.01333333	2.87840796	1.11259176	0.29468964	0.49521999	0.47494031	Rpa2	1
GO:0046323 D-glucose import	1/67	75/14464	0.01333333	2.87840796	1.11259176	0.29468964	0.49521999	0.47494031	Enpp1	1
GO:0050672 negative regulation of lymphocyte proliferation	1/67	75/14464	0.01333333	2.87840796	1.11259176	0.29468964	0.49521999	0.47494031	H2-T23	1
GO:0070050 neuron cellular homeostasis	1/67	75/14464	0.01333333	2.87840796	1.11259176	0.29468964	0.49521999	0.47494031	Aim2	1
GO:1990874 vascular associated smooth muscle cell proliferation	1/67	75/14464	0.01333333	2.87840796	1.11259176	0.29468964	0.49521999	0.47494031	Enpp1	1
GO:0016053 organic acid biosynthetic process	2/67	235/14464	0.00851064	1.83728168	0.88277275	0.29710245	0.49748388	0.47711149	Nr1d1/Pycr1	2
GO:0071900 regulation of protein serine/threonine kinase activity	2/67	235/14464	0.00851064	1.83728168	0.88277275	0.29710245	0.49748388	0.47711149	Ager/Pkia	2
GO:0032945 negative regulation of mononuclear cell proliferation	1/67	76/14464	0.01315789	2.84053417	1.09744069	0.2979738	0.49748388	0.47711149	H2-T23	1
GO:0050709 negative regulation of protein secretion	1/67	76/14464	0.01315789	2.84053417	1.09744069	0.2979738	0.49748388	0.47711149	Cd200	1
GO:0055092 sterol homeostasis	1/67	76/14464	0.01315789	2.84053417	1.09744069	0.2979738	0.49748388	0.47711149	Nr1d1	1
GO:0140056 organelle localization by membrane tethering	1/67	76/14464	0.01315789	2.84053417	1.09744069	0.2979738	0.49748388	0.47711149	Stx17	1
GO:0007249 canonical NF-kappaB signal transduction	2/67	236/14464	0.00847458	1.82949658	0.87645428	0.29881884	0.49781604	0.47743004	Nr1d1/Tnfsf10	2
GO:0033044 regulation of chromosome organization	2/67	236/14464	0.00847458	1.82949658	0.87645428	0.29881884	0.49781604	0.47743004	Cdk2/Hnmpd	2
GO:0000380 alternative mRNA splicing, via spliceosome	1/67	77/14464	0.01298701	2.80364412	1.08253434	0.30124289	0.500232	0.47974708	Arglu1	1
GO:0007589 body fluid secretion	1/67	77/14464	0.01298701	2.80364412	1.08253434	0.30124289	0.500232	0.47974708	Enpp1	1
GO:1901880 negative regulation of protein depolymerization	1/67	77/14464	0.01298701	2.80364412	1.08253434	0.30124289	0.500232	0.47974708	Ccdc88c	1
GO:0001818 negative regulation of cytokine production	2/67	239/14464	0.0083682	1.80653219	0.85767966	0.30396447	0.5040063	0.48336681	Ager/Cd200	2
GO:0008380 RNA splicing	3/67	417/14464	0.00719424	1.55309782	0.7818236	0.30446858	0.5040063	0.48336681	Srsf5/Arglu1/Luc7l2	3
GO:0044774 mitotic DNA integrity checkpoint signaling	1/67	78/14464	0.01282051	2.76769996	1.06786516	0.30449699	0.5040063	0.48336681	Rpa2	1
GO:0042113 B cell activation	2/67	240/14464	0.00833333	1.79900498	0.85148073	0.3056784	0.50441062	0.48375457	Enpp1/Fnip1	2
GO:0051896 regulation of phosphatidylinositol 3-kinase/protein kinase B signal transduction	2/67	240/14464	0.00833333	1.79900498	0.85148073	0.3056784	0.50441062	0.48375457	Aim2/Epha8	2
GO:0043410 positive regulation of MAPK cascade	3/67	419/14464	0.0071599	1.54568447	0.77324714	0.30701494	0.50441062	0.48375457	Ccdc88c/Ager/Epha8	3
GO:0031669 cellular response to nutrient levels	2/67	241/14464	0.00829876	1.79154022	0.84531096	0.30739163	0.50441062	0.48375457	Ehmt2/Fnip1	2
GO:0045444 fat cell differentiation	2/67	241/14464	0.00829876	1.79154022	0.84531096	0.30739163	0.50441062	0.48375457	Nr1d1/Enpp1	2
GO:0051965 positive regulation of synapse assembly	1/67	79/14464	0.01265823	2.73266578	1.05342593	0.30773616	0.50441062	0.48375457	Il1rapl1	1
GO:0071677 positive regulation of mononuclear cell migration	1/67	79/14464	0.01265823	2.73266578	1.05342593	0.30773616	0.50441062	0.48375457	Ager	1
GO:0097009 energy homeostasis	1/67	79/14464	0.01265823	2.73266578	1.05342593	0.30773616	0.50441062	0.48375457	Enpp1	1
GO:1904063 negative regulation of cation transmembrane transport	1/67	79/14464	0.01265823	2.73266578	1.05342593	0.30773616	0.50441062	0.48375457	Slc30a1	1
GO:0007264 small GTPase-mediated signal transduction	3/67	420/14464	0.00714286	1.54200426	0.76897555	0.30828854	0.50441062	0.48375457	Ccdc88c/Cdk2/Tiam1	3
GO:0071695 anatomical structure maturation	2/67	242/14464	0.00826446	1.78413717	0.83917005	0.30910413	0.50441062	0.48375457	Epha8/Enpp1	2
GO:0045785 positive regulation of cell adhesion	3/67	422/14464	0.007109	1.53469619	0.76046533	0.31083652	0.50441062	0.48375457	H2-T23/Ager/Abi3bp	3
GO:0010507 negative regulation of autophagy	1/67	80/14464	0.0125	2.69850746	1.03920974	0.31096047	0.50441062	0.48375457	Ehmt2	1
GO:0021766 hippocampus development	1/67	80/14464	0.0125	2.69850746	1.03920974	0.31096047	0.50441062	0.48375457	Xrn2	1
GO:0033627 cell adhesion mediated by integrin	1/67	80/14464	0.0125	2.69850746	1.03920974	0.31096047	0.50441062	0.48375457	Epha8	1
GO:0045685 regulation of glial cell differentiation	1/67	80/14464	0.0125	2.69850746	1.03920974	0.31096047	0.50441062	0.48375457	Nr1d1	1
GO:0070227 lymphocyte apoptotic process	1/67	80/14464	0.0125	2.69850746	1.03920974	0.31096047	0.50441062	0.48375457	Fnip1	1
GO:0071230 cellular response to amino acid stimulus	1/67	80/14464	0.0125	2.69850746	1.03920974	0.31096047	0.50441062	0.48375457	Hnmpd	1
GO:1902369 negative regulation of RNA catabolic process	1/67	80/14464	0.0125	2.69850746	1.03920974	0.31096047	0.50441062	0.48375457	Hnmpd	1
GO:0007283 spermatogenesis	3/67	423/14464	0.0070922	1.53106806	0.75622659	0.31211086	0.50574431	0.48503365	Spata24/Ehmt2/Xrn2	3
GO:0021510 spinal cord development	1/67	81/14464	0.01234568	2.66519256	1.02520997	0.31416999	0.5070568	0.48629239	Enpp1	1
GO:0045824 negative regulation of innate immune response	1/67	81/14464	0.01234568	2.66519256	1.02520997	0.31416999	0.5070568	0.48629239	H2-T23	1
GO:0070664 negative regulation of leukocyte proliferation	1/67	81/14464	0.01234568	2.66519256	1.02520997	0.31416999	0.5070568	0.48629239	H2-T23	1
GO:0060326 cell chemotaxis	2/67	245/14464	0.00816327	1.76229059	0.82091779	0.31423702	0.5070568	0.48629239	Slamf9/Tiam1	2
GO:0031346 positive regulation of cell projection organization	3/67	426/14464	0.00704225	1.52028589	0.74357508	0.31593514	0.50831362	0.48749775	Slc30a1/Tiam1/Il1rapl1	3
GO:0032642 regulation of chemokine production	1/67	82/14464	0.01219512	2.63269021	1.0114203	0.31736477	0.50831362	0.48749775	Ager	1
GO:0033209 tumor necrosis factor-mediated signaling pathway	1/67	82/14464	0.01219512	2.63269021	1.0114203	0.31736477	0.50831362	0.48749775	Aim2	1
GO:0061157 mRNA destabilization	1/67	82/14464	0.01219512	2.63269021	1.0114203	0.31736477	0.50831362	0.48749775	Hnmpd	1
GO:1901222 regulation of non-canonical NF-kappaB signal transduction	1/67	82/14464	0.01219512	2.63269021	1.0114203	0.31736477	0.50831362	0.48749775	Ager	1
GO:2000628 regulation of miRNA metabolic process	1/67	82/14464	0.01219512	2.63269021	1.0114203	0.31736477	0.50831362	0.48749775	Nfatc4	1
GO:0032984 protein-containing complex disassembly	2/67	247/14464	0.00809717	1.74802103	0.80888893	0.31765478	0.50831362	0.48749775	Ccdc88c/Stx17	2
GO:0097305 response to alcohol	2/67	247/14464	0.00809717	1.74802103	0.80888893	0.31765478	0.50831362	0.48749775	Hnmpd/Ehmt2	2
GO:0035282 segmentation	1/67	83/14464	0.01204819	2.60097105	0.99783463	0.3205449	0.51165177	0.49069919	Ppp2r3a	1
GO:1900076 regulation of cellular response to insulin stimulus	1/67	83/14464	0.01204819	2.60097105	0.99783463	0.3205449	0.51165177	0.49069919	Enpp1	1
GO:0033002 muscle cell proliferation	2/67	249/14464	0.0080321	1.7339807	0.79696908	0.32106896	0.51165177	0.49069919	Ager/Enpp1	2
GO:0070372 regulation of ERK1 and ERK2 cascade	2/67	249/14464	0.0080321	1.7339807	0.79696908	0.32106896	0.51165177	0.49069919	Ager/Tiam1	2
GO:0002253 activation of immune response	3/67	431/14464	0.00696056	1.50264917	0.72270146	0.32231223	0.51310243	0.49209044	Nr1d1/Aim2/Rftn1	3
GO:0034766 negative regulation of monoatomic ion transmembrane transport	1/67	84/14464	0.01190476	2.57000711	0.98444716	0.32371043	0.51418288	0.49312665	Slc30a1	1
GO:0071901 negative regulation of protein serine/threonine kinase activity	1/67	84/14464	0.01190476	2.57000711	0.98444716	0.32371043	0.51418288	0.49312665	Pkia	1
GO:0002683 negative regulation of immune system process	3/67	433/14464	0.00692841	1.49570852	0.71442495	0.32486388	0.51418288	0.49312665	Nr1d1/H2-T23/Cd200	3
GO:0010721 negative regulation of cell development	2/67	252/14464	0.00793651	1.71333807	0.77928922	0.326183	0.51418288	0.49312665	Nr1d1/Nfatc4	2
GO:0022409 positive regulation of cell-cell adhesion	2/67	252/14464	0.00793651	1.71333807	0.77928922	0.326183	0.51418288	0.49312665	H2-T23/Ager	2
GO:0019915 lipid storage	1/67	85/14464	0.01176471	2.53977173	0.97125229	0.32686143	0.51418288	0.49312665	Enpp1	1
GO:0022406 membrane docking	1/67	85/14464	0.01176471	2.53977173	0.97125229	0.32686143	0.51418288	0.49312665	Stx17	1
GO:0046651 lymphocyte proliferation	2/67	253/14464	0.00790514	1.70656598	0.77344825	0.32788564	0.51418288	0.49312665	H2-T23/Ager	2
GO:0060560 developmental growth involved in morphogenesis	2/67	254/14464	0.00787402	1.69984722	0.76763303	0.32958723	0.51418288	0.49312665	Ppp2r3a/Tiam1	2
GO:0048232 male gamete generation	3/67	437/14464	0.00686499	1.48201783	0.69799452	0.32996796	0.51418288	0.49312665	Spata24/Ehmt2/Xrn2	3
GO:0000041 transition metal ion transport	1/67	86/14464	0.01162791	2.5102395	0.95824465	0.32999797	0.51418288	0.49312665	Slc30a1	1
GO:0001570 vasculogenesis	1/67	86/14464	0.01162791	2.5102395	0.95824465	0.32999797	0.51418288	0.49312665	Enpp1	1
GO:0006821 chloride transport	1/67	86/14464	0.01162791	2.5102395	0.95824465	0.32999797	0.51418288	0.49312665	Gabrg1	1
GO:0007127 meiosis I	1/67	86/14464	0.01162791	2.5102395	0.95824465	0.32999797	0.51418288	0.49312665	Ehmt2	1
GO:0030168 platelet activation	1/67	86/14464	0.01162791	2.5102395	0.95824465	0.32999797	0.51418288	0.49312665	Fundc2	1
GO:0030512 negative regulation of transforming growth factor beta receptor signaling pathway	1/67	86/14464	0.01162791	2.5102395	0.95824465	0.32999797	0.51418288	0.49312665	Ldlrad4	1
GO:0032609 type II interferon production	1/67	86/14464	0.01162791	2.5102395	0.95824465	0.32999797	0.51418288	0.49312665	Enpp1	1
GO:0043242 negative regulation of protein-containing complex disassembly	1/67	86/14464	0.01162791	2.5102395	0.95824465	0.32999797	0.51418288	0.49312665	Ccdc88c	1
GO:0046849 bone remodeling	1/67	86/14464	0.01162791	2.5102395	0.95824465	0.32999797	0.51418288	0.49312665	Enpp1	1
GO:0070167 regulation of biomineral tissue development	1/67	86/14464	0.01162791	2.5102395	0.95824465	0.32999797	0.51418288	0.49312665	Enpp1	1
GO:0070252 actin-mediated cell contraction	1/67	86/14464	0.01162791	2.5102395	0.95824465	0.32999797	0.51418288	0.49312665	Ccdc88c	1
GO:0030072 peptide hormone secretion	2/67	256/14464	0.0078125	1.68656716	0.75607892	0.3329871	0.51488273	0.49379785	Nr1d1/Tiam1	2
GO:0050878 regulation of body fluid levels	2/67	256/14464	0.0078125	1.68656716	0.75607892	0.3329871	0.51488273	0.49379785	Fundc2/Enpp1	2
GO:0090150 establishment of protein localization to membrane	2/67	256/14464	0.0078125	1.68656716	0.75607892	0.3329871	0.51488273	0.49379785	Rftn1/Sys1	2
GO:0032755 positive regulation of interleukin-6 production	1/67	87/14464	0.01149425	2.48138617	0.94541909	0.33312011	0.51488273	0.49379785	Ager	1
GO:0048477 oogenesis	1/67	87/14464	0.01149425	2.48138617	0.94541909	0.33312011	0.51488273	0.49379785	Ehmt2	1
GO:0050779 RNA destabilization	1/67	87/14464	0.01149425	2.48138617	0.94541909	0.33312011	0.51488273	0.49379785	Hnmpd	1

GO:0051881	regulation of mitochondrial membrane potential	1/67	87/14464	0.01149425	2.48138617	0.94541909	0.33312011	0.51488273	0.49379785	Pycr1	1
GO:0061014	positive regulation of mRNA catabolic process	1/67	87/14464	0.01149425	2.48138617	0.94541909	0.33312011	0.51488273	0.49379785	Hnrnpd	1
GO:0021537	telencephalon development	2/67	257/14464	0.0077821	1.68000465	0.75033956	0.33468534	0.51678368	0.49562095	Bhlhe22/Xrn2	2
GO:0034644	cellular response to UV	1/67	88/14464	0.01136364	2.4531886	0.93277066	0.33622792	0.51760961	0.49641305	Nfatc4	1
GO:0043406	positive regulation of MAP kinase activity	1/67	88/14464	0.01136364	2.4531886	0.93277066	0.33622792	0.51760961	0.49641305	Ager	1
GO:2000106	regulation of leukocyte apoptotic process	1/67	88/14464	0.01136364	2.4531886	0.93277066	0.33622792	0.51760961	0.49641305	Fnip1	1
GO:0032943	mononuclear cell proliferation	2/67	259/14464	0.00772201	1.66703164	0.73893509	0.3380783	0.51977571	0.49849045	H2-T23/Ager	2
GO:0019218	regulation of steroid metabolic process	1/67	89/14464	0.01123596	2.42562469	0.9202946	0.33932145	0.51977571	0.49849045	Nr1d1	1
GO:0021954	central nervous system neuron development	1/67	89/14464	0.01123596	2.42562469	0.9202946	0.33932145	0.51977571	0.49849045	Bhlhe22	1
GO:0098661	inorganic anion transmembrane transport	1/67	89/14464	0.01123596	2.42562469	0.9202946	0.33932145	0.51977571	0.49849045	Gabrg1	1
GO:1903312	negative regulation of mRNA metabolic process	1/67	89/14464	0.01123596	2.42562469	0.9202946	0.33932145	0.51977571	0.49849045	Hnrnpd	1
GO:0051251	positive regulation of lymphocyte activation	2/67	260/14464	0.00769231	1.66061998	0.73326953	0.33977296	0.51995048	0.49865806	H2-T23/Ager	2
GO:0007409	axonogenesis	3/67	446/14464	0.00672646	1.45211164	0.6616079	0.34145184	0.52138302	0.50003194	Epha8/Tiam1/Bhlhe22	3
GO:0046883	regulation of hormone secretion	2/67	261/14464	0.00766284	1.65425745	0.72762813	0.34146638	0.52138302	0.50003194	Nr1d1/Tiam1	2
GO:0008589	regulation of smoothened signaling pathway	1/67	90/14464	0.01111111	2.3986733	0.90798633	0.34240079	0.52138302	0.50003194	Enpp1	1
GO:0010951	negative regulation of endopeptidase activity	1/67	90/14464	0.01111111	2.3986733	0.90798633	0.34240079	0.52138302	0.50003194	Fnip1	1
GO:0070555	response to interleukin-1	1/67	90/14464	0.01111111	2.3986733	0.90798633	0.34240079	0.52138302	0.50003194	Nr1d1	1
GO:0002790	peptide secretion	2/67	263/14464	0.00760456	1.64167754	0.71641694	0.3448494	0.5234642	0.50202789	Nr1d1/Tiam1	2
GO:0006091	generation of precursor metabolites and energy	3/67	449/14464	0.00668151	1.44240933	0.64965307	0.34527841	0.5234642	0.50202789	Nr1d1/Nfatc4/Enpp1	3
GO:0061982	meiosis I cell cycle process	1/67	91/14464	0.01098901	2.37231425	0.89584144	0.34546599	0.5234642	0.50202789	Ehmt2	1
GO:0071229	cellular response to acid chemical	1/67	91/14464	0.01098901	2.37231425	0.89584144	0.34546599	0.5234642	0.50202789	Hnrnpd	1
GO:1904019	epithelial cell apoptotic process	1/67	91/14464	0.01098901	2.37231425	0.89584144	0.34546599	0.5234642	0.50202789	Ager	1
GO:0007346	regulation of mitotic cell cycle	3/67	450/14464	0.00666667	1.43920398	0.64568703	0.34655367	0.52459647	0.50311379	Cdk2/Pkia/Rpa2	3
GO:0044344	cellular response to fibroblast growth factor stimulus	1/67	92/14464	0.01086957	2.34652823	0.88385569	0.34851711	0.52601848	0.50447757	Enpp1	1
GO:0046889	positive regulation of lipid biosynthetic process	1/67	92/14464	0.01086957	2.34652823	0.88385569	0.34851711	0.52601848	0.50447757	Nr1d1	1
GO:0048678	response to axon injury	1/67	92/14464	0.01086957	2.34652823	0.88385569	0.34851711	0.52601848	0.50447757	Enpp1	1
GO:0043280	positive regulation of cysteine-type endopeptidase activity involved in apoptotic process	1/67	93/14464	0.01075269	2.32129674	0.872025	0.35155422	0.52853176	0.50688794	Tnfsf10	1
GO:0098659	inorganic cation import across plasma membrane	1/67	93/14464	0.01075269	2.32129674	0.872025	0.35155422	0.52853176	0.50688794	Slc30a1	1
GO:0099587	inorganic ion import across plasma membrane	1/67	93/14464	0.01075269	2.32129674	0.872025	0.35155422	0.52853176	0.50688794	Slc30a1	1
GO:1901879	regulation of protein depolymerization	1/67	93/14464	0.01075269	2.32129674	0.872025	0.35155422	0.52853176	0.50688794	Ccdc88c	1
GO:0010817	regulation of hormone levels	3/67	455/14464	0.00659341	1.42338855	0.6259964	0.35292733	0.52952839	0.50784375	Nr1d1/Tiam1/Enpp1	3
GO:0031667	response to nutrient levels	3/67	455/14464	0.00659341	1.42338855	0.6259964	0.35292733	0.52952839	0.50784375	Ehmt2/Enpp1/Fnip1	3
GO:0006401	RNA catabolic process	2/67	268/14464	0.00746269	1.61104923	0.68879692	0.35328352	0.52952839	0.50784375	Hnrnpd/Xrn2	2
GO:0031110	regulation of microtubule polymerization or depolymerization	1/67	94/14464	0.0106383	2.2966021	0.86034543	0.35457738	0.52952839	0.50784375	Ccdc88c	1
GO:0032355	response to estradiol	1/67	94/14464	0.0106383	2.2966021	0.86034543	0.35457738	0.52952839	0.50784375	Hnrnpd	1
GO:0060349	bone morphogenesis	1/67	94/14464	0.0106383	2.2966021	0.86034543	0.35457738	0.52952839	0.50784375	Enpp1	1
GO:0060070	canonical Wnt signaling pathway	2/67	269/14464	0.00743494	1.6050602	0.68334122	0.35496614	0.52952839	0.50784375	Ccdc88c/Ppp2r3a	2
GO:1903311	regulation of mRNA metabolic process	2/67	269/14464	0.00743494	1.6050602	0.68334122	0.35496614	0.52952839	0.50784375	Hnrnpd/Arglu1	2
GO:0002708	positive regulation of lymphocyte mediated immunity	1/67	95/14464	0.01052632	2.27242734	0.84881318	0.35758666	0.53137999	0.50961952	H2-T23	1
GO:0010586	miRNA metabolic process	1/67	95/14464	0.01052632	2.27242734	0.84881318	0.35758666	0.53137999	0.50961952	Nfatc4	1
GO:0071482	cellular response to light stimulus	1/67	95/14464	0.01052632	2.27242734	0.84881318	0.35758666	0.53137999	0.50961952	Nfatc4	1
GO:1990542	mitochondrial transmembrane transport	1/67	95/14464	0.01052632	2.27242734	0.84881318	0.35758666	0.53137999	0.50961952	Tst	1
GO:0015833	peptide transport	2/67	272/14464	0.00735294	1.58735733	0.66710721	0.36000518	0.53445856	0.51257202	Nr1d1/Tiam1	2
GO:0050764	regulation of phagocytosis	1/67	96/14464	0.01041667	2.24875622	0.8374246	0.36058212	0.53479985	0.51289934	Ager	1
GO:0070371	ERK1 and ERK2 cascade	2/67	273/14464	0.00732601	1.58154284	0.6617396	0.36168184	0.53563562	0.51370089	Ager/Tiam1	2
GO:0071407	cellular response to organic cyclic compound	3/67	462/14464	0.00649351	1.40182206	0.59881176	0.36184081	0.53563562	0.51370089	Hnrnpd/Nfatc4/Ehmt2	3
GO:0008344	adult locomotory behavior	1/67	97/14464	0.01030928	2.22557317	0.82617616	0.36356381	0.53612616	0.51417133	Enpp1	1
GO:0016239	positive regulation of macroautophagy	1/67	97/14464	0.01030928	2.22557317	0.82617616	0.36356381	0.53612616	0.51417133	Gpsm1	1
GO:0050772	positive regulation of axonogenesis	1/67	97/14464	0.01030928	2.22557317	0.82617616	0.36356381	0.53612616	0.51417133	Tiam1	1
GO:0098656	monoatomic anion transmembrane transport	1/67	97/14464	0.01030928	2.22557317	0.82617616	0.36356381	0.53612616	0.51417133	Gabrg1	1
GO:0002718	regulation of cytokine production involved in immune response	1/67	98/14464	0.01020408	2.20286323	0.81506445	0.36653181	0.53690639	0.51491961	H2-T23	1
GO:0007040	lysosome organization	1/67	98/14464	0.01020408	2.20286323	0.81506445	0.36653181	0.53690639	0.51491961	Fnip1	1
GO:0032204	regulation of telomere maintenance	1/67	98/14464	0.01020408	2.20286323	0.81506445	0.36653181	0.53690639	0.51491961	Hnrnpd	1
GO:0043473	pigmentation	1/67	98/14464	0.01020408	2.20286323	0.81506445	0.36653181	0.53690639	0.51491961	Enpp1	1
GO:0071774	response to fibroblast growth factor	1/67	98/14464	0.01020408	2.20286323	0.81506445	0.36653181	0.53690639	0.51491961	Enpp1	1
GO:0080171	lytic vacuole organization	1/67	98/14464	0.01020408	2.20286323	0.81506445	0.36653181	0.53690639	0.51491961	Fnip1	1
GO:0090263	positive regulation of canonical Wnt signaling pathway	1/67	98/14464	0.01020408	2.20286323	0.81506445	0.36653181	0.53690639	0.51491961	Ppp2r3a	1
GO:0010608	post-transcriptional regulation of gene expression	3/67	468/14464	0.00641026	1.38384998	0.57585491	0.36946905	0.53765646	0.51563897	Hnrnpd/Pcif1/Zfp598	3
GO:0002444	myeloid leukocyte mediated immunity	1/67	99/14464	0.01010101	2.18061209	0.80408619	0.36948617	0.53765646	0.51563897	H2-T23	1
GO:0006096	glycolytic process	1/67	99/14464	0.01010101	2.18061209	0.80408619	0.36948617	0.53765646	0.51563897	Enpp1	1
GO:0033135	regulation of peptidyl-serine phosphorylation	1/67	99/14464	0.01010101	2.18061209	0.80408619	0.36948617	0.53765646	0.51563897	Fnip1	1
GO:0061041	regulation of wound healing	1/67	99/14464	0.01010101	2.18061209	0.80408619	0.36948617	0.53765646	0.51563897	Ajap1	1
GO:1903432	regulation of TORC1 signaling	1/67	99/14464	0.01010101	2.18061209	0.80408619	0.36948617	0.53765646	0.51563897	Fnip1	1
GO:2001243	negative regulation of intrinsic apoptotic signaling pathway	1/67	99/14464	0.01010101	2.18061209	0.80408619	0.36948617	0.53765646	0.51563897	Pycr1	1
GO:0043491	phosphatidylinositol 3-kinase/protein kinase B signal transduction	2/67	278/14464	0.00719424	1.55309782	0.63522116	0.37004158	0.53787249	0.51584615	Aim2/Epha8	2
GO:0006310	DNA recombination	2/67	279/14464	0.00716846	1.54753116	0.62998015	0.37170867	0.53787249	0.51584615	Rpa2/Xrn2	2
GO:0002009	morphogenesis of an epithelium	3/67	470/14464	0.00638298	1.37796126	0.56827149	0.37200887	0.53787249	0.51584615	Ajap1/Nfatc4/Enpp1	3
GO:0002698	negative regulation of immune effector process	1/67	100/14464	0.01	2.15880597	0.7932382	0.37242696	0.53787249	0.51584615	H2-T23	1
GO:0008645	hexose transmembrane transport	1/67	100/14464	0.01	2.15880597	0.7932382	0.37242696	0.53787249	0.51584615	Enpp1	1
GO:0014910	regulation of smooth muscle cell migration	1/67	100/14464	0.01	2.15880597	0.7932382	0.37242696	0.53787249	0.51584615	Ager	1
GO:0046928	regulation of neurotransmitter secretion	1/67	100/14464	0.01	2.15880597	0.7932382	0.37242696	0.53787249	0.51584615	Slc30a1	1
GO:1904659	D-glucose transmembrane transport	1/67	100/14464	0.01	2.15880597	0.7932382	0.37242696	0.53787249	0.51584615	Enpp1	1
GO:0034655	nucleobase-containing compound catabolic process	3/67	471/14464	0.00636943	1.37503565	0.56449251	0.37327818	0.53859707	0.51654106	Hnrnpd/Enpp1/Xrn2	3
GO:0003300	cardiac muscle hypertrophy	1/67	101/14464	0.00990099	2.13743165	0.78251741	0.37535424	0.53906885	0.51699352	Tiam1	1
GO:0021761	limbic system development	1/67	101/14464	0.00990099	2.13743165	0.78251741	0.37535424	0.53906885	0.51699352	Xrn2	1
GO:0035710	CD4-positive, alpha-beta T cell activation	1/67	101/14464	0.00990099	2.13743165	0.78251741	0.37535424	0.53906885	0.51699352	Ager	1
GO:0043255	regulation of carbohydrate biosynthetic process	1/67	101/14464	0.00990099	2.13743165	0.78251741	0.37535424	0.53906885	0.51699352	Enpp1	1
GO:0050852	T cell receptor signaling pathway	1/67	101/14464	0.00990099	2.13743165	0.78251741	0.37535424	0.53906885	0.51699352	Rftn1	1
GO:0000422	autophagy of mitochondrion	1/67	102/14464	0.00980392	2.11647644	0.77192087	0.37826806	0.54036414	0.51823577	Fundc2	1
GO:0002824	positive regulation of adaptive immune response based on somatic recombination of immune receptors b	1/67	102/14464	0.00980392	2.11647644	0.77192087	0.37826806	0.54036414	0.51823577	H2-T23	1
GO:0015749	monosaccharide transmembrane transport	1/67	102/14464	0.00980392	2.11647644	0.77192087	0.37826806	0.54036414	0.51823577	Enpp1	1
GO:0042476	odontogenesis	1/67	102/14464	0.00980392	2.11647644	0.77192087	0.37826806	0.54036414	0.51823577	Enpp1	1
GO:0099068	postsynapse assembly	1/67	102/14464	0.00980392	2.11647644	0.77192087	0.37826806	0.54036414	0.51823577	Abi3bp	1
GO:0002696	positive regulation of leukocyte activation	2/67	283/14464	0.00706714	1.52565793	0.60921923	0.3783601	0.54036414	0.51823577	H2-T23/Ager	2
GO:0048193	Golgi vesicle transport	2/67	284/14464	0.00704225	1.52028589	0.60407894	0.38001863	0.54186407	0.51967427	Sys1/Stx17	2
GO:0006892	post-Golgi vesicle-mediated transport	1/67	103/14464	0.00970874	2.09592813	0.7614457	0.3811685	0.54186407	0.51967427	Sys1	1
GO:0033555	multicellular organismal response to stress	1/67	103/14464	0.00970874	2.09592813	0.7614457	0.3811685	0.54186407	0.51967427	Gja4	1
GO:0046032	ADP catabolic process	1/67	103/14464	0.00970874	2.09592813	0.7614457	0.3811685	0.54186407	0.51967427	Enpp1	1
GO:2000278	regulation of DNA biosynthetic process	1/67	103/14464	0.00970874	2.09592813	0.7614457	0.3811685	0.54186407	0.51967427	Hnrnpd	1
GO:0014897	striated muscle hypertrophy	1/67	104/14464	0.00961538	2.07577497	0.75108913	0.3840556	0.54304031	0.52080235	Tiam1	1
GO:0031330	negative regulation of cellular catabolic process	1/67	104/14464	0.00961538	2.07577497	0.75108913	0.3840556	0.54304031	0.52080235	Ehmt2	1
GO:0043279	response to alkaloid	1/67	104/14464	0.00961538	2.07577497	0.75108913	0.3840556	0.54304031	0.52080235	Ehmt2	1
GO:0098754	detoxification	1/67	104/14464	0.00961538	2.07577497	0.75108913	0.3840556	0.54304031	0.52080235	Slc30a1	1
GO:0051054	positive regulation of DNA metabolic process	2/67	287/14464	0.00696864	1.5043944	0.58877537	0.38498354	0.54304031	0.52080235	Cdk2/Hnrnpd	2
GO:0070661	leukocyte proliferation	2/67	287/14464	0.00696864	1.5043944	0.58877537	0.38498354	0.54304031	0.52080235	H2-T23/Ager	2
GO:1901888	regulation of cell junction assembly	2/67	288/14464	0.00694444	1.49917081	0.58371273	0.38663488	0.54304031	0.52080235	Abi3bp/Il1rapl1	2
GO:0002832	negative regulation of response to biotic stimulus	1/67	105/14464	0.00952381	2.05600569	0.74084849	0.38692944	0.54304031	0.52080235	H2-T23	1
GO:0008630	intrinsic apoptotic signaling pathway in response to DNA damage	1/67	105/14464	0.00952381	2.05600569	0.74084849	0.38692944	0.54304031	0.52080235	Nfatc4	1
GO:0009137	purine nucleoside diphosphate catabolic process	1/67	105/14464	0.00952381	2.05600569	0.74084849	0.38692944	0.54304031	0.52080235	Enpp1	1
GO:0009181	purine ribonucleoside diphosphate catabolic process	1/67	105/14464	0.00952381	2.05600569	0.74084849	0.38692944	0.54304031	0.52080235	Enpp1	1
GO:0019364	pyridine nucleotide catabolic process	1/67	105/14464	0.00952381	2.05600569	0.74084849	0.38692944	0.54304031	0.52080235	Enpp1	1
GO:0036503	ERAD pathway	1/67	105/14464	0.00952381	2.05600569	0.74084849	0.38692944	0.54304031	0.52080235	Foxred2	1
GO:0038061	non-canonical NF-kappaB signal transduction	1/67	105/14464	0.00952381	2.05600569	0.74084849	0.38692944	0.54304031	0.52080235	Ager	1
GO:0014896	muscle hypertrophy	1/67	106/14464	0.00943396	2.03660941	0.73072118	0.38979006	0.54556447	0.52322314	Tiam1	1
GO:0048024	regulation of mRNA splicing, via spliceosome	1/67	106/14464	0.00943396	2.03660941	0.73072118	0.38979006	0.54556447	0.52322314	Arglu1	1
GO:0060840	artery development	1/67	106/14464	0.00943396	2.03660941	0.73072118	0.38979006	0.54556447	0.52322314	Enpp1	1
GO:0034329	cell junction assembly	3/67	485/14464	0.00618557	1.3353439	0.51245274	0.39099941	0.54657731	0.52419451	Gabrg1/Abi3bp/Il1rapl1	3
GO:0048511	rhythmic process	2/67	291/14464	0.00687285	1.48371544	0.56863835	0.39157778	0.54657731	0.52419451	Nr1d1/Hnrnpd	2
GO:0141188	nucleic acid catabolic process	2/67	291/14464	0.00687285	1.48371544	0.56863835	0.39157778	0.54657731	0.52419451	Hnrnpd/Xrn2	2
GO:0032963	collagen metabolic process	1/67	107/14464	0.00934579	2.01757567	0.72070469	0.39263754	0.54689038	0.52449475	Ager	1
GO:0042274	ribosomal small subunit biogenesis	1/67	107/14464	0.00934579	2.01757567	0.72070469	0.39263754	0.54689038	0.52449475	Rrp9	1
GO:0071375	cellular response to peptide hormone stimulus	2/67	292/14464	0.00684932	1.47863423	0.56365089	0.39322163	0.54689038	0.52449475	Cdk2/Enpp1	2
GO:1903037	regulation of leukocyte cell-cell adhesion	2/67	292/14464	0.00684932	1.47863423	0.56365089	0.39322163	0.54689038	0.52449475	H2-T23/Ager	2
GO:0030316	osteoclast differentiation	1/67	108/14464	0.00925926	1.99889442	0.71079658	0.39547192	0.54804158	0.52559881	Enpp1	1
GO:0042471	ear morphogenesis	1/67	108/14464	0.00925926	1.99889442	0.71079658	0.39547192	0.54804158	0.52559881	Enpp1	1
GO:0046031	ADP metabolic process	1/67	108/14464	0.00925926	1.99889442	0.71079658	0.39547192	0.54804158	0.52559881	Enpp1	1
GO:0072526	pyridine-containing compound catabolic process	1/67	108/14464	0.00925926	1.99889442	0.71079658	0.39547192	0.54804158	0.52559881	Enpp1	1
GO:0055074	calcium ion homeostasis	2/67	295/14464	0.00677966	1.46359727	0.54879844	0.39814164	0.54997307	0.5274512	Slc30a1/Enpp1	2
GO:0001578	microtubule bundle formation	1/67	109/14464	0.00917431	1.98055594	0.70099451	0.39829328	0.54997307	0.5274512	Ccdc88c	1
GO:0009191	ribonucleoside diphosphate catabolic process	1/67	109/14464	0.00917431	1.98055594	0.70099451	0.39829328	0.54997307	0.5274512	Enpp1	1
GO:0046683	response to organophosphorus	1/67	109/14464	0.00917431	1.98055594	0.70099451	0.39829328	0.54997307	0.5274512	Cdk2	1
GO:0002702	positive regulation of production of molecular mediator of immune response	1/67	110/14464	0.00909091	1.96255088	0.69129618	0.40110166	0.55131517	0.52873834	H2-T23	1
GO:0002821	positive regulation of adaptive immune response	1/67	110/14464	0.00909091	1.96255088	0.69129618	0.40110166	0.55131517	0.52873834	H2-T23	1
GO:0030048	actin filament-based movement	1/67	110/14464	0.00909091	1.96255088	0.69129618	0.40110166	0.55131517	0.52873834	Ccdc88c	1
GO:0034219	carbohydrate transmembrane transport	1/67	110/14464	0.00909091	1.96255088	0.69129618	0.40110166	0.55131517	0.52873834	Enpp1	1
GO:0050868	negative regulation of T cell activation	1/67	110/14464	0.00909091	1.96255088	0.69129618	0.40110166	0.55131517	0.52873834	H2-T23	1
GO:0050863	regulation of T cell activation	2/67	297/14464	0.00673401	1.45374139	0.53898698	0.40141182	0.55131517	0.52873834	H2-T23/Ager	2
GO:0009134	nucleoside diphosphate catabolic process	1/67	111/14464	0.00900901	1.94487024	0.6816994	0.40389713	0.55324932	0.53059329	Enpp1	1
GO:0034502	protein localization to chromosome	1/67	111/14464	0.00900901	1.94487024	0.6816994	0.40389713	0.55324932	0.53059329	Rpa2	1
GO:0048661	positive regulation of smooth muscle cell proliferation	1/67	111/14464	0.00900901	1.94487024	0.6816994	0.40389713	0.55324932	0.53059329	Ager	1
GO:0006413	translational initiation	1/67	112/14464	0.00892857	1.92750533	0.67220201	0.40667975	0.55459601	0.53188484	Zfp598	1
GO:0007265	Ras protein signal transduction	1/67	112/14464	0.00892857	1.92750533	0.67220201	0.40667975	0.55459601	0.53188484	Cdk2	1
GO:0009791	post-embryonic development	1/67	112/14464	0.00892857	1.92750533	0.67220201	0.40667975	0.55459601	0.53188484	Enpp1	1
GO:0048145	regulation of fibroblast proliferation	1/67	112/14464	0.00892857	1.92750533	0.67220201	0.40667975	0.55459601	0.53188484	Ager	1
GO:0071887	leukocyte apoptotic process	1/67	112/14464	0.00892857	1.92750533	0.67220201	0.40667975	0.55459601	0.53188484	Fnip1	1
GO:0044770	cell cycle phase transition	3/67	498/14464	0.0060241	1.30048552	0.46551406	0.40735451	0.55502502	0.53229628	Cdk2/Pkia/Rpa2	3
GO:0071396	cellular response to lipid	3/67	499/14464	0.00601202	1.29787934	0.46195618	0.40860789	0.55556301	0.53281223	Nr1d1/Hnrnpd/Nfatc4	3
GO:0009135	purine nucleoside diphosphate metabolic process	1/67	113/14464	0.00884956	1.91044776	0.66280193	0.40944957	0.55556301	0.53281223	Enpp1	1
GO:0009179	purine ribonucleoside diphosphate metabolic process	1/67	113/14464	0.00884956	1.91044776	0.66280193	0.40944957	0.55556301	0.53281223	Enpp1	1
GO:0038202	TORC1 signaling	1/67	113/14464	0.00884956	1.91044776	0.66280193	0.40944957	0.55556301	0.53281223	Fnip1	1
GO:0097193	intrinsic apoptotic signaling pathway	2/67	302/14464	0.00662252	1.42967283	0.51476577	0.40955197	0.55556301	0.53281223	Pycr1/Nfatc4	2
GO:0050867	positive regulation of cell activation	2/67	303/14464	0.00660066	1.42495444	0.50997315	0.41117381	0.55573968	0.53298166	H2-T23/Ager	2
GO:0000077	DNA damage checkpoint signaling	1/67	114/14464	0.00877193	1.89368945	0.65349715	0.41220665	0.55573968	0.53298166	Rpa2	1
GO:0001952	regulation of cell-matrix adhesion	1/67	114/14464	0.00877193	1.89368945	0.65349715	0.41220665	0.55573968	0.53298166	Ajap1	1
GO:0007259	cell surface receptor signaling pathway via JAK-STAT	1/67	114/14464	0.00877193	1.89368945	0.65349715	0.41220665	0.55573968	0.53298166	Ager	1
GO:0008637	apoptotic mitochondrial changes	1/67	114/14464	0.00877193	1.89368945	0.65349715	0.41220665	0.55573968	0.53298166	Tnfsf10	1
GO:0008643	carbohydrate transport	1/67	114/14464	0.00877193	1.89368945	0.65349715	0.41220665	0.55573968	0.53298166	Enpp1	1
GO:1990830	cellular response to leukemia inhibitory factor	1/67	114/14464	0.00877193	1.89368945	0.65349715	0.41220665	0.55573968	0.53298166	Gstt2	1
GO:0002705	positive regulation of leukocyte mediated immunity	1/67	115/14464	0.00869565	1.87722258	0.64428571	0.41495105	0.5579752	0.53512564	H2-T23	1
GO:0030282	bone mineralization	1/67	115/14464	0.00869565	1.87722258	0.64428571	0.41495105	0.5579752	0.53512564	Enpp1	1
GO:0043200	response to amino acid	1/67	115/14464	0.00869565	1.87722258	0.64428571	0.41495105	0.5579752	0.53512564	Hnrnpd	1
GO:0051347	positive regulation of transferase activity	2/67	306/14464	0.00653595	1.41098429	0.49569619	0.41602669	0.55893385	0.53604503	Ager/Cd200	2
GO:0048709	oligodendrocyte differentiation	1/67	116/14464	0.00862069	1.86103963	0.63516571	0.41768283	0.56018211	0.53724217	Enpp1	1
GO:1990823	response to leukemia inhibitory factor	1/67	116/14464	0.00862069	1.86103963	0.63516571	0.41768283	0.56018211	0.53724217	Gstt2	1
GO:0000018	regulation of DNA recombination	1/67	117/14464	0.00854701	1.84513331	0.6261353	0.42040204	0.56187298	0.5388638	Rpa2	1
GO:0008584	male gonad development	1/67	117/14464	0.00854701	1.84513331	0.6261353	0.42040204	0.56187298	0.5388638	Tnfsf10	1
GO:0014013	regulation of gliogenesis	1/67	117/14464	0.00854701	1.84513331	0.6261353	0.42040204	0.56187298	0.5388638	Nr1d1	1
GO:0021549	cerebellum development	1/67	117/14464	0.00854701	1.84513331	0.6261353	0.42040204	0.56187298	0.5388638	Hnrnpd	1
GO:0010821	regulation of mitochondrion organization	1/67	118/14464	0.00847458	1.82949658	0.61719271	0.42310875	0.5635355	0.54045824	Tnfsf10	1
GO:0043271	negative regulation of monoatomic ion transport	1/67	118/14464	0.00847458	1.82949658	0.61719271	0.42310875	0.5635355	0.54045824	Slc30a1	1
GO:0046546	development of primary male sexual characteristics	1/67	118/14464	0.00847458	1.82949658	0.61719271	0.42310875	0.5635355	0.54045824	Tnfsf10	1
GO:0051588	regulation of neurotransmitter transport	1/67	118/14464	0.00847458	1.82949658	0.61719271	0.42310875	0.5635355	0.54045824	Slc30a1	1
GO:0043010	camera-type eye development	2/67	311/14464	0.00643087	1.38829966	0.47222997	0.42407173	0.56433035	0.54122054	Bhlhe22/Xrn2	2
GO:0006888	endoplasmic reticulum to Golgi vesicle-mediated transport	1/67	119/14464	0.00840336	1.81412266	0.60833617	0.425803	0.56468366	0.54155939	Stx17	1
GO:0030278	regulation of ossification	1/67	119/14464	0.00840336	1.81412266	0.60833617	0.425803	0.56468366	0.54155939	Enpp1	1
GO:0048593	camera-type eye morphogenesis	1/67	119/14464	0.00840336	1.81412266	0.60833617	0.425803	0.56468366	0.54155939	Bhlhe22	1

GO:1903313	positive regulation of mRNA metabolic process	1/67	119/14464	0.00840336	1.81412266	0.60833617	0.425803	0.56468366	0.54155939	Hnrnpd	1
GO:0042886	amide transport	2/67	314/14464	0.00636943	1.37503565	0.4583421	0.42887227	0.56777678	0.54452584	Nr1d1/Tiam1	2
GO:0046879	hormone secretion	2/67	314/14464	0.00636943	1.37503565	0.4583421	0.42887227	0.56777678	0.54452584	Nr1d1/Tiam1	2
GO:0031570	DNA integrity checkpoint signaling	1/67	121/14464	0.00826446	1.78413717	0.59087459	0.43115436	0.56884322	0.5455486	Rpa2	1
GO:0043414	macromolecule methylation	1/67	121/14464	0.00826446	1.78413717	0.59087459	0.43115436	0.56884322	0.5455486	Ehmt2	1
GO:0051261	protein depolymerization	1/67	121/14464	0.00826446	1.78413717	0.59087459	0.43115436	0.56884322	0.5455486	Ccdc88c	1
GO:1901890	positive regulation of cell junction assembly	1/67	121/14464	0.00826446	1.78413717	0.59087459	0.43115436	0.56884322	0.5455486	Il1rapl1	1
GO:0045834	positive regulation of lipid metabolic process	1/67	122/14464	0.00819672	1.76951309	0.58226631	0.4338116	0.57185943	0.54844129	Nr1d1	1
GO:1903038	negative regulation of leukocyte cell-cell adhesion	1/67	123/14464	0.00813008	1.7551268	0.57373761	0.4364566	0.57439457	0.55087263	H2-T23	1
GO:0002697	regulation of immune effector process	2/67	320/14464	0.00625	1.34925373	0.43098337	0.43841201	0.57439457	0.55087263	H2-T23/Ager	2
GO:0003002	regionalization	2/67	320/14464	0.00625	1.34925373	0.43098337	0.43841201	0.57439457	0.55087263	Ppp2r3a/Bhlhe22	2
GO:0008286	insulin receptor signaling pathway	1/67	124/14464	0.00806452	1.74097256	0.56528698	0.43908943	0.57439457	0.55087263	Enpp1	1
GO:0009185	ribonucleoside diphosphate metabolic process	1/67	124/14464	0.00806452	1.74097256	0.56528698	0.43908943	0.57439457	0.55087263	Enpp1	1
GO:0021987	cerebral cortex development	1/67	124/14464	0.00806452	1.74097256	0.56528698	0.43908943	0.57439457	0.55087263	Bhlhe22	1
GO:0045471	response to ethanol	1/67	124/14464	0.00806452	1.74097256	0.56528698	0.43908943	0.57439457	0.55087263	Ehmt2	1
GO:0051100	negative regulation of binding	1/67	124/14464	0.00806452	1.74097256	0.56528698	0.43908943	0.57439457	0.55087263	Nfatc4	1
GO:0097696	cell surface receptor signaling pathway via STAT	1/67	124/14464	0.00806452	1.74097256	0.56528698	0.43908943	0.57439457	0.55087263	Ager	1
GO:0009914	hormone transport	2/67	321/14464	0.00623053	1.34505045	0.42647633	0.43999386	0.57460216	0.55107171	Nr1d1/Tiam1	2
GO:0042060	wound healing	2/67	321/14464	0.00623053	1.34505045	0.42647633	0.43999386	0.57460216	0.55107171	Ajap1/Fundc2	2
GO:0022037	metencephalon development	1/67	125/14464	0.008	1.72704478	0.55691296	0.44171014	0.57635506	0.55275283	Hnrnpd	1
GO:0006694	steroid biosynthetic process	1/67	126/14464	0.00793651	1.71333807	0.54861412	0.44431879	0.57682835	0.55320674	Nr1d1	1
GO:0006959	humoral immune response	1/67	126/14464	0.00793651	1.71333807	0.54861412	0.44431879	0.57682835	0.55320674	H2-T23	1
GO:0010466	negative regulation of peptidase activity	1/67	126/14464	0.00793651	1.71333807	0.54861412	0.44431879	0.57682835	0.55320674	Fnip1	1
GO:0010811	positive regulation of cell-substrate adhesion	1/67	126/14464	0.00793651	1.71333807	0.54861412	0.44431879	0.57682835	0.55320674	Abi3bp	1
GO:0015718	monocarboxylic acid transport	1/67	126/14464	0.00793651	1.71333807	0.54861412	0.44431879	0.57682835	0.55320674	Slco1a4	1
GO:0090101	negative regulation of transmembrane receptor protein serine/threonine kinase signaling pathway	1/67	126/14464	0.00793651	1.71333807	0.54861412	0.44431879	0.57682835	0.55320674	Ldlrad4	1
GO:0001936	regulation of endothelial cell proliferation	1/67	127/14464	0.00787402	1.69984722	0.54038906	0.44691543	0.57776567	0.55410567	Ager	1
GO:0010565	regulation of cellular ketone metabolic process	1/67	127/14464	0.00787402	1.69984722	0.54038906	0.44691543	0.57776567	0.55410567	Nr1d1	1
GO:0030177	positive regulation of Wnt signaling pathway	1/67	127/14464	0.00787402	1.69984722	0.54038906	0.44691543	0.57776567	0.55410567	Ppp2r3a	1
GO:0046822	regulation of nucleocytoplasmic transport	1/67	127/14464	0.00787402	1.69984722	0.54038906	0.44691543	0.57776567	0.55410567	Pkia	1
GO:1902652	secondary alcohol metabolic process	1/67	127/14464	0.00787402	1.69984722	0.54038906	0.44691543	0.57776567	0.55410567	Enpp1	1
GO:0014074	response to purine-containing compound	1/67	128/14464	0.0078125	1.68656716	0.53223645	0.44950012	0.58013374	0.55637676	Cdk2	1
GO:0030856	regulation of epithelial cell differentiation	1/67	128/14464	0.0078125	1.68656716	0.53223645	0.44950012	0.58013374	0.55637676	Ajap1	1
GO:0006090	pyruvate metabolic process	1/67	129/14464	0.00775194	1.673493	0.52415494	0.45207291	0.58102114	0.55722783	Enpp1	1
GO:0009132	nucleoside diphosphate metabolic process	1/67	129/14464	0.00775194	1.673493	0.52415494	0.45207291	0.58102114	0.55722783	Enpp1	1
GO:0016482	cytosolic transport	1/67	129/14464	0.00775194	1.673493	0.52415494	0.45207291	0.58102114	0.55722783	Sys1	1
GO:0017015	regulation of transforming growth factor beta receptor signaling pathway	1/67	129/14464	0.00775194	1.673493	0.52415494	0.45207291	0.58102114	0.55722783	Ldlrad4	1
GO:0050684	regulation of mRNA processing	1/67	129/14464	0.00775194	1.673493	0.52415494	0.45207291	0.58102114	0.55722783	Arglu1	1
GO:0009895	negative regulation of catabolic process	2/67	330/14464	0.00606061	1.30836725	0.38656566	0.45412336	0.58188601	0.55805729	Hnrnpd/Ehmt2	2
GO:0006906	vesicle fusion	1/67	130/14464	0.00769231	1.66061998	0.51614327	0.45463385	0.58188601	0.55805729	Stx17	1
GO:0018205	peptidyl-lysine modification	1/67	130/14464	0.00769231	1.66061998	0.51614327	0.45463385	0.58188601	0.55805729	Ehmt2	1
GO:0051592	response to calcium ion	1/67	130/14464	0.00769231	1.66061998	0.51614327	0.45463385	0.58188601	0.55805729	Hnrnpd	1
GO:0170039	proteinogenic amino acid metabolic process	1/67	130/14464	0.00769231	1.66061998	0.51614327	0.45463385	0.58188601	0.55805729	Pycr1	1
GO:0007030	Golgi organization	1/67	131/14464	0.00763359	1.64794349	0.50820019	0.457183	0.58224711	0.55840359	Stx17	1
GO:0007098	centrosome cycle	1/67	131/14464	0.00763359	1.64794349	0.50820019	0.457183	0.58224711	0.55840359	Cdk2	1
GO:0009154	purine ribonucleotide catabolic process	1/67	131/14464	0.00763359	1.64794349	0.50820019	0.457183	0.58224711	0.55840359	Enpp1	1
GO:0009566	fertilization	1/67	131/14464	0.00763359	1.64794349	0.50820019	0.457183	0.58224711	0.55840359	Ehmt2	1
GO:0043244	regulation of protein-containing complex disassembly	1/67	131/14464	0.00763359	1.64794349	0.50820019	0.457183	0.58224711	0.55840359	Ccdc88c	1
GO:1903844	regulation of cellular response to transforming growth factor beta stimulus	1/67	131/14464	0.00763359	1.64794349	0.50820019	0.457183	0.58224711	0.55840359	Ldlrad4	1
GO:0001101	response to acid chemical	1/67	132/14464	0.00757576	1.63545907	0.50032446	0.45972041	0.58403063	0.56011408	Hnrnpd	1
GO:0031109	microtubule polymerization or depolymerization	1/67	132/14464	0.00757576	1.63545907	0.50032446	0.45972041	0.58403063	0.56011408	Ccdc88c	1
GO:0090174	organelle membrane fusion	1/67	132/14464	0.00757576	1.63545907	0.50032446	0.45972041	0.58403063	0.56011408	Stx17	1
GO:0002687	positive regulation of leukocyte migration	1/67	133/14464	0.0075188	1.62316238	0.49251492	0.46224614	0.58579054	0.56180192	Ager	1
GO:0007292	female gamete generation	1/67	133/14464	0.0075188	1.62316238	0.49251492	0.46224614	0.58579054	0.56180192	Ehmt2	1
GO:2000779	regulation of double-strand break repair	1/67	133/14464	0.0075188	1.62316238	0.49251492	0.46224614	0.58579054	0.56180192	Rpa2	1
GO:0003158	endothelium development	1/67	134/14464	0.00746269	1.61104923	0.4847704	0.46476024	0.58800946	0.56392997	Gja4	1
GO:0046661	male sex differentiation	1/67	134/14464	0.00746269	1.61104923	0.4847704	0.46476024	0.58800946	0.56392997	Tnfsf10	1
GO:0008277	regulation of G protein-coupled receptor signaling pathway	1/67	135/14464	0.00740741	1.59911553	0.47708978	0.46726275	0.59020648	0.56603702	Gpsm1	1
GO:0071902	positive regulation of protein serine/threonine kinase activity	1/67	135/14464	0.00740741	1.59911553	0.47708978	0.46726275	0.59020648	0.56603702	Pkia	1
GO:0007093	mitotic cell cycle checkpoint signaling	1/67	136/14464	0.00735294	1.58735733	0.46947195	0.46975374	0.59286693	0.56858852	Rpa2	1
GO:0048144	fibroblast proliferation	1/67	138/14464	0.00724638	1.56435215	0.45442043	0.47470135	0.5976428	0.57316881	Ager	1
GO:0050851	antigen receptor-mediated signaling pathway	1/67	138/14464	0.00724638	1.56435215	0.45442043	0.47470135	0.5976428	0.57316881	Rftn1	1
GO:0060078	regulation of postsynaptic membrane potential	1/67	138/14464	0.00724638	1.56435215	0.45442043	0.47470135	0.5976428	0.57316881	Gabrg1	1
GO:0007584	response to nutrient	1/67	139/14464	0.00719424	1.55309782	0.44698467	0.47715807	0.59877898	0.57425847	Enpp1	1
GO:0035107	appendage morphogenesis	1/67	139/14464	0.00719424	1.55309782	0.44698467	0.47715807	0.59877898	0.57425847	Enpp1	1
GO:0035108	limb morphogenesis	1/67	139/14464	0.00719424	1.55309782	0.44698467	0.47715807	0.59877898	0.57425847	Enpp1	1
GO:0120161	regulation of cold-induced thermogenesis	1/67	139/14464	0.00719424	1.55309782	0.44698467	0.47715807	0.59877898	0.57425847	Nr1d1	1
GO:0050678	regulation of epithelial cell proliferation	2/67	346/14464	0.00578035	1.24786472	0.31834408	0.47874684	0.59989364	0.57532748	Nr1d1/Ager	2
GO:0009261	ribonucleotide catabolic process	1/67	140/14464	0.00714286	1.54200426	0.43960756	0.47960348	0.59989364	0.57532748	Enpp1	1
GO:0043405	regulation of MAP kinase activity	1/67	140/14464	0.00714286	1.54200426	0.43960756	0.47960348	0.59989364	0.57532748	Ager	1
GO:0051250	negative regulation of lymphocyte activation	1/67	140/14464	0.00714286	1.54200426	0.43960756	0.47960348	0.59989364	0.57532748	H2-T23	1
GO:0009410	response to xenobiotic stimulus	2/67	348/14464	0.00574713	1.24069309	0.31004734	0.48177853	0.60001613	0.57544495	Aim2/Ehmt2	2
GO:0006195	purine nucleotide catabolic process	1/67	141/14464	0.0070922	1.53106806	0.43228816	0.48203761	0.60001613	0.57544495	Enpp1	1
GO:0006261	DNA-templated DNA replication	1/67	141/14464	0.0070922	1.53106806	0.43228816	0.48203761	0.60001613	0.57544495	Cdk2	1
GO:0007605	sensory perception of sound	1/67	141/14464	0.0070922	1.53106806	0.43228816	0.48203761	0.60001613	0.57544495	Enpp1	1
GO:0031023	microtubule organizing center organization	1/67	141/14464	0.0070922	1.53106806	0.43228816	0.48203761	0.60001613	0.57544495	Cdk2	1
GO:2000377	regulation of reactive oxygen species metabolic process	1/67	141/14464	0.0070922	1.53106806	0.43228816	0.48203761	0.60001613	0.57544495	Ager	1
GO:0050680	negative regulation of epithelial cell proliferation	1/67	142/14464	0.00704225	1.52028589	0.42502549	0.48446053	0.60157428	0.5769393	Ager	1
GO:0140013	meiotic nuclear division	1/67	142/14464	0.00704225	1.52028589	0.42502549	0.48446053	0.60157428	0.5769393	Ehmt2	1
GO:1903034	regulation of response to wounding	1/67	142/14464	0.00704225	1.52028589	0.42502549	0.48446053	0.60157428	0.5769393	Ajap1	1
GO:0032006	regulation of TOR signaling	1/67	143/14464	0.00699301	1.50965452	0.41781865	0.48687229	0.60311109	0.57841318	Fnip1	1
GO:0043123	positive regulation of canonical NF-kappaB signal transduction	1/67	143/14464	0.00699301	1.50965452	0.41781865	0.48687229	0.60311109	0.57841318	Tnfsf10	1
GO:0106106	cold-induced thermogenesis	1/67	143/14464	0.00699301	1.50965452	0.41781865	0.48687229	0.60311109	0.57841318	Nr1d1	1
GO:0006887	exocytosis	2/67	352/14464	0.00568182	1.2265943	0.29360039	0.4878103	0.60378769	0.57906207	Stx17/Il1rapl1	2
GO:0043467	regulation of generation of precursor metabolites and energy	1/67	144/14464	0.00694444	1.49917081	0.41066672	0.48927293	0.60511202	0.58033217	Enpp1	1
GO:0007596	blood coagulation	1/67	145/14464	0.00689655	1.4888317	0.40356883	0.4916625	0.60757972	0.58269882	Fundc2	1
GO:0007389	pattern specification process	2/67	356/14464	0.00561798	1.21281234	0.27734458	0.49379947	0.60905382	0.58411255	Ppp2r3a/Bhlhe22	2
GO:0007286	spermatid development	1/67	146/14464	0.00684932	1.47863423	0.3965241	0.49404106	0.60905382	0.58411255	Ehmt2	1
GO:1900180	regulation of protein localization to nucleus	1/67	146/14464	0.00684932	1.47863423	0.3965241	0.49404106	0.60905382	0.58411255	Pkia	1
GO:0043488	regulation of mRNA stability	1/67	147/14464	0.00680272	1.46857549	0.38953171	0.49640866	0.61002053	0.58503967	Hnrnpd	1
GO:0043547	positive regulation of GTPase activity	1/67	147/14464	0.00680272	1.46857549	0.38953171	0.49640866	0.61002053	0.58503967	Tiam1	1
GO:0170033	L-amino acid metabolic process	1/67	147/14464	0.00680272	1.46857549	0.38953171	0.49640866	0.61002053	0.58503967	Pycr1	1
GO:2001236	regulation of extrinsic apoptotic signaling pathway	1/67	147/14464	0.00680272	1.46857549	0.38953171	0.49640866	0.61002053	0.58503967	Tnfsf10	1
GO:0030098	lymphocyte differentiation	2/67	359/14464	0.00557103	1.20267742	0.26527519	0.49826311	0.61048244	0.58548266	Enpp1/Fnip1	2
GO:0001935	endothelial cell proliferation	1/67	148/14464	0.00675676	1.45865268	0.38259082	0.49876534	0.61048244	0.58548266	Ager	1
GO:0002244	hematopoietic progenitor cell differentiation	1/67	148/14464	0.00675676	1.45865268	0.38259082	0.49876534	0.61048244	0.58548266	Fnip1	1
GO:0007599	hemostasis	1/67	148/14464	0.00675676	1.45865268	0.38259082	0.49876534	0.61048244	0.58548266	Fundc2	1
GO:0009411	response to UV	1/67	148/14464	0.00675676	1.45865268	0.38259082	0.49876534	0.61048244	0.58548266	Nfatc4	1
GO:0048872	homeostasis of number of cells	2/67	360/14464	0.00555556	1.19933665	0.261275	0.49974557	0.61119676	0.58616773	Aim2/Enpp1	2
GO:0001889	liver development	1/67	150/14464	0.00666667	1.43920398	0.36886036	0.50344616	0.61328896	0.58817425	Hnrnpd	1
GO:0010977	negative regulation of neuron projection development	1/67	150/14464	0.00666667	1.43920398	0.36886036	0.50344616	0.61328896	0.58817425	Ngef	1
GO:0031214	biomineral tissue development	1/67	150/14464	0.00666667	1.43920398	0.36886036	0.50344616	0.61328896	0.58817425	Enpp1	1
GO:0048754	branching morphogenesis of an epithelial tube	1/67	150/14464	0.00666667	1.43920398	0.36886036	0.50344616	0.61328896	0.58817425	Nfatc4	1
GO:0050817	coagulation	1/67	150/14464	0.00666667	1.43920398	0.36886036	0.50344616	0.61328896	0.58817425	Fundc2	1
GO:0007224	smoothened signaling pathway	1/67	151/14464	0.00662252	1.42967283	0.36206923	0.5057704	0.6146626	0.58949165	Enpp1	1
GO:1902850	microtubule cytoskeleton organization involved in mitosis	1/67	151/14464	0.00662252	1.42967283	0.36206923	0.5057704	0.6146626	0.58949165	Gpsm1	1
GO:2000045	regulation of G1/S transition of mitotic cell cycle	1/67	151/14464	0.00662252	1.42967283	0.36206923	0.5057704	0.6146626	0.58949165	Rpa2	1
GO:0042445	hormone metabolic process	1/67	152/14464	0.00657895	1.42026709	0.3553265	0.50808392	0.61698764	0.59172147	Enpp1	1
GO:0061008	hepaticobiliary system development	1/67	153/14464	0.00653595	1.41098429	0.34863143	0.51038676	0.61929607	0.59393536	Hnrnpd	1
GO:0048515	spermatid differentiation	1/67	154/14464	0.00649351	1.40182206	0.34198331	0.51267899	0.62061141	0.59519685	Ehmt2	1
GO:0062013	positive regulation of small molecule metabolic process	1/67	154/14464	0.00649351	1.40182206	0.34198331	0.51267899	0.62061141	0.59519685	Nr1d1	1
GO:0098876	vesicle-mediated transport to the plasma membrane	1/67	154/14464	0.00649351	1.40182206	0.34198331	0.51267899	0.62061141	0.59519685	Sys1	1
GO:0050777	negative regulation of immune response	1/67	155/14464	0.00645161	1.39277805	0.33538142	0.51496065	0.62288411	0.59737648	H2-T23	1
GO:0030010	establishment of cell polarity	1/67	156/14464	0.00641026	1.38384998	0.32882509	0.51723178	0.62395779	0.59840619	Gpsm1	1
GO:0072330	monocarboxylic acid biosynthetic process	1/67	156/14464	0.00641026	1.38384998	0.32882509	0.51723178	0.62395779	0.59840619	Nr1d1	1
GO:0030900	forebrain development	2/67	373/14464	0.00536193	1.15753671	0.21027899	0.51876745	0.62395779	0.59840619	Bhlhe22/Xm2	2
GO:0002700	regulation of production of molecular mediator of immune response	1/67	157/14464	0.00636943	1.37503565	0.32231364	0.51949244	0.62395779	0.59840619	H2-T23	1
GO:0002753	cytoplasmic pattern recognition receptor signaling pathway	1/67	157/14464	0.00636943	1.37503565	0.32231364	0.51949244	0.62395779	0.59840619	Rftn1	1
GO:0062207	regulation of pattern recognition receptor signaling pathway	1/67	157/14464	0.00636943	1.37503565	0.32231364	0.51949244	0.62395779	0.59840619	Nr1d1	1
GO:0071478	cellular response to radiation	1/67	157/14464	0.00636943	1.37503565	0.32231364	0.51949244	0.62395779	0.59840619	Nfatc4	1
GO:0072523	purine-containing compound catabolic process	1/67	157/14464	0.00636943	1.37503565	0.32231364	0.51949244	0.62395779	0.59840619	Enpp1	1
GO:1990845	adaptive thermogenesis	1/67	157/14464	0.00636943	1.37503565	0.32231364	0.51949244	0.62395779	0.59840619	Nr1d1	1
GO:0009166	nucleotide catabolic process	1/67	158/14464	0.00632911	1.36633289	0.31584641	0.52174267	0.62617247	0.60053017	Enpp1	1
GO:0003341	cilium movement	1/67	159/14464	0.00628931	1.3577396	0.30942275	0.52398251	0.62690765	0.60123525	Ccdc88c	1
GO:0010594	regulation of endothelial cell migration	1/67	159/14464	0.00628931	1.3577396	0.30942275	0.52398251	0.62690765	0.60123525	Ager	1
GO:0061025	membrane fusion	1/67	159/14464	0.00628931	1.3577396	0.30942275	0.52398251	0.62690765	0.60123525	Stx17	1
GO:0071897	DNA biosynthetic process	1/67	159/14464	0.00628931	1.3577396	0.30942275	0.52398251	0.62690765	0.60123525	Hnrnpd	1
GO:0006816	calcium ion transport	2/67	377/14464	0.00530504	1.14525516	0.19495015	0.52452567	0.62707064	0.60139156	Gja4/Slc30a1	2
GO:0002181	cytoplasmic translation	1/67	160/14464	0.00625	1.34925373	0.30304204	0.52621202	0.62811211	0.60239038	Hnrnpd	1
GO:0070507	regulation of microtubule cytoskeleton organization	1/67	160/14464	0.00625	1.34925373	0.30304204	0.52621202	0.62811211	0.60239038	Ccdc88c	1
GO:0006469	negative regulation of protein kinase activity	1/67	161/14464	0.00621118	1.34087327	0.29670367	0.52843125	0.62881278	0.60306237	Pkia	1
GO:0033157	regulation of intracellular protein transport	1/67	161/14464	0.00621118	1.34087327	0.29670367	0.52843125	0.62881278	0.60306237	Pkia	1
GO:0051897	positive regulation of phosphatidylinositol 3-kinase/protein kinase B signal transduction	1/67	161/14464	0.00621118	1.34087327	0.29670367	0.52843125	0.62881278	0.60306237	Epha8	1
GO:0061013	regulation of mRNA catabolic process	1/67	161/14464	0.00621118	1.34087327	0.29670367	0.52843125	0.62881278	0.60306237	Hnrnpd	1
GO:0043487	regulation of RNA stability	1/67	162/14464	0.00617284	1.33259628	0.29040701	0.53064023	0.6304677	0.60464951	Hnrnpd	1
GO:0070374	positive regulation of ERK1 and ERK2 cascade	1/67	162/14464	0.00617284	1.33259628	0.29040701	0.53064023	0.6304677	0.60464951	Ager	1
GO:0002285	lymphocyte activation involved in immune response	1/67	163/14464	0.00613497	1.32442084	0.28415149	0.53283903	0.63161918	0.60575384	Enpp1	1
GO:0006606	protein import into nucleus	1/67	163/14464	0.00613497	1.32442084	0.28415149	0.53283903	0.63161918	0.60575384	Pkia	1
GO:1901991	negative regulation of mitotic cell cycle phase transition	1/67	163/14464	0.00613497	1.32442084	0.28415149	0.53283903	0.63161918	0.60575384	Rpa2	1
GO:0000724	double-strand break repair via homologous recombination	1/67	164/14464	0.00609756	1.3163451	0.27793652	0.53502767	0.63372609	0.60777446	Rpa2	1
GO:1903046	meiotic cell cycle process	1/67	166/14464	0.0060241	1.30048552	0.26562598	0.5393747	0.63838434	0.61224195	Ehmt2	1
GO:0045807	positive regulation of endocytosis	1/67	167/14464	0.00598802	1.29269819	0.2595293	0.54153317	0.63946561	0.61327895	Ager	1
GO:0071248	cellular response to metal ion	1/67	167/14464	0.00598802	1.29269819	0.2595293	0.54153317	0.63946561	0.61327895	Nfatc4	1
GO:0098739	import across plasma membrane	1/67	167/14464	0.00598802	1.29269819	0.2595293	0.54153317	0.63946561	0.61327895	Slc30a1	1
GO:0000725	recombinational repair	1/67	169/14464	0.00591716	1.27739998	0.24745048	0.54582028	0.639778	0.61357855	Rpa2	1
GO:0009755	hormone-mediated signaling pathway	1/67	169/14464	0.00591716	1.27739998	0.24745048	0.54582028	0.639778	0.61357855	Nr1d1	1
GO:0009952	anterior/posterior pattern specification	1/67	169/14464	0.00591716	1.27739998	0.24745048	0.54582028	0.639778	0.61357855	Ppp2r3a	1
GO:0019362	pyridine nucleotide metabolic process	1/67	169/14464	0.00591716	1.27739998	0.24745048	0.54582028	0.639778	0.61357855	Enpp1	1
GO:0046496	nicotinamide nucleotide metabolic process	1/67	169/14464	0.00591716	1.27739998	0.24745048	0.54582028	0.639778	0.61357855	Enpp1	1
GO:0051170	import into nucleus	1/67	169/14464	0.00591716	1.27739998	0.24745048	0.54582028	0.639778	0.61357855	Pkia	1
GO:0010876	lipid localization	2/67	393/14464	0.00508906	1.09862899	0.13522873	0.54710389	0.639778	0.61357855	Slco1a4/Enpp1	2
GO:0007269	neurotransmitter secretion	1/67	170/14464	0.00588235	1.26988586	0.24146729	0.547949	0.639778	0.61357855	Slc30a1	1
GO:0030902	hindbrain development	1/67	170/14464	0.00588235	1.26988586	0.24146729	0.547949	0.639778	0.61357855	Hnrnpd	1
GO:0046890	regulation of lipid biosynthetic process	1/67	170/14464	0.00588235	1.26988586	0.24146729	0.547949	0.639778	0.61357855	Nr1d1	1
GO:0099643	signal release from synapse	1/67	170/14464	0.00588235	1.26988586	0.24146729	0.547949	0.639778	0.61357855	Slc30a1	1
GO:0022408	negative regulation of cell-cell adhesion	1/67	171/14464	0.00584795	1.26245963	0.23552091	0.55006789	0.639778	0.61357855	H2-T23	1
GO:0030308	negative regulation of cell growth	1/67	171/14464	0.00584795	1.26245963	0.23552091	0.55006789	0.639778	0.61357855	Enpp1	1
GO:0042552	myelination	1/67	171/14464	0.00584795	1.26245963	0.23552091	0.55006789	0.639778	0.61357855	Enpp1	1
GO:0050770	regulation of axonogenesis	1/67	171/14464	0.00584795	1.26245963	0.23552091	0.55006789	0.639778	0.61357855	Tiam1	1
GO:0051494	negative regulation of cytoskeleton organization	1/67	171/14464	0.00584795	1.26245963	0.23552091	0.55006789	0.639778	0.61357855	Ccdc88c	1
GO:1901605	alpha-amino acid metabolic process	1/67	171/14464	0.00584795	1.26245963	0.23552091	0.55006789	0.639778	0.61357855	Pycr1	1
GO:1902107	positive regulation of leukocyte differentiation	1/67	171/14464	0.00584795	1.26245963	0.23552091	0.55006789	0.639778	0.61357855	Ager	1
GO:1903708	positive regulation of hemopoiesis	1/67	171/14464	0.00584795	1.26245963	0.23552091	0.55006789	0.639778	0.61357855	Ager	1
GO:0031929	TOR signaling	1/67	172/14464	0.00581395	1.25511975	0.22961084	0.55217699	0.639778	0.61357855	Fnip1	1
GO:0048660	regulation of smooth muscle cell proliferation	1/67	172/14464	0.00581395	1.25511975	0.22961084	0.55217699	0.639778	0.61357855	Ager	1
GO:0048736	appendage development	1/67	172/14464	0.00581395	1.25511975	0.22961084	0.55217699	0.639778	0.61357855	Enpp1	1

GO:0060173 limb development	1/67	172/14464	0.00581395	1.25511975	0.22961084	0.55217699	0.639778	0.61357855	Enpp1	1
GO:1902904 negative regulation of supramolecular fiber organization	1/67	172/14464	0.00581395	1.25511975	0.22961084	0.55217699	0.639778	0.61357855	Ccdc88c	1
GO:2001242 regulation of intrinsic apoptotic signaling pathway	1/67	172/14464	0.00581395	1.25511975	0.22961084	0.55217699	0.639778	0.61357855	Pycr1	1
GO:0022407 regulation of cell-cell adhesion	2/67	398/14464	0.00502513	1.08482712	0.11706351	0.55400856	0.64076509	0.61452522	H2-T23/Ager	2
GO:0033673 negative regulation of kinase activity	1/67	173/14464	0.00578035	1.24786472	0.22373661	0.55427636	0.64076509	0.61452522	Pkia	1
GO:0071222 cellular response to lipopolysaccharide	1/67	173/14464	0.00578035	1.24786472	0.22373661	0.55427636	0.64076509	0.61452522	Nr1d1	1
GO:0001659 temperature homeostasis	1/67	174/14464	0.00574713	1.24069309	0.21789774	0.55636603	0.64077838	0.61453796	Nr1d1	1
GO:0007272 ensheathment of neurons	1/67	174/14464	0.00574713	1.24069309	0.21789774	0.55636603	0.64077838	0.61453796	Enpp1	1
GO:0008366 axon ensheathment	1/67	174/14464	0.00574713	1.24069309	0.21789774	0.55636603	0.64077838	0.61453796	Enpp1	1
GO:0048771 tissue remodeling	1/67	174/14464	0.00574713	1.24069309	0.21789774	0.55636603	0.64077838	0.61453796	Enpp1	1
GO:0140694 non-membrane-bounded organelle assembly	2/67	400/14464	0.005	1.07940299	0.10986086	0.55675011	0.64077838	0.61453796	Cdk2/Dazap2	2
GO:0006839 mitochondrial transport	1/67	175/14464	0.00571429	1.23360341	0.21209375	0.55844605	0.64077838	0.61453796	Tst	1
GO:0021953 central nervous system neuron differentiation	1/67	175/14464	0.00571429	1.23360341	0.21209375	0.55844605	0.64077838	0.61453796	Bhlhe22	1
GO:0042770 signal transduction in response to DNA damage	1/67	175/14464	0.00571429	1.23360341	0.21209375	0.55844605	0.64077838	0.61453796	Rpa2	1
GO:0048469 cell maturation	1/67	175/14464	0.00571429	1.23360341	0.21209375	0.55844605	0.64077838	0.61453796	Epha8	1
GO:1902806 regulation of cell cycle G1/S phase transition	1/67	175/14464	0.00571429	1.23360341	0.21209375	0.55844605	0.64077838	0.61453796	Rpa2	1
GO:0048284 organelle fusion	1/67	176/14464	0.00568182	1.2265943	0.20632421	0.56051646	0.64124415	0.61498465	Stx17	1
GO:0050954 sensory perception of mechanical stimulus	1/67	176/14464	0.00568182	1.2265943	0.20632421	0.56051646	0.64124415	0.61498465	Enpp1	1
GO:0072524 pyridine-containing compound metabolic process	1/67	176/14464	0.00568182	1.2265943	0.20632421	0.56051646	0.64124415	0.61498465	Enpp1	1
GO:1901292 nucleoside phosphate catabolic process	1/67	176/14464	0.00568182	1.2265943	0.20632421	0.56051646	0.64124415	0.61498465	Enpp1	1
GO:0022618 protein-RNA complex assembly	1/67	177/14464	0.00564972	1.21966439	0.20058865	0.56257731	0.64264762	0.61633065	Luc7l2	1
GO:0061138 morphogenesis of a branching epithelium	1/67	177/14464	0.00564972	1.21966439	0.20058865	0.56257731	0.64264762	0.61633065	Nfatc4	1
GO:0030595 leukocyte chemotaxis	1/67	179/14464	0.00558659	1.20603686	0.18921776	0.56667049	0.64684387	0.62035506	Slamf9	1
GO:0071219 cellular response to molecule of bacterial origin	1/67	180/14464	0.00555556	1.19933665	0.18358157	0.5687029	0.64847673	0.62192105	Nr1d1	1
GO:0051249 regulation of lymphocyte activation	2/67	409/14464	0.00488998	1.05565084	0.07788405	0.56894259	0.64847673	0.62192105	H2-T23/Ager	2
GO:0000075 cell cycle checkpoint signaling	1/67	181/14464	0.00552486	1.19271048	0.17797766	0.57072593	0.64906912	0.62248919	Rpa2	1
GO:0019722 calcium-mediated signaling	1/67	181/14464	0.00552486	1.19271048	0.17797766	0.57072593	0.64906912	0.62248919	Nfatc4	1
GO:0048839 inner ear development	1/67	181/14464	0.00552486	1.19271048	0.17797766	0.57072593	0.64906912	0.62248919	H2-T23	1
GO:0098742 cell-cell adhesion via plasma-membrane adhesion molecules	1/67	182/14464	0.00549451	1.18615713	0.17240563	0.57273961	0.65064269	0.62399832	Il1rapl1	1
GO:0043161 proteasome-mediated ubiquitin-dependent protein catabolic process	2/67	412/14464	0.00485437	1.04796406	0.06737924	0.57295401	0.65064269	0.62399832	Psmb9/Zfp598	2
GO:0002520 immune system development	1/67	183/14464	0.00546448	1.17967539	0.16686507	0.57474398	0.65075715	0.62410809	H2-T23	1
GO:0006109 regulation of carbohydrate metabolic process	1/67	183/14464	0.00546448	1.17967539	0.16686507	0.57474398	0.65075715	0.62410809	Enpp1	1
GO:0042180 cellular ketone metabolic process	1/67	183/14464	0.00546448	1.17967539	0.16686507	0.57474398	0.65075715	0.62410809	Nr1d1	1
GO:1990138 neuron projection extension	1/67	183/14464	0.00546448	1.17967539	0.16686507	0.57474398	0.65075715	0.62410809	Tiam1	1
GO:0045664 regulation of neuron differentiation	1/67	185/14464	0.00540541	1.16692215	0.15587681	0.57872498	0.6546169	0.62780978	Tiam1	1
GO:0050673 epithelial cell proliferation	2/67	417/14464	0.00479616	1.03539855	0.05003712	0.57958089	0.6546169	0.62780978	Nr1d1/Ager	2
GO:1901615 organic hydroxy compound metabolic process	2/67	417/14464	0.00479616	1.03539855	0.05003712	0.57958089	0.6546169	0.62780978	Nr1d1/Enpp1	2
GO:0001649 osteoblast differentiation	1/67	186/14464	0.00537634	1.16064837	0.15042835	0.58070169	0.6546169	0.62780978	Enpp1	1
GO:0051216 cartilage development	1/67	186/14464	0.00537634	1.16064837	0.15042835	0.58070169	0.6546169	0.62780978	Enpp1	1
GO:0071826 protein-RNA complex organization	1/67	186/14464	0.00537634	1.16064837	0.15042835	0.58070169	0.6546169	0.62780978	Luc7l2	1
GO:1901617 organic hydroxy compound biosynthetic process	1/67	187/14464	0.00534759	1.1544417	0.14500984	0.58266926	0.65635477	0.62947648	Nr1d1	1
GO:0007179 transforming growth factor beta receptor signaling pathway	1/67	190/14464	0.00526316	1.13621367	0.12893037	0.58851756	0.66245841	0.63533018	Ldlrad4	1
GO:0031099 regeneration	1/67	191/14464	0.0052356	1.13026491	0.12362807	0.590449	0.66414738	0.63694998	Enpp1	1
GO:0045859 regulation of protein kinase activity	2/67	426/14464	0.00469484	1.01352393	0.01932794	0.5913234	0.66437008	0.63716356	Ager/Pkia	2
GO:0031334 positive regulation of protein-containing complex assembly	1/67	192/14464	0.00520833	1.12437811	0.11835395	0.59237151	0.66437008	0.63716356	Fnip1	1
GO:0045732 positive regulation of protein catabolic process	1/67	192/14464	0.00520833	1.12437811	0.11835395	0.59237151	0.66437008	0.63716356	Ppp2r3a	1
GO:1901654 response to ketone	1/67	192/14464	0.00520833	1.12437811	0.11835395	0.59237151	0.66437008	0.63716356	Hnmpd	1
GO:0001763 morphogenesis of a branching structure	1/67	194/14464	0.00515464	1.11278658	0.10788894	0.59618989	0.66768068	0.64033859	Nfatc4	1
GO:0006909 phagocytosis	1/67	194/14464	0.00515464	1.11278658	0.10788894	0.59618989	0.66768068	0.64033859	Ager	1
GO:0051348 negative regulation of transferase activity	1/67	196/14464	0.00510204	1.10143162	0.09753274	0.59997303	0.67142951	0.6439339	Pkia	1
GO:0002064 epithelial cell development	1/67	198/14464	0.00505051	1.09030605	0.08728285	0.60372125	0.67456673	0.64694265	Ccdc88c	1
GO:0030705 cytoskeleton-dependent intracellular transport	1/67	198/14464	0.00505051	1.09030605	0.08728285	0.60372125	0.67456673	0.64694265	Ccdc88c	1
GO:0022613 ribonucleoprotein complex biogenesis	2/67	436/14464	0.00458716	0.99027797	-0.0140615	0.60408961	0.67456673	0.64694265	Luc7l2/Rrp9	2
GO:0042391 regulation of membrane potential	2/67	437/14464	0.00457666	0.98801189	-0.0173596	0.60534994	0.67548462	0.64782295	Pycr1/Gabrg1	2
GO:0008406 gonad development	1/67	200/14464	0.005	1.07940299	0.07713682	0.60743487	0.67732065	0.64958379	Tnfsf10	1
GO:0017157 regulation of exocytosis	1/67	202/14464	0.0049505	1.06871583	0.06709232	0.6111142	0.68043857	0.65257403	Il1rapl1	1
GO:0035264 multicellular organism growth	1/67	202/14464	0.0049505	1.06871583	0.06709232	0.6111142	0.68043857	0.65257403	Enpp1	1
GO:0045137 development of primary sexual characteristics	1/67	203/14464	0.00492611	1.06345122	0.06210743	0.61294111	0.68197996	0.6540523	Tnfsf10	1
GO:0071216 cellular response to biotic stimulus	1/67	205/14464	0.00487805	1.05307608	0.05221098	0.6165696	0.68552219	0.65744947	Nr1d1	1
GO:0034762 regulation of transmembrane transport	2/67	447/14464	0.00447427	0.96590871	-0.0499459	0.61779029	0.68638416	0.65827614	Slc30a1/Enpp1	2
GO:0003018 vascular process in circulatory system	1/67	207/14464	0.00483092	1.04290143	0.04241051	0.62016457	0.6870407	0.65890579	Ager	1
GO:0007411 axon guidance	1/67	207/14464	0.00483092	1.04290143	0.04241051	0.62016457	0.6870407	0.65890579	Epha8	1
GO:0060348 bone development	1/67	207/14464	0.00483092	1.04290143	0.04241051	0.62016457	0.6870407	0.65890579	Enpp1	1
GO:0097191 extrinsic apoptotic signaling pathway	1/67	207/14464	0.00483092	1.04290143	0.04241051	0.62016457	0.6870407	0.65890579	Tnfsf10	1
GO:0006282 regulation of DNA repair	1/67	208/14464	0.00480769	1.03788749	0.03754561	0.62194959	0.68802894	0.65985357	Rpa2	1
GO:0097485 neuron projection guidance	1/67	208/14464	0.00480769	1.03788749	0.03754561	0.62194959	0.68802894	0.65985357	Epha8	1
GO:0007163 establishment or maintenance of cell polarity	1/67	210/14464	0.0047619	1.02800284	0.02788521	0.62549487	0.69095885	0.66266349	Gpsm1	1
GO:0043542 endothelial cell migration	1/67	210/14464	0.0047619	1.02800284	0.02788521	0.62549487	0.69095885	0.66266349	Ager	1
GO:0098771 inorganic ion homeostasis	2/67	454/14464	0.00440529	0.95101585	-0.0723428	0.62632237	0.69137735	0.66306486	Slc30a1/Enpp1	2
GO:0043549 regulation of kinase activity	2/67	456/14464	0.00438596	0.94684472	-0.0786813	0.6287335	0.69186323	0.66353084	Ager/Pkia	2
GO:0002699 positive regulation of immune effector process	1/67	212/14464	0.00471698	1.0183047	0.01831569	0.62900739	0.69186323	0.66353084	H2-T23	1
GO:0006813 potassium ion transport	1/67	212/14464	0.00471698	1.0183047	0.01831569	0.62900739	0.69186323	0.66353084	Cdk2	1
GO:0099111 microtubule-based transport	1/67	212/14464	0.00471698	1.0183047	0.01831569	0.62900739	0.69186323	0.66353084	Ccdc88c	1
GO:1905475 regulation of protein localization to membrane	1/67	212/14464	0.00471698	1.0183047	0.01831569	0.62900739	0.69186323	0.66353084	Slc30a1	1
GO:0045930 negative regulation of mitotic cell cycle	1/67	213/14464	0.00469484	1.01352393	0.0135644	0.63075146	0.69279259	0.66442214	Rpa2	1
GO:0072593 reactive oxygen species metabolic process	1/67	213/14464	0.00469484	1.01352393	0.0135644	0.63075146	0.69279259	0.66442214	Ager	1
GO:0006364 rRNA processing	1/67	214/14464	0.0046729	1.00878784	0.00883511	0.63248745	0.69371044	0.66530241	Rrp9	1
GO:0048705 skeletal system morphogenesis	1/67	214/14464	0.0046729	1.00878784	0.00883511	0.63248745	0.69371044	0.66530241	Enpp1	1
GO:0045926 negative regulation of growth	1/67	216/14464	0.00462963	0.99944721	-0.0005584	0.63593536	0.69699601	0.66845343	Enpp1	1
GO:0048863 stem cell differentiation	1/67	218/14464	0.00458716	0.99027797	-0.0098666	0.63935139	0.69974467	0.67108953	Ccdc88c	1
GO:0055001 muscle cell development	1/67	218/14464	0.00458716	0.99027797	-0.0098666	0.63935139	0.69974467	0.67108953	Nfatc4	1
GO:0007005 mitochondrion organization	2/67	466/14464	0.00429185	0.92652617	-0.1099824	0.64061216	0.70060587	0.67191546	Tnfsf10/Enpp1	2
GO:0006836 neurotransmitter transport	1/67	219/14464	0.00456621	0.98575615	-0.0144893	0.64104755	0.70060587	0.67191546	Slc30a1	1
GO:0046034 ATP metabolic process	1/67	220/14464	0.00454545	0.98127544	-0.0190914	0.64273585	0.70195318	0.6732076	Enpp1	1
GO:0044283 small molecule biosynthetic process	2/67	470/14464	0.00425532	0.91864084	-0.122325	0.64528122	0.70411823	0.67528398	Nr1d1/Pycr1	2
GO:0043588 skin development	1/67	222/14464	0.0045045	0.97243512	-0.0282343	0.64608901	0.70411823	0.67528398	Enpp1	1
GO:0046434 organophosphate catabolic process	1/67	222/14464	0.0045045	0.97243512	-0.0282343	0.64608901	0.70411823	0.67528398	Enpp1	1
GO:0090092 regulation of transmembrane receptor protein serine/threonine kinase signaling pathway	1/67	223/14464	0.0044843	0.96807443	-0.0327757	0.64775395	0.70543381	0.67654569	Ldlrad4	1
GO:0045861 negative regulation of proteolysis	1/67	224/14464	0.00446429	0.96375267	-0.0372972	0.64941117	0.70624038	0.67731923	Fnip1	1
GO:1903522 regulation of blood circulation	1/67	224/14464	0.00446429	0.96375267	-0.0372972	0.64941117	0.70624038	0.67731923	Ager	1
GO:2001234 negative regulation of apoptotic signaling pathway	1/67	225/14464	0.00444444	0.95946932	-0.041799	0.65106071	0.70753495	0.67856079	Pycr1	1
GO:0016049 cell growth	2/67	476/14464	0.00420168	0.90706133	-0.1406542	0.65219685	0.70827015	0.67926588	Tiam1/Enpp1	2
GO:1901136 carbohydrate derivative catabolic process	1/67	229/14464	0.00436681	0.94271003	-0.059614	0.65758276	0.71361622	0.68439303	Enpp1	1
GO:0001894 tissue homeostasis	1/67	230/14464	0.00434783	0.93861129	-0.0640206	0.65919442	0.71385706	0.68462401	Enpp1	1
GO:0019318 hexose metabolic process	1/67	230/14464	0.00434783	0.93861129	-0.0640206	0.65919442	0.71385706	0.68462401	Man2b1	1
GO:0060249 anatomical structure homeostasis	1/67	230/14464	0.00434783	0.93861129	-0.0640206	0.65919442	0.71385706	0.68462401	Enpp1	1
GO:0001667 ameboidal-type cell migration	1/67	231/14464	0.004329	0.93454804	-0.0684087	0.66079861	0.71509175	0.68580813	Ager	1
GO:0002573 myeloid leukocyte differentiation	1/67	232/14464	0.00431034	0.93051981	-0.0727784	0.66239535	0.71631666	0.68698288	Enpp1	1
GO:0045333 cellular respiration	1/67	234/14464	0.0042735	0.92256665	-0.0814637	0.66556666	0.7192414	0.68978785	Nfatc4	1
GO:0015850 organic hydroxy compound transport	1/67	235/14464	0.00425532	0.91864084	-0.0857796	0.6671413	0.7204378	0.69093526	Slco1a4	1
GO:0007548 sex differentiation	1/67	237/14464	0.00421941	0.91088859	-0.0943586	0.67026869	0.72330816	0.69368807	Tnfsf10	1
GO:0006520 amino acid metabolic process	1/67	238/14464	0.00420168	0.90706133	-0.0986221	0.67182151	0.72447652	0.69480859	Pycr1	1
GO:0007626 locomotory behavior	1/67	240/14464	0.00416667	0.89950249	-0.1070978	0.67490557	0.72627758	0.69653589	Enpp1	1
GO:0051924 regulation of calcium ion transport	1/67	240/14464	0.00416667	0.89950249	-0.1070978	0.67490557	0.72627758	0.69653589	Slc30a1	1
GO:1901988 negative regulation of cell cycle phase transition	1/67	240/14464	0.00416667	0.89950249	-0.1070978	0.67490557	0.72627758	0.69653589	Rpa2	1
GO:0002366 leukocyte activation involved in immune response	1/67	241/14464	0.00414938	0.89577011	-0.1113104	0.67643688	0.72741747	0.6976291	Enpp1	1
GO:0071560 cellular response to transforming growth factor beta stimulus	1/67	242/14464	0.00413223	0.89206858	-0.1155063	0.67796108	0.72854814	0.69871347	Ldlrad4	1
GO:0000209 protein polyubiquitination	1/67	243/14464	0.00411523	0.88839752	-0.1196858	0.67947821	0.72865409	0.69881508	Zfp598	1
GO:0009205 purine ribonucleoside triphosphate metabolic process	1/67	243/14464	0.00411523	0.88839752	-0.1196858	0.67947821	0.72865409	0.69881508	Enpp1	1
GO:0034599 cellular response to oxidative stress	1/67	243/14464	0.00411523	0.88839752	-0.1196858	0.67947821	0.72865409	0.69881508	Pycr1	1
GO:0002263 cell activation involved in immune response	1/67	245/14464	0.00408163	0.88114529	-0.1279957	0.68249138	0.73086811	0.70093844	Enpp1	1
GO:0032886 regulation of microtubule-based process	1/67	245/14464	0.00408163	0.88114529	-0.1279957	0.68249138	0.73086811	0.70093844	Ccdc88c	1
GO:0045860 positive regulation of protein kinase activity	1/67	246/14464	0.00406504	0.8775634	-0.1321266	0.68398748	0.7319616	0.70198715	Ager	1
GO:0016072 rRNA metabolic process	1/67	247/14464	0.00404858	0.87401051	-0.1362415	0.68547664	0.73304615	0.70302728	Rrp9	1
GO:0009199 ribonucleoside triphosphate metabolic process	1/67	248/14464	0.00403226	0.87048628	-0.1403407	0.68695888	0.73361306	0.70357098	Enpp1	1
GO:0051607 defense response to virus	1/67	248/14464	0.00403226	0.87048628	-0.1403407	0.68695888	0.73361306	0.70357098	Aim2	1
GO:0071559 response to transforming growth factor beta	1/67	249/14464	0.00401606	0.86699035	-0.1444242	0.68843425	0.73467948	0.70459373	Ldlrad4	1
GO:0005996 monosaccharide metabolic process	1/67	250/14464	0.004	0.86352239	-0.1484923	0.68990276	0.73573713	0.70560807	Man2b1	1
GO:0009144 purine nucleoside triphosphate metabolic process	1/67	251/14464	0.00398406	0.86008206	-0.152545	0.69136445	0.73678604	0.70661403	Enpp1	1
GO:0048562 embryonic organ morphogenesis	1/67	254/14464	0.00393701	0.84992361	-0.1646125	0.69570893	0.7403919	0.71007222	Enpp1	1
GO:0048588 developmental cell growth	1/67	254/14464	0.00393701	0.84992361	-0.1646125	0.69570893	0.7403919	0.71007222	Tiam1	1
GO:0034976 response to endoplasmic reticulum stress	1/67	256/14464	0.00390625	0.84328358	-0.1725832	0.69857173	0.74292549	0.71250205	Foxred2	1
GO:0033674 positive regulation of kinase activity	1/67	258/14464	0.00387597	0.8367465	-0.1804958	0.70140799	0.74491365	0.7144088	Ager	1
GO:0045927 positive regulation of growth	1/67	258/14464	0.00387597	0.8367465	-0.1804958	0.70140799	0.74491365	0.7144088	Rftn1	1
GO:0030217 T cell differentiation	1/67	263/14464	0.00380228	0.82083877	-0.200029	0.70838413	0.75180437	0.72101734	Enpp1	1
GO:0098813 nuclear chromosome segregation	1/67	264/14464	0.00378788	0.81772953	-0.2038941	0.70975996	0.75274611	0.72192052	Ehmt2	1
GO:0009141 nucleoside triphosphate metabolic process	1/67	265/14464	0.00377358	0.81464376	-0.2077456	0.71112941	0.75367979	0.72281596	Enpp1	1
GO:0048608 reproductive structure development	1/67	268/14464	0.00373134	0.80552462	-0.2192197	0.71519966	0.75747263	0.72645348	Tnfsf10	1
GO:0061448 connective tissue development	1/67	272/14464	0.00367647	0.79367867	-0.2343351	0.72053889	0.76207991	0.73087209	Enpp1	1
GO:0061458 reproductive system development	1/67	272/14464	0.00367647	0.79367867	-0.2343351	0.72053889	0.76207991	0.73087209	Tnfsf10	1
GO:0006066 alcohol metabolic process	1/67	275/14464	0.00363636	0.78502035	-0.2455378	0.72447849	0.76499741	0.73367011	Enpp1	1
GO:0007281 germ cell development	1/67	276/14464	0.00362319	0.78217608	-0.2492471	0.7257795	0.76499741	0.73367011	Ehmt2	1
GO:0030198 extracellular matrix organization	1/67	276/14464	0.00362319	0.78217608	-0.2492471	0.7257795	0.76499741	0.73367011	Abi3bp	1
GO:0043062 extracellular structure organization	1/67	276/14464	0.00362319	0.78217608	-0.2492471	0.7257795	0.76499741	0.73367011	Abi3bp	1
GO:0045229 external encapsulating structure organization	1/67	276/14464	0.00362319	0.78217608	-0.2492471	0.7257795	0.76499741	0.73367011	Abi3bp	1
GO:0010948 negative regulation of cell cycle process	1/67	277/14464	0.00361011	0.77935234	-0.2529441	0.72707445	0.76531539	0.73397507	Rpa2	1
GO:0032386 regulation of intracellular transport	1/67	277/14464	0.00361011	0.77935234	-0.2529441	0.72707445	0.76531539	0.73397507	Pkia	1
GO:0006874 intracellular calcium ion homeostasis	1/67	278/14464	0.00359712	0.77654891	-0.2566289	0.72836338	0.76562617	0.73427313	Slc30a1	1
GO:0090287 regulation of cellular response to growth factor stimulus	1/67	278/14464	0.00359712	0.77654891	-0.2566289	0.72836338	0.76562617	0.73427313	Ldlrad4	1
GO:0006302 double-strand break repair	1/67	281/14464	0.00355872	0.76825835	-0.2676109	0.73219429	0.7686045	0.73712949	Rpa2	1
GO:0046942 carboxylic acid transport	1/67	281/14464	0.00355872	0.76825835	-0.2676109	0.73219429	0.7686045	0.73712949	Slco1a4	1
GO:0009416 response to light stimulus	1/67	283/14464	0.00353357	0.76282896	-0.274873	0.73471861	0.76968143	0.73816232	Nfatc4	1
GO:0015849 organic acid transport	1/67	283/14464	0.00353357	0.76282896	-0.274873	0.73471861	0.76968143	0.73816232	Slco1a4	1
GO:0032496 response to lipopolysaccharide	1/67	283/14464	0.00353357	0.76282896	-0.274873	0.73471861	0.76968143	0.73816232	Nr1d1	1
GO:1902105 regulation of leukocyte differentiation	1/67	286/14464	0.0034965	0.75482726	-0.285679	0.73846121	0.77307658	0.74141843	Ager	1
GO:0001666 response to hypoxia	1/67	289/14464	0.00346021	0.74699169	-0.2963826	0.74215178	0.77641269	0.74461793	Ager	1
GO:0072659 protein localization to plasma membrane	1/67	292/14464	0.00342466	0.73931711	-0.3069863	0.74579104	0.77916203	0.74725468	Sys1	1
GO:0141091 transforming growth factor beta receptor superfamily signaling pathway	1/67	292/14464	0.00342466	0.73931711	-0.3069863	0.74579104	0.77916203	0.74725468	Ldlrad4	1
GO:1904062 regulation of monoatomic cation transmembrane transport	1/67	294/14464	0.00340136	0.73428774	-0.3140009	0.74818906	0.78113776	0.7491495	Slc30a1	1
GO:0022412 cellular process involved in reproduction in multicellular organism	1/67	295/14464	0.00338983	0.73179863	-0.3174921	0.74937969	0.78185112	0.74983365	Ehmt2	1
GO:0002237 response to molecule of bacterial origin	1/67	297/14464	0.003367	0.7268707	-0.3244428	0.75174436	0.78378759	0.75169082	Nr1d1	1
GO:0019216 regulation of lipid metabolic process	1/67	298/14464	0.0033557	0.72443153	-0.3279024	0.75291843	0.78448094	0.75235577	Nr1d1	1
GO:0071214 cellular response to abiotic stimulus	1/67	301/14464	0.00332226	0.71721129	-0.3382192	0.75640794	0.78705242	0.75482195	Nfatc4	1
GO:0104004 cellular response to environmental stimulus	1/67	301/14464	0.00332226	0.71721129	-0.3382192	0.75640794	0.78705242	0.75482195	Nfatc4	1
GO:0001822 kidney development	1/67	303/14464	0.00330033	0.71247722	-0.3450462	0.75870726	0.7889122	0.75660558	Enpp1	1
GO:0036293 response to decreased oxygen levels	1/67	304/14464	0.00328947	0.71013354	-0.3484446	0.75984889	0.78956651	0.75723309	Ager	1
GO:0062197 cellular response to chemical stress	1/67	305/14464	0.00327869	0.70780524	-0.3518331	0.7609852	0.79021441	0.75785446	Pycr1	1
GO:0034504 protein localization to nucleus	1/67	306/14464	0.00326797	0.70549215	-0.3552117	0.76211621	0.79085594	0.75846972	Pkia	1
GO:0051235 maintenance of location	1/67	307/14464	0.00325733	0.70319413	-0.3585805	0.76324195	0.79149114	0.75907891	Enpp1	1
GO:0042254 ribosome biogenesis	1/67	309/14464	0.00323625	0.69864271	-0.365289	0.7654777	0.79327582	0.7607905	Rrp9	1
GO:0030100 regulation of endocytosis	1/67	310/14464	0.00322581	0.69638902	-0.3686288	0.76658777	0.79389231	0.76138174	Ager	1
GO:0009150 purine ribonucleotide metabolic process	1/67	311/14464	0.00321543	0.69414983	-0.3719592	0.76769266	0.79450261	0.76196705	Enpp1	1
GO:0009615 response to virus	1/67	312/14464	0.00320513	0.69192499	-0.37528	0.7687924	0.79510677	0.76254647	Aim2	1
GO:0007178 cell surface receptor protein serine/threonine kinase signaling pathway	1/67	313/14464	0.00319489	0.68971437	-0.3785915	0.76988701	0.79570482	0.76312003	Ldlrad4	1
GO:0072001 renal system development	1/67	315/14464	0.0031746	0.68533523	-0.3851866	0.77206094	0.79741682	0.76476193	Enpp1	1
GO:0030522 intracellular receptor signaling pathway	1/67	318/14464	0.00314465	0.6788698	-0.3950103	0.77528394	0.80020935	0.76744009	Rftn1	1

GO:0062012	regulation of small molecule metabolic process	1/67	319/14464	0.0031348	0.67674168	-0.3982669	0.77634827	0.80077154	0.76797927	Nr1d1	1
GO:0034765	regulation of monoatomic ion transmembrane transport	1/67	323/14464	0.00309598	0.66836098	-0.4112041	0.78055615	0.80403153	0.77110576	Slc30a1	1
GO:0060562	epithelial tube morphogenesis	1/67	323/14464	0.00309598	0.66836098	-0.4112041	0.78055615	0.80403153	0.77110576	Nfatc4	1
GO:0006913	nucleocytoplasmic transport	1/67	324/14464	0.00308642	0.66629814	-0.4144165	0.78159587	0.80403153	0.77110576	Pkia	1
GO:0051169	nuclear transport	1/67	324/14464	0.00308642	0.66629814	-0.4144165	0.78159587	0.80403153	0.77110576	Pkia	1
GO:0070482	response to oxygen levels	1/67	327/14464	0.0030581	0.66018531	-0.4240021	0.78468602	0.80667188	0.77363798	Ager	1
GO:0032956	regulation of actin cytoskeleton organization	1/67	328/14464	0.00304878	0.65817255	-0.4271803	0.78570646	0.80718244	0.77412763	Ngef	1
GO:0009259	ribonucleotide metabolic process	1/67	332/14464	0.00301205	0.65024276	-0.4398094	0.78974082	0.81078654	0.77758414	Enpp1	1
GO:0006631	fatty acid metabolic process	1/67	335/14464	0.00298507	0.64441969	-0.4491951	0.7927174	0.8133006	0.77999525	Enpp1	1
GO:0019693	ribose phosphate metabolic process	1/67	339/14464	0.00294985	0.63681592	-0.461597	0.79662168	0.81676248	0.78331536	Enpp1	1
GO:0015711	organic anion transport	1/67	341/14464	0.00293255	0.63308093	-0.4677509	0.79854657	0.81819166	0.78468602	Slco1a4	1
GO:0006869	lipid transport	1/67	342/14464	0.00292398	0.63122982	-0.4708161	0.79950227	0.81862658	0.78510312	Slco1a4	1
GO:0045786	negative regulation of cell cycle	1/67	343/14464	0.00291545	0.6293895	-0.4738737	0.80045351	0.81905634	0.78551529	Rpa2	1
GO:0042176	regulation of protein catabolic process	1/67	344/14464	0.00290698	0.62755988	-0.4769237	0.8014003	0.81948099	0.78592255	Ppp2r3a	1
GO:0016050	vesicle organization	1/67	351/14464	0.002849	0.61504444	-0.4980639	0.80790504	0.82558466	0.79177627	Stx17	1
GO:0019221	cytokine-mediated signaling pathway	1/67	353/14464	0.00283286	0.61155976	-0.504038	0.80972468	0.82689578	0.79303369	Aim2	1
GO:0001503	ossification	1/67	359/14464	0.00278552	0.60133871	-0.5217895	0.81508232	0.83126529	0.79722427	Enpp1	1
GO:0007018	microtubule-based movement	1/67	359/14464	0.00278552	0.60133871	-0.5217895	0.81508232	0.83126529	0.79722427	Ccdc88c	1
GO:0000280	nuclear division	1/67	360/14464	0.00277778	0.59966833	-0.5247237	0.8159607	0.83161074	0.79755557	Ehmt2	1
GO:0007600	sensory perception	1/67	362/14464	0.00276243	0.59635524	-0.5305714	0.81770513	0.83228772	0.79820483	Enpp1	1
GO:1903706	regulation of hemopoiesis	1/67	362/14464	0.00276243	0.59635524	-0.5305714	0.81770513	0.83228772	0.79820483	Ager	1
GO:0007059	chromosome segregation	1/67	364/14464	0.00274725	0.59307856	-0.536392	0.81943328	0.83349616	0.79936379	Ehmt2	1
GO:0032970	regulation of actin filament-based process	1/67	368/14464	0.00271739	0.58663206	-0.5479531	0.82284131	0.83641059	0.80215886	Ngef	1
GO:0044782	cilium organization	1/67	369/14464	0.00271003	0.58504227	-0.5508268	0.82368336	0.83671461	0.80245043	Ccdc88c	1
GO:1902903	regulation of supramolecular fiber organization	1/67	374/14464	0.0026738	0.57722085	-0.5650989	0.82783487	0.84037782	0.80596363	Ccdc88c	1
GO:0010959	regulation of metal ion transport	1/67	375/14464	0.00266667	0.57568159	-0.5679342	0.82865354	0.8406551	0.80622956	Slc30a1	1
GO:1990778	protein localization to cell periphery	1/67	378/14464	0.0026455	0.57111269	-0.5764025	0.83108661	0.84256872	0.80806482	Sys1	1
GO:0006979	response to oxidative stress	1/67	388/14464	0.00257732	0.55639329	-0.6042337	0.83895362	0.84998522	0.81517761	Pycr1	1
GO:0001558	regulation of cell growth	1/67	389/14464	0.00257069	0.55496297	-0.6069841	0.83972018	0.85020289	0.81538636	Enpp1	1
GO:0009314	response to radiation	1/67	394/14464	0.00253807	0.5479203	-0.6206499	0.8434994	0.85310229	0.81816702	Nfatc4	1
GO:0003012	muscle system process	1/67	395/14464	0.00253165	0.54653316	-0.6233659	0.84424464	0.85310229	0.81816702	Tiam1	1
GO:0006163	purine nucleotide metabolic process	1/67	395/14464	0.00253165	0.54653316	-0.6233659	0.84424464	0.85310229	0.81816702	Enpp1	1
GO:0048732	gland development	1/67	408/14464	0.00245098	0.52911911	-0.6581742	0.85362044	0.8620112	0.82671111	Hnrnpd	1
GO:0043254	regulation of protein-containing complex assembly	1/67	413/14464	0.00242131	0.52271331	-0.6713227	0.85707652	0.86493446	0.82951466	Fnip1	1
GO:0072594	establishment of protein localization to organelle	1/67	415/14464	0.00240964	0.52019421	-0.6765461	0.85843634	0.86573979	0.83028701	Pkia	1
GO:0048285	organelle fission	1/67	416/14464	0.00240385	0.51894374	-0.6791503	0.85911146	0.86585399	0.83039653	Ehmt2	1
GO:0048568	embryonic organ development	1/67	421/14464	0.0023753	0.51278052	-0.692096	0.86243977	0.86864032	0.83306876	Enpp1	1
GO:0042692	muscle cell differentiation	1/67	423/14464	0.00236407	0.51035602	-0.6972398	0.86374931	0.86939104	0.83378873	Nfatc4	1
GO:0030099	myeloid cell differentiation	1/67	428/14464	0.00233645	0.50439392	-0.7100148	0.86696965	0.87206281	0.8363511	Enpp1	1
GO:0043269	regulation of monoatomic ion transport	1/67	440/14464	0.00227273	0.49063772	-0.7401977	0.87439572	0.87895878	0.84296467	Slc30a1	1
GO:0051656	establishment of organelle localization	1/67	442/14464	0.00226244	0.48841764	-0.7451648	0.87559305	0.87958859	0.84356869	Gpsm1	1
GO:0008015	blood circulation	1/67	443/14464	0.00225734	0.48731512	-0.7476416	0.87618749	0.87961233	0.84359146	Ager	1
GO:0030003	intracellular monoatomic cation homeostasis	1/67	447/14464	0.00223714	0.48295436	-0.7575053	0.87853741	0.88139723	0.84530326	Slc30a1	1
GO:0009117	nucleotide metabolic process	1/67	450/14464	0.00222222	0.47973466	-0.7648578	0.88027096	0.88256184	0.84642018	Enpp1	1
GO:0006873	intracellular monoatomic ion homeostasis	1/67	454/14464	0.00220264	0.47550792	-0.7746016	0.8825445	0.88426598	0.84805453	Slc30a1	1
GO:0001701	in utero embryonic development	1/67	461/14464	0.0021692	0.46828763	-0.7914934	0.88642131	0.88757325	0.85122637	Slc30a1	1
GO:0001501	skeletal system development	1/67	466/14464	0.00214592	0.46326308	-0.8034374	0.889113	0.88969035	0.85325677	Enpp1	1
GO:0003013	circulatory system process	1/67	470/14464	0.00212766	0.45932042	-0.8129214	0.89122103	0.89122103	0.85472477	Ager	1

ID	Description	GeneRatio	BgRatio	RichFactor	FoldEnrichm	zScore	pvalue	p.adjust	qvalue	geneID	Count
GO:0048511	rhythmic process	13/95	291/14464	0.04467354	6.80166395	8.12916417	5.4063E-08	0.00010375	9.9191E-05	Dbp/Ciart/Per3/Per1/Nr1d2/Tef/Hlf/Bhlhe40/Fzd4/Klf9/Bhlhe41/Gc	13
GO:0007623	circadian rhythm	8/95	204/14464	0.03921569	5.97069143	5.81366293	5.8055E-05	0.04254121	0.04067322	Dbp/Ciart/Per3/Per1/Nr1d2/Bhlhe40/Bhlhe41/Agrn	8
GO:0032922	circadian regulation of gene expression	5/95	65/14464	0.07692308	11.7117409	7.03766021	6.6505E-05	0.04254121	0.04067322	Ciart/Per3/Per1/Bhlhe40/Bhlhe41	5
GO:0043153	entrainment of circadian clock by photoperiod	3/95	22/14464	0.13636364	20.7617225	7.54223725	0.00038611	0.14133952	0.13513331	Per3/Per1/Bhlhe40	3
GO:0009648	photoperiodism	3/95	23/14464	0.13043478	19.8590389	7.35974155	0.00044192	0.14133952	0.13513331	Per3/Per1/Bhlhe40	3
GO:0009649	entrainment of circadian clock	3/95	23/14464	0.13043478	19.8590389	7.35974155	0.00044192	0.14133952	0.13513331	Per3/Per1/Bhlhe40	3
GO:0034143	regulation of toll-like receptor 4 signaling pathway	3/95	25/14464	0.12	18.2703158	7.02715363	0.00056848	0.15584565	0.14900247	Trem2/Peli1/Lyn	3
GO:0071675	regulation of mononuclear cell migration	5/95	114/14464	0.04385965	6.677747	4.94857589	0.00091804	0.22021589	0.21054623	Trem2/Serpine1/Abl1/Lyn/Jam2	5
GO:0097028	dendritic cell differentiation	3/95	35/14464	0.08571429	13.0502256	5.80348418	0.00154268	0.24134334	0.23074597	Trem2/Gimap5/Lyn	3
GO:0002714	positive regulation of B cell mediated immunity	3/95	36/14464	0.08333333	12.6877193	5.70894264	0.00167497	0.24134334	0.23074597	Trem2/Gimap5/Paxip1	3
GO:0002891	positive regulation of immunoglobulin mediated immune response	3/95	36/14464	0.08333333	12.6877193	5.70894264	0.00167497	0.24134334	0.23074597	Trem2/Gimap5/Paxip1	3
GO:0007162	negative regulation of cell adhesion	7/95	263/14464	0.02661597	4.05235141	4.06191345	0.00171964	0.24134334	0.23074597	Fzd4/Serpine1/Gimap5/Acer2/Abl1/Peli1/Jam2	7
GO:0071677	positive regulation of mononuclear cell migration	4/95	79/14464	0.05063291	7.708994	4.8617625	0.00180111	0.24134334	0.23074597	Trem2/Serpine1/Abl1/Jam2	4
GO:0002922	positive regulation of humoral immune response	2/95	10/14464	0.2	30.4505263	7.57489747	0.00185616	0.24134334	0.23074597	Trem2/Gimap5	2
GO:0002532	production of molecular mediator involved in inflammatory response	4/95	80/14464	0.05	7.61263158	4.82233311	0.00188648	0.24134334	0.23074597	Per1/Trem2/Serpine1/Lyn	4
GO:0009068	aspartate family amino acid catabolic process	2/95	11/14464	0.18181818	27.6822967	7.19810658	0.00225895	0.25116102	0.24013255	Pipox/Tha1	2
GO:0070988	demethylation	2/95	11/14464	0.18181818	27.6822967	7.19810658	0.00225895	0.25116102	0.24013255	Por/Cyp3a13	2
GO:0002696	positive regulation of leukocyte activation	7/95	283/14464	0.02473498	3.76596615	3.82088858	0.00259914	0.25116102	0.24013255	Trem2/Gimap5/Il15ra/Abl1/Peli1/Paxip1/Arid2	7
GO:1903706	regulation of hemopoiesis	8/95	362/14464	0.02209945	3.36469904	3.70482355	0.00260984	0.25116102	0.24013255	Trem2/Tcim/Gimap5/Il15ra/Lrrc17/Abl1/Lyn/Arid2	8
GO:0033628	regulation of cell adhesion mediated by integrin	3/95	42/14464	0.07142857	10.875188	5.21116703	0.00261762	0.25116102	0.24013255	Serpine1/Acer2/Lyn	3
GO:1902105	regulation of leukocyte differentiation	7/95	286/14464	0.02447552	3.72646301	3.78662994	0.00275643	0.2518856	0.24082532	Trem2/Gimap5/Il15ra/Lrrc17/Abl1/Lyn/Arid2	7
GO:0002923	regulation of humoral immune response mediated by circulating immunoglobulin	2/95	13/14464	0.15384615	23.4234818	6.57663133	0.00317633	0.27706218	0.2648964	Trem2/Gimap5	2
GO:0034134	toll-like receptor 2 signaling pathway	2/95	14/14464	0.14285714	21.7503759	6.31587879	0.00368991	0.29200346	0.27918161	Trem2/Lyn	2
GO:0050867	positive regulation of cell activation	7/95	303/14464	0.02310231	3.51738753	3.60082614	0.00379071	0.29200346	0.27918161	Trem2/Gimap5/Il15ra/Abl1/Peli1/Paxip1/Arid2	7
GO:0002712	regulation of B cell mediated immunity	3/95	50/14464	0.06	9.13515789	4.68528974	0.00430326	0.29200346	0.27918161	Trem2/Gimap5/Paxip1	3
GO:0002889	regulation of immunoglobulin mediated immune response	3/95	50/14464	0.06	9.13515789	4.68528974	0.00430326	0.29200346	0.27918161	Trem2/Gimap5/Paxip1	3
GO:0071674	mononuclear cell migration	5/95	163/14464	0.03067485	4.67032612	3.83171304	0.00438377	0.29200346	0.27918161	Trem2/Serpine1/Abl1/Lyn/Jam2	5
GO:0000724	double-strand break repair via homologous recombination	5/95	164/14464	0.0304878	4.64184852	3.81376129	0.00449857	0.29200346	0.27918161	Abl1/Xrcc3/Peli1/Fan1/Arid2	5
GO:0042752	regulation of circadian rhythm	4/95	103/14464	0.03883495	5.91272356	4.06842506	0.0047035	0.29200346	0.27918161	Per3/Per1/Nr1d2/Bhlhe40	4
GO:0034142	toll-like receptor 4 signaling pathway	3/95	52/14464	0.05769231	8.78380567	4.57203191	0.00480655	0.29200346	0.27918161	Trem2/Peli1/Lyn	3
GO:0000725	recombinational repair	5/95	169/14464	0.0295858	4.50451573	3.72612147	0.0051049	0.29200346	0.27918161	Abl1/Xrcc3/Peli1/Fan1/Arid2	5
GO:0035567	non-canonical Wnt signaling pathway	3/95	54/14464	0.05555556	8.45847953	4.46470651	0.00534399	0.29200346	0.27918161	Fzd4/Abl1/Celsr3	3
GO:0048512	circadian behavior	3/95	54/14464	0.05555556	8.45847953	4.46470651	0.00534399	0.29200346	0.27918161	Ciart/Per3/Nr1d2	3
GO:0090030	regulation of steroid hormone biosynthetic process	2/95	17/14464	0.11764706	17.9120743	5.67296659	0.00544442	0.29200346	0.27918161	Por/Clcn2	2
GO:0007622	rhythmic behavior	3/95	55/14464	0.05454545	8.304689	4.41310111	0.0056257	0.29200346	0.27918161	Ciart/Per3/Nr1d2	3
GO:0010812	negative regulation of cell-substrate adhesion	3/95	55/14464	0.05454545	8.304689	4.41310111	0.0056257	0.29200346	0.27918161	Fzd4/Serpine1/Acer2	3
GO:0071222	cellular response to lipopolysaccharide	5/95	173/14464	0.02890173	4.40036507	3.65843221	0.00563008	0.29200346	0.27918161	Trem2/Serpine1/Abl1/Lyn/Cdk19	5
GO:0048145	regulation of fibroblast proliferation	4/95	112/14464	0.03571429	5.43759398	3.83334658	0.00631884	0.31910131	0.30508959	Serpine1/Inca1/Abl1/Brpf1	4
GO:0071219	cellular response to molecule of bacterial origin	5/95	180/14464	0.02777778	4.22923977	3.54478049	0.00663928	0.32668663	0.31234184	Trem2/Serpine1/Abl1/Lyn/Cdk19	5
GO:0140895	cell surface toll-like receptor signaling pathway	3/95	60/14464	0.05	7.61263158	4.17336259	0.00716712	0.32837844	0.31395937	Trem2/Peli1/Lyn	3
GO:0051251	positive regulation of lymphocyte activation	6/95	260/14464	0.02307692	3.51352227	3.325384	0.00730203	0.32837844	0.31395937	Gimap5/Il15ra/Abl1/Peli1/Paxip1/Arid2	6
GO:0000018	regulation of DNA recombination	4/95	117/14464	0.03418803	5.20521817	3.71345904	0.00735814	0.32837844	0.31395937	Abl1/Peli1/Paxip1/Arid2	4
GO:0021549	cerebellum development	4/95	117/14464	0.03418803	5.20521817	3.71345904	0.00735814	0.32837844	0.31395937	Fzd4/Gdf10/Rere/Abl1	4
GO:0002752	cell surface pattern recognition receptor signaling pathway	3/95	63/14464	0.04761905	7.25012531	4.04241036	0.00820057	0.35251909	0.33704	Trem2/Peli1/Lyn	3
GO:0046165	alcohol biosynthetic process	4/95	121/14464	0.03305785	5.03314485	3.62238185	0.00826647	0.35251909	0.33704	Acer2/Por/Lss/Clcn2	4
GO:0002685	regulation of leukocyte migration	5/95	191/14464	0.02617801	3.98567098	3.37737062	0.00847187	0.35342441	0.33790556	Trem2/Serpine1/Abl1/Lyn/Jam2	5
GO:0006909	phagocytosis	5/95	194/14464	0.0257732	3.9240369	3.33387606	0.00902696	0.36856872	0.35238489	Trem2/Il15ra/Rab34/Clcn2/Abl1	5
GO:0022037	metencephalon development	4/95	125/14464	0.032	4.87208421	3.5352334	0.00924551	0.36962778	0.35339745	Fzd4/Gdf10/Rere/Abl1	4
GO:0002220	innate immune response activating cell surface receptor signaling pathway	3/95	67/14464	0.04477612	6.81728201	3.88060323	0.0097084	0.37410191	0.35767512	Trem2/Peli1/Lyn	3
GO:0046885	regulation of hormone biosynthetic process	2/95	23/14464	0.08695652	13.2393593	4.77641101	0.00987267	0.37410191	0.35767512	Por/Clcn2	2
GO:0006310	DNA recombination	6/95	279/14464	0.02150538	3.27425014	3.1189139	0.01014172	0.37410191	0.35767512	Abl1/Xrcc3/Peli1/Fan1/Paxip1/Arid2	6
GO:0046475	glycerophospholipid catabolic process	2/95	24/14464	0.08333333	12.6877193	4.6593949	0.01072449	0.37410191	0.35767512	Pnpla7/Gpcpd1	2
GO:0090025	regulation of monocyte chemotaxis	2/95	24/14464	0.08333333	12.6877193	4.6593949	0.01072449	0.37410191	0.35767512	Serpine1/Lyn	2
GO:0032496	response to lipopolysaccharide	6/95	283/14464	0.02120141	3.22797099	3.07770538	0.01082751	0.37410191	0.35767512	Trem2/Serpine1/Abl1/Peli1/Lyn/Cdk19	6
GO:0071216	cellular response to biotic stimulus	5/95	205/14464	0.02439024	3.71347882	3.1815339	0.01127739	0.37410191	0.35767512	Trem2/Serpine1/Abl1/Lyn/Cdk19	5
GO:0045911	positive regulation of DNA recombination	3/95	71/14464	0.04225352	6.43320979	3.73153663	0.01136747	0.37410191	0.35767512	Peli1/Paxip1/Arid2	3
GO:0071347	cellular response to interleukin-1	3/95	71/14464	0.04225352	6.43320979	3.73153663	0.01136747	0.37410191	0.35767512	Serpine1/Rps6ka5/Rbmx	3
GO:0002687	positive regulation of leukocyte migration	4/95	133/14464	0.03007519	4.57902651	3.37155471	0.01142388	0.37410191	0.35767512	Trem2/Serpine1/Abl1/Jam2	4
GO:0002694	regulation of leukocyte activation	8/95	467/14464	0.01713062	2.60818213	2.87245949	0.01166267	0.37410191	0.35767512	Trem2/Gimap5/Il15ra/Abl1/Peli1/Lyn/Paxip1/Arid2	8
GO:0010959	regulation of metal ion transport	7/95	375/14464	0.01866667	2.84204912	2.93869477	0.01169678	0.37410191	0.35767512	Per1/Trem2/Serpine1/Gimap5/Abl1/Agrn/Lyn	7
GO:0006289	nucleotide-excision repair	3/95	73/14464	0.04109589	6.25695746	3.66123942	0.01225455	0.38063134	0.36391784	Pole/Fan1/Arid2	3
GO:0010720	positive regulation of cell development	8/95	473/14464	0.01691332	2.57509736	2.83198742	0.01252201	0.38063134	0.36391784	Fzd4/Trem2/Gimap5/Il15ra/Myo5b/Clcn2/Lyn/Arid2	8
GO:0048144	fibroblast proliferation	4/95	138/14464	0.02898551	4.41311976	3.27571659	0.01293999	0.38063134	0.36391784	Serpine1/Inca1/Abl1/Brpf1	4
GO:0043122	regulation of canonical NF-kappaB signal transduction	5/95	213/14464	0.02347418	3.57400544	3.07719002	0.01313667	0.38063134	0.36391784	Per1/Trem2/Alpk1/Abl1/Peli1	5
GO:0010569	regulation of double-strand break repair via homologous recombination	3/95	75/14464	0.04	6.09010526	3.5935179	0.01318035	0.38063134	0.36391784	Abl1/Peli1/Arid2	3
GO:0051353	positive regulation of oxidoreductase activity	2/95	27/14464	0.07407407	11.2779727	4.34638894	0.01346606	0.38063134	0.36391784	Por/Abl1	2
GO:2000352	negative regulation of endothelial cell apoptotic process	2/95	27/14464	0.07407407	11.2779727	4.34638894	0.01346606	0.38063134	0.36391784	Serpine1/Abl1	2
GO:0002237	response to molecule of bacterial origin	6/95	297/14464	0.02020202	3.07581074	2.93903498	0.01348772	0.38063134	0.36391784	Trem2/Serpine1/Abl1/Peli1/Lyn/Cdk19	6
GO:0042100	B cell proliferation	3/95	78/14464	0.03846154	5.85587045	3.49640786	0.01464219	0.40455877	0.38679462	Abl1/Peli1/Lyn	3
GO:0099173	postsynapse organization	6/95	303/14464	0.01980198	3.0149036	2.8820821	0.01475722	0.40455877	0.38679462	Trem2/Myo5b/Rps6ka5/Abl1/Agrn/Rbmx	6
GO:0002920	regulation of humoral immune response	2/95	29/14464	0.06896552	10.5001815	4.16389942	0.01544453	0.41181742	0.39373454	Trem2/Gimap5	2
GO:0002260	lymphocyte homeostasis	3/95	80/14464	0.0375	5.70947368	3.43443463	0.0156658	0.41181742	0.39373454	Gimap5/Abl1/Lyn	3
GO:0033627	cell adhesion mediated by integrin	3/95	80/14464	0.0375	5.70947368	3.43443463	0.0156658	0.41181742	0.39373454	Serpine1/Acer2/Lyn	3
GO:0002455	humoral immune response mediated by circulating immunoglobulin	2/95	30/14464	0.06666667	10.1501754	4.07919457	0.01647772	0.41945363	0.40103545	Trem2/Gimap5	2
GO:0060071	Wnt signaling pathway, planar cell polarity pathway	2/95	30/14464	0.06666667	10.1501754	4.07919457	0.01647772	0.41945363	0.40103545	Abl1/Celsr3	2
GO:0044770	cell cycle phase transition	8/95	498/14464	0.01606426	2.44582541	2.66976004	0.01661202	0.41945363	0.40103545	Tcim/Wee1/Ccdc57/Pole/Xrcc3/Lyn/Paxip1/Arid2	8
GO:1905244	regulation of modification of synaptic structure	2/95	31/14464	0.06451613	9.82275042	3.99838177	0.01753965	0.42113893	0.40264675	Myo5b/Abl1	2
GO:0071901	negative regulation of protein serine/threonine kinase activity	3/95	84/14464	0.03571429	5.43759398	3.31654189	0.01783147	0.42113893	0.40264675	Inca1/Abl1/Lyn	3
GO:0051249	regulation of lymphocyte activation	7/95	409/14464	0.01711491	2.60579076	2.6786311	0.0180508	0.42113893	0.40264675	Gimap5/Il15ra/Abl1/Peli1/Lyn/Paxip1/Arid2	7
GO:0031349	positive regulation of defense response	7/95	410/14464	0.01707317	2.59943517	2.67138399	0.01826838	0.42113893	0.40264675	Trem2/Serpine1/Gimap5/Rab34/Alpk1/Peli1/Lyn	7
GO:0030522	intracellular receptor signaling pathway	6/95	318/14464	0.01886792	2.87269116	2.74562119	0.01829142	0.42113893	0.40264675	Per1/Nr1d2/Trem2/Klf9/Alpk1/Peli1	6
GO:0018108	peptidyl-tyrosine phosphorylation	5/95	233/14464	0.02145923	3.26722385	2.8368239	0.01866719	0.42113893	0.40264675	Trem2/Wee1/Abl1/Agrn/Lyn	5
GO:0001570	vasculogenesis	3/95	86/14464	0.03488372	5.31113831	3.26039085	0.01897377	0.42113893	0.40264675	Fzd4/Brpf1/Paxip1	3
GO:0071900	regulation of protein serine/threonine kinase activity	5/95	235/14464	0.0212766	3.23941769	2.81422988	0.01929301	0.42113893	0.40264675	Fzd4/Tcim/Inca1/Abl1/Lyn	5
GO:0007249	canonical NF-kappaB signal transduction	5/95	236/14464	0.02118644	3.22569135	2.80302347	0.01961105	0.42113893	0.40264675	Per1/Trem2/Alpk1/Abl1/Peli1	5
GO:0002548	monocyte chemotaxis	2/95	33/14464	0.06060606	9.22743222	3.84725341	0.01974807	0.42113893	0.40264675	Serpine1/Lyn	2
GO:0043409	negative regulation of MAPK cascade	4/95	157/14464	0.02547771	3.87904794	2.94918596	0.01984866	0.42113893	0.40264675	Per1/Trem2/Abl1/Lyn	4
GO:0018212	peptidyl-tyrosine modification	5/95	237/14464	0.02109705	3.21208083	2.79187663	0.01993253	0.42113893	0.40264675	Trem2/Wee1/Abl1/Agrn/Lyn	5
GO:0050767	regulation of neurogenesis	7/95	418/14464	0.01674641	2.54968522	2.61416516	0.02007622	0.42113893	0.40264675	Bhlhe40/Fzd4/Trem2/Bhlhe41/Myo5b/Clcn2/Lyn	7
GO:0001953	negative regulation of cell-matrix adhesion	2/95	34/14464	0.05882353	8.95603715	3.77642461	0.02089375	0.42113893	0.40264675	Serpine1/Acer2	2
GO:2000403	positive regulation of lymphocyte migration	2/95	34/14464	0.05882353	8.95603715	3.77642461	0.02089375	0.42113893	0.40264675	Abl1/Jam2	2
GO:0070555	response to interleukin-1	3/95	90/14464	0.03333333	5.07508772	3.15316807	0.02137776	0.42113893	0.40264675	Serpine1/Rps6ka5/Rbmx	3
GO:0002833	positive regulation of response to biotic stimulus	6/95	331/14464	0.01812689	2.75986643	2.6336269	0.02179268	0.42113893	0.40264675	Trem2/Gimap5/Rab34/Alpk1/Peli1/Lyn	6
GO:0046785	microtubule polymerization	3/95	91/14464	0.03296703	5.01931752	3.12735385	0.02200366	0.42113893	0.40264675	Ccdc57/Abl1/Nav3	3
GO:0002263	cell activation involved in immune response	5/95	245/14464	0.02040816	3.10719656	2.7047759	0.02262964	0.42113893	0.40264675	Trem2/Tcim/Abl1/Lyn/Paxip1	5
GO:0009066	aspartate family amino acid metabolic process	2/95	36/14464	0.05555556	8.45847953	3.64314292	0.02326605	0.42113893	0.40264675	Pipox/Tha1	2
GO:0032350	regulation of hormone metabolic process	2/95	36/14464	0.05555556	8.45847953	3.64314292	0.02326605	0.42113893	0.40264675	Por/Clcn2	2
GO:0036297	interstrand cross-link repair	2/95	37/14464	0.05405405	8.22987198	3.58031449	0.02449187	0.42113893	0.40264675	Xrcc3/Fan1	2
GO:0060563	neuroepithelial cell differentiation	2/95	37/14464	0.05405405	8.22987198	3.58031449	0.02449187	0.42113893	0.40264675	Serpine1/Abl1	2
GO:0002708	positive regulation of lymphocyte mediated immunity	3/95	95/14464	0.03157895	4.80797784	3.02775455	0.02460695	0.42113893	0.40264675	Trem2/Gimap5/Paxip1	3
GO:0030902	hindbrain development	4/95	170/14464	0.02352941	3.58241486	2.75391992	0.02567574	0.42113893	0.40264675	Fzd4/Gdf10/Rere/Abl1	4
GO:0043269	regulation of monoatomic ion transport	7/95	440/14464	0.01590909	2.42220096	2.46336676	0.02568861	0.42113893	0.40264675	Per1/Trem2/Serpine1/Gimap5/Abl1/Agrn/Lyn	7
GO:1902107	positive regulation of leukocyte differentiation	4/95	171/14464	0.02339181	3.56146507	2.7396969	0.02616228	0.42113893	0.40264675	Trem2/Gimap5/Il15ra/Arid2	4
GO:1903708	positive regulation of hemopoiesis	4/95	171/14464	0.02339181	3.56146507	2.7396969	0.02616228	0.42113893	0.40264675	Trem2/Gimap5/Il15ra/Arid2	4
GO:0031929	TOR signaling	4/95	172/14464	0.02325581	3.54075887	2.72557975	0.02665435	0.42113893	0.40264675	Trem2/Sar1b/Rps6ka5/Peli1	4
GO:1900744	regulation of p38MAPK cascade	2/95	39/14464	0.05128205	7.80782726	3.46147057	0.02702092	0.42113893	0.40264675	Per1/Trem2	2
GO:0033674	positive regulation of kinase activity	5/95	258/14464	0.01937984	2.95063239	2.5705562	0.02749967	0.42113893	0.40264675	Fzd4/Trem2/Tcim/Abl1/Agrn	5
GO:0002065	columnar/cuboidal epithelial cell differentiation	3/95	100/14464	0.03	4.56757895	2.91080839	0.02808517	0.42113893	0.40264675	Serpine1/Clcn2/Abl1	3
GO:0042770	signal transduction in response to DNA damage	4/95	175/14464	0.02285714	3.48006015	2.68384879	0.02816388	0.42113893	0.40264675	Acer2/Abl1/Lyn/Paxip1	4
GO:0050920	regulation of chemotaxis	4/95	175/14464	0.02285714	3.48006015	2.68384879	0.02816388	0.42113893	0.40264675	Trem2/Serpine1/Agrn/Lyn	4
GO:0001782	B cell homeostasis	2/95	40/14464	0.05	7.61263158	3.40517306	0.02832338	0.42113893	0.40264675	Abl1/Lyn	2
GO:0045880	positive regulation of smoothened signaling pathway	2/95	40/14464	0.05	7.61263158	3.40517306	0.02832338	0.42113893	0.40264675	Por/Rab34	2
GO:1901016	regulation of potassium ion transmembrane transporter activity	2/95	40/14464	0.05	7.61263158	3.40517306	0.02832338	0.42113893	0.40264675	Trem2/Agrn	2
GO:0002824	positive regulation of adaptive immune response based on somatic recombination of immune receptors b	3/95	102/14464	0.02941176	4.47801858	2.86617198	0.02954599	0.42113893	0.40264675	Trem2/Gimap5/Paxip1	3
GO:0009395	phospholipid catabolic process	2/95	41/14464	0.04878049	7.42695764	3.35079059	0.02965062	0.42113893	0.40264675	Pnpla7/Gpcpd1	2
GO:0045619	regulation of lymphocyte differentiation	4/95	178/14464	0.02247191	3.42140745	2.64301894	0.02972359	0.42113893	0.40264675	Gimap5/Il15ra/Abl1/Arid2	4
GO:0050866	negative regulation of cell activation	4/95	178/14464	0.02247191	3.42140745	2.64301894	0.02972359	0.42113893	0.40264675	Trem2/Gimap5/Peli1/Lyn	4
GO:0043549	regulation of kinase activity	7/95	456/14464	0.01535088	2.33721145	2.35924023	0.03039266	0.42113893	0.40264675	Fzd4/Trem2/Tcim/Inca1/Abl1/Agrn/Lyn	7
GO:0042446	hormone biosynthetic process	2/95	42/14464	0.04761905	7.25012531	3.29821033	0.03100227	0.42113893	0.40264675	Por/Clcn2	2
GO:1905168	positive regulation of double-strand break repair via homologous recombination	2/95	42/14464	0.04761905	7.25012531	3.29821033	0.03100227	0.42113893	0.40264675	Peli1/Arid2	2
GO:0002520	immune system development	4/95	183/14464	0.02185792	3.32792637	2.57687409	0.0324353	0.42113893	0.40264675	Lrrc17/Abl1/Lyn/Paxip1	4
GO:0016064	immunoglobulin mediated immune response	3/95	107/14464	0.02803738	4.26876537	2.75944353	0.0333711	0.42113893	0.40264675	Trem2/Gimap5/Paxip1	3
GO:0042542	response to hydrogen peroxide	3/95	107/14464	0.02803738	4.26876537	2.75944353	0.0333711	0.42113893	0.40264675	Net1/ND5/Abl1	3
GO:1901617	organic hydroxy compound biosynthetic process	4/95	187/14464	0.02139037	3.25674078	2.52558346	0.03470614	0.42113893	0.40264675	Acer2/Por/Lss/Clcn2	4
GO:0050807	regulation of synapse organization	6/95	370/14464	0.01621622	2.46896159	2.32740462	0.03494856	0.42113893	0.40264675	Trem2/Myo5b/Rps6ka5/Abl1/Agrn/Rbmx	6
GO:1904262	negative regulation of TORC1 signaling	2/95	45/14464	0.04444444	6.76678363	3.1502866	0.03519998	0.42113893	0.40264675	Sar1b/Peli1	2
GO:0002821	positive regulation of adaptive immune response	3/95	110/14464	0.02727273	4.1523445	2.69849282	0.03578418	0.42113893	0.40264675	Trem2/Gimap5/Paxip1	3
GO:0019724	B cell mediated immunity	3/95	110/14464	0.02727273	4.1523445	2.69849282	0.03578418	0.42113893	0.40264675	Trem2/Gimap5/Paxip1	3
GO:0051928	positive regulation of calcium ion transport	3/95	111/14464	0.02702703	4.11493599	2.67865633	0.03660809	0.42113893	0.40264675	Serpine1/Gimap5/Abl1	3
GO:0046503	glycerolipid catabolic process	2/95	46/14464	0.04347826	6.61967963	3.10395693	0.0366456	0.42113893	0.40264675	Pnpla7/Gpcpd1	2
GO:1900015	regulation of cytokine production involved in inflammatory response	2/95	46/14464	0.04347826	6.61967963	3.10395693	0.0366456	0.42113893	0.40264675	Per1/Trem2	2
GO:2000351	regulation of endothelial cell apoptotic process	2/95	46/14464	0.04347826	6.61967963	3.10395693	0.0366456	0.42113893	0.40264675	Serpine1/Abl1	2
GO:0050803	regulation of synapse structure or activity	6/95	376/14464	0.01595745	2.42956327	2.28375944	0.03734611	0.42113893	0.40264675	Trem2/Myo5b/Rps6ka5/Abl1/Agrn/Rbmx	6
GO:0006302	double-strand break repair	5/95	281/14464	0.01779359	2.70912156	2.35244666	0.03765559	0.42113893	0.40264675	Abl1/Xrcc3/Peli1/Fan1/Arid2	5
GO:1902930	regulation of alcohol biosynthetic process	2/95	47/14464	0.04255319	6.47883539	3.05898576	0.03811381	0.42113893	0.40264675	Por/Clcn2	2
GO:0038202	TORC1 signaling	3/95	113/14464	0.02654867	4.04210526	2.63967282	0.0382851	0.42113893	0.40264675	Sar1b/Rps6ka5/Peli1	3
GO:1905477	positive regulation of protein localization to membrane	3/95	113/14464	0.02654867	4.04210526	2.63967282	0.0382851	0.42113893	0.40264675	Trem2/Myo5b/Agrn	3
GO:0006486	protein glycosylation	4/95	194/14464	0.02061856	3.13922952	2.43906843	0.03889822	0.42113893	0.40264675	Acer2/Galnt15/Tet3/Alg10b	4
GO:0043413	macromolecule glycosylation	4/95	194/14464	0.02061856	3.13922952	2.43906843	0.03889822	0.42113893	0.40264675	Acer2/Galnt15/Tet3/Alg10b	4
GO:0001952	regulation of cell-matrix adhesion	3/95	114/14464	0.02631579	4.0066482	2.62051599	0.03913816	0.42113893	0.40264675	Serpine1/Acer2/Abl1	3
GO:0045621	positive regulation of lymphocyte differentiation	3/95	114/14464	0.02631579	4.0066482	2.62051599	0.03913816	0.42113893	0.40264675	Gimap5/Il15ra/Arid2	3
GO:0051983	regulation of chromosome segregation	3/95	114/14464	0.02631579	4.0066482	2.62051599	0.03913816	0.42113893	0.40264675	Xrcc3/Ncapd3/Arid2	3
GO:0006911	phagocytosis, engulfment	2/95	48/14464	0.04166667	6.34385965	3.01530327	0.03960426	0.42113893	0.40264675	Trem2/Clcn2	2
GO:0051341	regulation of oxidoreductase activity	2/95	48/14464	0.04166667	6.34385965	3.01530327	0.03960426	0.42113893	0.40264675	Por/Abl1	2
GO:0140353	lipid export from cell	2/95	48/14464	0.04166667	6.34385965	3.01530327	0.03960426	0.42113893	0.40264675	Fzd4/Sar1b	2
GO:0002705	positive regulation of leukocyte mediated immunity	3/95	115/14464	0.02608696	3.97180778	2.60157614	0.04000088	0.42113893	0.40264675	Trem2/Gimap5/Paxip1	3
GO:0002703	regulation of leukocyte mediated immunity	4/95	197/14464	0.02030457	3.09142399	2.40318161	0.04078004	0.42113893	0.40264675	Trem2/Gimap5/Lyn/Paxip1	4
GO:0170040	proteinogenic amino acid catabolic process	2/95	49/14464	0.04081633	6.21439313	2.97284462	0.0411166	0.42113893	0.40264675	Pipox/Tha1	2
GO:0001776	leukocyte homeostasis	3/95	117/14464	0.02564103	3.90391363	2.56432933	0.04175522	0.42113893	0.40264675	Gimap5/Abl1/Lyn	3
GO:0014013	regulation of gliogenesis	3/95	117/14464	0.02564103	3.90391363	2.56432933	0.04175522	0.42113893	0.40264675	Trem2/Clcn2/Lyn	3
GO:0002762	negative regulation of myeloid leukocyte differentiation	2/95	50/14464	0.04	6.09010526	2.93154949	0.04265049	0.42113893	0.40264675	Lrrc17/Lyn	2
GO:0032663	regulation of interleukin-2 production	2/95	50/14464	0.04	6.09010526	2.93154949	0.04265049	0.42113893	0.40264675	Abl1/Nav3	2
GO:0072577	endothelial cell apoptotic process	2/95	50/14464	0.04	6.09010526	2.93154949	0.04265049	0.42113893	0.40264675	Serpine1/Abl1	2
GO:0002534	cytokine production involved in inflammatory response	2/95	51/14464	0.03921569	5.97069143	2.89136169	0.04420559	0.42113893	0.40264675	Per1/Trem2	2
GO:0006695	cholesterol biosynthetic process	2/95	51/14464	0.03921569	5.97069143	2.89136169	0.04420559	0.42113893	0.40264675	Por/Lss	2
GO:0034605	cellular response to heat	2/95	51/14464	0.03921569	5.97069143	2.89136169	0.04420559	0.42113893	0.40264675	Tcim/Lyn	2
GO:0050829	defense response to Gram-negative bacterium	2/95	51/14464	0.03921569	5.97069143	2.89136169	0.04420559	0.42113893	0.40264675	Trem2/Serpine1	2
GO:0050853	B cell receptor signaling pathway	2/95	51/14464	0.03921569	5.97069143	2.89136169	0.04420559	0.42113893	0.40264675	Abl1/Lyn	2
GO:0070098	chemokine-mediated signaling pathway	2/95	51/14464	0.03921569	5.97069143	2.89136169	0.04420559	0.42113893	0.40264675	Trem2/Lyn	2
GO:1902653	secondary alcohol biosynthetic process	2/95	51/14464	0.03921569	5.97069143	2.89136169	0.04420559	0.42113893	0.40264675	Por/Lss	2
GO:1904062	regulation of monoatomic cation transmembrane transport	5/95	294/14464	0.0170068	2.58933047	2.23862151	0.04429563	0.42113893	0.40264675	Trem2/Gimap5/Abl1/Agrn/Lyn	5
GO:0002761	regulation of myeloid leukocyte differentiation	3/95	121/14464	0.02479339	3.77485863	2.49224809	0.04537868	0.42113893	0.40264675	Trem2/Lrrc17/Lyn	3
GO:2000401	regulation of lymphocyte migration	2/95	52/14464	0.03846154	5.85587045	2.85222879	0.04578155	0.42113893	0.40264675	Abl1/Jam2	2
GO:0071396	cellular response to lipid	7/95	499/14464	0.01402806	2.13580846	2.09948427	0.0458324	0.42113893	0.40264675	Per1/Trem2/Serpine1/Klf9/Abl1/Lyn/Cdk19	7
GO:0019216	regulation of lipid metabolic process	5/95	298/14464	0.01677852	2.55457436	2.20482303	0.04647188	0.42113893	0.40264675	Nr1d2/Trem2/Gimap5/Por/Clcn2	5

GO:0010810	regulation of cell-substrate adhesion	4/95	206/14464	0.01941748	2.95636178	2.29948827	0.04673264	0.42113893	0.40264675	Fzd4/Serpine1/Acer2/Abl1	4
GO:0043270	positive regulation of monoatomic ion transport	4/95	206/14464	0.01941748	2.95636178	2.29948827	0.04673264	0.42113893	0.40264675	Trem2/Serpine1/Gimap5/Abl1	4
GO:0050864	regulation of B cell activation	3/95	123/14464	0.02439024	3.71347882	2.45734989	0.04724731	0.42113893	0.40264675	Peli1/Lyn/Paxip1	3
GO:0099563	modification of synaptic structure	2/95	53/14464	0.03773585	5.74538232	2.81410182	0.04737804	0.42113893	0.40264675	Myo5b/Abl1	2
GO:0170035	L-amino acid catabolic process	2/95	53/14464	0.03773585	5.74538232	2.81410182	0.04737804	0.42113893	0.40264675	Pipox/Tha1	2
GO:0071466	cellular response to xenobiotic stimulus	3/95	124/14464	0.02419355	3.68353141	2.44017331	0.04819574	0.42113893	0.40264675	Serpine1/Acer2/Gsta4	3
GO:1901987	regulation of cell cycle phase transition	6/95	402/14464	0.01492537	2.27242734	2.10377938	0.04894596	0.42113893	0.40264675	Tcim/Wee1/Xrcc3/Lyn/Paxip1/Arid2	6
GO:0031663	lipopolysaccharide-mediated signaling pathway	2/95	54/14464	0.03703704	5.63898635	2.77693496	0.04899474	0.42113893	0.40264675	Trem2/Lyn	2
GO:1901224	positive regulation of non-canonical NF-kappaB signal transduction	2/95	54/14464	0.03703704	5.63898635	2.77693496	0.04899474	0.42113893	0.40264675	Trem2/Tcim	2
GO:0070085	glycosylation	4/95	210/14464	0.01904762	2.90005013	2.25519475	0.04952598	0.42113893	0.40264675	Acer2/Galnt15/Tet3/Alg10b	4
GO:0050900	leukocyte migration	5/95	304/14464	0.01644737	2.50415512	2.15514014	0.04985454	0.42113893	0.40264675	Trem2/Serpine1/Abl1/Lyn/Jam2	5
GO:0006694	steroid biosynthetic process	3/95	126/14464	0.02380952	3.62506266	2.40634771	0.05012065	0.42113893	0.40264675	Por/Lss/Clcn2	3
GO:0000768	syncytium formation by plasma membrane fusion	2/95	55/14464	0.03636364	5.53645933	2.74068529	0.0506313	0.42113893	0.40264675	Trem2/Jam2	2
GO:0140253	cell-cell fusion	2/95	55/14464	0.03636364	5.53645933	2.74068529	0.0506313	0.42113893	0.40264675	Trem2/Jam2	2
GO:0051347	positive regulation of transferase activity	5/95	306/14464	0.01633987	2.4877881	2.13884131	0.0510137	0.42113893	0.40264675	Fzd4/Trem2/Tcim/Abl1/Agrn	5
GO:0051090	regulation of DNA-binding transcription factor activity	5/95	308/14464	0.01623377	2.47163363	2.12267013	0.05218869	0.42113893	0.40264675	Bhlhe40/Fzd4/Rps6ka5/Peli1/Paxip1	5
GO:0016126	sterol biosynthetic process	2/95	56/14464	0.03571429	5.43759398	2.70531257	0.05228742	0.42113893	0.40264675	Por/Lss	2
GO:0038066	p38MAPK cascade	2/95	56/14464	0.03571429	5.43759398	2.70531257	0.05228742	0.42113893	0.40264675	Per1/Trem2	2
GO:0043124	negative regulation of canonical NF-kappaB signal transduction	2/95	56/14464	0.03571429	5.43759398	2.70531257	0.05228742	0.42113893	0.40264675	Per1/Trem2	2
GO:0045089	positive regulation of innate immune response	5/95	310/14464	0.01612903	2.45568761	2.10662464	0.05337952	0.42113893	0.40264675	Trem2/Gimap5/Alpk1/Peli1/Lyn	5
GO:0002429	immune response-activating cell surface receptor signaling pathway	4/95	216/14464	0.01851852	2.81949318	2.19067563	0.05388586	0.42113893	0.40264675	Trem2/Abl1/Peli1/Lyn	4
GO:0006949	syncytium formation	2/95	57/14464	0.03508772	5.3421976	2.67077902	0.05396276	0.42113893	0.40264675	Trem2/Jam2	2
GO:0031113	regulation of microtubule polymerization	2/95	57/14464	0.03508772	5.3421976	2.67077902	0.05396276	0.42113893	0.40264675	Abl1/Nav3	2
GO:0032623	interleukin-2 production	2/95	57/14464	0.03508772	5.3421976	2.67077902	0.05396276	0.42113893	0.40264675	Abl1/Nav3	2
GO:0060038	cardiac muscle cell proliferation	2/95	57/14464	0.03508772	5.3421976	2.67077902	0.05396276	0.42113893	0.40264675	Abl1/Arid2	2
GO:0099024	plasma membrane invagination	2/95	57/14464	0.03508772	5.3421976	2.67077902	0.05396276	0.42113893	0.40264675	Trem2/Clcn2	2
GO:0043433	negative regulation of DNA-binding transcription factor activity	3/95	130/14464	0.02307692	3.51352227	2.34071445	0.0540817	0.42113893	0.40264675	Bhlhe40/Peli1/Paxip1	3
GO:0031109	microtubule polymerization or depolymerization	3/95	132/14464	0.02272727	3.46028708	2.30885721	0.05611724	0.42113893	0.40264675	Ccdc57/Abl1/Nav3	3
GO:0050731	positive regulation of peptidyl-tyrosine phosphorylation	3/95	133/14464	0.02255639	3.43426989	2.29315823	0.05714862	0.42113893	0.40264675	Trem2/Abl1/Agrn	3
GO:2000779	regulation of double-strand break repair	3/95	133/14464	0.02255639	3.43426989	2.29315823	0.05714862	0.42113893	0.40264675	Abl1/Peli1/Arid2	3
GO:1903078	positive regulation of protein localization to plasma membrane	2/95	59/14464	0.03389831	5.16110616	2.60408942	0.05736986	0.42113893	0.40264675	Trem2/Myo5b	2
GO:0051345	positive regulation of hydrolase activity	5/95	317/14464	0.01577287	2.40146107	2.0514277	0.0576722	0.42113893	0.40264675	Acer2/Net1/Adap2/Agrn/Lyn	5
GO:0050773	regulation of dendrite development	3/95	136/14464	0.02205882	3.35851393	2.24694704	0.06029676	0.42113893	0.40264675	Fzd4/Myo5b/Abl1	3
GO:1904064	positive regulation of cation transmembrane transport	3/95	136/14464	0.02205882	3.35851393	2.24694704	0.06029676	0.42113893	0.40264675	Trem2/Gimap5/Abl1	3
GO:0008202	steroid metabolic process	4/95	225/14464	0.01777778	2.70671345	2.09792447	0.06080555	0.42113893	0.40264675	Por/Lss/Clcn2/Cyp3a13	4
GO:0030888	regulation of B cell proliferation	2/95	61/14464	0.03278689	4.99188956	2.54035656	0.06085009	0.42113893	0.40264675	Peli1/Lyn	2
GO:1990868	response to chemokine	2/95	61/14464	0.03278689	4.99188956	2.54035656	0.06085009	0.42113893	0.40264675	Trem2/Lyn	2
GO:1990869	cellular response to chemokine	2/95	61/14464	0.03278689	4.99188956	2.54035656	0.06085009	0.42113893	0.40264675	Trem2/Lyn	2
GO:0045859	regulation of protein kinase activity	6/95	426/14464	0.01408451	2.14440326	1.94943604	0.06144226	0.42113893	0.40264675	Fzd4/Tcim/Inca1/Abl1/Agrn/Lyn	6
GO:0034765	regulation of monoatomic ion transmembrane transport	5/95	323/14464	0.01547988	2.35685188	2.00526311	0.0615061	0.42113893	0.40264675	Trem2/Gimap5/Abl1/Agrn/Lyn	5
GO:0002768	immune response-regulating cell surface receptor signaling pathway	4/95	227/14464	0.01762115	2.68286575	2.07792985	0.06240471	0.42113893	0.40264675	Trem2/Abl1/Peli1/Lyn	4
GO:0007159	leukocyte cell-cell adhesion	5/95	326/14464	0.01533742	2.33516306	1.9825623	0.06347646	0.42113893	0.40264675	Gimap5/Abl1/Peli1/Jam2/Arid2	5
GO:0008582	regulation of synaptic assembly at neuromuscular junction	1/95	10/14464	0.1	15.2252632	3.65884496	0.06379193	0.42113893	0.40264675	Agrn	1
GO:0010944	negative regulation of transcription by competitive promoter binding	1/95	10/14464	0.1	15.2252632	3.65884496	0.06379193	0.42113893	0.40264675	Bhlhe41	1
GO:0032341	aldosterone metabolic process	1/95	10/14464	0.1	15.2252632	3.65884496	0.06379193	0.42113893	0.40264675	Clcn2	1
GO:0036466	synaptic vesicle recycling via endosome	1/95	10/14464	0.1	15.2252632	3.65884496	0.06379193	0.42113893	0.40264675	Myo5b	1
GO:0044029	positive regulation of gene expression via chromosomal CpG island demethylation	1/95	10/14464	0.1	15.2252632	3.65884496	0.06379193	0.42113893	0.40264675	Tet3	1
GO:0060049	regulation of protein glycosylation	1/95	10/14464	0.1	15.2252632	3.65884496	0.06379193	0.42113893	0.40264675	Acer2	1
GO:0061043	regulation of vascular wound healing	1/95	10/14464	0.1	15.2252632	3.65884496	0.06379193	0.42113893	0.40264675	Serpine1	1
GO:0070344	regulation of fat cell proliferation	1/95	10/14464	0.1	15.2252632	3.65884496	0.06379193	0.42113893	0.40264675	Trem2	1
GO:0070391	response to lipoteichoic acid	1/95	10/14464	0.1	15.2252632	3.65884496	0.06379193	0.42113893	0.40264675	Trem2	1
GO:0070445	regulation of oligodendrocyte progenitor proliferation	1/95	10/14464	0.1	15.2252632	3.65884496	0.06379193	0.42113893	0.40264675	Lyn	1
GO:0071223	cellular response to lipoteichoic acid	1/95	10/14464	0.1	15.2252632	3.65884496	0.06379193	0.42113893	0.40264675	Trem2	1
GO:0099515	actin filament-based transport	1/95	10/14464	0.1	15.2252632	3.65884496	0.06379193	0.42113893	0.40264675	Myo5b	1
GO:0099639	neurotransmitter receptor transport, endosome to plasma membrane	1/95	10/14464	0.1	15.2252632	3.65884496	0.06379193	0.42113893	0.40264675	Myo5b	1
GO:1900426	positive regulation of defense response to bacterium	1/95	10/14464	0.1	15.2252632	3.65884496	0.06379193	0.42113893	0.40264675	Rab34	1
GO:1903867	extraembryonic membrane development	1/95	10/14464	0.1	15.2252632	3.65884496	0.06379193	0.42113893	0.40264675	Paxip1	1
GO:2000650	negative regulation of sodium ion transmembrane transporter activity	1/95	10/14464	0.1	15.2252632	3.65884496	0.06379193	0.42113893	0.40264675	Agrn	1
GO:0050775	positive regulation of dendrite morphogenesis	2/95	63/14464	0.03174603	4.83341688	2.47934934	0.064401	0.42113893	0.40264675	Fzd4/Myo5b	2
GO:0002706	regulation of lymphocyte mediated immunity	3/95	140/14464	0.02142857	3.26255639	2.18730324	0.06461865	0.42113893	0.40264675	Trem2/Gimap5/Paxip1	3
GO:0051250	negative regulation of lymphocyte activation	3/95	140/14464	0.02142857	3.26255639	2.18730324	0.06461865	0.42113893	0.40264675	Gimap5/Peli1/Lyn	3
GO:0044839	cell cycle G2/M phase transition	3/95	141/14464	0.0212766	3.23941769	2.17272814	0.06572105	0.42113893	0.40264675	Wee1/Ccdc57/Paxip1	3
GO:0055088	lipid homeostasis	3/95	141/14464	0.0212766	3.23941769	2.17272814	0.06572105	0.42113893	0.40264675	Nr1d2/Trem2/Sar1b	3
GO:0010770	positive regulation of cell morphogenesis	2/95	64/14464	0.03125	4.75789474	2.44980251	0.06620221	0.42113893	0.40264675	Fzd4/Myo5b	2
GO:0002221	pattern recognition receptor signaling pathway	4/95	232/14464	0.01724138	2.62504537	2.02887009	0.06649954	0.42113893	0.40264675	Trem2/Alpk1/Peli1/Lyn	4
GO:0002573	myeloid leukocyte differentiation	4/95	232/14464	0.01724138	2.62504537	2.02887009	0.06649954	0.42113893	0.40264675	Trem2/Gimap5/Lrrc17/Lyn	4
GO:0032006	regulation of TOR signaling	3/95	143/14464	0.02097902	3.19411115	2.14396502	0.06795183	0.42113893	0.40264675	Trem2/Sar1b/Peli1	3
GO:0043123	positive regulation of canonical NF-kappaB signal transduction	3/95	143/14464	0.02097902	3.19411115	2.14396502	0.06795183	0.42113893	0.40264675	Alpk1/Abl1/Peli1	3
GO:0010324	membrane invagination	2/95	65/14464	0.03076923	4.68469636	2.42086148	0.0680202	0.42113893	0.40264675	Trem2/Clcn2	2
GO:0032007	negative regulation of TOR signaling	2/95	65/14464	0.03076923	4.68469636	2.42086148	0.0680202	0.42113893	0.40264675	Sar1b/Peli1	2
GO:0050766	positive regulation of phagocytosis	2/95	65/14464	0.03076923	4.68469636	2.42086148	0.0680202	0.42113893	0.40264675	Trem2/Il15ra	2
GO:1905818	regulation of chromosome separation	2/95	65/14464	0.03076923	4.68469636	2.42086148	0.0680202	0.42113893	0.40264675	Xrcc3/Ncapd3	2
GO:0002758	innate immune response-activating signaling pathway	4/95	235/14464	0.01702128	2.59153415	2.00004804	0.06902249	0.42113893	0.40264675	Trem2/Alpk1/Peli1/Lyn	4
GO:0033044	regulation of chromosome organization	4/95	236/14464	0.01694915	2.58055308	1.99053987	0.06987441	0.42113893	0.40264675	Xrcc3/Ncapd3/Ylpm1/Arid2	4
GO:0002291	T cell activation via T cell receptor contact with antigen bound to MHC molecule on antigen presenting ce	1/95	11/14464	0.09090909	13.8411483	3.46416785	0.06994523	0.42113893	0.40264675	Trem2	1
GO:0006544	glycine metabolic process	1/95	11/14464	0.09090909	13.8411483	3.46416785	0.06994523	0.42113893	0.40264675	Tha1	1
GO:0008212	mineralocorticoid metabolic process	1/95	11/14464	0.09090909	13.8411483	3.46416785	0.06994523	0.42113893	0.40264675	Clcn2	1
GO:0010988	regulation of low-density lipoprotein particle clearance	1/95	11/14464	0.09090909	13.8411483	3.46416785	0.06994523	0.42113893	0.40264675	Trem2	1
GO:0016114	terpenoid biosynthetic process	1/95	11/14464	0.09090909	13.8411483	3.46416785	0.06994523	0.42113893	0.40264675	Lss	1
GO:0032825	positive regulation of natural killer cell differentiation	1/95	11/14464	0.09090909	13.8411483	3.46416785	0.06994523	0.42113893	0.40264675	Il15ra	1
GO:0034309	primary alcohol biosynthetic process	1/95	11/14464	0.09090909	13.8411483	3.46416785	0.06994523	0.42113893	0.40264675	Clcn2	1
GO:0034351	negative regulation of glial cell apoptotic process	1/95	11/14464	0.09090909	13.8411483	3.46416785	0.06994523	0.42113893	0.40264675	Trem2	1
GO:0035701	hematopoietic stem cell migration	1/95	11/14464	0.09090909	13.8411483	3.46416785	0.06994523	0.42113893	0.40264675	Jam2	1
GO:0042730	fibrinolysis	1/95	11/14464	0.09090909	13.8411483	3.46416785	0.06994523	0.42113893	0.40264675	Serpine1	1
GO:0043652	engulfment of apoptotic cell	1/95	11/14464	0.09090909	13.8411483	3.46416785	0.06994523	0.42113893	0.40264675	Trem2	1
GO:0045588	positive regulation of gamma-delta T cell differentiation	1/95	11/14464	0.09090909	13.8411483	3.46416785	0.06994523	0.42113893	0.40264675	Gimap5	1
GO:0046645	positive regulation of gamma-delta T cell activation	1/95	11/14464	0.09090909	13.8411483	3.46416785	0.06994523	0.42113893	0.40264675	Gimap5	1
GO:0048302	regulation of isotype switching to IgG isotypes	1/95	11/14464	0.09090909	13.8411483	3.46416785	0.06994523	0.42113893	0.40264675	Paxip1	1
GO:0061299	retina vasculature morphogenesis in camera-type eye	1/95	11/14464	0.09090909	13.8411483	3.46416785	0.06994523	0.42113893	0.40264675	Fzd4	1
GO:0070099	regulation of chemokine-mediated signaling pathway	1/95	11/14464	0.09090909	13.8411483	3.46416785	0.06994523	0.42113893	0.40264675	Trem2	1
GO:0070444	oligodendrocyte progenitor proliferation	1/95	11/14464	0.09090909	13.8411483	3.46416785	0.06994523	0.42113893	0.40264675	Lyn	1
GO:0071871	response to epinephrine	1/95	11/14464	0.09090909	13.8411483	3.46416785	0.06994523	0.42113893	0.40264675	Abl1	1
GO:0097501	stress response to metal ion	1/95	11/14464	0.09090909	13.8411483	3.46416785	0.06994523	0.42113893	0.40264675	Mt1	1
GO:0140052	cellular response to oxidised low-density lipoprotein particle stimulus	1/95	11/14464	0.09090909	13.8411483	3.46416785	0.06994523	0.42113893	0.40264675	Trem2	1
GO:0150078	positive regulation of neuroinflammatory response	1/95	11/14464	0.09090909	13.8411483	3.46416785	0.06994523	0.42113893	0.40264675	Trem2	1
GO:1902306	negative regulation of sodium ion transmembrane transport	1/95	11/14464	0.09090909	13.8411483	3.46416785	0.06994523	0.42113893	0.40264675	Agrn	1
GO:1903276	regulation of sodium ion export across plasma membrane	1/95	11/14464	0.09090909	13.8411483	3.46416785	0.06994523	0.42113893	0.40264675	Agrn	1
GO:1904395	positive regulation of skeletal muscle acetylcholine-gated channel clustering	1/95	11/14464	0.09090909	13.8411483	3.46416785	0.06994523	0.42113893	0.40264675	Agrn	1
GO:1905806	regulation of synapse pruning	1/95	11/14464	0.09090909	13.8411483	3.46416785	0.06994523	0.42113893	0.40264675	Trem2	1
GO:0030330	DNA damage response, signal transduction by p53 class mediator	2/95	67/14464	0.02985075	4.54485467	2.3647082	0.07170531	0.42113893	0.40264675	Acer2/Paxip1	2
GO:0050810	regulation of steroid biosynthetic process	2/95	67/14464	0.02985075	4.54485467	2.3647082	0.07170531	0.42113893	0.40264675	Por/Clcn2	2
GO:0061951	establishment of protein localization to plasma membrane	2/95	67/14464	0.02985075	4.54485467	2.3647082	0.07170531	0.42113893	0.40264675	Rab34/Myo5b	2
GO:0043547	positive regulation of GTPase activity	3/95	147/14464	0.02040816	3.10719656	2.08792756	0.07251606	0.42113893	0.40264675	Net1/Adap2/Agrn	3
GO:0060041	retina development in camera-type eye	3/95	147/14464	0.02040816	3.10719656	2.08792756	0.07251606	0.42113893	0.40264675	Per1/Fzd4/Clcn2	3
GO:0002831	regulation of response to biotic stimulus	6/95	446/14464	0.01345291	2.04824168	1.82836774	0.07318838	0.42113893	0.40264675	Trem2/Gimap5/Rab34/Alpk1/Peli1/Lyn	6
GO:0042113	B cell activation	4/95	240/14464	0.01666667	2.53754386	1.95298985	0.07333647	0.42113893	0.40264675	Abl1/Peli1/Lyn/Paxip1	4
GO:0051924	regulation of calcium ion transport	4/95	240/14464	0.01666667	2.53754386	1.95298985	0.07333647	0.42113893	0.40264675	Serpine1/Gimap5/Abl1/Lyn	4
GO:0048146	positive regulation of fibroblast proliferation	2/95	68/14464	0.02941176	4.47801858	2.33745446	0.07357187	0.42113893	0.40264675	Serpine1/Abl1	2
GO:1901606	alpha-amino acid catabolic process	2/95	68/14464	0.02941176	4.47801858	2.33745446	0.07357187	0.42113893	0.40264675	Pipox/Tha1	2
GO:1903131	mononuclear cell differentiation	6/95	447/14464	0.01342282	2.04365948	1.82248001	0.07380754	0.42113893	0.40264675	Trem2/Gimap5/Il15ra/Abl1/Lyn/Arid2	6
GO:0002366	leukocyte activation involved in immune response	4/95	241/14464	0.01659751	2.52701463	1.9437206	0.07421551	0.42113893	0.40264675	Trem2/Abl1/Lyn/Paxip1	4
GO:0045786	negative regulation of cell cycle	5/95	343/14464	0.01457726	2.21942612	1.85843574	0.07531109	0.42113893	0.40264675	Wee1/Inca1/Abl1/Xrcc3/Lyn	5
GO:0001767	establishment of lymphocyte polarity	1/95	12/14464	0.08333333	12.6877193	3.2933216	0.07605851	0.42113893	0.40264675	Abl1	1
GO:0001768	establishment of T cell polarity	1/95	12/14464	0.08333333	12.6877193	3.2933216	0.07605851	0.42113893	0.40264675	Abl1	1
GO:0002679	respiratory burst involved in defense response	1/95	12/14464	0.08333333	12.6877193	3.2933216	0.07605851	0.42113893	0.40264675	Trem2	1
GO:0008063	Toll signaling pathway	1/95	12/14464	0.08333333	12.6877193	3.2933216	0.07605851	0.42113893	0.40264675	Peli1	1
GO:0009070	serine family amino acid biosynthetic process	1/95	12/14464	0.08333333	12.6877193	3.2933216	0.07605851	0.42113893	0.40264675	Tha1	1
GO:0018342	protein prenylation	1/95	12/14464	0.08333333	12.6877193	3.2933216	0.07605851	0.42113893	0.40264675	Agrn	1
GO:0021924	cell proliferation in external granule layer	1/95	12/14464	0.08333333	12.6877193	3.2933216	0.07605851	0.42113893	0.40264675	Rere	1
GO:0021930	cerebellar granule cell precursor proliferation	1/95	12/14464	0.08333333	12.6877193	3.2933216	0.07605851	0.42113893	0.40264675	Rere	1
GO:0032490	detection of molecule of bacterial origin	1/95	12/14464	0.08333333	12.6877193	3.2933216	0.07605851	0.42113893	0.40264675	Trem2	1
GO:0032494	response to peptidoglycan	1/95	12/14464	0.08333333	12.6877193	3.2933216	0.07605851	0.42113893	0.40264675	Trem2	1
GO:0034139	regulation of toll-like receptor 3 signaling pathway	1/95	12/14464	0.08333333	12.6877193	3.2933216	0.07605851	0.42113893	0.40264675	Peli1	1
GO:0034145	positive regulation of toll-like receptor 4 signaling pathway	1/95	12/14464	0.08333333	12.6877193	3.2933216	0.07605851	0.42113893	0.40264675	Peli1	1
GO:0034638	phosphatidylcholine catabolic process	1/95	12/14464	0.08333333	12.6877193	3.2933216	0.07605851	0.42113893	0.40264675	Pnpla7	1
GO:0035791	platelet-derived growth factor receptor-beta signaling pathway	1/95	12/14464	0.08333333	12.6877193	3.2933216	0.07605851	0.42113893	0.40264675	Abl1	1
GO:0036006	cellular response to macrophage colony-stimulating factor stimulus	1/95	12/14464	0.08333333	12.6877193	3.2933216	0.07605851	0.42113893	0.40264675	Trem2	1
GO:0042118	endothelial cell activation	1/95	12/14464	0.08333333	12.6877193	3.2933216	0.07605851	0.42113893	0.40264675	Tcim	1
GO:0044725	epigenetic programming in the zygotic pronuclei	1/95	12/14464	0.08333333	12.6877193	3.2933216	0.07605851	0.42113893	0.40264675	Tet3	1
GO:0046512	sphingosine biosynthetic process	1/95	12/14464	0.08333333	12.6877193	3.2933216	0.07605851	0.42113893	0.40264675	Acer2	1
GO:0046520	sphingoid biosynthetic process	1/95	12/14464	0.08333333	12.6877193	3.2933216	0.07605851	0.42113893	0.40264675	Acer2	1
GO:0046886	positive regulation of hormone biosynthetic process	1/95	12/14464	0.08333333	12.6877193	3.2933216	0.07605851	0.42113893	0.40264675	Por	1
GO:0048291	isotype switching to IgG isotypes	1/95	12/14464	0.08333333	12.6877193	3.2933216	0.07605851	0.42113893	0.40264675	Paxip1	1
GO:0051549	positive regulation of keratinocyte migration	1/95	12/14464	0.08333333	12.6877193	3.2933216	0.07605851	0.42113893	0.40264675	Serpine1	1
GO:0060020	Bergmann glial cell differentiation	1/95	12/14464	0.08333333	12.6877193	3.2933216	0.07605851	0.42113893	0.40264675	Abl1	1
GO:0061517	macrophage proliferation	1/95	12/14464	0.08333333	12.6877193	3.2933216	0.07605851	0.42113893	0.40264675	Trem2	1
GO:0071294	cellular response to zinc ion	1/95	12/14464	0.08333333	12.6877193	3.2933216	0.07605851	0.42113893	0.40264675	Mt1	1
GO:0071476	cellular hypotonic response	1/95	12/14464	0.08333333	12.6877193	3.2933216	0.07605851	0.42113893	0.40264675	Clcn2	1
GO:0090232	positive regulation of spindle checkpoint	1/95	12/14464	0.08333333	12.6877193	3.2933216	0.07605851	0.42113893	0.40264675	Xrcc3	1
GO:0090267	positive regulation of mitotic cell cycle spindle assembly checkpoint	1/95	12/14464	0.08333333	12.6877193	3.2933216	0.07605851	0.42113893	0.40264675	Xrcc3	1
GO:0090385	phagosome-lysosome fusion	1/95	12/14464	0.08333333	12.6877193	3.2933216	0.07605851	0.42113893	0.40264675	Rab34	1
GO:0090399	replicative senescence	1/95	12/14464	0.08333333	12.6877193	3.2933216	0.07605851	0.42113893	0.40264675	Serpine1	1
GO:0097354	prenylation	1/95	12/14464	0.08333333	12.6877193	3.2933216	0.07605851	0.42113893	0.40264675	Agrn	1
GO:0150079	negative regulation of neuroinflammatory response	1/95	12/14464	0.08333333	12.6877193	3.2933216	0.07605851	0.42113893	0.40264675	Trem2	1
GO:1904396	regulation of neuromuscular junction development	1/95	12/14464	0.08333333	12.6877193	3.2933216	0.07605851	0.42113893	0.40264675	Agrn	1
GO:1905907	negative regulation of amyloid fibril formation	1/95	12/14464	0.08333333	12.6877193	3.2933216	0.07605851	0.42113893	0.40264675	Trem2	1
GO:1990253	cellular response to leucine starvation	1/95	12/14464	0.08333333	12.6877193	3.2933216	0.07605851	0.42113893	0.40264675	Sar1b	1
GO:2000322	regulation of glucocorticoid receptor signaling pathway	1/95	12/14464	0.08333333	12.6877193	3.2933216	0.07605851	0.42113893	0.40264675	Per1	1
GO:0045580	regulation of T cell differentiation	3/95	151/14464	0.01986755	3.02488672	2.03376886	0.07721464	0.42113893	0.40264675	Gimap5/Abl1/Arid2	3
GO:0042698	ovulation cycle	2/95	70/14464	0.02857143	4.35007519	2.28449677	0.07735157	0.42113893	0.40264675	Fzd4/Gdf10	2
GO:0045670	regulation of osteoclast differentiation	2/95	70/14464	0.02857143	4.35007519	2.28449677	0.07735157	0.42113893	0.40264675	Trem2/Lrrc17	2
GO:0032886	regulation of microtubule-based process	4/95	245/14464	0.01632653	2.48575725	1.90710277	0.07778541	0.42113893	0.40264675	Abl1/Agrn/Nav3/Xrcc3	4
GO:0045860	positive regulation of protein kinase activity	4/95	246/14464	0.01626016	2.47565255	1.89806086	0.07869124	0.42113893	0.40264675	Fzd4/Tcim/Abl1/Agrn	4
GO:0009410	response to xenobiotic stimulus	5/95	348/14464	0.01436782	2.18753781	1.8233035	0.07900682	0.42113893	0.40264675	Serpine1/Acer2/Gsta4/Abl1/Lyn	5
GO:1904377	positive regulation of protein localization to cell periphery	2/95	71/14464	0.02816901	4.28880652	2.25875725	0.07926416	0.42113893	0.40264675	Trem2/Myo5b	2
GO:0034767	positive regulation of monoatomic ion transmembrane transport	3/95	153/14464	0.01960784	2.98534572	2.00735688	0.07961334	0.42113893	0.40264675	Trem2/Gimap5/Abl1	3
GO:0051057	positive regulation of small GTPase mediated signal transduction	2/95	72/14464	0.02777778	4.22923977	2.23348833	0.08119153	0.42113893	0.40264675	Net1/Agrn	2
GO:0070373	negative regulation of ERK1 and ERK2 cascade	2/95	72/14464	0.02777778	4.22923977	2.23348833	0.08119153	0.42113893	0.40264675	Abl1/Lyn	2
GO:0051962	positive regulation of nervous system development	5/95	351/14464	0.01424501	2.16884091	1.80250722	0.08127071	0.42113893	0.40264675	Fzd4/Myo5b/Clcn2/Agrn/Lyn	5
GO:0001821	histamine secretion	1/95	13/14464	0.07692308	11.7117409	3.14166959	0.08213202	0.42113893	0.40264675	Lyn	1
GO:0006271	DNA strand elongation involved in DNA replication	1/95	13/14464	0.07692308	11.7117409	3.14166959	0.08213202	0.42113893	0.40264675	Pole	1
GO:0021534	cell proliferation in hindbrain	1/95	13/14464	0.07692308	11.7117409	3.14166959	0.08213202	0.42113893	0.40264675	Rere	1
GO:0023035	CD40 signaling pathway	1/95	13/14464	0.07692308	11.7117409	3.14166959	0.08213202	0.42113893	0.40264675	Trem2	1
GO:0032770	positive regulation of monooxygenase activity	1/95	13/14464	0.07692308	11.7117409	3.14166959	0.08213202	0.42113893	0.40264675	Por	1
GO:0032823	regulation of natural killer cell differentiation	1/95	13/14464	0.07692308	11.7117409	3.14166959	0.08213202	0.42113893	0.40264675	Il15ra	1
GO:0033690	positive regulation of osteoblast proliferation	1/95	13/14464	0.07692308	11.7117409	3.14166959	0.08213202	0.42113893	0.40264675	Abl1	1

GO:0036005	response to macrophage colony-stimulating factor	1/95	13/14464	0.07692308	11.7117409	3.14166959	0.08213202	0.42113893	0.40264675	Trem2	1
GO:0043605	amide catabolic process	1/95	13/14464	0.07692308	11.7117409	3.14166959	0.08213202	0.42113893	0.40264675	Pipox	1
GO:0050847	progesterone receptor signaling pathway	1/95	13/14464	0.07692308	11.7117409	3.14166959	0.08213202	0.42113893	0.40264675	Klf9	1
GO:0051547	regulation of keratinocyte migration	1/95	13/14464	0.07692308	11.7117409	3.14166959	0.08213202	0.42113893	0.40264675	Serpine1	1
GO:0060368	regulation of Fc receptor mediated stimulatory signaling pathway	1/95	13/14464	0.07692308	11.7117409	3.14166959	0.08213202	0.42113893	0.40264675	Lyn	1
GO:0060623	regulation of chromosome condensation	1/95	13/14464	0.07692308	11.7117409	3.14166959	0.08213202	0.42113893	0.40264675	Ncapd3	1
GO:0061687	detoxification of inorganic compound	1/95	13/14464	0.07692308	11.7117409	3.14166959	0.08213202	0.42113893	0.40264675	Mt1	1
GO:0070341	fat cell proliferation	1/95	13/14464	0.07692308	11.7117409	3.14166959	0.08213202	0.42113893	0.40264675	Trem2	1
GO:2000095	regulation of Wnt signaling pathway, planar cell polarity pathway	1/95	13/14464	0.07692308	11.7117409	3.14166959	0.08213202	0.42113893	0.40264675	Abl1	1
GO:0019221	cytokine-mediated signaling pathway	5/95	353/14464	0.01416431	2.15655286	1.78875831	0.08279924	0.42113893	0.40264675	Fzd4/Trem2/Il15ra/Rps6ka5/Lyn	5
GO:0032092	positive regulation of protein binding	2/95	73/14464	0.02739726	4.17130497	2.20867429	0.08313341	0.42113893	0.40264675	Abl1/Agrn	2
GO:0072676	lymphocyte migration	2/95	73/14464	0.02739726	4.17130497	2.20867429	0.08313341	0.42113893	0.40264675	Abl1/Jam2	2
GO:0002822	regulation of adaptive immune response based on somatic recombination of immune receptors built from	3/95	156/14464	0.01923077	2.92793522	1.96853434	0.08327201	0.42113893	0.40264675	Trem2/Gimap5/Paxip1	3
GO:0002753	cytoplasmic pattern recognition receptor signaling pathway	3/95	157/14464	0.01910828	2.90928595	1.95579908	0.08450753	0.42113893	0.40264675	Trem2/Alpk1/Peli1	3
GO:0062207	regulation of pattern recognition receptor signaling pathway	3/95	157/14464	0.01910828	2.90928595	1.95579908	0.08450753	0.42113893	0.40264675	Trem2/Peli1/Lyn	3
GO:0002312	B cell activation involved in immune response	2/95	74/14464	0.02702703	4.11493599	2.18430014	0.08508955	0.42113893	0.40264675	Abl1/Paxip1	2
GO:0014009	glial cell proliferation	2/95	74/14464	0.02702703	4.11493599	2.18430014	0.08508955	0.42113893	0.40264675	Trem2/Lyn	2
GO:0051279	regulation of release of sequestered calcium ion into cytosol	2/95	74/14464	0.02702703	4.11493599	2.18430014	0.08508955	0.42113893	0.40264675	Abl1/Lyn	2
GO:0051298	centrosome duplication	2/95	74/14464	0.02702703	4.11493599	2.18430014	0.08508955	0.42113893	0.40264675	Ccdc57/Xrcc3	2
GO:0051304	chromosome separation	2/95	74/14464	0.02702703	4.11493599	2.18430014	0.08508955	0.42113893	0.40264675	Xrcc3/Ncapd3	2
GO:0070301	cellular response to hydrogen peroxide	2/95	74/14464	0.02702703	4.11493599	2.18430014	0.08508955	0.42113893	0.40264675	Net1/Abl1	2
GO:0097553	calcium ion transmembrane import into cytosol	3/95	159/14464	0.01886792	2.87269116	1.93062762	0.0870022	0.42113893	0.40264675	Gimap5/Abl1/Lyn	3
GO:0006493	protein O-linked glycosylation	2/95	75/14464	0.02666667	4.06007018	2.16035158	0.08705966	0.42113893	0.40264675	Galnt15/Tet3	2
GO:0050672	negative regulation of lymphocyte proliferation	2/95	75/14464	0.02666667	4.06007018	2.16035158	0.08705966	0.42113893	0.40264675	Peli1/Lyn	2
GO:0150076	neuroinflammatory response	2/95	75/14464	0.02666667	4.06007018	2.16035158	0.08705966	0.42113893	0.40264675	Trem2/Lyn	2
GO:0002862	negative regulation of inflammatory response to antigenic stimulus	1/95	14/14464	0.07142857	10.875188	3.0057523	0.08816603	0.42113893	0.40264675	Trem2	1
GO:0006474	N-terminal protein amino acid acetylation	1/95	14/14464	0.07142857	10.875188	3.0057523	0.08816603	0.42113893	0.40264675	Naa50	1
GO:0006700	C21-steroid hormone biosynthetic process	1/95	14/14464	0.07142857	10.875188	3.0057523	0.08816603	0.42113893	0.40264675	Clcn2	1
GO:0006971	hypotonic response	1/95	14/14464	0.07142857	10.875188	3.0057523	0.08816603	0.42113893	0.40264675	Clcn2	1
GO:0010738	regulation of protein kinase A signaling	1/95	14/14464	0.07142857	10.875188	3.0057523	0.08816603	0.42113893	0.40264675	Tcim	1
GO:0010755	regulation of plasminogen activation	1/95	14/14464	0.07142857	10.875188	3.0057523	0.08816603	0.42113893	0.40264675	Serpine1	1
GO:0010832	negative regulation of myotube differentiation	1/95	14/14464	0.07142857	10.875188	3.0057523	0.08816603	0.42113893	0.40264675	Bhlhe41	1
GO:0010889	regulation of sequestering of triglyceride	1/95	14/14464	0.07142857	10.875188	3.0057523	0.08816603	0.42113893	0.40264675	Trem2	1
GO:0042541	hemoglobin biosynthetic process	1/95	14/14464	0.07142857	10.875188	3.0057523	0.08816603	0.42113893	0.40264675	Lyn	1
GO:0045586	regulation of gamma-delta T cell differentiation	1/95	14/14464	0.07142857	10.875188	3.0057523	0.08816603	0.42113893	0.40264675	Gimap5	1
GO:0046643	regulation of gamma-delta T cell activation	1/95	14/14464	0.07142857	10.875188	3.0057523	0.08816603	0.42113893	0.40264675	Gimap5	1
GO:0048102	autophagic cell death	1/95	14/14464	0.07142857	10.875188	3.0057523	0.08816603	0.42113893	0.40264675	Trem2	1
GO:0048755	branching morphogenesis of a nerve	1/95	14/14464	0.07142857	10.875188	3.0057523	0.08816603	0.42113893	0.40264675	Rere	1
GO:0051124	synaptic assembly at neuromuscular junction	1/95	14/14464	0.07142857	10.875188	3.0057523	0.08816603	0.42113893	0.40264675	Agrn	1
GO:0051451	myoblast migration	1/95	14/14464	0.07142857	10.875188	3.0057523	0.08816603	0.42113893	0.40264675	Net1	1
GO:0061762	CAMKK-AMPK signaling cascade	1/95	14/14464	0.07142857	10.875188	3.0057523	0.08816603	0.42113893	0.40264675	Trem2	1
GO:0071280	cellular response to copper ion	1/95	14/14464	0.07142857	10.875188	3.0057523	0.08816603	0.42113893	0.40264675	Mt1	1
GO:0071895	odontoblast differentiation	1/95	14/14464	0.07142857	10.875188	3.0057523	0.08816603	0.42113893	0.40264675	Serpine1	1
GO:0150011	regulation of neuron projection arborization	1/95	14/14464	0.07142857	10.875188	3.0057523	0.08816603	0.42113893	0.40264675	Fzd4	1
GO:1900221	regulation of amyloid-beta clearance	1/95	14/14464	0.07142857	10.875188	3.0057523	0.08816603	0.42113893	0.40264675	Trem2	1
GO:1900226	negative regulation of NLRP3 inflammasome complex assembly	1/95	14/14464	0.07142857	10.875188	3.0057523	0.08816603	0.42113893	0.40264675	Trem2	1
GO:1900272	negative regulation of long-term synaptic potentiation	1/95	14/14464	0.07142857	10.875188	3.0057523	0.08816603	0.42113893	0.40264675	Abl1	1
GO:1901018	positive regulation of potassium ion transmembrane transporter activity	1/95	14/14464	0.07142857	10.875188	3.0057523	0.08816603	0.42113893	0.40264675	Trem2	1
GO:1903977	positive regulation of glial cell migration	1/95	14/14464	0.07142857	10.875188	3.0057523	0.08816603	0.42113893	0.40264675	Trem2	1
GO:1904393	regulation of skeletal muscle acetylcholine-gated channel clustering	1/95	14/14464	0.07142857	10.875188	3.0057523	0.08816603	0.42113893	0.40264675	Agrn	1
GO:2001171	positive regulation of ATP biosynthetic process	1/95	14/14464	0.07142857	10.875188	3.0057523	0.08816603	0.42113893	0.40264675	Trem2	1
GO:0070507	regulation of microtubule cytoskeleton organization	3/95	160/14464	0.01875	2.85473684	1.91818841	0.08826125	0.42113893	0.40264675	Abl1/Agrn/Nav3	3
GO:0006809	nitric oxide biosynthetic process	2/95	76/14464	0.02631579	4.0066482	2.13681498	0.08904349	0.42113893	0.40264675	Gimap5/Por	2
GO:0032945	negative regulation of mononuclear cell proliferation	2/95	76/14464	0.02631579	4.0066482	2.13681498	0.08904349	0.42113893	0.40264675	Peli1/Lyn	2
GO:0002695	negative regulation of leukocyte activation	3/95	161/14464	0.01863354	2.83700556	1.90584493	0.08952803	0.42113893	0.40264675	Gimap5/Peli1/Lyn	3
GO:0006469	negative regulation of protein kinase activity	3/95	161/14464	0.01863354	2.83700556	1.90584493	0.08952803	0.42113893	0.40264675	Inca1/Abl1/Lyn	3
GO:0002218	activation of innate immune response	4/95	258/14464	0.01550388	2.36050592	1.79288365	0.08997122	0.42113893	0.40264675	Trem2/Alpk1/Peli1/Lyn	4
GO:0016042	lipid catabolic process	4/95	259/14464	0.01544402	2.35139199	1.78438403	0.09094488	0.42113893	0.40264675	Gimap5/Acer2/Pnpla7/Gpcpd1	4
GO:1901379	regulation of potassium ion transmembrane transport	2/95	77/14464	0.02597403	3.95461381	2.11367732	0.09104078	0.42113893	0.40264675	Trem2/Agrn	2
GO:0002285	lymphocyte activation involved in immune response	3/95	163/14464	0.01840491	2.80219567	1.88143953	0.0920846	0.42113893	0.40264675	Trem2/Abl1/Paxip1	3
GO:0032649	regulation of type II interferon production	2/95	78/14464	0.02564103	3.90391363	2.09092614	0.09305127	0.42113893	0.40264675	Gimap5/Abl1	2
GO:0035023	regulation of Rho protein signal transduction	2/95	78/14464	0.02564103	3.90391363	2.09092614	0.09305127	0.42113893	0.40264675	Net1/Abl1	2
GO:0050871	positive regulation of B cell activation	2/95	78/14464	0.02564103	3.90391363	2.09092614	0.09305127	0.42113893	0.40264675	Peli1/Paxip1	2
GO:0000722	telomere maintenance via recombination	1/95	15/14464	0.06666667	10.1501754	2.88292855	0.09416079	0.42113893	0.40264675	Xrcc3	1
GO:0001553	luteinization	1/95	15/14464	0.06666667	10.1501754	2.88292855	0.09416079	0.42113893	0.40264675	Fzd4	1
GO:0002431	Fc receptor mediated stimulatory signaling pathway	1/95	15/14464	0.06666667	10.1501754	2.88292855	0.09416079	0.42113893	0.40264675	Lyn	1
GO:0007076	mitotic chromosome condensation	1/95	15/14464	0.06666667	10.1501754	2.88292855	0.09416079	0.42113893	0.40264675	Ncapd3	1
GO:0010172	embryonic body morphogenesis	1/95	15/14464	0.06666667	10.1501754	2.88292855	0.09416079	0.42113893	0.40264675	Zfp281	1
GO:0010524	positive regulation of calcium ion transport into cytosol	1/95	15/14464	0.06666667	10.1501754	2.88292855	0.09416079	0.42113893	0.40264675	Gimap5	1
GO:0010885	regulation of cholesterol storage	1/95	15/14464	0.06666667	10.1501754	2.88292855	0.09416079	0.42113893	0.40264675	Trem2	1
GO:0010984	regulation of lipoprotein particle clearance	1/95	15/14464	0.06666667	10.1501754	2.88292855	0.09416079	0.42113893	0.40264675	Trem2	1
GO:0021932	hindbrain radial glia guided cell migration	1/95	15/14464	0.06666667	10.1501754	2.88292855	0.09416079	0.42113893	0.40264675	Rere	1
GO:0032488	Cdc42 protein signal transduction	1/95	15/14464	0.06666667	10.1501754	2.88292855	0.09416079	0.42113893	0.40264675	Abl1	1
GO:0042492	gamma-delta T cell differentiation	1/95	15/14464	0.06666667	10.1501754	2.88292855	0.09416079	0.42113893	0.40264675	Gimap5	1
GO:0045542	positive regulation of cholesterol biosynthetic process	1/95	15/14464	0.06666667	10.1501754	2.88292855	0.09416079	0.42113893	0.40264675	Por	1
GO:0051546	keratinocyte migration	1/95	15/14464	0.06666667	10.1501754	2.88292855	0.09416079	0.42113893	0.40264675	Serpine1	1
GO:0099532	synaptic vesicle endosomal processing	1/95	15/14464	0.06666667	10.1501754	2.88292855	0.09416079	0.42113893	0.40264675	Myo5b	1
GO:0106120	positive regulation of sterol biosynthetic process	1/95	15/14464	0.06666667	10.1501754	2.88292855	0.09416079	0.42113893	0.40264675	Por	1
GO:1900424	regulation of defense response to bacterium	1/95	15/14464	0.06666667	10.1501754	2.88292855	0.09416079	0.42113893	0.40264675	Rab34	1
GO:1901978	positive regulation of cell cycle checkpoint	1/95	15/14464	0.06666667	10.1501754	2.88292855	0.09416079	0.42113893	0.40264675	Xrcc3	1
GO:1903543	positive regulation of exosomal secretion	1/95	15/14464	0.06666667	10.1501754	2.88292855	0.09416079	0.42113893	0.40264675	Myo5b	1
GO:2000052	positive regulation of non-canonical Wnt signaling pathway	1/95	15/14464	0.06666667	10.1501754	2.88292855	0.09416079	0.42113893	0.40264675	Abl1	1
GO:0002028	regulation of sodium ion transport	2/95	79/14464	0.02531646	3.854497	2.06854954	0.09507471	0.42113893	0.40264675	Per1/Agrn	2
GO:0009063	amino acid catabolic process	2/95	79/14464	0.02531646	3.854497	2.06854954	0.09507471	0.42113893	0.40264675	Pipox/Tha1	2
GO:0014015	positive regulation of gliogenesis	2/95	79/14464	0.02531646	3.854497	2.06854954	0.09507471	0.42113893	0.40264675	Clcn2/Lyn	2
GO:0032418	lysosome localization	2/95	79/14464	0.02531646	3.854497	2.06854954	0.09507471	0.42113893	0.40264675	Rab34/Lyn	2
GO:0035914	skeletal muscle cell differentiation	2/95	79/14464	0.02531646	3.854497	2.06854954	0.09507471	0.42113893	0.40264675	Nr1d2/Hlf	2
GO:1990849	vacuolar localization	2/95	79/14464	0.02531646	3.854497	2.06854954	0.09507471	0.42113893	0.40264675	Rab34/Lyn	2
GO:0030071	regulation of mitotic metaphase/anaphase transition	2/95	80/14464	0.025	3.80631579	2.04653615	0.09711083	0.42113893	0.40264675	Xrcc3/Arid2	2
GO:0045685	regulation of glial cell differentiation	2/95	80/14464	0.025	3.80631579	2.04653615	0.09711083	0.42113893	0.40264675	Trem2/Clcn2	2
GO:0045807	positive regulation of endocytosis	3/95	167/14464	0.01796407	2.73507721	1.83371698	0.09728821	0.42113893	0.40264675	Trem2/Serpine1/Il15ra	3
GO:1903169	regulation of calcium ion transmembrane transport	3/95	167/14464	0.01796407	2.73507721	1.83371698	0.09728821	0.42113893	0.40264675	Gimap5/Abl1/Lyn	3
GO:0045638	negative regulation of myeloid cell differentiation	2/95	81/14464	0.02469136	3.75932424	2.02487505	0.0991594	0.42113893	0.40264675	Lrrc17/Lyn	2
GO:0046209	nitric oxide metabolic process	2/95	81/14464	0.02469136	3.75932424	2.02487505	0.0991594	0.42113893	0.40264675	Gimap5/Por	2
GO:0070664	negative regulation of leukocyte proliferation	2/95	81/14464	0.02469136	3.75932424	2.02487505	0.0991594	0.42113893	0.40264675	Peli1/Lyn	2
GO:1904427	positive regulation of calcium ion transmembrane transport	2/95	81/14464	0.02469136	3.75932424	2.02487505	0.0991594	0.42113893	0.40264675	Gimap5/Abl1	2
GO:0045088	regulation of innate immune response	5/95	374/14464	0.01336898	2.03546299	1.64965303	0.09976533	0.42113893	0.40264675	Trem2/Gimap5/Alpk1/Peli1/Lyn	5
GO:0009755	hormone-mediated signaling pathway	3/95	169/14464	0.01775148	2.70270944	1.81037915	0.09993438	0.42113893	0.40264675	Per1/Nr1d2/Klf9	3
GO:0002577	regulation of antigen processing and presentation	1/95	16/14464	0.0625	9.51578947	2.7711419	0.10011654	0.42113893	0.40264675	Trem2	1
GO:0006991	response to sterol depletion	1/95	16/14464	0.0625	9.51578947	2.7711419	0.10011654	0.42113893	0.40264675	Lyn	1
GO:0010766	negative regulation of sodium ion transport	1/95	16/14464	0.0625	9.51578947	2.7711419	0.10011654	0.42113893	0.40264675	Agrn	1
GO:0030449	regulation of complement activation	1/95	16/14464	0.0625	9.51578947	2.7711419	0.10011654	0.42113893	0.40264675	Trem2	1
GO:0030730	sequestering of triglyceride	1/95	16/14464	0.0625	9.51578947	2.7711419	0.10011654	0.42113893	0.40264675	Trem2	1
GO:0030810	positive regulation of nucleotide biosynthetic process	1/95	16/14464	0.0625	9.51578947	2.7711419	0.10011654	0.42113893	0.40264675	Trem2	1
GO:0031987	locomotion involved in locomotory behavior	1/95	16/14464	0.0625	9.51578947	2.7711419	0.10011654	0.42113893	0.40264675	Fzd4	1
GO:0032328	alanine transport	1/95	16/14464	0.0625	9.51578947	2.7711419	0.10011654	0.42113893	0.40264675	Slc6a6	1
GO:0034350	regulation of glial cell apoptotic process	1/95	16/14464	0.0625	9.51578947	2.7711419	0.10011654	0.42113893	0.40264675	Trem2	1
GO:0042921	nuclear receptor-mediated glucocorticoid signaling pathway	1/95	16/14464	0.0625	9.51578947	2.7711419	0.10011654	0.42113893	0.40264675	Per1	1
GO:0042953	lipoprotein transport	1/95	16/14464	0.0625	9.51578947	2.7711419	0.10011654	0.42113893	0.40264675	Sar1b	1
GO:0045954	positive regulation of natural killer cell mediated cytotoxicity	1/95	16/14464	0.0625	9.51578947	2.7711419	0.10011654	0.42113893	0.40264675	Gimap5	1
GO:0046514	ceramide catabolic process	1/95	16/14464	0.0625	9.51578947	2.7711419	0.10011654	0.42113893	0.40264675	Acer2	1
GO:0048712	negative regulation of astrocyte differentiation	1/95	16/14464	0.0625	9.51578947	2.7711419	0.10011654	0.42113893	0.40264675	Trem2	1
GO:0050855	regulation of B cell receptor signaling pathway	1/95	16/14464	0.0625	9.51578947	2.7711419	0.10011654	0.42113893	0.40264675	Lyn	1
GO:0051608	histamine transport	1/95	16/14464	0.0625	9.51578947	2.7711419	0.10011654	0.42113893	0.40264675	Lyn	1
GO:0060100	positive regulation of phagocytosis, engulfment	1/95	16/14464	0.0625	9.51578947	2.7711419	0.10011654	0.42113893	0.40264675	Trem2	1
GO:1900373	positive regulation of purine nucleotide biosynthetic process	1/95	16/14464	0.0625	9.51578947	2.7711419	0.10011654	0.42113893	0.40264675	Trem2	1
GO:1901017	negative regulation of potassium ion transmembrane transporter activity	1/95	16/14464	0.0625	9.51578947	2.7711419	0.10011654	0.42113893	0.40264675	Agrn	1
GO:1905155	positive regulation of membrane invagination	1/95	16/14464	0.0625	9.51578947	2.7711419	0.10011654	0.42113893	0.40264675	Trem2	1
GO:1905906	regulation of amyloid fibril formation	1/95	16/14464	0.0625	9.51578947	2.7711419	0.10011654	0.42113893	0.40264675	Trem2	1
GO:0014855	striated muscle cell proliferation	2/95	82/14464	0.02439024	3.71347882	2.0035558	0.10122017	0.42113893	0.40264675	Abl1/Arid2	2
GO:1901222	regulation of non-canonical NF-kappaB signal transduction	2/95	82/14464	0.02439024	3.71347882	2.0035558	0.10122017	0.42113893	0.40264675	Trem2/Tcim	2
GO:1902099	regulation of metaphase/anaphase transition of cell cycle	2/95	82/14464	0.02439024	3.71347882	2.0035558	0.10122017	0.42113893	0.40264675	Xrcc3/Arid2	2
GO:0002819	regulation of adaptive immune response	3/95	170/14464	0.01764706	2.68681115	1.79883677	0.10126837	0.42113893	0.40264675	Trem2/Gimap5/Paxip1	3
GO:0022408	negative regulation of cell-cell adhesion	3/95	171/14464	0.01754386	2.6710988	1.78737718	0.10260955	0.42113893	0.40264675	Gimap5/Abl1/Peli1	3
GO:2001057	reactive nitrogen species metabolic process	2/95	83/14464	0.02409639	3.66873811	1.98256842	0.10329288	0.42113893	0.40264675	Gimap5/Por	2
GO:0009101	glycoprotein biosynthetic process	4/95	272/14464	0.01470588	2.23900929	1.67731618	0.10405985	0.42113893	0.40264675	Acer2/Galnt15/Tet3/Alg10b	4
GO:0010001	glial cell differentiation	4/95	272/14464	0.01470588	2.23900929	1.67731618	0.10405985	0.42113893	0.40264675	Trem2/Clcn2/Abl1/Lyn	4
GO:0002757	immune response-activating signaling pathway	5/95	380/14464	0.01315789	2.0033241	1.61156474	0.10491422	0.42113893	0.40264675	Trem2/Alpk1/Abl1/Peli1/Lyn	5
GO:0033673	negative regulation of kinase activity	3/95	173/14464	0.01734104	2.64021904	1.76470175	0.10531327	0.42113893	0.40264675	Inca1/Abl1/Lyn	3
GO:0032526	response to retinoic acid	2/95	84/14464	0.02380952	3.62506266	1.9619033	0.10537731	0.42113893	0.40264675	Acer2/Lyn	2
GO:0001845	phagolysosome assembly	1/95	17/14464	0.05882353	8.95603715	2.66876387	0.10603355	0.42113893	0.40264675	Rab34	1
GO:0002281	macrophage activation involved in immune response	1/95	17/14464	0.05882353	8.95603715	2.66876387	0.10603355	0.42113893	0.40264675	Trem2	1
GO:0002327	immature B cell differentiation	1/95	17/14464	0.05882353	8.95603715	2.66876387	0.10603355	0.42113893	0.40264675	Abl1	1
GO:0006337	nucleosome disassembly	1/95	17/14464	0.05882353	8.95603715	2.66876387	0.10603355	0.42113893	0.40264675	Arid2	1
GO:0032352	positive regulation of hormone metabolic process	1/95	17/14464	0.05882353	8.95603715	2.66876387	0.10603355	0.42113893	0.40264675	Por	1
GO:0036376	sodium ion export across plasma membrane	1/95	17/14464	0.05882353	8.95603715	2.66876387	0.10603355	0.42113893	0.40264675	Agrn	1
GO:0044872	lipoprotein localization	1/95	17/14464	0.05882353	8.95603715	2.66876387	0.10603355	0.42113893	0.40264675	Sar1b	1
GO:0045019	negative regulation of nitric oxide biosynthetic process	1/95	17/14464	0.05882353	8.95603715	2.66876387	0.10603355	0.42113893	0.40264675	Gimap5	1
GO:0045187	regulation of circadian sleep/wake cycle, sleep	1/95	17/14464	0.05882353	8.95603715	2.66876387	0.10603355	0.42113893	0.40264675	Per3	1
GO:0051444	negative regulation of ubiquitin-protein transferase activity	1/95	17/14464	0.05882353	8.95603715	2.66876387	0.10603355	0.42113893	0.40264675	Abl1	1
GO:0060546	negative regulation of necroptotic process	1/95	17/14464	0.05882353	8.95603715	2.66876387	0.10603355	0.42113893	0.40264675	Peli1	1
GO:0060856	establishment of blood-brain barrier	1/95	17/14464	0.05882353	8.95603715	2.66876387	0.10603355	0.42113893	0.40264675	Fzd4	1
GO:0070486	leukocyte aggregation	1/95	17/14464	0.05882353	8.95603715	2.66876387	0.10603355	0.42113893	0.40264675	Jam2	1
GO:0098883	synapse pruning	1/95	17/14464	0.05882353	8.95603715	2.66876387	0.10603355	0.42113893	0.40264675	Trem2	1
GO:1903376	regulation of oxidative stress-induced neuron intrinsic apoptotic signaling pathway	1/95	17/14464	0.05882353	8.95603715	2.66876387	0.10603355	0.42113893	0.40264675	Trem2	1
GO:1903541	regulation of exosomal secretion	1/95	17/14464	0.05882353	8.95603715	2.66876387	0.10603355	0.42113893	0.40264675	Myo5b	1
GO:1904406	negative regulation of nitric oxide metabolic process	1/95	17/14464	0.05882353	8.95603715	2.66876387	0.10603355	0.42113893	0.40264675	Gimap5	1
GO:0006066	alcohol metabolic process	4/95	275/14464	0.01454545	2.21458373	1.6534674	0.10720433	0.42113893	0.40264675	Acer2/Por/Lss/Clcn2	4
GO:0007091	metaphase/anaphase transition of mitotic cell cycle	2/95	85/14464	0.02352941	3.58241486	1.94155124	0.1074732	0.42113893	0.40264675	Xrcc3/Arid2	2
GO:0048708	astrocyte differentiation	2/95	85/14464	0.02352941	3.58241486	1.94155124	0.1074732	0.42113893	0.40264675	Trem2/Abl1	2
GO:0032609	type II interferon production	2/95	86/14464	0.02325581	3.54075887	1.92150341	0.10958033	0.42113893	0.40264675	Gimap5/Abl1	2
GO:0001819	positive regulation of cytokine production	5/95	386/14464	0.01295337	1.97218435	1.57416234	0.11019361	0.42113893	0.40264675	Trem2/Serpine1/Gimap5/Abl1/Peli1	5
GO:0009408	response to heat	2/95	87/14464	0.02298851	3.5000605	1.90175134	0.11169846	0.42113893	0.40264675	Tcim/Lyn	2
GO:0044784	metaphase/anaphase transition of cell cycle	2/95	87/14464	0.02298851	3.5000605	1.90175134	0.11169846	0.42113893	0.40264675	Xrcc3/Arid2	2
GO:0002717	positive regulation of natural killer cell mediated immunity	1/95	18/14464	0.05555556	8.45847953	2.57448563	0.11191206	0.42113893	0.40264675	Gimap5	1
GO:0002902	regulation of B cell apoptotic process	1/95	18/14464	0.05555556	8.45847953	2.57448563	0.11191206	0.42113893	0.40264675	Lyn	1
GO:0006488	dolichol-linked oligosaccharide biosynthetic process	1/95	18/14464	0.05555556	8.45847953	2.57448563	0.11191206	0.42113893	0.40264675	Alg10b	1
GO:0006670	sphingosine metabolic process	1/95	18/14464	0.05555556	8.45847953	2.57448563	0.11191206	0.42113893	0.40264675	Acer2	1
GO:0010522	regulation of calcium ion transport into cytosol	1/95	18/14464	0.05555556	8.45847953	2.57448563	0.11191206	0.42113893	0.40264675	Gimap5	1
GO:0030889	negative regulation of B cell proliferation	1/95	18/14464	0.05555556	8.45847953	2.57448563	0.11191206	0.42113893	0.40264675	Lyn	1
GO:0031639	plasminogen activation	1/95	18/14464	0.05555556	8.45847953	2.57448563	0.11191206	0.42113893	0.40264675	Serpine1	1
GO:0031645	negative regulation of nervous system process	1/95	18/14464	0.05555556	8.45847953	2.57448563	0.11191206	0.42113893	0.40264675	Jam2	1
GO:0031958	nuclear receptor-mediated corticosteroid signaling pathway	1/95	18/14464	0.05555556	8.45847953	2.57448563	0.11191206	0.42113893	0.40264675	Per1	1
GO:0033003	regulation of mast cell activation	1/95	18/14464	0.05555556	8.45847953	2.57448563	0.11191206	0.42113893	0.40264675	Lyn	1
GO:0034383	low-density lipoprotein particle clearance	1/95	18/14464	0.05555556	8.45847953	2.57448563	0.11191206	0.42113893	0.40264675	Trem2	1
GO:0038083	peptidyl-tyrosine autophosphorylation	1/95	18/14464	0.05555556	8.45847953	2.57448563	0.11191206	0.42113893	0.40264675	Abl1	1

GO:0043011	myeloid dendritic cell differentiation	1/95	18/14464	0.05555556	8.45847953	2.57448563	0.11191206	0.42113893	0.40264675	Gimap5	1
GO:0045591	positive regulation of regulatory T cell differentiation	1/95	18/14464	0.05555556	8.45847953	2.57448563	0.11191206	0.42113893	0.40264675	Gimap5	1
GO:0060099	regulation of phagocytosis, engulfment	1/95	18/14464	0.05555556	8.45847953	2.57448563	0.11191206	0.42113893	0.40264675	Trem2	1
GO:0090026	positive regulation of monocyte chemotaxis	1/95	18/14464	0.05555556	8.45847953	2.57448563	0.11191206	0.42113893	0.40264675	Serpine1	1
GO:0090110	COPII-coated vesicle cargo loading	1/95	18/14464	0.05555556	8.45847953	2.57448563	0.11191206	0.42113893	0.40264675	Sar1b	1
GO:0098581	detection of external biotic stimulus	1/95	18/14464	0.05555556	8.45847953	2.57448563	0.11191206	0.42113893	0.40264675	Trem2	1
GO:1903911	positive regulation of receptor clustering	1/95	18/14464	0.05555556	8.45847953	2.57448563	0.11191206	0.42113893	0.40264675	Agrn	1
GO:1903978	regulation of microglial cell activation	1/95	18/14464	0.05555556	8.45847953	2.57448563	0.11191206	0.42113893	0.40264675	Trem2	1
GO:0035265	organ growth	3/95	180/14464	0.01666667	2.53754386	1.68778285	0.1149946	0.42113893	0.40264675	Por/Abl1/Arid2	3
GO:0043523	regulation of neuron apoptotic process	4/95	283/14464	0.01413428	2.15198066	1.59133898	0.11579864	0.42113893	0.40264675	Mt1/Trem2/Abl1/Agrn	4
GO:0002275	myeloid cell activation involved in immune response	2/95	89/14464	0.02247191	3.42140745	1.86310218	0.11596679	0.42113893	0.40264675	Trem2/Lyn	2
GO:0019218	regulation of steroid metabolic process	2/95	89/14464	0.02247191	3.42140745	1.86310218	0.11596679	0.42113893	0.40264675	Por/Clcn2	2
GO:0009314	response to radiation	5/95	394/14464	0.01269036	1.93213999	1.52531636	0.11743205	0.42113893	0.40264675	Per3/Per1/Bhlhe40/Net1/Paxip1	5
GO:0002507	tolerance induction	1/95	19/14464	0.05263158	8.0132964	2.48724144	0.11775232	0.42113893	0.40264675	Lyn	1
GO:0003417	growth plate cartilage development	1/95	19/14464	0.05263158	8.0132964	2.48724144	0.11775232	0.42113893	0.40264675	Por	1
GO:0010566	regulation of ketone biosynthetic process	1/95	19/14464	0.05263158	8.0132964	2.48724144	0.11775232	0.42113893	0.40264675	Clcn2	1
GO:0020027	hemoglobin metabolic process	1/95	19/14464	0.05263158	8.0132964	2.48724144	0.11775232	0.42113893	0.40264675	Lyn	1
GO:0030194	positive regulation of blood coagulation	1/95	19/14464	0.05263158	8.0132964	2.48724144	0.11775232	0.42113893	0.40264675	Serpine1	1
GO:0032332	positive regulation of chondrocyte differentiation	1/95	19/14464	0.05263158	8.0132964	2.48724144	0.11775232	0.42113893	0.40264675	Por	1
GO:0032703	negative regulation of interleukin-2 production	1/95	19/14464	0.05263158	8.0132964	2.48724144	0.11775232	0.42113893	0.40264675	Nav3	1
GO:0032780	negative regulation of ATP-dependent activity	1/95	19/14464	0.05263158	8.0132964	2.48724144	0.11775232	0.42113893	0.40264675	Agrn	1
GO:0035022	positive regulation of Rac protein signal transduction	1/95	19/14464	0.05263158	8.0132964	2.48724144	0.11775232	0.42113893	0.40264675	Agrn	1
GO:0035372	protein localization to microtubule	1/95	19/14464	0.05263158	8.0132964	2.48724144	0.11775232	0.42113893	0.40264675	Abl1	1
GO:0036480	neuron intrinsic apoptotic signaling pathway in response to oxidative stress	1/95	19/14464	0.05263158	8.0132964	2.48724144	0.11775232	0.42113893	0.40264675	Trem2	1
GO:0046629	gamma-delta T cell activation	1/95	19/14464	0.05263158	8.0132964	2.48724144	0.11775232	0.42113893	0.40264675	Gimap5	1
GO:0048207	vesicle targeting, rough ER to cis-Golgi	1/95	19/14464	0.05263158	8.0132964	2.48724144	0.11775232	0.42113893	0.40264675	Sar1b	1
GO:0048208	COPII vesicle coating	1/95	19/14464	0.05263158	8.0132964	2.48724144	0.11775232	0.42113893	0.40264675	Sar1b	1
GO:0061042	vascular wound healing	1/95	19/14464	0.05263158	8.0132964	2.48724144	0.11775232	0.42113893	0.40264675	Serpine1	1
GO:0062099	negative regulation of programmed necrotic cell death	1/95	19/14464	0.05263158	8.0132964	2.48724144	0.11775232	0.42113893	0.40264675	Peli1	1
GO:0090205	positive regulation of cholesterol metabolic process	1/95	19/14464	0.05263158	8.0132964	2.48724144	0.11775232	0.42113893	0.40264675	Por	1
GO:0090266	regulation of mitotic cell cycle spindle assembly checkpoint	1/95	19/14464	0.05263158	8.0132964	2.48724144	0.11775232	0.42113893	0.40264675	Xrcc3	1
GO:0090280	positive regulation of calcium ion import	1/95	19/14464	0.05263158	8.0132964	2.48724144	0.11775232	0.42113893	0.40264675	Serpine1	1
GO:1900006	positive regulation of dendrite development	1/95	19/14464	0.05263158	8.0132964	2.48724144	0.11775232	0.42113893	0.40264675	Abl1	1
GO:1900048	positive regulation of hemostasis	1/95	19/14464	0.05263158	8.0132964	2.48724144	0.11775232	0.42113893	0.40264675	Serpine1	1
GO:1903504	regulation of mitotic spindle checkpoint	1/95	19/14464	0.05263158	8.0132964	2.48724144	0.11775232	0.42113893	0.40264675	Xrcc3	1
GO:1905153	regulation of membrane invagination	1/95	19/14464	0.05263158	8.0132964	2.48724144	0.11775232	0.42113893	0.40264675	Trem2	1
GO:2000671	regulation of motor neuron apoptotic process	1/95	19/14464	0.05263158	8.0132964	2.48724144	0.11775232	0.42113893	0.40264675	Agrn	1
GO:0042742	defense response to bacterium	3/95	182/14464	0.01648352	2.50965876	1.66647075	0.11782119	0.42113893	0.40264675	Trem2/Serpine1/Rab34	3
GO:0071805	potassium ion transmembrane transport	3/95	182/14464	0.01648352	2.50965876	1.66647075	0.11782119	0.42113893	0.40264675	Trem2/Agrn/Alg10b	3
GO:0008589	regulation of smoothened signaling pathway	2/95	90/14464	0.02222222	3.38339181	1.84418974	0.11811655	0.42113893	0.40264675	Por/Rab34	2
GO:0048639	positive regulation of developmental growth	3/95	183/14464	0.01639344	2.49594478	1.65592065	0.11924429	0.42113893	0.40264675	Serpine1/Myo5b/Agrn	3
GO:0002764	immune response-regulating signaling pathway	5/95	397/14464	0.01259446	1.91753944	1.50729054	0.12020418	0.42113893	0.40264675	Trem2/Alpk1/Abl1/Peli1/Lyn	5
GO:0070661	leukocyte proliferation	4/95	287/14464	0.01393728	2.1219879	1.56104231	0.12020719	0.42113893	0.40264675	Trem2/Abl1/Peli1/Lyn	4
GO:0043525	positive regulation of neuron apoptotic process	2/95	91/14464	0.02197802	3.34621168	1.8255423	0.12027639	0.42113893	0.40264675	Abl1/Agrn	2
GO:0007519	skeletal muscle tissue development	3/95	184/14464	0.01630435	2.48237986	1.64543995	0.12067385	0.42113893	0.40264675	Nr1d2/Hlf/Gpcpd1	3
GO:0022407	regulation of cell-cell adhesion	5/95	398/14464	0.01256281	1.9127215	1.50131642	0.12113513	0.42113893	0.40264675	Gimap5/Abl1/Peli1/Lyn/Arid2	5
GO:0045787	positive regulation of cell cycle	4/95	288/14464	0.01388889	2.11461988	1.55354523	0.12132068	0.42113893	0.40264675	Ccdc57/Abl1/Xrcc3/Ncapd3	4
GO:0044772	mitotic cell cycle phase transition	5/95	399/14464	0.01253133	1.90792771	1.49535937	0.12206952	0.42113893	0.40264675	Wee1/Ccdc57/Pole/Xrcc3/Arid2	5
GO:0006814	sodium ion transport	3/95	185/14464	0.01621622	2.46896159	1.63502776	0.1221098	0.42113893	0.40264675	Per1/Slc6a6/Agrn	3
GO:0044242	cellular lipid catabolic process	3/95	185/14464	0.01621622	2.46896159	1.63502776	0.1221098	0.42113893	0.40264675	Acer2/Pnpla7/Gpcpd1	3
GO:0048678	response to axon injury	2/95	92/14464	0.02173913	3.30983982	1.80715289	0.12244609	0.42113893	0.40264675	Trem2/Lyn	2
GO:2000781	positive regulation of double-strand break repair	2/95	92/14464	0.02173913	3.30983982	1.80715289	0.12244609	0.42113893	0.40264675	Peli1/Arid2	2
GO:0048813	dendrite morphogenesis	3/95	186/14464	0.01612903	2.45568761	1.62468321	0.12355209	0.42113893	0.40264675	Fzd4/Myo5b/Rere	3
GO:0006958	complement activation, classical pathway	1/95	20/14464	0.05	7.61263158	2.40615338	0.12355457	0.42113893	0.40264675	Trem2	1
GO:0010878	cholesterol storage	1/95	20/14464	0.05	7.61263158	2.40615338	0.12355457	0.42113893	0.40264675	Trem2	1
GO:0010888	negative regulation of lipid storage	1/95	20/14464	0.05	7.61263158	2.40615338	0.12355457	0.42113893	0.40264675	Trem2	1
GO:0032986	protein-DNA complex disassembly	1/95	20/14464	0.05	7.61263158	2.40615338	0.12355457	0.42113893	0.40264675	Arid2	1
GO:0034312	diol biosynthetic process	1/95	20/14464	0.05	7.61263158	2.40615338	0.12355457	0.42113893	0.40264675	Acer2	1
GO:0042749	regulation of circadian sleep/wake cycle	1/95	20/14464	0.05	7.61263158	2.40615338	0.12355457	0.42113893	0.40264675	Per3	1
GO:0045736	negative regulation of cyclin-dependent protein serine/threonine kinase activity	1/95	20/14464	0.05	7.61263158	2.40615338	0.12355457	0.42113893	0.40264675	Inca1	1
GO:0046519	sphingoid metabolic process	1/95	20/14464	0.05	7.61263158	2.40615338	0.12355457	0.42113893	0.40264675	Acer2	1
GO:0048143	astrocyte activation	1/95	20/14464	0.05	7.61263158	2.40615338	0.12355457	0.42113893	0.40264675	Trem2	1
GO:0050802	circadian sleep/wake cycle, sleep	1/95	20/14464	0.05	7.61263158	2.40615338	0.12355457	0.42113893	0.40264675	Per3	1
GO:0060143	positive regulation of syncytium formation by plasma membrane fusion	1/95	20/14464	0.05	7.61263158	2.40615338	0.12355457	0.42113893	0.40264675	Trem2	1
GO:0071340	skeletal muscle acetylcholine-gated channel clustering	1/95	20/14464	0.05	7.61263158	2.40615338	0.12355457	0.42113893	0.40264675	Agrn	1
GO:0099638	endosome to plasma membrane protein transport	1/95	20/14464	0.05	7.61263158	2.40615338	0.12355457	0.42113893	0.40264675	Myo5b	1
GO:0050769	positive regulation of neurogenesis	4/95	291/14464	0.0137457	2.09281968	1.53123494	0.12468806	0.42276538	0.40420178	Fzd4/Myo5b/Clcn2/Lyn	4
GO:1903037	regulation of leukocyte cell-cell adhesion	4/95	292/14464	0.01369863	2.08565249	1.52385768	0.12581941	0.42276538	0.40420178	Gimap5/Abl1/Peli1/Arid2	4
GO:0050870	positive regulation of T cell activation	3/95	188/14464	0.01595745	2.42956327	1.60419353	0.12645547	0.42276538	0.40420178	Gimap5/Abl1/Arid2	3
GO:0030516	regulation of axon extension	2/95	94/14464	0.0212766	3.23941769	1.77112157	0.12681424	0.42276538	0.40420178	Myo5b/Abl1	2
GO:0031110	regulation of microtubule polymerization or depolymerization	2/95	94/14464	0.0212766	3.23941769	1.77112157	0.12681424	0.42276538	0.40420178	Abl1/Nav3	2
GO:0033045	regulation of sister chromatid segregation	2/95	94/14464	0.0212766	3.23941769	1.77112157	0.12681424	0.42276538	0.40420178	Xrcc3/Arid2	2
GO:0034446	substrate adhesion-dependent cell spreading	2/95	94/14464	0.0212766	3.23941769	1.77112157	0.12681424	0.42276538	0.40420178	Fzd4/Abl1	2
GO:0050727	regulation of inflammatory response	4/95	294/14464	0.01360544	2.07146438	1.50919097	0.12809529	0.42276538	0.40420178	Nr1d2/Trem2/Serpine1/Lyn	4
GO:0006754	ATP biosynthetic process	2/95	95/14464	0.02105263	3.20531856	1.75346696	0.12901224	0.42276538	0.40420178	Trem2/ND5	2
GO:0019882	antigen processing and presentation	2/95	95/14464	0.02105263	3.20531856	1.75346696	0.12901224	0.42276538	0.40420178	Trem2/Rab34	2
GO:0002495	antigen processing and presentation of peptide antigen via MHC class II	1/95	21/14464	0.04761905	7.25012531	2.33049078	0.12931906	0.42276538	0.40420178	Trem2	1
GO:0007263	nitric oxide mediated signal transduction	1/95	21/14464	0.04761905	7.25012531	2.33049078	0.12931906	0.42276538	0.40420178	Mt1	1
GO:0032768	regulation of monooxygenase activity	1/95	21/14464	0.04761905	7.25012531	2.33049078	0.12931906	0.42276538	0.40420178	Por	1
GO:0032816	positive regulation of natural killer cell activation	1/95	21/14464	0.04761905	7.25012531	2.33049078	0.12931906	0.42276538	0.40420178	Il15ra	1
GO:0042634	regulation of hair cycle	1/95	21/14464	0.04761905	7.25012531	2.33049078	0.12931906	0.42276538	0.40420178	Per1	1
GO:0045475	locomotor rhythm	1/95	21/14464	0.04761905	7.25012531	2.33049078	0.12931906	0.42276538	0.40420178	Ciart	1
GO:0045838	positive regulation of membrane potential	1/95	21/14464	0.04761905	7.25012531	2.33049078	0.12931906	0.42276538	0.40420178	Gimap5	1
GO:0090050	positive regulation of cell migration involved in sprouting angiogenesis	1/95	21/14464	0.04761905	7.25012531	2.33049078	0.12931906	0.42276538	0.40420178	Abl1	1
GO:0141086	negative regulation of inflammasome-mediated signaling pathway	1/95	21/14464	0.04761905	7.25012531	2.33049078	0.12931906	0.42276538	0.40420178	Trem2	1
GO:1900016	negative regulation of cytokine production involved in inflammatory response	1/95	21/14464	0.04761905	7.25012531	2.33049078	0.12931906	0.42276538	0.40420178	Trem2	1
GO:1901380	negative regulation of potassium ion transmembrane transport	1/95	21/14464	0.04761905	7.25012531	2.33049078	0.12931906	0.42276538	0.40420178	Agrn	1
GO:1990182	exosomal secretion	1/95	21/14464	0.04761905	7.25012531	2.33049078	0.12931906	0.42276538	0.40420178	Myo5b	1
GO:2000114	regulation of establishment of cell polarity	1/95	21/14464	0.04761905	7.25012531	2.33049078	0.12931906	0.42276538	0.40420178	Abl1	1
GO:2001169	regulation of ATP biosynthetic process	1/95	21/14464	0.04761905	7.25012531	2.33049078	0.12931906	0.42276538	0.40420178	Trem2	1
GO:0050730	regulation of peptidyl-tyrosine phosphorylation	3/95	191/14464	0.01570681	2.39140259	1.57394511	0.13085664	0.42607403	0.40736515	Trem2/Abl1/Agrn	3
GO:0048814	regulation of dendrite morphogenesis	2/95	96/14464	0.02083333	3.17192982	1.73604496	0.13121926	0.42607403	0.40736515	Fzd4/Myo5b	2
GO:0050764	regulation of phagocytosis	2/95	96/14464	0.02083333	3.17192982	1.73604496	0.13121926	0.42607403	0.40736515	Trem2/Il15ra	2
GO:1902106	negative regulation of leukocyte differentiation	2/95	96/14464	0.02083333	3.17192982	1.73604496	0.13121926	0.42607403	0.40736515	Lrrc17/Lyn	2
GO:0050863	regulation of T cell activation	4/95	297/14464	0.01346801	2.05054049	1.48740692	0.13154173	0.42639962	0.40767644	Gimap5/Abl1/Peli1/Arid2	4
GO:0043266	regulation of potassium ion transport	2/95	97/14464	0.02061856	3.13922952	1.7188498	0.13343506	0.42694125	0.40819429	Trem2/Agrn	2
GO:0006910	phagocytosis, recognition	1/95	22/14464	0.04545455	6.92057416	2.25963982	0.13504604	0.42694125	0.40819429	Trem2	1
GO:0008207	C21-steroid hormone metabolic process	1/95	22/14464	0.04545455	6.92057416	2.25963982	0.13504604	0.42694125	0.40819429	Clcn2	1
GO:0010839	negative regulation of keratinocyte proliferation	1/95	22/14464	0.04545455	6.92057416	2.25963982	0.13504604	0.42694125	0.40819429	Klf9	1
GO:0021535	cell migration in hindbrain	1/95	22/14464	0.04545455	6.92057416	2.25963982	0.13504604	0.42694125	0.40819429	Rere	1
GO:0022410	circadian sleep/wake cycle process	1/95	22/14464	0.04545455	6.92057416	2.25963982	0.13504604	0.42694125	0.40819429	Per3	1
GO:0032691	negative regulation of interleukin-1 beta production	1/95	22/14464	0.04545455	6.92057416	2.25963982	0.13504604	0.42694125	0.40819429	Trem2	1
GO:0034349	glial cell apoptotic process	1/95	22/14464	0.04545455	6.92057416	2.25963982	0.13504604	0.42694125	0.40819429	Trem2	1
GO:0035025	positive regulation of Rho protein signal transduction	1/95	22/14464	0.04545455	6.92057416	2.25963982	0.13504604	0.42694125	0.40819429	Net1	1
GO:0061298	retina vasculature development in camera-type eye	1/95	22/14464	0.04545455	6.92057416	2.25963982	0.13504604	0.42694125	0.40819429	Fzd4	1
GO:0090231	regulation of spindle checkpoint	1/95	22/14464	0.04545455	6.92057416	2.25963982	0.13504604	0.42694125	0.40819429	Xrcc3	1
GO:0097734	extracellular exosome biogenesis	1/95	22/14464	0.04545455	6.92057416	2.25963982	0.13504604	0.42694125	0.40819429	Myo5b	1
GO:0120255	olefinic compound biosynthetic process	1/95	22/14464	0.04545455	6.92057416	2.25963982	0.13504604	0.42694125	0.40819429	Clcn2	1
GO:1904030	negative regulation of cyclin-dependent protein kinase activity	1/95	22/14464	0.04545455	6.92057416	2.25963982	0.13504604	0.42694125	0.40819429	Inca1	1
GO:2000042	negative regulation of double-strand break repair via homologous recombination	1/95	22/14464	0.04545455	6.92057416	2.25963982	0.13504604	0.42694125	0.40819429	Abl1	1
GO:0045637	regulation of myeloid cell differentiation	3/95	194/14464	0.01546392	2.35442214	1.54426079	0.13531174	0.42707769	0.40832473	Trem2/Lrrc17/Lyn	3
GO:0014706	striated muscle tissue development	5/95	414/14464	0.01207729	1.8387999	1.40798524	0.13648925	0.43008682	0.41120173	Nr1d2/Hlf/Gpcpd1/Abl1/Arid2	5
GO:0045582	positive regulation of T cell differentiation	2/95	99/14464	0.02020202	3.07581074	1.68511778	0.13789224	0.43121469	0.41228008	Gimap5/Arid2	2
GO:1903432	regulation of TORC1 signaling	2/95	99/14464	0.02020202	3.07581074	1.68511778	0.13789224	0.43121469	0.41228008	Sar1b/Peli1	2
GO:0051348	negative regulation of transferase activity	3/95	196/14464	0.01530612	2.33039742	1.52477444	0.138311	0.43121469	0.41228008	Inca1/Abl1/Lyn	3
GO:0097529	myeloid leukocyte migration	3/95	196/14464	0.01530612	2.33039742	1.52477444	0.138311	0.43121469	0.41228008	Trem2/Serpine1/Lyn	3
GO:0060538	skeletal muscle organ development	3/95	197/14464	0.01522843	2.31856799	1.51512002	0.13981921	0.43121469	0.41228008	Nr1d2/Hlf/Gpcpd1	3
GO:0055017	cardiac muscle tissue growth	2/95	100/14464	0.02	3.04505263	1.66857032	0.1401332	0.43121469	0.41228008	Abl1/Arid2	2
GO:0002504	antigen processing and presentation of peptide or polysaccharide antigen via MHC class II	1/95	23/14464	0.04347826	6.61967963	2.19308046	0.14073573	0.43121469	0.41228008	Trem2	1
GO:0006312	mitotic recombination	1/95	23/14464	0.04347826	6.61967963	2.19308046	0.14073573	0.43121469	0.41228008	Xrcc3	1
GO:0007064	mitotic sister chromatid cohesion	1/95	23/14464	0.04347826	6.61967963	2.19308046	0.14073573	0.43121469	0.41228008	Naa50	1
GO:0010737	protein kinase A signaling	1/95	23/14464	0.04347826	6.61967963	2.19308046	0.14073573	0.43121469	0.41228008	Tcim	1
GO:0043032	positive regulation of macrophage activation	1/95	23/14464	0.04347826	6.61967963	2.19308046	0.14073573	0.43121469	0.41228008	Trem2	1
GO:2000773	negative regulation of cellular senescence	1/95	23/14464	0.04347826	6.61967963	2.19308046	0.14073573	0.43121469	0.41228008	Abl1	1
GO:0002688	regulation of leukocyte chemotaxis	2/95	101/14464	0.01980198	3.0149036	1.65222847	0.14238214	0.43121469	0.41228008	Serpine1/Lyn	2
GO:0048534	hematopoietic or lymphoid organ development	2/95	102/14464	0.01960784	2.98534572	1.63608737	0.14463887	0.43121469	0.41228008	Lrrc17/Abl1	2
GO:1903707	negative regulation of hemopoiesis	2/95	102/14464	0.01960784	2.98534572	1.63608737	0.14463887	0.43121469	0.41228008	Lrrc17/Lyn	2
GO:0016358	dendrite development	4/95	309/14464	0.01294498	1.97090785	1.40274923	0.14570637	0.43121469	0.41228008	Fzd4/Myo5b/Rere/Abl1	4
GO:0000302	response to reactive oxygen species	3/95	201/14464	0.01492537	2.27242734	1.47707757	0.14590791	0.43121469	0.41228008	Net1/ND5/Abl1	3
GO:0009636	response to toxic substance	3/95	201/14464	0.01492537	2.27242734	1.47707757	0.14590791	0.43121469	0.41228008	Inmt/Mt1/Lyn	3
GO:0001783	B cell apoptotic process	1/95	24/14464	0.04166667	6.34385965	2.13036879	0.1463884	0.43121469	0.41228008	Lyn	1
GO:0006882	intracellular zinc ion homeostasis	1/95	24/14464	0.04166667	6.34385965	2.13036879	0.1463884	0.43121469	0.41228008	Mt1	1
GO:0007413	axonal fasciculation	1/95	24/14464	0.04166667	6.34385965	2.13036879	0.1463884	0.43121469	0.41228008	Celsr3	1
GO:0008299	isoprenoid biosynthetic process	1/95	24/14464	0.04166667	6.34385965	2.13036879	0.1463884	0.43121469	0.41228008	Lss	1
GO:0015812	gamma-aminobutyric acid transport	1/95	24/14464	0.04166667	6.34385965	2.13036879	0.1463884	0.43121469	0.41228008	Slc6a6	1
GO:0030808	regulation of nucleotide biosynthetic process	1/95	24/14464	0.04166667	6.34385965	2.13036879	0.1463884	0.43121469	0.41228008	Trem2	1
GO:0031365	N-terminal protein amino acid modification	1/95	24/14464	0.04166667	6.34385965	2.13036879	0.1463884	0.43121469	0.41228008	Naa50	1
GO:0034138	toll-like receptor 3 signaling pathway	1/95	24/14464	0.04166667	6.34385965	2.13036879	0.1463884	0.43121469	0.41228008	Peli1	1
GO:0045540	regulation of cholesterol biosynthetic process	1/95	24/14464	0.04166667	6.34385965	2.13036879	0.1463884	0.43121469	0.41228008	Por	1
GO:0046184	aldehyde biosynthetic process	1/95	24/14464	0.04166667	6.34385965	2.13036879	0.1463884	0.43121469	0.41228008	Clcn2	1
GO:0046688	response to copper ion	1/95	24/14464	0.04166667	6.34385965	2.13036879	0.1463884	0.43121469	0.41228008	Mt1	1
GO:0050820	positive regulation of coagulation	1/95	24/14464	0.04166667	6.34385965	2.13036879	0.1463884	0.43121469	0.41228008	Serpine1	1
GO:0060544	regulation of necroptotic process	1/95	24/14464	0.04166667	6.34385965	2.13036879	0.1463884	0.43121469	0.41228008	Peli1	1
GO:0061098	positive regulation of protein tyrosine kinase activity	1/95	24/14464	0.04166667	6.34385965	2.13036879	0.1463884	0.43121469	0.41228008	Agrn	1
GO:0090330	regulation of platelet aggregation	1/95	24/14464	0.04166667	6.34385965	2.13036879	0.1463884	0.43121469	0.41228008	Lyn	1
GO:0097062	dendritic spine maintenance	1/95	24/14464	0.04166667	6.34385965	2.13036879	0.1463884	0.43121469	0.41228008	Trem2	1
GO:0106030	neuron projection fasciculation	1/95	24/14464	0.04166667	6.34385965	2.13036879	0.1463884	0.43121469	0.41228008	Celsr3	1
GO:0106118	regulation of sterol biosynthetic process	1/95	24/14464	0.04166667	6.34385965	2.13036879	0.1463884	0.43121469	0.41228008	Por	1
GO:0140112	extracellular vesicle biogenesis	1/95	24/14464	0.04166667	6.34385965	2.13036879	0.1463884	0.43121469	0.41228008	Myo5b	1
GO:1900371	regulation of purine nucleotide biosynthetic process	1/95	24/14464	0.04166667	6.34385965	2.13036879	0.1463884	0.43121469	0.41228008	Trem2	1
GO:1902993	positive regulation of amyloid precursor protein catabolic process	1/95	24/14464	0.04166667	6.34385965	2.13036879	0.1463884	0.43121469	0.41228008	Lyn	1
GO:2001014	regulation of skeletal muscle cell differentiation	1/95	24/14464	0.04166667	6.34385965	2.13036879	0.1463884	0.43121469	0.41228008	Nr1d2	1
GO:0031346	positive regulation of cell projection organization	5/95	426/14464	0.01173709	1.78700272	1.34062136	0.14855129	0.43121469	0.41228008	Fzd4/Myo5b/Abl1/Agrn/Lyn	5
GO:0031396	regulation of protein ubiquitination	3/95	204/14464	0.01470588	2.23900929	1.44913086	0.15053144	0.43121469	0.41228008	Abl1/Peli1/Paxip1	3
GO:0038061	non-canonical NF-kappaB signal transduction	2/95	105/14464	0.01904762	2.90005013	1.58882232	0.15145378	0.43121469	0.41228008	Trem2/Tcim	2
GO:0046632	alpha-beta T cell differentiation	2/95	105/14464	0.01904762	2.90005013	1.58882232	0.15145378	0.43121469	0.41228008	Gimap5/Abl1	2
GO:0003416	endochondral bone growth	1/95	25/14464	0.04	6.09010526	2.0711232	0.15200426	0.43121469	0.41228008	Por	1
GO:0009595	detection of biotic stimulus	1/95	25/14464	0.04	6.09010526	2.0711232	0.15200426	0.43121469	0.41228008	Trem2	1
GO:0010875	positive regulation of cholesterol efflux	1/95	25/14464	0.04	6.09010526	2.0711232	0.15200426	0.43121469	0.41228008	Trem2	1
GO:0010955	negative regulation of protein processing	1/95	25/14464	0.04	6.09010526	2.0711232	0.15200426	0.43121469	0.41228008	Serpine1	1
GO:0030431	sleep	1/95	25/14464	0.04	6.09010526	2.0711232	0.15200426	0.43121469	0.41228008	Per3	1
GO:0032689	negative regulation of type II interferon production	1/95	25/14464	0.04	6.09010526	2.0711232	0.15200426	0.43121469	0.41228008	Gimap5	1
GO:0035335	peptidyl-tyrosine dephosphorylation	1/95	25/14464	0.04	6.09010526	2.0711232	0.15200426	0.43121469	0.41228008	Tns2	1
GO:0045830	positive regulation of isotype switching	1/95	25/14464	0.04	6.09010526	2.0711232	0.15200426	0.43121469	0.41228008	Paxip1	1
GO:0048311	mitochondrion distribution	1/95	25/14464	0.04	6.09010526	2.0711232	0.15200426	0.43121469	0.41228008	Tspan4	1
GO:0098877	neurotransmitter receptor transport to plasma membrane	1/95	25/14464	0.04	6.09010526	2.0711232	0.15200426	0.43121469	0.41228008	Myo5b	1

GO:0099159	regulation of modification of postsynaptic structure	1/95	25/14464	0.04	6.09010526	2.0711232	0.15200426	0.43121469	0.41228008	Myo5b	1
GO:1903318	negative regulation of protein maturation	1/95	25/14464	0.04	6.09010526	2.0711232	0.15200426	0.43121469	0.41228008	Serpine1	1
GO:1903975	regulation of glial cell migration	1/95	25/14464	0.04	6.09010526	2.0711232	0.15200426	0.43121469	0.41228008	Trem2	1
GO:0000819	sister chromatid segregation	3/95	205/14464	0.01463415	2.22808729	1.43992329	0.15208317	0.43121469	0.41228008	Xrcc3/Ncapd3/Arid2	3
GO:0002253	activation of immune response	5/95	431/14464	0.01160093	1.76627183	1.31317908	0.15370915	0.43121469	0.41228008	Trem2/Alpk1/Abl1/Peli1/Lyn	5
GO:0009206	purine ribonucleoside triphosphate biosynthetic process	2/95	106/14464	0.01886792	2.87269116	1.57343875	0.15373969	0.43121469	0.41228008	Trem2/ND5	2
GO:0097061	dendritic spine organization	2/95	106/14464	0.01886792	2.87269116	1.57343875	0.15373969	0.43121469	0.41228008	Trem2/Myo5b	2
GO:1902749	regulation of cell cycle G2/M phase transition	2/95	106/14464	0.01886792	2.87269116	1.57343875	0.15373969	0.43121469	0.41228008	Wee1/Paxip1	2
GO:0043087	regulation of GTPase activity	3/95	207/14464	0.01449275	2.20655988	1.4216664	0.15520209	0.43121469	0.41228008	Net1/Adap2/Agrn	3
GO:1903039	positive regulation of leukocyte cell-cell adhesion	3/95	207/14464	0.01449275	2.20655988	1.4216664	0.15520209	0.43121469	0.41228008	Gimap5/Abl1/Arid2	3
GO:0002683	negative regulation of immune system process	5/95	433/14464	0.01154734	1.75811353	1.3023017	0.15579342	0.43121469	0.41228008	Trem2/Gimap5/Lrrc17/Peli1/Lyn	5
GO:0009145	purine nucleoside triphosphate biosynthetic process	2/95	107/14464	0.01869159	2.84584358	1.55823393	0.15603242	0.43121469	0.41228008	Trem2/ND5	2
GO:0030518	nuclear receptor-mediated steroid hormone signaling pathway	2/95	107/14464	0.01869159	2.84584358	1.55823393	0.15603242	0.43121469	0.41228008	Per1/Klf9	2
GO:0032963	collagen metabolic process	2/95	107/14464	0.01869159	2.84584358	1.55823393	0.15603242	0.43121469	0.41228008	Tns2/Serpine1	2
GO:0060419	heart growth	2/95	107/14464	0.01869159	2.84584358	1.55823393	0.15603242	0.43121469	0.41228008	Abl1/Arid2	2
GO:0006282	regulation of DNA repair	3/95	208/14464	0.01442308	2.19595142	1.41261588	0.15676918	0.43121469	0.41228008	Abl1/Peli1/Arid2	3
GO:0002861	regulation of inflammatory response to antigenic stimulus	1/95	26/14464	0.03846154	5.85587045	2.01501355	0.15758357	0.43121469	0.41228008	Trem2	1
GO:0010893	positive regulation of steroid biosynthetic process	1/95	26/14464	0.03846154	5.85587045	2.01501355	0.15758357	0.43121469	0.41228008	Por	1
GO:0030149	sphingolipid catabolic process	1/95	26/14464	0.03846154	5.85587045	2.01501355	0.15758357	0.43121469	0.41228008	Acer2	1
GO:0032878	regulation of establishment or maintenance of cell polarity	1/95	26/14464	0.03846154	5.85587045	2.01501355	0.15758357	0.43121469	0.41228008	Abl1	1
GO:0033688	regulation of osteoblast proliferation	1/95	26/14464	0.03846154	5.85587045	2.01501355	0.15758357	0.43121469	0.41228008	Abl1	1
GO:0043372	positive regulation of CD4-positive, alpha-beta T cell differentiation	1/95	26/14464	0.03846154	5.85587045	2.01501355	0.15758357	0.43121469	0.41228008	Gimap5	1
GO:0048714	positive regulation of oligodendrocyte differentiation	1/95	26/14464	0.03846154	5.85587045	2.01501355	0.15758357	0.43121469	0.41228008	Clcn2	1
GO:0050995	negative regulation of lipid catabolic process	1/95	26/14464	0.03846154	5.85587045	2.01501355	0.15758357	0.43121469	0.41228008	Gimap5	1
GO:0060055	angiogenesis involved in wound healing	1/95	26/14464	0.03846154	5.85587045	2.01501355	0.15758357	0.43121469	0.41228008	Serpine1	1
GO:0060142	regulation of syncytium formation by plasma membrane fusion	1/95	26/14464	0.03846154	5.85587045	2.01501355	0.15758357	0.43121469	0.41228008	Trem2	1
GO:0070269	pyroptotic inflammatory response	1/95	26/14464	0.03846154	5.85587045	2.01501355	0.15758357	0.43121469	0.41228008	Trem2	1
GO:0070498	interleukin-1-mediated signaling pathway	1/95	26/14464	0.03846154	5.85587045	2.01501355	0.15758357	0.43121469	0.41228008	Rps6ka5	1
GO:0071276	cellular response to cadmium ion	1/95	26/14464	0.03846154	5.85587045	2.01501355	0.15758357	0.43121469	0.41228008	Mt1	1
GO:0097049	motor neuron apoptotic process	1/95	26/14464	0.03846154	5.85587045	2.01501355	0.15758357	0.43121469	0.41228008	Agrn	1
GO:0150077	regulation of neuroinflammatory response	1/95	26/14464	0.03846154	5.85587045	2.01501355	0.15758357	0.43121469	0.41228008	Trem2	1
GO:1902042	negative regulation of extrinsic apoptotic signaling pathway via death domain receptors	1/95	26/14464	0.03846154	5.85587045	2.01501355	0.15758357	0.43121469	0.41228008	Serpine1	1
GO:1905523	positive regulation of macrophage migration	1/95	26/14464	0.03846154	5.85587045	2.01501355	0.15758357	0.43121469	0.41228008	Trem2	1
GO:0060537	muscle tissue development	5/95	435/14464	0.01149425	1.75003025	1.29148026	0.15788958	0.43121469	0.41228008	Nr1d2/Hlf/Gpcpd1/Abl1/Arid2	5
GO:0008585	female gonad development	2/95	108/14464	0.01851852	2.81949318	1.54320388	0.1583318	0.43121469	0.41228008	Fzd4/Serpine1	2
GO:0030316	osteoclast differentiation	2/95	108/14464	0.01851852	2.81949318	1.54320388	0.1583318	0.43121469	0.41228008	Trem2/Lrrc17	2
GO:0002697	regulation of immune effector process	4/95	320/14464	0.0125	1.90315789	1.3284043	0.15919557	0.43121469	0.41228008	Trem2/Gimap5/Lyn/Paxip1	4
GO:0007160	cell-matrix adhesion	3/95	210/14464	0.01428571	2.17503759	1.39466774	0.1599183	0.43121469	0.41228008	Serpine1/Acer2/Abl1	3
GO:0042391	regulation of membrane potential	5/95	437/14464	0.01144165	1.74202096	1.28071417	0.15999753	0.43121469	0.41228008	Trem2/Gimap5/Tspan4/Clcn2/Agrn	5
GO:0008037	cell recognition	2/95	109/14464	0.01834862	2.79362627	1.52834475	0.16063762	0.43121469	0.41228008	Trem2/Celsr3	2
GO:0039531	regulation of cytoplasmic pattern recognition receptor signaling pathway	2/95	109/14464	0.01834862	2.79362627	1.52834475	0.16063762	0.43121469	0.41228008	Trem2/Peli1	2
GO:0002702	positive regulation of production of molecular mediator of immune response	2/95	110/14464	0.01818182	2.76822967	1.5136528	0.16294972	0.43121469	0.41228008	Gimap5/Paxip1	2
GO:0046545	development of primary female sexual characteristics	2/95	110/14464	0.01818182	2.76822967	1.5136528	0.16294972	0.43121469	0.41228008	Fzd4/Serpine1	2
GO:0050868	negative regulation of T cell activation	2/95	110/14464	0.01818182	2.76822967	1.5136528	0.16294972	0.43121469	0.41228008	Gimap5/Peli1	2
GO:0061387	regulation of extent of cell growth	2/95	110/14464	0.01818182	2.76822967	1.5136528	0.16294972	0.43121469	0.41228008	Myo5b/Abl1	2
GO:0009100	glycoprotein metabolic process	4/95	323/14464	0.0123839	1.88548151	1.30863466	0.16295355	0.43121469	0.41228008	Acer2/Galnt15/Tet3/Alg10b	4
GO:0002699	positive regulation of immune effector process	3/95	212/14464	0.01415094	2.15451837	1.37691964	0.16308697	0.43121469	0.41228008	Trem2/Gimap5/Paxip1	3
GO:0006813	potassium ion transport	3/95	212/14464	0.01415094	2.15451837	1.37691964	0.16308697	0.43121469	0.41228008	Trem2/Agrn/Alg10b	3
GO:1905475	regulation of protein localization to membrane	3/95	212/14464	0.01415094	2.15451837	1.37691964	0.16308697	0.43121469	0.41228008	Trem2/Myo5b/Agrn	3
GO:0001773	myeloid dendritic cell activation	1/95	27/14464	0.03703704	5.63898635	1.96175253	0.16312655	0.43121469	0.41228008	Gimap5	1
GO:0001779	natural killer cell differentiation	1/95	27/14464	0.03703704	5.63898635	1.96175253	0.16312655	0.43121469	0.41228008	Il15ra	1
GO:0006376	mRNA splice site recognition	1/95	27/14464	0.03703704	5.63898635	1.96175253	0.16312655	0.43121469	0.41228008	Rbmx	1
GO:0007608	sensory perception of smell	1/95	27/14464	0.03703704	5.63898635	1.96175253	0.16312655	0.43121469	0.41228008	Or8b53	1
GO:0034311	diol metabolic process	1/95	27/14464	0.03703704	5.63898635	1.96175253	0.16312655	0.43121469	0.41228008	Acer2	1
GO:0035929	steroid hormone secretion	1/95	27/14464	0.03703704	5.63898635	1.96175253	0.16312655	0.43121469	0.41228008	Fzd4	1
GO:0045730	respiratory burst	1/95	27/14464	0.03703704	5.63898635	1.96175253	0.16312655	0.43121469	0.41228008	Trem2	1
GO:0051984	positive regulation of chromosome segregation	1/95	27/14464	0.03703704	5.63898635	1.96175253	0.16312655	0.43121469	0.41228008	Ncapd3	1
GO:0060252	positive regulation of glial cell proliferation	1/95	27/14464	0.03703704	5.63898635	1.96175253	0.16312655	0.43121469	0.41228008	Lyn	1
GO:0060977	coronary vasculature morphogenesis	1/95	27/14464	0.03703704	5.63898635	1.96175253	0.16312655	0.43121469	0.41228008	Arid2	1
GO:2000050	regulation of non-canonical Wnt signaling pathway	1/95	27/14464	0.03703704	5.63898635	1.96175253	0.16312655	0.43121469	0.41228008	Abl1	1
GO:0002274	myeloid leukocyte activation	3/95	213/14464	0.01408451	2.14440326	1.36811921	0.16467849	0.43121469	0.41228008	Trem2/Gimap5/Lyn	3
GO:0045930	negative regulation of mitotic cell cycle	3/95	213/14464	0.01408451	2.14440326	1.36811921	0.16467849	0.43121469	0.41228008	Wee1/Abl1/Xrcc3	3
GO:0009201	ribonucleoside triphosphate biosynthetic process	2/95	111/14464	0.01801802	2.74329066	1.4991244	0.16526792	0.43121469	0.41228008	Trem2/ND5	2
GO:0031398	positive regulation of protein ubiquitination	2/95	111/14464	0.01801802	2.74329066	1.4991244	0.16526792	0.43121469	0.41228008	Peli1/Paxip1	2
GO:0034764	positive regulation of transmembrane transport	3/95	214/14464	0.01401869	2.13438269	1.35936714	0.16627473	0.43121469	0.41228008	Trem2/Gimap5/Abl1	3
GO:0051052	regulation of DNA metabolic process	5/95	443/14464	0.01128668	1.71842699	1.24874204	0.16639085	0.43121469	0.41228008	Abl1/Peli1/Paxip1/Ylpm1/Arid2	5
GO:0001841	neural tube formation	2/95	112/14464	0.01785714	2.71879699	1.48475606	0.16759203	0.43121469	0.41228008	Abl1/Brpf1	2
GO:0051209	release of sequestered calcium ion into cytosol	2/95	112/14464	0.01785714	2.71879699	1.48475606	0.16759203	0.43121469	0.41228008	Abl1/Lyn	2
GO:0002443	leukocyte mediated immunity	4/95	327/14464	0.01223242	1.86241751	1.28259794	0.16801472	0.43121469	0.41228008	Trem2/Gimap5/Lyn/Paxip1	4
GO:0009069	serine family amino acid metabolic process	1/95	28/14464	0.03571429	5.43759398	1.9110888	0.16863344	0.43121469	0.41228008	Tha1	1
GO:0014850	response to muscle activity	1/95	28/14464	0.03571429	5.43759398	1.9110888	0.16863344	0.43121469	0.41228008	Tns2	1
GO:0032692	negative regulation of interleukin-1 production	1/95	28/14464	0.03571429	5.43759398	1.9110888	0.16863344	0.43121469	0.41228008	Trem2	1
GO:0034381	plasma lipoprotein particle clearance	1/95	28/14464	0.03571429	5.43759398	1.9110888	0.16863344	0.43121469	0.41228008	Trem2	1
GO:0035459	vesicle cargo loading	1/95	28/14464	0.03571429	5.43759398	1.9110888	0.16863344	0.43121469	0.41228008	Sar1b	1
GO:0043304	regulation of mast cell degranulation	1/95	28/14464	0.03571429	5.43759398	1.9110888	0.16863344	0.43121469	0.41228008	Lyn	1
GO:0045686	negative regulation of glial cell differentiation	1/95	28/14464	0.03571429	5.43759398	1.9110888	0.16863344	0.43121469	0.41228008	Trem2	1
GO:0048199	vesicle targeting, to, from or within Golgi	1/95	28/14464	0.03571429	5.43759398	1.9110888	0.16863344	0.43121469	0.41228008	Sar1b	1
GO:0050901	leukocyte tethering or rolling	1/95	28/14464	0.03571429	5.43759398	1.9110888	0.16863344	0.43121469	0.41228008	Jam2	1
GO:0060117	auditory receptor cell development	1/95	28/14464	0.03571429	5.43759398	1.9110888	0.16863344	0.43121469	0.41228008	Alg10b	1
GO:0060402	calcium ion transport into cytosol	1/95	28/14464	0.03571429	5.43759398	1.9110888	0.16863344	0.43121469	0.41228008	Gimap5	1
GO:0061036	positive regulation of cartilage development	1/95	28/14464	0.03571429	5.43759398	1.9110888	0.16863344	0.43121469	0.41228008	Por	1
GO:0099150	regulation of postsynaptic specialization assembly	1/95	28/14464	0.03571429	5.43759398	1.9110888	0.16863344	0.43121469	0.41228008	Abl1	1
GO:1902932	positive regulation of alcohol biosynthetic process	1/95	28/14464	0.03571429	5.43759398	1.9110888	0.16863344	0.43121469	0.41228008	Por	1
GO:1903055	positive regulation of extracellular matrix organization	1/95	28/14464	0.03571429	5.43759398	1.9110888	0.16863344	0.43121469	0.41228008	Abl1	1
GO:1903909	regulation of receptor clustering	1/95	28/14464	0.03571429	5.43759398	1.9110888	0.16863344	0.43121469	0.41228008	Agrn	1
GO:1905820	positive regulation of chromosome separation	1/95	28/14464	0.03571429	5.43759398	1.9110888	0.16863344	0.43121469	0.41228008	Ncapd3	1
GO:2000819	regulation of nucleotide-excision repair	1/95	28/14464	0.03571429	5.43759398	1.9110888	0.16863344	0.43121469	0.41228008	Arid2	1
GO:0022900	electron transport chain	2/95	113/14464	0.01769912	2.69473684	1.47054437	0.16992189	0.43121469	0.41228008	Por/ND5	2
GO:0051283	negative regulation of sequestering of calcium ion	2/95	113/14464	0.01769912	2.69473684	1.47054437	0.16992189	0.43121469	0.41228008	Abl1/Lyn	2
GO:0034762	regulation of transmembrane transport	5/95	447/14464	0.01118568	1.70304957	1.22769333	0.17070978	0.43121469	0.41228008	Trem2/Gimap5/Abl1/Agrn/Lyn	5
GO:1903076	regulation of protein localization to plasma membrane	2/95	114/14464	0.01754386	2.6710988	1.45648604	0.17225731	0.43121469	0.41228008	Trem2/Myo5b	2
GO:0052548	regulation of endopeptidase activity	3/95	218/14464	0.01376147	2.0952197	1.32483181	0.17270565	0.43121469	0.41228008	Serpine1/Acer2/Lyn	3
GO:0048638	regulation of developmental growth	4/95	331/14464	0.01208459	1.83991096	1.25692043	0.17313205	0.43121469	0.41228008	Serpine1/Myo5b/Abl1/Agrn	4
GO:0007346	regulation of mitotic cell cycle	5/95	450/14464	0.01111111	1.69169591	1.21204328	0.17397806	0.43121469	0.41228008	Wee1/Ccdc57/Abl1/Xrcc3/Arid2	5
GO:0006120	mitochondrial electron transport, NADH to ubiquinone	1/95	29/14464	0.03448276	5.25009074	1.86280129	0.17410447	0.43121469	0.41228008	ND5	1
GO:0006779	porphyrin-containing compound biosynthetic process	1/95	29/14464	0.03448276	5.25009074	1.86280129	0.17410447	0.43121469	0.41228008	Rsad1	1
GO:0032733	positive regulation of interleukin-10 production	1/95	29/14464	0.03448276	5.25009074	1.86280129	0.17410447	0.43121469	0.41228008	Trem2	1
GO:0033014	tetrapyrrole biosynthetic process	1/95	29/14464	0.03448276	5.25009074	1.86280129	0.17410447	0.43121469	0.41228008	Rsad1	1
GO:0042269	regulation of natural killer cell mediated cytotoxicity	1/95	29/14464	0.03448276	5.25009074	1.86280129	0.17410447	0.43121469	0.41228008	Gimap5	1
GO:0045671	negative regulation of osteoclast differentiation	1/95	29/14464	0.03448276	5.25009074	1.86280129	0.17410447	0.43121469	0.41228008	Lrrc17	1
GO:0045940	positive regulation of steroid metabolic process	1/95	29/14464	0.03448276	5.25009074	1.86280129	0.17410447	0.43121469	0.41228008	Por	1
GO:0046466	membrane lipid catabolic process	1/95	29/14464	0.03448276	5.25009074	1.86280129	0.17410447	0.43121469	0.41228008	Acer2	1
GO:0051450	myoblast proliferation	1/95	29/14464	0.03448276	5.25009074	1.86280129	0.17410447	0.43121469	0.41228008	Abl1	1
GO:0051894	positive regulation of focal adhesion assembly	1/95	29/14464	0.03448276	5.25009074	1.86280129	0.17410447	0.43121469	0.41228008	Abl1	1
GO:0062098	regulation of programmed necrotic cell death	1/95	29/14464	0.03448276	5.25009074	1.86280129	0.17410447	0.43121469	0.41228008	Peli1	1
GO:0086010	membrane depolarization during action potential	1/95	29/14464	0.03448276	5.25009074	1.86280129	0.17410447	0.43121469	0.41228008	Clcn2	1
GO:0090382	phagosome maturation	1/95	29/14464	0.03448276	5.25009074	1.86280129	0.17410447	0.43121469	0.41228008	Rab34	1
GO:0098868	bone growth	1/95	29/14464	0.03448276	5.25009074	1.86280129	0.17410447	0.43121469	0.41228008	Por	1
GO:0120178	steroid hormone biosynthetic process	1/95	29/14464	0.03448276	5.25009074	1.86280129	0.17410447	0.43121469	0.41228008	Clcn2	1
GO:2000406	positive regulation of T cell migration	1/95	29/14464	0.03448276	5.25009074	1.86280129	0.17410447	0.43121469	0.41228008	Abl1	1
GO:0031589	cell-substrate adhesion	4/95	332/14464	0.01204819	1.83436906	1.25055599	0.17441996	0.43121469	0.41228008	Fzd4/Serpine1/Acer2/Abl1	4
GO:0043200	response to amino acid	2/95	115/14464	0.0173913	2.64787185	1.44257786	0.17459813	0.43121469	0.41228008	Gdf10/Lyn	2
GO:0050729	positive regulation of inflammatory response	2/95	115/14464	0.0173913	2.64787185	1.44257786	0.17459813	0.43121469	0.41228008	Trem2/Serpine1	2
GO:0051282	regulation of sequestering of calcium ion	2/95	115/14464	0.0173913	2.64787185	1.44257786	0.17459813	0.43121469	0.41228008	Abl1/Lyn	2
GO:0043401	steroid hormone receptor signaling pathway	2/95	116/14464	0.01724138	2.62504537	1.42881672	0.17694418	0.43477066	0.41567991	Per1/Klf9	2
GO:0045931	positive regulation of mitotic cell cycle	2/95	116/14464	0.01724138	2.62504537	1.42881672	0.17694418	0.43477066	0.41567991	Ccdc57/Abl1	2
GO:0048709	oligodendrocyte differentiation	2/95	116/14464	0.01724138	2.62504537	1.42881672	0.17694418	0.43477066	0.41567991	Clcn2/Lyn	2
GO:0051208	sequestering of calcium ion	2/95	116/14464	0.01724138	2.62504537	1.42881672	0.17694418	0.43477066	0.41567991	Abl1/Lyn	2
GO:0106027	neuron projection organization	2/95	117/14464	0.01709402	2.60260909	1.41519963	0.17929529	0.43612282	0.41697269	Trem2/Myo5b	2
GO:0030261	chromosome condensation	1/95	30/14464	0.03333333	5.07508772	1.81669475	0.17953988	0.43612282	0.41697269	Ncapd3	1
GO:0032814	regulation of natural killer cell activation	1/95	30/14464	0.03333333	5.07508772	1.81669475	0.17953988	0.43612282	0.41697269	Il15ra	1
GO:0042745	circadian sleep/wake cycle	1/95	30/14464	0.03333333	5.07508772	1.81669475	0.17953988	0.43612282	0.41697269	Per3	1
GO:0045672	positive regulation of osteoclast differentiation	1/95	30/14464	0.03333333	5.07508772	1.81669475	0.17953988	0.43612282	0.41697269	Trem2	1
GO:0048026	positive regulation of mRNA splicing, via spliceosome	1/95	30/14464	0.03333333	5.07508772	1.81669475	0.17953988	0.43612282	0.41697269	Rbmx	1
GO:0071404	cellular response to low-density lipoprotein particle stimulus	1/95	30/14464	0.03333333	5.07508772	1.81669475	0.17953988	0.43612282	0.41697269	Trem2	1
GO:0150146	cell junction disassembly	1/95	30/14464	0.03333333	5.07508772	1.81669475	0.17953988	0.43612282	0.41697269	Trem2	1
GO:2000108	positive regulation of leukocyte apoptotic process	1/95	30/14464	0.03333333	5.07508772	1.81669475	0.17953988	0.43612282	0.41697269	Lyn	1
GO:0002250	adaptive immune response	4/95	337/14464	0.01186944	1.8071529	1.21905559	0.18090957	0.4388944	0.41962257	Trem2/Gimap5/Lyn/Paxip1	4
GO:0008203	cholesterol metabolic process	2/95	118/14464	0.01694915	2.58055308	1.40172364	0.1816513	0.43958241	0.42028038	Por/Lss	2
GO:0009142	nucleoside triphosphate biosynthetic process	2/95	118/14464	0.01694915	2.58055308	1.40172364	0.1816513	0.43958241	0.42028038	Trem2/ND5	2
GO:0051402	neuron apoptotic process	4/95	339/14464	0.01179941	1.79649123	1.20660284	0.18352828	0.44196717	0.42256042	Mt1/Trem2/Abl1/Agrn	4
GO:0060271	cilium assembly	4/95	339/14464	0.01179941	1.79649123	1.20660284	0.18352828	0.44196717	0.42256042	Ccdc57/Rab34/Alpk1/Celsr3	4
GO:0030195	negative regulation of blood coagulation	1/95	31/14464	0.03225806	4.91137521	1.77259591	0.18493989	0.44196717	0.42256042	Serpine1	1
GO:0032967	positive regulation of collagen biosynthetic process	1/95	31/14464	0.03225806	4.91137521	1.77259591	0.18493989	0.44196717	0.42256042	Serpine1	1
GO:0033687	osteoblast proliferation	1/95	31/14464	0.03225806	4.91137521	1.77259591	0.18493989	0.44196717	0.42256042	Abl1	1
GO:0043267	negative regulation of potassium ion transport	1/95	31/14464	0.03225806	4.91137521	1.77259591	0.18493989	0.44196717	0.42256042	Agrn	1
GO:0045191	regulation of isotype switching	1/95	31/14464	0.03225806	4.91137521	1.77259591	0.18493989	0.44196717	0.42256042	Paxip1	1
GO:0055094	response to lipoprotein particle	1/95	31/14464	0.03225806	4.91137521	1.77259591	0.18493989	0.44196717	0.42256042	Trem2	1
GO:1990000	amyloid fibril formation	1/95	31/14464	0.03225806	4.91137521	1.77259591	0.18493989	0.44196717	0.42256042	Trem2	1
GO:2000780	negative regulation of double-strand break repair	1/95	31/14464	0.03225806	4.91137521	1.77259591	0.18493989	0.44196717	0.42256042	Abl1	1
GO:0048675	axon extension	2/95	120/14464	0.01666667	2.53754386	1.37518372	0.18637736	0.44481701	0.42528512	Myo5b/Abl1	2
GO:0051056	regulation of small GTPase mediated signal transduction	3/95	227/14464	0.01321586	2.01214932	1.2497582	0.18742861	0.44481701	0.42528512	Net1/Abl1/Agrn	3
GO:0006869	lipid transport	4/95	342/14464	0.01169591	1.78073253	1.18807799	0.18748015	0.44481701	0.42528512	Fzd4/Trem2/Sar1b/Lyn	4
GO:1901890	positive regulation of cell junction assembly	2/95	121/14464	0.01652893	2.51657242	1.36211434	0.18874708	0.44481701	0.42528512	Abl1/Agrn	2
GO:0042110	T cell activation	5/95	464/14464	0.01077586	1.64065336	1.14050135	0.18954664	0.44481701	0.42528512	Trem2/Gimap5/Abl1/Peli1/Arid2	5
GO:0002715	regulation of natural killer cell mediated immunity	1/95	32/14464	0.03125	4.75789474	1.73035042	0.19030472	0.44481701	0.42528512	Gimap5	1
GO:0006901	vesicle coating	1/95	32/14464	0.03125	4.75789474	1.73035042	0.19030472	0.44481701	0.42528512	Sar1b	1
GO:0031116	positive regulation of microtubule polymerization	1/95	32/14464	0.03125	4.75789474	1.73035042	0.19030472	0.44481701	0.42528512	Nav3	1
GO:0032373	positive regulation of sterol transport	1/95	32/14464	0.03125	4.75789474	1.73035042	0.19030472	0.44481701	0.42528512	Trem2	1
GO:0032376	positive regulation of cholesterol transport	1/95	32/14464	0.03125	4.75789474	1.73035042	0.19030472	0.44481701	0.42528512	Trem2	1
GO:0032743	positive regulation of interleukin-2 production	1/95	32/14464	0.03125	4.75789474	1.73035042	0.19030472	0.44481701	0.42528512	Abl1	1
GO:0043045	epigenetic programming of gene expression	1/95	32/14464	0.03125	4.75789474	1.73035042	0.19030472	0.44481701	0.42528512	Tet3	1
GO:0045589	regulation of regulatory T cell differentiation	1/95	32/14464	0.03125	4.75789474	1.73035042	0.19030472	0.44481701	0.42528512	Gimap5	1
GO:0045738	negative regulation of DNA repair	1/95	32/14464	0.03125	4.75789474	1.73035042	0.19030472	0.44481701	0.42528512	Abl1	1
GO:0097242	amyloid-beta clearance	1/95	32/14464	0.03125	4.75789474	1.73035042	0.19030472	0.44481701	0.42528512	Trem2	1
GO:0140058	neuron projection arborization	1/95	32/14464	0.03125	4.75789474	1.73035042	0.19030472	0.44481701	0.42528512	Fzd4	1
GO:1900047	negative regulation of hemostasis	1/95	32/14464	0.03125	4.75789474	1.73035042	0.19030472	0.44481701	0.42528512	Serpine1	1
GO:1901381	positive regulation of potassium ion transmembrane transport	1/95	32/14464	0.03125	4.75789474	1.73035042	0.19030472	0.44481701	0.42528512	Trem2	1
GO:0046660	female sex differentiation	2/95	122/14464	0.01639344	2.49594478	1.34917518	0.19112106	0.44618166	0.42658985	Fzd4/Serpine1	2
GO:0016125	sterol metabolic process	2/95	123/14464	0.01626016	2.47565255	1.33636371	0.19349914	0.44746464	0.4278165	Por/Lss	2
GO:0032368	regulation of lipid transport	2/95	123/14464	0.01626016	2.47565255	1.33636371	0.19349914	0.44746464	0.4278165	Trem2/Sar1b	2
GO:0051384	response to glucocorticoid	2/95	123/14464	0.01626016	2.47565255	1.33636371	0.19349914	0.44746464	0.4278165	Por/Klf9	2
GO:0070665	positive regulation of leukocyte proliferation	2/95	123/14464	0.01626016	2.47565255	1.33636371	0.19349914	0.44746464	0.4278165	Peli1/Lyn	2
GO:1903038	negative regulation of leukocyte cell-cell adhesion	2/95	123/14464	0.01626016	2.47565255	1.33636371	0.19349914	0.44746464	0.4278165	Gimap5/Peli1	2
GO:0000462	maturation of SSU-rRNA from tricistronic rRNA transcript (SSU-rRNA, 5.8S rRNA, LSU-rRNA)	1/95	33/14464	0.03030303	4.61371611	1.68982026	0.19563462	0.44746464	0.4278165	Wdr46	1
GO:0000731	DNA synthesis involved in DNA repair	1/95	33/14464	0.03030303	4.61371611	1.68982026	0.19563462	0.44746464	0.4278165	Pole	1

GO:0030947 regulation of vascular endothelial growth factor receptor signaling pathway	1/95	33/14464	0.03030303	4.61371611	1.68982026	0.19563462	0.44746464	0.4278165	Fzd4	1
GO:0035020 regulation of Rac protein signal transduction	1/95	33/14464	0.03030303	4.61371611	1.68982026	0.19563462	0.44746464	0.4278165	Agrn	1
GO:0046329 negative regulation of JNK cascade	1/95	33/14464	0.03030303	4.61371611	1.68982026	0.19563462	0.44746464	0.4278165	Per1	1
GO:0048710 regulation of astrocyte differentiation	1/95	33/14464	0.03030303	4.61371611	1.68982026	0.19563462	0.44746464	0.4278165	Trem2	1
GO:0050685 positive regulation of mRNA processing	1/95	33/14464	0.03030303	4.61371611	1.68982026	0.19563462	0.44746464	0.4278165	Rbmx	1
GO:0050819 negative regulation of coagulation	1/95	33/14464	0.03030303	4.61371611	1.68982026	0.19563462	0.44746464	0.4278165	Serpine1	1
GO:0050869 negative regulation of B cell activation	1/95	33/14464	0.03030303	4.61371611	1.68982026	0.19563462	0.44746464	0.4278165	Lyn	1
GO:0051491 positive regulation of filopodium assembly	1/95	33/14464	0.03030303	4.61371611	1.68982026	0.19563462	0.44746464	0.4278165	Agrn	1
GO:0060351 cartilage development involved in endochondral bone morphogenesis	1/95	33/14464	0.03030303	4.61371611	1.68982026	0.19563462	0.44746464	0.4278165	Por	1
GO:2000516 positive regulation of CD4-positive, alpha-beta T cell activation	1/95	33/14464	0.03030303	4.61371611	1.68982026	0.19563462	0.44746464	0.4278165	Gimap5	1
GO:0044283 small molecule biosynthetic process	5/95	470/14464	0.0106383	1.61970885	1.11056355	0.1963712	0.44861469	0.42891605	Acer2/Por/Lss/Clcn2/Tha1	5
GO:0044843 cell cycle G1/S phase transition	3/95	233/14464	0.01287554	1.96033431	1.20160139	0.19742265	0.44992331	0.43016721	Tcim/Pole/Arid2	3
GO:0000086 G2/M transition of mitotic cell cycle	2/95	125/14464	0.016	2.43604211	1.31111405	0.19826698	0.44992331	0.43016721	Wee1/Ccdc57	2
GO:0010950 positive regulation of endopeptidase activity	2/95	125/14464	0.016	2.43604211	1.31111405	0.19826698	0.44992331	0.43016721	Acer2/Lyn	2
GO:0009743 response to carbohydrate	3/95	234/14464	0.01282051	1.95195682	1.19371445	0.19910118	0.44992331	0.43016721	Trem2/Rab34/Lyn	3
GO:0001838 embryonic epithelial tube formation	2/95	126/14464	0.01587302	2.41670844	1.29867114	0.20065644	0.44992331	0.43016721	Abl1/Brpf1	2
GO:0006959 humoral immune response	2/95	126/14464	0.01587302	2.41670844	1.29867114	0.20065644	0.44992331	0.43016721	Trem2/Gimap5	2
GO:0014812 muscle cell migration	2/95	126/14464	0.01587302	2.41670844	1.29867114	0.20065644	0.44992331	0.43016721	Serpine1/Net1	2
GO:0050728 negative regulation of inflammatory response	2/95	126/14464	0.01587302	2.41670844	1.29867114	0.20065644	0.44992331	0.43016721	Nr1d2/Trem2	2
GO:0007431 salivary gland development	1/95	34/14464	0.02941176	4.47801858	1.65088155	0.2009298	0.44992331	0.43016721	Clcn2	1
GO:0010043 response to zinc ion	1/95	34/14464	0.02941176	4.47801858	1.65088155	0.2009298	0.44992331	0.43016721	Mt1	1
GO:0010714 positive regulation of collagen metabolic process	1/95	34/14464	0.02941176	4.47801858	1.65088155	0.2009298	0.44992331	0.43016721	Serpine1	1
GO:0034110 regulation of homotypic cell-cell adhesion	1/95	34/14464	0.02941176	4.47801858	1.65088155	0.2009298	0.44992331	0.43016721	Lyn	1
GO:0045745 positive regulation of G protein-coupled receptor signaling pathway	1/95	34/14464	0.02941176	4.47801858	1.65088155	0.2009298	0.44992331	0.43016721	Trem2	1
GO:0051654 establishment of mitochondrion localization	1/95	34/14464	0.02941176	4.47801858	1.65088155	0.2009298	0.44992331	0.43016721	Tspan4	1
GO:0150117 positive regulation of cell-substrate junction organization	1/95	34/14464	0.02941176	4.47801858	1.65088155	0.2009298	0.44992331	0.43016721	Abl1	1
GO:1902175 regulation of oxidative stress-induced intrinsic apoptotic signaling pathway	1/95	34/14464	0.02941176	4.47801858	1.65088155	0.2009298	0.44992331	0.43016721	Trem2	1
GO:1905332 positive regulation of morphogenesis of an epithelium	1/95	34/14464	0.02941176	4.47801858	1.65088155	0.2009298	0.44992331	0.43016721	Abl1	1
GO:0002449 lymphocyte mediated immunity	3/95	236/14464	0.01271186	1.93541481	1.17805628	0.20246875	0.45284095	0.43295674	Trem2/Gimap5/Paxip1	3
GO:0050921 positive regulation of chemotaxis	2/95	127/14464	0.01574803	2.39767924	1.28634646	0.20304939	0.45308346	0.4331886	Trem2/Serpine1	2
GO:1902652 secondary alcohol metabolic process	2/95	127/14464	0.01574803	2.39767924	1.28634646	0.20304939	0.45308346	0.4331886	Por/Lss	2
GO:0014902 myotube differentiation	2/95	128/14464	0.015625	2.37894737	1.27413781	0.20544568	0.45343617	0.43352582	Bhlhe41/Jam2	2
GO:0006956 complement activation	1/95	35/14464	0.02857143	4.35007519	1.61342278	0.20619048	0.45343617	0.43352582	Trem2	1
GO:0010874 regulation of cholesterol efflux	1/95	35/14464	0.02857143	4.35007519	1.61342278	0.20619048	0.45343617	0.43352582	Trem2	1
GO:0014912 negative regulation of smooth muscle cell migration	1/95	35/14464	0.02857143	4.35007519	1.61342278	0.20619048	0.45343617	0.43352582	Serpine1	1
GO:0018279 protein N-linked glycosylation via asparagine	1/95	35/14464	0.02857143	4.35007519	1.61342278	0.20619048	0.45343617	0.43352582	Alg10b	1
GO:0021680 cerebellar Purkinje cell layer development	1/95	35/14464	0.02857143	4.35007519	1.61342278	0.20619048	0.45343617	0.43352582	Rere	1
GO:0022616 DNA strand elongation	1/95	35/14464	0.02857143	4.35007519	1.61342278	0.20619048	0.45343617	0.43352582	Pole	1
GO:0033144 negative regulation of intracellular steroid hormone receptor signaling pathway	1/95	35/14464	0.02857143	4.35007519	1.61342278	0.20619048	0.45343617	0.43352582	Per1	1
GO:0045066 regulatory T cell differentiation	1/95	35/14464	0.02857143	4.35007519	1.61342278	0.20619048	0.45343617	0.43352582	Gimap5	1
GO:0045746 negative regulation of Notch signaling pathway	1/95	35/14464	0.02857143	4.35007519	1.61342278	0.20619048	0.45343617	0.43352582	Tcim	1
GO:0051281 positive regulation of release of sequestered calcium ion into cytosol	1/95	35/14464	0.02857143	4.35007519	1.61342278	0.20619048	0.45343617	0.43352582	Abl1	1
GO:0070316 regulation of G0 to G1 transition	1/95	35/14464	0.02857143	4.35007519	1.61342278	0.20619048	0.45343617	0.43352582	Arid2	1
GO:0006935 chemotaxis	4/95	356/14464	0.01123596	1.71070373	1.10397335	0.2062792	0.45343617	0.43352582	Trem2/Serpine1/Agrn/Lyn	4
GO:0001818 negative regulation of cytokine production	3/95	239/14464	0.0125523	1.9111209	1.15485257	0.20754558	0.45487144	0.43489806	Trem2/Gimap5/Nav3	3
GO:1903320 regulation of protein modification by small protein conjugation or removal	3/95	239/14464	0.0125523	1.9111209	1.15485257	0.20754558	0.45487144	0.43489806	Abl1/Peli1/Paxip1	3
GO:0042330 taxis	4/95	357/14464	0.01120448	1.70591184	1.09810777	0.20764324	0.45487144	0.43489806	Trem2/Serpine1/Agrn/Lyn	4
GO:1901988 negative regulation of cell cycle phase transition	3/95	240/14464	0.0125	1.90315789	1.14719212	0.20924443	0.45785641	0.43775196	Wee1/Xrcc3/Lyn	3
GO:0007266 Rho protein signal transduction	2/95	130/14464	0.01538462	2.34234818	1.25006005	0.21024774	0.45791086	0.43780402	Net1/Abl1	2
GO:0170039 proteinogenic amino acid metabolic process	2/95	130/14464	0.01538462	2.34234818	1.25006005	0.21024774	0.45791086	0.43780402	Pipox/Tha1	2
GO:0030098 lymphocyte differentiation	4/95	359/14464	0.01114206	1.69640815	1.08643125	0.21037936	0.45791086	0.43780402	Gimap5/Il15ra/Abl1/Arid2	4
GO:0007026 negative regulation of microtubule depolymerization	1/95	36/14464	0.02777778	4.22923977	1.5773432	0.21141689	0.45791086	0.43780402	Nav3	1
GO:0014002 astrocyte development	1/95	36/14464	0.02777778	4.22923977	1.5773432	0.21141689	0.45791086	0.43780402	Trem2	1
GO:0050798 activated T cell proliferation	1/95	36/14464	0.02777778	4.22923977	1.5773432	0.21141689	0.45791086	0.43780402	Abl1	1
GO:0070266 necroptotic process	1/95	36/14464	0.02777778	4.22923977	1.5773432	0.21141689	0.45791086	0.43780402	Peli1	1
GO:0071402 cellular response to lipoprotein particle stimulus	1/95	36/14464	0.02777778	4.22923977	1.5773432	0.21141689	0.45791086	0.43780402	Trem2	1
GO:1900225 regulation of NLRP3 inflammasome complex assembly	1/95	36/14464	0.02777778	4.22923977	1.5773432	0.21141689	0.45791086	0.43780402	Trem2	1
GO:0007098 centrosome cycle	2/95	131/14464	0.01526718	2.32446766	1.23818682	0.21265321	0.46006934	0.43986772	Ccdc57/Xrcc3	2
GO:0001101 response to acid chemical	2/95	132/14464	0.01515152	2.30685805	1.22642137	0.21506145	0.46288771	0.44256234	Gdf10/Lyn	2
GO:0010952 positive regulation of peptidase activity	2/95	132/14464	0.01515152	2.30685805	1.22642137	0.21506145	0.46288771	0.44256234	Acer2/Lyn	2
GO:1901873 regulation of post-translational protein modification	3/95	244/14464	0.01229508	1.87195858	1.11691133	0.21607125	0.46288771	0.44256234	Abl1/Peli1/Paxip1	3
GO:0006084 acetyl-CoA metabolic process	1/95	37/14464	0.02702703	4.11493599	1.54255158	0.21660925	0.46288771	0.44256234	Pipox	1
GO:0010837 regulation of keratinocyte proliferation	1/95	37/14464	0.02702703	4.11493599	1.54255158	0.21660925	0.46288771	0.44256234	Klf9	1
GO:0043277 apoptotic cell clearance	1/95	37/14464	0.02702703	4.11493599	1.54255158	0.21660925	0.46288771	0.44256234	Trem2	1
GO:0045023 G0 to G1 transition	1/95	37/14464	0.02702703	4.11493599	1.54255158	0.21660925	0.46288771	0.44256234	Arid2	1
GO:0051154 negative regulation of striated muscle cell differentiation	1/95	37/14464	0.02702703	4.11493599	1.54255158	0.21660925	0.46288771	0.44256234	Bhlhe41	1
GO:0090114 COPII-coated vesicle budding	1/95	37/14464	0.02702703	4.11493599	1.54255158	0.21660925	0.46288771	0.44256234	Sar1b	1
GO:0099637 neurotransmitter receptor transport	1/95	37/14464	0.02702703	4.11493599	1.54255158	0.21660925	0.46288771	0.44256234	Myo5b	1
GO:1904646 cellular response to amyloid-beta	1/95	37/14464	0.02702703	4.11493599	1.54255158	0.21660925	0.46288771	0.44256234	Trem2	1
GO:0045739 positive regulation of DNA repair	2/95	134/14464	0.01492537	2.27242734	1.20320608	0.21988572	0.46714861	0.44663614	Peli1/Arid2	2
GO:0042063 gliogenesis	4/95	366/14464	0.01092896	1.66396319	1.04612484	0.22003718	0.46714861	0.44663614	Trem2/Clcn2/Abl1/Lyn	4
GO:0001569 branching involved in blood vessel morphogenesis	1/95	38/14464	0.02631579	4.0066482	1.50896502	0.22176779	0.46714861	0.44663614	Abl1	1
GO:0006778 porphyrin-containing compound metabolic process	1/95	38/14464	0.02631579	4.0066482	1.50896502	0.22176779	0.46714861	0.44663614	Rsad1	1
GO:0007020 microtubule nucleation	1/95	38/14464	0.02631579	4.0066482	1.50896502	0.22176779	0.46714861	0.44663614	Ccdc57	1
GO:0009593 detection of chemical stimulus	1/95	38/14464	0.02631579	4.0066482	1.50896502	0.22176779	0.46714861	0.44663614	Trem2	1
GO:0031112 positive regulation of microtubule polymerization or depolymerization	1/95	38/14464	0.02631579	4.0066482	1.50896502	0.22176779	0.46714861	0.44663614	Nav3	1
GO:0044546 NLRP3 inflammasome complex assembly	1/95	38/14464	0.02631579	4.0066482	1.50896502	0.22176779	0.46714861	0.44663614	Trem2	1
GO:0090049 regulation of cell migration involved in sprouting angiogenesis	1/95	38/14464	0.02631579	4.0066482	1.50896502	0.22176779	0.46714861	0.44663614	Abl1	1
GO:0140632 canonical inflammasome complex assembly	1/95	38/14464	0.02631579	4.0066482	1.50896502	0.22176779	0.46714861	0.44663614	Trem2	1
GO:1900026 positive regulation of substrate adhesion-dependent cell spreading	1/95	38/14464	0.02631579	4.0066482	1.50896502	0.22176779	0.46714861	0.44663614	Abl1	1
GO:2000404 regulation of T cell migration	1/95	38/14464	0.02631579	4.0066482	1.50896502	0.22176779	0.46714861	0.44663614	Abl1	1
GO:2000649 regulation of sodium ion transmembrane transporter activity	1/95	38/14464	0.02631579	4.0066482	1.50896502	0.22176779	0.46714861	0.44663614	Agrn	1
GO:0071902 positive regulation of protein serine/threonine kinase activity	2/95	135/14464	0.01481481	2.25559454	1.19175254	0.22230147	0.46775934	0.44722006	Fzd4/Tcim	2
GO:0044782 cilium organization	4/95	369/14464	0.01084011	1.65043503	1.02911116	0.22421335	0.47078234	0.45011032	Ccdc57/Rab34/Alpk1/Celsr3	4
GO:0070372 regulation of ERK1 and ERK2 cascade	3/95	249/14464	0.01204819	1.83436906	1.07984682	0.22467141	0.47078234	0.45011032	Trem2/Abl1/Lyn	3
GO:0071559 response to transforming growth factor beta	3/95	249/14464	0.01204819	1.83436906	1.07984682	0.22467141	0.47078234	0.45011032	Serpine1/Gdf10/Abl1	3
GO:0072175 epithelial tube formation	2/95	136/14464	0.01470588	2.23900929	1.18039933	0.22471945	0.47078234	0.45011032	Abl1/Brpf1	2
GO:0010559 regulation of glycoprotein biosynthetic process	1/95	39/14464	0.02564103	3.90391363	1.47650802	0.2268927	0.47173033	0.45101669	Acer2	1
GO:0010830 regulation of myotube differentiation	1/95	39/14464	0.02564103	3.90391363	1.47650802	0.2268927	0.47173033	0.45101669	Bhlhe41	1
GO:0030890 positive regulation of B cell proliferation	1/95	39/14464	0.02564103	3.90391363	1.47650802	0.2268927	0.47173033	0.45101669	Peli1	1
GO:0043001 Golgi to plasma membrane protein transport	1/95	39/14464	0.02564103	3.90391363	1.47650802	0.2268927	0.47173033	0.45101669	Rab34	1
GO:0090279 regulation of calcium ion import	1/95	39/14464	0.02564103	3.90391363	1.47650802	0.2268927	0.47173033	0.45101669	Serpine1	1
GO:1903580 positive regulation of ATP metabolic process	1/95	39/14464	0.02564103	3.90391363	1.47650802	0.2268927	0.47173033	0.45101669	Trem2	1
GO:1905521 regulation of macrophage migration	1/95	39/14464	0.02564103	3.90391363	1.47650802	0.2268927	0.47173033	0.45101669	Trem2	1
GO:0035725 sodium ion transmembrane transport	2/95	137/14464	0.01459854	2.22266615	1.1691447	0.22713953	0.47173242	0.45101868	Slc6a6/Agrn	2
GO:0031960 response to corticosteroid	2/95	138/14464	0.01449275	2.20655988	1.15798697	0.22956158	0.47431181	0.45348482	Por/Klf9	2
GO:0050851 antigen receptor-mediated signaling pathway	2/95	138/14464	0.01449275	2.20655988	1.15798697	0.22956158	0.47431181	0.45348482	Abl1/Lyn	2
GO:0051099 positive regulation of binding	2/95	138/14464	0.01449275	2.20655988	1.15798697	0.22956158	0.47431181	0.45348482	Abl1/Agrn	2
GO:1903322 positive regulation of protein modification by small protein conjugation or removal	2/95	138/14464	0.01449275	2.20655988	1.15798697	0.22956158	0.47431181	0.45348482	Peli1/Paxip1	2
GO:0010721 negative regulation of cell development	3/95	252/14464	0.01190476	1.81253133	1.05801179	0.22986451	0.47431181	0.45348482	Trem2/Lrrc17/Lyn	3
GO:0022409 positive regulation of cell-cell adhesion	3/95	252/14464	0.01190476	1.81253133	1.05801179	0.22986451	0.47431181	0.45348482	Gimap5/Abl1/Arid2	3
GO:0046651 lymphocyte proliferation	3/95	253/14464	0.01185771	1.80536717	1.05079885	0.23160072	0.47612847	0.4552217	Abl1/Peli1/Lyn	3
GO:0007099 centriole replication	1/95	40/14464	0.025	3.80631579	1.44511165	0.23198422	0.47612847	0.4552217	Ccdc57	1
GO:0090181 regulation of cholesterol metabolic process	1/95	40/14464	0.025	3.80631579	1.44511165	0.23198422	0.47612847	0.4552217	Por	1
GO:0140894 endolysosomal toll-like receptor signaling pathway	1/95	40/14464	0.025	3.80631579	1.44511165	0.23198422	0.47612847	0.4552217	Peli1	1
GO:1901875 positive regulation of post-translational protein modification	2/95	139/14464	0.01438849	2.19068535	1.14692447	0.23198547	0.47612847	0.4552217	Peli1/Paxip1	2
GO:0007204 positive regulation of cytosolic calcium ion concentration	2/95	140/14464	0.01428571	2.17503759	1.13595557	0.23441108	0.47681832	0.45588126	Gimap5/Abl1	2
GO:0021782 glial cell development	2/95	140/14464	0.01428571	2.17503759	1.13595557	0.23441108	0.47681832	0.45588126	Trem2/Lyn	2
GO:0043405 regulation of MAP kinase activity	2/95	140/14464	0.01428571	2.17503759	1.13595557	0.23441108	0.47681832	0.45588126	Fzd4/Lyn	2
GO:0006816 calcium ion transport	4/95	377/14464	0.01061008	1.61541254	0.98447679	0.23545131	0.47681832	0.45588126	Serpine1/Gimap5/Abl1/Lyn	4
GO:0006367 transcription initiation at RNA polymerase II promoter	2/95	141/14464	0.0141844	2.1596118	1.12507871	0.23683827	0.47681832	0.45588126	Tet3/Paxip1	2
GO:0007605 sensory perception of sound	2/95	141/14464	0.0141844	2.1596118	1.12507871	0.23683827	0.47681832	0.45588126	Fzd4/Alg10b	2
GO:0031023 microtubule organizing center organization	2/95	141/14464	0.0141844	2.1596118	1.12507871	0.23683827	0.47681832	0.45588126	Ccdc57/Xrcc3	2
GO:0006284 base-excision repair	1/95	41/14464	0.02439024	3.71347882	1.41471281	0.23704256	0.47681832	0.45588126	Pole	1
GO:0007094 mitotic spindle assembly checkpoint signaling	1/95	41/14464	0.02439024	3.71347882	1.41471281	0.23704256	0.47681832	0.45588126	Xrcc3	1
GO:0007520 myoblast fusion	1/95	41/14464	0.02439024	3.71347882	1.41471281	0.23704256	0.47681832	0.45588126	Jam2	1
GO:0010543 regulation of platelet activation	1/95	41/14464	0.02439024	3.71347882	1.41471281	0.23704256	0.47681832	0.45588126	Lyn	1
GO:0043268 positive regulation of potassium ion transport	1/95	41/14464	0.02439024	3.71347882	1.41471281	0.23704256	0.47681832	0.45588126	Trem2	1
GO:0043331 response to dsRNA	1/95	41/14464	0.02439024	3.71347882	1.41471281	0.23704256	0.47681832	0.45588126	Peli1	1
GO:0043507 positive regulation of JUN kinase activity	1/95	41/14464	0.02439024	3.71347882	1.41471281	0.23704256	0.47681832	0.45588126	Fzd4	1
GO:0045907 positive regulation of vasoconstriction	1/95	41/14464	0.02439024	3.71347882	1.41471281	0.23704256	0.47681832	0.45588126	Abl1	1
GO:0045910 negative regulation of DNA recombination	1/95	41/14464	0.02439024	3.71347882	1.41471281	0.23704256	0.47681832	0.45588126	Abl1	1
GO:0048536 spleen development	1/95	41/14464	0.02439024	3.71347882	1.41471281	0.23704256	0.47681832	0.45588126	Abl1	1
GO:0071173 spindle assembly checkpoint signaling	1/95	41/14464	0.02439024	3.71347882	1.41471281	0.23704256	0.47681832	0.45588126	Xrcc3	1
GO:1902991 regulation of amyloid precursor protein catabolic process	1/95	41/14464	0.02439024	3.71347882	1.41471281	0.23704256	0.47681832	0.45588126	Lyn	1
GO:0010212 response to ionizing radiation	2/95	142/14464	0.01408451	2.14440326	1.11429235	0.23926693	0.47840374	0.45739706	Net1/Paxip1	2
GO:0045927 positive regulation of growth	3/95	258/14464	0.01162791	1.77037944	1.01521109	0.24031833	0.47840374	0.45739706	Serpine1/Myo5b/Agrn	3
GO:0000723 telomere maintenance	2/95	143/14464	0.01398601	2.12940743	1.10359497	0.24169693	0.47840374	0.45739706	Xrcc3/Ylpm1	2
GO:0007009 plasma membrane organization	2/95	143/14464	0.01398601	2.12940743	1.10359497	0.24169693	0.47840374	0.45739706	Spaca6/Agrn	2
GO:0000079 regulation of cyclin-dependent protein serine/threonine kinase activity	1/95	42/14464	0.02380952	3.62506266	1.38525363	0.24206794	0.47840374	0.45739706	Inca1	1
GO:0030851 granulocyte differentiation	1/95	42/14464	0.02380952	3.62506266	1.38525363	0.24206794	0.47840374	0.45739706	Lyn	1
GO:0031114 regulation of microtubule depolymerization	1/95	42/14464	0.02380952	3.62506266	1.38525363	0.24206794	0.47840374	0.45739706	Nav3	1
GO:0045981 positive regulation of nucleotide metabolic process	1/95	42/14464	0.02380952	3.62506266	1.38525363	0.24206794	0.47840374	0.45739706	Trem2	1
GO:0046627 negative regulation of insulin receptor signaling pathway	1/95	42/14464	0.02380952	3.62506266	1.38525363	0.24206794	0.47840374	0.45739706	Tns2	1
GO:0046638 positive regulation of alpha-beta T cell differentiation	1/95	42/14464	0.02380952	3.62506266	1.38525363	0.24206794	0.47840374	0.45739706	Gimap5	1
GO:0048147 negative regulation of fibroblast proliferation	1/95	42/14464	0.02380952	3.62506266	1.38525363	0.24206794	0.47840374	0.45739706	Inca1	1
GO:0070192 chromosome organization involved in meiotic cell cycle	1/95	42/14464	0.02380952	3.62506266	1.38525363	0.24206794	0.47840374	0.45739706	Ncapd3	1
GO:0071174 mitotic spindle checkpoint signaling	1/95	42/14464	0.02380952	3.62506266	1.38525363	0.24206794	0.47840374	0.45739706	Xrcc3	1
GO:0098534 centriole assembly	1/95	42/14464	0.02380952	3.62506266	1.38525363	0.24206794	0.47840374	0.45739706	Ccdc57	1
GO:1900544 positive regulation of purine nucleotide metabolic process	1/95	42/14464	0.02380952	3.62506266	1.38525363	0.24206794	0.47840374	0.45739706	Trem2	1
GO:1904645 response to amyloid-beta	1/95	42/14464	0.02380952	3.62506266	1.38525363	0.24206794	0.47840374	0.45739706	Trem2	1
GO:0032943 mononuclear cell proliferation	3/95	259/14464	0.01158301	1.763544	1.00818691	0.2420688	0.47840374	0.45739706	Abl1/Peli1/Lyn	3
GO:0007596 blood coagulation	2/95	145/14464	0.0137931	2.1000363	1.08246134	0.24656048	0.48297854	0.46177099	Serpine1/Lyn	2
GO:0032653 regulation of interleukin-10 production	1/95	43/14464	0.02325581	3.54075887	1.35668091	0.24706056	0.48297854	0.46177099	Trem2	1
GO:0033046 negative regulation of sister chromatid segregation	1/95	43/14464	0.02325581	3.54075887	1.35668091	0.24706056	0.48297854	0.46177099	Xrcc3	1
GO:0033048 negative regulation of mitotic sister chromatid segregation	1/95	43/14464	0.02325581	3.54075887	1.35668091	0.24706056	0.48297854	0.46177099	Xrcc3	1
GO:0042181 ketone biosynthetic process	1/95	43/14464	0.02325581	3.54075887	1.35668091	0.24706056	0.48297854	0.46177099	Clcn2	1
GO:0045841 negative regulation of mitotic metaphase/anaphase transition	1/95	43/14464	0.02325581	3.54075887	1.35668091	0.24706056	0.48297854	0.46177099	Xrcc3	1
GO:0061756 leukocyte adhesion to vascular endothelial cell	1/95	43/14464	0.02325581	3.54075887	1.35668091	0.24706056	0.48297854	0.46177099	Jam2	1
GO:1902041 regulation of extrinsic apoptotic signaling pathway via death domain receptors	1/95	43/14464	0.02325581	3.54075887	1.35668091	0.24706056	0.48297854	0.46177099	Serpine1	1
GO:1903018 regulation of glycoprotein metabolic process	1/95	43/14464	0.02325581	3.54075887	1.35668091	0.24706056	0.48297854	0.46177099	Acer2	1
GO:2000816 negative regulation of mitotic sister chromatid separation	1/95	43/14464	0.02325581	3.54075887	1.35668091	0.24706056	0.48297854	0.46177099	Xrcc3	1
GO:0052547 regulation of peptidase activity	3/95	262/14464	0.01145038	1.74335074	0.98729604	0.24733319	0.48297854	0.46177099	Serpine1/Acer2/Lyn	3
GO:0002460 adaptive immune response based on somatic recombination of immune receptors built from immunoglob	3/95	263/14464	0.01140684	1.73672203	0.98039208	0.24909215	0.48297854	0.46177099	Trem2/Gimap5/Paxip1	3
GO:0030217 T cell differentiation	3/95	263/14464	0.01140684	1.73672203	0.98039208	0.24909215	0.48297854	0.46177099	Gimap5/Abl1/Arid2	3
GO:0098813 nuclear chromosome segregation	3/95	264/14464	0.01136364	1.73014354	0.9735175	0.2508531	0.48297854	0.46177099	Xrcc3/Ncapd3/Arid2	3
GO:0009152 purine ribonucleotide biosynthetic process	2/95	147/14464	0.01360544	2.07146438	1.0616665	0.25142796	0.48297854	0.46177099	Trem2/ND5	2
GO:0170033 L-amino acid metabolic process	2/95	147/14464	0.01360544	2.07146438	1.0616665	0.25142796	0.48297854	0.46177099	Pipox/Tha1	2
GO:0001774 microglial cell activation	1/95	44/14464	0.02272727	3.46028708	1.32894564	0.25202063	0.48297854	0.46177099	Trem2	1
GO:0030858 positive regulation of epithelial cell differentiation	1/95	44/14464	0.02272727	3.46028708	1.32894564	0.25202063	0.48297854	0.46177099	Serpine1	1
GO:0043300 regulation of leukocyte degranulation	1/95	44/14464	0.02272727	3.46028708	1.32894564	0.25202063	0.48297854	0.46177099	Lyn	1
GO:0043370 regulation of CD4-positive, alpha-beta T cell differentiation	1/95	44/14464	0.02272727	3.46028708	1.32894564	0.25202063	0.48297854	0.46177099	Gimap5	1
GO:0051438 regulation of ubiquitin-protein transferase activity	1/95	44/14464	0.02272727	3.46028708	1.32894564	0.25202063	0.48297854	0.46177099	Abl1	1
GO:0071300 cellular response to retinoic acid	1/95	44/14464	0.02272727	3.46028708	1.32894564	0.25202063	0.48297854	0.46177099	Lyn	1
GO:0141085 regulation of inflammasome-mediated signaling pathway	1/95	44/14464	0.02272727	3.46028708	1.32894564	0.25202063	0.48297854	0.46177099	Trem2	1
GO:1901976 regulation of cell cycle checkpoint	1/95	44/14464	0.02272727	3.46028708	1.32894564	0.25202063	0.48297854	0.46177099	Xrcc3	1

GO:1902100	negative regulation of metaphase/anaphase transition of cell cycle	1/95	44/14464	0.02272727	3.46028708	1.32894564	0.25202063	0.48297854	0.46177099	Xrcc3	1
GO:1905819	negative regulation of chromosome separation	1/95	44/14464	0.02272727	3.46028708	1.32894564	0.25202063	0.48297854	0.46177099	Xrcc3	1
GO:0002244	hematopoietic progenitor cell differentiation	2/95	148/14464	0.01351351	2.05746799	1.05139273	0.25386289	0.48297854	0.46177099	Arl11/Lyn	2
GO:0007599	hemostasis	2/95	148/14464	0.01351351	2.05746799	1.05139273	0.25386289	0.48297854	0.46177099	Serpine1/Lyn	2
GO:0034614	cellular response to reactive oxygen species	2/95	148/14464	0.01351351	2.05746799	1.05139273	0.25386289	0.48297854	0.46177099	Net1/Abl1	2
GO:0035148	tube formation	2/95	148/14464	0.01351351	2.05746799	1.05139273	0.25386289	0.48297854	0.46177099	Abl1/Brpf1	2
GO:0045766	positive regulation of angiogenesis	2/95	148/14464	0.01351351	2.05746799	1.05139273	0.25386289	0.48297854	0.46177099	Serpine1/Abl1	2
GO:0016331	morphogenesis of embryonic epithelium	2/95	149/14464	0.01342282	2.04365948	1.04119963	0.25629846	0.48297854	0.46177099	Abl1/Brpf1	2
GO:0032200	telomere organization	2/95	149/14464	0.01342282	2.04365948	1.04119963	0.25629846	0.48297854	0.46177099	Xrcc3/Ylpm1	2
GO:0046631	alpha-beta T cell activation	2/95	149/14464	0.01342282	2.04365948	1.04119963	0.25629846	0.48297854	0.46177099	Gimap5/Abl1	2
GO:0002204	somatic recombination of immunoglobulin genes involved in immune response	1/95	45/14464	0.02222222	3.38339181	1.3020026	0.25694837	0.48297854	0.46177099	Paxip1	1
GO:0002208	somatic diversification of immunoglobulins involved in immune response	1/95	45/14464	0.02222222	3.38339181	1.3020026	0.25694837	0.48297854	0.46177099	Paxip1	1
GO:0002639	positive regulation of immunoglobulin production	1/95	45/14464	0.02222222	3.38339181	1.3020026	0.25694837	0.48297854	0.46177099	Paxip1	1
GO:0006509	membrane protein ectodomain proteolysis	1/95	45/14464	0.02222222	3.38339181	1.3020026	0.25694837	0.48297854	0.46177099	Rbmx	1
GO:0007606	sensory perception of chemical stimulus	1/95	45/14464	0.02222222	3.38339181	1.3020026	0.25694837	0.48297854	0.46177099	Or8b53	1
GO:0008038	neuron recognition	1/95	45/14464	0.02222222	3.38339181	1.3020026	0.25694837	0.48297854	0.46177099	Celsr3	1
GO:0031638	zymogen activation	1/95	45/14464	0.02222222	3.38339181	1.3020026	0.25694837	0.48297854	0.46177099	Serpine1	1
GO:0043029	T cell homeostasis	1/95	45/14464	0.02222222	3.38339181	1.3020026	0.25694837	0.48297854	0.46177099	Gimap5	1
GO:0043407	negative regulation of MAP kinase activity	1/95	45/14464	0.02222222	3.38339181	1.3020026	0.25694837	0.48297854	0.46177099	Lyn	1
GO:0045190	isotype switching	1/95	45/14464	0.02222222	3.38339181	1.3020026	0.25694837	0.48297854	0.46177099	Paxip1	1
GO:0098698	postsynaptic specialization assembly	1/95	45/14464	0.02222222	3.38339181	1.3020026	0.25694837	0.48297854	0.46177099	Abl1	1
GO:0099010	modification of postsynaptic structure	1/95	45/14464	0.02222222	3.38339181	1.3020026	0.25694837	0.48297854	0.46177099	Myo5b	1
GO:1905953	negative regulation of lipid localization	1/95	45/14464	0.02222222	3.38339181	1.3020026	0.25694837	0.48297854	0.46177099	Trem2	1
GO:2001259	positive regulation of cation channel activity	1/95	45/14464	0.02222222	3.38339181	1.3020026	0.25694837	0.48297854	0.46177099	Trem2	1
GO:0010876	lipid localization	4/95	393/14464	0.01017812	1.54964511	0.89824308	0.25832268	0.48297854	0.46177099	Fzd4/Trem2/Sar1b/Lyn	4
GO:0050817	coagulation	2/95	150/14464	0.01333333	2.03003509	1.03108594	0.25873455	0.48297854	0.46177099	Serpine1/Lyn	2
GO:0007224	smoothened signaling pathway	2/95	151/14464	0.01324503	2.01659115	1.02105041	0.26117106	0.48297854	0.46177099	Por/Rab34	2
GO:0141193	nuclear receptor-mediated signaling pathway	2/95	151/14464	0.01324503	2.01659115	1.02105041	0.26117106	0.48297854	0.46177099	Per1/Klf9	2
GO:1904018	positive regulation of vasculature development	2/95	151/14464	0.01324503	2.01659115	1.02105041	0.26117106	0.48297854	0.46177099	Serpine1/Abl1	2
GO:0001912	positive regulation of leukocyte mediated cytotoxicity	1/95	46/14464	0.02173913	3.30983982	1.27580997	0.26184399	0.48297854	0.46177099	Gimap5	1
GO:0010824	regulation of centrosome duplication	1/95	46/14464	0.02173913	3.30983982	1.27580997	0.26184399	0.48297854	0.46177099	Xrcc3	1
GO:0014014	negative regulation of gliogenesis	1/95	46/14464	0.02173913	3.30983982	1.27580997	0.26184399	0.48297854	0.46177099	Trem2	1
GO:0031577	spindle checkpoint signaling	1/95	46/14464	0.02173913	3.30983982	1.27580997	0.26184399	0.48297854	0.46177099	Xrcc3	1
GO:0032613	interleukin-10 production	1/95	46/14464	0.02173913	3.30983982	1.27580997	0.26184399	0.48297854	0.46177099	Trem2	1
GO:0032965	regulation of collagen biosynthetic process	1/95	46/14464	0.02173913	3.30983982	1.27580997	0.26184399	0.48297854	0.46177099	Serpine1	1
GO:0033013	tetrapyrrole metabolic process	1/95	46/14464	0.02173913	3.30983982	1.27580997	0.26184399	0.48297854	0.46177099	Rsad1	1
GO:0035272	exocrine system development	1/95	46/14464	0.02173913	3.30983982	1.27580997	0.26184399	0.48297854	0.46177099	Clcn2	1
GO:0043303	mast cell degranulation	1/95	46/14464	0.02173913	3.30983982	1.27580997	0.26184399	0.48297854	0.46177099	Lyn	1
GO:0043616	keratinocyte proliferation	1/95	46/14464	0.02173913	3.30983982	1.27580997	0.26184399	0.48297854	0.46177099	Klf9	1
GO:0048713	regulation of oligodendrocyte differentiation	1/95	46/14464	0.02173913	3.30983982	1.27580997	0.26184399	0.48297854	0.46177099	Clcn2	1
GO:0051985	negative regulation of chromosome segregation	1/95	46/14464	0.02173913	3.30983982	1.27580997	0.26184399	0.48297854	0.46177099	Xrcc3	1
GO:1904029	regulation of cyclin-dependent protein kinase activity	1/95	46/14464	0.02173913	3.30983982	1.27580997	0.26184399	0.48297854	0.46177099	Inca1	1
GO:2000772	regulation of cellular senescence	1/95	46/14464	0.02173913	3.30983982	1.27580997	0.26184399	0.48297854	0.46177099	Abl1	1
GO:0030336	negative regulation of cell migration	3/95	271/14464	0.01107011	1.68545349	0.92619676	0.2632315	0.48297854	0.46177099	Serpine1/Nav3/Arid2	3
GO:0042445	hormone metabolic process	2/95	152/14464	0.01315789	2.0033241	1.01109182	0.26360788	0.48297854	0.46177099	Por/Clcn2	2
GO:0050714	positive regulation of protein secretion	2/95	152/14464	0.01315789	2.0033241	1.01109182	0.26360788	0.48297854	0.46177099	Trem2/Rab34	2
GO:1905952	regulation of lipid localization	2/95	152/14464	0.01315789	2.0033241	1.01109182	0.26360788	0.48297854	0.46177099	Trem2/Sar1b	2
GO:0043086	negative regulation of catalytic activity	4/95	398/14464	0.01005025	1.5301772	0.87207734	0.26556426	0.48297854	0.46177099	Serpine1/Inca1/Abl1/Lyn	4
GO:0002269	leukocyte activation involved in inflammatory response	1/95	47/14464	0.0212766	3.23941769	1.25032901	0.26670769	0.48297854	0.46177099	Trem2	1
GO:0007062	sister chromatid cohesion	1/95	47/14464	0.0212766	3.23941769	1.25032901	0.26670769	0.48297854	0.46177099	Naa50	1
GO:0007566	embryo implantation	1/95	47/14464	0.0212766	3.23941769	1.25032901	0.26670769	0.48297854	0.46177099	Klf9	1
GO:0010883	regulation of lipid storage	1/95	47/14464	0.0212766	3.23941769	1.25032901	0.26670769	0.48297854	0.46177099	Trem2	1
GO:0030514	negative regulation of BMP signaling pathway	1/95	47/14464	0.0212766	3.23941769	1.25032901	0.26670769	0.48297854	0.46177099	Abl1	1
GO:0032330	regulation of chondrocyte differentiation	1/95	47/14464	0.0212766	3.23941769	1.25032901	0.26670769	0.48297854	0.46177099	Por	1
GO:0033120	positive regulation of RNA splicing	1/95	47/14464	0.0212766	3.23941769	1.25032901	0.26670769	0.48297854	0.46177099	Rbmx	1
GO:0033344	cholesterol efflux	1/95	47/14464	0.0212766	3.23941769	1.25032901	0.26670769	0.48297854	0.46177099	Trem2	1
GO:0045646	regulation of erythrocyte differentiation	1/95	47/14464	0.0212766	3.23941769	1.25032901	0.26670769	0.48297854	0.46177099	Lyn	1
GO:0045663	positive regulation of myoblast differentiation	1/95	47/14464	0.0212766	3.23941769	1.25032901	0.26670769	0.48297854	0.46177099	Arid2	1
GO:0046686	response to cadmium ion	1/95	47/14464	0.0212766	3.23941769	1.25032901	0.26670769	0.48297854	0.46177099	Mt1	1
GO:0071526	semaphorin-plexin signaling pathway	1/95	47/14464	0.0212766	3.23941769	1.25032901	0.26670769	0.48297854	0.46177099	Trem2	1
GO:0141084	inflammasome-mediated signaling pathway	1/95	47/14464	0.0212766	3.23941769	1.25032901	0.26670769	0.48297854	0.46177099	Trem2	1
GO:0170038	proteinogenic amino acid biosynthetic process	1/95	47/14464	0.0212766	3.23941769	1.25032901	0.26670769	0.48297854	0.46177099	Tha1	1
GO:1900077	negative regulation of cellular response to insulin stimulus	1/95	47/14464	0.0212766	3.23941769	1.25032901	0.26670769	0.48297854	0.46177099	Tns2	1
GO:0051258	protein polymerization	3/95	273/14464	0.01098901	1.67310584	0.91292669	0.26678335	0.48297854	0.46177099	Ccdc57/Abl1/Nav3	3
GO:0070371	ERK1 and ERK2 cascade	3/95	273/14464	0.01098901	1.67310584	0.91292669	0.26678335	0.48297854	0.46177099	Trem2/Abl1/Lyn	3
GO:0062013	positive regulation of small molecule metabolic process	2/95	154/14464	0.01298701	1.9773069	0.99140073	0.26848199	0.48468198	0.46339963	Trem2/Por	2
GO:0098876	vesicle-mediated transport to the plasma membrane	2/95	154/14464	0.01298701	1.9773069	0.99140073	0.26848199	0.48468198	0.46339963	Rab34/Myo5b	2
GO:1904375	regulation of protein localization to cell periphery	2/95	154/14464	0.01298701	1.9773069	0.99140073	0.26848199	0.48468198	0.46339963	Trem2/Myo5b	2
GO:0071333	cellular response to glucose stimulus	2/95	155/14464	0.01290323	1.96455008	0.98166592	0.27091909	0.48518123	0.46387696	Trem2/Rab34	2
GO:0072331	signal transduction by p53 class mediator	2/95	155/14464	0.01290323	1.96455008	0.98166592	0.27091909	0.48518123	0.46387696	Acer2/Paxip1	2
GO:0002448	mast cell mediated immunity	1/95	48/14464	0.02083333	3.17192982	1.22552378	0.27153968	0.48518123	0.46387696	Lyn	1
GO:0015804	neutral amino acid transport	1/95	48/14464	0.02083333	3.17192982	1.22552378	0.27153968	0.48518123	0.46387696	Slc6a6	1
GO:0030193	regulation of blood coagulation	1/95	48/14464	0.02083333	3.17192982	1.22552378	0.27153968	0.48518123	0.46387696	Serpine1	1
GO:0042491	inner ear auditory receptor cell differentiation	1/95	48/14464	0.02083333	3.17192982	1.22552378	0.27153968	0.48518123	0.46387696	Alg10b	1
GO:0045687	positive regulation of glial cell differentiation	1/95	48/14464	0.02083333	3.17192982	1.22552378	0.27153968	0.48518123	0.46387696	Clcn2	1
GO:0045815	transcription initiation-coupled chromatin remodeling	1/95	48/14464	0.02083333	3.17192982	1.22552378	0.27153968	0.48518123	0.46387696	Tet3	1
GO:0061045	negative regulation of wound healing	1/95	48/14464	0.02083333	3.17192982	1.22552378	0.27153968	0.48518123	0.46387696	Serpine1	1
GO:0071548	response to dexamethasone	1/95	48/14464	0.02083333	3.17192982	1.22552378	0.27153968	0.48518123	0.46387696	Por	1
GO:0170034	L-amino acid biosynthetic process	1/95	48/14464	0.02083333	3.17192982	1.22552378	0.27153968	0.48518123	0.46387696	Tha1	1
GO:0030010	establishment of cell polarity	2/95	156/14464	0.01282051	1.95195682	0.97200344	0.27335606	0.48740511	0.46600319	Wee1/Abl1	2
GO:0010948	negative regulation of cell cycle process	3/95	277/14464	0.01083032	1.64894547	0.8867078	0.27390479	0.48740511	0.46600319	Wee1/Xrcc3/Lyn	3
GO:0002700	regulation of production of molecular mediator of immune response	2/95	157/14464	0.01273885	1.93952397	0.96241221	0.27579282	0.48740511	0.46600319	Gimap5/Paxip1	2
GO:0006721	terpenoid metabolic process	1/95	49/14464	0.02040816	3.10719656	1.20136089	0.27634016	0.48740511	0.46600319	Lss	1
GO:0022602	ovulation cycle process	1/95	49/14464	0.02040816	3.10719656	1.20136089	0.27634016	0.48740511	0.46600319	Fzd4	1
GO:0031111	negative regulation of microtubule polymerization or depolymerization	1/95	49/14464	0.02040816	3.10719656	1.20136089	0.27634016	0.48740511	0.46600319	Nav3	1
GO:0032729	positive regulation of type II interferon production	1/95	49/14464	0.02040816	3.10719656	1.20136089	0.27634016	0.48740511	0.46600319	Abl1	1
GO:0032757	positive regulation of interleukin-8 production	1/95	49/14464	0.02040816	3.10719656	1.20136089	0.27634016	0.48740511	0.46600319	Serpine1	1
GO:0042267	natural killer cell mediated cytotoxicity	1/95	49/14464	0.02040816	3.10719656	1.20136089	0.27634016	0.48740511	0.46600319	Gimap5	1
GO:0043030	regulation of macrophage activation	1/95	49/14464	0.02040816	3.10719656	1.20136089	0.27634016	0.48740511	0.46600319	Trem2	1
GO:0048010	vascular endothelial growth factor receptor signaling pathway	1/95	49/14464	0.02040816	3.10719656	1.20136089	0.27634016	0.48740511	0.46600319	Fzd4	1
GO:0050850	positive regulation of calcium-mediated signaling	1/95	49/14464	0.02040816	3.10719656	1.20136089	0.27634016	0.48740511	0.46600319	Trem2	1
GO:0061097	regulation of protein tyrosine kinase activity	1/95	49/14464	0.02040816	3.10719656	1.20136089	0.27634016	0.48740511	0.46600319	Agrn	1
GO:1900046	regulation of hemostasis	1/95	49/14464	0.02040816	3.10719656	1.20136089	0.27634016	0.48740511	0.46600319	Serpine1	1
GO:0071331	cellular response to hexose stimulus	2/95	158/14464	0.01265823	1.9272485	0.95289116	0.27822925	0.48774757	0.46633061	Trem2/Rab34	2
GO:0061025	membrane fusion	2/95	159/14464	0.01257862	1.91512744	0.94343923	0.28066526	0.48774757	0.46633061	Rab34/Spaca6	2
GO:0071326	cellular response to monosaccharide stimulus	2/95	159/14464	0.01257862	1.91512744	0.94343923	0.28066526	0.48774757	0.46633061	Trem2/Rab34	2
GO:0001933	negative regulation of protein phosphorylation	3/95	281/14464	0.01067616	1.62547294	0.86090525	0.28104736	0.48774757	0.46633061	Inca1/Abl1/Lyn	3
GO:0000245	spliceosomal complex assembly	1/95	50/14464	0.02	3.04505263	1.17780924	0.28110934	0.48774757	0.46633061	Rbmx	1
GO:0002279	mast cell activation involved in immune response	1/95	50/14464	0.02	3.04505263	1.17780924	0.28110934	0.48774757	0.46633061	Lyn	1
GO:0003254	regulation of membrane depolarization	1/95	50/14464	0.02	3.04505263	1.17780924	0.28110934	0.48774757	0.46633061	Clcn2	1
GO:0010634	positive regulation of epithelial cell migration	1/95	50/14464	0.02	3.04505263	1.17780924	0.28110934	0.48774757	0.46633061	Serpine1	1
GO:0030490	maturation of SSU-rRNA	1/95	50/14464	0.02	3.04505263	1.17780924	0.28110934	0.48774757	0.46633061	Wdr46	1
GO:0032371	regulation of sterol transport	1/95	50/14464	0.02	3.04505263	1.17780924	0.28110934	0.48774757	0.46633061	Trem2	1
GO:0032374	regulation of cholesterol transport	1/95	50/14464	0.02	3.04505263	1.17780924	0.28110934	0.48774757	0.46633061	Trem2	1
GO:0032413	negative regulation of ion transmembrane transporter activity	1/95	50/14464	0.02	3.04505263	1.17780924	0.28110934	0.48774757	0.46633061	Agrn	1
GO:0033047	regulation of mitotic sister chromatid segregation	1/95	50/14464	0.02	3.04505263	1.17780924	0.28110934	0.48774757	0.46633061	Xrcc3	1
GO:0048538	thymus development	1/95	50/14464	0.02	3.04505263	1.17780924	0.28110934	0.48774757	0.46633061	Abl1	1
GO:0060251	regulation of glial cell proliferation	1/95	50/14464	0.02	3.04505263	1.17780924	0.28110934	0.48774757	0.46633061	Lyn	1
GO:0072678	T cell migration	1/95	50/14464	0.02	3.04505263	1.17780924	0.28110934	0.48774757	0.46633061	Abl1	1
GO:0090303	positive regulation of wound healing	1/95	50/14464	0.02	3.04505263	1.17780924	0.28110934	0.48774757	0.46633061	Serpine1	1
GO:0097300	programmed necrotic cell death	1/95	50/14464	0.02	3.04505263	1.17780924	0.28110934	0.48774757	0.46633061	Peli1	1
GO:2000146	negative regulation of cell motility	3/95	282/14464	0.0106383	1.61970885	0.85451803	0.28283595	0.49030008	0.46877104	Serpine1/Nav3/Arid2	3
GO:0009416	response to light stimulus	3/95	283/14464	0.01060071	1.61398549	0.84815577	0.28462564	0.49284922	0.47120824	Per3/Per1/Bhlhe40	3
GO:0009266	response to temperature stimulus	2/95	161/14464	0.01242236	1.89133704	0.92473873	0.28553564	0.49284922	0.47120824	Tcim/Lyn	2
GO:0001961	positive regulation of cytokine-mediated signaling pathway	1/95	51/14464	0.01960784	2.98534572	1.15483985	0.28584741	0.49284922	0.47120824	Trem2	1
GO:0046470	phosphatidylcholine metabolic process	1/95	51/14464	0.01960784	2.98534572	1.15483985	0.28584741	0.49284922	0.47120824	Pnpla7	1
GO:0060986	endocrine hormone secretion	1/95	51/14464	0.01960784	2.98534572	1.15483985	0.28584741	0.49284922	0.47120824	Fzd4	1
GO:0141137	positive regulation of gene expression, epigenetic	1/95	51/14464	0.01960784	2.98534572	1.15483985	0.28584741	0.49284922	0.47120824	Tet3	1
GO:0043254	regulation of protein-containing complex assembly	4/95	413/14464	0.00968523	1.47460176	0.79566174	0.28751013	0.49299226	0.47134501	Trem2/Sar1b/Abl1/Nav3	4
GO:0009260	ribonucleotide biosynthetic process	2/95	162/14464	0.01234568	1.87966212	0.91548816	0.2879698	0.49299226	0.47134501	Trem2/ND5	2
GO:0021915	neural tube development	2/95	162/14464	0.01234568	1.87966212	0.91548816	0.2879698	0.49299226	0.47134501	Abl1/Brpf1	2
GO:0070374	positive regulation of ERK1 and ERK2 cascade	2/95	162/14464	0.01234568	1.87966212	0.91548816	0.2879698	0.49299226	0.47134501	Trem2/Abl1	2
GO:1901991	negative regulation of mitotic cell cycle phase transition	2/95	163/14464	0.01226994	1.86813045	0.90630278	0.29040316	0.49299226	0.47134501	Wee1/Xrcc3	2
GO:0002042	cell migration involved in sprouting angiogenesis	1/95	52/14464	0.01923077	2.92793522	1.13242567	0.29055458	0.49299226	0.47134501	Abl1	1
GO:0002228	natural killer cell mediated immunity	1/95	52/14464	0.01923077	2.92793522	1.13242567	0.29055458	0.49299226	0.47134501	Gimap5	1
GO:0006749	glutathione metabolic process	1/95	52/14464	0.01923077	2.92793522	1.13242567	0.29055458	0.49299226	0.47134501	Gsta4	1
GO:0010965	regulation of mitotic sister chromatid separation	1/95	52/14464	0.01923077	2.92793522	1.13242567	0.29055458	0.49299226	0.47134501	Xrcc3	1
GO:0021587	cerebellum morphogenesis	1/95	52/14464	0.01923077	2.92793522	1.13242567	0.29055458	0.49299226	0.47134501	Abl1	1
GO:0031343	positive regulation of cell killing	1/95	52/14464	0.01923077	2.92793522	1.13242567	0.29055458	0.49299226	0.47134501	Gimap5	1
GO:0035315	hair cell differentiation	1/95	52/14464	0.01923077	2.92793522	1.13242567	0.29055458	0.49299226	0.47134501	Alg10b	1
GO:0045005	DNA-templated DNA replication maintenance of fidelity	1/95	52/14464	0.01923077	2.92793522	1.13242567	0.29055458	0.49299226	0.47134501	Pole	1
GO:0045773	positive regulation of axon extension	1/95	52/14464	0.01923077	2.92793522	1.13242567	0.29055458	0.49299226	0.47134501	Myo5b	1
GO:0051489	regulation of filopodium assembly	1/95	52/14464	0.01923077	2.92793522	1.13242567	0.29055458	0.49299226	0.47134501	Agrn	1
GO:0089718	amino acid import across plasma membrane	1/95	52/14464	0.01923077	2.92793522	1.13242567	0.29055458	0.49299226	0.47134501	Slc6a6	1
GO:0097006	regulation of plasma lipoprotein particle levels	1/95	52/14464	0.01923077	2.92793522	1.13242567	0.29055458	0.49299226	0.47134501	Trem2	1
GO:1902305	regulation of sodium ion transmembrane transport	1/95	52/14464	0.01923077	2.92793522	1.13242567	0.29055458	0.49299226	0.47134501	Agrn	1
GO:0051054	positive regulation of DNA metabolic process	3/95	287/14464	0.01045296	1.59149092	0.82295215	0.29179437	0.49465848	0.47293806	Peli1/Paxip1/Arid2	3
GO:1901615	organic hydroxy compound metabolic process	4/95	417/14464	0.00959233	1.4604569	0.77578779	0.29341038	0.49564806	0.47388419	Acer2/Por/Lss/Clcn2	4
GO:0001941	postsynaptic membrane organization	1/95	53/14464	0.01886792	2.87269116	1.11054142	0.29523106	0.49564806	0.47388419	Agrn	1
GO:0002067	glandular epithelial cell differentiation	1/95	53/14464	0.01886792	2.87269116	1.11054142	0.29523106	0.49564806	0.47388419	Clcn2	1
GO:0008631	intrinsic apoptotic signaling pathway in response to oxidative stress	1/95	53/14464	0.01886792	2.87269116	1.11054142	0.29523106	0.49564806	0.47388419	Trem2	1
GO:0014003	oligodendrocyte development	1/95	53/14464	0.01886792	2.87269116	1.11054142	0.29523106	0.49564806	0.47388419	Lyn	1
GO:0045839	negative regulation of mitotic nuclear division	1/95	53/14464	0.01886792	2.87269116	1.11054142	0.29523106	0.49564806	0.47388419	Xrcc3	1
GO:0048002	antigen processing and presentation of peptide antigen	1/95	53/14464	0.01886792	2.87269116	1.11054142	0.29523106	0.49564806	0.47388419	Trem2	1
GO:0050994	regulation of lipid catabolic process	1/95	53/14464	0.01886792	2.87269116	1.11054142	0.29523106	0.49564806	0.47388419	Gimap5	1
GO:0070527	platelet aggregation	1/95	53/14464	0.01886792	2.87269116	1.11054142	0.29523106	0.49564806	0.47388419	Lyn	1
GO:1901607	alpha-amino acid biosynthetic process	1/95	53/14464	0.01886792	2.87269116	1.11054142	0.29523106	0.49564806	0.47388419	Tha1	1
GO:0001666	response to hypoxia	3/95	289/14464	0.01038062	1.58047714	0.81049481	0.29538412	0.49564806	0.47388419	Fzd4/Trem2/ND5	3
GO:0043410	positive regulation of MAPK cascade	4/95	419/14464	0.00954654	1.45348574	0.76592721	0.296367	0.49564806	0.47388419	Fzd4/Trem2/Abl1/Lyn	4
GO:0048545	response to steroid hormone	3/95	290/14464	0.01034483	1.57502722	0.80430157	0.29718017	0.49564806	0.47388419	Per1/Por/Klf9	3
GO:0070588	calcium ion transmembrane transport	3/95	290/14464	0.01034483	1.57502722	0.80430157	0.29718017	0.49564806	0.47388419	Gimap5/Abl1/Lyn	3
GO:0002381	immunoglobulin production involved in immunoglobulin-mediated immune response	1/95	54/14464	0.01851852	2.81949318	1.08916341	0.29987702	0.49564806	0.47388419	Paxip1	1
GO:0006998	nuclear envelope organization	1/95	54/14464	0.01851852	2.81949318	1.08916341	0.29987702	0.49564806	0.47388419	Cdan1	1
GO:0010712	regulation of collagen metabolic process	1/95	54/14464	0.01851852	2.81949318	1.08916341	0.29987702	0.49564806	0.47388419	Serpine1	1
GO:0016447	somatic recombination of immunoglobulin gene segments	1/95	54/14464	0.01851852	2.81949318	1.08916341	0.29987702	0.49564806	0.47388419	Paxip1	1
GO:0021575	hindbrain morphogenesis	1/95	54/14464	0.01851852	2.81949318	1.08916341	0.29987702	0.49564806	0.47388419	Abl1	1
GO:0031641	regulation of myelination	1/95	54/14464	0.01851852	2.81949318	1.08916341	0.29987702	0.49564806	0.47388419	Jam2	1
GO:0032720	negative regulation of tumor necrosis factor production	1/95	54/14464	0.01851852	2.81949318	1.08916341	0.29987702	0.49564806	0.47388419	Trem2	1
GO:0043506	regulation of JUN kinase activity	1/95	54/14464	0.01851852	2.81949318	1.08916341	0.29987702	0.49564806	0.47388419	Fzd4	1
GO:0046173	polyol biosynthetic process	1/95	54/14464	0.01851852	2.81949318	1.08916341	0.29987702	0.49564806	0.47388419	Acer2	1
GO:0050818	regulation of coagulation	1/95	54/14464	0.01851852	2.81949318	1.08916341	0.29987702	0.49564806	0.47388419	Serpine1	1
GO:0051496	positive regulation of stress fiber assembly	1/95	54/14464	0.01851852	2.81949318	1.08916341	0.29987702	0.49564806	0.47388419	Abl1	1
GO:0060119	inner ear receptor cell development	1/95	54/14464	0.01851852	2.81949318	1.08916341	0.29987702	0.49564806	0.47388419	Alg10b	1
GO:0061900	glial cell activation	1/95	54/14464	0.01851852	2.81949318	1.08916341	0.29987702	0.49564806	0.47388419	Trem2	1
GO:1900024	regulation of substrate adhesion-dependent cell spreading	1/95	54/14464	0.01851852	2.81949318	1.08916341	0.29987702	0.49564806	0.47388419	Abl1	1
GO:0071248	cellular response to metal ion	2/95	167/14464	0.01197605	1.82338481	0.87019453	0.30012665	0.49564806	0.47388419	Mt1/Cpne7	2

GO:1902905 positive regulation of supramolecular fiber organization	2/95	167/14464	0.01197605	1.82338481	0.87019453	0.30012665	0.49564806	0.47388419	Abl1/Nav3	2
GO:0072659 protein localization to plasma membrane	3/95	292/14464	0.01027397	1.56423937	0.79198501	0.30077445	0.49629078	0.47449868	Trem2/Rab34/Myo5b	3
GO:0000070 mitotic sister chromatid segregation	2/95	168/14464	0.01190476	1.81253133	0.86132134	0.30255459	0.49795955	0.47609418	Xrcc3/Ncapd3	2
GO:0046390 ribose phosphate biosynthetic process	2/95	168/14464	0.01190476	1.81253133	0.86132134	0.30255459	0.49795955	0.47609418	Trem2/ND5	2
GO:0046605 regulation of centrosome cycle	1/95	55/14464	0.01818182	2.76822967	1.06826948	0.30449269	0.49795955	0.47609418	Xrcc3	1
GO:0051306 mitotic sister chromatid separation	1/95	55/14464	0.01818182	2.76822967	1.06826948	0.30449269	0.49795955	0.47609418	Xrcc3	1
GO:0060688 regulation of morphogenesis of a branching structure	1/95	55/14464	0.01818182	2.76822967	1.06826948	0.30449269	0.49795955	0.47609418	Abl1	1
GO:1905517 macrophage migration	1/95	55/14464	0.01818182	2.76822967	1.06826948	0.30449269	0.49795955	0.47609418	Trem2	1
GO:0009952 anterior/posterior pattern specification	2/95	169/14464	0.01183432	1.80180629	0.85250798	0.30498118	0.49795955	0.47609418	Bhlhe40/Bhlhe41	2
GO:0022412 cellular process involved in reproduction in multicellular organism	3/95	295/14464	0.01016949	1.54833185	0.77368219	0.30617061	0.49795955	0.47609418	Fzd4/Spaca6/Jam2	3
GO:0046890 regulation of lipid biosynthetic process	2/95	170/14464	0.01176471	1.79120743	0.84375363	0.30740634	0.49795955	0.47609418	Por/Clcn2	2
GO:0045862 positive regulation of proteolysis	3/95	296/14464	0.01013514	1.543101	0.7676264	0.30797043	0.49795955	0.47609418	Trem2/Acer2/Lyn	3
GO:0002437 inflammatory response to antigenic stimulus	1/95	56/14464	0.01785714	2.71879699	1.04783879	0.30907824	0.49795955	0.47609418	Trem2	1
GO:0002886 regulation of myeloid leukocyte mediated immunity	1/95	56/14464	0.01785714	2.71879699	1.04783879	0.30907824	0.49795955	0.47609418	Lyn	1
GO:0007019 microtubule depolymerization	1/95	56/14464	0.01785714	2.71879699	1.04783879	0.30907824	0.49795955	0.47609418	Nav3	1
GO:0032964 collagen biosynthetic process	1/95	56/14464	0.01785714	2.71879699	1.04783879	0.30907824	0.49795955	0.47609418	Serpine1	1
GO:0034198 cellular response to amino acid starvation	1/95	56/14464	0.01785714	2.71879699	1.04783879	0.30907824	0.49795955	0.47609418	Sar1b	1
GO:0039532 negative regulation of cytoplasmic pattern recognition receptor signaling pathway	1/95	56/14464	0.01785714	2.71879699	1.04783879	0.30907824	0.49795955	0.47609418	Trem2	1
GO:0042987 amyloid precursor protein catabolic process	1/95	56/14464	0.01785714	2.71879699	1.04783879	0.30907824	0.49795955	0.47609418	Lyn	1
GO:0043462 regulation of ATP-dependent activity	1/95	56/14464	0.01785714	2.71879699	1.04783879	0.30907824	0.49795955	0.47609418	Agm	1
GO:0043536 positive regulation of blood vessel endothelial cell migration	1/95	56/14464	0.01785714	2.71879699	1.04783879	0.30907824	0.49795955	0.47609418	Abl1	1
GO:0048260 positive regulation of receptor-mediated endocytosis	1/95	56/14464	0.01785714	2.71879699	1.04783879	0.30907824	0.49795955	0.47609418	Serpine1	1
GO:0050854 regulation of antigen receptor-mediated signaling pathway	1/95	56/14464	0.01785714	2.71879699	1.04783879	0.30907824	0.49795955	0.47609418	Lyn	1
GO:0051646 mitochondrion localization	1/95	56/14464	0.01785714	2.71879699	1.04783879	0.30907824	0.49795955	0.47609418	Tspan4	1
GO:0051784 negative regulation of nuclear division	1/95	56/14464	0.01785714	2.71879699	1.04783879	0.30907824	0.49795955	0.47609418	Xrcc3	1
GO:0072348 sulfur compound transport	1/95	56/14464	0.01785714	2.71879699	1.04783879	0.30907824	0.49795955	0.47609418	Slc6a6	1
GO:0072698 protein localization to microtubule cytoskeleton	1/95	56/14464	0.01785714	2.71879699	1.04783879	0.30907824	0.49795955	0.47609418	Abl1	1
GO:1903556 negative regulation of tumor necrosis factor superfamily cytokine production	1/95	56/14464	0.01785714	2.71879699	1.04783879	0.30907824	0.49795955	0.47609418	Trem2	1
GO:0030099 myeloid cell differentiation	4/95	428/14464	0.00934579	1.42292179	0.72216758	0.30971904	0.49795955	0.47609418	Trem2/Gimap5/Lrrc17/Lyn	4
GO:0040013 negative regulation of locomotion	3/95	297/14464	0.01010101	1.53790537	0.76159289	0.30977072	0.49795955	0.47609418	Serpine1/Nav3/Arid2	3
GO:0001678 intracellular glucose homeostasis	2/95	171/14464	0.01169591	1.78073253	0.83505746	0.30982997	0.49795955	0.47609418	Trem2/Rab34	2
GO:0071322 cellular response to carbohydrate stimulus	2/95	171/14464	0.01169591	1.78073253	0.83505746	0.30982997	0.49795955	0.47609418	Trem2/Rab34	2
GO:1901605 alpha-amino acid metabolic process	2/95	171/14464	0.01169591	1.78073253	0.83505746	0.30982997	0.49795955	0.47609418	Pipox/Tha1	2
GO:1902904 negative regulation of supramolecular fiber organization	2/95	172/14464	0.01162791	1.77037944	0.82641868	0.31225199	0.49988654	0.47793656	Trem2/Nav3	2
GO:0001954 positive regulation of cell-matrix adhesion	1/95	57/14464	0.01754386	2.6710988	1.0278518	0.31363387	0.49988654	0.47793656	Abl1	1
GO:0006473 protein acetylation	1/95	57/14464	0.01754386	2.6710988	1.0278518	0.31363387	0.49988654	0.47793656	Naa50	1
GO:0016601 Rac protein signal transduction	1/95	57/14464	0.01754386	2.6710988	1.0278518	0.31363387	0.49988654	0.47793656	Agm	1
GO:0032410 negative regulation of transporter activity	1/95	57/14464	0.01754386	2.6710988	1.0278518	0.31363387	0.49988654	0.47793656	Agm	1
GO:0033619 membrane protein proteolysis	1/95	57/14464	0.01754386	2.6710988	1.0278518	0.31363387	0.49988654	0.47793656	Rbmx	1
GO:0046635 positive regulation of alpha-beta T cell activation	1/95	57/14464	0.01754386	2.6710988	1.0278518	0.31363387	0.49988654	0.47793656	Gimap5	1
GO:0046902 regulation of mitochondrial membrane permeability	1/95	57/14464	0.01754386	2.6710988	1.0278518	0.31363387	0.49988654	0.47793656	Gimap5	1
GO:0071470 cellular response to osmotic stress	1/95	57/14464	0.01754386	2.6710988	1.0278518	0.31363387	0.49988654	0.47793656	Clcn2	1
GO:1900271 regulation of long-term synaptic potentiation	1/95	57/14464	0.01754386	2.6710988	1.0278518	0.31363387	0.49988654	0.47793656	Abl1	1
GO:0043393 regulation of protein binding	2/95	173/14464	0.01156069	1.76014603	0.81783651	0.31467233	0.50112547	0.47912109	Abl1/Agm	2
GO:0042326 negative regulation of phosphorylation	3/95	301/14464	0.00996678	1.51746809	0.73767814	0.31697583	0.50375299	0.48163323	Inca1/Abl1/Lyn	3
GO:0006805 xenobiotic metabolic process	1/95	58/14464	0.01724138	2.62504537	1.00829011	0.31815978	0.50375299	0.48163323	Gsta4	1
GO:0010171 body morphogenesis	1/95	58/14464	0.01724138	2.62504537	1.00829011	0.31815978	0.50375299	0.48163323	Zfp281	1
GO:0046637 regulation of alpha-beta T cell differentiation	1/95	58/14464	0.01724138	2.62504537	1.00829011	0.31815978	0.50375299	0.48163323	Gimap5	1
GO:0055117 regulation of cardiac muscle contraction	1/95	58/14464	0.01724138	2.62504537	1.00829011	0.31815978	0.50375299	0.48163323	Agm	1
GO:0060760 positive regulation of response to cytokine stimulus	1/95	58/14464	0.01724138	2.62504537	1.00829011	0.31815978	0.50375299	0.48163323	Trem2	1
GO:0070613 regulation of protein processing	1/95	58/14464	0.01724138	2.62504537	1.00829011	0.31815978	0.50375299	0.48163323	Serpine1	1
GO:0046777 protein autophosphorylation	2/95	175/14464	0.01142857	1.74003008	0.80083897	0.31950761	0.50505362	0.48287675	Abl1/Lyn	2
GO:1902806 regulation of cell cycle G1/S phase transition	2/95	175/14464	0.01142857	1.74003008	0.80083897	0.31950761	0.50505362	0.48287675	Tcim/Arid2	2
GO:0006164 purine nucleotide biosynthetic process	2/95	176/14464	0.01136364	1.73014354	0.79242211	0.32192239	0.50669163	0.48444283	Trem2/ND5	2
GO:0050954 sensory perception of mechanical stimulus	2/95	176/14464	0.01136364	1.73014354	0.79242211	0.32192239	0.50669163	0.48444283	Fzd4/Alg10b	2
GO:0036293 response to decreased oxygen levels	3/95	304/14464	0.00986842	1.50249307	0.71996763	0.32238264	0.50669163	0.48444283	Fzd4/Trem2/ND5	3
GO:1901990 regulation of mitotic cell cycle phase transition	3/95	304/14464	0.00986842	1.50249307	0.71996763	0.32238264	0.50669163	0.48444283	Wee1/Xrcc3/Arid2	3
GO:0000381 regulation of alternative mRNA splicing, via spliceosome	1/95	59/14464	0.01694915	2.58055308	0.9891364	0.32265616	0.50669163	0.48444283	Rbmx	1
GO:0002763 positive regulation of myeloid leukocyte differentiation	1/95	59/14464	0.01694915	2.58055308	0.9891364	0.32265616	0.50669163	0.48444283	Trem2	1
GO:0006903 vesicle targeting	1/95	59/14464	0.01694915	2.58055308	0.9891364	0.32265616	0.50669163	0.48444283	Sar1b	1
GO:0035176 social behavior	1/95	59/14464	0.01694915	2.58055308	0.9891364	0.32265616	0.50669163	0.48444283	Trem2	1
GO:0062197 cellular response to chemical stress	3/95	305/14464	0.00983607	1.49756687	0.7141062	0.32418526	0.50788787	0.48558655	Net1/Clcn2/Abl1	3
GO:0006352 DNA-templated transcription initiation	2/95	177/14464	0.01129944	1.72036872	0.78405888	0.32433517	0.50788787	0.48558655	Tet3/Paxip1	2
GO:0032412 regulation of monoatomic ion transmembrane transporter activity	2/95	177/14464	0.01129944	1.72036872	0.78405888	0.32433517	0.50788787	0.48558655	Trem2/Agm	2
GO:0051495 positive regulation of cytoskeleton organization	2/95	178/14464	0.01123596	1.71070373	0.77574859	0.32674586	0.50788787	0.48558655	Abl1/Nav3	2
GO:0010257 NADH dehydrogenase complex assembly	1/95	60/14464	0.01666667	2.53754386	0.97037434	0.32712319	0.50788787	0.48558655	ND5	1
GO:0016445 somatic diversification of immunoglobulins	1/95	60/14464	0.01666667	2.53754386	0.97037434	0.32712319	0.50788787	0.48558655	Paxip1	1
GO:0030101 natural killer cell activation	1/95	60/14464	0.01666667	2.53754386	0.97037434	0.32712319	0.50788787	0.48558655	Il15ra	1
GO:0032147 activation of protein kinase activity	1/95	60/14464	0.01666667	2.53754386	0.97037434	0.32712319	0.50788787	0.48558655	Abl1	1
GO:0032981 mitochondrial respiratory chain complex I assembly	1/95	60/14464	0.01666667	2.53754386	0.97037434	0.32712319	0.50788787	0.48558655	ND5	1
GO:0048008 platelet-derived growth factor receptor signaling pathway	1/95	60/14464	0.01666667	2.53754386	0.97037434	0.32712319	0.50788787	0.48558655	Abl1	1
GO:0051703 biological process involved in intraspecies interaction between organisms	1/95	60/14464	0.01666667	2.53754386	0.97037434	0.32712319	0.50788787	0.48558655	Trem2	1
GO:0070228 regulation of lymphocyte apoptotic process	1/95	60/14464	0.01666667	2.53754386	0.97037434	0.32712319	0.50788787	0.48558655	Lyn	1
GO:0071385 cellular response to glucocorticoid stimulus	1/95	60/14464	0.01666667	2.53754386	0.97037434	0.32712319	0.50788787	0.48558655	Klf9	1
GO:1905330 regulation of morphogenesis of an epithelium	1/95	60/14464	0.01666667	2.53754386	0.97037434	0.32712319	0.50788787	0.48558655	Abl1	1
GO:0051235 maintenance of location	3/95	307/14464	0.00977199	1.48781073	0.70244552	0.32779078	0.50851294	0.48618417	Trem2/Abl1/Lyn	3
GO:0030595 leukocyte chemotaxis	2/95	179/14464	0.01117318	1.70114672	0.76749053	0.32915438	0.50926259	0.4869009	Serpine1/Lyn	2
GO:0051091 positive regulation of DNA-binding transcription factor activity	2/95	179/14464	0.01117318	1.70114672	0.76749053	0.32915438	0.50926259	0.4869009	Fzd4/Rps6ka5	2
GO:0032102 negative regulation of response to external stimulus	3/95	308/14464	0.00974026	1.48298018	0.69664594	0.32959361	0.50926259	0.4869009	Nr1d2/Trem2/Serpine1	3
GO:0051656 establishment of organelle localization	4/95	442/14464	0.00904977	1.37785187	0.65600244	0.3306092	0.50926259	0.4869009	Tspan4/Sar1b/Myo5b/Lyn	4
GO:0002931 response to ischemia	1/95	61/14464	0.01639344	2.49594478	0.95198855	0.33156108	0.50926259	0.4869009	Trem2	1
GO:0032677 regulation of interleukin-8 production	1/95	61/14464	0.01639344	2.49594478	0.95198855	0.33156108	0.50926259	0.4869009	Serpine1	1
GO:0034308 primary alcohol metabolic process	1/95	61/14464	0.01639344	2.49594478	0.95198855	0.33156108	0.50926259	0.4869009	Clcn2	1
GO:0060350 endochondral bone morphogenesis	1/95	61/14464	0.01639344	2.49594478	0.95198855	0.33156108	0.50926259	0.4869009	Por	1
GO:0099518 vesicle cytoskeletal trafficking	1/95	61/14464	0.01639344	2.49594478	0.95198855	0.33156108	0.50926259	0.4869009	Myo5b	1
GO:1903053 regulation of extracellular matrix organization	1/95	61/14464	0.01639344	2.49594478	0.95198855	0.33156108	0.50926259	0.4869009	Abl1	1
GO:1990928 response to amino acid starvation	1/95	61/14464	0.01639344	2.49594478	0.95198855	0.33156108	0.50926259	0.4869009	Sar1b	1
GO:0030100 regulation of endocytosis	3/95	310/14464	0.00967742	1.47341256	0.68510755	0.33319919	0.50926259	0.4869009	Trem2/Serpine1/Il15ra	3
GO:0000075 cell cycle checkpoint signaling	2/95	181/14464	0.01104972	1.68234952	0.7511284	0.33396465	0.50926259	0.4869009	Xrcc3/Lyn	2
GO:0043010 camera-type eye development	3/95	311/14464	0.0096463	1.4686749	0.67936843	0.33500187	0.50926259	0.4869009	Per1/Fzd4/Clcn2	3
GO:0002562 somatic diversification of immune receptors via germline recombination within a single locus	1/95	62/14464	0.01612903	2.45568761	0.93396446	0.33597	0.50926259	0.4869009	Paxip1	1
GO:0006487 protein N-linked glycosylation	1/95	62/14464	0.01612903	2.45568761	0.93396446	0.33597	0.50926259	0.4869009	Alg10b	1
GO:0006893 Golgi to plasma membrane transport	1/95	62/14464	0.01612903	2.45568761	0.93396446	0.33597	0.50926259	0.4869009	Rab34	1
GO:0008652 amino acid biosynthetic process	1/95	62/14464	0.01612903	2.45568761	0.93396446	0.33597	0.50926259	0.4869009	Tha1	1
GO:0016444 somatic cell DNA recombination	1/95	62/14464	0.01612903	2.45568761	0.93396446	0.33597	0.50926259	0.4869009	Paxip1	1
GO:0019646 aerobic electron transport chain	1/95	62/14464	0.01612903	2.45568761	0.93396446	0.33597	0.50926259	0.4869009	ND5	1
GO:0021695 cerebellar cortex development	1/95	62/14464	0.01612903	2.45568761	0.93396446	0.33597	0.50926259	0.4869009	Rere	1
GO:0032233 positive regulation of actin filament bundle assembly	1/95	62/14464	0.01612903	2.45568761	0.93396446	0.33597	0.50926259	0.4869009	Abl1	1
GO:0032637 interleukin-8 production	1/95	62/14464	0.01612903	2.45568761	0.93396446	0.33597	0.50926259	0.4869009	Serpine1	1
GO:0042130 negative regulation of T cell proliferation	1/95	62/14464	0.01612903	2.45568761	0.93396446	0.33597	0.50926259	0.4869009	Peli1	1
GO:0051148 negative regulation of muscle cell differentiation	1/95	62/14464	0.01612903	2.45568761	0.93396446	0.33597	0.50926259	0.4869009	Bhlhe41	1
GO:0051893 regulation of focal adhesion assembly	1/95	62/14464	0.01612903	2.45568761	0.93396446	0.33597	0.50926259	0.4869009	Abl1	1
GO:0090109 regulation of cell-substrate junction assembly	1/95	62/14464	0.01612903	2.45568761	0.93396446	0.33597	0.50926259	0.4869009	Abl1	1
GO:1903317 regulation of protein maturation	1/95	62/14464	0.01612903	2.45568761	0.93396446	0.33597	0.50926259	0.4869009	Serpine1	1
GO:2000514 regulation of CD4-positive, alpha-beta T cell activation	1/95	62/14464	0.01612903	2.45568761	0.93396446	0.33597	0.50926259	0.4869009	Gimap5	1
GO:0007409 axonogenesis	4/95	446/14464	0.00896861	1.36549445	0.637504	0.33659684	0.50981005	0.48742432	Myo5b/Abl1/Agm/Celsr3	4
GO:0071383 cellular response to steroid hormone stimulus	2/95	183/14464	0.01092896	1.66396319	0.7349672	0.33876538	0.51228587	0.48979143	Per1/Klf9	2
GO:1990138 neuron projection extension	2/95	183/14464	0.01092896	1.66396319	0.7349672	0.33876538	0.51228587	0.48979143	Myo5b/Abl1	2
GO:0006081 cellular aldehyde metabolic process	1/95	63/14464	0.01587302	2.41670844	0.91628833	0.34035015	0.51266243	0.49015145	Clcn2	1
GO:0006919 activation of cysteine-type endopeptidase activity involved in apoptotic process	1/95	63/14464	0.01587302	2.41670844	0.91628833	0.34035015	0.51266243	0.49015145	Acer2	1
GO:0045669 positive regulation of osteoblast differentiation	1/95	63/14464	0.01587302	2.41670844	0.91628833	0.34035015	0.51266243	0.49015145	Gdf10	1
GO:0051898 negative regulation of phosphatidylinositol 3-kinase/protein kinase B signal transduction	1/95	63/14464	0.01587302	2.41670844	0.91628833	0.34035015	0.51266243	0.49015145	Trem2	1
GO:0061035 regulation of cartilage development	1/95	63/14464	0.01587302	2.41670844	0.91628833	0.34035015	0.51266243	0.49015145	Por	1
GO:1903532 positive regulation of secretion by cell	3/95	315/14464	0.00952381	1.45002506	0.656609	0.34221094	0.51437213	0.49178609	Trem2/Rab34/Myo5b	3
GO:0042098 T cell proliferation	2/95	185/14464	0.01081081	1.6459744	0.71900183	0.34355601	0.51437213	0.49178609	Abl1/Peli1	2
GO:0002637 regulation of immunoglobulin production	1/95	64/14464	0.015625	2.37894737	0.89894714	0.3447017	0.51437213	0.49178609	Paxip1	1
GO:0003407 neural retina development	1/95	64/14464	0.015625	2.37894737	0.89894714	0.3447017	0.51437213	0.49178609	Per1	1
GO:0007528 neuromuscular junction development	1/95	64/14464	0.015625	2.37894737	0.89894714	0.3447017	0.51437213	0.49178609	Agm	1
GO:0010972 negative regulation of G2/M transition of mitotic cell cycle	1/95	64/14464	0.015625	2.37894737	0.89894714	0.3447017	0.51437213	0.49178609	Wee1	1
GO:0042776 proton motive force-driven mitochondrial ATP synthesis	1/95	64/14464	0.015625	2.37894737	0.89894714	0.3447017	0.51437213	0.49178609	ND5	1
GO:0044380 protein localization to cytoskeleton	1/95	64/14464	0.015625	2.37894737	0.89894714	0.3447017	0.51437213	0.49178609	Abl1	1
GO:0045428 regulation of nitric oxide biosynthetic process	1/95	64/14464	0.015625	2.37894737	0.89894714	0.3447017	0.51437213	0.49178609	Gimap5	1
GO:0050885 neuromuscular process controlling balance	1/95	64/14464	0.015625	2.37894737	0.89894714	0.3447017	0.51437213	0.49178609	Abl1	1
GO:0062208 positive regulation of pattern recognition receptor signaling pathway	1/95	64/14464	0.015625	2.37894737	0.89894714	0.3447017	0.51437213	0.49178609	Peli1	1
GO:2000179 positive regulation of neural precursor cell proliferation	1/95	64/14464	0.015625	2.37894737	0.89894714	0.3447017	0.51437213	0.49178609	Lyn	1
GO:0022898 regulation of transmembrane transporter activity	2/95	186/14464	0.01075269	1.63712507	0.71109103	0.34594735	0.51542932	0.49279686	Trem2/Agm	2
GO:0099175 regulation of postsynapse organization	2/95	186/14464	0.01075269	1.63712507	0.71109103	0.34594735	0.51542932	0.49279686	Rps6ka5/Rbmx	2
GO:0001910 regulation of leukocyte mediated cytotoxicity	1/95	65/14464	0.01538462	2.34234818	0.88192857	0.34902485	0.51840456	0.49564145	Gimap5	1
GO:0045576 mast cell activation	1/95	65/14464	0.01538462	2.34234818	0.88192857	0.34902485	0.51840456	0.49564145	Lyn	1
GO:0060261 positive regulation of transcription initiation by RNA polymerase II	1/95	65/14464	0.01538462	2.34234818	0.88192857	0.34902485	0.51840456	0.49564145	Paxip1	1
GO:0140115 export across plasma membrane	1/95	65/14464	0.01538462	2.34234818	0.88192857	0.34902485	0.51840456	0.49564145	Agm	1
GO:0062012 regulation of small molecule metabolic process	3/95	319/14464	0.00940439	1.43184293	0.63415789	0.34941559	0.51858353	0.49581257	Trem2/Por/Clcn2	3
GO:0010817 regulation of hormone levels	4/95	455/14464	0.00879121	1.33848467	0.59651156	0.35008746	0.51917917	0.49638205	Fzd4/Por/Clcn2/Lyn	4
GO:0061351 neural precursor cell proliferation	2/95	189/14464	0.01058201	1.61113896	0.68763906	0.35310472	0.52195585	0.4990368	Rere/Lyn	2
GO:0002720 positive regulation of cytokine production involved in immune response	1/95	66/14464	0.01515152	2.30685805	0.86522095	0.35331978	0.52195585	0.4990368	Gimap5	1
GO:0060976 coronary vasculature development	1/95	66/14464	0.01515152	2.30685805	0.86522095	0.35331978	0.52195585	0.4990368	Arid2	1
GO:0071384 cellular response to corticosteroid stimulus	1/95	66/14464	0.01515152	2.30685805	0.86522095	0.35331978	0.52195585	0.4990368	Klf9	1
GO:1902750 negative regulation of cell cycle G2/M phase transition	1/95	66/14464	0.01515152	2.30685805	0.86522095	0.35331978	0.52195585	0.4990368	Wee1	1
GO:0007517 muscle organ development	3/95	323/14464	0.00928793	1.41411113	0.61200621	0.35661343	0.52422368	0.50120506	Nr1d2/Hlf/Gpcpd1	3
GO:0032088 negative regulation of NF-kappaB transcription factor activity	1/95	67/14464	0.01492537	2.27242734	0.84881318	0.35758666	0.52422368	0.50120506	Peli1	1
GO:0042246 tissue regeneration	1/95	67/14464	0.01492537	2.27242734	0.84881318	0.35758666	0.52422368	0.50120506	Serpine1	1
GO:0045123 cellular extravasation	1/95	67/14464	0.01492537	2.27242734	0.84881318	0.35758666	0.52422368	0.50120506	Jam2	1
GO:0045132 meiotic chromosome segregation	1/95	67/14464	0.01492537	2.27242734	0.84881318	0.35758666	0.52422368	0.50120506	Ncapd3	1
GO:0060612 adipose tissue development	1/95	67/14464	0.01492537	2.27242734	0.84881318	0.35758666	0.52422368	0.50120506	Paxip1	1
GO:0070534 protein K63-linked ubiquitination	1/95	67/14464	0.01492537	2.27242734	0.84881318	0.35758666	0.52422368	0.50120506	Peli1	1
GO:0080164 regulation of nitric oxide metabolic process	1/95	67/14464	0.01492537	2.27242734	0.84881318	0.35758666	0.52422368	0.50120506	Gimap5	1
GO:0150116 regulation of cell-substrate junction organization	1/95	67/14464	0.01492537	2.27242734	0.84881318	0.35758666	0.52422368	0.50120506	Abl1	1
GO:1903036 positive regulation of response to wounding	1/95	67/14464	0.01492537	2.27242734	0.84881318	0.35758666	0.52422368	0.50120506	Serpine1	1
GO:0032271 regulation of protein polymerization	2/95	192/14464	0.01041667	1.58596491	0.66459571	0.36023571	0.52689964	0.50376351	Abl1/Nav3	2
GO:0050670 regulation of lymphocyte proliferation	2/95	192/14464	0.01041667	1.58596491	0.66459571	0.36023571	0.52689964	0.50376351	Peli1/Lyn	2
GO:1901654 response to ketone	2/95	192/14464	0.01041667	1.58596491	0.66459571	0.36023571	0.52689964	0.50376351	Por/Klf9	2
GO:0006720 isoprenoid metabolic process	1/95	68/14464	0.01470588	2.23900929	0.83269474	0.36182569	0.52841971	0.50521683	Lss	1
GO:0071806 protein transmembrane transport	1/95	68/14464	0.01470588	2.23900929	0.83269474	0.36182569	0.52841971	0.50521683	Myo5b	1
GO:0070482 response to oxygen levels	3/95	327/14464	0.00917431	1.39681313	0.59014538	0.36380219	0.53013213	0.50685406	Fzd4/Trem2/ND5	3
GO:0001763 morphogenesis of a branching structure	2/95	194/14464	0.01030928	1.56961476	0.64945316	0.3649742	0.53013213	0.50685406	Rere/Abl1	2
GO:0051650 establishment of vesicle localization	2/95	194/14464	0.01030928	1.56961476	0.64945316	0.3649742	0.53013213	0.50685406	Sar1b/Myo5b	2
GO:0009611 response to wounding	4/95	465/14464	0.00860215	1.30970006	0.55194649	0.3650904	0.53013213	0.50685406	Trem2/Serpine1/Mir22hg/Lyn	4
GO:0002200 somatic diversification of immune receptors	1/95	69/14464	0.01449275	2.20655988	0.81685564	0.36603704	0.53013213	0.50685406	Paxip1	1
GO:0008347 glial cell migration	1/95	69/14464	0.01449275	2.20655988	0.81685564	0.36603704	0.53013213	0.50685406	Trem2	1
GO:0010632 regulation of epithelial cell migration	1/95	69/14464	0.01449275	2.20655988	0.81685564	0.36603704	0.53013213	0.50685406	Serpine1	1
GO:0015986 proton motive force-driven ATP synthesis	1/95	69/14464	0.01449275	2.20655988	0.81685564	0.36603704	0.53013213	0.50685406	ND5	1
GO:0042475 odontogenesis of dentin-containing tooth	1/95	69/14464	0.01449275	2.20655988	0.81685564	0.36603704	0.53013213	0.50685406	Serpine1	1
GO:0048844 artery morphogenesis	1/95	69/14464	0.01449275	2.20655988	0.81685564	0.36603704	0.53013213	0.50685406	Arid2	1
GO:0070509 calcium ion import	1/95	69/14464	0.01449275	2.20655988	0.81685564	0.36603704	0.53013213	0.50685406	Serpine1	1
GO:0008361 regulation of cell size	2/95	195/14464	0.01025641	1.56156545	0.64194621	0.36733861	0.53161598	0.50827276	Myo5b/Abl1	2
GO:0032409 regulation of transporter activity	2/95	196/14464	0.01020408	1.55359828	0.63448147	0.36969972	0.53297365	0.50957081	Trem2/Agm	2

GO:0032944	regulation of mononuclear cell proliferation	2/95	196/14464	0.01020408	1.55359828	0.63448147	0.36969972	0.53297365	0.50957081	Peli1/Lyn	2
GO:0001960	negative regulation of cytokine-mediated signaling pathway	1/95	70/14464	0.01428571	2.17503759	0.80128637	0.37022088	0.53297365	0.50957081	Trem2	1
GO:0032008	positive regulation of TOR signaling	1/95	70/14464	0.01428571	2.17503759	0.80128637	0.37022088	0.53297365	0.50957081	Trem2	1
GO:0033143	regulation of intracellular steroid hormone receptor signaling pathway	1/95	70/14464	0.01428571	2.17503759	0.80128637	0.37022088	0.53297365	0.50957081	Per1	1
GO:0046626	regulation of insulin receptor signaling pathway	1/95	70/14464	0.01428571	2.17503759	0.80128637	0.37022088	0.53297365	0.50957081	Tns2	1
GO:1903035	negative regulation of response to wounding	1/95	70/14464	0.01428571	2.17503759	0.80128637	0.37022088	0.53297365	0.50957081	Serpine1	1
GO:0034728	nucleosome organization	1/95	71/14464	0.01408451	2.14440326	0.78597788	0.37437741	0.53734499	0.5137502	Arid2	1
GO:0046847	filopodium assembly	1/95	71/14464	0.01408451	2.14440326	0.78597788	0.37437741	0.53734499	0.5137502	Agm	1
GO:0060113	inner ear receptor cell differentiation	1/95	71/14464	0.01408451	2.14440326	0.78597788	0.37437741	0.53734499	0.5137502	Alg10b	1
GO:2000144	positive regulation of DNA-templated transcription initiation	1/95	71/14464	0.01408451	2.14440326	0.78597788	0.37437741	0.53734499	0.5137502	Paxip1	1
GO:0051047	positive regulation of secretion	3/95	334/14464	0.00898204	1.36753861	0.55256432	0.37635399	0.53977825	0.51607663	Trem2/Rab34/Myo5b	3
GO:0015800	acidic amino acid transport	1/95	72/14464	0.01388889	2.11461988	0.77092154	0.37850679	0.54004054	0.51632739	Slc6a6	1
GO:0042775	mitochondrial ATP synthesis coupled electron transport	1/95	72/14464	0.01388889	2.11461988	0.77092154	0.37850679	0.54004054	0.51632739	ND5	1
GO:0042982	amyloid precursor protein metabolic process	1/95	72/14464	0.01388889	2.11461988	0.77092154	0.37850679	0.54004054	0.51632739	Lyn	1
GO:0043299	leukocyte degranulation	1/95	72/14464	0.01388889	2.11461988	0.77092154	0.37850679	0.54004054	0.51632739	Lyn	1
GO:0051591	response to cAMP	1/95	72/14464	0.01388889	2.11461988	0.77092154	0.37850679	0.54004054	0.51632739	Per1	1
GO:0090559	regulation of membrane permeability	1/95	72/14464	0.01388889	2.11461988	0.77092154	0.37850679	0.54004054	0.51632739	Gimap5	1
GO:0099084	postsynaptic specialization organization	1/95	72/14464	0.01388889	2.11461988	0.77092154	0.37850679	0.54004054	0.51632739	Abl1	1
GO:0008406	gonad development	2/95	200/14464	0.01	1.52252632	0.60503462	0.37910998	0.5404993	0.51676601	Fzd4/Serpine1	2
GO:0009749	response to glucose	2/95	201/14464	0.00995025	1.51495156	0.59777354	0.38145372	0.54343704	0.51957476	Trem2/Rab34	2
GO:0042773	ATP synthesis coupled electron transport	1/95	73/14464	0.01369863	2.08565249	0.75610916	0.38260919	0.54387188	0.51999051	ND5	1
GO:0071277	cellular response to calcium ion	1/95	73/14464	0.01369863	2.08565249	0.75610916	0.38260919	0.54387188	0.51999051	Cpne7	1
GO:1902476	chloride transmembrane transport	1/95	73/14464	0.01369863	2.08565249	0.75610916	0.38260919	0.54387188	0.51999051	Clcn2	1
GO:0017157	regulation of exocytosis	2/95	202/14464	0.00990099	1.5074518	0.59055179	0.38379382	0.54515199	0.52121441	Myo5b/Lyn	2
GO:0045137	development of primary sexual characteristics	2/95	203/14464	0.00985222	1.50002593	0.5833689	0.38613023	0.54562363	0.52166534	Fzd4/Serpine1	2
GO:0006942	regulation of striated muscle contraction	1/95	74/14464	0.01351351	2.05746799	0.74153289	0.38668481	0.54562363	0.52166534	Agm	1
GO:0014823	response to activity	1/95	74/14464	0.01351351	2.05746799	0.74153289	0.38668481	0.54562363	0.52166534	Tns2	1
GO:0019229	regulation of vasoconstriction	1/95	74/14464	0.01351351	2.05746799	0.74153289	0.38668481	0.54562363	0.52166534	Abl1	1
GO:0032651	regulation of interleukin-1 beta production	1/95	74/14464	0.01351351	2.05746799	0.74153289	0.38668481	0.54562363	0.52166534	Trem2	1
GO:0034109	homotypic cell-cell adhesion	1/95	74/14464	0.01351351	2.05746799	0.74153289	0.38668481	0.54562363	0.52166534	Lyn	1
GO:0060260	regulation of transcription initiation by RNA polymerase II	1/95	74/14464	0.01351351	2.05746799	0.74153289	0.38668481	0.54562363	0.52166534	Paxip1	1
GO:0060761	negative regulation of response to cytokine stimulus	1/95	74/14464	0.01351351	2.05746799	0.74153289	0.38668481	0.54562363	0.52166534	Trem2	1
GO:0071479	cellular response to ionizing radiation	1/95	74/14464	0.01351351	2.05746799	0.74153289	0.38668481	0.54562363	0.52166534	Net1	1
GO:0016054	organic acid catabolic process	2/95	204/14464	0.00980392	1.49267286	0.57622445	0.38846289	0.54732767	0.52329455	Pipox/Tha1	2
GO:0046395	carboxylic acid catabolic process	2/95	204/14464	0.00980392	1.49267286	0.57622445	0.38846289	0.54732767	0.52329455	Pipox/Tha1	2
GO:0008625	extrinsic apoptotic signaling pathway via death domain receptors	1/95	75/14464	0.01333333	2.03003509	0.72718526	0.39073379	0.54939881	0.52527474	Serpine1	1
GO:0009746	response to hexose	2/95	205/14464	0.0097561	1.48539153	0.56911799	0.39079175	0.54939881	0.52527474	Trem2/Rab34	2
GO:0051651	maintenance of location in cell	2/95	205/14464	0.0097561	1.48539153	0.56911799	0.39079175	0.54939881	0.52527474	Abl1/Lyn	2
GO:0043113	receptor clustering	1/95	76/14464	0.01315789	2.0033241	0.71305914	0.39475633	0.55345031	0.52914834	Agm	1
GO:0045661	regulation of myoblast differentiation	1/95	76/14464	0.01315789	2.0033241	0.71305914	0.39475633	0.55345031	0.52914834	Arid2	1
GO:0050886	endocrine process	1/95	76/14464	0.01315789	2.0033241	0.71305914	0.39475633	0.55345031	0.52914834	Fzd4	1
GO:0000082	G1/S transition of mitotic cell cycle	2/95	207/14464	0.00966184	1.47103992	0.55501732	0.39543789	0.55345031	0.52914834	Pole/Arid2	2
GO:0007411	axon guidance	2/95	207/14464	0.00966184	1.47103992	0.55501732	0.39543789	0.55345031	0.52914834	Agm/Celsr3	2
GO:0060348	bone development	2/95	207/14464	0.00966184	1.47103992	0.55501732	0.39543789	0.55345031	0.52914834	Por/Lrrc17	2
GO:0010563	negative regulation of phosphorus metabolic process	3/95	345/14464	0.00869565	1.32393593	0.49515871	0.39598086	0.55345031	0.52914834	Inca1/Abl1/Lyn	3
GO:0045936	negative regulation of phosphate metabolic process	3/95	345/14464	0.00869565	1.32393593	0.49515871	0.39598086	0.55345031	0.52914834	Inca1/Abl1/Lyn	3
GO:1903829	positive regulation of protein localization	4/95	486/14464	0.00823045	1.25310808	0.46150812	0.39654756	0.55383898	0.52951995	Trem2/Rab34/Myo5b/Agm	4
GO:0097485	neuron projection guidance	2/95	208/14464	0.00961538	1.46396761	0.54802228	0.39775506	0.55449726	0.53014932	Agm/Celsr3	2
GO:0000380	alternative mRNA splicing, via spliceosome	1/95	77/14464	0.01298701	1.9773069	0.6991477	0.39875259	0.55449726	0.53014932	Rbmx	1
GO:0032370	positive regulation of lipid transport	1/95	77/14464	0.01298701	1.9773069	0.6991477	0.39875259	0.55449726	0.53014932	Trem2	1
GO:0032414	positive regulation of ion transmembrane transporter activity	1/95	77/14464	0.01298701	1.9773069	0.6991477	0.39875259	0.55449726	0.53014932	Trem2	1
GO:0042490	mechanoreceptor differentiation	1/95	77/14464	0.01298701	1.9773069	0.6991477	0.39875259	0.55449726	0.53014932	Alg10b	1
GO:1901880	negative regulation of protein depolymerization	1/95	77/14464	0.01298701	1.9773069	0.6991477	0.39875259	0.55449726	0.53014932	Nav3	1
GO:0007163	establishment or maintenance of cell polarity	2/95	210/14464	0.00952381	1.45002506	0.53414073	0.40237737	0.55719173	0.53272548	Wee1/Abl1	2
GO:0031348	negative regulation of defense response	2/95	210/14464	0.00952381	1.45002506	0.53414073	0.40237737	0.55719173	0.53272548	Nr1d2/Trem2	2
GO:0043542	endothelial cell migration	2/95	210/14464	0.00952381	1.45002506	0.53414073	0.40237737	0.55719173	0.53272548	Abl1/Paxip1	2
GO:0031341	regulation of cell killing	1/95	78/14464	0.01282051	1.95195682	0.68544442	0.40272274	0.55719173	0.53272548	Gimap5	1
GO:0043367	CD4-positive, alpha-beta T cell differentiation	1/95	78/14464	0.01282051	1.95195682	0.68544442	0.40272274	0.55719173	0.53272548	Gimap5	1
GO:0050795	regulation of behavior	1/95	78/14464	0.01282051	1.95195682	0.68544442	0.40272274	0.55719173	0.53272548	Per3	1
GO:0060997	dendritic spine morphogenesis	1/95	78/14464	0.01282051	1.95195682	0.68544442	0.40272274	0.55719173	0.53272548	Myo5b	1
GO:0034284	response to monosaccharide	2/95	211/14464	0.00947867	1.44315291	0.52725343	0.40468242	0.55942212	0.53485794	Trem2/Rab34	2
GO:0001654	eye development	3/95	351/14464	0.00854701	1.30130454	0.46465144	0.40662347	0.55942212	0.53485794	Per1/Fzd4/Clcn2	3
GO:0031397	negative regulation of protein ubiquitination	1/95	79/14464	0.01265823	1.9272485	0.67194306	0.40666694	0.55942212	0.53485794	Abl1	1
GO:0032611	interleukin-1 beta production	1/95	79/14464	0.01265823	1.9272485	0.67194306	0.40666694	0.55942212	0.53485794	Trem2	1
GO:0051965	positive regulation of synapse assembly	1/95	79/14464	0.01265823	1.9272485	0.67194306	0.40666694	0.55942212	0.53485794	Agm	1
GO:0097009	energy homeostasis	1/95	79/14464	0.01265823	1.9272485	0.67194306	0.40666694	0.55942212	0.53485794	Nr1d2	1
GO:1903578	regulation of ATP metabolic process	1/95	79/14464	0.01265823	1.9272485	0.67194306	0.40666694	0.55942212	0.53485794	Trem2	1
GO:1904063	negative regulation of cation transmembrane transport	1/95	79/14464	0.01265823	1.9272485	0.67194306	0.40666694	0.55942212	0.53485794	Agm	1
GO:0070663	regulation of leukocyte proliferation	2/95	213/14464	0.00938967	1.42960217	0.51358381	0.40928005	0.56261348	0.53790916	Peli1/Lyn	2
GO:0006900	vesicle budding from membrane	1/95	80/14464	0.0125	1.90315789	0.65863766	0.41058537	0.56319752	0.53846756	Sar1b	1
GO:0010507	negative regulation of autophagy	1/95	80/14464	0.0125	1.90315789	0.65863766	0.41058537	0.56319752	0.53846756	Trem2	1
GO:0070227	lymphocyte apoptotic process	1/95	80/14464	0.0125	1.90315789	0.65863766	0.41058537	0.56319752	0.53846756	Lyn	1
GO:0009165	nucleotide biosynthetic process	2/95	214/14464	0.00934579	1.42292179	0.50680074	0.41157256	0.56414838	0.53937667	Trem2/ND5	2
GO:0150063	visual system development	3/95	354/14464	0.00847458	1.29027654	0.44960153	0.41192545	0.56422907	0.53945381	Per1/Fzd4/Clcn2	3
GO:0051648	vesicle localization	2/95	215/14464	0.00930233	1.41630355	0.50005166	0.41386079	0.56570673	0.54086659	Sar1b/Myo5b	2
GO:0002690	positive regulation of leukocyte chemotaxis	1/95	81/14464	0.01234568	1.87966212	0.6455225	0.4144782	0.56570673	0.54086659	Serpine1	1
GO:0033077	T cell differentiation in thymus	1/95	81/14464	0.01234568	1.87966212	0.6455225	0.4144782	0.56570673	0.54086659	Abl1	1
GO:0071103	DNA conformation change	1/95	81/14464	0.01234568	1.87966212	0.6455225	0.4144782	0.56570673	0.54086659	Abl1	1
GO:2000142	regulation of DNA-templated transcription initiation	1/95	81/14464	0.01234568	1.87966212	0.6455225	0.4144782	0.56570673	0.54086659	Paxip1	1
GO:0032411	positive regulation of transporter activity	1/95	82/14464	0.01219512	1.85673941	0.63259208	0.41834558	0.57017413	0.54513782	Trem2	1
GO:0032642	regulation of chemokine production	1/95	82/14464	0.01219512	1.85673941	0.63259208	0.41834558	0.57017413	0.54513782	Trem2	1
GO:0048880	sensory system development	3/95	359/14464	0.00835655	1.27230611	0.42481104	0.42073078	0.57301801	0.54785683	Per1/Fzd4/Clcn2	3
GO:0006637	acyl-CoA metabolic process	1/95	83/14464	0.01204819	1.83436906	0.61984116	0.42218769	0.5733745	0.54819767	Pipox	1
GO:0030510	regulation of BMP signaling pathway	1/95	83/14464	0.01204819	1.83436906	0.61984116	0.42218769	0.5733745	0.54819767	Abl1	1
GO:0035383	thioester metabolic process	1/95	83/14464	0.01204819	1.83436906	0.61984116	0.42218769	0.5733745	0.54819767	Pipox	1
GO:1900076	regulation of cellular response to insulin stimulus	1/95	83/14464	0.01204819	1.83436906	0.61984116	0.42218769	0.5733745	0.54819767	Tns2	1
GO:0048872	homeostasis of number of cells	3/95	360/14464	0.00833333	1.26877193	0.419896	0.42248691	0.57337509	0.54819823	Gimap5/Abl1/Lyn	3
GO:0002440	production of molecular mediator of immune response	2/95	219/14464	0.00913242	1.39043499	0.47338805	0.42297019	0.57362529	0.54843745	Gimap5/Paxip1	2
GO:0010976	positive regulation of neuron projection development	2/95	220/14464	0.00909091	1.38411483	0.46680356	0.42523645	0.57570632	0.5504271	Abl1/Lyn	2
GO:0046034	ATP metabolic process	2/95	220/14464	0.00909091	1.38411483	0.46680356	0.42523645	0.57570632	0.5504271	Trem2/ND5	2
GO:0007600	sensory perception	3/95	362/14464	0.00828729	1.26176214	0.41010823	0.42599402	0.57570632	0.5504271	Fzd4/Or8b53/Alg10b	3
GO:0008593	regulation of Notch signaling pathway	1/95	84/14464	0.01190476	1.81253133	0.6072647	0.42600468	0.57570632	0.5504271	Tcim	1
GO:0034766	negative regulation of monoatomic ion transmembrane transport	1/95	84/14464	0.01190476	1.81253133	0.6072647	0.42600468	0.57570632	0.5504271	Agm	1
GO:0007059	chromosome segregation	3/95	364/14464	0.00824176	1.25482938	0.4003763	0.42949408	0.57879292	0.55337816	Xrcc3/Ncapd3/Arid2	3
GO:0046434	organophosphate catabolic process	2/95	222/14464	0.00900901	1.37164533	0.4537299	0.42975538	0.57879292	0.55337816	Pnpla7/Gpcpd1	2
GO:0090068	positive regulation of cell cycle process	2/95	222/14464	0.00900901	1.37164533	0.4537299	0.42975538	0.57879292	0.55337816	Xrcc3/Ncapd3	2
GO:0019915	lipid storage	1/95	85/14464	0.01176471	1.79120743	0.59485786	0.42979672	0.57879292	0.55337816	Trem2	1
GO:0048041	focal adhesion assembly	1/95	85/14464	0.01176471	1.79120743	0.59485786	0.42979672	0.57879292	0.55337816	Abl1	1
GO:0006672	ceramide metabolic process	1/95	86/14464	0.01162791	1.77037944	0.58261598	0.43356397	0.5806066	0.55511221	Acer2	1
GO:0006821	chloride transport	1/95	86/14464	0.01162791	1.77037944	0.58261598	0.43356397	0.5806066	0.55511221	Clcn2	1
GO:0030168	platelet activation	1/95	86/14464	0.01162791	1.77037944	0.58261598	0.43356397	0.5806066	0.55511221	Lyn	1
GO:0032273	positive regulation of protein polymerization	1/95	86/14464	0.01162791	1.77037944	0.58261598	0.43356397	0.5806066	0.55511221	Nav3	1
GO:0032456	endocytic recycling	1/95	86/14464	0.01162791	1.77037944	0.58261598	0.43356397	0.5806066	0.55511221	Myo5b	1
GO:0043242	negative regulation of protein-containing complex disassembly	1/95	86/14464	0.01162791	1.77037944	0.58261598	0.43356397	0.5806066	0.55511221	Nav3	1
GO:0050848	regulation of calcium-mediated signaling	1/95	86/14464	0.01162791	1.77037944	0.58261598	0.43356397	0.5806066	0.55511221	Trem2	1
GO:2001237	negative regulation of extrinsic apoptotic signaling pathway	1/95	86/14464	0.01162791	1.77037944	0.58261598	0.43356397	0.5806066	0.55511221	Serpine1	1
GO:1903522	regulation of blood circulation	2/95	224/14464	0.00892857	1.3593985	0.44078113	0.43425594	0.58112772	0.55561045	Abl1/Agm	2
GO:0015908	fatty acid transport	1/95	87/14464	0.01149425	1.75003025	0.57053461	0.43730659	0.58398842	0.55834553	Lyn	1
GO:0051881	regulation of mitochondrial membrane potential	1/95	87/14464	0.01149425	1.75003025	0.57053461	0.43730659	0.58398842	0.55834553	Tspan4	1
GO:0090288	negative regulation of cellular response to growth factor stimulus	1/95	87/14464	0.01149425	1.75003025	0.57053461	0.43730659	0.58398842	0.55834553	Abl1	1
GO:0002286	T cell activation involved in immune response	1/95	88/14464	0.01136364	1.73014354	0.55860945	0.44102475	0.58407625	0.5584295	Trem2	1
GO:0030148	sphingolipid biosynthetic process	1/95	88/14464	0.01136364	1.73014354	0.55860945	0.44102475	0.58407625	0.5584295	Acer2	1
GO:0030301	cholesterol transport	1/95	88/14464	0.01136364	1.73014354	0.55860945	0.44102475	0.58407625	0.5584295	Trem2	1
GO:0032652	regulation of interleukin-1 production	1/95	88/14464	0.01136364	1.73014354	0.55860945	0.44102475	0.58407625	0.5584295	Trem2	1
GO:0043406	positive regulation of MAP kinase activity	1/95	88/14464	0.01136364	1.73014354	0.55860945	0.44102475	0.58407625	0.5584295	Fzd4	1
GO:0043543	protein acylation	1/95	88/14464	0.01136364	1.73014354	0.55860945	0.44102475	0.58407625	0.5584295	Naa50	1
GO:0043954	cellular component maintenance	1/95	88/14464	0.01136364	1.73014354	0.55860945	0.44102475	0.58407625	0.5584295	Trem2	1
GO:0045833	negative regulation of lipid metabolic process	1/95	88/14464	0.01136364	1.73014354	0.55860945	0.44102475	0.58407625	0.5584295	Gimap5	1
GO:0051153	regulation of striated muscle cell differentiation	1/95	88/14464	0.01136364	1.73014354	0.55860945	0.44102475	0.58407625	0.5584295	Bhlhe41	1
GO:0120254	olefinic compound metabolic process	1/95	88/14464	0.01136364	1.73014354	0.55860945	0.44102475	0.58407625	0.5584295	Clcn2	1
GO:1900542	regulation of purine nucleotide metabolic process	1/95	88/14464	0.01136364	1.73014354	0.55860945	0.44102475	0.58407625	0.5584295	Trem2	1
GO:2000106	regulation of leukocyte apoptotic process	1/95	88/14464	0.01136364	1.73014354	0.55860945	0.44102475	0.58407625	0.5584295	Lyn	1
GO:0072522	purine-containing compound biosynthetic process	2/95	228/14464	0.00877193	1.3355494	0.41524807	0.44320079	0.5865395	0.56078459	Trem2/ND5	2
GO:0006140	regulation of nucleotide metabolic process	1/95	89/14464	0.01123596	1.71070373	0.54683637	0.44471859	0.5865395	0.56078459	Trem2	1
GO:0007156	homophilic cell adhesion via plasma membrane adhesion molecules	1/95	89/14464	0.01123596	1.71070373	0.54683637	0.44471859	0.5865395	0.56078459	Celsr3	1
GO:0098661	inorganic anion transmembrane transport	1/95	89/14464	0.01123596	1.71070373	0.54683637	0.44471859	0.5865395	0.56078459	Clcn2	1
GO:1903321	negative regulation of protein modification by small protein conjugation or removal	1/95	89/14464	0.01123596	1.71070373	0.54683637	0.44471859	0.5865395	0.56078459	Abl1	1
GO:2001251	negative regulation of chromosome organization	1/95	89/14464	0.01123596	1.71070373	0.54683637	0.44471859	0.5865395	0.56078459	Xrcc3	1
GO:1902903	regulation of supramolecular fiber organization	3/95	374/14464	0.00802139	1.22127779	0.35252982	0.4468817	0.58898762	0.56312522	Trem2/Abl1/Nav3	3
GO:0010951	negative regulation of endopeptidase activity	1/95	90/14464	0.01111111	1.69169591	0.53521141	0.44838827	0.58975812	0.56386188	Serpine1	1
GO:0043535	regulation of blood vessel endothelial cell migration	1/95	90/14464	0.01111111	1.69169591	0.53521141	0.44838827	0.58975812	0.56386188	Abl1	1
GO:0046634	regulation of alpha-beta T cell activation	1/95	90/14464	0.01111111	1.69169591	0.53521141	0.44838827	0.58975812	0.56386188	Gimap5	1
GO:0043603	amide metabolic process	3/95	376/14464	0.00797872	1.21478163	0.34311849	0.45033532	0.5905059	0.56457683	Acer2/Pipox/Gsta4	3
GO:0006970	response to osmotic stress	1/95	91/14464	0.01098901	1.67310584	0.52373074	0.45203396	0.5905059	0.56457683	Clcn2	1
GO:0010469	regulation of signaling receptor activity	1/95	91/14464	0.01098901	1.67310584	0.52373074	0.45203396	0.5905059	0.56457683	Serpine1	1
GO:0019751	polyol metabolic process	1/95	91/14464	0.01098901	1.67310584	0.52373074	0.45203396	0.5905059	0.56457683	Acer2	1
GO:0032602	chemokine production	1/95	91/14464	0.01098901	1.67310584	0.52373074	0.45203396	0.5905059	0.56457683	Trem2	1
GO:0042310	vasoconstriction	1/95	91/14464	0.01098901	1.67310584	0.52373074	0.45203396	0.5905059	0.56457683	Abl1	1
GO:0045921	positive regulation of exocytosis	1/95	91/14464	0.01098901	1.67310584	0.52373074	0.45203396	0.5905059	0.56457683	Myo5b	1
GO:0090398	cellular senescence	1/95	91/14464	0.01098901	1.67310584	0.52373074	0.45203396	0.5905059	0.56457683	Abl1	1
GO:1904019	epithelial cell apoptotic process	1/95	91/14464	0.01098901	1.67310584	0.52373074	0.45203396	0.5905059	0.56457683	Abl1	1
GO:2001257	regulation of cation channel activity	1/95	91/14464	0.01098901	1.67310584	0.52373074	0.45203396	0.5905059	0.56457683	Trem2	1
GO:0031400	negative regulation of protein modification process	3/95	378/14464	0.00793651	1.20835422	0.3337583	0.45378053	0.59198154	0.56598767	Inca1/Abl1/Lyn	3
GO:1990778	protein localization to cell periphery	3/95	378/14464	0.00793651	1.20835422	0.3337583	0.45378053	0.59198154	0.56598767	Trem2/Rab34/Myo5b	3
GO:2001233	regulation of apoptotic signaling pathway	3/95	379/14464	0.00791557	1.20516595	0.3290972	0.45549993	0.59241429	0.56640142	Trem2/Serpine1/Inca1	3
GO:0007173	epidermal growth factor receptor signaling pathway	1/95	92/14464	0.01086957	1.65491991	0.51239068	0.45565581	0.59241429	0.56640142	Abl1	1
GO:0015918	sterol transport	1/95	92/14464	0.01086957	1.65491991	0.51239068	0.45565581	0.59241429	0.56640142	Trem2	1
GO:0046889	positive regulation of lipid biosynthetic process	1/95	92/14464	0.01086957	1.65491991	0.51239068	0.45565581	0.59241429	0.56640142	Por	1
GO:0051899	membrane depolarization	1/95	92/14464	0.01086957	1.65491991	0.51239068	0.45565581	0.59241429	0.56640142	Clcn2	1
GO:0045333	cellular respiration	2/95	234/14464	0.00854701	1.30130454	0.37782343	0.45647323	0.59307524	0.56703335	ND5/Coq10b	2
GO:0010631	epithelial cell migration	1/95	93/14464	0.01075269	1.63712507	0.50118769	0.45925396	0.59387355	0.56779661	Serpine1	1
GO:0032612	interleukin-1 production	1/95	93/14464	0.01075269	1.63712507	0.50118769	0.45925396	0.59387355	0.56779661	Trem2	1
GO:0042116	macrophage activation	1/95	93/14464	0.01075269	1.63712507	0.50118769	0.45925396	0.59387355	0.56779661	Trem2	1
GO:0043280	positive regulation of cysteine-type endopeptidase activity involved in apoptotic process	1/95	93/14464	0.01075269	1.63712507	0.50118769	0.45925396	0.59387355	0.56779661	Acer2	1
GO:0090132	epithelium migration	1/95	93/14464	0.01075269	1.63712507	0.50118769	0.45925396	0.59387355	0.56779661	Serpine1	1
GO:1901874	negative regulation of post-translational protein modification	1/95	93/14464	0.01075269	1.63712507	0.50118769	0.45925396	0.59387355	0.56779661	Abl1	1
GO:1901879	regulation of protein depolymerization	1/95	93/14464	0.01075269	1.63712507	0.50118769	0.45925396	0.59387355	0.56779661	Nav3	1
GO:0001843	neural tube closure	1/95	94/14464	0.0106383	1.61970885	0.49011835	0.46282858	0.59516302	0.56902946	Abl1	1
GO:0007229	integrin-mediated signaling pathway	1/95	94/14464	0.0106383	1.61970885	0.49011835	0.46282858	0.59516302	0.56902946	Abl1	1
GO:0009308	amine metabolic process	1/95	94/14464	0.0106383	1.61970885	0.49011835	0.46282858	0.59516302	0.56902946	Inmt	1
GO:0010389	regulation of G2/M transition of mitotic cell cycle	1/95	94/14464	0.0106383	1.61970885	0.49011835	0.46282858	0.59516302	0.56902946	Wee1	1
GO:0051492	regulation of stress fiber assembly	1/95	94/14464	0.0106383	1.61970885	0.49011835	0.46282858	0.59516302	0.56902946	Abl1	1
GO:0060349	bone morphogenesis	1/95	94/14464	0.0106383	1.61970885	0.49011835	0.46282858	0.59516302	0.56902946	Por	1
GO:0060606	tube closure	1/95	94/14464	0.0106383	1.61970885	0.49011835	0.46282858	0.59516302	0.56902946	Abl1	1
GO:0007548	sex differentiation	2/95	237/14464	0.00843882	1.28483233	0.3594881	0.46304242	0.59516302	0.56902946	Fzd4/Serpine1	2
GO:0048738	cardiac muscle tissue development	2/95	237/14464	0.00843882	1.28483233	0.3594881	0.46304242	0.59516302	0.56902946	Abl1/Arid2	2

GO:0006520 amino acid metabolic process	2/95	238/14464	0.00840336	1.27943388	0.35343036	0.46522201	0.59745185	0.57121778 Pipox/Tha1	2
GO:0016055 Wnt signaling pathway	3/95	385/14464	0.00779221	1.18638414	0.3013916	0.46577001	0.59745185	0.57121778 Fzd4/Abl1/Celsr3	3
GO:0007044 cell-substrate junction assembly	1/95	95/14464	0.01052632	1.60265928	0.47917936	0.46637982	0.59745185	0.57121778 Abl1	1
GO:0045639 positive regulation of myeloid cell differentiation	1/95	95/14464	0.01052632	1.60265928	0.47917936	0.46637982	0.59745185	0.57121778 Trem2	1
GO:0070936 protein K48-linked ubiquitination	1/95	95/14464	0.01052632	1.60265928	0.47917936	0.46637982	0.59745185	0.57121778 Peli1	1
GO:0007626 locomotory behavior	2/95	240/14464	0.00833333	1.26877193	0.34139439	0.46956584	0.60113199	0.57473632 Ciart/Fzd4	2
GO:0003333 amino acid transmembrane transport	1/95	96/14464	0.01041667	1.58596491	0.46836754	0.46990783	0.60116874	0.57477147 Slc6a6	1
GO:0006979 response to oxidative stress	3/95	388/14464	0.00773196	1.17721107	0.28770327	0.47087432	0.60200387	0.57556992 Net1/ND5/Abl1	3
GO:0050772 positive regulation of axonogenesis	1/95	97/14464	0.01030928	1.56961476	0.45767983	0.47341275	0.60384662	0.57733175 Myo5b	1
GO:0090130 tissue migration	1/95	97/14464	0.01030928	1.56961476	0.45767983	0.47341275	0.60384662	0.57733175 Serpine1	1
GO:0098656 monoatomic anion transmembrane transport	1/95	97/14464	0.01030928	1.56961476	0.45767983	0.47341275	0.60384662	0.57733175 Clcn2	1
GO:0071560 cellular response to transforming growth factor beta stimulus	2/95	242/14464	0.00826446	1.25828621	0.32946273	0.47388901	0.60384662	0.57733175 Serpine1/Abl1	2
GO:0140014 mitotic nuclear division	2/95	242/14464	0.00826446	1.25828621	0.32946273	0.47388901	0.60384662	0.57733175 Xrcc3/Ncapd3	2
GO:0009205 purine ribonucleoside triphosphate metabolic process	2/95	243/14464	0.00823045	1.25310808	0.32353539	0.47604278	0.60446566	0.57792362 Trem2/ND5	2
GO:0034599 cellular response to oxidative stress	2/95	243/14464	0.00823045	1.25310808	0.32353539	0.47604278	0.60446566	0.57792362 Net1/Abl1	2
GO:0002718 regulation of cytokine production involved in immune response	1/95	98/14464	0.01020408	1.55359828	0.44711327	0.47689474	0.60446566	0.57792362 Gimap5	1
GO:0007040 lysosome organization	1/95	98/14464	0.01020408	1.55359828	0.44711327	0.47689474	0.60446566	0.57792362 Rab34	1
GO:0032204 regulation of telomere maintenance	1/95	98/14464	0.01020408	1.55359828	0.44711327	0.47689474	0.60446566	0.57792362 Ylpm1	1
GO:0036465 synaptic vesicle recycling	1/95	98/14464	0.01020408	1.55359828	0.44711327	0.47689474	0.60446566	0.57792362 Myo5b	1
GO:0080171 lytic vacuole organization	1/95	98/14464	0.01020408	1.55359828	0.44711327	0.47689474	0.60446566	0.57792362 Rab34	1
GO:1905954 positive regulation of lipid localization	1/95	98/14464	0.01020408	1.55359828	0.44711327	0.47689474	0.60446566	0.57792362 Trem2	1
GO:0060326 cell chemotaxis	2/95	245/14464	0.00816327	1.24287863	0.31175651	0.48033456	0.60684611	0.58019953 Serpine1/Lyn	2
GO:0002444 myeloid leukocyte mediated immunity	1/95	99/14464	0.01010101	1.53790537	0.436665	0.48035395	0.60684611	0.58019953 Lyn	1
GO:0014020 primary neural tube formation	1/95	99/14464	0.01010101	1.53790537	0.436665	0.48035395	0.60684611	0.58019953 Abl1	1
GO:0033108 mitochondrial respiratory chain complex assembly	1/95	99/14464	0.01010101	1.53790537	0.436665	0.48035395	0.60684611	0.58019953 ND5	1
GO:0061041 regulation of wound healing	1/95	99/14464	0.01010101	1.53790537	0.436665	0.48035395	0.60684611	0.58019953 Serpine1	1
GO:0007088 regulation of mitotic nuclear division	1/95	100/14464	0.01	1.52252632	0.42633226	0.48379053	0.61038397	0.58358205 Xrcc3	1
GO:0014910 regulation of smooth muscle cell migration	1/95	100/14464	0.01	1.52252632	0.42633226	0.48379053	0.61038397	0.58358205 Serpine1	1
GO:0006650 glycerophospholipid metabolic process	2/95	247/14464	0.00809717	1.23281483	0.3000771	0.48460517	0.61060888	0.58379709 Pnpla7/Gpcpd1	2
GO:0032984 protein-containing complex disassembly	2/95	247/14464	0.00809717	1.23281483	0.3000771	0.48460517	0.61060888	0.58379709 Nav3/Arid2	2
GO:0009199 ribonucleoside triphosphate metabolic process	2/95	248/14464	0.00806452	1.2278438	0.29427413	0.48673249	0.61187542	0.58500801 Trem2/ND5	2
GO:0007338 single fertilization	1/95	101/14464	0.00990099	1.5074518	0.41611237	0.48720461	0.61187542	0.58500801 Spaca6	1
GO:0035710 CD4-positive, alpha-beta T cell activation	1/95	101/14464	0.00990099	1.5074518	0.41611237	0.48720461	0.61187542	0.58500801 Gimap5	1
GO:0050852 T cell receptor signaling pathway	1/95	101/14464	0.00990099	1.5074518	0.41611237	0.48720461	0.61187542	0.58500801 Abl1	1
GO:0097306 cellular response to alcohol	1/95	101/14464	0.00990099	1.5074518	0.41611237	0.48720461	0.61187542	0.58500801 Klf9	1
GO:0033002 muscle cell proliferation	2/95	249/14464	0.00803213	1.2229127	0.28849533	0.48885444	0.61354589	0.58660513 Abl1/Arid2	2
GO:0001909 leukocyte mediated cytotoxicity	1/95	102/14464	0.00980392	1.49267286	0.40600276	0.49059635	0.61372516	0.58677653 Gimap5	1
GO:0010595 positive regulation of endothelial cell migration	1/95	102/14464	0.00980392	1.49267286	0.40600276	0.49059635	0.61372516	0.58677653 Abl1	1
GO:0042476 odontogenesis	1/95	102/14464	0.00980392	1.49267286	0.40600276	0.49059635	0.61372516	0.58677653 Serpine1	1
GO:0099068 postsynapse assembly	1/95	102/14464	0.00980392	1.49267286	0.40600276	0.49059635	0.61372516	0.58677653 Abl1	1
GO:0150115 cell-substrate junction organization	1/95	102/14464	0.00980392	1.49267286	0.40600276	0.49059635	0.61372516	0.58677653 Abl1	1
GO:0022411 cellular component disassembly	3/95	400/14464	0.0075	1.14189474	0.234002	0.49107396	0.61392243	0.58696513 Trem2/Nav3/Arid2	3
GO:0009144 purine nucleoside triphosphate metabolic process	2/95	251/14464	0.00796813	1.21316838	0.27700939	0.49308217	0.61553282	0.58850482 Trem2/ND5	2
GO:0006892 post-Golgi vesicle-mediated transport	1/95	103/14464	0.00970874	1.47818089	0.39600093	0.49396589	0.61553282	0.58850482 Rab34	1
GO:0042303 molting cycle	1/95	103/14464	0.00970874	1.47818089	0.39600093	0.49396589	0.61553282	0.58850482 Per1	1
GO:0042633 hair cycle	1/95	103/14464	0.00970874	1.47818089	0.39600093	0.49396589	0.61553282	0.58850482 Per1	1
GO:0140029 exocytic process	1/95	103/14464	0.00970874	1.47818089	0.39600093	0.49396589	0.61553282	0.58850482 Lyn	1
GO:0031330 negative regulation of cellular catabolic process	1/95	104/14464	0.00961538	1.46396761	0.38610448	0.49731338	0.61849927	0.59134101 Trem2	1
GO:0098754 detoxification	1/95	104/14464	0.00961538	1.46396761	0.38610448	0.49731338	0.61849927	0.59134101 Mt1	1
GO:1901655 cellular response to ketone	1/95	104/14464	0.00961538	1.46396761	0.38610448	0.49731338	0.61849927	0.59134101 Klf9	1
GO:0048588 developmental cell growth	2/95	254/14464	0.00787402	1.19883962	0.25995637	0.49938304	0.61902458	0.59184325 Myo5b/Abl1	2
GO:0060560 developmental growth involved in morphogenesis	2/95	254/14464	0.00787402	1.19883962	0.25995637	0.49938304	0.61902458	0.59184325 Myo5b/Abl1	2
GO:0002377 immunoglobulin production	1/95	105/14464	0.00952381	1.45002506	0.37631105	0.50063895	0.61902458	0.59184325 Paxip1	1
GO:0019395 fatty acid oxidation	1/95	105/14464	0.00952381	1.45002506	0.37631105	0.50063895	0.61902458	0.59184325 Por	1
GO:0022904 respiratory electron transport chain	1/95	105/14464	0.00952381	1.45002506	0.37631105	0.50063895	0.61902458	0.59184325 ND5	1
GO:0045445 myoblast differentiation	1/95	105/14464	0.00952381	1.45002506	0.37631105	0.50063895	0.61902458	0.59184325 Arid2	1
GO:0051092 positive regulation of NF-kappaB transcription factor activity	1/95	105/14464	0.00952381	1.45002506	0.37631105	0.50063895	0.61902458	0.59184325 Rps6ka5	1
GO:0110020 regulation of actomyosin structure organization	1/95	105/14464	0.00952381	1.45002506	0.37631105	0.50063895	0.61902458	0.59184325 Abl1	1
GO:1901800 positive regulation of proteasomal protein catabolic process	1/95	105/14464	0.00952381	1.45002506	0.37631105	0.50063895	0.61902458	0.59184325 Trem2	1
GO:0050878 regulation of body fluid levels	2/95	256/14464	0.0078125	1.18947368	0.24870249	0.50355623	0.62110863	0.59383579 Serpine1/Lyn	2
GO:0090150 establishment of protein localization to membrane	2/95	256/14464	0.0078125	1.18947368	0.24870249	0.50355623	0.62110863	0.59383579 Rab34/Myo5b	2
GO:0016525 negative regulation of angiogenesis	1/95	106/14464	0.00943396	1.43634558	0.3666184	0.50394275	0.62110863	0.59383579 Serpine1	1
GO:0048024 regulation of mRNA splicing, via spliceosome	1/95	106/14464	0.00943396	1.43634558	0.3666184	0.50394275	0.62110863	0.59383579 Rbmx	1
GO:0060840 artery development	1/95	106/14464	0.00943396	1.43634558	0.3666184	0.50394275	0.62110863	0.59383579 Arid2	1
GO:0002062 chondrocyte differentiation	1/95	107/14464	0.00934579	1.42292179	0.35702434	0.50722492	0.62355197	0.59617185 Por	1
GO:0008306 associative learning	1/95	107/14464	0.00934579	1.42292179	0.35702434	0.50722492	0.62355197	0.59617185 Abl1	1
GO:0042274 ribosomal small subunit biogenesis	1/95	107/14464	0.00934579	1.42292179	0.35702434	0.50722492	0.62355197	0.59617185 Wdr46	1
GO:0097250 mitochondrial respirasome assembly	1/95	107/14464	0.00934579	1.42292179	0.35702434	0.50722492	0.62355197	0.59617185 ND5	1
GO:0032231 regulation of actin filament bundle assembly	1/95	108/14464	0.00925926	1.40974659	0.34752674	0.51048561	0.62555676	0.5980886 Abl1	1
GO:0050671 positive regulation of lymphocyte proliferation	1/95	108/14464	0.00925926	1.40974659	0.34752674	0.51048561	0.62555676	0.5980886 Peli1	1
GO:0120034 positive regulation of plasma membrane bounded cell projection assembly	1/95	108/14464	0.00925926	1.40974659	0.34752674	0.51048561	0.62555676	0.5980886 Agrn	1
GO:2000181 negative regulation of blood vessel morphogenesis	1/95	108/14464	0.00925926	1.40974659	0.34752674	0.51048561	0.62555676	0.5980886 Serpine1	1
GO:2001056 positive regulation of cysteine-type endopeptidase activity	1/95	108/14464	0.00925926	1.40974659	0.34752674	0.51048561	0.62555676	0.5980886 Acer2	1
GO:0034440 lipid oxidation	1/95	109/14464	0.00917431	1.39681313	0.33812354	0.51372494	0.6279224	0.60035037 Por	1
GO:0038127 ERBB signaling pathway	1/95	109/14464	0.00917431	1.39681313	0.33812354	0.51372494	0.6279224	0.60035037 Abl1	1
GO:0046683 response to organophosphorus	1/95	109/14464	0.00917431	1.39681313	0.33812354	0.51372494	0.6279224	0.60035037 Per1	1
GO:1901343 negative regulation of vasculature development	1/95	109/14464	0.00917431	1.39681313	0.33812354	0.51372494	0.6279224	0.60035037 Serpine1	1
GO:0051222 positive regulation of protein transport	2/95	262/14464	0.00763359	1.16223383	0.21547408	0.51594273	0.63023177	0.60255833 Trem2/Rab34	2
GO:0030048 actin filament-based movement	1/95	110/14464	0.00909091	1.38411483	0.32881277	0.51694306	0.63065082	0.60295898 Myo5b	1
GO:0045446 endothelial cell differentiation	1/95	110/14464	0.00909091	1.38411483	0.32881277	0.51694306	0.63065082	0.60295898 Fzd4	1
GO:0050708 regulation of protein secretion	2/95	263/14464	0.00760456	1.15781469	0.21001173	0.51798757	0.63134021	0.6036181 Trem2/Rab34	2
GO:0007006 mitochondrial membrane organization	1/95	111/14464	0.00900901	1.37164533	0.31959248	0.52014011	0.63134021	0.6036181 Gimap5	1
GO:0014909 smooth muscle cell migration	1/95	111/14464	0.00900901	1.37164533	0.31959248	0.52014011	0.63134021	0.6036181 Serpine1	1
GO:0030038 contractile actin filament bundle assembly	1/95	111/14464	0.00900901	1.37164533	0.31959248	0.52014011	0.63134021	0.6036181 Abl1	1
GO:0032946 positive regulation of mononuclear cell proliferation	1/95	111/14464	0.00900901	1.37164533	0.31959248	0.52014011	0.63134021	0.6036181 Peli1	1
GO:0043149 stress fiber assembly	1/95	111/14464	0.00900901	1.37164533	0.31959248	0.52014011	0.63134021	0.6036181 Abl1	1
GO:0060291 long-term synaptic potentiation	1/95	111/14464	0.00900901	1.37164533	0.31959248	0.52014011	0.63134021	0.6036181 Abl1	1
GO:2000177 regulation of neural precursor cell proliferation	1/95	111/14464	0.00900901	1.37164533	0.31959248	0.52014011	0.63134021	0.6036181 Lyn	1
GO:0009141 nucleoside triphosphate metabolic process	2/95	265/14464	0.00754717	1.14907646	0.19915025	0.5220603	0.63247078	0.60469903 Trem2/ND5	2
GO:0032868 response to insulin	2/95	265/14464	0.00754717	1.14907646	0.19915025	0.5220603	0.63247078	0.60469903 Tns2/Lyn	2
GO:0042593 glucose homeostasis	2/95	265/14464	0.00754717	1.14907646	0.19915025	0.5220603	0.63247078	0.60469903 Trem2/Rab34	2
GO:0009791 post-embryonic development	1/95	112/14464	0.00892857	1.3593985	0.3104608	0.52331622	0.63319283	0.60538937 Abl1	1
GO:0071887 leukocyte apoptotic process	1/95	112/14464	0.00892857	1.3593985	0.3104608	0.52331622	0.63319283	0.60538937 Lyn	1
GO:0007264 small GTPase-mediated signal transduction	3/95	420/14464	0.00714286	1.0875188	0.14799884	0.5238988	0.63332822	0.60551882 Net1/Abl1/Agrn	3
GO:0033500 carbohydrate homeostasis	2/95	266/14464	0.0075188	1.14475653	0.19375076	0.52408818	0.63332822	0.60551882 Trem2/Rab34	2
GO:2001252 positive regulation of chromosome organization	1/95	113/14464	0.00884956	1.34736842	0.30141593	0.52647152	0.63580796	0.60788968 Ncapd3	1
GO:0045785 positive regulation of cell adhesion	3/95	422/14464	0.007109	1.08236468	0.13962413	0.5271194	0.63619001	0.60825495 Gimap5/Abl1/Arid2	3
GO:0022604 regulation of cell morphogenesis	2/95	268/14464	0.00746269	1.13621367	0.18301337	0.52812687	0.63660519	0.6086519 Fzd4/Myo5b	2
GO:0048608 reproductive structure development	2/95	268/14464	0.00746269	1.13621367	0.18301337	0.52812687	0.63660519	0.6086519 Fzd4/Serpine1	2
GO:0000077 DNA damage checkpoint signaling	1/95	114/14464	0.00877193	1.3355494	0.29245609	0.52960616	0.63678836	0.60882703 Lyn	1
GO:0007259 cell surface receptor signaling pathway via JAK-STAT	1/95	114/14464	0.00877193	1.3355494	0.29245609	0.52960616	0.63678836	0.60882703 Il15ra	1
GO:0060048 cardiac muscle contraction	1/95	114/14464	0.00877193	1.3355494	0.29245609	0.52960616	0.63678836	0.60882703 Agrn	1
GO:1990830 cellular response to leukemia inhibitory factor	1/95	114/14464	0.00877193	1.3355494	0.29245609	0.52960616	0.63678836	0.60882703 Fzd4	1
GO:0045765 regulation of angiogenesis	2/95	270/14464	0.00740741	1.12779727	0.17235694	0.53214274	0.63943764	0.61135998 Serpine1/Abl1	2
GO:0051053 negative regulation of DNA metabolic process	1/95	115/14464	0.00869565	1.32393593	0.28357957	0.53272027	0.63973104	0.61164049 Abl1	1
GO:0034763 negative regulation of transmembrane transport	1/95	116/14464	0.00862069	1.31252269	0.27478472	0.53581397	0.64222494	0.61402489 Agrn	1
GO:1990823 response to leukemia inhibitory factor	1/95	116/14464	0.00862069	1.31252269	0.27478472	0.53581397	0.64222494	0.61402489 Fzd4	1
GO:0061448 connective tissue development	2/95	272/14464	0.00735294	1.11950464	0.16178012	0.53613567	0.64222494	0.61402489 Por/Paxip1	2
GO:0061458 reproductive system development	2/95	272/14464	0.00735294	1.11950464	0.16178012	0.53613567	0.64222494	0.61402489 Fzd4/Serpine1	2
GO:0000375 RNA splicing, via transesterification reactions	2/95	273/14464	0.00732601	1.11540389	0.15652114	0.53812351	0.64271283	0.61449135 Srrm2/Rbmx	2
GO:0000377 RNA splicing, via transesterification reactions with bulged adenosine as nucleophile	2/95	273/14464	0.00732601	1.11540389	0.15652114	0.53812351	0.64271283	0.61449135 Srrm2/Rbmx	2
GO:0000398 mRNA splicing, via spliceosome	2/95	273/14464	0.00732601	1.11540389	0.15652114	0.53812351	0.64271283	0.61449135 Srrm2/Rbmx	2
GO:0006820 monoatomic anion transport	1/95	117/14464	0.00854701	1.30130454	0.26606992	0.53888741	0.64271283	0.61449135 Clcn2	1
GO:0015695 organic cation transport	1/95	117/14464	0.00854701	1.30130454	0.26606992	0.53888741	0.64271283	0.61449135 Slc6a6	1
GO:0015698 inorganic anion transport	1/95	117/14464	0.00854701	1.30130454	0.26606992	0.53888741	0.64271283	0.61449135 Clcn2	1
GO:0043534 blood vessel endothelial cell migration	1/95	117/14464	0.00854701	1.30130454	0.26606992	0.53888741	0.64271283	0.61449135 Abl1	1
GO:0002367 cytokine production involved in immune response	1/95	118/14464	0.00847458	1.29027654	0.25743361	0.54194071	0.64555197	0.61720583 Gimap5	1
GO:0043271 negative regulation of monoatomic ion transport	1/95	118/14464	0.00847458	1.29027654	0.25743361	0.54194071	0.64555197	0.61720583 Agrn	1
GO:0006888 endoplasmic reticulum to Golgi vesicle-mediated transport	1/95	119/14464	0.00840336	1.27943388	0.24887426	0.544974	0.64795856	0.61950674 Sar1b	1
GO:0030278 regulation of ossification	1/95	119/14464	0.00840336	1.27943388	0.24887426	0.544974	0.64795856	0.61950674 Gdf10	1
GO:1903313 positive regulation of mRNA metabolic process	1/95	119/14464	0.00840336	1.27943388	0.24887426	0.544974	0.64795856	0.61950674 Rbmx	1
GO:1901342 regulation of vasculature development	2/95	277/14464	0.00722022	1.09929698	0.13567766	0.5460171	0.64879679	0.62030817 Serpine1/Abl1	2
GO:0006874 intracellular calcium ion homeostasis	2/95	278/14464	0.00719424	1.09534267	0.13051411	0.547976	0.64993069	0.62139228 Abl1/Lyn	2
GO:0090287 regulation of cellular response to growth factor stimulus	2/95	278/14464	0.00719424	1.09534267	0.13051411	0.547976	0.64993069	0.62139228 Fzd4/Abl1	2
GO:0051783 regulation of nuclear division	1/95	120/14464	0.00833333	1.26877193	0.2403904	0.54798742	0.64993069	0.62139228 Xrcc3	1
GO:0030509 BMP signaling pathway	1/95	121/14464	0.00826446	1.25828621	0.23198058	0.55098109	0.65186974	0.62324618 Abl1	1
GO:0031570 DNA integrity checkpoint signaling	1/95	121/14464	0.00826446	1.25828621	0.23198058	0.55098109	0.65186974	0.62324618 Lyn	1
GO:0046467 membrane lipid biosynthetic process	1/95	121/14464	0.00826446	1.25828621	0.23198058	0.55098109	0.65186974	0.62324618 Acer2	1
GO:0051261 protein depolymerization	1/95	121/14464	0.00826446	1.25828621	0.23198058	0.55098109	0.65186974	0.62324618 Nav3	1
GO:0046942 carboxylic acid transport	2/95	281/14464	0.00711744	1.08364862	0.11513454	0.55381777	0.65458123	0.62583861 Slc6a6/Lyn	2
GO:0045834 positive regulation of lipid metabolic process	1/95	122/14464	0.00819672	1.24797239	0.22364341	0.55395514	0.65458123	0.62583861 Por	1
GO:0007416 synapse assembly	2/95	283/14464	0.00706714	1.07599033	0.10497257	0.55768309	0.6581758	0.62927535 Abl1/Agrn	2
GO:0015849 organic acid transport	2/95	283/14464	0.00706714	1.07599033	0.10497257	0.55768309	0.6581758	0.62927535 Slc6a6/Lyn	2
GO:0048193 Golgi vesicle transport	2/95	284/14464	0.00704225	1.07220163	0.09991847	0.55960697	0.658298	0.62939218 Sar1b/Rab34	2
GO:0002040 sprouting angiogenesis	1/95	124/14464	0.00806452	1.2278438	0.20718161	0.55984489	0.658298	0.62939218 Abl1	1
GO:0008286 insulin receptor signaling pathway	1/95	124/14464	0.00806452	1.2278438	0.20718161	0.55984489	0.658298	0.62939218 Tns2	1
GO:0045667 regulation of osteoblast differentiation	1/95	124/14464	0.00806452	1.2278438	0.20718161	0.55984489	0.658298	0.62939218 Gdf10	1
GO:0097696 cell surface receptor signaling pathway via STAT	1/95	124/14464	0.00806452	1.2278438	0.20718161	0.55984489	0.658298	0.62939218 Il15ra	1
GO:1903052 positive regulation of proteolysis involved in protein catabolic process	1/95	124/14464	0.00806452	1.2278438	0.20718161	0.55984489	0.658298	0.62939218 Trem2	1
GO:0006790 sulfur compound metabolic process	2/95	285/14464	0.00701754	1.06843952	0.0948821	0.56152498	0.65986922	0.63089441 Pipox/Gsta4	2
GO:1901293 nucleoside phosphate biosynthetic process	2/95	286/14464	0.00699301	1.06470372	0.08986333	0.56343713	0.66171104	0.63265536 Trem2/ND5	2
GO:0010466 negative regulation of peptidase activity	1/95	126/14464	0.00793651	1.20835422	0.19099456	0.56565768	0.66269663	0.63359767 Serpine1	1
GO:0010811 positive regulation of cell-substrate adhesion	1/95	126/14464	0.00793651	1.20835422	0.19099456	0.56565768	0.66269663	0.63359767 Abl1	1
GO:0015718 monocarboxylic acid transport	1/95	126/14464	0.00793651	1.20835422	0.19099456	0.56565768	0.66269663	0.63359767 Lyn	1
GO:0090101 negative regulation of transmembrane receptor protein serine/threonine kinase signaling pathway	1/95	126/14464	0.00793651	1.20835422	0.19099456	0.56565768	0.66269663	0.63359767 Abl1	1
GO:0030003 intracellular monoatomic cation homeostasis	3/95	447/14464	0.00671141	1.02182974	0.03811997	0.56635578	0.66310966	0.63399256 Mt1/Abl1/Lyn	3
GO:1901888 regulation of cell junction assembly	2/95	288/14464	0.00694444	1.05730994	0.07987798	0.56724377	0.66374439	0.63459942 Abl1/Agrn	2
GO:0007041 lysosomal transport	1/95	127/14464	0.00787402	1.19883962	0.18300096	0.56853552	0.66404118	0.63488318 Rab34	1
GO:0010565 regulation of cellular ketone metabolic process	1/95	127/14464	0.00787402	1.19883962	0.18300096	0.56853552	0.66404118	0.63488318 Clcn2	1
GO:0030177 positive regulation of Wnt signaling pathway	1/95	127/14464	0.00787402	1.19883962	0.18300096	0.56853552	0.66404118	0.63488318 Abl1	1
GO:0006091 generation of precursor metabolites and energy	3/95	449/14464	0.00668151	1.01727816	0.0302414	0.56941003	0.66465806	0.63547297 Por/ND5/Coq10b	3
GO:0014074 response to purine-containing compound	1/95	128/14464	0.0078125	1.18947368	0.17507237	0.57139449	0.66575958	0.63652613 Per1	1
GO:0030856 regulation of epithelial cell differentiation	1/95	128/14464	0.0078125	1.18947368	0.17507237	0.57139449	0.66575958	0.63652613 Serpine1	1
GO:0046328 regulation of JNK cascade	1/95	128/14464	0.0078125	1.18947368	0.17507237	0.57139449	0.66575958	0.63652613 Per1	1
GO:0006665 sphingolipid metabolic process	1/95	129/14464	0.00775194	1.18025296	0.16720764	0.57423472	0.66744787	0.63814028 Acer2	1
GO:0030183 B cell differentiation	1/95	129/14464	0.00775194	1.18025296	0.16720764	0.57423472	0.66744787	0.63814028 Abl1	1
GO:0048259 regulation of receptor-mediated endocytosis	1/95	129/14464	0.00775194	1.18025296	0.16720764	0.57423472	0.66744787	0.63814028 Serpine1	1
GO:0050684 regulation of mRNA processing	1/95	129/14464	0.00775194	1.18025296	0.16720764	0.57423472	0.66744787	0.63814028 Rbmx	1
GO:0006873 intracellular monoatomic ion homeostasis	3/95	454/14464	0.00660793	1.00607466	0.01069322	0.57698892	0.6691064	0.63972598 Mt1/Abl1/Lyn	3
GO:0098771 inorganic ion homeostasis	3/95	454/14464	0.00660793	1.00607466	0.01069322	0.57698892	0.6691064	0.63972598 Mt1/Abl1/Lyn	3
GO:0006906 vesicle fusion	1/95	130/14464	0.00769231	1.17117409	0.15940566	0.57705633	0.6691064	0.63972598 Rab34	1
GO:0051592 response to calcium ion	1/95	130/14464	0.00769231	1.17117409	0.15940566	0.57705633	0.6691064	0.63972598 Cpne7	1
GO:0009566 fertilization	1/95	131/14464	0.00763359	1.16223383	0.1516653	0.57985943	0.6705308	0.64108783 Spaca6	1
GO:0043244 regulation of protein-containing complex disassembly	1/95	131/14464	0.00763359	1.16223383	0.1516653	0.57985943	0.6705308	0.64108783 Nav3	1
GO:0071772 response to BMP	1/95	131/14464	0.00763359	1.16223383	0.1516653	0.57985943	0.6705308	0.64108783 Abl1	1
GO:0071773 cellular response to BMP stimulus	1/95	131/14464	0.00763359	1.16223383	0.1516653	0.57985943	0.6705308	0.64108783 Abl1	1

GO:0010506 regulation of autophagy	2/95	295/14464	0.00677966	1.03222123	0.04546333	0.58038127	0.6705308	0.64108783	Trem2/Acer2	2
GO:0055074 calcium ion homeostasis	2/95	295/14464	0.00677966	1.03222123	0.04546333	0.58038127	0.6705308	0.64108783	Abl1/Lyn	2
GO:0044282 small molecule catabolic process	2/95	296/14464	0.00675676	1.028734	0.04061303	0.5822344	0.67226704	0.64274784	Pipox/Tha1	2
GO:0090174 organelle membrane fusion	1/95	132/14464	0.00757576	1.15342903	0.14398552	0.58264415	0.67233561	0.6428134	Rab34	1
GO:0032675 regulation of interleukin-6 production	1/95	133/14464	0.0075188	1.14475663	0.13636526	0.5854106	0.67512196	0.6454774	Trem2	1
GO:0001959 regulation of cytokine-mediated signaling pathway	1/95	134/14464	0.00746269	1.13621367	0.12880352	0.58815891	0.67707075	0.64734062	Trem2	1
GO:0003158 endothelium development	1/95	134/14464	0.00746269	1.13621367	0.12880352	0.58815891	0.67707075	0.64734062	Fzd4	1
GO:0060996 dendritic spine development	1/95	134/14464	0.00746269	1.13621367	0.12880352	0.58815891	0.67707075	0.64734062	Myo5b	1
GO:0008277 regulation of G protein-coupled receptor signaling pathway	1/95	135/14464	0.00740741	1.12779727	0.12129929	0.59088919	0.67959164	0.64975081	Trem2	1
GO:0071214 cellular response to abiotic stimulus	2/95	301/14464	0.00664452	1.01164539	0.01660112	0.59141117	0.67959164	0.64975081	Net1/Clcn2	2
GO:0104004 cellular response to environmental stimulus	2/95	301/14464	0.00664452	1.01164539	0.01660112	0.59141117	0.67959164	0.64975081	Net1/Clcn2	2
GO:0007093 mitotic cell cycle checkpoint signaling	1/95	136/14464	0.00735294	1.11950464	0.11385161	0.59360155	0.68088547	0.65098783	Xrcc3	1
GO:0007565 female pregnancy	1/95	136/14464	0.00735294	1.11950464	0.11385161	0.59360155	0.68088547	0.65098783	Klf9	1
GO:1903825 organic acid transmembrane transport	1/95	136/14464	0.00735294	1.11950464	0.11385161	0.59360155	0.68088547	0.65098783	Slc6a6	1
GO:0007005 mitochondrion organization	3/95	466/14464	0.00643777	0.98016716	-0.0353853	0.59484222	0.68190097	0.65195875	Gimap5/Tspan4/ND5	3
GO:0006470 protein dephosphorylation	1/95	137/14464	0.00729927	1.11133308	0.10645954	0.59629613	0.68275195	0.65277236	Tns2	1
GO:1902600 proton transmembrane transport	1/95	137/14464	0.00729927	1.11133308	0.10645954	0.59629613	0.68275195	0.65277236	ND5	1
GO:0006119 oxidative phosphorylation	1/95	138/14464	0.00724638	1.10327994	0.09912216	0.59897302	0.68459156	0.65453119	ND5	1
GO:0050768 negative regulation of neurogenesis	1/95	138/14464	0.00724638	1.10327994	0.09912216	0.59897302	0.68459156	0.65453119	Trem2	1
GO:0051606 detection of stimulus	1/95	138/14464	0.00724638	1.10327994	0.09912216	0.59897302	0.68459156	0.65453119	Trem2	1
GO:0006997 nucleus organization	1/95	139/14464	0.00719424	1.09534267	0.09183857	0.60163235	0.6868129	0.65665499	Cdan1	1
GO:0032635 interleukin-6 production	1/95	139/14464	0.00719424	1.09534267	0.09183857	0.60163235	0.6868129	0.65665499	Trem2	1
GO:0007611 learning or memory	2/95	307/14464	0.00651466	0.99187382	-0.0117017	0.60222743	0.68708349	0.6569137	Trem2/Abl1	2
GO:0051098 regulation of binding	2/95	308/14464	0.00649351	0.98865345	-0.0163662	0.60400935	0.68859991	0.65836354	Abl1/Agrn	2
GO:0071456 cellular response to hypoxia	1/95	140/14464	0.00714286	1.0875188	0.0846079	0.60427423	0.68859991	0.65836354	Trem2	1
GO:0006261 DNA-templated DNA replication	1/95	141/14464	0.0070922	1.0798059	0.07742929	0.60689878	0.69046174	0.66014362	Pole	1
GO:0031644 regulation of nervous system process	1/95	141/14464	0.0070922	1.0798059	0.07742929	0.60689878	0.69046174	0.66014362	Jam2	1
GO:0009150 purine ribonucleotide metabolic process	2/95	311/14464	0.00643087	0.9791166	-0.0302716	0.60931947	0.69046174	0.66014362	Trem2/ND5	2
GO:0010038 response to metal ion	2/95	311/14464	0.00643087	0.9791166	-0.0302716	0.60931947	0.69046174	0.66014362	Mt1/Cpne7	2
GO:0051146 striated muscle cell differentiation	2/95	311/14464	0.00643087	0.9791166	-0.0302716	0.60931947	0.69046174	0.66014362	Bhlhe41/Jam2	2
GO:0006937 regulation of muscle contraction	1/95	142/14464	0.00704225	1.07220163	0.0703019	0.60950609	0.69046174	0.66014362	Agrn	1
GO:0009913 epidermal cell differentiation	1/95	142/14464	0.00704225	1.07220163	0.0703019	0.60950609	0.69046174	0.66014362	Alg10b	1
GO:0050680 negative regulation of epithelial cell proliferation	1/95	142/14464	0.00704225	1.07220163	0.0703019	0.60950609	0.69046174	0.66014362	Klf9	1
GO:0140013 meiotic nuclear division	1/95	142/14464	0.00704225	1.07220163	0.0703019	0.60950609	0.69046174	0.66014362	Ncapd3	1
GO:1903034 regulation of response to wounding	1/95	142/14464	0.00704225	1.07220163	0.0703019	0.60950609	0.69046174	0.66014362	Serpine1	1
GO:0001890 placenta development	1/95	143/14464	0.00699301	1.06470372	0.06322492	0.6120963	0.69217018	0.66177704	Serpine1	1
GO:0042129 regulation of T cell proliferation	1/95	143/14464	0.00699301	1.06470372	0.06322492	0.6120963	0.69217018	0.66177704	Peli1	1
GO:1903305 regulation of regulated secretory pathway	1/95	143/14464	0.00699301	1.06470372	0.06322492	0.6120963	0.69217018	0.66177704	Lyn	1
GO:0046879 hormone secretion	2/95	314/14464	0.00636943	0.96976198	-0.0440471	0.61457614	0.6938534	0.66338634	Fzd4/Lyn	2
GO:0043281 regulation of cysteine-type endopeptidase activity involved in apoptotic process	1/95	144/14464	0.00694444	1.05730994	0.05619756	0.6146695	0.6938534	0.66338634	Acer2	1
GO:0051147 regulation of muscle cell differentiation	1/95	144/14464	0.00694444	1.05730994	0.05619756	0.6146695	0.6938534	0.66338634	Bhlhe41	1
GO:0060759 regulation of response to cytokine stimulus	1/95	145/14464	0.00689655	1.05001815	0.04921902	0.61722582	0.69632942	0.66575364	Trem2	1
GO:1904951 positive regulation of establishment of protein localization	2/95	316/14464	0.00632911	0.96362425	-0.0531601	0.61805091	0.69685059	0.66625193	Trem2/Rab34	2
GO:0007286 spermatid development	1/95	146/14464	0.00684932	1.04282624	0.04228857	0.61976535	0.69796344	0.66731591	Jam2	1
GO:2001235 positive regulation of apoptotic signaling pathway	1/95	146/14464	0.00684932	1.04282624	0.04228857	0.61976535	0.69796344	0.66731591	Inca1	1
GO:0051961 negative regulation of nervous system development	1/95	147/14464	0.00680272	1.03573219	0.03540544	0.62228821	0.69998304	0.66924683	Trem2	1
GO:2001236 regulation of extrinsic apoptotic signaling pathway	1/95	147/14464	0.00680272	1.03573219	0.03540544	0.62228821	0.69998304	0.66924683	Serpine1	1
GO:0006644 phospholipid metabolic process	2/95	319/14464	0.00626959	0.95456195	-0.0667254	0.62321859	0.70061891	0.66985478	Pnpla7/Gpcpd1	2
GO:0003002 regionalization	2/95	320/14464	0.00625	0.95157895	-0.0712198	0.6249293	0.70213076	0.67130024	Bhlhe40/Bhlhe41	2
GO:0009914 hormone transport	2/95	321/14464	0.00623053	0.94861453	-0.0757007	0.62663409	0.70313005	0.67225566	Fzd4/Lyn	2
GO:0042060 wound healing	2/95	321/14464	0.00623053	0.94861453	-0.0757007	0.62663409	0.70313005	0.67225566	Serpine1/Lyn	2
GO:0030218 erythrocyte differentiation	1/95	149/14464	0.00671141	1.02182974	0.02177829	0.62728434	0.70313005	0.67225566	Lyn	1
GO:0036294 cellular response to decreased oxygen levels	1/95	149/14464	0.00671141	1.02182974	0.02177829	0.62728434	0.70313005	0.67225566	Trem2	1
GO:0006941 striated muscle contraction	1/95	150/14464	0.00666667	1.01501754	0.01503286	0.62975783	0.70455696	0.67361991	Agrn	1
GO:0010977 negative regulation of neuron projection development	1/95	150/14464	0.00666667	1.01501754	0.01503286	0.62975783	0.70455696	0.67361991	Il15ra	1
GO:0048754 branching morphogenesis of an epithelial tube	1/95	150/14464	0.00666667	1.01501754	0.01503286	0.62975783	0.70455696	0.67361991	Abl1	1
GO:0060562 epithelial tube morphogenesis	2/95	323/14464	0.00619195	0.94274075	-0.0846222	0.63002592	0.70455696	0.67361991	Abl1/Brpf1	2
GO:0031647 regulation of protein stability	2/95	324/14464	0.00617284	0.93983106	-0.0890631	0.63171296	0.70603214	0.67503031	Per3/Lss	2
GO:2000045 regulation of G1/S transition of mitotic cell cycle	1/95	151/14464	0.00662252	1.00829557	0.00833196	0.63221507	0.70618203	0.67517362	Arid2	1
GO:0046486 glycerolipid metabolic process	2/95	325/14464	0.00615385	0.93693927	-0.0934908	0.6333941	0.70708742	0.67603926	Pnpla7/Gpcpd1	2
GO:0044703 multi-organism reproductive process	1/95	152/14464	0.00657895	1.00166205	0.00167492	0.63465618	0.70808442	0.67699248	Klf9	1
GO:0031333 negative regulation of protein-containing complex assembly	1/95	153/14464	0.00653595	0.99511524	-0.0049389	0.63708125	0.70996453	0.67879004	Trem2	1
GO:0044706 multi-multicellular organism process	1/95	153/14464	0.00653595	0.99511524	-0.0049389	0.63708125	0.70996453	0.67879004	Klf9	1
GO:0006575 cellular modified amino acid metabolic process	1/95	154/14464	0.00649351	0.98865345	-0.0115102	0.6394904	0.71058603	0.67938425	Gsta4	1
GO:0035150 regulation of tube size	1/95	154/14464	0.00649351	0.98865345	-0.0115102	0.6394904	0.71058603	0.67938425	Abl1	1
GO:0035296 regulation of tube diameter	1/95	154/14464	0.00649351	0.98865345	-0.0115102	0.6394904	0.71058603	0.67938425	Abl1	1
GO:0048515 spermatid differentiation	1/95	154/14464	0.00649351	0.98865345	-0.0115102	0.6394904	0.71058603	0.67938425	Jam2	1
GO:0097746 blood vessel diameter maintenance	1/95	154/14464	0.00649351	0.98865345	-0.0115102	0.6394904	0.71058603	0.67938425	Abl1	1
GO:0009895 negative regulation of catabolic process	2/95	330/14464	0.00606061	0.92274322	-0.1154353	0.64171133	0.71159726	0.68035107	Trem2/Gimap5	2
GO:0015980 energy derivation by oxidation of organic compounds	2/95	330/14464	0.00606061	0.92274322	-0.1154353	0.64171133	0.71159726	0.68035107	ND5/Coq10b	2
GO:0001508 action potential	1/95	155/14464	0.00645161	0.98227504	-0.0180395	0.64188372	0.71159726	0.68035107	Clcn2	1
GO:0050777 negative regulation of immune response	1/95	155/14464	0.00645161	0.98227504	-0.0180395	0.64188372	0.71159726	0.68035107	Trem2	1
GO:0001906 cell killing	1/95	156/14464	0.00641026	0.97597841	-0.0245274	0.64426132	0.71299739	0.68168972	Gimap5	1
GO:0007254 JNK cascade	1/95	156/14464	0.00641026	0.97597841	-0.0245274	0.64426132	0.71299739	0.68168972	Per1	1
GO:0007613 memory	1/95	156/14464	0.00641026	0.97597841	-0.0245274	0.64426132	0.71299739	0.68168972	Trem2	1
GO:0009259 ribonucleotide metabolic process	2/95	332/14464	0.0060241	0.91718453	-0.1241242	0.64499703	0.71340017	0.68207482	Trem2/ND5	2
GO:0071478 cellular response to radiation	1/95	157/14464	0.00636943	0.96976198	-0.0309747	0.64662329	0.71478692	0.68340068	Net1	1
GO:0006865 amino acid transport	1/95	158/14464	0.00632911	0.96362425	-0.0373817	0.64896975	0.71696774	0.68548573	Slc6a6	1
GO:0010594 regulation of endothelial cell migration	1/95	159/14464	0.00628931	0.95756372	-0.0437492	0.65130079	0.71830243	0.68676182	Abl1	1
GO:0030041 actin filament polymerization	1/95	159/14464	0.00628931	0.95756372	-0.0437492	0.65130079	0.71830243	0.68676182	Abl1	1
GO:0071897 DNA biosynthetic process	1/95	159/14464	0.00628931	0.95756372	-0.0437492	0.65130079	0.71830243	0.68676182	Pole	1
GO:0032680 regulation of tumor necrosis factor production	1/95	161/14464	0.00621118	0.94566852	-0.0563675	0.65591702	0.72256301	0.69083532	Trem2	1
GO:0051897 positive regulation of phosphatidylinositol 3-kinase/protein kinase B signal transduction	1/95	161/14464	0.00621118	0.94566852	-0.0563675	0.65591702	0.72256301	0.69083532	Trem2	1
GO:0019693 ribose phosphate metabolic process	2/95	339/14464	0.00589971	0.89824561	-0.1541475	0.65631235	0.72258371	0.69085511	Trem2/ND5	2
GO:0007034 vacuolar transport	1/95	162/14464	0.00617284	0.93983106	-0.0626194	0.65820241	0.72341949	0.69165419	Rab34	1
GO:0007219 Notch signaling pathway	1/95	162/14464	0.00617284	0.93983106	-0.0626194	0.65820241	0.72341949	0.69165419	Tcim	1
GO:0034101 erythrocyte homeostasis	1/95	162/14464	0.00617284	0.93983106	-0.0626194	0.65820241	0.72341949	0.69165419	Lyn	1
GO:0015711 organic anion transport	2/95	341/14464	0.0058651	0.89297731	-0.1626175	0.65949276	0.72425557	0.69245356	Slc6a6/Lyn	2
GO:0051017 actin filament bundle assembly	1/95	163/14464	0.00613497	0.93406522	-0.068834	0.66047278	0.72425557	0.69245356	Abl1	1
GO:1903555 regulation of tumor necrosis factor superfamily cytokine production	1/95	163/14464	0.00613497	0.93406522	-0.068834	0.66047278	0.72425557	0.69245356	Trem2	1
GO:2000116 regulation of cysteine-type endopeptidase activity	1/95	163/14464	0.00613497	0.93406522	-0.068834	0.66047278	0.72425557	0.69245356	Acer2	1
GO:0050890 cognition	2/95	344/14464	0.00581395	0.88518972	-0.1752348	0.66421977	0.72793793	0.69597423	Trem2/Abl1	2
GO:0008016 regulation of heart contraction	1/95	165/14464	0.00606061	0.92274322	-0.0811528	0.66496883	0.72793793	0.69597423	Agrn	1
GO:0071453 cellular response to oxygen levels	1/95	165/14464	0.00606061	0.92274322	-0.0811528	0.66496883	0.72793793	0.69597423	Trem2	1
GO:0032640 tumor necrosis factor production	1/95	166/14464	0.0060241	0.91718453	-0.0872581	0.66719472	0.72912681	0.6971109	Trem2	1
GO:0061572 actin filament bundle organization	1/95	166/14464	0.0060241	0.91718453	-0.0872581	0.66719472	0.72912681	0.6971109	Abl1	1
GO:1903046 meiotic cell cycle process	1/95	166/14464	0.0060241	0.91718453	-0.0872581	0.66719472	0.72912681	0.6971109	Ncapd3	1
GO:0098739 import across plasma membrane	1/95	167/14464	0.00598802	0.9116924	-0.0933279	0.66940598	0.73112696	0.69902322	Slc6a6	1
GO:0071706 tumor necrosis factor superfamily cytokine production	1/95	168/14464	0.00595238	0.90626566	-0.0993628	0.67160269	0.73310897	0.7009182	Trem2	1
GO:0051170 import into nucleus	1/95	169/14464	0.00591716	0.90090315	-0.1053632	0.67378496	0.73507296	0.70279596	Cdan1	1
GO:0016050 vesicle organization	2/95	351/14464	0.00569801	0.86753636	-0.2042764	0.67504707	0.73603144	0.70371235	Sar1b/Rab34	2
GO:0006887 exocytosis	2/95	352/14464	0.00568182	0.86507177	-0.2083807	0.67657081	0.7368555	0.70450023	Myo5b/Lyn	2
GO:0009306 protein secretion	2/95	352/14464	0.00568182	0.86507177	-0.2083807	0.67657081	0.7368555	0.70450023	Trem2/Rab34	2
GO:0042552 myelination	1/95	171/14464	0.00584795	0.89036627	-0.1172623	0.67810655	0.73727278	0.70489919	Jam2	1
GO:0050770 regulation of axonogenesis	1/95	171/14464	0.00584795	0.89036627	-0.1172623	0.67810655	0.73727278	0.70489919	Myo5b	1
GO:0051494 negative regulation of cytoskeleton organization	1/95	171/14464	0.00584795	0.89036627	-0.1172623	0.67810655	0.73727278	0.70489919	Nav3	1
GO:0035592 establishment of protein localization to extracellular region	2/95	354/14464	0.00564972	0.86018436	-0.2165567	0.67960112	0.73834398	0.70592335	Trem2/Rab34	2
GO:0006643 membrane lipid metabolic process	1/95	172/14464	0.00581395	0.88518972	-0.1231619	0.68024605	0.73834398	0.70592335	Acer2	1
GO:2001242 regulation of intrinsic apoptotic signaling pathway	1/95	172/14464	0.00581395	0.88518972	-0.1231619	0.68024605	0.73834398	0.70592335	Trem2	1
GO:0010639 negative regulation of organelle organization	2/95	355/14464	0.0056338	0.8577613	-0.2206285	0.6811077	0.73886132	0.70641797	Nav3/Xrcc3	2
GO:0030307 positive regulation of cell growth	1/95	173/14464	0.00578035	0.88007301	-0.1290287	0.68237147	0.73917993	0.70672259	Myo5b	1
GO:0007389 pattern specification process	2/95	356/14464	0.00561798	0.85535186	-0.2246895	0.68260858	0.73917993	0.70672259	Bhlhe40/Bhlhe41	2
GO:0071692 protein localization to extracellular region	2/95	357/14464	0.00560224	0.85295592	-0.22874	0.68410376	0.73917993	0.70672259	Trem2/Rab34	2
GO:0001659 temperature homeostasis	1/95	174/14464	0.00574713	0.87501512	-0.1348633	0.68448292	0.73917993	0.70672259	Gimap5	1
GO:0007272 ensheathment of neurons	1/95	174/14464	0.00574713	0.87501512	-0.1348633	0.68448292	0.73917993	0.70672259	Jam2	1
GO:0007612 learning	1/95	174/14464	0.00574713	0.87501512	-0.1348633	0.68448292	0.73917993	0.70672259	Abl1	1
GO:0008366 axon ensheathment	1/95	174/14464	0.00574713	0.87501512	-0.1348633	0.68448292	0.73917993	0.70672259	Jam2	1
GO:0043484 regulation of RNA splicing	1/95	174/14464	0.00574713	0.87501512	-0.1348633	0.68448292	0.73917993	0.70672259	Rbmx	1
GO:0006839 mitochondrial transport	1/95	175/14464	0.00571429	0.87001504	-0.1406659	0.68658048	0.74102809	0.7084896	Gimap5	1
GO:0000280 nuclear division	2/95	360/14464	0.00555556	0.84584795	-0.2408281	0.68855524	0.74244196	0.70984138	Xrcc3/Ncapd3	2
GO:0048284 organelle fusion	1/95	176/14464	0.00568182	0.86507177	-0.1464371	0.68866424	0.74244196	0.70984138	Rab34	1
GO:0022618 protein-RNA complex assembly	1/95	177/14464	0.00564972	0.86018436	-0.1521772	0.69073429	0.74383789	0.71117602	Rbmx	1
GO:0061138 morphogenesis of a branching epithelium	1/95	177/14464	0.00564972	0.86018436	-0.1521772	0.69073429	0.74383789	0.71117602	Abl1	1
GO:0001764 neuron migration	1/95	178/14464	0.00561798	0.85535186	-0.1578866	0.69279073	0.74521603	0.71249365	Celsr3	1
GO:0030258 lipid modification	1/95	178/14464	0.00561798	0.85535186	-0.1578866	0.69279073	0.74521603	0.71249365	Por	1
GO:0051346 negative regulation of hydrolase activity	1/95	179/14464	0.00558659	0.85057336	-0.1635657	0.69483363	0.74699481	0.71419432	Serpine1	1
GO:0032535 regulation of cellular component size	2/95	365/14464	0.00547945	0.83426099	-0.2607683	0.69586137	0.74768084	0.71485022	Myo5b/Abl1	2
GO:0009060 aerobic respiration	1/95	180/14464	0.00555556	0.84584795	-0.1692148	0.69686309	0.74791961	0.71507851	ND5	1
GO:0071824 protein-DNA complex organization	1/95	180/14464	0.00555556	0.84584795	-0.1692148	0.69686309	0.74791961	0.71507851	Arid2	1
GO:0019722 calcium-mediated signaling	1/95	181/14464	0.00552486	0.84117476	-0.1748344	0.69887919	0.74924534	0.71634603	Trem2	1
GO:0048839 inner ear development	1/95	181/14464	0.00552486	0.84117476	-0.1748344	0.69887919	0.74924534	0.71634603	Alg10b	1
GO:0098742 cell-cell adhesion via plasma-membrane adhesion molecules	1/95	182/14464	0.00549451	0.83655292	-0.1804247	0.70088202	0.75097298	0.7179978	Celsr3	1
GO:0008154 actin polymerization or depolymerization	1/95	183/14464	0.00546448	0.83198159	-0.1859862	0.70287167	0.75184545	0.71883197	Abl1	1
GO:0009267 cellular response to starvation	1/95	183/14464	0.00546448	0.83198159	-0.1859862	0.70287167	0.75184545	0.71883197	Sar1b	1
GO:0042180 cellular ketone metabolic process	1/95	183/14464	0.00546448	0.83198159	-0.1859862	0.70287167	0.75184545	0.71883197	Clcn2	1
GO:0061136 regulation of proteasomal protein catabolic process	1/95	184/14464	0.00543478	0.82745995	-0.1915193	0.70484823	0.75353969	0.72045182	Trem2	1
GO:0001649 osteoblast differentiation	1/95	186/14464	0.00537634	0.81856254	-0.2025011	0.70876239	0.75645997	0.72324387	Gdf10	1
GO:0051216 cartilage development	1/95	186/14464	0.00537634	0.81856254	-0.2025011	0.70876239	0.75645997	0.72324387	Por	1
GO:0071826 protein-RNA complex organization	1/95	186/14464	0.00537634	0.81856254	-0.2025011	0.70876239	0.75645997	0.72324387	Rbmx	1
GO:0043524 negative regulation of neuron apoptotic process	1/95	188/14464	0.00531915	0.80985442	-0.2133731	0.71262519	0.75973763	0.7263776	Mt1	1
GO:0050905 neuromuscular process	1/95	188/14464	0.00531915	0.80985442	-0.2133731	0.71262519	0.75973763	0.7263776	Abl1	1
GO:0050806 positive regulation of synaptic transmission	1/95	189/14464	0.00529101	0.80556948	-0.2187686	0.71453753	0.76135343	0.72792245	Abl1	1
GO:0032259 methylation	1/95	190/14464	0.00526316	0.80132964	-0.2241376	0.71643728	0.76253086	0.72904818	Inmt	1
GO:0120032 regulation of plasma membrane bounded cell projection assembly	1/95	190/14464	0.00526316	0.80132964	-0.2241376	0.71643728	0.76253086	0.72904818	Agrn	1
GO:0031099 regeneration	1/95	191/14464	0.0052356	0.7971342	-0.2294804	0.71832452	0.76411572	0.73056345	Serpine1	1
GO:0031334 positive regulation of protein-containing complex assembly	1/95	192/14464	0.00520833	0.79298246	-0.2347973	0.72019933	0.76483814	0.73125415	Nav3	1
GO:0045732 positive regulation of protein catabolic process	1/95	192/14464	0.00520833	0.79298246	-0.2347973	0.72019933	0.76483814	0.73125415	Trem2	1
GO:0060491 regulation of cell projection assembly	1/95	192/14464	0.00520833	0.79298246	-0.2347973	0.72019933	0.76483814	0.73125415	Agrn	1
GO:0030324 lung development	1/95	194/14464	0.00515464	0.78480738	-0.2453545	0.72391199	0.7683557	0.73461725	Clcn2	1
GO:0031329 regulation of cellular catabolic process	2/95	388/14464	0.00515464	0.78480738	-0.3493666	0.72768489	0.77193328	0.73803775	Trem2/Acer2	2
GO:0001558 regulation of cell growth	2/95	389/14464	0.00514139	0.78278988	-0.3531087	0.72900331	0.77203472	0.73813473	Myo5b/Abl1	2
GO:0030323 respiratory tube development	1/95	197/14464	0.00507614	0.772856	-0.2610032	0.72938976	0.77203472	0.73813473	Clcn2	1
GO:0050821 protein stabilization	1/95	197/14464	0.00507614	0.772856	-0.2610032	0.72938976	0.77203472	0.73813473	Per3	1
GO:0051963 regulation of synapse assembly	1/95	197/14464	0.00507614	0.772856	-0.2610032	0.72938976	0.77203472	0.73813473	Agrn	1
GO:0030705 cytoskeleton-dependent intracellular transport	1/95	198/14464	0.00505051	0.76895268	-0.2661706	0.73119168	0.77351535	0.73955034	Myo5b	1
GO:0031345 negative regulation of cell projection organization	1/95	201/14464	0.00497512	0.75747578	-0.2815305	0.73652651	0.77859219	0.74440426	Il15ra	1
GO:0006163 purine nucleotide metabolic process	2/95	395/14464	0.00506329	0.7708994	-0.3753798	0.7368022	0.77859219	0.74440426	Trem2/ND5	2
GO:0002262 myeloid cell homeostasis	1/95	202/14464	0.0049505	0.7537259	-0.2866038	0.7382814	0.77929703	0.74507815	Lyn	1
GO:0035264 multicellular organism growth	1/95	202/14464	0.0049505	0.7537259	-0.2866038	0.7382814	0.77929703	0.74507815	Tns2	1
GO:0060047 heart contraction	1/95	203/14464	0.00492611	0.75001296	-0.2916542	0.74002472	0.78070777	0.74642695	Agrn	1
GO:0003018 vascular process in circulatory system	1/95	207/14464	0.00483092	0.73551996	-0.3116318	0.74688385	0.7866466	0.752105	Abl1	1
GO:0043583 ear development	1/95	207/14464	0.00483092	0.73551996	-0.3116318	0.74688385	0.7866466	0.752105	Alg10b	1
GO:0097191 extrinsic apoptotic signaling pathway	1/95	207/14464	0.00483092	0.73551996	-0.3116318	0.74688385	0.7866466	0.752105	Serpine1	1
GO:0016311 dephosphorylation	1/95	209/14464	0.00478469	0.72848149	-0.3214894	0.75024595	0.78975424	0.75507619	Tns2	1
GO:0048732 gland development	2/95	408/14464	0.00490196	0.74633643	-0.4226054	0.75305381	0.79227536	0.75748661	Clcn2/Abl1	2
GO:0051321 meiotic cell cycle	1/95	211/14464	0.00473934	0.72157645	-0.3312621	0.75356386	0.79237756	0.75758432	Ncapd3	1

GO:0031032	actomyosin structure organization	1/95	212/14464	0.00471698	0.71817279	-0.3361171	0.75520642	0.7930855	0.75826117	Abl1	1
GO:0032869	cellular response to insulin stimulus	1/95	212/14464	0.00471698	0.71817279	-0.3361171	0.75520642	0.7930855	0.75826117	Tns2	1
GO:0043434	response to peptide hormone	2/95	410/14464	0.00487805	0.74269576	-0.4297507	0.75547696	0.7930855	0.75826117	Tns2/Lyn	2
GO:0003015	heart process	1/95	213/14464	0.00469484	0.71480109	-0.3409516	0.75683815	0.79408005	0.75921205	Agrn	1
GO:0006364	rRNA processing	1/95	214/14464	0.0046729	0.7114609	-0.3457657	0.75845912	0.79491155	0.76000704	Wdr46	1
GO:0048705	skeletal system morphogenesis	1/95	214/14464	0.0046729	0.7114609	-0.3457657	0.75845912	0.79491155	0.76000704	Por	1
GO:0042594	response to starvation	1/95	216/14464	0.00462963	0.70487329	-0.3553335	0.76166904	0.79740474	0.76239075	Sar1b	1
GO:0045926	negative regulation of growth	1/95	216/14464	0.00462963	0.70487329	-0.3553335	0.76166904	0.79740474	0.76239075	Mt1	1
GO:0048285	organelle fission	2/95	416/14464	0.00480769	0.73198381	-0.4510017	0.76262533	0.79797056	0.76293172	Xrcc3/Ncapd3	2
GO:0008380	RNA splicing	2/95	417/14464	0.00479616	0.73022845	-0.454517	0.7637992	0.79876331	0.76368966	Srrm2/Rbmx	2
GO:0048863	stem cell differentiation	1/95	218/14464	0.00458716	0.69840657	-0.3648223	0.76483675	0.79897753	0.76389448	Zfp281	1
GO:1903050	regulation of proteolysis involved in protein catabolic process	1/95	218/14464	0.00458716	0.69840657	-0.3648223	0.76483675	0.79897753	0.76389448	Trem2	1
GO:0006836	neurotransmitter transport	1/95	219/14464	0.00456621	0.6952175	-0.3695376	0.76640494	0.80018013	0.76504428	Slc6a6	1
GO:0016485	protein processing	1/95	220/14464	0.00454545	0.69205742	-0.3742336	0.76796279	0.8009351	0.7657661	Serpine1	1
GO:0060541	respiratory system development	1/95	220/14464	0.00454545	0.69205742	-0.3742336	0.76796279	0.8009351	0.7657661	Clcn2	1
GO:0048568	embryonic organ development	2/95	421/14464	0.00475059	0.72329041	-0.4685037	0.76844515	0.80100285	0.76583087	Pole/Arid2	2
GO:0042692	muscle cell differentiation	2/95	423/14464	0.00472813	0.7198706	-0.475453	0.7707386	0.80295731	0.76769951	Bhlhe41/Jam2	2
GO:0007033	vacuole organization	1/95	223/14464	0.0044843	0.68274723	-0.3882088	0.77257491	0.80399743	0.76869396	Rab34	1
GO:0090092	regulation of transmembrane receptor protein serine/threonine kinase signaling pathway	1/95	223/14464	0.0044843	0.68274723	-0.3882088	0.77257491	0.80399743	0.76869396	Abl1	1
GO:0045861	negative regulation of proteolysis	1/95	224/14464	0.00446429	0.67969925	-0.39283	0.77409204	0.80513963	0.76978601	Serpine1	1
GO:2001234	negative regulation of apoptotic signaling pathway	1/95	225/14464	0.00444444	0.67667836	-0.3974331	0.77559915	0.80627019	0.77086692	Serpine1	1
GO:0007015	actin filament organization	2/95	429/14464	0.004662	0.70980248	-0.4961281	0.77750212	0.80781081	0.77233989	Myo5b/Abl1	2
GO:0040029	epigenetic regulation of gene expression	1/95	230/14464	0.00434783	0.66196796	-0.4201806	0.78298676	0.81262931	0.77694681	Tet3	1
GO:1902115	regulation of organelle assembly	1/95	230/14464	0.00434783	0.66196796	-0.4201806	0.78298676	0.81262931	0.77694681	Abl1	1
GO:0001667	ameboidal-type cell migration	1/95	231/14464	0.004329	0.6591023	-0.4246778	0.78443514	0.81325285	0.77754298	Serpine1	1
GO:0045055	regulated exocytosis	1/95	231/14464	0.004329	0.6591023	-0.4246778	0.78443514	0.81325285	0.77754298	Lyn	1
GO:0022613	ribonucleoprotein complex biogenesis	2/95	436/14464	0.00458716	0.69840657	-0.5199301	0.78517466	0.81358	0.77785576	Rbmx/Wdr46	2
GO:0046394	carboxylic acid biosynthetic process	1/95	232/14464	0.00431034	0.65626134	-0.4291579	0.78587395	0.81386515	0.77812838	Tha1	1
GO:0015850	organic hydroxy compound transport	1/95	235/14464	0.00425532	0.64788354	-0.4424975	0.79013363	0.8169539	0.78108151	Trem2	1
GO:0016053	organic acid biosynthetic process	1/95	235/14464	0.00425532	0.64788354	-0.4424975	0.79013363	0.8169539	0.78108151	Tha1	1
GO:0090257	regulation of muscle system process	1/95	235/14464	0.00425532	0.64788354	-0.4424975	0.79013363	0.8169539	0.78108151	Agrn	1
GO:0008015	blood circulation	2/95	443/14464	0.00451467	0.6873708	-0.5434	0.79261681	0.81908006	0.78311431	Abl1/Agrn	2
GO:0048167	regulation of synaptic plasticity	1/95	239/14464	0.0041841	0.6370403	-0.4600541	0.79568302	0.82180609	0.78572065	Abl1	1
GO:0051896	regulation of phosphatidylinositol 3-kinase/protein kinase B signal transduction	1/95	240/14464	0.00416667	0.63438596	-0.4644033	0.79704753	0.82277257	0.78664469	Trem2	1
GO:0031669	cellular response to nutrient levels	1/95	241/14464	0.00414938	0.63175366	-0.4687368	0.79840302	0.82328608	0.78713565	Sar1b	1
GO:0045444	fat cell differentiation	1/95	241/14464	0.00414938	0.63175366	-0.4687368	0.79840302	0.82328608	0.78713565	Gdf10	1
GO:0071695	anatomical structure maturation	1/95	242/14464	0.00413223	0.62914311	-0.4730548	0.79974955	0.82387574	0.78769941	Rere	1
GO:0009117	nucleotide metabolic process	2/95	450/14464	0.00444444	0.67667836	-0.5665493	0.7998335	0.82387574	0.78769941	Trem2/ND5	2
GO:0000209	protein polyubiquitination	1/95	243/14464	0.00411523	0.62655404	-0.4773573	0.80108718	0.8242822	0.78808803	Peli1	1
GO:0003007	heart morphogenesis	1/95	243/14464	0.00411523	0.62655404	-0.4773573	0.80108718	0.8242822	0.78808803	Arid2	1
GO:0051223	regulation of protein transport	2/95	452/14464	0.00442478	0.67368421	-0.573106	0.80185469	0.82462977	0.78842034	Trem2/Rab34	2
GO:0006260	DNA replication	1/95	245/14464	0.00408163	0.62143931	-0.4859166	0.80373598	0.82612177	0.78984682	Pole	1
GO:0006397	mRNA processing	2/95	455/14464	0.0043956	0.66924234	-0.5828942	0.80485298	0.82638463	0.79009813	Srrm2/Rbmx	2
GO:0031667	response to nutrient levels	2/95	455/14464	0.0043956	0.66924234	-0.5828942	0.80485298	0.82638463	0.79009813	Sar1b/Il15ra	2
GO:0016072	rRNA metabolic process	1/95	247/14464	0.00404858	0.61640742	-0.4944159	0.80634987	0.82703656	0.79072144	Wdr46	1
GO:0097305	response to alcohol	1/95	247/14464	0.00404858	0.61640742	-0.4944159	0.80634987	0.82703656	0.79072144	Klf9	1
GO:0099504	synaptic vesicle cycle	1/95	249/14464	0.00401606	0.61145635	-0.5028562	0.80892931	0.82923896	0.79282714	Myo5b	1
GO:0031503	protein-containing complex localization	1/95	251/14464	0.00398406	0.60658419	-0.5112385	0.81147474	0.83118573	0.79468842	Myo5b	1
GO:0071407	cellular response to organic cyclic compound	2/95	462/14464	0.004329	0.6591023	-0.605519	0.81169466	0.83118573	0.79468842	Per1/Klf9	2
GO:0006898	receptor-mediated endocytosis	1/95	254/14464	0.00393701	0.59941981	-0.5237054	0.81523012	0.83420105	0.79757134	Serpine1	1
GO:0001501	skeletal system development	2/95	466/14464	0.00429185	0.65344477	-0.6183155	0.81550869	0.83420105	0.79757134	Por/Lrrc17	2
GO:0034976	response to endoplasmic reticulum stress	1/95	256/14464	0.00390625	0.59473684	-0.531947	0.81769248	0.83598927	0.79928104	Abl1	1
GO:0002009	morphogenesis of an epithelium	2/95	470/14464	0.00425532	0.64788354	-0.6310184	0.81925441	0.83669463	0.79995543	Abl1/Brpf1	2
GO:0003013	circulatory system process	2/95	470/14464	0.00425532	0.64788354	-0.6310184	0.81925441	0.83669463	0.79995543	Abl1/Agrn	2
GO:0110053	regulation of actin filament organization	1/95	258/14464	0.00387597	0.59012648	-0.540134	0.82012237	0.83713554	0.80037698	Abl1	1
GO:0008544	epidermis development	1/95	259/14464	0.003861	0.587848	-0.5442073	0.82132526	0.83791769	0.80112478	Alg10b	1
GO:0016049	cell growth	2/95	476/14464	0.00420168	0.63971694	-0.649901	0.82474694	0.84096142	0.80403486	Myo5b/Abl1	2
GO:0034329	cell junction assembly	2/95	485/14464	0.00412371	0.6278459	-0.6778496	0.83270832	0.84794851	0.81071515	Abl1/Agrn	2
GO:0060070	canonical Wnt signaling pathway	1/95	269/14464	0.00371747	0.56599491	-0.5842261	0.83292493	0.84794851	0.81071515	Fzd4	1
GO:1903311	regulation of mRNA metabolic process	1/95	269/14464	0.00371747	0.56599491	-0.5842261	0.83292493	0.84794851	0.81071515	Rbmx	1
GO:0007281	germ cell development	1/95	276/14464	0.00362319	0.55163997	-0.611503	0.84059816	0.85349623	0.81601927	Jam2	1
GO:0016197	endosomal transport	1/95	276/14464	0.00362319	0.55163997	-0.611503	0.84059816	0.85349623	0.81601927	Myo5b	1
GO:0030198	extracellular matrix organization	1/95	276/14464	0.00362319	0.55163997	-0.611503	0.84059816	0.85349623	0.81601927	Abl1	1
GO:0043062	extracellular structure organization	1/95	276/14464	0.00362319	0.55163997	-0.611503	0.84059816	0.85349623	0.81601927	Abl1	1
GO:0045229	external encapsulating structure organization	1/95	276/14464	0.00362319	0.55163997	-0.611503	0.84059816	0.85349623	0.81601927	Abl1	1
GO:0043491	phosphatidylinositol 3-kinase/protein kinase B signal transduction	1/95	278/14464	0.00359712	0.54767134	-0.6191905	0.84272574	0.85520396	0.81765201	Trem2	1
GO:0051604	protein maturation	2/95	498/14464	0.00401606	0.61145635	-0.7174575	0.84363948	0.85567873	0.81810594	Serpine1/Naa50	2
GO:0006936	muscle contraction	1/95	281/14464	0.00355872	0.54182431	-0.6306362	0.84586452	0.85748231	0.81983032	Agrn	1
GO:0030111	regulation of Wnt signaling pathway	1/95	282/14464	0.0035461	0.53990295	-0.6344289	0.84689694	0.85807563	0.82039759	Abl1	1
GO:0071375	cellular response to peptide hormone stimulus	1/95	292/14464	0.00342466	0.52141312	-0.6717603	0.85685213	0.86724643	0.8291657	Tns2	1
GO:0141091	transforming growth factor beta receptor superfamily signaling pathway	1/95	292/14464	0.00342466	0.52141312	-0.6717603	0.85685213	0.86724643	0.8291657	Abl1	1
GO:0097193	intrinsic apoptotic signaling pathway	1/95	302/14464	0.00331126	0.50414779	-0.7080618	0.86616637	0.87619594	0.83772223	Trem2	1
GO:0001822	kidney development	1/95	303/14464	0.00330033	0.50248393	-0.7116381	0.86706414	0.87619594	0.83772223	Tns2	1
GO:0099003	vesicle-mediated transport in synapse	1/95	303/14464	0.00330033	0.50248393	-0.7116381	0.86706414	0.87619594	0.83772223	Myo5b	1
GO:0042254	ribosome biogenesis	1/95	309/14464	0.00323625	0.49272696	-0.7328985	0.87232696	0.88105023	0.84236338	Wdr46	1
GO:0007178	cell surface receptor protein serine/threonine kinase signaling pathway	1/95	313/14464	0.00319489	0.48643013	-0.7468887	0.87572043	0.88401237	0.84519545	Abl1	1
GO:0072001	renal system development	1/95	315/14464	0.0031746	0.48334169	-0.7538302	0.87738354	0.88522556	0.84635537	Tns2	1
GO:0021700	developmental maturation	1/95	323/14464	0.00309598	0.47137038	-0.7812507	0.88381861	0.89110011	0.85197197	Rere	1
GO:0006913	nucleocytoplasmic transport	1/95	324/14464	0.00308642	0.46991553	-0.7846403	0.88459912	0.89110011	0.85197197	Cdan1	1
GO:0051169	nuclear transport	1/95	324/14464	0.00308642	0.46991553	-0.7846403	0.88459912	0.89110011	0.85197197	Cdan1	1
GO:0032956	regulation of actin cytoskeleton organization	1/95	328/14464	0.00304878	0.46418485	-0.7981166	0.88766963	0.89372404	0.85448068	Abl1	1
GO:0006631	fatty acid metabolic process	1/95	335/14464	0.00298507	0.45448547	-0.8213925	0.89284971	0.89846806	0.85901639	Por	1
GO:0042176	regulation of protein catabolic process	1/95	344/14464	0.00290698	0.44259486	-0.8507669	0.89916384	0.9043477	0.86463786	Trem2	1
GO:0050678	regulation of epithelial cell proliferation	1/95	346/14464	0.00289017	0.44003651	-0.8572133	0.90051619	0.9052334	0.86548467	Klf9	1
GO:0001503	ossification	1/95	359/14464	0.00278552	0.42410204	-0.8984294	0.90887931	0.91316199	0.87306511	Gdf10	1
GO:0032970	regulation of actin filament-based process	1/95	368/14464	0.00271739	0.41372998	-0.9262994	0.91425775	0.9180851	0.87777205	Abl1	1
GO:0003012	muscle system process	1/95	395/14464	0.00253165	0.3854497	-1.0069365	0.92857885	0.93197846	0.89105535	Agrn	1
GO:0140694	non-membrane-bounded organelle assembly	1/95	400/14464	0.0025	0.38063158	-1.0214153	0.93095817	0.93387806	0.89287154	Ccdc57	1
GO:0043161	proteasome-mediated ubiquitin-dependent protein catabolic process	1/95	412/14464	0.00242718	0.36954522	-1.0556287	0.93635329	0.93879936	0.89757674	Peli1	1
GO:0051051	negative regulation of transport	1/95	415/14464	0.00240964	0.36687381	-1.0640679	0.93763555	0.93959405	0.89833654	Agrn	1
GO:0050673	epithelial cell proliferation	1/95	417/14464	0.00239808	0.36511422	-1.0696694	0.93847615	0.93994558	0.89867263	Klf9	1
GO:0007283	spermatogenesis	1/95	423/14464	0.00236407	0.3599353	-1.0863575	0.94093126	0.94191293	0.9005536	Jam2	1
GO:0048232	male gamete generation	1/95	437/14464	0.00228833	0.34840419	-1.1246421	0.94628925	0.94678263	0.90520947	Jam2	1
GO:0010608	post-transcriptional regulation of gene expression	1/95	468/14464	0.00213675	0.32532614	-1.2064034	0.95650177	0.95650177	0.91450184	Per1	1

ID	Description	GeneRatio	BgRatio	RichFactor	FoldEnrichm	zScore	pvalue	p.adjust	qvalue	geneID	Count
GO:1902475	L-alpha-amino acid transmembrane transport	4/47	68/14464	0.05882353	18.1026283	8.07118063	6.8433E-05	0.07876637	0.07318728	Per2/Slc1a5/Slc7a2/Slc25a15	4
GO:0015807	L-amino acid transport	4/47	87/14464	0.04597701	14.1491807	7.02368227	0.00017879	0.10021232	0.0931142	Per2/Slc1a5/Slc7a2/Slc25a15	4
GO:0003333	amino acid transmembrane transport	4/47	96/14464	0.04166667	12.822695	6.6358203	0.0002612	0.10021232	0.0931142	Per2/Slc1a5/Slc7a2/Slc25a15	4
GO:0089718	amino acid import across plasma membrane	3/47	52/14464	0.05769231	17.7545008	6.91053564	0.0006356	0.11052783	0.10269905	Per2/Slc1a5/Slc7a2	3
GO:2000107	negative regulation of leukocyte apoptotic process	3/47	53/14464	0.05660377	17.4195102	6.83741196	0.00067221	0.11052783	0.10269905	Tsc22d3/Mertk/Bcl2l1	3
GO:0009404	toxin metabolic process	2/47	13/14464	0.15384615	47.3453355	9.54485706	0.00078794	0.11052783	0.10269905	Fmo2/Fmo1	2
GO:0006805	xenobiotic metabolic process	3/47	58/14464	0.05172414	15.9178283	6.49962902	0.0008754	0.11052783	0.10269905	Fmo2/Fmo1/Cyp2f2	3
GO:1905039	carboxylic acid transmembrane transport	4/47	133/14464	0.03007519	9.25547912	5.46097928	0.00089727	0.11052783	0.10269905	Per2/Slc1a5/Slc7a2/Slc25a15	4
GO:1903826	L-arginine transmembrane transport	2/47	14/14464	0.14285714	43.9635258	9.18270605	0.00091736	0.11052783	0.10269905	Slc7a2/Slc25a15	2
GO:1903825	organic acid transmembrane transport	4/47	136/14464	0.02941176	9.05131414	5.3862203	0.00097505	0.11052783	0.10269905	Per2/Slc1a5/Slc7a2/Slc25a15	4
GO:1990822	basic amino acid transmembrane transport	2/47	15/14464	0.13333333	41.0326241	8.85689438	0.0010563	0.11052783	0.10269905	Slc7a2/Slc25a15	2
GO:0046322	negative regulation of fatty acid oxidation	2/47	16/14464	0.125	38.4680851	8.56166628	0.00120471	0.11555151	0.1073669	Fmo2/Fmo1	2
GO:0015802	basic amino acid transport	2/47	18/14464	0.11111111	34.1938534	8.04564363	0.00152966	0.12575951	0.11685186	Slc7a2/Slc25a15	2
GO:1902236	negative regulation of endoplasmic reticulum stress-induced intrinsic apoptotic signaling pathway	2/47	18/14464	0.11111111	34.1938534	8.04564363	0.00152966	0.12575951	0.11685186	Bcl2l1/Herpud1	2
GO:0006865	amino acid transport	4/47	158/14464	0.02531646	7.79100458	4.90054215	0.00169671	0.13019388	0.12097213	Per2/Slc1a5/Slc7a2/Slc25a15	4
GO:2000106	regulation of leukocyte apoptotic process	3/47	88/14464	0.03409091	10.4912959	5.09904086	0.00290845	0.20922667	0.19440697	Tsc22d3/Mertk/Bcl2l1	3
GO:1902235	regulation of endoplasmic reticulum stress-induced intrinsic apoptotic signaling pathway	2/47	29/14464	0.06896552	21.2237711	6.22434159	0.00396782	0.24805124	0.23048156	Bcl2l1/Herpud1	2
GO:1903077	negative regulation of protein localization to plasma membrane	2/47	30/14464	0.06666667	20.5163121	6.10950048	0.00424246	0.24805124	0.23048156	Bcl2l1/Ppfia1	2
GO:0006631	fatty acid metabolic process	5/47	335/14464	0.01492537	4.59320419	3.79917467	0.00447121	0.24805124	0.23048156	Fmo2/Per2/Fmo1/Cyp2f2/Acsf3	5
GO:0045922	negative regulation of fatty acid metabolic process	2/47	31/14464	0.06451613	19.8544955	6.00009506	0.0045257	0.24805124	0.23048156	Fmo2/Fmo1	2
GO:1904376	negative regulation of protein localization to cell periphery	2/47	31/14464	0.06451613	19.8544955	6.00009506	0.0045257	0.24805124	0.23048156	Bcl2l1/Ppfia1	2
GO:0071887	leukocyte apoptotic process	3/47	112/14464	0.02678571	8.24316109	4.39361113	0.00572162	0.29934452	0.27814169	Tsc22d3/Mertk/Bcl2l1	3
GO:0015695	organic cation transport	3/47	117/14464	0.02564103	7.89088925	4.27295506	0.00645688	0.29939616	0.27818967	Slc1a5/Slc7a2/Slc25a15	3
GO:1905476	negative regulation of protein localization to membrane	2/47	38/14464	0.05263158	16.1970885	5.3557451	0.006744	0.29939616	0.27818967	Bcl2l1/Ppfia1	2
GO:0016053	organic acid biosynthetic process	4/47	235/14464	0.01702128	5.23820733	3.73998287	0.00701172	0.29939616	0.27818967	Per2/Fmo1/Acsf3/Rdh10	4
GO:0046320	regulation of fatty acid oxidation	2/47	39/14464	0.05128205	15.7817785	5.27766422	0.00709391	0.29939616	0.27818967	Fmo2/Fmo1	2
GO:0051896	regulation of phosphatidylinositol 3-kinase/protein kinase B signal transduction	4/47	240/14464	0.01666667	5.12907801	3.68288799	0.00754259	0.29939616	0.27818967	Xdh/Mertk/Phlda3/Sesn1	4
GO:0071466	cellular response to xenobiotic stimulus	3/47	124/14464	0.02419355	7.44543583	4.11556276	0.00757717	0.29939616	0.27818967	Fmo2/Fmo1/Cyp2f2	3
GO:0019748	secondary metabolic process	2/47	41/14464	0.04878049	15.0119357	5.12982953	0.00781793	0.29939616	0.27818967	Fmo2/Fmo1	2
GO:0010565	regulation of cellular ketone metabolic process	3/47	127/14464	0.02362205	7.26955939	4.0518225	0.00809032	0.29939616	0.27818967	Fmo2/Fmo1/Rdh10	3
GO:1903573	negative regulation of response to endoplasmic reticulum stress	2/47	42/14464	0.04761905	14.6545086	5.05974521	0.00819194	0.29939616	0.27818967	Bcl2l1/Herpud1	2
GO:0001558	regulation of cell growth	5/47	389/14464	0.01285347	3.95558716	3.37392203	0.00832379	0.29939616	0.27818967	Fam107a/Trip10/Crlf3/Sesn1/Ip6k2	5
GO:0006739	NADP metabolic process	2/47	49/14464	0.04081633	12.5610074	4.62835932	0.01102898	0.36409233	0.33830335	Fmo2/Fmo1	2
GO:0043491	phosphatidylinositol 3-kinase/protein kinase B signal transduction	4/47	278/14464	0.01438849	4.42798102	3.29512202	0.01246126	0.36409233	0.33830335	Xdh/Mertk/Phlda3/Sesn1	4
GO:0046942	carboxylic acid transport	4/47	281/14464	0.01423488	4.3807072	3.26751312	0.01291964	0.36409233	0.33830335	Per2/Slc1a5/Slc7a2/Slc25a15	4
GO:0015849	organic acid transport	4/47	283/14464	0.01413428	4.34974814	3.24932099	0.01323117	0.36409233	0.33830335	Per2/Slc1a5/Slc7a2/Slc25a15	4
GO:0072330	monocarboxylic acid biosynthetic process	3/47	156/14464	0.01923077	5.91816694	3.52627982	0.01410922	0.36409233	0.33830335	Per2/Acsf3/Rdh10	3
GO:2000134	negative regulation of G1/S transition of mitotic cell cycle	2/47	59/14464	0.03389831	10.4320231	4.14490806	0.01572021	0.36409233	0.33830335	Fam107a/Crlf3	2
GO:0019216	regulation of lipid metabolic process	4/47	298/14464	0.01342282	4.13080109	3.11803267	0.01572185	0.36409233	0.33830335	Fmo2/Fmo1/Rdh10/Mtmr2	4
GO:0097193	intrinsic apoptotic signaling pathway	4/47	302/14464	0.01324503	4.07608849	3.08447087	0.01643277	0.36409233	0.33830335	Prodh/Phlda3/Bcl2l1/Herpud1	4
GO:0001701	in utero embryonic development	5/47	461/14464	0.01084599	3.3377948	2.91264114	0.01643344	0.36409233	0.33830335	Otud7b/Bcl2l1/Rdh10/Mxi1/Ints1	5
GO:0002931	response to ischemia	2/47	61/14464	0.03278689	10.0899895	4.06202408	0.01674472	0.36409233	0.33830335	Per2/Bcl2l1	2
GO:0098739	import across plasma membrane	3/47	167/14464	0.01796407	5.52834756	3.3605952	0.01690845	0.36409233	0.33830335	Per2/Slc1a5/Slc7a2	3
GO:0070059	intrinsic apoptotic signaling pathway in response to endoplasmic reticulum stress	2/47	62/14464	0.03225806	9.92724777	4.02200589	0.01726743	0.36409233	0.33830335	Bcl2l1/Herpud1	2
GO:0044283	small molecule biosynthetic process	5/47	470/14464	0.0106383	3.27387958	2.86144979	0.01772229	0.36409233	0.33830335	Per2/Fmo1/Acsf3/Ip6k2/Rdh10	5
GO:0051898	negative regulation of phosphatidylinositol 3-kinase/protein kinase B signal transduction	2/47	63/14464	0.03174603	9.76967241	3.98288707	0.01779706	0.36409233	0.33830335	Xdh/Phlda3	2
GO:0030308	negative regulation of cell growth	3/47	171/14464	0.01754386	5.39902949	3.30395333	0.01799817	0.36409233	0.33830335	Crlf3/Sesn1/Ip6k2	3
GO:2001242	regulation of intrinsic apoptotic signaling pathway	3/47	172/14464	0.01744186	5.36763978	3.29007051	0.01827661	0.36409233	0.33830335	Prodh/Bcl2l1/Herpud1	3
GO:0016049	cell growth	5/47	476/14464	0.0105042	3.23261219	2.82800146	0.01861806	0.36409233	0.33830335	Fam107a/Trip10/Crlf3/Sesn1/Ip6k2	5
GO:0062012	regulation of small molecule metabolic process	4/47	319/14464	0.01253918	3.85886747	2.94800735	0.01967947	0.36409233	0.33830335	Fmo2/Fmo1/Bcl2l1/Rdh10	4
GO:1902807	negative regulation of cell cycle G1/S phase transition	2/47	67/14464	0.02985075	9.18640838	3.83473606	0.01998368	0.36409233	0.33830335	Fam107a/Crlf3	2
GO:0030258	lipid modification	3/47	178/14464	0.01685393	5.1867081	3.20898757	0.01999791	0.36409233	0.33830335	Fmo2/Fmo1/Mtmr2	3
GO:0042180	cellular ketone metabolic process	3/47	183/14464	0.01639344	5.04499477	3.14416169	0.02149874	0.36409233	0.33830335	Fmo2/Fmo1/Rdh10	3
GO:0015800	acidic amino acid transport	2/47	72/14464	0.02777778	8.54846336	3.66610498	0.02286667	0.36409233	0.33830335	Per2/Slc1a5	2
GO:0015711	organic anion transport	4/47	341/14464	0.01173021	3.60990828	2.78470778	0.02443691	0.36409233	0.33830335	Per2/Slc1a5/Slc7a2/Slc25a15	4
GO:0097009	energy homeostasis	2/47	79/14464	0.02531646	7.79100458	3.45567832	0.02717188	0.36409233	0.33830335	Fmo2/Fmo1	2
GO:0071230	cellular response to amino acid stimulus	2/47	80/14464	0.025	7.69361702	3.42773066	0.02781173	0.36409233	0.33830335	Sesn1/Bcl2l1	2
GO:1905897	regulation of response to endoplasmic reticulum stress	2/47	81/14464	0.02469136	7.5986341	3.40026284	0.02845766	0.36409233	0.33830335	Bcl2l1/Herpud1	2
GO:0031396	regulation of protein ubiquitination	3/47	204/14464	0.01470588	4.52565707	2.89558329	0.02846311	0.36409233	0.33830335	Per2/Fam107a/Herpud1	3
GO:0043648	dicarboxylic acid metabolic process	2/47	83/14464	0.02409639	7.41553448	3.34670952	0.02976756	0.36409233	0.33830335	Prodh/Acsf3	2
GO:0048041	focal adhesion assembly	2/47	85/14464	0.02352941	7.24105131	3.29490993	0.03110122	0.36409233	0.33830335	Fam107a/Tesk2	2
GO:0035871	protein K11-linked deubiquitination	1/47	10/14464	0.1	30.7744681	5.3776268	0.03203323	0.36409233	0.33830335	Otud7b	1
GO:0036466	synaptic vesicle recycling via endosome	1/47	10/14464	0.1	30.7744681	5.3776268	0.03203323	0.36409233	0.33830335	Bcl2l1	1
GO:0070344	regulation of fat cell proliferation	1/47	10/14464	0.1	30.7744681	5.3776268	0.03203323	0.36409233	0.33830335	Per2	1
GO:1901031	regulation of response to reactive oxygen species	1/47	10/14464	0.1	30.7744681	5.3776268	0.03203323	0.36409233	0.33830335	Sesn1	1
GO:2000807	regulation of synaptic vesicle clustering	1/47	10/14464	0.1	30.7744681	5.3776268	0.03203323	0.36409233	0.33830335	Bcl2l1	1
GO:0090288	negative regulation of cellular response to growth factor stimulus	2/47	87/14464	0.02298851	7.07459036	3.24476433	0.03245829	0.36409233	0.33830335	Xdh/Fstl3	2
GO:0045926	negative regulation of growth	3/47	216/14464	0.01388889	4.27423168	2.76821617	0.032921	0.36409233	0.33830335	Crlf3/Sesn1/Ip6k2	3
GO:0006835	dicarboxylic acid transport	2/47	88/14464	0.02272727	6.99419729	3.22028278	0.0331455	0.36409233	0.33830335	Per2/Slc1a5	2
GO:0045833	negative regulation of lipid metabolic process	2/47	88/14464	0.02272727	6.99419729	3.22028278	0.0331455	0.36409233	0.33830335	Fmo2/Fmo1	2
GO:0120254	olefinic compound metabolic process	2/47	88/14464	0.02272727	6.99419729	3.22028278	0.0331455	0.36409233	0.33830335	Cyp2f2/Rdh10	2
GO:0019217	regulation of fatty acid metabolic process	2/47	89/14464	0.02247191	6.91561081	3.19618083	0.03383843	0.36409233	0.33830335	Fmo2/Fmo1	2
GO:2001233	regulation of apoptotic signaling pathway	4/47	379/14464	0.01055409	3.24796497	2.53204483	0.03418455	0.36409233	0.33830335	G0s2/Prodh/Bcl2l1/Herpud1	4
GO:1903828	negative regulation of protein localization	3/47	221/14464	0.01357466	4.1775296	2.71785121	0.03488047	0.36409233	0.33830335	Otud7b/Bcl2l1/Ppfia1	3
GO:0009120	deoxyribonucleoside metabolic process	1/47	11/14464	0.09090909	27.9767892	5.11032207	0.03518077	0.36409233	0.33830335	Xdh	1
GO:0009169	purine ribonucleoside monophosphate catabolic process	1/47	11/14464	0.09090909	27.9767892	5.11032207	0.03518077	0.36409233	0.33830335	Xdh	1
GO:0016114	terpenoid biosynthetic process	1/47	11/14464	0.09090909	27.9767892	5.11032207	0.03518077	0.36409233	0.33830335	Rdh10	1
GO:0042573	retinoic acid metabolic process	1/47	11/14464	0.09090909	27.9767892	5.11032207	0.03518077	0.36409233	0.33830335	Rdh10	1
GO:0045602	negative regulation of endothelial cell differentiation	1/47	11/14464	0.09090909	27.9767892	5.11032207	0.03518077	0.36409233	0.33830335	Xdh	1
GO:0075522	IRES-dependent viral translational initiation	1/47	11/14464	0.09090909	27.9767892	5.11032207	0.03518077	0.36409233	0.33830335	Pcbp2	1
GO:1902548	negative regulation of cellular response to vascular endothelial growth factor stimulus	1/47	11/14464	0.09090909	27.9767892	5.11032207	0.03518077	0.36409233	0.33830335	Xdh	1
GO:0071229	cellular response to acid chemical	2/47	91/14464	0.02197802	6.76361936	3.14907459	0.03524129	0.36409233	0.33830335	Sesn1/Bcl2l1	2
GO:0120162	positive regulation of cold-induced thermogenesis	2/47	91/14464	0.02197802	6.76361936	3.14907459	0.03524129	0.36409233	0.33830335	Per2/G0s2	2
GO:0007044	cell-substrate junction assembly	2/47	95/14464	0.02105263	6.47883539	3.05898576	0.03811381	0.36409233	0.33830335	Fam107a/Tesk2	2
GO:0009128	purine nucleoside monophosphate catabolic process	1/47	12/14464	0.08333333	25.6453901	4.87644147	0.03831828	0.36409233	0.33830335	Xdh	1
GO:0009155	purine deoxyribonucleotide catabolic process	1/47	12/14464	0.08333333	25.6453901	4.87644147	0.03831828	0.36409233	0.33830335	Xdh	1
GO:0009170	purine deoxyribonucleoside monophosphate metabolic process	1/47	12/14464	0.08333333	25.6453901	4.87644147	0.03831828	0.36409233	0.33830335	Xdh	1
GO:0032717	negative regulation of interleukin-8 production	1/47	12/14464	0.08333333	25.6453901	4.87644147	0.03831828	0.36409233	0.33830335	Otud7b	1
GO:0043584	nose development	1/47	12/14464	0.08333333	25.6453901	4.87644147	0.03831828	0.36409233	0.33830335	Rdh10	1
GO:0046085	adenosine metabolic process	1/47	12/14464	0.08333333	25.6453901	4.87644147	0.03831828	0.36409233	0.33830335	Xdh	1
GO:0071233	cellular response to L-leucine	1/47	12/14464	0.08333333	25.6453901	4.87644147	0.03831828	0.36409233	0.33830335	Sesn1	1
GO:0090394	negative regulation of excitatory postsynaptic potential	1/47	12/14464	0.08333333	25.6453901	4.87644147	0.03831828	0.36409233	0.33830335	Mtmr2	1
GO:1900244	positive regulation of synaptic vesicle endocytosis	1/47	12/14464	0.08333333	25.6453901	4.87644147	0.03831828	0.36409233	0.33830335	Bcl2l1	1
GO:1901029	negative regulation of mitochondrial outer membrane permeabilization involved in apoptotic signaling pat	1/47	12/14464	0.08333333	25.6453901	4.87644147	0.03831828	0.36409233	0.33830335	Bcl2l1	1
GO:1990253	cellular response to leucine starvation	1/47	12/14464	0.08333333	25.6453901	4.87644147	0.03831828	0.36409233	0.33830335	Sesn1	1
GO:2000644	regulation of receptor catabolic process	1/47	12/14464	0.08333333	25.6453901	4.87644147	0.03831828	0.36409233	0.33830335	Mtmr2	1
GO:2000756	regulation of peptidyl-lysine acetylation	1/47	12/14464	0.08333333	25.6453901	4.87644147	0.03831828	0.36409233	0.33830335	Klf15	1
GO:0006163	purine nucleotide metabolic process	4/47	395/14464	0.01012658	3.11640183	2.43503795	0.0388823	0.36409233	0.33830335	Fmo2/Fmo1/Xdh/Bcl2l1	4
GO:0046394	carboxylic acid biosynthetic process	3/47	232/14464	0.01293103	3.97945708	2.61209418	0.03940082	0.36409233	0.33830335	Per2/Acsf3/Rdh10	3
GO:0062014	negative regulation of small molecule metabolic process	2/47	98/14464	0.02040816	6.28050369	2.99475384	0.04032535	0.36409233	0.33830335	Fmo2/Fmo1	2
GO:2001243	negative regulation of intrinsic apoptotic signaling pathway	2/47	99/14464	0.02020202	6.21706426	2.97393616	0.04107319	0.36409233	0.33830335	Bcl2l1/Herpud1	2
GO:0006924	activation-induced cell death of T cells	1/47	13/14464	0.07692308	23.6726678	4.66945315	0.04144581	0.36409233	0.33830335	Tsc22d3	1
GO:0030540	female genitalia development	1/47	13/14464	0.07692308	23.6726678	4.66945315	0.04144581	0.36409233	0.33830335	Mertk	1
GO:0042454	ribonucleoside catabolic process	1/47	13/14464	0.07692308	23.6726678	4.66945315	0.04144581	0.36409233	0.33830335	Xdh	1
GO:0042754	negative regulation of circadian rhythm	1/47	13/14464	0.07692308	23.6726678	4.66945315	0.04144581	0.36409233	0.33830335	Per2	1
GO:0043605	amide catabolic process	1/47	13/14464	0.07692308	23.6726678	4.66945315	0.04144581	0.36409233	0.33830335	Xdh	1
GO:0055057	neuroblast division	1/47	13/14464	0.07692308	23.6726678	4.66945315	0.04144581	0.36409233	0.33830335	Tead3	1
GO:0070341	fat cell proliferation	1/47	13/14464	0.07692308	23.6726678	4.66945315	0.04144581	0.36409233	0.33830335	Per2	1
GO:0007548	sex differentiation	3/47	237/14464	0.01265823	3.89550229	2.56615032	0.04155005	0.36409233	0.33830335	Mertk/Bcl2l1/Rdh10	3
GO:1903320	regulation of protein modification by small protein conjugation or removal	3/47	239/14464	0.0125523	3.86290394	2.54812226	0.04242617	0.36409233	0.33830335	Per2/Fam107a/Herpud1	3
GO:0003300	cardiac muscle hypertrophy	2/47	101/14464	0.01980198	6.09395408	2.93314684	0.04258463	0.36409233	0.33830335	Klf15/Trip10	2
GO:0150115	cell-substrate junction organization	2/47	102/14464	0.01960784	6.03420943	2.9131616	0.04334815	0.36409233	0.33830335	Fam107a/Tesk2	2
GO:0002385	mucosal immune response	1/47	14/14464	0.07142857	21.9817629	4.48448672	0.04456338	0.36409233	0.33830335	Otud7b	1
GO:0035234	ectopic germ cell programmed cell death	1/47	14/14464	0.07142857	21.9817629	4.48448672	0.04456338	0.36409233	0.33830335	Bcl2l1	1
GO:0043201	response to L-leucine	1/47	14/14464	0.07142857	21.9817629	4.48448672	0.04456338	0.36409233	0.33830335	Sesn1	1
GO:0043649	dicarboxylic acid catabolic process	1/47	14/14464	0.07142857	21.9817629	4.48448672	0.04456338	0.36409233	0.33830335	Acsf3	1
GO:0060572	morphogenesis of an epithelial bud	1/47	14/14464	0.07142857	21.9817629	4.48448672	0.04456338	0.36409233	0.33830335	Rdh10	1
GO:0061051	positive regulation of cell growth involved in cardiac muscle cell development	1/47	14/14464	0.07142857	21.9817629	4.48448672	0.04456338	0.36409233	0.33830335	Trip10	1
GO:0070207	protein homotrimerization	1/47	14/14464	0.07142857	21.9817629	4.48448672	0.04456338	0.36409233	0.33830335	Slc1a5	1
GO:1900272	negative regulation of long-term synaptic potentiation	1/47	14/14464	0.07142857	21.9817629	4.48448672	0.04456338	0.36409233	0.33830335	Fam107a	1
GO:2001171	positive regulation of ATP biosynthetic process	1/47	14/14464	0.07142857	21.9817629	4.48448672	0.04456338	0.36409233	0.33830335	Bcl2l1	1
GO:1901873	regulation of post-translational protein modification	3/47	244/14464	0.01229508	3.78374608	2.50389092	0.04465729	0.36409233	0.33830335	Per2/Fam107a/Herpud1	3
GO:0014897	striated muscle hypertrophy	2/47	104/14464	0.01923077	5.91816694	2.87397766	0.04489057	0.36409233	0.33830335	Klf15/Trip10	2
GO:0008630	intrinsic apoptotic signaling pathway in response to DNA damage	2/47	105/14464	0.01904762	5.86180344	2.8547667	0.0456694	0.36409233	0.33830335	Phlda3/Bcl2l1	2
GO:0019395	fatty acid oxidation	2/47	105/14464	0.01904762	5.86180344	2.8547667	0.0456694	0.36409233	0.33830335	Fmo2/Fmo1	2
GO:0014896	muscle hypertrophy	2/47	106/14464	0.01886792	5.80650341	2.83580188	0.04645325	0.36409233	0.33830335	Klf15/Trip10	2
GO:0001833	inner cell mass cell proliferation	1/47	15/14464	0.06666667	20.5163121	4.31782623	0.04767103	0.36409233	0.33830335	Ints1	1
GO:0032926	negative regulation of activin receptor signaling pathway	1/47	15/14464	0.06666667	20.5163121	4.31782623	0.04767103	0.36409233	0.33830335	Fstl3	1
GO:0099532	synaptic vesicle endosomal processing	1/47	15/14464	0.06666667	20.5163121	4.31782623	0.04767103	0.36409233	0.33830335	Bcl2l1	1
GO:0160049	negative regulation of cGAS/STING signaling pathway	1/47	15/14464	0.06666667	20.5163121	4.31782623	0.04767103	0.36409233	0.33830335	Pcbp2	1
GO:1901653	cellular response to peptide	1/47	15/14464	0.06666667	20.5163121	4.31782623	0.04767103	0.36409233	0.33830335	Klf15	1
GO:2000811	negative regulation of anoikis	1/47	15/14464	0.06666667	20.5163121	4.31782623	0.04767103	0.36409233	0.33830335	Bcl2l1	1
GO:2001212	regulation of vasculogenesis	1/47	15/14464	0.06666667	20.5163121	4.31782623	0.04767103	0.36409233	0.33830335	Xdh	1
GO:0007283	spermatogenesis	4/47	423/14464	0.00945626	2.91011519	2.2765196	0.04796006	0.36409233	0.33830335	Cfap157/Mertk/Tesk2/Bcl2l1	4
GO:0034440	lipid oxidation	2/47	109/14464	0.01834862	5.64669139	2.78032877	0.04883456	0.36409233	0.33830335	Fmo2/Fmo1	2
GO:0032946	positive regulation of mononuclear cell proliferation	2/47	111/14464	0.01801802	5.5449492	2.74447863	0.05044651	0.36409233	0.33830335	Bcl2l1/Icosl	2
GO:0002251	organ or tissue specific immune response	1/47	16/14464	0.0625	19.2340426	4.16658035	0.05076879	0.36409233	0.33830335	Otud7b	1
GO:0009159	deoxyribonucleoside monophosphate catabolic process	1/47	16/14464	0.0625	19.2340426	4.16658035	0.05076879	0.36409233	0.33830335	Xdh	1
GO:0030810	positive regulation of nucleotide biosynthetic process	1/47	16/14464	0.0625	19.2340426	4.16658035	0.05076879	0.36409233	0.33830335	Bcl2l1	1
GO:0032753	positive regulation of interleukin-4 production	1/47	16/14464	0.0625	19.2340426	4.16658035	0.05076879	0.36409233	0.33830335	Icosl	1
GO:0051895	negative regulation of focal adhesion assembly	1/47	16/14464	0.0625	19.2340426	4.16658035	0.05076879	0.36409233	0.33830335	Fam107a	1
GO:0098712	L-glutamate import across plasma membrane	1/47	16/14464	0.0625	19.2340426	4.16658035	0.05076879	0.36409233	0.33830335	Per2	1
GO:0150118	negative regulation of cell-substrate junction organization	1/47	16/14464	0.0625	19.2340426	4.16658035	0.05076879	0.36409233	0.33830335	Fam107a	1
GO:1900373	positive regulation of purine nucleotide biosynthetic process	1/47	16/14464	0.0625	19.2340426	4.16658035	0.05076879	0.36409233	0.33830335	Bcl2l1	1
GO:1903423	positive regulation of synaptic vesicle recycling	1/47	16/14464	0.0625	19.2340426	4.16658035	0.05076879	0.36409233	0.33830335	Bcl2l1	1
GO:0002683	negative regulation of immune system process	4/47	433/14464	0.00923788	2.84290698	2.22302181	0.05146669	0.36409233	0.33830335	Tsc22d3/Mertk/Fstl3/Pcbp2	4
GO:1903076	regulation of protein localization to plasma membrane	2/47	114/14464	0.01754386	5.39902949	2.69230353	0.05290027	0.36409233	0.33830335	Bcl2l1/Ppfia1	2
GO:0048232	male gamete generation	4/47	437/14464	0.00915332	2.81688495	2.20204617	0.05290824	0.36409233	0.33830335	Cfap157/Mertk/Tesk2/Bcl2l1	4
GO:0043200	response to amino acid	2/47	115/14464	0.0173913	5.35208141	2.6753203	0.0537276	0.36409233	0.33830335	Sesn1/Bcl2l1	2
GO:0002091	negative regulation of receptor internalization	1/47	17/14464	0.05882353	18.1026283	4.02846091	0.05385668	0.36409233	0.33830335	Mtmr2	1
GO:0008356	asymmetric cell division	1/47	17/14464	0.05882353	18.1026283	4.02846091	0.05385668	0.36409233	0.33830335	Tead3	1
GO:0032352	positive regulation of hormone metabolic process	1/47	17/14464	0.05882353	18.1026283	4.02846091	0.05385668	0.36409233	0.33830335	Rdh10	1
GO:0050687	negative regulation of defense response to virus	1/47	17/14464	0.05882353	18.1026283	4.02846091	0.05385668	0.36409233	0.33830335	Pcbp2	1
GO:0060602	branch elongation of an epithelium	1/47	17/14464	0.05882353	18.1026283	4.02846091	0.05385668	0.36409233	0.33830335	Rdh10	1
GO:1903376	regulation of oxidative stress-induced neuron intrinsic apoptotic signaling pathway	1/47	17/14464	0.05882353	18.1026283	4.02846091	0.05385668	0.36409233	0.33830335	Prodh	1
GO:0001776	leukocyte homeostasis	2/47	117/14464	0.01709402	5.26059284	2.64194083	0.05539613	0.36409233	0.33830335	Tsc22d3/Mertk	2
GO:0048608	reproductive structure development	3/47	268/14464	0.01119403	3.44490314	2.30667673	0.0561655	0.36409233	0.33830335	Mertk/Bcl2l1/Rdh10	3
GO:0051588	regulation of neurotransmitter transport	2/47	118/14464	0.01694915	5.21601154	2.62553646	0.05623726	0.36409233	0.33830335	Per2/Bcl2l1	2
GO:0009158	ribonucleoside monophosphate catabolic process	1/47	18/14464	0.05555556	17.0969267	3.90163004	0.05693474	0.36409233	0.33830335	Xdh	1
GO:0009164	nucleoside catabolic process	1/47	18/14464	0.05555556	17.0969267	3.90163004	0.05693474	0.36409233	0.33830335	Xdh	1
GO:0010447	response to acidic pH	1/47	18/14464	0.05555556	17.0969267	3.90163004	0.05693474	0.36409233	0.33830335	Kcnk4	1
GO:0015810	aspartate transmembrane transport	1/47	18/14464	0.05555556	17.0969267	3.90163004	0.05693474	0.36409233	0.33830335	Slc1a5	1
GO:0031645	negative regulation of nervous system process	1/47	18/14464	0.05555556	17.0969267	3.90163004	0.05693474	0.36409233	0.33830335	Mtmr2	1
GO:0042104	positive regulation of activated T cell proliferation	1/47	18/14464	0.05555556	17.0969267	3.90163004	0.05693474	0.36409233	0.33830335	Icosl	1
GO:0051580	regulation of neurotransmitter uptake	1/47	18/14464	0.05555556	17.0969267	3.90163004	0.05693474	0.36409233	0.33830335	Per2	1

GO:0070206	protein trimerization	1/47	18/14464	0.05555556	17.0969267	3.90163004	0.05693474	0.36409233	0.33830335 Slc1a5	1
GO:0071467	cellular response to pH	1/47	18/14464	0.05555556	17.0969267	3.90163004	0.05693474	0.36409233	0.33830335 Kcnk4	1
GO:0009117	nucleotide metabolic process	4/47	450/14464	0.00888889	2.73550827	2.13546551	0.05774633	0.36409233	0.33830335 Fmo2/Fmo1/Xdh/Bcl2l1	4
GO:0061458	reproductive system development	3/47	272/14464	0.01102941	3.3942428	2.2759961	0.05820958	0.36409233	0.33830335 Mertk/Bcl2l1/Rdh10	3
GO:0046660	female sex differentiation	2/47	122/14464	0.01639344	5.04499477	2.561732	0.05964671	0.36409233	0.33830335 Mertk/Bcl2l1	2
GO:0006144	purine nucleobase metabolic process	1/47	19/14464	0.05263158	16.1970885	3.78459222	0.060003	0.36409233	0.33830335 Xdh	1
GO:0006817	phosphate ion transport	1/47	19/14464	0.05263158	16.1970885	3.78459222	0.060003	0.36409233	0.33830335 Ip6k2	1
GO:0009151	purine deoxyribonucleotide metabolic process	1/47	19/14464	0.05263158	16.1970885	3.78459222	0.060003	0.36409233	0.33830335 Xdh	1
GO:0036480	neuron intrinsic apoptotic signaling pathway in response to oxidative stress	1/47	19/14464	0.05263158	16.1970885	3.78459222	0.060003	0.36409233	0.33830335 Prodh	1
GO:0150104	transport across blood-brain barrier	1/47	19/14464	0.05263158	16.1970885	3.78459222	0.060003	0.36409233	0.33830335 Slc1a5	1
GO:1900452	regulation of long-term synaptic depression	1/47	19/14464	0.05263158	16.1970885	3.78459222	0.060003	0.36409233	0.33830335 Bcl2l1	1
GO:0070665	positive regulation of leukocyte proliferation	2/47	123/14464	0.01626016	5.00397855	2.54621606	0.06051012	0.36409233	0.33830335 Bcl2l1/Icosl	2
GO:0006089	lactate metabolic process	1/47	20/14464	0.05	15.387234	3.67611627	0.06306149	0.36409233	0.33830335 Per2	1
GO:0009065	glutamine family amino acid catabolic process	1/47	20/14464	0.05	15.387234	3.67611627	0.06306149	0.36409233	0.33830335 Prodh	1
GO:0009125	nucleoside monophosphate catabolic process	1/47	20/14464	0.05	15.387234	3.67611627	0.06306149	0.36409233	0.33830335 Xdh	1
GO:0010232	vascular transport	1/47	20/14464	0.05	15.387234	3.67611627	0.06306149	0.36409233	0.33830335 Slc1a5	1
GO:0010958	regulation of amino acid import across plasma membrane	1/47	20/14464	0.05	15.387234	3.67611627	0.06306149	0.36409233	0.33830335 Per2	1
GO:0017121	plasma membrane phospholipid scrambling	1/47	20/14464	0.05	15.387234	3.67611627	0.06306149	0.36409233	0.33830335 Plscr4	1
GO:0034472	snRNA 3'-end processing	1/47	20/14464	0.05	15.387234	3.67611627	0.06306149	0.36409233	0.33830335 Ints1	1
GO:0035795	negative regulation of mitochondrial membrane permeability	1/47	20/14464	0.05	15.387234	3.67611627	0.06306149	0.36409233	0.33830335 Bcl2l1	1
GO:0097091	synaptic vesicle clustering	1/47	20/14464	0.05	15.387234	3.67611627	0.06306149	0.36409233	0.33830335 Bcl2l1	1
GO:0140896	cGAS/STING signaling pathway	1/47	20/14464	0.05	15.387234	3.67611627	0.06306149	0.36409233	0.33830335 Pcbp2	1
GO:1900746	regulation of vascular endothelial growth factor signaling pathway	1/47	20/14464	0.05	15.387234	3.67611627	0.06306149	0.36409233	0.33830335 Xdh	1
GO:1901028	regulation of mitochondrial outer membrane permeabilization involved in apoptotic signaling pathway	1/47	20/14464	0.05	15.387234	3.67611627	0.06306149	0.36409233	0.33830335 Bcl2l1	1
GO:1903789	regulation of amino acid transmembrane transport	1/47	20/14464	0.05	15.387234	3.67611627	0.06306149	0.36409233	0.33830335 Per2	1
GO:1904294	positive regulation of ERAD pathway	1/47	20/14464	0.05	15.387234	3.67611627	0.06306149	0.36409233	0.33830335 Herpud1	1
GO:2000641	regulation of early endosome to late endosome transport	1/47	20/14464	0.05	15.387234	3.67611627	0.06306149	0.36409233	0.33830335 Mtmr2	1
GO:0043500	muscle adaptation	2/47	126/14464	0.01587302	4.8848362	2.5006584	0.06312627	0.36409233	0.33830335 Klf15/Trip10	2
GO:0001824	blastocyst development	2/47	127/14464	0.01574803	4.84637293	2.48579196	0.06400685	0.36409233	0.33830335 Mxi1/Ints1	2
GO:0019081	viral translation	1/47	21/14464	0.04761905	14.6545086	3.57517817	0.06611023	0.36409233	0.33830335 Pcbp2	1
GO:0034656	nucleobase-containing small molecule catabolic process	1/47	21/14464	0.04761905	14.6545086	3.57517817	0.06611023	0.36409233	0.33830335 Xdh	1
GO:0046040	IMP metabolic process	1/47	21/14464	0.04761905	14.6545086	3.57517817	0.06611023	0.36409233	0.33830335 Xdh	1
GO:0050872	white fat cell differentiation	1/47	21/14464	0.04761905	14.6545086	3.57517817	0.06611023	0.36409233	0.33830335 Per2	1
GO:0060571	morphogenesis of an epithelial fold	1/47	21/14464	0.04761905	14.6545086	3.57517817	0.06611023	0.36409233	0.33830335 Rdh10	1
GO:0090201	negative regulation of release of cytochrome c from mitochondria	1/47	21/14464	0.04761905	14.6545086	3.57517817	0.06611023	0.36409233	0.33830335 Bcl2l1	1
GO:2000209	regulation of anoikis	1/47	21/14464	0.04761905	14.6545086	3.57517817	0.06611023	0.36409233	0.33830335 Bcl2l1	1
GO:2001169	regulation of ATP biosynthetic process	1/47	21/14464	0.04761905	14.6545086	3.57517817	0.06611023	0.36409233	0.33830335 Bcl2l1	1
GO:0001101	response to acid chemical	2/47	132/14464	0.01515152	4.66279819	2.4137219	0.06847207	0.36409233	0.33830335 Sesn1/Bcl2l1	2
GO:0007288	sperm axoneme assembly	1/47	22/14464	0.04545455	13.9883946	3.48091819	0.06914927	0.36409233	0.33830335 Cfap157	1
GO:0010623	programmed cell death involved in cell development	1/47	22/14464	0.04545455	13.9883946	3.48091819	0.06914927	0.36409233	0.33830335 Bcl2l1	1
GO:0032673	regulation of interleukin-4 production	1/47	22/14464	0.04545455	13.9883946	3.48091819	0.06914927	0.36409233	0.33830335 Icosl	1
GO:0032925	regulation of activin receptor signaling pathway	1/47	22/14464	0.04545455	13.9883946	3.48091819	0.06914927	0.36409233	0.33830335 Fstl3	1
GO:0043153	entrainment of circadian clock by photoperiod	1/47	22/14464	0.04545455	13.9883946	3.48091819	0.06914927	0.36409233	0.33830335 Per2	1
GO:0061318	renal filtration cell differentiation	1/47	22/14464	0.04545455	13.9883946	3.48091819	0.06914927	0.36409233	0.33830335 Klf15	1
GO:0072112	podocyte differentiation	1/47	22/14464	0.04545455	13.9883946	3.48091819	0.06914927	0.36409233	0.33830335 Klf15	1
GO:1902547	regulation of cellular response to vascular endothelial growth factor stimulus	1/47	22/14464	0.04545455	13.9883946	3.48091819	0.06914927	0.36409233	0.33830335 Xdh	1
GO:1905709	negative regulation of membrane permeability	1/47	22/14464	0.04545455	13.9883946	3.48091819	0.06914927	0.36409233	0.33830335 Bcl2l1	1
GO:0044282	small molecule catabolic process	3/47	296/14464	0.01013514	3.11903393	2.10314959	0.07120337	0.36409233	0.33830335 Xdh/Prodh/Acsf3	3
GO:0009264	deoxyribonucleotide catabolic process	1/47	23/14464	0.04347826	13.3802035	3.39260846	0.07217863	0.36409233	0.33830335 Xdh	1
GO:0009648	photoperiodism	1/47	23/14464	0.04347826	13.3802035	3.39260846	0.07217863	0.36409233	0.33830335 Per2	1
GO:0009649	entrainment of circadian clock	1/47	23/14464	0.04347826	13.3802035	3.39260846	0.07217863	0.36409233	0.33830335 Per2	1
GO:0015740	C4-dicarboxylate transport	1/47	23/14464	0.04347826	13.3802035	3.39260846	0.07217863	0.36409233	0.33830335 Slc1a5	1
GO:0046037	GMP metabolic process	1/47	23/14464	0.04347826	13.3802035	3.39260846	0.07217863	0.36409233	0.33830335 Xdh	1
GO:0046386	deoxyribose phosphate catabolic process	1/47	23/14464	0.04347826	13.3802035	3.39260846	0.07217863	0.36409233	0.33830335 Xdh	1
GO:0072311	glomerular epithelial cell differentiation	1/47	23/14464	0.04347826	13.3802035	3.39260846	0.07217863	0.36409233	0.33830335 Klf15	1
GO:0046488	phosphatidylinositol metabolic process	2/47	137/14464	0.01459854	4.49262308	2.34517386	0.073038	0.36409233	0.33830335 Ip6k2/Mtmr2	2
GO:0120161	regulation of cold-induced thermogenesis	2/47	139/14464	0.01438849	4.42798102	2.31867119	0.07489162	0.36409233	0.33830335 Per2/G0s2	2
GO:0001956	positive regulation of neurotransmitter secretion	1/47	24/14464	0.04166667	12.822695	3.30962802	0.07519834	0.36409233	0.33830335 Bcl2l1	1
GO:0008299	isoprenoid biosynthetic process	1/47	24/14464	0.04166667	12.822695	3.30962802	0.07519834	0.36409233	0.33830335 Rdh10	1
GO:0030808	regulation of nucleotide biosynthetic process	1/47	24/14464	0.04166667	12.822695	3.30962802	0.07519834	0.36409233	0.33830335 Bcl2l1	1
GO:0032958	inositol phosphate biosynthetic process	1/47	24/14464	0.04166667	12.822695	3.30962802	0.07519834	0.36409233	0.33830335 Ip6k2	1
GO:0070233	negative regulation of T cell apoptotic process	1/47	24/14464	0.04166667	12.822695	3.30962802	0.07519834	0.36409233	0.33830335 Tsc22d3	1
GO:0097062	dendritic spine maintenance	1/47	24/14464	0.04166667	12.822695	3.30962802	0.07519834	0.36409233	0.33830335 Mtmr2	1
GO:1900371	regulation of purine nucleotide biosynthetic process	1/47	24/14464	0.04166667	12.822695	3.30962802	0.07519834	0.36409233	0.33830335 Bcl2l1	1
GO:1901658	glycosyl compound catabolic process	1/47	24/14464	0.04166667	12.822695	3.30962802	0.07519834	0.36409233	0.33830335 Xdh	1
GO:1901983	regulation of protein acetylation	1/47	24/14464	0.04166667	12.822695	3.30962802	0.07519834	0.36409233	0.33830335 Klf15	1
GO:0001832	blastocyst growth	1/47	25/14464	0.04	12.3097872	3.2314434	0.07820843	0.36409233	0.33830335 Ints1	1
GO:0006353	DNA-templated transcription termination	1/47	25/14464	0.04	12.3097872	3.2314434	0.07820843	0.36409233	0.33830335 Per2	1
GO:0039694	viral RNA genome replication	1/47	25/14464	0.04	12.3097872	3.2314434	0.07820843	0.36409233	0.33830335 Pcbp2	1
GO:0046033	AMP metabolic process	1/47	25/14464	0.04	12.3097872	3.2314434	0.07820843	0.36409233	0.33830335 Xdh	1
GO:0046128	purine ribonucleoside metabolic process	1/47	25/14464	0.04	12.3097872	3.2314434	0.07820843	0.36409233	0.33830335 Xdh	1
GO:0097284	hepatocyte apoptotic process	1/47	25/14464	0.04	12.3097872	3.2314434	0.07820843	0.36409233	0.33830335 Bcl2l1	1
GO:0098810	neurotransmitter reuptake	1/47	25/14464	0.04	12.3097872	3.2314434	0.07820843	0.36409233	0.33830335 Per2	1
GO:1900117	regulation of execution phase of apoptosis	1/47	25/14464	0.04	12.3097872	3.2314434	0.07820843	0.36409233	0.33830335 Bcl2l1	1
GO:1902230	negative regulation of intrinsic apoptotic signaling pathway in response to DNA damage	1/47	25/14464	0.04	12.3097872	3.2314434	0.07820843	0.36409233	0.33830335 Bcl2l1	1
GO:0106106	cold-induced thermogenesis	2/47	143/14464	0.01398601	4.30412141	2.26713831	0.07864408	0.36409233	0.33830335 Per2/G0s2	2
GO:0030100	regulation of endocytosis	3/47	310/14464	0.00967742	2.97817433	2.01023341	0.07934097	0.36409233	0.33830335 Mertk/Bcl2l1/Mtmr2	3
GO:0043281	regulation of cysteine-type endopeptidase activity involved in apoptotic process	2/47	144/14464	0.01388889	4.27423168	2.25454971	0.07959143	0.36409233	0.33830335 Xdh/Ints1	2
GO:0003299	muscle hypertrophy in response to stress	1/47	26/14464	0.03846154	11.8363339	3.1575934	0.08120892	0.36409233	0.33830335 Klf15	1
GO:0014898	cardiac muscle hypertrophy in response to stress	1/47	26/14464	0.03846154	11.8363339	3.1575934	0.08120892	0.36409233	0.33830335 Klf15	1
GO:0035115	embryonic forelimb morphogenesis	1/47	26/14464	0.03846154	11.8363339	3.1575934	0.08120892	0.36409233	0.33830335 Rdh10	1
GO:0070536	protein K63-linked deubiquitination	1/47	26/14464	0.03846154	11.8363339	3.1575934	0.08120892	0.36409233	0.33830335 Otud7b	1
GO:0071108	protein K48-linked deubiquitination	1/47	26/14464	0.03846154	11.8363339	3.1575934	0.08120892	0.36409233	0.33830335 Otud7b	1
GO:0072010	glomerular epithelium development	1/47	26/14464	0.03846154	11.8363339	3.1575934	0.08120892	0.36409233	0.33830335 Klf15	1
GO:1900745	positive regulation of p38MAPK cascade	1/47	26/14464	0.03846154	11.8363339	3.1575934	0.08120892	0.36409233	0.33830335 Xdh	1
GO:1902042	negative regulation of extrinsic apoptotic signaling pathway via death domain receptors	1/47	26/14464	0.03846154	11.8363339	3.1575934	0.08120892	0.36409233	0.33830335 Bcl2l1	1
GO:2001236	regulation of extrinsic apoptotic signaling pathway	2/47	147/14464	0.01360544	4.18700246	2.21745965	0.08245511	0.36409233	0.33830335 G0s2/Bcl2l1	2
GO:0001779	natural killer cell differentiation	1/47	27/14464	0.03703704	11.3979511	3.0876769	0.08419986	0.36409233	0.33830335 Mertk	1
GO:0006536	glutamate metabolic process	1/47	27/14464	0.03703704	11.3979511	3.0876769	0.08419986	0.36409233	0.33830335 Prodh	1
GO:0006953	acute-phase response	1/47	27/14464	0.03703704	11.3979511	3.0876769	0.08419986	0.36409233	0.33830335 Cd163	1
GO:0014887	cardiac muscle adaptation	1/47	27/14464	0.03703704	11.3979511	3.0876769	0.08419986	0.36409233	0.33830335 Klf15	1
GO:0018394	peptidyl-lysine acetylation	1/47	27/14464	0.03703704	11.3979511	3.0876769	0.08419986	0.36409233	0.33830335 Klf15	1
GO:0030970	retrograde protein transport, ER to cytosol	1/47	27/14464	0.03703704	11.3979511	3.0876769	0.08419986	0.36409233	0.33830335 Herpud1	1
GO:0032288	myelin assembly	1/47	27/14464	0.03703704	11.3979511	3.0876769	0.08419986	0.36409233	0.33830335 Mtmr2	1
GO:0032633	interleukin-4 production	1/47	27/14464	0.03703704	11.3979511	3.0876769	0.08419986	0.36409233	0.33830335 Icosl	1
GO:0032801	receptor catabolic process	1/47	27/14464	0.03703704	11.3979511	3.0876769	0.08419986	0.36409233	0.33830335 Mtmr2	1
GO:0042572	retinol metabolic process	1/47	27/14464	0.03703704	11.3979511	3.0876769	0.08419986	0.36409233	0.33830335 Rdh10	1
GO:1903513	endoplasmic reticulum to cytosol transport	1/47	27/14464	0.03703704	11.3979511	3.0876769	0.08419986	0.36409233	0.33830335 Herpud1	1
GO:1904292	regulation of ERAD pathway	1/47	27/14464	0.03703704	11.3979511	3.0876769	0.08419986	0.36409233	0.33830335 Herpud1	1
GO:2000045	regulation of G1/S transition of mitotic cell cycle	2/47	151/14464	0.01324503	4.07608849	2.16951489	0.08632262	0.36409233	0.33830335 Fam107a/Crlf3	2
GO:0001780	neutrophil homeostasis	1/47	28/14464	0.03571429	10.9908815	3.02134318	0.08718127	0.36409233	0.33830335 Mertk	1
GO:0003401	axis elongation	1/47	28/14464	0.03571429	10.9908815	3.02134318	0.08718127	0.36409233	0.33830335 Rdh10	1
GO:0009162	deoxyribonucleoside monophosphate metabolic process	1/47	28/14464	0.03571429	10.9908815	3.02134318	0.08718127	0.36409233	0.33830335 Xdh	1
GO:0016226	iron-sulfur cluster assembly	1/47	28/14464	0.03571429	10.9908815	3.02134318	0.08718127	0.36409233	0.33830335 Xdh	1
GO:0031163	metallo-sulfur cluster assembly	1/47	28/14464	0.03571429	10.9908815	3.02134318	0.08718127	0.36409233	0.33830335 Xdh	1
GO:0032469	endoplasmic reticulum calcium ion homeostasis	1/47	28/14464	0.03571429	10.9908815	3.02134318	0.08718127	0.36409233	0.33830335 Herpud1	1
GO:0035303	regulation of dephosphorylation	1/47	28/14464	0.03571429	10.9908815	3.02134318	0.08718127	0.36409233	0.33830335 Mtmr2	1
GO:0046856	phosphatidylinositol dephosphorylation	1/47	28/14464	0.03571429	10.9908815	3.02134318	0.08718127	0.36409233	0.33830335 Mtmr2	1
GO:0048261	negative regulation of receptor-mediated endocytosis	1/47	28/14464	0.03571429	10.9908815	3.02134318	0.08718127	0.36409233	0.33830335 Mtmr2	1
GO:0097345	mitochondrial outer membrane permeabilization	1/47	28/14464	0.03571429	10.9908815	3.02134318	0.08718127	0.36409233	0.33830335 Bcl2l1	1
GO:0062013	positive regulation of small molecule metabolic process	2/47	154/14464	0.01298701	3.99668417	2.13462794	0.0892591	0.36409233	0.33830335 Bcl2l1/Rdh10	2
GO:1904375	regulation of protein localization to cell periphery	2/47	154/14464	0.01298701	3.99668417	2.13462794	0.0892591	0.36409233	0.33830335 Bcl2l1/Ppfia1	2
GO:0015813	L-glutamate transmembrane transport	1/47	29/14464	0.03448276	10.6118855	2.95828403	0.09015318	0.36409233	0.33830335 Per2	1
GO:0019369	arachidonate metabolic process	1/47	29/14464	0.03448276	10.6118855	2.95828403	0.09015318	0.36409233	0.33830335 Cyp2f2	1
GO:0043276	anoikis	1/47	29/14464	0.03448276	10.6118855	2.95828403	0.09015318	0.36409233	0.33830335 Bcl2l1	1
GO:0045671	negative regulation of osteoclast differentiation	1/47	29/14464	0.03448276	10.6118855	2.95828403	0.09015318	0.36409233	0.33830335 Fstl3	1
GO:0046006	regulation of activated T cell proliferation	1/47	29/14464	0.03448276	10.6118855	2.95828403	0.09015318	0.36409233	0.33830335 Icosl	1
GO:0060259	regulation of feeding behavior	1/47	29/14464	0.03448276	10.6118855	2.95828403	0.09015318	0.36409233	0.33830335 Mchr1	1
GO:0060441	epithelial tube branching involved in lung morphogenesis	1/47	29/14464	0.03448276	10.6118855	2.95828403	0.09015318	0.36409233	0.33830335 Rdh10	1
GO:0071480	cellular response to gamma radiation	1/47	29/14464	0.03448276	10.6118855	2.95828403	0.09015318	0.36409233	0.33830335 Bcl2l1	1
GO:1990845	adaptive thermogenesis	2/47	157/14464	0.01273885	3.92031441	2.1006119	0.09222539	0.36957269	0.34339554 Per2/G0s2	2
GO:0031589	cell-substrate adhesion	3/47	332/14464	0.00903614	2.78082543	1.87425746	0.09291418	0.36957269	0.34339554 Fam107a/Mertk/Tesk2	3
GO:0046427	positive regulation of receptor signaling pathway via JAK-STAT	1/47	30/14464	0.03333333	10.258156	2.89822732	0.09311562	0.36957269	0.34339554 Crlf3	1
GO:0051590	positive regulation of neurotransmitter transport	1/47	30/14464	0.03333333	10.258156	2.89822732	0.09311562	0.36957269	0.34339554 Bcl2l1	1
GO:0061050	regulation of cell growth involved in cardiac muscle cell development	1/47	30/14464	0.03333333	10.258156	2.89822732	0.09311562	0.36957269	0.34339554 Trip10	1
GO:0009119	ribonucleoside metabolic process	1/47	31/14464	0.03225806	9.92724777	2.84093176	0.09606862	0.37868144	0.35185911 Xdh	1
GO:0042278	purine nucleoside metabolic process	1/47	31/14464	0.03225806	9.92724777	2.84093176	0.09606862	0.37868144	0.35185911 Xdh	1
GO:0051017	actin filament bundle assembly	2/47	163/14464	0.01226994	3.77600835	2.03503574	0.09824405	0.37896252	0.35212028 Fam107a/Ppfia1	2
GO:1901991	negative regulation of mitotic cell cycle phase transition	2/47	163/14464	0.01226994	3.77600835	2.03503574	0.09824405	0.37896252	0.35212028 Fam107a/Crlf3	2
GO:2000116	regulation of cysteine-type endopeptidase activity	2/47	163/14464	0.01226994	3.77600835	2.03503574	0.09824405	0.37896252	0.35212028 Xdh/Ints1	2
GO:0009112	nucleobase metabolic process	1/47	32/14464	0.03125	9.61702128	2.78618255	0.0990122	0.37896252	0.35212028 Xdh	1
GO:0016180	snRNA processing	1/47	32/14464	0.03125	9.61702128	2.78618255	0.0990122	0.37896252	0.35212028 Ints1	1
GO:1900242	regulation of synaptic vesicle endocytosis	1/47	32/14464	0.03125	9.61702128	2.78618255	0.0990122	0.37896252	0.35212028 Bcl2l1	1
GO:1902110	positive regulation of mitochondrial membrane permeability involved in apoptotic process	1/47	32/14464	0.03125	9.61702128	2.78618255	0.0990122	0.37896252	0.35212028 Bcl2l1	1
GO:2000677	regulation of transcription regulatory region DNA binding	1/47	32/14464	0.03125	9.61702128	2.78618255	0.0990122	0.37896252	0.35212028 Per2	1
GO:0061572	actin filament bundle organization	2/47	166/14464	0.01204819	3.70776724	2.00340337	0.10129482	0.37896252	0.35212028 Fam107a/Ppfia1	2
GO:0051497	negative regulation of stress fiber assembly	1/47	33/14464	0.03030303	9.32559639	2.73378768	0.10194641	0.37896252	0.35212028 Ppfia1	1
GO:1903725	regulation of phospholipid metabolic process	1/47	33/14464	0.03030303	9.32559639	2.73378768	0.10194641	0.37896252	0.35212028 Mtmr2	1
GO:0045807	positive regulation of endocytosis	2/47	167/14464	0.01197605	3.68556504	1.99302215	0.10231768	0.37896252	0.35212028 Mertk/Bcl2l1	2
GO:0009410	response to xenobiotic stimulus	3/47	348/14464	0.00862069	2.65297139	1.78213174	0.10335638	0.37896252	0.35212028 Fmo2/Fmo1/Cyp2f2	3
GO:0019362	pyridine nucleotide metabolic process	2/47	169/14464	0.01183432	3.64194889	1.97249702	0.10437215	0.37896252	0.35212028 Fmo2/Fmo1	2
GO:0046496	nicotinamide nucleotide metabolic process	2/47	169/14464	0.01183432	3.64194889	1.97249702	0.10437215	0.37896252	0.35212028 Fmo2/Fmo1	2
GO:0001953	negative regulation of cell-matrix adhesion	1/47	34/14464	0.02941176	9.05131414	2.683575	0.10487126	0.37896252	0.35212028 Fam107a	1
GO:0010667	negative regulation of cardiac muscle cell apoptotic process	1/47	34/14464	0.02941176	9.05131414	2.683575	0.10487126	0.37896252	0.35212028 Trip10	1
GO:0035136	forelimb morphogenesis	1/47	34/14464	0.02941176	9.05131414	2.683575	0.10487126	0.37896252	0.35212028 Rdh10	1
GO:0048806	genitalia development	1/47	34/14464	0.02941176	9.05131414	2.683575	0.10487126	0.37896252	0.35212028 Mertk	1
GO:0071398	cellular response to fatty acid	1/47	34/14464	0.02941176	9.05131414	2.683575	0.10487126	0.37896252	0.35212028 Kcnk4	1
GO:1902175	regulation of oxidative stress-induced intrinsic apoptotic signaling pathway	1/47	34/14464	0.02941176	9.05131414	2.683575	0.10487126	0.37896252	0.35212028 Prodh	1
GO:1902686	mitochondrial outer membrane permeabilization involved in programmed cell death	1/47	34/14464	0.02941176	9.05131414	2.683575	0.10487126	0.37896252	0.35212028 Bcl2l1	1
GO:0007595	lactation	1/47	35/14464	0.02857143	8.79270517	2.63538957	0.10778679	0.37896252	0.35212028 Xdh	1
GO:0009268	response to pH	1/47	35/14464	0.02857143	8.79270517	2.63538957	0.10778679	0.37896252	0.35212028 Kcnk4	1
GO:0031076	embryonic camera-type eye development	1/47	35/14464	0.02857143	8.79270517	2.63538957	0.10778679	0.37896252	0.35212028 Rdh10	1
GO:0043392	negative regulation of DNA binding	1/47	35/14464	0.02857143	8.79270517	2.63538957	0.10778679	0.37896252	0.35212028 Per2	1
GO:0070229	negative regulation of lymphocyte apoptotic process	1/47	35/14464	0.02857143	8.79270517	2.63538957	0.10778679	0.37896252	0.35212028 Tsc22d3	1
GO:1905898	positive regulation of response to endoplasmic reticulum stress	1/47	35/14464	0.02857143	8.79270517	2.63538957	0.10778679	0.37896252	0.35212028 Herpud1	1
GO:0001659	temperature homeostasis	2/47	174/14464	0.01149425	3.53729518	1.92251695	0.10955798	0.37896252	0.35212028 Per2/G0s2	2
GO:0006839	mitochondrial transport	2/47	175/14464	0.01142857	3.51708207	1.91274094	0.11060343	0.37896252	0.35212028 Bcl2l1/Slc25a15	2
GO:1902806	regulation of cell cycle G1/S phase transition	2/47	175/14464	0.01142857	3.51708207	1.91274094	0.11060343	0.37896252	0.35212028 Fam107a/Crlf3	2
GO:0001523	retinoid metabolic process	1/47	36/14464	0.02777778	8.54846336	2.58909155	0.11069302	0.37896252	0.35212028 Rdh10	1
GO:0009167	purine ribonucleoside monophosphate metabolic process	1/47	36/14464	0.02777778	8.54846336	2.58909155	0.11069302	0.37896252	0.35212028 Xdh	1
GO:0010664	negative regulation of striated muscle cell apoptotic process	1/47	36/14464	0.02777778	8.54846336	2.58909155	0.11069302	0.37896252	0.35212028 Trip10	1
GO:0032350	regulation of hormone metabolic process	1/47	36/14464	0.02777778	8.54846336	2.58909155	0.11069302	0.37896252	0.35212028 Rdh10	1
GO:0043647	inositol phosphate metabolic process	1/47	36/14464	0.02777778	8.54846336	2.58909155	0.11069302	0.37896252	0.35212028 Ip6k2	1
GO:0050798	activated T cell proliferation	1/47	36/14464	0.02777778	8.54846336	2.58909155	0.11069302	0.37896252	0.35212028 Icosl	1
GO:0050951	sensory perception of temperature stimulus	1/47	36/14464	0.02777778	8.54846336	2.58909155	0.11069302	0.37896252	0.35212028 Kcnk4	1
GO:0060292	long-term synaptic depression	1/47	36/14464	0.02777778	8.54846336	2.58909155	0.11069302	0.37896252	0.35212028 Bcl2l1	1

GO:1901889 negative regulation of cell junction assembly	1/47	36/14464	0.02777778	8.54846336	2.58909155	0.11069302	0.37896252	0.35212028	Fam107a	1
GO:1902229 regulation of intrinsic apoptotic signaling pathway in response to DNA damage	1/47	36/14464	0.02777778	8.54846336	2.58909155	0.11069302	0.37896252	0.35212028	Bcl2l1	1
GO:0048872 homeostasis of number of cells	3/47	360/14464	0.00833333	2.56453901	1.7163552	0.11148549	0.37896252	0.35212028	Tsc22d3/Slc1a5/Mertk	3
GO:0072524 pyridine-containing compound metabolic process	2/47	176/14464	0.01136364	3.49709865	1.90303592	0.11165158	0.37896252	0.35212028	Fmo2/Fmo1	2
GO:0001504 neurotransmitter uptake	1/47	37/14464	0.02702703	8.31742381	2.54455433	0.11358998	0.37896252	0.35212028	Per2	1
GO:0010613 positive regulation of cardiac muscle hypertrophy	1/47	37/14464	0.02702703	8.31742381	2.54455433	0.11358998	0.37896252	0.35212028	Trip10	1
GO:0014742 positive regulation of muscle hypertrophy	1/47	37/14464	0.02702703	8.31742381	2.54455433	0.11358998	0.37896252	0.35212028	Trip10	1
GO:0035794 positive regulation of mitochondrial membrane permeability	1/47	37/14464	0.02702703	8.31742381	2.54455433	0.11358998	0.37896252	0.35212028	Bcl2l1	1
GO:0038084 vascular endothelial growth factor signaling pathway	1/47	37/14464	0.02702703	8.31742381	2.54455433	0.11358998	0.37896252	0.35212028	Xdh	1
GO:0043277 apoptotic cell clearance	1/47	37/14464	0.02702703	8.31742381	2.54455433	0.11358998	0.37896252	0.35212028	Mertk	1
GO:0045601 regulation of endothelial cell differentiation	1/47	37/14464	0.02702703	8.31742381	2.54455433	0.11358998	0.37896252	0.35212028	Xdh	1
GO:0046326 positive regulation of D-glucose import	1/47	37/14464	0.02702703	8.31742381	2.54455433	0.11358998	0.37896252	0.35212028	Klf15	1
GO:0055023 positive regulation of cardiac muscle tissue growth	1/47	37/14464	0.02702703	8.31742381	2.54455433	0.11358998	0.37896252	0.35212028	Trip10	1
GO:1904894 positive regulation of receptor signaling pathway via STAT	1/47	37/14464	0.02702703	8.31742381	2.54455433	0.11358998	0.37896252	0.35212028	Crlf3	1
GO:0032924 activin receptor signaling pathway	1/47	38/14464	0.02631579	8.09854423	2.50166297	0.11647771	0.38414283	0.35693366	Fstl3	1
GO:0051938 L-glutamate import	1/47	38/14464	0.02631579	8.09854423	2.50166297	0.11647771	0.38414283	0.35693366	Per2	1
GO:0071312 cellular response to alkaloid	1/47	38/14464	0.02631579	8.09854423	2.50166297	0.11647771	0.38414283	0.35693366	Bcl2l1	1
GO:0120316 sperm flagellum assembly	1/47	38/14464	0.02631579	8.09854423	2.50166297	0.11647771	0.38414283	0.35693366	Cfap157	1
GO:0009126 purine nucleoside monophosphate metabolic process	1/47	39/14464	0.02564103	7.89088925	2.46031285	0.11935623	0.38589614	0.35856278	Xdh	1
GO:0032232 negative regulation of actin filament bundle assembly	1/47	39/14464	0.02564103	7.89088925	2.46031285	0.11935623	0.38589614	0.35856278	Ppfia1	1
GO:0060421 positive regulation of heart growth	1/47	39/14464	0.02564103	7.89088925	2.46031285	0.11935623	0.38589614	0.35856278	Trip10	1
GO:1900744 regulation of p38MAPK cascade	1/47	39/14464	0.02564103	7.89088925	2.46031285	0.11935623	0.38589614	0.35856278	Xdh	1
GO:1902108 regulation of mitochondrial membrane permeability involved in apoptotic process	1/47	39/14464	0.02564103	7.89088925	2.46031285	0.11935623	0.38589614	0.35856278	Bcl2l1	1
GO:1902882 regulation of response to oxidative stress	1/47	39/14464	0.02564103	7.89088925	2.46031285	0.11935623	0.38589614	0.35856278	Sesn1	1
GO:1903580 positive regulation of ATP metabolic process	1/47	39/14464	0.02564103	7.89088925	2.46031285	0.11935623	0.38589614	0.35856278	Bcl2l1	1
GO:0016101 diterpenoid metabolic process	1/47	40/14464	0.025	7.69361702	2.42040851	0.12222558	0.39017268	0.36253641	Rdh10	1
GO:1900181 negative regulation of protein localization to nucleus	1/47	40/14464	0.025	7.69361702	2.42040851	0.12222558	0.39017268	0.36253641	Otud7b	1
GO:1901617 organic hydroxy compound biosynthetic process	2/47	187/14464	0.01069519	3.29138696	1.80070029	0.12335143	0.39017268	0.36253641	Per2/Ip6k2	2
GO:0031400 negative regulation of protein modification process	3/47	378/14464	0.00793651	2.4424181	1.62249697	0.12412974	0.39017268	0.36253641	Per2/Klf15/Xdh	3
GO:0003298 physiological muscle hypertrophy	1/47	41/14464	0.02439024	7.50596783	2.38186263	0.12508577	0.39017268	0.36253641	Trip10	1
GO:0003301 physiological cardiac muscle hypertrophy	1/47	41/14464	0.02439024	7.50596783	2.38186263	0.12508577	0.39017268	0.36253641	Trip10	1
GO:0009394 2'-deoxyribonucleotide metabolic process	1/47	41/14464	0.02439024	7.50596783	2.38186263	0.12508577	0.39017268	0.36253641	Xdh	1
GO:0032527 protein exit from endoplasmic reticulum	1/47	41/14464	0.02439024	7.50596783	2.38186263	0.12508577	0.39017268	0.36253641	Herpud1	1
GO:0045047 protein targeting to ER	1/47	41/14464	0.02439024	7.50596783	2.38186263	0.12508577	0.39017268	0.36253641	Herpud1	1
GO:0046839 phospholipid dephosphorylation	1/47	41/14464	0.02439024	7.50596783	2.38186263	0.12508577	0.39017268	0.36253641	Mtmr2	1
GO:0048701 embryonic cranial skeleton morphogenesis	1/47	41/14464	0.02439024	7.50596783	2.38186263	0.12508577	0.39017268	0.36253641	Rdh10	1
GO:0061049 cell growth involved in cardiac muscle cell development	1/47	41/14464	0.02439024	7.50596783	2.38186263	0.12508577	0.39017268	0.36253641	Trip10	1
GO:1903421 regulation of synaptic vesicle recycling	1/47	41/14464	0.02439024	7.50596783	2.38186263	0.12508577	0.39017268	0.36253641	Bcl2l1	1
GO:0050806 positive regulation of synaptic transmission	2/47	189/14464	0.01058201	3.25655747	1.78291203	0.12551059	0.39043969	0.36278451	Fam107a/Bcl2l1	2
GO:0001937 negative regulation of endothelial cell proliferation	1/47	42/14464	0.02380952	7.32725431	2.34459515	0.12793684	0.39199586	0.36423046	Xdh	1
GO:0019692 deoxyribose phosphate metabolic process	1/47	42/14464	0.02380952	7.32725431	2.34459515	0.12793684	0.39199586	0.36423046	Xdh	1
GO:0045981 positive regulation of nucleotide metabolic process	1/47	42/14464	0.02380952	7.32725431	2.34459515	0.12793684	0.39199586	0.36423046	Bcl2l1	1
GO:0050974 detection of mechanical stimulus involved in sensory perception	1/47	42/14464	0.02380952	7.32725431	2.34459515	0.12793684	0.39199586	0.36423046	Kcnk4	1
GO:1900544 positive regulation of purine nucleotide metabolic process	1/47	42/14464	0.02380952	7.32725431	2.34459515	0.12793684	0.39199586	0.36423046	Bcl2l1	1
GO:0005978 glycogen biosynthetic process	1/47	43/14464	0.02325581	7.15685304	2.30853254	0.13077881	0.39199586	0.36423046	Per2	1
GO:0009250 glucan biosynthetic process	1/47	43/14464	0.02325581	7.15685304	2.30853254	0.13077881	0.39199586	0.36423046	Per2	1
GO:0009262 deoxyribonucleotide metabolic process	1/47	43/14464	0.02325581	7.15685304	2.30853254	0.13077881	0.39199586	0.36423046	Xdh	1
GO:0035850 epithelial cell differentiation involved in kidney development	1/47	43/14464	0.02325581	7.15685304	2.30853254	0.13077881	0.39199586	0.36423046	Klf15	1
GO:0042771 intrinsic apoptotic signaling pathway in response to DNA damage by p53 class mediator	1/47	43/14464	0.02325581	7.15685304	2.30853254	0.13077881	0.39199586	0.36423046	Phlda3	1
GO:0045022 early endosome to late endosome transport	1/47	43/14464	0.02325581	7.15685304	2.30853254	0.13077881	0.39199586	0.36423046	Mtmr2	1
GO:0070232 regulation of T cell apoptotic process	1/47	43/14464	0.02325581	7.15685304	2.30853254	0.13077881	0.39199586	0.36423046	Tsc22d3	1
GO:1902041 regulation of extrinsic apoptotic signaling pathway via death domain receptors	1/47	43/14464	0.02325581	7.15685304	2.30853254	0.13077881	0.39199586	0.36423046	Bcl2l1	1
GO:1905710 positive regulation of membrane permeability	1/47	43/14464	0.02325581	7.15685304	2.30853254	0.13077881	0.39199586	0.36423046	Bcl2l1	1
GO:0032944 regulation of mononuclear cell proliferation	2/47	196/14464	0.01020408	3.14025185	1.72247147	0.13313948	0.39738266	0.36923571	Bcl2l1/Icosl	2
GO:0010828 positive regulation of D-glucose transmembrane transport	1/47	44/14464	0.02272727	6.99419729	2.27360709	0.13361172	0.39738266	0.36923571	Klf15	1
GO:0030857 negative regulation of epithelial cell differentiation	1/47	44/14464	0.02272727	6.99419729	2.27360709	0.13361172	0.39738266	0.36923571	Xdh	1
GO:0002204 somatic recombination of immunoglobulin genes involved in immune response	1/47	45/14464	0.02222222	6.83877069	2.23975636	0.13643559	0.39756296	0.36940323	Icosl	1
GO:0002208 somatic diversification of immunoglobulins involved in immune response	1/47	45/14464	0.02222222	6.83877069	2.23975636	0.13643559	0.39756296	0.36940323	Icosl	1
GO:0043029 T cell homeostasis	1/47	45/14464	0.02222222	6.83877069	2.23975636	0.13643559	0.39756296	0.36940323	Tsc22d3	1
GO:0045190 isotype switching	1/47	45/14464	0.02222222	6.83877069	2.23975636	0.13643559	0.39756296	0.36940323	Icosl	1
GO:0045332 phospholipid translocation	1/47	45/14464	0.02222222	6.83877069	2.23975636	0.13643559	0.39756296	0.36940323	Plscr4	1
GO:0046854 phosphatidylinositol phosphate biosynthetic process	1/47	45/14464	0.02222222	6.83877069	2.23975636	0.13643559	0.39756296	0.36940323	Ip6k2	1
GO:0072599 establishment of protein localization to endoplasmic reticulum	1/47	45/14464	0.02222222	6.83877069	2.23975636	0.13643559	0.39756296	0.36940323	Herpud1	1
GO:1904262 negative regulation of TORC1 signaling	1/47	45/14464	0.02222222	6.83877069	2.23975636	0.13643559	0.39756296	0.36940323	Sesn1	1
GO:0008406 gonad development	2/47	200/14464	0.01	3.07744681	1.68913717	0.13754664	0.39978833	0.37147098	Bcl2l1/Rdh10	2
GO:0009636 response to toxic substance	2/47	201/14464	0.00995025	3.06213613	1.68093373	0.13865361	0.40075208	0.37236647	Cyp2f2/Sesn1	2
GO:0009116 nucleoside metabolic process	1/47	46/14464	0.02173913	6.69010176	2.20692265	0.13925046	0.40075208	0.37236647	Xdh	1
GO:0051155 positive regulation of striated muscle cell differentiation	1/47	46/14464	0.02173913	6.69010176	2.20692265	0.13925046	0.40075208	0.37236647	Trip10	1
GO:0002262 myeloid cell homeostasis	2/47	202/14464	0.00990099	3.04697704	1.6727811	0.13976261	0.40075208	0.37236647	Slc1a5/Mertk	2
GO:0045137 development of primary sexual characteristics	2/47	203/14464	0.00985222	3.0319673	1.66467868	0.1408736	0.40075208	0.37236647	Bcl2l1/Rdh10	2
GO:0016054 organic acid catabolic process	2/47	204/14464	0.00980392	3.01710471	1.65662587	0.14198658	0.40075208	0.37236647	Prodh/Acsf3	2
GO:0046395 carboxylic acid catabolic process	2/47	204/14464	0.00980392	3.01710471	1.65662587	0.14198658	0.40075208	0.37236647	Prodh/Acsf3	2
GO:0007566 embryo implantation	1/47	47/14464	0.0212766	6.54775917	2.17505254	0.14205634	0.40075208	0.37236647	Ints1	1
GO:0030514 negative regulation of BMP signaling pathway	1/47	47/14464	0.0212766	6.54775917	2.17505254	0.14205634	0.40075208	0.37236647	Fstl3	1
GO:0051955 regulation of amino acid transport	1/47	47/14464	0.0212766	6.54775917	2.17505254	0.14205634	0.40075208	0.37236647	Per2	1
GO:0090199 regulation of release of cytochrome c from mitochondria	1/47	47/14464	0.0212766	6.54775917	2.17505254	0.14205634	0.40075208	0.37236647	Bcl2l1	1
GO:1902459 positive regulation of stem cell population maintenance	1/47	47/14464	0.0212766	6.54775917	2.17505254	0.14205634	0.40075208	0.37236647	Tead3	1
GO:0015804 neutral amino acid transport	1/47	48/14464	0.02083333	6.41134752	2.14409651	0.14485327	0.40460741	0.37594872	Slc1a5	1
GO:0000082 G1/S transition of mitotic cell cycle	2/47	207/14464	0.00966184	2.97337856	1.63275934	0.14533713	0.40460741	0.37594872	Fam107a/Crlf3	2
GO:0003018 vascular process in circulatory system	2/47	207/14464	0.00966184	2.97337856	1.63275934	0.14533713	0.40460741	0.37594872	Per2/Slc1a5	2
GO:0097191 extrinsic apoptotic signaling pathway	2/47	207/14464	0.00966184	2.97337856	1.63275934	0.14533713	0.40460741	0.37594872	G0s2/Bcl2l1	2
GO:0001825 blastocyst formation	1/47	49/14464	0.02040816	6.28050369	2.11400855	0.14764128	0.40460741	0.37594872	Mxi1	1
GO:0006721 terpenoid metabolic process	1/47	49/14464	0.02040816	6.28050369	2.11400855	0.14764128	0.40460741	0.37594872	Rdh10	1
GO:0014888 striated muscle adaptation	1/47	49/14464	0.02040816	6.28050369	2.11400855	0.14764128	0.40460741	0.37594872	Klf15	1
GO:0034204 lipid translocation	1/47	49/14464	0.02040816	6.28050369	2.11400855	0.14764128	0.40460741	0.37594872	Plscr4	1
GO:0043030 regulation of macrophage activation	1/47	49/14464	0.02040816	6.28050369	2.11400855	0.14764128	0.40460741	0.37594872	Slc7a2	1
GO:0046622 positive regulation of organ growth	1/47	49/14464	0.02040816	6.28050369	2.11400855	0.14764128	0.40460741	0.37594872	Trip10	1
GO:0098927 vesicle-mediated transport between endosomal compartments	1/47	49/14464	0.02040816	6.28050369	2.11400855	0.14764128	0.40460741	0.37594872	Mtmr2	1
GO:0170040 proteinogenic amino acid catabolic process	1/47	49/14464	0.02040816	6.28050369	2.11400855	0.14764128	0.40460741	0.37594872	Prodh	1
GO:0007160 cell-matrix adhesion	2/47	210/14464	0.00952381	2.93090172	1.6093189	0.14870462	0.40655349	0.37775696	Fam107a/Tesk2	2
GO:0002762 negative regulation of myeloid leukocyte differentiation	1/47	50/14464	0.02	6.15489362	2.08474588	0.15042039	0.4073738	0.37851917	Fstl3	1
GO:0016073 snRNA metabolic process	1/47	50/14464	0.02	6.15489362	2.08474588	0.15042039	0.4073738	0.37851917	Ints1	1
GO:0043154 negative regulation of cysteine-type endopeptidase activity involved in apoptotic process	1/47	50/14464	0.02	6.15489362	2.08474588	0.15042039	0.4073738	0.37851917	Ints1	1
GO:0061005 cell differentiation involved in kidney development	1/47	50/14464	0.02	6.15489362	2.08474588	0.15042039	0.4073738	0.37851917	Klf15	1
GO:1905475 regulation of protein localization to membrane	2/47	212/14464	0.00943396	2.90325171	1.59392148	0.15095872	0.40787204	0.37898211	Bcl2l1/Ppfia1	2
GO:0045930 negative regulation of mitotic cell cycle	2/47	213/14464	0.00938967	2.88962142	1.58629008	0.15208843	0.40805079	0.3791482	Fam107a/Crlf3	2
GO:0070663 regulation of leukocyte proliferation	2/47	213/14464	0.00938967	2.88962142	1.58629008	0.15208843	0.40805079	0.3791482	Bcl2l1/Icosl	2
GO:0072593 reactive oxygen species metabolic process	2/47	213/14464	0.00938967	2.88962142	1.58629008	0.15208843	0.40805079	0.3791482	Xdh/Sesn1	2
GO:0048645 animal organ formation	1/47	51/14464	0.01960784	6.03420943	2.05626861	0.15319063	0.40914186	0.38016199	Rdh10	1
GO:1901615 organic hydroxy compound metabolic process	3/47	417/14464	0.00719424	2.21399051	1.43625714	0.15320603	0.40914186	0.38016199	Per2/Ip6k2/Rdh10	3
GO:2000242 negative regulation of reproductive process	1/47	52/14464	0.01923077	5.91816694	2.02853957	0.15595203	0.41551107	0.38608007	Bcl2l1	1
GO:0052548 regulation of endopeptidase activity	2/47	218/14464	0.00917431	2.8233457	1.54878618	0.15776272	0.41567466	0.38623207	Xdh/Ints1	2
GO:0008631 intrinsic apoptotic signaling pathway in response to oxidative stress	1/47	53/14464	0.01886792	5.80650341	2.00152401	0.15870461	0.41567466	0.38623207	Prodh	1
GO:0009161 ribonucleoside monophosphate metabolic process	1/47	53/14464	0.01886792	5.80650341	2.00152401	0.15870461	0.41567466	0.38623207	Xdh	1
GO:0042149 cellular response to glucose starvation	1/47	53/14464	0.01886792	5.80650341	2.00152401	0.15870461	0.41567466	0.38623207	Sesn1	1
GO:0050982 detection of mechanical stimulus	1/47	53/14464	0.01886792	5.80650341	2.00152401	0.15870461	0.41567466	0.38623207	Kcnk4	1
GO:0170035 L-amino acid catabolic process	1/47	53/14464	0.01886792	5.80650341	2.00152401	0.15870461	0.41567466	0.38623207	Prodh	1
GO:0006836 neurotransmitter transport	2/47	219/14464	0.00913242	2.81045371	1.54141268	0.15890256	0.41567466	0.38623207	Per2/Bcl2l1	2
GO:0048762 mesenchymal cell differentiation	2/47	219/14464	0.00913242	2.81045371	1.54141268	0.15890256	0.41567466	0.38623207	Trip10/Rdh10	2
GO:0002381 immunoglobulin production involved in immunoglobulin-mediated immune response	1/47	54/14464	0.01851852	5.69897557	1.97518942	0.16144841	0.41665274	0.38714087	Icosl	1
GO:0016447 somatic recombination of immunoglobulin gene segments	1/47	54/14464	0.01851852	5.69897557	1.97518942	0.16144841	0.41665274	0.38714087	Icosl	1
GO:0031641 regulation of myelination	1/47	54/14464	0.01851852	5.69897557	1.97518942	0.16144841	0.41665274	0.38714087	Mtmr2	1
GO:0035329 hippo signaling	1/47	54/14464	0.01851852	5.69897557	1.97518942	0.16144841	0.41665274	0.38714087	Tead3	1
GO:0046173 polyol biosynthetic process	1/47	54/14464	0.01851852	5.69897557	1.97518942	0.16144841	0.41665274	0.38714087	Ip6k2	1
GO:0097035 regulation of membrane lipid distribution	1/47	54/14464	0.01851852	5.69897557	1.97518942	0.16144841	0.41665274	0.38714087	Plscr4	1
GO:0010332 response to gamma radiation	1/47	55/14464	0.01818182	5.59535783	1.94950535	0.16418345	0.41673131	0.38721387	Bcl2l1	1
GO:0010812 negative regulation of cell-substrate adhesion	1/47	55/14464	0.01818182	5.59535783	1.94950535	0.16418345	0.41673131	0.38721387	Fam107a	1
GO:0019228 neuronal action potential	1/47	55/14464	0.01818182	5.59535783	1.94950535	0.16418345	0.41673131	0.38721387	Kcnk4	1
GO:0060425 lung morphogenesis	1/47	55/14464	0.01818182	5.59535783	1.94950535	0.16418345	0.41673131	0.38721387	Rdh10	1
GO:0070542 response to fatty acid	1/47	55/14464	0.01818182	5.59535783	1.94950535	0.16418345	0.41673131	0.38721387	Kcnk4	1
GO:0150052 regulation of postsynapse assembly	1/47	55/14464	0.01818182	5.59535783	1.94950535	0.16418345	0.41673131	0.38721387	Fam107a	1
GO:2001234 negative regulation of apoptotic signaling pathway	2/47	225/14464	0.00888889	2.73550827	1.49802436	0.16577471	0.41673131	0.38721387	Bcl2l1/Herpud1	2
GO:0034198 cellular response to amino acid starvation	1/47	56/14464	0.01785714	5.49544073	1.92444327	0.16690976	0.41673131	0.38721387	Sesn1	1
GO:0035924 cellular response to vascular endothelial growth factor stimulus	1/47	56/14464	0.01785714	5.49544073	1.92444327	0.16690976	0.41673131	0.38721387	Xdh	1
GO:0038066 p38MAPK cascade	1/47	56/14464	0.01785714	5.49544073	1.92444327	0.16690976	0.41673131	0.38721387	Xdh	1
GO:0039532 negative regulation of cytoplasmic pattern recognition receptor signaling pathway	1/47	56/14464	0.01785714	5.49544073	1.92444327	0.16690976	0.41673131	0.38721387	Pcbp2	1
GO:0043124 negative regulation of canonical NF-kappaB signal transduction	1/47	56/14464	0.01785714	5.49544073	1.92444327	0.16690976	0.41673131	0.38721387	Otud7b	1
GO:0050688 regulation of defense response to virus	1/47	56/14464	0.01785714	5.49544073	1.92444327	0.16690976	0.41673131	0.38721387	Pcbp2	1
GO:1903307 positive regulation of regulated secretory pathway	1/47	56/14464	0.01785714	5.49544073	1.92444327	0.16690976	0.41673131	0.38721387	Bcl2l1	1
GO:2001238 positive regulation of extrinsic apoptotic signaling pathway	1/47	56/14464	0.01785714	5.49544073	1.92444327	0.16690976	0.41673131	0.38721387	G0s2	1
GO:0042391 regulation of membrane potential	3/47	437/14464	0.00686499	2.11266371	1.34853716	0.16890421	0.41718183	0.38763248	Kcnk4/Bcl2l1/Mtmr2	3
GO:0006473 protein acetylation	1/47	57/14464	0.01754386	5.39902949	1.89997635	0.16962736	0.41718183	0.38763248	Klf15	1
GO:0009064 glutamine family amino acid metabolic process	1/47	57/14464	0.01754386	5.39902949	1.89997635	0.16962736	0.41718183	0.38763248	Prodh	1
GO:0010665 regulation of cardiac muscle cell apoptotic process	1/47	57/14464	0.01754386	5.39902949	1.89997635	0.16962736	0.41718183	0.38763248	Trip10	1
GO:0046902 regulation of mitochondrial membrane permeability	1/47	57/14464	0.01754386	5.39902949	1.89997635	0.16962736	0.41718183	0.38763248	Bcl2l1	1
GO:0098815 modulation of excitatory postsynaptic potential	1/47	57/14464	0.01754386	5.39902949	1.89997635	0.16962736	0.41718183	0.38763248	Mtmr2	1
GO:1900271 regulation of long-term synaptic potentiation	1/47	57/14464	0.01754386	5.39902949	1.89997635	0.16962736	0.41718183	0.38763248	Fam107a	1
GO:0046324 regulation of D-glucose import	1/47	58/14464	0.01724138	5.30594277	1.87607941	0.17233629	0.42204058	0.39214708	Klf15	1
GO:2000117 negative regulation of cysteine-type endopeptidase activity	1/47	58/14464	0.01724138	5.30594277	1.87607941	0.17233629	0.42204058	0.39214708	Ints1	1
GO:0044843 cell cycle G1/S phase transition	2/47	233/14464	0.00858369	2.64158524	1.44232909	0.17501983	0.42503605	0.39493039	Fam107a/Crlf3	2
GO:0010662 regulation of striated muscle cell apoptotic process	1/47	59/14464	0.01694915	5.21601154	1.85272874	0.17503657	0.42503605	0.39493039	Trip10	1
GO:0044458 motile cilium assembly	1/47	59/14464	0.01694915	5.21601154	1.85272874	0.17503657	0.42503605	0.39493039	Cfap157	1
GO:0070231 T cell apoptotic process	1/47	59/14464	0.01694915	5.21601154	1.85272874	0.17503657	0.42503605	0.39493039	Tsc22d3	1
GO:0007218 neuropeptide signaling pathway	1/47	60/14464	0.01666667	5.12907801	1.82990199	0.17772822	0.42617747	0.39599095	Mchr1	1
GO:0016445 somatic diversification of immunoglobulins	1/47	60/14464	0.01666667	5.12907801	1.82990199	0.17772822	0.42617747	0.39599095	Icosl	1
GO:0030101 natural killer cell activation	1/47	60/14464	0.01666667	5.12907801	1.82990199	0.17772822	0.42617747	0.39599095	Mertk	1
GO:0070228 regulation of lymphocyte apoptotic process	1/47	60/14464	0.01666667	5.12907801	1.82990199	0.17772822	0.42617747	0.39599095	Tsc22d3	1
GO:0071385 cellular response to glucocorticoid stimulus	1/47	60/14464	0.01666667	5.12907801	1.82990199	0.17772822	0.42617747	0.39599095	Fam107a	1
GO:2000300 regulation of synaptic vesicle exocytosis	1/47	60/14464	0.01666667	5.12907801	1.82990199	0.17772822	0.42617747	0.39599095	Bcl2l1	1
GO:0032677 regulation of interleukin-8 production	1/47	61/14464	0.01639344	5.04499477	1.80757811	0.18041128	0.42760045	0.39731315	Otud7b	1
GO:0034308 primary alcohol metabolic process	1/47	61/14464	0.01639344	5.04499477	1.80757811	0.18041128	0.42760045	0.39731315	Rdh10	1
GO:1990928 response to amino acid starvation	1/47	61/14464	0.01639344	5.04499477	1.80757811	0.18041128	0.42760045	0.39731315	Sesn1	1
GO:0001818 negative regulation of cytokine production	2/47	239/14464	0.0083682	2.5752693	1.40206547	0.18200955	0.42760045	0.39731315	Mertk/Otud7b	2
GO:0048167 regulation of synaptic plasticity	2/47	239/14464	0.0083682	2.5752693	1.40206547	0.18200955	0.42760045	0.39731315	Fam107a/Bcl2l1	2
GO:0000271 polysaccharide biosynthetic process	1/47	62/14464	0.01612903	4.96362388	1.78573722	0.18308577	0.42760045	0.39731315	Per2	1
GO:0002562 somatic diversification of immune receptors via germline recombination within a single locus	1/47	62/14464	0.01612903	4.96362388	1.78573722	0.18308577	0.42760045	0.39731315	Icosl	1
GO:0016444 somatic cell DNA recombination	1/47	62/14464	0.01612903	4.96362388	1.78573722	0.18308577	0.42760045	0.39731315	Icosl	1
GO:0032637 interleukin-8 production	1/47	62/14464	0.01612903	4.96362388	1.78573722	0.18308577	0.42760045	0.39731315	Otud7b	1
GO:0050805 negative regulation of synaptic transmission	1/47	62/14464	0.01612903	4.96362388	1.78573722	0.18308577	0.42760045	0.39731315	Bcl2l1	1
GO:0051893 regulation of focal adhesion assembly	1/47	62/14464	0.01612903	4.96362388	1.78573722	0.18308577	0.42760045	0.39731315	Fam107a	1
GO:0090109 regulation of cell-substrate junction assembly	1/47	62/14464	0.01612903	4.96362388	1.78573722	0.18308577	0.42760045	0.39731315	Fam107a	1
GO:1901988 negative regulation of cell cycle phase transition	2/47	240/14464	0.00833333	2.56453901	1.39547422	0.18317881	0.42760045	0.39731315	Fam107a/Crlf3	2
GO:0031669 cellular response to nutrient levels	2/47	241/14464	0.00829876	2.55389777	1.38891616	0.18434924	0.42760045	0.39731315	Fam107a/Sesn1	2
GO:0001836 release of cytochrome c from mitochondria	1/47	63/14464	0.01587302	4.8848362	1.76436053	0.18575172	0.42760045	0.39731315	Bcl2l1	1
GO:0006081 cellular aldehyde metabolic process	1/47	63/14464	0.01587302	4.8848362	1.76436053	0.18575172	0.42760045	0.39731315	Rdh10	1
GO:0006919 activation of cysteine-type endopeptidase activity involved in apoptotic process	1/47	63/14464	0.01587302	4.8848362	1.76436053	0.18575172	0.42760045	0.39731315	Xdh	1

GO:0010656	negative regulation of muscle cell apoptotic process	1/47	63/14464	0.01587302	4.8848362	1.76436053	0.18575172	0.42760045	0.39731315	Trip10	1
GO:0097192	extrinsic apoptotic signaling pathway in absence of ligand	1/47	63/14464	0.01587302	4.8848362	1.76436053	0.18575172	0.42760045	0.39731315	Bcl2l1	1
GO:1901657	glycosyl compound metabolic process	1/47	63/14464	0.01587302	4.8848362	1.76436053	0.18575172	0.42760045	0.39731315	Xdh	1
GO:0001541	ovarian follicle development	1/47	64/14464	0.015625	4.80851064	1.74343029	0.18840915	0.43280204	0.4021463	Bcl2l1	1
GO:0010659	cardiac muscle cell apoptotic process	1/47	65/14464	0.01538462	4.73453355	1.72292967	0.19105809	0.43280204	0.4021463	Trip10	1
GO:0032007	negative regulation of TOR signaling	1/47	65/14464	0.01538462	4.73453355	1.72292967	0.19105809	0.43280204	0.4021463	Sesn1	1
GO:0032922	circadian regulation of gene expression	1/47	65/14464	0.01538462	4.73453355	1.72292967	0.19105809	0.43280204	0.4021463	Per2	1
GO:0038034	signal transduction in absence of ligand	1/47	65/14464	0.01538462	4.73453355	1.72292967	0.19105809	0.43280204	0.4021463	Bcl2l1	1
GO:0046425	regulation of receptor signaling pathway via JAK-STAT	1/47	65/14464	0.01538462	4.73453355	1.72292967	0.19105809	0.43280204	0.4021463	Crlf3	1
GO:0050766	positive regulation of phagocytosis	1/47	65/14464	0.01538462	4.73453355	1.72292967	0.19105809	0.43280204	0.4021463	Mertk	1
GO:1904888	cranial skeletal system development	1/47	65/14464	0.01538462	4.73453355	1.72292967	0.19105809	0.43280204	0.4021463	Rdh10	1
GO:0006650	glycerophospholipid metabolic process	2/47	247/14464	0.00809717	2.49185976	1.35024684	0.19139552	0.43280204	0.4021463	Ip6k2/Mtmr2	2
GO:0051607	defense response to virus	2/47	248/14464	0.00806452	2.48181194	1.3439122	0.19257366	0.43459465	0.40381194	Bcl2l1/Pcbp2	2
GO:0002694	regulation of leukocyte activation	3/47	467/14464	0.00642398	1.97694656	1.22532823	0.19329829	0.43459465	0.40381194	Mertk/Slc7a2/Icosl	3
GO:0071384	cellular response to corticosteroid stimulus	1/47	66/14464	0.01515152	4.66279819	1.70284276	0.19369857	0.43459465	0.40381194	Fam107a	1
GO:0098869	cellular oxidant detoxification	1/47	66/14464	0.01515152	4.66279819	1.70284276	0.19369857	0.43459465	0.40381194	Sesn1	1
GO:0006766	vitamin metabolic process	1/47	67/14464	0.01492537	4.59320419	1.68315445	0.19633061	0.43624814	0.40534831	Rdh10	1
GO:0010658	striated muscle cell apoptotic process	1/47	67/14464	0.01492537	4.59320419	1.68315445	0.19633061	0.43624814	0.40534831	Trip10	1
GO:0097479	synaptic vesicle localization	1/47	67/14464	0.01492537	4.59320419	1.68315445	0.19633061	0.43624814	0.40534831	Bcl2l1	1
GO:0150116	regulation of cell-substrate junction organization	1/47	67/14464	0.01492537	4.59320419	1.68315445	0.19633061	0.43624814	0.40534831	Fam107a	1
GO:2000036	regulation of stem cell population maintenance	1/47	67/14464	0.01492537	4.59320419	1.68315445	0.19633061	0.43624814	0.40534831	Tead3	1
GO:0022409	positive regulation of cell-cell adhesion	2/47	252/14464	0.00793651	2.4424181	1.31887765	0.1972963	0.43701592	0.4060617	Fstl3/Icosl	2
GO:0006720	isoprenoid metabolic process	1/47	68/14464	0.01470588	4.52565707	1.66385041	0.19895425	0.43701592	0.4060617	Rdh10	1
GO:0033138	positive regulation of peptidyl-serine phosphorylation	1/47	68/14464	0.01470588	4.52565707	1.66385041	0.19895425	0.43701592	0.4060617	Ip6k2	1
GO:0045806	negative regulation of endocytosis	1/47	68/14464	0.01470588	4.52565707	1.66385041	0.19895425	0.43701592	0.4060617	Mtmr2	1
GO:0055021	regulation of cardiac muscle tissue growth	1/47	68/14464	0.01470588	4.52565707	1.66385041	0.19895425	0.43701592	0.4060617	Trip10	1
GO:1901606	alpha-amino acid catabolic process	1/47	68/14464	0.01470588	4.52565707	1.66385041	0.19895425	0.43701592	0.4060617	Prodh	1
GO:0002200	somatic diversification of immune receptors	1/47	69/14464	0.01449275	4.46006784	1.64491703	0.2015695	0.44041918	0.40922391	Icosl	1
GO:0071260	cellular response to mechanical stimulus	1/47	69/14464	0.01449275	4.46006784	1.64491703	0.2015695	0.44041918	0.40922391	Kcnk4	1
GO:0034976	response to endoplasmic reticulum stress	2/47	256/14464	0.0078125	2.40425532	1.29431582	0.20203417	0.44041918	0.40922391	Bcl2l1/Herpud1	2
GO:0050878	regulation of body fluid levels	2/47	256/14464	0.0078125	2.40425532	1.29431582	0.20203417	0.44041918	0.40922391	Xdh/Mertk	2
GO:0001676	long-chain fatty acid metabolic process	1/47	70/14464	0.01428571	4.39635258	1.62634138	0.20417639	0.44257444	0.41122651	Cyp2f2	1
GO:0032835	glomerulus development	1/47	70/14464	0.01428571	4.39635258	1.62634138	0.20417639	0.44257444	0.41122651	Klf15	1
GO:0045670	regulation of osteoclast differentiation	1/47	70/14464	0.01428571	4.39635258	1.62634138	0.20417639	0.44257444	0.41122651	Fstl3	1
GO:0032943	mononuclear cell proliferation	2/47	259/14464	0.00772201	2.3764068	1.27619414	0.20559687	0.44461137	0.41311916	Bcl2l1/Icosl	2
GO:0009123	nucleoside monophosphate metabolic process	1/47	71/14464	0.01408451	4.33443212	1.60811116	0.20677496	0.44461137	0.41311916	Xdh	1
GO:0071496	cellular response to external stimulus	1/47	71/14464	0.01408451	4.33443212	1.60811116	0.20677496	0.44461137	0.41311916	Kcnk4	1
GO:2000379	positive regulation of reactive oxygen species metabolic process	1/47	71/14464	0.01408451	4.33443212	1.60811116	0.20677496	0.44461137	0.41311916	Xdh	1
GO:0052547	regulation of peptidase activity	2/47	262/14464	0.00763359	2.34919604	1.25832151	0.20916702	0.44461137	0.41311916	Xdh/Ints1	2
GO:0014032	neural crest cell development	1/47	72/14464	0.01388889	4.27423168	1.59021466	0.20936521	0.44461137	0.41311916	Rdh10	1
GO:0019080	viral gene expression	1/47	72/14464	0.01388889	4.27423168	1.59021466	0.20936521	0.44461137	0.41311916	Pcbp2	1
GO:0030968	endoplasmic reticulum unfolded protein response	1/47	72/14464	0.01388889	4.27423168	1.59021466	0.20936521	0.44461137	0.41311916	Herpud1	1
GO:0051149	positive regulation of muscle cell differentiation	1/47	72/14464	0.01388889	4.27423168	1.59021466	0.20936521	0.44461137	0.41311916	Trip10	1
GO:0090559	regulation of membrane permeability	1/47	72/14464	0.01388889	4.27423168	1.59021466	0.20936521	0.44461137	0.41311916	Bcl2l1	1
GO:0097194	execution phase of apoptosis	1/47	72/14464	0.01388889	4.27423168	1.59021466	0.20936521	0.44461137	0.41311916	Bcl2l1	1
GO:0010611	regulation of cardiac muscle hypertrophy	1/47	73/14464	0.01369863	4.21568056	1.57264072	0.21194719	0.44619011	0.41458609	Trip10	1
GO:0032092	positive regulation of protein binding	1/47	73/14464	0.01369863	4.21568056	1.57264072	0.21194719	0.44619011	0.41458609	Ip6k2	1
GO:0060420	regulation of heart growth	1/47	73/14464	0.01369863	4.21568056	1.57264072	0.21194719	0.44619011	0.41458609	Trip10	1
GO:1904892	regulation of receptor signaling pathway via STAT	1/47	73/14464	0.01369863	4.21568056	1.57264072	0.21194719	0.44619011	0.41458609	Crlf3	1
GO:0002312	B cell activation involved in immune response	1/47	74/14464	0.01351351	4.1587119	1.55537872	0.21452092	0.44619011	0.41458609	Icosl	1
GO:0006690	icosanoid metabolic process	1/47	74/14464	0.01351351	4.1587119	1.55537872	0.21452092	0.44619011	0.41458609	Cyp2f2	1
GO:0019229	regulation of vasoconstriction	1/47	74/14464	0.01351351	4.1587119	1.55537872	0.21452092	0.44619011	0.41458609	Per2	1
GO:0042102	positive regulation of T cell proliferation	1/47	74/14464	0.01351351	4.1587119	1.55537872	0.21452092	0.44619011	0.41458609	Icosl	1
GO:0071479	cellular response to ionizing radiation	1/47	74/14464	0.01351351	4.1587119	1.55537872	0.21452092	0.44619011	0.41458609	Bcl2l1	1
GO:0060485	mesenchyme development	2/47	267/14464	0.00749064	2.3052036	1.22906903	0.21513253	0.44619011	0.41458609	Trip10/Rdh10	2
GO:0001656	metanephros development	1/47	75/14464	0.01333333	4.10326241	1.53841852	0.21708642	0.44619011	0.41458609	Rdh10	1
GO:0002526	acute inflammatory response	1/47	75/14464	0.01333333	4.10326241	1.53841852	0.21708642	0.44619011	0.41458609	Cd163	1
GO:0008625	extrinsic apoptotic signaling pathway via death domain receptors	1/47	75/14464	0.01333333	4.10326241	1.53841852	0.21708642	0.44619011	0.41458609	Bcl2l1	1
GO:0010827	regulation of D-glucose transmembrane transport	1/47	75/14464	0.01333333	4.10326241	1.53841852	0.21708642	0.44619011	0.41458609	Klf15	1
GO:0014743	regulation of muscle hypertrophy	1/47	75/14464	0.01333333	4.10326241	1.53841852	0.21708642	0.44619011	0.41458609	Trip10	1
GO:0032465	regulation of cytokinesis	1/47	75/14464	0.01333333	4.10326241	1.53841852	0.21708642	0.44619011	0.41458609	Bcl2l1	1
GO:0035082	axoneme assembly	1/47	75/14464	0.01333333	4.10326241	1.53841852	0.21708642	0.44619011	0.41458609	Cfap157	1
GO:0046323	D-glucose import	1/47	75/14464	0.01333333	4.10326241	1.53841852	0.21708642	0.44619011	0.41458609	Klf15	1
GO:0006809	nitric oxide biosynthetic process	1/47	76/14464	0.01315789	4.04927212	1.52175044	0.21964371	0.45064155	0.41872223	Slc7a2	1
GO:0071396	cellular response to lipid	3/47	499/14464	0.00601202	1.85016842	1.1035079	0.2202543	0.45104753	0.41909945	Fam107a/Kcnk4/Plscr4	3
GO:0007589	body fluid secretion	1/47	77/14464	0.01298701	3.99668417	1.50536525	0.22219283	0.45104753	0.41909945	Xdh	1
GO:0032890	regulation of organic acid transport	1/47	77/14464	0.01298701	3.99668417	1.50536525	0.22219283	0.45104753	0.41909945	Per2	1
GO:0033559	unsaturated fatty acid metabolic process	1/47	77/14464	0.01298701	3.99668417	1.50536525	0.22219283	0.45104753	0.41909945	Cyp2f2	1
GO:0043112	receptor metabolic process	1/47	77/14464	0.01298701	3.99668417	1.50536525	0.22219283	0.45104753	0.41909945	Mtmr2	1
GO:0070972	protein localization to endoplasmic reticulum	1/47	77/14464	0.01298701	3.99668417	1.50536525	0.22219283	0.45104753	0.41909945	Herpud1	1
GO:0006066	alcohol metabolic process	2/47	275/14464	0.00727273	2.23814313	1.18359189	0.2247124	0.45178568	0.41978532	Ip6k2/Rdh10	2
GO:0048864	stem cell development	1/47	78/14464	0.01282051	3.94544463	1.48925411	0.2247338	0.45178568	0.41978532	Rdh10	1
GO:0050795	regulation of behavior	1/47	78/14464	0.01282051	3.94544463	1.48925411	0.2247338	0.45178568	0.41978532	Mchr1	1
GO:0050906	detection of stimulus involved in sensory perception	1/47	78/14464	0.01282051	3.94544463	1.48925411	0.2247338	0.45178568	0.41978532	Kcnk4	1
GO:0007281	germ cell development	2/47	276/14464	0.00724638	2.23003392	1.17801742	0.22591257	0.45178568	0.41978532	Cfap157/Bcl2l1	2
GO:0016197	endosomal transport	2/47	276/14464	0.00724638	2.23003392	1.17801742	0.22591257	0.45178568	0.41978532	Bcl2l1/Mtmr2	2
GO:0010948	negative regulation of cell cycle process	2/47	277/14464	0.00722022	2.22198326	1.17246673	0.22711329	0.45178568	0.41978532	Fam107a/Crlf3	2
GO:0007405	neuroblast proliferation	1/47	79/14464	0.01265823	3.89550229	1.47340859	0.22726665	0.45178568	0.41978532	Tead3	1
GO:0009063	amino acid catabolic process	1/47	79/14464	0.01265823	3.89550229	1.47340859	0.22726665	0.45178568	0.41978532	Prodh	1
GO:0015914	phospholipid transport	1/47	79/14464	0.01265823	3.89550229	1.47340859	0.22726665	0.45178568	0.41978532	Plscr4	1
GO:0031397	negative regulation of protein ubiquitination	1/47	79/14464	0.01265823	3.89550229	1.47340859	0.22726665	0.45178568	0.41978532	Per2	1
GO:1903578	regulation of ATP metabolic process	1/47	79/14464	0.01265823	3.89550229	1.47340859	0.22726665	0.45178568	0.41978532	Bcl2l1	1
GO:0090287	regulation of cellular response to growth factor stimulus	2/47	278/14464	0.00719424	2.21399051	1.16693962	0.22831453	0.45308625	0.42099376	Xdh/Fstl3	2
GO:0002260	lymphocyte homeostasis	1/47	80/14464	0.0125	3.84680851	1.45782061	0.22979139	0.45367049	0.42153662	Tsc22d3	1
GO:0002532	production of molecular mediator involved in inflammatory response	1/47	80/14464	0.0125	3.84680851	1.45782061	0.22979139	0.45367049	0.42153662	Slc7a2	1
GO:0070227	lymphocyte apoptotic process	1/47	80/14464	0.0125	3.84680851	1.45782061	0.22979139	0.45367049	0.42153662	Tsc22d3	1
GO:0007601	visual perception	1/47	81/14464	0.01234568	3.79931705	1.44248243	0.23230806	0.4562911	0.42397161	Rdh10	1
GO:0045638	negative regulation of myeloid cell differentiation	1/47	81/14464	0.01234568	3.79931705	1.44248243	0.23230806	0.4562911	0.42397161	Fstl3	1
GO:0046209	nitric oxide metabolic process	1/47	81/14464	0.01234568	3.79931705	1.44248243	0.23230806	0.4562911	0.42397161	Slc7a2	1
GO:0032496	response to lipopolysaccharide	2/47	283/14464	0.00706714	2.17487407	1.13965075	0.23432812	0.45869331	0.42620367	Fmo1/Plscr4	2
GO:0043523	regulation of neuron apoptotic process	2/47	283/14464	0.00706714	2.17487407	1.13965075	0.23432812	0.45869331	0.42620367	Prodh/Bcl2l1	2
GO:0014033	neural crest cell differentiation	1/47	83/14464	0.01204819	3.70776724	1.41252618	0.23731729	0.46140574	0.42872397	Rdh10	1
GO:0030510	regulation of BMP signaling pathway	1/47	83/14464	0.01204819	3.70776724	1.41252618	0.23731729	0.46140574	0.42872397	Fstl3	1
GO:0050953	sensory perception of light stimulus	1/47	83/14464	0.01204819	3.70776724	1.41252618	0.23731729	0.46140574	0.42872397	Rdh10	1
GO:2001057	reactive nitrogen species metabolic process	1/47	83/14464	0.01204819	3.70776724	1.41252618	0.23731729	0.46140574	0.42872397	Slc7a2	1
GO:0070661	leukocyte proliferation	2/47	287/14464	0.00696864	2.14456224	1.11822414	0.23914692	0.46157388	0.42888021	Bcl2l1/Icosl	2
GO:0005977	glycogen metabolic process	1/47	84/14464	0.01190476	3.66362715	1.39789421	0.23980989	0.46157388	0.42888021	Per2	1
GO:0006094	gluconeogenesis	1/47	84/14464	0.01190476	3.66362715	1.39789421	0.23980989	0.46157388	0.42888021	Per2	1
GO:0016579	protein deubiquitination	1/47	84/14464	0.01190476	3.66362715	1.39789421	0.23980989	0.46157388	0.42888021	Otud7b	1
GO:0034620	cellular response to unfolded protein	1/47	84/14464	0.01190476	3.66362715	1.39789421	0.23980989	0.46157388	0.42888021	Herpud1	1
GO:0048488	synaptic vesicle endocytosis	1/47	84/14464	0.01190476	3.66362715	1.39789421	0.23980989	0.46157388	0.42888021	Bcl2l1	1
GO:0044042	glucan metabolic process	1/47	85/14464	0.01176471	3.62052566	1.38348422	0.24229451	0.46557761	0.43260034	Per2	1
GO:0001570	vasculogenesis	1/47	86/14464	0.01162791	3.57842652	1.36928994	0.24477119	0.46563304	0.43265185	Xdh	1
GO:0002090	regulation of receptor internalization	1/47	86/14464	0.01162791	3.57842652	1.36928994	0.24477119	0.46563304	0.43265185	Mtmr2	1
GO:0030168	platelet activation	1/47	86/14464	0.01162791	3.57842652	1.36928994	0.24477119	0.46563304	0.43265185	Mertk	1
GO:0043242	negative regulation of protein-containing complex disassembly	1/47	86/14464	0.01162791	3.57842652	1.36928994	0.24477119	0.46563304	0.43265185	Per2	1
GO:0072332	intrinsic apoptotic signaling pathway by p53 class mediator	1/47	86/14464	0.01162791	3.57842652	1.36928994	0.24477119	0.46563304	0.43265185	Phlda3	1
GO:2001237	negative regulation of extrinsic apoptotic signaling pathway	1/47	86/14464	0.01162791	3.57842652	1.36928994	0.24477119	0.46563304	0.43265185	Bcl2l1	1
GO:0072659	protein localization to plasma membrane	2/47	292/14464	0.00684932	2.10784028	1.09192718	0.24517875	0.46563304	0.43265185	Bcl2l1/Ppfia1	2
GO:0006661	phosphatidylinositol biosynthetic process	1/47	87/14464	0.01149425	3.53729518	1.35530537	0.24723995	0.46563304	0.43265185	Ip6k2	1
GO:0051881	regulation of mitochondrial membrane potential	1/47	87/14464	0.01149425	3.53729518	1.35530537	0.24723995	0.46563304	0.43265185	Bcl2l1	1
GO:0022412	cellular process involved in reproduction in multicellular organism	2/47	295/14464	0.00677966	2.08640462	1.07639998	0.24880159	0.46563304	0.43265185	Cfap157/Bcl2l1	2
GO:0019319	hexose biosynthetic process	1/47	88/14464	0.01136364	3.49709865	1.34152471	0.2497008	0.46563304	0.43265185	Per2	1
GO:0031123	RNA 3'-end processing	1/47	88/14464	0.01136364	3.49709865	1.34152471	0.2497008	0.46563304	0.43265185	Ints1	1
GO:0043543	protein acylation	1/47	88/14464	0.01136364	3.49709865	1.34152471	0.2497008	0.46563304	0.43265185	Klf15	1
GO:0043954	cellular component maintenance	1/47	88/14464	0.01136364	3.49709865	1.34152471	0.2497008	0.46563304	0.43265185	Mtmr2	1
GO:0048704	embryonic skeletal system morphogenesis	1/47	88/14464	0.01136364	3.49709865	1.34152471	0.2497008	0.46563304	0.43265185	Rdh10	1
GO:0051153	regulation of striated muscle cell differentiation	1/47	88/14464	0.01136364	3.49709865	1.34152471	0.2497008	0.46563304	0.43265185	Trip10	1
GO:1900542	regulation of purine nucleotide metabolic process	1/47	88/14464	0.01136364	3.49709865	1.34152471	0.2497008	0.46563304	0.43265185	Bcl2l1	1
GO:1990748	cellular detoxification	1/47	88/14464	0.01136364	3.49709865	1.34152471	0.2497008	0.46563304	0.43265185	Sesn1	1
GO:0045862	positive regulation of proteolysis	2/47	296/14464	0.00675676	2.07935595	1.07126507	0.25000975	0.46563304	0.43265185	Xdh/Herpud1	2
GO:0002237	response to molecule of bacterial origin	2/47	297/14464	0.00673401	2.07235475	1.0661503	0.25121814	0.46702295	0.43394331	Fmo1/Plscr4	2
GO:0006140	regulation of nucleotide metabolic process	1/47	89/14464	0.01123596	3.4578054	1.32794242	0.25215378	0.46702295	0.43394331	Bcl2l1	1
GO:1903321	negative regulation of protein modification by small protein conjugation or removal	1/47	89/14464	0.01123596	3.4578054	1.32794242	0.25215378	0.46702295	0.43394331	Per2	1
GO:0010951	negative regulation of endopeptidase activity	1/47	90/14464	0.01111111	3.41938534	1.31455316	0.25459892	0.46702295	0.43394331	Ints1	1
GO:0019233	sensory perception of pain	1/47	90/14464	0.01111111	3.41938534	1.31455316	0.25459892	0.46702295	0.43394331	Kcnk4	1
GO:0051101	regulation of DNA binding	1/47	90/14464	0.01111111	3.41938534	1.31455316	0.25459892	0.46702295	0.43394331	Per2	1
GO:0140238	presynaptic endocytosis	1/47	90/14464	0.01111111	3.41938534	1.31455316	0.25459892	0.46702295	0.43394331	Bcl2l1	1
GO:0071214	cellular response to abiotic stimulus	2/47	301/14464	0.00664452	2.04481516	1.04588939	0.2560539	0.46702295	0.43394331	Kcnk4/Bcl2l1	2
GO:0104004	cellular response to environmental stimulus	2/47	301/14464	0.00664452	2.04481516	1.04588939	0.2560539	0.46702295	0.43394331	Kcnk4/Bcl2l1	2
GO:0006970	response to osmotic stress	1/47	91/14464	0.01098901	3.38180968	1.30135179	0.25703622	0.46702295	0.43394331	Tsc22d3	1
GO:0019751	polyol metabolic process	1/47	91/14464	0.01098901	3.38180968	1.30135179	0.25703622	0.46702295	0.43394331	Ip6k2	1
GO:0042310	vasoconstriction	1/47	91/14464	0.01098901	3.38180968	1.30135179	0.25703622	0.46702295	0.43394331	Per2	1
GO:0045921	positive regulation of exocytosis	1/47	91/14464	0.01098901	3.38180968	1.30135179	0.25703622	0.46702295	0.43394331	Bcl2l1	1
GO:0051952	regulation of amine transport	1/47	91/14464	0.01098901	3.38180968	1.30135179	0.25703622	0.46702295	0.43394331	Per2	1
GO:1904019	epithelial cell apoptotic process	1/47	91/14464	0.01098901	3.38180968	1.30135179	0.25703622	0.46702295	0.43394331	Bcl2l1	1
GO:0001822	kidney development	2/47	303/14464	0.00660066	2.03131803	1.03587567	0.25847289	0.46702295	0.43394331	Klf15/Rdh10	2
GO:0099173	postsynapse organization	2/47	303/14464	0.00660066	2.03131803	1.03587567	0.25847289	0.46702295	0.43394331	Fam107a/Mtmr2	2
GO:0007631	feeding behavior	1/47	92/14464	0.01086957	3.34505088	1.28833336	0.25946573	0.46702295	0.43394331	Mchr1	1
GO:0009581	detection of external stimulus	1/47	92/14464	0.01086957	3.34505088	1.28833336	0.25946573	0.46702295	0.43394331	Kcnk4	1
GO:0009582	detection of abiotic stimulus	1/47	92/14464	0.01086957	3.34505088	1.28833336	0.25946573	0.46702295	0.43394331	Kcnk4	1
GO:0046889	positive regulation of lipid biosynthetic process	1/47	92/14464	0.01086957	3.34505088	1.28833336	0.25946573	0.46702295	0.43394331	Rdh10	1
GO:1901990	regulation of mitotic cell cycle phase transition	2/47	304/14464	0.00657895	2.02463606	1.03089747	0.25968261	0.46702295	0.43394331	Fam107a/Crlf3	2
GO:0006112	energy reserve metabolic process	1/47	93/14464	0.01075269	3.30908259	1.27549312	0.26188746	0.46733716	0.43423527	Per2	1
GO:0042116	macrophage activation	1/47	93/14464	0.01075269	3.30908259	1.27549312	0.26188746	0.46733716	0.43423527	Slc7a2	1
GO:0043280	positive regulation of cysteine-type endopeptidase activity involved in apoptotic process	1/47	93/14464	0.01075269	3.30908259	1.27549312	0.26188746	0.46733716	0.43423527	Xdh	1
GO:0046364	monosaccharide biosynthetic process	1/47	93/14464	0.01075269	3.30908259	1.27549312	0.26188746	0.46733716	0.43423527	Per2	1
GO:1901874	negative regulation of post-translational protein modification	1/47	93/14464	0.01075269	3.30908259	1.27549312	0.26188746	0.46733716	0.43423527	Per2	1
GO:0034446	substrate adhesion-dependent cell spreading	1/47	94/14464	0.0106383	3.27387958	1.26282647	0.26430144	0.46985442	0.43657423	Mertk	1
GO:0051492	regulation of stress fiber assembly	1/47	94/14464	0.0106383	3.27387958	1.26282647	0.26430144	0.46985442	0.43657423	Ppfia1	1
GO:0051098	regulation of binding	2/47	308/14464	0.00649351	1.99834208	1.01117223	0.26452273	0.46985442	0.43657423	Per2/Ip6k2	2
GO:0006754	ATP biosynthetic process	1/47	95/14464	0.01052632	3.23941769	1.25032901	0.26670769	0.47082907	0.43747984	Bcl2l1	1
GO:0019226	transmission of nerve impulse	1/47	95/14464	0.01052632	3.23941769	1.25032901	0.26670769	0.47082907	0.43747984	Kcnk4	1
GO:1990542	mitochondrial transmembrane transport	1/47	95/14464	0.01052632	3.23941769	1.25032901	0.26670769	0.47082907	0.43747984	Slc25a15	1
GO:2000060	positive regulation of ubiquitin-dependent protein catabolic process	1/47	95/14464	0.01052632	3.23941769	1.25032901	0.26670769	0.47082907	0.43747984	Herpud1	1
GO:0009150	purine ribonucleotide metabolic process	2/47	311/14464	0.00643087	1.97906547	0.99657124	0.26815373	0.47189979	0.43847472	Xdh/Bcl2l1	2
GO:0043010	camera-type eye development	2/47	311/14464	0.00643087	1.97906547	0.99657124	0.26815373	0.47189979	0.43847472	Mertk/Rdh10	2
GO:0050764	regulation of phagocytosis	1/47	96/14464	0.01041667	3.20567376	1.23799647	0.26910624	0.47189979	0.43847472	Mertk	1
GO:1902106	negative regulation of leukocyte differentiation	1/47	96/14464	0.01041667	3.20567376	1.23799647	0.26910624	0.47189979	0.43847472	Fstl3	1
GO:0009615	response to virus	2/47	312/14464	0.00641026	1.97272231	0.99174025	0.26936417	0.47189979	0.43847472	Bcl2l1/Pcbp2	2
GO:0016239	positive regulation of macroautophagy	1/47	97/14464	0.01030928	3.17262558	1.22582474	0.27149711	0.47491363	0.44127509	Sesn1	1
GO:0072001	renal system development	2/47	315/14464	0.00634921	1.95393448	0.97735339	0.27299562	0.47681025	0.44303737	Klf15/Rdh10	2
GO:0036465	synaptic vesicle recycling	1/47	98/14464	0.01020408	3.14025185	1.21380986	0.27388032	0.47690809	0.44312829	Bcl2l1	1
GO:0055013	cardiac muscle cell development	1/47	98/14464	0.01020408	3.14025185	1.21380986	0.27388032	0.47690809	0.44312829	Trip10	1
GO:0033135	regulation of peptidyl-serine phosphorylation	1/47	99/14464	0.01010101	3.10853213	1.201948	0.2762559	0.47793754	0.44408481	Ip6k2	1
GO:0046620	regulation of organ growth	1/47	99/14464	0.01010101	3.10853213	1.201948	0.2762559	0.47793754	0.44408481	Trip10	1

GO:1903432 regulation of TORC1 signaling	1/47	99/14464	0.01010101	3.10853213	1.201948	0.2762559	0.47793754	0.44408481	Sesn1	1
GO:0006644 phospholipid metabolic process	2/47	319/14464	0.00626959	1.92943374	0.95841361	0.27783739	0.47793754	0.44408481	Ip6k2/Mtmr2	2
GO:0008645 hexose transmembrane transport	1/47	100/14464	0.01	3.07744681	1.19023547	0.27862388	0.47793754	0.44408481	Klf15	1
GO:0035967 cellular response to topologically incorrect protein	1/47	100/14464	0.01	3.07744681	1.19023547	0.27862388	0.47793754	0.44408481	Herpud1	1
GO:0046928 regulation of neurotransmitter secretion	1/47	100/14464	0.01	3.07744681	1.19023547	0.27862388	0.47793754	0.44408481	Bcl2l1	1
GO:0055017 cardiac muscle tissue growth	1/47	100/14464	0.01	3.07744681	1.19023547	0.27862388	0.47793754	0.44408481	Trip10	1
GO:0060079 excitatory postsynaptic potential	1/47	100/14464	0.01	3.07744681	1.19023547	0.27862388	0.47793754	0.44408481	Mtmr2	1
GO:1904659 D-glucose transmembrane transport	1/47	100/14464	0.01	3.07744681	1.19023547	0.27862388	0.47793754	0.44408481	Klf15	1
GO:0010660 regulation of muscle cell apoptotic process	1/47	101/14464	0.00990099	3.04697704	1.1786687	0.28098427	0.47913022	0.44519302	Trip10	1
GO:0015837 amine transport	1/47	101/14464	0.00990099	3.04697704	1.1786687	0.28098427	0.47913022	0.44519302	Per2	1
GO:0050852 T cell receptor signaling pathway	1/47	101/14464	0.00990099	3.04697704	1.1786687	0.28098427	0.47913022	0.44519302	Icosl	1
GO:0097237 cellular response to toxic substance	1/47	101/14464	0.00990099	3.04697704	1.1786687	0.28098427	0.47913022	0.44519302	Sesn1	1
GO:0015749 monosaccharide transmembrane transport	1/47	102/14464	0.00980392	3.01710471	1.16724425	0.28333711	0.48100444	0.44693448	Klf15	1
GO:0099068 postsynapse assembly	1/47	102/14464	0.00980392	3.01710471	1.16724425	0.28333711	0.48100444	0.44693448	Fam107a	1
GO:1903707 negative regulation of hemopoiesis	1/47	102/14464	0.00980392	3.01710471	1.16724425	0.28333711	0.48100444	0.44693448	Fstl3	1
GO:0031647 regulation of protein stability	2/47	324/14464	0.00617284	1.89965852	0.93511747	0.28388825	0.4812303	0.44714435	Fam107a/Ip6k2	2
GO:0046486 glycerolipid metabolic process	2/47	325/14464	0.00615385	1.89381342	0.9305075	0.28509812	0.48157762	0.44746706	Ip6k2/Mtmr2	2
GO:0042752 regulation of circadian rhythm	1/47	103/14464	0.00970874	2.98781244	1.15595878	0.2856824	0.48157762	0.44746706	Per2	1
GO:0043502 regulation of muscle adaptation	1/47	103/14464	0.00970874	2.98781244	1.15595878	0.2856824	0.48157762	0.44746706	Trip10	1
GO:0031330 negative regulation of cellular catabolic process	1/47	104/14464	0.00961538	2.95908347	1.14480907	0.28802019	0.48157762	0.44746706	Mtmr2	1
GO:0043279 response to alkaloid	1/47	104/14464	0.00961538	2.95908347	1.14480907	0.28802019	0.48157762	0.44746706	Bcl2l1	1
GO:0070646 protein modification by small protein removal	1/47	104/14464	0.00961538	2.95908347	1.14480907	0.28802019	0.48157762	0.44746706	Otud7b	1
GO:0072009 nephron epithelium development	1/47	104/14464	0.00961538	2.95908347	1.14480907	0.28802019	0.48157762	0.44746706	Klf15	1
GO:0098754 detoxification	1/47	104/14464	0.00961538	2.95908347	1.14480907	0.28802019	0.48157762	0.44746706	Sesn1	1
GO:0032956 regulation of actin cytoskeleton organization	2/47	328/14464	0.00609756	1.87649196	0.91677394	0.28872691	0.48157762	0.44746706	Fam107a/Ppfia1	2
GO:0002377 immunoglobulin production	1/47	105/14464	0.00952381	2.93090172	1.13379202	0.29035048	0.48157762	0.44746706	Icosl	1
GO:0002832 negative regulation of response to biotic stimulus	1/47	105/14464	0.00952381	2.93090172	1.13379202	0.29035048	0.48157762	0.44746706	Pcbp2	1
GO:0036503 ERAD pathway	1/47	105/14464	0.00952381	2.93090172	1.13379202	0.29035048	0.48157762	0.44746706	Herpud1	1
GO:0110020 regulation of actomyosin structure organization	1/47	105/14464	0.00952381	2.93090172	1.13379202	0.29035048	0.48157762	0.44746706	Ppfia1	1
GO:1901800 positive regulation of proteasomal protein catabolic process	1/47	105/14464	0.00952381	2.93090172	1.13379202	0.29035048	0.48157762	0.44746706	Herpud1	1
GO:0009206 purine ribonucleoside triphosphate biosynthetic process	1/47	106/14464	0.00943396	2.90325171	1.12290459	0.29267331	0.48157762	0.44746706	Bcl2l1	1
GO:0097061 dendritic spine organization	1/47	106/14464	0.00943396	2.90325171	1.12290459	0.29267331	0.48157762	0.44746706	Mtmr2	1
GO:0009259 ribonucleotide metabolic process	2/47	332/14464	0.0060241	1.85388362	0.89868312	0.29356296	0.48157762	0.44746706	Xdh/Bcl2l1	2
GO:0005976 polysaccharide metabolic process	1/47	107/14464	0.00934579	2.87611851	1.11214389	0.2949887	0.48157762	0.44746706	Per2	1
GO:0009145 purine nucleoside triphosphate biosynthetic process	1/47	107/14464	0.00934579	2.87611851	1.11214389	0.2949887	0.48157762	0.44746706	Bcl2l1	1
GO:0016064 immunoglobulin mediated immune response	1/47	107/14464	0.00934579	2.87611851	1.11214389	0.2949887	0.48157762	0.44746706	Icosl	1
GO:0055006 cardiac cell development	1/47	107/14464	0.00934579	2.87611851	1.11214389	0.2949887	0.48157762	0.44746706	Trip10	1
GO:0060419 heart growth	1/47	107/14464	0.00934579	2.87611851	1.11214389	0.2949887	0.48157762	0.44746706	Trip10	1
GO:0008585 female gonad development	1/47	108/14464	0.00925926	2.84948779	1.10150708	0.29729667	0.48157762	0.44746706	Bcl2l1	1
GO:0019079 viral genome replication	1/47	108/14464	0.00925926	2.84948779	1.10150708	0.29729667	0.48157762	0.44746706	Pcbp2	1
GO:0030316 osteoclast differentiation	1/47	108/14464	0.00925926	2.84948779	1.10150708	0.29729667	0.48157762	0.44746706	Fstl3	1
GO:0032231 regulation of actin filament bundle assembly	1/47	108/14464	0.00925926	2.84948779	1.10150708	0.29729667	0.48157762	0.44746706	Ppfia1	1
GO:0050671 positive regulation of lymphocyte proliferation	1/47	108/14464	0.00925926	2.84948779	1.10150708	0.29729667	0.48157762	0.44746706	Icosl	1
GO:2001056 positive regulation of cysteine-type endopeptidase activity	1/47	108/14464	0.00925926	2.84948779	1.10150708	0.29729667	0.48157762	0.44746706	Xdh	1
GO:0001578 microtubule bundle formation	1/47	109/14464	0.00917431	2.8233457	1.09099143	0.29959725	0.48157762	0.44746706	Cfap157	1
GO:0030326 embryonic limb morphogenesis	1/47	109/14464	0.00917431	2.8233457	1.09099143	0.29959725	0.48157762	0.44746706	Rdh10	1
GO:0035113 embryonic appendage morphogenesis	1/47	109/14464	0.00917431	2.8233457	1.09099143	0.29959725	0.48157762	0.44746706	Rdh10	1
GO:0039531 regulation of cytoplasmic pattern recognition receptor signaling pathway	1/47	109/14464	0.00917431	2.8233457	1.09099143	0.29959725	0.48157762	0.44746706	Pcbp2	1
GO:0002250 adaptive immune response	2/47	337/14464	0.00593472	1.82637793	0.87641411	0.29960324	0.48157762	0.44746706	Otud7b/Icosl	2
GO:0006986 response to unfolded protein	1/47	110/14464	0.00909091	2.79767892	1.08059429	0.30189045	0.48157762	0.44746706	Herpud1	1
GO:0019724 B cell mediated immunity	1/47	110/14464	0.00909091	2.79767892	1.08059429	0.30189045	0.48157762	0.44746706	Icosl	1
GO:0034219 carbohydrate transmembrane transport	1/47	110/14464	0.00909091	2.79767892	1.08059429	0.30189045	0.48157762	0.44746706	Klf15	1
GO:0045446 endothelial cell differentiation	1/47	110/14464	0.00909091	2.79767892	1.08059429	0.30189045	0.48157762	0.44746706	Xdh	1
GO:0046545 development of primary female sexual characteristics	1/47	110/14464	0.00909091	2.79767892	1.08059429	0.30189045	0.48157762	0.44746706	Bcl2l1	1
GO:0019693 ribose phosphate metabolic process	2/47	339/14464	0.00589971	1.81560284	0.86761094	0.30201758	0.48157762	0.44746706	Xdh/Bcl2l1	2
GO:0051402 neuron apoptotic process	2/47	339/14464	0.00589971	1.81560284	0.86761094	0.30201758	0.48157762	0.44746706	Prodh/Bcl2l1	2
GO:0007006 mitochondrial membrane organization	1/47	111/14464	0.00900901	2.7724746	1.0703131	0.30417631	0.48157762	0.44746706	Bcl2l1	1
GO:0009201 ribonucleoside triphosphate biosynthetic process	1/47	111/14464	0.00900901	2.7724746	1.0703131	0.30417631	0.48157762	0.44746706	Bcl2l1	1
GO:0030038 contractile actin filament bundle assembly	1/47	111/14464	0.00900901	2.7724746	1.0703131	0.30417631	0.48157762	0.44746706	Ppfia1	1
GO:0031398 positive regulation of protein ubiquitination	1/47	111/14464	0.00900901	2.7724746	1.0703131	0.30417631	0.48157762	0.44746706	Fam107a	1
GO:0043149 stress fiber assembly	1/47	111/14464	0.00900901	2.7724746	1.0703131	0.30417631	0.48157762	0.44746706	Ppfia1	1
GO:0051928 positive regulation of calcium ion transport	1/47	111/14464	0.00900901	2.7724746	1.0703131	0.30417631	0.48157762	0.44746706	Mchr1	1
GO:0060291 long-term synaptic potentiation	1/47	111/14464	0.00900901	2.7724746	1.0703131	0.30417631	0.48157762	0.44746706	Fam107a	1
GO:0099565 chemical synaptic transmission, postsynaptic	1/47	111/14464	0.00900901	2.7724746	1.0703131	0.30417631	0.48157762	0.44746706	Mtmr2	1
GO:0010657 muscle cell apoptotic process	1/47	112/14464	0.00892857	2.74772036	1.06014536	0.30645483	0.48446641	0.45015124	Trip10	1
GO:0045786 negative regulation of cell cycle	2/47	343/14464	0.0058309	1.79442963	0.85017913	0.30684275	0.48446641	0.45015124	Fam107a/Crlf3	2
GO:0038202 TORC1 signaling	1/47	113/14464	0.00884956	2.72340426	1.05008867	0.30872606	0.48677218	0.45229369	Sesn1	1
GO:0001952 regulation of cell-matrix adhesion	1/47	114/14464	0.00877193	2.69951474	1.04014069	0.31099	0.48700612	0.45251106	Fam107a	1
GO:0007259 cell surface receptor signaling pathway via JAK-STAT	1/47	114/14464	0.00877193	2.69951474	1.04014069	0.31099	0.48700612	0.45251106	Crlf3	1
GO:0008637 apoptotic mitochondrial changes	1/47	114/14464	0.00877193	2.69951474	1.04014069	0.31099	0.48700612	0.45251106	Bcl2l1	1
GO:0008643 carbohydrate transport	1/47	114/14464	0.00877193	2.69951474	1.04014069	0.31099	0.48700612	0.45251106	Klf15	1
GO:1990830 cellular response to leukemia inhibitory factor	1/47	114/14464	0.00877193	2.69951474	1.04014069	0.31099	0.48700612	0.45251106	Mynn	1
GO:1990823 response to leukemia inhibitory factor	1/47	116/14464	0.00862069	2.65297139	1.02056183	0.31549615	0.49339139	0.45844406	Mynn	1
GO:0001654 eye development	2/47	351/14464	0.00569801	1.75353095	0.81599153	0.31647669	0.494212	0.45920654	Mertk/Rdh10	2
GO:0008584 male gonad development	1/47	117/14464	0.00854701	2.63029642	1.01092661	0.31773839	0.494212	0.45920654	Bcl2l1	1
GO:0015698 inorganic anion transport	1/47	117/14464	0.00854701	2.63029642	1.01092661	0.31773839	0.494212	0.45920654	Ip6k2	1
GO:0106027 neuron projection organization	1/47	117/14464	0.00854701	2.63029642	1.01092661	0.31773839	0.494212	0.45920654	Mtmr2	1
GO:0009142 nucleoside triphosphate biosynthetic process	1/47	118/14464	0.00847458	2.60800577	1.00139142	0.31997344	0.49518207	0.4601079	Bcl2l1	1
GO:0010821 regulation of mitochondrion organization	1/47	118/14464	0.00847458	2.60800577	1.00139142	0.31997344	0.49518207	0.4601079	Bcl2l1	1
GO:0046546 development of primary male sexual characteristics	1/47	118/14464	0.00847458	2.60800577	1.00139142	0.31997344	0.49518207	0.4601079	Bcl2l1	1
GO:0150063 visual system development	2/47	354/14464	0.00564972	1.73867051	0.80339581	0.32008293	0.49518207	0.4601079	Mertk/Rdh10	2
GO:0010639 negative regulation of organelle organization	2/47	355/14464	0.0056338	1.73377285	0.79922373	0.32128415	0.49637323	0.46121469	Bcl2l1/Ppfia1	2
GO:0016079 synaptic vesicle exocytosis	1/47	119/14464	0.00840336	2.58608976	0.99195424	0.32220132	0.49645746	0.46129296	Bcl2l1	1
GO:0030317 flagellated sperm motility	1/47	119/14464	0.00840336	2.58608976	0.99195424	0.32220132	0.49645746	0.46129296	Cfap157	1
GO:0048706 embryonic skeletal system development	1/47	120/14464	0.00833333	2.56453901	0.98261311	0.32442207	0.49921096	0.46385142	Rdh10	1
GO:0048880 sensory system development	2/47	359/14464	0.00557103	1.71445505	0.78266563	0.32608443	0.49927978	0.46391537	Mertk/Rdh10	2
GO:0002761 regulation of myeloid leukocyte differentiation	1/47	121/14464	0.00826446	2.54334447	0.97336613	0.32663569	0.49927978	0.46391537	Fstl3	1
GO:0030509 BMP signaling pathway	1/47	121/14464	0.00826446	2.54334447	0.97336613	0.32663569	0.49927978	0.46391537	Fstl3	1
GO:0046165 alcohol biosynthetic process	1/47	121/14464	0.00826446	2.54334447	0.97336613	0.32663569	0.49927978	0.46391537	Ip6k2	1
GO:0097722 sperm motility	1/47	121/14464	0.00826446	2.54334447	0.97336613	0.32663569	0.49927978	0.46391537	Cfap157	1
GO:0030879 mammary gland development	1/47	122/14464	0.00819672	2.52249738	0.96421145	0.32884221	0.50132103	0.46581203	Xdh	1
GO:0045834 positive regulation of lipid metabolic process	1/47	122/14464	0.00819672	2.52249738	0.96421145	0.32884221	0.50132103	0.46581203	Rdh10	1
GO:0007600 sensory perception	2/47	362/14464	0.00552486	1.70024686	0.77038137	0.32967956	0.50193277	0.46638045	Kcnk4/Rdh10	2
GO:0006633 fatty acid biosynthetic process	1/47	123/14464	0.00813008	2.50198928	0.95514728	0.33104165	0.5026767	0.46707168	Acsf3	1
GO:0051384 response to glucocorticoid	1/47	123/14464	0.00813008	2.50198928	0.95514728	0.33104165	0.5026767	0.46707168	Fam107a	1
GO:0051100 negative regulation of binding	1/47	124/14464	0.00806452	2.48181194	0.94617187	0.33323404	0.50401101	0.46831148	Per2	1
GO:0097696 cell surface receptor signaling pathway via STAT	1/47	124/14464	0.00806452	2.48181194	0.94617187	0.33323404	0.50401101	0.46831148	Crlf3	1
GO:1903052 positive regulation of proteolysis involved in protein catabolic process	1/47	124/14464	0.00806452	2.48181194	0.94617187	0.33323404	0.50401101	0.46831148	Herpud1	1
GO:0010950 positive regulation of endopeptidase activity	1/47	125/14464	0.008	2.46195745	0.93728352	0.33541939	0.50665055	0.47076406	Xdh	1
GO:0032970 regulation of actin filament-based process	2/47	368/14464	0.00543478	1.67252544	0.74614864	0.33685568	0.50794117	0.47196326	Fam107a/Ppfia1	2
GO:0010466 negative regulation of peptidase activity	1/47	126/14464	0.00793651	2.4424181	0.92848057	0.33759774	0.50794117	0.47196326	Ints1	1
GO:0090101 negative regulation of transmembrane receptor protein serine/threonine kinase signaling pathway	1/47	126/14464	0.00793651	2.4424181	0.92848057	0.33759774	0.50794117	0.47196326	Fstl3	1
GO:0001936 regulation of endothelial cell proliferation	1/47	127/14464	0.00787402	2.42318646	0.91976143	0.3397691	0.50987514	0.47376024	Xdh	1
GO:0035966 response to topologically incorrect protein	1/47	127/14464	0.00787402	2.42318646	0.91976143	0.3397691	0.50987514	0.47376024	Herpud1	1
GO:0010927 cellular component assembly involved in morphogenesis	1/47	128/14464	0.0078125	2.40425532	0.91112453	0.34193348	0.51046101	0.47430462	Mtmr2	1
GO:0030856 regulation of epithelial cell differentiation	1/47	128/14464	0.0078125	2.40425532	0.91112453	0.34193348	0.51046101	0.47430462	Xdh	1
GO:0032989 cellular anatomical entity morphogenesis	1/47	128/14464	0.0078125	2.40425532	0.91112453	0.34193348	0.51046101	0.47430462	Mtmr2	1
GO:0060294 cilium movement involved in cell motility	1/47	128/14464	0.0078125	2.40425532	0.91112453	0.34193348	0.51046101	0.47430462	Cfap157	1
GO:0015748 organophosphate ester transport	1/47	129/14464	0.00775194	2.38561768	0.90256834	0.34409093	0.51235273	0.47606235	Plscr4	1
GO:0048259 regulation of receptor-mediated endocytosis	1/47	129/14464	0.00775194	2.38561768	0.90256834	0.34409093	0.51235273	0.47606235	Mtmr2	1
GO:0018205 peptidyl-lysine modification	1/47	130/14464	0.00769231	2.36726678	0.89409138	0.34624145	0.51399953	0.4775925	Klf15	1
GO:0170039 proteinogenic amino acid metabolic process	1/47	130/14464	0.00769231	2.36726678	0.89409138	0.34624145	0.51399953	0.4775925	Prodh	1
GO:0009154 purine ribonucleotide catabolic process	1/47	131/14464	0.00763359	2.34919604	0.88569221	0.34838507	0.51399953	0.4775925	Xdh	1
GO:0009566 fertilization	1/47	131/14464	0.00763359	2.34919604	0.88569221	0.34838507	0.51399953	0.4775925	Bcl2l1	1
GO:0043244 regulation of protein-containing complex disassembly	1/47	131/14464	0.00763359	2.34919604	0.88569221	0.34838507	0.51399953	0.4775925	Per2	1
GO:0071772 response to BMP	1/47	131/14464	0.00763359	2.34919604	0.88569221	0.34838507	0.51399953	0.4775925	Fstl3	1
GO:0071773 cellular response to BMP stimulus	1/47	131/14464	0.00763359	2.34919604	0.88569221	0.34838507	0.51399953	0.4775925	Fstl3	1
GO:1990778 protein localization to cell periphery	2/47	378/14464	0.00529101	1.62827873	0.70671627	0.34876945	0.51399953	0.4775925	Bcl2l1/Ppfia1	2
GO:0001539 cilium or flagellum-dependent cell motility	1/47	132/14464	0.00757576	2.3313991	0.87736943	0.35052181	0.51460536	0.47815543	Cfap157	1
GO:0010952 positive regulation of peptidase activity	1/47	132/14464	0.00757576	2.3313991	0.87736943	0.35052181	0.51460536	0.47815543	Xdh	1
GO:0060285 cilium-dependent cell motility	1/47	132/14464	0.00757576	2.3313991	0.87736943	0.35052181	0.51460536	0.47815543	Cfap157	1
GO:0002757 immune response-activating signaling pathway	2/47	380/14464	0.00526316	1.61970885	0.69896778	0.35114461	0.51486299	0.47839481	Icosl/Pcbp2	2
GO:0055007 cardiac muscle cell differentiation	1/47	133/14464	0.0075188	2.31386978	0.86912165	0.35265169	0.51641489	0.47983678	Trip10	1
GO:0003158 endothelium development	1/47	134/14464	0.00746269	2.2966021	0.86094755	0.35477474	0.51820524	0.48150031	Xdh	1
GO:0046661 male sex differentiation	1/47	134/14464	0.00746269	2.2966021	0.86094755	0.35477474	0.51820524	0.48150031	Bcl2l1	1
GO:0071902 positive regulation of protein serine/threonine kinase activity	1/47	135/14464	0.00740741	2.27959023	0.85284583	0.35689097	0.52063562	0.48375856	Ip6k2	1
GO:0007565 female pregnancy	1/47	136/14464	0.00735294	2.26282854	0.84481522	0.35900041	0.52238871	0.48538747	Ints1	1
GO:2000241 regulation of reproductive process	1/47	136/14464	0.00735294	2.26282854	0.84481522	0.35900041	0.52238871	0.48538747	Bcl2l1	1
GO:0031329 regulation of cellular catabolic process	2/47	388/14464	0.00515464	1.58631279	0.66841513	0.36061781	0.52320655	0.48614738	Sesn1/Mtmr2	2
GO:0072073 kidney epithelium development	1/47	137/14464	0.00729927	2.24631154	0.83685447	0.36110307	0.52320655	0.48614738	Klf15	1
GO:0031960 response to corticosteroid	1/47	138/14464	0.00724638	2.23003392	0.8289624	0.36319899	0.52320655	0.48614738	Fam107a	1
GO:0050851 antigen receptor-mediated signaling pathway	1/47	138/14464	0.00724638	2.23003392	0.8289624	0.36319899	0.52320655	0.48614738	Icosl	1
GO:0051099 positive regulation of binding	1/47	138/14464	0.00724638	2.23003392	0.8289624	0.36319899	0.52320655	0.48614738	Ip6k2	1
GO:0051606 detection of stimulus	1/47	138/14464	0.00724638	2.23003392	0.8289624	0.36319899	0.52320655	0.48614738	Kcnk4	1
GO:0060078 regulation of postsynaptic membrane potential	1/47	138/14464	0.00724638	2.23003392	0.8289624	0.36319899	0.52320655	0.48614738	Mtmr2	1
GO:1903322 positive regulation of protein modification by small protein conjugation or removal	1/47	138/14464	0.00724638	2.23003392	0.8289624	0.36319899	0.52320655	0.48614738	Fam107a	1
GO:0035107 appendage morphogenesis	1/47	139/14464	0.00719424	2.21399051	0.82113781	0.36528817	0.52359487	0.4865082	Rdh10	1
GO:0035108 limb morphogenesis	1/47	139/14464	0.00719424	2.21399051	0.82113781	0.36528817	0.52359487	0.4865082	Rdh10	1
GO:0044272 sulfur compound biosynthetic process	1/47	139/14464	0.00719424	2.21399051	0.82113781	0.36528817	0.52359487	0.4865082	Fmo1	1
GO:1901875 positive regulation of post-translational protein modification	1/47	139/14464	0.00719424	2.21399051	0.82113781	0.36528817	0.52359487	0.4865082	Fam107a	1
GO:0007204 positive regulation of cytosolic calcium ion concentration	1/47	140/14464	0.00714286	2.19817629	0.81337956	0.36737065	0.52368577	0.48659266	Mchr1	1
GO:0009261 ribonucleotide catabolic process	1/47	140/14464	0.00714286	2.19817629	0.81337956	0.36737065	0.52368577	0.48659266	Xdh	1
GO:0051250 negative regulation of lymphocyte activation	1/47	140/14464	0.00714286	2.19817629	0.81337956	0.36737065	0.52368577	0.48659266	Mertk	1
GO:0071456 cellular response to hypoxia	1/47	140/14464	0.00714286	2.19817629	0.81337956	0.36737065	0.52368577	0.48659266	Hif3a	1
GO:0009314 response to radiation	2/47	394/14464	0.00507614	1.56215574	0.64594923	0.36769211	0.52368577	0.48659266	Per2/Bcl2l1	2
GO:0003012 muscle system process	2/47	395/14464	0.00506329	1.55820092	0.64224118	0.36886847	0.52368577	0.48659266	Klf15/Trip10	2
GO:0006195 purine nucleotide catabolic process	1/47	141/14464	0.0070922	2.18258639	0.80568654	0.36944643	0.52368577	0.48659266	Xdh	1
GO:0031644 regulation of nervous system process	1/47	141/14464	0.0070922	2.18258639	0.80568654	0.36944643	0.52368577	0.48659266	Mtmr2	1
GO:2000377 regulation of reactive oxygen species metabolic process	1/47	141/14464	0.0070922	2.18258639	0.80568654	0.36944643	0.52368577	0.48659266	Xdh	1
GO:0002764 immune response-regulating signaling pathway	2/47	397/14464	0.00503778	1.55035104	0.63485562	0.37121883	0.52373729	0.48664052	Icosl/Pcbp2	2
GO:0010212 response to ionizing radiation	1/47	142/14464	0.00704225	2.16721606	0.79805765	0.37151555	0.52373729	0.48664052	Bcl2l1	1
GO:0050680 negative regulation of epithelial cell proliferation	1/47	142/14464	0.00704225	2.16721606	0.79805765	0.37151555	0.52373729	0.48664052	Xdh	1
GO:0022407 regulation of cell-cell adhesion	2/47	398/14464	0.00502513	1.54645568	0.63117799	0.37239282	0.52373729	0.48664052	Fstl3/Icosl	2
GO:0044772 mitotic cell cycle phase transition	2/47	399/14464	0.00501253	1.54257985	0.62751038	0.37356599	0.52373729	0.48664052	Fam107a/Crlf3	2
GO:0007009 plasma membrane organization	1/47	143/14464	0.00699301	2.15206071	0.79049183	0.37357803	0.52373729	0.48664052	Plscr4	1
GO:0032006 regulation of TOR signaling	1/47	143/14464	0.00699301	2.15206071	0.79049183	0.37357803	0.52373729	0.48664052	Sesn1	1
GO:0042129 regulation of T cell proliferation	1/47	143/14464	0.00699301	2.15206071	0.79049183	0.37357803	0.52373729	0.48664052	Icosl	1
GO:1903305 regulation of regulated secretory pathway	1/47	143/14464	0.00699301	2.15206071	0.79049183	0.37357803	0.52373729	0.48664052	Bcl2l1	1
GO:0016241 regulation of macroautophagy	1/47	144/14464	0.00694444	2.13711584	0.78298802	0.37563388	0.52470218	0.48753707	Sesn1	1
GO:0051147 regulation of muscle cell differentiation	1/47	144/14464	0.00694444	2.13711584	0.78298802	0.37563388	0.52470218	0.48753707	Trip10	1
GO:0072006 nephron development	1/47	144/14464	0.00694444	2.13711584	0.78298802	0.37563388	0.52470218	0.48753707	Klf15	1
GO:1901987 regulation of cell cycle phase transition	2/47	402/14464	0.00497512	1.53106806	0.61656709	0.3770806	0.52565088	0.48841858	Fam107a/Crlf3	2
GO:0007596 blood coagulation	1/47	145/14464	0.00689655	2.12237711	0.77554523	0.37768313	0.52565088	0.48841858	Mertk	1
GO:0032388 positive regulation of intracellular transport	1/47	145/14464	0.00689655	2.12237711	0.77554523	0.37768313	0.52565088	0.48841858	Mtmr2	1
GO:0007286 spermatid development	1/47	146/14464	0.00684932	2.10784028	0.76816245	0.37972579	0.52658359	0.48928523	Cfap157	1
GO:1900180 regulation of protein localization to nucleus	1/47	146/14464	0.00684932	2.10784028	0.76816245	0.37972579	0.52658359	0.48928523	Otud7b	1

GO:2001235	positive regulation of apoptotic signaling pathway	1/47	146/14464	0.00684932	2.10784028	0.76816245	0.37972579	0.52658359	0.48928523	G0s2	1
GO:0009152	purine ribonucleotide biosynthetic process	1/47	147/14464	0.00680272	2.09350123	0.76083872	0.38176189	0.52750052	0.49013721	Bcl2l1	1
GO:0060041	retina development in camera-type eye	1/47	147/14464	0.00680272	2.09350123	0.76083872	0.38176189	0.52750052	0.49013721	Mertk	1
GO:0170033	L-amino acid metabolic process	1/47	147/14464	0.00680272	2.09350123	0.76083872	0.38176189	0.52750052	0.49013721	Prodh	1
GO:0001935	endothelial cell proliferation	1/47	148/14464	0.00675676	2.07935595	0.75357308	0.38379145	0.5280291	0.49062835	Xdh	1
GO:0002244	hematopoietic progenitor cell differentiation	1/47	148/14464	0.00675676	2.07935595	0.75357308	0.38379145	0.5280291	0.49062835	Fstl3	1
GO:0007599	hemostasis	1/47	148/14464	0.00675676	2.07935595	0.75357308	0.38379145	0.5280291	0.49062835	Mertk	1
GO:0051249	regulation of lymphocyte activation	2/47	409/14464	0.00488998	1.50486397	0.59137271	0.38525177	0.5280291	0.49062835	Mertk/Icosl	2
GO:0001837	epithelial to mesenchymal transition	1/47	149/14464	0.00671141	2.06540054	0.74636462	0.38581449	0.5280291	0.49062835	Trip10	1
GO:0016331	morphogenesis of embryonic epithelium	1/47	149/14464	0.00671141	2.06540054	0.74636462	0.38581449	0.5280291	0.49062835	Rdh10	1
GO:0030218	erythrocyte differentiation	1/47	149/14464	0.00671141	2.06540054	0.74636462	0.38581449	0.5280291	0.49062835	Slc1a5	1
GO:0036294	cellular response to decreased oxygen levels	1/47	149/14464	0.00671141	2.06540054	0.74636462	0.38581449	0.5280291	0.49062835	Hif3a	1
GO:0031623	receptor internalization	1/47	150/14464	0.00666667	2.05163121	0.73921243	0.38783102	0.52890226	0.49143966	Mtmr2	1
GO:0048754	branching morphogenesis of an epithelial tube	1/47	150/14464	0.00666667	2.05163121	0.73921243	0.38783102	0.52890226	0.49143966	Rdh10	1
GO:0050817	coagulation	1/47	150/14464	0.00666667	2.05163121	0.73921243	0.38783102	0.52890226	0.49143966	Mertk	1
GO:0042445	hormone metabolic process	1/47	152/14464	0.00657895	2.02463606	0.72507334	0.39184467	0.53248314	0.4947669	Rdh10	1
GO:0044703	multi-organism reproductive process	1/47	152/14464	0.00657895	2.02463606	0.72507334	0.39184467	0.53248314	0.4947669	Ints1	1
GO:0051302	regulation of cell division	1/47	152/14464	0.00657895	2.02463606	0.72507334	0.39184467	0.53248314	0.4947669	Bcl2l1	1
GO:0044706	multi-multicellular organism process	1/47	153/14464	0.00653595	2.01140314	0.71808474	0.39384183	0.53349331	0.49570552	Ints1	1
GO:0046474	glycerophospholipid biosynthetic process	1/47	153/14464	0.00653595	2.01140314	0.71808474	0.39384183	0.53349331	0.49570552	Ip6k2	1
GO:0050673	epithelial cell proliferation	2/47	417/14464	0.00479616	1.47599367	0.56314222	0.39453711	0.53349331	0.49570552	Xdh/Bcl2l1	2
GO:0035150	regulation of tube size	1/47	154/14464	0.00649351	1.99834208	0.71114899	0.39583257	0.53349331	0.49570552	Per2	1
GO:0035296	regulation of tube diameter	1/47	154/14464	0.00649351	1.99834208	0.71114899	0.39583257	0.53349331	0.49570552	Per2	1
GO:0048515	spermatid differentiation	1/47	154/14464	0.00649351	1.99834208	0.71114899	0.39583257	0.53349331	0.49570552	Cfap157	1
GO:0097746	blood vessel diameter maintenance	1/47	154/14464	0.00649351	1.99834208	0.71114899	0.39583257	0.53349331	0.49570552	Per2	1
GO:0001508	action potential	1/47	155/14464	0.00645161	1.98544955	0.70426528	0.3978169	0.53491502	0.49702653	Kcnk4	1
GO:0072331	signal transduction by p53 class mediator	1/47	155/14464	0.00645161	1.98544955	0.70426528	0.3978169	0.53491502	0.49702653	Phlda3	1
GO:0048568	embryonic organ development	2/47	421/14464	0.00475059	1.46196998	0.54924426	0.39915756	0.5358438	0.49788952	Tead3/Rdh10	2
GO:0045785	positive regulation of cell adhesion	2/47	422/14464	0.00473934	1.4585056	0.54579186	0.40031029	0.5358438	0.49788952	Fstl3/Icosl	2
GO:0002753	cytoplasmic pattern recognition receptor signaling pathway	1/47	157/14464	0.00636943	1.9601572	0.69065086	0.40176646	0.5358438	0.49788952	Pcbp2	1
GO:0062207	regulation of pattern recognition receptor signaling pathway	1/47	157/14464	0.00636943	1.9601572	0.69065086	0.40176646	0.5358438	0.49788952	Pcbp2	1
GO:0071478	cellular response to radiation	1/47	157/14464	0.00636943	1.9601572	0.69065086	0.40176646	0.5358438	0.49788952	Bcl2l1	1
GO:0072523	purine-containing compound catabolic process	1/47	157/14464	0.00636943	1.9601572	0.69065086	0.40176646	0.5358438	0.49788952	Xdh	1
GO:2000058	regulation of ubiquitin-dependent protein catabolic process	1/47	157/14464	0.00636943	1.9601572	0.69065086	0.40176646	0.5358438	0.49788952	Herpud1	1
GO:0009166	nucleotide catabolic process	1/47	158/14464	0.00632911	1.94775114	0.68391861	0.40373172	0.53784168	0.49974589	Xdh	1
GO:0003341	cilium movement	1/47	159/14464	0.00628931	1.93550114	0.67723536	0.40569066	0.53858126	0.50043309	Cfap157	1
GO:0030041	actin filament polymerization	1/47	159/14464	0.00628931	1.93550114	0.67723536	0.40569066	0.53858126	0.50043309	Fam107a	1
GO:0099072	regulation of postsynaptic membrane neurotransmitter receptor levels	1/47	159/14464	0.00628931	1.93550114	0.67723536	0.40569066	0.53858126	0.50043309	Ppfia1	1
GO:0030099	myeloid cell differentiation	2/47	428/14464	0.0046729	1.43805926	0.52525929	0.4072062	0.53992801	0.50168445	Slc1a5/Fstl3	2
GO:0070507	regulation of microtubule cytoskeleton organization	1/47	160/14464	0.00625	1.92340426	0.67060036	0.4076433	0.53992801	0.50168445	Fam107a	1
GO:0007015	actin filament organization	2/47	429/14464	0.004662	1.43470714	0.52186705	0.40835205	0.54002028	0.50177018	Fam107a/Ppfia1	2
GO:0002695	negative regulation of leukocyte activation	1/47	161/14464	0.00621118	1.91145765	0.66401293	0.40958967	0.54002028	0.50177018	Mertk	1
GO:0009266	response to temperature stimulus	1/47	161/14464	0.00621118	1.91145765	0.66401293	0.40958967	0.54002028	0.50177018	Kcnk4	1
GO:0051897	positive regulation of phosphatidylinositol 3-kinase/protein kinase B signal transduction	1/47	161/14464	0.00621118	1.91145765	0.66401293	0.40958967	0.54002028	0.50177018	Mertk	1
GO:0002253	activation of immune response	2/47	431/14464	0.00464037	1.42804956	0.51510777	0.41064074	0.54072005	0.50242039	Icosl/Pcbp2	2
GO:0009260	ribonucleotide biosynthetic process	1/47	162/14464	0.00617284	1.89965852	0.65747235	0.41152977	0.54072005	0.50242039	Bcl2l1	1
GO:0034101	erythrocyte homeostasis	1/47	162/14464	0.00617284	1.89965852	0.65747235	0.41152977	0.54072005	0.50242039	Slc1a5	1
GO:0002285	lymphocyte activation involved in immune response	1/47	163/14464	0.00613497	1.88800418	0.65097795	0.41346363	0.54202351	0.50363152	Icosl	1
GO:0010508	positive regulation of autophagy	1/47	163/14464	0.00613497	1.88800418	0.65097795	0.41346363	0.54202351	0.50363152	Sesn1	1
GO:0035051	cardiocyte differentiation	1/47	165/14464	0.00606061	1.86511928	0.63812506	0.41731271	0.54582606	0.50716473	Trip10	1
GO:0071453	cellular response to oxygen levels	1/47	165/14464	0.00606061	1.86511928	0.63812506	0.41731271	0.54582606	0.50716473	Hif3a	1
GO:0000910	cytokinesis	1/47	168/14464	0.00595238	1.83181358	0.61917591	0.42304003	0.55206244	0.51295939	Bcl2l1	1
GO:0046390	ribose phosphate biosynthetic process	1/47	168/14464	0.00595238	1.83181358	0.61917591	0.42304003	0.55206244	0.51295939	Bcl2l1	1
GO:0007269	neurotransmitter secretion	1/47	170/14464	0.00588235	1.81026283	0.60675611	0.42682759	0.55430435	0.5150425	Bcl2l1	1
GO:0046890	regulation of lipid biosynthetic process	1/47	170/14464	0.00588235	1.81026283	0.60675611	0.42682759	0.55430435	0.5150425	Rdh10	1
GO:0099643	signal release from synapse	1/47	170/14464	0.00588235	1.81026283	0.60675611	0.42682759	0.55430435	0.5150425	Bcl2l1	1
GO:0001678	intracellular glucose homeostasis	1/47	171/14464	0.00584795	1.7996765	0.60060835	0.42871223	0.55430435	0.5150425	Klf15	1
GO:0042552	myelination	1/47	171/14464	0.00584795	1.7996765	0.60060835	0.42871223	0.55430435	0.5150425	Mtmr2	1
GO:0051494	negative regulation of cytoskeleton organization	1/47	171/14464	0.00584795	1.7996765	0.60060835	0.42871223	0.55430435	0.5150425	Ppfia1	1
GO:1901605	alpha-amino acid metabolic process	1/47	171/14464	0.00584795	1.7996765	0.60060835	0.42871223	0.55430435	0.5150425	Prodh	1
GO:0034762	regulation of transmembrane transport	2/47	447/14464	0.00447427	1.3769337	0.46220204	0.42880026	0.55430435	0.5150425	Per2/Klf15	2
GO:0031929	TOR signaling	1/47	172/14464	0.00581395	1.78921326	0.59450124	0.43059081	0.55430435	0.5150425	Sesn1	1
GO:0048736	appendage development	1/47	172/14464	0.00581395	1.78921326	0.59450124	0.43059081	0.55430435	0.5150425	Rdh10	1
GO:0060173	limb development	1/47	172/14464	0.00581395	1.78921326	0.59450124	0.43059081	0.55430435	0.5150425	Rdh10	1
GO:1902904	negative regulation of supramolecular fiber organization	1/47	172/14464	0.00581395	1.78921326	0.59450124	0.43059081	0.55430435	0.5150425	Ppfia1	1
GO:0007346	regulation of mitotic cell cycle	2/47	450/14464	0.00444444	1.36775414	0.45250506	0.43217446	0.55430435	0.5150425	Fam107a/Crlf3	2
GO:0030307	positive regulation of cell growth	1/47	173/14464	0.00578035	1.77887099	0.58843425	0.43246334	0.55430435	0.5150425	Trip10	1
GO:0043393	regulation of protein binding	1/47	173/14464	0.00578035	1.77887099	0.58843425	0.43246334	0.55430435	0.5150425	Ip6k2	1
GO:0071222	cellular response to lipopolysaccharide	1/47	173/14464	0.00578035	1.77887099	0.58843425	0.43246334	0.55430435	0.5150425	Plscr4	1
GO:0007272	ensheathment of neurons	1/47	174/14464	0.00574713	1.76864759	0.58240682	0.43432985	0.55545962	0.51611594	Mtmr2	1
GO:0008366	axon ensheathment	1/47	174/14464	0.00574713	1.76864759	0.58240682	0.43432985	0.55545962	0.51611594	Mtmr2	1
GO:0010817	regulation of hormone levels	2/47	455/14464	0.0043956	1.35272387	0.43649338	0.43777579	0.55711561	0.51765463	Per2/Rdh10	2
GO:0031667	response to nutrient levels	2/47	455/14464	0.0043956	1.35272387	0.43649338	0.43777579	0.55711561	0.51765463	Fam107a/Sesn1	2
GO:0006164	purine nucleotide biosynthetic process	1/47	176/14464	0.00568182	1.74854932	0.57046855	0.43804485	0.55711561	0.51765463	Bcl2l1	1
GO:0050954	sensory perception of mechanical stimulus	1/47	176/14464	0.00568182	1.74854932	0.57046855	0.43804485	0.55711561	0.51765463	Kcnk4	1
GO:1901292	nucleoside phosphate catabolic process	1/47	176/14464	0.00568182	1.74854932	0.57046855	0.43804485	0.55711561	0.51765463	Xdh	1
GO:0061138	morphogenesis of a branching epithelium	1/47	177/14464	0.00564972	1.73867051	0.56455667	0.43989339	0.55884911	0.51926535	Rdh10	1
GO:0050866	negative regulation of cell activation	1/47	178/14464	0.00561798	1.7289027	0.5586823	0.44173598	0.56057123	0.52086549	Mertk	1
GO:0019827	stem cell population maintenance	1/47	179/14464	0.00558659	1.71924403	0.55284493	0.44357263	0.56104626	0.52130687	Tead3	1
GO:0050796	regulation of insulin secretion	1/47	179/14464	0.00558659	1.71924403	0.55284493	0.44357263	0.56104626	0.52130687	Per2	1
GO:0051346	negative regulation of hydrolase activity	1/47	179/14464	0.00558659	1.71924403	0.55284493	0.44357263	0.56104626	0.52130687	Ints1	1
GO:0035265	organ growth	1/47	180/14464	0.00555556	1.70969267	0.54704408	0.44540336	0.5621264	0.5223105	Trip10	1
GO:0071219	cellular response to molecule of bacterial origin	1/47	180/14464	0.00555556	1.70969267	0.54704408	0.44540336	0.5621264	0.5223105	Plscr4	1
GO:0051260	protein homooligomerization	1/47	181/14464	0.00552486	1.70024686	0.54127929	0.44722821	0.56381124	0.52387601	Slc1a5	1
GO:0071805	potassium ion transmembrane transport	1/47	182/14464	0.00549451	1.69090484	0.53555008	0.44904717	0.56406541	0.52411218	Kcnk4	1
GO:0002520	immune system development	1/47	183/14464	0.00546448	1.68166492	0.529856	0.45086028	0.56406541	0.52411218	Icosl	1
GO:0008154	actin polymerization or depolymerization	1/47	183/14464	0.00546448	1.68166492	0.529856	0.45086028	0.56406541	0.52411218	Fam107a	1
GO:0009267	cellular response to starvation	1/47	183/14464	0.00546448	1.68166492	0.529856	0.45086028	0.56406541	0.52411218	Sesn1	1
GO:0016051	carbohydrate biosynthetic process	1/47	183/14464	0.00546448	1.68166492	0.529856	0.45086028	0.56406541	0.52411218	Per2	1
GO:0048639	positive regulation of developmental growth	1/47	183/14464	0.00546448	1.68166492	0.529856	0.45086028	0.56406541	0.52411218	Trip10	1
GO:0071383	cellular response to steroid hormone stimulus	1/47	183/14464	0.00546448	1.68166492	0.529856	0.45086028	0.56406541	0.52411218	Fam107a	1
GO:0061136	regulation of proteasomal protein catabolic process	1/47	184/14464	0.00543478	1.67252544	0.5241966	0.45266754	0.56571156	0.52564172	Herpud1	1
GO:0003013	circulatory system process	2/47	470/14464	0.00425532	1.30955183	0.38953999	0.45440713	0.56611884	0.52602016	Per2/Slc1a5	2
GO:0042098	T cell proliferation	1/47	185/14464	0.00540541	1.66348476	0.51857144	0.45446899	0.56611884	0.52602016	Icosl	1
GO:0098727	maintenance of cell number	1/47	185/14464	0.00540541	1.66348476	0.51857144	0.45446899	0.56611884	0.52602016	Tead3	1
GO:0099175	regulation of postsynapse organization	1/47	186/14464	0.00537634	1.65454129	0.51298009	0.45626463	0.56774118	0.52752758	Fam107a	1
GO:0018105	peptidyl-serine phosphorylation	1/47	188/14464	0.00531915	1.63693979	0.50189713	0.45983857	0.56972465	0.52937056	Ip6k2	1
GO:0043524	negative regulation of neuron apoptotic process	1/47	188/14464	0.00531915	1.63693979	0.50189713	0.45983857	0.56972465	0.52937056	Bcl2l1	1
GO:0050870	positive regulation of T cell activation	1/47	188/14464	0.00531915	1.63693979	0.50189713	0.45983857	0.56972465	0.52937056	Icosl	1
GO:0055002	striated muscle cell development	1/47	188/14464	0.00531915	1.63693979	0.50189713	0.45983857	0.56972465	0.52937056	Trip10	1
GO:0061351	neural precursor cell proliferation	1/47	189/14464	0.00529101	1.62827873	0.49640469	0.46161691	0.57131298	0.53084639	Tead3	1
GO:0045732	positive regulation of protein catabolic process	1/47	192/14464	0.00520833	1.60283688	0.48011877	0.46691762	0.5760152	0.53521555	Herpud1	1
GO:0050670	regulation of lymphocyte proliferation	1/47	192/14464	0.00520833	1.60283688	0.48011877	0.46691762	0.5760152	0.53521555	Icosl	1
GO:1901654	response to ketone	1/47	192/14464	0.00520833	1.60283688	0.48011877	0.46691762	0.5760152	0.53521555	Bcl2l1	1
GO:0001763	morphogenesis of a branching structure	1/47	194/14464	0.00515464	1.58631279	0.46941711	0.47042302	0.57767027	0.53675339	Rdh10	1
GO:0006909	phagocytosis	1/47	194/14464	0.00515464	1.58631279	0.46941711	0.47042302	0.57767027	0.53675339	Mertk	1
GO:0030324	lung development	1/47	194/14464	0.00515464	1.58631279	0.46941711	0.47042302	0.57767027	0.53675339	Rdh10	1
GO:0045637	regulation of myeloid cell differentiation	1/47	194/14464	0.00515464	1.58631279	0.46941711	0.47042302	0.57767027	0.53675339	Fstl3	1
GO:0034329	cell junction assembly	2/47	485/14464	0.00412371	1.26905023	0.34411758	0.47076864	0.57767027	0.53675339	Fam107a/Tesk2	2
GO:0045165	cell fate commitment	1/47	195/14464	0.00512821	1.57817785	0.46411185	0.47216725	0.57876944	0.5377747	Tead3	1
GO:0045017	glycerolipid biosynthetic process	1/47	196/14464	0.00510204	1.57012592	0.45883649	0.47390585	0.57993677	0.53885936	Ip6k2	1
GO:0006006	glucose metabolic process	1/47	197/14464	0.00507614	1.56215574	0.45359066	0.47563885	0.57993677	0.53885936	Per2	1
GO:0030323	respiratory tube development	1/47	197/14464	0.00507614	1.56215574	0.45359066	0.47563885	0.57993677	0.53885936	Rdh10	1
GO:0050821	protein stabilization	1/47	197/14464	0.00507614	1.56215574	0.45359066	0.47563885	0.57993677	0.53885936	Ip6k2	1
GO:0051963	regulation of synapse assembly	1/47	197/14464	0.00507614	1.56215574	0.45359066	0.47563885	0.57993677	0.53885936	Fam107a	1
GO:0018209	peptidyl-serine modification	1/47	199/14464	0.00502513	1.54645568	0.44318624	0.4790881	0.58352424	0.54219271	Ip6k2	1
GO:0000302	response to reactive oxygen species	1/47	201/14464	0.00497512	1.53106806	0.43289587	0.48251514	0.58645716	0.5449179	Sesn1	1
GO:0008654	phospholipid biosynthetic process	1/47	201/14464	0.00497512	1.53106806	0.43289587	0.48251514	0.58645716	0.5449179	Ip6k2	1
GO:0009612	response to mechanical stimulus	1/47	202/14464	0.0049505	1.52348852	0.42779263	0.48422038	0.58727746	0.54568009	Kcnk4	1
GO:0017157	regulation of exocytosis	1/47	202/14464	0.0049505	1.52348852	0.42779263	0.48422038	0.58727746	0.54568009	Bcl2l1	1
GO:0044770	cell cycle phase transition	2/47	498/14464	0.00401606	1.23592241	0.30590637	0.48472075	0.58727746	0.54568009	Fam107a/Crlf3	2
GO:0007623	circadian rhythm	1/47	204/14464	0.00490196	1.50855236	0.41766844	0.48761437	0.59016208	0.5483604	Per2	1
GO:0071216	cellular response to biotic stimulus	1/47	205/14464	0.00487805	1.50119357	0.41264687	0.48930316	0.59158397	0.54968157	Plscr4	1
GO:0010810	regulation of cell-substrate adhesion	1/47	206/14464	0.00485437	1.49390622	0.4076519	0.4909865	0.5923747	0.55041629	Fam107a	1
GO:0043270	positive regulation of monoatomic ion transport	1/47	206/14464	0.00485437	1.49390622	0.4076519	0.4909865	0.5923747	0.55041629	Mchr1	1
GO:0043583	ear development	1/47	207/14464	0.00483092	1.48668928	0.40268324	0.49266441	0.59315558	0.55114186	Rdh10	1
GO:1903039	positive regulation of leukocyte cell-cell adhesion	1/47	207/14464	0.00483092	1.48668928	0.40268324	0.49266441	0.59315558	0.55114186	Icosl	1
GO:0016311	dephosphorylation	1/47	209/14464	0.00478469	1.47246259	0.39282365	0.496004	0.59655236	0.55429804	Mtmr2	1
GO:0031348	negative regulation of defense response	1/47	210/14464	0.0047619	1.46545086	0.38793216	0.49766572	0.59730266	0.5549952	Pcbp2	1
GO:0090276	regulation of peptide hormone secretion	1/47	210/14464	0.0047619	1.46545086	0.38793216	0.49766572	0.59730266	0.5549952	Per2	1
GO:0006813	potassium ion transport	1/47	212/14464	0.00471698	1.45162585	0.37822436	0.50097309	0.60002084	0.55752085	Kcnk4	1
GO:0031032	actomyosin structure organization	1/47	212/14464	0.00471698	1.45162585	0.37822436	0.50097309	0.60002084	0.55752085	Ppfia1	1
GO:0002274	myeloid leukocyte activation	1/47	213/14464	0.00469484	1.44481071	0.3734075	0.50261877	0.60011847	0.55761157	Slc7a2	1
GO:0030073	insulin secretion	1/47	213/14464	0.00469484	1.44481071	0.3734075	0.50261877	0.60011847	0.55761157	Per2	1
GO:0043122	regulation of canonical NF-kappaB signal transduction	1/47	213/14464	0.00469484	1.44481071	0.3734075	0.50261877	0.60011847	0.55761157	Otud7b	1
GO:0009165	nucleotide biosynthetic process	1/47	214/14464	0.0046729	1.43805926	0.36861499	0.50425914	0.60020918	0.55769585	Bcl2l1	1
GO:0034764	positive regulation of transmembrane transport	1/47	214/14464	0.0046729	1.43805926	0.36861499	0.50425914	0.60020918	0.55769585	Klf15	1
GO:0048705	skeletal system morphogenesis	1/47	214/14464	0.0046729	1.43805926	0.36861499	0.50425914	0.60020918	0.55769585	Rdh10	1
GO:0002791	regulation of peptide secretion	1/47	215/14464	0.00465116	1.43137061	0.36384655	0.50589422	0.60029304	0.55777377	Per2	1
GO:0051648	vesicle localization	1/47	215/14464	0.00465116	1.43137061	0.36384655	0.50589422	0.60029304	0.55777377	Bcl2l1	1
GO:0090087	regulation of peptide transport	1/47	215/14464	0.00465116	1.43137061	0.36384655	0.50589422	0.60029304	0.55777377	Per2	1
GO:0002429	immune response-activating cell surface receptor signaling pathway	1/47	216/14464	0.00462963	1.42474389	0.35910193	0.50752402	0.6009878	0.55841932	Icosl	1
GO:0042594	response to starvation	1/47	216/14464	0.00462963	1.42474389	0.35910193	0.50752402	0.6009878	0.55841932	Sesn1	1
GO:0048863	stem cell differentiation	1/47	218/14464	0.00458716	1.41167285	0.34968312	0.51076784	0.60296798	0.56025924	Rdh10	1
GO:0055001	muscle cell development	1/47	218/14464	0.00458716	1.41167285	0.34968312	0.51076784	0.60296798	0.56025924	Trip10	1
GO:1903050	regulation of proteolysis involved in protein catabolic process	1/47	218/14464	0.00458716	1.41167285	0.34968312	0.51076784	0.60296798	0.56025924	Herpud1	1
GO:0002440	production of molecular mediator of immune response	1/47	219/14464	0.00456621	1.40522685	0.34500844	0.5123819	0.60425366	0.56145385	Icosl	1
GO:0046034	ATP metabolic process	1/47	220/14464	0.00454545	1.39883946	0.34035658	0.51399075	0.6049114	0.56206501	Bcl2l1	1
GO:0060541	respiratory system development	1/47	220/14464	0.00454545	1.39883946	0.34035658	0.51399075	0.6049114	0.56206501	Rdh10	1
GO:0046434	organophosphate catabolic process	1/47	222/14464	0.0045045	1.3862373	0.33112036	0.51719288	0.60805822	0.56498894	Xdh	1
GO:0090092	regulation of transmembrane receptor protein serine/threonine kinase signaling pathway	1/47	223/14464	0.0044843	1.38002099	0.32653553	0.51878619	0.60930908	0.5661512	Fstl3	1
GO:0045861	negative regulation of proteolysis	1/47	224/14464	0.00446429	1.37386018	0.32197259	0.52037435	0.60992961	0.56672777	Ints1	1
GO:1903522	regulation of blood circulation	1/47	224/14464	0.00446429	1.37386018	0.32197259	0.52037435	0.60992961	0.56672777	Per2	1
GO:0002768	immune response-regulating cell surface receptor signaling pathway	1/47	227/14464	0.00440529	1.35570344	0.30841282	0.52510813	0.61422709	0.57072086	Icosl	1
GO:0019058	viral life cycle	1/47	227/14464	0.00440529	1.35570344	0.30841282	0.52510813	0.61422709	0.57072086	Pcbp2	1
GO:0072522	purine-containing compound biosynthetic process	1/47	228/14464	0.00438596	1.34975737	0.30393519	0.52667587	0.61543546	0.57184364	Bcl2l1	1
GO:1901136	carbohydrate derivative catabolic process	1/47	229/14464	0.00436681	1.34386324	0.29947834	0.52823854	0.61663546	0.57295864	Xdh	1
GO:0019318	hexose metabolic process	1/47	230/14464	0.00434783	1.33802035	0.29504208	0.52979617	0.61720181	0.57348488	Per2	1
GO:0031331	positive regulation of cellular catabolic process	1/47	230/14464	0.00434783	1.33802035	0.29504208	0.52979617	0.61720181	0.57348488	Sesn1	1
GO:0045055	regulated exocytosis	1/47	231/14464	0.004329	1.33222806	0.29062619	0.53134876	0.61838465	0.57458394	Bcl2l1	1
GO:0002221	pattern recognition receptor signaling pathway	1/47	232/14464	0.00431034	1.32648569	0.28623046	0.53289633	0.61893408	0.57509445	Pcbp2	1
GO:0002573	myeloid leukocyte differentiation	1/47	232/14464	0.00431034	1.32648569	0.28623046	0.53289633	0.61893408	0.57509445	Fstl3	1
GO:0002758	innate immune response-activating signaling pathway	1/47	235/14464	0.00425532	1.30955183	0.27316232	0.5375091	0.62240742	0.57832177	Pcbp2	1
GO:0071900	regulation of protein serine/threonine kinase activity	1/47	235/14464	0.00425532	1.30955183	0.27316232	0.5375091	0.62240742	0.57832177	Ip6k2	1
GO:0090257	regulation of muscle system process	1/47	235/14464	0.00425532	1.30955183	0.27316232	0.5375091	0.62240742	0.57832177	Trip10	1
GO:0002449	lymphocyte mediated immunity	1/47	236/14464	0.00423729	1.30400288	0.2688453	0.53903676	0.62292301	0.57880084	Icosl	1

GO:0007249	canonical NF-kappaB signal transduction	1/47	236/14464	0.00423729	1.30400288	0.2688453	0.53903676	0.62292301	0.57880084	Otud7b	1
GO:0048738	cardiac muscle tissue development	1/47	237/14464	0.00421941	1.29850076	0.26454748	0.54055948	0.62405613	0.5798537	Trip10	1
GO:0006520	amino acid metabolic process	1/47	238/14464	0.00420168	1.29304488	0.26026866	0.54207728	0.62518131	0.58089918	Prodh	1
GO:0042113	B cell activation	1/47	240/14464	0.00416667	1.2822695	0.25176733	0.54509817	0.62740799	0.58296815	Icosl	1
GO:0051924	regulation of calcium ion transport	1/47	240/14464	0.00416667	1.2822695	0.25176733	0.54509817	0.62740799	0.58296815	Mchr1	1
GO:0002366	leukocyte activation involved in immune response	1/47	241/14464	0.00414938	1.27694888	0.24754446	0.54660129	0.62788232	0.58340888	Icosl	1
GO:0045444	fat cell differentiation	1/47	241/14464	0.00414938	1.27694888	0.24754446	0.54660129	0.62788232	0.58340888	Per2	1
GO:0009205	purine ribonucleoside triphosphate metabolic process	1/47	243/14464	0.00411523	1.26643902	0.2391534	0.54959296	0.63068943	0.58601716	Bcl2l1	1
GO:0051259	protein complex oligomerization	1/47	244/14464	0.00409836	1.26124869	0.23498488	0.55108154	0.63176778	0.58701913	Slc1a5	1
GO:0002263	cell activation involved in immune response	1/47	245/14464	0.00408163	1.25610074	0.23083413	0.55256531	0.63220941	0.58742948	Icosl	1
GO:0032886	regulation of microtubule-based process	1/47	245/14464	0.00408163	1.25610074	0.23083413	0.55256531	0.63220941	0.58742948	Fam107a	1
GO:0045860	positive regulation of protein kinase activity	1/47	246/14464	0.00406504	1.25099464	0.22670099	0.55404428	0.63327206	0.58841686	Ip6k2	1
GO:0032984	protein-containing complex disassembly	1/47	247/14464	0.00404858	1.24592988	0.22258529	0.55551846	0.63369846	0.58881305	Per2	1
GO:0097305	response to alcohol	1/47	247/14464	0.00404858	1.24592988	0.22258529	0.55551846	0.63369846	0.58881305	Bcl2l1	1
GO:0009199	ribonucleoside triphosphate metabolic process	1/47	248/14464	0.00403226	1.24090597	0.21848688	0.55698787	0.63474558	0.58978601	Bcl2l1	1
GO:0099504	synaptic vesicle cycle	1/47	249/14464	0.00401606	1.23592241	0.2144056	0.55845253	0.63578522	0.59075201	Bcl2l1	1
GO:0005996	monosaccharide metabolic process	1/47	250/14464	0.004	1.23097872	0.21034127	0.55991245	0.63681742	0.59171109	Per2	1
GO:0009144	purine nucleoside triphosphate metabolic process	1/47	251/14464	0.00398406	1.22607443	0.20629376	0.56136764	0.6378422	0.59266329	Bcl2l1	1
GO:0010721	negative regulation of cell development	1/47	252/14464	0.00396825	1.22120905	0.2022629	0.56281812	0.63885962	0.59360865	Fstl3	1
GO:0046651	lymphocyte proliferation	1/47	253/14464	0.00395257	1.21638214	0.19824855	0.56426391	0.63898575	0.59372584	Icosl	1
GO:0006898	receptor-mediated endocytosis	1/47	254/14464	0.00393701	1.21159323	0.19425055	0.56570502	0.63898575	0.59372584	Mtmr2	1
GO:0048562	embryonic organ morphogenesis	1/47	254/14464	0.00393701	1.21159323	0.19425055	0.56570502	0.63898575	0.59372584	Rdh10	1
GO:0048588	developmental cell growth	1/47	254/14464	0.00393701	1.21159323	0.19425055	0.56570502	0.63898575	0.59372584	Trip10	1
GO:0060560	developmental growth involved in morphogenesis	1/47	254/14464	0.00393701	1.21159323	0.19425055	0.56570502	0.63898575	0.59372584	Rdh10	1
GO:0030072	peptide hormone secretion	1/47	256/14464	0.00390625	1.20212766	0.18630303	0.56857326	0.6415959	0.59615111	Per2	1
GO:0002218	activation of innate immune response	1/47	258/14464	0.00387597	1.19280884	0.17841919	0.57142295	0.64229279	0.59679864	Pcbp2	1
GO:0033674	positive regulation of kinase activity	1/47	258/14464	0.00387597	1.19280884	0.17841919	0.57142295	0.64229279	0.59679864	Ip6k2	1
GO:0045927	positive regulation of growth	1/47	258/14464	0.00387597	1.19280884	0.17841919	0.57142295	0.64229279	0.59679864	Trip10	1
GO:0110053	regulation of actin filament organization	1/47	258/14464	0.00387597	1.19280884	0.17841919	0.57142295	0.64229279	0.59679864	Ppfia1	1
GO:0051251	positive regulation of lymphocyte activation	1/47	260/14464	0.00384615	1.18363339	0.17059789	0.57425422	0.64484547	0.59917051	Icosl	1
GO:0046883	regulation of hormone secretion	1/47	261/14464	0.00383142	1.17909839	0.16671035	0.57566298	0.64579736	0.60005498	Per2	1
GO:0002460	adaptive immune response based on somatic recombination of immune receptors built from immunoglobu	1/47	263/14464	0.00380228	1.17013187	0.15898083	0.57846683	0.64642264	0.60063598	Icosl	1
GO:0002790	peptide secretion	1/47	263/14464	0.00380228	1.17013187	0.15898083	0.57846683	0.64642264	0.60063598	Per2	1
GO:0007162	negative regulation of cell adhesion	1/47	263/14464	0.00380228	1.17013187	0.15898083	0.57846683	0.64642264	0.60063598	Fam107a	1
GO:0050708	regulation of protein secretion	1/47	263/14464	0.00380228	1.17013187	0.15898083	0.57846683	0.64642264	0.60063598	Per2	1
GO:0009141	nucleoside triphosphate metabolic process	1/47	265/14464	0.00377358	1.16130068	0.15131115	0.58125255	0.64764926	0.6017757	Bcl2l1	1
GO:0032868	response to insulin	1/47	265/14464	0.00377358	1.16130068	0.15131115	0.58125255	0.64764926	0.6017757	Klf15	1
GO:0042593	glucose homeostasis	1/47	265/14464	0.00377358	1.16130068	0.15131115	0.58125255	0.64764926	0.6017757	Klf15	1
GO:0033500	carbohydrate homeostasis	1/47	266/14464	0.0037594	1.15693489	0.14749844	0.58263864	0.64856584	0.60262736	Klf15	1
GO:0010001	glial cell differentiation	1/47	272/14464	0.00367647	1.13141427	0.12492399	0.59086139	0.65644928	0.60995242	Klf15	1
GO:0015833	peptide transport	1/47	272/14464	0.00367647	1.13141427	0.12492399	0.59086139	0.65644928	0.60995242	Per2	1
GO:0051258	protein polymerization	1/47	273/14464	0.003663	1.12726989	0.12121076	0.59221634	0.65732016	0.61076161	Fam107a	1
GO:0032386	regulation of intracellular transport	1/47	277/14464	0.00361011	1.11099163	0.10649409	0.59759238	0.66264819	0.61571226	Mtmr2	1
GO:0006605	protein targeting	1/47	278/14464	0.00359712	1.10699525	0.10284842	0.59892551	0.66284929	0.61589911	Herpud1	1
GO:0006874	intracellular calcium ion homeostasis	1/47	278/14464	0.00359712	1.10699525	0.10284842	0.59892551	0.66284929	0.61589911	Herpud1	1
GO:0006310	DNA recombination	1/47	279/14464	0.00358423	1.10302753	0.09921593	0.60025432	0.66368177	0.61667262	Icosl	1
GO:0001933	negative regulation of protein phosphorylation	1/47	281/14464	0.00355872	1.0951768	0.09199006	0.60289903	0.6659662	0.61879524	Xdh	1
GO:0030111	regulation of Wnt signaling pathway	1/47	282/14464	0.0035461	1.09129319	0.08839646	0.60421495	0.66631083	0.61911546	Klf15	1
GO:0002696	positive regulation of leukocyte activation	1/47	283/14464	0.00353357	1.08743703	0.08481563	0.60552661	0.66631083	0.61911546	Icosl	1
GO:0007416	synapse assembly	1/47	283/14464	0.00353357	1.08743703	0.08481563	0.60552661	0.66631083	0.61911546	Fam107a	1
GO:0009416	response to light stimulus	1/47	283/14464	0.00353357	1.08743703	0.08481563	0.60552661	0.66631083	0.61911546	Per2	1
GO:0006790	sulfur compound metabolic process	1/47	285/14464	0.00350877	1.0798059	0.07769182	0.60813717	0.6685443	0.62119073	Fmo1	1
GO:1901293	nucleoside phosphate biosynthetic process	1/47	286/14464	0.0034965	1.07603035	0.07414864	0.6094361	0.6686949	0.62133067	Bcl2l1	1
GO:1902105	regulation of leukocyte differentiation	1/47	286/14464	0.0034965	1.07603035	0.07414864	0.6094361	0.6686949	0.62133067	Fstl3	1
GO:1901888	regulation of cell junction assembly	1/47	288/14464	0.00347222	1.06855792	0.06709925	0.61202133	0.67089196	0.6233721	Fam107a	1
GO:0001666	response to hypoxia	1/47	289/14464	0.00346021	1.06486049	0.06359285	0.61330766	0.67166234	0.62408792	Hif3a	1
GO:0048545	response to steroid hormone	1/47	290/14464	0.00344828	1.06118855	0.0600985	0.61458982	0.67242669	0.62479813	Fam107a	1
GO:0048511	rhythmic process	1/47	291/14464	0.00343643	1.05754186	0.05661613	0.61586781	0.67318504	0.62550277	Per2	1
GO:0141091	transforming growth factor beta receptor superfamily signaling pathway	1/47	292/14464	0.00342466	1.05392014	0.05314563	0.61714165	0.67329862	0.6256083	Fstl3	1
GO:1903037	regulation of leukocyte cell-cell adhesion	1/47	292/14464	0.00342466	1.05392014	0.05314563	0.61714165	0.67329862	0.6256083	Icosl	1
GO:0050727	regulation of inflammatory response	1/47	294/14464	0.00340136	1.04675062	0.04623988	0.61967695	0.67542441	0.62758352	Slc7a2	1
GO:0010506	regulation of autophagy	1/47	295/14464	0.00338983	1.04320231	0.04280446	0.62093844	0.67551998	0.62767232	Sesn1	1
GO:0055074	calcium ion homeostasis	1/47	295/14464	0.00338983	1.04320231	0.04280446	0.62093844	0.67551998	0.62767232	Herpud1	1
GO:0050863	regulation of T cell activation	1/47	297/14464	0.003367	1.03617738	0.03596806	0.62344912	0.6776109	0.62961514	Icosl	1
GO:0016032	viral process	1/47	301/14464	0.00332226	1.02240758	0.02243062	0.62842177	0.68172805	0.63344066	Pcbp2	1
GO:0042326	negative regulation of phosphorylation	1/47	301/14464	0.00332226	1.02240758	0.02243062	0.62842177	0.68172805	0.63344066	Xdh	1
GO:0050867	positive regulation of cell activation	1/47	303/14464	0.00330033	1.01565901	0.01572821	0.63088393	0.68311139	0.63472603	Icosl	1
GO:0099003	vesicle-mediated transport in synapse	1/47	303/14464	0.00330033	1.01565901	0.01572821	0.63088393	0.68311139	0.63472603	Bcl2l1	1
GO:0036293	response to decreased oxygen levels	1/47	304/14464	0.00328947	1.01231803	0.0123933	0.63210902	0.68379463	0.63536087	Hif3a	1
GO:0034504	protein localization to nucleus	1/47	306/14464	0.00326797	1.00570157	0.00575566	0.63454727	0.68514438	0.63661501	Otud7b	1
GO:0051347	positive regulation of transferase activity	1/47	306/14464	0.00326797	1.00570157	0.00575566	0.63454727	0.68514438	0.63661501	Ip6k2	1
GO:0032102	negative regulation of response to external stimulus	1/47	308/14464	0.00324675	0.99917104	-0.0008396	0.6369697	0.68711539	0.63844642	Pcbp2	1
GO:0016236	macroautophagy	1/47	309/14464	0.00323625	0.99593748	-0.0041216	0.63817501	0.68777101	0.6390556	Sesn1	1
GO:0045089	positive regulation of innate immune response	1/47	310/14464	0.00322581	0.99272478	-0.0073931	0.63937641	0.68842118	0.63965972	Pcbp2	1
GO:0010038	response to metal ion	1/47	311/14464	0.00321543	0.98953274	-0.0106544	0.6405739	0.68842256	0.639661	Xdh	1
GO:0051146	striated muscle cell differentiation	1/47	311/14464	0.00321543	0.98953274	-0.0106544	0.6405739	0.68842256	0.639661	Trip10	1
GO:0007178	cell surface receptor protein serine/threonine kinase signaling pathway	1/47	313/14464	0.00319489	0.98320984	-0.0171464	0.64295722	0.69032465	0.64142837	Fstl3	1
GO:0042886	amide transport	1/47	314/14464	0.00318471	0.9800786	-0.0203773	0.64414307	0.69032465	0.64142837	Per2	1
GO:0046879	hormone secretion	1/47	314/14464	0.00318471	0.9800786	-0.0203773	0.64414307	0.69032465	0.64142837	Per2	1
GO:1903532	positive regulation of secretion by cell	1/47	315/14464	0.0031746	0.97696724	-0.0235982	0.64532507	0.69094805	0.64200761	Bcl2l1	1
GO:0051345	positive regulation of hydrolase activity	1/47	317/14464	0.00315457	0.97080341	-0.0300103	0.64767755	0.69282236	0.64374916	Xdh	1
GO:0030522	intracellular receptor signaling pathway	1/47	318/14464	0.00314465	0.96775057	-0.0332016	0.64884806	0.69343001	0.64431376	Pcbp2	1
GO:0009914	hormone transport	1/47	321/14464	0.00311526	0.95870617	-0.0427177	0.65233679	0.69586621	0.64657741	Per2	1
GO:0042060	wound healing	1/47	321/14464	0.00311526	0.95870617	-0.0427177	0.65233679	0.69586621	0.64657741	Mertk	1
GO:0060562	epithelial tube morphogenesis	1/47	323/14464	0.00309598	0.95276991	-0.0490141	0.65464374	0.6976805	0.6482632	Rdh10	1
GO:0007159	leukocyte cell-cell adhesion	1/47	326/14464	0.00306748	0.94400209	-0.0583885	0.6580761	0.7006038	0.65097943	Icosl	1
GO:0002443	leukocyte mediated immunity	1/47	327/14464	0.0030581	0.94111523	-0.0614949	0.65921278	0.7006038	0.65097943	Icosl	1
GO:0070482	response to oxygen levels	1/47	327/14464	0.0030581	0.94111523	-0.0614949	0.65921278	0.7006038	0.65097943	Hif3a	1
GO:0009895	negative regulation of catabolic process	1/47	330/14464	0.0030303	0.93255964	-0.0707595	0.66260069	0.70280107	0.65302107	Mtmr2	1
GO:0015980	energy derivation by oxidation of organic compounds	1/47	330/14464	0.0030303	0.93255964	-0.0707595	0.66260069	0.70280107	0.65302107	Per2	1
GO:0002833	positive regulation of response to biotic stimulus	1/47	331/14464	0.00302115	0.92974224	-0.0738298	0.66372264	0.70280107	0.65302107	Pcbp2	1
GO:0048638	regulation of developmental growth	1/47	331/14464	0.00302115	0.92974224	-0.0738298	0.66372264	0.70280107	0.65302107	Trip10	1
GO:0051047	positive regulation of secretion	1/47	334/14464	0.00299401	0.92139126	-0.0829877	0.66706666	0.70569276	0.65570794	Bcl2l1	1
GO:0060271	cilium assembly	1/47	339/14464	0.00294985	0.90780142	-0.0980778	0.67256783	0.71085911	0.66050835	Cfap157	1
GO:0006869	lipid transport	1/47	342/14464	0.00292398	0.89983825	-0.1070305	0.67582572	0.71364716	0.66309892	Plscr4	1
GO:0042176	regulation of protein catabolic process	1/47	344/14464	0.00290698	0.89460663	-0.1129577	0.67798	0.71443209	0.66382826	Herpud1	1
GO:0050890	cognition	1/47	344/14464	0.00290698	0.89460663	-0.1129577	0.67798	0.71443209	0.66382826	Fam107a	1
GO:0010563	negative regulation of phosphorus metabolic process	1/47	345/14464	0.00289855	0.89201357	-0.1159091	0.67905188	0.71443209	0.66382826	Xdh	1
GO:0045936	negative regulation of phosphate metabolic process	1/47	345/14464	0.00289855	0.89201357	-0.1159091	0.67905188	0.71443209	0.66382826	Xdh	1
GO:0050678	regulation of epithelial cell proliferation	1/47	346/14464	0.00289017	0.88943549	-0.1188524	0.68012027	0.71490267	0.6642655	Xdh	1
GO:0006887	exocytosis	1/47	352/14464	0.00284091	0.87427466	-0.1363454	0.68645787	0.72024887	0.66923303	Bcl2l1	1
GO:0009306	protein secretion	1/47	352/14464	0.00284091	0.87427466	-0.1363454	0.68645787	0.72024887	0.66923303	Per2	1
GO:0035592	establishment of protein localization to extracellular region	1/47	354/14464	0.00282486	0.86933526	-0.1421141	0.68854297	0.72177865	0.67065445	Per2	1
GO:0006935	chemotaxis	1/47	356/14464	0.00280899	0.86445135	-0.1478523	0.6906145	0.72305506	0.67184045	Maco1	1
GO:0042330	taxis	1/47	357/14464	0.00280112	0.86202992	-0.1507101	0.6916452	0.72305506	0.67184045	Maco1	1
GO:0071692	protein localization to extracellular region	1/47	357/14464	0.00280112	0.86202992	-0.1507101	0.6916452	0.72305506	0.67184045	Per2	1
GO:0001503	ossification	1/47	359/14464	0.00278552	0.85722752	-0.1564033	0.69369653	0.7232289	0.67200198	Fstl3	1
GO:0007018	microtubule-based movement	1/47	359/14464	0.00278552	0.85722752	-0.1564033	0.69369653	0.7232289	0.67200198	Cfap157	1
GO:0030098	lymphocyte differentiation	1/47	359/14464	0.00278552	0.85722752	-0.1564033	0.69369653	0.7232289	0.67200198	Mertk	1
GO:1903706	regulation of hemopoiesis	1/47	362/14464	0.00276243	0.85012343	-0.1648877	0.6967485	0.72575342	0.67434768	Fstl3	1
GO:0042063	gliogenesis	1/47	366/14464	0.00273224	0.84083246	-0.176099	0.70077154	0.72928395	0.67762814	Klf15	1
GO:0044782	cilium organization	1/47	369/14464	0.00271003	0.83399643	-0.1844331	0.7037545	0.73172667	0.67989784	Cfap157	1
GO:0050807	regulation of synapse organization	1/47	370/14464	0.0027027	0.83174238	-0.1871971	0.70474233	0.73209244	0.6802377	Fam107a	1
GO:0045088	regulation of innate immune response	1/47	374/14464	0.0026738	0.82284674	-0.1981847	0.70866153	0.73483732	0.68278816	Pcbp2	1
GO:1902903	regulation of supramolecular fiber organization	1/47	374/14464	0.0026738	0.82284674	-0.1981847	0.70866153	0.73483732	0.68278816	Ppfia1	1
GO:0010959	regulation of metal ion transport	1/47	375/14464	0.00266667	0.82065248	-0.2009146	0.70963335	0.73486334	0.68281234	Mchr1	1
GO:0043603	amide metabolic process	1/47	376/14464	0.00265957	0.8184699	-0.2036379	0.710602	0.73486334	0.68281234	Xdh	1
GO:0050803	regulation of synapse structure or activity	1/47	376/14464	0.00265957	0.8184699	-0.2036379	0.710602	0.73486334	0.68281234	Fam107a	1
GO:0006816	calcium ion transport	1/47	377/14464	0.00265252	0.81629889	-0.2063545	0.71156748	0.73520123	0.68312629	Mchr1	1
GO:0016055	Wnt signaling pathway	1/47	385/14464	0.0025974	0.79933683	-0.227852	0.71917868	0.74239879	0.68981405	Klf15	1
GO:0001819	positive regulation of cytokine production	1/47	386/14464	0.00259067	0.79726601	-0.2305103	0.72011614	0.74270043	0.69009432	Icosl	1
GO:0006979	response to oxidative stress	1/47	388/14464	0.00257732	0.79315639	-0.2358083	0.7219819	0.74395807	0.69126288	Sesn1	1
GO:0010876	lipid localization	1/47	393/14464	0.00254453	0.78306535	-0.248945	0.72659319	0.74804004	0.69505572	Plscr4	1
GO:0043086	negative regulation of catalytic activity	1/47	398/14464	0.00251256	0.77322784	-0.2619308	0.7311296	0.75203769	0.69877021	Ints1	1
GO:0022411	cellular component disassembly	1/47	400/14464	0.0025	0.7693617	-0.2670839	0.73292346	0.75320974	0.69985925	Per2	1
GO:0048732	gland development	1/47	408/14464	0.00245098	0.75427618	-0.2874664	0.73998251	0.75978579	0.70596951	Xdh	1
GO:0031349	positive regulation of defense response	1/47	410/14464	0.00243902	0.75059678	-0.2925059	0.74171854	0.76021197	0.70636551	Pcbp2	1
GO:0043434	response to peptide hormone	1/47	410/14464	0.00243902	0.75059678	-0.2925059	0.74171854	0.76021197	0.70636551	Klf15	1
GO:0043161	proteasome-mediated ubiquitin-dependent protein catabolic process	1/47	412/14464	0.00242718	0.74695311	-0.2975235	0.74344323	0.76130174	0.70737809	Pcbp2	1
GO:0014706	striated muscle tissue development	1/47	414/14464	0.00241546	0.74334464	-0.3025193	0.74515664	0.76189576	0.70793003	Trip10	1
GO:0051051	negative regulation of transport	1/47	415/14464	0.00240964	0.74155345	-0.3050091	0.74600914	0.76189576	0.70793003	Mtmr2	1
GO:0072594	establishment of protein localization to organelle	1/47	415/14464	0.00240964	0.74155345	-0.3050091	0.74600914	0.76189576	0.70793003	Herpud1	1
GO:0050767	regulation of neurogenesis	1/47	418/14464	0.00239234	0.73623129	-0.3124466	0.74854993	0.76381292	0.70971139	Per2	1
GO:0043410	positive regulation of MAPK cascade	1/47	419/14464	0.00238663	0.73447418	-0.3149152	0.74939132	0.76399416	0.7098798	Xdh	1
GO:0042692	muscle cell differentiation	1/47	423/14464	0.00236407	0.7275288	-0.3247375	0.75272941	0.76671818	0.71241088	Trip10	1
GO:0045859	regulation of protein kinase activity	1/47	426/14464	0.00234742	0.72240535	-0.3320503	0.75520438	0.76855901	0.71412132	Ip6k2	1
GO:0060537	muscle tissue development	1/47	435/14464	0.00229885	0.70745904	-0.3537192	0.76248471	0.7752826	0.72036867	Trip10	1
GO:0043269	regulation of monoatomic ion transport	1/47	440/14464	0.00227273	0.69941973	-0.3655877	0.76643732	0.77861373	0.72346386	Mchr1	1
GO:0008015	blood circulation	1/47	443/14464	0.00225734	0.69468325	-0.3726522	0.76877791	0.7803028	0.72503329	Per2	1
GO:0002831	regulation of response to biotic stimulus	1/47	446/14464	0.00224215	0.6900105	-0.3796751	0.77109554	0.78136705	0.72602215	Pcbp2	1
GO:0030003	intracellular monoatomic cation homeostasis	1/47	447/14464	0.00223714	0.68846685	-0.3820069	0.77186302	0.78136705	0.72602215	Herpud1	1
GO:1903131	mononuclear cell differentiation	1/47	447/14464	0.00223714	0.68846685	-0.3820069	0.77186302	0.78136705	0.72602215	Mertk	1
GO:0006091	generation of precursor metabolites and energy	1/47	449/14464	0.00222717	0.68540018	-0.3866569	0.77339043	0.78222529	0.7268196	Per2	1
GO:0051223	regulation of protein transport	1/47	452/14464	0.00221239	0.68085106	-0.3935983	0.77566279	0.78383483	0.72831514	Per2	1
GO:0006873	intracellular monoatomic ion homeostasis	1/47	454/14464	0.00220264	0.67785172	-0.3982038	0.7771653	0.78397656	0.72844683	Herpud1	1
GO:0098771	inorganic ion homeostasis	1/47	454/14464	0.00220264	0.67785172	-0.3982038	0.7771653	0.78397656	0.72844683	Herpud1	1
GO:0043549	regulation of kinase activity	1/47	456/14464	0.00219298	0.67487869	-0.4027917	0.77865795	0.78479448	0.72920682	Ip6k2	1
GO:0071407	cellular response to organic cyclic compound	1/47	462/14464	0.0021645	0.66611403	-0.4164519	0.78307744	0.78855829	0.73270403	Fam107a	1
GO:0042110	T cell activation	1/47	464/14464	0.00215517	0.66324285	-0.4209713	0.78453132	0.78871664	0.73285116	Icosl	1
GO:0009611	response to wounding	1/47	465/14464	0.00215054	0.66181652	-0.4232247	0.78525467	0.78871664	0.73285116	Mertk	1
GO:0001501	skeletal system development	1/47	466/14464	0.00214592	0.66039631	-0.425474	0.78597566	0.78871664	0.73285116	Rdh10	1
GO:0007005	mitochondrion organization	1/47	466/14464	0.00214592	0.66039631	-0.425474	0.78597566	0.78871664	0.73285116	Bcl2l1	1
GO:0002009	morphogenesis of an epithelium	1/47	470/14464	0.00212766	0.65477592	-0.4344299	0.78883597	0.79089739	0.73487745	Rdh10	1
GO:0034655	nucleobase-containing compound catabolic process	1/47	471/14464	0.00212314	0.65338573	-0.4366587	0.78954518	0.7909195	0.73489799	Xdh	1
GO:0005975	carbohydrate metabolic process	1/47	491/14464	0.00203666	0.62677124	-0.4804096	0.80324965	0.80394813	0.74700379	Per2	1
GO:0051604	protein maturation	1/47	498/14464	0.00200803	0.61796121	-0.4953667	0.80783671	0.80783671	0.75061694	Pcbp2	1

ID	Description	GeneRatio	BgRatio	RichFactor	FoldEnrichment	zScore	pvalue	p.adjust	qvalue	geneID	Count
GO:0036499	PERK-mediated unfolded protein response	2/40	16/14464	0.125	45.2	9.31525453	0.00087319	0.34729843	0.32084266	Bok/Ptpn1	2
GO:0097193	intrinsic apoptotic signaling pathway	5/40	302/14464	0.01655629	5.98675497	4.61185386	0.00138771	0.34729843	0.32084266	Bok/Rrp8/Pnp/Ptpn1/Fbh1	5
GO:0035561	regulation of chromatin binding	2/40	21/14464	0.0952381	34.4380952	8.0749307	0.00151479	0.34729843	0.32084266	Ctbp2/Fbh1	2
GO:0140467	integrated stress response signaling	2/40	25/14464	0.08	28.928	7.35965767	0.00214891	0.34729843	0.32084266	Bok/Ptpn1	2
GO:1900101	regulation of endoplasmic reticulum unfolded protein response	2/40	26/14464	0.07692308	27.8153846	7.20665145	0.00232393	0.34729843	0.32084266	Bok/Ptpn1	2
GO:0097345	mitochondrial outer membrane permeabilization	2/40	28/14464	0.07142857	25.8285714	6.92506163	0.00269348	0.34729843	0.32084266	Bok/Rhot1	2
GO:1902235	regulation of endoplasmic reticulum stress-induced intrinsic apoptotic signaling pathway	2/40	29/14464	0.06896552	24.937931	6.79506395	0.00288795	0.34729843	0.32084266	Bok/Ptpn1	2
GO:0008630	intrinsic apoptotic signaling pathway in response to DNA damage	3/40	105/14464	0.02857143	10.3314286	5.05355527	0.0030219	0.34729843	0.32084266	Bok/Pnp/Fbh1	3
GO:1902110	positive regulation of mitochondrial membrane permeability involved in apoptotic process	2/40	32/14464	0.0625	22.6	6.44142289	0.00350972	0.34729843	0.32084266	Bok/Rhot1	2
GO:0008637	apoptotic mitochondrial changes	3/40	114/14464	0.02631579	9.51578947	4.80692918	0.00381044	0.34729843	0.32084266	Bok/Pnp/Rhot1	3
GO:1902686	mitochondrial outer membrane permeabilization involved in programmed cell death	2/40	34/14464	0.05882353	21.2705882	6.23144802	0.00395584	0.34729843	0.32084266	Bok/Rhot1	2
GO:0035794	positive regulation of mitochondrial membrane permeability	2/40	37/14464	0.05405405	19.5459459	5.94809872	0.00467174	0.34729843	0.32084266	Bok/Rhot1	2
GO:1902108	regulation of mitochondrial membrane permeability involved in apoptotic process	2/40	39/14464	0.05128205	18.5435897	5.77709072	0.00517975	0.34729843	0.32084266	Bok/Rhot1	2
GO:0006984	ER-nucleus signaling pathway	2/40	43/14464	0.04651163	16.8186047	5.47042469	0.00626834	0.34729843	0.32084266	Bok/Ptpn1	2
GO:1905710	positive regulation of membrane permeability	2/40	43/14464	0.04651163	16.8186047	5.47042469	0.00626834	0.34729843	0.32084266	Bok/Rhot1	2
GO:0046902	regulation of mitochondrial membrane permeability	2/40	57/14464	0.03508772	12.6877193	4.65582614	0.01081245	0.34729843	0.32084266	Bok/Rhot1	2
GO:1904035	regulation of epithelial cell apoptotic process	2/40	59/14464	0.03389831	12.2576271	4.56281191	0.01155136	0.34729843	0.32084266	Bok/Capn10	2
GO:2001242	regulation of intrinsic apoptotic signaling pathway	3/40	172/14464	0.01744186	6.30697674	3.68706875	0.01182761	0.34729843	0.32084266	Bok/Ptpn1/Fbh1	3
GO:1902806	regulation of cell cycle G1/S phase transition	3/40	175/14464	0.01714286	6.19885714	3.64369756	0.01239049	0.34729843	0.32084266	Plk5/Fam83d/Ctdspl	3
GO:0070059	intrinsic apoptotic signaling pathway in response to endoplasmic reticulum stress	2/40	62/14464	0.03225806	11.6645161	4.43140995	0.01270027	0.34729843	0.32084266	Bok/Ptpn1	2
GO:2001244	positive regulation of intrinsic apoptotic signaling pathway	2/40	62/14464	0.03225806	11.6645161	4.43140995	0.01270027	0.34729843	0.32084266	Bok/Fbh1	2
GO:0031102	neuron projection regeneration	2/40	71/14464	0.02816901	10.1859155	4.08594286	0.01643123	0.34729843	0.32084266	Matn2/Cntf	2
GO:0030968	endoplasmic reticulum unfolded protein response	2/40	72/14464	0.02777778	10.0444444	4.05138859	0.01687144	0.34729843	0.32084266	Bok/Ptpn1	2
GO:0090559	regulation of membrane permeability	2/40	72/14464	0.02777778	10.0444444	4.05138859	0.01687144	0.34729843	0.32084266	Bok/Rhot1	2
GO:0042063	gliogenesis	4/40	366/14464	0.01092896	3.95191257	3.01218479	0.01799287	0.34729843	0.32084266	Matn2/Bok/Cntf/Arhgef10	4
GO:0031400	negative regulation of protein modification process	4/40	378/14464	0.01058201	3.82645503	2.93231397	0.02000407	0.34729843	0.32084266	Cry1/Pkib/Ctdspl/Ptpn1	4
GO:1905897	regulation of response to endoplasmic reticulum stress	2/40	81/14464	0.02469136	8.92839506	3.76807366	0.02105576	0.34729843	0.32084266	Bok/Ptpn1	2
GO:0034620	cellular response to unfolded protein	2/40	84/14464	0.02380952	8.60952381	3.68327387	0.02253723	0.34729843	0.32084266	Bok/Ptpn1	2
GO:0071901	negative regulation of protein serine/threonine kinase activity	2/40	84/14464	0.02380952	8.60952381	3.68327387	0.02253723	0.34729843	0.32084266	Pkib/Ptpn1	2
GO:0072332	intrinsic apoptotic signaling pathway by p53 class mediator	2/40	86/14464	0.02325581	8.40930233	3.6290557	0.02354829	0.34729843	0.32084266	Bok/Rrp8	2
GO:1901987	regulation of cell cycle phase transition	4/40	402/14464	0.00995025	3.59800995	2.78193271	0.02443334	0.34729843	0.32084266	Plk5/Cry1/Fam83d/Ctdspl	4
GO:1904019	epithelial cell apoptotic process	2/40	91/14464	0.02197802	7.94725275	3.50087347	0.02615625	0.34729843	0.32084266	Bok/Capn10	2
GO:0044843	cell cycle G1/S phase transition	3/40	233/14464	0.01287554	4.65579399	2.96249958	0.02632204	0.34729843	0.32084266	Plk5/Fam83d/Ctdspl	3
GO:0048678	response to axon injury	2/40	92/14464	0.02173913	7.86086957	3.47640851	0.02669138	0.34729843	0.32084266	Matn2/Cntf	2
GO:0002357	defense response to tumor cell	1/40	10/14464	0.1	36.16	5.85694537	0.02732163	0.34729843	0.32084266	Plk5	1
GO:0032263	GMP salvage	1/40	10/14464	0.1	36.16	5.85694537	0.02732163	0.34729843	0.32084266	Pnp	1
GO:0042451	purine nucleoside biosynthetic process	1/40	10/14464	0.1	36.16	5.85694537	0.02732163	0.34729843	0.32084266	Pnp	1
GO:0042455	ribonucleoside biosynthetic process	1/40	10/14464	0.1	36.16	5.85694537	0.02732163	0.34729843	0.32084266	Pnp	1
GO:0046129	purine ribonucleoside biosynthetic process	1/40	10/14464	0.1	36.16	5.85694537	0.02732163	0.34729843	0.32084266	Pnp	1
GO:1902231	positive regulation of intrinsic apoptotic signaling pathway in response to DNA damage	1/40	10/14464	0.1	36.16	5.85694537	0.02732163	0.34729843	0.32084266	Fbh1	1
GO:1990700	nucleolar chromatin organization	1/40	10/14464	0.1	36.16	5.85694537	0.02732163	0.34729843	0.32084266	Rrp8	1
GO:0006183	GTP biosynthetic process	1/40	11/14464	0.09090909	32.8727273	5.56868817	0.03001342	0.34729843	0.32084266	Pnp	1
GO:0009120	deoxyribonucleoside metabolic process	1/40	11/14464	0.09090909	32.8727273	5.56868817	0.03001342	0.34729843	0.32084266	Pnp	1
GO:0009169	purine ribonucleoside monophosphate catabolic process	1/40	11/14464	0.09090909	32.8727273	5.56868817	0.03001342	0.34729843	0.32084266	Pnp	1
GO:0009215	purine deoxyribonucleoside triphosphate metabolic process	1/40	11/14464	0.09090909	32.8727273	5.56868817	0.03001342	0.34729843	0.32084266	Pnp	1
GO:0046015	regulation of transcription by glucose	1/40	11/14464	0.09090909	32.8727273	5.56868817	0.03001342	0.34729843	0.32084266	Rrp8	1
GO:0048012	hepatocyte growth factor receptor signaling pathway	1/40	11/14464	0.09090909	32.8727273	5.56868817	0.03001342	0.34729843	0.32084266	Ptpn1	1
GO:0070493	thrombin-activated receptor signaling pathway	1/40	11/14464	0.09090909	32.8727273	5.56868817	0.03001342	0.34729843	0.32084266	Iqgap2	1
GO:1902513	regulation of organelle transport along microtubule	1/40	11/14464	0.09090909	32.8727273	5.56868817	0.03001342	0.34729843	0.32084266	Rhot1	1
GO:1990709	presynaptic active zone organization	1/40	11/14464	0.09090909	32.8727273	5.56868817	0.03001342	0.34729843	0.32084266	Ctbp2	1
GO:0035967	cellular response to topologically incorrect protein	2/40	100/14464	0.02	7.232	3.29310879	0.03113038	0.34729843	0.32084266	Bok/Ptpn1	2
GO:0009128	purine nucleoside monophosphate catabolic process	1/40	12/14464	0.08333333	30.1333333	5.31658983	0.03269795	0.34729843	0.32084266	Pnp	1
GO:0009155	purine deoxyribonucleotide catabolic process	1/40	12/14464	0.08333333	30.1333333	5.31658983	0.03269795	0.34729843	0.32084266	Pnp	1
GO:0009170	purine deoxyribonucleoside monophosphate metabolic process	1/40	12/14464	0.08333333	30.1333333	5.31658983	0.03269795	0.34729843	0.32084266	Pnp	1
GO:0030948	negative regulation of vascular endothelial growth factor receptor signaling pathway	1/40	12/14464	0.08333333	30.1333333	5.31658983	0.03269795	0.34729843	0.32084266	Ptpn1	1
GO:0035563	positive regulation of chromatin binding	1/40	12/14464	0.08333333	30.1333333	5.31658983	0.03269795	0.34729843	0.32084266	Ctbp2	1
GO:0035791	platelet-derived growth factor receptor-beta signaling pathway	1/40	12/14464	0.08333333	30.1333333	5.31658983	0.03269795	0.34729843	0.32084266	Ptpn1	1
GO:0046085	adenosine metabolic process	1/40	12/14464	0.08333333	30.1333333	5.31658983	0.03269795	0.34729843	0.32084266	Pnp	1
GO:1901029	negative regulation of mitochondrial outer membrane permeabilization involved in apoptotic signaling pat	1/40	12/14464	0.08333333	30.1333333	5.31658983	0.03269795	0.34729843	0.32084266	Bok	1
GO:2000035	regulation of stem cell division	1/40	12/14464	0.08333333	30.1333333	5.31658983	0.03269795	0.34729843	0.32084266	Prdm15	1
GO:2000322	regulation of glucocorticoid receptor signaling pathway	1/40	12/14464	0.08333333	30.1333333	5.31658983	0.03269795	0.34729843	0.32084266	Cry1	1
GO:2000644	regulation of receptor catabolic process	1/40	12/14464	0.08333333	30.1333333	5.31658983	0.03269795	0.34729843	0.32084266	Ptpn1	1
GO:2000674	regulation of type B pancreatic cell apoptotic process	1/40	12/14464	0.08333333	30.1333333	5.31658983	0.03269795	0.34729843	0.32084266	Capn10	1
GO:2000849	regulation of glucocorticoid secretion	1/40	12/14464	0.08333333	30.1333333	5.31658983	0.03269795	0.34729843	0.32084266	Cry1	1
GO:0046883	regulation of hormone secretion	3/40	261/14464	0.01149425	4.15632184	2.70974125	0.03514751	0.34729843	0.32084266	Cry1/Cntf/Capn10	3
GO:0009200	deoxyribonucleoside triphosphate metabolic process	1/40	13/14464	0.07692308	27.8153846	5.09357949	0.03537523	0.34729843	0.32084266	Pnp	1
GO:0035933	glucocorticoid secretion	1/40	13/14464	0.07692308	27.8153846	5.09357949	0.03537523	0.34729843	0.32084266	Cry1	1
GO:0042454	ribonucleoside catabolic process	1/40	13/14464	0.07692308	27.8153846	5.09357949	0.03537523	0.34729843	0.32084266	Pnp	1
GO:0042754	negative regulation of circadian rhythm	1/40	13/14464	0.07692308	27.8153846	5.09357949	0.03537523	0.34729843	0.32084266	Cry1	1
GO:0043605	amide catabolic process	1/40	13/14464	0.07692308	27.8153846	5.09357949	0.03537523	0.34729843	0.32084266	Pnp	1
GO:0048385	regulation of retinoic acid receptor signaling pathway	1/40	13/14464	0.07692308	27.8153846	5.09357949	0.03537523	0.34729843	0.32084266	Ctbp2	1
GO:1904753	negative regulation of vascular associated smooth muscle cell migration	1/40	13/14464	0.07692308	27.8153846	5.09357949	0.03537523	0.34729843	0.32084266	Ptpn1	1
GO:0032868	response to insulin	3/40	265/14464	0.01132075	4.09358491	2.67653193	0.03651932	0.34729843	0.32084266	Cry1/Capn10/Ptpn1	3
GO:0006986	response to unfolded protein	2/40	110/14464	0.01818182	6.57454545	3.09054322	0.03705868	0.34729843	0.32084266	Bok/Ptpn1	2
GO:0007006	mitochondrial membrane organization	2/40	111/14464	0.01801802	6.51531532	3.07168605	0.03767379	0.34729843	0.32084266	Bok/Rhot1	2
GO:0015721	bile acid and bile salt transport	1/40	14/14464	0.07142857	25.8285714	4.89438533	0.03804529	0.34729843	0.32084266	Slc10a6	1
GO:0036498	IRE1-mediated unfolded protein response	1/40	14/14464	0.07142857	25.8285714	4.89438533	0.03804529	0.34729843	0.32084266	Ptpn1	1
GO:0048240	sperm capacitation	1/40	14/14464	0.07142857	25.8285714	4.89438533	0.03804529	0.34729843	0.32084266	Catsperz	1
GO:0048680	positive regulation of axon regeneration	1/40	14/14464	0.07142857	25.8285714	4.89438533	0.03804529	0.34729843	0.32084266	Cntf	1
GO:0010001	glial cell differentiation	3/40	272/14464	0.01102941	3.98823529	2.61995465	0.03898615	0.34729843	0.32084266	Bok/Cntf/Arhgef10	3
GO:0007005	mitochondrion organization	4/40	466/14464	0.00858369	3.10386266	2.39054505	0.03899502	0.34729843	0.32084266	Bok/Capn10/Pnp/Rhot1	4
GO:0001732	formation of cytoplasmic translation initiation complex	1/40	15/14464	0.06666667	24.1066667	4.71498554	0.04070814	0.34729843	0.32084266	Eif3j1	1
GO:0006167	AMP biosynthetic process	1/40	15/14464	0.06666667	24.1066667	4.71498554	0.04070814	0.34729843	0.32084266	Pnp	1
GO:0006188	IMP biosynthetic process	1/40	15/14464	0.06666667	24.1066667	4.71498554	0.04070814	0.34729843	0.32084266	Pnp	1
GO:0009163	nucleoside biosynthetic process	1/40	15/14464	0.06666667	24.1066667	4.71498554	0.04070814	0.34729843	0.32084266	Pnp	1
GO:0034404	nucleobase-containing small molecule biosynthetic process	1/40	15/14464	0.06666667	24.1066667	4.71498554	0.04070814	0.34729843	0.32084266	Pnp	1
GO:0070572	positive regulation of neuron projection regeneration	1/40	15/14464	0.06666667	24.1066667	4.71498554	0.04070814	0.34729843	0.32084266	Cntf	1
GO:0090308	regulation of DNA methylation-dependent heterochromatin formation	1/40	15/14464	0.06666667	24.1066667	4.71498554	0.04070814	0.34729843	0.32084266	Samd1	1
GO:0097050	type B pancreatic cell apoptotic process	1/40	15/14464	0.06666667	24.1066667	4.71498554	0.04070814	0.34729843	0.32084266	Capn10	1
GO:1900102	negative regulation of endoplasmic reticulum unfolded protein response	1/40	15/14464	0.06666667	24.1066667	4.71498554	0.04070814	0.34729843	0.32084266	Ptpn1	1
GO:0032386	regulation of intracellular transport	3/40	277/14464	0.01083032	3.91624549	2.58068509	0.04079948	0.34729843	0.32084266	Capn10/Rhot1/Ptpn1	3
GO:0010821	regulation of mitochondrion organization	2/40	118/14464	0.01694915	6.12881356	2.94583783	0.04208873	0.34729843	0.32084266	Bok/Rhot1	2
GO:0001933	negative regulation of protein phosphorylation	3/40	281/14464	0.01067616	3.86049822	2.54992333	0.04228077	0.34729843	0.32084266	Pkib/Ctdspl/Ptpn1	3
GO:0006177	GMP biosynthetic process	1/40	16/14464	0.0625	22.6	4.55225108	0.0433638	0.34729843	0.32084266	Pnp	1
GO:0009159	deoxyribonucleoside monophosphate catabolic process	1/40	16/14464	0.0625	22.6	4.55225108	0.0433638	0.34729843	0.32084266	Pnp	1
GO:0016081	synaptic vesicle docking	1/40	16/14464	0.0625	22.6	4.55225108	0.0433638	0.34729843	0.32084266	Ctbp2	1
GO:0042921	nuclear receptor-mediated glucocorticoid signaling pathway	1/40	16/14464	0.0625	22.6	4.55225108	0.0433638	0.34729843	0.32084266	Cry1	1
GO:0045721	negative regulation of gluconeogenesis	1/40	16/14464	0.0625	22.6	4.55225108	0.0433638	0.34729843	0.32084266	Cry1	1
GO:0070314	G1 to G0 transition	1/40	16/14464	0.0625	22.6	4.55225108	0.0433638	0.34729843	0.32084266	Rrp8	1
GO:0106380	purine ribonucleotide salvage	1/40	16/14464	0.0625	22.6	4.55225108	0.0433638	0.34729843	0.32084266	Pnp	1
GO:0140718	facultative heterochromatin formation	1/40	16/14464	0.0625	22.6	4.55225108	0.0433638	0.34729843	0.32084266	Rrp8	1
GO:1904355	positive regulation of telomere capping	1/40	16/14464	0.0625	22.6	4.55225108	0.0433638	0.34729843	0.32084266	Pkib	1
GO:0031507	heterochromatin formation	2/40	120/14464	0.01666667	6.02666667	2.91173538	0.04338447	0.34729843	0.32084266	Rrp8/Samd1	2
GO:0007052	mitotic spindle organization	2/40	121/14464	0.01652893	5.9768595	2.89497221	0.04403794	0.34729843	0.32084266	Plk5/Arhgef10	2
GO:0045579	positive regulation of B cell differentiation	1/40	17/14464	0.05882353	21.2705882	4.40370591	0.0460123	0.34729843	0.32084266	Pnp	1
GO:0060546	negative regulation of necroptotic process	1/40	17/14464	0.05882353	21.2705882	4.40370591	0.0460123	0.34729843	0.32084266	Bok	1
GO:0071732	cellular response to nitric oxide	1/40	17/14464	0.05882353	21.2705882	4.40370591	0.0460123	0.34729843	0.32084266	Ptpn1	1
GO:2000846	regulation of corticosteroid hormone secretion	1/40	17/14464	0.05882353	21.2705882	4.40370591	0.0460123	0.34729843	0.32084266	Cry1	1
GO:0015718	monocarboxylic acid transport	2/40	126/14464	0.01587302	5.73968254	2.81388646	0.04736003	0.34729843	0.32084266	Slc10a6/Slc43a3	2
GO:0044770	cell cycle phase transition	4/40	498/14464	0.00803213	2.90441767	2.27749371	0.04781458	0.34729843	0.32084266	Plk5/Cry1/Fam83d/Ctdspl	4
GO:0035966	response to topologically incorrect protein	2/40	127/14464	0.01574803	5.69448819	2.79819065	0.04803561	0.34729843	0.32084266	Bok/Ptpn1	2
GO:0006614	SRP-dependent cotranslational protein targeting to membrane	1/40	18/14464	0.05555556	20.0888889	4.26736145	0.04865364	0.34729843	0.32084266	Zfand2b	1
GO:0009158	ribonucleoside monophosphate catabolic process	1/40	18/14464	0.05555556	20.0888889	4.26736145	0.04865364	0.34729843	0.32084266	Pnp	1
GO:0009164	nucleoside catabolic process	1/40	18/14464	0.05555556	20.0888889	4.26736145	0.04865364	0.34729843	0.32084266	Pnp	1
GO:0031958	nuclear receptor-mediated corticosteroid signaling pathway	1/40	18/14464	0.05555556	20.0888889	4.26736145	0.04865364	0.34729843	0.32084266	Cry1	1
GO:0043567	regulation of insulin-like growth factor receptor signaling pathway	1/40	18/14464	0.05555556	20.0888889	4.26736145	0.04865364	0.34729843	0.32084266	Zfand2b	1
GO:0060081	membrane hyperpolarization	1/40	18/14464	0.05555556	20.0888889	4.26736145	0.04865364	0.34729843	0.32084266	Cntf	1
GO:0060221	retinal rod cell differentiation	1/40	18/14464	0.05555556	20.0888889	4.26736145	0.04865364	0.34729843	0.32084266	Cntf	1
GO:0060795	cell fate commitment involved in formation of primary germ layer	1/40	18/14464	0.05555556	20.0888889	4.26736145	0.04865364	0.34729843	0.32084266	Smad1	1
GO:1900119	positive regulation of execution phase of apoptosis	1/40	18/14464	0.05555556	20.0888889	4.26736145	0.04865364	0.34729843	0.32084266	Bok	1
GO:1902236	negative regulation of endoplasmic reticulum stress-induced intrinsic apoptotic signaling pathway	1/40	18/14464	0.05555556	20.0888889	4.26736145	0.04865364	0.34729843	0.32084266	Ptpn1	1
GO:1903232	melanosome assembly	1/40	18/14464	0.05555556	20.0888889	4.26736145	0.04865364	0.34729843	0.32084266	Hps5	1
GO:0042326	negative regulation of phosphorylation	3/40	301/14464	0.00996678	3.60398671	2.40414532	0.05009094	0.34729843	0.32084266	Pkib/Ctdspl/Ptpn1	3
GO:0003084	positive regulation of systemic arterial blood pressure	1/40	19/14464	0.05263158	19.0315789	4.14159967	0.05128785	0.34729843	0.32084266	Ptpn1	1
GO:0006144	purine nucleobase metabolic process	1/40	19/14464	0.05263158	19.0315789	4.14159967	0.05128785	0.34729843	0.32084266	Pnp	1
GO:0006613	cotranslational protein targeting to membrane	1/40	19/14464	0.05263158	19.0315789	4.14159967	0.05128785	0.34729843	0.32084266	Zfand2b	1
GO:0007000	nucleolus organization	1/40	19/14464	0.05263158	19.0315789	4.14159967	0.05128785	0.34729843	0.32084266	Rrp8	1
GO:0009151	purine deoxyribonucleotide metabolic process	1/40	19/14464	0.05263158	19.0315789	4.14159967	0.05128785	0.34729843	0.32084266	Pnp	1
GO:0032261	purine nucleotide salvage	1/40	19/14464	0.05263158	19.0315789	4.14159967	0.05128785	0.34729843	0.32084266	Pnp	1
GO:0035930	corticosteroid hormone secretion	1/40	19/14464	0.05263158	19.0315789	4.14159967	0.05128785	0.34729843	0.32084266	Cry1	1
GO:0048333	mesodermal cell differentiation	1/40	19/14464	0.05263158	19.0315789	4.14159967	0.05128785	0.34729843	0.32084266	Smad1	1
GO:0062099	negative regulation of programmed necrotic cell death	1/40	19/14464	0.05263158	19.0315789	4.14159967	0.05128785	0.34729843	0.32084266	Bok	1
GO:1902018	negative regulation of cilium assembly	1/40	19/14464	0.05263158	19.0315789	4.14159967	0.05128785	0.34729843	0.32084266	Tchp	1
GO:1902170	cellular response to reactive nitrogen species	1/40	19/14464	0.05263158	19.0315789	4.14159967	0.05128785	0.34729843	0.32084266	Ptpn1	1
GO:2000353	positive regulation of endothelial cell apoptotic process	1/40	19/14464	0.05263158	19.0315789	4.14159967	0.05128785	0.34729843	0.32084266	Ptpn1	1
GO:0050731	positive regulation of peptidyl-tyrosine phosphorylation	2/40	133/14464	0.01503759	5.43759398	2.70739404	0.05216129	0.34729843	0.32084266	Cntf/Ptpn1	2
GO:0009125	nucleoside monophosphate catabolic process	1/40	20/14464	0.05	18.08	4.02508871	0.05391496	0.34729843	0.32084266	Pnp	1
GO:0033762	response to glucagon	1/40	20/14464	0.05	18.08	4.02508871	0.05391496	0.34729843	0.32084266	Cry1	1
GO:0035795	negative regulation of mitochondrial membrane permeability	1/40	20/14464	0.05	18.08	4.02508871	0.05391496	0.34729843	0.32084266	Bok	1
GO:0048143	astrocyte activation	1/40	20/14464	0.05	18.08	4.02508871	0.05391496	0.34729843	0.32084266	Cntf	1
GO:0060155	platelet dense granule organization	1/40	20/14464	0.05	18.08	4.02508871	0.05391496	0.34729843	0.32084266	Hps5	1
GO:1901028	regulation of mitochondrial outer membrane permeabilization involved in apoptotic signaling pathway	1/40	20/14464	0.05	18.08	4.02508871	0.05391496	0.34729843	0.32084266	Bok	1
GO:1901659	glycosyl compound biosynthetic process	1/40	20/14464	0.05	18.08	4.02508871	0.05391496	0.34729843	0.32084266	Pnp	1
GO:0046879	hormone secretion	3/40	314/14464	0.00955414	3.45477707	2.31587436	0.05552242	0.34729843	0.32084266	Cry1/Cntf/Capn10	3
GO:0031445	regulation of heterochromatin formation	1/40	21/14464	0.04761905	17.2190476	3.91672087	0.05653497	0.34729843	0.32084266	Samd1	1
GO:0034656	nucleobase-containing small molecule catabolic process	1/40	21/14464	0.04761905	17.2190476	3.91672087	0.05653497	0.34729843	0.32084266	Pnp	1
GO:0043101	purine-containing compound salvage	1/40	21/14464	0.04761905	17.2190476	3.91672087	0.05653497	0.34729843	0.32084266	Pnp	1
GO:0046040	IMP metabolic process	1/40	21/14464	0.04761905	17.2190476	3.91672087	0.05653497	0.34729843	0.32084266	Pnp	1
GO:0050872	white fat cell differentiation	1/40	21/14464	0.04761905	17.2190476	3.91672087	0.05653497	0.34729843	0.32084266	Ctbp2	1
GO:0071731	response to nitric oxide	1/40	21/14464	0.04761905	17.2190476	3.91672087	0.05653497	0.34729843	0.32084266	Ptpn1	1
GO:2000001	regulation of DNA damage checkpoint	1/40	21/14464	0.04761905	17.2190476	3.91672087	0.05653497	0.34729843	0.32084266	Cry1	1
GO:0021782	glial cell development	2/40	140/14464	0.01428571	5.16571429	2.60818068	0.05712962	0.34729843	0.32084266	Cntf/Arhgef10	2
GO:0009914	hormone transport	3/40	321/14464	0.00934579	3.37943925	2.27024485	0.05856053	0.34729843	0.32084266	Cry1/Cntf/Capn10	3
GO:0010623	programmed cell death involved in cell development	1/40	22/14464	0.04545455	16.4363636	3.81556629	0.0591479	0.34729843	0.32084266	Cntf	1
GO:0043153	entrainment of circadian clock by photoperiod	1/40	22/14464	0.04545455	16.4363636	3.81556629	0.0591479	0.34729843	0.32084266	Cry1	1
GO:0051900	regulation of mitochondrial depolarization	1/40	22/14464	0.04545455	16.4363636	3.81556629	0.0591479	0.34729843	0.32084266	Bok	1
GO:1903672	positive regulation of sprouting angiogenesis	1/40	22/14464	0.04545455	16.4363636	3.81556629	0.0591479	0.34729843	0.32084266	Smad1	1
GO:1905709	negative regulation of membrane permeability	1/40	22/14464	0.04545455	16.4363636	3.81556629	0.0591479	0.34729843	0.32084266	Bok	1
GO:2000042	negative regulation of double-strand break repair via homologous recombination	1/40	22/14464	0.04545455	16.4363636	3.81556629	0.0591479	0.34729843	0.32084266	Fbh1	1
GO:2001235	positive regulation of apoptotic signaling pathway	2/40	146/14464	0.01369863	4.95342466	2.52827929	0.06151542	0.34729843	0.32084266	Bok/Fbh1	2
GO:0032956	regulation of actin cytoskeleton organization	3/40	328/14464	0.00914634	3.30731707	2.22585703	0.06167683	0.34729843	0.32084266	Iqgap2/Arhgef10/Capn10	3
GO:0009168	purine ribonucleoside monophosphate biosynthetic process	1/40	23/14464	0.04347826	15.7217391	3.72083776	0.06175378	0.34729843	0.32084266	Pnp	1
GO:0009264	deoxyribonucleotide catabolic process	1/40	23/14464	0.04347826	15.7217391	3.72083776	0.06175378	0.34729843	0.32084266	Pnp	1
GO:0009648	photoperiodism	1/40	23/14464	0.04347826	15.7217391	3.72083776	0.06175378	0.34729843	0.32084266	Cry1	1
GO:0009649	entrainment of circadian clock	1/40	23/14464	0.04347826	15.7217391	3.72083776	0.06175378	0.34729843	0.32084266	Cry1	1

GO:0010666	positive regulation of cardiac muscle cell apoptotic process	1/40	23/14464	0.04347826	15.7217391	3.72083776	0.06175378	0.34729843	0.32084266	Ptpn1	1
GO:0046037	GMP metabolic process	1/40	23/14464	0.04347826	15.7217391	3.72083776	0.06175378	0.34729843	0.32084266	Pnp	1
GO:0046386	deoxyribose phosphate catabolic process	1/40	23/14464	0.04347826	15.7217391	3.72083776	0.06175378	0.34729843	0.32084266	Pnp	1
GO:2000831	regulation of steroid hormone secretion	1/40	23/14464	0.04347826	15.7217391	3.72083776	0.06175378	0.34729843	0.32084266	Cry1	1
GO:0010663	positive regulation of striated muscle cell apoptotic process	1/40	24/14464	0.04166667	15.0666667	3.63186379	0.06435262	0.34729843	0.32084266	Ptpn1	1
GO:0060544	regulation of necroptotic process	1/40	24/14464	0.04166667	15.0666667	3.63186379	0.06435262	0.34729843	0.32084266	Bok	1
GO:0061098	positive regulation of protein tyrosine kinase activity	1/40	24/14464	0.04166667	15.0666667	3.63186379	0.06435262	0.34729843	0.32084266	Ptpn1	1
GO:0070233	negative regulation of T cell apoptotic process	1/40	24/14464	0.04166667	15.0666667	3.63186379	0.06435262	0.34729843	0.32084266	Pnp	1
GO:0072530	purine-containing compound transmembrane transport	1/40	24/14464	0.04166667	15.0666667	3.63186379	0.06435262	0.34729843	0.32084266	Slc43a3	1
GO:1901658	glycosyl compound catabolic process	1/40	24/14464	0.04166667	15.0666667	3.63186379	0.06435262	0.34729843	0.32084266	Pnp	1
GO:1904037	positive regulation of epithelial cell apoptotic process	1/40	24/14464	0.04166667	15.0666667	3.63186379	0.06435262	0.34729843	0.32084266	Capn10	1
GO:0141193	nuclear receptor-mediated signaling pathway	2/40	151/14464	0.01324503	4.78940397	2.46496284	0.0652563	0.34729843	0.32084266	Cry1/Ctbp2	2
GO:1902850	microtubule cytoskeleton organization involved in mitosis	2/40	151/14464	0.01324503	4.78940397	2.46496284	0.0652563	0.34729843	0.32084266	Plk5/Arhgef10	2
GO:2000045	regulation of G1/S transition of mitotic cell cycle	2/40	151/14464	0.01324503	4.78940397	2.46496284	0.0652563	0.34729843	0.32084266	Plk5/Ctdspl	2
GO:0045814	negative regulation of gene expression, epigenetic	2/40	153/14464	0.0130719	4.72679739	2.44041024	0.06677389	0.34729843	0.32084266	Rrp8/Samd1	2
GO:0046887	positive regulation of hormone secretion	2/40	153/14464	0.0130719	4.72679739	2.44041024	0.06677389	0.34729843	0.32084266	Cntf/Capn10	2
GO:0009127	purine nucleoside monophosphate biosynthetic process	1/40	25/14464	0.04	14.464	3.54806756	0.06694444	0.34729843	0.32084266	Pnp	1
GO:0035335	peptidyl-tyrosine dephosphorylation	1/40	25/14464	0.04	14.464	3.54806756	0.06694444	0.34729843	0.32084266	Ptpn1	1
GO:0046033	AMP metabolic process	1/40	25/14464	0.04	14.464	3.54806756	0.06694444	0.34729843	0.32084266	Pnp	1
GO:0046128	purine ribonucleoside metabolic process	1/40	25/14464	0.04	14.464	3.54806756	0.06694444	0.34729843	0.32084266	Pnp	1
GO:1900117	regulation of execution phase of apoptosis	1/40	25/14464	0.04	14.464	3.54806756	0.06694444	0.34729843	0.32084266	Bok	1
GO:1904353	regulation of telomere capping	1/40	25/14464	0.04	14.464	3.54806756	0.06694444	0.34729843	0.32084266	Pkib	1
GO:0006869	lipid transport	3/40	342/14464	0.00877193	3.17192982	2.14055835	0.06813997	0.34729843	0.32084266	Cry1/Slc10a6/Slc43a3	3
GO:0072331	signal transduction by p53 class mediator	2/40	155/14464	0.01290323	4.66580645	2.41627895	0.06830336	0.34729843	0.32084266	Bok/Rrp8	2
GO:0045786	negative regulation of cell cycle	3/40	343/14464	0.00874636	3.16268222	2.13463376	0.06861322	0.34729843	0.32084266	Cry1/Rrp8/Ctdspl	3
GO:0003299	muscle hypertrophy in response to stress	1/40	26/14464	0.03846154	13.9076923	3.46895041	0.06952926	0.34729843	0.32084266	Smad1	1
GO:0010460	positive regulation of heart rate	1/40	26/14464	0.03846154	13.9076923	3.46895041	0.06952926	0.34729843	0.32084266	Ptpn1	1
GO:0014898	cardiac muscle hypertrophy in response to stress	1/40	26/14464	0.03846154	13.9076923	3.46895041	0.06952926	0.34729843	0.32084266	Smad1	1
GO:0034643	establishment of mitochondrion localization, microtubule-mediated	1/40	26/14464	0.03846154	13.9076923	3.46895041	0.06952926	0.34729843	0.32084266	Rhot1	1
GO:0046039	GTP metabolic process	1/40	26/14464	0.03846154	13.9076923	3.46895041	0.06952926	0.34729843	0.32084266	Pnp	1
GO:0046628	positive regulation of insulin receptor signaling pathway	1/40	26/14464	0.03846154	13.9076923	3.46895041	0.06952926	0.34729843	0.32084266	Ptpn1	1
GO:0047497	mitochondrion transport along microtubule	1/40	26/14464	0.03846154	13.9076923	3.46895041	0.06952926	0.34729843	0.32084266	Rhot1	1
GO:0010563	negative regulation of phosphorus metabolic process	3/40	345/14464	0.00869565	3.14434783	2.12284919	0.06956429	0.34729843	0.32084266	Pkib/Ctdspl/Ptpn1	3
GO:0045936	negative regulation of phosphate metabolic process	3/40	345/14464	0.00869565	3.14434783	2.12284919	0.06956429	0.34729843	0.32084266	Pkib/Ctdspl/Ptpn1	3
GO:0043409	negative regulation of MAPK cascade	2/40	157/14464	0.01273885	4.60636943	2.39255584	0.06984454	0.34729843	0.32084266	Prdm15/Ptpn1	2
GO:0006308	DNA catabolic process	1/40	27/14464	0.03703704	13.3925926	3.3940787	0.0721071	0.34729843	0.32084266	Fbh1	1
GO:0006921	cellular component disassembly involved in execution phase of apoptosis	1/40	27/14464	0.03703704	13.3925926	3.3940787	0.0721071	0.34729843	0.32084266	Bok	1
GO:0014887	cardiac muscle adaptation	1/40	27/14464	0.03703704	13.3925926	3.3940787	0.0721071	0.34729843	0.32084266	Smad1	1
GO:0032801	receptor catabolic process	1/40	27/14464	0.03703704	13.3925926	3.3940787	0.0721071	0.34729843	0.32084266	Ptpn1	1
GO:0035929	steroid hormone secretion	1/40	27/14464	0.03703704	13.3925926	3.3940787	0.0721071	0.34729843	0.32084266	Cry1	1
GO:0036120	cellular response to platelet-derived growth factor stimulus	1/40	27/14464	0.03703704	13.3925926	3.3940787	0.0721071	0.34729843	0.32084266	Ptpn1	1
GO:0045722	positive regulation of gluconeogenesis	1/40	27/14464	0.03703704	13.3925926	3.3940787	0.0721071	0.34729843	0.32084266	Cry1	1
GO:0006469	negative regulation of protein kinase activity	2/40	161/14464	0.01242236	4.49192547	2.34628447	0.07296135	0.349549	0.32292179	Pkib/Ptpn1	2
GO:0001916	positive regulation of T cell mediated cytotoxicity	1/40	28/14464	0.03571429	12.9142857	3.3230733	0.07467797	0.349549	0.32292179	Pnp	1
GO:0009162	deoxyribonucleoside monophosphate metabolic process	1/40	28/14464	0.03571429	12.9142857	3.3230733	0.07467797	0.349549	0.32292179	Pnp	1
GO:0060219	camera-type eye photoreceptor cell differentiation	1/40	28/14464	0.03571429	12.9142857	3.3230733	0.07467797	0.349549	0.32292179	Cntf	1
GO:0061036	positive regulation of cartilage development	1/40	28/14464	0.03571429	12.9142857	3.3230733	0.07467797	0.349549	0.32292179	Smad1	1
GO:0090077	foam cell differentiation	1/40	28/14464	0.03571429	12.9142857	3.3230733	0.07467797	0.349549	0.32292179	Samd1	1
GO:0016339	calcium-dependent cell-cell adhesion via plasma membrane cell adhesion molecules	1/40	29/14464	0.03448276	12.4689655	3.25560108	0.0772419	0.35119634	0.32444364	Cdh19	1
GO:0030901	midbrain development	1/40	29/14464	0.03448276	12.4689655	3.25560108	0.0772419	0.35119634	0.32444364	Smad1	1
GO:0032369	negative regulation of lipid transport	1/40	29/14464	0.03448276	12.4689655	3.25560108	0.0772419	0.35119634	0.32444364	Cry1	1
GO:0043173	nucleotide salvage	1/40	29/14464	0.03448276	12.4689655	3.25560108	0.0772419	0.35119634	0.32444364	Pnp	1
GO:0051882	mitochondrial depolarization	1/40	29/14464	0.03448276	12.4689655	3.25560108	0.0772419	0.35119634	0.32444364	Bok	1
GO:0062098	regulation of programmed necrotic cell death	1/40	29/14464	0.03448276	12.4689655	3.25560108	0.0772419	0.35119634	0.32444364	Bok	1
GO:0009755	hormone-mediated signaling pathway	2/40	169/14464	0.01183432	4.27928994	2.2581221	0.07932819	0.35119634	0.32444364	Cry1/Ctbp2	2
GO:0036119	response to platelet-derived growth factor	1/40	30/14464	0.03333333	12.0533333	3.19136793	0.0797989	0.35119634	0.32444364	Ptpn1	1
GO:0046427	positive regulation of receptor signaling pathway via JAK-STAT	1/40	30/14464	0.03333333	12.0533333	3.19136793	0.0797989	0.35119634	0.32444364	Cntf	1
GO:0048384	retinoic acid receptor signaling pathway	1/40	30/14464	0.03333333	12.0533333	3.19136793	0.0797989	0.35119634	0.32444364	Ctbp2	1
GO:0007269	neurotransmitter secretion	2/40	170/14464	0.01176471	4.25411765	2.24748682	0.08013613	0.35119634	0.32444364	Ctbp2/Rhot1	2
GO:0099643	signal release from synapse	2/40	170/14464	0.01176471	4.25411765	2.24748682	0.08013613	0.35119634	0.32444364	Ctbp2/Rhot1	2
GO:0032970	regulation of actin filament-based process	3/40	368/14464	0.00815217	2.94782609	1.99316143	0.08093229	0.35119634	0.32444364	Iqgap2/Arhgef10/Capn10	3
GO:0009119	ribonucleoside metabolic process	1/40	31/14464	0.03225806	11.6645161	3.13011309	0.082349	0.35119634	0.32444364	Pnp	1
GO:0042278	purine nucleoside metabolic process	1/40	31/14464	0.03225806	11.6645161	3.13011309	0.082349	0.35119634	0.32444364	Pnp	1
GO:0065002	intracellular protein transmembrane transport	1/40	31/14464	0.03225806	11.6645161	3.13011309	0.082349	0.35119634	0.32444364	Zfand2b	1
GO:1900078	positive regulation of cellular response to insulin stimulus	1/40	31/14464	0.03225806	11.6645161	3.13011309	0.082349	0.35119634	0.32444364	Ptpn1	1
GO:2000780	negative regulation of double-strand break repair	1/40	31/14464	0.03225806	11.6645161	3.13011309	0.082349	0.35119634	0.32444364	Fbh1	1
GO:0033673	negative regulation of kinase activity	2/40	173/14464	0.01156069	4.18034682	2.21606532	0.08257561	0.35119634	0.32444364	Pkib/Ptpn1	2
GO:0006839	mitochondrial transport	2/40	175/14464	0.01142857	4.13257143	2.19551001	0.08421477	0.35119634	0.32444364	Bok/Rhot1	2
GO:0002347	response to tumor cell	1/40	32/14464	0.03125	11.3	3.07160443	0.0848922	0.35119634	0.32444364	Plk5	1
GO:0009112	nucleobase metabolic process	1/40	32/14464	0.03125	11.3	3.07160443	0.0848922	0.35119634	0.32444364	Pnp	1
GO:0032743	positive regulation of interleukin-2 production	1/40	32/14464	0.03125	11.3	3.07160443	0.0848922	0.35119634	0.32444364	Pnp	1
GO:0045577	regulation of B cell differentiation	1/40	32/14464	0.03125	11.3	3.07160443	0.0848922	0.35119634	0.32444364	Pnp	1
GO:0045738	negative regulation of DNA repair	1/40	32/14464	0.03125	11.3	3.07160443	0.0848922	0.35119634	0.32444364	Fbh1	1
GO:2001233	regulation of apoptotic signaling pathway	3/40	379/14464	0.00791557	2.86226913	1.93463908	0.08664146	0.35119634	0.32444364	Bok/Ptpn1/Fbh1	3
GO:0006346	DNA methylation-dependent heterochromatin formation	1/40	33/14464	0.03030303	10.9575758	3.01563453	0.08742853	0.35119634	0.32444364	Samd1	1
GO:0022011	myelination in peripheral nervous system	1/40	33/14464	0.03030303	10.9575758	3.01563453	0.08742853	0.35119634	0.32444364	Arhgef10	1
GO:0030947	regulation of vascular endothelial growth factor receptor signaling pathway	1/40	33/14464	0.03030303	10.9575758	3.01563453	0.08742853	0.35119634	0.32444364	Ptpn1	1
GO:0032292	peripheral nervous system axon ensheathment	1/40	33/14464	0.03030303	10.9575758	3.01563453	0.08742853	0.35119634	0.32444364	Arhgef10	1
GO:1902410	mitotic cytokinetic process	1/40	33/14464	0.03030303	10.9575758	3.01563453	0.08742853	0.35119634	0.32444364	Iqgap2	1
GO:1904385	cellular response to angiotensin	1/40	33/14464	0.03030303	10.9575758	3.01563453	0.08742853	0.35119634	0.32444364	Ptpn1	1
GO:0001914	regulation of T cell mediated cytotoxicity	1/40	34/14464	0.02941176	10.6352941	2.96201737	0.089958	0.35119634	0.32444364	Pnp	1
GO:0042531	positive regulation of tyrosine phosphorylation of STAT protein	1/40	34/14464	0.02941176	10.6352941	2.96201737	0.089958	0.35119634	0.32444364	Cntf	1
GO:0051654	establishment of mitochondrion localization	1/40	34/14464	0.02941176	10.6352941	2.96201737	0.089958	0.35119634	0.32444364	Rhot1	1
GO:1902275	regulation of chromatin organization	1/40	34/14464	0.02941176	10.6352941	2.96201737	0.089958	0.35119634	0.32444364	Samd1	1
GO:1904752	regulation of vascular associated smooth muscle cell migration	1/40	34/14464	0.02941176	10.6352941	2.96201737	0.089958	0.35119634	0.32444364	Ptpn1	1
GO:0007140	male meiotic nuclear division	1/40	35/14464	0.02857143	10.3314286	2.91058558	0.09248064	0.35119634	0.32444364	Catsperz	1
GO:0014912	negative regulation of smooth muscle cell migration	1/40	35/14464	0.02857143	10.3314286	2.91058558	0.09248064	0.35119634	0.32444364	Ptpn1	1
GO:0032212	positive regulation of telomere maintenance via telomerase	1/40	35/14464	0.02857143	10.3314286	2.91058558	0.09248064	0.35119634	0.32444364	Pkib	1
GO:0032438	melanosome organization	1/40	35/14464	0.02857143	10.3314286	2.91058558	0.09248064	0.35119634	0.32444364	Hps5	1
GO:0033144	negative regulation of intracellular steroid hormone receptor signaling pathway	1/40	35/14464	0.02857143	10.3314286	2.91058558	0.09248064	0.35119634	0.32444364	Cry1	1
GO:0048679	regulation of axon regeneration	1/40	35/14464	0.02857143	10.3314286	2.91058558	0.09248064	0.35119634	0.32444364	Cntf	1
GO:0070229	negative regulation of lymphocyte apoptotic process	1/40	35/14464	0.02857143	10.3314286	2.91058558	0.09248064	0.35119634	0.32444364	Pnp	1
GO:1905898	positive regulation of response to endoplasmic reticulum stress	1/40	35/14464	0.02857143	10.3314286	2.91058558	0.09248064	0.35119634	0.32444364	Bok	1
GO:0010876	lipid localization	3/40	393/14464	0.00763359	2.76030534	1.86310844	0.09415229	0.35119634	0.32444364	Cry1/Slc10a6/Slc43a3	3
GO:0007051	spindle organization	2/40	187/14464	0.01069519	3.86737968	2.07827904	0.09425619	0.35119634	0.32444364	Plk5/Arhgef10	2
GO:0009156	ribonucleoside monophosphate biosynthetic process	1/40	36/14464	0.02777778	10.0444444	2.86118811	0.09499646	0.35119634	0.32444364	Pnp	1
GO:0009167	purine ribonucleoside monophosphate metabolic process	1/40	36/14464	0.02777778	10.0444444	2.86118811	0.09499646	0.35119634	0.32444364	Pnp	1
GO:0014002	astrocyte development	1/40	36/14464	0.02777778	10.0444444	2.86118811	0.09499646	0.35119634	0.32444364	Cntf	1
GO:0042908	xenobiotic transport	1/40	36/14464	0.02777778	10.0444444	2.86118811	0.09499646	0.35119634	0.32444364	Slc43a3	1
GO:0045823	positive regulation of heart contraction	1/40	36/14464	0.02777778	10.0444444	2.86118811	0.09499646	0.35119634	0.32444364	Ptpn1	1
GO:0048753	pigment granule organization	1/40	36/14464	0.02777778	10.0444444	2.86118811	0.09499646	0.35119634	0.32444364	Hps5	1
GO:0070266	necroptotic process	1/40	36/14464	0.02777778	10.0444444	2.86118811	0.09499646	0.35119634	0.32444364	Bok	1
GO:0120033	negative regulation of plasma membrane bounded cell projection assembly	1/40	36/14464	0.02777778	10.0444444	2.86118811	0.09499646	0.35119634	0.32444364	Tchp	1
GO:1902229	regulation of intrinsic apoptotic signaling pathway in response to DNA damage	1/40	36/14464	0.02777778	10.0444444	2.86118811	0.09499646	0.35119634	0.32444364	Fbh1	1
GO:0043524	negative regulation of neuron apoptotic process	2/40	188/14464	0.0106383	3.84680851	2.06895118	0.09510834	0.35119634	0.32444364	Bok/Cntf	2
GO:0032259	methylation	2/40	190/14464	0.01052632	3.80631579	2.05048608	0.09681944	0.3528454	0.32596709	Rrp8/Prdm15	2
GO:0016233	telomere capping	1/40	37/14464	0.02702703	9.77297297	2.8136882	0.09750548	0.3528454	0.32596709	Pkib	1
GO:0046326	positive regulation of D-glucose import	1/40	37/14464	0.02702703	9.77297297	2.8136882	0.09750548	0.3528454	0.32596709	Capn10	1
GO:1904894	positive regulation of receptor signaling pathway via STAT	1/40	37/14464	0.02702703	9.77297297	2.8136882	0.09750548	0.3528454	0.32596709	Cntf	1
GO:0031099	regeneration	2/40	191/14464	0.0104712	3.78638743	2.04134724	0.09767837	0.3528454	0.32596709	Matn2/Cntf	2
GO:0050730	regulation of peptidyl-tyrosine phosphorylation	2/40	191/14464	0.0104712	3.78638743	2.04134724	0.09767837	0.3528454	0.32596709	Cntf/Ptpn1	2
GO:0044331	cell-cell adhesion mediated by cadherin	1/40	38/14464	0.02631579	9.51578947	2.76796174	0.10000772	0.35598503	0.32886755	Cdh19	1
GO:0045165	cell fate commitment	2/40	195/14464	0.01025641	3.70871795	2.00539835	0.10113609	0.35598503	0.32886755	Prdm15/Smad1	2
GO:0051348	negative regulation of transferase activity	2/40	196/14464	0.01020408	3.68979592	1.996559	0.10200594	0.35598503	0.32886755	Pkib/Ptpn1	2
GO:0006904	vesicle docking involved in exocytosis	1/40	39/14464	0.02564103	9.27179487	2.72389574	0.10250319	0.35598503	0.32886755	Ctbp2	1
GO:0009126	purine nucleoside monophosphate metabolic process	1/40	39/14464	0.02564103	9.27179487	2.72389574	0.10250319	0.35598503	0.32886755	Pnp	1
GO:0010661	positive regulation of muscle cell apoptotic process	1/40	39/14464	0.02564103	9.27179487	2.72389574	0.10250319	0.35598503	0.32886755	Ptpn1	1
GO:0017145	stem cell division	1/40	39/14464	0.02564103	9.27179487	2.72389574	0.10250319	0.35598503	0.32886755	Prdm15	1
GO:0043094	cellular metabolic compound salvage	1/40	39/14464	0.02564103	9.27179487	2.72389574	0.10250319	0.35598503	0.32886755	Pnp	1
GO:0060632	regulation of microtubule-based movement	1/40	39/14464	0.02564103	9.27179487	2.72389574	0.10250319	0.35598503	0.32886755	Rhot1	1
GO:1904358	positive regulation of telomere maintenance via telomere lengthening	1/40	39/14464	0.02564103	9.27179487	2.72389574	0.10250319	0.35598503	0.32886755	Pkib	1
GO:1904738	vascular associated smooth muscle cell migration	1/40	39/14464	0.02564103	9.27179487	2.72389574	0.10250319	0.35598503	0.32886755	Ptpn1	1
GO:0051963	regulation of synapse assembly	2/40	197/14464	0.01015228	3.67106599	1.98777732	0.10287793	0.35598503	0.32886755	Lrrn3/Ptpn1	2
GO:0043434	response to peptide hormone	3/40	410/14464	0.00731707	2.64585366	1.78032587	0.10362676	0.35598503	0.32886755	Cry1/Capn10/Ptpn1	3
GO:0099558	maintenance of synapse structure	1/40	40/14464	0.025	9.04	2.68138712	0.10499192	0.35598503	0.32886755	Ctbp2	1
GO:1903524	positive regulation of blood circulation	1/40	40/14464	0.025	9.04	2.68138712	0.10499192	0.35598503	0.32886755	Ptpn1	1
GO:0031345	negative regulation of cell projection organization	2/40	201/14464	0.00995025	3.59800995	1.95321356	0.10638683	0.35598503	0.32886755	Tchp/Ptpn1	2
GO:0001754	eye photoreceptor cell differentiation	1/40	41/14464	0.02439024	8.8195122	2.64034161	0.10747392	0.35598503	0.32886755	Cntf	1
GO:0009394	2'-deoxyribonucleotide metabolic process	1/40	41/14464	0.02439024	8.8195122	2.64034161	0.10747392	0.35598503	0.32886755	Pnp	1
GO:0030866	cortical actin cytoskeleton organization	1/40	41/14464	0.02439024	8.8195122	2.64034161	0.10747392	0.35598503	0.32886755	Iqgap2	1
GO:0034314	Arp2/3 complex-mediated actin nucleation	1/40	41/14464	0.02439024	8.8195122	2.64034161	0.10747392	0.35598503	0.32886755	Iqgap2	1
GO:0043507	positive regulation of JUN kinase activity	1/40	41/14464	0.02439024	8.8195122	2.64034161	0.10747392	0.35598503	0.32886755	Ptpn1	1
GO:0044060	regulation of endocrine process	1/40	41/14464	0.02439024	8.8195122	2.64034161	0.10747392	0.35598503	0.32886755	Cry1	1
GO:0045047	protein targeting to ER	1/40	41/14464	0.02439024	8.8195122	2.64034161	0.10747392	0.35598503	0.32886755	Zfand2b	1
GO:0045910	negative regulation of DNA recombination	1/40	41/14464	0.02439024	8.8195122	2.64034161	0.10747392	0.35598503	0.32886755	Fbh1	1
GO:1990776	response to angiotensin	1/40	41/14464	0.02439024	8.8195122	2.64034161	0.10747392	0.35598503	0.32886755	Ptpn1	1
GO:0019692	deoxyribose phosphate metabolic process	1/40	42/14464	0.02380952	8.60952381	2.60067278	0.1099492	0.3570663	0.32986645	Pnp	1
GO:0045777	positive regulation of blood pressure	1/40	42/14464	0.02380952	8.60952381	2.60067278	0.1099492	0.3570663	0.32986645	Ptpn1	1
GO:0046638	positive regulation of alpha-beta T cell differentiation	1/40	42/14464	0.02380952	8.60952381	2.60067278	0.1099492	0.3570663	0.32986645	Pnp	1
GO:0070570	regulation of neuron projection regeneration	1/40	42/14464	0.02380952	8.60952381	2.60067278	0.1099492	0.3570663	0.32986645	Cntf	1
GO:0090630	activation of GTPase activity	1/40	42/14464	0.02380952	8.60952381	2.60067278	0.1099492	0.3570663	0.32986645	Arhgef10	1
GO:1903573	negative regulation of response to endoplasmic reticulum stress	1/40	42/14464	0.02380952	8.60952381	2.60067278	0.1099492	0.3570663	0.32986645	Ptpn1	1
GO:0000082	G1/S transition of mitotic cell cycle	2/40	207/14464	0.00966184	3.49371981	1.90298377	0.11171118	0.35774606	0.33049443	Plk5/Ctdspl	2
GO:0009262	deoxyribonucleotide metabolic process	1/40	43/14464	0.02325581	8.40930233	2.56230124	0.11241779	0.35774606	0.33049443	Pnp	1
GO:0050732	negative regulation of peptidyl-tyrosine phosphorylation	1/40	43/14464	0.02325581	8.40930233	2.56230124	0.11241779	0.35774606	0.33049443	Ptpn1	1
GO:0070232	regulation of T cell apoptotic process	1/40	43/14464	0.02325581	8.40930233	2.56230124	0.11241779	0.35774606	0.33049443	Pnp	1
GO:0140719	constitutive heterochromatin formation	1/40	43/14464	0.02325581	8.40930233	2.56230124	0.11241779	0.35774606	0.33049443	Samd1	1
GO:0006282	regulation of DNA repair	2/40	208/14464	0.00961538	3.47692308	1.89479258	0.11260547	0.35774606	0.33049443	Pnp/Fbh1	2
GO:0001913	T cell mediated cytotoxicity	1/40	44/14464	0.02272727	8.21818182	2.52515393	0.11487971	0.35774606	0.33049443	Pnp	1
GO:0002183	cytoplasmic translational initiation	1/40	44/14464	0.02272727	8.21818182	2.52515393	0.11487971	0.35774606	0.33049443	Eif3j1	1
GO:0010828	positive regulation of D-glucose transmembrane transport	1/40	44/14464	0.02272727	8.21818182	2.52515393	0.11487971	0.35774606	0.33049443	Capn10	1
GO:0042509	regulation of tyrosine phosphorylation of STAT protein	1/40	44/14464	0.02272727	8.21818182	2.52515393	0.11487971	0.35774606	0.33049443	Cntf	1
GO:1901976	regulation of cell cycle checkpoint	1/40	44/14464	0.02272727	8.21818182	2.52515393	0.11487971	0.35774606	0.33049443	Cry1	1
GO:1902116	negative regulation of organelle assembly	1/40	44/14464	0.02272727	8.21818182	2.52515393	0.11487971	0.35774606	0.33049443	Tchp	1
GO:1903670	regulation of sprouting angiogenesis	1/40	44/14464	0.02272727	8.21818182	2.52515393	0.11487971	0.35774606	0.33049443	Smad1	1
GO:0031032	actomyosin structure organization	2/40	212/14464	0.00943396	3.41132075	1.86252169	0.1162017	0.35774606	0.33049443	Iqgap2/Arhgef10	2
GO:0032869	cellular response to insulin stimulus	2/40	212/14464	0.00943396	3.41132075	1.86252169	0.1162017	0.35774606	0.33049443	Capn10/Ptpn1	2
GO:0009124	nucleoside monophosphate biosynthetic process	1/40	45/14464	0.02222222	8.03555556	2.48916345	0.11733497	0.35774606	0.33049443	Pnp	1
GO:0043407	negative regulation of MAP kinase activity	1/40	45/14464	0.02222222	8.03555556	2.48916345	0.11733497	0.35774606	0.33049443	Ptpn1	1
GO:0048009	insulin-like growth factor receptor signaling pathway	1/40	45/14464	0.02222222	8.03555556	2.48916345	0.11733497	0.35774606	0.33049443	Zfand2b	1
GO:0072599	establishment of protein localization to endoplasmic reticulum	1/40	45/14464	0.02222222	8.03555556	2.48916345	0.11733497	0.35774606	0.33049443	Zfand2b	1
GO:1905953	negative regulation of lipid localization	1/40	45/14464	0.02222222	8.03555556	2.48916345	0.11733497	0.35774606	0.33049443	Cry1	1
GO:0001912	positive regulation of leukocyte mediated cytotoxicity	1/40	46/14464	0.02173913	7.86086957	2.45426752	0.11978358	0.36189161	0.33432419	Pnp	1
GO:0009116	nucleoside metabolic process	1/40	46/14464	0.02173913	7.86086957	2.45426752	0.11978358	0.36189161	0.33432419	Pnp	1
GO:2000351	regulation of endothelial cell apoptotic process	1/40	46/14464	0.02173913	7.86086957	2.45426752	0.11978358	0.36189161	0.33432419	Ptpn1	1
GO:0007260	tyrosine phosphorylation of STAT protein	1/40	47/14464	0.0212766	7.69361702	2.42040851	0.12222558	0.36477112	0.33698435	Cntf	1

GO:0014044	Schwann cell development	1/40	47/14464	0.0212766	7.69361702	2.42040851	0.12222558	0.36477112	0.33698435	Arhgef10	1
GO:0031297	replication fork processing	1/40	47/14464	0.0212766	7.69361702	2.42040851	0.12222558	0.36477112	0.33698435	Fbh1	1
GO:0048066	developmental pigmentation	1/40	47/14464	0.0212766	7.69361702	2.42040851	0.12222558	0.36477112	0.33698435	Hps5	1
GO:0006836	neurotransmitter transport	2/40	219/14464	0.00913242	3.30228311	1.80786455	0.12256602	0.36477112	0.33698435	Ctbp2/Rhot1	2
GO:0051052	regulation of DNA metabolic process	3/40	443/14464	0.00677201	2.44875847	1.63089006	0.12305726	0.36514311	0.337328	Pkib/Pnp/Fbh1	3
GO:0010976	positive regulation of neuron projection development	2/40	220/14464	0.00909091	3.28727273	1.80023683	0.12348232	0.36531712	0.33748876	Plk5/Cntf	2
GO:0032506	cytokinetic process	1/40	48/14464	0.02083333	7.53333333	2.38753299	0.12466096	0.36662826	0.33870002	Iqgap2	1
GO:0140353	lipid export from cell	1/40	48/14464	0.02083333	7.53333333	2.38753299	0.12466096	0.36662826	0.33870002	Cry1	1
GO:0014888	striated muscle adaptation	1/40	49/14464	0.02040816	7.37959184	2.3555913	0.12708976	0.36923085	0.34110435	Smad1	1
GO:0048010	vascular endothelial growth factor receptor signaling pathway	1/40	49/14464	0.02040816	7.37959184	2.3555913	0.12708976	0.36923085	0.34110435	Ptpn1	1
GO:0061097	regulation of protein tyrosine kinase activity	1/40	49/14464	0.02040816	7.37959184	2.3555913	0.12708976	0.36923085	0.34110435	Ptpn1	1
GO:1902895	positive regulation of miRNA transcription	1/40	49/14464	0.02040816	7.37959184	2.3555913	0.12708976	0.36923085	0.34110435	Smad1	1
GO:2001234	negative regulation of apoptotic signaling pathway	2/40	225/14464	0.00888889	3.21422222	1.76274321	0.1280894	0.36923085	0.34110435	Bok/Ptpn1	2
GO:0003254	regulation of membrane depolarization	1/40	50/14464	0.02	7.232	2.3245373	0.12951199	0.36923085	0.34110435	Bok	1
GO:0032663	regulation of interleukin-2 production	1/40	50/14464	0.02	7.232	2.3245373	0.12951199	0.36923085	0.34110435	Pnp	1
GO:0072577	endothelial cell apoptotic process	1/40	50/14464	0.02	7.232	2.3245373	0.12951199	0.36923085	0.34110435	Ptpn1	1
GO:0097300	programmed necrotic cell death	1/40	50/14464	0.02	7.232	2.3245373	0.12951199	0.36923085	0.34110435	Bok	1
GO:0010817	regulation of hormone levels	3/40	455/14464	0.00659341	2.38417582	1.57982769	0.13043999	0.36923085	0.34110435	Cry1/Cntf/Capn10	3
GO:0031667	response to nutrient levels	3/40	455/14464	0.00659341	2.38417582	1.57982769	0.13043999	0.36923085	0.34110435	Rrp8/Capn10/Ptpn1	3
GO:0002711	positive regulation of T cell mediated immunity	1/40	51/14464	0.01960784	7.09019608	2.29432798	0.13192766	0.36923085	0.34110435	Pnp	1
GO:0060986	endocrine hormone secretion	1/40	51/14464	0.01960784	7.09019608	2.29432798	0.13192766	0.36923085	0.34110435	Cry1	1
GO:0040029	epigenetic regulation of gene expression	2/40	230/14464	0.00869565	3.14434783	1.72628303	0.13273772	0.36923085	0.34110435	Rrp8/Samd1	2
GO:0001707	mesoderm formation	1/40	52/14464	0.01923077	6.95384615	2.26492323	0.1343368	0.36923085	0.34110435	Smad1	1
GO:0006111	regulation of gluconeogenesis	1/40	52/14464	0.01923077	6.95384615	2.26492323	0.1343368	0.36923085	0.34110435	Cry1	1
GO:0031343	positive regulation of cell killing	1/40	52/14464	0.01923077	6.95384615	2.26492323	0.1343368	0.36923085	0.34110435	Pnp	1
GO:0032210	regulation of telomere maintenance via telomerase	1/40	52/14464	0.01923077	6.95384615	2.26492323	0.1343368	0.36923085	0.34110435	Pkib	1
GO:0045005	DNA-templated DNA replication maintenance of fidelity	1/40	52/14464	0.01923077	6.95384615	2.26492323	0.1343368	0.36923085	0.34110435	Fbh1	1
GO:0018108	peptidyl-tyrosine phosphorylation	2/40	233/14464	0.00858369	3.10386266	1.7048806	0.13554571	0.36923085	0.34110435	Cntf/Ptpn1	2
GO:0009611	response to wounding	3/40	465/14464	0.00645161	2.33290323	1.53848378	0.13671302	0.36923085	0.34110435	Matn2/Cntf/Hps5	3
GO:0001881	receptor recycling	1/40	53/14464	0.01886792	6.82264151	2.23628556	0.13673942	0.36923085	0.34110435	Ptpn1	1
GO:0009161	ribonucleoside monophosphate metabolic process	1/40	53/14464	0.01886792	6.82264151	2.23628556	0.13673942	0.36923085	0.34110435	Pnp	1
GO:0033059	cellular pigmentation	1/40	53/14464	0.01886792	6.82264151	2.23628556	0.13673942	0.36923085	0.34110435	Hps5	1
GO:0042149	cellular response to glucose starvation	1/40	53/14464	0.01886792	6.82264151	2.23628556	0.13673942	0.36923085	0.34110435	Rrp8	1
GO:2000107	negative regulation of leukocyte apoptotic process	1/40	53/14464	0.01886792	6.82264151	2.23628556	0.13673942	0.36923085	0.34110435	Pnp	1
GO:0071900	regulation of protein serine/threonine kinase activity	2/40	235/14464	0.00851064	3.07744681	1.69080291	0.13742536	0.36923085	0.34110435	Pkib/Ptpn1	2
GO:0014037	Schwann cell differentiation	1/40	54/14464	0.01851852	6.6962963	2.20837994	0.13913553	0.36923085	0.34110435	Arhgef10	1
GO:0034332	adherens junction organization	1/40	54/14464	0.01851852	6.6962963	2.20837994	0.13913553	0.36923085	0.34110435	Cdh19	1
GO:0043506	regulation of JUN kinase activity	1/40	54/14464	0.01851852	6.6962963	2.20837994	0.13913553	0.36923085	0.34110435	Ptpn1	1
GO:0051496	positive regulation of stress fiber assembly	1/40	54/14464	0.01851852	6.6962963	2.20837994	0.13913553	0.36923085	0.34110435	Arhgef10	1
GO:0061900	glial cell activation	1/40	54/14464	0.01851852	6.6962963	2.20837994	0.13913553	0.36923085	0.34110435	Cntf	1
GO:1990090	cellular response to nerve growth factor stimulus	1/40	54/14464	0.01851852	6.6962963	2.20837994	0.13913553	0.36923085	0.34110435	Ptpn1	1
GO:0018212	peptidyl-tyrosine modification	2/40	237/14464	0.00843882	3.05147679	1.67687406	0.139311	0.36923085	0.34110435	Cntf/Ptpn1	2
GO:0010332	response to gamma radiation	1/40	55/14464	0.01818182	6.57454545	2.18117352	0.14152516	0.36923085	0.34110435	Pnp	1
GO:0010812	negative regulation of cell-substrate adhesion	1/40	55/14464	0.01818182	6.57454545	2.18117352	0.14152516	0.36923085	0.34110435	Ptpn1	1
GO:0010907	positive regulation of glucose metabolic process	1/40	55/14464	0.01818182	6.57454545	2.18117352	0.14152516	0.36923085	0.34110435	Cry1	1
GO:0030865	cortical cytoskeleton organization	1/40	55/14464	0.01818182	6.57454545	2.18117352	0.14152516	0.36923085	0.34110435	Iqgap2	1
GO:0150052	regulation of postsynapse assembly	1/40	55/14464	0.01818182	6.57454545	2.18117352	0.14152516	0.36923085	0.34110435	Ptpn1	1
GO:0051896	regulation of phosphatidylinositol 3-kinase/protein kinase B signal transduction	2/40	240/14464	0.00833333	3.01333333	1.65625333	0.14215042	0.36923085	0.34110435	Cntf/Ptpn1	2
GO:1901988	negative regulation of cell cycle phase transition	2/40	240/14464	0.00833333	3.01333333	1.65625333	0.14215042	0.36923085	0.34110435	Cry1/Ctdspl	2
GO:0048332	mesoderm morphogenesis	1/40	56/14464	0.01785714	6.45714286	2.15463554	0.14390833	0.36923085	0.34110435	Smad1	1
GO:0051646	mitochondrion localization	1/40	56/14464	0.01785714	6.45714286	2.15463554	0.14390833	0.36923085	0.34110435	Rhot1	1
GO:0060042	retina morphogenesis in camera-type eye	1/40	56/14464	0.01785714	6.45714286	2.15463554	0.14390833	0.36923085	0.34110435	Cntf	1
GO:0072698	protein localization to microtubule cytoskeleton	1/40	56/14464	0.01785714	6.45714286	2.15463554	0.14390833	0.36923085	0.34110435	Fam83d	1
GO:1990089	response to nerve growth factor	1/40	56/14464	0.01785714	6.45714286	2.15463554	0.14390833	0.36923085	0.34110435	Ptpn1	1
GO:2000630	positive regulation of miRNA metabolic process	1/40	56/14464	0.01785714	6.45714286	2.15463554	0.14390833	0.36923085	0.34110435	Smad1	1
GO:0051259	protein complex oligomerization	2/40	244/14464	0.00819672	2.96393443	1.62925263	0.14595618	0.36923085	0.34110435	Bok/Samd1	2
GO:0010665	regulation of cardiac muscle cell apoptotic process	1/40	57/14464	0.01754386	6.34385965	2.12873708	0.14628504	0.36923085	0.34110435	Ptpn1	1
GO:0032623	interleukin-2 production	1/40	57/14464	0.01754386	6.34385965	2.12873708	0.14628504	0.36923085	0.34110435	Pnp	1
GO:0033363	secretory granule organization	1/40	57/14464	0.01754386	6.34385965	2.12873708	0.14628504	0.36923085	0.34110435	Hps5	1
GO:0045010	actin nucleation	1/40	57/14464	0.01754386	6.34385965	2.12873708	0.14628504	0.36923085	0.34110435	Iqgap2	1
GO:0046635	positive regulation of alpha-beta T cell activation	1/40	57/14464	0.01754386	6.34385965	2.12873708	0.14628504	0.36923085	0.34110435	Pnp	1
GO:0048278	vesicle docking	1/40	57/14464	0.01754386	6.34385965	2.12873708	0.14628504	0.36923085	0.34110435	Ctbp2	1
GO:0060038	cardiac muscle cell proliferation	1/40	57/14464	0.01754386	6.34385965	2.12873708	0.14628504	0.36923085	0.34110435	Smad1	1
GO:1902808	positive regulation of cell cycle G1/S phase transition	1/40	57/14464	0.01754386	6.34385965	2.12873708	0.14628504	0.36923085	0.34110435	Fam83d	1
GO:0045912	negative regulation of carbohydrate metabolic process	1/40	58/14464	0.01724138	6.23448276	2.10345099	0.14865532	0.36994133	0.34176072	Cry1	1
GO:0046324	regulation of D-glucose import	1/40	58/14464	0.01724138	6.23448276	2.10345099	0.14865532	0.36994133	0.34176072	Capn10	1
GO:0046530	photoreceptor cell differentiation	1/40	58/14464	0.01724138	6.23448276	2.10345099	0.14865532	0.36994133	0.34176072	Cntf	1
GO:0046637	regulation of alpha-beta T cell differentiation	1/40	58/14464	0.01724138	6.23448276	2.10345099	0.14865532	0.36994133	0.34176072	Pnp	1
GO:0034329	cell junction assembly	3/40	485/14464	0.00618557	2.23670103	1.45886086	0.14957059	0.36994133	0.34176072	Cdh19/Lrrn3/Ptpn1	3
GO:0070372	regulation of ERK1 and ERK2 cascade	2/40	249/14464	0.00803213	2.90441767	1.59626421	0.15074399	0.36994133	0.34176072	Fam83d/Ptpn1	2
GO:0006278	RNA-templated DNA biosynthetic process	1/40	59/14464	0.01694915	6.12881356	2.07875171	0.15101918	0.36994133	0.34176072	Pkib	1
GO:0007004	telomere maintenance via telomerase	1/40	59/14464	0.01694915	6.12881356	2.07875171	0.15101918	0.36994133	0.34176072	Pkib	1
GO:0010662	regulation of striated muscle cell apoptotic process	1/40	59/14464	0.01694915	6.12881356	2.07875171	0.15101918	0.36994133	0.34176072	Ptpn1	1
GO:0031103	axon regeneration	1/40	59/14464	0.01694915	6.12881356	2.07875171	0.15101918	0.36994133	0.34176072	Cntf	1
GO:0070231	T cell apoptotic process	1/40	59/14464	0.01694915	6.12881356	2.07875171	0.15101918	0.36994133	0.34176072	Pnp	1
GO:2000134	negative regulation of G1/S transition of mitotic cell cycle	1/40	59/14464	0.01694915	6.12881356	2.07875171	0.15101918	0.36994133	0.34176072	Ctdspl	1
GO:0009880	embryonic pattern specification	1/40	60/14464	0.01666667	6.02666667	2.05461516	0.15337664	0.37205964	0.34371766	Smad1	1
GO:0034331	cell junction maintenance	1/40	60/14464	0.01666667	6.02666667	2.05461516	0.15337664	0.37205964	0.34371766	Ctbp2	1
GO:0048008	platelet-derived growth factor receptor signaling pathway	1/40	60/14464	0.01666667	6.02666667	2.05461516	0.15337664	0.37205964	0.34371766	Ptpn1	1
GO:0070228	regulation of lymphocyte apoptotic process	1/40	60/14464	0.01666667	6.02666667	2.05461516	0.15337664	0.37205964	0.34371766	Pnp	1
GO:1904356	regulation of telomere maintenance via telomere lengthening	1/40	61/14464	0.01639344	5.92786885	2.03101865	0.15572772	0.37684596	0.34813938	Pkib	1
GO:0034976	response to endoplasmic reticulum stress	2/40	256/14464	0.0078125	2.825	1.55143187	0.15750092	0.37808407	0.34928318	Bok/Ptpn1	2
GO:0032233	positive regulation of actin filament bundle assembly	1/40	62/14464	0.01612903	5.83225806	2.00794075	0.15807243	0.37808407	0.34928318	Arhgef10	1
GO:0045744	negative regulation of G protein-coupled receptor signaling pathway	1/40	62/14464	0.01612903	5.83225806	2.00794075	0.15807243	0.37808407	0.34928318	Cry1	1
GO:0051148	negative regulation of muscle cell differentiation	1/40	62/14464	0.01612903	5.83225806	2.00794075	0.15807243	0.37808407	0.34928318	Smad1	1
GO:0001836	release of cytochrome c from mitochondria	1/40	63/14464	0.01587302	5.73968254	1.98536122	0.16041079	0.37808407	0.34928318	Bok	1
GO:0006919	activation of cysteine-type endopeptidase activity involved in apoptotic process	1/40	63/14464	0.01587302	5.73968254	1.98536122	0.16041079	0.37808407	0.34928318	Bok	1
GO:0045669	positive regulation of osteoblast differentiation	1/40	63/14464	0.01587302	5.73968254	1.98536122	0.16041079	0.37808407	0.34928318	Smad1	1
GO:0051898	negative regulation of phosphatidylinositol 3-kinase/protein kinase B signal transduction	1/40	63/14464	0.01587302	5.73968254	1.98536122	0.16041079	0.37808407	0.34928318	Ptpn1	1
GO:0061035	regulation of cartilage development	1/40	63/14464	0.01587302	5.73968254	1.98536122	0.16041079	0.37808407	0.34928318	Smad1	1
GO:0097192	extrinsic apoptotic signaling pathway in absence of ligand	1/40	63/14464	0.01587302	5.73968254	1.98536122	0.16041079	0.37808407	0.34928318	Bok	1
GO:1901657	glycosyl compound metabolic process	1/40	63/14464	0.01587302	5.73968254	1.98536122	0.16041079	0.37808407	0.34928318	Pnp	1
GO:0003407	neural retina development	1/40	64/14464	0.015625	5.65	1.96326094	0.16274282	0.38177552	0.35269343	Cntf	1
GO:0044380	protein localization to cytoskeleton	1/40	64/14464	0.015625	5.65	1.96326094	0.16274282	0.38177552	0.35269343	Fam83d	1
GO:0001910	regulation of leukocyte mediated cytotoxicity	1/40	65/14464	0.01538462	5.56307692	1.94162178	0.16506854	0.38236397	0.35323705	Pnp	1
GO:0010659	cardiac muscle cell apoptotic process	1/40	65/14464	0.01538462	5.56307692	1.94162178	0.16506854	0.38236397	0.35323705	Ptpn1	1
GO:0032922	circadian regulation of gene expression	1/40	65/14464	0.01538462	5.56307692	1.94162178	0.16506854	0.38236397	0.35323705	Cry1	1
GO:0038034	signal transduction in absence of ligand	1/40	65/14464	0.01538462	5.56307692	1.94162178	0.16506854	0.38236397	0.35323705	Bok	1
GO:0046425	regulation of receptor signaling pathway via JAK-STAT	1/40	65/14464	0.01538462	5.56307692	1.94162178	0.16506854	0.38236397	0.35323705	Cntf	1
GO:0098813	nuclear chromosome segregation	2/40	264/14464	0.00757576	2.73939394	1.50200741	0.16529476	0.38236397	0.35323705	Arhgef10/Fam83d	2
GO:0032508	DNA duplex unwinding	1/40	66/14464	0.01515152	5.47878788	1.92042658	0.16738795	0.38541753	0.356058	Fbh1	1
GO:2000573	positive regulation of DNA biosynthetic process	1/40	66/14464	0.01515152	5.47878788	1.92042658	0.16738795	0.38541753	0.356058	Pkib	1
GO:0010658	striated muscle cell apoptotic process	1/40	67/14464	0.01492537	5.39701493	1.89965909	0.16970109	0.38805501	0.35849457	Ptpn1	1
GO:1902807	negative regulation of cell cycle G1/S phase transition	1/40	67/14464	0.01492537	5.39701493	1.89965909	0.16970109	0.38805501	0.35849457	Ctdspl	1
GO:1903036	positive regulation of response to wounding	1/40	67/14464	0.01492537	5.39701493	1.89965909	0.16970109	0.38805501	0.35849457	Cntf	1
GO:0060395	SMAD protein signal transduction	1/40	68/14464	0.01470588	5.31764706	1.87930387	0.17200795	0.39064221	0.36088469	Smad1	1
GO:0071806	protein transmembrane transport	1/40	68/14464	0.01470588	5.31764706	1.87930387	0.17200795	0.39064221	0.36088469	Zfand2b	1
GO:1902893	regulation of miRNA transcription	1/40	68/14464	0.01470588	5.31764706	1.87930387	0.17200795	0.39064221	0.36088469	Smad1	1
GO:0070371	ERK1 and ERK2 cascade	2/40	273/14464	0.00732601	2.64908425	1.4485517	0.17414603	0.39407175	0.36405299	Fam83d/Ptpn1	2
GO:0008347	glial cell migration	1/40	69/14464	0.01449275	5.24057971	1.85934626	0.17430857	0.39407175	0.36405299	Matn2	1
GO:0033143	regulation of intracellular steroid hormone receptor signaling pathway	1/40	70/14464	0.01428571	5.16571429	1.83977233	0.17660295	0.39478284	0.3647099	Cry1	1
GO:0046626	regulation of insulin receptor signaling pathway	1/40	70/14464	0.01428571	5.16571429	1.83977233	0.17660295	0.39478284	0.3647099	Ptpn1	1
GO:0051480	regulation of cytosolic calcium ion concentration	1/40	70/14464	0.01428571	5.16571429	1.83977233	0.17660295	0.39478284	0.3647099	Bok	1
GO:0061614	miRNA transcription	1/40	70/14464	0.01428571	5.16571429	1.83977233	0.17660295	0.39478284	0.3647099	Smad1	1
GO:1902017	regulation of cilium assembly	1/40	70/14464	0.01428571	5.16571429	1.83977233	0.17660295	0.39478284	0.3647099	Tchp	1
GO:0010948	negative regulation of cell cycle process	2/40	277/14464	0.00722022	2.61083032	1.42547869	0.17810588	0.39723157	0.3669721	Cry1/Ctdspl	2
GO:0009123	nucleoside monophosphate metabolic process	1/40	71/14464	0.01408451	5.09295775	1.82056884	0.17889112	0.39723157	0.3669721	Pnp	1
GO:0043491	phosphatidylinositol 3-kinase/protein kinase B signal transduction	2/40	278/14464	0.00719424	2.60143885	1.41977365	0.17909818	0.39723157	0.3669721	Cntf/Ptpn1	2
GO:0010833	telomere maintenance via telomere lengthening	1/40	72/14464	0.01388889	5.02222222	1.80172318	0.18117309	0.39723157	0.3669721	Pkib	1
GO:0032392	DNA geometric change	1/40	72/14464	0.01388889	5.02222222	1.80172318	0.18117309	0.39723157	0.3669721	Fbh1	1
GO:0070373	negative regulation of ERK1 and ERK2 cascade	1/40	72/14464	0.01388889	5.02222222	1.80172318	0.18117309	0.39723157	0.3669721	Ptpn1	1
GO:0090307	mitotic spindle assembly	1/40	72/14464	0.01388889	5.02222222	1.80172318	0.18117309	0.39723157	0.3669721	Arhgef10	1
GO:0097194	execution phase of apoptosis	1/40	72/14464	0.01388889	5.02222222	1.80172318	0.18117309	0.39723157	0.3669721	Bok	1
GO:0046942	carboxylic acid transport	2/40	281/14464	0.00711744	2.57366548	1.40280679	0.18208055	0.39723157	0.3669721	Slc10a6/Slc43a3	2
GO:0032206	positive regulation of telomere maintenance	1/40	73/14464	0.01369863	4.95342466	1.78322335	0.18344887	0.39723157	0.3669721	Pkib	1
GO:0045665	negative regulation of neuron differentiation	1/40	73/14464	0.01369863	4.95342466	1.78322335	0.18344887	0.39723157	0.3669721	Cntf	1
GO:0048644	muscle organ morphogenesis	1/40	73/14464	0.01369863	4.95342466	1.78322335	0.18344887	0.39723157	0.3669721	Cntf	1
GO:1904892	regulation of receptor signaling pathway via STAT	1/40	73/14464	0.01369863	4.95342466	1.78322335	0.18344887	0.39723157	0.3669721	Cntf	1
GO:0007416	synapse assembly	2/40	283/14464	0.00706714	2.55547703	1.39161699	0.18407321	0.39723157	0.3669721	Lrrn3/Ptpn1	2
GO:0015849	organic acid transport	2/40	283/14464	0.00706714	2.55547703	1.39161699	0.18407321	0.39723157	0.3669721	Slc10a6/Slc43a3	2
GO:0043523	regulation of neuron apoptotic process	2/40	283/14464	0.00706714	2.55547703	1.39161699	0.18407321	0.39723157	0.3669721	Bok/Cntf	2
GO:0014823	response to activity	1/40	74/14464	0.01351351	4.88648649	1.7650579	0.18571849	0.39809865	0.36777313	Cry1	1
GO:0042102	positive regulation of T cell proliferation	1/40	74/14464	0.01351351	4.88648649	1.7650579	0.18571849	0.39809865	0.36777313	Pnp	1
GO:0051298	centrosome duplication	1/40	74/14464	0.01351351	4.88648649	1.7650579	0.18571849	0.39809865	0.36777313	Arhgef10	1
GO:0002709	regulation of T cell mediated immunity	1/40	75/14464	0.01333333	4.82133333	1.74721593	0.18798195	0.39809865	0.36777313	Pnp	1
GO:0010569	regulation of double-strand break repair via homologous recombination	1/40	75/14464	0.01333333	4.82133333	1.74721593	0.18798195	0.39809865	0.36777313	Fbh1	1
GO:0010827	regulation of D-glucose transmembrane transport	1/40	75/14464	0.01333333	4.82133333	1.74721593	0.18798195	0.39809865	0.36777313	Capn10	1
GO:0046323	D-glucose import	1/40	75/14464	0.01333333	4.82133333	1.74721593	0.18798195	0.39809865	0.36777313	Capn10	1
GO:0150076	neuroinflammatory response	1/40	75/14464	0.01333333	4.82133333	1.74721593	0.18798195	0.39809865	0.36777313	Cntf	1
GO:0051054	positive regulation of DNA metabolic process	2/40	287/14464	0.00696864	2.51986063	1.36952156	0.18806867	0.39809865	0.36777313	Pkib/Pnp	2
GO:1901888	regulation of cell junction assembly	2/40	288/14464	0.00694444	2.51111111	1.36405576	0.18906958	0.39936944	0.36894711	Lrrn3/Ptpn1	2
GO:0050886	endocrine process	1/40	76/14464	0.01315789	4.75789474	1.72968701	0.19023928	0.40014465	0.36966327	Cry1	1
GO:0140056	organelle localization by membrane tethering	1/40	76/14464	0.01315789	4.75789474	1.72968701	0.19023928	0.40014465	0.36966327	Ctbp2	1
GO:0043112	receptor metabolic process	1/40	77/14464	0.01298701	4.6961039	1.71246121	0.19249049	0.40233338	0.37168527	Ptpn1	1
GO:0046888	negative regulation of hormone secretion	1/40	77/14464	0.01298701	4.6961039	1.71246121	0.19249049	0.40233338	0.37168527	Cry1	1
GO:0070972	protein localization to endoplasmic reticulum	1/40	77/14464	0.01298701	4.6961039	1.71246121	0.19249049	0.40233338	0.37168527	Zfand2b	1
GO:0071375	cellular response to peptide hormone stimulus	2/40	292/14464	0.00684932	2.47671233	1.34241899	0.19308107	0.40272349	0.37204566	Capn10/Ptpn1	2
GO:0007422	peripheral nervous system development	1/40	78/14464	0.01282051	4.63589744	1.69552903	0.1947356	0.40364116	0.37289343	Arhgef10	1
GO:0031341	regulation of cell killing	1/40	78/14464	0.01282051	4.63589744	1.69552903	0.1947356	0.40364116	0.37289343	Pnp	1
GO:0050871	positive regulation of B cell activation	1/40	78/14464	0.01282051	4.63589744	1.69552903	0.1947356	0.40364116	0.37289343	Pnp	1
GO:0031397	negative regulation of protein ubiquitination	1/40	79/14464	0.01265823	4.57721519	1.67888137	0.19697463	0.40575145	0.37484297	Cry1	1
GO:0051965	positive regulation of synapse assembly	1/40	79/14464	0.01265823	4.57721519	1.67888137	0.19697463	0.40575145	0.37484297	Lrrn3	1
GO:0097009	energy homeostasis	1/40	79/14464	0.01265823	4.57721519	1.67888137	0.19697463	0.40575145	0.37484297	Rrp8	1
GO:0070227	lymphocyte apoptotic process	1/40	80/14464	0.0125	4.52	1.66250956	0.19920758	0.40950506	0.37831065	Pnp	1
GO:0071103	DNA conformation change	1/40	81/14464	0.01234568	4.46419753	1.64640528	0.20143448	0.41323081	0.38175258	Fbh1	1
GO:0014855	striated muscle cell proliferation	1/40	82/14464	0.01219512	4.4097561	1.63056055	0.20365533	0.41607453	0.38437968	Smad1	1
GO:2000628	regulation of miRNA metabolic process	1/40	82/14464	0.01219512	4.4097561	1.63056055	0.20365533	0.41607453	0.38437968	Smad1	1
GO:0001822	kidney development	2/40	303/14464	0.00660066	2.38679868	1.28470704	0.20417227	0.41627761	0.38456729	Cys1/Smad1	2
GO:1901990	regulation of mitotic cell cycle phase transition	2/40	304/14464	0.00657895	2.37894737	1.27958514	0.20518454	0.41748772	0.38568522	Plk5/Ctdspl	2
GO:1900076	regulation of cellular response to insulin stimulus	1/40	83/14464	0.01204819	4.35662651	1.61496775	0.20587017	0.41802966	0.38618587	Ptpn1	1
GO:0006094	gluconeogenesis	1/40	84/14464	0.01190476	4.3047619	1.59961957	0.208079	0.42014228	0.38813756	Cry1	1
GO:0042157	lipoprotein metabolic process	1/40	84/14464	0.01190476	4.3047619	1.59961957	0.208079	0.42014228	0.38813756	Samd1	1
GO:0051098	regulation of binding	2/40	308/14464	0.00649351	2.34805195	1.25929663	0.20923965	0.42014228	0.38813756	Ctbp2/Fbh1	2
GO:0003073	regulation of systemic arterial blood pressure	1/40	85/14464	0.01176471	4.25411765	1.58450897	0.21028184	0.42014228	0.38813756	Ptpn1	1
GO:0019915	lipid storage	1/40	85/14464	0.01176471	4.25411765	1.58450897	0.21028184	0.42014228	0.38813756	Cry1	1
GO:0022406	membrane docking	1/40	85/14464	0.01176471	4.25411765	1.58450897	0.21028184	0.42014228	0.38813756	Ctbp2	1

GO:0048708 astrocyte differentiation	1/40	85/14464	0.01176471	4.25411765	1.58450897	0.21028184	0.42014228	0.38813756 Cntf	1
GO:0051310 metaphase chromosome alignment	1/40	85/14464	0.01176471	4.25411765	1.58450897	0.21028184	0.42014228	0.38813756 Fam83d	1
GO:0000281 mitotic cytokinesis	1/40	87/14464	0.01149425	4.15632184	1.55497389	0.21466961	0.42392944	0.39163623 Iqgap2	1
GO:0015908 fatty acid transport	1/40	87/14464	0.01149425	4.15632184	1.55497389	0.21466961	0.42392944	0.39163623 Slc43a3	1
GO:0051881 regulation of mitochondrial membrane potential	1/40	87/14464	0.01149425	4.15632184	1.55497389	0.21466961	0.42392944	0.39163623 Bok	1
GO:0072384 organelle transport along microtubule	1/40	87/14464	0.01149425	4.15632184	1.55497389	0.21466961	0.42392944	0.39163623 Rhot1	1
GO:0090288 negative regulation of cellular response to growth factor stimulus	1/40	87/14464	0.01149425	4.15632184	1.55497389	0.21466961	0.42392944	0.39163623 Ptpn1	1
GO:0072001 renal system development	2/40	315/14464	0.00634921	2.29587302	1.22453494	0.21635775	0.42392944	0.39163623 Cys1/Smad1	2
GO:1903532 positive regulation of secretion by cell	2/40	315/14464	0.00634921	2.29587302	1.22453494	0.21635775	0.42392944	0.39163623 Cntf/Capn10	2
GO:0019319 hexose biosynthetic process	1/40	88/14464	0.01136364	4.10909091	1.54053673	0.21685458	0.42392944	0.39163623 Cry1	1
GO:0043406 positive regulation of MAP kinase activity	1/40	88/14464	0.01136364	4.10909091	1.54053673	0.21685458	0.42392944	0.39163623 Ptpn1	1
GO:0043954 cellular component maintenance	1/40	88/14464	0.01136364	4.10909091	1.54053673	0.21685458	0.42392944	0.39163623 Ctbp2	1
GO:2000106 regulation of leukocyte apoptotic process	1/40	88/14464	0.01136364	4.10909091	1.54053673	0.21685458	0.42392944	0.39163623 Pnp	1
GO:0051345 positive regulation of hydrolase activity	2/40	317/14464	0.00630915	2.28138801	1.21477108	0.21839614	0.42559885	0.39317848 Bok/Arhgef10	2
GO:0045913 positive regulation of carbohydrate metabolic process	1/40	89/14464	0.01123596	4.06292135	1.52631177	0.21903361	0.42559885	0.39317848 Cry1	1
GO:1903321 negative regulation of protein modification by small protein conjugation or removal	1/40	89/14464	0.01123596	4.06292135	1.52631177	0.21903361	0.42559885	0.39317848 Cry1	1
GO:0030522 intracellular receptor signaling pathway	2/40	318/14464	0.00628931	2.27421384	1.20991645	0.21941606	0.42559885	0.39317848 Cry1/Ctbp2	2
GO:0001657 ureteric bud development	1/40	90/14464	0.01111111	4.01777778	1.51229328	0.22120674	0.42575891	0.39332635 Smad1	1
GO:0002027 regulation of heart rate	1/40	90/14464	0.01111111	4.01777778	1.51229328	0.22120674	0.42575891	0.39332635 Ptpn1	1
GO:0007498 mesoderm development	1/40	90/14464	0.01111111	4.01777778	1.51229328	0.22120674	0.42575891	0.39332635 Smad1	1
GO:0046634 regulation of alpha-beta T cell activation	1/40	90/14464	0.01111111	4.01777778	1.51229328	0.22120674	0.42575891	0.39332635 Pnp	1
GO:0072163 mesonephric epithelium development	1/40	91/14464	0.01098901	3.97362637	1.49847574	0.22337396	0.42827662	0.39565226 Smad1	1
GO:0072164 mesonephric tubule development	1/40	91/14464	0.01098901	3.97362637	1.49847574	0.22337396	0.42827662	0.39565226 Smad1	1
GO:0001823 mesonephros development	1/40	92/14464	0.01086957	3.93043478	1.48485382	0.22553531	0.42994016	0.39718909 Smad1	1
GO:0044344 cellular response to fibroblast growth factor stimulus	1/40	92/14464	0.01086957	3.93043478	1.48485382	0.22553531	0.42994016	0.39718909 Ptpn1	1
GO:0051899 membrane depolarization	1/40	92/14464	0.01086957	3.93043478	1.48485382	0.22553531	0.42994016	0.39718909 Bok	1
GO:0043280 positive regulation of cysteine-type endopeptidase activity involved in apoptotic process	1/40	93/14464	0.01075269	3.88817204	1.47142243	0.22769079	0.43075469	0.39794156 Bok	1
GO:0046364 monosaccharide biosynthetic process	1/40	93/14464	0.01075269	3.88817204	1.47142243	0.22769079	0.43075469	0.39794156 Cry1	1
GO:0051303 establishment of chromosome localization	1/40	93/14464	0.01075269	3.88817204	1.47142243	0.22769079	0.43075469	0.39794156 Fam83d	1
GO:1901874 negative regulation of post-translational protein modification	1/40	93/14464	0.01075269	3.88817204	1.47142243	0.22769079	0.43075469	0.39794156 Cry1	1
GO:0051492 regulation of stress fiber assembly	1/40	94/14464	0.0106383	3.84680851	1.45817662	0.22984043	0.43399793	0.40093774 Arhgef10	1
GO:0002708 positive regulation of lymphocyte mediated immunity	1/40	95/14464	0.01052632	3.80631579	1.44511165	0.23198422	0.43639297	0.40315034 Pnp	1
GO:0010586 miRNA metabolic process	1/40	95/14464	0.01052632	3.80631579	1.44511165	0.23198422	0.43639297	0.40315034 Smad1	1
GO:0001704 formation of primary germ layer	1/40	96/14464	0.01041667	3.76666667	1.43222294	0.2341222	0.43958538	0.40609957 Smad1	1
GO:0051047 positive regulation of secretion	2/40	334/14464	0.00598802	2.16526946	1.13460541	0.23579182	0.44175767	0.40810638 Cntf/Capn10	2
GO:0032024 positive regulation of insulin secretion	1/40	97/14464	0.01030928	3.72783505	1.41950609	0.23625438	0.44175767	0.40810638 Capn10	1
GO:0032204 regulation of telomere maintenance	1/40	98/14464	0.01020408	3.68979592	1.40695682	0.23838077	0.44175767	0.40810638 Pkib	1
GO:0043473 pigmentation	1/40	98/14464	0.01020408	3.68979592	1.40695682	0.23838077	0.44175767	0.40810638 Hps5	1
GO:0062014 negative regulation of small molecule metabolic process	1/40	98/14464	0.01020408	3.68979592	1.40695682	0.23838077	0.44175767	0.40810638 Cry1	1
GO:0071774 response to fibroblast growth factor	1/40	98/14464	0.01020408	3.68979592	1.40695682	0.23838077	0.44175767	0.40810638 Ptpn1	1
GO:0090263 positive regulation of canonical Wnt signaling pathway	1/40	98/14464	0.01020408	3.68979592	1.40695682	0.23838077	0.44175767	0.40810638 Prdm15	1
GO:0045582 positive regulation of T cell differentiation	1/40	99/14464	0.01010101	3.65252525	1.39457104	0.24050138	0.44207085	0.4083957 Pnp	1
GO:2001243 negative regulation of intrinsic apoptotic signaling pathway	1/40	99/14464	0.01010101	3.65252525	1.39457104	0.24050138	0.44207085	0.4083957 Ptpn1	1
GO:0051402 neuron apoptotic process	2/40	339/14464	0.00589971	2.13333333	1.11193555	0.24092755	0.44207085	0.4083957 Bok/Cntf	2
GO:0008645 hexose transmembrane transport	1/40	100/14464	0.01	3.616	1.38234477	0.24261624	0.44207085	0.4083957 Capn10	1
GO:0014910 regulation of smooth muscle cell migration	1/40	100/14464	0.01	3.616	1.38234477	0.24261624	0.44207085	0.4083957 Ptpn1	1
GO:0046928 regulation of neurotransmitter secretion	1/40	100/14464	0.01	3.616	1.38234477	0.24261624	0.44207085	0.4083957 Rhot1	1
GO:0050000 chromosome localization	1/40	100/14464	0.01	3.616	1.38234477	0.24261624	0.44207085	0.4083957 Fam83d	1
GO:0055017 cardiac muscle tissue growth	1/40	100/14464	0.01	3.616	1.38234477	0.24261624	0.44207085	0.4083957 Smad1	1
GO:1904659 D-glucose transmembrane transport	1/40	100/14464	0.01	3.616	1.38234477	0.24261624	0.44207085	0.4083957 Capn10	1
GO:0015711 organic anion transport	2/40	341/14464	0.0058651	2.12082111	1.10297672	0.24298377	0.44207085	0.4083957 Slc10a6/Slc43a3	2
GO:0003300 cardiac muscle hypertrophy	1/40	101/14464	0.00990099	3.58019802	1.37027419	0.24472536	0.44260479	0.40888897 Smad1	1
GO:0010660 regulation of muscle cell apoptotic process	1/40	101/14464	0.00990099	3.58019802	1.37027419	0.24472536	0.44260479	0.40888897 Ptpn1	1
GO:0043255 regulation of carbohydrate biosynthetic process	1/40	101/14464	0.00990099	3.58019802	1.37027419	0.24472536	0.44260479	0.40888897 Cry1	1
GO:0001909 leukocyte mediated cytotoxicity	1/40	102/14464	0.00980392	3.54509804	1.35835559	0.24682875	0.44260479	0.40888897 Pnp	1
GO:0002824 positive regulation of adaptive immune response based on somatic recombination of immune receptors b	1/40	102/14464	0.00980392	3.54509804	1.35835559	0.24682875	0.44260479	0.40888897 Pnp	1
GO:0015749 monosaccharide transmembrane transport	1/40	102/14464	0.00980392	3.54509804	1.35835559	0.24682875	0.44260479	0.40888897 Capn10	1
GO:0099068 postsynapse assembly	1/40	102/14464	0.00980392	3.54509804	1.35835559	0.24682875	0.44260479	0.40888897 Ptpn1	1
GO:1901989 positive regulation of cell cycle phase transition	1/40	102/14464	0.00980392	3.54509804	1.35835559	0.24682875	0.44260479	0.40888897 Fam83d	1
GO:0042752 regulation of circadian rhythm	1/40	103/14464	0.00970874	3.51067961	1.34658539	0.24892643	0.44397075	0.41015088 Cry1	1
GO:0140029 exocytic process	1/40	103/14464	0.00970874	3.51067961	1.34658539	0.24892643	0.44397075	0.41015088 Ctbp2	1
GO:2000278 regulation of DNA biosynthetic process	1/40	103/14464	0.00970874	3.51067961	1.34658539	0.24892643	0.44397075	0.41015088 Pkib	1
GO:0009410 response to xenobiotic stimulus	2/40	348/14464	0.00574713	2.07816092	1.0720961	0.25018796	0.44542393	0.41149336 Pnp/Smad1	2
GO:0014897 striated muscle hypertrophy	1/40	104/14464	0.00961538	3.47692308	1.33496015	0.25101841	0.4461058	0.41212329 Smad1	1
GO:0010906 regulation of glucose metabolic process	1/40	105/14464	0.00952381	3.44380952	1.32347651	0.25310471	0.44742091	0.41333822 Cry1	1
GO:0046632 alpha-beta T cell differentiation	1/40	105/14464	0.00952381	3.44380952	1.32347651	0.25310471	0.44742091	0.41333822 Pnp	1
GO:0110020 regulation of actomyosin structure organization	1/40	105/14464	0.00952381	3.44380952	1.32347651	0.25310471	0.44742091	0.41333822 Arhgef10	1
GO:0009206 purine ribonucleoside triphosphate biosynthetic process	1/40	106/14464	0.00943396	3.41132075	1.31213124	0.25518534	0.44943809	0.41520174 Pnp	1
GO:0014896 muscle hypertrophy	1/40	106/14464	0.00943396	3.41132075	1.31213124	0.25518534	0.44943809	0.41520174 Smad1	1
GO:0002456 T cell mediated immunity	1/40	107/14464	0.00934579	3.37943925	1.3009212	0.25726032	0.44943809	0.41520174 Pnp	1
GO:0009145 purine nucleoside triphosphate biosynthetic process	1/40	107/14464	0.00934579	3.37943925	1.3009212	0.25726032	0.44943809	0.41520174 Pnp	1
GO:0030518 nuclear receptor-mediated steroid hormone signaling pathway	1/40	107/14464	0.00934579	3.37943925	1.3009212	0.25726032	0.44943809	0.41520174 Cry1	1
GO:0060419 heart growth	1/40	107/14464	0.00934579	3.37943925	1.3009212	0.25726032	0.44943809	0.41520174 Smad1	1
GO:0010639 negative regulation of organelle organization	2/40	355/14464	0.0056338	2.0371831	1.04192827	0.25740135	0.44943809	0.41520174 Bok/Tchp	2
GO:0032231 regulation of actin filament bundle assembly	1/40	108/14464	0.00925926	3.34814815	1.28984338	0.25932967	0.44965509	0.41540222 Arhgef10	1
GO:0050671 positive regulation of lymphocyte proliferation	1/40	108/14464	0.00925926	3.34814815	1.28984338	0.25932967	0.44965509	0.41540222 Pnp	1
GO:0072526 pyridine-containing compound catabolic process	1/40	108/14464	0.00925926	3.34814815	1.28984338	0.25932967	0.44965509	0.41540222 Pnp	1
GO:2001056 positive regulation of cysteine-type endopeptidase activity	1/40	108/14464	0.00925926	3.34814815	1.28984338	0.25932967	0.44965509	0.41540222 Bok	1
GO:0034440 lipid oxidation	1/40	109/14464	0.00917431	3.31743119	1.27889483	0.26139339	0.45058839	0.41626442 Samd1	1
GO:0007018 microtubule-based movement	2/40	359/14464	0.00557103	2.01448468	1.02499679	0.26152629	0.45058839	0.41626442 Catsperz/Rhot1	2
GO:0000280 nuclear division	2/40	360/14464	0.00555556	2.00888889	1.02079791	0.26255778	0.45058839	0.41626442 Catsperz/Arhgef10	2
GO:0002821 positive regulation of adaptive immune response	1/40	110/14464	0.00909091	3.28727273	1.26807271	0.26345151	0.45058839	0.41626442 Pnp	1
GO:0034219 carbohydrate transmembrane transport	1/40	110/14464	0.00909091	3.28727273	1.26807271	0.26345151	0.45058839	0.41626442 Capn10	1
GO:0009201 ribonucleoside triphosphate biosynthetic process	1/40	111/14464	0.00900901	3.25765766	1.25737426	0.26550403	0.45058839	0.41626442 Pnp	1
GO:0014909 smooth muscle cell migration	1/40	111/14464	0.00900901	3.25765766	1.25737426	0.26550403	0.45058839	0.41626442 Ptpn1	1
GO:0030038 contractile actin filament bundle assembly	1/40	111/14464	0.00900901	3.25765766	1.25737426	0.26550403	0.45058839	0.41626442 Arhgef10	1
GO:0031398 positive regulation of protein ubiquitination	1/40	111/14464	0.00900901	3.25765766	1.25737426	0.26550403	0.45058839	0.41626442 Cry1	1
GO:0032946 positive regulation of mononuclear cell proliferation	1/40	111/14464	0.00900901	3.25765766	1.25737426	0.26550403	0.45058839	0.41626442 Pnp	1
GO:0043149 stress fiber assembly	1/40	111/14464	0.00900901	3.25765766	1.25737426	0.26550403	0.45058839	0.41626442 Arhgef10	1
GO:0007059 chromosome segregation	2/40	364/14464	0.00549451	1.98681319	1.00413571	0.26668452	0.45058839	0.41626442 Arhgef10/Fam83d	2
GO:0006413 translational initiation	1/40	112/14464	0.00892857	3.22857143	1.24679683	0.26755098	0.45058839	0.41626442 Eif3j1	1
GO:0006612 protein targeting to membrane	1/40	112/14464	0.00892857	3.22857143	1.24679683	0.26755098	0.45058839	0.41626442 Zfand2b	1
GO:0010657 muscle cell apoptotic process	1/40	112/14464	0.00892857	3.22857143	1.24679683	0.26755098	0.45058839	0.41626442 Ptpn1	1
GO:0061640 cytoskeleton-dependent cytokinesis	1/40	112/14464	0.00892857	3.22857143	1.24679683	0.26755098	0.45058839	0.41626442 Iqgap2	1
GO:0071887 leukocyte apoptotic process	1/40	112/14464	0.00892857	3.22857143	1.24679683	0.26755098	0.45058839	0.41626442 Pnp	1
GO:2001252 positive regulation of chromosome organization	1/40	113/14464	0.00884956	3.2	1.23633781	0.26959237	0.45286508	0.41836768 Pkib	1
GO:0000077 DNA damage checkpoint signaling	1/40	114/14464	0.00877193	3.17192982	1.22599471	0.2716282	0.45286508	0.41836768 Cry1	1
GO:0007259 cell surface receptor signaling pathway via JAK-STAT	1/40	114/14464	0.00877193	3.17192982	1.22599471	0.2716282	0.45286508	0.41836768 Cntf	1
GO:0008643 carbohydrate transport	1/40	114/14464	0.00877193	3.17192982	1.22599471	0.2716282	0.45286508	0.41836768 Capn10	1
GO:0045621 positive regulation of lymphocyte differentiation	1/40	114/14464	0.00877193	3.17192982	1.22599471	0.2716282	0.45286508	0.41836768 Pnp	1
GO:1990830 cellular response to leukemia inhibitory factor	1/40	114/14464	0.00877193	3.17192982	1.22599471	0.2716282	0.45286508	0.41836768 Ctbp2	1
GO:0050807 regulation of synapse organization	2/40	370/14464	0.00540541	1.95459459	0.97953291	0.2728761	0.4539726	0.41939083 Lrrn3/Ptpn1	2
GO:0002705 positive regulation of leukocyte mediated immunity	1/40	115/14464	0.00869565	3.14434783	1.21576508	0.27365851	0.4539726	0.41939083 Pnp	1
GO:0051053 negative regulation of DNA metabolic process	1/40	115/14464	0.00869565	3.14434783	1.21576508	0.27365851	0.4539726	0.41939083 Fbh1	1
GO:0043401 steroid hormone receptor signaling pathway	1/40	116/14464	0.00862069	3.11724138	1.20564657	0.27568329	0.45506001	0.4203954 Cry1	1
GO:0048709 oligodendrocyte differentiation	1/40	116/14464	0.00862069	3.11724138	1.20564657	0.27568329	0.45506001	0.4203954 Bok	1
GO:1990823 response to leukemia inhibitory factor	1/40	116/14464	0.00862069	3.11724138	1.20564657	0.27568329	0.45506001	0.4203954 Ctbp2	1
GO:0000018 regulation of DNA recombination	1/40	117/14464	0.00854701	3.09059829	1.19563689	0.27770258	0.45612763	0.4213817 Fbh1	1
GO:0008584 male gonad development	1/40	117/14464	0.00854701	3.09059829	1.19563689	0.27770258	0.45612763	0.4213817 Bok	1
GO:0015698 inorganic anion transport	1/40	117/14464	0.00854701	3.09059829	1.19563689	0.27770258	0.45612763	0.4213817 Slc25a30	1
GO:0050803 regulation of synapse structure or activity	2/40	376/14464	0.00531915	1.92340426	0.95538288	0.27906793	0.45642753	0.42165876 Lrrn3/Ptpn1	2
GO:0009142 nucleoside triphosphate biosynthetic process	1/40	118/14464	0.00847458	3.06440678	1.1857338	0.27971637	0.45642753	0.42165876 Pnp	1
GO:0046546 development of primary male sexual characteristics	1/40	118/14464	0.00847458	3.06440678	1.1857338	0.27971637	0.45642753	0.42165876 Bok	1
GO:0051588 regulation of neurotransmitter transport	1/40	118/14464	0.00847458	3.06440678	1.1857338	0.27971637	0.45642753	0.42165876 Rhot1	1
GO:0016079 synaptic vesicle exocytosis	1/40	119/14464	0.00840336	3.03865546	1.17593515	0.28172469	0.4574585	0.42261118 Ctbp2	1
GO:0030317 flagellated sperm motility	1/40	119/14464	0.00840336	3.03865546	1.17593515	0.28172469	0.4574585	0.42261118 Catsperz	1
GO:0048593 camera-type eye morphogenesis	1/40	119/14464	0.00840336	3.03865546	1.17593515	0.28172469	0.4574585	0.42261118 Cntf	1
GO:0007043 cell-cell junction assembly	1/40	121/14464	0.00826446	2.98842975	1.15664283	0.28572496	0.45946417	0.42446408 Cdh19	1
GO:0030509 BMP signaling pathway	1/40	121/14464	0.00826446	2.98842975	1.15664283	0.28572496	0.45946417	0.42446408 Smad1	1
GO:0031570 DNA integrity checkpoint signaling	1/40	121/14464	0.00826446	2.98842975	1.15664283	0.28572496	0.45946417	0.42446408 Cry1	1
GO:0090277 positive regulation of peptide hormone secretion	1/40	121/14464	0.00826446	2.98842975	1.15664283	0.28572496	0.45946417	0.42446408 Capn10	1
GO:0097722 sperm motility	1/40	121/14464	0.00826446	2.98842975	1.15664283	0.28572496	0.45946417	0.42446408 Catsperz	1
GO:1901890 positive regulation of cell junction assembly	1/40	121/14464	0.00826446	2.98842975	1.15664283	0.28572496	0.45946417	0.42446408 Lrrn3	1
GO:0032368 regulation of lipid transport	1/40	123/14464	0.00813008	2.9398374	1.13774385	0.28970351	0.46233644	0.42711755 Cry1	1
GO:0050864 regulation of B cell activation	1/40	123/14464	0.00813008	2.9398374	1.13774385	0.28970351	0.46233644	0.42711755 Pnp	1
GO:0070665 positive regulation of leukocyte proliferation	1/40	123/14464	0.00813008	2.9398374	1.13774385	0.28970351	0.46233644	0.42711755 Pnp	1
GO:0031329 regulation of cellular catabolic process	2/40	388/14464	0.00515464	1.86391753	0.90837432	0.29144578	0.46233644	0.42711755 Bok/Ptpn1	2
GO:0002040 sprouting angiogenesis	1/40	124/14464	0.00806452	2.91612903	1.12843708	0.29168467	0.46233644	0.42711755 Smad1	1
GO:0008286 insulin receptor signaling pathway	1/40	124/14464	0.00806452	2.91612903	1.12843708	0.29168467	0.46233644	0.42711755 Ptpn1	1
GO:0045667 regulation of osteoblast differentiation	1/40	124/14464	0.00806452	2.91612903	1.12843708	0.29168467	0.46233644	0.42711755 Smad1	1
GO:0051100 negative regulation of binding	1/40	124/14464	0.00806452	2.91612903	1.12843708	0.29168467	0.46233644	0.42711755 Fbh1	1
GO:0097696 cell surface receptor signaling pathway via STAT	1/40	124/14464	0.00806452	2.91612903	1.12843708	0.29168467	0.46233644	0.42711755 Cntf	1
GO:0001558 regulation of cell growth	2/40	389/14464	0.00514139	1.85912596	0.90453167	0.29247662	0.46285585	0.42759739 Cntf/Tchp	2
GO:0002793 positive regulation of peptide secretion	1/40	125/14464	0.008	2.8928	1.11922302	0.29366045	0.46325865	0.42796951 Capn10	1
GO:0010950 positive regulation of endopeptidase activity	1/40	125/14464	0.008	2.8928	1.11922302	0.29366045	0.46325865	0.42796951 Bok	1
GO:0014812 muscle cell migration	1/40	126/14464	0.00793651	2.86984127	1.11009988	0.29563085	0.46416371	0.42880563 Ptpn1	1
GO:0043500 muscle adaptation	1/40	126/14464	0.00793651	2.86984127	1.11009988	0.29563085	0.46416371	0.42880563 Smad1	1
GO:0051225 spindle assembly	1/40	126/14464	0.00793651	2.86984127	1.11009988	0.29563085	0.46416371	0.42880563 Arhgef10	1
GO:0030177 positive regulation of Wnt signaling pathway	1/40	127/14464	0.00787402	2.84724409	1.10106596	0.29759589	0.46583324	0.43034797 Prdm15	1
GO:0009314 response to radiation	2/40	394/14464	0.00507614	1.83553299	0.88548457	0.29762866	0.46583324	0.43034797 Cry1/Pnp	2
GO:0060294 cilium movement involved in cell motility	1/40	128/14464	0.0078125	2.825	1.09211957	0.29955559	0.4681143	0.43245527 Catsperz	1
GO:0030183 B cell differentiation	1/40	129/14464	0.00775194	2.80310078	1.08325908	0.30150995	0.47006573	0.43425805 Pnp	1
GO:0043086 negative regulation of catalytic activity	2/40	398/14464	0.00502513	1.81708543	0.87044238	0.30174731	0.47006573	0.43425805 Pkib/Ptpn1	2
GO:0044772 mitotic cell cycle phase transition	2/40	399/14464	0.00501253	1.81253133	0.86670844	0.3027765	0.47093318	0.43505943 Plk5/Ctdspl	2
GO:0043433 negative regulation of DNA-binding transcription factor activity	1/40	130/14464	0.00769231	2.78153846	1.07448291	0.303459	0.47125954	0.43536092 Cys1	1
GO:0007098 centrosome cycle	1/40	131/14464	0.00763359	2.76030534	1.0657895	0.30540275	0.47134139	0.43543654 Arhgef10	1
GO:0009154 purine ribonucleotide catabolic process	1/40	131/14464	0.00763359	2.76030534	1.0657895	0.30540275	0.47134139	0.43543654 Pnp	1
GO:0071772 response to BMP	1/40	131/14464	0.00763359	2.76030534	1.0657895	0.30540275	0.47134139	0.43543654 Smad1	1
GO:0071773 cellular response to BMP stimulus	1/40	131/14464	0.00763359	2.76030534	1.0657895	0.30540275	0.47134139	0.43543654 Smad1	1
GO:0001539 cilium or flagellum-dependent cell motility	1/40	132/14464	0.00757576	2.73939394	1.05717735	0.3073412	0.47214049	0.43617476 Catsperz	1
GO:0010952 positive regulation of peptidase activity	1/40	132/14464	0.00757576	2.73939394	1.05717735	0.3073412	0.47214049	0.43617476 Bok	1
GO:0060285 cilium-dependent cell motility	1/40	132/14464	0.00757576	2.73939394	1.05717735	0.3073412	0.47214049	0.43617476 Catsperz	1
GO:2000779 regulation of double-strand break repair	1/40	133/14464	0.0075188	2.71879699	1.048645	0.30927438	0.47437932	0.43824305 Fbh1	1
GO:0045739 positive regulation of DNA repair	1/40	134/14464	0.00746269	2.69850746	1.04019101	0.3112023	0.47587223	0.43962224 Pnp	1
GO:0046661 male sex differentiation	1/40	134/14464	0.00746269	2.69850746	1.04019101	0.3112023	0.47587223	0.43962224 Bok	1
GO:0008277 regulation of G protein-coupled receptor signaling pathway	1/40	135/14464	0.00740741	2.67851852	1.03181399	0.31312498	0.47734801	0.4409856 Cry1	1
GO:0071902 positive regulation of protein serine/threonine kinase activity	1/40	135/14464	0.00740741	2.67851852	1.03181399	0.31312498	0.47734801	0.4409856 Ptpn1	1
GO:0043161 proteasome-mediated ubiquitin-dependent protein catabolic process	2/40	412/14464	0.00485437	1.75533981	0.81910248	0.31613527	0.48097987	0.4443408 Lrrc75a/Zfand2b	2
GO:0006470 protein dephosphorylation	1/40	137/14464	0.00729927	2.63941606	1.01528547	0.31695464	0.48097987	0.4443408 Ptpn1	1
GO:0072073 kidney epithelium development	1/40	137/14464	0.00729927	2.63941606	1.01528547	0.31695464	0.48097987	0.4443408 Smad1	1
GO:0051099 positive regulation of binding	1/40	138/14464	0.00724638	2.62028986	1.00713135	0.31886165	0.48144767	0.44477296 Ctbp2	1
GO:1903322 positive regulation of protein modification by small protein conjugation or removal	1/40	138/14464	0.00724638	2.62028986	1.00713135	0.31886165	0.48144767	0.44477296 Cry1	1
GO:0048285 organelle fission	2/40	416/14464	0.00480769	1.73846154	0.80479196	0.32023661	0.48144767	0.44477296 Catsperz/Arhgef10	2
GO:0006997 nucleus organization	1/40	139/14464	0.00719424	2.60143885	0.99904897	0.32076348	0.48144767	0.44477296 Rrp8	1
GO:1901875 positive regulation of post-translational protein modification	1/40	139/14464	0.00719424	2.60143885	0.99904897	0.32076348	0.48144767	0.44477296 Cry1	1
GO:0002706 regulation of lymphocyte mediated immunity	1/40	140/14464	0.00714286	2.58285714	0.9910371	0.32266012	0.48144767	0.44477296 Pnp	1

GO:0009261 ribonucleotide catabolic process	1/40	140/14464	0.00714286	2.58285714	0.9910371	0.32266012	0.48144767	0.44477296	Pnp	1
GO:0043405 regulation of MAP kinase activity	1/40	140/14464	0.00714286	2.58285714	0.9910371	0.32266012	0.48144767	0.44477296	Ptpn1	1
GO:0071456 cellular response to hypoxia	1/40	140/14464	0.00714286	2.58285714	0.9910371	0.32266012	0.48144767	0.44477296	Ptpn1	1
GO:0043410 positive regulation of MAPK cascade	2/40	419/14464	0.00477327	1.72601432	0.79415936	0.32330939	0.48144767	0.44477296	Cntf/Ptpn1	2
GO:0007264 small GTPase-mediated signal transduction	2/40	420/14464	0.0047619	1.72190476	0.79063398	0.32433301	0.48144767	0.44477296	Sipa1l2/Rhot1	2
GO:0006195 purine nucleotide catabolic process	1/40	141/14464	0.0070922	2.56453901	0.98309455	0.32455161	0.48144767	0.44477296	Pnp	1
GO:0006261 DNA-templated DNA replication	1/40	141/14464	0.0070922	2.56453901	0.98309455	0.32455161	0.48144767	0.44477296	Fbh1	1
GO:0031023 microtubule organizing center organization	1/40	141/14464	0.0070922	2.56453901	0.98309455	0.32455161	0.48144767	0.44477296	Arhgef10	1
GO:0055088 lipid homeostasis	1/40	141/14464	0.0070922	2.56453901	0.98309455	0.32455161	0.48144767	0.44477296	Tlcd3a	1
GO:0010212 response to ionizing radiation	1/40	142/14464	0.00704225	2.54647887	0.97522014	0.32643794	0.48144767	0.44477296	Pnp	1
GO:0048592 eye morphogenesis	1/40	142/14464	0.00704225	2.54647887	0.97522014	0.32643794	0.48144767	0.44477296	Cntf	1
GO:0140013 meiotic nuclear division	1/40	142/14464	0.00704225	2.54647887	0.97522014	0.32643794	0.48144767	0.44477296	Catsperz	1
GO:1903034 regulation of response to wounding	1/40	142/14464	0.00704225	2.54647887	0.97522014	0.32643794	0.48144767	0.44477296	Cntf	1
GO:0000723 telomere maintenance	1/40	143/14464	0.00699301	2.52867133	0.96741273	0.32831913	0.48208273	0.44535965	Pkib	1
GO:0032006 regulation of TOR signaling	1/40	143/14464	0.00699301	2.52867133	0.96741273	0.32831913	0.48208273	0.44535965	Fam83d	1
GO:0042129 regulation of T cell proliferation	1/40	143/14464	0.00699301	2.52867133	0.96741273	0.32831913	0.48208273	0.44535965	Pnp	1
GO:0043281 regulation of cysteine-type endopeptidase activity involved in apoptotic process	1/40	144/14464	0.00694444	2.51111111	0.95967121	0.33019521	0.48239543	0.44564853	Bok	1
GO:0051147 regulation of muscle cell differentiation	1/40	144/14464	0.00694444	2.51111111	0.95967121	0.33019521	0.48239543	0.44564853	Smad1	1
GO:0031346 positive regulation of cell projection organization	2/40	426/14464	0.00469484	1.69765258	0.7696756	0.33046748	0.48239543	0.44564853	Plk5/Cntf	2
GO:0045859 regulation of protein kinase activity	2/40	426/14464	0.00469484	1.69765258	0.7696756	0.33046748	0.48239543	0.44564853	Pkib/Ptpn1	2
GO:0007596 blood coagulation	1/40	145/14464	0.00689655	2.4937931	0.9519945	0.33206617	0.48331383	0.44649696	Hps5	1
GO:0032388 positive regulation of intracellular transport	1/40	145/14464	0.00689655	2.4937931	0.9519945	0.33206617	0.48331383	0.44649696	Capn10	1
GO:0007015 actin filament organization	2/40	429/14464	0.004662	1.68578089	0.75931879	0.33352983	0.48461461	0.44769866	Iqgap2/Arhgef10	2
GO:0007286 spermatid development	1/40	146/14464	0.00684932	2.47671233	0.94438152	0.33393204	0.48461461	0.44769866	Catsperz	1
GO:0009152 purine ribonucleotide biosynthetic process	1/40	147/14464	0.00680272	2.45986395	0.93683125	0.33579282	0.48519629	0.44823603	Pnp	1
GO:0043547 positive regulation of GTPase activity	1/40	147/14464	0.00680272	2.45986395	0.93683125	0.33579282	0.48519629	0.44823603	Arhgef10	1
GO:0060041 retina development in camera-type eye	1/40	147/14464	0.00680272	2.45986395	0.93683125	0.33579282	0.48519629	0.44823603	Cntf	1
GO:0007599 hemostasis	1/40	148/14464	0.00675676	2.44324324	0.92934267	0.33764854	0.48632285	0.44927677	Hps5	1
GO:0045766 positive regulation of angiogenesis	1/40	148/14464	0.00675676	2.44324324	0.92934267	0.33764854	0.48632285	0.44927677	Smad1	1
GO:0001837 epithelial to mesenchymal transition	1/40	149/14464	0.00671141	2.42684564	0.92191479	0.3394992	0.48632285	0.44927677	Fam83d	1
GO:0032200 telomere organization	1/40	149/14464	0.00671141	2.42684564	0.92191479	0.3394992	0.48632285	0.44927677	Pkib	1
GO:0036294 cellular response to decreased oxygen levels	1/40	149/14464	0.00671141	2.42684564	0.92191479	0.3394992	0.48632285	0.44927677	Ptpn1	1
GO:0046631 alpha-beta T cell activation	1/40	149/14464	0.00671141	2.42684564	0.92191479	0.3394992	0.48632285	0.44927677	Pnp	1
GO:0022613 ribonucleoprotein complex biogenesis	2/40	436/14464	0.00458716	1.6587156	0.73546099	0.34066157	0.48664814	0.44957728	Rrp8/Eif3j1	2
GO:0010977 negative regulation of neuron projection development	1/40	150/14464	0.00666667	2.41066667	0.91454664	0.34134482	0.48664814	0.44957728	Ptpn1	1
GO:0050817 coagulation	1/40	150/14464	0.00666667	2.41066667	0.91454664	0.34134482	0.48664814	0.44957728	Hps5	1
GO:0042391 regulation of membrane potential	2/40	437/14464	0.00457666	1.65491991	0.73208725	0.34167874	0.48664814	0.44957728	Bok/Cntf	2
GO:0007224 smoothened signaling pathway	1/40	151/14464	0.00662252	2.39470199	0.90723728	0.34318541	0.48670818	0.44963275	Evc2	1
GO:0045580 regulation of T cell differentiation	1/40	151/14464	0.00662252	2.39470199	0.90723728	0.34318541	0.48670818	0.44963275	Pnp	1
GO:1904018 positive regulation of vasculature development	1/40	151/14464	0.00662252	2.39470199	0.90723728	0.34318541	0.48670818	0.44963275	Smad1	1
GO:0050714 positive regulation of protein secretion	1/40	152/14464	0.00657895	2.37894737	0.89998579	0.34502098	0.48723218	0.45011683	Capn10	1
GO:0051302 regulation of cell division	1/40	152/14464	0.00657895	2.37894737	0.89998579	0.34502098	0.48723218	0.45011683	Prdm15	1
GO:1905952 regulation of lipid localization	1/40	152/14464	0.00657895	2.37894737	0.89998579	0.34502098	0.48723218	0.45011683	Cry1	1
GO:0051656 establishment of organelle localization	2/40	442/14464	0.00452489	1.6361991	0.71534498	0.34675804	0.4889926	0.45174315	Fam83d/Rhot1	2
GO:0048515 spermatid differentiation	1/40	154/14464	0.00649351	2.34805195	0.88565281	0.34867714	0.48962128	0.45232394	Catsperz	1
GO:0062013 positive regulation of small molecule metabolic process	1/40	154/14464	0.00649351	2.34805195	0.88565281	0.34867714	0.48962128	0.45232394	Cry1	1
GO:1903531 negative regulation of secretion by cell	1/40	154/14464	0.00649351	2.34805195	0.88565281	0.34867714	0.48962128	0.45232394	Cry1	1
GO:0001906 cell killing	1/40	156/14464	0.00641026	2.31794872	0.87154071	0.3523134	0.49333772	0.45575727	Pnp	1
GO:0002822 regulation of adaptive immune response based on somatic recombination of immune receptors built from	1/40	156/14464	0.00641026	2.31794872	0.87154071	0.3523134	0.49333772	0.45575727	Pnp	1
GO:0072523 purine-containing compound catabolic process	1/40	157/14464	0.00636943	2.30318471	0.86456539	0.35412409	0.49517773	0.45745712	Pnp	1
GO:0007346 regulation of mitotic cell cycle	2/40	450/14464	0.00444444	1.60711111	0.68898492	0.35486099	0.49551317	0.45776701	Plk5/Ctdspl	2
GO:0009166 nucleotide catabolic process	1/40	158/14464	0.00632911	2.28860759	0.85764281	0.35592985	0.49631058	0.45850368	Pnp	1
GO:0051223 regulation of protein transport	2/40	452/14464	0.00442478	1.6	0.68247498	0.35688187	0.49673747	0.45889805	Capn10/Ptpn1	2
GO:0003341 cilium movement	1/40	159/14464	0.00628931	2.27421384	0.85077218	0.35773069	0.49673747	0.45889805	Catsperz	1
GO:0071897 DNA biosynthetic process	1/40	159/14464	0.00628931	2.27421384	0.85077218	0.35773069	0.49673747	0.45889805	Pkib	1
GO:0002181 cytoplasmic translation	1/40	160/14464	0.00625	2.26	0.84395272	0.35952662	0.49824855	0.46029402	Eif3j1	1
GO:0043549 regulation of kinase activity	2/40	456/14464	0.00438596	1.58596491	0.6695489	0.36091752	0.49824855	0.46029402	Pkib/Ptpn1	2
GO:0008217 regulation of blood pressure	1/40	161/14464	0.00621118	2.24596273	0.83718368	0.36131766	0.49824855	0.46029402	Ptpn1	1
GO:0033157 regulation of intracellular protein transport	1/40	161/14464	0.00621118	2.24596273	0.83718368	0.36131766	0.49824855	0.46029402	Ptpn1	1
GO:0051897 positive regulation of phosphatidylinositol 3-kinase/protein kinase B signal transduction	1/40	161/14464	0.00621118	2.24596273	0.83718368	0.36131766	0.49824855	0.46029402	Cntf	1
GO:0007369 gastrulation	1/40	162/14464	0.00617284	2.23209877	0.83046431	0.36310381	0.49933034	0.4612934	Smad1	1
GO:0009260 ribonucleotide biosynthetic process	1/40	162/14464	0.00617284	2.23209877	0.83046431	0.36310381	0.49933034	0.4612934	Pnp	1
GO:0051017 actin filament bundle assembly	1/40	163/14464	0.00613497	2.21840491	0.8237939	0.36488508	0.49971213	0.46164611	Arhgef10	1
GO:1901991 negative regulation of mitotic cell cycle phase transition	1/40	163/14464	0.00613497	2.21840491	0.8237939	0.36488508	0.49971213	0.46164611	Ctdspl	1
GO:2000116 regulation of cysteine-type endopeptidase activity	1/40	163/14464	0.00613497	2.21840491	0.8237939	0.36488508	0.49971213	0.46164611	Bok	1
GO:0000724 double-strand break repair via homologous recombination	1/40	164/14464	0.00609756	2.20487805	0.81717172	0.36666151	0.50117024	0.46299315	Fbh1	1
GO:0071407 cellular response to organic cyclic compound	2/40	462/14464	0.004329	1.56536797	0.65038944	0.36695514	0.50117024	0.46299315	Cry1/Smad1	2
GO:0008016 regulation of heart contraction	1/40	165/14464	0.00606061	2.19151515	0.81059709	0.36843308	0.50181391	0.46358779	Ptpn1	1
GO:0071453 cellular response to oxygen levels	1/40	165/14464	0.00606061	2.19151515	0.81059709	0.36843308	0.50181391	0.46358779	Ptpn1	1
GO:0061572 actin filament bundle organization	1/40	166/14464	0.0060241	2.17831325	0.80406932	0.37019983	0.50284636	0.46454159	Arhgef10	1
GO:1903046 meiotic cell cycle process	1/40	166/14464	0.0060241	2.17831325	0.80406932	0.37019983	0.50284636	0.46454159	Catsperz	1
GO:1902905 positive regulation of supramolecular fiber organization	1/40	167/14464	0.00598802	2.16526946	0.79758775	0.37196175	0.5045522	0.46611748	Arhgef10	1
GO:0000070 mitotic sister chromatid segregation	1/40	168/14464	0.00595238	2.15238095	0.79115172	0.37371887	0.50487495	0.46641565	Arhgef10	1
GO:0000910 cytokinesis	1/40	168/14464	0.00595238	2.15238095	0.79115172	0.37371887	0.50487495	0.46641565	Iqgap2	1
GO:0046390 ribose phosphate biosynthetic process	1/40	168/14464	0.00595238	2.15238095	0.79115172	0.37371887	0.50487495	0.46641565	Pnp	1
GO:0044283 small molecule biosynthetic process	2/40	470/14464	0.00425532	1.5387234	0.62525931	0.37497416	0.50511315	0.4666357	Cry1/Pnp	2
GO:0000725 recombinational repair	1/40	169/14464	0.00591716	2.13964497	0.78476059	0.3754712	0.50511315	0.4666357	Fbh1	1
GO:0034655 nucleobase-containing compound catabolic process	2/40	471/14464	0.00424628	1.53545648	0.62215063	0.37597393	0.50511315	0.4666357	Pnp/Fbh1	2
GO:0002819 regulation of adaptive immune response	1/40	170/14464	0.00588235	2.12705882	0.77841373	0.37721874	0.50511315	0.4666357	Pnp	1
GO:0030902 hindbrain development	1/40	170/14464	0.00588235	2.12705882	0.77841373	0.37721874	0.50511315	0.4666357	Smad1	1
GO:0051048 negative regulation of secretion	1/40	170/14464	0.00588235	2.12705882	0.77841373	0.37721874	0.50511315	0.4666357	Cry1	1
GO:0030308 negative regulation of cell growth	1/40	171/14464	0.00584795	2.11461988	0.77211053	0.37896152	0.50511315	0.4666357	Tchp	1
GO:0042552 myelination	1/40	171/14464	0.00584795	2.11461988	0.77211053	0.37896152	0.50511315	0.4666357	Arhgef10	1
GO:1902107 positive regulation of leukocyte differentiation	1/40	171/14464	0.00584795	2.11461988	0.77211053	0.37896152	0.50511315	0.4666357	Pnp	1
GO:1903708 positive regulation of hemopoiesis	1/40	171/14464	0.00584795	2.11461988	0.77211053	0.37896152	0.50511315	0.4666357	Pnp	1
GO:0031929 TOR signaling	1/40	172/14464	0.00581395	2.10232558	0.76585039	0.38069954	0.50642787	0.46785028	Fam83d	1
GO:0016049 cell growth	2/40	476/14464	0.00420168	1.51932773	0.60671329	0.3809638	0.50642787	0.46785028	Cntf/Tchp	2
GO:0030307 positive regulation of cell growth	1/40	173/14464	0.00578035	2.09017341	0.75963271	0.38243282	0.50770375	0.46902897	Cntf	1
GO:0007272 ensheathment of neurons	1/40	174/14464	0.00574713	2.07816092	0.75345692	0.38416137	0.50864393	0.46989753	Arhgef10	1
GO:0008366 axon ensheathment	1/40	174/14464	0.00574713	2.07816092	0.75345692	0.38416137	0.50864393	0.46989753	Arhgef10	1
GO:0042770 signal transduction in response to DNA damage	1/40	175/14464	0.00571429	2.06628571	0.74732245	0.3858852	0.5091456	0.47036098	Cry1	1
GO:0048469 cell maturation	1/40	175/14464	0.00571429	2.06628571	0.74732245	0.3858852	0.5091456	0.47036098	Catsperz	1
GO:0006164 purine nucleotide biosynthetic process	1/40	176/14464	0.00568182	2.05454545	0.74122874	0.38760432	0.5091456	0.47036098	Pnp	1
GO:0045216 cell-cell junction organization	1/40	176/14464	0.00568182	2.05454545	0.74122874	0.38760432	0.5091456	0.47036098	Cdh19	1
GO:0072524 pyridine-containing compound metabolic process	1/40	176/14464	0.00568182	2.05454545	0.74122874	0.38760432	0.5091456	0.47036098	Pnp	1
GO:1901292 nucleoside phosphate catabolic process	1/40	176/14464	0.00568182	2.05454545	0.74122874	0.38760432	0.5091456	0.47036098	Pnp	1
GO:0010970 transport along microtubule	1/40	177/14464	0.00564972	2.04293785	0.73517524	0.38931876	0.50961493	0.47079456	Rhot1	1
GO:0022618 protein-RNA complex assembly	1/40	177/14464	0.00564972	2.04293785	0.73517524	0.38931876	0.50961493	0.47079456	Eif3j1	1
GO:0001764 neuron migration	1/40	178/14464	0.00561798	2.03146067	0.72916142	0.39102851	0.50961493	0.47079456	Matn2	1
GO:0030258 lipid modification	1/40	178/14464	0.00561798	2.03146067	0.72916142	0.39102851	0.50961493	0.47079456	Samd1	1
GO:0045619 regulation of lymphocyte differentiation	1/40	178/14464	0.00561798	2.03146067	0.72916142	0.39102851	0.50961493	0.47079456	Pnp	1
GO:0051495 positive regulation of cytoskeleton organization	1/40	178/14464	0.00561798	2.03146067	0.72916142	0.39102851	0.50961493	0.47079456	Arhgef10	1
GO:0050796 regulation of insulin secretion	1/40	179/14464	0.00558659	2.02011173	0.72318676	0.3927336	0.51116892	0.47223017	Capn10	1
GO:0035265 organ growth	1/40	180/14464	0.00555556	2.00888889	0.71725074	0.39443403	0.51271281	0.47365645	Smad1	1
GO:0000075 cell cycle checkpoint signaling	1/40	181/14464	0.00552486	1.99779006	0.71135284	0.39612982	0.51291094	0.47383949	Cry1	1
GO:0048839 inner ear development	1/40	181/14464	0.00552486	1.99779006	0.71135284	0.39612982	0.51291094	0.47383949	Cys1	1
GO:0051260 protein homooligomerization	1/40	181/14464	0.00552486	1.99779006	0.71135284	0.39612982	0.51291094	0.47383949	Samd1	1
GO:0098742 cell-cell adhesion via plasma-membrane adhesion molecules	1/40	182/14464	0.00549451	1.98681319	0.70549259	0.39782097	0.51394708	0.4747967	Cdh19	1
GO:0006109 regulation of carbohydrate metabolic process	1/40	183/14464	0.00546448	1.97595628	0.69966948	0.39950751	0.51394708	0.4747967	Cry1	1
GO:0009267 cellular response to starvation	1/40	183/14464	0.00546448	1.97595628	0.69966948	0.39950751	0.51394708	0.4747967	Rrp8	1
GO:0016051 carbohydrate biosynthetic process	1/40	183/14464	0.00546448	1.97595628	0.69966948	0.39950751	0.51394708	0.4747967	Cry1	1
GO:0071383 cellular response to steroid hormone stimulus	1/40	183/14464	0.00546448	1.97595628	0.69966948	0.39950751	0.51394708	0.4747967	Cry1	1
GO:0006814 sodium ion transport	1/40	185/14464	0.00540541	1.95459459	0.68813282	0.40286679	0.51576206	0.47647342	Slc10a6	1
GO:0042098 T cell proliferation	1/40	185/14464	0.00540541	1.95459459	0.68813282	0.40286679	0.51576206	0.47647342	Pnp	1
GO:0045664 regulation of neuron differentiation	1/40	185/14464	0.00540541	1.95459459	0.68813282	0.40286679	0.51576206	0.47647342	Cntf	1
GO:0001649 osteoblast differentiation	1/40	186/14464	0.00537634	1.94408602	0.68241833	0.40453955	0.51576206	0.47647342	Smad1	1
GO:0051216 cartilage development	1/40	186/14464	0.00537634	1.94408602	0.68241833	0.40453955	0.51576206	0.47647342	Smad1	1
GO:0071826 protein-RNA complex organization	1/40	186/14464	0.00537634	1.94408602	0.68241833	0.40453955	0.51576206	0.47647342	Eif3j1	1
GO:0099175 regulation of postsynapse organization	1/40	186/14464	0.00537634	1.94408602	0.68241833	0.40453955	0.51576206	0.47647342	Ptpn1	1
GO:0050870 positive regulation of T cell activation	1/40	188/14464	0.00531915	1.92340426	0.67109478	0.40787137	0.51934579	0.47978416	Pnp	1
GO:0007179 transforming growth factor beta receptor signaling pathway	1/40	190/14464	0.00526316	1.90315789	0.65990887	0.41118501	0.52223116	0.48244973	Smad1	1
GO:0120032 regulation of plasma membrane bounded cell projection assembly	1/40	190/14464	0.00526316	1.90315789	0.65990887	0.41118501	0.52223116	0.48244973	Tchp	1
GO:0050670 regulation of lymphocyte proliferation	1/40	192/14464	0.00520833	1.88333333	0.64885723	0.41448058	0.52507895	0.48508059	Pnp	1
GO:0060491 regulation of cell projection assembly	1/40	192/14464	0.00520833	1.88333333	0.64885723	0.41448058	0.52507895	0.48508059	Tchp	1
GO:0032944 regulation of mononuclear cell proliferation	1/40	196/14464	0.00510204	1.84489796	0.62714372	0.42101783	0.53268373	0.49210607	Pnp	1
GO:0002703 regulation of leukocyte mediated immunity	1/40	197/14464	0.00507614	1.83553299	0.62179428	0.422641	0.53338364	0.49275266	Pnp	1
GO:0006006 glucose metabolic process	1/40	197/14464	0.00507614	1.83553299	0.62179428	0.422641	0.53338364	0.49275266	Cry1	1
GO:0030705 cytoskeleton-dependent intracellular transport	1/40	198/14464	0.00505051	1.82626263	0.61647565	0.42425972	0.53474961	0.49401458	Rhot1	1
GO:0008406 gonad development	1/40	200/14464	0.005	1.808	0.6059294	0.42748391	0.53813315	0.49714038	Bok	1
GO:0045137 development of primary sexual characteristics	1/40	203/14464	0.00492611	1.78128079	0.59033225	0.43228721	0.542809	0.50146004	Bok	1
GO:0060047 heart contraction	1/40	203/14464	0.00492611	1.78128079	0.59033225	0.43228721	0.542809	0.50146004	Ptpn1	1
GO:0007623 circadian rhythm	1/40	204/14464	0.00490196	1.77254902	0.58519108	0.43387956	0.54324799	0.50186559	Cry1	1
GO:0031396 regulation of protein ubiquitination	1/40	204/14464	0.00490196	1.77254902	0.58519108	0.43387956	0.54324799	0.50186559	Cry1	1
GO:0000819 sister chromatid segregation	1/40	205/14464	0.00487805	1.76390244	0.58007831	0.43546755	0.54324799	0.50186559	Arhgef10	1
GO:0071216 cellular response to biotic stimulus	1/40	205/14464	0.00487805	1.76390244	0.58007831	0.43546755	0.54324799	0.50186559	Fbh1	1
GO:0010810 regulation of cell-substrate adhesion	1/40	206/14464	0.00485437	1.75533981	0.5749936	0.43705121	0.54324799	0.50186559	Ptpn1	1
GO:0007411 axon guidance	1/40	207/14464	0.00483092	1.7468599	0.56993664	0.43863053	0.54324799	0.50186559	Matn2	1
GO:0043087 regulation of GTPase activity	1/40	207/14464	0.00483092	1.7468599	0.56993664	0.43863053	0.54324799	0.50186559	Arhgef10	1
GO:0043583 ear development	1/40	207/14464	0.00483092	1.7468599	0.56993664	0.43863053	0.54324799	0.50186559	Cys1	1
GO:0060348 bone development	1/40	207/14464	0.00483092	1.7468599	0.56993664	0.43863053	0.54324799	0.50186559	Smad1	1
GO:0097191 extrinsic apoptotic signaling pathway	1/40	207/14464	0.00483092	1.7468599	0.56993664	0.43863053	0.54324799	0.50186559	Bok	1
GO:1903039 positive regulation of leukocyte cell-cell adhesion	1/40	207/14464	0.00483092	1.7468599	0.56993664	0.43863053	0.54324799	0.50186559	Pnp	1
GO:0097485 neuron projection guidance	1/40	208/14464	0.00480769	1.73846154	0.5649071	0.44020553	0.54452222	0.50304275	Matn2	1
GO:0016311 dephosphorylation	1/40	209/14464	0.00478469	1.73014354	0.5599047	0.44177622	0.54578797	0.50421208	Ptpn1	1
GO:0090276 regulation of peptide hormone secretion	1/40	210/14464	0.0047619	1.72190476	0.55492911	0.44334261	0.54704528	0.50537362	Capn10	1
GO:0051321 meiotic cell cycle	1/40	211/14464	0.00473934	1.71374408	0.54998005	0.44490472	0.54794224	0.50620225	Catsperz	1
GO:0002699 positive regulation of immune effector process	1/40	212/14464	0.00471698	1.70566038	0.54505721	0.44646256	0.54794224	0.50620225	Pnp	1
GO:0099111 microtubule-based transport	1/40	212/14464	0.00471698	1.70566038	0.54505721	0.44646256	0.54794224	0.50620225	Rhot1	1
GO:0003015 heart process	1/40	213/14464	0.00469484	1.69765258	0.54016031	0.44801613	0.54794224	0.50620225	Ptpn1	1
GO:0030073 insulin secretion	1/40	213/14464	0.00469484	1.69765258	0.54016031	0.44801613	0.54794224	0.50620225	Capn10	1
GO:0045930 negative regulation of mitotic cell cycle	1/40	213/14464	0.00469484	1.69765258	0.54016031	0.44801613	0.54794224	0.50620225	Ctdspl	1
GO:0070663 regulation of leukocyte proliferation	1/40	213/14464	0.00469484	1.69765258	0.54016031	0.44801613	0.54794224	0.50620225	Pnp	1
GO:0006364 rRNA processing	1/40	214/14464	0.0046729	1.68971963	0.53528906	0.44956545	0.54794224	0.50620225	Rrp8	1
GO:0009165 nucleotide biosynthetic process	1/40	214/14464	0.0046729	1.68971963	0.53528906	0.44956545	0.54794224	0.50620225	Pnp	1
GO:0034764 positive regulation of transmembrane transport	1/40	214/14464	0.0046729	1.68971963	0.53528906	0.44956545	0.54794224	0.50620225	Capn10	1
GO:0002791 regulation of peptide secretion	1/40	215/14464	0.00465116	1.68186047	0.53044319	0.45111053	0.54848438	0.50670309	Capn10	1
GO:0090087 regulation of peptide transport	1/40	215/14464	0.00465116	1.68186047	0.53044319	0.45111053	0.54848438	0.50670309	Capn10	1
GO:0042594 response to starvation	1/40	216/14464	0.00462963	1.67407407	0.52562242	0.45265138	0.54901876	0.50719677	Rrp8	1
GO:0045926 negative regulation of growth	1/40	216/14464	0.00462963	1.67407407	0.52562242	0.45265138	0.54901876	0.50719677	Tchp	1
GO:0052548 regulation of endopeptidase activity	1/40	218/14464	0.00458716	1.6587156	0.51605508	0.45572044	0.55206959	0.5100152	Bok	1
GO:0048762 mesenchymal cell differentiation	1/40	219/14464	0.00456621	1.65114155	0.51130798	0.45724867	0.55324869	0.51110448	Fam83d	1
GO:0046434 organophosphate catabolic process	1/40	222/14464	0.0045045	1.62882883	0.49720991	0.4618083	0.55741268	0.51495127	Pnp	1
GO:0090068 positive regulation of cell cycle process	1/40	222/14464	0.0045045	1.62882883	0.49720991	0.4618083	0.55741268	0.51495127	Fam83d	1
GO:1903522 regulation of blood circulation	1/40	224/14464	0.00446429	1.61428571	0.48792802	0.46482729	0.56037824	0.51769093	Ptpn1	1
GO:0060828 regulation of canonical Wnt signaling pathway	1/40	226/14464	0.00442478	1.6	0.47873735	0.46782976	0.56331675	0.52040559	Prdm15	1
GO:0051056 regulation of small GTPase mediated signal transduction	1/40	227/14464	0.00440529	1.59295154	0.47417562	0.46932483	0.56443529	0.52143892	Sipa1l2	1

GO:0072522 purine-containing compound biosynthetic process	1/40	228/14464	0.00438596	1.58596491	0.469636	0.4708158	0.56554621	0.52246522	Pnp	1
GO:1901136 carbohydrate derivative catabolic process	1/40	229/14464	0.00436681	1.5790393	0.46511825	0.47230269	0.56638388	0.52323908	Pnp	1
GO:0019318 hexose metabolic process	1/40	230/14464	0.00434783	1.57217391	0.46062215	0.47378551	0.56638388	0.52323908	Cry1	1
GO:0031331 positive regulation of cellular catabolic process	1/40	230/14464	0.00434783	1.57217391	0.46062215	0.47378551	0.56638388	0.52323908	Ptpn1	1
GO:1902115 regulation of organelle assembly	1/40	230/14464	0.00434783	1.57217391	0.46062215	0.47378551	0.56638388	0.52323908	Tchp	1
GO:0045055 regulated exocytosis	1/40	231/14464	0.004329	1.56536797	0.4561475	0.47526426	0.56747122	0.52424359	Ctbp2	1
GO:0015850 organic hydroxy compound transport	1/40	235/14464	0.00425532	1.5387234	0.43845893	0.48113887	0.57379838	0.53008878	Slc10a6	1
GO:0002449 lymphocyte mediated immunity	1/40	236/14464	0.00423729	1.53220339	0.43408826	0.48259747	0.57416429	0.53042681	Pnp	1
GO:0033044 regulation of chromosome organization	1/40	236/14464	0.00423729	1.53220339	0.43408826	0.48259747	0.57416429	0.53042681	Pkib	1
GO:0007548 sex differentiation	1/40	237/14464	0.00421941	1.5257384	0.42973778	0.48405207	0.57452371	0.53075885	Bok	1
GO:0048738 cardiac muscle tissue development	1/40	237/14464	0.00421941	1.5257384	0.42973778	0.48405207	0.57452371	0.53075885	Smad1	1
GO:1903320 regulation of protein modification by small protein conjugation or removal	1/40	239/14464	0.0041841	1.51297071	0.42109658	0.48694933	0.57727524	0.53330078	Cry1	1
GO:0042113 B cell activation	1/40	240/14464	0.00416667	1.50666667	0.41680547	0.488392	0.57758455	0.53358653	Pnp	1
GO:0031669 cellular response to nutrient levels	1/40	241/14464	0.00414938	1.50041494	0.41253378	0.48983072	0.57758455	0.53358653	Rrp8	1
GO:0045444 fat cell differentiation	1/40	241/14464	0.00414938	1.50041494	0.41253378	0.48983072	0.57758455	0.53358653	Ctbp2	1
GO:0071560 cellular response to transforming growth factor beta stimulus	1/40	242/14464	0.00413223	1.49421488	0.4082813	0.49126549	0.57758455	0.53358653	Smad1	1
GO:0071695 anatomical structure maturation	1/40	242/14464	0.00413223	1.49421488	0.4082813	0.49126549	0.57758455	0.53358653	Catsperz	1
GO:0090596 sensory organ morphogenesis	1/40	242/14464	0.00413223	1.49421488	0.4082813	0.49126549	0.57758455	0.53358653	Cntf	1
GO:0140014 mitotic nuclear division	1/40	242/14464	0.00413223	1.49421488	0.4082813	0.49126549	0.57758455	0.53358653	Arhgef10	1
GO:0009205 purine ribonucleoside triphosphate metabolic process	1/40	243/14464	0.00411523	1.48806584	0.40404786	0.49269633	0.5785845	0.53451031	Pnp	1
GO:1901873 regulation of post-translational protein modification	1/40	244/14464	0.00409836	1.48196721	0.39983328	0.49412324	0.5795775	0.53542766	Cry1	1
GO:0006260 DNA replication	1/40	245/14464	0.00408163	1.47591837	0.39563738	0.49554624	0.57988217	0.53570912	Fbh1	1
GO:0032886 regulation of microtubule-based process	1/40	245/14464	0.00408163	1.47591837	0.39563738	0.49554624	0.57988217	0.53570912	Rhot1	1
GO:0045860 positive regulation of protein kinase activity	1/40	246/14464	0.00406504	1.4699187	0.39145997	0.49696534	0.58086101	0.53661341	Ptpn1	1
GO:0016072 rRNA metabolic process	1/40	247/14464	0.00404858	1.46396761	0.38730089	0.49838055	0.58183303	0.53751137	Rrp8	1
GO:0009199 ribonucleoside triphosphate metabolic process	1/40	248/14464	0.00403226	1.45806452	0.38315996	0.49979187	0.58239595	0.53803141	Pnp	1
GO:0033002 muscle cell proliferation	1/40	249/14464	0.00401606	1.45220884	0.37903701	0.50119932	0.58239595	0.53803141	Smad1	1
GO:0071559 response to transforming growth factor beta	1/40	249/14464	0.00401606	1.45220884	0.37903701	0.50119932	0.58239595	0.53803141	Smad1	1
GO:0099504 synaptic vesicle cycle	1/40	249/14464	0.00401606	1.45220884	0.37903701	0.50119932	0.58239595	0.53803141	Ctbp2	1
GO:0005996 monosaccharide metabolic process	1/40	250/14464	0.004	1.4464	0.37493188	0.50260291	0.58334703	0.53891005	Cry1	1
GO:0009144 purine nucleoside triphosphate metabolic process	1/40	251/14464	0.00398406	1.44063745	0.3708444	0.50400265	0.58429144	0.53978252	Pnp	1
GO:0022409 positive regulation of cell-cell adhesion	1/40	252/14464	0.00396825	1.43492063	0.3667744	0.50539855	0.58522921	0.54064885	Pnp	1
GO:0046651 lymphocyte proliferation	1/40	253/14464	0.00395257	1.42924901	0.36272173	0.50679061	0.58616037	0.54150908	Pnp	1
GO:0030072 peptide hormone secretion	1/40	256/14464	0.00390625	1.4125	0.35066611	0.51094393	0.58891457	0.54405347	Capn10	1
GO:0050878 regulation of body fluid levels	1/40	256/14464	0.00390625	1.4125	0.35066611	0.51094393	0.58891457	0.54405347	Hps5	1
GO:0090150 establishment of protein localization to membrane	1/40	256/14464	0.00390625	1.4125	0.35066611	0.51094393	0.58891457	0.54405347	Zfand2b	1
GO:0033674 positive regulation of kinase activity	1/40	258/14464	0.00387597	1.40155039	0.3427128	0.51369385	0.59003775	0.5450911	Ptpn1	1
GO:0045927 positive regulation of growth	1/40	258/14464	0.00387597	1.40155039	0.3427128	0.51369385	0.59003775	0.5450911	Cntf	1
GO:0110053 regulation of actin filament organization	1/40	258/14464	0.00387597	1.40155039	0.3427128	0.51369385	0.59003775	0.5450911	Arhgef10	1
GO:0032943 mononuclear cell proliferation	1/40	259/14464	0.003861	1.396139	0.33876083	0.51506315	0.59092976	0.54591515	Pnp	1
GO:0051251 positive regulation of lymphocyte activation	1/40	260/14464	0.00384615	1.39076923	0.33482512	0.51642869	0.5917233	0.54664825	Pnp	1
GO:0051222 positive regulation of protein transport	1/40	262/14464	0.00381679	1.38015267	0.3270019	0.51914852	0.5917233	0.54664825	Capn10	1
GO:0052547 regulation of peptidase activity	1/40	262/14464	0.00381679	1.38015267	0.3270019	0.51914852	0.5917233	0.54664825	Bok	1
GO:0002460 adaptive immune response based on somatic recombination of immune receptors built from immunoglobu	1/40	263/14464	0.00380228	1.37490494	0.3231141	0.52050284	0.5917233	0.54664825	Pnp	1
GO:0002790 peptide secretion	1/40	263/14464	0.00380228	1.37490494	0.3231141	0.52050284	0.5917233	0.54664825	Capn10	1
GO:0007162 negative regulation of cell adhesion	1/40	263/14464	0.00380228	1.37490494	0.3231141	0.52050284	0.5917233	0.54664825	Ptpn1	1
GO:0030217 T cell differentiation	1/40	263/14464	0.00380228	1.37490494	0.3231141	0.52050284	0.5917233	0.54664825	Pnp	1
GO:0050708 regulation of protein secretion	1/40	263/14464	0.00380228	1.37490494	0.3231141	0.52050284	0.5917233	0.54664825	Capn10	1
GO:0009141 nucleoside triphosphate metabolic process	1/40	265/14464	0.00377358	1.3645283	0.31538544	0.52320034	0.59343656	0.548231	Pnp	1
GO:0042593 glucose homeostasis	1/40	265/14464	0.00377358	1.3645283	0.31538544	0.52320034	0.59343656	0.548231	Cry1	1
GO:0033500 carbohydrate homeostasis	1/40	266/14464	0.0037594	1.3593985	0.31154431	0.52454353	0.59428398	0.54901386	Cry1	1
GO:0060485 mesenchyme development	1/40	267/14464	0.00374532	1.35430712	0.30771846	0.52588303	0.59512529	0.54979109	Fam83d	1
GO:0048608 reproductive structure development	1/40	268/14464	0.00373134	1.34925373	0.30390776	0.52721886	0.59596054	0.55056271	Bok	1
GO:0060070 canonical Wnt signaling pathway	1/40	269/14464	0.00371747	1.34423792	0.30011208	0.52855101	0.59678976	0.55132876	Prdm15	1
GO:0045765 regulation of angiogenesis	1/40	270/14464	0.0037037	1.33925926	0.2963313	0.5298795	0.59761297	0.55208926	Smad1	1
GO:0030336 negative regulation of cell migration	1/40	271/14464	0.00369004	1.33431734	0.29256528	0.53120434	0.59789186	0.55234691	Ptpn1	1
GO:0015833 peptide transport	1/40	272/14464	0.00367647	1.32941176	0.2888139	0.53252555	0.59789186	0.55234691	Capn10	1
GO:0061448 connective tissue development	1/40	272/14464	0.00367647	1.32941176	0.2888139	0.53252555	0.59789186	0.55234691	Smad1	1
GO:0061458 reproductive system development	1/40	272/14464	0.00367647	1.32941176	0.2888139	0.53252555	0.59789186	0.55234691	Bok	1
GO:0007281 germ cell development	1/40	276/14464	0.00362319	1.31014493	0.27395231	0.53777415	0.60107716	0.55528957	Catsperz	1
GO:0030198 extracellular matrix organization	1/40	276/14464	0.00362319	1.31014493	0.27395231	0.53777415	0.60107716	0.55528957	Matn2	1
GO:0043062 extracellular structure organization	1/40	276/14464	0.00362319	1.31014493	0.27395231	0.53777415	0.60107716	0.55528957	Matn2	1
GO:0045229 external encapsulating structure organization	1/40	276/14464	0.00362319	1.31014493	0.27395231	0.53777415	0.60107716	0.55528957	Matn2	1
GO:1901342 regulation of vasculature development	1/40	277/14464	0.00361011	1.30541516	0.27027229	0.53907729	0.60128987	0.55548608	Smad1	1
GO:0006605 protein targeting	1/40	278/14464	0.00359712	1.30071942	0.26660619	0.54037686	0.60128987	0.55548608	Zfand2b	1
GO:0006874 intracellular calcium ion homeostasis	1/40	278/14464	0.00359712	1.30071942	0.26660619	0.54037686	0.60128987	0.55548608	Bok	1
GO:0090287 regulation of cellular response to growth factor stimulus	1/40	278/14464	0.00359712	1.30071942	0.26660619	0.54037686	0.60128987	0.55548608	Ptpn1	1
GO:0006310 DNA recombination	1/40	279/14464	0.00358423	1.29605735	0.26295389	0.54167285	0.60206001	0.55619755	Fbh1	1
GO:0006302 double-strand break repair	1/40	281/14464	0.00355872	1.28683274	0.25569025	0.54425415	0.60425544	0.55822574	Fbh1	1
GO:0030111 regulation of Wnt signaling pathway	1/40	282/14464	0.0035461	1.2822695	0.25207867	0.54553948	0.60433651	0.55830064	Prdm15	1
GO:2000146 negative regulation of cell motility	1/40	282/14464	0.0035461	1.2822695	0.25207867	0.54553948	0.60433651	0.55830064	Ptpn1	1
GO:0002696 positive regulation of leukocyte activation	1/40	283/14464	0.00353357	1.27773852	0.24848045	0.54682127	0.60441331	0.55837159	Pnp	1
GO:0009416 response to light stimulus	1/40	283/14464	0.00353357	1.27773852	0.24848045	0.54682127	0.60441331	0.55837159	Cry1	1
GO:1901293 nucleoside phosphate biosynthetic process	1/40	286/14464	0.0034965	1.26433566	0.23776484	0.55064555	0.60729382	0.56103267	Pnp	1
GO:1902105 regulation of leukocyte differentiation	1/40	286/14464	0.0034965	1.26433566	0.23776484	0.55064555	0.60729382	0.56103267	Pnp	1
GO:0070661 leukocyte proliferation	1/40	287/14464	0.00348432	1.25993031	0.23421896	0.5519133	0.6080194	0.56170298	Pnp	1
GO:0045787 positive regulation of cell cycle	1/40	288/14464	0.00347222	1.25555556	0.2306859	0.55317756	0.60873955	0.56236827	Fam83d	1
GO:0001666 response to hypoxia	1/40	289/14464	0.00346021	1.25121107	0.22716556	0.55443835	0.60945428	0.56302855	Ptpn1	1
GO:0048545 response to steroid hormone	1/40	290/14464	0.00344828	1.24689655	0.22365785	0.55569566	0.61016363	0.56368387	Cry1	1
GO:0048511 rhythmic process	1/40	291/14464	0.00343643	1.24261168	0.22016265	0.55694952	0.61019634	0.56371409	Cry1	1
GO:0141188 nucleic acid catabolic process	1/40	291/14464	0.00343643	1.24261168	0.22016265	0.55694952	0.61019634	0.56371409	Fbh1	1
GO:0141091 transforming growth factor beta receptor superfamily signaling pathway	1/40	292/14464	0.00342466	1.23835616	0.21667987	0.55819993	0.61022514	0.56374069	Smad1	1
GO:1903037 regulation of leukocyte cell-cell adhesion	1/40	292/14464	0.00342466	1.23835616	0.21667987	0.55819993	0.61022514	0.56374069	Pnp	1
GO:0010506 regulation of autophagy	1/40	295/14464	0.00338983	1.22576271	0.20630509	0.56193054	0.61228934	0.56564765	Bok	1
GO:0022412 cellular process involved in reproduction in multicellular organism	1/40	295/14464	0.00338983	1.22576271	0.20630509	0.56193054	0.61228934	0.56564765	Catsperz	1
GO:0055074 calcium ion homeostasis	1/40	295/14464	0.00338983	1.22576271	0.20630509	0.56193054	0.61228934	0.56564765	Bok	1
GO:0044282 small molecule catabolic process	1/40	296/14464	0.00337838	1.22162162	0.20287103	0.56316724	0.61229851	0.56565613	Pnp	1
GO:0045862 positive regulation of proteolysis	1/40	296/14464	0.00337838	1.22162162	0.20287103	0.56316724	0.61229851	0.56565613	Bok	1
GO:0040013 negative regulation of locomotion	1/40	297/14464	0.003367	1.21750842	0.19944892	0.56440053	0.61230395	0.56566115	Ptpn1	1
GO:0050863 regulation of T cell activation	1/40	297/14464	0.003367	1.21750842	0.19944892	0.56440053	0.61230395	0.56566115	Pnp	1
GO:0050867 positive regulation of cell activation	1/40	303/14464	0.00330033	1.19339934	0.17916191	0.57172932	0.61823658	0.57114186	Pnp	1
GO:0099003 vesicle-mediated transport in synapse	1/40	303/14464	0.00330033	1.19339934	0.17916191	0.57172932	0.61823658	0.57114186	Ctbp2	1
GO:0099173 postsynapse organization	1/40	303/14464	0.00330033	1.19339934	0.17916191	0.57172932	0.61823658	0.57114186	Ptpn1	1
GO:0036293 response to decreased oxygen levels	1/40	304/14464	0.00328947	1.18947368	0.17582086	0.57293904	0.61887348	0.57173024	Ptpn1	1
GO:0051347 positive regulation of transferase activity	1/40	306/14464	0.00326797	1.18169935	0.16917238	0.57534849	0.62080351	0.57351325	Ptpn1	1
GO:0051235 maintenance of location	1/40	307/14464	0.00325733	1.17785016	0.16586478	0.57654824	0.62142551	0.57408786	Cry1	1
GO:0051090 regulation of DNA-binding transcription factor activity	1/40	308/14464	0.00324675	1.17402597	0.16256816	0.57774468	0.62198386	0.57460368	Cys1	1
GO:0016358 dendrite development	1/40	309/14464	0.00323625	1.17022654	0.15928244	0.57893783	0.62198386	0.57460368	Matn2	1
GO:0042254 ribosome biogenesis	1/40	309/14464	0.00323625	1.17022654	0.15928244	0.57893783	0.62198386	0.57460368	Rrp8	1
GO:0030100 regulation of endocytosis	1/40	310/14464	0.00322581	1.16645161	0.15600754	0.58012769	0.62252452	0.57510316	Ptpn1	1
GO:0009150 purine ribonucleotide metabolic process	1/40	311/14464	0.00321543	1.16270096	0.15274338	0.58131428	0.62252452	0.57510316	Pnp	1
GO:0043010 camera-type eye development	1/40	311/14464	0.00321543	1.16270096	0.15274338	0.58131428	0.62252452	0.57510316	Cntf	1
GO:0007178 cell surface receptor protein serine/threonine kinase signaling pathway	1/40	313/14464	0.00319489	1.15527157	0.14624697	0.58367764	0.62438477	0.5768217	Smad1	1
GO:0042886 amide transport	1/40	314/14464	0.00318471	1.15159236	0.14301456	0.58485444	0.62497307	0.57736519	Capn10	1
GO:1904951 positive regulation of establishment of protein localization	1/40	316/14464	0.00316456	1.1443038	0.13658094	0.58719832	0.62680591	0.57905841	Capn10	1
GO:0062012 regulation of small molecule metabolic process	1/40	319/14464	0.0031348	1.13354232	0.12700743	0.59068996	0.62985871	0.58187866	Cry1	1
GO:0002697 regulation of immune effector process	1/40	320/14464	0.003125	1.13	0.12383647	0.59184743	0.63041868	0.58239598	Pnp	1
GO:0042060 wound healing	1/40	321/14464	0.00311526	1.12647975	0.12067549	0.59300171	0.63097407	0.58290905	Hps5	1
GO:0007517 muscle organ development	1/40	323/14464	0.00309598	1.11950464	0.11438322	0.59530072	0.63207116	0.58392257	Cntf	1
GO:0021700 developmental maturation	1/40	323/14464	0.00309598	1.11950464	0.11438322	0.59530072	0.63207116	0.58392257	Catsperz	1
GO:0007159 leukocyte cell-cell adhesion	1/40	326/14464	0.00306748	1.10920245	0.10501795	0.59872552	0.63488455	0.58652165	Pnp	1
GO:0002443 leukocyte mediated immunity	1/40	327/14464	0.0030581	1.1058104	0.10191543	0.59986083	0.63488455	0.58652165	Pnp	1
GO:0070482 response to oxygen levels	1/40	327/14464	0.0030581	1.1058104	0.10191543	0.59986083	0.63488455	0.58652165	Ptpn1	1
GO:0009259 ribonucleotide metabolic process	1/40	332/14464	0.00301205	1.08915663	0.08654404	0.60549056	0.63948526	0.5907719	Pnp	1
GO:0031589 cell-substrate adhesion	1/40	332/14464	0.00301205	1.08915663	0.08654404	0.60549056	0.63948526	0.5907719	Ptpn1	1
GO:0002250 adaptive immune response	1/40	337/14464	0.00296736	1.07299703	0.07140219	0.61104303	0.64466656	0.59555851	Pnp	1
GO:0019693 ribose phosphate metabolic process	1/40	339/14464	0.00294985	1.06666667	0.06540797	0.61324262	0.64562079	0.59644005	Pnp	1
GO:0060271 cilium assembly	1/40	339/14464	0.00294985	1.06666667	0.06540797	0.61324262	0.64562079	0.59644005	Tchp	1
GO:0042176 regulation of protein catabolic process	1/40	344/14464	0.00290698	1.05116279	0.0505746	0.61868867	0.6506673	0.60110214	Fam83d	1
GO:0001654 eye development	1/40	351/14464	0.002849	1.03019943	0.03016192	0.6261877	0.65620718	0.60622001	Cntf	1
GO:0016050 vesicle organization	1/40	351/14464	0.002849	1.03019943	0.03016192	0.6261877	0.65620718	0.60622001	Hps5	1
GO:0051962 positive regulation of nervous system development	1/40	351/14464	0.002849	1.03019943	0.03016192	0.6261877	0.65620718	0.60622001	Lrrn3	1
GO:0006887 exocytosis	1/40	352/14464	0.00284091	1.02727273	0.02727859	0.62724719	0.65620718	0.60622001	Ctbp2	1
GO:0009306 protein secretion	1/40	352/14464	0.00284091	1.02727273	0.02727859	0.62724719	0.65620718	0.60622001	Capn10	1
GO:0019221 cytokine-mediated signaling pathway	1/40	353/14464	0.00283286	1.02436261	0.0244033	0.62830374	0.65634865	0.6063507	Cntf	1
GO:0035592 establishment of protein localization to extracellular region	1/40	354/14464	0.00282486	1.02146893	0.02153599	0.62935738	0.65634865	0.6063507	Capn10	1
GO:0150063 visual system development	1/40	354/14464	0.00282486	1.02146893	0.02153599	0.62935738	0.65634865	0.6063507	Cntf	1
GO:0007389 pattern specification process	1/40	356/14464	0.00280899	1.01573034	0.01582511	0.63145592	0.65784906	0.60773682	Smad1	1
GO:0071692 protein localization to extracellular region	1/40	357/14464	0.00280112	1.01288515	0.01298144	0.63250084	0.65824983	0.60810706	Capn10	1
GO:0001503 ossification	1/40	359/14464	0.00278552	1.00724234	0.00731739	0.63458203	0.65835409	0.60820338	Smad1	1
GO:0030098 lymphocyte differentiation	1/40	359/14464	0.00278552	1.00724234	0.00731739	0.63458203	0.65835409	0.60820338	Pnp	1
GO:0048880 sensory system development	1/40	359/14464	0.00278552	1.00724234	0.00731739	0.63458203	0.65835409	0.60820338	Cntf	1
GO:1903706 regulation of hemopoiesis	1/40	362/14464	0.00276243	0.99889503	-0.0011212	0.63768228	0.66088278	0.61053944	Pnp	1
GO:0044782 cilium organization	1/40	369/14464	0.00271003	0.9799458	-0.0205496	0.6448168	0.66758291	0.61672919	Tchp	1
GO:1902903 regulation of supramolecular fiber organization	1/40	374/14464	0.0026738	0.96684492	-0.0342095	0.64982883	0.672074	0.62087816	Arhgef10	1
GO:0043603 amide metabolic process	1/40	376/14464	0.00265957	0.96170213	-0.0396242	0.65181427	0.67342884	0.62212979	Pnp	1
GO:0016055 Wnt signaling pathway	1/40	385/14464	0.0025974	0.93922078	-0.0636526	0.66061378	0.6818136	0.62987584	Prdm15	1
GO:0001819 positive regulation of cytokine production	1/40	386/14464	0.00259067	0.93678756	-0.0662891	0.66157801	0.68210267	0.63014288	Pnp	1
GO:0003012 muscle system process	1/40	395/14464	0.00253165	0.91544304	-0.089729	0.67013678	0.68950089	0.63697754	Smad1	1
GO:0006163 purine nucleotide metabolic process	1/40	395/14464	0.00253165	0.91544304	-0.089729	0.67013678	0.68950089	0.63697754	Pnp	1
GO:0022407 regulation of cell-cell adhesion	1/40	398/14464	0.00251256	0.90854271	-0.0974296	0.67294251	0.6916739	0.63898502	Pnp	1
GO:0022411 cellular component disassembly	1/40	400/14464	0.0025	0.904	-0.1025329	0.67480007	0.69215604	0.63943043	Bok	1
GO:0140694 non-membrane-bounded organelle assembly	1/40	400/14464	0.0025	0.904	-0.1025329	0.67480007	0.69215604	0.63943043	Arhgef10	1
GO:0051249 regulation of lymphocyte activation	1/40	409/14464	0.00244499	0.88410758	-0.1252038	0.68303254	0.6998802	0.6465662	Pnp	1
GO:0014706 striated muscle tissue development	1/40	414/14464	0.00241546	0.87342995	-0.1375971	0.68751796	0.70321964	0.64965125	Smad1	1
GO:0051051 negative regulation of transport	1/40	415/14464	0.00240964	0.8713253	-0.1400589	0.68840759	0.70321964	0.64965125	Cry1	1
GO:0072594 establishment of protein localization to organelle	1/40	415/14464	0.00240964	0.8713253	-0.1400589	0.68840759	0.70321964	0.64965125	Zfand2b	1
GO:0045785 positive regulation of cell adhesion	1/40	422/14464	0.00236967	0.85687204	-0.1571385	0.69456621	0.70822264	0.65427314	Pnp	1
GO:0007283 spermatogenesis	1/40	423/14464	0.00236407	0.85484634	-0.1595569	0.69543627	0.70822264	0.65427314	Catsperz	1
GO:0042692 muscle cell differentiation	1/40	423/14464	0.00236407	0.85484634	-0.1595569	0.69543627	0.70822264	0.65427314	Smad1	1
GO:0060537 muscle tissue development	1/40	435/14464	0.00229885	0.83126437	-0.1881717	0.70569032	0.71793189	0.66324278	Smad1	1
GO:0048232 male gamete generation	1/40	437/14464	0.00228833	0.82745995	-0.1928699	0.70736628	0.71890335	0.66414024	Catsperz	1
GO:0008015 blood circulation	1/40	443/14464	0.00225734	0.81625282	-0.2068471	0.71233852	0.72321946	0.66812757	Ptpn1	1
GO:0007409 axonogenesis	1/40	446/14464	0.00224215	0.81076233	-0.2137708	0.71479366	0.72359094	0.66847075	Matn2	1
GO:0030003 intracellular monoatomic cation homeostasis	1/40	447/14464	0.00223714	0.80894855	-0.2160692	0.71560749	0.72359094	0.66847075	Bok	1
GO:0034762 regulation of transmembrane transport	1/40	447/14464	0.00223714	0.80894855	-0.2160692	0.71560749	0.72359094	0.66847075	Capn10	1
GO:1903131 mononuclear cell differentiation	1/40	447/14464	0.00223714	0.80894855	-0.2160692	0.71560749	0.72359094	0.66847075	Pnp	1
GO:0009117 nucleotide metabolic process	1/40	450/14464	0.00222222	0.80355556	-0.2229366	0.71803541	0.72531034	0.67005918	Pnp	1
GO:0006873 intracellular monoatomic ion homeostasis	1/40	454/14464	0.00220264	0.79647577	-0.2320285	0.72124123	0.72707534	0.67168972	Bok	1
GO:0098771 inorganic ion homeostasis	1/40	454/14464	0.00220264	0.79647577	-0.2320285	0.72124123	0.72707534	0.67168972	Bok	1
GO:0042110 T cell activation	1/40	464/14464	0.00215517	0.77931034	-0.2544446	0.72910109	0.73425635	0.67832372	Pnp	1
GO:0001501 skeletal system development	1/40	466/14464	0.00214592	0.77596567	-0.2588755	0.73064693	0.73507063	0.67907596	Smad1	1
GO:0002694 regulation of leukocyte activation	1/40	467/14464	0.00214133	0.77430407	-0.2610845	0.73141662	0.7351032	0.67910605	Pnp	1
GO:0003013 circulatory system process	1/40	470/14464	0.00212766	0.7693617	-0.2676861	0.73371285	0.73666839	0.68055201	Ptpn1	1
GO:0010720 positive regulation of cell development	1/40	473/14464	0.00211416	0.76448203	-0.27425	0.73598993	0.73821123	0.68197733	Pnp	1
GO:1903829 positive regulation of protein localization	1/40	486/14464	0.00205761	0.74403292	-0.3022707	0.74563983	0.7471386	0.69022465	Capn10	1

GO:0005975	carbohydrate metabolic process	1/40	491/14464	0.00203666	0.73645621	-0.3128708	0.74925901	0.75001127	0.69287849	Cry1	1
GO:0071396	cellular response to lipid	1/40	499/14464	0.00200401	0.7246493	-0.3296342	0.75494551	0.75494551	0.69743686	Cry1	1

ID	Description	GeneRatio	BgRatio	RichFactor	FoldEnrichm	zScore	pvalue	p.adjust	qvalue	geneID	Count
GO:0033152	immunoglobulin V(D)J recombination	2/97	10/14464	0.2	29.8226804	7.49156161	0.00193413	0.50874395	0.5048838	Lig4/Nhej1	2
GO:0033197	response to vitamin E	2/97	10/14464	0.2	29.8226804	7.49156161	0.00193413	0.50874395	0.5048838	Fkbp1b/Becn1	2
GO:0050821	protein stabilization	6/97	197/14464	0.03045685	4.54152494	4.11234082	0.00209363	0.50874395	0.5048838	Hspa1b/Ptges3/Atp1b3/Ctnnd1/Caml/Cct7	6
GO:0071838	cell proliferation in bone marrow	2/97	11/14464	0.18181818	27.1115276	7.11838767	0.00235363	0.50874395	0.5048838	Shc1/Lipa	2
GO:1903265	positive regulation of tumor necrosis factor-mediated signaling pathway	2/97	11/14464	0.18181818	27.1115276	7.11838767	0.00235363	0.50874395	0.5048838	Hspa1b/Traf2	2
GO:0051084	'de novo' post-translational protein folding	3/97	41/14464	0.07317073	10.9107367	5.22158174	0.00259125	0.50874395	0.5048838	Hspa1b/Ptges3/Cct7	3
GO:0006458	'de novo' protein folding	3/97	43/14464	0.06976744	10.4032606	5.07395985	0.00297101	0.50874395	0.5048838	Hspa1b/Ptges3/Cct7	3
GO:0006266	DNA ligation	2/97	13/14464	0.15384615	22.9405234	6.50281986	0.00330884	0.50874395	0.5048838	Lig4/Nhej1	2
GO:0007040	lysosome organization	4/97	98/14464	0.04081633	6.08626131	4.15122038	0.0042438	0.50874395	0.5048838	Rab39/Lipa/Cln5/Becn1	4
GO:0080171	lytic vacuole organization	4/97	98/14464	0.04081633	6.08626131	4.15122038	0.0042438	0.50874395	0.5048838	Rab39/Lipa/Cln5/Becn1	4
GO:0006644	phospholipid metabolic process	7/97	319/14464	0.02194357	3.27208092	3.37170582	0.00561474	0.50874395	0.5048838	Pcsk9/Pmvk/Smpd4/Sphk2/Becn1/Pip4k2c/Pigc	7
GO:0051604	protein maturation	9/97	498/14464	0.01807229	2.69482052	3.16252801	0.00618475	0.50874395	0.5048838	Hspa1b/Tspan14/Pcsk9/Ptges3/Ctnnd1/Cln5/Cct7/Aph1b/Pigc	9
GO:0033151	V(D)J recombination	2/97	18/14464	0.11111111	16.5681558	5.43039762	0.00635046	0.50874395	0.5048838	Lig4/Nhej1	2
GO:0006352	DNA-templated transcription initiation	5/97	177/14464	0.02824859	4.212243	3.53310477	0.00675494	0.50874395	0.5048838	Sphk2/Ubtf/Taf4b/Maz/Eloa	5
GO:0032412	regulation of monoatomic ion transmembrane transporter activity	5/97	177/14464	0.02824859	4.212243	3.53310477	0.00675494	0.50874395	0.5048838	Gpr35/Fkbp1b/Pcsk9/Atp1b3/Sphk2	5
GO:0034612	response to tumor necrosis factor	5/97	180/14464	0.02777778	4.14203895	3.48541832	0.00724004	0.50874395	0.5048838	Hspa1b/Commd7/Smpd4/Traf2/Tuba1a	5
GO:2000786	positive regulation of autophagosome assembly	2/97	20/14464	0.1	14.9113402	5.11531337	0.00781784	0.50874395	0.5048838	Becn1/Pip4k2c	2
GO:0022898	regulation of transmembrane transporter activity	5/97	186/14464	0.02688172	4.00842479	3.39307888	0.00828243	0.50874395	0.5048838	Gpr35/Fkbp1b/Pcsk9/Atp1b3/Sphk2	5
GO:0061077	chaperone-mediated protein folding	3/97	62/14464	0.0483871	7.21516462	4.02966638	0.00830821	0.50874395	0.5048838	Hspa1b/Ptges3/Cct7	3
GO:0007339	binding of sperm to zona pellucida	2/97	22/14464	0.09090909	13.5557638	4.8425356	0.00942255	0.50874395	0.5048838	Hspa1b/Cct7	2
GO:0032409	regulation of transporter activity	5/97	196/14464	0.0255102	3.80391332	3.24745456	0.01024426	0.50874395	0.5048838	Gpr35/Fkbp1b/Pcsk9/Atp1b3/Sphk2	5
GO:0033273	response to vitamin	3/97	67/14464	0.04477612	6.6767195	3.82677666	0.01027399	0.50874395	0.5048838	Fkbp1b/Lipa/Becn1	3
GO:0008654	phospholipid biosynthetic process	5/97	201/14464	0.02487562	3.70928861	3.17819012	0.01133576	0.50874395	0.5048838	Pmvk/Sphk2/Becn1/Pip4k2c/Pigc	5
GO:0035036	sperm-egg recognition	2/97	26/14464	0.07692308	11.4702617	4.3905882	0.01302862	0.50874395	0.5048838	Hspa1b/Cct7	2
GO:0070536	protein K63-linked deubiquitination	2/97	26/14464	0.07692308	11.4702617	4.3905882	0.01302862	0.50874395	0.5048838	Stambpl1/Otub2	2
GO:0032801	receptor catabolic process	2/97	27/14464	0.07407407	11.0454372	4.29283564	0.01401004	0.50874395	0.5048838	Pcsk9/Becn1	2
GO:0044090	positive regulation of vacuole organization	2/97	27/14464	0.07407407	11.0454372	4.29283564	0.01401004	0.50874395	0.5048838	Becn1/Pip4k2c	2
GO:0006367	transcription initiation at RNA polymerase II promoter	4/97	141/14464	0.02836879	4.23016743	3.16701127	0.01491489	0.50874395	0.5048838	Sphk2/Taf4b/Maz/Eloa	4
GO:0043112	receptor metabolic process	3/97	77/14464	0.03896104	5.80961307	3.47698161	0.01495428	0.50874395	0.5048838	Pcsk9/Becn1/Caml	3
GO:0034381	plasma lipoprotein particle clearance	2/97	28/14464	0.07142857	10.6509573	4.2000841	0.0150225	0.50874395	0.5048838	Pcsk9/Lipa	2
GO:0045648	positive regulation of erythrocyte differentiation	2/97	28/14464	0.07142857	10.6509573	4.2000841	0.0150225	0.50874395	0.5048838	Hspa1b/Ankrd54	2
GO:1904063	negative regulation of cation transmembrane transport	3/97	79/14464	0.03797468	5.66253426	3.41438636	0.01601248	0.50874395	0.5048838	Gpr35/Fkbp1b/Pcsk9	3
GO:0009314	response to radiation	7/97	394/14464	0.0177665	2.64922288	2.72717136	0.01666341	0.50874395	0.5048838	Cdkn1a/Mettl3/Tuba1a/Lig4/Nhej1/Pde6d/Smc1a	7
GO:0007033	vacuole organization	5/97	223/14464	0.02242152	3.34334982	2.89768604	0.01708352	0.50874395	0.5048838	Rab39/Lipa/Cln5/Becn1/Pip4k2c	5
GO:0051085	chaperone cofactor-dependent protein refolding	2/97	30/14464	0.06666667	9.94089347	4.02791421	0.01713873	0.50874395	0.5048838	Hspa1b/Ptges3	2
GO:0002244	hematopoietic progenitor cell differentiation	4/97	148/14464	0.02702703	4.03009195	3.04444291	0.01751425	0.50874395	0.5048838	Lipa/Mettl3/Lig4/Dnai4	4
GO:0033209	tumor necrosis factor-mediated signaling pathway	3/97	82/14464	0.03658537	5.45536837	3.32439726	0.01767703	0.50874395	0.5048838	Hspa1b/Commd7/Traf2	3
GO:0034766	negative regulation of monoatomic ion transmembrane transport	3/97	84/14464	0.03571429	5.32547865	3.26682888	0.01883842	0.50874395	0.5048838	Gpr35/Fkbp1b/Pcsk9	3
GO:0001964	startle response	2/97	32/14464	0.0625	9.31958763	3.87119942	0.01937377	0.50874395	0.5048838	Tuba1a/Aph1b	2
GO:0006661	phosphatidylinositol biosynthetic process	3/97	87/14464	0.03448276	5.14184145	3.18383809	0.02065827	0.50874395	0.5048838	Becn1/Pip4k2c/Pigc	3
GO:0030301	cholesterol transport	3/97	88/14464	0.03409091	5.08341143	3.15702091	0.02128566	0.50874395	0.5048838	Pcsk9/Lipa/Stard3	3
GO:0031647	regulation of protein stability	6/97	324/14464	0.01851852	2.7613593	2.63467937	0.02176333	0.50874395	0.5048838	Hspa1b/Ptges3/Atp1b3/Ctnnd1/Caml/Cct7	6
GO:0046486	glycerolipid metabolic process	6/97	325/14464	0.01846154	2.75286281	2.62610613	0.02205603	0.50874395	0.5048838	Pcsk9/Lipa/Smpd4/Becn1/Pip4k2c/Pigc	6
GO:0071897	DNA biosynthetic process	4/97	159/14464	0.02515723	3.75128056	2.86629921	0.02214075	0.50874395	0.5048838	Cdkn1a/Ptges3/Lig4/Cct7	4
GO:0071356	cellular response to tumor necrosis factor	4/97	160/14464	0.025	3.72783505	2.85089591	0.02259491	0.50874395	0.5048838	Hspa1b/Commd7/Smpd4/Traf2	4
GO:0032212	positive regulation of telomere maintenance via telomerase	2/97	35/14464	0.05714286	8.52076583	3.66024357	0.02294147	0.50874395	0.5048838	Ptges3/Cct7	2
GO:0007173	epidermal growth factor receptor signaling pathway	3/97	92/14464	0.0326087	4.86239355	3.05368226	0.02389915	0.50874395	0.5048838	Shc1/Nrg2/Caml	3
GO:0015918	sterol transport	3/97	92/14464	0.0326087	4.86239355	3.05368226	0.02389915	0.50874395	0.5048838	Pcsk9/Lipa/Stard3	3
GO:0016239	positive regulation of macroautophagy	3/97	97/14464	0.03092784	4.6117547	2.9326017	0.02739978	0.50874395	0.5048838	Becn1/Pafah1b2/Pip4k2c	3
GO:1904358	positive regulation of telomere maintenance via telomere lengthening	2/97	39/14464	0.05128205	7.64684113	3.41524375	0.02808226	0.50874395	0.5048838	Ptges3/Cct7	2
GO:0034976	response to endoplasmic reticulum stress	5/97	256/14464	0.01953125	2.91237113	2.53663338	0.02888869	0.50874395	0.5048838	Pdia6/Edem1/Uggt1/Nhlrc1/Rnf103	5
GO:0051452	intracellular pH reduction	2/97	40/14464	0.05	7.4556701	3.35939044	0.02943326	0.50874395	0.5048838	Rab39/Cln5	2
GO:0007338	single fertilization	3/97	101/14464	0.02970297	4.42911095	2.84152616	0.0303868	0.50874395	0.5048838	Hspa1b/Antxr2/Cct7	3
GO:0009988	cell-cell recognition	2/97	41/14464	0.04878049	7.27382449	3.30543412	0.03080977	0.50874395	0.5048838	Hspa1b/Cct7	2
GO:0019221	cytokine-mediated signaling pathway	6/97	353/14464	0.01699717	2.53450542	2.39832976	0.0313654	0.50874395	0.5048838	Hspa1b/Gpr35/Mettl3/Commd7/Gpr75/Traf2	6
GO:2000278	regulation of DNA biosynthetic process	3/97	103/14464	0.02912621	4.3431088	2.7977493	0.03194222	0.50874395	0.5048838	Cdkn1a/Ptges3/Cct7	3
GO:0000079	regulation of cyclin-dependent protein serine/threonine kinase activity	2/97	42/14464	0.04761905	7.10063819	3.25326307	0.03221141	0.50874395	0.5048838	Cdkn1a/Sfn	2
GO:0019827	stem cell population maintenance	4/97	179/14464	0.02234637	3.33214306	2.57972609	0.0323163	0.50874395	0.5048838	Mettl3/Lig4/Sap30l/Smc1a	4
GO:0002377	immunoglobulin production	3/97	105/14464	0.02857143	4.26038292	2.75507341	0.03353874	0.50874395	0.5048838	Traf2/Lig4/Nhej1	3
GO:0036503	ERAD pathway	3/97	105/14464	0.02857143	4.26038292	2.75507341	0.03353874	0.50874395	0.5048838	Edem1/Uggt1/Rnf103	3
GO:0005978	glycogen biosynthetic process	2/97	43/14464	0.04651163	6.93550707	3.20277464	0.03363777	0.50874395	0.5048838	Ptges3/Nhlrc1	2
GO:0009250	glucan biosynthetic process	2/97	43/14464	0.04651163	6.93550707	3.20277464	0.03363777	0.50874395	0.5048838	Ptges3/Nhlrc1	2
GO:0010803	regulation of tumor necrosis factor-mediated signaling pathway	2/97	43/14464	0.04651163	6.93550707	3.20277464	0.03363777	0.50874395	0.5048838	Hspa1b/Traf2	2
GO:0009206	purine ribonucleoside triphosphate biosynthetic process	3/97	106/14464	0.02830189	4.22019062	2.73413248	0.03435237	0.50874395	0.5048838	Lipa/Sphk2/Nme1	3
GO:0048873	homeostasis of number of cells within a tissue	2/97	44/14464	0.04545455	6.77788191	3.15387433	0.03508847	0.50874395	0.5048838	Lipa/Tuba1a	2
GO:0009145	purine nucleoside triphosphate biosynthetic process	3/97	107/14464	0.02803738	4.18074959	2.71344819	0.03517622	0.50874395	0.5048838	Lipa/Sphk2/Nme1	3
GO:0098727	maintenance of cell number	4/97	185/14464	0.02162162	3.22407356	2.5016015	0.03582419	0.50874395	0.5048838	Mettl3/Lig4/Sap30l/Smc1a	4
GO:0046854	phosphatidylinositol phosphate biosynthetic process	2/97	45/14464	0.04444444	6.62726231	3.10647498	0.03656313	0.50874395	0.5048838	Becn1/Pip4k2c	2
GO:0038127	ERBB signaling pathway	3/97	109/14464	0.02752294	4.10403859	2.67282665	0.03685448	0.50874395	0.5048838	Shc1/Nrg2/Caml	3
GO:0006066	alcohol metabolic process	5/97	275/14464	0.01818182	2.71115276	2.35402597	0.03757541	0.50874395	0.5048838	Pcsk9/Lipa/Pmvk/Sphk2/Nudt3	5
GO:0043616	keratinocyte proliferation	2/97	46/14464	0.04347826	6.48319139	3.06049606	0.03806137	0.50874395	0.5048838	Cdkn1a/Sfn	2
GO:1904029	regulation of cyclin-dependent protein kinase activity	2/97	46/14464	0.04347826	6.48319139	3.06049606	0.03806137	0.50874395	0.5048838	Cdkn1a/Sfn	2
GO:2000785	regulation of autophagosome assembly	2/97	46/14464	0.04347826	6.48319139	3.06049606	0.03806137	0.50874395	0.5048838	Becn1/Pip4k2c	2
GO:0009201	ribonucleoside triphosphate biosynthetic process	3/97	111/14464	0.02702703	4.03009195	2.63344016	0.03857333	0.50874395	0.5048838	Lipa/Sphk2/Nme1	3
GO:0034655	nucleobase-containing compound catabolic process	7/97	471/14464	0.014862	2.21612275	2.20477595	0.03887305	0.50874395	0.5048838	Hspa1b/Dnase1l3/Lipa/Mettl3/Traf2/Nudt3/Hbs1l	7
GO:0045646	regulation of erythrocyte differentiation	2/97	47/14464	0.04255319	6.34525115	3.015863	0.03958281	0.50874395	0.5048838	Hspa1b/Ankrd54	2
GO:2001252	positive regulation of chromosome organization	3/97	113/14464	0.02654867	3.95876289	2.5944202	0.04033256	0.50874395	0.5048838	Ptges3/Lig4/Cct7	3
GO:0072089	stem cell proliferation	3/97	114/14464	0.02631579	3.9240369	2.57538011	0.04122726	0.50874395	0.5048838	Sfn/Lig4/Taf4b	3
GO:1903170	negative regulation of calcium ion transmembrane transport	2/97	49/14464	0.04081633	6.08626131	2.93036285	0.04269382	0.50874395	0.5048838	Gpr35/Fkbp1b	2
GO:0034763	negative regulation of transmembrane transport	3/97	116/14464	0.02586207	3.85638109	2.53794072	0.04304665	0.50874395	0.5048838	Gpr35/Fkbp1b/Pcsk9	3
GO:0032413	negative regulation of ion transmembrane transporter activity	2/97	50/14464	0.04	5.96453608	2.8893718	0.04428265	0.50874395	0.5048838	Gpr35/Pcsk9	2
GO:0008203	cholesterol metabolic process	3/97	118/14464	0.02542373	3.7910187	2.50132631	0.04490583	0.50874395	0.5048838	Pcsk9/Lipa/Pmvk	3
GO:0009142	nucleoside triphosphate biosynthetic process	3/97	118/14464	0.02542373	3.7910187	2.50132631	0.04490583	0.50874395	0.5048838	Lipa/Sphk2/Nme1	3
GO:0043271	negative regulation of monoatomic ion transport	3/97	118/14464	0.02542373	3.7910187	2.50132631	0.04490583	0.50874395	0.5048838	Gpr35/Fkbp1b/Pcsk9	3
GO:0001961	positive regulation of cytokine-mediated signaling pathway	2/97	51/14464	0.03921569	5.84758439	2.84947787	0.04589323	0.50874395	0.5048838	Hspa1b/Traf2	2
GO:0006695	cholesterol biosynthetic process	2/97	51/14464	0.03921569	5.84758439	2.84947787	0.04589323	0.50874395	0.5048838	Lipa/Pmvk	2
GO:0070098	chemokine-mediated signaling pathway	2/97	51/14464	0.03921569	5.84758439	2.84947787	0.04589323	0.50874395	0.5048838	Gpr35/Gpr75	2
GO:1902653	secondary alcohol biosynthetic process	2/97	51/14464	0.03921569	5.84758439	2.84947787	0.04589323	0.50874395	0.5048838	Lipa/Pmvk	2
GO:0141188	nucleic acid catabolic process	5/97	291/14464	0.01718213	2.56208595	2.21183405	0.04601688	0.50874395	0.5048838	Hspa1b/Dnase1l3/Mettl3/Traf2/Hbs1l	5
GO:0032210	regulation of telomere maintenance via telomerase	2/97	52/14464	0.03846154	5.73513085	2.81062914	0.04752519	0.50874395	0.5048838	Ptges3/Cct7	2
GO:0097006	regulation of plasma lipoprotein particle levels	2/97	52/14464	0.03846154	5.73513085	2.81062914	0.04752519	0.50874395	0.5048838	Pcsk9/Lipa	2
GO:1902305	regulation of sodium ion transmembrane transport	2/97	52/14464	0.03846154	5.73513085	2.81062914	0.04752519	0.50874395	0.5048838	Pcsk9/Atp1b3	2
GO:1904062	regulation of monoatomic cation transmembrane transport	5/97	294/14464	0.0170068	2.53594221	2.1862289	0.04771621	0.50874395	0.5048838	Gpr35/Fkbp1b/Pcsk9/Atp1b3/Sphk2	5
GO:0007052	mitotic spindle organization	3/97	121/14464	0.02479339	3.6970265	2.44787881	0.04776866	0.50874395	0.5048838	Hspa1b/Efhc1/Smc1a	3
GO:0046165	alcohol biosynthetic process	3/97	121/14464	0.02479339	3.6970265	2.44787881	0.04776866	0.50874395	0.5048838	Lipa/Pmvk/Sphk2	3
GO:0046467	membrane lipid biosynthetic process	3/97	121/14464	0.02479339	3.6970265	2.44787881	0.04776866	0.50874395	0.5048838	Smpd4/Sphk2/Pigc	3
GO:0001738	morphogenesis of a polarized epithelium	2/97	53/14464	0.03773585	5.62692083	2.7727771	0.04917818	0.50874395	0.5048838	Sfrp5/Ctnnd1	2
GO:0001881	receptor recycling	2/97	53/14464	0.03773585	5.62692083	2.7727771	0.04917818	0.50874395	0.5048838	Pcsk9/Caml	2
GO:0050435	amyloid-beta metabolic process	2/97	53/14464	0.03773585	5.62692083	2.7727771	0.04917818	0.50874395	0.5048838	Becn1/Aph1b	2
GO:0016125	sterol metabolic process	3/97	123/14464	0.02439024	3.63691225	2.41318451	0.04972615	0.50874395	0.5048838	Pcsk9/Lipa/Pmvk	3
GO:0016447	somatic recombination of immunoglobulin gene segments	2/97	54/14464	0.03703704	5.52271859	2.73587638	0.05085185	0.50874395	0.5048838	Lig4/Nhej1	2
GO:0043506	regulation of JUN kinase activity	2/97	54/14464	0.03703704	5.52271859	2.73587638	0.05085185	0.50874395	0.5048838	Sfrp5/Traf2	2
GO:0010332	response to gamma radiation	2/97	55/14464	0.03636364	5.42230553	2.69988448	0.05254586	0.50874395	0.5048838	Cdkn1a/Lig4	2
GO:0070085	glycosylation	4/97	210/14464	0.01904762	2.84025528	2.20724914	0.05273437	0.50874395	0.5048838	Mlec/Uggt1/Cln5/Fut11	4
GO:0006694	steroid biosynthetic process	3/97	126/14464	0.02380952	3.5503191	2.36247663	0.05273509	0.50874395	0.5048838	Lipa/Pmvk/Stard3	3
GO:0007041	lysosomal transport	3/97	127/14464	0.02362205	3.52236383	2.34591603	0.05375729	0.50874395	0.5048838	Pcsk9/Lipa/Becn1	3
GO:1902652	secondary alcohol metabolic process	3/97	127/14464	0.02362205	3.52236383	2.34591603	0.05375729	0.50874395	0.5048838	Pcsk9/Lipa/Pmvk	3
GO:0002886	regulation of myeloid leukocyte mediated immunity	2/97	56/14464	0.03571429	5.32547865	2.66476153	0.05425986	0.50874395	0.5048838	Dnase1l3/Sphk2	2
GO:0016126	sterol biosynthetic process	2/97	56/14464	0.03571429	5.32547865	2.66476153	0.05425986	0.50874395	0.5048838	Lipa/Pmvk	2
GO:0034198	cellular response to amino acid starvation	2/97	56/14464	0.03571429	5.32547865	2.66476153	0.05425986	0.50874395	0.5048838	Cdkn1a/Becn1	2
GO:1903409	reactive oxygen species biosynthetic process	2/97	56/14464	0.03571429	5.32547865	2.66476153	0.05425986	0.50874395	0.5048838	Lipa/Sphk2	2
GO:0072593	reactive oxygen species metabolic process	4/97	213/14464	0.01877934	2.80025168	2.17486534	0.05501184	0.50874395	0.5048838	Shc1/Cdkn1a/Lipa/Sphk2	4
GO:0032410	negative regulation of transporter activity	2/97	57/14464	0.03508772	5.2320492	2.63047011	0.05599352	0.50874395	0.5048838	Gpr35/Pcsk9	2
GO:0044088	regulation of vacuole organization	2/97	58/14464	0.03448276	5.14184145	2.59697504	0.0577465	0.50874395	0.5048838	Becn1/Pip4k2c	2
GO:0060760	positive regulation of response to cytokine stimulus	2/97	58/14464	0.03448276	5.14184145	2.59697504	0.0577465	0.50874395	0.5048838	Hspa1b/Traf2	2
GO:0009566	fertilization	3/97	131/14464	0.02290076	3.41481073	2.281299	0.05794115	0.50874395	0.5048838	Hspa1b/Antxr2/Cct7	3
GO:0009150	purine ribonucleotide metabolic process	5/97	311/14464	0.01607717	2.39732158	2.046842	0.05804645	0.50874395	0.5048838	Hspa1b/Lipa/Sphk2/Nudt3/Nme1	5
GO:0006278	RNA-templated DNA biosynthetic process	2/97	59/14464	0.03389831	5.0546916	2.56424322	0.05951848	0.50874395	0.5048838	Ptges3/Cct7	2
GO:0007004	telomere maintenance via telomerase	2/97	59/14464	0.03389831	5.0546916	2.56424322	0.05951848	0.50874395	0.5048838	Ptges3/Cct7	2
GO:0002440	production of molecular mediator of immune response	4/97	219/14464	0.01826484	2.72353246	2.11174907	0.05972495	0.50874395	0.5048838	Traf2/Sphk2/Lig4/Nhej1	4
GO:0016485	protein processing	4/97	220/14464	0.01818182	2.71115276	2.10143594	0.06053087	0.50874395	0.5048838	Pcsk9/Ctnnd1/Cln5/Aph1b	4
GO:0001959	regulation of cytokine-mediated signaling pathway	3/97	134/14464	0.02238806	3.33835975	2.23446031	0.06117764	0.50874395	0.5048838	Hspa1b/Mettl3/Traf2	3
GO:0016445	somatic diversification of immunoglobulins	2/97	60/14464	0.03333333	4.97044674	2.53224346	0.06130911	0.50874395	0.5048838	Lig4/Nhej1	2
GO:0046513	ceramide biosynthetic process	2/97	60/14464	0.03333333	4.97044674	2.53224346	0.06130911	0.50874395	0.5048838	Smpd4/Sphk2	2
GO:1904356	regulation of telomere maintenance via telomere lengthening	2/97	61/14464	0.03278689	4.888964	2.50094635	0.06311809	0.50874395	0.5048838	Ptges3/Cct7	2
GO:1990868	response to chemokine	2/97	61/14464	0.03278689	4.888964	2.50094635	0.06311809	0.50874395	0.5048838	Gpr35/Gpr75	2
GO:1990869	cellular response to chemokine	2/97	61/14464	0.03278689	4.888964	2.50094635	0.06311809	0.50874395	0.5048838	Gpr35/Gpr75	2
GO:1990928	response to amino acid starvation	2/97	61/14464	0.03278689	4.888964	2.50094635	0.06311809	0.50874395	0.5048838	Cdkn1a/Becn1	2
GO:0046488	phosphatidylinositol metabolic process	3/97	137/14464	0.02189781	3.26525698	2.18893137	0.06449729	0.50874395	0.5048838	Becn1/Pip4k2c/Pigc	3
GO:0008202	steroid metabolic process	4/97	225/14464	0.01777778	2.65090493	2.05071634	0.06464741	0.50874395	0.5048838	Pcsk9/Lipa/Pmvk/Stard3	4
GO:0000271	polysaccharide biosynthetic process	2/97	62/14464	0.03225806	4.81010974	2.47032413	0.06494509	0.50874395	0.5048838	Ptges3/Nhlrc1	2
GO:0002562	somatic diversification of immune receptors via germline recombination within a single locus	2/97	62/14464	0.03225806	4.81010974	2.47032413	0.06494509	0.50874395	0.5048838	Lig4/Nhej1	2
GO:0006487	protein N-linked glycosylation	2/97	62/14464	0.03225806	4.81010974	2.47032413	0.06494509	0.50874395	0.5048838	Mlec/Uggt1	2
GO:0016444	somatic cell DNA recombination	2/97	62/14464	0.03225806	4.81010974	2.47032413	0.06494509	0.50874395	0.5048838	Lig4/Nhej1	2
GO:0030199	collagen fibril organization	2/97	62/14464	0.03225806	4.81010974	2.47032413	0.06494509	0.50874395	0.5048838	P4ha1/Antxr2	2
GO:0006517	protein deglycosylation	1/97	10/14464	0.1	14.9113402	3.61582132	0.06509462	0.50874395	0.5048838	Edem1	1
GO:0007220	Notch receptor processing	1/97	10/14464	0.1	14.9113402	3.61582132	0.06509462	0.50874395	0.5048838	Aph1b	1
GO:0007602	phototransduction	1/97	10/14464	0.1	14.9113402	3.61582132	0.06509462	0.50874395	0.5048838	Pde6d	1
GO:0009221	pyrimidine deoxyribonucleotide biosynthetic process	1/97	10/14464	0.1	14.9113402	3.61582132	0.06509462	0.50874395	0.5048838	Nme1	1
GO:0010881	regulation of cardiac muscle contraction by regulation of the release of sequestered calcium ion	1/97	10/14464	0.1	14.9113402	3.61582132	0.06509462	0.50874395	0.5048838	Fkbp1b	1
GO:0019511	peptidyl-proline hydroxylation	1/97	10/14464	0.1	14.9113402	3.61582132	0.06509462	0.50874395	0.5048838	P4ha1	1
GO:0031424	keratinization	1/97	10/14464	0.1	14.9113402	3.61582132	0.06509462	0.50874395	0.5048838	Sfn	1
GO:0033189	response to vitamin A	1/97	10/14464	0.1	14.9113402	3.61582132	0.06509462	0.50874395	0.5048838	Lipa	1
GO:0035871	protein K11-linked deubiquitination	1/97	10/14464	0.1	14.9113402	3.61582132	0.06509462	0.50874395	0.5048838	Otub2	1
GO:0046051	UTP metabolic process	1/97	10/14464	0.1	14.9113402	3.61582132	0.06509462	0.50874395	0.5048838	Nme1	1
GO:0048853	forebrain morphogenesis	1/97	10/14464	0.1	14.9113402	3.61582132	0.06509462	0.50874395	0.5048838	Tuba1a	1
GO:0051775	response to redox state	1/97	10/14464	0.1	14.9113402	3.61582132	0.06509462	0.50874395	0.5048838	Fkbp1b	1
GO:0070424	regulation of nucleotide-binding domain, leucine rich repeat containing receptor signaling pathway	1/97	10/14464	0.1	14.9113402	3.61582132	0.06509462	0.50874395	0.5048838	Hspa1b	1
GO:0070863	positive regulation of protein exit from endoplasmic reticulum	1/97	10/14464	0.1	14.9113402	3.61582132	0.06509462	0.50874395	0.5048838	Edem1	1
GO:0070942	neutrophil mediated cytotoxicity	1/97	10/14464	0.1	14.9113402	3.61582132	0.06509462	0.50874395	0.5048838	Dnase1l3	1
GO:0072102	glomerulus morphogenesis	1/97	10/14464	0.1	14.9113402	3.61582132	0.06509462	0.50874395	0.5048838	Ctnnd1	1
GO:0090649	response to oxygen-glucose deprivation	1/97	10/14464	0.1	14.9113402	3.61582132	0.06509462	0.50874395	0.5048838	Becn1	1
GO:0097400	interleukin-17-mediated signaling pathway	1/97	10/14464	0.1	14.9113402	3.61582132	0.06509462	0.50874395	0.5048838	Traf2	1
GO:0098908	regulation of neuronal action potential	1/97	10/14464	0.1	14.9113402	3.61582132	0.06509462	0.50874395	0.5048838	Gpr35	1
GO:1902423	regulation of attachment of mitotic spindle microtubules to kinetochore	1/97	10/14464	0.1	14.9113402	3.61582132	0.06509462	0.50874395	0.5048838	Becn1	1
GO:1903278	positive regulation of sodium ion export across plasma membrane	1/97	10/14464	0.1	14.9113402	3.61582132	0.06509462	0.50874395	0.5048838	Atp1b3	1
GO:1904627	response to phorbol 13-acetate 12-myristate	1/97	10/14464	0.1	14.9113402	3.61582132	0.06509462	0.50874395	0.5048838	Sphk2	1
GO:1904628	cellular response to phorbol 13-acetate 12-myristate	1/97	10/14464	0.1	14.9113402	3.61582132	0.06509462	0.50874395	0.5048838	Sphk2	1
GO:1904851	positive regulation of establishment of protein localization to telomere	1/97	10/14464	0.1	14.9113402	3.61582132	0.06509462	0.50874395	0.5048838	Cct7	1
GO:2000650	negative regulation of sodium ion transmembrane transporter activity	1/97	10/14464	0.1	14.9113402	3.61582132	0.06509462	0.50874395	0.5048838	Pcsk9	1
GO:0006402	mRNA catabolic process	4/97	226/14464	0.01769912	2.63917526	2.04073743	0.06548802	0.50874395	0.5048838	Hspa1b/Mettl3/Traf2/Hbs1l	4
GO:0048144	fibroblast proliferation	3/97	138/14464	0.02173913	3.2415957	2.17403418	0.06562211	0.50874395	0.5048838	Cdkn1a/Ptges3/Lig4	3
GO:0034765	regulation of monoatomic ion transmembrane transport	5/97	323/14464	0.01547988	2.308257	1.95382778	0.06605639	0.50874395	0.5048838	Gpr35/Fkbp1b/Pcsk9/Atp1b3/Sphk2	5
GO:0045859	regulation of protein kinase activity	6/97	426/14464	0.01408451	2.10018876	1.89387538	0.06660823	0.50874395	0.5048838	Cdkn1a/Sfrp5/Sfn/Ankrd54/Traf2/Nhlrc1	6
GO:0007584	response to nutrient	3/97	139/14464	0.02158273	3.21827486	2.15927252	0.06675597	0.50874395	0.5048838	Fkbp1b/Lipa/Becn1	3

GO:0051453 regulation of intracellular pH	2/97	63/14464	0.03174603	4.7337588	2.44035059	0.06678979	0.50874395	0.5048838	Rab39/Cln5	2
GO:1901019 regulation of calcium ion transmembrane transporter activity	2/97	63/14464	0.03174603	4.7337588	2.44035059	0.06678979	0.50874395	0.5048838	Gpr35/Fkbp1b	2
GO:0031331 positive regulation of cellular catabolic process	4/97	230/14464	0.0173913	2.59327656	2.00135264	0.06890781	0.50874395	0.5048838	Pcsk9/Becn1/Pafah1b2/Pip4k2c	4
GO:2000377 regulation of reactive oxygen species metabolic process	3/97	141/14464	0.0212766	3.17262558	2.13014645	0.06905067	0.50874395	0.5048838	Shc1/Cdkn1a/Sphk2	3
GO:0010212 response to ionizing radiation	3/97	142/14464	0.02112676	3.15028314	2.11577754	0.0702114	0.50874395	0.5048838	Cdkn1a/Lig4/Nhej1	3
GO:0006303 double-strand break repair via nonhomologous end joining	2/97	65/14464	0.03076923	4.58810468	2.38225162	0.07053103	0.50874395	0.5048838	Lig4/Nhej1	2
GO:0051926 negative regulation of calcium ion transport	2/97	65/14464	0.03076923	4.58810468	2.38225162	0.07053103	0.50874395	0.5048838	Gpr35/Fkbp1b	2
GO:0140115 export across plasma membrane	2/97	65/14464	0.03076923	4.58810468	2.38225162	0.07053103	0.50874395	0.5048838	Abcc5/Atp1b3	2
GO:0003334 keratinocyte development	1/97	11/14464	0.09090909	13.5557638	3.42288646	0.07136872	0.50874395	0.5048838	Sfn	1
GO:0006183 GTP biosynthetic process	1/97	11/14464	0.09090909	13.5557638	3.42288646	0.07136872	0.50874395	0.5048838	Nme1	1
GO:0006241 CTP biosynthetic process	1/97	11/14464	0.09090909	13.5557638	3.42288646	0.07136872	0.50874395	0.5048838	Nme1	1
GO:0006361 transcription initiation at RNA polymerase I promoter	1/97	11/14464	0.09090909	13.5557638	3.42288646	0.07136872	0.50874395	0.5048838	Ubtf	1
GO:0006977 DNA damage response, signal transduction by p53 class mediator resulting in cell cycle arrest	1/97	11/14464	0.09090909	13.5557638	3.42288646	0.07136872	0.50874395	0.5048838	Cdkn1a	1
GO:0009157 deoxyribonucleoside monophosphate biosynthetic process	1/97	11/14464	0.09090909	13.5557638	3.42288646	0.07136872	0.50874395	0.5048838	Nme1	1
GO:0010968 regulation of microtubule nucleation	1/97	11/14464	0.09090909	13.5557638	3.42288646	0.07136872	0.50874395	0.5048838	Hspa1b	1
GO:0010988 regulation of low-density lipoprotein particle clearance	1/97	11/14464	0.09090909	13.5557638	3.42288646	0.07136872	0.50874395	0.5048838	Pcsk9	1
GO:0030007 intracellular potassium ion homeostasis	1/97	11/14464	0.09090909	13.5557638	3.42288646	0.07136872	0.50874395	0.5048838	Atp1b3	1
GO:0031293 membrane protein intracellular domain proteolysis	1/97	11/14464	0.09090909	13.5557638	3.42288646	0.07136872	0.50874395	0.5048838	Aph1b	1
GO:0034351 negative regulation of glial cell apoptotic process	1/97	11/14464	0.09090909	13.5557638	3.42288646	0.07136872	0.50874395	0.5048838	Traf2	1
GO:0034635 glutathione transport	1/97	11/14464	0.09090909	13.5557638	3.42288646	0.07136872	0.50874395	0.5048838	Abcc5	1
GO:0036065 fucosylation	1/97	11/14464	0.09090909	13.5557638	3.42288646	0.07136872	0.50874395	0.5048838	Fut11	1
GO:0043508 negative regulation of JUN kinase activity	1/97	11/14464	0.09090909	13.5557638	3.42288646	0.07136872	0.50874395	0.5048838	Sfrp5	1
GO:0043652 engulfment of apoptotic cell	1/97	11/14464	0.09090909	13.5557638	3.42288646	0.07136872	0.50874395	0.5048838	Becn1	1
GO:0051987 positive regulation of attachment of spindle microtubules to kinetochore	1/97	11/14464	0.09090909	13.5557638	3.42288646	0.07136872	0.50874395	0.5048838	Becn1	1
GO:0060837 blood vessel endothelial cell differentiation	1/97	11/14464	0.09090909	13.5557638	3.42288646	0.07136872	0.50874395	0.5048838	Lipa	1
GO:0062028 regulation of stress granule assembly	1/97	11/14464	0.09090909	13.5557638	3.42288646	0.07136872	0.50874395	0.5048838	Becn1	1
GO:0071493 cellular response to UV-B	1/97	11/14464	0.09090909	13.5557638	3.42288646	0.07136872	0.50874395	0.5048838	Cdkn1a	1
GO:1900121 negative regulation of receptor binding	1/97	11/14464	0.09090909	13.5557638	3.42288646	0.07136872	0.50874395	0.5048838	Pcsk9	1
GO:1901678 iron coordination entity transport	1/97	11/14464	0.09090909	13.5557638	3.42288646	0.07136872	0.50874395	0.5048838	Abcc5	1
GO:1902306 negative regulation of sodium ion transmembrane transport	1/97	11/14464	0.09090909	13.5557638	3.42288646	0.07136872	0.50874395	0.5048838	Pcsk9	1
GO:1903276 regulation of sodium ion export across plasma membrane	1/97	11/14464	0.09090909	13.5557638	3.42288646	0.07136872	0.50874395	0.5048838	Atp1b3	1
GO:0002720 positive regulation of cytokine production involved in immune response	2/97	66/14464	0.03030303	4.51858794	2.35408049	0.07242697	0.50874395	0.5048838	Traf2/Sphk2	2
GO:2000573 positive regulation of DNA biosynthetic process	2/97	66/14464	0.03030303	4.51858794	2.35408049	0.07242697	0.50874395	0.5048838	Ptges3/Cct7	2
GO:0009259 ribonucleotide metabolic process	5/97	332/14464	0.01506024	2.24568377	1.88671823	0.07245211	0.50874395	0.5048838	Hspa1b/Lipa/Sphk2/Nudt3/Nme1	5
GO:0016241 regulation of macroautophagy	3/97	144/14464	0.02083333	3.10652921	2.0874171	0.07255937	0.50874395	0.5048838	Becn1/Pafah1b2/Pip4k2c	3
GO:0071900 regulation of protein serine/threonine kinase activity	4/97	235/14464	0.01702128	2.53810046	1.9532753	0.07331059	0.50874395	0.5048838	Cdkn1a/Sfrp5/Sfn/Traf2	4
GO:0060759 regulation of response to cytokine stimulus	3/97	145/14464	0.02068966	3.08510487	2.07342139	0.07374651	0.50874395	0.5048838	Hspa1b/Mettl3/Traf2	3
GO:0033044 regulation of chromosome organization	4/97	236/14464	0.01694915	2.5273458	1.94380843	0.07420809	0.50874395	0.5048838	Ptges3/Lig4/Becn1/Cct7	4
GO:0006766 vitamin metabolic process	2/97	67/14464	0.02985075	4.45114633	2.32646641	0.07433937	0.50874395	0.5048838	Lipa/Mmaa	2
GO:0043269 regulation of monoatomic ion transport	6/97	440/14464	0.01363636	2.03336457	1.80873928	0.07527238	0.50874395	0.5048838	Gpr35/Trpv2/Fkbp1b/Pcsk9/Atp1b3/Sphk2	6
GO:0009152 purine ribonucleotide biosynthetic process	3/97	147/14464	0.02040816	3.04313065	2.04578886	0.07614685	0.50874395	0.5048838	Lipa/Sphk2/Nme1	3
GO:0006720 isoprenoid metabolic process	2/97	68/14464	0.02941176	4.3856883	2.29938939	0.07626793	0.50874395	0.5048838	Lipa/Pmvk	2
GO:0035019 somatic stem cell population maintenance	2/97	68/14464	0.02941176	4.3856883	2.29938939	0.07626793	0.50874395	0.5048838	Lig4/Smc1a	2
GO:0048146 positive regulation of fibroblast proliferation	2/97	68/14464	0.02941176	4.3856883	2.29938939	0.07626793	0.50874395	0.5048838	Cdkn1a/Lig4	2
GO:0000012 single strand break repair	1/97	12/14464	0.08333333	12.4261168	3.25354877	0.07760114	0.50874395	0.5048838	Lig4	1
GO:0002679 respiratory burst involved in defense response	1/97	12/14464	0.08333333	12.4261168	3.25354877	0.07760114	0.50874395	0.5048838	Lipa	1
GO:0009209 pyrimidine ribonucleoside triphosphate biosynthetic process	1/97	12/14464	0.08333333	12.4261168	3.25354877	0.07760114	0.50874395	0.5048838	Nme1	1
GO:0019227 neuronal action potential propagation	1/97	12/14464	0.08333333	12.4261168	3.25354877	0.07760114	0.50874395	0.5048838	Fkbp1b	1
GO:0032736 positive regulation of interleukin-13 production	1/97	12/14464	0.08333333	12.4261168	3.25354877	0.07760114	0.50874395	0.5048838	Sphk2	1
GO:0042939 tripeptide transport	1/97	12/14464	0.08333333	12.4261168	3.25354877	0.07760114	0.50874395	0.5048838	Abcc5	1
GO:0046036 CTP metabolic process	1/97	12/14464	0.08333333	12.4261168	3.25354877	0.07760114	0.50874395	0.5048838	Nme1	1
GO:0046073 dTMP metabolic process	1/97	12/14464	0.08333333	12.4261168	3.25354877	0.07760114	0.50874395	0.5048838	Nme1	1
GO:0046512 sphingosine biosynthetic process	1/97	12/14464	0.08333333	12.4261168	3.25354877	0.07760114	0.50874395	0.5048838	Sphk2	1
GO:0046520 sphingoid biosynthetic process	1/97	12/14464	0.08333333	12.4261168	3.25354877	0.07760114	0.50874395	0.5048838	Sphk2	1
GO:0070203 regulation of establishment of protein localization to telomere	1/97	12/14464	0.08333333	12.4261168	3.25354877	0.07760114	0.50874395	0.5048838	Cct7	1
GO:0071233 cellular response to L-leucine	1/97	12/14464	0.08333333	12.4261168	3.25354877	0.07760114	0.50874395	0.5048838	Stambpl1	1
GO:0090385 phagosome-lysosome fusion	1/97	12/14464	0.08333333	12.4261168	3.25354877	0.07760114	0.50874395	0.5048838	Rab39	1
GO:1904152 regulation of retrograde protein transport, ER to cytosol	1/97	12/14464	0.08333333	12.4261168	3.25354877	0.07760114	0.50874395	0.5048838	Edem1	1
GO:1904424 regulation of GTP binding	1/97	12/14464	0.08333333	12.4261168	3.25354877	0.07760114	0.50874395	0.5048838	Cln5	1
GO:2000303 regulation of ceramide biosynthetic process	1/97	12/14464	0.08333333	12.4261168	3.25354877	0.07760114	0.50874395	0.5048838	Sphk2	1
GO:2000644 regulation of receptor catabolic process	1/97	12/14464	0.08333333	12.4261168	3.25354877	0.07760114	0.50874395	0.5048838	Pcsk9	1
GO:0019693 ribose phosphate metabolic process	5/97	339/14464	0.01474926	2.19931271	1.83598899	0.07765489	0.50874395	0.5048838	Hspa1b/Lipa/Sphk2/Nudt3/Nme1	5
GO:0002200 somatic diversification of immune receptors	2/97	69/14464	0.02898551	4.3221276	2.27283042	0.07821236	0.50874395	0.5048838	Lig4/Nhej1	2
GO:0030641 regulation of cellular pH	2/97	69/14464	0.02898551	4.3221276	2.27283042	0.07821236	0.50874395	0.5048838	Rab39/Cln5	2
GO:0009205 purine ribonucleoside triphosphate metabolic process	4/97	243/14464	0.01646091	2.4545416	1.87886782	0.08064691	0.50874395	0.5048838	Hspa1b/Lipa/Sphk2/Nme1	4
GO:1902850 microtubule cytoskeleton organization involved in mitosis	3/97	151/14464	0.01986755	2.96251792	1.99190574	0.08105025	0.50874395	0.5048838	Hspa1b/Efhc1/Smc1a	3
GO:0051302 regulation of cell division	3/97	152/14464	0.01973684	2.94302767	1.97871219	0.08229719	0.50874395	0.5048838	Sfn/Efhc1/Becn1	3
GO:0046474 glycerophospholipid biosynthetic process	3/97	153/14464	0.01960784	2.9237922	1.96562604	0.08355245	0.50874395	0.5048838	Becn1/Pip4k2c/Pigc	3
GO:0002328 pro-B cell differentiation	1/97	13/14464	0.07692308	11.4702617	3.10321794	0.08379217	0.50874395	0.5048838	Lig4	1
GO:0003376 sphingosine-1-phosphate receptor signaling pathway	1/97	13/14464	0.07692308	11.4702617	3.10321794	0.08379217	0.50874395	0.5048838	Sphk2	1
GO:0010459 negative regulation of heart rate	1/97	13/14464	0.07692308	11.4702617	3.10321794	0.08379217	0.50874395	0.5048838	Fkbp1b	1
GO:0023035 CD40 signaling pathway	1/97	13/14464	0.07692308	11.4702617	3.10321794	0.08379217	0.50874395	0.5048838	Traf2	1
GO:0038133 ERBB2-ERBB3 signaling pathway	1/97	13/14464	0.07692308	11.4702617	3.10321794	0.08379217	0.50874395	0.5048838	Nrg2	1
GO:0051280 negative regulation of release of sequestered calcium ion into cytosol	1/97	13/14464	0.07692308	11.4702617	3.10321794	0.08379217	0.50874395	0.5048838	Fkbp1b	1
GO:0070202 regulation of establishment of protein localization to chromosome	1/97	13/14464	0.07692308	11.4702617	3.10321794	0.08379217	0.50874395	0.5048838	Cct7	1
GO:0070341 fat cell proliferation	1/97	13/14464	0.07692308	11.4702617	3.10321794	0.08379217	0.50874395	0.5048838	Lipa	1
GO:0097384 cellular lipid biosynthetic process	1/97	13/14464	0.07692308	11.4702617	3.10321794	0.08379217	0.50874395	0.5048838	Lipa	1
GO:0098870 action potential propagation	1/97	13/14464	0.07692308	11.4702617	3.10321794	0.08379217	0.50874395	0.5048838	Fkbp1b	1
GO:1901386 negative regulation of voltage-gated calcium channel activity	1/97	13/14464	0.07692308	11.4702617	3.10321794	0.08379217	0.50874395	0.5048838	Gpr35	1
GO:1902902 negative regulation of autophagosome assembly	1/97	13/14464	0.07692308	11.4702617	3.10321794	0.08379217	0.50874395	0.5048838	Becn1	1
GO:1904816 positive regulation of protein localization to chromosome, telomeric region	1/97	13/14464	0.07692308	11.4702617	3.10321794	0.08379217	0.50874395	0.5048838	Cct7	1
GO:1905671 regulation of lysosome organization	1/97	13/14464	0.07692308	11.4702617	3.10321794	0.08379217	0.50874395	0.5048838	Becn1	1
GO:0010833 telomere maintenance via telomere lengthening	2/97	72/14464	0.02777778	4.14203895	2.19608553	0.08413794	0.50874395	0.5048838	Ptges3/Cct7	2
GO:0030968 endoplasmic reticulum unfolded protein response	2/97	72/14464	0.02777778	4.14203895	2.19608553	0.08413794	0.50874395	0.5048838	Pdia6/Edem1	2
GO:0032091 negative regulation of protein binding	2/97	72/14464	0.02777778	4.14203895	2.19608553	0.08413794	0.50874395	0.5048838	Cdkn1a/Pcsk9	2
GO:0090307 mitotic spindle assembly	2/97	72/14464	0.02777778	4.14203895	2.19608553	0.08413794	0.50874395	0.5048838	Hspa1b/Smc1a	2
GO:0006650 glycerophospholipid metabolic process	4/97	247/14464	0.01619433	2.41479194	1.84276131	0.08444769	0.50874395	0.5048838	Smpd4/Becn1/Pip4k2c/Pigc	4
GO:0009199 ribonucleoside triphosphate metabolic process	4/97	248/14464	0.01612903	2.40505487	1.83384419	0.08541152	0.50874395	0.5048838	Hspa1b/Lipa/Sphk2/Nme1	4
GO:0043549 regulation of kinase activity	6/97	456/14464	0.01315789	1.96201845	1.71518026	0.08593023	0.50874395	0.5048838	Cdkn1a/Sfrp5/Sfn/Ankrd54/Traf2/Nhlrc1	6
GO:0032206 positive regulation of telomere maintenance	2/97	73/14464	0.02739726	4.08529869	2.17142669	0.08614293	0.50874395	0.5048838	Ptges3/Cct7	2
GO:0007254 JNK cascade	3/97	156/14464	0.01923077	2.86756542	1.92699538	0.08736766	0.50874395	0.5048838	Sfrp5/Traf2/Becn1	3
GO:0006690 icosanoid metabolic process	2/97	74/14464	0.02702703	4.03009195	2.14720389	0.08816235	0.50874395	0.5048838	Cyp2j13/Ptges3	2
GO:0071479 cellular response to ionizing radiation	2/97	74/14464	0.02702703	4.03009195	2.14720389	0.08816235	0.50874395	0.5048838	Cdkn1a/Lig4	2
GO:0009144 purine nucleoside triphosphate metabolic process	4/97	251/14464	0.01593625	2.3763092	1.80734894	0.08833546	0.50874395	0.5048838	Hspa1b/Lipa/Sphk2/Nme1	4
GO:0071478 cellular response to radiation	3/97	157/14464	0.01910828	2.84930068	1.9143224	0.08865568	0.50874395	0.5048838	Cdkn1a/Mettl3/Lig4	3
GO:0006700 C21-steroid hormone biosynthetic process	1/97	14/14464	0.07142857	10.6509573	2.96846889	0.08994206	0.50874395	0.5048838	Stard3	1
GO:0009208 pyrimidine ribonucleoside triphosphate metabolic process	1/97	14/14464	0.07142857	10.6509573	2.96846889	0.08994206	0.50874395	0.5048838	Nme1	1
GO:0023019 signal transduction involved in regulation of gene expression	1/97	14/14464	0.07142857	10.6509573	2.96846889	0.08994206	0.50874395	0.5048838	Traf2	1
GO:0030213 hyaluronan biosynthetic process	1/97	14/14464	0.07142857	10.6509573	2.96846889	0.08994206	0.50874395	0.5048838	Abcc5	1
GO:0036498 IRE1-mediated unfolded protein response	1/97	14/14464	0.07142857	10.6509573	2.96846889	0.08994206	0.50874395	0.5048838	Pdia6	1
GO:0038129 ERBB3 signaling pathway	1/97	14/14464	0.07142857	10.6509573	2.96846889	0.08994206	0.50874395	0.5048838	Nrg2	1
GO:0043201 response to L-leucine	1/97	14/14464	0.07142857	10.6509573	2.96846889	0.08994206	0.50874395	0.5048838	Stambpl1	1
GO:0048680 positive regulation of axon regeneration	1/97	14/14464	0.07142857	10.6509573	2.96846889	0.08994206	0.50874395	0.5048838	Fkbp1b	1
GO:0071280 cellular response to copper ion	1/97	14/14464	0.07142857	10.6509573	2.96846889	0.08994206	0.50874395	0.5048838	Becn1	1
GO:0071285 cellular response to lithium ion	1/97	14/14464	0.07142857	10.6509573	2.96846889	0.08994206	0.50874395	0.5048838	Lig4	1
GO:0072393 microtubule anchoring at microtubule organizing center	1/97	14/14464	0.07142857	10.6509573	2.96846889	0.08994206	0.50874395	0.5048838	Bicd2	1
GO:0090153 regulation of sphingolipid biosynthetic process	1/97	14/14464	0.07142857	10.6509573	2.96846889	0.08994206	0.50874395	0.5048838	Sphk2	1
GO:1901018 positive regulation of potassium ion transmembrane transporter activity	1/97	14/14464	0.07142857	10.6509573	2.96846889	0.08994206	0.50874395	0.5048838	Atp1b3	1
GO:0002526 acute inflammatory response	2/97	75/14464	0.02666667	3.97635739	2.12340296	0.09019592	0.50874395	0.5048838	Dnase1l3/Lipa	2
GO:0003341 cilium movement	3/97	159/14464	0.01886792	2.81346042	1.88927303	0.0912558	0.50874395	0.5048838	Efhc1/Tuba1a/Dnai4	3
GO:0042110 T cell activation	6/97	464/14464	0.01293103	1.92819054	1.66979873	0.0915589	0.50874395	0.5048838	Fkbp1b/Lipa/Mettl3/Traf2/Lig4/Nhej1	6
GO:0009266 response to temperature stimulus	3/97	161/14464	0.01863354	2.7785106	1.86460928	0.09388766	0.50874395	0.5048838	Hspa1b/Trpv2/Lipa	3
GO:0033559 unsaturated fatty acid metabolic process	2/97	77/14464	0.02597403	3.87307538	2.07701329	0.09430441	0.50874395	0.5048838	Cyp2j13/Ptges3	2
GO:0061178 regulation of insulin secretion involved in cellular response to glucose stimulus	2/97	77/14464	0.02597403	3.87307538	2.07701329	0.09430441	0.50874395	0.5048838	Myt1/Fkbp1b	2
GO:0048872 homeostasis of number of cells	5/97	360/14464	0.01388889	2.07101947	1.69086713	0.09444245	0.50874395	0.5048838	Hspa1b/Lipa/Ankrd54/Tuba1a/Caml	5
GO:0007034 vacuolar transport	3/97	162/14464	0.01851852	2.7613593	1.85241842	0.09521535	0.50874395	0.5048838	Pcsk9/Lipa/Becn1	3
GO:0007219 Notch signaling pathway	3/97	162/14464	0.01851852	2.7613593	1.85241842	0.09521535	0.50874395	0.5048838	Tspan14/Mettl3/Aph1b	3
GO:0009260 ribonucleotide biosynthetic process	3/97	162/14464	0.01851852	2.7613593	1.85241842	0.09521535	0.50874395	0.5048838	Lipa/Sphk2/Nme1	3
GO:0006465 signal peptide processing	1/97	15/14464	0.06666667	9.94089347	2.84668668	0.0960511	0.50874395	0.5048838	Cln5	1
GO:0007042 lysosomal lumen acidification	1/97	15/14464	0.06666667	9.94089347	2.84668668	0.0960511	0.50874395	0.5048838	Cln5	1
GO:0009176 pyrimidine deoxyribonucleoside monophosphate metabolic process	1/97	15/14464	0.06666667	9.94089347	2.84668668	0.0960511	0.50874395	0.5048838	Nme1	1
GO:0010635 regulation of mitochondrial fusion	1/97	15/14464	0.06666667	9.94089347	2.84668668	0.0960511	0.50874395	0.5048838	Mief1	1
GO:0010882 regulation of cardiac muscle contraction by calcium ion signaling	1/97	15/14464	0.06666667	9.94089347	2.84668668	0.0960511	0.50874395	0.5048838	Fkbp1b	1
GO:0010984 regulation of lipoprotein particle clearance	1/97	15/14464	0.06666667	9.94089347	2.84668668	0.0960511	0.50874395	0.5048838	Pcsk9	1
GO:0042159 lipoprotein catabolic process	1/97	15/14464	0.06666667	9.94089347	2.84668668	0.0960511	0.50874395	0.5048838	Lipa	1
GO:0051284 positive regulation of sequestering of calcium ion	1/97	15/14464	0.06666667	9.94089347	2.84668668	0.0960511	0.50874395	0.5048838	Fkbp1b	1
GO:0060026 convergent extension	1/97	15/14464	0.06666667	9.94089347	2.84668668	0.0960511	0.50874395	0.5048838	Sfrp5	1
GO:0070572 positive regulation of neuron projection regeneration	1/97	15/14464	0.06666667	9.94089347	2.84668668	0.0960511	0.50874395	0.5048838	Fkbp1b	1
GO:0090136 epithelial cell-cell adhesion	1/97	15/14464	0.06666667	9.94089347	2.84668668	0.0960511	0.50874395	0.5048838	Serpinb8	1
GO:0090235 regulation of metaphase plate congression	1/97	15/14464	0.06666667	9.94089347	2.84668668	0.0960511	0.50874395	0.5048838	Becn1	1
GO:0090520 sphingolipid mediated signaling pathway	1/97	15/14464	0.06666667	9.94089347	2.84668668	0.0960511	0.50874395	0.5048838	Sphk2	1
GO:1900102 negative regulation of endoplasmic reticulum unfolded protein response	1/97	15/14464	0.06666667	9.94089347	2.84668668	0.0960511	0.50874395	0.5048838	Pdia6	1
GO:1902036 regulation of hematopoietic stem cell differentiation	1/97	15/14464	0.06666667	9.94089347	2.84668668	0.0960511	0.50874395	0.5048838	Mettl3	1
GO:0010508 positive regulation of autophagy	3/97	163/14464	0.01840491	2.74441844	1.84031973	0.0965508	0.50874395	0.5048838	Becn1/Pafah1b2/Pip4k2c	3
GO:0002028 regulation of sodium ion transport	2/97	79/14464	0.02531646	3.77502284	2.03215668	0.09846622	0.50874395	0.5048838	Pcsk9/Atp1b3	2
GO:0030216 keratinocyte differentiation	2/97	79/14464	0.02531646	3.77502284	2.03215668	0.09846622	0.50874395	0.5048838	Cdkn1a/Sfn	2
GO:0031397 negative regulation of protein ubiquitination	2/97	79/14464	0.02531646	3.77502284	2.03215668	0.09846622	0.50874395	0.5048838	Hspa1b/Caml	2
GO:0016049 cell growth	6/97	476/14464	0.01260504	1.8795807	1.60336858	0.10037177	0.50874395	0.5048838	Hspa1b/Cdkn1a/Sfn/Trpv2/Sphk2/Raph1	6
GO:0030217 T cell differentiation	4/97	263/14464	0.01520913	2.26788444	1.70502243	0.10050887	0.50874395	0.5048838	Lipa/Mettl3/Lig4/Nhej1	4
GO:0006885 regulation of pH	2/97	80/14464	0.025	3.72783505	2.01027409	0.10056645	0.50874395	0.5048838	Rab39/Cln5	2
GO:0006457 protein folding	3/97	166/14464	0.01807229	2.69482052	1.80456332	0.10060312	0.50874395	0.5048838	Hspa1b/Ptges3/Cct7	3
GO:0071248 cellular response to metal ion	3/97	167/14464	0.01796407	2.67868387	1.79282008	0.10196902	0.50874395	0.5048838	Tuba1a/Lig4/Becn1	3
GO:0006309 apoptotic DNA fragmentation	1/97	16/14464	0.0625	9.31958763	2.73583524	0.10211955	0.50874395	0.5048838	Dnase1l3	1
GO:0006883 intracellular sodium ion homeostasis	1/97	16/14464	0.0625	9.31958763	2.73583524	0.10211955	0.50874395	0.5048838	Atp1b3	1
GO:0006978 DNA damage response, signal transduction by p53 class mediator resulting in transcription of p21 class r	1/97	16/14464	0.0625	9.31958763	2.73583524	0.10211955	0.50874395	0.5048838	Cdkn1a	1
GO:0009148 pyrimidine nucleoside triphosphate biosynthetic process	1/97	16/14464	0.0625	9.31958763	2.73583524	0.10211955	0.50874395	0.5048838	Nme1	1
GO:0010766 negative regulation of sodium ion transport	1/97	16/14464	0.0625	9.31958763	2.73583524	0.10211955	0.50874395	0.5048838	Pcsk9	1
GO:0031053 primary miRNA processing	1/97	16/14464	0.0625	9.31958763	2.73583524	0.10211955	0.50874395	0.5048838	Mettl3	1
GO:0034350 regulation of glial cell apoptotic process	1/97	16/14464	0.0625	9.31958763	2.73583524	0.10211955	0.50874395	0.5048838	Traf2	1
GO:0035461 vitamin transmembrane transport	1/97	16/14464	0.0625	9.31958763	2.73583524	0.10211955	0.50874395	0.5048838	Abcc5	1
GO:0035672 oligopeptide transmembrane transport	1/97	16/14464	0.0625	9.31958763	2.73583524	0.10211955	0.50874395	0.5048838	Abcc5	1
GO:0042921 nuclear receptor-mediated glucocorticoid signaling pathway	1/97	16/14464	0.0625	9.31958763	2.73583524	0.10211955	0.50874395	0.5048838	Ptges3	1
GO:0050862 positive regulation of T cell receptor signaling pathway	1/97	16/14464	0.0625	9.31958763	2.73583524	0.10211955	0.50874395	0.5048838	Lipa	1
GO:0060134 prepulse inhibition	1/97	16/14464	0.0625	9.31958763	2.73583524	0.10211955	0.50874395	0.5048838	Aph1b	1
GO:1902065 response to L-glutamate	1/97	16/14464	0.0625	9.31958763	2.73583524	0.10211955	0.50874395	0.5048838	Tuba1a	1
GO:1904814 regulation of protein localization to chromosome, telomeric region	1/97	16/14464	0.0625	9.31958763	2.73583524	0.10211955	0.50874395	0.5048838	Cct7	1
GO:0009141 nucleoside triphosphate metabolic process	4/97	265/14464	0.01509434	2.25076833	1.68850732	0.1026105	0.50874395	0.5048838	Hspa1b/Lipa/Sphk2/Nme1	4
GO:0007601 visual perception	2/97	81/14464	0.02469136	3.6818124	1.98874077	0.10267923	0.50874395	0.5048838	Cln5/Pde6d	2
GO:0045824 negative regulation of innate immune response	2/97	81/14464	0.02469136	3.6818124	1.98874077	0.10267923	0.50874395	0.5048838	Sfn/Mettl3	2
GO:0072091 regulation of stem cell proliferation	2/97	81/14464	0.02469136	3.6818124	1.98874077	0.10267923	0.50874395	0.5048838	Sfn/Taf4b	2
GO:0000070 mitotic sister chromatid segregation	3/97	168/14464	0.01785714	2.66273932	1.78116254	0.1033424	0.50874395	0.5048838	Hspa1b/Becn1/Smc1a	3
GO:0046390 ribose phosphate biosynthetic process	3/97	168/14464	0.01785714	2.66273932	1.78116254	0.1033424	0.50874395	0.5048838	Lipa/Sphk2/Nme1	3
GO:0006401 RNA catabolic process	4/97	268/14464	0.01492537	2.22557317	1.66400896	0.105801	0.50874395	0.5048838	Hspa1b/Mettl3/Traf2/Hbs1l	4
GO:0050953 sensory perception of light stimulus	2/97	83/14464	0.02409639	3.59309403	1.94668096	0.10694136	0.50874395	0.5048838	Cln5/Pde6d	2
GO:0010959 regulation of metal ion transport	5/97	375/14464	0.01333333	1.98817869	1.59310004	0.107488	0.50874395	0.5048838	Gpr35/Trpv2/Fkbp1b/Pcsk9/Atp1b3	5
GO:0030308 negative regulation of cell growth	3/97	171/14464	0.01754386	2.6160246	1.74669203	0.10750672	0.50874395	0.5048838	Hspa1b/Cdkn1a/Sphk2	3

GO:0001845	phagolysosome assembly	1/97	17/14464	0.05882353	8.77137659	2.63430219	0.10814768	0.50874395	0.5048838	Rab39	1
GO:0002091	negative regulation of receptor internalization	1/97	17/14464	0.05882353	8.77137659	2.63430219	0.10814768	0.50874395	0.5048838	Pcsk9	1
GO:0002726	positive regulation of T cell cytokine production	1/97	17/14464	0.05882353	8.77137659	2.63430219	0.10814768	0.50874395	0.5048838	Traf2	1
GO:0007614	short-term memory	1/97	17/14464	0.05882353	8.77137659	2.63430219	0.10814768	0.50874395	0.5048838	Aph1b	1
GO:0009130	pyrimidine nucleoside monophosphate biosynthetic process	1/97	17/14464	0.05882353	8.77137659	2.63430219	0.10814768	0.50874395	0.5048838	Nme1	1
GO:0009263	deoxyribonucleotide biosynthetic process	1/97	17/14464	0.05882353	8.77137659	2.63430219	0.10814768	0.50874395	0.5048838	Nme1	1
GO:0009265	2'-deoxyribonucleotide biosynthetic process	1/97	17/14464	0.05882353	8.77137659	2.63430219	0.10814768	0.50874395	0.5048838	Nme1	1
GO:0010224	response to UV-B	1/97	17/14464	0.05882353	8.77137659	2.63430219	0.10814768	0.50874395	0.5048838	Cdkn1a	1
GO:0010226	response to lithium ion	1/97	17/14464	0.05882353	8.77137659	2.63430219	0.10814768	0.50874395	0.5048838	Lig4	1
GO:0032656	regulation of interleukin-13 production	1/97	17/14464	0.05882353	8.77137659	2.63430219	0.10814768	0.50874395	0.5048838	Sphk2	1
GO:0036092	phosphatidylinositol-3-phosphate biosynthetic process	1/97	17/14464	0.05882353	8.77137659	2.63430219	0.10814768	0.50874395	0.5048838	Becn1	1
GO:0036376	sodium ion export across plasma membrane	1/97	17/14464	0.05882353	8.77137659	2.63430219	0.10814768	0.50874395	0.5048838	Atp1b3	1
GO:0042772	DNA damage response, signal transduction resulting in transcription	1/97	17/14464	0.05882353	8.77137659	2.63430219	0.10814768	0.50874395	0.5048838	Cdkn1a	1
GO:0045472	response to ether	1/97	17/14464	0.05882353	8.77137659	2.63430219	0.10814768	0.50874395	0.5048838	Lipa	1
GO:0045606	positive regulation of epidermal cell differentiation	1/97	17/14464	0.05882353	8.77137659	2.63430219	0.10814768	0.50874395	0.5048838	Sfn	1
GO:0046385	deoxyribose phosphate biosynthetic process	1/97	17/14464	0.05882353	8.77137659	2.63430219	0.10814768	0.50874395	0.5048838	Nme1	1
GO:0051969	regulation of transmission of nerve impulse	1/97	17/14464	0.05882353	8.77137659	2.63430219	0.10814768	0.50874395	0.5048838	Gpr35	1
GO:0060019	radial glial cell differentiation	1/97	17/14464	0.05882353	8.77137659	2.63430219	0.10814768	0.50874395	0.5048838	Mettl3	1
GO:0060314	regulation of ryanodine-sensitive calcium-release channel activity	1/97	17/14464	0.05882353	8.77137659	2.63430219	0.10814768	0.50874395	0.5048838	Fkbp1b	1
GO:0061179	negative regulation of insulin secretion involved in cellular response to glucose stimulus	1/97	17/14464	0.05882353	8.77137659	2.63430219	0.10814768	0.50874395	0.5048838	Fkbp1b	1
GO:0070131	positive regulation of mitochondrial translation	1/97	17/14464	0.05882353	8.77137659	2.63430219	0.10814768	0.50874395	0.5048838	Mrps27	1
GO:0071732	cellular response to nitric oxide	1/97	17/14464	0.05882353	8.77137659	2.63430219	0.10814768	0.50874395	0.5048838	Traf2	1
GO:0071816	tail-anchored membrane protein insertion into ER membrane	1/97	17/14464	0.05882353	8.77137659	2.63430219	0.10814768	0.50874395	0.5048838	Caml	1
GO:0043603	amide metabolic process	5/97	376/14464	0.01329787	1.98289098	1.58674309	0.10838807	0.50874395	0.5048838	Pmvk/Smpd4/Sphk2/Becn1/Aph1b	5
GO:0006643	membrane lipid metabolic process	3/97	172/14464	0.01744186	2.60081515	1.73536535	0.10890937	0.50874395	0.5048838	Smpd4/Sphk2/Pigc	3
GO:0031929	TOR signaling	3/97	172/14464	0.01744186	2.60081515	1.73536535	0.10890937	0.50874395	0.5048838	Stambpl1/Lipa/Tbck	3
GO:0005977	glycogen metabolic process	2/97	84/14464	0.02380952	3.5503191	1.92613506	0.10909021	0.50874395	0.5048838	Ptges3/Nhlrc1	2
GO:0008593	regulation of Notch signaling pathway	2/97	84/14464	0.02380952	3.5503191	1.92613506	0.10909021	0.50874395	0.5048838	Tspan14/Mettl3	2
GO:0016579	protein deubiquitination	2/97	84/14464	0.02380952	3.5503191	1.92613506	0.10909021	0.50874395	0.5048838	Stambpl1/Otub2	2
GO:0034620	cellular response to unfolded protein	2/97	84/14464	0.02380952	3.5503191	1.92613506	0.10909021	0.50874395	0.5048838	Pdia6/Edem1	2
GO:0042157	lipoprotein metabolic process	2/97	84/14464	0.02380952	3.5503191	1.92613506	0.10909021	0.50874395	0.5048838	Pcsk9/Lipa	2
GO:0071901	negative regulation of protein serine/threonine kinase activity	2/97	84/14464	0.02380952	3.5503191	1.92613506	0.10909021	0.50874395	0.5048838	Cdkn1a/Sfrp5	2
GO:0044042	glucan metabolic process	2/97	85/14464	0.02352941	3.50855064	1.90589955	0.11125059	0.50874395	0.5048838	Ptges3/Nhlrc1	2
GO:0006672	ceramide metabolic process	2/97	86/14464	0.02325581	3.46775354	1.88596567	0.11342224	0.50874395	0.5048838	Smpd4/Sphk2	2
GO:0006670	sphingosine metabolic process	1/97	18/14464	0.05555556	8.28407789	2.54079159	0.11413575	0.50874395	0.5048838	Sphk2	1
GO:0021859	pyramidal neuron differentiation	1/97	18/14464	0.05555556	8.28407789	2.54079159	0.11413575	0.50874395	0.5048838	Tuba1a	1
GO:0031645	negative regulation of nervous system process	1/97	18/14464	0.05555556	8.28407789	2.54079159	0.11413575	0.50874395	0.5048838	Gpr35	1
GO:0031958	nuclear receptor-mediated corticosteroid signaling pathway	1/97	18/14464	0.05555556	8.28407789	2.54079159	0.11413575	0.50874395	0.5048838	Ptges3	1
GO:0032616	interleukin-13 production	1/97	18/14464	0.05555556	8.28407789	2.54079159	0.11413575	0.50874395	0.5048838	Sphk2	1
GO:0033003	regulation of mast cell activation	1/97	18/14464	0.05555556	8.28407789	2.54079159	0.11413575	0.50874395	0.5048838	Sphk2	1
GO:0034383	low-density lipoprotein particle clearance	1/97	18/14464	0.05555556	8.28407789	2.54079159	0.11413575	0.50874395	0.5048838	Pcsk9	1
GO:0038083	peptidyl-tyrosine autophosphorylation	1/97	18/14464	0.05555556	8.28407789	2.54079159	0.11413575	0.50874395	0.5048838	Ctnnd1	1
GO:0043306	positive regulation of mast cell degranulation	1/97	18/14464	0.05555556	8.28407789	2.54079159	0.11413575	0.50874395	0.5048838	Sphk2	1
GO:0070200	establishment of protein localization to telomere	1/97	18/14464	0.05555556	8.28407789	2.54079159	0.11413575	0.50874395	0.5048838	Cct7	1
GO:0090083	regulation of inclusion body assembly	1/97	18/14464	0.05555556	8.28407789	2.54079159	0.11413575	0.50874395	0.5048838	Hspa1b	1
GO:0097398	cellular response to interleukin-17	1/97	18/14464	0.05555556	8.28407789	2.54079159	0.11413575	0.50874395	0.5048838	Traf2	1
GO:0110154	RNA decapping	1/97	18/14464	0.05555556	8.28407789	2.54079159	0.11413575	0.50874395	0.5048838	Nudt3	1
GO:0141156	cAMP/PKA signal transduction	1/97	18/14464	0.05555556	8.28407789	2.54079159	0.11413575	0.50874395	0.5048838	Sfn	1
GO:1905038	regulation of membrane lipid metabolic process	1/97	18/14464	0.05555556	8.28407789	2.54079159	0.11413575	0.50874395	0.5048838	Sphk2	1
GO:0030198	extracellular matrix organization	4/97	276/14464	0.01449275	2.1610638	1.60022868	0.11452812	0.50874395	0.5048838	P4ha1/Vwa1/Adamts8/Antxr2	4
GO:0043062	extracellular structure organization	4/97	276/14464	0.01449275	2.1610638	1.60022868	0.11452812	0.50874395	0.5048838	P4ha1/Vwa1/Adamts8/Antxr2	4
GO:0045229	external encapsulating structure organization	4/97	276/14464	0.01449275	2.1610638	1.60022868	0.11452812	0.50874395	0.5048838	P4ha1/Vwa1/Adamts8/Antxr2	4
GO:0006164	purine nucleotide biosynthetic process	3/97	176/14464	0.01704545	2.54170572	1.69084622	0.11459102	0.50874395	0.5048838	Lipa/Sphk2/Nme1	3
GO:0035773	insulin secretion involved in cellular response to glucose stimulus	2/97	87/14464	0.02298851	3.4278943	1.86632503	0.11560492	0.50874395	0.5048838	Myt1/Fkbp1b	2
GO:0048477	oogenesis	2/97	87/14464	0.02298851	3.4278943	1.86632503	0.11560492	0.50874395	0.5048838	Mettl3/Taf4b	2
GO:0072384	organelle transport along microtubule	2/97	87/14464	0.02298851	3.4278943	1.86632503	0.11560492	0.50874395	0.5048838	Bicd2/Tuba1a	2
GO:1902117	positive regulation of organelle assembly	2/97	87/14464	0.02298851	3.4278943	1.86632503	0.11560492	0.50874395	0.5048838	Becn1/Pip4k2c	2
GO:0006641	triglyceride metabolic process	2/97	88/14464	0.02272727	3.38894096	1.84696955	0.11779839	0.50874395	0.5048838	Pcsk9/Lipa	2
GO:0030148	sphingolipid biosynthetic process	2/97	88/14464	0.02272727	3.38894096	1.84696955	0.11779839	0.50874395	0.5048838	Smpd4/Sphk2	2
GO:0034644	cellular response to UV	2/97	88/14464	0.02272727	3.38894096	1.84696955	0.11779839	0.50874395	0.5048838	Cdkn1a/Mettl3	2
GO:0120254	olefinic compound metabolic process	2/97	88/14464	0.02272727	3.38894096	1.84696955	0.11779839	0.50874395	0.5048838	Cyp2j13/Stard3	2
GO:0031329	regulation of cellular catabolic process	5/97	388/14464	0.0128866	1.92156446	1.51193794	0.11947716	0.50874395	0.5048838	Mtcl3/Pcsk9/Becn1/Pafah1b2/Pip4k2c	5
GO:0002275	myeloid cell activation involved in immune response	2/97	89/14464	0.02247191	3.35086297	1.82789147	0.12000241	0.50874395	0.5048838	Dnase1l3/Sphk2	2
GO:1903321	negative regulation of protein modification by small protein conjugation or removal	2/97	89/14464	0.02247191	3.35086297	1.82789147	0.12000241	0.50874395	0.5048838	Hspa1b/Caml	2
GO:0002283	neutrophil activation involved in immune response	1/97	19/14464	0.05263158	7.84807379	2.45424816	0.12008403	0.50874395	0.5048838	Dnase1l3	1
GO:0006857	oligopeptide transport	1/97	19/14464	0.05263158	7.84807379	2.45424816	0.12008403	0.50874395	0.5048838	Abcc5	1
GO:0010288	response to lead ion	1/97	19/14464	0.05263158	7.84807379	2.45424816	0.12008403	0.50874395	0.5048838	Becn1	1
GO:0051988	regulation of attachment of spindle microtubules to kinetochore	1/97	19/14464	0.05263158	7.84807379	2.45424816	0.12008403	0.50874395	0.5048838	Becn1	1
GO:0070431	nucleotide-binding oligomerization domain containing 2 signaling pathway	1/97	19/14464	0.05263158	7.84807379	2.45424816	0.12008403	0.50874395	0.5048838	Hspa1b	1
GO:0090280	positive regulation of calcium ion import	1/97	19/14464	0.05263158	7.84807379	2.45424816	0.12008403	0.50874395	0.5048838	Trpv2	1
GO:1900120	regulation of receptor binding	1/97	19/14464	0.05263158	7.84807379	2.45424816	0.12008403	0.50874395	0.5048838	Pcsk9	1
GO:1902170	cellular response to reactive nitrogen species	1/97	19/14464	0.05263158	7.84807379	2.45424816	0.12008403	0.50874395	0.5048838	Traf2	1
GO:0001558	regulation of cell growth	5/97	389/14464	0.01285347	1.91662471	1.50582385	0.12042494	0.50874395	0.5048838	Hspa1b/Cdkn1a/Sfn/Trpv2/Sphk2	5
GO:0010951	negative regulation of endopeptidase activity	2/97	90/14464	0.02222222	3.31363116	1.80908333	0.12221675	0.50874395	0.5048838	Hspa1b/Serpinb8	2
GO:0009416	response to light stimulus	4/97	283/14464	0.01413428	2.10760992	1.54617498	0.12241848	0.50874395	0.5048838	Cdkn1a/Mettl3/Tuba1a/Pde6d	4
GO:0019751	polyol metabolic process	2/97	91/14464	0.02197802	3.27721763	1.79053797	0.12444117	0.50874395	0.5048838	Sphk2/Nudt3	2
GO:0046785	microtubule polymerization	2/97	91/14464	0.02197802	3.27721763	1.79053797	0.12444117	0.50874395	0.5048838	Hspa1b/Tuba1a	2
GO:2001257	regulation of cation channel activity	2/97	91/14464	0.02197802	3.27721763	1.79053797	0.12444117	0.50874395	0.5048838	Gpr35/Fkbp1b	2
GO:0002520	immune system development	3/97	183/14464	0.01639344	2.444482	1.61581035	0.12479817	0.50874395	0.5048838	Lipa/Lig4/Nhej1	3
GO:0009267	cellular response to starvation	3/97	183/14464	0.01639344	2.444482	1.61581035	0.12479817	0.50874395	0.5048838	Cdkn1a/Pcsk9/Becn1	3
GO:0006684	sphingomyelin metabolic process	1/97	20/14464	0.05	7.4556701	2.3738026	0.12599278	0.50874395	0.5048838	Smpd4	1
GO:0006775	fat-soluble vitamin metabolic process	1/97	20/14464	0.05	7.4556701	2.3738026	0.12599278	0.50874395	0.5048838	Lipa	1
GO:0007289	spermatid nucleus differentiation	1/97	20/14464	0.05	7.4556701	2.3738026	0.12599278	0.50874395	0.5048838	Agfg1	1
GO:0009147	pyrimidine nucleoside triphosphate metabolic process	1/97	20/14464	0.05	7.4556701	2.3738026	0.12599278	0.50874395	0.5048838	Nme1	1
GO:0009219	pyrimidine deoxyribonucleotide metabolic process	1/97	20/14464	0.05	7.4556701	2.3738026	0.12599278	0.50874395	0.5048838	Nme1	1
GO:0010155	regulation of proton transport	1/97	20/14464	0.05	7.4556701	2.3738026	0.12599278	0.50874395	0.5048838	Sphk2	1
GO:0010878	cholesterol storage	1/97	20/14464	0.05	7.4556701	2.3738026	0.12599278	0.50874395	0.5048838	Lipa	1
GO:0031954	positive regulation of protein autophosphorylation	1/97	20/14464	0.05	7.4556701	2.3738026	0.12599278	0.50874395	0.5048838	Ctnnd1	1
GO:0034312	diol biosynthetic process	1/97	20/14464	0.05	7.4556701	2.3738026	0.12599278	0.50874395	0.5048838	Sphk2	1
GO:0035751	regulation of lysosomal lumen pH	1/97	20/14464	0.05	7.4556701	2.3738026	0.12599278	0.50874395	0.5048838	Cln5	1
GO:0036342	post-anal tail morphogenesis	1/97	20/14464	0.05	7.4556701	2.3738026	0.12599278	0.50874395	0.5048838	Sfrp5	1
GO:0045736	negative regulation of cyclin-dependent protein serine/threonine kinase activity	1/97	20/14464	0.05	7.4556701	2.3738026	0.12599278	0.50874395	0.5048838	Cdkn1a	1
GO:0046519	sphingoid metabolic process	1/97	20/14464	0.05	7.4556701	2.3738026	0.12599278	0.50874395	0.5048838	Sphk2	1
GO:0051131	chaperone-mediated protein complex assembly	1/97	20/14464	0.05	7.4556701	2.3738026	0.12599278	0.50874395	0.5048838	Ptges3	1
GO:0006163	purine nucleotide metabolic process	5/97	395/14464	0.01265823	1.88751142	1.46951119	0.12618647	0.50874395	0.5048838	Hspa1b/Lipa/Sphk2/Nudt3/Nme1	5
GO:0006112	energy reserve metabolic process	2/97	93/14464	0.02150538	3.20673983	1.75420816	0.12891932	0.50874395	0.5048838	Ptges3/Nhlrc1	2
GO:0098659	inorganic cation import across plasma membrane	2/97	93/14464	0.02150538	3.20673983	1.75420816	0.12891932	0.50874395	0.5048838	Trpv2/Atp1b3	2
GO:0099587	inorganic ion import across plasma membrane	2/97	93/14464	0.02150538	3.20673983	1.75420816	0.12891932	0.50874395	0.5048838	Trpv2/Atp1b3	2
GO:1901874	negative regulation of post-translational protein modification	2/97	93/14464	0.02150538	3.20673983	1.75420816	0.12891932	0.50874395	0.5048838	Hspa1b/Caml	2
GO:0007051	spindle organization	3/97	187/14464	0.01604278	2.39219362	1.5744685	0.1307751	0.50874395	0.5048838	Hspa1b/Efhc1/Smc1a	3
GO:1901617	organic hydroxy compound biosynthetic process	3/97	187/14464	0.01604278	2.39219362	1.5744685	0.1307751	0.50874395	0.5048838	Lipa/Pmvk/Sphk2	3
GO:0009220	pyrimidine ribonucleotide biosynthetic process	1/97	21/14464	0.04761905	7.10063819	2.29873139	0.13186225	0.50874395	0.5048838	Nme1	1
GO:0009583	detection of light stimulus	1/97	21/14464	0.04761905	7.10063819	2.29873139	0.13186225	0.50874395	0.5048838	Pde6d	1
GO:0010954	positive regulation of protein processing	1/97	21/14464	0.04761905	7.10063819	2.29873139	0.13186225	0.50874395	0.5048838	Ctnnd1	1
GO:0021542	dentate gyrus development	1/97	21/14464	0.04761905	7.10063819	2.29873139	0.13186225	0.50874395	0.5048838	Tuba1a	1
GO:0035641	locomotory exploration behavior	1/97	21/14464	0.04761905	7.10063819	2.29873139	0.13186225	0.50874395	0.5048838	Tuba1a	1
GO:0038128	ERBB2 signaling pathway	1/97	21/14464	0.04761905	7.10063819	2.29873139	0.13186225	0.50874395	0.5048838	Nrg2	1
GO:0045684	positive regulation of epidermis development	1/97	21/14464	0.04761905	7.10063819	2.29873139	0.13186225	0.50874395	0.5048838	Sfn	1
GO:0045948	positive regulation of translational initiation	1/97	21/14464	0.04761905	7.10063819	2.29873139	0.13186225	0.50874395	0.5048838	Mettl3	1
GO:0046827	positive regulation of protein export from nucleus	1/97	21/14464	0.04761905	7.10063819	2.29873139	0.13186225	0.50874395	0.5048838	Sfn	1
GO:0060065	uterus development	1/97	21/14464	0.04761905	7.10063819	2.29873139	0.13186225	0.50874395	0.5048838	Antxr2	1
GO:0070861	regulation of protein exit from endoplasmic reticulum	1/97	21/14464	0.04761905	7.10063819	2.29873139	0.13186225	0.50874395	0.5048838	Edem1	1
GO:0071731	response to nitric oxide	1/97	21/14464	0.04761905	7.10063819	2.29873139	0.13186225	0.50874395	0.5048838	Traf2	1
GO:0097396	response to interleukin-17	1/97	21/14464	0.04761905	7.10063819	2.29873139	0.13186225	0.50874395	0.5048838	Traf2	1
GO:1902455	negative regulation of stem cell population maintenance	1/97	21/14464	0.04761905	7.10063819	2.29873139	0.13186225	0.50874395	0.5048838	Sap30l	1
GO:2000647	negative regulation of stem cell proliferation	1/97	21/14464	0.04761905	7.10063819	2.29873139	0.13186225	0.50874395	0.5048838	Sfn	1
GO:2001169	regulation of ATP biosynthetic process	1/97	21/14464	0.04761905	7.10063819	2.29873139	0.13186225	0.50874395	0.5048838	Sphk2	1
GO:0072659	protein localization to plasma membrane	4/97	292/14464	0.01369863	2.04264934	1.47892497	0.13289776	0.50874395	0.5048838	Tspan14/Sfn/Nhlrc1/Atp1b3	4
GO:0006754	ATP biosynthetic process	2/97	95/14464	0.02105263	3.13922952	1.7188498	0.13343506	0.50874395	0.5048838	Lipa/Sphk2	2
GO:0019226	transmission of nerve impulse	2/97	95/14464	0.02105263	3.13922952	1.7188498	0.13343506	0.50874395	0.5048838	Gpr35/Fkbp1b	2
GO:0045639	positive regulation of myeloid cell differentiation	2/97	95/14464	0.02105263	3.13922952	1.7188498	0.13343506	0.50874395	0.5048838	Hspa1b/Ankrd54	2
GO:0071482	cellular response to light stimulus	2/97	95/14464	0.02105263	3.13922952	1.7188498	0.13343506	0.50874395	0.5048838	Cdkn1a/Mettl3	2
GO:0010506	regulation of autophagy	4/97	295/14464	0.01355932	2.02187664	1.4570414	0.1364716	0.50874395	0.5048838	Mtcl3/Becn1/Pafah1b2/Pip4k2c	4
GO:0001675	acrosome assembly	1/97	22/14464	0.04545455	6.77788191	2.22842666	0.13769272	0.50874395	0.5048838	Agfg1	1
GO:0008207	C21-steroid hormone metabolic process	1/97	22/14464	0.04545455	6.77788191	2.22842666	0.13769272	0.50874395	0.5048838	Stard3	1
GO:0010623	programmed cell death involved in cell development	1/97	22/14464	0.04545455	6.77788191	2.22842666	0.13769272	0.50874395	0.5048838	Dnase1l3	1
GO:0010839	negative regulation of keratinocyte proliferation	1/97	22/14464	0.04545455	6.77788191	2.22842666	0.13769272	0.50874395	0.5048838	Sfn	1
GO:0016556	mRNA modification	1/97	22/14464	0.04545455	6.77788191	2.22842666	0.13769272	0.50874395	0.5048838	Mettl3	1
GO:0031571	mitotic G1 DNA damage checkpoint signaling	1/97	22/14464	0.04545455	6.77788191	2.22842666	0.13769272	0.50874395	0.5048838	Cdkn1a	1
GO:0034349	glial cell apoptotic process	1/97	22/14464	0.04545455	6.77788191	2.22842666	0.13769272	0.50874395	0.5048838	Traf2	1
GO:0035872	nucleotide-binding domain, leucine rich repeat containing receptor signaling pathway	1/97	22/14464	0.04545455	6.77788191	2.22842666	0.13769272	0.50874395	0.5048838	Hspa1b	1
GO:0045822	negative regulation of heart contraction	1/97	22/14464	0.04545455	6.77788191	2.22842666	0.13769272	0.50874395	0.5048838	Fkbp1b	1
GO:0046579	positive regulation of Ras protein signal transduction	1/97	22/14464	0.04545455	6.77788191	2.22842666	0.13769272	0.50874395	0.5048838	Rasgef1a	1
GO:0050849	negative regulation of calcium-mediated signaling	1/97	22/14464	0.04545455	6.77788191	2.22842666	0.13769272	0.50874395	0.5048838	Fkbp1b	1
GO:0120255	olefinic compound biosynthetic process	1/97	22/14464	0.04545455	6.77788191	2.22842666	0.13769272	0.50874395	0.5048838	Stard3	1
GO:0140354	lipid import into cell	1/97	22/14464	0.04545455	6.77788191	2.22842666	0.13769272	0.50874395	0.5048838	Lipa	1
GO:1904030	negative regulation of cyclin-dependent protein kinase activity	1/97	22/14464	0.04545455	6.77788191	2.22842666	0.13769272	0.50874395	0.5048838	Cdkn1a	1
GO:0007029	endoplasmic reticulum organization	2/97	97/14464	0.02061856	3.07450314	1.68441434	0.13798661	0.50874395	0.5048838	Smpd4/Caml	2
GO:0002718	regulation of cytokine production involved in immune response	2/97	98/14464	0.02040816	3.04313065	1.66752848	0.14027527	0.50874395	0.5048838	Traf2/Sphk2	2
GO:0032204	regulation of telomere maintenance	2/97	98/14464	0.02040816	3.04313065	1.66752848	0.14027527	0.50874395	0.5048838	Ptges3/Cct7	2
GO:0006486	protein glycosylation	3/97	194/14464	0.01546392	2.30587735	1.50460771	0.14147292	0.50874395	0.5048838	Mlec/Uggt1/Fut11	3
GO:0043413	macromolecule glycosylation	3/97	194/14464	0.01546392	2.30587735	1.50460771	0.14147292	0.50874395	0.5048838	Mlec/Uggt1/Fut11	3
GO:0045637	regulation of myeloid cell differentiation	3/97	194/14464	0.01546392	2.30587735	1.50460771	0.14147292	0.50874395	0.5048838	Hspa1b/Ankrd54/Nme1	3
GO:0002444	myeloid leukocyte mediated immunity	2/97	99/14464	0.02020202	3.01239196	1.65085668	0.14257223	0.50874395	0.5048838	Dnase1l3/Sphk2	2
GO:0001963	synaptic transmission, dopaminergic	1/97	23/14464	0.04347826	6.48319139	2.16237347	0.14348442	0.50874395	0.5048838	Aph1b	1
GO:0002320	lymphoid progenitor cell differentiation	1/97	23/14464	0.04347826	6.48319139	2.16237347	0.14348442	0.50874395	0.5048838	Lig4	1
GO:0009048	dosage compensation by inactivation of X chromosome	1/97	23/14464	0.04347826	6.48319139	2.16237347	0.14348442	0.50874395	0.5048838	Mettl3	1
GO:0009129	pyrimidine nucleoside monophosphate metabolic process	1/97	23/14464	0.04347826	6.48319139	2.16237347	0.14348442	0.50874395	0.5048838	Nme1	1
GO:0010165	response to X-ray	1/97	23/14464	0.04347826	6.48319139	2.16237347	0.14348442	0.50874395	0.5048838	Lig4	1
GO:0016540	protein autoprocessing	1/97	23/14464	0.04347826	6.48319139	2.16237347	0.14348442	0.50874395	0.5048838	Pcsk9	1
GO:0032482	Rab protein signal transduction	1/97	23/14464	0.04347826	6.48319139	2.16237347	0.14348442	0.50874395	0.5048838	Rab39	1
GO:0033617	mitochondrial cytochrome c oxidase assembly	1/97	23/14464	0.04347826	6.48319139	2.16237347	0.14348442	0.50874395	0.5048838	Coa4	1
GO:0042026	protein refolding	1/97	23/14464	0.04347826	6.48319139	2.16237347	0.14348442	0.50874395	0.5048838	Hspa1b	1
GO:0044819	mitotic G1/S transition checkpoint signaling	1/97	23/14464	0.04347826	6.48319139	2.16237347	0.14348442	0.50874395	0.5048838	Cdkn1a	1
GO:0045109	intermediate filament organization	1/97	23/14464	0.04347826	6.48319139	2.16237347	0.14348442	0.50874395	0.5048838	Agfg1	1
GO:0046164	alcohol catabolic process	1/97	23/14464	0.04347826	6.48319139	2.16237347	0.14348442	0.50874395	0.5048838	Nudt3	1
GO:0050857	positive regulation of antigen receptor-mediated signaling pathway	1/97	23/14464	0.04347826	6.48319139	2.16237347	0.14348442	0.50874395	0.5048838	Lipa	1
GO:0051315	attachment of mitotic spindle microtubules to kinetochore	1/97	23/14464	0.04347826	6.48319139	2.16237347	0.14348442	0.50874395	0.5048838	Becn1	1
GO:0060339	negative regulation of type I interferon-mediated signaling pathway	1/97	23/14464	0.04347826	6.48319139	2.16237347	0.14348442	0.50874395	0.5048838	Mettl3	1
GO:1901673	regulation of mitotic spindle assembly	1/97	23/14464	0.04347826	6.48319139	2.16237347	0.14348442	0.50874395	0.5048838	Hspa1b	1
GO:1902307	positive regulation of sodium ion transmembrane transport	1/97	23/14464	0.04347826	6.48319139	2.16237347	0.14348442	0.50874395	0.5048838	Atp1b3	1
GO:1903319	positive regulation of protein maturation	1/97	23/14464	0.04347826	6.48319139	2.16237347	0.14348442	0.50874395	0.5048838	Ctnnd1	1
GO:0045017	glycerolipid biosynthetic process	3/97	196/14464	0.01530612	2.28234799	1.48519814	0.1445827	0.50944436	0.50557889	Becn1/Pip4k2c/Pigc	3
GO:0035967	cellular response to topologically incorrect protein	2/97	100/14464	0.02	2.98226804	1.63439376	0.14487729	0.50944436	0.50557889	Pdia6/Edem1	2
GO:0002703	regulation of leukocyte mediated immunity	3/97	197/14464	0.01522843	2.27076247	1.47558141	0.14614616	0.50944436	0.50557889	Dnase1l3/Traf2/Sphk2	3
GO:1901615	organic hydroxy compound metabolic process	5/97	417/14464	0.01199041	1.78793048	1.34151691	0.14837165	0.50944436	0.50557889	Pcsk9/Lipa/Pmvk/Sphk2/Nudt3	5

GO:0001516 prostaglandin biosynthetic process	1/97	24/14464	0.04166667	6.21305842	2.10013218	0.14923763	0.50944436	0.50557889	Ptges3	1
GO:0007035 vacuolar acidification	1/97	24/14464	0.04166667	6.21305842	2.10013218	0.14923763	0.50944436	0.50557889	Cln5	1
GO:0008299 isoprenoid biosynthetic process	1/97	24/14464	0.04166667	6.21305842	2.10013218	0.14923763	0.50944436	0.50557889	Pmvk	1
GO:0010880 regulation of release of sequestered calcium ion into cytosol by sarcoplasmic reticulum	1/97	24/14464	0.04166667	6.21305842	2.10013218	0.14923763	0.50944436	0.50557889	Fkbp1b	1
GO:0018126 protein hydroxylation	1/97	24/14464	0.04166667	6.21305842	2.10013218	0.14923763	0.50944436	0.50557889	P4ha1	1
GO:0030808 regulation of nucleotide biosynthetic process	1/97	24/14464	0.04166667	6.21305842	2.10013218	0.14923763	0.50944436	0.50557889	Sphk2	1
GO:0034453 microtubule anchoring	1/97	24/14464	0.04166667	6.21305842	2.10013218	0.14923763	0.50944436	0.50557889	Bicd2	1
GO:0046457 prostanoid biosynthetic process	1/97	24/14464	0.04166667	6.21305842	2.10013218	0.14923763	0.50944436	0.50557889	Ptges3	1
GO:0046475 glycerophospholipid catabolic process	1/97	24/14464	0.04166667	6.21305842	2.10013218	0.14923763	0.50944436	0.50557889	Smpd4	1
GO:0046688 response to copper ion	1/97	24/14464	0.04166667	6.21305842	2.10013218	0.14923763	0.50944436	0.50557889	Becn1	1
GO:0055075 potassium ion homeostasis	1/97	24/14464	0.04166667	6.21305842	2.10013218	0.14923763	0.50944436	0.50557889	Atp1b3	1
GO:0070723 response to cholesterol	1/97	24/14464	0.04166667	6.21305842	2.10013218	0.14923763	0.50944436	0.50557889	Pmvk	1
GO:0090141 positive regulation of mitochondrial fission	1/97	24/14464	0.04166667	6.21305842	2.10013218	0.14923763	0.50944436	0.50557889	Mief1	1
GO:1900371 regulation of purine nucleotide biosynthetic process	1/97	24/14464	0.04166667	6.21305842	2.10013218	0.14923763	0.50944436	0.50557889	Sphk2	1
GO:1903523 negative regulation of blood circulation	1/97	24/14464	0.04166667	6.21305842	2.10013218	0.14923763	0.50944436	0.50557889	Fkbp1b	1
GO:0006939 smooth muscle contraction	2/97	102/14464	0.01960784	2.9237922	1.60207483	0.14951084	0.50944436	0.50557889	Shc1/Fkbp1b	2
GO:0051098 regulation of binding	4/97	308/14464	0.01298701	1.93653769	1.36509336	0.15240194	0.51519346	0.51128436	Cdkn1a/Pcsk9/Cln5/Nme1	4
GO:0002262 myeloid cell homeostasis	3/97	202/14464	0.01485149	2.21455548	1.42835066	0.15404666	0.51519346	0.51128436	Hspa1b/Lipa/Ankrd54	3
GO:0070646 protein modification by small protein removal	2/97	104/14464	0.01923077	2.86756542	1.57053381	0.1541743	0.51519346	0.51128436	Stambpl1/Otub2	2
GO:0090316 positive regulation of intracellular protein transport	2/97	104/14464	0.01923077	2.86756542	1.57053381	0.1541743	0.51519346	0.51128436	Sfn/Edem1	2
GO:0002724 regulation of T cell cytokine production	1/97	25/14464	0.04	5.96453608	2.04132489	0.15495258	0.51519346	0.51128436	Traf2	1
GO:0006353 DNA-templated transcription termination	1/97	25/14464	0.04	5.96453608	2.04132489	0.15495258	0.51519346	0.51128436	Maz	1
GO:0007549 sex-chromosome dosage compensation	1/97	25/14464	0.04	5.96453608	2.04132489	0.15495258	0.51519346	0.51128436	Mettl3	1
GO:0010039 response to iron ion	1/97	25/14464	0.04	5.96453608	2.04132489	0.15495258	0.51519346	0.51128436	Becn1	1
GO:0034755 iron ion transmembrane transport	1/97	25/14464	0.04	5.96453608	2.04132489	0.15495258	0.51519346	0.51128436	Abcc5	1
GO:0070199 establishment of protein localization to chromosome	1/97	25/14464	0.04	5.96453608	2.04132489	0.15495258	0.51519346	0.51128436	Cct7	1
GO:1901099 negative regulation of signal transduction in absence of ligand	1/97	25/14464	0.04	5.96453608	2.04132489	0.15495258	0.51519346	0.51128436	Hspa1b	1
GO:2001240 negative regulation of extrinsic apoptotic signaling pathway in absence of ligand	1/97	25/14464	0.04	5.96453608	2.04132489	0.15495258	0.51519346	0.51128436	Hspa1b	1
GO:0002832 negative regulation of response to biotic stimulus	2/97	105/14464	0.01904762	2.84025528	1.55504384	0.15651674	0.51519346	0.51128436	Sfn/Mettl3	2
GO:0008630 intrinsic apoptotic signaling pathway in response to DNA damage	2/97	105/14464	0.01904762	2.84025528	1.55504384	0.15651674	0.51519346	0.51128436	Cdkn1a/Sfn	2
GO:0051092 positive regulation of NF-kappaB transcription factor activity	2/97	105/14464	0.01904762	2.84025528	1.55504384	0.15651674	0.51519346	0.51128436	Hspa1b/Traf2	2
GO:0031396 regulation of protein ubiquitination	3/97	204/14464	0.01470588	2.19284415	1.40984393	0.15724452	0.51519346	0.51128436	Hspa1b/Nhlrc1/Caml	3
GO:0000819 sister chromatid segregation	3/97	205/14464	0.01463415	2.18214735	1.40067063	0.15885127	0.51519346	0.51128436	Hspa1b/Becn1/Smc1a	3
GO:0008535 respiratory chain complex IV assembly	1/97	26/14464	0.03846154	5.73513085	1.98562464	0.16062954	0.51519346	0.51128436	Coa4	1
GO:0015868 purine ribonucleotide transport	1/97	26/14464	0.03846154	5.73513085	1.98562464	0.16062954	0.51519346	0.51128436	Abcc5	1
GO:0030149 sphingolipid catabolic process	1/97	26/14464	0.03846154	5.73513085	1.98562464	0.16062954	0.51519346	0.51128436	Smpd4	1
GO:0030262 apoptotic nuclear changes	1/97	26/14464	0.03846154	5.73513085	1.98562464	0.16062954	0.51519346	0.51128436	Dnase1l3	1
GO:0042790 nucleolar large rRNA transcription by RNA polymerase I	1/97	26/14464	0.03846154	5.73513085	1.98562464	0.16062954	0.51519346	0.51128436	Ubtf	1
GO:0043302 positive regulation of leukocyte degranulation	1/97	26/14464	0.03846154	5.73513085	1.98562464	0.16062954	0.51519346	0.51128436	Sphk2	1
GO:0045324 late endosome to vacuole transport	1/97	26/14464	0.03846154	5.73513085	1.98562464	0.16062954	0.51519346	0.51128436	Becn1	1
GO:0046039 GTP metabolic process	1/97	26/14464	0.03846154	5.73513085	1.98562464	0.16062954	0.51519346	0.51128436	Nme1	1
GO:0061436 establishment of skin barrier	1/97	26/14464	0.03846154	5.73513085	1.98562464	0.16062954	0.51519346	0.51128436	Sfn	1
GO:0070129 regulation of mitochondrial translation	1/97	26/14464	0.03846154	5.73513085	1.98562464	0.16062954	0.51519346	0.51128436	Mrps27	1
GO:0071108 protein K48-linked deubiquitination	1/97	26/14464	0.03846154	5.73513085	1.98562464	0.16062954	0.51519346	0.51128436	Otub2	1
GO:0098885 modification of postsynaptic actin cytoskeleton	1/97	26/14464	0.03846154	5.73513085	1.98562464	0.16062954	0.51519346	0.51128436	Cap1	1
GO:1900101 regulation of endoplasmic reticulum unfolded protein response	1/97	26/14464	0.03846154	5.73513085	1.98562464	0.16062954	0.51519346	0.51128436	Pdia6	1
GO:1901020 negative regulation of calcium ion transmembrane transporter activity	1/97	26/14464	0.03846154	5.73513085	1.98562464	0.16062954	0.51519346	0.51128436	Gpr35	1
GO:1901385 regulation of voltage-gated calcium channel activity	1/97	26/14464	0.03846154	5.73513085	1.98562464	0.16062954	0.51519346	0.51128436	Gpr35	1
GO:0005976 polysaccharide metabolic process	2/97	107/14464	0.01869159	2.78716639	1.52460396	0.16122205	0.51520319	0.51129402	Ptges3/Nhlrc1	2
GO:0042542 response to hydrogen peroxide	2/97	107/14464	0.01869159	2.78716639	1.52460396	0.16122205	0.51520319	0.51129402	Fkbp1b/Becn1	2
GO:1904951 positive regulation of establishment of protein localization	4/97	316/14464	0.01265823	1.88751142	1.31069602	0.16254527	0.51779006	0.51386127	Sfn/Edem1/Cct7/Mief1	4
GO:0008037 cell recognition	2/97	109/14464	0.01834862	2.73602573	1.49485759	0.16595332	0.51779006	0.51386127	Hspa1b/Cct7	2
GO:0006308 DNA catabolic process	1/97	27/14464	0.03703704	5.52271859	1.9327469	0.16626875	0.51779006	0.51386127	Dnase1l3	1
GO:0006516 glycoprotein catabolic process	1/97	27/14464	0.03703704	5.52271859	1.9327469	0.16626875	0.51779006	0.51386127	Edem1	1
GO:0006921 cellular component disassembly involved in execution phase of apoptosis	1/97	27/14464	0.03703704	5.52271859	1.9327469	0.16626875	0.51779006	0.51386127	Dnase1l3	1
GO:0014808 release of sequestered calcium ion into cytosol by sarcoplasmic reticulum	1/97	27/14464	0.03703704	5.52271859	1.9327469	0.16626875	0.51779006	0.51386127	Fkbp1b	1
GO:0030970 retrograde protein transport, ER to cytosol	1/97	27/14464	0.03703704	5.52271859	1.9327469	0.16626875	0.51779006	0.51386127	Edem1	1
GO:0034311 diol metabolic process	1/97	27/14464	0.03703704	5.52271859	1.9327469	0.16626875	0.51779006	0.51386127	Sphk2	1
GO:0045730 respiratory burst	1/97	27/14464	0.03703704	5.52271859	1.9327469	0.16626875	0.51779006	0.51386127	Lipa	1
GO:0051503 adenine nucleotide transport	1/97	27/14464	0.03703704	5.52271859	1.9327469	0.16626875	0.51779006	0.51386127	Abcc5	1
GO:0051984 positive regulation of chromosome segregation	1/97	27/14464	0.03703704	5.52271859	1.9327469	0.16626875	0.51779006	0.51386127	Becn1	1
GO:0070841 inclusion body assembly	1/97	27/14464	0.03703704	5.52271859	1.9327469	0.16626875	0.51779006	0.51386127	Hspa1b	1
GO:1903513 endoplasmic reticulum to cytosol transport	1/97	27/14464	0.03703704	5.52271859	1.9327469	0.16626875	0.51779006	0.51386127	Edem1	1
GO:2000050 regulation of non-canonical Wnt signaling pathway	1/97	27/14464	0.03703704	5.52271859	1.9327469	0.16626875	0.51779006	0.51386127	Sfrp5	1
GO:0002702 positive regulation of production of molecular mediator of immune response	2/97	110/14464	0.01818182	2.71115276	1.480235	0.16832822	0.51779006	0.51386127	Traf2/Sphk2	2
GO:0006986 response to unfolded protein	2/97	110/14464	0.01818182	2.71115276	1.480235	0.16832822	0.51779006	0.51386127	Pdia6/Edem1	2
GO:0032869 cellular response to insulin stimulus	3/97	212/14464	0.01415094	2.11009531	1.33789585	0.17023885	0.51779006	0.51386127	Shc1/Pcsk9/Pip4k2c	3
GO:1905475 regulation of protein localization to membrane	3/97	212/14464	0.01415094	2.11009531	1.33789585	0.17023885	0.51779006	0.51386127	Sfn/Nhlrc1/Mief1	3
GO:0009100 glycoprotein metabolic process	4/97	323/14464	0.0123839	1.8466056	1.26437049	0.17161951	0.51779006	0.51386127	Mlec/Edem1/Uggt1/Fut11	4
GO:0003401 axis elongation	1/97	28/14464	0.03571429	5.32547865	1.8824427	0.17187046	0.51779006	0.51386127	Sfrp5	1
GO:0006221 pyrimidine nucleotide biosynthetic process	1/97	28/14464	0.03571429	5.32547865	1.8824427	0.17187046	0.51779006	0.51386127	Nme1	1
GO:0006622 protein targeting to lysosome	1/97	28/14464	0.03571429	5.32547865	1.8824427	0.17187046	0.51779006	0.51386127	Becn1	1
GO:0009162 deoxyribonucleoside monophosphate metabolic process	1/97	28/14464	0.03571429	5.32547865	1.8824427	0.17187046	0.51779006	0.51386127	Nme1	1
GO:0015865 purine nucleotide transport	1/97	28/14464	0.03571429	5.32547865	1.8824427	0.17187046	0.51779006	0.51386127	Abcc5	1
GO:0036314 response to sterol	1/97	28/14464	0.03571429	5.32547865	1.8824427	0.17187046	0.51779006	0.51386127	Pmvk	1
GO:0043304 regulation of mast cell degranulation	1/97	28/14464	0.03571429	5.32547865	1.8824427	0.17187046	0.51779006	0.51386127	Sphk2	1
GO:0048261 negative regulation of receptor-mediated endocytosis	1/97	28/14464	0.03571429	5.32547865	1.8824427	0.17187046	0.51779006	0.51386127	Pcsk9	1
GO:0055078 sodium ion homeostasis	1/97	28/14464	0.03571429	5.32547865	1.8824427	0.17187046	0.51779006	0.51386127	Atp1b3	1
GO:0070286 axonemal dynein complex assembly	1/97	28/14464	0.03571429	5.32547865	1.8824427	0.17187046	0.51779006	0.51386127	Dnai4	1
GO:1903514 release of sequestered calcium ion into cytosol by endoplasmic reticulum	1/97	28/14464	0.03571429	5.32547865	1.8824427	0.17187046	0.51779006	0.51386127	Fkbp1b	1
GO:0007265 Ras protein signal transduction	2/97	112/14464	0.01785714	2.66273932	1.45147306	0.17309561	0.51779006	0.51386127	Cdkn1a/Rasgef1a	2
GO:0048145 regulation of fibroblast proliferation	2/97	112/14464	0.01785714	2.66273932	1.45147306	0.17309561	0.51779006	0.51386127	Cdkn1a/Lig4	2
GO:0009165 nucleotide biosynthetic process	3/97	214/14464	0.01401869	2.0903748	1.32040534	0.17353569	0.51779006	0.51386127	Lipa/Sphk2/Nme1	3
GO:0051656 establishment of organelle localization	5/97	442/14464	0.01131222	1.68680319	1.20495401	0.1754542	0.51779006	0.51386127	Bicd2/Sphk2/Tuba1a/Stard3/Becn1	5
GO:0006417 regulation of translation	4/97	326/14464	0.01226994	1.8296123	1.24486428	0.17556271	0.51779006	0.51386127	Mettl3/Nhlrc1/Mrps27/Hbs1l	4
GO:0042594 response to starvation	3/97	216/14464	0.01388889	2.07101947	1.30310468	0.17685086	0.51779006	0.51386127	Cdkn1a/Pcsk9/Becn1	3
GO:0045926 negative regulation of growth	3/97	216/14464	0.01388889	2.07101947	1.30310468	0.17685086	0.51779006	0.51386127	Hspa1b/Cdkn1a/Sphk2	3
GO:0002443 leukocyte mediated immunity	4/97	327/14464	0.01223242	1.82401715	1.23840733	0.17688413	0.51779006	0.51386127	Dnase1l3/Traf2/Sphk2/Lig4	4
GO:0001919 regulation of receptor recycling	1/97	29/14464	0.03448276	5.14184145	1.83449313	0.17743492	0.51779006	0.51386127	Pcsk9	1
GO:0002092 positive regulation of receptor internalization	1/97	29/14464	0.03448276	5.14184145	1.83449313	0.17743492	0.51779006	0.51386127	Pcsk9	1
GO:0006506 GPI anchor biosynthetic process	1/97	29/14464	0.03448276	5.14184145	1.83449313	0.17743492	0.51779006	0.51386127	Pigc	1
GO:0009218 pyrimidine ribonucleotide metabolic process	1/97	29/14464	0.03448276	5.14184145	1.83449313	0.17743492	0.51779006	0.51386127	Nme1	1
GO:0019369 arachidonate metabolic process	1/97	29/14464	0.03448276	5.14184145	1.83449313	0.17743492	0.51779006	0.51386127	Cyp2j13	1
GO:0032369 negative regulation of lipid transport	1/97	29/14464	0.03448276	5.14184145	1.83449313	0.17743492	0.51779006	0.51386127	Pcsk9	1
GO:0045048 protein insertion into ER membrane	1/97	29/14464	0.03448276	5.14184145	1.83449313	0.17743492	0.51779006	0.51386127	Caml	1
GO:0046466 membrane lipid catabolic process	1/97	29/14464	0.03448276	5.14184145	1.83449313	0.17743492	0.51779006	0.51386127	Smpd4	1
GO:0048266 behavioral response to pain	1/97	29/14464	0.03448276	5.14184145	1.83449313	0.17743492	0.51779006	0.51386127	Vwa1	1
GO:0071480 cellular response to gamma radiation	1/97	29/14464	0.03448276	5.14184145	1.83449313	0.17743492	0.51779006	0.51386127	Cdkn1a	1
GO:0072576 liver morphogenesis	1/97	29/14464	0.03448276	5.14184145	1.83449313	0.17743492	0.51779006	0.51386127	Lipa	1
GO:0090382 phagosome maturation	1/97	29/14464	0.03448276	5.14184145	1.83449313	0.17743492	0.51779006	0.51386127	Rab39	1
GO:0120178 steroid hormone biosynthetic process	1/97	29/14464	0.03448276	5.14184145	1.83449313	0.17743492	0.51779006	0.51386127	Stard3	1
GO:1901258 negative regulation of cation channel activity	1/97	29/14464	0.03448276	5.14184145	1.83449313	0.17743492	0.51779006	0.51386127	Gpr35	1
GO:1903076 regulation of protein localization to plasma membrane	2/97	114/14464	0.01754386	2.6160246	1.4233328	0.17788525	0.51823902	0.51430682	Sfn/Nhlrc1	2
GO:0052548 regulation of endopeptidase activity	3/97	218/14464	0.01376147	2.05201929	1.28598972	0.18018386	0.51827196	0.51433951	Hspa1b/Serpinb8/Aph1b	3
GO:0006639 acylglycerol metabolic process	2/97	115/14464	0.0173913	2.59327656	1.40948768	0.18028796	0.51827196	0.51433951	Pcsk9/Lipa	2
GO:0043200 response to amino acid	2/97	115/14464	0.0173913	2.59327656	1.40948768	0.18028796	0.51827196	0.51433951	Stambpl1/Tuba1a	2
GO:0030003 intracellular monoatomic cation homeostasis	5/97	447/14464	0.01118568	1.66793515	1.17867083	0.18108954	0.51827196	0.51433951	Rab39/Efhc1/Fkbp1b/Atp1b3/Cln5	5
GO:0034762 regulation of transmembrane transport	5/97	447/14464	0.01118568	1.66793515	1.17867083	0.18108954	0.51827196	0.51433951	Gpr35/Fkbp1b/Pcsk9/Atp1b3/Sphk2	5
GO:0006638 neutral lipid metabolic process	2/97	116/14464	0.01724138	2.57092073	1.39578843	0.1826957	0.51827196	0.51433951	Pcsk9/Lipa	2
GO:0006505 GPI anchor metabolic process	1/97	30/14464	0.03333333	4.97044674	1.78870477	0.18296238	0.51827196	0.51433951	Pigc	1
GO:0007588 excretion	1/97	30/14464	0.03333333	4.97044674	1.78870477	0.18296238	0.51827196	0.51433951	Lipa	1
GO:0034250 positive regulation of amide metabolic process	1/97	30/14464	0.03333333	4.97044674	1.78870477	0.18296238	0.51827196	0.51433951	Sphk2	1
GO:0090128 regulation of synapse maturation	1/97	30/14464	0.03333333	4.97044674	1.78870477	0.18296238	0.51827196	0.51433951	Nrg2	1
GO:0090314 positive regulation of protein targeting to membrane	1/97	30/14464	0.03333333	4.97044674	1.78870477	0.18296238	0.51827196	0.51433951	Mief1	1
GO:0099022 vesicle tethering	1/97	30/14464	0.03333333	4.97044674	1.78870477	0.18296238	0.51827196	0.51433951	Stard3	1
GO:1903077 negative regulation of protein localization to plasma membrane	1/97	30/14464	0.03333333	4.97044674	1.78870477	0.18296238	0.51827196	0.51433951	Sfn	1
GO:1904706 negative regulation of vascular associated smooth muscle cell proliferation	1/97	30/14464	0.03333333	4.97044674	1.78870477	0.18296238	0.51827196	0.51433951	Cdkn1a	1
GO:0046034 ATP metabolic process	3/97	220/14464	0.01363636	2.03336457	1.26905645	0.18353422	0.51827196	0.51433951	Hspa1b/Lipa/Sphk2	3
GO:0060541 respiratory system development	3/97	220/14464	0.01363636	2.03336457	1.26905645	0.18353422	0.51827196	0.51433951	Myt1/Lipa/Ptges3	3
GO:0009117 nucleotide metabolic process	5/97	450/14464	0.01111111	1.65681558	1.16305609	0.18450358	0.51827196	0.51433951	Hspa1b/Lipa/Sphk2/Nudt3/Nme1	5
GO:0001776 leukocyte homeostasis	2/97	117/14464	0.01709402	2.54894704	1.38223207	0.1851083	0.51827196	0.51433951	Lipa/Caml	2
GO:0006354 DNA-templated transcription elongation	2/97	117/14464	0.01709402	2.54894704	1.38223207	0.1851083	0.51827196	0.51433951	Ubtf/Eloa	2
GO:0043588 skin development	3/97	222/14464	0.01351351	2.01504597	1.25230099	0.18690145	0.51827196	0.51433951	Cdkn1a/Sfn/Ptges3	3
GO:0046434 organophosphate catabolic process	3/97	222/14464	0.01351351	2.01504597	1.25230099	0.18690145	0.51827196	0.51433951	Lipa/Smpd4/Nudt3	3
GO:0000045 autophagosome assembly	2/97	118/14464	0.01694915	2.5273458	1.3688157	0.18752558	0.51827196	0.51433951	Becn1/Pip4k2c	2
GO:0002367 cytokine production involved in immune response	2/97	118/14464	0.01694915	2.5273458	1.3688157	0.18752558	0.51827196	0.51433951	Traf2/Sphk2	2
GO:0001736 establishment of planar polarity	1/97	31/14464	0.03225806	4.81010974	1.74490602	0.18845307	0.51827196	0.51433951	Sfrp5	1
GO:0007164 establishment of tissue polarity	1/97	31/14464	0.03225806	4.81010974	1.74490602	0.18845307	0.51827196	0.51433951	Sfrp5	1
GO:0030212 hyaluronan metabolic process	1/97	31/14464	0.03225806	4.81010974	1.74490602	0.18845307	0.51827196	0.51433951	Abcc5	1
GO:0032367 intracellular cholesterol transport	1/97	31/14464	0.03225806	4.81010974	1.74490602	0.18845307	0.51827196	0.51433951	Pcsk9	1
GO:0034067 protein localization to Golgi apparatus	1/97	31/14464	0.03225806	4.81010974	1.74490602	0.18845307	0.51827196	0.51433951	Bicd2	1
GO:0060218 hematopoietic stem cell differentiation	1/97	31/14464	0.03225806	4.81010974	1.74490602	0.18845307	0.51827196	0.51433951	Mettl3	1
GO:0072337 modified amino acid transport	1/97	31/14464	0.03225806	4.81010974	1.74490602	0.18845307	0.51827196	0.51433951	Abcc5	1
GO:0098703 calcium ion import across plasma membrane	1/97	31/14464	0.03225806	4.81010974	1.74490602	0.18845307	0.51827196	0.51433951	Trpv2	1
GO:1902656 calcium ion import into cytosol	1/97	31/14464	0.03225806	4.81010974	1.74490602	0.18845307	0.51827196	0.51433951	Trpv2	1
GO:1904376 negative regulation of protein localization to cell periphery	1/97	31/14464	0.03225806	4.81010974	1.74490602	0.18845307	0.51827196	0.51433951	Sfn	1
GO:2000279 negative regulation of DNA biosynthetic process	1/97	31/14464	0.03225806	4.81010974	1.74490602	0.18845307	0.51827196	0.51433951	Cdkn1a	1
GO:0006873 intracellular monoatomic ion homeostasis	5/97	454/14464	0.01101322	1.64221808	1.1424137	0.18909301	0.51827196	0.51433951	Rab39/Efhc1/Fkbp1b/Atp1b3/Cln5	5
GO:0030317 flagellated sperm motility	2/97	119/14464	0.01680672	2.5061076	1.35553651	0.18994738	0.51827196	0.51433951	Efhc1/Tuba1a	2
GO:0090090 negative regulation of canonical Wnt signaling pathway	2/97	119/14464	0.01680672	2.5061076	1.35553651	0.18994738	0.51827196	0.51433951	Sfrp5/Ctnnd1	2
GO:0031667 response to nutrient levels	5/97	455/14464	0.01098901	1.63860881	1.13728432	0.19024692	0.51827196	0.51433951	Cdkn1a/Fkbp1b/Pcsk9/Lipa/Becn1	5
GO:0045861 negative regulation of proteolysis	3/97	224/14464	0.01339286	1.99705449	1.23571957	0.19028507	0.51827196	0.51433951	Hspa1b/Serpinb8/Caml	3
GO:0048675 axon extension	2/97	120/14464	0.01666667	2.48522337	1.34239174	0.19237353	0.51827196	0.51433951	Trpv2/Raph1	2
GO:0051402 neuron apoptotic process	4/97	339/14464	0.01179941	1.75945017	1.16261767	0.19300287	0.51827196	0.51433951	Pcsk9/Ptges3/Tuba1a/Lig4	4
GO:0002021 response to dietary excess	1/97	32/14464	0.03125	4.65979381	1.70294398	0.19390724	0.51827196	0.51433951	Lipa	1
GO:0002446 neutrophil mediated immunity	1/97	32/14464	0.03125	4.65979381	1.70294398	0.19390724	0.51827196	0.51433951	Dnase1l3	1
GO:0007435 salivary gland morphogenesis	1/97	32/14464	0.03125	4.65979381	1.70294398	0.19390724	0.51827196	0.51433951	Ctnnd1	1
GO:0031116 positive regulation of microtubule polymerization	1/97	32/14464	0.03125	4.65979381	1.70294398	0.19390724	0.51827196	0.51433951	Hspa1b	1
GO:0032743 positive regulation of interleukin-2 production	1/97	32/14464	0.03125	4.65979381	1.70294398	0.19390724	0.51827196	0.51433951	Traf2	1
GO:0051180 vitamin transport	1/97	32/14464	0.03125	4.65979381	1.70294398	0.19390724	0.51827196	0.51433951	Abcc5	1
GO:0070198 protein localization to chromosome, telomeric region	1/97	32/14464	0.03125	4.65979381	1.70294398	0.19390724	0.51827196	0.51433951	Cct7	1
GO:0090169 regulation of spindle assembly	1/97	32/14464	0.03125	4.65979381	1.70294398	0.19390724	0.51827196	0.51433951	Hspa1b	1
GO:0090322 regulation of superoxide metabolic process	1/97	32/14464	0.03125	4.65979381	1.70294398	0.19390724	0.51827196	0.51433951	Shc1	1
GO:0140058 neuron projection arborization	1/97	32/14464	0.03125	4.65979381	1.70294398	0.19390724	0.51827196	0.51433951	Tuba1a	1
GO:0180046 GPI anchored protein biosynthesis	1/97	32/14464	0.03125	4.65979381	1.70294398	0.19390724	0.51827196	0.51433951	Pigc	1
GO:1901381 positive regulation of potassium ion transmembrane transport	1/97	32/14464	0.03125	4.65979381	1.70294398	0.19390724	0.51827196	0.51433951	Atp1b3	1
GO:2000765 regulation of cytoplasmic translation	1/97	32/14464	0.03125	4.65979381	1.70294398	0.19390724	0.51827196	0.51433951	Mettl3	1
GO:0032434 regulation of proteasomal ubiquitin-dependent protein catabolic process	2/97	121/14464	0.01652893	2.46468433	1.32937876	0.19480387	0.51908104	0.51514245	Hspa1b/Caml	2
GO:0097722 sperm motility	2/97	121/14464	0.01652893	2.46468433	1.32937876	0.19480387	0.51908104	0.51514245	Efhc1/Tuba1a	2
GO:0072522 purine-containing compound biosynthetic process	3/97	228/14464	0.01315789	1.96201845	1.20306432	0.19709957	0.52158732	0.51762972	Lipa/Sphk2/Nme1	3
GO:1901136 carbohydrate derivative catabolic process	3/97	229/14464	0.01310044	1.95345068	1.19500371	0.19881269	0.52158732	0.51762972	Lipa/Edem1/Nudt3	3
GO:0006862 nucleotide transport	1/97	33/14464	0.03030303	4.51858794	1.66268195	0.19932513	0.52158732	0.51762972	Abcc5	1
GO:0018208 peptidyl-proline modification	1/97	33/14464	0.03030303	4.51858794	1.66268195	0.19932513	0.52158732	0.51762972	P4ha1	1
GO:0031018 endocrine pancreas development	1/97	33/14464	0.03030303	4.51858794	1.66268195	0.19932513	0.52158732	0.51762972	Myt1	1
GO:0032781 positive regulation of ATP-dependent activity	1/97	33/14464	0.03030303	4.51858794	1.66268195	0.19932513	0.52158732	0.51762972	Atp1b3	1
GO:0045943 positive regulation of transcription by RNA polymerase I	1/97	33/14464	0.03030303	4.51858794	1.66268195	0.19932513	0.52158732	0.51762972	Ubtf	1

GO:0046329	negative regulation of JNK cascade	1/97	33/14464	0.03030303	4.51858794	1.66268195	0.19932513	0.52158732	0.51762972	Sfrp5	1
GO:0046456	icosanoid biosynthetic process	1/97	33/14464	0.03030303	4.51858794	1.66268195	0.19932513	0.52158732	0.51762972	Ptges3	1
GO:0048854	brain morphogenesis	1/97	33/14464	0.03030303	4.51858794	1.66268195	0.19932513	0.52158732	0.51762972	Tuba1a	1
GO:0051642	centrosome localization	1/97	33/14464	0.03030303	4.51858794	1.66268195	0.19932513	0.52158732	0.51762972	Bicd2	1
GO:0070296	sarcoplasmic reticulum calcium ion transport	1/97	33/14464	0.03030303	4.51858794	1.66268195	0.19932513	0.52158732	0.51762972	Fkbp1b	1
GO:1902115	regulation of organelle assembly	3/97	230/14464	0.01304348	1.94495742	1.18698352	0.2005295	0.5239545	0.51997893	Hspa1b/Becn1/Pip4k2c	3
GO:0008286	insulin receptor signaling pathway	2/97	124/14464	0.01612903	2.40505487	1.29110501	0.20211836	0.52453241	0.52055246	Shc1/Pip4k2c	2
GO:0021987	cerebral cortex development	2/97	124/14464	0.01612903	2.40505487	1.29110501	0.20211836	0.52453241	0.52055246	Efhc1/Tuba1a	2
GO:0051100	negative regulation of binding	2/97	124/14464	0.01612903	2.40505487	1.29110501	0.20211836	0.52453241	0.52055246	Cdkn1a/Pcsk9	2
GO:0045727	positive regulation of translation	2/97	125/14464	0.016	2.38581443	1.27859402	0.20456382	0.52453241	0.52055246	Mettl3/Mrps27	2
GO:0002369	T cell cytokine production	1/97	34/14464	0.02941176	4.3856883	1.62399721	0.20470698	0.52453241	0.52055246	Traf2	1
GO:0007431	salivary gland development	1/97	34/14464	0.02941176	4.3856883	1.62399721	0.20470698	0.52453241	0.52055246	Ctnnd1	1
GO:0032366	intracellular sterol transport	1/97	34/14464	0.02941176	4.3856883	1.62399721	0.20470698	0.52453241	0.52055246	Pcsk9	1
GO:0034063	stress granule assembly	1/97	34/14464	0.02941176	4.3856883	1.62399721	0.20470698	0.52453241	0.52055246	Becn1	1
GO:0042119	neutrophil activation	1/97	34/14464	0.02941176	4.3856883	1.62399721	0.20470698	0.52453241	0.52055246	Dnase1l3	1
GO:0061842	microtubule organizing center localization	1/97	34/14464	0.02941176	4.3856883	1.62399721	0.20470698	0.52453241	0.52055246	Bicd2	1
GO:0010608	post-transcriptional regulation of gene expression	5/97	468/14464	0.01068376	1.5930919	1.07170429	0.2054771	0.52453241	0.52055246	Mettl3/Traf2/Nhlrc1/Mrps27/Hbs1l	5
GO:0010466	negative regulation of peptidase activity	2/97	126/14464	0.01587302	2.3668794	1.26620259	0.20701267	0.52453241	0.52055246	Hspa1b/Serpinb8	2
GO:0051225	spindle assembly	2/97	126/14464	0.01587302	2.3668794	1.26620259	0.20701267	0.52453241	0.52055246	Hspa1b/Smc1a	2
GO:1905037	autophagosome organization	2/97	126/14464	0.01587302	2.3668794	1.26620259	0.20701267	0.52453241	0.52055246	Becn1/Pip4k2c	2
GO:0044283	small molecule biosynthetic process	5/97	470/14464	0.0106383	1.58631279	1.06179182	0.20785659	0.52453241	0.52055246	Lipa/Ptges3/Pmvk/Sphk2/Stard3	5
GO:0015850	organic hydroxy compound transport	3/97	235/14464	0.01276596	1.90357535	1.14747472	0.20916669	0.52453241	0.52055246	Pcsk9/Lipa/Stard3	3
GO:0090257	regulation of muscle system process	3/97	235/14464	0.01276596	1.90357535	1.14747472	0.20916669	0.52453241	0.52055246	Shc1/Fkbp1b/Becn1	3
GO:0035966	response to topologically incorrect protein	2/97	127/14464	0.01574803	2.34824255	1.25392847	0.20946475	0.52453241	0.52055246	Pdia6/Edem1	2
GO:0002673	regulation of acute inflammatory response	1/97	35/14464	0.02857143	4.26038292	1.58677931	0.21005303	0.52453241	0.52055246	Dnase1l3	1
GO:0007595	lactation	1/97	35/14464	0.02857143	4.26038292	1.58677931	0.21005303	0.52453241	0.52055246	Nme1	1
GO:0008053	mitochondrial fusion	1/97	35/14464	0.02857143	4.26038292	1.58677931	0.21005303	0.52453241	0.52055246	Mief1	1
GO:0016242	negative regulation of macroautophagy	1/97	35/14464	0.02857143	4.26038292	1.58677931	0.21005303	0.52453241	0.52055246	Becn1	1
GO:0018279	protein N-linked glycosylation via asparagine	1/97	35/14464	0.02857143	4.26038292	1.58677931	0.21005303	0.52453241	0.52055246	Uggt1	1
GO:0045746	negative regulation of Notch signaling pathway	1/97	35/14464	0.02857143	4.26038292	1.58677931	0.21005303	0.52453241	0.52055246	Mettl3	1
GO:0048546	digestive tract morphogenesis	1/97	35/14464	0.02857143	4.26038292	1.58677931	0.21005303	0.52453241	0.52055246	Sfrp5	1
GO:0048679	regulation of axon regeneration	1/97	35/14464	0.02857143	4.26038292	1.58677931	0.21005303	0.52453241	0.52055246	Fkbp1b	1
GO:0062125	regulation of mitochondrial gene expression	1/97	35/14464	0.02857143	4.26038292	1.58677931	0.21005303	0.52453241	0.52055246	Mrps27	1
GO:0072344	rescue of stalled ribosome	1/97	35/14464	0.02857143	4.26038292	1.58677931	0.21005303	0.52453241	0.52055246	Hbs1l	1
GO:0072528	pyrimidine-containing compound biosynthetic process	1/97	35/14464	0.02857143	4.26038292	1.58677931	0.21005303	0.52453241	0.52055246	Nme1	1
GO:0090313	regulation of protein targeting to membrane	1/97	35/14464	0.02857143	4.26038292	1.58677931	0.21005303	0.52453241	0.52055246	Mief1	1
GO:1901616	organic hydroxy compound catabolic process	1/97	35/14464	0.02857143	4.26038292	1.58677931	0.21005303	0.52453241	0.52055246	Nudt3	1
GO:0022612	gland morphogenesis	2/97	128/14464	0.015625	2.32989691	1.24176947	0.21191992	0.52693603	0.52293784	Lipa/Ctnnd1	2
GO:0046328	regulation of JNK cascade	2/97	128/14464	0.015625	2.32989691	1.24176947	0.21191992	0.52693603	0.52293784	Sfrp5/Traf2	2
GO:0060294	cilium movement involved in cell motility	2/97	128/14464	0.015625	2.32989691	1.24176947	0.21191992	0.52693603	0.52293784	Efhc1/Tuba1a	2
GO:0006665	sphingolipid metabolic process	2/97	129/14464	0.01550388	2.31183569	1.22972347	0.21437803	0.52741674	0.5234149	Smpd4/Sphk2	2
GO:0001523	retinoid metabolic process	1/97	36/14464	0.02777778	4.14203895	1.55092845	0.2153635	0.52741674	0.5234149	Lipa	1
GO:0031952	regulation of protein autophosphorylation	1/97	36/14464	0.02777778	4.14203895	1.55092845	0.2153635	0.52741674	0.5234149	Ctnnd1	1
GO:0032350	regulation of hormone metabolic process	1/97	36/14464	0.02777778	4.14203895	1.55092845	0.2153635	0.52741674	0.5234149	Myt1	1
GO:0042908	xenobiotic transport	1/97	36/14464	0.02777778	4.14203895	1.55092845	0.2153635	0.52741674	0.5234149	Abcc5	1
GO:0043647	inositol phosphate metabolic process	1/97	36/14464	0.02777778	4.14203895	1.55092845	0.2153635	0.52741674	0.5234149	Nudt3	1
GO:0045747	positive regulation of Notch signaling pathway	1/97	36/14464	0.02777778	4.14203895	1.55092845	0.2153635	0.52741674	0.5234149	Tspan14	1
GO:0061744	motor behavior	1/97	36/14464	0.02777778	4.14203895	1.55092845	0.2153635	0.52741674	0.5234149	Tuba1a	1
GO:0090140	regulation of mitochondrial fission	1/97	36/14464	0.02777778	4.14203895	1.55092845	0.2153635	0.52741674	0.5234149	Mief1	1
GO:1903320	regulation of protein modification by small protein conjugation or removal	3/97	239/14464	0.0125523	1.87171634	1.11655473	0.21613676	0.52741674	0.5234149	Hspa1b/Nhlrc1/Caml	3
GO:0042113	B cell activation	3/97	240/14464	0.0125	1.86391753	1.10891702	0.21788717	0.52741674	0.5234149	Cdkn1a/Lig4/Nhej1	3
GO:0051924	regulation of calcium ion transport	3/97	240/14464	0.0125	1.86391753	1.10891702	0.21788717	0.52741674	0.5234149	Gpr35/Trpv2/Fkbp1b	3
GO:0009154	purine ribonucleotide catabolic process	2/97	131/14464	0.01526718	2.27654049	1.20596223	0.21930245	0.52741674	0.5234149	Lipa/Nudt3	2
GO:0002366	leukocyte activation involved in immune response	3/97	241/14464	0.01244813	1.85618343	1.10131549	0.21964063	0.52741674	0.5234149	Dnase1l3/Sphk2/Lig4	3
GO:0031669	cellular response to nutrient levels	3/97	241/14464	0.01244813	1.85618343	1.10131549	0.21964063	0.52741674	0.5234149	Cdkn1a/Pcsk9/Becn1	3
GO:0006084	acetyl-CoA metabolic process	1/97	37/14464	0.02702703	4.03009195	1.51635423	0.22063864	0.52741674	0.5234149	Pmvk	1
GO:0007095	mitotic G2 DNA damage checkpoint signaling	1/97	37/14464	0.02702703	4.03009195	1.51635423	0.22063864	0.52741674	0.5234149	Cdkn1a	1
GO:0010613	positive regulation of cardiac muscle hypertrophy	1/97	37/14464	0.02702703	4.03009195	1.51635423	0.22063864	0.52741674	0.5234149	Becn1	1
GO:0010837	regulation of keratinocyte proliferation	1/97	37/14464	0.02702703	4.03009195	1.51635423	0.22063864	0.52741674	0.5234149	Sfn	1
GO:0014742	positive regulation of muscle hypertrophy	1/97	37/14464	0.02702703	4.03009195	1.51635423	0.22063864	0.52741674	0.5234149	Becn1	1
GO:0017004	cytochrome complex assembly	1/97	37/14464	0.02702703	4.03009195	1.51635423	0.22063864	0.52741674	0.5234149	Coa4	1
GO:0043277	apoptotic cell clearance	1/97	37/14464	0.02702703	4.03009195	1.51635423	0.22063864	0.52741674	0.5234149	Becn1	1
GO:0046825	regulation of protein export from nucleus	1/97	37/14464	0.02702703	4.03009195	1.51635423	0.22063864	0.52741674	0.5234149	Sfn	1
GO:0048246	macrophage chemotaxis	1/97	37/14464	0.02702703	4.03009195	1.51635423	0.22063864	0.52741674	0.5234149	Gpr35	1
GO:1990573	potassium ion import across plasma membrane	1/97	37/14464	0.02702703	4.03009195	1.51635423	0.22063864	0.52741674	0.5234149	Atp1b3	1
GO:0007018	microtubule-based movement	4/97	359/14464	0.01114206	1.66143066	1.04274236	0.2208377	0.52741674	0.5234149	Bicd2/Efhc1/Tuba1a/Dnai4	4
GO:0030098	lymphocyte differentiation	4/97	359/14464	0.01114206	1.66143066	1.04274236	0.2208377	0.52741674	0.5234149	Lipa/Mettl3/Lig4/Nhej1	4
GO:0140014	mitotic nuclear division	3/97	242/14464	0.01239669	1.84851325	1.0937498	0.22139708	0.52741674	0.5234149	Hspa1b/Becn1/Smc1a	3
GO:0001101	response to acid chemical	2/97	132/14464	0.01515152	2.25929397	1.194243	0.22176848	0.52741674	0.5234149	Stambpl1/Tuba1a	2
GO:0001539	cilium or flagellum-dependent cell motility	2/97	132/14464	0.01515152	2.25929397	1.194243	0.22176848	0.52741674	0.5234149	Efhc1/Tuba1a	2
GO:0031109	microtubule polymerization or depolymerization	2/97	132/14464	0.01515152	2.25929397	1.194243	0.22176848	0.52741674	0.5234149	Hspa1b/Tuba1a	2
GO:0060285	cilium-dependent cell motility	2/97	132/14464	0.01515152	2.25929397	1.194243	0.22176848	0.52741674	0.5234149	Efhc1/Tuba1a	2
GO:0007292	female gamete generation	2/97	133/14464	0.01503759	2.2423068	1.18262879	0.22423686	0.52998113	0.52595983	Mettl3/Taf4b	2
GO:1901873	regulation of post-translational protein modification	3/97	244/14464	0.01229508	1.8333615	1.07872455	0.2249187	0.52998113	0.52595983	Hspa1b/Nhlrc1/Caml	3
GO:1903706	regulation of hemopoiesis	4/97	362/14464	0.01104972	1.6476619	1.02540232	0.22510566	0.52998113	0.52595983	Hspa1b/Mettl3/Ankrd54/Nme1	4
GO:0007020	microtubule nucleation	1/97	38/14464	0.02631579	3.9240369	1.48297455	0.22587868	0.52998113	0.52595983	Hspa1b	1
GO:0009303	rRNA transcription	1/97	38/14464	0.02631579	3.9240369	1.48297455	0.22587868	0.52998113	0.52595983	Ubtf	1
GO:0031112	positive regulation of microtubule polymerization or depolymerization	1/97	38/14464	0.02631579	3.9240369	1.48297455	0.22587868	0.52998113	0.52595983	Hspa1b	1
GO:0044331	cell-cell adhesion mediated by cadherin	1/97	38/14464	0.02631579	3.9240369	1.48297455	0.22587868	0.52998113	0.52595983	Ctnnd1	1
GO:1905476	negative regulation of protein localization to membrane	1/97	38/14464	0.02631579	3.9240369	1.48297455	0.22587868	0.52998113	0.52595983	Sfn	1
GO:2000649	regulation of sodium ion transmembrane transporter activity	1/97	38/14464	0.02631579	3.9240369	1.48297455	0.22587868	0.52998113	0.52595983	Pcsk9	1
GO:2001239	regulation of extrinsic apoptotic signaling pathway in absence of ligand	1/97	38/14464	0.02631579	3.9240369	1.48297455	0.22587868	0.52998113	0.52595983	Hspa1b	1
GO:0002263	cell activation involved in immune response	3/97	245/14464	0.0122449	1.82587839	1.0712643	0.22668378	0.53115716	0.52712694	Dnase1l3/Sphk2/Lig4	3
GO:0043604	amide biosynthetic process	2/97	135/14464	0.01481481	2.20908744	1.15970804	0.22918012	0.53628762	0.53221848	Smpd4/Sphk2	2
GO:0097305	response to alcohol	3/97	247/14464	0.01214575	1.81109395	1.0564469	0.23022215	0.5364337	0.53236344	Lipa/Pmvk/Sphk2	3
GO:0006693	prostaglandin metabolic process	1/97	39/14464	0.02564103	3.82342057	1.4507146	0.23108385	0.5364337	0.53236344	Ptges3	1
GO:0030890	positive regulation of B cell proliferation	1/97	39/14464	0.02564103	3.82342057	1.4507146	0.23108385	0.5364337	0.53236344	Cdkn1a	1
GO:0032435	negative regulation of proteasomal ubiquitin-dependent protein catabolic process	1/97	39/14464	0.02564103	3.82342057	1.4507146	0.23108385	0.5364337	0.53236344	Caml	1
GO:0033028	myeloid cell apoptotic process	1/97	39/14464	0.02564103	3.82342057	1.4507146	0.23108385	0.5364337	0.53236344	Lipa	1
GO:0090279	regulation of calcium ion import	1/97	39/14464	0.02564103	3.82342057	1.4507146	0.23108385	0.5364337	0.53236344	Trpv2	1
GO:0035725	sodium ion transmembrane transport	2/97	137/14464	0.01459854	2.17683799	1.13718558	0.23413113	0.53791601	0.5338345	Pcsk9/Atp1b3	2
GO:0001782	B cell homeostasis	1/97	40/14464	0.025	3.72783505	1.41950609	0.23625438	0.53791601	0.5338345	Caml	1
GO:0006220	pyrimidine nucleotide metabolic process	1/97	40/14464	0.025	3.72783505	1.41950609	0.23625438	0.53791601	0.5338345	Nme1	1
GO:0006636	unsaturated fatty acid biosynthetic process	1/97	40/14464	0.025	3.72783505	1.41950609	0.23625438	0.53791601	0.5338345	Ptges3	1
GO:0006692	prostanoid metabolic process	1/97	40/14464	0.025	3.72783505	1.41950609	0.23625438	0.53791601	0.5338345	Ptges3	1
GO:0016101	diterpenoid metabolic process	1/97	40/14464	0.025	3.72783505	1.41950609	0.23625438	0.53791601	0.5338345	Lipa	1
GO:0035196	miRNA processing	1/97	40/14464	0.025	3.72783505	1.41950609	0.23625438	0.53791601	0.5338345	Mettl3	1
GO:0036230	granulocyte activation	1/97	40/14464	0.025	3.72783505	1.41950609	0.23625438	0.53791601	0.5338345	Dnase1l3	1
GO:0043388	positive regulation of DNA binding	1/97	40/14464	0.025	3.72783505	1.41950609	0.23625438	0.53791601	0.5338345	Nme1	1
GO:0051445	regulation of meiotic cell cycle	1/97	40/14464	0.025	3.72783505	1.41950609	0.23625438	0.53791601	0.5338345	Mettl3	1
GO:0060338	regulation of type I interferon-mediated signaling pathway	1/97	40/14464	0.025	3.72783505	1.41950609	0.23625438	0.53791601	0.5338345	Mettl3	1
GO:1901016	regulation of potassium ion transmembrane transporter activity	1/97	40/14464	0.025	3.72783505	1.41950609	0.23625438	0.53791601	0.5338345	Atp1b3	1
GO:1901532	regulation of hematopoietic progenitor cell differentiation	1/97	40/14464	0.025	3.72783505	1.41950609	0.23625438	0.53791601	0.5338345	Mettl3	1
GO:0051099	positive regulation of binding	2/97	138/14464	0.01449275	2.1610638	1.12606943	0.23660921	0.53791601	0.5338345	Cln5/Nme1	2
GO:0006997	nucleus organization	2/97	139/14464	0.01438849	2.14551658	1.11504778	0.23908882	0.53791601	0.5338345	Agfg1/Golm1	2
GO:0046651	lymphocyte proliferation	3/97	253/14464	0.01185771	1.76814311	1.01279627	0.24089914	0.53791601	0.5338345	Cdkn1a/Fkbp1b/Lipa	3
GO:0008608	attachment of spindle microtubules to kinetochore	1/97	41/14464	0.02439024	3.63691225	1.38928651	0.2413905	0.53791601	0.5338345	Becn1	1
GO:0009394	2'-deoxyribonucleotide metabolic process	1/97	41/14464	0.02439024	3.63691225	1.38928651	0.2413905	0.53791601	0.5338345	Nme1	1
GO:0009395	phospholipid catabolic process	1/97	41/14464	0.02439024	3.63691225	1.38928651	0.2413905	0.53791601	0.5338345	Smpd4	1
GO:0010765	positive regulation of sodium ion transport	1/97	41/14464	0.02439024	3.63691225	1.38928651	0.2413905	0.53791601	0.5338345	Atp1b3	1
GO:0032527	protein exit from endoplasmic reticulum	1/97	41/14464	0.02439024	3.63691225	1.38928651	0.2413905	0.53791601	0.5338345	Edem1	1
GO:0043268	positive regulation of potassium ion transport	1/97	41/14464	0.02439024	3.63691225	1.38928651	0.2413905	0.53791601	0.5338345	Atp1b3	1
GO:0043507	positive regulation of JUN kinase activity	1/97	41/14464	0.02439024	3.63691225	1.38928651	0.2413905	0.53791601	0.5338345	Traf2	1
GO:0045047	protein targeting to ER	1/97	41/14464	0.02439024	3.63691225	1.38928651	0.2413905	0.53791601	0.5338345	Edem1	1
GO:0045104	intermediate filament cytoskeleton organization	1/97	41/14464	0.02439024	3.63691225	1.38928651	0.2413905	0.53791601	0.5338345	Agfg1	1
GO:0045907	positive regulation of vasoconstriction	1/97	41/14464	0.02439024	3.63691225	1.38928651	0.2413905	0.53791601	0.5338345	Shc1	1
GO:0048536	spleen development	1/97	41/14464	0.02439024	3.63691225	1.38928651	0.2413905	0.53791601	0.5338345	Lipa	1
GO:0051205	protein insertion into membrane	1/97	41/14464	0.02439024	3.63691225	1.38928651	0.2413905	0.53791601	0.5338345	Caml	1
GO:0060236	regulation of mitotic spindle organization	1/97	41/14464	0.02439024	3.63691225	1.38928651	0.2413905	0.53791601	0.5338345	Hspa1b	1
GO:0007204	positive regulation of cytosolic calcium ion concentration	2/97	140/14464	0.01428571	2.13019146	1.10411903	0.24156983	0.53791601	0.5338345	Gpr35/Fkbp1b	2
GO:0009261	ribonucleotide catabolic process	2/97	140/14464	0.01428571	2.13019146	1.10411903	0.24156983	0.53791601	0.5338345	Lipa/Nudt3	2
GO:0043405	regulation of MAP kinase activity	2/97	140/14464	0.01428571	2.13019146	1.10411903	0.24156983	0.53791601	0.5338345	Sfrp5/Traf2	2
GO:0045088	regulation of innate immune response	4/97	374/14464	0.01069519	1.59479574	0.95759125	0.24238845	0.53800769	0.53392549	Hspa1b/Sfn/Mettl3/Becn1	4
GO:0060560	developmental growth involved in morphogenesis	3/97	254/14464	0.01181102	1.76118191	1.0056348	0.24268708	0.53800769	0.53392549	Sfrp5/Trpv2/Raph1	3
GO:0006195	purine nucleotide catabolic process	2/97	141/14464	0.0141844	2.11508372	1.09328162	0.24405212	0.53800769	0.53392549	Lipa/Nudt3	2
GO:0055088	lipid homeostasis	2/97	141/14464	0.0141844	2.11508372	1.09328162	0.24405212	0.53800769	0.53392549	Pcsk9/Lipa	2
GO:0009225	nucleotide-sugar metabolic process	1/97	42/14464	0.02380952	3.5503191	1.35999852	0.24649242	0.53800769	0.53392549	Uggt1	1
GO:0019692	deoxyribose phosphate metabolic process	1/97	42/14464	0.02380952	3.5503191	1.35999852	0.24649242	0.53800769	0.53392549	Nme1	1
GO:0032790	ribosome disassembly	1/97	42/14464	0.02380952	3.5503191	1.35999852	0.24649242	0.53800769	0.53392549	Hbs1l	1
GO:0042446	hormone biosynthetic process	1/97	42/14464	0.02380952	3.5503191	1.35999852	0.24649242	0.53800769	0.53392549	Stard3	1
GO:0045103	intermediate filament-based process	1/97	42/14464	0.02380952	3.5503191	1.35999852	0.24649242	0.53800769	0.53392549	Agfg1	1
GO:0045604	regulation of epidermal cell differentiation	1/97	42/14464	0.02380952	3.5503191	1.35999852	0.24649242	0.53800769	0.53392549	Sfn	1
GO:0046627	negative regulation of insulin receptor signaling pathway	1/97	42/14464	0.02380952	3.5503191	1.35999852	0.24649242	0.53800769	0.53392549	Pip4k2c	1
GO:0070570	regulation of neuron projection regeneration	1/97	42/14464	0.02380952	3.5503191	1.35999852	0.24649242	0.53800769	0.53392549	Fkbp1b	1
GO:0086009	membrane repolarization	1/97	42/14464	0.02380952	3.5503191	1.35999852	0.24649242	0.53800769	0.53392549	Atp1b3	1
GO:1903573	negative regulation of response to endoplasmic reticulum stress	1/97	42/14464	0.02380952	3.5503191	1.35999852	0.24649242	0.53800769	0.53392549	Pdia6	1
GO:0006937	regulation of muscle contraction	2/97	142/14464	0.01408451	2.10018876	1.08253403	0.24653556	0.53800769	0.53392549	Shc1/Fkbp1b	2
GO:0009913	epidermal cell differentiation	2/97	142/14464	0.01408451	2.10018876	1.08253403	0.24653556	0.53800769	0.53392549	Cdkn1a/Sfn	2
GO:0031400	negative regulation of protein modification process	4/97	378/14464	0.01058201	1.5779196	0.93551659	0.24821846	0.53843486	0.53434942	Hspa1b/Cdkn1a/Sfrp5/Caml	4
GO:1990778	protein localization to cell periphery	4/97	378/14464	0.01058201	1.5779196	0.93551659	0.24821846	0.53843486	0.53434942	Tspan14/Sfn/Nhlrc1/Atp1b3	4
GO:0000723	telomere maintenance	2/97	143/14464	0.01398601	2.08550213	1.07187475	0.24902002	0.53843486	0.53434942	Ptges3/Cct7	2
GO:0032006	regulation of TOR signaling	2/97	143/14464	0.01398601	2.08550213	1.07187475	0.24902002	0.53843486	0.53434942	Stambpl1/Tbck	2
GO:2001233	regulation of apoptotic signaling pathway	4/97	379/14464	0.01055409	1.57375622	0.9300378	0.24968094	0.53843486	0.53434942	Hspa1b/Traf2/Becn1/Maz	4
GO:0030178	negative regulation of Wnt signaling pathway	2/97	144/14464	0.01388889	2.07101947	1.06130233	0.25150538	0.53843486	0.53434942	Sfrp5/Ctnnd1	2
GO:0009262	deoxyribonucleotide metabolic process	1/97	43/14464	0.02325581	3.46775354	1.33158942	0.25156039	0.53843486	0.53434942	Nme1	1
GO:0021696	cerebellar cortex morphogenesis	1/97	43/14464	0.02325581	3.46775354	1.33158942	0.25156039	0.53843486	0.53434942	Tuba1a	1
GO:0021872	forebrain generation of neurons	1/97	43/14464	0.02325581	3.46775354	1.33158942	0.25156039	0.53843486	0.53434942	Mettl3	1
GO:0032365	intracellular lipid transport	1/97	43/14464	0.02325581	3.46775354	1.33158942	0.25156039	0.53843486	0.53434942	Pcsk9	1
GO:0042181	ketone biosynthetic process	1/97	43/14464	0.02325581	3.46775354	1.33158942	0.25156039	0.53843486	0.53434942	Stard3	1
GO:0042771	intrinsic apoptotic signaling pathway in response to DNA damage by p53 class mediator	1/97	43/14464	0.02325581	3.46775354	1.33158942	0.25156039	0.53843486	0.53434942	Cdkn1a	1
GO:0045022	early endosome to late endosome transport	1/97	43/14464	0.02325581	3.46775354	1.33158942	0.25156039	0.53843486	0.53434942	Becn1	1
GO:0050856	regulation of T cell receptor signaling pathway	1/97	43/14464	0.02325581	3.46775354	1.33158942	0.25156039	0.53843486	0.53434942	Lipa	1
GO:0016042	lipid catabolic process	3/97	259/14464	0.01158301	1.72718226	0.9702967	0.25165977	0.53843486	0.53434942	Lipa/Smpd4/Pafah1b2	3
GO:0032943	mononuclear cell proliferation	3/97	259/14464	0.01158301	1.72718226	0.9702967	0.25165977	0.53843486	0.53434942	Cdkn1a/Fkbp1b/Lipa	3
GO:0032388	positive regulation of intracellular transport	2/97	145/14464	0.0137931	2.05673658	1.05081536	0.25399152	0.54114633	0.53704032	Sfn/Edem1	2
GO:2001235	positive regulation of apoptotic signaling pathway	2/97	146/14464	0.01369863	2.04264934	1.04041244	0.25647832	0.54114633	0.53704032	Traf2/Becn1	2
GO:0002183	cytoplasmic translational initiation	1/97	44/14464	0.02272727	3.38894096	1.30401065	0.25659462	0.54114633	0.53704032	Mettl3	1
GO:0006356	regulation of transcription by RNA polymerase I	1/97	44/14464	0.02272727	3.38894096	1.30401065	0.25659462	0.54114633	0.53704032	Ubtf	1
GO:0006767	water-soluble vitamin metabolic process	1/97	44/14464	0.02272727	3.38894096	1.30401065	0.25659462	0.54114633	0.53704032	Mmaa	1
GO:0030858	positive regulation of epithelial cell differentiation	1/97	44/14464	0.02272727	3.38894096	1.30401065	0.25659462	0.54114633	0.53704032	Sfn	1
GO:0034205	amyloid-beta formation	1/97	44/14464	0.02272727	3.38894096	1.30401065	0.25659462	0.54114633	0.53704032	Aph1b	1
GO:0043300	regulation of leukocyte degranulation	1/97	44/14464	0.02272727	3.38894096	1.30401065	0.25659462	0.54114633	0.53704032	Sphk2	1
GO:0048265	response to pain	1/97	44/14464	0.02272727	3.38894096	1.30401065	0.25659462	0.54114633	0.53704032	Vwa1	1
GO:1902116	negative regulation of organelle assembly	1/97	44/14464	0.02272727	3.38894096	1.30401065	0.25659462	0.54114633	0.53704032	Becn1	1
GO:0052547	regulation of peptidase activity	3/97	262/14464	0.01145038	1.70740537	0.94945909	0.25706784	0.54114633	0.53704032	Hspa1b/Serpinb8/Aph1b	3
GO:0043488	regulation of mRNA stability	2/97	147/14464	0.01360544	2.02875377	1.03009222	0.25896565	0.54114633	0.53704032	Mettl3/Traf2	2

GO:2001236 regulation of extrinsic apoptotic signaling pathway	2/97	147/14464	0.01360544	2.02875377	1.03009222	0.25896565	0.54114633	0.53704032 Hspa1b/Traf2	2
GO:0098813 nuclear chromosome segregation	3/97	264/14464	0.01136364	1.69447048	0.93571501	0.26068253	0.54114633	0.53704032 Hspa1b/Becn1/Smc1a	3
GO:0009411 response to UV	2/97	148/14464	0.01351351	2.01504597	1.01985338	0.2614534	0.54114633	0.53704032 Cdkn1a/Mettl3	2
GO:0002204 somatic recombination of immunoglobulin genes involved in immune response	1/97	45/14464	0.02222222	3.31363116	1.27721739	0.26159534	0.54114633	0.53704032 Lig4	1
GO:0002208 somatic diversification of immunoglobulins involved in immune response	1/97	45/14464	0.02222222	3.31363116	1.27721739	0.26159534	0.54114633	0.53704032 Lig4	1
GO:0006509 membrane protein ectodomain proteolysis	1/97	45/14464	0.02222222	3.31363116	1.27721739	0.26159534	0.54114633	0.53704032 Aph1b	1
GO:0006623 protein targeting to vacuole	1/97	45/14464	0.02222222	3.31363116	1.27721739	0.26159534	0.54114633	0.53704032 Becn1	1
GO:0009124 nucleoside monophosphate biosynthetic process	1/97	45/14464	0.02222222	3.31363116	1.27721739	0.26159534	0.54114633	0.53704032 Nme1	1
GO:0043407 negative regulation of MAP kinase activity	1/97	45/14464	0.02222222	3.31363116	1.27721739	0.26159534	0.54114633	0.53704032 Sfrp5	1
GO:0045190 isotype switching	1/97	45/14464	0.02222222	3.31363116	1.27721739	0.26159534	0.54114633	0.53704032 Lig4	1
GO:0048009 insulin-like growth factor receptor signaling pathway	1/97	45/14464	0.02222222	3.31363116	1.27721739	0.26159534	0.54114633	0.53704032 Shc1	1
GO:0072599 establishment of protein localization to endoplasmic reticulum	1/97	45/14464	0.02222222	3.31363116	1.27721739	0.26159534	0.54114633	0.53704032 Edem1	1
GO:0090224 regulation of spindle organization	1/97	45/14464	0.02222222	3.31363116	1.27721739	0.26159534	0.54114633	0.53704032 Hspa1b	1
GO:0099010 modification of postsynaptic structure	1/97	45/14464	0.02222222	3.31363116	1.27721739	0.26159534	0.54114633	0.53704032 Cap1	1
GO:1905953 negative regulation of lipid localization	1/97	45/14464	0.02222222	3.31363116	1.27721739	0.26159534	0.54114633	0.53704032 Pcsk9	1
GO:2000648 positive regulation of stem cell proliferation	1/97	45/14464	0.02222222	3.31363116	1.27721739	0.26159534	0.54114633	0.53704032 Taf4b	1
GO:0032868 response to insulin	3/97	265/14464	0.01132075	1.68807625	0.92888647	0.26249252	0.54236043	0.53824521 Shc1/Pcsk9/Pip4k2c	3
GO:0030218 erythrocyte differentiation	2/97	149/14464	0.01342282	2.00152218	1.00969462	0.26394145	0.54306726	0.53894667 Hspa1b/Ankrd54	2
GO:0032200 telomere organization	2/97	149/14464	0.01342282	2.00152218	1.00969462	0.26394145	0.54306726	0.53894667 Ptges3/Cct7	2
GO:0036294 cellular response to decreased oxygen levels	2/97	149/14464	0.01342282	2.00152218	1.00969462	0.26394145	0.54306726	0.53894667 Becn1/Mief1	2
GO:0001889 liver development	2/97	150/14464	0.01333333	1.98817869	0.99961468	0.26642969	0.54306726	0.53894667 Pcsk9/Lipa	2
GO:0006890 retrograde vesicle-mediated transport, Golgi to endoplasmic reticulum	1/97	46/14464	0.02173913	3.2415957	1.25116822	0.26656276	0.54306726	0.53894667 Bicd2	1
GO:0008340 determination of adult lifespan	1/97	46/14464	0.02173913	3.2415957	1.25116822	0.26656276	0.54306726	0.53894667 Lipa	1
GO:0033013 tetrapyrrole metabolic process	1/97	46/14464	0.02173913	3.2415957	1.25116822	0.26656276	0.54306726	0.53894667 Mmaa	1
GO:0035272 exocrine system development	1/97	46/14464	0.02173913	3.2415957	1.25116822	0.26656276	0.54306726	0.53894667 Ctnnd1	1
GO:0043303 mast cell degranulation	1/97	46/14464	0.02173913	3.2415957	1.25116822	0.26656276	0.54306726	0.53894667 Sphk2	1
GO:0046503 glycerolipid catabolic process	1/97	46/14464	0.02173913	3.2415957	1.25116822	0.26656276	0.54306726	0.53894667 Smpd4	1
GO:0071364 cellular response to epidermal growth factor stimulus	1/97	46/14464	0.02173913	3.2415957	1.25116822	0.26656276	0.54306726	0.53894667 Becn1	1
GO:1903426 regulation of reactive oxygen species biosynthetic process	1/97	46/14464	0.02173913	3.2415957	1.25116822	0.26656276	0.54306726	0.53894667 Sphk2	1
GO:0042445 hormone metabolic process	2/97	152/14464	0.01315789	1.96201845	0.97968632	0.27140628	0.54801033	0.54385223 Myt1/Stard3	2
GO:0007062 sister chromatid cohesion	1/97	47/14464	0.0212766	3.17262558	1.22582474	0.27149711	0.54801033	0.54385223 Smc1a	1
GO:0021795 cerebral cortex cell migration	1/97	47/14464	0.0212766	3.17262558	1.22582474	0.27149711	0.54801033	0.54385223 Efhc1	1
GO:0033344 cholesterol efflux	1/97	47/14464	0.0212766	3.17262558	1.22582474	0.27149711	0.54801033	0.54385223 Lipa	1
GO:0035640 exploration behavior	1/97	47/14464	0.0212766	3.17262558	1.22582474	0.27149711	0.54801033	0.54385223 Tuba1a	1
GO:0042789 mRNA transcription by RNA polymerase II	1/97	47/14464	0.0212766	3.17262558	1.22582474	0.27149711	0.54801033	0.54385223 Taf4b	1
GO:1900077 negative regulation of cellular response to insulin stimulus	1/97	47/14464	0.0212766	3.17262558	1.22582474	0.27149711	0.54801033	0.54385223 Pip4k2c	1
GO:1902459 positive regulation of stem cell population maintenance	1/97	47/14464	0.0212766	3.17262558	1.22582474	0.27149711	0.54801033	0.54385223 Sap30l	1
GO:0061008 hepaticobiliary system development	2/97	153/14464	0.0130719	1.9491948	0.96983553	0.27389441	0.55216543	0.54797581 Pcsk9/Lipa	2
GO:0009101 glycoprotein biosynthetic process	3/97	272/14464	0.01102941	1.64463311	0.88187549	0.27520691	0.55216543	0.54797581 Mlec/Uggt1/Fut11	3
GO:1904375 regulation of protein localization to cell periphery	2/97	154/14464	0.01298701	1.93653769	0.96005877	0.27638228	0.55216543	0.54797581 Sfn/Nhlrc1	2
GO:0002448 mast cell mediated immunity	1/97	48/14464	0.02083333	3.10652921	1.20115134	0.2763986	0.55216543	0.54797581 Sphk2	1
GO:0006911 phagocytosis, engulfment	1/97	48/14464	0.02083333	3.10652921	1.20115134	0.2763986	0.55216543	0.54797581 Becn1	1
GO:0009409 response to cold	1/97	48/14464	0.02083333	3.10652921	1.20115134	0.2763986	0.55216543	0.54797581 Lipa	1
GO:0045682 regulation of epidermis development	1/97	48/14464	0.02083333	3.10652921	1.20115134	0.2763986	0.55216543	0.54797581 Sfn	1
GO:0045815 transcription initiation-coupled chromatin remodeling	1/97	48/14464	0.02083333	3.10652921	1.20115134	0.2763986	0.55216543	0.54797581 Sphk2	1
GO:0051341 regulation of oxidoreductase activity	1/97	48/14464	0.02083333	3.10652921	1.20115134	0.2763986	0.55216543	0.54797581 Sphk2	1
GO:0043086 negative regulation of catalytic activity	4/97	398/14464	0.01005025	1.49862716	0.82882754	0.27780308	0.55243184	0.5482402 Hspa1b/Cdkn1a/Sfrp5/Serpinb8	4
GO:0001508 action potential	2/97	155/14464	0.01290323	1.9240439	0.95035491	0.27886979	0.55243184	0.5482402 Gpr35/Fkbp1b	2
GO:0050777 negative regulation of immune response	2/97	155/14464	0.01290323	1.9240439	0.95035491	0.27886979	0.55243184	0.5482402 Sfn/Mettl3	2
GO:0071333 cellular response to glucose stimulus	2/97	155/14464	0.01290323	1.9240439	0.95035491	0.27886979	0.55243184	0.5482402 Myt1/Fkbp1b	2
GO:0000266 mitochondrial fission	1/97	49/14464	0.02040816	3.04313065	1.17711494	0.28126745	0.55243184	0.5482402 Mief1	1
GO:0006721 terpenoid metabolic process	1/97	49/14464	0.02040816	3.04313065	1.17711494	0.28126745	0.55243184	0.5482402 Lipa	1
GO:0032757 positive regulation of interleukin-8 production	1/97	49/14464	0.02040816	3.04313065	1.17711494	0.28126745	0.55243184	0.5482402 Hspa1b	1
GO:0060074 synapse maturation	1/97	49/14464	0.02040816	3.04313065	1.17711494	0.28126745	0.55243184	0.5482402 Nrg2	1
GO:0098927 vesicle-mediated transport between endosomal compartments	1/97	49/14464	0.02040816	3.04313065	1.17711494	0.28126745	0.55243184	0.5482402 Becn1	1
GO:0007613 memory	2/97	156/14464	0.01282051	1.91171028	0.94072287	0.28135683	0.55243184	0.5482402 Tuba1a/Aph1b	2
GO:0007281 germ cell development	3/97	276/14464	0.01086957	1.62079785	0.85561062	0.28250281	0.55243184	0.5482402 Agfg1/Mettl3/Taf4b	3
GO:0002700 regulation of production of molecular mediator of immune response	2/97	157/14464	0.01273885	1.89953378	0.93116154	0.2838433	0.55243184	0.5482402 Traf2/Sphk2	2
GO:0002753 cytoplasmic pattern recognition receptor signaling pathway	2/97	157/14464	0.01273885	1.89953378	0.93116154	0.2838433	0.55243184	0.5482402 Hspa1b/Becn1	2
GO:0072523 purine-containing compound catabolic process	2/97	157/14464	0.01273885	1.89953378	0.93116154	0.2838433	0.55243184	0.5482402 Lipa/Nudt3	2
GO:1990845 adaptive thermogenesis	2/97	157/14464	0.01273885	1.89953378	0.93116154	0.2838433	0.55243184	0.5482402 Trpv2/Lipa	2
GO:2000058 regulation of ubiquitin-dependent protein catabolic process	2/97	157/14464	0.01273885	1.89953378	0.93116154	0.2838433	0.55243184	0.5482402 Hspa1b/Caml	2
GO:0032386 regulation of intracellular transport	3/97	277/14464	0.01083032	1.61494659	0.84911011	0.28432977	0.55243184	0.5482402 Sfn/Pcsk9/Edem1	3
GO:0002279 mast cell activation involved in immune response	1/97	50/14464	0.02	2.98226804	1.15368471	0.28610387	0.55243184	0.5482402 Sphk2	1
GO:0002762 negative regulation of myeloid leukocyte differentiation	1/97	50/14464	0.02	2.98226804	1.15368471	0.28610387	0.55243184	0.5482402 Nme1	1
GO:0006826 iron ion transport	1/97	50/14464	0.02	2.98226804	1.15368471	0.28610387	0.55243184	0.5482402 Abcc5	1
GO:0032371 regulation of sterol transport	1/97	50/14464	0.02	2.98226804	1.15368471	0.28610387	0.55243184	0.5482402 Pcsk9	1
GO:0032374 regulation of cholesterol transport	1/97	50/14464	0.02	2.98226804	1.15368471	0.28610387	0.55243184	0.5482402 Pcsk9	1
GO:0032663 regulation of interleukin-2 production	1/97	50/14464	0.02	2.98226804	1.15368471	0.28610387	0.55243184	0.5482402 Traf2	1
GO:0033047 regulation of mitotic sister chromatid segregation	1/97	50/14464	0.02	2.98226804	1.15368471	0.28610387	0.55243184	0.5482402 Becn1	1
GO:0043154 negative regulation of cysteine-type endopeptidase activity involved in apoptotic process	1/97	50/14464	0.02	2.98226804	1.15368471	0.28610387	0.55243184	0.5482402 Hspa1b	1
GO:0070849 response to epidermal growth factor	1/97	50/14464	0.02	2.98226804	1.15368471	0.28610387	0.55243184	0.5482402 Becn1	1
GO:0070918 regulatory ncRNA processing	1/97	50/14464	0.02	2.98226804	1.15368471	0.28610387	0.55243184	0.5482402 Mettl3	1
GO:0097300 programmed necrotic cell death	1/97	50/14464	0.02	2.98226804	1.15368471	0.28610387	0.55243184	0.5482402 Traf2	1
GO:0006605 protein targeting	3/97	278/14464	0.01079137	1.60913743	0.84263544	0.28615782	0.55243184	0.5482402 Edem1/Becn1/Mief1	3
GO:0009166 nucleotide catabolic process	2/97	158/14464	0.01265823	1.88751142	0.92166988	0.28632909	0.55243184	0.5482402 Lipa/Nudt3	2
GO:0071331 cellular response to hexose stimulus	2/97	158/14464	0.01265823	1.88751142	0.92166988	0.28632909	0.55243184	0.5482402 Myt1/Fkbp1b	2
GO:0071326 cellular response to monosaccharide stimulus	2/97	159/14464	0.01257862	1.87564028	0.91224686	0.2888141	0.55599895	0.55178024 Myt1/Fkbp1b	2
GO:0097553 calcium ion transmembrane import into cytosol	2/97	159/14464	0.01257862	1.87564028	0.91224686	0.2888141	0.55599895	0.55178024 Trpv2/Fkbp1b	2
GO:0002711 positive regulation of T cell mediated immunity	1/97	51/14464	0.01960784	2.9237922	1.13083194	0.29090808	0.55696312	0.5527371 Traf2	1
GO:0009299 mRNA transcription	1/97	51/14464	0.01960784	2.9237922	1.13083194	0.29090808	0.55696312	0.5527371 Taf4b	1
GO:0034605 cellular response to heat	1/97	51/14464	0.01960784	2.9237922	1.13083194	0.29090808	0.55696312	0.5527371 Hspa1b	1
GO:0046676 negative regulation of insulin secretion	1/97	51/14464	0.01960784	2.9237922	1.13083194	0.29090808	0.55696312	0.5527371 Fkbp1b	1
GO:0141137 positive regulation of gene expression, epigenetic	1/97	51/14464	0.01960784	2.9237922	1.13083194	0.29090808	0.55696312	0.5527371 Sphk2	1
GO:0070507 regulation of microtubule cytoskeleton organization	2/97	160/14464	0.0125	1.86391753	0.90289145	0.29129823	0.5570999	0.55287284 Hspa1b/Bicd2	2
GO:0048732 gland development	4/97	408/14464	0.00980392	1.4618961	0.77763449	0.29281873	0.55939578	0.5551513 Pcsk9/Lipa/Ctnnd1/Nme1	4
GO:0006469 negative regulation of protein kinase activity	2/97	161/14464	0.01242236	1.8523404	0.89360267	0.29378138	0.55940072	0.5551562 Cdkn1a/Sfrp5	2
GO:0033157 regulation of intracellular protein transport	2/97	161/14464	0.01242236	1.8523404	0.89360267	0.29378138	0.55940072	0.5551562 Sfn/Edem1	2
GO:0061013 regulation of mRNA catabolic process	2/97	161/14464	0.01242236	1.8523404	0.89360267	0.29378138	0.55940072	0.5551562 Mettl3/Traf2	2
GO:0021587 cerebellum morphogenesis	1/97	52/14464	0.01923077	2.86756542	1.10852983	0.2956803	0.56046377	0.55621119 Tuba1a	1
GO:0032387 negative regulation of intracellular transport	1/97	52/14464	0.01923077	2.86756542	1.10852983	0.2956803	0.56046377	0.55621119 Pcsk9	1
GO:0044818 mitotic G2/M transition checkpoint	1/97	52/14464	0.01923077	2.86756542	1.10852983	0.2956803	0.56046377	0.55621119 Cdkn1a	1
GO:0045773 positive regulation of axon extension	1/97	52/14464	0.01923077	2.86756542	1.10852983	0.2956803	0.56046377	0.55621119 Trpv2	1
GO:0034101 erythrocyte homeostasis	2/97	162/14464	0.01234568	1.8409062	0.88437954	0.29626346	0.56046377	0.55621119 Hspa1b/Ankrd54	2
GO:0043487 regulation of RNA stability	2/97	162/14464	0.01234568	1.8409062	0.88437954	0.29626346	0.56046377	0.55621119 Mettl3/Traf2	2
GO:0006024 glycosaminoglycan biosynthetic process	1/97	53/14464	0.01886792	2.81346042	1.08675331	0.30042072	0.56526956	0.56098051 Abcc5	1
GO:0042149 cellular response to glucose starvation	1/97	53/14464	0.01886792	2.81346042	1.08675331	0.30042072	0.56526956	0.56098051 Becn1	1
GO:0046578 regulation of Ras protein signal transduction	1/97	53/14464	0.01886792	2.81346042	1.08675331	0.30042072	0.56526956	0.56098051 Rasgef1a	1
GO:0070527 platelet aggregation	1/97	53/14464	0.01886792	2.81346042	1.08675331	0.30042072	0.56526956	0.56098051 Pdia6	1
GO:0099563 modification of synaptic structure	1/97	53/14464	0.01886792	2.81346042	1.08675331	0.30042072	0.56526956	0.56098051 Cap1	1
GO:1901293 nucleoside phosphate biosynthetic process	3/97	286/14464	0.01048951	1.5641266	0.79174279	0.30081587	0.56540445	0.56111438 Lipa/Sphk2/Nme1	3
GO:0070661 leukocyte proliferation	3/97	287/14464	0.01045296	1.55867668	0.78549122	0.30265163	0.56801543	0.56370555 Cdkn1a/Fkbp1b/Lipa	3
GO:0072594 establishment of protein localization to organelle	4/97	415/14464	0.00963855	1.43723761	0.74259303	0.30339787	0.56801543	0.56370555 Cdkn1a/Edem1/Becn1/Cct7	4
GO:0071453 cellular response to oxygen levels	2/97	165/14464	0.01212121	1.80743518	0.85709472	0.30370227	0.56801543	0.56370555 Becn1/Mief1	2
GO:0048285 organelle fission	4/97	416/14464	0.00961538	1.43378271	0.73763866	0.3049131	0.56801543	0.56370555 Hspa1b/Becn1/Mief1/Smc1a	4
GO:0002381 immunoglobulin production involved in immunoglobulin-mediated immune response	1/97	54/14464	0.01851852	2.7613593	1.06547892	0.30512957	0.56801543	0.56370555 Lig4	1
GO:0021575 hindbrain morphogenesis	1/97	54/14464	0.01851852	2.7613593	1.06547892	0.30512957	0.56801543	0.56370555 Tuba1a	1
GO:0035567 non-canonical Wnt signaling pathway	1/97	54/14464	0.01851852	2.7613593	1.06547892	0.30512957	0.56801543	0.56370555 Sfrp5	1
GO:0046173 polyol biosynthetic process	1/97	54/14464	0.01851852	2.7613593	1.06547892	0.30512957	0.56801543	0.56370555 Sphk2	1
GO:0061462 protein localization to lysosome	1/97	54/14464	0.01851852	2.7613593	1.06547892	0.30512957	0.56801543	0.56370555 Becn1	1
GO:0050673 epithelial cell proliferation	4/97	417/14464	0.00959233	1.43034438	0.73269696	0.30642924	0.569828	0.56550436 Cdkn1a/Sfn/Lipa/Nme1	4
GO:0070588 calcium ion transmembrane transport	3/97	290/14464	0.01034483	1.54255244	0.76687842	0.30816249	0.57004497	0.56571968 Gpr35/Trpv2/Fkbp1b	3
GO:0098739 import across plasma membrane	2/97	167/14464	0.01197605	1.78578925	0.83921623	0.30865436	0.57004497	0.56571968 Trpv2/Atp1b3	2
GO:1903169 regulation of calcium ion transmembrane transport	2/97	167/14464	0.01197605	1.78578925	0.83921623	0.30865436	0.57004497	0.56571968 Gpr35/Fkbp1b	2
GO:0007080 mitotic metaphase chromosome alignment	1/97	55/14464	0.01818182	2.71115276	1.04468467	0.30980705	0.57004497	0.56571968 Becn1	1
GO:0019228 neuronal action potential	1/97	55/14464	0.01818182	2.71115276	1.04468467	0.30980705	0.57004497	0.56571968 Gpr35	1
GO:0072527 pyrimidine-containing compound metabolic process	1/97	55/14464	0.01818182	2.71115276	1.04468467	0.30980705	0.57004497	0.56571968 Nme1	1
GO:1901799 negative regulation of proteasomal protein catabolic process	1/97	55/14464	0.01818182	2.71115276	1.04468467	0.30980705	0.57004497	0.56571968 Caml	1
GO:1904263 positive regulation of TORC1 signaling	1/97	55/14464	0.01818182	2.71115276	1.04468467	0.30980705	0.57004497	0.56571968 Stambpl1	1
GO:1905517 macrophage migration	1/97	55/14464	0.01818182	2.71115276	1.04468467	0.30980705	0.57004497	0.56571968 Gpr35	1
GO:2000059 negative regulation of ubiquitin-dependent protein catabolic process	1/97	55/14464	0.01818182	2.71115276	1.04468467	0.30980705	0.57004497	0.56571968 Caml	1
GO:0007264 small GTPase-mediated signal transduction	4/97	420/14464	0.00952381	1.42012764	0.71794719	0.31098281	0.57127284	0.56693824 Cdkn1a/Rab39/Lipa/Rasgef1a	4
GO:0000910 cytokinesis	2/97	168/14464	0.01190476	1.77515955	0.83036777	0.311128	0.57127284	0.56693824 Efhc1/Becn1	2
GO:0071375 cellular response to peptide hormone stimulus	3/97	292/14464	0.01027397	1.53198701	0.75458626	0.31183891	0.57137679	0.5670414 Shc1/Pcsk9/Pip4k2c	3
GO:0038066 p38MAPK cascade	1/97	56/14464	0.01785714	2.66273932	1.02434994	0.31445336	0.57137679	0.5670414 Becn1	1
GO:0042987 amyloid precursor protein catabolic process	1/97	56/14464	0.01785714	2.66273932	1.02434994	0.31445336	0.57137679	0.5670414 Aph1b	1
GO:0043462 regulation of ATP-dependent activity	1/97	56/14464	0.01785714	2.66273932	1.02434994	0.31445336	0.57137679	0.5670414 Atp1b3	1
GO:0048260 positive regulation of receptor-mediated endocytosis	1/97	56/14464	0.01785714	2.66273932	1.02434994	0.31445336	0.57137679	0.5670414 Pcsk9	1
GO:0050854 regulation of antigen receptor-mediated signaling pathway	1/97	56/14464	0.01785714	2.66273932	1.02434994	0.31445336	0.57137679	0.5670414 Lipa	1
GO:0072348 sulfur compound transport	1/97	56/14464	0.01785714	2.66273932	1.02434994	0.31445336	0.57137679	0.5670414 Abcc5	1
GO:1903307 positive regulation of regulated secretory pathway	1/97	56/14464	0.01785714	2.66273932	1.02434994	0.31445336	0.57137679	0.5670414 Sphk2	1
GO:1903533 regulation of protein targeting	1/97	56/14464	0.01785714	2.66273932	1.02434994	0.31445336	0.57137679	0.5670414 Mief1	1
GO:2001238 positive regulation of extrinsic apoptotic signaling pathway	1/97	56/14464	0.01785714	2.66273932	1.02434994	0.31445336	0.57137679	0.5670414 Traf2	1
GO:0007283 spermatogenesis	4/97	423/14464	0.00945626	1.41005581	0.70330896	0.31554355	0.57203841	0.567698 Agfg1/Mettl3/Pafah1b2/Taf4b	4
GO:0022412 cellular process involved in reproduction in multicellular organism	3/97	295/14464	0.01016949	1.51640748	0.7363189	0.31735645	0.57203841	0.567698 Agfg1/Mettl3/Taf4b	3
GO:0001678 intracellular glucose homeostasis	2/97	171/14464	0.01169591	1.7440164	0.80417558	0.31853822	0.57203841	0.567698 Myt1/Fkbp1b	2
GO:0071322 cellular response to carbohydrate stimulus	2/97	171/14464	0.01169591	1.7440164	0.80417558	0.31853822	0.57203841	0.567698 Myt1/Fkbp1b	2
GO:0031113 regulation of microtubule polymerization	1/97	57/14464	0.01754386	2.6160246	1.00445534	0.31906871	0.57203841	0.567698 Hspa1b	1
GO:0032623 interleukin-2 production	1/97	57/14464	0.01754386	2.6160246	1.00445534	0.31906871	0.57203841	0.567698 Traf2	1
GO:0033363 secretory granule organization	1/97	57/14464	0.01754386	2.6160246	1.00445534	0.31906871	0.57203841	0.567698 Agfg1	1
GO:0033619 membrane protein proteolysis	1/97	57/14464	0.01754386	2.6160246	1.00445534	0.31906871	0.57203841	0.567698 Aph1b	1
GO:0048286 lung alveolus development	1/97	57/14464	0.01754386	2.6160246	1.00445534	0.31906871	0.57203841	0.567698 Ptges3	1
GO:0051123 RNA polymerase II preinitiation complex assembly	1/97	57/14464	0.01754386	2.6160246	1.00445534	0.31906871	0.57203841	0.567698 Taf4b	1
GO:0099024 plasma membrane invagination	1/97	57/14464	0.01754386	2.6160246	1.00445534	0.31906871	0.57203841	0.567698 Becn1	1
GO:0045862 positive regulation of proteolysis	3/97	296/14464	0.01013514	1.51128448	0.73027462	0.31919623	0.57203841	0.567698 Hspa1b/Ctnnd1/Aph1b	3
GO:0048660 regulation of smooth muscle cell proliferation	2/97	172/14464	0.01162791	1.73387677	0.79555988	0.32100444	0.57203841	0.567698 Shc1/Cdkn1a	2
GO:0030099 myeloid cell differentiation	4/97	428/14464	0.00934579	1.3935832	0.67915479	0.3231588	0.57203841	0.567698 Hspa1b/Lipa/Ankrd54/Nme1	4
GO:0030307 positive regulation of cell growth	2/97	173/14464	0.01156069	1.72385436	0.7870004	0.3234686	0.57203841	0.567698 Sfn/Trpv2	2
GO:0033673 negative regulation of kinase activity	2/97	173/14464	0.01156069	1.72385436	0.7870004	0.3234686	0.57203841	0.567698 Cdkn1a/Sfrp5	2
GO:0043393 regulation of protein binding	2/97	173/14464	0.01156069	1.72385436	0.7870004	0.3234686	0.57203841	0.567698 Cdkn1a/Pcsk9	2
GO:0006023 aminoglycan biosynthetic process	1/97	58/14464	0.01724138	2.57092073	0.98498264	0.32365331	0.57203841	0.567698 Abcc5	1
GO:0006805 xenobiotic metabolic process	1/97	58/14464	0.01724138	2.57092073	0.98498264	0.32365331	0.57203841	0.567698 Cyp2j13	1
GO:0006940 regulation of smooth muscle contraction	1/97	58/14464	0.01724138	2.57092073	0.98498264	0.32365331	0.57203841	0.567698 Shc1	1
GO:0055117 regulation of cardiac muscle contraction	1/97	58/14464	0.01724138	2.57092073	0.98498264	0.32365331	0.57203841	0.567698 Fkbp1b	1
GO:0060337 type I interferon-mediated signaling pathway	1/97	58/14464	0.01724138	2.57092073	0.98498264	0.32365331	0.57203841	0.567698 Mettl3	1
GO:0070613 regulation of protein processing	1/97	58/14464	0.01724138	2.57092073	0.98498264	0.32365331	0.57203841	0.567698 Ctnnd1	1
GO:0071357 cellular response to type I interferon	1/97	58/14464	0.01724138	2.57092073	0.98498264	0.32365331	0.57203841	0.567698 Mettl3	1
GO:0072666 establishment of protein localization to vacuole	1/97	58/14464	0.01724138	2.57092073	0.98498264	0.32365331	0.57203841	0.567698 Becn1	1
GO:0090278 negative regulation of peptide hormone secretion	1/97	58/14464	0.01724138	2.57092073	0.98498264	0.32365331	0.57203841	0.567698 Fkbp1b	1
GO:2000117 negative regulation of cysteine-type endopeptidase activity	1/97	58/14464	0.01724138	2.57092073	0.98498264	0.32365331	0.57203841	0.567698 Hspa1b	1
GO:0006903 vesicle targeting	1/97	59/14464	0.01694915	2.5273458	0.96591467	0.32820736	0.5746137	0.57025375 Stard3	1
GO:0022029 telencephalon cell migration	1/97	59/14464	0.01694915	2.5273458	0.96591467	0.32820736	0.5746137	0.57025375 Efhc1	1
GO:0031103 axon regeneration	1/97	59/14464	0.01694915	2.5273458	0.96591467	0.32820736	0.5746137	0.57025375 Fkbp1b	1
GO:0048255 mRNA stabilization	1/97	59/14464	0.01694915	2.5273458	0.96591467	0.32820736	0.5746137	0.57025375 Traf2	1
GO:0070231 T cell apoptotic process	1/97	59/14464	0.01694915	2.5273458	0.96591467	0.32820736	0.5746137	0.57025375 Lipa	1
GO:0098900 regulation of action potential	1/97	59/14464	0.01694915	2.5273458	0.96591467	0.32820736	0.5746137	0.57025375 Gpr35	1

GO:2000134	negative regulation of G1/S transition of mitotic cell cycle	1/97	59/14464	0.01694915	2.5273458	0.96591467	0.32820736	0.5746137	0.57025375	Cdkn1a	1
GO:0021543	pallium development	2/97	175/14464	0.01142857	1.70415317	0.77004704	0.32839038	0.5746137	0.57025375	Efhc1/Tuba1a	2
GO:0071214	cellular response to abiotic stimulus	3/97	301/14464	0.00996678	1.48618009	0.70038172	0.32839765	0.5746137	0.57025375	Cdkn1a/Mettl3/Lig4	3
GO:0104004	cellular response to environmental stimulus	3/97	301/14464	0.00996678	1.48618009	0.70038172	0.32839765	0.5746137	0.57025375	Cdkn1a/Mettl3/Lig4	3
GO:0097193	intrinsic apoptotic signaling pathway	3/97	302/14464	0.00993377	1.48125896	0.69446765	0.33023814	0.57716769	0.57278836	Cdkn1a/Sfn/Becn1	3
GO:0048284	organelle fusion	2/97	176/14464	0.01136364	1.69447048	0.7616517	0.33084784	0.57716769	0.57278836	Rab39/Mief1	2
GO:1901292	nucleoside phosphate catabolic process	2/97	176/14464	0.01136364	1.69447048	0.7616517	0.33084784	0.57716769	0.57278836	Lipa/Nudt3	2
GO:0002792	negative regulation of peptide secretion	1/97	60/14464	0.01666667	2.48522337	0.94723526	0.33273106	0.57814503	0.57375828	Fkbp1b	1
GO:0019933	cAMP-mediated signaling	1/97	60/14464	0.01666667	2.48522337	0.94723526	0.33273106	0.57814503	0.57375828	Cap1	1
GO:0032147	activation of protein kinase activity	1/97	60/14464	0.01666667	2.48522337	0.94723526	0.33273106	0.57814503	0.57375828	Traf2	1
GO:0048662	negative regulation of smooth muscle cell proliferation	1/97	60/14464	0.01666667	2.48522337	0.94723526	0.33273106	0.57814503	0.57375828	Cdkn1a	1
GO:0010970	transport along microtubule	2/97	177/14464	0.01129944	1.6848972	0.75330962	0.33330292	0.57856355	0.57417363	Bicd2/Tuba1a	2
GO:0042391	regulation of membrane potential	4/97	437/14464	0.00915332	1.3648824	0.63641916	0.33690122	0.58187338	0.57745835	Gpr35/Fkbp1b/Lipa/Atp1b3	4
GO:0048232	male gamete generation	4/97	437/14464	0.00915332	1.3648824	0.63641916	0.33690122	0.58187338	0.57745835	Agfg1/Mettl3/Pafah1b2/Taf4b	4
GO:0009247	glycolipid biosynthetic process	1/97	61/14464	0.01639344	2.444482	0.92892914	0.33722461	0.58187338	0.57745835	Pigc	1
GO:0021885	forebrain cell migration	1/97	61/14464	0.01639344	2.444482	0.92892914	0.33722461	0.58187338	0.57745835	Efhc1	1
GO:0030888	regulation of B cell proliferation	1/97	61/14464	0.01639344	2.444482	0.92892914	0.33722461	0.58187338	0.57745835	Cdkn1a	1
GO:0032677	regulation of interleukin-8 production	1/97	61/14464	0.01639344	2.444482	0.92892914	0.33722461	0.58187338	0.57745835	Hspa1b	1
GO:0050796	regulation of insulin secretion	2/97	179/14464	0.01117318	1.66607153	0.73678246	0.33820558	0.58187338	0.57745835	Myt1/Fkbp1b	2
GO:0051091	positive regulation of DNA-binding transcription factor activity	2/97	179/14464	0.01117318	1.66607153	0.73678246	0.33820558	0.58187338	0.57745835	Hspa1b/Traf2	2
GO:0051346	negative regulation of hydrolase activity	2/97	179/14464	0.01117318	1.66607153	0.73678246	0.33820558	0.58187338	0.57745835	Hspa1b/Serpinb8	2
GO:0048659	smooth muscle cell proliferation	2/97	180/14464	0.01111111	1.65681558	0.72859601	0.34065302	0.58213547	0.57771845	Shc1/Cdkn1a	2
GO:0032102	negative regulation of response to external stimulus	3/97	308/14464	0.00974026	1.45240327	0.65942108	0.34127908	0.58213547	0.57771845	Sfn/Mettl3/Aph1b	3
GO:0051090	regulation of DNA-binding transcription factor activity	3/97	308/14464	0.00974026	1.45240327	0.65942108	0.34127908	0.58213547	0.57771845	Hspa1b/Commd7/Traf2	3
GO:0006611	protein export from nucleus	1/97	62/14464	0.01612903	2.40505487	0.91098188	0.34168821	0.58213547	0.57771845	Sfn	1
GO:0006801	superoxide metabolic process	1/97	62/14464	0.01612903	2.40505487	0.91098188	0.34168821	0.58213547	0.57771845	Shc1	1
GO:0021695	cerebellar cortex development	1/97	62/14464	0.01612903	2.40505487	0.91098188	0.34168821	0.58213547	0.57771845	Tuba1a	1
GO:0031016	pancreas development	1/97	62/14464	0.01612903	2.40505487	0.91098188	0.34168821	0.58213547	0.57771845	Myt1	1
GO:0032637	interleukin-8 production	1/97	62/14464	0.01612903	2.40505487	0.91098188	0.34168821	0.58213547	0.57771845	Hspa1b	1
GO:1903317	regulation of protein maturation	1/97	62/14464	0.01612903	2.40505487	0.91098188	0.34168821	0.58213547	0.57771845	Ctnnd1	1
GO:2001244	positive regulation of intrinsic apoptotic signaling pathway	1/97	62/14464	0.01612903	2.40505487	0.91098188	0.34168821	0.58213547	0.57771845	Becn1	1
GO:0016236	macroautophagy	3/97	309/14464	0.00970874	1.44770293	0.65365149	0.34311852	0.5840031	0.5795719	Becn1/Pafah1b2/Pip4k2c	3
GO:0071805	potassium ion transmembrane transport	2/97	182/14464	0.01098901	1.63860881	0.71237407	0.34553974	0.58569347	0.58124945	Kcnk10/Atp1b3	2
GO:0001836	release of cytochrome c from mitochondria	1/97	63/14464	0.01587302	2.3668794	0.89337987	0.34612206	0.58569347	0.58124945	Sfn	1
GO:0051898	negative regulation of phosphatidylinositol 3-kinase/protein kinase B signal transduction	1/97	63/14464	0.01587302	2.3668794	0.89337987	0.34612206	0.58569347	0.58124945	Sfrp5	1
GO:0097192	extrinsic apoptotic signaling pathway in absence of ligand	1/97	63/14464	0.01587302	2.3668794	0.89337987	0.34612206	0.58569347	0.58124945	Hspa1b	1
GO:0097352	autophagosome maturation	1/97	63/14464	0.01587302	2.3668794	0.89337987	0.34612206	0.58569347	0.58124945	Becn1	1
GO:0170041	non-proteinogenic amino acid metabolic process	1/97	63/14464	0.01587302	2.3668794	0.89337987	0.34612206	0.58569347	0.58124945	P4ha1	1
GO:0010038	response to metal ion	3/97	311/14464	0.0096463	1.43839295	0.64217222	0.34679643	0.58626708	0.58181871	Tuba1a/Lig4/Becn1	3
GO:0016051	carbohydrate biosynthetic process	2/97	183/14464	0.01092896	1.62965467	0.70433728	0.34797887	0.58713038	0.58267546	Ptges3/Nhlrc1	2
GO:1990138	neuron projection extension	2/97	183/14464	0.01092896	1.62965467	0.70433728	0.34797887	0.58713038	0.58267546	Trpv2/Raph1	2
GO:0061136	regulation of proteasomal protein catabolic process	2/97	184/14464	0.01086957	1.62079785	0.69634911	0.35041509	0.58883415	0.5843663	Hspa1b/Caml	2
GO:0002637	regulation of immunoglobulin production	1/97	64/14464	0.015625	2.32989691	0.87611019	0.35052634	0.58883415	0.5843663	Traf2	1
GO:0010972	negative regulation of G2/M transition of mitotic cell cycle	1/97	64/14464	0.015625	2.32989691	0.87611019	0.35052634	0.58883415	0.5843663	Cdkn1a	1
GO:0062208	positive regulation of pattern recognition receptor signaling pathway	1/97	64/14464	0.015625	2.32989691	0.87611019	0.35052634	0.58883415	0.5843663	Hspa1b	1
GO:0002831	regulation of response to biotic stimulus	4/97	446/14464	0.00896861	1.33733993	0.59459899	0.35067297	0.58883415	0.5843663	Hspa1b/Sfn/Mettl3/Becn1	4
GO:1903131	mononuclear cell differentiation	4/97	447/14464	0.00894855	1.33434812	0.59000694	0.3522042	0.59021903	0.58574067	Lipa/Mettl3/Lig4/Nhej1	4
GO:0042886	amide transport	3/97	314/14464	0.00955414	1.42465034	0.62510085	0.35231027	0.59021903	0.58574067	Myt1/Abcc5/Fkbp1b	3
GO:0006814	sodium ion transport	2/97	185/14464	0.01081081	1.61203678	0.68840894	0.35284833	0.59021903	0.58574067	Pcsk9/Atp1b3	2
GO:0042098	T cell proliferation	2/97	185/14464	0.01081081	1.61203678	0.68840894	0.35284833	0.59021903	0.58574067	Fkbp1b/Lipa	2
GO:0001910	regulation of leukocyte mediated cytotoxicity	1/97	65/14464	0.01538462	2.29405234	0.85916063	0.35490127	0.59026396	0.58578526	Dnase1l3	1
GO:0010324	membrane invagination	1/97	65/14464	0.01538462	2.29405234	0.85916063	0.35490127	0.59026396	0.58578526	Becn1	1
GO:0034340	response to type I interferon	1/97	65/14464	0.01538462	2.29405234	0.85916063	0.35490127	0.59026396	0.58578526	Mettl3	1
GO:0038034	signal transduction in absence of ligand	1/97	65/14464	0.01538462	2.29405234	0.85916063	0.35490127	0.59026396	0.58578526	Hspa1b	1
GO:0045576	mast cell activation	1/97	65/14464	0.01538462	2.29405234	0.85916063	0.35490127	0.59026396	0.58578526	Sphk2	1
GO:0060261	positive regulation of transcription initiation by RNA polymerase II	1/97	65/14464	0.01538462	2.29405234	0.85916063	0.35490127	0.59026396	0.58578526	Taf4b	1
GO:0006091	generation of precursor metabolites and energy	4/97	449/14464	0.00890869	1.32840447	0.58085495	0.35526698	0.59031054	0.58583149	Lipa/Ptges3/Nhlrc1/Sphk2	4
GO:0034248	regulation of amide metabolic process	1/97	66/14464	0.01515152	2.25929397	0.84251962	0.35924703	0.59282607	0.58832793	Sphk2	1
GO:0060976	coronary vasculature development	1/97	66/14464	0.01515152	2.25929397	0.84251962	0.35924703	0.59282607	0.58832793	Dctn5	1
GO:0070897	transcription preinitiation complex assembly	1/97	66/14464	0.01515152	2.25929397	0.84251962	0.35924703	0.59282607	0.58832793	Taf4b	1
GO:0140962	multicellular organismal-level chemical homeostasis	1/97	66/14464	0.01515152	2.25929397	0.84251962	0.35924703	0.59282607	0.58832793	Lipa	1
GO:1902373	negative regulation of mRNA catabolic process	1/97	66/14464	0.01515152	2.25929397	0.84251962	0.35924703	0.59282607	0.58832793	Traf2	1
GO:1902750	negative regulation of cell cycle G2/M phase transition	1/97	66/14464	0.01515152	2.25929397	0.84251962	0.35924703	0.59282607	0.58832793	Cdkn1a	1
GO:0030522	intracellular receptor signaling pathway	3/97	318/14464	0.00943396	1.40673021	0.60260839	0.35965479	0.59282607	0.58832793	Hspa1b/Ptges3/Becn1	3
GO:0051223	regulation of protein transport	4/97	452/14464	0.00884956	1.31958763	0.56720641	0.35986163	0.59282607	0.58832793	Sfn/Myt1/Fkbp1b/Edem1	4
GO:0050905	neuromuscular process	2/97	188/14464	0.0106383	1.58631279	0.66487046	0.36012946	0.59282607	0.58832793	Tuba1a/Aph1b	2
GO:0002697	regulation of immune effector process	3/97	320/14464	0.009375	1.39793814	0.59147508	0.36332326	0.59282607	0.58832793	Dnase1l3/Traf2/Sphk2	3
GO:0001510	RNA methylation	1/97	67/14464	0.01492537	2.22557317	0.82617616	0.36356381	0.59282607	0.58832793	Mettl3	1
GO:0030330	DNA damage response, signal transduction by p53 class mediator	1/97	67/14464	0.01492537	2.22557317	0.82617616	0.36356381	0.59282607	0.58832793	Cdkn1a	1
GO:0032088	negative regulation of NF-kappaB transcription factor activity	1/97	67/14464	0.01492537	2.22557317	0.82617616	0.36356381	0.59282607	0.58832793	Commd7	1
GO:0035904	aorta development	1/97	67/14464	0.01492537	2.22557317	0.82617616	0.36356381	0.59282607	0.58832793	Dctn5	1
GO:0042246	tissue regeneration	1/97	67/14464	0.01492537	2.22557317	0.82617616	0.36356381	0.59282607	0.58832793	Cdkn1a	1
GO:0060612	adipose tissue development	1/97	67/14464	0.01492537	2.22557317	0.82617616	0.36356381	0.59282607	0.58832793	Lipa	1
GO:0070534	protein K63-linked ubiquitination	1/97	67/14464	0.01492537	2.22557317	0.82617616	0.36356381	0.59282607	0.58832793	Traf2	1
GO:1902807	negative regulation of cell cycle G1/S phase transition	1/97	67/14464	0.01492537	2.22557317	0.82617616	0.36356381	0.59282607	0.58832793	Cdkn1a	1
GO:1903036	positive regulation of response to wounding	1/97	67/14464	0.01492537	2.22557317	0.82617616	0.36356381	0.59282607	0.58832793	Fkbp1b	1
GO:2000036	regulation of stem cell population maintenance	1/97	67/14464	0.01492537	2.22557317	0.82617616	0.36356381	0.59282607	0.58832793	Sap30l	1
GO:0031099	regeneration	2/97	191/14464	0.0104712	1.56139688	0.64174288	0.3673813	0.59592675	0.59140509	Cdkn1a/Fkbp1b	2
GO:0006414	translational elongation	1/97	68/14464	0.01470588	2.19284415	0.81011982	0.36785181	0.59592675	0.59140509	Hbs1l	1
GO:0006446	regulation of translational initiation	1/97	68/14464	0.01470588	2.19284415	0.81011982	0.36785181	0.59592675	0.59140509	Mettl3	1
GO:0008542	visual learning	1/97	68/14464	0.01470588	2.19284415	0.81011982	0.36785181	0.59592675	0.59140509	Tuba1a	1
GO:0045806	negative regulation of endocytosis	1/97	68/14464	0.01470588	2.19284415	0.81011982	0.36785181	0.59592675	0.59140509	Pcsk9	1
GO:0051865	protein autoubiquitination	1/97	68/14464	0.01470588	2.19284415	0.81011982	0.36785181	0.59592675	0.59140509	Traf2	1
GO:0060395	SMAD protein signal transduction	1/97	68/14464	0.01470588	2.19284415	0.81011982	0.36785181	0.59592675	0.59140509	Becn1	1
GO:0015931	nucleobase-containing compound transport	2/97	192/14464	0.01041667	1.5532646	0.6341224	0.36979177	0.59740852	0.59287561	Bicd2/Abcc5	2
GO:0045732	positive regulation of protein catabolic process	2/97	192/14464	0.01041667	1.5532646	0.6341224	0.36979177	0.59740852	0.59287561	Hspa1b/Pcsk9	2
GO:1901654	response to ketone	2/97	192/14464	0.01041667	1.5532646	0.6341224	0.36979177	0.59740852	0.59287561	Lipa/Sphk2	2
GO:0006913	nucleocytoplasmic transport	3/97	324/14464	0.00925926	1.38067965	0.56942886	0.37065115	0.59769207	0.59315701	Cdkn1a/Sfn/Ankrd54	3
GO:0051169	nuclear transport	3/97	324/14464	0.00925926	1.38067965	0.56942886	0.37065115	0.59769207	0.59315701	Cdkn1a/Sfn/Ankrd54	3
GO:0006360	transcription by RNA polymerase I	1/97	69/14464	0.01449275	2.1610638	0.79434069	0.37211121	0.59784044	0.59330426	Ubtf	1
GO:0008333	endosome to lysosome transport	1/97	69/14464	0.01449275	2.1610638	0.79434069	0.37211121	0.59784044	0.59330426	Lipa	1
GO:0070509	calcium ion import	1/97	69/14464	0.01449275	2.1610638	0.79434069	0.37211121	0.59784044	0.59330426	Trpv2	1
GO:1901264	carbohydrate derivative transport	1/97	69/14464	0.01449275	2.1610638	0.79434069	0.37211121	0.59784044	0.59330426	Abcc5	1
GO:0001701	in utero embryonic development	4/97	461/14464	0.00867679	1.29382561	0.52681919	0.37364308	0.59967747	0.59512735	Cdkn1a/Smpd4/Lig4/Rundc1	4
GO:0006909	phagocytosis	2/97	194/14464	0.01030928	1.53725157	0.61901131	0.37460213	0.59967747	0.59512735	Rab39/Becn1	2
GO:0030324	lung development	2/97	194/14464	0.01030928	1.53725157	0.61901131	0.37460213	0.59967747	0.59512735	Lipa/Ptges3	2
GO:0001676	long-chain fatty acid metabolic process	1/97	70/14464	0.01428571	2.13019146	0.77882934	0.37634221	0.59967747	0.59512735	Cyp2j13	1
GO:0001960	negative regulation of cytokine-mediated signaling pathway	1/97	70/14464	0.01428571	2.13019146	0.77882934	0.37634221	0.59967747	0.59512735	Mettl3	1
GO:0032008	positive regulation of TOR signaling	1/97	70/14464	0.01428571	2.13019146	0.77882934	0.37634221	0.59967747	0.59512735	Stambpl1	1
GO:0032835	glomerulus development	1/97	70/14464	0.01428571	2.13019146	0.77882934	0.37634221	0.59967747	0.59512735	Ctnnd1	1
GO:0043489	RNA stabilization	1/97	70/14464	0.01428571	2.13019146	0.77882934	0.37634221	0.59967747	0.59512735	Traf2	1
GO:0046626	regulation of insulin receptor signaling pathway	1/97	70/14464	0.01428571	2.13019146	0.77882934	0.37634221	0.59967747	0.59512735	Pip4k2c	1
GO:0051348	negative regulation of transferase activity	2/97	196/14464	0.01020408	1.52156533	0.60406992	0.37939797	0.60144	0.59687651	Cdkn1a/Sfrp5	2
GO:0003281	ventricular septum development	1/97	71/14464	0.01408451	2.10018876	0.76357681	0.38054499	0.60144	0.59687651	Dctn5	1
GO:0007632	visual behavior	1/97	71/14464	0.01408451	2.10018876	0.76357681	0.38054499	0.60144	0.59687651	Tuba1a	1
GO:0009123	nucleoside monophosphate metabolic process	1/97	71/14464	0.01408451	2.10018876	0.76357681	0.38054499	0.60144	0.59687651	Nme1	1
GO:0031102	neuron projection regeneration	1/97	71/14464	0.01408451	2.10018876	0.76357681	0.38054499	0.60144	0.59687651	Fkbp1b	1
GO:1903051	negative regulation of proteolysis involved in protein catabolic process	1/97	71/14464	0.01408451	2.10018876	0.76357681	0.38054499	0.60144	0.59687651	Caml	1
GO:1904705	regulation of vascular associated smooth muscle cell proliferation	1/97	71/14464	0.01408451	2.10018876	0.76357681	0.38054499	0.60144	0.59687651	Cdkn1a	1
GO:2000144	positive regulation of DNA-templated transcription initiation	1/97	71/14464	0.01408451	2.10018876	0.76357681	0.38054499	0.60144	0.59687651	Taf4b	1
GO:2000379	positive regulation of reactive oxygen species metabolic process	1/97	71/14464	0.01408451	2.10018876	0.76357681	0.38054499	0.60144	0.59687651	Cdkn1a	1
GO:0007005	mitochondrion organization	4/97	466/14464	0.00858369	1.27994337	0.50473353	0.38129329	0.60177587	0.59720982	Sfn/Coa4/Lipa/Mief1	4
GO:0009895	negative regulation of catabolic process	3/97	330/14464	0.00909091	1.35557638	0.53689499	0.38161603	0.60177587	0.59720982	Traf2/Becn1/Caml	3
GO:0030323	respiratory tube development	2/97	197/14464	0.01015228	1.51384165	0.59666161	0.38179029	0.60177587	0.59720982	Lipa/Ptges3	2
GO:0030705	cytoskeleton-dependent intracellular transport	2/97	198/14464	0.01010101	1.50619598	0.58929423	0.38417882	0.60205023	0.5974821	Bicd2/Tuba1a	2
GO:0022600	digestive system process	1/97	72/14464	0.01388889	2.07101947	0.74857454	0.38471974	0.60205023	0.5974821	Lipa	1
GO:0042982	amyloid precursor protein metabolic process	1/97	72/14464	0.01388889	2.07101947	0.74857454	0.38471974	0.60205023	0.5974821	Aph1b	1
GO:0043299	leukocyte degranulation	1/97	72/14464	0.01388889	2.07101947	0.74857454	0.38471974	0.60205023	0.5974821	Sphk2	1
GO:0051057	positive regulation of small GTPase mediated signal transduction	1/97	72/14464	0.01388889	2.07101947	0.74857454	0.38471974	0.60205023	0.5974821	Rasgef1a	1
GO:0097194	execution phase of apoptosis	1/97	72/14464	0.01388889	2.07101947	0.74857454	0.38471974	0.60205023	0.5974821	Dnase1l3	1
GO:1903008	organelle disassembly	1/97	72/14464	0.01388889	2.07101947	0.74857454	0.38471974	0.60205023	0.5974821	Hbs1l	1
GO:2000027	regulation of animal organ morphogenesis	1/97	72/14464	0.01388889	2.07101947	0.74857454	0.38471974	0.60205023	0.5974821	Sfrp5	1
GO:0006289	nucleotide-excision repair	1/97	73/14464	0.01369863	2.04264934	0.7338144	0.38886664	0.60523287	0.60064059	Lig4	1
GO:0010611	regulation of cardiac muscle hypertrophy	1/97	73/14464	0.01369863	2.04264934	0.7338144	0.38886664	0.60523287	0.60064059	Becn1	1
GO:0046824	positive regulation of nucleocytoplasmic transport	1/97	73/14464	0.01369863	2.04264934	0.7338144	0.38886664	0.60523287	0.60064059	Sfn	1
GO:0071277	cellular response to calcium ion	1/97	73/14464	0.01369863	2.04264934	0.7338144	0.38886664	0.60523287	0.60064059	Tuba1a	1
GO:0140888	interferon-mediated signaling pathway	1/97	73/14464	0.01369863	2.04264934	0.7338144	0.38886664	0.60523287	0.60064059	Mettl3	1
GO:1903510	mucopolysaccharide metabolic process	1/97	73/14464	0.01369863	2.04264934	0.7338144	0.38886664	0.60523287	0.60064059	Abcc5	1
GO:0006631	fatty acid metabolic process	3/97	335/14464	0.00895522	1.3353439	0.51025717	0.39072397	0.60523287	0.60064059	Cyp2j13/Lipa/Ptges3	3
GO:0000302	response to reactive oxygen species	2/97	201/14464	0.00995025	1.48371544	0.56743296	0.39132098	0.60523287	0.60064059	Fkbp1b/Becn1	2
GO:0009749	response to glucose	2/97	201/14464	0.00995025	1.48371544	0.56743296	0.39132098	0.60523287	0.60064059	Myt1/Fkbp1b	2
GO:0002312	B cell activation involved in immune response	1/97	74/14464	0.01351351	2.01504597	0.71928862	0.39298587	0.60523287	0.60064059	Lig4	1
GO:0006942	regulation of striated muscle contraction	1/97	74/14464	0.01351351	2.01504597	0.71928862	0.39298587	0.60523287	0.60064059	Fkbp1b	1
GO:0019229	regulation of vasoconstriction	1/97	74/14464	0.01351351	2.01504597	0.71928862	0.39298587	0.60523287	0.60064059	Shc1	1
GO:0032436	positive regulation of proteasomal ubiquitin-dependent protein catabolic process	1/97	74/14464	0.01351351	2.01504597	0.71928862	0.39298587	0.60523287	0.60064059	Hspa1b	1
GO:0034109	homotypic cell-cell adhesion	1/97	74/14464	0.01351351	2.01504597	0.71928862	0.39298587	0.60523287	0.60064059	Pdia6	1
GO:0051279	regulation of release of sequestered calcium ion into cytosol	1/97	74/14464	0.01351351	2.01504597	0.71928862	0.39298587	0.60523287	0.60064059	Fkbp1b	1
GO:0060260	regulation of transcription initiation by RNA polymerase II	1/97	74/14464	0.01351351	2.01504597	0.71928862	0.39298587	0.60523287	0.60064059	Taf4b	1
GO:0060761	negative regulation of response to cytokine stimulus	1/97	74/14464	0.01351351	2.01504597	0.71928862	0.39298587	0.60523287	0.60064059	Mettl3	1
GO:0070301	cellular response to hydrogen peroxide	1/97	74/14464	0.01351351	2.01504597	0.71928862	0.39298587	0.60523287	0.60064059	Becn1	1
GO:0002709	regulation of T cell mediated immunity	1/97	75/14464	0.01333333	1.98817869	0.70498979	0.39707763	0.60778608	0.60317443	Traf2	1
GO:0014743	regulation of muscle hypertrophy	1/97	75/14464	0.01333333	1.98817869	0.70498979	0.39707763	0.60778608	0.60317443	Becn1	1
GO:0032465	regulation of cytokinesis	1/97	75/14464	0.01333333	1.98817869	0.70498979	0.39707763	0.60778608	0.60317443	Becn1	1
GO:0035082	axoneme assembly	1/97	75/14464	0.01333333	1.98817869	0.70498979	0.39707763	0.60778608	0.60317443	Dnai4	1
GO:0042632	cholesterol homeostasis	1/97	75/14464	0.01333333	1.98817869	0.70498979	0.39707763	0.60778608	0.60317443	Pcsk9	1
GO:0044773	mitotic DNA damage checkpoint signaling	1/97	75/14464	0.01333333	1.98817869	0.70498979	0.39707763	0.60778608	0.60317443	Cdkn1a	1
GO:1990874	vascular associated smooth muscle cell proliferation	1/97	75/14464	0.01333333	1.98817869	0.70498979	0.39707763	0.60778608	0.60317443	Cdkn1a	1
GO:0009746	response to hexose	2/97	205/14464	0.0097561	1.4547649	0.53882948	0.40078728	0.61239858	0.60775193	Myt1/Fkbp1b	2
GO:0050709	negative regulation of protein secretion	1/97	76/14464	0.01315789	1.96201845	0.69091082	0.40114209	0.61239858	0.60775193	Fkbp1b	1
GO:0055092	sterol homeostasis	1/97	76/14464	0.01315789	1.96201845	0.69091082	0.40114209	0.61239858	0.60775193	Pcsk9	1
GO:0043270	positive regulation of monoatomic ion transport	2/97	206/14464	0.00970874	1.44770293	0.53177297	0.4031434	0.61368456	0.60902816	Trpv2/Atp1b3	2
GO:0006869	lipid transport	3/97	342/14464	0.00877193	1.3080123	0.47365816	0.40342172	0.61368456	0.60902816	Pcsk9/Lipa/Stard3	3
GO:0007586	digestion	1/97	77/14464	0.01298701	1.93653769	0.67704497	0.40517943	0.61368456	0.60902816	Lipa	1
GO:0007589	body fluid secretion	1/97	77/14464	0.01298701	1.93653769	0.67704497	0.40517943	0.61368456	0.60902816	Nme1	1
GO:0032414	positive regulation of ion transmembrane transporter activity	1/97	77/14464	0.01298701	1.93653769	0.67704497	0.40517943	0.61368456	0.60902816	Atp1b3	1
GO:0046888	negative regulation of hormone secretion	1/97	77/14464	0.01298701	1.93653769	0.67704497	0.40517943	0.61368456	0.60902816	Fkbp1b	1
GO:0070972	protein localization to endoplasmic reticulum	1/97	77/14464	0.01298701	1.93653769	0.67704497	0.40517943	0.61368456	0.60902816	Edem1	1
GO:1901379	regulation of potassium ion transmembrane transport	1/97	77/14464	0.01298701	1.93653769	0.67704497	0.40517943	0.61368456	0.60902816	Atp1b3	1
GO:2000736	regulation of stem cell differentiation	1/97	77/14464	0.01298701	1.93653769	0.67704497	0.40517943	0.61368456	0.60902816	Mettl3	1
GO:0097191	extrinsic apoptotic signaling pathway	2/97	207/14464	0.00966184	1.4407092	0.52475336	0.40549523	0.61368456	0.60902816	Hspa1b/Traf2	2
GO:0042176	regulation of protein catabolic process	3/97	344/14464	0.00872093	1.30040758	0.46334531	0.40703688	0.61548483	0.61081477	Hspa1b/Pcsk9/Caml	3
GO:0031341	regulation of cell killing	1/97	78/14464	0.01282051	1.91171028	0.66338575	0.40918982	0.61607563	0.61140109	Dnase1l3	1
GO:0042100	B cell proliferation	1/97	78/14464	0.01282051	1.91171028	0.66338575	0.40918982	0.61607563	0.61140109	Cdkn1a	1
GO:0044774	mitotic DNA integrity checkpoint signaling	1/97	78/14464	0.01282051	1.91171028	0.66338575	0.40918982	0.61607563	0.61140109	Cdkn1a	1
GO:0050871	positive regulation of B cell activation	1/97	78/14464	0.01282051	1.91171028	0.66338575	0.40918982	0.61607563	0.61140109	Cdkn1a	1
GO:0072665	protein localization to vacuole	1/97	78/14464	0.01282051	1.91171028	0.66338575	0.40918982	0.61607563	0.61140109	Becn1	1

GO:1903829	positive regulation of protein localization	4/97	486/14464	0.00823045	1.2272708	0.41876586	0.41178813	0.61748139	0.61279618	Sfn/Edem1/Cct7/Mief1	4
GO:0031348	negative regulation of defense response	2/97	210/14464	0.00952381	1.42012764	0.50391181	0.41252457	0.61748139	0.61279618	Sfn/Mettl3	2
GO:0090276	regulation of peptide hormone secretion	2/97	210/14464	0.00952381	1.42012764	0.50391181	0.41252457	0.61748139	0.61279618	Myt1/Fkbp1b	2
GO:0032418	lysosome localization	1/97	79/14464	0.01265823	1.88751142	0.649927	0.41317346	0.61748139	0.61279618	Sphk2	1
GO:0097009	energy homeostasis	1/97	79/14464	0.01265823	1.88751142	0.649927	0.41317346	0.61748139	0.61279618	Lipa	1
GO:1903578	regulation of ATP metabolic process	1/97	79/14464	0.01265823	1.88751142	0.649927	0.41317346	0.61748139	0.61279618	Sphk2	1
GO:1990849	vacuolar localization	1/97	79/14464	0.01265823	1.88751142	0.649927	0.41317346	0.61748139	0.61279618	Sphk2	1
GO:0009410	response to xenobiotic stimulus	3/97	348/14464	0.00862069	1.28546036	0.44290606	0.41424856	0.61748139	0.61279618	Cyp2j13/Lipa/Becn1	3
GO:0034284	response to monosaccharide	2/97	211/14464	0.00947867	1.41339718	0.49703572	0.4148588	0.61748139	0.61279618	Myt1/Fkbp1b	2
GO:0051321	meiotic cell cycle	2/97	211/14464	0.00947867	1.41339718	0.49703572	0.4148588	0.61748139	0.61279618	Mettl3/Smc1a	2
GO:0002260	lymphocyte homeostasis	1/97	80/14464	0.0125	1.86391753	0.63666277	0.41713051	0.61748139	0.61279618	Caml	1
GO:0002532	production of molecular mediator involved in inflammatory response	1/97	80/14464	0.0125	1.86391753	0.63666277	0.41713051	0.61748139	0.61279618	Lipa	1
GO:0010507	negative regulation of autophagy	1/97	80/14464	0.0125	1.86391753	0.63666277	0.41713051	0.61748139	0.61279618	Becn1	1
GO:0021766	hippocampus development	1/97	80/14464	0.0125	1.86391753	0.63666277	0.41713051	0.61748139	0.61279618	Tuba1a	1
GO:0070227	lymphocyte apoptotic process	1/97	80/14464	0.0125	1.86391753	0.63666277	0.41713051	0.61748139	0.61279618	Lipa	1
GO:0071230	cellular response to amino acid stimulus	1/97	80/14464	0.0125	1.86391753	0.63666277	0.41713051	0.61748139	0.61279618	Stambpl1	1
GO:1902369	negative regulation of RNA catabolic process	1/97	80/14464	0.0125	1.86391753	0.63666277	0.41713051	0.61748139	0.61279618	Traf2	1
GO:0002699	positive regulation of immune effector process	2/97	212/14464	0.00943396	1.40673021	0.49019454	0.41718851	0.61748139	0.61279618	Traf2/Sphk2	2
GO:0006813	potassium ion transport	2/97	212/14464	0.00943396	1.40673021	0.49019454	0.41718851	0.61748139	0.61279618	Kcnk10/Atp1b3	2
GO:0099111	microtubule-based transport	2/97	212/14464	0.00943396	1.40673021	0.49019454	0.41718851	0.61748139	0.61279618	Bicd2/Tuba1a	2
GO:0005975	carbohydrate metabolic process	4/97	491/14464	0.00814664	1.21477313	0.39783948	0.41937231	0.61882691	0.61413149	Lipa/Ptges3/Edem1/Nhlrc1	4
GO:0002274	myeloid leukocyte activation	2/97	213/14464	0.00938967	1.40012584	0.48338787	0.41951367	0.61882691	0.61413149	Dnase1l3/Sphk2	2
GO:0030073	insulin secretion	2/97	213/14464	0.00938967	1.40012584	0.48338787	0.41951367	0.61882691	0.61413149	Myt1/Fkbp1b	2
GO:0043122	regulation of canonical NF-kappaB signal transduction	2/97	213/14464	0.00938967	1.40012584	0.48338787	0.41951367	0.61882691	0.61413149	Traf2/Sphk2	2
GO:0033077	T cell differentiation in thymus	1/97	81/14464	0.01234568	1.8409062	0.62358741	0.42106115	0.61902009	0.61432321	Lig4	1
GO:0045638	negative regulation of myeloid cell differentiation	1/97	81/14464	0.01234568	1.8409062	0.62358741	0.42106115	0.61902009	0.61432321	Nme1	1
GO:1905897	regulation of response to endoplasmic reticulum stress	1/97	81/14464	0.01234568	1.8409062	0.62358741	0.42106115	0.61902009	0.61432321	Pdia6	1
GO:2000142	regulation of DNA-templated transcription initiation	1/97	81/14464	0.01234568	1.8409062	0.62358741	0.42106115	0.61902009	0.61432321	Taf4b	1
GO:0002791	regulation of peptide secretion	2/97	215/14464	0.00930233	1.38710141	0.46987659	0.42415011	0.62251419	0.61779079	Myt1/Fkbp1b	2
GO:0090087	regulation of peptide transport	2/97	215/14464	0.00930233	1.38710141	0.46987659	0.42415011	0.62251419	0.61779079	Myt1/Fkbp1b	2
GO:0032411	positive regulation of transporter activity	1/97	82/14464	0.01219512	1.81845612	0.61069547	0.42496556	0.62266538	0.61794083	Atp1b3	1
GO:0061157	mRNA destabilization	1/97	82/14464	0.01219512	1.81845612	0.61069547	0.42496556	0.62266538	0.61794083	Mettl3	1
GO:0006637	acyl-CoA metabolic process	1/97	83/14464	0.01204819	1.79654701	0.59798175	0.4288439	0.62677185	0.62201615	Pmvk	1
GO:0035383	thioester metabolic process	1/97	83/14464	0.01204819	1.79654701	0.59798175	0.4288439	0.62677185	0.62201615	Pmvk	1
GO:1900076	regulation of cellular response to insulin stimulus	1/97	83/14464	0.01204819	1.79654701	0.59798175	0.4288439	0.62677185	0.62201615	Pip4k2c	1
GO:1903050	regulation of proteolysis involved in protein catabolic process	2/97	218/14464	0.00917431	1.36801286	0.44985939	0.4310695	0.62949832	0.62472192	Hspa1b/Caml	2
GO:0010976	positive regulation of neuron projection development	2/97	220/14464	0.00909091	1.35557638	0.43667696	0.43565843	0.63428293	0.62947023	Fkbp1b/Nme1	2
GO:0000280	nuclear division	3/97	360/14464	0.00833333	1.24261168	0.38302209	0.43571779	0.63428293	0.62947023	Hspa1b/Becn1/Smc1a	3
GO:0006664	glycolipid metabolic process	1/97	85/14464	0.01176471	1.75427532	0.57306915	0.43652309	0.63428293	0.62947023	Pigc	1
GO:0019915	lipid storage	1/97	85/14464	0.01176471	1.75427532	0.57306915	0.43652309	0.63428293	0.62947023	Lipa	1
GO:0030203	glycosaminoglycan metabolic process	1/97	85/14464	0.01176471	1.75427532	0.57306915	0.43652309	0.63428293	0.62947023	Abcc5	1
GO:0051310	metaphase chromosome alignment	1/97	85/14464	0.01176471	1.75427532	0.57306915	0.43652309	0.63428293	0.62947023	Becn1	1
GO:1903828	negative regulation of protein localization	2/97	221/14464	0.00904977	1.34944255	0.43013344	0.43794557	0.6345316	0.62971702	Sfn/Fkbp1b	2
GO:0000041	transition metal ion transport	1/97	86/14464	0.01162791	1.73387677	0.56086087	0.44032428	0.6345316	0.62971702	Abcc5	1
GO:0002090	regulation of receptor internalization	1/97	86/14464	0.01162791	1.73387677	0.56086087	0.44032428	0.6345316	0.62971702	Pcsk9	1
GO:0030168	platelet activation	1/97	86/14464	0.01162791	1.73387677	0.56086087	0.44032428	0.6345316	0.62971702	Pdia6	1
GO:0030512	negative regulation of transforming growth factor beta receptor signaling pathway	1/97	86/14464	0.01162791	1.73387677	0.56086087	0.44032428	0.6345316	0.62971702	Sap30l	1
GO:0032273	positive regulation of protein polymerization	1/97	86/14464	0.01162791	1.73387677	0.56086087	0.44032428	0.6345316	0.62971702	Hspa1b	1
GO:0050848	regulation of calcium-mediated signaling	1/97	86/14464	0.01162791	1.73387677	0.56086087	0.44032428	0.6345316	0.62971702	Fkbp1b	1
GO:0072332	intrinsic apoptotic signaling pathway by p53 class mediator	1/97	86/14464	0.01162791	1.73387677	0.56086087	0.44032428	0.6345316	0.62971702	Cdkn1a	1
GO:1903509	liposaccharide metabolic process	1/97	86/14464	0.01162791	1.73387677	0.56086087	0.44032428	0.6345316	0.62971702	Pigc	1
GO:2001237	negative regulation of extrinsic apoptotic signaling pathway	1/97	86/14464	0.01162791	1.73387677	0.56086087	0.44032428	0.6345316	0.62971702	Hspa1b	1
GO:0007059	chromosome segregation	3/97	364/14464	0.00824176	1.22895661	0.36351835	0.44281302	0.63577964	0.63095559	Hspa1b/Becn1/Smc1a	3
GO:0000281	mitotic cytokinesis	1/97	87/14464	0.01149425	1.71394715	0.54881196	0.44410008	0.63577964	0.63095559	Efhc1	1
GO:0000423	mitophagy	1/97	87/14464	0.01149425	1.71394715	0.54881196	0.44410008	0.63577964	0.63095559	Becn1	1
GO:0009408	response to heat	1/97	87/14464	0.01149425	1.71394715	0.54881196	0.44410008	0.63577964	0.63095559	Hspa1b	1
GO:0032755	positive regulation of interleukin-6 production	1/97	87/14464	0.01149425	1.71394715	0.54881196	0.44410008	0.63577964	0.63095559	Sphk2	1
GO:0050779	RNA destabilization	1/97	87/14464	0.01149425	1.71394715	0.54881196	0.44410008	0.63577964	0.63095559	Mettl3	1
GO:0051881	regulation of mitochondrial membrane potential	1/97	87/14464	0.01149425	1.71394715	0.54881196	0.44410008	0.63577964	0.63095559	Lipa	1
GO:0061014	positive regulation of mRNA catabolic process	1/97	87/14464	0.01149425	1.71394715	0.54881196	0.44410008	0.63577964	0.63095559	Mettl3	1
GO:1903522	regulation of blood circulation	2/97	224/14464	0.00892857	1.33136966	0.41068966	0.4447773	0.63622809	0.63140063	Shc1/Fkbp1b	2
GO:2001234	negative regulation of apoptotic signaling pathway	2/97	225/14464	0.00888889	1.32545246	0.40426961	0.44704451	0.63853424	0.63368929	Hspa1b/Maz	2
GO:0006835	dicarboxylic acid transport	1/97	88/14464	0.01136364	1.69447048	0.53691818	0.44785068	0.63853424	0.63368929	Abcc5	1
GO:0043406	positive regulation of MAP kinase activity	1/97	88/14464	0.01136364	1.69447048	0.53691818	0.44785068	0.63853424	0.63368929	Traf2	1
GO:1900542	regulation of purine nucleotide metabolic process	1/97	88/14464	0.01136364	1.69447048	0.53691818	0.44785068	0.63853424	0.63368929	Sphk2	1
GO:0060828	regulation of canonical Wnt signaling pathway	2/97	226/14464	0.00884956	1.31958763	0.39787962	0.44930666	0.64008805	0.6352313	Sfrp5/Ctnnd1	2
GO:0006140	regulation of nucleotide metabolic process	1/97	89/14464	0.01123596	1.67543148	0.52517542	0.45157623	0.6422744	0.63740107	Sphk2	1
GO:1903312	negative regulation of mRNA metabolic process	1/97	89/14464	0.01123596	1.67543148	0.52517542	0.45157623	0.6422744	0.63740107	Traf2	1
GO:0002027	regulation of heart rate	1/97	90/14464	0.01111111	1.65681558	0.51357975	0.45527689	0.64593364	0.64103254	Fkbp1b	1
GO:0051101	regulation of DNA binding	1/97	90/14464	0.01111111	1.65681558	0.51357975	0.45527689	0.64593364	0.64103254	Nme1	1
GO:0001894	tissue homeostasis	2/97	230/14464	0.00869565	1.29663828	0.37261441	0.45830382	0.64593364	0.64103254	Lipa/Tuba1a	2
GO:0040029	epigenetic regulation of gene expression	2/97	230/14464	0.00869565	1.29663828	0.37261441	0.45830382	0.64593364	0.64103254	Mettl3/Sphk2	2
GO:0060249	anatomical structure homeostasis	2/97	230/14464	0.00869565	1.29663828	0.37261441	0.45830382	0.64593364	0.64103254	Lipa/Tuba1a	2
GO:0030900	forebrain development	3/97	373/14464	0.0080429	1.1993035	0.32042799	0.4586528	0.64593364	0.64103254	Efhc1/Mettl3/Tuba1a	3
GO:0010469	regulation of signaling receptor activity	1/97	91/14464	0.01098901	1.63860881	0.50212738	0.45895285	0.64593364	0.64103254	Pcsk9	1
GO:0042310	vasoconstriction	1/97	91/14464	0.01098901	1.63860881	0.50212738	0.45895285	0.64593364	0.64103254	Shc1	1
GO:0043525	positive regulation of neuron apoptotic process	1/97	91/14464	0.01098901	1.63860881	0.50212738	0.45895285	0.64593364	0.64103254	Pcsk9	1
GO:0045921	positive regulation of exocytosis	1/97	91/14464	0.01098901	1.63860881	0.50212738	0.45895285	0.64593364	0.64103254	Sphk2	1
GO:0071229	cellular response to acid chemical	1/97	91/14464	0.01098901	1.63860881	0.50212738	0.45895285	0.64593364	0.64103254	Stambpl1	1
GO:0090398	cellular senescence	1/97	91/14464	0.01098901	1.63860881	0.50212738	0.45895285	0.64593364	0.64103254	Cdkn1a	1
GO:0120162	positive regulation of cold-induced thermogenesis	1/97	91/14464	0.01098901	1.63860881	0.50212738	0.45895285	0.64593364	0.64103254	Trpv2	1
GO:0009581	detection of external stimulus	1/97	92/14464	0.01086957	1.62079785	0.49081466	0.46260425	0.64765725	0.64274307	Pde6d	1
GO:0009582	detection of abiotic stimulus	1/97	92/14464	0.01086957	1.62079785	0.49081466	0.46260425	0.64765725	0.64274307	Pde6d	1
GO:0042147	retrograde transport, endosome to Golgi	1/97	92/14464	0.01086957	1.62079785	0.49081466	0.46260425	0.64765725	0.64274307	Cln5	1
GO:0046889	positive regulation of lipid biosynthetic process	1/97	92/14464	0.01086957	1.62079785	0.49081466	0.46260425	0.64765725	0.64274307	Sphk2	1
GO:0048565	digestive tract development	1/97	92/14464	0.01086957	1.62079785	0.49081466	0.46260425	0.64765725	0.64274307	Sfrp5	1
GO:0048678	response to axon injury	1/97	92/14464	0.01086957	1.62079785	0.49081466	0.46260425	0.64765725	0.64274307	Fkbp1b	1
GO:0002221	pattern recognition receptor signaling pathway	2/97	232/14464	0.00862069	1.28546036	0.36015451	0.46277111	0.64765725	0.64274307	Hspa1b/Becn1	2
GO:0006816	calcium ion transport	3/97	377/14464	0.00795756	1.1865788	0.30161693	0.46563382	0.65114233	0.64620171	Gpr35/Trpv2/Fkbp1b	3
GO:0051303	establishment of chromosome localization	1/97	93/14464	0.01075269	1.60336991	0.47963807	0.46623126	0.65145662	0.64651362	Becn1	1
GO:0009743	response to carbohydrate	2/97	234/14464	0.00854701	1.27447352	0.3478067	0.46721721	0.65231284	0.64736334	Myt1/Fkbp1b	2
GO:0002758	innate immune response-activating signaling pathway	2/97	235/14464	0.00851064	1.26905023	0.34167415	0.46943223	0.65335712	0.64839969	Hspa1b/Becn1	2
GO:0010389	regulation of G2/M transition of mitotic cell cycle	1/97	94/14464	0.0106383	1.58631279	0.46859422	0.46983404	0.65335712	0.64839969	Cdkn1a	1
GO:0030516	regulation of axon extension	1/97	94/14464	0.0106383	1.58631279	0.46859422	0.46983404	0.65335712	0.64839969	Trpv2	1
GO:0031110	regulation of microtubule polymerization or depolymerization	1/97	94/14464	0.0106383	1.58631279	0.46859422	0.46983404	0.65335712	0.64839969	Hspa1b	1
GO:0033045	regulation of sister chromatid segregation	1/97	94/14464	0.0106383	1.58631279	0.46859422	0.46983404	0.65335712	0.64839969	Becn1	1
GO:0002757	immune response-activating signaling pathway	3/97	380/14464	0.00789474	1.17721107	0.28764157	0.47084452	0.65424182	0.64927769	Hspa1b/Lipa/Becn1	3
GO:0002449	lymphocyte mediated immunity	2/97	236/14464	0.00847458	1.2636729	0.33556881	0.47164187	0.65430952	0.64934487	Traf2/Lig4	2
GO:0007249	canonical NF-kappaB signal transduction	2/97	236/14464	0.00847458	1.2636729	0.33556881	0.47164187	0.65430952	0.64934487	Traf2/Sphk2	2
GO:0002708	positive regulation of lymphocyte mediated immunity	1/97	95/14464	0.01052632	1.56961476	0.45767983	0.47341275	0.65572543	0.65075003	Traf2	1
GO:2000060	positive regulation of ubiquitin-dependent protein catabolic process	1/97	95/14464	0.01052632	1.56961476	0.45767983	0.47341275	0.65572543	0.65075003	Hspa1b	1
GO:0006022	aminoglycan metabolic process	1/97	96/14464	0.01041667	1.5532646	0.44689177	0.47696755	0.65960386	0.65459904	Abcc5	1
GO:1902106	negative regulation of leukocyte differentiation	1/97	96/14464	0.01041667	1.5532646	0.44689177	0.47696755	0.65960386	0.65459904	Nme1	1
GO:0007626	locomotory behavior	2/97	240/14464	0.00833333	1.24261168	0.31141425	0.48042603	0.66186884	0.65684683	Tuba1a/Aph1b	2
GO:0051896	regulation of phosphatidylinositol 3-kinase/protein kinase B signal transduction	2/97	240/14464	0.00833333	1.24261168	0.31141425	0.48042603	0.66186884	0.65684683	Sfrp5/Maz	2
GO:0008344	adult locomotory behavior	1/97	97/14464	0.01030928	1.53725157	0.43622698	0.4804986	0.66186884	0.65684683	Tuba1a	1
GO:0043266	regulation of potassium ion transport	1/97	97/14464	0.01030928	1.53725157	0.43622698	0.4804986	0.66186884	0.65684683	Atp1b3	1
GO:0050772	positive regulation of axonogenesis	1/97	97/14464	0.01030928	1.53725157	0.43622698	0.4804986	0.66186884	0.65684683	Trpv2	1
GO:0001819	positive regulation of cytokine production	3/97	386/14464	0.00777202	1.15891245	0.26002381	0.48119891	0.66231157	0.6572862	Hspa1b/Traf2/Sphk2	3
GO:0000209	protein polyubiquitination	2/97	243/14464	0.00823045	1.2272708	0.29357138	0.48695628	0.66781556	0.66274843	Traf2/Nhlrc1	2
GO:0006096	glycolytic process	1/97	99/14464	0.01010101	1.50619598	0.41525559	0.48749008	0.66781556	0.66274843	Lipa	1
GO:0032760	positive regulation of tumor necrosis factor production	1/97	99/14464	0.01010101	1.50619598	0.41525559	0.48749008	0.66781556	0.66274843	Sphk2	1
GO:0033108	mitochondrial respiratory chain complex assembly	1/97	99/14464	0.01010101	1.50619598	0.41525559	0.48749008	0.66781556	0.66274843	Coa4	1
GO:0048640	negative regulation of developmental growth	1/97	99/14464	0.01010101	1.50619598	0.41525559	0.48749008	0.66781556	0.66274843	Cdkn1a	1
GO:1903432	regulation of TORC1 signaling	1/97	99/14464	0.01010101	1.50619598	0.41525559	0.48749008	0.66781556	0.66274843	Stambpl1	1
GO:0035270	endocrine system development	1/97	100/14464	0.01	1.49113402	0.40494341	0.49095082	0.6709068	0.66581622	Myt1	1
GO:0050000	chromosome localization	1/97	100/14464	0.01	1.49113402	0.40494341	0.49095082	0.6709068	0.66581622	Becn1	1
GO:1903557	positive regulation of tumor necrosis factor superfamily cytokine production	1/97	100/14464	0.01	1.49113402	0.40494341	0.49095082	0.6709068	0.66581622	Sphk2	1
GO:0032886	regulation of microtubule-based process	2/97	245/14464	0.00816327	1.21725226	0.2818025	0.49128187	0.6709068	0.66581622	Hspa1b/Bicd2	2
GO:0010876	lipid localization	3/97	393/14464	0.00763359	1.13827024	0.22834659	0.49316114	0.67147705	0.66638214	Pcsk9/Lipa/Stard3	3
GO:0045860	positive regulation of protein kinase activity	2/97	246/14464	0.00813008	1.21230408	0.27595525	0.49343621	0.67147705	0.66638214	Cdkn1a/Traf2	2
GO:0003300	cardiac muscle hypertrophy	1/97	101/14464	0.00990099	1.47637032	0.39474334	0.49438842	0.67147705	0.66638214	Becn1	1
GO:0006368	transcription elongation by RNA polymerase II	1/97	101/14464	0.00990099	1.47637032	0.39474334	0.49438842	0.67147705	0.66638214	Eloa	1
GO:0021761	limbic system development	1/97	101/14464	0.00990099	1.47637032	0.39474334	0.49438842	0.67147705	0.66638214	Tuba1a	1
GO:0050852	T cell receptor signaling pathway	1/97	101/14464	0.00990099	1.47637032	0.39474334	0.49438842	0.67147705	0.66638214	Lipa	1
GO:0097306	cellular response to alcohol	1/97	101/14464	0.00990099	1.47637032	0.39474334	0.49438842	0.67147705	0.66638214	Sphk2	1
GO:0003012	muscle system process	3/97	395/14464	0.00759494	1.13250685	0.21940033	0.49655472	0.67297736	0.66787107	Shc1/Fkbp1b/Becn1	3
GO:0000422	autophagy of mitochondrion	1/97	102/14464	0.00980392	1.4618961	0.38465283	0.49780305	0.67297736	0.66787107	Becn1	1
GO:0001909	leukocyte mediated cytotoxicity	1/97	102/14464	0.00980392	1.4618961	0.38465283	0.49780305	0.67297736	0.66787107	Dnase1l3	1
GO:0002824	positive regulation of adaptive immune response based on somatic recombination of immune receptors b	1/97	102/14464	0.00980392	1.4618961	0.38465283	0.49780305	0.67297736	0.66787107	Traf2	1
GO:0048534	hematopoietic or lymphoid organ development	1/97	102/14464	0.00980392	1.4618961	0.38465283	0.49780305	0.67297736	0.66787107	Lipa	1
GO:1903707	negative regulation of hemopoiesis	1/97	102/14464	0.00980392	1.4618961	0.38465283	0.49780305	0.67297736	0.66787107	Nme1	1
GO:0033002	muscle cell proliferation	2/97	249/14464	0.00803213	1.19769801	0.25855953	0.49986521	0.67481881	0.66969854	Shc1/Cdkn1a	2
GO:0002764	immune response-regulating signaling pathway	3/97	397/14464	0.00755668	1.12680153	0.21049947	0.49993727	0.67481881	0.66969854	Hspa1b/Lipa/Becn1	3
GO:0033555	multicellular organismal response to stress	1/97	103/14464	0.00970874	1.44770293	0.3746694	0.50119486	0.6749527	0.66983142	Vwa1	1
GO:0043502	regulation of muscle adaptation	1/97	103/14464	0.00970874	1.44770293	0.3746694	0.50119486	0.6749527	0.66983142	Becn1	1
GO:0046032	ADP catabolic process	1/97	103/14464	0.00970874	1.44770293	0.3746694	0.50119486	0.6749527	0.66983142	Lipa	1
GO:0014897	striated muscle hypertrophy	1/97	104/14464	0.00961538	1.43378271	0.36479065	0.50456399	0.6769346	0.67179827	Becn1	1
GO:0031330	negative regulation of cellular catabolic process	1/97	104/14464	0.00961538	1.43378271	0.36479065	0.50456399	0.6769346	0.67179827	Becn1	1
GO:0055123	digestive system development	1/97	104/14464	0.00961538	1.43378271	0.36479065	0.50456399	0.6769346	0.67179827	Sfrp5	1
GO:1901655	cellular response to ketone	1/97	104/14464	0.00961538	1.43378271	0.36479065	0.50456399	0.6769346	0.67179827	Sphk2	1
GO:0022411	cellular component disassembly	3/97	400/14464	0.0075	1.11835052	0.19723216	0.50499011	0.6769346	0.67179827	Dnase1l3/Becn1/Hbs1l	3
GO:0140694	non-membrane-bounded organelle assembly	3/97	400/14464	0.0075	1.11835052	0.19723216	0.50499011	0.6769346	0.67179827	Hspa1b/Becn1/Smc1a	3
GO:0003279	cardiac septum development	1/97	105/14464	0.00952381	1.42012764	0.35501428	0.5079106	0.67721413	0.67207568	Dctn5	1
GO:0009137	purine nucleoside diphosphate catabolic process	1/97	105/14464	0.00952381	1.42012764	0.35501428	0.5079106	0.67721413	0.67207568	Lipa	1
GO:0009181	purine ribonucleoside diphosphate catabolic process	1/97	105/14464	0.00952381	1.42012764	0.35501428	0.5079106	0.67721413	0.67207568	Lipa	1
GO:0019364	pyridine nucleotide catabolic process	1/97	105/14464	0.00952381	1.42012764	0.35501428	0.5079106	0.67721413	0.67207568	Lipa	1
GO:0031047	regulatory ncRNA-mediated gene silencing	1/97	105/14464	0.00952381	1.42012764	0.35501428	0.5079106	0.67721413	0.67207568	Mettl3	1
GO:0038061	non-canonical NF-kappaB signal transduction	1/97	105/14464	0.00952381	1.42012764	0.35501428	0.5079106	0.67721413	0.67207568	Traf2	1
GO:1901800	positive regulation of proteasomal protein catabolic process	1/97	105/14464	0.00952381	1.42012764	0.35501428	0.5079106	0.67721413	0.67207568	Hspa1b	1
GO:0006898	receptor-mediated endocytosis	2/97	254/14464	0.00787402	1.17412128	0.23004017	0.51046548	0.67845731	0.67330944	Pcsk9/Cap1	2
GO:0048588	developmental cell growth	2/97	254/14464	0.00787402	1.17412128	0.23004017	0.51046548	0.67845731	0.67330944	Trpv2/Raph1	2
GO:0014896	muscle hypertrophy	1/97	106/14464	0.00943396	1.40673021	0.34533803	0.51123483	0.67845731	0.67330944	Becn1	1
GO:0016525	negative regulation of angiogenesis	1/97	106/14464	0.00943396	1.40673021	0.34533803	0.51123483	0.67845731	0.67330944	Shc1	1
GO:0060840	artery development	1/97	106/14464	0.00943396	1.40673021	0.34533803	0.51123483	0.67845731	0.67330944	Dctn5	1
GO:1902749	regulation of cell cycle G2/M phase transition	1/97	106/14464	0.00943396	1.40673021	0.34533803	0.51123483	0.67845731	0.67330944	Cdkn1a	1
GO:0002456	T cell mediated immunity	1/97	107/14464	0.00934579	1.3935832	0.33575973	0.51453684	0.67845731	0.67330944	Traf2	1
GO:0008306	associative learning	1/97	107/14464	0.00934579	1.3935832	0.33575973	0.51453684	0.67845731	0.67330944	Tuba1a	1
GO:0016064	immunoglobulin mediated immune response	1/97	107/14464	0.00934579	1.3935832	0.33575973	0.51453684	0.67845731	0.67330944	Lig4	1
GO:0030518	nuclear receptor-mediated steroid hormone signaling pathway	1/97	107/14464	0.00934579	1.3935832	0.33575973	0.51453684	0.67845731	0.67330944	Ptges3	1
GO:0042274	ribosomal small subunit biogenesis	1/97	107/14464	0.00934579	1.3935832	0.33575973	0.51453684	0.67845731	0.67330944	Rps19bp1	1
GO:0097250	mitochondrial respirasome assembly	1/97	107/14464	0.00934579	1.3935832	0.33575973	0.51453684	0.67845731	0.67330944	Coa4	1
GO:0030072	peptide hormone secretion	2/97	256/14464	0.0078125	1.16494845	0.21879318	0.51466499	0.67845731	0.67330944	Myt1/Fkbp1b	2
GO:0050878	regulation of body fluid levels	2/97	256/14464	0.0078125	1.16494845	0.21879318	0.51466499	0.67845731	0.67330944	Pdia6/Nme1	2
GO:0090150	establishment of protein localization to membrane	2/97	256/14464	0.0078125	1.16494845	0.21879318	0.51466499	0.67845731	0.67330944	Caml/Mief1	2
GO:0021537	telencephalon development	2/97	257/14464	0.0077821	1.16041558	0.2132033	0.51675597	0.67913331	0.6739803	Efhc1/Tuba1a	2

GO:0042177 negative regulation of protein catabolic process	1/97	108/14464	0.00925926	1.38067965	0.32627728	0.51781676	0.67913331	0.6739803	Caml	1
GO:0046031 ADP metabolic process	1/97	108/14464	0.00925926	1.38067965	0.32627728	0.51781676	0.67913331	0.6739803	Lipa	1
GO:0050671 positive regulation of lymphocyte proliferation	1/97	108/14464	0.00925926	1.38067965	0.32627728	0.51781676	0.67913331	0.6739803	Cdkn1a	1
GO:0072526 pyridine-containing compound catabolic process	1/97	108/14464	0.00925926	1.38067965	0.32627728	0.51781676	0.67913331	0.6739803	Lipa	1
GO:2000181 negative regulation of blood vessel morphogenesis	1/97	108/14464	0.00925926	1.38067965	0.32627728	0.51781676	0.67913331	0.6739803	Shc1	1
GO:0002218 activation of innate immune response	2/97	258/14464	0.00775194	1.15591785	0.20763559	0.51884109	0.67913331	0.6739803	Hspa1b/Becn1	2
GO:0033674 positive regulation of kinase activity	2/97	258/14464	0.00775194	1.15591785	0.20763559	0.51884109	0.67913331	0.6739803	Cdkn1a/Traf2	2
GO:0045927 positive regulation of growth	2/97	258/14464	0.00775194	1.15591785	0.20763559	0.51884109	0.67913331	0.6739803	Sfn/Trpv2	2
GO:0008544 epidermis development	2/97	259/14464	0.00772201	1.15145484	0.20208982	0.52092032	0.67913331	0.6739803	Cdkn1a/Sfn	2
GO:0001578 microtubule bundle formation	1/97	109/14464	0.00917431	1.36801286	0.31688863	0.52107476	0.67913331	0.6739803	Dnai4	1
GO:0009191 ribonucleoside diphosphate catabolic process	1/97	109/14464	0.00917431	1.36801286	0.31688863	0.52107476	0.67913331	0.6739803	Lipa	1
GO:0039531 regulation of cytoplasmic pattern recognition receptor signaling pathway	1/97	109/14464	0.00917431	1.36801286	0.31688863	0.52107476	0.67913331	0.6739803	Hspa1b	1
GO:1901343 negative regulation of vasculature development	1/97	109/14464	0.00917431	1.36801286	0.31688863	0.52107476	0.67913331	0.6739803	Shc1	1
GO:0043434 response to peptide hormone	3/97	410/14464	0.00731707	1.09107367	0.1537152	0.52164544	0.67913331	0.6739803	Shc1/Pcsk9/Pip4k2c	3
GO:0002821 positive regulation of adaptive immune response	1/97	110/14464	0.00909091	1.35557638	0.30759183	0.52431097	0.67913331	0.6739803	Traf2	1
GO:0019724 B cell mediated immunity	1/97	110/14464	0.00909091	1.35557638	0.30759183	0.52431097	0.67913331	0.6739803	Lig4	1
GO:0045446 endothelial cell differentiation	1/97	110/14464	0.00909091	1.35557638	0.30759183	0.52431097	0.67913331	0.6739803	Lipa	1
GO:0061387 regulation of extent of cell growth	1/97	110/14464	0.00909091	1.35557638	0.30759183	0.52431097	0.67913331	0.6739803	Trpv2	1
GO:0043161 proteasome-mediated ubiquitin-dependent protein catabolic process	3/97	412/14464	0.00728155	1.0857772	0.14513876	0.52494094	0.67913331	0.6739803	Hspa1b/Nhlrc1/Caml	3
GO:0046883 regulation of hormone secretion	2/97	261/14464	0.00766284	1.14263143	0.19106339	0.52506107	0.67913331	0.6739803	Myt1/Fkbp1b	2
GO:0051222 positive regulation of protein transport	2/97	262/14464	0.00763359	1.13827024	0.18558234	0.52712256	0.67913331	0.6739803	Sfn/Edem1	2
GO:0009134 nucleoside diphosphate catabolic process	1/97	111/14464	0.00900901	1.34336398	0.29838494	0.52752553	0.67913331	0.6739803	Lipa	1
GO:0031398 positive regulation of protein ubiquitination	1/97	111/14464	0.00900901	1.34336398	0.29838494	0.52752553	0.67913331	0.6739803	Nhlrc1	1
GO:0032946 positive regulation of mononuclear cell proliferation	1/97	111/14464	0.00900901	1.34336398	0.29838494	0.52752553	0.67913331	0.6739803	Cdkn1a	1
GO:0034502 protein localization to chromosome	1/97	111/14464	0.00900901	1.34336398	0.29838494	0.52752553	0.67913331	0.6739803	Cct7	1
GO:0048661 positive regulation of smooth muscle cell proliferation	1/97	111/14464	0.00900901	1.34336398	0.29838494	0.52752553	0.67913331	0.6739803	Shc1	1
GO:0051028 mRNA transport	1/97	111/14464	0.00900901	1.34336398	0.29838494	0.52752553	0.67913331	0.6739803	Bicd2	1
GO:0051928 positive regulation of calcium ion transport	1/97	111/14464	0.00900901	1.34336398	0.29838494	0.52752553	0.67913331	0.6739803	Trpv2	1
GO:0002460 adaptive immune response based on somatic recombination of immune receptors built from immunoglobu	2/97	263/14464	0.00760456	1.13394222	0.18012248	0.5291781	0.67913331	0.6739803	Traf2/Lig4	2
GO:0002790 peptide secretion	2/97	263/14464	0.00760456	1.13394222	0.18012248	0.5291781	0.67913331	0.6739803	Myt1/Fkbp1b	2
GO:0050708 regulation of protein secretion	2/97	263/14464	0.00760456	1.13394222	0.18012248	0.5291781	0.67913331	0.6739803	Myt1/Fkbp1b	2
GO:0051051 negative regulation of transport	3/97	415/14464	0.00722892	1.07792821	0.13235125	0.52986137	0.67913331	0.6739803	Gpr35/Fkbp1b/Pcsk9	3
GO:0006413 translational initiation	1/97	112/14464	0.00892857	1.33136966	0.28926611	0.53071859	0.67913331	0.6739803	Mettl3	1
GO:0006612 protein targeting to membrane	1/97	112/14464	0.00892857	1.33136966	0.28926611	0.53071859	0.67913331	0.6739803	Mief1	1
GO:0009791 post-embryonic development	1/97	112/14464	0.00892857	1.33136966	0.28926611	0.53071859	0.67913331	0.6739803	Myt1	1
GO:0035249 synaptic transmission, glutamatergic	1/97	112/14464	0.00892857	1.33136966	0.28926611	0.53071859	0.67913331	0.6739803	Aph1b	1
GO:0051209 release of sequestered calcium ion into cytosol	1/97	112/14464	0.00892857	1.33136966	0.28926611	0.53071859	0.67913331	0.6739803	Fkbp1b	1
GO:0061640 cytoskeleton-dependent cytokinesis	1/97	112/14464	0.00892857	1.33136966	0.28926611	0.53071859	0.67913331	0.6739803	Efhc1	1
GO:0071887 leukocyte apoptotic process	1/97	112/14464	0.00892857	1.33136966	0.28926611	0.53071859	0.67913331	0.6739803	Lipa	1
GO:0008380 RNA splicing	3/97	417/14464	0.00719424	1.07275829	0.12387701	0.53312629	0.67970884	0.67455147	Mettl3/Fam50a/Sugp2	3
GO:0042593 glucose homeostasis	2/97	265/14464	0.00754717	1.12538417	0.16926562	0.5332713	0.67970884	0.67455147	Myt1/Fkbp1b	2
GO:0009135 purine nucleoside diphosphate metabolic process	1/97	113/14464	0.00884956	1.31958763	0.28023354	0.5338903	0.67970884	0.67455147	Lipa	1
GO:0009179 purine ribonucleoside diphosphate metabolic process	1/97	113/14464	0.00884956	1.31958763	0.28023354	0.5338903	0.67970884	0.67455147	Lipa	1
GO:0038202 TORC1 signaling	1/97	113/14464	0.00884956	1.31958763	0.28023354	0.5338903	0.67970884	0.67455147	Stambpl1	1
GO:0051283 negative regulation of sequestering of calcium ion	1/97	113/14464	0.00884956	1.31958763	0.28023354	0.5338903	0.67970884	0.67455147	Fkbp1b	1
GO:1905477 positive regulation of protein localization to membrane	1/97	113/14464	0.00884956	1.31958763	0.28023354	0.5338903	0.67970884	0.67455147	Mief1	1
GO:0033500 carbohydrate homeostasis	2/97	266/14464	0.0075188	1.1211534	0.16386826	0.53530893	0.68074495	0.67557972	Myt1/Fkbp1b	2
GO:0000077 DNA damage checkpoint signaling	1/97	114/14464	0.00877193	1.3080123	0.27128548	0.53704078	0.68074495	0.67557972	Cdkn1a	1
GO:0008637 apoptotic mitochondrial changes	1/97	114/14464	0.00877193	1.3080123	0.27128548	0.53704078	0.68074495	0.67557972	Sfn	1
GO:0051983 regulation of chromosome segregation	1/97	114/14464	0.00877193	1.3080123	0.27128548	0.53704078	0.68074495	0.67557972	Becn1	1
GO:0060048 cardiac muscle contraction	1/97	114/14464	0.00877193	1.3080123	0.27128548	0.53704078	0.68074495	0.67557972	Fkbp1b	1
GO:1990830 cellular response to leukemia inhibitory factor	1/97	114/14464	0.00877193	1.3080123	0.27128548	0.53704078	0.68074495	0.67557972	Ubtf	1
GO:0000956 nuclear-transcribed mRNA catabolic process	1/97	115/14464	0.00869565	1.29663828	0.26242023	0.54017019	0.68273138	0.67755107	Hbs1l	1
GO:0002705 positive regulation of leukocyte mediated immunity	1/97	115/14464	0.00869565	1.29663828	0.26242023	0.54017019	0.68273138	0.67755107	Traf2	1
GO:0051053 negative regulation of DNA metabolic process	1/97	115/14464	0.00869565	1.29663828	0.26242023	0.54017019	0.68273138	0.67755107	Cdkn1a	1
GO:0051282 regulation of sequestering of calcium ion	1/97	115/14464	0.00869565	1.29663828	0.26242023	0.54017019	0.68273138	0.67755107	Fkbp1b	1
GO:0060070 canonical Wnt signaling pathway	2/97	269/14464	0.00743494	1.10864983	0.14779832	0.54138579	0.68327968	0.67809522	Sfrp5/Ctnnd1	2
GO:1903311 regulation of mRNA metabolic process	2/97	269/14464	0.00743494	1.10864983	0.14779832	0.54138579	0.68327968	0.67809522	Mettl3/Traf2	2
GO:0043401 steroid hormone receptor signaling pathway	1/97	116/14464	0.00862069	1.28546036	0.25363614	0.54327867	0.68418668	0.67899533	Ptges3	1
GO:0051208 sequestering of calcium ion	1/97	116/14464	0.00862069	1.28546036	0.25363614	0.54327867	0.68418668	0.67899533	Fkbp1b	1
GO:1990823 response to leukemia inhibitory factor	1/97	116/14464	0.00862069	1.28546036	0.25363614	0.54327867	0.68418668	0.67899533	Ubtf	1
GO:0017148 negative regulation of translation	1/97	117/14464	0.00854701	1.27447352	0.24493162	0.54636634	0.68708516	0.68187182	Nhlrc1	1
GO:0021549 cerebellum development	1/97	117/14464	0.00854701	1.27447352	0.24493162	0.54636634	0.68708516	0.68187182	Tuba1a	1
GO:0010001 glial cell differentiation	2/97	272/14464	0.00735294	1.09642207	0.131908	0.54740838	0.6872327	0.68201825	Mettl3/Tuba1a	2
GO:0015833 peptide transport	2/97	272/14464	0.00735294	1.09642207	0.131908	0.54740838	0.6872327	0.68201825	Myt1/Fkbp1b	2
GO:0031346 positive regulation of cell projection organization	3/97	426/14464	0.00704225	1.05009438	0.08623294	0.54766313	0.6872327	0.68201825	Trpv2/Fkbp1b/Nme1	3
GO:0051258 protein polymerization	2/97	273/14464	0.00732601	1.09240588	0.12665036	0.5494038	0.68797243	0.68275236	Hspa1b/Tuba1a	2
GO:0010821 regulation of mitochondrion organization	1/97	118/14464	0.00847458	1.2636729	0.2363051	0.54943336	0.68797243	0.68275236	Mief1	1
GO:0098773 skin epidermis development	1/97	118/14464	0.00847458	1.2636729	0.2363051	0.54943336	0.68797243	0.68275236	Sfn	1
GO:1903313 positive regulation of mRNA metabolic process	1/97	119/14464	0.00840336	1.2530538	0.22775508	0.55247985	0.69129189	0.68604664	Mettl3	1
GO:0016197 endosomal transport	2/97	276/14464	0.00724638	1.0805319	0.11099257	0.5553536	0.69324479	0.68798471	Cln5/Becn1	2
GO:0031507 heterochromatin formation	1/97	120/14464	0.00833333	1.24261168	0.2192801	0.55550595	0.69324479	0.68798471	Mettl3	1
GO:0051224 negative regulation of protein transport	1/97	120/14464	0.00833333	1.24261168	0.2192801	0.55550595	0.69324479	0.68798471	Fkbp1b	1
GO:0002253 activation of immune response	3/97	431/14464	0.00696056	1.03791231	0.06565619	0.55562697	0.69324479	0.68798471	Hspa1b/Lipa/Becn1	3
GO:0002761 regulation of myeloid leukocyte differentiation	1/97	121/14464	0.00826446	1.23234217	0.21087871	0.5585118	0.69483889	0.68956672	Nme1	1
GO:0031570 DNA integrity checkpoint signaling	1/97	121/14464	0.00826446	1.23234217	0.21087871	0.5585118	0.69483889	0.68956672	Cdkn1a	1
GO:0043414 macromolecule methylation	1/97	121/14464	0.00826446	1.23234217	0.21087871	0.5585118	0.69483889	0.68956672	Mettl3	1
GO:0002683 negative regulation of immune system process	3/97	433/14464	0.00692841	1.03311826	0.05749091	0.55878963	0.69483889	0.68956672	Sfn/Mettl3/Nme1	3
GO:0006874 intracellular calcium ion homeostasis	2/97	278/14464	0.00719424	1.07275829	0.10064841	0.55928966	0.69483889	0.68956672	Efhc1/Fkbp1b	2
GO:0043491 phosphatidylinositol 3-kinase/protein kinase B signal transduction	2/97	278/14464	0.00719424	1.07275829	0.10064841	0.55928966	0.69483889	0.68956672	Sfrp5/Maz	2
GO:0006310 DNA recombination	2/97	279/14464	0.00716846	1.06891328	0.09550417	0.56124852	0.69560432	0.69032634	Lig4/Nhej1	2
GO:0003231 cardiac ventricle development	1/97	122/14464	0.00819672	1.222241	0.20254954	0.56149754	0.69560432	0.69032634	Dctn5	1
GO:0030879 mammary gland development	1/97	122/14464	0.00819672	1.222241	0.20254954	0.56149754	0.69560432	0.69032634	Nme1	1
GO:0045834 positive regulation of lipid metabolic process	1/97	122/14464	0.00819672	1.222241	0.20254954	0.56149754	0.69560432	0.69032634	Sphk2	1
GO:0006633 fatty acid biosynthetic process	1/97	123/14464	0.00813008	1.21230408	0.19429123	0.56446328	0.69667031	0.69138424	Ptges3	1
GO:0032368 regulation of lipid transport	1/97	123/14464	0.00813008	1.21230408	0.19429123	0.56446328	0.69667031	0.69138424	Pcsk9	1
GO:0050864 regulation of B cell activation	1/97	123/14464	0.00813008	1.21230408	0.19429123	0.56446328	0.69667031	0.69138424	Cdkn1a	1
GO:0070665 positive regulation of leukocyte proliferation	1/97	123/14464	0.00813008	1.21230408	0.19429123	0.56446328	0.69667031	0.69138424	Cdkn1a	1
GO:0001933 negative regulation of protein phosphorylation	2/97	281/14464	0.00711744	1.06130535	0.08527064	0.56514788	0.69667031	0.69138424	Cdkn1a/Sfrp5	2
GO:0006302 double-strand break repair	2/97	281/14464	0.00711744	1.06130535	0.08527064	0.56514788	0.69667031	0.69138424	Lig4/Nhej1	2
GO:0006936 muscle contraction	2/97	281/14464	0.00711744	1.06130535	0.08527064	0.56514788	0.69667031	0.69138424	Shc1/Fkbp1b	2
GO:0030111 regulation of Wnt signaling pathway	2/97	282/14464	0.0070922	1.05754186	0.08018105	0.56708837	0.69749032	0.69219803	Sfrp5/Ctnnd1	2
GO:0009185 ribonucleoside diphosphate metabolic process	1/97	124/14464	0.00806452	1.20252744	0.18610247	0.56740918	0.69749032	0.69219803	Lipa	1
GO:0071466 cellular response to xenobiotic stimulus	1/97	124/14464	0.00806452	1.20252744	0.18610247	0.56740918	0.69749032	0.69219803	Cyp2j13	1
GO:1903052 positive regulation of proteolysis involved in protein catabolic process	1/97	124/14464	0.00806452	1.20252744	0.18610247	0.56740918	0.69749032	0.69219803	Hspa1b	1
GO:0002696 positive regulation of leukocyte activation	2/97	283/14464	0.00706714	1.05380496	0.07510937	0.56902271	0.69849137	0.69319148	Cdkn1a/Sphk2	2
GO:0043523 regulation of neuron apoptotic process	2/97	283/14464	0.00706714	1.05380496	0.07510937	0.56902271	0.69849137	0.69319148	Pcsk9/Lig4	2
GO:0000086 G2/M transition of mitotic cell cycle	1/97	125/14464	0.008	1.19290722	0.17798199	0.57033535	0.69863083	0.69332988	Cdkn1a	1
GO:0010950 positive regulation of endopeptidase activity	1/97	125/14464	0.008	1.19290722	0.17798199	0.57033535	0.69863083	0.69332988	Aph1b	1
GO:0022037 metencephalon development	1/97	125/14464	0.008	1.19290722	0.17798199	0.57033535	0.69863083	0.69332988	Tuba1a	1
GO:0043500 muscle adaptation	1/97	126/14464	0.00793651	1.1834397	0.16992854	0.57324193	0.70120846	0.69588796	Becn1	1
GO:0090101 negative regulation of transmembrane receptor protein serine/threonine kinase signaling pathway	1/97	126/14464	0.00793651	1.1834397	0.16992854	0.57324193	0.70120846	0.69588796	Sap30l	1
GO:0051052 regulation of DNA metabolic process	3/97	443/14464	0.00677201	1.00979731	0.01720882	0.57440315	0.70211805	0.69679064	Cdkn1a/Ptges3/Cct7	3
GO:1902105 regulation of leukocyte differentiation	2/97	286/14464	0.00699301	1.04275106	0.06000044	0.57478886	0.70211805	0.69679064	Mettl3/Nme1	2
GO:0046822 regulation of nucleocytoplasmic transport	1/97	127/14464	0.00787402	1.17412128	0.16194091	0.57612905	0.70326367	0.69792758	Sfn	1
GO:0051054 positive regulation of DNA metabolic process	2/97	287/14464	0.00696864	1.03911778	0.05499903	0.5766986	0.70346765	0.69813001	Ptges3/Cct7	2
GO:0010927 cellular component assembly involved in morphogenesis	1/97	128/14464	0.0078125	1.16494845	0.15401792	0.57899684	0.70385176	0.69851121	Agfg1	1
GO:0030856 regulation of epithelial cell differentiation	1/97	128/14464	0.0078125	1.16494845	0.15401792	0.57899684	0.70385176	0.69851121	Sfn	1
GO:0032989 cellular anatomical entity morphogenesis	1/97	128/14464	0.0078125	1.16494845	0.15401792	0.57899684	0.70385176	0.69851121	Agfg1	1
GO:0001666 response to hypoxia	2/97	289/14464	0.00692042	1.03192666	0.04504772	0.58049957	0.70385176	0.69851121	Becn1/Mief1	2
GO:0006090 pyruvate metabolic process	1/97	129/14464	0.00775194	1.15591785	0.14615842	0.58184542	0.70385176	0.69851121	Lipa	1
GO:0009132 nucleoside diphosphate metabolic process	1/97	129/14464	0.00775194	1.15591785	0.14615842	0.58184542	0.70385176	0.69851121	Lipa	1
GO:0015748 organophosphate ester transport	1/97	129/14464	0.00775194	1.15591785	0.14615842	0.58184542	0.70385176	0.69851121	Abcc5	1
GO:0016482 cytosolic transport	1/97	129/14464	0.00775194	1.15591785	0.14615842	0.58184542	0.70385176	0.69851121	Cln5	1
GO:0017015 regulation of transforming growth factor beta receptor signaling pathway	1/97	129/14464	0.00775194	1.15591785	0.14615842	0.58184542	0.70385176	0.69851121	Sap30l	1
GO:0030183 B cell differentiation	1/97	129/14464	0.00775194	1.15591785	0.14615842	0.58184542	0.70385176	0.69851121	Nhej1	1
GO:0032543 mitochondrial translation	1/97	129/14464	0.00775194	1.15591785	0.14615842	0.58184542	0.70385176	0.69851121	Mrps27	1
GO:0048259 regulation of receptor-mediated endocytosis	1/97	129/14464	0.00775194	1.15591785	0.14615842	0.58184542	0.70385176	0.69851121	Pcsk9	1
GO:0050769 positive regulation of neurogenesis	2/97	291/14464	0.00687285	1.02483438	0.03516421	0.58427585	0.70532214	0.69997042	Trpv2/Lig4	2
GO:0006906 vesicle fusion	1/97	130/14464	0.00769231	1.14702617	0.1383613	0.58467493	0.70532214	0.69997042	Rab39	1
GO:0043433 negative regulation of DNA-binding transcription factor activity	1/97	130/14464	0.00769231	1.14702617	0.1383613	0.58467493	0.70532214	0.69997042	Commd7	1
GO:0051592 response to calcium ion	1/97	130/14464	0.00769231	1.14702617	0.1383613	0.58467493	0.70532214	0.69997042	Tuba1a	1
GO:0007098 centrosome cycle	1/97	131/14464	0.00763359	1.13827024	0.13062546	0.58748549	0.70773579	0.70236576	Tuba1a	1
GO:1903844 regulation of cellular response to transforming growth factor beta stimulus	1/97	131/14464	0.00763359	1.13827024	0.13062546	0.58748549	0.70773579	0.70236576	Sap30l	1
GO:0010952 positive regulation of peptidase activity	1/97	132/14464	0.00757576	1.12964699	0.12294985	0.59027722	0.71003551	0.70464803	Aph1b	1
GO:0090174 organelle membrane fusion	1/97	132/14464	0.00757576	1.12964699	0.12294985	0.59027722	0.71003551	0.70464803	Rab39	1
GO:0098771 inorganic ion homeostasis	3/97	454/14464	0.00660793	0.98533085	-0.0260943	0.59118505	0.71003551	0.70464803	Efhc1/Fkbp1b/Atp1b3	3
GO:0055074 calcium ion homeostasis	2/97	295/14464	0.00677966	1.01093832	0.01559639	0.59175426	0.71003551	0.70464803	Efhc1/Fkbp1b	2
GO:0006397 mRNA processing	3/97	455/14464	0.00659341	0.98316529	-0.0299806	0.59268989	0.71003551	0.70464803	Mettl3/Fam50a/Sugp2	3
GO:0010817 regulation of hormone levels	3/97	455/14464	0.00659341	0.98316529	-0.0299806	0.59268989	0.71003551	0.70464803	Myt1/Fkbp1b/Stard3	3
GO:0032675 regulation of interleukin-6 production	1/97	133/14464	0.0075188	1.1211534	0.11533343	0.59305025	0.71003551	0.70464803	Sphk2	1
GO:0050731 positive regulation of peptidyl-tyrosine phosphorylation	1/97	133/14464	0.0075188	1.1211534	0.11533343	0.59305025	0.71003551	0.70464803	Ctnnd1	1
GO:1905039 carboxylic acid transmembrane transport	1/97	133/14464	0.0075188	1.1211534	0.11533343	0.59305025	0.71003551	0.70464803	Abcc5	1
GO:0003158 endothelium development	1/97	134/14464	0.00746269	1.11278658	0.10777518	0.59580471	0.71284506	0.70743627	Lipa	1
GO:0019216 regulation of lipid metabolic process	2/97	298/14464	0.00671141	1.00076109	0.00109082	0.59729809	0.714143	0.70872436	Sphk2/Golm1	2
GO:0071902 positive regulation of protein serine/threonine kinase activity	1/97	135/14464	0.00740741	1.10454372	0.10027413	0.59854072	0.71465107	0.70922857	Traf2	1
GO:1904950 negative regulation of establishment of protein localization	1/97	135/14464	0.00740741	1.10454372	0.10027413	0.59854072	0.71465107	0.70922857	Fkbp1b	1
GO:0007093 mitotic cell cycle checkpoint signaling	1/97	136/14464	0.00735294	1.09642207	0.09282932	0.60125839	0.71496576	0.70954087	Cdkn1a	1
GO:0050657 nucleic acid transport	1/97	136/14464	0.00735294	1.09642207	0.09282932	0.60125839	0.71496576	0.70954087	Bicd2	1
GO:0050658 RNA transport	1/97	136/14464	0.00735294	1.09642207	0.09282932	0.60125839	0.71496576	0.70954087	Bicd2	1
GO:1903825 organic acid transmembrane transport	1/97	136/14464	0.00735294	1.09642207	0.09282932	0.60125839	0.71496576	0.70954087	Abcc5	1
GO:1904064 positive regulation of cation transmembrane transport	1/97	136/14464	0.00735294	1.09642207	0.09282932	0.60125839	0.71496576	0.70954087	Atp1b3	1
GO:2000241 regulation of reproductive process	1/97	136/14464	0.00735294	1.09642207	0.09282932	0.60125839	0.71496576	0.70954087	Mettl3	1
GO:0042326 negative regulation of phosphorylation	2/97	301/14464	0.00664452	0.99078672	-0.0132725	0.60278621	0.71629524	0.71086026	Cdkn1a/Sfrp5	2
GO:1902600 proton transmembrane transport	1/97	137/14464	0.00729927	1.08841899	0.0854398	0.60395785	0.71719995	0.71175811	Sphk2	1
GO:0001822 kidney development	2/97	303/14464	0.00660066	0.98424688	-0.0227705	0.60641399	0.71794541	0.71249792	Pcsk9/Ctnnd1	2
GO:0050867 positive regulation of cell activation	2/97	303/14464	0.00660066	0.98424688	-0.0227705	0.60641399	0.71794541	0.71249792	Cdkn1a/Sphk2	2
GO:0050851 antigen receptor-mediated signaling pathway	1/97	138/14464	0.00724638	1.0805319	0.07810467	0.60663923	0.71794541	0.71249792	Lipa	1
GO:0051606 detection of stimulus	1/97	138/14464	0.00724638	1.0805319	0.07810467	0.60663923	0.71794541	0.71249792	Pde6d	1
GO:1903322 positive regulation of protein modification by small protein conjugation or removal	1/97	138/14464	0.00724638	1.0805319	0.07810467	0.60663923	0.71794541	0.71249792	Nhlrc1	1
GO:0009611 response to wounding	3/97	465/14464	0.00645161	0.96202195	-0.0683979	0.60754474	0.71818005	0.71273077	Pdia6/Cdkn1a/Fkbp1b	3
GO:0036293 response to decreased oxygen levels	2/97	304/14464	0.00657895	0.98100922	-0.0274967	0.6082186	0.71818005	0.71273077	Becn1/Mief1	2
GO:0032635 interleukin-6 production	1/97	139/14464	0.00719424	1.07275829	0.07082304	0.60930264	0.71818005	0.71273077	Sphk2	1
GO:0051236 establishment of RNA localization	1/97	139/14464	0.00719424	1.07275829	0.07082304	0.60930264	0.71818005	0.71273077	Bicd2	1
GO:0120161 regulation of cold-induced thermogenesis	1/97	139/14464	0.00719424	1.07275829	0.07082304	0.60930264	0.71818005	0.71273077	Trpv2	1
GO:1901875 positive regulation of post-translational protein modification	1/97	139/14464	0.00719424	1.07275829	0.07082304	0.60930264	0.71818005	0.71273077	Nhlrc1	1
GO:0002694 regulation of leukocyte activation	3/97	467/14464	0.00642398	0.95790194	-0.0759863	0.61047316	0.71907485	0.71361879	Cdkn1a/Mettl3/Sphk2	3
GO:0051347 positive regulation of transferase activity	2/97	306/14464	0.00653595	0.9745974	-0.0369037	0.61180923	0.71935807	0.71389986	Cdkn1a/Traf2	2
GO:0002706 regulation of lymphocyte mediated immunity	1/97	140/14464	0.00714286	1.06509573	0.06359403	0.6119482	0.71935807	0.71389986	Traf2	1
GO:0071456 cellular response to hypoxia	1/97	140/14464	0.00714286	1.06509573	0.06359403	0.6119482	0.71935807	0.71389986	Mief1	1
GO:0007611 learning or memory	2/97	307/14464	0.00651466	0.97142281	-0.0415848	0.61359526	0.72002607	0.71456279	Tuba1a/Aph1b	2
GO:0051235 maintenance of location	2/97	307/14464	0.00651466	0.97142281	-0.0415848	0.61359526	0.72002607	0.71456279	Fkbp1b/Lipa	2
GO:0031023 microtubule organizing center organization	1/97	141/14464	0.0070922	1.05754186	0.0564168	0.61457603	0.72002607	0.71456279	Tuba1a	1
GO:0031644 regulation of nervous system process	1/97	141/14464	0.0070922	1.05754186	0.0564168	0.61457603	0.72002607	0.71456279	Gpr35	1
GO:0044839 cell cycle G2/M phase transition	1/97	141/14464	0.0070922	1.05754186	0.0564168	0.61457603	0.72002607	0.71456279	Cdkn1a	1
GO:0050680 negative regulation of epithelial cell proliferation	1/97	142/14464	0.00704225	1.05009438	0.04929052	0.61718625	0.72211617	0.71663703	Sfn	1

GO:1903034 regulation of response to wounding	1/97	142/14464	0.00704225	1.05009438	0.04929052	0.61718625	0.72211617	0.71663703	Fkbp1b	1
GO:0045089 positive regulation of innate immune response	2/97	310/14464	0.00645161	0.96202195	-0.05554	0.61891624	0.72273091	0.7172471	Hspa1b/Becn1	2
GO:0009451 RNA modification	1/97	143/14464	0.00699301	1.04275106	0.04221437	0.61977897	0.72273091	0.7172471	Mettl3	1
GO:0043123 positive regulation of canonical NF-kappaB signal transduction	1/97	143/14464	0.00699301	1.04275106	0.04221437	0.61977897	0.72273091	0.7172471	Traf2	1
GO:0106106 cold-induced thermogenesis	1/97	143/14464	0.00699301	1.04275106	0.04221437	0.61977897	0.72273091	0.7172471	Trpv2	1
GO:1903305 regulation of regulated secretory pathway	1/97	143/14464	0.00699301	1.04275106	0.04221437	0.61977897	0.72273091	0.7172471	Sphk2	1
GO:0043281 regulation of cysteine-type endopeptidase activity involved in apoptotic process	1/97	144/14464	0.00694444	1.03550974	0.03518756	0.62235431	0.7242845	0.71878891	Hspa1b	1
GO:0043467 regulation of generation of precursor metabolites and energy	1/97	144/14464	0.00694444	1.03550974	0.03518756	0.62235431	0.7242845	0.71878891	Sphk2	1
GO:0072006 nephron development	1/97	144/14464	0.00694444	1.03550974	0.03518756	0.62235431	0.7242845	0.71878891	Ctnnd1	1
GO:0007178 cell surface receptor protein serine/threonine kinase signaling pathway	2/97	313/14464	0.00638978	0.95280129	-0.0693651	0.62418158	0.72592775	0.72041969	Becn1/Sap30l	2
GO:0007596 blood coagulation	1/97	145/14464	0.00689655	1.02836829	0.02820933	0.62491238	0.72629444	0.7207836	Pdia6	1
GO:0046879 hormone secretion	2/97	314/14464	0.00636943	0.94976689	-0.073945	0.62592435	0.72698722	0.72147111	Myt1/Fkbp1b	2
GO:0007286 spermatid development	1/97	146/14464	0.00684932	1.02132467	0.02127892	0.6274533	0.72803671	0.72251265	Agfg1	1
GO:0072001 renal system development	2/97	315/14464	0.00634921	0.94675176	-0.0785109	0.62766094	0.72803671	0.72251265	Pcsk9/Ctnnd1	2
GO:0170033 L-amino acid metabolic process	1/97	147/14464	0.00680272	1.01437688	0.01439559	0.62997719	0.73023882	0.72469804	P4ha1	1
GO:0001935 endothelial cell proliferation	1/97	148/14464	0.00675676	1.00752299	0.00755862	0.63248416	0.73168915	0.72613737	Lipa	1
GO:0007599 hemostasis	1/97	148/14464	0.00675676	1.00752299	0.00755862	0.63248416	0.73168915	0.72613737	Pdia6	1
GO:0034614 cellular response to reactive oxygen species	1/97	148/14464	0.00675676	1.00752299	0.00755862	0.63248416	0.73168915	0.72613737	Becn1	1
GO:0006941 striated muscle contraction	1/97	150/14464	0.00666667	0.99408935	-0.0059791	0.63744776	0.73557956	0.72999826	Fkbp1b	1
GO:0031623 receptor internalization	1/97	150/14464	0.00666667	0.99408935	-0.0059791	0.63744776	0.73557956	0.72999826	Pcsk9	1
GO:0050817 coagulation	1/97	150/14464	0.00666667	0.99408935	-0.0059791	0.63744776	0.73557956	0.72999826	Pdia6	1
GO:0009914 hormone transport	2/97	321/14464	0.00623053	0.92905546	-0.1056167	0.63795115	0.73557956	0.72999826	Myt1/Fkbp1b	2
GO:0042060 wound healing	2/97	321/14464	0.00623053	0.92905546	-0.1056167	0.63795115	0.73557956	0.72999826	Pdia6/Cdkn1a	2
GO:0007224 smoothened signaling pathway	1/97	151/14464	0.00662252	0.98750597	-0.0126811	0.63990463	0.73589032	0.73030667	Tuba1a	1
GO:0045580 regulation of T cell differentiation	1/97	151/14464	0.00662252	0.98750597	-0.0126811	0.63990463	0.73589032	0.73030667	Mettl3	1
GO:0141193 nuclear receptor-mediated signaling pathway	1/97	151/14464	0.00662252	0.98750597	-0.0126811	0.63990463	0.73589032	0.73030667	Ptges3	1
GO:2000045 regulation of G1/S transition of mitotic cell cycle	1/97	151/14464	0.00662252	0.98750597	-0.0126811	0.63990463	0.73589032	0.73030667	Cdkn1a	1
GO:1905952 regulation of lipid localization	1/97	152/14464	0.00657895	0.98100922	-0.0193395	0.64234502	0.7382111	0.73260984	Pcsk9	1
GO:0034767 positive regulation of monoatomic ion transmembrane transport	1/97	153/14464	0.00653595	0.9745974	-0.025955	0.64476903	0.73987248	0.73425861	Atp1b3	1
GO:0045814 negative regulation of gene expression, epigenetic	1/97	153/14464	0.00653595	0.9745974	-0.025955	0.64476903	0.73987248	0.73425861	Mettl3	1
GO:0006575 cellular modified amino acid metabolic process	1/97	154/14464	0.00649351	0.96826884	-0.0325281	0.64717679	0.73987248	0.73425861	P4ha1	1
GO:0035150 regulation of tube size	1/97	154/14464	0.00649351	0.96826884	-0.0325281	0.64717679	0.73987248	0.73425861	Shc1	1
GO:0035296 regulation of tube diameter	1/97	154/14464	0.00649351	0.96826884	-0.0325281	0.64717679	0.73987248	0.73425861	Shc1	1
GO:0048515 spermatid differentiation	1/97	154/14464	0.00649351	0.96826884	-0.0325281	0.64717679	0.73987248	0.73425861	Agfg1	1
GO:0097746 blood vessel diameter maintenance	1/97	154/14464	0.00649351	0.96826884	-0.0325281	0.64717679	0.73987248	0.73425861	Shc1	1
GO:1903531 negative regulation of secretion by cell	1/97	154/14464	0.00649351	0.96826884	-0.0325281	0.64717679	0.73987248	0.73425861	Fkbp1b	1
GO:0070482 response to oxygen levels	2/97	327/14464	0.00611621	0.91200858	-0.1322415	0.64802031	0.74035261	0.7347351	Becn1/Mief1	2
GO:0072331 signal transduction by p53 class mediator	1/97	155/14464	0.00645161	0.96202195	-0.0390594	0.64956839	0.74163655	0.73600929	Cdkn1a	1
GO:0001906 cell killing	1/97	156/14464	0.00641026	0.95585514	-0.0455496	0.65194395	0.74289311	0.73725632	Dnase1l3	1
GO:0002822 regulation of adaptive immune response based on somatic recombination of immune receptors built from	1/97	156/14464	0.00641026	0.95585514	-0.0455496	0.65194395	0.74289311	0.73725632	Traf2	1
GO:0072330 monocarboxylic acid biosynthetic process	1/97	156/14464	0.00641026	0.95585514	-0.0455496	0.65194395	0.74289311	0.73725632	Ptges3	1
GO:0015980 energy derivation by oxidation of organic compounds	2/97	330/14464	0.00606061	0.90371759	-0.1453796	0.65297235	0.74302582	0.73738802	Ptges3/Nhlrc1	2
GO:0043409 negative regulation of MAPK cascade	1/97	157/14464	0.00636943	0.94976689	-0.0519993	0.65430357	0.74302582	0.73738802	Sfrp5	1
GO:0051168 nuclear export	1/97	157/14464	0.00636943	0.94976689	-0.0519993	0.65430357	0.74302582	0.73738802	Sfn	1
GO:0062207 regulation of pattern recognition receptor signaling pathway	1/97	157/14464	0.00636943	0.94976689	-0.0519993	0.65430357	0.74302582	0.73738802	Hspa1b	1
GO:0002833 positive regulation of response to biotic stimulus	2/97	331/14464	0.0060423	0.90098732	-0.1497337	0.65461085	0.74302582	0.73738802	Hspa1b/Becn1	2
GO:0048638 regulation of developmental growth	2/97	331/14464	0.0060423	0.90098732	-0.1497337	0.65461085	0.74302582	0.73738802	Cdkn1a/Trpv2	2
GO:0061025 membrane fusion	1/97	159/14464	0.00628931	0.93782014	-0.0647793	0.65897542	0.74700975	0.74134172	Rab39	1
GO:0099072 regulation of postsynaptic membrane neurotransmitter receptor levels	1/97	159/14464	0.00628931	0.93782014	-0.0647793	0.65897542	0.74700975	0.74134172	Ctnnd1	1
GO:0002181 cytoplasmic translation	1/97	160/14464	0.00625	0.93195876	-0.0711108	0.66128785	0.74866008	0.74297953	Mettl3	1
GO:0065004 protein-DNA complex assembly	1/97	160/14464	0.00625	0.93195876	-0.0711108	0.66128785	0.74866008	0.74297953	Taf4b	1
GO:0032680 regulation of tumor necrosis factor production	1/97	161/14464	0.00621118	0.9261702	-0.0774039	0.66358477	0.7502886	0.74459569	Sphk2	1
GO:0051897 positive regulation of phosphatidylinositol 3-kinase/protein kinase B signal transduction	1/97	161/14464	0.00621118	0.9261702	-0.0774039	0.66358477	0.7502886	0.74459569	Maz	1
GO:0002250 adaptive immune response	2/97	337/14464	0.00593472	0.88494601	-0.1755997	0.66431434	0.75057553	0.74488045	Traf2/Lig4	2
GO:0003205 cardiac chamber development	1/97	162/14464	0.00617284	0.9204531	-0.0836593	0.66586627	0.75057553	0.74488045	Dctn5	1
GO:0070374 positive regulation of ERK1 and ERK2 cascade	1/97	162/14464	0.00617284	0.9204531	-0.0836593	0.66586627	0.75057553	0.74488045	Shc1	1
GO:0002285 lymphocyte activation involved in immune response	1/97	163/14464	0.00613497	0.91480615	-0.0898775	0.66813245	0.75057553	0.74488045	Lig4	1
GO:0006403 RNA localization	1/97	163/14464	0.00613497	0.91480615	-0.0898775	0.66813245	0.75057553	0.74488045	Bicd2	1
GO:0006606 protein import into nucleus	1/97	163/14464	0.00613497	0.91480615	-0.0898775	0.66813245	0.75057553	0.74488045	Cdkn1a	1
GO:0071674 mononuclear cell migration	1/97	163/14464	0.00613497	0.91480615	-0.0898775	0.66813245	0.75057553	0.74488045	Gpr35	1
GO:1901991 negative regulation of mitotic cell cycle phase transition	1/97	163/14464	0.00613497	0.91480615	-0.0898775	0.66813245	0.75057553	0.74488045	Cdkn1a	1
GO:1903555 regulation of tumor necrosis factor superfamily cytokine production	1/97	163/14464	0.00613497	0.91480615	-0.0898775	0.66813245	0.75057553	0.74488045	Sphk2	1
GO:2000116 regulation of cysteine-type endopeptidase activity	1/97	163/14464	0.00613497	0.91480615	-0.0898775	0.66813245	0.75057553	0.74488045	Hspa1b	1
GO:0030534 adult behavior	1/97	164/14464	0.00609756	0.90922806	-0.0960589	0.67038342	0.75262057	0.74690997	Tuba1a	1
GO:0008016 regulation of heart contraction	1/97	165/14464	0.00606061	0.90371759	-0.1022041	0.67261928	0.75464603	0.74892006	Fkbp1b	1
GO:0032640 tumor necrosis factor production	1/97	166/14464	0.0060241	0.89827351	-0.1083135	0.67484013	0.75665205	0.75091087	Sphk2	1
GO:0050890 cognition	2/97	344/14464	0.00581395	0.86693838	-0.2052328	0.67536053	0.75669855	0.75095701	Tuba1a/Aph1b	2
GO:0010563 negative regulation of phosphorus metabolic process	2/97	345/14464	0.0057971	0.86442552	-0.2094197	0.67691457	0.75669855	0.75095701	Cdkn1a/Sfrp5	2
GO:0045936 negative regulation of phosphate metabolic process	2/97	345/14464	0.0057971	0.86442552	-0.2094197	0.67691457	0.75669855	0.75095701	Cdkn1a/Sfrp5	2
GO:0045807 positive regulation of endocytosis	1/97	167/14464	0.00598802	0.89289462	-0.1143876	0.67704607	0.75669855	0.75095701	Pcsk9	1
GO:1902905 positive regulation of supramolecular fiber organization	1/97	167/14464	0.00598802	0.89289462	-0.1143876	0.67704607	0.75669855	0.75095701	Hspa1b	1
GO:0050678 regulation of epithelial cell proliferation	2/97	346/14464	0.00578035	0.86192718	-0.2135953	0.67846264	0.75721086	0.75146543	Sfn/Nme1	2
GO:0016052 carbohydrate catabolic process	1/97	168/14464	0.00595238	0.88757977	-0.120427	0.6792372	0.75721086	0.75146543	Lipa	1
GO:0071706 tumor necrosis factor superfamily cytokine production	1/97	168/14464	0.00595238	0.88757977	-0.120427	0.6792372	0.75721086	0.75146543	Sphk2	1
GO:0140053 mitochondrial gene expression	1/97	168/14464	0.00595238	0.88757977	-0.120427	0.6792372	0.75721086	0.75146543	Mrps27	1
GO:0009755 hormone-mediated signaling pathway	1/97	169/14464	0.00591716	0.88232782	-0.126432	0.68141361	0.75770419	0.75195502	Ptges3	1
GO:0019362 pyridine nucleotide metabolic process	1/97	169/14464	0.00591716	0.88232782	-0.126432	0.68141361	0.75770419	0.75195502	Lipa	1
GO:0046496 nicotinamide nucleotide metabolic process	1/97	169/14464	0.00591716	0.88232782	-0.126432	0.68141361	0.75770419	0.75195502	Lipa	1
GO:0051170 import into nucleus	1/97	169/14464	0.00591716	0.88232782	-0.126432	0.68141361	0.75770419	0.75195502	Cdkn1a	1
GO:0002819 regulation of adaptive immune response	1/97	170/14464	0.00588235	0.87713766	-0.1324032	0.68357541	0.75810804	0.75235581	Traf2	1
GO:0030902 hindbrain development	1/97	170/14464	0.00588235	0.87713766	-0.1324032	0.68357541	0.75810804	0.75235581	Tuba1a	1
GO:0046890 regulation of lipid biosynthetic process	1/97	170/14464	0.00588235	0.87713766	-0.1324032	0.68357541	0.75810804	0.75235581	Sphk2	1
GO:0051048 negative regulation of secretion	1/97	170/14464	0.00588235	0.87713766	-0.1324032	0.68357541	0.75810804	0.75235581	Fkbp1b	1
GO:0022408 negative regulation of cell-cell adhesion	1/97	171/14464	0.00584795	0.8720082	-0.1383409	0.68572268	0.75810804	0.75235581	Mettl3	1
GO:0050770 regulation of axonogenesis	1/97	171/14464	0.00584795	0.8720082	-0.1383409	0.68572268	0.75810804	0.75235581	Trpv2	1
GO:0051494 negative regulation of cytoskeleton organization	1/97	171/14464	0.00584795	0.8720082	-0.1383409	0.68572268	0.75810804	0.75235581	Togaram2	1
GO:1901605 alpha-amino acid metabolic process	1/97	171/14464	0.00584795	0.8720082	-0.1383409	0.68572268	0.75810804	0.75235581	P4ha1	1
GO:0016050 vesicle organization	2/97	351/14464	0.00569801	0.84964901	-0.2343053	0.6861138	0.75810804	0.75235581	Rab39/Agfg1	2
GO:0051962 positive regulation of nervous system development	2/97	351/14464	0.00569801	0.84964901	-0.2343053	0.6861138	0.75810804	0.75235581	Trpv2/Lig4	2
GO:0009306 protein secretion	2/97	352/14464	0.00568182	0.84723524	-0.2384143	0.68762626	0.758594	0.75283807	Myt1/Fkbp1b	2
GO:1902904 negative regulation of supramolecular fiber organization	1/97	172/14464	0.00581395	0.86693838	-0.1442456	0.68785554	0.758594	0.75283807	Togaram2	1
GO:2001242 regulation of intrinsic apoptotic signaling pathway	1/97	172/14464	0.00581395	0.86693838	-0.1442456	0.68785554	0.758594	0.75283807	Becn1	1
GO:0035592 establishment of protein localization to extracellular region	2/97	354/14464	0.00564972	0.8424486	-0.2465996	0.69063348	0.76042753	0.7546577	Myt1/Fkbp1b	2
GO:0001659 temperature homeostasis	1/97	174/14464	0.00574713	0.85697358	-0.1559577	0.69207837	0.76042753	0.7546577	Trpv2	1
GO:0007612 learning	1/97	174/14464	0.00574713	0.85697358	-0.1559577	0.69207837	0.76042753	0.7546577	Tuba1a	1
GO:0043484 regulation of RNA splicing	1/97	174/14464	0.00574713	0.85697358	-0.1559577	0.69207837	0.76042753	0.7546577	Fam50a	1
GO:0048771 tissue remodeling	1/97	174/14464	0.00574713	0.85697358	-0.1559577	0.69207837	0.76042753	0.7546577	Lipa	1
GO:0010639 negative regulation of organelle organization	2/97	355/14464	0.0056338	0.8400755	-0.2506762	0.69212826	0.76042753	0.7546577	Becn1/Togaram2	2
GO:0042770 signal transduction in response to DNA damage	1/97	175/14464	0.00571429	0.85207658	-0.1617659	0.69416853	0.76123374	0.75545779	Cdkn1a	1
GO:0046777 protein autophosphorylation	1/97	175/14464	0.00571429	0.85207658	-0.1617659	0.69416853	0.76123374	0.75545779	Ctnnd1	1
GO:1902806 regulation of cell cycle G1/S phase transition	1/97	175/14464	0.00571429	0.85207658	-0.1617659	0.69416853	0.76123374	0.75545779	Cdkn1a	1
GO:0071692 protein localization to extracellular region	2/97	357/14464	0.00560224	0.8353692	-0.2587974	0.69510022	0.76177755	0.75599747	Myt1/Fkbp1b	2
GO:0072524 pyridine-containing compound metabolic process	1/97	176/14464	0.00568182	0.84723524	-0.1675428	0.69624464	0.76255366	0.75676769	Lipa	1
GO:0022618 protein-RNA complex assembly	1/97	177/14464	0.00564972	0.8424486	-0.1732888	0.69830681	0.76433332	0.75853385	Ptges3	1
GO:0001764 neuron migration	1/97	178/14464	0.00561798	0.83771574	-0.1790041	0.70035513	0.76513798	0.7593324	Tuba1a	1
GO:0045619 regulation of lymphocyte differentiation	1/97	178/14464	0.00561798	0.83771574	-0.1790041	0.70035513	0.76513798	0.7593324	Mettl3	1
GO:0051495 positive regulation of cytoskeleton organization	1/97	178/14464	0.00561798	0.83771574	-0.1790041	0.70035513	0.76513798	0.7593324	Hspa1b	1
GO:0030595 leukocyte chemotaxis	1/97	179/14464	0.00558659	0.83303577	-0.1846894	0.70238967	0.76644449	0.760629	Gpr35	1
GO:0007600 sensory perception	2/97	362/14464	0.00552486	0.82383095	-0.2789174	0.70242796	0.76644449	0.760629	Cln5/Pde6d	2
GO:0071824 protein-DNA complex organization	1/97	180/14464	0.00555556	0.82840779	-0.1903448	0.70441055	0.76812828	0.76230002	Taf4b	1
GO:0000075 cell cycle checkpoint signaling	1/97	181/14464	0.00552486	0.82383095	-0.1959707	0.70641784	0.7688782	0.76304424	Cdkn1a	1
GO:0019722 calcium-mediated signaling	1/97	181/14464	0.00552486	0.82383095	-0.1959707	0.70641784	0.7688782	0.76304424	Fkbp1b	1
GO:0051260 protein homooligomerization	1/97	181/14464	0.00552486	0.82383095	-0.1959707	0.70641784	0.7688782	0.76304424	Vwa1	1
GO:0042063 gliogenesis	2/97	366/14464	0.00546448	0.81482733	-0.2948293	0.70818579	0.77008927	0.76424613	Mettl3/Tuba1a	2
GO:0042742 defense response to bacterium	1/97	182/14464	0.00549451	0.81930441	-0.2015676	0.70841164	0.77008927	0.76424613	Shc1	1
GO:0042180 cellular ketone metabolic process	1/97	183/14464	0.00546448	0.81482733	-0.2071358	0.71039204	0.77080403	0.76495546	Stard3	1
GO:0048639 positive regulation of developmental growth	1/97	183/14464	0.00546448	0.81482733	-0.2071358	0.71039204	0.77080403	0.76495546	Trpv2	1
GO:0071383 cellular response to steroid hormone stimulus	1/97	183/14464	0.00546448	0.81482733	-0.2071358	0.71039204	0.77080403	0.76495546	Ptges3	1
GO:0050807 regulation of synapse organization	2/97	370/14464	0.00540541	0.80601839	-0.310582	0.71385166	0.77407735	0.76820395	Nrg2/Tuba1a	2
GO:0044242 cellular lipid catabolic process	1/97	185/14464	0.00540541	0.80601839	-0.2181873	0.71431299	0.7740974	0.76822385	Smpd4	1
GO:0071826 protein-RNA complex organization	1/97	186/14464	0.00537634	0.80168496	-0.2236714	0.71625372	0.77571964	0.76983378	Ptges3	1
GO:0050679 positive regulation of epithelial cell proliferation	1/97	187/14464	0.00534759	0.79739787	-0.2291281	0.7181814	0.77732575	0.7714277	Nme1	1
GO:1902903 regulation of supramolecular fiber organization	2/97	374/14464	0.00534759	0.79739787	-0.3261792	0.71942632	0.77819134	0.77228672	Hspa1b/Togaram2	2
GO:0043524 negative regulation of neuron apoptotic process	1/97	188/14464	0.00531915	0.79315639	-0.2345577	0.72009612	0.77843414	0.77252768	Lig4	1
GO:0050803 regulation of synapse structure or activity	2/97	376/14464	0.00531915	0.79315639	-0.3339207	0.7221797	0.78020403	0.77428414	Nrg2/Tuba1a	2
GO:0007179 transforming growth factor beta receptor signaling pathway	1/97	190/14464	0.00526316	0.78480738	-0.2453372	0.72388701	0.78108302	0.77515646	Sap30l	1
GO:0032259 methylation	1/97	190/14464	0.00526316	0.78480738	-0.2453372	0.72388701	0.78108302	0.77515646	Mettl3	1
GO:0050730 regulation of peptidyl-tyrosine phosphorylation	1/97	191/14464	0.0052356	0.78069844	-0.2506876	0.72576335	0.78262452	0.77668626	Ctnnd1	1
GO:0031334 positive regulation of protein-containing complex assembly	1/97	192/14464	0.00520833	0.7766323	-0.2560122	0.72762708	0.78318481	0.7772423	Hspa1b	1
GO:0032271 regulation of protein polymerization	1/97	192/14464	0.00520833	0.7766323	-0.2560122	0.72762708	0.78318481	0.7772423	Hspa1b	1
GO:0050670 regulation of lymphocyte proliferation	1/97	192/14464	0.00520833	0.7766323	-0.2560122	0.72762708	0.78318481	0.7772423	Cdkn1a	1
GO:0051650 establishment of vesicle localization	1/97	194/14464	0.00515464	0.76862578	-0.2665851	0.731317	0.78667208	0.78070311	Stard3	1
GO:0008361 regulation of cell size	1/97	195/14464	0.00512821	0.76468411	-0.271834	0.73314337	0.78815167	0.78217147	Trpv2	1
GO:0016055 Wnt signaling pathway	2/97	385/14464	0.00519481	0.77461508	-0.3682997	0.73429293	0.78864679	0.78266284	Sfrp5/Ctnnd1	2
GO:0032944 regulation of mononuclear cell proliferation	1/97	196/14464	0.00510204	0.76078266	-0.2770583	0.73495745	0.78864679	0.78266284	Cdkn1a	1
GO:0097529 myeloid leukocyte migration	1/97	196/14464	0.00510204	0.76078266	-0.2770583	0.73495745	0.78864679	0.78266284	Gpr35	1
GO:0006006 glucose metabolic process	1/97	197/14464	0.00507614	0.75692082	-0.2822582	0.73675932	0.78912702	0.78313942	Lipa	1
GO:0051963 regulation of synapse assembly	1/97	197/14464	0.00507614	0.75692082	-0.2822582	0.73675932	0.78912702	0.78313942	Nrg2	1
GO:0060538 skeletal muscle organ development	1/97	197/14464	0.00507614	0.75692082	-0.2822582	0.73675932	0.78912702	0.78313942	Myt1	1
GO:0006979 response to oxidative stress	2/97	388/14464	0.00515464	0.76862578	-0.3795974	0.73823105	0.79007575	0.78408096	Fkbp1b/Becn1	2
GO:0002064 epithelial cell development	1/97	198/14464	0.00505051	0.75309799	-0.287434	0.73854907	0.79007575	0.78408096	Sfn	1
GO:0009612 response to mechanical stimulus	1/97	202/14464	0.0049505	0.73818516	-0.3079015	0.74558843	0.79663116	0.79058662	Tuba1a	1
GO:0017157 regulation of exocytosis	1/97	202/14464	0.0049505	0.73818516	-0.3079015	0.74558843	0.79663116	0.79058662	Sphk2	1
GO:0060047 heart contraction	1/97	203/14464	0.00492611	0.73454878	-0.3129607	0.74731876	0.79799217	0.79193731	Fkbp1b	1
GO:0007623 circadian rhythm	1/97	204/14464	0.00490196	0.73094805	-0.3179973	0.74903743	0.79933909	0.79327401	Mettl3	1
GO:0051651 maintenance of location in cell	1/97	205/14464	0.00487805	0.72738245	-0.3230117	0.75074454	0.79997346	0.79390357	Fkbp1b	1
GO:0071216 cellular response to biotic stimulus	1/97	205/14464	0.00487805	0.72738245	-0.3230117	0.75074454	0.79997346	0.79390357	Smc1a	1
GO:0022407 regulation of cell-cell adhesion	2/97	398/14464	0.00502513	0.74931358	-0.4166957	0.75100483	0.79997346	0.79390357	Sfn/Mettl3	2
GO:0000082 G1/S transition of mitotic cell cycle	1/97	207/14464	0.00483092	0.7203546	-0.3329744	0.75412436	0.80183052	0.79574654	Cdkn1a	1
GO:0003018 vascular process in circulatory system	1/97	207/14464	0.00483092	0.7203546	-0.3329744	0.75412436	0.80183052	0.79574654	Shc1	1
GO:0060348 bone development	1/97	207/14464	0.00483092	0.7203546	-0.3329744	0.75412436	0.80183052	0.79574654	Lipa	1
GO:0003015 heart process	1/97	213/14464	0.00469484	0.70006292	-0.3623509	0.76399402	0.81082715	0.8046749	Fkbp1b	1
GO:0045930 negative regulation of mitotic cell cycle	1/97	213/14464	0.00469484	0.70006292	-0.3623509	0.76399402	0.81082715	0.8046749	Cdkn1a	1
GO:0070663 regulation of leukocyte proliferation	1/97	213/14464	0.00469484	0.70006292	-0.3623509	0.76399402	0.81082715	0.8046749	Cdkn1a	1
GO:0051249 regulation of lymphocyte activation	2/97	409/14464	0.00488998	0.72916089	-0.4565513	0.76444116	0.81082715	0.8046749	Cdkn1a/Mettl3	2
GO:0034764 positive regulation of transmembrane transport	1/97	214/14464	0.0046729	0.6967916	-0.3671747	0.76560041	0.81110519	0.80495084	Atp1b3	1
GO:0031349 positive regulation of defense response	2/97	410/14464	0.00487805	0.72738245	-0.4601271	0.76563133	0.81110519	0.80495084	Hspa1b/Becn1	2
GO:0051648 vesicle localization	1/97	215/14464	0.00465116	0.69355071	-0.3719784	0.76719597	0.81227048	0.80610728	Stard3	1
GO:0002429 immune response-activating cell surface receptor signaling pathway	1/97	216/14464	0.00462963	0.69033982	-0.3767622	0.76878078	0.81337622	0.80720463	Lipa	1
GO:0043254 regulation of protein-containing complex assembly	2/97	413/14464	0.00484262	0.7220988	-0.470808	0.76917099	0.81337622	0.80720463	Hspa1b/Traf2	2
GO:0048863 stem cell differentiation	1/97	218/14464	0.00458716	0.68400643	-0.3862709	0.77191844	0.81578805	0.80959816	Mettl3	1
GO:0050767 regulation of neurogenesis	2/97	418/14464	0.00478469	0.71346125	-0.4884579	0.7749684	0.81851647	0.81230588	Trpv2/Lig4	2
GO:0043410 positive regulation of MAPK cascade	2/97	419/14464	0.00477327	0.71175848	-0.4919655	0.77611271	0.81923009	0.81301408	Shc1/Traf2	2
GO:0090068 positive regulation of cell cycle process	1/97	222/14464	0.0045045	0.67168199	-0.4050585	0.77806789	0.82079823	0.81457033	Becn1	1
GO:0090092 regulation of transmembrane receptor protein serine/threonine kinase signaling pathway	1/97	223/14464	0.0044843	0.66866996	-0.4097085	0.77957944	0.82189678	0.81566054	Sap30l	1
GO:0002768 immune response-regulating cell surface receptor signaling pathway	1/97	227/14464	0.00440529	0.65688723	-0.428127	0.7855244	0.82716665	0.82089043	Lipa	1

GO:0051056	regulation of small GTPase mediated signal transduction	1/97	227/14464	0.00440529	0.65688723	-0.428127	0.7855244	0.82716665	0.82089043	Rasgef1a	1
GO:0019318	hexose metabolic process	1/97	230/14464	0.00434783	0.64831914	-0.4417547	0.78987872	0.83125105	0.82494384	Lipa	1
GO:0001667	ameboidal-type cell migration	1/97	231/14464	0.004329	0.64551256	-0.4462627	0.79131062	0.83175645	0.8254454	Cap1	1
GO:0045055	regulated exocytosis	1/97	231/14464	0.004329	0.64551256	-0.4462627	0.79131062	0.83175645	0.8254454	Sphk2	1
GO:0002573	myeloid leukocyte differentiation	1/97	232/14464	0.00431034	0.64273018	-0.4507538	0.79273287	0.83225049	0.82593569	Nme1	1
GO:0046394	carboxylic acid biosynthetic process	1/97	232/14464	0.00431034	0.64273018	-0.4507538	0.79273287	0.83225049	0.82593569	Ptges3	1
GO:0018108	peptidyl-tyrosine phosphorylation	1/97	233/14464	0.00429185	0.63997168	-0.455228	0.79414553	0.83273328	0.82641482	Ctnnd1	1
GO:0044843	cell cycle G1/S phase transition	1/97	233/14464	0.00429185	0.63997168	-0.455228	0.79414553	0.83273328	0.82641482	Cdkn1a	1
GO:0022613	ribonucleoprotein complex biogenesis	2/97	436/14464	0.00458716	0.68400643	-0.5504978	0.79481017	0.83293057	0.82661061	Rps19bp1/Ptges3	2
GO:0016053	organic acid biosynthetic process	1/97	235/14464	0.00425532	0.63452512	-0.4641264	0.79694231	0.83466457	0.82833145	Ptges3	1
GO:0018212	peptidyl-tyrosine modification	1/97	237/14464	0.00421941	0.62917047	-0.4729594	0.79970149	0.83705282	0.83070158	Ctnnd1	1
GO:0006520	amino acid metabolic process	1/97	238/14464	0.00420168	0.6265269	-0.4773516	0.80106713	0.83798045	0.83162218	P4ha1	1
GO:0008015	blood circulation	2/97	443/14464	0.00451467	0.6731982	-0.5740224	0.80210478	0.83856409	0.83220139	Shc1/Fkbp1b	2
GO:1901988	negative regulation of cell cycle phase transition	1/97	240/14464	0.00416667	0.62130584	-0.4860885	0.80377082	0.83980358	0.83343147	Cdkn1a	1
GO:0007409	axonogenesis	2/97	446/14464	0.0044843	0.66866996	-0.5840059	0.80516091	0.84075345	0.83437413	Trpv2/Raph1	2
GO:0071560	cellular response to transforming growth factor beta stimulus	1/97	242/14464	0.00413223	0.61617108	-0.4947628	0.80643814	0.8415844	0.83519878	Sap30l	1
GO:0034599	cellular response to oxidative stress	1/97	243/14464	0.00411523	0.6136354	-0.4990768	0.80775832	0.84245915	0.83606689	Becn1	1
GO:0051259	protein complex oligomerization	1/97	244/14464	0.00409836	0.6111205	-0.5033756	0.80906958	0.84332357	0.83692475	Vwa1	1
GO:0060326	cell chemotaxis	1/97	245/14464	0.00408163	0.60862613	-0.5076593	0.81037199	0.84417773	0.83777243	Gpr35	1
GO:0016072	rRNA metabolic process	1/97	247/14464	0.00404858	0.60369798	-0.5161819	0.81295048	0.84585562	0.83943759	Ubtf	1
GO:0032984	protein-containing complex disassembly	1/97	247/14464	0.00404858	0.60369798	-0.5161819	0.81295048	0.84585562	0.83943759	Becn1	1
GO:0051607	defense response to virus	1/97	248/14464	0.00403226	0.60126372	-0.5204211	0.81422669	0.84667951	0.84025522	Becn1	1
GO:0070372	regulation of ERK1 and ERK2 cascade	1/97	249/14464	0.00401606	0.598849	-0.5246457	0.81549427	0.84698989	0.84056326	Shc1	1
GO:0071559	response to transforming growth factor beta	1/97	249/14464	0.00401606	0.598849	-0.5246457	0.81549427	0.84698989	0.84056326	Sap30l	1
GO:0005996	monosaccharide metabolic process	1/97	250/14464	0.004	0.59645361	-0.5288559	0.8167533	0.84779381	0.84136107	Lipa	1
GO:0010721	negative regulation of cell development	1/97	252/14464	0.00396825	0.59171985	-0.5372333	0.8192459	0.84987646	0.84342792	Nme1	1
GO:0002009	morphogenesis of an epithelium	2/97	470/14464	0.00425532	0.63452512	-0.6618622	0.82815153	0.85809654	0.85158563	Sfrp5/Ctnnd1	2
GO:0003013	circulatory system process	2/97	470/14464	0.00425532	0.63452512	-0.6618622	0.82815153	0.85809654	0.85158563	Shc1/Fkbp1b	2
GO:0051251	positive regulation of lymphocyte activation	1/97	260/14464	0.00384615	0.57351308	-0.5701893	0.82888513	0.85834787	0.85183506	Cdkn1a	1
GO:0010720	positive regulation of cell development	2/97	473/14464	0.00422833	0.63050064	-0.6713545	0.83084929	0.85987244	0.85334806	Trpv2/Lig4	2
GO:0007162	negative regulation of cell adhesion	1/97	263/14464	0.00380228	0.56697111	-0.5823275	0.83236715	0.8609336	0.85440116	Mettl3	1
GO:0048608	reproductive structure development	1/97	268/14464	0.00373134	0.55639329	-0.6023023	0.83801534	0.86626305	0.85969018	Antxr2	1
GO:0045765	regulation of angiogenesis	1/97	270/14464	0.0037037	0.55227186	-0.6102052	0.84022151	0.86780136	0.86121682	Shc1	1
GO:0030336	negative regulation of cell migration	1/97	271/14464	0.00369004	0.55023396	-0.6141384	0.84131342	0.86780136	0.86121682	Sap30l	1
GO:0061448	connective tissue development	1/97	272/14464	0.00367647	0.54821104	-0.6180595	0.84239794	0.86780136	0.86121682	Lipa	1
GO:0061458	reproductive system development	1/97	272/14464	0.00367647	0.54821104	-0.6180595	0.84239794	0.86780136	0.86121682	Antxr2	1
GO:0000375	RNA splicing, via transesterification reactions	1/97	273/14464	0.003663	0.54620294	-0.6219687	0.84347512	0.86780136	0.86121682	Mettl3	1
GO:0000377	RNA splicing, via transesterification reactions with bulged adenosine as nucleophile	1/97	273/14464	0.003663	0.54620294	-0.6219687	0.84347512	0.86780136	0.86121682	Mettl3	1
GO:0000398	mRNA splicing, via spliceosome	1/97	273/14464	0.003663	0.54620294	-0.6219687	0.84347512	0.86780136	0.86121682	Mettl3	1
GO:0070371	ERK1 and ERK2 cascade	1/97	273/14464	0.003663	0.54620294	-0.6219687	0.84347512	0.86780136	0.86121682	Shc1	1
GO:0010948	negative regulation of cell cycle process	1/97	277/14464	0.00361011	0.53831553	-0.6374878	0.84771148	0.87113443	0.86452459	Cdkn1a	1
GO:1901342	regulation of vasculature development	1/97	277/14464	0.00361011	0.53831553	-0.6374878	0.84771148	0.87113443	0.86452459	Shc1	1
GO:0090287	regulation of cellular response to growth factor stimulus	1/97	278/14464	0.00359712	0.53637914	-0.6413386	0.84875271	0.87169197	0.86507791	Sap30l	1
GO:0046942	carboxylic acid transport	1/97	281/14464	0.00355872	0.53065268	-0.652823	0.85183432	0.87434316	0.86770897	Abcc5	1
GO:0071396	cellular response to lipid	2/97	499/14464	0.00400802	0.59764891	-0.7515658	0.85269257	0.87435642	0.86772214	Ptges3/Sphk2	2
GO:2000146	negative regulation of cell motility	1/97	282/14464	0.0035461	0.52877093	-0.6566288	0.85284765	0.87435642	0.86772214	Sap30l	1
GO:0007416	synapse assembly	1/97	283/14464	0.00353357	0.52690248	-0.6604234	0.85385412	0.87436263	0.8677283	Nrg2	1
GO:0015849	organic acid transport	1/97	283/14464	0.00353357	0.52690248	-0.6604234	0.85385412	0.87436263	0.8677283	Abcc5	1
GO:0048193	Golgi vesicle transport	1/97	284/14464	0.00352113	0.52504719	-0.6642071	0.85485378	0.87487378	0.86823557	Bicd2	1
GO:0006790	sulfur compound metabolic process	1/97	285/14464	0.00350877	0.52320492	-0.6679799	0.85584667	0.8753774	0.86873537	Pmvk	1
GO:0045787	positive regulation of cell cycle	1/97	288/14464	0.00347222	0.51775487	-0.6792338	0.85878519	0.87735623	0.87069919	Becn1	1
GO:1901888	regulation of cell junction assembly	1/97	288/14464	0.00347222	0.51775487	-0.6792338	0.85878519	0.87735623	0.87069919	Nrg2	1
GO:0048545	response to steroid hormone	1/97	290/14464	0.00344828	0.51418415	-0.6866833	0.86071118	0.87881025	0.87214217	Ptges3	1
GO:0048511	rhythmic process	1/97	291/14464	0.00343643	0.51241719	-0.6903924	0.86166441	0.87926993	0.87259836	Mettl3	1
GO:0141091	transforming growth factor beta receptor superfamily signaling pathway	1/97	292/14464	0.00342466	0.51066234	-0.6940912	0.86261118	0.87972248	0.87304749	Sap30l	1
GO:0050727	regulation of inflammatory response	1/97	294/14464	0.00340136	0.50718844	-0.7014579	0.86448552	0.88111993	0.87443433	Dnase1l3	1
GO:0044282	small molecule catabolic process	1/97	296/14464	0.00337838	0.50376149	-0.7087842	0.86633455	0.88239373	0.87569847	Nudt3	1
GO:0040013	negative regulation of locomotion	1/97	297/14464	0.003367	0.50206533	-0.7124324	0.86724968	0.88239373	0.87569847	Sap30l	1
GO:0050863	regulation of T cell activation	1/97	297/14464	0.003367	0.50206533	-0.7124324	0.86724968	0.88239373	0.87569847	Mettl3	1
GO:0050900	leukocyte migration	1/97	304/14464	0.00328947	0.49050461	-0.737696	0.87348426	0.88770377	0.88096821	Gpr35	1
GO:1901990	regulation of mitotic cell cycle phase transition	1/97	304/14464	0.00328947	0.49050461	-0.737696	0.87348426	0.88770377	0.88096821	Cdkn1a	1
GO:0062197	cellular response to chemical stress	1/97	305/14464	0.00327869	0.4888964	-0.7412669	0.87435092	0.88806823	0.8813299	Becn1	1
GO:0034504	protein localization to nucleus	1/97	306/14464	0.00326797	0.4872987	-0.7448283	0.87521172	0.8884263	0.88168526	Cdkn1a	1
GO:0042254	ribosome biogenesis	1/97	309/14464	0.00323625	0.48256764	-0.7554573	0.87775923	0.89049515	0.88373841	Rps19bp1	1
GO:0030100	regulation of endocytosis	1/97	310/14464	0.00322581	0.48101097	-0.7589819	0.87859691	0.89082796	0.88406869	Pcsk9	1
GO:0009615	response to virus	1/97	312/14464	0.00320513	0.47792757	-0.7660042	0.88025526	0.891992	0.8852239	Becn1	1
GO:1903532	positive regulation of secretion by cell	1/97	315/14464	0.0031746	0.47337588	-0.7764709	0.88270084	0.89395196	0.88716899	Sphk2	1
GO:0051345	positive regulation of hydrolase activity	1/97	317/14464	0.00315457	0.47038928	-0.7834048	0.8843037	0.89505667	0.88826532	Aph1b	1
GO:0062012	regulation of small molecule metabolic process	1/97	319/14464	0.0031348	0.46744013	-0.7903042	0.88588488	0.89613818	0.88933862	Sphk2	1
GO:0007517	muscle organ development	1/97	323/14464	0.00309598	0.4616514	-0.8040014	0.88898335	0.89823289	0.89141744	Myt1	1
GO:0021700	developmental maturation	1/97	323/14464	0.00309598	0.4616514	-0.8040014	0.88898335	0.89823289	0.89141744	Nrg2	1
GO:0051047	positive regulation of secretion	1/97	334/14464	0.00299401	0.44644731	-0.8409945	0.89708134	0.9058915	0.89901794	Sphk2	1
GO:0060271	cilium assembly	1/97	339/14464	0.00294985	0.43986254	-0.8574963	0.90056625	0.90888557	0.90198929	Dnai4	1
GO:0015711	organic anion transport	1/97	341/14464	0.00293255	0.4372827	-0.8640443	0.90192728	0.90973393	0.90283121	Abcc5	1
GO:0045786	negative regulation of cell cycle	1/97	343/14464	0.00291545	0.43473295	-0.8705626	0.90326988	0.91056271	0.90365371	Cdkn1a	1
GO:0006887	exocytosis	1/97	352/14464	0.00284091	0.42361762	-0.8995385	0.9090899	0.91590153	0.90895201	Sphk2	1
GO:0006935	chemotaxis	1/97	356/14464	0.00280899	0.41885787	-0.9122348	0.91156402	0.91786515	0.91090074	Gpr35	1
GO:0042330	taxis	1/97	357/14464	0.00280112	0.4176846	-0.9153919	0.91217207	0.91794863	0.91098359	Gpr35	1
GO:0032535	regulation of cellular component size	1/97	365/14464	0.00273973	0.40852987	-0.9404097	0.91688978	0.92216532	0.91516828	Trpv2	1
GO:0044782	cilium organization	1/97	369/14464	0.00271003	0.40410136	-0.9527634	0.91915369	0.92391066	0.91690038	Dnai4	1
GO:0044772	mitotic cell cycle phase transition	1/97	399/14464	0.00250627	0.3737178	-1.0423622	0.9342956	0.93859121	0.93146954	Cdkn1a	1
GO:1901987	regulation of cell cycle phase transition	1/97	402/14464	0.00248756	0.37092886	-1.0510449	0.93564574	0.93940767	0.9322798	Cdkn1a	1
GO:0045785	positive regulation of cell adhesion	1/97	422/14464	0.00236967	0.35334929	-1.1077545	0.94397093	0.94722226	0.9400351	Sfn	1
GO:0007015	actin filament organization	1/97	429/14464	0.002331	0.34758369	-1.1271436	0.94662526	0.94934077	0.94213753	Cap1	1
GO:0007346	regulation of mitotic cell cycle	1/97	450/14464	0.00222222	0.33136312	-1.1839887	0.95386406	0.95605182	0.94879766	Cdkn1a	1
GO:0071407	cellular response to organic cyclic compound	1/97	462/14464	0.0021645	0.32275628	-1.2156343	0.95755492	0.95920115	0.9519231	Ptges3	1
GO:0001501	skeletal system development	1/97	466/14464	0.00214592	0.31998584	-1.2260549	0.95871905	0.95981724	0.95253451	Lipa	1
GO:0034329	cell junction assembly	1/97	485/14464	0.00206186	0.30745031	-1.2747228	0.96383124	0.96438294	0.95706557	Nrg2	1
GO:0044770	cell cycle phase transition	1/97	498/14464	0.00200803	0.2994245	-1.307271	0.96696275	0.96696275	0.95962581	Cdkn1a	1

ID	Description	GeneRatio	BgRatio	RichFactor	FoldEnrichm	zScore	pvalue	p.adjust	qvalue	geneID	Count
GO:0006457	protein folding	14/138	166/14464	0.08433735	8.83953204	9.970405	6.0978E-10	1.2293E-06	1.2074E-06	Sdf2l1/Hspa5/Calr/Chordc1/Hsp90b1/Pdia3/Hspd1/St13/Fkbp2/D	14
GO:0051604	protein maturation	17/138	498/14464	0.03413655	3.57790583	5.7458193	5.2371E-06	0.00527896	0.00518469	Sdf2l1/Hspa5/Calr/Mmp14/Chordc1/Hsp90b1/Pdia3/Hspd1/St13/I	17
GO:0098761	cellular response to interleukin-7	4/138	19/14464	0.21052632	22.0655988	9.01774808	2.751E-05	0.01720348	0.01689628	Stip1/Pdia3/Hspd1/P4hb	4
GO:0098760	response to interleukin-7	4/138	20/14464	0.2	20.9623188	8.76775708	3.4134E-05	0.01720348	0.01689628	Stip1/Pdia3/Hspd1/P4hb	4
GO:0016556	mRNA modification	4/138	22/14464	0.18181818	19.0566535	8.31842676	5.0777E-05	0.02047346	0.02010786	Dnajb11/Hnrnpc/Mettl14/Virma	4
GO:0042026	protein refolding	4/138	23/14464	0.17391304	18.2281033	8.11538289	6.1014E-05	0.02050065	0.02013457	Hspa5/Hspd1/St13/Bag5	4
GO:1903332	regulation of protein folding	3/138	11/14464	0.27272727	28.5849802	8.98248046	0.00013258	0.03818247	0.03750064	Pdia3/St13/Bag5	3
GO:0061077	chaperone-mediated protein folding	5/138	62/14464	0.08064516	8.45254792	5.77159012	0.00030749	0.07748673	0.07610303	Sdf2l1/Hspa5/Chordc1/St13/Fkbp2	5
GO:0034976	response to endoplasmic reticulum stress	9/138	256/14464	0.03515625	3.68478261	4.25371998	0.00078065	0.1748665	0.17174388	Sdf2l1/Hspa5/Manf/Calr/Hsp90b1/Pdia3/Dnajc3/Ppp2cb/P4hb	9
GO:1901570	fatty acid derivative biosynthetic process	3/138	21/14464	0.14285714	14.9730849	6.2889697	0.00099652	0.2008982	0.19731073	Far2/Hmgcll1/Elovl7	3
GO:1900024	regulation of substrate adhesion-dependent cell spreading	4/138	54/14464	0.07407407	7.76382179	4.88723598	0.00173378	0.31009531	0.30455789	Calr/Has2/Pkp2/P4hb	4
GO:0045185	maintenance of protein location	5/138	93/14464	0.05376344	5.63503195	4.40105681	0.00195139	0.31009531	0.30455789	Hspa5/Dand5/Scin/Pkp2/Sri	5
GO:0032410	negative regulation of transporter activity	4/138	57/14464	0.07017544	7.35519959	4.71830605	0.0021184	0.31009531	0.30455789	Rrad/Sri/Ndfip2/Oxsr1	4
GO:0044272	sulfur compound biosynthetic process	6/138	139/14464	0.04316547	4.52424148	4.09762307	0.00215344	0.31009531	0.30455789	Mettl16/Gclm/Ppcs/Elovl7/Pdk3/Enoph1	6
GO:0009451	RNA modification	6/138	143/14464	0.04195804	4.39768927	4.00747911	0.00248306	0.33372349	0.32776415	Mettl16/Dnajb11/Hnrnpc/Tfb1m/Mettl14/Virma	6
GO:0051085	chaperone cofactor-dependent protein refolding	3/138	30/14464	0.1	10.4811594	5.10193579	0.00285729	0.36001825	0.35358935	Sdf2l1/Hspa5/St13	3
GO:1902373	negative regulation of mRNA catabolic process	4/138	66/14464	0.06060606	6.35221783	4.27721348	0.00361852	0.41132523	0.40398013	Mettl16/Hnrnpc/Pkp3/Mettl14	4
GO:0002396	MHC protein complex assembly	2/138	11/14464	0.18181818	19.0566535	5.87977718	0.00469793	0.41132523	0.40398013	Calr/Pdia3	2
GO:0002501	peptide antigen assembly with MHC protein complex	2/138	11/14464	0.18181818	19.0566535	5.87977718	0.00469793	0.41132523	0.40398013	Calr/Pdia3	2
GO:0035384	thioester biosynthetic process	3/138	36/14464	0.08333333	8.73429952	4.5601112	0.00482002	0.41132523	0.40398013	Ppcs/Elovl7/Pdk3	3
GO:0071616	acyl-CoA biosynthetic process	3/138	36/14464	0.08333333	8.73429952	4.5601112	0.00482002	0.41132523	0.40398013	Ppcs/Elovl7/Pdk3	3
GO:0010837	regulation of keratinocyte proliferation	3/138	37/14464	0.08108108	8.49823737	4.48206648	0.00520911	0.41132523	0.40398013	Has2/Ift172/Ift80	3
GO:0038092	nodal signaling pathway	2/138	12/14464	0.16666667	17.468599	5.60130944	0.00560241	0.41132523	0.40398013	Dand5/Smad2	2
GO:0048368	lateral mesoderm development	2/138	12/14464	0.16666667	17.468599	5.60130944	0.00560241	0.41132523	0.40398013	Dand5/Smad2	2
GO:1901841	regulation of high voltage-gated calcium channel activity	2/138	12/14464	0.16666667	17.468599	5.60130944	0.00560241	0.41132523	0.40398013	Rrad/Sri	2
GO:1900026	positive regulation of substrate adhesion-dependent cell spreading	3/138	38/14464	0.07894737	8.27459954	4.40691007	0.00561659	0.41132523	0.40398013	Calr/Has2/P4hb	3
GO:0006790	sulfur compound metabolic process	8/138	285/14464	0.02807018	2.94207984	3.24992225	0.00602309	0.41132523	0.40398013	Mettl16/Hexa/Gclm/Far2/Ppcs/Elovl7/Pdk3/Enoph1	8
GO:0048385	regulation of retinoic acid receptor signaling pathway	2/138	13/14464	0.15384615	16.1248606	5.35451785	0.00657983	0.41132523	0.40398013	Dhrs3/Calr	2
GO:0071677	positive regulation of mononuclear cell migration	4/138	79/14464	0.05063291	5.30691616	3.76730944	0.00686046	0.41132523	0.40398013	Calr/Mmp14/Cd99l2/Oxsr1	4
GO:0045047	protein targeting to ER	3/138	41/14464	0.07317073	7.66914104	4.19701278	0.00695154	0.41132523	0.40398013	Hspa5/Ubl4a/Srprb	3
GO:0051084	'de novo' post-translational protein folding	3/138	41/14464	0.07317073	7.66914104	4.19701278	0.00695154	0.41132523	0.40398013	Sdf2l1/Hspa5/St13	3
GO:1902369	negative regulation of RNA catabolic process	4/138	80/14464	0.05	5.24057971	3.73281658	0.0071691	0.41132523	0.40398013	Mettl16/Hnrnpc/Pkp3/Mettl14	4
GO:0035966	response to topologically incorrect protein	5/138	127/14464	0.03937008	4.12644072	3.47319509	0.00738544	0.41132523	0.40398013	Sdf2l1/Hspa5/Manf/Hspd1/Dnajc3	5
GO:0023019	signal transduction involved in regulation of gene expression	2/138	14/14464	0.14285714	14.9730849	5.13367835	0.00762874	0.41132523	0.40398013	Dand5/Smad2	2
GO:0071895	odontoblast differentiation	2/138	14/14464	0.14285714	14.9730849	5.13367835	0.00762874	0.41132523	0.40398013	Smad2/Ift80	2
GO:0017015	regulation of transforming growth factor beta receptor signaling pathway	5/138	129/14464	0.03875969	4.06246489	3.42904661	0.00787625	0.41132523	0.40398013	Hspa5/Dand5/Smad2/Brms1/Spred3	5
GO:0006458	'de novo' protein folding	3/138	43/14464	0.06976744	7.3124368	4.06855176	0.0079373	0.41132523	0.40398013	Sdf2l1/Hspa5/St13	3
GO:0006637	acyl-CoA metabolic process	4/138	83/14464	0.04819277	5.05116117	3.6327063	0.0081497	0.41132523	0.40398013	Far2/Ppcs/Elovl7/Pdk3	4
GO:0035383	thioester metabolic process	4/138	83/14464	0.04819277	5.05116117	3.6327063	0.0081497	0.41132523	0.40398013	Far2/Ppcs/Elovl7/Pdk3	4
GO:1903844	regulation of cellular response to transforming growth factor beta stimulus	5/138	131/14464	0.03816794	4.00044253	3.38577951	0.00838932	0.41132523	0.40398013	Hspa5/Dand5/Smad2/Brms1/Spred3	5
GO:1903670	regulation of sprouting angiogenesis	3/138	44/14464	0.06818182	7.14624506	4.0073737	0.00845947	0.41132523	0.40398013	Rhoj/Stard13/S2bpcox16	3
GO:0072599	establishment of protein localization to endoplasmic reticulum	3/138	45/14464	0.06666667	6.98743961	3.94808137	0.00900138	0.41132523	0.40398013	Hspa5/Ubl4a/Srprb	3
GO:0030512	negative regulation of transforming growth factor beta receptor signaling pathway	4/138	86/14464	0.04651163	4.87495787	3.53731091	0.00921447	0.41132523	0.40398013	Hspa5/Dand5/Brms1/Spred3	4
GO:0043604	amide biosynthetic process	5/138	135/14464	0.03703704	3.8819109	3.30176192	0.00948418	0.41132523	0.40398013	Gclm/Ppcs/Degs1/Elovl7/Pdk3	5
GO:0043616	keratinocyte proliferation	3/138	46/14464	0.06521739	6.83553875	3.89057351	0.00956319	0.41132523	0.40398013	Has2/Ift172/Ift80	3
GO:1901568	fatty acid derivative metabolic process	3/138	46/14464	0.06521739	6.83553875	3.89057351	0.00956319	0.41132523	0.40398013	Far2/Hmgcll1/Elovl7	3
GO:0035269	protein O-linked mannosylation	2/138	16/14464	0.125	13.1014493	4.75334977	0.0099353	0.41132523	0.40398013	Fktn/Crppa	2
GO:1903671	negative regulation of sprouting angiogenesis	2/138	16/14464	0.125	13.1014493	4.75334977	0.0099353	0.41132523	0.40398013	Stard13/S2bpcox16	2
GO:0043603	amide metabolic process	9/138	376/14464	0.02393617	2.50878816	2.90940232	0.00999749	0.41132523	0.40398013	Hsp90b1/Hexa/Gclm/Far2/Ppcs/Degs1/Elovl7/Pdk3/P4hb	9
GO:1903312	negative regulation of mRNA metabolic process	4/138	89/14464	0.04494382	4.71063345	3.44623901	0.01036611	0.4177655	0.4103054	Mettl16/Hnrnpc/Pkp3/Mettl14	4
GO:0006620	post-translational protein targeting to endoplasmic reticulum membrane	2/138	17/14464	0.11764706	12.3307758	4.5877689	0.01119015	0.4177655	0.4103054	Hspa5/Ubl4a	2
GO:0043171	peptide catabolic process	2/138	17/14464	0.11764706	12.3307758	4.5877689	0.01119015	0.4177655	0.4103054	Lta4h/Trhde	2
GO:0046500	S-adenosylmethionine metabolic process	2/138	17/14464	0.11764706	12.3307758	4.5877689	0.01119015	0.4177655	0.4103054	Mettl16/Enoph1	2
GO:1901142	insulin metabolic process	2/138	17/14464	0.11764706	12.3307758	4.5877689	0.01119015	0.4177655	0.4103054	Hsp90b1/P4hb	2
GO:0032413	negative regulation of ion transmembrane transporter activity	3/138	50/14464	0.06	6.28869565	3.67661015	0.01201206	0.42036537	0.41285884	Rrad/Sri/Oxsr1	3
GO:0034446	substrate adhesion-dependent cell spreading	4/138	94/14464	0.04255319	4.46006784	3.30313573	0.01248538	0.42036537	0.41285884	Calr/Has2/Pkp2/P4hb	4
GO:0060349	bone morphogenesis	4/138	94/14464	0.04255319	4.46006784	3.30313573	0.01248538	0.42036537	0.41285884	Dhrs3/Mmp14/Has2/Ift80	4
GO:0035268	protein mannosylation	2/138	18/14464	0.11111111	11.6457327	4.43551719	0.01251087	0.42036537	0.41285884	Fktn/Crppa	2
GO:0035336	long-chain fatty-acyl-CoA metabolic process	2/138	18/14464	0.11111111	11.6457327	4.43551719	0.01251087	0.42036537	0.41285884	Far2/Elovl7	2
GO:2000561	regulation of CD4-positive, alpha-beta T cell proliferation	2/138	18/14464	0.11111111	11.6457327	4.43551719	0.01251087	0.42036537	0.41285884	Card11/Itch	2
GO:0006518	peptide metabolic process	3/138	52/14464	0.05769231	6.04682274	3.5781938	0.0133591	0.43772795	0.42991138	Gclm/Lta4h/Trhde	3
GO:0035720	intraciliary anterograde transport	2/138	19/14464	0.10526316	11.0327994	4.29483436	0.01389613	0.43772795	0.42991138	Ift172/Ift80	2
GO:0035739	CD4-positive, alpha-beta T cell proliferation	2/138	19/14464	0.10526316	11.0327994	4.29483436	0.01389613	0.43772795	0.42991138	Card11/Itch	2
GO:0097502	mannosylation	2/138	19/14464	0.10526316	11.0327994	4.29483436	0.01389613	0.43772795	0.42991138	Fktn/Crppa	2
GO:0010810	regulation of cell-substrate adhesion	6/138	206/14464	0.02912621	3.05276488	2.91239165	0.01413155	0.43829537	0.43046866	Calr/Mmp14/Has2/Pkp2/Sema3e/P4hb	6
GO:0006958	complement activation, classical pathway	2/138	20/14464	0.1	10.4811594	4.16427086	0.01534457	0.45495979	0.44683551	Ighg2c/Cr1l	2
GO:0035967	cellular response to topologically incorrect protein	4/138	100/14464	0.04	4.19246377	3.1440869	0.01537132	0.45495979	0.44683551	Sdf2l1/Hspa5/Hspd1/Dnajc3	4
GO:0000375	RNA splicing, via transesterification reactions	7/138	273/14464	0.02564103	2.68747677	2.76260562	0.01579721	0.45495979	0.44683551	Mettl16/Obi1/Srpk1/Prpf31/Hnrnpc/Mettl14/Prpf18	7
GO:0000377	RNA splicing, via transesterification reactions with bulged adenosine as nucleophile	7/138	273/14464	0.02564103	2.68747677	2.76260562	0.01579721	0.45495979	0.44683551	Mettl16/Obi1/Srpk1/Prpf31/Hnrnpc/Mettl14/Prpf18	7
GO:0000398	mRNA splicing, via spliceosome	7/138	273/14464	0.02564103	2.68747677	2.76260562	0.01579721	0.45495979	0.44683551	Mettl16/Obi1/Srpk1/Prpf31/Hnrnpc/Mettl14/Prpf18	7
GO:0006085	acetyl-CoA biosynthetic process	2/138	21/14464	0.0952381	9.98205659	4.04262066	0.01685489	0.46547201	0.45716001	Ppcs/Pdk3	2
GO:0042634	regulation of hair cycle	2/138	21/14464	0.0952381	9.98205659	4.04262066	0.01685489	0.46547201	0.45716001	Bmal1/Pkp3	2
GO:0070935	3'-UTR-mediated mRNA stabilization	2/138	21/14464	0.0952381	9.98205659	4.04262066	0.01685489	0.46547201	0.45716001	Mettl16/Hnrnpc	2
GO:0036503	ERAD pathway	4/138	105/14464	0.03809524	3.99282264	3.02078485	0.01807297	0.4762359	0.46773169	Sdf2l1/Hspa5/Calr/Hsp90b1	4
GO:0010594	regulation of endothelial cell migration	5/138	159/14464	0.03144654	3.29596208	2.85710108	0.01816589	0.4762359	0.46773169	Calr/Rhoj/Sash1/Stard13/S2bpcox16	5
GO:0010839	negative regulation of keratinocyte proliferation	2/138	22/14464	0.09090909	9.52832675	3.92887095	0.01842579	0.4762359	0.46773169	Ift172/Ift80	2
GO:0016486	peptide hormone processing	2/138	22/14464	0.09090909	9.52832675	3.92887095	0.01842579	0.4762359	0.46773169	Hsp90b1/P4hb	2
GO:0031167	rRNA methylation	2/138	22/14464	0.09090909	9.52832675	3.92887095	0.01842579	0.4762359	0.46773169	Mettl16/Tfb1m	2
GO:0048255	mRNA stabilization	3/138	59/14464	0.05084746	5.3294031	3.27042098	0.01872904	0.47794615	0.46941139	Mettl16/Hnrnpc/Mettl14	3
GO:0006986	response to unfolded protein	4/138	110/14464	0.03636364	3.8113307	2.90487915	0.02105297	0.53053488	0.52106104	Hspa5/Manf/Hspd1/Dnajc3	4
GO:0071675	regulation of mononuclear cell migration	4/138	114/14464	0.03508772	3.6775998	2.81694524	0.02364195	0.58842181	0.57791427	Calr/Mmp14/Cd99l2/Oxsr1	4
GO:0000096	sulfur amino acid metabolic process	2/138	26/14464	0.07692308	8.06243032	3.53747675	0.02529	0.59981933	0.58910827	Gclm/Enoph1	2
GO:0035337	fatty-acyl-CoA metabolic process	2/138	26/14464	0.07692308	8.06243032	3.53747675	0.02529	0.59981933	0.58910827	Far2/Elovl7	2
GO:1901020	negative regulation of calcium ion transmembrane transporter activity	2/138	26/14464	0.07692308	8.06243032	3.53747675	0.02529	0.59981933	0.58910827	Rrad/Sri	2
GO:1901385	regulation of voltage-gated calcium channel activity	2/138	26/14464	0.07692308	8.06243032	3.53747675	0.02529	0.59981933	0.58910827	Rrad/Sri	2
GO:0001510	RNA methylation	3/138	67/14464	0.04477612	4.69305646	2.97367573	0.026129	0.60547195	0.59465995	Mettl16/Tfb1m/Mettl14	3
GO:0032088	negative regulation of NF-kappaB transcription factor activity	3/138	67/14464	0.04477612	4.69305646	2.97367573	0.026129	0.60547195	0.59465995	Ppp2cb/Brms1/Itch	3
GO:0070293	renal absorption	2/138	27/14464	0.07407407	7.76382179	3.45256468	0.02714505	0.61497013	0.60398852	Has2/Oxsr1	2
GO:0051865	protein autoubiquitination	3/138	68/14464	0.04411765	4.62404092	2.93990227	0.02714898	0.61497013	0.60398852	Sash1/Obi1/Itch	3
GO:0032507	maintenance of protein location in cell	3/138	69/14464	0.04347826	4.55702583	2.90677886	0.02819001	0.63095922	0.6196921	Hspa5/Scin/Pkp2	3
GO:0071560	cellular response to transforming growth factor beta stimulus	6/138	242/14464	0.02479339	2.59863457	2.46140347	0.02854658	0.63095922	0.6196921	Hspa5/Dand5/Smad2/Stk16/Brms1/Spred3	6
GO:0043489	RNA stabilization	3/138	70/14464	0.04285714	4.49192547	2.87428282	0.02925207	0.63095922	0.6196921	Mettl16/Hnrnpc/Mettl14	3
GO:1904377	positive regulation of protein localization to cell periphery	3/138	71/14464	0.04225352	4.42865891	2.84239256	0.03033511	0.63095922	0.6196921	Arhgef16/Rab38/Cnpy4	3
GO:0002040	sprouting angiogenesis	4/138	124/14464	0.03225806	3.38101917	2.6133972	0.03093035	0.63095922	0.6196921	Rhoj/Stard13/S2bpcox16/Sema3e	4
GO:0140448	signaling receptor ligand precursor processing	2/138	29/14464	0.06896552	7.22838581	3.29512864	0.03101358	0.63095922	0.6196921	Hsp90b1/P4hb	2
GO:2000406	positive regulation of T cell migration	2/138	29/14464	0.06896552	7.22838581	3.29512864	0.03101358	0.63095922	0.6196921	Cd99l2/Oxsr1	2
GO:2001258	negative regulation of cation channel activity	2/138	29/14464	0.06896552	7.22838581	3.29512864	0.03101358	0.63095922	0.6196921	Rrad/Sri	2
GO:0071559	response to transforming growth factor beta	6/138	249/14464	0.02409639	2.52558058	2.38323933	0.03217806	0.63095922	0.6196921	Hspa5/Dand5/Smad2/Stk16/Brms1/Spred3	6
GO:0090101	negative regulation of transmembrane receptor protein serine/threonine kinase signaling pathway	4/138	126/14464	0.03174603	3.3273522	2.57519047	0.03252997	0.63095922	0.6196921	Hspa5/Dand5/Brms1/Spred3	4
GO:0002455	humoral immune response mediated by circulating immunoglobulin	2/138	30/14464	0.06666667	6.98743961	3.22191951	0.03302475	0.63095922	0.6196921	Ighg2c/Cr1l	2
GO:0048384	retinoic acid receptor signaling pathway	2/138	30/14464	0.06666667	6.98743961	3.22191951	0.03302475	0.63095922	0.6196921	Dhrs3/Calr	2
GO:0086004	regulation of cardiac muscle cell contraction	2/138	30/14464	0.06666667	6.98743961	3.22191951	0.03302475	0.63095922	0.6196921	Pkp2/Sri	2
GO:0086065	cell communication involved in cardiac conduction	2/138	30/14464	0.06666667	6.98743961	3.22191951	0.03302475	0.63095922	0.6196921	Pkp2/Sri	2
GO:0000154	rRNA modification	2/138	31/14464	0.06451613	6.76203834	3.15199084	0.03508566	0.63095922	0.6196921	Mettl16/Tfb1m	2
GO:0030212	hyaluronan metabolic process	2/138	31/14464	0.06451613	6.76203834	3.15199084	0.03508566	0.63095922	0.6196921	Has2/Hexa	2
GO:0007179	transforming growth factor beta receptor signaling pathway	5/138	190/14464	0.02631579	2.75819985	2.39429939	0.03551923	0.63095922	0.6196921	Hspa5/Dand5/Smad2/Brms1/Spred3	5
GO:0043433	negative regulation of DNA-binding transcription factor activity	4/138	130/14464	0.03076923	3.22497213	2.5010295	0.0358726	0.63095922	0.6196921	Sri/Ppp2cb/Brms1/Itch	4
GO:0016180	snRNA processing	2/138	32/14464	0.0625	6.55072464	3.08508884	0.0371952	0.63095922	0.6196921	Mettl16/Ints10	2
GO:0045577	regulation of B cell differentiation	2/138	32/14464	0.0625	6.55072464	3.08508884	0.0371952	0.63095922	0.6196921	Card11/Mmp14	2
GO:0070972	protein localization to endoplasmic reticulum	3/138	77/14464	0.03896104	4.08356861	2.66268701	0.03727119	0.63095922	0.6196921	Hspa5/Ubl4a/Srprb	3
GO:0002687	positive regulation of leukocyte migration	4/138	133/14464	0.03007519	3.1522284	2.44727173	0.03850547	0.63095922	0.6196921	Calr/Mmp14/Cd99l2/Oxsr1	4
GO:0002474	antigen processing and presentation of peptide antigen via MHC class I	2/138	33/14464	0.06060606	6.35221783	3.02098661	0.03935227	0.63095922	0.6196921	Calr/Pdia3	2
GO:0018208	peptidyl-proline modification	2/138	33/14464	0.06060606	6.35221783	3.02098661	0.03935227	0.63095922	0.6196921	Fkbp2/P4hb	2
GO:0046329	negative regulation of JNK cascade	2/138	33/14464	0.06060606	6.35221783	3.02098661	0.03935227	0.63095922	0.6196921	Fktn/Itch	2
GO:1904063	negative regulation of cation transmembrane transport	3/138	79/14464	0.03797468	3.98018712	2.60680418	0.03974852	0.63095922	0.6196921	Rrad/Sri/Oxsr1	3
GO:0032409	regulation of transporter activity	5/138	196/14464	0.0255102	2.67376516	2.31551325	0.03975579	0.63095922	0.6196921	Slc5a3/Rrad/Sri/Ndfip2/Oxsr1	5
GO:0001953	negative regulation of cell-matrix adhesion	2/138	34/14464	0.05882353	6.16538789	2.95948059	0.04155578	0.63095922	0.6196921	Mmp14/Sema3e	2
GO:0001958	endochondral ossification	2/138	34/14464	0.05882353	6.16538789	2.95948059	0.04155578	0.63095922	0.6196921	Mmp14/Ift80	2
GO:0036075	replacement ossification	2/138	34/14464	0.05882353	6.16538789	2.95948059	0.04155578	0.63095922	0.6196921	Mmp14/Ift80	2
GO:1902175	regulation of oxidative stress-induced intrinsic apoptotic signaling pathway	2/138	34/14464	0.05882353	6.16538789	2.95948059	0.04155578	0.63095922	0.6196921	Bag5/P4hb	2
GO:2000403	positive regulation of lymphocyte migration	2/138	34/14464	0.05882353	6.16538789	2.95948059	0.04155578	0.63095922	0.6196921	Cd99l2/Oxsr1	2
GO:0006956	complement activation	2/138	35/14464	0.05714286	5.98923395	2.90038759	0.04380468	0.63095922	0.6196921	Ighg2c/Cr1l	2
GO:0033144	negative regulation of intracellular steroid hormone receptor signaling pathway	2/138	35/14464	0.05714286	5.98923395	2.90038759	0.04380468	0.63095922	0.6196921	Bmal1/Calr	2
GO:0046640	regulation of alpha-beta T cell proliferation	2/138	35/14464	0.05714286	5.98923395	2.90038759	0.04380468	0.63095922	0.6196921	Card11/Itch	2
GO:1903311	regulation of mRNA metabolic process	6/138	269/14464	0.02230483	2.33780508	2.17373973	0.04416945	0.63095922	0.6196921	Mettl16/Obi1/Srpk1/Hnrnpc/Pkp3/Mettl14	6
GO:0045765	regulation of angiogenesis	6/138	270/14464	0.02222222	2.32914654	2.16375761	0.04483347	0.63095922	0.6196921	Rhoj/Sash1/Stard13/S2bpcox16/Sema3e/Aggf1	6
GO:0034620	cellular response to unfolded protein	3/138	84/14464	0.03571429	3.74327122	2.4747721	0.04629753	0.63095922	0.6196921	Hspa5/Hspd1/Dnajc3	3
GO:0034766	negative regulation of monoatomic ion transmembrane transport	3/138	84/14464	0.03571429	3.74327122	2.4747721	0.04629753	0.63095922	0.6196921	Rrad/Sri/Oxsr1	3
GO:0008380	RNA splicing	8/138	417/14464	0.01918465	2.01077399	2.05559029	0.04646287	0.63095922	0.6196921	Mettl16/Obi1/Srpk1/Prpf31/Hnrnpc/Mettl14/Virma/Prpf18	8
GO:0051651	maintenance of location in cell	5/138	205/14464	0.02439024	2.55638035	2.20269516	0.04666638	0.63095922	0.6196921	Hspa5/Scin/Hexa/Pkp2/Sri	5
GO:0060348	bone development	5/138	207/14464	0.02415459	2.53168102	2.17844044	0.04829336	0.63095922	0.6196921	Dhrs3/Mmp14/Has2/Ift172/Ift80	5
GO:0006084	acetyl-CoA metabolic process	2/138	37/14464	0.05405405	5.66549158	2.78879454	0.04843439	0.63095922	0.6196921	Ppcs/Pdk3	2
GO:0060563	neuroepithelial cell differentiation	2/138	37/14464	0.05405405	5.66549158	2.78879454	0.04843439	0.63095922	0.6196921	Smad2/Ift80	2
GO:0060021	roof of mouth development	3/138	86/14464	0.03488372	3.6562184	2.4247671	0.04905743	0.63095922	0.6196921	Dhrs3/Smad2/Ift172	3
GO:1901342	regulation of vasculature development	6/138	277/14464	0.02166065	2.27028724	2.09509055	0.04965684	0.63095922	0.6196921	Rhoj/Sash1/Stard13/S2bpcox16/Sema3e/Aggf1	6
GO:0043542	endothelial cell migration	5/138	210/14464	0.02380952	2.49551415	2.14258499	0.05079633	0.63095922	0.6196921	Calr/Rhoj/Sash1/Stard13/S2bpcox16	5
GO:0032924	activin receptor signaling pathway	2/138	38/14464	0.05263158	5.51639969	2.73600865	0.05081316	0.63095922	0.6196921	Dand5/Smad2	2
GO:0090049	regulation of cell migration involved in sprouting angiogenesis	2/138	38/14464	0.05263158	5.51639969	2.73600865	0.05081316	0.63095922	0.6196921	Rhoj/Stard13	2
GO:2000404	regulation of T cell migration	2/138	38/14464	0.05263158	5.51639969	2.73600865	0.05081316	0.63095922	0.6196921	Cd99l2/Oxsr1	2
GO:0170033	L-amino acid metabolic process	4/138	147/14464	0.02721088	2.85201617	2.2150508	0.0522221	0.63095922	0.6196921	Gclm/Hmgcll1/Enoph1/P4hb	4
GO:1903115	regulation of actin filament-based movement	2/138	39/14464	0.05128205	5.37495355	2.68506066	0.05323319	0.63095922	0.6196921	Pkp2/Sri	2
GO:0048705	skeletal system morphogenesis	5/138	214/14464	0.02336449	2.44886902	2.09572766	0.0542505	0.63095922	0.6196921	Dhrs3/Mmp14/Has2/Smad2/Ift80	5
GO:0046633	alpha-beta T cell proliferation	2/138	40/14464	0.05	5.24057971	2.6358375	0.05569348	0.63095922	0.6196921	Card11/Itch	2
GO:0141193	nuclear receptor-mediated signaling pathway	4/138	151/14464	0.02649007	2.77646607	2.15370551	0.05657199	0.63095922	0.6196921	Bmal1/Dhrs3/Calr/Pdk3	4
GO:0044703	multi-organism reproductive process	4/138	152/14464	0.02631579	2.75819985	2.13868159	0.05768914	0.63095922	0.6196921	Bmal1/Calr/Cr1l/Tppp3	4
GO:0001706	endoderm formation	2/138	41/14464	0.04878049	5.11276069	2.58823574	0.05819307	0.63095922	0.6196921	Mmp14/Smad2	2
GO:0019748	secondary metabolic process	2/138	41/14464	0.04878049	5.11276069	2.58823574	0.05819307	0.63095922	0.6196921	Rab38/Akr1a1	2
GO:0043507	positive regulation of JUN kinase activity	2/138	41/14464	0.04878049	5.11276069	2.58823574	0.05819307	0.63095922	0.6196921	Fzd8/Sash1	2
GO:0048701	embryonic cranial skeleton morphogenesis	2/138	41/14464	0.04878049	5.11276069	2.58823574	0.05819307	0.63095922	0.6196921	Mmp14/Smad2	2
GO:0044706	multi-multicellular organism process	4/138	153/14464	0.02614379	2.7401724	2.12377871	0.05881811	0.63095922	0.6196921	Bmal1/Calr/Cr1l/Tppp3	4
GO:0046474	glycerophospholipid biosynthetic process	4/138	153/14464	0.02614379	2.7401724	2.12377871	0.05881811	0.63095922	0.6196921	Rab38/Dgkk/Pigg/Selenoi	4
GO:1904375	regulation of protein localization to cell periphery	4/138	154/14464	0.02597403	2.72237907	2.10899498	0.05995887	0.63095922	0.6196921	Mmp14/Arhgef16/Rab38/Cnpy4	4
GO:0071333	cellular response to glucose stimulus	4/138	155/14464	0.02580645	2.70481533	2.09432855	0.06111141	0.63095922	0.6196921	Smad2/Gclm/Sri/Pdk3	4
GO:0070936	protein K48-linked ubiquitination	3/138	95/14464	0.03157895	3.30983982	2.21685178	0.06244008	0.63095922	0.6196921	Dtx4/Itch/Ube3c	3
GO:0007033	vacuole organization	5/138	223/14464	0.02242152	2.35003574	1.99403932	0.06251093	0.63095922	0.6196921	Hexa/Snx18/Aktip/Wdr45b/Sec22b	5
GO:0090092	regulation of transmembrane receptor protein serine/threonine kinase signaling pathway	5/138	223/14464	0.02242152	2.35003574	1.99403932	0.06251093	0.63095922	0.6196921	Hspa5/Dand5/Smad2/Brms1/Spred3	5
GO:0021872	forebrain generation of neurons	2/138	43/14464	0.04651163	4.87495787	2.49752467	0.06330629	0.63095922	0.6196921	Sema3e/Mettl14	2
GO:0043409	negative regulation of MAPK cascade	4/138	157/14464	0.02547771	2.67035909	2.06534042	0.06345167	0.63095922	0.6196921	Fktn/S2bpcox16/Itch/Spred3	4
GO:0071331	cellular response to hexose stimulus	4/138	158/14464	0.02531646	2.65345808	2.05101523	0.06463934	0.63095922	0.6196921	Smad2/Gclm/Sri/Pdk3	4
GO:0071326	cellular response to monosaccharide stimulus	4/138	159/14464	0.02515723	2.63676967	2.03680035	0.06583865	0.63095922	0.6196921	Smad2/Gclm/Sri/Pdk3	4
GO:0061013	regulation of mRNA catabolic process	4/138	161/14464	0.02484472	2.60401476	2.00869497	0.06827209	0.63095922	0.6196921	Mettl16/Hnrnpc/Pkp3/Mettl14	4
GO:0007219	Notch signaling pathway	4/138	162/14464	0.02469136	2.5879406	1.99480127	0.06950614	0.63095922	0.6196921	Mmp14/Dtx4/Ift172/S2bpcox16	4
GO:0006397	mRNA processing	8/138	455/14464	0.01758242	1.84284122	1.79289212	0.06994329	0.63095922	0.6196921	Mettl16/Obi1/Srpk1/Prpf31/Hnrnpc/Mettl14/Virma/Prpf18	8
GO:1990778	protein localization to cell periphery	7/138	378/14464	0.01851852	1.94095545	1.81939661	0.07023276	0.63095922	0.6196921	Mmp14/Arhgef16/Rab38/Cnpy4/Pkp3/Pkp2/Ift80	7
GO:0002065	columnar/cuboidal epithelial cell differentiation	3/138	100/14464	0.03	3.14434783	2.11185354	0.0705323	0.63095922	0.6196921	Bmal1/Smad2/Ift80	3

GO:0071674	mononuclear cell migration	4/138	163/14464	0.02453988	2.57206366	1.98101147	0.07075169	0.63095922	0.6196921	Calr/Mmp14/Cd99l2/Oxsr1	4
GO:0006890	retrograde vesicle-mediated transport, Golgi to endoplasmic reticulum	2/138	46/14464	0.04347826	4.55702583	2.37148121	0.07124724	0.63095922	0.6196921	Lman2/Sec22b	2
GO:1903426	regulation of reactive oxygen species biosynthetic process	2/138	46/14464	0.04347826	4.55702583	2.37148121	0.07124724	0.63095922	0.6196921	Slc5a3/Hspd1	2
GO:2000785	regulation of autophagosome assembly	2/138	46/14464	0.04347826	4.55702583	2.37148121	0.07124724	0.63095922	0.6196921	Snx18/Sec22b	2
GO:0046394	carboxylic acid biosynthetic process	5/138	232/14464	0.02155172	2.25887056	1.89713557	0.07144553	0.63095922	0.6196921	Degs1/Elovl7/Lta4h/Enoph1/Akr1a1	5
GO:0007368	determination of left/right symmetry	3/138	101/14464	0.02970297	3.11321567	2.09164604	0.07220504	0.63095922	0.6196921	Dand5/Smad2/Ift172	3
GO:0051235	maintenance of location	6/138	307/14464	0.01954397	2.04843507	1.82234824	0.07384839	0.63095922	0.6196921	Hspa5/Dand5/Scin/Hexa/Pkp2/Sri	6
GO:0010595	positive regulation of endothelial cell migration	3/138	102/14464	0.02941176	3.08269395	2.07168796	0.07389552	0.63095922	0.6196921	Calr/Rhoj/Sash1	3
GO:0007566	embryo implantation	2/138	47/14464	0.04255319	4.46006784	2.33185937	0.0739629	0.63095922	0.6196921	Calr/Tppp3	2
GO:0042073	intraciliary transport	2/138	47/14464	0.04255319	4.46006784	2.33185937	0.0739629	0.63095922	0.6196921	Ift172/Ift80	2
GO:0016053	organic acid biosynthetic process	5/138	235/14464	0.0212766	2.23003392	1.86582149	0.07457248	0.63095922	0.6196921	Degs1/Elovl7/Lta4h/Enoph1/Akr1a1	5
GO:0009409	response to cold	2/138	48/14464	0.04166667	4.36714976	2.29333198	0.07671141	0.63095922	0.6196921	Hspd1/Dnajc3	2
GO:0090102	cochlea development	2/138	48/14464	0.04166667	4.36714976	2.29333198	0.07671141	0.63095922	0.6196921	Grhl3/Slc17a8	2
GO:0006520	amino acid metabolic process	5/138	238/14464	0.0210084	2.20192425	1.8349762	0.07777324	0.63095922	0.6196921	Gclm/Hmgcll1/Yars2/Enoph1/P4hb	5
GO:0001501	skeletal system development	8/138	466/14464	0.01716738	1.79934067	1.72146477	0.07789467	0.63095922	0.6196921	Dhrs3/Mmp14/Has2/Smad2/Scin/Hexa/Ift172/Ift80	8
GO:0006979	response to oxidative stress	7/138	388/14464	0.01804124	1.89093082	1.74592861	0.07824586	0.63095922	0.6196921	Bmal1/Mmp14/Dgkk/Gclm/Ppp2cb/Msrb2/Oxsr1	7
GO:1903320	regulation of protein modification by small protein conjugation or removal	5/138	239/14464	0.0209205	2.19271118	1.82479619	0.0788565	0.63095922	0.6196921	Hspa5/Sash1/Bag5/Itch/Ndfip2	5
GO:0010596	negative regulation of endothelial cell migration	2/138	49/14464	0.04081633	4.27802425	2.25584441	0.07949192	0.63095922	0.6196921	Stard13/S2bpcox16	2
GO:0061371	determination of heart left/right asymmetry	2/138	49/14464	0.04081633	4.27802425	2.25584441	0.07949192	0.63095922	0.6196921	Dand5/Ift172	2
GO:1903170	negative regulation of calcium ion transmembrane transport	2/138	49/14464	0.04081633	4.27802425	2.25584441	0.07949192	0.63095922	0.6196921	Rrad/Sri	2
GO:0016525	negative regulation of angiogenesis	3/138	106/14464	0.02830189	2.96636587	1.99423599	0.08083193	0.63095922	0.6196921	Stard13/S2bpcox16/Sema3e	3
GO:0001678	intracellular glucose homeostasis	4/138	171/14464	0.02339181	2.4517332	1.87425824	0.08112528	0.63095922	0.6196921	Smad2/Gclm/Sri/Pdk3	4
GO:0071322	cellular response to carbohydrate stimulus	4/138	171/14464	0.02339181	2.4517332	1.87425824	0.08112528	0.63095922	0.6196921	Smad2/Gclm/Sri/Pdk3	4
GO:1901605	alpha-amino acid metabolic process	4/138	171/14464	0.02339181	2.4517332	1.87425824	0.08112528	0.63095922	0.6196921	Gclm/Hmgcll1/Enoph1/P4hb	4
GO:0000959	mitochondrial RNA metabolic process	2/138	50/14464	0.04	4.19246377	2.21934584	0.08230359	0.63095922	0.6196921	Tfb1m/Yars2	2
GO:0016073	snRNA metabolic process	2/138	50/14464	0.04	4.19246377	2.21934584	0.08230359	0.63095922	0.6196921	Mettl16/Ints10	2
GO:0072678	T cell migration	2/138	50/14464	0.04	4.19246377	2.21934584	0.08230359	0.63095922	0.6196921	Cd99l2/Oxsr1	2
GO:0006643	membrane lipid metabolic process	4/138	172/14464	0.02325581	2.43747893	1.86133898	0.08247246	0.63095922	0.6196921	Pigg/Hexa/Degs1/Elovl7	4
GO:0016064	immunoglobulin mediated immune response	3/138	107/14464	0.02803738	2.93864283	1.97544119	0.08260888	0.63095922	0.6196921	Ighg2c/Hspd1/Cr1l	3
GO:0003007	heart morphogenesis	5/138	243/14464	0.02057613	2.15661716	1.78457155	0.0832707	0.63095922	0.6196921	Dhrs3/Has2/Smad2/Ift172/Pkp2	5
GO:1901873	regulation of post-translational protein modification	5/138	244/14464	0.0204918	2.14777857	1.77463683	0.08439446	0.63095922	0.6196921	Hspa5/Sash1/Bag5/Itch/Ndfip2	5
GO:0060972	left/right pattern formation	3/138	108/14464	0.02777778	2.91143317	1.95686351	0.08440264	0.63095922	0.6196921	Dand5/Smad2/Ift172	3
GO:2000181	negative regulation of blood vessel morphogenesis	3/138	108/14464	0.02777778	2.91143317	1.95686351	0.08440264	0.63095922	0.6196921	Stard13/S2bpcox16/Sema3e	3
GO:0009799	specification of symmetry	3/138	109/14464	0.02752294	2.88472278	1.93849816	0.08621307	0.63095922	0.6196921	Dand5/Smad2/Ift172	3
GO:0009855	determination of bilateral symmetry	3/138	109/14464	0.02752294	2.88472278	1.93849816	0.08621307	0.63095922	0.6196921	Dand5/Smad2/Ift172	3
GO:1901343	negative regulation of vasculature development	3/138	109/14464	0.02752294	2.88472278	1.93849816	0.08621307	0.63095922	0.6196921	Stard13/S2bpcox16/Sema3e	3
GO:0002042	cell migration involved in sprouting angiogenesis	2/138	52/14464	0.03846154	4.03121516	2.14912944	0.08801701	0.63095922	0.6196921	Rhoj/Stard13	2
GO:0061337	cardiac conduction	2/138	52/14464	0.03846154	4.03121516	2.14912944	0.08801701	0.63095922	0.6196921	Pkp2/Sri	2
GO:2000401	regulation of lymphocyte migration	2/138	52/14464	0.03846154	4.03121516	2.14912944	0.08801701	0.63095922	0.6196921	Cd99l2/Oxsr1	2
GO:0019724	B cell mediated immunity	3/138	110/14464	0.02727273	2.85849802	1.92034049	0.08803999	0.63095922	0.6196921	Ighg2c/Hspd1/Cr1l	3
GO:0006024	glycosaminoglycan biosynthetic process	2/138	53/14464	0.03773585	3.9551545	2.11532611	0.09091715	0.63095922	0.6196921	Has2/Hexa	2
GO:0006513	protein monoubiquitination	2/138	53/14464	0.03773585	3.9551545	2.11532611	0.09091715	0.63095922	0.6196921	Obi1/Itch	2
GO:0008631	intrinsic apoptotic signaling pathway in response to oxidative stress	2/138	53/14464	0.03773585	3.9551545	2.11532611	0.09091715	0.63095922	0.6196921	Bag5/P4hb	2
GO:0048002	antigen processing and presentation of peptide antigen	2/138	53/14464	0.03773585	3.9551545	2.11532611	0.09091715	0.63095922	0.6196921	Calr/Pdia3	2
GO:0000301	retrograde transport, vesicle recycling within Golgi	1/138	10/14464	0.1	10.4811594	2.94356538	0.09144271	0.63095922	0.6196921	Cog6	1
GO:0002713	negative regulation of B cell mediated immunity	1/138	10/14464	0.1	10.4811594	2.94356538	0.09144271	0.63095922	0.6196921	Cr1l	1
GO:0002836	positive regulation of response to tumor cell	1/138	10/14464	0.1	10.4811594	2.94356538	0.09144271	0.63095922	0.6196921	Hspd1	1
GO:0002839	positive regulation of immune response to tumor cell	1/138	10/14464	0.1	10.4811594	2.94356538	0.09144271	0.63095922	0.6196921	Hspd1	1
GO:0002890	negative regulation of immunoglobulin mediated immune response	1/138	10/14464	0.1	10.4811594	2.94356538	0.09144271	0.63095922	0.6196921	Cr1l	1
GO:0006517	protein deglycosylation	1/138	10/14464	0.1	10.4811594	2.94356538	0.09144271	0.63095922	0.6196921	Fktn	1
GO:0006534	cysteine metabolic process	1/138	10/14464	0.1	10.4811594	2.94356538	0.09144271	0.63095922	0.6196921	Gclm	1
GO:0006551	L-leucine metabolic process	1/138	10/14464	0.1	10.4811594	2.94356538	0.09144271	0.63095922	0.6196921	Hmgcll1	1
GO:0007440	foregut morphogenesis	1/138	10/14464	0.1	10.4811594	2.94356538	0.09144271	0.63095922	0.6196921	Smad2	1
GO:0009086	methionine biosynthetic process	1/138	10/14464	0.1	10.4811594	2.94356538	0.09144271	0.63095922	0.6196921	Enoph1	1
GO:0009221	pyrimidine deoxyribonucleotide biosynthetic process	1/138	10/14464	0.1	10.4811594	2.94356538	0.09144271	0.63095922	0.6196921	Tbpl1	1
GO:0010819	regulation of T cell chemotaxis	1/138	10/14464	0.1	10.4811594	2.94356538	0.09144271	0.63095922	0.6196921	Oxsr1	1
GO:0019370	leukotriene biosynthetic process	1/138	10/14464	0.1	10.4811594	2.94356538	0.09144271	0.63095922	0.6196921	Lta4h	1
GO:0019511	peptidyl-proline hydroxylation	1/138	10/14464	0.1	10.4811594	2.94356538	0.09144271	0.63095922	0.6196921	P4hb	1
GO:0021979	hypothalamus cell differentiation	1/138	10/14464	0.1	10.4811594	2.94356538	0.09144271	0.63095922	0.6196921	Sema3e	1
GO:0035338	long-chain fatty-acyl-CoA biosynthetic process	1/138	10/14464	0.1	10.4811594	2.94356538	0.09144271	0.63095922	0.6196921	Elovl7	1
GO:0035646	endosome to melanosome transport	1/138	10/14464	0.1	10.4811594	2.94356538	0.09144271	0.63095922	0.6196921	Rab38	1
GO:0043485	endosome to pigment granule transport	1/138	10/14464	0.1	10.4811594	2.94356538	0.09144271	0.63095922	0.6196921	Rab38	1
GO:0045654	positive regulation of megakaryocyte differentiation	1/138	10/14464	0.1	10.4811594	2.94356538	0.09144271	0.63095922	0.6196921	Scin	1
GO:0045916	negative regulation of complement activation	1/138	10/14464	0.1	10.4811594	2.94356538	0.09144271	0.63095922	0.6196921	Cr1l	1
GO:0048757	pigment granule maturation	1/138	10/14464	0.1	10.4811594	2.94356538	0.09144271	0.63095922	0.6196921	Rab38	1
GO:0051775	response to redox state	1/138	10/14464	0.1	10.4811594	2.94356538	0.09144271	0.63095922	0.6196921	Bmal1	1
GO:0060049	regulation of protein glycosylation	1/138	10/14464	0.1	10.4811594	2.94356538	0.09144271	0.63095922	0.6196921	Fktn	1
GO:0060509	type I pneumocyte differentiation	1/138	10/14464	0.1	10.4811594	2.94356538	0.09144271	0.63095922	0.6196921	Lta4h	1
GO:0071073	positive regulation of phospholipid biosynthetic process	1/138	10/14464	0.1	10.4811594	2.94356538	0.09144271	0.63095922	0.6196921	Rab38	1
GO:0097067	cellular response to thyroid hormone stimulus	1/138	10/14464	0.1	10.4811594	2.94356538	0.09144271	0.63095922	0.6196921	Gclm	1
GO:0097499	protein localization to non-motile cilium	1/138	10/14464	0.1	10.4811594	2.94356538	0.09144271	0.63095922	0.6196921	Ift80	1
GO:0098700	neurotransmitter loading into synaptic vesicle	1/138	10/14464	0.1	10.4811594	2.94356538	0.09144271	0.63095922	0.6196921	Slc17a8	1
GO:0150172	regulation of phosphatidylcholine metabolic process	1/138	10/14464	0.1	10.4811594	2.94356538	0.09144271	0.63095922	0.6196921	Rab38	1
GO:2000562	negative regulation of CD4-positive, alpha-beta T cell proliferation	1/138	10/14464	0.1	10.4811594	2.94356538	0.09144271	0.63095922	0.6196921	Itch	1
GO:0006612	protein targeting to membrane	3/138	112/14464	0.02678571	2.80745342	1.88463032	0.09174265	0.63095922	0.6196921	Hspa5/Ubl4a/Srprb	3
GO:1905477	positive regulation of protein localization to membrane	3/138	113/14464	0.02654867	2.7826087	1.86706924	0.09361807	0.63095922	0.6196921	Slc5a3/Arhgef16/Cnpy4	3
GO:0034332	adherens junction organization	2/138	54/14464	0.03703704	3.8819109	2.08234029	0.09384516	0.63095922	0.6196921	Pkp2/Cdh10	2
GO:0035567	non-canonical Wnt signaling pathway	2/138	54/14464	0.03703704	3.8819109	2.08234029	0.09384516	0.63095922	0.6196921	Fzd8/Ift80	2
GO:0043506	regulation of JUN kinase activity	2/138	54/14464	0.03703704	3.8819109	2.08234029	0.09384516	0.63095922	0.6196921	Fzd8/Sash1	2
GO:0048524	positive regulation of viral process	2/138	54/14464	0.03703704	3.8819109	2.08234029	0.09384516	0.63095922	0.6196921	Srpk1/P4hb	2
GO:1901224	positive regulation of non-canonical NF-kappaB signal transduction	2/138	54/14464	0.03703704	3.8819109	2.08234029	0.09384516	0.63095922	0.6196921	Calr/Sash1	2
GO:0008637	apoptotic mitochondrial changes	3/138	114/14464	0.02631579	2.75819985	1.84969863	0.09550931	0.63095922	0.6196921	Hspd1/Gclm/Ppp2cb	3
GO:1903076	regulation of protein localization to plasma membrane	3/138	114/14464	0.02631579	2.75819985	1.84969863	0.09550931	0.63095922	0.6196921	Mmp14/Arhgef16/Cnpy4	3
GO:0009895	negative regulation of catabolic process	6/138	330/14464	0.01818182	1.90566535	1.63342215	0.09626365	0.63095922	0.6196921	Mettl16/Hnrnpc/Pkp3/Bag5/Mettl14/Sec22b	6
GO:0071805	potassium ion transmembrane transport	4/138	182/14464	0.02197802	2.30355152	1.73690331	0.09654382	0.63095922	0.6196921	Kcnn1/Kcnk13/Kcnip2/Oxsr1	4
GO:0010812	negative regulation of cell-substrate adhesion	2/138	55/14464	0.03636364	3.8113307	2.05013576	0.09680026	0.63095922	0.6196921	Mmp14/Sema3e	2
GO:0031589	cell-substrate adhesion	6/138	332/14464	0.01807229	1.89418544	1.61771154	0.09836867	0.63095922	0.6196921	Calr/Mmp14/Has2/Pkp2/Sema3e/P4hb	6
GO:0034763	negative regulation of transmembrane transport	3/138	116/14464	0.02586207	2.71064468	1.81551309	0.09933862	0.63095922	0.6196921	Rrad/Sri/Oxsr1	3
GO:0043161	proteasome-mediated ubiquitin-dependent protein catabolic process	7/138	412/14464	0.01699029	1.78077951	1.5780262	0.09957379	0.63095922	0.6196921	Bmal1/Dtx4/Klhl5/Ppp2cb/Bag5/Itch/Cul2	7
GO:1903409	reactive oxygen species biosynthetic process	2/138	56/14464	0.03571429	3.74327122	2.01867858	0.09978167	0.63095922	0.6196921	Slc5a3/Hspd1	2
GO:0000244	spliceosomal tri-snRNP complex assembly	1/138	11/14464	0.09090909	9.52832675	2.77707389	0.10011719	0.63095922	0.6196921	Prpf31	1
GO:0002407	dendritic cell chemotaxis	1/138	11/14464	0.09090909	9.52832675	2.77707389	0.10011719	0.63095922	0.6196921	Calr	1
GO:0002424	T cell mediated immune response to tumor cell	1/138	11/14464	0.09090909	9.52832675	2.77707389	0.10011719	0.63095922	0.6196921	Hspd1	1
GO:0002755	MyD88-dependent toll-like receptor signaling pathway	1/138	11/14464	0.09090909	9.52832675	2.77707389	0.10011719	0.63095922	0.6196921	Hspd1	1
GO:0002840	regulation of T cell mediated immune response to tumor cell	1/138	11/14464	0.09090909	9.52832675	2.77707389	0.10011719	0.63095922	0.6196921	Hspd1	1
GO:0003096	renal sodium ion transport	1/138	11/14464	0.09090909	9.52832675	2.77707389	0.10011719	0.63095922	0.6196921	Oxsr1	1
GO:0006555	methionine metabolic process	1/138	11/14464	0.09090909	9.52832675	2.77707389	0.10011719	0.63095922	0.6196921	Enoph1	1
GO:0006596	polyamine biosynthetic process	1/138	11/14464	0.09090909	9.52832675	2.77707389	0.10011719	0.63095922	0.6196921	Sat1	1
GO:0009950	dorsal/ventral axis specification	1/138	11/14464	0.09090909	9.52832675	2.77707389	0.10011719	0.63095922	0.6196921	Smad2	1
GO:0035630	bone mineralization involved in bone maturation	1/138	11/14464	0.09090909	9.52832675	2.77707389	0.10011719	0.63095922	0.6196921	Ift80	1
GO:0042402	cellular biogenic amine catabolic process	1/138	11/14464	0.09090909	9.52832675	2.77707389	0.10011719	0.63095922	0.6196921	Sat1	1
GO:0042989	sequestering of actin monomers	1/138	11/14464	0.09090909	9.52832675	2.77707389	0.10011719	0.63095922	0.6196921	Scin	1
GO:0048340	paraxial mesoderm morphogenesis	1/138	11/14464	0.09090909	9.52832675	2.77707389	0.10011719	0.63095922	0.6196921	Smad2	1
GO:0050655	dermatan sulfate proteoglycan metabolic process	1/138	11/14464	0.09090909	9.52832675	2.77707389	0.10011719	0.63095922	0.6196921	Hexa	1
GO:0061299	retina vasculature morphogenesis in camera-type eye	1/138	11/14464	0.09090909	9.52832675	2.77707389	0.10011719	0.63095922	0.6196921	Rhoj	1
GO:0071907	left/right axis specification	1/138	11/14464	0.09090909	9.52832675	2.77707389	0.10011719	0.63095922	0.6196921	Ift172	1
GO:0071257	cellular response to electrical stimulus	1/138	11/14464	0.09090909	9.52832675	2.77707389	0.10011719	0.63095922	0.6196921	Calr	1
GO:0090085	regulation of protein deubiquitination	1/138	11/14464	0.09090909	9.52832675	2.77707389	0.10011719	0.63095922	0.6196921	Itch	1
GO:1903441	protein localization to ciliary membrane	1/138	11/14464	0.09090909	9.52832675	2.77707389	0.10011719	0.63095922	0.6196921	Ift80	1
GO:1903727	positive regulation of phospholipid metabolic process	1/138	11/14464	0.09090909	9.52832675	2.77707389	0.10011719	0.63095922	0.6196921	Rab38	1
GO:0033363	secretory granule organization	2/138	57/14464	0.03508772	3.6775998	1.98793683	0.10278864	0.63095922	0.6196921	Rab38/Tbpl1	2
GO:0000045	autophagosome assembly	3/138	118/14464	0.02542373	2.66470155	1.78204324	0.10322922	0.63095922	0.6196921	Snx18/Wdr45b/Sec22b	3
GO:0043271	negative regulation of monoatomic ion transport	3/138	118/14464	0.02542373	2.66470155	1.78204324	0.10322922	0.63095922	0.6196921	Rrad/Sri/Oxsr1	3
GO:0006888	endoplasmic reticulum to Golgi vesicle-mediated transport	3/138	119/14464	0.02521008	2.6423091	1.76556766	0.10519706	0.63095922	0.6196921	Lman2/P4hb/Sec22b	3
GO:1903313	positive regulation of mRNA metabolic process	3/138	119/14464	0.02521008	2.6423091	1.76556766	0.10519706	0.63095922	0.6196921	Mettl16/Obi1/Mettl14	3
GO:0006023	aminoglycan biosynthetic process	2/138	58/14464	0.03448276	3.6141929	1.95788052	0.10582041	0.63095922	0.6196921	Has2/Hexa	2
GO:0044088	regulation of vacuole organization	2/138	58/14464	0.03448276	3.6141929	1.95788052	0.10582041	0.63095922	0.6196921	Snx18/Sec22b	2
GO:0055117	regulation of cardiac muscle contraction	2/138	58/14464	0.03448276	3.6141929	1.95788052	0.10582041	0.63095922	0.6196921	Pkp2/Sri	2
GO:0086003	cardiac muscle cell contraction	2/138	58/14464	0.03448276	3.6141929	1.95788052	0.10582041	0.63095922	0.6196921	Pkp2/Sri	2
GO:0006983	ER overload response	1/138	12/14464	0.08333333	8.73429952	2.63059443	0.10870944	0.63095922	0.6196921	Hspa5	1
GO:0008611	ether lipid biosynthetic process	1/138	12/14464	0.08333333	8.73429952	2.63059443	0.10870944	0.63095922	0.6196921	Selenoi	1
GO:0009310	amine catabolic process	1/138	12/14464	0.08333333	8.73429952	2.63059443	0.10870944	0.63095922	0.6196921	Sat1	1
GO:0010998	regulation of translational initiation by eIF2 alpha phosphorylation	1/138	12/14464	0.08333333	8.73429952	2.63059443	0.10870944	0.63095922	0.6196921	Dnajc3	1
GO:0015937	coenzyme A biosynthetic process	1/138	12/14464	0.08333333	8.73429952	2.63059443	0.10870944	0.63095922	0.6196921	Ppcs	1
GO:0016137	glycoside metabolic process	1/138	12/14464	0.08333333	8.73429952	2.63059443	0.10870944	0.63095922	0.6196921	Akr1a1	1
GO:0019852	L-ascorbic acid metabolic process	1/138	12/14464	0.08333333	8.73429952	2.63059443	0.10870944	0.63095922	0.6196921	Akr1a1	1
GO:0036490	regulation of translation in response to endoplasmic reticulum stress	1/138	12/14464	0.08333333	8.73429952	2.63059443	0.10870944	0.63095922	0.6196921	Dnajc3	1
GO:0042364	water-soluble vitamin biosynthetic process	1/138	12/14464	0.08333333	8.73429952	2.63059443	0.10870944	0.63095922	0.6196921	Akr1a1	1
GO:0042761	very long-chain fatty acid biosynthetic process	1/138	12/14464	0.08333333	8.73429952	2.63059443	0.10870944	0.63095922	0.6196921	Elovl7	1
GO:0043476	pigment accumulation	1/138	12/14464	0.08333333	8.73429952	2.63059443	0.10870944	0.63095922	0.6196921	Rab38	1
GO:0043482	cellular pigment accumulation	1/138	12/14464	0.08333333	8.73429952	2.63059443	0.10870944	0.63095922	0.6196921	Rab38	1
GO:0046479	glycosphingolipid catabolic process	1/138	12/14464	0.08333333	8.73429952	2.63059443	0.10870944	0.63095922	0.6196921	Hexa	1
GO:0046504	glycerol ether biosynthetic process	1/138	12/14464	0.08333333	8.73429952	2.63059443	0.10870944	0.63095922	0.6196921	Selenoi	1
GO:0046949	fatty-acyl-CoA biosynthetic process	1/138	12/14464	0.08333333	8.73429952	2.63059443	0.10870944	0.63095922	0.6196921	Elovl7	1
GO:0048291	isotype switching to IgG isotypes	1/138	12/14464	0.08333333	8.73429952	2.63059443	0.10870944	0.63095922	0.6196921	Hspd1	1
GO:0048312	intracellular distribution of mitochondria	1/138	12/14464	0.08333333	8.73429952	2.63059443	0.10870944	0.63095922	0.6196921	S2bpcox16	1
GO:0051549	positive regulation of keratinocyte migration	1/138	12/14464	0.08333333	8.73429952	2.63059443	0.10870944	0.63095922	0.6196921	Has2	1
GO:0061723	glycophagy	1/138	12/14464	0.08333333	8.73429952	2.63059443	0.10870944	0.63095922	0.6196921	Wdr45b	1
GO:0071166	ribonucleoprotein complex localization	1/138	12/14464	0.08333333	8.73429952	2.63059443	0.10870944	0.63095922	0.6196921	Prpf31	1
GO:0071476	cellular hypotonic response	1/138	12/14464	0.08333333	8.73429952	2.63059443	0.10870944	0.63095922	0.6196921	Oxsr1	1
GO:0072683	T cell extravasation	1/138	12/14464	0.08333333	8.73429952	2.63059443	0.10870944	0.63095922	0.6196921	Cd99l2	1
GO:0140627	ubiquitin-dependent protein catabolic process via the C-end degron rule pathway	1/138	12/14464	0.08333333	8.73429952	2.63059443	0.10870944	0.63095922	0.6196921	Cul2	1
GO:1901163	regulation of trophoblast cell migration	1/138	12/14464	0.08333333	8.73429952	2.63059443	0.10870944	0.63095922	0.6196921	Calr	1
GO:1902644	tertiary alcohol metabolic process	1/138	12/14464	0.08333333	8.73429952	2.63059443	0.10870944	0.63095922	0.6196921	Akr1a1	1
GO:2000322	regulation of glucocorticoid receptor signaling pathway	1/138	12/14464	0.08333333	8.73429952	2.63059443	0.10870944	0.63095922	0.6196921	Bmal1	1
GO:2000644	regulation of receptor catabolic process	1/138	12/14464	0.08333333	8.73429952	2.63059443	0.10870944	0.63095922	0.6196921	Itch	1
GO:1903078	positive regulation of protein localization to plasma membrane	2/138	59/14464	0.03389831	3.5529354	1.92848143	0.10887625	0.63095922	0.6196921	Arhgef16/Cnpy4	2
GO:0007043	cell-cell junction assembly	3/138	121/14464	0.02479339	2.59863457	1.73311806	0.10917699	0.63095922	0.6196921	Pkp3/Pkp2/Cdh10	3
GO:0043414	macromolecule methylation	3/138	121/14464	0.02479339	2.59863457	1.73311806	0.10917699	0.63095922	0.6196921	Mettl16/Tfb1m/Mettl14	3
GO:0046467	membrane lipid biosynthetic process	3/138	121/14464	0.02479339	2.59863457	1.73311806	0.10917699	0.63095922	0.6196921	Pigg/Degs1/Elovl7	3
GO:0045785	positive regulation of cell adhesion	7/138	422/14464	0.01658768	1.73858095	1.51128407	0.10932157	0.63095922	0.6196921	Card11/Calr/Has2/Hspd1/Pkp3/Pkp2/P4hb	7
GO:0002685	regulation of leukocyte migration	4/138	191/14464	0.02094241	2.19500721	1.63168319	0.1101076	0.63095922	0.6196921	Calr/Mmp14/Cd99l2/Oxsr1	4
GO:0060485	mesenchyme development	5/138	267/14464	0.01872659	1.96276394	1.55841606	0.11241274	0.63095922	0.6196921	Has2/Dand5/Smad2/Sema3e/Spred3	5
GO:0034308	primary alcohol metabolic process	2/138	61/14464	0.03278689	3.43644571	1.87155002	0.11505723	0.63095922	0.6196921	Dhrs3/Akr1a1	2
GO:0060350	endochondral bone morphogenesis	2/138	61/14464	0.03278689	3.43644571	1.87155002	0.11505723	0.63095922	0.6196921	Mmp14/Ift80	2
GO:0000097	sulfur amino acid biosynthetic process	1/138	13/14464	0.07692308	8.06243032	2.50024844	0.11722025	0.63095922	0.6196921	Enoph1	1
GO:0002643	regulation of tolerance induction	1/138	13/14464	0.07692308	8.06243032	2.50024844	0.11722025	0.63095922	0.6196921	Itch	1
GO:0002921	negative regulation of humoral immune response	1/138	13/14464	0.07692308	8.06243032	2.50024844	0.11722025	0.63095922	0.6196921	Cr1l	1
GO:0002923	regulation of humoral immune response mediated by circulating immunoglobulin	1/138	13/14464	0.07692308	8.06243032	2.50024844	0.11722025	0.63095922	0.6196921	Cr1l	1
GO:0003091	renal water homeostasis	1/138	13/14464	0.07692308	8.06243032	2.50024844	0.11722025	0.63095922	0.6196921	Has2	1
GO:0003413	chondrocyte differentiation involved in endochondral bone morphogenesis	1/138	13/14464	0.07692308	8.06243032	2.50024844	0.11722025	0.63095922	0.6196921	Ift80	1
GO:0006750	glutathione biosynthetic process	1/138	13/14464	0.07692308	8.06243032	2.50024844	0.11722025	0.63095922	0.6196921	Gclm	1
GO:0006833	water transport	1/138	13/14464	0.07692308	8.06243032	2.50024844	0.11722025	0.63095922	0.6196921	Has2	1
GO:0009078	pyruvate family amino acid metabolic process	1/138	13/14464	0.07692308	8.06243032	2.50024844	0.11722025	0.63095922	0.6196921	Hmgcll1	1
GO:0010459	negative regulation of heart rate	1/138	13/14464	0.07692308	8.06243032	2.50024844	0.11722025	0.63095922	0.6196921	Sri	1
GO:0016264	gap junction assembly	1/138	13/14464	0.07692308	8.06243032	2.50024844	0.11722025	0.63095922	0.6196921	Pkp2	1

GO:0019377 glycolipid catabolic process	1/138	13/14464	0.07692308	8.06243032	2.50024844	0.11722025	0.63095922	0.6196921	Hexa	1
GO:0030214 hyaluronan catabolic process	1/138	13/14464	0.07692308	8.06243032	2.50024844	0.11722025	0.63095922	0.6196921	Hexa	1
GO:0035437 maintenance of protein localization in endoplasmic reticulum	1/138	13/14464	0.07692308	8.06243032	2.50024844	0.11722025	0.63095922	0.6196921	Hspa5	1
GO:0046185 aldehyde catabolic process	1/138	13/14464	0.07692308	8.06243032	2.50024844	0.11722025	0.63095922	0.6196921	Akr1a1	1
GO:0046642 negative regulation of alpha-beta T cell proliferation	1/138	13/14464	0.07692308	8.06243032	2.50024844	0.11722025	0.63095922	0.6196921	Itch	1
GO:0051127 positive regulation of actin nucleation	1/138	13/14464	0.07692308	8.06243032	2.50024844	0.11722025	0.63095922	0.6196921	Scin	1
GO:0051280 negative regulation of release of sequestered calcium ion into cytosol	1/138	13/14464	0.07692308	8.06243032	2.50024844	0.11722025	0.63095922	0.6196921	Sri	1
GO:0051547 regulation of keratinocyte migration	1/138	13/14464	0.07692308	8.06243032	2.50024844	0.11722025	0.63095922	0.6196921	Has2	1
GO:0055062 phosphate ion homeostasis	1/138	13/14464	0.07692308	8.06243032	2.50024844	0.11722025	0.63095922	0.6196921	Slc17a8	1
GO:0061450 trophoblast cell migration	1/138	13/14464	0.07692308	8.06243032	2.50024844	0.11722025	0.63095922	0.6196921	Calr	1
GO:0071372 cellular response to follicle-stimulating hormone stimulus	1/138	13/14464	0.07692308	8.06243032	2.50024844	0.11722025	0.63095922	0.6196921	Gclm	1
GO:0072672 neutrophil extravasation	1/138	13/14464	0.07692308	8.06243032	2.50024844	0.11722025	0.63095922	0.6196921	Cd99l2	1
GO:0097384 cellular lipid biosynthetic process	1/138	13/14464	0.07692308	8.06243032	2.50024844	0.11722025	0.63095922	0.6196921	Selenoi	1
GO:0140131 positive regulation of lymphocyte chemotaxis	1/138	13/14464	0.07692308	8.06243032	2.50024844	0.11722025	0.63095922	0.6196921	Oxsr1	1
GO:1900044 regulation of protein K63-linked ubiquitination	1/138	13/14464	0.07692308	8.06243032	2.50024844	0.11722025	0.63095922	0.6196921	Sash1	1
GO:1901386 negative regulation of voltage-gated calcium channel activity	1/138	13/14464	0.07692308	8.06243032	2.50024844	0.11722025	0.63095922	0.6196921	Rrad	1
GO:1901503 ether biosynthetic process	1/138	13/14464	0.07692308	8.06243032	2.50024844	0.11722025	0.63095922	0.6196921	Selenoi	1
GO:1901533 negative regulation of hematopoietic progenitor cell differentiation	1/138	13/14464	0.07692308	8.06243032	2.50024844	0.11722025	0.63095922	0.6196921	Mettl14	1
GO:1902902 negative regulation of autophagosome assembly	1/138	13/14464	0.07692308	8.06243032	2.50024844	0.11722025	0.63095922	0.6196921	Sec22b	1
GO:1902950 regulation of dendritic spine maintenance	1/138	13/14464	0.07692308	8.06243032	2.50024844	0.11722025	0.63095922	0.6196921	Zfp804a	1
GO:0030336 negative regulation of cell migration	5/138	271/14464	0.01845018	1.93379325	1.52301625	0.11769657	0.63095922	0.6196921	Calr/Stard13/S2bpcox16/Pkp2/Brms1	5
GO:0045017 glycerolipid biosynthetic process	4/138	196/14464	0.02040816	2.13901213	1.57572743	0.11799377	0.63095922	0.6196921	Rab38/Dgkk/Pigg/Selenoi	4
GO:0006611 protein export from nucleus	2/138	62/14464	0.03225806	3.38101917	1.84396892	0.11818095	0.63095922	0.6196921	Calr/Frat2	2
GO:0021695 cerebellar cortex development	2/138	62/14464	0.03225806	3.38101917	1.84396892	0.11818095	0.63095922	0.6196921	Hspa5/Fktn	2
GO:0031016 pancreas development	2/138	62/14464	0.03225806	3.38101917	1.84396892	0.11818095	0.63095922	0.6196921	Bmal1/Smad2	2
GO:2000514 regulation of CD4-positive, alpha-beta T cell activation	2/138	62/14464	0.03225806	3.38101917	1.84396892	0.11818095	0.63095922	0.6196921	Card11/Itch	2
GO:0010811 positive regulation of cell-substrate adhesion	3/138	126/14464	0.02380952	2.49551415	1.65477051	0.11937665	0.63095922	0.6196921	Calr/Has2/P4hb	3
GO:1905037 autophagosome organization	3/138	126/14464	0.02380952	2.49551415	1.65477051	0.11937665	0.63095922	0.6196921	Snx18/Wdr45b/Sec22b	3
GO:0050821 protein stabilization	4/138	197/14464	0.02030457	2.1281542	1.56473756	0.11960005	0.63095922	0.6196921	Calr/Hspd1/Bag5/Ift80	4
GO:1901019 regulation of calcium ion transmembrane transporter activity	2/138	63/14464	0.03174603	3.3273522	1.81694726	0.12132589	0.63095922	0.6196921	Rrad/Sri	2
GO:0009953 dorsal/ventral pattern formation	2/138	64/14464	0.03125	3.27536232	1.79046389	0.12449139	0.63095922	0.6196921	Smad2/Ift172	2
GO:0006086 acetyl-CoA biosynthetic process from pyruvate	1/138	14/14464	0.07142857	7.48654244	2.38314036	0.12565036	0.63095922	0.6196921	Pdk3	1
GO:0006971 hypotonic response	1/138	14/14464	0.07142857	7.48654244	2.38314036	0.12565036	0.63095922	0.6196921	Oxsr1	1
GO:0010818 T cell chemotaxis	1/138	14/14464	0.07142857	7.48654244	2.38314036	0.12565036	0.63095922	0.6196921	Oxsr1	1
GO:0010831 positive regulation of myotube differentiation	1/138	14/14464	0.07142857	7.48654244	2.38314036	0.12565036	0.63095922	0.6196921	Mmp14	1
GO:0015721 bile acid and bile salt transport	1/138	14/14464	0.07142857	7.48654244	2.38314036	0.12565036	0.63095922	0.6196921	Slc51a	1
GO:0030213 hyaluronan biosynthetic process	1/138	14/14464	0.07142857	7.48654244	2.38314036	0.12565036	0.63095922	0.6196921	Has2	1
GO:0030242 autophagy of peroxisome	1/138	14/14464	0.07142857	7.48654244	2.38314036	0.12565036	0.63095922	0.6196921	Wdr45b	1
GO:0035729 cellular response to hepatocyte growth factor stimulus	1/138	14/14464	0.07142857	7.48654244	2.38314036	0.12565036	0.63095922	0.6196921	Gclm	1
GO:0035810 positive regulation of urine volume	1/138	14/14464	0.07142857	7.48654244	2.38314036	0.12565036	0.63095922	0.6196921	Has2	1
GO:0036498 IRE1-mediated unfolded protein response	1/138	14/14464	0.07142857	7.48654244	2.38314036	0.12565036	0.63095922	0.6196921	Hspa5	1
GO:0043455 regulation of secondary metabolic process	1/138	14/14464	0.07142857	7.48654244	2.38314036	0.12565036	0.63095922	0.6196921	Rab38	1
GO:0044849 estrous cycle	1/138	14/14464	0.07142857	7.48654244	2.38314036	0.12565036	0.63095922	0.6196921	Has2	1
GO:0045161 neuronal ion channel clustering	1/138	14/14464	0.07142857	7.48654244	2.38314036	0.12565036	0.63095922	0.6196921	Kcnip2	1
GO:0048021 regulation of melanin biosynthetic process	1/138	14/14464	0.07142857	7.48654244	2.38314036	0.12565036	0.63095922	0.6196921	Rab38	1
GO:0051797 regulation of hair follicle development	1/138	14/14464	0.07142857	7.48654244	2.38314036	0.12565036	0.63095922	0.6196921	Pkp3	1
GO:0071285 cellular response to lithium ion	1/138	14/14464	0.07142857	7.48654244	2.38314036	0.12565036	0.63095922	0.6196921	Calr	1
GO:0071318 cellular response to ATP	1/138	14/14464	0.07142857	7.48654244	2.38314036	0.12565036	0.63095922	0.6196921	Hsp90b1	1
GO:1900376 regulation of secondary metabolite biosynthetic process	1/138	14/14464	0.07142857	7.48654244	2.38314036	0.12565036	0.63095922	0.6196921	Rab38	1
GO:0006665 sphingolipid metabolic process	3/138	129/14464	0.02325581	2.43747893	1.60954735	0.12566068	0.63095922	0.6196921	Hexa/Degs1/Elovl7	3
GO:0050684 regulation of mRNA processing	3/138	129/14464	0.02325581	2.43747893	1.60954735	0.12566068	0.63095922	0.6196921	Mettl16/Obi1/Srpk1	3
GO:0008654 phospholipid biosynthetic process	4/138	201/14464	0.0199005	2.08580287	1.5214225	0.12611966	0.63095922	0.6196921	Rab38/Dgkk/Pigg/Selenoi	4
GO:0009749 response to glucose	4/138	201/14464	0.0199005	2.08580287	1.5214225	0.12611966	0.63095922	0.6196921	Smad2/Gclm/Sri/Pdk3	4
GO:0090287 regulation of cellular response to growth factor stimulus	5/138	278/14464	0.01798561	1.88510062	1.46248468	0.12722237	0.63095922	0.6196921	Hspa5/Dand5/Smad2/Brms1/Spred3	5
GO:0050766 positive regulation of phagocytosis	2/138	65/14464	0.03076923	3.22497213	1.76449873	0.12767675	0.63095922	0.6196921	Calr/Lman2	2
GO:0051926 negative regulation of calcium ion transport	2/138	65/14464	0.03076923	3.22497213	1.76449873	0.12767675	0.63095922	0.6196921	Rrad/Sri	2
GO:1904888 cranial skeletal system development	2/138	65/14464	0.03076923	3.22497213	1.76449873	0.12767675	0.63095922	0.6196921	Mmp14/Smad2	2
GO:0170039 proteinogenic amino acid metabolic process	3/138	130/14464	0.02307692	2.4187291	1.59475405	0.12778166	0.63095922	0.6196921	Gclm/Hmgcll1/Enoph1	3
GO:0007030 Golgi organization	3/138	131/14464	0.02290076	2.40026552	1.58009702	0.12991549	0.63095922	0.6196921	Bag5/Cog6/Sec22b	3
GO:1905515 non-motile cilium assembly	2/138	66/14464	0.03030303	3.17610892	1.7390328	0.13088134	0.63095922	0.6196921	Ift172/Ift80	2
GO:0031396 regulation of protein ubiquitination	4/138	204/14464	0.01960784	2.0551293	1.48959178	0.13110653	0.63095922	0.6196921	Hspa5/Sash1/Bag5/Ndfip2	4
GO:0009746 response to hexose	4/138	205/14464	0.0195122	2.04510428	1.47910254	0.13278693	0.63095922	0.6196921	Smad2/Gclm/Sri/Pdk3	4
GO:2000146 negative regulation of cell motility	5/138	282/14464	0.0177305	1.8583616	1.42867142	0.13282116	0.63095922	0.6196921	Calr/Stard13/S2bpcox16/Pkp2/Brms1	5
GO:0006491 N-glycan processing	1/138	15/14464	0.06666667	6.98743961	2.27705826	0.13400055	0.63095922	0.6196921	Hexa	1
GO:0006595 polyamine metabolic process	1/138	15/14464	0.06666667	6.98743961	2.27705826	0.13400055	0.63095922	0.6196921	Sat1	1
GO:0019184 nonribosomal peptide biosynthetic process	1/138	15/14464	0.06666667	6.98743961	2.27705826	0.13400055	0.63095922	0.6196921	Gclm	1
GO:0030497 fatty acid elongation	1/138	15/14464	0.06666667	6.98743961	2.27705826	0.13400055	0.63095922	0.6196921	Elovl7	1
GO:0032488 Cdc42 protein signal transduction	1/138	15/14464	0.06666667	6.98743961	2.27705826	0.13400055	0.63095922	0.6196921	Rhoj	1
GO:0032926 negative regulation of activin receptor signaling pathway	1/138	15/14464	0.06666667	6.98743961	2.27705826	0.13400055	0.63095922	0.6196921	Dand5	1
GO:0035733 hepatic stellate cell activation	1/138	15/14464	0.06666667	6.98743961	2.27705826	0.13400055	0.63095922	0.6196921	Gclm	1
GO:0043558 regulation of translational initiation in response to stress	1/138	15/14464	0.06666667	6.98743961	2.27705826	0.13400055	0.63095922	0.6196921	Dnajc3	1
GO:0051284 positive regulation of sequestering of calcium ion	1/138	15/14464	0.06666667	6.98743961	2.27705826	0.13400055	0.63095922	0.6196921	Sri	1
GO:0051546 keratinocyte migration	1/138	15/14464	0.06666667	6.98743961	2.27705826	0.13400055	0.63095922	0.6196921	Has2	1
GO:0071305 cellular response to vitamin D	1/138	15/14464	0.06666667	6.98743961	2.27705826	0.13400055	0.63095922	0.6196921	Pdia3	1
GO:0090136 epithelial cell-cell adhesion	1/138	15/14464	0.06666667	6.98743961	2.27705826	0.13400055	0.63095922	0.6196921	Pkp3	1
GO:0097094 craniofacial suture morphogenesis	1/138	15/14464	0.06666667	6.98743961	2.27705826	0.13400055	0.63095922	0.6196921	Mmp14	1
GO:0097623 potassium ion export across plasma membrane	1/138	15/14464	0.06666667	6.98743961	2.27705826	0.13400055	0.63095922	0.6196921	Kcnip2	1
GO:0098528 skeletal muscle fiber differentiation	1/138	15/14464	0.06666667	6.98743961	2.27705826	0.13400055	0.63095922	0.6196921	Fktn	1
GO:0110096 cellular response to aldehyde	1/138	15/14464	0.06666667	6.98743961	2.27705826	0.13400055	0.63095922	0.6196921	Akr1a1	1
GO:1900102 negative regulation of endoplasmic reticulum unfolded protein response	1/138	15/14464	0.06666667	6.98743961	2.27705826	0.13400055	0.63095922	0.6196921	Hspa5	1
GO:1901334 lactone metabolic process	1/138	15/14464	0.06666667	6.98743961	2.27705826	0.13400055	0.63095922	0.6196921	Akr1a1	1
GO:0070534 protein K63-linked ubiquitination	2/138	67/14464	0.02985075	3.1287043	1.71404808	0.13410449	0.63095922	0.6196921	Sash1/Itch	2
GO:0006720 isoprenoid metabolic process	2/138	68/14464	0.02941176	3.08269395	1.68952746	0.13734556	0.63095922	0.6196921	Dhrs3/Crppa	2
GO:0007492 endoderm development	2/138	68/14464	0.02941176	3.08269395	1.68952746	0.13734556	0.63095922	0.6196921	Mmp14/Smad2	2
GO:0071806 protein transmembrane transport	2/138	68/14464	0.02941176	3.08269395	1.68952746	0.13734556	0.63095922	0.6196921	Hspa5/Hspd1	2
GO:0006360 transcription by RNA polymerase I	2/138	69/14464	0.02898551	3.03801722	1.6654547	0.14060394	0.63095922	0.6196921	Rasl11a/Taf1a	2
GO:0010632 regulation of epithelial cell migration	2/138	69/14464	0.02898551	3.03801722	1.6654547	0.14060394	0.63095922	0.6196921	Has2/Sash1	2
GO:0007565 female pregnancy	3/138	136/14464	0.02205882	2.31202046	1.50877173	0.14077114	0.63095922	0.6196921	Calr/Cr1l/Tppp3	3
GO:0002837 regulation of immune response to tumor cell	1/138	16/14464	0.0625	6.55072464	2.180279	0.14227157	0.63095922	0.6196921	Hspd1	1
GO:0007398 ectoderm development	1/138	16/14464	0.0625	6.55072464	2.180279	0.14227157	0.63095922	0.6196921	Grhl3	1
GO:0009148 pyrimidine nucleoside triphosphate biosynthetic process	1/138	16/14464	0.0625	6.55072464	2.180279	0.14227157	0.63095922	0.6196921	Tbpl1	1
GO:0010644 cell communication by electrical coupling	1/138	16/14464	0.0625	6.55072464	2.180279	0.14227157	0.63095922	0.6196921	Sri	1
GO:0018410 C-terminal protein amino acid modification	1/138	16/14464	0.0625	6.55072464	2.180279	0.14227157	0.63095922	0.6196921	Wdr45b	1
GO:0030449 regulation of complement activation	1/138	16/14464	0.0625	6.55072464	2.180279	0.14227157	0.63095922	0.6196921	Cr1l	1
GO:0034497 protein localization to phagophore assembly site	1/138	16/14464	0.0625	6.55072464	2.180279	0.14227157	0.63095922	0.6196921	Wdr45b	1
GO:0035728 response to hepatocyte growth factor	1/138	16/14464	0.0625	6.55072464	2.180279	0.14227157	0.63095922	0.6196921	Gclm	1
GO:0042921 nuclear receptor-mediated glucocorticoid signaling pathway	1/138	16/14464	0.0625	6.55072464	2.180279	0.14227157	0.63095922	0.6196921	Bmal1	1
GO:0046514 ceramide catabolic process	1/138	16/14464	0.0625	6.55072464	2.180279	0.14227157	0.63095922	0.6196921	Hexa	1
GO:0050862 positive regulation of T cell receptor signaling pathway	1/138	16/14464	0.0625	6.55072464	2.180279	0.14227157	0.63095922	0.6196921	Card11	1
GO:0050891 multicellular organismal-level water homeostasis	1/138	16/14464	0.0625	6.55072464	2.180279	0.14227157	0.63095922	0.6196921	Has2	1
GO:0051014 actin filament severing	1/138	16/14464	0.0625	6.55072464	2.180279	0.14227157	0.63095922	0.6196921	Scin	1
GO:0051895 negative regulation of focal adhesion assembly	1/138	16/14464	0.0625	6.55072464	2.180279	0.14227157	0.63095922	0.6196921	Mmp14	1
GO:0061029 eyelid development in camera-type eye	1/138	16/14464	0.0625	6.55072464	2.180279	0.14227157	0.63095922	0.6196921	Grhl3	1
GO:0090051 negative regulation of cell migration involved in sprouting angiogenesis	1/138	16/14464	0.0625	6.55072464	2.180279	0.14227157	0.63095922	0.6196921	Stard13	1
GO:0098712 L-glutamate import across plasma membrane	1/138	16/14464	0.0625	6.55072464	2.180279	0.14227157	0.63095922	0.6196921	Slc17a8	1
GO:0150118 negative regulation of cell-substrate junction organization	1/138	16/14464	0.0625	6.55072464	2.180279	0.14227157	0.63095922	0.6196921	Mmp14	1
GO:1901017 negative regulation of potassium ion transmembrane transporter activity	1/138	16/14464	0.0625	6.55072464	2.180279	0.14227157	0.63095922	0.6196921	Oxsr1	1
GO:1901077 regulation of relaxation of muscle	1/138	16/14464	0.0625	6.55072464	2.180279	0.14227157	0.63095922	0.6196921	Sri	1
GO:1901985 positive regulation of protein acetylation	1/138	16/14464	0.0625	6.55072464	2.180279	0.14227157	0.63095922	0.6196921	Bmal1	1
GO:0001666 response to hypoxia	5/138	289/14464	0.01730104	1.81334938	1.37078957	0.14288216	0.63095922	0.6196921	Mmp14/Hsp90b1/Cr1l/Pdk3/P4hb	5
GO:0034284 response to monosaccharide	4/138	211/14464	0.01895735	1.98694965	1.41738994	0.14305419	0.63095922	0.6196921	Smad2/Gclm/Sri/Pdk3	4
GO:0033143 regulation of intracellular steroid hormone receptor signaling pathway	2/138	70/14464	0.02857143	2.99461698	1.64181434	0.14387899	0.63095922	0.6196921	Bmal1/Calr	2
GO:0006813 potassium ion transport	4/138	212/14464	0.01886792	1.97757725	1.40730222	0.14479549	0.63095922	0.6196921	Kcnn1/Kcnk13/Kcnip2/Oxsr1	4
GO:1905475 regulation of protein localization to membrane	4/138	212/14464	0.01886792	1.97757725	1.40730222	0.14479549	0.63095922	0.6196921	Slc5a3/Mmp14/Arhgef16/Cnpy4	4
GO:0003015 heart process	4/138	213/14464	0.01877934	1.96829285	1.39726931	0.14654518	0.63095922	0.6196921	Kcnip2/Ppcs/Pkp2/Sri	4
GO:0072593 reactive oxygen species metabolic process	4/138	213/14464	0.01877934	1.96829285	1.39726931	0.14654518	0.63095922	0.6196921	Slc5a3/Hspd1/Sh3pxd2a/Pdk3	4
GO:0072659 protein localization to plasma membrane	5/138	292/14464	0.01712329	1.79471908	1.34646713	0.14729367	0.63095922	0.6196921	Mmp14/Arhgef16/Cnpy4/Pkp3/Pkp2	5
GO:0141091 transforming growth factor beta receptor superfamily signaling pathway	5/138	292/14464	0.01712329	1.79471908	1.34646713	0.14729367	0.63095922	0.6196921	Hspa5/Dand5/Smad2/Brms1/Spred3	5
GO:0071456 cellular response to hypoxia	3/138	140/14464	0.02142857	2.24596273	1.45392893	0.14966866	0.63095922	0.6196921	Cr1l/Pdk3/P4hb	3
GO:0002834 regulation of response to tumor cell	1/138	17/14464	0.05882353	6.16538789	2.0914372	0.15046416	0.63095922	0.6196921	Hspd1	1
GO:0003184 pulmonary valve morphogenesis	1/138	17/14464	0.05882353	6.16538789	2.0914372	0.15046416	0.63095922	0.6196921	Smad2	1
GO:0003323 type B pancreatic cell development	1/138	17/14464	0.05882353	6.16538789	2.0914372	0.15046416	0.63095922	0.6196921	Bmal1	1
GO:0009263 deoxyribonucleotide biosynthetic process	1/138	17/14464	0.05882353	6.16538789	2.0914372	0.15046416	0.63095922	0.6196921	Tbpl1	1
GO:0009265 2'-deoxyribonucleotide biosynthetic process	1/138	17/14464	0.05882353	6.16538789	2.0914372	0.15046416	0.63095922	0.6196921	Tbpl1	1
GO:0010226 response to lithium ion	1/138	17/14464	0.05882353	6.16538789	2.0914372	0.15046416	0.63095922	0.6196921	Calr	1
GO:0021854 hypothalamus development	1/138	17/14464	0.05882353	6.16538789	2.0914372	0.15046416	0.63095922	0.6196921	Sema3e	1
GO:0036336 dendritic cell migration	1/138	17/14464	0.05882353	6.16538789	2.0914372	0.15046416	0.63095922	0.6196921	Calr	1
GO:0040019 positive regulation of embryonic development	1/138	17/14464	0.05882353	6.16538789	2.0914372	0.15046416	0.63095922	0.6196921	Hspa5	1
GO:0042762 regulation of sulfur metabolic process	1/138	17/14464	0.05882353	6.16538789	2.0914372	0.15046416	0.63095922	0.6196921	Pdk3	1
GO:0043217 myelin maintenance	1/138	17/14464	0.05882353	6.16538789	2.0914372	0.15046416	0.63095922	0.6196921	Degs1	1
GO:0045579 positive regulation of B cell differentiation	1/138	17/14464	0.05882353	6.16538789	2.0914372	0.15046416	0.63095922	0.6196921	Mmp14	1
GO:0046385 deoxyribose phosphate biosynthetic process	1/138	17/14464	0.05882353	6.16538789	2.0914372	0.15046416	0.63095922	0.6196921	Tbpl1	1
GO:0048532 anatomical structure arrangement	1/138	17/14464	0.05882353	6.16538789	2.0914372	0.15046416	0.63095922	0.6196921	Hspa5	1
GO:0050687 negative regulation of defense response to virus	1/138	17/14464	0.05882353	6.16538789	2.0914372	0.15046416	0.63095922	0.6196921	Itch	1
GO:0051220 cytoplasmic sequestering of protein	1/138	17/14464	0.05882353	6.16538789	2.0914372	0.15046416	0.63095922	0.6196921	Sri	1
GO:0051444 negative regulation of ubiquitin-protein transferase activity	1/138	17/14464	0.05882353	6.16538789	2.0914372	0.15046416	0.63095922	0.6196921	Bag5	1
GO:0060019 radial glial cell differentiation	1/138	17/14464	0.05882353	6.16538789	2.0914372	0.15046416	0.63095922	0.6196921	Mettl14	1
GO:0060314 regulation of ryanodine-sensitive calcium-release channel activity	1/138	17/14464	0.05882353	6.16538789	2.0914372	0.15046416	0.63095922	0.6196921	Sri	1
GO:0070417 cellular response to cold	1/138	17/14464	0.05882353	6.16538789	2.0914372	0.15046416	0.63095922	0.6196921	Dnajc3	1
GO:0070486 leukocyte aggregation	1/138	17/14464	0.05882353	6.16538789	2.0914372	0.15046416	0.63095922	0.6196921	Has2	1
GO:0071816 tail-anchored membrane protein insertion into ER membrane	1/138	17/14464	0.05882353	6.16538789	2.0914372	0.15046416	0.63095922	0.6196921	Ubl4a	1
GO:0141198 protein branched polyubiquitination	1/138	17/14464	0.05882353	6.16538789	2.0914372	0.15046416	0.63095922	0.6196921	Itch	1
GO:1901623 regulation of lymphocyte chemotaxis	1/138	17/14464	0.05882353	6.16538789	2.0914372	0.15046416	0.63095922	0.6196921	Oxsr1	1
GO:1903902 positive regulation of viral life cycle	1/138	17/14464	0.05882353	6.16538789	2.0914372	0.15046416	0.63095922	0.6196921	P4hb	1
GO:0009798 axis specification	2/138	72/14464	0.02777778	2.91143317	1.59577279	0.15047672	0.63095922	0.6196921	Smad2/Ift172	2
GO:0030968 endoplasmic reticulum unfolded protein response	2/138	72/14464	0.02777778	2.91143317	1.59577279	0.15047672	0.63095922	0.6196921	Hspa5/Dnajc3	2
GO:0070373 negative regulation of ERK1 and ERK2 cascade	2/138	72/14464	0.02777778	2.91143317	1.59577279	0.15047672	0.63095922	0.6196921	S2bpcox16/Spred3	2
GO:2000377 regulation of reactive oxygen species metabolic process	3/138	141/14464	0.0212766	2.23003392	1.44050878	0.15192112	0.63095922	0.6196921	Slc5a3/Hspd1/Pdk3	3
GO:0072676 lymphocyte migration	2/138	73/14464	0.02739726	2.87155053	1.57334427	0.15379821	0.63095922	0.6196921	Cd99l2/Oxsr1	2
GO:1903510 mucopolysaccharide metabolic process	2/138	73/14464	0.02739726	2.87155053	1.57334427	0.15379821	0.63095922	0.6196921	Has2/Hexa	2
GO:0050680 negative regulation of epithelial cell proliferation	3/138	142/14464	0.02112676	2.21432945	1.42720096	0.15418445	0.63095922	0.6196921	Ift172/S2bpcox16/Ift80	3
GO:0040013 negative regulation of locomotion	5/138	297/14464	0.01683502	1.76450495	1.30654943	0.15477481	0.63095922	0.6196921	Calr/Stard13/S2bpcox16/Pkp2/Brms1	5
GO:0006942 regulation of striated muscle contraction	2/138	74/14464	0.02702703	2.83274579	1.55129344	0.157134	0.63095922	0.6196921	Pkp2/Sri	2
GO:0034109 homotypic cell-cell adhesion	2/138	74/14464	0.02702703	2.83274579	1.55129344	0.157134	0.63095922	0.6196921	Pdia3/Pkp2	2
GO:0048762 mesenchymal cell differentiation	4/138	219/14464	0.01826484	1.91436702	1.33818959	0.15721497	0.63095922	0.6196921	Has2/Smad2/Sema3e/Spred3	4
GO:0001976 nervous system process involved in regulation of systemic arterial blood pressure	1/138	18/14464	0.05555556	5.82286634	2.00943488	0.15857906	0.63095922	0.6196921	Manf	1
GO:0002418 immune response to tumor cell	1/138	18/14464	0.05555556	5.82286634	2.00943488	0.15857906	0.63095922	0.6196921	Hspd1	1
GO:0003159 morphogenesis of an endothelium	1/138	18/14464	0.05555556	5.82286634	2.00943488	0.15857906	0.63095922	0.6196921	Stard13	1
GO:0003283 atrial septum development	1/138	18/14464	0.05555556	5.82286634	2.00943488	0.15857906	0.63095922	0.6196921	Dand5	1
GO:0006614 SRP-dependent cotranslational protein targeting to membrane	1/138	18/14464	0.05555556	5.82286634	2.00943488	0.15857906	0.63095922	0.6196921	Srprb	1
GO:0009067 aspartate family amino acid biosynthetic process	1/138	18/14464	0.05555556	5.82286634	2.00943488	0.15857906	0.63095922	0.6196921	Enoph1	1
GO:0031958 nuclear receptor-mediated corticosteroid signaling pathway	1/138	18/14464	0.05555556	5.82286634	2.00943488	0.15857906	0.63095922	0.6196921	Bmal1	1
GO:0032354 response to follicle-stimulating hormone	1/138	18/14464	0.05555556	5.82286634	2.00943488	0.15857906	0.63095922	0.6196921	Gclm	1
GO:0042438 melanin biosynthetic process	1/138	18/14464	0.05555556	5.82286634	2.00943488	0.15857906	0.63095922	0.6196921	Rab38	1
GO:0045061 thymic T cell selection	1/138	18/14464	0.05555556	5.82286634	2.00943488	0.15857906	0.63095922	0.6196921	Card11	1
GO:0051580 regulation of neurotransmitter uptake	1/138	18/14464	0.05555556	5.82286634	2.00943488	0.15857906	0.63095922	0.6196921	Slc17a8	1

GO:0061154	endothelial tube morphogenesis	1/138	18/14464	0.05555556	5.82286634	2.00943488	0.15857906	0.63095922	0.6196921	Stard13	1
GO:0071071	regulation of phospholipid biosynthetic process	1/138	18/14464	0.05555556	5.82286634	2.00943488	0.15857906	0.63095922	0.6196921	Rab38	1
GO:0071218	cellular response to misfolded protein	1/138	18/14464	0.05555556	5.82286634	2.00943488	0.15857906	0.63095922	0.6196921	Sdf2l1	1
GO:0071498	cellular response to fluid shear stress	1/138	18/14464	0.05555556	5.82286634	2.00943488	0.15857906	0.63095922	0.6196921	Has2	1
GO:0090083	regulation of inclusion body assembly	1/138	18/14464	0.05555556	5.82286634	2.00943488	0.15857906	0.63095922	0.6196921	Bag5	1
GO:1903232	melanosome assembly	1/138	18/14464	0.05555556	5.82286634	2.00943488	0.15857906	0.63095922	0.6196921	Rab38	1
GO:0016485	protein processing	4/138	220/14464	0.01818182	1.90566535	1.3285239	0.15902113	0.63095922	0.6196921	Mmp14/Hsp90b1/Ift172/P4hb	4
GO:0006493	protein O-linked glycosylation	2/138	75/14464	0.02666667	2.79497585	1.52960818	0.16048353	0.63095922	0.6196921	Fktn/Crppa	2
GO:0044283	small molecule biosynthetic process	7/138	470/14464	0.01489362	1.56102374	1.21357186	0.16276294	0.63095922	0.6196921	Degs1/Hmgcll1/Elovl7/Lta4h/Arpp19/Enoph1/Akr1a1	7
GO:0036293	response to decreased oxygen levels	5/138	304/14464	0.01644737	1.7238749	1.2519153	0.1655092	0.63095922	0.6196921	Mmp14/Hsp90b1/Cr1l/Pdk3/P4hb	5
GO:0043488	regulation of mRNA stability	3/138	147/14464	0.02040816	2.13901213	1.36228278	0.16565797	0.63095922	0.6196921	Mettl16/Hnrnpc/Mettl14	3
GO:0043547	positive regulation of GTPase activity	3/138	147/14464	0.02040816	2.13901213	1.36228278	0.16565797	0.63095922	0.6196921	Arhgef16/Grhl3/Snx18	3
GO:0002507	tolerance induction	1/138	19/14464	0.05263158	5.51639969	1.9333775	0.166617	0.63095922	0.6196921	Itch	1
GO:0003084	positive regulation of systemic arterial blood pressure	1/138	19/14464	0.05263158	5.51639969	1.9333775	0.166617	0.63095922	0.6196921	Manf	1
GO:0003417	growth plate cartilage development	1/138	19/14464	0.05263158	5.51639969	1.9333775	0.166617	0.63095922	0.6196921	Ift80	1
GO:0006613	cotranslational protein targeting to membrane	1/138	19/14464	0.05263158	5.51639969	1.9333775	0.166617	0.63095922	0.6196921	Srprb	1
GO:0006817	phosphate ion transport	1/138	19/14464	0.05263158	5.51639969	1.9333775	0.166617	0.63095922	0.6196921	Slc17a8	1
GO:0010288	response to lead ion	1/138	19/14464	0.05263158	5.51639969	1.9333775	0.166617	0.63095922	0.6196921	Ppp2cb	1
GO:0010804	negative regulation of tumor necrosis factor-mediated signaling pathway	1/138	19/14464	0.05263158	5.51639969	1.9333775	0.166617	0.63095922	0.6196921	Ppp2cb	1
GO:0015936	coenzyme A metabolic process	1/138	19/14464	0.05263158	5.51639969	1.9333775	0.166617	0.63095922	0.6196921	Ppcs	1
GO:0044550	secondary metabolite biosynthetic process	1/138	19/14464	0.05263158	5.51639969	1.9333775	0.166617	0.63095922	0.6196921	Rab38	1
GO:0045217	cell-cell junction maintenance	1/138	19/14464	0.05263158	5.51639969	1.9333775	0.166617	0.63095922	0.6196921	Pkp2	1
GO:0046641	positive regulation of alpha-beta T cell proliferation	1/138	19/14464	0.05263158	5.51639969	1.9333775	0.166617	0.63095922	0.6196921	Card11	1
GO:0048339	paraxial mesoderm development	1/138	19/14464	0.05263158	5.51639969	1.9333775	0.166617	0.63095922	0.6196921	Smad2	1
GO:0050884	neuromuscular process controlling posture	1/138	19/14464	0.05263158	5.51639969	1.9333775	0.166617	0.63095922	0.6196921	Hexa	1
GO:0051788	response to misfolded protein	1/138	19/14464	0.05263158	5.51639969	1.9333775	0.166617	0.63095922	0.6196921	Sdf2l1	1
GO:0051930	regulation of sensory perception of pain	1/138	19/14464	0.05263158	5.51639969	1.9333775	0.166617	0.63095922	0.6196921	Smr2l	1
GO:0071371	cellular response to gonadotropin stimulus	1/138	19/14464	0.05263158	5.51639969	1.9333775	0.166617	0.63095922	0.6196921	Gclm	1
GO:0071474	cellular hyperosmotic response	1/138	19/14464	0.05263158	5.51639969	1.9333775	0.166617	0.63095922	0.6196921	Oxsr1	1
GO:0097320	plasma membrane tubulation	1/138	19/14464	0.05263158	5.51639969	1.9333775	0.166617	0.63095922	0.6196921	Snx18	1
GO:1901379	regulation of potassium ion transmembrane transport	2/138	77/14464	0.02597403	2.72237907	1.48728858	0.16722157	0.63095922	0.6196921	Kcnip2/Oxsr1	2
GO:0001935	endothelial cell proliferation	3/138	148/14464	0.02027027	2.12455934	1.34961114	0.16798279	0.63095922	0.6196921	Mmp14/S2bpcox16/Aggf1	3
GO:0006402	mRNA catabolic process	4/138	226/14464	0.01769912	1.85507246	1.27156601	0.17001782	0.63095922	0.6196921	Mettl16/Hnrnpc/Pkp3/Mettl14	4
GO:0001837	epithelial to mesenchymal transition	3/138	149/14464	0.02013423	2.11030055	1.33703962	0.17031723	0.63095922	0.6196921	Has2/Smad2/Spred3	3
GO:0036294	cellular response to decreased oxygen levels	3/138	149/14464	0.02013423	2.11030055	1.33703962	0.17031723	0.63095922	0.6196921	Cr1l/Pdk3/P4hb	3
GO:0042100	B cell proliferation	2/138	78/14464	0.02564103	2.68747677	1.46663262	0.17060899	0.63095922	0.6196921	Card11/Hspd1	2
GO:0050871	positive regulation of B cell activation	2/138	78/14464	0.02564103	2.68747677	1.46663262	0.17060899	0.63095922	0.6196921	Card11/Mmp14	2
GO:0051090	regulation of DNA-binding transcription factor activity	5/138	308/14464	0.01623377	1.70148692	1.22132408	0.17177439	0.63095922	0.6196921	Card11/Sri/Ppp2cb/Brms1/Itch	5
GO:0035914	skeletal muscle cell differentiation	2/138	79/14464	0.02531646	2.65345808	1.44629893	0.17400796	0.63095922	0.6196921	Bmal1/Fktn	2
GO:0006027	glycosaminoglycan catabolic process	1/138	20/14464	0.05	5.24057971	1.86252775	0.1745787	0.63095922	0.6196921	Hexa	1
GO:0006582	melanin metabolic process	1/138	20/14464	0.05	5.24057971	1.86252775	0.1745787	0.63095922	0.6196921	Rab38	1
GO:0006656	phosphatidylcholine biosynthetic process	1/138	20/14464	0.05	5.24057971	1.86252775	0.1745787	0.63095922	0.6196921	Rab38	1
GO:0007289	spermatid nucleus differentiation	1/138	20/14464	0.05	5.24057971	1.86252775	0.1745787	0.63095922	0.6196921	Tbpl1	1
GO:0009110	vitamin biosynthetic process	1/138	20/14464	0.05	5.24057971	1.86252775	0.1745787	0.63095922	0.6196921	Akr1a1	1
GO:0009147	pyrimidine nucleoside triphosphate metabolic process	1/138	20/14464	0.05	5.24057971	1.86252775	0.1745787	0.63095922	0.6196921	Tbpl1	1
GO:0009219	pyrimidine deoxyribonucleotide metabolic process	1/138	20/14464	0.05	5.24057971	1.86252775	0.1745787	0.63095922	0.6196921	Tbpl1	1
GO:0010958	regulation of amino acid import across plasma membrane	1/138	20/14464	0.05	5.24057971	1.86252775	0.1745787	0.63095922	0.6196921	Slc17a8	1
GO:0032211	negative regulation of telomere maintenance via telomerase	1/138	20/14464	0.05	5.24057971	1.86252775	0.1745787	0.63095922	0.6196921	Hnrnpc	1
GO:0042044	fluid transport	1/138	20/14464	0.05	5.24057971	1.86252775	0.1745787	0.63095922	0.6196921	Has2	1
GO:0042753	positive regulation of circadian rhythm	1/138	20/14464	0.05	5.24057971	1.86252775	0.1745787	0.63095922	0.6196921	Bmal1	1
GO:0051931	regulation of sensory perception	1/138	20/14464	0.05	5.24057971	1.86252775	0.1745787	0.63095922	0.6196921	Smr2l	1
GO:0060155	platelet dense granule organization	1/138	20/14464	0.05	5.24057971	1.86252775	0.1745787	0.63095922	0.6196921	Rab38	1
GO:0071295	cellular response to vitamin	1/138	20/14464	0.05	5.24057971	1.86252775	0.1745787	0.63095922	0.6196921	Pdia3	1
GO:0097066	response to thyroid hormone	1/138	20/14464	0.05	5.24057971	1.86252775	0.1745787	0.63095922	0.6196921	Gclm	1
GO:1901201	regulation of extracellular matrix assembly	1/138	20/14464	0.05	5.24057971	1.86252775	0.1745787	0.63095922	0.6196921	Has2	1
GO:1903427	negative regulation of reactive oxygen species biosynthetic process	1/138	20/14464	0.05	5.24057971	1.86252775	0.1745787	0.63095922	0.6196921	Hspd1	1
GO:1903428	positive regulation of reactive oxygen species biosynthetic process	1/138	20/14464	0.05	5.24057971	1.86252775	0.1745787	0.63095922	0.6196921	Slc5a3	1
GO:1903789	regulation of amino acid transmembrane transport	1/138	20/14464	0.05	5.24057971	1.86252775	0.1745787	0.63095922	0.6196921	Slc17a8	1
GO:2000786	positive regulation of autophagosome assembly	1/138	20/14464	0.05	5.24057971	1.86252775	0.1745787	0.63095922	0.6196921	Snx18	1
GO:0043010	camera-type eye development	5/138	311/14464	0.01607717	1.68507386	1.19866991	0.17653363	0.63095922	0.6196921	Rhoj/Grhl3/Slc17a8/Ift172/Spred3	5
GO:0042445	hormone metabolic process	3/138	152/14464	0.01973684	2.06864989	1.29991016	0.17737652	0.63095922	0.6196921	Dhrs3/Hsp90b1/P4hb	3
GO:0001667	ameboidal-type cell migration	4/138	231/14464	0.01731602	1.81491938	1.22540225	0.17938181	0.63095922	0.6196921	Calr/Has2/Sash1/Sema3e	4
GO:0007178	cell surface receptor protein serine/threonine kinase signaling pathway	5/138	313/14464	0.01597444	1.67430662	1.18370174	0.17973447	0.63095922	0.6196921	Hspa5/Dand5/Smad2/Brms1/Spred3	5
GO:0002690	positive regulation of leukocyte chemotaxis	2/138	81/14464	0.02469136	2.5879406	1.40656007	0.18083848	0.63095922	0.6196921	Calr/Oxsr1	2
GO:0021510	spinal cord development	2/138	81/14464	0.02469136	2.5879406	1.40656007	0.18083848	0.63095922	0.6196921	Ift172/Ift80	2
GO:0033077	T cell differentiation in thymus	2/138	81/14464	0.02469136	2.5879406	1.40656007	0.18083848	0.63095922	0.6196921	Card11/Fzd8	2
GO:1905897	regulation of response to endoplasmic reticulum stress	2/138	81/14464	0.02469136	2.5879406	1.40656007	0.18083848	0.63095922	0.6196921	Hspa5/Manf	2
GO:0000413	protein peptidyl-prolyl isomerization	1/138	21/14464	0.04761905	4.9910283	1.79627163	0.18246489	0.63095922	0.6196921	Fkbp2	1
GO:0003177	pulmonary valve development	1/138	21/14464	0.04761905	4.9910283	1.79627163	0.18246489	0.63095922	0.6196921	Smad2	1
GO:0003309	type B pancreatic cell differentiation	1/138	21/14464	0.04761905	4.9910283	1.79627163	0.18246489	0.63095922	0.6196921	Bmal1	1
GO:0006654	phosphatidic acid biosynthetic process	1/138	21/14464	0.04761905	4.9910283	1.79627163	0.18246489	0.63095922	0.6196921	Dgkk	1
GO:0006662	glycerol ether metabolic process	1/138	21/14464	0.04761905	4.9910283	1.79627163	0.18246489	0.63095922	0.6196921	Selenoi	1
GO:0006691	leukotriene metabolic process	1/138	21/14464	0.04761905	4.9910283	1.79627163	0.18246489	0.63095922	0.6196921	Lta4h	1
GO:0010954	positive regulation of protein processing	1/138	21/14464	0.04761905	4.9910283	1.79627163	0.18246489	0.63095922	0.6196921	Mmp14	1
GO:0043576	regulation of respiratory gaseous exchange	1/138	21/14464	0.04761905	4.9910283	1.79627163	0.18246489	0.63095922	0.6196921	Slc5a3	1
GO:0045116	protein neddylation	1/138	21/14464	0.04761905	4.9910283	1.79627163	0.18246489	0.63095922	0.6196921	Aktip	1
GO:0045652	regulation of megakaryocyte differentiation	1/138	21/14464	0.04761905	4.9910283	1.79627163	0.18246489	0.63095922	0.6196921	Scin	1
GO:0045948	positive regulation of translational initiation	1/138	21/14464	0.04761905	4.9910283	1.79627163	0.18246489	0.63095922	0.6196921	Dnajc3	1
GO:0046485	ether lipid metabolic process	1/138	21/14464	0.04761905	4.9910283	1.79627163	0.18246489	0.63095922	0.6196921	Selenoi	1
GO:0072537	fibroblast activation	1/138	21/14464	0.04761905	4.9910283	1.79627163	0.18246489	0.63095922	0.6196921	Gclm	1
GO:0086019	cell-cell signaling involved in cardiac conduction	1/138	21/14464	0.04761905	4.9910283	1.79627163	0.18246489	0.63095922	0.6196921	Pkp2	1
GO:0090050	positive regulation of cell migration involved in sprouting angiogenesis	1/138	21/14464	0.04761905	4.9910283	1.79627163	0.18246489	0.63095922	0.6196921	Rhoj	1
GO:0090103	cochlea morphogenesis	1/138	21/14464	0.04761905	4.9910283	1.79627163	0.18246489	0.63095922	0.6196921	Grhl3	1
GO:1901380	negative regulation of potassium ion transmembrane transport	1/138	21/14464	0.04761905	4.9910283	1.79627163	0.18246489	0.63095922	0.6196921	Oxsr1	1
GO:1902455	negative regulation of stem cell population maintenance	1/138	21/14464	0.04761905	4.9910283	1.79627163	0.18246489	0.63095922	0.6196921	Brms1	1
GO:2000209	regulation of anoikis	1/138	21/14464	0.04761905	4.9910283	1.79627163	0.18246489	0.63095922	0.6196921	Brms1	1
GO:0061157	mRNA destabilization	2/138	82/14464	0.02439024	2.55638035	1.38713677	0.18426901	0.63208191	0.62079474	Mettl16/Mettl14	2
GO:1901222	regulation of non-canonical NF-kappaB signal transduction	2/138	82/14464	0.02439024	2.55638035	1.38713677	0.18426901	0.63208191	0.62079474	Calr/Sash1	2
GO:0009743	response to carbohydrate	4/138	234/14464	0.01709402	1.79165118	1.19824495	0.18508281	0.63208191	0.62079474	Smad2/Gclm/Sri/Pdk3	4
GO:0030510	regulation of BMP signaling pathway	2/138	83/14464	0.02409639	2.52558058	1.36799944	0.18770905	0.63208191	0.62079474	Dand5/Smad2	2
GO:0030522	intracellular receptor signaling pathway	5/138	318/14464	0.01572327	1.64798104	1.14674088	0.1878318	0.63208191	0.62079474	Bmal1/Dhrs3/Calr/Itch/Pdk3	5
GO:0001675	acrosome assembly	1/138	22/14464	0.04545455	4.76416337	1.73409305	0.19027628	0.63208191	0.62079474	Tbpl1	1
GO:0002693	positive regulation of cellular extravasation	1/138	22/14464	0.04545455	4.76416337	1.73409305	0.19027628	0.63208191	0.62079474	Cd99l2	1
GO:0009083	branched-chain amino acid catabolic process	1/138	22/14464	0.04545455	4.76416337	1.73409305	0.19027628	0.63208191	0.62079474	Hmgcll1	1
GO:0032925	regulation of activin receptor signaling pathway	1/138	22/14464	0.04545455	4.76416337	1.73409305	0.19027628	0.63208191	0.62079474	Dand5	1
GO:0035357	peroxisome proliferator activated receptor signaling pathway	1/138	22/14464	0.04545455	4.76416337	1.73409305	0.19027628	0.63208191	0.62079474	Pdk3	1
GO:0035809	regulation of urine volume	1/138	22/14464	0.04545455	4.76416337	1.73409305	0.19027628	0.63208191	0.62079474	Has2	1
GO:0045822	negative regulation of heart contraction	1/138	22/14464	0.04545455	4.76416337	1.73409305	0.19027628	0.63208191	0.62079474	Sri	1
GO:0046337	phosphatidylethanolamine metabolic process	1/138	22/14464	0.04545455	4.76416337	1.73409305	0.19027628	0.63208191	0.62079474	Selenoi	1
GO:0051900	regulation of mitochondrial depolarization	1/138	22/14464	0.04545455	4.76416337	1.73409305	0.19027628	0.63208191	0.62079474	Gclm	1
GO:0060039	pericardium development	1/138	22/14464	0.04545455	4.76416337	1.73409305	0.19027628	0.63208191	0.62079474	Smad2	1
GO:0061298	retina vasculature development in camera-type eye	1/138	22/14464	0.04545455	4.76416337	1.73409305	0.19027628	0.63208191	0.62079474	Rhoj	1
GO:2000810	regulation of bicellular tight junction assembly	1/138	22/14464	0.04545455	4.76416337	1.73409305	0.19027628	0.63208191	0.62079474	Pkp2	1
GO:0008593	regulation of Notch signaling pathway	2/138	84/14464	0.02380952	2.49551415	1.34913991	0.19115811	0.63208191	0.62079474	Mmp14/S2bpcox16	2
GO:0006664	glycolipid metabolic process	2/138	85/14464	0.02352941	2.46615516	1.33055036	0.19461572	0.63208191	0.62079474	Pigg/Hexa	2
GO:0030203	glycosaminoglycan metabolic process	2/138	85/14464	0.02352941	2.46615516	1.33055036	0.19461572	0.63208191	0.62079474	Has2/Hexa	2
GO:0060562	epithelial tube morphogenesis	5/138	323/14464	0.01547988	1.6224705	1.11041734	0.19606023	0.63208191	0.62079474	Mmp14/Grhl3/Stard13/Ift172/Sema3e	5
GO:0031647	regulation of protein stability	5/138	324/14464	0.0154321	1.61746287	1.10322708	0.1977211	0.63208191	0.62079474	Calr/Hspd1/Ubl4a/Bag5/Ift80	5
GO:0006390	mitochondrial transcription	1/138	23/14464	0.04347826	4.55702583	1.67555453	0.19801357	0.63208191	0.62079474	Tfb1m	1
GO:0010669	epithelial structure maintenance	1/138	23/14464	0.04347826	4.55702583	1.67555453	0.19801357	0.63208191	0.62079474	Pkp3	1
GO:0021884	forebrain neuron development	1/138	23/14464	0.04347826	4.55702583	1.67555453	0.19801357	0.63208191	0.62079474	Sema3e	1
GO:0031295	T cell costimulation	1/138	23/14464	0.04347826	4.55702583	1.67555453	0.19801357	0.63208191	0.62079474	Card11	1
GO:0032482	Rab protein signal transduction	1/138	23/14464	0.04347826	4.55702583	1.67555453	0.19801357	0.63208191	0.62079474	Rab9b	1
GO:0032727	positive regulation of interferon-alpha production	1/138	23/14464	0.04347826	4.55702583	1.67555453	0.19801357	0.63208191	0.62079474	Hspd1	1
GO:0035988	chondrocyte proliferation	1/138	23/14464	0.04347826	4.55702583	1.67555453	0.19801357	0.63208191	0.62079474	Mmp14	1
GO:0043032	positive regulation of macrophage activation	1/138	23/14464	0.04347826	4.55702583	1.67555453	0.19801357	0.63208191	0.62079474	Hspd1	1
GO:0043043	peptide biosynthetic process	1/138	23/14464	0.04347826	4.55702583	1.67555453	0.19801357	0.63208191	0.62079474	Gclm	1
GO:0043555	regulation of translation in response to stress	1/138	23/14464	0.04347826	4.55702583	1.67555453	0.19801357	0.63208191	0.62079474	Dnajc3	1
GO:0045109	intermediate filament organization	1/138	23/14464	0.04347826	4.55702583	1.67555453	0.19801357	0.63208191	0.62079474	Pkp2	1
GO:0050857	positive regulation of antigen receptor-mediated signaling pathway	1/138	23/14464	0.04347826	4.55702583	1.67555453	0.19801357	0.63208191	0.62079474	Card11	1
GO:0086005	ventricular cardiac muscle cell action potential	1/138	23/14464	0.04347826	4.55702583	1.67555453	0.19801357	0.63208191	0.62079474	Pkp2	1
GO:0098901	regulation of cardiac muscle cell action potential	1/138	23/14464	0.04347826	4.55702583	1.67555453	0.19801357	0.63208191	0.62079474	Pkp2	1
GO:1903319	positive regulation of protein maturation	1/138	23/14464	0.04347826	4.55702583	1.67555453	0.19801357	0.63208191	0.62079474	Mmp14	1
GO:0006672	ceramide metabolic process	2/138	86/14464	0.02325581	2.43747893	1.31222328	0.19808139	0.63208191	0.62079474	Hexa/Degs1	2
GO:0070252	actin-mediated cell contraction	2/138	86/14464	0.02325581	2.43747893	1.31222328	0.19808139	0.63208191	0.62079474	Pkp2/Sri	2
GO:1903509	liposaccharide metabolic process	2/138	86/14464	0.02325581	2.43747893	1.31222328	0.19808139	0.63208191	0.62079474	Pigg/Hexa	2
GO:0009266	response to temperature stimulus	3/138	161/14464	0.01863354	1.95301107	1.19344821	0.19901714	0.63208191	0.62079474	Chordc1/Hspd1/Dnajc3	3
GO:0003205	cardiac chamber development	3/138	162/14464	0.01851852	1.94095545	1.18204618	0.20146075	0.63208191	0.62079474	Dhrs3/Dand5/Pkp2	3
GO:0043487	regulation of RNA stability	3/138	162/14464	0.01851852	1.94095545	1.18204618	0.20146075	0.63208191	0.62079474	Mettl16/Hnrnpc/Mettl14	3
GO:0050779	RNA destabilization	2/138	87/14464	0.02298851	2.40946194	1.29415145	0.20155467	0.63208191	0.62079474	Mettl16/Mettl14	2
GO:0061014	positive regulation of mRNA catabolic process	2/138	87/14464	0.02298851	2.40946194	1.29415145	0.20155467	0.63208191	0.62079474	Mettl16/Mettl14	2
GO:0000209	protein polyubiquitination	4/138	243/14464	0.01646091	1.72529373	1.11907258	0.20253173	0.63208191	0.62079474	Sash1/Dtx4/Itch/Ube3c	4
GO:0070482	response to oxygen levels	5/138	327/14464	0.01529052	1.60262376	1.08180196	0.20273317	0.63208191	0.62079474	Mmp14/Hsp90b1/Cr1l/Pdk3/P4hb	5
GO:0030148	sphingolipid biosynthetic process	2/138	88/14464	0.02272727	2.38208169	1.27632795	0.2050351	0.63208191	0.62079474	Degs1/Elovl7	2
GO:0043406	positive regulation of MAP kinase activity	2/138	88/14464	0.02272727	2.38208169	1.27632795	0.2050351	0.63208191	0.62079474	Fzd8/Sash1	2
GO:0043954	cellular component maintenance	2/138	88/14464	0.02272727	2.38208169	1.27632795	0.2050351	0.63208191	0.62079474	Zfp804a/Pkp2	2
GO:0048704	embryonic skeletal system morphogenesis	2/138	88/14464	0.02272727	2.38208169	1.27632795	0.2050351	0.63208191	0.62079474	Mmp14/Smad2	2
GO:0001573	ganglioside metabolic process	1/138	24/14464	0.04166667	4.36714976	1.62028242	0.20567745	0.63208191	0.62079474	Hexa	1
GO:0008299	isoprenoid biosynthetic process	1/138	24/14464	0.04166667	4.36714976	1.62028242	0.20567745	0.63208191	0.62079474	Crppa	1
GO:0018126	protein hydroxylation	1/138	24/14464	0.04166667	4.36714976	1.62028242	0.20567745	0.63208191	0.62079474	P4hb	1
GO:0021522	spinal cord motor neuron differentiation	1/138	24/14464	0.04166667	4.36714976	1.62028242	0.20567745	0.63208191	0.62079474	Ift172	1
GO:0031664	regulation of lipopolysaccharide-mediated signaling pathway	1/138	24/14464	0.04166667	4.36714976	1.62028242	0.20567745	0.63208191	0.62079474	Sash1	1
GO:0035883	enteroendocrine cell differentiation	1/138	24/14464	0.04166667	4.36714976	1.62028242	0.20567745	0.63208191	0.62079474	Bmal1	1
GO:0040020	regulation of meiotic nuclear division	1/138	24/14464	0.04166667	4.36714976	1.62028242	0.20567745	0.63208191	0.62079474	Calr	1
GO:0043931	ossification involved in bone maturation	1/138	24/14464	0.04166667	4.36714976	1.62028242	0.20567745	0.63208191	0.62079474	Ift80	1
GO:0044804	nucleophagy	1/138	24/14464	0.04166667	4.36714976	1.62028242	0.20567745	0.63208191	0.62079474	Wdr45b	1
GO:0046473	phosphatidic acid metabolic process	1/138	24/14464	0.04166667	4.36714976	1.62028242	0.20567745	0.63208191	0.62079474	Dgkk	1
GO:0046580	negative regulation of Ras protein signal transduction	1/138	24/14464	0.04166667	4.36714976	1.62028242	0.20567745	0.63208191	0.62079474	Ppp2cb	1
GO:0048596	embryonic camera-type eye morphogenesis	1/138	24/14464	0.04166667	4.36714976	1.62028242	0.20567745	0.63208191	0.62079474	Ift172	1
GO:0051016	barbed-end actin filament capping	1/138	24/14464	0.04166667	4.36714976	1.62028242	0.20567745	0.63208191	0.62079474	Scin	1
GO:0062009	secondary palate development	1/138	24/14464	0.04166667	4.36714976	1.62028242	0.20567745	0.63208191	0.62079474	Smad2	1
GO:0070723	response to cholesterol	1/138	24/14464	0.04166667	4.36714976	1.62028242	0.20567745	0.63208191	0.62079474	Smad2	1
GO:0071542	dopaminergic neuron differentiation	1/138	24/14464	0.04166667	4.36714976	1.62028242	0.20567745	0.63208191	0.62079474	Manf	1
GO:0072530	purine-containing compound transmembrane transport	1/138	24/14464	0.04166667	4.36714976	1.62028242	0.20567745	0.63208191	0.62079474	Slc33a1	1
GO:0097062	dendritic spine maintenance	1/138	24/14464	0.04166667	4.36714976	1.62028242	0.20567745	0.63208191	0.62079474	Zfp804a	1
GO:1901983	regulation of protein acetylation	1/138	24/14464	0.04166667	4.36714976	1.62028242	0.20567745	0.63208191	0.62079474	Bmal1	1
GO:1902176	negative regulation of oxidative stress-induced intrinsic apoptotic signaling pathway	1/138	24/14464	0.04166667	4.36714976	1.62028242	0.20567745	0.63208191	0.62079474	Bag5	1
GO:1903523	negative regulation of blood circulation	1/138	24/14464	0.04166667	4.36714976	1.62028242	0.20567745	0.63208191	0.62079474	Sri	1
GO:2001014	regulation of skeletal muscle cell differentiation	1/138	24/14464	0.04166667	4.36714976	1.62028242	0.20567745	0.63208191	0.62079474	Bmal1	1
GO:0050673	epithelial cell proliferation	6/138	417/14464	0.01438849	1.50808049	1.03327285	0.20838761	0.63696228	0.62558795	Mmp14/Has2/Ift172/S2bpcox16/Ift80/Aggf1	6
GO:0045913	positive regulation of carbohydrate metabolic process	2/138	89/14464	0.02247191	2.35531672	1.2587461	0.20852223	0.63696228	0.62558795	Has2/Arpp19	2
GO:0008016	regulation of heart contraction	3/138	165/14464	0.01818182	1.90566535	1.14832059	0.20883403	0.63696228	0.62558795	Kcnip2/Pkp2/Sri	3
GO:0071453	cellular response to oxygen levels	3/138	165/14464	0.01818182	1.90566535	1.14832059	0.20883403	0.63696228	0.62558795	Cr1l/Pdk3/P4hb	3
GO:0006650	glycerophospholipid metabolic process	4/138	247/14464	0.01619433	1.69735375	1.08493545	0.21044155	0.63696228	0.62558795	Rab38/Dgkk/Pigg/Selenoi	4
GO:0002027	regulation of heart rate	2/138	90/14464	0.02222222	2.32914654	1.24139949	0.21201562	0.63696228	0.62558795	Pkp2/Sri	2
GO:0007498	mesoderm development	2/138	90/14464	0.02222222	2.32914654	1.24139949	0.21201562	0.63696228	0.62558795	Dand5/Smad2	2

GO:0043535	regulation of blood vessel endothelial cell migration	2/138	90/14464	0.02222222	2.32914654	1.24139949	0.21201562	0.63696228	0.62558795	Rhoj/Stard13	2
GO:0046634	regulation of alpha-beta T cell activation	2/138	90/14464	0.02222222	2.32914654	1.24139949	0.21201562	0.63696228	0.62558795	Card11/Itch	2
GO:0003180	aortic valve morphogenesis	1/138	25/14464	0.04	4.19246377	1.56795533	0.21326862	0.63696228	0.62558795	Smad2	1
GO:0003416	endochondral bone growth	1/138	25/14464	0.04	4.19246377	1.56795533	0.21326862	0.63696228	0.62558795	Ift80	1
GO:0018904	ether metabolic process	1/138	25/14464	0.04	4.19246377	1.56795533	0.21326862	0.63696228	0.62558795	Selenoi	1
GO:0030325	adrenal gland development	1/138	25/14464	0.04	4.19246377	1.56795533	0.21326862	0.63696228	0.62558795	Smad2	1
GO:0031294	lymphocyte costimulation	1/138	25/14464	0.04	4.19246377	1.56795533	0.21326862	0.63696228	0.62558795	Card11	1
GO:0033280	response to vitamin D	1/138	25/14464	0.04	4.19246377	1.56795533	0.21326862	0.63696228	0.62558795	Pdia3	1
GO:0045070	positive regulation of viral genome replication	1/138	25/14464	0.04	4.19246377	1.56795533	0.21326862	0.63696228	0.62558795	Srpk1	1
GO:0046697	decidualization	1/138	25/14464	0.04	4.19246377	1.56795533	0.21326862	0.63696228	0.62558795	Tppp3	1
GO:0048311	mitochondrion distribution	1/138	25/14464	0.04	4.19246377	1.56795533	0.21326862	0.63696228	0.62558795	S2bpcox16	1
GO:0098810	neurotransmitter reuptake	1/138	25/14464	0.04	4.19246377	1.56795533	0.21326862	0.63696228	0.62558795	Slc17a8	1
GO:0090398	cellular senescence	2/138	91/14464	0.02197802	2.30355152	1.22428195	0.21551483	0.63952318	0.62810312	Bmal1/Calr	2
GO:2001257	regulation of cation channel activity	2/138	91/14464	0.02197802	2.30355152	1.22428195	0.21551483	0.63952318	0.62810312	Rrad/Sri	2
GO:0140053	mitochondrial gene expression	3/138	168/14464	0.01785714	1.87163561	1.11529203	0.21626749	0.63952318	0.62810312	Mrps6/Tfb1m/Yars2	3
GO:0009755	hormone-mediated signaling pathway	3/138	169/14464	0.01775148	1.86056084	1.10443234	0.21875791	0.63952318	0.62810312	Bmal1/Dhrs3/Calr	3
GO:0044344	cellular response to fibroblast growth factor stimulus	2/138	92/14464	0.02173913	2.27851292	1.20738755	0.21901945	0.63952318	0.62810312	Gclm/Ift80	2
GO:0048565	digestive tract development	2/138	92/14464	0.02173913	2.27851292	1.20738755	0.21901945	0.63952318	0.62810312	Smad2/Ift172	2
GO:0022409	positive regulation of cell-cell adhesion	4/138	252/14464	0.01587302	1.6636761	1.04312201	0.22045124	0.63952318	0.62810312	Card11/Has2/Hspd1/Pkp3	4
GO:0006026	aminoglycan catabolic process	1/138	26/14464	0.03846154	4.03121516	1.51829507	0.22078776	0.63952318	0.62810312	Hexa	1
GO:0006972	hyperosmotic response	1/138	26/14464	0.03846154	4.03121516	1.51829507	0.22078776	0.63952318	0.62810312	Oxsr1	1
GO:0009081	branched-chain amino acid metabolic process	1/138	26/14464	0.03846154	4.03121516	1.51829507	0.22078776	0.63952318	0.62810312	Hmgcll1	1
GO:0030149	sphingolipid catabolic process	1/138	26/14464	0.03846154	4.03121516	1.51829507	0.22078776	0.63952318	0.62810312	Hexa	1
GO:0045932	negative regulation of muscle contraction	1/138	26/14464	0.03846154	4.03121516	1.51829507	0.22078776	0.63952318	0.62810312	Sri	1
GO:0046339	diacylglycerol metabolic process	1/138	26/14464	0.03846154	4.03121516	1.51829507	0.22078776	0.63952318	0.62810312	Dgkk	1
GO:0060479	lung cell differentiation	1/138	26/14464	0.03846154	4.03121516	1.51829507	0.22078776	0.63952318	0.62810312	Lta4h	1
GO:0060487	lung epithelial cell differentiation	1/138	26/14464	0.03846154	4.03121516	1.51829507	0.22078776	0.63952318	0.62810312	Lta4h	1
GO:0061436	establishment of skin barrier	1/138	26/14464	0.03846154	4.03121516	1.51829507	0.22078776	0.63952318	0.62810312	Grhl3	1
GO:0070977	bone maturation	1/138	26/14464	0.03846154	4.03121516	1.51829507	0.22078776	0.63952318	0.62810312	Ift80	1
GO:1900101	regulation of endoplasmic reticulum unfolded protein response	1/138	26/14464	0.03846154	4.03121516	1.51829507	0.22078776	0.63952318	0.62810312	Hspa5	1
GO:1900745	positive regulation of p38MAPK cascade	1/138	26/14464	0.03846154	4.03121516	1.51829507	0.22078776	0.63952318	0.62810312	Sash1	1
GO:1904357	negative regulation of telomere maintenance via telomere lengthening	1/138	26/14464	0.03846154	4.03121516	1.51829507	0.22078776	0.63952318	0.62810312	Hnrnpc	1
GO:1905523	positive regulation of macrophage migration	1/138	26/14464	0.03846154	4.03121516	1.51829507	0.22078776	0.63952318	0.62810312	Mmp14	1
GO:0010631	epithelial cell migration	2/138	93/14464	0.02150538	2.25401278	1.19071056	0.22252905	0.64173346	0.63027393	Has2/Sash1	2
GO:0046364	monosaccharide biosynthetic process	2/138	93/14464	0.02150538	2.25401278	1.19071056	0.22252905	0.64173346	0.63027393	Arpp19/Akr1a1	2
GO:0090132	epithelium migration	2/138	93/14464	0.02150538	2.25401278	1.19071056	0.22252905	0.64173346	0.63027393	Has2/Sash1	2
GO:0042552	myelination	3/138	171/14464	0.01754386	1.8387999	1.08293151	0.22375666	0.64173346	0.63027393	Hexa/Selenoi/Degs1	3
GO:0048562	embryonic organ morphogenesis	4/138	254/14464	0.01574803	1.65057629	1.02665449	0.22449102	0.64173346	0.63027393	Mmp14/Grhl3/Smad2/Ift172	4
GO:0001843	neural tube closure	2/138	94/14464	0.0212766	2.23003392	1.17424547	0.22604323	0.64173346	0.63027393	Grhl3/Ift172	2
GO:0010717	regulation of epithelial to mesenchymal transition	2/138	94/14464	0.0212766	2.23003392	1.17424547	0.22604323	0.64173346	0.63027393	Smad2/Spred3	2
GO:0060606	tube closure	2/138	94/14464	0.0212766	2.23003392	1.17424547	0.22604323	0.64173346	0.63027393	Grhl3/Ift172	2
GO:0006536	glutamate metabolic process	1/138	27/14464	0.03703704	3.8819109	1.47105942	0.22823556	0.64173346	0.63027393	Gclm	1
GO:0007608	sensory perception of smell	1/138	27/14464	0.03703704	3.8819109	1.47105942	0.22823556	0.64173346	0.63027393	Ric8b	1
GO:0030970	retrograde protein transport, ER to cytosol	1/138	27/14464	0.03703704	3.8819109	1.47105942	0.22823556	0.64173346	0.63027393	Hsp90b1	1
GO:0032647	regulation of interferon-alpha production	1/138	27/14464	0.03703704	3.8819109	1.47105942	0.22823556	0.64173346	0.63027393	Hspd1	1
GO:0032801	receptor catabolic process	1/138	27/14464	0.03703704	3.8819109	1.47105942	0.22823556	0.64173346	0.63027393	Itch	1
GO:0036120	cellular response to platelet-derived growth factor stimulus	1/138	27/14464	0.03703704	3.8819109	1.47105942	0.22823556	0.64173346	0.63027393	Has2	1
GO:0042572	retinol metabolic process	1/138	27/14464	0.03703704	3.8819109	1.47105942	0.22823556	0.64173346	0.63027393	Dhrs3	1
GO:0044090	positive regulation of vacuole organization	1/138	27/14464	0.03703704	3.8819109	1.47105942	0.22823556	0.64173346	0.63027393	Snx18	1
GO:0045722	positive regulation of gluconeogenesis	1/138	27/14464	0.03703704	3.8819109	1.47105942	0.22823556	0.64173346	0.63027393	Arpp19	1
GO:0051957	positive regulation of amino acid transport	1/138	27/14464	0.03703704	3.8819109	1.47105942	0.22823556	0.64173346	0.63027393	Slc17a8	1
GO:0070841	inclusion body assembly	1/138	27/14464	0.03703704	3.8819109	1.47105942	0.22823556	0.64173346	0.63027393	Bag5	1
GO:1903513	endoplasmic reticulum to cytosol transport	1/138	27/14464	0.03703704	3.8819109	1.47105942	0.22823556	0.64173346	0.63027393	Hsp90b1	1
GO:2000050	regulation of non-canonical Wnt signaling pathway	1/138	27/14464	0.03703704	3.8819109	1.47105942	0.22823556	0.64173346	0.63027393	Ift80	1
GO:0019882	antigen processing and presentation	2/138	95/14464	0.02105263	2.20655988	1.15798697	0.22956158	0.64456287	0.63305282	Calr/Pdia3	2
GO:0007272	ensheathment of neurons	3/138	174/14464	0.01724138	1.80709645	1.05121176	0.23129712	0.64625193	0.63471171	Hexa/Selenoi/Degs1	3
GO:0008366	axon ensheathment	3/138	174/14464	0.01724138	1.80709645	1.05121176	0.23129712	0.64625193	0.63471171	Hexa/Selenoi/Degs1	3
GO:0001704	formation of primary germ layer	2/138	96/14464	0.02083333	2.18357488	1.14192994	0.2330837	0.64625193	0.63471171	Mmp14/Smad2	2
GO:0006022	aminoglycan metabolic process	2/138	96/14464	0.02083333	2.18357488	1.14192994	0.2330837	0.64625193	0.63471171	Has2/Hexa	2
GO:0050764	regulation of phagocytosis	2/138	96/14464	0.02083333	2.18357488	1.14192994	0.2330837	0.64625193	0.63471171	Calr/Lman2	2
GO:0050678	regulation of epithelial cell proliferation	5/138	346/14464	0.01445087	1.51461841	0.95091795	0.2354202	0.64625193	0.63471171	Has2/Ift172/S2bpcox16/Ift80/Aggf1	5
GO:0000038	very long-chain fatty acid metabolic process	1/138	28/14464	0.03571429	3.74327122	1.42603633	0.23561268	0.64625193	0.63471171	Elovl7	1
GO:0002068	glandular epithelial cell development	1/138	28/14464	0.03571429	3.74327122	1.42603633	0.23561268	0.64625193	0.63471171	Bmal1	1
GO:0003230	cardiac atrium development	1/138	28/14464	0.03571429	3.74327122	1.42603633	0.23561268	0.64625193	0.63471171	Dand5	1
GO:0006221	pyrimidine nucleotide biosynthetic process	1/138	28/14464	0.03571429	3.74327122	1.42603633	0.23561268	0.64625193	0.63471171	Tbpl1	1
GO:0032735	positive regulation of interleukin-12 production	1/138	28/14464	0.03571429	3.74327122	1.42603633	0.23561268	0.64625193	0.63471171	Hspd1	1
GO:0034698	response to gonadotropin	1/138	28/14464	0.03571429	3.74327122	1.42603633	0.23561268	0.64625193	0.63471171	Gclm	1
GO:0036314	response to sterol	1/138	28/14464	0.03571429	3.74327122	1.42603633	0.23561268	0.64625193	0.63471171	Smad2	1
GO:0048799	animal organ maturation	1/138	28/14464	0.03571429	3.74327122	1.42603633	0.23561268	0.64625193	0.63471171	Ift80	1
GO:0060317	cardiac epithelial to mesenchymal transition	1/138	28/14464	0.03571429	3.74327122	1.42603633	0.23561268	0.64625193	0.63471171	Has2	1
GO:0086091	regulation of heart rate by cardiac conduction	1/138	28/14464	0.03571429	3.74327122	1.42603633	0.23561268	0.64625193	0.63471171	Pkp2	1
GO:1902914	regulation of protein polyubiquitination	1/138	28/14464	0.03571429	3.74327122	1.42603633	0.23561268	0.64625193	0.63471171	Sash1	1
GO:0045216	cell-cell junction organization	3/138	176/14464	0.01704545	1.78656126	1.0304085	0.23635045	0.6463471	0.63480519	Pkp3/Pkp2/Cdh10	3
GO:0043266	regulation of potassium ion transport	2/138	97/14464	0.02061856	2.1610638	1.12606943	0.23660921	0.6463471	0.63480519	Kcnip2/Oxsr1	2
GO:0090130	tissue migration	2/138	97/14464	0.02061856	2.1610638	1.12606943	0.23660921	0.6463471	0.63480519	Has2/Sash1	2
GO:0022618	protein-RNA complex assembly	3/138	177/14464	0.01694915	1.7764677	1.02010709	0.23888454	0.64778611	0.6362185	Srpk1/Prpf31/Prpf18	3
GO:0032412	regulation of monoatomic ion transmembrane transporter activity	3/138	177/14464	0.01694915	1.7764677	1.02010709	0.23888454	0.64778611	0.6362185	Rrad/Sri/Oxsr1	3
GO:0003014	renal system process	2/138	98/14464	0.02040816	2.13901213	1.11040068	0.24013772	0.64778611	0.6362185	Has2/Oxsr1	2
GO:0007040	lysosome organization	2/138	98/14464	0.02040816	2.13901213	1.11040068	0.24013772	0.64778611	0.6362185	Hexa/Aktip	2
GO:0071774	response to fibroblast growth factor	2/138	98/14464	0.02040816	2.13901213	1.11040068	0.24013772	0.64778611	0.6362185	Gclm/Ift80	2
GO:0080171	lytic vacuole organization	2/138	98/14464	0.02040816	2.13901213	1.11040068	0.24013772	0.64778611	0.6362185	Hexa/Aktip	2
GO:0002920	regulation of humoral immune response	1/138	29/14464	0.03448276	3.6141929	1.38303923	0.24291979	0.64778611	0.6362185	Cr1l	1
GO:0006506	GPI anchor biosynthetic process	1/138	29/14464	0.03448276	3.6141929	1.38303923	0.24291979	0.64778611	0.6362185	Pigg	1
GO:0015813	L-glutamate transmembrane transport	1/138	29/14464	0.03448276	3.6141929	1.38303923	0.24291979	0.64778611	0.6362185	Slc17a8	1
GO:0016339	calcium-dependent cell-cell adhesion via plasma membrane cell adhesion molecules	1/138	29/14464	0.03448276	3.6141929	1.38303923	0.24291979	0.64778611	0.6362185	Cdh10	1
GO:0032733	positive regulation of interleukin-10 production	1/138	29/14464	0.03448276	3.6141929	1.38303923	0.24291979	0.64778611	0.6362185	Hspd1	1
GO:0043276	anoikis	1/138	29/14464	0.03448276	3.6141929	1.38303923	0.24291979	0.64778611	0.6362185	Brms1	1
GO:0045048	protein insertion into ER membrane	1/138	29/14464	0.03448276	3.6141929	1.38303923	0.24291979	0.64778611	0.6362185	Ubl4a	1
GO:0046466	membrane lipid catabolic process	1/138	29/14464	0.03448276	3.6141929	1.38303923	0.24291979	0.64778611	0.6362185	Hexa	1
GO:0048247	lymphocyte chemotaxis	1/138	29/14464	0.03448276	3.6141929	1.38303923	0.24291979	0.64778611	0.6362185	Oxsr1	1
GO:0051882	mitochondrial depolarization	1/138	29/14464	0.03448276	3.6141929	1.38303923	0.24291979	0.64778611	0.6362185	Gclm	1
GO:0090382	phagosome maturation	1/138	29/14464	0.03448276	3.6141929	1.38303923	0.24291979	0.64778611	0.6362185	Rab38	1
GO:0098868	bone growth	1/138	29/14464	0.03448276	3.6141929	1.38303923	0.24291979	0.64778611	0.6362185	Ift80	1
GO:0014020	primary neural tube formation	2/138	99/14464	0.02020202	2.11740594	1.09491908	0.24366886	0.64892525	0.6373373	Grhl3/Ift172	2
GO:0001654	eye development	5/138	351/14464	0.01424501	1.49304265	0.91777166	0.24426501	0.64965471	0.63805373	Rhoj/Grhl3/Slc17a8/Ift172/Spred3	5
GO:0042593	glucose homeostasis	4/138	265/14464	0.01509434	1.5820618	0.93857594	0.24704003	0.65140066	0.6397685	Smad2/Gclm/Sri/Pdk3	4
GO:0035270	endocrine system development	2/138	100/14464	0.02	2.09623188	1.07962018	0.24720226	0.65140066	0.6397685	Bmal1/Smad2	2
GO:0033500	carbohydrate homeostasis	4/138	266/14464	0.01503759	1.5761142	0.93076935	0.24911529	0.65140066	0.6397685	Smad2/Gclm/Sri/Pdk3	4
GO:0150063	visual system development	5/138	354/14464	0.01412429	1.48038975	0.89812776	0.24961484	0.65140066	0.6397685	Rhoj/Grhl3/Slc17a8/Ift172/Spred3	5
GO:0003176	aortic valve development	1/138	30/14464	0.03333333	3.49371981	1.34190322	0.25015755	0.65140066	0.6397685	Smad2	1
GO:0006505	GPI anchor metabolic process	1/138	30/14464	0.03333333	3.49371981	1.34190322	0.25015755	0.65140066	0.6397685	Pigg	1
GO:0006891	intra-Golgi vesicle-mediated transport	1/138	30/14464	0.03333333	3.49371981	1.34190322	0.25015755	0.65140066	0.6397685	Cog6	1
GO:0021515	cell differentiation in spinal cord	1/138	30/14464	0.03333333	3.49371981	1.34190322	0.25015755	0.65140066	0.6397685	Ift172	1
GO:0032607	interferon-alpha production	1/138	30/14464	0.03333333	3.49371981	1.34190322	0.25015755	0.65140066	0.6397685	Hspd1	1
GO:0033198	response to ATP	1/138	30/14464	0.03333333	3.49371981	1.34190322	0.25015755	0.65140066	0.6397685	Hsp90b1	1
GO:0036119	response to platelet-derived growth factor	1/138	30/14464	0.03333333	3.49371981	1.34190322	0.25015755	0.65140066	0.6397685	Has2	1
GO:0048026	positive regulation of mRNA splicing, via spliceosome	1/138	30/14464	0.03333333	3.49371981	1.34190322	0.25015755	0.65140066	0.6397685	Obi1	1
GO:0048048	embryonic eye morphogenesis	1/138	30/14464	0.03333333	3.49371981	1.34190322	0.25015755	0.65140066	0.6397685	Ift172	1
GO:0051590	positive regulation of neurotransmitter transport	1/138	30/14464	0.03333333	3.49371981	1.34190322	0.25015755	0.65140066	0.6397685	Slc17a8	1
GO:0071353	cellular response to interleukin-4	1/138	30/14464	0.03333333	3.49371981	1.34190322	0.25015755	0.65140066	0.6397685	Hspa5	1
GO:0002688	regulation of leukocyte chemotaxis	2/138	101/14464	0.01980198	2.07547711	1.0644997	0.25073755	0.65140066	0.6397685	Calr/Oxsr1	2
GO:0035710	CD4-positive, alpha-beta T cell activation	2/138	101/14464	0.01980198	2.07547711	1.0644997	0.25073755	0.65140066	0.6397685	Card11/Itch	2
GO:0043255	regulation of carbohydrate biosynthetic process	2/138	101/14464	0.01980198	2.07547711	1.0644997	0.25073755	0.65140066	0.6397685	Has2/Arpp19	2
GO:0006401	RNA catabolic process	4/138	268/14464	0.01492537	1.56435215	0.91525234	0.25327732	0.65377341	0.64209888	Mettl16/Hnrnpc/Pkp3/Mettl14	4
GO:0006109	regulation of carbohydrate metabolic process	3/138	183/14464	0.01639344	1.71822286	0.95964746	0.25418324	0.65377341	0.64209888	Has2/Pdk3/Arpp19	3
GO:0016051	carbohydrate biosynthetic process	3/138	183/14464	0.01639344	1.71822286	0.95964746	0.25418324	0.65377341	0.64209888	Has2/Arpp19/Akr1a1	3
GO:0000578	embryonic axis specification	1/138	31/14464	0.03225806	3.38101917	1.3024819	0.25732662	0.65377341	0.64209888	Smad2	1
GO:0001736	establishment of planar polarity	1/138	31/14464	0.03225806	3.38101917	1.3024819	0.25732662	0.65377341	0.64209888	Grhl3	1
GO:0005980	glycogen catabolic process	1/138	31/14464	0.03225806	3.38101917	1.3024819	0.25732662	0.65377341	0.64209888	Wdr45b	1
GO:0007164	establishment of tissue polarity	1/138	31/14464	0.03225806	3.38101917	1.3024819	0.25732662	0.65377341	0.64209888	Grhl3	1
GO:0009251	glucan catabolic process	1/138	31/14464	0.03225806	3.38101917	1.3024819	0.25732662	0.65377341	0.64209888	Wdr45b	1
GO:0030513	positive regulation of BMP signaling pathway	1/138	31/14464	0.03225806	3.38101917	1.3024819	0.25732662	0.65377341	0.64209888	Smad2	1
GO:0030574	collagen catabolic process	1/138	31/14464	0.03225806	3.38101917	1.3024819	0.25732662	0.65377341	0.64209888	Mmp14	1
GO:0033687	osteoblast proliferation	1/138	31/14464	0.03225806	3.38101917	1.3024819	0.25732662	0.65377341	0.64209888	Ift80	1
GO:0034405	response to fluid shear stress	1/138	31/14464	0.03225806	3.38101917	1.3024819	0.25732662	0.65377341	0.64209888	Has2	1
GO:0043267	negative regulation of potassium ion transport	1/138	31/14464	0.03225806	3.38101917	1.3024819	0.25732662	0.65377341	0.64209888	Oxsr1	1
GO:0065002	intracellular protein transmembrane transport	1/138	31/14464	0.03225806	3.38101917	1.3024819	0.25732662	0.65377341	0.64209888	Hspa5	1
GO:0090311	regulation of protein deacetylation	1/138	31/14464	0.03225806	3.38101917	1.3024819	0.25732662	0.65377341	0.64209888	Brms1	1
GO:2000279	negative regulation of DNA biosynthetic process	1/138	31/14464	0.03225806	3.38101917	1.3024819	0.25732662	0.65377341	0.64209888	Hnrnpc	1
GO:2000515	negative regulation of CD4-positive, alpha-beta T cell activation	1/138	31/14464	0.03225806	3.38101917	1.3024819	0.25732662	0.65377341	0.64209888	Itch	1
GO:0042303	molting cycle	2/138	103/14464	0.01941748	2.03517659	1.03477749	0.25781243	0.65377341	0.64209888	Bmal1/Pkp3	2
GO:0042633	hair cycle	2/138	103/14464	0.01941748	2.03517659	1.03477749	0.25781243	0.65377341	0.64209888	Bmal1/Pkp3	2
GO:0048880	sensory system development	5/138	359/14464	0.01392758	1.45977151	0.86578288	0.25859823	0.65494225	0.64324685	Rhoj/Grhl3/Slc17a8/Ift172/Spred3	5
GO:0006814	sodium ion transport	3/138	185/14464	0.01621622	1.69964747	0.93998841	0.25931499	0.65593353	0.64422043	Slc5a3/Slc17a8/Oxsr1	3
GO:0055123	digestive system development	2/138	104/14464	0.01923077	2.01560758	1.02016788	0.26135132	0.65604385	0.64432879	Smad2/Ift172	2
GO:0022898	regulation of transmembrane transporter activity	3/138	186/14464	0.01612903	1.69050958	0.93024803	0.26188623	0.65604385	0.64432879	Rrad/Sri/Oxsr1	3
GO:0071826	protein-RNA complex organization	3/138	186/14464	0.01612903	1.69050958	0.93024803	0.26188623	0.65604385	0.64432879	Srpk1/Prpf31/Prpf18	3
GO:0007600	sensory perception	5/138	362/14464	0.01381215	1.44767395	0.84660732	0.26402616	0.65604385	0.64432879	Rbp1/Slc17a8/Hexa/Smr2l/Ric8b	5
GO:0002347	response to tumor cell	1/138	32/14464	0.03125	3.27536232	1.26464477	0.26442763	0.65604385	0.64432879	Hspd1	1
GO:0009309	amine biosynthetic process	1/138	32/14464	0.03125	3.27536232	1.26464477	0.26442763	0.65604385	0.64432879	Sat1	1
GO:0032743	positive regulation of interleukin-2 production	1/138	32/14464	0.03125	3.27536232	1.26464477	0.26442763	0.65604385	0.64432879	Card11	1
GO:0042401	biogenic amine biosynthetic process	1/138	32/14464	0.03125	3.27536232	1.26464477	0.26442763	0.65604385	0.64432879	Sat1	1
GO:0043537	negative regulation of blood vessel endothelial cell migration	1/138	32/14464	0.03125	3.27536232	1.26464477	0.26442763	0.65604385	0.64432879	Stard13	1
GO:0180046	GPI anchored protein biosynthesis	1/138	32/14464	0.03125	3.27536232	1.26464477	0.26442763	0.65604385	0.64432879	Pigg	1
GO:1901381	positive regulation of potassium ion transmembrane transport	1/138	32/14464	0.03125	3.27536232	1.26464477	0.26442763	0.65604385	0.64432879	Kcnip2	1
GO:2000677	regulation of transcription regulatory region DNA binding	1/138	32/14464	0.03125	3.27536232	1.26464477	0.26442763	0.65604385	0.64432879	Sri	1
GO:0002377	immunoglobulin production	2/138	105/14464	0.01904762	1.99641132	1.00572088	0.26489072	0.65604385	0.64432879	Card11/Hspd1	2
GO:0002832	negative regulation of response to biotic stimulus	2/138	105/14464	0.01904762	1.99641132	1.00572088	0.26489072	0.65604385	0.64432879	Dtx4/Itch	2
GO:0003279	cardiac septum development	2/138	105/14464	0.01904762	1.99641132	1.00572088	0.26489072	0.65604385	0.64432879	Dhrs3/Dand5	2
GO:0010906	regulation of glucose metabolic process	2/138	105/14464	0.01904762	1.99641132	1.00572088	0.26489072	0.65604385	0.64432879	Pdk3/Arpp19	2
GO:0038061	non-canonical NF-kappaB signal transduction	2/138	105/14464	0.01904762	1.99641132	1.00572088	0.26489072	0.65604385	0.64432879	Calr/Sash1	2
GO:0043524	negative regulation of neuron apoptotic process	3/138	188/14464	0.01595745	1.67252544	0.91094181	0.26703866	0.65720011	0.64546439	Hspd1/Gclm/Sema3e	3
GO:0010817	regulation of hormone levels	6/138	455/14464	0.01318681	1.38213091	0.81286901	0.26784113	0.65720011	0.64546439	Bmal1/Dhrs3/Hsp90b1/Smad2/Sri/P4hb	6
GO:0048024	regulation of mRNA splicing, via spliceosome	2/138	106/14464	0.01886792	1.97757725	0.99143285	0.26843032	0.65720011	0.64546439	Mettl16/Obi1	2
GO:0003203	endocardial cushion morphogenesis	1/138	33/14464	0.03030303	3.17610892	1.2282751	0.27146123	0.65720011	0.64546439	Smad2	1
GO:0010719	negative regulation of epithelial to mesenchymal transition	1/138	33/14464	0.03030303	3.17610892	1.2282751	0.27146123	0.65720011	0.64546439	Spred3	1
GO:0019731	antibacterial humoral response	1/138	33/14464	0.03030303	3.17610892	1.2282751	0.27146123	0.65720011	0.64546439	Ighg2c	1
GO:0031018	endocrine pancreas development	1/138	33/14464	0.03030303	3.17610892	1.2282751	0.27146123	0.65720011	0.64546439	Bmal1	1
GO:0032781	positive regulation of ATP-dependent activity	1/138	33/14464	0.03030303	3.17610892	1.2282751	0.27146123	0.65720011	0.64546439	Dnajb11	1
GO:0045943	positive regulation of transcription by RNA polymerase I	1/138	33/14464	0.03030303	3.17610892	1.2282751	0.27146123	0.65720011	0.64546439	Rasl11a	1
GO:0046456	icosanoid biosynthetic process	1/138	33/14464	0.03030303	3.17610892	1.2282751	0.27146123	0.65720011	0.64546439	Lta4h	1
GO:0050685	positive regulation of mRNA processing	1/138	33/14464	0.03030303	3.17610892	1.2282751	0.27146123	0.65720011	0.64546439	Obi1	1
GO:0060351	cartilage development involved in endochondral bone morphogenesis	1/138	33/14464	0.03030303	3.17610892	1.2282751	0.27146123	0.65720011	0.64546439	Ift80	1
GO:0070670	response to interleukin-4	1/138	33/14464	0.03030303	3.17610892	1.2282751	0.27146123	0.65720011	0.64546439	Hspa5	1
GO:0090075	relaxation of muscle	1/138	33/14464	0.03030303	3.17610892	1.2282751	0.27146123	0.65720011	0.64546439	Sri	1
GO:1903725	regulation of phospholipid metabolic process	1/138	33/14464	0.03030303	3.17610892	1.2282751	0.27146123	0.65720011	0.64546439	Rab38	1

GO:2000516	positive regulation of CD4-positive, alpha-beta T cell activation	1/138	33/14464	0.03030303	3.17610892	1.2282751	0.27146123	0.65720011	0.64546439	Card11	1
GO:0002062	chondrocyte differentiation	2/138	107/14464	0.01869159	1.95909522	0.97730029	0.27196977	0.65720011	0.64546439	Scin/Ift80	2
GO:0002456	T cell mediated immunity	2/138	107/14464	0.01869159	1.95909522	0.97730029	0.27196977	0.65720011	0.64546439	Hspd1/Cr1l	2
GO:0005976	polysaccharide metabolic process	2/138	107/14464	0.01869159	1.95909522	0.97730029	0.27196977	0.65720011	0.64546439	Has2/Wdr45b	2
GO:0030518	nuclear receptor-mediated steroid hormone signaling pathway	2/138	107/14464	0.01869159	1.95909522	0.97730029	0.27196977	0.65720011	0.64546439	Bmal1/Calr	2
GO:0032259	methylation	3/138	190/14464	0.01578947	1.65491991	0.89186354	0.27220342	0.65720011	0.64546439	Mettl16/Tfb1m/Mettl14	3
GO:0006605	protein targeting	4/138	278/14464	0.01438849	1.50808049	0.8395203	0.27429608	0.66146041	0.64964862	Hspa5/Hspd1/Ubl4a/Srprb	4
GO:0032970	regulation of actin filament-based process	5/138	368/14464	0.01358696	1.42407057	0.8087613	0.2749607	0.66192333	0.65010327	Arhgef16/Grhl3/Scin/Pkp2/Sri	5
GO:0030316	osteoclast differentiation	2/138	108/14464	0.01851852	1.94095545	0.96331978	0.27550877	0.66192333	0.65010327	Sh3pxd2a/Ift80	2
GO:0000272	polysaccharide catabolic process	1/138	34/14464	0.02941176	3.08269395	1.19326805	0.27842807	0.66192333	0.65010327	Wdr45b	1
GO:0001893	maternal placenta development	1/138	34/14464	0.02941176	3.08269395	1.19326805	0.27842807	0.66192333	0.65010327	Tppp3	1
GO:0006884	cell volume homeostasis	1/138	34/14464	0.02941176	3.08269395	1.19326805	0.27842807	0.66192333	0.65010327	Oxsr1	1
GO:0010043	response to zinc ion	1/138	34/14464	0.02941176	3.08269395	1.19326805	0.27842807	0.66192333	0.65010327	Lta4h	1
GO:0021517	ventral spinal cord development	1/138	34/14464	0.02941176	3.08269395	1.19326805	0.27842807	0.66192333	0.65010327	Ift172	1
GO:0035987	endodermal cell differentiation	1/138	34/14464	0.02941176	3.08269395	1.19326805	0.27842807	0.66192333	0.65010327	Mmp14	1
GO:0051125	regulation of actin nucleation	1/138	34/14464	0.02941176	3.08269395	1.19326805	0.27842807	0.66192333	0.65010327	Scin	1
GO:0070306	lens fiber cell differentiation	1/138	34/14464	0.02941176	3.08269395	1.19326805	0.27842807	0.66192333	0.65010327	Spred3	1
GO:0071398	cellular response to fatty acid	1/138	34/14464	0.02941176	3.08269395	1.19326805	0.27842807	0.66192333	0.65010327	Pdk3	1
GO:0086002	cardiac muscle cell action potential involved in contraction	1/138	34/14464	0.02941176	3.08269395	1.19326805	0.27842807	0.66192333	0.65010327	Pkp2	1
GO:0030324	lung development	3/138	194/14464	0.01546392	1.62079785	0.85436873	0.28256528	0.66499742	0.65312247	Mmp14/Smad2/Lta4h	3
GO:0030048	actin filament-based movement	2/138	110/14464	0.01818182	1.90566535	0.93580182	0.28258418	0.66499742	0.65312247	Pkp2/Sri	2
GO:0046545	development of primary female sexual characteristics	2/138	110/14464	0.01818182	1.90566535	0.93580182	0.28258418	0.66499742	0.65312247	Mmp14/Lhfpl2	2
GO:0000387	spliceosomal snRNP assembly	1/138	35/14464	0.02857143	2.99461698	1.15952917	0.28532875	0.66499742	0.65312247	Prpf31	1
GO:0016242	negative regulation of macroautophagy	1/138	35/14464	0.02857143	2.99461698	1.15952917	0.28532875	0.66499742	0.65312247	Sec22b	1
GO:0021680	cerebellar Purkinje cell layer development	1/138	35/14464	0.02857143	2.99461698	1.15952917	0.28532875	0.66499742	0.65312247	Hspa5	1
GO:0031076	embryonic camera-type eye development	1/138	35/14464	0.02857143	2.99461698	1.15952917	0.28532875	0.66499742	0.65312247	Ift172	1
GO:0032205	negative regulation of telomere maintenance	1/138	35/14464	0.02857143	2.99461698	1.15952917	0.28532875	0.66499742	0.65312247	Hnrnpc	1
GO:0032438	melanosome organization	1/138	35/14464	0.02857143	2.99461698	1.15952917	0.28532875	0.66499742	0.65312247	Rab38	1
GO:0033574	response to testosterone	1/138	35/14464	0.02857143	2.99461698	1.15952917	0.28532875	0.66499742	0.65312247	Calr	1
GO:0043392	negative regulation of DNA binding	1/138	35/14464	0.02857143	2.99461698	1.15952917	0.28532875	0.66499742	0.65312247	Sri	1
GO:0045746	negative regulation of Notch signaling pathway	1/138	35/14464	0.02857143	2.99461698	1.15952917	0.28532875	0.66499742	0.65312247	Mmp14	1
GO:0046717	acid secretion	1/138	35/14464	0.02857143	2.99461698	1.15952917	0.28532875	0.66499742	0.65312247	Slc51a	1
GO:0048546	digestive tract morphogenesis	1/138	35/14464	0.02857143	2.99461698	1.15952917	0.28532875	0.66499742	0.65312247	Smad2	1
GO:0051281	positive regulation of release of sequestered calcium ion into cytosol	1/138	35/14464	0.02857143	2.99461698	1.15952917	0.28532875	0.66499742	0.65312247	Sri	1
GO:0072528	pyrimidine-containing compound biosynthetic process	1/138	35/14464	0.02857143	2.99461698	1.15952917	0.28532875	0.66499742	0.65312247	Tbpl1	1
GO:1905314	semi-lunar valve development	1/138	35/14464	0.02857143	2.99461698	1.15952917	0.28532875	0.66499742	0.65312247	Smad2	1
GO:0031398	positive regulation of protein ubiquitination	2/138	111/14464	0.01801802	1.88849719	0.92225808	0.28611998	0.66607144	0.65417731	Hspa5/Ndfip2	2
GO:0048193	Golgi vesicle transport	4/138	284/14464	0.01408451	1.47621964	0.7954898	0.28705073	0.66746744	0.65554838	Lman2/Cog6/P4hb/Sec22b	4
GO:0051348	negative regulation of transferase activity	3/138	196/14464	0.01530612	1.60425909	0.83594161	0.28776012	0.66834609	0.65641133	Chordc1/Dnajc3/Bag5	3
GO:0001841	neural tube formation	2/138	112/14464	0.01785714	1.87163561	0.9088538	0.28965411	0.6691946	0.6572447	Grhl3/Ift172	2
GO:0030323	respiratory tube development	3/138	197/14464	0.01522843	1.59611565	0.8268059	0.29036053	0.6691946	0.6572447	Mmp14/Smad2/Lta4h	3
GO:1901293	nucleoside phosphate biosynthetic process	4/138	286/14464	0.01398601	1.46589642	0.78103621	0.29132232	0.6691946	0.6572447	Tbpl1/Ppcs/Elovl7/Pdk3	4
GO:0001523	retinoid metabolic process	1/138	36/14464	0.02777778	2.91143317	1.12697314	0.29216392	0.6691946	0.6572447	Dhrs3	1
GO:0009066	aspartate family amino acid metabolic process	1/138	36/14464	0.02777778	2.91143317	1.12697314	0.29216392	0.6691946	0.6572447	Enoph1	1
GO:0021879	forebrain neuron differentiation	1/138	36/14464	0.02777778	2.91143317	1.12697314	0.29216392	0.6691946	0.6572447	Sema3e	1
GO:0045879	negative regulation of smoothened signaling pathway	1/138	36/14464	0.02777778	2.91143317	1.12697314	0.29216392	0.6691946	0.6572447	Ift172	1
GO:0048753	pigment granule organization	1/138	36/14464	0.02777778	2.91143317	1.12697314	0.29216392	0.6691946	0.6572447	Rab38	1
GO:0050931	pigment cell differentiation	1/138	36/14464	0.02777778	2.91143317	1.12697314	0.29216392	0.6691946	0.6572447	Rab38	1
GO:0051693	actin filament capping	1/138	36/14464	0.02777778	2.91143317	1.12697314	0.29216392	0.6691946	0.6572447	Scin	1
GO:1901889	negative regulation of cell junction assembly	1/138	36/14464	0.02777778	2.91143317	1.12697314	0.29216392	0.6691946	0.6572447	Mmp14	1
GO:1902624	positive regulation of neutrophil migration	1/138	36/14464	0.02777778	2.91143317	1.12697314	0.29216392	0.6691946	0.6572447	Cd99l2	1
GO:0003013	circulatory system process	6/138	470/14464	0.01276596	1.33802035	0.73118472	0.2924407	0.6691946	0.6572447	Manf/Kcnip2/Gclm/Ppcs/Pkp2/Sri	6
GO:0001952	regulation of cell-matrix adhesion	2/138	114/14464	0.01754386	1.8387999	0.88245203	0.29671624	0.67744046	0.66534331	Mmp14/Sema3e	2
GO:0060048	cardiac muscle contraction	2/138	114/14464	0.01754386	1.8387999	0.88245203	0.29671624	0.67744046	0.66534331	Pkp2/Sri	2
GO:0001504	neurotransmitter uptake	1/138	37/14464	0.02702703	2.83274579	1.0955226	0.29893419	0.67942652	0.6672939	Slc17a8	1
GO:0042554	superoxide anion generation	1/138	37/14464	0.02702703	2.83274579	1.0955226	0.29893419	0.67942652	0.6672939	Sh3pxd2a	1
GO:0044743	protein transmembrane import into intracellular organelle	1/138	37/14464	0.02702703	2.83274579	1.0955226	0.29893419	0.67942652	0.6672939	Hspd1	1
GO:0046825	regulation of protein export from nucleus	1/138	37/14464	0.02702703	2.83274579	1.0955226	0.29893419	0.67942652	0.6672939	Frat2	1
GO:0141188	nucleic acid catabolic process	4/138	291/14464	0.0137457	1.4407092	0.74537355	0.30203918	0.68571057	0.67346574	Mettl16/Hnrnpc/Pkp3/Mettl14	4
GO:0043401	steroid hormone receptor signaling pathway	2/138	116/14464	0.01724138	1.80709645	0.85657424	0.30376832	0.68769036	0.67541017	Bmal1/Calr	2
GO:1903037	regulation of leukocyte cell-cell adhesion	4/138	292/14464	0.01369863	1.43577526	0.7383201	0.30418842	0.68769036	0.67541017	Card11/Has2/Hspd1/Itch	4
GO:0001569	branching involved in blood vessel morphogenesis	1/138	38/14464	0.02631579	2.75819985	1.06510724	0.30564016	0.68769036	0.67541017	Sema3e	1
GO:0002691	regulation of cellular extravasation	1/138	38/14464	0.02631579	2.75819985	1.06510724	0.30564016	0.68769036	0.67541017	Cd99l2	1
GO:0044273	sulfur compound catabolic process	1/138	38/14464	0.02631579	2.75819985	1.06510724	0.30564016	0.68769036	0.67541017	Hexa	1
GO:0044331	cell-cell adhesion mediated by cadherin	1/138	38/14464	0.02631579	2.75819985	1.06510724	0.30564016	0.68769036	0.67541017	Cdh10	1
GO:0046636	negative regulation of alpha-beta T cell activation	1/138	38/14464	0.02631579	2.75819985	1.06510724	0.30564016	0.68769036	0.67541017	Itch	1
GO:0051938	L-glutamate import	1/138	38/14464	0.02631579	2.75819985	1.06510724	0.30564016	0.68769036	0.67541017	Slc17a8	1
GO:0060047	heart contraction	3/138	203/14464	0.01477833	1.54893982	0.77304366	0.30599638	0.68772429	0.6754435	Kcnip2/Pkp2/Sri	3
GO:0003206	cardiac chamber morphogenesis	2/138	117/14464	0.01709402	1.79165118	0.84382524	0.30728992	0.68804526	0.67575874	Dhrs3/Pkp2	2
GO:0021549	cerebellum development	2/138	117/14464	0.01709402	1.79165118	0.84382524	0.30728992	0.68804526	0.67575874	Hspa5/Fktn	2
GO:0043534	blood vessel endothelial cell migration	2/138	117/14464	0.01709402	1.79165118	0.84382524	0.30728992	0.68804526	0.67575874	Rhoj/Stard13	2
GO:1904062	regulation of monoatomic cation transmembrane transport	4/138	294/14464	0.01360544	1.42600808	0.72429059	0.30849217	0.68804526	0.67575874	Rrad/Kcnip2/Sri/Oxsr1	4
GO:0016054	organic acid catabolic process	3/138	204/14464	0.01470588	1.54134697	0.76425325	0.30860667	0.68804526	0.67575874	Hexa/Hmgcll1/Akr1a1	3
GO:0046395	carboxylic acid catabolic process	3/138	204/14464	0.01470588	1.54134697	0.76425325	0.30860667	0.68804526	0.67575874	Hexa/Hmgcll1/Akr1a1	3
GO:0046546	development of primary male sexual characteristics	2/138	118/14464	0.01694915	1.7764677	0.83119945	0.31080821	0.68804526	0.67575874	Mmp14/Lhfpl2	2
GO:0098773	skin epidermis development	2/138	118/14464	0.01694915	1.7764677	0.83119945	0.31080821	0.68804526	0.67575874	Grhl3/Pkp3	2
GO:0010559	regulation of glycoprotein biosynthetic process	1/138	39/14464	0.02564103	2.68747677	1.035663	0.31228245	0.68804526	0.67575874	Fktn	1
GO:0010830	regulation of myotube differentiation	1/138	39/14464	0.02564103	2.68747677	1.035663	0.31228245	0.68804526	0.67575874	Mmp14	1
GO:0030890	positive regulation of B cell proliferation	1/138	39/14464	0.02564103	2.68747677	1.035663	0.31228245	0.68804526	0.67575874	Card11	1
GO:0032435	negative regulation of proteasomal ubiquitin-dependent protein catabolic process	1/138	39/14464	0.02564103	2.68747677	1.035663	0.31228245	0.68804526	0.67575874	Bag5	1
GO:0032885	regulation of polysaccharide biosynthetic process	1/138	39/14464	0.02564103	2.68747677	1.035663	0.31228245	0.68804526	0.67575874	Has2	1
GO:0043094	cellular metabolic compound salvage	1/138	39/14464	0.02564103	2.68747677	1.035663	0.31228245	0.68804526	0.67575874	Enoph1	1
GO:0046189	phenol-containing compound biosynthetic process	1/138	39/14464	0.02564103	2.68747677	1.035663	0.31228245	0.68804526	0.67575874	Rab38	1
GO:0099170	postsynaptic modulation of chemical synaptic transmission	1/138	39/14464	0.02564103	2.68747677	1.035663	0.31228245	0.68804526	0.67575874	Snx14	1
GO:1900744	regulation of p38MAPK cascade	1/138	39/14464	0.02564103	2.68747677	1.035663	0.31228245	0.68804526	0.67575874	Sash1	1
GO:1905521	regulation of macrophage migration	1/138	39/14464	0.02564103	2.68747677	1.035663	0.31228245	0.68804526	0.67575874	Mmp14	1
GO:0043087	regulation of GTPase activity	3/138	207/14464	0.01449275	1.51900861	0.73816241	0.31644238	0.69115161	0.67880962	Arhgef16/Grhl3/Snx18	3
GO:1903039	positive regulation of leukocyte cell-cell adhesion	3/138	207/14464	0.01449275	1.51900861	0.73816241	0.31644238	0.69115161	0.67880962	Card11/Has2/Hspd1	3
GO:0048706	embryonic skeletal system development	2/138	120/14464	0.01666667	1.7468599	0.80630786	0.31783383	0.69115161	0.67880962	Mmp14/Smad2	2
GO:1903900	regulation of viral life cycle	2/138	120/14464	0.01666667	1.7468599	0.80630786	0.31783383	0.69115161	0.67880962	Srpk1/P4hb	2
GO:0002823	negative regulation of adaptive immune response based on somatic recombination of immune receptors	1/138	40/14464	0.025	2.62028986	1.00713135	0.31886165	0.69115161	0.67880962	Cr1l	1
GO:0006220	pyrimidine nucleotide metabolic process	1/138	40/14464	0.025	2.62028986	1.00713135	0.31886165	0.69115161	0.67880962	Tbpl1	1
GO:0006636	unsaturated fatty acid biosynthetic process	1/138	40/14464	0.025	2.62028986	1.00713135	0.31886165	0.69115161	0.67880962	Elovl7	1
GO:0016101	diterpenoid metabolic process	1/138	40/14464	0.025	2.62028986	1.00713135	0.31886165	0.69115161	0.67880962	Dhrs3	1
GO:0042398	cellular modified amino acid biosynthetic process	1/138	40/14464	0.025	2.62028986	1.00713135	0.31886165	0.69115161	0.67880962	Gclm	1
GO:0045880	positive regulation of smoothened signaling pathway	1/138	40/14464	0.025	2.62028986	1.00713135	0.31886165	0.69115161	0.67880962	Ift172	1
GO:0051445	regulation of meiotic cell cycle	1/138	40/14464	0.025	2.62028986	1.00713135	0.31886165	0.69115161	0.67880962	Calr	1
GO:0051452	intracellular pH reduction	1/138	40/14464	0.025	2.62028986	1.00713135	0.31886165	0.69115161	0.67880962	Rab38	1
GO:0071711	basement membrane organization	1/138	40/14464	0.025	2.62028986	1.00713135	0.31886165	0.69115161	0.67880962	Fktn	1
GO:1901016	regulation of potassium ion transmembrane transporter activity	1/138	40/14464	0.025	2.62028986	1.00713135	0.31886165	0.69115161	0.67880962	Oxsr1	1
GO:1901532	regulation of hematopoietic progenitor cell differentiation	1/138	40/14464	0.025	2.62028986	1.00713135	0.31886165	0.69115161	0.67880962	Mettl14	1
GO:1903829	positive regulation of protein localization	6/138	486/14464	0.01234568	1.2939703	0.64700157	0.31917765	0.69115161	0.67880962	Slc5a3/Arhgef16/Rab38/Cnpy4/Pkp3/Sri	6
GO:0030509	BMP signaling pathway	2/138	121/14464	0.01652893	1.73242304	0.79403738	0.32134067	0.69312881	0.68075151	Dand5/Smad2	2
GO:0072329	monocarboxylic acid catabolic process	2/138	121/14464	0.01652893	1.73242304	0.79403738	0.32134067	0.69312881	0.68075151	Hexa/Akr1a1	2
GO:0070085	glycosylation	3/138	210/14464	0.01428571	1.49730849	0.71248119	0.32428267	0.69312881	0.68075151	Fktn/Cog6/Crppa	3
GO:0003231	cardiac ventricle development	2/138	122/14464	0.01639344	1.71822286	0.78188079	0.3248432	0.69312881	0.68075151	Dand5/Pkp2	2
GO:0046660	female sex differentiation	2/138	122/14464	0.01639344	1.71822286	0.78188079	0.3248432	0.69312881	0.68075151	Mmp14/Lhfpl2	2
GO:0002707	negative regulation of lymphocyte mediated immunity	1/138	41/14464	0.02439024	2.55638035	0.9794587	0.32537837	0.69312881	0.68075151	Cr1l	1
GO:0006418	tRNA aminoacylation for protein translation	1/138	41/14464	0.02439024	2.55638035	0.9794587	0.32537837	0.69312881	0.68075151	Yars2	1
GO:0009394	2'-deoxyribonucleotide metabolic process	1/138	41/14464	0.02439024	2.55638035	0.9794587	0.32537837	0.69312881	0.68075151	Tbpl1	1
GO:0030866	cortical actin cytoskeleton organization	1/138	41/14464	0.02439024	2.55638035	0.9794587	0.32537837	0.69312881	0.68075151	Calr	1
GO:0032527	protein exit from endoplasmic reticulum	1/138	41/14464	0.02439024	2.55638035	0.9794587	0.32537837	0.69312881	0.68075151	Hsp90b1	1
GO:0043268	positive regulation of potassium ion transport	1/138	41/14464	0.02439024	2.55638035	0.9794587	0.32537837	0.69312881	0.68075151	Kcnip2	1
GO:0045058	T cell selection	1/138	41/14464	0.02439024	2.55638035	0.9794587	0.32537837	0.69312881	0.68075151	Card11	1
GO:0045104	intermediate filament cytoskeleton organization	1/138	41/14464	0.02439024	2.55638035	0.9794587	0.32537837	0.69312881	0.68075151	Pkp2	1
GO:0046596	regulation of viral entry into host cell	1/138	41/14464	0.02439024	2.55638035	0.9794587	0.32537837	0.69312881	0.68075151	P4hb	1
GO:0051205	protein insertion into membrane	1/138	41/14464	0.02439024	2.55638035	0.9794587	0.32537837	0.69312881	0.68075151	Ubl4a	1
GO:0005975	carbohydrate metabolic process	6/138	491/14464	0.01221996	1.28079341	0.62128246	0.32761534	0.69312881	0.68075151	Has2/Hexa/Pdk3/Arpp19/Wdr45b/Akr1a1	6
GO:0006633	fatty acid biosynthetic process	2/138	123/14464	0.01626016	1.70425356	0.76983589	0.32834119	0.69312881	0.68075151	Degs1/Elovl7	2
GO:0050864	regulation of B cell activation	2/138	123/14464	0.01626016	1.70425356	0.76983589	0.32834119	0.69312881	0.68075151	Card11/Mmp14	2
GO:0050900	leukocyte migration	4/138	304/14464	0.01315789	1.37909992	0.65563952	0.33009645	0.69312881	0.68075151	Calr/Mmp14/Cd99l2/Oxsr1	4
GO:0022407	regulation of cell-cell adhesion	5/138	398/14464	0.01256281	1.31672857	0.62885386	0.33084366	0.69312881	0.68075151	Card11/Has2/Hspd1/Pkp3/Itch	5
GO:0001937	negative regulation of endothelial cell proliferation	1/138	42/14464	0.02380952	2.49551415	0.95259586	0.33183318	0.69312881	0.68075151	S2bpcox16	1
GO:0019692	deoxyribose phosphate metabolic process	1/138	42/14464	0.02380952	2.49551415	0.95259586	0.33183318	0.69312881	0.68075151	Tbpl1	1
GO:0030835	negative regulation of actin filament depolymerization	1/138	42/14464	0.02380952	2.49551415	0.95259586	0.33183318	0.69312881	0.68075151	Scin	1
GO:0031670	cellular response to nutrient	1/138	42/14464	0.02380952	2.49551415	0.95259586	0.33183318	0.69312881	0.68075151	Pdia3	1
GO:0032655	regulation of interleukin-12 production	1/138	42/14464	0.02380952	2.49551415	0.95259586	0.33183318	0.69312881	0.68075151	Hspd1	1
GO:0035774	positive regulation of insulin secretion involved in cellular response to glucose stimulus	1/138	42/14464	0.02380952	2.49551415	0.95259586	0.33183318	0.69312881	0.68075151	Sri	1
GO:0045103	intermediate filament-based process	1/138	42/14464	0.02380952	2.49551415	0.95259586	0.33183318	0.69312881	0.68075151	Pkp2	1
GO:0045777	positive regulation of blood pressure	1/138	42/14464	0.02380952	2.49551415	0.95259586	0.33183318	0.69312881	0.68075151	Manf	1
GO:0046677	response to antibiotic	1/138	42/14464	0.02380952	2.49551415	0.95259586	0.33183318	0.69312881	0.68075151	Ppp2cb	1
GO:0060428	lung epithelium development	1/138	42/14464	0.02380952	2.49551415	0.95259586	0.33183318	0.69312881	0.68075151	Lta4h	1
GO:0072595	maintenance of protein localization in organelle	1/138	42/14464	0.02380952	2.49551415	0.95259586	0.33183318	0.69312881	0.68075151	Hspa5	1
GO:0086009	membrane repolarization	1/138	42/14464	0.02380952	2.49551415	0.95259586	0.33183318	0.69312881	0.68075151	Kcnip2	1
GO:0090630	activation of GTPase activity	1/138	42/14464	0.02380952	2.49551415	0.95259586	0.33183318	0.69312881	0.68075151	Arhgef16	1
GO:1903573	negative regulation of response to endoplasmic reticulum stress	1/138	42/14464	0.02380952	2.49551415	0.95259586	0.33183318	0.69312881	0.68075151	Hspa5	1
GO:0030073	insulin secretion	3/138	213/14464	0.01408451	1.47621964	0.68719611	0.33212422	0.69312881	0.68075151	Bmal1/Smad2/Sri	3
GO:0034764	positive regulation of transmembrane transport	3/138	214/14464	0.01401869	1.46932141	0.67885354	0.33473779	0.69343365	0.68105091	Slc17a8/Kcnip2/Sri	3
GO:0022037	metencephalon development	2/138	125/14464	0.016	1.67698551	0.7460728	0.33532263	0.69343365	0.68105091	Hspa5/Fktn	2
GO:0045727	positive regulation of translation	2/138	125/14464	0.016	1.67698551	0.7460728	0.33532263	0.69343365	0.68105091	Dnajc3/Mettl14	2
GO:0002820	negative regulation of adaptive immune response	1/138	43/14464	0.02325581	2.43747893	0.92649758	0.33822668	0.69343365	0.68105091	Cr1l	1
GO:0003197	endocardial cushion development	1/138	43/14464	0.02325581	2.43747893	0.92649758	0.33822668	0.69343365	0.68105091	Smad2	1
GO:0006984	ER-nucleus signaling pathway	1/138	43/14464	0.02325581	2.43747893	0.92649758	0.33822668	0.69343365	0.68105091	Hspa5	1
GO:0009262	deoxyribonucleotide metabolic process	1/138	43/14464	0.02325581	2.43747893	0.92649758	0.33822668	0.69343365	0.68105091	Tbpl1	1
GO:0010803	regulation of tumor necrosis factor-mediated signaling pathway	1/138	43/14464	0.02325581	2.43747893	0.92649758	0.33822668	0.69343365	0.68105091	Ppp2cb	1
GO:0031146	SCF-dependent proteasomal ubiquitin-dependent protein catabolic process	1/138	43/14464	0.02325581	2.43747893	0.92649758	0.33822668	0.69343365	0.68105091	Cul2	1
GO:0032653	regulation of interleukin-10 production	1/138	43/14464	0.02325581	2.43747893	0.92649758	0.33822668	0.69343365	0.68105091	Hspd1	1
GO:0045022	early endosome to late endosome transport	1/138	43/14464	0.02325581	2.43747893	0.92649758	0.33822668	0.69343365	0.68105091	Aktip	1
GO:0050856	regulation of T cell receptor signaling pathway	1/138	43/14464	0.02325581	2.43747893	0.92649758	0.33822668	0.69343365	0.68105091	Card11	1
GO:0098586	cellular response to virus	1/138	43/14464	0.02325581	2.43747893	0.92649758	0.33822668	0.69343365	0.68105091	Calr	1
GO:0099560	synaptic membrane adhesion	1/138	43/14464	0.02325581	2.43747893	0.92649758	0.33822668	0.69343365	0.68105091	Cdh10	1
GO:1903018	regulation of glycoprotein metabolic process	1/138	43/14464	0.02325581	2.43747893	0.92649758	0.33822668	0.69343365	0.68105091	Fktn	1
GO:2000378	negative regulation of reactive oxygen species metabolic process	1/138	43/14464	0.02325581	2.43747893	0.92649758	0.33822668	0.69343365	0.68105091	Hspd1	1
GO:0051098	regulation of binding	4/138	308/14464	0.01298701	1.36118954	0.62884919	0.33876709	0.69343365	0.68105091	Smad2/Fktn/Sri/Aktip	4
GO:0001838	embryonic epithelial tube formation	2/138	126/14464	0.01587302	1.6636761	0.73435055	0.33880563	0.69343365	0.68105091	Grhl3/Ift172	2
GO:0006959	humoral immune response	2/138	126/14464	0.01587302	1.6636761	0.73435055	0.33880563	0.69343365	0.68105091	Ighg2c/Cr1l	2
GO:0016236	macroautophagy	4/138	309/14464	0.01294498	1.35678439	0.62220918	0.34093642	0.69393789	0.68154614	Snx14/Snx18/Wdr45b/Sec22b	4
GO:0001936	regulation of endothelial cell proliferation	2/138	127/14464	0.01574803	1.65057629	0.72273188	0.34228319	0.69393789	0.68154614	S2bpcox16/Aggf1	2
GO:0050921	positive regulation of chemotaxis	2/138	127/14464	0.01574803	1.65057629	0.72273188	0.34228319	0.69393789	0.68154614	Calr/Oxsr1	2
GO:0002066	columnar/cuboidal epithelial cell development	1/138	44/14464	0.02272727	2.38208169	0.90112219	0.34455944	0.69393789	0.68154614	Bmal1	1
GO:0006356	regulation of transcription by RNA polymerase I	1/138	44/14464	0.02272727	2.38208169	0.90112219	0.34455944	0.69393789	0.68154614	Rasl11a	1
GO:0006476	protein deacetylation	1/138	44/14464	0.02272727	2.38208169	0.90112219	0.34455944	0.69393789	0.68154614	Brms1	1
GO:0006767	water-soluble vitamin metabolic process	1/138	44/14464	0.02272727	2.38208169	0.90112219	0.34455944	0.69393789	0.68154614	Akr1a1	1
GO:0030857	negative regulation of epithelial cell differentiation	1/138	44/14464	0.02272727	2.38208169	0.90112219	0.34455944	0.69393789	0.68154614	Spred3	1
GO:0032615	interleukin-12 production	1/138	44/14464	0.02272727	2.38208169	0.90112219	0.34455944	0.69393789	0.68154614	Hspd1	1
GO:0034113	heterotypic cell-cell adhesion	1/138	44/14464	0.02272727	2.38208169	0.90112219	0.34455944	0.69393789	0.68154614	Pkp2	1

GO:0043039	tRNA aminoacylation	1/138	44/14464	0.02272727	2.38208169	0.90112219	0.34455944	0.69393789	0.68154614	Yars2	1
GO:0046148	pigment biosynthetic process	1/138	44/14464	0.02272727	2.38208169	0.90112219	0.34455944	0.69393789	0.68154614	Rab38	1
GO:0050919	negative chemotaxis	1/138	44/14464	0.02272727	2.38208169	0.90112219	0.34455944	0.69393789	0.68154614	Sema3e	1
GO:0051438	regulation of ubiquitin-protein transferase activity	1/138	44/14464	0.02272727	2.38208169	0.90112219	0.34455944	0.69393789	0.68154614	Bag5	1
GO:0051602	response to electrical stimulus	1/138	44/14464	0.02272727	2.38208169	0.90112219	0.34455944	0.69393789	0.68154614	Calr	1
GO:1902116	negative regulation of organelle assembly	1/138	44/14464	0.02272727	2.38208169	0.90112219	0.34455944	0.69393789	0.68154614	Sec22b	1
GO:0014902	myotube differentiation	2/138	128/14464	0.015625	1.63768116	0.71121488	0.3457551	0.69495741	0.68254746	Mmp14/Fktn	2
GO:0046328	regulation of JNK cascade	2/138	128/14464	0.015625	1.63768116	0.71121488	0.3457551	0.69495741	0.68254746	Fktn/Itch	2
GO:0002440	production of molecular mediator of immune response	3/138	219/14464	0.01369863	1.43577526	0.63776351	0.34779827	0.696825	0.6843817	Card11/Hspd1/Pkp3	3
GO:0030183	B cell differentiation	2/138	129/14464	0.01550388	1.62498596	0.69979773	0.34922116	0.696825	0.6843817	Card11/Mmp14	2
GO:0032543	mitochondrial translation	2/138	129/14464	0.01550388	1.62498596	0.69979773	0.34922116	0.696825	0.6843817	Mrps6/Yars2	2
GO:0060541	respiratory system development	3/138	220/14464	0.01363636	1.42924901	0.62966699	0.35040809	0.696825	0.6843817	Mmp14/Smad2/Lta4h	3
GO:0002204	somatic recombination of immunoglobulin genes involved in immune response	1/138	45/14464	0.02222222	2.32914654	0.87643117	0.35083203	0.696825	0.6843817	Hspd1	1
GO:0002208	somatic diversification of immunoglobulins involved in immune response	1/138	45/14464	0.02222222	2.32914654	0.87643117	0.35083203	0.696825	0.6843817	Hspd1	1
GO:0007606	sensory perception of chemical stimulus	1/138	45/14464	0.02222222	2.32914654	0.87643117	0.35083203	0.696825	0.6843817	Ric8b	1
GO:0007628	adult walking behavior	1/138	45/14464	0.02222222	2.32914654	0.87643117	0.35083203	0.696825	0.6843817	Hexa	1
GO:0031638	zymogen activation	1/138	45/14464	0.02222222	2.32914654	0.87643117	0.35083203	0.696825	0.6843817	Mmp14	1
GO:0043038	amino acid activation	1/138	45/14464	0.02222222	2.32914654	0.87643117	0.35083203	0.696825	0.6843817	Yars2	1
GO:0045190	isotype switching	1/138	45/14464	0.02222222	2.32914654	0.87643117	0.35083203	0.696825	0.6843817	Hspd1	1
GO:1902622	regulation of neutrophil migration	1/138	45/14464	0.02222222	2.32914654	0.87643117	0.35083203	0.696825	0.6843817	Cd99l2	1
GO:0042886	amide transport	4/138	314/14464	0.01273885	1.33517954	0.58934604	0.35178967	0.69803935	0.68557436	Bmal1/Smad2/Sri/Slc33a1	4
GO:0007266	Rho protein signal transduction	2/138	130/14464	0.01538462	1.61248606	0.68847861	0.35268115	0.69912015	0.68663586	Rhoj/Stard13	2
GO:0071772	response to BMP	2/138	131/14464	0.01526718	1.60017701	0.67725577	0.35613488	0.69951679	0.68702542	Dand5/Smad2	2
GO:0071773	cellular response to BMP stimulus	2/138	131/14464	0.01526718	1.60017701	0.67725577	0.35613488	0.69951679	0.68702542	Dand5/Smad2	2
GO:0001947	heart looping	1/138	46/14464	0.02173913	2.27851292	0.85238891	0.35704503	0.69951679	0.68702542	Ift172	1
GO:0010824	regulation of centrosome duplication	1/138	46/14464	0.02173913	2.27851292	0.85238891	0.35704503	0.69951679	0.68702542	Chordc1	1
GO:0032613	interleukin-10 production	1/138	46/14464	0.02173913	2.27851292	0.85238891	0.35704503	0.69951679	0.68702542	Hspd1	1
GO:0032892	positive regulation of organic acid transport	1/138	46/14464	0.02173913	2.27851292	0.85238891	0.35704503	0.69951679	0.68702542	Slc17a8	1
GO:0051155	positive regulation of striated muscle cell differentiation	1/138	46/14464	0.02173913	2.27851292	0.85238891	0.35704503	0.69951679	0.68702542	Mmp14	1
GO:0051954	positive regulation of amine transport	1/138	46/14464	0.02173913	2.27851292	0.85238891	0.35704503	0.69951679	0.68702542	Slc17a8	1
GO:0052372	modulation by symbiont of entry into host	1/138	46/14464	0.02173913	2.27851292	0.85238891	0.35704503	0.69951679	0.68702542	P4hb	1
GO:0055010	ventricular cardiac muscle tissue morphogenesis	1/138	46/14464	0.02173913	2.27851292	0.85238891	0.35704503	0.69951679	0.68702542	Pkp2	1
GO:0120163	negative regulation of cold-induced thermogenesis	1/138	46/14464	0.02173913	2.27851292	0.85238891	0.35704503	0.69951679	0.68702542	Bmal1	1
GO:2000772	regulation of cellular senescence	1/138	46/14464	0.02173913	2.27851292	0.85238891	0.35704503	0.69951679	0.68702542	Bmal1	1
GO:0051345	positive regulation of hydrolase activity	4/138	317/14464	0.0126183	1.32254378	0.56989177	0.35830439	0.70130257	0.68877931	Arhgef16/Grhl3/Hspd1/Snx18	4
GO:1903522	regulation of blood circulation	3/138	224/14464	0.01339286	1.40372671	0.59767179	0.3608361	0.70337095	0.69081075	Kcnip2/Pkp2/Sri	3
GO:0006644	phospholipid metabolic process	4/138	319/14464	0.01253918	1.31425196	0.55702942	0.36264755	0.70337095	0.69081075	Rab38/Dgkk/Pigg/Selenoi	4
GO:0072594	establishment of protein localization to organelle	5/138	415/14464	0.01204819	1.26279029	0.53311011	0.36305114	0.70337095	0.69081075	Bmal1/Hspa5/Hspd1/Ubl4a/Srprb	5
GO:0030514	negative regulation of BMP signaling pathway	1/138	47/14464	0.0212766	2.23003392	0.8289624	0.36319899	0.70337095	0.69081075	Dand5	1
GO:0032330	regulation of chondrocyte differentiation	1/138	47/14464	0.0212766	2.23003392	0.8289624	0.36319899	0.70337095	0.69081075	Scin	1
GO:0033120	positive regulation of RNA splicing	1/138	47/14464	0.0212766	2.23003392	0.8289624	0.36319899	0.70337095	0.69081075	Obi1	1
GO:0048066	developmental pigmentation	1/138	47/14464	0.0212766	2.23003392	0.8289624	0.36319899	0.70337095	0.69081075	Rab38	1
GO:0051955	regulation of amino acid transport	1/138	47/14464	0.0212766	2.23003392	0.8289624	0.36319899	0.70337095	0.69081075	Slc17a8	1
GO:0071526	semaphorin-plexin signaling pathway	1/138	47/14464	0.0212766	2.23003392	0.8289624	0.36319899	0.70337095	0.69081075	Sema3e	1
GO:0170038	proteinogenic amino acid biosynthetic process	1/138	47/14464	0.0212766	2.23003392	0.8289624	0.36319899	0.70337095	0.69081075	Enoph1	1
GO:1902459	positive regulation of stem cell population maintenance	1/138	47/14464	0.0212766	2.23003392	0.8289624	0.36319899	0.70337095	0.69081075	Brms1	1
GO:0046661	male sex differentiation	2/138	134/14464	0.01492537	1.56435215	0.64414813	0.36645658	0.70899852	0.69633784	Mmp14/Lhfpl2	2
GO:0030219	megakaryocyte differentiation	1/138	48/14464	0.02083333	2.18357488	0.806121	0.36929447	0.71107702	0.69837922	Scin	1
GO:0032506	cytokinetic process	1/138	48/14464	0.02083333	2.18357488	0.806121	0.36929447	0.71107702	0.69837922	Snx18	1
GO:0045071	negative regulation of viral genome replication	1/138	48/14464	0.02083333	2.18357488	0.806121	0.36929447	0.71107702	0.69837922	Srpk1	1
GO:0085029	extracellular matrix assembly	1/138	48/14464	0.02083333	2.18357488	0.806121	0.36929447	0.71107702	0.69837922	Has2	1
GO:0170034	L-amino acid biosynthetic process	1/138	48/14464	0.02083333	2.18357488	0.806121	0.36929447	0.71107702	0.69837922	Enoph1	1
GO:0071902	positive regulation of protein serine/threonine kinase activity	2/138	135/14464	0.01481481	1.55276436	0.6332938	0.36988336	0.71153135	0.69882543	Fzd8/Sash1	2
GO:0072522	purine-containing compound biosynthetic process	3/138	228/14464	0.01315789	1.37909992	0.56628283	0.37124093	0.71246793	0.69974528	Ppcs/Elovl7/Pdk3	3
GO:0034765	regulation of monoatomic ion transmembrane transport	4/138	323/14464	0.0123839	1.2979764	0.53155637	0.37133155	0.71246793	0.69974528	Rrad/Kcnip2/Sri/Oxsr1	4
GO:0072175	epithelial tube formation	2/138	136/14464	0.01470588	1.54134697	0.62252765	0.37330294	0.71246793	0.69974528	Grhl3/Ift172	2
GO:1904064	positive regulation of cation transmembrane transport	2/138	136/14464	0.01470588	1.54134697	0.62252765	0.37330294	0.71246793	0.69974528	Kcnip2/Sri	2
GO:2000241	regulation of reproductive process	2/138	136/14464	0.01470588	1.54134697	0.62252765	0.37330294	0.71246793	0.69974528	Calr/Lhfpl2	2
GO:0001755	neural crest cell migration	1/138	49/14464	0.02040816	2.13901213	0.78383622	0.37533202	0.71246793	0.69974528	Sema3e	1
GO:0003179	heart valve morphogenesis	1/138	49/14464	0.02040816	2.13901213	0.78383622	0.37533202	0.71246793	0.69974528	Smad2	1
GO:0006687	glycosphingolipid metabolic process	1/138	49/14464	0.02040816	2.13901213	0.78383622	0.37533202	0.71246793	0.69974528	Hexa	1
GO:0006721	terpenoid metabolic process	1/138	49/14464	0.02040816	2.13901213	0.78383622	0.37533202	0.71246793	0.69974528	Dhrs3	1
GO:0032729	positive regulation of type II interferon production	1/138	49/14464	0.02040816	2.13901213	0.78383622	0.37533202	0.71246793	0.69974528	Hspd1	1
GO:0032881	regulation of polysaccharide metabolic process	1/138	49/14464	0.02040816	2.13901213	0.78383622	0.37533202	0.71246793	0.69974528	Has2	1
GO:0043030	regulation of macrophage activation	1/138	49/14464	0.02040816	2.13901213	0.78383622	0.37533202	0.71246793	0.69974528	Hspd1	1
GO:0098927	vesicle-mediated transport between endosomal compartments	1/138	49/14464	0.02040816	2.13901213	0.78383622	0.37533202	0.71246793	0.69974528	Aktip	1
GO:0170040	proteinogenic amino acid catabolic process	1/138	49/14464	0.02040816	2.13901213	0.78383622	0.37533202	0.71246793	0.69974528	Hmgcll1	1
GO:0046486	glycerolipid metabolic process	4/138	325/14464	0.01230769	1.28998885	0.51894329	0.37567133	0.71246793	0.69974528	Rab38/Dgkk/Pigg/Selenoi	4
GO:0007159	leukocyte cell-cell adhesion	4/138	326/14464	0.01226994	1.28603183	0.51266709	0.37784045	0.71509338	0.70232385	Card11/Has2/Hspd1/Itch	4
GO:1903322	positive regulation of protein modification by small protein conjugation or removal	2/138	138/14464	0.01449275	1.51900861	0.60125389	0.38011984	0.71509338	0.70232385	Hspa5/Ndfip2	2
GO:0000245	spliceosomal complex assembly	1/138	50/14464	0.02	2.09623188	0.76208153	0.38131219	0.71509338	0.70232385	Srpk1	1
GO:0002712	regulation of B cell mediated immunity	1/138	50/14464	0.02	2.09623188	0.76208153	0.38131219	0.71509338	0.70232385	Cr1l	1
GO:0002889	regulation of immunoglobulin mediated immune response	1/138	50/14464	0.02	2.09623188	0.76208153	0.38131219	0.71509338	0.70232385	Cr1l	1
GO:0003254	regulation of membrane depolarization	1/138	50/14464	0.02	2.09623188	0.76208153	0.38131219	0.71509338	0.70232385	Gclm	1
GO:0010634	positive regulation of epithelial cell migration	1/138	50/14464	0.02	2.09623188	0.76208153	0.38131219	0.71509338	0.70232385	Has2	1
GO:0010718	positive regulation of epithelial to mesenchymal transition	1/138	50/14464	0.02	2.09623188	0.76208153	0.38131219	0.71509338	0.70232385	Smad2	1
GO:0030834	regulation of actin filament depolymerization	1/138	50/14464	0.02	2.09623188	0.76208153	0.38131219	0.71509338	0.70232385	Scin	1
GO:0032663	regulation of interleukin-2 production	1/138	50/14464	0.02	2.09623188	0.76208153	0.38131219	0.71509338	0.70232385	Card11	1
GO:0043903	regulation of biological process involved in symbiotic interaction	1/138	50/14464	0.02	2.09623188	0.76208153	0.38131219	0.71509338	0.70232385	P4hb	1
GO:0090497	mesenchymal cell migration	1/138	50/14464	0.02	2.09623188	0.76208153	0.38131219	0.71509338	0.70232385	Sema3e	1
GO:0007584	response to nutrient	2/138	139/14464	0.01438849	1.50808049	0.59074339	0.38351681	0.71789219	0.70507269	Pdia3/Gclm	2
GO:1901875	positive regulation of post-translational protein modification	2/138	139/14464	0.01438849	1.50808049	0.59074339	0.38351681	0.71789219	0.70507269	Hspa5/Ndfip2	2
GO:0002706	regulation of lymphocyte mediated immunity	2/138	140/14464	0.01428571	1.49730849	0.58031527	0.38690591	0.71884606	0.70600952	Hspd1/Cr1l	2
GO:0008360	regulation of cell shape	2/138	140/14464	0.01428571	1.49730849	0.58031527	0.38690591	0.71884606	0.70600952	Rhoj/Sema3e	2
GO:0043405	regulation of MAP kinase activity	2/138	140/14464	0.01428571	1.49730849	0.58031527	0.38690591	0.71884606	0.70600952	Fzd8/Sash1	2
GO:0002711	positive regulation of T cell mediated immunity	1/138	51/14464	0.01960784	2.0551293	0.74083218	0.38723552	0.71884606	0.70600952	Hspd1	1
GO:0034605	cellular response to heat	1/138	51/14464	0.01960784	2.0551293	0.74083218	0.38723552	0.71884606	0.70600952	Chordc1	1
GO:0046470	phosphatidylcholine metabolic process	1/138	51/14464	0.01960784	2.0551293	0.74083218	0.38723552	0.71884606	0.70600952	Rab38	1
GO:0070098	chemokine-mediated signaling pathway	1/138	51/14464	0.01960784	2.0551293	0.74083218	0.38723552	0.71884606	0.70600952	Oxsr1	1
GO:0072132	mesenchyme morphogenesis	1/138	51/14464	0.01960784	2.0551293	0.74083218	0.38723552	0.71884606	0.70600952	Smad2	1
GO:1902692	regulation of neuroblast proliferation	1/138	51/14464	0.01960784	2.0551293	0.74083218	0.38723552	0.71884606	0.70600952	Tafa1	1
GO:0071900	regulation of protein serine/threonine kinase activity	3/138	235/14464	0.01276596	1.33802035	0.51273841	0.38937384	0.7214712	0.70858779	Chordc1/Fzd8/Sash1	3
GO:0007605	sensory perception of sound	2/138	141/14464	0.0141844	1.48668928	0.56996817	0.39028699	0.7214712	0.70858779	Slc17a8/Hexa	2
GO:0002449	lymphocyte mediated immunity	3/138	236/14464	0.01271186	1.33235077	0.50522756	0.39195484	0.7214712	0.70858779	Ighg2c/Hspd1/Cr1l	3
GO:0001707	mesoderm formation	1/138	52/14464	0.01923077	2.01560758	0.72006507	0.39310255	0.7214712	0.70858779	Smad2	1
GO:0002704	negative regulation of leukocyte mediated immunity	1/138	52/14464	0.01923077	2.01560758	0.72006507	0.39310255	0.7214712	0.70858779	Cr1l	1
GO:0006111	regulation of gluconeogenesis	1/138	52/14464	0.01923077	2.01560758	0.72006507	0.39310255	0.7214712	0.70858779	Arpp19	1
GO:0006749	glutathione metabolic process	1/138	52/14464	0.01923077	2.01560758	0.72006507	0.39310255	0.7214712	0.70858779	Gclm	1
GO:0021587	cerebellum morphogenesis	1/138	52/14464	0.01923077	2.01560758	0.72006507	0.39310255	0.7214712	0.70858779	Hspa5	1
GO:0032210	regulation of telomere maintenance via telomerase	1/138	52/14464	0.01923077	2.01560758	0.72006507	0.39310255	0.7214712	0.70858779	Hnrnpc	1
GO:0086001	cardiac muscle cell action potential	1/138	52/14464	0.01923077	2.01560758	0.72006507	0.39310255	0.7214712	0.70858779	Pkp2	1
GO:0089718	amino acid import across plasma membrane	1/138	52/14464	0.01923077	2.01560758	0.72006507	0.39310255	0.7214712	0.70858779	Slc17a8	1
GO:2000242	negative regulation of reproductive process	1/138	52/14464	0.01923077	2.01560758	0.72006507	0.39310255	0.7214712	0.70858779	Calr	1
GO:0002253	activation of immune response	5/138	431/14464	0.01160093	1.21591177	0.446628	0.39345616	0.7214712	0.70858779	Card11/Ighg2c/Hspd1/Cr1l/Itch	5
GO:0006937	regulation of muscle contraction	2/138	142/14464	0.01408451	1.47621964	0.55970076	0.39365988	0.7214712	0.70858779	Pkp2/Sri	2
GO:0007009	plasma membrane organization	2/138	143/14464	0.01398601	1.46589642	0.54951175	0.39702444	0.72256087	0.70965799	Degs1/Snx18	2
GO:0042129	regulation of T cell proliferation	2/138	143/14464	0.01398601	1.46589642	0.54951175	0.39702444	0.72256087	0.70965799	Card11/Itch	2
GO:0001738	morphogenesis of a polarized epithelium	1/138	53/14464	0.01886792	1.97757725	0.69975857	0.39891381	0.72256087	0.70965799	Grhl3	1
GO:0002067	glandular epithelial cell differentiation	1/138	53/14464	0.01886792	1.97757725	0.69975857	0.39891381	0.72256087	0.70965799	Bmal1	1
GO:0003143	embryonic heart tube morphogenesis	1/138	53/14464	0.01886792	1.97757725	0.69975857	0.39891381	0.72256087	0.70965799	Ift172	1
GO:0033059	cellular pigmentation	1/138	53/14464	0.01886792	1.97757725	0.69975857	0.39891381	0.72256087	0.70965799	Rab38	1
GO:0042149	cellular response to glucose starvation	1/138	53/14464	0.01886792	1.97757725	0.69975857	0.39891381	0.72256087	0.70965799	Hspa5	1
GO:0045599	negative regulation of fat cell differentiation	1/138	53/14464	0.01886792	1.97757725	0.69975857	0.39891381	0.72256087	0.70965799	Bmal1	1
GO:0046578	regulation of Ras protein signal transduction	1/138	53/14464	0.01886792	1.97757725	0.69975857	0.39891381	0.72256087	0.70965799	Ppp2cb	1
GO:0060135	maternal process involved in female pregnancy	1/138	53/14464	0.01886792	1.97757725	0.69975857	0.39891381	0.72256087	0.70965799	Tppp3	1
GO:0070527	platelet aggregation	1/138	53/14464	0.01886792	1.97757725	0.69975857	0.39891381	0.72256087	0.70965799	Pdia3	1
GO:0170035	L-amino acid catabolic process	1/138	53/14464	0.01886792	1.97757725	0.69975857	0.39891381	0.72256087	0.70965799	Hmgcll1	1
GO:1901607	alpha-amino acid biosynthetic process	1/138	53/14464	0.01886792	1.97757725	0.69975857	0.39891381	0.72256087	0.70965799	Enoph1	1
GO:0016241	regulation of macroautophagy	2/138	144/14464	0.01388889	1.45571659	0.53939987	0.40038052	0.72456654	0.71162785	Snx18/Sec22b	2
GO:0002250	adaptive immune response	4/138	337/14464	0.01186944	1.24405453	0.44492122	0.40164728	0.72522947	0.71227895	Ighg2c/Pdia3/Hspd1/Cr1l	4
GO:0042113	B cell activation	3/138	240/14464	0.0125	1.31014493	0.47551661	0.40225152	0.72522947	0.71227895	Card11/Mmp14/Hspd1	3
GO:0022613	ribonucleoprotein complex biogenesis	5/138	436/14464	0.01146789	1.20196782	0.42027525	0.40294627	0.72522947	0.71227895	Mettl16/Srpk1/Prpf31/Tfb1m/Prpf18	5
GO:0002381	immunoglobulin production involved in immunoglobulin-mediated immune response	1/138	54/14464	0.01851852	1.94095545	0.67989244	0.40466982	0.72522947	0.71227895	Hspd1	1
GO:0016447	somatic recombination of immunoglobulin gene segments	1/138	54/14464	0.01851852	1.94095545	0.67989244	0.40466982	0.72522947	0.71227895	Hspd1	1
GO:0021575	hindbrain morphogenesis	1/138	54/14464	0.01851852	1.94095545	0.67989244	0.40466982	0.72522947	0.71227895	Hspa5	1
GO:0030042	actin filament depolymerization	1/138	54/14464	0.01851852	1.94095545	0.67989244	0.40466982	0.72522947	0.71227895	Scin	1
GO:0031663	lipopolysaccharide-mediated signaling pathway	1/138	54/14464	0.01851852	1.94095545	0.67989244	0.40466982	0.72522947	0.71227895	Sash1	1
GO:0035601	protein deacylation	1/138	54/14464	0.01851852	1.94095545	0.67989244	0.40466982	0.72522947	0.71227895	Brms1	1
GO:0090659	walking behavior	1/138	54/14464	0.01851852	1.94095545	0.67989244	0.40466982	0.72522947	0.71227895	Hexa	1
GO:0031669	cellular response to nutrient levels	3/138	241/14464	0.01244813	1.30470864	0.4681704	0.40481841	0.72522947	0.71227895	Hspa5/Pdia3/Wdr45b	3
GO:0000768	syncytium formation by plasma membrane fusion	1/138	55/14464	0.01818182	1.90566535	0.66044771	0.41037111	0.72522947	0.71227895	Sh3pxd2a	1
GO:0010907	positive regulation of glucose metabolic process	1/138	55/14464	0.01818182	1.90566535	0.66044771	0.41037111	0.72522947	0.71227895	Arpp19	1
GO:0030865	cortical cytoskeleton organization	1/138	55/14464	0.01818182	1.90566535	0.66044771	0.41037111	0.72522947	0.71227895	Calr	1
GO:0046605	regulation of centrosome cycle	1/138	55/14464	0.01818182	1.90566535	0.66044771	0.41037111	0.72522947	0.71227895	Chordc1	1
GO:0070542	response to fatty acid	1/138	55/14464	0.01818182	1.90566535	0.66044771	0.41037111	0.72522947	0.71227895	Pdk3	1
GO:0072527	pyrimidine-containing compound metabolic process	1/138	55/14464	0.01818182	1.90566535	0.66044771	0.41037111	0.72522947	0.71227895	Tbpl1	1
GO:0140253	cell-cell fusion	1/138	55/14464	0.01818182	1.90566535	0.66044771	0.41037111	0.72522947	0.71227895	Sh3pxd2a	1
GO:1901799	negative regulation of proteasomal protein catabolic process	1/138	55/14464	0.01818182	1.90566535	0.66044771	0.41037111	0.72522947	0.71227895	Bag5	1
GO:1905517	macrophage migration	1/138	55/14464	0.01818182	1.90566535	0.66044771	0.41037111	0.72522947	0.71227895	Mmp14	1
GO:2000059	negative regulation of ubiquitin-dependent protein catabolic process	1/138	55/14464	0.01818182	1.90566535	0.66044771	0.41037111	0.72522947	0.71227895	Bag5	1
GO:0060041	retina development in camera-type eye	2/138	147/14464	0.01360544	1.42600808	0.50951475	0.41039642	0.72522947	0.71227895	Rhoj/Slc17a8	2
GO:2001236	regulation of extrinsic apoptotic signaling pathway	2/138	147/14464	0.01360544	1.42600808	0.50951475	0.41039642	0.72522947	0.71227895	Pdia3/Gclm	2
GO:0035148	tube formation	2/138	148/14464	0.01351351	1.41637289	0.49969928	0.41371716	0.72522947	0.71227895	Grhl3/Ift172	2
GO:0045766	positive regulation of angiogenesis	2/138	148/14464	0.01351351	1.41637289	0.49969928	0.41371716	0.72522947	0.71227895	Sash1/Aggf1	2
GO:0060326	cell chemotaxis	3/138	245/14464	0.0122449	1.28340728	0.43910236	0.41505425	0.72522947	0.71227895	Calr/Arhgef16/Oxsr1	3
GO:0006497	protein lipidation	1/138	56/14464	0.01785714	1.87163561	0.64140655	0.41601819	0.72522947	0.71227895	Wdr45b	1
GO:0021536	diencephalon development	1/138	56/14464	0.01785714	1.87163561	0.64140655	0.41601819	0.72522947	0.71227895	Sema3e	1
GO:0038066	p38MAPK cascade	1/138	56/14464	0.01785714	1.87163561	0.64140655	0.41601819	0.72522947	0.71227895	Sash1	1
GO:0039532	negative regulation of cytoplasmic pattern recognition receptor signaling pathway	1/138	56/14464	0.01785714	1.87163561	0.64140655	0.41601819	0.72522947	0.71227895	Itch	1
GO:0043462	regulation of ATP-dependent activity	1/138	56/14464	0.01785714	1.87163561	0.64140655	0.41601819	0.72522947	0.71227895	Dnajb11	1
GO:0043536	positive regulation of blood vessel endothelial cell migration	1/138	56/14464	0.01785714	1.87163561	0.64140655	0.41601819	0.72522947	0.71227895	Rhoj	1
GO:0048332	mesoderm morphogenesis	1/138	56/14464	0.01785714	1.87163561	0.64140655	0.41601819	0.72522947	0.71227895	Smad2	1
GO:0050688	regulation of defense response to virus	1/138	56/14464	0.01785714	1.87163561	0.64140655	0.41601819	0.72522947	0.71227895	Itch	1
GO:0050854	regulation of antigen receptor-mediated signaling pathway	1/138	56/14464	0.01785714	1.87163561	0.64140655	0.41601819	0.72522947	0.71227895	Card11	1
GO:0051646	mitochondrion localization	1/138	56/14464	0.01785714	1.87163561	0.64140655	0.41601819	0.72522947	0.71227895	S2bpcox16	1
GO:0070830	bicellular tight junction assembly	1/138	56/14464	0.01785714	1.87163561	0.64140655	0.41601819	0.72522947	0.71227895	Pkp2	1
GO:0072348	sulfur compound transport	1/138	56/14464	0.01785714	1.87163561	0.64140655	0.41601819	0.72522947	0.71227895	Slc33a1	1
GO:2001238	positive regulation of extrinsic apoptotic signaling pathway	1/138	56/14464	0.01785714	1.87163561	0.64140655	0.41601819	0.72522947	0.71227895	Pdia3	1
GO:0008015	blood circulation	5/138	443/14464	0.01128668	1.1829751	0.38389339	0.41620664	0.72522947	0.71227895	Manf/Kcnip2/Gclm/Pkp2/Sri	5
GO:0042176	regulation of protein catabolic process	4/138	344/14464	0.01162791	1.21873947	0.40299094	0.41672213	0.72522947	0.71227895	Bmal1/Bag5/Itch/Sec22b	4
GO:0016331	morphogenesis of embryonic epithelium	2/138	149/14464	0.01342282	1.40686704	0.48995504	0.41702872	0.72522947	0.71227895	Grhl3/Ift172	2
GO:0046631	alpha-beta T cell activation	2/138	149/14464	0.01342282	1.40686704	0.48995504	0.41702872	0.72522947	0.71227895	Card11/Itch	2
GO:0050792	regulation of viral process	2/138	149/14464	0.01342282	1.40686704	0.48995504	0.41702872	0.72522947	0.71227895	Srpk1/P4hb	2
GO:0006941	striated muscle contraction	2/138	150/14464	0.01333333	1.39748792	0.48028091	0.42033099	0.72522947	0.71227895	Pkp2/Sri	2
GO:0048754	branching morphogenesis of an epithelial tube	2/138	150/14464	0.01333333	1.39748792	0.48028091	0.42033099	0.72522947	0.71227895	Mmp14/Sema3e	2
GO:0003229	ventricular cardiac muscle tissue development	1/138	57/14464	0.01754386	1.8387999	0.62275222	0.42161158	0.72522947	0.71227895	Pkp2	1

GO:0006473	protein acetylation	1/138	57/14464	0.01754386	1.8387999	0.62275222	0.42161158	0.72522947	0.71227895	Bmal1	1
GO:0006949	syncytium formation	1/138	57/14464	0.01754386	1.8387999	0.62275222	0.42161158	0.72522947	0.71227895	Sh3pxd2a	1
GO:0009064	glutamine family amino acid metabolic process	1/138	57/14464	0.01754386	1.8387999	0.62275222	0.42161158	0.72522947	0.71227895	Gclm	1
GO:0032623	interleukin-2 production	1/138	57/14464	0.01754386	1.8387999	0.62275222	0.42161158	0.72522947	0.71227895	Card11	1
GO:0042440	pigment metabolic process	1/138	57/14464	0.01754386	1.8387999	0.62275222	0.42161158	0.72522947	0.71227895	Rab38	1
GO:0045010	actin nucleation	1/138	57/14464	0.01754386	1.8387999	0.62275222	0.42161158	0.72522947	0.71227895	Scin	1
GO:0046635	positive regulation of alpha-beta T cell activation	1/138	57/14464	0.01754386	1.8387999	0.62275222	0.42161158	0.72522947	0.71227895	Card11	1
GO:0055008	cardiac muscle tissue morphogenesis	1/138	57/14464	0.01754386	1.8387999	0.62275222	0.42161158	0.72522947	0.71227895	Pkp2	1
GO:0071470	cellular response to osmotic stress	1/138	57/14464	0.01754386	1.8387999	0.62275222	0.42161158	0.72522947	0.71227895	Oxsr1	1
GO:0099024	plasma membrane invagination	1/138	57/14464	0.01754386	1.8387999	0.62275222	0.42161158	0.72522947	0.71227895	Snx18	1
GO:1902808	positive regulation of cell cycle G1/S phase transition	1/138	57/14464	0.01754386	1.8387999	0.62275222	0.42161158	0.72522947	0.71227895	Pkp3	1
GO:0007224	smoothened signaling pathway	2/138	151/14464	0.01324503	1.38823304	0.47067582	0.42362383	0.72707377	0.71409031	Ift172/Ift80	2
GO:1904018	positive regulation of vasculature development	2/138	151/14464	0.01324503	1.38823304	0.47067582	0.42362383	0.72707377	0.71409031	Sash1/Aggf1	2
GO:0034762	regulation of transmembrane transport	5/138	447/14464	0.01118568	1.1723892	0.36336453	0.42376571	0.72707377	0.71409031	Rrad/Slc17a8/Kcnip2/Sri/Oxsr1	5
GO:0070613	regulation of protein processing	1/138	58/14464	0.01724138	1.80709645	0.60446894	0.42715177	0.73226018	0.71918411	Mmp14	1
GO:0005996	monosaccharide metabolic process	3/138	250/14464	0.012	1.25773913	0.40345813	0.42777134	0.73245964	0.71938	Pdk3/Arpp19/Akr1a1	3
GO:0031333	negative regulation of protein-containing complex assembly	2/138	153/14464	0.0130719	1.3700862	0.45166858	0.43018076	0.73245964	0.71938	Hspa5/Scin	2
GO:0034767	positive regulation of monoatomic ion transmembrane transport	2/138	153/14464	0.0130719	1.3700862	0.45166858	0.43018076	0.73245964	0.71938	Kcnip2/Sri	2
GO:0016050	vesicle organization	4/138	351/14464	0.01139601	1.19443412	0.36192838	0.43171536	0.73245964	0.71938	Rab38/Tbpl1/Aktip/Sec22b	4
GO:0003170	heart valve development	1/138	59/14464	0.01694915	1.7764677	0.58654189	0.43263928	0.73245964	0.71938	Smad2	1
GO:0006278	RNA-templated DNA biosynthetic process	1/138	59/14464	0.01694915	1.7764677	0.58654189	0.43263928	0.73245964	0.71938	Hnrnpc	1
GO:0007004	telomere maintenance via telomerase	1/138	59/14464	0.01694915	1.7764677	0.58654189	0.43263928	0.73245964	0.71938	Hnrnpc	1
GO:0035176	social behavior	1/138	59/14464	0.01694915	1.7764677	0.58654189	0.43263928	0.73245964	0.71938	Gnb1l	1
GO:0042158	lipoprotein biosynthetic process	1/138	59/14464	0.01694915	1.7764677	0.58654189	0.43263928	0.73245964	0.71938	Wdr45b	1
GO:0043627	response to estrogen	1/138	59/14464	0.01694915	1.7764677	0.58654189	0.43263928	0.73245964	0.71938	Mmp14	1
GO:0098732	macromolecule deacylation	1/138	59/14464	0.01694915	1.7764677	0.58654189	0.43263928	0.73245964	0.71938	Brms1	1
GO:0098900	regulation of action potential	1/138	59/14464	0.01694915	1.7764677	0.58654189	0.43263928	0.73245964	0.71938	Pkp2	1
GO:0051223	regulation of protein transport	5/138	452/14464	0.01106195	1.15942029	0.33796289	0.43319112	0.73245964	0.71938	Bmal1/Frat2/Sri/Gapvd1/Ndfip2	5
GO:0006575	cellular modified amino acid metabolic process	2/138	154/14464	0.01298701	1.36118954	0.44226438	0.43344462	0.73245964	0.71938	Gclm/P4hb	2
GO:0035150	regulation of tube size	2/138	154/14464	0.01298701	1.36118954	0.44226438	0.43344462	0.73245964	0.71938	Manf/Gclm	2
GO:0035296	regulation of tube diameter	2/138	154/14464	0.01298701	1.36118954	0.44226438	0.43344462	0.73245964	0.71938	Manf/Gclm	2
GO:0097746	blood vessel diameter maintenance	2/138	154/14464	0.01298701	1.36118954	0.44226438	0.43344462	0.73245964	0.71938	Manf/Gclm	2
GO:0046651	lymphocyte proliferation	3/138	253/14464	0.01185771	1.24282523	0.38242653	0.43535628	0.73460294	0.72148503	Card11/Hspd1/Itch	3
GO:0001508	action potential	2/138	155/14464	0.01290323	1.35240767	0.43292515	0.43669859	0.73460294	0.72148503	Kcnip2/Pkp2	2
GO:0050777	negative regulation of immune response	2/138	155/14464	0.01290323	1.35240767	0.43292515	0.43669859	0.73460294	0.72148503	Dtx4/Cr1l	2
GO:0009880	embryonic pattern specification	1/138	60/14464	0.01666667	1.7468599	0.56895704	0.4380746	0.73460294	0.72148503	Smad2	1
GO:0016445	somatic diversification of immunoglobulins	1/138	60/14464	0.01666667	1.7468599	0.56895704	0.4380746	0.73460294	0.72148503	Hspd1	1
GO:0034331	cell junction maintenance	1/138	60/14464	0.01666667	1.7468599	0.56895704	0.4380746	0.73460294	0.72148503	Pkp2	1
GO:0046513	ceramide biosynthetic process	1/138	60/14464	0.01666667	1.7468599	0.56895704	0.4380746	0.73460294	0.72148503	Degs1	1
GO:0051703	biological process involved in intraspecies interaction between organisms	1/138	60/14464	0.01666667	1.7468599	0.56895704	0.4380746	0.73460294	0.72148503	Gnb1l	1
GO:0002822	regulation of adaptive immune response based on somatic recombination of immune receptors built from	2/138	156/14464	0.01282051	1.34373839	0.42364992	0.43994255	0.73460294	0.72148503	Hspd1/Cr1l	2
GO:0007254	JNK cascade	2/138	156/14464	0.01282051	1.34373839	0.42364992	0.43994255	0.73460294	0.72148503	Fktn/Itch	2
GO:0072330	monocarboxylic acid biosynthetic process	2/138	156/14464	0.01282051	1.34373839	0.42364992	0.43994255	0.73460294	0.72148503	Degs1/Elovl7	2
GO:0006935	chemotaxis	4/138	356/14464	0.01123596	1.17765836	0.33310733	0.44236464	0.73460294	0.72148503	Calr/Arhgef16/Sema3e/Oxsr1	4
GO:0007389	pattern specification process	4/138	356/14464	0.01123596	1.17765836	0.33310733	0.44236464	0.73460294	0.72148503	Dand5/Grhl3/Smad2/Ift172	4
GO:0030072	peptide hormone secretion	3/138	256/14464	0.01171875	1.22826087	0.36165231	0.44290472	0.73460294	0.72148503	Bmal1/Smad2/Sri	3
GO:0090150	establishment of protein localization to membrane	3/138	256/14464	0.01171875	1.22826087	0.36165231	0.44290472	0.73460294	0.72148503	Hspa5/Ubl4a/Srprb	3
GO:0051168	nuclear export	2/138	157/14464	0.01273885	1.33517954	0.41443775	0.44317642	0.73460294	0.72148503	Calr/Frat2	2
GO:0002224	toll-like receptor signaling pathway	1/138	61/14464	0.01639344	1.71822286	0.55170119	0.44345823	0.73460294	0.72148503	Hspd1	1
GO:0009247	glycolipid biosynthetic process	1/138	61/14464	0.01639344	1.71822286	0.55170119	0.44345823	0.73460294	0.72148503	Pigg	1
GO:0030888	regulation of B cell proliferation	1/138	61/14464	0.01639344	1.71822286	0.55170119	0.44345823	0.73460294	0.72148503	Card11	1
GO:0120192	tight junction assembly	1/138	61/14464	0.01639344	1.71822286	0.55170119	0.44345823	0.73460294	0.72148503	Pkp2	1
GO:1903053	regulation of extracellular matrix organization	1/138	61/14464	0.01639344	1.71822286	0.55170119	0.44345823	0.73460294	0.72148503	Has2	1
GO:1904356	regulation of telomere maintenance via telomere lengthening	1/138	61/14464	0.01639344	1.71822286	0.55170119	0.44345823	0.73460294	0.72148503	Hnrnpc	1
GO:1990868	response to chemokine	1/138	61/14464	0.01639344	1.71822286	0.55170119	0.44345823	0.73460294	0.72148503	Oxsr1	1
GO:1990869	cellular response to chemokine	1/138	61/14464	0.01639344	1.71822286	0.55170119	0.44345823	0.73460294	0.72148503	Oxsr1	1
GO:0042330	taxis	4/138	357/14464	0.01120448	1.1743596	0.32739263	0.4444878	0.73510042	0.72197363	Calr/Arhgef16/Sema3e/Oxsr1	4
GO:0071692	protein localization to extracellular region	4/138	357/14464	0.01120448	1.1743596	0.32739263	0.4444878	0.73510042	0.72197363	Bmal1/Dand5/Smad2/Sri	4
GO:0000271	polysaccharide biosynthetic process	1/138	62/14464	0.01612903	1.69050958	0.53476185	0.44879064	0.73526893	0.72213913	Has2	1
GO:0002562	somatic diversification of immune receptors via germline recombination within a single locus	1/138	62/14464	0.01612903	1.69050958	0.53476185	0.44879064	0.73526893	0.72213913	Hspd1	1
GO:0006801	superoxide metabolic process	1/138	62/14464	0.01612903	1.69050958	0.53476185	0.44879064	0.73526893	0.72213913	Sh3pxd2a	1
GO:0008652	amino acid biosynthetic process	1/138	62/14464	0.01612903	1.69050958	0.53476185	0.44879064	0.73526893	0.72213913	Enoph1	1
GO:0016444	somatic cell DNA recombination	1/138	62/14464	0.01612903	1.69050958	0.53476185	0.44879064	0.73526893	0.72213913	Hspd1	1
GO:0042130	negative regulation of T cell proliferation	1/138	62/14464	0.01612903	1.69050958	0.53476185	0.44879064	0.73526893	0.72213913	Itch	1
GO:0042551	neuron maturation	1/138	62/14464	0.01612903	1.69050958	0.53476185	0.44879064	0.73526893	0.72213913	Kcnip2	1
GO:0051893	regulation of focal adhesion assembly	1/138	62/14464	0.01612903	1.69050958	0.53476185	0.44879064	0.73526893	0.72213913	Mmp14	1
GO:0090109	regulation of cell-substrate junction assembly	1/138	62/14464	0.01612903	1.69050958	0.53476185	0.44879064	0.73526893	0.72213913	Mmp14	1
GO:1903317	regulation of protein maturation	1/138	62/14464	0.01612903	1.69050958	0.53476185	0.44879064	0.73526893	0.72213913	Mmp14	1
GO:0030041	actin filament polymerization	2/138	159/14464	0.01257862	1.31838483	0.3961989	0.44961343	0.73526893	0.72213913	Scin/Msrb2	2
GO:0008544	epidermis development	3/138	259/14464	0.01158301	1.21403391	0.34112858	0.45041468	0.73526893	0.72213913	Grhl3/Ift172/Pkp3	3
GO:0032943	mononuclear cell proliferation	3/138	259/14464	0.01158301	1.21403391	0.34112858	0.45041468	0.73526893	0.72213913	Card11/Hspd1/Itch	3
GO:0071407	cellular response to organic cyclic compound	5/138	462/14464	0.01082251	1.13432461	0.2879969	0.45194584	0.73526893	0.72213913	Bmal1/Calr/Hsp90b1/Pdia3/Gclm	5
GO:0071356	cellular response to tumor necrosis factor	2/138	160/14464	0.0125	1.31014493	0.38717043	0.45281637	0.73526893	0.72213913	Has2/Ppp2cb	2
GO:0051251	positive regulation of lymphocyte activation	3/138	260/14464	0.01153846	1.20936455	0.33434185	0.45290912	0.73526893	0.72213913	Card11/Mmp14/Hspd1	3
GO:0003208	cardiac ventricle morphogenesis	1/138	63/14464	0.01587302	1.6636761	0.5181272	0.45407233	0.73526893	0.72213913	Pkp2	1
GO:0006081	cellular aldehyde metabolic process	1/138	63/14464	0.01587302	1.6636761	0.5181272	0.45407233	0.73526893	0.72213913	Akr1a1	1
GO:0006919	activation of cysteine-type endopeptidase activity involved in apoptotic process	1/138	63/14464	0.01587302	1.6636761	0.5181272	0.45407233	0.73526893	0.72213913	Hspd1	1
GO:0007585	respiratory gaseous exchange by respiratory system	1/138	63/14464	0.01587302	1.6636761	0.5181272	0.45407233	0.73526893	0.72213913	Slc5a3	1
GO:0030837	negative regulation of actin filament polymerization	1/138	63/14464	0.01587302	1.6636761	0.5181272	0.45407233	0.73526893	0.72213913	Scin	1
GO:0051453	regulation of intracellular pH	1/138	63/14464	0.01587302	1.6636761	0.5181272	0.45407233	0.73526893	0.72213913	Rab38	1
GO:0061035	regulation of cartilage development	1/138	63/14464	0.01587302	1.6636761	0.5181272	0.45407233	0.73526893	0.72213913	Scin	1
GO:0097352	autophagosome maturation	1/138	63/14464	0.01587302	1.6636761	0.5181272	0.45407233	0.73526893	0.72213913	Snx14	1
GO:0170041	non-proteinogenic amino acid metabolic process	1/138	63/14464	0.01587302	1.6636761	0.5181272	0.45407233	0.73526893	0.72213913	P4hb	1
GO:1901657	glycosyl compound metabolic process	1/138	63/14464	0.01587302	1.6636761	0.5181272	0.45407233	0.73526893	0.72213913	Akr1a1	1
GO:0006469	negative regulation of protein kinase activity	2/138	161/14464	0.01242236	1.30200738	0.37820145	0.45600881	0.73781201	0.7246368	Chordc1/Dnajc3	2
GO:0007369	gastrulation	2/138	162/14464	0.01234568	1.2939703	0.3692911	0.45919066	0.73892953	0.72573436	Mmp14/Smad2	2
GO:0021915	neural tube development	2/138	162/14464	0.01234568	1.2939703	0.3692911	0.45919066	0.73892953	0.72573436	Grhl3/Ift172	2
GO:0001541	ovarian follicle development	1/138	64/14464	0.015625	1.63768116	0.50178606	0.45930377	0.73892953	0.72573436	Mmp14	1
GO:0003407	neural retina development	1/138	64/14464	0.015625	1.63768116	0.50178606	0.45930377	0.73892953	0.72573436	Slc17a8	1
GO:0050885	neuromuscular process controlling balance	1/138	64/14464	0.015625	1.63768116	0.50178606	0.45930377	0.73892953	0.72573436	Hexa	1
GO:0060411	cardiac septum morphogenesis	1/138	64/14464	0.015625	1.63768116	0.50178606	0.45930377	0.73892953	0.72573436	Dhrs3	1
GO:0007005	mitochondrion organization	5/138	466/14464	0.01072961	1.12458792	0.26831328	0.45940536	0.73892953	0.72573436	Rab38/Hspd1/Gclm/S2bpcox16/Ppp2cb	5
GO:0002460	adaptive immune response based on somatic recombination of immune receptors built from immunoglob	3/138	263/14464	0.01140684	1.19556952	0.31414188	0.46036483	0.73892953	0.72573436	Ighg2c/Hspd1/Cr1l	3
GO:0002790	peptide secretion	3/138	263/14464	0.01140684	1.19556952	0.31414188	0.46036483	0.73892953	0.72573436	Bmal1/Smad2/Sri	3
GO:0007162	negative regulation of cell adhesion	3/138	263/14464	0.01140684	1.19556952	0.31414188	0.46036483	0.73892953	0.72573436	Mmp14/Itch/Sema3e	3
GO:0010608	post-transcriptional regulation of gene expression	5/138	468/14464	0.01068376	1.11978199	0.25853464	0.46312492	0.74082489	0.72759587	Calr/Mettl16/Dnajc3/Hnrnpc/Mettl14	5
GO:0010324	membrane invagination	1/138	65/14464	0.01538462	1.61248606	0.48572783	0.46448545	0.74082489	0.72759587	Snx18	1
GO:0032007	negative regulation of TOR signaling	1/138	65/14464	0.01538462	1.61248606	0.48572783	0.46448545	0.74082489	0.72759587	Bmal1	1
GO:0032922	circadian regulation of gene expression	1/138	65/14464	0.01538462	1.61248606	0.48572783	0.46448545	0.74082489	0.72759587	Bmal1	1
GO:0043297	apical junction assembly	1/138	65/14464	0.01538462	1.61248606	0.48572783	0.46448545	0.74082489	0.72759587	Pkp2	1
GO:0051058	negative regulation of small GTPase mediated signal transduction	1/138	65/14464	0.01538462	1.61248606	0.48572783	0.46448545	0.74082489	0.72759587	Ppp2cb	1
GO:0061512	protein localization to cilium	1/138	65/14464	0.01538462	1.61248606	0.48572783	0.46448545	0.74082489	0.72759587	Ift80	1
GO:0140115	export across plasma membrane	1/138	65/14464	0.01538462	1.61248606	0.48572783	0.46448545	0.74082489	0.72759587	Kcnip2	1
GO:0002009	morphogenesis of an epithelium	5/138	470/14464	0.0106383	1.11501696	0.24879757	0.46683732	0.74398737	0.73070189	Mmp14/Grhl3/Stard13/Ift172/Sema3e	5
GO:0014911	positive regulation of smooth muscle cell migration	1/138	66/14464	0.01515152	1.58805446	0.46994247	0.46961782	0.74605951	0.73273702	Has2	1
GO:0031122	cytoplasmic microtubule organization	1/138	66/14464	0.01515152	1.58805446	0.46994247	0.46961782	0.74605951	0.73273702	Ift172	1
GO:0034248	regulation of amide metabolic process	1/138	66/14464	0.01515152	1.58805446	0.46994247	0.46961782	0.74605951	0.73273702	Pdk3	1
GO:0140962	multicellular organismal-level chemical homeostasis	1/138	66/14464	0.01515152	1.58805446	0.46994247	0.46961782	0.74605951	0.73273702	Has2	1
GO:0006766	vitamin metabolic process	1/138	67/14464	0.01492537	1.56435215	0.45442043	0.47470135	0.74894039	0.73556646	Akr1a1	1
GO:0033273	response to vitamin	1/138	67/14464	0.01492537	1.56435215	0.45442043	0.47470135	0.74894039	0.73556646	Pdia3	1
GO:0045123	cellular extravasation	1/138	67/14464	0.01492537	1.56435215	0.45442043	0.47470135	0.74894039	0.73556646	Cd99l2	1
GO:0060415	muscle tissue morphogenesis	1/138	67/14464	0.01492537	1.56435215	0.45442043	0.47470135	0.74894039	0.73556646	Pkp2	1
GO:0120193	tight junction organization	1/138	67/14464	0.01492537	1.56435215	0.45442043	0.47470135	0.74894039	0.73556646	Pkp2	1
GO:0150116	regulation of cell-substrate junction organization	1/138	67/14464	0.01492537	1.56435215	0.45442043	0.47470135	0.74894039	0.73556646	Mmp14	1
GO:2000036	regulation of stem cell population maintenance	1/138	67/14464	0.01492537	1.56435215	0.45442043	0.47470135	0.74894039	0.73556646	Brms1	1
GO:0045807	positive regulation of endocytosis	2/138	167/14464	0.01197605	1.25522867	0.32559033	0.474938	0.74894039	0.73556646	Calr/Lman2	2
GO:1903169	regulation of calcium ion transmembrane transport	2/138	167/14464	0.01197605	1.25522867	0.32559033	0.474938	0.74894039	0.73556646	Rrad/Sri	2
GO:0060070	canonical Wnt signaling pathway	3/138	269/14464	0.01115242	1.16890254	0.27444219	0.47514621	0.74894039	0.73556646	Bmal1/Fzd8/Frat2	3
GO:0006446	regulation of translational initiation	1/138	68/14464	0.01470588	1.54134697	0.43915266	0.47973651	0.75381825	0.74035721	Dnajc3	1
GO:0060395	SMAD protein signal transduction	1/138	68/14464	0.01470588	1.54134697	0.43915266	0.47973651	0.75381825	0.74035721	Smad2	1
GO:1901606	alpha-amino acid catabolic process	1/138	68/14464	0.01470588	1.54134697	0.43915266	0.47973651	0.75381825	0.74035721	Hmgcll1	1
GO:1902475	L-alpha-amino acid transmembrane transport	1/138	68/14464	0.01470588	1.54134697	0.43915266	0.47973651	0.75381825	0.74035721	Slc17a8	1
GO:0010959	regulation of metal ion transport	4/138	375/14464	0.01066667	1.11799034	0.22721043	0.48225799	0.75545283	0.7419626	Rrad/Kcnip2/Sri/Oxsr1	4
GO:0015833	peptide transport	3/138	272/14464	0.01102941	1.15601023	0.25493061	0.48246854	0.75545283	0.7419626	Bmal1/Smad2/Sri	3
GO:0002819	regulation of adaptive immune response	2/138	170/14464	0.01176471	1.23307758	0.30002285	0.48425397	0.75545283	0.7419626	Hspd1/Cr1l	2
GO:0030902	hindbrain development	2/138	170/14464	0.01176471	1.23307758	0.30002285	0.48425397	0.75545283	0.7419626	Hspa5/Fktn	2
GO:0002200	somatic diversification of immune receptors	1/138	69/14464	0.01449275	1.51900861	0.42413053	0.48472376	0.75545283	0.7419626	Hspd1	1
GO:0008333	endosome to lysosome transport	1/138	69/14464	0.01449275	1.51900861	0.42413053	0.48472376	0.75545283	0.7419626	Aktip	1
GO:0008347	glial cell migration	1/138	69/14464	0.01449275	1.51900861	0.42413053	0.48472376	0.75545283	0.7419626	Mmp14	1
GO:0008543	fibroblast growth factor receptor signaling pathway	1/138	69/14464	0.01449275	1.51900861	0.42413053	0.48472376	0.75545283	0.7419626	Ift80	1
GO:0030641	regulation of cellular pH	1/138	69/14464	0.01449275	1.51900861	0.42413053	0.48472376	0.75545283	0.7419626	Rab38	1
GO:2000243	positive regulation of reproductive process	1/138	69/14464	0.01449275	1.51900861	0.42413053	0.48472376	0.75545283	0.7419626	Lhfpl2	1
GO:0051258	protein polymerization	3/138	273/14464	0.01098901	1.15177576	0.24847546	0.48489879	0.75545283	0.7419626	Scin/Tppp3/Msrb2	3
GO:0001960	negative regulation of cytokine-mediated signaling pathway	1/138	70/14464	0.01428571	1.49730849	0.40934586	0.48966354	0.7593492	0.74578939	Ppp2cb	1
GO:0042698	ovulation cycle	1/138	70/14464	0.01428571	1.49730849	0.40934586	0.48966354	0.7593492	0.74578939	Has2	1
GO:0006066	alcohol metabolic process	3/138	275/14464	0.01090909	1.14339921	0.23563699	0.48974318	0.7593492	0.74578939	Dhrs3/Slc5a3/Akr1a1	3
GO:0031929	TOR signaling	2/138	172/14464	0.01162791	1.21873947	0.28323775	0.49040822	0.7593492	0.74578939	Bmal1/Card11	2
GO:0048736	appendage development	2/138	172/14464	0.01162791	1.21873947	0.28323775	0.49040822	0.7593492	0.74578939	Ift172/Ift80	2
GO:0060173	limb development	2/138	172/14464	0.01162791	1.21873947	0.28323775	0.49040822	0.7593492	0.74578939	Ift172/Ift80	2
GO:2001242	regulation of intrinsic apoptotic signaling pathway	2/138	172/14464	0.01162791	1.21873947	0.28323775	0.49040822	0.7593492	0.74578939	Bag5/P4hb	2
GO:2001233	regulation of apoptotic signaling pathway	4/138	379/14464	0.01055409	1.10619097	0.20560564	0.49052126	0.7593492	0.74578939	Pdia3/Gclm/Bag5/P4hb	4
GO:0016197	endosomal transport	3/138	276/14464	0.01086957	1.13925646	0.22925328	0.49215721	0.7593492	0.74578939	Rab9b/Snx18/Aktip	3
GO:0030198	extracellular matrix organization	3/138	276/14464	0.01086957	1.13925646	0.22925328	0.49215721	0.7593492	0.74578939	Mmp14/Has2/Fktn	3
GO:0043062	extracellular structure organization	3/138	276/14464	0.01086957	1.13925646	0.22925328	0.49215721	0.7593492	0.74578939	Mmp14/Has2/Fktn	3
GO:0045229	external encapsulating structure organization	3/138	276/14464	0.01086957	1.13925646	0.22925328	0.49215721	0.7593492	0.74578939	Mmp14/Has2/Fktn	3
GO:0033673	negative regulation of kinase activity	2/138	173/14464	0.01156069	1.21169473	0.27492109	0.49346822	0.7593492	0.74578939	Chordc1/Dnajc3	2
GO:0043393	regulation of protein binding	2/138	173/14464	0.01156069	1.21169473	0.27492109	0.49346822	0.7593492	0.74578939	Fktn/Aktip	2
GO:0034329	cell junction assembly	5/138	485/14464	0.01030928	1.0805319	0.17705452	0.49442538	0.7593492	0.74578939	Mmp14/Lrrn1/Pkp3/Pkp2/Cdh10	5
GO:0003281	ventricular septum development	1/138	71/14464	0.01408451	1.47621964	0.39479085	0.4945563	0.7593492	0.74578939	Dand5	1
GO:0071347	cellular response to interleukin-1	1/138	71/14464	0.01408451	1.47621964	0.39479085	0.4945563	0.7593492	0.74578939	Has2	1
GO:1903051	negative regulation of proteolysis involved in protein catabolic process	1/138	71/14464	0.01408451	1.47621964	0.39479085	0.4945563	0.7593492	0.74578939	Bag5	1
GO:2000379	positive regulation of reactive oxygen species metabolic process	1/138	71/14464	0.01408451	1.47621964	0.39479085	0.4945563	0.7593492	0.74578939	Slc5a3	1
GO:0043484	regulation of RNA splicing	2/138	174/14464	0.01149425	1.20473097	0.26665413	0.49651672	0.76060574	0.74702349	Mettl16/Obi1	2
GO:0003151	outflow tract morphogenesis	1/138	72/14464	0.01388889	1.45571659	0.38045804	0.49940249	0.76060574	0.74702349	Dhrs3	1
GO:0007032	endosome organization	1/138	72/14464	0.01388889	1.45571659	0.38045804	0.49940249	0.76060574	0.74702349	Aktip	1
GO:0010833	telomere maintenance via telomere lengthening	1/138	72/14464	0.01388889	1.45571659	0.38045804	0.49940249	0.76060574	0.74702349	Hnrnpc	1
GO:0014032	neural crest cell development	1/138	72/14464	0.01388889	1.45571659	0.38045804	0.49940249	0.76060574	0.74702349	Sema3e	1
GO:0015800	acidic amino acid transport	1/138	72/14464	0.01388889	1.45571659	0.38045804	0.49940249	0.76060574	0.74702349	Slc17a8	1
GO:0019730	antimicrobial humoral response	1/138	72/14464	0.01388889	1.45571659	0.38045804	0.49940249	0.76060574	0.74702349	Ighg2c	1
GO:0051149	positive regulation of muscle cell differentiation	1/138	72/14464	0.01388889	1.45571659	0.38045804	0.49940249	0.76060574	0.74702349	Mmp14	1
GO:0021953	central nervous system neuron differentiation	2/138	175/14464	0.01142857	1.19784679	0.25843622	0.49955366	0.76060574	0.74702349	Ift172/Sema3e	2
GO:0046777	protein autophosphorylation	2/138	175/14464	0.01142857	1.19784679	0.25843622	0.49955366	0.76060574	0.74702349	Stk16/Oxsr1	2
GO:0050920	regulation of chemotaxis	2/138	175/14464	0.01142857	1.19784679	0.25843622	0.49955366	0.76060574	0.74702349	Calr/Oxsr1	2
GO:0050954	sensory perception of mechanical stimulus	2/138	176/14464	0.01136364	1.19104084	0.25026672	0.50257898	0.76060574	0.74702349	Slc17a8/Hexa	2
GO:0016055	Wnt signaling pathway	4/138	385/14464	0.01038961	1.08895163	0.17362195	0.50281582	0.76060574	0.74702349	Bmal1/Fzd8/Frat2/Ift80	4
GO:0006936	muscle contraction	3/138	281/14464	0.01067616	1.11898499	0.1976822	0.50414356	0.76060574	0.74702349	Kcnip2/Pkp2/Sri	3

GO:0032092	positive regulation of protein binding	1/138	73/14464	0.01369863	1.43577526	0.36634037	0.50420255	0.76060574	0.74702349	Aktip	1
GO:0032481	positive regulation of type I interferon production	1/138	73/14464	0.01369863	1.43577526	0.36634037	0.50420255	0.76060574	0.74702349	Hspd1	1
GO:0035050	embryonic heart tube development	1/138	73/14464	0.01369863	1.43577526	0.36634037	0.50420255	0.76060574	0.74702349	Ift172	1
GO:0045665	negative regulation of neuron differentiation	1/138	73/14464	0.01369863	1.43577526	0.36634037	0.50420255	0.76060574	0.74702349	Calr	1
GO:0048644	muscle organ morphogenesis	1/138	73/14464	0.01369863	1.43577526	0.36634037	0.50420255	0.76060574	0.74702349	Pkp2	1
GO:1902476	chloride transmembrane transport	1/138	73/14464	0.01369863	1.43577526	0.36634037	0.50420255	0.76060574	0.74702349	Slc17a8	1
GO:0006352	DNA-templated transcription initiation	2/138	177/14464	0.01129944	1.1843118	0.242145	0.50559263	0.76060574	0.74702349	Tbpl1/Tfb1m	2
GO:0010970	transport along microtubule	2/138	177/14464	0.01129944	1.1843118	0.242145	0.50559263	0.76060574	0.74702349	Ift172/Ift80	2
GO:0061138	morphogenesis of a branching epithelium	2/138	177/14464	0.01129944	1.1843118	0.242145	0.50559263	0.76060574	0.74702349	Mmp14/Sema3e	2
GO:0001764	neuron migration	2/138	178/14464	0.01123596	1.17765836	0.23407045	0.50859456	0.76060574	0.74702349	Fktn/Sema3e	2
GO:0045619	regulation of lymphocyte differentiation	2/138	178/14464	0.01123596	1.17765836	0.23407045	0.50859456	0.76060574	0.74702349	Card11/Mmp14	2
GO:0002696	positive regulation of leukocyte activation	3/138	283/14464	0.01060071	1.11107697	0.18521241	0.50889806	0.76060574	0.74702349	Card11/Mmp14/Hspd1	3
GO:0043523	regulation of neuron apoptotic process	3/138	283/14464	0.01060071	1.11107697	0.18521241	0.50889806	0.76060574	0.74702349	Hspd1/Gclm/Sema3e	3
GO:0031329	regulation of cellular catabolic process	4/138	388/14464	0.01030928	1.0805319	0.15781578	0.50891571	0.76060574	0.74702349	Snx18/Itch/Rmc1/Sec22b	4
GO:0002312	B cell activation involved in immune response	1/138	74/14464	0.01351351	1.41637289	0.35243106	0.50895691	0.76060574	0.74702349	Hspd1	1
GO:0006690	icosanoid metabolic process	1/138	74/14464	0.01351351	1.41637289	0.35243106	0.50895691	0.76060574	0.74702349	Lta4h	1
GO:0014823	response to activity	1/138	74/14464	0.01351351	1.41637289	0.35243106	0.50895691	0.76060574	0.74702349	Gclm	1
GO:0042102	positive regulation of T cell proliferation	1/138	74/14464	0.01351351	1.41637289	0.35243106	0.50895691	0.76060574	0.74702349	Card11	1
GO:0045069	regulation of viral genome replication	1/138	74/14464	0.01351351	1.41637289	0.35243106	0.50895691	0.76060574	0.74702349	Srpk1	1
GO:0051279	regulation of release of sequestered calcium ion into cytosol	1/138	74/14464	0.01351351	1.41637289	0.35243106	0.50895691	0.76060574	0.74702349	Sri	1
GO:0051298	centrosome duplication	1/138	74/14464	0.01351351	1.41637289	0.35243106	0.50895691	0.76060574	0.74702349	Chordc1	1
GO:0060761	negative regulation of response to cytokine stimulus	1/138	74/14464	0.01351351	1.41637289	0.35243106	0.50895691	0.76060574	0.74702349	Ppp2cb	1
GO:0019827	stem cell population maintenance	2/138	179/14464	0.01117318	1.17107926	0.22604246	0.5115847	0.76283636	0.74921428	Brms1/Mettl14	2
GO:0030595	leukocyte chemotaxis	2/138	179/14464	0.01117318	1.17107926	0.22604246	0.5115847	0.76283636	0.74921428	Calr/Oxsr1	2
GO:0050796	regulation of insulin secretion	2/138	179/14464	0.01117318	1.17107926	0.22604246	0.5115847	0.76283636	0.74921428	Bmal1/Sri	2
GO:0002088	lens development in camera-type eye	1/138	75/14464	0.01333333	1.39748792	0.33872365	0.51366601	0.76368044	0.75004329	Spred3	1
GO:0002709	regulation of T cell mediated immunity	1/138	75/14464	0.01333333	1.39748792	0.33872365	0.51366601	0.76368044	0.75004329	Hspd1	1
GO:0006626	protein targeting to mitochondrion	1/138	75/14464	0.01333333	1.39748792	0.33872365	0.51366601	0.76368044	0.75004329	Hspd1	1
GO:0050672	negative regulation of lymphocyte proliferation	1/138	75/14464	0.01333333	1.39748792	0.33872365	0.51366601	0.76368044	0.75004329	Itch	1
GO:0034612	response to tumor necrosis factor	2/138	180/14464	0.01111111	1.16457327	0.21806044	0.51456302	0.76388737	0.75024652	Has2/Ppp2cb	2
GO:0035265	organ growth	2/138	180/14464	0.01111111	1.16457327	0.21806044	0.51456302	0.76388737	0.75024652	Smad2/Ift80	2
GO:0048839	inner ear development	2/138	181/14464	0.01104972	1.15813916	0.21012381	0.51752946	0.76772582	0.75401643	Grhl3/Slc17a8	2
GO:0032945	negative regulation of mononuclear cell proliferation	1/138	76/14464	0.01315789	1.37909992	0.32521198	0.51833028	0.76779282	0.75408223	Itch	1
GO:0070661	leukocyte proliferation	3/138	287/14464	0.01045296	1.09559158	0.16053682	0.51833632	0.76779282	0.75408223	Card11/Hspd1/Itch	3
GO:0071396	cellular response to lipid	5/138	499/14464	0.01002004	1.05021637	0.11204215	0.51970414	0.76855635	0.75483213	Bmal1/Calr/Pdia3/Sash1/Pdk3	5
GO:1901888	regulation of cell junction assembly	3/138	288/14464	0.01041667	1.09178744	0.15442191	0.52068088	0.76855635	0.75483213	Mmp14/Lrrn1/Pkp2	3
GO:0032890	regulation of organic acid transport	1/138	77/14464	0.01298701	1.36118954	0.31189015	0.52295013	0.76855635	0.75483213	Slc17a8	1
GO:0033559	unsaturated fatty acid metabolic process	1/138	77/14464	0.01298701	1.36118954	0.31189015	0.52295013	0.76855635	0.75483213	Elovl7	1
GO:0043112	receptor metabolic process	1/138	77/14464	0.01298701	1.36118954	0.31189015	0.52295013	0.76855635	0.75483213	Itch	1
GO:0061178	regulation of insulin secretion involved in cellular response to glucose stimulus	1/138	77/14464	0.01298701	1.36118954	0.31189015	0.52295013	0.76855635	0.75483213	Sri	1
GO:1901880	negative regulation of protein depolymerization	1/138	77/14464	0.01298701	1.36118954	0.31189015	0.52295013	0.76855635	0.75483213	Scin	1
GO:0002520	immune system development	2/138	183/14464	0.01092896	1.1454819	0.19438443	0.52342652	0.76855635	0.75483213	Hspd1/Itch	2
GO:0008154	actin polymerization or depolymerization	2/138	183/14464	0.01092896	1.1454819	0.19438443	0.52342652	0.76855635	0.75483213	Scin/Msrb2	2
GO:0009267	cellular response to starvation	2/138	183/14464	0.01092896	1.1454819	0.19438443	0.52342652	0.76855635	0.75483213	Hspa5/Wdr45b	2
GO:0042180	cellular ketone metabolic process	2/138	183/14464	0.01092896	1.1454819	0.19438443	0.52342652	0.76855635	0.75483213	Pdk3/Akr1a1	2
GO:0071383	cellular response to steroid hormone stimulus	2/138	183/14464	0.01092896	1.1454819	0.19438443	0.52342652	0.76855635	0.75483213	Bmal1/Calr	2
GO:0007519	skeletal muscle tissue development	2/138	184/14464	0.01086957	1.13925646	0.18658057	0.52635706	0.77064666	0.75688511	Bmal1/Fktn	2
GO:0007422	peripheral nervous system development	1/138	78/14464	0.01282051	1.34373839	0.29875252	0.52752599	0.77064666	0.75688511	Slc5a3	1
GO:0031341	regulation of cell killing	1/138	78/14464	0.01282051	1.34373839	0.29875252	0.52752599	0.77064666	0.75688511	Cr1l	1
GO:0032649	regulation of type II interferon production	1/138	78/14464	0.01282051	1.34373839	0.29875252	0.52752599	0.77064666	0.75688511	Hspd1	1
GO:0035023	regulation of Rho protein signal transduction	1/138	78/14464	0.01282051	1.34373839	0.29875252	0.52752599	0.77064666	0.75688511	Stard13	1
GO:0045995	regulation of embryonic development	1/138	78/14464	0.01282051	1.34373839	0.29875252	0.52752599	0.77064666	0.75688511	Hspa5	1
GO:0048864	stem cell development	1/138	78/14464	0.01282051	1.34373839	0.29875252	0.52752599	0.77064666	0.75688511	Sema3e	1
GO:0042098	T cell proliferation	2/138	185/14464	0.01081081	1.13309832	0.17881988	0.52927556	0.77152532	0.75774808	Card11/Itch	2
GO:0045664	regulation of neuron differentiation	2/138	185/14464	0.01081081	1.13309832	0.17881988	0.52927556	0.77152532	0.75774808	Calr/Mettl14	2
GO:0098727	maintenance of cell number	2/138	185/14464	0.01081081	1.13309832	0.17881988	0.52927556	0.77152532	0.75774808	Brms1/Mettl14	2
GO:0007405	neuroblast proliferation	1/138	79/14464	0.01265823	1.32672904	0.28579367	0.53205827	0.77185528	0.75807215	Tafa1	1
GO:0009063	amino acid catabolic process	1/138	79/14464	0.01265823	1.32672904	0.28579367	0.53205827	0.77185528	0.75807215	Hmgcll1	1
GO:0031397	negative regulation of protein ubiquitination	1/138	79/14464	0.01265823	1.32672904	0.28579367	0.53205827	0.77185528	0.75807215	Bag5	1
GO:0032272	negative regulation of protein polymerization	1/138	79/14464	0.01265823	1.32672904	0.28579367	0.53205827	0.77185528	0.75807215	Scin	1
GO:0051965	positive regulation of synapse assembly	1/138	79/14464	0.01265823	1.32672904	0.28579367	0.53205827	0.77185528	0.75807215	Lrrn1	1
GO:0097009	energy homeostasis	1/138	79/14464	0.01265823	1.32672904	0.28579367	0.53205827	0.77185528	0.75807215	Bmal1	1
GO:0051216	cartilage development	2/138	186/14464	0.01075269	1.12700639	0.17110181	0.53218196	0.77185528	0.75807215	Scin/Ift80	2
GO:0050679	positive regulation of epithelial cell proliferation	2/138	187/14464	0.01069519	1.12097962	0.16342586	0.53507624	0.77549512	0.76164699	Has2/Aggf1	2
GO:0006885	regulation of pH	1/138	80/14464	0.0125	1.31014493	0.27300846	0.53654739	0.77593606	0.76208006	Rab38	1
GO:0010507	negative regulation of autophagy	1/138	80/14464	0.0125	1.31014493	0.27300846	0.53654739	0.77593606	0.76208006	Sec22b	1
GO:0071230	cellular response to amino acid stimulus	1/138	80/14464	0.0125	1.31014493	0.27300846	0.53654739	0.77593606	0.76208006	Gclm	1
GO:0010506	regulation of autophagy	3/138	295/14464	0.01016949	1.06588062	0.11220323	0.53692004	0.77593606	0.76208006	Snx18/Rmc1/Sec22b	3
GO:0050870	positive regulation of T cell activation	2/138	188/14464	0.0106383	1.11501696	0.15579152	0.53795837	0.77687971	0.76300685	Card11/Hspd1	2
GO:0044282	small molecule catabolic process	3/138	296/14464	0.01013514	1.06227967	0.10625372	0.53921476	0.77813669	0.7642414	Hexa/Hmgcll1/Akr1a1	3
GO:0007601	visual perception	1/138	81/14464	0.01234568	1.2939703	0.26039191	0.54099375	0.77847494	0.76457361	Rbp1	1
GO:0045824	negative regulation of innate immune response	1/138	81/14464	0.01234568	1.2939703	0.26039191	0.54099375	0.77847494	0.76457361	Dtx4	1
GO:0070664	negative regulation of leukocyte proliferation	1/138	81/14464	0.01234568	1.2939703	0.26039191	0.54099375	0.77847494	0.76457361	Itch	1
GO:1904427	positive regulation of calcium ion transmembrane transport	1/138	81/14464	0.01234568	1.2939703	0.26039191	0.54099375	0.77847494	0.76457361	Sri	1
GO:0050863	regulation of T cell activation	3/138	297/14464	0.01010101	1.05870297	0.10032418	0.54150308	0.77865207	0.76474757	Card11/Hspd1/Itch	3
GO:0006029	proteoglycan metabolic process	1/138	82/14464	0.01219512	1.27819017	0.24793928	0.54539776	0.78257785	0.76860325	Hexa	1
GO:0006576	biogenic amine metabolic process	1/138	82/14464	0.01219512	1.27819017	0.24793928	0.54539776	0.78257785	0.76860325	Sat1	1
GO:0033209	tumor necrosis factor-mediated signaling pathway	1/138	82/14464	0.01219512	1.27819017	0.24793928	0.54539776	0.78257785	0.76860325	Ppp2cb	1
GO:0032271	regulation of protein polymerization	2/138	192/14464	0.01041667	1.09178744	0.12566019	0.54936465	0.78548249	0.77145601	Scin/Tppp3	2
GO:0045732	positive regulation of protein catabolic process	2/138	192/14464	0.01041667	1.09178744	0.12566019	0.54936465	0.78548249	0.77145601	Itch/Sec22b	2
GO:0050670	regulation of lymphocyte proliferation	2/138	192/14464	0.01041667	1.09178744	0.12566019	0.54936465	0.78548249	0.77145601	Card11/Itch	2
GO:0014033	neural crest cell differentiation	1/138	83/14464	0.01204819	1.26279029	0.23564601	0.54975981	0.78548249	0.77145601	Sema3e	1
GO:0043648	dicarboxylic acid metabolic process	1/138	83/14464	0.01204819	1.26279029	0.23564601	0.54975981	0.78548249	0.77145601	Gclm	1
GO:0050953	sensory perception of light stimulus	1/138	83/14464	0.01204819	1.26279029	0.23564601	0.54975981	0.78548249	0.77145601	Rbp1	1
GO:0051249	regulation of lymphocyte activation	4/138	409/14464	0.00977995	1.02505227	0.05044284	0.55064307	0.78618727	0.77214822	Card11/Mmp14/Hspd1/Itch	4
GO:0005977	glycogen metabolic process	1/138	84/14464	0.01190476	1.24775707	0.22350772	0.55408032	0.78641539	0.77237225	Wdr45b	1
GO:0006094	gluconeogenesis	1/138	84/14464	0.01190476	1.24775707	0.22350772	0.55408032	0.78641539	0.77237225	Arpp19	1
GO:0016579	protein deubiquitination	1/138	84/14464	0.01190476	1.24775707	0.22350772	0.55408032	0.78641539	0.77237225	Itch	1
GO:0018107	peptidyl-threonine phosphorylation	1/138	84/14464	0.01190476	1.24775707	0.22350772	0.55408032	0.78641539	0.77237225	Oxsr1	1
GO:0042157	lipoprotein metabolic process	1/138	84/14464	0.01190476	1.24775707	0.22350772	0.55408032	0.78641539	0.77237225	Wdr45b	1
GO:0071901	negative regulation of protein serine/threonine kinase activity	1/138	84/14464	0.01190476	1.24775707	0.22350772	0.55408032	0.78641539	0.77237225	Chordc1	1
GO:0001763	morphogenesis of a branching structure	2/138	194/14464	0.01030928	1.0805319	0.11083147	0.55499407	0.78641539	0.77237225	Mmp14/Sema3e	2
GO:0006486	protein glycosylation	2/138	194/14464	0.01030928	1.0805319	0.11083147	0.55499407	0.78641539	0.77237225	Fktn/Crppa	2
GO:0006909	phagocytosis	2/138	194/14464	0.01030928	1.0805319	0.11083147	0.55499407	0.78641539	0.77237225	Calr/Lman2	2
GO:0043413	macromolecule glycosylation	2/138	194/14464	0.01030928	1.0805319	0.11083147	0.55499407	0.78641539	0.77237225	Fktn/Crppa	2
GO:0050867	positive regulation of cell activation	3/138	303/14464	0.00990099	1.03773856	0.06515772	0.55509654	0.78641539	0.77237225	Card11/Mmp14/Hspd1	3
GO:0003073	regulation of systemic arterial blood pressure	1/138	85/14464	0.01176471	1.23307758	0.21152022	0.55835966	0.78641539	0.77237225	Manf	1
GO:0019915	lipid storage	1/138	85/14464	0.01176471	1.23307758	0.21152022	0.55835966	0.78641539	0.77237225	Hexa	1
GO:0044042	glucan metabolic process	1/138	85/14464	0.01176471	1.23307758	0.21152022	0.55835966	0.78641539	0.77237225	Wdr45b	1
GO:0048041	focal adhesion assembly	1/138	85/14464	0.01176471	1.23307758	0.21152022	0.55835966	0.78641539	0.77237225	Mmp14	1
GO:0014706	striated muscle tissue development	4/138	414/14464	0.00966184	1.01267241	0.025676	0.56030805	0.78641539	0.77237225	Bmal1/Calr/Fktn/Pkp2	4
GO:0032944	regulation of mononuclear cell proliferation	2/138	196/14464	0.01020408	1.06950606	0.09615579	0.56057408	0.78641539	0.77237225	Card11/Itch	2
GO:0097529	myeloid leukocyte migration	2/138	196/14464	0.01020408	1.06950606	0.09615579	0.56057408	0.78641539	0.77237225	Mmp14/Cd99l2	2
GO:0051051	negative regulation of transport	4/138	415/14464	0.00963855	1.01023223	0.02075764	0.56222782	0.78641539	0.77237225	Rrad/Sri/Ndfip2/Oxsr1	4
GO:0001570	vasculogenesis	1/138	86/14464	0.01162791	1.21873947	0.19967946	0.56259823	0.78641539	0.77237225	Has2	1
GO:0001938	positive regulation of endothelial cell proliferation	1/138	86/14464	0.01162791	1.21873947	0.19967946	0.56259823	0.78641539	0.77237225	Aggf1	1
GO:0006821	chloride transport	1/138	86/14464	0.01162791	1.21873947	0.19967946	0.56259823	0.78641539	0.77237225	Slc17a8	1
GO:0030168	platelet activation	1/138	86/14464	0.01162791	1.21873947	0.19967946	0.56259823	0.78641539	0.77237225	Pdia3	1
GO:0032273	positive regulation of protein polymerization	1/138	86/14464	0.01162791	1.21873947	0.19967946	0.56259823	0.78641539	0.77237225	Tppp3	1
GO:0032609	type II interferon production	1/138	86/14464	0.01162791	1.21873947	0.19967946	0.56259823	0.78641539	0.77237225	Hspd1	1
GO:0043242	negative regulation of protein-containing complex disassembly	1/138	86/14464	0.01162791	1.21873947	0.19967946	0.56259823	0.78641539	0.77237225	Scin	1
GO:2001237	negative regulation of extrinsic apoptotic signaling pathway	1/138	86/14464	0.01162791	1.21873947	0.19967946	0.56259823	0.78641539	0.77237225	Gclm	1
GO:0002703	regulation of leukocyte mediated immunity	2/138	197/14464	0.01015228	1.0640771	0.08887424	0.5633455	0.78641539	0.77237225	Hspd1/Cr1l	2
GO:0006006	glucose metabolic process	2/138	197/14464	0.01015228	1.0640771	0.08887424	0.5633455	0.78641539	0.77237225	Pdk3/Arpp19	2
GO:0060538	skeletal muscle organ development	2/138	197/14464	0.01015228	1.0640771	0.08887424	0.5633455	0.78641539	0.77237225	Bmal1/Fktn	2
GO:1901615	organic hydroxy compound metabolic process	4/138	417/14464	0.00959233	1.00538699	0.01095542	0.56605395	0.78641539	0.77237225	Dhrs3/Slc5a3/Rab38/Akr1a1	4
GO:0030705	cytoskeleton-dependent intracellular transport	2/138	198/14464	0.01010101	1.05870297	0.08162963	0.56610451	0.78641539	0.77237225	Ift172/Ift80	2
GO:0000281	mitotic cytokinesis	1/138	87/14464	0.01149425	1.20473097	0.18798158	0.56679641	0.78641539	0.77237225	Snx18	1
GO:0006661	phosphatidylinositol biosynthetic process	1/138	87/14464	0.01149425	1.20473097	0.18798158	0.56679641	0.78641539	0.77237225	Pigg	1
GO:0009408	response to heat	1/138	87/14464	0.01149425	1.20473097	0.18798158	0.56679641	0.78641539	0.77237225	Chordc1	1
GO:0015807	L-amino acid transport	1/138	87/14464	0.01149425	1.20473097	0.18798158	0.56679641	0.78641539	0.77237225	Slc17a8	1
GO:0032755	positive regulation of interleukin-6 production	1/138	87/14464	0.01149425	1.20473097	0.18798158	0.56679641	0.78641539	0.77237225	Hspd1	1
GO:0035773	insulin secretion involved in cellular response to glucose stimulus	1/138	87/14464	0.01149425	1.20473097	0.18798158	0.56679641	0.78641539	0.77237225	Sri	1
GO:0051881	regulation of mitochondrial membrane potential	1/138	87/14464	0.01149425	1.20473097	0.18798158	0.56679641	0.78641539	0.77237225	Gclm	1
GO:0090288	negative regulation of cellular response to growth factor stimulus	1/138	87/14464	0.01149425	1.20473097	0.18798158	0.56679641	0.78641539	0.77237225	Dand5	1
GO:1902117	positive regulation of organelle assembly	1/138	87/14464	0.01149425	1.20473097	0.18798158	0.56679641	0.78641539	0.77237225	Snx18	1
GO:0006835	dicarboxylic acid transport	1/138	88/14464	0.01136364	1.19104084	0.17642283	0.57095458	0.78748612	0.77342387	Slc17a8	1
GO:0019319	hexose biosynthetic process	1/138	88/14464	0.01136364	1.19104084	0.17642283	0.57095458	0.78748612	0.77342387	Arpp19	1
GO:0043543	protein acylation	1/138	88/14464	0.01136364	1.19104084	0.17642283	0.57095458	0.78748612	0.77342387	Bmal1	1
GO:0051153	regulation of striated muscle cell differentiation	1/138	88/14464	0.01136364	1.19104084	0.17642283	0.57095458	0.78748612	0.77342387	Mmp14	1
GO:0120254	olefinic compound metabolic process	1/138	88/14464	0.01136364	1.19104084	0.17642283	0.57095458	0.78748612	0.77342387	Dhrs3	1
GO:1990748	cellular detoxification	1/138	88/14464	0.01136364	1.19104084	0.17642283	0.57095458	0.78748612	0.77342387	Akr1a1	1
GO:0007264	small GTPase-mediated signal transduction	4/138	420/14464	0.00952381	0.99820566	-0.0036626	0.57175924	0.78748612	0.77342387	Rhoj/Stard13/Rab9b/Ppp2cb	4
GO:0010038	response to metal ion	3/138	311/14464	0.0096463	1.01104432	0.01932418	0.57284834	0.78748612	0.77342387	Calr/Lta4h/Ppp2cb	3
GO:0051146	striated muscle cell differentiation	3/138	311/14464	0.0096463	1.01104432	0.01932418	0.57284834	0.78748612	0.77342387	Calr/Mmp14/Fktn	3
GO:0048568	embryonic organ development	4/138	421/14464	0.00950119	0.99583462	-0.0085128	0.57365186	0.78748612	0.77342387	Mmp14/Grhl3/Smad2/Ift172	4
GO:0007156	homophilic cell adhesion via plasma membrane adhesion molecules	1/138	89/14464	0.01123596	1.17765836	0.16499964	0.57507313	0.78748612	0.77342387	Cdh10	1
GO:0019217	regulation of fatty acid metabolic process	1/138	89/14464	0.01123596	1.17765836	0.16499964	0.57507313	0.78748612	0.77342387	Pdk3	1
GO:0021954	central nervous system neuron development	1/138	89/14464	0.01123596	1.17765836	0.16499964	0.57507313	0.78748612	0.77342387	Sema3e	1
GO:0042472	inner ear morphogenesis	1/138	89/14464	0.01123596	1.17765836	0.16499964	0.57507313	0.78748612	0.77342387	Grhl3	1
GO:0098661	inorganic anion transmembrane transport	1/138	89/14464	0.01123596	1.17765836	0.16499964	0.57507313	0.78748612	0.77342387	Slc17a8	1
GO:1903321	negative regulation of protein modification by small protein conjugation or removal	1/138	89/14464	0.01123596	1.17765836	0.16499964	0.57507313	0.78748612	0.77342387	Bag5	1
GO:2001251	negative regulation of chromosome organization	1/138	89/14464	0.01123596	1.17765836	0.16499964	0.57507313	0.78748612	0.77342387	Hnrnpc	1
GO:0007283	spermatogenesis	4/138	423/14464	0.00945626	0.99112619	-0.0181797	0.57742327	0.78748612	0.77342387	Bmal1/Calr/Tbpl1/Mettl14	4
GO:0001657	ureteric bud development	1/138	90/14464	0.01111111	1.16457327	0.15370854	0.57915243	0.78748612	0.77342387	Smad2	1
GO:0001942	hair follicle development	1/138	90/14464	0.01111111	1.16457327	0.15370854	0.57915243	0.78748612	0.77342387	Pkp3	1
GO:0008589	regulation of smoothened signaling pathway	1/138	90/14464	0.01111111	1.16457327	0.15370854	0.57915243	0.78748612	0.77342387	Ift172	1
GO:0019233	sensory perception of pain	1/138	90/14464	0.01111111	1.16457327	0.15370854	0.57915243	0.78748612	0.77342387	Smr2l	1
GO:0048525	negative regulation of viral process	1/138	90/14464	0.01111111	1.16457327	0.15370854	0.57915243	0.78748612	0.77342387	Srpk1	1
GO:0051101	regulation of DNA binding	1/138	90/14464	0.01111111	1.16457327	0.15370854	0.57915243	0.78748612	0.77342387	Sri	1
GO:0051702	biological process involved in interaction with symbiont	1/138	90/14464	0.01111111	1.16457327	0.15370854	0.57915243	0.78748612	0.77342387	Hspd1	1
GO:0070555	response to interleukin-1	1/138	90/14464	0.01111111	1.16457327	0.15370854	0.57915243	0.78748612	0.77342387	Has2	1
GO:0046879	hormone secretion	3/138	314/14464	0.00955414	1.00138466	0.00243464	0.57939271	0.78748612	0.77342387	Bmal1/Smad2/Sri	3
GO:0045137	development of primary sexual characteristics	2/138	203/14464	0.00985222	1.03262654	0.04594628	0.5797128	0.78748612	0.77342387	Mmp14/Lhfpl2	2
GO:0045859	regulation of protein kinase activity	4/138	426/14464	0.00938967	0.98414642	-0.0325976	0.58304547	0.78748612	0.77342387	Chordc1/Fzd8/Sash1/Dnajc3	4
GO:0006970	response to osmotic stress	1/138	91/14464	0.01098901	1.15177576	0.14254621	0.58319285	0.78748612	0.77342387	Oxsr1	1
GO:0010469	regulation of signaling receptor activity	1/138	91/14464	0.01098901	1.15177576	0.14254621	0.58319285	0.78748612	0.77342387	Tafa1	1
GO:0019751	polyol metabolic process	1/138	91/14464	0.01098901	1.15177576	0.14254621	0.58319285	0.78748612	0.77342387	Slc5a3	1
GO:0042310	vasoconstriction	1/138	91/14464	0.01098901	1.15177576	0.14254621	0.58319285	0.78748612	0.77342387	Manf	1
GO:0046718	symbiont entry into host cell	1/138	91/14464	0.01098901	1.15177576	0.14254621	0.58319285	0.78748612	0.77342387	P4hb	1
GO:0046785	microtubule polymerization	1/138	91/14464	0.01098901	1.15177576	0.14254621	0.58319285	0.78748612	0.77342387	Tppp3	1
GO:0051952	regulation of amine transport	1/138	91/14464	0.01098901	1.15177576	0.14254621	0.58319285	0.78748612	0.77342387	Slc17a8	1
GO:0071229	cellular response to acid chemical	1/138	91/14464	0.01098901	1.15177576	0.14254621	0.58319285	0.78748612	0.77342387	Gclm	1
GO:0072163	mesonephric epithelium development	1/138	91/14464	0.01098901	1.15177576	0.14254621	0.58319285	0.78748612	0.77342387	Smad2	1
GO:0072164	mesonephric tubule development	1/138	91/14464	0.01098901	1.15177576	0.14254621	0.58319285	0.78748612	0.77342387	Smad2	1
GO:0090100	positive regulation of transmembrane receptor protein serine/threonine kinase signaling pathway	1/138	91/14464	0.01098901	1.15177576	0.14254621	0.58319285	0.78748612	0.77342387	Smad2	1

GO:0071216 cellular response to biotic stimulus	2/138	205/14464	0.0097561	1.02255214	0.03191732	0.58506873	0.78769039	0.77362449	Hspa5/Sash1	2
GO:0001823 mesonephros development	1/138	92/14464	0.01086957	1.13925646	0.13150944	0.58719475	0.78769039	0.77362449	Smad2	1
GO:0042147 retrograde transport, endosome to Golgi	1/138	92/14464	0.01086957	1.13925646	0.13150944	0.58719475	0.78769039	0.77362449	Rab9b	1
GO:0046889 positive regulation of lipid biosynthetic process	1/138	92/14464	0.01086957	1.13925646	0.13150944	0.58719475	0.78769039	0.77362449	Rab38	1
GO:0051899 membrane depolarization	1/138	92/14464	0.01086957	1.13925646	0.13150944	0.58719475	0.78769039	0.77362449	Gclm	1
GO:1990266 neutrophil migration	1/138	92/14464	0.01086957	1.13925646	0.13150944	0.58719475	0.78769039	0.77362449	Cd99l2	1
GO:0043270 positive regulation of monoatomic ion transport	2/138	206/14464	0.00970874	1.01758829	0.02495366	0.58772794	0.78769039	0.77362449	Kcnip2/Sri	2
GO:0007015 actin filament organization	4/138	429/14464	0.00932401	0.97726428	-0.0469178	0.58862525	0.78769039	0.77362449	Rhoj/Hsp90b1/Scin/Msrb2	4
GO:0003018 vascular process in circulatory system	2/138	207/14464	0.00966184	1.01267241	0.01802339	0.59037463	0.78769039	0.77362449	Manf/Gclm	2
GO:0007411 axon guidance	2/138	207/14464	0.00966184	1.01267241	0.01802339	0.59037463	0.78769039	0.77362449	Sema3e/Crppa	2
GO:0043583 ear development	2/138	207/14464	0.00966184	1.01267241	0.01802339	0.59037463	0.78769039	0.77362449	Grhl3/Slc17a8	2
GO:0097191 extrinsic apoptotic signaling pathway	2/138	207/14464	0.00966184	1.01267241	0.01802339	0.59037463	0.78769039	0.77362449	Pdia3/Gclm	2
GO:0006112 energy reserve metabolic process	1/138	93/14464	0.01075269	1.12700639	0.12059514	0.59115851	0.78769039	0.77362449	Wdr45b	1
GO:0018210 peptidyl-threonine modification	1/138	93/14464	0.01075269	1.12700639	0.12059514	0.59115851	0.78769039	0.77362449	Oxsr1	1
GO:0022404 molting cycle process	1/138	93/14464	0.01075269	1.12700639	0.12059514	0.59115851	0.78769039	0.77362449	Pkp3	1
GO:0022405 hair cycle process	1/138	93/14464	0.01075269	1.12700639	0.12059514	0.59115851	0.78769039	0.77362449	Pkp3	1
GO:0042116 macrophage activation	1/138	93/14464	0.01075269	1.12700639	0.12059514	0.59115851	0.78769039	0.77362449	Hspd1	1
GO:0043280 positive regulation of cysteine-type endopeptidase activity involved in apoptotic process	1/138	93/14464	0.01075269	1.12700639	0.12059514	0.59115851	0.78769039	0.77362449	Hspd1	1
GO:1901874 negative regulation of post-translational protein modification	1/138	93/14464	0.01075269	1.12700639	0.12059514	0.59115851	0.78769039	0.77362449	Bag5	1
GO:1901879 regulation of protein depolymerization	1/138	93/14464	0.01075269	1.12700639	0.12059514	0.59115851	0.78769039	0.77362449	Scin	1
GO:0002697 regulation of immune effector process	3/138	320/14464	0.009375	0.9826087	-0.0308764	0.59229349	0.78816084	0.77408654	Hspd1/Cr1l/Pkp3	3
GO:0003002 regionalization	3/138	320/14464	0.009375	0.9826087	-0.0308764	0.59229349	0.78816084	0.77408654	Dand5/Smad2/Ift172	3
GO:0097485 neuron projection guidance	2/138	208/14464	0.00961538	1.00780379	0.01112614	0.59300879	0.78820471	0.77412962	Sema3e/Crppa	2
GO:0009914 hormone transport	3/138	321/14464	0.00934579	0.97954761	-0.0363691	0.59441899	0.78820471	0.77412962	Bmal1/Smad2/Sri	3
GO:0009308 amine metabolic process	1/138	94/14464	0.0106383	1.11501696	0.10980034	0.59508448	0.78820471	0.77412962	Sat1	1
GO:0032355 response to estradiol	1/138	94/14464	0.0106383	1.11501696	0.10980034	0.59508448	0.78820471	0.77412962	Calr	1
GO:0044409 symbiont entry into host	1/138	94/14464	0.0106383	1.11501696	0.10980034	0.59508448	0.78820471	0.77412962	P4hb	1
GO:0072655 establishment of protein localization to mitochondrion	1/138	94/14464	0.0106383	1.11501696	0.10980034	0.59508448	0.78820471	0.77412962	Hspd1	1
GO:0007160 cell-matrix adhesion	2/138	210/14464	0.00952381	0.99820566	-0.0025707	0.59823955	0.78820471	0.77412962	Mmp14/Sema3e	2
GO:0007163 establishment or maintenance of cell polarity	2/138	210/14464	0.00952381	0.99820566	-0.0025707	0.59823955	0.78820471	0.77412962	Rhoj/Ift80	2
GO:0031348 negative regulation of defense response	2/138	210/14464	0.00952381	0.99820566	-0.0025707	0.59823955	0.78820471	0.77412962	Dtx4/Itch	2
GO:0090276 regulation of peptide hormone secretion	2/138	210/14464	0.00952381	0.99820566	-0.0025707	0.59823955	0.78820471	0.77412962	Bmal1/Sri	2
GO:0007517 muscle organ development	3/138	323/14464	0.00928793	0.9734823	-0.0473046	0.59864871	0.78820471	0.77412962	Bmal1/Fktn/Pkp2	3
GO:0009100 glycoprotein metabolic process	3/138	323/14464	0.00928793	0.9734823	-0.0473046	0.59864871	0.78820471	0.77412962	Fktn/Hexa/Crppa	3
GO:0021700 developmental maturation	3/138	323/14464	0.00928793	0.9734823	-0.0473046	0.59864871	0.78820471	0.77412962	Rab38/Kcnip2/Ift80	3
GO:0002708 positive regulation of lymphocyte mediated immunity	1/138	95/14464	0.01052632	1.10327994	0.09912216	0.59897302	0.78820471	0.77412962	Hspd1	1
GO:0007044 cell-substrate junction assembly	1/138	95/14464	0.01052632	1.10327994	0.09912216	0.59897302	0.78820471	0.77412962	Mmp14	1
GO:0045639 positive regulation of myeloid cell differentiation	1/138	95/14464	0.01052632	1.10327994	0.09912216	0.59897302	0.78820471	0.77412962	Scin	1
GO:1990542 mitochondrial transmembrane transport	1/138	95/14464	0.01052632	1.10327994	0.09912216	0.59897302	0.78820471	0.77412962	Hspd1	1
GO:0060537 muscle tissue development	4/138	435/14464	0.0091954	0.96378477	-0.0752712	0.59965544	0.78858798	0.77450605	Bmal1/Calr/Fktn/Pkp2	4
GO:0006913 nucleocytoplasmic transport	3/138	324/14464	0.00925926	0.97047772	-0.0527477	0.60075289	0.78900184	0.77491252	Bmal1/Calr/Frat2	3
GO:0051169 nuclear transport	3/138	324/14464	0.00925926	0.97047772	-0.0527477	0.60075289	0.78900184	0.77491252	Bmal1/Calr/Frat2	3
GO:0003333 amino acid transmembrane transport	1/138	96/14464	0.01041667	1.09178744	0.08855783	0.60282449	0.7909591	0.77683483	Slc17a8	1
GO:0048232 male gamete generation	4/138	437/14464	0.00915332	0.95937386	-0.0846389	0.60329331	0.7909591	0.77683483	Bmal1/Calr/Tbpl1/Mettl14	4
GO:0099111 microtubule-based transport	2/138	212/14464	0.00943396	0.98878862	-0.0161397	0.60342019	0.7909591	0.77683483	Ift172/Ift80	2
GO:0006417 regulation of translation	3/138	326/14464	0.00920245	0.96452387	-0.0635854	0.60493979	0.7915636	0.77742854	Calr/Dnajc3/Mettl14	3
GO:0070663 regulation of leukocyte proliferation	2/138	213/14464	0.00938967	0.98414642	-0.0228771	0.60599171	0.7915636	0.77742854	Card11/Itch	2
GO:0007029 endoplasmic reticulum organization	1/138	97/14464	0.01030928	1.0805319	0.07810467	0.60663923	0.7915636	0.77742854	Ubl4a	1
GO:0008344 adult locomotory behavior	1/138	97/14464	0.01030928	1.0805319	0.07810467	0.60663923	0.7915636	0.77742854	Hexa	1
GO:0016239 positive regulation of macroautophagy	1/138	97/14464	0.01030928	1.0805319	0.07810467	0.60663923	0.7915636	0.77742854	Snx18	1
GO:0032024 positive regulation of insulin secretion	1/138	97/14464	0.01030928	1.0805319	0.07810467	0.60663923	0.7915636	0.77742854	Sri	1
GO:0098656 monoatomic anion transmembrane transport	1/138	97/14464	0.01030928	1.0805319	0.07810467	0.60663923	0.7915636	0.77742854	Slc17a8	1
GO:0002443 leukocyte mediated immunity	3/138	327/14464	0.00917431	0.96157426	-0.0689801	0.60702248	0.7915636	0.77742854	Ighg2c/Hspd1/Cr1l	3
GO:0006364 rRNA processing	2/138	214/14464	0.00934579	0.97954761	-0.0295835	0.60855071	0.79175574	0.77761724	Mettl16/Tfb1m	2
GO:0043269 regulation of monoatomic ion transport	4/138	440/14464	0.00909091	0.95283267	-0.0986138	0.60871319	0.79175574	0.77761724	Rrad/Kcnip2/Sri/Oxsr1	4
GO:0032956 regulation of actin cytoskeleton organization	3/138	328/14464	0.00914634	0.95864263	-0.0743589	0.60909798	0.79175574	0.77761724	Arhgef16/Grhl3/Scin	3
GO:0002718 regulation of cytokine production involved in immune response	1/138	98/14464	0.01020408	1.06950606	0.0677601	0.6104176	0.79175574	0.77761724	Pkp3	1
GO:0018958 phenol-containing compound metabolic process	1/138	98/14464	0.01020408	1.06950606	0.0677601	0.6104176	0.79175574	0.77761724	Rab38	1
GO:0032204 regulation of telomere maintenance	1/138	98/14464	0.01020408	1.06950606	0.0677601	0.6104176	0.79175574	0.77761724	Hnrnpc	1
GO:0043473 pigmentation	1/138	98/14464	0.01020408	1.06950606	0.0677601	0.6104176	0.79175574	0.77761724	Rab38	1
GO:0090263 positive regulation of canonical Wnt signaling pathway	1/138	98/14464	0.01020408	1.06950606	0.0677601	0.6104176	0.79175574	0.77761724	Bmal1	1
GO:0002791 regulation of peptide secretion	2/138	215/14464	0.00930233	0.97499157	-0.0362593	0.61109719	0.79175574	0.77761724	Bmal1/Sri	2
GO:0090087 regulation of peptide transport	2/138	215/14464	0.00930233	0.97499157	-0.0362593	0.61109719	0.79175574	0.77761724	Bmal1/Sri	2
GO:0042594 response to starvation	2/138	216/14464	0.00925926	0.97047772	-0.0429048	0.61363114	0.7936836	0.77951068	Hspa5/Wdr45b	2
GO:0044403 biological process involved in symbiotic interaction	2/138	216/14464	0.00925926	0.97047772	-0.0429048	0.61363114	0.7936836	0.77951068	Hspd1/P4hb	2
GO:0032760 positive regulation of tumor necrosis factor production	1/138	99/14464	0.01010101	1.05870297	0.05752162	0.61415993	0.7936836	0.77951068	Hspd1	1
GO:2001243 negative regulation of intrinsic apoptotic signaling pathway	1/138	99/14464	0.01010101	1.05870297	0.05752162	0.61415993	0.7936836	0.77951068	Bag5	1
GO:0002833 positive regulation of response to biotic stimulus	3/138	331/14464	0.00906344	0.94995403	-0.0904008	0.61528116	0.7946232	0.7804335	Sash1/Hspd1/Itch	3
GO:0002698 negative regulation of immune effector process	1/138	100/14464	0.01	1.04811594	0.04738682	0.61786658	0.79541444	0.78121061	Cr1l	1
GO:0008645 hexose transmembrane transport	1/138	100/14464	0.01	1.04811594	0.04738682	0.61786658	0.79541444	0.78121061	Slc5a3	1
GO:0014910 regulation of smooth muscle cell migration	1/138	100/14464	0.01	1.04811594	0.04738682	0.61786658	0.79541444	0.78121061	Has2	1
GO:1903557 positive regulation of tumor necrosis factor superfamily cytokine production	1/138	100/14464	0.01	1.04811594	0.04738682	0.61786658	0.79541444	0.78121061	Hspd1	1
GO:1904659 D-glucose transmembrane transport	1/138	100/14464	0.01	1.04811594	0.04738682	0.61786658	0.79541444	0.78121061	Slc5a3	1
GO:0048863 stem cell differentiation	2/138	218/14464	0.00917431	0.96157426	-0.0561061	0.61866152	0.79592956	0.78171654	Sema3e/Ift80	2
GO:0002831 regulation of response to biotic stimulus	4/138	446/14464	0.00896861	0.9400143	-0.1262927	0.6194185	0.79639521	0.78217387	Sash1/Dtx4/Hspd1/Itch	4
GO:0007338 single fertilization	1/138	101/14464	0.00990099	1.03773856	0.03735336	0.62153787	0.79658	0.78235536	Lhfpl2	1
GO:0015837 amine transport	1/138	101/14464	0.00990099	1.03773856	0.03735336	0.62153787	0.79658	0.78235536	Slc17a8	1
GO:0021761 limbic system development	1/138	101/14464	0.00990099	1.03773856	0.03735336	0.62153787	0.79658	0.78235536	Sema3e	1
GO:0050852 T cell receptor signaling pathway	1/138	101/14464	0.00990099	1.03773856	0.03735336	0.62153787	0.79658	0.78235536	Card11	1
GO:0097237 cellular response to toxic substance	1/138	101/14464	0.00990099	1.03773856	0.03735336	0.62153787	0.79658	0.78235536	Akr1a1	1
GO:0006631 fatty acid metabolic process	3/138	335/14464	0.00895522	0.93861129	-0.1115736	0.62342381	0.797186	0.78295053	Degs1/Elovl7/Pdk3	3
GO:0010976 positive regulation of neuron projection development	2/138	220/14464	0.00909091	0.95283267	-0.0691899	0.6236419	0.797186	0.78295053	Hspa5/Zfp804a	2
GO:0000422 autophagy of mitochondrion	1/138	102/14464	0.00980392	1.02756465	0.02741898	0.62517414	0.797186	0.78295053	Wdr45b	1
GO:0002824 positive regulation of adaptive immune response based on somatic recombination of immune receptors b	1/138	102/14464	0.00980392	1.02756465	0.02741898	0.62517414	0.797186	0.78295053	Hspd1	1
GO:0015749 monosaccharide transmembrane transport	1/138	102/14464	0.00980392	1.02756465	0.02741898	0.62517414	0.797186	0.78295053	Slc5a3	1
GO:0042476 odontogenesis	1/138	102/14464	0.00980392	1.02756465	0.02741898	0.62517414	0.797186	0.78295053	Ift80	1
GO:0150115 cell-substrate junction organization	1/138	102/14464	0.00980392	1.02756465	0.02741898	0.62517414	0.797186	0.78295053	Mmp14	1
GO:1901989 positive regulation of cell cycle phase transition	1/138	102/14464	0.00980392	1.02756465	0.02741898	0.62517414	0.797186	0.78295053	Pkp3	1
GO:0043588 skin development	2/138	222/14464	0.00900901	0.9442486	-0.0821586	0.62857235	0.79975511	0.78547377	Grhl3/Pkp3	2
GO:0042752 regulation of circadian rhythm	1/138	103/14464	0.00970874	1.01758829	0.01758151	0.62877572	0.79975511	0.78547377	Bmal1	1
GO:0070585 protein localization to mitochondrion	1/138	103/14464	0.00970874	1.01758829	0.01758151	0.62877572	0.79975511	0.78547377	Hspd1	1
GO:2000278 regulation of DNA biosynthetic process	1/138	103/14464	0.00970874	1.01758829	0.01758151	0.62877572	0.79975511	0.78547377	Hnrnpc	1
GO:0051402 neuron apoptotic process	3/138	339/14464	0.00884956	0.92753623	-0.1325052	0.63144965	0.80226771	0.7879415	Hspd1/Gclm/Sema3e	3
GO:0031330 negative regulation of cellular catabolic process	1/138	104/14464	0.00961538	1.00780379	0.00783883	0.63234295	0.80226771	0.7879415	Sec22b	1
GO:0070646 protein modification by small protein removal	1/138	104/14464	0.00961538	1.00780379	0.00783883	0.63234295	0.80226771	0.7879415	Itch	1
GO:0098754 detoxification	1/138	104/14464	0.00961538	1.00780379	0.00783883	0.63234295	0.80226771	0.7879415	Akr1a1	1
GO:0031667 response to nutrient levels	4/138	455/14464	0.00879121	0.92142061	-0.1671541	0.6351346	0.80472461	0.79035453	Hspa5/Pdia3/Gclm/Wdr45b	4
GO:0015711 organic anion transport	3/138	341/14464	0.00879765	0.92209614	-0.1428825	0.63541855	0.80472461	0.79035453	Slc51a/Slc17a8/Slc33a1	3
GO:2001234 negative regulation of apoptotic signaling pathway	2/138	225/14464	0.00888889	0.93165862	-0.1014008	0.6358746	0.80472461	0.79035453	Gclm/Bag5	2
GO:0051092 positive regulation of NF-kappaB transcription factor activity	1/138	105/14464	0.00952381	0.99820566	-0.0018111	0.63587614	0.80472461	0.79035453	Card11	1
GO:0043549 regulation of kinase activity	4/138	456/14464	0.00877193	0.91939995	-0.1716469	0.63685517	0.80545799	0.79107481	Chordc1/Fzd8/Sash1/Dnajc3	4
GO:0006275 regulation of DNA replication	1/138	106/14464	0.00943396	0.98878862	-0.0113703	0.63937563	0.80763237	0.79321037	Obi1	1
GO:0097061 dendritic spine organization	1/138	106/14464	0.00943396	0.98878862	-0.0113703	0.63937563	0.80763237	0.79321037	Zfp804a	1
GO:0019058 viral life cycle	2/138	227/14464	0.00881057	0.92345017	-0.1140917	0.64068064	0.80826794	0.79383458	Srpk1/P4hb	2
GO:0051056 regulation of small GTPase mediated signal transduction	2/138	227/14464	0.00881057	0.92345017	-0.1140917	0.64068064	0.80826794	0.79383458	Stard13/Ppp2cb	2
GO:0032963 collagen metabolic process	1/138	107/14464	0.00934579	0.97954761	-0.0208406	0.64284172	0.80998057	0.79551663	Mmp14	1
GO:0042542 response to hydrogen peroxide	1/138	107/14464	0.00934579	0.97954761	-0.0208406	0.64284172	0.80998057	0.79551663	Ppp2cb	1
GO:0008585 female gonad development	1/138	108/14464	0.00925926	0.97047772	-0.030224	0.64627474	0.81065074	0.79617483	Mmp14	1
GO:0019079 viral genome replication	1/138	108/14464	0.00925926	0.97047772	-0.030224	0.64627474	0.81065074	0.79617483	Srpk1	1
GO:0042177 negative regulation of protein catabolic process	1/138	108/14464	0.00925926	0.97047772	-0.030224	0.64627474	0.81065074	0.79617483	Bag5	1
GO:0042471 ear morphogenesis	1/138	108/14464	0.00925926	0.97047772	-0.030224	0.64627474	0.81065074	0.79617483	Grhl3	1
GO:0050671 positive regulation of lymphocyte proliferation	1/138	108/14464	0.00925926	0.97047772	-0.030224	0.64627474	0.81065074	0.79617483	Card11	1
GO:2001056 positive regulation of cysteine-type endopeptidase activity	1/138	108/14464	0.00925926	0.97047772	-0.030224	0.64627474	0.81065074	0.79617483	Hspd1	1
GO:0001894 tissue homeostasis	2/138	230/14464	0.00869565	0.91140517	-0.1329276	0.64779679	0.81065074	0.79617483	Pkp3/Ift80	2
GO:0019318 hexose metabolic process	2/138	230/14464	0.00869565	0.91140517	-0.1329276	0.64779679	0.81065074	0.79617483	Pdk3/Arpp19	2
GO:0031331 positive regulation of cellular catabolic process	2/138	230/14464	0.00869565	0.91140517	-0.1329276	0.64779679	0.81065074	0.79617483	Snx18/Itch	2
GO:0060249 anatomical structure homeostasis	2/138	230/14464	0.00869565	0.91140517	-0.1329276	0.64779679	0.81065074	0.79617483	Pkp3/Ift80	2
GO:1902115 regulation of organelle assembly	2/138	230/14464	0.00869565	0.91140517	-0.1329276	0.64779679	0.81065074	0.79617483	Snx18/Sec22b	2
GO:0009410 response to xenobiotic stimulus	3/138	348/14464	0.00862069	0.90354823	-0.1787517	0.6490775	0.81098749	0.79650557	Calr/Gclm/Oxsr1	3
GO:0001578 microtubule bundle formation	1/138	109/14464	0.00917431	0.96157426	-0.0395221	0.649675	0.81098749	0.79650557	Tppp3	1
GO:0039531 regulation of cytoplasmic pattern recognition receptor signaling pathway	1/138	109/14464	0.00917431	0.96157426	-0.0395221	0.649675	0.81098749	0.79650557	Itch	1
GO:0046683 response to organophosphorus	1/138	109/14464	0.00917431	0.96157426	-0.0395221	0.649675	0.81098749	0.79650557	Hsp90b1	1
GO:0042110 T cell activation	4/138	464/14464	0.00862069	0.90354823	-0.2072581	0.65043269	0.81143088	0.79694104	Card11/Fzd8/Hspd1/Itch	4
GO:0002221 pattern recognition receptor signaling pathway	2/138	232/14464	0.00862069	0.90354823	-0.1453542	0.65247917	0.81217415	0.79767104	Hspd1/Itch	2
GO:0002573 myeloid leukocyte differentiation	2/138	232/14464	0.00862069	0.90354823	-0.1453542	0.65247917	0.81217415	0.79767104	Sh3pxd2a/Ift80	2
GO:0002821 positive regulation of adaptive immune response	1/138	110/14464	0.00909091	0.95283267	-0.0487368	0.6530428	0.81217415	0.79767104	Hspd1	1
GO:0034219 carbohydrate transmembrane transport	1/138	110/14464	0.00909091	0.95283267	-0.0487368	0.6530428	0.81217415	0.79767104	Slc5a3	1
GO:0050868 negative regulation of T cell activation	1/138	110/14464	0.00909091	0.95283267	-0.0487368	0.6530428	0.81217415	0.79767104	Itch	1
GO:0002694 regulation of leukocyte activation	4/138	467/14464	0.00856531	0.89774385	-0.2204635	0.6554379	0.81290922	0.79839299	Card11/Mmp14/Hspd1/Itch	4
GO:0014909 smooth muscle cell migration	1/138	111/14464	0.00900901	0.9442486	-0.0578698	0.65637846	0.81290922	0.79839299	Has2	1
GO:0032946 positive regulation of mononuclear cell proliferation	1/138	111/14464	0.00900901	0.9442486	-0.0578698	0.65637846	0.81290922	0.79839299	Card11	1
GO:0051928 positive regulation of calcium ion transport	1/138	111/14464	0.00900901	0.9442486	-0.0578698	0.65637846	0.81290922	0.79839299	Sri	1
GO:2000177 regulation of neural precursor cell proliferation	1/138	111/14464	0.00900901	0.9442486	-0.0578698	0.65637846	0.81290922	0.79839299	Tafa1	1
GO:0009306 protein secretion	3/138	352/14464	0.00852273	0.89328063	-0.198942	0.65671984	0.81290922	0.79839299	Bmal1/Smad2/Sri	3
GO:0002758 innate immune response-activating signaling pathway	2/138	235/14464	0.00851064	0.89201357	-0.1638031	0.65941053	0.81290922	0.79839299	Hspd1/Itch	2
GO:0015850 organic hydroxy compound transport	2/138	235/14464	0.00851064	0.89201357	-0.1638031	0.65941053	0.81290922	0.79839299	Slc5a3/Slc51a	2
GO:0090257 regulation of muscle system process	2/138	235/14464	0.00851064	0.89201357	-0.1638031	0.65941053	0.81290922	0.79839299	Pkp2/Sri	2
GO:0006413 translational initiation	1/138	112/14464	0.00892857	0.93581781	-0.0669227	0.65968229	0.81290922	0.79839299	Dnajc3	1
GO:0007265 Ras protein signal transduction	1/138	112/14464	0.00892857	0.93581781	-0.0669227	0.65968229	0.81290922	0.79839299	Ppp2cb	1
GO:0009791 post-embryonic development	1/138	112/14464	0.00892857	0.93581781	-0.0669227	0.65968229	0.81290922	0.79839299	Smad2	1
GO:0035249 synaptic transmission, glutamatergic	1/138	112/14464	0.00892857	0.93581781	-0.0669227	0.65968229	0.81290922	0.79839299	Slc17a8	1
GO:0051209 release of sequestered calcium ion into cytosol	1/138	112/14464	0.00892857	0.93581781	-0.0669227	0.65968229	0.81290922	0.79839299	Sri	1
GO:0061640 cytoskeleton-dependent cytokinesis	1/138	112/14464	0.00892857	0.93581781	-0.0669227	0.65968229	0.81290922	0.79839299	Snx18	1
GO:0035592 establishment of protein localization to extracellular region	3/138	354/14464	0.00847458	0.88823385	-0.2089559	0.6604965	0.81341537	0.79889009	Bmal1/Smad2/Sri	3
GO:0007249 canonical NF-kappaB signal transduction	2/138	236/14464	0.00847458	0.88823385	-0.1699028	0.66169647	0.81395641	0.79942147	Card11/Ift80	2
GO:0034655 nucleobase-containing compound catabolic process	4/138	471/14464	0.00849257	0.8901197	-0.2379475	0.66203768	0.81395641	0.79942147	Mettl16/Hnrnpc/Pkp3/Mettl14	4
GO:0010639 negative regulation of organelle organization	3/138	355/14464	0.0084507	0.88573178	-0.2139429	0.6623737	0.81395641	0.79942147	Scin/Hnrnpc/Sec22b	3
GO:0038202 TORC1 signaling	1/138	113/14464	0.00884956	0.92753623	-0.0758971	0.66295457	0.81395641	0.79942147	Card11	1
GO:0051283 negative regulation of sequestering of calcium ion	1/138	113/14464	0.00884956	0.92753623	-0.0758971	0.66295457	0.81395641	0.79942147	Sri	1
GO:0007548 sex differentiation	2/138	237/14464	0.00843882	0.88448603	-0.175978	0.6639702	0.81421164	0.79967214	Mmp14/Lhfpl2	2
GO:0048738 cardiac muscle tissue development	2/138	237/14464	0.00843882	0.88448603	-0.175978	0.6639702	0.81421164	0.79967214	Calr/Pkp2	2
GO:0000077 DNA damage checkpoint signaling	1/138	114/14464	0.00877193	0.91939995	-0.0847946	0.66619562	0.81446354	0.79991954	Gnb1l	1
GO:0008643 carbohydrate transport	1/138	114/14464	0.00877193	0.91939995	-0.0847946	0.66619562	0.81446354	0.79991954	Slc5a3	1
GO:0045621 positive regulation of lymphocyte differentiation	1/138	114/14464	0.00877193	0.91939995	-0.0847946	0.66619562	0.81446354	0.79991954	Mmp14	1
GO:0072089 stem cell proliferation	1/138	114/14464	0.00877193	0.91939995	-0.0847946	0.66619562	0.81446354	0.79991954	Ift80	1
GO:1990830 cellular response to leukemia inhibitory factor	1/138	114/14464	0.00877193	0.91939995	-0.0847946	0.66619562	0.81446354	0.79991954	Gclm	1
GO:0002705 positive regulation of leukocyte mediated immunity	1/138	115/14464	0.00869565	0.91140517	-0.0936166	0.66940573	0.81492655	0.80037429	Hspd1	1
GO:0006639 acylglycerol metabolic process	1/138	115/14464	0.00869565	0.91140517	-0.0936166	0.66940573	0.81492655	0.80037429	Dgkk	1
GO:0030282 bone mineralization	1/138	115/14464	0.00869565	0.91140517	-0.0936166	0.66940573	0.81492655	0.80037429	Ift80	1
GO:0043200 response to amino acid	1/138	115/14464	0.00869565	0.91140517	-0.0936166	0.66940573	0.81492655	0.80037429	Gclm	1
GO:0051053 negative regulation of DNA metabolic process	1/138	115/14464	0.00869565	0.91140517	-0.0936166	0.66940573	0.81492655	0.80037429	Hnrnpc	1
GO:0051282 regulation of sequestering of calcium ion	1/138	115/14464	0.00869565	0.91140517	-0.0936166	0.66940573	0.81492655	0.80037429	Sri	1
GO:0001503 ossification	3/138	359/14464	0.00835655	0.8758629	-0.2337591	0.66980818	0.81492655	0.80037429	Dhrs3/Mmp14/Ift80	3
GO:0030098 lymphocyte differentiation	3/138	359/14464	0.00835655	0.8758629	-0.2337591	0.66980818	0.81492655	0.80037429	Card11/Mmp14/Fzd8	3
GO:0051924 regulation of calcium ion transport	2/138	240/14464	0.00833333	0.87342995	-0.1940582	0.67071833	0.81554171	0.80097847	Rrad/Sri	2
GO:0006638 neutral lipid metabolic process	1/138	116/14464	0.00862069	0.90354823	-0.1023646	0.67258518	0.81573671	0.80116998	Dgkk	1

GO:0051208 sequestering of calcium ion	1/138	116/14464	0.00862069	0.90354823	-0.1023646	0.67258518	0.81573671	0.80116998	Sri	1
GO:1990823 response to leukemia inhibitory factor	1/138	116/14464	0.00862069	0.90354823	-0.1023646	0.67258518	0.81573671	0.80116998	Gclm	1
GO:0071695 anatomical structure maturation	2/138	242/14464	0.00826446	0.86621152	-0.2059929	0.67515645	0.81573671	0.80116998	Kcnip2/Ift80	2
GO:0090596 sensory organ morphogenesis	2/138	242/14464	0.00826446	0.86621152	-0.2059929	0.67515645	0.81573671	0.80116998	Grhl3/Ift172	2
GO:1903706 regulation of hemopoiesis	3/138	362/14464	0.00828729	0.86860437	-0.2484855	0.67530602	0.81573671	0.80116998	Card11/Mmp14/Scin	3
GO:0006820 monoatomic anion transport	1/138	117/14464	0.00854701	0.89582559	-0.1110401	0.67573428	0.81573671	0.80116998	Slc17a8	1
GO:0008584 male gonad development	1/138	117/14464	0.00854701	0.89582559	-0.1110401	0.67573428	0.81573671	0.80116998	Mmp14	1
GO:0015695 organic cation transport	1/138	117/14464	0.00854701	0.89582559	-0.1110401	0.67573428	0.81573671	0.80116998	Slc17a8	1
GO:0015698 inorganic anion transport	1/138	117/14464	0.00854701	0.89582559	-0.1110401	0.67573428	0.81573671	0.80116998	Slc17a8	1
GO:0017148 negative regulation of translation	1/138	117/14464	0.00854701	0.89582559	-0.1110401	0.67573428	0.81573671	0.80116998	Calr	1
GO:0106027 neuron projection organization	1/138	117/14464	0.00854701	0.89582559	-0.1110401	0.67573428	0.81573671	0.80116998	Zfp804a	1
GO:0002367 cytokine production involved in immune response	1/138	118/14464	0.00847458	0.88823385	-0.1196443	0.6788533	0.81754376	0.80294477	Pkp3	1
GO:0009142 nucleoside triphosphate biosynthetic process	1/138	118/14464	0.00847458	0.88823385	-0.1196443	0.6788533	0.81754376	0.80294477	Tbpl1	1
GO:0032479 regulation of type I interferon production	1/138	118/14464	0.00847458	0.88823385	-0.1196443	0.6788533	0.81754376	0.80294477	Hspd1	1
GO:0051588 regulation of neurotransmitter transport	1/138	118/14464	0.00847458	0.88823385	-0.1196443	0.6788533	0.81754376	0.80294477	Slc17a8	1
GO:0030278 regulation of ossification	1/138	119/14464	0.00840336	0.8807697	-0.1281788	0.68194254	0.82028411	0.80563618	Dhrs3	1
GO:0048593 camera-type eye morphogenesis	1/138	119/14464	0.00840336	0.8807697	-0.1281788	0.68194254	0.82028411	0.80563618	Ift172	1
GO:0045860 positive regulation of protein kinase activity	2/138	246/14464	0.00813008	0.85212678	-0.2295853	0.68388808	0.82200274	0.80732412	Fzd8/Sash1	2
GO:0031507 heterochromatin formation	1/138	120/14464	0.00833333	0.87342995	-0.1366447	0.68500228	0.82200274	0.80732412	Cbx1	1
GO:0051224 negative regulation of protein transport	1/138	120/14464	0.00833333	0.87342995	-0.1366447	0.68500228	0.82200274	0.80732412	Ndfip2	1
GO:0051783 regulation of nuclear division	1/138	120/14464	0.00833333	0.87342995	-0.1366447	0.68500228	0.82200274	0.80732412	Calr	1
GO:0016072 rRNA metabolic process	2/138	247/14464	0.00809717	0.84867688	-0.2354269	0.68604103	0.82221346	0.80753108	Mettl16/Tfb1m	2
GO:0032984 protein-containing complex disassembly	2/138	247/14464	0.00809717	0.84867688	-0.2354269	0.68604103	0.82221346	0.80753108	Scin/Snx14	2
GO:0031570 DNA integrity checkpoint signaling	1/138	121/14464	0.00826446	0.86621152	-0.1450433	0.6880328	0.82221346	0.80753108	Gnb1l	1
GO:0032434 regulation of proteasomal ubiquitin-dependent protein catabolic process	1/138	121/14464	0.00826446	0.86621152	-0.1450433	0.6880328	0.82221346	0.80753108	Bag5	1
GO:0051261 protein depolymerization	1/138	121/14464	0.00826446	0.86621152	-0.1450433	0.6880328	0.82221346	0.80753108	Scin	1
GO:0090277 positive regulation of peptide hormone secretion	1/138	121/14464	0.00826446	0.86621152	-0.1450433	0.6880328	0.82221346	0.80753108	Sri	1
GO:1901890 positive regulation of cell junction assembly	1/138	121/14464	0.00826446	0.86621152	-0.1450433	0.6880328	0.82221346	0.80753108	Lrrn1	1
GO:0070372 regulation of ERK1 and ERK2 cascade	2/138	249/14464	0.00803213	0.84186019	-0.2470437	0.69031118	0.82433448	0.80961422	S2bpcox16/Spred3	2
GO:0032606 type I interferon production	1/138	122/14464	0.00819672	0.85911143	-0.1533759	0.69103436	0.82433448	0.80961422	Hspd1	1
GO:0045834 positive regulation of lipid metabolic process	1/138	122/14464	0.00819672	0.85911143	-0.1533759	0.69103436	0.82433448	0.80961422	Rab38	1
GO:0070665 positive regulation of leukocyte proliferation	1/138	123/14464	0.00813008	0.85212678	-0.1616436	0.69400726	0.82690226	0.81213615	Card11	1
GO:1903038 negative regulation of leukocyte cell-cell adhesion	1/138	123/14464	0.00813008	0.85212678	-0.1616436	0.69400726	0.82690226	0.81213615	Itch	1
GO:0031503 protein-containing complex localization	2/138	251/14464	0.00796813	0.83515215	-0.2585732	0.69453385	0.82698023	0.81221273	Ift172/Ift80	2
GO:0030900 forebrain development	3/138	373/14464	0.0080429	0.84298869	-0.3015232	0.69489311	0.82698023	0.81221273	Fktn/Sema3e/Mettl14	3
GO:0045088 regulation of innate immune response	3/138	374/14464	0.00802139	0.84073471	-0.3062723	0.6966293	0.82747628	0.81269992	Dtx4/Hspd1/Itch	3
GO:0021987 cerebral cortex development	1/138	124/14464	0.00806452	0.84525479	-0.1698477	0.69695175	0.82747628	0.81269992	Fktn	1
GO:0051100 negative regulation of binding	1/138	124/14464	0.00806452	0.84525479	-0.1698477	0.69695175	0.82747628	0.81269992	Sri	1
GO:0097530 granulocyte migration	1/138	124/14464	0.00806452	0.84525479	-0.1698477	0.69695175	0.82747628	0.81269992	Cd99l2	1
GO:0000086 G2/M transition of mitotic cell cycle	1/138	125/14464	0.008	0.83849275	-0.1779893	0.69986812	0.82923638	0.81442858	Arpp19	1
GO:0002793 positive regulation of peptide secretion	1/138	125/14464	0.008	0.83849275	-0.1779893	0.69986812	0.82923638	0.81442858	Sri	1
GO:0010950 positive regulation of endopeptidase activity	1/138	125/14464	0.008	0.83849275	-0.1779893	0.69986812	0.82923638	0.81442858	Hspd1	1
GO:0050803 regulation of synapse structure or activity	3/138	376/14464	0.00797872	0.83626272	-0.3157353	0.70007952	0.82923638	0.81442858	Lrrn1/Slc17a8/Zfp804a	3
GO:0014812 muscle cell migration	1/138	126/14464	0.00793651	0.83183805	-0.1860694	0.70275662	0.83143036	0.81658339	Has2	1
GO:0015718 monocarboxylic acid transport	1/138	126/14464	0.00793651	0.83183805	-0.1860694	0.70275662	0.83143036	0.81658339	Slc5a1	1
GO:0031400 negative regulation of protein modification process	3/138	378/14464	0.00793651	0.83183805	-0.3251517	0.70350022	0.83182197	0.816968	Chordc1/Dnajc3/Bag5	3
GO:0050878 regulation of body fluid levels	2/138	256/14464	0.0078125	0.81884058	-0.2870256	0.70488452	0.83188591	0.81703081	Has2/Pdia3	2
GO:0007041 lysosomal transport	1/138	127/14464	0.00787402	0.82528814	-0.1940892	0.70561752	0.83188591	0.81703081	Aktip	1
GO:0010565 regulation of cellular ketone metabolic process	1/138	127/14464	0.00787402	0.82528814	-0.1940892	0.70561752	0.83188591	0.81703081	Pdk3	1
GO:0030177 positive regulation of Wnt signaling pathway	1/138	127/14464	0.00787402	0.82528814	-0.1940892	0.70561752	0.83188591	0.81703081	Bmal1	1
GO:0046822 regulation of nucleocytoplasmic transport	1/138	127/14464	0.00787402	0.82528814	-0.1940892	0.70561752	0.83188591	0.81703081	Frat2	1
GO:0002757 immune response-activating signaling pathway	3/138	380/14464	0.00789474	0.82745995	-0.3345222	0.70689147	0.83239908	0.81753481	Card11/Hspd1/Itch	3
GO:0010927 cellular component assembly involved in morphogenesis	1/138	128/14464	0.0078125	0.81884058	-0.2020497	0.70845108	0.83239908	0.81753481	Tbpl1	1
GO:0014074 response to purine-containing compound	1/138	128/14464	0.0078125	0.81884058	-0.2020497	0.70845108	0.83239908	0.81753481	Hsp90b1	1
GO:0030856 regulation of epithelial cell differentiation	1/138	128/14464	0.0078125	0.81884058	-0.2020497	0.70845108	0.83239908	0.81753481	Spred3	1
GO:0032989 cellular anatomical entity morphogenesis	1/138	128/14464	0.0078125	0.81884058	-0.2020497	0.70845108	0.83239908	0.81753481	Tbpl1	1
GO:0002218 activation of innate immune response	2/138	258/14464	0.00775194	0.81249298	-0.2982619	0.70894307	0.83239908	0.81753481	Hspd1/Itch	2
GO:0033674 positive regulation of kinase activity	2/138	258/14464	0.00775194	0.81249298	-0.2982619	0.70894307	0.83239908	0.81753481	Fzd8/Sash1	2
GO:0006090 pyruvate metabolic process	1/138	129/14464	0.00775194	0.81249298	-0.2099519	0.71125756	0.83366003	0.81877324	Pdk3	1
GO:0015748 organophosphate ester transport	1/138	129/14464	0.00775194	0.81249298	-0.2099519	0.71125756	0.83366003	0.81877324	Slc33a1	1
GO:0016482 cytosolic transport	1/138	129/14464	0.00775194	0.81249298	-0.2099519	0.71125756	0.83366003	0.81877324	Rab9b	1
GO:0006906 vesicle fusion	1/138	130/14464	0.00769231	0.80624303	-0.2177968	0.71403722	0.83643175	0.82149547	Sec22b	1
GO:0046883 regulation of hormone secretion	2/138	261/14464	0.00766284	0.80315398	-0.3149656	0.71494417	0.83700781	0.82206124	Bmal1/Sri	2
GO:0007098 centrosome cycle	1/138	131/14464	0.00763359	0.80008851	-0.2255855	0.71679032	0.83722438	0.82227395	Chordc1	1
GO:0009566 fertilization	1/138	131/14464	0.00763359	0.80008851	-0.2255855	0.71679032	0.83722438	0.82227395	Lhfpl2	1
GO:0030833 regulation of actin filament polymerization	1/138	131/14464	0.00763359	0.80008851	-0.2255855	0.71679032	0.83722438	0.82227395	Scin	1
GO:0043244 regulation of protein-containing complex disassembly	1/138	131/14464	0.00763359	0.80008851	-0.2255855	0.71679032	0.83722438	0.82227395	Scin	1
GO:0030217 T cell differentiation	2/138	263/14464	0.00760456	0.79704634	-0.326003	0.7188875	0.83749796	0.82254264	Card11/Fzd8	2
GO:0050708 regulation of protein secretion	2/138	263/14464	0.00760456	0.79704634	-0.326003	0.7188875	0.83749796	0.82254264	Bmal1/Sri	2
GO:0001101 response to acid chemical	1/138	132/14464	0.00757576	0.79402723	-0.2333187	0.7195171	0.83749796	0.82254264	Gclm	1
GO:0010952 positive regulation of peptidase activity	1/138	132/14464	0.00757576	0.79402723	-0.2333187	0.7195171	0.83749796	0.82254264	Hspd1	1
GO:0031109 microtubule polymerization or depolymerization	1/138	132/14464	0.00757576	0.79402723	-0.2333187	0.7195171	0.83749796	0.82254264	Tppp3	1
GO:0090174 organelle membrane fusion	1/138	132/14464	0.00757576	0.79402723	-0.2333187	0.7195171	0.83749796	0.82254264	Sec22b	1
GO:0032675 regulation of interleukin-6 production	1/138	133/14464	0.0075188	0.7880571	-0.2409976	0.72221781	0.83918796	0.82420246	Hspd1	1
GO:0055007 cardiac muscle cell differentiation	1/138	133/14464	0.0075188	0.7880571	-0.2409976	0.72221781	0.83918796	0.82420246	Calr	1
GO:1905039 carboxylic acid transmembrane transport	1/138	133/14464	0.0075188	0.7880571	-0.2409976	0.72221781	0.83918796	0.82420246	Slc17a8	1
GO:0001959 regulation of cytokine-mediated signaling pathway	1/138	134/14464	0.00746269	0.78217608	-0.2486229	0.72489271	0.84084218	0.82582714	Ppp2cb	1
GO:0003158 endothelium development	1/138	134/14464	0.00746269	0.78217608	-0.2486229	0.72489271	0.84084218	0.82582714	Stard13	1
GO:0045598 regulation of fat cell differentiation	1/138	134/14464	0.00746269	0.78217608	-0.2486229	0.72489271	0.84084218	0.82582714	Bmal1	1
GO:0051701 biological process involved in interaction with host	1/138	135/14464	0.00740741	0.77638218	-0.2561956	0.72754203	0.84294525	0.82789266	P4hb	1
GO:1904950 negative regulation of establishment of protein localization	1/138	135/14464	0.00740741	0.77638218	-0.2561956	0.72754203	0.84294525	0.82789266	Ndfip2	1
GO:0022604 regulation of cell morphogenesis	2/138	268/14464	0.00746269	0.78217608	-0.3532614	0.72854706	0.84362486	0.82856013	Rhoj/Sema3e	2
GO:1903825 organic acid transmembrane transport	1/138	136/14464	0.00735294	0.77067349	-0.2637164	0.73016603	0.84501418	0.82992465	Slc17a8	1
GO:0003012 muscle system process	3/138	395/14464	0.00759494	0.79603742	-0.4033887	0.73139313	0.84559479	0.83049488	Kcnip2/Pkp2/Sri	3
GO:0035725 sodium ion transmembrane transport	1/138	137/14464	0.00729927	0.76504813	-0.2711863	0.73276493	0.84559479	0.83049488	Slc17a8	1
GO:0046488 phosphatidylinositol metabolic process	1/138	137/14464	0.00729927	0.76504813	-0.2711863	0.73276493	0.84559479	0.83049488	Pigg	1
GO:0072073 kidney epithelium development	1/138	137/14464	0.00729927	0.76504813	-0.2711863	0.73276493	0.84559479	0.83049488	Smad2	1
GO:1902600 proton transmembrane transport	1/138	137/14464	0.00729927	0.76504813	-0.2711863	0.73276493	0.84559479	0.83049488	Atp6v0b	1
GO:0002764 immune response-regulating signaling pathway	3/138	397/14464	0.00755668	0.79202716	-0.4123893	0.73453669	0.8460507	0.83094265	Card11/Hspd1/Itch	3
GO:0050768 negative regulation of neurogenesis	1/138	138/14464	0.00724638	0.75950431	-0.2786061	0.73533898	0.8460507	0.83094265	Dnajb11	1
GO:0050851 antigen receptor-mediated signaling pathway	1/138	138/14464	0.00724638	0.75950431	-0.2786061	0.73533898	0.8460507	0.83094265	Card11	1
GO:0051099 positive regulation of binding	1/138	138/14464	0.00724638	0.75950431	-0.2786061	0.73533898	0.8460507	0.83094265	Aktip	1
GO:0009101 glycoprotein biosynthetic process	2/138	272/14464	0.00735294	0.77067349	-0.3747341	0.73607273	0.8460507	0.83094265	Fktn/Crppa	2
GO:0061448 connective tissue development	2/138	272/14464	0.00735294	0.77067349	-0.3747341	0.73607273	0.8460507	0.83094265	Scin/Ift80	2
GO:0043086 negative regulation of catalytic activity	3/138	398/14464	0.00753769	0.79003714	-0.4168742	0.73609768	0.8460507	0.83094265	Chordc1/Dnajc3/Bag5	3
GO:0006997 nucleus organization	1/138	139/14464	0.00719424	0.75404025	-0.2859765	0.73788842	0.84622278	0.83111166	Tbpl1	1
GO:0032635 interleukin-6 production	1/138	139/14464	0.00719424	0.75404025	-0.2859765	0.73788842	0.84622278	0.83111166	Hspd1	1
GO:0120161 regulation of cold-induced thermogenesis	1/138	139/14464	0.00719424	0.75404025	-0.2859765	0.73788842	0.84622278	0.83111166	Bmal1	1
GO:0070371 ERK1 and ERK2 cascade	2/138	273/14464	0.00732601	0.76785051	-0.3800571	0.73792641	0.84622278	0.83111166	S2bpcox16/Spred3	2
GO:0051250 negative regulation of lymphocyte activation	1/138	140/14464	0.00714286	0.74865424	-0.2932984	0.74041347	0.84859213	0.8334387	Itch	1
GO:0031023 microtubule organizing center organization	1/138	141/14464	0.0070922	0.74334464	-0.3005724	0.74291438	0.84952659	0.83435647	Chordc1	1
GO:0031644 regulation of nervous system process	1/138	141/14464	0.0070922	0.74334464	-0.3005724	0.74291438	0.84952659	0.83435647	Smr2l	1
GO:0044839 cell cycle G2/M phase transition	1/138	141/14464	0.0070922	0.74334464	-0.3005724	0.74291438	0.84952659	0.83435647	Arpp19	1
GO:0055088 lipid homeostasis	1/138	141/14464	0.0070922	0.74334464	-0.3005724	0.74291438	0.84952659	0.83435647	Pkp2	1
GO:0048592 eye morphogenesis	1/138	142/14464	0.00704225	0.73810982	-0.3077994	0.74539136	0.85130141	0.8360996	Ift172	1
GO:0140013 meiotic nuclear division	1/138	142/14464	0.00704225	0.73810982	-0.3077994	0.74539136	0.85130141	0.8360996	Calr	1
GO:0043491 phosphatidylinositol 3-kinase/protein kinase B signal transduction	2/138	278/14464	0.00719424	0.75404025	-0.4064085	0.74703039	0.85130141	0.8360996	Sema3e/Ift80	2
GO:0000723 telomere maintenance	1/138	143/14464	0.00699301	0.73294821	-0.3149801	0.74784465	0.85130141	0.8360996	Hnrnpc	1
GO:0001890 placenta development	1/138	143/14464	0.00699301	0.73294821	-0.3149801	0.74784465	0.85130141	0.8360996	Tppp3	1
GO:0032006 regulation of TOR signaling	1/138	143/14464	0.00699301	0.73294821	-0.3149801	0.74784465	0.85130141	0.8360996	Bmal1	1
GO:0043123 positive regulation of canonical NF-kappaB signal transduction	1/138	143/14464	0.00699301	0.73294821	-0.3149801	0.74784465	0.85130141	0.8360996	Card11	1
GO:0106106 cold-induced thermogenesis	1/138	143/14464	0.00699301	0.73294821	-0.3149801	0.74784465	0.85130141	0.8360996	Bmal1	1
GO:0030178 negative regulation of Wnt signaling pathway	1/138	144/14464	0.00694444	0.72785829	-0.3221151	0.75027446	0.85262307	0.83739765	Ift80	1
GO:0043281 regulation of cysteine-type endopeptidase activity involved in apoptotic process	1/138	144/14464	0.00694444	0.72785829	-0.3221151	0.75027446	0.85262307	0.83739765	Hspd1	1
GO:0051147 regulation of muscle cell differentiation	1/138	144/14464	0.00694444	0.72785829	-0.3221151	0.75027446	0.85262307	0.83739765	Mmp14	1
GO:0001933 negative regulation of protein phosphorylation	2/138	281/14464	0.00711744	0.74598999	-0.4220134	0.75236268	0.85343362	0.83819373	Chordc1/Dnajc3	2
GO:0046942 carboxylic acid transport	2/138	281/14464	0.00711744	0.74598999	-0.4220134	0.75236268	0.85343362	0.83819373	Slc51a/Slc17a8	2
GO:0007596 blood coagulation	1/138	145/14464	0.00689655	0.72283858	-0.3292052	0.75268104	0.85343362	0.83819373	Pdia3	1
GO:0060759 regulation of response to cytokine stimulus	1/138	145/14464	0.00689655	0.72283858	-0.3292052	0.75268104	0.85343362	0.83819373	Ppp2cb	1
GO:0030111 regulation of Wnt signaling pathway	2/138	282/14464	0.0070922	0.74334464	-0.4271815	0.75411867	0.85458305	0.83932264	Bmal1/Ift80	2
GO:0007286 spermatid development	1/138	146/14464	0.00684932	0.71788763	-0.336251	0.75506459	0.85469411	0.83943171	Tbpl1	1
GO:2001235 positive regulation of apoptotic signaling pathway	1/138	146/14464	0.00684932	0.71788763	-0.336251	0.75506459	0.85469411	0.83943171	Pdia3	1
GO:0015849 organic acid transport	2/138	283/14464	0.00706714	0.74071798	-0.4323331	0.75586402	0.85511889	0.83984891	Slc51a/Slc17a8	2
GO:0051961 negative regulation of nervous system development	1/138	147/14464	0.00680272	0.71300404	-0.3432533	0.75742533	0.85640463	0.84111169	Dnajb11	1
GO:0043254 regulation of protein-containing complex assembly	3/138	413/14464	0.00726392	0.7613433	-0.482949	0.75865906	0.85731876	0.8420095	Hspa5/Scin/Tppp3	3
GO:0002244 hematopoietic progenitor cell differentiation	1/138	148/14464	0.00675676	0.70818645	-0.3502126	0.75976348	0.85760536	0.84229098	Mettl14	1
GO:0007599 hemostasis	1/138	148/14464	0.00675676	0.70818645	-0.3502126	0.75976348	0.85760536	0.84229098	Pdia3	1
GO:1902105 regulation of leukocyte differentiation	2/138	286/14464	0.00699301	0.73294821	-0.4476899	0.76103656	0.85856167	0.84323021	Card11/Mmp14	2
GO:0032200 telomere organization	1/138	149/14464	0.00671141	0.70343352	-0.3571295	0.76207925	0.85902776	0.84368798	Hnrnpc	1
GO:0008064 regulation of actin polymerization or depolymerization	1/138	150/14464	0.00666667	0.69874396	-0.3640048	0.76437286	0.85902776	0.84368798	Scin	1
GO:0010977 negative regulation of neuron projection development	1/138	150/14464	0.00666667	0.69874396	-0.3640048	0.76437286	0.85902776	0.84368798	Bag5	1
GO:0031214 biomineral tissue development	1/138	150/14464	0.00666667	0.69874396	-0.3640048	0.76437286	0.85902776	0.84368798	Ift80	1
GO:0031623 receptor internalization	1/138	150/14464	0.00666667	0.69874396	-0.3640048	0.76437286	0.85902776	0.84368798	Itch	1
GO:0050817 coagulation	1/138	150/14464	0.00666667	0.69874396	-0.3640048	0.76437286	0.85902776	0.84368798	Pdia3	1
GO:0045787 positive regulation of cell cycle	2/138	288/14464	0.00694444	0.72785829	-0.4578474	0.76443244	0.85902776	0.84368798	Calr/Pkp3	2
GO:0030832 regulation of actin filament length	1/138	151/14464	0.00662252	0.69411652	-0.370839	0.76664452	0.86055421	0.84518717	Scin	1
GO:0045580 regulation of T cell differentiation	1/138	151/14464	0.00662252	0.69411652	-0.370839	0.76664452	0.86055421	0.84518717	Card11	1
GO:0048545 response to steroid hormone	2/138	290/14464	0.00689655	0.72283858	-0.4679418	0.76778673	0.86087766	0.84550485	Bmal1/Calr	2
GO:0070588 calcium ion transmembrane transport	2/138	290/14464	0.00689655	0.72283858	-0.4679418	0.76778673	0.86087766	0.84550485	Rrad/Sri	2
GO:0050714 positive regulation of protein secretion	1/138	152/14464	0.00657895	0.68954996	-0.3776327	0.76889444	0.86164046	0.84625403	Sri	1
GO:0048511 rhythmic process	2/138	291/14464	0.00687285	0.7203546	-0.4729656	0.76944838	0.86178218	0.84639322	Bmal1/Has2	2
GO:0045814 negative regulation of gene expression, epigenetic	1/138	153/14464	0.00653595	0.6850431	-0.3843865	0.77112282	0.862699	0.84729367	Cbx1	1
GO:0046887 positive regulation of hormone secretion	1/138	153/14464	0.00653595	0.6850431	-0.3843865	0.77112282	0.862699	0.84729367	Sri	1
GO:0042692 muscle cell differentiation	3/138	423/14464	0.0070922	0.74334464	-0.5258087	0.77282637	0.8637302	0.84830645	Calr/Mmp14/Fktn	3
GO:0048515 spermatid differentiation	1/138	154/14464	0.00649351	0.68059477	-0.3911009	0.77332987	0.8637302	0.84830645	Tbpl1	1
GO:0062013 positive regulation of small molecule metabolic process	1/138	154/14464	0.00649351	0.68059477	-0.3911009	0.77332987	0.8637302	0.84830645	Arpp19	1
GO:0045862 positive regulation of proteolysis	2/138	296/14464	0.00675676	0.70818645	-0.4978555	0.77760346	0.86666332	0.85118719	Mmp14/Hspd1	2
GO:0001906 cell killing	1/138	156/14464	0.00641026	0.67186919	-0.4044139	0.77768077	0.86666332	0.85118719	Cr1l	1
GO:0030010 establishment of cell polarity	1/138	156/14464	0.00641026	0.67186919	-0.4044139	0.77768077	0.86666332	0.85118719	Rhoj	1
GO:0030099 myeloid cell differentiation	3/138	428/14464	0.00700935	0.73466071	-0.5469001	0.77965384	0.86666332	0.85118719	Scin/Sh3pxd2a/Ift80	3
GO:0002700 regulation of production of molecular mediator of immune response	1/138	157/14464	0.00636943	0.66758977	-0.4110136	0.77982503	0.86666332	0.85118719	Pkp3	1
GO:0002753 cytoplasmic pattern recognition receptor signaling pathway	1/138	157/14464	0.00636943	0.66758977	-0.4110136	0.77982503	0.86666332	0.85118719	Itch	1
GO:0062207 regulation of pattern recognition receptor signaling pathway	1/138	157/14464	0.00636943	0.66758977	-0.4110136	0.77982503	0.86666332	0.85118719	Itch	1
GO:1990845 adaptive thermogenesis	1/138	157/14464	0.00636943	0.66758977	-0.4110136	0.77982503	0.86666332	0.85118719	Bmal1	1
GO:2000058 regulation of ubiquitin-dependent protein catabolic process	1/138	157/14464	0.00636943	0.66758977	-0.4110136	0.77982503	0.86666332	0.85118719	Bag5	1
GO:0019216 regulation of lipid metabolic process	2/138	298/14464	0.00671141	0.70343352	-0.5077066	0.78079481	0.86726299	0.85177615	Rab38/Pdk3	2
GO:0006865 amino acid transport	1/138	158/14464	0.00632911	0.66336452	-0.4175761	0.78194876	0.86804814	0.85254728	Slc17a8	1
GO:0061025 membrane fusion	1/138	159/14464	0.00628931	0.65919242	-0.4241018	0.78405215	0.86804814	0.85254728	Sec22b	1
GO:0071897 DNA biosynthetic process	1/138	159/14464	0.00628931	0.65919242	-0.4241018	0.78405215	0.86804814	0.85254728	Hnrnpc	1
GO:0097553 calcium ion transmembrane import into cytosol	1/138	159/14464	0.00628931	0.65919242	-0.4241018	0.78405215	0.86804814	0.85254728	Sri	1
GO:0016032 viral process	2/138	301/14464	0.00664452	0.69642255	-0.5223738	0.78550719	0.86804814	0.85254728	Srpk1/P4hb	2
GO:0042326 negative regulation of phosphorylation	2/138	301/14464	0.00664452	0.69642255	-0.5223738	0.78550719	0.86804814	0.85254728	Chordc1/Dnajc3	2
GO:0071214 cellular response to abiotic stimulus	2/138	301/14464	0.00664452	0.69642255	-0.5223738	0.78550719	0.86804814	0.85254728	Calr/Oxsr1	2
GO:0104004 cellular response to environmental stimulus	2/138	301/14464	0.00664452	0.69642255	-0.5223738	0.78550719	0.86804814	0.85254728	Calr/Oxsr1	2
GO:0002683 negative regulation of immune system process	3/138	433/14464	0.00692841	0.72617733	-0.5677737	0.78631338	0.86804814	0.85254728	Dtx4/Cr1l/Itch	3
GO:0097193 intrinsic apoptotic signaling pathway	2/138	302/14464	0.00662252	0.69411652	-0.5272341	0.78705826	0.86804814	0.85254728	Bag5/P4hb	2

GO:0002695 negative regulation of leukocyte activation	1/138	161/14464	0.00621118	0.65100369	-0.4370453	0.78819868	0.86804814	0.85254728	Itch	1
GO:0008217 regulation of blood pressure	1/138	161/14464	0.00621118	0.65100369	-0.4370453	0.78819868	0.86804814	0.85254728	Manf	1
GO:0032680 regulation of tumor necrosis factor production	1/138	161/14464	0.00621118	0.65100369	-0.4370453	0.78819868	0.86804814	0.85254728	Hspd1	1
GO:0033157 regulation of intracellular protein transport	1/138	161/14464	0.00621118	0.65100369	-0.4370453	0.78819868	0.86804814	0.85254728	Frat2	1
GO:0051897 positive regulation of phosphatidylinositol 3-kinase/protein kinase B signal transduction	1/138	161/14464	0.00621118	0.65100369	-0.4370453	0.78819868	0.86804814	0.85254728	Sema3e	1
GO:0001822 kidney development	2/138	303/14464	0.00660066	0.6918257	-0.5320801	0.78859953	0.86804814	0.85254728	Has2/Smad2	2
GO:0007034 vacuolar transport	1/138	162/14464	0.00617284	0.64698515	-0.443464	0.79024221	0.86804814	0.85254728	Aktip	1
GO:0042391 regulation of membrane potential	3/138	437/14464	0.00686499	0.71953039	-0.5843195	0.79152162	0.86804814	0.85254728	Kcnip2/Gclm/Pkp2	3
GO:0062197 cellular response to chemical stress	2/138	305/14464	0.00655738	0.68728914	-0.54173	0.79165288	0.86804814	0.85254728	Bmal1/Oxsr1	2
GO:0002285 lymphocyte activation involved in immune response	1/138	163/14464	0.00613497	0.64301592	-0.4498479	0.79226616	0.86804814	0.85254728	Hspd1	1
GO:0006606 protein import into nucleus	1/138	163/14464	0.00613497	0.64301592	-0.4498479	0.79226616	0.86804814	0.85254728	Bmal1	1
GO:0010508 positive regulation of autophagy	1/138	163/14464	0.00613497	0.64301592	-0.4498479	0.79226616	0.86804814	0.85254728	Snx18	1
GO:0051017 actin filament bundle assembly	1/138	163/14464	0.00613497	0.64301592	-0.4498479	0.79226616	0.86804814	0.85254728	Hsp90b1	1
GO:1903555 regulation of tumor necrosis factor superfamily cytokine production	1/138	163/14464	0.00613497	0.64301592	-0.4498479	0.79226616	0.86804814	0.85254728	Hspd1	1
GO:2000116 regulation of cysteine-type endopeptidase activity	1/138	163/14464	0.00613497	0.64301592	-0.4498479	0.79226616	0.86804814	0.85254728	Hspd1	1
GO:0034504 protein localization to nucleus	2/138	306/14464	0.00653595	0.6850431	-0.546534	0.79316504	0.86808943	0.85258783	Bmal1/Calr	2
GO:0051347 positive regulation of transferase activity	2/138	306/14464	0.00653595	0.6850431	-0.546534	0.79316504	0.86808943	0.85258783	Fzd8/Sash1	2
GO:0030534 adult behavior	1/138	164/14464	0.00609756	0.63909509	-0.4561976	0.79427073	0.86882788	0.8533131	Hexa	1
GO:0032102 negative regulation of response to external stimulus	2/138	308/14464	0.00649351	0.68059477	-0.5561006	0.79616057	0.87055543	0.85451873	Dtx4/Itch	2
GO:0035051 cardiocyte differentiation	1/138	165/14464	0.00606061	0.63522178	-0.4625134	0.79625609	0.87055543	0.85451873	Calr	1
GO:0042254 ribosome biogenesis	2/138	309/14464	0.00647249	0.6783922	-0.5608635	0.79764402	0.87008768	0.8545504	Mettl16/Tfb1m	2
GO:0032640 tumor necrosis factor production	1/138	166/14464	0.0060241	0.63139515	-0.4687958	0.79822242	0.87008768	0.8545504	Hspd1	1
GO:0061572 actin filament bundle organization	1/138	166/14464	0.0060241	0.63139515	-0.4687958	0.79822242	0.87008768	0.8545504	Hsp90b1	1
GO:1903046 meiotic cell cycle process	1/138	166/14464	0.0060241	0.63139515	-0.4687958	0.79822242	0.87008768	0.8545504	Calr	1
GO:0030100 regulation of endocytosis	2/138	310/14464	0.00645161	0.67620383	-0.5656128	0.79911798	0.87008768	0.8545504	Calr/Lman2	2
GO:0045089 positive regulation of innate immune response	2/138	310/14464	0.00645161	0.67620383	-0.5656128	0.79911798	0.87008768	0.8545504	Hspd1/Itch	2
GO:0071248 cellular response to metal ion	1/138	167/14464	0.00598802	0.62761434	-0.4750453	0.80016992	0.87008768	0.8545504	Calr	1
GO:0098739 import across plasma membrane	1/138	167/14464	0.00598802	0.62761434	-0.4750453	0.80016992	0.87008768	0.8545504	Slc17a8	1
GO:1902905 positive regulation of supramolecular fiber organization	1/138	167/14464	0.00598802	0.62761434	-0.4750453	0.80016992	0.87008768	0.8545504	Scin	1
GO:0009615 response to virus	2/138	312/14464	0.00641026	0.67186919	-0.5750712	0.80203765	0.87030737	0.85476617	Calr/Itch	2
GO:0000910 cytokinesis	1/138	168/14464	0.00595238	0.62387854	-0.4812622	0.80209875	0.87030737	0.85476617	Snx18	1
GO:0016052 carbohydrate catabolic process	1/138	168/14464	0.00595238	0.62387854	-0.4812622	0.80209875	0.87030737	0.85476617	Wdr45b	1
GO:0071706 tumor necrosis factor superfamily cytokine production	1/138	168/14464	0.00595238	0.62387854	-0.4812622	0.80209875	0.87030737	0.85476617	Hspd1	1
GO:0009952 anterior/posterior pattern specification	1/138	169/14464	0.00591716	0.62018695	-0.487447	0.80400911	0.87105743	0.85550283	Smad2	1
GO:0051170 import into nucleus	1/138	169/14464	0.00591716	0.62018695	-0.487447	0.80400911	0.87105743	0.85550283	Bmal1	1
GO:1903131 mononuclear cell differentiation	3/138	447/14464	0.00671141	0.70343352	-0.6251073	0.80408625	0.87105743	0.85550283	Card11/Mmp14/Fzd8	3
GO:0046890 regulation of lipid biosynthetic process	1/138	170/14464	0.00588235	0.61653879	-0.4936001	0.80590115	0.87177436	0.85620696	Rab38	1
GO:0072001 renal system development	2/138	315/14464	0.00634921	0.66547044	-0.5891599	0.80634713	0.87177436	0.85620696	Has2/Smad2	2
GO:0022408 negative regulation of cell-cell adhesion	1/138	171/14464	0.00584795	0.6129333	-0.499722	0.80777506	0.87177436	0.85620696	Itch	1
GO:0030308 negative regulation of cell growth	1/138	171/14464	0.00584795	0.6129333	-0.499722	0.80777506	0.87177436	0.85620696	Rrad	1
GO:0051494 negative regulation of cytoskeleton organization	1/138	171/14464	0.00584795	0.6129333	-0.499722	0.80777506	0.87177436	0.85620696	Scin	1
GO:1902107 positive regulation of leukocyte differentiation	1/138	171/14464	0.00584795	0.6129333	-0.499722	0.80777506	0.87177436	0.85620696	Mmp14	1
GO:1903708 positive regulation of hemopoiesis	1/138	171/14464	0.00584795	0.6129333	-0.499722	0.80777506	0.87177436	0.85620696	Mmp14	1
GO:1902904 negative regulation of supramolecular fiber organization	1/138	172/14464	0.00581395	0.60936973	-0.5058129	0.809631	0.87330984	0.85771503	Scin	1
GO:0030307 positive regulation of cell growth	1/138	173/14464	0.00578035	0.60584737	-0.5118733	0.81146916	0.87405693	0.85844877	Mmp14	1
GO:0071222 cellular response to lipopolysaccharide	1/138	173/14464	0.00578035	0.60584737	-0.5118733	0.81146916	0.87405693	0.85844877	Sash1	1
GO:0062012 regulation of small molecule metabolic process	2/138	319/14464	0.00626959	0.65712598	-0.6077636	0.81196427	0.87405693	0.85844877	Pdk3/Arpp19	2
GO:0001659 temperature homeostasis	1/138	174/14464	0.00574713	0.60236548	-0.5179035	0.81328969	0.87405693	0.85844877	Bmal1	1
GO:0048771 tissue remodeling	1/138	174/14464	0.00574713	0.60236548	-0.5179035	0.81328969	0.87405693	0.85844877	Mmp14	1
GO:0042060 wound healing	2/138	321/14464	0.00623053	0.65303174	-0.6169895	0.81471843	0.87405693	0.85844877	Pdia3/Grhl3	2
GO:0006839 mitochondrial transport	1/138	175/14464	0.00571429	0.5989234	-0.523904	0.81509277	0.87405693	0.85844877	Hspd1	1
GO:0021543 pallium development	1/138	175/14464	0.00571429	0.5989234	-0.523904	0.81509277	0.87405693	0.85844877	Fktn	1
GO:0042770 signal transduction in response to DNA damage	1/138	175/14464	0.00571429	0.5989234	-0.523904	0.81509277	0.87405693	0.85844877	Gnb1l	1
GO:0048469 cell maturation	1/138	175/14464	0.00571429	0.5989234	-0.523904	0.81509277	0.87405693	0.85844877	Kcnip2	1
GO:1902806 regulation of cell cycle G1/S phase transition	1/138	175/14464	0.00571429	0.5989234	-0.523904	0.81509277	0.87405693	0.85844877	Pkp3	1
GO:0048284 organelle fusion	1/138	176/14464	0.00568182	0.59552042	-0.5298751	0.81687857	0.87550621	0.85987217	Sec22b	1
GO:0050866 negative regulation of cell activation	1/138	178/14464	0.00561798	0.58882918	-0.5417304	0.82039895	0.87810828	0.86242778	Itch	1
GO:0051495 positive regulation of cytoskeleton organization	1/138	178/14464	0.00561798	0.58882918	-0.5417304	0.82039895	0.87810828	0.86242778	Scin	1
GO:0001701 in utero embryonic development	3/138	461/14464	0.00650759	0.68207111	-0.6808888	0.8206131	0.87810828	0.86242778	Smad2/Cr1l/Sh3pxd2a	3
GO:0051091 positive regulation of DNA-binding transcription factor activity	1/138	179/14464	0.00558659	0.58553963	-0.5476154	0.82213386	0.87926889	0.86356766	Card11	1
GO:0071219 cellular response to molecule of bacterial origin	1/138	180/14464	0.00555556	0.58228663	-0.5534724	0.82385213	0.88063939	0.86491369	Sash1	1
GO:0000075 cell cycle checkpoint signaling	1/138	181/14464	0.00552486	0.57906958	-0.5593017	0.82555392	0.88152368	0.86578219	Gnb1l	1
GO:0051260 protein homooligomerization	1/138	181/14464	0.00552486	0.57906958	-0.5593017	0.82555392	0.88152368	0.86578219	Card11	1
GO:0042742 defense response to bacterium	1/138	182/14464	0.00549451	0.57588788	-0.5651037	0.82723939	0.88238869	0.86663174	Ighg2c	1
GO:0098742 cell-cell adhesion via plasma-membrane adhesion molecules	1/138	182/14464	0.00549451	0.57588788	-0.5651037	0.82723939	0.88238869	0.86663174	Cdh10	1
GO:0061136 regulation of proteasomal protein catabolic process	1/138	184/14464	0.00543478	0.56962823	-0.5766268	0.83056198	0.88546428	0.86965242	Bag5	1
GO:0044242 cellular lipid catabolic process	1/138	185/14464	0.00540541	0.56654916	-0.5823487	0.83219941	0.88674102	0.87090636	Hexa	1
GO:0001649 osteoblast differentiation	1/138	186/14464	0.00537634	0.56350319	-0.5880444	0.83382112	0.88753083	0.87168206	Ift80	1
GO:0099175 regulation of postsynapse organization	1/138	186/14464	0.00537634	0.56350319	-0.5880444	0.83382112	0.88753083	0.87168206	Zfp804a	1
GO:0006399 tRNA metabolic process	1/138	187/14464	0.00534759	0.56048981	-0.5937143	0.83542728	0.88830242	0.87243988	Yars2	1
GO:1901617 organic hydroxy compound biosynthetic process	1/138	187/14464	0.00534759	0.56048981	-0.5937143	0.83542728	0.88830242	0.87243988	Rab38	1
GO:0018105 peptidyl-serine phosphorylation	1/138	188/14464	0.00531915	0.55750848	-0.5993588	0.83701802	0.88905602	0.87318002	Pdk3	1
GO:0050905 neuromuscular process	1/138	188/14464	0.00531915	0.55750848	-0.5993588	0.83701802	0.88905602	0.87318002	Hexa	1
GO:0060271 cilium assembly	2/138	339/14464	0.00589971	0.61835749	-0.6978605	0.83793712	0.88956358	0.87367851	Ift172/Ift80	2
GO:0061351 neural precursor cell proliferation	1/138	189/14464	0.00529101	0.5545587	-0.604978	0.8385935	0.88979184	0.8739027	Tafa1	1
GO:0015931 nucleobase-containing compound transport	1/138	192/14464	0.00520833	0.54589372	-0.6216873	0.84322978	0.89330071	0.87734891	Slc33a1	1
GO:0031334 positive regulation of protein-containing complex assembly	1/138	192/14464	0.00520833	0.54589372	-0.6216873	0.84322978	0.89330071	0.87734891	Tppp3	1
GO:1901654 response to ketone	1/138	192/14464	0.00520833	0.54589372	-0.6216873	0.84322978	0.89330071	0.87734891	Calr	1
GO:0010563 negative regulation of phosphorus metabolic process	2/138	345/14464	0.0057971	0.60760344	-0.7240008	0.84507657	0.89431725	0.8783473	Chordc1/Dnajc3	2
GO:0045936 negative regulation of phosphate metabolic process	2/138	345/14464	0.0057971	0.60760344	-0.7240008	0.84507657	0.89431725	0.8783473	Chordc1/Dnajc3	2
GO:0045637 regulation of myeloid cell differentiation	1/138	194/14464	0.00515464	0.54026595	-0.6327058	0.84624694	0.89508596	0.87910228	Scin	1
GO:0008361 regulation of cell size	1/138	195/14464	0.00512821	0.53749535	-0.6381796	0.84773383	0.89571877	0.87972379	Oxsr1	1
GO:0045165 cell fate commitment	1/138	195/14464	0.00512821	0.53749535	-0.6381796	0.84773383	0.89571877	0.87972379	Smad2	1
GO:0051963 regulation of synapse assembly	1/138	197/14464	0.00507614	0.53203855	-0.6490574	0.85066492	0.89834494	0.88230306	Lrrn1	1
GO:0002064 epithelial cell development	1/138	198/14464	0.00505051	0.52935149	-0.654462	0.85210939	0.89939923	0.88333853	Bmal1	1
GO:0018209 peptidyl-serine modification	1/138	199/14464	0.00502513	0.52669143	-0.659844	0.85353999	0.90043779	0.88435854	Pdk3	1
GO:0019221 cytokine-mediated signaling pathway	2/138	353/14464	0.00566572	0.59383339	-0.7582614	0.85415496	0.90061528	0.88453286	Ppp2cb/Oxsr1	2
GO:0008406 gonad development	1/138	200/14464	0.005	0.52405797	-0.6652037	0.85495685	0.90098955	0.88490045	Mmp14	1
GO:0000302 response to reactive oxygen species	1/138	201/14464	0.00497512	0.52145072	-0.6705413	0.8563601	0.9010553	0.88496503	Ppp2cb	1
GO:0009636 response to toxic substance	1/138	201/14464	0.00497512	0.52145072	-0.6705413	0.8563601	0.9010553	0.88496503	Akr1a1	1
GO:0031345 negative regulation of cell projection organization	1/138	201/14464	0.00497512	0.52145072	-0.6705413	0.8563601	0.9010553	0.88496503	Bag5	1
GO:0009612 response to mechanical stimulus	1/138	202/14464	0.0049505	0.51886928	-0.675857	0.85774987	0.9015765	0.88547692	Mmp14	1
GO:0035264 multicellular organism growth	1/138	202/14464	0.0049505	0.51886928	-0.675857	0.85774987	0.9015765	0.88547692	Ift80	1
GO:0044770 cell cycle phase transition	3/138	498/14464	0.0060241	0.63139515	-0.8215726	0.85871626	0.90212193	0.88601261	Gnb1l/Pkp3/Arpp19	3
GO:0007623 circadian rhythm	1/138	204/14464	0.00490196	0.51378232	-0.6864238	0.86048949	0.90320685	0.88707815	Bmal1	1
GO:0007018 microtubule-based movement	2/138	359/14464	0.00557103	0.5839086	-0.7835301	0.86064502	0.90320685	0.88707815	Ift172/Ift80	2
GO:0032535 regulation of cellular component size	2/138	365/14464	0.00547945	0.57431011	-0.8084476	0.8668719	0.90926834	0.89303141	Scin/Oxsr1	2
GO:0042063 gliogenesis	2/138	366/14464	0.00546448	0.57274095	-0.8125673	0.86788476	0.90985734	0.89360989	Mmp14/Mettl14	2
GO:0051321 meiotic cell cycle	1/138	211/14464	0.00473934	0.49673741	-0.7227515	0.86967208	0.91125723	0.89498478	Calr	1
GO:0044782 cilium organization	2/138	369/14464	0.00542005	0.56808452	-0.8248703	0.87088134	0.9115201	0.89524296	Ift172/Ift80	2
GO:0002699 positive regulation of immune effector process	1/138	212/14464	0.00471698	0.49439431	-0.7278606	0.87093394	0.9115201	0.89524296	Hspd1	1
GO:0050807 regulation of synapse organization	2/138	370/14464	0.00540541	0.56654916	-0.8289529	0.87186635	0.9115201	0.89524296	Lrrn1/Zfp804a	2
GO:0002274 myeloid leukocyte activation	1/138	213/14464	0.00469484	0.49207321	-0.7329503	0.87218367	0.9115201	0.89524296	Hspd1	1
GO:0043122 regulation of canonical NF-kappaB signal transduction	1/138	213/14464	0.00469484	0.49207321	-0.7329503	0.87218367	0.9115201	0.89524296	Card11	1
GO:0009165 nucleotide biosynthetic process	1/138	214/14464	0.0046729	0.4897738	-0.7380206	0.87342138	0.91234068	0.89604888	Tbpl1	1
GO:0002429 immune response-activating cell surface receptor signaling pathway	1/138	216/14464	0.00462963	0.48523886	-0.7481039	0.87586122	0.91394215	0.89762175	Card11	1
GO:0045926 negative regulation of growth	1/138	216/14464	0.00462963	0.48523886	-0.7481039	0.87586122	0.91394215	0.89762175	Rrad	1
GO:0052548 regulation of endopeptidase activity	1/138	218/14464	0.00458716	0.48078713	-0.7581122	0.87825437	0.91534893	0.89900341	Hspd1	1
GO:1903050 regulation of proteolysis involved in protein catabolic process	1/138	218/14464	0.00458716	0.48078713	-0.7581122	0.87825437	0.91534893	0.89900341	Bag5	1
GO:0006816 calcium ion transport	2/138	377/14464	0.00530504	0.55602968	-0.8572779	0.87857151	0.91534893	0.89900341	Rrad/Sri	2
GO:0006836 neurotransmitter transport	1/138	219/14464	0.00456621	0.47859175	-0.7630886	0.87943371	0.91577395	0.89942084	Slc17a8	1
GO:1903828 negative regulation of protein localization	1/138	221/14464	0.00452489	0.47426061	-0.772987	0.88175848	0.91772075	0.90133288	Ndfip2	1
GO:0090068 positive regulation of cell cycle process	1/138	222/14464	0.0045045	0.4721243	-0.7779092	0.88290412	0.91843896	0.90203826	Pkp3	1
GO:0045861 negative regulation of proteolysis	1/138	224/14464	0.00446429	0.4679089	-0.7877008	0.88516244	0.92031329	0.90387913	Bag5	1
GO:0001819 positive regulation of cytokine production	2/138	386/14464	0.00518135	0.54306525	-0.8930661	0.88672023	0.92145772	0.90500312	Card11/Hspd1	2
GO:0060828 regulation of canonical Wnt signaling pathway	1/138	226/14464	0.00442478	0.46376812	-0.7974227	0.88737751	0.92166567	0.90520735	Bmal1	1
GO:0002768 immune response-regulating cell surface receptor signaling pathway	1/138	227/14464	0.00440529	0.46172508	-0.8022581	0.88846909	0.92232425	0.90585417	Card11	1
GO:0001558 regulation of cell growth	2/138	389/14464	0.00514139	0.53887709	-0.9048437	0.88932282	0.92273536	0.90625794	Mmp14/Rrad	2
GO:1901136 carbohydrate derivative catabolic process	1/138	229/14464	0.00436681	0.45769255	-0.8118781	0.89062084	0.9236068	0.90711382	Hexa	1
GO:0040029 epigenetic regulation of gene expression	1/138	230/14464	0.00434783	0.45570258	-0.8166632	0.89168121	0.92423101	0.90772688	Cbx1	1
GO:0010876 lipid localization	2/138	393/14464	0.00508906	0.53339234	-0.9204325	0.89270737	0.92481915	0.90830452	Slc51a/Hexa	2
GO:0044843 cell cycle G1/S phase transition	1/138	233/14464	0.00429185	0.44983517	-0.8309202	0.89480146	0.92651245	0.90996759	Pkp3	1
GO:0033044 regulation of chromosome organization	1/138	236/14464	0.00423729	0.44411692	-0.8450332	0.89783247	0.92917364	0.91258125	Hnrnpc	1
GO:0022411 cellular component disassembly	2/138	400/14464	0.005	0.52405797	-0.9474055	0.89840167	0.92928567	0.91269128	Scin/Snx14	2
GO:1901987 regulation of cell cycle phase transition	2/138	402/14464	0.00497512	0.52145072	-0.9550419	0.89997658	0.93043732	0.91382237	Gnb1l/Pkp3	2
GO:0007626 locomotory behavior	1/138	240/14464	0.00416667	0.43671498	-0.863633	0.90173934	0.93082771	0.91420578	Hexa	1
GO:0051896 regulation of phosphatidylinositol 3-kinase/protein kinase B signal transduction	1/138	240/14464	0.00416667	0.43671498	-0.863633	0.90173934	0.93082771	0.91420578	Sema3e	1
GO:1901988 negative regulation of cell cycle phase transition	1/138	240/14464	0.00416667	0.43671498	-0.863633	0.90173934	0.93082771	0.91420578	Gnb1l	1
GO:0002366 leukocyte activation involved in immune response	1/138	241/14464	0.00414938	0.43490288	-0.868245	0.90269266	0.93085852	0.91423604	Hspd1	1
GO:0045444 fat cell differentiation	1/138	241/14464	0.00414938	0.43490288	-0.868245	0.90269266	0.93085852	0.91423604	Bmal1	1
GO:0034599 cellular response to oxidative stress	1/138	243/14464	0.00411523	0.43132343	-0.8774243	0.90457183	0.93183712	0.91519717	Bmal1	1
GO:0051259 protein complex oligomerization	1/138	244/14464	0.00409836	0.42955571	-0.8819919	0.90549786	0.93183712	0.91519717	Card11	1
GO:0031349 positive regulation of defense response	2/138	410/14464	0.00487805	0.51127607	-0.9852849	0.90605298	0.93183712	0.91519717	Hspd1/Itch	2
GO:0002263 cell activation involved in immune response	1/138	245/14464	0.00408163	0.42780243	-0.886545	0.90641497	0.93183712	0.91519717	Hspd1	1
GO:0006260 DNA replication	1/138	245/14464	0.00408163	0.42780243	-0.886545	0.90641497	0.93183712	0.91519717	Obi1	1
GO:0032886 regulation of microtubule-based process	1/138	245/14464	0.00408163	0.42780243	-0.886545	0.90641497	0.93183712	0.91519717	Chordc1	1
GO:0097305 response to alcohol	1/138	247/14464	0.00404858	0.42433844	-0.8956081	0.90822277	0.93321972	0.91655508	Smad2	1
GO:0051607 defense response to virus	1/138	248/14464	0.00403226	0.4226274	-0.9001183	0.90911362	0.93365922	0.91698674	Itch	1
GO:0099504 synaptic vesicle cycle	1/138	249/14464	0.00401606	0.4209301	-0.9046144	0.90999589	0.93408946	0.9174093	Slc17a8	1
GO:0050767 regulation of neurogenesis	2/138	418/14464	0.00478469	0.50149088	-1.0150587	0.91178553	0.93532561	0.91862336	Bmal1/Dnajb11	2
GO:0043410 positive regulation of MAPK cascade	2/138	419/14464	0.00477327	0.50029401	-1.0187485	0.91247879	0.93532561	0.91862336	Fzd8/Sash1	2
GO:0010721 negative regulation of cell development	1/138	252/14464	0.00396825	0.41591902	-0.9180197	0.912592	0.93532561	0.91862336	Dnajb11	1
GO:0006898 receptor-mediated endocytosis	1/138	254/14464	0.00393701	0.41264407	-0.9268884	0.9142813	0.93658084	0.91985618	Itch	1
GO:0021537 telencephalon development	1/138	257/14464	0.00389105	0.40782721	-0.9400914	0.91675466	0.93803628	0.92128563	Fktn	1
GO:0031346 positive regulation of cell projection organization	2/138	426/14464	0.00469484	0.49207321	-1.0443824	0.91719141	0.93803628	0.92128563	Hspa5/Zfp804a	2
GO:0045927 positive regulation of growth	1/138	258/14464	0.00387597	0.40624649	-0.9444662	0.91756326	0.93803628	0.92128563	Mmp14	1
GO:0110053 regulation of actin filament organization	1/138	258/14464	0.00387597	0.40624649	-0.9444662	0.91756326	0.93803628	0.92128563	Scin	1
GO:0016042 lipid catabolic process	1/138	259/14464	0.003861	0.40467797	-0.948828	0.91836407	0.9383791	0.92162233	Hexa	1
GO:0051222 positive regulation of protein transport	1/138	262/14464	0.00381679	0.40004425	-0.9618368	0.92072045	0.93983414	0.92305139	Sri	1
GO:0052547 regulation of peptidase activity	1/138	262/14464	0.00381679	0.40004425	-0.9618368	0.92072045	0.93983414	0.92305139	Hspd1	1
GO:0009141 nucleoside triphosphate metabolic process	1/138	265/14464	0.00377358	0.39551545	-0.9747327	0.92300929	0.94169369	0.92487773	Tbpl1	1
GO:0048608 reproductive structure development	1/138	268/14464	0.00373134	0.39108804	-0.9875183	0.92523251	0.94348444	0.92663651	Mmp14	1
GO:0051052 regulation of DNA metabolic process	2/138	443/14464	0.00451467	0.47319004	-1.1052808	0.92766902	0.94497247	0.92809797	Obi1/Hnrnpc	2
GO:0010001 glial cell differentiation	1/138	272/14464	0.00367647	0.38533674	-1.0043988	0.92809797	0.94497247	0.92809797	Mettl14	1
GO:0061458 reproductive system development	1/138	272/14464	0.00367647	0.38533674	-1.0043988	0.92809797	0.94497247	0.92809797	Mmp14	1
GO:0007409 axonogenesis	2/138	446/14464	0.0044843	0.47000715	-1.1158368	0.929384	0.94520812	0.9283294	Sema3e/Crppa	2
GO:0030003 intracellular monoatomic cation homeostasis	2/138	447/14464	0.00447427	0.46895568	-1.1193432	0.92994713	0.94520812	0.9283294	Rab38/Sri	2
GO:0007281 germ cell development	1/138	276/14464	0.00362319	0.37975215	-1.0210934	0.93085437	0.94520812	0.9283294	Tbpl1	1
GO:0006091 generation of precursor metabolites and energy	2/138	449/14464	0.00445434	0.46686679	-1.1263377	0.93106079	0.94520812	0.9283294	Hmgcll1/Wdr45b	2
GO:0010948 negative regulation of cell cycle process	1/138	277/14464	0.00361011	0.37838121	-1.0252387	0.93152691	0.94520812	0.9283294	Gnb1l	1
GO:0032386 regulation of intracellular transport	1/138	277/14464	0.00361011	0.37838121	-1.0252387	0.93152691	0.94520812	0.9283294	Frat2	1
GO:0009117 nucleotide metabolic process	2/138	450/14464	0.00444444	0.46582931	-1.1298259	0.93161137	0.94520812	0.9283294	Tbpl1/Nt5c3b	2
GO:0006874 intracellular calcium ion homeostasis	1/138	278/14464	0.00359712	0.37702012	-1.0293728	0.93219297	0.94532244	0.92844169	Sri	1
GO:0006310 DNA recombination	1/138	279/14464	0.00358423	0.3756688	-1.0334958	0.93285259	0.9454976	0.92861372	Hspd1	1
GO:0006873 intracellular monoatomic ion homeostasis	2/138	454/14464	0.00440529	0.46172508	-1.1437188	0.93377268	0.9454976	0.92861372	Rab38/Sri	2
GO:0098771 inorganic ion homeostasis	2/138	454/14464	0.00440529	0.46172508	-1.1437188	0.93377268	0.9454976	0.92861372	Slc17a8/Sri	2

GO:0007416	synapse assembly	1/138	283/14464	0.00353357	0.37035899	-1.0498785	0.93542797	0.94622317	0.92932633	Lrrn1	1
GO:0032496	response to lipopolysaccharide	1/138	283/14464	0.00353357	0.37035899	-1.0498785	0.93542797	0.94622317	0.92932633	Sash1	1
GO:0009611	response to wounding	2/138	465/14464	0.00430108	0.45080256	-1.1814429	0.93938997	0.94975436	0.93279446	Pdia3/Grhl3	2
GO:0022412	cellular process involved in reproduction in multicellular organism	1/138	295/14464	0.00338983	0.35529354	-1.0980186	0.94258028	0.95202498	0.93502453	Tbpl1	1
GO:0055074	calcium ion homeostasis	1/138	295/14464	0.00338983	0.35529354	-1.0980186	0.94258028	0.95202498	0.93502453	Sri	1
GO:0002237	response to molecule of bacterial origin	1/138	297/14464	0.003367	0.35290099	-1.1059011	0.94369336	0.95267192	0.93565992	Sash1	1
GO:0016049	cell growth	2/138	476/14464	0.00420168	0.44038485	-1.2184884	0.94455624	0.95306576	0.93604672	Mmp14/Rrad	2
GO:0099003	vesicle-mediated transport in synapse	1/138	303/14464	0.00330033	0.34591285	-1.129318	0.94690569	0.95448093	0.93743663	Slc17a8	1
GO:0099173	postsynapse organization	1/138	303/14464	0.00330033	0.34591285	-1.129318	0.94690569	0.95448093	0.93743663	Zfp804a	1
GO:1903532	positive regulation of secretion by cell	1/138	315/14464	0.0031746	0.33273522	-1.1751596	0.95279454	0.95992107	0.94277963	Sri	1
GO:1904951	positive regulation of establishment of protein localization	1/138	316/14464	0.00316456	0.33168226	-1.1789225	0.95325495	0.95992107	0.94277963	Sri	1
GO:0015980	energy derivation by oxidation of organic compounds	1/138	330/14464	0.0030303	0.31761089	-1.23073	0.95925154	0.96547733	0.94823667	Wdr45b	1
GO:0051047	positive regulation of secretion	1/138	334/14464	0.00299401	0.31380717	-1.2452444	0.96081997	0.96657338	0.94931314	Sri	1
GO:0006869	lipid transport	1/138	342/14464	0.00292398	0.30646665	-1.2739095	0.96377925	0.96893945	0.95163696	Slc51a	1
GO:0045786	negative regulation of cell cycle	1/138	343/14464	0.00291545	0.30557316	-1.2774594	0.9641332	0.96893945	0.95163696	Gnb1l	1
GO:0051962	positive regulation of nervous system development	1/138	351/14464	0.002849	0.29860853	-1.3056015	0.96684391	0.97117953	0.95383704	Lrrn1	1
GO:0000280	nuclear division	1/138	360/14464	0.00277778	0.29114332	-1.3367334	0.96965098	0.97302956	0.95565403	Calr	1
GO:0048872	homeostasis of number of cells	1/138	360/14464	0.00277778	0.29114332	-1.3367334	0.96965098	0.97302956	0.95565403	Card11	1
GO:0007059	chromosome segregation	1/138	364/14464	0.00274725	0.28794394	-1.3503974	0.97082158	0.97371955	0.9563317	Srpk1	1
GO:1902903	regulation of supramolecular fiber organization	1/138	374/14464	0.0026738	0.2802449	-1.3841123	0.97355563	0.9759762	0.95854806	Scin	1
GO:0044772	mitotic cell cycle phase transition	1/138	399/14464	0.00250627	0.2626857	-1.4658026	0.97932819	0.98127516	0.96375239	Arpp19	1
GO:0048732	gland development	1/138	408/14464	0.00245098	0.25689116	-1.4943692	0.98108405	0.9824283	0.96488494	Smad2	1
GO:0043434	response to peptide hormone	1/138	410/14464	0.00243902	0.25563803	-1.5006603	0.98145367	0.9824283	0.96488494	Lta4h	1
GO:0048285	organelle fission	1/138	416/14464	0.00240385	0.25195095	-1.5194127	0.98252005	0.98300765	0.96545394	Calr	1
GO:0010720	positive regulation of cell development	1/138	473/14464	0.00211416	0.22158899	-1.6893569	0.99005352	0.99005352	0.97237399	Mmp14	1

ID	Description	GeneRatio	BgRatio	RichFactor	FoldEnrichm	zScore	pvalue	p.adjust	qvalue	geneID	Count
GO:0005504	fatty acid binding	2/23	39/14323	0.05128205	31.93534	7.75818284	0.00176293	0.08015468	0.04256673	Fabp7/Crabp1	2
GO:1901682	sulfur compound transmembrane transporter activity	2/23	52/14323	0.03846154	23.951505	6.64941398	0.00311498	0.08015468	0.04256673	Slc6a13/Slc47a1	2
GO:0033293	monocarboxylic acid binding	2/23	53/14323	0.03773585	23.4995898	6.58109687	0.00323398	0.08015468	0.04256673	Fabp7/Crabp1	2
GO:0022853	active monoatomic ion transmembrane transporter activity	3/23	194/14323	0.01546392	9.62998655	4.85347733	0.00354866	0.08015468	0.04256673	Slc6a13/Slc47a1/Slc9b2	3
GO:0015291	secondary active transmembrane transporter activity	3/23	218/14323	0.01376147	8.56980455	4.5167297	0.00491913	0.08015468	0.04256673	Slc6a13/Slc47a1/Slc9b2	3
GO:0019003	GDP binding	2/23	79/14323	0.02531646	15.7655476	5.27770189	0.00704995	0.08015468	0.04256673	Rab8a/Arl3	2
GO:0015171	amino acid transmembrane transporter activity	2/23	86/14323	0.02325581	14.4823054	5.02923439	0.00830664	0.08015468	0.04256673	Slc6a13/Slc47a1	2
GO:0003924	GTPase activity	3/23	275/14323	0.01090909	6.79351779	3.89044407	0.00933109	0.08015468	0.04256673	Rab8a/Rab13/Arl3	3
GO:0015297	antiporter activity	2/23	107/14323	0.01869159	11.6399837	4.4303892	0.0126281	0.08015468	0.04256673	Slc47a1/Slc9b2	2
GO:0005525	GTP binding	3/23	329/14323	0.00911854	5.67847231	3.44293491	0.01513081	0.08015468	0.04256673	Rab8a/Rab13/Arl3	3
GO:0015078	proton transmembrane transporter activity	2/23	118/14323	0.01694915	10.5549005	4.17969208	0.01520832	0.08015468	0.04256673	Slc47a1/Slc9b2	2
GO:0015081	sodium ion transmembrane transporter activity	2/23	118/14323	0.01694915	10.5549005	4.17969208	0.01520832	0.08015468	0.04256673	Slc6a13/Slc9b2	2
GO:0015203	polyamine transmembrane transporter activity	1/23	10/14323	0.1	62.273913	7.77334627	0.01594752	0.08015468	0.04256673	Slc47a1	1
GO:0019841	retinol binding	1/23	10/14323	0.1	62.273913	7.77334627	0.01594752	0.08015468	0.04256673	Crabp1	1
GO:0140161	monocarboxylate:sodium symporter activity	1/23	10/14323	0.1	62.273913	7.77334627	0.01594752	0.08015468	0.04256673	Slc6a13	1
GO:0022804	active transmembrane transporter activity	3/23	336/14323	0.00892857	5.56017081	3.39223707	0.01600329	0.08015468	0.04256673	Slc6a13/Slc47a1/Slc9b2	3
GO:0019001	guanyl nucleotide binding	3/23	345/14323	0.00869565	5.41512287	3.3291055	0.01716645	0.08015468	0.04256673	Rab8a/Rab13/Arl3	3
GO:0032561	guanyl ribonucleotide binding	3/23	345/14323	0.00869565	5.41512287	3.3291055	0.01716645	0.08015468	0.04256673	Rab8a/Rab13/Arl3	3
GO:1901474	azole transmembrane transporter activity	1/23	11/14323	0.09090909	56.6126482	7.39975753	0.01752882	0.08015468	0.04256673	Slc47a1	1
GO:0015378	sodium:chloride symporter activity	1/23	12/14323	0.08333333	51.8949275	7.07339427	0.0191077	0.08015468	0.04256673	Slc6a13	1
GO:0015385	sodium:proton antiporter activity	1/23	12/14323	0.08333333	51.8949275	7.07339427	0.0191077	0.08015468	0.04256673	Slc9b2	1
GO:0016918	retinal binding	1/23	12/14323	0.08333333	51.8949275	7.07339427	0.0191077	0.08015468	0.04256673	Crabp1	1
GO:0015355	secondary active monocarboxylate transmembrane transporter activity	1/23	13/14323	0.07692308	47.90301	6.78500712	0.02068414	0.08015468	0.04256673	Slc6a13	1
GO:0015373	monoatomic anion:sodium symporter activity	1/23	13/14323	0.07692308	47.90301	6.78500712	0.02068414	0.08015468	0.04256673	Slc6a13	1
GO:0061459	L-arginine transmembrane transporter activity	1/23	13/14323	0.07692308	47.90301	6.78500712	0.02068414	0.08015468	0.04256673	Slc47a1	1
GO:0031406	carboxylic acid binding	2/23	141/14323	0.0141844	8.83317916	3.74866481	0.0212658	0.08015468	0.04256673	Fabp7/Crabp1	2
GO:0046943	carboxylic acid transmembrane transporter activity	2/23	144/14323	0.01388889	8.64915459	3.69972647	0.02211932	0.08015468	0.04256673	Slc6a13/Slc47a1	2
GO:0001846	opsonin binding	1/23	14/14323	0.07142857	44.4813665	6.52770161	0.02225817	0.08015468	0.04256673	Megf10	1
GO:0015929	hexosaminidase activity	1/23	14/14323	0.07142857	44.4813665	6.52770161	0.02225817	0.08015468	0.04256673	Hexb	1
GO:0046703	natural killer cell lectin-like receptor binding	1/23	14/14323	0.07142857	44.4813665	6.52770161	0.02225817	0.08015468	0.04256673	Mill2	1
GO:0005342	organic acid transmembrane transporter activity	2/23	145/14323	0.0137931	8.58950525	3.68372935	0.02240699	0.08015468	0.04256673	Slc6a13/Slc47a1	2
GO:0043177	organic acid binding	2/23	149/14323	0.01342282	8.3589145	3.62124919	0.02357327	0.08015468	0.04256673	Fabp7/Crabp1	2
GO:0034236	protein kinase A catalytic subunit binding	1/23	15/14323	0.06666667	41.515942	6.29621919	0.02382977	0.08015468	0.04256673	Rab13	1
GO:0015174	basic amino acid transmembrane transporter activity	1/23	16/14323	0.0625	38.9211957	6.08646962	0.02539896	0.08292012	0.04403534	Slc47a1	1
GO:0031489	myosin V binding	1/23	17/14323	0.05882353	36.6317136	5.89521696	0.02696573	0.08551988	0.04541597	Rab8a	1
GO:0001848	complement binding	1/23	18/14323	0.05555556	34.5966184	5.71986293	0.02853009	0.08559028	0.04545335	Megf10	1
GO:0015377	chloride:monoatomic cation symporter activity	1/23	18/14323	0.05555556	34.5966184	5.71986293	0.02853009	0.08559028	0.04545335	Slc6a13	1
GO:0005283	amino acid:sodium symporter activity	1/23	20/14323	0.05	31.1369565	5.4087705	0.03165161	0.08636412	0.0458643	Slc6a13	1
GO:0015296	monoatomic anion:monoatomic cation symporter activity	1/23	20/14323	0.05	31.1369565	5.4087705	0.03165161	0.08636412	0.0458643	Slc6a13	1
GO:0051139	metal cation:proton antiporter activity	1/23	20/14323	0.05	31.1369565	5.4087705	0.03165161	0.08636412	0.0458643	Slc9b2	1
GO:0005044	scavenger receptor activity	1/23	21/14323	0.04761905	29.6542443	5.26984636	0.03320876	0.08636412	0.0458643	Megf10	1
GO:0019956	chemokine binding	1/23	21/14323	0.04761905	29.6542443	5.26984636	0.03320876	0.08636412	0.0458643	Plp2	1
GO:0022890	inorganic cation transmembrane transporter activity	3/23	446/14323	0.00672646	4.18882823	2.74377501	0.03345637	0.08636412	0.0458643	Slc6a13/Slc47a1/Slc9b2	3
GO:0005501	retinoid binding	1/23	22/14323	0.04545455	28.3063241	5.14030764	0.03476352	0.08756484	0.04650195	Crabp1	1
GO:0005112	Notch binding	1/23	23/14323	0.04347826	27.0756144	5.01912698	0.03631589	0.08756484	0.04650195	Megf10	1
GO:0019840	isoprenoid binding	1/23	23/14323	0.04347826	27.0756144	5.01912698	0.03631589	0.08756484	0.04650195	Crabp1	1
GO:0005416	amino acid:monoatomic cation symporter activity	1/23	24/14323	0.04166667	25.9474638	4.90542819	0.03786588	0.08756484	0.04650195	Slc6a13	1
GO:0090482	vitamin transmembrane transporter activity	1/23	24/14323	0.04166667	25.9474638	4.90542819	0.03786588	0.08756484	0.04650195	Slc47a1	1
GO:0005343	organic acid:sodium symporter activity	1/23	25/14323	0.04	24.9095652	4.79845881	0.03941347	0.08802377	0.04674567	Slc6a13	1
GO:0008324	monoatomic cation transmembrane transporter activity	3/23	477/14323	0.00628931	3.9165983	2.59819282	0.03965035	0.08802377	0.04674567	Slc6a13/Slc47a1/Slc9b2	3
GO:0072349	modified amino acid transmembrane transporter activity	1/23	26/14323	0.03846154	23.951505	4.69756847	0.04095869	0.08901263	0.04727081	Slc6a13	1
GO:0008514	organic anion transmembrane transporter activity	2/23	204/14323	0.00980392	6.10528559	2.94530918	0.04197956	0.08901263	0.04727081	Slc6a13/Slc47a1	2
GO:0042910	xenobiotic transmembrane transporter activity	1/23	27/14323	0.03703704	23.0644122	4.60219175	0.04250153	0.08901263	0.04727081	Slc47a1	1
GO:0016757	glycosyltransferase activity	2/23	217/14323	0.00921659	5.73953116	2.82137649	0.0469234	0.09645365	0.05122242	Glt1d1/Hexb	2
GO:0042887	amide transmembrane transporter activity	1/23	31/14323	0.03225806	20.088359	4.26678414	0.04864917	0.09818287	0.05214074	Slc47a1	1
GO:0017080	sodium channel regulator activity	1/23	33/14323	0.03030303	18.8708827	4.12177798	0.05170881	0.10069611	0.05347541	Fxyd3	1
GO:0140828	metal cation:monoatomic cation antiporter activity	1/23	33/14323	0.03030303	18.8708827	4.12177798	0.05170881	0.10069611	0.05347541	Slc9b2	1
GO:0008375	acetylglucosaminyltransferase activity	1/23	36/14323	0.02777778	17.2983092	3.92663935	0.05628064	0.1077095	0.05719993	Hexb	1
GO:0030291	protein serine/threonine kinase inhibitor activity	1/23	38/14323	0.02631579	16.3878719	3.80914909	0.0593168	0.11159602	0.05926389	Inka1	1
GO:0051879	Hsp90 protein binding	1/23	46/14323	0.02173913	13.5378072	3.41570029	0.07136828	0.13203133	0.07011621	Npas2	1
GO:0015370	solute:sodium symporter activity	1/23	49/14323	0.02040816	12.7089618	3.29261778	0.0758494	0.13590416	0.07217291	Slc6a13	1
GO:0004857	enzyme inhibitor activity	2/23	285/14323	0.00701754	4.37009916	2.30467783	0.07591043	0.13590416	0.07217291	Angptl4/Inka1	2
GO:0016597	amino acid binding	1/23	50/14323	0.02	12.4547826	3.25395809	0.0773385	0.13626308	0.07236351	Slc6a13	1
GO:0001786	phosphatidylserine binding	1/23	53/14323	0.01886792	11.7497949	3.14429917	0.08179206	0.13967567	0.07417579	Marcks	1
GO:0051018	protein kinase A binding	1/23	53/14323	0.01886792	11.7497949	3.14429917	0.08179206	0.13967567	0.07417579	Rab13	1
GO:0038024	cargo receptor activity	1/23	54/14323	0.01851852	11.5322061	3.1096907	0.083272	0.14004836	0.07437372	Megf10	1
GO:0008028	monocarboxylic acid transmembrane transporter activity	1/23	56/14323	0.01785714	11.1203416	3.04313059	0.08622504	0.14285044	0.07586178	Slc6a13	1
GO:0005080	protein kinase C binding	1/23	58/14323	0.01724138	10.7368816	2.9798591	0.08916899	0.14555526	0.07729819	Marcks	1
GO:0015179	L-amino acid transmembrane transporter activity	1/23	60/14323	0.01666667	10.3789855	2.91960288	0.09210386	0.14816707	0.07868522	Slc47a1	1
GO:0004553	hydrolase activity, hydrolyzing O-glycosyl compounds	1/23	67/14323	0.01492537	9.29461389	2.7291818	0.10230483	0.16212696	0.08609872	Hexb	1
GO:0017022	myosin binding	1/23	68/14323	0.01470588	9.15792839	2.70426015	0.10375313	0.16212696	0.08609872	Rab8a	1
GO:0046873	metal ion transmembrane transporter activity	2/23	346/14323	0.00578035	3.59964815	1.9631043	0.10562165	0.16212696	0.08609872	Slc6a13/Slc9b2	2
GO:0015101	organic cation transmembrane transporter activity	1/23	70/14323	0.01428571	8.89627329	2.65592485	0.10664303	0.16212696	0.08609872	Slc47a1	1
GO:0004860	protein kinase inhibitor activity	1/23	71/14323	0.01408451	8.77097367	2.63247611	0.10808464	0.16212696	0.08609872	Inka1	1
GO:0019210	kinase inhibitor activity	1/23	74/14323	0.01351351	8.41539365	2.56481244	0.11239612	0.16634626	0.08833941	Inka1	1
GO:0015294	solute:monoatomic cation symporter activity	1/23	77/14323	0.01298701	8.08752117	2.5008693	0.11668766	0.17026788	0.09042201	Slc6a13	1
GO:0043178	alcohol binding	1/23	78/14323	0.01282051	7.98383501	2.48032036	0.11811376	0.17026788	0.09042201	Crabp1	1
GO:0015108	chloride transmembrane transporter activity	1/23	80/14323	0.0125	7.78423913	2.44029954	0.12095935	0.17213445	0.09141327	Slc6a13	1
GO:0072341	modified amino acid binding	1/23	86/14323	0.01162791	7.24115268	2.32810479	0.12944352	0.18187634	0.09658677	Marcks	1
GO:0140678	molecular function inhibitor activity	2/23	399/14323	0.00501253	3.1214994	1.72363414	0.13361652	0.18539292	0.09845427	Angptl4/Inka1	2
GO:0051117	ATPase binding	1/23	93/14323	0.01075269	6.69611968	2.21012005	0.13924273	0.19081411	0.10133324	Fxyd3	1
GO:0008509	monoatomic anion transmembrane transporter activity	1/23	100/14323	0.01	6.2273913	2.10371615	0.14893639	0.19680879	0.10451676	Slc6a13	1
GO:0016798	hydrolase activity, acting on glycosyl bonds	1/23	100/14323	0.01	6.2273913	2.10371615	0.14893639	0.19680879	0.10451676	Hexb	1
GO:0019955	cytokine binding	1/23	100/14323	0.01	6.2273913	2.10371615	0.14893639	0.19680879	0.10451676	Plp2	1
GO:0015103	inorganic anion transmembrane transporter activity	1/23	103/14323	0.00970874	6.04601098	2.0611744	0.15305876	0.19987674	0.10614602	Slc6a13	1
GO:0015293	symporter activity	1/23	106/14323	0.00943396	5.87489746	2.02028282	0.15716204	0.20284868	0.10772429	Slc6a13	1
GO:1990782	protein tyrosine kinase binding	1/23	108/14323	0.00925926	5.76610306	1.99388272	0.15988698	0.20399374	0.10833238	Rab8a	1
GO:0019842	vitamin binding	1/23	110/14323	0.00909091	5.66126482	1.96813541	0.1626035	0.20510214	0.10892101	Crabp1	1
GO:0008194	UDP-glycosyltransferase activity	1/23	114/14323	0.00877193	5.46262395	1.91848627	0.16801135	0.20954225	0.11127896	Hexb	1
GO:0099106	ion channel regulator activity	1/23	138/14323	0.00724638	4.51260239	1.66284435	0.19976361	0.24637511	0.13083932	Fxyd3	1
GO:0031072	heat shock protein binding	1/23	144/14323	0.00694444	4.32457729	1.60802432	0.20751865	0.25312714	0.13442503	Npas2	1
GO:0016247	channel regulator activity	1/23	148/14323	0.00675676	4.20769683	1.57311464	0.21264868	0.25412124	0.13495296	Fxyd3	1
GO:0016758	hexosyltransferase activity	1/23	150/14323	0.00666667	4.1515942	1.55611885	0.21520177	0.25412124	0.13495296	Hexb	1
GO:0141108	transporter regulator activity	1/23	150/14323	0.00666667	4.1515942	1.55611885	0.21520177	0.25412124	0.13495296	Fxyd3	1
GO:0005516	calmodulin binding	1/23	178/14323	0.00561798	3.49853444	1.34521468	0.25012403	0.29225018	0.15520161	Marcks	1
GO:0051015	actin filament binding	1/23	201/14323	0.00497512	3.09820463	1.20142178	0.27769329	0.32108287	0.17051342	Marcks	1
GO:0030246	carbohydrate binding	1/23	204/14323	0.00490196	3.0526428	1.18419774	0.28121698	0.321805	0.17089692	Hexb	1
GO:0019887	protein kinase regulator activity	1/23	210/14323	0.0047619	2.96542443	1.15067866	0.2882151	0.32574944	0.17299164	Inka1	1
GO:0000287	magnesium ion binding	1/23	212/14323	0.00471698	2.93744873	1.13976938	0.29053329	0.32574944	0.17299164	Arl3	1
GO:0019207	kinase regulator activity	1/23	242/14323	0.00413223	2.57330219	0.98992163	0.32445129	0.36014093	0.1912555	Inka1	1
GO:0008017	microtubule binding	1/23	248/14323	0.00403226	2.51104488	0.96266845	0.33104615	0.363823	0.19321089	Arl3	1
GO:0031267	small GTPase binding	1/23	284/14323	0.00352113	2.19274342	0.8142089	0.36934065	0.3980273	0.21137533	Rab8a	1
GO:0048018	receptor ligand activity	1/23	284/14323	0.00352113	2.19274342	0.8142089	0.36934065	0.3980273	0.21137533	Mill2	1
GO:0030546	signaling receptor activator activity	1/23	289/14323	0.00346021	2.15480668	0.79536268	0.37449051	0.3996966	0.21226183	Mill2	1
GO:0030545	signaling receptor regulator activity	1/23	319/14323	0.0031348	1.95216028	0.68972678	0.40455507	0.4276725	0.22711864	Mill2	1
GO:0051020	GTPase binding	1/23	324/14323	0.00308642	1.92203435	0.67323833	0.40942949	0.4287422	0.22768671	Rab8a	1
GO:0015631	tubulin binding	1/23	353/14323	0.00283286	1.76413351	0.58298332	0.43695711	0.45289675	0.24051416	Arl3	1
GO:0001228	DNA-binding transcription activator activity, RNA polymerase II-specific	1/23	357/14323	0.00280112	1.74436731	0.57119332	0.4406563	0.45289675	0.24051416	Pbx3	1
GO:0001216	DNA-binding transcription activator activity	1/23	363/14323	0.00275482	1.71553479	0.55378233	0.44616153	0.45434798	0.24128485	Pbx3	1
GO:0003779	actin binding	1/23	402/14323	0.00248756	1.54910231	0.44784612	0.48070231	0.48507233	0.25760124	Marcks	1
GO:0005543	phospholipid binding	1/23	428/14323	0.00233645	1.45499797	0.38326556	0.50257419	0.50257419	0.26689573	Marcks	1

ID	Description	GeneRatio	BgRatio	RichFactor	FoldEnrichm	zScore	pvalue	p.adjust	qvalue	geneID	Count
GO:0031492	nucleosomal DNA binding	2/29	14/14323	0.14285714	70.5566502	11.7279466	0.00035482	0.04259452	0.02756829	H3f3b/H1f0	2
GO:1901681	sulfur compound binding	4/29	225/14323	0.01777778	8.78038314	5.29831085	0.00103442	0.04259452	0.02756829	Fbn1/Gstm7/Ccn3/Comp	4
GO:0005201	extracellular matrix structural constituent	3/29	121/14323	0.02479339	12.2453691	5.59519966	0.00183059	0.04259452	0.02756829	Fbn1/Comp/Col6a1	3
GO:0019838	growth factor binding	3/29	124/14323	0.02419355	11.9491101	5.515498	0.00196334	0.04259452	0.02756829	Igfbp5/Col6a1/Kl	3
GO:0001968	fibronectin binding	2/29	33/14323	0.06060606	29.9331243	7.49480148	0.00201023	0.04259452	0.02756829	Igfbp5/Comp	2
GO:0008201	heparin binding	3/29	129/14323	0.02325581	11.4859663	5.38858649	0.00219769	0.04259452	0.02756829	Fbn1/Ccn3/Comp	3
GO:0005178	integrin binding	3/29	133/14323	0.02255639	11.1405237	5.29198925	0.0023972	0.04259452	0.02756829	Fbn1/Ccn3/Comp	3
GO:0048018	receptor ligand activity	4/29	284/14323	0.01408451	6.95628946	4.5665774	0.00243158	0.04259452	0.02756829	Fbn1/Igfbp5/Ccn3/Bmp6	4
GO:0030546	signaling receptor activator activity	4/29	289/14323	0.01384083	6.83593843	4.5143251	0.00259021	0.04259452	0.02756829	Fbn1/Igfbp5/Ccn3/Bmp6	4
GO:0030545	signaling receptor regulator activity	4/29	319/14323	0.01253918	6.19306021	4.22490155	0.00369466	0.04995604	0.03233286	Fbn1/Igfbp5/Ccn3/Bmp6	4
GO:0031491	nucleosome binding	2/29	45/14323	0.04444444	21.9509579	6.3401626	0.00371295	0.04995604	0.03233286	H3f3b/H1f0	2
GO:0005539	glycosaminoglycan binding	3/29	178/14323	0.01685393	8.32409919	4.42880631	0.00543801	0.06706878	0.04340867	Fbn1/Ccn3/Comp	3
GO:0005179	hormone activity	2/29	61/14323	0.03278689	16.1933296	5.35613611	0.00672733	0.07658807	0.0495698	Fbn1/Ccn3	2
GO:0005518	collagen binding	2/29	64/14323	0.03125	15.4342672	5.2127169	0.00738336	0.07805268	0.05051774	Comp/Col6a1	2
GO:0050839	cell adhesion molecule binding	3/29	257/14323	0.01167315	5.7653294	3.47214718	0.01478761	0.10738273	0.06950091	Fbn1/Ccn3/Comp	3
GO:0008083	growth factor activity	2/29	92/14323	0.02173913	10.7368816	4.22007371	0.01480449	0.10738273	0.06950091	Ccn3/Bmp6	2
GO:0031490	chromatin DNA binding	2/29	96/14323	0.02083333	10.2895115	4.11335102	0.01604708	0.10738273	0.06950091	H3f3b/H1f0	2
GO:0019955	cytokine binding	2/29	100/14323	0.02	9.87793103	4.01273155	0.01733302	0.10738273	0.06950091	Cx3cr1/Comp	2
GO:0016493	C-C chemokine receptor activity	1/29	10/14323	0.1	49.3896552	6.89463273	0.02006992	0.10738273	0.06950091	Cx3cr1	1
GO:0019870	potassium channel inhibitor activity	1/29	10/14323	0.1	49.3896552	6.89463273	0.02006992	0.10738273	0.06950091	Kcnk2	1
GO:0001637	G protein-coupled chemoattractant receptor activity	1/29	11/14323	0.09090909	44.8996865	6.56041847	0.02205539	0.10738273	0.06950091	Cx3cr1	1
GO:0004950	chemokine receptor activity	1/29	11/14323	0.09090909	44.8996865	6.56041847	0.02205539	0.10738273	0.06950091	Cx3cr1	1
GO:0019957	C-C chemokine binding	1/29	11/14323	0.09090909	44.8996865	6.56041847	0.02205539	0.10738273	0.06950091	Cx3cr1	1
GO:0031994	insulin-like growth factor I binding	1/29	11/14323	0.09090909	44.8996865	6.56041847	0.02205539	0.10738273	0.06950091	Igfbp5	1
GO:0070700	BMP receptor binding	1/29	11/14323	0.09090909	44.8996865	6.56041847	0.02205539	0.10738273	0.06950091	Bmp6	1
GO:0022841	potassium ion leak channel activity	1/29	12/14323	0.08333333	41.158046	6.26833429	0.02403697	0.10738273	0.06950091	Kcnk2	1
GO:0048407	platelet-derived growth factor binding	1/29	12/14323	0.08333333	41.158046	6.26833429	0.02403697	0.10738273	0.06950091	Col6a1	1
GO:0005520	insulin-like growth factor binding	1/29	14/14323	0.07142857	35.2783251	5.7796683	0.02798851	0.10738273	0.06950091	Igfbp5	1
GO:0015149	hexose transmembrane transporter activity	1/29	14/14323	0.07142857	35.2783251	5.7796683	0.02798851	0.10738273	0.06950091	Slc2a10	1
GO:0016641	oxidoreductase activity, acting on the CH-NH2 group of donors, oxygen as acceptor	1/29	14/14323	0.07142857	35.2783251	5.7796683	0.02798851	0.10738273	0.06950091	Loxl1	1
GO:0016805	dipeptidase activity	1/29	14/14323	0.07142857	35.2783251	5.7796683	0.02798851	0.10738273	0.06950091	Cpq	1
GO:0036122	BMP binding	1/29	14/14323	0.07142857	35.2783251	5.7796683	0.02798851	0.10738273	0.06950091	Comp	1
GO:0055056	D-glucose transmembrane transporter activity	1/29	14/14323	0.07142857	35.2783251	5.7796683	0.02798851	0.10738273	0.06950091	Slc2a10	1
GO:0008381	mechanosensitive monoatomic ion channel activity	1/29	15/14323	0.06666667	32.9264368	5.57224957	0.02995848	0.10738273	0.06950091	Kcnk2	1
GO:0015145	monosaccharide transmembrane transporter activity	1/29	15/14323	0.06666667	32.9264368	5.57224957	0.02995848	0.10738273	0.06950091	Slc2a10	1
GO:0022840	leak channel activity	1/29	15/14323	0.06666667	32.9264368	5.57224957	0.02995848	0.10738273	0.06950091	Kcnk2	1
GO:0022842	narrow pore channel activity	1/29	15/14323	0.06666667	32.9264368	5.57224957	0.02995848	0.10738273	0.06950091	Kcnk2	1
GO:0030527	structural constituent of chromatin	1/29	15/14323	0.06666667	32.9264368	5.57224957	0.02995848	0.10738273	0.06950091	H1f0	1
GO:0042813	Wnt receptor activity	1/29	15/14323	0.06666667	32.9264368	5.57224957	0.02995848	0.10738273	0.06950091	Fzd7	1
GO:0043295	glutathione binding	1/29	15/14323	0.06666667	32.9264368	5.57224957	0.02995848	0.10738273	0.06950091	Gstm7	1
GO:0055105	ubiquitin-protein transferase inhibitor activity	1/29	15/14323	0.06666667	32.9264368	5.57224957	0.02995848	0.10738273	0.06950091	Gbp4	1
GO:0016638	oxidoreductase activity, acting on the CH-NH2 group of donors	1/29	16/14323	0.0625	30.8685345	5.38422948	0.0319246	0.10738273	0.06950091	Loxl1	1
GO:0051119	sugar transmembrane transporter activity	1/29	16/14323	0.0625	30.8685345	5.38422948	0.0319246	0.10738273	0.06950091	Slc2a10	1
GO:1900750	oligopeptide binding	1/29	16/14323	0.0625	30.8685345	5.38422948	0.0319246	0.10738273	0.06950091	Gstm7	1
GO:0005104	fibroblast growth factor receptor binding	1/29	17/14323	0.05882353	29.0527383	5.2127219	0.03388687	0.11145013	0.07213344	Kl	1
GO:0043395	heparan sulfate proteoglycan binding	1/29	18/14323	0.05555556	27.4386973	5.05540925	0.03584529	0.11417109	0.07389452	Comp	1
GO:0004602	glutathione peroxidase activity	1/29	19/14323	0.05263158	25.9945554	4.91040609	0.03779989	0.11417109	0.07389452	Gstm7	1
GO:0005537	D-mannose binding	1/29	19/14323	0.05263158	25.9945554	4.91040609	0.03779989	0.11417109	0.07389452	Asgr1	1
GO:0070696	transmembrane receptor protein serine/threonine kinase binding	1/29	19/14323	0.05263158	25.9945554	4.91040609	0.03779989	0.11417109	0.07389452	Bmp6	1
GO:0015271	outward rectifier potassium channel activity	1/29	21/14323	0.04761905	23.5188834	4.65138253	0.04169761	0.12100483	0.07831749	Kcnk2	1
GO:0019956	chemokine binding	1/29	21/14323	0.04761905	23.5188834	4.65138253	0.04169761	0.12100483	0.07831749	Cx3cr1	1
GO:0015144	carbohydrate transmembrane transporter activity	1/29	22/14323	0.04545455	22.4498433	4.53498865	0.04364075	0.12265186	0.07938349	Slc2a10	1
GO:0000979	RNA polymerase II core promoter sequence-specific DNA binding	1/29	23/14323	0.04347826	21.4737631	4.42606226	0.04558008	0.12265186	0.07938349	H3f3b	1
GO:0005112	Notch binding	1/29	23/14323	0.04347826	21.4737631	4.42606226	0.04558008	0.12265186	0.07938349	Ccn3	1
GO:0017134	fibroblast growth factor binding	1/29	23/14323	0.04347826	21.4737631	4.42606226	0.04558008	0.12265186	0.07938349	Kl	1
GO:0004364	glutathione transferase activity	1/29	24/14323	0.04166667	20.579023	4.32382142	0.04751562	0.12337389	0.07985081	Gstm7	1
GO:0090482	vitamin transmembrane transporter activity	1/29	24/14323	0.04166667	20.579023	4.32382142	0.04751562	0.12337389	0.07985081	Slc2a10	1
GO:0033612	receptor serine/threonine kinase binding	1/29	26/14323	0.03846154	18.9960212	4.13680126	0.05137533	0.13109567	0.08484855	Bmp6	1
GO:0017147	Wnt-protein binding	1/29	28/14323	0.03571429	17.6391626	3.96955977	0.05521994	0.13851781	0.08965235	Fzd7	1
GO:0005109	frizzled binding	1/29	31/14323	0.03225806	15.9321468	3.74869874	0.06095865	0.15036468	0.09731996	Fzd7	1
GO:0004180	carboxypeptidase activity	1/29	32/14323	0.03125	15.4342672	3.68181842	0.06286407	0.15214811	0.09847424	Cpq	1
GO:0000217	DNA secondary structure binding	1/29	33/14323	0.03030303	14.9665622	3.61788135	0.06476575	0.15214811	0.09847424	H1f0	1
GO:0055106	ubiquitin-protein transferase regulator activity	1/29	33/14323	0.03030303	14.9665622	3.61788135	0.06476575	0.15214811	0.09847424	Gbp4	1
GO:0042379	chemokine receptor binding	1/29	35/14323	0.02857143	14.11133	3.49799348	0.06855794	0.15797536	0.10224578	Cx3cr1	1
GO:0038187	pattern recognition receptor activity	1/29	36/14323	0.02777778	13.7193487	3.44167253	0.07044847	0.15797536	0.10224578	Asgr1	1
GO:0043394	proteoglycan binding	1/29	36/14323	0.02777778	13.7193487	3.44167253	0.07044847	0.15797536	0.10224578	Comp	1
GO:0042162	telomeric DNA binding	1/29	37/14323	0.02702703	13.3485555	3.38754942	0.07233529	0.15970299	0.10336396	Telo2	1
GO:0030020	extracellular matrix structural constituent conferring tensile strength	1/29	38/14323	0.02631579	12.9972777	3.33547993	0.07421841	0.15970299	0.10336396	Col6a1	1
GO:0001664	G protein-coupled receptor binding	2/29	223/14323	0.00896861	4.42956549	2.32490879	0.07445613	0.15970299	0.10336396	Cx3cr1/Fzd7	2
GO:0005504	fatty acid binding	1/29	39/14323	0.02564103	12.6640141	3.28533277	0.07609784	0.16064495	0.10397362	Gstm7	1
GO:0004930	G protein-coupled receptor activity	2/29	231/14323	0.00865801	4.27616062	2.2610429	0.07912684	0.16064495	0.10397362	Cx3cr1/Fzd7	2
GO:0004601	peroxidase activity	1/29	41/14323	0.02439024	12.0462574	3.19033667	0.07984566	0.16064495	0.10397362	Gstm7	1
GO:0008200	ion channel inhibitor activity	1/29	41/14323	0.02439024	12.0462574	3.19033667	0.07984566	0.16064495	0.10397362	Kcnk2	1
GO:0016248	channel inhibitor activity	1/29	42/14323	0.02380952	11.7594417	3.14527786	0.08171406	0.16064495	0.10397362	Kcnk2	1
GO:0141110	transporter inhibitor activity	1/29	42/14323	0.02380952	11.7594417	3.14527786	0.08171406	0.16064495	0.10397362	Kcnk2	1
GO:0003777	microtubule motor activity	1/29	43/14323	0.02325581	11.4859663	3.10171955	0.08357879	0.16064495	0.10397362	Kifc3	1
GO:0016684	oxidoreductase activity, acting on peroxide as acceptor	1/29	43/14323	0.02325581	11.4859663	3.10171955	0.08357879	0.16064495	0.10397362	Gstm7	1
GO:0001046	core promoter sequence-specific DNA binding	1/29	45/14323	0.02222222	10.9754789	3.01877169	0.0872973	0.16564103	0.10720721	H3f3b	1
GO:0051879	Hsp90 protein binding	1/29	46/14323	0.02173913	10.7368816	2.97923162	0.08915109	0.16627387	0.1076168	Telo2	1
GO:0046982	protein heterodimerization activity	2/29	249/14323	0.00803213	3.96704058	2.12734698	0.08996402	0.16627387	0.1076168	Bmp6/Kcnk2	2
GO:0005507	copper ion binding	1/29	47/14323	0.0212766	10.5084373	2.94088979	0.09100124	0.16627387	0.1076168	Loxl1	1
GO:0008235	metalloexopeptidase activity	1/29	48/14323	0.02083333	10.2895115	2.9036842	0.09284776	0.16757889	0.10846144	Cpq	1
GO:0033293	monocarboxylic acid binding	1/29	53/14323	0.01886792	9.31880286	2.7328181	0.10202619	0.18082306	0.11703342	Gstm7	1
GO:0015459	potassium channel regulator activity	1/29	54/14323	0.01851852	9.14623244	2.70134998	0.10385108	0.18082306	0.11703342	Kcnk2	1
GO:0038024	cargo receptor activity	1/29	54/14323	0.01851852	9.14623244	2.70134998	0.10385108	0.18082306	0.11703342	Asgr1	1
GO:0003924	GTPase activity	2/29	275/14323	0.00727273	3.59197492	1.9548518	0.10634614	0.18285678	0.11834969	Gbp4/Gpn3	2
GO:0016765	transferase activity, transferring alkyl or aryl (other than methyl) groups	1/29	56/14323	0.01785714	8.81958128	2.64079766	0.10749013	0.18285678	0.11834969	Gstm7	1
GO:0004896	cytokine receptor activity	1/29	59/14323	0.01694915	8.371128	2.55542558	0.11292197	0.18991423	0.12291746	Cx3cr1	1
GO:0016209	antioxidant activity	1/29	63/14323	0.01587302	7.83962781	2.45056895	0.1201148	0.19974146	0.1292779	Gstm7	1
GO:0004553	hydrolase activity, hydrolyzing O-glycosyl compounds	1/29	67/14323	0.01492537	7.37159032	2.35456317	0.12725129	0.20925768	0.13543705	Kl	1
GO:0048029	monosaccharide binding	1/29	69/14323	0.01449275	7.15792104	2.30948012	0.13079855	0.21272731	0.13768268	Asgr1	1
GO:0005546	phosphatidylinositol-4,5-bisphosphate binding	1/29	72/14323	0.01388889	6.85967433	2.24512746	0.13609335	0.21657866	0.14017538	Fzd7	1
GO:0019843	rRNA binding	1/29	72/14323	0.01388889	6.85967433	2.24512746	0.13609335	0.21657866	0.14017538	Rpl8	1
GO:0005525	GTP binding	2/29	329/14323	0.00607903	3.00241065	1.65502327	0.14263883	0.2236626	0.14476029	Gbp4/Gpn3	2
GO:0003774	cytoskeletal motor activity	1/29	77/14323	0.01298701	6.41424093	2.14565758	0.14484888	0.2236626	0.14476029	Kifc3	1
GO:0005249	voltage-gated potassium channel activity	1/29	78/14323	0.01282051	6.33200707	2.12682	0.14658968	0.2236626	0.14476029	Kcnk2	1
GO:0008238	exopeptidase activity	1/29	78/14323	0.01282051	6.33200707	2.12682	0.14658968	0.2236626	0.14476029	Cpq	1
GO:0140375	immune receptor activity	1/29	80/14323	0.0125	6.1737069	2.09011423	0.15006101	0.22662275	0.14667618	Cx3cr1	1
GO:0019001	guanyl nucleotide binding	2/29	345/14323	0.0057971	2.86316842	1.57784046	0.15386824	0.22772499	0.14738958	Gbp4/Gpn3	2
GO:0032561	guanyl ribonucleotide binding	2/29	345/14323	0.0057971	2.86316842	1.57784046	0.15386824	0.22772499	0.14738958	Gbp4/Gpn3	2
GO:0005496	steroid binding	1/29	86/14323	0.01162791	5.74298316	1.98708072	0.16039341	0.23272769	0.15062745	Comp	1
GO:0072341	modified amino acid binding	1/29	86/14323	0.01162791	5.74298316	1.98708072	0.16039341	0.23272769	0.15062745	Gstm7	1
GO:1902936	phosphatidylinositol bisphosphate binding	1/29	97/14323	0.01030928	5.09171703	1.8212686	0.17902202	0.25723552	0.16648956	Fzd7	1
GO:0016798	hydrolase activity, acting on glycosyl bonds	1/29	100/14323	0.01	4.93896552	1.78037103	0.18403288	0.26159285	0.16930974	Kl	1
GO:0005267	potassium channel activity	1/29	101/14323	0.00990099	4.89006487	1.76710005	0.1856966	0.26159285	0.16930974	Kcnk2	1
GO:0005125	cytokine activity	1/29	102/14323	0.00980392	4.84212306	1.7540026	0.18735704	0.26159285	0.16930974	Bmp6	1
GO:0140678	molecular function inhibitor activity	2/29	399/14323	0.00501253	2.47567194	1.34653673	0.19294078	0.26559935	0.17190285	Gbp4/Kcnk2	2
GO:0015293	symporter activity	1/29	106/14323	0.00943396	4.65940143	1.70326785	0.19396618	0.26559935	0.17190285	Slc2a10	1
GO:0070851	growth factor receptor binding	1/29	107/14323	0.00934579	4.61585562	1.69097897	0.19561034	0.26559935	0.17190285	Kl	1
GO:0030165	PDZ domain binding	1/29	109/14323	0.00917431	4.53116102	1.66685016	0.19888894	0.26736453	0.17304532	Fzd7	1
GO:0019842	vitamin binding	1/29	110/14323	0.00909091	4.48996865	1.65500357	0.2005234	0.26736453	0.17304532	Comp	1
GO:0022843	voltage-gated monoatomic cation channel activity	1/29	117/14323	0.00854701	4.22133805	1.5758587	0.21187481	0.27997742	0.18120872	Kcnk2	1
GO:0022834	ligand-gated channel activity	1/29	121/14323	0.00826446	4.08178968	1.53336262	0.21829129	0.28590364	0.18504432	Kcnk2	1
GO:0002020	protease binding	1/29	130/14323	0.00769231	3.79920424	1.44408617	0.23254449	0.30129274	0.19500455	Comp	1
GO:0015079	potassium ion transmembrane transporter activity	1/29	131/14323	0.00763359	3.77020268	1.43466115	0.23411261	0.30129274	0.19500455	Kcnk2	1
GO:0008237	metallopeptidase activity	1/29	136/14323	0.00735294	3.63159229	1.38888638	0.24190687	0.30532587	0.1976149	Cpq	1
GO:0005509	calcium ion binding	2/29	466/14323	0.00429185	2.11972769	1.10686515	0.24305842	0.30532587	0.1976149	Fbn1/Comp	2
GO:0099106	ion channel regulator activity	1/29	138/14323	0.00724638	3.57896052	1.37117697	0.24500309	0.30532587	0.1976149	Kcnk2	1
GO:0005244	voltage-gated monoatomic ion channel activity	1/29	139/14323	0.00719424	3.5532126	1.36244495	0.24654662	0.30532587	0.1976149	Kcnk2	1
GO:0022832	voltage-gated channel activity	1/29	140/14323	0.00714286	3.52783251	1.35379282	0.24808709	0.30532587	0.1976149	Kcnk2	1
GO:0031406	carboxylic acid binding	1/29	141/14323	0.0070922	3.50281242	1.34521922	0.24962453	0.30532587	0.1976149	Gstm7	1
GO:0031072	heat shock protein binding	1/29	144/14323	0.00694444	3.42983716	1.31995632	0.25421865	0.3083964	0.19960222	Telo2	1
GO:0016247	channel regulator activity	1/29	148/14323	0.00675676	3.33713886	1.28729412	0.26030192	0.31177747	0.20179054	Kcnk2	1
GO:0043177	organic acid binding	1/29	149/14323	0.00671141	3.31474196	1.27930309	0.26181524	0.31177747	0.20179054	Gstm7	1
GO:0141108	transporter regulator activity	1/29	150/14323	0.00666667	3.29264368	1.27137961	0.26332557	0.31177747	0.20179054	Kcnk2	1
GO:0003735	structural constituent of ribosome	1/29	164/14323	0.00609756	3.01156434	1.16698025	0.2841594	0.33377454	0.21602762	Rpl8	1
GO:1901981	phosphatidylinositol phosphate binding	1/29	170/14323	0.00588235	2.90527383	1.12559344	0.29291299	0.34134742	0.22092898	Fzd7	1
GO:0005126	cytokine receptor binding	1/29	172/14323	0.00581395	2.87149158	1.11219895	0.29580781	0.34202778	0.22136933	Cx3cr1	1
GO:0033218	amide binding	1/29	193/14323	0.00518135	2.55904949	0.98218075	0.32552081	0.37346574	0.2417168	Gstm7	1
GO:0008514	organic anion transmembrane transporter activity	1/29	204/14323	0.00490196	2.42106153	0.92076741	0.34059789	0.38479761	0.24905109	Slc2a10	1
GO:0030246	carbohydrate binding	1/29	204/14323	0.00490196	2.42106153	0.92076741	0.34059789	0.38479761	0.24905109	Asgr1	1
GO:0015291	secondary active transmembrane transporter activity	1/29	218/14323	0.00458716	2.26558051	0.84811633	0.3593168	0.40287035	0.26074823	Slc2a10	1
GO:0022836	gated channel activity	1/29	231/14323	0.004329	2.13808031	0.78544635	0.3762385	0.41867141	0.2709751	Kcnk2	1
GO:0008017	microtubule binding	1/29	248/14323	0.00403226	1.99151835	0.70945743	0.39771682	0.43926932	0.2843066	Kifc3	1
GO:0005261	monoatomic cation channel activity	1/29	257/14323	0.00389105	1.92177647	0.67163113	0.40879679	0.4481624	0.29006244	Kcnk2	1
GO:0035091	phosphatidylinositol binding	1/29	265/14323	0.00377358	1.86376057	0.63926141	0.41848022	0.45540494	0.29475	Fzd7	1
GO:0004857	enzyme inhibitor activity	1/29	285/14323	0.00350877	1.73297036	0.56296341	0.44202354	0.47751449	0.30905987	Gbp4	1
GO:0005216	monoatomic ion channel activity	1/29	325/14323	0.00307692	1.5196817	0.42684486	0.48638025	0.5216252	0.33760948	Kcnk2	1
GO:0022804	active transmembrane transporter activity	1/29	336/14323	0.00297619	1.46993021	0.39261299	0.49796872	0.53021129	0.34316663	Slc2a10	1
GO:0046873	metal ion transmembrane transporter activity	1/29	346/14323	0.00289017	1.42744668	0.36252417	0.50828431	0.53732913	0.34777347	Kcnk2	1
GO:0015631	tubulin binding	1/29	353/14323	0.00283286	1.39914037	0.34200996	0.51538313	0.54096953	0.35012964	Kifc3	1
GO:0015267	channel activity	1/29	362/14323	0.00276243	1.36435512	0.31626042	0.52436491	0.54269935	0.35124922	Kcnk2	1
GO:0022803	passive transmembrane transporter activity	1/29	362/14323	0.00276243	1.36435512	0.31626042	0.52436491	0.54269935	0.35124922	Kcnk2	1
GO:0008233	peptidase activity	1/29	416/14323	0.00240385	1.18725133	0.17457369	0.57497382	0.59094532	0.38247528	Cpq	1
GO:0005543	phospholipid binding	1/29	428/14323	0.00233645	1.15396391	0.14565838	0.58549239	0.59760602	0.38678626	Fzd7	1
GO:0022890	inorganic cation transmembrane transporter activity	1/29	446/14323	0.00224215	1.10739137	0.10377983	0.60080039	0.60903053	0.3941805	Kcnk2	1
GO:0008324	monoatomic cation transmembrane transporter activity	1/29	477/14323	0.00209644	1.03542254	0.03544057	0.62589368	0.63015146	0.40785052	Kcnk2	1
GO:0140657	ATP-dependent activity	1/29	491/14323	0.00203666	1.00589929	0.00599131	0.63672034	0.63672034	0.41210207	Kifc3	1

ID	Description	GeneRatio	BgRatio	RichFactor	FoldEnrichm	zScore	pvalue	p.adjust	qvalue	geneID	Count
GO:0004364	glutathione transferase activity	2/69	24/14323	0.08333333	17.2983092	5.55972541	0.00589508	0.35746543	0.34623427	Gstt2/Gstt3	2
GO:0046875	ephrin receptor binding	2/69	31/14323	0.06451613	13.3922394	4.80553937	0.00971893	0.35746543	0.34623427	Ngef/Tiam1	2
GO:0001965	G-protein alpha-subunit binding	2/69	33/14323	0.06060606	12.5805885	4.63371681	0.01096765	0.35746543	0.34623427	Ccdc88c/Gpsm1	2
GO:0016798	hydrolase activity, acting on glycosyl bonds	3/69	100/14323	0.03	6.2273913	3.64961799	0.01240349	0.35746543	0.34623427	Hexd/Il1rapl1/Man2b1	3
GO:0042162	telomeric DNA binding	2/69	37/14323	0.05405405	11.2205249	4.33088589	0.01366432	0.35746543	0.34623427	Hnrnpd/Rpa2	2
GO:0070888	E-box binding	2/69	37/14323	0.05405405	11.2205249	4.33088589	0.01366432	0.35746543	0.34623427	Nr1d1/Bhlhe22	2
GO:0005085	guanyl-nucleotide exchange factor activity	4/69	197/14323	0.02030457	4.21481645	3.16109608	0.01497882	0.35746543	0.34623427	Ccdc88c/Ngef/Dennd6b/Tiam1	4
GO:0098631	cell adhesion mediator activity	2/69	41/14323	0.04878049	10.1258395	4.07125443	0.01661765	0.35746543	0.34623427	Ager/Cd200	2
GO:0001227	DNA-binding transcription repressor activity, RNA polymerase II-specific	4/69	224/14323	0.01785714	3.70678054	2.84080159	0.02283132	0.35746543	0.34623427	Nr1d1/Nfatc4/Nfe2l3/Nfx1	4
GO:0001217	DNA-binding transcription repressor activity	4/69	230/14323	0.0173913	3.61008192	2.77635075	0.02486072	0.35746543	0.34623427	Nr1d1/Nfatc4/Nfe2l3/Nfx1	4
GO:0001222	transcription corepressor binding	2/69	56/14323	0.03571429	7.41356108	3.34568401	0.02980239	0.35746543	0.34623427	Nr1d1/Ehmt2	2
GO:0016765	transferase activity, transferring alkyl or aryl (other than methyl) groups	2/69	56/14323	0.03571429	7.41356108	3.34568401	0.02980239	0.35746543	0.34623427	Gstt2/Gstt3	2
GO:0004553	hydrolase activity, hydrolyzing O-glycosyl compounds	2/69	67/14323	0.02985075	6.19640926	2.96619644	0.04137168	0.35746543	0.34623427	Hexd/Man2b1	2
GO:0002039	p53 binding	2/69	70/14323	0.02857143	5.93084886	2.87723657	0.04477706	0.35746543	0.34623427	Ehmt2/Dazap2	2
GO:0004527	exonuclease activity	2/69	72/14323	0.02777778	5.76610306	2.82075268	0.04710328	0.35746543	0.34623427	Enpp1/Xrn2	2
GO:0030971	receptor tyrosine kinase binding	2/69	72/14323	0.02777778	5.76610306	2.82075268	0.04710328	0.35746543	0.34623427	Tiam1/Dazap2	2
GO:0005005	transmembrane-ephrin receptor activity	1/69	10/14323	0.1	20.757971	4.34845531	0.04715772	0.35746543	0.34623427	Epha8	1
GO:0008503	benzodiazepine receptor activity	1/69	10/14323	0.1	20.757971	4.34845531	0.04715772	0.35746543	0.34623427	Gabrg1	1
GO:0015347	sodium-independent organic anion transmembrane transporter activity	1/69	10/14323	0.1	20.757971	4.34845531	0.04715772	0.35746543	0.34623427	Slco1a4	1
GO:0030695	GTPase regulator activity	5/69	414/14323	0.01207729	2.50700133	2.16483029	0.04908528	0.35746543	0.34623427	Ccdc88c/Ngef/Dennd6b/Tiam1/Gpsm1	5
GO:0060589	nucleoside-triphosphatase regulator activity	5/69	414/14323	0.01207729	2.50700133	2.16483029	0.04908528	0.35746543	0.34623427	Ccdc88c/Ngef/Dennd6b/Tiam1/Gpsm1	5
GO:0016783	sulfurtransferase activity	1/69	11/14323	0.09090909	18.8708827	4.12524918	0.05175118	0.35746543	0.34623427	Tst	1
GO:0034511	U3 snoRNA binding	1/69	11/14323	0.09090909	18.8708827	4.12524918	0.05175118	0.35746543	0.34623427	Rrp9	1
GO:0050815	phosphoserine residue binding	1/69	11/14323	0.09090909	18.8708827	4.12524918	0.05175118	0.35746543	0.34623427	Pcif1	1
GO:0061809	NAD+ nucleotidase, cyclic ADP-ribose generating	1/69	11/14323	0.09090909	18.8708827	4.12524918	0.05175118	0.35746543	0.34623427	Il1rapl1	1
GO:0070003	threonine-type peptidase activity	1/69	11/14323	0.09090909	18.8708827	4.12524918	0.05175118	0.35746543	0.34623427	Psmb9	1
GO:0098505	G-rich strand telomeric DNA binding	1/69	11/14323	0.09090909	18.8708827	4.12524918	0.05175118	0.35746543	0.34623427	Rpa2	1
GO:0099122	RNA polymerase II C-terminal domain binding	1/69	11/14323	0.09090909	18.8708827	4.12524918	0.05175118	0.35746543	0.34623427	Pcif1	1
GO:0004890	GABA-A receptor activity	1/69	12/14323	0.08333333	17.2983092	3.92967096	0.05632281	0.35746543	0.34623427	Gabrg1	1
GO:0005243	gap junction channel activity	1/69	12/14323	0.08333333	17.2983092	3.92967096	0.05632281	0.35746543	0.34623427	Gja4	1
GO:0015125	bile acid transmembrane transporter activity	1/69	12/14323	0.08333333	17.2983092	3.92967096	0.05632281	0.35746543	0.34623427	Slco1a4	1
GO:0022851	GABA-gated chloride ion channel activity	1/69	12/14323	0.08333333	17.2983092	3.92967096	0.05632281	0.35746543	0.34623427	Gabrg1	1
GO:0008174	mRNA methyltransferase activity	1/69	13/14323	0.07692308	15.96767	3.75633279	0.06087272	0.35746543	0.34623427	Pcif1	1
GO:0019855	calcium channel inhibitor activity	1/69	13/14323	0.07692308	15.96767	3.75633279	0.06087272	0.35746543	0.34623427	Slc30a1	1
GO:0030881	beta-2-microglobulin binding	1/69	13/14323	0.07692308	15.96767	3.75633279	0.06087272	0.35746543	0.34623427	H2-T23	1
GO:0043047	single-stranded telomeric DNA binding	1/69	13/14323	0.07692308	15.96767	3.75633279	0.06087272	0.35746543	0.34623427	Rpa2	1
GO:0046974	histone H3K9 methyltransferase activity	1/69	13/14323	0.07692308	15.96767	3.75633279	0.06087272	0.35746543	0.34623427	Ehmt2	1
GO:0004559	alpha-mannosidase activity	1/69	14/14323	0.07142857	14.8271222	3.60121606	0.065401	0.35746543	0.34623427	Man2b1	1
GO:0008097	5S rRNA binding	1/69	14/14323	0.07142857	14.8271222	3.60121606	0.065401	0.35746543	0.34623427	Tst	1
GO:0015929	hexosaminidase activity	1/69	14/14323	0.07142857	14.8271222	3.60121606	0.065401	0.35746543	0.34623427	Hexd	1
GO:0044548	S100 protein binding	1/69	14/14323	0.07142857	14.8271222	3.60121606	0.065401	0.35746543	0.34623427	Ager	1
GO:0046703	natural killer cell lectin-like receptor binding	1/69	14/14323	0.07142857	14.8271222	3.60121606	0.065401	0.35746543	0.34623427	H2-T23	1
GO:0070742	C2H2 zinc finger domain binding	1/69	14/14323	0.07142857	14.8271222	3.60121606	0.065401	0.35746543	0.34623427	Ehmt2	1
GO:0005003	ephrin receptor activity	1/69	15/14323	0.06666667	13.8386473	3.46125373	0.06990777	0.35746543	0.34623427	Epha8	1
GO:0015923	mannosidase activity	1/69	15/14323	0.06666667	13.8386473	3.46125373	0.06990777	0.35746543	0.34623427	Man2b1	1
GO:0016917	GABA receptor activity	1/69	15/14323	0.06666667	13.8386473	3.46125373	0.06990777	0.35746543	0.34623427	Gabrg1	1
GO:0034236	protein kinase A catalytic subunit binding	1/69	15/14323	0.06666667	13.8386473	3.46125373	0.06990777	0.35746543	0.34623427	Pkia	1
GO:0098847	sequence-specific single stranded DNA binding	1/69	15/14323	0.06666667	13.8386473	3.46125373	0.06990777	0.35746543	0.34623427	Rpa2	1
GO:0005092	GDP-dissociation inhibitor activity	1/69	16/14323	0.0625	12.9737319	3.33405865	0.07439312	0.35746543	0.34623427	Gpsm1	1
GO:0016646	oxidoreductase activity, acting on the CH-NH group of donors, NAD or NADP as acceptor	1/69	16/14323	0.0625	12.9737319	3.33405865	0.07439312	0.35746543	0.34623427	Pycr1	1
GO:0008409	5'-3' exonuclease activity	1/69	17/14323	0.05882353	12.2105712	3.21774107	0.07885715	0.35746543	0.34623427	Xrn2	1
GO:0043422	protein kinase B binding	1/69	17/14323	0.05882353	12.2105712	3.21774107	0.07885715	0.35746543	0.34623427	Srsf5	1
GO:0061133	endopeptidase activator activity	1/69	17/14323	0.05882353	12.2105712	3.21774107	0.07885715	0.35746543	0.34623427	Aim2	1
GO:0050811	GABA receptor binding	1/69	18/14323	0.05555556	11.5322061	3.11078269	0.08329996	0.35746543	0.34623427	Gabrg1	1
GO:0004115	3',5'-cyclic-AMP phosphodiesterase activity	1/69	19/14323	0.05263158	10.9252479	3.01194762	0.08772165	0.35746543	0.34623427	Enpp1	1
GO:0004602	glutathione peroxidase activity	1/69	19/14323	0.05263158	10.9252479	3.01194762	0.08772165	0.35746543	0.34623427	Gstt2	1
GO:0005537	D-mannose binding	1/69	19/14323	0.05263158	10.9252479	3.01194762	0.08772165	0.35746543	0.34623427	Man2b1	1
GO:0042608	T cell receptor binding	1/69	19/14323	0.05263158	10.9252479	3.01194762	0.08772165	0.35746543	0.34623427	H2-T23	1
GO:0050699	WW domain binding	1/69	19/14323	0.05263158	10.9252479	3.01194762	0.08772165	0.35746543	0.34623427	Dazap2	1
GO:0051959	dynein light intermediate chain binding	1/69	19/14323	0.05263158	10.9252479	3.01194762	0.08772165	0.35746543	0.34623427	Ccdc88c	1
GO:0004114	3',5'-cyclic-nucleotide phosphodiesterase activity	1/69	20/14323	0.05	10.3789855	2.92021815	0.09212232	0.35746543	0.34623427	Enpp1	1
GO:0030247	polysaccharide binding	1/69	20/14323	0.05	10.3789855	2.92021815	0.09212232	0.35746543	0.34623427	Enpp1	1
GO:0042975	peroxisome proliferator activated receptor binding	1/69	20/14323	0.05	10.3789855	2.92021815	0.09212232	0.35746543	0.34623427	Nfatc4	1
GO:0051139	metal cation:proton antiporter activity	1/69	20/14323	0.05	10.3789855	2.92021815	0.09212232	0.35746543	0.34623427	Slc30a1	1
GO:0070628	proteasome binding	1/69	20/14323	0.05	10.3789855	2.92021815	0.09212232	0.35746543	0.34623427	Psmb9	1
GO:0140517	protein-RNA adaptor activity	1/69	20/14323	0.05	10.3789855	2.92021815	0.09212232	0.35746543	0.34623427	Zfp598	1
GO:1904047	S-adenosyl-L-methionine binding	1/69	20/14323	0.05	10.3789855	2.92021815	0.09212232	0.35746543	0.34623427	Pcif1	1
GO:1990782	protein tyrosine kinase binding	2/69	108/14323	0.01851852	3.84406871	2.06412756	0.09548603	0.35746543	0.34623427	Tiam1/Dazap2	2
GO:0005044	scavenger receptor activity	1/69	21/14323	0.04761905	9.8847481	2.83474751	0.09650207	0.35746543	0.34623427	Enpp1	1
GO:0005164	tumor necrosis factor receptor binding	1/69	21/14323	0.04761905	9.8847481	2.83474751	0.09650207	0.35746543	0.34623427	Tnfsf10	1
GO:0022829	wide pore channel activity	1/69	21/14323	0.04761905	9.8847481	2.83474751	0.09650207	0.35746543	0.34623427	Gja4	1
GO:0004112	cyclic-nucleotide phosphodiesterase activity	1/69	22/14323	0.04545455	9.43544137	2.75482459	0.100861	0.35846544	0.34720287	Enpp1	1
GO:0005385	zinc ion transmembrane transporter activity	1/69	22/14323	0.04545455	9.43544137	2.75482459	0.100861	0.35846544	0.34720287	Slc30a1	1
GO:0008143	poly(A) binding	1/69	22/14323	0.04545455	9.43544137	2.75482459	0.100861	0.35846544	0.34720287	Hnrnpdl	1
GO:0005158	insulin receptor binding	1/69	23/14323	0.04347826	9.02520479	2.67984716	0.1051992	0.36003638	0.34872445	Enpp1	1
GO:0120283	protein serine/threonine kinase binding	1/69	23/14323	0.04347826	9.02520479	2.67984716	0.1051992	0.36003638	0.34872445	Dazap2	1
GO:0016645	oxidoreductase activity, acting on the CH-NH group of donors	1/69	24/14323	0.04166667	8.64915459	2.60930129	0.10951677	0.36003638	0.34872445	Pycr1	1
GO:0042288	MHC class I protein binding	1/69	24/14323	0.04166667	8.64915459	2.60930129	0.10951677	0.36003638	0.34872445	H2-T23	1
GO:0042605	peptide antigen binding	1/69	24/14323	0.04166667	8.64915459	2.60930129	0.10951677	0.36003638	0.34872445	H2-T23	1
GO:0070412	R-SMAD binding	1/69	24/14323	0.04166667	8.64915459	2.60930129	0.10951677	0.36003638	0.34872445	Ldlrad4	1
GO:0003697	single-stranded DNA binding	2/69	119/14323	0.01680672	3.48873462	1.89672663	0.11226127	0.36450263	0.35305038	Hnrnpdl/Rpa2	2
GO:0031435	mitogen-activated protein kinase kinase kinase binding	1/69	26/14323	0.03846154	7.98383501	2.47979733	0.11809041	0.37561894	0.36381743	Dazap2	1
GO:0001221	transcription coregulator binding	2/69	123/14323	0.01626016	3.37527984	1.84069219	0.11854134	0.37561894	0.36381743	Nr1d1/Ehmt2	2
GO:0099095	ligand-gated monoatomic anion channel activity	1/69	27/14323	0.03703704	7.68813741	2.42012503	0.12234667	0.38306159	0.37102624	Gabrg1	1
GO:0000175	3'-5'-RNA exonuclease activity	1/69	29/14323	0.03448276	7.15792104	2.30948012	0.13079855	0.38651706	0.37437314	Xrn2	1
GO:0004693	cyclin-dependent protein serine/threonine kinase activity	1/69	29/14323	0.03448276	7.15792104	2.30948012	0.13079855	0.38651706	0.37437314	Cdk2	1
GO:0070717	poly-purine tract binding	1/69	29/14323	0.03448276	7.15792104	2.30948012	0.13079855	0.38651706	0.37437314	Hnrnpdl	1
GO:0097472	cyclin-dependent protein kinase activity	1/69	29/14323	0.03448276	7.15792104	2.30948012	0.13079855	0.38651706	0.37437314	Cdk2	1
GO:0098632	cell-cell adhesion mediator activity	1/69	29/14323	0.03448276	7.15792104	2.30948012	0.13079855	0.38651706	0.37437314	Cd200	1
GO:0035925	mRNA 3'-UTR AU-rich region binding	1/69	30/14323	0.03333333	6.91932367	2.25802643	0.13499436	0.39174352	0.37943539	Hnrnpd	1
GO:0005109	frizzled binding	1/69	31/14323	0.03225806	6.69611968	2.20887659	0.13917021	0.39174352	0.37943539	Ccdc88c	1
GO:0016896	RNA exonuclease activity, producing 5'-phosphomonoesters	1/69	32/14323	0.03125	6.48686594	2.16185236	0.14332619	0.39174352	0.37943539	Xrn2	1
GO:0042287	MHC protein binding	1/69	32/14323	0.03125	6.48686594	2.16185236	0.14332619	0.39174352	0.37943539	H2-T23	1
GO:0000217	DNA secondary structure binding	1/69	33/14323	0.03030303	6.29029425	2.11679444	0.14746239	0.39174352	0.37943539	Hnrnpd	1
GO:0004532	RNA exonuclease activity	1/69	33/14323	0.03030303	6.29029425	2.11679444	0.14746239	0.39174352	0.37943539	Xrn2	1
GO:0005484	SNAP receptor activity	1/69	33/14323	0.03030303	6.29029425	2.11679444	0.14746239	0.39174352	0.37943539	Stx17	1
GO:0016799	hydrolase activity, hydrolyzing N-glycosyl compounds	1/69	33/14323	0.03030303	6.29029425	2.11679444	0.14746239	0.39174352	0.37943539	Il1rapl1	1
GO:0030515	snoRNA binding	1/69	33/14323	0.03030303	6.29029425	2.11679444	0.14746239	0.39174352	0.37943539	Rrp9	1
GO:0140828	metal cation:monoatomic cation antiporter activity	1/69	33/14323	0.03030303	6.29029425	2.11679444	0.14746239	0.39174352	0.37943539	Slc30a1	1
GO:1901618	organic hydroxy compound transmembrane transporter activity	1/69	34/14323	0.02941176	6.10528559	2.0735599	0.15157891	0.39769868	0.38520345	Slco1a4	1
GO:0030332	cyclin binding	1/69	35/14323	0.02857143	5.93084886	2.03202008	0.15567584	0.39769868	0.38520345	Cdk2	1
GO:0032813	tumor necrosis factor receptor superfamily binding	1/69	35/14323	0.02857143	5.93084886	2.03202008	0.15567584	0.39769868	0.38520345	Tnfsf10	1
GO:0038187	pattern recognition receptor activity	1/69	36/14323	0.02777778	5.76610306	1.99205883	0.15975328	0.39769868	0.38520345	Aim2	1
GO:0008757	S-adenosylmethionine-dependent methyltransferase activity	2/69	149/14323	0.01342282	2.78630483	1.52496718	0.16124246	0.39769868	0.38520345	Pcif1/Ehmt2	2
GO:0046915	transition metal ion transmembrane transporter activity	1/69	37/14323	0.02702703	5.61026244	1.95357096	0.1638113	0.39769868	0.38520345	Slc30a1	1
GO:0000993	RNA polymerase II complex binding	1/69	38/14323	0.02631579	5.46262395	1.91646093	0.16785001	0.39769868	0.38520345	Pcif1	1
GO:0022824	transmitter-gated monoatomic ion channel activity	1/69	38/14323	0.02631579	5.46262395	1.91646093	0.16785001	0.39769868	0.38520345	Gabrg1	1
GO:0022835	transmitter-gated channel activity	1/69	38/14323	0.02631579	5.46262395	1.91646093	0.16785001	0.39769868	0.38520345	Gabrg1	1
GO:0030291	protein serine/threonine kinase inhibitor activity	1/69	38/14323	0.02631579	5.46262395	1.91646093	0.16785001	0.39769868	0.38520345	Pkia	1
GO:0036002	pre-mRNA binding	1/69	38/14323	0.02631579	5.46262395	1.91646093	0.16785001	0.39769868	0.38520345	Arglu1	1
GO:0170055	lipid transmembrane transporter activity	1/69	38/14323	0.02631579	5.46262395	1.91646093	0.16785001	0.39769868	0.38520345	Slco1a4	1
GO:0004601	peroxidase activity	1/69	41/14323	0.02439024	5.06291976	1.8125653	0.17985115	0.41131177	0.39838883	Gstt2	1
GO:0005246	calcium channel regulator activity	1/69	41/14323	0.02439024	5.06291976	1.8125653	0.17985115	0.41131177	0.39838883	Slc30a1	1
GO:0008200	ion channel inhibitor activity	1/69	41/14323	0.02439024	5.06291976	1.8125653	0.17985115	0.41131177	0.39838883	Slc30a1	1
GO:0140938	histone H3 methyltransferase activity	1/69	41/14323	0.02439024	5.06291976	1.8125653	0.17985115	0.41131177	0.39838883	Ehmt2	1
GO:0016248	channel inhibitor activity	1/69	42/14323	0.02380952	4.94237405	1.78016872	0.1838135	0.41318762	0.40020574	Slc30a1	1
GO:0141110	transporter inhibitor activity	1/69	42/14323	0.02380952	4.94237405	1.78016872	0.1838135	0.41318762	0.40020574	Slc30a1	1
GO:0016684	oxidoreductase activity, acting on peroxide as acceptor	1/69	43/14323	0.02325581	4.82743512	1.74878317	0.18775699	0.41495872	0.40192119	Gstt2	1
GO:0043175	RNA polymerase core enzyme binding	1/69	43/14323	0.02325581	4.82743512	1.74878317	0.18775699	0.41495872	0.40192119	Pcif1	1
GO:0004879	nuclear receptor activity	1/69	44/14323	0.02272727	4.71772069	1.71835227	0.19168169	0.41663045	0.4035404	Nr1d1	1
GO:0005547	phosphatidylinositol-3,4,5-trisphosphate binding	1/69	44/14323	0.02272727	4.71772069	1.71835227	0.19168169	0.41663045	0.4035404	Fundc2	1
GO:0098531	ligand-activated transcription factor activity	1/69	45/14323	0.02222222	4.61288245	1.68882404	0.1955877	0.41969564	0.40650928	Nr1d1	1
GO:0019903	protein phosphatase binding	2/69	171/14323	0.01169591	2.42783287	1.30684633	0.19922513	0.41969564	0.40650928	Stx17/Rpa2	2
GO:0005230	extracellular ligand-gated monoatomic ion channel activity	1/69	46/14323	0.02173913	4.51260239	1.66015039	0.19947511	0.41969564	0.40650928	Gabrg1	1
GO:0060590	ATPase regulator activity	1/69	46/14323	0.02173913	4.51260239	1.66015039	0.19947511	0.41969564	0.40650928	Fnip1	1
GO:0008408	3'-5' exonuclease activity	1/69	47/14323	0.0212766	4.41658958	1.63228682	0.20334401	0.42444027	0.41110485	Xrn2	1
GO:0004518	nuclease activity	2/69	175/14323	0.01142857	2.37233954	1.2708406	0.20625236	0.42712103	0.41370138	Enpp1/Xrn2	2
GO:0003823	antigen binding	1/69	49/14323	0.02040816	4.23632062	1.5788277	0.2110266	0.43132094	0.41776933	H2-T23	1
GO:0008168	methyltransferase activity	2/69	179/14323	0.01117318	2.31932637	1.23580696	0.21330731	0.43132094	0.41776933	Pcif1/Ehmt2	2
GO:0004714	transmembrane receptor protein tyrosine kinase activity	1/69	50/14323	0.02	4.1515942	1.5531581	0.21484047	0.43132094	0.41776933	Epha8	1
GO:0016796	exonuclease activity, active with either ribo- or deoxyribonucleic acids and producing 5'-phosphomonoest	1/69	50/14323	0.02	4.1515942	1.5531581	0.21484047	0.43132094	0.41776933	Xrn2	1
GO:0005254	chloride channel activity	1/69	51/14323	0.01960784	4.07019039	1.52814998	0.21863617	0.43234069	0.41875705	Gabrg1	1
GO:0016504	peptidase activator activity	1/69	51/14323	0.01960784	4.07019039	1.52814998	0.21863617	0.43234069	0.41875705	Aim2	1
GO:0030374	nuclear receptor coactivator activity	1/69	53/14323	0.01886792	3.9165983	1.4799961	0.2261734	0.43958983	0.42577842	Arglu1	1
GO:0051018	protein kinase A binding	1/69	53/14323	0.01886792	3.9165983	1.4799961	0.2261734	0.43958983	0.42577842	Pkia	1
GO:0016741	transferase activity, transferring one-carbon groups	2/69	188/14323	0.0106383	2.20829479	1.16027764	0.22926403	0.43958983	0.42577842	Pcif1/Ehmt2	2
GO:0038024	cargo receptor activity	1/69	54/14323	0.01851852	3.84406871	1.45679418	0.22991509	0.43958983	0.42577842	Enpp1	1
GO:0016782	transferase activity, transferring sulphur-containing groups	1/69	55/14323	0.01818182	3.77417655	1.43414114	0.23363896	0.43958983	0.42577842	Tst	1
GO:0140299	molecular sensor activity	1/69	55/14323	0.01818182	3.77417655	1.43414114	0.23363896	0.43958983	0.42577842	Zfp598	1
GO:0008028	monocarboxylic acid transmembrane transporter activity	1/69	56/14323	0.01785714	3.70678054	1.41201312	0.23734508	0.43958983	0.42577842	Slco1a4	1
GO:0016278	lysine N-methyltransferase activity	1/69	56/14323	0.01785714	3.70678054	1.41201312	0.23734508	0.43958983	0.42577842	Ehmt2	1
GO:0016279	protein-lysine N-methyltransferase activity	1/69	56/14323	0.01785714	3.70678054	1.41201312	0.23734508	0.43958983	0.42577842	Ehmt2	1
GO:0045309	protein phosphorylated amino acid binding	1/69	59/14323	0.01694915	3.51830017	1.34856175	0.24835778	0.44906414	0.43495506	Pcif1	1
GO:0042277	peptide binding	2/69	200/14323	0.01	2.0757971	1.0659575	0.25066425	0.44906414	0.43495506	H2-T23/Ager	2
GO:0008173	RNA methyltransferase activity	1/69	61/14323	0.01639344	3.40294607	1.30850904	0.25561237	0.44906414	0.43495506	Pcif1	1
GO:0030246	carbohydrate binding	2/69	204/14323	0.00980392	2.0350952	1.03598009	0.25781637	0.44906414	0.43495506	Enpp1/Man2b1	2
GO:0001540	amyloid-beta binding	1/69	62/14323	0.01612903	3.34805984	1.28910419	0.25921374	0.44906414	0.43495506	Ager	1
GO:0070063	RNA polymerase binding	1/69	62/14323	0.01612903	3.34805984	1.28910419	0.25921374	0.44906414	0.43495506	Pcif1	1
GO:0001098	basal transcription machinery binding	1/69	63/14323	0.01587302	3.29491603	1.27009239	0.26279794	0.44906414	0.43495506	Pcif1	1
GO:0001099	basal RNA polymerase II transcription machinery binding	1/69	63/14323	0.01587302	3.29491603	1.27009239	0.26279794	0.44906414	0.43495506	Pcif1	1
GO:0004497	monooxygenase activity	1/69	63/14323	0.01587302	3.29491603	1.27009239	0.26279794	0.44906414	0.43495506	Foxred2	1
GO:0016209	antioxidant activity	1/69	63/14323	0.01587302	3.29491603	1.27009239	0.26279794	0.44906414	0.43495506	Gstt2	1
GO:0030594	neurotransmitter receptor activity	1/69	63/14323	0.01587302	3.29491603	1.27009239	0.26279794	0.44906414	0.43495506	Gabrg1	1
GO:0005253	monoatomic anion channel activity	1/69	64/14323	0.015625	3.24343297	1.25145879	0.26636504	0.44906414	0.43495506	Gabrg1	1
GO:0005518	collagen binding	1/69	64/14323	0.015625	3.24343297	1.25145879	0.26636504	0.44906414	0.43495506	Abi3bp	1
GO:0043539	protein serine/threonine kinase activator activity	1/69	64/14323	0.015625	3.24343297	1.25145879	0.26636504	0.44906414	0.43495506	Dazap2	1
GO:0019887	protein kinase regulator activity	2/69	210/14323	0.00952381	1.97694962	0.99227066	0.26855214	0.44928912	0.43517297	Pkia/Dazap2	2
GO:0042054	histone methyltransferase activity	1/69	65/14323	0.01538462	3.193534	1.2331893	0.26991514	0.44928912	0.43517297	Ehmt2	1
GO:0019199	transmembrane receptor protein kinase activity	1/69	66/14323	0.01515152	3.14514712	1.21527057	0.2734483	0.45230757	0.43809658	Epha8	1
GO:0015291	secondary active transmembrane transporter activity	2/69	218/14323	0.00917431	1.90440101	0.93618298	0.2828665	0.45814762	0.44375314	Slco1a4/Slc30a1	2
GO:0019902	phosphatase binding	2/69	218/14323	0.00917431	1.90440101	0.93618298	0.2828665	0.45814762	0.44375314	Stx17/Rpa2	2
GO:0003725	double-stranded RNA binding	1/69	69/14323	0.01449275	3.0084016	1.16349556	0.283947	0.45814762	0.44375314	Rftn1	1
GO:0048029	monosaccharide binding	1/69	69/14323	0.01449275	3.0084016	1.16349556	0.283947	0.45814762	0.44375314	Man2b1	1
GO:0004860	protein kinase inhibitor activity	1/69	71/14323	0.01408451	2.92365789	1.13051703	0.29086293	0.46361788	0.44905153	Pkia	1
GO:1990841	promoter-specific chromatin binding	1/69	71/14323	0.01408451	2.92365789	1.13051703	0.29086293	0.46361788	0.44905153	Ehmt2	1

GO:0019843 rRNA binding	1/69	72/14323	0.01388889	2.88305153	1.1144582	0.29429616	0.4651745	0.45055925	Tst	1
GO:1901681 sulfur compound binding	2/69	225/14323	0.00888889	1.84515298	0.88900834	0.29537696	0.4651745	0.45055925	Pcif1/Abi3bp	2
GO:0003684 damaged DNA binding	1/69	74/14323	0.01351351	2.80513122	1.0831546	0.30111356	0.46701844	0.45234525	Rpa2	1
GO:0019210 kinase inhibitor activity	1/69	74/14323	0.01351351	2.80513122	1.0831546	0.30111356	0.46701844	0.45234525	Pkia	1
GO:0140678 molecular function inhibitor activity	3/69	399/14323	0.0075188	1.5607497	0.7903719	0.30187504	0.46701844	0.45234525	Pkia/Slc30a1/Fnip1	3
GO:0008081 phosphoric diester hydrolase activity	1/69	76/14323	0.01315789	2.73131198	1.05287889	0.30786605	0.47350158	0.4586247	Enpp1	1
GO:0046332 SMAD binding	1/69	77/14323	0.01298701	2.69584039	1.0381064	0.31121814	0.47587425	0.46092282	Ldlrad4	1
GO:0015108 chloride transmembrane transporter activity	1/69	80/14323	0.0125	2.59474638	0.99516223	0.32117871	0.48545977	0.47020719	Gabrg1	1
GO:0050660 flavin adenine dinucleotide binding	1/69	80/14323	0.0125	2.59474638	0.99516223	0.32117871	0.48545977	0.47020719	Foxred2	1
GO:0019207 kinase regulator activity	2/69	242/14323	0.00826446	1.71553479	0.78105154	0.32562368	0.48936587	0.47399056	Pkia/Dazap2	2
GO:0003727 single-stranded RNA binding	1/69	83/14323	0.01204819	2.50096036	0.95413819	0.3309973	0.49461528	0.47907504	Hnrnpdl	1
GO:0020037 heme binding	1/69	84/14323	0.01190476	2.47118703	0.94086169	0.33423895	0.49663754	0.48103376	Nr1d1	1
GO:0008017 microtubule binding	2/69	248/14323	0.00806452	1.67402992	0.74497006	0.33623021	0.49678958	0.48118102	Ccdc88c/Tiam1	2
GO:0019888 protein phosphatase regulator activity	1/69	88/14323	0.01136364	2.35886034	0.88960134	0.3470515	0.50991365	0.49389275	Ppp2r3a	1
GO:0050839 cell adhesion molecule binding	2/69	257/14323	0.0077821	1.6154063	0.69262984	0.35205113	0.51063376	0.49459024	Ager/Cd200	2
GO:0008170 N-methyltransferase activity	1/69	90/14323	0.01111111	2.30644122	0.86500956	0.35336633	0.51063376	0.49459024	Ehmt2	1
GO:0008276 protein methyltransferase activity	1/69	90/14323	0.01111111	2.30644122	0.86500956	0.35336633	0.51063376	0.49459024	Ehmt2	1
GO:0046906 tetrapyrrole binding	1/69	91/14323	0.01098901	2.28109572	0.85295734	0.35650114	0.51234863	0.49625123	Nr1d1	1
GO:0019208 phosphatase regulator activity	1/69	96/14323	0.01041667	2.16228865	0.79497038	0.37195191	0.53164866	0.51494487	Ppp2r3a	1
GO:0004540 RNA nuclease activity	1/69	99/14323	0.01010101	2.09676475	0.76186533	0.38104628	0.5401328	0.52316245	Xrn2	1
GO:0008509 monoatomic anion transmembrane transporter activity	1/69	100/14323	0.01	2.0757971	0.75109136	0.3840488	0.5401328	0.52316245	Gabrg1	1
GO:0019955 cytokine binding	1/69	100/14323	0.01	2.0757971	0.75109136	0.3840488	0.5401328	0.52316245	Ager	1
GO:0003730 mRNA 3'-UTR binding	1/69	101/14323	0.00990099	2.05524465	0.7404428	0.38703696	0.54110596	0.52410503	Hnrnpd	1
GO:0005125 cytokine activity	1/69	102/14323	0.00980392	2.0350952	0.72991672	0.39001083	0.54110596	0.52410503	Tnfsf10	1
GO:0015103 inorganic anion transmembrane transporter activity	1/69	103/14323	0.00970874	2.01533699	0.71951032	0.39297049	0.54110596	0.52410503	Gabrg1	1
GO:0051219 phosphoprotein binding	1/69	103/14323	0.00970874	2.01533699	0.71951032	0.39297049	0.54110596	0.52410503	Pcif1	1
GO:0008013 beta-catenin binding	1/69	104/14323	0.00961538	1.99595875	0.70922085	0.39591599	0.54232242	0.52528328	Ajap1	1
GO:0015297 antiporter activity	1/69	107/14323	0.00934579	1.93999729	0.67902816	0.40466824	0.55073478	0.53343133	Slc30a1	1
GO:0035591 signaling adaptor activity	1/69	108/14323	0.00925926	1.92203435	0.66918092	0.4075578	0.55073478	0.53343133	Aim2	1
GO:0030165 PDZ domain binding	1/69	109/14323	0.00917431	1.90440101	0.65943825	0.41043353	0.55073478	0.53343133	Ccdc88c	1
GO:0061135 endopeptidase regulator activity	1/69	109/14323	0.00917431	1.90440101	0.65943825	0.41043353	0.55073478	0.53343133	Aim2	1
GO:0000149 SNARE binding	1/69	110/14323	0.00909091	1.88708827	0.6497979	0.4132955	0.55175998	0.53442432	Stx17	1
GO:0043022 ribosome binding	1/69	112/14323	0.00892857	1.85339027	0.63081554	0.41897843	0.55312732	0.5357487	Zfp598	1
GO:0031625 ubiquitin protein ligase binding	2/69	297/14323	0.00673401	1.39784317	0.48203793	0.42062914	0.55312732	0.5357487	Rpa2/Dazap2	2
GO:0061630 ubiquitin protein ligase activity	2/69	297/14323	0.00673401	1.39784317	0.48203793	0.42062914	0.55312732	0.5357487	Nfx1/Zfp598	2
GO:0004713 protein tyrosine kinase activity	1/69	115/14323	0.00869565	1.80504096	0.60305709	0.42740126	0.55923648	0.54166591	Epha8	1
GO:0015276 ligand-gated monoatomic ion channel activity	1/69	116/14323	0.00862069	1.78948026	0.59398719	0.43018204	0.5600885	0.54249116	Gabrg1	1
GO:0030295 protein kinase activator activity	1/69	117/14323	0.00854701	1.77418556	0.58500569	0.43294951	0.56091488	0.54329158	Dazap2	1
GO:0015078 proton transmembrane transporter activity	1/69	118/14323	0.00847458	1.75915009	0.57611082	0.43570373	0.56171609	0.54406761	Slc30a1	1
GO:0042826 histone deacetylase binding	1/69	119/14323	0.00840336	1.74436731	0.56730086	0.43844477	0.56249256	0.54481969	Hnrnpd	1
GO:0022834 ligand-gated channel activity	1/69	121/14323	0.00826446	1.71553479	0.54992909	0.44388754	0.56601903	0.54823536	Gabrg1	1
GO:0044389 ubiquitin-like protein ligase binding	2/69	313/14323	0.00638978	1.32638792	0.40620493	0.44706809	0.56601903	0.54823536	Rpa2/Dazap2	2
GO:0061659 ubiquitin-like protein ligase activity	2/69	314/14323	0.00636943	1.32216376	0.40160207	0.448699	0.56601903	0.54823536	Nfx1/Zfp598	2
GO:0003729 mRNA binding	2/69	316/14323	0.00632911	1.31379563	0.39244236	0.45195297	0.56601903	0.54823536	Hnrnpd/Luc7l2	2
GO:0019838 growth factor binding	1/69	124/14323	0.00806452	1.67402992	0.52446818	0.45195436	0.56601903	0.54823536	Epha8	1
GO:0005319 lipid transporter activity	1/69	125/14323	0.008	1.66063768	0.51613435	0.45461758	0.56665604	0.54885236	Slco1a4	1
GO:0019209 kinase activator activity	1/69	126/14323	0.00793651	1.64745802	0.50787472	0.45726805	0.56727122	0.54944821	Dazap2	1
GO:0008201 heparin binding	1/69	129/14323	0.00775194	1.60914504	0.48352749	0.46514354	0.57433216	0.5562873	Abi3bp	1
GO:0022804 active transmembrane transporter activity	2/69	336/14323	0.00595238	1.23559351	0.30403857	0.48389642	0.59430747	0.57563501	Slco1a4/Slc30a1	2
GO:0051087 protein-folding chaperone binding	1/69	138/14323	0.00724638	1.5042008	0.41408205	0.48810043	0.59430747	0.57563501	Fnip1	1
GO:0099106 ion channel regulator activity	1/69	138/14323	0.00724638	1.5042008	0.41408205	0.48810043	0.59430747	0.57563501	Slc30a1	1
GO:0140098 catalytic activity, acting on RNA	2/69	343/14323	0.0058309	1.21037732	0.2743787	0.49481075	0.59970151	0.58085958	Pcif1/Xrn2	2
GO:0046943 carboxylic acid transmembrane transporter activity	1/69	144/14323	0.00694444	1.44152576	0.3704866	0.50286267	0.60666459	0.58760389	Slco1a4	1
GO:0005342 organic acid transmembrane transporter activity	1/69	145/14323	0.00689655	1.43158421	0.36341267	0.50528191	0.60679974	0.58773479	Slco1a4	1
GO:0061134 peptidase regulator activity	1/69	146/14323	0.00684932	1.42177884	0.35639126	0.50768955	0.60691979	0.58785106	Aim2	1
GO:0015631 tubulin binding	2/69	353/14323	0.00566572	1.17608901	0.23306626	0.51015392	0.60710625	0.58803167	Ccdc88c/Tiam1	2
GO:0016247 channel regulator activity	1/69	148/14323	0.00675676	1.40256561	0.3425027	0.51247025	0.60711565	0.58804078	Slc30a1	1
GO:0141108 transporter regulator activity	1/69	150/14323	0.00666667	1.38386473	0.32881449	0.51720518	0.60997741	0.59081262	Slc30a1	1
GO:0015267 channel activity	2/69	362/14323	0.00552486	1.14684923	0.19689092	0.52370704	0.61215534	0.59292212	Gja4/Gabrg1	2
GO:0022803 passive transmembrane transporter activity	2/69	362/14323	0.00552486	1.14684923	0.19689092	0.52370704	0.61215534	0.59292212	Gja4/Gabrg1	2
GO:0004842 ubiquitin-protein transferase activity	2/69	376/14323	0.00531915	1.10414739	0.14238359	0.54429722	0.63340783	0.61350688	Nfx1/Zfp598	2
GO:1901981 phosphatidylinositol phosphate binding	1/69	170/14323	0.00588235	1.22105712	0.20172639	0.56213087	0.65127937	0.63081692	Fundc2	1
GO:0005126 cytokine receptor binding	1/69	172/14323	0.00581395	1.20685878	0.18989023	0.56639009	0.65333593	0.63280887	Tnfsf10	1
GO:0005539 glycosaminoglycan binding	1/69	178/14323	0.00561798	1.16617815	0.15521741	0.57892428	0.66269107	0.64187008	Abi3bp	1
GO:0019787 ubiquitin-like protein transferase activity	2/69	401/14323	0.00498753	1.03531028	0.04989769	0.57953972	0.66269107	0.64187008	Nfx1/Zfp598	2
GO:0140993 histone modifying activity	2/69	404/14323	0.0049505	1.02762233	0.03918365	0.58363557	0.66448552	0.64360815	Cdk2/Ehmt2	2
GO:0016755 aminoacyltransferase activity	2/69	411/14323	0.00486618	1.01012024	0.01448355	0.59308076	0.67232862	0.65120483	Nfx1/Zfp598	2
GO:0043021 ribonucleoprotein complex binding	1/69	190/14323	0.00526316	1.09252479	0.08932556	0.60293104	0.68056165	0.65917918	Zfp598	1
GO:0033218 amide binding	1/69	193/14323	0.00518135	1.07554254	0.07351182	0.6087188	0.68338537	0.66191419	Ager	1
GO:0022853 active monoatomic ion transmembrane transporter activity	1/69	194/14323	0.00515464	1.06999851	0.06829547	0.61062952	0.68338537	0.66191419	Slc30a1	1
GO:0005543 phospholipid binding	2/69	428/14323	0.0046729	0.96999865	-0.0438421	0.61536602	0.68576806	0.66422202	Fundc2/Tiam1	2
GO:0051015 actin filament binding	1/69	201/14323	0.00497512	1.03273488	0.03251761	0.62374946	0.69217767	0.67043025	Abi3bp	1
GO:0008514 organic anion transmembrane transporter activity	1/69	204/14323	0.00490196	1.0175476	0.0175626	0.62923802	0.69533445	0.67348784	Slco1a4	1
GO:0000287 magnesium ion binding	1/69	212/14323	0.00471698	0.97914958	-0.0212795	0.64349137	0.70810975	0.68586175	Cdk2	1
GO:0005509 calcium ion binding	2/69	466/14323	0.00429185	0.89090004	-0.1665869	0.66184397	0.7226625	0.69995728	Ppp2r3a/Enpp1	2
GO:0001664 G protein-coupled receptor binding	1/69	223/14323	0.0044843	0.93085072	-0.0724083	0.66221165	0.7226625	0.69995728	Ccdc88c	1
GO:0035173 histone kinase activity	1/69	226/14323	0.00442478	0.91849429	-0.0859284	0.66714679	0.72503969	0.70225978	Cdk2	1
GO:0022836 gated channel activity	1/69	231/14323	0.004329	0.89861346	-0.1080831	0.67521458	0.7307878	0.70782728	Gabrg1	1
GO:0016791 phosphatase activity	1/69	239/14323	0.0041841	0.86853435	-0.1425956	0.68772395	0.74127623	0.71798618	Enpp1	1
GO:0004672 protein kinase activity	2/69	497/14323	0.00402414	0.83533083	-0.2599562	0.69640233	0.74756658	0.7240789	Epha8/Cdk2	2
GO:0004175 endopeptidase activity	1/69	255/14323	0.00392157	0.81403808	-0.2084663	0.71133521	0.7604214	0.73652983	Psmb9	1
GO:0003713 transcription coactivator activity	1/69	257/14323	0.00389105	0.80770315	-0.216427	0.71416002	0.7604214	0.73652983	Arglu1	1
GO:0035091 phosphatidylinositol binding	1/69	265/14323	0.00377358	0.78331966	-0.2477072	0.72518939	0.76905165	0.74488893	Fundc2	1
GO:0048018 receptor ligand activity	1/69	284/14323	0.00352113	0.73091447	-0.3186687	0.74973108	0.7900111	0.76518986	Tnfsf10	1
GO:0004857 enzyme inhibitor activity	1/69	285/14323	0.00350877	0.72834986	-0.3222833	0.75096112	0.7900111	0.76518986	Pkia	1
GO:0030546 signaling receptor activator activity	1/69	289/14323	0.00346021	0.71826889	-0.3366286	0.75582199	0.79195691	0.76707453	Tnfsf10	1
GO:0042578 phosphoric ester hydrolase activity	1/69	318/14323	0.00314465	0.65276638	-0.4356641	0.78837403	0.82061892	0.79483601	Enpp1	1
GO:0030545 signaling receptor regulator activity	1/69	319/14323	0.0031348	0.65072009	-0.4389357	0.78941668	0.82061892	0.79483601	Tnfsf10	1
GO:0005216 monoatomic ion channel activity	1/69	325/14323	0.00307692	0.6387068	-0.4583809	0.79556705	0.82259961	0.79675447	Gabrg1	1
GO:0106310 protein serine kinase activity	1/69	327/14323	0.0030581	0.63480034	-0.4647938	0.79757757	0.82259961	0.79675447	Cdk2	1
GO:0046873 metal ion transmembrane transporter activity	1/69	346/14323	0.00289017	0.59994136	-0.5240983	0.81573132	0.83803647	0.81170633	Slc30a1	1
GO:0004674 protein serine/threonine kinase activity	1/69	388/14323	0.00257732	0.53499925	-0.6460618	0.85035968	0.87021243	0.84287136	Cdk2	1
GO:0003779 actin binding	1/69	402/14323	0.00248756	0.51636744	-0.6843076	0.86041021	0.87708483	0.84952783	Abi3bp	1
GO:0008233 peptidase activity	1/69	416/14323	0.00240385	0.49898969	-0.7214971	0.86979484	0.88322797	0.85547796	Psmb9	1
GO:0022890 inorganic cation transmembrane transporter activity	1/69	446/14323	0.00224215	0.46542536	-0.7979689	0.88786105	0.8981056	0.86988815	Slc30a1	1
GO:0140297 DNA-binding transcription factor binding	1/69	471/14323	0.00212314	0.44072125	-0.8586982	0.90101047	0.90393293	0.87553239	Dazap2	1
GO:0003712 transcription coregulator activity	1/69	473/14323	0.00211416	0.43885774	-0.8634489	0.90199422	0.90393293	0.87553239	Arglu1	1
GO:0008324 monoatomic cation transmembrane transporter activity	1/69	477/14323	0.00209644	0.43517759	-0.8729049	0.90393293	0.90393293	0.87553239	Slc30a1	1

ID	Description	GeneRatio	BgRatio	RichFactor	FoldEnrichm	zScore	pvalue	p.adjust	qvalue	geneID	Count
GO:0070888	E-box binding	4/96	37/14323	0.10810811	16.1295045	7.56921828	0.00010564	0.03422717	0.03347102	Ciart/Per1/Bhlhe40/Bhlhe41	4
GO:0004715	non-membrane spanning protein tyrosine kinase activity	3/96	34/14323	0.08823529	13.1645221	5.83330086	0.00150189	0.24330629	0.2379311	Wee1/Abl1/Lyn	3
GO:0001227	DNA-binding transcription repressor activity, RNA polymerase II-specific	6/96	224/14323	0.02678571	3.99637277	3.71284211	0.00394003	0.34408649	0.33648484	Nr1d2/Bhlhe40/Bhlhe41/Zbtb49/Zfp281/Ylpm1	6
GO:0001217	DNA-binding transcription repressor activity	6/96	230/14323	0.02608696	3.89211957	3.63211191	0.00448008	0.34408649	0.33648484	Nr1d2/Bhlhe40/Bhlhe41/Zbtb49/Zfp281/Ylpm1	6
GO:0000400	four-way junction DNA binding	2/96	17/14323	0.11764706	17.5526961	5.6093802	0.00566275	0.34408649	0.33648484	Abl1/Xrcc3	2
GO:0004536	DNA nuclease activity	3/96	57/14323	0.05263158	7.85252193	4.25811648	0.00657239	0.34408649	0.33648484	Pole/Xrcc3/Fan1	3
GO:0004713	protein tyrosine kinase activity	4/96	115/14323	0.03478261	5.18949275	3.70531108	0.00743397	0.34408649	0.33648484	Wee1/Rps6ka5/Abl1/Lyn	4
GO:0010485	histone H4 acetyltransferase activity	2/96	21/14323	0.0952381	14.2093254	4.97591193	0.00859316	0.34802291	0.3403343	Brpf1/Naa50	2
GO:0051861	glycolipid binding	2/96	27/14323	0.07407407	11.0516975	4.29432401	0.01399385	0.39245647	0.38378622	Trem2/Lyn	2
GO:0043425	bHLH transcription factor binding	2/96	29/14323	0.06896552	10.2895115	4.11335102	0.01604708	0.39245647	0.38378622	Bhlhe40/Bhlhe41	2
GO:0046875	ephrin receptor binding	2/96	31/14323	0.06451613	9.62567204	3.94919038	0.01822077	0.39245647	0.38378622	Abl1/Lyn	2
GO:0000217	DNA secondary structure binding	2/96	33/14323	0.06060606	9.04229798	3.79928437	0.02051141	0.39245647	0.38378622	Abl1/Xrcc3	2
GO:0004520	DNA endonuclease activity	2/96	37/14323	0.05405405	8.06475225	3.53446155	0.02542982	0.39245647	0.38378622	Xrcc3/Fan1	2
GO:0004402	histone acetyltransferase activity	2/96	41/14323	0.04878049	7.27794715	3.30670807	0.03077557	0.39245647	0.38378622	Brpf1/Naa50	2
GO:0051219	phosphoprotein binding	3/96	103/14323	0.02912621	4.34557039	2.79910875	0.03189074	0.39245647	0.38378622	Abl1/Dpysl4/Lyn	3
GO:0051539	4 iron, 4 sulfur cluster binding	2/96	43/14323	0.04651163	6.93943798	3.2040277	0.03360064	0.39245647	0.38378622	Pole/Rsad1	2
GO:0061733	peptide-lysine-N-acetyltransferase activity	2/96	44/14323	0.04545455	6.78172348	3.15511755	0.03504985	0.39245647	0.38378622	Brpf1/Naa50	2
GO:0009055	electron transfer activity	2/96	47/14323	0.04255319	6.34884752	3.01707886	0.03953962	0.39245647	0.38378622	Por/ND5	2
GO:0034212	peptide N-acetyltransferase activity	2/96	49/14323	0.04081633	6.08971088	2.93156206	0.04264751	0.39245647	0.38378622	Brpf1/Naa50	2
GO:0030246	carbohydrate binding	4/96	204/14323	0.01960784	2.92544935	2.27523363	0.04824302	0.39245647	0.38378622	Galnt15/Clec1a/Alpk1/Gpcpd1	4
GO:0016651	oxidoreductase activity, acting on NAD(P)H	2/96	53/14323	0.03773585	5.63011006	2.77394629	0.04912545	0.39245647	0.38378622	Por/ND5	2
GO:0004672	protein kinase activity	7/96	497/14323	0.01408451	2.10137911	2.05280425	0.04927351	0.39245647	0.38378622	Mylk4/Wee1/Alpk1/Rps6ka5/Abl1/Lyn/Cdk19	7
GO:0001221	transcription coregulator binding	3/96	123/14323	0.02439024	3.63897358	2.41448333	0.0496498	0.39245647	0.38378622	Per3/Per1/Zbtb49	3
GO:0140299	molecular sensor activity	2/96	55/14323	0.03636364	5.42537879	2.70104009	0.05248986	0.39245647	0.38378622	Sar1b/Xrcc3	2
GO:0001222	transcription corepressor binding	2/96	56/14323	0.03571429	5.32849702	2.66591066	0.0542022	0.39245647	0.38378622	Per3/Per1	2
GO:0004896	cytokine receptor activity	2/96	59/14323	0.03389831	5.0575565	2.56537408	0.05945579	0.39245647	0.38378622	Fzd4/Il15ra	2
GO:0005178	integrin binding	3/96	133/14323	0.02255639	3.36536654	2.25119885	0.05999947	0.39245647	0.38378622	Tspan4/Lyn/Jam2	3
GO:0016705	oxidoreductase activity, acting on paired donors, with incorporation or reduction of molecular oxygen	3/96	134/14323	0.02238806	3.34025187	2.23573276	0.0610862	0.39245647	0.38378622	Por/Cyp3a13/Tet3	3
GO:0001540	amyloid-beta binding	2/96	62/14323	0.03225806	4.81283602	2.47143827	0.06487729	0.39245647	0.38378622	Fzd4/Trem2	2
GO:0005225	volume-sensitive anion channel activity	1/96	10/14323	0.1	14.9197917	3.61699731	0.06505904	0.39245647	0.38378622	Clcn2	1
GO:0005247	voltage-gated chloride channel activity	1/96	10/14323	0.1	14.9197917	3.61699731	0.06505904	0.39245647	0.38378622	Clcn2	1
GO:0008035	high-density lipoprotein particle binding	1/96	10/14323	0.1	14.9197917	3.61699731	0.06505904	0.39245647	0.38378622	Trem2	1
GO:0016653	oxidoreductase activity, acting on NAD(P)H, heme protein as acceptor	1/96	10/14323	0.1	14.9197917	3.61699731	0.06505904	0.39245647	0.38378622	Por	1
GO:0016832	aldehyde-lyase activity	1/96	10/14323	0.1	14.9197917	3.61699731	0.06505904	0.39245647	0.38378622	Tha1	1
GO:0033691	sialic acid binding	1/96	10/14323	0.1	14.9197917	3.61699731	0.06505904	0.39245647	0.38378622	Agrn	1
GO:0070097	delta-catenin binding	1/96	10/14323	0.1	14.9197917	3.61699731	0.06505904	0.39245647	0.38378622	Abl1	1
GO:0140161	monocarboxylate:sodium symporter activity	1/96	10/14323	0.1	14.9197917	3.61699731	0.06505904	0.39245647	0.38378622	Slc6a6	1
GO:0008080	N-acetyltransferase activity	2/96	65/14323	0.03076923	4.59070513	2.38335042	0.07045806	0.39245647	0.38378622	Brpf1/Naa50	2
GO:0008395	steroid hydroxylase activity	1/96	11/14323	0.09090909	13.563447	3.42401592	0.07132987	0.39245647	0.38378622	Cyp3a13	1
GO:0010181	FMN binding	1/96	11/14323	0.09090909	13.563447	3.42401592	0.07132987	0.39245647	0.38378622	Por	1
GO:0016894	endonuclease activity, active with either ribo- or deoxyribonucleic acids and producing 3'-phosphomonoe	1/96	11/14323	0.09090909	13.563447	3.42401592	0.07132987	0.39245647	0.38378622	Xrcc3	1
GO:0043995	histone H4K5 acetyltransferase activity	1/96	11/14323	0.09090909	13.563447	3.42401592	0.07132987	0.39245647	0.38378622	Brpf1	1
GO:0043996	histone H4K8 acetyltransferase activity	1/96	11/14323	0.09090909	13.563447	3.42401592	0.07132987	0.39245647	0.38378622	Brpf1	1
GO:0004861	cyclin-dependent protein serine/threonine kinase inhibitor activity	1/96	12/14323	0.08333333	12.4331597	3.25463798	0.07755907	0.39245647	0.38378622	Inca1	1
GO:0015378	sodium:chloride symporter activity	1/96	12/14323	0.08333333	12.4331597	3.25463798	0.07755907	0.39245647	0.38378622	Slc6a6	1
GO:0015643	toxic substance binding	1/96	12/14323	0.08333333	12.4331597	3.25463798	0.07755907	0.39245647	0.38378622	Gsta4	1
GO:0016888	endodeoxyribonuclease activity, producing 5'-phosphomonoesters	1/96	12/14323	0.08333333	12.4331597	3.25463798	0.07755907	0.39245647	0.38378622	Fan1	1
GO:0043997	histone H4K12 acetyltransferase activity	1/96	12/14323	0.08333333	12.4331597	3.25463798	0.07755907	0.39245647	0.38378622	Brpf1	1
GO:0140031	phosphorylation-dependent protein binding	1/96	12/14323	0.08333333	12.4331597	3.25463798	0.07755907	0.39245647	0.38378622	Lyn	1
GO:0140996	histone H3 kinase activity	1/96	12/14323	0.08333333	12.4331597	3.25463798	0.07755907	0.39245647	0.38378622	Rps6ka5	1
GO:0051536	iron-sulfur cluster binding	2/96	69/14323	0.02898551	4.32457729	2.27391061	0.0781324	0.39245647	0.38378622	Pole/Rsad1	2
GO:0051540	metal cluster binding	2/96	70/14323	0.02857143	4.26279762	2.24784727	0.08009065	0.39245647	0.38378622	Pole/Rsad1	2
GO:0005545	1-phosphatidylinositol binding	1/96	13/14323	0.07692308	11.4767628	3.10427192	0.08374692	0.39245647	0.38378622	Adap2	1
GO:0008296	3'-5'-DNA exonuclease activity	1/96	13/14323	0.07692308	11.4767628	3.10427192	0.08374692	0.39245647	0.38378622	Pole	1
GO:0015355	secondary active monocarboxylate transmembrane transporter activity	1/96	13/14323	0.07692308	11.4767628	3.10427192	0.08374692	0.39245647	0.38378622	Slc6a6	1
GO:0015373	monoatomic anion:sodium symporter activity	1/96	13/14323	0.07692308	11.4767628	3.10427192	0.08374692	0.39245647	0.38378622	Slc6a6	1
GO:0016812	hydrolase activity, acting on carbon-nitrogen (but not peptide) bonds, in cyclic amides	1/96	13/14323	0.07692308	11.4767628	3.10427192	0.08374692	0.39245647	0.38378622	Dpysl4	1
GO:0017154	semaphorin receptor activity	1/96	13/14323	0.07692308	11.4767628	3.10427192	0.08374692	0.39245647	0.38378622	Trem2	1
GO:0140664	ATP-dependent DNA damage sensor activity	1/96	13/14323	0.07692308	11.4767628	3.10427192	0.08374692	0.39245647	0.38378622	Xrcc3	1
GO:0004527	exonuclease activity	2/96	72/14323	0.02777778	4.14438657	2.19715298	0.08405269	0.39245647	0.38378622	Pole/Fan1	2
GO:0005161	platelet-derived growth factor receptor binding	1/96	14/14323	0.07142857	10.656994	2.96949176	0.08989369	0.39245647	0.38378622	Lyn	1
GO:0008308	voltage-gated monoatomic anion channel activity	1/96	14/14323	0.07142857	10.656994	2.96949176	0.08989369	0.39245647	0.38378622	Clcn2	1
GO:0008429	phosphatidylethanolamine binding	1/96	14/14323	0.07142857	10.656994	2.96949176	0.08989369	0.39245647	0.38378622	Trem2	1
GO:0022858	alanine transmembrane transporter activity	1/96	14/14323	0.07142857	10.656994	2.96949176	0.08989369	0.39245647	0.38378622	Slc6a6	1
GO:0034450	ubiquitin-ubiquitin ligase activity	1/96	14/14323	0.07142857	10.656994	2.96949176	0.08989369	0.39245647	0.38378622	Peli1	1
GO:0036122	BMP binding	1/96	14/14323	0.07142857	10.656994	2.96949176	0.08989369	0.39245647	0.38378622	Agrn	1
GO:0048038	quinone binding	1/96	14/14323	0.07142857	10.656994	2.96949176	0.08989369	0.39245647	0.38378622	Coq10b	1
GO:0070742	C2H2 zinc finger domain binding	1/96	14/14323	0.07142857	10.656994	2.96949176	0.08989369	0.39245647	0.38378622	Srrm2	1
GO:0008081	phosphoric diester hydrolase activity	2/96	76/14323	0.02631579	3.92626096	2.10106227	0.09215102	0.39245647	0.38378622	Gpcpd1/Fan1	2
GO:0051213	dioxygenase activity	2/96	77/14323	0.02597403	3.87527056	2.0780615	0.09421028	0.39245647	0.38378622	Por/Tet3	2
GO:0004622	lysophospholipase activity	1/96	15/14323	0.06666667	9.94652778	2.84768183	0.09599966	0.39245647	0.38378622	Pnpla7	1
GO:0030169	low-density lipoprotein particle binding	1/96	15/14323	0.06666667	9.94652778	2.84768183	0.09599966	0.39245647	0.38378622	Trem2	1
GO:0030548	acetylcholine receptor regulator activity	1/96	15/14323	0.06666667	9.94652778	2.84768183	0.09599966	0.39245647	0.38378622	Agrn	1
GO:0031005	filamin binding	1/96	15/14323	0.06666667	9.94652778	2.84768183	0.09599966	0.39245647	0.38378622	Dpysl4	1
GO:0035014	phosphatidylinositol 3-kinase regulator activity	1/96	15/14323	0.06666667	9.94652778	2.84768183	0.09599966	0.39245647	0.38378622	Lyn	1
GO:0042813	Wnt receptor activity	1/96	15/14323	0.06666667	9.94652778	2.84768183	0.09599966	0.39245647	0.38378622	Fzd4	1
GO:0097110	scaffold protein binding	2/96	78/14323	0.02564103	3.82558761	2.05544394	0.09628287	0.39245647	0.38378622	Trem2/Lyn	2
GO:0015108	chloride transmembrane transporter activity	2/96	80/14323	0.025	3.72994792	2.01131181	0.10046698	0.39245647	0.38378622	Slc6a6/Clcn2	2
GO:0016410	N-acyltransferase activity	2/96	80/14323	0.025	3.72994792	2.01131181	0.10046698	0.39245647	0.38378622	Brpf1/Naa50	2
GO:0050660	flavin adenine dinucleotide binding	2/96	80/14323	0.025	3.72994792	2.01131181	0.10046698	0.39245647	0.38378622	Por/Pipox	2
GO:0140375	immune receptor activity	2/96	80/14323	0.025	3.72994792	2.01131181	0.10046698	0.39245647	0.38378622	Fzd4/Il15ra	2
GO:0099602	neurotransmitter receptor regulator activity	1/96	16/14323	0.0625	9.32486979	2.73680552	0.10206508	0.39245647	0.38378622	Agrn	1
GO:0140612	DNA damage sensor activity	1/96	16/14323	0.0625	9.32486979	2.73680552	0.10206508	0.39245647	0.38378622	Xrcc3	1
GO:0004653	polypeptide N-acetylgalactosaminyltransferase activity	1/96	17/14323	0.05882353	8.77634804	2.63525002	0.10809022	0.39245647	0.38378622	Galnt15	1
GO:0008137	NADH dehydrogenase (ubiquinone) activity	1/96	17/14323	0.05882353	8.77634804	2.63525002	0.10809022	0.39245647	0.38378622	ND5	1
GO:0008409	5'-3' exonuclease activity	1/96	17/14323	0.05882353	8.77634804	2.63525002	0.10809022	0.39245647	0.38378622	Fan1	1
GO:0140036	ubiquitin-modified protein reader activity	1/96	17/14323	0.05882353	8.77634804	2.63525002	0.10809022	0.39245647	0.38378622	Fan1	1
GO:0020037	heme binding	2/96	84/14323	0.02380952	3.55233135	1.92715985	0.10898361	0.39245647	0.38378622	Cyp3a13/Rsad1	2
GO:0004518	nuclease activity	3/96	175/14323	0.01714286	2.55767857	1.70306071	0.11300749	0.39245647	0.38378622	Pole/Xrcc3/Fan1	3
GO:0015377	chloride:monoatomic cation symporter activity	1/96	18/14323	0.05555556	8.28877315	2.54171903	0.11407536	0.39245647	0.38378622	Slc6a6	1
GO:0034185	apolipoprotein binding	1/96	18/14323	0.05555556	8.28877315	2.54171903	0.11407536	0.39245647	0.38378622	Trem2	1
GO:0043395	heparan sulfate proteoglycan binding	1/96	18/14323	0.05555556	8.28877315	2.54171903	0.11407536	0.39245647	0.38378622	Agrn	1
GO:0046527	glucosyltransferase activity	1/96	18/14323	0.05555556	8.28877315	2.54171903	0.11407536	0.39245647	0.38378622	Alg10b	1
GO:0016407	acetyltransferase activity	2/96	88/14323	0.02272727	3.39086174	1.84798248	0.11768467	0.39245647	0.38378622	Brpf1/Naa50	2
GO:0046906	tetrapyrrole binding	2/96	91/14323	0.02197802	3.27907509	1.79154264	0.12432213	0.39245647	0.38378622	Cyp3a13/Rsad1	2
GO:0005283	amino acid:sodium symporter activity	1/96	20/14323	0.05	7.45989583	2.3746944	0.12592664	0.39245647	0.38378622	Slc6a6	1
GO:0015296	monoatomic anion:monoatomic cation symporter activity	1/96	20/14323	0.05	7.45989583	2.3746944	0.12592664	0.39245647	0.38378622	Slc6a6	1
GO:0030247	polysaccharide binding	1/96	20/14323	0.05	7.45989583	2.3746944	0.12592664	0.39245647	0.38378622	Gpcpd1	1
GO:0070064	proline-rich region binding	1/96	20/14323	0.05	7.45989583	2.3746944	0.12592664	0.39245647	0.38378622	Abl1	1
GO:0140035	ubiquitin-like protein reader activity	1/96	20/14323	0.05	7.45989583	2.3746944	0.12592664	0.39245647	0.38378622	Fan1	1
GO:0003714	transcription corepressor activity	3/96	184/14323	0.01630435	2.43257473	1.60656254	0.12611646	0.39245647	0.38378622	Bhlhe41/Rere/Eid1	3
GO:0140030	modification-dependent protein binding	3/96	184/14323	0.01630435	2.43257473	1.60656254	0.12611646	0.39245647	0.38378622	Ncapd3/Fan1/Lyn	3
GO:0003887	DNA-directed DNA polymerase activity	1/96	21/14323	0.04761905	7.1046627	2.29960751	0.13179332	0.39245647	0.38378622	Pole	1
GO:0016895	DNA exonuclease activity, producing 5'-phosphomonoesters	1/96	21/14323	0.04761905	7.1046627	2.29960751	0.13179332	0.39245647	0.38378622	Pole	1
GO:0038191	neuropilin binding	1/96	21/14323	0.04761905	7.1046627	2.29960751	0.13179332	0.39245647	0.38378622	Abl1	1
GO:0140993	histone modifying activity	5/96	404/14323	0.01237624	1.84650887	1.41774774	0.13480656	0.39245647	0.38378622	Mylk4/Alpk1/Rps6ka5/Brpf1/Naa50	5
GO:0003950	NAD+-protein poly-ADP-ribosyltransferase activity	1/96	22/14323	0.04545455	6.78172348	2.22928832	0.13762102	0.39245647	0.38378622	Art5	1
GO:0004529	DNA exonuclease activity	1/96	22/14323	0.04545455	6.78172348	2.22928832	0.13762102	0.39245647	0.38378622	Pole	1
GO:0008327	methyl-CpG binding	1/96	22/14323	0.04545455	6.78172348	2.22928832	0.13762102	0.39245647	0.38378622	Tet3	1
GO:0030215	semaphorin receptor binding	1/96	22/14323	0.04545455	6.78172348	2.22928832	0.13762102	0.39245647	0.38378622	Trem2	1
GO:0031625	ubiquitin protein ligase binding	4/96	297/14323	0.01346801	2.00939955	1.44396012	0.13865317	0.39245647	0.38378622	Per3/Per1/Fzd4/Lyn	4
GO:0005112	Notch binding	1/96	23/14323	0.04347826	6.48686594	2.16322172	0.14341002	0.39245647	0.38378622	Tcim	1
GO:0050431	transforming growth factor beta binding	1/96	23/14323	0.04347826	6.48686594	2.16322172	0.14341002	0.39245647	0.38378622	Agrn	1
GO:0008509	monoatomic anion transmembrane transporter activity	2/96	100/14323	0.02	2.98395833	1.6353764	0.14474248	0.39245647	0.38378622	Slc6a6/Clcn2	2
GO:0019955	cytokine binding	2/96	100/14323	0.02	2.98395833	1.6353764	0.14474248	0.39245647	0.38378622	Fzd4/Agrn	2
GO:0004364	glutathione transferase activity	1/96	24/14323	0.04166667	6.21657986	2.10096798	0.14916055	0.39245647	0.38378622	Gsta4	1
GO:0005416	amino acid:monoatomic cation symporter activity	1/96	24/14323	0.04166667	6.21657986	2.10096798	0.14916055	0.39245647	0.38378622	Slc6a6	1
GO:0016645	oxidoreductase activity, acting on the CH-NH group of donors	1/96	24/14323	0.04166667	6.21657986	2.10096798	0.14916055	0.39245647	0.38378622	Pipox	1
GO:0016712	oxidoreductase activity, acting on paired donors, with incorporation or reduction of molecular oxygen, red	1/96	24/14323	0.04166667	6.21657986	2.10096798	0.14916055	0.39245647	0.38378622	Cyp3a13	1
GO:0030742	GTP-dependent protein binding	1/96	24/14323	0.04166667	6.21657986	2.10096798	0.14916055	0.39245647	0.38378622	Rab34	1
GO:0004519	endonuclease activity	2/96	103/14323	0.01941748	2.89704693	1.58718545	0.15169896	0.39245647	0.38378622	Xrcc3/Fan1	2
GO:0015103	inorganic anion transmembrane transporter activity	2/96	103/14323	0.01941748	2.89704693	1.58718545	0.15169896	0.39245647	0.38378622	Slc6a6/Clcn2	2
GO:0005343	organic acid:sodium symporter activity	1/96	25/14323	0.04	5.96791667	2.04214909	0.15487288	0.39245647	0.38378622	Slc6a6	1
GO:0016866	intramolecular transferase activity	1/96	25/14323	0.04	5.96791667	2.04214909	0.15487288	0.39245647	0.38378622	Lss	1
GO:0035035	histone acetyltransferase binding	1/96	25/14323	0.04	5.96791667	2.04214909	0.15487288	0.39245647	0.38378622	Eid1	1
GO:1990404	NAD+-protein ADP-ribosyltransferase activity	1/96	25/14323	0.04	5.96791667	2.04214909	0.15487288	0.39245647	0.38378622	Art5	1
GO:0044389	ubiquitin-like protein ligase binding	4/96	313/14323	0.01277955	1.90668264	1.33226061	0.15846714	0.39245647	0.38378622	Per3/Per1/Fzd4/Lyn	4
GO:0034061	DNA polymerase activity	1/96	26/14323	0.03846154	5.73838141	1.98643801	0.16054725	0.39245647	0.38378622	Pole	1
GO:0071813	lipoprotein particle binding	1/96	26/14323	0.03846154	5.73838141	1.98643801	0.16054725	0.39245647	0.38378622	Trem2	1
GO:0071814	protein-lipid complex binding	1/96	26/14323	0.03846154	5.73838141	1.98643801	0.16054725	0.39245647	0.38378622	Trem2	1
GO:0016810	hydrolase activity, acting on carbon-nitrogen (but not peptide) bonds	2/96	107/14323	0.01869159	2.78874611	1.52557186	0.16107526	0.39245647	0.38378622	Acer2/Dpysl4	2
GO:0043325	phosphatidylinositol-3,4-bisphosphate binding	1/96	27/14323	0.03703704	5.52584877	1.93355013	0.16618392	0.39245647	0.38378622	Adap2	1
GO:0046625	sphingolipid binding	1/96	27/14323	0.03703704	5.52584877	1.93355013	0.16618392	0.39245647	0.38378622	Lyn	1
GO:0000287	magnesium ion binding	3/96	212/14323	0.01415094	2.11129127	1.33905287	0.17003049	0.39245647	0.38378622	Wee1/Rps6ka5/Abl1	3
GO:0017147	Wnt-protein binding	1/96	28/14323	0.03571429	5.32849702	1.88323642	0.17178314	0.39245647	0.38378622	Fzd4	1
GO:0106310	protein serine kinase activity	4/96	327/14323	0.01223242	1.82505097	1.23974816	0.17661973	0.39245647	0.38378622	Mylk4/Alpk1/Rps6ka5/Cdk19	4
GO:0044024	histone H2AS1 kinase activity	3/96	216/14323	0.01388889	2.07219329	1.30425771	0.17663671	0.39245647	0.38378622	Mylk4/Alpk1/Rps6ka5	3
GO:0140097	catalytic activity, acting on DNA	3/96	216/14323	0.01388889	2.07219329	1.30425771	0.17663671	0.39245647	0.38378622	Pole/Xrcc3/Fan1	3
GO:0140995	histone H2A kinase activity	3/96	216/14323	0.01388889	2.07219329	1.30425771	0.17663671	0.39245647	0.38378622	Mylk4/Alpk1/Rps6ka5	3
GO:0000146	microfilament motor activity	1/96	29/14323	0.03448276	5.14475575	1.83527791	0.17734514	0.39245647	0.38378622	Myo5b	1
GO:0001530	lipopolysaccharide binding	1/96	29/14323	0.03448276	5.14475575	1.83527791	0.17734514	0.39245647	0.38378622	Trem2	1
GO:0004693	cyclin-dependent protein serine/threonine kinase activity	1/96	29/14323	0.03448276	5.14475575	1.83527791	0.17734514	0.39245647	0.38378622	Cdk19	1
GO:0008376	acetylgalactosaminyltransferase activity	1/96	29/14323	0.03448276	5.14475575	1.83527791	0.17734514	0.39245647	0.38378622	Galnt15	1
GO:0016655	oxidoreductase activity, acting on NAD(P)H, quinone or similar compound as acceptor	1/96	29/14323	0.03448276	5.14475575	1.83527791	0.17734514	0.39245647	0.38378622	ND5	1
GO:0043236	laminin binding	1/96	29/14323	0.03448276	5.14475575	1.83527791	0.17734514	0.39245647	0.38378622	Agrn	1
GO:0097472	cyclin-dependent protein kinase activity	1/96	29/14323	0.03448276	5.14475575	1.83527791	0.17734514	0.39245647	0.38378622	Cdk19	1
GO:0016757	glycosyltransferase activity	3/96	217/14323	0.01382488	2.06264401	1.29567629	0.17829957	0.39245647	0.38378622	Art5/Galnt15/Alg10b	3
GO:0005525	GTP binding	4/96	329/14323	0.01215805	1.81395643	1.22689942	0.17927024	0.39245647	0.38378622	Gimap5/Sar1b/Rab34/Arl11	4
GO:0035173	histone kinase activity	3/96	226/14323	0.01327434	1.98050332	1.22045206	0.19345628	0.41762554	0.40839924	Mylk4/Alpk1/Rps6ka5	3
GO:0015453	oxidoreduction-driven active transmembrane transporter activity	1/96	32/14323	0.03125	4.6624349	1.70370492	0.19381032	0.41762554	0.40839924	ND5	1
GO:0017124	SH3 domain binding	2/96	121/14323	0.01652893	2.46608127	1.33032196	0.19463412	0.41762554	0.40839924	Abl1/Lyn	2
GO:0043015	gamma-tubulin binding	1/96	33/14323	0.03030303	4.52114899	1.66343579	0.19922591	0.42004067	0.41076102	Lyn	1
GO:0051019	mitogen-activated protein kinase binding	1/96	33/14323	0.03030303	4.52114899	1.66343579	0.19922591	0.42004067	0.41076102	Abl1	1
GO:0019001	guanyl nucleotide binding	4/96	345/14323	0.0115942	1.72983092	1.12717169	0.20094538	0.42004067	0.41076102	Gimap5/Sar1b/Rab34/Arl11	4
GO:0032561	guanyl ribonucleotide binding	4/96	345/14323	0.0115942	1.72983092	1.12717169	0.20094538	0.42004067	0.41076102	Gimap5/Sar1b/Rab34/Arl11	4
GO:0016779	nucleotidyltransferase activity	2/96	127/14323	0.01574803	2.34957349	1.25486264	0.20928568	0.43052898	0.42101762	Art5/Pole	2
GO:0016893	endonuclease activity, active with either ribo- or deoxyribonucleic acids and producing 5'-phosphomonoe	1/96	35/14323	0.02857143	4.26279762	1.58752007	0.20994932	0.43052898	0.42101762	Fan1	1
GO:0030332	cyclin binding	1/96	35/14323	0.02857143	4.26279762	1.58752007	0.20994932	0.43052898	0.42101762	Inca1	1
GO:0015175	neutral L-amino acid transmembrane transporter activity	1/96	36/14323	0.02777778	4.14438657	1.55166315	0.21525761	0.43589666	0.42626672	Slc6a6	1
GO:0043394	proteoglycan binding	1/96	36/14323	0.02777778	4.14438657	1.55166315	0.21525761	0.43589666	0.42626672	Agrn	1
GO:0001228	DNA-binding transcription activator activity, RNA polymerase II-specific	4/96	357/14323	0.01120448	1.67168534	1.05571027	0.21770196	0.43810829	0.42842949	Dbp/Tef/Hlf/Klf9	4
GO:0019207	kinase regulator activity	3/96	242/14323	0.01239669	1.84956095	1.09487934	0.22114721	0.44229443	0.43252314	Trem2/Inca1/Lyn	3
GO:0030291	protein serine/threonine kinase inhibitor activity	1/96	38/14323	0.02631579	3.92626096	1.48369801	0.22576853	0.44692837	0.4370547	Inca1	1
GO:0001216	DNA-binding transcription activator activity	4/96	363/14323	0.01101928	1.64405418	1.0209719	0.226223	0.44692837	0.4370547	Dbp/Tef/Hlf/Klf9	4
GO:0046982	protein heterodimerization activity	3/96	249/14323	0.01204819	1.79756526	1.04288859	0.23351234	0.45853332	0.44840328	Bhlhe40/Fzd4/Bhlhe41	3

GO:0016538	cyclin-dependent protein serine/threonine kinase regulator activity	1/96	40/14323	0.025	3.72994792	1.42021935	0.23614012	0.4609	0.45071767	Inca1	1
GO:0050839	cell adhesion molecule binding	3/96	257/14323	0.01167315	1.74160992	0.98545676	0.24779574	0.47958663	0.46899147	Tspan4/Lyn/Jam2	3
GO:0042169	SH2 domain binding	1/96	43/14323	0.02325581	3.46971899	1.33228903	0.25144024	0.47958663	0.46899147	Abl1	1
GO:0001784	phosphotyrosine residue binding	1/96	44/14323	0.02272727	3.39086174	1.30470609	0.25647257	0.47958663	0.46899147	Abl1	1
GO:0004879	nuclear receptor activity	1/96	44/14323	0.02272727	3.39086174	1.30470609	0.25647257	0.47958663	0.46899147	Nr1d2	1
GO:0005547	phosphatidylinositol-3,4,5-trisphosphate binding	1/96	44/14323	0.02272727	3.39086174	1.30470609	0.25647257	0.47958663	0.46899147	Adap2	1
GO:0001223	transcription coactivator binding	1/96	45/14323	0.02222222	3.31550926	1.27790884	0.26147142	0.47958663	0.46899147	Zbtb49	1
GO:0050661	NADP binding	1/96	45/14323	0.02222222	3.31550926	1.27790884	0.26147142	0.47958663	0.46899147	Por	1
GO:0098531	ligand-activated transcription factor activity	1/96	45/14323	0.02222222	3.31550926	1.27790884	0.26147142	0.47958663	0.46899147	Nr1d2	1
GO:0004674	protein serine/threonine kinase activity	4/96	388/14323	0.01030928	1.53812285	0.88272331	0.26258871	0.47958663	0.46899147	Mylk4/Alpk1/Rps6ka5/Cdk19	4
GO:0043177	organic acid binding	2/96	149/14323	0.01342282	2.0026566	1.01060146	0.26373122	0.47958663	0.46899147	Slc6a6/Agrn	2
GO:0016758	hexosyltransferase activity	2/96	150/14323	0.01333333	1.98930556	1.00052045	0.26621817	0.47958663	0.46899147	Galnt15/Alg10b	2
GO:0005544	calcium-dependent phospholipid binding	1/96	46/14323	0.02173913	3.24343297	1.25185584	0.26643702	0.47958663	0.46899147	Cpne7	1
GO:0016830	carbon-carbon lyase activity	1/96	46/14323	0.02173913	3.24343297	1.25185584	0.26643702	0.47958663	0.46899147	Tha1	1
GO:0060590	ATPase regulator activity	1/96	46/14323	0.02173913	3.24343297	1.25185584	0.26643702	0.47958663	0.46899147	Agrn	1
GO:0005507	copper ion binding	1/96	47/14323	0.0212766	3.17442376	1.22650869	0.27136957	0.48309748	0.47242476	Mt1	1
GO:0008408	3'-5' exonuclease activity	1/96	47/14323	0.0212766	3.17442376	1.22650869	0.27136957	0.48309748	0.47242476	Pole	1
GO:0016763	pentosyltransferase activity	1/96	48/14323	0.02083333	3.10828993	1.20183177	0.2762693	0.48913253	0.47832648	Art5	1
GO:0003924	GTPase activity	3/96	275/14323	0.01090909	1.62761364	0.86324139	0.2803881	0.4897215	0.47890244	Sar1b/Rab34/Arl11	3
GO:0003823	antigen binding	1/96	49/14323	0.02040816	3.04485544	1.17779198	0.28113642	0.4897215	0.47890244	Tspan4	1
GO:0015370	solute:sodium symporter activity	1/96	49/14323	0.02040816	3.04485544	1.17779198	0.28113642	0.4897215	0.47890244	Slc6a6	1
GO:0016796	exonuclease activity, active with either ribo- or deoxyribonucleic acids and producing 5'-phosphomonoest	1/96	50/14323	0.02	2.98395833	1.15435849	0.28597115	0.49547942	0.48453316	Pole	1
GO:0005254	chloride channel activity	1/96	51/14323	0.01960784	2.92544935	1.13150259	0.29077369	0.50112062	0.49004973	Clcn2	1
GO:0035255	ionotropic glutamate receptor binding	1/96	52/14323	0.01923077	2.86919071	1.10919746	0.29554427	0.50217851	0.49108425	Myo5b	1
GO:1901682	sulfur compound transmembrane transporter activity	1/96	52/14323	0.01923077	2.86919071	1.10919746	0.29554427	0.50217851	0.49108425	Slc6a6	1
GO:0044325	transmembrane transporter binding	2/96	162/14323	0.01234568	1.84194959	0.88527341	0.29603733	0.50217851	0.49108425	Agrn/Lyn	2
GO:0001786	phosphatidylserine binding	1/96	53/14323	0.01886792	2.81505503	1.08741803	0.3002831	0.5041022	0.49296544	Trem2	1
GO:0004867	serine-type endopeptidase inhibitor activity	1/96	53/14323	0.01886792	2.81505503	1.08741803	0.3002831	0.5041022	0.49296544	Serpine1	1
GO:0003925	G protein activity	1/96	54/14323	0.01851852	2.76292438	1.06614084	0.30499037	0.50936537	0.49811233	Sar1b	1
GO:0008028	monocarboxylic acid transmembrane transporter activity	1/96	56/14323	0.01785714	2.66424851	1.02500656	0.31431111	0.51693807	0.50551774	Slc6a6	1
GO:0016627	oxidoreductase activity, acting on the CH-CH group of donors	1/96	56/14323	0.01785714	2.66424851	1.02500656	0.31431111	0.51693807	0.50551774	Rsad1	1
GO:0016765	transferase activity, transferring alkyl or aryl (other than methyl) groups	1/96	56/14323	0.01785714	2.66424851	1.02500656	0.31431111	0.51693807	0.50551774	Gsta4	1
GO:0005543	phospholipid binding	4/96	428/14323	0.00934579	1.39437305	0.68042557	0.3227839	0.52671676	0.51508039	Trem2/Cpne7/Syt16/Adap2	4
GO:0005080	protein kinase C binding	1/96	58/14323	0.01724138	2.57237787	0.98563432	0.32350813	0.52671676	0.51508039	Abl1	1
GO:0016706	2-oxoglutarate-dependent dioxygenase activity	1/96	59/14323	0.01694915	2.52877825	0.96656401	0.32806076	0.52881436	0.51713165	Tet3	1
GO:0045309	protein phosphorylated amino acid binding	1/96	59/14323	0.01694915	2.52877825	0.96656401	0.32806076	0.52881436	0.51713165	Abl1	1
GO:0005539	glycosaminoglycan binding	2/96	178/14323	0.01123596	1.67638109	0.74590027	0.33551255	0.53814884	0.52625991	Trem2/Agrn	2
GO:0004497	monooxygenase activity	1/96	63/14323	0.01587302	2.3682209	0.89402058	0.34597005	0.54680144	0.53472136	Cyp3a13	1
GO:0016811	hydrolase activity, acting on carbon-nitrogen (but not peptide) bonds, in linear amides	1/96	63/14323	0.01587302	2.3682209	0.89402058	0.34597005	0.54680144	0.53472136	Acer2	1
GO:0050840	extracellular matrix binding	1/96	63/14323	0.01587302	2.3682209	0.89402058	0.34597005	0.54680144	0.53472136	Agrn	1
GO:0005253	monoatomic anion channel activity	1/96	64/14323	0.015625	2.33121745	0.87674892	0.35037305	0.54841	0.53629438	Clcn2	1
GO:0030145	manganese ion binding	1/96	64/14323	0.015625	2.33121745	0.87674892	0.35037305	0.54841	0.53629438	Abl1	1
GO:0042578	phosphoric ester hydrolase activity	3/96	318/14323	0.00943396	1.40752752	0.60368408	0.35932797	0.55972241	0.54735687	Tns2/Gpcpd1/Fan1	3
GO:0016747	acyltransferase activity, transferring groups other than amino-acyl groups	2/96	189/14323	0.01058201	1.57881393	0.65798822	0.36229728	0.56164746	0.5492394	Brpf1/Naa50	2
GO:0019905	syntaxin binding	1/96	68/14323	0.01470588	2.19408701	0.81075121	0.36769366	0.56578601	0.55328652	Abl1	1
GO:0033218	amide binding	2/96	193/14323	0.01036269	1.5460924	0.62741441	0.37194272	0.56578601	0.55328652	Fzd4/Trem2	2
GO:0004620	phospholipase activity	1/96	69/14323	0.01449275	2.16228865	0.79497038	0.37195191	0.56578601	0.55328652	Pnpla7	1
GO:0048029	monosaccharide binding	1/96	69/14323	0.01449275	2.16228865	0.79497038	0.37195191	0.56578601	0.55328652	Alpk1	1
GO:0022853	active monoatomic ion transmembrane transporter activity	2/96	194/14323	0.01030928	1.53812285	0.61987964	0.37434534	0.56676584	0.5542447	Slc6a6/ND5	2
GO:0015101	organic cation transmembrane transporter activity	1/96	70/14323	0.01428571	2.13139881	0.77945739	0.37618179	0.56689721	0.55437316	Slc6a6	1
GO:0004860	protein kinase inhibitor activity	1/96	71/14323	0.01408451	2.10137911	0.76420326	0.38038347	0.5705752	0.5579699	Inca1	1
GO:0005546	phosphatidylinositol-4,5-bisphosphate binding	1/96	72/14323	0.01388889	2.07219329	0.74919945	0.38455714	0.57330662	0.56064098	Adap2	1
GO:0140297	DNA-binding transcription factor binding	4/96	471/14323	0.00849257	1.2670736	0.48413442	0.38853355	0.57330662	0.56064098	Per1/Bhlhe40/Bhlhe41/Zbtb49	4
GO:0042277	peptide binding	2/96	200/14323	0.01	1.49197917	0.57554464	0.38868302	0.57330662	0.56064098	Fzd4/Trem2	2
GO:0051015	actin filament binding	2/96	201/14323	0.00995025	1.48455638	0.56829656	0.39105909	0.57330662	0.56064098	Myo5b/Abl1	2
GO:0019210	kinase inhibitor activity	1/96	74/14323	0.01351351	2.01618806	0.71991056	0.3928212	0.57330662	0.56064098	Inca1	1
GO:0035064	methylated histone binding	1/96	74/14323	0.01351351	2.01618806	0.71991056	0.3928212	0.57330662	0.56064098	Ncapd3	1
GO:0140034	methylation-dependent protein binding	1/96	76/14323	0.01315789	1.96313048	0.69152996	0.40097544	0.58258315	0.56971256	Ncapd3	1
GO:0003774	cytoskeletal motor activity	1/96	77/14323	0.01298701	1.93763528	0.67766276	0.40501182	0.58321702	0.57033244	Myo5b	1
GO:0015294	solute:monoatomic cation symporter activity	1/96	77/14323	0.01298701	1.93763528	0.67766276	0.40501182	0.58321702	0.57033244	Slc6a6	1
GO:0035254	glutamate receptor binding	1/96	78/14323	0.01282051	1.9127938	0.66400224	0.40902129	0.58380131	0.57090381	Myo5b	1
GO:0043178	alcohol binding	1/96	78/14323	0.01282051	1.9127938	0.66400224	0.40902129	0.58380131	0.57090381	Adap2	1
GO:0003727	single-stranded RNA binding	1/96	83/14323	0.01204819	1.79756526	0.59859222	0.42867105	0.60650401	0.59310496	Rbmx	1
GO:0004252	serine-type endopeptidase activity	1/96	83/14323	0.01204819	1.79756526	0.59859222	0.42867105	0.60650401	0.59310496	Tmprss5	1
GO:0016298	lipase activity	1/96	85/14323	0.01176471	1.75526961	0.57367743	0.43634866	0.61205281	0.59853118	Pnpla7	1
GO:0005496	steroid binding	1/96	86/14323	0.01162791	1.7348595	0.56146809	0.44014909	0.61205281	0.59853118	Cyp3a13	1
GO:0015171	amino acid transmembrane transporter activity	1/96	86/14323	0.01162791	1.7348595	0.56146809	0.44014909	0.61205281	0.59853118	Slc6a6	1
GO:0072341	modified amino acid binding	1/96	86/14323	0.01162791	1.7348595	0.56146809	0.44014909	0.61205281	0.59853118	Trem2	1
GO:0004725	protein tyrosine phosphatase activity	1/96	87/14323	0.01149425	1.71491858	0.54941816	0.44392416	0.61466422	0.6010849	Tns2	1
GO:1901681	sulfur compound binding	2/96	225/14323	0.00888889	1.3262037	0.4051186	0.44676932	0.61597132	0.60236311	Slc6a6/Agrn	2
GO:0004866	endopeptidase inhibitor activity	1/96	90/14323	0.01111111	1.65775463	0.51418302	0.45509889	0.62216051	0.60841557	Serpine1	1
GO:0008170	N-methyltransferase activity	1/96	90/14323	0.01111111	1.65775463	0.51418302	0.45509889	0.62216051	0.60841557	Inmt	1
GO:0004930	G protein-coupled receptor activity	2/96	231/14323	0.00865801	1.29175685	0.36721594	0.46026255	0.62657591	0.61273343	Fzd4/Celsr3	2
GO:0008083	growth factor activity	1/96	92/14323	0.01086957	1.62171649	0.49141609	0.46242495	0.62688571	0.61303638	Gdf10	1
GO:0008236	serine-type peptidase activity	1/96	95/14323	0.01052632	1.57050439	0.45827867	0.47323166	0.63621186	0.6221565	Tmprss5	1
GO:0030414	peptidase inhibitor activity	1/96	95/14323	0.01052632	1.57050439	0.45827867	0.47323166	0.63621186	0.6221565	Serpine1	1
GO:0031490	chromatin DNA binding	1/96	96/14323	0.01041667	1.55414497	0.44748979	0.4767859	0.63834146	0.62423905	Per1	1
GO:1902936	phosphatidylinositol bisphosphate binding	1/96	97/14323	0.01030928	1.53812285	0.4368242	0.48031641	0.64042188	0.62627351	Adap2	1
GO:0017171	serine hydrolase activity	1/96	99/14323	0.01010101	1.50704966	0.41585125	0.48730686	0.6470796	0.63278414	Tmprss5	1
GO:0005125	cytokine activity	1/96	102/14323	0.00980392	1.46272467	0.38524629	0.49761841	0.65807496	0.64353659	Gdf10	1
GO:0140678	molecular function inhibitor activity	3/96	399/14323	0.0075188	1.12178885	0.20267153	0.50295232	0.66161389	0.64699734	Serpine1/Inca1/Agrn	3
GO:0008013	beta-catenin binding	1/96	104/14323	0.00961538	1.43459535	0.36538272	0.50437849	0.66161389	0.64699734	Trem2	1
GO:0015293	symporter activity	1/96	106/14323	0.00943396	1.40752752	0.34592876	0.51104854	0.66594487	0.65123264	Slc6a6	1
GO:0070851	growth factor receptor binding	1/96	107/14323	0.00934579	1.39437305	0.33634982	0.51435017	0.66594487	0.65123264	Lyn	1
GO:1990782	protein tyrosine kinase binding	1/96	108/14323	0.00925926	1.38146219	0.32686674	0.51762974	0.66594487	0.65123264	Trem2	1
GO:0008094	ATP-dependent activity, acting on DNA	1/96	109/14323	0.00917431	1.36878823	0.31747748	0.5208874	0.66594487	0.65123264	Xrcc3	1
GO:0030165	PDZ domain binding	1/96	109/14323	0.00917431	1.36878823	0.31747748	0.5208874	0.66594487	0.65123264	Fzd4	1
GO:0061135	endopeptidase regulator activity	1/96	109/14323	0.00917431	1.36878823	0.31747748	0.5208874	0.66594487	0.65123264	Serpine1	1
GO:0000149	SNARE binding	1/96	110/14323	0.00909091	1.3563447	0.30818007	0.52412328	0.66594487	0.65123264	Abl1	1
GO:0005506	iron ion binding	1/96	110/14323	0.00909091	1.3563447	0.30818007	0.52412328	0.66594487	0.65123264	Cyp3a13	1
GO:0008194	UDP-glycosyltransferase activity	1/96	114/14323	0.00877193	1.30875365	0.27187142	0.53685194	0.67945324	0.66444257	Galnt15	1
GO:0015399	primary active transmembrane transporter activity	1/96	115/14323	0.00869565	1.29737319	0.26300563	0.5399811	0.68075438	0.66571497	ND5	1
GO:0015078	proton transmembrane transporter activity	1/96	118/14323	0.00847458	1.26438912	0.23688892	0.54924359	0.68708465	0.67190539	ND5	1
GO:0015081	sodium ion transmembrane transporter activity	1/96	118/14323	0.00847458	1.26438912	0.23688892	0.54924359	0.68708465	0.67190539	Slc6a6	1
GO:0042826	histone deacetylase binding	1/96	119/14323	0.00840336	1.25376401	0.2283384	0.55228989	0.68823816	0.67303342	Bhlhe41	1
GO:0005201	extracellular matrix structural constituent	1/96	121/14323	0.00826446	1.23304063	0.21146104	0.55832149	0.69308874	0.67777684	Agrn	1
GO:0019838	growth factor binding	1/96	124/14323	0.00806452	1.20320901	0.1866834	0.56721843	0.69800191	0.68258147	Agrn	1
GO:0031267	small GTPase binding	2/96	284/14323	0.00704225	1.05068955	0.07087455	0.57066728	0.69800191	0.68258147	Rab34/Myo5b	2
GO:0048018	receptor ligand activity	2/96	284/14323	0.00704225	1.05068955	0.07087455	0.57066728	0.69800191	0.68258147	Gdf10/Agrn	2
GO:0004857	enzyme inhibitor activity	2/96	285/14323	0.00701754	1.04700292	0.06583782	0.57258944	0.69800191	0.68258147	Serpine1/Inca1	2
GO:0019209	kinase activator activity	1/96	126/14323	0.00793651	1.18411045	0.17050857	0.57305095	0.69800191	0.68258147	Trem2	1
GO:0030546	signaling receptor activator activity	2/96	289/14323	0.00692042	1.03251153	0.04586448	0.58021655	0.704083	0.68852821	Gdf10/Agrn	2
GO:0002020	protease binding	1/96	130/14323	0.00769231	1.14767628	0.13893962	0.58448365	0.70661456	0.69100384	Serpine1	1
GO:0099106	ion channel regulator activity	1/96	138/14323	0.00724638	1.08114432	0.0786799	0.60644788	0.7304428	0.71430566	Clcn2	1
GO:0005244	voltage-gated monoatomic ion channel activity	1/96	139/14323	0.00719424	1.07336631	0.07139791	0.60911133	0.73093359	0.71478561	Clcn2	1
GO:0022832	voltage-gated channel activity	1/96	140/14323	0.00714286	1.0656994	0.06416854	0.61175694	0.73139944	0.71524116	Clcn2	1
GO:0031406	carboxylic acid binding	1/96	141/14323	0.0070922	1.05814125	0.05699096	0.61438482	0.73184075	0.71567272	Agrn	1
GO:0003712	transcription coregulator activity	3/96	473/14323	0.00634249	0.946287	-0.0975811	0.61882971	0.73192167	0.71575186	Bhlhe41/Rere/Eid1	3
GO:0052689	carboxylic ester hydrolase activity	1/96	143/14323	0.00699301	1.04334207	0.04278784	0.61958791	0.73192167	0.71575186	Pnpla7	1
GO:0046943	carboxylic acid transmembrane transporter activity	1/96	144/14323	0.00694444	1.03609664	0.0357607	0.62216333	0.73192167	0.71575186	Slc6a6	1
GO:0005342	organic acid transmembrane transporter activity	1/96	145/14323	0.00689655	1.02895115	0.02878214	0.62472151	0.73192167	0.71575186	Slc6a6	1
GO:0061134	peptidase regulator activity	1/96	146/14323	0.00684932	1.02190354	0.0218514	0.62726254	0.73192167	0.71575186	Serpine1	1
GO:0003729	mRNA binding	2/96	316/14323	0.00632911	0.94429061	-0.0822584	0.62911454	0.73192167	0.71575186	Srrm2/Rbmx	2
GO:0016247	channel regulator activity	1/96	148/14323	0.00675676	1.00809403	0.00813046	0.63229363	0.73192167	0.71575186	Clcn2	1
GO:0030545	signaling receptor regulator activity	2/96	319/14323	0.00626959	0.93541014	-0.0958329	0.63426982	0.73192167	0.71575186	Gdf10/Agrn	2
GO:0008757	S-adenosylmethionine-dependent methyltransferase activity	1/96	149/14323	0.00671141	1.0013283	0.00133882	0.63478391	0.73192167	0.71575186	Inmt	1
GO:0141108	transporter regulator activity	1/96	150/14323	0.00666667	0.99465278	-0.0054078	0.63725751	0.7321682	0.71599295	Clcn2	1
GO:0051020	GTPase binding	2/96	324/14323	0.00617284	0.92097479	-0.1181872	0.64273911	0.73487941	0.71864426	Rab34/Myo5b	2
GO:0140657	ATP-dependent activity	3/96	491/14323	0.00610998	0.91159623	-0.1637381	0.64415356	0.73487941	0.71864426	Myo5b/Nav3/Xrcc3	3
GO:0004721	phosphoprotein phosphatase activity	1/96	155/14323	0.00645161	0.9625672	-0.0384897	0.64937897	0.73824136	0.72193193	Tns2	1
GO:0016853	isomerase activity	1/96	157/14323	0.00636943	0.9503052	-0.0514302	0.65411454	0.74102487	0.72465395	Lss	1
GO:0022804	active transmembrane transporter activity	2/96	336/14323	0.00595238	0.88808284	-0.1705237	0.66244263	0.74524796	0.72878375	Slc6a6/ND5	2
GO:0061629	RNA polymerase II-specific DNA-binding transcription factor binding	2/96	336/14323	0.00595238	0.88808284	-0.1705237	0.66244263	0.74524796	0.72878375	Bhlhe40/Bhlhe41	2
GO:0016887	ATP hydrolysis activity	2/96	342/14323	0.00584795	0.87250244	-0.1960318	0.67196743	0.75334757	0.73670442	Myo5b/Nav3	2
GO:1901981	phosphatidylinositol phosphate binding	1/96	170/14323	0.00588235	0.8776348	-0.1318375	0.68338961	0.76175472	0.74492583	Adap2	1
GO:0016829	lyase activity	1/96	171/14323	0.00584795	0.87250244	-0.1377754	0.68553719	0.76175472	0.74492583	Tha1	1
GO:0005126	cytokine receptor binding	1/96	172/14323	0.00581395	0.86742975	-0.1436804	0.68767034	0.76175472	0.74492583	Lyn	1
GO:0015631	tubulin binding	2/96	353/14323	0.00566572	0.84531398	-0.2417247	0.68887078	0.76175472	0.74492583	Nav3/Lyn	2
GO:0005516	calmodulin binding	1/96	178/14323	0.00561798	0.83819054	-0.1784404	0.70017186	0.77123724	0.75419886	Myo5b	1
GO:0008168	methyltransferase activity	1/96	179/14323	0.00558659	0.83350791	-0.1841259	0.70220676	0.77123724	0.75419886	Inmt	1
GO:0016741	transferase activity, transferring one-carbon groups	1/96	188/14323	0.00531915	0.79360594	-0.2339963	0.71991648	0.78801669	0.77060761	Inmt	1
GO:0005085	guanyl-nucleotide exchange factor activity	1/96	197/14323	0.00507614	0.75734983	-0.2816987	0.73658334	0.80354546	0.78579332	Net1	1
GO:0008514	organic anion transmembrane transporter activity	1/96	204/14323	0.00490196	0.73136234	-0.3174394	0.7488645	0.81420167	0.79621411	Slc6a6	1
GO:0003779	actin binding	2/96	402/14323	0.00497512	0.74227819	-0.4305363	0.75572926	0.81891732	0.80082558	Myo5b/Abl1	2
GO:0019887	protein kinase regulator activity	1/96	210/14323	0.0047619	0.71046627	-0.3472002	0.75893902	0.81965414	0.80154612	Inca1	1
GO:0030695	GTPase regulator activity	2/96	414/14323	0.00483092	0.72076288	-0.473594	0.77011289	0.82523982	0.8070084	Net1/Adap2	2
GO:0060589	nucleoside-triphosphatase regulator activity	2/96	414/14323	0.00483092	0.72076288	-0.473594	0.77011289	0.82523982	0.8070084	Net1/Adap2	2
GO:0015291	secondary active transmembrane transporter activity	1/96	218/14323	0.00458716	0.68439411	-0.3857159	0.77175206	0.82523982	0.8070084	Slc6a6	1
GO:0005096	GTPase activator activity	1/96	225/14323	0.00444444	0.66310185	-0.4184002	0.78240909	0.83114933	0.81278735	Adap2	1
GO:0042393	histone binding	1/96	225/14323	0.00444444	0.66310185	-0.4184002	0.78240909	0.83114933	0.81278735	Ncapd3	1
GO:0022836	gated channel activity	1/96	231/14323	0.004329	0.64587843	-0.4457105	0.79115072	0.837689	0.81918255	Clcn2	1
GO:0016791	phosphatase activity	1/96	239/14323	0.0041841	0.62425907	-0.4811774	0.80226777	0.84669302	0.82798765	Tns2	1
GO:0022890	inorganic cation transmembrane transporter activity	2/96	446/14323	0.0044843	0.66904895	-0.5832627	0.80495352	0.84676929	0.82806223	Slc6a6/ND5	2
GO:0008017	microtubule binding	1/96	248/14323	0.00403226	0.6016045	-0.5198725	0.81407564	0.85359387	0.83473605	Nav3	1
GO:0004175	endopeptidase activity	1/96	255/14323	0.00392157	0.58508987	-0.5491472	0.82277468	0.85692318	0.83799181	Tmprss5	1
GO:0005509	calcium ion binding	2/96	466/14323	0.00429185	0.64033441	-0.6483926	0.82430064	0.85692318	0.83799181	Agrn/Celsr3	2
GO:0003713	transcription coactivator activity	1/96	257/14323	0.00389105	0.58053664	-0.557386	0.82518529	0.85692318	0.83799181	Rere	1
GO:0008324	monoatomic cation transmembrane transporter activity	2/96	477/14323	0.00419287	0.62556778	-0.683205	0.83420033	0.86108415	0.84206084	Slc6a6/ND5	2
GO:0035091	phosphatidylinositol binding	1/96	265/14323	0.00377358	0.56301101	-0.5898104	0.83450747	0.86108415	0.84206084	Adap2	1
GO:0061630	ubiquitin protein ligase activity	1/96	297/14323	0.003367	0.50234989	-0.7118954	0.86712481	0.8918998	0.87219572	Peli1	1
GO:0061659	ubiquitin-like protein ligase activity	1/96	314/14323	0.00318471	0.4751526	-0.7724584	0.88177518	0.90409861	0.88412502	Peli1	1
GO:0005216	monoatomic ion channel activity	1/96	325/14323	0.00307692	0.45907051	-0.8102707	0.8903908	0.91005242	0.8899473	Clcn2	1
GO:0046873	metal ion transmembrane transporter activity	1/96	346/14323	0.00289017	0.43120785	-0.8797625	0.9051496	0.92222789	0.90185379	Slc6a6	1
GO:0015267	channel activity	1/96	362/14323	0.00276243	0.41214894	-0.9305594	0.91505809	0.92649632	0.90602792	Clcn2	1
GO:0022803	passive transmembrane transporter activity	1/96	362/14323	0.00276243	0.41214894	-0.9305594	0.91505809	0.92649632	0.90602792	Clcn2	1
GO:0004842	ubiquitin-protein transferase activity	1/96	376/14323	0.00265957	0.39680297	-0.973629	0.92288316	0.93150823	0.9109291	Peli1	1
GO:0019787	ubiquitin-like protein transferase activity	1/96	401/14323	0.00249377	0.37206463	-1.0476521	0.93512272	0.94093094	0.92014364	Peli1	1
GO:0016755	aminoacyltransferase activity	1/96	411/14323	0.00243309	0.36301196	-1.076312	0.93946144	0.94152184	0.92072148	Peli1	1
GO:0008233	peptidase activity	1/96	416/14323	0.00240385	0.35864884	-1.0904521	0.94152184	0.94152184	0.92072148	Tmprss5	1

ID	Description	GeneRatio	BgRatio	RichFactor	FoldEnrichm	zScore	pvalue	p.adjust	qvalue	geneID	Count
GO:0050660	flavin adenine dinucleotide binding	4/46	80/14323	0.05	15.5684783	7.41687086	0.0001232	0.01971189	0.01530265	Fmo2/Fmo1/Xdh/Prodh	4
GO:0061459	L-arginine transmembrane transporter activity	2/46	13/14323	0.15384615	47.90301	9.60317504	0.00076956	0.03765425	0.02923159	Slc7a2/Slc25a15	2
GO:0015179	L-amino acid transmembrane transporter activity	3/46	60/14323	0.05	15.5684783	6.4186936	0.00093332	0.03765425	0.02923159	Slc1a5/Slc7a2/Slc25a15	3
GO:0004497	monooxygenase activity	3/46	63/14323	0.04761905	14.8271222	6.24316168	0.00107582	0.03765425	0.02923159	Fmo2/Fmo1/Cyp2f2	3
GO:0015174	basic amino acid transmembrane transporter activity	2/46	16/14323	0.125	38.9211957	8.61449842	0.0011767	0.03765425	0.02923159	Slc7a2/Slc25a15	2
GO:0015101	organic cation transmembrane transporter activity	3/46	70/14323	0.04285714	13.3444099	5.87663184	0.0014599	0.03893061	0.03022245	Slc1a5/Slc7a2/Slc25a15	3
GO:0016709	oxidoreductase activity, acting on paired donors, with incorporation or reduction of molecular oxygen, NA	2/46	23/14323	0.08695652	27.0756144	7.10383261	0.00244564	0.05266738	0.04088652	Fmo2/Fmo1	2
GO:0015171	amino acid transmembrane transporter activity	3/46	86/14323	0.03488372	10.861729	5.20661324	0.00263337	0.05266738	0.04088652	Slc1a5/Slc7a2/Slc25a15	3
GO:0071949	FAD binding	2/46	39/14323	0.05128205	15.96767	5.31280472	0.00693308	0.1232547	0.09568457	Xdh/Prodh	2
GO:0016705	oxidoreductase activity, acting on paired donors, with incorporation or reduction of molecular oxygen	3/46	134/14323	0.02238806	6.97096042	3.94169456	0.00906462	0.12830889	0.09960822	Fmo2/Fmo1/Cyp2f2	3
GO:0050661	NADP binding	2/46	45/14323	0.04444444	13.8386473	4.89614317	0.00915048	0.12830889	0.09960822	Fmo2/Fmo1	2
GO:0046943	carboxylic acid transmembrane transporter activity	3/46	144/14323	0.02083333	6.48686594	3.75616849	0.0110192	0.12830889	0.09960822	Slc1a5/Slc7a2/Slc25a15	3
GO:0016597	amino acid binding	2/46	50/14323	0.04	12.4547826	4.60549687	0.01120816	0.12830889	0.09960822	Prodh/Sesn1	2
GO:0005342	organic acid transmembrane transporter activity	3/46	145/14323	0.02068966	6.44212894	3.73858804	0.01122703	0.12830889	0.09960822	Slc1a5/Slc7a2/Slc25a15	3
GO:0008514	organic anion transmembrane transporter activity	3/46	204/14323	0.01470588	4.57896419	2.92235343	0.02760117	0.18391686	0.14277756	Slc1a5/Slc7a2/Slc25a15	3
GO:0008392	arachidonate epoxygenase activity	1/46	10/14323	0.1	31.1369565	5.41123415	0.03166578	0.18391686	0.14277756	Cyp2f2	1
GO:0015183	L-aspartate transmembrane transporter activity	1/46	10/14323	0.1	31.1369565	5.41123415	0.03166578	0.18391686	0.14277756	Slc1a5	1
GO:0042609	CD4 receptor binding	1/46	10/14323	0.1	31.1369565	5.41123415	0.03166578	0.18391686	0.14277756	Plscr4	1
GO:0048185	activin binding	1/46	10/14323	0.1	31.1369565	5.41123415	0.03166578	0.18391686	0.14277756	Fstl3	1
GO:0180030	inositol phosphate kinase activity	1/46	10/14323	0.1	31.1369565	5.41123415	0.03166578	0.18391686	0.14277756	Ip6k2	1
GO:0008391	arachidonate monooxygenase activity	1/46	11/14323	0.09090909	28.3063241	5.14246909	0.03477788	0.18391686	0.14277756	Cyp2f2	1
GO:0052650	all-trans-retinol dehydrogenase (NADP+) activity	1/46	11/14323	0.09090909	28.3063241	5.14246909	0.03477788	0.18391686	0.14277756	Rdh10	1
GO:0097371	MDM2/MDM4 family protein binding	1/46	11/14323	0.09090909	28.3063241	5.14246909	0.03477788	0.18391686	0.14277756	Bcl2l1	1
GO:0106018	phosphatidylinositol-3,5-bisphosphate phosphatase activity	1/46	11/14323	0.09090909	28.3063241	5.14246909	0.03477788	0.18391686	0.14277756	Mtmr2	1
GO:0004022	alcohol dehydrogenase (NAD+) activity	1/46	12/14323	0.08333333	25.9474638	4.90731915	0.03788018	0.18391686	0.14277756	Rdh10	1
GO:0004438	phosphatidylinositol-3-phosphate phosphatase activity	1/46	12/14323	0.08333333	25.9474638	4.90731915	0.03788018	0.18391686	0.14277756	Mtmr2	1
GO:0016661	oxidoreductase activity, acting on other nitrogenous compounds as donors	1/46	12/14323	0.08333333	25.9474638	4.90731915	0.03788018	0.18391686	0.14277756	Xdh	1
GO:0022841	potassium ion leak channel activity	1/46	12/14323	0.08333333	25.9474638	4.90731915	0.03788018	0.18391686	0.14277756	Kcnk4	1
GO:0070513	death domain binding	1/46	12/14323	0.08333333	25.9474638	4.90731915	0.03788018	0.18391686	0.14277756	Bcl2l1	1
GO:0015556	C4-dicarboxylate transmembrane transporter activity	1/46	13/14323	0.07692308	23.951505	4.69921482	0.04097274	0.18391686	0.14277756	Slc1a5	1
GO:0019825	oxygen binding	1/46	13/14323	0.07692308	23.951505	4.69921482	0.04097274	0.18391686	0.14277756	Cyp2f2	1
GO:0016500	protein-hormone receptor activity	1/46	14/14323	0.07142857	22.2406832	4.51325767	0.04405556	0.18391686	0.14277756	Mchr1	1
GO:0052744	phosphatidylinositol monophosphate phosphatase activity	1/46	14/14323	0.07142857	22.2406832	4.51325767	0.04405556	0.18391686	0.14277756	Mtmr2	1
GO:0015297	antiporter activity	2/46	107/14323	0.01869159	5.81999187	2.84061096	0.0462503	0.18391686	0.14277756	Slc1a5/Slc25a15	2
GO:0008381	mechanosensitive monoatomic ion channel activity	1/46	15/14323	0.06666667	20.757971	4.34571037	0.04712869	0.18391686	0.14277756	Kcnk4	1
GO:0016530	metallochaperone activity	1/46	15/14323	0.06666667	20.757971	4.34571037	0.04712869	0.18391686	0.14277756	Pcbp2	1
GO:0017128	phospholipid scramblase activity	1/46	15/14323	0.06666667	20.757971	4.34571037	0.04712869	0.18391686	0.14277756	Plscr4	1
GO:0022840	leak channel activity	1/46	15/14323	0.06666667	20.757971	4.34571037	0.04712869	0.18391686	0.14277756	Kcnk4	1
GO:0022842	narrow pore channel activity	1/46	15/14323	0.06666667	20.757971	4.34571037	0.04712869	0.18391686	0.14277756	Kcnk4	1
GO:0051400	BH domain binding	1/46	15/14323	0.06666667	20.757971	4.34571037	0.04712869	0.18391686	0.14277756	Bcl2l1	1
GO:1990226	histone methyltransferase binding	1/46	15/14323	0.06666667	20.757971	4.34571037	0.04712869	0.18391686	0.14277756	Per2	1
GO:0005506	iron ion binding	2/46	110/14323	0.01818182	5.66126482	2.78560487	0.04859964	0.18514147	0.14372824	Xdh/Cyp2f2	2
GO:0004713	protein tyrosine kinase activity	2/46	115/14323	0.0173913	5.41512287	2.69828309	0.05261004	0.18931908	0.14697139	Mertk/Tesk2	2
GO:0015645	fatty acid ligase activity	1/46	17/14323	0.05882353	18.3158568	4.05482024	0.05324599	0.18931908	0.14697139	Acsf3	1
GO:0120515	fatty acid-CoA ligase activity	1/46	17/14323	0.05882353	18.3158568	4.05482024	0.05324599	0.18931908	0.14697139	Acsf3	1
GO:0016405	CoA-ligase activity	1/46	18/14323	0.05555556	17.2983092	3.92732773	0.05629022	0.19371385	0.15038312	Acsf3	1
GO:0010314	phosphatidylinositol-5-phosphate binding	1/46	19/14323	0.05263158	16.3878719	3.80968336	0.05932487	0.19371385	0.15038312	Phlda3	1
GO:0015172	acidic amino acid transmembrane transporter activity	1/46	19/14323	0.05263158	16.3878719	3.80968336	0.05932487	0.19371385	0.15038312	Slc1a5	1
GO:0043027	cysteine-type endopeptidase inhibitor activity involved in apoptotic process	1/46	19/14323	0.05263158	16.3878719	3.80968336	0.05932487	0.19371385	0.15038312	Bcl2l1	1
GO:1990380	K48-linked deubiquitinase activity	1/46	20/14323	0.05	15.5684783	3.70064895	0.06234997	0.19732997	0.15319037	Otud7b	1
GO:0005044	scavenger receptor activity	1/46	21/14323	0.04761905	14.8271222	3.59919462	0.06536555	0.19732997	0.15319037	Cd163	1
GO:0015271	outward rectifier potassium channel activity	1/46	21/14323	0.04761905	14.8271222	3.59919462	0.06536555	0.19732997	0.15319037	Kcnk4	1
GO:0042923	neuropeptide binding	1/46	21/14323	0.04761905	14.8271222	3.59919462	0.06536555	0.19732997	0.15319037	Mchr1	1
GO:0005310	dicarboxylic acid transmembrane transporter activity	1/46	22/14323	0.04545455	14.1531621	3.50445574	0.06837165	0.19889934	0.1544087	Slc1a5	1
GO:0016878	acid-thiol ligase activity	1/46	22/14323	0.04545455	14.1531621	3.50445574	0.06837165	0.19889934	0.1544087	Acsf3	1
GO:0008106	alcohol dehydrogenase (NADP+) activity	1/46	23/14323	0.04347826	13.5378072	3.41570029	0.07136828	0.20075433	0.15584876	Rdh10	1
GO:0016645	oxidoreductase activity, acting on the CH-NH group of donors	1/46	24/14323	0.04166667	12.9737319	3.3323038	0.07435549	0.20075433	0.15584876	Prodh	1
GO:0016712	oxidoreductase activity, acting on paired donors, with incorporation or reduction of molecular oxygen, red	1/46	24/14323	0.04166667	12.9737319	3.3323038	0.07435549	0.20075433	0.15584876	Cyp2f2	1
GO:0034593	phosphatidylinositol bisphosphate phosphatase activity	1/46	24/14323	0.04166667	12.9737319	3.3323038	0.07435549	0.20075433	0.15584876	Mtmr2	1
GO:0004033	aldo-keto reductase (NADPH) activity	1/46	25/14323	0.04	12.4547826	3.25372976	0.0773333	0.20075433	0.15584876	Rdh10	1
GO:0070530	K63-linked polyubiquitin modification-dependent protein binding	1/46	25/14323	0.04	12.4547826	3.25372976	0.0773333	0.20075433	0.15584876	Otud7b	1
GO:0051537	2 iron, 2 sulfur cluster binding	1/46	26/14323	0.03846154	11.9757525	3.17951437	0.08030173	0.20075433	0.15584876	Xdh	1
GO:0052866	phosphatidylinositol phosphate phosphatase activity	1/46	26/14323	0.03846154	11.9757525	3.17951437	0.08030173	0.20075433	0.15584876	Mtmr2	1
GO:0106222	lncRNA binding	1/46	26/14323	0.03846154	11.9757525	3.17951437	0.08030173	0.20075433	0.15584876	Pcbp2	1
GO:0043325	phosphatidylinositol-3,4-bisphosphate binding	1/46	27/14323	0.03703704	11.5322061	3.10925422	0.08326082	0.20494972	0.1591057	Phlda3	1
GO:0080025	phosphatidylinositol-3,5-bisphosphate binding	1/46	28/14323	0.03571429	11.1203416	3.04259662	0.0862106	0.20899539	0.16224642	Phlda3	1
GO:0016776	phosphotransferase activity, phosphate group as acceptor	1/46	30/14323	0.03333333	10.3789855	2.91888555	0.09208232	0.21989808	0.17071035	Ip6k2	1
GO:0016877	ligase activity, forming carbon-sulfur bonds	1/46	32/14323	0.03125	9.73029891	2.80630625	0.09791713	0.22705422	0.17626577	Acsf3	1
GO:0018455	alcohol dehydrogenase [NAD(P)+] activity	1/46	32/14323	0.03125	9.73029891	2.80630625	0.09791713	0.22705422	0.17626577	Rdh10	1
GO:0001968	fibronectin binding	1/46	33/14323	0.03030303	9.43544137	2.75366394	0.10082076	0.22720172	0.17638028	Fstl3	1
GO:0043028	cysteine-type endopeptidase regulator activity involved in apoptotic process	1/46	33/14323	0.03030303	9.43544137	2.75366394	0.10082076	0.22720172	0.17638028	Bcl2l1	1
GO:0015175	neutral L-amino acid transmembrane transporter activity	1/46	36/14323	0.02777778	8.64915459	2.60829337	0.10947691	0.2376652	0.18450325	Slc1a5	1
GO:0015267	channel activity	3/46	362/14323	0.00828729	2.58041076	1.72873439	0.10992015	0.2376652	0.18450325	Slc1a5/Kcnk4/Bcl2l1	3
GO:0022803	passive transmembrane transporter activity	3/46	362/14323	0.00828729	2.58041076	1.72873439	0.10992015	0.2376652	0.18450325	Slc1a5/Kcnk4/Bcl2l1	3
GO:0004712	protein serine/threonine/tyrosine kinase activity	1/46	38/14323	0.02631579	8.19393593	2.52046408	0.11520233	0.23938147	0.18583562	Tesk2	1
GO:0036002	pre-mRNA binding	1/46	38/14323	0.02631579	8.19393593	2.52046408	0.11520233	0.23938147	0.18583562	Per2	1
GO:0140303	intramembrane lipid transporter activity	1/46	38/14323	0.02631579	8.19393593	2.52046408	0.11520233	0.23938147	0.18583562	Plscr4	1
GO:0016684	oxidoreductase activity, acting on peroxide as acceptor	1/46	43/14323	0.02325581	7.24115268	2.32646864	0.1293588	0.26432678	0.20520105	Sesn1	1
GO:0005547	phosphatidylinositol-3,4,5-trisphosphate binding	1/46	44/14323	0.02272727	7.07658103	2.29138996	0.13216339	0.26432678	0.20520105	Phlda3	1
GO:0032266	phosphatidylinositol-3-phosphate binding	1/46	44/14323	0.02272727	7.07658103	2.29138996	0.13216339	0.26432678	0.20520105	Phlda3	1
GO:0030276	clathrin binding	1/46	48/14323	0.02083333	6.48686594	2.16132054	0.14329362	0.28304913	0.21973551	Bcl2l1	1
GO:0004714	transmembrane receptor protein tyrosine kinase activity	1/46	50/14323	0.02	6.2273913	2.101719	0.14880625	0.29035366	0.22540613	Mertk	1
GO:0015291	secondary active transmembrane transporter activity	2/46	218/14323	0.00917431	2.85660152	1.56791557	0.15486151	0.29852821	0.23175216	Slc1a5/Slc25a15	2
GO:0038024	cargo receptor activity	1/46	54/14323	0.01851852	5.76610306	1.99170866	0.1597276	0.30024863	0.23308775	Cd163	1
GO:0001227	DNA-binding transcription repressor activity, RNA polymerase II-specific	2/46	224/14323	0.00892857	2.7800854	1.52416844	0.16162047	0.30024863	0.23308775	Mynn/Mxi1	2
GO:0005548	phospholipid transporter activity	1/46	55/14323	0.01818182	5.66126482	1.96592001	0.16243645	0.30024863	0.23308775	Plscr4	1
GO:0001222	transcription corepressor binding	1/46	56/14323	0.01785714	5.56017081	1.94075656	0.16513675	0.30024863	0.23308775	Per2	1
GO:0031593	polyubiquitin modification-dependent protein binding	1/46	56/14323	0.01785714	5.56017081	1.94075656	0.16513675	0.30024863	0.23308775	Otud7b	1
GO:0001217	DNA-binding transcription repressor activity	2/46	230/14323	0.00869565	2.70756144	1.48183825	0.16843435	0.30146672	0.23403337	Mynn/Mxi1	2
GO:0022836	gated channel activity	2/46	231/14323	0.00865801	2.69584039	1.47491475	0.16957503	0.30146672	0.23403337	Slc1a5/Kcnk4	2
GO:0070063	RNA polymerase binding	1/46	62/14323	0.01612903	5.02208976	1.80150256	0.18116062	0.31852417	0.24727535	Per2	1
GO:0042562	hormone binding	1/46	64/14323	0.015625	4.86514946	1.75903311	0.18643475	0.32423435	0.25170825	Mchr1	1
GO:0008017	microtubule binding	2/46	248/14323	0.00806452	2.51104488	1.36251494	0.18916202	0.32544004	0.25264424	Cfap157/Maco1	2
GO:0019199	transmembrane receptor protein kinase activity	1/46	66/14323	0.01515152	4.71772069	1.71829182	0.19167565	0.32625642	0.25327801	Mertk	1
GO:0051536	iron-sulfur cluster binding	1/46	69/14323	0.01449275	4.51260239	1.66015039	0.19947511	0.33595809	0.26080957	Xdh	1
GO:0051540	metal cluster binding	1/46	70/14323	0.01428571	4.44813665	1.64150654	0.20205854	0.33676423	0.26143539	Xdh	1
GO:0005546	phosphatidylinositol-4,5-bisphosphate binding	1/46	72/14323	0.01388889	4.32457729	1.60524854	0.20720095	0.34177476	0.26532514	Phlda3	1
GO:0005249	voltage-gated potassium channel activity	1/46	78/14323	0.01282051	3.9919175	1.50393117	0.22243441	0.35948996	0.27907773	Kcnk4	1
GO:0097110	scaffold protein binding	1/46	78/14323	0.01282051	3.9919175	1.50393117	0.22243441	0.35948996	0.27907773	Cd163	1
GO:0004843	cysteine-type deubiquitinase activity	1/46	79/14323	0.01265823	3.9413869	1.48803103	0.22494533	0.35991253	0.27940578	Otud7b	1
GO:0020037	heme binding	1/46	84/14323	0.01190476	3.70678054	1.41226181	0.23738142	0.37604977	0.29193338	Cyp2f2	1
GO:0101005	deubiquitinase activity	1/46	87/14323	0.01149425	3.57896052	1.36953331	0.24474915	0.38392023	0.29804334	Otud7b	1
GO:0046906	tetrapyrrole binding	1/46	91/14323	0.01098901	3.42164357	1.31540722	0.25446456	0.39148393	0.30391516	Cyp2f2	1
GO:1990756	ubiquitin-like ligase-substrate adaptor activity	1/46	91/14323	0.01098901	3.42164357	1.31540722	0.25446456	0.39148393	0.30391516	Herpud1	1
GO:0140104	molecular carrier activity	1/46	94/14323	0.0106383	3.31244218	1.27676183	0.26167077	0.39805234	0.30901432	Pcbp2	1
GO:0016616	oxidoreductase activity, acting on the CH-OH group of donors, NAD or NADP as acceptor	1/46	95/14323	0.01052632	3.27757437	1.26422598	0.26405767	0.39805234	0.30901432	Rdh10	1
GO:0031490	chromatin DNA binding	1/46	96/14323	0.01041667	3.24343297	1.25185584	0.26643702	0.39805234	0.30901432	Pcbp2	1
GO:1902936	phosphatidylinositol bisphosphate binding	1/46	97/14323	0.01030928	3.20999552	1.23964727	0.26880884	0.39805234	0.30901432	Phlda3	1
GO:0019783	ubiquitin-like protein peptidase activity	1/46	98/14323	0.01020408	3.17724046	1.22759629	0.27117316	0.39805234	0.30901432	Otud7b	1
GO:0005267	potassium channel activity	1/46	101/14323	0.00990099	3.08286698	1.1923511	0.27822133	0.4007941	0.31114279	Kcnk4	1
GO:0140767	enzyme-substrate adaptor activity	1/46	101/14323	0.00990099	3.08286698	1.1923511	0.27822133	0.4007941	0.31114279	Herpud1	1
GO:0016614	oxidoreductase activity, acting on CH-OH group of donors	1/46	102/14323	0.00980392	3.0526428	1.18089333	0.28055587	0.4007941	0.31114279	Rdh10	1
GO:0015293	symporter activity	1/46	106/14323	0.00943396	2.93744873	1.13642673	0.28982038	0.40859257	0.31719686	Slc1a5	1
GO:0022804	active transmembrane transporter activity	2/46	336/14323	0.00595238	1.85339027	0.8984984	0.29367591	0.40859257	0.31719686	Slc1a5/Slc25a15	2
GO:0061629	RNA polymerase II-specific DNA-binding transcription factor binding	2/46	336/14323	0.00595238	1.85339027	0.8984984	0.29367591	0.40859257	0.31719686	Per2/Tead3	2
GO:0015631	tubulin binding	2/46	353/14323	0.00566572	1.76413351	0.82512675	0.31395013	0.429084	0.33310469	Cfap157/Maco1	2
GO:0022843	voltage-gated monoatomic cation channel activity	1/46	117/14323	0.00854701	2.66127834	1.02414559	0.31470016	0.429084	0.33310469	Kcnk4	1
GO:0003697	single-stranded DNA binding	1/46	119/14323	0.00840336	2.61655097	1.00512441	0.31913123	0.429084	0.33310469	Pcbp2	1
GO:0042826	histone deacetylase binding	1/46	119/14323	0.00840336	2.61655097	1.00512441	0.31913123	0.429084	0.33310469	Per2	1
GO:0022834	ligand-gated channel activity	1/46	121/14323	0.00826446	2.57330219	0.98648923	0.32353426	0.43137902	0.33488634	Slc1a5	1
GO:0001221	transcription coregulator binding	1/46	123/14323	0.00813008	2.53145988	0.96822497	0.32790944	0.43359926	0.33660995	Per2	1
GO:0005319	lipid transporter activity	1/46	125/14323	0.008	2.49095652	0.95031738	0.33225693	0.43574679	0.33827711	Plscr4	1
GO:0015079	potassium ion transmembrane transporter activity	1/46	131/14323	0.00763359	2.37686691	0.89860345	0.34513499	0.44895608	0.34853169	Kcnk4	1
GO:0005244	voltage-gated monoatomic ion channel activity	1/46	139/14323	0.00719424	2.24006881	0.83390407	0.36192841	0.46014588	0.35721851	Kcnk4	1
GO:0022832	voltage-gated channel activity	1/46	140/14323	0.00714286	2.22406832	0.82612905	0.36399773	0.46014588	0.35721851	Kcnk4	1
GO:0008234	cysteine-type peptidase activity	1/46	141/14323	0.0070922	2.20829479	0.81841954	0.36606049	0.46014588	0.35721851	Otud7b	1
GO:0031406	carboxylic acid binding	1/46	141/14323	0.0070922	2.20829479	0.81841954	0.36606049	0.46014588	0.35721851	Sesn1	1
GO:0016922	nuclear receptor binding	1/46	142/14323	0.00704225	2.19274342	0.81077443	0.3681167	0.46014588	0.35721851	Per2	1
GO:0016874	ligase activity	1/46	149/14323	0.00671141	2.08972863	0.75897514	0.38232863	0.47055831	0.36530185	Acsf3	1
GO:0043177	organic acid binding	1/46	149/14323	0.00671141	2.08972863	0.75897514	0.38232863	0.47055831	0.36530185	Sesn1	1
GO:0044325	transmembrane transporter binding	1/46	162/14323	0.00617284	1.92203435	0.66991533	0.4078973	0.49819518	0.38675678	Herpud1	1
GO:1901981	phosphatidylinositol phosphate binding	1/46	170/14323	0.00588235	1.83158568	0.61911237	0.42311411	0.51286559	0.39814565	Phlda3	1
GO:0140030	modification-dependent protein binding	1/46	184/14323	0.00543478	1.6922259	0.53642583	0.44882813	0.53674296	0.41668203	Otud7b	1
GO:0140297	DNA-binding transcription factor binding	2/46	471/14323	0.00424628	1.32216376	0.40354611	0.44952223	0.53674296	0.41668203	Per2/Tead3	2
GO:0042277	peptide binding	1/46	200/14323	0.005	1.55684783	0.45014281	0.47684411	0.56180541	0.43613841	Mchr1	1
GO:0004672	protein kinase activity	2/46	497/14323	0.00402414	1.25299624	0.32584139	0.4775346	0.56180541	0.43613841	Mertk/Tesk2	2
GO:0030246	carbohydrate binding	1/46	204/14323	0.00490196	1.5263214	0.42976042	0.48362748	0.56482041	0.438479	Lgalsl	1
GO:0004930	G protein-coupled receptor activity	1/46	231/14323	0.004329	1.3479202	0.30259488	0.52721681	0.61126587	0.47453535	Mchr1	1
GO:0016791	phosphatase activity	1/46	239/14323	0.0041841	1.30280153	0.26795155	0.53942598	0.620922	0.48203155	Mtmr2	1
GO:0003713	transcription coactivator activity	1/46	257/14323	0.00389105	1.21155473	0.19425234	0.56577987	0.64201971	0.49841004	Per2	1
GO:0005261	monoatomic cation channel activity	1/46	257/14323	0.00389105	1.21155473	0.19425234	0.56577987	0.64201971	0.49841004	Kcnk4	1
GO:0035091	phosphatidylinositol binding	1/46	265/14323	0.00377358	1.17497949	0.16319641	0.57701368	0.65015626	0.50472657	Phlda3	1
GO:0031625	ubiquitin protein ligase binding	1/46	297/14323	0.003367	1.04838237	0.04782564	0.61917381	0.69278189	0.53781752	Pcbp2	1
GO:0044389	ubiquitin-like protein ligase binding	1/46	313/14323	0.00319489	0.99479094	-0.005289	0.63868336	0.70330159	0.54598413	Pcbp2	1
GO:0003729	mRNA binding	1/46	316/14323	0.00316456	0.98534673	-0.0149509	0.64223095	0.70330159	0.54598413	Pcbp2	1
GO:0042578	phosphoric ester hydrolase activity	1/46	318/14323	0.00314465	0.97914958	-0.0213427	0.64457705	0.70330159	0.54598413	Mtmr2	1
GO:0051020	GTPase binding	1/46	324/14323	0.00308642	0.96101718	-0.0402865	0.65152543	0.70330159	0.54598413	Bcl2l1	1
GO:0005216	monoatomic ion channel activity	1/46	325/14323	0.00307692	0.9580602	-0.0434108	0.6526705	0.70330159	0.54598413	Kcnk4	1
GO:0106310	protein serine kinase activity	1/46	327/14323	0.0030581	0.95220051	-0.0496315	0.6549496	0.70330159	0.54598413	Tesk2	1
GO:0046873	metal ion transmembrane transporter activity	1/46	346/14323	0.00289017	0.89991204	-0.1069732	0.67588419	0.72094313	0.55967954	Kcnk4	1
GO:0001228	DNA-binding transcription activator activity, RNA polymerase II-specific	1/46	357/14323	0.00280112	0.87218366	-0.1388183	0.68743081	0.7284035	0.56547114	Klf15	1
GO:0001216	DNA-binding transcription activator activity	1/46	363/14323	0.00275482	0.8577674	-0.1558016	0.69355833	0.7300614	0.56675819	Klf15	1
GO:0004674	protein serine/threonine kinase activity	1/46	388/14323	0.00257732	0.80249888	-0.2238691	0.7178491	0.75069187	0.58277395	Tesk2	1
GO:0003779	actin binding	1/46	402/14323	0.00248756	0.77455116	-0.2602484	0.73061829	0.75908393	0.58928884	Fam107a	1
GO:0008233	peptidase activity	1/46	416/14323	0.00240385	0.74848453	-0.2954996	0.74282159	0.76678357	0.5952662	Otud7b	1
GO:0005543	phospholipid binding	1/46	428/14323	0.00233645	0.72749898	-0.32488	0.7528498	0.77215364	0.59943506	Phlda3	1
GO:0022890	inorganic cation transmembrane transporter activity	1/46	446/14323	0.00224215	0.69813804	-0.3676125	0.76717874	0.78183821	0.60695335	Kcnk4	1
GO:0003712	transcription coregulator activity	1/46	473/14323	0.00211416	0.65828661	-0.428973	0.78716068	0.79494293	0.61712675	Per2	1
GO:0008324	monoatomic cation transmembrane transporter activity	1/46	477/14323	0.00209644	0.65276638	-0.4378053	0.78997454	0.79494293	0.61712675	Kcnk4	1
GO:0140657	ATP-dependent activity	1/46	491/14323	0.00203666	0.6341539	-0.4682297	0.79953939	0.79953939	0.62069505	Acsf3	1

ID	Description	GeneRatio	BgRatio	RichFactor	FoldEnrichm	zScore	pvalue	p.adjust	qvalue	geneID	Count
GO:0170055	lipid transmembrane transporter activity	2/40	38/14323	0.05263158	18.8460526	5.82929398	0.00501699	0.3393819	0.30722993	Slc10a6/Slc43a3	2
GO:0019894	kinesin binding	2/40	49/14323	0.04081633	14.6153061	5.05213232	0.00823184	0.3393819	0.30722993	Arhgef10/Fam83d	2
GO:0008028	monocarboxylic acid transmembrane transporter activity	2/40	56/14323	0.03571429	12.7883929	4.67739876	0.01064815	0.3393819	0.30722993	Slc10a6/Slc43a3	2
GO:0001882	nucleoside binding	1/40	10/14323	0.1	35.8075	5.82679325	0.02758731	0.3393819	0.30722993	Pnp	1
GO:0004726	non-membrane spanning protein tyrosine phosphatase activity	1/40	10/14323	0.1	35.8075	5.82679325	0.02758731	0.3393819	0.30722993	Ptpn1	1
GO:0036435	K48-linked polyubiquitin modification-dependent protein binding	1/40	10/14323	0.1	35.8075	5.82679325	0.02758731	0.3393819	0.30722993	Zfand2b	1
GO:0042301	phosphate ion binding	1/40	10/14323	0.1	35.8075	5.82679325	0.02758731	0.3393819	0.30722993	Pnp	1
GO:0098882	structural constituent of presynaptic active zone	1/40	10/14323	0.1	35.8075	5.82679325	0.02758731	0.3393819	0.30722993	Ctbp2	1
GO:0140161	monocarboxylate:sodium symporter activity	1/40	10/14323	0.1	35.8075	5.82679325	0.02758731	0.3393819	0.30722993	Slc10a6	1
GO:0004198	calcium-dependent cysteine-type endopeptidase activity	1/40	11/14323	0.09090909	32.5522727	5.5398619	0.03030487	0.3393819	0.30722993	Capn10	1
GO:0070878	primary miRNA binding	1/40	11/14323	0.09090909	32.5522727	5.5398619	0.03030487	0.3393819	0.30722993	Smad1	1
GO:0015125	bile acid transmembrane transporter activity	1/40	12/14323	0.08333333	29.8395833	5.28891669	0.03301503	0.3393819	0.30722993	Slc10a6	1
GO:0015355	secondary active monocarboxylate transmembrane transporter activity	1/40	13/14323	0.07692308	27.5442308	5.0669208	0.0357178	0.3393819	0.30722993	Slc10a6	1
GO:0015245	fatty acid transmembrane transporter activity	1/40	15/14323	0.06666667	23.8716667	4.69003506	0.04110127	0.3393819	0.30722993	Slc43a3	1
GO:0030169	low-density lipoprotein particle binding	1/40	15/14323	0.06666667	23.8716667	4.69003506	0.04110127	0.3393819	0.30722993	Samd1	1
GO:0051400	BH domain binding	1/40	15/14323	0.06666667	23.8716667	4.69003506	0.04110127	0.3393819	0.30722993	Bok	1
GO:0071933	Arp2/3 complex binding	1/40	15/14323	0.06666667	23.8716667	4.69003506	0.04110127	0.3393819	0.30722993	Iqgap2	1
GO:0043138	3'-5' DNA helicase activity	1/40	16/14323	0.0625	22.3796875	4.52802867	0.04378201	0.3393819	0.30722993	Fbh1	1
GO:0070411	I-SMAD binding	1/40	16/14323	0.0625	22.3796875	4.52802867	0.04378201	0.3393819	0.30722993	Smad1	1
GO:0140994	RNA polymerase II CTD heptapeptide repeat modifying activity	1/40	17/14323	0.05882353	21.0632353	4.38014441	0.04645544	0.3393819	0.30722993	Ctdspl	1
GO:0005319	lipid transporter activity	2/40	125/14323	0.016	5.7292	2.81029049	0.04751347	0.3393819	0.30722993	Slc10a6/Slc43a3	2
GO:0050699	WW domain binding	1/40	19/14323	0.05263158	18.8460526	4.11919481	0.05178044	0.33963206	0.30745639	Wbp1	1
GO:0016922	nuclear receptor binding	2/40	142/14323	0.01408451	5.04330986	2.56241744	0.05959706	0.33963206	0.30745639	Cry1/Ctbp2	2
GO:0046943	carboxylic acid transmembrane transporter activity	2/40	144/14323	0.01388889	4.97326389	2.53587573	0.06108246	0.33963206	0.30745639	Slc10a6/Slc43a3	2
GO:0005342	organic acid transmembrane transporter activity	2/40	145/14323	0.0137931	4.93896552	2.52278829	0.06182997	0.33963206	0.30745639	Slc10a6/Slc43a3	2
GO:0005158	insulin receptor binding	1/40	23/14323	0.04347826	15.5684783	3.70025967	0.06234362	0.33963206	0.30745639	Ptpn1	1
GO:0005452	solute:inorganic anion antiporter activity	1/40	23/14323	0.04347826	15.5684783	3.70025967	0.06234362	0.33963206	0.30745639	Slc25a30	1
GO:0005343	organic acid:sodium symporter activity	1/40	25/14323	0.04	14.323	3.52822683	0.0675821	0.33963206	0.30745639	Slc10a6	1
GO:0004721	phosphoprotein phosphatase activity	2/40	155/14323	0.01290323	4.62032258	2.39817547	0.06947591	0.33963206	0.30745639	Ctdspl/Ptpn1	2
GO:0071813	lipoprotein particle binding	1/40	26/14323	0.03846154	13.7721154	3.44944449	0.07019063	0.33963206	0.30745639	Samd1	1
GO:0071814	protein-lipid complex binding	1/40	26/14323	0.03846154	13.7721154	3.44944449	0.07019063	0.33963206	0.30745639	Samd1	1
GO:0042910	xenobiotic transmembrane transporter activity	1/40	27/14323	0.03703704	13.262037	3.37488787	0.07279204	0.34121267	0.30888726	Slc43a3	1
GO:0042974	nuclear retinoic acid receptor binding	1/40	30/14323	0.03333333	11.9358333	3.17302085	0.08055377	0.3530082	0.31956532	Ctbp2	1
GO:0046875	ephrin receptor binding	1/40	31/14323	0.03225806	11.5508065	3.11201808	0.0831269	0.3530082	0.31956532	Ptpn1	1
GO:0008168	methyltransferase activity	2/40	179/14323	0.01117318	4.00083799	2.13798696	0.08899102	0.3530082	0.31956532	Rrp8/Prdm15	2
GO:1901618	organic hydroxy compound transmembrane transporter activity	1/40	34/14323	0.02941176	10.5316176	2.94460649	0.09080426	0.3530082	0.31956532	Slc10a6	1
GO:0003714	transcription corepressor activity	2/40	184/14323	0.01086957	3.89211957	2.08947856	0.09324418	0.3530082	0.31956532	Cys1/Ctbp2	2
GO:0140030	modification-dependent protein binding	2/40	184/14323	0.01086957	3.89211957	2.08947856	0.09324418	0.3530082	0.31956532	Rrp8/Zfand2b	2
GO:0016741	transferase activity, transferring one-carbon groups	2/40	188/14323	0.0106383	3.80930851	2.05188287	0.09668935	0.3530082	0.31956532	Rrp8/Prdm15	2
GO:0070888	E-box binding	1/40	37/14323	0.02702703	9.6777027	2.79687089	0.09841892	0.3530082	0.31956532	Cry1	1
GO:0030291	protein serine/threonine kinase inhibitor activity	1/40	38/14323	0.02631579	9.42302632	2.75132533	0.1009433	0.3530082	0.31956532	Pkib	1
GO:0098918	structural constituent of synapse	1/40	38/14323	0.02631579	9.42302632	2.75132533	0.1009433	0.3530082	0.31956532	Ctbp2	1
GO:0071949	FAD binding	1/40	39/14323	0.02564103	9.18141026	2.70743267	0.10346079	0.3530082	0.31956532	Cry1	1
GO:0051721	protein phosphatase 2A binding	1/40	40/14323	0.025	8.951875	2.66509034	0.1059714	0.3530082	0.31956532	Ptpn1	1
GO:0030695	GTPase regulator activity	3/40	414/14323	0.00724638	2.59474638	1.74247258	0.10825585	0.3530082	0.31956532	Iqgap2/Arhgef10/Sipa1l2	3
GO:0060589	nucleoside-triphosphatase regulator activity	3/40	414/14323	0.00724638	2.59474638	1.74247258	0.10825585	0.3530082	0.31956532	Iqgap2/Arhgef10/Sipa1l2	3
GO:0008514	organic anion transmembrane transporter activity	2/40	204/14323	0.00980392	3.51053922	1.91118284	0.11082422	0.35369431	0.32018643	Slc10a6/Slc43a3	2
GO:0005547	phosphatidylinositol-3,4,5-trisphosphate binding	1/40	44/14323	0.02272727	8.13806818	2.50945982	0.11594547	0.36150506	0.32725721	Iqgap2	1
GO:0015291	secondary active transmembrane transporter activity	2/40	218/14323	0.00917431	3.28509174	1.79915034	0.12361445	0.36150506	0.32725721	Slc10a6/Slc25a30	2
GO:0019902	phosphatase binding	2/40	218/14323	0.00917431	3.28509174	1.79915034	0.12361445	0.36150506	0.32725721	Cry1/Ptpn1	2
GO:0003743	translation initiation factor activity	1/40	48/14323	0.02083333	7.45989583	2.37235779	0.12581102	0.36150506	0.32725721	Eif3j1	1
GO:0016763	pentosyltransferase activity	1/40	48/14323	0.02083333	7.45989583	2.37235779	0.12581102	0.36150506	0.32725721	Pnp	1
GO:0015370	solute:sodium symporter activity	1/40	49/14323	0.02040816	7.30765306	2.34053481	0.12826059	0.36150506	0.32725721	Slc10a6	1
GO:0005096	GTPase activator activity	2/40	225/14323	0.00888889	3.18288889	1.74649058	0.13014182	0.36150506	0.32725721	Iqgap2/Sipa1l2	2
GO:0003678	DNA helicase activity	1/40	54/14323	0.01851852	6.63101852	2.19386136	0.14040838	0.37557193	0.33999144	Fbh1	1
GO:0016791	phosphatase activity	2/40	239/14323	0.0083682	2.99644351	1.64708147	0.14343337	0.37557193	0.33999144	Ctdspl/Ptpn1	2
GO:0001222	transcription corepressor binding	1/40	56/14323	0.01785714	6.39419643	2.14030937	0.14522115	0.37557193	0.33999144	Ctbp2	1
GO:0031593	polyubiquitin modification-dependent protein binding	1/40	56/14323	0.01785714	6.39419643	2.14030937	0.14522115	0.37557193	0.33999144	Zfand2b	1
GO:0045296	cadherin binding	1/40	59/14323	0.01694915	6.0690678	2.06469332	0.15239107	0.38691997	0.3502644	Cdh19	1
GO:0051287	NAD binding	1/40	60/14323	0.01666667	5.96791667	2.04064096	0.15476799	0.38691997	0.3502644	Ctbp2	1
GO:0004197	cysteine-type endopeptidase activity	1/40	66/14323	0.01515152	5.42537879	1.90691114	0.16889362	0.40254077	0.36440533	Capn10	1
GO:0017018	myosin phosphatase activity	1/40	68/14323	0.01470588	5.26580882	1.8659257	0.17355081	0.40254077	0.36440533	Ctdspl	1
GO:0003924	GTPase activity	2/40	275/14323	0.00727273	2.60418182	1.42146051	0.17880629	0.40254077	0.36440533	Rhot1/Rab24	2
GO:0004860	protein kinase inhibitor activity	1/40	71/14323	0.01408451	5.04330986	1.80738388	0.18048892	0.40254077	0.36440533	Pkib	1
GO:1990841	promoter-specific chromatin binding	1/40	71/14323	0.01408451	5.04330986	1.80738388	0.18048892	0.40254077	0.36440533	Prdm15	1
GO:0030971	receptor tyrosine kinase binding	1/40	72/14323	0.01388889	4.97326389	1.78859948	0.18278898	0.40254077	0.36440533	Ptpn1	1
GO:0019210	kinase inhibitor activity	1/40	74/14323	0.01351351	4.83885135	1.75205233	0.18737023	0.40254077	0.36440533	Pkib	1
GO:0035064	methylated histone binding	1/40	74/14323	0.01351351	4.83885135	1.75205233	0.18737023	0.40254077	0.36440533	Rrp8	1
GO:0140034	methylation-dependent protein binding	1/40	76/14323	0.01315789	4.71151316	1.71679406	0.19192644	0.40254077	0.36440533	Rrp8	1
GO:0008135	translation factor activity, RNA binding	1/40	77/14323	0.01298701	4.65032468	1.69962261	0.19419519	0.40254077	0.36440533	Eif3j1	1
GO:0015294	solute:monoatomic cation symporter activity	1/40	77/14323	0.01298701	4.65032468	1.69962261	0.19419519	0.40254077	0.36440533	Slc10a6	1
GO:0046332	SMAD binding	1/40	77/14323	0.01298701	4.65032468	1.69962261	0.19419519	0.40254077	0.36440533	Smad1	1
GO:0050660	flavin adenine dinucleotide binding	1/40	80/14323	0.0125	4.4759375	1.6498267	0.20096426	0.40254077	0.36440533	Cry1	1
GO:0031625	ubiquitin protein ligase binding	2/40	297/14323	0.00673401	2.41127946	1.30060806	0.2010613	0.40254077	0.36440533	Bok/Smad1	2
GO:0004722	protein serine/threonine phosphatase activity	1/40	82/14323	0.01219512	4.36676829	1.61797581	0.20544613	0.40254077	0.36440533	Ctdspl	1
GO:0004725	protein tyrosine phosphatase activity	1/40	87/14323	0.01149425	4.1158046	1.54261647	0.21654385	0.40254077	0.36440533	Ptpn1	1
GO:0044389	ubiquitin-like protein ligase binding	2/40	313/14323	0.00638978	2.28801917	1.21926341	0.21745878	0.40254077	0.36440533	Bok/Smad1	2
GO:0042578	phosphoric ester hydrolase activity	2/40	318/14323	0.00628931	2.25204403	1.1948509	0.22261121	0.40254077	0.36440533	Ctdspl/Ptpn1	2
GO:1990756	ubiquitin-like ligase-substrate adaptor activity	1/40	91/14323	0.01098901	3.93489011	1.4862836	0.22531308	0.40254077	0.36440533	Lrrc75a	1
GO:0008083	growth factor activity	1/40	92/14323	0.01086957	3.89211957	1.47270093	0.22749039	0.40254077	0.36440533	Cntf	1
GO:0090079	translation regulator activity, nucleic acid binding	1/40	93/14323	0.01075269	3.85026882	1.45930798	0.22966173	0.40254077	0.36440533	Eif3j1	1
GO:0005525	GTP binding	2/40	329/14323	0.00607903	2.17674772	1.1427007	0.23398406	0.40254077	0.36440533	Rhot1/Rab24	2
GO:0016616	oxidoreductase activity, acting on the CH-OH group of donors, NAD or NADP as acceptor	1/40	95/14323	0.01052632	3.76921053	1.43307183	0.23398658	0.40254077	0.36440533	Ctbp2	1
GO:0022804	active transmembrane transporter activity	2/40	336/14323	0.00595238	2.13139881	1.11056807	0.24124338	0.40254077	0.36440533	Slc10a6/Slc25a30	2
GO:0061629	RNA polymerase II-specific DNA-binding transcription factor binding	2/40	336/14323	0.00595238	2.13139881	1.11056807	0.24124338	0.40254077	0.36440533	Cry1/Ctbp2	2
GO:0043130	ubiquitin binding	1/40	99/14323	0.01010101	3.61691919	1.38267194	0.24256538	0.40254077	0.36440533	Zfand2b	1
GO:0140767	enzyme-substrate adaptor activity	1/40	101/14323	0.00990099	3.54529703	1.35844148	0.24681957	0.40254077	0.36440533	Lrrc75a	1
GO:0005125	cytokine activity	1/40	102/14323	0.00980392	3.51053922	1.34655513	0.24893793	0.40254077	0.36440533	Cntf	1
GO:0016614	oxidoreductase activity, acting on CH-OH group of donors	1/40	102/14323	0.00980392	3.51053922	1.34655513	0.24893793	0.40254077	0.36440533	Ctbp2	1
GO:0019001	guanyl nucleotide binding	2/40	345/14323	0.0057971	2.0757971	1.07038376	0.2505958	0.40254077	0.36440533	Rhot1/Rab24	2
GO:0032561	guanyl ribonucleotide binding	2/40	345/14323	0.0057971	2.0757971	1.07038376	0.2505958	0.40254077	0.36440533	Rhot1/Rab24	2
GO:0015103	inorganic anion transmembrane transporter activity	1/40	103/14323	0.00970874	3.47645631	1.33481659	0.25105047	0.40254077	0.36440533	Slc25a30	1
GO:0008013	beta-catenin binding	1/40	104/14323	0.00961538	3.44302885	1.32322242	0.25315722	0.40254077	0.36440533	Cdh19	1
GO:0015293	symporter activity	1/40	106/14323	0.00943396	3.37806604	1.30045394	0.2573534	0.40254077	0.36440533	Slc10a6	1
GO:0015297	antiporter activity	1/40	107/14323	0.00934579	3.34649533	1.2892733	0.25944286	0.40254077	0.36440533	Slc25a30	1
GO:0070851	growth factor receptor binding	1/40	107/14323	0.00934579	3.34649533	1.2892733	0.25944286	0.40254077	0.36440533	Cntf	1
GO:1990782	protein tyrosine kinase binding	1/40	108/14323	0.00925926	3.31550926	1.27822433	0.26152659	0.40254077	0.36440533	Ptpn1	1
GO:0008094	ATP-dependent activity, acting on DNA	1/40	109/14323	0.00917431	3.28509174	1.26730412	0.2636046	0.40254077	0.36440533	Fbh1	1
GO:0000149	SNARE binding	1/40	110/14323	0.00909091	3.25522727	1.25650983	0.26567691	0.40254077	0.36440533	Capn10	1
GO:0015081	sodium ion transmembrane transporter activity	1/40	118/14323	0.00847458	3.0345339	1.1743767	0.28205196	0.41157193	0.3725809	Slc10a6	1
GO:0003697	single-stranded DNA binding	1/40	119/14323	0.00840336	3.00903361	1.16460182	0.28407363	0.41157193	0.3725809	Fbh1	1
GO:0042826	histone deacetylase binding	1/40	119/14323	0.00840336	3.00903361	1.16460182	0.28407363	0.41157193	0.3725809	Cry1	1
GO:0032182	ubiquitin-like protein binding	1/40	120/14323	0.00833333	2.98395833	1.15492887	0.28608976	0.41157193	0.3725809	Zfand2b	1
GO:0005201	extracellular matrix structural constituent	1/40	121/14323	0.00826446	2.95929752	1.14535583	0.28810035	0.41157193	0.3725809	Matn2	1
GO:0045182	translation regulator activity	1/40	121/14323	0.00826446	2.95929752	1.14535583	0.28810035	0.41157193	0.3725809	Eif3j1	1
GO:0001221	transcription coregulator binding	1/40	123/14323	0.00813008	2.91117886	1.12650164	0.29210498	0.4133561	0.37419605	Ctbp2	1
GO:0004386	helicase activity	1/40	139/14323	0.00719424	2.57607914	0.98811655	0.323361	0.45330981	0.41036467	Fbh1	1
GO:0008234	cysteine-type peptidase activity	1/40	141/14323	0.0070922	2.53953901	0.97219554	0.3271721	0.4544057	0.41135674	Capn10	1
GO:0016853	isomerase activity	1/40	157/14323	0.00636943	2.28073248	0.85389947	0.35691672	0.4911698	0.44463792	Fbh1	1
GO:0005509	calcium ion binding	2/40	466/14323	0.00429185	1.53680258	0.62344056	0.37556286	0.50039423	0.45298846	Matn2/Rhot1	2
GO:1901981	phosphatidylinositol phosphate binding	1/40	170/14323	0.00588235	2.10632353	0.76789965	0.38013662	0.50039423	0.45298846	Iqgap2	1
GO:0140297	DNA-binding transcription factor binding	2/40	471/14323	0.00424628	1.52048832	0.60783725	0.3806031	0.50039423	0.45298846	Cry1/Ctbp2	2
GO:0019903	protein phosphatase binding	1/40	171/14323	0.00584795	2.09400585	0.76160695	0.38188851	0.50039423	0.45298846	Ptpn1	1
GO:0003712	transcription coregulator activity	2/40	473/14323	0.00422833	1.5140592	0.60164586	0.38261484	0.50039423	0.45298846	Cys1/Ctbp2	2
GO:0005126	cytokine receptor binding	1/40	172/14323	0.00581395	2.0818314	0.75535716	0.38363558	0.50039423	0.45298846	Cntf	1
GO:0005516	calmodulin binding	1/40	178/14323	0.00561798	2.0116573	0.71872716	0.3940173	0.50950513	0.46123623	Iqgap2	1
GO:0022853	active monoatomic ion transmembrane transporter activity	1/40	194/14323	0.00515464	1.84574742	0.62763615	0.42087544	0.53958389	0.48846542	Slc10a6	1
GO:0005085	guanyl-nucleotide exchange factor activity	1/40	197/14323	0.00507614	1.81763959	0.6115156	0.42578047	0.54124637	0.48997039	Arhgef10	1
GO:0051015	actin filament binding	1/40	201/14323	0.00497512	1.78146766	0.59044989	0.43225756	0.54486247	0.49324392	Iqgap2	1
GO:0019887	protein kinase regulator activity	1/40	210/14323	0.0047619	1.70511905	0.54473407	0.44657164	0.55821455	0.50533107	Pkib	1
GO:0140097	catalytic activity, acting on DNA	1/40	216/14323	0.00462963	1.65775463	0.51546071	0.45591823	0.562452	0.50916707	Fbh1	1
GO:0016757	glycosyltransferase activity	1/40	217/14323	0.00460829	1.65011521	0.51067002	0.45746096	0.562452	0.50916707	Pnp	1
GO:0042393	histone binding	1/40	225/14323	0.00444444	1.59144444	0.47320418	0.4696502	0.57274414	0.51848417	Rrp8	1
GO:0019207	kinase regulator activity	1/40	242/14323	0.00413223	1.47964876	0.3982322	0.49467238	0.598394	0.54170405	Pkib	1
GO:0008017	microtubule binding	1/40	248/14323	0.00403226	1.44385081	0.37313051	0.50322588	0.59975819	0.542939	Fam83d	1
GO:0046982	protein heterodimerization activity	1/40	249/14323	0.00401606	1.43805221	0.36901064	0.50463767	0.59975819	0.542939	Bok	1
GO:0004175	endopeptidase activity	1/40	255/14323	0.00392157	1.40421569	0.34465865	0.51302663	0.59975819	0.542939	Capn10	1
GO:0003713	transcription coactivator activity	1/40	257/14323	0.00389105	1.39328794	0.33667742	0.51579205	0.59975819	0.542939	Ctbp2	1
GO:0050839	cell adhesion molecule binding	1/40	257/14323	0.00389105	1.39328794	0.33667742	0.51579205	0.59975819	0.542939	Cdh19	1
GO:0035091	phosphatidylinositol binding	1/40	265/14323	0.00377358	1.35122642	0.305401	0.52670139	0.60773238	0.55015773	Iqgap2	1
GO:0031267	small GTPase binding	1/40	284/14323	0.00352113	1.26082746	0.23494517	0.55165952	0.62361308	0.56453395	Iqgap2	1
GO:0048018	receptor ligand activity	1/40	284/14323	0.00352113	1.26082746	0.23494517	0.55165952	0.62361308	0.56453395	Cntf	1
GO:0004857	enzyme inhibitor activity	1/40	285/14323	0.00350877	1.25640351	0.23137471	0.55293693	0.62361308	0.56453395	Pkib	1
GO:0030546	signaling receptor activator activity	1/40	289/14323	0.00346021	1.23901384	0.21722177	0.5580112	0.6246394	0.56546303	Cntf	1
GO:0030545	signaling receptor regulator activity	1/40	319/14323	0.0031348	1.12249216	0.11708458	0.59431901	0.66035446	0.59779456	Cntf	1
GO:0051020	GTPase binding	1/40	324/14323	0.00308642	1.10516975	0.10132975	0.60008061	0.66185362	0.59915169	Iqgap2	1
GO:0016887	ATP hydrolysis activity	1/40	342/14323	0.00292398	1.04700292	0.04655764	0.62016924	0.67380866	0.60997415	Fbh1	1
GO:0046873	metal ion transmembrane transporter activity	1/40	346/14323	0.00289017	1.03489884	0.03477477	0.6244979	0.67380866	0.60997415	Slc10a6	1
GO:0015631	tubulin binding	1/40	353/14323	0.00283286	1.01437677	0.01447347	0.63195762	0.67380866	0.60997415	Fam83d	1
GO:0001228	DNA-binding transcription activator activity, RNA polymerase II-specific	1/40	357/14323	0.00280112	1.0030112	0.00304902	0.63615523	0.67380866	0.60997415	Smad1	1
GO:0015267	channel activity	1/40	362/14323	0.00276243	0.98915746	-0.0110573	0.64133664	0.67380866	0.60997415	Bok	1
GO:0022803	passive transmembrane transporter activity	1/40	362/14323	0.00276243	0.98915746	-0.0110573	0.64133664	0.67380866	0.60997415	Bok	1
GO:0001216	DNA-binding transcription activator activity	1/40	363/14323	0.00275482	0.98643251	-0.0138558	0.64236425	0.67380866	0.60997415	Smad1	1
GO:0140678	molecular function inhibitor activity	1/40	399/14323	0.00250627	0.89743108	-0.1099619	0.67750579	0.70373518	0.63706553	Pkib	1
GO:0003779	actin binding	1/40	402/14323	0.00248756	0.89073383	-0.1175941	0.68027734	0.70373518	0.63706553	Iqgap2	1
GO:0008233	peptidase activity	1/40	416/14323	0.00240385	0.86075721	-0.1525193	0.69290714	0.7118909	0.6444486	Capn10	1
GO:0005543	phospholipid binding	1/40	428/14323	0.00233645	0.83662383	-0.1815948	0.70334443	0.7176984	0.64970592	Iqgap2	1
GO:0022890	inorganic cation transmembrane transporter activity	1/40	446/14323	0.00224215	0.80285874	-0.2238305	0.71835483	0.72806233	0.659088	Slc10a6	1
GO:0008324	monoatomic cation transmembrane transporter activity	1/40	477/14323	0.00209644	0.75068134	-0.2930717	0.74248821	0.74747136	0.67665828	Slc10a6	1
GO:0140657	ATP-dependent activity	1/40	491/14323	0.00203666	0.72927699	-0.323032	0.75271463	0.75271463	0.68140482	Fbh1	1

ID	Description	GeneRatio	BgRatio	RichFactor	FoldEnrichm	zScore	pvalue	p.adjust	qvalue	geneID	Count
GO:0140311	protein sequestering activity	3/94	26/14323	0.11538462	17.5814239	6.87803861	0.00063794	0.14884586	0.14386059	Cdkn1a/Sfn/Ctnnd1	3
GO:0051059	NF-kappaB binding	3/94	35/14323	0.08571429	13.0604863	5.80619602	0.00153879	0.14884586	0.14386059	Hspa1b/Commd7/Taf4b	3
GO:0051082	unfolded protein binding	4/94	80/14323	0.05	7.61861702	4.82491959	0.00188014	0.14884586	0.14386059	Hspa1b/Ptges3/Uggt1/Cct7	4
GO:0001637	G protein-coupled chemoattractant receptor activity	2/94	11/14323	0.18181818	27.7040619	7.20115363	0.00225524	0.14884586	0.14386059	Gpr35/Gpr75	2
GO:0004950	chemokine receptor activity	2/94	11/14323	0.18181818	27.7040619	7.20115363	0.00225524	0.14884586	0.14386059	Gpr35/Gpr75	2
GO:0140313	molecular sequestering activity	3/94	44/14323	0.06818182	10.3890232	5.06963189	0.00298399	0.16411961	0.15862279	Cdkn1a/Sfn/Ctnnd1	3
GO:0015216	purine nucleotide transmembrane transporter activity	2/94	19/14323	0.10526316	16.0391937	5.33152163	0.00677651	0.31946383	0.30876409	Abcc5/Slc25a53	2
GO:0015215	nucleotide transmembrane transporter activity	2/94	22/14323	0.09090909	13.8520309	4.90318717	0.00903814	0.33139858	0.32029911	Abcc5/Slc25a53	2
GO:0070182	DNA polymerase binding	2/94	22/14323	0.09090909	13.8520309	4.90318717	0.00903814	0.33139858	0.32029911	Ptges3/Nhej1	2
GO:0140662	ATP-dependent protein folding chaperone	2/94	29/14323	0.06896552	10.5084373	4.16590947	0.01542032	0.39733168	0.38402392	Hspa1b/Cct7	2
GO:0016776	phosphotransferase activity, phosphate group as acceptor	2/94	30/14323	0.06666667	10.158156	4.08117804	0.01645195	0.39733168	0.38402392	Pmvk/Nme1	2
GO:0005154	epidermal growth factor receptor binding	2/94	32/14323	0.0625	9.52327128	3.92309263	0.01860092	0.39733168	0.38402392	Shc1/Nrg2	2
GO:0101005	deubiquitinase activity	3/94	87/14323	0.03448276	5.25421864	3.23492349	0.01951562	0.39733168	0.38402392	Stambpl1/Otub2/Usp31	3
GO:0016853	isomerase activity	4/94	157/14323	0.02547771	3.88209785	2.95130097	0.01979074	0.39733168	0.38402392	Pdia6/Cyp2j13/Fkbp1b/Ptges3	4
GO:0046982	protein heterodimerization activity	5/94	249/14323	0.02008032	3.05968555	2.66483543	0.02397944	0.39733168	0.38402392	Mettl3/Tuba1a/Pafah1b2/Taf4b/Smc1a	5
GO:0001727	lipid kinase activity	2/94	38/14323	0.05263158	8.01959686	3.52161941	0.02570422	0.39733168	0.38402392	Sphk2/Pip4k2c	2
GO:0019001	guanyl nucleotide binding	6/94	345/14323	0.0173913	2.64995375	2.52137459	0.02593956	0.39733168	0.38402392	Rab39/Tuba1a/Nme1/Mmaa/Mief1/Hbs1l	6
GO:0032561	guanyl ribonucleotide binding	6/94	345/14323	0.0173913	2.64995375	2.52137459	0.02593956	0.39733168	0.38402392	Rab39/Tuba1a/Nme1/Mmaa/Mief1/Hbs1l	6
GO:0019783	ubiquitin-like protein peptidase activity	3/94	98/14323	0.03061224	4.6644594	2.95852597	0.0266052	0.39733168	0.38402392	Stambpl1/Otub2/Usp31	3
GO:0008200	ion channel inhibitor activity	2/94	41/14323	0.04878049	7.43279709	3.35255444	0.0296056	0.39733168	0.38402392	Fkbp1b/Pcsk9	2
GO:0015605	organophosphate ester transmembrane transporter activity	2/94	42/14323	0.04761905	7.25582573	3.29995904	0.03095533	0.39733168	0.38402392	Abcc5/Slc25a53	2
GO:0016248	channel inhibitor activity	2/94	42/14323	0.04761905	7.25582573	3.29995904	0.03095533	0.39733168	0.38402392	Fkbp1b/Pcsk9	2
GO:0016860	intramolecular oxidoreductase activity	2/94	42/14323	0.04761905	7.25582573	3.29995904	0.03095533	0.39733168	0.38402392	Pdia6/Ptges3	2
GO:0141110	transporter inhibitor activity	2/94	42/14323	0.04761905	7.25582573	3.29995904	0.03095533	0.39733168	0.38402392	Fkbp1b/Pcsk9	2
GO:0140223	general transcription initiation factor activity	2/94	43/14323	0.04651163	7.0870856	3.24906279	0.03232907	0.39733168	0.38402392	Ubtf/Taf4b	2
GO:0015932	nucleobase-containing compound transmembrane transporter activity	2/94	47/14323	0.04255319	6.48392938	3.06066703	0.03805688	0.39733168	0.38402392	Abcc5/Slc25a53	2
GO:0031625	ubiquitin protein ligase binding	5/94	297/14323	0.01683502	2.56519092	2.21542574	0.04577516	0.39733168	0.38402392	Hspa1b/Cdkn1a/Traf2/Becn1/Caml	5
GO:0140097	catalytic activity, acting on DNA	4/94	216/14323	0.01851852	2.82171001	2.19264013	0.05374554	0.39733168	0.38402392	Dnase1l3/Ptges3/Lig4/Nme1	4
GO:0004536	DNA nuclease activity	2/94	57/14323	0.03508772	5.34639791	2.67235637	0.05388433	0.39733168	0.38402392	Dnase1l3/Nme1	2
GO:0044389	ubiquitin-like protein ligase binding	5/94	313/14323	0.01597444	2.43406295	2.08497037	0.05502404	0.39733168	0.38402392	Hspa1b/Cdkn1a/Traf2/Becn1/Caml	5
GO:0004896	cytokine receptor activity	2/94	59/14323	0.03389831	5.16516408	2.60564969	0.05728693	0.39733168	0.38402392	Gpr35/Gpr75	2
GO:0044183	protein folding chaperone	2/94	59/14323	0.03389831	5.16516408	2.60564969	0.05728693	0.39733168	0.38402392	Hspa1b/Cct7	2
GO:0045309	protein phosphorylated amino acid binding	2/94	59/14323	0.03389831	5.16516408	2.60564969	0.05728693	0.39733168	0.38402392	Shc1/Sfn	2
GO:0008093	cytoskeletal anchor activity	1/94	10/14323	0.1	15.237234	3.66048693	0.06374348	0.39733168	0.38402392	Bicd2	1
GO:0008392	arachidonate epoxygenase activity	1/94	10/14323	0.1	15.237234	3.66048693	0.06374348	0.39733168	0.38402392	Cyp2j13	1
GO:0016493	C-C chemokine receptor activity	1/94	10/14323	0.1	15.237234	3.66048693	0.06374348	0.39733168	0.38402392	Gpr75	1
GO:0019215	intermediate filament binding	1/94	10/14323	0.1	15.237234	3.66048693	0.06374348	0.39733168	0.38402392	Nme1	1
GO:0031996	thioesterase binding	1/94	10/14323	0.1	15.237234	3.66048693	0.06374348	0.39733168	0.38402392	Traf2	1
GO:0034452	dynactin binding	1/94	10/14323	0.1	15.237234	3.66048693	0.06374348	0.39733168	0.38402392	Bicd2	1
GO:0045125	bioactive lipid receptor activity	1/94	10/14323	0.1	15.237234	3.66048693	0.06374348	0.39733168	0.38402392	Sphk2	1
GO:0097177	mitochondrial ribosome binding	1/94	10/14323	0.1	15.237234	3.66048693	0.06374348	0.39733168	0.38402392	Mrps27	1
GO:0005525	GTP binding	5/94	329/14323	0.01519757	2.31568906	1.96227039	0.06528477	0.39733168	0.38402392	Rab39/Tuba1a/Nme1/Mmaa/Hbs1l	5
GO:0052689	carboxylic ester hydrolase activity	3/94	143/14323	0.02097902	3.19662253	2.14567484	0.06781629	0.39733168	0.38402392	Lipa/Smpd4/Pafah1b2	3
GO:0008391	arachidonate monooxygenase activity	1/94	11/14323	0.09090909	13.8520309	3.46574427	0.0698923	0.39733168	0.38402392	Cyp2j13	1
GO:0045503	dynein light chain binding	1/94	11/14323	0.09090909	13.8520309	3.46574427	0.0698923	0.39733168	0.38402392	Dnai4	1
GO:0050815	phosphoserine residue binding	1/94	11/14323	0.09090909	13.8520309	3.46574427	0.0698923	0.39733168	0.38402392	Sfn	1
GO:0016887	ATP hydrolysis activity	5/94	342/14323	0.01461988	2.2276658	1.86768084	0.07436384	0.39733168	0.38402392	Hspa1b/Abcc5/Cct7/Mmaa/Smc1a	5
GO:0048029	monosaccharide binding	2/94	69/14323	0.02898551	4.41658958	2.31221147	0.07534796	0.39733168	0.38402392	P4ha1/Cln5	2
GO:0004706	JUN kinase kinase kinase activity	1/94	12/14323	0.08333333	12.697695	3.29484128	0.07600117	0.39733168	0.38402392	Map3k21	1
GO:0004861	cyclin-dependent protein serine/threonine kinase inhibitor activity	1/94	12/14323	0.08333333	12.697695	3.29484128	0.07600117	0.39733168	0.38402392	Cdkn1a	1
GO:0005159	insulin-like growth factor receptor binding	1/94	12/14323	0.08333333	12.697695	3.29484128	0.07600117	0.39733168	0.38402392	Shc1	1
GO:0008559	ABC-type xenobiotic transporter activity	1/94	12/14323	0.08333333	12.697695	3.29484128	0.07600117	0.39733168	0.38402392	Abcc5	1
GO:0016888	endodeoxyribonuclease activity, producing 5'-phosphomonoesters	1/94	12/14323	0.08333333	12.697695	3.29484128	0.07600117	0.39733168	0.38402392	Dnase1l3	1
GO:0022841	potassium ion leak channel activity	1/94	12/14323	0.08333333	12.697695	3.29484128	0.07600117	0.39733168	0.38402392	Kcnk10	1
GO:0031543	peptidyl-proline dioxygenase activity	1/94	12/14323	0.08333333	12.697695	3.29484128	0.07600117	0.39733168	0.38402392	P4ha1	1
GO:0035251	UDP-glucosyltransferase activity	1/94	12/14323	0.08333333	12.697695	3.29484128	0.07600117	0.39733168	0.38402392	Uggt1	1
GO:0035673	oligopeptide transmembrane transporter activity	1/94	12/14323	0.08333333	12.697695	3.29484128	0.07600117	0.39733168	0.38402392	Abcc5	1
GO:0140776	protein-containing complex destabilizing activity	1/94	12/14323	0.08333333	12.697695	3.29484128	0.07600117	0.39733168	0.38402392	Hspa1b	1
GO:0008174	mRNA methyltransferase activity	1/94	13/14323	0.07692308	11.7209493	3.14313961	0.08207034	0.39733168	0.38402392	Mettl3	1
GO:0019855	calcium channel inhibitor activity	1/94	13/14323	0.07692308	11.7209493	3.14313961	0.08207034	0.39733168	0.38402392	Fkbp1b	1
GO:0052816	long-chain fatty acyl-CoA hydrolase activity	1/94	13/14323	0.07692308	11.7209493	3.14313961	0.08207034	0.39733168	0.38402392	Cln5	1
GO:0061578	K63-linked deubiquitinase activity	1/94	13/14323	0.07692308	11.7209493	3.14313961	0.08207034	0.39733168	0.38402392	Stambpl1	1
GO:0004559	alpha-mannosidase activity	1/94	14/14323	0.07142857	10.8837386	3.00717844	0.08810007	0.39733168	0.38402392	Edem1	1
GO:0005068	transmembrane receptor protein tyrosine kinase adaptor activity	1/94	14/14323	0.07142857	10.8837386	3.00717844	0.08810007	0.39733168	0.38402392	Shc1	1
GO:0008081	phosphoric diester hydrolase activity	2/94	76/14323	0.02631579	4.00979843	2.13826274	0.08892072	0.39733168	0.38402392	Smpd4/Pde6d	2
GO:0003756	protein disulfide isomerase activity	1/94	15/14323	0.06666667	10.158156	2.88431557	0.0940906	0.39733168	0.38402392	Pdia6	1
GO:0005165	neurotrophin receptor binding	1/94	15/14323	0.06666667	10.158156	2.88431557	0.0940906	0.39733168	0.38402392	Shc1	1
GO:0008179	adenylate cyclase binding	1/94	15/14323	0.06666667	10.158156	2.88431557	0.0940906	0.39733168	0.38402392	Cap1	1
GO:0008301	DNA binding, bending	1/94	15/14323	0.06666667	10.158156	2.88431557	0.0940906	0.39733168	0.38402392	Sap30l	1
GO:0015562	efflux transmembrane transporter activity	1/94	15/14323	0.06666667	10.158156	2.88431557	0.0940906	0.39733168	0.38402392	Abcc5	1
GO:0015923	mannosidase activity	1/94	15/14323	0.06666667	10.158156	2.88431557	0.0940906	0.39733168	0.38402392	Edem1	1
GO:0016864	intramolecular oxidoreductase activity, transposing S-S bonds	1/94	15/14323	0.06666667	10.158156	2.88431557	0.0940906	0.39733168	0.38402392	Pdia6	1
GO:0022840	leak channel activity	1/94	15/14323	0.06666667	10.158156	2.88431557	0.0940906	0.39733168	0.38402392	Kcnk10	1
GO:0022842	narrow pore channel activity	1/94	15/14323	0.06666667	10.158156	2.88431557	0.0940906	0.39733168	0.38402392	Kcnk10	1
GO:0030169	low-density lipoprotein particle binding	1/94	15/14323	0.06666667	10.158156	2.88431557	0.0940906	0.39733168	0.38402392	Pcsk9	1
GO:0045504	dynein heavy chain binding	1/94	15/14323	0.06666667	10.158156	2.88431557	0.0940906	0.39733168	0.38402392	Dnai4	1
GO:0051787	misfolded protein binding	1/94	15/14323	0.06666667	10.158156	2.88431557	0.0940906	0.39733168	0.38402392	Edem1	1
GO:0004843	cysteine-type deubiquitinase activity	2/94	79/14323	0.02531646	3.85752761	2.069982	0.09494471	0.39733168	0.38402392	Otub2/Usp31	2
GO:0140375	immune receptor activity	2/94	80/14323	0.025	3.80930851	2.04796374	0.09697843	0.39733168	0.38402392	Gpr35/Gpr75	2
GO:0004550	nucleoside diphosphate kinase activity	1/94	16/14323	0.0625	9.52327128	2.77249381	0.1000422	0.39733168	0.38402392	Nme1	1
GO:0120020	cholesterol transfer activity	1/94	16/14323	0.0625	9.52327128	2.77249381	0.1000422	0.39733168	0.38402392	Stard3	1
GO:0019903	protein phosphatase binding	3/94	171/14323	0.01754386	2.67319895	1.78902044	0.10241779	0.39733168	0.38402392	Shc1/Traf2/Ctnnd1	3
GO:0042626	ATPase-coupled transmembrane transporter activity	2/94	83/14323	0.02409639	3.67162266	1.98398207	0.10315323	0.39733168	0.38402392	Abcc5/Atp1b3	2
GO:0140657	ATP-dependent activity	6/94	491/14323	0.01221996	1.86198379	1.57970477	0.10368935	0.39733168	0.38402392	Hspa1b/Abcc5/Atp1b3/Cct7/Mmaa/Smc1a	6
GO:0000295	adenine nucleotide transmembrane transporter activity	1/94	17/14323	0.05882353	8.96307885	2.67008409	0.10595511	0.39733168	0.38402392	Slc25a53	1
GO:0003746	translation elongation factor activity	1/94	17/14323	0.05882353	8.96307885	2.67008409	0.10595511	0.39733168	0.38402392	Hbs1l	1
GO:0061133	endopeptidase activator activity	1/94	17/14323	0.05882353	8.96307885	2.67008409	0.10595511	0.39733168	0.38402392	Aph1b	1
GO:0120015	sterol transfer activity	1/94	17/14323	0.05882353	8.96307885	2.67008409	0.10595511	0.39733168	0.38402392	Stard3	1
GO:0016298	lipase activity	2/94	85/14323	0.02352941	3.58523154	1.94295609	0.10732872	0.39796041	0.38463159	Lipa/Smpd4	2
GO:0034185	apolipoprotein binding	1/94	18/14323	0.05555556	8.46513002	2.57577705	0.11182959	0.40030657	0.38689917	Pcsk9	1
GO:0043024	ribosomal small subunit binding	1/94	18/14323	0.05555556	8.46513002	2.57577705	0.11182959	0.40030657	0.38689917	Nme1	1
GO:0046527	glucosyltransferase activity	1/94	18/14323	0.05555556	8.46513002	2.57577705	0.11182959	0.40030657	0.38689917	Uggt1	1
GO:0005537	D-mannose binding	1/94	19/14323	0.05263158	8.01959686	2.48850658	0.11766587	0.40030657	0.38689917	Cln5	1
GO:0010314	phosphatidylinositol-5-phosphate binding	1/94	19/14323	0.05263158	8.01959686	2.48850658	0.11766587	0.40030657	0.38689917	Sap30l	1
GO:0031418	L-ascorbic acid binding	1/94	19/14323	0.05263158	8.01959686	2.48850658	0.11766587	0.40030657	0.38689917	P4ha1	1
GO:0047555	3',5'-cyclic-GMP phosphodiesterase activity	1/94	19/14323	0.05263158	8.01959686	2.48850658	0.11766587	0.40030657	0.38689917	Pde6d	1
GO:0051959	dynein light intermediate chain binding	1/94	19/14323	0.05263158	8.01959686	2.48850658	0.11766587	0.40030657	0.38689917	Bicd2	1
GO:0140678	molecular function inhibitor activity	5/94	399/14323	0.01253133	1.90942782	1.49744628	0.12174642	0.40144351	0.38799803	Cdkn1a/Serpinb8/Fkbp1b/Pcsk9/Pde6d	5
GO:0004114	3',5'-cyclic-nucleotide phosphodiesterase activity	1/94	20/14323	0.05	7.61861702	2.40739443	0.12346421	0.40144351	0.38799803	Pde6d	1
GO:0070840	dynein complex binding	1/94	20/14323	0.05	7.61861702	2.40739443	0.12346421	0.40144351	0.38799803	Bicd2	1
GO:1904047	S-adenosyl-L-methionine binding	1/94	20/14323	0.05	7.61861702	2.40739443	0.12346421	0.40144351	0.38799803	Mettl3	1
GO:0005164	tumor necrosis factor receptor binding	1/94	21/14323	0.04761905	7.25582573	2.33170967	0.12922484	0.40144351	0.38799803	Traf2	1
GO:0015271	outward rectifier potassium channel activity	1/94	21/14323	0.04761905	7.25582573	2.33170967	0.12922484	0.40144351	0.38799803	Kcnk10	1
GO:0004112	cyclic-nucleotide phosphodiesterase activity	1/94	22/14323	0.04545455	6.92601547	2.26083824	0.13494802	0.40144351	0.38799803	Pde6d	1
GO:0016289	acyl-CoA hydrolase activity	1/94	22/14323	0.04545455	6.92601547	2.26083824	0.13494802	0.40144351	0.38799803	Cln5	1
GO:0047617	fatty acyl-CoA hydrolase activity	1/94	22/14323	0.04545455	6.92601547	2.26083824	0.13494802	0.40144351	0.38799803	Cln5	1
GO:0008233	peptidase activity	5/94	416/14323	0.01201923	1.83139832	1.39867771	0.13811325	0.40144351	0.38799803	Stambpl1/Otub2/Adamts8/Pcsk9/Usp31	5
GO:0016798	hydrolase activity, acting on glycosyl bonds	2/94	100/14323	0.02	3.04744681	1.66991973	0.13995269	0.40144351	0.38799803	Edem1/Cln5	2
GO:0004709	MAP kinase kinase kinase activity	1/94	23/14323	0.04347826	6.62488437	2.19425992	0.14063398	0.40144351	0.38799803	Map3k21	1
GO:0005158	insulin receptor binding	1/94	23/14323	0.04347826	6.62488437	2.19425992	0.14063398	0.40144351	0.38799803	Shc1	1
GO:0052742	phosphatidylinositol kinase activity	1/94	23/14323	0.04347826	6.62488437	2.19425992	0.14063398	0.40144351	0.38799803	Pip4k2c	1
GO:0120283	protein serine/threonine kinase binding	1/94	23/14323	0.04347826	6.62488437	2.19425992	0.14063398	0.40144351	0.38799803	Cdkn1a	1
GO:0016712	oxidoreductase activity, acting on paired donors, with incorporation or reduction of molecular oxygen, red	1/94	24/14323	0.04166667	6.34884752	2.13153063	0.14628296	0.40144351	0.38799803	Cyp2j13	1
GO:0050750	low-density lipoprotein particle receptor binding	1/94	24/14323	0.04166667	6.34884752	2.13153063	0.14628296	0.40144351	0.38799803	Pcsk9	1
GO:0051219	phosphoprotein binding	2/94	103/14323	0.01941748	2.95868622	1.62148246	0.14671557	0.40144351	0.38799803	Shc1/Sfn	2
GO:0030246	carbohydrate binding	3/94	204/14323	0.01470588	2.24076971	1.45071757	0.15027151	0.40144351	0.38799803	P4ha1/Mlec/Cln5	3
GO:0022884	macromolecule transmembrane transporter activity	1/94	25/14323	0.04	6.09489362	2.07226862	0.15189519	0.40144351	0.38799803	Abcc5	1
GO:0030547	signaling receptor inhibitor activity	1/94	25/14323	0.04	6.09489362	2.07226862	0.15189519	0.40144351	0.38799803	Pcsk9	1
GO:0070851	growth factor receptor binding	2/94	107/14323	0.01869159	2.84808113	1.55956251	0.15583545	0.40144351	0.38799803	Shc1/Nrg2	2
GO:0001671	ATPase activator activity	1/94	26/14323	0.03846154	5.86047463	2.01614363	0.15747093	0.40144351	0.38799803	Atp1b3	1
GO:0031435	mitogen-activated protein kinase kinase kinase binding	1/94	26/14323	0.03846154	5.86047463	2.01614363	0.15747093	0.40144351	0.38799803	Traf2	1
GO:0034061	DNA polymerase activity	1/94	26/14323	0.03846154	5.86047463	2.01614363	0.15747093	0.40144351	0.38799803	Ptges3	1
GO:0070325	lipoprotein particle receptor binding	1/94	26/14323	0.03846154	5.86047463	2.01614363	0.15747093	0.40144351	0.38799803	Pcsk9	1
GO:0071813	lipoprotein particle binding	1/94	26/14323	0.03846154	5.86047463	2.01614363	0.15747093	0.40144351	0.38799803	Pcsk9	1
GO:0071814	protein-lipid complex binding	1/94	26/14323	0.03846154	5.86047463	2.01614363	0.15747093	0.40144351	0.38799803	Pcsk9	1
GO:0072349	modified amino acid transmembrane transporter activity	1/94	26/14323	0.03846154	5.86047463	2.01614363	0.15747093	0.40144351	0.38799803	Abcc5	1
GO:0035591	signaling adaptor activity	2/94	108/14323	0.01851852	2.82171001	1.54452969	0.15813249	0.40144351	0.38799803	Shc1/Traf2	2
GO:1990782	protein tyrosine kinase binding	2/94	108/14323	0.01851852	2.82171001	1.54452969	0.15813249	0.40144351	0.38799803	Shc1/Ctnnd1	2
GO:0061135	endopeptidase regulator activity	2/94	109/14323	0.01834862	2.79582276	1.52966784	0.160436	0.40144351	0.38799803	Serpinb8/Aph1b	2
GO:0000287	magnesium ion binding	3/94	212/14323	0.01415094	2.15621236	1.37849507	0.16281082	0.40144351	0.38799803	Lig4/Nudt3/Nme1	3
GO:0015662	P-type ion transporter activity	1/94	27/14323	0.03703704	5.64342002	1.96286826	0.16301039	0.40144351	0.38799803	Atp1b3	1
GO:0042910	xenobiotic transmembrane transporter activity	1/94	27/14323	0.03703704	5.64342002	1.96286826	0.16301039	0.40144351	0.38799803	Abcc5	1
GO:0046625	sphingolipid binding	1/94	27/14323	0.03703704	5.64342002	1.96286826	0.16301039	0.40144351	0.38799803	Traf2	1
GO:0140358	P-type transmembrane transporter activity	1/94	27/14323	0.03703704	5.64342002	1.96286826	0.16301039	0.40144351	0.38799803	Atp1b3	1
GO:0043022	ribosome binding	2/94	112/14323	0.01785714	2.72093465	1.48607128	0.16738351	0.40590921	0.39231416	Nme1/Mrps27	2
GO:0017147	Wnt-protein binding	1/94	28/14323	0.03571429	5.4418693	1.91219106	0.16851382	0.40590921	0.39231416	Sfrp5	1
GO:0070273	phosphatidylinositol-4-phosphate binding	1/94	28/14323	0.03571429	5.4418693	1.91219106	0.16851382	0.40590921	0.39231416	Sap30l	1
GO:0019902	phosphatase binding	3/94	218/14323	0.01376147	2.09686707	1.32639926	0.1724175	0.40812991	0.39446049	Shc1/Traf2/Ctnnd1	3
GO:0030371	translation repressor activity	1/94	29/14323	0.03448276	5.25421864	1.8638909	0.17398145	0.40812991	0.39446049	Nhlrc1	1
GO:0030551	cyclic nucleotide binding	1/94	29/14323	0.03448276	5.25421864	1.8638909	0.17398145	0.40812991	0.39446049	Fkbp1b	1
GO:0015399	primary active transmembrane transporter activity	2/94	115/14323	0.0173913	2.64995375	1.44388561	0.17438278	0.40812991	0.39446049	Abcc5/Atp1b3	2
GO:0015035	protein-disulfide reductase activity	1/94	30/14323	0.03333333	5.07907801	1.81777244	0.1794135	0.41694686	0.40298214	Pdia6	1
GO:0042887	amide transmembrane transporter activity	1/94	31/14323	0.03225806	4.91523679	1.77366235	0.18481021	0.42352339	0.4093384	Abcc5	1
GO:0046875	ephrin receptor binding	1/94	31/14323	0.03225806	4.91523679	1.77366235	0.18481021	0.42352339	0.4093384	Shc1	1
GO:0045182	translation regulator activity	2/94	121/14323	0.01652893	2.51855108	1.36340822	0.18851833	0.42904173	0.41467191	Nhlrc1/Hbs1l	2
GO:0004930	G protein-coupled receptor activity	3/94	231/14323	0.01298701	1.97886156	1.21904439	0.19376287	0.43298324	0.41848141	Gpr35/Gpr75/Sphk2	3
GO:0003755	peptidyl-prolyl cis-trans isomerase activity	1/94	33/14323	0.03030303	4.61734365	1.69086602	0.19549849	0.43298324	0.41848141	Fkbp1b	1
GO:0017080	sodium channel regulator activity	1/94	33/14323	0.03030303	4.61734365	1.69086602	0.19549849	0.43298324	0.41848141	Pcsk9	1
GO:0043015	gamma-tubulin binding	1/94	33/14323	0.03030303	4.61734365	1.69086602	0.19549849	0.43298324	0.41848141	Nme1	1
GO:0016251	RNA polymerase II general transcription initiation factor activity	1/94	35/14323	0.02857143	4.35349544	1.61444998	0.20604812	0.44153168	0.42674354	Taf4b	1
GO:0016859	cis-trans isomerase activity	1/94	35/14323	0.02857143	4.35349544	1.61444998	0.20604812	0.44153168	0.42674354	Fkbp1b	1
GO:0016893	endonuclease activity, active with either ribo- or deoxyribonucleic acids and producing 5'-phosphomonoe	1/94	35/14323	0.02857143	4.35349544	1.61444998	0.20604812	0.44153168	0.42674354	Dnase1l3	1
GO:0030332	cyclin binding	1/94	35/14323	0.02857143	4.35349544	1.61444998	0.20604812	0.44153168	0.42674354	Cdkn1a	1
GO:0032813	tumor necrosis factor receptor superfamily binding	1/94	35/14323	0.02857143	4.35349544	1.61444998	0.20604812	0.44153168	0.42674354	Traf2	1
GO:0140359	ABC-type transporter activity	1/94	36/14323	0.02777778	4.23256501	1.57836182	0.21127148	0.44931399	0.4342652	Abcc5	1
GO:0015079	potassium ion transmembrane transporter activity	2/94	131/14323	0.01526718	2.32629527	1.23946036	0.21240298	0.44931399	0.4342652	Kcnk10/Atp1b3	2
GO:0004520	DNA endonuclease activity	1/94	37/14323	0.02702703	4.11817136	1.54356204	0.21646085	0.45210178	0.43695961	Dnase1l3	1
GO:0015036	disulfide oxidoreductase activity	1/94	37/14323	0.02702703	4.11817136	1.54356204	0.21646085	0.45210178	0.43695961	Pdia6	1
GO:0016705	oxidoreductase activity, acting on paired donors, with incorporation or reduction of molecular oxygen	2/94	134/14323	0.01492537	2.27421404	1.20447409	0.21962927	0.45268019	0.43751865	P4ha1/Cyp2j13	2
GO:0019205	nucleobase-containing compound kinase activity	1/94	38/14323	0.02631579	4.00979843	1.50996771	0.22161643	0.45268019	0.43751865	Nme1	1
GO:0030291	protein serine/threonine kinase inhibitor activity	1/94	38/14323	0.02631579	4.00979843	1.50996771	0.22161643	0.45268019	0.43751865	Cdkn1a	1
GO:0008237	metallopeptidase activity	2/94	136/14323	0.01470588	2.24076971	1.18166378	0.22445892	0.45268019	0.43751865	Stambpl1/Adamts8	2
GO:0030159	signaling receptor complex adaptor activity	1/94	39/14323	0.02564103	3.90698309	1.4775033	0.22673845	0.45268019	0.43751865	Shc1	1
GO:0043531	ADP binding	1/94	39/14323	0.02564103	3.90698309	1.4775033	0.22673845	0.45268019	0.43751865	Mief1	1
GO:0043548	phosphatidylinositol 3-kinase binding	1/94	39/14323	0.02564103	3.90698309	1.4775033	0.22673845	0.45268019	0.43751865	Becn1	1

GO:0099106	ion channel regulator activity	2/94	138/14323	0.01449275	2.20829479	1.15924798	0.22929702	0.45268019	0.43751865	Fkbp1b/Pcsk9	2
GO:0016538	cyclin-dependent protein serine/threonine kinase regulator activity	1/94	40/14323	0.025	3.80930851	1.44609986	0.23182713	0.45268019	0.43751865	Cdkn1a	1
GO:0016790	thiolester hydrolase activity	1/94	40/14323	0.025	3.80930851	1.44609986	0.23182713	0.45268019	0.43751865	Cln5	1
GO:0051721	protein phosphatase 2A binding	1/94	40/14323	0.025	3.80930851	1.44609986	0.23182713	0.45268019	0.43751865	Shc1	1
GO:0008234	cysteine-type peptidase activity	2/94	141/14323	0.0141844	2.16130979	1.12633473	0.23656777	0.45714198	0.44183101	Otub2/Usp31	2
GO:0005246	calcium channel regulator activity	1/94	41/14323	0.02439024	3.71639855	1.41569427	0.23688266	0.45714198	0.44183101	Fkbp1b	1
GO:0031072	heat shock protein binding	2/94	144/14323	0.01388889	2.11628251	1.09423634	0.24385183	0.4642546	0.4487054	Hspa1b/Ptges3	2
GO:1901505	carbohydrate derivative transmembrane transporter activity	1/94	43/14323	0.02325581	3.5435428	1.35764976	0.24689519	0.4642546	0.4487054	Abcc5	1
GO:0061134	peptidase regulator activity	2/94	146/14323	0.01369863	2.08729233	1.07327041	0.24871365	0.4642546	0.4487054	Serpinb8/Aph1b	2
GO:0001784	phosphotyrosine residue binding	1/94	44/14323	0.02272727	3.46300774	1.32990858	0.2518526	0.4642546	0.4487054	Shc1	1
GO:0032266	phosphatidylinositol-3-phosphate binding	1/94	44/14323	0.02272727	3.46300774	1.32990858	0.2518526	0.4642546	0.4487054	Sap30l	1
GO:0120013	lipid transfer activity	1/94	44/14323	0.02272727	3.46300774	1.32990858	0.2518526	0.4642546	0.4487054	Stard3	1
GO:0016247	channel regulator activity	2/94	148/14323	0.01351351	2.05908568	1.05263787	0.25357899	0.4642546	0.4487054	Fkbp1b/Pcsk9	2
GO:0001046	core promoter sequence-specific DNA binding	1/94	45/14323	0.02222222	3.38605201	1.30295989	0.25677773	0.4642546	0.4487054	Ubtf	1
GO:0015485	cholesterol binding	1/94	45/14323	0.02222222	3.38605201	1.30295989	0.25677773	0.4642546	0.4487054	Stard3	1
GO:0031491	nucleosome binding	1/94	45/14323	0.02222222	3.38605201	1.30295989	0.25677773	0.4642546	0.4487054	Sap30l	1
GO:0016758	hexosyltransferase activity	2/94	150/14323	0.01333333	2.03163121	1.03232817	0.25844694	0.4642546	0.4487054	Uggt1/Fut11	2
GO:0141108	transporter regulator activity	2/94	150/14323	0.01333333	2.03163121	1.03232817	0.25844694	0.4642546	0.4487054	Fkbp1b/Pcsk9	2
GO:0043014	alpha-tubulin binding	1/94	46/14323	0.02173913	3.31244218	1.27676183	0.26167077	0.4642546	0.4487054	Efhc1	1
GO:0051879	Hsp90 protein binding	1/94	46/14323	0.02173913	3.31244218	1.27676183	0.26167077	0.4642546	0.4487054	Ptges3	1
GO:0060590	ATPase regulator activity	1/94	46/14323	0.02173913	3.31244218	1.27676183	0.26167077	0.4642546	0.4487054	Atp1b3	1
GO:0008408	3'-5' exonuclease activity	1/94	47/14323	0.0212766	3.24196469	1.25127567	0.26653195	0.46784863	0.45217906	Nme1	1
GO:0019829	ATPase-coupled monoatomic cation transmembrane transporter activity	1/94	47/14323	0.0212766	3.24196469	1.25127567	0.26653195	0.46784863	0.45217906	Atp1b3	1
GO:0003924	GTPase activity	3/94	275/14323	0.01090909	1.66224371	0.90127124	0.26995094	0.47134291	0.45555631	Rab39/Mmaa/Hbs1l	3
GO:0004714	transmembrane receptor protein tyrosine kinase activity	1/94	50/14323	0.02	3.04744681	1.17874149	0.28092627	0.48536999	0.46911358	Nrg2	1
GO:0016667	oxidoreductase activity, acting on a sulfur group of donors	1/94	50/14323	0.02	3.04744681	1.17874149	0.28092627	0.48536999	0.46911358	Pdia6	1
GO:0016504	peptidase activator activity	1/94	51/14323	0.01960784	2.98769295	1.15576766	0.28566199	0.48822025	0.47186838	Aph1b	1
GO:0004857	enzyme inhibitor activity	3/94	285/14323	0.01052632	1.60391937	0.83700364	0.28780277	0.48822025	0.47186838	Cdkn1a/Serpinb8/Pde6d	3
GO:0030695	GTPase regulator activity	4/94	414/14323	0.00966184	1.47219653	0.79241662	0.28849378	0.48822025	0.47186838	Agfg1/Tbck/Pde6d/Rasgef1a	4
GO:0060589	nucleoside-triphosphatase regulator activity	4/94	414/14323	0.00966184	1.47219653	0.79241662	0.28849378	0.48822025	0.47186838	Agfg1/Tbck/Pde6d/Rasgef1a	4
GO:1901682	sulfur compound transmembrane transporter activity	1/94	52/14323	0.01923077	2.93023732	1.13334921	0.29036685	0.48888296	0.47250889	Abcc5	1
GO:0004867	serine-type endopeptidase inhibitor activity	1/94	53/14323	0.01886792	2.87494982	1.11146085	0.29504105	0.4942312	0.477678	Serpinb8	1
GO:0008028	monocarboxylic acid transmembrane transporter activity	1/94	56/14323	0.01785714	2.72093465	1.04874676	0.30888166	0.51480277	0.49756057	Slc16a5	1
GO:0005126	cytokine receptor binding	2/94	172/14323	0.01162791	1.7717714	0.82763321	0.31192749	0.51716806	0.49984664	Shc1/Traf2	2
GO:0032934	sterol binding	1/94	57/14323	0.01754386	2.67319895	1.02875672	0.31343519	0.51716806	0.49984664	Stard3	1
GO:0004518	nuclease activity	2/94	175/14323	0.01142857	1.74139818	0.80205026	0.31917864	0.52402463	0.50647357	Dnase1l3/Nme1	2
GO:0016706	2-oxoglutarate-dependent dioxygenase activity	1/94	59/14323	0.01694915	2.58258204	0.99003408	0.32245337	0.52418528	0.50662884	P4ha1	1
GO:0045296	cadherin binding	1/94	59/14323	0.01694915	2.58258204	0.99003408	0.32245337	0.52418528	0.50662884	Ctnnd1	1
GO:0008173	RNA methyltransferase activity	1/94	61/14323	0.01639344	2.49790722	0.95287995	0.33135434	0.53601438	0.51806175	Mettl3	1
GO:0004497	monooxygenase activity	1/94	63/14323	0.01587302	2.41860858	0.91717385	0.34013961	0.54393051	0.52571274	Cyp2j13	1
GO:0050840	extracellular matrix binding	1/94	63/14323	0.01587302	2.41860858	0.91717385	0.34013961	0.54393051	0.52571274	Vwa1	1
GO:0005200	structural constituent of cytoskeleton	1/94	64/14323	0.015625	2.38081782	0.89982987	0.34448932	0.54393051	0.52571274	Tuba1a	1
GO:0030145	manganese ion binding	1/94	64/14323	0.015625	2.38081782	0.89982987	0.34448932	0.54393051	0.52571274	Nudt3	1
GO:0042562	hormone binding	1/94	64/14323	0.015625	2.38081782	0.89982987	0.34448932	0.54393051	0.52571274	Shc1	1
GO:0016747	acyltransferase activity, transferring groups other than amino-acyl groups	2/94	189/14323	0.01058201	1.61240572	0.68883653	0.35275673	0.55199069	0.53350297	Cln5/Pafah1b2	2
GO:0019199	transmembrane receptor protein kinase activity	1/94	66/14323	0.01515152	2.30867182	0.86609836	0.35310382	0.55199069	0.53350297	Nrg2	1
GO:0043021	ribonucleoprotein complex binding	2/94	190/14323	0.01052632	1.60391937	0.68110984	0.3551355	0.55199069	0.53350297	Nme1/Mrps27	2
GO:0004553	hydrolase activity, hydrolyzing O-glycosyl compounds	1/94	67/14323	0.01492537	2.27421404	0.84968806	0.35736897	0.55199069	0.53350297	Edem1	1
GO:0051020	GTPase binding	3/94	324/14323	0.00925926	1.410855	0.6079826	0.3579576	0.55199069	0.53350297	Bicd2/Becn1/Pde6d	3
GO:0004620	phospholipase activity	1/94	69/14323	0.01449275	2.20829479	0.81772569	0.36581598	0.56148499	0.54267927	Smpd4	1
GO:0002039	p53 binding	1/94	70/14323	0.01428571	2.17674772	0.80215411	0.36999819	0.56407203	0.54517967	Ptges3	1
GO:0004860	protein kinase inhibitor activity	1/94	71/14323	0.01408451	2.1460893	0.78684338	0.37415312	0.56407203	0.54517967	Cdkn1a	1
GO:0004527	exonuclease activity	1/94	72/14323	0.01388889	2.11628251	0.77178488	0.37828093	0.56407203	0.54517967	Nme1	1
GO:0019843	rRNA binding	1/94	72/14323	0.01388889	2.11628251	0.77178488	0.37828093	0.56407203	0.54517967	Mrps27	1
GO:0030971	receptor tyrosine kinase binding	1/94	72/14323	0.01388889	2.11628251	0.77178488	0.37828093	0.56407203	0.54517967	Shc1	1
GO:0022804	active transmembrane transporter activity	3/94	336/14323	0.00892857	1.36046733	0.54344028	0.37946664	0.56407203	0.54517967	Abcc5/Atp1b3/Slc16a5	3
GO:0061629	RNA polymerase II-specific DNA-binding transcription factor binding	3/94	336/14323	0.00892857	1.36046733	0.54344028	0.37946664	0.56407203	0.54517967	Hspa1b/Commd7/Taf4b	3
GO:0019210	kinase inhibitor activity	1/94	74/14323	0.01351351	2.05908568	0.74239209	0.38645592	0.57004763	0.55095513	Cdkn1a	1
GO:0008514	organic anion transmembrane transporter activity	2/94	204/14323	0.00980392	1.49384647	0.57740913	0.38809785	0.57004763	0.55095513	Abcc5/Slc16a5	2
GO:0000049	tRNA binding	1/94	76/14323	0.01315789	2.00489922	0.71391443	0.39452455	0.57004763	0.55095513	Mrps27	1
GO:0004222	metalloendopeptidase activity	1/94	76/14323	0.01315789	2.00489922	0.71391443	0.39452455	0.57004763	0.55095513	Adamts8	1
GO:0046873	metal ion transmembrane transporter activity	3/94	346/14323	0.00867052	1.32114746	0.49148937	0.39728394	0.57004763	0.55095513	Trpv2/Kcnk10/Atp1b3	3
GO:0008135	translation factor activity, RNA binding	1/94	77/14323	0.01298701	1.97886156	0.70000113	0.3985194	0.57004763	0.55095513	Hbs1l	1
GO:0051213	dioxygenase activity	1/94	77/14323	0.01298701	1.97886156	0.70000113	0.3985194	0.57004763	0.55095513	P4ha1	1
GO:0019887	protein kinase regulator activity	2/94	210/14323	0.00952381	1.45116515	0.53532077	0.40200647	0.57004763	0.55095513	Cdkn1a/Ankrd54	2
GO:0005249	voltage-gated potassium channel activity	1/94	78/14323	0.01282051	1.95349154	0.68629604	0.40248818	0.57004763	0.55095513	Kcnk10	1
GO:0043178	alcohol binding	1/94	78/14323	0.01282051	1.95349154	0.68629604	0.40248818	0.57004763	0.55095513	Stard3	1
GO:0097110	scaffold protein binding	1/94	78/14323	0.01282051	1.95349154	0.68629604	0.40248818	0.57004763	0.55095513	Ubtf	1
GO:0019003	GDP binding	1/94	79/14323	0.01265823	1.9287638	0.67279293	0.40643104	0.57317198	0.55397484	Mief1	1
GO:0015631	tubulin binding	3/94	353/14323	0.00849858	1.29494907	0.45605241	0.40968027	0.5752957	0.55602743	Efhc1/Togaram2/Nme1	3
GO:0016757	glycosyltransferase activity	2/94	217/14323	0.00921659	1.40435337	0.48782894	0.4180472	0.58455753	0.56497905	Uggt1/Fut11	2
GO:0004252	serine-type endopeptidase activity	1/94	83/14323	0.01204819	1.83581133	0.62068447	0.42194673	0.58752076	0.56784303	Pcsk9	1
GO:0020037	heme binding	1/94	84/14323	0.01190476	1.81395643	0.60810648	0.42576253	0.590343	0.57057075	Cyp2j13	1
GO:0005496	steroid binding	1/94	86/14323	0.01162791	1.7717714	0.58345483	0.43331953	0.59470361	0.57478531	Stard3	1
GO:0005096	GTPase activator activity	2/94	225/14323	0.00888889	1.3544208	0.43552219	0.43611598	0.59470361	0.57478531	Agfg1/Tbck	2
GO:0042393	histone binding	2/94	225/14323	0.00888889	1.3544208	0.43552219	0.43611598	0.59470361	0.57478531	Sphk2/Sap30l	2
GO:1901681	sulfur compound binding	2/94	225/14323	0.00888889	1.3544208	0.43552219	0.43611598	0.59470361	0.57478531	Adamts8/Mettl3	2
GO:0016407	acetyltransferase activity	1/94	88/14323	0.01136364	1.73150387	0.55944552	0.44077812	0.59858756	0.57853918	Pafah1b2	1
GO:0004866	endopeptidase inhibitor activity	1/94	90/14323	0.01111111	1.693026	0.53604486	0.44813957	0.60591007	0.58561643	Serpinb8	1
GO:0004842	ubiquitin-protein transferase activity	3/94	376/14323	0.00797872	1.21573676	0.34455316	0.44984233	0.60591007	0.58561643	Traf2/Nhlrc1/Rnf103	3
GO:0046906	tetrapyrrole binding	1/94	91/14323	0.01098901	1.67442132	0.52456293	0.45178426	0.60605205	0.58575366	Cyp2j13	1
GO:0008083	growth factor activity	1/94	92/14323	0.01086957	1.65622109	0.51322164	0.45540513	0.60831636	0.58794213	Nrg2	1
GO:0051117	ATPase binding	1/94	93/14323	0.01075269	1.63841226	0.50201745	0.45900234	0.60831636	0.58794213	Atp1b3	1
GO:0090079	translation regulator activity, nucleic acid binding	1/94	93/14323	0.01075269	1.63841226	0.50201745	0.45900234	0.60831636	0.58794213	Hbs1l	1
GO:0140104	molecular carrier activity	1/94	94/14323	0.0106383	1.62098234	0.49094695	0.46257604	0.6104036	0.58995947	Mmaa	1
GO:0008236	serine-type peptidase activity	1/94	95/14323	0.01052632	1.60391937	0.48000682	0.46612639	0.6104036	0.58995947	Pcsk9	1
GO:0030414	peptidase inhibitor activity	1/94	95/14323	0.01052632	1.60391937	0.48000682	0.46612639	0.6104036	0.58995947	Serpinb8	1
GO:0005262	calcium channel activity	1/94	96/14323	0.01041667	1.58721188	0.4691939	0.46965353	0.61259156	0.59207414	Trpv2	1
GO:0019207	kinase regulator activity	2/94	242/14323	0.00826446	1.25927554	0.33062147	0.47349557	0.61517142	0.59456759	Cdkn1a/Ankrd54	2
GO:0017171	serine hydrolase activity	1/94	99/14323	0.01010101	1.53911455	0.43748821	0.4800972	0.61887529	0.59814741	Pcsk9	1
GO:0043130	ubiquitin binding	1/94	99/14323	0.01010101	1.53911455	0.43748821	0.4800972	0.61887529	0.59814741	Otub2	1
GO:0019955	cytokine binding	1/94	100/14323	0.01	1.5237234	0.42715446	0.483533	0.62087895	0.60008396	Traf2	1
GO:0005267	potassium channel activity	1/94	101/14323	0.00990099	1.50863703	0.4169336	0.48694634	0.62283834	0.60197773	Kcnk10	1
GO:0019787	ubiquitin-like protein transferase activity	3/94	401/14323	0.0074813	1.1399427	0.23102032	0.49223931	0.6266271	0.60563959	Traf2/Nhlrc1/Rnf103	3
GO:0004519	endonuclease activity	1/94	103/14323	0.00970874	1.47934311	0.3968203	0.4937062	0.6266271	0.60563959	Dnase1l3	1
GO:0008013	beta-catenin binding	1/94	104/14323	0.00961538	1.46511866	0.38692294	0.49705301	0.62845782	0.607409	Ctnnd1	1
GO:0004175	endopeptidase activity	2/94	255/14323	0.00784314	1.19507718	0.25546944	0.50107377	0.62916097	0.60808859	Adamts8/Pcsk9	2
GO:0015293	symporter activity	1/94	106/14323	0.00943396	1.43747491	0.36743513	0.50368109	0.62916097	0.60808859	Slc16a5	1
GO:0005261	monoatomic cation channel activity	2/94	257/14323	0.0077821	1.18577697	0.24425963	0.50523532	0.62916097	0.60808859	Trpv2/Kcnk10	2
GO:0050839	cell adhesion molecule binding	2/94	257/14323	0.0077821	1.18577697	0.24425963	0.50523532	0.62916097	0.60808859	Ctnnd1/Caml	2
GO:0016755	aminoacyltransferase activity	3/94	411/14323	0.00729927	1.11220686	0.18759624	0.50876084	0.63116946	0.61002981	Traf2/Nhlrc1/Rnf103	3
GO:0005506	iron ion binding	1/94	110/14323	0.00909091	1.38520309	0.32962626	0.51667909	0.63620933	0.61490088	P4ha1	1
GO:0019842	vitamin binding	1/94	110/14323	0.00909091	1.38520309	0.32962626	0.51667909	0.63620933	0.61490088	P4ha1	1
GO:0008194	UDP-glycosyltransferase activity	1/94	114/14323	0.00877193	1.33659948	0.29326665	0.52934022	0.64697138	0.62530248	Uggt1	1
GO:0015085	calcium ion transmembrane transporter activity	1/94	114/14323	0.00877193	1.33659948	0.29326665	0.52934022	0.64697138	0.62530248	Trpv2	1
GO:0004713	protein tyrosine kinase activity	1/94	115/14323	0.00869565	1.32497687	0.28438944	0.53245388	0.64837557	0.62665964	Nrg2	1
GO:0022843	voltage-gated monoatomic cation channel activity	1/94	117/14323	0.00854701	1.3023277	0.26687847	0.53862019	0.65107935	0.62927287	Kcnk10	1
GO:0030295	protein kinase activator activity	1/94	117/14323	0.00854701	1.3023277	0.26687847	0.53862019	0.65107935	0.62927287	Cdkn1a	1
GO:0015081	sodium ion transmembrane transporter activity	1/94	118/14323	0.00847458	1.29129102	0.25824151	0.54167311	0.65127895	0.62946578	Atp1b3	1
GO:0003697	single-stranded DNA binding	1/94	119/14323	0.00840336	1.28043984	0.24968153	0.54470603	0.65127895	0.62946578	Nme1	1
GO:0042826	histone deacetylase binding	1/94	119/14323	0.00840336	1.28043984	0.24968153	0.54470603	0.65127895	0.62946578	Hspa1b	1
GO:0032182	ubiquitin-like protein binding	1/94	120/14323	0.00833333	1.2697695	0.24119706	0.5477191	0.65251734	0.6306627	Otub2	1
GO:0005201	extracellular matrix structural constituent	1/94	121/14323	0.00826446	1.25927554	0.23278664	0.55071244	0.6537234	0.63182836	Vwa1	1
GO:0031267	small GTPase binding	2/94	284/14323	0.00704225	1.07304465	0.10105506	0.55920406	0.65928595	0.6372046	Bicd2/Pde6d	2
GO:0019838	growth factor binding	1/94	124/14323	0.00806452	1.2288092	0.20798596	0.55957536	0.65928595	0.6372046	Shc1	1
GO:0005319	lipid transporter activity	1/94	125/14323	0.008	1.21897872	0.19985817	0.56249105	0.65928595	0.6372046	Stard3	1
GO:0022890	inorganic cation transmembrane transporter activity	3/94	446/14323	0.00672646	1.02492606	0.04346621	0.56432164	0.65928595	0.6372046	Trpv2/Kcnk10/Atp1b3	3
GO:0019209	kinase activator activity	1/94	126/14323	0.00793651	1.20930429	0.19179783	0.56538765	0.65928595	0.6372046	Cdkn1a	1
GO:0016779	nucleotidyltransferase activity	1/94	127/14323	0.00787402	1.19978221	0.1838037	0.56826527	0.66030823	0.63819264	Ptges3	1
GO:0008201	heparin binding	1/94	129/14323	0.00775194	1.18118093	0.16800938	0.57396407	0.66458998	0.64233098	Adamts8	1
GO:0002020	protease binding	1/94	130/14323	0.00769231	1.17209493	0.1602069	0.5767855	0.66552173	0.64323153	Hspa1b	1
GO:0061630	ubiquitin protein ligase activity	2/94	297/14323	0.00673401	1.02607637	0.0369094	0.58367971	0.67112998	0.64865194	Traf2/Nhlrc1	2
GO:0051087	protein-folding chaperone binding	1/94	138/14323	0.00724638	1.10414739	0.09991987	0.59870136	0.68462871	0.66169856	Ptges3	1
GO:0005244	voltage-gated monoatomic ion channel activity	1/94	139/14323	0.00719424	1.09620389	0.09263588	0.60136066	0.68462871	0.66169856	Kcnk10	1
GO:0140297	DNA-binding transcription factor binding	3/94	471/14323	0.00636943	0.97052446	-0.0528682	0.60164341	0.68462871	0.66169856	Hspa1b/Commd7/Taf4b	3
GO:0022832	voltage-gated channel activity	1/94	140/14323	0.00714286	1.08837386	0.08540482	0.60400251	0.6849513	0.66201035	Kcnk10	1
GO:0031406	carboxylic acid binding	1/94	141/14323	0.0070922	1.0806549	0.07822582	0.60662705	0.68557166	0.66260993	P4ha1	1
GO:0008324	monoatomic cation transmembrane transporter activity	3/94	477/14323	0.00628931	0.95831661	-0.0752556	0.61028943	0.68729249	0.66427312	Trpv2/Kcnk10/Atp1b3	3
GO:0061659	ubiquitin-like protein ligase activity	2/94	314/14323	0.00636943	0.97052446	-0.0429241	0.61417792	0.68729249	0.66427312	Traf2/Nhlrc1	2
GO:0046943	carboxylic acid transmembrane transporter activity	1/94	144/14323	0.00694444	1.05814125	0.05699298	0.61439783	0.68729249	0.66427312	Slc16a5	1
GO:0005342	organic acid transmembrane transporter activity	1/94	145/14323	0.00689655	1.05084373	0.0500141	0.61695419	0.68742292	0.66439918	Slc16a5	1
GO:0042578	phosphoric ester hydrolase activity	2/94	318/14323	0.00628931	0.95831661	-0.0610961	0.62110496	0.68742292	0.66439918	Smpd4/Pde6d	2
GO:0030545	signaling receptor regulator activity	2/94	319/14323	0.00626959	0.95531248	-0.0656046	0.62282192	0.68742292	0.66439918	Nrg2/Pcsk9	2
GO:0008757	S-adenosylmethionine-dependent methyltransferase activity	1/94	149/14323	0.00671141	1.02263316	0.02257203	0.62701302	0.68742292	0.66439918	Mettl3	1
GO:0016874	ligase activity	1/94	149/14323	0.00671141	1.02263316	0.02257203	0.62701302	0.68742292	0.66439918	Lig4	1
GO:0043177	organic acid binding	1/94	149/14323	0.00671141	1.02263316	0.02257203	0.62701302	0.68742292	0.66439918	P4ha1	1
GO:0005216	monoatomic ion channel activity	2/94	325/14323	0.00615385	0.93767594	-0.0923726	0.63299955	0.69168825	0.66852166	Trpv2/Kcnk10	2
GO:0004672	protein kinase activity	3/94	497/14323	0.00603622	0.91975256	-0.1479925	0.63821418	0.69508475	0.6718044	Cdkn1a/Nrg2/Map3k21	3
GO:0044325	transmembrane transporter binding	1/94	162/14323	0.00617284	0.94057	-0.0618294	0.65793342	0.71420404	0.69028333	Fkbp1b	1
GO:1901981	phosphatidylinositol phosphate binding	1/94	170/14323	0.00588235	0.89630788	-0.1105413	0.6756862	0.73107032	0.70658471	Sap30l	1
GO:0001228	DNA-binding transcription activator activity, RNA polymerase II-specific	2/94	357/14323	0.00560224	0.85362656	-0.227635	0.68372467	0.73700379	0.71231945	Myt1/Rfxap	2
GO:0015267	channel activity	2/94	362/14323	0.00552486	0.84183613	-0.2477319	0.69111847	0.73700379	0.71231945	Trpv2/Kcnk10	2
GO:0022803	passive transmembrane transporter activity	2/94	362/14323	0.00552486	0.84183613	-0.2477319	0.69111847	0.73700379	0.71231945	Trpv2/Kcnk10	2
GO:0005539	glycosaminoglycan binding	1/94	178/14323	0.00561798	0.85602438	-0.1570999	0.69252694	0.73700379	0.71231945	Adamts8	1
GO:0001216	DNA-binding transcription activator activity	2/94	363/14323	0.00550964	0.83951703	-0.2517202	0.6925803	0.73700379	0.71231945	Myt1/Rfxap	2
GO:0008168	methyltransferase activity	1/94	179/14323	0.00558659	0.85124213	-0.1627792	0.69457024	0.73700379	0.71231945	Mettl3	1
GO:0016741	transferase activity, transferring one-carbon groups	1/94	188/14323	0.00531915	0.81049117	-0.2125881	0.71236572	0.75346374	0.72822811	Mettl3	1
GO:0033218	amide binding	1/94	193/14323	0.00518135	0.78949399	-0.2393044	0.72180474	0.7605296	0.73505732	Fkbp1b	1
GO:0022853	active monoatomic ion transmembrane transporter activity	1/94	194/14323	0.00515464	0.78542444	-0.2445705	0.72365544	0.7605296	0.73505732	Atp1b3	1
GO:0005085	guanyl-nucleotide exchange factor activity	1/94	197/14323	0.00507614	0.77346366	-0.2602196	0.72913476	0.76385546	0.73827179	Rasgef1a	1
GO:0015291	secondary active transmembrane transporter activity	1/94	218/14323	0.00458716	0.69895569	-0.3640413	0.76459383	0.79846824	0.77172529	Slc16a5	1
GO:0001664	G protein-coupled receptor binding	1/94	223/14323	0.0044843	0.68328404	-0.3874284	0.77233515	0.8040082	0.7770797	Hspa1b	1
GO:0022836	gated channel activity	1/94	231/14323	0.004329	0.65962052	-0.4238982	0.78420058	0.81379306	0.78653683	Kcnk10	1
GO:0008017	microtubule binding	1/94	248/14323	0.00403226	0.6144046	-0.4978655	0.80742091	0.83526301	0.80728769	Togaram2	1
GO:0005509	calcium ion binding	2/94	466/14323	0.00429185	0.65395854	-0.6172556	0.81521328	0.8406887	0.81253166	Efhc1/Edem1	2
GO:0035091	phosphatidylinositol binding	1/94	265/14323	0.00377358	0.57498996	-0.5675955	0.82816642	0.85138604	0.82287072	Sap30l	1
GO:0048018	receptor ligand activity	1/94	284/14323	0.00352113	0.53652233	-0.6412073	0.84874415	0.86983096	0.84069787	Nrg2	1
GO:0030546	signaling receptor activator activity	1/94	289/14323	0.00346021	0.52723993	-0.6598991	0.85374127	0.8722434	0.84302951	Nrg2	1
GO:0003729	mRNA binding	1/94	316/14323	0.00316456	0.48219095	-0.7565179	0.87803264	0.89429251	0.86434013	Mettl3	1
GO:0106310	protein serine kinase activity	1/94	327/14323	0.0030581	0.46597046	-0.7939913	0.88674319	0.9003854	0.87022895	Map3k21	1
GO:0140098	catalytic activity, acting on RNA	1/94	343/14323	0.00291545	0.44423423	-0.8467668	0.89832534	0.90934774	0.87889112	Mettl3	1
GO:0004674	protein serine/threonine kinase activity	1/94	388/14323	0.00257732	0.39271222	-0.985679	0.92498405	0.93347014	0.90220559	Map3k21	1
GO:0003779	actin binding	1/94	402/14323	0.00248756	0.37903567	-1.0264151	0.93176907	0.93745059	0.90605272	Cap1	1
GO:0005543	phospholipid binding	1/94	428/14323	0.00233645	0.35601014	-1.099386	0.94279842	0.94566407	0.91399111	Sap30l	1
GO:0003712	transcription coregulator activity	1/94	473/14323	0.00211416	0.32214025	-1.218496	0.95787528	0.95787528	0.92579333	Sap30l	1

ID	Description	GeneRatio	BgRatio	RichFactor	FoldEnrichm	zScore	pvalue	p.adjust	qvalue	geneID	Count
GO:0051787	misfolded protein binding	4/135	15/14323	0.26666667	28.2923457	10.3158533	9.5059E-06	0.00331756	0.00296184	Sdf2l1/Hspa5/Dnajc3/Dnajb11	4
GO:0070006	metalloaminopeptidase activity	4/135	20/14323	0.2	21.2192593	8.82622102	3.2528E-05	0.00417356	0.00372605	Mmp14/Lta4h/Lap3/Trhde	4
GO:1902936	phosphatidylinositol bisphosphate binding	7/135	97/14323	0.07216495	7.65643375	6.41644104	3.5876E-05	0.00417356	0.00372605	Rlbp1/Snx21/Scin/Sh3pxd2a/Snx14/Snx18/Wdr45b	7
GO:0004177	aminopeptidase activity	4/135	33/14323	0.12121212	12.8601571	6.65333556	0.00024984	0.02179837	0.01946107	Mmp14/Lta4h/Lap3/Trhde	4
GO:0051087	protein-folding chaperone binding	7/135	138/14323	0.05072464	5.38169619	5.04516372	0.00032878	0.02294887	0.02048821	Sdf2l1/Stip1/Hspd1/St13/Dnajc3/Ubl4a/Bag5	7
GO:0044389	ubiquitin-like protein ligase binding	10/135	313/14323	0.03194888	3.38965803	4.16962488	0.00076175	0.03968681	0.03543144	Hspa5/Calr/Fzd8/Hspd1/Smad2/Bag5/Itch/Srprb/Arih1/Cul2	10
GO:0051082	unfolded protein binding	5/135	80/14323	0.0625	6.63101852	4.92650969	0.00094267	0.03968681	0.03543144	Hspa5/Calr/Hsp90b1/St13/Dnajb11	5
GO:0016780	phosphotransferase activity, for other substituted phosphate groups	3/135	21/14323	0.14285714	15.1566138	6.33255212	0.00096181	0.03968681	0.03543144	Fktn/Pigg/Selenoi	3
GO:0008235	metalloexopeptidase activity	4/135	48/14323	0.08333333	8.84135802	5.30801294	0.00106516	0.03968681	0.03543144	Mmp14/Lta4h/Lap3/Trhde	4
GO:1901981	phosphatidylinositol phosphate binding	7/135	170/14323	0.04117647	4.36867102	4.30989746	0.00113716	0.03968681	0.03543144	Rlbp1/Snx21/Scin/Sh3pxd2a/Snx14/Snx18/Wdr45b	7
GO:0031625	ubiquitin protein ligase binding	9/135	297/14323	0.03030303	3.21503928	3.76271105	0.00200806	0.06234736	0.05566225	Hspa5/Calr/Fzd8/Hspd1/Smad2/Bag5/Srprb/Arih1/Cul2	9
GO:0044183	protein folding chaperone	4/135	59/14323	0.06779661	7.19296924	4.64956574	0.00230044	0.06234736	0.05566225	Hspa5/Hsp90b1/Hspd1/Ric8b	4
GO:0031072	heat shock protein binding	6/135	144/14323	0.04166667	4.42067901	4.02419389	0.00241737	0.06234736	0.05566225	Bmal1/Hspa5/Chordc1/Stip1/St13/Lman2	6
GO:0140662	ATP-dependent protein folding chaperone	3/135	29/14323	0.10344828	10.9754789	5.24522346	0.00250104	0.06234736	0.05566225	Hspa5/Hsp90b1/Hspd1	3
GO:0016853	isomerase activity	6/135	157/14323	0.03821656	4.05463553	3.75399539	0.00371031	0.08632648	0.07707024	Pdia3/Hspd1/Fkbp2/Degs1/Enoph1/P4hb	6
GO:0016671	oxidoreductase activity, acting on a sulfur group of donors, disulfide as acceptor	2/135	11/14323	0.18181818	19.2902357	5.91934441	0.00458732	0.09178291	0.08194161	Pdia3/Msrb2	2
GO:0045294	alpha-catenin binding	2/135	11/14323	0.18181818	19.2902357	5.91934441	0.00458732	0.09178291	0.08194161	Pkp3/Pkp2	2
GO:0005546	phosphatidylinositol-4,5-bisphosphate binding	4/135	72/14323	0.05555556	5.89423868	4.06103612	0.00473379	0.09178291	0.08194161	Snx21/Scin/Sh3pxd2a/Snx18	4
GO:0008238	exopeptidase activity	4/135	78/14323	0.05128205	5.44083571	3.83609108	0.00628571	0.1121321	0.10010889	Mmp14/Lta4h/Lap3/Trhde	4
GO:0008174	mRNA methyltransferase activity	2/135	13/14323	0.15384615	16.3225071	5.39125433	0.00642591	0.1121321	0.10010889	Mettl16/Mettl14	2
GO:0016860	intramolecular oxidoreductase activity	3/135	42/14323	0.07142857	7.57830688	4.16454533	0.00718881	0.11819759	0.10552401	Pdia3/Enoph1/P4hb	3
GO:0001846	opsonin binding	2/135	14/14323	0.14285714	15.1566138	5.16924228	0.00745085	0.11819759	0.10552401	Calr/Cr1l	2
GO:0032266	phosphatidylinositol-3-phosphate binding	3/135	44/14323	0.06818182	7.23383838	4.03962554	0.00818106	0.11927948	0.1064899	Snx21/Sh3pxd2a/Wdr45b	3
GO:0003756	protein disulfide isomerase activity	2/135	15/14323	0.13333333	14.1461728	4.9689386	0.00854438	0.11927948	0.1064899	Pdia3/P4hb	2
GO:0016864	intramolecular oxidoreductase activity, transposing S-S bonds	2/135	15/14323	0.13333333	14.1461728	4.9689386	0.00854438	0.11927948	0.1064899	Pdia3/P4hb	2
GO:0051879	Hsp90 protein binding	3/135	46/14323	0.06521739	6.91932367	3.9222986	0.00925003	0.12416392	0.11085061	Bmal1/Chordc1/Stip1	3
GO:0016667	oxidoreductase activity, acting on a sulfur group of donors	3/135	50/14323	0.06	6.36577778	3.7073892	0.01162267	0.15023371	0.13412511	Pdia3/P4hb/Msrb2	3
GO:0001848	complement binding	2/135	18/14323	0.11111111	11.7884774	4.46745931	0.0122229	0.15234968	0.13601419	Calr/Cr1l	2
GO:0035091	phosphatidylinositol binding	7/135	265/14323	0.02641509	2.80254368	2.88905023	0.01279334	0.15396127	0.13745298	Rlbp1/Snx21/Scin/Sh3pxd2a/Snx14/Snx18/Wdr45b	7
GO:0015459	potassium channel regulator activity	3/135	54/14323	0.05555556	5.89423868	3.51474147	0.01431433	0.16352454	0.14599085	Slc5a3/Kcnip2/Arpp19	3
GO:0008649	rRNA methyltransferase activity	2/135	20/14323	0.1	10.6096296	4.19484779	0.01499365	0.16352454	0.14599085	Mettl16/Tfb1m	2
GO:0016881	acid-amino acid ligase activity	2/135	20/14323	0.1	10.6096296	4.19484779	0.01499365	0.16352454	0.14599085	Gclm/Ppcs	2
GO:0015271	outward rectifier potassium channel activity	2/135	21/14323	0.0952381	10.1044092	4.07259438	0.01647069	0.1690668	0.15093884	Kcnk13/Kcnip2	2
GO:0140102	catalytic activity, acting on a rRNA	2/135	21/14323	0.0952381	10.1044092	4.07259438	0.01647069	0.1690668	0.15093884	Mettl16/Tfb1m	2
GO:0045296	cadherin binding	3/135	59/14323	0.05084746	5.39472693	3.29947897	0.01813566	0.18083844	0.16144828	Pkp3/Pkp2/Cdh10	3
GO:0008106	alcohol dehydrogenase (NADP+) activity	2/135	23/14323	0.08695652	9.22576469	3.85106032	0.01960182	0.18683449	0.16680142	Dhrs3/Akr1a1	2
GO:0008173	RNA methyltransferase activity	3/135	61/14323	0.04918033	5.21785064	3.22013461	0.01980767	0.18683449	0.16680142	Mettl16/Tfb1m/Mettl14	3
GO:0004033	aldo-keto reductase (NADPH) activity	2/135	25/14323	0.08	8.4877037	3.65501478	0.02296088	0.21087753	0.18826647	Dhrs3/Akr1a1	2
GO:0080025	phosphatidylinositol-3,5-bisphosphate binding	2/135	28/14323	0.07142857	7.57830688	3.39867154	0.02840608	0.25419804	0.226942	Snx14/Wdr45b	2
GO:0015035	protein-disulfide reductase activity	2/135	30/14323	0.06666667	7.07308642	3.24800374	0.03229391	0.28176436	0.25155255	Pdia3/P4hb	2
GO:0050839	cell adhesion molecule binding	6/135	257/14323	0.0233463	2.47695633	2.33054257	0.03486316	0.29525005	0.26359226	Calr/Mmp14/Pkp3/Pkp2/Cdh10/P4hb	6
GO:0005525	GTP binding	7/135	329/14323	0.0212766	2.25736801	2.25059603	0.0362528	0.29525005	0.26359226	Rhoj/Rasl10a/Rab38/Rrad/Rasl11a/Rab9b/Srprb	7
GO:0018455	alcohol dehydrogenase [NAD(P)+] activity	2/135	32/14323	0.0625	6.63101852	3.1105613	0.03637751	0.29525005	0.26359226	Dhrs3/Akr1a1	2
GO:0005249	voltage-gated potassium channel activity	3/135	78/14323	0.03846154	4.08062678	2.66111284	0.03733869	0.29616371	0.26440796	Kcnn1/Kcnk13/Kcnip2	3
GO:0001965	G-protein alpha-subunit binding	2/135	33/14323	0.06060606	6.43007856	3.04617674	0.03849004	0.29851161	0.26650411	Sash1/Ric8b	2
GO:0008237	metallopeptidase activity	4/135	136/14323	0.02941176	3.1204793	2.4236301	0.03972084	0.30136025	0.2690473	Mmp14/Lta4h/Lap3/Trhde	4
GO:1901618	organic hydroxy compound transmembrane transporter activity	2/135	34/14323	0.05882353	6.24095861	2.98440253	0.0406483	0.30183524	0.26947137	Slc5a3/Slc51a	2
GO:0099106	ion channel regulator activity	4/135	138/14323	0.02898551	3.07525497	2.38948585	0.04154567	0.30207167	0.26968244	Slc5a3/Rrad/Kcnip2/Arpp19	4
GO:0016859	cis-trans isomerase activity	2/135	35/14323	0.05714286	6.0626455	2.92505441	0.04285127	0.30520596	0.27248066	Fkbp2/Degs1	2
GO:0019001	guanyl nucleotide binding	7/135	345/14323	0.02028986	2.15267848	2.11399201	0.04490544	0.30729406	0.27434487	Rhoj/Rasl10a/Rab38/Rrad/Rasl11a/Rab9b/Srprb	7
GO:0032561	guanyl ribonucleotide binding	7/135	345/14323	0.02028986	2.15267848	2.11399201	0.04490544	0.30729406	0.27434487	Rhoj/Rasl10a/Rab38/Rrad/Rasl11a/Rab9b/Srprb	7
GO:0003924	GTPase activity	6/135	275/14323	0.02181818	2.31482828	2.14751529	0.04592945	0.3082573	0.27520483	Rhoj/Rasl10a/Rab38/Rrad/Rasl11a/Rab9b	6
GO:0015036	disulfide oxidoreductase activity	2/135	37/14323	0.05405405	5.73493493	2.81298673	0.04738725	0.31204055	0.27858242	Pdia3/P4hb	2
GO:0016247	channel regulator activity	4/135	148/14323	0.02702703	2.86746747	2.22756558	0.0513734	0.33202436	0.2964235	Slc5a3/Rrad/Kcnip2/Arpp19	4
GO:0016874	ligase activity	4/135	149/14323	0.02684564	2.84822272	2.21212342	0.05242047	0.33263173	0.29696574	Gclm/Ppcs/Itch/Yars2	4
GO:0141108	transporter regulator activity	4/135	150/14323	0.02666667	2.82923457	2.19680883	0.05347919	0.33328994	0.29755338	Slc5a3/Rrad/Kcnip2/Arpp19	4
GO:0004864	protein phosphatase inhibitor activity	2/135	41/14323	0.04878049	5.17542909	2.61159997	0.05695174	0.34870454	0.31131517	Hsp90b1/Arpp19	2
GO:0017069	snRNA binding	2/135	43/14323	0.04651163	4.93471146	2.52052577	0.06196499	0.37285829	0.33287906	Mettl16/Prpf31	2
GO:0016879	ligase activity, forming carbon-nitrogen bonds	2/135	45/14323	0.04444444	4.71539095	2.43492291	0.06712242	0.39704616	0.35447343	Gclm/Ppcs	2
GO:0019212	phosphatase inhibitor activity	2/135	46/14323	0.04347826	4.61288245	2.39399035	0.06975298	0.40139727	0.35835799	Hsp90b1/Arpp19	2
GO:0005267	potassium channel activity	3/135	101/14323	0.02970297	3.15137514	2.11643349	0.07015826	0.40139727	0.35835799	Kcnn1/Kcnk13/Kcnip2	3
GO:0015370	solute:sodium symporter activity	2/135	49/14323	0.04081633	4.33046107	2.27791589	0.07784171	0.41670987	0.37202872	Slc5a3/Slc17a8	2
GO:0019787	ubiquitin-like protein transferase activity	7/135	401/14323	0.01745636	1.85205505	1.68809605	0.08510044	0.41670987	0.37202872	Dtx4/Obi1/Pja1/Itch/Ube3c/Aktip/Arih1	7
GO:0046982	protein heterodimerization activity	5/135	249/14323	0.02008032	2.13044772	1.75529255	0.08662182	0.41670987	0.37202872	Bmal1/Slc51a/Hexa/Sri/P4hb	5
GO:0008035	high-density lipoprotein particle binding	1/135	10/14323	0.1	10.6096296	2.96516895	0.09038233	0.41670987	0.37202872	Hspd1	1
GO:0019215	intermediate filament binding	1/135	10/14323	0.1	10.6096296	2.96516895	0.09038233	0.41670987	0.37202872	Pkp2	1
GO:0019841	retinol binding	1/135	10/14323	0.1	10.6096296	2.96516895	0.09038233	0.41670987	0.37202872	Rlbp1	1
GO:1990763	arrestin family protein binding	1/135	10/14323	0.1	10.6096296	2.96516895	0.09038233	0.41670987	0.37202872	Itch	1
GO:0003925	G protein activity	2/135	54/14323	0.03703704	3.92949246	2.10378146	0.09193013	0.41670987	0.37202872	Rasl10a/Rasl11a	2
GO:0016755	aminoacyltransferase activity	7/135	411/14323	0.01703163	1.80699288	1.61921291	0.09393281	0.41670987	0.37202872	Dtx4/Obi1/Pja1/Itch/Ube3c/Aktip/Arih1	7
GO:0022843	voltage-gated monoatomic cation channel activity	3/135	117/14323	0.02564103	2.72041785	1.82263244	0.09852989	0.41670987	0.37202872	Kcnn1/Kcnk13/Kcnip2	3
GO:0004143	ATP-dependent diacylglycerol kinase activity	1/135	11/14323	0.09090909	9.64511785	2.79785514	0.09896183	0.41670987	0.37202872	Dgkk	1
GO:0005049	nuclear export signal receptor activity	1/135	11/14323	0.09090909	9.64511785	2.79785514	0.09896183	0.41670987	0.37202872	Calr	1
GO:0008253	5'-nucleotidase activity	1/135	11/14323	0.09090909	9.64511785	2.79785514	0.09896183	0.41670987	0.37202872	Nt5c3b	1
GO:0015269	calcium-activated potassium channel activity	1/135	11/14323	0.09090909	9.64511785	2.79785514	0.09896183	0.41670987	0.37202872	Kcnn1	1
GO:0016670	oxidoreductase activity, acting on a sulfur group of donors, oxygen as acceptor	1/135	11/14323	0.09090909	9.64511785	2.79785514	0.09896183	0.41670987	0.37202872	P4hb	1
GO:0016717	oxidoreductase activity, acting on paired donors, with oxidation of a pair of donors resulting in the reduct	1/135	11/14323	0.09090909	9.64511785	2.79785514	0.09896183	0.41670987	0.37202872	Degs1	1
GO:0017070	U6 snRNA binding	1/135	11/14323	0.09090909	9.64511785	2.79785514	0.09896183	0.41670987	0.37202872	Mettl16	1
GO:0052650	all-trans-retinol dehydrogenase (NADP+) activity	1/135	11/14323	0.09090909	9.64511785	2.79785514	0.09896183	0.41670987	0.37202872	Dhrs3	1
GO:1990247	N6-methyladenosine-containing RNA reader activity	1/135	11/14323	0.09090909	9.64511785	2.79785514	0.09896183	0.41670987	0.37202872	Hnrnpc	1
GO:0030544	Hsp70 protein binding	2/135	57/14323	0.03508772	3.72267706	2.00904923	0.10071273	0.41670987	0.37202872	Stip1/St13	2
GO:0043021	ribonucleoprotein complex binding	4/135	190/14323	0.02105263	2.23360624	1.66972253	0.10502004	0.41670987	0.37202872	Hspa5/Prpf31/Hnrnpc/Itch	4
GO:0004022	alcohol dehydrogenase (NAD+) activity	1/135	12/14323	0.08333333	8.84135802	2.65066623	0.107461	0.41670987	0.37202872	Dhrs3	1
GO:0005326	neurotransmitter transmembrane transporter activity	1/135	12/14323	0.08333333	8.84135802	2.65066623	0.107461	0.41670987	0.37202872	Slc17a8	1
GO:0015125	bile acid transmembrane transporter activity	1/135	12/14323	0.08333333	8.84135802	2.65066623	0.107461	0.41670987	0.37202872	Slc51a	1
GO:0016918	retinal binding	1/135	12/14323	0.08333333	8.84135802	2.65066623	0.107461	0.41670987	0.37202872	Rlbp1	1
GO:0022841	potassium ion leak channel activity	1/135	12/14323	0.08333333	8.84135802	2.65066623	0.107461	0.41670987	0.37202872	Kcnk13	1
GO:0031543	peptidyl-proline dioxygenase activity	1/135	12/14323	0.08333333	8.84135802	2.65066623	0.107461	0.41670987	0.37202872	P4hb	1
GO:0034713	type I transforming growth factor beta receptor binding	1/135	12/14323	0.08333333	8.84135802	2.65066623	0.107461	0.41670987	0.37202872	Smad2	1
GO:0034987	immunoglobulin receptor binding	1/135	12/14323	0.08333333	8.84135802	2.65066623	0.107461	0.41670987	0.37202872	Ighg2c	1
GO:0005543	phospholipid binding	7/135	428/14323	0.01635514	1.7352198	1.50632239	0.11008206	0.42218283	0.37691485	Rlbp1/Snx21/Scin/Sh3pxd2a/Snx14/Snx18/Wdr45b	7
GO:0008252	nucleotidase activity	1/135	13/14323	0.07692308	8.16125356	2.51970118	0.11588059	0.42419182	0.37870843	Nt5c3b	1
GO:0050700	CARD domain binding	1/135	13/14323	0.07692308	8.16125356	2.51970118	0.11588059	0.42419182	0.37870843	Card11	1
GO:0000774	adenyl-nucleotide exchange factor activity	1/135	14/14323	0.07142857	7.57830688	2.40204774	0.12422134	0.42419182	0.37870843	Bag5	1
GO:0005313	L-glutamate transmembrane transporter activity	1/135	14/14323	0.07142857	7.57830688	2.40204774	0.12422134	0.42419182	0.37870843	Slc17a8	1
GO:0015149	hexose transmembrane transporter activity	1/135	14/14323	0.07142857	7.57830688	2.40204774	0.12422134	0.42419182	0.37870843	Slc5a3	1
GO:0015929	hexosaminidase activity	1/135	14/14323	0.07142857	7.57830688	2.40204774	0.12422134	0.42419182	0.37870843	Hexa	1
GO:0034450	ubiquitin-ubiquitin ligase activity	1/135	14/14323	0.07142857	7.57830688	2.40204774	0.12422134	0.42419182	0.37870843	Itch	1
GO:0055056	D-glucose transmembrane transporter activity	1/135	14/14323	0.07142857	7.57830688	2.40204774	0.12422134	0.42419182	0.37870843	Slc5a3	1
GO:0070990	snRNP binding	1/135	14/14323	0.07142857	7.57830688	2.40204774	0.12422134	0.42419182	0.37870843	Prpf31	1
GO:0008080	N-acetyltransferase activity	2/135	65/14323	0.03076923	3.26450142	1.78487621	0.12516913	0.42419182	0.37870843	Sat1/Nat9	2
GO:0015079	potassium ion transmembrane transporter activity	3/135	131/14323	0.02290076	2.42968617	1.60347685	0.12652937	0.42419182	0.37870843	Kcnn1/Kcnk13/Kcnip2	3
GO:0005178	integrin binding	3/135	133/14323	0.02255639	2.39314954	1.57449211	0.13074266	0.42419182	0.37870843	Calr/Mmp14/P4hb	3
GO:0004857	enzyme inhibitor activity	5/135	285/14323	0.01754386	1.86133853	1.43268863	0.13215221	0.42419182	0.37870843	Hsp90b1/Dnajc3/Hnrnpc/Smr2l/Arpp19	5
GO:0015145	monosaccharide transmembrane transporter activity	1/135	15/14323	0.06666667	7.07308642	2.29548127	0.13248398	0.42419182	0.37870843	Slc5a3	1
GO:0022840	leak channel activity	1/135	15/14323	0.06666667	7.07308642	2.29548127	0.13248398	0.42419182	0.37870843	Kcnk13	1
GO:0022842	narrow pore channel activity	1/135	15/14323	0.06666667	7.07308642	2.29548127	0.13248398	0.42419182	0.37870843	Kcnk13	1
GO:0042813	Wnt receptor activity	1/135	15/14323	0.06666667	7.07308642	2.29548127	0.13248398	0.42419182	0.37870843	Fzd8	1
GO:1990226	histone methyltransferase binding	1/135	15/14323	0.06666667	7.07308642	2.29548127	0.13248398	0.42419182	0.37870843	Cbx1	1
GO:0000287	magnesium ion binding	4/135	212/14323	0.01886792	2.00181691	1.43345981	0.14032316	0.42690055	0.38112672	Srpk1/Enoph1/Nt5c3b/Oxsr1	4
GO:0005315	phosphate transmembrane transporter activity	1/135	16/14323	0.0625	6.63101852	2.19826876	0.14066924	0.42690055	0.38112672	Slc17a8	1
GO:0035613	RNA stem-loop binding	1/135	16/14323	0.0625	6.63101852	2.19826876	0.14066924	0.42690055	0.38112672	Mettl16	1
GO:0043176	amine binding	1/135	16/14323	0.0625	6.63101852	2.19826876	0.14066924	0.42690055	0.38112672	Sat1	1
GO:0051119	sugar transmembrane transporter activity	1/135	16/14323	0.0625	6.63101852	2.19826876	0.14066924	0.42690055	0.38112672	Slc5a3	1
GO:0070411	I-SMAD binding	1/135	16/14323	0.0625	6.63101852	2.19826876	0.14066924	0.42690055	0.38112672	Smad2	1
GO:0005244	voltage-gated monoatomic ion channel activity	3/135	139/14323	0.02158273	2.28984812	1.49057812	0.14367224	0.43142196	0.38516333	Kcnn1/Kcnk13/Kcnip2	3
GO:0004842	ubiquitin-protein transferase activity	6/135	376/14323	0.01595745	1.693026	1.32834591	0.14467474	0.43142196	0.38516333	Dtx4/Obi1/Pja1/Itch/Ube3c/Arih1	6
GO:0022832	voltage-gated channel activity	3/135	140/14323	0.02142857	2.27349206	1.47701307	0.1458676	0.43142196	0.38516333	Kcnn1/Kcnk13/Kcnip2	3
GO:0061630	ubiquitin protein ligase activity	5/135	297/14323	0.01683502	1.78613293	1.33541247	0.149345	0.43799499	0.39103157	Dtx4/Pja1/Itch/Ube3c/Arih1	5
GO:0034185	apolipoprotein binding	1/135	18/14323	0.05555556	5.89423868	2.02668193	0.15681047	0.44233836	0.39490923	Hspd1	1
GO:0042625	ATPase-coupled ion transmembrane transporter activity	1/135	18/14323	0.05555556	5.89423868	2.02668193	0.15681047	0.44233836	0.39490923	Atp6v0b	1
GO:0044769	ATPase activity, coupled to transmembrane movement of ions, rotational mechanism	1/135	18/14323	0.05555556	5.89423868	2.02668193	0.15681047	0.44233836	0.39490923	Atp6v0b	1
GO:0046961	proton-transporting ATPase activity, rotational mechanism	1/135	18/14323	0.05555556	5.89423868	2.02668193	0.15681047	0.44233836	0.39490923	Atp6v0b	1
GO:0015294	solute:monoatomic cation symporter activity	2/135	77/14323	0.02597403	2.75574796	1.50684712	0.164074	0.44233836	0.39490923	Slc5a3/Slc17a8	2
GO:0005242	inward rectifier potassium channel activity	1/135	19/14323	0.05263158	5.58401559	1.95030379	0.16476787	0.44233836	0.39490923	Kcnn1	1
GO:0005537	D-mannose binding	1/135	19/14323	0.05263158	5.58401559	1.95030379	0.16476787	0.44233836	0.39490923	Lman2	1
GO:0010314	phosphatidylinositol-5-phosphate binding	1/135	19/14323	0.05263158	5.58401559	1.95030379	0.16476787	0.44233836	0.39490923	Sh3pxd2a	1
GO:0015172	acidic amino acid transmembrane transporter activity	1/135	19/14323	0.05263158	5.58401559	1.95030379	0.16476787	0.44233836	0.39490923	Slc17a8	1
GO:0032794	GTPase activating protein binding	1/135	19/14323	0.05263158	5.58401559	1.95030379	0.16476787	0.44233836	0.39490923	Gapvd1	1
GO:0050699	WW domain binding	1/135	19/14323	0.05263158	5.58401559	1.95030379	0.16476787	0.44233836	0.39490923	Ndfip2	1
GO:0008757	S-adenosylmethionine-dependent methyltransferase activity	3/135	149/14323	0.02013423	2.13616704	1.35986951	0.16610406	0.4425215	0.39507273	Mettl16/Tfb1m/Mettl14	3
GO:0005227	calcium-activated cation channel activity	1/135	20/14323	0.05	5.30481481	1.87916118	0.17265073	0.44965383	0.40144031	Kcnn1	1
GO:0140517	protein-RNA adaptor activity	1/135	20/14323	0.05	5.30481481	1.87916118	0.17265073	0.44965383	0.40144031	Hnrnpc	1
GO:1904047	S-adenosyl-L-methionine binding	1/135	20/14323	0.05	5.30481481	1.87916118	0.17265073	0.44965383	0.40144031	Tfb1m	1
GO:0016410	N-acyltransferase activity	2/135	80/14323	0.025	2.65240741	1.44567116	0.17411422	0.44965383	0.40144031	Sat1/Nat9	2
GO:0061659	ubiquitin-like protein ligase activity	5/135	314/14323	0.01592357	1.68943147	1.20492631	0.17522327	0.44965383	0.40144031	Dtx4/Pja1/Itch/Ube3c/Arih1	5
GO:0038191	neuropilin binding	1/135	21/14323	0.04761905	5.05220459	1.81263664	0.18045973	0.45638004	0.4074453	Sema3e	1
GO:0070034	telomerase RNA binding	1/135	21/14323	0.04761905	5.05220459	1.81263664	0.18045973	0.45638004	0.4074453	Hnrnpc	1
GO:0005501	retinoid binding	1/135	22/14323	0.04545455	4.82255892	1.75021118	0.18819556	0.4610663	0.41162909	Rlbp1	1
GO:0008266	poly(U) RNA binding	1/135	22/14323	0.04545455	4.82255892	1.75021118	0.18819556	0.4610663	0.41162909	Hnrnpc	1
GO:0015144	carbohydrate transmembrane transporter activity	1/135	22/14323	0.04545455	4.82255892	1.75021118	0.18819556	0.4610663	0.41162909	Slc5a3	1
GO:0030215	semaphorin receptor binding	1/135	22/14323	0.04545455	4.82255892	1.75021118	0.18819556	0.4610663	0.41162909	Sema3e	1
GO:0000979	RNA polymerase II core promoter sequence-specific DNA binding	1/135	23/14323	0.04347826	4.61288245	1.69144492	0.19585892	0.4610663	0.41162909	Tbpl1	1
GO:0019840	isoprenoid binding	1/135	23/14323	0.04347826	4.61288245	1.69144492	0.19585892	0.4610663	0.41162909	Rlbp1	1
GO:0048156	tau protein binding	1/135	23/14323	0.04347826	4.61288245	1.69144492	0.19585892	0.4610663	0.41162909	Smad2	1
GO:0044325	transmembrane transporter binding	3/135	162/14323	0.01851852	1.96474623	1.20456923	0.19666604	0.4610663	0.41162909	Slc5a3/Kcnip2/Ppp2cb	3
GO:0008233	peptidase activity	6/135	416/14323	0.01442308	1.53023504	1.07054886	0.19936868	0.4610663	0.41162909	Mmp14/Pgpep1/Pdia3/Lta4h/Lap3/Trhde	6
GO:0016407	acetyltransferase activity	2/135	88/14323	0.02272727	2.41127946	1.29533992	0.20132605	0.4610663	0.41162909	Sat1/Nat9	2
GO:0019888	protein phosphatase regulator activity	2/135	88/14323	0.02272727	2.41127946	1.29533992	0.20132605	0.4610663	0.41162909	Hsp90b1/Arpp19	2
GO:0022839	monoatomic ion-gated channel activity	1/135	24/14323	0.04166667	4.42067901	1.63596211	0.20345046	0.4610663	0.41162909	Kcnn1	1
GO:0030742	GTP-dependent protein binding	1/135	24/14323	0.04166667	4.42067901	1.63596211	0.20345046	0.4610663	0.41162909	Rab38	1
GO:0042288	MHC class I protein binding	1/135	24/14323	0.04166667	4.42067901	1.63596211	0.20345046	0.4610663	0.41162909	Pdia3	1
GO:0050750	low-density lipoprotein particle receptor binding	1/135	24/14323	0.04166667	4.42067901	1.63596211	0.20345046	0.4610663	0.41162909	Hsp90b1	1
GO:0070412	R-SMAD binding	1/135	24/14323	0.04166667	4.42067901	1.63596211	0.20345046	0.4610663	0.41162909	Smad2	1
GO:0008170	N-methyltransferase activity	2/135	90/14323	0.02222222	2.35769547	1.26032784	0.20820817	0.46880421	0.41853731	Mettl16/Tfb1m	2
GO:0005160	transforming growth factor beta receptor binding	1/135	25/14323	0.04	4.24385185	1.58343959	0.21097086	0.47188325	0.42128621	Smad2	1
GO:0008187	poly-pyrimidine tract binding	1/135	26/14323	0.03846154	4.08062678	1.53359764	0.21842078	0.47188325	0.42128621	Hnrnpc	1
GO:0031435	mitogen-activated protein kinase kinase kinase binding	1/135	26/14323	0.03846154	4.08062678	1.53359764	0.21842078	0.47188325	0.42128621	Sash1	1
GO:0070325	lipoprotein particle receptor binding	1/135	26/14323	0.03846154	4.08062678	1.53359764	0.21842078	0.47188325	0.42128621	Hsp90b1	1
GO:0071813	lipoprotein particle binding	1/135	26/14323	0.03846154	4.08062678	1.53359764	0.21842078	0.47188325	0.42128621	Hspd1	1
GO:0071814	protein-lipid complex binding	1/135	26/14323	0.03846154	4.08062678	1.53359764	0.21842078	0.47188325	0.42128621	Hspd1	1
GO:0016616	oxidoreductase activity, acting on the CH-OH group of donors, NAD or NADP as acceptor	2/135	95/14323	0.02105263	2.23360624	1.17672386	0.22551399	0.47188325	0.42128621	Dhrs3/Akr1a1	2
GO:0015662	P-type ion transporter activity	1/135	27/14323	0.03703704	3.92949246	1.48619271	0.22580087	0.47188325	0.42128621	Atp6v0b	1
GO:0043325	phosphatidylinositol-3,4-bisphosphate binding	1/135	27/14323	0.03703704	3.92949246	1.48619271	0.22580087	0.47188325	0.42128621	Sh3pxd2a	1
GO:0045499	chemorepellent activity	1/135	27/14323	0.03703704	3.92949246	1.48619271	0.22580087	0.47188325	0.42128621	Sema3e	1

GO:0051861	glycolipid binding	1/135	27/14323	0.03703704	3.92949246	1.48619271	0.22580087	0.47188325	0.42128621	Manf	1
GO:0140358	P-type transmembrane transporter activity	1/135	27/14323	0.03703704	3.92949246	1.48619271	0.22580087	0.47188325	0.42128621	Atp6v0b	1
GO:0046873	metal ion transmembrane transporter activity	5/135	346/14323	0.01445087	1.53318347	0.97930027	0.22805267	0.47288288	0.42217866	Slc5a3/Kcnn1/Kcnk13/Slc17a8/Kcnip2	5
GO:0019208	phosphatase regulator activity	2/135	96/14323	0.02083333	2.21033951	1.16063133	0.22898913	0.47288288	0.42217866	Hsp90b1/Arpp19	2
GO:0017147	Wnt-protein binding	1/135	28/14323	0.03571429	3.78915344	1.44101158	0.23311179	0.47576616	0.42475278	Fzd8	1
GO:0070273	phosphatidylinositol-4-phosphate binding	1/135	28/14323	0.03571429	3.78915344	1.44101158	0.23311179	0.47576616	0.42475278	Sh3pxd2a	1
GO:0008168	methyltransferase activity	3/135	179/14323	0.01675978	1.77815022	1.02190839	0.23845292	0.48208969	0.43039828	Mettl16/Tfb1m/Mettl14	3
GO:0001530	lipopolysaccharide binding	1/135	29/14323	0.03448276	3.65849298	1.39786669	0.24035417	0.48208969	0.43039828	Hspd1	1
GO:0043425	bHLH transcription factor binding	1/135	29/14323	0.03448276	3.65849298	1.39786669	0.24035417	0.48208969	0.43039828	Bmal1	1
GO:0022890	inorganic cation transmembrane transporter activity	6/135	446/14323	0.01345291	1.42730443	0.8942628	0.24472706	0.48805568	0.43572457	Slc5a3/Kcnn1/Kcnk13/Slc17a8/Kcnip2/Atp6v0b	6
GO:0140142	nucleocytoplasmic carrier activity	1/135	30/14323	0.03333333	3.53654321	1.35659223	0.24752866	0.49083809	0.43820864	Calr	1
GO:0016614	oxidoreductase activity, acting on CH-OH group of donors	2/135	102/14323	0.01960784	2.08031954	1.06805933	0.24990585	0.49275221	0.43991752	Dhrs3/Akr1a1	2
GO:0042887	amide transmembrane transporter activity	1/135	31/14323	0.03225806	3.42246117	1.31704101	0.25463589	0.49646887	0.44323566	Slc33a1	1
GO:0050681	nuclear androgen receptor binding	1/135	31/14323	0.03225806	3.42246117	1.31704101	0.25463589	0.49646887	0.44323566	Calr	1
GO:0016741	transferase activity, transferring one-carbon groups	3/135	188/14323	0.01595745	1.693026	0.93301511	0.26116944	0.49781926	0.44444127	Mettl16/Tfb1m/Mettl14	3
GO:0004180	carboxypeptidase activity	1/135	32/14323	0.03125	3.31550926	1.27908183	0.26167649	0.49781926	0.44444127	Lap3	1
GO:0042287	MHC protein binding	1/135	32/14323	0.03125	3.31550926	1.27908183	0.26167649	0.49781926	0.44444127	Pdia3	1
GO:0097718	disordered domain specific binding	1/135	32/14323	0.03125	3.31550926	1.27908183	0.26167649	0.49781926	0.44444127	Smad2	1
GO:0016747	acyltransferase activity, transferring groups other than amino-acyl groups	3/135	189/14323	0.01587302	1.6840682	0.92343405	0.2637121	0.49781926	0.44444127	Sat1/Nat9/Elovl7	3
GO:0015293	symporter activity	2/135	106/14323	0.01886792	2.00181691	1.00982342	0.263887	0.49781926	0.44444127	Slc5a3/Slc17a8	2
GO:0003755	peptidyl-prolyl cis-trans isomerase activity	1/135	33/14323	0.03030303	3.21503928	1.24259733	0.26865106	0.50138621	0.44762575	Fkbp2	1
GO:0005484	SNAP receptor activity	1/135	33/14323	0.03030303	3.21503928	1.24259733	0.26865106	0.50138621	0.44762575	Sec22b	1
GO:0030165	PDZ domain binding	2/135	109/14323	0.01834862	1.94672103	0.96779918	0.27437696	0.50543186	0.45123761	Arhgef16/Fzd8	2
GO:0004715	non-membrane spanning protein tyrosine kinase activity	1/135	34/14323	0.02941176	3.1204793	1.20748212	0.27556024	0.50543186	0.45123761	Stk16	1
GO:0022853	active monoatomic ion transmembrane transporter activity	3/135	194/14323	0.01546392	1.64066438	0.87636264	0.27647054	0.50543186	0.45123761	Slc5a3/Slc17a8/Atp6v0b	3
GO:0048018	receptor ligand activity	4/135	284/14323	0.01408451	1.49431403	0.82073348	0.27971656	0.50543186	0.45123761	Manf/Dand5/Tafa1/Sema3e	4
GO:0016251	RNA polymerase II general transcription initiation factor activity	1/135	35/14323	0.02857143	3.03132275	1.17364124	0.28240462	0.50543186	0.45123761	Tbpl1	1
GO:0031624	ubiquitin conjugating enzyme binding	1/135	35/14323	0.02857143	3.03132275	1.17364124	0.28240462	0.50543186	0.45123761	Arih1	1
GO:0042379	chemokine receptor binding	1/135	35/14323	0.02857143	3.03132275	1.17364124	0.28240462	0.50543186	0.45123761	Itch	1
GO:0051059	NF-kappaB binding	1/135	35/14323	0.02857143	3.03132275	1.17364124	0.28240462	0.50543186	0.45123761	Brms1	1
GO:0005085	guanyl-nucleotide exchange factor activity	3/135	197/14323	0.01522843	1.61567964	0.84876299	0.2841567	0.5059729	0.45172064	Arhgef16/Gapvd1/Ric8b	3
GO:0008375	acetylglucosaminyltransferase activity	1/135	36/14323	0.02777778	2.94711934	1.14098892	0.28918481	0.51159162	0.4567369	Hexa	1
GO:0030546	signaling receptor activator activity	4/135	289/14323	0.01384083	1.46846085	0.78476498	0.29024395	0.51159162	0.4567369	Manf/Dand5/Tafa1/Sema3e	4
GO:0008194	UDP-glycosyltransferase activity	2/135	114/14323	0.01754386	1.86133853	0.90064299	0.29184352	0.51182607	0.45694621	Has2/Hexa	2
GO:0008324	monoatomic cation transmembrane transporter activity	6/135	477/14323	0.01257862	1.33454461	0.72486844	0.29444107	0.51377907	0.4586898	Slc5a3/Kcnn1/Kcnk13/Slc17a8/Kcnip2/Atp6v0b	6
GO:0070888	E-box binding	1/135	37/14323	0.02702703	2.86746747	1.10944739	0.29590141	0.51377907	0.4586898	Bmal1	1
GO:0008514	organic anion transmembrane transporter activity	3/135	204/14323	0.01470588	1.56023965	0.7861313	0.30215818	0.51760636	0.46210672	Slc51a/Slc17a8/Slc33a1	3
GO:0001727	lipid kinase activity	1/135	38/14323	0.02631579	2.7920078	1.07894598	0.30255501	0.51760636	0.46210672	Dgkk	1
GO:0170055	lipid transmembrane transporter activity	1/135	38/14323	0.02631579	2.7920078	1.07894598	0.30255501	0.51760636	0.46210672	Slc51a	1
GO:0015081	sodium ion transmembrane transporter activity	2/135	118/14323	0.01694915	1.79824231	0.84930417	0.30578126	0.52057395	0.46475612	Slc5a3/Slc17a8	2
GO:0016620	oxidoreductase activity, acting on the aldehyde or oxo group of donors, NAD or NADP as acceptor	1/135	39/14323	0.02564103	2.72041785	1.04942028	0.30914619	0.5212175	0.46533066	Far2	1
GO:0043531	ADP binding	1/135	39/14323	0.02564103	2.72041785	1.04942028	0.30914619	0.5212175	0.46533066	Chordc1	1
GO:0051721	protein phosphatase 2A binding	1/135	40/14323	0.025	2.65240741	1.02081147	0.31567555	0.52966714	0.4728743	Arpp19	1
GO:0004812	aminoacyl-tRNA ligase activity	1/135	41/14323	0.02439024	2.58771454	0.99306566	0.32214364	0.53023236	0.47337891	Yars2	1
GO:0005246	calcium channel regulator activity	1/135	41/14323	0.02439024	2.58771454	0.99306566	0.32214364	0.53023236	0.47337891	Rrad	1
GO:0016875	ligase activity, forming carbon-oxygen bonds	1/135	41/14323	0.02439024	2.58771454	0.99306566	0.32214364	0.53023236	0.47337891	Yars2	1
GO:0044390	ubiquitin-like protein conjugating enzyme binding	1/135	41/14323	0.02439024	2.58771454	0.99306566	0.32214364	0.53023236	0.47337891	Arih1	1
GO:0140678	molecular function inhibitor activity	5/135	399/14323	0.01253133	1.32952752	0.65118481	0.32360886	0.53023236	0.47337891	Hsp90b1/Dnajc3/Hnrnpc/Smr2l/Arpp19	5
GO:0015605	organophosphate ester transmembrane transporter activity	1/135	42/14323	0.02380952	2.52610229	0.96613343	0.32855105	0.53581456	0.47836257	Slc33a1	1
GO:0044024	histone H2AS1 kinase activity	3/135	216/14323	0.01388889	1.47355967	0.68405714	0.33312985	0.53825147	0.48053819	Srpk1/Stk16/Oxsr1	3
GO:0140995	histone H2A kinase activity	3/135	216/14323	0.01388889	1.47355967	0.68405714	0.33312985	0.53825147	0.48053819	Srpk1/Stk16/Oxsr1	3
GO:0140223	general transcription initiation factor activity	1/135	43/14323	0.02325581	2.46735573	0.93996929	0.33489834	0.5386153	0.480863	Tbpl1	1
GO:0019902	phosphatase binding	3/135	218/14323	0.01376147	1.46004077	0.66764585	0.33829368	0.54158024	0.48351004	Hsp90b1/Smad2/Arpp19	3
GO:0140313	molecular sequestering activity	1/135	44/14323	0.02272727	2.41127946	0.91453134	0.34118606	0.5437166	0.48541733	Calr	1
GO:0002020	protease binding	2/135	130/14323	0.01538462	1.63225071	0.70636965	0.34723505	0.54863239	0.48980603	Sh3pxd2a/Sri	2
GO:0001046	core promoter sequence-specific DNA binding	1/135	45/14323	0.02222222	2.35769547	0.88978088	0.34741478	0.54863239	0.48980603	Tbpl1	1
GO:0016830	carbon-carbon lyase activity	1/135	46/14323	0.02173913	2.30644122	0.86568211	0.35358504	0.54949499	0.49057613	Hmgcll1	1
GO:0016903	oxidoreductase activity, acting on the aldehyde or oxo group of donors	1/135	46/14323	0.02173913	2.30644122	0.86568211	0.35358504	0.54949499	0.49057613	Far2	1
GO:0060590	ATPase regulator activity	1/135	46/14323	0.02173913	2.30644122	0.86568211	0.35358504	0.54949499	0.49057613	Bag5	1
GO:0030545	signaling receptor regulator activity	4/135	319/14323	0.01253918	1.33036108	0.58205805	0.35425895	0.54949499	0.49057613	Manf/Dand5/Tafa1/Sema3e	4
GO:0035173	histone kinase activity	3/135	226/14323	0.01327434	1.40835792	0.60358714	0.35892107	0.54999582	0.49102326	Srpk1/Stk16/Oxsr1	3
GO:0015932	nucleobase-containing compound transmembrane transporter activity	1/135	47/14323	0.0212766	2.25736801	0.84220186	0.35969739	0.54999582	0.49102326	Slc33a1	1
GO:0019829	ATPase-coupled monoatomic cation transmembrane transporter activity	1/135	47/14323	0.0212766	2.25736801	0.84220186	0.35969739	0.54999582	0.49102326	Atp6v0b	1
GO:0016705	oxidoreductase activity, acting on paired donors, with incorporation or reduction of molecular oxygen	2/135	134/14323	0.01492537	1.58352681	0.66198088	0.36088551	0.54999582	0.49102326	Degs1/P4hb	2
GO:0005216	monoatomic ion channel activity	4/135	325/14323	0.01230769	1.30580057	0.54394515	0.3671326	0.55708381	0.49735126	Kcnn1/Kcnk13/Slc17a8/Kcnip2	4
GO:0003823	antigen binding	1/135	49/14323	0.02040816	2.16523054	0.79697588	0.37175051	0.55926236	0.49929621	Ighg2c	1
GO:0022836	gated channel activity	3/135	231/14323	0.01298701	1.37787398	0.56477412	0.37177326	0.55926236	0.49929621	Kcnn1/Kcnk13/Kcnip2	3
GO:0016597	amino acid binding	1/135	50/14323	0.02	2.12192593	0.77517487	0.37769234	0.56572801	0.50506859	Yars2	1
GO:0005254	chloride channel activity	1/135	51/14323	0.01960784	2.08031954	0.7538814	0.38357839	0.57104579	0.50981618	Slc17a8	1
GO:0008234	cysteine-type peptidase activity	2/135	141/14323	0.0141844	1.504912	0.58771211	0.38451508	0.57104579	0.50981618	Pgpep1/Trhde	2
GO:1901682	sulfur compound transmembrane transporter activity	1/135	52/14323	0.01923077	2.04031339	0.73307226	0.38940917	0.57517024	0.51349839	Slc33a1	1
GO:0061629	RNA polymerase II-specific DNA-binding transcription factor binding	4/135	336/14323	0.01190476	1.26305115	0.47594381	0.39069892	0.57517024	0.51349839	Bmal1/Calr/Smad2/Brms1	4
GO:0016791	phosphatase activity	3/135	239/14323	0.0125523	1.33175267	0.5044969	0.39223644	0.57517024	0.51349839	Ppp2cb/Enoph1/Nt5c3b	3
GO:0046943	carboxylic acid transmembrane transporter activity	2/135	144/14323	0.01388889	1.47355967	0.55711042	0.39452946	0.57611206	0.51433923	Slc51a/Slc17a8	2
GO:0005342	organic acid transmembrane transporter activity	2/135	145/14323	0.0137931	1.46339719	0.54706388	0.39785145	0.57854232	0.5165089	Slc51a/Slc17a8	2
GO:0140098	catalytic activity, acting on RNA	4/135	343/14323	0.01166181	1.23727459	0.43386322	0.40564133	0.5874225	0.52443692	Mettl16/Tfb1m/Mettl14/Yars2	4
GO:0008028	monocarboxylic acid transmembrane transporter activity	1/135	56/14323	0.01785714	1.89457672	0.65426457	0.41218988	0.59443913	0.5307012	Slc51a	1
GO:0016758	hexosyltransferase activity	2/135	150/14323	0.01333333	1.41461728	0.49793226	0.41433403	0.59507233	0.5312665	Has2/Hexa	2
GO:0005080	protein kinase C binding	1/135	58/14323	0.01724138	1.82924649	0.61726248	0.42326182	0.60540318	0.54048964	Pkp2	1
GO:0016706	2-oxoglutarate-dependent dioxygenase activity	1/135	59/14323	0.01694915	1.79824231	0.59930543	0.42871991	0.61070714	0.54522489	P4hb	1
GO:0015179	L-amino acid transmembrane transporter activity	1/135	60/14323	0.01666667	1.7682716	0.58169201	0.43412673	0.61589523	0.5498567	Slc17a8	1
GO:0005261	monoatomic cation channel activity	3/135	257/14323	0.01167315	1.23847817	0.37630328	0.43760977	0.61832311	0.55202426	Kcnn1/Kcnk13/Kcnip2	3
GO:0015267	channel activity	4/135	362/14323	0.01104972	1.17233477	0.3239493	0.44581553	0.62485791	0.55785837	Kcnn1/Kcnk13/Slc17a8/Kcnip2	4
GO:0022803	passive transmembrane transporter activity	4/135	362/14323	0.01104972	1.17233477	0.3239493	0.44581553	0.62485791	0.55785837	Kcnn1/Kcnk13/Slc17a8/Kcnip2	4
GO:0005253	monoatomic anion channel activity	1/135	64/14323	0.015625	1.65775463	0.51441959	0.45525074	0.63048614	0.56288312	Slc17a8	1
GO:0030145	manganese ion binding	1/135	64/14323	0.015625	1.65775463	0.51441959	0.45525074	0.63048614	0.56288312	Lap3	1
GO:0042562	hormone binding	1/135	64/14323	0.015625	1.65775463	0.51441959	0.45525074	0.63048614	0.56288312	Calr	1
GO:0140297	DNA-binding transcription factor binding	5/135	471/14323	0.01061571	1.12628765	0.27184617	0.45812147	0.63195413	0.5641937	Bmal1/Calr/Smad2/Sri/Brms1	5
GO:0004553	hydrolase activity, hydrolyzing O-glycosyl compounds	1/135	67/14323	0.01492537	1.58352681	0.46698991	0.47057835	0.64658206	0.57725318	Hexa	1
GO:0017018	myosin phosphatase activity	1/135	68/14323	0.01470588	1.56023965	0.45170284	0.47559181	0.64808249	0.57859272	Ppp2cb	1
GO:0019905	syntaxin binding	1/135	68/14323	0.01470588	1.56023965	0.45170284	0.47559181	0.64808249	0.57859272	Sec22b	1
GO:0003725	double-stranded RNA binding	1/135	69/14323	0.01449275	1.53762748	0.43666236	0.48055815	0.64808249	0.57859272	Hspd1	1
GO:0048029	monosaccharide binding	1/135	69/14323	0.01449275	1.53762748	0.43666236	0.48055815	0.64808249	0.57859272	Lman2	1
GO:0019903	protein phosphatase binding	2/135	171/14323	0.01169591	1.24089235	0.30911515	0.4809552	0.64808249	0.57859272	Hsp90b1/Arpp19	2
GO:0005126	cytokine receptor binding	2/135	172/14323	0.01162791	1.23367786	0.30074358	0.48401303	0.64916379	0.57955808	Smad2/Itch	2
GO:0002039	p53 binding	1/135	70/14323	0.01428571	1.51566138	0.42186025	0.48547779	0.64916379	0.57955808	Hspd1	1
GO:0004860	protein kinase inhibitor activity	1/135	71/14323	0.01408451	1.49431403	0.40728868	0.49035119	0.65317773	0.58314163	Dnajc3	1
GO:0019843	rRNA binding	1/135	72/14323	0.01388889	1.47355967	0.39294016	0.49517876	0.65461132	0.58442151	Mrps6	1
GO:0030971	receptor tyrosine kinase binding	1/135	72/14323	0.01388889	1.47355967	0.39294016	0.49517876	0.65461132	0.58442151	Arhgef16	1
GO:0004674	protein serine/threonine kinase activity	4/135	388/14323	0.01030928	1.09377625	0.18266838	0.49938219	0.65723752	0.58676612	Srpk1/Stk16/Pdk3/Oxsr1	4
GO:0005516	calmodulin binding	2/135	178/14323	0.01123596	1.19209322	0.25155253	0.50212625	0.65723752	0.58676612	Kcnn1/Rrad	2
GO:0019210	kinase inhibitor activity	1/135	74/14323	0.01351351	1.43373373	0.36488415	0.50469816	0.65723752	0.58676612	Dnajc3	1
GO:0035064	methylated histone binding	1/135	74/14323	0.01351351	1.43373373	0.36488415	0.50469816	0.65723752	0.58676612	Cbx1	1
GO:0000049	tRNA binding	1/135	76/14323	0.01315789	1.3960039	0.33763905	0.51403935	0.66199163	0.59101048	Yars2	1
GO:0004222	metalloendopeptidase activity	1/135	76/14323	0.01315789	1.3960039	0.33763905	0.51403935	0.66199163	0.59101048	Mmp14	1
GO:0140034	methylation-dependent protein binding	1/135	76/14323	0.01315789	1.3960039	0.33763905	0.51403935	0.66199163	0.59101048	Cbx1	1
GO:0046332	SMAD binding	1/135	77/14323	0.01298701	1.37787398	0.32430527	0.51864415	0.66211911	0.59112428	Smad2	1
GO:0051213	dioxygenase activity	1/135	77/14323	0.01298701	1.37787398	0.32430527	0.51864415	0.66211911	0.59112428	P4hb	1
GO:0140030	modification-dependent protein binding	2/135	184/14323	0.01086957	1.15322061	0.2040445	0.5198299	0.66211911	0.59112428	Hspd1/Cbx1	2
GO:0043178	alcohol binding	1/135	78/14323	0.01282051	1.36020893	0.31115635	0.52320565	0.66399553	0.59279951	Rlbp1	1
GO:0003779	actin binding	4/135	402/14323	0.00995025	1.05568454	0.11046395	0.52731903	0.66489443	0.59360203	Scin/P4hb/Msrb2/Fbxo25	4
GO:0019003	GDP binding	1/135	79/14323	0.01265823	1.34299109	0.29818688	0.52772423	0.66489443	0.59360203	Rab9b	1
GO:0015108	chloride transmembrane transporter activity	1/135	80/14323	0.0125	1.3262037	0.28539166	0.53220031	0.66572727	0.59434556	Slc17a8	1
GO:0140375	immune receptor activity	1/135	80/14323	0.0125	1.3262037	0.28539166	0.53220031	0.66572727	0.59434556	Cr1l	1
GO:0004722	protein serine/threonine phosphatase activity	1/135	82/14323	0.01219512	1.29385727	0.26030425	0.54102652	0.67435091	0.60204455	Ppp2cb	1
GO:0003727	single-stranded RNA binding	1/135	83/14323	0.01204819	1.27826863	0.24800271	0.54537744	0.67495293	0.60258201	Hnrnpc	1
GO:0042626	ATPase-coupled transmembrane transporter activity	1/135	83/14323	0.01204819	1.27826863	0.24800271	0.54537744	0.67495293	0.60258201	Atp6v0b	1
GO:0030695	GTPase regulator activity	4/135	414/14323	0.00966184	1.02508499	0.05052127	0.5506655	0.6766981	0.60414006	Arhgef16/Stard13/Gapvd1/Ric8b	4
GO:0060589	nucleoside-triphosphatase regulator activity	4/135	414/14323	0.00966184	1.02508499	0.05052127	0.5506655	0.6766981	0.60414006	Arhgef16/Stard13/Gapvd1/Ric8b	4
GO:0015171	amino acid transmembrane transporter activity	1/135	86/14323	0.01162791	1.23367786	0.21201456	0.55818606	0.68353311	0.61024219	Slc17a8	1
GO:0042277	peptide binding	2/135	200/14323	0.01	1.06096296	0.08468875	0.56496648	0.68891078	0.61504325	Calr/Trhde	2
GO:0099094	ligand-gated monoatomic cation channel activity	1/135	88/14323	0.01136364	1.20563973	0.188746	0.56652548	0.68891078	0.61504325	Kcnn1	1
GO:0004866	endopeptidase inhibitor activity	1/135	90/14323	0.01111111	1.17884774	0.1660216	0.57470864	0.69290855	0.61861237	Smr2l	1
GO:0003729	mRNA binding	3/135	316/14323	0.00949367	1.00724332	0.01270038	0.57545711	0.69290855	0.61861237	Calr/Hnrnpc/Mettl14	3
GO:0030246	carbohydrate binding	2/135	204/14323	0.00980392	1.04015977	0.0563524	0.57576928	0.69290855	0.61861237	Calr/Lman2	2
GO:1990756	ubiquitin-like ligase-substrate adaptor activity	1/135	91/14323	0.01098901	1.16589337	0.15485487	0.57874253	0.69293121	0.6186326	Klhl5	1
GO:0042578	phosphoric ester hydrolase activity	3/135	318/14323	0.00943396	1.00090846	0.00159803	0.57975906	0.69293121	0.6186326	Ppp2cb/Enoph1/Nt5c3b	3
GO:0008083	growth factor activity	1/135	92/14323	0.01086957	1.15322061	0.14381412	0.58273843	0.69411506	0.61968952	Manf	1
GO:0051117	ATPase binding	1/135	93/14323	0.01075269	1.14082039	0.13289624	0.58669672	0.6964529	0.62177668	Sdf2l1	1
GO:0140104	molecular carrier activity	1/135	94/14323	0.0106383	1.128684	0.12209825	0.59061772	0.6987308	0.62381034	Calr	1
GO:0030414	peptidase inhibitor activity	1/135	95/14323	0.01052632	1.11680312	0.11141725	0.59450181	0.70094977	0.62579138	Smr2l	1
GO:0031490	chromatin DNA binding	1/135	96/14323	0.01041667	1.10516975	0.10085047	0.59834931	0.7012557	0.6260645	Grhl3	1
GO:0106310	protein serine kinase activity	3/135	327/14323	0.00917431	0.97336052	-0.0475342	0.59877994	0.7012557	0.6260645	Srpk1/Stk16/Oxsr1	3
GO:0016757	glycosyltransferase activity	2/135	217/14323	0.00921659	0.97784605	-0.0320765	0.60953045	0.70628169	0.63055159	Has2/Hexa	2
GO:0015291	secondary active transmembrane transporter activity	2/135	218/14323	0.00917431	0.97336052	-0.0386612	0.6120419	0.70628169	0.63055159	Slc5a3/Slc17a8	2
GO:0008509	monoatomic anion transmembrane transporter activity	1/135	100/14323	0.01	1.06096296	0.0596731	0.6133803	0.70628169	0.63055159	Slc17a8	1
GO:0016798	hydrolase activity, acting on glycosyl bonds	1/135	100/14323	0.01	1.06096296	0.0596731	0.6133803	0.70628169	0.63055159	Hexa	1
GO:0003730	mRNA 3'-UTR binding	1/135	101/14323	0.00990099	1.05045838	0.04963886	0.61704997	0.70628169	0.63055159	Hnrnpc	1
GO:0140767	enzyme-substrate adaptor activity	1/135	101/14323	0.00990099	1.05045838	0.04963886	0.61704997	0.70628169	0.63055159	Klhl5	1
GO:0022804	active transmembrane transporter activity	3/135	336/14323	0.00892857	0.94728836	-0.0953722	0.61723758	0.70628169	0.63055159	Slc5a3/Slc17a8/Atp6v0b	3
GO:0015103	inorganic anion transmembrane transporter activity	1/135	103/14323	0.00970874	1.03006113	0.02986636	0.6242859	0.71201235	0.63566779	Slc17a8	1
GO:0008013	beta-catenin binding	1/135	104/14323	0.00961538	1.0201567	0.02012378	0.6278528	0.7130482	0.63659257	Cdh10	1
GO:0005096	GTPase activator activity	2/135	225/14323	0.00888889	0.94307819	-0.0839459	0.62928036	0.7130482	0.63659257	Stard13/Gapvd1	2
GO:1990782	protein tyrosine kinase binding	1/135	108/14323	0.00925926	0.98237311	-0.0179359	0.64178741	0.72085087	0.64355861	Arhgef16	1
GO:0005509	calcium ion binding	4/135	466/14323	0.00858369	0.91069782	-0.1911739	0.64436646	0.72085087	0.64355861	Calr/Kcnip2/Sri/Cdh10	4
GO:0061135	endopeptidase regulator activity	1/135	109/14323	0.00917431	0.97336052	-0.0272326	0.64518936	0.72085087	0.64355861	Smr2l	1
GO:0000149	SNARE binding	1/135	110/14323	0.00909091	0.96451178	-0.0364457	0.64855924	0.72085087	0.64355861	Sec22b	1
GO:0005506	iron ion binding	1/135	110/14323	0.00909091	0.96451178	-0.0364457	0.64855924	0.72085087	0.64355861	Calr	1
GO:0019842	vitamin binding	1/135	110/14323	0.00909091	0.96451178	-0.0364457	0.64855924	0.72085087	0.64355861	Rlbp1	1
GO:0043022	ribosome binding	1/135	112/14323	0.00892857	0.94728836	-0.0546275	0.65520398	0.72592441	0.64808814	Hspa5	1
GO:0001228	DNA-binding transcription activator activity, RNA polymerase II-specific	3/135	357/14323	0.00840336	0.89156552	-0.2023828	0.65806038	0.72678188	0.64885367	Bmal1/Grhl3/Smad2	3
GO:0004713	protein tyrosine kinase activity	1/135	115/14323	0.00869565	0.92257649	-0.0813136	0.66493796	0.72975896	0.65151154	Stk16	1
GO:0015399	primary active transmembrane transporter activity	1/135	115/14323	0.00869565	0.92257649	-0.0813136	0.66493796	0.72975896	0.65151154	Atp6v0b	1
GO:0015276	ligand-gated monoatomic ion channel activity	1/135	116/14323	0.00862069	0.91462324	-0.0900587	0.66812162	0.72977997	0.6515303	Kcnn1	1
GO:0001216	DNA-binding transcription activator activity	3/135	363/14323	0.00826446	0.8768289	-0.2318609	0.66913922	0.72977997	0.6515303	Bmal1/Grhl3/Smad2	3
GO:0015078	proton transmembrane transporter activity	1/135	118/14323	0.00847458	0.89912116	-0.1073318	0.67439912	0.73322522	0.65460614	Atp6v0b	1
GO:0042826	histone deacetylase binding	1/135	119/14323	0.00840336	0.89156552	-0.1158627	0.67749354	0.734302	0.65556746	Brms1	1
GO:0022834	ligand-gated channel activity	1/135	121/14323	0.00826446	0.8768289	-0.1327195	0.68359506	0.7374654	0.65839167	Kcnn1	1
GO:0140657	ATP-dependent activity	4/135	491/14323	0.00814664	0.86432828	-0.2983979	0.68463836	0.7374654	0.65839167	Hspa5/Hsp90b1/Hspd1/Atp6v0b	4
GO:0004672	protein kinase activity	4/135	497/14323	0.00804829	0.85389373	-0.3233753	0.69381917	0.74452239	0.66469198	Srpk1/Stk16/Pdk3/Oxsr1	4
GO:0005319	lipid transporter activity	1/135	125/14323	0.008	0.84877037	-0.1656481	0.69545644	0.74452239	0.66469198	Slc51a	1
GO:0016779	nucleotidyltransferase activity	1/135	127/14323	0.00787402	0.83540391	-0.1817383	0.70122054	0.74839746	0.66815156	Crppa	1
GO:0140101	catalytic activity, acting on a tRNA	1/135	134/14323	0.00746269	0.79176341	-0.2362336	0.72055517	0.76668827	0.68448116	Yars2	1
GO:0140993	histone modifying activity	3/135	404/14323	0.00742574	0.78784378	-0.4219399	0.73789336	0.78117545	0.69741497	Srpk1/Stk16/Oxsr1	3
GO:0031406	carboxylic acid binding	1/135	141/14323	0.0070922	0.752456	-0.2881385	0.73864727	0.78117545	0.69741497	Yars2	1
GO:0016922	nuclear receptor binding	1/135	142/14323	0.00704225	0.74715702	-0.2953587	0.74113512	0.78143854	0.69764985	Calr	1

GO:0061134	peptidase regulator activity	1/135	146/14323	0.00684932	0.72668696	-0.323782	0.75085361	0.78775287	0.70328713	Smr2l	1
GO:0031267	small GTPase binding	2/135	284/14323	0.00704225	0.74715702	-0.4198074	0.75163812	0.78775287	0.70328713	Arhgef16/Gapvd1	2
GO:0043177	organic acid binding	1/135	149/14323	0.00671141	0.71205568	-0.3446383	0.75790401	0.79194161	0.70702674	Yars2	1
GO:0004721	phosphoprotein phosphatase activity	1/135	155/14323	0.00645161	0.68449223	-0.3852386	0.77141609	0.80365438	0.71748362	Ppp2cb	1
GO:0003735	structural constituent of ribosome	1/135	164/14323	0.00609756	0.64692864	-0.4435844	0.79029305	0.82086986	0.7328532	Mrps6	1
GO:0016829	lyase activity	1/135	171/14323	0.00584795	0.62044618	-0.4870467	0.80389807	0.83252352	0.74325731	Hmgcll1	1
GO:0051020	GTPase binding	2/135	324/14323	0.00617284	0.65491541	-0.6128549	0.81350801	0.83998313	0.74991707	Arhgef16/Gapvd1	2
GO:0016887	ATP hydrolysis activity	2/135	342/14323	0.00584795	0.62044618	-0.6929876	0.83660459	0.86128319	0.76893327	Hspa5/Hsp90b1	2
GO:0051015	actin filament binding	1/135	201/14323	0.00497512	0.52784227	-0.6575748	0.85294807	0.87552611	0.78164901	Scin	1
GO:0019887	protein kinase regulator activity	1/135	210/14323	0.0047619	0.50522046	-0.704563	0.86513	0.88542631	0.79048767	Dnajc3	1
GO:0001664	G protein-coupled receptor binding	1/135	223/14323	0.0044843	0.47576814	-0.7696166	0.88097829	0.89608449	0.80000304	Itch	1
GO:0042393	histone binding	1/135	225/14323	0.00444444	0.47153909	-0.7793517	0.8832466	0.89608449	0.80000304	Cbx1	1
GO:1901681	sulfur compound binding	1/135	225/14323	0.00444444	0.47153909	-0.7793517	0.8832466	0.89608449	0.80000304	Tfb1m	1
GO:0004930	G protein-coupled receptor activity	1/135	231/14323	0.004329	0.45929133	-0.8081484	0.88979724	0.90011373	0.80360026	Fzd8	1
GO:0019207	kinase regulator activity	1/135	242/14323	0.00413223	0.43841445	-0.8594389	0.90087346	0.9086845	0.81125204	Dnajc3	1
GO:0004175	endopeptidase activity	1/135	255/14323	0.00392157	0.41606391	-0.9177563	0.91254659	0.91780622	0.81939569	Mmp14	1
GO:0015631	tubulin binding	1/135	353/14323	0.00283286	0.30055608	-1.2979267	0.96611936	0.96889557	0.86500705	Tppp3	1
GO:0003712	transcription coregulator activity	1/135	473/14323	0.00211416	0.22430507	-1.6734193	0.98948755	0.98948755	0.88339108	Bmal1	1

ID	Description	GeneRatio	BgRatio	RichFactor	FoldEnrichm	zScore	pvalue	p.adjust	qvalue	geneID	Count
GO:0055038	recycling endosome membrane	3/23	92/14612	0.0326087	20.7164461	7.53234785	0.00039045	0.0433403	0.03164725	Slc9b2/Rab8a/Rab13	3
GO:0030140	trans-Golgi network transport vesicle	2/23	34/14612	0.05882353	37.370844	8.43014396	0.00128948	0.07156612	0.05225786	Rab8a/Rab13	2
GO:0055037	recycling endosome	3/23	187/14612	0.01604278	10.1920484	5.02303633	0.00302504	0.11192633	0.08172904	Slc9b2/Rab8a/Rab13	3
GO:0016323	basolateral plasma membrane	3/23	235/14612	0.01276596	8.11026827	4.36291657	0.0057346	0.12507276	0.09132862	Slc6a13/Slc47a1/Slc9b2	3
GO:0009925	basal plasma membrane	3/23	258/14612	0.01162791	7.38725986	4.10987955	0.00742059	0.12507276	0.09132862	Slc6a13/Slc47a1/Slc9b2	3
GO:0045178	basal part of cell	3/23	275/14612	0.01090909	6.93059289	3.94208916	0.00883776	0.12507276	0.09132862	Slc6a13/Slc47a1/Slc9b2	3
GO:0005798	Golgi-associated vesicle	2/23	91/14612	0.02197802	13.9627329	4.9250442	0.008914	0.12507276	0.09132862	Rab8a/Rab13	2
GO:0008021	synaptic vesicle	3/23	277/14612	0.01083032	6.8805525	3.92328894	0.00901425	0.12507276	0.09132862	Slc9b2/Rab8a/Rab13	3
GO:0070382	exocytic vesicle	3/23	293/14612	0.01023891	6.50482267	3.77930486	0.01050145	0.12951788	0.09457446	Slc9b2/Rab8a/Rab13	3
GO:0097730	non-motile cilium	2/23	117/14612	0.01709402	10.8599034	4.25155309	0.01441071	0.139728	0.10202993	Rab8a/Arl3	2
GO:0032593	insulin-responsive compartment	1/23	10/14612	0.1	63.5304348	7.85373788	0.01563424	0.139728	0.10202993	Rab13	1
GO:0005766	primary lysosome	1/23	11/14612	0.09090909	57.7549407	7.47652591	0.01718474	0.139728	0.10202993	Hexb	1
GO:0042582	azurophil granule	1/23	11/14612	0.09090909	57.7549407	7.47652591	0.01718474	0.139728	0.10202993	Hexb	1
GO:0001674	female germ cell nucleus	1/23	12/14612	0.08333333	52.942029	7.1470074	0.01873291	0.139728	0.10202993	Marcks	1
GO:0036126	sperm flagellum	2/23	135/14612	0.01481481	9.41191626	3.89864476	0.01888216	0.139728	0.10202993	Slc9b2/Marcks	2
GO:0033391	chromatoid body	1/23	13/14612	0.07692308	48.8695652	6.85584091	0.02027874	0.14068375	0.10272782	Marcks	1
GO:0097729	9+2 motile cilium	2/23	152/14612	0.01315789	8.35926773	3.6212906	0.02357368	0.14456815	0.10556423	Slc9b2/Marcks	2
GO:0030136	clathrin-coated vesicle	2/23	155/14612	0.01290323	8.19747546	3.57682817	0.0244469	0.14456815	0.10556423	Rab8a/Rab13	2
GO:0030133	transport vesicle	3/23	411/14612	0.00729927	4.63725801	2.96979644	0.02577254	0.14456815	0.10556423	Slc9b2/Rab8a/Rab13	3
GO:0036064	ciliary basal body	2/23	170/14612	0.01176471	7.4741688	3.371211	0.02900907	0.14456815	0.10556423	Rab8a/Arl3	2
GO:0005686	U2 snRNP	1/23	21/14612	0.04761905	30.252588	5.32626217	0.03256181	0.14456815	0.10556423	Phf5a	1
GO:0097228	sperm principal piece	1/23	21/14612	0.04761905	30.252588	5.32626217	0.03256181	0.14456815	0.10556423	Slc9b2	1
GO:0010008	endosome membrane	3/23	456/14612	0.00657895	4.17963387	2.73892654	0.03364635	0.14456815	0.10556423	Slc9b2/Rab8a/Rab13	3
GO:0030496	midbody	2/23	190/14612	0.01052632	6.68741419	3.13306957	0.03557902	0.14456815	0.10556423	Rab8a/Arl3	2
GO:0099571	postsynaptic cytoskeleton	1/23	25/14612	0.04	25.4121739	4.85047862	0.03864797	0.14456815	0.10556423	Marcks	1
GO:0000323	lytic vacuole	3/23	487/14612	0.00616016	3.91357914	2.59649923	0.03972983	0.14456815	0.10556423	Slc9b2/Rab8a/Hexb	3
GO:0005764	lysosome	3/23	487/14612	0.00616016	3.91357914	2.59649923	0.03972983	0.14456815	0.10556423	Slc9b2/Rab8a/Hexb	3
GO:0030139	endocytic vesicle	2/23	203/14612	0.00985222	6.25915614	2.99597572	0.04013194	0.14456815	0.10556423	Rab8a/Rab13	2
GO:0005689	U12-type spliceosomal complex	1/23	26/14612	0.03846154	24.4347826	4.74865507	0.04016378	0.14456815	0.10556423	Phf5a	1
GO:0031514	motile cilium	2/23	204/14612	0.00980392	6.228474	2.98592787	0.04049102	0.14456815	0.10556423	Slc9b2/Marcks	2
GO:0001891	phagocytic cup	1/23	27/14612	0.03703704	23.5297907	4.65239894	0.0416773	0.14456815	0.10556423	Megf10	1
GO:0043202	lysosomal lumen	1/23	27/14612	0.03703704	23.5297907	4.65239894	0.0416773	0.14456815	0.10556423	Hexb	1
GO:0005775	vacuolar lumen	1/23	31/14612	0.03225806	20.4936886	4.31392255	0.0477086	0.16024509	0.11701159	Hexb	1
GO:0043197	dendritic spine	2/23	227/14612	0.00881057	5.59739514	2.77179402	0.04908408	0.16024509	0.11701159	Rab8a/Marcks	2
GO:0044309	neuron spine	2/23	232/14612	0.00862069	5.47676162	2.72910123	0.05103399	0.16185064	0.11818397	Rab8a/Marcks	2
GO:0030135	coated vesicle	2/23	240/14612	0.00833333	5.2942029	2.66330377	0.054212	0.16715366	0.12205626	Rab8a/Rab13	2
GO:0032391	photoreceptor connecting cilium	1/23	39/14612	0.02564103	16.2898551	3.79622825	0.05966243	0.1789873	0.13069722	Arl3	1
GO:0097546	ciliary base	1/23	42/14612	0.02380952	15.126294	3.64010772	0.06410801	0.18726287	0.13674007	Rab8a	1
GO:0099523	presynaptic cytosol	1/23	49/14612	0.02040816	12.9653949	3.33112382	0.07440297	0.21176231	0.15462966	Marcks	1
GO:0030670	phagocytic vesicle membrane	1/23	52/14612	0.01923077	12.2173913	3.21739202	0.07878186	0.21461962	0.15671608	Rab8a	1
GO:0071005	U2-type precatalytic spliceosome	1/23	53/14612	0.01886792	11.9868745	3.18154046	0.08023708	0.21461962	0.15671608	Phf5a	1
GO:0060170	ciliary membrane	1/23	54/14612	0.01851852	11.7648953	3.14663928	0.08169011	0.21461962	0.15671608	Rab8a	1
GO:0071011	precatalytic spliceosome	1/23	55/14612	0.01818182	11.5509881	3.11264552	0.08314093	0.21461962	0.15671608	Phf5a	1
GO:0030141	secretory granule	2/23	318/14612	0.00628931	3.99562483	2.14444652	0.08854654	0.21958725	0.16034346	Rab13/Hexb	2
GO:0016324	apical plasma membrane	2/23	319/14612	0.00626959	3.98309936	2.1389099	0.08902186	0.21958725	0.16034346	Slc47a1/Slc9b2	2
GO:0016328	lateral plasma membrane	1/23	63/14612	0.01587302	10.084196	2.86900513	0.09466887	0.22357968	0.16325875	Rab13	1
GO:0097525	spliceosomal snRNP complex	1/23	63/14612	0.01587302	10.084196	2.86900513	0.09466887	0.22357968	0.16325875	Phf5a	1
GO:0043073	germ cell nucleus	1/23	66/14612	0.01515152	9.62582345	2.78863624	0.098956	0.22883575	0.16709675	Marcks	1
GO:0030532	small nuclear ribonucleoprotein complex	1/23	71/14612	0.01408451	8.94794856	2.66549708	0.10605809	0.23083232	0.16855466	Phf5a	1
GO:0035869	ciliary transition zone	1/23	71/14612	0.01408451	8.94794856	2.66549708	0.10605809	0.23083232	0.16855466	Arl3	1
GO:0099522	cytosolic region	1/23	71/14612	0.01408451	8.94794856	2.66549708	0.10605809	0.23083232	0.16855466	Marcks	1
GO:0030666	endocytic vesicle membrane	1/23	73/14612	0.01369863	8.70279929	2.61959329	0.10888391	0.23242527	0.16971784	Rab8a	1
GO:0005876	spindle microtubule	1/23	77/14612	0.01298701	8.25070582	2.53284874	0.11450994	0.2398227	0.17511947	Arl3	1
GO:0097733	photoreceptor cell cilium	1/23	79/14612	0.01265823	8.04182719	2.4917955	0.11731019	0.24113762	0.17607962	Arl3	1
GO:0120114	Sm-like protein family complex	1/23	83/14612	0.01204819	7.65426925	2.41386335	0.12288531	0.2480049	0.18109414	Phf5a	1
GO:0045177	apical part of cell	2/23	393/14612	0.00508906	3.23310101	1.78180938	0.12617851	0.25010384	0.1826268	Slc47a1/Slc9b2	2
GO:0097731	9+0 non-motile cilium	1/23	92/14612	0.01086957	6.90548204	2.25609345	0.13530645	0.2604016	0.19014626	Arl3	1
GO:0005881	cytoplasmic microtubule	1/23	94/14612	0.0106383	6.75855689	2.22390033	0.13804376	0.2604016	0.19014626	Arl3	1
GO:0005874	microtubule	2/23	416/14612	0.00480769	3.05434783	1.68783028	0.13841166	0.2604016	0.19014626	Marcks/Arl3	2
GO:0005684	U2-type spliceosomal complex	1/23	96/14612	0.01041667	6.61775362	2.19263303	0.14077278	0.26042964	0.19016674	Phf5a	1
GO:0001669	acrosomal vesicle	1/23	99/14612	0.01010101	6.41721563	2.14736687	0.14485082	0.26358101	0.19246788	Hexb	1
GO:0005923	bicellular tight junction	1/23	104/14612	0.00961538	6.10869565	2.07593644	0.15160645	0.27142445	0.19819519	Rab13	1
GO:0032432	actin filament bundle	1/23	109/14612	0.00917431	5.82848026	2.00902764	0.15831101	0.2752096	0.20095912	Marcks	1
GO:0005667	transcription regulator complex	2/23	458/14612	0.00436681	2.77425479	1.53179362	0.16140811	0.2752096	0.20095912	Npas2/Pbx3	2
GO:0070160	tight junction	1/23	112/14612	0.00892857	5.67236025	1.97084494	0.16230939	0.2752096	0.20095912	Rab13	1
GO:0019897	extrinsic component of plasma membrane	1/23	113/14612	0.00884956	5.62216237	1.95842309	0.16363814	0.2752096	0.20095912	Rab13	1
GO:0005911	cell-cell junction	2/23	467/14612	0.00428266	2.7207895	1.50063765	0.16643178	0.27573027	0.20133931	Fabp7/Rab13	2
GO:0045335	phagocytic vesicle	1/23	117/14612	0.00854701	5.42995169	1.91017843	0.16893301	0.2757583	0.20135978	Rab8a	1
GO:0016363	nuclear matrix	1/23	121/14612	0.00826446	5.25044916	1.86410108	0.17419581	0.28022804	0.2046236	Phf5a	1
GO:0043296	apical junction complex	1/23	127/14612	0.00787402	5.00239644	1.79867973	0.18203027	0.28864801	0.2107719	Rab13	1
GO:0005930	axoneme	1/23	134/14612	0.00746269	4.74107722	1.72737215	0.19108063	0.29656378	0.21655203	Arl3	1
GO:0097014	ciliary plasm	1/23	135/14612	0.00740741	4.70595813	1.71758893	0.1923657	0.29656378	0.21655203	Arl3	1
GO:0005814	centriole	1/23	148/14612	0.00675676	4.29259694	1.59851365	0.20889484	0.31334226	0.22880373	Rab8a	1
GO:0034399	nuclear periphery	1/23	148/14612	0.00675676	4.29259694	1.59851365	0.20889484	0.31334226	0.22880373	Phf5a	1
GO:0030672	synaptic vesicle membrane	1/23	164/14612	0.00609756	3.873807	1.4694905	0.22879444	0.3341603	0.24400515	Slc9b2	1
GO:0099501	exocytic vesicle membrane	1/23	164/14612	0.00609756	3.873807	1.4694905	0.22879444	0.3341603	0.24400515	Slc9b2	1
GO:0019898	extrinsic component of membrane	1/23	174/14612	0.00574713	3.65117441	1.39685244	0.24098758	0.34739768	0.25367113	Rab13	1
GO:0043679	axon terminus	1/23	182/14612	0.00549451	3.49068323	1.34249338	0.2506091	0.35663603	0.26041702	Marcks	1
GO:0005681	spliceosomal complex	1/23	194/14612	0.00515464	3.27476468	1.26641474	0.26482295	0.372093	0.27170376	Phf5a	1
GO:0044306	neuron projection terminus	1/23	204/14612	0.00490196	3.114237	1.20741906	0.27647048	0.38360279	0.28010825	Marcks	1
GO:0030027	lamellipodium	1/23	212/14612	0.00471698	2.99671862	1.1627723	0.28566121	0.39146165	0.28584682	Rab13	1
GO:0090575	RNA polymerase II transcription regulator complex	1/23	227/14612	0.00440529	2.79869757	1.08444435	0.30259368	0.3977311	0.2904248	Npas2	1
GO:0036464	cytoplasmic ribonucleoprotein granule	1/23	229/14612	0.00436681	2.77425479	1.0744844	0.30482213	0.3977311	0.2904248	Marcks	1
GO:0030658	transport vesicle membrane	1/23	231/14612	0.004329	2.75023527	1.06463075	0.30704376	0.3977311	0.2904248	Slc9b2	1
GO:0030426	growth cone	1/23	232/14612	0.00431034	2.73838081	1.05974309	0.30815202	0.3977311	0.2904248	Marcks	1
GO:0032838	plasma membrane bounded cell projection cytoplasm	1/23	232/14612	0.00431034	2.73838081	1.05974309	0.30815202	0.3977311	0.2904248	Arl3	1
GO:0030427	site of polarized growth	1/23	238/14612	0.00420168	2.669346	1.03094891	0.31476611	0.40159814	0.29324852	Marcks	1
GO:0005802	trans-Golgi network	1/23	243/14612	0.00411523	2.61442118	1.00762246	0.3202316	0.4039285	0.29495016	Rab13	1
GO:0035770	ribonucleoprotein granule	1/23	248/14612	0.00403226	2.56171108	0.98487238	0.32565539	0.40430305	0.29522366	Marcks	1
GO:0042734	presynaptic membrane	1/23	250/14612	0.004	2.54121739	0.97592753	0.32781329	0.40430305	0.29522366	Marcks	1
GO:0099568	cytoplasmic region	1/23	270/14612	0.0037037	2.35297907	0.89096209	0.34903214	0.4257425	0.31087883	Arl3	1
GO:0005765	lysosomal membrane	1/23	280/14612	0.00357143	2.2689441	0.85125413	0.35939981	0.42896106	0.31322903	Slc9b2	1
GO:0098852	lytic vacuole membrane	1/23	280/14612	0.00357143	2.2689441	0.85125413	0.35939981	0.42896106	0.31322903	Slc9b2	1
GO:0009897	external side of plasma membrane	1/23	298/14612	0.0033557	2.13189378	0.78383459	0.37766486	0.44596595	0.32564607	Mill2	1
GO:0062023	collagen-containing extracellular matrix	1/23	306/14612	0.00326797	2.076158	0.75538571	0.38562179	0.45056861	0.32900695	Angptl4	1
GO:0031253	cell projection membrane	1/23	324/14612	0.00308642	1.96081589	0.69441339	0.40317061	0.46574766	0.34009075	Rab8a	1
GO:0005938	cell cortex	1/23	328/14612	0.00304878	1.9369035	0.6813935	0.40700471	0.46574766	0.34009075	Marcks	1
GO:0005774	vacuolar membrane	1/23	334/14612	0.00299401	1.90210883	0.66220065	0.41271172	0.46745919	0.34134052	Slc9b2	1
GO:0098791	Golgi apparatus subcompartment	1/23	347/14612	0.00288184	1.83084826	0.62193027	0.4248972	0.47574341	0.34738969	Rab13	1
GO:0005759	mitochondrial matrix	1/23	351/14612	0.002849	1.8099839	0.60988237	0.42859767	0.47574341	0.34738969	Hmgcs2	1
GO:0045211	postsynaptic membrane	1/23	365/14612	0.00273973	1.74055986	0.5689001	0.44137078	0.48507086	0.35420063	Marcks	1
GO:0016607	nuclear speck	1/23	389/14612	0.00257069	1.63317313	0.50256565	0.46263461	0.49259616	0.35969563	Phf5a	1
GO:0150034	distal axon	1/23	393/14612	0.00254453	1.6165505	0.49195064	0.46610244	0.49259616	0.35969563	Marcks	1
GO:0005819	spindle	1/23	394/14612	0.00253807	1.61244758	0.48931543	0.46696605	0.49259616	0.35969563	Arl3	1
GO:0030312	external encapsulating structure	1/23	398/14612	0.00251256	1.59624208	0.4788474	0.47040714	0.49259616	0.35969563	Angptl4	1
GO:0031012	extracellular matrix	1/23	398/14612	0.00251256	1.59624208	0.4788474	0.47040714	0.49259616	0.35969563	Angptl4	1
GO:0014069	postsynaptic density	1/23	443/14612	0.00225734	1.43409559	0.36839079	0.50768395	0.52666279	0.38457121	Rab8a	1
GO:0031252	cell leading edge	1/23	454/14612	0.00220264	1.39934878	0.34321832	0.51640674	0.53075137	0.38755671	Rab13	1
GO:0032279	asymmetric synapse	1/23	465/14612	0.00215054	1.36624591	0.31868249	0.52498158	0.53461427	0.39037742	Rab8a	1
GO:0099572	postsynaptic specialization	1/23	487/14612	0.00205339	1.30452638	0.27138529	0.54169699	0.54662151	0.39914515	Rab8a	1
GO:0015629	actin cytoskeleton	1/23	495/14612	0.0020202	1.28344313	0.2547349	0.54763465	0.54763465	0.39988495	Marcks	1

ID	Description	GeneRatio	BgRatio	RichFactor	FoldEnrichm	zScore	pvalue	p.adjust	qvalue	geneID	Count
GO:0062023	collagen-containing extracellular matrix	7/30	306/14612	0.02287582	11.1420479	8.13241087	2.2199E-06	0.00014652	0.00012385	Fmod/Fbn1/Cilp/Ccn3/Loxl1/Comp/Col6a1	7
GO:0030312	external encapsulating structure	7/30	398/14612	0.01758794	8.56649916	6.94177319	1.2492E-05	0.00027483	0.00023231	Fmod/Fbn1/Cilp/Ccn3/Loxl1/Comp/Col6a1	7
GO:0031012	extracellular matrix	7/30	398/14612	0.01758794	8.56649916	6.94177319	1.2492E-05	0.00027483	0.00023231	Fmod/Fbn1/Cilp/Ccn3/Loxl1/Comp/Col6a1	7
GO:0005604	basement membrane	3/30	98/14612	0.03061224	14.9102041	6.2667884	0.00104144	0.01718368	0.01452528	Fbn1/Loxl1/Col6a1	3
GO:0000786	nucleosome	2/30	55/14612	0.03636364	17.7115152	5.63187856	0.00565604	0.07465976	0.06310953	H3f3b/H1f0	2
GO:0016529	sarcoplasmic reticulum	2/30	80/14612	0.025	12.1766667	4.54659896	0.01165984	0.12825824	0.10841606	Gstm7/Col6a1	2
GO:0016528	sarcoplasm	2/30	108/14612	0.01851852	9.01975309	3.79420708	0.02057765	0.17569698	0.14851579	Gstm7/Col6a1	2
GO:0000781	chromosome, telomeric region	2/30	110/14612	0.01818182	8.85575758	3.75113346	0.0212966	0.17569698	0.14851579	H3f3b/Telo2	2
GO:0001527	microfibril	1/30	12/14612	0.08333333	40.5888889	6.22270996	0.02437004	0.17871366	0.15106577	Fbn1	1
GO:0032993	protein-DNA complex	2/30	154/14612	0.01298701	6.32554113	3.0134365	0.03959424	0.24218844	0.20472069	H3f3b/H1f0	2
GO:0005921	gap junction	1/30	21/14612	0.04761905	23.1936508	4.61623509	0.04226979	0.24218844	0.20472069	Ccn3	1
GO:0097449	astrocyte projection	1/30	23/14612	0.04347826	21.1768116	4.39233793	0.04620419	0.24218844	0.20472069	Kcnk2	1
GO:0033268	node of Ranvier	1/30	24/14612	0.04166667	20.2944444	4.29073875	0.04816552	0.24218844	0.20472069	Kcnk2	1
GO:0042383	sarcolemma	2/30	178/14612	0.01123596	5.47265918	2.72316845	0.0513733	0.24218844	0.20472069	Col6a1/Kcnk2	2
GO:0032809	neuronal cell body membrane	1/30	35/14612	0.02857143	13.9161905	3.46997573	0.06948448	0.28277988	0.23903244	Cx3cr1	1
GO:0044305	calyx of Held	1/30	36/14612	0.02777778	13.5296296	3.41399087	0.07139951	0.28277988	0.23903244	Kcnk2	1
GO:0044298	cell body membrane	1/30	38/14612	0.02631579	12.8175439	3.30842801	0.07521816	0.28277988	0.23903244	Cx3cr1	1
GO:0005871	kinesin complex	1/30	39/14612	0.02564103	12.4888889	3.25857635	0.07712179	0.28277988	0.23903244	Kifc3	1
GO:0097386	glial cell projection	1/30	43/14612	0.02325581	11.3271318	3.07603396	0.08469848	0.29421579	0.24869915	Kcnk2	1
GO:0000791	euchromatin	1/30	61/14612	0.01639344	7.98469945	2.47946665	0.11805594	0.34567708	0.29219913	H1f0	1
GO:0022625	cytosolic large ribosomal subunit	1/30	62/14612	0.01612903	7.85591398	2.45370167	0.11987425	0.34567708	0.29219913	Rpl8	1
GO:0009897	external side of plasma membrane	2/30	298/14612	0.00671141	3.2689038	1.79488141	0.12431799	0.34567708	0.29219913	Asgr1/Cx3cr1	2
GO:0005581	collagen trimer	1/30	68/14612	0.01470588	7.1627451	2.31035533	0.13070829	0.34567708	0.29219913	Col6a1	1
GO:0008076	voltage-gated potassium channel complex	1/30	71/14612	0.01408451	6.8600939	2.24506367	0.13607685	0.34567708	0.29219913	Kcnk2	1
GO:0016324	apical plasma membrane	2/30	319/14612	0.00626959	3.05370951	1.68214996	0.13885049	0.34567708	0.29219913	Kcnk2/Kl	2
GO:0098687	chromosomal region	2/30	321/14612	0.00623053	3.03468328	1.67189914	0.14025444	0.34567708	0.29219913	H3f3b/Telo2	2
GO:0017053	transcription repressor complex	1/30	74/14612	0.01351351	6.58198198	2.18345381	0.14141335	0.34567708	0.29219913	H1f0	1
GO:0045171	intercellular bridge	1/30	82/14612	0.01219512	5.9398374	2.03459899	0.15548871	0.35356035	0.29886281	Gstm7	1
GO:0034705	potassium channel complex	1/30	85/14612	0.01176471	5.73019608	1.98377626	0.16070925	0.35356035	0.29886281	Kcnk2	1
GO:0044304	main axon	1/30	85/14612	0.01176471	5.73019608	1.98377626	0.16070925	0.35356035	0.29886281	Kcnk2	1
GO:0055038	recycling endosome membrane	1/30	92/14612	0.01086957	5.2942029	1.87406745	0.17276947	0.36783177	0.31092638	Fzd7	1
GO:0001669	acrosomal vesicle	1/30	99/14612	0.01010101	4.91986532	1.77501569	0.18466209	0.38086555	0.32194377	Loxl1	1
GO:0045177	apical part of cell	2/30	393/14612	0.00508906	2.47871077	1.34783595	0.19261507	0.38523014	0.32563313	Kcnk2/Kl	2
GO:0015934	large ribosomal subunit	1/30	120/14612	0.00833333	4.05888889	1.52609726	0.21935667	0.3874078	0.3274739	Rpl8	1
GO:0005875	microtubule associated complex	1/30	121/14612	0.00826446	4.02534435	1.51568995	0.22097269	0.3874078	0.3274739	Kifc3	1
GO:0022626	cytosolic ribosome	1/30	123/14612	0.00813008	3.9598916	1.4952066	0.22419502	0.3874078	0.3274739	Rpl8	1
GO:0014069	postsynaptic density	2/30	443/14612	0.00451467	2.19894658	1.16231581	0.23041301	0.3874078	0.3274739	Kcnk2/Rpl8	2
GO:0043296	apical junction complex	1/30	127/14612	0.00787402	3.8351706	1.45550553	0.23060109	0.3874078	0.3274739	Kifc3	1
GO:0032279	asymmetric synapse	2/30	465/14612	0.00430108	2.09491039	1.08834085	0.24724536	0.3874078	0.3274739	Kcnk2/Rpl8	2
GO:0005911	cell-cell junction	2/30	467/14612	0.00428266	2.08593862	1.08181822	0.24877932	0.3874078	0.3274739	Ccn3/Kifc3	2
GO:0098685	Schaffer collateral - CA1 synapse	1/30	144/14612	0.00694444	3.38240741	1.303122	0.25726098	0.3874078	0.3274739	Kcnk2	1
GO:0098839	postsynaptic density membrane	1/30	147/14612	0.00680272	3.31337868	1.27861039	0.26187203	0.3874078	0.3274739	Kcnk2	1
GO:0000323	lytic vacuole	2/30	487/14612	0.00410678	2.00027379	1.01831277	0.26414168	0.3874078	0.3274739	Col6a1/Cpq	2
GO:0005764	lysosome	2/30	487/14612	0.00410678	2.00027379	1.01831277	0.26414168	0.3874078	0.3274739	Col6a1/Cpq	2
GO:0099572	postsynaptic specialization	2/30	487/14612	0.00410678	2.00027379	1.01831277	0.26414168	0.3874078	0.3274739	Kcnk2/Rpl8	2
GO:0005912	adherens junction	1/30	161/14612	0.00621118	3.0252588	1.17202416	0.28302686	0.40608201	0.34325912	Kifc3	1
GO:0034703	cation channel complex	1/30	176/14612	0.00568182	2.76742424	1.0699556	0.30504217	0.42248629	0.35712557	Kcnk2	1
GO:0099634	postsynaptic specialization membrane	1/30	180/14612	0.00555556	2.70592593	1.04454033	0.31080165	0.42248629	0.35712557	Kcnk2	1
GO:0043679	axon terminus	1/30	182/14612	0.00549451	2.67619048	1.0320909	0.31366407	0.42248629	0.35712557	Kcnk2	1
GO:0055037	recycling endosome	1/30	187/14612	0.00534759	2.60463458	1.00168482	0.32076991	0.42341628	0.35791169	Fzd7	1
GO:0044391	ribosomal subunit	1/30	193/14612	0.00518135	2.52366149	0.96647722	0.32920308	0.42602752	0.36011896	Rpl8	1
GO:0044306	neuron projection terminus	1/30	204/14612	0.00490196	2.3875817	0.90524023	0.34440176	0.43712531	0.36949986	Kcnk2	1
GO:0005840	ribosome	1/30	240/14612	0.00416667	2.02944444	0.72936007	0.39185078	0.48048028	0.4061476	Rpl8	1
GO:0030016	myofibril	1/30	241/14612	0.00414938	2.02102351	0.72492457	0.39312023	0.48048028	0.4061476	Col6a1	1
GO:0043292	contractile muscle fiber	1/30	249/14612	0.00401606	1.95609103	0.69018956	0.40318399	0.48382079	0.40897132	Col6a1	1
GO:0034702	monoatomic ion channel complex	1/30	271/14612	0.00369004	1.79729397	0.6009045	0.43003457	0.50682645	0.42841789	Kcnk2	1
GO:0030141	secretory granule	1/30	318/14612	0.00314465	1.53165618	0.43477002	0.48354153	0.55989019	0.47327241	Loxl1	1
GO:0045211	postsynaptic membrane	1/30	365/14612	0.00273973	1.33442922	0.29348168	0.53217747	0.60558125	0.51189484	Kcnk2	1
GO:1902495	transmembrane transporter complex	1/30	388/14612	0.00257732	1.25532646	0.23120268	0.55433426	0.61491642	0.51978581	Kcnk2	1
GO:0150034	distal axon	1/30	393/14612	0.00254453	1.23935539	0.21817099	0.55901493	0.61491642	0.51978581	Kcnk2	1
GO:1990351	transporter complex	1/30	412/14612	0.00242718	1.18220065	0.17015566	0.57637187	0.61735641	0.52184832	Kcnk2	1
GO:0005874	microtubule	1/30	416/14612	0.00240385	1.17083333	0.160335	0.57994087	0.61735641	0.52184832	Kifc3	1
GO:0010008	endosome membrane	1/30	456/14612	0.00219298	1.06812865	0.06704	0.614069	0.62929385	0.53193898	Fzd7	1
GO:0005667	transcription regulator complex	1/30	458/14612	0.00218341	1.06346434	0.06259144	0.61570308	0.62929385	0.53193898	H1f0	1
GO:0098797	plasma membrane protein complex	1/30	463/14612	0.00215983	1.05197984	0.05155308	0.61975909	0.62929385	0.53193898	Kcnk2	1
GO:0015629	actin cytoskeleton	1/30	495/14612	0.0020202	0.98397306	-0.0164541	0.64475451	0.64475451	0.5450078	H1f0	1

ID	Description	GeneRatio	BgRatio	RichFactor	FoldEnrichm	zScore	pvalue	p.adjust	qvalue	geneID	Count
GO:0009925	basal plasma membrane	5/69	258/14612	0.01937984	4.10403325	3.46487252	0.00740463	0.40527483	0.38692089	Slco1a4/Ajap1/Ager/Slc30a1/Enpp1	5
GO:0045178	basal part of cell	5/69	275/14612	0.01818182	3.85032938	3.28677343	0.00960597	0.40527483	0.38692089	Slco1a4/Ajap1/Ager/Slc30a1/Enpp1	5
GO:0016607	nuclear speck	6/69	389/14612	0.01542416	3.26634626	3.12063499	0.01002119	0.40527483	0.38692089	Srsf5/Arglu1/Nfatc4/Luc7l2/Ehmt2/Dazap2	6
GO:0014069	postsynaptic density	6/69	443/14612	0.01354402	2.86819119	2.75036983	0.01803228	0.40527483	0.38692089	Hnrnpd/Cd200/Slc30a1/Tiam1/Abi3bp/Il1rapl1	6
GO:0032279	asymmetric synapse	6/69	465/14612	0.01290323	2.73249182	2.61518855	0.02229592	0.40527483	0.38692089	Hnrnpd/Cd200/Slc30a1/Tiam1/Abi3bp/Il1rapl1	6
GO:0016323	basolateral plasma membrane	4/69	235/14612	0.01702128	3.60456368	2.77250468	0.02498972	0.40527483	0.38692089	Slco1a4/Ajap1/Slc30a1/Enpp1	4
GO:0001673	male germ cell nucleus	2/69	54/14612	0.03703704	7.84326355	3.47013845	0.02685684	0.40527483	0.38692089	Spata24/Cdk2	2
GO:0099572	postsynaptic specialization	6/69	487/14612	0.01232033	2.60905276	2.4875857	0.02719833	0.40527483	0.38692089	Hnrnpd/Cd200/Slc30a1/Tiam1/Abi3bp/Il1rapl1	6
GO:0098839	postsynaptic density membrane	3/69	147/14612	0.02040816	4.32179828	2.78811202	0.03227004	0.40527483	0.38692089	Slc30a1/Tiam1/Il1rapl1	3
GO:0043073	germ cell nucleus	2/69	66/14612	0.03030303	6.41721563	3.03818219	0.03883279	0.40527483	0.38692089	Spata24/Cdk2	2
GO:0005922	connexin complex	1/69	10/14612	0.1	21.1768116	4.39626517	0.0462445	0.40527483	0.38692089	Gja4	1
GO:0019774	proteasome core complex, beta-subunit complex	1/69	10/14612	0.1	21.1768116	4.39626517	0.0462445	0.40527483	0.38692089	Psmb9	1
GO:0061702	canonical inflammasome complex	1/69	10/14612	0.1	21.1768116	4.39626517	0.0462445	0.40527483	0.38692089	Aim2	1
GO:0097441	basal dendrite	1/69	10/14612	0.1	21.1768116	4.39626517	0.0462445	0.40527483	0.38692089	Enpp1	1
GO:0098553	lumenal side of endoplasmic reticulum membrane	1/69	10/14612	0.1	21.1768116	4.39626517	0.0462445	0.40527483	0.38692089	H2-T23	1
GO:0099147	extrinsic component of postsynaptic density membrane	1/69	10/14612	0.1	21.1768116	4.39626517	0.0462445	0.40527483	0.38692089	Tiam1	1
GO:0032398	MHC class Ib protein complex	1/69	11/14612	0.09090909	19.2516469	4.17104249	0.05075135	0.40527483	0.38692089	H2-T23	1
GO:0099634	postsynaptic specialization membrane	3/69	180/14612	0.01666667	3.5294686	2.35201344	0.0533517	0.40527483	0.38692089	Slc30a1/Tiam1/Il1rapl1	3
GO:0042612	MHC class I protein complex	1/69	12/14612	0.08333333	17.647343	3.97371427	0.05523722	0.40527483	0.38692089	H2-T23	1
GO:0098892	extrinsic component of postsynaptic specialization membrane	1/69	12/14612	0.08333333	17.647343	3.97371427	0.05523722	0.40527483	0.38692089	Tiam1	1
GO:1902711	GABA-A receptor complex	1/69	12/14612	0.08333333	17.647343	3.97371427	0.05523722	0.40527483	0.38692089	Gabrg1	1
GO:0044291	cell-cell contact zone	2/69	82/14612	0.02439024	5.165076	2.60516097	0.05731743	0.40527483	0.38692089	Ajap1/Tiam1	2
GO:0005662	DNA replication factor A complex	1/69	13/14612	0.07692308	16.2898551	3.79884002	0.0597022	0.40527483	0.38692089	Rpa2	1
GO:0030897	HOPS complex	1/69	13/14612	0.07692308	16.2898551	3.79884002	0.0597022	0.40527483	0.38692089	Stx17	1
GO:1902710	GABA receptor complex	1/69	14/14612	0.07142857	15.126294	3.64236188	0.06414637	0.40527483	0.38692089	Gabrg1	1
GO:0005614	interstitial matrix	1/69	15/14612	0.06666667	14.1178744	3.50118297	0.06856985	0.40527483	0.38692089	Abi3bp	1
GO:0035861	site of double-strand break	2/69	93/14612	0.02150538	4.55415303	2.36835477	0.07145968	0.40527483	0.38692089	Aim2/Rpa2	2
GO:0005839	proteasome core complex	1/69	17/14612	0.05882353	12.456948	3.25558371	0.07735508	0.40527483	0.38692089	Psmb9	1
GO:0071004	U2-type prespliceosome	1/69	17/14612	0.05882353	12.456948	3.25558371	0.07735508	0.40527483	0.38692089	Luc7l2	1
GO:0071010	prespliceosome	1/69	17/14612	0.05882353	12.456948	3.25558371	0.07735508	0.40527483	0.38692089	Luc7l2	1
GO:0098576	lumenal side of membrane	1/69	17/14612	0.05882353	12.456948	3.25558371	0.07735508	0.40527483	0.38692089	H2-T23	1
GO:0000159	protein phosphatase type 2A complex	1/69	18/14612	0.05555556	11.7648953	3.14772227	0.08171701	0.40527483	0.38692089	Ppp2r3a	1
GO:0005685	U1 snRNP	1/69	18/14612	0.05555556	11.7648953	3.14772227	0.08171701	0.40527483	0.38692089	Luc7l2	1
GO:0042611	MHC protein complex	1/69	20/14612	0.05	10.5884058	2.95557175	0.09038001	0.40527483	0.38692089	H2-T23	1
GO:0043197	dendritic spine	3/69	227/14612	0.01321586	2.79869757	1.88128094	0.09211894	0.40527483	0.38692089	Nr1d1/Tiam1/Abi3bp	3
GO:0045211	postsynaptic membrane	4/69	365/14612	0.0109589	2.32074648	1.76011764	0.09380662	0.40527483	0.38692089	Slc30a1/Gabrg1/Tiam1/Il1rapl1	4
GO:0005921	gap junction	1/69	21/14612	0.04761905	10.084196	2.86940023	0.09468126	0.40527483	0.38692089	Gja4	1
GO:0043601	nuclear replisome	1/69	21/14612	0.04761905	10.084196	2.86940023	0.09468126	0.40527483	0.38692089	Rpa2	1
GO:0044233	mitochondria-associated endoplasmic reticulum membrane contact site	1/69	21/14612	0.04761905	10.084196	2.86940023	0.09468126	0.40527483	0.38692089	Stx17	1
GO:0000781	chromosome, telomeric region	2/69	110/14612	0.01818182	3.85032938	2.06687854	0.09523184	0.40527483	0.38692089	Cdk2/Rpa2	2
GO:0044309	neuron spine	3/69	232/14612	0.01293103	2.73838081	1.83842951	0.09678774	0.40527483	0.38692089	Nr1d1/Tiam1/Abi3bp	3
GO:0030894	replisome	1/69	22/14612	0.04545455	9.62582345	2.78882828	0.09896246	0.40527483	0.38692089	Rpa2	1
GO:0005732	sno(s)RNA-containing ribonucleoprotein complex	1/69	23/14612	0.04347826	9.20730939	2.7132479	0.10322371	0.41075546	0.39215331	Rrp9	1
GO:0000242	pericentriolar material	1/69	24/14612	0.04166667	8.8236715	2.64214023	0.10746509	0.41075546	0.39215331	Tiam1	1
GO:0098890	extrinsic component of postsynaptic membrane	1/69	24/14612	0.04166667	8.8236715	2.64214023	0.10746509	0.41075546	0.39215331	Tiam1	1
GO:0090734	site of DNA damage	2/69	122/14612	0.01639344	3.47160846	1.88826485	0.11318405	0.42320992	0.40404374	Aim2/Rpa2	2
GO:0043202	lysosomal lumen	1/69	27/14612	0.03703704	7.84326355	2.45148616	0.12007098	0.43940871	0.41950893	Man2b1	1
GO:0012507	ER to Golgi transport vesicle membrane	1/69	29/14612	0.03448276	7.30234883	2.33999814	0.12837727	0.46001853	0.43918538	Stx17	1
GO:0097440	apical dendrite	1/69	30/14612	0.03333333	7.0589372	2.28815819	0.13250138	0.46510689	0.44404329	Enpp1	1
GO:0005775	vacuolar lumen	1/69	31/14612	0.03225806	6.83122955	2.23864301	0.13660626	0.46992555	0.44864373	Man2b1	1
GO:0016235	aggresome	1/69	33/14612	0.03030303	6.41721563	2.14588646	0.14475869	0.48820577	0.46609608	Xrn2	1
GO:0005790	smooth endoplasmic reticulum	1/69	34/14612	0.02941176	6.228474	2.10233999	0.1488064	0.49220579	0.46991496	Stx17	1
GO:0034707	chloride channel complex	1/69	35/14612	0.02857143	6.0505176	2.06050346	0.15283524	0.4959936	0.47353122	Gabrg1	1
GO:0000803	sex chromosome	1/69	36/14612	0.02777778	5.88244767	2.02025961	0.15684528	0.49958126	0.4769564	Cdk2	1
GO:0043596	nuclear replication fork	1/69	38/14612	0.02631579	5.57284516	1.94413503	0.16480932	0.50620007	0.48327546	Rpa2	1
GO:0099243	extrinsic component of synaptic membrane	1/69	38/14612	0.02631579	5.57284516	1.94413503	0.16480932	0.50620007	0.48327546	Tiam1	1
GO:0005667	transcription regulator complex	4/69	458/14612	0.00873362	1.8495032	1.27231801	0.17034622	0.51402719	0.49074811	Cdk2/Nfatc4/Nfe2l3/Dazap2	4
GO:0005911	cell-cell junction	4/69	467/14612	0.00856531	1.81385967	1.23124365	0.17870715	0.52624905	0.50241647	Ccdc88c/Ajap1/Gja4/Tiam1	4
GO:0033116	endoplasmic reticulum-Golgi intermediate compartment membrane	1/69	42/14612	0.02380952	5.042098	1.80692123	0.18051566	0.52624905	0.50241647	Stx17	1
GO:0031201	SNARE complex	1/69	45/14612	0.02222222	4.70595813	1.71498063	0.19210404	0.53041384	0.50639265	Stx17	1
GO:0031901	early endosome membrane	2/69	174/14612	0.01149425	2.43411628	1.31081977	0.19845484	0.53041384	0.50639265	Epha8/Ldlrad4	2
GO:0044232	organelle membrane contact site	1/69	47/14612	0.0212766	4.50570459	1.65808395	0.19973975	0.53041384	0.50639265	Stx17	1
GO:0044295	axonal growth cone	1/69	47/14612	0.0212766	4.50570459	1.65808395	0.19973975	0.53041384	0.50639265	Tiam1	1
GO:0008287	protein serine/threonine phosphatase complex	1/69	48/14612	0.02083333	4.41183575	1.63081956	0.20353089	0.53041384	0.50639265	Ppp2r3a	1
GO:0099092	postsynaptic density, intracellular component	1/69	48/14612	0.02083333	4.41183575	1.63081956	0.20353089	0.53041384	0.50639265	Slc30a1	1
GO:1903293	phosphatase complex	1/69	48/14612	0.02083333	4.41183575	1.63081956	0.20353089	0.53041384	0.50639265	Ppp2r3a	1
GO:0000307	cyclin-dependent protein kinase holoenzyme complex	1/69	51/14612	0.01960784	4.152316	1.55330502	0.21479843	0.53543957	0.51119078	Cdk2	1
GO:0000421	autophagosome membrane	1/69	51/14612	0.01960784	4.152316	1.55330502	0.21479843	0.53543957	0.51119078	Stx17	1
GO:0032590	dendrite membrane	1/69	51/14612	0.01960784	4.152316	1.55330502	0.21479843	0.53543957	0.51119078	Gabrg1	1
GO:0030670	phagocytic vesicle membrane	1/69	52/14612	0.01923077	4.07246377	1.52878097	0.21851926	0.53693303	0.5126166	H2-T23	1
GO:0015030	Cajal body	1/69	54/14612	0.01851852	3.92163178	1.48152511	0.22590885	0.54727215	0.52248748	Cdk2	1
GO:0031256	leading edge membrane	2/69	192/14612	0.01041667	2.20591787	1.15857254	0.22962621	0.5485515	0.52370889	Gabrg1/Tiam1	2
GO:0099091	postsynaptic specialization, intracellular component	1/69	56/14612	0.01785714	3.7815735	1.43648483	0.23322957	0.54952721	0.52464042	Slc30a1	1
GO:0030134	COPII-coated ER to Golgi transport vesicle	1/69	57/14612	0.01754386	3.7152301	1.41473623	0.23686431	0.55054947	0.52561638	Stx17	1
GO:0032421	stereocilium bundle	1/69	59/14612	0.01694915	3.5892901	1.37267576	0.2440829	0.55976346	0.53441309	Tiam1	1
GO:0097525	spliceosomal snRNP complex	1/69	63/14612	0.01587302	3.36139867	1.29378029	0.25831882	0.57915626	0.55292764	Luc7l2	1
GO:0000502	proteasome complex	1/69	64/14612	0.015625	3.30887681	1.27504824	0.26183631	0.57915626	0.55292764	Psmb9	1
GO:0005581	collagen trimer	1/69	68/14612	0.01470588	3.114237	1.20365966	0.27574262	0.57915626	0.55292764	C1qtnf7	1
GO:0005657	replication fork	1/69	70/14612	0.01428571	3.0252588	1.16991684	0.28259863	0.57915626	0.55292764	Rpa2	1
GO:0099023	vesicle tethering complex	1/69	70/14612	0.01428571	3.0252588	1.16991684	0.28259863	0.57915626	0.55292764	Stx17	1
GO:0030532	small nuclear ribonucleoprotein complex	1/69	71/14612	0.01408451	2.98264952	1.15349443	0.28600261	0.57915626	0.55292764	Luc7l2	1
GO:0043198	dendritic shaft	1/69	71/14612	0.01408451	2.98264952	1.15349443	0.28600261	0.57915626	0.55292764	Abi3bp	1
GO:0005769	early endosome	3/69	399/14612	0.0075188	1.59224147	0.82618994	0.29145163	0.57915626	0.55292764	Rftn1/Epha8/Ldlrad4	3
GO:0016234	inclusion body	1/69	73/14612	0.01369863	2.9009331	1.12149801	0.29276289	0.57915626	0.55292764	Xrn2	1
GO:0030666	endocytic vesicle membrane	1/69	73/14612	0.01369863	2.9009331	1.12149801	0.29276289	0.57915626	0.55292764	H2-T23	1
GO:0032589	neuron projection membrane	1/69	74/14612	0.01351351	2.8617313	1.10590556	0.29611934	0.57915626	0.55292764	Gabrg1	1
GO:0030315	T-tubule	1/69	75/14612	0.01333333	2.82357488	1.09057202	0.29946009	0.57915626	0.55292764	Slc30a1	1
GO:0032040	small-subunit processome	1/69	75/14612	0.01333333	2.82357488	1.09057202	0.29946009	0.57915626	0.55292764	Rrp9	1
GO:0030426	growth cone	2/69	232/14612	0.00862069	1.82558721	0.87310207	0.29967969	0.57915626	0.55292764	Ngef/Tiam1	2
GO:0030427	site of polarized growth	2/69	238/14612	0.00840336	1.779564	0.83519704	0.3101601	0.59275041	0.56590614	Ngef/Tiam1	2
GO:0045171	intercellular bridge	1/69	82/14612	0.01219512	2.582538	0.98984178	0.32241176	0.60113065	0.57390686	Pcif1	1
GO:1903561	extracellular vesicle	1/69	82/14612	0.01219512	2.582538	0.98984178	0.32241176	0.60113065	0.57390686	Hexd	1
GO:0120114	Sm-like protein family complex	1/69	83/14612	0.01204819	2.55142308	0.97631272	0.32562949	0.60113065	0.57390686	Luc7l2	1
GO:0010494	cytoplasmic stress granule	1/69	85/14612	0.01176471	2.4913896	0.94983926	0.33201984	0.60113065	0.57390686	Dazap2	1
GO:0044304	main axon	1/69	85/14612	0.01176471	2.4913896	0.94983926	0.33201984	0.60113065	0.57390686	Tiam1	1
GO:0043230	extracellular organelle	1/69	90/14612	0.01111111	2.35297907	0.88682235	0.34773578	0.61660364	0.58867912	Hexd	1
GO:0065010	extracellular membrane-bounded organelle	1/69	90/14612	0.01111111	2.35297907	0.88682235	0.34773578	0.61660364	0.58867912	Hexd	1
GO:1905369	endopeptidase complex	1/69	92/14612	0.01086957	2.30182735	0.86278286	0.35391961	0.62116504	0.59303394	Psmb9	1
GO:0031252	cell leading edge	3/69	454/14612	0.00660793	1.39934878	0.595411	0.36286405	0.62971689	0.6011985	Gabrg1/Tiam1/Abi3bp	3
GO:0005684	U2-type spliceosomal complex	1/69	96/14612	0.01041667	2.20591787	0.81652105	0.36611447	0.62971689	0.6011985	Luc7l2	1
GO:0005793	endoplasmic reticulum-Golgi intermediate compartment	1/69	99/14612	0.01010101	2.13907188	0.78329913	0.37511148	0.63254092	0.60389464	Stx17	1
GO:0032587	ruffle membrane	1/69	99/14612	0.01010101	2.13907188	0.78329913	0.37511148	0.63254092	0.60389464	Tiam1	1
GO:0005788	endoplasmic reticulum lumen	1/69	105/14612	0.00952381	2.0168392	0.72027052	0.3927296	0.65276918	0.6232068	Foxred2	1
GO:0016605	PML body	1/69	107/14612	0.00934579	1.97914127	0.70018998	0.39849281	0.65276918	0.6232068	Rpa2	1
GO:0030684	preribosome	1/69	107/14612	0.00934579	1.97914127	0.70018998	0.39849281	0.65276918	0.6232068	Rrp9	1
GO:0005776	autophagosome	1/69	111/14612	0.00900901	1.90782086	0.66130253	0.409858	0.65558979	0.62589967	Stx17	1
GO:1902554	serine/threonine protein kinase complex	1/69	111/14612	0.00900901	1.90782086	0.66130253	0.409858	0.65558979	0.62589967	Cdk2	1
GO:0009897	external side of plasma membrane	2/69	298/14612	0.00671141	1.42126252	0.50607859	0.41241189	0.65558979	0.62589967	Slamf9/H2-T23	2
GO:0019897	extrinsic component of plasma membrane	1/69	113/14612	0.00884956	1.87405412	0.64245992	0.41546097	0.65558979	0.62589967	Tiam1	1
GO:0045335	phagocytic vesicle	1/69	117/14612	0.00854701	1.8099839	0.60589549	0.42651009	0.65696365	0.62721132	H2-T23	1
GO:0097730	non-motile cilium	1/69	117/14612	0.00854701	1.8099839	0.60589549	0.42651009	0.65696365	0.62721132	Tiam1	1
GO:1905368	peptidase complex	1/69	122/14612	0.00819672	1.73580423	0.56214133	0.44003241	0.65696365	0.62721132	Psmb9	1
GO:0022626	cytosolic ribosome	1/69	123/14612	0.00813008	1.721692	0.553634	0.44269893	0.65696365	0.62721132	Zfp598	1
GO:0030662	coated vesicle membrane	1/69	123/14612	0.00813008	1.721692	0.553634	0.44269893	0.65696365	0.62721132	Stx17	1
GO:1902911	protein kinase complex	1/69	123/14612	0.00813008	1.721692	0.553634	0.44269893	0.65696365	0.62721132	Cdk2	1
GO:0016324	apical plasma membrane	2/69	319/14612	0.00626959	1.32769979	0.40761233	0.44655533	0.65696365	0.62721132	Ajap1/Ager	2
GO:0098687	chromosomal region	2/69	321/14612	0.00623053	1.31942751	0.39859426	0.44975164	0.65696365	0.62721132	Cdk2/Rpa2	2
GO:0043296	apical junction complex	1/69	127/14612	0.00787402	1.66746548	0.52036603	0.45324041	0.65696365	0.62721132	Ccdc88c	1
GO:0031253	cell projection membrane	2/69	324/14612	0.00617284	1.30721059	0.38517712	0.45452718	0.65696365	0.62721132	Gabrg1/Tiam1	2
GO:0005774	vacuolar membrane	2/69	334/14612	0.00598802	1.26807255	0.34137319	0.47027784	0.67406491	0.6435381	Fnip1/Stx17	2
GO:0048786	presynaptic active zone	1/69	136/14612	0.00735294	1.5571185	0.4496041	0.47624557	0.67697717	0.64631848	Cd200	1
GO:0001650	fibrillar center	1/69	144/14612	0.00694444	1.47061192	0.39091058	0.49589206	0.69344255	0.66203818	Ager	1
GO:0098685	Schaffer collateral - CA1 synapse	1/69	144/14612	0.00694444	1.47061192	0.39091058	0.49589206	0.69344255	0.66203818	Slc30a1	1
GO:0009898	cytoplasmic side of plasma membrane	1/69	147/14612	0.00680272	1.44059943	0.36981191	0.5030707	0.69780775	0.66620568	Ajap1	1
GO:0032993	protein-DNA complex	1/69	154/14612	0.00649351	1.37511764	0.32233781	0.51943141	0.71219589	0.67994223	Rpa2	1
GO:0098862	cluster of actin-based cell projections	1/69	155/14612	0.00645161	1.36624591	0.31574542	0.5217249	0.71219589	0.67994223	Tiam1	1
GO:0005912	adherens junction	1/69	161/14612	0.00621118	1.31532991	0.27711924	0.53526102	0.72492044	0.6920905	Ajap1	1
GO:0045177	apical part of cell	2/69	393/14612	0.00508906	1.07770034	0.10755291	0.55752277	0.7433637	0.70969851	Ajap1/Ager	2
GO:0150034	distal axon	2/69	393/14612	0.00508906	1.07770034	0.10755291	0.55752277	0.7433637	0.70969851	Ngef/Tiam1	2
GO:0019898	extrinsic component of membrane	1/69	174/14612	0.00574713	1.21705814	0.19839681	0.5633074	0.74529903	0.71154619	Tiam1	1
GO:0042383	sarcolemma	1/69	178/14612	0.00561798	1.18970852	0.17540461	0.57159652	0.75049314	0.71650508	Slc30a1	1
GO:0001726	ruffle	1/69	187/14612	0.00534759	1.13244982	0.12556013	0.58968428	0.75690819	0.7226296	Tiam1	1
GO:0005741	mitochondrial outer membrane	1/69	187/14612	0.00534759	1.13244982	0.12556013	0.58968428	0.75690819	0.7226296	Fundc2	1
GO:0055037	recycling endosome	1/69	187/14612	0.00534759	1.13244982	0.12556013	0.58968428	0.75690819	0.7226296	Dennd6b	1
GO:0098562	cytoplasmic side of membrane	1/69	190/14612	0.00526316	1.11456903	0.10948858	0.59554463	0.75876798	0.72440517	Ajap1	1
GO:0005681	spliceosomal complex	1/69	194/14612	0.00515464	1.09158823	0.08845564	0.60323029	0.7629089	0.72835856	Luc7l2	1
GO:0030139	endocytic vesicle	1/69	203/14612	0.00492611	1.04319269	0.04268535	0.62000068	0.77163485	0.73668933	H2-T23	1
GO:0000228	nuclear chromosome	1/69	204/14612	0.00490196	1.038079	0.03772561	0.62182037	0.77163485	0.73668933	Rpa2	1
GO:0030027	lamellipodium	1/69	212/14612	0.00471698	0.99890621	-0.001105	0.63607207	0.77163485	0.73668933	Abi3bp	1
GO:0010008	endosome membrane	2/69	456/14612	0.00438596	0.92880753	-0.1063858	0.63914203	0.77163485	0.73668933	Epha8/Ldlrad4	2
GO:0019867	outer membrane	1/69	214/14612	0.0046729	0.98957064	-0.0105865	0.63955148	0.77163485	0.73668933	Fundc2	1
GO:0031968	organelle outer membrane	1/69	214/14612	0.0046729	0.98957064	-0.0105865	0.63955148	0.77163485	0.73668933	Fundc2	1
GO:0005635	nuclear envelope	2/69	458/14612	0.00436681	0.9247516	-0.112701	0.64153362	0.77163485	0.73668933	Cdk2/Slc30a1	2
GO:0098797	plasma membrane protein complex	2/69	463/14612	0.00431965	0.91476508	-0.1283757	0.64745922	0.77328824	0.73826784	H2-T23/Gja4	2
GO:0090575	RNA polymerase II transcription regulator complex	1/69	227/14612	0.00440529	0.93289919	-0.0701816	0.66138188	0.77328824	0.73826784	Nfe2l3	1
GO:0036464	cytoplasmic ribonucleoprotein granule	1/69	229/14612	0.00436681	0.9247516	-0.0790547	0.66462268	0.77328824	0.73826784	Dazap2	1
GO:0030658	transport vesicle membrane	1/69	231/14612	0.004329	0.91674509	-0.0878534	0.6678329	0.77328824	0.73826784	Stx17	1
GO:0000323	lytic vacuole	2/69	487/14612	0.00410678	0.86968425	-0.2014673	0.67484803	0.77328824	0.73826784	Fnip1/Man2b1	2
GO:0005764	lysosome	2/69	487/14612	0.00410678	0.86968425	-0.2014673	0.67484803	0.77328824	0.73826784	Fnip1/Man2b1	2
GO:0005840	ribosome	1/69	240/14612	0.00416667	0.88236715	-0.1265649	0.68190815	0.77328824	0.73826784	Zfp598	1
GO:0030135	coated vesicle	1/69	240/14612	0.00416667	0.88236715	-0.1265649	0.68190815	0.77328824	0.73826784	Stx17	1
GO:0005802	trans-Golgi network	1/69	243/14612	0.00411523	0.87147373	-0.1391616	0.68646799	0.77328824	0.73826784	Sys1	1
GO:0000793	condensed chromosome	1/69	248/14612	0.00403226	0.85390369	-0.1598324	0.69392497	0.77328824	0.73826784	Cdk2	1
GO:0035770	ribonucleoprotein granule	1/69	248/14612	0.00403226	0.85390369	-0.1598324	0.69392497	0.77328824	0.73826784	Dazap2	1
GO:0061695	transferase complex, transferring phosphorus-containing groups	1/69	250/14612	0.004	0.84707246	-0.1679909	0.69685858	0.77328824	0.73826784	Cdk2	1
GO:0034702	monoatomic ion channel complex	1/69	271/14612	0.00369004	0.78143216	-0.2501605	0.72603664	0.80050193	0.76424909	Gabrg1	1
GO:0005765	lysosomal membrane	1/69	280/14612	0.00357143	0.7563147	-0.283591	0.73767733	0.80206971	0.76574586	Fnip1	1
GO:0098852	lytic vacuole membrane	1/69	280/14612	0.00357143	0.7563147	-0.283591	0.73767733	0.80206971	0.76574586	Fnip1	1
GO:0031965	nuclear membrane	1/69	283/14612	0.00353357	0.74829723	-0.2945173	0.74144816	0.80206971	0.76574586	Slc30a1	1
GO:0045121	membrane raft	1/69	299/14612	0.00334448	0.70825457	-0.3510846	0.76067487	0.8134263	0.77658815	Rftn1	1
GO:0098857	membrane microdomain	1/69	301/14612	0.00332226	0.70354856	-0.357964	0.76297686	0.8134263	0.77658815	Rftn1	1
GO:0062023	collagen-containing extracellular matrix	1/69	306/14612	0.00326797	0.69205267	-0.3749864	0.7686368	0.8134263	0.77658815	Abi3bp	1
GO:0043235	receptor complex	1/69	308/14612	0.00324675	0.68755882	-0.3817266	0.7708633	0.8134263	0.77658815	Gabrg1	1
GO:0005938	cell cortex	1/69	328/14612	0.00304878	0.6456345	-0.4470962	0.79199869	0.83063277	0.79301538	Gpsm1	1
GO:0098791	Golgi apparatus subcompartment	1/69	347/14612	0.00288184	0.61028275	-0.5060763	0.81029261	0.84335632	0.80516271	Sys1	1

GO:0005759	mitochondrial matrix	1/69	351/14612	0.002849	0.60332797	-0.5181406	0.81393692	0.84335632	0.80516271	Tst	1
GO:1902495	transmembrane transporter complex	1/69	388/14612	0.00257732	0.54579411	-0.6245903	0.84453756	0.8643447	0.82520057	Gabrg1	1
GO:0030312	external encapsulating structure	1/69	398/14612	0.00251256	0.53208069	-0.6519163	0.85191875	0.8643447	0.82520057	Abi3bp	1
GO:0031012	extracellular matrix	1/69	398/14612	0.00251256	0.53208069	-0.6519163	0.85191875	0.8643447	0.82520057	Abi3bp	1
GO:0030133	transport vesicle	1/69	411/14612	0.00243309	0.51525089	-0.6866193	0.86100001	0.8643447	0.82520057	Stx17	1
GO:1990351	transporter complex	1/69	412/14612	0.00242718	0.51400028	-0.6892519	0.86167539	0.8643447	0.82520057	Gabrg1	1
GO:0005874	microtubule	1/69	416/14612	0.00240385	0.50905797	-0.6997315	0.8643447	0.8643447	0.82520057	Tiam1	1

ID	Description	GeneRatio	BgRatio	RichFactor	FoldEnrichm	zScore	pvalue	p.adjust	qvalue	geneID	Count
GO:0098636	protein complex involved in cell adhesion	2/101	49/14612	0.04081633	5.90503132	2.86923676	0.04508586	0.83742825	0.83742825	Lyn/Jam2	2
GO:0031414	N-terminal protein acetyltransferase complex	1/101	10/14612	0.1	14.4673267	3.5540801	0.06703044	0.83742825	0.83742825	Naa50	1
GO:0097450	astrocyte end-foot	1/101	11/14612	0.09090909	13.1521152	3.36363534	0.07348366	0.83742825	0.83742825	Clcn2	1
GO:1904090	peptidase inhibitor complex	1/101	11/14612	0.09090909	13.1521152	3.36363534	0.07348366	0.83742825	0.83742825	Serpine1	1
GO:1990062	RPAP3/R2TP/prefoldin-like complex	1/101	11/14612	0.09090909	13.1521152	3.36363534	0.07348366	0.83742825	0.83742825	Rpap3	1
GO:0043073	germ cell nucleus	2/101	66/14612	0.03030303	4.3840384	2.29872726	0.07631587	0.83742825	0.83742825	Ankrd37/Ncapd3	2
GO:0001674	female germ cell nucleus	1/101	12/14612	0.08333333	12.0561056	3.19645327	0.07989269	0.83742825	0.83742825	Ncapd3	1
GO:0044666	MLL3/4 complex	1/101	12/14612	0.08333333	12.0561056	3.19645327	0.07989269	0.83742825	0.83742825	Paxip1	1
GO:0098895	postsynaptic endosome membrane	1/101	12/14612	0.08333333	12.0561056	3.19645327	0.07989269	0.83742825	0.83742825	Myo5b	1
GO:0070062	extracellular exosome	2/101	68/14612	0.02941176	4.2550961	2.24454504	0.08034241	0.83742825	0.83742825	Serpine1/Rbmx	2
GO:0030061	mitochondrial crista	1/101	14/14612	0.07142857	10.3338048	2.91492939	0.09257934	0.83742825	0.83742825	Lyn	1
GO:0042583	chromaffin granule	1/101	14/14612	0.07142857	10.3338048	2.91492939	0.09257934	0.83742825	0.83742825	Serpine1	1
GO:0098837	postsynaptic recycling endosome	1/101	14/14612	0.07142857	10.3338048	2.91492939	0.09257934	0.83742825	0.83742825	Myo5b	1
GO:0016586	RSC-type complex	1/101	15/14612	0.06666667	9.64488449	2.79463444	0.09885756	0.83742825	0.83742825	Arid2	1
GO:0030127	COPII vesicle coat	1/101	15/14612	0.06666667	9.64488449	2.79463444	0.09885756	0.83742825	0.83742825	Sar1b	1
GO:1903561	extracellular vesicle	2/101	82/14612	0.02439024	3.52861628	1.91561855	0.11020676	0.83742825	0.83742825	Serpine1/Rbmx	2
GO:0032591	dendritic spine membrane	1/101	18/14612	0.05555556	8.03740374	2.49237585	0.11743533	0.83742825	0.83742825	Clcn2	1
GO:0042575	DNA polymerase complex	1/101	18/14612	0.05555556	8.03740374	2.49237585	0.11743533	0.83742825	0.83742825	Pole	1
GO:0045120	pronucleus	1/101	18/14612	0.05555556	8.03740374	2.49237585	0.11743533	0.83742825	0.83742825	Tet3	1
GO:0098871	postsynaptic actin cytoskeleton	1/101	18/14612	0.05555556	8.03740374	2.49237585	0.11743533	0.83742825	0.83742825	Myo5b	1
GO:0071013	catalytic step 2 spliceosome	2/101	89/14612	0.02247191	3.25108466	1.77709502	0.12607754	0.83742825	0.83742825	Srrm2/Rbmx	2
GO:0043230	extracellular organelle	2/101	90/14612	0.02222222	3.2149615	1.75843454	0.12838795	0.83742825	0.83742825	Serpine1/Rbmx	2
GO:0065010	extracellular membrane-bounded organelle	2/101	90/14612	0.02222222	3.2149615	1.75843454	0.12838795	0.83742825	0.83742825	Serpine1/Rbmx	2
GO:0005811	lipid droplet	2/101	93/14612	0.02150538	3.11125306	1.70398356	0.1353784	0.83742825	0.83742825	Pnpla7/Lss	2
GO:0031528	microvillus membrane	1/101	22/14612	0.04545455	6.57605761	2.18354895	0.14161703	0.83742825	0.83742825	Slc6a6	1
GO:0031248	protein acetyltransferase complex	2/101	96/14612	0.02083333	3.0140264	1.65169283	0.14245261	0.83742825	0.83742825	Brpf1/Naa50	2
GO:0097449	astrocyte projection	1/101	23/14612	0.04347826	6.29014206	2.11821713	0.14755923	0.83742825	0.83742825	Clcn2	1
GO:1902493	acetyltransferase complex	2/101	100/14612	0.02	2.89346535	1.58506002	0.15200429	0.83742825	0.83742825	Brpf1/Naa50	2
GO:0002080	acrosomal membrane	1/101	24/14612	0.04166667	6.02805281	2.05664607	0.15346069	0.83742825	0.83742825	Spaca6	1
GO:0031985	Golgi cisterna	2/101	101/14612	0.01980198	2.86481717	1.56891806	0.15441203	0.83742825	0.83742825	Sar1b/Rab34	2
GO:0005796	Golgi lumen	1/101	25/14612	0.04	5.78693069	1.99846312	0.15932171	0.83742825	0.83742825	Agrn	1
GO:0099571	postsynaptic cytoskeleton	1/101	25/14612	0.04	5.78693069	1.99846312	0.15932171	0.83742825	0.83742825	Myo5b	1
GO:0032585	multivesicular body membrane	1/101	26/14612	0.03846154	5.56435644	1.94334589	0.16514254	0.83742825	0.83742825	Gimap5	1
GO:0016605	PML body	2/101	107/14612	0.01869159	2.70417322	1.4760421	0.16900822	0.83742825	0.83742825	Ciart/Rere	2
GO:0098845	postsynaptic endosome	1/101	27/14612	0.03703704	5.35826916	1.89101381	0.17092347	0.83742825	0.83742825	Myo5b	1
GO:0016514	SWI/SNF complex	1/101	28/14612	0.03571429	5.1669024	1.84122135	0.17666476	0.83742825	0.83742825	Arid2	1
GO:0005640	nuclear outer membrane	1/101	29/14612	0.03448276	4.98873336	1.79375264	0.18236668	0.83742825	0.83742825	Nav3	1
GO:0012507	ER to Golgi transport vesicle membrane	1/101	29/14612	0.03448276	4.98873336	1.79375264	0.18236668	0.83742825	0.83742825	Sar1b	1
GO:0043194	axon initial segment	1/101	29/14612	0.03448276	4.98873336	1.79375264	0.18236668	0.83742825	0.83742825	Clcn2	1
GO:0005721	pericentric heterochromatin	1/101	30/14612	0.03333333	4.82244224	1.74841691	0.18802951	0.83742825	0.83742825	Ncapd3	1
GO:0008305	integrin complex	1/101	30/14612	0.03333333	4.82244224	1.74841691	0.18802951	0.83742825	0.83742825	Lyn	1
GO:0070971	endoplasmic reticulum exit site	1/101	30/14612	0.03333333	4.82244224	1.74841691	0.18802951	0.83742825	0.83742825	Sar1b	1
GO:0071007	U2-type catalytic step 2 spliceosome	1/101	30/14612	0.03333333	4.82244224	1.74841691	0.18802951	0.83742825	0.83742825	Srrm2	1
GO:1990752	microtubule end	1/101	31/14612	0.03225806	4.66687959	1.70504493	0.1936535	0.83742825	0.83742825	Nav3	1
GO:0016363	nuclear matrix	2/101	121/14612	0.01652893	2.39129367	1.28207757	0.20386736	0.83742825	0.83742825	Paxip1/Arid2	2
GO:0034707	chloride channel complex	1/101	35/14612	0.02857143	4.13352192	1.54840537	0.21576636	0.83742825	0.83742825	Clcn2	1
GO:0101031	protein folding chaperone complex	1/101	35/14612	0.02857143	4.13352192	1.54840537	0.21576636	0.83742825	0.83742825	Rpap3	1
GO:0005795	Golgi stack	2/101	134/14612	0.01492537	2.1593025	1.12472635	0.23689089	0.83742825	0.83742825	Sar1b/Rab34	2
GO:0032391	photoreceptor connecting cilium	1/101	39/14612	0.02564103	3.70957096	1.41354727	0.23727865	0.83742825	0.83742825	Alpk1	1
GO:0099738	cell cortex region	1/101	40/14612	0.025	3.61683168	1.38260521	0.24256479	0.83742825	0.83742825	Myo5b	1
GO:0097386	glial cell projection	1/101	43/14612	0.02325581	3.36449459	1.29541722	0.25820652	0.83742825	0.83742825	Clcn2	1
GO:0005814	centriole	2/101	148/14612	0.01351351	1.95504415	0.97423132	0.27275485	0.83742825	0.83742825	Ccdc57/Rab34	2
GO:0034399	nuclear periphery	2/101	148/14612	0.01351351	1.95504415	0.97423132	0.27275485	0.83742825	0.83742825	Paxip1/Arid2	2
GO:0016459	myosin complex	1/101	46/14612	0.02173913	3.14507103	1.21563285	0.27352837	0.83742825	0.83742825	Myo5b	1
GO:0044295	axonal growth cone	1/101	47/14612	0.0212766	3.07815462	1.19048391	0.2785657	0.83742825	0.83742825	Agrn	1
GO:0016607	nuclear speck	4/101	389/14612	0.01028278	1.48764285	0.8132668	0.28223606	0.83742825	0.83742825	Tcim/Fam76a/Srrm2/Ylpm1	4
GO:0030964	NADH dehydrogenase complex	1/101	48/14612	0.02083333	3.0140264	1.16599699	0.28356843	0.83742825	0.83742825	ND5	1
GO:0045271	respiratory chain complex I	1/101	48/14612	0.02083333	3.0140264	1.16599699	0.28356843	0.83742825	0.83742825	ND5	1
GO:0030312	external encapsulating structure	4/101	398/14612	0.01005025	1.45400269	0.76611469	0.29618878	0.83742825	0.83742825	Angptl2/Serpine1/Lrrc17/Agrn	4
GO:0031012	extracellular matrix	4/101	398/14612	0.01005025	1.45400269	0.76611469	0.29618878	0.83742825	0.83742825	Angptl2/Serpine1/Lrrc17/Agrn	4
GO:0000776	kinetochore	2/101	158/14612	0.01265823	1.83130718	0.87649216	0.29836637	0.83742825	0.83742825	Ncapd3/Arid2	2
GO:0032590	dendrite membrane	1/101	51/14612	0.01960784	2.83673073	1.09619285	0.29837148	0.83742825	0.83742825	Clcn2	1
GO:0005771	multivesicular body	1/101	52/14612	0.01923077	2.78217822	1.07404902	0.30323821	0.83742825	0.83742825	Gimap5	1
GO:0030670	phagocytic vesicle membrane	1/101	52/14612	0.01923077	2.78217822	1.07404902	0.30323821	0.83742825	0.83742825	Rab34	1
GO:0071005	U2-type precatalytic spliceosome	1/101	53/14612	0.01886792	2.72968429	1.05242459	0.30807152	0.83742825	0.83742825	Srrm2	1
GO:0001673	male germ cell nucleus	1/101	54/14612	0.01851852	2.67913458	1.03129643	0.31287162	0.83742825	0.83742825	Ankrd37	1
GO:0015030	Cajal body	1/101	54/14612	0.01851852	2.67913458	1.03129643	0.31287162	0.83742825	0.83742825	Srrm2	1
GO:0071011	precatalytic spliceosome	1/101	55/14612	0.01818182	2.63042304	1.01064282	0.31763876	0.83742825	0.83742825	Srrm2	1
GO:0099091	postsynaptic specialization, intracellular component	1/101	56/14612	0.01785714	2.5834512	0.9904434	0.32237315	0.83742825	0.83742825	Lyn	1
GO:0000779	condensed chromosome, centromeric region	2/101	168/14612	0.01190476	1.7223008	0.78556293	0.32384525	0.83742825	0.83742825	Ncapd3/Arid2	2
GO:0030134	COPII-coated ER to Golgi transport vesicle	1/101	57/14612	0.01754386	2.5381275	0.97067902	0.32707501	0.83742825	0.83742825	Sar1b	1
GO:0030120	vesicle coat	1/101	59/14612	0.01694915	2.45208928	0.9323845	0.33638204	0.83742825	0.83742825	Sar1b	1
GO:0000794	condensed nuclear chromosome	1/101	60/14612	0.01666667	2.41122112	0.91382143	0.34098765	0.83742825	0.83742825	Ncapd3	1
GO:0000791	euchromatin	1/101	61/14612	0.01639344	2.37169291	0.89562741	0.34556161	0.83742825	0.83742825	Rbmx	1
GO:0032580	Golgi cisterna membrane	1/101	63/14612	0.01587302	2.29640107	0.86029039	0.35461544	0.83742825	0.83742825	Sar1b	1
GO:0062023	collagen-containing extracellular matrix	3/101	306/14612	0.00980392	1.41836537	0.61703431	0.35485639	0.83742825	0.83742825	Angptl2/Serpine1/Agrn	3
GO:0001726	ruffle	2/101	187/14612	0.01069519	1.54730767	0.62841481	0.37155455	0.83742825	0.83742825	Rab34/Abl1	2
GO:0005657	replication fork	1/101	70/14612	0.01428571	2.06676096	0.74637322	0.38533807	0.83742825	0.83742825	Xrcc3	1
GO:0005681	spliceosomal complex	2/101	194/14612	0.01030928	1.49147698	0.57491485	0.38881427	0.83742825	0.83742825	Srrm2/Rbmx	2
GO:0035097	histone methyltransferase complex	1/101	71/14612	0.01408451	2.03765165	0.73119903	0.38960715	0.83742825	0.83742825	Paxip1	1
GO:0035869	ciliary transition zone	1/101	71/14612	0.01408451	2.03765165	0.73119903	0.38960715	0.83742825	0.83742825	Alpk1	1
GO:0048787	presynaptic active zone membrane	1/101	71/14612	0.01408451	2.03765165	0.73119903	0.38960715	0.83742825	0.83742825	Celsr3	1
GO:0005667	transcription regulator complex	4/101	458/14612	0.00873362	1.26352199	0.47803752	0.39052941	0.83742825	0.83742825	Dbp/Hlf/Bhlhe41/Arid2	4
GO:0030666	endocytic vesicle membrane	1/101	73/14612	0.01369863	1.98182558	0.70158532	0.39805742	0.83742825	0.83742825	Rab34	1
GO:0017053	transcription repressor complex	1/101	74/14612	0.01351351	1.95504415	0.68713009	0.40223901	0.83742825	0.83742825	Bhlhe41	1
GO:0032589	neuron projection membrane	1/101	74/14612	0.01351351	1.95504415	0.68713009	0.40223901	0.83742825	0.83742825	Clcn2	1
GO:0032040	small-subunit processome	1/101	75/14612	0.01333333	1.9289769	0.67289939	0.40639184	0.83742825	0.83742825	Wdr46	1
GO:0000228	nuclear chromosome	2/101	204/14612	0.00980392	1.41836537	0.50201991	0.41310767	0.83742825	0.83742825	Pole/Ncapd3	2
GO:0005876	spindle microtubule	1/101	77/14612	0.01298701	1.8788736	0.64508397	0.414612	0.83742825	0.83742825	Ccdc57	1
GO:0030667	secretory granule membrane	1/101	77/14612	0.01298701	1.8788736	0.64508397	0.414612	0.83742825	0.83742825	Spaca6	1
GO:0097733	photoreceptor cell cilium	1/101	79/14612	0.01265823	1.83130718	0.61808674	0.42271943	0.83742825	0.83742825	Alpk1	1
GO:0000118	histone deacetylase complex	1/101	81/14612	0.01234568	1.78608972	0.59185982	0.43071568	0.83742825	0.83742825	Rere	1
GO:0044291	cell-cell contact zone	1/101	82/14612	0.01219512	1.76430814	0.57902137	0.43467258	0.83742825	0.83742825	Jam2	1
GO:0045171	intercellular bridge	1/101	82/14612	0.01219512	1.76430814	0.57902137	0.43467258	0.83742825	0.83742825	Fan1	1
GO:0099572	postsynaptic specialization	4/101	487/14612	0.00821355	1.18828146	0.35255736	0.43585237	0.83742825	0.83742825	Abl1/Rbmx/Celsr3/Lyn	4
GO:0044304	main axon	1/101	85/14612	0.01176471	1.70203844	0.54154478	0.44638062	0.83742825	0.83742825	Clcn2	1
GO:0000123	histone acetyltransferase complex	1/101	86/14612	0.01162791	1.68224729	0.52938303	0.4502297	0.83742825	0.83742825	Brpf1	1
GO:0005902	microvillus	1/101	87/14612	0.01149425	1.66291112	0.51737906	0.45405228	0.83742825	0.83742825	Slc6a6	1
GO:0000775	chromosome, centromeric region	2/101	225/14612	0.00888889	1.2859846	0.36066232	0.46252058	0.83742825	0.83742825	Ncapd3/Arid2	2
GO:0045211	postsynaptic membrane	3/101	365/14612	0.00821918	1.18909535	0.30522321	0.46418795	0.83742825	0.83742825	Slc6a6/Clcn2/Celsr3	3
GO:0000792	heterochromatin	1/101	90/14612	0.01111111	1.60748075	0.48227255	0.46536284	0.83742825	0.83742825	Ncapd3	1
GO:0043197	dendritic spine	2/101	227/14612	0.00881057	1.27465434	0.34793366	0.46710249	0.83742825	0.83742825	Myo5b/Clcn2	2
GO:0090575	RNA polymerase II transcription regulator complex	2/101	227/14612	0.00881057	1.27465434	0.34793366	0.46710249	0.83742825	0.83742825	Dbp/Hlf	2
GO:0097731	9+0 non-motile cilium	1/101	92/14612	0.01086957	1.57253551	0.45958413	0.472774	0.83742825	0.83742825	Alpk1	1
GO:0035861	site of double-strand break	1/101	93/14612	0.01075269	1.55562653	0.44844386	0.47644134	0.83742825	0.83742825	Peli1	1
GO:0030426	growth cone	2/101	232/14612	0.00862069	1.24718334	0.31661809	0.47845712	0.83742825	0.83742825	Abl1/Agrn	2
GO:0044309	neuron spine	2/101	232/14612	0.00862069	1.24718334	0.31661809	0.47845712	0.83742825	0.83742825	Myo5b/Clcn2	2
GO:0070603	SWI/SNF superfamily-type complex	1/101	94/14612	0.0106383	1.53907731	0.43743504	0.48008342	0.83742825	0.83742825	Arid2	1
GO:0016323	basolateral plasma membrane	2/101	235/14612	0.00851064	1.23126185	0.29816439	0.48520004	0.83742825	0.83742825	Slc6a6/Clcn2	2
GO:0005684	U2-type spliceosomal complex	1/101	96/14612	0.01041667	1.5070132	0.41579895	0.4872925	0.83742825	0.83742825	Srrm2	1
GO:0030117	membrane coat	1/101	96/14612	0.01041667	1.5070132	0.41579895	0.4872925	0.83742825	0.83742825	Sar1b	1
GO:0048475	coated membrane	1/101	96/14612	0.01041667	1.5070132	0.41579895	0.4872925	0.83742825	0.83742825	Sar1b	1
GO:0030427	site of polarized growth	2/101	238/14612	0.00840336	1.21574174	0.27995345	0.49188966	0.83742825	0.83742825	Abl1/Agrn	2
GO:0005604	basement membrane	1/101	98/14612	0.01020408	1.47625783	0.39465135	0.4944026	0.83742825	0.83742825	Agrn	1
GO:0001669	acrosomal vesicle	1/101	99/14612	0.01010101	1.46134613	0.38425354	0.49792095	0.83742825	0.83742825	Spaca6	1
GO:0005903	brush border	1/101	102/14612	0.00980392	1.41836537	0.35373179	0.5083312	0.83742825	0.83742825	Myo5b	1
GO:0098803	respiratory chain complex	1/101	103/14612	0.00970874	1.40459483	0.34377333	0.51175357	0.83742825	0.83742825	ND5	1
GO:0000793	condensed chromosome	2/101	248/14612	0.00806452	1.1667199	0.2209162	0.51379509	0.83742825	0.83742825	Ncapd3/Arid2	2
GO:0005923	bicellular tight junction	1/101	104/14612	0.00961538	1.39108911	0.33391859	0.51515235	0.83742825	0.83742825	Jam2	1
GO:0034708	methyltransferase complex	1/101	104/14612	0.00961538	1.39108911	0.33391859	0.51515235	0.83742825	0.83742825	Paxip1	1
GO:0030684	preribosome	1/101	107/14612	0.00934579	1.35208661	0.30495413	0.52520877	0.83742825	0.83742825	Wdr46	1
GO:0009925	basal plasma membrane	2/101	258/14612	0.00775194	1.1214982	0.16426506	0.53507988	0.83742825	0.83742825	Slc6a6/Clcn2	2
GO:0034451	centriolar satellite	1/101	112/14612	0.00892857	1.2917256	0.25855417	0.54151251	0.83742825	0.83742825	Ccdc57	1
GO:0070160	tight junction	1/101	112/14612	0.00892857	1.2917256	0.25855417	0.54151251	0.83742825	0.83742825	Jam2	1
GO:0045335	phagocytic vesicle	1/101	117/14612	0.00854701	1.23652365	0.21429418	0.55726176	0.83742825	0.83742825	Rab34	1
GO:0097730	non-motile cilium	1/101	117/14612	0.00854701	1.23652365	0.21429418	0.55726176	0.83742825	0.83742825	Alpk1	1
GO:0005770	late endosome	2/101	270/14612	0.00740741	1.07165383	0.09914446	0.55978039	0.83742825	0.83742825	Gimap5/Rab34	2
GO:0045178	basal part of cell	2/101	275/14612	0.00727273	1.05216922	0.07286241	0.56979716	0.83742825	0.83742825	Slc6a6/Clcn2	2
GO:0090734	site of DNA damage	1/101	122/14612	0.00819672	1.18584645	0.17196972	0.57247519	0.83742825	0.83742825	Peli1	1
GO:0030662	coated vesicle membrane	1/101	123/14612	0.00813008	1.17620543	0.16372108	0.57545517	0.83742825	0.83742825	Sar1b	1
GO:1904949	ATPase complex	1/101	123/14612	0.00813008	1.17620543	0.16372108	0.57545517	0.83742825	0.83742825	Arid2	1
GO:0005765	lysosomal membrane	2/101	280/14612	0.00714286	1.03338048	0.04705114	0.5796507	0.83742825	0.83742825	Gimap5/Sar1b	2
GO:0098852	lytic vacuole membrane	2/101	280/14612	0.00714286	1.03338048	0.04705114	0.5796507	0.83742825	0.83742825	Gimap5/Sar1b	2
GO:0031965	nuclear membrane	2/101	283/14612	0.00706714	1.02242592	0.03178246	0.58548422	0.83742825	0.83742825	Il15ra/Nav3	2
GO:0043296	apical junction complex	1/101	127/14612	0.00787402	1.13915959	0.13140374	0.58717085	0.83742825	0.83742825	Jam2	1
GO:0014069	postsynaptic density	3/101	443/14612	0.00677201	0.97972867	-0.0361465	0.59495811	0.83742825	0.83742825	Abl1/Rbmx/Celsr3	3
GO:1990204	oxidoreductase complex	1/101	130/14612	0.00769231	1.11287129	0.10784323	0.595747	0.83742825	0.83742825	ND5	1
GO:0005777	peroxisome	1/101	131/14612	0.00763359	1.10437609	0.10011274	0.59856633	0.83742825	0.83742825	Pipox	1
GO:0042579	microbody	1/101	131/14612	0.00763359	1.10437609	0.10011274	0.59856633	0.83742825	0.83742825	Pipox	1
GO:0044853	plasma membrane raft	1/101	131/14612	0.00763359	1.10437609	0.10011274	0.59856633	0.83742825	0.83742825	Trem2	1
GO:0031252	cell leading edge	3/101	454/14612	0.00660793	0.95599075	-0.0794736	0.61169955	0.83742825	0.83742825	Rab34/Clcn2/Abl1	3
GO:0048786	presynaptic active zone	1/101	136/14612	0.00735294	1.06377402	0.06233629	0.6123736	0.83742825	0.83742825	Celsr3	1
GO:0045121	membrane raft	2/101	299/14612	0.00668896	0.96771416	-0.0470581	0.61559745	0.83742825	0.83742825	Trem2/Lyn	2
GO:0098857	membrane microdomain	2/101	301/14612	0.00664452	0.96128417	-0.0566225	0.61924328	0.83742825	0.83742825	Trem2/Lyn	2
GO:0032279	asymmetric synapse	3/101	465/14612	0.00645161	0.93337592	-0.1218085	0.62798146	0.83742825	0.83742825	Abl1/Rbmx/Celsr3	3
GO:0005911	cell-cell junction	3/101	467/14612	0.00642398	0.92937859	-0.1294033	0.63089189	0.83742825	0.83742825	Fzd4/Lyn/Jam2	3
GO:0098685	Schaffer collateral - CA1 synapse	1/101	144/14612	0.00694444	1.00467547	0.00470386	0.6334935	0.83742825	0.83742825	Myo5b	1
GO:0009898	cytoplasmic side of plasma membrane	1/101	147/14612	0.00680272	0.98417189	-0.0160909	0.64111623	0.83742825	0.83742825	Lyn	1
GO:0098802	plasma membrane signaling receptor complex	1/101	147/14612	0.00680272	0.98417189	-0.0160909	0.64111623	0.83742825	0.83742825	Lyn	1
GO:0098839	postsynaptic density membrane	1/101	147/14612	0.00680272	0.98417189	-0.0160909	0.64111623	0.83742825	0.83742825	Celsr3	1
GO:0030141	secretory granule	2/101	318/14612	0.00628931	0.90989476	-0.135531	0.64917581	0.83742825	0.83742825	Serpine1/Spaca6	2
GO:0031902	late endosome membrane	1/101	152/14612	0.00657895	0.95179781	-0.0498375	0.65347346	0.83742825	0.83742825	Gimap5	1
GO:0098687	chromosomal region	2/101	321/14612	0.00623053	0.90139107	-0.1490354	0.65426276	0.83742825	0.83742825	Ncapd3/Arid2	2
GO:0000323	lytic vacuole	3/101	487/14612	0.00616016	0.89121109	-0.2037074	0.65914257	0.83742825	0.83742825	Mt1/Gimap5/Sar1b	3
GO:0005764	lysosome	3/101	487/14612	0.00616016	0.89121109	-0.2037074	0.65914257	0.83742825	0.83742825	Mt1/Gimap5/Sar1b	3
GO:0031253	cell projection membrane	2/101	324/14612	0.00617284	0.89304486	-0.1624203	0.65929158	0.83742825	0.83742825	Slc6a6/Clcn2	2
GO:0098862	cluster of actin-based cell projections	1/101	155/14612	0.00645161	0.93337592	-0.0695681	0.6606846	0.83742825	0.83742825	Myo5b	1
GO:0098982	GABA-ergic synapse	1/101	158/14612	0.00632911	0.91565359	-0.088931	0.66774713	0.84032384	0.84032384	Slc6a6	1
GO:0005912	adherens junction	1/101	161/14612	0.00621118	0.89859172	-0.1079417	0.67466408	0.84032384	0.84032384	Lyn	1
GO:0005774	vacuolar membrane	2/101	334/14612	0.00598802	0.86630699	-0.2062055	0.67563726	0.84032384	0.84032384	Gimap5/Sar1b	2
GO:0000922	spindle pole	1/101	167/14612	0.00598802	0.86630699	-0.144964	0.68807309	0.85047544	0.85047544	Alpk1	1
GO:0098791	Golgi apparatus subcompartment	2/101	347/14612	0.00576369	0.83385168	-0.2613225	0.69593974	0.85488894	0.85488894	Sar1b/Rab34	2
GO:0042383	sarcolemma	1/101	178/14612	0.00561798	0.81277116	-0.2096725	0.71125051	0.86012934	0.86012934	Agrn	1
GO:0099634	postsynaptic specialization membrane	1/101	180/14612	0.00555556	0.80374037	-0.2210324	0.71527748	0.86012934	0.86012934	Celsr3	1
GO:0005925	focal adhesion	1/101	187/14612	0.00534759	0.77365384	-0.2598891	0.7289389	0.86012934	0.86012934	Tns2	1

GO:0055037	recycling endosome	1/101	187/14612	0.00534759	0.77365384	-0.2598891	0.7289389	0.86012934	0.86012934	Myo5b	1
GO:0098562	cytoplasmic side of membrane	1/101	190/14612	0.00526316	0.76143825	-0.2761321	0.73459321	0.86012934	0.86012934	Lyn	1
GO:0031256	leading edge membrane	1/101	192/14612	0.00520833	0.7535066	-0.2868305	0.73829771	0.86012934	0.86012934	Clcn2	1
GO:0043204	perikaryon	1/101	196/14612	0.00510204	0.73812891	-0.3079252	0.74555381	0.86012934	0.86012934	Clcn2	1
GO:0030055	cell-substrate junction	1/101	199/14612	0.00502513	0.72700134	-0.3234907	0.75086482	0.86012934	0.86012934	Tns2	1
GO:1902495	transmembrane transporter complex	2/101	388/14612	0.00515464	0.74573849	-0.4234846	0.75324046	0.86012934	0.86012934	ND5/Clcn2	2
GO:0030139	endocytic vesicle	1/101	203/14612	0.00492611	0.71267619	-0.3439178	0.75777579	0.86012934	0.86012934	Rab34	1
GO:0045177	apical part of cell	2/101	393/14612	0.00508906	0.73625072	-0.442186	0.75956052	0.86012934	0.86012934	Slc6a6/Myo5b	2
GO:0150034	distal axon	2/101	393/14612	0.00508906	0.73625072	-0.442186	0.75956052	0.86012934	0.86012934	Abl1/Agrn	2
GO:0005819	spindle	2/101	394/14612	0.00507614	0.73438207	-0.4459008	0.76080782	0.86012934	0.86012934	Ccdc57/Alpk1	2
GO:0043209	myelin sheath	1/101	210/14612	0.0047619	0.68892032	-0.3788104	0.76941625	0.86012934	0.86012934	Clcn2	1
GO:0019867	outer membrane	1/101	214/14612	0.0046729	0.67604331	-0.3982857	0.77581744	0.86012934	0.86012934	Nav3	1
GO:0031968	organelle outer membrane	1/101	214/14612	0.0046729	0.67604331	-0.3982857	0.77581744	0.86012934	0.86012934	Nav3	1
GO:0098858	actin-based cell projection	1/101	216/14612	0.00462963	0.66978365	-0.4079026	0.77895173	0.86012934	0.86012934	Slc6a6	1
GO:0030133	transport vesicle	2/101	411/14612	0.00486618	0.70400617	-0.507804	0.78117852	0.86012934	0.86012934	Sar1b/Galnt15	2
GO:1990351	transporter complex	2/101	412/14612	0.00485437	0.70229741	-0.5113745	0.7823287	0.86012934	0.86012934	ND5/Clcn2	2
GO:0005874	microtubule	2/101	416/14612	0.00480769	0.69554455	-0.5255807	0.78687719	0.8603767	0.8603767	Ccdc57/Nav3	2
GO:0036464	cytoplasmic ribonucleoprotein granule	1/101	229/14612	0.00436681	0.63176099	-0.4685703	0.79829314	0.86642452	0.86642452	Eid1	1
GO:0030658	transport vesicle membrane	1/101	231/14612	0.004329	0.6262912	-0.4776357	0.80111614	0.86642452	0.86642452	Sar1b	1
GO:0030135	coated vesicle	1/101	240/14612	0.00416667	0.60280528	-0.5176098	0.81334315	0.86915271	0.86915271	Sar1b	1
GO:0035770	ribonucleoprotein granule	1/101	248/14612	0.00403226	0.58335995	-0.5520789	0.82358499	0.86915271	0.86915271	Eid1	1
GO:0042734	presynaptic membrane	1/101	250/14612	0.004	0.57869307	-0.5605484	0.82605727	0.86915271	0.86915271	Celsr3	1
GO:0061695	transferase complex, transferring phosphorus-containing groups	1/101	250/14612	0.004	0.57869307	-0.5605484	0.82605727	0.86915271	0.86915271	Pole	1
GO:0010008	endosome membrane	2/101	456/14612	0.00438596	0.63453187	-0.6614753	0.8279868	0.86915271	0.86915271	Gimap5/Myo5b	2
GO:0005635	nuclear envelope	2/101	458/14612	0.00436681	0.63176099	-0.6679976	0.8298443	0.86915271	0.86915271	Il15ra/Nav3	2
GO:0098797	plasma membrane protein complex	2/101	463/14612	0.00431965	0.62493852	-0.6841984	0.83441017	0.86935928	0.86935928	Slc6a6/Lyn	2
GO:0099568	cytoplasmic region	1/101	270/14612	0.0037037	0.53582692	-0.6422572	0.84896879	0.87645826	0.87645826	Myo5b	1
GO:0034702	monoatomic ion channel complex	1/101	271/14612	0.00369004	0.53384969	-0.6462088	0.85003239	0.87645826	0.87645826	Clcn2	1
GO:0015629	actin cytoskeleton	2/101	495/14612	0.0040404	0.58453845	-0.7845384	0.8611242	0.88331813	0.88331813	Myo5b/Abl1	2
GO:0043235	receptor complex	1/101	308/14612	0.00324675	0.4697184	-0.7847027	0.88457905	0.90272426	0.90272426	Lyn	1
GO:0016324	apical plasma membrane	1/101	319/14612	0.0031348	0.45352121	-0.8233016	0.89323689	0.90690888	0.90690888	Slc6a6	1
GO:0005938	cell cortex	1/101	328/14612	0.00304878	0.44107703	-0.8541142	0.89983977	0.9089752	0.9089752	Myo5b	1
GO:0005759	mitochondrial matrix	1/101	351/14612	0.002849	0.41217455	-0.9299914	0.91493312	0.919554	0.919554	Xrcc3	1
GO:0005769	early endosome	1/101	399/14612	0.00250627	0.36258964	-1.0769974	0.93955616	0.93955616	0.93955616	Rab34	1

ID	Description	GeneRatio	BgRatio	RichFactor	FoldEnrichm	zScore	pvalue	p.adjust	qvalue	geneID	Count
GO:0097136	Bcl-2 family protein complex	1/48	10/14612	0.1	30.4416667	5.34658497	0.03237817	0.49346845	0.49346845	Bcl2l1	1
GO:0061700	GATOR2 complex	1/48	11/14612	0.09090909	27.6742424	5.08062791	0.03555896	0.49346845	0.49346845	Sesn1	1
GO:0044322	endoplasmic reticulum quality control compartment	1/48	13/14612	0.07692308	23.4166667	4.64196017	0.04188985	0.49346845	0.49346845	Herpud1	1
GO:0035859	Seh1-associated complex	1/48	15/14612	0.06666667	20.2944444	4.29206566	0.04818004	0.49346845	0.49346845	Sesn1	1
GO:0097730	non-motile cilium	2/48	117/14612	0.01709402	5.2037037	2.62081383	0.05648398	0.49346845	0.49346845	Mchr1/Mertk	2
GO:0098871	postsynaptic actin cytoskeleton	1/48	18/14612	0.05555556	16.912037	3.8778867	0.05753958	0.49346845	0.49346845	Fam107a	1
GO:0032039	integrator complex	1/48	19/14612	0.05263158	16.0219298	3.76140842	0.06063935	0.49346845	0.49346845	Ints1	1
GO:0031965	nuclear membrane	3/48	283/14612	0.01060071	3.2270318	2.17186061	0.06572639	0.49346845	0.49346845	Bcl2l1/Ints1/Maco1	3
GO:0005777	peroxisome	2/48	131/14612	0.01526718	4.6475827	2.40747492	0.06887554	0.49346845	0.49346845	Xdh/Pxmp2	2
GO:0042579	microbody	2/48	131/14612	0.01526718	4.6475827	2.40747492	0.06887554	0.49346845	0.49346845	Xdh/Pxmp2	2
GO:0033268	node of Ranvier	1/48	24/14612	0.04166667	12.6840278	3.28867213	0.0759891	0.49346845	0.49346845	Kcnk4	1
GO:0099571	postsynaptic cytoskeleton	1/48	25/14612	0.04	12.1766667	3.21084666	0.07902944	0.49346845	0.49346845	Fam107a	1
GO:0001650	fibrillar center	2/48	144/14612	0.01388889	4.22800926	2.23476583	0.08110414	0.49346845	0.49346845	Sesn1/Ip6k2	2
GO:0001891	phagocytic cup	1/48	27/14612	0.03703704	11.2746914	3.06773403	0.08508077	0.49346845	0.49346845	Trip10	1
GO:0030867	rough endoplasmic reticulum membrane	1/48	27/14612	0.03703704	11.2746914	3.06773403	0.08508077	0.49346845	0.49346845	Maco1	1
GO:0005925	focal adhesion	2/48	187/14612	0.01069519	3.25579323	1.78230668	0.12557448	0.53676081	0.53676081	Fam107a/Ppfia1	2
GO:0016607	nuclear speck	3/48	389/14612	0.00771208	2.34768638	1.54663661	0.1352878	0.53676081	0.53676081	Hif3a/Fam107a/Klf15	3
GO:0030055	cell-substrate junction	2/48	199/14612	0.01005025	3.05946399	1.67928265	0.13886389	0.53676081	0.53676081	Fam107a/Ppfia1	2
GO:0044306	neuron projection terminus	2/48	204/14612	0.00980392	2.98447712	1.63862503	0.14449134	0.53676081	0.53676081	Fstl3/Maco1	2
GO:0060170	ciliary membrane	1/48	54/14612	0.01851852	5.63734568	1.95990828	0.16306093	0.53676081	0.53676081	Mchr1	1
GO:0001750	photoreceptor outer segment	1/48	55/14612	0.01818182	5.53484848	1.93432052	0.16582045	0.53676081	0.53676081	Mertk	1
GO:0005778	peroxisomal membrane	1/48	56/14612	0.01785714	5.4360119	1.90935176	0.16857106	0.53676081	0.53676081	Pxmp2	1
GO:0005791	rough endoplasmic reticulum	1/48	56/14612	0.01785714	5.4360119	1.90935176	0.16857106	0.53676081	0.53676081	Maco1	1
GO:0031903	microbody membrane	1/48	56/14612	0.01785714	5.4360119	1.90935176	0.16857106	0.53676081	0.53676081	Pxmp2	1
GO:0043197	dendritic spine	2/48	227/14612	0.00881057	2.68208517	1.46631147	0.17095703	0.53676081	0.53676081	Ppfia1/Mtmr2	2
GO:0044309	neuron spine	2/48	232/14612	0.00862069	2.62428161	1.43168057	0.17681889	0.53676081	0.53676081	Ppfia1/Mtmr2	2
GO:0005635	nuclear envelope	3/48	458/14612	0.00655022	1.99399563	1.24078883	0.19004102	0.53676081	0.53676081	Bcl2l1/Ints1/Maco1	3
GO:0005667	transcription regulator complex	3/48	458/14612	0.00655022	1.99399563	1.24078883	0.19004102	0.53676081	0.53676081	Per2/Tead3/Mxi1	3
GO:0005905	clathrin-coated pit	1/48	68/14612	0.01470588	4.47671569	1.64968853	0.20089358	0.53676081	0.53676081	Bcl2l1	1
GO:0016234	inclusion body	1/48	73/14612	0.01369863	4.17009132	1.5587849	0.21399519	0.53676081	0.53676081	Herpud1	1
GO:0017053	transcription repressor complex	1/48	74/14612	0.01351351	4.11373874	1.54157959	0.21659016	0.53676081	0.53676081	Per2	1
GO:0097733	photoreceptor cell cilium	1/48	79/14612	0.01265823	3.85337553	1.45987306	0.22943968	0.53676081	0.53676081	Mertk	1
GO:0008021	synaptic vesicle	2/48	277/14612	0.00722022	2.19795427	1.15558403	0.23075946	0.53676081	0.53676081	Bcl2l1/Mtmr2	2
GO:0016529	sarcoplasmic reticulum	1/48	80/14612	0.0125	3.80520833	1.44433413	0.23198471	0.53676081	0.53676081	Xdh	1
GO:0034705	potassium channel complex	1/48	85/14612	0.01176471	3.58137255	1.37022673	0.24458691	0.53676081	0.53676081	Kcnk4	1
GO:0044304	main axon	1/48	85/14612	0.01176471	3.58137255	1.37022673	0.24458691	0.53676081	0.53676081	Kcnk4	1
GO:0070382	exocytic vesicle	2/48	293/14612	0.00682594	2.07792947	1.07001056	0.25025875	0.53676081	0.53676081	Bcl2l1/Mtmr2	2
GO:0009897	external side of plasma membrane	2/48	298/14612	0.00671141	2.04306488	1.04438157	0.25636805	0.53676081	0.53676081	Cd163/Icosl	2
GO:0097731	9+0 non-motile cilium	1/48	92/14612	0.01086957	3.30887681	1.27535656	0.26189041	0.53676081	0.53676081	Mertk	1
GO:0005811	lipid droplet	1/48	93/14612	0.01075269	3.27329749	1.26255309	0.26433044	0.53676081	0.53676081	Rdh10	1
GO:0042470	melanosome	1/48	98/14612	0.01020408	3.10629252	1.20104262	0.27641262	0.53676081	0.53676081	Slc1a5	1
GO:0048770	pigment granule	1/48	98/14612	0.01020408	3.10629252	1.20104262	0.27641262	0.53676081	0.53676081	Slc1a5	1
GO:0032587	ruffle membrane	1/48	99/14612	0.01010101	3.07491582	1.18921324	0.27880563	0.53676081	0.53676081	Fam107a	1
GO:0001725	stress fiber	1/48	100/14612	0.01	3.04416667	1.17753255	0.2811909	0.53676081	0.53676081	Fam107a	1
GO:0097517	contractile actin filament bundle	1/48	100/14612	0.01	3.04416667	1.17753255	0.2811909	0.53676081	0.53676081	Fam107a	1
GO:0031253	cell projection membrane	2/48	324/14612	0.00617284	1.87911523	0.91865584	0.28816855	0.53676081	0.53676081	Fam107a/Mchr1	2
GO:0016528	sarcoplasm	1/48	108/14612	0.00925926	2.81867284	1.08903746	0.29999695	0.53676081	0.53676081	Xdh	1
GO:0032432	actin filament bundle	1/48	109/14612	0.00917431	2.79281346	1.07854851	0.30231356	0.53676081	0.53676081	Fam107a	1
GO:0042641	actomyosin	1/48	109/14612	0.00917431	2.79281346	1.07854851	0.30231356	0.53676081	0.53676081	Fam107a	1
GO:0005759	mitochondrial matrix	2/48	351/14612	0.00569801	1.7345679	0.79970676	0.32107577	0.55867184	0.55867184	Prodh/Bcl2l1	2
GO:0048786	presynaptic active zone	1/48	136/14612	0.00735294	2.23835784	0.8329359	0.36210079	0.61770135	0.61770135	Ppfia1	1
GO:0098685	Schaffer collateral - CA1 synapse	1/48	144/14612	0.00694444	2.11400463	0.77123059	0.37883105	0.63381348	0.63381348	Ppfia1	1
GO:0030133	transport vesicle	2/48	411/14612	0.00486618	1.48134631	0.56824987	0.39275762	0.64471534	0.64471534	Bcl2l1/Mtmr2	2
GO:0030672	synaptic vesicle membrane	1/48	164/14612	0.00609756	1.85619919	0.63301372	0.41879955	0.64780735	0.64780735	Bcl2l1	1
GO:0099501	exocytic vesicle membrane	1/48	164/14612	0.00609756	1.85619919	0.63301372	0.41879955	0.64780735	0.64780735	Bcl2l1	1
GO:0014069	postsynaptic density	2/48	443/14612	0.00451467	1.37434161	0.45932557	0.42970113	0.64780735	0.64780735	Pcbp2/Mtmr2	2
GO:0036064	ciliary basal body	1/48	170/14612	0.00588235	1.79068627	0.59529913	0.43029111	0.64780735	0.64780735	Cfap157	1
GO:0031901	early endosome membrane	1/48	174/14612	0.00574713	1.74952107	0.57098567	0.43782824	0.64780735	0.64780735	Mtmr2	1
GO:0034703	cation channel complex	1/48	176/14612	0.00568182	1.72964015	0.55906445	0.4415601	0.64780735	0.64780735	Kcnk4	1
GO:0032279	asymmetric synapse	2/48	465/14612	0.00430108	1.309319	0.38915368	0.45444123	0.64780735	0.64780735	Pcbp2/Mtmr2	2
GO:0001726	ruffle	1/48	187/14612	0.00534759	1.62789661	0.49610235	0.46165581	0.64780735	0.64780735	Fam107a	1
GO:0005741	mitochondrial outer membrane	1/48	187/14612	0.00534759	1.62789661	0.49610235	0.46165581	0.64780735	0.64780735	Bcl2l1	1
GO:0031256	leading edge membrane	1/48	192/14612	0.00520833	1.58550347	0.4688325	0.47055448	0.64981333	0.64981333	Fam107a	1
GO:0099572	postsynaptic specialization	2/48	487/14612	0.00410678	1.25017112	0.32235	0.47858822	0.65058086	0.65058086	Pcbp2/Mtmr2	2
GO:0019867	outer membrane	1/48	214/14612	0.0046729	1.42250779	0.35744627	0.50802744	0.66967254	0.66967254	Bcl2l1	1
GO:0031968	organelle outer membrane	1/48	214/14612	0.0046729	1.42250779	0.35744627	0.50802744	0.66967254	0.66967254	Bcl2l1	1
GO:0090575	RNA polymerase II transcription regulator complex	1/48	227/14612	0.00440529	1.34104258	0.29729449	0.52893643	0.68473232	0.68473232	Mxi1	1
GO:0030658	transport vesicle membrane	1/48	231/14612	0.004329	1.31782107	0.27952096	0.53519308	0.68473232	0.68473232	Bcl2l1	1
GO:0009925	basal plasma membrane	1/48	258/14612	0.00387597	1.17990956	0.16737781	0.57534774	0.72543846	0.72543846	Slc1a5	1
GO:0034702	monoatomic ion channel complex	1/48	271/14612	0.00369004	1.12330873	0.11762754	0.59344984	0.73382185	0.73382185	Kcnk4	1
GO:0045178	basal part of cell	1/48	275/14612	0.00363636	1.1069697	0.10280594	0.5988661	0.73382185	0.73382185	Slc1a5	1
GO:0062023	collagen-containing extracellular matrix	1/48	306/14612	0.00326797	0.99482571	-0.0052514	0.63851352	0.76385438	0.76385438	Lgalsl	1
GO:0043235	receptor complex	1/48	308/14612	0.00324675	0.9883658	-0.0118469	0.64093528	0.76385438	0.76385438	Mertk	1
GO:0030141	secretory granule	1/48	318/14612	0.00314465	0.95728512	-0.0442116	0.65280778	0.76749023	0.76749023	Fstl3	1
GO:0005938	cell cortex	1/48	328/14612	0.00304878	0.92809959	-0.0756073	0.66429563	0.76812849	0.76812849	Trip10	1
GO:0005774	vacuolar membrane	1/48	334/14612	0.00299401	0.91142715	-0.0940071	0.6710088	0.76812849	0.76812849	Mtmr2	1
GO:1902495	transmembrane transporter complex	1/48	388/14612	0.00257732	0.78457904	-0.2468955	0.72580261	0.7963545	0.7963545	Kcnk4	1
GO:0030312	external encapsulating structure	1/48	398/14612	0.00251256	0.764866	-0.2730355	0.73491921	0.7963545	0.7963545	Lgalsl	1
GO:0031012	extracellular matrix	1/48	398/14612	0.00251256	0.764866	-0.2730355	0.73491921	0.7963545	0.7963545	Lgalsl	1
GO:0005769	early endosome	1/48	399/14612	0.00250627	0.76294904	-0.2756167	0.73581437	0.7963545	0.7963545	Mtmr2	1
GO:1990351	transporter complex	1/48	412/14612	0.00242718	0.7388754	-0.3086544	0.7471856	0.7963545	0.7963545	Kcnk4	1
GO:0005874	microtubule	1/48	416/14612	0.00240385	0.73177083	-0.3186324	0.750587	0.7963545	0.7963545	Trip10	1
GO:0031252	cell leading edge	1/48	454/14612	0.00220264	0.67052129	-0.4094252	0.78074461	0.80928103	0.80928103	Fam107a	1
GO:0010008	endosome membrane	1/48	456/14612	0.00219298	0.66758041	-0.4140178	0.78222882	0.80928103	0.80928103	Mtmr2	1
GO:0000323	lytic vacuole	1/48	487/14612	0.00205339	0.62508556	-0.483084	0.80401466	0.80928103	0.80928103	Trip10	1
GO:0005764	lysosome	1/48	487/14612	0.00205339	0.62508556	-0.483084	0.80401466	0.80928103	0.80928103	Trip10	1
GO:0015629	actin cytoskeleton	1/48	495/14612	0.0020202	0.61498316	-0.500301	0.80928103	0.80928103	0.80928103	Fam107a	1

ID	Description	GeneRatio	BgRatio	RichFactor	FoldEnrichm	zScore	pvalue	p.adjust	qvalue	geneID	Count
GO:0099738	cell cortex region	2/40	40/14612	0.05	18.265	5.72858527	0.00533676	0.2946151	0.26916176	Ctbp2/Tchp	2
GO:0099568	cytoplasmic region	4/40	270/14612	0.01481481	5.41185185	3.83361352	0.00617917	0.2946151	0.26916176	Cys1/Cntf/Ctbp2/Tchp	4
GO:0060170	ciliary membrane	2/40	54/14612	0.03703704	13.5296296	4.83275346	0.00955762	0.2946151	0.26916176	Cys1/Evc2	2
GO:0005741	mitochondrial outer membrane	3/40	187/14612	0.01604278	5.86042781	3.50467519	0.01440068	0.2946151	0.26916176	Bok/Capn10/Rhot1	3
GO:0019867	outer membrane	3/40	214/14612	0.01401869	5.12102804	3.18179178	0.02056102	0.2946151	0.26916176	Bok/Capn10/Rhot1	3
GO:0031968	organelle outer membrane	3/40	214/14612	0.01401869	5.12102804	3.18179178	0.02056102	0.2946151	0.26916176	Bok/Capn10/Rhot1	3
GO:0031904	endosome lumen	1/40	11/14612	0.09090909	33.2090909	5.59878634	0.02971348	0.2946151	0.26916176	Ptpn1	1
GO:0005677	chromatin silencing complex	1/40	13/14612	0.07692308	28.1	5.12141298	0.03502265	0.2946151	0.26916176	Rrp8	1
GO:0030061	mitochondrial crista	1/40	14/14612	0.07142857	26.0928571	4.9212773	0.0376666	0.2946151	0.26916176	Ptpn1	1
GO:0005852	eukaryotic translation initiation factor 3 complex	1/40	15/14612	0.06666667	24.3533333	4.7410342	0.04030349	0.2946151	0.26916176	Eif3j1	1
GO:0033106	cis-Golgi network membrane	1/40	16/14612	0.0625	22.83125	4.57753899	0.04293334	0.2946151	0.26916176	Bok	1
GO:0098684	photoreceptor ribbon synapse	1/40	16/14612	0.0625	22.83125	4.57753899	0.04293334	0.2946151	0.26916176	Ctbp2	1
GO:0030057	desmosome	1/40	18/14612	0.05555556	20.2944444	4.29132834	0.04817197	0.2946151	0.26916176	Tchp	1
GO:0033290	eukaryotic 48S preinitiation complex	1/40	18/14612	0.05555556	20.2944444	4.29132834	0.04817197	0.2946151	0.26916176	Eif3j1	1
GO:0016282	eukaryotic 43S preinitiation complex	1/40	19/14612	0.05263158	19.2263158	4.16498829	0.05078079	0.2946151	0.26916176	Eif3j1	1
GO:0031082	BLOC complex	1/40	20/14612	0.05	18.265	4.04794455	0.05338264	0.2946151	0.26916176	Hps5	1
GO:0070993	translation preinitiation complex	1/40	20/14612	0.05	18.265	4.04794455	0.05338264	0.2946151	0.26916176	Eif3j1	1
GO:0098554	cytoplasmic side of endoplasmic reticulum membrane	1/40	20/14612	0.05	18.265	4.04794455	0.05338264	0.2946151	0.26916176	Ptpn1	1
GO:0097228	sperm principal piece	1/40	21/14612	0.04761905	17.3952381	3.9390839	0.05597753	0.2946151	0.26916176	Catsperz	1
GO:0031462	Cul2-RING ubiquitin ligase complex	1/40	22/14612	0.04545455	16.6045455	3.83747184	0.05856549	0.2946151	0.26916176	Lrrc75a	1
GO:0005938	cell cortex	3/40	328/14612	0.00914634	3.34115854	2.24674203	0.0601907	0.2946151	0.26916176	Iqgap2/Ctbp2/Tchp	3
GO:0098831	presynaptic active zone cytoplasmic component	1/40	23/14612	0.04347826	15.8826087	3.74231727	0.06114653	0.2946151	0.26916176	Ctbp2	1
GO:0030131	clathrin adaptor complex	1/40	26/14612	0.03846154	14.05	3.48930928	0.06884833	0.30871731	0.2820456	Lrrn3	1
GO:0016342	catenin complex	1/40	27/14612	0.03703704	13.5296296	3.41410822	0.07140187	0.30871731	0.2820456	Cdh19	1
GO:0000922	spindle pole	2/40	167/14612	0.01197605	4.3748503	2.29807586	0.07637094	0.30871731	0.2820456	Plk5/Fam83d	2
GO:0005640	nuclear outer membrane	1/40	29/14612	0.03448276	12.5965517	3.27502648	0.07648851	0.30871731	0.2820456	Bok	1
GO:0097470	ribbon synapse	1/40	30/14612	0.03333333	12.1766667	3.21051545	0.07902162	0.30871731	0.2820456	Ctbp2	1
GO:0005891	voltage-gated calcium channel complex	1/40	31/14612	0.03225806	11.783871	3.14899709	0.08154797	0.30871731	0.2820456	Catsperz	1
GO:0031461	cullin-RING ubiquitin ligase complex	2/40	190/14612	0.01052632	3.84526316	2.0682207	0.09517496	0.33646876	0.30739946	Lrrc75a/Fbh1	2
GO:0005769	early endosome	3/40	399/14612	0.0075188	2.74661654	1.85332474	0.09522701	0.33646876	0.30739946	Bok/Hps5/Ptpn1	3
GO:0097431	mitotic spindle pole	1/40	39/14612	0.02564103	9.36666667	2.74107345	0.10151693	0.34257945	0.31298222	Fam83d	1
GO:0005637	nuclear inner membrane	1/40	41/14612	0.02439024	8.9097561	2.65717848	0.10644264	0.34257945	0.31298222	Smad1	1
GO:0030119	AP-type membrane coat adaptor complex	1/40	43/14612	0.02325581	8.49534884	2.57882333	0.11134202	0.34257945	0.31298222	Lrrn3	1
GO:0097386	glial cell projection	1/40	43/14612	0.02325581	8.49534884	2.57882333	0.11134202	0.34257945	0.31298222	Cntf	1
GO:0030118	clathrin coat	1/40	48/14612	0.02083333	7.61041667	2.40336373	0.12347618	0.34257945	0.31298222	Lrrn3	1
GO:0008023	transcription elongation factor complex	1/40	49/14612	0.02040816	7.45510204	2.37129787	0.12588355	0.34257945	0.31298222	Aff1	1
GO:0099523	presynaptic cytosol	1/40	49/14612	0.02040816	7.45510204	2.37129787	0.12588355	0.34257945	0.31298222	Ctbp2	1
GO:0034704	calcium channel complex	1/40	50/14612	0.02	7.306	2.34012383	0.12828447	0.34257945	0.31298222	Catsperz	1
GO:0032588	trans-Golgi network membrane	1/40	51/14612	0.01960784	7.1627451	2.3097984	0.13067897	0.34257945	0.31298222	Bok	1
GO:0032838	plasma membrane bounded cell projection cytoplasm	2/40	232/14612	0.00862069	3.14913793	1.72877799	0.13241299	0.34257945	0.31298222	Cys1/Cntf	2
GO:0098797	plasma membrane protein complex	3/40	463/14612	0.00647948	2.36695464	1.56600128	0.13250715	0.34257945	0.31298222	Catsperz/Cdh19/Evc2	3
GO:0005801	cis-Golgi network	1/40	58/14612	0.01724138	6.29827586	2.1182039	0.14726178	0.3687948	0.33693269	Bok	1
GO:0019005	SCF ubiquitin ligase complex	1/40	59/14612	0.01694915	6.19152542	2.09341394	0.14960544	0.3687948	0.33693269	Fbh1	1
GO:0005882	intermediate filament	1/40	61/14612	0.01639344	5.98852459	2.04550714	0.15427392	0.37165989	0.33955025	Tchp	1
GO:0000502	proteasome complex	1/40	64/14612	0.015625	5.7078125	1.97750633	0.16122982	0.3797858	0.34697412	Zfand2b	1
GO:0099522	cytosolic region	1/40	71/14612	0.01408451	5.14507042	1.83431676	0.17724404	0.40636793	0.37125967	Ctbp2	1
GO:0031965	nuclear membrane	2/40	283/14612	0.00706714	2.58162544	1.40766497	0.18121965	0.40636793	0.37125967	Bok/Smad1	2
GO:0017053	transcription repressor complex	1/40	74/14612	0.01351351	4.93648649	1.77861798	0.18401566	0.40636793	0.37125967	Ctbp2	1
GO:0045171	intercellular bridge	1/40	82/14612	0.01219512	4.45487805	1.64367983	0.20180875	0.42407425	0.38743626	Fam83d	1
GO:0000151	ubiquitin ligase complex	2/40	306/14612	0.00653595	2.3875817	1.28519951	0.20407266	0.42407425	0.38743626	Lrrc75a/Fbh1	2
GO:0005902	microvillus	1/40	87/14612	0.01149425	4.19885057	1.567855	0.21273673	0.42407425	0.38743626	Iqgap2	1
GO:0000792	heterochromatin	1/40	90/14612	0.01111111	4.05888889	1.52504313	0.21922335	0.42407425	0.38743626	Rrp8	1
GO:0031253	cell projection membrane	2/40	324/14612	0.00617284	2.25493827	1.19679436	0.22219157	0.42407425	0.38743626	Cys1/Evc2	2
GO:0055038	recycling endosome membrane	1/40	92/14612	0.01086957	3.97065217	1.49752056	0.22351878	0.42407425	0.38743626	Bok	1
GO:1905369	endopeptidase complex	1/40	92/14612	0.01086957	3.97065217	1.49752056	0.22351878	0.42407425	0.38743626	Zfand2b	1
GO:0045111	intermediate filament cytoskeleton	1/40	94/14612	0.0106383	3.88617021	1.47076354	0.22779117	0.42407425	0.38743626	Tchp	1
GO:0030117	membrane coat	1/40	96/14612	0.01041667	3.80520833	1.44473316	0.23204063	0.42407425	0.38743626	Lrrn3	1
GO:0048475	coated membrane	1/40	96/14612	0.01041667	3.80520833	1.44473316	0.23204063	0.42407425	0.38743626	Lrrn3	1
GO:0005604	basement membrane	1/40	98/14612	0.01020408	3.72755102	1.41939328	0.23626729	0.42448022	0.38780715	Matn2	1
GO:0005759	mitochondrial matrix	2/40	351/14612	0.00569801	2.08148148	1.07450401	0.24961657	0.44098927	0.4028899	Capn10/Ptpn1	2
GO:0030175	filopodium	1/40	109/14612	0.00917431	3.35137615	1.29097199	0.25911162	0.44966265	0.41081394	Iqgap2	1
GO:0005776	autophagosome	1/40	111/14612	0.00900901	3.29099099	1.26939353	0.26319294	0.44966265	0.41081394	Rab24	1
GO:0019897	extrinsic component of plasma membrane	1/40	113/14612	0.00884956	3.23274336	1.2483012	0.26725233	0.44966265	0.41081394	Cdh19	1
GO:1905368	peptidase complex	1/40	122/14612	0.00819672	2.9942623	1.15887978	0.28525137	0.46639439	0.42610014	Zfand2b	1
GO:0005819	spindle	2/40	394/14612	0.00507614	1.85431472	0.90065223	0.29351665	0.46639439	0.42610014	Plk5/Fam83d	2
GO:0030312	external encapsulating structure	2/40	398/14612	0.00502513	1.83567839	0.88559048	0.29759619	0.46639439	0.42610014	Matn2/Lrrn3	2
GO:0031012	extracellular matrix	2/40	398/14612	0.00502513	1.83567839	0.88559048	0.29759619	0.46639439	0.42610014	Matn2/Lrrn3	2
GO:0005930	axoneme	1/40	134/14612	0.00746269	2.7261194	1.05166963	0.30858087	0.46639439	0.42610014	Cys1	1
GO:0036126	sperm flagellum	1/40	135/14612	0.00740741	2.70592593	1.04327344	0.31049113	0.46639439	0.42610014	Catsperz	1
GO:0097014	ciliary plasm	1/40	135/14612	0.00740741	2.70592593	1.04327344	0.31049113	0.46639439	0.42610014	Cys1	1
GO:0048786	presynaptic active zone	1/40	136/14612	0.00735294	2.68602941	1.03495317	0.31239624	0.46639439	0.42610014	Ctbp2	1
GO:0097729	9+2 motile cilium	1/40	152/14612	0.00657895	2.40328947	0.91116137	0.34218914	0.48524319	0.44332049	Catsperz	1
GO:0000776	kinetochore	1/40	158/14612	0.00632911	2.31202532	0.8687346	0.3530338	0.48524319	0.44332049	Plk5	1
GO:0098982	GABA-ergic synapse	1/40	158/14612	0.00632911	2.31202532	0.8687346	0.3530338	0.48524319	0.44332049	Ctbp2	1
GO:0005635	nuclear envelope	2/40	458/14612	0.00436681	1.59519651	0.67805341	0.35825486	0.48524319	0.44332049	Bok/Smad1	2
GO:0005667	transcription regulator complex	2/40	458/14612	0.00436681	1.59519651	0.67805341	0.35825486	0.48524319	0.44332049	Ctbp2/Smad1	2
GO:0005912	adherens junction	1/40	161/14612	0.00621118	2.2689441	0.84823632	0.35839056	0.48524319	0.44332049	Cdh19	1
GO:0005911	cell-cell junction	2/40	467/14612	0.00428266	1.56445396	0.649525	0.36722503	0.48524319	0.44332049	Cdh19/Tchp	2
GO:0000779	condensed chromosome, centromeric region	1/40	168/14612	0.00595238	2.17440476	0.80211953	0.37072205	0.48524319	0.44332049	Plk5	1
GO:0036064	ciliary basal body	1/40	170/14612	0.00588235	2.14882353	0.78935888	0.37420268	0.48524319	0.44332049	Cys1	1
GO:0072686	mitotic spindle	1/40	170/14612	0.00588235	2.14882353	0.78935888	0.37420268	0.48524319	0.44332049	Fam83d	1
GO:0019898	extrinsic component of membrane	1/40	174/14612	0.00574713	2.09942529	0.7643587	0.38110773	0.48524319	0.44332049	Cdh19	1
GO:0031901	early endosome membrane	1/40	174/14612	0.00574713	2.09942529	0.7643587	0.38110773	0.48524319	0.44332049	Bok	1
GO:0034703	cation channel complex	1/40	176/14612	0.00568182	2.07556818	0.75210977	0.38453234	0.48524319	0.44332049	Catsperz	1
GO:0055037	recycling endosome	1/40	187/14612	0.00534759	1.95347594	0.68751632	0.40303997	0.50261456	0.45919105	Bok	1
GO:0098562	cytoplasmic side of membrane	1/40	190/14612	0.00526316	1.92263158	0.67066054	0.4079926	0.50287461	0.45942864	Ptpn1	1
GO:0031514	motile cilium	1/40	204/14612	0.00490196	1.79068627	0.59583748	0.43058014	0.52461488	0.47929066	Catsperz	1
GO:0030027	lamellipodium	1/40	212/14612	0.00471698	1.72311321	0.55565262	0.4431077	0.53374336	0.48763048	Iqgap2	1
GO:0098858	actin-based cell projection	1/40	216/14612	0.00462963	1.6912037	0.53619455	0.44927029	0.53508597	0.48885709	Iqgap2	1
GO:0000775	chromosome, centromeric region	1/40	225/14612	0.00444444	1.62355556	0.49384615	0.46289403	0.54266944	0.49578539	Plk5	1
GO:0090575	RNA polymerase II transcription regulator complex	1/40	227/14612	0.00440529	1.6092511	0.48469072	0.4658766	0.54266944	0.49578539	Smad1	1
GO:0016323	basolateral plasma membrane	1/40	235/14612	0.00425532	1.55446809	0.44893838	0.47764622	0.55033152	0.5027855	Slc43a3	1
GO:0005802	trans-Golgi network	1/40	243/14612	0.00411523	1.50329218	0.41449608	0.48916284	0.55754045	0.50937161	Bok	1
GO:0000793	condensed chromosome	1/40	248/14612	0.00403226	1.47298387	0.39359073	0.49623461	0.55958371	0.51123835	Plk5	1
GO:0009925	basal plasma membrane	1/40	258/14612	0.00387597	1.41589147	0.35311306	0.51009296	0.56915635	0.51998396	Slc43a3	1
GO:0034702	monoatomic ion channel complex	1/40	271/14612	0.00369004	1.34797048	0.30293379	0.52755419	0.58223911	0.53193643	Catsperz	1
GO:0045178	basal part of cell	1/40	275/14612	0.00363636	1.32836364	0.28800674	0.53280371	0.58223911	0.53193643	Slc43a3	1
GO:0062023	collagen-containing extracellular matrix	1/40	306/14612	0.00326797	1.19379085	0.17949207	0.5716039	0.61826545	0.56485026	Matn2	1
GO:0098687	chromosomal region	1/40	321/14612	0.00311526	1.13800623	0.13098763	0.58923205	0.63089493	0.57638861	Plk5	1
GO:0098791	Golgi apparatus subcompartment	1/40	347/14612	0.00288184	1.05273775	0.05209078	0.61812649	0.65521408	0.5986067	Bok	1
GO:1902495	transmembrane transporter complex	1/40	388/14612	0.00257732	0.94149485	-0.0611941	0.65970963	0.69052577	0.63086763	Catsperz	1
GO:0045177	apical part of cell	1/40	393/14612	0.00254453	0.92951654	-0.0742095	0.66446819	0.69052577	0.63086763	Tchp	1
GO:1990351	transporter complex	1/40	412/14612	0.00242718	0.88665049	-0.1222741	0.68196613	0.69871824	0.63835231	Catsperz	1
GO:0005874	microtubule	1/40	416/14612	0.00240385	0.878125	-0.1321261	0.68553488	0.69871824	0.63835231	Iqgap2	1
GO:0031252	cell leading edge	1/40	454/14612	0.00220264	0.80462555	-0.221567	0.71754777	0.71914157	0.65701117	Iqgap2	1
GO:0010008	endosome membrane	1/40	456/14612	0.00219298	0.80109649	-0.2260815	0.71914157	0.71914157	0.65701117	Bok	1

ID	Description	GeneRatio	BgRatio	RichFactor	FoldEnrichm	zScore	pvalue	p.adjust	qvalue	geneID	Count
GO:0140534	endoplasmic reticulum protein-containing complex	6/98	119/14612	0.05042017	7.51774996	5.86612724	0.00014701	0.03043037	0.0300203	P4ha1/Pdia6/Mlec/Traf2/Caml/Pigc	6
GO:0001650	fibrillar center	6/98	144/14612	0.04166667	6.21258503	5.1652292	0.00041276	0.04272032	0.04214463	Lipa/Ubtf/Pafah1b2/Sap30l/Nhej1/Taf4b	6
GO:0005788	endoplasmic reticulum lumen	4/98	105/14612	0.03809524	5.68007775	3.95474386	0.00542384	0.37424488	0.36920166	P4ha1/Pdia6/Pcsk9/Uggt1	4
GO:1990391	DNA repair complex	2/98	22/14612	0.09090909	13.554731	4.84228732	0.00942475	0.48773092	0.48115839	Lig4/Nhej1	2
GO:0005879	axonemal microtubule	2/98	32/14612	0.0625	9.31887755	3.87097537	0.01937805	0.58253279	0.57468275	Efhc1/Tuba1a	2
GO:0016235	aggresome	2/98	33/14612	0.06060606	9.03648732	3.79768391	0.02053926	0.58253279	0.57468275	Hspa1b/Edem1	2
GO:0101031	protein folding chaperone complex	2/98	35/14612	0.05714286	8.52011662	3.66002431	0.02294647	0.58253279	0.57468275	Ptges3/Cct7	2
GO:0005881	cytoplasmic microtubule	3/98	94/14612	0.03191489	4.75857577	3.00395408	0.02527799	0.58253279	0.57468275	Efhc1/Tuba1a/Togaram2	3
GO:0042470	melanosome	3/98	98/14612	0.03061224	4.56434819	2.90910267	0.02814168	0.58253279	0.57468275	Pdia6/Trpv2/Atp1b3	3
GO:0048770	pigment granule	3/98	98/14612	0.03061224	4.56434819	2.90910267	0.02814168	0.58253279	0.57468275	Pdia6/Trpv2/Atp1b3	3
GO:0045335	phagocytic vesicle	3/98	117/14612	0.02564103	3.82312925	2.51926604	0.04398664	0.58482147	0.57694058	Rab39/Becn1/Cd82	3
GO:0031514	motile cilium	4/98	204/14612	0.01960784	2.92356943	2.27342457	0.04836244	0.58482147	0.57694058	Efhc1/Atp1b3/Tuba1a/Dnai4	4
GO:0090734	site of DNA damage	3/98	122/14612	0.02459016	3.66644363	2.43017516	0.04875919	0.58482147	0.57694058	Spindoc/Nhej1/Eloa	3
GO:0045121	membrane raft	5/98	299/14612	0.01672241	2.49334516	2.14382784	0.05066362	0.58482147	0.57694058	Hspa1b/Traf2/Atp1b3/Tuba1a/Ctnnd1	5
GO:0098857	membrane microdomain	5/98	301/14612	0.0166113	2.47677809	2.12727162	0.05185883	0.58482147	0.57694058	Hspa1b/Traf2/Atp1b3/Tuba1a/Ctnnd1	5
GO:0019867	outer membrane	4/98	214/14612	0.01869159	2.78695403	2.16385738	0.0558094	0.58482147	0.57694058	Smpd4/Becn1/Nme1/Mief1	4
GO:0031968	organelle outer membrane	4/98	214/14612	0.01869159	2.78695403	2.16385738	0.0558094	0.58482147	0.57694058	Smpd4/Becn1/Nme1/Mief1	4
GO:0005930	axoneme	3/98	134/14612	0.02238806	3.33810539	2.23419793	0.06119745	0.58482147	0.57694058	Efhc1/Tuba1a/Dnai4	3
GO:0036126	sperm flagellum	3/98	135/14612	0.02222222	3.31337868	2.2188798	0.06229509	0.58482147	0.57694058	Efhc1/Atp1b3/Tuba1a	3
GO:0097014	ciliary plasm	3/98	135/14612	0.02222222	3.31337868	2.2188798	0.06229509	0.58482147	0.57694058	Efhc1/Tuba1a/Dnai4	3
GO:0035631	CD40 receptor complex	1/98	10/14612	0.1	14.9102041	3.61565329	0.06509923	0.58482147	0.57694058	Traf2	1
GO:0034663	endoplasmic reticulum chaperone complex	1/98	11/14612	0.09090909	13.554731	3.42272417	0.07137373	0.58482147	0.57694058	Pdia6	1
GO:0016323	basolateral plasma membrane	4/98	235/14612	0.01702128	2.53790708	1.95294197	0.07334287	0.58482147	0.57694058	Hspa1b/Abcc5/Atp1b3/Slc16a5	4
GO:0000800	lateral element	1/98	12/14612	0.08333333	12.4251701	3.25339142	0.07760655	0.58482147	0.57694058	Smc1a	1
GO:0097729	9+2 motile cilium	3/98	152/14612	0.01973684	2.94280344	1.97845401	0.08232172	0.58482147	0.57694058	Efhc1/Atp1b3/Tuba1a	3
GO:0044322	endoplasmic reticulum quality control compartment	1/98	13/14612	0.07692308	11.4693878	3.10306488	0.08379796	0.58482147	0.57694058	Edem1	1
GO:0000793	condensed chromosome	4/98	248/14612	0.01612903	2.40487163	1.83351401	0.08544722	0.58482147	0.57694058	Tuba1a/Lig4/Dctn5/Smc1a	4
GO:0016234	inclusion body	2/98	73/14612	0.02739726	4.08498742	2.17123588	0.08615813	0.58482147	0.57694058	Hspa1b/Edem1	2
GO:0008250	oligosaccharyltransferase complex	1/98	14/14612	0.07142857	10.6501458	2.96831961	0.08994823	0.58482147	0.57694058	Mlec	1
GO:0160111	axonemal A tubule inner sheath	1/98	14/14612	0.07142857	10.6501458	2.96831961	0.08994823	0.58482147	0.57694058	Efhc1	1
GO:0009925	basal plasma membrane	4/98	258/14612	0.01550388	2.31165955	1.74664223	0.09538312	0.58482147	0.57694058	Hspa1b/Abcc5/Atp1b3/Slc16a5	4
GO:0005614	interstitial matrix	1/98	15/14612	0.06666667	9.94013605	2.84654073	0.09605763	0.58482147	0.57694058	Vwa1	1
GO:0005858	axonemal dynein complex	1/98	15/14612	0.06666667	9.94013605	2.84654073	0.09605763	0.58482147	0.57694058	Dnai4	1
GO:0005942	phosphatidylinositol 3-kinase complex	1/98	15/14612	0.06666667	9.94013605	2.84654073	0.09605763	0.58482147	0.57694058	Becn1	1
GO:0120293	dynein axonemal particle	1/98	16/14612	0.0625	9.31887755	2.73569228	0.10212644	0.58915965	0.5812203	Dnai4	1
GO:0072686	mitotic spindle	3/98	170/14612	0.01764706	2.63121248	1.7578458	0.10614032	0.58915965	0.5812203	Efhc1/Tbck/Smc1a	3
GO:0099568	cytoplasmic region	4/98	270/14612	0.01481481	2.20891912	1.64753147	0.10799431	0.58915965	0.5812203	Efhc1/Tuba1a/Ctnnd1/Dnai4	4
GO:0016327	apicolateral plasma membrane	1/98	17/14612	0.05882353	8.77070828	2.63416191	0.10815491	0.58915965	0.5812203	Ctnnd1	1
GO:0045178	basal part of cell	4/98	275/14612	0.01454545	2.16875696	1.60775868	0.11346242	0.60222362	0.59410822	Hspa1b/Abcc5/Atp1b3/Slc16a5	4
GO:0005697	telomerase holoenzyme complex	1/98	20/14612	0.05	7.45510204	2.37366895	0.12600099	0.61881588	0.61047689	Ptges3	1
GO:0005741	mitochondrial outer membrane	3/98	187/14612	0.01604278	2.39201135	1.57421888	0.13080796	0.61881588	0.61047689	Becn1/Nme1/Mief1	3
GO:0070822	Sin3-type complex	1/98	21/14612	0.04761905	7.10009718	2.29859957	0.13187078	0.61881588	0.61047689	Sap30l	1
GO:0030496	midbody	3/98	190/14612	0.01578947	2.35424275	1.54390304	0.13535712	0.61881588	0.61047689	Ankrd54/Ctnnd1/Tbck	3
GO:0005901	caveola	2/98	98/14612	0.02040816	3.04289879	1.66734618	0.14029656	0.61881588	0.61047689	Atp1b3/Ctnnd1	2
GO:0005793	endoplasmic reticulum-Golgi intermediate compartment	2/98	99/14612	0.02020202	3.01216244	1.65067467	0.14259374	0.61881588	0.61047689	Pdia6/Uggt1	2
GO:0097649	A axonemal microtubule	1/98	23/14612	0.04347826	6.48269743	2.16224491	0.14349354	0.61881588	0.61047689	Efhc1	1
GO:0098831	presynaptic active zone cytoplasmic component	1/98	23/14612	0.04347826	6.48269743	2.16224491	0.14349354	0.61881588	0.61047689	Ctnnd1	1
GO:0160110	axonemal microtubule doublet inner sheath	1/98	23/14612	0.04347826	6.48269743	2.16224491	0.14349354	0.61881588	0.61047689	Efhc1	1
GO:0005796	Golgi lumen	1/98	25/14612	0.04	5.96408163	2.04119914	0.15496226	0.62740326	0.61894855	Abcc5	1
GO:0097545	axonemal doublet microtubule	1/98	25/14612	0.04	5.96408163	2.04119914	0.15496226	0.62740326	0.61894855	Efhc1	1
GO:0030139	endocytic vesicle	3/98	203/14612	0.01477833	2.20347844	1.41882509	0.15567897	0.62740326	0.61894855	Rab39/Becn1/Cd82	3
GO:0000795	synaptonemal complex	1/98	26/14612	0.03846154	5.73469388	1.98550014	0.16063948	0.62740326	0.61894855	Smc1a	1
GO:0099086	synaptonemal structure	1/98	26/14612	0.03846154	5.73469388	1.98550014	0.16063948	0.62740326	0.61894855	Smc1a	1
GO:0016342	catenin complex	1/98	27/14612	0.03703704	5.52229781	1.93262357	0.16627895	0.63740266	0.6288132	Ctnnd1	1
GO:0005776	autophagosome	2/98	111/14612	0.01801802	2.68652326	1.46559609	0.17073291	0.64257659	0.63391741	Becn1/Pip4k2c	2
GO:0005640	nuclear outer membrane	1/98	29/14612	0.03448276	5.14144968	1.83437194	0.17744562	0.65209772	0.64331024	Smpd4	1
GO:0005669	transcription factor TFIID complex	1/98	31/14612	0.03225806	4.80974325	1.74478673	0.18846424	0.65209772	0.64331024	Taf4b	1
GO:0005775	vacuolar lumen	1/98	31/14612	0.03225806	4.80974325	1.74478673	0.18846424	0.65209772	0.64331024	Cln5	1
GO:0000314	organellar small ribosomal subunit	1/98	32/14612	0.03125	4.65943878	1.70282556	0.19391863	0.65209772	0.64331024	Mrps27	1
GO:0005763	mitochondrial small ribosomal subunit	1/98	32/14612	0.03125	4.65943878	1.70282556	0.19391863	0.65209772	0.64331024	Mrps27	1
GO:0005875	microtubule associated complex	2/98	121/14612	0.01652893	2.46449654	1.32920298	0.19482938	0.65209772	0.64331024	Dnai4/Dctn5	2
GO:0098797	plasma membrane protein complex	5/98	463/14612	0.01079914	1.61017323	1.09632728	0.19963862	0.65209772	0.64331024	Shc1/Traf2/Atp1b3/Ctnnd1/Aph1b	5
GO:0032838	plasma membrane bounded cell projection cytoplasm	3/98	232/14612	0.01293103	1.92804363	1.17082609	0.2040142	0.65209772	0.64331024	Efhc1/Tuba1a/Dnai4	3
GO:0005790	smooth endoplasmic reticulum	1/98	34/14612	0.02941176	4.38535414	1.62388041	0.20471879	0.65209772	0.64331024	Pdia6	1
GO:0005840	ribosome	3/98	240/14612	0.0125	1.86377551	1.10868291	0.21792828	0.65209772	0.64331024	Nufip2/Mrps27/Hbs1l	3
GO:0044853	plasma membrane raft	2/98	131/14612	0.01526718	2.27636704	1.20578923	0.2193293	0.65209772	0.64331024	Atp1b3/Ctnnd1	2
GO:0000407	phagophore assembly site	1/98	37/14612	0.02702703	4.02978489	1.51623956	0.22065103	0.65209772	0.64331024	Becn1	1
GO:0090533	cation-transporting ATPase complex	1/98	37/14612	0.02702703	4.02978489	1.51623956	0.22065103	0.65209772	0.64331024	Atp1b3	1
GO:0098533	ATPase dependent transmembrane transport complex	1/98	37/14612	0.02702703	4.02978489	1.51623956	0.22065103	0.65209772	0.64331024	Atp1b3	1
GO:0000323	lytic vacuole	5/98	487/14612	0.01026694	1.53082177	0.97899039	0.22851371	0.65209772	0.64331024	Rab39/Pcsk9/Lipa/Sphk2/Cln5	5
GO:0005764	lysosome	5/98	487/14612	0.01026694	1.53082177	0.97899039	0.22851371	0.65209772	0.64331024	Rab39/Pcsk9/Lipa/Sphk2/Cln5	5
GO:0033017	sarcoplasmic reticulum membrane	1/98	39/14612	0.02564103	3.82312925	1.4506012	0.2310966	0.65209772	0.64331024	Fkbp1b	1
GO:0097431	mitotic spindle pole	1/98	39/14612	0.02564103	3.82312925	1.4506012	0.2310966	0.65209772	0.64331024	Smc1a	1
GO:0061695	transferase complex, transferring phosphorus-containing groups	3/98	250/14612	0.012	1.78922449	1.03424289	0.23559136	0.65209772	0.64331024	Cdkn1a/Becn1/Taf4b	3
GO:0099738	cell cortex region	1/98	40/14612	0.025	3.72755102	1.41939328	0.23626729	0.65209772	0.64331024	Ctnnd1	1
GO:0030286	dynein complex	1/98	44/14612	0.02272727	3.38868275	1.30390002	0.25660814	0.69891953	0.68950109	Dnai4	1
GO:0044232	organelle membrane contact site	1/98	47/14612	0.0212766	3.17238385	1.22571556	0.27151101	0.72054845	0.71083854	Stard3	1
GO:0044295	axonal growth cone	1/98	47/14612	0.0212766	3.17238385	1.22571556	0.27151101	0.72054845	0.71083854	Trpv2	1
GO:0099092	postsynaptic density, intracellular component	1/98	48/14612	0.02083333	3.10629252	1.20104262	0.27641262	0.7228793	0.71313798	Ctnnd1	1
GO:0008023	transcription elongation factor complex	1/98	49/14612	0.02040816	3.04289879	1.17700665	0.28128158	0.7228793	0.71313798	Eloa	1
GO:0034704	calcium channel complex	1/98	50/14612	0.02	2.98204082	1.15357685	0.28611811	0.7228793	0.71313798	Fkbp1b	1
GO:0000776	kinetochore	2/98	158/14612	0.01265823	1.88736761	0.92150466	0.28635798	0.7228793	0.71313798	Dctn5/Smc1a	2
GO:0000307	cyclin-dependent protein kinase holoenzyme complex	1/98	51/14612	0.01960784	2.92356943	1.1307245	0.29092243	0.72555352	0.71577617	Cdkn1a	1
GO:0030670	phagocytic vesicle membrane	1/98	52/14612	0.01923077	2.86734694	1.10842279	0.29569474	0.72867631	0.71885688	Rab39	1
GO:0005874	microtubule	4/98	416/14612	0.00961538	1.43367347	0.73737086	0.30496889	0.7314034	0.72154722	Efhc1/Tuba1a/Togaram2/Cct7	4
GO:0000922	spindle pole	2/98	167/14612	0.01197605	1.78565318	0.83905376	0.30868344	0.7314034	0.72154722	Efhc1/Smc1a	2
GO:0000786	nucleosome	1/98	55/14612	0.01818182	2.7109462	1.04457879	0.30982174	0.7314034	0.72154722	Sphk2	1
GO:0000779	condensed chromosome, centromeric region	2/98	168/14612	0.01190476	1.7750243	0.83020562	0.31115709	0.7314034	0.72154722	Dctn5/Smc1a	2
GO:0099091	postsynaptic specialization, intracellular component	1/98	56/14612	0.01785714	2.66253644	1.02424443	0.31446813	0.7314034	0.72154722	Ctnnd1	1
GO:0030134	COPII-coated ER to Golgi transport vesicle	1/98	57/14612	0.01754386	2.61582528	1.0043502	0.31908355	0.73389217	0.72400245	Pcsk9	1
GO:0019898	extrinsic component of membrane	2/98	174/14612	0.01149425	1.71381656	0.7783361	0.32595967	0.7414687	0.73147688	Ctnnd1/Becn1	2
GO:0000794	condensed nuclear chromosome	1/98	60/14612	0.01666667	2.48503401	0.94713118	0.33274609	0.74867869	0.73858971	Smc1a	1
GO:0005882	intermediate filament	1/98	61/14612	0.01639344	2.44429575	0.92882539	0.33723969	0.75063027	0.74051499	Nme1	1
GO:0098686	hippocampal mossy fiber to CA3 synapse	1/98	62/14612	0.01612903	2.40487163	0.91087847	0.34170333	0.75100257	0.74088228	Ctnnd1	1
GO:0032580	Golgi cisterna membrane	1/98	63/14612	0.01587302	2.36669906	0.89327679	0.34613722	0.75100257	0.74088228	Fut11	1
GO:0016324	apical plasma membrane	3/98	319/14612	0.00940439	1.40221355	0.59682883	0.36153001	0.75100257	0.74088228	Hspa1b/Abcc5/Atp1b3	3
GO:0031252	cell leading edge	4/98	454/14612	0.00881057	1.31367437	0.55791168	0.36297669	0.75100257	0.74088228	Trpv2/Raph1/Ctnnd1/Nme1	4
GO:0098562	cytoplasmic side of membrane	2/98	190/14612	0.01052632	1.56949517	0.64925237	0.36499592	0.75100257	0.74088228	Traf2/Becn1	2
GO:0010008	endosome membrane	4/98	456/14612	0.00877193	1.30791264	0.54890743	0.36603936	0.75100257	0.74088228	Shc1/Abcc5/Stard3/Becn1	4
GO:0016591	RNA polymerase II, holoenzyme	1/98	68/14612	0.01470588	2.19267707	0.81001832	0.36786712	0.75100257	0.74088228	Taf4b	1
GO:0005938	cell cortex	3/98	328/14612	0.00914634	1.36373818	0.54747001	0.3780047	0.75100257	0.74088228	Traf2/Ctnnd1/Cap1	3
GO:0030666	endocytic vesicle membrane	1/98	73/14612	0.01369863	2.04249371	0.73371442	0.388882	0.75100257	0.74088228	Rab39	1
GO:0032040	small-subunit processome	1/98	75/14612	0.01333333	1.98802721	0.70489039	0.39709299	0.75100257	0.74088228	Rps19bp1	1
GO:0000228	nuclear chromosome	2/98	204/14612	0.00980392	1.46178471	0.54577324	0.39845449	0.75100257	0.74088228	Sap30l/Smc1a	2
GO:0005643	nuclear pore	1/98	77/14612	0.01298701	1.93639014	0.67694615	0.40519476	0.75100257	0.74088228	Bicd2	1
GO:0015935	small ribosomal subunit	1/98	77/14612	0.01298701	1.93639014	0.67694615	0.40519476	0.75100257	0.74088228	Mrps27	1
GO:0098791	Golgi apparatus subcompartment	3/98	347/14612	0.00864553	1.28906664	0.44780224	0.41248514	0.75100257	0.74088228	Smpd4/Becn1/Fut11	3
GO:0043209	myelin sheath	2/98	210/14612	0.00952381	1.42001944	0.50376394	0.41255158	0.75100257	0.74088228	Tuba1a/Nme1	2
GO:0016529	sarcoplasmic reticulum	1/98	80/14612	0.0125	1.86377551	0.63656462	0.41714579	0.75100257	0.74088228	Fkbp1b	1
GO:0030027	lamellipodium	2/98	212/14612	0.00943396	1.40662303	0.49004741	0.41721532	0.75100257	0.74088228	Trpv2/Ctnnd1	2
GO:0005759	mitochondrial matrix	3/98	351/14612	0.00854701	1.27437642	0.42754792	0.41967656	0.75100257	0.74088228	Hspa1b/Shc1/Mrps27	3
GO:0000118	histone deacetylase complex	1/98	81/14612	0.01234568	1.84076594	0.62348974	0.42107641	0.75100257	0.74088228	Sap30l	1
GO:0005758	mitochondrial intermembrane space	1/98	82/14612	0.01219512	1.81831757	0.61059809	0.42498079	0.75100257	0.74088228	Coa4	1
GO:0010494	cytoplasmic stress granule	1/98	85/14612	0.01176471	1.75414166	0.57297263	0.43653822	0.75100257	0.74088228	Nufip2	1
GO:0030864	cortical actin cytoskeleton	1/98	86/14612	0.01162791	1.73374466	0.56076462	0.44033936	0.75100257	0.74088228	Cap1	1
GO:0000775	chromosome, centromeric region	2/98	225/14612	0.00888889	1.32535147	0.40412746	0.44706983	0.75100257	0.74088228	Dctn5/Smc1a	2
GO:0000313	organellar ribosome	1/98	88/14612	0.01136364	1.69434137	0.5368225	0.44786568	0.75100257	0.74088228	Mrps27	1
GO:0005761	mitochondrial ribosome	1/98	88/14612	0.01136364	1.69434137	0.5368225	0.44786568	0.75100257	0.74088228	Mrps27	1
GO:0090575	RNA polymerase II transcription regulator complex	2/98	227/14612	0.00881057	1.31367437	0.39137806	0.45158875	0.75100257	0.74088228	Rfxap/Taf4b	2
GO:0036464	cytoplasmic ribonucleoprotein granule	2/98	229/14612	0.00873362	1.30220123	0.37874654	0.45608711	0.75100257	0.74088228	Nufip2/Tuba1a	2
GO:0055029	nuclear DNA-directed RNA polymerase complex	1/98	91/14612	0.01098901	1.63848397	0.50203255	0.45896769	0.75100257	0.74088228	Taf4b	1
GO:0030426	growth cone	2/98	232/14612	0.00862069	1.28536242	0.36001515	0.46279551	0.75100257	0.74088228	Trpv2/Ctnnd1	2
GO:0035861	site of double-strand break	1/98	93/14612	0.01075269	1.60324775	0.4795438	0.46624599	0.75100257	0.74088228	Nhej1	1
GO:0000428	DNA-directed RNA polymerase complex	1/98	94/14612	0.0106383	1.58619192	0.46850023	0.46984871	0.75100257	0.74088228	Taf4b	1
GO:0045111	intermediate filament cytoskeleton	1/98	94/14612	0.0106383	1.58619192	0.46850023	0.46984871	0.75100257	0.74088228	Nme1	1
GO:0030880	RNA polymerase complex	1/98	95/14612	0.01052632	1.56949517	0.45758613	0.47342736	0.75100257	0.74088228	Taf4b	1
GO:0031970	organelle envelope lumen	1/98	95/14612	0.01052632	1.56949517	0.45758613	0.47342736	0.75100257	0.74088228	Coa4	1
GO:0030427	site of polarized growth	2/98	238/14612	0.00840336	1.25295833	0.32330177	0.47606842	0.75100257	0.74088228	Trpv2/Ctnnd1	2
GO:0005604	basement membrane	1/98	98/14612	0.01020408	1.5214494	0.42558969	0.48402046	0.75100257	0.74088228	Vwa1	1
GO:0016607	nuclear speck	3/98	389/14612	0.00771208	1.1498872	0.24620859	0.48637088	0.75100257	0.74088228	Hspa1b/Rfxap/Mettl3	3
GO:0005802	trans-Golgi network	2/98	243/14612	0.00823045	1.22717729	0.29343656	0.48697907	0.75100257	0.74088228	Smpd4/Becn1	2
GO:0032587	ruffle membrane	1/98	99/14612	0.01010101	1.50608122	0.41516303	0.48750441	0.75100257	0.74088228	Nme1	1
GO:0045177	apical part of cell	3/98	393/14612	0.00763359	1.13818352	0.22817993	0.49318968	0.75100257	0.74088228	Hspa1b/Abcc5/Atp1b3	3
GO:0031985	Golgi cisterna	1/98	101/14612	0.00990099	1.47625783	0.39465135	0.4944026	0.75100257	0.74088228	Fut11	1
GO:0005819	spindle	3/98	394/14612	0.00761421	1.13529473	0.22370164	0.49488763	0.75100257	0.74088228	Efhc1/Tbck/Smc1a	3
GO:0035770	ribonucleoprotein granule	2/98	248/14612	0.00806452	1.20243581	0.26420129	0.49774991	0.75100257	0.74088228	Nufip2/Tuba1a	2
GO:0005769	early endosome	3/98	399/14612	0.0075188	1.12106798	0.20148017	0.50333589	0.75100257	0.74088228	Pcsk9/Nme1/Tbck	3
GO:0005923	bicellular tight junction	1/98	104/14612	0.00961538	1.43367347	0.36469953	0.50457792	0.75100257	0.74088228	Ctnnd1	1
GO:0034708	methyltransferase complex	1/98	104/14612	0.00961538	1.43367347	0.36469953	0.50457792	0.75100257	0.74088228	Mettl3	1
GO:0031594	neuromuscular junction	1/98	105/14612	0.00952381	1.42001944	0.35492344	0.50792445	0.75100257	0.74088228	Tuba1a	1
GO:0030684	preribosome	1/98	107/14612	0.00934579	1.39347702	0.33566947	0.51455051	0.75486537	0.74469302	Rps19bp1	1
GO:0016528	sarcoplasm	1/98	108/14612	0.00925926	1.38057445	0.32618731	0.51783035	0.75486537	0.74469302	Fkbp1b	1
GO:1902554	serine/threonine protein kinase complex	1/98	111/14612	0.00900901	1.34326163	0.29829586	0.52753884	0.75697264	0.74677189	Cdkn1a	1
GO:0030863	cortical cytoskeleton	1/98	112/14612	0.00892857	1.33126822	0.28917732	0.5307318	0.75697264	0.74677189	Cap1	1
GO:0070160	tight junction	1/98	112/14612	0.00892857	1.33126822	0.28917732	0.5307318	0.75697264	0.74677189	Ctnnd1	1
GO:0019897	extrinsic component of plasma membrane	1/98	113/14612	0.00884956	1.31948709	0.28014505	0.53390341	0.75697264	0.74677189	Ctnnd1	1
GO:0005770	late endosome	2/98	270/14612	0.00740741	1.10445956	0.14235892	0.54341759	0.76522069	0.75490879	Pcsk9/Stard3	2
GO:0016363	nuclear matrix	1/98	121/14612	0.00826446	1.23224827	0.21079264	0.55852408	0.76872628	0.75836714	Smc1a	1
GO:0005765	lysosomal membrane	2/98	280/14612	0.00714286	1.06501458	0.0902601	0.56321763	0.76872628	0.75836714	Sphk2/Cln5	2
GO:0098852	lytic vacuole membrane	2/98	280/14612	0.00714286	1.06501458	0.0902601	0.56321763	0.76872628	0.75836714	Sphk2/Cln5	2
GO:0022626	cytosolic ribosome	1/98	123/14612	0.00813008	1.21221171	0.19420578	0.56447533	0.76872628	0.75836714	Hbs1l	1
GO:1902911	protein kinase complex	1/98	123/14612	0.00813008	1.21221171	0.19420578	0.56447533	0.76872628	0.75836714	Cdkn1a	1
GO:0031965	nuclear membrane	2/98	283/14612	0.00706714	1.05372467	0.07499261	0.56903851	0.76969492	0.75932273	Smpd4/Dctn5	2
GO:0043296	apical junction complex	1/98	127/14612	0.00787402	1.17403182	0.16185671	0.57614064	0.76969492	0.75932273	Ctnnd1	1
GO:0005884	actin filament	1/98	128/14612	0.0078125	1.16485969	0.15393404	0.57900831	0.76969492	0.75932273	Ptges3	1
GO:1990204	oxidoreductase complex	1/98	130/14612	0.00769231	1.14693878	0.13827806	0.58468617	0.76969492	0.75932273	P4ha1	1
GO:0005777	peroxisome	1/98	131/14612	0.00763359	1.13818352	0.13054254	0.58749661	0.76969492	0.75932273	Pmvk	1
GO:0042579	microbody	1/98	131/14612	0.00763359	1.13818352	0.13054254	0.58749661	0.76969492	0.75932273	Pmvk	1
GO:0005795	Golgi stack	1/98	134/14612	0.00746269	1.1127018	0.10769323	0.59581546	0.77262296	0.76221131	Fut11	1
GO:0005635	nuclear envelope	3/98	458/14612	0.00655022	0.97665092	-0.0417179	0.59719649	0.77262296	0.76221131	Bicd2/Smpd4/Dctn5	3
GO:0048786	presynaptic active zone	1/98	136/14612	0.00735294	1.09633854	0.09274802	0.60126889	0.77306001	0.76264247	Ctnnd1	1
GO:0062023	collagen-containing extracellular matrix	2/98	306/14612	0.00653595	0.97452314	-0.037009	0.61182053	0.77769506	0.76721506	Vwa1/Adamts8	2
GO:0030018	Z disc	1/98	141/14612	0.0070922	1.05746128	0.05633717	0.6145859	0.77769506	0.76721506	Fkbp1b	1
GO:0098685	Schaffer collateral - CA1 synapse	1/98	144/14612	0.00694444	1.03543084	0.03510895	0.62236379	0.77769506	0.76721506	Ctnnd1	1
GO:0009898	cytoplasmic side of plasma membrane	1/98	147/14612	0.00680272	1.0142996	0.01431801	0.62998629	0.77769506	0.76721506	Traf2	1

GO:0098802 plasma membrane signaling receptor complex	1/98	147/14612	0.00680272	1.0142996	0.01431801	0.62998629	0.77769506	0.76721506	Traf2	1
GO:0005814 centriole	1/98	148/14612	0.00675676	1.00744622	0.00748139	0.63249312	0.77769506	0.76721506	Hspa1b	1
GO:0034399 nuclear periphery	1/98	148/14612	0.00675676	1.00744622	0.00748139	0.63249312	0.77769506	0.76721506	Smc1a	1
GO:0098687 chromosomal region	2/98	321/14612	0.00623053	0.92898468	-0.1057141	0.63795945	0.77769506	0.76721506	Dctn5/Smc1a	2
GO:0031902 late endosome membrane	1/98	152/14612	0.00657895	0.98093448	-0.0194154	0.64235345	0.77769506	0.76721506	Stard3	1
GO:0032993 protein-DNA complex	1/98	154/14612	0.00649351	0.96819507	-0.0326032	0.64718496	0.77769506	0.76721506	Sphk2	1
GO:0031674 I band	1/98	155/14612	0.00645161	0.96194865	-0.0391342	0.64957643	0.77769506	0.76721506	Fkbp1b	1
GO:0015629 actin cytoskeleton	3/98	495/14612	0.00606061	0.90364873	-0.1792042	0.64995771	0.77769506	0.76721506	Ptges3/Cap1/Dctn5	3
GO:0098982 GABA-ergic synapse	1/98	158/14612	0.00632911	0.9436838	-0.0584827	0.65665499	0.78008905	0.76957679	Nrg2	1
GO:0005774 vacuolar membrane	2/98	334/14612	0.00598802	0.89282659	-0.1628119	0.65949557	0.78008905	0.76957679	Sphk2/Cln5	2
GO:0005912 adherens junction	1/98	161/14612	0.00621118	0.92609963	-0.0774765	0.66359199	0.78047467	0.76995722	Ctnnd1	1
GO:0098798 mitochondrial protein-containing complex	1/98	172/14612	0.00581395	0.86687233	-0.144314	0.68786127	0.80444793	0.79360742	Mrps27	1
GO:0034703 cation channel complex	1/98	176/14612	0.00568182	0.84717069	-0.1676096	0.69624984	0.8096838	0.79877273	Fkbp1b	1
GO:0042383 sarcolemma	1/98	178/14612	0.00561798	0.83765191	-0.1790702	0.70036005	0.80991357	0.79899941	Smpd4	1
GO:0001726 ruffle	1/98	187/14612	0.00534759	0.79733712	-0.2291905	0.71818511	0.81683691	0.80582945	Nme1	1
GO:0005925 focal adhesion	1/98	187/14612	0.00534759	0.79733712	-0.2291905	0.71818511	0.81683691	0.80582945	Shc1	1
GO:0055037 recycling endosome	1/98	187/14612	0.00534759	0.79733712	-0.2291905	0.71818511	0.81683691	0.80582945	Tuba1a	1
GO:0031256 leading edge membrane	1/98	192/14612	0.00520833	0.77657313	-0.2560724	0.72763013	0.82066633	0.80960726	Nme1	1
GO:0044391 ribosomal subunit	1/98	193/14612	0.00518135	0.77254943	-0.2613711	0.72948118	0.82066633	0.80960726	Mrps27	1
GO:1902495 transmembrane transporter complex	2/98	388/14612	0.00515464	0.76856722	-0.3796552	0.73822654	0.82252228	0.8114382	Fkbp1b/Atp1b3	2
GO:0030055 cell-substrate junction	1/98	199/14612	0.00502513	0.74925649	-0.2926432	0.74032891	0.82252228	0.8114382	Shc1	1
GO:0150034 distal axon	2/98	393/14612	0.00508906	0.75878901	-0.3983073	0.74468005	0.82252228	0.8114382	Trpv2/Ctnnd1	2
GO:0030312 external encapsulating structure	2/98	398/14612	0.00502513	0.74925649	-0.4167471	0.7509986	0.82252228	0.8114382	Vwa1/Adamts8	2
GO:0031012 extracellular matrix	2/98	398/14612	0.00502513	0.74925649	-0.4167471	0.7509986	0.82252228	0.8114382	Vwa1/Adamts8	2
GO:1990351 transporter complex	2/98	412/14612	0.00485437	0.72379631	-0.4672974	0.7679877	0.83670239	0.82542723	Fkbp1b/Atp1b3	2
GO:0030017 sarcomere	1/98	221/14612	0.00452489	0.67466987	-0.4004372	0.77654548	0.84159641	0.83025529	Fkbp1b	1
GO:0043197 dendritic spine	1/98	227/14612	0.00440529	0.65683718	-0.4281714	0.78552308	0.84689207	0.83547959	Ctnnd1	1
GO:0044309 neuron spine	1/98	232/14612	0.00431034	0.64268121	-0.4507958	0.79273098	0.85023478	0.83877726	Ctnnd1	1
GO:0030135 coated vesicle	1/98	240/14612	0.00416667	0.6212585	-0.4861266	0.80376805	0.85465111	0.84313407	Pcsk9	1
GO:0030016 myofibril	1/98	241/14612	0.00414938	0.61868067	-0.490471	0.80510612	0.85465111	0.84313407	Fkbp1b	1
GO:0043292 contractile muscle fiber	1/98	249/14612	0.00401606	0.59880338	-0.5246792	0.81549055	0.85842978	0.84686182	Fkbp1b	1
GO:0005667 transcription regulator complex	2/98	458/14612	0.00436681	0.65110061	-0.6233799	0.81695974	0.85842978	0.84686182	Rfxap/Taf4b	2
GO:0034702 monoatomic ion channel complex	1/98	271/14612	0.00369004	0.55019203	-0.61416	0.8413076	0.87954886	0.86769631	Fkbp1b	1
GO:0009897 external side of plasma membrane	1/98	298/14612	0.0033557	0.50034242	-0.7160767	0.86815067	0.9030512	0.89088193	Antxr2	1
GO:0000151 ubiquitin ligase complex	1/98	306/14612	0.00326797	0.48726157	-0.7448296	0.87520325	0.90308357	0.89091387	Traf2	1
GO:0043235 receptor complex	1/98	308/14612	0.00324675	0.48409754	-0.7519235	0.87690723	0.90308357	0.89091387	Traf2	1
GO:0031253 cell projection membrane	1/98	324/14612	0.00308642	0.46019148	-0.8073948	0.88973534	0.9117585	0.8994719	Nme1	1
GO:0030133 transport vesicle	1/98	411/14612	0.00243309	0.36277869	-1.0767408	0.93952153	0.95803427	0.94512407	Aph1b	1
GO:0014069 postsynaptic density	1/98	443/14612	0.00225734	0.33657346	-1.1651573	0.95155402	0.96364839	0.95066254	Ctnnd1	1
GO:0032279 asymmetric synapse	1/98	465/14612	0.00215054	0.32064955	-1.2233412	0.95841898	0.96364839	0.95066254	Ctnnd1	1
GO:0005911 cell-cell junction	1/98	467/14612	0.00214133	0.31927632	-1.2285343	0.95899309	0.96364839	0.95066254	Ctnnd1	1
GO:0099572 postsynaptic specialization	1/98	487/14612	0.00205339	0.30616435	-1.2796356	0.96431966	0.96431966	0.95132476	Ctnnd1	1

ID	Description	GeneRatio	BgRatio	RichFactor	FoldEnrichm	zScore	pvalue	p.adjust	qvalue	geneID	Count
GO:0034663	endoplasmic reticulum chaperone complex	5/140	11/14612	0.45454545	47.4415584	15.1548464	3.3154E-08	8.9515E-06	8.5502E-06	Sdf2l1/Hspa5/Hsp90b1/Dnajb11/P4hb	5
GO:0005788	endoplasmic reticulum lumen	9/140	105/14612	0.08571429	8.94612245	8.03712658	7.2458E-07	9.7819E-05	9.3433E-05	Sdf2l1/Hspa5/Manf/Calr/Hsp90b1/Pdia3/Dnajc3/Dnajb11/P4hb	9
GO:0005790	smooth endoplasmic reticulum	5/140	34/14612	0.14705882	15.3487395	8.23842036	1.6724E-05	0.00125774	0.00120135	Hspa5/Calr/Hsp90b1/Pdia3/Dnajc3	5
GO:0140534	endoplasmic reticulum protein-containing complex	8/140	119/14612	0.06722689	7.01656663	6.48160821	1.8633E-05	0.00125774	0.00120135	Sdf2l1/Hspa5/Calr/Hsp90b1/Pdia3/Dnajb11/Srprb/P4hb	8
GO:0042470	melanosome	7/140	98/14612	0.07142857	7.45510204	6.30612103	4.2679E-05	0.00192055	0.00183444	Hspa5/Mmp14/Hsp90b1/Pdia3/Rab38/P4hb/Sec22b	7
GO:0048770	pigment granule	7/140	98/14612	0.07142857	7.45510204	6.30612103	4.2679E-05	0.00192055	0.00183444	Hspa5/Mmp14/Hsp90b1/Pdia3/Rab38/P4hb/Sec22b	7
GO:0016528	sarcoplasm	5/140	108/14612	0.0462963	4.83201058	3.93128377	0.00380604	0.14680425	0.14022239	Manf/Calr/Hsp90b1/Sri/Bag5	5
GO:0101031	protein folding chaperone complex	3/140	35/14612	0.08571429	8.94612245	4.62908238	0.00450248	0.15195859	0.14514563	Sdf2l1/Stip1/Dnajb11	3
GO:0042824	MHC class I peptide loading complex	2/140	12/14612	0.16666667	17.3952381	5.58819605	0.00564879	0.16946356	0.16186578	Calr/Pdia3	2
GO:0016529	sarcoplasmic reticulum	4/140	80/14612	0.05	5.21857143	3.72123774	0.00727671	0.19465482	0.18592761	Manf/Calr/Hsp90b1/Sri	4
GO:1903561	extracellular vesicle	4/140	82/14612	0.04878049	5.0912892	3.65404599	0.00793038	0.19465482	0.18592761	Has2/Hspd1/Snx18/Sri	4
GO:0043230	extracellular organelle	4/140	90/14612	0.04444444	4.63873016	3.40563108	0.01092783	0.21283359	0.20329134	Has2/Hspd1/Snx18/Sri	4
GO:0065010	extracellular membrane-bounded organelle	4/140	90/14612	0.04444444	4.63873016	3.40563108	0.01092783	0.21283359	0.20329134	Has2/Hspd1/Snx18/Sri	4
GO:0030057	desmosome	2/140	18/14612	0.11111111	11.5968254	4.42450379	0.01261236	0.21283359	0.20329134	Pkp3/Pkp2	2
GO:0030992	intraciliary transport particle B	2/140	18/14612	0.11111111	11.5968254	4.42450379	0.01261236	0.21283359	0.20329134	Ift172/Ift80	2
GO:0045120	pronucleus	2/140	18/14612	0.11111111	11.5968254	4.42450379	0.01261236	0.21283359	0.20329134	Hnrnpc/Cbx1	2
GO:0005793	endoplasmic reticulum-Golgi intermediate compartment	4/140	99/14612	0.04040404	4.21702742	3.15888251	0.01507751	0.23946641	0.2287301	Hspa5/Lman2/P4hb/Sec22b	4
GO:0031462	Cul2-RING ubiquitin ligase complex	2/140	22/14612	0.09090909	9.48831169	3.91872443	0.01857325	0.26914696	0.25707994	Arih1/Cul2	2
GO:0019005	SCF ubiquitin ligase complex	3/140	59/14612	0.05084746	5.30702179	3.26036725	0.01893997	0.26914696	0.25707994	Arih1/Cul2/Fbxo25	3
GO:0030990	intraciliary transport particle	2/140	24/14612	0.08333333	8.69761905	3.71196497	0.0219171	0.29588079	0.28261518	Ift172/Ift80	2
GO:0070062	extracellular exosome	3/140	68/14612	0.04411765	4.60462185	2.93029906	0.02744735	0.34451115	0.32906523	Hspd1/Snx18/Sri	3
GO:0030135	coated vesicle	6/140	240/14612	0.025	2.60928571	2.47240654	0.02807128	0.34451115	0.32906523	Slc17a8/Hspd1/Lman2/Snx18/Atp6v0b/Sec22b	6
GO:0046540	U4/U6 x U5 tri-snRNP complex	2/140	37/14612	0.05405405	5.64169884	2.78043823	0.04880248	0.52706679	0.50343612	Prpf31/Prpf18	2
GO:0097225	sperm midpiece	2/140	37/14612	0.05405405	5.64169884	2.78043823	0.04880248	0.52706679	0.50343612	Hspd1/Ift172	2
GO:0097526	spliceosomal tri-snRNP complex	2/140	37/14612	0.05405405	5.64169884	2.78043823	0.04880248	0.52706679	0.50343612	Prpf31/Prpf18	2
GO:0005912	adherens junction	4/140	161/14612	0.02484472	2.59307897	1.99912985	0.0691203	0.55737159	0.53238222	Pkp3/Pkp2/Cdh10/Cd99l2	4
GO:0002102	podosome	2/140	48/14612	0.04166667	4.34880952	2.28565375	0.07727228	0.55737159	0.53238222	Scin/Sh3pxd2a	2
GO:0034708	methyltransferase complex	3/140	104/14612	0.02884615	3.01071429	2.02396487	0.0780973	0.55737159	0.53238222	Prpf31/Mettl14/Virma	3
GO:0030141	secretory granule	6/140	318/14612	0.01886792	1.96927224	1.71878738	0.08549159	0.55737159	0.53238222	Calr/Fzd8/Pdia3/Hspd1/Hexa/Sri	6
GO:0030670	phagocytic vesicle membrane	2/140	52/14612	0.03846154	4.01428571	2.1416333	0.08865145	0.55737159	0.53238222	Rab38/Rab9b	2
GO:0000153	cytoplasmic ubiquitin ligase complex	1/140	10/14612	0.1	10.4371429	2.93612181	0.09181158	0.55737159	0.53238222	Cul2	1
GO:0005687	U4 snRNP	1/140	10/14612	0.1	10.4371429	2.93612181	0.09181158	0.55737159	0.53238222	Prpf31	1
GO:0017119	Golgi transport complex	1/140	10/14612	0.1	10.4371429	2.93612181	0.09181158	0.55737159	0.53238222	Cog6	1
GO:0044194	cytolytic granule	1/140	10/14612	0.1	10.4371429	2.93612181	0.09181158	0.55737159	0.53238222	Calr	1
GO:0097441	basal dendrite	1/140	10/14612	0.1	10.4371429	2.93612181	0.09181158	0.55737159	0.53238222	Slc17a8	1
GO:0015030	Cajal body	2/140	54/14612	0.03703704	3.86560847	2.07492587	0.09451678	0.55737159	0.53238222	Prpf31/Arih1	2
GO:0000323	lytic vacuole	8/140	487/14612	0.0164271	1.71452039	1.57733736	0.09630711	0.55737159	0.53238222	Calr/Has2/Rab38/Hexa/Rab9b/Snx14/Rmc1/Wdr45b	8
GO:0005764	lysosome	8/140	487/14612	0.0164271	1.71452039	1.57733736	0.09630711	0.55737159	0.53238222	Calr/Has2/Rab38/Hexa/Rab9b/Snx14/Rmc1/Wdr45b	8
GO:0031463	Cul3-RING ubiquitin ligase complex	2/140	55/14612	0.03636364	3.79532468	2.04276013	0.09749055	0.55737159	0.53238222	Klhl5/Arih1	2
GO:0005766	primary lysosome	1/140	11/14612	0.09090909	9.48831169	2.76991264	0.10051905	0.55737159	0.53238222	Hexa	1
GO:0042582	azurophil granule	1/140	11/14612	0.09090909	9.48831169	2.76991264	0.10051905	0.55737159	0.53238222	Hexa	1
GO:0097450	astrocyte end-foot	1/140	11/14612	0.09090909	9.48831169	2.76991264	0.10051905	0.55737159	0.53238222	Slc17a8	1
GO:0045335	phagocytic vesicle	3/140	117/14612	0.02564103	2.67619048	1.79038694	0.10224504	0.55737159	0.53238222	Calr/Rab38/Rab9b	3
GO:0030134	COPII-coated ER to Golgi transport vesicle	2/140	57/14612	0.03508772	3.66215539	1.98063494	0.10351639	0.55737159	0.53238222	Lman2/Sec22b	2
GO:0005801	cis-Golgi network	2/140	58/14612	0.03448276	3.59901478	1.95061371	0.10656694	0.55737159	0.53238222	Fktn/Phtf1	2
GO:0072379	ER membrane insertion complex	1/140	12/14612	0.08333333	8.69761905	2.62367671	0.10914361	0.55737159	0.53238222	Ubl4a	1
GO:0031461	cullin-RING ubiquitin ligase complex	4/140	190/14612	0.02105263	2.19729323	1.63382032	0.10981391	0.55737159	0.53238222	Klhl5/Arih1/Cul2/Fbxo25	4
GO:0030662	coated vesicle membrane	3/140	123/14612	0.02439024	2.5456446	1.69310036	0.11427861	0.55737159	0.53238222	Slc17a8/Atp6v0b/Sec22b	3
GO:0030897	HOPS complex	1/140	13/14612	0.07692308	8.02857143	2.4935432	0.11768607	0.55737159	0.53238222	Aktip	1
GO:0031464	Cul4A-RING E3 ubiquitin ligase complex	1/140	13/14612	0.07692308	8.02857143	2.4935432	0.11768607	0.55737159	0.53238222	Arih1	1
GO:0033391	chromatoid body	1/140	13/14612	0.07692308	8.02857143	2.4935432	0.11768607	0.55737159	0.53238222	Bmal1	1
GO:0044322	endoplasmic reticulum quality control compartment	1/140	13/14612	0.07692308	8.02857143	2.4935432	0.11768607	0.55737159	0.53238222	Calr	1
GO:0097525	spliceosomal snRNP complex	2/140	63/14612	0.03174603	3.31337868	1.80983984	0.12216646	0.55737159	0.53238222	Prpf31/Prpf18	2
GO:0030061	mitochondrial crista	1/140	14/14612	0.07142857	7.45510204	2.37662226	0.1261472	0.55737159	0.53238222	Hspd1	1
GO:0042583	chromaffin granule	1/140	14/14612	0.07142857	7.45510204	2.37662226	0.1261472	0.55737159	0.53238222	Sri	1
GO:0051286	cell tip	1/140	14/14612	0.07142857	7.45510204	2.37662226	0.1261472	0.55737159	0.53238222	Pkp2	1
GO:0030139	endocytic vesicle	4/140	203/14612	0.01970443	2.05657987	1.49098455	0.1308809	0.55737159	0.53238222	Calr/Rab38/Rab9b/Gapvd1	4
GO:0010369	chromocenter	1/140	15/14612	0.06666667	6.95809524	2.27070637	0.13452776	0.55737159	0.53238222	Cbx1	1
GO:0019814	immunoglobulin complex	1/140	15/14612	0.06666667	6.95809524	2.27070637	0.13452776	0.55737159	0.53238222	Ighg2c	1
GO:0033106	cis-Golgi network membrane	1/140	16/14612	0.0625	6.52321429	2.17407573	0.14282851	0.55737159	0.53238222	Phtf1	1
GO:0033162	melanosome membrane	1/140	16/14612	0.0625	6.52321429	2.17407573	0.14282851	0.55737159	0.53238222	Rab38	1
GO:0045009	chitosome	1/140	16/14612	0.0625	6.52321429	2.17407573	0.14282851	0.55737159	0.53238222	Rab38	1
GO:0090741	pigment granule membrane	1/140	16/14612	0.0625	6.52321429	2.17407573	0.14282851	0.55737159	0.53238222	Rab38	1
GO:0120293	dynein axonemal particle	1/140	16/14612	0.0625	6.52321429	2.17407573	0.14282851	0.55737159	0.53238222	Stip1	1
GO:0043209	myelin sheath	4/140	210/14612	0.01904762	1.98802721	1.41842658	0.14287122	0.55737159	0.53238222	Hspa5/Stip1/Pdia3/Hspd1	4
GO:0099023	vesicle tethering complex	2/140	70/14612	0.02857143	2.98204082	1.6348927	0.14485065	0.55737159	0.53238222	Cog6/Aktip	2
GO:0008076	voltage-gated potassium channel complex	2/140	71/14612	0.02816901	2.94004024	1.61169362	0.14816035	0.55737159	0.53238222	Slc5a3/Kcnip2	2
GO:0030532	small nuclear ribonucleoprotein complex	2/140	71/14612	0.02816901	2.94004024	1.61169362	0.14816035	0.55737159	0.53238222	Prpf31/Prpf18	2
GO:0043198	dendritic shaft	2/140	71/14612	0.02816901	2.94004024	1.61169362	0.14816035	0.55737159	0.53238222	Hspa5/Zfp804a	2
GO:0005682	U5 snRNP	1/140	17/14612	0.05882353	6.1394958	2.08536768	0.15105022	0.55737159	0.53238222	Prpf18	1
GO:0042588	zymogen granule	1/140	17/14612	0.05882353	6.1394958	2.08536768	0.15105022	0.55737159	0.53238222	Hspd1	1
GO:0060187	cell pole	1/140	17/14612	0.05882353	6.1394958	2.08536768	0.15105022	0.55737159	0.53238222	Pkp2	1
GO:0090443	FAR/SIN/STRIPAK complex	1/140	17/14612	0.05882353	6.1394958	2.08536768	0.15105022	0.55737159	0.53238222	Ppp2cb	1
GO:0016234	inclusion body	2/140	73/14612	0.02739726	2.85949119	1.56649134	0.15482544	0.55737159	0.53238222	Bag5/Arih1	2
GO:0030666	endocytic vesicle membrane	2/140	73/14612	0.02739726	2.85949119	1.56649134	0.15482544	0.55737159	0.53238222	Rab38/Rab9b	2
GO:0030665	clathrin-coated vesicle membrane	2/140	74/14612	0.02702703	2.82084942	1.54446216	0.15817965	0.55821139	0.53318437	Slc17a8/Atp6v0b	2
GO:0000159	protein phosphatase type 2A complex	1/140	18/14612	0.05555556	5.7984127	2.00348636	0.15919362	0.55821139	0.53318437	Ppp2cb	1
GO:0030315	T-tubule	2/140	75/14612	0.02666667	2.7832381	1.52279794	0.16154752	0.55920296	0.53413149	Rrad/Sri	2
GO:0001533	cornified envelope	1/140	19/14612	0.05263158	5.49323308	1.92753898	0.16725946	0.56450068	0.53919168	Pkp3	1
GO:0032039	integrator complex	1/140	19/14612	0.05263158	5.49323308	1.92753898	0.16725946	0.56450068	0.53919168	Ints10	1
GO:0000151	ubiquitin ligase complex	5/140	306/14612	0.01633987	1.7054155	1.22655298	0.17068055	0.56843189	0.54294664	Klhl5/Itch/Arih1/Cul2/Fbxo25	5
GO:0005697	telomerase holoenzyme complex	1/140	20/14612	0.05	5.21857143	1.85678965	0.17524847	0.56843189	0.54294664	Hnrnpc	1
GO:0033177	proton-transporting two-sector ATPase complex, proton-transporting domain	1/140	20/14612	0.05	5.21857143	1.85678965	0.17524847	0.56843189	0.54294664	Atp6v0b	1
GO:0034045	phagophore assembly site membrane	1/140	21/14612	0.04761905	4.97006803	1.79062556	0.18316139	0.56843189	0.54294664	Wdr45b	1
GO:0044233	mitochondria-associated endoplasmic reticulum membrane contact site	1/140	21/14612	0.04761905	4.97006803	1.79062556	0.18316139	0.56843189	0.54294664	Rab38	1
GO:0070822	Sin3-type complex	1/140	21/14612	0.04761905	4.97006803	1.79062556	0.18316139	0.56843189	0.54294664	Brms1	1
GO:0097228	sperm principal piece	1/140	21/14612	0.04761905	4.97006803	1.79062556	0.18316139	0.56843189	0.54294664	Ift172	1
GO:0030136	clathrin-coated vesicle	3/140	155/14612	0.01935484	2.02009217	1.25575779	0.18608957	0.57095663	0.54535819	Slc17a8/Snx18/Atp6v0b	3
GO:0120114	Sm-like protein family complex	2/140	83/14612	0.02409639	2.51497418	1.3613385	0.18891701	0.57299675	0.54730684	Prpf31/Prpf18	2
GO:0016471	vacuolar proton-transporting V-type ATPase complex	1/140	22/14612	0.04545455	4.74415584	1.7285316	0.19099892	0.57299675	0.54730684	Atp6v0b	1
GO:0034705	potassium channel complex	2/140	85/14612	0.02352941	2.45579832	1.32392206	0.19585875	0.57705032	0.55117867	Slc5a3/Kcnip2	2
GO:0032045	guanyl-nucleotide exchange factor complex	1/140	23/14612	0.04347826	4.5378882	1.67007114	0.19876178	0.57705032	0.55117867	Rmc1	1
GO:0097449	astrocyte projection	1/140	23/14612	0.04347826	4.5378882	1.67007114	0.19876178	0.57705032	0.55117867	Slc17a8	1
GO:0033176	proton-transporting V-type ATPase complex	1/140	25/14612	0.04	4.17485714	1.56261113	0.21406631	0.60638973	0.57920267	Atp6v0b	1
GO:0005798	Golgi-associated vesicle	2/140	91/14612	0.02197802	2.29387755	1.21774211	0.2168605	0.60638973	0.57920267	Stk16/Sec22b	2
GO:0005811	lipid droplet	2/140	93/14612	0.02150538	2.24454685	1.18419735	0.223908	0.60638973	0.57920267	Dhrs3/Stard13	2
GO:0045111	intermediate filament cytoskeleton	2/140	94/14612	0.0212766	2.22066869	1.16774509	0.22743863	0.60638973	0.57920267	Mmp14/Pkp2	2
GO:0005782	peroxisomal matrix	1/140	27/14612	0.03703704	3.86560847	1.46583548	0.22908056	0.60638973	0.57920267	Hspd1	1
GO:0016342	catenin complex	1/140	27/14612	0.03703704	3.86560847	1.46583548	0.22908056	0.60638973	0.57920267	Cdh10	1
GO:0030867	rough endoplasmic reticulum membrane	1/140	27/14612	0.03703704	3.86560847	1.46583548	0.22908056	0.60638973	0.57920267	Srprb	1
GO:0031907	microbody lumen	1/140	27/14612	0.03703704	3.86560847	1.46583548	0.22908056	0.60638973	0.57920267	Hspd1	1
GO:0043202	lysosomal lumen	1/140	27/14612	0.03703704	3.86560847	1.46583548	0.22908056	0.60638973	0.57920267	Hexa	1
GO:0005684	U2-type spliceosomal complex	2/140	96/14612	0.02083333	2.17440476	1.13545428	0.23451161	0.61241958	0.58496218	Prpf31/Prpf18	2
GO:0012507	ER to Golgi transport vesicle membrane	1/140	29/14612	0.03448276	3.59901478	1.37792006	0.24381	0.61241958	0.58496218	Sec22b	1
GO:0001669	acrosomal vesicle	2/140	99/14612	0.02020202	2.10851371	1.08847831	0.24514454	0.61241958	0.58496218	Calr/Pdia3	2
GO:0005759	mitochondrial matrix	5/140	351/14612	0.01424501	1.48677249	0.90791314	0.24692024	0.61241958	0.58496218	Hspd1/Mrps6/Tfb1m/Pdk3/Yars2	5
GO:0005721	pericentric heterochromatin	1/140	30/14612	0.03333333	3.47904762	1.33683147	0.25106959	0.61241958	0.58496218	Cbx1	1
GO:0097440	apical dendrite	1/140	30/14612	0.03333333	3.47904762	1.33683147	0.25106959	0.61241958	0.58496218	Slc17a8	1
GO:0005775	vacuolar lumen	1/140	31/14612	0.03225806	3.36682028	1.29745464	0.25825998	0.61241958	0.58496218	Hexa	1
GO:0030137	COPI-coated vesicle	1/140	31/14612	0.03225806	3.36682028	1.29745464	0.25825998	0.61241958	0.58496218	Sec22b	1
GO:0005770	late endosome	4/140	270/14612	0.01481481	1.54624339	0.89105175	0.25984945	0.61241958	0.58496218	Slc17a8/Rab9b/Snx14/Rmc1	4
GO:0034702	monoatomic ion channel complex	4/140	271/14612	0.01476015	1.54053769	0.88340656	0.26195579	0.61241958	0.58496218	Slc5a3/Kcnk13/Slc17a8/Kcnip2	4
GO:0000314	organellar small ribosomal subunit	1/140	32/14612	0.03125	3.26160714	1.25965932	0.26538182	0.61241958	0.58496218	Mrps6	1
GO:0005763	mitochondrial small ribosomal subunit	1/140	32/14612	0.03125	3.26160714	1.25965932	0.26538182	0.61241958	0.58496218	Mrps6	1
GO:0008180	COP9 signalosome	1/140	32/14612	0.03125	3.26160714	1.25965932	0.26538182	0.61241958	0.58496218	Hspa5	1
GO:0071339	MLL1 complex	1/140	32/14612	0.03125	3.26160714	1.25965932	0.26538182	0.61241958	0.58496218	Prpf31	1
GO:0080008	Cul4-RING E3 ubiquitin ligase complex	1/140	32/14612	0.03125	3.26160714	1.25965932	0.26538182	0.61241958	0.58496218	Arih1	1
GO:0044665	MLL1/2 complex	1/140	33/14612	0.03030303	3.16277056	1.22332898	0.27243577	0.62336998	0.59542161	Prpf31	1
GO:0005681	spliceosomal complex	3/140	194/14612	0.01546392	1.61399116	0.84674021	0.28469262	0.63894615	0.61029944	Prpf31/Hnrnpc/Prpf18	3
GO:0034707	chloride channel complex	1/140	35/14612	0.02857143	2.98204082	1.15465503	0.28634253	0.63894615	0.61029944	Slc17a8	1
GO:0098992	neuronal dense core vesicle	1/140	35/14612	0.02857143	2.98204082	1.15465503	0.28634253	0.63894615	0.61029944	Fzd8	1
GO:0001772	immunological synapse	1/140	36/14612	0.02777778	2.89920635	1.12213197	0.29319662	0.64796835	0.61891714	Card11	1
GO:0000407	phagophore assembly site	1/140	37/14612	0.02702703	2.82084942	1.09071258	0.29998535	0.64796835	0.61891714	Wdr45b	1
GO:0090533	cation-transporting ATPase complex	1/140	37/14612	0.02702703	2.82084942	1.09071258	0.29998535	0.64796835	0.61891714	Atp6v0b	1
GO:0098533	ATPase dependent transmembrane transport complex	1/140	37/14612	0.02702703	2.82084942	1.09071258	0.29998535	0.64796835	0.61891714	Atp6v0b	1
GO:0033017	sarcoplasmic reticulum membrane	1/140	39/14612	0.02564103	2.67619048	1.03091035	0.31336918	0.66992809	0.63989233	Sri	1
GO:1902495	transmembrane transporter complex	5/140	388/14612	0.0128866	1.34499264	0.67741456	0.31511432	0.66992809	0.63989233	Slc5a3/Kcnk13/Slc17a8/Kcnip2/Atp6v0b	5
GO:0033116	endoplasmic reticulum-Golgi intermediate compartment membrane	1/140	42/14612	0.02380952	2.48503401	0.94791846	0.33297001	0.70235861	0.67086884	Sec22b	1
GO:0097386	glial cell projection	1/140	43/14612	0.02325581	2.42724252	0.92184275	0.33937935	0.70699381	0.67529624	Slc17a8	1
GO:0016469	proton-transporting two-sector ATPase complex	1/140	44/14612	0.02272727	2.37207792	0.89648878	0.34572755	0.70699381	0.67529624	Atp6v0b	1
GO:0031201	SNARE complex	1/140	45/14612	0.02222222	2.31936508	0.87181812	0.35201518	0.70699381	0.67529624	Sec22b	1
GO:0005777	peroxisome	2/140	131/14612	0.01526718	1.59345692	0.67106608	0.35803622	0.70699381	0.67529624	Hspd1/Far2	2
GO:0042579	microbody	2/140	131/14612	0.01526718	1.59345692	0.67106608	0.35803622	0.70699381	0.67529624	Hspd1/Far2	2
GO:0044853	plasma membrane raft	2/140	131/14612	0.01526718	1.59345692	0.67106608	0.35803622	0.70699381	0.67529624	Has2/Cr1l	2
GO:1990351	transporter complex	5/140	412/14612	0.01213592	1.26664355	0.53997696	0.36065588	0.70699381	0.67529624	Slc5a3/Kcnk13/Slc17a8/Kcnip2/Atp6v0b	5
GO:0009295	nucleoid	1/140	47/14612	0.0212766	2.22066869	0.82438713	0.36441101	0.70699381	0.67529624	Tfb1m	1
GO:0042645	mitochondrial nucleoid	1/140	47/14612	0.0212766	2.22066869	0.82438713	0.36441101	0.70699381	0.67529624	Tfb1m	1
GO:0044232	organelle membrane contact site	1/140	47/14612	0.0212766	2.22066869	0.82438713	0.36441101	0.70699381	0.67529624	Rab38	1
GO:0008287	protein serine/threonine phosphatase complex	1/140	48/14612	0.02083333	2.17440476	0.80156325	0.37052034	0.70699381	0.67529624	Ppp2cb	1
GO:1903293	phosphatase complex	1/140	48/14612	0.02083333	2.17440476	0.80156325	0.37052034	0.70699381	0.67529624	Ppp2cb	1
GO:0090575	RNA polymerase II transcription regulator complex	3/140	227/14612	0.01321586	1.37935809	0.56656149	0.37112594	0.70699381	0.67529624	Bmal1/Smad2/Tbpl1	3
GO:0036126	sperm flagellum	2/140	135/14612	0.01481481	1.54624339	0.62712411	0.37182638	0.70699381	0.67529624	Hspd1/Ift172	2
GO:0016323	basolateral plasma membrane	3/140	235/14612	0.01276596	1.33240122	0.5052451	0.39192547	0.72933306	0.69663392	Slc5a3/Slc51a/Cr1l	3
GO:0030018	Z disc	2/140	141/14612	0.0141844	1.4804458	0.56382508	0.39228762	0.72933306	0.69663392	Kcnn1/Sri	2
GO:0005771	multivesicular body	1/140	52/14612	0.01923077	2.00714286	0.71556942	0.3943801	0.72933306	0.69663392	Slc17a8	1
GO:0031045	dense core granule	1/140	52/14612	0.01923077	2.00714286	0.71556942	0.3943801	0.72933306	0.69663392	Fzd8	1
GO:0071005	U2-type precatalytic spliceosome	1/140	53/14612	0.01886792	1.96927224	0.69527665	0.40020337	0.73506741	0.70211117	Prpf31	1
GO:0001673	male germ cell nucleus	1/140	54/14612	0.01851852	1.93280423	0.67542362	0.40597104	0.74062284	0.70741753	Tbpl1	1
GO:0071011	precatalytic spliceosome	1/140	55/14612	0.01818182	1.89766234	0.65599136	0.41168364	0.74133064	0.7080936	Prpf31	1
GO:0005778	peroxisomal membrane	1/140	56/14612	0.01785714	1.86377551	0.63696209	0.4173417	0.74133064	0.7080936	Far2	1
GO:0005791	rough endoplasmic reticulum	1/140	56/14612	0.01785714	1.86377551	0.63696209	0.4173417	0.74133064	0.7080936	Srprb	1
GO:0031903	microbody membrane	1/140	56/14612	0.01785714	1.86377551	0.63696209	0.4173417	0.74133064	0.7080936	Far2	1
GO:0014704	intercalated disc	1/140	58/14612	0.01724138	1.79950739	0.60004664	0.42849622	0.74728724	0.71378313	Pkp2	1
GO:0031902	late endosome membrane	2/140	152/14612	0.01315789	1.37330827	0.45503518	0.42899823	0.74728724	0.71378313	Snx14/Rmc1	2
GO:0097729	9+2 motile cilium	2/140	152/14612	0.01315789	1.37330827	0.45503518	0.42899823	0.74728724	0.71378313	Hspd1/Ift172	2
GO:0031674	I band	2/140	155/14612	0.01290323	1.34672811	0.42683058	0.43881102	0.75948061	0.72542983	Kcnn1/Sri	2
GO:0010008	endosome membrane	5/140	456/14612	0.01096491	1.14442356	0.30816967	0.44430211	0.7601554	0.72607436	Snx21/Snx14/Snx18/Itch/Rmc1	5
GO:0005882	intermediate filament	1/140	61/14612	0.01639344	1.71100703	0.54730838	0.44483168	0.7601554	0.72607436	Pkp2	1
GO:0009925	basal plasma membrane	3/140	258/14612	0.01162791	1.21362126	0.34049252	0.45062135	0.76520607	0.73089858	Slc5a3/Slc51a/Cr1l	3
GO:0043073	germ cell nucleus	1/140	66/14612	0.01515152	1.58138528	0.4655903	0.47103351	0.79486904	0.75923164	Tbpl1	1
GO:0005905	clathrin-coated pit	1/140	68/14612	0.01470588	1.53487395	0.43481416	0.48116708	0.80194513	0.76599047	Hspd1	1
GO:0016591	RNA polymerase II, holoenzyme	1/140	68/14612	0.01470588	1.53487395	0.43481416	0.48116708	0.80194513	0.76599047	Tbpl1	1
GO:0036064	ciliary basal body	2/140	170/14612	0.01176471	1.22789916	0.29396338	0.48645238	0.80578001	0.76965342	Ift172/Ift80	2
GO:0045178	basal part of cell	3/140	275/14612	0.01090909	1.1385974	0.2282086	0.49252375	0.80633387	0.77018245	Slc5a3/Slc51a/Cr1l	3
GO:0035097	histone methyltransferase complex	1/140	71/14612	0.01408451	1.47002012	0.39047047	0.49600712	0.80633387	0.77018245	Prpf31	1

GO:0048787	presynaptic active zone membrane	1/140	71/14612	0.01408451	1.47002012	0.39047047	0.49600712	0.80633387	0.77018245	Cdh10	1
GO:0031901	early endosome membrane	2/140	174/14612	0.01149425	1.19967159	0.26060161	0.49873243	0.80633387	0.77018245	Snx21/Itch	2
GO:0034703	cation channel complex	2/140	176/14612	0.01136364	1.18603896	0.24421735	0.5048025	0.81128973	0.77491612	Slc5a3/Kcnip2	2
GO:0042383	sarcolemma	2/140	178/14612	0.01123596	1.17271268	0.22802401	0.51082532	0.81377775	0.77729259	Rrad/Sri	2
GO:0016607	nuclear speck	4/140	389/14612	0.01028278	1.07322806	0.14397809	0.51423077	0.81377775	0.77729259	Srpk1/Prpf31/Virma/Prpf18	4
GO:0150034	distal axon	4/140	393/14612	0.01017812	1.06230462	0.12314639	0.52230499	0.81377775	0.77729259	Slc17a8/Zfp804a/Snx18/Sri	4
GO:0043679	axon terminus	2/140	182/14612	0.01098901	1.14693878	0.19619081	0.52272758	0.81377775	0.77729259	Slc17a8/Sri	2
GO:0015935	small ribosomal subunit	1/140	77/14612	0.01298701	1.3554731	0.30759856	0.52443455	0.81377775	0.77729259	Mrps6	1
GO:0030667	secretory granule membrane	1/140	77/14612	0.01298701	1.3554731	0.30759856	0.52443455	0.81377775	0.77729259	Sri	1
GO:0005769	early endosome	4/140	399/14612	0.01002506	1.04633011	0.09228829	0.53429819	0.82334082	0.78642691	Rab38/Hspd1/Snx21/Itch	4
GO:0000118	histone deacetylase complex	1/140	81/14612	0.01234568	1.28853616	0.25611482	0.5424958	0.82334082	0.78642691	Brms1	1
GO:0030496	midbody	2/140	190/14612	0.01052632	1.09864662	0.13461268	0.54594894	0.82334082	0.78642691	Hspa5/Hsp90b1	2
GO:0009897	external side of plasma membrane	3/140	298/14612	0.01006711	1.05071908	0.0870039	0.54661744	0.82334082	0.78642691	Calr/Cr1l/P4hb	3
GO:0044291	cell-cell contact zone	1/140	82/14612	0.01219512	1.2728223	0.24366531	0.54690366	0.82334082	0.78642691	Pkp2	1
GO:0045121	membrane raft	3/140	299/14612	0.01003344	1.04720497	0.08111435	0.54889388	0.82334082	0.78642691	Card11/Has2/Cr1l	3
GO:0098857	membrane microdomain	3/140	301/14612	0.00996678	1.0402468	0.06939358	0.55342691	0.82555395	0.78854081	Card11/Has2/Cr1l	3
GO:0043235	receptor complex	3/140	308/14612	0.00974026	1.01660482	0.02896814	0.56908071	0.83034853	0.79312042	Bmal1/Hspd1/Cr1l	3
GO:0000313	organellar ribosome	1/140	88/14612	0.01136364	1.18603896	0.1721638	0.57247918	0.83034853	0.79312042	Mrps6	1
GO:0005761	mitochondrial ribosome	1/140	88/14612	0.01136364	1.18603896	0.1721638	0.57247918	0.83034853	0.79312042	Mrps6	1
GO:0071013	catalytic step 2 spliceosome	1/140	89/14612	0.01123596	1.17271268	0.16074252	0.57660015	0.83034853	0.79312042	Hnrnpc	1
GO:0000792	heterochromatin	1/140	90/14612	0.01111111	1.15968254	0.14945319	0.58068167	0.83034853	0.79312042	Cbx1	1
GO:0031514	motile cilium	2/140	204/14612	0.00980392	1.0232493	0.03288992	0.58467467	0.83034853	0.79312042	Hspd1/Ift172	2
GO:0044306	neuron projection terminus	2/140	204/14612	0.00980392	1.0232493	0.03288992	0.58467467	0.83034853	0.79312042	Slc17a8/Sri	2
GO:0055029	nuclear DNA-directed RNA polymerase complex	1/140	91/14612	0.01098901	1.14693878	0.13829248	0.58472413	0.83034853	0.79312042	Tbpl1	1
GO:0055038	recycling endosome membrane	1/140	92/14612	0.01086957	1.13447205	0.12725719	0.58872789	0.83034853	0.79312042	Snx18	1
GO:0097731	9+0 non-motile cilium	1/140	92/14612	0.01086957	1.13447205	0.12725719	0.58872789	0.83034853	0.79312042	Ift80	1
GO:0016324	apical plasma membrane	3/140	319/14612	0.00940439	0.98154948	-0.0327704	0.59299549	0.83034853	0.79312042	Slc5a3/Pdia3/Akr1a1	3
GO:0000428	DNA-directed RNA polymerase complex	1/140	94/14612	0.0106383	1.11033435	0.10555066	0.59662079	0.83034853	0.79312042	Tbpl1	1
GO:0005881	cytoplasmic microtubule	1/140	94/14612	0.0106383	1.11033435	0.10555066	0.59662079	0.83034853	0.79312042	Srprb	1
GO:0030880	RNA polymerase complex	1/140	95/14612	0.01052632	1.09864662	0.09487358	0.60051066	0.8314763	0.79419763	Tbpl1	1
GO:0060076	excitatory synapse	1/140	99/14612	0.01010101	1.05425685	0.05327621	0.6157012	0.84178744	0.80404648	Slc17a8	1
GO:0031985	Golgi cisterna	1/140	101/14612	0.00990099	1.03338048	0.03310887	0.62307998	0.84178744	0.80404648	Hspd1	1
GO:0005774	vacuolar membrane	3/140	334/14612	0.00898204	0.93746792	-0.1137057	0.62421167	0.84178744	0.80404648	Snx14/Rmc1/Atp6v0b	3
GO:0005903	brush border	1/140	102/14612	0.00980392	1.0232493	0.0231748	0.62671645	0.84178744	0.80404648	Scin	1
GO:0030017	sarcomere	2/140	221/14612	0.00904977	0.94453782	-0.0817123	0.62837979	0.84178744	0.80404648	Kcnn1/Sri	2
GO:0000775	chromosome, centromeric region	2/140	225/14612	0.00888889	0.92774603	-0.1074255	0.63813426	0.84178744	0.80404648	Ppp2cb/Cbx1	2
GO:0043197	dendritic spine	2/140	227/14612	0.00881057	0.91957206	-0.1201171	0.64293636	0.84178744	0.80404648	Zfp804a/Sri	2
GO:0005667	transcription regulator complex	4/140	458/14612	0.00873362	0.91154086	-0.1891801	0.64351737	0.84178744	0.80404648	Bmal1/Smad2/Tbpl1/Taf1a	4
GO:0016605	PML body	1/140	107/14612	0.00934579	0.97543391	-0.0250847	0.64438284	0.84178744	0.80404648	Bmal1	1
GO:0098791	Golgi apparatus subcompartment	3/140	347/14612	0.00864553	0.90234664	-0.1810738	0.64992793	0.84178744	0.80404648	Rab38/Hspd1/Lap3	3
GO:0098797	plasma membrane protein complex	4/140	463/14612	0.00863931	0.90169701	-0.2114142	0.65197349	0.84178744	0.80404648	Slc5a3/Fzd8/Kcnip2/Cdh10	4
GO:0030658	transport vesicle membrane	2/140	231/14612	0.00865801	0.90364873	-0.1451806	0.65239097	0.84178744	0.80404648	Slc17a8/Sec22b	2
GO:0000781	chromosome, telomeric region	1/140	110/14612	0.00909091	0.94883117	-0.0529819	0.65458155	0.84178744	0.80404648	Cbx1	1
GO:0030426	growth cone	2/140	232/14612	0.00862069	0.89975369	-0.1513814	0.65472356	0.84178744	0.80404648	Zfp804a/Snx18	2
GO:0044309	neuron spine	2/140	232/14612	0.00862069	0.89975369	-0.1513814	0.65472356	0.84178744	0.80404648	Zfp804a/Sri	2
GO:0005911	cell-cell junction	4/140	467/14612	0.00856531	0.89397369	-0.2290395	0.65864307	0.84281341	0.80502645	Pkp3/Pkp2/Cdh10/Cd99l2	4
GO:0019897	extrinsic component of plasma membrane	1/140	113/14612	0.00884956	0.92364096	-0.0801439	0.66448981	0.84554761	0.80763807	Cdh10	1
GO:0030427	site of polarized growth	2/140	238/14612	0.00840336	0.87707083	-0.1880591	0.66845981	0.84554761	0.80763807	Zfp804a/Snx18	2
GO:0005840	ribosome	2/140	240/14612	0.00833333	0.8697619	-0.2000897	0.67294034	0.84554761	0.80763807	Calr/Mrps6	2
GO:0030016	myofibril	2/140	241/14612	0.00829876	0.86615293	-0.2060694	0.6751623	0.84554761	0.80763807	Kcnn1/Sri	2
GO:0097730	non-motile cilium	1/140	117/14612	0.00854701	0.89206349	-0.1152901	0.67726316	0.84554761	0.80763807	Ift80	1
GO:0005802	trans-Golgi network	2/140	243/14612	0.00823045	0.8590241	-0.2179588	0.67956975	0.84554761	0.80763807	Rab38/Lap3	2
GO:0016363	nuclear matrix	1/140	121/14612	0.00826446	0.86257379	-0.1492976	0.68955355	0.84973438	0.81163713	Srpk1	1
GO:0043292	contractile muscle fiber	2/140	249/14612	0.00803213	0.83832473	-0.2530815	0.69250232	0.84973438	0.81163713	Kcnn1/Sri	2
GO:0090734	site of DNA damage	1/140	122/14612	0.00819672	0.85550351	-0.1576314	0.69255282	0.84973438	0.81163713	Cbx1	1
GO:1904949	ATPase complex	1/140	123/14612	0.00813008	0.8485482	-0.1659004	0.69552333	0.84973438	0.81163713	Atp6v0b	1
GO:1990204	oxidoreductase complex	1/140	130/14612	0.00769231	0.80285714	-0.222064	0.71553395	0.87023604	0.83121961	P4hb	1
GO:0005795	Golgi stack	1/140	134/14612	0.00746269	0.77889126	-0.252897	0.72637651	0.87023604	0.83121961	Hspd1	1
GO:0005930	axoneme	1/140	134/14612	0.00746269	0.77889126	-0.252897	0.72637651	0.87023604	0.83121961	Ift172	1
GO:0097014	ciliary plasm	1/140	135/14612	0.00740741	0.77312169	-0.2604715	0.7290224	0.87023604	0.83121961	Ift172	1
GO:0045177	apical part of cell	3/140	393/14612	0.00763359	0.79672846	-0.4017705	0.73079982	0.87023604	0.83121961	Slc5a3/Pdia3/Akr1a1	3
GO:0048786	presynaptic active zone	1/140	136/14612	0.00735294	0.76743697	-0.2679943	0.73164289	0.87023604	0.83121961	Cdh10	1
GO:0030312	external encapsulating structure	3/140	398/14612	0.00753769	0.78671931	-0.4243016	0.73864879	0.87089595	0.83184993	Calr/Mmp14/Lrrn1	3
GO:0031012	extracellular matrix	3/140	398/14612	0.00753769	0.78671931	-0.4243016	0.73864879	0.87089595	0.83184993	Calr/Mmp14/Lrrn1	3
GO:0008021	synaptic vesicle	2/140	277/14612	0.00722022	0.75358432	-0.4072387	0.74729516	0.87592173	0.83665038	Slc17a8/Sec22b	2
GO:0005765	lysosomal membrane	2/140	280/14612	0.00714286	0.7455102	-0.4228981	0.75264385	0.87592173	0.83665038	Snx14/Rmc1	2
GO:0098852	lytic vacuole membrane	2/140	280/14612	0.00714286	0.7455102	-0.4228981	0.75264385	0.87592173	0.83665038	Snx14/Rmc1	2
GO:0009898	cytoplasmic side of plasma membrane	1/140	147/14612	0.00680272	0.71000972	-0.3475543	0.75885927	0.8782998	0.83892183	Snx18	1
GO:0034399	nuclear periphery	1/140	148/14612	0.00675676	0.70521236	-0.3545158	0.76119316	0.8782998	0.83892183	Srpk1	1
GO:0005874	microtubule	3/140	416/14612	0.00721154	0.75267857	-0.5033443	0.76542477	0.8794242	0.83999582	Zfp804a/Tppp3/Srprb	3
GO:0070382	exocytic vesicle	2/140	293/14612	0.00682594	0.71243296	-0.4890534	0.7747159	0.88509214	0.84540965	Slc17a8/Sec22b	2
GO:0098862	cluster of actin-based cell projections	1/140	155/14612	0.00645161	0.67336406	-0.4020966	0.77691422	0.88509214	0.84540965	Scin	1
GO:0098982	GABA-ergic synapse	1/140	158/14612	0.00632911	0.66057866	-0.4219037	0.78333311	0.88865521	0.84881297	Cdh10	1
GO:0030672	synaptic vesicle membrane	1/140	164/14612	0.00609756	0.63641115	-0.460541	0.79562587	0.89507911	0.85494886	Slc17a8	1
GO:0099501	exocytic vesicle membrane	1/140	164/14612	0.00609756	0.63641115	-0.460541	0.79562587	0.89507911	0.85494886	Slc17a8	1
GO:0000922	spindle pole	1/140	167/14612	0.00598802	0.62497861	-0.4793969	0.80151	0.89795726	0.85769797	Ppp2cb	1
GO:0098798	mitochondrial protein-containing complex	1/140	172/14612	0.00581395	0.60681063	-0.5101784	0.81094539	0.90352714	0.86301812	Mrps6	1
GO:0019898	extrinsic component of membrane	1/140	174/14612	0.00574713	0.5998358	-0.5222748	0.81459363	0.90352714	0.86301812	Cdh10	1
GO:0098687	chromosomal region	2/140	321/14612	0.00623053	0.65028927	-0.6231174	0.81652082	0.90352714	0.86301812	Ppp2cb/Cbx1	2
GO:0099634	postsynaptic specialization membrane	1/140	180/14612	0.00555556	0.57984127	-0.5578611	0.82512418	0.90640478	0.86576675	Cdh10	1
GO:0005938	cell cortex	2/140	328/14612	0.00609756	0.63641115	-0.6550316	0.82583547	0.90640478	0.86576675	Itch/Ric8b	2
GO:0005741	mitochondrial outer membrane	1/140	187/14612	0.00534759	0.55813598	-0.598124	0.83666134	0.9108813	0.87004256	S2bpcox16	1
GO:0055037	recycling endosome	1/140	187/14612	0.00534759	0.55813598	-0.598124	0.83666134	0.9108813	0.87004256	Snx18	1
GO:0098562	cytoplasmic side of membrane	1/140	190/14612	0.00526316	0.54932331	-0.6149911	0.84137144	0.91233048	0.87142678	Snx18	1
GO:0044391	ribosomal subunit	1/140	193/14612	0.00518135	0.5407846	-0.6316365	0.84594667	0.9136224	0.87266077	Mrps6	1
GO:0043204	perikaryon	1/140	196/14612	0.00510204	0.53250729	-0.6480674	0.85039085	0.91476306	0.87375029	Slc17a8	1
GO:0015629	actin cytoskeleton	3/140	495/14612	0.00606061	0.63255411	-0.8180211	0.85780634	0.91907823	0.87787199	Scin/Hnrnpc/Sh3pxd2a	3
GO:0000228	nuclear chromosome	1/140	204/14612	0.00490196	0.51162465	-0.6908865	0.86162934	0.91952538	0.8782991	Brms1	1
GO:0030027	lamellipodium	1/140	212/14612	0.00471698	0.49231806	-0.732349	0.87202918	0.92233001	0.88097799	P4hb	1
GO:0019867	outer membrane	1/140	214/14612	0.0046729	0.48771696	-0.7425155	0.87450549	0.92233001	0.88097799	S2bpcox16	1
GO:0031968	organelle outer membrane	1/140	214/14612	0.0046729	0.48771696	-0.7425155	0.87450549	0.92233001	0.88097799	S2bpcox16	1
GO:0036464	cytoplasmic ribonucleoprotein granule	1/140	229/14612	0.00436681	0.45577043	-0.8164223	0.89162223	0.93286981	0.89104523	Bmal1	1
GO:0032838	plasma membrane bounded cell projection cytoplasm	1/140	232/14612	0.00431034	0.44987685	-0.8307382	0.8947565	0.93286981	0.89104523	Ift172	1
GO:0005819	spindle	2/140	394/14612	0.00507614	0.52980421	-0.9305663	0.894864	0.93286981	0.89104523	Ppp2cb/Cbx1	2
GO:0030133	transport vesicle	2/140	411/14612	0.00486618	0.50789016	-0.9953211	0.90800783	0.9360265	0.8940604	Slc17a8/Sec22b	2
GO:0035770	ribonucleoprotein granule	1/140	248/14612	0.00403226	0.42085253	-0.9047259	0.91001351	0.9360265	0.8940604	Bmal1	1
GO:0042734	presynaptic membrane	1/140	250/14612	0.004	0.41748571	-0.913711	0.91175915	0.9360265	0.8940604	Cdh10	1
GO:0061695	transferase complex, transferring phosphorus-containing groups	1/140	250/14612	0.004	0.41748571	-0.913711	0.91175915	0.9360265	0.8940604	Tbpl1	1
GO:0099568	cytoplasmic region	1/140	270/14612	0.0037037	0.38656085	-1.0006639	0.92746826	0.94854708	0.90601963	Ift172	1
GO:0031965	nuclear membrane	1/140	283/14612	0.00353357	0.36880363	-1.0546042	0.9361557	0.95090929	0.90827593	Phf11b	1
GO:0005635	nuclear envelope	2/140	458/14612	0.00436681	0.45577043	-1.1638982	0.93682174	0.95090929	0.90827593	Phf11b/Calr	2
GO:0062023	collagen-containing extracellular matrix	1/140	306/14612	0.00326797	0.3410831	-1.1457028	0.94907017	0.95973388	0.91670488	Calr	1
GO:0045211	postsynaptic membrane	1/140	365/14612	0.00273973	0.28594912	-1.3587939	0.97152463	0.97877481	0.93489212	Cdh10	1
GO:0031252	cell leading edge	1/140	454/14612	0.00220264	0.22989301	-1.6395239	0.98820819	0.99150829	0.9470547	P4hb	1
GO:0099572	postsynaptic specialization	1/140	487/14612	0.00205339	0.21431505	-1.7344365	0.99150829	0.99150829	0.9470547	Cdh10	1

gene_id	gene_short_name	RAIN_q	JTK_CYCLE_q	ARSER_q	CircaCompare_q	RAIN_call_q015	JTK_CYCLE_call_q015	ARSER_call_q015	CircaCompare_call_q015	n_methods_q015	method_combination_q015	canonical_core_clock_gene
ENSMUSG00000055116	Arntl	1.5518E-03	5.8549E-02	6.8078E-04	2.6275E-03	TRUE	TRUE	TRUE	TRUE	4	All four	TRUE
ENSMUSG00000028957	Per3	9.7544E-03	3.8352E-02	3.6646E-03	2.6275E-03	TRUE	TRUE	TRUE	TRUE	4	All four	TRUE
ENSMUSG00000022389	Tef	9.7544E-03	1.0697E-01	4.4407E-03	2.6275E-03	TRUE	TRUE	TRUE	TRUE	4	All four	FALSE
ENSMUSG00000105881	4932422M17Rik	4.9770E-02	3.3893E-01	5.6204E-02	8.0364E-03	TRUE	FALSE	TRUE	TRUE	3	RAIN + ARSER + CircaCompare	FALSE
ENSMUSG00000015452	Ager	1.9409E-02	4.1828E-01	4.7906E-02	4.3517E-03	TRUE	FALSE	TRUE	TRUE	3	RAIN + ARSER + CircaCompare	FALSE
ENSMUSG00000035086	Becn1	7.1344E-02	5.0222E-01	1.2252E-01	1.0642E-01	TRUE	FALSE	TRUE	TRUE	3	RAIN + ARSER + CircaCompare	FALSE
ENSMUSG00000030103	Bhlhe40	3.1913E-02	5.0222E-01	4.7906E-02	6.6791E-03	TRUE	FALSE	TRUE	TRUE	3	RAIN + ARSER + CircaCompare	FALSE
ENSMUSG00000105741	C79130	2.9373E-02	2.3355E-01	1.4064E-01	4.1268E-02	TRUE	FALSE	TRUE	TRUE	3	RAIN + ARSER + CircaCompare	FALSE
ENSMUSG00000050623	Catsperz	9.7544E-03	1.6084E-01	3.6646E-03	2.6756E-03	TRUE	FALSE	TRUE	TRUE	3	RAIN + ARSER + CircaCompare	FALSE
ENSMUSG00000048445	Ccdc57	6.8281E-03	7.5273E-01	1.1726E-01	6.1787E-02	TRUE	FALSE	TRUE	TRUE	3	RAIN + ARSER + CircaCompare	FALSE
ENSMUSG00000038550	Ciart	7.6198E-02	7.5273E-01	1.2252E-01	6.8645E-02	TRUE	FALSE	TRUE	TRUE	3	RAIN + ARSER + CircaCompare	FALSE
ENSMUSG00000025981	Coq10b	3.1913E-02	5.9891E-01	4.7906E-02	6.6791E-03	TRUE	FALSE	TRUE	TRUE	3	RAIN + ARSER + CircaCompare	FALSE
ENSMUSG00000062563	Cys1	9.7544E-03	3.3893E-01	1.4184E-01	3.6368E-02	TRUE	FALSE	TRUE	TRUE	3	RAIN + ARSER + CircaCompare	FALSE
ENSMUSG00000059824	Dbp	1.5518E-03	3.3893E-01	1.0812E-02	2.6756E-03	TRUE	FALSE	TRUE	TRUE	3	RAIN + ARSER + CircaCompare	FALSE
ENSMUSG00000013787	Ehmt2	1.4178E-01	9.1146E-01	1.1483E-01	1.2542E-01	TRUE	FALSE	TRUE	TRUE	3	RAIN + ARSER + CircaCompare	FALSE
ENSMUSG00000019874	Fabp7	1.5518E-03	1.6084E-01	1.0288E-01	4.9398E-03	TRUE	FALSE	TRUE	TRUE	3	RAIN + ARSER + CircaCompare	FALSE
ENSMUSG00000030303	Far2	7.2298E-02	5.9891E-01	3.4160E-02	1.0642E-01	TRUE	FALSE	TRUE	TRUE	3	RAIN + ARSER + CircaCompare	FALSE
ENSMUSG00000027204	Fbn1	9.7544E-03	3.3893E-01	4.7906E-02	8.0364E-03	TRUE	FALSE	TRUE	TRUE	3	RAIN + ARSER + CircaCompare	FALSE
ENSMUSG00000040170	Fmo2	4.9770E-02	5.0222E-01	4.1138E-03	2.6275E-03	TRUE	FALSE	TRUE	TRUE	3	RAIN + ARSER + CircaCompare	FALSE
ENSMUSG00000007415	Gatad1	9.7544E-03	5.0222E-01	9.6005E-02	1.3666E-02	TRUE	FALSE	TRUE	TRUE	3	RAIN + ARSER + CircaCompare	FALSE
ENSMUSG00000109904	Gm45819	2.9373E-02	1.0000E+00	1.0806E-01	2.1157E-02	TRUE	FALSE	TRUE	TRUE	3	RAIN + ARSER + CircaCompare	FALSE
ENSMUSG00000112850	Gm9176	1.0262E-01	5.9891E-01	8.9878E-02	2.0523E-02	TRUE	FALSE	TRUE	TRUE	3	RAIN + ARSER + CircaCompare	FALSE
ENSMUSG00000033318	Gstt2	4.9770E-02	5.0222E-01	1.1483E-01	6.5276E-02	TRUE	FALSE	TRUE	TRUE	3	RAIN + ARSER + CircaCompare	FALSE
ENSMUSG00000021676	Iqgap2	2.0408E-02	4.1828E-01	8.9878E-02	7.0437E-02	TRUE	FALSE	TRUE	TRUE	3	RAIN + ARSER + CircaCompare	FALSE
ENSMUSG00000024957	Kcnk4	1.4318E-02	7.5273E-01	1.0288E-01	6.5276E-02	TRUE	FALSE	TRUE	TRUE	3	RAIN + ARSER + CircaCompare	FALSE
ENSMUSG00000022324	Matn2	7.1344E-02	5.9891E-01	6.7400E-02	7.9030E-02	TRUE	FALSE	TRUE	TRUE	3	RAIN + ARSER + CircaCompare	FALSE
ENSMUSG00000000957	Mmp14	4.9770E-02	8.5840E-01	1.4064E-01	4.3892E-02	TRUE	FALSE	TRUE	TRUE	3	RAIN + ARSER + CircaCompare	FALSE
ENSMUSG00000020889	Nr1d1	1.4060E-01	5.9891E-01	2.7236E-02	6.6791E-03	TRUE	FALSE	TRUE	TRUE	3	RAIN + ARSER + CircaCompare	TRUE
ENSMUSG00000021775	Nr1d2	2.9373E-02	2.3355E-01	3.6646E-03	1.3745E-03	TRUE	FALSE	TRUE	TRUE	3	RAIN + ARSER + CircaCompare	TRUE
ENSMUSG00000061039	Olfr920	2.3823E-02	5.0222E-01	7.9983E-02	9.0861E-03	TRUE	FALSE	TRUE	TRUE	3	RAIN + ARSER + CircaCompare	FALSE
ENSMUSG00000038495	Otud7b	3.1913E-02	5.9891E-01	1.1403E-01	6.3465E-02	TRUE	FALSE	TRUE	TRUE	3	RAIN + ARSER + CircaCompare	FALSE
ENSMUSG00000020134	Peli1	2.9373E-02	5.9891E-01	1.4272E-01	1.0862E-01	TRUE	FALSE	TRUE	TRUE	3	RAIN + ARSER + CircaCompare	FALSE
ENSMUSG00000020893	Per1	2.3823E-02	5.0222E-01	1.1483E-01	5.6768E-02	TRUE	FALSE	TRUE	TRUE	3	RAIN + ARSER + CircaCompare	TRUE
ENSMUSG00000055866	Per2	3.1913E-02	7.5273E-01	6.2735E-02	3.6368E-02	TRUE	FALSE	TRUE	TRUE	3	RAIN + ARSER + CircaCompare	TRUE
ENSMUSG00000031482	Slc25a15	2.9373E-02	4.1828E-01	4.7906E-02	9.0861E-03	TRUE	FALSE	TRUE	TRUE	3	RAIN + ARSER + CircaCompare	FALSE
ENSMUSG00000069808	Tlcd3a	1.4856E-02	5.0222E-01	4.7906E-02	6.1787E-02	TRUE	FALSE	TRUE	TRUE	3	RAIN + ARSER + CircaCompare	FALSE
ENSMUSG00000037824	Tspan14	2.4133E-02	5.9891E-01	1.4165E-01	6.1810E-02	TRUE	FALSE	TRUE	TRUE	3	RAIN + ARSER + CircaCompare	FALSE
ENSMUSG00000064367	mt-Nd5	7.7948E-02	5.0222E-01	8.9878E-02	1.0851E-01	TRUE	FALSE	TRUE	TRUE	3	RAIN + ARSER + CircaCompare	FALSE
ENSMUSG00000030247	Kcnj8	2.5635E-01	1.0000E+00	1.3467E-01	1.4350E-01	FALSE	FALSE	TRUE	TRUE	2	ARSER + CircaCompare only	FALSE
ENSMUSG00000097462	9530026P05Rik	1.4060E-01	9.1146E-01	5.1951E-01	1.4199E-01	TRUE	FALSE	FALSE	TRUE	2	RAIN + CircaCompare only	FALSE
ENSMUSG00000114780	AI197445	1.4060E-01	8.5840E-01	4.2492E-01	1.1922E-01	TRUE	FALSE	FALSE	TRUE	2	RAIN + CircaCompare only	FALSE
ENSMUSG00000026842	Abl1	1.0026E-01	8.5840E-01	4.2743E-01	1.1797E-01	TRUE	FALSE	FALSE	TRUE	2	RAIN + CircaCompare only	FALSE
ENSMUSG00000038007	Acer2	7.3586E-02	1.0000E+00	2.9333E-01	1.0862E-01	TRUE	FALSE	FALSE	TRUE	2	RAIN + CircaCompare only	FALSE
ENSMUSG00000015016	Acsf3	7.1344E-02	8.5840E-01	3.3650E-01	1.2542E-01	TRUE	FALSE	FALSE	TRUE	2	RAIN + CircaCompare only	FALSE
ENSMUSG00000031994	Adamts8	1.4060E-01	9.9933E-01	4.2743E-01	1.2542E-01	TRUE	FALSE	FALSE	TRUE	2	RAIN + CircaCompare only	FALSE
ENSMUSG00000020709	Adap2	2.9373E-02	9.1146E-01	4.9045E-01	1.3897E-01	TRUE	FALSE	FALSE	TRUE	2	RAIN + CircaCompare only	FALSE
ENSMUSG00000026159	Agfg1	7.1344E-02	5.0222E-01	3.3650E-01	1.2542E-01	TRUE	FALSE	FALSE	TRUE	2	RAIN + CircaCompare only	FALSE
ENSMUSG00000041936	Agrn	1.4060E-01	1.0000E+00	4.6937E-01	1.3926E-01	TRUE	FALSE	FALSE	TRUE	2	RAIN + CircaCompare only	FALSE
ENSMUSG00000028692	Akr1a1	1.4060E-01	9.1146E-01	4.7003E-01	1.3822E-01	TRUE	FALSE	FALSE	TRUE	2	RAIN + CircaCompare only	FALSE
ENSMUSG00000075470	Alg10b	1.0026E-01	1.0000E+00	5.0297E-01	1.4953E-01	TRUE	FALSE	FALSE	TRUE	2	RAIN + CircaCompare only	FALSE
ENSMUSG00000004105	Angptl2	1.4060E-01	9.1146E-01	3.3650E-01	7.9649E-02	TRUE	FALSE	FALSE	TRUE	2	RAIN + CircaCompare only	FALSE
ENSMUSG00000029338	Antxr2	1.4034E-01	1.0000E+00	4.7811E-01	1.2976E-01	TRUE	FALSE	FALSE	TRUE	2	RAIN + CircaCompare only	FALSE
ENSMUSG00000032375	Aph1b	7.1344E-02	8.5840E-01	3.9571E-01	1.0642E-01	TRUE	FALSE	FALSE	TRUE	2	RAIN + CircaCompare only	FALSE
ENSMUSG00000033237	Arid2	1.0026E-01	9.1146E-01	4.2643E-01	1.1797E-01	TRUE	FALSE	FALSE	TRUE	2	RAIN + CircaCompare only	FALSE
ENSMUSG00000025234	Arih1	1.0026E-01	1.0000E+00	5.0397E-01	1.4852E-01	TRUE	FALSE	FALSE	TRUE	2	RAIN + CircaCompare only	FALSE
ENSMUSG00000025035	Arl3	2.9373E-02	9.9933E-01	4.7063E-01	1.3897E-01	TRUE	FALSE	FALSE	TRUE	2	RAIN + CircaCompare only	FALSE
ENSMUSG00000060268	Armh1	7.1344E-02	1.0000E+00	4.7081E-01	1.2976E-01	TRUE	FALSE	FALSE	TRUE	2	RAIN + CircaCompare only	FALSE
ENSMUSG00000007656	Arpp19	2.9373E-02	9.1146E-01	4.2743E-01	1.4852E-01	TRUE	FALSE	FALSE	TRUE	2	RAIN + CircaCompare only	FALSE
ENSMUSG00000032412	Atp1b3	1.0026E-01	5.0222E-01	3.6298E-01	1.3270E-01	TRUE	FALSE	FALSE	TRUE	2	RAIN + CircaCompare only	FALSE
ENSMUSG00000049792	Bag5	7.1344E-02	5.9891E-01	3.3650E-01	1.0642E-01	TRUE	FALSE	FALSE	TRUE	2	RAIN + CircaCompare only	FALSE
ENSMUSG00000030256	Bhlhe41	1.4328E-01	1.0000E+00	4.2743E-01	1.2542E-01	TRUE	FALSE	FALSE	TRUE	2	RAIN + CircaCompare only	FALSE

ENSMUSG00000026278	Bok	2.9373E-02	8.5840E-01	4.1458E-01	1.0946E-01	TRUE	FALSE	FALSE	TRUE	2	RAIN + CircaCompare only	FALSE
ENSMUSG00000000202	Btbd17	7.1344E-02	5.0222E-01	3.9631E-01	1.0642E-01	TRUE	FALSE	FALSE	TRUE	2	RAIN + CircaCompare only	FALSE
ENSMUSG00000028568	Btf3l4	4.9770E-02	1.0000E+00	4.2061E-01	1.4852E-01	TRUE	FALSE	FALSE	TRUE	2	RAIN + CircaCompare only	FALSE
ENSMUSG00000003814	Calr	1.0026E-01	8.5840E-01	2.9333E-01	1.2976E-01	TRUE	FALSE	FALSE	TRUE	2	RAIN + CircaCompare only	FALSE
ENSMUSG00000021501	Caml	2.9373E-02	9.1146E-01	4.3324E-01	1.1540E-01	TRUE	FALSE	FALSE	TRUE	2	RAIN + CircaCompare only	FALSE
ENSMUSG00000026270	Capn10	8.5813E-02	1.0000E+00	4.1458E-01	1.2976E-01	TRUE	FALSE	FALSE	TRUE	2	RAIN + CircaCompare only	FALSE
ENSMUSG00000036526	Card11	9.0771E-02	5.9891E-01	3.9571E-01	1.2542E-01	TRUE	FALSE	FALSE	TRUE	2	RAIN + CircaCompare only	FALSE
ENSMUSG00000021182	Ccdc88c	1.0411E-01	8.5840E-01	4.7177E-01	1.2779E-01	TRUE	FALSE	FALSE	TRUE	2	RAIN + CircaCompare only	FALSE
ENSMUSG00000096257	Ccer2	1.4060E-01	8.5840E-01	3.8474E-01	1.1707E-01	TRUE	FALSE	FALSE	TRUE	2	RAIN + CircaCompare only	FALSE
ENSMUSG00000037362	Ccn3	5.0644E-02	1.0000E+00	4.1458E-01	1.0862E-01	TRUE	FALSE	FALSE	TRUE	2	RAIN + CircaCompare only	FALSE
ENSMUSG00000030007	Cct7	1.0651E-01	8.5840E-01	3.3650E-01	1.2542E-01	TRUE	FALSE	FALSE	TRUE	2	RAIN + CircaCompare only	FALSE
ENSMUSG00000035776	Cd99l2	1.4060E-01	1.0000E+00	4.4245E-01	1.4852E-01	TRUE	FALSE	FALSE	TRUE	2	RAIN + CircaCompare only	FALSE
ENSMUSG00000022321	Cdh10	1.4060E-01	1.0000E+00	5.3067E-01	1.4989E-01	TRUE	FALSE	FALSE	TRUE	2	RAIN + CircaCompare only	FALSE
ENSMUSG00000038481	Cdk19	7.5485E-02	1.0000E+00	4.3401E-01	1.2542E-01	TRUE	FALSE	FALSE	TRUE	2	RAIN + CircaCompare only	FALSE
ENSMUSG00000023067	Cdkn1a	4.9770E-02	1.0000E+00	4.9054E-01	1.4544E-01	TRUE	FALSE	FALSE	TRUE	2	RAIN + CircaCompare only	FALSE
ENSMUSG00000042254	Cilp	1.0026E-01	1.0000E+00	4.2743E-01	1.2542E-01	TRUE	FALSE	FALSE	TRUE	2	RAIN + CircaCompare only	FALSE
ENSMUSG00000033082	Clec1a	2.9373E-02	5.9891E-01	5.2528E-01	1.4852E-01	TRUE	FALSE	FALSE	TRUE	2	RAIN + CircaCompare only	FALSE
ENSMUSG00000022125	Cln5	1.0026E-01	1.0000E+00	4.0285E-01	1.4366E-01	TRUE	FALSE	FALSE	TRUE	2	RAIN + CircaCompare only	FALSE
ENSMUSG00000044881	Coa4	2.9373E-02	9.9933E-01	3.3650E-01	7.2596E-02	TRUE	FALSE	FALSE	TRUE	2	RAIN + CircaCompare only	FALSE
ENSMUSG00000027742	Cog6	1.4060E-01	9.9933E-01	4.4245E-01	1.2766E-01	TRUE	FALSE	FALSE	TRUE	2	RAIN + CircaCompare only	FALSE
ENSMUSG00000056941	Commd7	1.4060E-01	8.5840E-01	4.1458E-01	1.0642E-01	TRUE	FALSE	FALSE	TRUE	2	RAIN + CircaCompare only	FALSE
ENSMUSG00000016481	Cr1l	1.4060E-01	1.0000E+00	5.2128E-01	1.4852E-01	TRUE	FALSE	FALSE	TRUE	2	RAIN + CircaCompare only	FALSE
ENSMUSG00000032291	Crabp1	7.2888E-02	8.5840E-01	3.1904E-01	1.0662E-01	TRUE	FALSE	FALSE	TRUE	2	RAIN + CircaCompare only	FALSE
ENSMUSG00000017561	Crlf3	1.4060E-01	1.0000E+00	3.9629E-01	1.2976E-01	TRUE	FALSE	FALSE	TRUE	2	RAIN + CircaCompare only	FALSE
ENSMUSG00000043153	Crppa	1.4060E-01	1.0000E+00	4.7736E-01	1.4852E-01	TRUE	FALSE	FALSE	TRUE	2	RAIN + CircaCompare only	FALSE
ENSMUSG00000020038	Cry1	1.4060E-01	1.0000E+00	3.3650E-01	1.2542E-01	TRUE	FALSE	FALSE	TRUE	2	RAIN + CircaCompare only	TRUE
ENSMUSG00000101523	Csnk2a1-ps3	2.3823E-02	5.0222E-01	3.1904E-01	6.3465E-02	TRUE	FALSE	FALSE	TRUE	2	RAIN + CircaCompare only	FALSE
ENSMUSG00000034101	Ctnnd1	7.1344E-02	9.1146E-01	4.2061E-01	1.2003E-01	TRUE	FALSE	FALSE	TRUE	2	RAIN + CircaCompare only	FALSE
ENSMUSG00000024231	Cul2	1.4060E-01	1.0000E+00	5.0397E-01	1.4544E-01	TRUE	FALSE	FALSE	TRUE	2	RAIN + CircaCompare only	FALSE
ENSMUSG00000052974	Cyp2f2	4.9770E-02	9.9933E-01	3.5815E-01	1.1707E-01	TRUE	FALSE	FALSE	TRUE	2	RAIN + CircaCompare only	FALSE
ENSMUSG00000028571	Cyp2j13	2.9373E-02	5.9891E-01	4.2176E-01	1.3822E-01	TRUE	FALSE	FALSE	TRUE	2	RAIN + CircaCompare only	FALSE
ENSMUSG00000053226	Dand5	2.9373E-02	5.0222E-01	1.8264E-01	6.5928E-02	TRUE	FALSE	FALSE	TRUE	2	RAIN + CircaCompare only	FALSE
ENSMUSG00000000346	Dazap2	5.4040E-02	9.9933E-01	4.1458E-01	1.0862E-01	TRUE	FALSE	FALSE	TRUE	2	RAIN + CircaCompare only	FALSE
ENSMUSG00000066026	Dhrs3	1.4060E-01	1.0000E+00	4.9607E-01	1.4865E-01	TRUE	FALSE	FALSE	TRUE	2	RAIN + CircaCompare only	FALSE
ENSMUSG00000025279	Dnase1l3	7.1344E-02	9.1146E-01	4.1458E-01	1.2542E-01	TRUE	FALSE	FALSE	TRUE	2	RAIN + CircaCompare only	FALSE
ENSMUSG00000039982	Dtx4	5.2946E-02	9.1146E-01	4.1458E-01	1.4350E-01	TRUE	FALSE	FALSE	TRUE	2	RAIN + CircaCompare only	FALSE
ENSMUSG00000030104	Edem1	1.0680E-01	9.1146E-01	5.0297E-01	1.4989E-01	TRUE	FALSE	FALSE	TRUE	2	RAIN + CircaCompare only	FALSE
ENSMUSG00000091337	Eid1	1.4060E-01	1.0000E+00	5.0397E-01	1.4199E-01	TRUE	FALSE	FALSE	TRUE	2	RAIN + CircaCompare only	FALSE
ENSMUSG00000021696	Elovl7	1.4060E-01	9.9933E-01	5.2528E-01	1.4852E-01	TRUE	FALSE	FALSE	TRUE	2	RAIN + CircaCompare only	FALSE
ENSMUSG00000037370	Enpp1	1.0026E-01	1.0000E+00	4.1458E-01	1.0642E-01	TRUE	FALSE	FALSE	TRUE	2	RAIN + CircaCompare only	FALSE
ENSMUSG00000028661	Epha8	1.4531E-01	1.0000E+00	3.4683E-01	1.1540E-01	TRUE	FALSE	FALSE	TRUE	2	RAIN + CircaCompare only	FALSE
ENSMUSG00000021750	Fam107a	1.0026E-01	9.1146E-01	2.6539E-01	6.5276E-02	TRUE	FALSE	FALSE	TRUE	2	RAIN + CircaCompare only	FALSE
ENSMUSG00000057497	Fam136a	1.0026E-01	1.0000E+00	5.0297E-01	1.3287E-01	TRUE	FALSE	FALSE	TRUE	2	RAIN + CircaCompare only	FALSE
ENSMUSG00000001962	Fam50a	2.9373E-02	5.9891E-01	3.9697E-01	1.0862E-01	TRUE	FALSE	FALSE	TRUE	2	RAIN + CircaCompare only	FALSE
ENSMUSG00000028878	Fam76a	1.4072E-01	1.0000E+00	3.9697E-01	1.0642E-01	TRUE	FALSE	FALSE	TRUE	2	RAIN + CircaCompare only	FALSE
ENSMUSG00000027654	Fam83d	1.4060E-01	1.0000E+00	3.9571E-01	1.2105E-01	TRUE	FALSE	FALSE	TRUE	2	RAIN + CircaCompare only	FALSE
ENSMUSG00000033458	Fan1	1.4060E-01	9.1146E-01	2.2798E-01	1.0946E-01	TRUE	FALSE	FALSE	TRUE	2	RAIN + CircaCompare only	FALSE
ENSMUSG00000038365	Fbxo25	2.9373E-02	9.1146E-01	4.4517E-01	1.2542E-01	TRUE	FALSE	FALSE	TRUE	2	RAIN + CircaCompare only	FALSE
ENSMUSG00000056629	Fkbp2	7.1344E-02	5.9891E-01	3.1904E-01	1.1206E-01	TRUE	FALSE	FALSE	TRUE	2	RAIN + CircaCompare only	FALSE
ENSMUSG00000028414	Fktn	7.1344E-02	7.5273E-01	2.9333E-01	6.3465E-02	TRUE	FALSE	FALSE	TRUE	2	RAIN + CircaCompare only	FALSE
ENSMUSG00000040181	Fmo1	7.1344E-02	1.0000E+00	4.1458E-01	1.2542E-01	TRUE	FALSE	FALSE	TRUE	2	RAIN + CircaCompare only	FALSE
ENSMUSG00000047604	Frat2	2.9373E-02	1.0000E+00	4.5563E-01	1.4199E-01	TRUE	FALSE	FALSE	TRUE	2	RAIN + CircaCompare only	FALSE
ENSMUSG00000031198	Fundc2	7.1344E-02	9.9933E-01	4.1458E-01	1.0642E-01	TRUE	FALSE	FALSE	TRUE	2	RAIN + CircaCompare only	FALSE
ENSMUSG00000039357	Fut11	4.9770E-02	1.0000E+00	4.7003E-01	1.2766E-01	TRUE	FALSE	FALSE	TRUE	2	RAIN + CircaCompare only	FALSE
ENSMUSG00000049791	Fzd4	4.9770E-02	4.1828E-01	2.9648E-01	7.9030E-02	TRUE	FALSE	FALSE	TRUE	2	RAIN + CircaCompare only	FALSE
ENSMUSG00000041075	Fzd7	1.0026E-01	7.5273E-01	2.7623E-01	7.0437E-02	TRUE	FALSE	FALSE	TRUE	2	RAIN + CircaCompare only	FALSE
ENSMUSG00000036904	Fzd8	3.1913E-02	5.0222E-01	4.2743E-01	1.2542E-01	TRUE	FALSE	FALSE	TRUE	2	RAIN + CircaCompare only	FALSE
ENSMUSG00000009633	G0s2	1.0026E-01	1.0000E+00	3.7495E-01	1.4852E-01	TRUE	FALSE	FALSE	TRUE	2	RAIN + CircaCompare only	FALSE
ENSMUSG00000021903	Galnt15	1.0026E-01	1.0000E+00	4.2743E-01	1.2466E-01	TRUE	FALSE	FALSE	TRUE	2	RAIN + CircaCompare only	FALSE
ENSMUSG00000021943	Gdf10	2.9373E-02	5.0222E-01	2.9648E-01	7.3769E-02	TRUE	FALSE	FALSE	TRUE	2	RAIN + CircaCompare only	FALSE
ENSMUSG00000043505	Gimap5	5.4040E-02	9.9933E-01	4.2743E-01	1.2542E-01	TRUE	FALSE	FALSE	TRUE	2	RAIN + CircaCompare only	FALSE
ENSMUSG00000040006	Ginm1	1.4060E-01	9.1146E-01	3.3650E-01	1.4852E-01	TRUE	FALSE	FALSE	TRUE	2	RAIN + CircaCompare only	FALSE

ENSMUSG00000097649	Gm10561	7.1344E-02	5.0222E-01	4.1761E-01	1.4199E-01	TRUE	FALSE	FALSE	TRUE	2	RAIN + CircaCompare only	FALSE
ENSMUSG00000074385	Gm10684	1.4060E-01	1.0000E+00	4.4245E-01	1.2542E-01	TRUE	FALSE	FALSE	TRUE	2	RAIN + CircaCompare only	FALSE
ENSMUSG00000078867	Gm14418	7.1344E-02	9.9933E-01	4.9701E-01	1.4852E-01	TRUE	FALSE	FALSE	TRUE	2	RAIN + CircaCompare only	FALSE
ENSMUSG00000086288	Gm15265	2.3823E-02	5.0222E-01	3.5815E-01	1.0642E-01	TRUE	FALSE	FALSE	TRUE	2	RAIN + CircaCompare only	FALSE
ENSMUSG00000100801	Gm15459	7.1344E-02	9.1146E-01	4.6937E-01	1.4745E-01	TRUE	FALSE	FALSE	TRUE	2	RAIN + CircaCompare only	FALSE
ENSMUSG00000086914	Gm16124	4.9770E-02	8.5840E-01	5.1757E-01	1.2542E-01	TRUE	FALSE	FALSE	TRUE	2	RAIN + CircaCompare only	FALSE
ENSMUSG00000083892	Gm1848	7.4545E-02	5.0222E-01	3.8464E-01	1.2976E-01	TRUE	FALSE	FALSE	TRUE	2	RAIN + CircaCompare only	FALSE
ENSMUSG00000111052	Gm20139	7.1344E-02	9.9933E-01	4.4245E-01	1.2542E-01	TRUE	FALSE	FALSE	TRUE	2	RAIN + CircaCompare only	FALSE
ENSMUSG00000097840	Gm26756	6.4393E-02	5.9891E-01	3.9631E-01	1.0642E-01	TRUE	FALSE	FALSE	TRUE	2	RAIN + CircaCompare only	FALSE
ENSMUSG00000103042	Gm2719	4.9770E-02	1.0000E+00	4.1458E-01	1.0862E-01	TRUE	FALSE	FALSE	TRUE	2	RAIN + CircaCompare only	FALSE
ENSMUSG00000114662	Gm31683	1.0026E-01	8.5840E-01	4.4845E-01	1.2766E-01	TRUE	FALSE	FALSE	TRUE	2	RAIN + CircaCompare only	FALSE
ENSMUSG00000101439	Gm336	2.9373E-02	7.5273E-01	4.7736E-01	1.2741E-01	TRUE	FALSE	FALSE	TRUE	2	RAIN + CircaCompare only	FALSE
ENSMUSG00000104168	Gm38250	1.0815E-01	9.1146E-01	5.0297E-01	1.4199E-01	TRUE	FALSE	FALSE	TRUE	2	RAIN + CircaCompare only	FALSE
ENSMUSG00000106535	Gm43031	1.4060E-01	1.0000E+00	5.0297E-01	1.3763E-01	TRUE	FALSE	FALSE	TRUE	2	RAIN + CircaCompare only	FALSE
ENSMUSG00000105957	Gm43254	1.0026E-01	1.0000E+00	4.9934E-01	1.2766E-01	TRUE	FALSE	FALSE	TRUE	2	RAIN + CircaCompare only	FALSE
ENSMUSG00000116614	Gm46610	1.4060E-01	1.0000E+00	4.7736E-01	1.4953E-01	TRUE	FALSE	FALSE	TRUE	2	RAIN + CircaCompare only	FALSE
ENSMUSG00000114019	Gm47155	1.0026E-01	8.5840E-01	2.2456E-01	6.3465E-02	TRUE	FALSE	FALSE	TRUE	2	RAIN + CircaCompare only	FALSE
ENSMUSG00000111681	Gm47640	1.4178E-01	9.1146E-01	4.2743E-01	1.2542E-01	TRUE	FALSE	FALSE	TRUE	2	RAIN + CircaCompare only	FALSE
ENSMUSG00000111394	Gm49759	4.9770E-02	9.1146E-01	4.6172E-01	1.2542E-01	TRUE	FALSE	FALSE	TRUE	2	RAIN + CircaCompare only	FALSE
ENSMUSG00000111374	Gm50490	1.4836E-01	7.5273E-01	5.0297E-01	1.4919E-01	TRUE	FALSE	FALSE	TRUE	2	RAIN + CircaCompare only	FALSE
ENSMUSG00000106416	Gm5857	7.1344E-02	5.0222E-01	3.1904E-01	6.9560E-02	TRUE	FALSE	FALSE	TRUE	2	RAIN + CircaCompare only	FALSE
ENSMUSG00000111662	Gm7435	4.9770E-02	1.0000E+00	4.1761E-01	1.1450E-01	TRUE	FALSE	FALSE	TRUE	2	RAIN + CircaCompare only	FALSE
ENSMUSG00000049230	Gm9833	7.1344E-02	8.5840E-01	4.1450E-01	1.0862E-01	TRUE	FALSE	FALSE	TRUE	2	RAIN + CircaCompare only	FALSE
ENSMUSG00000000884	Gnb1l	1.4060E-01	7.5273E-01	3.1904E-01	7.2596E-02	TRUE	FALSE	FALSE	TRUE	2	RAIN + CircaCompare only	FALSE
ENSMUSG00000027346	Gpcpd1	2.9373E-02	5.0222E-01	2.9648E-01	6.5276E-02	TRUE	FALSE	FALSE	TRUE	2	RAIN + CircaCompare only	FALSE
ENSMUSG00000029464	Gpn3	1.4060E-01	1.0000E+00	3.9631E-01	1.3926E-01	TRUE	FALSE	FALSE	TRUE	2	RAIN + CircaCompare only	FALSE
ENSMUSG00000097715	Gpr137b-ps	1.4060E-01	1.0000E+00	3.9631E-01	1.3805E-01	TRUE	FALSE	FALSE	TRUE	2	RAIN + CircaCompare only	FALSE
ENSMUSG00000071531	Gprin2	2.9373E-02	7.5273E-01	3.9697E-01	1.0862E-01	TRUE	FALSE	FALSE	TRUE	2	RAIN + CircaCompare only	FALSE
ENSMUSG00000026930	Gpsm1	7.4545E-02	5.9891E-01	2.5798E-01	6.3465E-02	TRUE	FALSE	FALSE	TRUE	2	RAIN + CircaCompare only	FALSE
ENSMUSG00000004035	Gstm7	1.0026E-01	1.0000E+00	4.2643E-01	1.1797E-01	TRUE	FALSE	FALSE	TRUE	2	RAIN + CircaCompare only	FALSE
ENSMUSG00000001665	Gstt3	7.1344E-02	5.9891E-01	3.1904E-01	8.5869E-02	TRUE	FALSE	FALSE	TRUE	2	RAIN + CircaCompare only	FALSE
ENSMUSG00000022367	Has2	2.9373E-02	9.1146E-01	3.3650E-01	1.0642E-01	TRUE	FALSE	FALSE	TRUE	2	RAIN + CircaCompare only	FALSE
ENSMUSG00000021665	Hexb	1.4060E-01	1.0000E+00	4.1458E-01	1.3522E-01	TRUE	FALSE	FALSE	TRUE	2	RAIN + CircaCompare only	FALSE
ENSMUSG00000004328	Hif3a	5.4040E-02	8.5840E-01	3.5437E-01	1.2542E-01	TRUE	FALSE	FALSE	TRUE	2	RAIN + CircaCompare only	FALSE
ENSMUSG00000003949	Hlf	9.7544E-03	9.1146E-01	3.3650E-01	7.9030E-02	TRUE	FALSE	FALSE	TRUE	2	RAIN + CircaCompare only	FALSE
ENSMUSG00000007908	Hmgcll1	1.0026E-01	8.5840E-01	4.2743E-01	1.1540E-01	TRUE	FALSE	FALSE	TRUE	2	RAIN + CircaCompare only	FALSE
ENSMUSG00000027875	Hmgcs2	3.1913E-02	9.1146E-01	2.3437E-01	7.9030E-02	TRUE	FALSE	FALSE	TRUE	2	RAIN + CircaCompare only	FALSE
ENSMUSG00000084844	Hoxb3os	7.1344E-02	1.0000E+00	4.6937E-01	1.2741E-01	TRUE	FALSE	FALSE	TRUE	2	RAIN + CircaCompare only	FALSE
ENSMUSG00000020048	Hsp90b1	7.9869E-02	9.9933E-01	4.7736E-01	1.4852E-01	TRUE	FALSE	FALSE	TRUE	2	RAIN + CircaCompare only	FALSE
ENSMUSG00000000732	Icosl	1.0026E-01	9.9933E-01	4.1458E-01	1.1065E-01	TRUE	FALSE	FALSE	TRUE	2	RAIN + CircaCompare only	FALSE
ENSMUSG00000038564	Ift172	1.3559E-01	9.1146E-01	4.1458E-01	1.0862E-01	TRUE	FALSE	FALSE	TRUE	2	RAIN + CircaCompare only	FALSE
ENSMUSG00000076612	Ighg2c	2.9373E-02	9.1146E-01	4.2569E-01	1.1707E-01	TRUE	FALSE	FALSE	TRUE	2	RAIN + CircaCompare only	FALSE
ENSMUSG00000023206	Il15ra	3.1913E-02	4.1828E-01	4.3401E-01	1.4199E-01	TRUE	FALSE	FALSE	TRUE	2	RAIN + CircaCompare only	FALSE
ENSMUSG00000052372	Il1rapl1	4.9770E-02	1.0000E+00	5.1668E-01	1.4852E-01	TRUE	FALSE	FALSE	TRUE	2	RAIN + CircaCompare only	FALSE
ENSMUSG00000042106	Inka1	1.0026E-01	9.1146E-01	3.9571E-01	1.0642E-01	TRUE	FALSE	FALSE	TRUE	2	RAIN + CircaCompare only	FALSE
ENSMUSG00000003477	Inmt	1.0026E-01	5.9891E-01	3.9571E-01	1.4199E-01	TRUE	FALSE	FALSE	TRUE	2	RAIN + CircaCompare only	FALSE
ENSMUSG00000032599	Ip6k2	7.1344E-02	8.5840E-01	3.5815E-01	1.0585E-01	TRUE	FALSE	FALSE	TRUE	2	RAIN + CircaCompare only	FALSE
ENSMUSG00000033854	Kcnk10	7.6198E-02	9.1146E-01	4.7736E-01	1.3897E-01	TRUE	FALSE	FALSE	TRUE	2	RAIN + CircaCompare only	FALSE
ENSMUSG00000045404	Kcnk13	9.7544E-03	1.0000E+00	4.4245E-01	1.2542E-01	TRUE	FALSE	FALSE	TRUE	2	RAIN + CircaCompare only	FALSE
ENSMUSG00000002908	Kcnn1	2.9373E-02	5.9891E-01	3.8474E-01	9.3457E-02	TRUE	FALSE	FALSE	TRUE	2	RAIN + CircaCompare only	FALSE
ENSMUSG00000051727	Kctd14	7.1344E-02	5.9891E-01	3.9629E-01	1.1413E-01	TRUE	FALSE	FALSE	TRUE	2	RAIN + CircaCompare only	FALSE
ENSMUSG00000030087	Klf15	1.4060E-01	1.0000E+00	4.6937E-01	1.4199E-01	TRUE	FALSE	FALSE	TRUE	2	RAIN + CircaCompare only	FALSE
ENSMUSG00000033863	Klf9	4.9770E-02	9.1146E-01	3.9631E-01	1.0946E-01	TRUE	FALSE	FALSE	TRUE	2	RAIN + CircaCompare only	FALSE
ENSMUSG00000090799	Klhl33	7.6198E-02	1.0000E+00	4.4245E-01	1.4852E-01	TRUE	FALSE	FALSE	TRUE	2	RAIN + CircaCompare only	FALSE
ENSMUSG00000054920	Klhl5	4.9770E-02	8.5840E-01	4.1636E-01	1.1795E-01	TRUE	FALSE	FALSE	TRUE	2	RAIN + CircaCompare only	FALSE
ENSMUSG00000035545	Leng8	7.9869E-02	7.5273E-01	3.1904E-01	1.4199E-01	TRUE	FALSE	FALSE	TRUE	2	RAIN + CircaCompare only	FALSE
ENSMUSG00000045312	Lhfpl2	3.1913E-02	8.5840E-01	4.2743E-01	1.2542E-01	TRUE	FALSE	FALSE	TRUE	2	RAIN + CircaCompare only	FALSE
ENSMUSG00000049717	Lig4	1.4060E-01	1.0000E+00	5.0397E-01	1.3897E-01	TRUE	FALSE	FALSE	TRUE	2	RAIN + CircaCompare only	FALSE
ENSMUSG00000021484	Lman2	2.9373E-02	9.1146E-01	4.0184E-01	1.1707E-01	TRUE	FALSE	FALSE	TRUE	2	RAIN + CircaCompare only	FALSE
ENSMUSG00000039883	Lrrc17	2.9373E-02	8.5840E-01	2.9648E-01	1.0642E-01	TRUE	FALSE	FALSE	TRUE	2	RAIN + CircaCompare only	FALSE
ENSMUSG00000036295	Lrrn3	1.4060E-01	1.0000E+00	4.1458E-01	1.2542E-01	TRUE	FALSE	FALSE	TRUE	2	RAIN + CircaCompare only	FALSE
ENSMUSG00000033105	Lss	2.9373E-02	5.0222E-01	3.5437E-01	1.1795E-01	TRUE	FALSE	FALSE	TRUE	2	RAIN + CircaCompare only	FALSE

ENSMUSG00000015889	Lta4h	7.1344E-02	7.5273E-01	3.5474E-01	1.1797E-01	TRUE	FALSE	FALSE	TRUE	2	RAIN + CircaCompare only	FALSE
ENSMUSG00000029823	Luc7l2	7.6131E-02	8.5840E-01	5.0770E-01	1.4350E-01	TRUE	FALSE	FALSE	TRUE	2	RAIN + CircaCompare only	FALSE
ENSMUSG00000028826	Maco1	3.1913E-02	9.9933E-01	5.0297E-01	1.4199E-01	TRUE	FALSE	FALSE	TRUE	2	RAIN + CircaCompare only	FALSE
ENSMUSG00000031853	Map3k21	7.1344E-02	9.1146E-01	2.9333E-01	1.0675E-01	TRUE	FALSE	FALSE	TRUE	2	RAIN + CircaCompare only	FALSE
ENSMUSG00000050164	Mchr1	9.7544E-03	5.0222E-01	3.3650E-01	7.9030E-02	TRUE	FALSE	FALSE	TRUE	2	RAIN + CircaCompare only	FALSE
ENSMUSG00000024593	Megf10	7.1344E-02	8.5840E-01	4.2743E-01	1.2976E-01	TRUE	FALSE	FALSE	TRUE	2	RAIN + CircaCompare only	FALSE
ENSMUSG00000085148	Mir22hg	7.1344E-02	1.0000E+00	5.0297E-01	1.2542E-01	TRUE	FALSE	FALSE	TRUE	2	RAIN + CircaCompare only	FALSE
ENSMUSG00000048578	Mlec	7.1344E-02	1.0000E+00	4.1636E-01	1.4852E-01	TRUE	FALSE	FALSE	TRUE	2	RAIN + CircaCompare only	FALSE
ENSMUSG00000031765	Mt1	5.2628E-02	9.9933E-01	3.3650E-01	1.0946E-01	TRUE	FALSE	FALSE	TRUE	2	RAIN + CircaCompare only	FALSE
ENSMUSG00000025885	Myo5b	1.4060E-01	9.9933E-01	5.0297E-01	1.4852E-01	TRUE	FALSE	FALSE	TRUE	2	RAIN + CircaCompare only	FALSE
ENSMUSG00000015542	Nat9	9.7544E-03	4.1828E-01	3.1904E-01	6.5276E-02	TRUE	FALSE	FALSE	TRUE	2	RAIN + CircaCompare only	FALSE
ENSMUSG00000029832	Nfe2l3	1.4060E-01	1.0000E+00	5.0297E-01	1.4199E-01	TRUE	FALSE	FALSE	TRUE	2	RAIN + CircaCompare only	FALSE
ENSMUSG00000028423	Nfx1	1.4060E-01	8.5840E-01	5.2528E-01	1.4852E-01	TRUE	FALSE	FALSE	TRUE	2	RAIN + CircaCompare only	FALSE
ENSMUSG00000044231	Nhlrc1	7.1344E-02	9.1146E-01	3.1410E-01	6.5276E-02	TRUE	FALSE	FALSE	TRUE	2	RAIN + CircaCompare only	FALSE
ENSMUSG00000026077	Npas2	1.0026E-01	1.0000E+00	3.1904E-01	1.0642E-01	TRUE	FALSE	FALSE	TRUE	2	RAIN + CircaCompare only	TRUE
ENSMUSG00000024213	Nudt3	1.4708E-01	9.9933E-01	4.4517E-01	1.3822E-01	TRUE	FALSE	FALSE	TRUE	2	RAIN + CircaCompare only	FALSE
ENSMUSG00000022120	Obi1	1.0026E-01	1.0000E+00	4.7081E-01	1.2976E-01	TRUE	FALSE	FALSE	TRUE	2	RAIN + CircaCompare only	FALSE
ENSMUSG00000021203	Otub2	1.0026E-01	9.1146E-01	4.4235E-01	1.4199E-01	TRUE	FALSE	FALSE	TRUE	2	RAIN + CircaCompare only	FALSE
ENSMUSG00000025130	P4hb	1.4060E-01	8.5840E-01	3.8318E-01	1.2542E-01	TRUE	FALSE	FALSE	TRUE	2	RAIN + CircaCompare only	FALSE
ENSMUSG00000003131	Pafah1b2	1.0026E-01	9.9933E-01	2.2798E-01	1.2976E-01	TRUE	FALSE	FALSE	TRUE	2	RAIN + CircaCompare only	FALSE
ENSMUSG00000102206	Pcdha11	4.9770E-02	9.1146E-01	4.2743E-01	1.2542E-01	TRUE	FALSE	FALSE	TRUE	2	RAIN + CircaCompare only	FALSE
ENSMUSG00000027248	Pdia3	3.1913E-02	5.0222E-01	1.7778E-01	7.2596E-02	TRUE	FALSE	FALSE	TRUE	2	RAIN + CircaCompare only	FALSE
ENSMUSG00000056204	Pgpep1	1.0026E-01	9.9933E-01	4.4245E-01	1.4852E-01	TRUE	FALSE	FALSE	TRUE	2	RAIN + CircaCompare only	FALSE
ENSMUSG00000091649	Phf11b	2.9373E-02	5.0222E-01	3.4090E-01	7.2596E-02	TRUE	FALSE	FALSE	TRUE	2	RAIN + CircaCompare only	FALSE
ENSMUSG00000041801	Phlda3	1.4060E-01	9.9933E-01	3.7495E-01	1.0862E-01	TRUE	FALSE	FALSE	TRUE	2	RAIN + CircaCompare only	FALSE
ENSMUSG00000029263	Pigg	4.9770E-02	7.5273E-01	4.1636E-01	1.2741E-01	TRUE	FALSE	FALSE	TRUE	2	RAIN + CircaCompare only	FALSE
ENSMUSG00000017453	Pipox	4.9770E-02	1.0000E+00	2.9356E-01	1.1795E-01	TRUE	FALSE	FALSE	TRUE	2	RAIN + CircaCompare only	FALSE
ENSMUSG00000023795	Pisd-ps2	7.1344E-02	7.5273E-01	4.7736E-01	1.2976E-01	TRUE	FALSE	FALSE	TRUE	2	RAIN + CircaCompare only	FALSE
ENSMUSG00000035486	Plk5	2.9373E-02	5.0222E-01	2.3437E-01	6.3465E-02	TRUE	FALSE	FALSE	TRUE	2	RAIN + CircaCompare only	FALSE
ENSMUSG00000032377	Plscr4	1.4060E-01	1.0000E+00	3.3650E-01	1.0515E-01	TRUE	FALSE	FALSE	TRUE	2	RAIN + CircaCompare only	FALSE
ENSMUSG00000027952	Pmvk	7.6198E-02	9.9933E-01	3.9571E-01	1.0862E-01	TRUE	FALSE	FALSE	TRUE	2	RAIN + CircaCompare only	FALSE
ENSMUSG00000005514	Por	1.9409E-02	2.3355E-01	1.9153E-01	5.6768E-02	TRUE	FALSE	FALSE	TRUE	2	RAIN + CircaCompare only	FALSE
ENSMUSG00000037519	Ppfia1	4.9770E-02	1.0000E+00	3.9629E-01	1.0862E-01	TRUE	FALSE	FALSE	TRUE	2	RAIN + CircaCompare only	FALSE
ENSMUSG00000008373	Prpf31	1.4178E-01	1.0000E+00	4.3401E-01	1.2542E-01	TRUE	FALSE	FALSE	TRUE	2	RAIN + CircaCompare only	FALSE
ENSMUSG00000029499	Pxmp2	4.9770E-02	8.5840E-01	4.2569E-01	1.0862E-01	TRUE	FALSE	FALSE	TRUE	2	RAIN + CircaCompare only	FALSE
ENSMUSG00000027935	Rab13	7.1344E-02	9.9933E-01	4.2643E-01	1.2003E-01	TRUE	FALSE	FALSE	TRUE	2	RAIN + CircaCompare only	FALSE
ENSMUSG00000003037	Rab8a	1.0026E-01	9.1146E-01	3.9571E-01	1.0642E-01	TRUE	FALSE	FALSE	TRUE	2	RAIN + CircaCompare only	FALSE
ENSMUSG00000034209	Rasl10a	2.9373E-02	1.0000E+00	4.7063E-01	1.4199E-01	TRUE	FALSE	FALSE	TRUE	2	RAIN + CircaCompare only	FALSE
ENSMUSG00000078184	Rbm8a2	1.0026E-01	1.0000E+00	3.4683E-01	1.1922E-01	TRUE	FALSE	FALSE	TRUE	2	RAIN + CircaCompare only	FALSE
ENSMUSG00000039852	Rere	1.4060E-01	8.5840E-01	4.1761E-01	1.2542E-01	TRUE	FALSE	FALSE	TRUE	2	RAIN + CircaCompare only	FALSE
ENSMUSG00000025978	Rftn2	3.5513E-03	5.0222E-01	1.7778E-01	6.5276E-02	TRUE	FALSE	FALSE	TRUE	2	RAIN + CircaCompare only	FALSE
ENSMUSG00000046768	Rhoj	2.9373E-02	7.5273E-01	5.0132E-01	1.4199E-01	TRUE	FALSE	FALSE	TRUE	2	RAIN + CircaCompare only	FALSE
ENSMUSG00000017686	Rhot1	1.4060E-01	1.0000E+00	4.2743E-01	1.4852E-01	TRUE	FALSE	FALSE	TRUE	2	RAIN + CircaCompare only	FALSE
ENSMUSG00000039194	Rlbp1	3.2908E-02	7.5273E-01	3.7495E-01	1.0642E-01	TRUE	FALSE	FALSE	TRUE	2	RAIN + CircaCompare only	FALSE
ENSMUSG00000024410	Rmc1	2.3984E-02	7.5273E-01	3.5979E-01	9.9173E-02	TRUE	FALSE	FALSE	TRUE	2	RAIN + CircaCompare only	FALSE
ENSMUSG00000028884	Rpa2	7.1344E-02	1.0000E+00	5.3099E-01	1.4852E-01	TRUE	FALSE	FALSE	TRUE	2	RAIN + CircaCompare only	FALSE
ENSMUSG00000059835	Rpl13-ps3	1.4943E-01	1.0000E+00	5.0297E-01	1.4852E-01	TRUE	FALSE	FALSE	TRUE	2	RAIN + CircaCompare only	FALSE
ENSMUSG00000021180	Rps6ka5	7.1344E-02	1.0000E+00	3.4683E-01	1.0862E-01	TRUE	FALSE	FALSE	TRUE	2	RAIN + CircaCompare only	FALSE
ENSMUSG00000039096	Rsad1	1.4060E-01	1.0000E+00	4.4245E-01	1.2542E-01	TRUE	FALSE	FALSE	TRUE	2	RAIN + CircaCompare only	FALSE
ENSMUSG00000020386	Sar1b	2.9373E-02	7.5273E-01	4.1458E-01	1.1797E-01	TRUE	FALSE	FALSE	TRUE	2	RAIN + CircaCompare only	FALSE
ENSMUSG00000015305	Sash1	1.0026E-01	8.5840E-01	5.0297E-01	1.3763E-01	TRUE	FALSE	FALSE	TRUE	2	RAIN + CircaCompare only	FALSE
ENSMUSG00000025283	Sat1	2.9373E-02	5.0222E-01	3.4751E-01	7.2596E-02	TRUE	FALSE	FALSE	TRUE	2	RAIN + CircaCompare only	FALSE
ENSMUSG00000022769	Sdf2l1	1.0026E-01	9.1146E-01	4.2743E-01	1.4852E-01	TRUE	FALSE	FALSE	TRUE	2	RAIN + CircaCompare only	FALSE
ENSMUSG00000037411	Serpine1	7.1344E-02	9.1146E-01	3.1904E-01	7.2596E-02	TRUE	FALSE	FALSE	TRUE	2	RAIN + CircaCompare only	FALSE
ENSMUSG00000038332	Sesn1	1.4060E-01	1.0000E+00	4.2643E-01	1.2542E-01	TRUE	FALSE	FALSE	TRUE	2	RAIN + CircaCompare only	FALSE
ENSMUSG00000047281	Sfn	7.6131E-02	5.0222E-01	4.3348E-01	1.4852E-01	TRUE	FALSE	FALSE	TRUE	2	RAIN + CircaCompare only	FALSE
ENSMUSG00000018822	Sfrp5	5.4040E-02	9.9933E-01	3.6260E-01	1.0642E-01	TRUE	FALSE	FALSE	TRUE	2	RAIN + CircaCompare only	FALSE
ENSMUSG00000029321	Slc10a6	1.4060E-01	7.5273E-01	3.9629E-01	1.2344E-01	TRUE	FALSE	FALSE	TRUE	2	RAIN + CircaCompare only	FALSE
ENSMUSG00000045775	Slc16a5	7.1344E-02	1.0000E+00	5.3874E-01	1.4852E-01	TRUE	FALSE	FALSE	TRUE	2	RAIN + CircaCompare only	FALSE
ENSMUSG00000001918	Slc1a5	1.4060E-01	9.1146E-01	3.9571E-01	1.1866E-01	TRUE	FALSE	FALSE	TRUE	2	RAIN + CircaCompare only	FALSE
ENSMUSG00000037434	Slc30a1	1.4178E-01	1.0000E+00	5.0297E-01	1.3623E-01	TRUE	FALSE	FALSE	TRUE	2	RAIN + CircaCompare only	FALSE
ENSMUSG00000027074	Slc43a3	1.4060E-01	9.9933E-01	3.6622E-01	1.2542E-01	TRUE	FALSE	FALSE	TRUE	2	RAIN + CircaCompare only	FALSE

ENSMUSG00000035699	Slc51a	1.0680E-01	1.0000E+00	4.2743E-01	1.1707E-01	TRUE	FALSE	FALSE	TRUE	2	RAIN + CircaCompare only	FALSE
ENSMUSG00000030096	Slc6a6	1.4060E-01	1.0000E+00	4.2743E-01	1.2542E-01	TRUE	FALSE	FALSE	TRUE	2	RAIN + CircaCompare only	FALSE
ENSMUSG00000031596	Slc7a2	7.1344E-02	9.9933E-01	3.9571E-01	1.4199E-01	TRUE	FALSE	FALSE	TRUE	2	RAIN + CircaCompare only	FALSE
ENSMUSG00000037994	Slc9b2	7.1344E-02	8.5840E-01	3.9571E-01	1.0642E-01	TRUE	FALSE	FALSE	TRUE	2	RAIN + CircaCompare only	FALSE
ENSMUSG00000030237	Slco1a4	9.7544E-03	5.0222E-01	4.4517E-01	1.0862E-01	TRUE	FALSE	FALSE	TRUE	2	RAIN + CircaCompare only	FALSE
ENSMUSG00000024563	Smad2	1.0026E-01	1.0000E+00	4.7736E-01	1.4953E-01	TRUE	FALSE	FALSE	TRUE	2	RAIN + CircaCompare only	FALSE
ENSMUSG00000005899	Smpd4	1.4531E-01	1.0000E+00	3.7495E-01	1.3745E-01	TRUE	FALSE	FALSE	TRUE	2	RAIN + CircaCompare only	FALSE
ENSMUSG00000090302	Smr2l	1.0026E-01	1.0000E+00	4.8681E-01	1.4852E-01	TRUE	FALSE	FALSE	TRUE	2	RAIN + CircaCompare only	FALSE
ENSMUSG00000032422	Snx14	7.3586E-02	9.1146E-01	3.9571E-01	1.0662E-01	TRUE	FALSE	FALSE	TRUE	2	RAIN + CircaCompare only	FALSE
ENSMUSG00000050373	Snx21	1.4060E-01	7.5273E-01	5.0397E-01	1.3897E-01	TRUE	FALSE	FALSE	TRUE	2	RAIN + CircaCompare only	FALSE
ENSMUSG00000080316	Spaca6	4.9770E-02	5.0222E-01	4.1450E-01	1.1707E-01	TRUE	FALSE	FALSE	TRUE	2	RAIN + CircaCompare only	FALSE
ENSMUSG00000024352	Spata24	1.0026E-01	9.9933E-01	3.9571E-01	1.0642E-01	TRUE	FALSE	FALSE	TRUE	2	RAIN + CircaCompare only	FALSE
ENSMUSG00000003161	Sri	1.0411E-01	9.9933E-01	5.0297E-01	1.4852E-01	TRUE	FALSE	FALSE	TRUE	2	RAIN + CircaCompare only	FALSE
ENSMUSG00000039218	Srrm2	2.9373E-02	4.1828E-01	2.8297E-01	6.5276E-02	TRUE	FALSE	FALSE	TRUE	2	RAIN + CircaCompare only	FALSE
ENSMUSG00000021134	Srsf5	1.0026E-01	8.5840E-01	3.3650E-01	7.6429E-02	TRUE	FALSE	FALSE	TRUE	2	RAIN + CircaCompare only	FALSE
ENSMUSG00000022403	St13	1.0026E-01	9.1146E-01	3.9571E-01	1.3897E-01	TRUE	FALSE	FALSE	TRUE	2	RAIN + CircaCompare only	FALSE
ENSMUSG00000024776	Stambpl1	1.0026E-01	9.1146E-01	3.9571E-01	1.3763E-01	TRUE	FALSE	FALSE	TRUE	2	RAIN + CircaCompare only	FALSE
ENSMUSG00000026201	Stk16	7.1344E-02	9.1146E-01	3.4683E-01	1.0642E-01	TRUE	FALSE	FALSE	TRUE	2	RAIN + CircaCompare only	FALSE
ENSMUSG00000045503	Sys1	3.1913E-02	5.9891E-01	3.3650E-01	1.0642E-01	TRUE	FALSE	FALSE	TRUE	2	RAIN + CircaCompare only	FALSE
ENSMUSG00000059187	Tafa1	2.9373E-02	7.5273E-01	5.0132E-01	1.3926E-01	TRUE	FALSE	FALSE	TRUE	2	RAIN + CircaCompare only	FALSE
ENSMUSG00000071359	Tbpl1	4.9770E-02	7.5273E-01	3.9631E-01	1.2542E-01	TRUE	FALSE	FALSE	TRUE	2	RAIN + CircaCompare only	FALSE
ENSMUSG00000056313	Tcim	7.1344E-02	8.5840E-01	3.5437E-01	7.9030E-02	TRUE	FALSE	FALSE	TRUE	2	RAIN + CircaCompare only	FALSE
ENSMUSG00000002249	Tead3	7.1344E-02	1.0000E+00	4.3401E-01	1.2741E-01	TRUE	FALSE	FALSE	TRUE	2	RAIN + CircaCompare only	FALSE
ENSMUSG00000033985	Tesk2	1.4060E-01	1.0000E+00	4.2743E-01	1.2527E-01	TRUE	FALSE	FALSE	TRUE	2	RAIN + CircaCompare only	FALSE
ENSMUSG00000034832	Tet3	1.4060E-01	7.5273E-01	3.9571E-01	1.2741E-01	TRUE	FALSE	FALSE	TRUE	2	RAIN + CircaCompare only	FALSE
ENSMUSG00000036983	Tfb1m	3.1913E-02	5.0222E-01	3.1904E-01	7.2596E-02	TRUE	FALSE	FALSE	TRUE	2	RAIN + CircaCompare only	FALSE
ENSMUSG00000002489	Tiam1	1.0026E-01	9.1146E-01	5.1150E-01	1.4199E-01	TRUE	FALSE	FALSE	TRUE	2	RAIN + CircaCompare only	FALSE
ENSMUSG00000041143	Tmco4	1.0026E-01	9.9933E-01	4.7736E-01	1.3897E-01	TRUE	FALSE	FALSE	TRUE	2	RAIN + CircaCompare only	FALSE
ENSMUSG00000048572	Tmem252	7.1344E-02	9.9933E-01	4.2743E-01	1.1797E-01	TRUE	FALSE	FALSE	TRUE	2	RAIN + CircaCompare only	FALSE
ENSMUSG00000037003	Tns2	9.7544E-03	5.0222E-01	2.2798E-01	7.2596E-02	TRUE	FALSE	FALSE	TRUE	2	RAIN + CircaCompare only	FALSE
ENSMUSG00000014846	Tppp3	1.4060E-01	8.5840E-01	4.4245E-01	1.4199E-01	TRUE	FALSE	FALSE	TRUE	2	RAIN + CircaCompare only	FALSE
ENSMUSG00000019487	Trip10	7.1344E-02	8.5840E-01	4.2743E-01	1.3522E-01	TRUE	FALSE	FALSE	TRUE	2	RAIN + CircaCompare only	FALSE
ENSMUSG00000031431	Tsc22d3	3.1913E-02	8.5840E-01	3.3650E-01	1.0862E-01	TRUE	FALSE	FALSE	TRUE	2	RAIN + CircaCompare only	FALSE
ENSMUSG00000025511	Tspan4	2.9373E-02	5.0222E-01	1.8167E-01	6.5276E-02	TRUE	FALSE	FALSE	TRUE	2	RAIN + CircaCompare only	FALSE
ENSMUSG00000044986	Tst	5.4040E-02	8.5840E-01	3.8276E-01	1.0585E-01	TRUE	FALSE	FALSE	TRUE	2	RAIN + CircaCompare only	FALSE
ENSMUSG00000037470	Uggt1	1.0026E-01	9.1146E-01	4.7063E-01	1.2741E-01	TRUE	FALSE	FALSE	TRUE	2	RAIN + CircaCompare only	FALSE
ENSMUSG00000063317	Usp31	2.9373E-02	5.0222E-01	4.7273E-01	1.3926E-01	TRUE	FALSE	FALSE	TRUE	2	RAIN + CircaCompare only	FALSE
ENSMUSG00000040720	Virma	7.1344E-02	9.1146E-01	4.2728E-01	1.2766E-01	TRUE	FALSE	FALSE	TRUE	2	RAIN + CircaCompare only	FALSE
ENSMUSG00000042116	Vwa1	6.4393E-02	5.9891E-01	3.3650E-01	7.2596E-02	TRUE	FALSE	FALSE	TRUE	2	RAIN + CircaCompare only	FALSE
ENSMUSG00000030035	Wbp1	4.9770E-02	1.0000E+00	4.3802E-01	1.2542E-01	TRUE	FALSE	FALSE	TRUE	2	RAIN + CircaCompare only	FALSE
ENSMUSG00000031016	Wee1	1.0026E-01	9.1146E-01	3.9820E-01	1.0946E-01	TRUE	FALSE	FALSE	TRUE	2	RAIN + CircaCompare only	FALSE
ENSMUSG00000024066	Xdh	7.1344E-02	5.0222E-01	3.1904E-01	1.0862E-01	TRUE	FALSE	FALSE	TRUE	2	RAIN + CircaCompare only	FALSE
ENSMUSG00000021244	Ylpm1	1.4060E-01	1.0000E+00	5.0297E-01	1.4350E-01	TRUE	FALSE	FALSE	TRUE	2	RAIN + CircaCompare only	FALSE
ENSMUSG00000026197	Zfand2b	1.0026E-01	1.0000E+00	3.5437E-01	1.2200E-01	TRUE	FALSE	FALSE	TRUE	2	RAIN + CircaCompare only	FALSE
ENSMUSG00000042213	Zfand4	1.4060E-01	9.1146E-01	5.0397E-01	1.4852E-01	TRUE	FALSE	FALSE	TRUE	2	RAIN + CircaCompare only	FALSE
ENSMUSG00000074283	Zfp109	7.1344E-02	9.9933E-01	3.9571E-01	1.0585E-01	TRUE	FALSE	FALSE	TRUE	2	RAIN + CircaCompare only	FALSE
ENSMUSG00000057101	Zfp180	1.0302E-01	1.0000E+00	4.7811E-01	1.4852E-01	TRUE	FALSE	FALSE	TRUE	2	RAIN + CircaCompare only	FALSE
ENSMUSG00000070866	Zfp804a	4.9770E-02	1.0000E+00	5.2528E-01	1.4852E-01	TRUE	FALSE	FALSE	TRUE	2	RAIN + CircaCompare only	FALSE
ENSMUSG00000022680	Pdxdc1	1.9276E-01	1.0000E+00	1.3467E-01	1.5982E-01	FALSE	FALSE	TRUE	FALSE	1	ARSER only	FALSE
ENSMUSG00000070509	Rgma	3.2439E-01	1.0000E+00	1.3280E-01	1.8681E-01	FALSE	FALSE	TRUE	FALSE	1	ARSER only	FALSE
ENSMUSG00000030062	Rpn1	1.8138E-01	1.0000E+00	1.1483E-01	1.6940E-01	FALSE	FALSE	TRUE	FALSE	1	ARSER only	FALSE
ENSMUSG00000043629	1700019D03Rik	2.5767E-01	1.0000E+00	2.3437E-01	1.3897E-01	FALSE	FALSE	FALSE	TRUE	1	CircaCompare only	FALSE
ENSMUSG00000106951	5930430L01Rik	2.7850E-01	9.9933E-01	3.5437E-01	1.4199E-01	FALSE	FALSE	FALSE	TRUE	1	CircaCompare only	FALSE
ENSMUSG00000047878	A4galt	2.0484E-01	9.1146E-01	3.9629E-01	1.0662E-01	FALSE	FALSE	FALSE	TRUE	1	CircaCompare only	FALSE
ENSMUSG00000033453	Adamts15	1.9276E-01	9.9933E-01	4.1450E-01	1.0642E-01	FALSE	FALSE	FALSE	TRUE	1	CircaCompare only	FALSE
ENSMUSG00000030790	Adm	2.5340E-01	1.0000E+00	3.9571E-01	1.0642E-01	FALSE	FALSE	FALSE	TRUE	1	CircaCompare only	FALSE
ENSMUSG00000054204	Alkal2	1.9276E-01	1.0000E+00	3.1904E-01	1.4852E-01	FALSE	FALSE	FALSE	TRUE	1	CircaCompare only	FALSE
ENSMUSG00000079036	Alkbh1	3.3244E-01	1.0000E+00	5.0297E-01	1.4852E-01	FALSE	FALSE	FALSE	TRUE	1	CircaCompare only	FALSE
ENSMUSG00000031382	Asb11	2.4509E-01	9.9933E-01	4.2743E-01	1.4852E-01	FALSE	FALSE	FALSE	TRUE	1	CircaCompare only	FALSE
ENSMUSG00000037685	Atp8a1	2.5340E-01	1.0000E+00	5.0397E-01	1.4199E-01	FALSE	FALSE	FALSE	TRUE	1	CircaCompare only	FALSE
ENSMUSG00000023175	Bsg	1.9276E-01	9.9933E-01	4.2643E-01	1.2542E-01	FALSE	FALSE	FALSE	TRUE	1	CircaCompare only	FALSE
ENSMUSG00000030666	Calcb	2.5340E-01	1.0000E+00	4.9701E-01	1.4801E-01	FALSE	FALSE	FALSE	TRUE	1	CircaCompare only	FALSE

ENSMUSG00000053931	Cnn3	2.5942E-01	1.0000E+00	4.2327E-01	1.4852E-01	FALSE	FALSE	FALSE	TRUE	1	CircaCompare only	FALSE
ENSMUSG00000027230	Creb3l1	1.9276E-01	1.0000E+00	5.3792E-01	1.4953E-01	FALSE	FALSE	FALSE	TRUE	1	CircaCompare only	FALSE
ENSMUSG00000036748	Cuedc2	1.9276E-01	9.9933E-01	4.9701E-01	1.4953E-01	FALSE	FALSE	FALSE	TRUE	1	CircaCompare only	FALSE
ENSMUSG00000028447	Dctn3	1.9276E-01	1.0000E+00	4.2743E-01	1.2542E-01	FALSE	FALSE	FALSE	TRUE	1	CircaCompare only	FALSE
ENSMUSG00000061589	Dot1l	1.7116E-01	5.9891E-01	3.3650E-01	1.0642E-01	FALSE	FALSE	FALSE	TRUE	1	CircaCompare only	FALSE
ENSMUSG00000020537	Drg2	1.9472E-01	9.1146E-01	4.4245E-01	1.4199E-01	FALSE	FALSE	FALSE	TRUE	1	CircaCompare only	FALSE
ENSMUSG00000038619	Ensa	1.9276E-01	1.0000E+00	5.1150E-01	1.4852E-01	FALSE	FALSE	FALSE	TRUE	1	CircaCompare only	FALSE
ENSMUSG00000034152	Exoc3	2.5758E-01	1.0000E+00	4.2743E-01	1.2542E-01	FALSE	FALSE	FALSE	TRUE	1	CircaCompare only	FALSE
ENSMUSG00000021461	Fancc	1.9276E-01	1.0000E+00	5.0297E-01	1.4820E-01	FALSE	FALSE	FALSE	TRUE	1	CircaCompare only	FALSE
ENSMUSG00000047632	Fgfbp3	2.5635E-01	1.0000E+00	4.5128E-01	1.2542E-01	FALSE	FALSE	FALSE	TRUE	1	CircaCompare only	FALSE
ENSMUSG00000033739	Fkbpl	1.9472E-01	1.0000E+00	4.7736E-01	1.4953E-01	FALSE	FALSE	FALSE	TRUE	1	CircaCompare only	FALSE
ENSMUSG00000086825	Gm15675	1.9276E-01	1.0000E+00	4.5302E-01	1.4953E-01	FALSE	FALSE	FALSE	TRUE	1	CircaCompare only	FALSE
ENSMUSG00000092085	Gm17224	2.5121E-01	9.9933E-01	4.1458E-01	1.3926E-01	FALSE	FALSE	FALSE	TRUE	1	CircaCompare only	FALSE
ENSMUSG00000098739	Gm27151	1.9276E-01	9.1146E-01	4.7713E-01	1.3715E-01	FALSE	FALSE	FALSE	TRUE	1	CircaCompare only	FALSE
ENSMUSG00000101335	Gm28229	2.1700E-01	1.0000E+00	5.1757E-01	1.4852E-01	FALSE	FALSE	FALSE	TRUE	1	CircaCompare only	FALSE
ENSMUSG00000111027	Gm2981	1.9472E-01	1.0000E+00	5.2913E-01	1.4953E-01	FALSE	FALSE	FALSE	TRUE	1	CircaCompare only	FALSE
ENSMUSG00000094942	Gm3604	1.9276E-01	1.0000E+00	5.2913E-01	1.4852E-01	FALSE	FALSE	FALSE	TRUE	1	CircaCompare only	FALSE
ENSMUSG00000117484	Gm5500	1.9472E-01	1.0000E+00	4.2743E-01	1.4852E-01	FALSE	FALSE	FALSE	TRUE	1	CircaCompare only	FALSE
ENSMUSG00000091269	Gm6682	3.1661E-01	1.0000E+00	4.9045E-01	1.4374E-01	FALSE	FALSE	FALSE	TRUE	1	CircaCompare only	FALSE
ENSMUSG00000022546	Gpt	1.9276E-01	9.9933E-01	5.2528E-01	1.4199E-01	FALSE	FALSE	FALSE	TRUE	1	CircaCompare only	FALSE
ENSMUSG00000029864	Gstk1	1.9276E-01	9.1146E-01	4.2728E-01	1.4852E-01	FALSE	FALSE	FALSE	TRUE	1	CircaCompare only	FALSE
ENSMUSG00000028980	H6pd	1.9472E-01	1.0000E+00	4.1458E-01	1.4199E-01	FALSE	FALSE	FALSE	TRUE	1	CircaCompare only	FALSE
ENSMUSG00000064109	Hcst	2.3840E-01	1.0000E+00	5.0397E-01	1.4852E-01	FALSE	FALSE	FALSE	TRUE	1	CircaCompare only	FALSE
ENSMUSG00000025757	Hspa4l	1.7740E-01	1.0000E+00	4.6443E-01	1.4852E-01	FALSE	FALSE	FALSE	TRUE	1	CircaCompare only	FALSE
ENSMUSG00000039745	Htatip2	1.9472E-01	1.0000E+00	5.0624E-01	1.4199E-01	FALSE	FALSE	FALSE	TRUE	1	CircaCompare only	FALSE
ENSMUSG00000032968	Inha	2.5340E-01	1.0000E+00	4.2743E-01	1.3897E-01	FALSE	FALSE	FALSE	TRUE	1	CircaCompare only	FALSE
ENSMUSG00000026223	Itm2c	1.9287E-01	1.0000E+00	5.0397E-01	1.4852E-01	FALSE	FALSE	FALSE	TRUE	1	CircaCompare only	FALSE
ENSMUSG00000051401	Kctd16	1.9276E-01	1.0000E+00	4.4235E-01	1.3673E-01	FALSE	FALSE	FALSE	TRUE	1	CircaCompare only	FALSE
ENSMUSG00000024395	Lims2	2.4760E-01	1.0000E+00	4.4235E-01	1.2542E-01	FALSE	FALSE	FALSE	TRUE	1	CircaCompare only	FALSE
ENSMUSG00000020782	Llgl2	2.5340E-01	1.0000E+00	3.7495E-01	1.4852E-01	FALSE	FALSE	FALSE	TRUE	1	CircaCompare only	FALSE
ENSMUSG00000033192	Lpcat2	1.5209E-01	1.0000E+00	5.0132E-01	1.4199E-01	FALSE	FALSE	FALSE	TRUE	1	CircaCompare only	FALSE
ENSMUSG00000028080	Lrba	2.5340E-01	1.0000E+00	5.0297E-01	1.3822E-01	FALSE	FALSE	FALSE	TRUE	1	CircaCompare only	FALSE
ENSMUSG00000079018	Ly6c1	1.9276E-01	1.0000E+00	4.4245E-01	1.3606E-01	FALSE	FALSE	FALSE	TRUE	1	CircaCompare only	FALSE
ENSMUSG00000030621	Me3	1.9276E-01	9.9933E-01	3.9631E-01	1.3926E-01	FALSE	FALSE	FALSE	TRUE	1	CircaCompare only	FALSE
ENSMUSG00000055660	Mettl4	2.5340E-01	1.0000E+00	5.0297E-01	1.4852E-01	FALSE	FALSE	FALSE	TRUE	1	CircaCompare only	FALSE
ENSMUSG00000029049	Morn1	1.9472E-01	9.9933E-01	4.4517E-01	1.2542E-01	FALSE	FALSE	FALSE	TRUE	1	CircaCompare only	FALSE
ENSMUSG00000045934	Mtmr11	1.9276E-01	1.0000E+00	3.1904E-01	1.0642E-01	FALSE	FALSE	FALSE	TRUE	1	CircaCompare only	FALSE
ENSMUSG00000025402	Nab2	1.9276E-01	7.5273E-01	4.4237E-01	1.2542E-01	FALSE	FALSE	FALSE	TRUE	1	CircaCompare only	FALSE
ENSMUSG00000043924	Ncmap	2.5340E-01	1.0000E+00	4.2743E-01	1.2779E-01	FALSE	FALSE	FALSE	TRUE	1	CircaCompare only	FALSE
ENSMUSG00000028923	Necap2	2.5340E-01	1.0000E+00	4.9607E-01	1.3897E-01	FALSE	FALSE	FALSE	TRUE	1	CircaCompare only	FALSE
ENSMUSG00000071547	Nt5dc2	2.5340E-01	1.0000E+00	5.0297E-01	1.4350E-01	FALSE	FALSE	FALSE	TRUE	1	CircaCompare only	FALSE
ENSMUSG00000026784	Pdss1	2.1345E-01	1.0000E+00	5.0297E-01	1.4852E-01	FALSE	FALSE	FALSE	TRUE	1	CircaCompare only	FALSE
ENSMUSG00000025271	Pfkfb1	1.9276E-01	9.1146E-01	4.2743E-01	1.3693E-01	FALSE	FALSE	FALSE	TRUE	1	CircaCompare only	FALSE
ENSMUSG00000018572	Phf23	2.5612E-01	1.0000E+00	5.2528E-01	1.4852E-01	FALSE	FALSE	FALSE	TRUE	1	CircaCompare only	FALSE
ENSMUSG00000041417	Pik3r1	2.5325E-01	1.0000E+00	3.9571E-01	1.2542E-01	FALSE	FALSE	FALSE	TRUE	1	CircaCompare only	FALSE
ENSMUSG00000035828	Pim3	2.5635E-01	1.0000E+00	4.1458E-01	1.4199E-01	FALSE	FALSE	FALSE	TRUE	1	CircaCompare only	FALSE
ENSMUSG00000022525	Plaat1	2.9314E-01	1.0000E+00	4.7063E-01	1.4199E-01	FALSE	FALSE	FALSE	TRUE	1	CircaCompare only	FALSE
ENSMUSG00000052468	Pmp2	1.5781E-01	9.9933E-01	4.7063E-01	1.4199E-01	FALSE	FALSE	FALSE	TRUE	1	CircaCompare only	FALSE
ENSMUSG00000042496	Prdm10	3.3854E-01	1.0000E+00	4.2743E-01	1.3926E-01	FALSE	FALSE	FALSE	TRUE	1	CircaCompare only	FALSE
ENSMUSG00000045725	Prr15	2.4105E-01	1.0000E+00	4.2643E-01	1.1707E-01	FALSE	FALSE	FALSE	TRUE	1	CircaCompare only	FALSE
ENSMUSG00000117310	Ptp4a1	1.5388E-01	1.0000E+00	4.3401E-01	1.2542E-01	FALSE	FALSE	FALSE	TRUE	1	CircaCompare only	FALSE
ENSMUSG00000038042	Ptpdc1	1.9122E-01	1.0000E+00	3.8276E-01	1.0642E-01	FALSE	FALSE	FALSE	TRUE	1	CircaCompare only	FALSE
ENSMUSG00000029528	Pxn	4.1032E-01	1.0000E+00	5.0397E-01	1.4556E-01	FALSE	FALSE	FALSE	TRUE	1	CircaCompare only	FALSE
ENSMUSG00000032202	Rab27a	3.1661E-01	1.0000E+00	4.1458E-01	1.3799E-01	FALSE	FALSE	FALSE	TRUE	1	CircaCompare only	FALSE
ENSMUSG00000029191	Rfc1	1.9276E-01	9.9933E-01	5.2528E-01	1.4852E-01	FALSE	FALSE	FALSE	TRUE	1	CircaCompare only	FALSE
ENSMUSG00000038530	Rgs4	1.6480E-01	8.5840E-01	4.7273E-01	1.4366E-01	FALSE	FALSE	FALSE	TRUE	1	CircaCompare only	FALSE
ENSMUSG00000029130	Rnf32	1.9276E-01	9.1146E-01	3.5815E-01	1.0642E-01	FALSE	FALSE	FALSE	TRUE	1	CircaCompare only	FALSE
ENSMUSG00000068874	Selenbp1	2.5635E-01	1.0000E+00	5.0397E-01	1.4852E-01	FALSE	FALSE	FALSE	TRUE	1	CircaCompare only	FALSE
ENSMUSG00000019970	Sgk1	3.2439E-01	1.0000E+00	5.0971E-01	1.4199E-01	FALSE	FALSE	FALSE	TRUE	1	CircaCompare only	FALSE
ENSMUSG00000042594	Sh2b3	1.9276E-01	1.0000E+00	4.4235E-01	1.2525E-01	FALSE	FALSE	FALSE	TRUE	1	CircaCompare only	FALSE
ENSMUSG00000044813	Shb	3.1661E-01	1.0000E+00	3.1904E-01	1.0642E-01	FALSE	FALSE	FALSE	TRUE	1	CircaCompare only	FALSE
ENSMUSG00000036432	Siah2	1.9472E-01	1.0000E+00	4.9701E-01	1.4953E-01	FALSE	FALSE	FALSE	TRUE	1	CircaCompare only	FALSE

ENSMUSG00000034908	Sidt2	2.6465E-01	1.0000E+00	4.6887E-01	1.2542E-01	FALSE	FALSE	FALSE	TRUE	1	CircaCompare only	FALSE
ENSMUSG00000024042	Sik1	3.2491E-01	1.0000E+00	4.6915E-01	1.4199E-01	FALSE	FALSE	FALSE	TRUE	1	CircaCompare only	FALSE
ENSMUSG00000031622	Sin3b	1.9921E-01	1.0000E+00	5.0297E-01	1.4852E-01	FALSE	FALSE	FALSE	TRUE	1	CircaCompare only	FALSE
ENSMUSG00000031105	Slc25a14	1.9276E-01	1.0000E+00	4.6937E-01	1.4852E-01	FALSE	FALSE	FALSE	TRUE	1	CircaCompare only	FALSE
ENSMUSG00000026435	Slc45a3	2.0456E-01	1.0000E+00	4.3401E-01	1.4199E-01	FALSE	FALSE	FALSE	TRUE	1	CircaCompare only	FALSE
ENSMUSG00000019558	Slc6a8	2.5340E-01	9.1146E-01	4.4517E-01	1.2003E-01	FALSE	FALSE	FALSE	TRUE	1	CircaCompare only	FALSE
ENSMUSG00000025880	Smad7	1.9276E-01	9.1146E-01	3.8318E-01	1.1510E-01	FALSE	FALSE	FALSE	TRUE	1	CircaCompare only	FALSE
ENSMUSG00000024378	Stard4	2.0682E-01	1.0000E+00	5.0397E-01	1.3897E-01	FALSE	FALSE	FALSE	TRUE	1	CircaCompare only	FALSE
ENSMUSG00000028832	Stmn1	1.9276E-01	1.0000E+00	3.4683E-01	8.2711E-02	FALSE	FALSE	FALSE	TRUE	1	CircaCompare only	FALSE
ENSMUSG00000039033	Tasp1	1.9276E-01	1.0000E+00	5.0297E-01	1.4852E-01	FALSE	FALSE	FALSE	TRUE	1	CircaCompare only	FALSE
ENSMUSG00000039759	Thap3	2.5635E-01	1.0000E+00	3.3650E-01	1.4852E-01	FALSE	FALSE	FALSE	TRUE	1	CircaCompare only	FALSE
ENSMUSG00000023885	Thbs2	2.2022E-01	1.0000E+00	5.2528E-01	1.4852E-01	FALSE	FALSE	FALSE	TRUE	1	CircaCompare only	FALSE
ENSMUSG00000013495	Tmem175	2.5635E-01	1.0000E+00	4.7713E-01	1.4852E-01	FALSE	FALSE	FALSE	TRUE	1	CircaCompare only	FALSE
ENSMUSG00000050270	Tmem220	3.2439E-01	1.0000E+00	5.2528E-01	1.4953E-01	FALSE	FALSE	FALSE	TRUE	1	CircaCompare only	FALSE
ENSMUSG00000027940	Tpm3	2.3344E-01	9.1146E-01	5.2913E-01	1.4852E-01	FALSE	FALSE	FALSE	TRUE	1	CircaCompare only	FALSE
ENSMUSG00000032010	Usp2	3.8640E-01	1.0000E+00	4.2643E-01	1.2003E-01	FALSE	FALSE	FALSE	TRUE	1	CircaCompare only	FALSE
ENSMUSG00000032612	Usp4	2.0456E-01	1.0000E+00	4.1761E-01	1.2542E-01	FALSE	FALSE	FALSE	TRUE	1	CircaCompare only	FALSE
ENSMUSG00000024924	Vldlr	1.9276E-01	8.5840E-01	3.1904E-01	1.2542E-01	FALSE	FALSE	FALSE	TRUE	1	CircaCompare only	FALSE
ENSMUSG00000029158	Yipf7	2.5612E-01	9.1146E-01	4.1458E-01	1.4989E-01	FALSE	FALSE	FALSE	TRUE	1	CircaCompare only	FALSE
ENSMUSG00000075600	Zc3h3	2.5340E-01	1.0000E+00	5.0297E-01	1.4199E-01	FALSE	FALSE	FALSE	TRUE	1	CircaCompare only	FALSE
ENSMUSG00000034538	Zfp418	1.9276E-01	1.0000E+00	4.7736E-01	1.4350E-01	FALSE	FALSE	FALSE	TRUE	1	CircaCompare only	FALSE
ENSMUSG00000100891	2810049E08Rik	1.0026E-01	1.0000E+00	5.7199E-01	1.9396E-01	TRUE	FALSE	FALSE	FALSE	1	RAIN only	FALSE
ENSMUSG00000086629	2810403D21Rik	1.4060E-01	9.9933E-01	4.1458E-01	1.6850E-01	TRUE	FALSE	FALSE	FALSE	1	RAIN only	FALSE
ENSMUSG00000043833	2900005J15Rik	1.0411E-01	1.0000E+00	5.8654E-01	1.8204E-01	TRUE	FALSE	FALSE	FALSE	1	RAIN only	FALSE
ENSMUSG00000062822	4833420G17Rik	1.4178E-01	1.0000E+00	5.6778E-01	1.6940E-01	TRUE	FALSE	FALSE	FALSE	1	RAIN only	FALSE
ENSMUSG00000118087	4833438C02Rik	1.0026E-01	1.0000E+00	5.7420E-01	1.6940E-01	TRUE	FALSE	FALSE	FALSE	1	RAIN only	FALSE
ENSMUSG00000053574	4930563E22Rik	1.0026E-01	8.5840E-01	6.0849E-01	1.8407E-01	TRUE	FALSE	FALSE	FALSE	1	RAIN only	FALSE
ENSMUSG00000059939	9430015G10Rik	1.4060E-01	1.0000E+00	6.5830E-01	2.3733E-01	TRUE	FALSE	FALSE	FALSE	1	RAIN only	FALSE
ENSMUSG00000087528	9830144P21Rik	1.4060E-01	1.0000E+00	7.3302E-01	2.7420E-01	TRUE	FALSE	FALSE	FALSE	1	RAIN only	FALSE
ENSMUSG00000084846	A730011C13Rik	1.4060E-01	1.0000E+00	5.2913E-01	1.6850E-01	TRUE	FALSE	FALSE	FALSE	1	RAIN only	FALSE
ENSMUSG00000097415	AU020206	7.1239E-02	9.9933E-01	3.4683E-01	1.5378E-01	TRUE	FALSE	FALSE	FALSE	1	RAIN only	FALSE
ENSMUSG00000022822	Abcc5	1.0026E-01	1.0000E+00	5.6511E-01	1.7615E-01	TRUE	FALSE	FALSE	FALSE	1	RAIN only	FALSE
ENSMUSG00000035258	Abi3bp	1.4060E-01	1.0000E+00	6.5526E-01	2.1212E-01	TRUE	FALSE	FALSE	FALSE	1	RAIN only	FALSE
ENSMUSG00000029313	Aff1	5.4040E-02	9.9933E-01	5.2783E-01	1.6911E-01	TRUE	FALSE	FALSE	FALSE	1	RAIN only	FALSE
ENSMUSG00000021681	Aggf1	4.9770E-02	1.0000E+00	6.0659E-01	1.8320E-01	TRUE	FALSE	FALSE	FALSE	1	RAIN only	FALSE
ENSMUSG00000037860	Aim2	2.9373E-02	1.0000E+00	5.2528E-01	1.6911E-01	TRUE	FALSE	FALSE	FALSE	1	RAIN only	FALSE
ENSMUSG00000039546	Ajap1	1.4943E-01	1.0000E+00	5.8654E-01	1.7172E-01	TRUE	FALSE	FALSE	FALSE	1	RAIN only	FALSE
ENSMUSG00000031667	Aktip	1.4060E-01	9.9933E-01	5.1910E-01	1.6702E-01	TRUE	FALSE	FALSE	FALSE	1	RAIN only	FALSE
ENSMUSG00000028028	Alpk1	1.4060E-01	1.0000E+00	5.2913E-01	1.6850E-01	TRUE	FALSE	FALSE	FALSE	1	RAIN only	FALSE
ENSMUSG00000002289	Angptl4	9.9807E-02	1.0000E+00	5.0397E-01	1.9664E-01	TRUE	FALSE	FALSE	FALSE	1	RAIN only	FALSE
ENSMUSG00000050914	Ankrd37	7.1344E-02	1.0000E+00	5.3397E-01	1.5952E-01	TRUE	FALSE	FALSE	FALSE	1	RAIN only	FALSE
ENSMUSG00000033055	Ankrd54	2.9373E-02	1.0000E+00	5.3792E-01	1.6987E-01	TRUE	FALSE	FALSE	FALSE	1	RAIN only	FALSE
ENSMUSG00000040459	Arglu1	4.9770E-02	1.0000E+00	5.4720E-01	1.6911E-01	TRUE	FALSE	FALSE	FALSE	1	RAIN only	FALSE
ENSMUSG00000071176	Arhgef10	7.5945E-02	9.1146E-01	4.2728E-01	1.6094E-01	TRUE	FALSE	FALSE	FALSE	1	RAIN only	FALSE
ENSMUSG00000029032	Arhgef16	5.2946E-02	1.0000E+00	5.0297E-01	1.5363E-01	TRUE	FALSE	FALSE	FALSE	1	RAIN only	FALSE
ENSMUSG00000043157	Arl11	1.0026E-01	1.0000E+00	5.8197E-01	1.6940E-01	TRUE	FALSE	FALSE	FALSE	1	RAIN only	FALSE
ENSMUSG00000070424	Art5	1.0922E-01	8.5840E-01	4.1636E-01	1.6850E-01	TRUE	FALSE	FALSE	FALSE	1	RAIN only	FALSE
ENSMUSG00000020884	Asgr1	1.0330E-01	1.0000E+00	6.2843E-01	1.9664E-01	TRUE	FALSE	FALSE	FALSE	1	RAIN only	FALSE
ENSMUSG00000033379	Atp6v0b	1.4178E-01	1.0000E+00	7.3788E-01	2.7501E-01	TRUE	FALSE	FALSE	FALSE	1	RAIN only	FALSE
ENSMUSG00000093606	B130034C11Rik	1.4060E-01	1.0000E+00	5.5981E-01	1.7172E-01	TRUE	FALSE	FALSE	FALSE	1	RAIN only	FALSE
ENSMUSG00000081137	BC022960	1.4060E-01	1.0000E+00	6.2893E-01	1.9691E-01	TRUE	FALSE	FALSE	FALSE	1	RAIN only	FALSE
ENSMUSG00000007659	Bcl2l1	1.4060E-01	1.0000E+00	6.3837E-01	2.2769E-01	TRUE	FALSE	FALSE	FALSE	1	RAIN only	FALSE
ENSMUSG00000092060	Bend4	1.0680E-01	1.0000E+00	5.7440E-01	2.1461E-01	TRUE	FALSE	FALSE	FALSE	1	RAIN only	FALSE
ENSMUSG00000072964	Bhlhb9	7.3965E-02	1.0000E+00	5.8482E-01	1.7809E-01	TRUE	FALSE	FALSE	FALSE	1	RAIN only	FALSE
ENSMUSG00000025128	Bhlhe22	1.0026E-01	9.1146E-01	5.8654E-01	1.9388E-01	TRUE	FALSE	FALSE	FALSE	1	RAIN only	FALSE
ENSMUSG00000037933	Bicd2	1.4060E-01	1.0000E+00	4.7811E-01	1.9122E-01	TRUE	FALSE	FALSE	FALSE	1	RAIN only	FALSE
ENSMUSG00000039004	Bmp6	1.0026E-01	1.0000E+00	5.8482E-01	1.6940E-01	TRUE	FALSE	FALSE	FALSE	1	RAIN only	FALSE
ENSMUSG00000080268	Brms1	1.0411E-01	1.0000E+00	5.3424E-01	1.6850E-01	TRUE	FALSE	FALSE	FALSE	1	RAIN only	FALSE
ENSMUSG00000001632	Brpf1	1.4060E-01	9.1146E-01	5.0297E-01	1.6911E-01	TRUE	FALSE	FALSE	FALSE	1	RAIN only	FALSE
ENSMUSG00000052951	C130021I20Rik	1.4943E-01	1.0000E+00	6.6107E-01	2.1651E-01	TRUE	FALSE	FALSE	FALSE	1	RAIN only	FALSE
ENSMUSG00000085154	C130046K22Rik	1.4060E-01	1.0000E+00	6.0659E-01	2.1351E-01	TRUE	FALSE	FALSE	FALSE	1	RAIN only	FALSE
ENSMUSG00000061535	C1qtnf7	7.1344E-02	9.9933E-01	5.8496E-01	1.8836E-01	TRUE	FALSE	FALSE	FALSE	1	RAIN only	FALSE

ENSMUSG00000028656	Cap1	1.4060E-01	1.0000E+00	6.7229E-01	2.2794E-01	TRUE	FALSE	FALSE	FALSE	1	RAIN only	FALSE
ENSMUSG00000018666	Cbx1	1.0026E-01	1.0000E+00	6.1140E-01	1.9388E-01	TRUE	FALSE	FALSE	FALSE	1	RAIN only	FALSE
ENSMUSG00000008845	Cd163	1.3830E-01	9.9933E-01	5.3965E-01	1.7993E-01	TRUE	FALSE	FALSE	FALSE	1	RAIN only	FALSE
ENSMUSG00000022661	Cd200	4.9770E-02	1.0000E+00	4.7081E-01	1.5549E-01	TRUE	FALSE	FALSE	FALSE	1	RAIN only	FALSE
ENSMUSG00000027215	Cd82	7.1344E-02	9.9933E-01	5.3786E-01	1.7172E-01	TRUE	FALSE	FALSE	FALSE	1	RAIN only	FALSE
ENSMUSG00000027284	Cdan1	7.1344E-02	1.0000E+00	7.1324E-01	2.4684E-01	TRUE	FALSE	FALSE	FALSE	1	RAIN only	FALSE
ENSMUSG00000047216	Cdh19	1.4060E-01	1.0000E+00	5.0397E-01	1.6850E-01	TRUE	FALSE	FALSE	FALSE	1	RAIN only	FALSE
ENSMUSG00000025358	Cdk2	1.0026E-01	8.5840E-01	6.2696E-01	2.1637E-01	TRUE	FALSE	FALSE	FALSE	1	RAIN only	FALSE
ENSMUSG00000023473	Celsr3	7.0645E-02	1.0000E+00	5.2913E-01	1.5378E-01	TRUE	FALSE	FALSE	FALSE	1	RAIN only	FALSE
ENSMUSG00000038987	Cfap157	1.0026E-01	1.0000E+00	6.0855E-01	2.1461E-01	TRUE	FALSE	FALSE	FALSE	1	RAIN only	FALSE
ENSMUSG00000001774	Chordc1	1.4060E-01	1.0000E+00	5.7440E-01	1.8204E-01	TRUE	FALSE	FALSE	FALSE	1	RAIN only	FALSE
ENSMUSG00000022843	Clcn2	1.0918E-01	1.0000E+00	5.4410E-01	1.7052E-01	TRUE	FALSE	FALSE	FALSE	1	RAIN only	FALSE
ENSMUSG00000036968	Cnpy4	1.0026E-01	1.0000E+00	6.1692E-01	2.0935E-01	TRUE	FALSE	FALSE	FALSE	1	RAIN only	FALSE
ENSMUSG00000079415	Cntf	1.4060E-01	1.0000E+00	6.6196E-01	2.1331E-01	TRUE	FALSE	FALSE	FALSE	1	RAIN only	FALSE
ENSMUSG00000001119	Col6a1	1.4060E-01	1.0000E+00	7.6804E-01	3.1822E-01	TRUE	FALSE	FALSE	FALSE	1	RAIN only	FALSE
ENSMUSG00000031849	Comp	1.4060E-01	1.0000E+00	7.1548E-01	2.9549E-01	TRUE	FALSE	FALSE	FALSE	1	RAIN only	FALSE
ENSMUSG00000034796	Cpne7	1.4060E-01	9.1146E-01	5.2913E-01	1.6911E-01	TRUE	FALSE	FALSE	FALSE	1	RAIN only	FALSE
ENSMUSG00000039007	Cpq	1.4060E-01	1.0000E+00	6.5559E-01	2.1527E-01	TRUE	FALSE	FALSE	FALSE	1	RAIN only	FALSE
ENSMUSG00000030970	Ctbp2	1.0026E-01	1.0000E+00	6.0918E-01	2.0350E-01	TRUE	FALSE	FALSE	FALSE	1	RAIN only	FALSE
ENSMUSG00000047409	Ctdspl	5.2430E-02	7.5273E-01	4.7053E-01	1.5607E-01	TRUE	FALSE	FALSE	FALSE	1	RAIN only	FALSE
ENSMUSG00000052336	Cx3cr1	1.4060E-01	1.0000E+00	6.5526E-01	2.2025E-01	TRUE	FALSE	FALSE	FALSE	1	RAIN only	FALSE
ENSMUSG00000029727	Cyp3a13	4.9770E-02	1.0000E+00	5.7199E-01	1.6940E-01	TRUE	FALSE	FALSE	FALSE	1	RAIN only	FALSE
ENSMUSG00000102553	D930036K23Rik	2.9373E-02	1.0000E+00	5.3408E-01	1.5703E-01	TRUE	FALSE	FALSE	FALSE	1	RAIN only	FALSE
ENSMUSG00000030868	Dctn5	1.4060E-01	1.0000E+00	5.4410E-01	1.5519E-01	TRUE	FALSE	FALSE	FALSE	1	RAIN only	FALSE
ENSMUSG00000038633	Degs1	1.0026E-01	1.0000E+00	5.8197E-01	2.0584E-01	TRUE	FALSE	FALSE	FALSE	1	RAIN only	FALSE
ENSMUSG00000015377	Dennd6b	1.0026E-01	1.0000E+00	6.5885E-01	2.0853E-01	TRUE	FALSE	FALSE	FALSE	1	RAIN only	FALSE
ENSMUSG00000062393	Dgkk	7.1344E-02	9.9933E-01	5.3319E-01	1.5023E-01	TRUE	FALSE	FALSE	FALSE	1	RAIN only	FALSE
ENSMUSG00000022543	Dnaaf8	1.0026E-01	1.0000E+00	7.6804E-01	3.3140E-01	TRUE	FALSE	FALSE	FALSE	1	RAIN only	FALSE
ENSMUSG00000035126	Dnai4	1.4060E-01	1.0000E+00	6.6388E-01	2.4666E-01	TRUE	FALSE	FALSE	FALSE	1	RAIN only	FALSE
ENSMUSG00000004460	Dnajb11	1.0026E-01	1.0000E+00	5.5309E-01	1.6911E-01	TRUE	FALSE	FALSE	FALSE	1	RAIN only	FALSE
ENSMUSG00000022136	Dnajc3	1.4060E-01	1.0000E+00	5.2783E-01	1.8989E-01	TRUE	FALSE	FALSE	FALSE	1	RAIN only	FALSE
ENSMUSG00000025478	Dpysl4	1.4060E-01	1.0000E+00	5.6471E-01	1.7809E-01	TRUE	FALSE	FALSE	FALSE	1	RAIN only	FALSE
ENSMUSG00000041809	Efhc1	1.4943E-01	1.0000E+00	5.8654E-01	1.7227E-01	TRUE	FALSE	FALSE	FALSE	1	RAIN only	FALSE
ENSMUSG00000027236	Eif3j1	1.1178E-01	1.0000E+00	5.4410E-01	1.6911E-01	TRUE	FALSE	FALSE	FALSE	1	RAIN only	FALSE
ENSMUSG00000028668	Eloa	1.0026E-01	9.9933E-01	5.6471E-01	1.6911E-01	TRUE	FALSE	FALSE	FALSE	1	RAIN only	FALSE
ENSMUSG00000029326	Enoph1	1.4060E-01	1.0000E+00	5.9022E-01	2.0090E-01	TRUE	FALSE	FALSE	FALSE	1	RAIN only	FALSE
ENSMUSG00000050248	Evc2	1.4060E-01	1.0000E+00	6.1140E-01	2.0175E-01	TRUE	FALSE	FALSE	FALSE	1	RAIN only	FALSE
ENSMUSG00000040339	Fam102b	7.8208E-02	9.1146E-01	5.0297E-01	1.6094E-01	TRUE	FALSE	FALSE	FALSE	1	RAIN only	FALSE
ENSMUSG00000029185	Fam114a1	1.4060E-01	1.0000E+00	6.2843E-01	2.1243E-01	TRUE	FALSE	FALSE	FALSE	1	RAIN only	FALSE
ENSMUSG00000025262	Fam120c	1.4178E-01	1.0000E+00	6.0210E-01	1.8204E-01	TRUE	FALSE	FALSE	FALSE	1	RAIN only	FALSE
ENSMUSG00000058594	Fbh1	1.1258E-01	1.0000E+00	5.8654E-01	1.8204E-01	TRUE	FALSE	FALSE	FALSE	1	RAIN only	FALSE
ENSMUSG00000020635	Fkbp1b	1.4060E-01	9.9933E-01	5.2528E-01	1.5952E-01	TRUE	FALSE	FALSE	FALSE	1	RAIN only	FALSE
ENSMUSG00000041559	Fmod	1.4060E-01	1.0000E+00	5.8197E-01	1.7809E-01	TRUE	FALSE	FALSE	FALSE	1	RAIN only	FALSE
ENSMUSG00000035992	Fnip1	1.4060E-01	1.0000E+00	5.5250E-01	1.5503E-01	TRUE	FALSE	FALSE	FALSE	1	RAIN only	FALSE
ENSMUSG00000016552	Foxred2	1.4178E-01	9.9933E-01	5.6382E-01	1.7918E-01	TRUE	FALSE	FALSE	FALSE	1	RAIN only	FALSE
ENSMUSG00000020325	Fstl3	5.2946E-02	1.0000E+00	6.0554E-01	1.9495E-01	TRUE	FALSE	FALSE	FALSE	1	RAIN only	FALSE
ENSMUSG00000057092	Fxyd3	1.4060E-01	1.0000E+00	6.4124E-01	2.1212E-01	TRUE	FALSE	FALSE	FALSE	1	RAIN only	FALSE
ENSMUSG00000001260	Gabrg1	1.4060E-01	1.0000E+00	6.1321E-01	1.9664E-01	TRUE	FALSE	FALSE	FALSE	1	RAIN only	FALSE
ENSMUSG00000026867	Gapvd1	5.0193E-02	1.0000E+00	5.6065E-01	1.6850E-01	TRUE	FALSE	FALSE	FALSE	1	RAIN only	FALSE
ENSMUSG00000079363	Gbp4	1.4060E-01	1.0000E+00	7.1547E-01	2.6738E-01	TRUE	FALSE	FALSE	FALSE	1	RAIN only	FALSE
ENSMUSG00000028124	Gclm	7.1344E-02	1.0000E+00	5.6222E-01	1.6911E-01	TRUE	FALSE	FALSE	FALSE	1	RAIN only	FALSE
ENSMUSG00000050234	Gja4	1.4060E-01	1.0000E+00	6.0918E-01	1.8204E-01	TRUE	FALSE	FALSE	FALSE	1	RAIN only	FALSE
ENSMUSG00000049971	Glt1d1	7.1344E-02	1.0000E+00	5.8440E-01	1.8204E-01	TRUE	FALSE	FALSE	FALSE	1	RAIN only	FALSE
ENSMUSG00000046341	Gm11223	1.4178E-01	1.0000E+00	7.1176E-01	2.3723E-01	TRUE	FALSE	FALSE	FALSE	1	RAIN only	FALSE
ENSMUSG00000081151	Gm11448	1.4060E-01	1.0000E+00	5.9090E-01	2.0312E-01	TRUE	FALSE	FALSE	FALSE	1	RAIN only	FALSE
ENSMUSG00000081871	Gm11488	1.0026E-01	1.0000E+00	6.6388E-01	2.0853E-01	TRUE	FALSE	FALSE	FALSE	1	RAIN only	FALSE
ENSMUSG00000087523	Gm12319	1.4060E-01	1.0000E+00	5.3099E-01	1.6940E-01	TRUE	FALSE	FALSE	FALSE	1	RAIN only	FALSE
ENSMUSG00000084159	Gm12696	1.0026E-01	1.0000E+00	6.5830E-01	2.3728E-01	TRUE	FALSE	FALSE	FALSE	1	RAIN only	FALSE
ENSMUSG00000086368	Gm13830	7.6198E-02	1.0000E+00	5.2528E-01	1.5378E-01	TRUE	FALSE	FALSE	FALSE	1	RAIN only	FALSE
ENSMUSG00000083055	Gm14140	4.9770E-02	1.0000E+00	5.5100E-01	1.6911E-01	TRUE	FALSE	FALSE	FALSE	1	RAIN only	FALSE
ENSMUSG00000083396	Gm15542	1.0026E-01	9.9933E-01	5.6511E-01	1.7326E-01	TRUE	FALSE	FALSE	FALSE	1	RAIN only	FALSE
ENSMUSG00000082088	Gm15753	1.4060E-01	9.9933E-01	5.6065E-01	1.6940E-01	TRUE	FALSE	FALSE	FALSE	1	RAIN only	FALSE

ENSMUSG00000091509	Gm17066	1.0026E-01	1.0000E+00	6.5610E-01	1.9664E-01	TRUE	FALSE	FALSE	FALSE	1	RAIN only	FALSE
ENSMUSG00000097613	Gm17597	1.4060E-01	1.0000E+00	5.8654E-01	1.7541E-01	TRUE	FALSE	FALSE	FALSE	1	RAIN only	FALSE
ENSMUSG00000118240	Gm1859	7.1344E-02	1.0000E+00	5.8654E-01	1.8320E-01	TRUE	FALSE	FALSE	FALSE	1	RAIN only	FALSE
ENSMUSG00000021139	Gm20498	7.1344E-02	1.0000E+00	5.0397E-01	1.6940E-01	TRUE	FALSE	FALSE	FALSE	1	RAIN only	FALSE
ENSMUSG00000093502	Gm20700	1.4060E-01	1.0000E+00	7.2485E-01	2.6321E-01	TRUE	FALSE	FALSE	FALSE	1	RAIN only	FALSE
ENSMUSG00000097789	Gm2115	1.0026E-01	9.1146E-01	6.1692E-01	1.9664E-01	TRUE	FALSE	FALSE	FALSE	1	RAIN only	FALSE
ENSMUSG00000101037	Gm28424	1.0026E-01	1.0000E+00	5.9941E-01	1.9581E-01	TRUE	FALSE	FALSE	FALSE	1	RAIN only	FALSE
ENSMUSG00000115138	Gm36899	1.4178E-01	1.0000E+00	7.4834E-01	2.7610E-01	TRUE	FALSE	FALSE	FALSE	1	RAIN only	FALSE
ENSMUSG00000102813	Gm37795	1.0026E-01	1.0000E+00	5.3792E-01	1.6850E-01	TRUE	FALSE	FALSE	FALSE	1	RAIN only	FALSE
ENSMUSG00000110924	Gm40518	1.0026E-01	1.0000E+00	6.1692E-01	2.2026E-01	TRUE	FALSE	FALSE	FALSE	1	RAIN only	FALSE
ENSMUSG00000105161	Gm42595	7.1344E-02	9.1146E-01	4.2743E-01	1.6911E-01	TRUE	FALSE	FALSE	FALSE	1	RAIN only	FALSE
ENSMUSG00000103009	Gm4430	1.4060E-01	1.0000E+00	5.2783E-01	1.5519E-01	TRUE	FALSE	FALSE	FALSE	1	RAIN only	FALSE
ENSMUSG00000107451	Gm44421	9.9807E-02	1.0000E+00	4.7273E-01	1.7172E-01	TRUE	FALSE	FALSE	FALSE	1	RAIN only	FALSE
ENSMUSG00000108880	Gm44560	1.0026E-01	1.0000E+00	5.8654E-01	1.8204E-01	TRUE	FALSE	FALSE	FALSE	1	RAIN only	FALSE
ENSMUSG00000108037	Gm44597	2.9373E-02	7.5273E-01	5.2128E-01	1.5587E-01	TRUE	FALSE	FALSE	FALSE	1	RAIN only	FALSE
ENSMUSG00000110344	Gm45716	1.4328E-01	1.0000E+00	6.8637E-01	2.7764E-01	TRUE	FALSE	FALSE	FALSE	1	RAIN only	FALSE
ENSMUSG00000111325	Gm47140	1.4060E-01	1.0000E+00	5.6511E-01	1.7587E-01	TRUE	FALSE	FALSE	FALSE	1	RAIN only	FALSE
ENSMUSG00000111737	Gm47248	7.1344E-02	1.0000E+00	7.5458E-01	2.7198E-01	TRUE	FALSE	FALSE	FALSE	1	RAIN only	FALSE
ENSMUSG00000117819	Gm50253	1.4178E-01	1.0000E+00	6.8394E-01	2.4156E-01	TRUE	FALSE	FALSE	FALSE	1	RAIN only	FALSE
ENSMUSG00000055676	Gm5069	1.4060E-01	1.0000E+00	5.2046E-01	1.6965E-01	TRUE	FALSE	FALSE	FALSE	1	RAIN only	FALSE
ENSMUSG00000099998	Gm6501	1.4060E-01	1.0000E+00	5.9789E-01	1.9664E-01	TRUE	FALSE	FALSE	FALSE	1	RAIN only	FALSE
ENSMUSG00000111357	Gm6980	1.4060E-01	1.0000E+00	5.2913E-01	1.7498E-01	TRUE	FALSE	FALSE	FALSE	1	RAIN only	FALSE
ENSMUSG00000083064	Gm7785	1.4178E-01	1.0000E+00	6.5625E-01	2.0001E-01	TRUE	FALSE	FALSE	FALSE	1	RAIN only	FALSE
ENSMUSG00000101431	Gm7901	1.4060E-01	8.5840E-01	5.0297E-01	2.0210E-01	TRUE	FALSE	FALSE	FALSE	1	RAIN only	FALSE
ENSMUSG00000093798	Gm8355	7.1344E-02	1.0000E+00	5.3009E-01	1.5378E-01	TRUE	FALSE	FALSE	FALSE	1	RAIN only	FALSE
ENSMUSG00000081226	Gm8802	1.4060E-01	1.0000E+00	6.5830E-01	2.0913E-01	TRUE	FALSE	FALSE	FALSE	1	RAIN only	FALSE
ENSMUSG00000021556	Golm1	1.0680E-01	1.0000E+00	6.0918E-01	2.0685E-01	TRUE	FALSE	FALSE	FALSE	1	RAIN only	FALSE
ENSMUSG00000026271	Gpr35	1.0026E-01	5.9891E-01	4.2743E-01	1.5378E-01	TRUE	FALSE	FALSE	FALSE	1	RAIN only	FALSE
ENSMUSG00000043999	Gpr75	1.4178E-01	5.9891E-01	4.4245E-01	1.7809E-01	TRUE	FALSE	FALSE	FALSE	1	RAIN only	FALSE
ENSMUSG00000037188	Grhl3	1.4060E-01	1.0000E+00	7.3869E-01	3.0367E-01	TRUE	FALSE	FALSE	FALSE	1	RAIN only	FALSE
ENSMUSG00000032348	Gsta4	1.4328E-01	1.0000E+00	7.1176E-01	2.2976E-01	TRUE	FALSE	FALSE	FALSE	1	RAIN only	FALSE
ENSMUSG00000096210	H1f0	7.1344E-02	9.1146E-01	4.7736E-01	1.8836E-01	TRUE	FALSE	FALSE	FALSE	1	RAIN only	FALSE
ENSMUSG00000067212	H2-T23	1.4060E-01	1.0000E+00	4.7811E-01	1.6850E-01	TRUE	FALSE	FALSE	FALSE	1	RAIN only	FALSE
ENSMUSG00000016559	H3f3b	1.4060E-01	1.0000E+00	5.3792E-01	1.6994E-01	TRUE	FALSE	FALSE	FALSE	1	RAIN only	FALSE
ENSMUSG00000019977	Hbs1l	1.0026E-01	8.5840E-01	6.0659E-01	1.9664E-01	TRUE	FALSE	FALSE	FALSE	1	RAIN only	FALSE
ENSMUSG00000031770	Herpud1	1.4060E-01	1.0000E+00	7.5494E-01	2.8148E-01	TRUE	FALSE	FALSE	FALSE	1	RAIN only	FALSE
ENSMUSG00000025232	Hexa	4.9770E-02	1.0000E+00	4.9701E-01	1.5378E-01	TRUE	FALSE	FALSE	FALSE	1	RAIN only	FALSE
ENSMUSG00000039307	Hexdc	1.0026E-01	1.0000E+00	5.0397E-01	1.8204E-01	TRUE	FALSE	FALSE	FALSE	1	RAIN only	FALSE
ENSMUSG00000060373	Hnrnpc	1.0026E-01	1.0000E+00	5.8654E-01	1.8992E-01	TRUE	FALSE	FALSE	FALSE	1	RAIN only	FALSE
ENSMUSG00000000568	Hnrnpd	1.0026E-01	1.0000E+00	5.6511E-01	1.6911E-01	TRUE	FALSE	FALSE	FALSE	1	RAIN only	FALSE
ENSMUSG00000029328	Hnrnpdl	1.4060E-01	9.9933E-01	5.9022E-01	1.7172E-01	TRUE	FALSE	FALSE	FALSE	1	RAIN only	FALSE
ENSMUSG00000014418	Hps5	8.2397E-02	1.0000E+00	5.9941E-01	1.9388E-01	TRUE	FALSE	FALSE	FALSE	1	RAIN only	FALSE
ENSMUSG00000090877	Hspa1b	1.4060E-01	1.0000E+00	6.7690E-01	2.8449E-01	TRUE	FALSE	FALSE	FALSE	1	RAIN only	FALSE
ENSMUSG00000026864	Hspa5	7.1344E-02	8.5840E-01	5.1150E-01	1.8204E-01	TRUE	FALSE	FALSE	FALSE	1	RAIN only	FALSE
ENSMUSG00000025980	Hspd1	1.0026E-01	1.0000E+00	5.4410E-01	1.6330E-01	TRUE	FALSE	FALSE	FALSE	1	RAIN only	FALSE
ENSMUSG00000058809	Hspd1-ps3	1.0026E-01	1.0000E+00	5.0297E-01	1.6689E-01	TRUE	FALSE	FALSE	FALSE	1	RAIN only	FALSE
ENSMUSG00000027778	Ift80	7.1344E-02	1.0000E+00	5.3067E-01	1.6136E-01	TRUE	FALSE	FALSE	FALSE	1	RAIN only	FALSE
ENSMUSG00000026185	Igfbp5	1.4060E-01	1.0000E+00	6.5535E-01	2.2039E-01	TRUE	FALSE	FALSE	FALSE	1	RAIN only	FALSE
ENSMUSG00000057054	Inca1	7.1344E-02	1.0000E+00	5.4410E-01	1.9837E-01	TRUE	FALSE	FALSE	FALSE	1	RAIN only	FALSE
ENSMUSG00000029547	Ints1	1.4060E-01	9.1146E-01	6.5830E-01	2.1461E-01	TRUE	FALSE	FALSE	FALSE	1	RAIN only	FALSE
ENSMUSG00000031864	Ints10	1.0026E-01	1.0000E+00	5.4447E-01	1.6702E-01	TRUE	FALSE	FALSE	FALSE	1	RAIN only	FALSE
ENSMUSG00000027598	Itch	1.0026E-01	9.9933E-01	4.2671E-01	1.6030E-01	TRUE	FALSE	FALSE	FALSE	1	RAIN only	FALSE
ENSMUSG00000053062	Jam2	1.4060E-01	1.0000E+00	5.2528E-01	1.5434E-01	TRUE	FALSE	FALSE	FALSE	1	RAIN only	FALSE
ENSMUSG00000025221	Kcnip2	1.4060E-01	1.0000E+00	7.0594E-01	2.4219E-01	TRUE	FALSE	FALSE	FALSE	1	RAIN only	FALSE
ENSMUSG00000037624	Kcnk2	1.4060E-01	1.0000E+00	6.3681E-01	2.0620E-01	TRUE	FALSE	FALSE	FALSE	1	RAIN only	FALSE
ENSMUSG00000031788	Kifc3	5.7900E-02	9.9933E-01	5.5981E-01	1.6940E-01	TRUE	FALSE	FALSE	FALSE	1	RAIN only	FALSE
ENSMUSG00000058488	Kl	1.4060E-01	1.0000E+00	5.2528E-01	1.5378E-01	TRUE	FALSE	FALSE	FALSE	1	RAIN only	FALSE
ENSMUSG00000039682	Lap3	1.4060E-01	1.0000E+00	6.0918E-01	1.9388E-01	TRUE	FALSE	FALSE	FALSE	1	RAIN only	FALSE
ENSMUSG00000024544	Ldlrad4	7.1344E-02	1.0000E+00	5.4410E-01	1.6330E-01	TRUE	FALSE	FALSE	FALSE	1	RAIN only	FALSE
ENSMUSG00000042363	Lgalsl	1.0026E-01	1.0000E+00	6.1862E-01	2.2930E-01	TRUE	FALSE	FALSE	FALSE	1	RAIN only	FALSE
ENSMUSG00000024781	Lipa	1.0026E-01	1.0000E+00	6.0659E-01	1.8204E-01	TRUE	FALSE	FALSE	FALSE	1	RAIN only	FALSE
ENSMUSG00000032334	Loxl1	1.0026E-01	1.0000E+00	5.8654E-01	1.9122E-01	TRUE	FALSE	FALSE	FALSE	1	RAIN only	FALSE

ENSMUSG00000046417	Lrrc75a	1.4060E-01	1.0000E+00	6.2940E-01	2.4872E-01	TRUE	FALSE	FALSE	FALSE	1	RAIN only	FALSE
ENSMUSG00000034648	Lrrn1	7.1344E-02	1.0000E+00	5.9090E-01	1.9568E-01	TRUE	FALSE	FALSE	FALSE	1	RAIN only	FALSE
ENSMUSG00000042228	Lyn	1.4060E-01	1.0000E+00	5.0821E-01	1.7313E-01	TRUE	FALSE	FALSE	FALSE	1	RAIN only	FALSE
ENSMUSG00000005142	Man2b1	8.7679E-02	1.0000E+00	6.6196E-01	2.0913E-01	TRUE	FALSE	FALSE	FALSE	1	RAIN only	FALSE
ENSMUSG00000032575	Manf	1.4060E-01	1.0000E+00	5.8654E-01	1.9664E-01	TRUE	FALSE	FALSE	FALSE	1	RAIN only	FALSE
ENSMUSG00000069662	Marcks	1.0026E-01	1.0000E+00	6.2370E-01	1.9664E-01	TRUE	FALSE	FALSE	FALSE	1	RAIN only	FALSE
ENSMUSG00000030678	Maz	1.4060E-01	1.0000E+00	6.5088E-01	2.0165E-01	TRUE	FALSE	FALSE	FALSE	1	RAIN only	FALSE
ENSMUSG00000014361	Mertk	1.0084E-01	1.0000E+00	4.1458E-01	1.9664E-01	TRUE	FALSE	FALSE	FALSE	1	RAIN only	FALSE
ENSMUSG00000028114	Mettl14	1.4060E-01	1.0000E+00	6.1140E-01	1.9664E-01	TRUE	FALSE	FALSE	FALSE	1	RAIN only	FALSE
ENSMUSG00000010554	Mettl16	7.1344E-02	1.0000E+00	4.9419E-01	1.5378E-01	TRUE	FALSE	FALSE	FALSE	1	RAIN only	FALSE
ENSMUSG00000022160	Mettl3	7.8208E-02	9.1146E-01	5.2528E-01	1.6850E-01	TRUE	FALSE	FALSE	FALSE	1	RAIN only	FALSE
ENSMUSG00000022412	Mief1	1.4060E-01	1.0000E+00	7.8302E-01	3.8563E-01	TRUE	FALSE	FALSE	FALSE	1	RAIN only	FALSE
ENSMUSG00000040987	Mill2	7.5485E-02	1.0000E+00	6.0918E-01	2.1231E-01	TRUE	FALSE	FALSE	FALSE	1	RAIN only	FALSE
ENSMUSG00000037022	Mmaa	1.4060E-01	1.0000E+00	7.4797E-01	2.7336E-01	TRUE	FALSE	FALSE	FALSE	1	RAIN only	FALSE
ENSMUSG00000037221	Mospd3	7.2888E-02	1.0000E+00	6.0679E-01	1.9405E-01	TRUE	FALSE	FALSE	FALSE	1	RAIN only	FALSE
ENSMUSG00000026566	Mpzl1	7.1344E-02	1.0000E+00	7.4071E-01	2.7528E-01	TRUE	FALSE	FALSE	FALSE	1	RAIN only	FALSE
ENSMUSG00000041632	Mrps27	1.4178E-01	1.0000E+00	6.9904E-01	2.3786E-01	TRUE	FALSE	FALSE	FALSE	1	RAIN only	FALSE
ENSMUSG00000039680	Mrps6	1.4060E-01	1.0000E+00	4.2743E-01	1.5378E-01	TRUE	FALSE	FALSE	FALSE	1	RAIN only	FALSE
ENSMUSG00000023094	Msrb2	1.4060E-01	1.0000E+00	6.9086E-01	2.3127E-01	TRUE	FALSE	FALSE	FALSE	1	RAIN only	FALSE
ENSMUSG00000031918	Mtmr2	7.1344E-02	9.1146E-01	5.0329E-01	1.5378E-01	TRUE	FALSE	FALSE	FALSE	1	RAIN only	FALSE
ENSMUSG00000025025	Mxi1	1.0026E-01	8.5840E-01	5.2913E-01	1.6940E-01	TRUE	FALSE	FALSE	FALSE	1	RAIN only	FALSE
ENSMUSG00000044951	Mylk4	7.1344E-02	8.5840E-01	4.2743E-01	1.5349E-01	TRUE	FALSE	FALSE	FALSE	1	RAIN only	FALSE
ENSMUSG00000037730	Mynn	5.0644E-02	1.0000E+00	5.9789E-01	1.9703E-01	TRUE	FALSE	FALSE	FALSE	1	RAIN only	FALSE
ENSMUSG00000010505	Myt1	7.2888E-02	9.1146E-01	5.2913E-01	1.6850E-01	TRUE	FALSE	FALSE	FALSE	1	RAIN only	FALSE
ENSMUSG00000022698	Naa50	1.4060E-01	1.0000E+00	5.8654E-01	1.7615E-01	TRUE	FALSE	FALSE	FALSE	1	RAIN only	FALSE
ENSMUSG00000020181	Nav3	1.0026E-01	1.0000E+00	5.8654E-01	2.2025E-01	TRUE	FALSE	FALSE	FALSE	1	RAIN only	FALSE
ENSMUSG00000035024	Ncapd3	1.4060E-01	1.0000E+00	6.2893E-01	1.9664E-01	TRUE	FALSE	FALSE	FALSE	1	RAIN only	FALSE
ENSMUSG00000053253	Ndfip2	1.0262E-01	1.0000E+00	6.2444E-01	2.0475E-01	TRUE	FALSE	FALSE	FALSE	1	RAIN only	FALSE
ENSMUSG00000068184	Ndufaf2	1.4060E-01	1.0000E+00	6.6196E-01	2.1894E-01	TRUE	FALSE	FALSE	FALSE	1	RAIN only	FALSE
ENSMUSG00000021215	Net1	1.4060E-01	1.0000E+00	5.9425E-01	1.8204E-01	TRUE	FALSE	FALSE	FALSE	1	RAIN only	FALSE
ENSMUSG00000023411	Nfatc4	7.1344E-02	1.0000E+00	5.9665E-01	1.9987E-01	TRUE	FALSE	FALSE	FALSE	1	RAIN only	FALSE
ENSMUSG00000026259	Ngef	1.4034E-01	1.0000E+00	6.5610E-01	2.0473E-01	TRUE	FALSE	FALSE	FALSE	1	RAIN only	FALSE
ENSMUSG00000026162	Nhej1	1.4060E-01	1.0000E+00	5.3099E-01	1.9388E-01	TRUE	FALSE	FALSE	FALSE	1	RAIN only	FALSE
ENSMUSG00000037601	Nme1	1.0918E-01	9.9933E-01	5.4410E-01	1.7541E-01	TRUE	FALSE	FALSE	FALSE	1	RAIN only	FALSE
ENSMUSG00000061411	Nol4l	1.0026E-01	9.9933E-01	7.6804E-01	3.3483E-01	TRUE	FALSE	FALSE	FALSE	1	RAIN only	FALSE
ENSMUSG00000060275	Nrg2	1.0026E-01	1.0000E+00	5.0397E-01	1.9664E-01	TRUE	FALSE	FALSE	FALSE	1	RAIN only	FALSE
ENSMUSG00000017176	Nt5c3b	1.4060E-01	1.0000E+00	5.2625E-01	1.8105E-01	TRUE	FALSE	FALSE	FALSE	1	RAIN only	FALSE
ENSMUSG00000037857	Nufip2	1.4178E-01	1.0000E+00	5.9090E-01	2.0350E-01	TRUE	FALSE	FALSE	FALSE	1	RAIN only	FALSE
ENSMUSG00000044229	Nxpe4	1.0411E-01	1.0000E+00	7.0790E-01	2.4156E-01	TRUE	FALSE	FALSE	FALSE	1	RAIN only	FALSE
ENSMUSG00000046719	Nxph3	1.0026E-01	1.0000E+00	7.2333E-01	2.7019E-01	TRUE	FALSE	FALSE	FALSE	1	RAIN only	FALSE
ENSMUSG00000036737	Oxsr1	8.2397E-02	1.0000E+00	6.2843E-01	1.9866E-01	TRUE	FALSE	FALSE	FALSE	1	RAIN only	FALSE
ENSMUSG00000019916	P4ha1	1.4060E-01	1.0000E+00	4.1432E-01	1.7172E-01	TRUE	FALSE	FALSE	FALSE	1	RAIN only	FALSE
ENSMUSG00000002221	Paxip1	1.4060E-01	1.0000E+00	5.9022E-01	1.7809E-01	TRUE	FALSE	FALSE	FALSE	1	RAIN only	FALSE
ENSMUSG00000038718	Pbx3	1.4060E-01	1.0000E+00	6.0918E-01	1.9664E-01	TRUE	FALSE	FALSE	FALSE	1	RAIN only	FALSE
ENSMUSG00000056851	Pcbp2	1.0026E-01	1.0000E+00	5.2625E-01	1.6850E-01	TRUE	FALSE	FALSE	FALSE	1	RAIN only	FALSE
ENSMUSG00000039849	Pcif1	1.3960E-01	1.0000E+00	6.1140E-01	1.9664E-01	TRUE	FALSE	FALSE	FALSE	1	RAIN only	FALSE
ENSMUSG00000044254	Pcsk9	1.4060E-01	1.0000E+00	5.8654E-01	1.7172E-01	TRUE	FALSE	FALSE	FALSE	1	RAIN only	FALSE
ENSMUSG00000026239	Pde6d	1.0026E-01	1.0000E+00	6.0918E-01	1.9006E-01	TRUE	FALSE	FALSE	FALSE	1	RAIN only	FALSE
ENSMUSG00000020571	Pdia6	1.4060E-01	9.9933E-01	4.7736E-01	1.6295E-01	TRUE	FALSE	FALSE	FALSE	1	RAIN only	FALSE
ENSMUSG00000035232	Pdk3	1.0026E-01	1.0000E+00	5.8654E-01	1.8681E-01	TRUE	FALSE	FALSE	FALSE	1	RAIN only	FALSE
ENSMUSG00000061360	Phf5a	1.4060E-01	1.0000E+00	5.1350E-01	1.5378E-01	TRUE	FALSE	FALSE	FALSE	1	RAIN only	FALSE
ENSMUSG00000058388	Phtf1	2.9373E-02	1.0000E+00	5.9679E-01	1.9664E-01	TRUE	FALSE	FALSE	FALSE	1	RAIN only	FALSE
ENSMUSG00000026698	Pigc	1.0026E-01	9.9933E-01	5.8654E-01	1.9388E-01	TRUE	FALSE	FALSE	FALSE	1	RAIN only	FALSE
ENSMUSG00000025417	Pip4k2c	1.4060E-01	1.0000E+00	7.6804E-01	3.6295E-01	TRUE	FALSE	FALSE	FALSE	1	RAIN only	FALSE
ENSMUSG00000034403	Pja1	1.0026E-01	1.0000E+00	4.7273E-01	1.7227E-01	TRUE	FALSE	FALSE	FALSE	1	RAIN only	FALSE
ENSMUSG00000027499	Pkia	1.4328E-01	1.0000E+00	5.3792E-01	1.7918E-01	TRUE	FALSE	FALSE	FALSE	1	RAIN only	FALSE
ENSMUSG00000019876	Pkib	7.1344E-02	1.0000E+00	5.8654E-01	2.0396E-01	TRUE	FALSE	FALSE	FALSE	1	RAIN only	FALSE
ENSMUSG00000041957	Pkp2	1.0026E-01	5.9891E-01	6.0659E-01	1.9664E-01	TRUE	FALSE	FALSE	FALSE	1	RAIN only	FALSE
ENSMUSG00000054065	Pkp3	1.4060E-01	1.0000E+00	6.9707E-01	2.6372E-01	TRUE	FALSE	FALSE	FALSE	1	RAIN only	FALSE
ENSMUSG00000031146	Plp2	1.0026E-01	1.0000E+00	5.6471E-01	1.6940E-01	TRUE	FALSE	FALSE	FALSE	1	RAIN only	FALSE
ENSMUSG00000115338	Pnp	1.0026E-01	1.0000E+00	6.8566E-01	2.3777E-01	TRUE	FALSE	FALSE	FALSE	1	RAIN only	FALSE
ENSMUSG00000036833	Pnpla7	4.9770E-02	1.0000E+00	5.6222E-01	1.6114E-01	TRUE	FALSE	FALSE	FALSE	1	RAIN only	FALSE

ENSMUSG00000033152	Podxl2	1.4531E-01	1.0000E+00	6.0011E-01	1.8836E-01	TRUE	FALSE	FALSE	FALSE	1	RAIN only	FALSE
ENSMUSG00000007080	Pole	1.4060E-01	1.0000E+00	5.8496E-01	1.8681E-01	TRUE	FALSE	FALSE	FALSE	1	RAIN only	FALSE
ENSMUSG00000028636	Ppcs	7.1344E-02	9.1146E-01	5.5981E-01	1.7809E-01	TRUE	FALSE	FALSE	FALSE	1	RAIN only	FALSE
ENSMUSG00000009630	Ppp2cb	1.0411E-01	1.0000E+00	6.0918E-01	2.2976E-01	TRUE	FALSE	FALSE	FALSE	1	RAIN only	FALSE
ENSMUSG00000043154	Ppp2r3a	1.4178E-01	1.0000E+00	4.7063E-01	1.8204E-01	TRUE	FALSE	FALSE	FALSE	1	RAIN only	FALSE
ENSMUSG00000014039	Prdm15	1.4060E-01	1.0000E+00	6.7178E-01	2.7420E-01	TRUE	FALSE	FALSE	FALSE	1	RAIN only	FALSE
ENSMUSG00000003526	Prodh	1.4060E-01	9.9933E-01	6.2843E-01	2.1493E-01	TRUE	FALSE	FALSE	FALSE	1	RAIN only	FALSE
ENSMUSG00000039449	Prpf18	1.0026E-01	1.0000E+00	6.0659E-01	1.9581E-01	TRUE	FALSE	FALSE	FALSE	1	RAIN only	FALSE
ENSMUSG00000096727	Psmb9	1.4060E-01	9.9933E-01	6.6388E-01	2.1461E-01	TRUE	FALSE	FALSE	FALSE	1	RAIN only	FALSE
ENSMUSG00000071072	Ptges3	5.4040E-02	9.1146E-01	5.1829E-01	1.7172E-01	TRUE	FALSE	FALSE	FALSE	1	RAIN only	FALSE
ENSMUSG00000027540	Ptpn1	1.4060E-01	9.9933E-01	6.5625E-01	2.6664E-01	TRUE	FALSE	FALSE	FALSE	1	RAIN only	FALSE
ENSMUSG00000025140	Pycr1	1.0026E-01	1.0000E+00	5.1951E-01	1.6940E-01	TRUE	FALSE	FALSE	FALSE	1	RAIN only	FALSE
ENSMUSG00000034789	Rab24	1.1258E-01	1.0000E+00	5.2913E-01	1.6911E-01	TRUE	FALSE	FALSE	FALSE	1	RAIN only	FALSE
ENSMUSG00000002059	Rab34	2.9373E-02	1.0000E+00	5.8654E-01	1.7541E-01	TRUE	FALSE	FALSE	FALSE	1	RAIN only	FALSE
ENSMUSG00000030559	Rab38	1.4060E-01	1.0000E+00	6.5535E-01	2.2746E-01	TRUE	FALSE	FALSE	FALSE	1	RAIN only	FALSE
ENSMUSG00000055069	Rab39	1.0918E-01	1.0000E+00	5.4410E-01	1.8204E-01	TRUE	FALSE	FALSE	FALSE	1	RAIN only	FALSE
ENSMUSG00000043463	Rab9b	1.4060E-01	1.0000E+00	5.9789E-01	2.0299E-01	TRUE	FALSE	FALSE	FALSE	1	RAIN only	FALSE
ENSMUSG00000026014	Raph1	1.0680E-01	8.5840E-01	5.2913E-01	1.6940E-01	TRUE	FALSE	FALSE	FALSE	1	RAIN only	FALSE
ENSMUSG00000030134	Rasgef1a	1.4060E-01	1.0000E+00	6.5535E-01	2.0978E-01	TRUE	FALSE	FALSE	FALSE	1	RAIN only	FALSE
ENSMUSG00000029641	Rasl11a	5.6892E-02	1.0000E+00	6.5830E-01	2.0350E-01	TRUE	FALSE	FALSE	FALSE	1	RAIN only	FALSE
ENSMUSG00000099875	Rbm3-ps	1.4060E-01	1.0000E+00	5.2913E-01	1.5378E-01	TRUE	FALSE	FALSE	FALSE	1	RAIN only	FALSE
ENSMUSG00000031134	Rbmx	2.9373E-02	1.0000E+00	5.3480E-01	1.5916E-01	TRUE	FALSE	FALSE	FALSE	1	RAIN only	FALSE
ENSMUSG00000025921	Rdh10	1.4060E-01	1.0000E+00	6.0659E-01	1.9456E-01	TRUE	FALSE	FALSE	FALSE	1	RAIN only	FALSE
ENSMUSG00000039316	Rftn1	1.0918E-01	1.0000E+00	6.3899E-01	1.9664E-01	TRUE	FALSE	FALSE	FALSE	1	RAIN only	FALSE
ENSMUSG00000036615	Rfxap	7.8208E-02	1.0000E+00	5.6860E-01	1.8600E-01	TRUE	FALSE	FALSE	FALSE	1	RAIN only	FALSE
ENSMUSG00000035620	Ric8b	1.4060E-01	1.0000E+00	6.1692E-01	1.9043E-01	TRUE	FALSE	FALSE	FALSE	1	RAIN only	FALSE
ENSMUSG00000052656	Rnf103	4.9770E-02	1.0000E+00	6.3386E-01	2.0913E-01	TRUE	FALSE	FALSE	FALSE	1	RAIN only	FALSE
ENSMUSG00000022466	Rpap3	1.4943E-01	1.0000E+00	5.2625E-01	1.5567E-01	TRUE	FALSE	FALSE	FALSE	1	RAIN only	FALSE
ENSMUSG00000058603	Rpl28-ps1	1.4060E-01	1.0000E+00	6.4173E-01	2.0396E-01	TRUE	FALSE	FALSE	FALSE	1	RAIN only	FALSE
ENSMUSG00000003970	Rpl8	1.4060E-01	1.0000E+00	6.5053E-01	2.3884E-01	TRUE	FALSE	FALSE	FALSE	1	RAIN only	FALSE
ENSMUSG00000051518	Rps19bp1	1.4405E-01	1.0000E+00	5.6511E-01	1.6940E-01	TRUE	FALSE	FALSE	FALSE	1	RAIN only	FALSE
ENSMUSG00000031880	Rrad	1.4060E-01	1.0000E+00	6.0659E-01	1.8681E-01	TRUE	FALSE	FALSE	FALSE	1	RAIN only	FALSE
ENSMUSG00000030888	Rrp8	8.7679E-02	1.0000E+00	6.3351E-01	2.0810E-01	TRUE	FALSE	FALSE	FALSE	1	RAIN only	FALSE
ENSMUSG00000041506	Rrp9	1.0026E-01	1.0000E+00	5.6125E-01	1.6863E-01	TRUE	FALSE	FALSE	FALSE	1	RAIN only	FALSE
ENSMUSG00000035007	Rundc1	1.4178E-01	1.0000E+00	6.5559E-01	2.0913E-01	TRUE	FALSE	FALSE	FALSE	1	RAIN only	FALSE
ENSMUSG00000079003	Samd1	4.9770E-02	9.9933E-01	4.2743E-01	1.5378E-01	TRUE	FALSE	FALSE	FALSE	1	RAIN only	FALSE
ENSMUSG00000020519	Sap30l	1.0026E-01	1.0000E+00	6.4767E-01	2.0685E-01	TRUE	FALSE	FALSE	FALSE	1	RAIN only	FALSE
ENSMUSG00000002565	Scin	2.9373E-02	1.0000E+00	5.1951E-01	1.5681E-01	TRUE	FALSE	FALSE	FALSE	1	RAIN only	FALSE
ENSMUSG00000027879	Sec22b	7.1344E-02	1.0000E+00	5.2913E-01	1.5587E-01	TRUE	FALSE	FALSE	FALSE	1	RAIN only	FALSE
ENSMUSG00000075703	Selenoi	1.4060E-01	9.1146E-01	5.2913E-01	1.5378E-01	TRUE	FALSE	FALSE	FALSE	1	RAIN only	FALSE
ENSMUSG00000063531	Sema3e	1.4060E-01	1.0000E+00	5.4481E-01	2.0316E-01	TRUE	FALSE	FALSE	FALSE	1	RAIN only	FALSE
ENSMUSG00000026315	Serpinb8	1.0918E-01	1.0000E+00	6.3851E-01	2.2193E-01	TRUE	FALSE	FALSE	FALSE	1	RAIN only	FALSE
ENSMUSG00000053617	Sh3pxd2a	1.4060E-01	1.0000E+00	7.2082E-01	2.5377E-01	TRUE	FALSE	FALSE	FALSE	1	RAIN only	FALSE
ENSMUSG00000042626	Shc1	1.4178E-01	1.0000E+00	5.0297E-01	1.8836E-01	TRUE	FALSE	FALSE	FALSE	1	RAIN only	FALSE
ENSMUSG00000001995	Sipa1l2	1.0026E-01	1.0000E+00	5.8654E-01	1.8992E-01	TRUE	FALSE	FALSE	FALSE	1	RAIN only	FALSE
ENSMUSG00000026548	Slamf9	1.4440E-01	9.9933E-01	5.0624E-01	1.6330E-01	TRUE	FALSE	FALSE	FALSE	1	RAIN only	FALSE
ENSMUSG00000019935	Slc17a8	4.9770E-02	9.9933E-01	6.0855E-01	2.0412E-01	TRUE	FALSE	FALSE	FALSE	1	RAIN only	FALSE
ENSMUSG00000022003	Slc25a30	1.4060E-01	1.0000E+00	7.1176E-01	2.5120E-01	TRUE	FALSE	FALSE	FALSE	1	RAIN only	FALSE
ENSMUSG00000044348	Slc25a53	1.4060E-01	7.5273E-01	4.7811E-01	1.5766E-01	TRUE	FALSE	FALSE	FALSE	1	RAIN only	FALSE
ENSMUSG00000027661	Slc2a10	1.4060E-01	1.0000E+00	6.2679E-01	1.9664E-01	TRUE	FALSE	FALSE	FALSE	1	RAIN only	FALSE
ENSMUSG00000027822	Slc33a1	1.0026E-01	1.0000E+00	5.8197E-01	1.7163E-01	TRUE	FALSE	FALSE	FALSE	1	RAIN only	FALSE
ENSMUSG00000010122	Slc47a1	1.4060E-01	1.0000E+00	7.1176E-01	2.5523E-01	TRUE	FALSE	FALSE	FALSE	1	RAIN only	FALSE
ENSMUSG00000089774	Slc5a3	1.0411E-01	9.9933E-01	5.8654E-01	2.0810E-01	TRUE	FALSE	FALSE	FALSE	1	RAIN only	FALSE
ENSMUSG00000030108	Slc6a13	1.4060E-01	1.0000E+00	6.0208E-01	1.8407E-01	TRUE	FALSE	FALSE	FALSE	1	RAIN only	FALSE
ENSMUSG00000054404	Slfn5	1.0026E-01	9.9933E-01	5.6807E-01	1.7172E-01	TRUE	FALSE	FALSE	FALSE	1	RAIN only	FALSE
ENSMUSG00000031681	Smad1	1.0918E-01	1.0000E+00	7.9772E-01	4.1355E-01	TRUE	FALSE	FALSE	FALSE	1	RAIN only	FALSE
ENSMUSG00000041133	Smc1a	1.0026E-01	1.0000E+00	5.9022E-01	1.8204E-01	TRUE	FALSE	FALSE	FALSE	1	RAIN only	FALSE
ENSMUSG00000042364	Snx18	1.4060E-01	1.0000E+00	5.2829E-01	1.9344E-01	TRUE	FALSE	FALSE	FALSE	1	RAIN only	FALSE
ENSMUSG00000038916	Soga3	1.0026E-01	9.9933E-01	5.2913E-01	1.6911E-01	TRUE	FALSE	FALSE	FALSE	1	RAIN only	FALSE
ENSMUSG00000057342	Sphk2	1.4178E-01	1.0000E+00	7.6804E-01	3.2287E-01	TRUE	FALSE	FALSE	FALSE	1	RAIN only	FALSE
ENSMUSG00000024970	Spindoc	1.4943E-01	1.0000E+00	4.3828E-01	1.6911E-01	TRUE	FALSE	FALSE	FALSE	1	RAIN only	FALSE
ENSMUSG00000037239	Spred3	1.4178E-01	1.0000E+00	7.1321E-01	2.7954E-01	TRUE	FALSE	FALSE	FALSE	1	RAIN only	FALSE

ENSMUSG00000004865	Srpk1	1.4060E-01	1.0000E+00	5.7929E-01	1.7809E-01	TRUE	FALSE	FALSE	FALSE	1	RAIN only	FALSE
ENSMUSG00000032553	Srprb	1.4060E-01	1.0000E+00	6.6196E-01	2.1631E-01	TRUE	FALSE	FALSE	FALSE	1	RAIN only	FALSE
ENSMUSG00000016128	Stard13	1.0262E-01	1.0000E+00	6.1874E-01	1.9960E-01	TRUE	FALSE	FALSE	FALSE	1	RAIN only	FALSE
ENSMUSG00000018167	Stard3	1.0411E-01	1.0000E+00	6.1248E-01	2.0279E-01	TRUE	FALSE	FALSE	FALSE	1	RAIN only	FALSE
ENSMUSG00000024966	Stip1	7.1344E-02	7.5273E-01	4.9045E-01	1.6911E-01	TRUE	FALSE	FALSE	FALSE	1	RAIN only	FALSE
ENSMUSG00000061455	Stx17	1.0918E-01	1.0000E+00	6.5610E-01	2.0955E-01	TRUE	FALSE	FALSE	FALSE	1	RAIN only	FALSE
ENSMUSG00000036054	Sugp2	1.4060E-01	8.5840E-01	6.1874E-01	2.1331E-01	TRUE	FALSE	FALSE	FALSE	1	RAIN only	FALSE
ENSMUSG00000044912	Syt16	1.0026E-01	1.0000E+00	6.2501E-01	1.9664E-01	TRUE	FALSE	FALSE	FALSE	1	RAIN only	FALSE
ENSMUSG00000072258	Taf1a	1.4060E-01	9.9933E-01	6.0918E-01	1.9558E-01	TRUE	FALSE	FALSE	FALSE	1	RAIN only	FALSE
ENSMUSG00000054321	Taf4b	1.4440E-01	1.0000E+00	5.8482E-01	1.8204E-01	TRUE	FALSE	FALSE	FALSE	1	RAIN only	FALSE
ENSMUSG00000028030	Tbck	1.4060E-01	1.0000E+00	6.0679E-01	1.9664E-01	TRUE	FALSE	FALSE	FALSE	1	RAIN only	FALSE
ENSMUSG00000051224	Tceanc	1.4060E-01	9.1146E-01	5.0297E-01	1.7172E-01	TRUE	FALSE	FALSE	FALSE	1	RAIN only	FALSE
ENSMUSG00000002486	Tchp	1.4060E-01	1.0000E+00	7.1176E-01	2.7501E-01	TRUE	FALSE	FALSE	FALSE	1	RAIN only	FALSE
ENSMUSG00000024170	Telo2	1.0411E-01	1.0000E+00	7.4016E-01	2.6851E-01	TRUE	FALSE	FALSE	FALSE	1	RAIN only	FALSE
ENSMUSG00000017713	Tha1	1.4178E-01	1.0000E+00	5.8654E-01	2.8935E-01	TRUE	FALSE	FALSE	FALSE	1	RAIN only	FALSE
ENSMUSG00000044390	Tigd3	1.4178E-01	1.0000E+00	6.9940E-01	2.6203E-01	TRUE	FALSE	FALSE	FALSE	1	RAIN only	FALSE
ENSMUSG00000031617	Tmem184c	1.4060E-01	1.0000E+00	5.6511E-01	1.9348E-01	TRUE	FALSE	FALSE	FALSE	1	RAIN only	FALSE
ENSMUSG00000010307	Tmem86a	1.0026E-01	1.0000E+00	5.8197E-01	1.7235E-01	TRUE	FALSE	FALSE	FALSE	1	RAIN only	FALSE
ENSMUSG00000032268	Tmprss5	1.0026E-01	9.1146E-01	5.5050E-01	1.6911E-01	TRUE	FALSE	FALSE	FALSE	1	RAIN only	FALSE
ENSMUSG00000039304	Tnfsf10	1.4060E-01	1.0000E+00	5.9789E-01	1.8490E-01	TRUE	FALSE	FALSE	FALSE	1	RAIN only	FALSE
ENSMUSG00000045761	Togaram2	1.4060E-01	1.0000E+00	6.9303E-01	2.3613E-01	TRUE	FALSE	FALSE	FALSE	1	RAIN only	FALSE
ENSMUSG00000015363	Trabd	1.4060E-01	1.0000E+00	6.5830E-01	2.2224E-01	TRUE	FALSE	FALSE	FALSE	1	RAIN only	FALSE
ENSMUSG00000076928	Trac	1.4060E-01	1.0000E+00	6.0855E-01	2.1774E-01	TRUE	FALSE	FALSE	FALSE	1	RAIN only	FALSE
ENSMUSG00000026942	Traf2	7.9869E-02	1.0000E+00	5.8654E-01	1.8204E-01	TRUE	FALSE	FALSE	FALSE	1	RAIN only	FALSE
ENSMUSG00000023992	Trem2	1.4060E-01	1.0000E+00	6.0659E-01	1.9388E-01	TRUE	FALSE	FALSE	FALSE	1	RAIN only	FALSE
ENSMUSG00000050663	Trhde	1.4060E-01	1.0000E+00	6.1771E-01	1.9960E-01	TRUE	FALSE	FALSE	FALSE	1	RAIN only	FALSE
ENSMUSG00000018507	Trpv2	1.4060E-01	9.1146E-01	5.9396E-01	1.7993E-01	TRUE	FALSE	FALSE	FALSE	1	RAIN only	FALSE
ENSMUSG00000072235	Tuba1a	1.0026E-01	9.9933E-01	4.9419E-01	1.5378E-01	TRUE	FALSE	FALSE	FALSE	1	RAIN only	FALSE
ENSMUSG00000039000	Ube3c	7.2888E-02	1.0000E+00	5.5250E-01	1.7172E-01	TRUE	FALSE	FALSE	FALSE	1	RAIN only	FALSE
ENSMUSG00000015290	Ubl4a	1.4060E-01	1.0000E+00	5.3792E-01	1.6940E-01	TRUE	FALSE	FALSE	FALSE	1	RAIN only	FALSE
ENSMUSG00000020923	Ubtf	1.0026E-01	1.0000E+00	4.4673E-01	1.8072E-01	TRUE	FALSE	FALSE	FALSE	1	RAIN only	FALSE
ENSMUSG00000025173	Wdr45b	1.0026E-01	1.0000E+00	5.8197E-01	1.7498E-01	TRUE	FALSE	FALSE	FALSE	1	RAIN only	FALSE
ENSMUSG00000024312	Wdr46	7.1344E-02	1.0000E+00	5.6807E-01	1.8204E-01	TRUE	FALSE	FALSE	FALSE	1	RAIN only	FALSE
ENSMUSG00000021287	Xrcc3	4.9770E-02	5.9891E-01	5.7411E-01	1.9388E-01	TRUE	FALSE	FALSE	FALSE	1	RAIN only	FALSE
ENSMUSG00000027433	Xrn2	1.4060E-01	9.9933E-01	6.3351E-01	1.9664E-01	TRUE	FALSE	FALSE	FALSE	1	RAIN only	FALSE
ENSMUSG00000022792	Yars2	1.4060E-01	9.9933E-01	5.2528E-01	1.5587E-01	TRUE	FALSE	FALSE	FALSE	1	RAIN only	FALSE
ENSMUSG00000071661	Zbtb3	1.4060E-01	1.0000E+00	7.1783E-01	2.5874E-01	TRUE	FALSE	FALSE	FALSE	1	RAIN only	FALSE
ENSMUSG00000029127	Zbtb49	1.4060E-01	1.0000E+00	5.8654E-01	1.7546E-01	TRUE	FALSE	FALSE	FALSE	1	RAIN only	FALSE
ENSMUSG00000039634	Zfp189	7.1344E-02	7.5273E-01	6.5535E-01	2.0620E-01	TRUE	FALSE	FALSE	FALSE	1	RAIN only	FALSE
ENSMUSG00000041483	Zfp281	1.4060E-01	1.0000E+00	7.0257E-01	2.2419E-01	TRUE	FALSE	FALSE	FALSE	1	RAIN only	FALSE
ENSMUSG00000041130	Zfp598	7.1344E-02	9.9933E-01	6.0452E-01	1.9920E-01	TRUE	FALSE	FALSE	FALSE	1	RAIN only	FALSE
ENSMUSG00000059689	Zfp637	1.4060E-01	1.0000E+00	7.3327E-01	2.5128E-01	TRUE	FALSE	FALSE	FALSE	1	RAIN only	FALSE
ENSMUSG00000072653	Zfp783	1.4328E-01	1.0000E+00	6.7352E-01	2.6712E-01	TRUE	FALSE	FALSE	FALSE	1	RAIN only	FALSE

ID	Description	GeneRatio	BgRatio	RichFactor	FoldEnrichm	zScore	pvalue	p.adjust	qvalue	geneID	Count
GO:0071375	cellular response to peptide hormone stimulus	5/22	292/14464	0.01712329	11.2577833	6.91124419	6.4361E-05	0.03520548	0.02770911	Hmgcs2/Rab8a/Rab13/Marcks/Fam114a1	5
GO:0032869	cellular response to insulin stimulus	4/22	212/14464	0.01886792	12.4048027	6.52896988	0.00026674	0.05755167	0.04529709	Hmgcs2/Rab8a/Rab13/Marcks	4
GO:0043434	response to peptide hormone	5/22	410/14464	0.01219512	8.01773836	5.62621066	0.00031564	0.05755167	0.04529709	Hmgcs2/Rab8a/Rab13/Marcks/Fam114a1	5
GO:0032868	response to insulin	4/22	265/14464	0.01509434	9.9238422	5.72232872	0.00062115	0.06881916	0.05416538	Hmgcs2/Rab8a/Rab13/Marcks	4
GO:0046942	carboxylic acid transport	4/22	281/14464	0.01423488	9.35878357	5.52254231	0.00077384	0.06881916	0.05416538	Fabp7/Slc6a13/Slc47a1/Crabp1	4
GO:0015849	organic acid transport	4/22	283/14464	0.01413428	9.29264375	5.49869538	0.00079464	0.06881916	0.05416538	Fabp7/Slc6a13/Slc47a1/Crabp1	4
GO:0015718	monocarboxylic acid transport	3/22	126/14464	0.02380952	15.6536797	6.44784429	0.00088068	0.06881916	0.05416538	Fabp7/Slc6a13/Crabp1	3
GO:0035725	sodium ion transmembrane transport	3/22	137/14464	0.02189781	14.3968149	6.14909315	0.00112204	0.07671952	0.0603835	Slc6a13/Slc9b2/Fxyd3	3
GO:0098876	vesicle-mediated transport to the plasma membrane	3/22	154/14464	0.01948052	12.8075561	5.74946486	0.0015711	0.08677652	0.06829904	Rab8a/Rab13/Arl3	3
GO:0015711	organic anion transport	4/22	341/14464	0.01173021	7.71207678	4.89550489	0.00158641	0.08677652	0.06829904	Fabp7/Slc6a13/Slc47a1/Crabp1	4
GO:0006814	sodium ion transport	3/22	185/14464	0.01621622	10.6614251	5.16184572	0.0026506	0.12748147	0.10033661	Slc6a13/Slc9b2/Fxyd3	3
GO:0089718	amino acid import across plasma membrane	2/22	52/14464	0.03846154	25.2867133	6.84754909	0.00279667	0.12748147	0.10033661	Slc6a13/Slc47a1	2
GO:0072348	sulfur compound transport	2/22	56/14464	0.03571429	23.4805195	6.57847713	0.00323609	0.13616489	0.10717106	Slc6a13/Slc47a1	2
GO:0006893	Golgi to plasma membrane transport	2/22	62/14464	0.03225806	21.2082111	6.22356422	0.00395182	0.148197	0.11664115	Rab13/Arl3	2
GO:0061512	protein localization to cilium	2/22	65/14464	0.03076923	20.2293706	6.06432469	0.00433483	0.148197	0.11664115	Rab8a/Arl3	2
GO:0140115	export across plasma membrane	2/22	65/14464	0.03076923	20.2293706	6.06432469	0.00433483	0.148197	0.11664115	Slc47a1/Fxyd3	2
GO:0007626	locomotory behavior	3/22	240/14464	0.0125	8.21818182	4.40098035	0.00550308	0.1770698	0.13936602	Npas2/Pbx3/Hexb	3
GO:0032456	endocytic recycling	2/22	86/14464	0.02325581	15.2896406	5.18738109	0.00747178	0.18634746	0.14666817	Rab8a/Rab13	2
GO:0015908	fatty acid transport	2/22	87/14464	0.02298851	15.1138976	5.15346481	0.00764057	0.18634746	0.14666817	Fabp7/Crabp1	2
GO:0048193	Golgi vesicle transport	3/22	284/14464	0.01056338	6.94494238	3.94907669	0.00874989	0.18634746	0.14666817	Rab8a/Rab13/Arl3	3
GO:0003333	amino acid transmembrane transport	2/22	96/14464	0.02083333	13.6969697	4.87151903	0.00923674	0.18634746	0.14666817	Slc6a13/Slc47a1	2
GO:0006892	post-Golgi vesicle-mediated transport	2/22	103/14464	0.01941748	12.7661077	4.67720013	0.01057243	0.18634746	0.14666817	Rab13/Arl3	2
GO:0033555	multicellular organismal response to stress	2/22	103/14464	0.01941748	12.7661077	4.67720013	0.01057243	0.18634746	0.14666817	Hmgcs2/Npas2	2
GO:0006855	xenobiotic transmembrane transport	1/22	10/14464	0.1	65.7454545	7.99359466	0.01511116	0.18634746	0.14666817	Slc47a1	1
GO:0009313	oligosaccharide catabolic process	1/22	10/14464	0.1	65.7454545	7.99359466	0.01511116	0.18634746	0.14666817	Hexb	1
GO:0046618	xenobiotic export from cell	1/22	10/14464	0.1	65.7454545	7.99359466	0.01511116	0.18634746	0.14666817	Slc47a1	1
GO:0051775	response to redox state	1/22	10/14464	0.1	65.7454545	7.99359466	0.01511116	0.18634746	0.14666817	Npas2	1
GO:0060179	male mating behavior	1/22	10/14464	0.1	65.7454545	7.99359466	0.01511116	0.18634746	0.14666817	Hexb	1
GO:0099639	neurotransmitter receptor transport, endosome to plasma membrane	1/22	10/14464	0.1	65.7454545	7.99359466	0.01511116	0.18634746	0.14666817	Rab8a	1
GO:1903278	positive regulation of sodium ion export across plasma membrane	1/22	10/14464	0.1	65.7454545	7.99359466	0.01511116	0.18634746	0.14666817	Fxyd3	1
GO:1903365	regulation of fear response	1/22	10/14464	0.1	65.7454545	7.99359466	0.01511116	0.18634746	0.14666817	Npas2	1
GO:0016114	terpenoid biosynthetic process	1/22	11/14464	0.09090909	59.768595	7.61008509	0.01661023	0.18634746	0.14666817	Hmgcs2	1
GO:0043652	engulfment of apoptotic cell	1/22	11/14464	0.09090909	59.768595	7.61008509	0.01661023	0.18634746	0.14666817	Megf10	1
GO:0045117	azole transmembrane transport	1/22	11/14464	0.09090909	59.768595	7.61008509	0.01661023	0.18634746	0.14666817	Slc47a1	1
GO:0050655	dermatan sulfate proteoglycan metabolic process	1/22	11/14464	0.09090909	59.768595	7.61008509	0.01661023	0.18634746	0.14666817	Hexb	1
GO:0072531	pyrimidine-containing compound transmembrane transport	1/22	11/14464	0.09090909	59.768595	7.61008509	0.01661023	0.18634746	0.14666817	Slc47a1	1
GO:1903276	regulation of sodium ion export across plasma membrane	1/22	11/14464	0.09090909	59.768595	7.61008509	0.01661023	0.18634746	0.14666817	Fxyd3	1
GO:1903441	protein localization to ciliary membrane	1/22	11/14464	0.09090909	59.768595	7.61008509	0.01661023	0.18634746	0.14666817	Arl3	1
GO:1905039	carboxylic acid transmembrane transport	2/22	133/14464	0.01503759	9.88653452	4.01834173	0.01718847	0.18634746	0.14666817	Slc6a13/Slc47a1	2
GO:1903825	organic acid transmembrane transport	2/22	136/14464	0.01470588	9.6684492	3.96410308	0.01792641	0.18634746	0.14666817	Slc6a13/Slc47a1	2
GO:0002087	regulation of respiratory gaseous exchange by nervous system process	1/22	12/14464	0.08333333	54.7878788	7.27508258	0.01810712	0.18634746	0.14666817	Pbx3	1
GO:0046479	glycosphingolipid catabolic process	1/22	12/14464	0.08333333	54.7878788	7.27508258	0.01810712	0.18634746	0.14666817	Hexb	1
GO:1902600	proton transmembrane transport	2/22	137/14464	0.01459854	9.59787658	3.94639655	0.01817536	0.18634746	0.14666817	Slc47a1/Slc9b2	2
GO:1990778	protein localization to cell periphery	3/22	378/14464	0.00793651	5.21789322	3.24320575	0.01886336	0.18634746	0.14666817	Rab8a/Rab13/Arl3	3
GO:0014816	skeletal muscle satellite cell differentiation	1/22	13/14464	0.07692308	50.5734266	6.97908564	0.01960184	0.18634746	0.14666817	Megf10	1
GO:0015846	polyamine transport	1/22	13/14464	0.07692308	50.5734266	6.97908564	0.01960184	0.18634746	0.14666817	Slc47a1	1
GO:0019377	glycolipid catabolic process	1/22	13/14464	0.07692308	50.5734266	6.97908564	0.01960184	0.18634746	0.14666817	Hexb	1
GO:0030214	hyaluronan catabolic process	1/22	13/14464	0.07692308	50.5734266	6.97908564	0.01960184	0.18634746	0.14666817	Hexb	1
GO:0051764	actin crosslink formation	1/22	13/14464	0.07692308	50.5734266	6.97908564	0.01960184	0.18634746	0.14666817	Marcks	1
GO:0097709	connective tissue replacement	1/22	13/14464	0.07692308	50.5734266	6.97908564	0.01960184	0.18634746	0.14666817	Fam114a1	1
GO:0010876	lipid localization	3/22	393/14464	0.00763359	5.01873699	3.15246476	0.0208963	0.18634746	0.14666817	Fabp7/Crabp1/Hexb	3
GO:0051451	myoblast migration	1/22	14/14464	0.07142857	46.961039	6.71501203	0.02109438	0.18634746	0.14666817	Megf10	1
GO:1903826	L-arginine transmembrane transport	1/22	14/14464	0.07142857	46.961039	6.71501203	0.02109438	0.18634746	0.14666817	Slc47a1	1
GO:1905274	regulation of modification of postsynaptic actin cytoskeleton	1/22	14/14464	0.07142857	46.961039	6.71501203	0.02109438	0.18634746	0.14666817	Marcks	1
GO:0002486	antigen processing and presentation of endogenous peptide antigen via MHC class I via ER pathway, T.	1/22	15/14464	0.06666667	43.830303	6.47746007	0.02258476	0.18634746	0.14666817	Mill2	1
GO:0006044	N-acetylglucosamine metabolic process	1/22	15/14464	0.06666667	43.830303	6.47746007	0.02258476	0.18634746	0.14666817	Hexb	1
GO:0006491	N-glycan processing	1/22	15/14464	0.06666667	43.830303	6.47746007	0.02258476	0.18634746	0.14666817	Hexb	1
GO:0044065	regulation of respiratory system process	1/22	15/14464	0.06666667	43.830303	6.47746007	0.02258476	0.18634746	0.14666817	Pbx3	1
GO:1990822	basic amino acid transmembrane transport	1/22	15/14464	0.06666667	43.830303	6.47746007	0.02258476	0.18634746	0.14666817	Slc47a1	1
GO:2000288	positive regulation of myoblast proliferation	1/22	15/14464	0.06666667	43.830303	6.47746007	0.02258476	0.18634746	0.14666817	Megf10	1
GO:0006865	amino acid transport	2/22	158/14464	0.01265823	8.32220944	3.61192267	0.02373829	0.18634746	0.14666817	Slc6a13/Slc47a1	2
GO:0002476	antigen processing and presentation of endogenous peptide antigen via MHC class Ib	1/22	16/14464	0.0625	41.0909091	6.26222824	0.02407297	0.18634746	0.14666817	Mill2	1
GO:0002484	antigen processing and presentation of endogenous peptide antigen via MHC class I via ER pathway	1/22	16/14464	0.0625	41.0909091	6.26222824	0.02407297	0.18634746	0.14666817	Mill2	1
GO:0035461	vitamin transmembrane transport	1/22	16/14464	0.0625	41.0909091	6.26222824	0.02407297	0.18634746	0.14666817	Slc47a1	1
GO:0036035	osteoclast development	1/22	16/14464	0.0625	41.0909091	6.26222824	0.02407297	0.18634746	0.14666817	Slc9b2	1
GO:0046514	ceramide catabolic process	1/22	16/14464	0.0625	41.0909091	6.26222824	0.02407297	0.18634746	0.14666817	Hexb	1
GO:0060134	prepulse inhibition	1/22	16/14464	0.0625	41.0909091	6.26222824	0.02407297	0.18634746	0.14666817	Fabp7	1
GO:0098751	bone cell development	1/22	16/14464	0.0625	41.0909091	6.26222824	0.02407297	0.18634746	0.14666817	Slc9b2	1
GO:0021915	neural tube development	2/22	162/14464	0.01234568	8.11672278	3.55521634	0.02486842	0.18634746	0.14666817	Inka1/Marcks	2
GO:0002428	antigen processing and presentation of peptide antigen via MHC class Ib	1/22	17/14464	0.05882353	38.6737968	6.06599263	0.02555901	0.18634746	0.14666817	Mill2	1
GO:0036376	sodium ion export across plasma membrane	1/22	17/14464	0.05882353	38.6737968	6.06599263	0.02555901	0.18634746	0.14666817	Fxyd3	1
GO:0098739	import across plasma membrane	2/22	167/14464	0.01197605	7.87370713	3.48701379	0.02631181	0.18634746	0.14666817	Slc6a13/Slc47a1	2
GO:0015802	basic amino acid transport	1/22	18/14464	0.05555556	36.5252525	5.88608439	0.0270429	0.18634746	0.14666817	Slc47a1	1
GO:0014841	skeletal muscle satellite cell proliferation	1/22	19/14464	0.05263158	34.6028708	5.72033249	0.02852463	0.18634746	0.14666817	Megf10	1
GO:0060009	Sertoli cell development	1/22	19/14464	0.05263158	34.6028708	5.72033249	0.02852463	0.18634746	0.14666817	Rab13	1
GO:0002475	antigen processing and presentation via MHC class Ib	1/22	20/14464	0.05	32.8727273	5.56695035	0.0300042	0.18634746	0.14666817	Mill2	1
GO:0002483	antigen processing and presentation of endogenous peptide antigen	1/22	20/14464	0.05	32.8727273	5.56695035	0.0300042	0.18634746	0.14666817	Mill2	1
GO:0006027	glycosaminoglycan catabolic process	1/22	20/14464	0.05	32.8727273	5.56695035	0.0300042	0.18634746	0.14666817	Hexb	1
GO:0019885	antigen processing and presentation of endogenous peptide antigen via MHC class I	1/22	20/14464	0.05	32.8727273	5.56695035	0.0300042	0.18634746	0.14666817	Mill2	1
GO:0033762	response to glucagon	1/22	20/14464	0.05	32.8727273	5.56695035	0.0300042	0.18634746	0.14666817	Hmgcs2	1
GO:0038166	angiotensin-activated signaling pathway	1/22	20/14464	0.05	32.8727273	5.56695035	0.0300042	0.18634746	0.14666817	Fam114a1	1
GO:0099638	endosome to plasma membrane protein transport	1/22	20/14464	0.05	32.8727273	5.56695035	0.0300042	0.18634746	0.14666817	Rab8a	1
GO:2000291	regulation of myoblast proliferation	1/22	20/14464	0.05	32.8727273	5.56695035	0.0300042	0.18634746	0.14666817	Megf10	1
GO:0043576	regulation of respiratory gaseous exchange	1/22	21/14464	0.04761905	31.3073593	5.4244524	0.03148163	0.18634746	0.14666817	Pbx3	1
GO:0045475	locomotor rhythm	1/22	21/14464	0.04761905	31.3073593	5.4244524	0.03148163	0.18634746	0.14666817	Npas2	1
GO:0072537	fibroblast activation	1/22	21/14464	0.04761905	31.3073593	5.4244524	0.03148163	0.18634746	0.14666817	Fam114a1	1
GO:0098969	neurotransmitter receptor transport to postsynaptic membrane	1/22	21/14464	0.04761905	31.3073593	5.4244524	0.03148163	0.18634746	0.14666817	Rab8a	1
GO:1901570	fatty acid derivative biosynthetic process	1/22	21/14464	0.04761905	31.3073593	5.4244524	0.03148163	0.18634746	0.14666817	Hmgcs2	1
GO:0050905	neuromuscular process	2/22	188/14464	0.0106383	6.99419729	3.22874355	0.03273501	0.18634746	0.14666817	Fabp7/Hexb	2
GO:0006910	phagocytosis, recognition	1/22	22/14464	0.04545455	29.8842975	5.2915919	0.0329569	0.18634746	0.14666817	Megf10	1
GO:0019883	antigen processing and presentation of endogenous antigen	1/22	22/14464	0.04545455	29.8842975	5.2915919	0.0329569	0.18634746	0.14666817	Mill2	1
GO:1903540	establishment of protein localization to postsynaptic membrane	1/22	22/14464	0.04545455	29.8842975	5.2915919	0.0329569	0.18634746	0.14666817	Rab8a	1
GO:0010737	protein kinase A signaling	1/22	23/14464	0.04347826	28.5849802	5.16731366	0.03443003	0.18634746	0.14666817	Rab13	1
GO:1901071	glucosamine-containing compound metabolic process	1/22	23/14464	0.04347826	28.5849802	5.16731366	0.03443003	0.18634746	0.14666817	Hexb	1
GO:1902307	positive regulation of sodium ion transmembrane transport	1/22	23/14464	0.04347826	28.5849802	5.16731366	0.03443003	0.18634746	0.14666817	Fxyd3	1
GO:0001573	ganglioside metabolic process	1/22	24/14464	0.04166667	27.3939394	5.05071779	0.03590102	0.18634746	0.14666817	Hexb	1
GO:0008299	isoprenoid biosynthetic process	1/22	24/14464	0.04166667	27.3939394	5.05071779	0.03590102	0.18634746	0.14666817	Hmgcs2	1
GO:0015812	gamma-aminobutyric acid transport	1/22	24/14464	0.04166667	27.3939394	5.05071779	0.03590102	0.18634746	0.14666817	Slc6a13	1
GO:0008654	phospholipid biosynthetic process	2/22	201/14464	0.00995025	6.54183627	3.08797049	0.03699128	0.18634746	0.14666817	Hmgcs2/Hexb	2
GO:0030204	chondroitin sulfate metabolic process	1/22	25/14464	0.04	26.2981818	4.94103146	0.03736987	0.18634746	0.14666817	Hexb	1
GO:0048641	regulation of skeletal muscle tissue development	1/22	25/14464	0.04	26.2981818	4.94103146	0.03736987	0.18634746	0.14666817	Megf10	1
GO:0055090	acylglycerol homeostasis	1/22	25/14464	0.04	26.2981818	4.94103146	0.03736987	0.18634746	0.14666817	Angptl4	1
GO:0060008	Sertoli cell differentiation	1/22	25/14464	0.04	26.2981818	4.94103146	0.03736987	0.18634746	0.14666817	Rab13	1
GO:0070328	triglyceride homeostasis	1/22	25/14464	0.04	26.2981818	4.94103146	0.03736987	0.18634746	0.14666817	Angptl4	1
GO:0098877	neurotransmitter receptor transport to plasma membrane	1/22	25/14464	0.04	26.2981818	4.94103146	0.03736987	0.18634746	0.14666817	Rab8a	1
GO:0099159	regulation of modification of postsynaptic structure	1/22	25/14464	0.04	26.2981818	4.94103146	0.03736987	0.18634746	0.14666817	Marcks	1
GO:0006026	aminoglycan catabolic process	1/22	26/14464	0.03846154	25.2867133	4.83758673	0.03883658	0.18634746	0.14666817	Hexb	1
GO:0014856	skeletal muscle cell proliferation	1/22	26/14464	0.03846154	25.2867133	4.83758673	0.03883658	0.18634746	0.14666817	Megf10	1
GO:0021846	cell proliferation in forebrain	1/22	26/14464	0.03846154	25.2867133	4.83758673	0.03883658	0.18634746	0.14666817	Fabp7	1
GO:0030149	sphingolipid catabolic process	1/22	26/14464	0.03846154	25.2867133	4.83758673	0.03883658	0.18634746	0.14666817	Hexb	1
GO:0034694	response to prostaglandin	1/22	26/14464	0.03846154	25.2867133	4.83758673	0.03883658	0.18634746	0.14666817	Hmgcs2	1
GO:0046628	positive regulation of insulin receptor signaling pathway	1/22	26/14464	0.03846154	25.2867133	4.83758673	0.03883658	0.18634746	0.14666817	Marcks	1
GO:0060416	response to growth hormone	1/22	26/14464	0.03846154	25.2867133	4.83758673	0.03883658	0.18634746	0.14666817	Hmgcs2	1
GO:0098885	modification of postsynaptic actin cytoskeleton	1/22	26/14464	0.03846154	25.2867133	4.83758673	0.03883658	0.18634746	0.14666817	Marcks	1
GO:0048520	positive regulation of behavior	1/22	27/14464	0.03703704	24.3501684	4.73980291	0.04030116	0.18889745	0.14867518	Npas2	1
GO:2000352	negative regulation of endothelial cell apoptotic process	1/22	27/14464	0.03703704	24.3501684	4.73980291	0.04030116	0.18889745	0.14867518	Angptl4	1
GO:0034284	response to monosaccharide	2/22	211/14464	0.00947867	6.23179664	2.98789824	0.04040403	0.18889745	0.14867518	Hmgcs2/Slc9b2	2
GO:0001916	positive regulation of T cell mediated cytotoxicity	1/22	28/14464	0.03571429	23.4805195	4.64717241	0.04176361	0.19037246	0.14983611	Mill2	1
GO:0035767	endothelial cell chemotaxis	1/22	28/14464	0.03571429	23.4805195	4.64717241	0.04176361	0.19037246	0.14983611	Rab13	1
GO:0055078	sodium ion homeostasis	1/22	28/14464	0.03571429	23.4805195	4.64717241	0.04176361	0.19037246	0.14983611	Slc9b2	1
GO:0042594	response to starvation	2/22	216/14464	0.00925926	6.08754209	2.94025367	0.04215421	0.19056492	0.14998759	Angptl4/Hmgcs2	2
GO:0046466	membrane lipid catabolic process	1/22	29/14464	0.03448276	22.6708464	4.55924932	0.04322393	0.19119392	0.15048266	Hexb	1
GO:0051450	myoblast proliferation	1/22	29/14464	0.03448276	22.6708464	4.55924932	0.04322393	0.19119392	0.15048266	Megf10	1
GO:0042745	circadian sleep/wake cycle	1/22	30/14464	0.03333333	21.9151515	4.47564009	0.04468213	0.19119392	0.15048266	Npas2	1
GO:0045672	positive regulation of osteoclast differentiation	1/22	30/14464	0.03333333	21.9151515	4.47564009	0.04468213	0.19119392	0.15048266	Slc9b2	1
GO:0060191	regulation of lipase activity	1/22	30/14464	0.03333333	21.9151515	4.47564009	0.04468213	0.19119392	0.15048266	Angptl4	1
GO:0030212	hyaluronan metabolic process	1/22	31/14464	0.03225806	21.2082111	4.39599586	0.0461382	0.19119392	0.15048266	Hexb	1
GO:0050654	chondroitin sulfate proteoglycan metabolic process	1/22	31/14464	0.03225806	21.2082111	4.39599586	0.0461382	0.19119392	0.15048266	Hexb	1
GO:0061003	positive regulation of dendritic spine morphogenesis	1/22	31/14464	0.03225806	21.2082111	4.39599586	0.0461382	0.19119392	0.15048266	Marcks	1
GO:0072337	modified amino acid transport	1/22	31/14464	0.03225806	21.2082111	4.39599586	0.0461382	0.19119392	0.15048266	Slc6a13	1
GO:1900078	positive regulation of cellular response to insulin stimulus	1/22	31/14464	0.03225806	21.2082111	4.39599586	0.0461382	0.19119392	0.15048266	Marcks	1
GO:1905244	regulation of modification of synaptic structure	1/22	31/14464	0.03225806	21.2082111	4.39599586	0.0461382	0.19119392	0.15048266	Marcks	1
GO:0001964	startle response	1/22	32/14464	0.03125	20.5454545	4.32000617	0.04759216	0.19427547	0.15290804	Fabp7	1
GO:0051180	vitamin transport	1/22	32/14464	0.03125	20.5454545	4.32000617	0.04759216	0.19427547	0.15290804	Slc47a1	1
GO:0009743	response to carbohydrate	2/22	234/14464	0.00854701	5.61926962	2.78038959	0.04868777	0.19439905	0.15300532	Hmgcs2/Slc9b2	2
GO:0002474	antigen processing and presentation of peptide antigen via MHC class I	1/22	33/14464	0.03030303	19.922865	4.24739363	0.049044	0.19439905	0.15300532	Mill2	1
GO:0043330	response to exogenous dsRNA	1/22	33/14464	0.03030303	19.922865	4.24739363	0.049044	0.19439905	0.15300532	Rftn2	1
GO:1904385	cellular response to angiotensin	1/22	33/14464	0.03030303	19.922865	4.24739363	0.049044	0.19439905	0.15300532	Fam114a1	1
GO:0001914	regulation of T cell mediated cytotoxicity	1/22	34/14464	0.02941176	19.3368984	4.17790956	0.05049373	0.19588703	0.15417645	Mill2	1
GO:0009311	oligosaccharide metabolic process	1/22	34/14464	0.02941176	19.3368984	4.17790956	0.05049373	0.19588703	0.15417645	Hexb	1
GO:0071398	cellular response to fatty acid	1/22	34/14464	0.02941176	19.3368984	4.17790956	0.05049373	0.19588703	0.15417645	Hmgcs2	1
GO:0007617	mating behavior	1/22	35/14464	0.02857143	18.7844156	4.11133028	0.05194135	0.19868474	0.15637845	Hexb	1
GO:0033574	response to testosterone	1/22	35/14464	0.02857143	18.7844156	4.11133028	0.05194135	0.19868474	0.15637845	Hmgcs2	1
GO:0042908	xenobiotic transport	1/22	36/14464	0.02777778	18.2626263	4.04745394	0.05338686	0.2012151	0.15837001	Slc47a1	1
GO:1902742	apoptotic process involved in development	1/22	36/14464	0.02777778	18.2626263	4.04745394	0.05338686	0.2012151	0.15837001	Megf10	1
GO:0006084	acetyl-CoA metabolic process	1/22	37/14464	0.02702703	17.7690418	3.98609785	0.05483027	0.2012151	0.15837001	Hmgcs2	1
GO:0043277	apoptotic cell clearance	1/22	37/14464	0.02702703	17.7690418	3.98609785	0.05483027	0.2012151	0.15837001	Megf10	1
GO:0048169	regulation of long-term neuronal synaptic plasticity	1/22	37/14464	0.02702703	17.7690418	3.98609785	0.05483027	0.2012151	0.15837001	Rab8a	1
GO:0099637	neurotransmitter receptor transport	1/22	37/14464	0.02702703	17.7690418	3.98609785	0.05483027	0.2012151	0.15837001	Rab8a	1
GO:0031503	protein-containing complex localization	2/22	251/14464	0.00796813	5.23868164	2.6439403	0.05517781	0.2012151	0.15837001	Rab8a/Arl3	2
GO:0044273	sulfur compound catabolic process	1/22	38/14464	0.02631579	17.3014354	3.92709624	0.05627158	0.20384472	0.1604397	Hexb	1
GO:0006904	vesicle docking involved in exocytosis	1/22	39/14464	0.02564103	16.8578089	3.87029826	0.05771079	0.20632549	0.16239223	Rab8a	1
GO:0042461	photoreceptor cell development	1/22	39/14464	0.02564103	16.8578089	3.87029826	0.05771079	0.20632549	0.16239223	Arl3	1
GO:0010765	positive regulation of sodium ion transport	1/22	41/14464	0.02439024	16.0354767	3.7627745	0.06058292	0.21107552	0.16613084	Fxyd3	1
GO:0030866	cortical actin cytoskeleton organization	1/22	41/14464	0.02439024	16.0354767	3.7627745	0.06058292	0.21107552	0.16613084	Rab13	1
GO:0043331	response to dsRNA	1/22	41/14464	0.02439024	16.0354767	3.7627745	0.06058292	0.21107552	0.16613084	Rftn2	1
GO:1990776	response to angiotensin	1/22	41/14464	0.02439024	16.0354767	3.7627745	0.06058292	0.21107552	0.16613084	Fam114a1	1
GO:0022604	regulation of cell morphogenesis	2/22	268/14464	0.00746269	4.9063772	2.51933223	0.06195953	0.21335013	0.16792111	Marcks/Hexb	2
GO:0006040	amino sugar metabolic process	1/22	42/14464	0.02380952	15.6536797	3.71180759	0.06201585	0.21335013	0.16792111	Hexb	1
GO:0016202	regulation of striated muscle tissue development	1/22	43/14464	0.02325581	15.2896406	3.66255966	0.0634467	0.21587661	0.16990962	Megf10	1
GO:0001662	behavioral fear response	1/22	44/14464	0.02272727	14.9421488	3.61493326	0.06487546	0.21587661	0.16990962	Npas2	1
GO:0001913	T cell mediated cytotoxicity	1/22	44/14464	0.02272727	14.9421488	3.61493326	0.06487546	0.21587661	0.16990962	Mill2	1
GO:1901861	regulation of muscle tissue development	1/22	44/14464	0.02272727	14.9421488	3.61493326	0.06487546	0.21587661	0.16990962	Megf10	1
GO:0016197	endosomal transport	2/22	276/14464	0.00724638	4.76416337	2.46427561	0.0652468	0.21587661	0.16990962	Rab8a/Rab13	2
GO:0002209	behavioral defense response	1/22	45/14464	0.02222222	14.610101	3.56883852	0.06630214	0.21587661	0.16990962	Npas2	1

GO:0003016	respiratory system process	1/22	45/14464	0.02222222	14.610101	3.56883852	0.06630214	0.21587661	0.16990962	Pbx3	1
GO:0048634	regulation of muscle organ development	1/22	45/14464	0.02222222	14.610101	3.56883852	0.06630214	0.21587661	0.16990962	Megf10	1
GO:0099010	modification of postsynaptic structure	1/22	45/14464	0.02222222	14.610101	3.56883852	0.06630214	0.21587661	0.16990962	Marcks	1
GO:0001912	positive regulation of leukocyte mediated cytotoxicity	1/22	46/14464	0.02173913	14.2924901	3.52419243	0.06772674	0.21664637	0.17051547	Mill2	1
GO:1901568	fatty acid derivative metabolic process	1/22	46/14464	0.02173913	14.2924901	3.52419243	0.06772674	0.21664637	0.17051547	Hmgcs2	1
GO:2000351	regulation of endothelial cell apoptotic process	1/22	46/14464	0.02173913	14.2924901	3.52419243	0.06772674	0.21664637	0.17051547	Angptl4	1
GO:0006790	sulfur compound metabolic process	2/22	285/14464	0.00701754	4.61371611	2.40480864	0.06901528	0.21738305	0.17109529	Hmgcs2/Hexb	2
GO:0019098	reproductive behavior	1/22	47/14464	0.0212766	13.9883946	3.48091819	0.06914927	0.21738305	0.17109529	Hexb	1
GO:0042073	intraciliary transport	1/22	47/14464	0.0212766	13.9883946	3.48091819	0.06914927	0.21738305	0.17109529	Arl3	1
GO:0006911	phagocytosis, engulfment	1/22	48/14464	0.02083333	13.6969697	3.43894459	0.07056973	0.2187886	0.17220155	Megf10	1
GO:0072583	clathrin-dependent endocytosis	1/22	48/14464	0.02083333	13.6969697	3.43894459	0.07056973	0.2187886	0.17220155	Slc9b2	1
GO:0003179	heart valve morphogenesis	1/22	49/14464	0.02040816	13.4174397	3.39820551	0.07198811	0.2187886	0.17220155	Fam114a1	1
GO:0006687	glycosphingolipid metabolic process	1/22	49/14464	0.02040816	13.4174397	3.39820551	0.07198811	0.2187886	0.17220155	Hexb	1
GO:0006721	terpenoid metabolic process	1/22	49/14464	0.02040816	13.4174397	3.39820551	0.07198811	0.2187886	0.17220155	Hmgcs2	1
GO:0072659	protein localization to plasma membrane	2/22	292/14464	0.00684932	4.50311333	2.36024401	0.07199625	0.2187886	0.17220155	Rab8a/Rab13	2
GO:0042596	fear response	1/22	50/14464	0.02	13.1490909	3.35863948	0.07340443	0.22061663	0.17364033	Npas2	1
GO:0072577	endothelial cell apoptotic process	1/22	50/14464	0.02	13.1490909	3.35863948	0.07340443	0.22061663	0.17364033	Angptl4	1
GO:0002711	positive regulation of T cell mediated immunity	1/22	51/14464	0.01960784	12.8912656	3.32018924	0.07481869	0.22122067	0.17411576	Mill2	1
GO:0006695	cholesterol biosynthetic process	1/22	51/14464	0.01960784	12.8912656	3.32018924	0.07481869	0.22122067	0.17411576	Hmgcs2	1
GO:1902653	secondary alcohol biosynthetic process	1/22	51/14464	0.01960784	12.8912656	3.32018924	0.07481869	0.22122067	0.17411576	Hmgcs2	1
GO:0031343	positive regulation of cell killing	1/22	52/14464	0.01923077	12.6433566	3.28280139	0.07623089	0.22298554	0.17550483	Mill2	1
GO:1902305	regulation of sodium ion transmembrane transport	1/22	52/14464	0.01923077	12.6433566	3.28280139	0.07623089	0.22298554	0.17550483	Fxyd3	1
GO:0001822	kidney development	2/22	303/14464	0.00660066	4.33963396	2.29297904	0.07676589	0.22335608	0.17579647	Hmgcs2/Arl3	2
GO:0048002	antigen processing and presentation of peptide antigen	1/22	53/14464	0.01886792	12.4048027	3.24642607	0.07764103	0.22352443	0.17592898	Mill2	1
GO:0099563	modification of synaptic structure	1/22	53/14464	0.01886792	12.4048027	3.24642607	0.07764103	0.22352443	0.17592898	Marcks	1
GO:0048512	circadian behavior	1/22	54/14464	0.01851852	12.1750842	3.21101664	0.07904911	0.22638672	0.17818179	Npas2	1
GO:0007622	rhythmic behavior	1/22	55/14464	0.01818182	11.953719	3.17652943	0.08045514	0.22685033	0.17854668	Npas2	1
GO:0030865	cortical cytoskeleton organization	1/22	55/14464	0.01818182	11.953719	3.17652943	0.08045514	0.22685033	0.17854668	Rab13	1
GO:0070542	response to fatty acid	1/22	55/14464	0.01818182	11.953719	3.17652943	0.08045514	0.22685033	0.17854668	Hmgcs2	1
GO:0016126	sterol biosynthetic process	1/22	56/14464	0.01785714	11.7402597	3.14292351	0.08185913	0.22791699	0.17938622	Hmgcs2	1
GO:0070830	bicellular tight junction assembly	1/22	56/14464	0.01785714	11.7402597	3.14292351	0.08185913	0.22791699	0.17938622	Rab13	1
GO:0072001	renal system development	2/22	315/14464	0.00634921	4.17431457	2.22315237	0.08208345	0.22791699	0.17938622	Hmgcs2/Arl3	2
GO:0048278	vesicle docking	1/22	57/14464	0.01754386	11.5342903	3.11016045	0.08326106	0.22886332	0.18013105	Rab8a	1
GO:0099024	plasma membrane invagination	1/22	57/14464	0.01754386	11.5342903	3.11016045	0.08326106	0.22886332	0.18013105	Megf10	1
GO:0006644	phospholipid metabolic process	2/22	319/14464	0.00626959	4.12197207	2.20064375	0.08388154	0.22941603	0.18056606	Hmgcs2/Hexb	2
GO:0046530	photoreceptor cell differentiation	1/22	58/14464	0.01724138	11.3354232	3.07820415	0.08466096	0.23039573	0.18133716	Arl3	1
GO:0002763	positive regulation of myeloid leukocyte differentiation	1/22	59/14464	0.01694915	11.1432974	3.04702064	0.08605881	0.23189245	0.18251518	Slc9b2	1
GO:0003170	heart valve development	1/22	59/14464	0.01694915	11.1432974	3.04702064	0.08605881	0.23189245	0.18251518	Fam114a1	1
GO:0071385	cellular response to glucocorticoid stimulus	1/22	60/14464	0.01666667	10.9575758	3.01657794	0.08745462	0.23449842	0.18456626	Hmgcs2	1
GO:0061001	regulation of dendritic spine morphogenesis	1/22	61/14464	0.01639344	10.7779434	2.98684591	0.0888484	0.2359227	0.18568726	Marcks	1
GO:0120192	tight junction assembly	1/22	61/14464	0.01639344	10.7779434	2.98684591	0.0888484	0.2359227	0.18568726	Rab13	1
GO:0007585	respiratory gaseous exchange by respiratory system	1/22	63/14464	0.01587302	10.4357864	2.92940161	0.09162987	0.2380989	0.18740008	Pbx3	1
GO:0050775	positive regulation of dendrite morphogenesis	1/22	63/14464	0.01587302	10.4357864	2.92940161	0.09162987	0.2380989	0.18740008	Marcks	1
GO:0010770	positive regulation of cell morphogenesis	1/22	64/14464	0.015625	10.2727273	2.90163707	0.09301756	0.2380989	0.18740008	Marcks	1
GO:0050885	neuromuscular process controlling balance	1/22	64/14464	0.015625	10.2727273	2.90163707	0.09301756	0.2380989	0.18740008	Hexb	1
GO:0060271	cilium assembly	2/22	339/14464	0.00589971	3.87878788	2.09335146	0.09305476	0.2380989	0.18740008	Rab8a/Arl3	2
GO:0001910	regulation of leukocyte mediated cytotoxicity	1/22	65/14464	0.01538462	10.1146853	2.87447844	0.09440323	0.2380989	0.18740008	Mill2	1
GO:0010324	membrane invagination	1/22	65/14464	0.01538462	10.1146853	2.87447844	0.09440323	0.2380989	0.18740008	Megf10	1
GO:0032922	circadian regulation of gene expression	1/22	65/14464	0.01538462	10.1146853	2.87447844	0.09440323	0.2380989	0.18740008	Npas2	1
GO:0043297	apical junction assembly	1/22	65/14464	0.01538462	10.1146853	2.87447844	0.09440323	0.2380989	0.18740008	Rab13	1
GO:0060999	positive regulation of dendritic spine development	1/22	65/14464	0.01538462	10.1146853	2.87447844	0.09440323	0.2380989	0.18740008	Marcks	1
GO:0006869	lipid transport	2/22	342/14464	0.00584795	3.84476342	2.07796375	0.09445605	0.2380989	0.18740008	Fabp7/Crabp1	2
GO:0071384	cellular response to corticosteroid stimulus	1/22	66/14464	0.01515152	9.96143251	2.84790294	0.09578687	0.23968866	0.18865132	Hmgcs2	1
GO:0060612	adipose tissue development	1/22	67/14464	0.01492537	9.81275441	2.82188902	0.0971685	0.23968866	0.18865132	Hmgcs2	1
GO:0061951	establishment of protein localization to plasma membrane	1/22	67/14464	0.01492537	9.81275441	2.82188902	0.0971685	0.23968866	0.18865132	Rab8a	1
GO:0120193	tight junction organization	1/22	67/14464	0.01492537	9.81275441	2.82188902	0.0971685	0.23968866	0.18865132	Rab13	1
GO:0009410	response to xenobiotic stimulus	2/22	348/14464	0.00574713	3.7784744	2.04770344	0.09727766	0.23968866	0.18865132	Hmgcs2/Npas2	2
GO:0006720	isoprenoid metabolic process	1/22	68/14464	0.01470588	9.6684492	2.7964162	0.09854811	0.24065097	0.18940873	Hmgcs2	1
GO:1902475	L-alpha-amino acid transmembrane transport	1/22	68/14464	0.01470588	9.6684492	2.7964162	0.09854811	0.24065097	0.18940873	Slc47a1	1
GO:0006887	exocytosis	2/22	352/14464	0.00568182	3.73553719	2.0279	0.09917257	0.24079016	0.18951828	Rab8a/Rab13	2
GO:0008347	glial cell migration	1/22	69/14464	0.01449275	9.52832675	2.77146504	0.09992571	0.24079016	0.18951828	Hexb	1
GO:0010761	fibroblast migration	1/22	69/14464	0.01449275	9.52832675	2.77146504	0.09992571	0.24079016	0.18951828	Fam114a1	1
GO:0045670	regulation of osteoclast differentiation	1/22	70/14464	0.01428571	9.39220779	2.7470171	0.10130131	0.24092093	0.18962121	Slc9b2	1
GO:0046626	regulation of insulin receptor signaling pathway	1/22	70/14464	0.01428571	9.39220779	2.7470171	0.10130131	0.24092093	0.18962121	Marcks	1
GO:1903539	protein localization to postsynaptic membrane	1/22	70/14464	0.01428571	9.39220779	2.7470171	0.10130131	0.24092093	0.18962121	Rab8a	1
GO:0007018	microtubule-based movement	2/22	359/14464	0.00557103	3.66269942	1.99392898	0.10251463	0.2427511	0.19106167	Slc9b2/Arl3	2
GO:0015800	acidic amino acid transport	1/22	72/14464	0.01388889	9.13131313	2.69956148	0.10404647	0.2442636	0.19225212	Slc6a13	1
GO:0051591	response to cAMP	1/22	72/14464	0.01388889	9.13131313	2.69956148	0.10404647	0.2442636	0.19225212	Hmgcs2	1
GO:0048168	regulation of neuronal synaptic plasticity	1/22	73/14464	0.01369863	9.00622665	2.6765212	0.10541605	0.24433296	0.19230671	Rab8a	1
GO:0062237	protein localization to postsynapse	1/22	73/14464	0.01369863	9.00622665	2.6765212	0.10541605	0.24433296	0.19230671	Rab8a	1
GO:1903510	mucopolysaccharide metabolic process	1/22	73/14464	0.01369863	9.00622665	2.6765212	0.10541605	0.24433296	0.19230671	Hexb	1
GO:0034109	homotypic cell-cell adhesion	1/22	74/14464	0.01351351	8.88452088	2.65391882	0.10678363	0.24645842	0.19397959	Megf10	1
GO:0044782	cilium organization	2/22	369/14464	0.00542005	3.56343927	1.94684125	0.10734466	0.24649008	0.19400451	Rab8a/Arl3	2
GO:0002709	regulation of T cell mediated immunity	1/22	75/14464	0.01333333	8.76606061	2.63173989	0.10814921	0.24649008	0.19400451	Mill2	1
GO:0070050	neuron cellular homeostasis	1/22	75/14464	0.01333333	8.76606061	2.63173989	0.10814921	0.24649008	0.19400451	Hexb	1
GO:0140056	organelle localization by membrane tethering	1/22	76/14464	0.01315789	8.6507177	2.60997064	0.1095128	0.24856226	0.19563545	Rab8a	1
GO:0043603	amide metabolic process	2/22	376/14464	0.00531915	3.49709865	1.91483701	0.11076327	0.24956101	0.19642154	Hmgcs2/Hexb	2
GO:0061178	regulation of insulin secretion involved in cellular response to glucose stimulus	1/22	77/14464	0.01298701	8.53837072	2.5885979	0.11087441	0.24956101	0.19642154	Slc9b2	1
GO:0031341	regulation of cell killing	1/22	78/14464	0.01282051	8.42890443	2.56760911	0.11223402	0.24956101	0.19642154	Mill2	1
GO:0050795	regulation of behavior	1/22	78/14464	0.01282051	8.42890443	2.56760911	0.11223402	0.24956101	0.19642154	Npas2	1
GO:0060997	dendritic spine morphogenesis	1/22	78/14464	0.01282051	8.42890443	2.56760911	0.11223402	0.24956101	0.19642154	Marcks	1
GO:0002028	regulation of sodium ion transport	1/22	79/14464	0.01265823	8.32220944	2.54699224	0.11359165	0.25054287	0.19719433	Fxyd3	1
GO:0035914	skeletal muscle cell differentiation	1/22	79/14464	0.01265823	8.32220944	2.54699224	0.11359165	0.25054287	0.19719433	Megf10	1
GO:0071230	cellular response to amino acid stimulus	1/22	80/14464	0.0125	8.21818182	2.52673582	0.1149473	0.25251474	0.19874633	Hmgcs2	1
GO:0021510	spinal cord development	1/22	81/14464	0.01234568	8.11672278	2.50682883	0.11630096	0.25446651	0.2002825	Pbx3	1
GO:0006029	proteoglycan metabolic process	1/22	82/14464	0.01219512	8.01773836	2.48726077	0.11765266	0.25527176	0.20091629	Hexb	1
GO:0014855	striated muscle cell proliferation	1/22	82/14464	0.01219512	8.01773836	2.48726077	0.11765266	0.25527176	0.20091629	Megf10	1
GO:0006637	acyl-CoA metabolic process	1/22	83/14464	0.01204819	7.9211391	2.46802153	0.11900237	0.25527176	0.20091629	Hmgcs2	1
GO:0035383	thioester metabolic process	1/22	83/14464	0.01204819	7.9211391	2.46802153	0.11900237	0.25527176	0.20091629	Hmgcs2	1
GO:1900076	regulation of cellular response to insulin stimulus	1/22	83/14464	0.01204819	7.9211391	2.46802153	0.11900237	0.25527176	0.20091629	Marcks	1
GO:0060998	regulation of dendritic spine development	1/22	84/14464	0.01190476	7.82683983	2.44910146	0.12035012	0.25577712	0.20131404	Marcks	1
GO:0006664	glycolipid metabolic process	1/22	85/14464	0.01176471	7.73475936	2.43049128	0.1216959	0.25577712	0.20131404	Hexb	1
GO:0019915	lipid storage	1/22	85/14464	0.01176471	7.73475936	2.43049128	0.1216959	0.25577712	0.20131404	Hexb	1
GO:0022406	membrane docking	1/22	85/14464	0.01176471	7.73475936	2.43049128	0.1216959	0.25577712	0.20131404	Rab8a	1
GO:0030203	glycosaminoglycan metabolic process	1/22	85/14464	0.01176471	7.73475936	2.43049128	0.1216959	0.25577712	0.20131404	Hexb	1
GO:0006672	ceramide metabolic process	1/22	86/14464	0.01162791	7.6448203	2.41218209	0.12303971	0.25577712	0.20131404	Hexb	1
GO:1903509	liposaccharide metabolic process	1/22	86/14464	0.01162791	7.6448203	2.41218209	0.12303971	0.25577712	0.20131404	Hexb	1
GO:0000281	mitotic cytokinesis	1/22	87/14464	0.01149425	7.5569488	2.39416537	0.12438156	0.25577712	0.20131404	Arl3	1
GO:0015807	L-amino acid transport	1/22	87/14464	0.01149425	7.5569488	2.39416537	0.12438156	0.25577712	0.20131404	Slc47a1	1
GO:0035773	insulin secretion involved in cellular response to glucose stimulus	1/22	87/14464	0.01149425	7.5569488	2.39416537	0.12438156	0.25577712	0.20131404	Slc9b2	1
GO:0048477	oogenesis	1/22	87/14464	0.01149425	7.5569488	2.39416537	0.12438156	0.25577712	0.20131404	Hexb	1
GO:0061515	myeloid cell development	1/22	91/14464	0.01098901	7.22477522	2.32486383	0.12972939	0.2647835	0.20840268	Slc9b2	1
GO:0071229	cellular response to acid chemical	1/22	91/14464	0.01098901	7.22477522	2.32486383	0.12972939	0.2647835	0.20840268	Hmgcs2	1
GO:0048565	digestive tract development	1/22	92/14464	0.01086957	7.14624506	2.3081926	0.13106147	0.2665079	0.20975989	Hmgcs2	1
GO:0002708	positive regulation of lymphocyte mediated immunity	1/22	95/14464	0.01052632	6.92057416	2.25963982	0.13504604	0.27158155	0.21375321	Mill2	1
GO:0019882	antigen processing and presentation	1/22	95/14464	0.01052632	6.92057416	2.25963982	0.13504604	0.27158155	0.21375321	Mill2	1
GO:0045639	positive regulation of myeloid cell differentiation	1/22	95/14464	0.01052632	6.92057416	2.25963982	0.13504604	0.27158155	0.21375321	Slc9b2	1
GO:0006022	aminoglycan metabolic process	1/22	96/14464	0.01041667	6.84848485	2.24392165	0.13637034	0.27224299	0.2142738	Hexb	1
GO:0048814	regulation of dendrite morphogenesis	1/22	96/14464	0.01041667	6.84848485	2.24392165	0.13637034	0.27224299	0.2142738	Marcks	1
GO:0008344	adult locomotory behavior	1/22	97/14464	0.01030928	6.77788191	2.22842666	0.13769272	0.27352589	0.21528353	Pbx3	1
GO:0007040	lysosome organization	1/22	98/14464	0.01020408	6.70871985	2.21314924	0.13901316	0.27352589	0.21528353	Hexb	1
GO:0080171	lytic vacuole organization	1/22	98/14464	0.01020408	6.70871985	2.21314924	0.13901316	0.27352589	0.21528353	Hexb	1
GO:0097120	receptor localization to synapse	1/22	98/14464	0.01020408	6.70871985	2.21314924	0.13901316	0.27352589	0.21528353	Rab8a	1
GO:0007338	single fertilization	1/22	101/14464	0.00990099	6.50945095	2.16856923	0.14296291	0.28028928	0.22060679	Hexb	1
GO:0001909	leukocyte mediated cytotoxicity	1/22	102/14464	0.00980392	6.4456328	2.15410982	0.14427564	0.28084974	0.2210479	Mill2	1
GO:0002824	positive regulation of adaptive immune response based on somatic recombination of immune receptors b	1/22	102/14464	0.00980392	6.4456328	2.15410982	0.14427564	0.28084974	0.2210479	Mill2	1
GO:0042752	regulation of circadian rhythm	1/22	103/14464	0.00970874	6.38305384	2.13984274	0.14558646	0.28139856	0.22147986	Npas2	1
GO:0140029	exocytic process	1/22	103/14464	0.00970874	6.38305384	2.13984274	0.14558646	0.28139856	0.22147986	Rab8a	1
GO:0055123	digestive system development	1/22	104/14464	0.00961538	6.32167832	2.12576343	0.14689536	0.281936	0.22190286	Hmgcs2	1
GO:0098754	detoxification	1/22	104/14464	0.00961538	6.32167832	2.12576343	0.14689536	0.281936	0.22190286	Slc47a1	1
GO:0035418	protein localization to synapse	1/22	105/14464	0.00952381	6.26147186	2.1118675	0.14820234	0.2824623	0.2223171	Rab8a	1
GO:0045445	myoblast differentiation	1/22	105/14464	0.00952381	6.26147186	2.1118675	0.14820234	0.2824623	0.2223171	Megf10	1
GO:0097061	dendritic spine organization	1/22	106/14464	0.00943396	6.20240137	2.09815069	0.14950741	0.28317074	0.22287469	Marcks	1
GO:0051223	regulation of protein transport	2/22	452/14464	0.00442478	2.90909091	1.60943198	0.1496094	0.28317074	0.22287469	Slc9b2/Rab8a	2
GO:0098771	inorganic ion homeostasis	2/22	454/14464	0.00440529	2.89627553	1.60227539	0.15066881	0.28323948	0.2229288	Slc9b2/Hexb	2
GO:0002456	T cell mediated immunity	1/22	107/14464	0.00934579	6.144435	2.08460889	0.15081058	0.28323948	0.2229288	Mill2	1
GO:0031667	response to nutrient levels	2/22	455/14464	0.0043956	2.88991009	1.59871164	0.15119914	0.28323948	0.2229288	Angptl4/Hmgcs2	2
GO:0030316	osteoclast differentiation	1/22	108/14464	0.00925926	6.08754209	2.07123812	0.15211184	0.28397671	0.22350904	Slc9b2	1
GO:0008037	cell recognition	1/22	109/14464	0.00917431	6.03169308	2.05803452	0.15341119	0.28446075	0.22389002	Megf10	1
GO:0046683	response to organophosphorus	1/22	109/14464	0.00917431	6.03169308	2.05803452	0.15341119	0.28446075	0.22389002	Hmgcs2	1
GO:0002821	positive regulation of adaptive immune response	1/22	110/14464	0.00909091	5.9768595	2.04499437	0.15470865	0.2858974	0.22502075	Mill2	1
GO:0001501	skeletal system development	2/22	466/14464	0.00429185	2.82169333	1.56013469	0.15705922	0.28873131	0.22725124	Slc9b2/Hexb	2
GO:0061640	cytoskeleton-dependent cytokinesis	1/22	112/14464	0.00892857	5.87012987	2.01939001	0.15729786	0.28873131	0.22725124	Arl3	1
GO:0010720	positive regulation of cell development	2/22	473/14464	0.00422833	2.77993465	1.53616234	0.16081277	0.29288578	0.23052109	Slc9b2/Marcks	2
GO:0002705	positive regulation of leukocyte mediated immunity	1/22	115/14464	0.00869565	5.71699605	1.98212244	0.1611675	0.29288578	0.23052109	Mill2	1
GO:0043200	response to amino acid	1/22	115/14464	0.00869565	5.71699605	1.98212244	0.1611675	0.29288578	0.23052109	Hmgcs2	1
GO:0008584	male gonad development	1/22	117/14464	0.00854701	5.61926962	1.95799941	0.16373782	0.29462036	0.23188632	Rab13	1
GO:0015695	organic cation transport	1/22	117/14464	0.00854701	5.61926962	1.95799941	0.16373782	0.29462036	0.23188632	Slc47a1	1
GO:0106027	neuron projection organization	1/22	117/14464	0.00854701	5.61926962	1.95799941	0.16373782	0.29462036	0.23188632	Marcks	1
GO:0008203	cholesterol metabolic process	1/22	118/14464	0.00847458	5.57164869	1.94614549	0.16502017	0.29498703	0.23217492	Hmgcs2	1
GO:0046546	development of primary male sexual characteristics	1/22	118/14464	0.00847458	5.57164869	1.94614549	0.16502017	0.29498703	0.23217492	Rab13	1
GO:0030317	flagellated sperm motility	1/22	119/14464	0.00840336	5.52482811	1.93442615	0.16630063	0.2960391	0.23300297	Slc9b2	1
GO:0002761	regulation of myeloid leukocyte differentiation	1/22	121/14464	0.00826446	5.43350864	1.91138031	0.16885594	0.2960391	0.23300297	Slc9b2	1
GO:0007043	cell-cell junction assembly	1/22	121/14464	0.00826446	5.43350864	1.91138031	0.16885594	0.2960391	0.23300297	Rab13	1
GO:0046165	alcohol biosynthetic process	1/22	121/14464	0.00826446	5.43350864	1.91138031	0.16885594	0.2960391	0.23300297	Hmgcs2	1
GO:0072329	monocarboxylic acid catabolic process	1/22	121/14464	0.00826446	5.43350864	1.91138031	0.16885594	0.2960391	0.23300297	Hexb	1
GO:0097722	sperm motility	1/22	121/14464	0.00826446	5.43350864	1.91138031	0.16885594	0.2960391	0.23300297	Slc9b2	1
GO:0045834	positive regulation of lipid metabolic process	1/22	122/14464	0.00819672	5.38897168	1.90004855	0.17013079	0.29732122	0.23401208	Angptl4	1
GO:0016125	sterol metabolic process	1/22	123/14464	0.00813008	5.34515891	1.88884084	0.17140377	0.29764401	0.23426614	Hmgcs2	1
GO:0051384	response to glucocorticoid	1/22	123/14464	0.00813008	5.34515891	1.88884084	0.17140377	0.29764401	0.23426614	Hmgcs2	1
GO:0008286	insulin receptor signaling pathway	1/22	124/14464	0.00806452	5.30205279	1.87775473	0.17267489	0.29795952	0.23451447	Marcks	1
GO:0045471	response to ethanol	1/22	124/14464	0.00806452	5.30205279	1.87775473	0.17267489	0.29795952	0.23451447	Hmgcs2	1
GO:0006694	steroid biosynthetic process	1/22	126/14464	0.00793651	5.21789322	1.85593785	0.17521155	0.30044112	0.23646766	Hmgcs2	1
GO:0014812	muscle cell migration	1/22	126/14464	0.00793651	5.21789322	1.85593785	0.17521155	0.30044112	0.23646766	Megf10	1
GO:1902652	secondary alcohol metabolic process	1/22	127/14464	0.00787402	5.17680744	1.8452025	0.17647709	0.30166553	0.23743135	Hmgcs2	1
GO:0014074	response to purine-containing compound	1/22	128/14464	0.0078125	5.13636364	1.83457961	0.17774078	0.30193853	0.23764622	Hmgcs2	1
GO:0060294	cilium movement involved in cell motility	1/22	128/14464	0.0078125	5.13636364	1.83457961	0.17774078	0.30193853	0.23764622	Slc9b2	1
GO:0006665	sphingolipid metabolic process	1/22	129/14464	0.00775194	5.09654686	1.82406705	0.17900262	0.30296681	0.23845554	Hexb	1
GO:0006906	vesicle fusion	1/22	130/14464	0.00769231	5.05734266	1.81366272	0.18026261	0.30296681	0.23845554	Rab8a	1
GO:0007030	Golgi organization	1/22	131/14464	0.00763359	5.01873699	1.80336462	0.18152075	0.30296681	0.23845554	Rab8a	1
GO:0009566	fertilization	1/22	131/14464	0.00763359	5.01873699	1.80336462	0.18152075	0.30296681	0.23845554	Hexb	1
GO:0001101	response to acid chemical	1/22	132/14464	0.00757576	4.98071625	1.79317077	0.18277705	0.30296681	0.23845554	Hmgcs2	1
GO:0001539	cilium or flagellum-dependent cell motility	1/22	132/14464	0.00757576	4.98071625	1.79317077	0.18277705	0.30296681	0.23845554	Slc9b2	1
GO:0060285	cilium-dependent cell motility	1/22	132/14464	0.00757576	4.98071625	1.79317077	0.18277705	0.30296681	0.23845554	Slc9b2	1
GO:0090174	organelle membrane fusion	1/22	132/14464	0.00757576	4.98071625	1.79317077	0.18277705	0.30296681	0.23845554	Rab8a	1
GO:0007292	female gamete generation	1/22	133/14464	0.0075188	4.94326726	1.78307926	0.18403151	0.30344437	0.23883142	Hexb	1

GO:0045739 positive regulation of DNA repair	1/22	134/14464	0.00746269	4.9063772	1.77308822	0.18528413	0.30344437	0.23883142	Npas2	1
GO:0046661 male sex differentiation	1/22	134/14464	0.00746269	4.9063772	1.77308822	0.18528413	0.30344437	0.23883142	Rab13	1
GO:0060996 dendritic spine development	1/22	134/14464	0.00746269	4.9063772	1.77308822	0.18528413	0.30344437	0.23883142	Marcks	1
GO:0050657 nucleic acid transport	1/22	136/14464	0.00735294	4.8342246	1.75340031	0.18778387	0.30389874	0.23918904	Rftn2	1
GO:0050658 RNA transport	1/22	136/14464	0.00735294	4.8342246	1.75340031	0.18778387	0.30389874	0.23918904	Rftn2	1
GO:0050773 regulation of dendrite development	1/22	136/14464	0.00735294	4.8342246	1.75340031	0.18778387	0.30389874	0.23918904	Marcks	1
GO:1904064 positive regulation of cation transmembrane transport	1/22	136/14464	0.00735294	4.8342246	1.75340031	0.18778387	0.30389874	0.23918904	Fxyd3	1
GO:1902414 protein localization to cell junction	1/22	137/14464	0.00729927	4.79893829	1.74369995	0.18903099	0.3050146	0.2400673	Rab8a	1
GO:0031960 response to corticosteroid	1/22	138/14464	0.00724638	4.76416337	1.73409305	0.19027628	0.30522324	0.24023151	Hmgcs2	1
GO:0048144 fibroblast proliferation	1/22	138/14464	0.00724638	4.76416337	1.73409305	0.19027628	0.30522324	0.24023151	Fam114a1	1
GO:0007584 response to nutrient	1/22	139/14464	0.00719424	4.72988882	1.72457797	0.19151975	0.30542654	0.24039152	Hmgcs2	1
GO:0051236 establishment of RNA localization	1/22	139/14464	0.00719424	4.72988882	1.72457797	0.19151975	0.30542654	0.24039152	Rftn2	1
GO:0002706 regulation of lymphocyte mediated immunity	1/22	140/14464	0.00714286	4.6961039	1.71515311	0.19276139	0.30562459	0.2405474	Mill2	1
GO:0008360 regulation of cell shape	1/22	140/14464	0.00714286	4.6961039	1.71515311	0.19276139	0.30562459	0.2405474	Hexb	1
GO:0007605 sensory perception of sound	1/22	141/14464	0.0070922	4.66279819	1.70581691	0.19400122	0.30581748	0.24069922	Hexb	1
GO:0055088 lipid homeostasis	1/22	141/14464	0.0070922	4.66279819	1.70581691	0.19400122	0.30581748	0.24069922	Angptl4	1
GO:0051147 regulation of muscle cell differentiation	1/22	144/14464	0.00694444	4.56565657	1.67832518	0.1977098	0.31076798	0.2445956	Megf10	1
GO:0001889 liver development	1/22	150/14464	0.00666667	4.3830303	1.6255408	0.20507814	0.3214262	0.25298435	Hmgcs2	1
GO:0007224 smoothened signaling pathway	1/22	151/14464	0.00662252	4.35400361	1.61701306	0.2062999	0.32241728	0.25376439	Arl3	1
GO:0034767 positive regulation of monoatomic ion transmembrane transport	1/22	153/14464	0.00653595	4.29708853	1.60017764	0.20873805	0.32345528	0.25458137	Fxyd3	1
GO:0044706 multi-multicellular organism process	1/22	153/14464	0.00653595	4.29708853	1.60017764	0.20873805	0.32345528	0.25458137	Hexb	1
GO:0061008 hepaticobiliary system development	1/22	153/14464	0.00653595	4.29708853	1.60017764	0.20873805	0.32345528	0.25458137	Hmgcs2	1
GO:0071333 cellular response to glucose stimulus	1/22	155/14464	0.00645161	4.24164223	1.58362793	0.21116905	0.32629793	0.25681873	Slc9b2	1
GO:0001906 cell killing	1/22	156/14464	0.00641026	4.21445221	1.57545742	0.21238187	0.32632832	0.25684265	Mill2	1
GO:0002822 regulation of adaptive immune response based on somatic recombination of immune receptors built from	1/22	156/14464	0.00641026	4.21445221	1.57545742	0.21238187	0.32632832	0.25684265	Mill2	1
GO:0071331 cellular response to hexose stimulus	1/22	158/14464	0.00632911	4.16110472	1.55931975	0.21480218	0.32730551	0.25761176	Slc9b2	1
GO:0003341 cilium movement	1/22	159/14464	0.00628931	4.13493425	1.5513505	0.21600967	0.32730551	0.25761176	Slc9b2	1
GO:0061025 membrane fusion	1/22	159/14464	0.00628931	4.13493425	1.5513505	0.21600967	0.32730551	0.25761176	Rab8a	1
GO:0071326 cellular response to monosaccharide stimulus	1/22	159/14464	0.00628931	4.13493425	1.5513505	0.21600967	0.32730551	0.25761176	Slc9b2	1
GO:0099072 regulation of postsynaptic membrane neurotransmitter receptor levels	1/22	159/14464	0.00628931	4.13493425	1.5513505	0.21600967	0.32730551	0.25761176	Rab8a	1
GO:0009266 response to temperature stimulus	1/22	161/14464	0.00621118	4.08356861	1.53560617	0.21841933	0.33004247	0.25976594	Hmgcs2	1
GO:0006403 RNA localization	1/22	163/14464	0.00613497	4.03346347	1.52011425	0.22082193	0.33183954	0.26118036	Rftn2	1
GO:0051017 actin filament bundle assembly	1/22	163/14464	0.00613497	4.03346347	1.52011425	0.22082193	0.33183954	0.26118036	Marcks	1
GO:0030534 adult behavior	1/22	164/14464	0.00609756	4.00886918	1.51246061	0.22202058	0.33272673	0.26187863	Pbx3	1
GO:0061572 actin filament bundle organization	1/22	166/14464	0.0060241	3.96056955	1.49733346	0.2244126	0.3353926	0.26397686	Marcks	1
GO:0000910 cytokinesis	1/22	168/14464	0.00595238	3.91341991	1.48244066	0.22679761	0.33691981	0.26517887	Arl3	1
GO:0016052 carbohydrate catabolic process	1/22	168/14464	0.00595238	3.91341991	1.48244066	0.22679761	0.33691981	0.26517887	Hexb	1
GO:0002819 regulation of adaptive immune response	1/22	170/14464	0.00588235	3.86737968	1.46777551	0.22917561	0.33691981	0.26517887	Mill2	1
GO:0001678 intracellular glucose homeostasis	1/22	171/14464	0.00584795	3.84476342	1.46052628	0.23036199	0.33691981	0.26517887	Slc9b2	1
GO:0042552 myelination	1/22	171/14464	0.00584795	3.84476342	1.46052628	0.23036199	0.33691981	0.26517887	Hexb	1
GO:0071322 cellular response to carbohydrate stimulus	1/22	171/14464	0.00584795	3.84476342	1.46052628	0.23036199	0.33691981	0.26517887	Slc9b2	1
GO:1902107 positive regulation of leukocyte differentiation	1/22	171/14464	0.00584795	3.84476342	1.46052628	0.23036199	0.33691981	0.26517887	Slc9b2	1
GO:1903708 positive regulation of hemopoiesis	1/22	171/14464	0.00584795	3.84476342	1.46052628	0.23036199	0.33691981	0.26517887	Slc9b2	1
GO:0006643 membrane lipid metabolic process	1/22	172/14464	0.00581395	3.82241015	1.45333157	0.23154663	0.33759669	0.26571162	Hexb	1
GO:0071222 cellular response to lipopolysaccharide	1/22	173/14464	0.00578035	3.80031529	1.44619061	0.23272953	0.33759669	0.26571162	Hmgcs2	1
GO:0007272 ensheathment of neurons	1/22	174/14464	0.00574713	3.7784744	1.43910265	0.23391068	0.33759669	0.26571162	Hexb	1
GO:0008366 axon ensheathment	1/22	174/14464	0.00574713	3.7784744	1.43910265	0.23391068	0.33759669	0.26571162	Hexb	1
GO:0048771 tissue remodeling	1/22	174/14464	0.00574713	3.7784744	1.43910265	0.23391068	0.33759669	0.26571162	Fam114a1	1
GO:0045216 cell-cell junction organization	1/22	176/14464	0.00568182	3.73553719	1.4250828	0.2362678	0.33832064	0.26628143	Rab13	1
GO:0048284 organelle fusion	1/22	176/14464	0.00568182	3.73553719	1.4250828	0.2362678	0.33832064	0.26628143	Rab8a	1
GO:0050954 sensory perception of mechanical stimulus	1/22	176/14464	0.00568182	3.73553719	1.4250828	0.2362678	0.33832064	0.26628143	Hexb	1
GO:0010970 transport along microtubule	1/22	177/14464	0.00564972	3.71443246	1.41814949	0.23744376	0.3391168	0.26690806	Arl3	1
GO:0050796 regulation of insulin secretion	1/22	179/14464	0.00558659	3.67293042	1.40443263	0.23979049	0.34068935	0.26814576	Slc9b2	1
GO:0051346 negative regulation of hydrolase activity	1/22	179/14464	0.00558659	3.67293042	1.40443263	0.23979049	0.34068935	0.26814576	Angptl4	1
GO:0071219 cellular response to molecule of bacterial origin	1/22	180/14464	0.00555556	3.65252525	1.39764773	0.24096128	0.34146585	0.26875692	Hmgcs2	1
GO:0009267 cellular response to starvation	1/22	183/14464	0.00546448	3.59264779	1.37757934	0.2444633	0.34464285	0.27125743	Angptl4	1
GO:0071383 cellular response to steroid hormone stimulus	1/22	183/14464	0.00546448	3.59264779	1.37757934	0.2444633	0.34464285	0.27125743	Hmgcs2	1
GO:0007519 skeletal muscle tissue development	1/22	184/14464	0.00543478	3.57312253	1.37098322	0.24562721	0.34539353	0.27184827	Megf10	1
GO:0044242 cellular lipid catabolic process	1/22	185/14464	0.00540541	3.55380835	1.36443275	0.24678941	0.34599131	0.27231877	Hexb	1
GO:0048813 dendrite morphogenesis	1/22	186/14464	0.00537634	3.53470186	1.35792733	0.2479499	0.34599131	0.27231877	Marcks	1
GO:0099175 regulation of postsynapse organization	1/22	186/14464	0.00537634	3.53470186	1.35792733	0.2479499	0.34599131	0.27231877	Marcks	1
GO:1901617 organic hydroxy compound biosynthetic process	1/22	187/14464	0.00534759	3.51579971	1.35146639	0.24910868	0.34672379	0.27289528	Hmgcs2	1
GO:0061351 neural precursor cell proliferation	1/22	189/14464	0.00529101	3.47859548	1.33867564	0.25142114	0.34905422	0.27472948	Fabp7	1
GO:0015931 nucleobase-containing compound transport	1/22	192/14464	0.00520833	3.42424242	1.31980906	0.25487708	0.35206506	0.27709922	Rftn2	1
GO:1901654 response to ketone	1/22	192/14464	0.00520833	3.42424242	1.31980906	0.25487708	0.35206506	0.27709922	Hmgcs2	1
GO:0006909 phagocytosis	1/22	194/14464	0.00515464	3.38894096	1.30743816	0.25717257	0.3525649	0.27749263	Megf10	1
GO:0030324 lung development	1/22	194/14464	0.00515464	3.38894096	1.30743816	0.25717257	0.3525649	0.27749263	Hmgcs2	1
GO:0045637 regulation of myeloid cell differentiation	1/22	194/14464	0.00515464	3.38894096	1.30743816	0.25717257	0.3525649	0.27749263	Slc9b2	1
GO:0002703 regulation of leukocyte mediated immunity	1/22	197/14464	0.00507614	3.33733272	1.28918185	0.26060316	0.3543901	0.27892918	Mill2	1
GO:0030323 respiratory tube development	1/22	197/14464	0.00507614	3.33733272	1.28918185	0.26060316	0.3543901	0.27892918	Hmgcs2	1
GO:0060538 skeletal muscle organ development	1/22	197/14464	0.00507614	3.33733272	1.28918185	0.26060316	0.3543901	0.27892918	Megf10	1
GO:0002064 epithelial cell development	1/22	198/14464	0.00505051	3.3204775	1.28317447	0.26174332	0.3543901	0.27892918	Rab13	1
GO:0030705 cytoskeleton-dependent intracellular transport	1/22	198/14464	0.00505051	3.3204775	1.28317447	0.26174332	0.3543901	0.27892918	Arl3	1
GO:0008406 gonad development	1/22	200/14464	0.005	3.28727273	1.27127395	0.26401862	0.35631318	0.28044278	Rab13	1
GO:0009636 response to toxic substance	1/22	201/14464	0.00497512	3.27091814	1.26537989	0.26515376	0.35631318	0.28044278	Slc47a1	1
GO:0009749 response to glucose	1/22	201/14464	0.00497512	3.27091814	1.26537989	0.26515376	0.35631318	0.28044278	Slc9b2	1
GO:0045137 development of primary sexual characteristics	1/22	203/14464	0.00492611	3.23869234	1.25370196	0.26741902	0.35631318	0.28044278	Rab13	1
GO:0007623 circadian rhythm	1/22	204/14464	0.00490196	3.2228164	1.24791722	0.26854915	0.35631318	0.28044278	Npas2	1
GO:0016054 organic acid catabolic process	1/22	204/14464	0.00490196	3.2228164	1.24791722	0.26854915	0.35631318	0.28044278	Hexb	1
GO:0046395 carboxylic acid catabolic process	1/22	204/14464	0.00490196	3.2228164	1.24791722	0.26854915	0.35631318	0.28044278	Hexb	1
GO:0009746 response to hexose	1/22	205/14464	0.00487805	3.20709534	1.24216805	0.26967762	0.35631318	0.28044278	Slc9b2	1
GO:0051651 maintenance of location in cell	1/22	205/14464	0.00487805	3.20709534	1.24216805	0.26967762	0.35631318	0.28044278	Hexb	1
GO:0071216 cellular response to biotic stimulus	1/22	205/14464	0.00487805	3.20709534	1.24216805	0.26967762	0.35631318	0.28044278	Hmgcs2	1
GO:0043270 positive regulation of monoatomic ion transport	1/22	206/14464	0.00485437	3.19152692	1.23645404	0.27080442	0.3569398	0.28093597	Fxyd3	1
GO:0060348 bone development	1/22	207/14464	0.00483092	3.17610892	1.23077478	0.27192956	0.35756123	0.28142508	Slc9b2	1
GO:0006282 regulation of DNA repair	1/22	208/14464	0.00480769	3.16083916	1.22512987	0.27305305	0.3581775	0.28191013	Npas2	1
GO:0043542 endothelial cell migration	1/22	210/14464	0.0047619	3.13073593	1.21394151	0.27529506	0.35939474	0.28286818	Rab13	1
GO:0090276 regulation of peptide hormone secretion	1/22	210/14464	0.0047619	3.13073593	1.21394151	0.27529506	0.35939474	0.28286818	Slc9b2	1
GO:0002699 positive regulation of immune effector process	1/22	212/14464	0.00471698	3.10120069	1.20288586	0.27753047	0.35973736	0.28313785	Mill2	1
GO:0006813 potassium ion transport	1/22	212/14464	0.00471698	3.10120069	1.20288586	0.27753047	0.35973736	0.28313785	Fxyd3	1
GO:0099111 microtubule-based transport	1/22	212/14464	0.00471698	3.10120069	1.20288586	0.27753047	0.35973736	0.28313785	Arl3	1
GO:0030073 insulin secretion	1/22	213/14464	0.00469484	3.08664106	1.19740686	0.27864571	0.36032908	0.28360357	Slc9b2	1
GO:0034764 positive regulation of transmembrane transport	1/22	214/14464	0.0046729	3.0722175	1.19195993	0.2797593	0.36064923	0.28385555	Fxyd3	1
GO:0002791 regulation of peptide secretion	1/22	215/14464	0.00465116	3.05792812	1.18654469	0.28087125	0.36064923	0.28385555	Slc9b2	1
GO:0090087 regulation of peptide transport	1/22	215/14464	0.00465116	3.05792812	1.18654469	0.28087125	0.36064923	0.28385555	Slc9b2	1
GO:0048863 stem cell differentiation	1/22	218/14464	0.00458716	3.01584654	1.17048569	0.28419728	0.36321474	0.28587478	Phf5a	1
GO:0055001 muscle cell development	1/22	218/14464	0.00458716	3.01584654	1.17048569	0.28419728	0.36321474	0.28587478	Megf10	1
GO:0006836 neurotransmitter transport	1/22	219/14464	0.00456621	3.00207555	1.16519377	0.28530268	0.36349034	0.28609169	Slc6a13	1
GO:0010976 positive regulation of neuron projection development	1/22	220/14464	0.00454545	2.98842975	1.15993185	0.28640646	0.36349034	0.28609169	Marcks	1
GO:0060541 respiratory system development	1/22	220/14464	0.00454545	2.98842975	1.15993185	0.28640646	0.36349034	0.28609169	Hmgcs2	1
GO:0007033 vacuole organization	1/22	223/14464	0.0044843	2.94822666	1.14432275	0.28970805	0.3668294	0.28871977	Hexb	1
GO:0008202 steroid metabolic process	1/22	225/14464	0.00444444	2.92202202	1.13406076	0.291901	0.36875253	0.2902334	Hmgcs2	1
GO:1901136 carbohydrate derivative catabolic process	1/22	229/14464	0.00436681	2.87098055	1.11387163	0.29626753	0.37340631	0.29389624	Hexb	1
GO:0001667 ameboidal-type cell migration	1/22	231/14464	0.004329	2.84612357	1.10393981	0.29844114	0.37528116	0.29537187	Fam114a1	1
GO:0002573 myeloid leukocyte differentiation	1/22	232/14464	0.00431034	2.8338558	1.09901361	0.29952554	0.3757809	0.2957652	Slc9b2	1
GO:0002449 lymphocyte mediated immunity	1/22	236/14464	0.00423729	2.78582435	1.07956719	0.30384717	0.38033044	0.299346	Mill2	1
GO:0007548 sex differentiation	1/22	237/14464	0.00421941	2.77406981	1.07476886	0.3049236	0.38080641	0.29972062	Rab13	1
GO:0048167 regulation of synaptic plasticity	1/22	239/14464	0.0041841	2.75085584	1.06524637	0.30707168	0.38261551	0.30114451	Rab8a	1
GO:0031669 cellular response to nutrient levels	1/22	241/14464	0.00414938	2.72802716	1.05582112	0.30921343	0.38440851	0.30255572	Angptl4	1
GO:0060326 cell chemotaxis	1/22	245/14464	0.00408163	2.68348794	1.03725464	0.31347798	0.38882642	0.30603292	Rab13	1
GO:0097305 response to alcohol	1/22	247/14464	0.00404858	2.66175929	1.02810974	0.31560082	0.39057386	0.30740827	Hmgcs2	1
GO:0033002 muscle cell proliferation	1/22	249/14464	0.00401606	2.6403797	1.01905469	0.31771739	0.39230567	0.30877133	Megf10	1
GO:0022409 positive regulation of cell-cell adhesion	1/22	252/14464	0.00396825	2.60894661	1.00563682	0.32088054	0.39531904	0.31114305	Megf10	1
GO:0006898 receptor-mediated endocytosis	1/22	254/14464	0.00393701	2.58840372	0.99679897	0.32298152	0.39691236	0.3123971	Slc9b2	1
GO:0030072 peptide hormone secretion	1/22	256/14464	0.00390625	2.56818182	0.98804514	0.3250763	0.39691236	0.3123971	Slc9b2	1
GO:0034976 response to endoplasmic reticulum stress	1/22	256/14464	0.00390625	2.56818182	0.98804514	0.3250763	0.39691236	0.3123971	Marcks	1
GO:0090150 establishment of protein localization to membrane	1/22	256/14464	0.00390625	2.56818182	0.98804514	0.3250763	0.39691236	0.3123971	Rab8a	1
GO:0016042 lipid catabolic process	1/22	259/14464	0.003861	2.53843454	0.97506855	0.32820687	0.39984222	0.31470311	Hexb	1
GO:0046883 regulation of hormone secretion	1/22	261/14464	0.00383142	2.51898293	0.96651805	0.33028621	0.40132582	0.3158708	Slc9b2	1
GO:0002460 adaptive immune response based on somatic recombination of immune receptors built from immunoglob	1/22	263/14464	0.00380228	2.49982717	0.95804628	0.33235941	0.40132582	0.3158708	Mill2	1
GO:0002790 peptide secretion	1/22	263/14464	0.00380228	2.49982717	0.95804628	0.33235941	0.40132582	0.3158708	Slc9b2	1
GO:0050708 regulation of protein secretion	1/22	263/14464	0.00380228	2.49982717	0.95804628	0.33235941	0.40132582	0.3158708	Slc9b2	1
GO:0042593 glucose homeostasis	1/22	265/14464	0.00377358	2.48096055	0.94965183	0.33442648	0.40293234	0.31713524	Slc9b2	1
GO:0033500 carbohydrate homeostasis	1/22	266/14464	0.0037594	2.47163363	0.94548316	0.33545772	0.40328654	0.31741402	Slc9b2	1
GO:0048608 reproductive structure development	1/22	268/14464	0.00373134	2.4531886	0.93720209	0.33751563	0.40487073	0.31866088	Rab13	1
GO:0015833 peptide transport	1/22	272/14464	0.00367647	2.4171123	0.92086037	0.34161322	0.40567253	0.31929195	Slc9b2	1
GO:0061448 connective tissue development	1/22	272/14464	0.00367647	2.4171123	0.92086037	0.34161322	0.40567253	0.31929195	Hmgcs2	1
GO:0061458 reproductive system development	1/22	272/14464	0.00367647	2.4171123	0.92086037	0.34161322	0.40567253	0.31929195	Rab13	1
GO:0000375 RNA splicing, via transesterification reactions	1/22	273/14464	0.003663	2.40825841	0.91681992	0.34263383	0.40567253	0.31929195	Phf5a	1
GO:0000377 RNA splicing, via transesterification reactions with bulged adenosine as nucleophile	1/22	273/14464	0.003663	2.40825841	0.91681992	0.34263383	0.40567253	0.31929195	Phf5a	1
GO:0000398 mRNA splicing, via spliceosome	1/22	273/14464	0.003663	2.40825841	0.91681992	0.34263383	0.40567253	0.31929195	Phf5a	1
GO:0006066 alcohol metabolic process	1/22	275/14464	0.00363636	2.3907438	0.9087919	0.34467053	0.40720255	0.32049618	Hmgcs2	1
GO:0007281 germ cell development	1/22	276/14464	0.00362319	2.38208169	0.90480403	0.34568661	0.4075228	0.32074824	Hexb	1
GO:0006874 intracellular calcium ion homeostasis	1/22	278/14464	0.00359712	2.36494441	0.89687981	0.34771428	0.40903163	0.3219358	Hexb	1
GO:0032496 response to lipopolysaccharide	1/22	283/14464	0.00353357	2.32316094	0.87736302	0.35275723	0.4140734	0.32590401	Hmgcs2	1
GO:1902105 regulation of leukocyte differentiation	1/22	286/14464	0.0034965	2.29879212	0.86584876	0.35576511	0.41670988	0.3279791	Slc9b2	1
GO:0051054 positive regulation of DNA metabolic process	1/22	287/14464	0.00348432	2.29078239	0.86204249	0.35676477	0.41698788	0.32819791	Npas2	1
GO:0001666 response to hypoxia	1/22	289/14464	0.00346021	2.27492922	0.8544769	0.35875965	0.41807096	0.32905037	Angptl4	1
GO:0048545 response to steroid hormone	1/22	290/14464	0.00344828	2.26708464	0.85071732	0.35975487	0.41807096	0.32905037	Hmgcs2	1
GO:0048511 rhythmic process	1/22	291/14464	0.00343643	2.25929397	0.84697304	0.36074862	0.41807096	0.32905037	Npas2	1
GO:0050769 positive regulation of neurogenesis	1/22	291/14464	0.00343643	2.25929397	0.84697304	0.36074862	0.41807096	0.32905037	Marcks	1
GO:1904062 regulation of monoatomic cation transmembrane transport	1/22	294/14464	0.00340136	2.23623995	0.83583081	0.36372104	0.41910877	0.32986719	Fxyd3	1
GO:0010506 regulation of autophagy	1/22	295/14464	0.00338983	2.22865948	0.83214653	0.36470891	0.41910877	0.32986719	Rab8a	1
GO:0022412 cellular process involved in reproduction in multicellular organism	1/22	295/14464	0.00338983	2.22865948	0.83214653	0.36470891	0.41910877	0.32986719	Hexb	1
GO:0055074 calcium ion homeostasis	1/22	295/14464	0.00338983	2.22865948	0.83214653	0.36470891	0.41910877	0.32986719	Hexb	1
GO:0044282 small molecule catabolic process	1/22	296/14464	0.00337838	2.22113022	0.82847694	0.36569532	0.4193613	0.33006595	Hexb	1
GO:0002237 response to molecule of bacterial origin	1/22	297/14464	0.003367	2.21365167	0.82482193	0.36668026	0.41961109	0.33026256	Hmgcs2	1
GO:0019216 regulation of lipid metabolic process	1/22	298/14464	0.0033557	2.20622331	0.82118138	0.36766375	0.41985818	0.33045703	Angptl4	1
GO:0099003 vesicle-mediated transport in synapse	1/22	303/14464	0.00330033	2.16981698	0.80319156	0.37255934	0.42367975	0.33346487	Rab8a	1
GO:0099173 postsynapse organization	1/22	303/14464	0.00330033	2.16981698	0.80319156	0.37255934	0.42367975	0.33346487	Marcks	1
GO:0036293 response to decreased oxygen levels	1/22	304/14464	0.00328947	2.16267943	0.79963541	0.37353411	0.42390697	0.3336437	Angptl4	1
GO:0051235 maintenance of location	1/22	307/14464	0.00325733	2.14154575	0.78904861	0.37644975	0.42633129	0.33555181	Hexb	1
GO:0032102 negative regulation of response to external stimulus	1/22	308/14464	0.00324675	2.13459268	0.78554654	0.37741875	0.42654557	0.33572046	Fabp7	1
GO:0016358 dendrite development	1/22	309/14464	0.00323625	2.12768461	0.78205774	0.37838631	0.42675734	0.33588714	Marcks	1
GO:0010038 response to metal ion	1/22	311/14464	0.00321543	2.11400175	0.77511953	0.38031713	0.4280524	0.33690644	Hmgcs2	1
GO:0042886 amide transport	1/22	314/14464	0.00318471	2.09380428	0.76480917	0.38320262	0.42953245	0.33807134	Slc9b2	1
GO:0046879 hormone secretion	1/22	314/14464	0.00318471	2.09380428	0.76480917	0.38320262	0.42953245	0.33807134	Slc9b2	1
GO:0002697 regulation of immune effector process	1/22	320/14464	0.003125	2.05454545	0.744528	0.38893518	0.43469564	0.34213512	Mill2	1
GO:0009914 hormone transport	1/22	321/14464	0.00311526	2.048145	0.74119073	0.38988565	0.43469564	0.34213512	Slc9b2	1
GO:0007517 muscle organ development	1/22	323/14464	0.00309598	2.03546299	0.73455217	0.39178236	0.43469564	0.34213512	Megf10	1
GO:0009100 glycoprotein metabolic process	1/22	323/14464	0.00309598	2.03546299	0.73455217	0.39178236	0.43469564	0.34213512	Hexb	1
GO:0034765 regulation of monoatomic ion transmembrane transport	1/22	323/14464	0.00309598	2.03546299	0.73455217	0.39178236	0.43469564	0.34213512	Fxyd3	1
GO:0002443 leukocyte mediated immunity	1/22	327/14464	0.0030581	2.01056436	0.72141653	0.39555889	0.43711256	0.3440374	Mill2	1
GO:0070482 response to oxygen levels	1/22	327/14464	0.0030581	2.01056436	0.72141653	0.39555889	0.43711256	0.3440374	Angptl4	1
GO:0031589 cell-substrate adhesion	1/22	332/14464	0.00301205	1.98028478	0.70525528	0.4002481	0.44140264	0.34741399	Marcks	1
GO:0002250 adaptive immune response	1/22	337/14464	0.00296736	1.9509037	0.68937158	0.40490256	0.44563723	0.3507469	Mill2	1

GO:0001654	eye development	1/22	351/14464	0.002849	1.87308987	0.64629337	0.41775234	0.45702106	0.35970675	Pbx3	1
GO:0016050	vesicle organization	1/22	351/14464	0.002849	1.87308987	0.64629337	0.41775234	0.45702106	0.35970675	Rab8a	1
GO:0051962	positive regulation of nervous system development	1/22	351/14464	0.002849	1.87308987	0.64629337	0.41775234	0.45702106	0.35970675	Marcks	1
GO:0009306	protein secretion	1/22	352/14464	0.00284091	1.8677686	0.64329154	0.41865998	0.45709982	0.35976874	Slc9b2	1
GO:0035592	establishment of protein localization to extracellular region	1/22	354/14464	0.00282486	1.85721623	0.63731681	0.4204712	0.45725198	0.3598885	Slc9b2	1
GO:0150063	visual system development	1/22	354/14464	0.00282486	1.85721623	0.63731681	0.4204712	0.45725198	0.3598885	Pbx3	1
GO:0006935	chemotaxis	1/22	356/14464	0.00280899	1.84678243	0.63138022	0.42227703	0.45746705	0.36005778	Rab13	1
GO:0042330	taxis	1/22	357/14464	0.00280112	1.84160937	0.62842608	0.42317793	0.45746705	0.36005778	Rab13	1
GO:0071692	protein localization to extracellular region	1/22	357/14464	0.00280112	1.84160937	0.62842608	0.42317793	0.45746705	0.36005778	Slc9b2	1
GO:0048880	sensory system development	1/22	359/14464	0.00278552	1.83134971	0.62254578	0.42497571	0.45850437	0.36087421	Pbx3	1
GO:0007600	sensory perception	1/22	362/14464	0.00276243	1.81617278	0.61379437	0.42766235	0.45959	0.36172868	Hexb	1
GO:1903706	regulation of hemopoiesis	1/22	362/14464	0.00276243	1.81617278	0.61379437	0.42766235	0.45959	0.36172868	Slc9b2	1
GO:0042063	gliogenesis	1/22	366/14464	0.00273224	1.79632389	0.60225221	0.43122591	0.46251093	0.36402765	Hexb	1
GO:0050807	regulation of synapse organization	1/22	370/14464	0.0027027	1.77690418	0.5908511	0.43476829	0.46539776	0.36629978	Marcks	1
GO:0030900	forebrain development	1/22	373/14464	0.00268097	1.76261272	0.58239068	0.43741124	0.4673124	0.36780674	Fabp7	1
GO:0010959	regulation of metal ion transport	1/22	375/14464	0.00266667	1.75321212	0.57679264	0.43916665	0.46827321	0.36856296	Fxyd3	1
GO:0050803	regulation of synapse structure or activity	1/22	376/14464	0.00265957	1.74854932	0.57400614	0.44004239	0.46829414	0.36857943	Marcks	1
GO:0031329	regulation of cellular catabolic process	1/22	388/14464	0.00257732	1.69447048	0.54119888	0.45045	0.47843913	0.37656423	Rab8a	1
GO:0022407	regulation of cell-cell adhesion	1/22	398/14464	0.00251256	1.65189584	0.51470846	0.45898171	0.48561508	0.3822122	Megf10	1
GO:0043086	negative regulation of catalytic activity	1/22	398/14464	0.00251256	1.65189584	0.51470846	0.45898171	0.48561508	0.3822122	Angptl4	1
GO:0048732	gland development	1/22	408/14464	0.00245098	1.6114082	0.48894198	0.4673869	0.49355335	0.38846015	Hmgcs2	1
GO:0031349	positive regulation of defense response	1/22	410/14464	0.00243902	1.60354767	0.48387189	0.46905291	0.49435827	0.38909368	Npas2	1
GO:0014706	striated muscle tissue development	1/22	414/14464	0.00241546	1.58805446	0.4738124	0.47237002	0.49654588	0.39081548	Megf10	1
GO:0008380	RNA splicing	1/22	417/14464	0.00239808	1.57662961	0.46633717	0.47484487	0.49654588	0.39081548	Phf5a	1
GO:0050673	epithelial cell proliferation	1/22	417/14464	0.00239808	1.57662961	0.46633717	0.47484487	0.49654588	0.39081548	Fabp7	1
GO:1901615	organic hydroxy compound metabolic process	1/22	417/14464	0.00239808	1.57662961	0.46633717	0.47484487	0.49654588	0.39081548	Hmgcs2	1
GO:0050767	regulation of neurogenesis	1/22	418/14464	0.00239234	1.57285776	0.46385845	0.47566735	0.49654588	0.39081548	Marcks	1
GO:0007264	small GTPase-mediated signal transduction	1/22	420/14464	0.00238095	1.56536797	0.45892033	0.47730863	0.49702549	0.39119297	Arl3	1
GO:0048568	embryonic organ development	1/22	421/14464	0.0023753	1.56164975	0.45646085	0.47812743	0.49702549	0.39119297	Pbx3	1
GO:0045785	positive regulation of cell adhesion	1/22	422/14464	0.00236967	1.55794916	0.45400771	0.478945	0.49702549	0.39119297	Megf10	1
GO:0042692	muscle cell differentiation	1/22	423/14464	0.00236407	1.55426606	0.45156089	0.47976135	0.49702549	0.39119297	Megf10	1
GO:0031346	positive regulation of cell projection organization	1/22	426/14464	0.00234742	1.54332053	0.4442579	0.48220309	0.49861075	0.39244068	Marcks	1
GO:0030099	myeloid cell differentiation	1/22	428/14464	0.00233645	1.53610875	0.43942015	0.48382483	0.49923679	0.39293341	Slc9b2	1
GO:0007015	actin filament organization	1/22	429/14464	0.002331	1.53252808	0.43701044	0.48463388	0.49923679	0.39293341	Marcks	1
GO:0060537	muscle tissue development	1/22	435/14464	0.00229885	1.51138976	0.4226785	0.48946283	0.50326348	0.39610269	Megf10	1
GO:0043269	regulation of monoatomic ion transport	1/22	440/14464	0.00227273	1.49421488	0.41089709	0.49345394	0.5064152	0.39858331	Fxyd3	1
GO:0051052	regulation of DNA metabolic process	1/22	443/14464	0.00225734	1.48409604	0.40389712	0.49583428	0.50790516	0.39975601	Npas2	1
GO:0007409	axonogenesis	1/22	446/14464	0.00224215	1.47411333	0.39694783	0.49820395	0.50828368	0.40005393	Rab8a	1
GO:0030003	intracellular monoatomic cation homeostasis	1/22	447/14464	0.00223714	1.47081554	0.39464252	0.49899148	0.50828368	0.40005393	Hexb	1
GO:0034762	regulation of transmembrane transport	1/22	447/14464	0.00223714	1.47081554	0.39464252	0.49899148	0.50828368	0.40005393	Fxyd3	1
GO:0006091	generation of precursor metabolites and energy	1/22	449/14464	0.00222717	1.46426402	0.39004841	0.50056299	0.50893672	0.40056792	Hmgcs2	1
GO:0006873	intracellular monoatomic ion homeostasis	1/22	454/14464	0.00220264	1.44813777	0.37865811	0.5044712	0.51085284	0.40207604	Hexb	1
GO:0006397	mRNA processing	1/22	455/14464	0.0021978	1.44495504	0.37639611	0.50524934	0.51085284	0.40207604	Phf5a	1
GO:0010817	regulation of hormone levels	1/22	455/14464	0.0021978	1.44495504	0.37639611	0.50524934	0.51085284	0.40207604	Slc9b2	1
GO:0071407	cellular response to organic cyclic compound	1/22	462/14464	0.0021645	1.42306179	0.3607087	0.51066369	0.51537461	0.40563498	Hmgcs2	1
GO:0007005	mitochondrion organization	1/22	466/14464	0.00214592	1.41084666	0.35185732	0.51373217	0.51751657	0.40732085	Marcks	1
GO:0044283	small molecule biosynthetic process	1/22	470/14464	0.00212766	1.39883946	0.34308598	0.51678228	0.51963218	0.40898597	Hmgcs2	1
GO:0034329	cell junction assembly	1/22	485/14464	0.00206186	1.35557638	0.31087986	0.52805832	0.52999615	0.41714313	Rab13	1
GO:0005975	carbohydrate metabolic process	1/22	491/14464	0.00203666	1.33901129	0.2982888	0.53249804	0.53347331	0.41987989	Hexb	1
GO:0071396	cellular response to lipid	1/22	499/14464	0.00200401	1.31754418	0.28174799	0.53835567	0.53835567	0.42372264	Hmgcs2	1

ID	Description	GeneRatio	BgRatio	RichFactor	FoldEnrichm	zScore	pvalue	p.adjust	qvalue	geneID	Count
GO:0001503	ossification	7/28	359/14464	0.01949861	10.0724234	7.66599905	4.133E-06	0.00418854	0.00322131	Igfbp5/Ccn3/Bmp6/Comp/Col6a1/H3f3b/Kl	7
GO:0042246	tissue regeneration	4/28	67/14464	0.05970149	30.8400853	10.7816749	7.9178E-06	0.00418854	0.00322131	Ccn3/Fzd7/Col6a1/Cpq	4
GO:0007178	cell surface receptor protein serine/threonine kinase signaling pathway	6/28	313/14464	0.01916933	9.9023277	7.01240438	2.4673E-05	0.00870126	0.00669195	Fbn1/Cilp/Ccn3/Bmp6/Comp/Slc2a10	6
GO:0090092	regulation of transmembrane receptor protein serine/threonine kinase signaling pathway	5/28	223/14464	0.02242152	11.582319	7.01371009	6.1289E-05	0.01071373	0.00823969	Fbn1/Cilp/Ccn3/Bmp6/Slc2a10	5
GO:0030282	bone mineralization	4/28	115/14464	0.03478261	17.9677019	8.04536773	6.7003E-05	0.01071373	0.00823969	Bmp6/Comp/Col6a1/Kl	4
GO:0030278	regulation of ossification	4/28	119/14464	0.03361345	17.3637455	7.89388314	7.6552E-05	0.01071373	0.00823969	Ccn3/Bmp6/Comp/Kl	4
GO:0051224	negative regulation of protein transport	4/28	120/14464	0.03333333	17.2190476	7.85716016	7.9087E-05	0.01071373	0.00823969	Fbn1/Ccn3/Gbp4/Ndufaf2	4
GO:0048009	insulin-like growth factor receptor signaling pathway	3/28	45/14464	0.06666667	34.4380952	9.89384241	8.7308E-05	0.01071373	0.00823969	Cilp/Igfbp5/Col6a1	3
GO:0090101	negative regulation of transmembrane receptor protein serine/threonine kinase signaling pathway	4/28	126/14464	0.03174603	16.399093	7.64576416	9.5599E-05	0.01071373	0.00823969	Fbn1/Cilp/Ccn3/Slc2a10	4
GO:0060390	regulation of SMAD protein signal transduction	3/28	49/14464	0.06122449	31.6268222	9.45752342	0.00011277	0.01071373	0.00823969	Cilp/Ccn3/Bmp6	3
GO:1904950	negative regulation of establishment of protein localization	4/28	135/14464	0.02962963	15.3058201	7.35455626	0.00012489	0.01071373	0.00823969	Fbn1/Ccn3/Gbp4/Ndufaf2	4
GO:0046676	negative regulation of insulin secretion	3/28	51/14464	0.05882353	30.3865546	9.25851507	0.00012714	0.01071373	0.00823969	Fbn1/Ccn3/Ndufaf2	3
GO:0042692	muscle cell differentiation	6/28	423/14464	0.0141844	7.32725431	5.81662715	0.00013164	0.01071373	0.00823969	Igfbp5/Ccn3/Comp/Fzd7/Col6a1/H3f3b	6
GO:0048286	lung alveolus development	3/28	57/14464	0.05263158	27.1879699	8.72443387	0.00017725	0.01146817	0.00881992	Fbn1/Igfbp5/Col6a1	3
GO:0045778	positive regulation of ossification	3/28	58/14464	0.05172414	26.7192118	8.64340213	0.00018668	0.01146817	0.00881992	Ccn3/Bmp6/Kl	3
GO:0090278	negative regulation of peptide hormone secretion	3/28	58/14464	0.05172414	26.7192118	8.64340213	0.00018668	0.01146817	0.00881992	Fbn1/Ccn3/Ndufaf2	3
GO:0031214	biomineral tissue development	4/28	150/14464	0.02666667	13.7752381	6.92658069	0.00018744	0.01146817	0.00881992	Bmp6/Comp/Col6a1/Kl	4
GO:0002792	negative regulation of peptide secretion	3/28	60/14464	0.05	25.8285714	8.48731993	0.0002065	0.01146817	0.00881992	Fbn1/Ccn3/Ndufaf2	3
GO:0141091	transforming growth factor beta receptor superfamily signaling pathway	5/28	292/14464	0.01712329	8.84540117	5.96452389	0.00021751	0.01146817	0.00881992	Fbn1/Cilp/Bmp6/Comp/Slc2a10	5
GO:0001501	skeletal system development	6/28	466/14464	0.01287554	6.65113427	5.46110293	0.00022305	0.01146817	0.00881992	Fbn1/Ccn3/Bmp6/Comp/Col6a1/Slc2a10	6
GO:0030199	collagen fibril organization	3/28	62/14464	0.0483871	24.9953917	8.33867519	0.00022763	0.01146817	0.00881992	Loxl1/Comp/Col6a1	3
GO:0060395	SMAD protein signal transduction	3/28	68/14464	0.04411765	22.789916	7.93183853	0.0002993	0.01439367	0.01106985	Cilp/Ccn3/Bmp6	3
GO:0030500	regulation of bone mineralization	3/28	72/14464	0.04166667	21.5238095	7.68862042	0.00035434	0.01575341	0.0121156	Bmp6/Comp/Kl	3
GO:0090136	epithelial cell-cell adhesion	2/28	15/14464	0.13333333	68.8761905	11.583211	0.00037359	0.01575341	0.0121156	Ccn3/Kifc3	2
GO:0014823	response to activity	3/28	74/14464	0.04054054	20.9420849	7.57427025	0.00038414	0.01575341	0.0121156	Bmp6/Col6a1/Kl	3
GO:0050709	negative regulation of protein secretion	3/28	76/14464	0.03947368	20.3909774	7.46433384	0.00041552	0.01575341	0.0121156	Fbn1/Ccn3/Ndufaf2	3
GO:0051216	cartilage development	4/28	186/14464	0.02150538	11.109063	6.11108687	0.00042577	0.01575341	0.0121156	Ccn3/Bmp6/Comp/Col6a1	4
GO:0060392	negative regulation of SMAD protein signal transduction	2/28	16/14464	0.125	64.5714286	11.204768	0.00042645	0.01575341	0.0121156	Cilp/Ccn3	2
GO:0046888	negative regulation of hormone secretion	3/28	77/14464	0.03896104	20.1261596	7.4109313	0.0004318	0.01575341	0.0121156	Fbn1/Ccn3/Ndufaf2	3
GO:0031099	regeneration	4/28	191/14464	0.02094241	10.8182498	6.01558546	0.0004707	0.01660003	0.01276671	Ccn3/Fzd7/Col6a1/Cpq	4
GO:0043567	regulation of insulin-like growth factor receptor signaling pathway	2/28	18/14464	0.11111111	57.3968254	10.5439145	0.00054242	0.01851242	0.01423749	Cilp/Igfbp5	2
GO:0030512	negative regulation of transforming growth factor beta receptor signaling pathway	3/28	86/14464	0.03488372	18.0199336	6.97176305	0.00059712	0.01914395	0.01472318	Fbn1/Cilp/Slc2a10	3
GO:0070167	regulation of biomineral tissue development	3/28	86/14464	0.03488372	18.0199336	6.97176305	0.00059712	0.01914395	0.01472318	Bmp6/Comp/Kl	3
GO:0060348	bone development	4/28	207/14464	0.01932367	9.98205659	5.73233624	0.00063728	0.01983052	0.01525121	Fbn1/Bmp6/Comp/Col6a1	4
GO:1903828	negative regulation of protein localization	4/28	221/14464	0.01809955	9.34970911	5.50873039	0.00081422	0.02433584	0.01871616	Fbn1/Ccn3/Gbp4/Ndufaf2	4
GO:0043588	skin development	4/28	222/14464	0.01801802	9.30759331	5.49352362	0.00082806	0.02433584	0.01871616	Igfbp5/Comp/Col6a1/Slc2a10	4
GO:0035988	chondrocyte proliferation	2/28	23/14464	0.08695652	44.9192547	9.28335626	0.0008916	0.02549487	0.01960753	Ccn3/Comp	2
GO:0090025	regulation of monocyte chemotaxis	2/28	24/14464	0.08333333	43.047619	9.07921301	0.00097149	0.02704828	0.02080223	Cx3cr1/Ccn3	2
GO:0051051	negative regulation of transport	5/28	415/14464	0.01204819	6.22375215	4.75519879	0.00108129	0.0293334	0.02255966	Fbn1/Gstm7/Ccn3/Gbp4/Ndufaf2	5
GO:0002062	chondrocyte differentiation	3/28	107/14464	0.02803738	14.4833111	6.1651176	0.00112706	0.029448	0.0226478	Ccn3/Bmp6/Comp	3
GO:0071560	cellular response to transforming growth factor beta stimulus	4/28	242/14464	0.01652893	8.53837072	5.20822346	0.00114118	0.029448	0.0226478	Fbn1/Cx3cr1/Cilp/Slc2a10	4
GO:0071559	response to transforming growth factor beta	4/28	249/14464	0.01606426	8.2983362	5.11605116	0.00126811	0.03194432	0.02456766	Fbn1/Cx3cr1/Cilp/Slc2a10	4
GO:0046883	regulation of hormone secretion	4/28	261/14464	0.01532567	7.9168035	4.96615672	0.00150829	0.03655387	0.02811277	Fbn1/Ccn3/Bmp6/Ndufaf2	4
GO:0030261	chromosome condensation	2/28	30/14464	0.06666667	34.4380952	8.07408987	0.0015202	0.03655387	0.02811277	H3f3b/H1f0	2
GO:0030509	BMP signaling pathway	3/28	121/14464	0.02479339	12.8075561	5.74404026	0.00160591	0.0374276	0.02878473	Fbn1/Bmp6/Comp	3
GO:0010817	regulation of hormone levels	5/28	455/14464	0.01098901	5.67660911	4.46393615	0.00162729	0.0374276	0.02878473	Fbn1/Ccn3/Bmp6/Cpq/Ndufaf2	5
GO:0044342	type B pancreatic cell proliferation	2/28	32/14464	0.0625	32.2857143	7.80265808	0.00172924	0.03766485	0.0289672	Igfbp5/Ccn3	2
GO:0061448	connective tissue development	4/28	272/14464	0.01470588	7.59663866	4.83693352	0.00175493	0.03766485	0.0289672	Ccn3/Bmp6/Comp/Col6a1	4
GO:0002548	monocyte chemotaxis	2/28	33/14464	0.06060606	31.3073593	7.67611772	0.0018386	0.03766485	0.0289672	Cx3cr1/Ccn3	2
GO:0030198	extracellular matrix organization	4/28	276/14464	0.01449275	7.48654244	4.79172612	0.0018512	0.03766485	0.0289672	Loxl1/Comp/Col6a1/Slc2a10	4
GO:0043062	extracellular structure organization	4/28	276/14464	0.01449275	7.48654244	4.79172612	0.0018512	0.03766485	0.0289672	Loxl1/Comp/Col6a1/Slc2a10	4
GO:0045229	external encapsulating structure organization	4/28	276/14464	0.01449275	7.48654244	4.79172612	0.0018512	0.03766485	0.0289672	Loxl1/Comp/Col6a1/Slc2a10	4
GO:0017015	regulation of transforming growth factor beta receptor signaling pathway	3/28	129/14464	0.02325581	12.013289	5.53347326	0.00192895	0.03808013	0.02928658	Fbn1/Cilp/Slc2a10	3
GO:0071772	response to BMP	3/28	131/14464	0.02290076	11.82988	5.48372308	0.00201558	0.03808013	0.02928658	Fbn1/Bmp6/Comp	3
GO:0071773	cellular response to BMP stimulus	3/28	131/14464	0.02290076	11.82988	5.48372308	0.00201558	0.03808013	0.02928658	Fbn1/Bmp6/Comp	3
GO:1903844	regulation of cellular response to transforming growth factor beta stimulus	3/28	131/14464	0.02290076	11.82988	5.48372308	0.00201558	0.03808013	0.02928658	Fbn1/Cilp/Slc2a10	3
GO:0051154	negative regulation of striated muscle cell differentiation	2/28	37/14464	0.05405405	27.9227799	7.22133545	0.00230808	0.04284119	0.03294821	Ccn3/Fzd7	2
GO:0051146	striated muscle cell differentiation	4/28	311/14464	0.01286174	6.64400551	4.43126928	0.0028559	0.05209561	0.04006557	Igfbp5/Ccn3/Fzd7/Col6a1	4
GO:0046879	hormone secretion	4/28	314/14464	0.01273885	6.58052775	4.40297935	0.00295645	0.05301572	0.04077321	Fbn1/Ccn3/Bmp6/Ndufaf2	4
GO:0030501	positive regulation of bone mineralization	2/28	43/14464	0.04651163	24.0265781	6.65963941	0.00310707	0.05461502	0.04200319	Bmp6/Kl	2
GO:1903531	negative regulation of secretion by cell	3/28	154/14464	0.01948052	10.0630798	4.97967637	0.00318973	0.05461502	0.04200319	Fbn1/Ccn3/Ndufaf2	3
GO:0009914	hormone transport	4/28	321/14464	0.01246106	6.43702715	4.33838429	0.0032005	0.05461502	0.04200319	Fbn1/Ccn3/Bmp6/Ndufaf2	4
GO:0043403	skeletal muscle tissue regeneration	2/28	45/14464	0.04444444	22.9587302	6.49726649	0.00339829	0.05706977	0.04389109	Fzd7/Col6a1	2
GO:0070169	positive regulation of biomineral tissue development	2/28	48/14464	0.04166667	21.5238095	6.27250447	0.00385815	0.06378007	0.04905183	Bmp6/Kl	2
GO:0051048	negative regulation of secretion	3/28	170/14464	0.01764706	9.11596639	4.68783788	0.00421092	0.06854088	0.05271326	Fbn1/Ccn3/Ndufaf2	3
GO:0009306	protein secretion	4/28	352/14464	0.01136364	5.87012987	4.07382494	0.0044485	0.07131082	0.05484356	Fbn1/Ccn3/Comp/Ndufaf2	4
GO:0035592	establishment of protein localization to extracellular region	4/28	354/14464	0.01129944	5.83696529	4.05784886	0.00453884	0.07167307	0.05512216	Fbn1/Ccn3/Comp/Ndufaf2	4
GO:0071692	protein localization to extracellular region	4/28	357/14464	0.01120448	5.78791517	4.03411239	0.00467668	0.07276363	0.05596089	Fbn1/Ccn3/Comp/Ndufaf2	4
GO:0034332	adherens junction organization	2/28	54/14464	0.03703704	19.1322751	5.87897972	0.00485959	0.07350646	0.05653218	Bmp6/Kifc3	2
GO:0050796	regulation of insulin secretion	3/28	179/14464	0.01675978	8.65762171	4.54009639	0.00486338	0.07350646	0.05653218	Fbn1/Ccn3/Ndufaf2	3
GO:0050879	multicellular organismal movement	2/28	56/14464	0.03571429	18.4489796	5.76165129	0.00521729	0.07774494	0.0597919	Comp/Col6a1	2
GO:0001649	osteoblast differentiation	3/28	186/14464	0.01612903	8.33179724	4.43218624	0.00541096	0.07951111	0.06115022	Igfbp5/Bmp6/H3f3b	3
GO:0007179	transforming growth factor beta receptor signaling pathway	3/28	190/14464	0.01578947	8.15639098	4.37300331	0.00573992	0.08318959	0.06397926	Fbn1/Cilp/Slc2a10	3
GO:0030324	lung development	3/28	194/14464	0.01546392	7.98821797	4.31558915	0.00608071	0.08579415	0.06598237	Fbn1/Igfbp5/Col6a1	3
GO:0060350	endochondral bone morphogenesis	2/28	61/14464	0.03278689	16.9367681	5.49317537	0.00616291	0.08579415	0.06598237	Bmp6/Comp	2
GO:1903053	regulation of extracellular matrix organization	2/28	61/14464	0.03278689	16.9367681	5.49317537	0.00616291	0.08579415	0.06598237	Col6a1/Slc2a10	2
GO:0030323	respiratory tube development	3/28	197/14464	0.01522843	7.86656998	4.27357574	0.00634413	0.08627786	0.06635438	Fbn1/Igfbp5/Col6a1	3
GO:0051148	negative regulation of muscle cell differentiation	2/28	62/14464	0.03225806	16.6635945	5.44327962	0.00636075	0.08627786	0.06635438	Ccn3/Fzd7	2
GO:0009612	response to mechanical stimulus	3/28	202/14464	0.01485149	7.6718529	4.20549095	0.00679821	0.09104445	0.07002025	Bmp6/Col6a1/Kcnk2	3
GO:0090276	regulation of peptide hormone secretion	3/28	210/14464	0.01428571	7.37959184	4.10127543	0.00756434	0.09926615	0.07634338	Fbn1/Ccn3/Ndufaf2	3
GO:0043434	response to peptide hormone	4/28	410/14464	0.0097561	5.03972125	3.65450134	0.00759977	0.09926615	0.07634338	Fbn1/Igfbp5/Col6a1/Kl	4
GO:0030073	insulin secretion	3/28	213/14464	0.01408451	7.27565392	4.06359942	0.00786434	0.10042564	0.07723512	Fbn1/Ccn3/Ndufaf2	3
GO:0050673	epithelial cell proliferation	4/28	417/14464	0.00959233	4.95512162	3.60928229	0.00805903	0.10042564	0.07723512	Igfbp5/Ccn3/Bmp6/Fzd7	4
GO:0002791	regulation of peptide secretion	3/28	215/14464	0.01395349	7.20797342	4.03888658	0.00806822	0.10042564	0.07723512	Fbn1/Ccn3/Ndufaf2	3
GO:0090087	regulation of peptide transport	3/28	215/14464	0.01395349	7.20797342	4.03888658	0.00806822	0.10042564	0.07723512	Fbn1/Ccn3/Ndufaf2	3
GO:0045926	negative regulation of growth	3/28	216/14464	0.01388889	7.17460317	4.02664874	0.00817133	0.10052638	0.0773126	Igfbp5/Ccn3/Kcnk2	3
GO:0060541	respiratory system development	3/28	220/14464	0.01363636	7.04415584	3.97846722	0.00859159	0.10448166	0.08035452	Fbn1/Igfbp5/Col6a1	3
GO:0060537	muscle tissue development	4/28	435/14464	0.0091954	4.7500821	3.49749272	0.00932362	0.11209534	0.08621002	Igfbp5/Fzd7/Col6a1/Kcnk2	4
GO:0033627	cell adhesion mediated by integrin	2/28	80/14464	0.025	12.9142857	4.70606519	0.01040383	0.12367704	0.09511725	Fbn1/Ccn3	2
GO:0051223	regulation of protein transport	4/28	452/14464	0.00884956	4.57142857	3.39739383	0.01063199	0.12498494	0.09612313	Fbn1/Ccn3/Gbp4/Ndufaf2	4
GO:0001818	negative regulation of cytokine production	3/28	239/14464	0.0125523	6.48416019	3.76502372	0.01076107	0.12511217	0.09622098	Cx3cr1/Gbp4/Slc2a10	3
GO:0009611	response to wounding	4/28	465/14464	0.00860215	4.44362519	3.32413473	0.01170973	0.13466188	0.10356545	Comp/Fzd7/Col6a1/Kcnk2	4
GO:0033002	muscle cell proliferation	3/28	249/14464	0.01204819	6.22375215	3.6617912	0.01201973	0.13674064	0.10516418	Igfbp5/Ccn3/Kcnk2	3
GO:0051153	regulation of striated muscle cell differentiation	2/28	88/14464	0.02272727	11.7402597	4.45063379	0.01248377	0.13902982	0.10692473	Ccn3/Fzd7	2
GO:0120254	olefinic compound metabolic process	2/28	88/14464	0.02272727	11.7402597	4.45063379	0.01248377	0.13902982	0.10692473	Gstm7/Bmp6	2
GO:0030072	peptide hormone secretion	3/28	256/14464	0.01171875	6.05357143	3.59283017	0.01294949	0.14212087	0.10930199	Fbn1/Ccn3/Ndufaf2	3
GO:0001942	hair follicle development	2/28	90/14464	0.02222222	11.4793651	4.39189731	0.01302999	0.14212087	0.10930199	Igfbp5/Col6a1	2
GO:0090100	positive regulation of transmembrane receptor protein serine/threonine kinase signaling pathway	2/28	91/14464	0.02197802	11.3532182	4.36322012	0.01330697	0.14278557	0.1098132	Bmp6/Slc2a10	2
GO:0006112	energy reserve metabolic process	2/28	93/14464	0.02150538	11.109063	4.30718627	0.01386864	0.14278557	0.1098132	Col6a1/Kl	2
GO:0022404	molting cycle process	2/28	93/14464	0.02150538	11.109063	4.30718627	0.01386864	0.14278557	0.1098132	Igfbp5/Col6a1	2
GO:0022405	hair cycle process	2/28	93/14464	0.02150538	11.109063	4.30718627	0.01386864	0.14278557	0.1098132	Igfbp5/Col6a1	2
GO:0002790	peptide secretion	3/28	263/14464	0.01140684	5.89244976	3.52638363	0.01391968	0.14278557	0.1098132	Fbn1/Ccn3/Ndufaf2	3
GO:0050708	regulation of protein secretion	3/28	263/14464	0.01140684	5.89244976	3.52638363	0.01391968	0.14278557	0.1098132	Fbn1/Ccn3/Ndufaf2	3
GO:0060349	bone morphogenesis	2/28	94/14464	0.0212766	10.9908815	4.27980637	0.0141533	0.14278557	0.1098132	Bmp6/Comp	2
GO:0042593	glucose homeostasis	3/28	265/14464	0.01132075	5.84797844	3.50783784	0.01420434	0.14278557	0.1098132	Fbn1/Igfbp5/Ndufaf2	3
GO:0033500	carbohydrate homeostasis	3/28	266/14464	0.0112782	5.82599356	3.49863585	0.01434792	0.14278557	0.1098132	Fbn1/Igfbp5/Ndufaf2	3
GO:0019226	transmission of nerve impulse	2/28	95/14464	0.02105263	10.875188	4.25283637	0.01444051	0.14278557	0.1098132	Col6a1/Kcnk2	2
GO:0048608	reproductive structure development	3/28	268/14464	0.01119403	5.78251599	3.48037151	0.01463758	0.14297795	0.10996115	Bmp6/Col6a1/H3f3b	3
GO:0001704	formation of primary germ layer	2/28	96/14464	0.02083333	10.7619048	4.22626568	0.01473024	0.14297795	0.10996115	Fzd7/Col6a1	2
GO:0030336	negative regulation of cell migration	3/28	271/14464	0.01107011	5.7185029	3.45331776	0.01507833	0.14341296	0.11029571	Cx3cr1/Igfbp5/Ccn3	3
GO:0015833	peptide transport	3/28	272/14464	0.01102941	5.69747899	3.4443896	0.01522692	0.14341296	0.11029571	Fbn1/Ccn3/Ndufaf2	3
GO:0061458	reproductive system development	3/28	272/14464	0.01102941	5.69747899	3.4443896	0.01522692	0.14341296	0.11029571	Bmp6/Col6a1/H3f3b	3
GO:0018958	phenol-containing compound metabolic process	2/28	98/14464	0.02040816	10.5422741	4.1742818	0.01531726	0.14341296	0.11029571	Cpq/Kl	2
GO:0043491	phosphatidylinositol 3-kinase/protein kinase B signal transduction	3/28	278/14464	0.01079137	5.57451182	3.39173526	0.01613605	0.14664168	0.11277884	Cx3cr1/Igfbp5/Col6a1	3
GO:0090287	regulation of cellular response to growth factor stimulus	3/28	278/14464	0.01079137	5.57451182	3.39173526	0.01613605	0.14664168	0.11277884	Fbn1/Cilp/Slc2a10	3
GO:0002688	regulation of leukocyte chemotaxis	2/28	101/14464	0.01980198	10.2291372	4.09905722	0.01621652	0.14664168	0.11277884	Cx3cr1/Ccn3	2
GO:0007338	single fertilization	2/28	101/14464	0.01980198	10.2291372	4.09905722	0.01621652	0.14664168	0.11277884	Col6a1/H3f3b	2
GO:2000146	negative regulation of cell motility	3/28	282/14464	0.0106383	5.49544073	3.35747557	0.01675893	0.14791678	0.1137595	Cx3cr1/Igfbp5/Ccn3	3
GO:0042303	molting cycle	2/28	103/14464	0.01941748	10.0305132	4.05063839	0.01682839	0.14791678	0.1137595	Igfbp5/Col6a1	2
GO:0042633	hair cycle	2/28	103/14464	0.01941748	10.0305132	4.05063839	0.01682839	0.14791678	0.1137595	Igfbp5/Col6a1	2
GO:0032496	response to lipopolysaccharide	3/28	283/14464	0.01060071	5.47602221	3.3490127	0.01691676	0.14791678	0.1137595	Cx3cr1/Bmp6/Col6a1	3
GO:0060840	artery development	2/28	106/14464	0.01886792	9.74663073	3.98044403	0.01776454	0.15405642	0.11848136	Comp/Slc2a10	2
GO:0032341	aldosterone metabolic process	1/28	10/14464	0.1	51.6571429	7.05718396	0.01919656	0.15882451	0.12214839	Bmp6	1
GO:0048630	skeletal muscle tissue growth	1/28	10/14464	0.1	51.6571429	7.05718396	0.01919656	0.15882451	0.12214839	Col6a1	1
GO:0071281	cellular response to iron ion	1/28	10/14464	0.1	51.6571429	7.05718396	0.01919656	0.15882451	0.12214839	Bmp6	1
GO:1905208	negative regulation of cardiocyte differentiation	1/28	10/14464	0.1	51.6571429	7.05718396	0.01919656	0.15882451	0.12214839	Fzd7	1
GO:0002237	response to molecule of bacterial origin	3/28	297/14464	0.01010101	5.21789322	3.23459291	0.01921506	0.15882451	0.12214839	Cx3cr1/Bmp6/Col6a1	3
GO:0040013	negative regulation of locomotion	3/28	297/14464	0.01010101	5.21789322	3.23459291	0.01921506	0.15882451	0.12214839	Cx3cr1/Igfbp5/Ccn3	3
GO:0014909	smooth muscle cell migration	2/28	111/14464	0.01801802	9.30759331	3.86945809	0.01937307	0.15888919	0.12219813	Igfbp5/Ccn3	2
GO:0071675	regulation of mononuclear cell migration	2/28	114/14464	0.01754386	9.06265664	3.80618078	0.02036672	0.16411877	0.12622008	Cx3cr1/Ccn3	2
GO:0051053	negative regulation of DNA metabolic process	2/28	115/14464	0.0173913	8.98385093	3.78560498	0.02070263	0.16411877	0.12622008	H1f0/Kcnk2	2
GO:0007611	learning or memory	3/28	307/14464	0.00977199	5.04792927	3.15719393	0.0209585	0.16411877	0.12622008	Cx3cr1/Kcnk2/Bhlhb9	3
GO:0008212	mineralocorticoid metabolic process	1/28	11/14464	0.09090909	46.961039	6.71571026	0.02109655	0.16411877	0.12622008	Bmp6	1
GO:0034309	primary alcohol biosynthetic process	1/28	11/14464	0.09090909	46.961039	6.71571026	0.02109655	0.16411877	0.12622008	Bmp6	1
GO:0060231	mesenchymal to epithelial transition	1/28	11/14464	0.09090909	46.961039	6.71571026	0.02109655	0.16411877	0.12622008	Fzd7	1
GO:0071313	cellular response to caffeine	1/28	11/14464	0.09090909	46.961039	6.71571026	0.02109655	0.16411877	0.12622008	Gstm7	1
GO:0098773	skin epidermis development	2/28	118/14464	0.01694915	8.75544794	3.72535144	0.02172433	0.16762263	0.12891483	Igfbp5/Col6a1	2
GO:0042886	amide transport	3/28	314/14464	0.00955414	4.93539581	3.1049787	0.02222957	0.16762263	0.12891483	Fbn1/Ccn3/Ndufaf2	3
GO:0031507	heterochromatin formation	2/28	120/14464	0.01666667	8.60952381	3.68635993	0.022417	0.16762263	0.12891483	H3f3b/H1f0	2
GO:0002689	negative regulation of leukocyte chemotaxis	1/28	12/14464	0.08333333	43.047619	6.41730717	0.022993	0.16762263	0.12891483	Ccn3	1
GO:0010561	negative regulation of glycoprotein biosynthetic process	1/28	12/14464	0.08333333	43.047619	6.41730717	0.022993	0.16762263	0.12891483	Slc2a10	1
GO:0046886	positive regulation of hormone biosynthetic process	1/28	12/14464	0.08333333	43.047619	6.41730717	0.022993	0.16762263	0.12891483	Bmp6	1
GO:1990314	cellular response to insulin-like growth factor stimulus	1/28	12/14464	0.08333333	43.047619	6.41730717	0.022993	0.16762263	0.12891483	Fbn1	1
GO:2000833	positive regulation of steroid hormone secretion	1/28	12/14464	0.08333333	43.047619	6.41730717	0.022993	0.16762263	0.12891483	Bmp6	1
GO:0045667	regulation of osteoblast differentiation	2/28	124/14464	0.01612903	8.33179724	3.61103323	0.02382968	0.16762263	0.12891483	Igfbp5/Bmp6	2
GO:0014812	muscle cell migration	2/28	126/14464	0.01587302	8.19954649	3.57462836	0.02454954	0.16762263	0.12891483	Igfbp5/Ccn3	2
GO:0050728	negative regulation of inflammatory response	2/28	126/14464	0.01587302	8.19954649	3.57462836	0.02454954	0.16762263	0.12891483	Cx3cr1/Ccn3	2
GO:0001765	membrane raft assembly	1/28	13/14464	0.07692308	39.7362637	6.15354282	0.0248859	0.16762263	0.12891483	Col6a1	1
GO:0006103	2-oxoglutarate metabolic process	1/28	13/14464	0.07692308	39.7362637	6.15354282	0.0248859	0.16762263	0.12891483	Col6a1	1
GO:0014745	negative regulation of muscle adaptation	1/28	13/14464	0.07692308	39.7362637	6.15354282	0.0248859	0.16762263	0.12891483	Igfbp5	1
GO:0044857	plasma membrane raft organization	1/28	13/14464	0.07692308	39.7362637	6.15354282	0.0248859	0.16762263	0.12891483	Col6a1	1
GO:0051280	negative regulation of release of sequestered calcium ion into cytosol	1/28	13/14464	0.07692308	39.7362637	6.15354282	0.0248859	0.16762263	0.12891483	Gstm7	1
GO:0060623	regulation of chromosome condensation	1/28	13/14464	0.07692308	39.7362637	6.15354282	0.0248859	0.16762263	0.12891483	H3f3b	1
GO:0070341	fat cell proliferation	1/28	13/14464	0.07692308	39.7362637	6.15354282	0.0248859	0.16762263	0.12891483	Col6a1	1
GO:0071415	cellular response to purine-containing compound	1/28	13/14464	0.07692308	39.7362637	6.15354282	0.0248859	0.16762263	0.12891483	Gstm7	1
GO:0014074	response to purine-containing compound	2/28	128/14464	0.015625	8.07142857	3.53901921	0.02527831	0.16762263	0.12891483	Gstm7/Igfbp5	2
GO:0014902	myotube differentiation	2/28	128/14464	0.015625	8.07142857	3.53901921	0.02527831	0.16762263	0.12891483	Ccn3/Col6a1	2
GO:0009566	fertilization	2/28	131/14464	0.01526718	7.8865867	3.48703165	0.02638802	0.16762263	0.12891483	Col6a1/H3f3b	2
GO:0006590	thyroid hormone generation	1/28	14/14464	0.07142857	36.8979592	5.9181317	0.02677526	0.16762263	0.12891483	Cpq	1
GO:0006700	C21-steroid hormone biosynthetic process	1/28	14/14464	0.07142857	36.8979592	5.9181317	0.02677526	0.16762263	0.12891483	Bmp6	1
GO:0010832	negative regulation of myotube differentiation	1/28	14/14464	0.07142857	36.8979592	5.9181317	0.02677526	0.16762263	0.12891483	Ccn3	1
GO:0014733	regulation of skeletal muscle adaptation	1/28	14/14464	0.07142857	36.8979592	5.9181317	0.02677526	0.16762263	0.12891483	Igfbp5	1
GO:0030539	male genitalia development	1/28	14/14464	0.07142857	36.8979592	5.9181317	0.02677526	0.16762263	0.12891483	Bmp6	1
GO:0048103	somatic stem cell division	1/28	14/14464	0.07142857	36.8979592	5.9181317	0.02677526	0.16762263	0.12891483	Fzd7	1
GO:0060044	negative regulation of cardiac muscle cell proliferation	1/28	14/14464	0.07142857	36.8979592	5.9181317	0.02677526	0.16762263	0.12891483	Kcnk2	1

GO:0072498 embryonic skeletal joint development	1/28	14/14464	0.07142857	36.8979592	5.9181317	0.02677526	0.16762263	0.12891483	Slc2a10	1
GO:1900272 negative regulation of long-term synaptic potentiation	1/28	14/14464	0.07142857	36.8979592	5.9181317	0.02677526	0.16762263	0.12891483	Cx3cr1	1
GO:1903019 negative regulation of glycoprotein metabolic process	1/28	14/14464	0.07142857	36.8979592	5.9181317	0.02677526	0.16762263	0.12891483	Slc2a10	1
GO:1903977 positive regulation of glial cell migration	1/28	14/14464	0.07142857	36.8979592	5.9181317	0.02677526	0.16762263	0.12891483	Cx3cr1	1
GO:0051402 neuron apoptotic process	3/28	339/14464	0.00884956	4.57142857	2.93043681	0.02711046	0.1681504	0.12932072	Cx3cr1/Col6a1/Bhlhb9	3
GO:0046661 male sex differentiation	2/28	134/14464	0.01492537	7.71002132	3.43667114	0.02751738	0.1681504	0.12932072	Bmp6/H3f3b	2
GO:0050890 cognition	3/28	344/14464	0.00872093	4.50498339	2.89756114	0.02815067	0.1681504	0.12932072	Cx3cr1/Kcnk2/Bhlhb9	3
GO:0007565 female pregnancy	2/28	136/14464	0.01470588	7.59663866	3.40395755	0.02828107	0.1681504	0.12932072	Igfbp5/H3f3b	2
GO:0042178 xenobiotic catabolic process	1/28	15/14464	0.06666667	34.4380952	5.70627945	0.02866109	0.1681504	0.12932072	Gstm7	1
GO:0051284 positive regulation of sequestering of calcium ion	1/28	15/14464	0.06666667	34.4380952	5.70627945	0.02866109	0.1681504	0.12932072	Gstm7	1
GO:0097084 vascular associated smooth muscle cell development	1/28	15/14464	0.06666667	34.4380952	5.70627945	0.02866109	0.1681504	0.12932072	Comp	1
GO:0098528 skeletal muscle fiber differentiation	1/28	15/14464	0.06666667	34.4380952	5.70627945	0.02866109	0.1681504	0.12932072	Col6a1	1
GO:2000047 regulation of cell-cell adhesion mediated by cadherin	1/28	15/14464	0.06666667	34.4380952	5.70627945	0.02866109	0.1681504	0.12932072	Bmp6	1
GO:2000679 positive regulation of transcription regulatory region DNA binding	1/28	15/14464	0.06666667	34.4380952	5.70627945	0.02866109	0.1681504	0.12932072	H1f0	1
GO:2000725 regulation of cardiac muscle cell differentiation	1/28	15/14464	0.06666667	34.4380952	5.70627945	0.02866109	0.1681504	0.12932072	Fzd7	1
GO:0002544 chronic inflammatory response	1/28	16/14464	0.0625	32.2857143	5.5142566	0.0305434	0.1681504	0.12932072	Cx3cr1	1
GO:0007252 I-kappaB phosphorylation	1/28	16/14464	0.0625	32.2857143	5.5142566	0.0305434	0.1681504	0.12932072	Cx3cr1	1
GO:0007398 ectoderm development	1/28	16/14464	0.0625	32.2857143	5.5142566	0.0305434	0.1681504	0.12932072	Fzd7	1
GO:0036035 osteoclast development	1/28	16/14464	0.0625	32.2857143	5.5142566	0.0305434	0.1681504	0.12932072	Fbn1	1
GO:0042308 negative regulation of protein import into nucleus	1/28	16/14464	0.0625	32.2857143	5.5142566	0.0305434	0.1681504	0.12932072	Gbp4	1
GO:0043117 positive regulation of vascular permeability	1/28	16/14464	0.0625	32.2857143	5.5142566	0.0305434	0.1681504	0.12932072	Bmp6	1
GO:0098751 bone cell development	1/28	16/14464	0.0625	32.2857143	5.5142566	0.0305434	0.1681504	0.12932072	Fbn1	1
GO:1901862 negative regulation of muscle tissue development	1/28	16/14464	0.0625	32.2857143	5.5142566	0.0305434	0.1681504	0.12932072	Igfbp5	1
GO:0051147 regulation of muscle cell differentiation	2/28	144/14464	0.01388889	7.17460317	3.27946923	0.03142056	0.1681504	0.12932072	Ccn3/Fzd7	2
GO:0048872 homeostasis of number of cells	3/28	360/14464	0.00833333	4.3047619	2.79643706	0.03162257	0.1681504	0.12932072	Cx3cr1/Ccn3/Col6a1	3
GO:0007600 sensory perception	3/28	362/14464	0.00828729	4.28097869	2.78421079	0.03207188	0.1681504	0.12932072	Ccn3/Col6a1/Kcnk2	3
GO:0002281 macrophage activation involved in immune response	1/28	17/14464	0.05882353	30.3865546	5.33911244	0.03242219	0.1681504	0.12932072	Cx3cr1	1
GO:0003323 type B pancreatic cell development	1/28	17/14464	0.05882353	30.3865546	5.33911244	0.03242219	0.1681504	0.12932072	Bmp6	1
GO:0032095 regulation of response to food	1/28	17/14464	0.05882353	30.3865546	5.33911244	0.03242219	0.1681504	0.12932072	Fbn1	1
GO:0032352 positive regulation of hormone metabolic process	1/28	17/14464	0.05882353	30.3865546	5.33911244	0.03242219	0.1681504	0.12932072	Bmp6	1
GO:0042415 norepinephrine metabolic process	1/28	17/14464	0.05882353	30.3865546	5.33911244	0.03242219	0.1681504	0.12932072	Kl	1
GO:0042759 long-chain fatty acid biosynthetic process	1/28	17/14464	0.05882353	30.3865546	5.33911244	0.03242219	0.1681504	0.12932072	Gstm7	1
GO:0043171 peptide catabolic process	1/28	17/14464	0.05882353	30.3865546	5.33911244	0.03242219	0.1681504	0.12932072	Cpq	1
GO:0060314 regulation of ryanodine-sensitive calcium-release channel activity	1/28	17/14464	0.05882353	30.3865546	5.33911244	0.03242219	0.1681504	0.12932072	Gstm7	1
GO:0061179 negative regulation of insulin secretion involved in cellular response to glucose stimulus	1/28	17/14464	0.05882353	30.3865546	5.33911244	0.03242219	0.1681504	0.12932072	Ndufaf2	1
GO:0061760 antifungal innate immune response	1/28	17/14464	0.05882353	30.3865546	5.33911244	0.03242219	0.1681504	0.12932072	Cx3cr1	1
GO:0090030 regulation of steroid hormone biosynthetic process	1/28	17/14464	0.05882353	30.3865546	5.33911244	0.03242219	0.1681504	0.12932072	Bmp6	1
GO:0098883 synapse pruning	1/28	17/14464	0.05882353	30.3865546	5.33911244	0.03242219	0.1681504	0.12932072	Cx3cr1	1
GO:2000846 regulation of corticosteroid hormone secretion	1/28	17/14464	0.05882353	30.3865546	5.33911244	0.03242219	0.1681504	0.12932072	Bmp6	1
GO:0001911 negative regulation of leukocyte mediated cytotoxicity	1/28	18/14464	0.05555556	28.6984127	5.17847759	0.03429747	0.1716012	0.13197466	Cx3cr1	1
GO:0031000 response to caffeine	1/28	18/14464	0.05555556	28.6984127	5.17847759	0.03429747	0.1716012	0.13197466	Gstm7	1
GO:0031645 negative regulation of nervous system process	1/28	18/14464	0.05555556	28.6984127	5.17847759	0.03429747	0.1716012	0.13197466	Ccn3	1
GO:0032026 response to magnesium ion	1/28	18/14464	0.05555556	28.6984127	5.17847759	0.03429747	0.1716012	0.13197466	Bmp6	1
GO:0042659 regulation of cell fate specification	1/28	18/14464	0.05555556	28.6984127	5.17847759	0.03429747	0.1716012	0.13197466	Fzd7	1
GO:0060795 cell fate commitment involved in formation of primary germ layer	1/28	18/14464	0.05555556	28.6984127	5.17847759	0.03429747	0.1716012	0.13197466	Fzd7	1
GO:0090026 positive regulation of monocyte chemotaxis	1/28	18/14464	0.05555556	28.6984127	5.17847759	0.03429747	0.1716012	0.13197466	Cx3cr1	1
GO:0042445 hormone metabolic process	2/28	152/14464	0.01315789	6.79699248	3.16416512	0.03469219	0.1716012	0.13197466	Bmp6/Cpq	2
GO:0044703 multi-organism reproductive process	2/28	152/14464	0.01315789	6.79699248	3.16416512	0.03469219	0.1716012	0.13197466	Igfbp5/H3f3b	2
GO:0044706 multi-multicellular organism process	2/28	153/14464	0.0130719	6.75256769	3.1503386	0.03511021	0.1716012	0.13197466	Igfbp5/H3f3b	2
GO:0045814 negative regulation of gene expression, epigenetic	2/28	153/14464	0.0130719	6.75256769	3.1503386	0.03511021	0.1716012	0.13197466	H3f3b/H1f0	2
GO:0006575 cellular modified amino acid metabolic process	2/28	154/14464	0.01298701	6.70871985	3.13663545	0.03553022	0.1716012	0.13197466	Gstm7/Cpq	2
GO:0003417 growth plate cartilage development	1/28	19/14464	0.05263158	27.1879699	5.03042447	0.03616925	0.1716012	0.13197466	Comp	1
GO:0010566 regulation of ketone biosynthetic process	1/28	19/14464	0.05263158	27.1879699	5.03042447	0.03616925	0.1716012	0.13197466	Bmp6	1
GO:0032098 regulation of appetite	1/28	19/14464	0.05263158	27.1879699	5.03042447	0.03616925	0.1716012	0.13197466	Fbn1	1
GO:0032332 positive regulation of chondrocyte differentiation	1/28	19/14464	0.05263158	27.1879699	5.03042447	0.03616925	0.1716012	0.13197466	Bmp6	1
GO:0035930 corticosteroid hormone secretion	1/28	19/14464	0.05263158	27.1879699	5.03042447	0.03616925	0.1716012	0.13197466	Bmp6	1
GO:0045217 cell-cell junction maintenance	1/28	19/14464	0.05263158	27.1879699	5.03042447	0.03616925	0.1716012	0.13197466	Kifc3	1
GO:0051930 regulation of sensory perception of pain	1/28	19/14464	0.05263158	27.1879699	5.03042447	0.03616925	0.1716012	0.13197466	Ccn3	1
GO:0007613 memory	2/28	156/14464	0.01282051	6.62271062	3.10959148	0.03637615	0.17181237	0.13213706	Cx3cr1/Kcnk2	2
GO:0042403 thyroid hormone metabolic process	1/28	20/14464	0.05	25.8285714	4.8933667	0.03803753	0.17598593	0.13534686	Cpq	1
GO:0042537 benzene-containing compound metabolic process	1/28	20/14464	0.05	25.8285714	4.8933667	0.03803753	0.17598593	0.13534686	Gstm7	1
GO:0051931 regulation of sensory perception	1/28	20/14464	0.05	25.8285714	4.8933667	0.03803753	0.17598593	0.13534686	Ccn3	1
GO:2001044 regulation of integrin-mediated signaling pathway	1/28	20/14464	0.05	25.8285714	4.8933667	0.03803753	0.17598593	0.13534686	Slc2a10	1
GO:0065004 protein-DNA complex assembly	2/28	160/14464	0.0125	6.45714286	3.05690073	0.03809147	0.17598593	0.13534686	H3f3b/H1f0	2
GO:0051897 positive regulation of phosphatidylinositol 3-kinase/protein kinase B signal transduction	2/28	161/14464	0.01242236	6.41703638	3.04400873	0.03852514	0.17721564	0.13629259	Cx3cr1/Igfbp5	2
GO:0007369 gastrulation	2/28	162/14464	0.01234568	6.37742504	3.03122559	0.03896072	0.177698	0.13666357	Fzd7/Col6a1	2
GO:0071674 mononuclear cell migration	2/28	163/14464	0.01226994	6.33829974	3.01854967	0.03939821	0.177698	0.13666357	Cx3cr1/Ccn3	2
GO:0003309 type B pancreatic cell differentiation	1/28	21/14464	0.04761905	24.5986395	4.76598509	0.03990231	0.177698	0.13666357	Bmp6	1
GO:0009713 catechol-containing compound biosynthetic process	1/28	21/14464	0.04761905	24.5986395	4.76598509	0.03990231	0.177698	0.13666357	Kl	1
GO:0010560 positive regulation of glycoprotein biosynthetic process	1/28	21/14464	0.04761905	24.5986395	4.76598509	0.03990231	0.177698	0.13666357	Slc2a10	1
GO:0042423 catecholamine biosynthetic process	1/28	21/14464	0.04761905	24.5986395	4.76598509	0.03990231	0.177698	0.13666357	Kl	1
GO:0060065 uterus development	1/28	21/14464	0.04761905	24.5986395	4.76598509	0.03990231	0.177698	0.13666357	Col6a1	1
GO:0003012 muscle system process	3/28	395/14464	0.00759494	3.92332731	2.59435348	0.03997365	0.177698	0.13666357	Igfbp5/Comp/Col6a1	3
GO:0008207 C21-steroid hormone metabolic process	1/28	22/14464	0.04545455	23.4805195	4.64717241	0.04176361	0.18011366	0.1385214	Bmp6	1
GO:0031342 negative regulation of cell killing	1/28	22/14464	0.04545455	23.4805195	4.64717241	0.04176361	0.18011366	0.1385214	Cx3cr1	1
GO:0032691 negative regulation of interleukin-1 beta production	1/28	22/14464	0.04545455	23.4805195	4.64717241	0.04176361	0.18011366	0.1385214	Cx3cr1	1
GO:0071676 negative regulation of mononuclear cell migration	1/28	22/14464	0.04545455	23.4805195	4.64717241	0.04176361	0.18011366	0.1385214	Ccn3	1
GO:0120255 olefinic compound biosynthetic process	1/28	22/14464	0.04545455	23.4805195	4.64717241	0.04176361	0.18011366	0.1385214	Bmp6	1
GO:1904754 positive regulation of vascular associated smooth muscle cell migration	1/28	22/14464	0.04545455	23.4805195	4.64717241	0.04176361	0.18011366	0.1385214	Igfbp5	1
GO:1905207 regulation of cardiocyte differentiation	1/28	22/14464	0.04545455	23.4805195	4.64717241	0.04176361	0.18011366	0.1385214	Fzd7	1
GO:0048736 appendage development	2/28	172/14464	0.01162791	6.00664452	2.90903702	0.04342014	0.18011366	0.1385214	Comp/Col6a1	2
GO:0060173 limb development	2/28	172/14464	0.01162791	6.00664452	2.90903702	0.04342014	0.18011366	0.1385214	Comp/Col6a1	2
GO:0010737 protein kinase A signaling	1/28	23/14464	0.04347826	22.4596273	4.5359915	0.04362143	0.18011366	0.1385214	Fbn1	1
GO:0046885 regulation of hormone biosynthetic process	1/28	23/14464	0.04347826	22.4596273	4.5359915	0.04362143	0.18011366	0.1385214	Bmp6	1
GO:1901223 negative regulation of non-canonical NF-kappaB signal transduction	1/28	23/14464	0.04347826	22.4596273	4.5359915	0.04362143	0.18011366	0.1385214	Ccn3	1
GO:1903020 positive regulation of glycoprotein metabolic process	1/28	23/14464	0.04347826	22.4596273	4.5359915	0.04362143	0.18011366	0.1385214	Slc2a10	1
GO:2000831 regulation of steroid hormone secretion	1/28	23/14464	0.04347826	22.4596273	4.5359915	0.04362143	0.18011366	0.1385214	Bmp6	1
GO:0071222 cellular response to lipopolysaccharide	2/28	173/14464	0.01156069	5.97192403	2.89735025	0.04387626	0.18011366	0.1385214	Cx3cr1/Bmp6	2
GO:0048771 tissue remodeling	2/28	174/14464	0.01149425	5.93760263	2.88575474	0.04433419	0.18011366	0.1385214	Igfbp5/Col6a1	2
GO:0050920 regulation of chemotaxis	2/28	175/14464	0.01142857	5.90367347	2.87424922	0.04479394	0.18011366	0.1385214	Cx3cr1/Ccn3	2
GO:0014706 striated muscle tissue development	3/28	414/14464	0.00724638	3.74327122	2.49410957	0.04493683	0.18011366	0.1385214	Fzd7/Col6a1/Kcnk2	3
GO:0045216 cell-cell junction organization	2/28	176/14464	0.01136364	5.87012987	2.86283243	0.04525548	0.18011366	0.1385214	Bmp6/Kifc3	2
GO:0050954 sensory perception of mechanical stimulus	2/28	176/14464	0.01136364	5.87012987	2.86283243	0.04525548	0.18011366	0.1385214	Col6a1/Kcnk2	2
GO:0003085 negative regulation of systemic arterial blood pressure	1/28	24/14464	0.04166667	21.5238095	4.43164304	0.04547577	0.18011366	0.1385214	Kl	1
GO:0010880 regulation of release of sequestered calcium ion into cytosol by sarcoplasmic reticulum	1/28	24/14464	0.04166667	21.5238095	4.43164304	0.04547577	0.18011366	0.1385214	Gstm7	1
GO:0031664 regulation of lipopolysaccharide-mediated signaling pathway	1/28	24/14464	0.04166667	21.5238095	4.43164304	0.04547577	0.18011366	0.1385214	Bmp6	1
GO:0035883 enteroendocrine cell differentiation	1/28	24/14464	0.04166667	21.5238095	4.43164304	0.04547577	0.18011366	0.1385214	Bmp6	1
GO:0043501 skeletal muscle adaptation	1/28	24/14464	0.04166667	21.5238095	4.43164304	0.04547577	0.18011366	0.1385214	Igfbp5	1
GO:0046184 aldehyde biosynthetic process	1/28	24/14464	0.04166667	21.5238095	4.43164304	0.04547577	0.18011366	0.1385214	Bmp6	1
GO:0090335 regulation of brown fat cell differentiation	1/28	24/14464	0.04166667	21.5238095	4.43164304	0.04547577	0.18011366	0.1385214	Fbn1	1
GO:1901615 organic hydroxy compound metabolic process	3/28	417/14464	0.00719424	3.71634121	2.47882194	0.04574779	0.18011366	0.1385214	Bmp6/Cpq/Kl	3
GO:0030595 leukocyte chemotaxis	2/28	179/14464	0.01117318	5.77174781	2.82910227	0.04665084	0.18011366	0.1385214	Cx3cr1/Ccn3	2
GO:0035265 organ growth	2/28	180/14464	0.01111111	5.73968254	2.81802835	0.04711951	0.18011366	0.1385214	Comp/Kcnk2	2
GO:0048659 smooth muscle cell proliferation	2/28	180/14464	0.01111111	5.73968254	2.81802835	0.04711951	0.18011366	0.1385214	Igfbp5/Ccn3	2
GO:0071219 cellular response to molecule of bacterial origin	2/28	180/14464	0.01111111	5.73968254	2.81802835	0.04711951	0.18011366	0.1385214	Cx3cr1/Bmp6	2
GO:0071824 protein-DNA complex organization	2/28	180/14464	0.01111111	5.73968254	2.81802835	0.04711951	0.18011366	0.1385214	H3f3b/H1f0	2
GO:0003416 endochondral bone growth	1/28	25/14464	0.04	20.6628571	4.33344054	0.04732665	0.18011366	0.1385214	Comp	1
GO:0010039 response to iron ion	1/28	25/14464	0.04	20.6628571	4.33344054	0.04732665	0.18011366	0.1385214	Bmp6	1
GO:0014829 vascular associated smooth muscle contraction	1/28	25/14464	0.04	20.6628571	4.33344054	0.04732665	0.18011366	0.1385214	Comp	1
GO:0033280 response to vitamin D	1/28	25/14464	0.04	20.6628571	4.33344054	0.04732665	0.18011366	0.1385214	Kl	1
GO:0046823 negative regulation of nucleocytoplasmic transport	1/28	25/14464	0.04	20.6628571	4.33344054	0.04732665	0.18011366	0.1385214	Gbp4	1
GO:1900037 regulation of cellular response to hypoxia	1/28	25/14464	0.04	20.6628571	4.33344054	0.04732665	0.18011366	0.1385214	Kcnk2	1
GO:1903975 regulation of glial cell migration	1/28	25/14464	0.04	20.6628571	4.33344054	0.04732665	0.18011366	0.1385214	Cx3cr1	1
GO:0010893 positive regulation of steroid biosynthetic process	1/28	26/14464	0.03846154	19.8681319	4.24079062	0.04917407	0.18157254	0.13964339	Bmp6	1
GO:0030262 apoptotic nuclear changes	1/28	26/14464	0.03846154	19.8681319	4.24079062	0.04917407	0.18157254	0.13964339	Col6a1	1
GO:0060416 response to growth hormone	1/28	26/14464	0.03846154	19.8681319	4.24079062	0.04917407	0.18157254	0.13964339	Igfbp5	1
GO:0060479 lung cell differentiation	1/28	26/14464	0.03846154	19.8681319	4.24079062	0.04917407	0.18157254	0.13964339	Col6a1	1
GO:0060487 lung epithelial cell differentiation	1/28	26/14464	0.03846154	19.8681319	4.24079062	0.04917407	0.18157254	0.13964339	Col6a1	1
GO:1901020 negative regulation of calcium ion transmembrane transporter activity	1/28	26/14464	0.03846154	19.8681319	4.24079062	0.04917407	0.18157254	0.13964339	Gstm7	1
GO:1905523 positive regulation of macrophage migration	1/28	26/14464	0.03846154	19.8681319	4.24079062	0.04917407	0.18157254	0.13964339	Cx3cr1	1
GO:0002683 negative regulation of immune system process	3/28	433/14464	0.00692841	3.57901683	2.39959851	0.05019735	0.18157254	0.13964339	Fbn1/Cx3cr1/Ccn3	3
GO:0050679 positive regulation of epithelial cell proliferation	2/28	187/14464	0.01069519	5.52482811	2.74276868	0.05044903	0.18157254	0.13964339	Bmp6/Fzd7	2
GO:1901617 organic hydroxy compound biosynthetic process	2/28	187/14464	0.01069519	5.52482811	2.74276868	0.05044903	0.18157254	0.13964339	Bmp6/Kl	2
GO:0043524 negative regulation of neuron apoptotic process	2/28	188/14464	0.0106383	5.49544073	2.73232719	0.05093155	0.18157254	0.13964339	Cx3cr1/Bhlhb9	2
GO:0006921 cellular component disassembly involved in execution phase of apoptosis	1/28	27/14464	0.03703704	19.1322751	4.15317734	0.05101803	0.18157254	0.13964339	Col6a1	1
GO:0014808 release of sequestered calcium ion into cytosol by sarcoplasmic reticulum	1/28	27/14464	0.03703704	19.1322751	4.15317734	0.05101803	0.18157254	0.13964339	Gstm7	1
GO:0031069 hair follicle morphogenesis	1/28	27/14464	0.03703704	19.1322751	4.15317734	0.05101803	0.18157254	0.13964339	Igfbp5	1
GO:0031579 membrane raft organization	1/28	27/14464	0.03703704	19.1322751	4.15317734	0.05101803	0.18157254	0.13964339	Col6a1	1
GO:0032647 regulation of interferon-alpha production	1/28	27/14464	0.03703704	19.1322751	4.15317734	0.05101803	0.18157254	0.13964339	Gbp4	1
GO:0034508 centromere complex assembly	1/28	27/14464	0.03703704	19.1322751	4.15317734	0.05101803	0.18157254	0.13964339	H3f3b	1
GO:0035929 steroid hormone secretion	1/28	27/14464	0.03703704	19.1322751	4.15317734	0.05101803	0.18157254	0.13964339	Bmp6	1
GO:0002685 regulation of leukocyte migration	2/28	191/14464	0.0104712	5.40912491	2.70144559	0.05238929	0.18157254	0.13964339	Cx3cr1/Ccn3	2
GO:0002068 glandular epithelial cell development	1/28	28/14464	0.03571429	18.4489796	4.07014973	0.05285855	0.18157254	0.13964339	Bmp6	1
GO:0014850 response to muscle activity	1/28	28/14464	0.03571429	18.4489796	4.07014973	0.05285855	0.18157254	0.13964339	Col6a1	1
GO:0032692 negative regulation of interleukin-1 production	1/28	28/14464	0.03571429	18.4489796	4.07014973	0.05285855	0.18157254	0.13964339	Cx3cr1	1
GO:0035767 endothelial cell chemotaxis	1/28	28/14464	0.03571429	18.4489796	4.07014973	0.05285855	0.18157254	0.13964339	Ccn3	1
GO:0042832 defense response to protozoan	1/28	28/14464	0.03571429	18.4489796	4.07014973	0.05285855	0.18157254	0.13964339	Gbp4	1
GO:0050901 leukocyte tethering or rolling	1/28	28/14464	0.03571429	18.4489796	4.07014973	0.05285855	0.18157254	0.13964339	Cx3cr1	1
GO:0055022 negative regulation of cardiac muscle tissue growth	1/28	28/14464	0.03571429	18.4489796	4.07014973	0.05285855	0.18157254	0.13964339	Kcnk2	1
GO:0061036 positive regulation of cartilage development	1/28	28/14464	0.03571429	18.4489796	4.07014973	0.05285855	0.18157254	0.13964339	Bmp6	1
GO:0061117 negative regulation of heart growth	1/28	28/14464	0.03571429	18.4489796	4.07014973	0.05285855	0.18157254	0.13964339	Kcnk2	1
GO:1902932 positive regulation of alcohol biosynthetic process	1/28	28/14464	0.03571429	18.4489796	4.07014973	0.05285855	0.18157254	0.13964339	Bmp6	1
GO:1903514 release of sequestered calcium ion into cytosol by endoplasmic reticulum	1/28	28/14464	0.03571429	18.4489796	4.07014973	0.05285855	0.18157254	0.13964339	Gstm7	1
GO:0008015 blood circulation	3/28	443/14464	0.00677201	3.49822638	2.35195458	0.05308382	0.18175625	0.13978468	Bmp6/Comp/Kl	3
GO:0045671 negative regulation of osteoclast differentiation	1/28	29/14464	0.03448276	17.8128079	3.99131154	0.05469562	0.18218623	0.14011537	Fbn1	1
GO:0045940 positive regulation of steroid metabolic process	1/28	29/14464	0.03448276	17.8128079	3.99131154	0.05469562	0.18218623	0.14011537	Bmp6	1
GO:0051882 mitochondrial depolarization	1/28	29/14464	0.03448276	17.8128079	3.99131154	0.05469562	0.18218623	0.14011537	Col6a1	1
GO:0060586 multicellular organismal-level iron ion homeostasis	1/28	29/14464	0.03448276	17.8128079	3.99131154	0.05469562	0.18218623	0.14011537	Bmp6	1
GO:0061484 hematopoietic stem cell homeostasis	1/28	29/14464	0.03448276	17.8128079	3.99131154	0.05469562	0.18218623	0.14011537	Ccn3	1
GO:0098868 bone growth	1/28	29/14464	0.03448276	17.8128079	3.99131154	0.05469562	0.18218623	0.14011537	Comp	1
GO:0120178 steroid hormone biosynthetic process	1/28	29/14464	0.03448276	17.8128079	3.99131154	0.05469562	0.18218623	0.14011537	Bmp6	1
GO:2001258 negative regulation of cation channel activity	1/28	29/14464	0.03448276	17.8128079	3.99131154	0.05469562	0.18218623	0.14011537	Gstm7	1
GO:0097529 myeloid leukocyte migration	2/28	196/14464	0.01020408	5.27113703	2.65139701	0.0548522	0.18218623	0.14011537	Cx3cr1/Ccn3	2
GO:0098771 inorganic ion homeostasis	3/28	454/14464	0.00660793	3.41346759	2.30109793	0.05635154	0.18218623	0.14011537	Gstm7/Bmp6/Kl	3
GO:0001562 response to protozoan	1/28	30/14464	0.03333333	17.2190476	3.91631303	0.05652925	0.18218623	0.14011537	Gbp4	1
GO:0002063 chondrocyte development	1/28	30/14464	0.03333333	17.2190476	3.91631303	0.05652925	0.18218623	0.14011537	Comp	1
GO:0010453 regulation of cell fate commitment	1/28	30/14464	0.03333333	17.2190476	3.91631303	0.05652925	0.18218623	0.14011537	Fzd7	1
GO:0030511 positive regulation of transforming growth factor beta receptor signaling pathway	1/28	30/14464	0.03333333	17.2190476	3.91631303	0.05652925	0.18218623	0.14011537	Slc2a10	1
GO:0032107 regulation of response to nutrient levels	1/28	30/14464	0.03333333	17.2190476	3.91631303	0.05652925	0.18218623	0.14011537	Fbn1	1
GO:0032607 interferon-alpha production	1/28	30/14464	0.03333333	17.2190476	3.91631303	0.05652925	0.18218623	0.14011537	Gbp4	1
GO:0048048 embryonic eye morphogenesis	1/28	30/14464	0.03333333	17.2190476	3.91631303	0.05652925	0.18218623	0.14011537	Fbn1	1
GO:0150146 cell junction disassembly	1/28	30/14464	0.03333333	17.2190476	3.91631303	0.05652925	0.18218623	0.14011537	Cx3cr1	1
GO:1903846 positive regulation of cellular response to transforming growth factor beta stimulus	1/28	30/14464	0.03333333	17.2190476	3.91631303	0.05652925	0.18218623	0.14011537	Slc2a10	1
GO:0031667 response to nutrient levels	3/28	455/14464	0.00659341	3.40596546	2.29655204	0.05665337	0.18218623	0.14011537	Fbn1/Col6a1/Kl	3
GO:0009636 response to toxic substance	2/28	201/14464	0.00995025	5.14001421	2.60303011	0.05735599	0.18321752	0.14090851	Gstm7/Col6a1	2
GO:0035264 multicellular organism growth	2/28	202/14464	0.00990099	5.1145686	2.59354954	0.05786157	0.18321752	0.14090851	Comp/H3f3b	2

GO:0006099 tricarboxylic acid cycle	1/28	31/14464	0.03225806	16.6635945	3.84484418	0.05835946	0.18321752	0.14090851	Col6a1	1
GO:0045454 cell redox homeostasis	1/28	31/14464	0.03225806	16.6635945	3.84484418	0.05835946	0.18321752	0.14090851	Slc2a10	1
GO:0048730 epidermis morphogenesis	1/28	31/14464	0.03225806	16.6635945	3.84484418	0.05835946	0.18321752	0.14090851	Igfbp5	1
GO:0061003 positive regulation of dendritic spine morphogenesis	1/28	31/14464	0.03225806	16.6635945	3.84484418	0.05835946	0.18321752	0.14090851	Bhlhb9	1
GO:1901021 positive regulation of calcium ion transmembrane transporter activity	1/28	31/14464	0.03225806	16.6635945	3.84484418	0.05835946	0.18321752	0.14090851	Gstm7	1
GO:2000279 negative regulation of DNA biosynthetic process	1/28	31/14464	0.03225806	16.6635945	3.84484418	0.05835946	0.18321752	0.14090851	Kcnk2	1
GO:0071216 cellular response to biotic stimulus	2/28	205/14464	0.0097561	5.03972125	2.56547961	0.05938781	0.18377075	0.14133399	Cx3cr1/Bmp6	2
GO:0002021 response to dietary excess	1/28	32/14464	0.03125	16.1428571	3.77662902	0.06018624	0.18377075	0.14133399	Col6a1	1
GO:0009309 amine biosynthetic process	1/28	32/14464	0.03125	16.1428571	3.77662902	0.06018624	0.18377075	0.14133399	Kl	1
GO:0032094 response to food	1/28	32/14464	0.03125	16.1428571	3.77662902	0.06018624	0.18377075	0.14133399	Fbn1	1
GO:0042401 biogenic amine biosynthetic process	1/28	32/14464	0.03125	16.1428571	3.77662902	0.06018624	0.18377075	0.14133399	Kl	1
GO:0050922 negative regulation of chemotaxis	1/28	32/14464	0.03125	16.1428571	3.77662902	0.06018624	0.18377075	0.14133399	Ccn3	1
GO:0051180 vitamin transport	1/28	32/14464	0.03125	16.1428571	3.77662902	0.06018624	0.18377075	0.14133399	Slc2a10	1
GO:1900047 negative regulation of hemostasis	1/28	32/14464	0.03125	16.1428571	3.77662902	0.06018624	0.18377075	0.14133399	Comp	1
GO:2000677 regulation of transcription regulatory region DNA binding	1/28	32/14464	0.03125	16.1428571	3.77662902	0.06018624	0.18377075	0.14133399	H1f0	1
GO:0003018 vascular process in circulatory system	2/28	207/14464	0.00966184	4.9910283	2.5470688	0.06041314	0.18377075	0.14133399	Bmp6/Comp	2
GO:0003013 circulatory system process	3/28	470/14464	0.00638298	3.29726444	2.22984028	0.06127541	0.18377075	0.14133399	Bmp6/Comp/Kl	3
GO:0031348 negative regulation of defense response	2/28	210/14464	0.00952381	4.91972789	2.5198922	0.06196277	0.18377075	0.14133399	Cx3cr1/Ccn3	2
GO:0014047 glutamate secretion	1/28	33/14464	0.03030303	15.6536797	3.71142101	0.0620096	0.18377075	0.14133399	Kcnk2	1
GO:0022011 myelination in peripheral nervous system	1/28	33/14464	0.03030303	15.6536797	3.71142101	0.0620096	0.18377075	0.14133399	Col6a1	1
GO:0031018 endocrine pancreas development	1/28	33/14464	0.03030303	15.6536797	3.71142101	0.0620096	0.18377075	0.14133399	Bmp6	1
GO:0032292 peripheral nervous system axon ensheathment	1/28	33/14464	0.03030303	15.6536797	3.71142101	0.0620096	0.18377075	0.14133399	Col6a1	1
GO:0060351 cartilage development involved in endochondral bone morphogenesis	1/28	33/14464	0.03030303	15.6536797	3.71142101	0.0620096	0.18377075	0.14133399	Comp	1
GO:0060391 positive regulation of SMAD protein signal transduction	1/28	33/14464	0.03030303	15.6536797	3.71142101	0.0620096	0.18377075	0.14133399	Bmp6	1
GO:0070296 sarcoplasmic reticulum calcium ion transport	1/28	33/14464	0.03030303	15.6536797	3.71142101	0.0620096	0.18377075	0.14133399	Gstm7	1
GO:0090317 negative regulation of intracellular protein transport	1/28	33/14464	0.03030303	15.6536797	3.71142101	0.0620096	0.18377075	0.14133399	Gbp4	1
GO:0001958 endochondral ossification	1/28	34/14464	0.02941176	15.1932773	3.64899907	0.06382955	0.18552656	0.14268434	Bmp6	1
GO:0002052 positive regulation of neuroblast proliferation	1/28	34/14464	0.02941176	15.1932773	3.64899907	0.06382955	0.18552656	0.14268434	Cx3cr1	1
GO:0035987 endodermal cell differentiation	1/28	34/14464	0.02941176	15.1932773	3.64899907	0.06382955	0.18552656	0.14268434	Col6a1	1
GO:0036075 replacement ossification	1/28	34/14464	0.02941176	15.1932773	3.64899907	0.06382955	0.18552656	0.14268434	Bmp6	1
GO:0048806 genitalia development	1/28	34/14464	0.02941176	15.1932773	3.64899907	0.06382955	0.18552656	0.14268434	Bmp6	1
GO:0071398 cellular response to fatty acid	1/28	34/14464	0.02941176	15.1932773	3.64899907	0.06382955	0.18552656	0.14268434	Kcnk2	1
GO:1904752 regulation of vascular associated smooth muscle cell migration	1/28	34/14464	0.02941176	15.1932773	3.64899907	0.06382955	0.18552656	0.14268434	Igfbp5	1
GO:0048705 skeletal system morphogenesis	2/28	214/14464	0.00934579	4.82777036	2.48444909	0.06405031	0.18565815	0.14278555	Bmp6/Comp	2
GO:0014912 negative regulation of smooth muscle cell migration	1/28	35/14464	0.02857143	14.7591837	3.58916435	0.0656461	0.1892468	0.14554549	Igfbp5	1
GO:0051281 positive regulation of release of sequestered calcium ion into cytosol	1/28	35/14464	0.02857143	14.7591837	3.58916435	0.0656461	0.1892468	0.14554549	Gstm7	1
GO:0055001 muscle cell development	2/28	218/14464	0.00917431	4.73918742	2.44987419	0.06616185	0.19021531	0.14629036	Comp/Col6a1	2
GO:0014741 negative regulation of muscle hypertrophy	1/28	36/14464	0.02777778	14.3492063	3.53173738	0.06745925	0.19237705	0.1479529	Igfbp5	1
GO:0032350 regulation of hormone metabolic process	1/28	36/14464	0.02777778	14.3492063	3.53173738	0.06745925	0.19237705	0.1479529	Bmp6	1
GO:0045747 positive regulation of Notch signaling pathway	1/28	36/14464	0.02777778	14.3492063	3.53173738	0.06745925	0.19237705	0.1479529	Ccn3	1
GO:0005975 carbohydrate metabolic process	3/28	491/14464	0.00610998	3.15624091	2.14080924	0.06803941	0.19350994	0.14882418	Fbn1/Col6a1/Kl	3
GO:0002686 negative regulation of leukocyte migration	1/28	37/14464	0.02702703	13.96139	3.47655573	0.069269	0.19543095	0.15030159	Ccn3	1
GO:0045601 regulation of endothelial cell differentiation	1/28	37/14464	0.02702703	13.96139	3.47655573	0.069269	0.19543095	0.15030159	Bmp6	1
GO:0046621 negative regulation of organ growth	1/28	37/14464	0.02702703	13.96139	3.47655573	0.069269	0.19543095	0.15030159	Kcnk2	1
GO:0071396 cellular response to lipid	3/28	499/14464	0.00601202	3.10563985	2.1081362	0.07070441	0.1973694	0.15179241	Cx3cr1/Bmp6/Kcnk2	3
GO:0035886 vascular associated smooth muscle cell differentiation	1/28	38/14464	0.02631579	13.593985	3.42347197	0.07107537	0.1973694	0.15179241	Comp	1
GO:0042755 eating behavior	1/28	38/14464	0.02631579	13.593985	3.42347197	0.07107537	0.1973694	0.15179241	Col6a1	1
GO:0044331 cell-cell adhesion mediated by cadherin	1/28	38/14464	0.02631579	13.593985	3.42347197	0.07107537	0.1973694	0.15179241	Bmp6	1
GO:0050832 defense response to fungus	1/28	38/14464	0.02631579	13.593985	3.42347197	0.07107537	0.1973694	0.15179241	Cx3cr1	1
GO:0071312 cellular response to alkaloid	1/28	38/14464	0.02631579	13.593985	3.42347197	0.07107537	0.1973694	0.15179241	Gstm7	1
GO:0040029 epigenetic regulation of gene expression	2/28	230/14464	0.00869565	4.49192547	2.35098671	0.07263585	0.19821415	0.15244208	H3f3b/H1f0	2
GO:0010559 regulation of glycoprotein biosynthetic process	1/28	39/14464	0.02564103	13.2454212	3.37235196	0.07287836	0.19821415	0.15244208	Slc2a10	1
GO:0010830 regulation of myotube differentiation	1/28	39/14464	0.02564103	13.2454212	3.37235196	0.07287836	0.19821415	0.15244208	Ccn3	1
GO:0017145 stem cell division	1/28	39/14464	0.02564103	13.2454212	3.37235196	0.07287836	0.19821415	0.15244208	Fzd7	1
GO:0032228 regulation of synaptic transmission, GABAergic	1/28	39/14464	0.02564103	13.2454212	3.37235196	0.07287836	0.19821415	0.15244208	Kcnk2	1
GO:0046189 phenol-containing compound biosynthetic process	1/28	39/14464	0.02564103	13.2454212	3.37235196	0.07287836	0.19821415	0.15244208	Kl	1
GO:1904738 vascular associated smooth muscle cell migration	1/28	39/14464	0.02564103	13.2454212	3.37235196	0.07287836	0.19821415	0.15244208	Igfbp5	1
GO:1905521 regulation of macrophage migration	1/28	39/14464	0.02564103	13.2454212	3.37235196	0.07287836	0.19821415	0.15244208	Cx3cr1	1
GO:0043114 regulation of vascular permeability	1/28	40/14464	0.025	12.9142857	3.3230733	0.07467797	0.2005312	0.15422408	Bmp6	1
GO:0043388 positive regulation of DNA binding	1/28	40/14464	0.025	12.9142857	3.3230733	0.07467797	0.2005312	0.15422408	H1f0	1
GO:0060043 regulation of cardiac muscle cell proliferation	1/28	40/14464	0.025	12.9142857	3.3230733	0.07467797	0.2005312	0.15422408	Kcnk2	1
GO:0071711 basement membrane organization	1/28	40/14464	0.025	12.9142857	3.3230733	0.07467797	0.2005312	0.15422408	Col6a1	1
GO:1900181 negative regulation of protein localization to nucleus	1/28	40/14464	0.025	12.9142857	3.3230733	0.07467797	0.2005312	0.15422408	Gbp4	1
GO:0001706 endoderm formation	1/28	41/14464	0.02439024	12.5993031	3.27552408	0.07647421	0.20134977	0.15485362	Col6a1	1
GO:0032480 negative regulation of type I interferon production	1/28	41/14464	0.02439024	12.5993031	3.27552408	0.07647421	0.20134977	0.15485362	Gbp4	1
GO:0044060 regulation of endocrine process	1/28	41/14464	0.02439024	12.5993031	3.27552408	0.07647421	0.20134977	0.15485362	Bmp6	1
GO:0045910 negative regulation of DNA recombination	1/28	41/14464	0.02439024	12.5993031	3.27552408	0.07647421	0.20134977	0.15485362	H1f0	1
GO:0048741 skeletal muscle fiber development	1/28	41/14464	0.02439024	12.5993031	3.27552408	0.07647421	0.20134977	0.15485362	Col6a1	1
GO:1990776 response to angiotensin	1/28	41/14464	0.02439024	12.5993031	3.27552408	0.07647421	0.20134977	0.15485362	Kl	1
GO:0007548 sex differentiation	2/28	237/14464	0.00843882	4.35925256	2.29638624	0.0765053	0.20134977	0.15485362	Bmp6/H3f3b	2
GO:0048738 cardiac muscle tissue development	2/28	237/14464	0.00843882	4.35925256	2.29638624	0.0765053	0.20134977	0.15485362	Fzd7/Kcnk2	2
GO:0051896 regulation of phosphatidylinositol 3-kinase/protein kinase B signal transduction	2/28	240/14464	0.00833333	4.3047619	2.27362951	0.07818384	0.20345599	0.15647346	Cx3cr1/Igfbp5	2
GO:0042446 hormone biosynthetic process	1/28	42/14464	0.02380952	12.2993197	3.22960173	0.0782671	0.20345599	0.15647346	Bmp6	1
GO:0050974 detection of mechanical stimulus involved in sensory perception	1/28	42/14464	0.02380952	12.2993197	3.22960173	0.0782671	0.20345599	0.15647346	Kcnk2	1
GO:0060428 lung epithelium development	1/28	42/14464	0.02380952	12.2993197	3.22960173	0.0782671	0.20345599	0.15647346	Col6a1	1
GO:1904707 positive regulation of vascular associated smooth muscle cell proliferation	1/28	42/14464	0.02380952	12.2993197	3.22960173	0.0782671	0.20345599	0.15647346	Igfbp5	1
GO:0006458 'de novo' protein folding	1/28	43/14464	0.02325581	12.013289	3.18521204	0.08005662	0.20558229	0.15810876	Telo2	1
GO:0042181 ketone biosynthetic process	1/28	43/14464	0.02325581	12.013289	3.18521204	0.08005662	0.20558229	0.15810876	Bmp6	1
GO:0061756 leukocyte adhesion to vascular endothelial cell	1/28	43/14464	0.02325581	12.013289	3.18521204	0.08005662	0.20558229	0.15810876	Cx3cr1	1
GO:0140719 constitutive heterochromatin formation	1/28	43/14464	0.02325581	12.013289	3.18521204	0.08005662	0.20558229	0.15810876	H3f3b	1
GO:1903018 regulation of glycoprotein metabolic process	1/28	43/14464	0.02325581	12.013289	3.18521204	0.08005662	0.20558229	0.15810876	Slc2a10	1
GO:0051259 protein complex oligomerization	2/28	244/14464	0.00819672	4.23419204	2.24385966	0.08044029	0.20606738	0.15848183	Comp/Col6a1	2
GO:0060326 cell chemotaxis	2/28	245/14464	0.00816327	4.21690962	2.23651671	0.08100765	0.20616589	0.15855759	Cx3cr1/Ccn3	2
GO:0001774 microglial cell activation	1/28	44/14464	0.02272727	11.7402597	3.1422683	0.08184279	0.20616589	0.15855759	Cx3cr1	1
GO:0002066 columnar/cuboidal epithelial cell development	1/28	44/14464	0.02272727	11.7402597	3.1422683	0.08184279	0.20616589	0.15855759	Bmp6	1
GO:0030858 positive regulation of epithelial cell differentiation	1/28	44/14464	0.02272727	11.7402597	3.1422683	0.08184279	0.20616589	0.15855759	Bmp6	1
GO:0048265 response to pain	1/28	44/14464	0.02272727	11.7402597	3.1422683	0.08184279	0.20616589	0.15855759	Col6a1	1
GO:0071320 cellular response to cAMP	1/28	44/14464	0.02272727	11.7402597	3.1422683	0.08184279	0.20616589	0.15855759	Igfbp5	1
GO:1901861 regulation of muscle tissue development	1/28	44/14464	0.02272727	11.7402597	3.1422683	0.08184279	0.20616589	0.15855759	Igfbp5	1
GO:0003016 respiratory system process	1/28	45/14464	0.02222222	11.4793651	3.10069057	0.08362562	0.20965855	0.16124372	Col6a1	1
GO:2001259 positive regulation of cation channel activity	1/28	45/14464	0.02222222	11.4793651	3.10069057	0.08362562	0.20965855	0.16124372	Gstm7	1
GO:0008340 determination of adult lifespan	1/28	46/14464	0.02173913	11.2298137	3.06040498	0.08540511	0.21361374	0.16428556	Kl	1
GO:0002269 leukocyte activation involved in inflammatory response	1/28	47/14464	0.0212766	10.9908815	3.02134318	0.08718127	0.21400879	0.16458939	Cx3cr1	1
GO:0007566 embryo implantation	1/28	47/14464	0.0212766	10.9908815	3.02134318	0.08718127	0.21400879	0.16458939	H3f3b	1
GO:0014044 Schwann cell development	1/28	47/14464	0.0212766	10.9908815	3.02134318	0.08718127	0.21400879	0.16458939	Col6a1	1
GO:0030514 negative regulation of BMP signaling pathway	1/28	47/14464	0.0212766	10.9908815	3.02134318	0.08718127	0.21400879	0.16458939	Fbn1	1
GO:0032330 regulation of chondrocyte differentiation	1/28	47/14464	0.0212766	10.9908815	3.02134318	0.08718127	0.21400879	0.16458939	Bmp6	1
GO:0045776 negative regulation of blood pressure	1/28	47/14464	0.0212766	10.9908815	3.02134318	0.08718127	0.21400879	0.16458939	Kl	1
GO:0140236 translation at presynapse	1/28	47/14464	0.0212766	10.9908815	3.02134318	0.08718127	0.21400879	0.16458939	Rpl8	1
GO:1902930 regulation of alcohol biosynthetic process	1/28	47/14464	0.0212766	10.9908815	3.02134318	0.08718127	0.21400879	0.16458939	Bmp6	1
GO:0009620 response to fungus	1/28	48/14464	0.02083333	10.7619048	2.9834418	0.08895411	0.21469062	0.16511377	Cx3cr1	1
GO:0014904 myotube cell development	1/28	48/14464	0.02083333	10.7619048	2.9834418	0.08895411	0.21469062	0.16511377	Col6a1	1
GO:0085029 extracellular matrix assembly	1/28	48/14464	0.02083333	10.7619048	2.9834418	0.08895411	0.21469062	0.16511377	Col6a1	1
GO:0090102 cochlea development	1/28	48/14464	0.02083333	10.7619048	2.9834418	0.08895411	0.21469062	0.16511377	Kcnk2	1
GO:0140241 translation at synapse	1/28	48/14464	0.02083333	10.7619048	2.9834418	0.08895411	0.21469062	0.16511377	Rpl8	1
GO:0140242 translation at postsynapse	1/28	48/14464	0.02083333	10.7619048	2.9834418	0.08895411	0.21469062	0.16511377	Rpl8	1
GO:0140353 lipid export from cell	1/28	48/14464	0.02083333	10.7619048	2.9834418	0.08895411	0.21469062	0.16511377	Bmp6	1
GO:0008544 epidermis development	2/28	259/14464	0.00772201	3.98896856	2.13764401	0.0890824	0.21469062	0.16511377	Igfbp5/Col6a1	2
GO:0014888 striated muscle adaptation	1/28	49/14464	0.02040816	10.5422741	2.94664199	0.09072362	0.21618375	0.16626211	Igfbp5	1
GO:0045668 negative regulation of osteoblast differentiation	1/28	49/14464	0.02040816	10.5422741	2.94664199	0.09072362	0.21618375	0.16626211	Igfbp5	1
GO:0060074 synapse maturation	1/28	49/14464	0.02040816	10.5422741	2.94664199	0.09072362	0.21618375	0.16626211	Cx3cr1	1
GO:1900046 regulation of hemostasis	1/28	49/14464	0.02040816	10.5422741	2.94664199	0.09072362	0.21618375	0.16626211	Comp	1
GO:1903170 negative regulation of calcium ion transmembrane transport	1/28	49/14464	0.02040816	10.5422741	2.94664199	0.09072362	0.21618375	0.16626211	Gstm7	1
GO:0007162 negative regulation of cell adhesion	2/28	263/14464	0.00760456	3.92829984	2.11066217	0.09143317	0.21738492	0.1671859	Bmp6/Fzd7	2
GO:0002762 negative regulation of myeloid leukocyte differentiation	1/28	50/14464	0.02	10.3314286	2.91088904	0.09248982	0.21842461	0.1679855	Fbn1	1
GO:0032413 negative regulation of ion transmembrane transporter activity	1/28	50/14464	0.02	10.3314286	2.91088904	0.09248982	0.21842461	0.1679855	Gstm7	1
GO:0060443 mammary gland morphogenesis	1/28	50/14464	0.02	10.3314286	2.91088904	0.09248982	0.21842461	0.1679855	Igfbp5	1
GO:0060986 endocrine hormone secretion	1/28	51/14464	0.01960784	10.1288515	2.87613198	0.0942527	0.22110723	0.17004864	Bmp6	1
GO:0070098 chemokine-mediated signaling pathway	1/28	51/14464	0.01960784	10.1288515	2.87613198	0.0942527	0.22110723	0.17004864	Cx3cr1	1
GO:1902692 regulation of neuroblast proliferation	1/28	51/14464	0.01960784	10.1288515	2.87613198	0.0942527	0.22110723	0.17004864	Cx3cr1	1
GO:0060070 canonical Wnt signaling pathway	2/28	269/14464	0.00743494	3.84067977	2.07116878	0.09499418	0.22227791	0.17094898	Fzd7/Col6a1	2
GO:0002704 negative regulation of leukocyte mediated immunity	1/28	52/14464	0.01923077	9.93406593	2.84232327	0.09601229	0.22227791	0.17094898	Cx3cr1	1
GO:0006518 peptide metabolic process	1/28	52/14464	0.01923077	9.93406593	2.84232327	0.09601229	0.22227791	0.17094898	Cpq	1
GO:0006749 glutathione metabolic process	1/28	52/14464	0.01923077	9.93406593	2.84232327	0.09601229	0.22227791	0.17094898	Gstm7	1
GO:0032387 negative regulation of intracellular transport	1/28	52/14464	0.01923077	9.93406593	2.84232327	0.09601229	0.22227791	0.17094898	Gbp4	1
GO:0050881 musculoskeletal movement	1/28	52/14464	0.01923077	9.93406593	2.84232327	0.09601229	0.22227791	0.17094898	Comp	1
GO:0002067 glandular epithelial cell differentiation	1/28	53/14464	0.01886792	9.74663073	2.80941851	0.09776858	0.22389428	0.1721921	Bmp6	1
GO:0006334 nucleosome assembly	1/28	53/14464	0.01886792	9.74663073	2.80941851	0.09776858	0.22389428	0.1721921	H1f0	1
GO:0045599 negative regulation of fat cell differentiation	1/28	53/14464	0.01886792	9.74663073	2.80941851	0.09776858	0.22389428	0.1721921	Fbn1	1
GO:0050982 detection of mechanical stimulus	1/28	53/14464	0.01886792	9.74663073	2.80941851	0.09776858	0.22389428	0.1721921	Kcnk2	1
GO:0070527 platelet aggregation	1/28	53/14464	0.01886792	9.74663073	2.80941851	0.09776858	0.22389428	0.1721921	Comp	1
GO:0006584 catecholamine metabolic process	1/28	54/14464	0.01851852	9.56613757	2.77737618	0.09952158	0.22498682	0.17303235	Kl	1
GO:0009712 catechol-containing compound metabolic process	1/28	54/14464	0.01851852	9.56613757	2.77737618	0.09952158	0.22498682	0.17303235	Kl	1
GO:0014037 Schwann cell differentiation	1/28	54/14464	0.01851852	9.56613757	2.77737618	0.09952158	0.22498682	0.17303235	Col6a1	1
GO:0031663 lipopolysaccharide-mediated signaling pathway	1/28	54/14464	0.01851852	9.56613757	2.77737618	0.09952158	0.22498682	0.17303235	Bmp6	1
GO:0035567 non-canonical Wnt signaling pathway	1/28	54/14464	0.01851852	9.56613757	2.77737618	0.09952158	0.22498682	0.17303235	Fzd7	1
GO:0061900 glial cell activation	1/28	54/14464	0.01851852	9.56613757	2.77737618	0.09952158	0.22498682	0.17303235	Cx3cr1	1
GO:0010812 negative regulation of cell-substrate adhesion	1/28	55/14464	0.01818182	9.39220779	2.74615739	0.1012713	0.22652227	0.17421322	Fzd7	1
GO:0019228 neuronal action potential	1/28	55/14464	0.01818182	9.39220779	2.74615739	0.1012713	0.22652227	0.17421322	Kcnk2	1
GO:0060425 lung morphogenesis	1/28	55/14464	0.01818182	9.39220779	2.74615739	0.1012713	0.22652227	0.17421322	Col6a1	1
GO:0070542 response to fatty acid	1/28	55/14464	0.01818182	9.39220779	2.74615739	0.1012713	0.22652227	0.17421322	Kcnk2	1
GO:1905517 macrophage migration	1/28	55/14464	0.01818182	9.39220779	2.74615739	0.1012713	0.22652227	0.17421322	Cx3cr1	1
GO:0002886 regulation of myeloid leukocyte mediated immunity	1/28	56/14464	0.01785714	9.2244898	2.7157257	0.10301773	0.22897639	0.17610064	Cx3cr1	1
GO:0006497 protein lipidation	1/28	56/14464	0.01785714	9.2244898	2.7157257	0.10301773	0.22897639	0.17610064	Col6a1	1
GO:0050688 regulation of defense response to virus	1/28	56/14464	0.01785714	9.2244898	2.7157257	0.10301773	0.22897639	0.17610064	Gbp4	1
GO:0043523 regulation of neuron apoptotic process	2/28	283/14464	0.00706714	3.65068147	1.98327119	0.10345896	0.229475	0.17648411	Cx3cr1/Bhlhb9	2
GO:0032410 negative regulation of transporter activity	1/28	57/14464	0.01754386	9.06265664	2.68604687	0.1047609	0.23091048	0.1775881	Gstm7	1
GO:0060038 cardiac muscle cell proliferation	1/28	57/14464	0.01754386	9.06265664	2.68604687	0.1047609	0.23091048	0.1775881	Kcnk2	1
GO:1900271 regulation of long-term synaptic potentiation	1/28	57/14464	0.01754386	9.06265664	2.68604687	0.1047609	0.23091048	0.1775881	Cx3cr1	1
GO:0006805 xenobiotic metabolic process	1/28	58/14464	0.01724138	8.90640394	2.65708876	0.10650079	0.23377145	0.17978841	Gstm7	1
GO:0071709 membrane assembly	1/28	58/14464	0.01724138	8.90640394	2.65708876	0.10650079	0.23377145	0.17978841	Col6a1	1
GO:0030166 proteoglycan biosynthetic process	1/28	59/14464	0.01694915	8.75544794	2.62882111	0.10823743	0.23504872	0.18077073	Slc2a10	1
GO:0035176 social behavior	1/28	59/14464	0.01694915	8.75544794	2.62882111	0.10823743	0.23504872	0.18077073	Cx3cr1	1
GO:0042158 lipoprotein biosynthetic process	1/28	59/14464	0.01694915	8.75544794	2.62882111	0.10823743	0.23504872	0.18077073	Col6a1	1
GO:0050769 positive regulation of neurogenesis	2/28	291/14464	0.00687285	3.5503191	1.93550767	0.10838887	0.23504872	0.18077073	Cx3cr1/Bhlhb9	2
GO:0071375 cellular response to peptide hormone stimulus	2/28	292/14464	0.00684932	3.53816047	1.92965515	0.10900965	0.23504872	0.18077073	Fbn1/Col6a1	2
GO:0006879 intracellular iron ion homeostasis	1/28	60/14464	0.01666667	8.60952381	2.60121543	0.10997081	0.23504872	0.18077073	Bmp6	1
GO:0010257 NADH dehydrogenase complex assembly	1/28	60/14464	0.01666667	8.60952381	2.60121543	0.10997081	0.23504872	0.18077073	Ndufaf2	1
GO:0032147 activation of protein kinase activity	1/28	60/14464	0.01666667	8.60952381	2.60121543	0.10997081	0.23504872	0.18077073	Fbn1	1
GO:0032981 mitochondrial respiratory chain complex I assembly	1/28	60/14464	0.01666667	8.60952381	2.60121543	0.10997081	0.23504872	0.18077073	Ndufaf2	1
GO:0034331 cell junction maintenance	1/28	60/14464	0.01666667	8.60952381	2.60121543	0.10997081	0.23504872	0.18077073	Kifc3	1
GO:0048662 negative regulation of smooth muscle cell proliferation	1/28	60/14464	0.01666667	8.60952381	2.60121543	0.10997081	0.23504872	0.18077073	Igfbp5	1
GO:0050873 brown fat cell differentiation	1/28	60/14464	0.01666667	8.60952381	2.60121543	0.10997081	0.23504872	0.18077073	Fbn1	1
GO:0051703 biological process involved in intraspecies interaction between organisms	1/28	60/14464	0.01666667	8.60952381	2.60121543	0.10997081	0.23504872	0.18077073	Cx3cr1	1
GO:0050727 regulation of inflammatory response	2/28	294/14464	0.00680272	3.51409135	1.91802634	0.11025419	0.23517929	0.18087115	Cx3cr1/Ccn3	2
GO:0055074 calcium ion homeostasis	2/28	295/14464	0.00677966	3.50217918	1.91224963	0.11087792	0.23541752	0.18105437	Gstm7/Kl	2

GO:0002931	response to ischemia	1/28	61/14464	0.01639344	8.46838407	2.57424486	0.11170094	0.23541752	0.18105437 Cx3cr1	1
GO:0034308	primary alcohol metabolic process	1/28	61/14464	0.01639344	8.46838407	2.57424486	0.11170094	0.23541752	0.18105437 Bmp6	1
GO:0061001	regulation of dendritic spine morphogenesis	1/28	61/14464	0.01639344	8.46838407	2.57424486	0.11170094	0.23541752	0.18105437 Bhlhb9	1
GO:1990868	response to chemokine	1/28	61/14464	0.01639344	8.46838407	2.57424486	0.11170094	0.23541752	0.18105437 Cx3cr1	1
GO:1990869	cellular response to chemokine	1/28	61/14464	0.01639344	8.46838407	2.57424486	0.11170094	0.23541752	0.18105437 Cx3cr1	1
GO:0031016	pancreas development	1/28	62/14464	0.01612903	8.33179724	2.54788405	0.11342783	0.2385818	0.18348794 Bmp6	1
GO:0006081	cellular aldehyde metabolic process	1/28	63/14464	0.01587302	8.19954649	2.52210906	0.11515148	0.23888288	0.18371949 Bmp6	1
GO:0007585	respiratory gaseous exchange by respiratory system	1/28	63/14464	0.01587302	8.19954649	2.52210906	0.11515148	0.23888288	0.18371949 Col6a1	1
GO:0045669	positive regulation of osteoblast differentiation	1/28	63/14464	0.01587302	8.19954649	2.52210906	0.11515148	0.23888288	0.18371949 Bmp6	1
GO:0050775	positive regulation of dendrite morphogenesis	1/28	63/14464	0.01587302	8.19954649	2.52210906	0.11515148	0.23888288	0.18371949 Bhlhb9	1
GO:0051932	synaptic transmission, GABAergic	1/28	63/14464	0.01587302	8.19954649	2.52210906	0.11515148	0.23888288	0.18371949 Kcnk2	1
GO:0061035	regulation of cartilage development	1/28	63/14464	0.01587302	8.19954649	2.52210906	0.11515148	0.23888288	0.18371949 Bmp6	1
GO:1901019	regulation of calcium ion transmembrane transporter activity	1/28	63/14464	0.01587302	8.19954649	2.52210906	0.11515148	0.23888288	0.18371949 Gstm7	1
GO:0001822	kidney development	2/28	303/14464	0.00660066	3.4097124	1.86691411	0.11590238	0.2396327	0.18429616 Fbn1/Bmp6	2
GO:0036293	response to decreased oxygen levels	2/28	304/14464	0.00657895	3.39849624	1.86135401	0.11653467	0.2396327	0.18429616 Col6a1/Kcnk2	2
GO:0050900	leukocyte migration	2/28	304/14464	0.00657895	3.39849624	1.86135401	0.11653467	0.2396327	0.18429616 Cx3cr1/Ccn3	2
GO:0010770	positive regulation of cell morphogenesis	1/28	64/14464	0.015625	8.07142857	2.49689726	0.1168719	0.2396327	0.18429616 Bhlhb9	1
GO:0045428	regulation of nitric oxide biosynthetic process	1/28	64/14464	0.015625	8.07142857	2.49689726	0.1168719	0.2396327	0.18429616 Cx3cr1	1
GO:2000179	positive regulation of neural precursor cell proliferation	1/28	64/14464	0.015625	8.07142857	2.49689726	0.1168719	0.2396327	0.18429616 Cx3cr1	1
GO:0051235	maintenance of location	2/28	307/14464	0.00651466	3.36528618	1.84481167	0.11843705	0.2412832	0.18556553 Fbn1/Gstm7	2
GO:0001910	regulation of leukocyte mediated cytotoxicity	1/28	65/14464	0.01538462	7.94725275	2.47222722	0.11858909	0.2412832	0.18556553 Cx3cr1	1
GO:0051926	negative regulation of calcium ion transport	1/28	65/14464	0.01538462	7.94725275	2.47222722	0.11858909	0.2412832	0.18556553 Gstm7	1
GO:0060999	positive regulation of dendritic spine development	1/28	65/14464	0.01538462	7.94725275	2.47222722	0.11858909	0.2412832	0.18556553 Bhlhb9	1
GO:0032102	negative regulation of response to external stimulus	2/28	308/14464	0.00649351	3.35435993	1.83934294	0.11907299	0.24180274	0.18596509 Cx3cr1/Ccn3	2
GO:0014911	positive regulation of smooth muscle cell migration	1/28	66/14464	0.01515152	7.82683983	2.44807866	0.12030307	0.24243933	0.18645468 Igfbp5	1
GO:0044091	membrane biogenesis	1/28	66/14464	0.01515152	7.82683983	2.44807866	0.12030307	0.24243933	0.18645468 Col6a1	1
GO:0098869	cellular oxidant detoxification	1/28	66/14464	0.01515152	7.82683983	2.44807866	0.12030307	0.24243933	0.18645468 Gstm7	1
GO:0140962	multicellular organismal-level chemical homeostasis	1/28	66/14464	0.01515152	7.82683983	2.44807866	0.12030307	0.24243933	0.18645468 Bmp6	1
GO:0001708	cell fate specification	1/28	67/14464	0.01492537	7.71002132	2.42443236	0.12201383	0.24310853	0.18696935 Fzd7	1
GO:0033273	response to vitamin	1/28	67/14464	0.01492537	7.71002132	2.42443236	0.12201383	0.24310853	0.18696935 Kl	1
GO:0045123	cellular extravasation	1/28	67/14464	0.01492537	7.71002132	2.42443236	0.12201383	0.24310853	0.18696935 Cx3cr1	1
GO:0050810	regulation of steroid biosynthetic process	1/28	67/14464	0.01492537	7.71002132	2.42443236	0.12201383	0.24310853	0.18696935 Bmp6	1
GO:0060612	adipose tissue development	1/28	67/14464	0.01492537	7.71002132	2.42443236	0.12201383	0.24310853	0.18696935 Col6a1	1
GO:0080164	regulation of nitric oxide metabolic process	1/28	67/14464	0.01492537	7.71002132	2.42443236	0.12201383	0.24310853	0.18696935 Cx3cr1	1
GO:0072001	renal system development	2/28	315/14464	0.00634921	3.27981859	1.80167921	0.12354949	0.2446677	0.18816847 Fbn1/Bmp6	2
GO:0007492	endoderm development	1/28	68/14464	0.01470588	7.59663866	2.40127005	0.12372138	0.2446677	0.18816847 Col6a1	1
GO:0050830	defense response to Gram-positive bacterium	1/28	68/14464	0.01470588	7.59663866	2.40127005	0.12372138	0.2446677	0.18816847 Gbp4	1
GO:0055021	regulation of cardiac muscle tissue growth	1/28	68/14464	0.01470588	7.59663866	2.40127005	0.12372138	0.2446677	0.18816847 Kcnk2	1
GO:0008347	glial cell migration	1/28	69/14464	0.01449275	7.48654244	2.37857444	0.12542573	0.24574152	0.18899432 Cx3cr1	1
GO:0008543	fibroblast growth factor receptor signaling pathway	1/28	69/14464	0.01449275	7.48654244	2.37857444	0.12542573	0.24574152	0.18899432 Kl	1
GO:0010761	fibroblast migration	1/28	69/14464	0.01449275	7.48654244	2.37857444	0.12542573	0.24574152	0.18899432 Ccn3	1
GO:0048844	artery morphogenesis	1/28	69/14464	0.01449275	7.48654244	2.37857444	0.12542573	0.24574152	0.18899432 Comp	1
GO:0071260	cellular response to mechanical stimulus	1/28	69/14464	0.01449275	7.48654244	2.37857444	0.12542573	0.24574152	0.18899432 Bmp6	1
GO:0001676	long-chain fatty acid metabolic process	1/28	70/14464	0.01428571	7.37959184	2.35632905	0.12712688	0.24815542	0.1908508 Gstm7	1
GO:0045670	regulation of osteoclast differentiation	1/28	70/14464	0.01428571	7.37959184	2.35632905	0.12712688	0.24815542	0.1908508 Fbn1	1
GO:0042060	wound healing	2/28	321/14464	0.00623053	3.21851357	1.77022556	0.12742014	0.24826982	0.19093878 Comp/Fzd7	2
GO:0034728	nucleosome organization	1/28	71/14464	0.01408451	7.27565392	2.33451823	0.12882484	0.24917126	0.19163206 H1f0	1
GO:0042306	regulation of protein import into nucleus	1/28	71/14464	0.01408451	7.27565392	2.33451823	0.12882484	0.24917126	0.19163206 Gbp4	1
GO:0071496	cellular response to external stimulus	1/28	71/14464	0.01408451	7.27565392	2.33451823	0.12882484	0.24917126	0.19163206 Bmp6	1
GO:1904705	regulation of vascular associated smooth muscle cell proliferation	1/28	71/14464	0.01408451	7.27565392	2.33451823	0.12882484	0.24917126	0.19163206 Igfbp5	1
GO:0015800	acidic amino acid transport	1/28	72/14464	0.01388889	7.17460317	2.3131271	0.13051961	0.25107228	0.19309409 Kcnk2	1
GO:0051591	response to cAMP	1/28	72/14464	0.01388889	7.17460317	2.3131271	0.13051961	0.25107228	0.19309409 Igfbp5	1
GO:0097194	execution phase of apoptosis	1/28	72/14464	0.01388889	7.17460317	2.3131271	0.13051961	0.25107228	0.19309409 Col6a1	1
GO:0070482	response to oxygen levels	2/28	327/14464	0.00611621	3.15945828	1.73950162	0.13132065	0.25215471	0.19392656 Col6a1/Kcnk2	2
GO:0060420	regulation of heart growth	1/28	73/14464	0.01369863	7.07632094	2.29214148	0.13221121	0.25294658	0.19453558 Kcnk2	1
GO:1902476	chloride transmembrane transport	1/28	73/14464	0.01369863	7.07632094	2.29214148	0.13221121	0.25294658	0.19453558 Kcnk2	1
GO:0015980	energy derivation by oxidation of organic compounds	2/28	330/14464	0.00606061	3.13073593	1.72440323	0.13328173	0.25342721	0.19490522 Col6a1/Kl	2
GO:0032651	regulation of interleukin-1 beta production	1/28	74/14464	0.01351351	6.98069498	2.27154784	0.13389963	0.25342721	0.19490522 Cx3cr1	1
GO:0034109	homotypic cell-cell adhesion	1/28	74/14464	0.01351351	6.98069498	2.27154784	0.13389963	0.25342721	0.19490522 Comp	1
GO:0051145	smooth muscle cell differentiation	1/28	74/14464	0.01351351	6.98069498	2.27154784	0.13389963	0.25342721	0.19490522 Comp	1
GO:0051279	regulation of release of sequestered calcium ion into cytosol	1/28	74/14464	0.01351351	6.98069498	2.27154784	0.13389963	0.25342721	0.19490522 Gstm7	1
GO:0051851	modulation by host of symbiont process	1/28	74/14464	0.01351351	6.98069498	2.27154784	0.13389963	0.25342721	0.19490522 Cx3cr1	1
GO:0001656	metanephros development	1/28	75/14464	0.01333333	6.88761905	2.25133332	0.13558489	0.25479363	0.1959561 Fbn1	1
GO:0014743	regulation of muscle hypertrophy	1/28	75/14464	0.01333333	6.88761905	2.25133332	0.13558489	0.25479363	0.1959561 Igfbp5	1
GO:0150076	neuroinflammatory response	1/28	75/14464	0.01333333	6.88761905	2.25133332	0.13558489	0.25479363	0.1959561 Cx3cr1	1
GO:1990874	vascular associated smooth muscle cell proliferation	1/28	75/14464	0.01333333	6.88761905	2.25133332	0.13558489	0.25479363	0.1959561 Igfbp5	1
GO:0006809	nitric oxide biosynthetic process	1/28	76/14464	0.01315789	6.79699248	2.23148561	0.13726698	0.25704153	0.19768491 Cx3cr1	1
GO:0050886	endocrine process	1/28	76/14464	0.01315789	6.79699248	2.23148561	0.13726698	0.25704153	0.19768491 Bmp6	1
GO:0032370	positive regulation of lipid transport	1/28	77/14464	0.01298701	6.70871985	2.21199298	0.13894592	0.25835638	0.19869613 Bmp6	1
GO:0032414	positive regulation of ion transmembrane transporter activity	1/28	77/14464	0.01298701	6.70871985	2.21199298	0.13894592	0.25835638	0.19869613 Gstm7	1
GO:0033559	unsaturated fatty acid metabolic process	1/28	77/14464	0.01298701	6.70871985	2.21199298	0.13894592	0.25835638	0.19869613 Gstm7	1
GO:0061178	regulation of insulin secretion involved in cellular response to glucose stimulus	1/28	77/14464	0.01298701	6.70871985	2.21199298	0.13894592	0.25835638	0.19869613 Ndufaf2	1
GO:0015711	organic anion transport	2/28	341/14464	0.0058651	3.02974445	1.67047448	0.14053152	0.25867253	0.19893927 Kcnk2/Slc2a10	2
GO:0007422	peripheral nervous system development	1/28	78/14464	0.01282051	6.62271062	2.19284421	0.1406217	0.25867253	0.19893927 Col6a1	1
GO:0031341	regulation of cell killing	1/28	78/14464	0.01282051	6.62271062	2.19284421	0.1406217	0.25867253	0.19893927 Cx3cr1	1
GO:0050906	detection of stimulus involved in sensory perception	1/28	78/14464	0.01282051	6.62271062	2.19284421	0.1406217	0.25867253	0.19893927 Kcnk2	1
GO:0060997	dendritic spine morphogenesis	1/28	78/14464	0.01282051	6.62271062	2.19284421	0.1406217	0.25867253	0.19893927 Bhlhb9	1
GO:0007405	neuroblast proliferation	1/28	79/14464	0.01265823	6.53887884	2.17402859	0.14229434	0.25867253	0.19893927 Cx3cr1	1
GO:0031397	negative regulation of protein ubiquitination	1/28	79/14464	0.01265823	6.53887884	2.17402859	0.14229434	0.25867253	0.19893927 Gbp4	1
GO:0032611	interleukin-1 beta production	1/28	79/14464	0.01265823	6.53887884	2.17402859	0.14229434	0.25867253	0.19893927 Cx3cr1	1
GO:0035914	skeletal muscle cell differentiation	1/28	79/14464	0.01265823	6.53887884	2.17402859	0.14229434	0.25867253	0.19893927 Col6a1	1
GO:0051965	positive regulation of synapse assembly	1/28	79/14464	0.01265823	6.53887884	2.17402859	0.14229434	0.25867253	0.19893927 Bhlhb9	1
GO:0071677	positive regulation of mononuclear cell migration	1/28	79/14464	0.01265823	6.53887884	2.17402859	0.14229434	0.25867253	0.19893927 Cx3cr1	1
GO:0097009	energy homeostasis	1/28	79/14464	0.01265823	6.53887884	2.17402859	0.14229434	0.25867253	0.19893927 Col6a1	1
GO:1904063	negative regulation of cation transmembrane transport	1/28	79/14464	0.01265823	6.53887884	2.17402859	0.14229434	0.25867253	0.19893927 Gstm7	1
GO:0050678	regulation of epithelial cell proliferation	2/28	346/14464	0.00578035	2.98596201	1.64667226	0.14385631	0.26059609	0.20041865 Bmp6/Fzd7	2
GO:0071230	cellular response to amino acid stimulus	1/28	80/14464	0.0125	6.45714286	2.15553586	0.14396384	0.26059609	0.20041865 Col6a1	1
GO:0009410	response to xenobiotic stimulus	2/28	348/14464	0.00574713	2.96880131	1.63727059	0.14519116	0.26059609	0.20041865 Gstm7/Col6a1	2
GO:0002690	positive regulation of leukocyte chemotaxis	1/28	81/14464	0.01234568	6.37742504	2.13735621	0.14563021	0.26059609	0.20041865 Cx3cr1	1
GO:0033077	T cell differentiation in thymus	1/28	81/14464	0.01234568	6.37742504	2.13735621	0.14563021	0.26059609	0.20041865 Fzd7	1
GO:0045638	negative regulation of myeloid cell differentiation	1/28	81/14464	0.01234568	6.37742504	2.13735621	0.14563021	0.26059609	0.20041865 Fbn1	1
GO:0045666	positive regulation of neuron differentiation	1/28	81/14464	0.01234568	6.37742504	2.13735621	0.14563021	0.26059609	0.20041865 Bmp6	1
GO:0046209	nitric oxide metabolic process	1/28	81/14464	0.01234568	6.37742504	2.13735621	0.14563021	0.26059609	0.20041865 Cx3cr1	1
GO:1904427	positive regulation of calcium ion transmembrane transport	1/28	81/14464	0.01234568	6.37742504	2.13735621	0.14563021	0.26059609	0.20041865 Gstm7	1
GO:0001654	eye development	2/28	351/14464	0.00569801	2.94342694	1.62329286	0.14719862	0.26059609	0.20041865 Fbn1/Bmp6	2
GO:0051962	positive regulation of nervous system development	2/28	351/14464	0.00569801	2.94342694	1.62329286	0.14719862	0.26059609	0.20041865 Cx3cr1/Bhlhb9	2
GO:0006029	proteoglycan metabolic process	1/28	82/14464	0.01219512	6.29965157	2.11948025	0.14729344	0.26059609	0.20041865 Slc2a10	1
GO:0006576	biogenic amine metabolic process	1/28	82/14464	0.01219512	6.29965157	2.11948025	0.14729344	0.26059609	0.20041865 Kl	1
GO:0014855	striated muscle cell proliferation	1/28	82/14464	0.01219512	6.29965157	2.11948025	0.14729344	0.26059609	0.20041865 Kcnk2	1
GO:0032411	positive regulation of transporter activity	1/28	82/14464	0.01219512	6.29965157	2.11948025	0.14729344	0.26059609	0.20041865 Gstm7	1
GO:1901222	regulation of non-canonical NF-kappaB signal transduction	1/28	82/14464	0.01219512	6.29965157	2.11948025	0.14729344	0.26059609	0.20041865 Ccn3	1
GO:0030510	regulation of BMP signaling pathway	1/28	83/14464	0.01204819	6.22375215	2.10189897	0.14895356	0.26208219	0.20156157 Fbn1	1
GO:0043648	dicarboxylic acid metabolic process	1/28	83/14464	0.01204819	6.22375215	2.10189897	0.14895356	0.26208219	0.20156157 Col6a1	1
GO:2001057	reactive nitrogen species metabolic process	1/28	83/14464	0.01204819	6.22375215	2.10189897	0.14895356	0.26208219	0.20156157 Cx3cr1	1
GO:0150063	visual system development	2/28	354/14464	0.00564972	2.91848265	1.60946216	0.14921216	0.26208219	0.20156157 Fbn1/Bmp6	2
GO:0006935	chemotaxis	2/28	356/14464	0.00561798	2.90208668	1.6003219	0.15055783	0.26208219	0.20156157 Cx3cr1/Ccn3	2
GO:0008593	regulation of Notch signaling pathway	1/28	84/14464	0.01190476	6.14965986	2.08460376	0.15061056	0.26208219	0.20156157 Ccn3	1
GO:0032526	response to retinoic acid	1/28	84/14464	0.01190476	6.14965986	2.08460376	0.15061056	0.26208219	0.20156157 Bmp6	1
GO:0034766	negative regulation of monoatomic ion transmembrane transport	1/28	84/14464	0.01190476	6.14965986	2.08460376	0.15061056	0.26208219	0.20156157 Gstm7	1
GO:0042157	lipoprotein metabolic process	1/28	84/14464	0.01190476	6.14965986	2.08460376	0.15061056	0.26208219	0.20156157 Col6a1	1
GO:0060998	regulation of dendritic spine development	1/28	84/14464	0.01190476	6.14965986	2.08460376	0.15061056	0.26208219	0.20156157 Bhlhb9	1
GO:0042330	taxis	2/28	357/14464	0.00560224	2.89395758	1.59577551	0.15123165	0.26273085	0.20206044 Cx3cr1/Ccn3	2
GO:0003073	regulation of systemic arterial blood pressure	1/28	85/14464	0.01176471	6.07731092	2.06758633	0.15226445	0.26365922	0.20277443 Kl	1
GO:0051262	protein tetramerization	1/28	85/14464	0.01176471	6.07731092	2.06758633	0.15226445	0.26365922	0.20277443 Col6a1	1
GO:0048880	sensory system development	2/28	359/14464	0.00557103	2.87783526	1.58672966	0.15258123	0.26377604	0.20286427 Fbn1/Bmp6	2
GO:0001938	positive regulation of endothelial cell proliferation	1/28	86/14464	0.01162791	6.00664452	2.05083875	0.15391523	0.26478425	0.20363966 Bmp6	1
GO:0006821	chloride transport	1/28	86/14464	0.01162791	6.00664452	2.05083875	0.15391523	0.26478425	0.20363966 Kcnk2	1
GO:0030168	platelet activation	1/28	86/14464	0.01162791	6.00664452	2.05083875	0.15391523	0.26478425	0.20363966 Comp	1
GO:0035773	insulin secretion involved in cellular response to glucose stimulus	1/28	87/14464	0.01149425	5.93760263	2.03435339	0.15556291	0.26588943	0.20448963 Ndufaf2	1
GO:0048477	oogenesis	1/28	87/14464	0.01149425	5.93760263	2.03435339	0.15556291	0.26588943	0.20448963 H3f3b	1
GO:0051881	regulation of mitochondrial membrane potential	1/28	87/14464	0.01149425	5.93760263	2.03435339	0.15556291	0.26588943	0.20448963 Col6a1	1
GO:0090288	negative regulation of cellular response to growth factor stimulus	1/28	87/14464	0.01149425	5.93760263	2.03435339	0.15556291	0.26588943	0.20448963 Fbn1	1
GO:0006835	dicarboxylic acid transport	1/28	88/14464	0.01136364	5.87012987	2.01812294	0.1572075	0.26674585	0.20514828 Kcnk2	1
GO:0032652	regulation of interleukin-1 production	1/28	88/14464	0.01136364	5.87012987	2.01812294	0.1572075	0.26674585	0.20514828 Cx3cr1	1
GO:0043954	cellular component maintenance	1/28	88/14464	0.01136364	5.87012987	2.01812294	0.1572075	0.26674585	0.20514828 Kifc3	1
GO:1990748	cellular detoxification	1/28	88/14464	0.01136364	5.87012987	2.01812294	0.1572075	0.26674585	0.20514828 Gstm7	1
GO:0042063	gliogenesis	2/28	366/14464	0.00546448	2.82279469	1.55555122	0.15732458	0.26674585	0.20514828 Cx3cr1/Col6a1	2
GO:0002275	myeloid cell activation involved in immune response	1/28	89/14464	0.01123596	5.80417335	2.00214035	0.158849	0.26718957	0.20548954 Cx3cr1	1
GO:0019218	regulation of steroid metabolic process	1/28	89/14464	0.01123596	5.80417335	2.00214035	0.158849	0.26718957	0.20548954 Bmp6	1
GO:0098661	inorganic anion transmembrane transport	1/28	89/14464	0.01123596	5.80417335	2.00214035	0.158849	0.26718957	0.20548954 Kcnk2	1
GO:1903321	negative regulation of protein modification by small protein conjugation or removal	1/28	89/14464	0.01123596	5.80417335	2.00214035	0.158849	0.26718957	0.20548954 Gbp4	1
GO:2001251	negative regulation of chromosome organization	1/28	89/14464	0.01123596	5.80417335	2.00214035	0.158849	0.26718957	0.20548954 H3f3b	1
GO:0019233	sensory perception of pain	1/28	90/14464	0.01111111	5.73968254	1.98639887	0.16048741	0.26866406	0.20662353 Ccn3	1
GO:0051101	regulation of DNA binding	1/28	90/14464	0.01111111	5.73968254	1.98639887	0.16048741	0.26866406	0.20662353 H1f0	1
GO:0051702	biological process involved in interaction with symbiont	1/28	90/14464	0.01111111	5.73968254	1.98639887	0.16048741	0.26866406	0.20662353 Cx3cr1	1
GO:0042310	vasoconstriction	1/28	91/14464	0.01098901	5.67660911	1.970892	0.16212275	0.2690261	0.20690198 Comp	1
GO:0061515	myeloid cell development	1/28	91/14464	0.01098901	5.67660911	1.970892	0.16212275	0.2690261	0.20690198 Fbn1	1
GO:0071229	cellular response to acid chemical	1/28	91/14464	0.01098901	5.67660911	1.970892	0.16212275	0.2690261	0.20690198 Col6a1	1
GO:0090398	cellular senescence	1/28	91/14464	0.01098901	5.67660911	1.970892	0.16212275	0.2690261	0.20690198 Comp	1
GO:2001257	regulation of cation channel activity	1/28	91/14464	0.01098901	5.67660911	1.970892	0.16212275	0.2690261	0.20690198 Gstm7	1
GO:0007631	feeding behavior	1/28	92/14464	0.01086957	5.61490683	1.95561347	0.16375502	0.2690261	0.20690198 Col6a1	1
GO:0009581	detection of external stimulus	1/28	92/14464	0.01086957	5.61490683	1.95561347	0.16375502	0.2690261	0.20690198 Kcnk2	1
GO:0009582	detection of abiotic stimulus	1/28	92/14464	0.01086957	5.61490683	1.95561347	0.16375502	0.2690261	0.20690198 Kcnk2	1
GO:0044344	cellular response to fibroblast growth factor stimulus	1/28	92/14464	0.01086957	5.61490683	1.95561347	0.16375502	0.2690261	0.20690198 Kl	1
GO:0046889	positive regulation of lipid biosynthetic process	1/28	92/14464	0.01086957	5.61490683	1.95561347	0.16375502	0.2690261	0.20690198 Bmp6	1
GO:0048678	response to axon injury	1/28	92/14464	0.01086957	5.61490683	1.95561347	0.16375502	0.2690261	0.20690198 Kcnk2	1
GO:0051899	membrane depolarization	1/28	92/14464	0.01086957	5.61490683	1.95561347	0.16375502	0.2690261	0.20690198 Col6a1	1
GO:0032612	interleukin-1 production	1/28	93/14464	0.01075269	5.55453149	1.94055725	0.16538422	0.27002547	0.20767057 Cx3cr1	1
GO:0042116	macrophage activation	1/28	93/14464	0.01075269	5.55453149	1.94055725	0.16538422	0.27002547	0.20767057 Cx3cr1	1
GO:0045185	maintenance of protein location	1/28	93/14464	0.01075269	5.55453149	1.94055725	0.16538422	0.27002547	0.20767057 Fbn1	1
GO:1901874	negative regulation of post-translational protein modification	1/28	93/14464	0.01075269	5.55453149	1.94055725	0.16538422	0.27002547	0.20767057 Gbp4	1
GO:0007229	integrin-mediated signaling pathway	1/28	94/14464	0.0106383	5.49544073	1.92571756	0.16701036	0.2714239	0.20874607 Slc2a10	1
GO:0009308	amine metabolic process	1/28	94/14464	0.0106383	5.49544073	1.92571756	0.16701036	0.2714239	0.20874607 Kl	1
GO:0034446	substrate adhesion-dependent cell spreading	1/28	94/14464	0.0106383	5.49544073	1.92571756	0.16701036	0.2714239	0.20874607 Fzd7	1
GO:1990542	mitochondrial transmembrane transport	1/28	95/14464	0.01052632	5.43759398	1.9110888	0.16863344	0.27364138	0.21045148 Col6a1	1
GO:0048814	regulation of dendrite morphogenesis	1/28	96/14464	0.01041667	5.38095238	1.89666557	0.17025347	0.27515164	0.21161299 Bhlhb9	1
GO:1902106	negative regulation of leukocyte differentiation	1/28	96/14464	0.01041667	5.38095238	1.89666557	0.17025347	0.27515164	0.21161299 Fbn1	1
GO:0016055	Wnt signaling pathway	2/28	385/14464	0.00519481	2.68348794	1.47448121	0.17034435	0.27515164	0.21161299 Fzd7/Col6a1	2
GO:0071346	cellular response to type II interferon	1/28	97/14464	0.01030928	5.32547865	1.8824427	0.17187046	0.27677161	0.21285888 Gbp4	1
GO:0098656	monoatomic anion transmembrane transport	1/28	97/14464	0.01030928	5.32547865	1.8824427	0.17187046	0.27677161	0.21285888 Kcnk2	1
GO:0001558	regulation of cell growth	2/28	389/14464	0.00514139	2.65589423	1.45803505	0.17311027	0.27810077	0.2138811 Igfbp5/Ccn3	2
GO:0071774	response to fibroblast growth factor	1/28	98/14464	0.01020408	5.27113703	1.86841516	0.17348441	0.27810077	0.2138811 Kl	1
GO:1905954	positive regulation of lipid localization	1/28	98/14464	0.01020408	5.27113703	1.86841516	0.17348441	0.27810077	0.2138811 Bmp6	1
GO:0002444	myeloid leukocyte mediated immunity	1/28	99/14464	0.01010101	5.21789322	1.85457813	0.17509533	0.27820239	0.21395926 Cx3cr1	1
GO:0006096	glycolytic process	1/28	99/14464	0.01010101	5.21789322	1.85457813	0.17509533	0.27820239	0.21395926 Col6a1	1
GO:0033108	mitochondrial respiratory chain complex assembly	1/28	99/14464	0.01010101	5.21789322	1.85457813	0.17509533	0.27820239	0.21395926 Ndufaf2	1

GO:0046620 regulation of organ growth	1/28	99/14464	0.01010101	5.21789322	1.85457813	0.17509533	0.27820239	0.21395926 Kcnk2	1
GO:0048640 negative regulation of developmental growth	1/28	99/14464	0.01010101	5.21789322	1.85457813	0.17509533	0.27820239	0.21395926 Kcnk2	1
GO:0002065 columnar/cuboidal epithelial cell differentiation	1/28	100/14464	0.01	5.16571429	1.84092694	0.17670322	0.27820239	0.21395926 Bmp6	1
GO:0002698 negative regulation of immune effector process	1/28	100/14464	0.01	5.16571429	1.84092694	0.17670322	0.27820239	0.21395926 Cx3cr1	1
GO:0008645 hexose transmembrane transport	1/28	100/14464	0.01	5.16571429	1.84092694	0.17670322	0.27820239	0.21395926 Slc2a10	1
GO:0014910 regulation of smooth muscle cell migration	1/28	100/14464	0.01	5.16571429	1.84092694	0.17670322	0.27820239	0.21395926 Igfbp5	1
GO:0035270 endocrine system development	1/28	100/14464	0.01	5.16571429	1.84092694	0.17670322	0.27820239	0.21395926 Bmp6	1
GO:0055017 cardiac muscle tissue growth	1/28	100/14464	0.01	5.16571429	1.84092694	0.17670322	0.27820239	0.21395926 Kcnk2	1
GO:1904659 D-glucose transmembrane transport	1/28	100/14464	0.01	5.16571429	1.84092694	0.17670322	0.27820239	0.21395926 Slc2a10	1
GO:0007200 phospholipase C-activating G protein-coupled receptor signaling pathway	1/28	101/14464	0.00990099	5.1145686	1.82745708	0.17830809	0.27989608	0.21526183 Cx3cr1	1
GO:0097237 cellular response to toxic substance	1/28	101/14464	0.00990099	5.1145686	1.82745708	0.17830809	0.27989608	0.21526183 Gstm7	1
GO:0001909 leukocyte mediated cytotoxicity	1/28	102/14464	0.00980392	5.06442577	1.8141642	0.17990994	0.28074442	0.21591427 Cx3cr1	1
GO:0006939 smooth muscle contraction	1/28	102/14464	0.00980392	5.06442577	1.8141642	0.17990994	0.28074442	0.21591427 Comp	1
GO:0015749 monosaccharide transmembrane transport	1/28	102/14464	0.00980392	5.06442577	1.8141642	0.17990994	0.28074442	0.21591427 Slc2a10	1
GO:1903707 negative regulation of hemopoiesis	1/28	102/14464	0.00980392	5.06442577	1.8141642	0.17990994	0.28074442	0.21591427 Fbn1	1
GO:0022411 cellular component disassembly	2/28	400/14464	0.005	2.58285714	1.41384584	0.18075716	0.28116586	0.2162384 Cx3cr1/Col6a1	2
GO:0033555 multicellular organismal response to stress	1/28	103/14464	0.00970874	5.01525659	1.8010441	0.18150878	0.28116586	0.2162384 Col6a1	1
GO:0043502 regulation of muscle adaptation	1/28	103/14464	0.00970874	5.01525659	1.8010441	0.18150878	0.28116586	0.2162384 Igfbp5	1
GO:0046032 ADP catabolic process	1/28	103/14464	0.00970874	5.01525659	1.8010441	0.18150878	0.28116586	0.2162384 Col6a1	1
GO:2000278 regulation of DNA biosynthetic process	1/28	103/14464	0.00970874	5.01525659	1.8010441	0.18150878	0.28116586	0.2162384 Kcnk2	1
GO:0014897 striated muscle hypertrophy	1/28	104/14464	0.00961538	4.96703297	1.7880927	0.18310461	0.28239749	0.21718561 Igfbp5	1
GO:0043279 response to alkaloid	1/28	104/14464	0.00961538	4.96703297	1.7880927	0.18310461	0.28239749	0.21718561 Gstm7	1
GO:0098754 detoxification	1/28	104/14464	0.00961538	4.96703297	1.7880927	0.18310461	0.28239749	0.21718561 Gstm7	1
GO:0002377 immunoglobulin production	1/28	105/14464	0.00952381	4.91972789	1.77530609	0.18469744	0.2827929	0.21748971 Fbn1	1
GO:0009137 purine nucleoside diphosphate catabolic process	1/28	105/14464	0.00952381	4.91972789	1.77530609	0.18469744	0.2827929	0.21748971 Col6a1	1
GO:0009181 purine ribonucleoside diphosphate catabolic process	1/28	105/14464	0.00952381	4.91972789	1.77530609	0.18469744	0.2827929	0.21748971 Col6a1	1
GO:0019364 pyridine nucleotide catabolic process	1/28	105/14464	0.00952381	4.91972789	1.77530609	0.18469744	0.2827929	0.21748971 Col6a1	1
GO:0038061 non-canonical NF-kappaB signal transduction	1/28	105/14464	0.00952381	4.91972789	1.77530609	0.18469744	0.2827929	0.21748971 Ccn3	1
GO:0014896 muscle hypertrophy	1/28	106/14464	0.00943396	4.87331536	1.76268045	0.18628728	0.28440395	0.21872874 Igfbp5	1
GO:0097061 dendritic spine organization	1/28	106/14464	0.00943396	4.87331536	1.76268045	0.18628728	0.28440395	0.21872874 Bhlhb9	1
GO:0032963 collagen metabolic process	1/28	107/14464	0.00934579	4.82777036	1.75021212	0.18787413	0.28559027	0.21964111 Col6a1	1
GO:0060419 heart growth	1/28	107/14464	0.00934579	4.82777036	1.75021212	0.18787413	0.28559027	0.21964111 Kcnk2	1
GO:0097250 mitochondrial respirasome assembly	1/28	107/14464	0.00934579	4.82777036	1.75021212	0.18787413	0.28559027	0.21964111 Ndufaf2	1
GO:0030316 osteoclast differentiation	1/28	108/14464	0.00925926	4.78306878	1.73789752	0.18945799	0.28676188	0.22054217 Fbn1	1
GO:0046031 ADP metabolic process	1/28	108/14464	0.00925926	4.78306878	1.73789752	0.18945799	0.28676188	0.22054217 Col6a1	1
GO:0072526 pyridine-containing compound catabolic process	1/28	108/14464	0.00925926	4.78306878	1.73789752	0.18945799	0.28676188	0.22054217 Col6a1	1
GO:0009191 ribonucleoside diphosphate catabolic process	1/28	109/14464	0.00917431	4.73918742	1.72573321	0.19103887	0.28832971	0.22174795 Col6a1	1
GO:0046683 response to organophosphorus	1/28	109/14464	0.00917431	4.73918742	1.72573321	0.19103887	0.28832971	0.22174795 Igfbp5	1
GO:0006986 response to unfolded protein	1/28	110/14464	0.00909091	4.6961039	1.71371585	0.19261678	0.28856018	0.2219252 Comp	1
GO:0034219 carbohydrate transmembrane transport	1/28	110/14464	0.00909091	4.6961039	1.71371585	0.19261678	0.28856018	0.2219252 Slc2a10	1
GO:0045446 endothelial cell differentiation	1/28	110/14464	0.00909091	4.6961039	1.71371585	0.19261678	0.28856018	0.2219252 Bmp6	1
GO:0050767 regulation of neurogenesis	2/28	418/14464	0.00478469	2.47163363	1.34460984	0.19338664	0.28856018	0.2219252 Cx3cr1/Bhlhb9	2
GO:0043410 positive regulation of MAPK cascade	2/28	419/14464	0.00477327	2.46573474	1.34086883	0.19409213	0.28856018	0.2219252 Fzd7/Kl	2
GO:0009134 nucleoside diphosphate catabolic process	1/28	111/14464	0.00900901	4.65379665	1.70184222	0.19419173	0.28856018	0.2219252 Col6a1	1
GO:0048661 positive regulation of smooth muscle cell proliferation	1/28	111/14464	0.00900901	4.65379665	1.70184222	0.19419173	0.28856018	0.2219252 Igfbp5	1
GO:0051928 positive regulation of calcium ion transport	1/28	111/14464	0.00900901	4.65379665	1.70184222	0.19419173	0.28856018	0.2219252 Gstm7	1
GO:0060291 long-term synaptic potentiation	1/28	111/14464	0.00900901	4.65379665	1.70184222	0.19419173	0.28856018	0.2219252 Cx3cr1	1
GO:0099565 chemical synaptic transmission, postsynaptic	1/28	111/14464	0.00900901	4.65379665	1.70184222	0.19419173	0.28856018	0.2219252 Kcnk2	1
GO:2000177 regulation of neural precursor cell proliferation	1/28	111/14464	0.00900901	4.65379665	1.70184222	0.19419173	0.28856018	0.2219252 Cx3cr1	1
GO:0010657 muscle cell apoptotic process	1/28	112/14464	0.00892857	4.6122449	1.69010917	0.19576371	0.29008124	0.22309501 Col6a1	1
GO:0051209 release of sequestered calcium ion into cytosol	1/28	112/14464	0.00892857	4.6122449	1.69010917	0.19576371	0.29008124	0.22309501 Gstm7	1
GO:0009135 purine nucleoside diphosphate metabolic process	1/28	113/14464	0.00884956	4.57142857	1.67851366	0.19733273	0.29118275	0.22394216 Col6a1	1
GO:0009179 purine ribonucleoside diphosphate metabolic process	1/28	113/14464	0.00884956	4.57142857	1.67851366	0.19733273	0.29118275	0.22394216 Col6a1	1
GO:0051283 negative regulation of sequestering of calcium ion	1/28	113/14464	0.00884956	4.57142857	1.67851366	0.19733273	0.29118275	0.22394216 Gstm7	1
GO:0008643 carbohydrate transport	1/28	114/14464	0.00877193	4.53132832	1.66705276	0.1988988	0.29267723	0.22509154 Slc2a10	1
GO:0034341 response to type II interferon	1/28	114/14464	0.00877193	4.53132832	1.66705276	0.1988988	0.29267723	0.22509154 Gbp4	1
GO:0043200 response to amino acid	1/28	115/14464	0.00869565	4.49192547	1.6557236	0.20046193	0.29415911	0.22623121 Col6a1	1
GO:0051282 regulation of sequestering of calcium ion	1/28	115/14464	0.00869565	4.49192547	1.6557236	0.20046193	0.29415911	0.22623121 Gstm7	1
GO:0034763 negative regulation of transmembrane transport	1/28	116/14464	0.00862069	4.45320197	1.64452342	0.20202211	0.29545536	0.22722813 Gstm7	1
GO:0051208 sequestering of calcium ion	1/28	116/14464	0.00862069	4.45320197	1.64452342	0.20202211	0.29545536	0.22722813 Gstm7	1
GO:0000018 regulation of DNA recombination	1/28	117/14464	0.00854701	4.41514042	1.63344952	0.20357936	0.29545536	0.22722813 H1f0	1
GO:0006820 monoatomic anion transport	1/28	117/14464	0.00854701	4.41514042	1.63344952	0.20357936	0.29545536	0.22722813 Kcnk2	1
GO:0008584 male gonad development	1/28	117/14464	0.00854701	4.41514042	1.63344952	0.20357936	0.29545536	0.22722813 H3f3b	1
GO:0015698 inorganic anion transport	1/28	117/14464	0.00854701	4.41514042	1.63344952	0.20357936	0.29545536	0.22722813 Kcnk2	1
GO:0017148 negative regulation of translation	1/28	117/14464	0.00854701	4.41514042	1.63344952	0.20357936	0.29545536	0.22722813 Igfbp5	1
GO:0106027 neuron projection organization	1/28	117/14464	0.00854701	4.41514042	1.63344952	0.20357936	0.29545536	0.22722813 Bhlhb9	1
GO:0032479 regulation of type I interferon production	1/28	118/14464	0.00847458	4.37772397	1.6224993	0.20513367	0.29649102	0.22802463 Gbp4	1
GO:0043271 negative regulation of monoatomic ion transport	1/28	118/14464	0.00847458	4.37772397	1.6224993	0.20513367	0.29649102	0.22802463 Gstm7	1
GO:0046546 development of primary male sexual characteristics	1/28	118/14464	0.00847458	4.37772397	1.6224993	0.20513367	0.29649102	0.22802463 H3f3b	1
GO:0042391 regulation of membrane potential	2/28	437/14464	0.00457666	2.3641713	1.27529894	0.20685191	0.2985666	0.22962091 Col6a1/Kcnk2	2
GO:0048706 embryonic skeletal system development	1/28	120/14464	0.00833333	4.3047619	1.60095982	0.20823353	0.30015133	0.2308397 Slc2a10	1
GO:0002761 regulation of myeloid leukocyte differentiation	1/28	121/14464	0.00826446	4.26918536	1.59036571	0.20977909	0.30114827	0.23160642 Fbn1	1
GO:0046165 alcohol biosynthetic process	1/28	121/14464	0.00826446	4.26918536	1.59036571	0.20977909	0.30114827	0.23160642 Bmp6	1
GO:1901890 positive regulation of cell junction assembly	1/28	121/14464	0.00826446	4.26918536	1.59036571	0.20977909	0.30114827	0.23160642 Bhlhb9	1
GO:0051052 regulation of DNA metabolic process	2/28	443/14464	0.00451467	2.33215092	1.25415314	0.21112858	0.3013186	0.23173741 H1f0/Kcnk2	2
GO:0003231 cardiac ventricle development	1/28	122/14464	0.00819672	4.23419204	1.57988557	0.21132174	0.3013186	0.23173741 Kcnk2	1
GO:0030879 mammary gland development	1/28	122/14464	0.00819672	4.23419204	1.57988557	0.21132174	0.3013186	0.23173741 Igfbp5	1
GO:0032606 type I interferon production	1/28	122/14464	0.00819672	4.23419204	1.57988557	0.21132174	0.3013186	0.23173741 Gbp4	1
GO:0045834 positive regulation of lipid metabolic process	1/28	122/14464	0.00819672	4.23419204	1.57988557	0.21132174	0.3013186	0.23173741 Bmp6	1
GO:0006633 fatty acid biosynthetic process	1/28	123/14464	0.00813008	4.19976771	1.56951714	0.21286148	0.30229187	0.23248594 Gstm7	1
GO:0032368 regulation of lipid transport	1/28	123/14464	0.00813008	4.19976771	1.56951714	0.21286148	0.30229187	0.23248594 Bmp6	1
GO:0051384 response to glucocorticoid	1/28	123/14464	0.00813008	4.19976771	1.56951714	0.21286148	0.30229187	0.23248594 Bmp6	1
GO:0002831 regulation of response to biotic stimulus	2/28	446/14464	0.0044843	2.31646381	1.24370705	0.21327085	0.30244457	0.23260337 Bmp6/Gbp4	2
GO:0030003 intracellular monoatomic cation homeostasis	2/28	447/14464	0.00447427	2.31128156	1.24024347	0.2139855	0.30244457	0.23260337 Gstm7/Bmp6	2
GO:0008286 insulin receptor signaling pathway	1/28	124/14464	0.00806452	4.16589862	1.55925822	0.21439832	0.30244457	0.23260337 Col6a1	1
GO:0009185 ribonucleoside diphosphate metabolic process	1/28	124/14464	0.00806452	4.16589862	1.55925822	0.21439832	0.30244457	0.23260337 Col6a1	1
GO:0071466 cellular response to xenobiotic stimulus	1/28	124/14464	0.00806452	4.16589862	1.55925822	0.21439832	0.30244457	0.23260337 Gstm7	1
GO:0006091 generation of precursor metabolites and energy	2/28	449/14464	0.00445434	2.30098632	1.2333437	0.21541563	0.30347501	0.23339586 Col6a1/Kl	2
GO:0006694 steroid biosynthetic process	1/28	126/14464	0.00793651	4.09977324	1.53906046	0.21746334	0.3052665	0.23477366 Bmp6	1
GO:0043500 muscle adaptation	1/28	126/14464	0.00793651	4.09977324	1.53906046	0.21746334	0.3052665	0.23477366 Igfbp5	1
GO:0001936 regulation of endothelial cell proliferation	1/28	127/14464	0.00787402	4.06749156	1.52911753	0.21899151	0.3052665	0.23477366 Bmp6	1
GO:0010565 regulation of cellular ketone metabolic process	1/28	127/14464	0.00787402	4.06749156	1.52911753	0.21899151	0.3052665	0.23477366 Bmp6	1
GO:0035966 response to topologically incorrect protein	1/28	127/14464	0.00787402	4.06749156	1.52911753	0.21899151	0.3052665	0.23477366 Comp	1
GO:0046822 regulation of nucleocytoplasmic transport	1/28	127/14464	0.00787402	4.06749156	1.52911753	0.21899151	0.3052665	0.23477366 Gbp4	1
GO:0050921 positive regulation of chemotaxis	1/28	127/14464	0.00787402	4.06749156	1.52911753	0.21899151	0.3052665	0.23477366 Cx3cr1	1
GO:0006873 intracellular monoatomic ion homeostasis	2/28	454/14464	0.00440529	2.27564506	1.21625176	0.21899553	0.3052665	0.23477366 Gstm7/Bmp6	2
GO:0022612 gland morphogenesis	1/28	128/14464	0.0078125	4.03571429	1.51927592	0.22051682	0.30657923	0.23578325 Igfbp5	1
GO:0030856 regulation of epithelial cell differentiation	1/28	128/14464	0.0078125	4.03571429	1.51927592	0.22051682	0.30657923	0.23578325 Bmp6	1
GO:0006090 pyruvate metabolic process	1/28	129/14464	0.00775194	4.00442968	1.50953373	0.22203924	0.30788666	0.23678876 Col6a1	1
GO:0009132 nucleoside diphosphate metabolic process	1/28	129/14464	0.00775194	4.00442968	1.50953373	0.22203924	0.30788666	0.23678876 Col6a1	1
GO:0071407 cellular response to organic cyclic compound	2/28	462/14464	0.004329	2.23623995	1.18936046	0.22473615	0.31087452	0.23908666 Gstm7/Igfbp5	2
GO:0007030 Golgi organization	1/28	131/14464	0.00763359	3.94329335	1.49034021	0.2250755	0.31087452	0.23908666 Kifc3	1
GO:0009154 purine ribonucleotide catabolic process	1/28	131/14464	0.00763359	3.94329335	1.49034021	0.2250755	0.31087452	0.23908666 Col6a1	1
GO:0001101 response to acid chemical	1/28	132/14464	0.00757576	3.91341991	1.4808853	0.22658934	0.3125574	0.24038093 Col6a1	1
GO:0007005 mitochondrion organization	2/28	466/14464	0.00429185	2.21704476	1.17611905	0.22761189	0.31260382	0.24041663 Col6a1/Ndufaf2	2
GO:0002687 positive regulation of leukocyte migration	1/28	133/14464	0.0075188	3.8839957	1.47152266	0.22810033	0.31260382	0.24041663 Cx3cr1	1
GO:0007292 female gamete generation	1/28	133/14464	0.0075188	3.8839957	1.47152266	0.22810033	0.31260382	0.24041663 H3f3b	1
GO:0050731 positive regulation of peptidyl-tyrosine phosphorylation	1/28	133/14464	0.0075188	3.8839957	1.47152266	0.22810033	0.31260382	0.24041663 Bmp6	1
GO:0055007 cardiac muscle cell differentiation	1/28	133/14464	0.0075188	3.8839957	1.47152266	0.22810033	0.31260382	0.24041663 Fzd7	1
GO:0003158 endothelium development	1/28	134/14464	0.00746269	3.85501066	1.46225061	0.22960848	0.3134526	0.24106941 Bmp6	1
GO:0045598 regulation of fat cell differentiation	1/28	134/14464	0.00746269	3.85501066	1.46225061	0.22960848	0.3134526	0.24106941 Fbn1	1
GO:0060996 dendritic spine development	1/28	134/14464	0.00746269	3.85501066	1.46225061	0.22960848	0.3134526	0.24106941 Bhlhb9	1
GO:0044283 small molecule biosynthetic process	2/28	470/14464	0.00425532	2.19817629	1.16301011	0.23049095	0.31425184	0.24168408 Gstm7/Bmp6	2
GO:0050773 regulation of dendrite development	1/28	136/14464	0.00735294	3.79831933	1.4439718	0.23261624	0.31597707	0.24301092 Bhlhb9	1
GO:1904064 positive regulation of cation transmembrane transport	1/28	136/14464	0.00735294	3.79831933	1.4439718	0.23261624	0.31597707	0.24301092 Gstm7	1
GO:0010720 positive regulation of cell development	2/28	473/14464	0.00422833	2.18423437	1.15326371	0.2326523	0.31597707	0.24301092 Cx3cr1/Bhlhb9	2
GO:0016049 cell growth	2/28	476/14464	0.00420168	2.17046819	1.14358921	0.23481532	0.31836298	0.24484587 Igfbp5/Ccn3	2
GO:0031960 response to corticosteroid	1/28	138/14464	0.00724638	3.74327122	1.42603633	0.23561268	0.31836298	0.24484587 Bmp6	1
GO:0051099 positive regulation of binding	1/28	138/14464	0.00724638	3.74327122	1.42603633	0.23561268	0.31836298	0.24484587 H1f0	1
GO:0051606 detection of stimulus	1/28	138/14464	0.00724638	3.74327122	1.42603633	0.23561268	0.31836298	0.24484587 Kcnk2	1
GO:0006997 nucleus organization	1/28	139/14464	0.00719424	3.71634121	1.41719359	0.23710667	0.31875331	0.24514606 H3f3b	1
GO:0007584 response to nutrient	1/28	139/14464	0.00719424	3.71634121	1.41719359	0.23710667	0.31875331	0.24514606 Kl	1
GO:0035107 appendage morphogenesis	1/28	139/14464	0.00719424	3.71634121	1.41719359	0.23710667	0.31875331	0.24514606 Col6a1	1
GO:0035108 limb morphogenesis	1/28	139/14464	0.00719424	3.71634121	1.41719359	0.23710667	0.31875331	0.24514606 Col6a1	1
GO:0007204 positive regulation of cytosolic calcium ion concentration	1/28	140/14464	0.00714286	3.68979592	1.40843226	0.23859784	0.31913592	0.24544032 Cx3cr1	1
GO:0009261 ribonucleotide catabolic process	1/28	140/14464	0.00714286	3.68979592	1.40843226	0.23859784	0.31913592	0.24544032 Col6a1	1
GO:0021782 glial cell development	1/28	140/14464	0.00714286	3.68979592	1.40843226	0.23859784	0.31913592	0.24544032 Col6a1	1
GO:0071456 cellular response to hypoxia	1/28	140/14464	0.00714286	3.68979592	1.40843226	0.23859784	0.31913592	0.24544032 Kcnk2	1
GO:0006195 purine nucleotide catabolic process	1/28	141/14464	0.0070922	3.66362715	1.39975093	0.2400862	0.32031677	0.24634848 Col6a1	1
GO:0031644 regulation of nervous system process	1/28	141/14464	0.0070922	3.66362715	1.39975093	0.2400862	0.32031677	0.24634848 Ccn3	1
GO:0048592 eye morphogenesis	1/28	142/14464	0.00704225	3.63782696	1.39114825	0.24157175	0.32189284	0.2475606 Fbn1	1
GO:0007009 plasma membrane organization	1/28	143/14464	0.00699301	3.61238761	1.38262286	0.2430545	0.32305486	0.24845429 Col6a1	1
GO:0043123 positive regulation of canonical NF-kappaB signal transduction	1/28	143/14464	0.00699301	3.61238761	1.38262286	0.2430545	0.32305486	0.24845429 Cx3cr1	1
GO:0007596 blood coagulation	1/28	145/14464	0.00689655	3.56256158	1.36579884	0.24601163	0.32657504	0.25116158 Comp	1
GO:0007286 spermatid development	1/28	146/14464	0.00684932	3.53816047	1.35749768	0.24748601	0.32770989	0.25203437 H3f3b	1
GO:1900180 regulation of protein localization to nucleus	1/28	146/14464	0.00684932	3.53816047	1.35749768	0.24748601	0.32770989	0.25203437 Gbp4	1
GO:0001935 endothelial cell proliferation	1/28	148/14464	0.00675676	3.49034749	1.34111103	0.25042644	0.32992757	0.25373994 Bmp6	1
GO:0007599 hemostasis	1/28	148/14464	0.00675676	3.49034749	1.34111103	0.25042644	0.32992757	0.25373994 Comp	1
GO:0009411 response to UV	1/28	148/14464	0.00675676	3.49034749	1.34111103	0.25042644	0.32992757	0.25373994 Col6a1	1
GO:0045766 positive regulation of angiogenesis	1/28	148/14464	0.00675676	3.49034749	1.34111103	0.25042644	0.32992757	0.25373994 Cx3cr1	1
GO:0051604 protein maturation	2/28	498/14464	0.00401606	2.07458405	1.07474099	0.25072001	0.32992757	0.25373994 Comp/Telo2	2
GO:0032200 telomere organization	1/28	149/14464	0.00671141	3.46692234	1.3330232	0.2518925	0.33064797	0.25429398 H3f3b	1
GO:0036294 cellular response to decreased oxygen levels	1/28	149/14464	0.00671141	3.46692234	1.3330232	0.2518925	0.33064797	0.25429398 Kcnk2	1
GO:0050817 coagulation	1/28	150/14464	0.00666667	3.44380952	1.32500418	0.25335579	0.33215666	0.25545428 Comp	1
GO:1904018 positive regulation of vasculature development	1/28	151/14464	0.00662252	3.42100284	1.31705287	0.25481632	0.333658	0.25660893 Cx3cr1	1
GO:1905952 regulation of lipid localization	1/28	152/14464	0.00657895	3.39849624	1.30916819	0.25627409	0.33515203	0.25775795 Bmp6	1
GO:0034767 positive regulation of monoatomic ion transmembrane transport	1/28	153/14464	0.00653595	3.37628385	1.30134909	0.25772912	0.33604647	0.25844585 Gstm7	1
GO:0046887 positive regulation of hormone secretion	1/28	153/14464	0.00653595	3.37628385	1.30134909	0.25772912	0.33604647	0.25844585 Bmp6	1
GO:0035150 regulation of tube size	1/28	154/14464	0.00649351	3.35435993	1.29359454	0.2591814	0.33604647	0.25844585 Comp	1
GO:0035296 regulation of tube diameter	1/28	154/14464	0.00649351	3.35435993	1.29359454	0.2591814	0.33604647	0.25844585 Comp	1
GO:0048515 spermatid differentiation	1/28	154/14464	0.00649351	3.35435993	1.29359454	0.2591814	0.33604647	0.25844585 H3f3b	1
GO:0062013 positive regulation of small molecule metabolic process	1/28	154/14464	0.00649351	3.35435993	1.29359454	0.2591814	0.33604647	0.25844585 Bmp6	1
GO:0097746 blood vessel diameter maintenance	1/28	154/14464	0.00649351	3.35435993	1.29359454	0.2591814	0.33604647	0.25844585 Comp	1
GO:0001508 action potential	1/28	155/14464	0.00645161	3.33271889	1.28590352	0.26063094	0.33709967	0.25925584 Kcnk2	1
GO:0071333 cellular response to glucose stimulus	1/28	155/14464	0.00645161	3.33271889	1.28590352	0.26063094	0.33709967	0.25925584 Ndufaf2	1
GO:0001906 cell killing	1/28	156/14464	0.00641026	3.31135531	1.27827507	0.26207774	0.33814421	0.26005917 Cx3cr1	1
GO:0072330 monocarboxylic acid biosynthetic process	1/28	156/14464	0.00641026	3.31135531	1.27827507	0.26207774	0.33814421	0.26005917 Gstm7	1
GO:0072523 purine-containing compound catabolic process	1/28	157/14464	0.00636943	3.29026388	1.2707082	0.26352182	0.33959328	0.26117362 Col6a1	1
GO:0006865 amino acid transport	1/28	158/14464	0.00632911	3.26943942	1.26320199	0.26496317	0.34020756	0.26164605 Kcnk2	1
GO:0009166 nucleotide catabolic process	1/28	158/14464	0.00632911	3.26943942	1.26320199	0.26496317	0.34020756	0.26164605 Col6a1	1
GO:0071331 cellular response to hexose stimulus	1/28	158/14464	0.00632911	3.26943942	1.26320199	0.26496317	0.34020756	0.26164605 Ndufaf2	1
GO:0071326 cellular response to monosaccharide stimulus	1/28	159/14464	0.00628931	3.24887691	1.2557555	0.2664018	0.34081391	0.26211238 Ndufaf2	1
GO:0071897 DNA biosynthetic process	1/28	159/14464	0.00628931	3.24887691	1.2557555	0.2664018	0.34081391	0.26211238 Kcnk2	1
GO:0097553 calcium ion transmembrane import into cytosol	1/28	159/14464	0.00628931	3.24887691	1.2557555	0.2664018	0.34081391	0.26211238 Gstm7	1
GO:0002181 cytoplasmic translation	1/28	160/14464	0.00625	3.22857143	1.24836784	0.26783771	0.34182424	0.26288941 Rpl8	1
GO:0071356 cellular response to tumor necrosis factor	1/28	160/14464	0.00625	3.22857143	1.24836784	0.26783771	0.34182424	0.26288941 Cx3cr1	1

GO:0008217	regulation of blood pressure	1/28	161/14464	0.00621118	3.20851819	1.24103812	0.26927092	0.34241422	0.26334314	Kl	1
GO:0032680	regulation of tumor necrosis factor production	1/28	161/14464	0.00621118	3.20851819	1.24103812	0.26927092	0.34241422	0.26334314	Cx3cr1	1
GO:0033157	regulation of intracellular protein transport	1/28	161/14464	0.00621118	3.20851819	1.24103812	0.26927092	0.34241422	0.26334314	Gbp4	1
GO:0003205	cardiac chamber development	1/28	162/14464	0.00617284	3.18871252	1.23376548	0.27070141	0.34340779	0.26410727	Kcnk2	1
GO:0007219	Notch signaling pathway	1/28	162/14464	0.00617284	3.18871252	1.23376548	0.27070141	0.34340779	0.26410727	Ccn3	1
GO:0006606	protein import into nucleus	1/28	163/14464	0.00613497	3.16914987	1.22654908	0.27212921	0.34439319	0.26486512	Gbp4	1
GO:1903555	regulation of tumor necrosis factor superfamily cytokine production	1/28	163/14464	0.00613497	3.16914987	1.22654908	0.27212921	0.34439319	0.26486512	Cx3cr1	1
GO:0035051	cardiocyte differentiation	1/28	165/14464	0.00606061	3.13073593	1.21228168	0.27497672	0.34716632	0.26699787	Fzd7	1
GO:0071453	cellular response to oxygen levels	1/28	165/14464	0.00606061	3.13073593	1.21228168	0.27497672	0.34716632	0.26699787	Kcnk2	1
GO:0006457	protein folding	1/28	166/14464	0.0060241	3.11187608	1.20522908	0.27639645	0.34812791	0.26773741	Telo2	1
GO:0032640	tumor necrosis factor production	1/28	166/14464	0.0060241	3.11187608	1.20522908	0.27639645	0.34812791	0.26773741	Cx3cr1	1
GO:0071248	cellular response to metal ion	1/28	167/14464	0.00598802	3.09324209	1.1982295	0.27781349	0.34866747	0.26815238	Bmp6	1
GO:0098739	import across plasma membrane	1/28	167/14464	0.00598802	3.09324209	1.1982295	0.27781349	0.34866747	0.26815238	Slc2a10	1
GO:1903169	regulation of calcium ion transmembrane transport	1/28	167/14464	0.00598802	3.09324209	1.1982295	0.27781349	0.34866747	0.26815238	Gstm7	1
GO:0016052	carbohydrate catabolic process	1/28	168/14464	0.00595238	3.07482993	1.19128219	0.27922786	0.34961311	0.26887965	Col6a1	1
GO:0071706	tumor necrosis factor superfamily cytokine production	1/28	168/14464	0.00595238	3.07482993	1.19128219	0.27922786	0.34961311	0.26887965	Cx3cr1	1
GO:0019362	pyridine nucleotide metabolic process	1/28	169/14464	0.00591716	3.05663567	1.18438639	0.28063956	0.35013756	0.26928299	Col6a1	1
GO:0046496	nicotinamide nucleotide metabolic process	1/28	169/14464	0.00591716	3.05663567	1.18438639	0.28063956	0.35013756	0.26928299	Col6a1	1
GO:0051170	import into nucleus	1/28	169/14464	0.00591716	3.05663567	1.18438639	0.28063956	0.35013756	0.26928299	Gbp4	1
GO:0046890	regulation of lipid biosynthetic process	1/28	170/14464	0.00588235	3.03865546	1.17754138	0.28204859	0.35116551	0.27007356	Bmp6	1
GO:0001678	intracellular glucose homeostasis	1/28	171/14464	0.00584795	3.02088555	1.17074645	0.28345496	0.35116551	0.27007356	Ndufaf2	1
GO:0022408	negative regulation of cell-cell adhesion	1/28	171/14464	0.00584795	3.02088555	1.17074645	0.28345496	0.35116551	0.27007356	Bmp6	1
GO:0030308	negative regulation of cell growth	1/28	171/14464	0.00584795	3.02088555	1.17074645	0.28345496	0.35116551	0.27007356	Ccn3	1
GO:0042552	myelination	1/28	171/14464	0.00584795	3.02088555	1.17074645	0.28345496	0.35116551	0.27007356	Col6a1	1
GO:0071322	cellular response to carbohydrate stimulus	1/28	171/14464	0.00584795	3.02088555	1.17074645	0.28345496	0.35116551	0.27007356	Ndufaf2	1
GO:0048660	regulation of smooth muscle cell proliferation	1/28	172/14464	0.00581395	3.00332226	1.16400088	0.28485867	0.35249178	0.27109357	Igfbp5	1
GO:0007272	ensheathment of neurons	1/28	174/14464	0.00574713	2.96880131	1.15065511	0.28765814	0.35512522	0.27311889	Col6a1	1
GO:0008366	axon ensheathment	1/28	174/14464	0.00574713	2.96880131	1.15065511	0.28765814	0.35512522	0.27311889	Col6a1	1
GO:0006839	mitochondrial transport	1/28	175/14464	0.00571429	2.95183673	1.14405358	0.28905392	0.35643245	0.27412425	Col6a1	1
GO:0072524	pyridine-containing compound metabolic process	1/28	176/14464	0.00568182	2.93506494	1.13749875	0.29044705	0.35731742	0.27480486	Col6a1	1
GO:1901292	nucleoside phosphate catabolic process	1/28	176/14464	0.00568182	2.93506494	1.13749875	0.29044705	0.35731742	0.27480486	Col6a1	1
GO:0032412	regulation of monoatomic ion transmembrane transporter activity	1/28	177/14464	0.00564972	2.91848265	1.13098998	0.29183755	0.35861107	0.27579978	Gstm7	1
GO:0019827	stem cell population maintenance	1/28	179/14464	0.00558659	2.8858739	1.11810816	0.29461068	0.36159872	0.27809751	Fzd7	1
GO:0009060	aerobic respiration	1/28	180/14464	0.00555556	2.86984127	1.11173389	0.29599331	0.36245477	0.27875588	Col6a1	1
GO:0034612	response to tumor necrosis factor	1/28	180/14464	0.00555556	2.86984127	1.11173389	0.29599331	0.36245477	0.27875588	Cx3cr1	1
GO:0019722	calcium-mediated signaling	1/28	181/14464	0.00552486	2.85398579	1.10540327	0.29737333	0.36288464	0.27908648	Cx3cr1	1
GO:0048839	inner ear development	1/28	181/14464	0.00552486	2.85398579	1.10540327	0.29737333	0.36288464	0.27908648	Kcnk2	1
GO:0051260	protein homooligomerization	1/28	181/14464	0.00552486	2.85398579	1.10540327	0.29737333	0.36288464	0.27908648	Comp	1
GO:0042742	defense response to bacterium	1/28	182/14464	0.00549451	2.83830455	1.09911571	0.29875074	0.36372644	0.2797339	Gbp4	1
GO:0071805	potassium ion transmembrane transport	1/28	182/14464	0.00549451	2.83830455	1.09911571	0.29875074	0.36372644	0.2797339	Kcnk2	1
GO:0042180	cellular ketone metabolic process	1/28	183/14464	0.00546448	2.82279469	1.09287065	0.30012554	0.36498026	0.28069818	Bmp6	1
GO:0007519	skeletal muscle tissue development	1/28	184/14464	0.00543478	2.80745342	1.08666754	0.30149775	0.36622803	0.28165782	Col6a1	1
GO:0045664	regulation of neuron differentiation	1/28	185/14464	0.00540541	2.79227799	1.08050582	0.30286736	0.36704888	0.28228911	Bmp6	1
GO:0098727	maintenance of cell number	1/28	185/14464	0.00540541	2.79227799	1.08050582	0.30286736	0.36704888	0.28228911	Fzd7	1
GO:0022898	regulation of transmembrane transporter activity	1/28	186/14464	0.00537634	2.77726575	1.07438497	0.30423438	0.3674429	0.28259214	Gstm7	1
GO:0048813	dendrite morphogenesis	1/28	186/14464	0.00537634	2.77726575	1.07438497	0.30423438	0.3674429	0.28259214	Bhlhb9	1
GO:0099175	regulation of postsynapse organization	1/28	186/14464	0.00537634	2.77726575	1.07438497	0.30423438	0.3674429	0.28259214	Bhlhb9	1
GO:0050905	neuromuscular process	1/28	188/14464	0.00531915	2.74772036	1.06226378	0.30696068	0.36989111	0.28447501	Comp	1
GO:0055002	striated muscle cell development	1/28	188/14464	0.00531915	2.74772036	1.06226378	0.30696068	0.36989111	0.28447501	Col6a1	1
GO:0050806	positive regulation of synaptic transmission	1/28	189/14464	0.00529101	2.73318216	1.05626241	0.30831996	0.37068468	0.28508532	Cx3cr1	1
GO:0061351	neural precursor cell proliferation	1/28	189/14464	0.00529101	2.73318216	1.05626241	0.30831996	0.37068468	0.28508532	Cx3cr1	1
GO:0050730	regulation of peptidyl-tyrosine phosphorylation	1/28	191/14464	0.0052356	2.70456245	1.04437566	0.31103081	0.37351941	0.28726545	Bmp6	1
GO:0045637	regulation of myeloid cell differentiation	1/28	194/14464	0.00515464	2.66273932	1.02682827	0.3150779	0.37795058	0.29067337	Fbn1	1
GO:0008361	regulation of cell size	1/28	195/14464	0.00512821	2.64908425	1.02105269	0.31642182	0.37870394	0.29125276	Col6a1	1
GO:0045165	cell fate commitment	1/28	195/14464	0.00512821	2.64908425	1.02105269	0.31642182	0.37870394	0.29125276	Fzd7	1
GO:0032409	regulation of transporter activity	1/28	196/14464	0.00510204	2.63556851	1.01531312	0.3177632	0.37933704	0.29173966	Gstm7	1
GO:0002703	regulation of leukocyte mediated immunity	1/28	197/14464	0.00507614	2.62218999	1.00960914	0.31910205	0.37933704	0.29173966	Cx3cr1	1
GO:0006006	glucose metabolic process	1/28	197/14464	0.00507614	2.62218999	1.00960914	0.31910205	0.37933704	0.29173966	Fbn1	1
GO:0050821	protein stabilization	1/28	197/14464	0.00507614	2.62218999	1.00960914	0.31910205	0.37933704	0.29173966	Telo2	1
GO:0051963	regulation of synapse assembly	1/28	197/14464	0.00507614	2.62218999	1.00960914	0.31910205	0.37933704	0.29173966	Bhlhb9	1
GO:0060538	skeletal muscle organ development	1/28	197/14464	0.00507614	2.62218999	1.00960914	0.31910205	0.37933704	0.29173966	Col6a1	1
GO:0002064	epithelial cell development	1/28	198/14464	0.00505051	2.60894661	1.00394031	0.32043836	0.38049807	0.29263258	Bmp6	1
GO:0008406	gonad development	1/28	200/14464	0.005	2.58285714	0.99270641	0.3231034	0.3832325	0.29473557	H3f3b	1
GO:0000302	response to reactive oxygen species	1/28	201/14464	0.00497512	2.57000711	0.98714052	0.32443213	0.38394765	0.29528558	Col6a1	1
GO:0009749	response to glucose	1/28	201/14464	0.00497512	2.57000711	0.98714052	0.32443213	0.38394765	0.29528558	Ndufaf2	1
GO:0045137	development of primary sexual characteristics	1/28	203/14464	0.00492611	2.54468684	0.97610884	0.32708207	0.3866512	0.29736482	H3f3b	1
GO:0007623	circadian rhythm	1/28	204/14464	0.00490196	2.53221289	0.97064228	0.32840327	0.38734745	0.29790029	Col6a1	1
GO:0031396	regulation of protein ubiquitination	1/28	204/14464	0.00490196	2.53221289	0.97064228	0.32840327	0.38734745	0.29790029	Gbp4	1
GO:0009746	response to hexose	1/28	205/14464	0.00487805	2.51986063	0.96520804	0.32972197	0.38803765	0.29843111	Ndufaf2	1
GO:0051651	maintenance of location in cell	1/28	205/14464	0.00487805	2.51986063	0.96520804	0.32972197	0.38803765	0.29843111	Gstm7	1
GO:0010810	regulation of cell-substrate adhesion	1/28	206/14464	0.00485437	2.50762829	0.95980577	0.33103818	0.38872186	0.29895732	Fzd7	1
GO:0043270	positive regulation of monoatomic ion transport	1/28	206/14464	0.00485437	2.50762829	0.95980577	0.33103818	0.38872186	0.29895732	Gstm7	1
GO:0043583	ear development	1/28	207/14464	0.00483092	2.49551415	0.95443509	0.33235189	0.38983182	0.29981096	Kcnk2	1
GO:0043542	endothelial cell migration	1/28	210/14464	0.0047619	2.45986395	0.93850896	0.33627813	0.39400029	0.30301683	Ccn3	1
GO:0034284	response to monosaccharide	1/28	211/14464	0.00473934	2.44820582	0.93326106	0.33758192	0.39509034	0.30385517	Ndufaf2	1
GO:0006813	potassium ion transport	1/28	212/14464	0.00471698	2.43665768	0.92804299	0.33888324	0.39573782	0.30435313	Kcnk2	1
GO:0032869	cellular response to insulin stimulus	1/28	212/14464	0.00471698	2.43665768	0.92804299	0.33888324	0.39573782	0.30435313	Col6a1	1
GO:0002274	myeloid leukocyte activation	1/28	213/14464	0.00469484	2.42521797	0.92285442	0.34018209	0.39594351	0.30451133	Cx3cr1	1
GO:0043122	regulation of canonical NF-kappaB signal transduction	1/28	213/14464	0.00469484	2.42521797	0.92285442	0.34018209	0.39594351	0.30451133	Cx3cr1	1
GO:0072593	reactive oxygen species metabolic process	1/28	213/14464	0.00469484	2.42521797	0.92285442	0.34018209	0.39594351	0.30451133	Col6a1	1
GO:0034764	positive regulation of transmembrane transport	1/28	214/14464	0.0046729	2.41388518	0.91769501	0.34147848	0.39701564	0.30533588	Gstm7	1
GO:0044403	biological process involved in symbiotic interaction	1/28	216/14464	0.00462963	2.39153439	0.9074624	0.34406391	0.39958245	0.30730995	Cx3cr1	1
GO:0002440	production of molecular mediator of immune response	1/28	219/14464	0.00456621	2.35877365	0.8923243	0.34792369	0.403622	0.31041668	Fbn1	1
GO:0010976	positive regulation of neuron projection development	1/28	220/14464	0.00454545	2.34805195	0.88733326	0.34920542	0.40378069	0.31053873	Bhlhb9	1
GO:0016485	protein processing	1/28	220/14464	0.00454545	2.34805195	0.88733326	0.34920542	0.40378069	0.31053873	Comp	1
GO:0046034	ATP metabolic process	1/28	220/14464	0.00454545	2.34805195	0.88733326	0.34920542	0.40378069	0.31053873	Col6a1	1
GO:0046434	organophosphate catabolic process	1/28	222/14464	0.0045045	2.32689833	0.87743189	0.35176158	0.4062923	0.31247035	Col6a1	1
GO:0008202	steroid metabolic process	1/28	225/14464	0.00444444	2.29587302	0.86277734	0.35557767	0.40980521	0.31517205	Bmp6	1
GO:2001234	negative regulation of apoptotic signaling pathway	1/28	225/14464	0.00444444	2.29587302	0.86277734	0.35557767	0.40980521	0.31517205	Cx3cr1	1
GO:0060828	regulation of canonical Wnt signaling pathway	1/28	226/14464	0.00442478	2.28571429	0.85794405	0.35684489	0.41081816	0.31595109	Fzd7	1
GO:1901136	carbohydrate derivative catabolic process	1/28	229/14464	0.00436681	2.25577043	0.84359506	0.36063212	0.41472694	0.31895724	Col6a1	1
GO:0001894	tissue homeostasis	1/28	230/14464	0.00434783	2.24596273	0.8388615	0.36188975	0.41482053	0.31902922	Comp	1
GO:0019318	hexose metabolic process	1/28	230/14464	0.00434783	2.24596273	0.8388615	0.36188975	0.41482053	0.31902922	Fbn1	1
GO:0060249	anatomical structure homeostasis	1/28	230/14464	0.00434783	2.24596273	0.8388615	0.36188975	0.41482053	0.31902922	Comp	1
GO:0001667	ameboidal-type cell migration	1/28	231/14464	0.004329	2.23623995	0.83415222	0.36314499	0.41580887	0.31978933	Ccn3	1
GO:0002573	myeloid leukocyte differentiation	1/28	232/14464	0.00431034	2.22660099	0.82946699	0.36439784	0.41634225	0.32019954	Fbn1	1
GO:0046394	carboxylic acid biosynthetic process	1/28	232/14464	0.00431034	2.22660099	0.82946699	0.36439784	0.41634225	0.32019954	Gstm7	1
GO:0018108	peptidyl-tyrosine phosphorylation	1/28	233/14464	0.00429185	2.21704476	0.82480555	0.36564833	0.41732031	0.32095175	Bmp6	1
GO:0009743	response to carbohydrate	1/28	234/14464	0.0042735	2.20757021	0.82016766	0.36689644	0.4178433	0.32135397	Ndufaf2	1
GO:0045333	cellular respiration	1/28	234/14464	0.0042735	2.20757021	0.82016766	0.36689644	0.4178433	0.32135397	Col6a1	1
GO:0015850	organic hydroxy compound transport	1/28	235/14464	0.00425532	2.19817629	0.81555309	0.36814218	0.41791247	0.32140716	Bmp6	1
GO:0016053	organic acid biosynthetic process	1/28	235/14464	0.00425532	2.19817629	0.81555309	0.36814218	0.41791247	0.32140716	Gstm7	1
GO:0090257	regulation of muscle system process	1/28	235/14464	0.00425532	2.19817629	0.81555309	0.36814218	0.41791247	0.32140716	Igfbp5	1
GO:0007249	canonical NF-kappaB signal transduction	1/28	236/14464	0.00423729	2.18886199	0.81096159	0.36938555	0.41842604	0.32180213	Cx3cr1	1
GO:0033044	regulation of chromosome organization	1/28	236/14464	0.00423729	2.18886199	0.81096159	0.36938555	0.41842604	0.32180213	H3f3b	1
GO:0018212	peptidyl-tyrosine modification	1/28	237/14464	0.00421941	2.17962628	0.80639294	0.37062657	0.4193828	0.32253796	Bmp6	1
GO:0048167	regulation of synaptic plasticity	1/28	239/14464	0.0041841	2.16138673	0.79732328	0.37310155	0.42128222	0.32399876	Cx3cr1	1
GO:1903320	regulation of protein modification by small protein conjugation or removal	1/28	239/14464	0.0041841	2.16138673	0.79732328	0.37310155	0.42128222	0.32399876	Gbp4	1
GO:0051924	regulation of calcium ion transport	1/28	240/14464	0.00416667	2.15238095	0.79282182	0.37433551	0.42222492	0.32472377	Gstm7	1
GO:0002366	leukocyte activation involved in immune response	1/28	241/14464	0.00414938	2.14344991	0.78834231	0.37556714	0.4227128	0.32509899	Cx3cr1	1
GO:0045444	fat cell differentiation	1/28	241/14464	0.00414938	2.14344991	0.78834231	0.37556714	0.4227128	0.32509899	Fbn1	1
GO:0071695	anatomical structure maturation	1/28	242/14464	0.00413223	2.13459268	0.78388453	0.37679642	0.42319598	0.32547059	Cx3cr1	1
GO:0090596	sensory organ morphogenesis	1/28	242/14464	0.00413223	2.13459268	0.78388453	0.37679642	0.42319598	0.32547059	Fbn1	1
GO:0009205	purine ribonucleoside triphosphate metabolic process	1/28	243/14464	0.00411523	2.12580835	0.77944828	0.37802337	0.42412378	0.32618415	Col6a1	1
GO:1901873	regulation of post-translational protein modification	1/28	244/14464	0.00409836	2.11709602	0.77503335	0.37924799	0.42504701	0.32689418	Gbp4	1
GO:0002263	cell activation involved in immune response	1/28	245/14464	0.00408163	2.10845481	0.77063953	0.38047029	0.42596568	0.32760071	Cx3cr1	1
GO:0045860	positive regulation of protein kinase activity	1/28	246/14464	0.00406504	2.09988386	0.7662666	0.38169027	0.42687981	0.32830374	Fbn1	1
GO:0009199	ribonucleoside triphosphate metabolic process	1/28	248/14464	0.00403226	2.08294931	0.75758267	0.38412327	0.42869454	0.32969941	Col6a1	1
GO:0051607	defense response to virus	1/28	248/14464	0.00403226	2.08294931	0.75758267	0.38412327	0.42869454	0.32969941	Gbp4	1
GO:0005996	monosaccharide metabolic process	1/28	250/14464	0.004	2.06628571	0.74897998	0.38654704	0.43094496	0.33143016	Fbn1	1
GO:0009144	purine nucleoside triphosphate metabolic process	1/28	251/14464	0.00398406	2.0580535	0.74470862	0.38775548	0.43183715	0.33211633	Col6a1	1
GO:0010721	negative regulation of cell development	1/28	252/14464	0.00396825	2.04988662	0.740457	0.38896162	0.43272491	0.33279908	Fbn1	1
GO:0048562	embryonic organ morphogenesis	1/28	254/14464	0.00393701	2.03374578	0.73201224	0.39136702	0.4349436	0.33450543	Fbn1	1
GO:0050878	regulation of body fluid levels	1/28	256/14464	0.00390625	2.01785714	0.72364424	0.3937633	0.4371475	0.3362004	Comp	1
GO:0033674	positive regulation of kinase activity	1/28	258/14464	0.00387597	2.00221484	0.71535159	0.39615047	0.43933669	0.33788405	Fbn1	1
GO:0030217	T cell differentiation	1/28	263/14464	0.00380228	1.96414992	0.69494071	0.40207882	0.44544439	0.34258135	Fzd7	1
GO:0009141	nucleoside triphosphate metabolic process	1/28	265/14464	0.00377358	1.94932615	0.68690119	0.40443442	0.44711767	0.34386823	Col6a1	1
GO:0032868	response to insulin	1/28	265/14464	0.00377358	1.94932615	0.68690119	0.40443442	0.44711767	0.34386823	Col6a1	1
GO:0022604	regulation of cell morphogenesis	1/28	268/14464	0.00373134	1.92750533	0.67497174	0.40795103	0.45053465	0.34649615	Bhlhb9	1
GO:0045765	regulation of angiogenesis	1/28	270/14464	0.0037037	1.91322751	0.66710353	0.41028431	0.452639	0.34811456	Cx3cr1	1
GO:0009101	glycoprotein biosynthetic process	1/28	272/14464	0.00367647	1.89915966	0.65930177	0.41260872	0.45425601	0.34935817	Slc2a10	1
GO:0010001	glial cell differentiation	1/28	272/14464	0.00367647	1.89915966	0.65930177	0.41260872	0.45425601	0.34935817	Col6a1	1
GO:0006066	alcohol metabolic process	1/28	275/14464	0.00363636	1.87844156	0.64772118	0.41607878	0.45760015	0.35193007	Bmp6	1
GO:0007281	germ cell development	1/28	276/14464	0.00362319	1.87163561	0.64389298	0.41723106	0.45839093	0.35253824	H3f3b	1
GO:0032386	regulation of intracellular transport	1/28	277/14464	0.00361011	1.8648788	0.64008054	0.41838116	0.45870183	0.35277735	Gbp4	1
GO:1901342	regulation of vasculature development	1/28	277/14464	0.00361011	1.8648788	0.64008054	0.41838116	0.45870183	0.35277735	Cx3cr1	1
GO:0006874	intracellular calcium ion homeostasis	1/28	278/14464	0.00359712	1.85817061	0.63628374	0.41952906	0.45948421	0.35337906	Gstm7	1
GO:0006310	DNA recombination	1/28	279/14464	0.00358423	1.8515105	0.63250245	0.42067478	0.46026258	0.35397769	H1f0	1
GO:0001933	negative regulation of protein phosphorylation	1/28	281/14464	0.00355872	1.83833249	0.62498584	0.42295968	0.46133128	0.3547996	Gbp4	1
GO:0006936	muscle contraction	1/28	281/14464	0.00355872	1.83833249	0.62498584	0.42295968	0.46133128	0.3547996	Comp	1
GO:0046942	carboxylic acid transport	1/28	281/14464	0.00355872	1.83833249	0.62498584	0.42295968	0.46133128	0.3547996	Kcnk2	1
GO:0030111	regulation of Wnt signaling pathway	1/28	282/14464	0.0035461	1.83181358	0.62125027	0.42409887	0.46190921	0.35524408	Fzd7	1
GO:0007416	synapse assembly	1/28	283/14464	0.00353357	1.82534074	0.61752969	0.42523589	0.46190921	0.35524408	Bhlhb9	1
GO:0009416	response to light stimulus	1/28	283/14464	0.00353357	1.82534074	0.61752969	0.42523589	0.46190921	0.35524408	Col6a1	1
GO:0015849	organic acid transport	1/28	283/14464	0.00353357	1.82534074	0.61752969	0.42523589	0.46190921	0.35524408	Kcnk2	1
GO:0006790	sulfur compound metabolic process	1/28	285/14464	0.00350877	1.81253133	0.610133	0.42750344	0.46389605	0.35677211	Gstm7	1
GO:1902105	regulation of leukocyte differentiation	1/28	286/14464	0.0034965	1.80619381	0.60645665	0.42863398	0.46464626	0.35734908	Fbn1	1
GO:1901888	regulation of cell junction assembly	1/28	288/14464	0.00347222	1.79365079	0.59914732	0.43088861	0.46661223	0.35886106	Bhlhb9	1
GO:0001666	response to hypoxia	1/28	289/14464	0.00346021	1.78744439	0.59551411	0.4320127	0.46735116	0.35942936	Kcnk2	1
GO:0048545	response to steroid hormone	1/28	290/14464	0.00344828	1.78128079	0.59189504	0.43313465	0.46760863	0.35962737	Bmp6	1
GO:0070588	calcium ion transmembrane transport	1/28	290/14464	0.00344828	1.78128079	0.59189504	0.43313465	0.46760863	0.35962737	Gstm7	1
GO:0048511	rhythmic process	1/28	291/14464	0.00343643	1.77515955	0.58829001	0.43425446	0.46833967	0.3601896	Col6a1	1
GO:1904062	regulation of monoatomic cation transmembrane transport	1/28	294/14464	0.00340136	1.75704568	0.57755799	0.43760111	0.47146841	0.36259584	Gstm7	1
GO:0022412	cellular process involved in reproduction in multicellular organism	1/28	295/14464	0.00338983	1.75108959	0.57400797	0.43871242	0.47218488	0.36314686	H3f3b	1
GO:0019216	regulation of lipid metabolic process	1/28	298/14464	0.0033557	1.73346117	0.56343836	0.44203364	0.475276	0.36552418	Bmp6	1
GO:0042326	negative regulation of phosphorylation	1/28	301/14464	0.00332226	1.71618415	0.55298722	0.4453359	0.47737121	0.36713555	Gbp4	1
GO:0071214	cellular response to abiotic stimulus	1/28	301/14464	0.00332226	1.71618415	0.55298722	0.4453359	0.47737121	0.36713555	Bmp6	1
GO:0104004	cellular response to environmental stimulus	1/28	301/14464	0.00332226	1.71618415	0.55298722	0.4453359	0.47737121	0.36713555	Bmp6	1
GO:0099173	postsynapse organization	1/28	303/14464	0.00330033	1.7048562	0.54608425	0.44752693	0.47923431	0.36856842	Bhlhb9	1
GO:0034504	protein localization to nucleus	1/28	306/14464	0.00326797	1.68814192	0.53582448	0.45079784	0.48176173	0.37051221	Gbp4	1
GO:0051347	positive regulation of transferase activity	1/28	306/14464	0.00326797	1.68814192	0.53582448	0.45079784	0.48176173	0.37051221	Fbn1	1
GO:0051098	regulation of binding	1/28	308/14464	0.00324675	1.67717996	0.52904663	0.45296806	0.48359254	0.37192024	H1f0	1
GO:0016358	dendrite development	1/28	309/14464	0.00323625	1.6717522	0.52567602	0.45405007	0.48425905	0.37243283	Bhlhb9	1
GO:0009150	purine ribonucleotide metabolic process	1/28	311/14464	0.00321543	1.66100138	0.51897099	0.4562079	0.48509342	0.37307453	Col6a1	1
GO:0010038	response to metal ion	1/28	311/14464	0.00321543	1.66100138	0.51897099	0.4562079	0.48509342	0.37307453	Bmp6	1
GO:0043010	camera-type eye development	1/28	311/14464	0.00321543	1.66100138	0.51897099	0.4562079	0.48509342	0.37307453	Fbn1	1

GO:0009615	response to virus	1/28	312/14464	0.00320513	1.65567766	0.51563637	0.45728372	0.48574918	0.37357886	Gbp4	1
GO:1903532	positive regulation of secretion by cell	1/28	315/14464	0.0031746	1.6399093	0.50570308	0.4604989	0.48867386	0.37582817	Bmp6	1
GO:0062012	regulation of small molecule metabolic process	1/28	319/14464	0.0031348	1.61934617	0.49262006	0.46475725	0.49269857	0.37892348	Bmp6	1
GO:0002697	regulation of immune effector process	1/28	320/14464	0.003125	1.61428571	0.48937756	0.46581676	0.49332746	0.37940715	Cx3cr1	1
GO:0007517	muscle organ development	1/28	323/14464	0.00309598	1.59929235	0.47971655	0.46898318	0.49433065	0.38017868	Col6a1	1
GO:0009100	glycoprotein metabolic process	1/28	323/14464	0.00309598	1.59929235	0.47971655	0.46898318	0.49433065	0.38017868	Slc2a10	1
GO:0021700	developmental maturation	1/28	323/14464	0.00309598	1.59929235	0.47971655	0.46898318	0.49433065	0.38017868	Cx3cr1	1
GO:0034765	regulation of monoatomic ion transmembrane transport	1/28	323/14464	0.00309598	1.59929235	0.47971655	0.46898318	0.49433065	0.38017868	Gstm7	1
GO:0006913	nucleocytoplasmic transport	1/28	324/14464	0.00308642	1.59435626	0.47651811	0.47003462	0.49433065	0.38017868	Gbp4	1
GO:0031647	regulation of protein stability	1/28	324/14464	0.00308642	1.59435626	0.47651811	0.47003462	0.49433065	0.38017868	Telo2	1
GO:0051169	nuclear transport	1/28	324/14464	0.00308642	1.59435626	0.47651811	0.47003462	0.49433065	0.38017868	Gbp4	1
GO:0006417	regulation of translation	1/28	326/14464	0.00306748	1.58457493	0.47015361	0.47213149	0.49555071	0.381117	Igfbp5	1
GO:0007159	leukocyte cell-cell adhesion	1/28	326/14464	0.00306748	1.58457493	0.47015361	0.47213149	0.49555071	0.381117	Cx3cr1	1
GO:0002443	leukocyte mediated immunity	1/28	327/14464	0.0030581	1.57972914	0.46698739	0.47317692	0.49615578	0.38158235	Cx3cr1	1
GO:0002833	positive regulation of response to biotic stimulus	1/28	331/14464	0.00302115	1.56063876	0.45442761	0.47733872	0.49952954	0.38417703	Bmp6	1
GO:0048638	regulation of developmental growth	1/28	331/14464	0.00302115	1.56063876	0.45442761	0.47733872	0.49952954	0.38417703	Kcnk2	1
GO:0009259	ribonucleotide metabolic process	1/28	332/14464	0.00301205	1.55593804	0.45131357	0.47837421	0.49962479	0.38425029	Col6a1	1
GO:0031589	cell-substrate adhesion	1/28	332/14464	0.00301205	1.55593804	0.45131357	0.47837421	0.49962479	0.38425029	Fzd7	1
GO:0051047	positive regulation of secretion	1/28	334/14464	0.00299401	1.54662104	0.44511607	0.48043925	0.50128671	0.38552843	Bmp6	1
GO:0006631	fatty acid metabolic process	1/28	335/14464	0.00298507	1.54200426	0.44203246	0.48146881	0.50186601	0.38597396	Gstm7	1
GO:0002250	adaptive immune response	1/28	337/14464	0.00296736	1.5328529	0.4358952	0.48352203	0.50351015	0.38723843	Cx3cr1	1
GO:0019693	ribose phosphate metabolic process	1/28	339/14464	0.00294985	1.52380952	0.4297974	0.48556742	0.5051429	0.38849414	Col6a1	1
GO:0006869	lipid transport	1/28	342/14464	0.00292398	1.51044277	0.42072351	0.48862085	0.5078201	0.39055312	Bmp6	1
GO:0010563	negative regulation of phosphorus metabolic process	1/28	345/14464	0.00289855	1.49730849	0.41173541	0.49165681	0.50997343	0.3922092	Gbp4	1
GO:0045936	negative regulation of phosphate metabolic process	1/28	345/14464	0.00289855	1.49730849	0.41173541	0.49165681	0.50997343	0.3922092	Gbp4	1
GO:0019221	cytokine-mediated signaling pathway	1/28	353/14464	0.00283286	1.46337515	0.38817358	0.49966796	0.51777542	0.39820953	Cx3cr1	1
GO:0010639	negative regulation of organelle organization	1/28	355/14464	0.0028169	1.45513078	0.38237285	0.50165165	0.51932235	0.39939924	H3f3b	1
GO:0007018	microtubule-based movement	1/28	359/14464	0.00278552	1.43891763	0.37087578	0.50559629	0.52238367	0.40175363	Kifc3	1
GO:0030098	lymphocyte differentiation	1/28	359/14464	0.00278552	1.43891763	0.37087578	0.50559629	0.52238367	0.40175363	Fzd7	1
GO:1903706	regulation of hemopoiesis	1/28	362/14464	0.00276243	1.4269929	0.36234256	0.508535	0.52490734	0.40369453	Fbn1	1
GO:0032535	regulation of cellular component size	1/28	365/14464	0.00273973	1.41526419	0.35388449	0.51145686	0.52740873	0.40561829	Col6a1	1
GO:0050807	regulation of synapse organization	1/28	370/14464	0.0027027	1.396139	0.33995082	0.51628944	0.53187364	0.40905215	Bhlhb9	1
GO:1902903	regulation of supramolecular fiber organization	1/28	374/14464	0.0026738	1.38120703	0.32894703	0.52012228	0.53530095	0.41168802	Col6a1	1
GO:0010959	regulation of metal ion transport	1/28	375/14464	0.00266667	1.37752381	0.32621556	0.5210759	0.53569865	0.41199389	Gstm7	1
GO:0043603	amide metabolic process	1/28	376/14464	0.00265957	1.37386018	0.32349177	0.5220277	0.53569865	0.41199389	Gstm7	1
GO:0050803	regulation of synapse structure or activity	1/28	376/14464	0.00265957	1.37386018	0.32349177	0.5220277	0.53569865	0.41199389	Bhlhb9	1
GO:0006816	calcium ion transport	1/28	377/14464	0.00265252	1.37021599	0.32077563	0.52297768	0.53615347	0.41234368	Gstm7	1
GO:0031400	negative regulation of protein modification process	1/28	378/14464	0.0026455	1.36659108	0.31806708	0.52392583	0.53660554	0.41269136	Gbp4	1
GO:2001233	regulation of apoptotic signaling pathway	1/28	379/14464	0.00263852	1.3629853	0.31536609	0.52487216	0.53705488	0.41303694	Cx3cr1	1
GO:0006979	response to oxidative stress	1/28	388/14464	0.00257732	1.33136966	0.2913894	0.53330795	0.54515923	0.41926981	Col6a1	1
GO:0010876	lipid localization	1/28	393/14464	0.00254453	1.31443112	0.27831979	0.53793189	0.54935516	0.4224968	Bmp6	1
GO:0009314	response to radiation	1/28	394/14464	0.00253807	1.311095	0.27572672	0.53885137	0.5497635	0.42281085	Col6a1	1
GO:0006163	purine nucleotide metabolic process	1/28	395/14464	0.00253165	1.30777577	0.27314052	0.53976908	0.55016925	0.4231229	Col6a1	1
GO:0022407	regulation of cell-cell adhesion	1/28	398/14464	0.00251256	1.29791816	0.26542265	0.54251165	0.55243246	0.42486349	Bmp6	1
GO:0048732	gland development	1/28	408/14464	0.00245098	1.26610644	0.2401261	0.5515402	0.56108609	0.4315188	Igfbp5	1
GO:0043254	regulation of protein-containing complex assembly	1/28	413/14464	0.00242131	1.25077828	0.22771734	0.5559898	0.56506937	0.43458225	H3f3b	1
GO:0072594	establishment of protein localization to organelle	1/28	415/14464	0.00240964	1.24475043	0.22279712	0.5577577	0.56632211	0.43554571	Gbp4	1
GO:0048568	embryonic organ development	1/28	421/14464	0.0023753	1.22701052	0.20818137	0.56302075	0.57111788	0.43923403	Fbn1	1
GO:0007283	spermatogenesis	1/28	423/14464	0.00236407	1.22120905	0.20335686	0.56476164	0.57233507	0.44017014	H3f3b	1
GO:0031346	positive regulation of cell projection organization	1/28	426/14464	0.00234742	1.21260899	0.19616367	0.56736044	0.57386935	0.44135012	Bhlhb9	1
GO:0045859	regulation of protein kinase activity	1/28	426/14464	0.00234742	1.21260899	0.19616367	0.56736044	0.57386935	0.44135012	Fbn1	1
GO:0030099	myeloid cell differentiation	1/28	428/14464	0.00233645	1.20694259	0.19139688	0.56908465	0.57506357	0.44226857	Fbn1	1
GO:0048232	male gamete generation	1/28	437/14464	0.00228833	1.18208565	0.17022322	0.57676196	0.58226541	0.44780734	H3f3b	1
GO:0043269	regulation of monoatomic ion transport	1/28	440/14464	0.00227273	1.17402597	0.16326355	0.57929162	0.58426171	0.44934265	Gstm7	1
GO:0034762	regulation of transmembrane transport	1/28	447/14464	0.00223714	1.15564078	0.14720901	0.5851376	0.58903481	0.45301354	Gstm7	1
GO:1903131	mononuclear cell differentiation	1/28	447/14464	0.00223714	1.15564078	0.14720901	0.5851376	0.58903481	0.45301354	Fzd7	1
GO:0009117	nucleotide metabolic process	1/28	450/14464	0.00222222	1.14793651	0.14040589	0.58761897	0.59097041	0.45450216	Col6a1	1
GO:0043549	regulation of kinase activity	1/28	456/14464	0.00219298	1.13283208	0.12693521	0.59253884	0.59535242	0.45787227	Fbn1	1
GO:0042110	T cell activation	1/28	464/14464	0.00215517	1.11330049	0.10924751	0.5990107	0.60128399	0.46243411	Fzd7	1
GO:0010608	post-transcriptional regulation of gene expression	1/28	468/14464	0.00213675	1.1037851	0.10051729	0.60220935	0.60392179	0.46446278	Igfbp5	1
GO:0002009	morphogenesis of an epithelium	1/28	470/14464	0.00212766	1.09908815	0.09617993	0.60379943	0.60494299	0.46524816	Igfbp5	1
GO:0034655	nucleobase-containing compound catabolic process	1/28	471/14464	0.00212314	1.09675463	0.09401812	0.60459217	0.60516416	0.46541826	Col6a1	1
GO:0034329	cell junction assembly	1/28	485/14464	0.00206186	1.06509573	0.06421997	0.61553122	0.61553122	0.47339134	Bhlhb9	1

ID	Description	GeneRatio	BgRatio	RichFactor	FoldEnrichm	zScore	pvalue	p.adjust	qvalue	geneID	Count
GO:0061001	regulation of dendritic spine morphogenesis	5/67	61/14464	0.08196721	17.6951309	8.91371733	8.921E-06	0.01374724	0.01318428	Ngef/Slc30a1/Tiam1/Abi3bp/Il1rapl1	5
GO:0060997	dendritic spine morphogenesis	5/67	78/14464	0.06410256	13.8384998	7.75572041	2.9793E-05	0.02295572	0.02201567	Ngef/Slc30a1/Tiam1/Abi3bp/Il1rapl1	5
GO:0097061	dendritic spine organization	5/67	106/14464	0.04716981	10.183047	6.47326459	0.00012943	0.06648488	0.06376227	Ngef/Slc30a1/Tiam1/Abi3bp/Il1rapl1	5
GO:0106027	neuron projection organization	5/67	117/14464	0.04273504	9.22566654	6.0941631	0.00020577	0.06693338	0.0641924	Ngef/Slc30a1/Tiam1/Abi3bp/Il1rapl1	5
GO:0048813	dendrite morphogenesis	6/67	186/14464	0.03225806	6.96389023	5.58448356	0.00021718	0.06693338	0.0641924	Ngef/Slc30a1/Nfatc4/Tiam1/Abi3bp/Il1rapl1	6
GO:0060996	dendritic spine development	5/67	134/14464	0.03731343	8.05524616	5.597216	0.00038595	0.08567481	0.08216635	Ngef/Slc30a1/Tiam1/Abi3bp/Il1rapl1	5
GO:0061003	positive regulation of dendritic spine morphogenesis	3/67	31/14464	0.09677419	20.8916707	7.56317926	0.00038918	0.08567481	0.08216635	Slc30a1/Tiam1/Il1rapl1	3
GO:0060998	regulation of dendritic spine development	4/67	84/14464	0.04761905	10.2800284	5.81888935	0.00061421	0.1183119	0.11346692	Ngef/Slc30a1/Tiam1/Il1rapl1	4
GO:0048013	ephrin receptor signaling pathway	3/67	39/14464	0.07692308	16.6061998	6.6573616	0.0007706	0.13194354	0.12654034	Ngef/Epha8/Tiam1	3
GO:0048814	regulation of dendrite morphogenesis	4/67	96/14464	0.04166667	8.99502488	5.36151923	0.00101442	0.15632145	0.14991995	Slc30a1/Nfatc4/Tiam1/Il1rapl1	4
GO:0002269	leukocyte activation involved in inflammatory response	3/67	47/14464	0.06382979	13.7796126	5.98631768	0.0013316	0.16049506	0.15392265	Nr1d1/Ager/Enpp1	3
GO:0043031	negative regulation of macrophage activation	2/67	12/14464	0.16666667	35.9800995	8.26948149	0.00135395	0.16049506	0.15392265	Nr1d1/Cd200	2
GO:0150079	negative regulation of neuroinflammatory response	2/67	12/14464	0.16666667	35.9800995	8.26948149	0.00135395	0.16049506	0.15392265	Nr1d1/Cd200	2
GO:0099175	regulation of postsynapse organization	5/67	186/14464	0.02688172	5.80324186	4.49767252	0.00168894	0.18590385	0.17829093	Ngef/Slc30a1/Tiam1/Abi3bp/Il1rapl1	5
GO:0031648	protein destabilization	3/67	57/14464	0.05263158	11.3621367	5.34725085	0.00232494	0.23884874	0.22906768	Nr1d1/Ccdc88c/Dazap2	3
GO:0045472	response to ether	2/67	17/14464	0.11764706	25.3977173	6.86618358	0.00274857	0.24806575	0.23790725	Hnrnpd/Nfatc4	2
GO:0016358	dendrite development	6/67	309/14464	0.01941748	4.19185625	3.86899545	0.00302864	0.24806575	0.23790725	Ngef/Slc30a1/Nfatc4/Tiam1/Abi3bp/Il1rapl1	6
GO:0050775	positive regulation of dendrite morphogenesis	3/67	63/14464	0.04761905	10.2800284	5.03563042	0.00309364	0.24806575	0.23790725	Slc30a1/Tiam1/Il1rapl1	3
GO:0010770	positive regulation of cell morphogenesis	3/67	64/14464	0.046875	10.119403	4.98776221	0.00323512	0.24806575	0.23790725	Slc30a1/Tiam1/Il1rapl1	3
GO:0052548	regulation of endopeptidase activity	5/67	218/14464	0.02293578	4.95138984	4.01016505	0.00336191	0.24806575	0.23790725	Aim2/Ager/Psmb9/Tnfsf10/Fnip1	5
GO:0060999	positive regulation of dendritic spine development	3/67	65/14464	0.04615385	9.96371986	4.94093772	0.00338052	0.24806575	0.23790725	Slc30a1/Tiam1/Il1rapl1	3
GO:0050773	regulation of dendrite development	4/67	136/14464	0.02941176	6.34942932	4.27577372	0.00362609	0.25399113	0.24358998	Slc30a1/Nfatc4/Tiam1/Il1rapl1	4
GO:0048143	astrocyte activation	2/67	20/14464	0.1	21.5880597	6.28517348	0.00380568	0.25498038	0.24453871	Nr1d1/Ager	2
GO:1902074	response to salt	2/67	22/14464	0.09090909	19.6255088	5.96398315	0.00459938	0.29531873	0.28322518	Hnrnpd/Enpp1	2
GO:0150076	neuroinflammatory response	3/67	75/14464	0.04	8.63522388	4.52238668	0.00505701	0.3117138	0.29894886	Nr1d1/Ager/Cd200	3
GO:0032611	interleukin-1 beta production	3/67	79/14464	0.03797468	8.19799735	4.37623754	0.00584505	0.34173761	0.32774316	Aim2/Slamf9/Ager	3
GO:0150077	regulation of neuroinflammatory response	2/67	26/14464	0.07692308	16.6061998	5.43326527	0.00639432	0.34173761	0.32774316	Nr1d1/Cd200	2
GO:0071674	mononuclear cell migration	4/67	163/14464	0.02453988	5.29768336	3.76422527	0.00686918	0.34173761	0.32774316	Slamf9/Ager/Cd200/Enpp1	4
GO:2000116	regulation of cysteine-type endopeptidase activity	4/67	163/14464	0.02453988	5.29768336	3.76422527	0.00686918	0.34173761	0.32774316	Aim2/Psmb9/Tnfsf10/Fnip1	4
GO:1905874	regulation of postsynaptic density organization	2/67	27/14464	0.07407407	15.9911553	5.31874416	0.00688534	0.34173761	0.32774316	Slc30a1/Abi3bp	2
GO:0050807	regulation of synapse organization	6/67	370/14464	0.01621622	3.50076644	3.32420235	0.00722988	0.34173761	0.32774316	Ngef/Slc30a1/Nfatc4/Tiam1/Abi3bp/Il1rapl1	6
GO:0052547	regulation of peptidase activity	5/67	262/14464	0.01908397	4.11985872	3.47649342	0.00727254	0.34173761	0.32774316	Aim2/Ager/Psmb9/Tnfsf10/Fnip1	5
GO:0032640	tumor necrosis factor production	4/67	166/14464	0.02409639	5.2019421	3.71447169	0.0073182	0.34173761	0.32774316	Slamf9/H2-T23/Ager/Nfatc4	4
GO:0071706	tumor necrosis factor superfamily cytokine production	4/67	168/14464	0.02380952	5.14001421	3.68196618	0.00762808	0.34334168	0.32928155	Slamf9/H2-T23/Ager/Nfatc4	4
GO:0050803	regulation of synapse structure or activity	6/67	376/14464	0.01595745	3.44490314	3.27688728	0.00779816	0.34334168	0.32928155	Ngef/Slc30a1/Nfatc4/Tiam1/Abi3bp/Il1rapl1	6
GO:0001819	positive regulation of cytokine production	6/67	386/14464	0.01554404	3.35565695	3.20011665	0.00881621	0.37152315	0.35630896	Aim2/H2-T23/Ager/Rftn1/Cd200/Nfatc4	6
GO:0032612	interleukin-1 production	3/67	93/14464	0.03225806	6.96389023	3.9360283	0.00916151	0.37152315	0.35630896	Aim2/Slamf9/Ager	3
GO:0042116	macrophage activation	3/67	93/14464	0.03225806	6.96389023	3.9360283	0.00916151	0.37152315	0.35630896	Nr1d1/Ager/Cd200	3
GO:0010717	regulation of epithelial to mesenchymal transition	3/67	94/14464	0.03191489	6.88980629	3.9081133	0.00943262	0.37270943	0.35744666	Ager/Tiam1/Ldlrad4	3
GO:0019882	antigen processing and presentation	3/67	95/14464	0.03157895	6.81728201	3.88060323	0.0097084	0.37401593	0.35869966	H2-T23/Rftn1/Psmb9	3
GO:0001914	regulation of T cell mediated cytotoxicity	2/67	34/14464	0.05882353	12.6988586	4.65886771	0.0107781	0.39752612	0.38124709	H2-T23/Ager	2
GO:0032760	positive regulation of tumor necrosis factor production	3/67	99/14464	0.03030303	6.54183627	3.77440992	0.01085841	0.39752612	0.38124709	H2-T23/Ager/Nfatc4	3
GO:1903557	positive regulation of tumor necrosis factor superfamily cytokine production	3/67	100/14464	0.03	6.47641791	3.74877587	0.01115771	0.39752612	0.38124709	H2-T23/Ager/Nfatc4	3
GO:0097028	dendritic cell differentiation	2/67	35/14464	0.05714286	12.3360341	4.58044467	0.01139743	0.39752612	0.38124709	Slamf9/Ager	2
GO:0050727	regulation of inflammatory response	5/67	294/14464	0.0170068	3.67143872	3.15692707	0.01160849	0.39752612	0.38124709	Nr1d1/Aim2/H2-T23/Ager/Cd200	5
GO:0014002	astrocyte development	2/67	36/14464	0.05555556	11.9933665	4.50515236	0.0120321	0.40307531	0.38656904	Nr1d1/Ager	2
GO:0099173	postsynapse organization	5/67	303/14464	0.01650165	3.56238609	3.0750311	0.01309017	0.42098983	0.40374994	Ngef/Slc30a1/Tiam1/Abi3bp/Il1rapl1	5
GO:0050900	leukocyte migration	5/67	304/14464	0.01644737	3.55066771	3.06612349	0.01326235	0.42098983	0.40374994	Slamf9/Ager/Podxl2/Cd200/Enpp1	5
GO:0002456	T cell mediated immunity	3/67	107/14464	0.02803738	6.05272702	3.57862726	0.01338644	0.42098983	0.40374994	H2-T23/Ager/Rftn1	3
GO:0051098	regulation of binding	5/67	308/14464	0.01623377	3.50455515	3.03086276	0.01396645	0.43044596	0.41281883	Ager/Nfatc4/Tiam1/Gpsm1/Dazap2	5
GO:0060291	long-term synaptic potentiation	3/67	111/14464	0.02702703	5.83461073	3.48804638	0.01476595	0.4307297	0.41309095	Ager/Nfatc4/Enpp1	3
GO:0051345	positive regulation of hydrolase activity	5/67	317/14464	0.01577287	3.40505674	2.95361151	0.01564221	0.4307297	0.41309095	Aim2/Ager/Cd200/Tnfsf10/Tiam1	5
GO:0005978	glycogen biosynthetic process	2/67	43/14464	0.04651163	10.040958	4.05024769	0.01689185	0.4307297	0.41309095	Nr1d1/Enpp1	2
GO:0007616	long-term memory	2/67	43/14464	0.04651163	10.040958	4.05024769	0.01689185	0.4307297	0.41309095	Nfatc4/Ehmt2	2
GO:0009250	glucan biosynthetic process	2/67	43/14464	0.04651163	10.040958	4.05024769	0.01689185	0.4307297	0.41309095	Nr1d1/Enpp1	2
GO:0001774	microglial cell activation	2/67	44/14464	0.04545455	9.81275441	3.99379683	0.01764392	0.4307297	0.41309095	Nr1d1/Ager	2
GO:0001913	T cell mediated cytotoxicity	2/67	44/14464	0.04545455	9.81275441	3.99379683	0.01764392	0.4307297	0.41309095	H2-T23/Ager	2
GO:0034113	heterotypic cell-cell adhesion	2/67	44/14464	0.04545455	9.81275441	3.99379683	0.01764392	0.4307297	0.41309095	Ager/Cd200	2
GO:0051602	response to electrical stimulus	2/67	44/14464	0.04545455	9.81275441	3.99379683	0.01764392	0.4307297	0.41309095	Cdk2/Hnrnpd	2
GO:0002443	leukocyte mediated immunity	5/67	327/14464	0.01529052	3.30092656	2.87096962	0.01765688	0.4307297	0.41309095	Slamf9/H2-T23/Ager/Rftn1/Enpp1	5
GO:0032731	positive regulation of interleukin-1 beta production	2/67	46/14464	0.04347826	9.38611291	3.88613339	0.01918997	0.4307297	0.41309095	Aim2/Ager	2
GO:0010950	positive regulation of endopeptidase activity	3/67	125/14464	0.024	5.18113433	3.2027297	0.02020956	0.4307297	0.41309095	Aim2/Ager/Tnfsf10	3
GO:0043030	regulation of macrophage activation	2/67	49/14464	0.04081633	8.81145294	3.73639799	0.02161174	0.4307297	0.41309095	Nr1d1/Cd200	2
GO:0010952	positive regulation of peptidase activity	3/67	132/14464	0.02272727	4.9063772	3.07566007	0.02329322	0.4307297	0.41309095	Aim2/Ager/Tnfsf10	3
GO:0002449	lymphocyte mediated immunity	4/67	236/14464	0.01694915	3.65899317	2.80951844	0.02378725	0.4307297	0.41309095	H2-T23/Ager/Rftn1/Enpp1	4
GO:0006749	glutathione metabolic process	2/67	52/14464	0.03846154	8.30309989	3.59896297	0.02415341	0.4307297	0.41309095	Gstt2/Gstt3	2
GO:0048663	neuron fate commitment	2/67	52/14464	0.03846154	8.30309989	3.59896297	0.02415341	0.4307297	0.41309095	Ehmt2/Bhlhe22	2
GO:0032732	positive regulation of interleukin-1 production	2/67	53/14464	0.03773585	8.14643762	3.55558508	0.02502664	0.4307297	0.41309095	Aim2/Ager	2
GO:0035567	non-canonical Wnt signaling pathway	2/67	54/14464	0.03703704	7.99557767	3.51333097	0.02591265	0.4307297	0.41309095	Ccdc88c/Tiam1	2
GO:0061900	glial cell activation	2/67	54/14464	0.03703704	7.99557767	3.51333097	0.02591265	0.4307297	0.41309095	Nr1d1/Ager	2
GO:0051099	positive regulation of binding	3/67	138/14464	0.02173913	4.69305646	2.97367573	0.026129	0.4307297	0.41309095	Tiam1/Gpsm1/Dazap2	3
GO:1905517	macrophage migration	2/67	55/14464	0.03636364	7.85020353	3.4721501	0.0268113	0.4307297	0.41309095	Cd200/Enpp1	2
GO:0021782	glial cell development	3/67	140/14464	0.02142857	4.62601279	2.94097807	0.02711374	0.4307297	0.41309095	Nr1d1/Ager/Enpp1	3
GO:0002437	inflammatory response to antigenic stimulus	2/67	56/14464	0.03571429	7.71002132	3.43199507	0.02772247	0.4307297	0.41309095	H2-T23/Enpp1	2
GO:0000723	telomere maintenance	3/67	143/14464	0.02097902	4.52896357	2.89307115	0.02862782	0.4307297	0.41309095	Cdk2/Hnrnpd/Rpa2	3
GO:0030178	negative regulation of Wnt signaling pathway	3/67	144/14464	0.02083333	4.49751244	2.87739574	0.02914235	0.4307297	0.41309095	Ccdc88c/Ppp2r3a/Nfatc4	3
GO:0044088	regulation of vacuole organization	2/67	58/14464	0.03448276	7.44415852	3.3545873	0.02958181	0.4307297	0.41309095	Ehmt2/Fnip1	2
GO:0071709	membrane assembly	2/67	58/14464	0.03448276	7.44415852	3.3545873	0.02958181	0.4307297	0.41309095	Rftn1/Il1rapl1	2
GO:0097106	postsynaptic density organization	2/67	58/14464	0.03448276	7.44415852	3.3545873	0.02958181	0.4307297	0.41309095	Slc30a1/Abi3bp	2
GO:0001837	epithelial to mesenchymal transition	3/67	149/14464	0.02013423	4.3465892	2.80111188	0.03178866	0.4307297	0.41309095	Ager/Tiam1/Ldlrad4	3
GO:0032200	telomere organization	3/67	149/14464	0.02013423	4.3465892	2.80111188	0.03178866	0.4307297	0.41309095	Cdk2/Hnrnpd/Rpa2	3
GO:0016055	Wnt signaling pathway	5/67	385/14464	0.01298701	2.80364412	2.44695455	0.03275679	0.4307297	0.41309095	Ccdc88c/Ppp2r3a/Nfatc4/Tiam1/Enpp1	5
GO:0000271	polysaccharide biosynthetic process	2/67	62/14464	0.03225806	6.96389023	3.21029302	0.03344493	0.4307297	0.41309095	Nr1d1/Enpp1	2
GO:0002460	adaptive immune response based on somatic recombination of immune receptors built from immunoglobu	4/67	263/14464	0.01520913	3.28335509	2.54930887	0.03355808	0.4307297	0.41309095	H2-T23/Ager/Rftn1/Enpp1	4
GO:0032868	response to insulin	4/67	265/14464	0.01509434	3.25857505	2.53139067	0.03436257	0.4307297	0.41309095	Srsf5/Cdk2/Tnfsf10/Enpp1	4
GO:0042593	glucose homeostasis	4/67	265/14464	0.01509434	3.25857505	2.53139067	0.03436257	0.4307297	0.41309095	Nr1d1/Ager/Tiam1/Enpp1	4
GO:1903531	negative regulation of secretion by cell	3/67	154/14464	0.01948052	4.20546618	2.72811287	0.03455723	0.4307297	0.41309095	Cd200/Slc30a1/Il1rapl1	3
GO:0033500	carbohydrate homeostasis	4/67	266/14464	0.01503759	3.24632477	2.52249533	0.03476902	0.4307297	0.41309095	Nr1d1/Ager/Tiam1/Enpp1	4
GO:0071333	cellular response to glucose stimulus	3/67	155/14464	0.01935484	4.17833414	2.71388444	0.03512555	0.4307297	0.41309095	Nr1d1/Ager/Tiam1	3
GO:0001910	regulation of leukocyte mediated cytotoxicity	2/67	65/14464	0.03076923	6.64247991	3.11022012	0.03646463	0.4307297	0.41309095	H2-T23/Ager	2
GO:0071331	cellular response to hexose stimulus	3/67	158/14464	0.01898734	4.09899868	2.67190748	0.03685962	0.4307297	0.41309095	Nr1d1/Ager/Tiam1	3
GO:0071326	cellular response to monosaccharide stimulus	3/67	159/14464	0.01886792	4.07321881	2.65814524	0.03744731	0.4307297	0.41309095	Nr1d1/Ager/Tiam1	3
GO:0044091	membrane biogenesis	2/67	66/14464	0.03030303	6.54183627	3.07825906	0.03749385	0.4307297	0.41309095	Rftn1/Il1rapl1	2
GO:0032088	negative regulation of NF-kappaB transcription factor activity	2/67	67/14464	0.02985075	6.44419693	3.04695343	0.03853417	0.4307297	0.41309095	Aim2/Cd200	2
GO:0045123	cellular extravasation	2/67	67/14464	0.02985075	6.44419693	3.04695343	0.03853417	0.4307297	0.41309095	Ager/Podxl2	2
GO:0002695	negative regulation of leukocyte activation	3/67	161/14464	0.01863354	4.02261982	2.6309554	0.03863716	0.4307297	0.41309095	Nr1d1/H2-T23/Cd200	3
GO:0032680	regulation of tumor necrosis factor production	3/67	161/14464	0.01863354	4.02261982	2.6309554	0.03863716	0.4307297	0.41309095	H2-T23/Ager/Nfatc4	3
GO:1903555	regulation of tumor necrosis factor superfamily cytokine production	3/67	163/14464	0.01840491	3.97326252	2.60420068	0.03984625	0.4307297	0.41309095	H2-T23/Ager/Nfatc4	3
GO:0008347	glial cell migration	2/67	69/14464	0.02898551	6.25740861	2.98621474	0.04064769	0.4307297	0.41309095	Tiam1/Enpp1	2
GO:0030111	regulation of Wnt signaling pathway	4/67	282/14464	0.0141844	3.06213613	2.38563357	0.04165578	0.4307297	0.41309095	Ccdc88c/Ppp2r3a/Nfatc4/Tiam1	4
GO:0051051	negative regulation of transport	5/67	415/14464	0.01204819	2.60097105	2.25743582	0.04301483	0.4307297	0.41309095	Pkia/Cd200/Slc30a1/Enpp1/Il1rapl1	5
GO:0099084	postsynaptic specialization organization	2/67	72/14464	0.02777778	5.99668325	2.89946643	0.04389832	0.4307297	0.41309095	Slc30a1/Abi3bp	2
GO:0050767	regulation of neurogenesis	5/67	418/14464	0.01196172	2.58230379	2.23940307	0.04413646	0.4307297	0.41309095	Nr1d1/Slc30a1/Nfatc4/Tiam1/Il1rapl1	5
GO:0051048	negative regulation of secretion	3/67	170/14464	0.01764706	3.80965759	2.51380486	0.04422841	0.4307297	0.41309095	Cd200/Slc30a1/Il1rapl1	3
GO:0001678	intracellular glucose homeostasis	3/67	171/14464	0.01754386	3.78737889	2.50128373	0.0448734	0.4307297	0.41309095	Nr1d1/Ager/Tiam1	3
GO:0071322	cellular response to carbohydrate stimulus	3/67	171/14464	0.01754386	3.78737889	2.50128373	0.0448734	0.4307297	0.41309095	Nr1d1/Ager/Tiam1	3
GO:0001787	natural killer cell proliferation	1/67	10/14464	0.1	21.5880597	4.44275113	0.04538199	0.4307297	0.41309095	H2-T23	1
GO:0009313	oligosaccharide catabolic process	1/67	10/14464	0.1	21.5880597	4.44275113	0.04538199	0.4307297	0.41309095	Man2b1	1
GO:0021871	forebrain regionalization	1/67	10/14464	0.1	21.5880597	4.44275113	0.04538199	0.4307297	0.41309095	Bhlhe22	1
GO:0021957	corticospinal tract morphogenesis	1/67	10/14464	0.1	21.5880597	4.44275113	0.04538199	0.4307297	0.41309095	Bhlhe22	1
GO:0031953	negative regulation of protein autophosphorylation	1/67	10/14464	0.1	21.5880597	4.44275113	0.04538199	0.4307297	0.41309095	Enpp1	1
GO:0043252	sodium-independent organic anion transport	1/67	10/14464	0.1	21.5880597	4.44275113	0.04538199	0.4307297	0.41309095	Slco1a4	1
GO:0070874	negative regulation of glycogen metabolic process	1/67	10/14464	0.1	21.5880597	4.44275113	0.04538199	0.4307297	0.41309095	Enpp1	1
GO:0070934	CRD-mediated mRNA stabilization	1/67	10/14464	0.1	21.5880597	4.44275113	0.04538199	0.4307297	0.41309095	Hnrnpd	1
GO:0099545	trans-synaptic signaling by trans-synaptic complex	1/67	10/14464	0.1	21.5880597	4.44275113	0.04538199	0.4307297	0.41309095	Il1rapl1	1
GO:0140970	AIM2 inflammasome complex assembly	1/67	10/14464	0.1	21.5880597	4.44275113	0.04538199	0.4307297	0.41309095	Aim2	1
GO:2000402	negative regulation of lymphocyte migration	1/67	10/14464	0.1	21.5880597	4.44275113	0.04538199	0.4307297	0.41309095	Cd200	1
GO:0032651	regulation of interleukin-1 beta production	2/67	74/14464	0.02702703	5.83461073	2.84431418	0.0461176	0.4307297	0.41309095	Aim2/Ager	2
GO:0042102	positive regulation of T cell proliferation	2/67	74/14464	0.02702703	5.83461073	2.84431418	0.0461176	0.4307297	0.41309095	H2-T23/Ager	2
GO:0002709	regulation of T cell mediated immunity	2/67	75/14464	0.02666667	5.75681592	2.81748922	0.04724255	0.4307297	0.41309095	H2-T23/Ager	2
GO:0050866	negative regulation of cell activation	3/67	178/14464	0.01685393	3.63843703	2.41619512	0.04951961	0.4307297	0.41309095	Nr1d1/H2-T23/Cd200	3
GO:0061178	regulation of insulin secretion involved in cellular response to glucose stimulus	2/67	77/14464	0.02597403	5.60728823	2.76526155	0.04952249	0.4307297	0.41309095	Nr1d1/Tiam1	2
GO:0002407	dendritic cell chemotaxis	1/67	11/14464	0.09090909	19.6255088	4.21556781	0.04980702	0.4307297	0.41309095	Slamf9	1
GO:0002863	positive regulation of inflammatory response to antigenic stimulus	1/67	11/14464	0.09090909	19.6255088	4.21556781	0.04980702	0.4307297	0.41309095	H2-T23	1
GO:0032604	granulocyte macrophage colony-stimulating factor production	1/67	11/14464	0.09090909	19.6255088	4.21556781	0.04980702	0.4307297	0.41309095	Slamf9	1
GO:0032793	positive regulation of CREB transcription factor activity	1/67	11/14464	0.09090909	19.6255088	4.21556781	0.04980702	0.4307297	0.41309095	Cd200	1
GO:0035630	bone mineralization involved in bone maturation	1/67	11/14464	0.09090909	19.6255088	4.21556781	0.04980702	0.4307297	0.41309095	Enpp1	1
GO:0035701	hematopoietic stem cell migration	1/67	11/14464	0.09090909	19.6255088	4.21556781	0.04980702	0.4307297	0.41309095	Enpp1	1
GO:0050774	negative regulation of dendrite morphogenesis	1/67	11/14464	0.09090909	19.6255088	4.21556781	0.04980702	0.4307297	0.41309095	Nfatc4	1
GO:0097090	presynaptic membrane organization	1/67	11/14464	0.09090909	19.6255088	4.21556781	0.04980702	0.4307297	0.41309095	Il1rapl1	1
GO:0097501	stress response to metal ion	1/67	11/14464	0.09090909	19.6255088	4.21556781	0.04980702	0.4307297	0.41309095	Slc30a1	1
GO:1900147	regulation of Schwann cell migration	1/67	11/14464	0.09090909	19.6255088	4.21556781	0.04980702	0.4307297	0.41309095	Tiam1	1
GO:1900152	negative regulation of nuclear-transcribed mRNA catabolic process, deadenylation-dependent decay	1/67	11/14464	0.09090909	19.6255088	4.21556781	0.04980702	0.4307297	0.41309095	Hnrnpd	1
GO:1903044	protein localization to membrane raft	1/67	11/14464	0.09090909	19.6255088	4.21556781	0.04980702	0.4307297	0.41309095	Rftn1	1
GO:1903377	negative regulation of oxidative stress-induced neuron intrinsic apoptotic signaling pathway	1/67	11/14464	0.09090909	19.6255088	4.21556781	0.04980702	0.4307297	0.41309095	Pycr1	1
GO:1905456	regulation of lymphoid progenitor cell differentiation	1/67	11/14464	0.09090909	19.6255088	4.21556781	0.04980702	0.4307297	0.41309095	Fnip1	1
GO:1905806	regulation of synapse pruning	1/67	11/14464	0.09090909	19.6255088	4.21556781	0.04980702	0.4307297	0.41309095	Ngef	1
GO:0051091	positive regulation of DNA-binding transcription factor activity	3/67	179/14464	0.01675978	3.61811056	2.40439031	0.05020198	0.4307297	0.41309095	Aim2/Ager/Cd200	3
GO:0031341	regulation of cell killing	2/67	78/14464	0.02564103	5.53539992	2.73982897	0.05067725	0.4307297	0.41309095	H2-T23/Ager	2
GO:0050795	regulation of behavior	2/67	78/14464	0.02564103	5.53539992	2.73982897	0.05067725	0.4307297	0.41309095	Nr1d1/Aim2	2
GO:0042742	defense response to bacterium	3/67	182/14464	0.01648352	3.55847138	2.36947611	0.05227672	0.4307297	0.41309095	Slamf9/H2-T23/Slc30a1	3
GO:0002260	lymphocyte homeostasis	2/67	80/14464	0.025	5.39701493	2.69025649	0.05301579	0.4307297	0.41309095	Aim2/Enpp1	2
GO:0007611	learning or memory	4/67	307/14464	0.01302932	2.81277651	2.19007218	0.05386432	0.4307297	0.41309095	Ager/Nfatc4/Ehmt2/Man2b1	4
GO:0002524	hypersensitivity	1/67	12/14464	0.08333333	17.9900498	4.01653808	0.05421184	0.4307297	0.41309095	H2-T23	1
GO:0006013	mannose metabolic process	1/67	12/14464	0.08333333	17.9900498	4.01653808	0.05421184	0.4307297	0.41309095	Man2b1	1
GO:0007130	synaptonemal complex assembly	1/67	12/14464	0.08333333	17.9900498	4.01653808	0.05421184	0.4307297	0.41309095	Ehmt2	1
GO:0009143	nucleoside triphosphate catabolic process	1/67	12/14464	0.08333333	17.9900498	4.01653808	0.05421184	0.4307297	0.41309095	Enpp1	1
GO:0031453	positive regulation of heterochromatin formation	1/67	12/14464	0.08333333	17.9900498	4.01653808	0.05421184	0.4307297	0.41309095	Cdk2	1
GO:0032736	positive regulation of interleukin-13 production	1/67	12/14464	0.08333333	17.9900498	4.01653808	0.05421184	0.4307297	0.41309095	H2-T23	1
GO:0036135	Schwann cell migration	1/67	12/14464	0.08333333	17.9900498	4.01653808	0.05421184	0.4307297	0.41309095	Tiam1	1
GO:0051589	negative regulation of neurotransmitter transport	1/67	12/14464	0.08333333	17.9900498	4.01653808	0.05421184	0.4307297	0.41309095	Slc30a1	1
GO:0061430	bone trabecula morphogenesis	1/67	12/14464	0.08333333	17.9900498	4.01653808	0.05421184	0.4307297	0.41309095	Enpp1	1
GO:0071636	positive regulation of transforming growth factor beta production	1/67	12/14464	0.08333333	17.9900498	4.01653808	0.05421184	0.4307297	0.41309095	Cd200	1
GO:0090281	negative regulation of calcium ion import	1/67	12/14464	0.08333333	17.9900498	4.01653808	0.05421184	0.4307297	0.41309095	Slc30a1	1
GO:0097119	postsynaptic density protein 95 clustering	1/67	12/14464	0.08333333	17.9900498	4.01653808	0.05421184	0.4307297	0.41309095	Slc30a1	1
GO:0141161	regulation of cAMP/PKA signal transduction	1/67	12/14464	0.08333333	17.9900498	4.01653808	0.05421184	0.4307297	0.41309095	Pkia	1
GO:1904424	regulation of GTP binding	1/67	12/14464	0.08333333	17.9900498	4.01653808	0.05421184	0.4307297	0.41309095	Gpsm1	1
GO:2000105	positive regulation of DNA-templated DNA replication	1/67	12/14464	0.08333333	17.9900498	4.01653808	0.05421184	0.4307297	0.41309095	Cdk2	1
GO:2000489	regulation of hepatic stellate cell activation	1/67	12/14464	0.08333333	17.9900498	4.01653808	0.05421184	0.4307297	0.41309095	Nr1d1	1
GO:2000674	regulation of type B pancreatic cell apoptotic process	1/67	12/14464	0.08333333	17.9900498	4.01653808	0.05421184	0.4307297	0.41309095	Ager	1
GO:0032102	negative regulation of response to external stimulus	4/67	308/14464	0.01298701	2.80364412	2.18266218	0.05438929	0.4307297	0.41309095	Nr1d1/Ajap1/H2-T23/Cd200	4
GO:0051090	regulation of DNA-binding transcription factor activity	4/67	308/14464	0.01298701	2.80364412	2.18266218	0.05438929	0.4307297	0.41309095	Aim2/Ager/Cd200/Enpp1	4
GO:0046330	positive regulation of JNK cascade	2/67	82/14464	0.02439024	5.26538041	2.64232143	0.0553923	0.4307297	0.41309095	Ccdc88c/Ager	2
GO:0045089	positive regulation of innate immune response	4/67	310/14464	0.01290323	2.78555609	2.16793043	0.05544764	0.4307297	0.41309095	Nr1d1/Aim2/H2-T23/Rftn1	4
GO:0010038	response to metal ion	4/67	311/14464	0.01286174	2.77659932	2.16060822	0.05598102	0.4307297	0.41309095	Hnrnpd/Slc30a1/Nfatc4/Enpp1	4

GO:0050806	positive regulation of synaptic transmission	3/67	189/14464	0.01587302	3.42667614	2.29078988	0.05727752	0.4307297	0.41309095	Ager/Nfatc4/Enpp1	3
GO:0005977	glycogen metabolic process	2/67	84/14464	0.02380952	5.14001421	2.59592789	0.05780588	0.4307297	0.41309095	Nr1d1/Enpp1	2
GO:0001765	membrane raft assembly	1/67	13/14464	0.07692308	16.6061998	3.84017026	0.05859655	0.4307297	0.41309095	Rftn1	1
GO:0002328	pro-B cell differentiation	1/67	13/14464	0.07692308	16.6061998	3.84017026	0.05859655	0.4307297	0.41309095	Fnip1	1
GO:0009084	glutamine family amino acid biosynthetic process	1/67	13/14464	0.07692308	16.6061998	3.84017026	0.05859655	0.4307297	0.41309095	Pycr1	1
GO:0030502	negative regulation of bone mineralization	1/67	13/14464	0.07692308	16.6061998	3.84017026	0.05859655	0.4307297	0.41309095	Enpp1	1
GO:0045346	regulation of MHC class II biosynthetic process	1/67	13/14464	0.07692308	16.6061998	3.84017026	0.05859655	0.4307297	0.41309095	Nfx1	1
GO:0055062	phosphate ion homeostasis	1/67	13/14464	0.07692308	16.6061998	3.84017026	0.05859655	0.4307297	0.41309095	Enpp1	1
GO:0061687	detoxification of inorganic compound	1/67	13/14464	0.07692308	16.6061998	3.84017026	0.05859655	0.4307297	0.41309095	Slc30a1	1
GO:0071372	cellular response to follicle-stimulating hormone stimulus	1/67	13/14464	0.07692308	16.6061998	3.84017026	0.05859655	0.4307297	0.41309095	Epha8	1
GO:0097154	GABAergic neuron differentiation	1/67	13/14464	0.07692308	16.6061998	3.84017026	0.05859655	0.4307297	0.41309095	Bhlhe22	1
GO:1902902	negative regulation of autophagosome assembly	1/67	13/14464	0.07692308	16.6061998	3.84017026	0.05859655	0.4307297	0.41309095	Ehmt2	1
GO:1905269	positive regulation of chromatin organization	1/67	13/14464	0.07692308	16.6061998	3.84017026	0.05859655	0.4307297	0.41309095	Cdk2	1
GO:1905671	regulation of lysosome organization	1/67	13/14464	0.07692308	16.6061998	3.84017026	0.05859655	0.4307297	0.41309095	Fnip1	1
GO:2000767	positive regulation of cytoplasmic translation	1/67	13/14464	0.07692308	16.6061998	3.84017026	0.05859655	0.4307297	0.41309095	Hnrnpd	1
GO:0044042	glucan metabolic process	2/67	85/14464	0.02352941	5.07954346	2.57328138	0.0590263	0.4307297	0.41309095	Nr1d1/Enpp1	2
GO:0048708	astrocyte differentiation	2/67	85/14464	0.02352941	5.07954346	2.57328138	0.0590263	0.4307297	0.41309095	Nr1d1/Ager	2
GO:0006397	mRNA processing	5/67	455/14464	0.01098901	2.37231425	2.02901985	0.05942544	0.4307297	0.41309095	Srsf5/Arglu1/Luc7l2/Pcif1/Xrn2	5
GO:0035773	insulin secretion involved in cellular response to glucose stimulus	2/67	87/14464	0.02298851	4.96277235	2.52903713	0.06149388	0.4307297	0.41309095	Nr1d1/Tiam1	2
GO:0097529	myeloid leukocyte migration	3/67	196/14464	0.01530612	3.30429485	2.21572127	0.06249816	0.4307297	0.41309095	Ager/Cd200/Enpp1	3
GO:0032652	regulation of interleukin-1 production	2/67	88/14464	0.02272727	4.9063772	2.50741993	0.06274083	0.4307297	0.41309095	Aim2/Ager	2
GO:0015721	bile acid and bile salt transport	1/67	14/14464	0.07142857	15.4200426	3.68236837	0.06296124	0.4307297	0.41309095	Slco1a4	1
GO:0032693	negative regulation of interleukin-10 production	1/67	14/14464	0.07142857	15.4200426	3.68236837	0.06296124	0.4307297	0.41309095	Ager	1
GO:0045342	MHC class II biosynthetic process	1/67	14/14464	0.07142857	15.4200426	3.68236837	0.06296124	0.4307297	0.41309095	Nfx1	1
GO:0045953	negative regulation of natural killer cell mediated cytotoxicity	1/67	14/14464	0.07142857	15.4200426	3.68236837	0.06296124	0.4307297	0.41309095	H2-T23	1
GO:0050930	induction of positive chemotaxis	1/67	14/14464	0.07142857	15.4200426	3.68236837	0.06296124	0.4307297	0.41309095	Ager	1
GO:0071285	cellular response to lithium ion	1/67	14/14464	0.07142857	15.4200426	3.68236837	0.06296124	0.4307297	0.41309095	Nfatc4	1
GO:0071639	positive regulation of monocyte chemotactic protein-1 production	1/67	14/14464	0.07142857	15.4200426	3.68236837	0.06296124	0.4307297	0.41309095	Ager	1
GO:1900272	negative regulation of long-term synaptic potentiation	1/67	14/14464	0.07142857	15.4200426	3.68236837	0.06296124	0.4307297	0.41309095	Ager	1
GO:1903977	positive regulation of glial cell migration	1/67	14/14464	0.07142857	15.4200426	3.68236837	0.06296124	0.4307297	0.41309095	Tiam1	1
GO:1905274	regulation of modification of postsynaptic actin cytoskeleton	1/67	14/14464	0.07142857	15.4200426	3.68236837	0.06296124	0.4307297	0.41309095	Tiam1	1
GO:0009611	response to wounding	5/67	465/14464	0.01075269	2.32129674	1.97564507	0.06402163	0.4307297	0.41309095	2810403D21Rik/Ajap1/Srsf5/Fundc2/Enpp1	5
GO:0046634	regulation of alpha-beta T cell activation	2/67	90/14464	0.02222222	4.7973466	2.46514952	0.0652605	0.4307297	0.41309095	H2-T23/Ager	2
GO:0051101	regulation of DNA binding	2/67	90/14464	0.02222222	4.7973466	2.46514952	0.0652605	0.4307297	0.41309095	Ager/Dazap2	2
GO:0009749	response to glucose	3/67	201/14464	0.01492537	3.22209846	2.16414561	0.06635903	0.4307297	0.41309095	Nr1d1/Ager/Tiam1	3
GO:0032602	chemokine production	2/67	91/14464	0.02197802	4.74462851	2.44447902	0.06653303	0.4307297	0.41309095	Slamf9/Ager	2
GO:0043525	positive regulation of neuron apoptotic process	2/67	91/14464	0.02197802	4.74462851	2.44447902	0.06653303	0.4307297	0.41309095	Ager/Nfatc4	2
GO:0002833	positive regulation of response to biotic stimulus	4/67	331/14464	0.01208459	2.60882897	2.0199345	0.06723259	0.4307297	0.41309095	Nr1d1/Aim2/H2-T23/Rftn1	4
GO:0002486	antigen processing and presentation of endogenous peptide antigen via MHC class I via ER pathway, T,	1/67	15/14464	0.06666667	14.3920398	3.54000648	0.06730598	0.4307297	0.41309095	H2-T23	1
GO:0002716	negative regulation of natural killer cell mediated immunity	1/67	15/14464	0.06666667	14.3920398	3.54000648	0.06730598	0.4307297	0.41309095	H2-T23	1
GO:0002864	regulation of acute inflammatory response to antigenic stimulus	1/67	15/14464	0.06666667	14.3920398	3.54000648	0.06730598	0.4307297	0.41309095	H2-T23	1
GO:0006699	bile acid biosynthetic process	1/67	15/14464	0.06666667	14.3920398	3.54000648	0.06730598	0.4307297	0.41309095	Nr1d1	1
GO:0006895	Golgi to endosome transport	1/67	15/14464	0.06666667	14.3920398	3.54000648	0.06730598	0.4307297	0.41309095	Sys1	1
GO:0032966	negative regulation of collagen biosynthetic process	1/67	15/14464	0.06666667	14.3920398	3.54000648	0.06730598	0.4307297	0.41309095	Ager	1
GO:0035733	hepatic stellate cell activation	1/67	15/14464	0.06666667	14.3920398	3.54000648	0.06730598	0.4307297	0.41309095	Nr1d1	1
GO:0038065	collagen-activated signaling pathway	1/67	15/14464	0.06666667	14.3920398	3.54000648	0.06730598	0.4307297	0.41309095	Enpp1	1
GO:0061158	3'-UTR-mediated mRNA destabilization	1/67	15/14464	0.06666667	14.3920398	3.54000648	0.06730598	0.4307297	0.41309095	Hnrnpd	1
GO:0061469	regulation of type B pancreatic cell proliferation	1/67	15/14464	0.06666667	14.3920398	3.54000648	0.06730598	0.4307297	0.41309095	Nr1d1	1
GO:0070193	synaptonemal complex organization	1/67	15/14464	0.06666667	14.3920398	3.54000648	0.06730598	0.4307297	0.41309095	Ehmt2	1
GO:0097050	type B pancreatic cell apoptotic process	1/67	15/14464	0.06666667	14.3920398	3.54000648	0.06730598	0.4307297	0.41309095	Ager	1
GO:0097084	vascular associated smooth muscle cell development	1/67	15/14464	0.06666667	14.3920398	3.54000648	0.06730598	0.4307297	0.41309095	Nfatc4	1
GO:2000679	positive regulation of transcription regulatory region DNA binding	1/67	15/14464	0.06666667	14.3920398	3.54000648	0.06730598	0.4307297	0.41309095	Dazap2	1
GO:0006112	energy reserve metabolic process	2/67	93/14464	0.02150538	4.64259348	2.40402665	0.06910294	0.4307297	0.41309095	Nr1d1/Enpp1	2
GO:0009746	response to hexose	3/67	205/14464	0.01463415	3.15922825	2.12403438	0.06952539	0.4307297	0.41309095	Nr1d1/Ager/Tiam1	3
GO:0010389	regulation of G2/M transition of mitotic cell cycle	2/67	94/14464	0.0212766	4.59320419	2.38422936	0.07040012	0.4307297	0.41309095	Cdk2/Pkia	2
GO:0002250	adaptive immune response	4/67	337/14464	0.01186944	2.56238097	1.97973705	0.07082276	0.4307297	0.41309095	H2-T23/Ager/Rftn1/Enpp1	4
GO:0043087	regulation of GTPase activity	3/67	207/14464	0.01449275	3.1287043	2.10434542	0.0711341	0.4307297	0.41309095	Ngef/Tiam1/Gpsm1	3
GO:0002371	dendritic cell cytokine production	1/67	16/14464	0.0625	13.4925373	3.41065171	0.07163088	0.4307297	0.41309095	Slamf9	1
GO:0002476	antigen processing and presentation of endogenous peptide antigen via MHC class Ib	1/67	16/14464	0.0625	13.4925373	3.41065171	0.07163088	0.4307297	0.41309095	H2-T23	1
GO:0002484	antigen processing and presentation of endogenous peptide antigen via MHC class I via ER pathway	1/67	16/14464	0.0625	13.4925373	3.41065171	0.07163088	0.4307297	0.41309095	H2-T23	1
GO:0010713	negative regulation of collagen metabolic process	1/67	16/14464	0.0625	13.4925373	3.41065171	0.07163088	0.4307297	0.41309095	Ager	1
GO:0010960	magnesium ion homeostasis	1/67	16/14464	0.0625	13.4925373	3.41065171	0.07163088	0.4307297	0.41309095	Enpp1	1
GO:0030730	sequestering of triglyceride	1/67	16/14464	0.0625	13.4925373	3.41065171	0.07163088	0.4307297	0.41309095	Enpp1	1
GO:0032753	positive regulation of interleukin-4 production	1/67	16/14464	0.0625	13.4925373	3.41065171	0.07163088	0.4307297	0.41309095	H2-T23	1
GO:0034497	protein localization to phagophore assembly site	1/67	16/14464	0.0625	13.4925373	3.41065171	0.07163088	0.4307297	0.41309095	Stx17	1
GO:0042308	negative regulation of protein import into nucleus	1/67	16/14464	0.0625	13.4925373	3.41065171	0.07163088	0.4307297	0.41309095	Pkia	1
GO:0045954	positive regulation of natural killer cell mediated cytotoxicity	1/67	16/14464	0.0625	13.4925373	3.41065171	0.07163088	0.4307297	0.41309095	H2-T23	1
GO:0048712	negative regulation of astrocyte differentiation	1/67	16/14464	0.0625	13.4925373	3.41065171	0.07163088	0.4307297	0.41309095	Nr1d1	1
GO:0060100	positive regulation of phagocytosis, engulfment	1/67	16/14464	0.0625	13.4925373	3.41065171	0.07163088	0.4307297	0.41309095	Ager	1
GO:0060392	negative regulation of SMAD protein signal transduction	1/67	16/14464	0.0625	13.4925373	3.41065171	0.07163088	0.4307297	0.41309095	Ldlrad4	1
GO:0061000	negative regulation of dendritic spine development	1/67	16/14464	0.0625	13.4925373	3.41065171	0.07163088	0.4307297	0.41309095	Ngef	1
GO:1904355	positive regulation of telomere capping	1/67	16/14464	0.0625	13.4925373	3.41065171	0.07163088	0.4307297	0.41309095	Hnrnpd	1
GO:1905155	positive regulation of membrane invagination	1/67	16/14464	0.0625	13.4925373	3.41065171	0.07163088	0.4307297	0.41309095	Ager	1
GO:0051402	neuron apoptotic process	4/67	339/14464	0.01179941	2.54726368	1.96652986	0.07204123	0.4307297	0.41309095	Ager/Pycr1/Nfatc4/Enpp1	4
GO:0031348	negative regulation of defense response	3/67	210/14464	0.01428571	3.08400853	2.07525445	0.07357871	0.4307297	0.41309095	Nr1d1/H2-T23/Cd200	3
GO:0034284	response to monosaccharide	3/67	211/14464	0.01421801	3.06939237	2.06567276	0.07440193	0.4307297	0.41309095	Nr1d1/Ager/Tiam1	3
GO:0050890	cognition	4/67	344/14464	0.01162791	2.5102395	1.93391904	0.07513464	0.4307297	0.41309095	Ager/Nfatc4/Ehmt2/Man2b1	4
GO:0002327	immature B cell differentiation	1/67	17/14464	0.05882353	12.6988586	3.29237814	0.07593603	0.4307297	0.41309095	Fnip1	1
GO:0002428	antigen processing and presentation of peptide antigen via MHC class Ib	1/67	17/14464	0.05882353	12.6988586	3.29237814	0.07593603	0.4307297	0.41309095	H2-T23	1
GO:0010226	response to lithium ion	1/67	17/14464	0.05882353	12.6988586	3.29237814	0.07593603	0.4307297	0.41309095	Nfatc4	1
GO:0032656	regulation of interleukin-13 production	1/67	17/14464	0.05882353	12.6988586	3.29237814	0.07593603	0.4307297	0.41309095	H2-T23	1
GO:0036336	dendritic cell migration	1/67	17/14464	0.05882353	12.6988586	3.29237814	0.07593603	0.4307297	0.41309095	Slamf9	1
GO:0045056	transcytosis	1/67	17/14464	0.05882353	12.6988586	3.29237814	0.07593603	0.4307297	0.41309095	Ager	1
GO:0046325	negative regulation of D-glucose import	1/67	17/14464	0.05882353	12.6988586	3.29237814	0.07593603	0.4307297	0.41309095	Enpp1	1
GO:0070254	mucus secretion	1/67	17/14464	0.05882353	12.6988586	3.29237814	0.07593603	0.4307297	0.41309095	Enpp1	1
GO:0071732	cellular response to nitric oxide	1/67	17/14464	0.05882353	12.6988586	3.29237814	0.07593603	0.4307297	0.41309095	Hnrnpd	1
GO:0098814	spontaneous synaptic transmission	1/67	17/14464	0.05882353	12.6988586	3.29237814	0.07593603	0.4307297	0.41309095	Ager	1
GO:0098883	synapse pruning	1/67	17/14464	0.05882353	12.6988586	3.29237814	0.07593603	0.4307297	0.41309095	Ngef	1
GO:1903376	regulation of oxidative stress-induced neuron intrinsic apoptotic signaling pathway	1/67	17/14464	0.05882353	12.6988586	3.29237814	0.07593603	0.4307297	0.41309095	Pycr1	1
GO:2001185	regulation of CD8-positive, alpha-beta T cell activation	1/67	17/14464	0.05882353	12.6988586	3.29237814	0.07593603	0.4307297	0.41309095	H2-T23	1
GO:0002274	myeloid leukocyte activation	3/67	213/14464	0.01408451	3.04057179	2.04667838	0.07606079	0.4307297	0.41309095	Nr1d1/Ager/Cd200	3
GO:0001911	negative regulation of leukocyte mediated cytotoxicity	1/67	18/14464	0.05555556	11.9933665	3.18363849	0.08022151	0.4307297	0.41309095	H2-T23	1
GO:0002675	positive regulation of acute inflammatory response	1/67	18/14464	0.05555556	11.9933665	3.18363849	0.08022151	0.4307297	0.41309095	H2-T23	1
GO:0002717	positive regulation of natural killer cell mediated immunity	1/67	18/14464	0.05555556	11.9933665	3.18363849	0.08022151	0.4307297	0.41309095	H2-T23	1
GO:0002902	regulation of B cell apoptotic process	1/67	18/14464	0.05555556	11.9933665	3.18363849	0.08022151	0.4307297	0.41309095	Fnip1	1
GO:0014004	microglia differentiation	1/67	18/14464	0.05555556	11.9933665	3.18363849	0.08022151	0.4307297	0.41309095	Enpp1	1
GO:0032026	response to magnesium ion	1/67	18/14464	0.05555556	11.9933665	3.18363849	0.08022151	0.4307297	0.41309095	Enpp1	1
GO:0032354	response to follicle-stimulating hormone	1/67	18/14464	0.05555556	11.9933665	3.18363849	0.08022151	0.4307297	0.41309095	Epha8	1
GO:0032616	interleukin-13 production	1/67	18/14464	0.05555556	11.9933665	3.18363849	0.08022151	0.4307297	0.41309095	H2-T23	1
GO:0034114	regulation of heterotypic cell-cell adhesion	1/67	18/14464	0.05555556	11.9933665	3.18363849	0.08022151	0.4307297	0.41309095	Ager	1
GO:0042104	positive regulation of activated T cell proliferation	1/67	18/14464	0.05555556	11.9933665	3.18363849	0.08022151	0.4307297	0.41309095	Ager	1
GO:0044027	negative regulation of gene expression via chromosomal CpG island methylation	1/67	18/14464	0.05555556	11.9933665	3.18363849	0.08022151	0.4307297	0.41309095	Ehmt2	1
GO:0048148	behavioral response to cocaine	1/67	18/14464	0.05555556	11.9933665	3.18363849	0.08022151	0.4307297	0.41309095	Ehmt2	1
GO:0060099	regulation of phagocytosis, engulfment	1/67	18/14464	0.05555556	11.9933665	3.18363849	0.08022151	0.4307297	0.41309095	Ager	1
GO:0141156	cAMP/PKA signal transduction	1/67	18/14464	0.05555556	11.9933665	3.18363849	0.08022151	0.4307297	0.41309095	Pkia	1
GO:1903978	regulation of microglial cell activation	1/67	18/14464	0.05555556	11.9933665	3.18363849	0.08022151	0.4307297	0.41309095	Nr1d1	1
GO:1905809	negative regulation of synapse organization	1/67	18/14464	0.05555556	11.9933665	3.18363849	0.08022151	0.4307297	0.41309095	Nfatc4	1
GO:0001909	leukocyte mediated cytotoxicity	2/67	102/14464	0.01960784	4.23295288	2.23523193	0.08105793	0.4307297	0.41309095	H2-T23/Ager	2
GO:0002440	production of molecular mediator of immune response	3/67	219/14464	0.01369863	2.95726845	1.99100256	0.08113551	0.4307297	0.41309095	Slamf9/H2-T23/Enpp1	3
GO:0048762	mesenchymal cell differentiation	3/67	219/14464	0.01369863	2.95726845	1.99100256	0.08113551	0.4307297	0.41309095	Ager/Tiam1/Ldlrad4	3
GO:0010976	positive regulation of neuron projection development	3/67	220/14464	0.01363636	2.94382632	1.98190763	0.08199542	0.4307297	0.41309095	Slc30a1/Tiam1/Il1rapl1	3
GO:0042752	regulation of circadian rhythm	2/67	103/14464	0.01941748	4.19185625	2.21768668	0.08242375	0.4307297	0.41309095	Nr1d1/Hnrnpd	2
GO:0002507	tolerance induction	1/67	19/14464	0.05263158	11.3621367	3.0831733	0.0844874	0.4307297	0.41309095	H2-T23	1
GO:0015936	coenzyme A metabolic process	1/67	19/14464	0.05263158	11.3621367	3.0831733	0.0844874	0.4307297	0.41309095	Enpp1	1
GO:0016322	neuron remodeling	1/67	19/14464	0.05263158	11.3621367	3.0831733	0.0844874	0.4307297	0.41309095	Epha8	1
GO:0036480	neuron intrinsic apoptotic signaling pathway in response to oxidative stress	1/67	19/14464	0.05263158	11.3621367	3.0831733	0.0844874	0.4307297	0.41309095	Pycr1	1
GO:0045947	negative regulation of translational initiation	1/67	19/14464	0.05263158	11.3621367	3.0831733	0.0844874	0.4307297	0.41309095	Zfp598	1
GO:0046641	positive regulation of alpha-beta T cell proliferation	1/67	19/14464	0.05263158	11.3621367	3.0831733	0.0844874	0.4307297	0.41309095	H2-T23	1
GO:0070230	positive regulation of lymphocyte apoptotic process	1/67	19/14464	0.05263158	11.3621367	3.0831733	0.0844874	0.4307297	0.41309095	Fnip1	1
GO:0071371	cellular response to gonadotropin stimulus	1/67	19/14464	0.05263158	11.3621367	3.0831733	0.0844874	0.4307297	0.41309095	Epha8	1
GO:0150104	transport across blood-brain barrier	1/67	19/14464	0.05263158	11.3621367	3.0831733	0.0844874	0.4307297	0.41309095	Ager	1
GO:1900452	regulation of long-term synaptic depression	1/67	19/14464	0.05263158	11.3621367	3.0831733	0.0844874	0.4307297	0.41309095	Ager	1
GO:1902170	cellular response to reactive nitrogen species	1/67	19/14464	0.05263158	11.3621367	3.0831733	0.0844874	0.4307297	0.41309095	Hnrnpd	1
GO:1905153	regulation of membrane invagination	1/67	19/14464	0.05263158	11.3621367	3.0831733	0.0844874	0.4307297	0.41309095	Ager	1
GO:2000353	positive regulation of endothelial cell apoptotic process	1/67	19/14464	0.05263158	11.3621367	3.0831733	0.0844874	0.4307297	0.41309095	Ager	1
GO:0007033	vacuole organization	3/67	223/14464	0.01345291	2.90422328	1.95492747	0.08459903	0.4307297	0.41309095	Ehmt2/Fnip1/Stx17	3
GO:0002377	immunoglobulin production	2/67	105/14464	0.01904762	4.11201137	2.18325427	0.08517673	0.4307297	0.41309095	H2-T23/Enpp1	2
GO:0051092	positive regulation of NF-kappaB transcription factor activity	2/67	105/14464	0.01904762	4.11201137	2.18325427	0.08517673	0.4307297	0.41309095	Aim2/Ager	2
GO:0006275	regulation of DNA replication	2/67	106/14464	0.01886792	4.07321881	2.16635703	0.08656371	0.4307297	0.41309095	Cdk2/Ehmt2	2
GO:1902749	regulation of cell cycle G2/M phase transition	2/67	106/14464	0.01886792	4.07321881	2.16635703	0.08656371	0.4307297	0.41309095	Cdk2/Pkia	2
GO:0005976	polysaccharide metabolic process	2/67	107/14464	0.01869159	4.03515135	2.14966598	0.08795757	0.4307297	0.41309095	Nr1d1/Enpp1	2
GO:0016064	immunoglobulin mediated immune response	2/67	107/14464	0.01869159	4.03515135	2.14966598	0.08795757	0.4307297	0.41309095	H2-T23/Enpp1	2
GO:0002475	antigen processing and presentation via MHC class Ib	1/67	20/14464	0.05	10.7940299	2.98994551	0.08873381	0.4307297	0.41309095	H2-T23	1
GO:0002483	antigen processing and presentation of endogenous peptide antigen	1/67	20/14464	0.05	10.7940299	2.98994551	0.08873381	0.4307297	0.41309095	H2-T23	1
GO:0006775	fat-soluble vitamin metabolic process	1/67	20/14464	0.05	10.7940299	2.98994551	0.08873381	0.4307297	0.41309095	Enpp1	1
GO:0010232	vascular transport	1/67	20/14464	0.05	10.7940299	2.98994551	0.08873381	0.4307297	0.41309095	Ager	1
GO:0019885	antigen processing and presentation of endogenous peptide antigen via MHC class I	1/67	20/14464	0.05	10.7940299	2.98994551	0.08873381	0.4307297	0.41309095	H2-T23	1
GO:0032740	positive regulation of interleukin-17 production	1/67	20/14464	0.05	10.7940299	2.98994551	0.08873381	0.4307297	0.41309095	Rftn1	1
GO:0042749	regulation of circadian sleep/wake cycle	1/67	20/14464	0.05	10.7940299	2.98994551	0.08873381	0.4307297	0.41309095	Nr1d1	1
GO:0042753	positive regulation of circadian rhythm	1/67	20/14464	0.05	10.7940299	2.98994551	0.08873381	0.4307297	0.41309095	Nr1d1	1
GO:0044321	response to leptin	1/67	20/14464	0.05	10.7940299	2.98994551	0.08873381	0.4307297	0.41309095	Nr1d1	1
GO:0070168	negative regulation of biomineral tissue development	1/67	20/14464	0.05	10.7940299	2.98994551	0.08873381	0.4307297	0.41309095	Enpp1	1
GO:1905962	glutamatergic neuron differentiation	1/67	20/14464	0.05	10.7940299	2.98994551	0.08873381	0.4307297	0.41309095	Bhlhe22	1
GO:0050671	positive regulation of lymphocyte proliferation	2/67	108/14464	0.01851852	3.99778883	2.13317646	0.0893582	0.4307297	0.41309095	H2-T23/Ager	2
GO:2001056	positive regulation of cysteine-type endopeptidase activity	2/67	108/14464	0.01851852	3.99778883	2.13317646	0.0893582	0.4307297	0.41309095	Aim2/Tnfsf10	2
GO:0042063	gliogenesis	4/67	366/14464	0.01092896	2.35935079	1.79690007	0.08953349	0.4307297	0.41309095	Nr1d1/Ager/Tiam1/Enpp1	4
GO:0019724	B cell mediated immunity	2/67	110/14464	0.01818182	3.92510176	2.10078421	0.09217944	0.4307297	0.41309095	H2-T23/Enpp1	2
GO:0002221	pattern recognition receptor signaling pathway	3/67	232/14464	0.01293103	2.79155944	1.87660487	0.09262047	0.4307297	0.41309095	Nr1d1/Aim2/Rftn1	3
GO:0018022	peptidyl-lysine methylation	1/67	21/14464	0.04761905	10.2800284	2.90309228	0.09296081	0.4307297	0.41309095	Ehmt2	1
GO:0030859	polarized epithelial cell differentiation	1/67	21/14464	0.04761905	10.2800284	2.90309228	0.09296081	0.4307297	0.41309095	Ajap1	1
GO:0031445	regulation of heterochromatin formation	1/67	21/14464	0.04761905	10.2800284	2.90309228	0.09296081	0.4307297	0.41309095	Cdk2	1
GO:0032816	positive regulation of natural killer cell activation	1/67	21/14464	0.04761905	10.2800284	2.90309228	0.09296081	0.4307297	0.41309095	H2-T23	1
GO:0035561	regulation of chromatin binding	1/67	21/14464	0.04761905	10.2800284	2.90309228	0.09296081	0.4307297	0.41309095	Nfatc4	1
GO:0050927	positive regulation of positive chemotaxis	1/67	21/14464	0.04761905	10.2800284	2.90309228	0.09296081	0.4307297	0.41309095	Ager	1
GO:0060343	trabecula formation	1/67	21/14464	0.04761905	10.2800284	2.90309228	0.09296081	0.4307297	0.41309095	Enpp1	1
GO:0071605	monocyte chemotactic protein-1 production	1/67	21/14464	0.04761905	10.2800284	2.90309228	0.09296081	0.4307297	0.41309095	Ager	1
GO:0071637	regulation of monocyte chemotactic protein-1 production	1/67	21/14464	0.04761905	10.2800284	2.90309228	0.09296081	0.4307297	0.41309095	Ager	1
GO:0071731	response to nitric oxide	1/67	21/14464	0.04761905	10.2800284	2.90309228	0.09296081	0.4307297	0.41309095	Hnrnpd	1
GO:0072537	fibroblast activation	1/67	21/14464	0.04761905	10.2800284	2.90309228	0.09296081	0.4307297	0.41309095	Nr1d1	1
GO:2000001	regulation of DNA damage checkpoint	1/67	21/14464	0.04761905	10.2800284	2.90309228	0.09296081	0.4307297	0.41309095	Rpa2	1
GO:0014909	smooth muscle cell migration	2/67	111/14464	0.01801802	3.88974049	2.08487289	0.09359987	0.4307297	0.41309095	Ager/Enpp1	2
GO:0032946	positive regulation of mononuclear cell proliferation	2/67	111/14464	0.01801802	3.88974049	2.08487289	0.09359987	0.4307297	0.41309095	H2-T23/Ager	2
GO:0009743	response to carbohydrate	3/67	234/14464	0.01282051	2.76769996	1.85970742	0.09444496	0.4307297	0.41309095	Nr1d1/Ager/Tiam1	3
GO:0045088	regulation of innate immune response	4/67	374/14464	0.01069519	2.30888339	1.74949157	0.09508016	0.4307297	0.41309095	Nr1d1/Aim2/H2-T23/Rftn1	4
GO:0002758	innate immune response-activating signaling pathway	3/67	235/14464	0.01276596	2.75592252	1.85132507	0.09536282	0.4307297	0.41309095	Nr1d1/Aim2/Rftn1	3
GO:2001252	positive regulation of chromosome organization	2/67	113/14464	0.01769912	3.82089552	2.05359942	0.09645991	0.4307297	0.41309095	Cdk2/Hnrnpd	2

GO:0002693	positive regulation of cellular extravasation	1/67	22/14464	0.04545455	9.81275441	2.82188902	0.0971685	0.4307297	0.41309095	Ager	1
GO:0002888	positive regulation of myeloid leukocyte mediated immunity	1/67	22/14464	0.04545455	9.81275441	2.82188902	0.0971685	0.4307297	0.41309095	H2-T23	1
GO:0010829	negative regulation of D-glucose transmembrane transport	1/67	22/14464	0.04545455	9.81275441	2.82188902	0.0971685	0.4307297	0.41309095	Enpp1	1
GO:0019883	antigen processing and presentation of endogenous antigen	1/67	22/14464	0.04545455	9.81275441	2.82188902	0.0971685	0.4307297	0.41309095	H2-T23	1
GO:0031342	negative regulation of cell killing	1/67	22/14464	0.04545455	9.81275441	2.82188902	0.0971685	0.4307297	0.41309095	H2-T23	1
GO:0031571	mitotic G1 DNA damage checkpoint signaling	1/67	22/14464	0.04545455	9.81275441	2.82188902	0.0971685	0.4307297	0.41309095	Rpa2	1
GO:0032673	regulation of interleukin-4 production	1/67	22/14464	0.04545455	9.81275441	2.82188902	0.0971685	0.4307297	0.41309095	H2-T23	1
GO:0034260	negative regulation of GTPase activity	1/67	22/14464	0.04545455	9.81275441	2.82188902	0.0971685	0.4307297	0.41309095	Gpsm1	1
GO:0034349	glial cell apoptotic process	1/67	22/14464	0.04545455	9.81275441	2.82188902	0.0971685	0.4307297	0.41309095	Enpp1	1
GO:0042474	middle ear morphogenesis	1/67	22/14464	0.04545455	9.81275441	2.82188902	0.0971685	0.4307297	0.41309095	Enpp1	1
GO:0071676	negative regulation of mononuclear cell migration	1/67	22/14464	0.04545455	9.81275441	2.82188902	0.0971685	0.4307297	0.41309095	Cd200	1
GO:0099151	regulation of postsynaptic density assembly	1/67	22/14464	0.04545455	9.81275441	2.82188902	0.0971685	0.4307297	0.41309095	Abi3bp	1
GO:0071675	regulation of mononuclear cell migration	2/67	114/14464	0.01754386	3.78737889	2.03822946	0.09789934	0.4307297	0.41309095	Ager/Cd200	2
GO:0048167	regulation of synaptic plasticity	3/67	239/14464	0.0125523	2.70979829	1.81822776	0.09907122	0.4307297	0.41309095	Ager/Nfatc4/Enpp1	3
GO:0000956	nuclear-transcribed mRNA catabolic process	2/67	115/14464	0.0173913	3.75444517	2.02303234	0.09934494	0.4307297	0.41309095	Hnrnpd/Xrn2	2
GO:0050729	positive regulation of inflammatory response	2/67	115/14464	0.0173913	3.75444517	2.02303234	0.09934494	0.4307297	0.41309095	Aim2/H2-T23	2
GO:0034763	negative regulation of transmembrane transport	2/67	116/14464	0.01724138	3.72207926	2.00800444	0.10079661	0.4307297	0.41309095	Slc30a1/Enpp1	2
GO:0002320	lymphoid progenitor cell differentiation	1/67	23/14464	0.04347826	9.38611291	2.74572212	0.10135696	0.4307297	0.41309095	Fnip1	1
GO:0044819	mitotic G1/S transition checkpoint signaling	1/67	23/14464	0.04347826	9.38611291	2.74572212	0.10135696	0.4307297	0.41309095	Rpa2	1
GO:0048665	neuron fate specification	1/67	23/14464	0.04347826	9.38611291	2.74572212	0.10135696	0.4307297	0.41309095	Ehmt2	1
GO:0050926	regulation of positive chemotaxis	1/67	23/14464	0.04347826	9.38611291	2.74572212	0.10135696	0.4307297	0.41309095	Ager	1
GO:1900151	regulation of nuclear-transcribed mRNA catabolic process, deadenylation-dependent decay	1/67	23/14464	0.04347826	9.38611291	2.74572212	0.10135696	0.4307297	0.41309095	Hnrnpd	1
GO:0090596	sensory organ morphogenesis	3/67	242/14464	0.01239669	2.67620575	1.79384644	0.10189079	0.4307297	0.41309095	Ppp2r3a/Enpp1/Bhlhe22	3
GO:0001776	leukocyte homeostasis	2/67	117/14464	0.01709402	3.69026662	1.99314225	0.10225427	0.4307297	0.41309095	Aim2/Enpp1	2
GO:0006820	monoatomic anion transport	2/67	117/14464	0.01709402	3.69026662	1.99314225	0.10225427	0.4307297	0.41309095	Slco1a4/Gabrg1	2
GO:0015698	inorganic anion transport	2/67	117/14464	0.01709402	3.69026662	1.99314225	0.10225427	0.4307297	0.41309095	Gabrg1/Enpp1	2
GO:0017148	negative regulation of translation	2/67	117/14464	0.01709402	3.69026662	1.99314225	0.10225427	0.4307297	0.41309095	Pcif1/Zfp598	2
GO:0000045	autophagosome assembly	2/67	118/14464	0.01694915	3.65899317	1.97844237	0.10371783	0.4307297	0.41309095	Ehmt2/Stx17	2
GO:0002367	cytokine production involved in immune response	2/67	118/14464	0.01694915	3.65899317	1.97844237	0.10371783	0.4307297	0.41309095	Slamf9/H2-T23	2
GO:0006260	DNA replication	3/67	245/14464	0.0122449	2.64343588	1.76938313	0.10474259	0.4307297	0.41309095	Cdk2/Ehmt2/Rpa2	3
GO:0090090	negative regulation of canonical Wnt signaling pathway	2/67	119/14464	0.01680672	3.62824533	1.96390148	0.10518722	0.4307297	0.41309095	Ccdc88c/Ppp2r3a	2
GO:0001783	B cell apoptotic process	1/67	24/14464	0.04166667	8.99502488	2.67406793	0.10552627	0.4307297	0.41309095	Fnip1	1
GO:0006298	mismatch repair	1/67	24/14464	0.04166667	8.99502488	2.67406793	0.10552627	0.4307297	0.41309095	Rpa2	1
GO:0006882	intracellular zinc ion homeostasis	1/67	24/14464	0.04166667	8.99502488	2.67406793	0.10552627	0.4307297	0.41309095	Slc30a1	1
GO:0008206	bile acid metabolic process	1/67	24/14464	0.04166667	8.99502488	2.67406793	0.10552627	0.4307297	0.41309095	Nr1d1	1
GO:0010763	positive regulation of fibroblast migration	1/67	24/14464	0.04166667	8.99502488	2.67406793	0.10552627	0.4307297	0.41309095	Ager	1
GO:0034138	toll-like receptor 3 signaling pathway	1/67	24/14464	0.04166667	8.99502488	2.67406793	0.10552627	0.4307297	0.41309095	Rftn1	1
GO:0043931	ossification involved in bone maturation	1/67	24/14464	0.04166667	8.99502488	2.67406793	0.10552627	0.4307297	0.41309095	Enpp1	1
GO:0046716	muscle cell cellular homeostasis	1/67	24/14464	0.04166667	8.99502488	2.67406793	0.10552627	0.4307297	0.41309095	Enpp1	1
GO:0071542	dopaminergic neuron differentiation	1/67	24/14464	0.04166667	8.99502488	2.67406793	0.10552627	0.4307297	0.41309095	Tiam1	1
GO:0071634	regulation of transforming growth factor beta production	1/67	24/14464	0.04166667	8.99502488	2.67406793	0.10552627	0.4307297	0.41309095	Cd200	1
GO:1902176	negative regulation of oxidative stress-induced intrinsic apoptotic signaling pathway	1/67	24/14464	0.04166667	8.99502488	2.67406793	0.10552627	0.4307297	0.41309095	Pycr1	1
GO:1902993	positive regulation of amyloid precursor protein catabolic process	1/67	24/14464	0.04166667	8.99502488	2.67406793	0.10552627	0.4307297	0.41309095	Ager	1
GO:1903523	negative regulation of blood circulation	1/67	24/14464	0.04166667	8.99502488	2.67406793	0.10552627	0.4307297	0.41309095	Ager	1
GO:1904037	positive regulation of epithelial cell apoptotic process	1/67	24/14464	0.04166667	8.99502488	2.67406793	0.10552627	0.4307297	0.41309095	Ager	1
GO:0031507	heterochromatin formation	2/67	120/14464	0.01666667	3.59800995	1.94951637	0.10666234	0.4307297	0.41309095	Cdk2/Ehmt2	2
GO:0051224	negative regulation of protein transport	2/67	120/14464	0.01666667	3.59800995	1.94951637	0.10666234	0.4307297	0.41309095	Pkia/Cd200	2
GO:0002230	positive regulation of defense response to virus by host	1/67	25/14464	0.04	8.63522388	2.6064765	0.10967653	0.4307297	0.41309095	Aim2	1
GO:0002438	acute inflammatory response to antigenic stimulus	1/67	25/14464	0.04	8.63522388	2.6064765	0.10967653	0.4307297	0.41309095	H2-T23	1
GO:0006353	DNA-templated transcription termination	1/67	25/14464	0.04	8.63522388	2.6064765	0.10967653	0.4307297	0.41309095	Xrn2	1
GO:0030318	melanocyte differentiation	1/67	25/14464	0.04	8.63522388	2.6064765	0.10967653	0.4307297	0.41309095	Enpp1	1
GO:0034143	regulation of toll-like receptor 4 signaling pathway	1/67	25/14464	0.04	8.63522388	2.6064765	0.10967653	0.4307297	0.41309095	Nr1d1	1
GO:0035235	ionotropic glutamate receptor signaling pathway	1/67	25/14464	0.04	8.63522388	2.6064765	0.10967653	0.4307297	0.41309095	Tiam1	1
GO:0046823	negative regulation of nucleocytoplasmic transport	1/67	25/14464	0.04	8.63522388	2.6064765	0.10967653	0.4307297	0.41309095	Pkia	1
GO:0055090	acylglycerol homeostasis	1/67	25/14464	0.04	8.63522388	2.6064765	0.10967653	0.4307297	0.41309095	Fundc2	1
GO:0070328	triglyceride homeostasis	1/67	25/14464	0.04	8.63522388	2.6064765	0.10967653	0.4307297	0.41309095	Fundc2	1
GO:0071577	zinc ion transmembrane transport	1/67	25/14464	0.04	8.63522388	2.6064765	0.10967653	0.4307297	0.41309095	Slc30a1	1
GO:0099159	regulation of modification of postsynaptic structure	1/67	25/14464	0.04	8.63522388	2.6064765	0.10967653	0.4307297	0.41309095	Tiam1	1
GO:1903975	regulation of glial cell migration	1/67	25/14464	0.04	8.63522388	2.6064765	0.10967653	0.4307297	0.41309095	Tiam1	1
GO:1904353	regulation of telomere capping	1/67	25/14464	0.04	8.63522388	2.6064765	0.10967653	0.4307297	0.41309095	Hnrnpd	1
GO:1904862	inhibitory synapse assembly	1/67	25/14464	0.04	8.63522388	2.6064765	0.10967653	0.4307297	0.41309095	Gabrg1	1
GO:0070665	positive regulation of leukocyte proliferation	2/67	123/14464	0.01626016	3.51025361	1.90726496	0.11112131	0.4307297	0.41309095	H2-T23/Ager	2
GO:0071466	cellular response to xenobiotic stimulus	2/67	124/14464	0.01612903	3.48194511	1.89347263	0.11261857	0.4307297	0.41309095	Aim2/Ehmt2	2
GO:0002313	mature B cell differentiation involved in immune response	1/67	26/14464	0.03846154	8.30309989	2.5425587	0.11380782	0.4307297	0.41309095	Enpp1	1
GO:0002861	regulation of inflammatory response to antigenic stimulus	1/67	26/14464	0.03846154	8.30309989	2.5425587	0.11380782	0.4307297	0.41309095	H2-T23	1
GO:0010893	positive regulation of steroid biosynthetic process	1/67	26/14464	0.03846154	8.30309989	2.5425587	0.11380782	0.4307297	0.41309095	Nr1d1	1
GO:0036037	CD8-positive, alpha-beta T cell activation	1/67	26/14464	0.03846154	8.30309989	2.5425587	0.11380782	0.4307297	0.41309095	H2-T23	1
GO:0070269	pyroptotic inflammatory response	1/67	26/14464	0.03846154	8.30309989	2.5425587	0.11380782	0.4307297	0.41309095	Aim2	1
GO:0070977	bone maturation	1/67	26/14464	0.03846154	8.30309989	2.5425587	0.11380782	0.4307297	0.41309095	Enpp1	1
GO:0090200	positive regulation of release of cytochrome c from mitochondria	1/67	26/14464	0.03846154	8.30309989	2.5425587	0.11380782	0.4307297	0.41309095	Tnfsf10	1
GO:0098885	modification of postsynaptic actin cytoskeleton	1/67	26/14464	0.03846154	8.30309989	2.5425587	0.11380782	0.4307297	0.41309095	Tiam1	1
GO:1900745	positive regulation of p38MAPK cascade	1/67	26/14464	0.03846154	8.30309989	2.5425587	0.11380782	0.4307297	0.41309095	Ager	1
GO:0000086	G2/M transition of mitotic cell cycle	2/67	125/14464	0.016	3.45408955	1.87982133	0.11412115	0.4307297	0.41309095	Cdk2/Pkia	2
GO:0045727	positive regulation of translation	2/67	125/14464	0.016	3.45408955	1.87982133	0.11412115	0.4307297	0.41309095	Hnrnpd/Pcif1	2
GO:0022411	cellular component disassembly	4/67	400/14464	0.01	2.15880597	1.60330772	0.11419951	0.4307297	0.41309095	Ccdc88c/Ngef/Stx17/Zfp598	4
GO:0014812	muscle cell migration	2/67	126/14464	0.01587302	3.42667614	1.86630834	0.11562899	0.4307297	0.41309095	Ager/Enpp1	2
GO:0050728	negative regulation of inflammatory response	2/67	126/14464	0.01587302	3.42667614	1.86630834	0.11562899	0.4307297	0.41309095	Nr1d1/Cd200	2
GO:1905037	autophagosome organization	2/67	126/14464	0.01587302	3.42667614	1.86630834	0.11562899	0.4307297	0.41309095	Ehmt2/Stx17	2
GO:0050921	positive regulation of chemotaxis	2/67	127/14464	0.01574803	3.39969444	1.85293104	0.117142	0.4307297	0.41309095	Ager/Tiam1	2
GO:0002218	activation of innate immune response	3/67	258/14464	0.01162791	2.5102395	1.66974582	0.11746	0.4307297	0.41309095	Nr1d1/Aim2/Rftn1	3
GO:0006376	mRNA splice site recognition	1/67	27/14464	0.03703704	7.99557767	2.48197601	0.11792022	0.4307297	0.41309095	Luc7l2	1
GO:0006829	zinc ion transport	1/67	27/14464	0.03703704	7.99557767	2.48197601	0.11792022	0.4307297	0.41309095	Slc30a1	1
GO:0031579	membrane raft organization	1/67	27/14464	0.03703704	7.99557767	2.48197601	0.11792022	0.4307297	0.41309095	Rftn1	1
GO:0032633	interleukin-4 production	1/67	27/14464	0.03703704	7.99557767	2.48197601	0.11792022	0.4307297	0.41309095	H2-T23	1
GO:0071604	transforming growth factor beta production	1/67	27/14464	0.03703704	7.99557767	2.48197601	0.11792022	0.4307297	0.41309095	Cd200	1
GO:1904889	regulation of excitatory synapse assembly	1/67	27/14464	0.03703704	7.99557767	2.48197601	0.11792022	0.4307297	0.41309095	Abi3bp	1
GO:2000050	regulation of non-canonical Wnt signaling pathway	1/67	27/14464	0.03703704	7.99557767	2.48197601	0.11792022	0.4307297	0.41309095	Tiam1	1
GO:0046328	regulation of JNK cascade	2/67	128/14464	0.015625	3.37313433	1.83968684	0.1186601	0.4307297	0.41309095	Ccdc88c/Ager	2
GO:0030183	B cell differentiation	2/67	129/14464	0.01550388	3.346986	1.82657327	0.12018322	0.4307297	0.41309095	Enpp1/Fnip1	2
GO:0043433	negative regulation of DNA-binding transcription factor activity	2/67	130/14464	0.01538462	3.32123995	1.81358788	0.12171128	0.4307297	0.41309095	Aim2/Cd200	2
GO:0031349	positive regulation of defense response	4/67	410/14464	0.0097561	2.10615217	1.55002042	0.12197935	0.4307297	0.41309095	Nr1d1/Aim2/H2-T23/Rftn1	4
GO:0043434	response to peptide hormone	4/67	410/14464	0.0097561	2.10615217	1.55002042	0.12197935	0.4307297	0.41309095	Srsf5/Cdk2/Tnfsf10/Enpp1	4
GO:0001916	positive regulation of T cell mediated cytotoxicity	1/67	28/14464	0.03571429	7.71002132	2.42443236	0.12201383	0.4307297	0.41309095	H2-T23	1
GO:0002478	antigen processing and presentation of exogenous peptide antigen	1/67	28/14464	0.03571429	7.71002132	2.42443236	0.12201383	0.4307297	0.41309095	H2-T23	1
GO:0003401	axis elongation	1/67	28/14464	0.03571429	7.71002132	2.42443236	0.12201383	0.4307297	0.41309095	Ppp2r3a	1
GO:0021952	central nervous system projection neuron axonogenesis	1/67	28/14464	0.03571429	7.71002132	2.42443236	0.12201383	0.4307297	0.41309095	Bhlhe22	1
GO:0032735	positive regulation of interleukin-12 production	1/67	28/14464	0.03571429	7.71002132	2.42443236	0.12201383	0.4307297	0.41309095	Ager	1
GO:0034505	tooth mineralization	1/67	28/14464	0.03571429	7.71002132	2.42443236	0.12201383	0.4307297	0.41309095	Enpp1	1
GO:0034698	response to gonadotropin	1/67	28/14464	0.03571429	7.71002132	2.42443236	0.12201383	0.4307297	0.41309095	Epha8	1
GO:0042832	defense response to protozoan	1/67	28/14464	0.03571429	7.71002132	2.42443236	0.12201383	0.4307297	0.41309095	Enpp1	1
GO:0045686	negative regulation of glial cell differentiation	1/67	28/14464	0.03571429	7.71002132	2.42443236	0.12201383	0.4307297	0.41309095	Nr1d1	1
GO:0048799	animal organ maturation	1/67	28/14464	0.03571429	7.71002132	2.42443236	0.12201383	0.4307297	0.41309095	Enpp1	1
GO:0050901	leukocyte tethering or rolling	1/67	28/14464	0.03571429	7.71002132	2.42443236	0.12201383	0.4307297	0.41309095	Podxl2	1
GO:0099150	regulation of postsynaptic specialization assembly	1/67	28/14464	0.03571429	7.71002132	2.42443236	0.12201383	0.4307297	0.41309095	Abi3bp	1
GO:1902894	negative regulation of miRNA transcription	1/67	28/14464	0.03571429	7.71002132	2.42443236	0.12201383	0.4307297	0.41309095	Nfatc4	1
GO:0007162	negative regulation of cell adhesion	3/67	263/14464	0.01140684	2.46251632	1.6328629	0.1225007	0.4307297	0.41309095	Ajap1/H2-T23/Ager	3
GO:0050708	regulation of protein secretion	3/67	263/14464	0.01140684	2.46251632	1.6328629	0.1225007	0.4307297	0.41309095	Nr1d1/Cd200/Tiam1	3
GO:0006270	DNA replication initiation	1/67	29/14464	0.03448276	7.44415852	2.3696675	0.12608871	0.4307297	0.41309095	Cdk2	1
GO:0006929	substrate-dependent cell migration	1/67	29/14464	0.03448276	7.44415852	2.3696675	0.12608871	0.4307297	0.41309095	Epha8	1
GO:0007129	homologous chromosome pairing at meiosis	1/67	29/14464	0.03448276	7.44415852	2.3696675	0.12608871	0.4307297	0.41309095	Ehmt2	1
GO:0042269	regulation of natural killer cell mediated cytotoxicity	1/67	29/14464	0.03448276	7.44415852	2.3696675	0.12608871	0.4307297	0.41309095	H2-T23	1
GO:0045940	positive regulation of steroid metabolic process	1/67	29/14464	0.03448276	7.44415852	2.3696675	0.12608871	0.4307297	0.41309095	Nr1d1	1
GO:0046006	regulation of activated T cell proliferation	1/67	29/14464	0.03448276	7.44415852	2.3696675	0.12608871	0.4307297	0.41309095	Ager	1
GO:0071392	cellular response to estradiol stimulus	1/67	29/14464	0.03448276	7.44415852	2.3696675	0.12608871	0.4307297	0.41309095	Hnrnpd	1
GO:0098868	bone growth	1/67	29/14464	0.03448276	7.44415852	2.3696675	0.12608871	0.4307297	0.41309095	Enpp1	1
GO:0032675	regulation of interleukin-6 production	2/67	133/14464	0.01503759	3.24632477	1.77537749	0.12632433	0.4307297	0.41309095	Ager/Cd200	2
GO:0060485	mesenchyme development	3/67	267/14464	0.01123596	2.42562469	1.60395854	0.12659039	0.4307297	0.41309095	Ager/Tiam1/Ldlrad4	3
GO:0022604	regulation of cell morphogenesis	3/67	268/14464	0.01119403	2.41657385	1.59681357	0.12762058	0.4307297	0.41309095	Slc30a1/Tiam1/Il1rapl1	3
GO:0045598	regulation of fat cell differentiation	2/67	134/14464	0.01492537	3.22209846	1.7628818	0.12787138	0.4307297	0.41309095	Nr1d1/Enpp1	2
GO:1904950	negative regulation of establishment of protein localization	2/67	135/14464	0.01481481	3.19823107	1.75050307	0.129423	0.4307297	0.41309095	Pkia/Cd200	2
GO:0001562	response to protozoan	1/67	30/14464	0.03333333	7.1960199	2.3174516	0.13014497	0.4307297	0.41309095	Enpp1	1
GO:0005979	regulation of glycogen biosynthetic process	1/67	30/14464	0.03333333	7.1960199	2.3174516	0.13014497	0.4307297	0.41309095	Enpp1	1
GO:0007214	gamma-aminobutyric acid signaling pathway	1/67	30/14464	0.03333333	7.1960199	2.3174516	0.13014497	0.4307297	0.41309095	Gabrg1	1
GO:0010962	regulation of glucan biosynthetic process	1/67	30/14464	0.03333333	7.1960199	2.3174516	0.13014497	0.4307297	0.41309095	Enpp1	1
GO:0032814	regulation of natural killer cell activation	1/67	30/14464	0.03333333	7.1960199	2.3174516	0.13014497	0.4307297	0.41309095	H2-T23	1
GO:0036119	response to platelet-derived growth factor	1/67	30/14464	0.03333333	7.1960199	2.3174516	0.13014497	0.4307297	0.41309095	Enpp1	1
GO:0042745	circadian sleep/wake cycle	1/67	30/14464	0.03333333	7.1960199	2.3174516	0.13014497	0.4307297	0.41309095	Nr1d1	1
GO:0060071	Wnt signaling pathway, planar cell polarity pathway	1/67	30/14464	0.03333333	7.1960199	2.3174516	0.13014497	0.4307297	0.41309095	Ccdc88c	1
GO:0090128	regulation of synapse maturation	1/67	30/14464	0.03333333	7.1960199	2.3174516	0.13014497	0.4307297	0.41309095	Nfatc4	1
GO:0150146	cell junction disassembly	1/67	30/14464	0.03333333	7.1960199	2.3174516	0.13014497	0.4307297	0.41309095	Ngef	1
GO:2000108	positive regulation of leukocyte apoptotic process	1/67	30/14464	0.03333333	7.1960199	2.3174516	0.13014497	0.4307297	0.41309095	Fnip1	1
GO:0030336	negative regulation of cell migration	3/67	271/14464	0.01107011	2.38982211	1.57556873	0.13072946	0.4307297	0.41309095	Ager/Cd200/Ldlrad4	3
GO:0050657	nucleic acid transport	2/67	136/14464	0.01470588	3.17471466	1.73823921	0.1309791	0.4307297	0.41309095	Tst/Rftn1	2
GO:0050658	RNA transport	2/67	136/14464	0.01470588	3.17471466	1.73823921	0.1309791	0.4307297	0.41309095	Tst/Rftn1	2
GO:0010001	glial cell differentiation	3/67	272/14464	0.01102941	2.381036	1.56854955	0.1317718	0.4307297	0.41309095	Nr1d1/Ager/Enpp1	3
GO:0000375	RNA splicing, via transesterification reactions	3/67	273/14464	0.01098901	2.37231425	1.56156113	0.13281712	0.4307297	0.41309095	Srsf5/Arglu1/Luc7l2	3
GO:0000377	RNA splicing, via transesterification reactions with bulged adenosine as nucleophile	3/67	273/14464	0.01098901	2.37231425	1.56156113	0.13281712	0.4307297	0.41309095	Srsf5/Arglu1/Luc7l2	3
GO:0000398	mRNA splicing, via spliceosome	3/67	273/14464	0.01098901	2.37231425	1.56156113	0.13281712	0.4307297	0.41309095	Srsf5/Arglu1/Luc7l2	3
GO:0050768	negative regulation of neurogenesis	2/67	138/14464	0.01449275	3.1287043	1.71404808	0.13410449	0.4307297	0.41309095	Nr1d1/Nfatc4	2
GO:0022038	corpus callosum development	1/67	31/14464	0.03225806	6.96389023	2.26758082	0.13418268	0.4307297	0.41309095	Bhlhe22	1
GO:0032660	regulation of interleukin-17 production	1/67	31/14464	0.03225806	6.96389023	2.26758082	0.13418268	0.4307297	0.41309095	Rftn1	1
GO:0034067	protein localization to Golgi apparatus	1/67	31/14464	0.03225806	6.96389023	2.26758082	0.13418268	0.4307297	0.41309095	Sys1	1
GO:1905244	regulation of modification of synaptic structure	1/67	31/14464	0.03225806	6.96389023	2.26758082	0.13418268	0.4307297	0.41309095	Tiam1	1
GO:2000629	negative regulation of miRNA metabolic process	1/67	31/14464	0.03225806	6.96389023	2.26758082	0.13418268	0.4307297	0.41309095	Nfatc4	1
GO:0032635	interleukin-6 production	2/67	139/14464	0.01438849	3.10619564	1.70211691	0.13567362	0.4307297	0.41309095	Ager/Cd200	2
GO:0051236	establishment of RNA localization	2/67	139/14464	0.01438849	3.10619564	1.70211691	0.13567362	0.4307297	0.41309095	Tst/Rftn1	2
GO:0002706	regulation of lymphocyte mediated immunity	2/67	140/14464	0.01428571	3.08400853	1.69029282	0.13724695	0.4307297	0.41309095	H2-T23/Ager	2
GO:0002021	response to dietary excess	1/67	32/14464	0.03125	6.74626866	2.21987361	0.13820192	0.4307297	0.41309095	Enpp1	1
GO:0002715	regulation of natural killer cell mediated immunity	1/67	32/14464	0.03125	6.74626866	2.21987361	0.13820192	0.4307297	0.41309095	H2-T23	1
GO:0022010	central nervous system myelination	1/67	32/14464	0.03125	6.74626866	2.21987361	0.13820192	0.4307297	0.41309095	Enpp1	1
GO:0032291	axon ensheathment in central nervous system	1/67	32/14464	0.03125	6.74626866	2.21987361	0.13820192	0.4307297	0.41309095	Enpp1	1
GO:0032620	interleukin-17 production	1/67	32/14464	0.03125	6.74626866	2.21987361	0.13820192	0.4307297	0.41309095	Rftn1	1
GO:0044342	type B pancreatic cell proliferation	1/67	32/14464	0.03125	6.74626866	2.21987361	0.13820192	0.4307297	0.41309095	Nr1d1	1
GO:2000677	regulation of transcription regulatory region DNA binding	1/67	32/14464	0.03125	6.74626866	2.21987361	0.13820192	0.4307297	0.41309095	Dazap2	1
GO:2000765	regulation of cytoplasmic translation	1/67	32/14464	0.03125	6.74626866	2.21987361	0.13820192	0.4307297	0.41309095	Hnrnpd	1
GO:0044839	cell cycle G2/M phase transition	2/67	141/14464	0.0141844	3.06213613	1.67857397	0.13882441	0.4307297	0.41309095	Cdk2/Pkia	2
GO:0055088	lipid homeostasis	2/67	141/14464	0.0141844	3.06213613	1.67857397	0.13882441	0.4307297	0.41309095	Nr1d1/Fundc2	2
GO:0048592	eye morphogenesis	2/67	142/14464	0.01408451	3.04057179	1.66695858	0.14040593	0.4307297	0.41309095	Ppp2r3a/Bhlhe22	2
GO:0001933	negative regulation of protein phosphorylation	3/67	281/14464	0.01067616	2.30477506	1.50672969	0.14128473	0.4307297	0.41309095	Ager/Pkia/Enpp1	3
GO:0042129	regulation of T cell proliferation	2/67	143/14464	0.01398601	3.01930905	1.6554449	0.14199143	0.4307297	0.41309095	H2-T23/Ager	2
GO:0002474	antigen processing and presentation of peptide antigen via MHC class I	1/67	33/14464	0.03030303	6.54183627	2.1741677	0.14220279	0.4307297	0.41309095	H2-T23	1
GO:0006346	DNA methylation-dependent heterochromatin formation	1/67	33/14464	0.03030303	6.54183627	2.1741677	0.14220279	0.4307297	0.41309095	Ehmt2	1
GO:0010719	negative regulation of epithelial to mesenchymal transition	1/67	33/14464	0.03030303	6.54183627	2.1741677	0.14220279	0.4307297	0.41309095	Ldlrad4	1
GO:0019731	antibacterial humoral response	1/67	33/14464	0.03030303	6.54183627	2.1741677	0.14220279	0.4307297	0.41309095	H2-T23	1
GO:0030279	negative regulation of ossification	1/67	33/14464	0.03030303	6.54183627	2.1741677	0.14220279	0.4307297	0.41309095	Enpp1	1
GO:0043247	telomere maintenance in response to DNA damage	1/67	33/14464	0.03030303	6.54183627	2.1741677	0.14220279	0.4307297	0.41309095	Cdk2	1

GO:0043330	response to exogenous dsRNA	1/67	33/14464	0.03030303	6.54183627	2.1741677	0.14220279	0.4307297	0.41309095	Rftn1	1
GO:0048710	regulation of astrocyte differentiation	1/67	33/14464	0.03030303	6.54183627	2.1741677	0.14220279	0.4307297	0.41309095	Nr1d1	1
GO:0050691	regulation of defense response to virus by host	1/67	33/14464	0.03030303	6.54183627	2.1741677	0.14220279	0.4307297	0.41309095	Aim2	1
GO:0090317	negative regulation of intracellular protein transport	1/67	33/14464	0.03030303	6.54183627	2.1741677	0.14220279	0.4307297	0.41309095	Pkia	1
GO:2000146	negative regulation of cell motility	3/67	282/14464	0.0106383	2.2966021	1.50000644	0.142356	0.4307297	0.41309095	Ager/Cd200/Ldlrad4	3
GO:0007416	synapse assembly	3/67	283/14464	0.01060071	2.28848689	1.49331142	0.14343007	0.4307297	0.41309095	Gabrg1/Abi3bp/Il1rapl1	3
GO:0043523	regulation of neuron apoptotic process	3/67	283/14464	0.01060071	2.28848689	1.49331142	0.14343007	0.4307297	0.41309095	Ager/Pycr1/Nfatc4	3
GO:0016241	regulation of macroautophagy	2/67	144/14464	0.01388889	2.99834163	1.64403123	0.14358086	0.4307297	0.41309095	Ehmt2/Gpsm1	2
GO:0043281	regulation of cysteine-type endopeptidase activity involved in apoptotic process	2/67	144/14464	0.01388889	2.99834163	1.64403123	0.14358086	0.4307297	0.41309095	Tnfsf10/Fnip1	2
GO:0006790	sulfur compound metabolic process	3/67	285/14464	0.01052632	2.27242734	1.4800051	0.14558646	0.4307297	0.41309095	Gstt2/Gstt3/Enpp1	3
GO:0000132	establishment of mitotic spindle orientation	1/67	34/14464	0.02941176	6.34942932	2.13031752	0.14618535	0.4307297	0.41309095	Gpsm1	1
GO:0001953	negative regulation of cell-matrix adhesion	1/67	34/14464	0.02941176	6.34942932	2.13031752	0.14618535	0.4307297	0.41309095	Ajap1	1
GO:0001958	endochondral ossification	1/67	34/14464	0.02941176	6.34942932	2.13031752	0.14618535	0.4307297	0.41309095	Enpp1	1
GO:0002335	mature B cell differentiation	1/67	34/14464	0.02941176	6.34942932	2.13031752	0.14618535	0.4307297	0.41309095	Enpp1	1
GO:0009311	oligosaccharide metabolic process	1/67	34/14464	0.02941176	6.34942932	2.13031752	0.14618535	0.4307297	0.41309095	Man2b1	1
GO:0010043	response to zinc ion	1/67	34/14464	0.02941176	6.34942932	2.13031752	0.14618535	0.4307297	0.41309095	Slc30a1	1
GO:0019884	antigen processing and presentation of exogenous antigen	1/67	34/14464	0.02941176	6.34942932	2.13031752	0.14618535	0.4307297	0.41309095	H2-T23	1
GO:0034063	stress granule assembly	1/67	34/14464	0.02941176	6.34942932	2.13031752	0.14618535	0.4307297	0.41309095	Dazap2	1
GO:0035136	forelimb morphogenesis	1/67	34/14464	0.02941176	6.34942932	2.13031752	0.14618535	0.4307297	0.41309095	Enpp1	1
GO:0036075	replacement ossification	1/67	34/14464	0.02941176	6.34942932	2.13031752	0.14618535	0.4307297	0.41309095	Enpp1	1
GO:0045920	negative regulation of exocytosis	1/67	34/14464	0.02941176	6.34942932	2.13031752	0.14618535	0.4307297	0.41309095	Il1rapl1	1
GO:0071398	cellular response to fatty acid	1/67	34/14464	0.02941176	6.34942932	2.13031752	0.14618535	0.4307297	0.41309095	Nfatc4	1
GO:0097107	postsynaptic density assembly	1/67	34/14464	0.02941176	6.34942932	2.13031752	0.14618535	0.4307297	0.41309095	Abi3bp	1
GO:1902175	regulation of oxidative stress-induced intrinsic apoptotic signaling pathway	1/67	34/14464	0.02941176	6.34942932	2.13031752	0.14618535	0.4307297	0.41309095	Pycr1	1
GO:1902275	regulation of chromatin organization	1/67	34/14464	0.02941176	6.34942932	2.13031752	0.14618535	0.4307297	0.41309095	Cdk2	1
GO:1905606	regulation of presynapse assembly	1/67	34/14464	0.02941176	6.34942932	2.13031752	0.14618535	0.4307297	0.41309095	Il1rapl1	1
GO:2001235	positive regulation of apoptotic signaling pathway	2/67	146/14464	0.01369863	2.95726845	1.62149733	0.14677118	0.43163052	0.41395489	Nfatc4/Tnfsf10	2
GO:0051961	negative regulation of nervous system development	2/67	147/14464	0.01360544	2.93715098	1.61037388	0.14837195	0.43329718	0.41555329	Nr1d1/Nfatc4	2
GO:0060041	retina development in camera-type eye	2/67	147/14464	0.01360544	2.93715098	1.61037388	0.14837195	0.43329718	0.41555329	Bhlhe22/Xrn2	2
GO:0051052	regulation of DNA metabolic process	4/67	443/14464	0.00902935	1.94926047	1.38429406	0.1492043	0.43329718	0.41555329	Cdk2/Hnrnpd/Ehmt2/Rpa2	4
GO:0002673	regulation of acute inflammatory response	1/67	35/14464	0.02857143	6.16801706	2.08819204	0.15014971	0.43329718	0.41555329	H2-T23	1
GO:0003351	epithelial cilium movement involved in extracellular fluid movement	1/67	35/14464	0.02857143	6.16801706	2.08819204	0.15014971	0.43329718	0.41555329	Ccdc88c	1
GO:0016242	negative regulation of macroautophagy	1/67	35/14464	0.02857143	6.16801706	2.08819204	0.15014971	0.43329718	0.41555329	Ehmt2	1
GO:0032212	positive regulation of telomere maintenance via telomerase	1/67	35/14464	0.02857143	6.16801706	2.08819204	0.15014971	0.43329718	0.41555329	Hnrnpd	1
GO:0046640	regulation of alpha-beta T cell proliferation	1/67	35/14464	0.02857143	6.16801706	2.08819204	0.15014971	0.43329718	0.41555329	H2-T23	1
GO:0072344	rescue of stalled ribosome	1/67	35/14464	0.02857143	6.16801706	2.08819204	0.15014971	0.43329718	0.41555329	Zfp598	1
GO:0090329	regulation of DNA-templated DNA replication	1/67	35/14464	0.02857143	6.16801706	2.08819204	0.15014971	0.43329718	0.41555329	Cdk2	1
GO:0046631	alpha-beta T cell activation	2/67	149/14464	0.01342282	2.89772613	1.58840626	0.15158437	0.43332448	0.41557948	H2-T23/Ager	2
GO:0002831	regulation of response to biotic stimulus	4/67	446/14464	0.00896861	1.93614885	1.36993481	0.15179035	0.43332448	0.41557948	Nr1d1/Aim2/H2-T23/Rftn1	4
GO:0050769	positive regulation of neurogenesis	3/67	291/14464	0.01030928	2.22557317	1.44073941	0.15212032	0.43332448	0.41557948	Slc30a1/Tiam1/Il1rapl1	3
GO:1903131	mononuclear cell differentiation	4/67	447/14464	0.00894855	1.93181742	1.36517285	0.15265626	0.43332448	0.41557948	Slamf9/Ager/Enpp1/Fnip1	4
GO:0002714	positive regulation of B cell mediated immunity	1/67	36/14464	0.02777778	5.99668325	2.04767296	0.15409592	0.43332448	0.41557948	H2-T23	1
GO:0002891	positive regulation of immunoglobulin mediated immune response	1/67	36/14464	0.02777778	5.99668325	2.04767296	0.15409592	0.43332448	0.41557948	H2-T23	1
GO:0007026	negative regulation of microtubule depolymerization	1/67	36/14464	0.02777778	5.99668325	2.04767296	0.15409592	0.43332448	0.41557948	Ccdc88c	1
GO:0031952	regulation of protein autophosphorylation	1/67	36/14464	0.02777778	5.99668325	2.04767296	0.15409592	0.43332448	0.41557948	Enpp1	1
GO:0045879	negative regulation of smoothened signaling pathway	1/67	36/14464	0.02777778	5.99668325	2.04767296	0.15409592	0.43332448	0.41557948	Enpp1	1
GO:0050798	activated T cell proliferation	1/67	36/14464	0.02777778	5.99668325	2.04767296	0.15409592	0.43332448	0.41557948	Ager	1
GO:0050931	pigment cell differentiation	1/67	36/14464	0.02777778	5.99668325	2.04767296	0.15409592	0.43332448	0.41557948	Enpp1	1
GO:0050951	sensory perception of temperature stimulus	1/67	36/14464	0.02777778	5.99668325	2.04767296	0.15409592	0.43332448	0.41557948	Enpp1	1
GO:0060292	long-term synaptic depression	1/67	36/14464	0.02777778	5.99668325	2.04767296	0.15409592	0.43332448	0.41557948	Ager	1
GO:1902742	apoptotic process involved in development	1/67	36/14464	0.02777778	5.99668325	2.04767296	0.15409592	0.43332448	0.41557948	Enpp1	1
GO:0010506	regulation of autophagy	3/67	295/14464	0.01016949	2.1953959	1.41508902	0.1565285	0.43805097	0.42011241	Ager/Ehmt2/Gpsm1	3
GO:0051223	regulation of protein transport	4/67	452/14464	0.00884956	1.91044776	1.34154356	0.15701462	0.43805097	0.42011241	Nr1d1/Pkia/Cd200/Tiam1	4
GO:0045862	positive regulation of proteolysis	3/67	296/14464	0.01013514	2.18797902	1.40874032	0.1576369	0.43805097	0.42011241	Aim2/Ager/Tnfsf10	3
GO:0002686	negative regulation of leukocyte migration	1/67	37/14464	0.02702703	5.83461073	2.00865315	0.15802409	0.43805097	0.42011241	Cd200	1
GO:0016233	telomere capping	1/67	37/14464	0.02702703	5.83461073	2.00865315	0.15802409	0.43805097	0.42011241	Hnrnpd	1
GO:1904646	cellular response to amyloid-beta	1/67	37/14464	0.02702703	5.83461073	2.00865315	0.15802409	0.43805097	0.42011241	Ager	1
GO:1990806	ligand-gated ion channel signaling pathway	1/67	37/14464	0.02702703	5.83461073	2.00865315	0.15802409	0.43805097	0.42011241	Tiam1	1
GO:0045814	negative regulation of gene expression, epigenetic	2/67	153/14464	0.0130719	2.82196859	1.54554695	0.15805084	0.43805097	0.42011241	Cdk2/Ehmt2	2
GO:0040013	negative regulation of locomotion	3/67	297/14464	0.01010101	2.18061209	1.40241677	0.15874779	0.43831694	0.42036748	Ager/Cd200/Ldlrad4	3
GO:0006575	cellular modified amino acid metabolic process	2/67	154/14464	0.01298701	2.80364412	1.53504809	0.15967575	0.43831694	0.42036748	Gstt2/Gstt3	2
GO:0001569	branching involved in blood vessel morphogenesis	1/67	38/14464	0.02631579	5.68106834	1.97103533	0.16193428	0.43831694	0.42036748	Nfatc4	1
GO:0002691	regulation of cellular extravasation	1/67	38/14464	0.02631579	5.68106834	1.97103533	0.16193428	0.43831694	0.42036748	Ager	1
GO:0006858	extracellular transport	1/67	38/14464	0.02631579	5.68106834	1.97103533	0.16193428	0.43831694	0.42036748	Ccdc88c	1
GO:0021955	central nervous system neuron axonogenesis	1/67	38/14464	0.02631579	5.68106834	1.97103533	0.16193428	0.43831694	0.42036748	Bhlhe22	1
GO:0035886	vascular associated smooth muscle cell differentiation	1/67	38/14464	0.02631579	5.68106834	1.97103533	0.16193428	0.43831694	0.42036748	Nfatc4	1
GO:0040001	establishment of mitotic spindle localization	1/67	38/14464	0.02631579	5.68106834	1.97103533	0.16193428	0.43831694	0.42036748	Gpsm1	1
GO:0044273	sulfur compound catabolic process	1/67	38/14464	0.02631579	5.68106834	1.97103533	0.16193428	0.43831694	0.42036748	Enpp1	1
GO:0071312	cellular response to alkaloid	1/67	38/14464	0.02631579	5.68106834	1.97103533	0.16193428	0.43831694	0.42036748	Ehmt2	1
GO:2000404	regulation of T cell migration	1/67	38/14464	0.02631579	5.68106834	1.97103533	0.16193428	0.43831694	0.42036748	Cd200	1
GO:0001906	cell killing	2/67	156/14464	0.01282051	2.76769996	1.51430019	0.1629351	0.43831694	0.42036748	H2-T23/Ager	2
GO:0002822	regulation of adaptive immune response based on somatic recombination of immune receptors built from	2/67	156/14464	0.01282051	2.76769996	1.51430019	0.1629351	0.43831694	0.42036748	H2-T23/Ager	2
GO:0007254	JNK cascade	2/67	156/14464	0.01282051	2.76769996	1.51430019	0.1629351	0.43831694	0.42036748	Ccdc88c/Ager	2
GO:0007613	memory	2/67	156/14464	0.01282051	2.76769996	1.51430019	0.1629351	0.43831694	0.42036748	Nfatc4/Ehmt2	2
GO:0042326	negative regulation of phosphorylation	3/67	301/14464	0.00996678	2.15163386	1.37737005	0.16321596	0.43831694	0.42036748	Ager/Pkia/Enpp1	3
GO:0003382	epithelial cell morphogenesis	1/67	39/14464	0.02564103	5.53539992	1.93473092	0.16582659	0.43831694	0.42036748	Ccdc88c	1
GO:0007157	heterophilic cell-cell adhesion via plasma membrane cell adhesion molecules	1/67	39/14464	0.02564103	5.53539992	1.93473092	0.16582659	0.43831694	0.42036748	Il1rapl1	1
GO:0032715	negative regulation of interleukin-6 production	1/67	39/14464	0.02564103	5.53539992	1.93473092	0.16582659	0.43831694	0.42036748	Cd200	1
GO:0032885	regulation of polysaccharide biosynthetic process	1/67	39/14464	0.02564103	5.53539992	1.93473092	0.16582659	0.43831694	0.42036748	Enpp1	1
GO:0043001	Golgi to plasma membrane protein transport	1/67	39/14464	0.02564103	5.53539992	1.93473092	0.16582659	0.43831694	0.42036748	Sys1	1
GO:0090279	regulation of calcium ion import	1/67	39/14464	0.02564103	5.53539992	1.93473092	0.16582659	0.43831694	0.42036748	Slc30a1	1
GO:0099174	regulation of presynapse organization	1/67	39/14464	0.02564103	5.53539992	1.93473092	0.16582659	0.43831694	0.42036748	Il1rapl1	1
GO:1900744	regulation of p38MAPK cascade	1/67	39/14464	0.02564103	5.53539992	1.93473092	0.16582659	0.43831694	0.42036748	Ager	1
GO:1904358	positive regulation of telomere maintenance via telomere lengthening	1/67	39/14464	0.02564103	5.53539992	1.93473092	0.16582659	0.43831694	0.42036748	Hnrnpd	1
GO:1904738	vascular associated smooth muscle cell migration	1/67	39/14464	0.02564103	5.53539992	1.93473092	0.16582659	0.43831694	0.42036748	Enpp1	1
GO:1905521	regulation of macrophage migration	1/67	39/14464	0.02564103	5.53539992	1.93473092	0.16582659	0.43831694	0.42036748	Cd200	1
GO:1901990	regulation of mitotic cell cycle phase transition	3/67	304/14464	0.00986842	2.13040063	1.35883945	0.16659233	0.43877412	0.42080594	Cdk2/Pkia/Rpa2	3
GO:0042110	T cell activation	4/67	464/14464	0.00862069	1.86103963	1.28602291	0.16766495	0.43877412	0.42080594	Slamf9/H2-T23/Ager/Enpp1	4
GO:0071356	cellular response to tumor necrosis factor	2/67	160/14464	0.0125	2.69850746	1.47376858	0.16949008	0.43877412	0.42080594	Nr1d1/Aim2	2
GO:0001782	B cell homeostasis	1/67	40/14464	0.025	5.39701493	1.89965909	0.16970109	0.43877412	0.42080594	Enpp1	1
GO:0007099	centriole replication	1/67	40/14464	0.025	5.39701493	1.89965909	0.16970109	0.43877412	0.42080594	Cdk2	1
GO:0032941	secretion by tissue	1/67	40/14464	0.025	5.39701493	1.89965909	0.16970109	0.43877412	0.42080594	Enpp1	1
GO:0043388	positive regulation of DNA binding	1/67	40/14464	0.025	5.39701493	1.89965909	0.16970109	0.43877412	0.42080594	Dazap2	1
GO:0045740	positive regulation of DNA replication	1/67	40/14464	0.025	5.39701493	1.89965909	0.16970109	0.43877412	0.42080594	Cdk2	1
GO:0046633	alpha-beta T cell proliferation	1/67	40/14464	0.025	5.39701493	1.89965909	0.16970109	0.43877412	0.42080594	H2-T23	1
GO:0070873	regulation of glycogen metabolic process	1/67	40/14464	0.025	5.39701493	1.89965909	0.16970109	0.43877412	0.42080594	Enpp1	1
GO:0140894	endolysosomal toll-like receptor signaling pathway	1/67	40/14464	0.025	5.39701493	1.89965909	0.16970109	0.43877412	0.42080594	Rftn1	1
GO:1900181	negative regulation of protein localization to nucleus	1/67	40/14464	0.025	5.39701493	1.89965909	0.16970109	0.43877412	0.42080594	Pkia	1
GO:1901532	regulation of hematopoietic progenitor cell differentiation	1/67	40/14464	0.025	5.39701493	1.89965909	0.16970109	0.43877412	0.42080594	Fnip1	1
GO:0002694	regulation of leukocyte activation	4/67	467/14464	0.00856531	1.84908434	1.27239626	0.17036812	0.43949958	0.4215017	Nr1d1/H2-T23/Ager/Cd200	4
GO:0016236	macroautophagy	3/67	309/14464	0.00970874	2.09592813	1.32842478	0.17226611	0.43949958	0.4215017	Ehmt2/Stx17/Gpsm1	3
GO:0001754	eye photoreceptor cell differentiation	1/67	41/14464	0.02439024	5.26538041	1.86574587	0.17355785	0.43949958	0.4215017	Ppp2r3a	1
GO:0002707	negative regulation of lymphocyte mediated immunity	1/67	41/14464	0.02439024	5.26538041	1.86574587	0.17355785	0.43949958	0.4215017	H2-T23	1
GO:0006284	base-excision repair	1/67	41/14464	0.02439024	5.26538041	1.86574587	0.17355785	0.43949958	0.4215017	Rpa2	1
GO:0010543	regulation of platelet activation	1/67	41/14464	0.02439024	5.26538041	1.86574587	0.17355785	0.43949958	0.4215017	Fundc2	1
GO:0043331	response to dsRNA	1/67	41/14464	0.02439024	5.26538041	1.86574587	0.17355785	0.43949958	0.4215017	Rftn1	1
GO:0043507	positive regulation of JUN kinase activity	1/67	41/14464	0.02439024	5.26538041	1.86574587	0.17355785	0.43949958	0.4215017	Ager	1
GO:0045143	homologous chromosome segregation	1/67	41/14464	0.02439024	5.26538041	1.86574587	0.17355785	0.43949958	0.4215017	Ehmt2	1
GO:0051294	establishment of spindle orientation	1/67	41/14464	0.02439024	5.26538041	1.86574587	0.17355785	0.43949958	0.4215017	Gpsm1	1
GO:1902991	regulation of amyloid precursor protein catabolic process	1/67	41/14464	0.02439024	5.26538041	1.86574587	0.17355785	0.43949958	0.4215017	Ager	1
GO:0006403	RNA localization	2/67	163/14464	0.01226994	2.64884168	1.44417608	0.17443614	0.43949958	0.4215017	Tst/Rftn1	2
GO:0010508	positive regulation of autophagy	2/67	163/14464	0.01226994	2.64884168	1.44417608	0.17443614	0.43949958	0.4215017	Ager/Gpsm1	2
GO:0010720	positive regulation of cell development	4/67	473/14464	0.00845666	1.82562873	1.24543647	0.17582137	0.43949958	0.4215017	Ager/Slc30a1/Tiam1/Il1rapl1	4
GO:0030534	adult behavior	2/67	164/14464	0.01219512	2.63269021	1.43445948	0.1760902	0.43949958	0.4215017	Ehmt2/Enpp1	2
GO:0001937	negative regulation of endothelial cell proliferation	1/67	42/14464	0.02380952	5.14001421	1.83292344	0.17739697	0.43949958	0.4215017	Ager	1
GO:0031114	regulation of microtubule depolymerization	1/67	42/14464	0.02380952	5.14001421	1.83292344	0.17739697	0.43949958	0.4215017	Ccdc88c	1
GO:0032655	regulation of interleukin-12 production	1/67	42/14464	0.02380952	5.14001421	1.83292344	0.17739697	0.43949958	0.4215017	Ager	1
GO:0032790	ribosome disassembly	1/67	42/14464	0.02380952	5.14001421	1.83292344	0.17739697	0.43949958	0.4215017	Zfp598	1
GO:0033628	regulation of cell adhesion mediated by integrin	1/67	42/14464	0.02380952	5.14001421	1.83292344	0.17739697	0.43949958	0.4215017	Epha8	1
GO:0046627	negative regulation of insulin receptor signaling pathway	1/67	42/14464	0.02380952	5.14001421	1.83292344	0.17739697	0.43949958	0.4215017	Enpp1	1
GO:0046677	response to antibiotic	1/67	42/14464	0.02380952	5.14001421	1.83292344	0.17739697	0.43949958	0.4215017	Ehmt2	1
GO:0070192	chromosome organization involved in meiotic cell cycle	1/67	42/14464	0.02380952	5.14001421	1.83292344	0.17739697	0.43949958	0.4215017	Ehmt2	1
GO:0090630	activation of GTPase activity	1/67	42/14464	0.02380952	5.14001421	1.83292344	0.17739697	0.43949958	0.4215017	Tiam1	1
GO:0098534	centriole assembly	1/67	42/14464	0.02380952	5.14001421	1.83292344	0.17739697	0.43949958	0.4215017	Cdk2	1
GO:1904645	response to amyloid-beta	1/67	42/14464	0.02380952	5.14001421	1.83292344	0.17739697	0.43949958	0.4215017	Ager	1
GO:0032653	regulation of interleukin-10 production	1/67	43/14464	0.02325581	5.020479	1.80112946	0.18121852	0.44609864	0.42783052	Ager	1
GO:0061756	leukocyte adhesion to vascular endothelial cell	1/67	43/14464	0.02325581	5.020479	1.80112946	0.18121852	0.44609864	0.42783052	Podxl2	1
GO:0099560	synaptic membrane adhesion	1/67	43/14464	0.02325581	5.020479	1.80112946	0.18121852	0.44609864	0.42783052	Il1rapl1	1
GO:0140719	constitutive heterochromatin formation	1/67	43/14464	0.02325581	5.020479	1.80112946	0.18121852	0.44609864	0.42783052	Ehmt2	1
GO:0016052	carbohydrate catabolic process	2/67	168/14464	0.01190476	2.57000711	1.39630271	0.18273165	0.44910602	0.43071475	Enpp1/Man2b1	2
GO:0032615	interleukin-12 production	1/67	44/14464	0.02272727	4.9063772	1.77030655	0.18502259	0.45113893	0.43266441	Ager	1
GO:0048265	response to pain	1/67	44/14464	0.02272727	4.9063772	1.77030655	0.18502259	0.45113893	0.43266441	Gja4	1
GO:1901976	regulation of cell cycle checkpoint	1/67	44/14464	0.02272727	4.9063772	1.77030655	0.18502259	0.45113893	0.43266441	Rpa2	1
GO:1902116	negative regulation of organelle assembly	1/67	44/14464	0.02272727	4.9063772	1.77030655	0.18502259	0.45113893	0.43266441	Ehmt2	1
GO:1904861	excitatory synapse assembly	1/67	44/14464	0.02272727	4.9063772	1.77030655	0.18502259	0.45113893	0.43266441	Abi3bp	1
GO:0002819	regulation of adaptive immune response	2/67	170/14464	0.01176471	2.53977173	1.37763607	0.18606673	0.45296813	0.4344187	H2-T23/Ager	2
GO:0021700	developmental maturation	3/67	323/14464	0.00928793	2.00508294	1.24621707	0.18844233	0.45350884	0.43493727	Epha8/Nfatc4/Enpp1	3
GO:0002639	positive regulation of immunoglobulin production	1/67	45/14464	0.02222222	4.7973466	1.74040177	0.18880923	0.45350884	0.43493727	H2-T23	1
GO:0003016	respiratory system process	1/67	45/14464	0.02222222	4.7973466	1.74040177	0.18880923	0.45350884	0.43493727	Ccdc88c	1
GO:0007628	adult walking behavior	1/67	45/14464	0.02222222	4.7973466	1.74040177	0.18880923	0.45350884	0.43493727	Enpp1	1
GO:0043029	T cell homeostasis	1/67	45/14464	0.02222222	4.7973466	1.74040177	0.18880923	0.45350884	0.43493727	Aim2	1
GO:0061383	trabecula morphogenesis	1/67	45/14464	0.02222222	4.7973466	1.74040177	0.18880923	0.45350884	0.43493727	Enpp1	1
GO:0098698	postsynaptic specialization assembly	1/67	45/14464	0.02222222	4.7973466	1.74040177	0.18880923	0.45350884	0.43493727	Abi3bp	1
GO:0099010	modification of postsynaptic structure	1/67	45/14464	0.02222222	4.7973466	1.74040177	0.18880923	0.45350884	0.43493727	Tiam1	1
GO:0031647	regulation of protein stability	3/67	324/14464	0.00925926	1.99889442	1.24050346	0.18961311	0.45350884	0.43493727	Nr1d1/Ccdc88c/Dazap2	3
GO:0043393	regulation of protein binding	2/67	173/14464	0.01156069	2.49572945	1.35012935	0.19108601	0.45350884	0.43493727	Nfatc4/Tiam1	2
GO:0006417	regulation of translation	3/67	326/14464	0.00920245	1.98663126	1.22913695	0.19196047	0.45350884	0.43493727	Hnrnpd/Pcif1/Zfp598	3
GO:0007159	leukocyte cell-cell adhesion	3/67	326/14464	0.00920245	1.98663126	1.22913695	0.19196047	0.45350884	0.43493727	H2-T23/Ager/Podxl2	3
GO:0005975	carbohydrate metabolic process	4/67	491/14464	0.00814664	1.7587014	1.16680262	0.19253802	0.45350884	0.43493727	Nr1d1/Enpp1/Hexd/Man2b1	4
GO:0001912	positive regulation of leukocyte mediated cytotoxicity	1/67	46/14464	0.02173913	4.69305646	1.71136618	0.19257855	0.45350884	0.43493727	H2-T23	1
GO:0008340	determination of adult lifespan	1/67	46/14464	0.02173913	4.69305646	1.71136618	0.19257855	0.45350884	0.43493727	Enpp1	1
GO:0014014	negative regulation of gliogenesis	1/67	46/14464	0.02173913	4.69305646	1.71136618	0.19257855	0.45350884	0.43493727	Nr1d1	1
GO:0032613	interleukin-10 production	1/67	46/14464	0.02173913	4.69305646	1.71136618	0.19257855	0.45350884	0.43493727	Ager	1
GO:0032965	regulation of collagen biosynthetic process	1/67	46/14464	0.02173913	4.69305646	1.71136618	0.19257855	0.45350884	0.43493727	Ager	1
GO:0120163	negative regulation of cold-induced thermogenesis	1/67	46/14464	0.02173913	4.69305646	1.71136618	0.19257855	0.45350884	0.43493727	Nr1d1	1
GO:2000351	regulation of endothelial cell apoptotic process	1/67	46/14464	0.02173913	4.69305646	1.71136618	0.19257855	0.45350884	0.43493727	Ager	1
GO:2000785	regulation of autophagosome assembly	1/67	46/14464	0.02173913	4.69305646	1.71136618	0.19257855	0.45350884	0.43493727	Ehmt2	1
GO:0043484	regulation of RNA splicing	2/67	174/14464	0.01149425	2.48138617	1.34108835	0.19276333	0.45350884	0.43493727	Srsf5/Arglu1	2
GO:0021543	pallium development	2/67	175/14464	0.01142857	2.46720682	1.33210984	0.19444267	0.45359142	0.43501646	Bhlhe22/Xrn2	2
GO:0046777	protein autophosphorylation	2/67	175/14464	0.01142857	2.46720682	1.33210984	0.19444267	0.45359142	0.43501646	Epha8/Enpp1	2
GO:0050920	regulation of chemotaxis	2/67	175/14464	0.01142857	2.46720682	1.33210984	0.19444267	0.45359142	0.43501646	Ager/Tiam1	2
GO:0033120	positive regulation of RNA splicing	1/67	47/14464	0.0212766	4.59320419	1.68315445	0.19633061	0.45359142	0.43501646	Srsf5	1
GO:0033173	calcineurin-NFAT signaling cascade	1/67	47/14464	0.0212766	4.59320419	1.68315445	0.19633061	0.45359142	0.43501646	Nfatc4	1
GO:0035458	cellular response to interferon-beta	1/67	47/14464	0.0212766	4.59320419	1.68315445	0.19633061	0.45359142	0.43501646	Aim2	1
GO:0042789	mRNA transcription by RNA polymerase II	1/67	47/14464	0.0212766	4.59320419	1.68315445	0.19633061	0.45359142	0.43501646	Enpp1	1
GO:0046686	response to cadmium ion	1/67	47/14464	0.0212766	4.59320419	1.68315445	0.19633061	0.45359142	0.43501646	Slc30a1	1

GO:0048066 developmental pigmentation	1/67	47/14464	0.0212766	4.59320419	1.68315445	0.19633061	0.45359142	0.43501646	Enpp1	1
GO:0090199 regulation of release of cytochrome c from mitochondria	1/67	47/14464	0.0212766	4.59320419	1.68315445	0.19633061	0.45359142	0.43501646	Tnfsf10	1
GO:0170038 proteinogenic amino acid biosynthetic process	1/67	47/14464	0.0212766	4.59320419	1.68315445	0.19633061	0.45359142	0.43501646	Pycr1	1
GO:1900077 negative regulation of cellular response to insulin stimulus	1/67	47/14464	0.0212766	4.59320419	1.68315445	0.19633061	0.45359142	0.43501646	Enpp1	1
GO:0015980 energy derivation by oxidation of organic compounds	3/67	330/14464	0.00909091	1.96255088	1.20664277	0.19667785	0.45371343	0.43513348	Nr1d1/Nfatc4/Enpp1	3
GO:0031589 cell-substrate adhesion	3/67	332/14464	0.00903614	1.95072829	1.19551281	0.19904756	0.45511601	0.43647862	Ajap1/Tiam1/Abi3bp	3
GO:0000288 nuclear-transcribed mRNA catabolic process, deadenylation-dependent decay	1/67	48/14464	0.02083333	4.49751244	1.65572456	0.2000655	0.45511601	0.43647862	Hnrnpd	1
GO:0006911 phagocytosis, engulfment	1/67	48/14464	0.02083333	4.49751244	1.65572456	0.2000655	0.45511601	0.43647862	Ager	1
GO:0010762 regulation of fibroblast migration	1/67	48/14464	0.02083333	4.49751244	1.65572456	0.2000655	0.45511601	0.43647862	Ager	1
GO:0050918 positive chemotaxis	1/67	48/14464	0.02083333	4.49751244	1.65572456	0.2000655	0.45511601	0.43647862	Ager	1
GO:0061045 negative regulation of wound healing	1/67	48/14464	0.02083333	4.49751244	1.65572456	0.2000655	0.45511601	0.43647862	Ajap1	1
GO:0097720 calcineurin-mediated signaling	1/67	48/14464	0.02083333	4.49751244	1.65572456	0.2000655	0.45511601	0.43647862	Nfatc4	1
GO:0170034 L-amino acid biosynthetic process	1/67	48/14464	0.02083333	4.49751244	1.65572456	0.2000655	0.45511601	0.43647862	Pycr1	1
GO:0050796 regulation of insulin secretion	2/67	179/14464	0.01117318	2.41207371	1.29680403	0.20117918	0.45511601	0.43647862	Nr1d1/Tiam1	2
GO:0051346 negative regulation of hydrolase activity	2/67	179/14464	0.01117318	2.41207371	1.29680403	0.20117918	0.45511601	0.43647862	Fnip1/Gpsm1	2
GO:0034612 response to tumor necrosis factor	2/67	180/14464	0.01111111	2.3986733	1.28812559	0.20286784	0.45511601	0.43647862	Nr1d1/Aim2	2
GO:0035265 organ growth	2/67	180/14464	0.01111111	2.3986733	1.28812559	0.20286784	0.45511601	0.43647862	Ehmt2/Enpp1	2
GO:0048659 smooth muscle cell proliferation	2/67	180/14464	0.01111111	2.3986733	1.28812559	0.20286784	0.45511601	0.43647862	Ager/Enpp1	2
GO:0007215 glutamate receptor signaling pathway	1/67	49/14464	0.02040816	4.40572647	1.62903747	0.20378329	0.45511601	0.43647862	Tiam1	1
GO:0010596 negative regulation of endothelial cell migration	1/67	49/14464	0.02040816	4.40572647	1.62903747	0.20378329	0.45511601	0.43647862	Ager	1
GO:0031111 negative regulation of microtubule polymerization or depolymerization	1/67	49/14464	0.02040816	4.40572647	1.62903747	0.20378329	0.45511601	0.43647862	Ccdc88c	1
GO:0032881 regulation of polysaccharide metabolic process	1/67	49/14464	0.02040816	4.40572647	1.62903747	0.20378329	0.45511601	0.43647862	Enpp1	1
GO:0042267 natural killer cell mediated cytotoxicity	1/67	49/14464	0.02040816	4.40572647	1.62903747	0.20378329	0.45511601	0.43647862	H2-T23	1
GO:0048599 oocyte development	1/67	49/14464	0.02040816	4.40572647	1.62903747	0.20378329	0.45511601	0.43647862	Ehmt2	1
GO:0060074 synapse maturation	1/67	49/14464	0.02040816	4.40572647	1.62903747	0.20378329	0.45511601	0.43647862	Nfatc4	1
GO:0060390 regulation of SMAD protein signal transduction	1/67	49/14464	0.02040816	4.40572647	1.62903747	0.20378329	0.45511601	0.43647862	Ldlrad4	1
GO:0099054 presynapse assembly	1/67	49/14464	0.02040816	4.40572647	1.62903747	0.20378329	0.45511601	0.43647862	Il1rapl1	1
GO:0000245 spliceosomal complex assembly	1/67	50/14464	0.02	4.31761194	1.60305684	0.20748405	0.45777351	0.43902729	Luc7l2	1
GO:0002712 regulation of B cell mediated immunity	1/67	50/14464	0.02	4.31761194	1.60305684	0.20748405	0.45777351	0.43902729	H2-T23	1
GO:0002889 regulation of immunoglobulin mediated immune response	1/67	50/14464	0.02	4.31761194	1.60305684	0.20748405	0.45777351	0.43902729	H2-T23	1
GO:0009994 oocyte differentiation	1/67	50/14464	0.02	4.31761194	1.60305684	0.20748405	0.45777351	0.43902729	Ehmt2	1
GO:0010718 positive regulation of epithelial to mesenchymal transition	1/67	50/14464	0.02	4.31761194	1.60305684	0.20748405	0.45777351	0.43902729	Ager	1
GO:0043154 negative regulation of cysteine-type endopeptidase activity involved in apoptotic process	1/67	50/14464	0.02	4.31761194	1.60305684	0.20748405	0.45777351	0.43902729	Fnip1	1
GO:0072577 endothelial cell apoptotic process	1/67	50/14464	0.02	4.31761194	1.60305684	0.20748405	0.45777351	0.43902729	Ager	1
GO:0072678 T cell migration	1/67	50/14464	0.02	4.31761194	1.60305684	0.20748405	0.45777351	0.43902729	Cd200	1
GO:0009267 cellular response to starvation	2/67	183/14464	0.01092896	2.35935079	1.26243309	0.20794384	0.45777351	0.43902729	Ehmt2/Fnip1	2
GO:0016051 carbohydrate biosynthetic process	2/67	183/14464	0.01092896	2.35935079	1.26243309	0.20794384	0.45777351	0.43902729	Nr1d1/Enpp1	2
GO:0001756 somitogenesis	1/67	51/14464	0.01960784	4.23295288	1.57774881	0.21116787	0.46136544	0.44247213	Ppp2r3a	1
GO:0002711 positive regulation of T cell mediated immunity	1/67	51/14464	0.01960784	4.23295288	1.57774881	0.21116787	0.46136544	0.44247213	H2-T23	1
GO:0009299 mRNA transcription	1/67	51/14464	0.01960784	4.23295288	1.57774881	0.21116787	0.46136544	0.44247213	Enpp1	1
GO:0050853 B cell receptor signaling pathway	1/67	51/14464	0.01960784	4.23295288	1.57774881	0.21116787	0.46136544	0.44247213	Rftn1	1
GO:0042098 T cell proliferation	2/67	185/14464	0.01081081	2.33384429	1.24558306	0.21133569	0.46136544	0.44247213	H2-T23/Ager	2
GO:0010563 negative regulation of phosphorus metabolic process	3/67	345/14464	0.00869565	1.87722258	1.12498822	0.21461769	0.46136544	0.44247213	Ager/Pkia/Enpp1	3
GO:0045936 negative regulation of phosphate metabolic process	3/67	345/14464	0.00869565	1.87722258	1.12498822	0.21461769	0.46136544	0.44247213	Ager/Pkia/Enpp1	3
GO:0002228 natural killer cell mediated immunity	1/67	52/14464	0.01923077	4.15154994	1.5530818	0.21483482	0.46136544	0.44247213	H2-T23	1
GO:0002704 negative regulation of leukocyte mediated immunity	1/67	52/14464	0.01923077	4.15154994	1.5530818	0.21483482	0.46136544	0.44247213	H2-T23	1
GO:0031343 positive regulation of cell killing	1/67	52/14464	0.01923077	4.15154994	1.5530818	0.21483482	0.46136544	0.44247213	H2-T23	1
GO:0032210 regulation of telomere maintenance via telomerase	1/67	52/14464	0.01923077	4.15154994	1.5530818	0.21483482	0.46136544	0.44247213	Hnrnpd	1
GO:0032387 negative regulation of intracellular transport	1/67	52/14464	0.01923077	4.15154994	1.5530818	0.21483482	0.46136544	0.44247213	Pkia	1
GO:0034142 toll-like receptor 4 signaling pathway	1/67	52/14464	0.01923077	4.15154994	1.5530818	0.21483482	0.46136544	0.44247213	Nr1d1	1
GO:0042220 response to cocaine	1/67	52/14464	0.01923077	4.15154994	1.5530818	0.21483482	0.46136544	0.44247213	Ehmt2	1
GO:0170036 import into the mitochondrion	1/67	52/14464	0.01923077	4.15154994	1.5530818	0.21483482	0.46136544	0.44247213	Tst	1
GO:2000401 regulation of lymphocyte migration	1/67	52/14464	0.01923077	4.15154994	1.5530818	0.21483482	0.46136544	0.44247213	Cd200	1
GO:0018105 peptidyl-serine phosphorylation	2/67	188/14464	0.0106383	2.2966021	1.2207113	0.21643428	0.46136544	0.44247213	Cdk2/Fnip1	2
GO:0043524 negative regulation of neuron apoptotic process	2/67	188/14464	0.0106383	2.2966021	1.2207113	0.21643428	0.46136544	0.44247213	Pycr1/Nfatc4	2
GO:0050870 positive regulation of T cell activation	2/67	188/14464	0.0106383	2.2966021	1.2207113	0.21643428	0.46136544	0.44247213	H2-T23/Ager	2
GO:0001738 morphogenesis of a polarized epithelium	1/67	53/14464	0.01886792	4.07321881	1.52902628	0.21848498	0.46136544	0.44247213	Ajap1	1
GO:0001941 postsynaptic membrane organization	1/67	53/14464	0.01886792	4.07321881	1.52902628	0.21848498	0.46136544	0.44247213	Slc30a1	1
GO:0006513 protein monoubiquitination	1/67	53/14464	0.01886792	4.07321881	1.52902628	0.21848498	0.46136544	0.44247213	Zfp598	1
GO:0008631 intrinsic apoptotic signaling pathway in response to oxidative stress	1/67	53/14464	0.01886792	4.07321881	1.52902628	0.21848498	0.46136544	0.44247213	Pycr1	1
GO:0014003 oligodendrocyte development	1/67	53/14464	0.01886792	4.07321881	1.52902628	0.21848498	0.46136544	0.44247213	Enpp1	1
GO:0045599 negative regulation of fat cell differentiation	1/67	53/14464	0.01886792	4.07321881	1.52902628	0.21848498	0.46136544	0.44247213	Enpp1	1
GO:0048002 antigen processing and presentation of peptide antigen	1/67	53/14464	0.01886792	4.07321881	1.52902628	0.21848498	0.46136544	0.44247213	H2-T23	1
GO:0051293 establishment of spindle localization	1/67	53/14464	0.01886792	4.07321881	1.52902628	0.21848498	0.46136544	0.44247213	Gpsm1	1
GO:0099563 modification of synaptic structure	1/67	53/14464	0.01886792	4.07321881	1.52902628	0.21848498	0.46136544	0.44247213	Tiam1	1
GO:1901607 alpha-amino acid biosynthetic process	1/67	53/14464	0.01886792	4.07321881	1.52902628	0.21848498	0.46136544	0.44247213	Pycr1	1
GO:0032259 methylation	2/67	190/14464	0.01052632	2.27242734	1.2043911	0.21983996	0.46136544	0.44247213	Pcif1/Ehmt2	2
GO:0002685 regulation of leukocyte migration	2/67	191/14464	0.0104712	2.26052981	1.19630733	0.22154465	0.46136544	0.44247213	Ager/Cd200	2
GO:0001654 eye development	3/67	351/14464	0.00854701	1.84513331	1.09345194	0.22189395	0.46136544	0.44247213	Ppp2r3a/Bhlhe22/Xrn2	3
GO:0051962 positive regulation of nervous system development	3/67	351/14464	0.00854701	1.84513331	1.09345194	0.22189395	0.46136544	0.44247213	Slc30a1/Tiam1/Il1rapl1	3
GO:0002381 immunoglobulin production involved in immunoglobulin-mediated immune response	1/67	54/14464	0.01851852	3.99778883	1.50555463	0.22211842	0.46136544	0.44247213	Enpp1	1
GO:0010712 regulation of collagen metabolic process	1/67	54/14464	0.01851852	3.99778883	1.50555463	0.22211842	0.46136544	0.44247213	Ager	1
GO:0043506 regulation of JUN kinase activity	1/67	54/14464	0.01851852	3.99778883	1.50555463	0.22211842	0.46136544	0.44247213	Ager	1
GO:0048512 circadian behavior	1/67	54/14464	0.01851852	3.99778883	1.50555463	0.22211842	0.46136544	0.44247213	Nr1d1	1
GO:0090659 walking behavior	1/67	54/14464	0.01851852	3.99778883	1.50555463	0.22211842	0.46136544	0.44247213	Enpp1	1
GO:1901224 positive regulation of non-canonical NF-kappaB signal transduction	1/67	54/14464	0.01851852	3.99778883	1.50555463	0.22211842	0.46136544	0.44247213	Ager	1
GO:0009306 protein secretion	3/67	352/14464	0.00852273	1.83989145	1.08825533	0.22311175	0.46136544	0.44247213	Nr1d1/Cd200/Tiam1	3
GO:0015931 nucleobase-containing compound transport	2/67	192/14464	0.01041667	2.24875622	1.18827361	0.22325051	0.46136544	0.44247213	Tst/Rftn1	2
GO:0050670 regulation of lymphocyte proliferation	2/67	192/14464	0.01041667	2.24875622	1.18827361	0.22325051	0.46136544	0.44247213	H2-T23/Ager	2
GO:0035592 establishment of protein localization to extracellular region	3/67	354/14464	0.00847458	1.82949658	1.07791203	0.22555154	0.46136544	0.44247213	Nr1d1/Cd200/Tiam1	3
GO:0150063 visual system development	3/67	354/14464	0.00847458	1.82949658	1.07791203	0.22555154	0.46136544	0.44247213	Ppp2r3a/Bhlhe22/Xrn2	3
GO:0006479 protein methylation	1/67	55/14464	0.01818182	3.92510176	1.48264097	0.22573522	0.46136544	0.44247213	Ehmt2	1
GO:0007622 rhythmic behavior	1/67	55/14464	0.01818182	3.92510176	1.48264097	0.22573522	0.46136544	0.44247213	Nr1d1	1
GO:0008213 protein alkylation	1/67	55/14464	0.01818182	3.92510176	1.48264097	0.22573522	0.46136544	0.44247213	Ehmt2	1
GO:0010812 negative regulation of cell-substrate adhesion	1/67	55/14464	0.01818182	3.92510176	1.48264097	0.22573522	0.46136544	0.44247213	Ajap1	1
GO:0035456 response to interferon-beta	1/67	55/14464	0.01818182	3.92510176	1.48264097	0.22573522	0.46136544	0.44247213	Aim2	1
GO:0070542 response to fatty acid	1/67	55/14464	0.01818182	3.92510176	1.48264097	0.22573522	0.46136544	0.44247213	Nfatc4	1
GO:1904263 positive regulation of TORC1 signaling	1/67	55/14464	0.01818182	3.92510176	1.48264097	0.22573522	0.46136544	0.44247213	Fnip1	1
GO:0010639 negative regulation of organelle organization	3/67	355/14464	0.0084507	1.82434307	1.07276512	0.2267735	0.46136544	0.44247213	Ccdc88c/Ehmt2/Fnip1	3
GO:0006935 chemotaxis	3/67	356/14464	0.00842697	1.81921851	1.06763456	0.2279968	0.46136544	0.44247213	Slamf9/Ager/Tiam1	3
GO:0045165 cell fate commitment	2/67	195/14464	0.01025641	2.21415997	1.16446661	0.22837462	0.46136544	0.44247213	Ehmt2/Bhlhe22	2
GO:0042330 taxis	3/67	357/14464	0.00840336	1.81412266	1.06252024	0.22922144	0.46136544	0.44247213	Slamf9/Ager/Tiam1	3
GO:0071692 protein localization to extracellular region	3/67	357/14464	0.00840336	1.81412266	1.06252024	0.22922144	0.46136544	0.44247213	Nr1d1/Cd200/Tiam1	3
GO:0002886 regulation of myeloid leukocyte mediated immunity	1/67	56/14464	0.01785714	3.85501066	1.46026101	0.22933545	0.46136544	0.44247213	H2-T23	1
GO:0007019 microtubule depolymerization	1/67	56/14464	0.01785714	3.85501066	1.46026101	0.22933545	0.46136544	0.44247213	Ccdc88c	1
GO:0032964 collagen biosynthetic process	1/67	56/14464	0.01785714	3.85501066	1.46026101	0.22933545	0.46136544	0.44247213	Ager	1
GO:0038066 p38MAPK cascade	1/67	56/14464	0.01785714	3.85501066	1.46026101	0.22933545	0.46136544	0.44247213	Ager	1
GO:0042987 amyloid precursor protein catabolic process	1/67	56/14464	0.01785714	3.85501066	1.46026101	0.22933545	0.46136544	0.44247213	Ager	1
GO:0043124 negative regulation of canonical NF-kappaB signal transduction	1/67	56/14464	0.01785714	3.85501066	1.46026101	0.22933545	0.46136544	0.44247213	Nr1d1	1
GO:0050688 regulation of defense response to virus	1/67	56/14464	0.01785714	3.85501066	1.46026101	0.22933545	0.46136544	0.44247213	Aim2	1
GO:0051653 spindle localization	1/67	56/14464	0.01785714	3.85501066	1.46026101	0.22933545	0.46136544	0.44247213	Gpsm1	1
GO:0060042 retina morphogenesis in camera-type eye	1/67	56/14464	0.01785714	3.85501066	1.46026101	0.22933545	0.46136544	0.44247213	Bhlhe22	1
GO:2001238 positive regulation of extrinsic apoptotic signaling pathway	1/67	56/14464	0.01785714	3.85501066	1.46026101	0.22933545	0.46136544	0.44247213	Tnfsf10	1
GO:0032944 regulation of mononuclear cell proliferation	2/67	196/14464	0.01020408	2.20286323	1.15662701	0.23008469	0.4619414	0.44302451	H2-T23/Ager	2
GO:0048880 sensory system development	3/67	359/14464	0.00835655	1.80401613	1.05233989	0.23167463	0.4619414	0.44302451	Ppp2r3a/Bhlhe22/Xrn2	3
GO:0002703 regulation of leukocyte mediated immunity	2/67	197/14464	0.01015228	2.19168119	1.14883447	0.23179569	0.4619414	0.44302451	H2-T23/Ager	2
GO:0051963 regulation of synapse assembly	2/67	197/14464	0.01015228	2.19168119	1.14883447	0.23179569	0.4619414	0.44302451	Abi3bp/Il1rapl1	2
GO:0009064 glutamine family amino acid metabolic process	1/67	57/14464	0.01754386	3.78737889	1.43839197	0.23291919	0.4619414	0.44302451	Pycr1	1
GO:0016601 Rac protein signal transduction	1/67	57/14464	0.01754386	3.78737889	1.43839197	0.23291919	0.4619414	0.44302451	Tiam1	1
GO:0030225 macrophage differentiation	1/67	57/14464	0.01754386	3.78737889	1.43839197	0.23291919	0.4619414	0.44302451	Enpp1	1
GO:0046635 positive regulation of alpha-beta T cell activation	1/67	57/14464	0.01754386	3.78737889	1.43839197	0.23291919	0.4619414	0.44302451	H2-T23	1
GO:0048278 vesicle docking	1/67	57/14464	0.01754386	3.78737889	1.43839197	0.23291919	0.4619414	0.44302451	Stx17	1
GO:0099024 plasma membrane invagination	1/67	57/14464	0.01754386	3.78737889	1.43839197	0.23291919	0.4619414	0.44302451	Ager	1
GO:1900271 regulation of long-term synaptic potentiation	1/67	57/14464	0.01754386	3.78737889	1.43839197	0.23291919	0.4619414	0.44302451	Ager	1
GO:0018209 peptidyl-serine modification	2/67	199/14464	0.01005025	2.16965424	1.13338834	0.23522034	0.46542237	0.44636292	Cdk2/Fnip1	2
GO:0032722 positive regulation of chemokine production	1/67	58/14464	0.01724138	3.72207926	1.4170124	0.23648651	0.46542237	0.44636292	Ager	1
GO:0045912 negative regulation of carbohydrate metabolic process	1/67	58/14464	0.01724138	3.72207926	1.4170124	0.23648651	0.46542237	0.44636292	Enpp1	1
GO:0046324 regulation of D-glucose import	1/67	58/14464	0.01724138	3.72207926	1.4170124	0.23648651	0.46542237	0.44636292	Enpp1	1
GO:0046530 photoreceptor cell differentiation	1/67	58/14464	0.01724138	3.72207926	1.4170124	0.23648651	0.46542237	0.44636292	Ppp2r3a	1
GO:2000117 negative regulation of cysteine-type endopeptidase activity	1/67	58/14464	0.01724138	3.72207926	1.4170124	0.23648651	0.46542237	0.44636292	Fnip1	1
GO:0009636 response to toxic substance	2/67	201/14464	0.00995025	2.14806564	1.11812382	0.23864822	0.46704264	0.44791685	Slc30a1/Ehmt2	2
GO:0031345 negative regulation of cell projection organization	2/67	201/14464	0.00995025	2.14806564	1.11812382	0.23864822	0.46704264	0.44791685	Ngef/Nfatc4	2
GO:0000381 regulation of alternative mRNA splicing, via spliceosome	1/67	59/14464	0.01694915	3.65899317	1.39610213	0.24003749	0.46704264	0.44791685	Arglu1	1
GO:0006278 RNA-templated DNA biosynthetic process	1/67	59/14464	0.01694915	3.65899317	1.39610213	0.24003749	0.46704264	0.44791685	Hnrnpd	1
GO:0007004 telomere maintenance via telomerase	1/67	59/14464	0.01694915	3.65899317	1.39610213	0.24003749	0.46704264	0.44791685	Hnrnpd	1
GO:0031103 axon regeneration	1/67	59/14464	0.01694915	3.65899317	1.39610213	0.24003749	0.46704264	0.44791685	Enpp1	1
GO:0048255 mRNA stabilization	1/67	59/14464	0.01694915	3.65899317	1.39610213	0.24003749	0.46704264	0.44791685	Hnrnpd	1
GO:1904035 regulation of epithelial cell apoptotic process	1/67	59/14464	0.01694915	3.65899317	1.39610213	0.24003749	0.46704264	0.44791685	Ager	1
GO:2000134 negative regulation of G1/S transition of mitotic cell cycle	1/67	59/14464	0.01694915	3.65899317	1.39610213	0.24003749	0.46704264	0.44791685	Rpa2	1
GO:0030101 natural killer cell activation	1/67	60/14464	0.01666667	3.59800995	1.37564214	0.2435722	0.47197061	0.45264301	H2-T23	1
GO:0070228 regulation of lymphocyte apoptotic process	1/67	60/14464	0.01666667	3.59800995	1.37564214	0.2435722	0.47197061	0.45264301	Fnip1	1
GO:0140895 cell surface toll-like receptor signaling pathway	1/67	60/14464	0.01666667	3.59800995	1.37564214	0.2435722	0.47197061	0.45264301	Nr1d1	1
GO:0007623 circadian rhythm	2/67	204/14464	0.00980392	2.11647644	1.09555826	0.24379533	0.47197061	0.45264301	Nr1d1/Hnrnpd	2
GO:0060350 endochondral bone morphogenesis	1/67	61/14464	0.01639344	3.53902618	1.35561451	0.24709071	0.47682237	0.45729609	Enpp1	1
GO:1904356 regulation of telomere maintenance via telomere lengthening	1/67	61/14464	0.01639344	3.53902618	1.35561451	0.24709071	0.47682237	0.45729609	Hnrnpd	1
GO:0010810 regulation of cell-substrate adhesion	2/67	206/14464	0.00970874	2.09592813	1.08072941	0.24722977	0.47682237	0.45729609	Ajap1/Abi3bp	2
GO:0000082 G1/S transition of mitotic cell cycle	2/67	207/14464	0.00966184	2.08580287	1.07337796	0.24894778	0.47792571	0.45835424	Cdk2/Rpa2	2
GO:0043583 ear development	2/67	207/14464	0.00966184	2.08580287	1.07337796	0.24894778	0.47792571	0.45835424	H2-T23/Enpp1	2
GO:1903039 positive regulation of leukocyte cell-cell adhesion	2/67	207/14464	0.00966184	2.08580287	1.07337796	0.24894778	0.47792571	0.45835424	H2-T23/Ager	2
GO:0006893 Golgi to plasma membrane transport	1/67	62/14464	0.01612903	3.48194511	1.3360023	0.2505931	0.47792571	0.45835424	Sys1	1
GO:0008652 amino acid biosynthetic process	1/67	62/14464	0.01612903	3.48194511	1.3360023	0.2505931	0.47792571	0.45835424	Pycr1	1
GO:0042130 negative regulation of T cell proliferation	1/67	62/14464	0.01612903	3.48194511	1.3360023	0.2505931	0.47792571	0.45835424	H2-T23	1
GO:0042551 neuron maturation	1/67	62/14464	0.01612903	3.48194511	1.3360023	0.2505931	0.47792571	0.45835424	Epha8	1
GO:0050805 negative regulation of synaptic transmission	1/67	62/14464	0.01612903	3.48194511	1.3360023	0.2505931	0.47792571	0.45835424	Ager	1
GO:2000514 regulation of CD4-positive, alpha-beta T cell activation	1/67	62/14464	0.01612903	3.48194511	1.3360023	0.2505931	0.47792571	0.45835424	Ager	1
GO:0043603 amide metabolic process	3/67	376/14464	0.00797872	1.72245157	0.96829699	0.25271822	0.47869799	0.4590949	Gstt2/Gstt3/Enpp1	3
GO:0001836 release of cytochrome c from mitochondria	1/67	63/14464	0.01587302	3.42667614	1.31678952	0.25407944	0.47869799	0.4590949	Tnfsf10	1
GO:0002752 cell surface pattern recognition receptor signaling pathway	1/67	63/14464	0.01587302	3.42667614	1.31678952	0.25407944	0.47869799	0.4590949	Nr1d1	1
GO:0007585 respiratory gaseous exchange by respiratory system	1/67	63/14464	0.01587302	3.42667614	1.31678952	0.25407944	0.47869799	0.4590949	Ccdc88c	1
GO:0051898 negative regulation of phosphatidylinositol 3-kinase/protein kinase B signal transduction	1/67	63/14464	0.01587302	3.42667614	1.31678952	0.25407944	0.47869799	0.4590949	Aim2	1
GO:0051932 synaptic transmission, GABAergic	1/67	63/14464	0.01587302	3.42667614	1.31678952	0.25407944	0.47869799	0.4590949	Gabrg1	1
GO:0097352 autophagosome maturation	1/67	63/14464	0.01587302	3.42667614	1.31678952	0.25407944	0.47869799	0.4590949	Stx17	1
GO:0099172 presynapse organization	1/67	63/14464	0.01587302	3.42667614	1.31678952	0.25407944	0.47869799	0.4590949	Il1rapl1	1
GO:0007160 cell-matrix adhesion	2/67	210/14464	0.00952381	2.05600569	1.05156983	0.25410445	0.47869799	0.4590949	Ajap1/Tiam1	2
GO:0090276 regulation of peptide hormone secretion	2/67	210/14464	0.00952381	2.05600569	1.05156983	0.25410445	0.47869799	0.4590949	Nr1d1/Tiam1	2
GO:0031400 negative regulation of protein modification process	3/67	378/14464	0.00793651	1.71333807	0.95868968	0.25521409	0.48020136	0.46053671	Ager/Pkia/Enpp1	3
GO:0051321 meiotic cell cycle	2/67	211/14464	0.00947867	2.04626158	1.04438099	0.25582409	0.4807621	0.46107449	Cdk2/Ehmt2	2
GO:2001233 regulation of apoptotic signaling pathway	3/67	379/14464	0.00791557	1.70881739	0.95390722	0.25646344	0.4807952	0.46110623	Pycr1/Nfatc4/Tnfsf10	3
GO:0032869 cellular response to insulin stimulus	2/67	212/14464	0.00943396	2.03660941	1.03723165	0.25754404	0.4807952	0.46110623	Cdk2/Enpp1	2
GO:0002637 regulation of immunoglobulin production	1/67	64/14464	0.015625	3.37313433	1.29796103	0.2575498	0.4807952	0.46110623	H2-T23	1
GO:0003407 neural retina development	1/67	64/14464	0.015625	3.37313433	1.29796103	0.2575498	0.4807952	0.46110623	Bhlhe22	1
GO:0045453 bone resorption	1/67	64/14464	0.015625	3.37313433	1.29796103	0.2575498	0.4807952	0.46110623	Enpp1	1
GO:0002757 immune response-activating signaling pathway	3/67	380/14464	0.00789474	1.7043205	0.94913877	0.25771372	0.4807952	0.46110623	Nr1d1/Aim2/Rftn1	3
GO:0030073 insulin secretion	2/67	213/14464	0.00938967	2.02704786	1.03012139	0.25926426	0.48165823	0.46193392	Nr1d1/Tiam1	2
GO:0043122 regulation of canonical NF-kappaB signal transduction	2/67	213/14464	0.00938967	2.02704786	1.03012139	0.25926426	0.48165823	0.46193392	Nr1d1/Tnfsf10	2
GO:0070663 regulation of leukocyte proliferation	2/67	213/14464	0.00938967	2.02704786	1.03012139	0.25926426	0.48165823	0.46193392	H2-T23/Ager	2

GO:0010324	membrane invagination	1/67	65/14464	0.01538462	3.32123995	1.27950251	0.26100426	0.48165823	0.46193392	Ager	1
GO:0032007	negative regulation of TOR signaling	1/67	65/14464	0.01538462	3.32123995	1.27950251	0.26100426	0.48165823	0.46193392	Fnip1	1
GO:0032922	circadian regulation of gene expression	1/67	65/14464	0.01538462	3.32123995	1.27950251	0.26100426	0.48165823	0.46193392	Nr1d1	1
GO:0050766	positive regulation of phagocytosis	1/67	65/14464	0.01538462	3.32123995	1.27950251	0.26100426	0.48165823	0.46193392	Ager	1
GO:0051926	negative regulation of calcium ion transport	1/67	65/14464	0.01538462	3.32123995	1.27950251	0.26100426	0.48165823	0.46193392	Slc30a1	1
GO:0140115	export across plasma membrane	1/67	65/14464	0.01538462	3.32123995	1.27950251	0.26100426	0.48165823	0.46193392	Slc30a1	1
GO:0002791	regulation of peptide secretion	2/67	215/14464	0.00930233	2.0081916	1.01601637	0.26270536	0.48165823	0.46193392	Nr1d1/Tiam1	2
GO:0090087	regulation of peptide transport	2/67	215/14464	0.00930233	2.0081916	1.01601637	0.26270536	0.48165823	0.46193392	Nr1d1/Tiam1	2
GO:0002429	immune response-activating cell surface receptor signaling pathway	2/67	216/14464	0.00925926	1.99889442	1.00902076	0.26442616	0.48165823	0.46193392	Nr1d1/Rftn1	2
GO:0042594	response to starvation	2/67	216/14464	0.00925926	1.99889442	1.00902076	0.26442616	0.48165823	0.46193392	Ehmt2/Fnip1	2
GO:0002720	positive regulation of cytokine production involved in immune response	1/67	66/14464	0.01515152	3.27091814	1.26140037	0.26444288	0.48165823	0.46193392	H2-T23	1
GO:0014911	positive regulation of smooth muscle cell migration	1/67	66/14464	0.01515152	3.27091814	1.26140037	0.26444288	0.48165823	0.46193392	Ager	1
GO:0031122	cytoplasmic microtubule organization	1/67	66/14464	0.01515152	3.27091814	1.26140037	0.26444288	0.48165823	0.46193392	Ccdc88c	1
GO:0061053	somite development	1/67	66/14464	0.01515152	3.27091814	1.26140037	0.26444288	0.48165823	0.46193392	Ppp2r3a	1
GO:1902373	negative regulation of mRNA catabolic process	1/67	66/14464	0.01515152	3.27091814	1.26140037	0.26444288	0.48165823	0.46193392	Hnrnpd	1
GO:2000573	positive regulation of DNA biosynthetic process	1/67	66/14464	0.01515152	3.27091814	1.26140037	0.26444288	0.48165823	0.46193392	Hnrnpd	1
GO:0031329	regulation of cellular catabolic process	3/67	388/14464	0.00773196	1.66917987	0.91148536	0.26774699	0.48165823	0.46193392	Ager/Ehmt2/Gpsm1	3
GO:0001708	cell fate specification	1/67	67/14464	0.01492537	3.22209846	1.24364174	0.26786574	0.48165823	0.46193392	Ehmt2	1
GO:0002220	innate immune response activating cell surface receptor signaling pathway	1/67	67/14464	0.01492537	3.22209846	1.24364174	0.26786574	0.48165823	0.46193392	Nr1d1	1
GO:0006766	vitamin metabolic process	1/67	67/14464	0.01492537	3.22209846	1.24364174	0.26786574	0.48165823	0.46193392	Enpp1	1
GO:0033273	response to vitamin	1/67	67/14464	0.01492537	3.22209846	1.24364174	0.26786574	0.48165823	0.46193392	Enpp1	1
GO:0035904	aorta development	1/67	67/14464	0.01492537	3.22209846	1.24364174	0.26786574	0.48165823	0.46193392	Enpp1	1
GO:0045132	meiotic chromosome segregation	1/67	67/14464	0.01492537	3.22209846	1.24364174	0.26786574	0.48165823	0.46193392	Ehmt2	1
GO:0050810	regulation of steroid biosynthetic process	1/67	67/14464	0.01492537	3.22209846	1.24364174	0.26786574	0.48165823	0.46193392	Nr1d1	1
GO:0060612	adipose tissue development	1/67	67/14464	0.01492537	3.22209846	1.24364174	0.26786574	0.48165823	0.46193392	Enpp1	1
GO:0061951	establishment of protein localization to plasma membrane	1/67	67/14464	0.01492537	3.22209846	1.24364174	0.26786574	0.48165823	0.46193392	Sys1	1
GO:0070534	protein K63-linked ubiquitination	1/67	67/14464	0.01492537	3.22209846	1.24364174	0.26786574	0.48165823	0.46193392	Zfp598	1
GO:1902807	negative regulation of cell cycle G1/S phase transition	1/67	67/14464	0.01492537	3.22209846	1.24364174	0.26786574	0.48165823	0.46193392	Rpa2	1
GO:0006414	translational elongation	1/67	68/14464	0.01470588	3.17471466	1.2262144	0.27127291	0.48327347	0.46348301	Zfp598	1
GO:0006446	regulation of translational initiation	1/67	68/14464	0.01470588	3.17471466	1.2262144	0.27127291	0.48327347	0.46348301	Zfp598	1
GO:0033138	positive regulation of peptidyl-serine phosphorylation	1/67	68/14464	0.01470588	3.17471466	1.2262144	0.27127291	0.48327347	0.46348301	Fnip1	1
GO:0048146	positive regulation of fibroblast proliferation	1/67	68/14464	0.01470588	3.17471466	1.2262144	0.27127291	0.48327347	0.46348301	Ager	1
GO:0050830	defense response to Gram-positive bacterium	1/67	68/14464	0.01470588	3.17471466	1.2262144	0.27127291	0.48327347	0.46348301	H2-T23	1
GO:0051865	protein autoubiquitination	1/67	68/14464	0.01470588	3.17471466	1.2262144	0.27127291	0.48327347	0.46348301	Nfx1	1
GO:0060395	SMAD protein signal transduction	1/67	68/14464	0.01470588	3.17471466	1.2262144	0.27127291	0.48327347	0.46348301	Ldlrad4	1
GO:1902893	regulation of miRNA transcription	1/67	68/14464	0.01470588	3.17471466	1.2262144	0.27127291	0.48327347	0.46348301	Nfatc4	1
GO:1903828	negative regulation of protein localization	2/67	221/14464	0.00904977	1.95367056	0.97459543	0.27303116	0.48584413	0.4659484	Pkia/Cd200	2
GO:0008543	fibroblast growth factor receptor signaling pathway	1/67	69/14464	0.01449275	3.1287043	1.20910674	0.27466446	0.48706321	0.46711755	Enpp1	1
GO:0010761	fibroblast migration	1/67	69/14464	0.01449275	3.1287043	1.20910674	0.27466446	0.48706321	0.46711755	Ager	1
GO:0070509	calcium ion import	1/67	69/14464	0.01449275	3.1287043	1.20910674	0.27466446	0.48706321	0.46711755	Slc30a1	1
GO:0032008	positive regulation of TOR signaling	1/67	70/14464	0.01428571	3.08400853	1.19230772	0.27804045	0.49022922	0.47015391	Fnip1	1
GO:0043489	RNA stabilization	1/67	70/14464	0.01428571	3.08400853	1.19230772	0.27804045	0.49022922	0.47015391	Hnrnpd	1
GO:0046626	regulation of insulin receptor signaling pathway	1/67	70/14464	0.01428571	3.08400853	1.19230772	0.27804045	0.49022922	0.47015391	Enpp1	1
GO:0061614	miRNA transcription	1/67	70/14464	0.01428571	3.08400853	1.19230772	0.27804045	0.49022922	0.47015391	Nfatc4	1
GO:1903035	negative regulation of response to wounding	1/67	70/14464	0.01428571	3.08400853	1.19230772	0.27804045	0.49022922	0.47015391	Ajap1	1
GO:0002764	immune response-regulating signaling pathway	3/67	397/14464	0.00755668	1.63133952	0.87013789	0.27909279	0.49094884	0.47084406	Nr1d1/Aim2/Rftn1	3
GO:0008202	steroid metabolic process	2/67	225/14464	0.00888889	1.91893864	0.94769507	0.27991418	0.49094884	0.47084406	Nr1d1/Enpp1	2
GO:0043086	negative regulation of catalytic activity	3/67	398/14464	0.00753769	1.62724068	0.86560756	0.28035675	0.49094884	0.47084406	Pkia/Fnip1/Gpsm1	3
GO:0031102	neuron projection regeneration	1/67	71/14464	0.01408451	3.04057179	1.17580684	0.28140097	0.49094884	0.47084406	Enpp1	1
GO:0042306	regulation of protein import into nucleus	1/67	71/14464	0.01408451	3.04057179	1.17580684	0.28140097	0.49094884	0.47084406	Pkia	1
GO:0071347	cellular response to interleukin-1	1/67	71/14464	0.01408451	3.04057179	1.17580684	0.28140097	0.49094884	0.47084406	Nr1d1	1
GO:2000379	positive regulation of reactive oxygen species metabolic process	1/67	71/14464	0.01408451	3.04057179	1.17580684	0.28140097	0.49094884	0.47084406	Ager	1
GO:0044772	mitotic cell cycle phase transition	3/67	399/14464	0.0075188	1.62316238	0.86108971	0.28162132	0.49094884	0.47084406	Cdk2/Pkia/Rpa2	3
GO:0006402	mRNA catabolic process	2/67	226/14464	0.00884956	1.91044776	0.94105574	0.28163451	0.49094884	0.47084406	Hnrnpd/Xrn2	2
GO:0060828	regulation of canonical Wnt signaling pathway	2/67	226/14464	0.00884956	1.91044776	0.94105574	0.28163451	0.49094884	0.47084406	Ccdc88c/Ppp2r3a	2
GO:0002768	immune response-regulating cell surface receptor signaling pathway	2/67	227/14464	0.00881057	1.90203169	0.93445001	0.28335458	0.49137033	0.4712483	Nr1d1/Rftn1	2
GO:0010833	telomere maintenance via telomere lengthening	1/67	72/14464	0.01388889	2.99834163	1.15959411	0.28474608	0.49137033	0.4712483	Hnrnpd	1
GO:0019730	antimicrobial humoral response	1/67	72/14464	0.01388889	2.99834163	1.15959411	0.28474608	0.49137033	0.4712483	H2-T23	1
GO:0030500	regulation of bone mineralization	1/67	72/14464	0.01388889	2.99834163	1.15959411	0.28474608	0.49137033	0.4712483	Enpp1	1
GO:0032091	negative regulation of protein binding	1/67	72/14464	0.01388889	2.99834163	1.15959411	0.28474608	0.49137033	0.4712483	Nfatc4	1
GO:0042982	amyloid precursor protein metabolic process	1/67	72/14464	0.01388889	2.99834163	1.15959411	0.28474608	0.49137033	0.4712483	Ager	1
GO:0051591	response to cAMP	1/67	72/14464	0.01388889	2.99834163	1.15959411	0.28474608	0.49137033	0.4712483	Cdk2	1
GO:1903008	organelle disassembly	1/67	72/14464	0.01388889	2.99834163	1.15959411	0.28474608	0.49137033	0.4712483	Zfp598	1
GO:2000027	regulation of animal organ morphogenesis	1/67	72/14464	0.01388889	2.99834163	1.15959411	0.28474608	0.49137033	0.4712483	Ajap1	1
GO:1901987	regulation of cell cycle phase transition	3/67	402/14464	0.00746269	1.61104923	0.84761039	0.28541847	0.49197972	0.47183273	Cdk2/Pkia/Rpa2	3
GO:0006289	nucleotide-excision repair	1/67	73/14464	0.01369863	2.95726845	1.14366	0.28807585	0.49290308	0.47271828	Rpa2	1
GO:0032092	positive regulation of protein binding	1/67	73/14464	0.01369863	2.95726845	1.14366	0.28807585	0.49290308	0.47271828	Tiam1	1
GO:0032206	positive regulation of telomere maintenance	1/67	73/14464	0.01369863	2.95726845	1.14366	0.28807585	0.49290308	0.47271828	Hnrnpd	1
GO:0072676	lymphocyte migration	1/67	73/14464	0.01369863	2.95726845	1.14366	0.28807585	0.49290308	0.47271828	Cd200	1
GO:1902476	chloride transmembrane transport	1/67	73/14464	0.01369863	2.95726845	1.14366	0.28807585	0.49290308	0.47271828	Gabrg1	1
GO:0031331	positive regulation of cellular catabolic process	2/67	230/14464	0.00869565	1.87722258	0.91483092	0.28851303	0.49290308	0.47271828	Ager/Gpsm1	2
GO:0040029	epigenetic regulation of gene expression	2/67	230/14464	0.00869565	1.87722258	0.91483092	0.28851303	0.49290308	0.47271828	Cdk2/Ehmt2	2
GO:1902115	regulation of organelle assembly	2/67	230/14464	0.00869565	1.87722258	0.91483092	0.28851303	0.49290308	0.47271828	Ehmt2/Abi3bp	2
GO:0002312	B cell activation involved in immune response	1/67	74/14464	0.01351351	2.91730537	1.12799544	0.29139034	0.49521999	0.47494031	Enpp1	1
GO:0051145	smooth muscle cell differentiation	1/67	74/14464	0.01351351	2.91730537	1.12799544	0.29139034	0.49521999	0.47494031	Nfatc4	1
GO:0051298	centrosome duplication	1/67	74/14464	0.01351351	2.91730537	1.12799544	0.29139034	0.49521999	0.47494031	Cdk2	1
GO:0046394	carboxylic acid biosynthetic process	2/67	232/14464	0.00862069	1.86103963	0.90191323	0.29195016	0.49521999	0.47494031	Nr1d1/Pycr1	2
GO:0044843	cell cycle G1/S phase transition	2/67	233/14464	0.00858369	1.85305233	0.89550192	0.29366807	0.49521999	0.47494031	Cdk2/Rpa2	2
GO:0002526	acute inflammatory response	1/67	75/14464	0.01333333	2.87840796	1.11259176	0.29468964	0.49521999	0.47494031	H2-T23	1
GO:0008625	extrinsic apoptotic signaling pathway via death domain receptors	1/67	75/14464	0.01333333	2.87840796	1.11259176	0.29468964	0.49521999	0.47494031	Tnfsf10	1
GO:0010569	regulation of double-strand break repair via homologous recombination	1/67	75/14464	0.01333333	2.87840796	1.11259176	0.29468964	0.49521999	0.47494031	Rpa2	1
GO:0010827	regulation of D-glucose transmembrane transport	1/67	75/14464	0.01333333	2.87840796	1.11259176	0.29468964	0.49521999	0.47494031	Enpp1	1
GO:0042632	cholesterol homeostasis	1/67	75/14464	0.01333333	2.87840796	1.11259176	0.29468964	0.49521999	0.47494031	Nr1d1	1
GO:0044773	mitotic DNA damage checkpoint signaling	1/67	75/14464	0.01333333	2.87840796	1.11259176	0.29468964	0.49521999	0.47494031	Rpa2	1
GO:0046323	D-glucose import	1/67	75/14464	0.01333333	2.87840796	1.11259176	0.29468964	0.49521999	0.47494031	Enpp1	1
GO:0050672	negative regulation of lymphocyte proliferation	1/67	75/14464	0.01333333	2.87840796	1.11259176	0.29468964	0.49521999	0.47494031	H2-T23	1
GO:0070050	neuron cellular homeostasis	1/67	75/14464	0.01333333	2.87840796	1.11259176	0.29468964	0.49521999	0.47494031	Aim2	1
GO:1990874	vascular associated smooth muscle cell proliferation	1/67	75/14464	0.01333333	2.87840796	1.11259176	0.29468964	0.49521999	0.47494031	Enpp1	1
GO:0016053	organic acid biosynthetic process	2/67	235/14464	0.00851064	1.83728168	0.88277275	0.29710245	0.49748388	0.47711149	Nr1d1/Pycr1	2
GO:0071900	regulation of protein serine/threonine kinase activity	2/67	235/14464	0.00851064	1.83728168	0.88277275	0.29710245	0.49748388	0.47711149	Ager/Pkia	2
GO:0032945	negative regulation of mononuclear cell proliferation	1/67	76/14464	0.01315789	2.84053417	1.09744069	0.2979738	0.49748388	0.47711149	H2-T23	1
GO:0050709	negative regulation of protein secretion	1/67	76/14464	0.01315789	2.84053417	1.09744069	0.2979738	0.49748388	0.47711149	Cd200	1
GO:0055092	sterol homeostasis	1/67	76/14464	0.01315789	2.84053417	1.09744069	0.2979738	0.49748388	0.47711149	Nr1d1	1
GO:0140056	organelle localization by membrane tethering	1/67	76/14464	0.01315789	2.84053417	1.09744069	0.2979738	0.49748388	0.47711149	Stx17	1
GO:0007249	canonical NF-kappaB signal transduction	2/67	236/14464	0.00847458	1.82949658	0.87645428	0.29881884	0.49781604	0.47743004	Nr1d1/Tnfsf10	2
GO:0033044	regulation of chromosome organization	2/67	236/14464	0.00847458	1.82949658	0.87645428	0.29881884	0.49781604	0.47743004	Cdk2/Hnrnpd	2
GO:0000380	alternative mRNA splicing, via spliceosome	1/67	77/14464	0.01298701	2.80364412	1.08253434	0.30124289	0.500232	0.47974708	Arglu1	1
GO:0007589	body fluid secretion	1/67	77/14464	0.01298701	2.80364412	1.08253434	0.30124289	0.500232	0.47974708	Enpp1	1
GO:1901880	negative regulation of protein depolymerization	1/67	77/14464	0.01298701	2.80364412	1.08253434	0.30124289	0.500232	0.47974708	Ccdc88c	1
GO:0001818	negative regulation of cytokine production	2/67	239/14464	0.0083682	1.80653219	0.85767966	0.30396447	0.5040063	0.48336681	Ager/Cd200	2
GO:0008380	RNA splicing	3/67	417/14464	0.00719424	1.55309782	0.7818236	0.30446858	0.5040063	0.48336681	Srsf5/Arglu1/Luc7l2	3
GO:0044774	mitotic DNA integrity checkpoint signaling	1/67	78/14464	0.01282051	2.76769996	1.06786516	0.30449699	0.5040063	0.48336681	Rpa2	1
GO:0042113	B cell activation	2/67	240/14464	0.00833333	1.79900498	0.85148073	0.3056784	0.50441062	0.48375457	Enpp1/Fnip1	2
GO:0051896	regulation of phosphatidylinositol 3-kinase/protein kinase B signal transduction	2/67	240/14464	0.00833333	1.79900498	0.85148073	0.3056784	0.50441062	0.48375457	Aim2/Epha8	2
GO:0043410	positive regulation of MAPK cascade	3/67	419/14464	0.0071599	1.54568447	0.77324714	0.30701494	0.50441062	0.48375457	Ccdc88c/Ager/Epha8	3
GO:0031669	cellular response to nutrient levels	2/67	241/14464	0.00829876	1.79154022	0.84531096	0.30739163	0.50441062	0.48375457	Ehmt2/Fnip1	2
GO:0045444	fat cell differentiation	2/67	241/14464	0.00829876	1.79154022	0.84531096	0.30739163	0.50441062	0.48375457	Nr1d1/Enpp1	2
GO:0051965	positive regulation of synapse assembly	1/67	79/14464	0.01265823	2.73266578	1.05342593	0.30773616	0.50441062	0.48375457	Il1rapl1	1
GO:0071677	positive regulation of mononuclear cell migration	1/67	79/14464	0.01265823	2.73266578	1.05342593	0.30773616	0.50441062	0.48375457	Ager	1
GO:0097009	energy homeostasis	1/67	79/14464	0.01265823	2.73266578	1.05342593	0.30773616	0.50441062	0.48375457	Enpp1	1
GO:1904063	negative regulation of cation transmembrane transport	1/67	79/14464	0.01265823	2.73266578	1.05342593	0.30773616	0.50441062	0.48375457	Slc30a1	1
GO:0007264	small GTPase-mediated signal transduction	3/67	420/14464	0.00714286	1.54200426	0.76897555	0.30828854	0.50441062	0.48375457	Ccdc88c/Cdk2/Tiam1	3
GO:0071695	anatomical structure maturation	2/67	242/14464	0.00826446	1.78413717	0.83917005	0.30910413	0.50441062	0.48375457	Epha8/Enpp1	2
GO:0045785	positive regulation of cell adhesion	3/67	422/14464	0.007109	1.53469619	0.76046533	0.31083652	0.50441062	0.48375457	H2-T23/Ager/Abi3bp	3
GO:0010507	negative regulation of autophagy	1/67	80/14464	0.0125	2.69850746	1.03920974	0.31096047	0.50441062	0.48375457	Ehmt2	1
GO:0021766	hippocampus development	1/67	80/14464	0.0125	2.69850746	1.03920974	0.31096047	0.50441062	0.48375457	Xrn2	1
GO:0033627	cell adhesion mediated by integrin	1/67	80/14464	0.0125	2.69850746	1.03920974	0.31096047	0.50441062	0.48375457	Epha8	1
GO:0045685	regulation of glial cell differentiation	1/67	80/14464	0.0125	2.69850746	1.03920974	0.31096047	0.50441062	0.48375457	Nr1d1	1
GO:0070227	lymphocyte apoptotic process	1/67	80/14464	0.0125	2.69850746	1.03920974	0.31096047	0.50441062	0.48375457	Fnip1	1
GO:0071230	cellular response to amino acid stimulus	1/67	80/14464	0.0125	2.69850746	1.03920974	0.31096047	0.50441062	0.48375457	Hnrnpd	1
GO:1902369	negative regulation of RNA catabolic process	1/67	80/14464	0.0125	2.69850746	1.03920974	0.31096047	0.50441062	0.48375457	Hnrnpd	1
GO:0007283	spermatogenesis	3/67	423/14464	0.0070922	1.53106806	0.75622659	0.31211086	0.50574431	0.48503365	Spata24/Ehmt2/Xrn2	3
GO:0021510	spinal cord development	1/67	81/14464	0.01234568	2.66519256	1.02520997	0.31416999	0.5070568	0.48629239	Enpp1	1
GO:0045824	negative regulation of innate immune response	1/67	81/14464	0.01234568	2.66519256	1.02520997	0.31416999	0.5070568	0.48629239	H2-T23	1
GO:0070664	negative regulation of leukocyte proliferation	1/67	81/14464	0.01234568	2.66519256	1.02520997	0.31416999	0.5070568	0.48629239	H2-T23	1
GO:0060326	cell chemotaxis	2/67	245/14464	0.00816327	1.76229059	0.82091779	0.31423702	0.5070568	0.48629239	Slamf9/Tiam1	2
GO:0031346	positive regulation of cell projection organization	3/67	426/14464	0.00704225	1.52028589	0.74357508	0.31593514	0.50831362	0.48749775	Slc30a1/Tiam1/Il1rapl1	3
GO:0032642	regulation of chemokine production	1/67	82/14464	0.01219512	2.63269021	1.0114203	0.31736477	0.50831362	0.48749775	Ager	1
GO:0033209	tumor necrosis factor-mediated signaling pathway	1/67	82/14464	0.01219512	2.63269021	1.0114203	0.31736477	0.50831362	0.48749775	Aim2	1
GO:0061157	mRNA destabilization	1/67	82/14464	0.01219512	2.63269021	1.0114203	0.31736477	0.50831362	0.48749775	Hnrnpd	1
GO:1901222	regulation of non-canonical NF-kappaB signal transduction	1/67	82/14464	0.01219512	2.63269021	1.0114203	0.31736477	0.50831362	0.48749775	Ager	1
GO:2000628	regulation of miRNA metabolic process	1/67	82/14464	0.01219512	2.63269021	1.0114203	0.31736477	0.50831362	0.48749775	Nfatc4	1
GO:0032984	protein-containing complex disassembly	2/67	247/14464	0.00809717	1.74802103	0.80888893	0.31765478	0.50831362	0.48749775	Ccdc88c/Stx17	2
GO:0097305	response to alcohol	2/67	247/14464	0.00809717	1.74802103	0.80888893	0.31765478	0.50831362	0.48749775	Hnrnpd/Ehmt2	2
GO:0035282	segmentation	1/67	83/14464	0.01204819	2.60097105	0.99783463	0.3205449	0.51165177	0.49069919	Ppp2r3a	1
GO:1900076	regulation of cellular response to insulin stimulus	1/67	83/14464	0.01204819	2.60097105	0.99783463	0.3205449	0.51165177	0.49069919	Enpp1	1
GO:0033002	muscle cell proliferation	2/67	249/14464	0.00803213	1.7339807	0.79696908	0.32106896	0.51165177	0.49069919	Ager/Enpp1	2
GO:0070372	regulation of ERK1 and ERK2 cascade	2/67	249/14464	0.00803213	1.7339807	0.79696908	0.32106896	0.51165177	0.49069919	Ager/Tiam1	2
GO:0002253	activation of immune response	3/67	431/14464	0.00696056	1.50264917	0.72270146	0.32231223	0.51310243	0.49209044	Nr1d1/Aim2/Rftn1	3
GO:0034766	negative regulation of monoatomic ion transmembrane transport	1/67	84/14464	0.01190476	2.57000711	0.98444716	0.32371043	0.51418288	0.49312665	Slc30a1	1
GO:0071901	negative regulation of protein serine/threonine kinase activity	1/67	84/14464	0.01190476	2.57000711	0.98444716	0.32371043	0.51418288	0.49312665	Pkia	1
GO:0002683	negative regulation of immune system process	3/67	433/14464	0.00692841	1.49570852	0.71442495	0.32486388	0.51418288	0.49312665	Nr1d1/H2-T23/Cd200	3
GO:0010721	negative regulation of cell development	2/67	252/14464	0.00793651	1.71333807	0.77928922	0.326183	0.51418288	0.49312665	Nr1d1/Nfatc4	2
GO:0022409	positive regulation of cell-cell adhesion	2/67	252/14464	0.00793651	1.71333807	0.77928922	0.326183	0.51418288	0.49312665	H2-T23/Ager	2
GO:0019915	lipid storage	1/67	85/14464	0.01176471	2.53977173	0.97125229	0.32686143	0.51418288	0.49312665	Enpp1	1
GO:0022406	membrane docking	1/67	85/14464	0.01176471	2.53977173	0.97125229	0.32686143	0.51418288	0.49312665	Stx17	1
GO:0046651	lymphocyte proliferation	2/67	253/14464	0.00790514	1.70656598	0.77344825	0.32788564	0.51418288	0.49312665	H2-T23/Ager	2
GO:0060560	developmental growth involved in morphogenesis	2/67	254/14464	0.00787402	1.69984722	0.76763303	0.32958723	0.51418288	0.49312665	Ppp2r3a/Tiam1	2
GO:0048232	male gamete generation	3/67	437/14464	0.00686499	1.48201783	0.69799452	0.32996796	0.51418288	0.49312665	Spata24/Ehmt2/Xrn2	3
GO:0000041	transition metal ion transport	1/67	86/14464	0.01162791	2.5102395	0.95824465	0.32999797	0.51418288	0.49312665	Slc30a1	1
GO:0001570	vasculogenesis	1/67	86/14464	0.01162791	2.5102395	0.95824465	0.32999797	0.51418288	0.49312665	Enpp1	1
GO:0006821	chloride transport	1/67	86/14464	0.01162791	2.5102395	0.95824465	0.32999797	0.51418288	0.49312665	Gabrg1	1
GO:0007127	meiosis I	1/67	86/14464	0.01162791	2.5102395	0.95824465	0.32999797	0.51418288	0.49312665	Ehmt2	1
GO:0030168	platelet activation	1/67	86/14464	0.01162791	2.5102395	0.95824465	0.32999797	0.51418288	0.49312665	Fundc2	1
GO:0030512	negative regulation of transforming growth factor beta receptor signaling pathway	1/67	86/14464	0.01162791	2.5102395	0.95824465	0.32999797	0.51418288	0.49312665	Ldlrad4	1
GO:0032609	type II interferon production	1/67	86/14464	0.01162791	2.5102395	0.95824465	0.32999797	0.51418288	0.49312665	Enpp1	1
GO:0043242	negative regulation of protein-containing complex disassembly	1/67	86/14464	0.01162791	2.5102395	0.95824465	0.32999797	0.51418288	0.49312665	Ccdc88c	1
GO:0046849	bone remodeling	1/67	86/14464	0.01162791	2.5102395	0.95824465	0.32999797	0.51418288	0.49312665	Enpp1	1
GO:0070167	regulation of biomineral tissue development	1/67	86/14464	0.01162791	2.5102395	0.95824465	0.32999797	0.51418288	0.49312665	Enpp1	1
GO:0070252	actin-mediated cell contraction	1/67	86/14464	0.01162791	2.5102395	0.95824465	0.32999797	0.51418288	0.49312665	Ccdc88c	1
GO:0030072	peptide hormone secretion	2/67	256/14464	0.0078125	1.68656716	0.75607892	0.3329871	0.51488273	0.49379785	Nr1d1/Tiam1	2
GO:0050878	regulation of body fluid levels	2/67	256/14464	0.0078125	1.68656716	0.75607892	0.3329871	0.51488273	0.49379785	Fundc2/Enpp1	2
GO:0090150	establishment of protein localization to membrane	2/67	256/14464	0.0078125	1.68656716	0.75607892	0.3329871	0.51488273	0.49379785	Rftn1/Sys1	2
GO:0032755	positive regulation of interleukin-6 production	1/67	87/14464	0.01149425	2.48138617	0.94541909	0.33312011	0.51488273	0.49379785	Ager	1
GO:0048477	oogenesis	1/67	87/14464	0.01149425	2.48138617	0.94541909	0.33312011	0.51488273	0.49379785	Ehmt2	1
GO:0050779	RNA destabilization	1/67	87/14464	0.01149425	2.48138617	0.94541909	0.33312011	0.51488273	0.49379785	Hnrnpd	1

GO:0051881	regulation of mitochondrial membrane potential	1/67	87/14464	0.01149425	2.48138617	0.94541909	0.33312011	0.51488273	0.49379785	Pycr1	1
GO:0061014	positive regulation of mRNA catabolic process	1/67	87/14464	0.01149425	2.48138617	0.94541909	0.33312011	0.51488273	0.49379785	Hnrnpd	1
GO:0021537	telencephalon development	2/67	257/14464	0.0077821	1.68000465	0.75033956	0.33468534	0.51678368	0.49562095	Bhlhe22/Xrn2	2
GO:0034644	cellular response to UV	1/67	88/14464	0.01136364	2.4531886	0.93277066	0.33622792	0.51760961	0.49641305	Nfatc4	1
GO:0043406	positive regulation of MAP kinase activity	1/67	88/14464	0.01136364	2.4531886	0.93277066	0.33622792	0.51760961	0.49641305	Ager	1
GO:2000106	regulation of leukocyte apoptotic process	1/67	88/14464	0.01136364	2.4531886	0.93277066	0.33622792	0.51760961	0.49641305	Fnip1	1
GO:0032943	mononuclear cell proliferation	2/67	259/14464	0.00772201	1.66703164	0.73893509	0.3380783	0.51977571	0.49849045	H2-T23/Ager	2
GO:0019218	regulation of steroid metabolic process	1/67	89/14464	0.01123596	2.42562469	0.9202946	0.33932145	0.51977571	0.49849045	Nr1d1	1
GO:0021954	central nervous system neuron development	1/67	89/14464	0.01123596	2.42562469	0.9202946	0.33932145	0.51977571	0.49849045	Bhlhe22	1
GO:0098661	inorganic anion transmembrane transport	1/67	89/14464	0.01123596	2.42562469	0.9202946	0.33932145	0.51977571	0.49849045	Gabrg1	1
GO:1903312	negative regulation of mRNA metabolic process	1/67	89/14464	0.01123596	2.42562469	0.9202946	0.33932145	0.51977571	0.49849045	Hnrnpd	1
GO:0051251	positive regulation of lymphocyte activation	2/67	260/14464	0.00769231	1.66061998	0.73326953	0.33977296	0.51995048	0.49865806	H2-T23/Ager	2
GO:0007409	axonogenesis	3/67	446/14464	0.00672646	1.45211164	0.6616079	0.34145184	0.52138302	0.50003194	Epha8/Tiam1/Bhlhe22	3
GO:0046883	regulation of hormone secretion	2/67	261/14464	0.00766284	1.65425745	0.72762813	0.34146638	0.52138302	0.50003194	Nr1d1/Tiam1	2
GO:0008589	regulation of smoothened signaling pathway	1/67	90/14464	0.01111111	2.3986733	0.90798633	0.34240079	0.52138302	0.50003194	Enpp1	1
GO:0010951	negative regulation of endopeptidase activity	1/67	90/14464	0.01111111	2.3986733	0.90798633	0.34240079	0.52138302	0.50003194	Fnip1	1
GO:0070555	response to interleukin-1	1/67	90/14464	0.01111111	2.3986733	0.90798633	0.34240079	0.52138302	0.50003194	Nr1d1	1
GO:0002790	peptide secretion	2/67	263/14464	0.00760456	1.64167754	0.71641694	0.3448494	0.5234642	0.50202789	Nr1d1/Tiam1	2
GO:0006091	generation of precursor metabolites and energy	3/67	449/14464	0.00668151	1.44240933	0.64965307	0.34527841	0.5234642	0.50202789	Nr1d1/Nfatc4/Enpp1	3
GO:0061982	meiosis I cell cycle process	1/67	91/14464	0.01098901	2.37231425	0.89584144	0.34546599	0.5234642	0.50202789	Ehmt2	1
GO:0071229	cellular response to acid chemical	1/67	91/14464	0.01098901	2.37231425	0.89584144	0.34546599	0.5234642	0.50202789	Hnrnpd	1
GO:1904019	epithelial cell apoptotic process	1/67	91/14464	0.01098901	2.37231425	0.89584144	0.34546599	0.5234642	0.50202789	Ager	1
GO:0007346	regulation of mitotic cell cycle	3/67	450/14464	0.00666667	1.43920398	0.64568703	0.34655367	0.52459647	0.50311379	Cdk2/Pkia/Rpa2	3
GO:0044344	cellular response to fibroblast growth factor stimulus	1/67	92/14464	0.01086957	2.34652823	0.88385569	0.34851711	0.52601848	0.50447757	Enpp1	1
GO:0046889	positive regulation of lipid biosynthetic process	1/67	92/14464	0.01086957	2.34652823	0.88385569	0.34851711	0.52601848	0.50447757	Nr1d1	1
GO:0048678	response to axon injury	1/67	92/14464	0.01086957	2.34652823	0.88385569	0.34851711	0.52601848	0.50447757	Enpp1	1
GO:0043280	positive regulation of cysteine-type endopeptidase activity involved in apoptotic process	1/67	93/14464	0.01075269	2.32129674	0.872025	0.35155422	0.52853176	0.50688794	Tnfsf10	1
GO:0098659	inorganic cation import across plasma membrane	1/67	93/14464	0.01075269	2.32129674	0.872025	0.35155422	0.52853176	0.50688794	Slc30a1	1
GO:0099587	inorganic ion import across plasma membrane	1/67	93/14464	0.01075269	2.32129674	0.872025	0.35155422	0.52853176	0.50688794	Slc30a1	1
GO:1901879	regulation of protein depolymerization	1/67	93/14464	0.01075269	2.32129674	0.872025	0.35155422	0.52853176	0.50688794	Ccdc88c	1
GO:0010817	regulation of hormone levels	3/67	455/14464	0.00659341	1.42338855	0.6259964	0.35292733	0.52952839	0.50784375	Nr1d1/Tiam1/Enpp1	3
GO:0031667	response to nutrient levels	3/67	455/14464	0.00659341	1.42338855	0.6259964	0.35292733	0.52952839	0.50784375	Ehmt2/Enpp1/Fnip1	3
GO:0006401	RNA catabolic process	2/67	268/14464	0.00746269	1.61104923	0.68879692	0.35328352	0.52952839	0.50784375	Hnrnpd/Xrn2	2
GO:0031110	regulation of microtubule polymerization or depolymerization	1/67	94/14464	0.0106383	2.2966021	0.86034543	0.35457738	0.52952839	0.50784375	Ccdc88c	1
GO:0032355	response to estradiol	1/67	94/14464	0.0106383	2.2966021	0.86034543	0.35457738	0.52952839	0.50784375	Hnrnpd	1
GO:0060349	bone morphogenesis	1/67	94/14464	0.0106383	2.2966021	0.86034543	0.35457738	0.52952839	0.50784375	Enpp1	1
GO:0060070	canonical Wnt signaling pathway	2/67	269/14464	0.00743494	1.6050602	0.68334122	0.35496614	0.52952839	0.50784375	Ccdc88c/Ppp2r3a	2
GO:1903311	regulation of mRNA metabolic process	2/67	269/14464	0.00743494	1.6050602	0.68334122	0.35496614	0.52952839	0.50784375	Hnrnpd/Arglu1	2
GO:0002708	positive regulation of lymphocyte mediated immunity	1/67	95/14464	0.01052632	2.27242734	0.84881318	0.35758666	0.53137999	0.50961952	H2-T23	1
GO:0010586	miRNA metabolic process	1/67	95/14464	0.01052632	2.27242734	0.84881318	0.35758666	0.53137999	0.50961952	Nfatc4	1
GO:0071482	cellular response to light stimulus	1/67	95/14464	0.01052632	2.27242734	0.84881318	0.35758666	0.53137999	0.50961952	Nfatc4	1
GO:1990542	mitochondrial transmembrane transport	1/67	95/14464	0.01052632	2.27242734	0.84881318	0.35758666	0.53137999	0.50961952	Tst	1
GO:0015833	peptide transport	2/67	272/14464	0.00735294	1.58735733	0.66710721	0.36000518	0.53445856	0.51257202	Nr1d1/Tiam1	2
GO:0050764	regulation of phagocytosis	1/67	96/14464	0.01041667	2.24875622	0.8374246	0.36058212	0.53479985	0.51289934	Ager	1
GO:0070371	ERK1 and ERK2 cascade	2/67	273/14464	0.00732601	1.58154284	0.6617396	0.36168184	0.53563562	0.51370089	Ager/Tiam1	2
GO:0071407	cellular response to organic cyclic compound	3/67	462/14464	0.00649351	1.40182206	0.59881176	0.36184081	0.53563562	0.51370089	Hnrnpd/Nfatc4/Ehmt2	3
GO:0008344	adult locomotory behavior	1/67	97/14464	0.01030928	2.22557317	0.82617616	0.36356381	0.53612616	0.51417133	Enpp1	1
GO:0016239	positive regulation of macroautophagy	1/67	97/14464	0.01030928	2.22557317	0.82617616	0.36356381	0.53612616	0.51417133	Gpsm1	1
GO:0050772	positive regulation of axonogenesis	1/67	97/14464	0.01030928	2.22557317	0.82617616	0.36356381	0.53612616	0.51417133	Tiam1	1
GO:0098656	monoatomic anion transmembrane transport	1/67	97/14464	0.01030928	2.22557317	0.82617616	0.36356381	0.53612616	0.51417133	Gabrg1	1
GO:0002718	regulation of cytokine production involved in immune response	1/67	98/14464	0.01020408	2.20286323	0.81506445	0.36653181	0.53690639	0.51491961	H2-T23	1
GO:0007040	lysosome organization	1/67	98/14464	0.01020408	2.20286323	0.81506445	0.36653181	0.53690639	0.51491961	Fnip1	1
GO:0032204	regulation of telomere maintenance	1/67	98/14464	0.01020408	2.20286323	0.81506445	0.36653181	0.53690639	0.51491961	Hnrnpd	1
GO:0043473	pigmentation	1/67	98/14464	0.01020408	2.20286323	0.81506445	0.36653181	0.53690639	0.51491961	Enpp1	1
GO:0071774	response to fibroblast growth factor	1/67	98/14464	0.01020408	2.20286323	0.81506445	0.36653181	0.53690639	0.51491961	Enpp1	1
GO:0080171	lytic vacuole organization	1/67	98/14464	0.01020408	2.20286323	0.81506445	0.36653181	0.53690639	0.51491961	Fnip1	1
GO:0090263	positive regulation of canonical Wnt signaling pathway	1/67	98/14464	0.01020408	2.20286323	0.81506445	0.36653181	0.53690639	0.51491961	Ppp2r3a	1
GO:0010608	post-transcriptional regulation of gene expression	3/67	468/14464	0.00641026	1.38384998	0.57585491	0.36946905	0.53765646	0.51563897	Hnrnpd/Pcif1/Zfp598	3
GO:0002444	myeloid leukocyte mediated immunity	1/67	99/14464	0.01010101	2.18061209	0.80408619	0.36948617	0.53765646	0.51563897	H2-T23	1
GO:0006096	glycolytic process	1/67	99/14464	0.01010101	2.18061209	0.80408619	0.36948617	0.53765646	0.51563897	Enpp1	1
GO:0033135	regulation of peptidyl-serine phosphorylation	1/67	99/14464	0.01010101	2.18061209	0.80408619	0.36948617	0.53765646	0.51563897	Fnip1	1
GO:0061041	regulation of wound healing	1/67	99/14464	0.01010101	2.18061209	0.80408619	0.36948617	0.53765646	0.51563897	Ajap1	1
GO:1903432	regulation of TORC1 signaling	1/67	99/14464	0.01010101	2.18061209	0.80408619	0.36948617	0.53765646	0.51563897	Fnip1	1
GO:2001243	negative regulation of intrinsic apoptotic signaling pathway	1/67	99/14464	0.01010101	2.18061209	0.80408619	0.36948617	0.53765646	0.51563897	Pycr1	1
GO:0043491	phosphatidylinositol 3-kinase/protein kinase B signal transduction	2/67	278/14464	0.00719424	1.55309782	0.63522116	0.37004158	0.53787249	0.51584615	Aim2/Epha8	2
GO:0006310	DNA recombination	2/67	279/14464	0.00716846	1.54753116	0.62998015	0.37170867	0.53787249	0.51584615	Rpa2/Xrn2	2
GO:0002009	morphogenesis of an epithelium	3/67	470/14464	0.00638298	1.37796126	0.56827149	0.37200887	0.53787249	0.51584615	Ajap1/Nfatc4/Enpp1	3
GO:0002698	negative regulation of immune effector process	1/67	100/14464	0.01	2.15880597	0.7932382	0.37242696	0.53787249	0.51584615	H2-T23	1
GO:0008645	hexose transmembrane transport	1/67	100/14464	0.01	2.15880597	0.7932382	0.37242696	0.53787249	0.51584615	Enpp1	1
GO:0014910	regulation of smooth muscle cell migration	1/67	100/14464	0.01	2.15880597	0.7932382	0.37242696	0.53787249	0.51584615	Ager	1
GO:0046928	regulation of neurotransmitter secretion	1/67	100/14464	0.01	2.15880597	0.7932382	0.37242696	0.53787249	0.51584615	Slc30a1	1
GO:1904659	D-glucose transmembrane transport	1/67	100/14464	0.01	2.15880597	0.7932382	0.37242696	0.53787249	0.51584615	Enpp1	1
GO:0034655	nucleobase-containing compound catabolic process	3/67	471/14464	0.00636943	1.37503565	0.56449251	0.37327818	0.53859707	0.51654106	Hnrnpd/Enpp1/Xrn2	3
GO:0003300	cardiac muscle hypertrophy	1/67	101/14464	0.00990099	2.13743165	0.78251741	0.37535424	0.53906885	0.51699352	Tiam1	1
GO:0021761	limbic system development	1/67	101/14464	0.00990099	2.13743165	0.78251741	0.37535424	0.53906885	0.51699352	Xrn2	1
GO:0035710	CD4-positive, alpha-beta T cell activation	1/67	101/14464	0.00990099	2.13743165	0.78251741	0.37535424	0.53906885	0.51699352	Ager	1
GO:0043255	regulation of carbohydrate biosynthetic process	1/67	101/14464	0.00990099	2.13743165	0.78251741	0.37535424	0.53906885	0.51699352	Enpp1	1
GO:0050852	T cell receptor signaling pathway	1/67	101/14464	0.00990099	2.13743165	0.78251741	0.37535424	0.53906885	0.51699352	Rftn1	1
GO:0000422	autophagy of mitochondrion	1/67	102/14464	0.00980392	2.11647644	0.77192087	0.37826806	0.54036414	0.51823577	Fundc2	1
GO:0002824	positive regulation of adaptive immune response based on somatic recombination of immune receptors b	1/67	102/14464	0.00980392	2.11647644	0.77192087	0.37826806	0.54036414	0.51823577	H2-T23	1
GO:0015749	monosaccharide transmembrane transport	1/67	102/14464	0.00980392	2.11647644	0.77192087	0.37826806	0.54036414	0.51823577	Enpp1	1
GO:0042476	odontogenesis	1/67	102/14464	0.00980392	2.11647644	0.77192087	0.37826806	0.54036414	0.51823577	Enpp1	1
GO:0099068	postsynapse assembly	1/67	102/14464	0.00980392	2.11647644	0.77192087	0.37826806	0.54036414	0.51823577	Abi3bp	1
GO:0002696	positive regulation of leukocyte activation	2/67	283/14464	0.00706714	1.52565793	0.60921923	0.3783601	0.54036414	0.51823577	H2-T23/Ager	2
GO:0048193	Golgi vesicle transport	2/67	284/14464	0.00704225	1.52028589	0.60407894	0.38001863	0.54186407	0.51967427	Sys1/Stx17	2
GO:0006892	post-Golgi vesicle-mediated transport	1/67	103/14464	0.00970874	2.09592813	0.7614457	0.3811685	0.54186407	0.51967427	Sys1	1
GO:0033555	multicellular organismal response to stress	1/67	103/14464	0.00970874	2.09592813	0.7614457	0.3811685	0.54186407	0.51967427	Gja4	1
GO:0046032	ADP catabolic process	1/67	103/14464	0.00970874	2.09592813	0.7614457	0.3811685	0.54186407	0.51967427	Enpp1	1
GO:2000278	regulation of DNA biosynthetic process	1/67	103/14464	0.00970874	2.09592813	0.7614457	0.3811685	0.54186407	0.51967427	Hnrnpd	1
GO:0014897	striated muscle hypertrophy	1/67	104/14464	0.00961538	2.07577497	0.75108913	0.3840556	0.54304031	0.52080235	Tiam1	1
GO:0031330	negative regulation of cellular catabolic process	1/67	104/14464	0.00961538	2.07577497	0.75108913	0.3840556	0.54304031	0.52080235	Ehmt2	1
GO:0043279	response to alkaloid	1/67	104/14464	0.00961538	2.07577497	0.75108913	0.3840556	0.54304031	0.52080235	Ehmt2	1
GO:0098754	detoxification	1/67	104/14464	0.00961538	2.07577497	0.75108913	0.3840556	0.54304031	0.52080235	Slc30a1	1
GO:0051054	positive regulation of DNA metabolic process	2/67	287/14464	0.00696864	1.5043944	0.58877537	0.38498354	0.54304031	0.52080235	Cdk2/Hnrnpd	2
GO:0070661	leukocyte proliferation	2/67	287/14464	0.00696864	1.5043944	0.58877537	0.38498354	0.54304031	0.52080235	H2-T23/Ager	2
GO:1901888	regulation of cell junction assembly	2/67	288/14464	0.00694444	1.49917081	0.58371273	0.38663488	0.54304031	0.52080235	Abi3bp/Il1rapl1	2
GO:0002832	negative regulation of response to biotic stimulus	1/67	105/14464	0.00952381	2.05600569	0.74084849	0.38692944	0.54304031	0.52080235	H2-T23	1
GO:0008630	intrinsic apoptotic signaling pathway in response to DNA damage	1/67	105/14464	0.00952381	2.05600569	0.74084849	0.38692944	0.54304031	0.52080235	Nfatc4	1
GO:0009137	purine nucleoside diphosphate catabolic process	1/67	105/14464	0.00952381	2.05600569	0.74084849	0.38692944	0.54304031	0.52080235	Enpp1	1
GO:0009181	purine ribonucleoside diphosphate catabolic process	1/67	105/14464	0.00952381	2.05600569	0.74084849	0.38692944	0.54304031	0.52080235	Enpp1	1
GO:0019364	pyridine nucleotide catabolic process	1/67	105/14464	0.00952381	2.05600569	0.74084849	0.38692944	0.54304031	0.52080235	Enpp1	1
GO:0036503	ERAD pathway	1/67	105/14464	0.00952381	2.05600569	0.74084849	0.38692944	0.54304031	0.52080235	Foxred2	1
GO:0038061	non-canonical NF-kappaB signal transduction	1/67	105/14464	0.00952381	2.05600569	0.74084849	0.38692944	0.54304031	0.52080235	Ager	1
GO:0014896	muscle hypertrophy	1/67	106/14464	0.00943396	2.03660941	0.73072118	0.38979006	0.54556447	0.52322314	Tiam1	1
GO:0048024	regulation of mRNA splicing, via spliceosome	1/67	106/14464	0.00943396	2.03660941	0.73072118	0.38979006	0.54556447	0.52322314	Arglu1	1
GO:0060840	artery development	1/67	106/14464	0.00943396	2.03660941	0.73072118	0.38979006	0.54556447	0.52322314	Enpp1	1
GO:0034329	cell junction assembly	3/67	485/14464	0.00618557	1.3353439	0.51245274	0.39099941	0.54657731	0.52419451	Gabrg1/Abi3bp/Il1rapl1	3
GO:0048511	rhythmic process	2/67	291/14464	0.00687285	1.48371544	0.56863835	0.39157778	0.54657731	0.52419451	Nr1d1/Hnrnpd	2
GO:0141188	nucleic acid catabolic process	2/67	291/14464	0.00687285	1.48371544	0.56863835	0.39157778	0.54657731	0.52419451	Hnrnpd/Xrn2	2
GO:0032963	collagen metabolic process	1/67	107/14464	0.00934579	2.01757567	0.72070469	0.39263754	0.54689038	0.52449475	Ager	1
GO:0042274	ribosomal small subunit biogenesis	1/67	107/14464	0.00934579	2.01757567	0.72070469	0.39263754	0.54689038	0.52449475	Rrp9	1
GO:0071375	cellular response to peptide hormone stimulus	2/67	292/14464	0.00684932	1.47863423	0.56365089	0.39322163	0.54689038	0.52449475	Cdk2/Enpp1	2
GO:1903037	regulation of leukocyte cell-cell adhesion	2/67	292/14464	0.00684932	1.47863423	0.56365089	0.39322163	0.54689038	0.52449475	H2-T23/Ager	2
GO:0030316	osteoclast differentiation	1/67	108/14464	0.00925926	1.99889442	0.71079658	0.39547192	0.54804158	0.52559881	Enpp1	1
GO:0042471	ear morphogenesis	1/67	108/14464	0.00925926	1.99889442	0.71079658	0.39547192	0.54804158	0.52559881	Enpp1	1
GO:0046031	ADP metabolic process	1/67	108/14464	0.00925926	1.99889442	0.71079658	0.39547192	0.54804158	0.52559881	Enpp1	1
GO:0072526	pyridine-containing compound catabolic process	1/67	108/14464	0.00925926	1.99889442	0.71079658	0.39547192	0.54804158	0.52559881	Enpp1	1
GO:0055074	calcium ion homeostasis	2/67	295/14464	0.00677966	1.46359727	0.54879844	0.39814164	0.54997307	0.5274512	Slc30a1/Enpp1	2
GO:0001578	microtubule bundle formation	1/67	109/14464	0.00917431	1.98055594	0.70099451	0.39829328	0.54997307	0.5274512	Ccdc88c	1
GO:0009191	ribonucleoside diphosphate catabolic process	1/67	109/14464	0.00917431	1.98055594	0.70099451	0.39829328	0.54997307	0.5274512	Enpp1	1
GO:0046683	response to organophosphorus	1/67	109/14464	0.00917431	1.98055594	0.70099451	0.39829328	0.54997307	0.5274512	Cdk2	1
GO:0002702	positive regulation of production of molecular mediator of immune response	1/67	110/14464	0.00909091	1.96255088	0.69129618	0.40110166	0.55131517	0.52873834	H2-T23	1
GO:0002821	positive regulation of adaptive immune response	1/67	110/14464	0.00909091	1.96255088	0.69129618	0.40110166	0.55131517	0.52873834	H2-T23	1
GO:0030048	actin filament-based movement	1/67	110/14464	0.00909091	1.96255088	0.69129618	0.40110166	0.55131517	0.52873834	Ccdc88c	1
GO:0034219	carbohydrate transmembrane transport	1/67	110/14464	0.00909091	1.96255088	0.69129618	0.40110166	0.55131517	0.52873834	Enpp1	1
GO:0050868	negative regulation of T cell activation	1/67	110/14464	0.00909091	1.96255088	0.69129618	0.40110166	0.55131517	0.52873834	H2-T23	1
GO:0050863	regulation of T cell activation	2/67	297/14464	0.00673401	1.45374139	0.53898698	0.40141182	0.55131517	0.52873834	H2-T23/Ager	2
GO:0009134	nucleoside diphosphate catabolic process	1/67	111/14464	0.00900901	1.94487024	0.6816994	0.40389713	0.55324932	0.53059329	Enpp1	1
GO:0034502	protein localization to chromosome	1/67	111/14464	0.00900901	1.94487024	0.6816994	0.40389713	0.55324932	0.53059329	Rpa2	1
GO:0048661	positive regulation of smooth muscle cell proliferation	1/67	111/14464	0.00900901	1.94487024	0.6816994	0.40389713	0.55324932	0.53059329	Ager	1
GO:0006413	translational initiation	1/67	112/14464	0.00892857	1.92750533	0.67220201	0.40667975	0.55459601	0.53188484	Zfp598	1
GO:0007265	Ras protein signal transduction	1/67	112/14464	0.00892857	1.92750533	0.67220201	0.40667975	0.55459601	0.53188484	Cdk2	1
GO:0009791	post-embryonic development	1/67	112/14464	0.00892857	1.92750533	0.67220201	0.40667975	0.55459601	0.53188484	Enpp1	1
GO:0048145	regulation of fibroblast proliferation	1/67	112/14464	0.00892857	1.92750533	0.67220201	0.40667975	0.55459601	0.53188484	Ager	1
GO:0071887	leukocyte apoptotic process	1/67	112/14464	0.00892857	1.92750533	0.67220201	0.40667975	0.55459601	0.53188484	Fnip1	1
GO:0044770	cell cycle phase transition	3/67	498/14464	0.0060241	1.30048552	0.46551406	0.40735451	0.55502502	0.53229628	Cdk2/Pkia/Rpa2	3
GO:0071396	cellular response to lipid	3/67	499/14464	0.00601202	1.29787934	0.46195618	0.40860789	0.55556301	0.53281223	Nr1d1/Hnrnpd/Nfatc4	3
GO:0009135	purine nucleoside diphosphate metabolic process	1/67	113/14464	0.00884956	1.91044776	0.66280193	0.40944957	0.55556301	0.53281223	Enpp1	1
GO:0009179	purine ribonucleoside diphosphate metabolic process	1/67	113/14464	0.00884956	1.91044776	0.66280193	0.40944957	0.55556301	0.53281223	Enpp1	1
GO:0038202	TORC1 signaling	1/67	113/14464	0.00884956	1.91044776	0.66280193	0.40944957	0.55556301	0.53281223	Fnip1	1
GO:0097193	intrinsic apoptotic signaling pathway	2/67	302/14464	0.00662252	1.42967283	0.51476577	0.40955197	0.55556301	0.53281223	Pycr1/Nfatc4	2
GO:0050867	positive regulation of cell activation	2/67	303/14464	0.00660066	1.42495444	0.50997315	0.41117381	0.55573968	0.53298166	H2-T23/Ager	2
GO:0000077	DNA damage checkpoint signaling	1/67	114/14464	0.00877193	1.89368945	0.65349715	0.41220665	0.55573968	0.53298166	Rpa2	1
GO:0001952	regulation of cell-matrix adhesion	1/67	114/14464	0.00877193	1.89368945	0.65349715	0.41220665	0.55573968	0.53298166	Ajap1	1
GO:0007259	cell surface receptor signaling pathway via JAK-STAT	1/67	114/14464	0.00877193	1.89368945	0.65349715	0.41220665	0.55573968	0.53298166	Ager	1
GO:0008637	apoptotic mitochondrial changes	1/67	114/14464	0.00877193	1.89368945	0.65349715	0.41220665	0.55573968	0.53298166	Tnfsf10	1
GO:0008643	carbohydrate transport	1/67	114/14464	0.00877193	1.89368945	0.65349715	0.41220665	0.55573968	0.53298166	Enpp1	1
GO:1990830	cellular response to leukemia inhibitory factor	1/67	114/14464	0.00877193	1.89368945	0.65349715	0.41220665	0.55573968	0.53298166	Gstt2	1
GO:0002705	positive regulation of leukocyte mediated immunity	1/67	115/14464	0.00869565	1.87722258	0.64428571	0.41495105	0.5579752	0.53512564	H2-T23	1
GO:0030282	bone mineralization	1/67	115/14464	0.00869565	1.87722258	0.64428571	0.41495105	0.5579752	0.53512564	Enpp1	1
GO:0043200	response to amino acid	1/67	115/14464	0.00869565	1.87722258	0.64428571	0.41495105	0.5579752	0.53512564	Hnrnpd	1
GO:0051347	positive regulation of transferase activity	2/67	306/14464	0.00653595	1.41098429	0.49569619	0.41602669	0.55893385	0.53604503	Ager/Cd200	2
GO:0048709	oligodendrocyte differentiation	1/67	116/14464	0.00862069	1.86103963	0.63516571	0.41768283	0.56018211	0.53724217	Enpp1	1
GO:1990823	response to leukemia inhibitory factor	1/67	116/14464	0.00862069	1.86103963	0.63516571	0.41768283	0.56018211	0.53724217	Gstt2	1
GO:0000018	regulation of DNA recombination	1/67	117/14464	0.00854701	1.84513331	0.6261353	0.42040204	0.56187298	0.5388638	Rpa2	1
GO:0008584	male gonad development	1/67	117/14464	0.00854701	1.84513331	0.6261353	0.42040204	0.56187298	0.5388638	Tnfsf10	1
GO:0014013	regulation of gliogenesis	1/67	117/14464	0.00854701	1.84513331	0.6261353	0.42040204	0.56187298	0.5388638	Nr1d1	1
GO:0021549	cerebellum development	1/67	117/14464	0.00854701	1.84513331	0.6261353	0.42040204	0.56187298	0.5388638	Hnrnpd	1
GO:0010821	regulation of mitochondrion organization	1/67	118/14464	0.00847458	1.82949658	0.61719271	0.42310875	0.5635355	0.54045824	Tnfsf10	1
GO:0043271	negative regulation of monoatomic ion transport	1/67	118/14464	0.00847458	1.82949658	0.61719271	0.42310875	0.5635355	0.54045824	Slc30a1	1
GO:0046546	development of primary male sexual characteristics	1/67	118/14464	0.00847458	1.82949658	0.61719271	0.42310875	0.5635355	0.54045824	Tnfsf10	1
GO:0051588	regulation of neurotransmitter transport	1/67	118/14464	0.00847458	1.82949658	0.61719271	0.42310875	0.5635355	0.54045824	Slc30a1	1
GO:0043010	camera-type eye development	2/67	311/14464	0.00643087	1.38829966	0.47222997	0.42407173	0.56433035	0.54122054	Bhlhe22/Xrn2	2
GO:0006888	endoplasmic reticulum to Golgi vesicle-mediated transport	1/67	119/14464	0.00840336	1.81412266	0.60833617	0.425803	0.56468366	0.54155939	Stx17	1
GO:0030278	regulation of ossification	1/67	119/14464	0.00840336	1.81412266	0.60833617	0.425803	0.56468366	0.54155939	Enpp1	1
GO:0048593	camera-type eye morphogenesis	1/67	119/14464	0.00840336	1.81412266	0.60833617	0.425803	0.56468366	0.54155939	Bhlhe22	1

GO:1903313	positive regulation of mRNA metabolic process	1/67	119/14464	0.00840336	1.81412266	0.60833617	0.425803	0.56468366	0.54155939	Hnrnpd	1
GO:0042886	amide transport	2/67	314/14464	0.00636943	1.37503565	0.4583421	0.42887227	0.56777678	0.54452584	Nr1d1/Tiam1	2
GO:0046879	hormone secretion	2/67	314/14464	0.00636943	1.37503565	0.4583421	0.42887227	0.56777678	0.54452584	Nr1d1/Tiam1	2
GO:0031570	DNA integrity checkpoint signaling	1/67	121/14464	0.00826446	1.78413717	0.59087459	0.43115436	0.56884322	0.5455486	Rpa2	1
GO:0043414	macromolecule methylation	1/67	121/14464	0.00826446	1.78413717	0.59087459	0.43115436	0.56884322	0.5455486	Ehmt2	1
GO:0051261	protein depolymerization	1/67	121/14464	0.00826446	1.78413717	0.59087459	0.43115436	0.56884322	0.5455486	Ccdc88c	1
GO:1901890	positive regulation of cell junction assembly	1/67	121/14464	0.00826446	1.78413717	0.59087459	0.43115436	0.56884322	0.5455486	Il1rapl1	1
GO:0045834	positive regulation of lipid metabolic process	1/67	122/14464	0.00819672	1.76951309	0.58226631	0.4338116	0.57185943	0.54844129	Nr1d1	1
GO:1903038	negative regulation of leukocyte cell-cell adhesion	1/67	123/14464	0.00813008	1.7551268	0.57373761	0.4364566	0.57439457	0.55087263	H2-T23	1
GO:0002697	regulation of immune effector process	2/67	320/14464	0.00625	1.34925373	0.43098337	0.43841201	0.57439457	0.55087263	H2-T23/Ager	2
GO:0003002	regionalization	2/67	320/14464	0.00625	1.34925373	0.43098337	0.43841201	0.57439457	0.55087263	Ppp2r3a/Bhlhe22	2
GO:0008286	insulin receptor signaling pathway	1/67	124/14464	0.00806452	1.74097256	0.56528698	0.43908943	0.57439457	0.55087263	Enpp1	1
GO:0009185	ribonucleoside diphosphate metabolic process	1/67	124/14464	0.00806452	1.74097256	0.56528698	0.43908943	0.57439457	0.55087263	Enpp1	1
GO:0021987	cerebral cortex development	1/67	124/14464	0.00806452	1.74097256	0.56528698	0.43908943	0.57439457	0.55087263	Bhlhe22	1
GO:0045471	response to ethanol	1/67	124/14464	0.00806452	1.74097256	0.56528698	0.43908943	0.57439457	0.55087263	Ehmt2	1
GO:0051100	negative regulation of binding	1/67	124/14464	0.00806452	1.74097256	0.56528698	0.43908943	0.57439457	0.55087263	Nfatc4	1
GO:0097696	cell surface receptor signaling pathway via STAT	1/67	124/14464	0.00806452	1.74097256	0.56528698	0.43908943	0.57439457	0.55087263	Ager	1
GO:0009914	hormone transport	2/67	321/14464	0.00623053	1.34505045	0.42647633	0.43999386	0.57460216	0.55107171	Nr1d1/Tiam1	2
GO:0042060	wound healing	2/67	321/14464	0.00623053	1.34505045	0.42647633	0.43999386	0.57460216	0.55107171	Ajap1/Fundc2	2
GO:0022037	metencephalon development	1/67	125/14464	0.008	1.72704478	0.55691296	0.44171014	0.57635506	0.55275283	Hnrnpd	1
GO:0006694	steroid biosynthetic process	1/67	126/14464	0.00793651	1.71333807	0.54861412	0.44431879	0.57682835	0.55320674	Nr1d1	1
GO:0006959	humoral immune response	1/67	126/14464	0.00793651	1.71333807	0.54861412	0.44431879	0.57682835	0.55320674	H2-T23	1
GO:0010466	negative regulation of peptidase activity	1/67	126/14464	0.00793651	1.71333807	0.54861412	0.44431879	0.57682835	0.55320674	Fnip1	1
GO:0010811	positive regulation of cell-substrate adhesion	1/67	126/14464	0.00793651	1.71333807	0.54861412	0.44431879	0.57682835	0.55320674	Abi3bp	1
GO:0015718	monocarboxylic acid transport	1/67	126/14464	0.00793651	1.71333807	0.54861412	0.44431879	0.57682835	0.55320674	Slco1a4	1
GO:0090101	negative regulation of transmembrane receptor protein serine/threonine kinase signaling pathway	1/67	126/14464	0.00793651	1.71333807	0.54861412	0.44431879	0.57682835	0.55320674	Ldlrad4	1
GO:0001936	regulation of endothelial cell proliferation	1/67	127/14464	0.00787402	1.69984722	0.54038906	0.44691543	0.57776567	0.55410567	Ager	1
GO:0010565	regulation of cellular ketone metabolic process	1/67	127/14464	0.00787402	1.69984722	0.54038906	0.44691543	0.57776567	0.55410567	Nr1d1	1
GO:0030177	positive regulation of Wnt signaling pathway	1/67	127/14464	0.00787402	1.69984722	0.54038906	0.44691543	0.57776567	0.55410567	Ppp2r3a	1
GO:0046822	regulation of nucleocytoplasmic transport	1/67	127/14464	0.00787402	1.69984722	0.54038906	0.44691543	0.57776567	0.55410567	Pkia	1
GO:1902652	secondary alcohol metabolic process	1/67	127/14464	0.00787402	1.69984722	0.54038906	0.44691543	0.57776567	0.55410567	Enpp1	1
GO:0014074	response to purine-containing compound	1/67	128/14464	0.0078125	1.68656716	0.53223645	0.44950012	0.58013374	0.55637676	Cdk2	1
GO:0030856	regulation of epithelial cell differentiation	1/67	128/14464	0.0078125	1.68656716	0.53223645	0.44950012	0.58013374	0.55637676	Ajap1	1
GO:0006090	pyruvate metabolic process	1/67	129/14464	0.00775194	1.673493	0.52415494	0.45207291	0.58102114	0.55722783	Enpp1	1
GO:0009132	nucleoside diphosphate metabolic process	1/67	129/14464	0.00775194	1.673493	0.52415494	0.45207291	0.58102114	0.55722783	Enpp1	1
GO:0016482	cytosolic transport	1/67	129/14464	0.00775194	1.673493	0.52415494	0.45207291	0.58102114	0.55722783	Sys1	1
GO:0017015	regulation of transforming growth factor beta receptor signaling pathway	1/67	129/14464	0.00775194	1.673493	0.52415494	0.45207291	0.58102114	0.55722783	Ldlrad4	1
GO:0050684	regulation of mRNA processing	1/67	129/14464	0.00775194	1.673493	0.52415494	0.45207291	0.58102114	0.55722783	Arglu1	1
GO:0009895	negative regulation of catabolic process	2/67	330/14464	0.00606061	1.30836725	0.38656566	0.45412336	0.58188601	0.55805729	Hnrnpd/Ehmt2	2
GO:0006906	vesicle fusion	1/67	130/14464	0.00769231	1.66061998	0.51614327	0.45463385	0.58188601	0.55805729	Stx17	1
GO:0018205	peptidyl-lysine modification	1/67	130/14464	0.00769231	1.66061998	0.51614327	0.45463385	0.58188601	0.55805729	Ehmt2	1
GO:0051592	response to calcium ion	1/67	130/14464	0.00769231	1.66061998	0.51614327	0.45463385	0.58188601	0.55805729	Hnrnpd	1
GO:0170039	proteinogenic amino acid metabolic process	1/67	130/14464	0.00769231	1.66061998	0.51614327	0.45463385	0.58188601	0.55805729	Pycr1	1
GO:0007030	Golgi organization	1/67	131/14464	0.00763359	1.64794349	0.50820019	0.457183	0.58224711	0.55840359	Stx17	1
GO:0007098	centrosome cycle	1/67	131/14464	0.00763359	1.64794349	0.50820019	0.457183	0.58224711	0.55840359	Cdk2	1
GO:0009154	purine ribonucleotide catabolic process	1/67	131/14464	0.00763359	1.64794349	0.50820019	0.457183	0.58224711	0.55840359	Enpp1	1
GO:0009566	fertilization	1/67	131/14464	0.00763359	1.64794349	0.50820019	0.457183	0.58224711	0.55840359	Ehmt2	1
GO:0043244	regulation of protein-containing complex disassembly	1/67	131/14464	0.00763359	1.64794349	0.50820019	0.457183	0.58224711	0.55840359	Ccdc88c	1
GO:1903844	regulation of cellular response to transforming growth factor beta stimulus	1/67	131/14464	0.00763359	1.64794349	0.50820019	0.457183	0.58224711	0.55840359	Ldlrad4	1
GO:0001101	response to acid chemical	1/67	132/14464	0.00757576	1.63545907	0.50032446	0.45972041	0.58403063	0.56011408	Hnrnpd	1
GO:0031109	microtubule polymerization or depolymerization	1/67	132/14464	0.00757576	1.63545907	0.50032446	0.45972041	0.58403063	0.56011408	Ccdc88c	1
GO:0090174	organelle membrane fusion	1/67	132/14464	0.00757576	1.63545907	0.50032446	0.45972041	0.58403063	0.56011408	Stx17	1
GO:0002687	positive regulation of leukocyte migration	1/67	133/14464	0.0075188	1.62316238	0.49251492	0.46224614	0.58579054	0.56180192	Ager	1
GO:0007292	female gamete generation	1/67	133/14464	0.0075188	1.62316238	0.49251492	0.46224614	0.58579054	0.56180192	Ehmt2	1
GO:2000779	regulation of double-strand break repair	1/67	133/14464	0.0075188	1.62316238	0.49251492	0.46224614	0.58579054	0.56180192	Rpa2	1
GO:0003158	endothelium development	1/67	134/14464	0.00746269	1.61104923	0.4847704	0.46476024	0.58800946	0.56392997	Gja4	1
GO:0046661	male sex differentiation	1/67	134/14464	0.00746269	1.61104923	0.4847704	0.46476024	0.58800946	0.56392997	Tnfsf10	1
GO:0008277	regulation of G protein-coupled receptor signaling pathway	1/67	135/14464	0.00740741	1.59911553	0.47708978	0.46726275	0.59020648	0.56603702	Gpsm1	1
GO:0071902	positive regulation of protein serine/threonine kinase activity	1/67	135/14464	0.00740741	1.59911553	0.47708978	0.46726275	0.59020648	0.56603702	Ager	1
GO:0007093	mitotic cell cycle checkpoint signaling	1/67	136/14464	0.00735294	1.58735733	0.46947195	0.46975374	0.59286693	0.56858852	Rpa2	1
GO:0048144	fibroblast proliferation	1/67	138/14464	0.00724638	1.56435215	0.45442043	0.47470135	0.5976428	0.57316881	Ager	1
GO:0050851	antigen receptor-mediated signaling pathway	1/67	138/14464	0.00724638	1.56435215	0.45442043	0.47470135	0.5976428	0.57316881	Rftn1	1
GO:0060078	regulation of postsynaptic membrane potential	1/67	138/14464	0.00724638	1.56435215	0.45442043	0.47470135	0.5976428	0.57316881	Gabrg1	1
GO:0007584	response to nutrient	1/67	139/14464	0.00719424	1.55309782	0.44698467	0.47715807	0.59877898	0.57425847	Enpp1	1
GO:0035107	appendage morphogenesis	1/67	139/14464	0.00719424	1.55309782	0.44698467	0.47715807	0.59877898	0.57425847	Enpp1	1
GO:0035108	limb morphogenesis	1/67	139/14464	0.00719424	1.55309782	0.44698467	0.47715807	0.59877898	0.57425847	Enpp1	1
GO:0120161	regulation of cold-induced thermogenesis	1/67	139/14464	0.00719424	1.55309782	0.44698467	0.47715807	0.59877898	0.57425847	Nr1d1	1
GO:0050678	regulation of epithelial cell proliferation	2/67	346/14464	0.00578035	1.24786472	0.31834408	0.47874684	0.59989364	0.57532748	Nr1d1/Ager	2
GO:0009261	ribonucleotide catabolic process	1/67	140/14464	0.00714286	1.54200426	0.43960756	0.47960348	0.59989364	0.57532748	Enpp1	1
GO:0043405	regulation of MAP kinase activity	1/67	140/14464	0.00714286	1.54200426	0.43960756	0.47960348	0.59989364	0.57532748	Ager	1
GO:0051250	negative regulation of lymphocyte activation	1/67	140/14464	0.00714286	1.54200426	0.43960756	0.47960348	0.59989364	0.57532748	H2-T23	1
GO:0009410	response to xenobiotic stimulus	2/67	348/14464	0.00574713	1.24069309	0.31004734	0.48177853	0.60001613	0.57544495	Aim2/Ehmt2	2
GO:0006195	purine nucleotide catabolic process	1/67	141/14464	0.0070922	1.53106806	0.43228816	0.48203761	0.60001613	0.57544495	Enpp1	1
GO:0006261	DNA-templated DNA replication	1/67	141/14464	0.0070922	1.53106806	0.43228816	0.48203761	0.60001613	0.57544495	Cdk2	1
GO:0007605	sensory perception of sound	1/67	141/14464	0.0070922	1.53106806	0.43228816	0.48203761	0.60001613	0.57544495	Enpp1	1
GO:0031023	microtubule organizing center organization	1/67	141/14464	0.0070922	1.53106806	0.43228816	0.48203761	0.60001613	0.57544495	Cdk2	1
GO:2000377	regulation of reactive oxygen species metabolic process	1/67	141/14464	0.0070922	1.53106806	0.43228816	0.48203761	0.60001613	0.57544495	Ager	1
GO:0050680	negative regulation of epithelial cell proliferation	1/67	142/14464	0.00704225	1.52028589	0.42502549	0.48446053	0.60157428	0.5769393	Ager	1
GO:0140013	meiotic nuclear division	1/67	142/14464	0.00704225	1.52028589	0.42502549	0.48446053	0.60157428	0.5769393	Ehmt2	1
GO:1903034	regulation of response to wounding	1/67	142/14464	0.00704225	1.52028589	0.42502549	0.48446053	0.60157428	0.5769393	Ajap1	1
GO:0032006	regulation of TOR signaling	1/67	143/14464	0.00699301	1.50965452	0.41781865	0.48687229	0.60311109	0.57841318	Fnip1	1
GO:0043123	positive regulation of canonical NF-kappaB signal transduction	1/67	143/14464	0.00699301	1.50965452	0.41781865	0.48687229	0.60311109	0.57841318	Tnfsf10	1
GO:0106106	cold-induced thermogenesis	1/67	143/14464	0.00699301	1.50965452	0.41781865	0.48687229	0.60311109	0.57841318	Nr1d1	1
GO:0006887	exocytosis	2/67	352/14464	0.00568182	1.2265943	0.29360039	0.4878103	0.60378769	0.57906207	Stx17/Il1rapl1	2
GO:0043467	regulation of generation of precursor metabolites and energy	1/67	144/14464	0.00694444	1.49917081	0.41066672	0.48927293	0.60511202	0.58033217	Enpp1	1
GO:0007596	blood coagulation	1/67	145/14464	0.00689655	1.4888317	0.40356883	0.4916625	0.60757972	0.58269882	Fundc2	1
GO:0007389	pattern specification process	2/67	356/14464	0.00561798	1.21281234	0.27734458	0.49379947	0.60905382	0.58411255	Ppp2r3a/Bhlhe22	2
GO:0007286	spermatid development	1/67	146/14464	0.00684932	1.47863423	0.3965241	0.49404106	0.60905382	0.58411255	Ehmt2	1
GO:1900180	regulation of protein localization to nucleus	1/67	146/14464	0.00684932	1.47863423	0.3965241	0.49404106	0.60905382	0.58411255	Pkia	1
GO:0043488	regulation of mRNA stability	1/67	147/14464	0.00680272	1.46857549	0.38953171	0.49640866	0.61002053	0.58503967	Hnrnpd	1
GO:0043547	positive regulation of GTPase activity	1/67	147/14464	0.00680272	1.46857549	0.38953171	0.49640866	0.61002053	0.58503967	Tiam1	1
GO:0170033	L-amino acid metabolic process	1/67	147/14464	0.00680272	1.46857549	0.38953171	0.49640866	0.61002053	0.58503967	Pycr1	1
GO:2001236	regulation of extrinsic apoptotic signaling pathway	1/67	147/14464	0.00680272	1.46857549	0.38953171	0.49640866	0.61002053	0.58503967	Tnfsf10	1
GO:0030098	lymphocyte differentiation	2/67	359/14464	0.00557103	1.20267742	0.26527519	0.49826311	0.61048244	0.58548266	Enpp1/Fnip1	2
GO:0001935	endothelial cell proliferation	1/67	148/14464	0.00675676	1.45865268	0.38259082	0.49876534	0.61048244	0.58548266	Ager	1
GO:0002244	hematopoietic progenitor cell differentiation	1/67	148/14464	0.00675676	1.45865268	0.38259082	0.49876534	0.61048244	0.58548266	Fnip1	1
GO:0007599	hemostasis	1/67	148/14464	0.00675676	1.45865268	0.38259082	0.49876534	0.61048244	0.58548266	Fundc2	1
GO:0009411	response to UV	1/67	148/14464	0.00675676	1.45865268	0.38259082	0.49876534	0.61048244	0.58548266	Nfatc4	1
GO:0048872	homeostasis of number of cells	2/67	360/14464	0.00555556	1.19933665	0.261275	0.49974557	0.61119676	0.58616773	Aim2/Enpp1	2
GO:0001889	liver development	1/67	150/14464	0.00666667	1.43920398	0.36886036	0.50344616	0.61328896	0.58817425	Hnrnpd	1
GO:0010977	negative regulation of neuron projection development	1/67	150/14464	0.00666667	1.43920398	0.36886036	0.50344616	0.61328896	0.58817425	Ngef	1
GO:0031214	biomineral tissue development	1/67	150/14464	0.00666667	1.43920398	0.36886036	0.50344616	0.61328896	0.58817425	Enpp1	1
GO:0048754	branching morphogenesis of an epithelial tube	1/67	150/14464	0.00666667	1.43920398	0.36886036	0.50344616	0.61328896	0.58817425	Nfatc4	1
GO:0050817	coagulation	1/67	150/14464	0.00666667	1.43920398	0.36886036	0.50344616	0.61328896	0.58817425	Fundc2	1
GO:0007224	smoothened signaling pathway	1/67	151/14464	0.00662252	1.42967283	0.36206923	0.5057704	0.6146626	0.58949165	Enpp1	1
GO:1902850	microtubule cytoskeleton organization involved in mitosis	1/67	151/14464	0.00662252	1.42967283	0.36206923	0.5057704	0.6146626	0.58949165	Gpsm1	1
GO:2000045	regulation of G1/S transition of mitotic cell cycle	1/67	151/14464	0.00662252	1.42967283	0.36206923	0.5057704	0.6146626	0.58949165	Rpa2	1
GO:0042445	hormone metabolic process	1/67	152/14464	0.00657895	1.42026709	0.3553265	0.50808392	0.61698764	0.59172147	Enpp1	1
GO:0061008	hepaticobiliary system development	1/67	153/14464	0.00653595	1.41098429	0.34863143	0.51038676	0.61929607	0.59393536	Hnrnpd	1
GO:0048515	spermatid differentiation	1/67	154/14464	0.00649351	1.40182206	0.34198331	0.51267899	0.62061141	0.59519685	Ehmt2	1
GO:0062013	positive regulation of small molecule metabolic process	1/67	154/14464	0.00649351	1.40182206	0.34198331	0.51267899	0.62061141	0.59519685	Nr1d1	1
GO:0098876	vesicle-mediated transport to the plasma membrane	1/67	154/14464	0.00649351	1.40182206	0.34198331	0.51267899	0.62061141	0.59519685	Sys1	1
GO:0050777	negative regulation of immune response	1/67	155/14464	0.00645161	1.39277805	0.33538142	0.51496065	0.62288411	0.59737648	H2-T23	1
GO:0030010	establishment of cell polarity	1/67	156/14464	0.00641026	1.38384998	0.32882509	0.51723178	0.62395779	0.59840619	Gpsm1	1
GO:0072330	monocarboxylic acid biosynthetic process	1/67	156/14464	0.00641026	1.38384998	0.32882509	0.51723178	0.62395779	0.59840619	Nr1d1	1
GO:0030900	forebrain development	2/67	373/14464	0.00536193	1.15753671	0.21027899	0.51876745	0.62395779	0.59840619	Bhlhe22/Xm2	2
GO:0002700	regulation of production of molecular mediator of immune response	1/67	157/14464	0.00636943	1.37503565	0.32231364	0.51949244	0.62395779	0.59840619	H2-T23	1
GO:0002753	cytoplasmic pattern recognition receptor signaling pathway	1/67	157/14464	0.00636943	1.37503565	0.32231364	0.51949244	0.62395779	0.59840619	Rftn1	1
GO:0062207	regulation of pattern recognition receptor signaling pathway	1/67	157/14464	0.00636943	1.37503565	0.32231364	0.51949244	0.62395779	0.59840619	Nr1d1	1
GO:0071478	cellular response to radiation	1/67	157/14464	0.00636943	1.37503565	0.32231364	0.51949244	0.62395779	0.59840619	Nfatc4	1
GO:0072523	purine-containing compound catabolic process	1/67	157/14464	0.00636943	1.37503565	0.32231364	0.51949244	0.62395779	0.59840619	Enpp1	1
GO:1990845	adaptive thermogenesis	1/67	157/14464	0.00636943	1.37503565	0.32231364	0.51949244	0.62395779	0.59840619	Nr1d1	1
GO:0009166	nucleotide catabolic process	1/67	158/14464	0.00632911	1.36633289	0.31584641	0.52174267	0.62617247	0.60053017	Enpp1	1
GO:0003341	cilium movement	1/67	159/14464	0.00628931	1.3577396	0.30942275	0.52398251	0.62690765	0.60123525	Ccdc88c	1
GO:0010594	regulation of endothelial cell migration	1/67	159/14464	0.00628931	1.3577396	0.30942275	0.52398251	0.62690765	0.60123525	Ager	1
GO:0061025	membrane fusion	1/67	159/14464	0.00628931	1.3577396	0.30942275	0.52398251	0.62690765	0.60123525	Stx17	1
GO:0071897	DNA biosynthetic process	1/67	159/14464	0.00628931	1.3577396	0.30942275	0.52398251	0.62690765	0.60123525	Hnrnpd	1
GO:0006816	calcium ion transport	2/67	377/14464	0.00530504	1.14525516	0.19495015	0.52452567	0.62707064	0.60139156	Gja4/Slc30a1	2
GO:0002181	cytoplasmic translation	1/67	160/14464	0.00625	1.34925373	0.30304204	0.52621202	0.62811211	0.60239038	Hnrnpd	1
GO:0070507	regulation of microtubule cytoskeleton organization	1/67	160/14464	0.00625	1.34925373	0.30304204	0.52621202	0.62811211	0.60239038	Ccdc88c	1
GO:0006469	negative regulation of protein kinase activity	1/67	161/14464	0.00621118	1.34087327	0.29670367	0.52843125	0.62881278	0.60306237	Pkia	1
GO:0033157	regulation of intracellular protein transport	1/67	161/14464	0.00621118	1.34087327	0.29670367	0.52843125	0.62881278	0.60306237	Pkia	1
GO:0051897	positive regulation of phosphatidylinositol 3-kinase/protein kinase B signal transduction	1/67	161/14464	0.00621118	1.34087327	0.29670367	0.52843125	0.62881278	0.60306237	Epha8	1
GO:0061013	regulation of mRNA catabolic process	1/67	161/14464	0.00621118	1.34087327	0.29670367	0.52843125	0.62881278	0.60306237	Hnrnpd	1
GO:0043487	regulation of RNA stability	1/67	162/14464	0.00617284	1.33259628	0.29040701	0.53064023	0.6304677	0.60464951	Hnrnpd	1
GO:0070374	positive regulation of ERK1 and ERK2 cascade	1/67	162/14464	0.00617284	1.33259628	0.29040701	0.53064023	0.6304677	0.60464951	Ager	1
GO:0002285	lymphocyte activation involved in immune response	1/67	163/14464	0.00613497	1.32442084	0.28415149	0.53283903	0.63161918	0.60575384	Enpp1	1
GO:0006606	protein import into nucleus	1/67	163/14464	0.00613497	1.32442084	0.28415149	0.53283903	0.63161918	0.60575384	Pkia	1
GO:1901991	negative regulation of mitotic cell cycle phase transition	1/67	163/14464	0.00613497	1.32442084	0.28415149	0.53283903	0.63161918	0.60575384	Rpa2	1
GO:0000724	double-strand break repair via homologous recombination	1/67	164/14464	0.00609756	1.3163451	0.27793652	0.53502767	0.63372609	0.60777446	Rpa2	1
GO:1903046	meiotic cell cycle process	1/67	166/14464	0.0060241	1.30048552	0.26562598	0.5393747	0.63838434	0.61224195	Ehmt2	1
GO:0045807	positive regulation of endocytosis	1/67	167/14464	0.00598802	1.29269819	0.2595293	0.54153317	0.63946561	0.61327895	Ager	1
GO:0071248	cellular response to metal ion	1/67	167/14464	0.00598802	1.29269819	0.2595293	0.54153317	0.63946561	0.61327895	Nfatc4	1
GO:0098739	import across plasma membrane	1/67	167/14464	0.00598802	1.29269819	0.2595293	0.54153317	0.63946561	0.61327895	Slc30a1	1
GO:0000725	recombinational repair	1/67	169/14464	0.00591716	1.27739998	0.24745048	0.54582028	0.639778	0.61357855	Rpa2	1
GO:0009755	hormone-mediated signaling pathway	1/67	169/14464	0.00591716	1.27739998	0.24745048	0.54582028	0.639778	0.61357855	Nr1d1	1
GO:0009952	anterior/posterior pattern specification	1/67	169/14464	0.00591716	1.27739998	0.24745048	0.54582028	0.639778	0.61357855	Ppp2r3a	1
GO:0019362	pyridine nucleotide metabolic process	1/67	169/14464	0.00591716	1.27739998	0.24745048	0.54582028	0.639778	0.61357855	Enpp1	1
GO:0046496	nicotinamide nucleotide metabolic process	1/67	169/14464	0.00591716	1.27739998	0.24745048	0.54582028	0.639778	0.61357855	Enpp1	1
GO:0051170	import into nucleus	1/67	169/14464	0.00591716	1.27739998	0.24745048	0.54582028	0.639778	0.61357855	Pkia	1
GO:0010876	lipid localization	2/67	393/14464	0.00508906	1.09862899	0.13522873	0.54710389	0.639778	0.61357855	Slco1a4/Enpp1	2
GO:0007269	neurotransmitter secretion	1/67	170/14464	0.00588235	1.26988586	0.24146729	0.547949	0.639778	0.61357855	Slc30a1	1
GO:0030902	hindbrain development	1/67	170/14464	0.00588235	1.26988586	0.24146729	0.547949	0.639778	0.61357855	Hnrnpd	1
GO:0046890	regulation of lipid biosynthetic process	1/67	170/14464	0.00588235	1.26988586	0.24146729	0.547949	0.639778	0.61357855	Nr1d1	1
GO:0099643	signal release from synapse	1/67	170/14464	0.00588235	1.26988586	0.24146729	0.547949	0.639778	0.61357855	Slc30a1	1
GO:0022408	negative regulation of cell-cell adhesion	1/67	171/14464	0.00584795	1.26245963	0.23552091	0.55006789	0.639778	0.61357855	H2-T23	1
GO:0030308	negative regulation of cell growth	1/67	171/14464	0.00584795	1.26245963	0.23552091	0.55006789	0.639778	0.61357855	Enpp1	1
GO:0042552	myelination	1/67	171/14464	0.00584795	1.26245963	0.23552091	0.55006789	0.639778	0.61357855	Enpp1	1
GO:0050770	regulation of axonogenesis	1/67	171/14464	0.00584795	1.26245963	0.23552091	0.55006789	0.639778	0.61357855	Tiam1	1
GO:0051494	negative regulation of cytoskeleton organization	1/67	171/14464	0.00584795	1.26245963	0.23552091	0.55006789	0.639778	0.61357855	Ccdc88c	1
GO:1901605	alpha-amino acid metabolic process	1/67	171/14464	0.00584795	1.26245963	0.23552091	0.55006789	0.639778	0.61357855	Pycr1	1
GO:1902107	positive regulation of leukocyte differentiation	1/67	171/14464	0.00584795	1.26245963	0.23552091	0.55006789	0.639778	0.61357855	Ager	1
GO:1903708	positive regulation of hemopoiesis	1/67	171/14464	0.00584795	1.26245963	0.23552091	0.55006789	0.639778	0.61357855	Ager	1
GO:0031929	TOR signaling	1/67	172/14464	0.00581395	1.25511975	0.22961084	0.55217699	0.639778	0.61357855	Fnip1	1
GO:0048660	regulation of smooth muscle cell proliferation	1/67	172/14464	0.00581395	1.25511975	0.22961084	0.55217699	0.639778	0.61357855	Ager	1
GO:0048736	appendage development	1/67	172/14464	0.00581395	1.25511975	0.22961084	0.55217699	0.639778	0.61357855	Enpp1	1

GO:0060173 limb development	1/67	172/14464	0.00581395	1.25511975	0.22961084	0.55217699	0.639778	0.61357855	Enpp1	1
GO:1902904 negative regulation of supramolecular fiber organization	1/67	172/14464	0.00581395	1.25511975	0.22961084	0.55217699	0.639778	0.61357855	Ccdc88c	1
GO:2001242 regulation of intrinsic apoptotic signaling pathway	1/67	172/14464	0.00581395	1.25511975	0.22961084	0.55217699	0.639778	0.61357855	Pycr1	1
GO:0022407 regulation of cell-cell adhesion	2/67	398/14464	0.00502513	1.08482712	0.11706351	0.55400856	0.64076509	0.61452522	H2-T23/Ager	2
GO:0033673 negative regulation of kinase activity	1/67	173/14464	0.00578035	1.24786472	0.22373661	0.55427636	0.64076509	0.61452522	Pkia	1
GO:0071222 cellular response to lipopolysaccharide	1/67	173/14464	0.00578035	1.24786472	0.22373661	0.55427636	0.64076509	0.61452522	Nr1d1	1
GO:0001659 temperature homeostasis	1/67	174/14464	0.00574713	1.24069309	0.21789774	0.55636603	0.64077838	0.61453796	Nr1d1	1
GO:0007272 ensheathment of neurons	1/67	174/14464	0.00574713	1.24069309	0.21789774	0.55636603	0.64077838	0.61453796	Enpp1	1
GO:0008366 axon ensheathment	1/67	174/14464	0.00574713	1.24069309	0.21789774	0.55636603	0.64077838	0.61453796	Enpp1	1
GO:0048771 tissue remodeling	1/67	174/14464	0.00574713	1.24069309	0.21789774	0.55636603	0.64077838	0.61453796	Enpp1	1
GO:0140694 non-membrane-bounded organelle assembly	2/67	400/14464	0.005	1.07940299	0.10986086	0.55675011	0.64077838	0.61453796	Cdk2/Dazap2	2
GO:0006839 mitochondrial transport	1/67	175/14464	0.00571429	1.23360341	0.21209375	0.55844605	0.64077838	0.61453796	Tst	1
GO:0021953 central nervous system neuron differentiation	1/67	175/14464	0.00571429	1.23360341	0.21209375	0.55844605	0.64077838	0.61453796	Bhlhe22	1
GO:0042770 signal transduction in response to DNA damage	1/67	175/14464	0.00571429	1.23360341	0.21209375	0.55844605	0.64077838	0.61453796	Rpa2	1
GO:0048469 cell maturation	1/67	175/14464	0.00571429	1.23360341	0.21209375	0.55844605	0.64077838	0.61453796	Epha8	1
GO:1902806 regulation of cell cycle G1/S phase transition	1/67	175/14464	0.00571429	1.23360341	0.21209375	0.55844605	0.64077838	0.61453796	Rpa2	1
GO:0048284 organelle fusion	1/67	176/14464	0.00568182	1.2265943	0.20632421	0.56051646	0.64124415	0.61498465	Stx17	1
GO:0050954 sensory perception of mechanical stimulus	1/67	176/14464	0.00568182	1.2265943	0.20632421	0.56051646	0.64124415	0.61498465	Enpp1	1
GO:0072524 pyridine-containing compound metabolic process	1/67	176/14464	0.00568182	1.2265943	0.20632421	0.56051646	0.64124415	0.61498465	Enpp1	1
GO:1901292 nucleoside phosphate catabolic process	1/67	176/14464	0.00568182	1.2265943	0.20632421	0.56051646	0.64124415	0.61498465	Enpp1	1
GO:0022618 protein-RNA complex assembly	1/67	177/14464	0.00564972	1.21966439	0.20058865	0.56257731	0.64264762	0.61633065	Luc7l2	1
GO:0061138 morphogenesis of a branching epithelium	1/67	177/14464	0.00564972	1.21966439	0.20058865	0.56257731	0.64264762	0.61633065	Nfatc4	1
GO:0030595 leukocyte chemotaxis	1/67	179/14464	0.00558659	1.20603685	0.18921776	0.56667049	0.64684387	0.62035506	Slamf9	1
GO:0071219 cellular response to molecule of bacterial origin	1/67	180/14464	0.00555556	1.19933665	0.18358157	0.5687029	0.64847673	0.62192105	Nr1d1	1
GO:0051249 regulation of lymphocyte activation	2/67	409/14464	0.00488998	1.05565084	0.07788405	0.56894259	0.64847673	0.62192105	H2-T23/Ager	2
GO:0000075 cell cycle checkpoint signaling	1/67	181/14464	0.00552486	1.19271048	0.17797766	0.57072593	0.64906912	0.62248919	Rpa2	1
GO:0019722 calcium-mediated signaling	1/67	181/14464	0.00552486	1.19271048	0.17797766	0.57072593	0.64906912	0.62248919	Nfatc4	1
GO:0048839 inner ear development	1/67	181/14464	0.00552486	1.19271048	0.17797766	0.57072593	0.64906912	0.62248919	H2-T23	1
GO:0098742 cell-cell adhesion via plasma-membrane adhesion molecules	1/67	182/14464	0.00549451	1.18615713	0.17240563	0.57273961	0.65064269	0.62399832	Il1rapl1	1
GO:0043161 proteasome-mediated ubiquitin-dependent protein catabolic process	2/67	412/14464	0.00485437	1.04796406	0.06737924	0.57295401	0.65064269	0.62399832	Psmb9/Zfp598	2
GO:0002520 immune system development	1/67	183/14464	0.00546448	1.17967539	0.16686507	0.57474398	0.65075715	0.62410809	H2-T23	1
GO:0006109 regulation of carbohydrate metabolic process	1/67	183/14464	0.00546448	1.17967539	0.16686507	0.57474398	0.65075715	0.62410809	Enpp1	1
GO:0042180 cellular ketone metabolic process	1/67	183/14464	0.00546448	1.17967539	0.16686507	0.57474398	0.65075715	0.62410809	Nr1d1	1
GO:1990138 neuron projection extension	1/67	183/14464	0.00546448	1.17967539	0.16686507	0.57474398	0.65075715	0.62410809	Tiam1	1
GO:0045664 regulation of neuron differentiation	1/67	185/14464	0.00540541	1.16692215	0.15587681	0.57872498	0.6546169	0.62780978	Tiam1	1
GO:0050673 epithelial cell proliferation	2/67	417/14464	0.00479616	1.03539855	0.05003712	0.57958089	0.6546169	0.62780978	Nr1d1/Ager	2
GO:1901615 organic hydroxy compound metabolic process	2/67	417/14464	0.00479616	1.03539855	0.05003712	0.57958089	0.6546169	0.62780978	Nr1d1/Enpp1	2
GO:0001649 osteoblast differentiation	1/67	186/14464	0.00537634	1.16064837	0.15042835	0.58070169	0.6546169	0.62780978	Enpp1	1
GO:0051216 cartilage development	1/67	186/14464	0.00537634	1.16064837	0.15042835	0.58070169	0.6546169	0.62780978	Enpp1	1
GO:0071826 protein-RNA complex organization	1/67	186/14464	0.00537634	1.16064837	0.15042835	0.58070169	0.6546169	0.62780978	Luc7l2	1
GO:1901617 organic hydroxy compound biosynthetic process	1/67	187/14464	0.00534759	1.1544417	0.14500984	0.58266926	0.65635477	0.62947648	Nr1d1	1
GO:0007179 transforming growth factor beta receptor signaling pathway	1/67	190/14464	0.00526316	1.13621367	0.12893037	0.58851756	0.66245841	0.63533018	Ldlrad4	1
GO:0031099 regeneration	1/67	191/14464	0.0052356	1.13026491	0.12362807	0.590449	0.66414738	0.63694998	Enpp1	1
GO:0045859 regulation of protein kinase activity	2/67	426/14464	0.00469484	1.01352393	0.01932794	0.5913234	0.66437008	0.63716356	Ager/Pkia	2
GO:0031334 positive regulation of protein-containing complex assembly	1/67	192/14464	0.00520833	1.12437811	0.11835395	0.59237151	0.66437008	0.63716356	Fnip1	1
GO:0045732 positive regulation of protein catabolic process	1/67	192/14464	0.00520833	1.12437811	0.11835395	0.59237151	0.66437008	0.63716356	Ppp2r3a	1
GO:1901654 response to ketone	1/67	192/14464	0.00520833	1.12437811	0.11835395	0.59237151	0.66437008	0.63716356	Hnrnpd	1
GO:0001763 morphogenesis of a branching structure	1/67	194/14464	0.00515464	1.11278658	0.10788894	0.59618989	0.66768068	0.64033859	Nfatc4	1
GO:0006909 phagocytosis	1/67	194/14464	0.00515464	1.11278658	0.10788894	0.59618989	0.66768068	0.64033859	Ager	1
GO:0051348 negative regulation of transferase activity	1/67	196/14464	0.00510204	1.10143162	0.09753274	0.59997303	0.67142951	0.6439339	Pkia	1
GO:0002064 epithelial cell development	1/67	198/14464	0.00505051	1.09030605	0.08728285	0.60372125	0.67456673	0.64694265	Ccdc88c	1
GO:0030705 cytoskeleton-dependent intracellular transport	1/67	198/14464	0.00505051	1.09030605	0.08728285	0.60372125	0.67456673	0.64694265	Ccdc88c	1
GO:0022613 ribonucleoprotein complex biogenesis	2/67	436/14464	0.00458716	0.99027797	-0.0140615	0.60408961	0.67456673	0.64694265	Luc7l2/Rrp9	2
GO:0042391 regulation of membrane potential	2/67	437/14464	0.00457666	0.98801189	-0.0173596	0.60534994	0.67548462	0.64782295	Pycr1/Gabrg1	2
GO:0008406 gonad development	1/67	200/14464	0.005	1.07940299	0.07713682	0.60743487	0.67732065	0.64958379	Tnfsf10	1
GO:0017157 regulation of exocytosis	1/67	202/14464	0.0049505	1.06871583	0.06709232	0.6111142	0.68043857	0.65257403	Il1rapl1	1
GO:0035264 multicellular organism growth	1/67	202/14464	0.0049505	1.06871583	0.06709232	0.6111142	0.68043857	0.65257403	Enpp1	1
GO:0045137 development of primary sexual characteristics	1/67	203/14464	0.00492611	1.06345122	0.06210743	0.61294111	0.68197996	0.6540523	Tnfsf10	1
GO:0071216 cellular response to biotic stimulus	1/67	205/14464	0.00487805	1.05307608	0.05221098	0.6165696	0.68552219	0.65744947	Nr1d1	1
GO:0034762 regulation of transmembrane transport	2/67	447/14464	0.00447427	0.96590871	-0.0499459	0.61779029	0.68638416	0.65827614	Slc30a1/Enpp1	2
GO:0003018 vascular process in circulatory system	1/67	207/14464	0.00483092	1.04290143	0.04241051	0.62016457	0.6870407	0.65890579	Ager	1
GO:0007411 axon guidance	1/67	207/14464	0.00483092	1.04290143	0.04241051	0.62016457	0.6870407	0.65890579	Epha8	1
GO:0060348 bone development	1/67	207/14464	0.00483092	1.04290143	0.04241051	0.62016457	0.6870407	0.65890579	Enpp1	1
GO:0097191 extrinsic apoptotic signaling pathway	1/67	207/14464	0.00483092	1.04290143	0.04241051	0.62016457	0.6870407	0.65890579	Tnfsf10	1
GO:0006282 regulation of DNA repair	1/67	208/14464	0.00480769	1.03788749	0.03754561	0.62194959	0.68802894	0.65985357	Rpa2	1
GO:0097485 neuron projection guidance	1/67	208/14464	0.00480769	1.03788749	0.03754561	0.62194959	0.68802894	0.65985357	Epha8	1
GO:0007163 establishment or maintenance of cell polarity	1/67	210/14464	0.0047619	1.02800284	0.02788521	0.62549487	0.69095885	0.66266349	Gpsm1	1
GO:0043542 endothelial cell migration	1/67	210/14464	0.0047619	1.02800284	0.02788521	0.62549487	0.69095885	0.66266349	Ager	1
GO:0098771 inorganic ion homeostasis	2/67	454/14464	0.00440529	0.95101585	-0.0723428	0.62632237	0.69137735	0.66306486	Slc30a1/Enpp1	2
GO:0043549 regulation of kinase activity	2/67	456/14464	0.00438596	0.94684472	-0.0786813	0.6287335	0.69186323	0.66353084	Ager/Pkia	2
GO:0002699 positive regulation of immune effector process	1/67	212/14464	0.00471698	1.0183047	0.01831569	0.62900739	0.69186323	0.66353084	H2-T23	1
GO:0006813 potassium ion transport	1/67	212/14464	0.00471698	1.0183047	0.01831569	0.62900739	0.69186323	0.66353084	Cdk2	1
GO:0099111 microtubule-based transport	1/67	212/14464	0.00471698	1.0183047	0.01831569	0.62900739	0.69186323	0.66353084	Ccdc88c	1
GO:1905475 regulation of protein localization to membrane	1/67	212/14464	0.00471698	1.0183047	0.01831569	0.62900739	0.69186323	0.66353084	Slc30a1	1
GO:0045930 negative regulation of mitotic cell cycle	1/67	213/14464	0.00469484	1.01352393	0.0135644	0.63075146	0.69279259	0.66442214	Rpa2	1
GO:0072593 reactive oxygen species metabolic process	1/67	213/14464	0.00469484	1.01352393	0.0135644	0.63075146	0.69279259	0.66442214	Ager	1
GO:0006364 rRNA processing	1/67	214/14464	0.0046729	1.00878784	0.00883511	0.63248745	0.69371044	0.66530241	Rrp9	1
GO:0048705 skeletal system morphogenesis	1/67	214/14464	0.0046729	1.00878784	0.00883511	0.63248745	0.69371044	0.66530241	Enpp1	1
GO:0045926 negative regulation of growth	1/67	216/14464	0.00462963	0.99944721	-0.0005584	0.63593536	0.69699601	0.66845343	Enpp1	1
GO:0048863 stem cell differentiation	1/67	218/14464	0.00458716	0.99027797	-0.0098666	0.63935139	0.69974467	0.67108953	Ccdc88c	1
GO:0055001 muscle cell development	1/67	218/14464	0.00458716	0.99027797	-0.0098666	0.63935139	0.69974467	0.67108953	Nfatc4	1
GO:0007005 mitochondrion organization	2/67	466/14464	0.00429185	0.92652617	-0.1099824	0.64061216	0.70060587	0.67191546	Tnfsf10/Enpp1	2
GO:0006836 neurotransmitter transport	1/67	219/14464	0.00456621	0.98575615	-0.0144893	0.64104755	0.70060587	0.67191546	Slc30a1	1
GO:0046034 ATP metabolic process	1/67	220/14464	0.00454545	0.98127544	-0.0190914	0.64273585	0.70195318	0.6732076	Enpp1	1
GO:0044283 small molecule biosynthetic process	2/67	470/14464	0.00425532	0.91864084	-0.122325	0.64528122	0.70411823	0.67528398	Nr1d1/Pycr1	2
GO:0043588 skin development	1/67	222/14464	0.0045045	0.97243512	-0.0282343	0.64608901	0.70411823	0.67528398	Enpp1	1
GO:0046434 organophosphate catabolic process	1/67	222/14464	0.0045045	0.97243512	-0.0282343	0.64608901	0.70411823	0.67528398	Enpp1	1
GO:0090092 regulation of transmembrane receptor protein serine/threonine kinase signaling pathway	1/67	223/14464	0.0044843	0.96807443	-0.0327757	0.64775395	0.70543381	0.67654569	Ldlrad4	1
GO:0045861 negative regulation of proteolysis	1/67	224/14464	0.00446429	0.96375267	-0.0372972	0.64941117	0.70624038	0.67731923	Fnip1	1
GO:1903522 regulation of blood circulation	1/67	224/14464	0.00446429	0.96375267	-0.0372972	0.64941117	0.70624038	0.67731923	Ager	1
GO:2001234 negative regulation of apoptotic signaling pathway	1/67	225/14464	0.00444444	0.95946932	-0.041799	0.65106071	0.70753495	0.67856079	Pycr1	1
GO:0016049 cell growth	2/67	476/14464	0.00420168	0.90706133	-0.1406542	0.65219685	0.70827015	0.67926588	Tiam1/Enpp1	2
GO:1901136 carbohydrate derivative catabolic process	1/67	229/14464	0.00436681	0.94271003	-0.059614	0.65758276	0.71361622	0.68439303	Enpp1	1
GO:0001894 tissue homeostasis	1/67	230/14464	0.00434783	0.93861129	-0.0640206	0.65919442	0.71385706	0.68462401	Enpp1	1
GO:0019318 hexose metabolic process	1/67	230/14464	0.00434783	0.93861129	-0.0640206	0.65919442	0.71385706	0.68462401	Man2b1	1
GO:0060249 anatomical structure homeostasis	1/67	230/14464	0.00434783	0.93861129	-0.0640206	0.65919442	0.71385706	0.68462401	Enpp1	1
GO:0001667 ameboidal-type cell migration	1/67	231/14464	0.004329	0.93454804	-0.0684087	0.66079861	0.71509175	0.68580813	Ager	1
GO:0002573 myeloid leukocyte differentiation	1/67	232/14464	0.00431034	0.93051981	-0.0727784	0.66239535	0.71631666	0.68698288	Enpp1	1
GO:0045333 cellular respiration	1/67	234/14464	0.0042735	0.92256665	-0.0814637	0.66556666	0.7192414	0.68978785	Nfatc4	1
GO:0015850 organic hydroxy compound transport	1/67	235/14464	0.00425532	0.91864084	-0.0857796	0.6671413	0.7204378	0.69093526	Slco1a4	1
GO:0007548 sex differentiation	1/67	237/14464	0.00421941	0.91088859	-0.0943586	0.67026869	0.72330816	0.69368807	Tnfsf10	1
GO:0006520 amino acid metabolic process	1/67	238/14464	0.00420168	0.90706133	-0.0986221	0.67182151	0.72447652	0.69480859	Pycr1	1
GO:0007626 locomotory behavior	1/67	240/14464	0.00416667	0.89950249	-0.1070978	0.67490557	0.72627758	0.69653589	Enpp1	1
GO:0051924 regulation of calcium ion transport	1/67	240/14464	0.00416667	0.89950249	-0.1070978	0.67490557	0.72627758	0.69653589	Slc30a1	1
GO:1901988 negative regulation of cell cycle phase transition	1/67	240/14464	0.00416667	0.89950249	-0.1070978	0.67490557	0.72627758	0.69653589	Rpa2	1
GO:0002366 leukocyte activation involved in immune response	1/67	241/14464	0.00414938	0.89577011	-0.1113104	0.67643688	0.72741747	0.6976291	Enpp1	1
GO:0071560 cellular response to transforming growth factor beta stimulus	1/67	242/14464	0.00413223	0.89206858	-0.1155063	0.67796108	0.72854814	0.69871347	Ldlrad4	1
GO:0000209 protein polyubiquitination	1/67	243/14464	0.00411523	0.88839752	-0.1196858	0.67947821	0.72865409	0.69881508	Zfp598	1
GO:0009205 purine ribonucleoside triphosphate metabolic process	1/67	243/14464	0.00411523	0.88839752	-0.1196858	0.67947821	0.72865409	0.69881508	Enpp1	1
GO:0034599 cellular response to oxidative stress	1/67	243/14464	0.00411523	0.88839752	-0.1196858	0.67947821	0.72865409	0.69881508	Pycr1	1
GO:0002263 cell activation involved in immune response	1/67	245/14464	0.00408163	0.88114529	-0.1279957	0.68249138	0.73086811	0.70093844	Enpp1	1
GO:0032886 regulation of microtubule-based process	1/67	245/14464	0.00408163	0.88114529	-0.1279957	0.68249138	0.73086811	0.70093844	Ccdc88c	1
GO:0045860 positive regulation of protein kinase activity	1/67	246/14464	0.00406504	0.8775634	-0.1321266	0.68398748	0.7319616	0.70198715	Ager	1
GO:0016072 rRNA metabolic process	1/67	247/14464	0.00404858	0.87401051	-0.1362415	0.68547664	0.73304615	0.70302728	Rrp9	1
GO:0009199 ribonucleoside triphosphate metabolic process	1/67	248/14464	0.00403226	0.87048628	-0.1403407	0.68695888	0.73361306	0.70357098	Enpp1	1
GO:0051607 defense response to virus	1/67	248/14464	0.00403226	0.87048628	-0.1403407	0.68695888	0.73361306	0.70357098	Aim2	1
GO:0071559 response to transforming growth factor beta	1/67	249/14464	0.00401606	0.86699035	-0.1444242	0.68843425	0.73467948	0.70459373	Ldlrad4	1
GO:0005996 monosaccharide metabolic process	1/67	250/14464	0.004	0.86352239	-0.1484923	0.68990276	0.73573713	0.70560807	Man2b1	1
GO:0009144 purine nucleoside triphosphate metabolic process	1/67	251/14464	0.00398406	0.86008206	-0.152545	0.69136445	0.73678604	0.70661403	Enpp1	1
GO:0048562 embryonic organ morphogenesis	1/67	254/14464	0.00393701	0.84992361	-0.1646125	0.69570893	0.7403919	0.71007222	Enpp1	1
GO:0048588 developmental cell growth	1/67	254/14464	0.00393701	0.84992361	-0.1646125	0.69570893	0.7403919	0.71007222	Tiam1	1
GO:0034976 response to endoplasmic reticulum stress	1/67	256/14464	0.00390625	0.84328358	-0.1725832	0.69857173	0.74292549	0.71250205	Foxred2	1
GO:0033674 positive regulation of kinase activity	1/67	258/14464	0.00387597	0.8367465	-0.1804958	0.70140799	0.74491365	0.7144088	Ager	1
GO:0045927 positive regulation of growth	1/67	258/14464	0.00387597	0.8367465	-0.1804958	0.70140799	0.74491365	0.7144088	Rftn1	1
GO:0030217 T cell differentiation	1/67	263/14464	0.00380228	0.82083877	-0.200029	0.70838413	0.75180437	0.72101734	Enpp1	1
GO:0098813 nuclear chromosome segregation	1/67	264/14464	0.00378788	0.81772953	-0.2038941	0.70975996	0.75274611	0.72192052	Ehmt2	1
GO:0009141 nucleoside triphosphate metabolic process	1/67	265/14464	0.00377358	0.81464376	-0.2077456	0.71112941	0.75367979	0.72281596	Enpp1	1
GO:0048608 reproductive structure development	1/67	268/14464	0.00373134	0.80552462	-0.2192197	0.71519966	0.75747263	0.72645348	Tnfsf10	1
GO:0061448 connective tissue development	1/67	272/14464	0.00367647	0.79367867	-0.2343351	0.72053889	0.76207991	0.73087209	Enpp1	1
GO:0061458 reproductive system development	1/67	272/14464	0.00367647	0.79367867	-0.2343351	0.72053889	0.76207991	0.73087209	Tnfsf10	1
GO:0006066 alcohol metabolic process	1/67	275/14464	0.00363636	0.78502035	-0.2455378	0.72447849	0.76499741	0.73367011	Enpp1	1
GO:0007281 germ cell development	1/67	276/14464	0.00362319	0.78217608	-0.2492471	0.7257795	0.76499741	0.73367011	Ehmt2	1
GO:0030198 extracellular matrix organization	1/67	276/14464	0.00362319	0.78217608	-0.2492471	0.7257795	0.76499741	0.73367011	Abi3bp	1
GO:0043062 extracellular structure organization	1/67	276/14464	0.00362319	0.78217608	-0.2492471	0.7257795	0.76499741	0.73367011	Abi3bp	1
GO:0045229 external encapsulating structure organization	1/67	276/14464	0.00362319	0.78217608	-0.2492471	0.7257795	0.76499741	0.73367011	Abi3bp	1
GO:0010948 negative regulation of cell cycle process	1/67	277/14464	0.00361011	0.77935234	-0.2529441	0.72707445	0.76531539	0.73397507	Rpa2	1
GO:0032386 regulation of intracellular transport	1/67	277/14464	0.00361011	0.77935234	-0.2529441	0.72707445	0.76531539	0.73397507	Pkia	1
GO:0006874 intracellular calcium ion homeostasis	1/67	278/14464	0.00359712	0.77654891	-0.2566289	0.72836338	0.76562617	0.73427313	Slc30a1	1
GO:0090287 regulation of cellular response to growth factor stimulus	1/67	278/14464	0.00359712	0.77654891	-0.2566289	0.72836338	0.76562617	0.73427313	Ldlrad4	1
GO:0006302 double-strand break repair	1/67	281/14464	0.00355872	0.76825835	-0.2676109	0.73219429	0.7686045	0.73712949	Rpa2	1
GO:0046942 carboxylic acid transport	1/67	281/14464	0.00355872	0.76825835	-0.2676109	0.73219429	0.7686045	0.73712949	Slco1a4	1
GO:0009416 response to light stimulus	1/67	283/14464	0.00353357	0.76282896	-0.274873	0.73471861	0.76968143	0.73816232	Nfatc4	1
GO:0015849 organic acid transport	1/67	283/14464	0.00353357	0.76282896	-0.274873	0.73471861	0.76968143	0.73816232	Slco1a4	1
GO:0032496 response to lipopolysaccharide	1/67	283/14464	0.00353357	0.76282896	-0.274873	0.73471861	0.76968143	0.73816232	Nr1d1	1
GO:1902105 regulation of leukocyte differentiation	1/67	286/14464	0.0034965	0.75482726	-0.285679	0.73846121	0.77307658	0.74141843	Ager	1
GO:0001666 response to hypoxia	1/67	289/14464	0.00346021	0.74699169	-0.2963826	0.74215178	0.77641269	0.74461793	Ager	1
GO:0072659 protein localization to plasma membrane	1/67	292/14464	0.00342466	0.73931711	-0.3069863	0.74579104	0.77916203	0.74725468	Sys1	1
GO:0141091 transforming growth factor beta receptor superfamily signaling pathway	1/67	292/14464	0.00342466	0.73931711	-0.3069863	0.74579104	0.77916203	0.74725468	Ldlrad4	1
GO:1904062 regulation of monoatomic cation transmembrane transport	1/67	294/14464	0.00340136	0.73428774	-0.3140009	0.74818906	0.78113776	0.7491495	Slc30a1	1
GO:0022412 cellular process involved in reproduction in multicellular organism	1/67	295/14464	0.00338983	0.73179863	-0.3174921	0.74937969	0.78185112	0.74983365	Ehmt2	1
GO:0002237 response to molecule of bacterial origin	1/67	297/14464	0.003367	0.7268707	-0.3244428	0.75174436	0.78378759	0.75169082	Nr1d1	1
GO:0019216 regulation of lipid metabolic process	1/67	298/14464	0.0033557	0.72443153	-0.3279024	0.75291843	0.78448094	0.75235577	Nr1d1	1
GO:0071214 cellular response to abiotic stimulus	1/67	301/14464	0.00332226	0.71721129	-0.3382192	0.75640794	0.78705242	0.75482195	Nfatc4	1
GO:0104004 cellular response to environmental stimulus	1/67	301/14464	0.00332226	0.71721129	-0.3382192	0.75640794	0.78705242	0.75482195	Nfatc4	1
GO:0001822 kidney development	1/67	303/14464	0.00330033	0.71247722	-0.3450462	0.75870726	0.7889122	0.75660558	Enpp1	1
GO:0036293 response to decreased oxygen levels	1/67	304/14464	0.00328947	0.71013354	-0.3484446	0.75984889	0.78956651	0.75723309	Ager	1
GO:0062197 cellular response to chemical stress	1/67	305/14464	0.00327869	0.70780524	-0.3518331	0.7609852	0.79021441	0.75785446	Pycr1	1
GO:0034504 protein localization to nucleus	1/67	306/14464	0.00326797	0.70549215	-0.3552117	0.76211621	0.79085594	0.75846972	Pkia	1
GO:0051235 maintenance of location	1/67	307/14464	0.00325733	0.70319413	-0.3585805	0.76324195	0.79149114	0.75907891	Enpp1	1
GO:0042254 ribosome biogenesis	1/67	309/14464	0.00323625	0.69864271	-0.365289	0.7654777	0.79327582	0.7607905	Rrp9	1
GO:0030100 regulation of endocytosis	1/67	310/14464	0.00322581	0.69638902	-0.3686288	0.76658777	0.79389231	0.76138174	Ager	1
GO:0009150 purine ribonucleotide metabolic process	1/67	311/14464	0.00321543	0.69414983	-0.3719592	0.76769266	0.79450261	0.76196705	Enpp1	1
GO:0009615 response to virus	1/67	312/14464	0.00320513	0.69192499	-0.37528	0.7687924	0.79510677	0.76254647	Aim2	1
GO:0007178 cell surface receptor protein serine/threonine kinase signaling pathway	1/67	313/14464	0.00319489	0.68971437	-0.3785915	0.76988701	0.79570482	0.76312003	Ldlrad4	1
GO:0072001 renal system development	1/67	315/14464	0.0031746	0.68533523	-0.3851866	0.77206094	0.79741682	0.76476193	Enpp1	1
GO:0030522 intracellular receptor signaling pathway	1/67	318/14464	0.00314465	0.6788698	-0.3950103	0.77528394	0.80020935	0.76744009	Rftn1	1

GO:0062012	regulation of small molecule metabolic process	1/67	319/14464	0.0031348	0.67674168	-0.3982669	0.77634827	0.80077154	0.76797927	Nr1d1	1
GO:0034765	regulation of monoatomic ion transmembrane transport	1/67	323/14464	0.00309598	0.66836098	-0.4112041	0.78055615	0.80403153	0.77110576	Slc30a1	1
GO:0060562	epithelial tube morphogenesis	1/67	323/14464	0.00309598	0.66836098	-0.4112041	0.78055615	0.80403153	0.77110576	Nfatc4	1
GO:0006913	nucleocytoplasmic transport	1/67	324/14464	0.00308642	0.66629814	-0.4144165	0.78159587	0.80403153	0.77110576	Pkia	1
GO:0051169	nuclear transport	1/67	324/14464	0.00308642	0.66629814	-0.4144165	0.78159587	0.80403153	0.77110576	Pkia	1
GO:0070482	response to oxygen levels	1/67	327/14464	0.0030581	0.66018531	-0.4240021	0.78468602	0.80667188	0.77363798	Ager	1
GO:0032956	regulation of actin cytoskeleton organization	1/67	328/14464	0.00304878	0.65817255	-0.4271803	0.78570646	0.80718244	0.77412763	Ngef	1
GO:0009259	ribonucleotide metabolic process	1/67	332/14464	0.00301205	0.65024276	-0.4398094	0.78974082	0.81078654	0.77758414	Enpp1	1
GO:0006631	fatty acid metabolic process	1/67	335/14464	0.00298507	0.64441969	-0.4491951	0.7927174	0.8133006	0.77999525	Enpp1	1
GO:0019693	ribose phosphate metabolic process	1/67	339/14464	0.00294985	0.63681592	-0.461597	0.79662168	0.81676248	0.78331536	Enpp1	1
GO:0015711	organic anion transport	1/67	341/14464	0.00293255	0.63308093	-0.4677509	0.79854657	0.81819166	0.78468602	Slco1a4	1
GO:0006869	lipid transport	1/67	342/14464	0.00292398	0.63122982	-0.4708161	0.79950227	0.81862658	0.78510312	Slco1a4	1
GO:0045786	negative regulation of cell cycle	1/67	343/14464	0.00291545	0.6293895	-0.4738737	0.80045351	0.81905634	0.78551529	Rpa2	1
GO:0042176	regulation of protein catabolic process	1/67	344/14464	0.00290698	0.62755988	-0.4769237	0.8014003	0.81948099	0.78592255	Ppp2r3a	1
GO:0016050	vesicle organization	1/67	351/14464	0.002849	0.61504444	-0.4980639	0.80790504	0.82558466	0.79177627	Stx17	1
GO:0019221	cytokine-mediated signaling pathway	1/67	353/14464	0.00283286	0.61155976	-0.504038	0.80972468	0.82689578	0.79303369	Aim2	1
GO:0001503	ossification	1/67	359/14464	0.00278552	0.60133871	-0.5217895	0.81508232	0.83126529	0.79722427	Enpp1	1
GO:0007018	microtubule-based movement	1/67	359/14464	0.00278552	0.60133871	-0.5217895	0.81508232	0.83126529	0.79722427	Ccdc88c	1
GO:0000280	nuclear division	1/67	360/14464	0.00277778	0.59966833	-0.5247237	0.8159607	0.83161074	0.79755557	Ehmt2	1
GO:0007600	sensory perception	1/67	362/14464	0.00276243	0.59635524	-0.5305714	0.81770513	0.83228772	0.79820483	Enpp1	1
GO:1903706	regulation of hemopoiesis	1/67	362/14464	0.00276243	0.59635524	-0.5305714	0.81770513	0.83228772	0.79820483	Ager	1
GO:0007059	chromosome segregation	1/67	364/14464	0.00274725	0.59307856	-0.536392	0.81943328	0.83349616	0.79936379	Ehmt2	1
GO:0032970	regulation of actin filament-based process	1/67	368/14464	0.00271739	0.58663206	-0.5479531	0.82284131	0.83641059	0.80215886	Ngef	1
GO:0044782	cilium organization	1/67	369/14464	0.00271003	0.58504227	-0.5508268	0.82368336	0.83671461	0.80245043	Ccdc88c	1
GO:1902903	regulation of supramolecular fiber organization	1/67	374/14464	0.0026738	0.57722085	-0.5650989	0.82783487	0.84037782	0.80596363	Ccdc88c	1
GO:0010959	regulation of metal ion transport	1/67	375/14464	0.00266667	0.57568159	-0.5679342	0.82865354	0.8406551	0.80622956	Slc30a1	1
GO:1990778	protein localization to cell periphery	1/67	378/14464	0.0026455	0.57111269	-0.5764025	0.83108661	0.84256872	0.80806482	Sys1	1
GO:0006979	response to oxidative stress	1/67	388/14464	0.00257732	0.55639329	-0.6042337	0.83895362	0.84998522	0.81517761	Pycr1	1
GO:0001558	regulation of cell growth	1/67	389/14464	0.00257069	0.55496297	-0.6069841	0.83972018	0.85020289	0.81538636	Enpp1	1
GO:0009314	response to radiation	1/67	394/14464	0.00253807	0.5479203	-0.6206499	0.8434994	0.85310229	0.81816702	Nfatc4	1
GO:0003012	muscle system process	1/67	395/14464	0.00253165	0.54653316	-0.6233659	0.84424464	0.85310229	0.81816702	Tiam1	1
GO:0006163	purine nucleotide metabolic process	1/67	395/14464	0.00253165	0.54653316	-0.6233659	0.84424464	0.85310229	0.81816702	Enpp1	1
GO:0048732	gland development	1/67	408/14464	0.00245098	0.52911911	-0.6581742	0.85362044	0.8620112	0.82671111	Hnrnpd	1
GO:0043254	regulation of protein-containing complex assembly	1/67	413/14464	0.00242131	0.52271331	-0.6713227	0.85707652	0.86493446	0.82951466	Fnip1	1
GO:0072594	establishment of protein localization to organelle	1/67	415/14464	0.00240964	0.52019421	-0.6765461	0.85843634	0.86573979	0.83028701	Pkia	1
GO:0048285	organelle fission	1/67	416/14464	0.00240385	0.51894374	-0.6791503	0.85911146	0.86585399	0.83039653	Ehmt2	1
GO:0048568	embryonic organ development	1/67	421/14464	0.0023753	0.51278052	-0.692096	0.86243977	0.86864032	0.83306876	Enpp1	1
GO:0042692	muscle cell differentiation	1/67	423/14464	0.00236407	0.51035602	-0.6972398	0.86374931	0.86939104	0.83378873	Nfatc4	1
GO:0030099	myeloid cell differentiation	1/67	428/14464	0.00233645	0.50439392	-0.7100148	0.86696965	0.87206281	0.8363511	Enpp1	1
GO:0043269	regulation of monoatomic ion transport	1/67	440/14464	0.00227273	0.49063772	-0.7401977	0.87439572	0.87895878	0.84296467	Slc30a1	1
GO:0051656	establishment of organelle localization	1/67	442/14464	0.00226244	0.48841764	-0.7451648	0.87559305	0.87958859	0.84356869	Gpsm1	1
GO:0008015	blood circulation	1/67	443/14464	0.00225734	0.48731512	-0.7476416	0.87618749	0.87961233	0.84359146	Ager	1
GO:0030003	intracellular monoatomic cation homeostasis	1/67	447/14464	0.00223714	0.48295436	-0.7575053	0.87853741	0.88139723	0.84530326	Slc30a1	1
GO:0009117	nucleotide metabolic process	1/67	450/14464	0.00222222	0.47973466	-0.7648578	0.88027096	0.88256184	0.84642018	Enpp1	1
GO:0006873	intracellular monoatomic ion homeostasis	1/67	454/14464	0.00220264	0.47550792	-0.7746016	0.8825445	0.88426598	0.84805453	Slc30a1	1
GO:0001701	in utero embryonic development	1/67	461/14464	0.0021692	0.46828763	-0.7914934	0.88642131	0.88757325	0.85122637	Slc30a1	1
GO:0001501	skeletal system development	1/67	466/14464	0.00214592	0.46326308	-0.8034374	0.889113	0.88969035	0.85325677	Enpp1	1
GO:0003013	circulatory system process	1/67	470/14464	0.00212766	0.45932042	-0.8129214	0.89122103	0.89122103	0.85472477	Ager	1

ID	Description	GeneRatio	BgRatio	RichFactor	FoldEnrichm	zScore	pvalue	p.adjust	qvalue	geneID	Count
GO:0048511	rhythmic process	13/95	291/14464	0.04467354	6.80166395	8.12916417	5.4063E-08	0.00010375	9.9191E-05	Dbp/Ciart/Per3/Per1/Nr1d2/Tef/Hlf/Bhlhe40/Fzd4/Klf9/Bhlhe41/Gc	13
GO:0007623	circadian rhythm	8/95	204/14464	0.03921569	5.97069143	5.81366293	5.8055E-05	0.04254121	0.04067322	Dbp/Ciart/Per3/Per1/Nr1d2/Bhlhe40/Bhlhe41/Agrn	8
GO:0032922	circadian regulation of gene expression	5/95	65/14464	0.07692308	11.7117409	7.03766021	6.6505E-05	0.04254121	0.04067322	Ciart/Per3/Per1/Bhlhe40/Bhlhe41	5
GO:0043153	entrainment of circadian clock by photoperiod	3/95	22/14464	0.13636364	20.7617225	7.54223725	0.00038611	0.14133952	0.13513331	Per3/Per1/Bhlhe40	3
GO:0009648	photoperiodism	3/95	23/14464	0.13043478	19.8590389	7.35974155	0.00044192	0.14133952	0.13513331	Per3/Per1/Bhlhe40	3
GO:0009649	entrainment of circadian clock	3/95	23/14464	0.13043478	19.8590389	7.35974155	0.00044192	0.14133952	0.13513331	Per3/Per1/Bhlhe40	3
GO:0034143	regulation of toll-like receptor 4 signaling pathway	3/95	25/14464	0.12	18.2703158	7.02715363	0.00056848	0.15584565	0.14900247	Trem2/Peli1/Lyn	3
GO:0071675	regulation of mononuclear cell migration	5/95	114/14464	0.04385965	6.677747	4.94857589	0.00091804	0.22021589	0.21054623	Trem2/Serpine1/Abl1/Lyn/Jam2	5
GO:0097028	dendritic cell differentiation	3/95	35/14464	0.08571429	13.0502256	5.80348418	0.00154268	0.24134334	0.23074597	Trem2/Gimap5/Lyn	3
GO:0002714	positive regulation of B cell mediated immunity	3/95	36/14464	0.08333333	12.6877193	5.70894264	0.00167497	0.24134334	0.23074597	Trem2/Gimap5/Paxip1	3
GO:0002891	positive regulation of immunoglobulin mediated immune response	3/95	36/14464	0.08333333	12.6877193	5.70894264	0.00167497	0.24134334	0.23074597	Trem2/Gimap5/Paxip1	3
GO:0007162	negative regulation of cell adhesion	7/95	263/14464	0.02661597	4.05235141	4.06191345	0.00171964	0.24134334	0.23074597	Fzd4/Serpine1/Gimap5/Acer2/Abl1/Peli1/Jam2	7
GO:0071677	positive regulation of mononuclear cell migration	4/95	79/14464	0.05063291	7.708994	4.8617625	0.00180111	0.24134334	0.23074597	Trem2/Abl1/Jam2	4
GO:0002922	positive regulation of humoral immune response	2/95	10/14464	0.2	30.4505263	7.57489747	0.00185616	0.24134334	0.23074597	Trem2/Gimap5	2
GO:0002532	production of molecular mediator involved in inflammatory response	4/95	80/14464	0.05	7.61263158	4.82233311	0.00188648	0.24134334	0.23074597	Per1/Trem2/Serpine1/Lyn	4
GO:0009068	aspartate family amino acid catabolic process	2/95	11/14464	0.18181818	27.6822967	7.19810658	0.00225895	0.25116102	0.24013255	Pipox/Tha1	2
GO:0070988	demethylation	2/95	11/14464	0.18181818	27.6822967	7.19810658	0.00225895	0.25116102	0.24013255	Por/Cyp3a13	2
GO:0002696	positive regulation of leukocyte activation	7/95	283/14464	0.02473498	3.76596615	3.82088858	0.00259914	0.25116102	0.24013255	Trem2/Gimap5/Il15ra/Abl1/Peli1/Paxip1/Arid2	7
GO:1903706	regulation of hemopoiesis	8/95	362/14464	0.02209945	3.36469904	3.70482355	0.00260984	0.25116102	0.24013255	Trem2/Tcim/Gimap5/Il15ra/Lrrc17/Abl1/Lyn/Arid2	8
GO:0033628	regulation of cell adhesion mediated by integrin	3/95	42/14464	0.07142857	10.875188	5.21116703	0.00261762	0.25116102	0.24013255	Serpine1/Acer2/Lyn	3
GO:1902105	regulation of leukocyte differentiation	7/95	286/14464	0.02447552	3.72646301	3.78662994	0.00275643	0.2518856	0.24082532	Trem2/Gimap5/Il15ra/Lrrc17/Abl1/Lyn/Arid2	7
GO:0002923	regulation of humoral immune response mediated by circulating immunoglobulin	2/95	13/14464	0.15384615	23.4234818	6.57663133	0.00317633	0.27706218	0.2648964	Trem2/Gimap5	2
GO:0034134	toll-like receptor 2 signaling pathway	2/95	14/14464	0.14285714	21.7503759	6.31587879	0.00368991	0.29200346	0.27918161	Trem2/Lyn	2
GO:0050867	positive regulation of cell activation	7/95	303/14464	0.02310231	3.51738753	3.60082614	0.00379071	0.29200346	0.27918161	Trem2/Gimap5/Il15ra/Abl1/Peli1/Paxip1/Arid2	7
GO:0002712	regulation of B cell mediated immunity	3/95	50/14464	0.06	9.13515789	4.68528974	0.00430326	0.29200346	0.27918161	Trem2/Gimap5/Paxip1	3
GO:0002889	regulation of immunoglobulin mediated immune response	3/95	50/14464	0.06	9.13515789	4.68528974	0.00430326	0.29200346	0.27918161	Trem2/Gimap5/Paxip1	3
GO:0071674	mononuclear cell migration	5/95	163/14464	0.03067485	4.67032612	3.83171304	0.00438377	0.29200346	0.27918161	Trem2/Serpine1/Abl1/Lyn/Jam2	5
GO:0000724	double-strand break repair via homologous recombination	5/95	164/14464	0.0304878	4.64184852	3.81376129	0.00449857	0.29200346	0.27918161	Abl1/Xrcc3/Peli1/Fan1/Arid2	5
GO:0042752	regulation of circadian rhythm	4/95	103/14464	0.03883495	5.91272356	4.06842506	0.00470035	0.29200346	0.27918161	Per3/Per1/Nr1d2/Bhlhe40	4
GO:0034142	toll-like receptor 4 signaling pathway	3/95	52/14464	0.05769231	8.78380567	4.57203191	0.00480655	0.29200346	0.27918161	Trem2/Peli1/Lyn	3
GO:0000725	recombinational repair	5/95	169/14464	0.0295858	4.50451573	3.72612147	0.0051049	0.29200346	0.27918161	Abl1/Xrcc3/Peli1/Fan1/Arid2	5
GO:0035567	non-canonical Wnt signaling pathway	3/95	54/14464	0.05555556	8.45847953	4.46470651	0.00534399	0.29200346	0.27918161	Fzd4/Abl1/Celsr3	3
GO:0048512	circadian behavior	3/95	54/14464	0.05555556	8.45847953	4.46470651	0.00534399	0.29200346	0.27918161	Ciart/Per3/Nr1d2	3
GO:0090030	regulation of steroid hormone biosynthetic process	2/95	17/14464	0.11764706	17.9120743	5.67296659	0.00544442	0.29200346	0.27918161	Por/Clcn2	2
GO:0007622	rhythmic behavior	3/95	55/14464	0.05454545	8.304689	4.41310111	0.0056257	0.29200346	0.27918161	Ciart/Per3/Nr1d2	3
GO:0010812	negative regulation of cell-substrate adhesion	3/95	55/14464	0.05454545	8.304689	4.41310111	0.0056257	0.29200346	0.27918161	Fzd4/Serpine1/Acer2	3
GO:0071222	cellular response to lipopolysaccharide	5/95	173/14464	0.02890173	4.40036507	3.65843221	0.00563008	0.29200346	0.27918161	Trem2/Serpine1/Abl1/Lyn/Cdk19	5
GO:0048145	regulation of fibroblast proliferation	4/95	112/14464	0.03571429	5.43759398	3.83334658	0.00631884	0.31910131	0.30508959	Serpine1/Inca1/Abl1/Brpf1	4
GO:0071219	cellular response to molecule of bacterial origin	5/95	180/14464	0.02777778	4.22923977	3.54478049	0.00663928	0.32668663	0.31234184	Trem2/Serpine1/Abl1/Lyn/Cdk19	5
GO:0140895	cell surface toll-like receptor signaling pathway	3/95	60/14464	0.05	7.61263158	4.17336259	0.00716712	0.32837844	0.31395937	Trem2/Peli1/Lyn	3
GO:0051251	positive regulation of lymphocyte activation	6/95	260/14464	0.02307692	3.51352227	3.325384	0.00730203	0.32837844	0.31395937	Gimap5/Il15ra/Abl1/Peli1/Paxip1/Arid2	6
GO:0000018	regulation of DNA recombination	4/95	117/14464	0.03418803	5.20521817	3.71345904	0.00735814	0.32837844	0.31395937	Abl1/Peli1/Paxip1/Arid2	4
GO:0021549	cerebellum development	4/95	117/14464	0.03418803	5.20521817	3.71345904	0.00735814	0.32837844	0.31395937	Fzd4/Gdf10/Rere/Abl1	4
GO:0002752	cell surface pattern recognition receptor signaling pathway	3/95	63/14464	0.04761905	7.25012531	4.04241036	0.00820057	0.35251909	0.33704	Trem2/Peli1/Lyn	3
GO:0046165	alcohol biosynthetic process	4/95	121/14464	0.03305785	5.03314485	3.62238185	0.00826647	0.35251909	0.33704	Acer2/Por/Lss/Clcn2	4
GO:0002685	regulation of leukocyte migration	5/95	191/14464	0.02617801	3.98567098	3.37737062	0.00847187	0.35342441	0.33790556	Trem2/Serpine1/Abl1/Lyn/Jam2	5
GO:0006909	phagocytosis	5/95	194/14464	0.0257732	3.9240369	3.33387606	0.00902696	0.36856872	0.35238489	Trem2/Il15ra/Rab34/Clcn2/Abl1	5
GO:0022037	metencephalon development	4/95	125/14464	0.032	4.87208421	3.5352334	0.00924551	0.36962778	0.35339745	Fzd4/Gdf10/Rere/Abl1	4
GO:0002220	innate immune response activating cell surface receptor signaling pathway	3/95	67/14464	0.04477612	6.81728201	3.88060323	0.0097084	0.37410191	0.35767512	Trem2/Peli1/Lyn	3
GO:0046885	regulation of hormone biosynthetic process	2/95	23/14464	0.08695652	13.2393593	4.77641101	0.00987267	0.37410191	0.35767512	Por/Clcn2	2
GO:0006310	DNA recombination	6/95	279/14464	0.02150538	3.27425014	3.1189139	0.01014172	0.37410191	0.35767512	Abl1/Xrcc3/Peli1/Fan1/Paxip1/Arid2	6
GO:0046475	glycerophospholipid catabolic process	2/95	24/14464	0.08333333	12.6877193	4.6593949	0.01072449	0.37410191	0.35767512	Pnpla7/Gpcpd1	2
GO:0090025	regulation of monocyte chemotaxis	2/95	24/14464	0.08333333	12.6877193	4.6593949	0.01072449	0.37410191	0.35767512	Serpine1/Lyn	2
GO:0032496	response to lipopolysaccharide	6/95	283/14464	0.02120141	3.22797099	3.07770538	0.01082751	0.37410191	0.35767512	Trem2/Serpine1/Abl1/Peli1/Lyn/Cdk19	6
GO:0071216	cellular response to biotic stimulus	5/95	205/14464	0.02439024	3.71347882	3.1815339	0.01127739	0.37410191	0.35767512	Trem2/Serpine1/Abl1/Lyn/Cdk19	5
GO:0045911	positive regulation of DNA recombination	3/95	71/14464	0.04225352	6.43320979	3.73153663	0.01136747	0.37410191	0.35767512	Peli1/Paxip1/Arid2	3
GO:0071347	cellular response to interleukin-1	3/95	71/14464	0.04225352	6.43320979	3.73153663	0.01136747	0.37410191	0.35767512	Serpine1/Rps6ka5/Rbmx	3
GO:0002687	positive regulation of leukocyte migration	4/95	133/14464	0.03007519	4.57902651	3.37155471	0.01142388	0.37410191	0.35767512	Trem2/Serpine1/Abl1/Jam2	4
GO:0002694	regulation of leukocyte activation	8/95	467/14464	0.01713062	2.60818213	2.87245949	0.01166267	0.37410191	0.35767512	Trem2/Gimap5/Il15ra/Abl1/Peli1/Lyn/Paxip1/Arid2	8
GO:0010959	regulation of metal ion transport	7/95	375/14464	0.01866667	2.84204912	2.93869477	0.01169678	0.37410191	0.35767512	Per1/Trem2/Serpine1/Gimap5/Abl1/Agrn/Lyn	7
GO:0006289	nucleotide-excision repair	3/95	73/14464	0.04109589	6.25695746	3.66123942	0.01225455	0.38063134	0.36391784	Pole/Fan1/Arid2	3
GO:0010720	positive regulation of cell development	8/95	473/14464	0.01691332	2.57509736	2.83198742	0.01252201	0.38063134	0.36391784	Fzd4/Trem2/Gimap5/Il15ra/Myo5b/Clcn2/Lyn/Arid2	8
GO:0048144	fibroblast proliferation	4/95	138/14464	0.02898551	4.41311976	3.27571659	0.01293999	0.38063134	0.36391784	Serpine1/Inca1/Abl1/Brpf1	4
GO:0043122	regulation of canonical NF-kappaB signal transduction	5/95	213/14464	0.02347418	3.57400544	3.07719002	0.01313667	0.38063134	0.36391784	Per1/Trem2/Alpk1/Abl1/Peli1	5
GO:0010569	regulation of double-strand break repair via homologous recombination	3/95	75/14464	0.04	6.09010526	3.5935179	0.01318035	0.38063134	0.36391784	Abl1/Peli1/Arid2	3
GO:0051353	positive regulation of oxidoreductase activity	2/95	27/14464	0.07407407	11.2779727	4.34638894	0.01346606	0.38063134	0.36391784	Por/Abl1	2
GO:2000352	negative regulation of endothelial cell apoptotic process	2/95	27/14464	0.07407407	11.2779727	4.34638894	0.01346606	0.38063134	0.36391784	Serpine1/Abl1	2
GO:0002237	response to molecule of bacterial origin	6/95	297/14464	0.02020202	3.07581074	2.93903498	0.01348772	0.38063134	0.36391784	Trem2/Serpine1/Abl1/Peli1/Lyn/Cdk19	6
GO:0042100	B cell proliferation	3/95	78/14464	0.03846154	5.85587045	3.49640786	0.01464219	0.40455877	0.38679462	Abl1/Peli1/Lyn	3
GO:0099173	postsynapse organization	6/95	303/14464	0.01980198	3.0149036	2.8820821	0.01475722	0.40455877	0.38679462	Trem2/Myo5b/Rps6ka5/Abl1/Agrn/Rbmx	6
GO:0002920	regulation of humoral immune response	2/95	29/14464	0.06896552	10.5001815	4.16389942	0.01544453	0.41181742	0.39373454	Trem2/Gimap5	2
GO:0002260	lymphocyte homeostasis	3/95	80/14464	0.0375	5.70947368	3.43443463	0.0156658	0.41181742	0.39373454	Gimap5/Abl1/Lyn	3
GO:0033627	cell adhesion mediated by integrin	3/95	80/14464	0.0375	5.70947368	3.43443463	0.0156658	0.41181742	0.39373454	Serpine1/Acer2/Lyn	3
GO:0002455	humoral immune response mediated by circulating immunoglobulin	2/95	30/14464	0.06666667	10.1501754	4.07919457	0.01647772	0.41945363	0.40103545	Trem2/Gimap5	2
GO:0060071	Wnt signaling pathway, planar cell polarity pathway	2/95	30/14464	0.06666667	10.1501754	4.07919457	0.01647772	0.41945363	0.40103545	Abl1/Celsr3	2
GO:0044770	cell cycle phase transition	8/95	498/14464	0.01606426	2.44582541	2.66976004	0.01661202	0.41945363	0.40103545	Tcim/Wee1/Ccdc57/Pole/Xrcc3/Lyn/Paxip1/Arid2	8
GO:1905244	regulation of modification of synaptic structure	2/95	31/14464	0.06451613	9.82275042	3.99838177	0.01753965	0.42113893	0.40264675	Myo5b/Abl1	2
GO:0071901	negative regulation of protein serine/threonine kinase activity	3/95	84/14464	0.03571429	5.43759398	3.31654189	0.01783147	0.42113893	0.40264675	Inca1/Abl1/Lyn	3
GO:0051249	regulation of lymphocyte activation	7/95	409/14464	0.01711491	2.60579076	2.6786311	0.0180508	0.42113893	0.40264675	Gimap5/Il15ra/Abl1/Peli1/Lyn/Paxip1/Arid2	7
GO:0031349	positive regulation of defense response	7/95	410/14464	0.01707317	2.59943517	2.67138399	0.01826838	0.42113893	0.40264675	Trem2/Serpine1/Gimap5/Rab34/Alpk1/Peli1/Lyn	7
GO:0030522	intracellular receptor signaling pathway	6/95	318/14464	0.01886792	2.87269116	2.74562119	0.01829142	0.42113893	0.40264675	Per1/Nr1d2/Trem2/Klf9/Alpk1/Peli1	6
GO:0018108	peptidyl-tyrosine phosphorylation	5/95	233/14464	0.02145923	3.26722385	2.8368239	0.01866719	0.42113893	0.40264675	Trem2/Wee1/Abl1/Agrn/Lyn	5
GO:0001570	vasculogenesis	3/95	86/14464	0.03488372	5.31113831	3.26039085	0.01897377	0.42113893	0.40264675	Fzd4/Brpf1/Paxip1	3
GO:0071900	regulation of protein serine/threonine kinase activity	5/95	235/14464	0.0212766	3.23941769	2.81422988	0.01929301	0.42113893	0.40264675	Fzd4/Tcim/Inca1/Abl1/Lyn	5
GO:0007249	canonical NF-kappaB signal transduction	5/95	236/14464	0.02118644	3.22569135	2.80302347	0.01961105	0.42113893	0.40264675	Per1/Trem2/Alpk1/Abl1/Peli1	5
GO:0002548	monocyte chemotaxis	2/95	33/14464	0.06060606	9.22743222	3.84725341	0.01974807	0.42113893	0.40264675	Serpine1/Lyn	2
GO:0043409	negative regulation of MAPK cascade	4/95	157/14464	0.02547771	3.87904794	2.94918596	0.01984866	0.42113893	0.40264675	Per1/Trem2/Abl1/Lyn	4
GO:0018212	peptidyl-tyrosine modification	5/95	237/14464	0.02109705	3.21208083	2.79187663	0.01993253	0.42113893	0.40264675	Trem2/Wee1/Abl1/Agrn/Lyn	5
GO:0050767	regulation of neurogenesis	7/95	418/14464	0.01674641	2.54968522	2.61416516	0.02007622	0.42113893	0.40264675	Bhlhe40/Fzd4/Trem2/Bhlhe41/Myo5b/Clcn2/Lyn	7
GO:0001953	negative regulation of cell-matrix adhesion	2/95	34/14464	0.05882353	8.95603715	3.77642461	0.02089375	0.42113893	0.40264675	Serpine1/Acer2	2
GO:2000403	positive regulation of lymphocyte migration	2/95	34/14464	0.05882353	8.95603715	3.77642461	0.02089375	0.42113893	0.40264675	Abl1/Jam2	2
GO:0070555	response to interleukin-1	3/95	90/14464	0.03333333	5.07508772	3.15316807	0.02137776	0.42113893	0.40264675	Serpine1/Rps6ka5/Rbmx	3
GO:0002833	positive regulation of response to biotic stimulus	6/95	331/14464	0.01812689	2.75986643	2.6336269	0.02179268	0.42113893	0.40264675	Trem2/Gimap5/Rab34/Alpk1/Peli1/Lyn	6
GO:0046785	microtubule polymerization	3/95	91/14464	0.03296703	5.01931752	3.12735385	0.02200366	0.42113893	0.40264675	Ccdc57/Abl1/Nav3	3
GO:0002263	cell activation involved in immune response	5/95	245/14464	0.02040816	3.10719656	2.7047759	0.02262964	0.42113893	0.40264675	Trem2/Tcim/Abl1/Lyn/Paxip1	5
GO:0009066	aspartate family amino acid metabolic process	2/95	36/14464	0.05555556	8.45847953	3.64314292	0.02326605	0.42113893	0.40264675	Pipox/Tha1	2
GO:0032350	regulation of hormone metabolic process	2/95	36/14464	0.05555556	8.45847953	3.64314292	0.02326605	0.42113893	0.40264675	Por/Clcn2	2
GO:0036297	interstrand cross-link repair	2/95	37/14464	0.05405405	8.22987198	3.58031449	0.02449187	0.42113893	0.40264675	Xrcc3/Fan1	2
GO:0060563	neuroepithelial cell differentiation	2/95	37/14464	0.05405405	8.22987198	3.58031449	0.02449187	0.42113893	0.40264675	Serpine1/Abl1	2
GO:0002708	positive regulation of lymphocyte mediated immunity	3/95	95/14464	0.03157895	4.80797784	3.02775455	0.02460695	0.42113893	0.40264675	Trem2/Gimap5/Paxip1	3
GO:0030902	hindbrain development	4/95	170/14464	0.02352941	3.58241486	2.75391992	0.02567574	0.42113893	0.40264675	Fzd4/Gdf10/Rere/Abl1	4
GO:0043269	regulation of monoatomic ion transport	7/95	440/14464	0.01590909	2.42220096	2.46336676	0.02568861	0.42113893	0.40264675	Per1/Trem2/Serpine1/Gimap5/Abl1/Agrn/Lyn	7
GO:1902107	positive regulation of leukocyte differentiation	4/95	171/14464	0.02339181	3.56146507	2.7396969	0.02616228	0.42113893	0.40264675	Trem2/Gimap5/Il15ra/Arid2	4
GO:1903708	positive regulation of hemopoiesis	4/95	171/14464	0.02339181	3.56146507	2.7396969	0.02616228	0.42113893	0.40264675	Trem2/Gimap5/Il15ra/Arid2	4
GO:0031929	TOR signaling	4/95	172/14464	0.02325581	3.54075887	2.72557975	0.02665435	0.42113893	0.40264675	Trem2/Sar1b/Rps6ka5/Peli1	4
GO:1900744	regulation of p38MAPK cascade	2/95	39/14464	0.05128205	7.80782726	3.46147057	0.02702092	0.42113893	0.40264675	Per1/Trem2	2
GO:0033674	positive regulation of kinase activity	5/95	258/14464	0.01937984	2.95063239	2.5705562	0.02749967	0.42113893	0.40264675	Fzd4/Trem2/Tcim/Abl1/Agrn	5
GO:0002065	columnar/cuboidal epithelial cell differentiation	3/95	100/14464	0.03	4.56757895	2.91080839	0.02808517	0.42113893	0.40264675	Serpine1/Clcn2/Abl1	3
GO:0042770	signal transduction in response to DNA damage	4/95	175/14464	0.02285714	3.48006015	2.68384879	0.02816388	0.42113893	0.40264675	Acer2/Abl1/Lyn/Paxip1	4
GO:0050920	regulation of chemotaxis	4/95	175/14464	0.02285714	3.48006015	2.68384879	0.02816388	0.42113893	0.40264675	Trem2/Serpine1/Agrn/Lyn	4
GO:0001782	B cell homeostasis	2/95	40/14464	0.05	7.61263158	3.40517306	0.02832338	0.42113893	0.40264675	Abl1/Lyn	2
GO:0045880	positive regulation of smoothened signaling pathway	2/95	40/14464	0.05	7.61263158	3.40517306	0.02832338	0.42113893	0.40264675	Por/Rab34	2
GO:1901016	regulation of potassium ion transmembrane transporter activity	2/95	40/14464	0.05	7.61263158	3.40517306	0.02832338	0.42113893	0.40264675	Trem2/Agrn	2
GO:0002824	positive regulation of adaptive immune response based on somatic recombination of immune receptors b	3/95	102/14464	0.02941176	4.47801858	2.86617198	0.02954599	0.42113893	0.40264675	Trem2/Gimap5/Paxip1	3
GO:0009395	phospholipid catabolic process	2/95	41/14464	0.04878049	7.42695764	3.35079059	0.02965062	0.42113893	0.40264675	Pnpla7/Gpcpd1	2
GO:0045619	regulation of lymphocyte differentiation	4/95	178/14464	0.02247191	3.42140745	2.64301894	0.02972359	0.42113893	0.40264675	Gimap5/Il15ra/Abl1/Arid2	4
GO:0050866	negative regulation of cell activation	4/95	178/14464	0.02247191	3.42140745	2.64301894	0.02972359	0.42113893	0.40264675	Trem2/Gimap5/Peli1/Lyn	4
GO:0043549	regulation of kinase activity	7/95	456/14464	0.01535088	2.33721145	2.35924023	0.03039266	0.42113893	0.40264675	Fzd4/Trem2/Tcim/Inca1/Abl1/Agrn/Lyn	7
GO:0042446	hormone biosynthetic process	2/95	42/14464	0.04761905	7.25012531	3.29821033	0.03100227	0.42113893	0.40264675	Por/Clcn2	2
GO:1905168	positive regulation of double-strand break repair via homologous recombination	2/95	42/14464	0.04761905	7.25012531	3.29821033	0.03100227	0.42113893	0.40264675	Peli1/Arid2	2
GO:0002520	immune system development	4/95	183/14464	0.02185792	3.32792637	2.57687409	0.0324353	0.42113893	0.40264675	Lrrc17/Abl1/Lyn/Paxip1	4
GO:0016064	immunoglobulin mediated immune response	3/95	107/14464	0.02803738	4.26876537	2.75944353	0.0333711	0.42113893	0.40264675	Trem2/Gimap5/Paxip1	3
GO:0042542	response to hydrogen peroxide	3/95	107/14464	0.02803738	4.26876537	2.75944353	0.0333711	0.42113893	0.40264675	Net1/ND5/Abl1	3
GO:1901617	organic hydroxy compound biosynthetic process	4/95	187/14464	0.02139037	3.25674078	2.52558346	0.03470614	0.42113893	0.40264675	Acer2/Por/Lss/Clcn2	4
GO:0050807	regulation of synapse organization	6/95	370/14464	0.01621622	2.46896159	2.32740462	0.03494856	0.42113893	0.40264675	Trem2/Myo5b/Rps6ka5/Abl1/Agrn/Rbmx	6
GO:1904262	negative regulation of TORC1 signaling	2/95	45/14464	0.04444444	6.76678363	3.1502866	0.03519998	0.42113893	0.40264675	Sar1b/Peli1	2
GO:0002821	positive regulation of adaptive immune response	3/95	110/14464	0.02727273	4.1523445	2.69849282	0.03578418	0.42113893	0.40264675	Trem2/Gimap5/Paxip1	3
GO:0019724	B cell mediated immunity	3/95	110/14464	0.02727273	4.1523445	2.69849282	0.03578418	0.42113893	0.40264675	Trem2/Gimap5/Paxip1	3
GO:0051928	positive regulation of calcium ion transport	3/95	111/14464	0.02702703	4.11493599	2.67865633	0.03660809	0.42113893	0.40264675	Serpine1/Gimap5/Abl1	3
GO:0046503	glycerolipid catabolic process	2/95	46/14464	0.04347826	6.61967963	3.10395693	0.0366456	0.42113893	0.40264675	Pnpla7/Gpcpd1	2
GO:1900015	regulation of cytokine production involved in inflammatory response	2/95	46/14464	0.04347826	6.61967963	3.10395693	0.0366456	0.42113893	0.40264675	Per1/Trem2	2
GO:2000351	regulation of endothelial cell apoptotic process	2/95	46/14464	0.04347826	6.61967963	3.10395693	0.0366456	0.42113893	0.40264675	Serpine1/Abl1	2
GO:0050803	regulation of synapse structure or activity	6/95	376/14464	0.01595745	2.42956327	2.28375944	0.03734611	0.42113893	0.40264675	Trem2/Myo5b/Rps6ka5/Abl1/Agrn/Rbmx	6
GO:0006302	double-strand break repair	5/95	281/14464	0.01779359	2.70912156	2.35244666	0.03765559	0.42113893	0.40264675	Abl1/Xrcc3/Peli1/Fan1/Arid2	5
GO:1902930	regulation of alcohol biosynthetic process	2/95	47/14464	0.04255319	6.47883539	3.05898576	0.03811381	0.42113893	0.40264675	Por/Clcn2	2
GO:0038202	TORC1 signaling	3/95	113/14464	0.02654867	4.04210526	2.63967282	0.0382851	0.42113893	0.40264675	Sar1b/Rps6ka5/Peli1	3
GO:1905477	positive regulation of protein localization to membrane	3/95	113/14464	0.02654867	4.04210526	2.63967282	0.0382851	0.42113893	0.40264675	Trem2/Myo5b/Agrn	3
GO:0006486	protein glycosylation	4/95	194/14464	0.02061856	3.13922952	2.43906843	0.03889822	0.42113893	0.40264675	Acer2/Galnt15/Tet3/Alg10b	4
GO:0043413	macromolecule glycosylation	4/95	194/14464	0.02061856	3.13922952	2.43906843	0.03889822	0.42113893	0.40264675	Acer2/Galnt15/Tet3/Alg10b	4
GO:0001952	regulation of cell-matrix adhesion	3/95	114/14464	0.02631579	4.0066482	2.62051599	0.03913816	0.42113893	0.40264675	Serpine1/Acer2/Abl1	3
GO:0045621	positive regulation of lymphocyte differentiation	3/95	114/14464	0.02631579	4.0066482	2.62051599	0.03913816	0.42113893	0.40264675	Gimap5/Il15ra/Arid2	3
GO:0051983	regulation of chromosome segregation	3/95	114/14464	0.02631579	4.0066482	2.62051599	0.03913816	0.42113893	0.40264675	Xrcc3/Ncapd3/Arid2	3
GO:0006911	phagocytosis, engulfment	2/95	48/14464	0.04166667	6.34385965	3.01530327	0.03960426	0.42113893	0.40264675	Trem2/Clcn2	2
GO:0051341	regulation of oxidoreductase activity	2/95	48/14464	0.04166667	6.34385965	3.01530327	0.03960426	0.42113893	0.40264675	Por/Abl1	2
GO:0140353	lipid export from cell	2/95	48/14464	0.04166667	6.34385965	3.01530327	0.03960426	0.42113893	0.40264675	Fzd4/Sar1b	2
GO:0002705	positive regulation of leukocyte mediated immunity	3/95	115/14464	0.02608696	3.97180778	2.60157614	0.04000088	0.42113893	0.40264675	Trem2/Gimap5/Paxip1	3
GO:0002703	regulation of leukocyte mediated immunity	4/95	197/14464	0.02030457	3.09142399	2.40318161	0.04078004	0.42113893	0.40264675	Trem2/Gimap5/Lyn/Paxip1	4
GO:0170040	proteinogenic amino acid catabolic process	2/95	49/14464	0.04081633	6.21439313	2.97284462	0.0411166	0.42113893	0.40264675	Pipox/Tha1	2
GO:0001776	leukocyte homeostasis	3/95	117/14464	0.02564103	3.90391363	2.56432933	0.04175522	0.42113893	0.40264675	Gimap5/Abl1/Lyn	3
GO:0014013	regulation of gliogenesis	3/95	117/14464	0.02564103	3.90391363	2.56432933	0.04175522	0.42113893	0.40264675	Trem2/Clcn2/Lyn	3
GO:0002762	negative regulation of myeloid leukocyte differentiation	2/95	50/14464	0.04	6.09010526	2.93154949	0.04265049	0.42113893	0.40264675	Lrrc17/Lyn	2
GO:0032663	regulation of interleukin-2 production	2/95	50/14464	0.04	6.09010526	2.93154949	0.04265049	0.42113893	0.40264675	Abl1/Nav3	2
GO:0072577	endothelial cell apoptotic process	2/95	50/14464	0.04	6.09010526	2.93154949	0.04265049	0.42113893	0.40264675	Serpine1/Abl1	2
GO:0002534	cytokine production involved in inflammatory response	2/95	51/14464	0.03921569	5.97069143	2.89136169	0.04420559	0.42113893	0.40264675	Per1/Trem2	2
GO:0006695	cholesterol biosynthetic process	2/95	51/14464	0.03921569	5.97069143	2.89136169	0.04420559	0.42113893	0.40264675	Por/Lss	2
GO:0034605	cellular response to heat	2/95	51/14464	0.03921569	5.97069143	2.89136169	0.04420559	0.42113893	0.40264675	Tcim/Lyn	2
GO:0050829	defense response to Gram-negative bacterium	2/95	51/14464	0.03921569	5.97069143	2.89136169	0.04420559	0.42113893	0.40264675	Trem2/Serpine1	2
GO:0050853	B cell receptor signaling pathway	2/95	51/14464	0.03921569	5.97069143	2.89136169	0.04420559	0.42113893	0.40264675	Abl1/Lyn	2
GO:0070098	chemokine-mediated signaling pathway	2/95	51/14464	0.03921569	5.97069143	2.89136169	0.04420559	0.42113893	0.40264675	Trem2/Lyn	2
GO:1902653	secondary alcohol biosynthetic process	2/95	51/14464	0.03921569	5.97069143	2.89136169	0.04420559	0.42113893	0.40264675	Por/Lss	2
GO:1904062	regulation of monoatomic cation transmembrane transport	5/95	294/14464	0.0170068	2.58933047	2.23862151	0.04429563	0.42113893	0.40264675	Trem2/Gimap5/Abl1/Agrn/Lyn	5
GO:0002761	regulation of myeloid leukocyte differentiation	3/95	121/14464	0.02479339	3.77485863	2.49224809	0.04537868	0.42113893	0.40264675	Trem2/Lrrc17/Lyn	3
GO:2000401	regulation of lymphocyte migration	2/95	52/14464	0.03846154	5.85587045	2.85222879	0.04578155	0.42113893	0.40264675	Abl1/Jam2	2
GO:0071396	cellular response to lipid	7/95	499/14464	0.01402806	2.13580846	2.09948427	0.0458324	0.42113893	0.40264675	Per1/Trem2/Serpine1/Klf9/Abl1/Lyn/Cdk19	7
GO:0019216	regulation of lipid metabolic process	5/95	298/14464	0.01677852	2.55457436	2.20482303	0.04647188	0.42113893	0.40264675	Nr1d2/Trem2/Gimap5/Por/Clcn2	5

GO:0010810	regulation of cell-substrate adhesion	4/95	206/14464	0.01941748	2.95636178	2.29948827	0.04673264	0.42113893	0.40264675	Fzd4/Serpine1/Acer2/Abl1	4
GO:0043270	positive regulation of monoatomic ion transport	4/95	206/14464	0.01941748	2.95636178	2.29948827	0.04673264	0.42113893	0.40264675	Trem2/Serpine1/Gimap5/Abl1	4
GO:0050864	regulation of B cell activation	3/95	123/14464	0.02439024	3.71347882	2.45734989	0.04724731	0.42113893	0.40264675	Peli1/Lyn/Paxip1	3
GO:0099563	modification of synaptic structure	2/95	53/14464	0.03773585	5.74538232	2.81410182	0.04737804	0.42113893	0.40264675	Myo5b/Abl1	2
GO:0170035	L-amino acid catabolic process	2/95	53/14464	0.03773585	5.74538232	2.81410182	0.04737804	0.42113893	0.40264675	Pipox/Tha1	2
GO:0071466	cellular response to xenobiotic stimulus	3/95	124/14464	0.02419355	3.68353141	2.44017331	0.04819574	0.42113893	0.40264675	Serpine1/Acer2/Gsta4	3
GO:1901987	regulation of cell cycle phase transition	6/95	402/14464	0.01492537	2.27242734	2.10377938	0.04894596	0.42113893	0.40264675	Tcim/Wee1/Xrcc3/Lyn/Paxip1/Arid2	6
GO:0031663	lipopolysaccharide-mediated signaling pathway	2/95	54/14464	0.03703704	5.63898635	2.77693496	0.04899474	0.42113893	0.40264675	Trem2/Lyn	2
GO:1901224	positive regulation of non-canonical NF-kappaB signal transduction	2/95	54/14464	0.03703704	5.63898635	2.77693496	0.04899474	0.42113893	0.40264675	Trem2/Tcim	2
GO:0070085	glycosylation	4/95	210/14464	0.01904762	2.90005013	2.25519475	0.04952598	0.42113893	0.40264675	Acer2/Galnt15/Tet3/Alg10b	4
GO:0050900	leukocyte migration	5/95	304/14464	0.01644737	2.50415512	2.15514014	0.04985454	0.42113893	0.40264675	Trem2/Serpine1/Abl1/Lyn/Jam2	5
GO:0006694	steroid biosynthetic process	3/95	126/14464	0.02380952	3.62506266	2.40634771	0.05012065	0.42113893	0.40264675	Por/Lss/Clcn2	3
GO:0000768	syncytium formation by plasma membrane fusion	2/95	55/14464	0.03636364	5.53645933	2.74068529	0.0506313	0.42113893	0.40264675	Trem2/Jam2	2
GO:0140253	cell-cell fusion	2/95	55/14464	0.03636364	5.53645933	2.74068529	0.0506313	0.42113893	0.40264675	Trem2/Jam2	2
GO:0051347	positive regulation of transferase activity	5/95	306/14464	0.01633987	2.4877881	2.13884131	0.0510137	0.42113893	0.40264675	Fzd4/Trem2/Tcim/Abl1/Agrn	5
GO:0051090	regulation of DNA-binding transcription factor activity	5/95	308/14464	0.01623377	2.47163363	2.12267013	0.05218869	0.42113893	0.40264675	Bhlhe40/Fzd4/Rps6ka5/Peli1/Paxip1	5
GO:0016126	sterol biosynthetic process	2/95	56/14464	0.03571429	5.43759398	2.70531257	0.05228742	0.42113893	0.40264675	Por/Lss	2
GO:0038066	p38MAPK cascade	2/95	56/14464	0.03571429	5.43759398	2.70531257	0.05228742	0.42113893	0.40264675	Per1/Trem2	2
GO:0043124	negative regulation of canonical NF-kappaB signal transduction	2/95	56/14464	0.03571429	5.43759398	2.70531257	0.05228742	0.42113893	0.40264675	Per1/Trem2	2
GO:0045089	positive regulation of innate immune response	5/95	310/14464	0.01612903	2.45568761	2.10662464	0.05337952	0.42113893	0.40264675	Trem2/Gimap5/Alpk1/Peli1/Lyn	5
GO:0002429	immune response-activating cell surface receptor signaling pathway	4/95	216/14464	0.01851852	2.81949318	2.19067563	0.05388586	0.42113893	0.40264675	Trem2/Abl1/Peli1/Lyn	4
GO:0006949	syncytium formation	2/95	57/14464	0.03508772	5.3421976	2.67077902	0.05396276	0.42113893	0.40264675	Trem2/Jam2	2
GO:0031113	regulation of microtubule polymerization	2/95	57/14464	0.03508772	5.3421976	2.67077902	0.05396276	0.42113893	0.40264675	Abl1/Nav3	2
GO:0032623	interleukin-2 production	2/95	57/14464	0.03508772	5.3421976	2.67077902	0.05396276	0.42113893	0.40264675	Abl1/Nav3	2
GO:0060038	cardiac muscle cell proliferation	2/95	57/14464	0.03508772	5.3421976	2.67077902	0.05396276	0.42113893	0.40264675	Abl1/Arid2	2
GO:0099024	plasma membrane invagination	2/95	57/14464	0.03508772	5.3421976	2.67077902	0.05396276	0.42113893	0.40264675	Trem2/Clcn2	2
GO:0043433	negative regulation of DNA-binding transcription factor activity	3/95	130/14464	0.02307692	3.51352227	2.34071445	0.0540817	0.42113893	0.40264675	Bhlhe40/Peli1/Paxip1	3
GO:0031109	microtubule polymerization or depolymerization	3/95	132/14464	0.02272727	3.46028708	2.30885721	0.05611724	0.42113893	0.40264675	Ccdc57/Abl1/Nav3	3
GO:0050731	positive regulation of peptidyl-tyrosine phosphorylation	3/95	133/14464	0.02255639	3.43426989	2.29315823	0.05714862	0.42113893	0.40264675	Trem2/Abl1/Agrn	3
GO:2000779	regulation of double-strand break repair	3/95	133/14464	0.02255639	3.43426989	2.29315823	0.05714862	0.42113893	0.40264675	Abl1/Peli1/Arid2	3
GO:1903078	positive regulation of protein localization to plasma membrane	2/95	59/14464	0.03389831	5.16110616	2.60408942	0.05736986	0.42113893	0.40264675	Trem2/Myo5b	2
GO:0051345	positive regulation of hydrolase activity	5/95	317/14464	0.01577287	2.40146107	2.0514277	0.0576722	0.42113893	0.40264675	Acer2/Net1/Adap2/Agrn/Lyn	5
GO:0050773	regulation of dendrite development	3/95	136/14464	0.02205882	3.35851393	2.24694704	0.06029676	0.42113893	0.40264675	Fzd4/Myo5b/Abl1	3
GO:1904064	positive regulation of cation transmembrane transport	3/95	136/14464	0.02205882	3.35851393	2.24694704	0.06029676	0.42113893	0.40264675	Trem2/Gimap5/Abl1	3
GO:0008202	steroid metabolic process	4/95	225/14464	0.01777778	2.70671345	2.09792447	0.06080555	0.42113893	0.40264675	Por/Lss/Clcn2/Cyp3a13	4
GO:0030888	regulation of B cell proliferation	2/95	61/14464	0.03278689	4.99188956	2.54035656	0.06085009	0.42113893	0.40264675	Peli1/Lyn	2
GO:1990868	response to chemokine	2/95	61/14464	0.03278689	4.99188956	2.54035656	0.06085009	0.42113893	0.40264675	Trem2/Lyn	2
GO:1990869	cellular response to chemokine	2/95	61/14464	0.03278689	4.99188956	2.54035656	0.06085009	0.42113893	0.40264675	Trem2/Lyn	2
GO:0045859	regulation of protein kinase activity	6/95	426/14464	0.01408451	2.14440326	1.94943604	0.06144226	0.42113893	0.40264675	Fzd4/Tcim/Inca1/Abl1/Agrn/Lyn	6
GO:0034765	regulation of monoatomic ion transmembrane transport	5/95	323/14464	0.01547988	2.35685188	2.00526311	0.0615061	0.42113893	0.40264675	Trem2/Gimap5/Abl1/Agrn/Lyn	5
GO:0002768	immune response-regulating cell surface receptor signaling pathway	4/95	227/14464	0.01762115	2.68286575	2.07792985	0.06240471	0.42113893	0.40264675	Trem2/Abl1/Peli1/Lyn	4
GO:0007159	leukocyte cell-cell adhesion	5/95	326/14464	0.01533742	2.33516306	1.9825623	0.06347646	0.42113893	0.40264675	Gimap5/Abl1/Peli1/Jam2/Arid2	5
GO:0008582	regulation of synaptic assembly at neuromuscular junction	1/95	10/14464	0.1	15.2252632	3.65884496	0.06379193	0.42113893	0.40264675	Agrn	1
GO:0010944	negative regulation of transcription by competitive promoter binding	1/95	10/14464	0.1	15.2252632	3.65884496	0.06379193	0.42113893	0.40264675	Bhlhe41	1
GO:0032341	aldosterone metabolic process	1/95	10/14464	0.1	15.2252632	3.65884496	0.06379193	0.42113893	0.40264675	Clcn2	1
GO:0036466	synaptic vesicle recycling via endosome	1/95	10/14464	0.1	15.2252632	3.65884496	0.06379193	0.42113893	0.40264675	Myo5b	1
GO:0044029	positive regulation of gene expression via chromosomal CpG island demethylation	1/95	10/14464	0.1	15.2252632	3.65884496	0.06379193	0.42113893	0.40264675	Tet3	1
GO:0060049	regulation of protein glycosylation	1/95	10/14464	0.1	15.2252632	3.65884496	0.06379193	0.42113893	0.40264675	Acer2	1
GO:0061043	regulation of vascular wound healing	1/95	10/14464	0.1	15.2252632	3.65884496	0.06379193	0.42113893	0.40264675	Serpine1	1
GO:0070344	regulation of fat cell proliferation	1/95	10/14464	0.1	15.2252632	3.65884496	0.06379193	0.42113893	0.40264675	Trem2	1
GO:0070391	response to lipoteichoic acid	1/95	10/14464	0.1	15.2252632	3.65884496	0.06379193	0.42113893	0.40264675	Trem2	1
GO:0070445	regulation of oligodendrocyte progenitor proliferation	1/95	10/14464	0.1	15.2252632	3.65884496	0.06379193	0.42113893	0.40264675	Lyn	1
GO:0071223	cellular response to lipoteichoic acid	1/95	10/14464	0.1	15.2252632	3.65884496	0.06379193	0.42113893	0.40264675	Trem2	1
GO:0099515	actin filament-based transport	1/95	10/14464	0.1	15.2252632	3.65884496	0.06379193	0.42113893	0.40264675	Myo5b	1
GO:0099639	neurotransmitter receptor transport, endosome to plasma membrane	1/95	10/14464	0.1	15.2252632	3.65884496	0.06379193	0.42113893	0.40264675	Myo5b	1
GO:1900426	positive regulation of defense response to bacterium	1/95	10/14464	0.1	15.2252632	3.65884496	0.06379193	0.42113893	0.40264675	Rab34	1
GO:1903867	extraembryonic membrane development	1/95	10/14464	0.1	15.2252632	3.65884496	0.06379193	0.42113893	0.40264675	Paxip1	1
GO:2000650	negative regulation of sodium ion transmembrane transporter activity	1/95	10/14464	0.1	15.2252632	3.65884496	0.06379193	0.42113893	0.40264675	Agrn	1
GO:0050775	positive regulation of dendrite morphogenesis	2/95	63/14464	0.03174603	4.83341688	2.47934934	0.064401	0.42113893	0.40264675	Fzd4/Myo5b	2
GO:0002706	regulation of lymphocyte mediated immunity	3/95	140/14464	0.02142857	3.26255639	2.18730324	0.06461865	0.42113893	0.40264675	Trem2/Gimap5/Paxip1	3
GO:0051250	negative regulation of lymphocyte activation	3/95	140/14464	0.02142857	3.26255639	2.18730324	0.06461865	0.42113893	0.40264675	Gimap5/Peli1/Lyn	3
GO:0044839	cell cycle G2/M phase transition	3/95	141/14464	0.0212766	3.23941769	2.17272814	0.06572105	0.42113893	0.40264675	Wee1/Ccdc57/Paxip1	3
GO:0055088	lipid homeostasis	3/95	141/14464	0.0212766	3.23941769	2.17272814	0.06572105	0.42113893	0.40264675	Nr1d2/Trem2/Sar1b	3
GO:0010770	positive regulation of cell morphogenesis	2/95	64/14464	0.03125	4.75789474	2.44980251	0.06620221	0.42113893	0.40264675	Fzd4/Myo5b	2
GO:0002221	pattern recognition receptor signaling pathway	4/95	232/14464	0.01724138	2.62504537	2.02887009	0.06649954	0.42113893	0.40264675	Trem2/Alpk1/Peli1/Lyn	4
GO:0002573	myeloid leukocyte differentiation	4/95	232/14464	0.01724138	2.62504537	2.02887009	0.06649954	0.42113893	0.40264675	Trem2/Gimap5/Lrrc17/Lyn	4
GO:0032006	regulation of TOR signaling	3/95	143/14464	0.02097902	3.19411115	2.14396502	0.06795183	0.42113893	0.40264675	Trem2/Sar1b/Peli1	3
GO:0043123	positive regulation of canonical NF-kappaB signal transduction	3/95	143/14464	0.02097902	3.19411115	2.14396502	0.06795183	0.42113893	0.40264675	Alpk1/Abl1/Peli1	3
GO:0010324	membrane invagination	2/95	65/14464	0.03076923	4.68469636	2.42086148	0.0680202	0.42113893	0.40264675	Trem2/Clcn2	2
GO:0032007	negative regulation of TOR signaling	2/95	65/14464	0.03076923	4.68469636	2.42086148	0.0680202	0.42113893	0.40264675	Sar1b/Peli1	2
GO:0050766	positive regulation of phagocytosis	2/95	65/14464	0.03076923	4.68469636	2.42086148	0.0680202	0.42113893	0.40264675	Trem2/Il15ra	2
GO:1905818	regulation of chromosome separation	2/95	65/14464	0.03076923	4.68469636	2.42086148	0.0680202	0.42113893	0.40264675	Xrcc3/Ncapd3	2
GO:0002758	innate immune response-activating signaling pathway	4/95	235/14464	0.01702128	2.59153415	2.00004804	0.06902249	0.42113893	0.40264675	Trem2/Alpk1/Peli1/Lyn	4
GO:0033044	regulation of chromosome organization	4/95	236/14464	0.01694915	2.58055308	1.99053987	0.06987441	0.42113893	0.40264675	Xrcc3/Ncapd3/Ylpm1/Arid2	4
GO:0002291	T cell activation via T cell receptor contact with antigen bound to MHC molecule on antigen presenting ce	1/95	11/14464	0.09090909	13.8411483	3.46416785	0.06994523	0.42113893	0.40264675	Trem2	1
GO:0006544	glycine metabolic process	1/95	11/14464	0.09090909	13.8411483	3.46416785	0.06994523	0.42113893	0.40264675	Tha1	1
GO:0008212	mineralocorticoid metabolic process	1/95	11/14464	0.09090909	13.8411483	3.46416785	0.06994523	0.42113893	0.40264675	Clcn2	1
GO:0010988	regulation of low-density lipoprotein particle clearance	1/95	11/14464	0.09090909	13.8411483	3.46416785	0.06994523	0.42113893	0.40264675	Trem2	1
GO:0016114	terpenoid biosynthetic process	1/95	11/14464	0.09090909	13.8411483	3.46416785	0.06994523	0.42113893	0.40264675	Lss	1
GO:0032825	positive regulation of natural killer cell differentiation	1/95	11/14464	0.09090909	13.8411483	3.46416785	0.06994523	0.42113893	0.40264675	Il15ra	1
GO:0034309	primary alcohol biosynthetic process	1/95	11/14464	0.09090909	13.8411483	3.46416785	0.06994523	0.42113893	0.40264675	Clcn2	1
GO:0034351	negative regulation of glial cell apoptotic process	1/95	11/14464	0.09090909	13.8411483	3.46416785	0.06994523	0.42113893	0.40264675	Trem2	1
GO:0035701	hematopoietic stem cell migration	1/95	11/14464	0.09090909	13.8411483	3.46416785	0.06994523	0.42113893	0.40264675	Jam2	1
GO:0042730	fibrinolysis	1/95	11/14464	0.09090909	13.8411483	3.46416785	0.06994523	0.42113893	0.40264675	Serpine1	1
GO:0043652	engulfment of apoptotic cell	1/95	11/14464	0.09090909	13.8411483	3.46416785	0.06994523	0.42113893	0.40264675	Trem2	1
GO:0045588	positive regulation of gamma-delta T cell differentiation	1/95	11/14464	0.09090909	13.8411483	3.46416785	0.06994523	0.42113893	0.40264675	Gimap5	1
GO:0046645	positive regulation of gamma-delta T cell activation	1/95	11/14464	0.09090909	13.8411483	3.46416785	0.06994523	0.42113893	0.40264675	Gimap5	1
GO:0048302	regulation of isotype switching to IgG isotypes	1/95	11/14464	0.09090909	13.8411483	3.46416785	0.06994523	0.42113893	0.40264675	Paxip1	1
GO:0061299	retina vasculature morphogenesis in camera-type eye	1/95	11/14464	0.09090909	13.8411483	3.46416785	0.06994523	0.42113893	0.40264675	Fzd4	1
GO:0070099	regulation of chemokine-mediated signaling pathway	1/95	11/14464	0.09090909	13.8411483	3.46416785	0.06994523	0.42113893	0.40264675	Trem2	1
GO:0070444	oligodendrocyte progenitor proliferation	1/95	11/14464	0.09090909	13.8411483	3.46416785	0.06994523	0.42113893	0.40264675	Lyn	1
GO:0071871	response to epinephrine	1/95	11/14464	0.09090909	13.8411483	3.46416785	0.06994523	0.42113893	0.40264675	Abl1	1
GO:0097501	stress response to metal ion	1/95	11/14464	0.09090909	13.8411483	3.46416785	0.06994523	0.42113893	0.40264675	Mt1	1
GO:0140052	cellular response to oxidised low-density lipoprotein particle stimulus	1/95	11/14464	0.09090909	13.8411483	3.46416785	0.06994523	0.42113893	0.40264675	Trem2	1
GO:0150078	positive regulation of neuroinflammatory response	1/95	11/14464	0.09090909	13.8411483	3.46416785	0.06994523	0.42113893	0.40264675	Trem2	1
GO:1902306	negative regulation of sodium ion transmembrane transport	1/95	11/14464	0.09090909	13.8411483	3.46416785	0.06994523	0.42113893	0.40264675	Agrn	1
GO:1903276	regulation of sodium ion export across plasma membrane	1/95	11/14464	0.09090909	13.8411483	3.46416785	0.06994523	0.42113893	0.40264675	Agrn	1
GO:1904395	positive regulation of skeletal muscle acetylcholine-gated channel clustering	1/95	11/14464	0.09090909	13.8411483	3.46416785	0.06994523	0.42113893	0.40264675	Agrn	1
GO:1905806	regulation of synapse pruning	1/95	11/14464	0.09090909	13.8411483	3.46416785	0.06994523	0.42113893	0.40264675	Trem2	1
GO:0030330	DNA damage response, signal transduction by p53 class mediator	2/95	67/14464	0.02985075	4.54485467	2.3647082	0.07170531	0.42113893	0.40264675	Acer2/Paxip1	2
GO:0050810	regulation of steroid biosynthetic process	2/95	67/14464	0.02985075	4.54485467	2.3647082	0.07170531	0.42113893	0.40264675	Por/Clcn2	2
GO:0061951	establishment of protein localization to plasma membrane	2/95	67/14464	0.02985075	4.54485467	2.3647082	0.07170531	0.42113893	0.40264675	Rab34/Myo5b	2
GO:0043547	positive regulation of GTPase activity	3/95	147/14464	0.02040816	3.10719656	2.08792756	0.07251606	0.42113893	0.40264675	Net1/Adap2/Agrn	3
GO:0060041	retina development in camera-type eye	3/95	147/14464	0.02040816	3.10719656	2.08792756	0.07251606	0.42113893	0.40264675	Per1/Fzd4/Clcn2	3
GO:0002831	regulation of response to biotic stimulus	6/95	446/14464	0.01345291	2.04824168	1.82836774	0.07318838	0.42113893	0.40264675	Trem2/Gimap5/Rab34/Alpk1/Peli1/Lyn	6
GO:0042113	B cell activation	4/95	240/14464	0.01666667	2.53754386	1.95298985	0.07333647	0.42113893	0.40264675	Abl1/Peli1/Lyn/Paxip1	4
GO:0051924	regulation of calcium ion transport	4/95	240/14464	0.01666667	2.53754386	1.95298985	0.07333647	0.42113893	0.40264675	Serpine1/Gimap5/Abl1/Lyn	4
GO:0048146	positive regulation of fibroblast proliferation	2/95	68/14464	0.02941176	4.47801858	2.33745446	0.07357187	0.42113893	0.40264675	Serpine1/Abl1	2
GO:1901606	alpha-amino acid catabolic process	2/95	68/14464	0.02941176	4.47801858	2.33745446	0.07357187	0.42113893	0.40264675	Pipox/Tha1	2
GO:1903131	mononuclear cell differentiation	6/95	447/14464	0.01342282	2.04365948	1.82248001	0.07380754	0.42113893	0.40264675	Trem2/Gimap5/Il15ra/Abl1/Lyn/Arid2	6
GO:0002366	leukocyte activation involved in immune response	4/95	241/14464	0.01659751	2.52701463	1.9437206	0.07421551	0.42113893	0.40264675	Trem2/Abl1/Lyn/Paxip1	4
GO:0045786	negative regulation of cell cycle	5/95	343/14464	0.01457726	2.21942612	1.85843574	0.07531109	0.42113893	0.40264675	Wee1/Inca1/Abl1/Xrcc3/Lyn	5
GO:0001767	establishment of lymphocyte polarity	1/95	12/14464	0.08333333	12.6877193	3.2933216	0.07605851	0.42113893	0.40264675	Abl1	1
GO:0001768	establishment of T cell polarity	1/95	12/14464	0.08333333	12.6877193	3.2933216	0.07605851	0.42113893	0.40264675	Abl1	1
GO:0002679	respiratory burst involved in defense response	1/95	12/14464	0.08333333	12.6877193	3.2933216	0.07605851	0.42113893	0.40264675	Trem2	1
GO:0008063	Toll signaling pathway	1/95	12/14464	0.08333333	12.6877193	3.2933216	0.07605851	0.42113893	0.40264675	Peli1	1
GO:0009070	serine family amino acid biosynthetic process	1/95	12/14464	0.08333333	12.6877193	3.2933216	0.07605851	0.42113893	0.40264675	Tha1	1
GO:0018342	protein prenylation	1/95	12/14464	0.08333333	12.6877193	3.2933216	0.07605851	0.42113893	0.40264675	Agrn	1
GO:0021924	cell proliferation in external granule layer	1/95	12/14464	0.08333333	12.6877193	3.2933216	0.07605851	0.42113893	0.40264675	Rere	1
GO:0021930	cerebellar granule cell precursor proliferation	1/95	12/14464	0.08333333	12.6877193	3.2933216	0.07605851	0.42113893	0.40264675	Rere	1
GO:0032490	detection of molecule of bacterial origin	1/95	12/14464	0.08333333	12.6877193	3.2933216	0.07605851	0.42113893	0.40264675	Trem2	1
GO:0032494	response to peptidoglycan	1/95	12/14464	0.08333333	12.6877193	3.2933216	0.07605851	0.42113893	0.40264675	Trem2	1
GO:0034139	regulation of toll-like receptor 3 signaling pathway	1/95	12/14464	0.08333333	12.6877193	3.2933216	0.07605851	0.42113893	0.40264675	Peli1	1
GO:0034145	positive regulation of toll-like receptor 4 signaling pathway	1/95	12/14464	0.08333333	12.6877193	3.2933216	0.07605851	0.42113893	0.40264675	Peli1	1
GO:0034638	phosphatidylcholine catabolic process	1/95	12/14464	0.08333333	12.6877193	3.2933216	0.07605851	0.42113893	0.40264675	Pnpla7	1
GO:0035791	platelet-derived growth factor receptor-beta signaling pathway	1/95	12/14464	0.08333333	12.6877193	3.2933216	0.07605851	0.42113893	0.40264675	Abl1	1
GO:0036006	cellular response to macrophage colony-stimulating factor stimulus	1/95	12/14464	0.08333333	12.6877193	3.2933216	0.07605851	0.42113893	0.40264675	Trem2	1
GO:0042118	endothelial cell activation	1/95	12/14464	0.08333333	12.6877193	3.2933216	0.07605851	0.42113893	0.40264675	Tcim	1
GO:0044725	epigenetic programming in the zygotic pronuclei	1/95	12/14464	0.08333333	12.6877193	3.2933216	0.07605851	0.42113893	0.40264675	Tet3	1
GO:0046512	sphingosine biosynthetic process	1/95	12/14464	0.08333333	12.6877193	3.2933216	0.07605851	0.42113893	0.40264675	Acer2	1
GO:0046520	sphingoid biosynthetic process	1/95	12/14464	0.08333333	12.6877193	3.2933216	0.07605851	0.42113893	0.40264675	Acer2	1
GO:0046886	positive regulation of hormone biosynthetic process	1/95	12/14464	0.08333333	12.6877193	3.2933216	0.07605851	0.42113893	0.40264675	Por	1
GO:0048291	isotype switching to IgG isotypes	1/95	12/14464	0.08333333	12.6877193	3.2933216	0.07605851	0.42113893	0.40264675	Paxip1	1
GO:0051549	positive regulation of keratinocyte migration	1/95	12/14464	0.08333333	12.6877193	3.2933216	0.07605851	0.42113893	0.40264675	Serpine1	1
GO:0060020	Bergmann glial cell differentiation	1/95	12/14464	0.08333333	12.6877193	3.2933216	0.07605851	0.42113893	0.40264675	Abl1	1
GO:0061517	macrophage proliferation	1/95	12/14464	0.08333333	12.6877193	3.2933216	0.07605851	0.42113893	0.40264675	Trem2	1
GO:0071294	cellular response to zinc ion	1/95	12/14464	0.08333333	12.6877193	3.2933216	0.07605851	0.42113893	0.40264675	Mt1	1
GO:0071476	cellular hypotonic response	1/95	12/14464	0.08333333	12.6877193	3.2933216	0.07605851	0.42113893	0.40264675	Clcn2	1
GO:0090232	positive regulation of spindle checkpoint	1/95	12/14464	0.08333333	12.6877193	3.2933216	0.07605851	0.42113893	0.40264675	Xrcc3	1
GO:0090267	positive regulation of mitotic cell cycle spindle assembly checkpoint	1/95	12/14464	0.08333333	12.6877193	3.2933216	0.07605851	0.42113893	0.40264675	Xrcc3	1
GO:0090385	phagosome-lysosome fusion	1/95	12/14464	0.08333333	12.6877193	3.2933216	0.07605851	0.42113893	0.40264675	Rab34	1
GO:0090399	replicative senescence	1/95	12/14464	0.08333333	12.6877193	3.2933216	0.07605851	0.42113893	0.40264675	Serpine1	1
GO:0097354	prenylation	1/95	12/14464	0.08333333	12.6877193	3.2933216	0.07605851	0.42113893	0.40264675	Agrn	1
GO:0150079	negative regulation of neuroinflammatory response	1/95	12/14464	0.08333333	12.6877193	3.2933216	0.07605851	0.42113893	0.40264675	Trem2	1
GO:1904396	regulation of neuromuscular junction development	1/95	12/14464	0.08333333	12.6877193	3.2933216	0.07605851	0.42113893	0.40264675	Agrn	1
GO:1905907	negative regulation of amyloid fibril formation	1/95	12/14464	0.08333333	12.6877193	3.2933216	0.07605851	0.42113893	0.40264675	Trem2	1
GO:1990253	cellular response to leucine starvation	1/95	12/14464	0.08333333	12.6877193	3.2933216	0.07605851	0.42113893	0.40264675	Sar1b	1
GO:2000322	regulation of glucocorticoid receptor signaling pathway	1/95	12/14464	0.08333333	12.6877193	3.2933216	0.07605851	0.42113893	0.40264675	Per1	1
GO:0045580	regulation of T cell differentiation	3/95	151/14464	0.01986755	3.02488672	2.03376886	0.07721464	0.42113893	0.40264675	Gimap5/Abl1/Arid2	3
GO:0042698	ovulation cycle	2/95	70/14464	0.02857143	4.35007519	2.28449677	0.07735157	0.42113893	0.40264675	Fzd4/Gdf10	2
GO:0045670	regulation of osteoclast differentiation	2/95	70/14464	0.02857143	4.35007519	2.28449677	0.07735157	0.42113893	0.40264675	Trem2/Lrrc17	2
GO:0032886	regulation of microtubule-based process	4/95	245/14464	0.01632653	2.48575725	1.90710277	0.07778541	0.42113893	0.40264675	Abl1/Agrn/Nav3/Xrcc3	4
GO:0045860	positive regulation of protein kinase activity	4/95	246/14464	0.01626016	2.47565255	1.89806086	0.07869124	0.42113893	0.40264675	Fzd4/Tcim/Abl1/Agrn	4
GO:0009410	response to xenobiotic stimulus	5/95	348/14464	0.01436782	2.18753781	1.8233035	0.07900682	0.42113893	0.40264675	Serpine1/Acer2/Gsta4/Abl1/Lyn	5
GO:1904377	positive regulation of protein localization to cell periphery	2/95	71/14464	0.02816901	4.28880652	2.25875725	0.07926416	0.42113893	0.40264675	Trem2/Myo5b	2
GO:0034767	positive regulation of monoatomic ion transmembrane transport	3/95	153/14464	0.01960784	2.98534572	2.00735688	0.07961334	0.42113893	0.40264675	Trem2/Gimap5/Abl1	3
GO:0051057	positive regulation of small GTPase mediated signal transduction	2/95	72/14464	0.02777778	4.22923977	2.23348833	0.08119153	0.42113893	0.40264675	Net1/Agrn	2
GO:0070373	negative regulation of ERK1 and ERK2 cascade	2/95	72/14464	0.02777778	4.22923977	2.23348833	0.08119153	0.42113893	0.40264675	Abl1/Lyn	2
GO:0051962	positive regulation of nervous system development	5/95	351/14464	0.01424501	2.16884091	1.80250722	0.08127071	0.42113893	0.40264675	Fzd4/Myo5b/Clcn2/Agrn/Lyn	5
GO:0001821	histamine secretion	1/95	13/14464	0.07692308	11.7117409	3.14166959	0.08213202	0.42113893	0.40264675	Lyn	1
GO:0006271	DNA strand elongation involved in DNA replication	1/95	13/14464	0.07692308	11.7117409	3.14166959	0.08213202	0.42113893	0.40264675	Pole	1
GO:0021534	cell proliferation in hindbrain	1/95	13/14464	0.07692308	11.7117409	3.14166959	0.08213202	0.42113893	0.40264675	Rere	1
GO:0023035	CD40 signaling pathway	1/95	13/14464	0.07692308	11.7117409	3.14166959	0.08213202	0.42113893	0.40264675	Trem2	1
GO:0032770	positive regulation of monooxygenase activity	1/95	13/14464	0.07692308	11.7117409	3.14166959	0.08213202	0.42113893	0.40264675	Por	1
GO:0032823	regulation of natural killer cell differentiation	1/95	13/14464	0.07692308	11.7117409	3.14166959	0.08213202	0.42113893	0.40264675	Il15ra	1
GO:0033690	positive regulation of osteoblast proliferation	1/95	13/14464	0.07692308	11.7117409	3.14166959	0.08213202	0.42113893	0.40264675	Abl1	1

GO:0036005	response to macrophage colony-stimulating factor	1/95	13/14464	0.07692308	11.7117409	3.14166959	0.08213202	0.42113893	0.40264675	Trem2	1
GO:0043605	amide catabolic process	1/95	13/14464	0.07692308	11.7117409	3.14166959	0.08213202	0.42113893	0.40264675	Pipox	1
GO:0050847	progesterone receptor signaling pathway	1/95	13/14464	0.07692308	11.7117409	3.14166959	0.08213202	0.42113893	0.40264675	Klf9	1
GO:0051547	regulation of keratinocyte migration	1/95	13/14464	0.07692308	11.7117409	3.14166959	0.08213202	0.42113893	0.40264675	Serpine1	1
GO:0060368	regulation of Fc receptor mediated stimulatory signaling pathway	1/95	13/14464	0.07692308	11.7117409	3.14166959	0.08213202	0.42113893	0.40264675	Lyn	1
GO:0060623	regulation of chromosome condensation	1/95	13/14464	0.07692308	11.7117409	3.14166959	0.08213202	0.42113893	0.40264675	Ncapd3	1
GO:0061687	detoxification of inorganic compound	1/95	13/14464	0.07692308	11.7117409	3.14166959	0.08213202	0.42113893	0.40264675	Mt1	1
GO:0070341	fat cell proliferation	1/95	13/14464	0.07692308	11.7117409	3.14166959	0.08213202	0.42113893	0.40264675	Trem2	1
GO:2000095	regulation of Wnt signaling pathway, planar cell polarity pathway	1/95	13/14464	0.07692308	11.7117409	3.14166959	0.08213202	0.42113893	0.40264675	Abl1	1
GO:0019221	cytokine-mediated signaling pathway	5/95	353/14464	0.01416431	2.15655286	1.78875831	0.08279924	0.42113893	0.40264675	Fzd4/Trem2/Il15ra/Rps6ka5/Lyn	5
GO:0032092	positive regulation of protein binding	2/95	73/14464	0.02739726	4.17130497	2.20867429	0.08313341	0.42113893	0.40264675	Abl1/Agrn	2
GO:0072676	lymphocyte migration	2/95	73/14464	0.02739726	4.17130497	2.20867429	0.08313341	0.42113893	0.40264675	Abl1/Jam2	2
GO:0002822	regulation of adaptive immune response based on somatic recombination of immune receptors built from	3/95	156/14464	0.01923077	2.92793522	1.96853434	0.08327201	0.42113893	0.40264675	Trem2/Gimap5/Paxip1	3
GO:0002753	cytoplasmic pattern recognition receptor signaling pathway	3/95	157/14464	0.01910828	2.90928595	1.95579908	0.08450753	0.42113893	0.40264675	Trem2/Alpk1/Peli1	3
GO:0062207	regulation of pattern recognition receptor signaling pathway	3/95	157/14464	0.01910828	2.90928595	1.95579908	0.08450753	0.42113893	0.40264675	Trem2/Peli1/Lyn	3
GO:0002312	B cell activation involved in immune response	2/95	74/14464	0.02702703	4.11493599	2.18430014	0.08508955	0.42113893	0.40264675	Abl1/Paxip1	2
GO:0014009	glial cell proliferation	2/95	74/14464	0.02702703	4.11493599	2.18430014	0.08508955	0.42113893	0.40264675	Trem2/Lyn	2
GO:0051279	regulation of release of sequestered calcium ion into cytosol	2/95	74/14464	0.02702703	4.11493599	2.18430014	0.08508955	0.42113893	0.40264675	Abl1/Lyn	2
GO:0051298	centrosome duplication	2/95	74/14464	0.02702703	4.11493599	2.18430014	0.08508955	0.42113893	0.40264675	Ccdc57/Xrcc3	2
GO:0051304	chromosome separation	2/95	74/14464	0.02702703	4.11493599	2.18430014	0.08508955	0.42113893	0.40264675	Xrcc3/Ncapd3	2
GO:0070301	cellular response to hydrogen peroxide	2/95	74/14464	0.02702703	4.11493599	2.18430014	0.08508955	0.42113893	0.40264675	Net1/Abl1	2
GO:0097553	calcium ion transmembrane import into cytosol	3/95	159/14464	0.01886792	2.87269116	1.93062762	0.0870022	0.42113893	0.40264675	Gimap5/Abl1/Lyn	3
GO:0006493	protein O-linked glycosylation	2/95	75/14464	0.02666667	4.06007018	2.16035158	0.08705966	0.42113893	0.40264675	Galnt15/Tet3	2
GO:0050672	negative regulation of lymphocyte proliferation	2/95	75/14464	0.02666667	4.06007018	2.16035158	0.08705966	0.42113893	0.40264675	Peli1/Lyn	2
GO:0150076	neuroinflammatory response	2/95	75/14464	0.02666667	4.06007018	2.16035158	0.08705966	0.42113893	0.40264675	Trem2/Lyn	2
GO:0002862	negative regulation of inflammatory response to antigenic stimulus	1/95	14/14464	0.07142857	10.875188	3.0057523	0.08816603	0.42113893	0.40264675	Trem2	1
GO:0006474	N-terminal protein amino acid acetylation	1/95	14/14464	0.07142857	10.875188	3.0057523	0.08816603	0.42113893	0.40264675	Naa50	1
GO:0006700	C21-steroid hormone biosynthetic process	1/95	14/14464	0.07142857	10.875188	3.0057523	0.08816603	0.42113893	0.40264675	Clcn2	1
GO:0006971	hypotonic response	1/95	14/14464	0.07142857	10.875188	3.0057523	0.08816603	0.42113893	0.40264675	Clcn2	1
GO:0010738	regulation of protein kinase A signaling	1/95	14/14464	0.07142857	10.875188	3.0057523	0.08816603	0.42113893	0.40264675	Tcim	1
GO:0010755	regulation of plasminogen activation	1/95	14/14464	0.07142857	10.875188	3.0057523	0.08816603	0.42113893	0.40264675	Serpine1	1
GO:0010832	negative regulation of myotube differentiation	1/95	14/14464	0.07142857	10.875188	3.0057523	0.08816603	0.42113893	0.40264675	Bhlhe41	1
GO:0010889	regulation of sequestering of triglyceride	1/95	14/14464	0.07142857	10.875188	3.0057523	0.08816603	0.42113893	0.40264675	Trem2	1
GO:0042541	hemoglobin biosynthetic process	1/95	14/14464	0.07142857	10.875188	3.0057523	0.08816603	0.42113893	0.40264675	Lyn	1
GO:0045586	regulation of gamma-delta T cell differentiation	1/95	14/14464	0.07142857	10.875188	3.0057523	0.08816603	0.42113893	0.40264675	Gimap5	1
GO:0046643	regulation of gamma-delta T cell activation	1/95	14/14464	0.07142857	10.875188	3.0057523	0.08816603	0.42113893	0.40264675	Gimap5	1
GO:0048102	autophagic cell death	1/95	14/14464	0.07142857	10.875188	3.0057523	0.08816603	0.42113893	0.40264675	Trem2	1
GO:0048755	branching morphogenesis of a nerve	1/95	14/14464	0.07142857	10.875188	3.0057523	0.08816603	0.42113893	0.40264675	Rere	1
GO:0051124	synaptic assembly at neuromuscular junction	1/95	14/14464	0.07142857	10.875188	3.0057523	0.08816603	0.42113893	0.40264675	Agrn	1
GO:0051451	myoblast migration	1/95	14/14464	0.07142857	10.875188	3.0057523	0.08816603	0.42113893	0.40264675	Net1	1
GO:0061762	CAMKK-AMPK signaling cascade	1/95	14/14464	0.07142857	10.875188	3.0057523	0.08816603	0.42113893	0.40264675	Trem2	1
GO:0071280	cellular response to copper ion	1/95	14/14464	0.07142857	10.875188	3.0057523	0.08816603	0.42113893	0.40264675	Mt1	1
GO:0071895	odontoblast differentiation	1/95	14/14464	0.07142857	10.875188	3.0057523	0.08816603	0.42113893	0.40264675	Serpine1	1
GO:0150011	regulation of neuron projection arborization	1/95	14/14464	0.07142857	10.875188	3.0057523	0.08816603	0.42113893	0.40264675	Fzd4	1
GO:1900221	regulation of amyloid-beta clearance	1/95	14/14464	0.07142857	10.875188	3.0057523	0.08816603	0.42113893	0.40264675	Trem2	1
GO:1900226	negative regulation of NLRP3 inflammasome complex assembly	1/95	14/14464	0.07142857	10.875188	3.0057523	0.08816603	0.42113893	0.40264675	Trem2	1
GO:1900272	negative regulation of long-term synaptic potentiation	1/95	14/14464	0.07142857	10.875188	3.0057523	0.08816603	0.42113893	0.40264675	Abl1	1
GO:1901018	positive regulation of potassium ion transmembrane transporter activity	1/95	14/14464	0.07142857	10.875188	3.0057523	0.08816603	0.42113893	0.40264675	Trem2	1
GO:1903977	positive regulation of glial cell migration	1/95	14/14464	0.07142857	10.875188	3.0057523	0.08816603	0.42113893	0.40264675	Trem2	1
GO:1904393	regulation of skeletal muscle acetylcholine-gated channel clustering	1/95	14/14464	0.07142857	10.875188	3.0057523	0.08816603	0.42113893	0.40264675	Agrn	1
GO:2001171	positive regulation of ATP biosynthetic process	1/95	14/14464	0.07142857	10.875188	3.0057523	0.08816603	0.42113893	0.40264675	Trem2	1
GO:0070507	regulation of microtubule cytoskeleton organization	3/95	160/14464	0.01875	2.85473684	1.91818841	0.08826125	0.42113893	0.40264675	Abl1/Agrn/Nav3	3
GO:0006809	nitric oxide biosynthetic process	2/95	76/14464	0.02631579	4.0066482	2.13681498	0.08904349	0.42113893	0.40264675	Gimap5/Por	2
GO:0032945	negative regulation of mononuclear cell proliferation	2/95	76/14464	0.02631579	4.0066482	2.13681498	0.08904349	0.42113893	0.40264675	Peli1/Lyn	2
GO:0002695	negative regulation of leukocyte activation	3/95	161/14464	0.01863354	2.83700556	1.90584493	0.08952803	0.42113893	0.40264675	Gimap5/Peli1/Lyn	3
GO:0006469	negative regulation of protein kinase activity	3/95	161/14464	0.01863354	2.83700556	1.90584493	0.08952803	0.42113893	0.40264675	Inca1/Abl1/Lyn	3
GO:0002218	activation of innate immune response	4/95	258/14464	0.01550388	2.36050592	1.79288365	0.08997122	0.42113893	0.40264675	Trem2/Alpk1/Peli1/Lyn	4
GO:0016042	lipid catabolic process	4/95	259/14464	0.01544402	2.35139199	1.78438403	0.09094488	0.42113893	0.40264675	Gimap5/Acer2/Pnpla7/Gpcpd1	4
GO:1901379	regulation of potassium ion transmembrane transport	2/95	77/14464	0.02597403	3.95461381	2.11367732	0.09104078	0.42113893	0.40264675	Trem2/Agrn	2
GO:0002285	lymphocyte activation involved in immune response	3/95	163/14464	0.01840491	2.80219567	1.88143953	0.0920846	0.42113893	0.40264675	Trem2/Abl1/Paxip1	3
GO:0032649	regulation of type II interferon production	2/95	78/14464	0.02564103	3.90391363	2.09092614	0.09305127	0.42113893	0.40264675	Gimap5/Abl1	2
GO:0035023	regulation of Rho protein signal transduction	2/95	78/14464	0.02564103	3.90391363	2.09092614	0.09305127	0.42113893	0.40264675	Net1/Abl1	2
GO:0050871	positive regulation of B cell activation	2/95	78/14464	0.02564103	3.90391363	2.09092614	0.09305127	0.42113893	0.40264675	Peli1/Paxip1	2
GO:0000722	telomere maintenance via recombination	1/95	15/14464	0.06666667	10.1501754	2.88292855	0.09416079	0.42113893	0.40264675	Xrcc3	1
GO:0001553	luteinization	1/95	15/14464	0.06666667	10.1501754	2.88292855	0.09416079	0.42113893	0.40264675	Fzd4	1
GO:0002431	Fc receptor mediated stimulatory signaling pathway	1/95	15/14464	0.06666667	10.1501754	2.88292855	0.09416079	0.42113893	0.40264675	Lyn	1
GO:0007076	mitotic chromosome condensation	1/95	15/14464	0.06666667	10.1501754	2.88292855	0.09416079	0.42113893	0.40264675	Ncapd3	1
GO:0010172	embryonic body morphogenesis	1/95	15/14464	0.06666667	10.1501754	2.88292855	0.09416079	0.42113893	0.40264675	Zfp281	1
GO:0010524	positive regulation of calcium ion transport into cytosol	1/95	15/14464	0.06666667	10.1501754	2.88292855	0.09416079	0.42113893	0.40264675	Gimap5	1
GO:0010885	regulation of cholesterol storage	1/95	15/14464	0.06666667	10.1501754	2.88292855	0.09416079	0.42113893	0.40264675	Trem2	1
GO:0010984	regulation of lipoprotein particle clearance	1/95	15/14464	0.06666667	10.1501754	2.88292855	0.09416079	0.42113893	0.40264675	Trem2	1
GO:0021932	hindbrain radial glia guided cell migration	1/95	15/14464	0.06666667	10.1501754	2.88292855	0.09416079	0.42113893	0.40264675	Rere	1
GO:0032488	Cdc42 protein signal transduction	1/95	15/14464	0.06666667	10.1501754	2.88292855	0.09416079	0.42113893	0.40264675	Abl1	1
GO:0042492	gamma-delta T cell differentiation	1/95	15/14464	0.06666667	10.1501754	2.88292855	0.09416079	0.42113893	0.40264675	Gimap5	1
GO:0045542	positive regulation of cholesterol biosynthetic process	1/95	15/14464	0.06666667	10.1501754	2.88292855	0.09416079	0.42113893	0.40264675	Por	1
GO:0051546	keratinocyte migration	1/95	15/14464	0.06666667	10.1501754	2.88292855	0.09416079	0.42113893	0.40264675	Serpine1	1
GO:0099532	synaptic vesicle endosomal processing	1/95	15/14464	0.06666667	10.1501754	2.88292855	0.09416079	0.42113893	0.40264675	Myo5b	1
GO:0106120	positive regulation of sterol biosynthetic process	1/95	15/14464	0.06666667	10.1501754	2.88292855	0.09416079	0.42113893	0.40264675	Por	1
GO:1900424	regulation of defense response to bacterium	1/95	15/14464	0.06666667	10.1501754	2.88292855	0.09416079	0.42113893	0.40264675	Rab34	1
GO:1901978	positive regulation of cell cycle checkpoint	1/95	15/14464	0.06666667	10.1501754	2.88292855	0.09416079	0.42113893	0.40264675	Xrcc3	1
GO:1903543	positive regulation of exosomal secretion	1/95	15/14464	0.06666667	10.1501754	2.88292855	0.09416079	0.42113893	0.40264675	Myo5b	1
GO:2000052	positive regulation of non-canonical Wnt signaling pathway	1/95	15/14464	0.06666667	10.1501754	2.88292855	0.09416079	0.42113893	0.40264675	Abl1	1
GO:0002028	regulation of sodium ion transport	2/95	79/14464	0.02531646	3.854497	2.06854954	0.09507471	0.42113893	0.40264675	Per1/Agrn	2
GO:0009063	amino acid catabolic process	2/95	79/14464	0.02531646	3.854497	2.06854954	0.09507471	0.42113893	0.40264675	Pipox/Tha1	2
GO:0014015	positive regulation of gliogenesis	2/95	79/14464	0.02531646	3.854497	2.06854954	0.09507471	0.42113893	0.40264675	Clcn2/Lyn	2
GO:0032418	lysosome localization	2/95	79/14464	0.02531646	3.854497	2.06854954	0.09507471	0.42113893	0.40264675	Rab34/Lyn	2
GO:0035914	skeletal muscle cell differentiation	2/95	79/14464	0.02531646	3.854497	2.06854954	0.09507471	0.42113893	0.40264675	Nr1d2/Hlf	2
GO:1990849	vacuolar localization	2/95	79/14464	0.02531646	3.854497	2.06854954	0.09507471	0.42113893	0.40264675	Rab34/Lyn	2
GO:0030071	regulation of mitotic metaphase/anaphase transition	2/95	80/14464	0.025	3.80631579	2.04653615	0.09711083	0.42113893	0.40264675	Xrcc3/Arid2	2
GO:0045685	regulation of glial cell differentiation	2/95	80/14464	0.025	3.80631579	2.04653615	0.09711083	0.42113893	0.40264675	Trem2/Clcn2	2
GO:0045807	positive regulation of endocytosis	3/95	167/14464	0.01796407	2.73507721	1.83371698	0.09728821	0.42113893	0.40264675	Trem2/Serpine1/Il15ra	3
GO:1903169	regulation of calcium ion transmembrane transport	3/95	167/14464	0.01796407	2.73507721	1.83371698	0.09728821	0.42113893	0.40264675	Gimap5/Abl1/Lyn	3
GO:0045638	negative regulation of myeloid cell differentiation	2/95	81/14464	0.02469136	3.75932424	2.02487505	0.0991594	0.42113893	0.40264675	Lrrc17/Lyn	2
GO:0046209	nitric oxide metabolic process	2/95	81/14464	0.02469136	3.75932424	2.02487505	0.0991594	0.42113893	0.40264675	Gimap5/Por	2
GO:0070664	negative regulation of leukocyte proliferation	2/95	81/14464	0.02469136	3.75932424	2.02487505	0.0991594	0.42113893	0.40264675	Peli1/Lyn	2
GO:1904427	positive regulation of calcium ion transmembrane transport	2/95	81/14464	0.02469136	3.75932424	2.02487505	0.0991594	0.42113893	0.40264675	Gimap5/Abl1	2
GO:0045088	regulation of innate immune response	5/95	374/14464	0.01336898	2.03546299	1.64965303	0.09976533	0.42113893	0.40264675	Trem2/Gimap5/Alpk1/Peli1/Lyn	5
GO:0009755	hormone-mediated signaling pathway	3/95	169/14464	0.01775148	2.70270944	1.81037915	0.09993438	0.42113893	0.40264675	Per1/Nr1d2/Klf9	3
GO:0002577	regulation of antigen processing and presentation	1/95	16/14464	0.0625	9.51578947	2.7711419	0.10011654	0.42113893	0.40264675	Trem2	1
GO:0006991	response to sterol depletion	1/95	16/14464	0.0625	9.51578947	2.7711419	0.10011654	0.42113893	0.40264675	Lyn	1
GO:0010766	negative regulation of sodium ion transport	1/95	16/14464	0.0625	9.51578947	2.7711419	0.10011654	0.42113893	0.40264675	Agrn	1
GO:0030449	regulation of complement activation	1/95	16/14464	0.0625	9.51578947	2.7711419	0.10011654	0.42113893	0.40264675	Trem2	1
GO:0030730	sequestering of triglyceride	1/95	16/14464	0.0625	9.51578947	2.7711419	0.10011654	0.42113893	0.40264675	Trem2	1
GO:0030810	positive regulation of nucleotide biosynthetic process	1/95	16/14464	0.0625	9.51578947	2.7711419	0.10011654	0.42113893	0.40264675	Trem2	1
GO:0031987	locomotion involved in locomotory behavior	1/95	16/14464	0.0625	9.51578947	2.7711419	0.10011654	0.42113893	0.40264675	Fzd4	1
GO:0032328	alanine transport	1/95	16/14464	0.0625	9.51578947	2.7711419	0.10011654	0.42113893	0.40264675	Slc6a6	1
GO:0034350	regulation of glial cell apoptotic process	1/95	16/14464	0.0625	9.51578947	2.7711419	0.10011654	0.42113893	0.40264675	Trem2	1
GO:0042921	nuclear receptor-mediated glucocorticoid signaling pathway	1/95	16/14464	0.0625	9.51578947	2.7711419	0.10011654	0.42113893	0.40264675	Per1	1
GO:0042953	lipoprotein transport	1/95	16/14464	0.0625	9.51578947	2.7711419	0.10011654	0.42113893	0.40264675	Sar1b	1
GO:0045954	positive regulation of natural killer cell mediated cytotoxicity	1/95	16/14464	0.0625	9.51578947	2.7711419	0.10011654	0.42113893	0.40264675	Gimap5	1
GO:0046514	ceramide catabolic process	1/95	16/14464	0.0625	9.51578947	2.7711419	0.10011654	0.42113893	0.40264675	Acer2	1
GO:0048712	negative regulation of astrocyte differentiation	1/95	16/14464	0.0625	9.51578947	2.7711419	0.10011654	0.42113893	0.40264675	Trem2	1
GO:0050855	regulation of B cell receptor signaling pathway	1/95	16/14464	0.0625	9.51578947	2.7711419	0.10011654	0.42113893	0.40264675	Lyn	1
GO:0051608	histamine transport	1/95	16/14464	0.0625	9.51578947	2.7711419	0.10011654	0.42113893	0.40264675	Lyn	1
GO:0060100	positive regulation of phagocytosis, engulfment	1/95	16/14464	0.0625	9.51578947	2.7711419	0.10011654	0.42113893	0.40264675	Trem2	1
GO:1900373	positive regulation of purine nucleotide biosynthetic process	1/95	16/14464	0.0625	9.51578947	2.7711419	0.10011654	0.42113893	0.40264675	Trem2	1
GO:1901017	negative regulation of potassium ion transmembrane transporter activity	1/95	16/14464	0.0625	9.51578947	2.7711419	0.10011654	0.42113893	0.40264675	Agrn	1
GO:1905155	positive regulation of membrane invagination	1/95	16/14464	0.0625	9.51578947	2.7711419	0.10011654	0.42113893	0.40264675	Trem2	1
GO:1905906	regulation of amyloid fibril formation	1/95	16/14464	0.0625	9.51578947	2.7711419	0.10011654	0.42113893	0.40264675	Trem2	1
GO:0014855	striated muscle cell proliferation	2/95	82/14464	0.02439024	3.71347882	2.0035558	0.10122017	0.42113893	0.40264675	Abl1/Arid2	2
GO:1901222	regulation of non-canonical NF-kappaB signal transduction	2/95	82/14464	0.02439024	3.71347882	2.0035558	0.10122017	0.42113893	0.40264675	Trem2/Tcim	2
GO:1902099	regulation of metaphase/anaphase transition of cell cycle	2/95	82/14464	0.02439024	3.71347882	2.0035558	0.10122017	0.42113893	0.40264675	Xrcc3/Arid2	2
GO:0002819	regulation of adaptive immune response	3/95	170/14464	0.01764706	2.68681115	1.79883677	0.10126837	0.42113893	0.40264675	Trem2/Gimap5/Paxip1	3
GO:0022408	negative regulation of cell-cell adhesion	3/95	171/14464	0.01754386	2.6710988	1.78737718	0.10260955	0.42113893	0.40264675	Gimap5/Abl1/Peli1	3
GO:2001057	reactive nitrogen species metabolic process	2/95	83/14464	0.02409639	3.66873811	1.98256842	0.10329288	0.42113893	0.40264675	Gimap5/Por	2
GO:0009101	glycoprotein biosynthetic process	4/95	272/14464	0.01470588	2.23900929	1.67731618	0.10405985	0.42113893	0.40264675	Acer2/Galnt15/Tet3/Alg10b	4
GO:0010001	glial cell differentiation	4/95	272/14464	0.01470588	2.23900929	1.67731618	0.10405985	0.42113893	0.40264675	Trem2/Clcn2/Abl1/Lyn	4
GO:0002757	immune response-activating signaling pathway	5/95	380/14464	0.01315789	2.0033241	1.61156474	0.10491422	0.42113893	0.40264675	Trem2/Alpk1/Abl1/Peli1/Lyn	5
GO:0033673	negative regulation of kinase activity	3/95	173/14464	0.01734104	2.64021904	1.76470175	0.10531327	0.42113893	0.40264675	Inca1/Abl1/Lyn	3
GO:0032526	response to retinoic acid	2/95	84/14464	0.02380952	3.62506266	1.9619033	0.10537731	0.42113893	0.40264675	Acer2/Lyn	2
GO:0001845	phagolysosome assembly	1/95	17/14464	0.05882353	8.95603715	2.66876387	0.10603355	0.42113893	0.40264675	Rab34	1
GO:0002281	macrophage activation involved in immune response	1/95	17/14464	0.05882353	8.95603715	2.66876387	0.10603355	0.42113893	0.40264675	Trem2	1
GO:0002327	immature B cell differentiation	1/95	17/14464	0.05882353	8.95603715	2.66876387	0.10603355	0.42113893	0.40264675	Abl1	1
GO:0006337	nucleosome disassembly	1/95	17/14464	0.05882353	8.95603715	2.66876387	0.10603355	0.42113893	0.40264675	Arid2	1
GO:0032352	positive regulation of hormone metabolic process	1/95	17/14464	0.05882353	8.95603715	2.66876387	0.10603355	0.42113893	0.40264675	Por	1
GO:0036376	sodium ion export across plasma membrane	1/95	17/14464	0.05882353	8.95603715	2.66876387	0.10603355	0.42113893	0.40264675	Agrn	1
GO:0044872	lipoprotein localization	1/95	17/14464	0.05882353	8.95603715	2.66876387	0.10603355	0.42113893	0.40264675	Sar1b	1
GO:0045019	negative regulation of nitric oxide biosynthetic process	1/95	17/14464	0.05882353	8.95603715	2.66876387	0.10603355	0.42113893	0.40264675	Gimap5	1
GO:0045187	regulation of circadian sleep/wake cycle, sleep	1/95	17/14464	0.05882353	8.95603715	2.66876387	0.10603355	0.42113893	0.40264675	Per3	1
GO:0051444	negative regulation of ubiquitin-protein transferase activity	1/95	17/14464	0.05882353	8.95603715	2.66876387	0.10603355	0.42113893	0.40264675	Abl1	1
GO:0060546	negative regulation of necroptotic process	1/95	17/14464	0.05882353	8.95603715	2.66876387	0.10603355	0.42113893	0.40264675	Peli1	1
GO:0060856	establishment of blood-brain barrier	1/95	17/14464	0.05882353	8.95603715	2.66876387	0.10603355	0.42113893	0.40264675	Fzd4	1
GO:0070486	leukocyte aggregation	1/95	17/14464	0.05882353	8.95603715	2.66876387	0.10603355	0.42113893	0.40264675	Jam2	1
GO:0098883	synapse pruning	1/95	17/14464	0.05882353	8.95603715	2.66876387	0.10603355	0.42113893	0.40264675	Trem2	1
GO:1903376	regulation of oxidative stress-induced neuron intrinsic apoptotic signaling pathway	1/95	17/14464	0.05882353	8.95603715	2.66876387	0.10603355	0.42113893	0.40264675	Trem2	1
GO:1903541	regulation of exosomal secretion	1/95	17/14464	0.05882353	8.95603715	2.66876387	0.10603355	0.42113893	0.40264675	Myo5b	1
GO:1904406	negative regulation of nitric oxide metabolic process	1/95	17/14464	0.05882353	8.95603715	2.66876387	0.10603355	0.42113893	0.40264675	Gimap5	1
GO:0006066	alcohol metabolic process	4/95	275/14464	0.01454545	2.21458373	1.6534674	0.10720433	0.42113893	0.40264675	Acer2/Por/Lss/Clcn2	4
GO:0007091	metaphase/anaphase transition of mitotic cell cycle	2/95	85/14464	0.02352941	3.58241486	1.94155124	0.1074732	0.42113893	0.40264675	Xrcc3/Arid2	2
GO:0048708	astrocyte differentiation	2/95	85/14464	0.02352941	3.58241486	1.94155124	0.1074732	0.42113893	0.40264675	Trem2/Abl1	2
GO:0032609	type II interferon production	2/95	86/14464	0.02325581	3.54075887	1.92150341	0.10958033	0.42113893	0.40264675	Gimap5/Abl1	2
GO:0001819	positive regulation of cytokine production	5/95	386/14464	0.01295337	1.97218435	1.57416234	0.11019361	0.42113893	0.40264675	Trem2/Serpine1/Gimap5/Abl1/Peli1	5
GO:0009408	response to heat	2/95	87/14464	0.02298851	3.5000605	1.90175134	0.11169846	0.42113893	0.40264675	Tcim/Lyn	2
GO:0044784	metaphase/anaphase transition of cell cycle	2/95	87/14464	0.02298851	3.5000605	1.90175134	0.11169846	0.42113893	0.40264675	Xrcc3/Arid2	2
GO:0002717	positive regulation of natural killer cell mediated immunity	1/95	18/14464	0.05555556	8.45847953	2.57448563	0.11191206	0.42113893	0.40264675	Gimap5	1
GO:0002902	regulation of B cell apoptotic process	1/95	18/14464	0.05555556	8.45847953	2.57448563	0.11191206	0.42113893	0.40264675	Lyn	1
GO:0006488	dolichol-linked oligosaccharide biosynthetic process	1/95	18/14464	0.05555556	8.45847953	2.57448563	0.11191206	0.42113893	0.40264675	Alg10b	1
GO:0006670	sphingosine metabolic process	1/95	18/14464	0.05555556	8.45847953	2.57448563	0.11191206	0.42113893	0.40264675	Acer2	1
GO:0010522	regulation of calcium ion transport into cytosol	1/95	18/14464	0.05555556	8.45847953	2.57448563	0.11191206	0.42113893	0.40264675	Gimap5	1
GO:0030889	negative regulation of B cell proliferation	1/95	18/14464	0.05555556	8.45847953	2.57448563	0.11191206	0.42113893	0.40264675	Lyn	1
GO:0031639	plasminogen activation	1/95	18/14464	0.05555556	8.45847953	2.57448563	0.11191206	0.42113893	0.40264675	Serpine1	1
GO:0031645	negative regulation of nervous system process	1/95	18/14464	0.05555556	8.45847953	2.57448563	0.11191206	0.42113893	0.40264675	Jam2	1
GO:0031958	nuclear receptor-mediated corticosteroid signaling pathway	1/95	18/14464	0.05555556	8.45847953	2.57448563	0.11191206	0.42113893	0.40264675	Per1	1
GO:0033003	regulation of mast cell activation	1/95	18/14464	0.05555556	8.45847953	2.57448563	0.11191206	0.42113893	0.40264675	Lyn	1
GO:0034383	low-density lipoprotein particle clearance	1/95	18/14464	0.05555556	8.45847953	2.57448563	0.11191206	0.42113893	0.40264675	Trem2	1
GO:0038083	peptidyl-tyrosine autophosphorylation	1/95	18/14464	0.05555556	8.45847953	2.57448563	0.11191206	0.42113893	0.40264675	Abl1	1

GO:0043011	myeloid dendritic cell differentiation	1/95	18/14464	0.05555556	8.45847953	2.57448563	0.11191206	0.42113893	0.40264675	Gimap5	1
GO:0045591	positive regulation of regulatory T cell differentiation	1/95	18/14464	0.05555556	8.45847953	2.57448563	0.11191206	0.42113893	0.40264675	Gimap5	1
GO:0060099	regulation of phagocytosis, engulfment	1/95	18/14464	0.05555556	8.45847953	2.57448563	0.11191206	0.42113893	0.40264675	Trem2	1
GO:0090026	positive regulation of monocyte chemotaxis	1/95	18/14464	0.05555556	8.45847953	2.57448563	0.11191206	0.42113893	0.40264675	Serpine1	1
GO:0090110	COPII-coated vesicle cargo loading	1/95	18/14464	0.05555556	8.45847953	2.57448563	0.11191206	0.42113893	0.40264675	Sar1b	1
GO:0098581	detection of external biotic stimulus	1/95	18/14464	0.05555556	8.45847953	2.57448563	0.11191206	0.42113893	0.40264675	Trem2	1
GO:1903911	positive regulation of receptor clustering	1/95	18/14464	0.05555556	8.45847953	2.57448563	0.11191206	0.42113893	0.40264675	Agrn	1
GO:1903978	regulation of microglial cell activation	1/95	18/14464	0.05555556	8.45847953	2.57448563	0.11191206	0.42113893	0.40264675	Trem2	1
GO:0035265	organ growth	3/95	180/14464	0.01666667	2.53754386	1.68778285	0.1149946	0.42113893	0.40264675	Por/Abl1/Arid2	3
GO:0043523	regulation of neuron apoptotic process	4/95	283/14464	0.01413428	2.15198066	1.59133898	0.11579864	0.42113893	0.40264675	Mt1/Trem2/Abl1/Agrn	4
GO:0002275	myeloid cell activation involved in immune response	2/95	89/14464	0.02247191	3.42140745	1.86310218	0.11596679	0.42113893	0.40264675	Trem2/Lyn	2
GO:0019218	regulation of steroid metabolic process	2/95	89/14464	0.02247191	3.42140745	1.86310218	0.11596679	0.42113893	0.40264675	Por/Clcn2	2
GO:0009314	response to radiation	5/95	394/14464	0.01269036	1.93213999	1.52531636	0.11743205	0.42113893	0.40264675	Per3/Per1/Bhlhe40/Net1/Paxip1	5
GO:0002507	tolerance induction	1/95	19/14464	0.05263158	8.0132964	2.48724144	0.11775232	0.42113893	0.40264675	Lyn	1
GO:0003417	growth plate cartilage development	1/95	19/14464	0.05263158	8.0132964	2.48724144	0.11775232	0.42113893	0.40264675	Por	1
GO:0010566	regulation of ketone biosynthetic process	1/95	19/14464	0.05263158	8.0132964	2.48724144	0.11775232	0.42113893	0.40264675	Clcn2	1
GO:0020027	hemoglobin metabolic process	1/95	19/14464	0.05263158	8.0132964	2.48724144	0.11775232	0.42113893	0.40264675	Lyn	1
GO:0030194	positive regulation of blood coagulation	1/95	19/14464	0.05263158	8.0132964	2.48724144	0.11775232	0.42113893	0.40264675	Serpine1	1
GO:0032332	positive regulation of chondrocyte differentiation	1/95	19/14464	0.05263158	8.0132964	2.48724144	0.11775232	0.42113893	0.40264675	Por	1
GO:0032703	negative regulation of interleukin-2 production	1/95	19/14464	0.05263158	8.0132964	2.48724144	0.11775232	0.42113893	0.40264675	Nav3	1
GO:0032780	negative regulation of ATP-dependent activity	1/95	19/14464	0.05263158	8.0132964	2.48724144	0.11775232	0.42113893	0.40264675	Agrn	1
GO:0035022	positive regulation of Rac protein signal transduction	1/95	19/14464	0.05263158	8.0132964	2.48724144	0.11775232	0.42113893	0.40264675	Agrn	1
GO:0035372	protein localization to microtubule	1/95	19/14464	0.05263158	8.0132964	2.48724144	0.11775232	0.42113893	0.40264675	Abl1	1
GO:0036480	neuron intrinsic apoptotic signaling pathway in response to oxidative stress	1/95	19/14464	0.05263158	8.0132964	2.48724144	0.11775232	0.42113893	0.40264675	Trem2	1
GO:0046629	gamma-delta T cell activation	1/95	19/14464	0.05263158	8.0132964	2.48724144	0.11775232	0.42113893	0.40264675	Gimap5	1
GO:0048207	vesicle targeting, rough ER to cis-Golgi	1/95	19/14464	0.05263158	8.0132964	2.48724144	0.11775232	0.42113893	0.40264675	Sar1b	1
GO:0048208	COPII vesicle coating	1/95	19/14464	0.05263158	8.0132964	2.48724144	0.11775232	0.42113893	0.40264675	Sar1b	1
GO:0061042	vascular wound healing	1/95	19/14464	0.05263158	8.0132964	2.48724144	0.11775232	0.42113893	0.40264675	Serpine1	1
GO:0062099	negative regulation of programmed necrotic cell death	1/95	19/14464	0.05263158	8.0132964	2.48724144	0.11775232	0.42113893	0.40264675	Peli1	1
GO:0090205	positive regulation of cholesterol metabolic process	1/95	19/14464	0.05263158	8.0132964	2.48724144	0.11775232	0.42113893	0.40264675	Por	1
GO:0090266	regulation of mitotic cell cycle spindle assembly checkpoint	1/95	19/14464	0.05263158	8.0132964	2.48724144	0.11775232	0.42113893	0.40264675	Xrcc3	1
GO:0090280	positive regulation of calcium ion import	1/95	19/14464	0.05263158	8.0132964	2.48724144	0.11775232	0.42113893	0.40264675	Serpine1	1
GO:1900006	positive regulation of dendrite development	1/95	19/14464	0.05263158	8.0132964	2.48724144	0.11775232	0.42113893	0.40264675	Abl1	1
GO:1900048	positive regulation of hemostasis	1/95	19/14464	0.05263158	8.0132964	2.48724144	0.11775232	0.42113893	0.40264675	Serpine1	1
GO:1903504	regulation of mitotic spindle checkpoint	1/95	19/14464	0.05263158	8.0132964	2.48724144	0.11775232	0.42113893	0.40264675	Xrcc3	1
GO:1905153	regulation of membrane invagination	1/95	19/14464	0.05263158	8.0132964	2.48724144	0.11775232	0.42113893	0.40264675	Trem2	1
GO:2000671	regulation of motor neuron apoptotic process	1/95	19/14464	0.05263158	8.0132964	2.48724144	0.11775232	0.42113893	0.40264675	Agrn	1
GO:0042742	defense response to bacterium	3/95	182/14464	0.01648352	2.50965876	1.66647075	0.11782119	0.42113893	0.40264675	Trem2/Serpine1/Rab34	3
GO:0071805	potassium ion transmembrane transport	3/95	182/14464	0.01648352	2.50965876	1.66647075	0.11782119	0.42113893	0.40264675	Trem2/Agrn/Alg10b	3
GO:0008589	regulation of smoothened signaling pathway	2/95	90/14464	0.02222222	3.38339181	1.84418974	0.11811655	0.42113893	0.40264675	Por/Rab34	2
GO:0048639	positive regulation of developmental growth	3/95	183/14464	0.01639344	2.49594478	1.65592065	0.11924429	0.42113893	0.40264675	Serpine1/Myo5b/Agrn	3
GO:0002764	immune response-regulating signaling pathway	5/95	397/14464	0.01259446	1.91753944	1.50729054	0.12020418	0.42113893	0.40264675	Trem2/Alpk1/Abl1/Peli1/Lyn	5
GO:0070661	leukocyte proliferation	4/95	287/14464	0.01393728	2.1219879	1.56104231	0.12020719	0.42113893	0.40264675	Trem2/Abl1/Peli1/Lyn	4
GO:0043525	positive regulation of neuron apoptotic process	2/95	91/14464	0.02197802	3.34621168	1.8255423	0.12027639	0.42113893	0.40264675	Abl1/Agrn	2
GO:0007519	skeletal muscle tissue development	3/95	184/14464	0.01630435	2.48237986	1.64543995	0.12067385	0.42113893	0.40264675	Nr1d2/Hlf/Gpcpd1	3
GO:0022407	regulation of cell-cell adhesion	5/95	398/14464	0.01256281	1.9127215	1.50131642	0.12113513	0.42113893	0.40264675	Gimap5/Abl1/Peli1/Lyn/Arid2	5
GO:0045787	positive regulation of cell cycle	4/95	288/14464	0.01388889	2.11461988	1.55354523	0.12132068	0.42113893	0.40264675	Ccdc57/Abl1/Xrcc3/Ncapd3	4
GO:0044772	mitotic cell cycle phase transition	5/95	399/14464	0.01253133	1.90792771	1.49535937	0.12206952	0.42113893	0.40264675	Wee1/Ccdc57/Pole/Xrcc3/Arid2	5
GO:0006814	sodium ion transport	3/95	185/14464	0.01621622	2.46896159	1.63502776	0.1221098	0.42113893	0.40264675	Per1/Slc6a6/Agrn	3
GO:0044242	cellular lipid catabolic process	3/95	185/14464	0.01621622	2.46896159	1.63502776	0.1221098	0.42113893	0.40264675	Acer2/Pnpla7/Gpcpd1	3
GO:0048678	response to axon injury	2/95	92/14464	0.02173913	3.30983982	1.80715289	0.12244609	0.42113893	0.40264675	Trem2/Lyn	2
GO:2000781	positive regulation of double-strand break repair	2/95	92/14464	0.02173913	3.30983982	1.80715289	0.12244609	0.42113893	0.40264675	Peli1/Arid2	2
GO:0048813	dendrite morphogenesis	3/95	186/14464	0.01612903	2.45568761	1.62468321	0.12355209	0.42113893	0.40264675	Fzd4/Myo5b/Rere	3
GO:0006958	complement activation, classical pathway	1/95	20/14464	0.05	7.61263158	2.40615338	0.12355457	0.42113893	0.40264675	Trem2	1
GO:0010878	cholesterol storage	1/95	20/14464	0.05	7.61263158	2.40615338	0.12355457	0.42113893	0.40264675	Trem2	1
GO:0010888	negative regulation of lipid storage	1/95	20/14464	0.05	7.61263158	2.40615338	0.12355457	0.42113893	0.40264675	Trem2	1
GO:0032986	protein-DNA complex disassembly	1/95	20/14464	0.05	7.61263158	2.40615338	0.12355457	0.42113893	0.40264675	Arid2	1
GO:0034312	diol biosynthetic process	1/95	20/14464	0.05	7.61263158	2.40615338	0.12355457	0.42113893	0.40264675	Acer2	1
GO:0042749	regulation of circadian sleep/wake cycle	1/95	20/14464	0.05	7.61263158	2.40615338	0.12355457	0.42113893	0.40264675	Per3	1
GO:0045736	negative regulation of cyclin-dependent protein serine/threonine kinase activity	1/95	20/14464	0.05	7.61263158	2.40615338	0.12355457	0.42113893	0.40264675	Inca1	1
GO:0046519	sphingoid metabolic process	1/95	20/14464	0.05	7.61263158	2.40615338	0.12355457	0.42113893	0.40264675	Acer2	1
GO:0048143	astrocyte activation	1/95	20/14464	0.05	7.61263158	2.40615338	0.12355457	0.42113893	0.40264675	Trem2	1
GO:0050802	circadian sleep/wake cycle, sleep	1/95	20/14464	0.05	7.61263158	2.40615338	0.12355457	0.42113893	0.40264675	Per3	1
GO:0060143	positive regulation of syncytium formation by plasma membrane fusion	1/95	20/14464	0.05	7.61263158	2.40615338	0.12355457	0.42113893	0.40264675	Trem2	1
GO:0071340	skeletal muscle acetylcholine-gated channel clustering	1/95	20/14464	0.05	7.61263158	2.40615338	0.12355457	0.42113893	0.40264675	Agrn	1
GO:0099638	endosome to plasma membrane protein transport	1/95	20/14464	0.05	7.61263158	2.40615338	0.12355457	0.42113893	0.40264675	Myo5b	1
GO:0050769	positive regulation of neurogenesis	4/95	291/14464	0.0137457	2.09281968	1.53123494	0.12468806	0.42276538	0.40420178	Fzd4/Myo5b/Clcn2/Lyn	4
GO:1903037	regulation of leukocyte cell-cell adhesion	4/95	292/14464	0.01369863	2.08565249	1.52385768	0.12581941	0.42276538	0.40420178	Gimap5/Abl1/Peli1/Arid2	4
GO:0050870	positive regulation of T cell activation	3/95	188/14464	0.01595745	2.42956327	1.60419353	0.12645547	0.42276538	0.40420178	Gimap5/Abl1/Arid2	3
GO:0030516	regulation of axon extension	2/95	94/14464	0.0212766	3.23941769	1.77112157	0.12681424	0.42276538	0.40420178	Myo5b/Abl1	2
GO:0031110	regulation of microtubule polymerization or depolymerization	2/95	94/14464	0.0212766	3.23941769	1.77112157	0.12681424	0.42276538	0.40420178	Abl1/Nav3	2
GO:0033045	regulation of sister chromatid segregation	2/95	94/14464	0.0212766	3.23941769	1.77112157	0.12681424	0.42276538	0.40420178	Xrcc3/Arid2	2
GO:0034446	substrate adhesion-dependent cell spreading	2/95	94/14464	0.0212766	3.23941769	1.77112157	0.12681424	0.42276538	0.40420178	Fzd4/Abl1	2
GO:0050727	regulation of inflammatory response	4/95	294/14464	0.01360544	2.07146438	1.50919097	0.12809529	0.42276538	0.40420178	Nr1d2/Trem2/Serpine1/Lyn	4
GO:0006754	ATP biosynthetic process	2/95	95/14464	0.02105263	3.20531856	1.75346696	0.12901224	0.42276538	0.40420178	Trem2/ND5	2
GO:0019882	antigen processing and presentation	2/95	95/14464	0.02105263	3.20531856	1.75346696	0.12901224	0.42276538	0.40420178	Trem2/Rab34	2
GO:0002495	antigen processing and presentation of peptide antigen via MHC class II	1/95	21/14464	0.04761905	7.25012531	2.33049078	0.12931906	0.42276538	0.40420178	Trem2	1
GO:0007263	nitric oxide mediated signal transduction	1/95	21/14464	0.04761905	7.25012531	2.33049078	0.12931906	0.42276538	0.40420178	Mt1	1
GO:0032768	regulation of monooxygenase activity	1/95	21/14464	0.04761905	7.25012531	2.33049078	0.12931906	0.42276538	0.40420178	Por	1
GO:0032816	positive regulation of natural killer cell activation	1/95	21/14464	0.04761905	7.25012531	2.33049078	0.12931906	0.42276538	0.40420178	Il15ra	1
GO:0042634	regulation of hair cycle	1/95	21/14464	0.04761905	7.25012531	2.33049078	0.12931906	0.42276538	0.40420178	Per1	1
GO:0045475	locomotor rhythm	1/95	21/14464	0.04761905	7.25012531	2.33049078	0.12931906	0.42276538	0.40420178	Ciart	1
GO:0045838	positive regulation of membrane potential	1/95	21/14464	0.04761905	7.25012531	2.33049078	0.12931906	0.42276538	0.40420178	Gimap5	1
GO:0090050	positive regulation of cell migration involved in sprouting angiogenesis	1/95	21/14464	0.04761905	7.25012531	2.33049078	0.12931906	0.42276538	0.40420178	Abl1	1
GO:0141086	negative regulation of inflammasome-mediated signaling pathway	1/95	21/14464	0.04761905	7.25012531	2.33049078	0.12931906	0.42276538	0.40420178	Trem2	1
GO:1900016	negative regulation of cytokine production involved in inflammatory response	1/95	21/14464	0.04761905	7.25012531	2.33049078	0.12931906	0.42276538	0.40420178	Trem2	1
GO:1901380	negative regulation of potassium ion transmembrane transport	1/95	21/14464	0.04761905	7.25012531	2.33049078	0.12931906	0.42276538	0.40420178	Agrn	1
GO:1990182	exosomal secretion	1/95	21/14464	0.04761905	7.25012531	2.33049078	0.12931906	0.42276538	0.40420178	Myo5b	1
GO:2000114	regulation of establishment of cell polarity	1/95	21/14464	0.04761905	7.25012531	2.33049078	0.12931906	0.42276538	0.40420178	Abl1	1
GO:2001169	regulation of ATP biosynthetic process	1/95	21/14464	0.04761905	7.25012531	2.33049078	0.12931906	0.42276538	0.40420178	Trem2	1
GO:0050730	regulation of peptidyl-tyrosine phosphorylation	3/95	191/14464	0.01570681	2.39140259	1.57394511	0.13085664	0.42607403	0.40736515	Trem2/Abl1/Agrn	3
GO:0048814	regulation of dendrite morphogenesis	2/95	96/14464	0.02083333	3.17192982	1.73604496	0.13121926	0.42607403	0.40736515	Fzd4/Myo5b	2
GO:0050764	regulation of phagocytosis	2/95	96/14464	0.02083333	3.17192982	1.73604496	0.13121926	0.42607403	0.40736515	Trem2/Il15ra	2
GO:1902106	negative regulation of leukocyte differentiation	2/95	96/14464	0.02083333	3.17192982	1.73604496	0.13121926	0.42607403	0.40736515	Lrrc17/Lyn	2
GO:0050863	regulation of T cell activation	4/95	297/14464	0.01346801	2.05054049	1.48740692	0.13154173	0.42639962	0.40767644	Gimap5/Abl1/Peli1/Arid2	4
GO:0043266	regulation of potassium ion transport	2/95	97/14464	0.02061856	3.13922952	1.7188498	0.13343506	0.42694125	0.40819429	Trem2/Agrn	2
GO:0006910	phagocytosis, recognition	1/95	22/14464	0.04545455	6.92057416	2.25963982	0.13504604	0.42694125	0.40819429	Trem2	1
GO:0008207	C21-steroid hormone metabolic process	1/95	22/14464	0.04545455	6.92057416	2.25963982	0.13504604	0.42694125	0.40819429	Clcn2	1
GO:0010839	negative regulation of keratinocyte proliferation	1/95	22/14464	0.04545455	6.92057416	2.25963982	0.13504604	0.42694125	0.40819429	Klf9	1
GO:0021535	cell migration in hindbrain	1/95	22/14464	0.04545455	6.92057416	2.25963982	0.13504604	0.42694125	0.40819429	Rere	1
GO:0022410	circadian sleep/wake cycle process	1/95	22/14464	0.04545455	6.92057416	2.25963982	0.13504604	0.42694125	0.40819429	Per3	1
GO:0032691	negative regulation of interleukin-1 beta production	1/95	22/14464	0.04545455	6.92057416	2.25963982	0.13504604	0.42694125	0.40819429	Trem2	1
GO:0034349	glial cell apoptotic process	1/95	22/14464	0.04545455	6.92057416	2.25963982	0.13504604	0.42694125	0.40819429	Trem2	1
GO:0035025	positive regulation of Rho protein signal transduction	1/95	22/14464	0.04545455	6.92057416	2.25963982	0.13504604	0.42694125	0.40819429	Net1	1
GO:0061298	retina vasculature development in camera-type eye	1/95	22/14464	0.04545455	6.92057416	2.25963982	0.13504604	0.42694125	0.40819429	Fzd4	1
GO:0090231	regulation of spindle checkpoint	1/95	22/14464	0.04545455	6.92057416	2.25963982	0.13504604	0.42694125	0.40819429	Xrcc3	1
GO:0097734	extracellular exosome biogenesis	1/95	22/14464	0.04545455	6.92057416	2.25963982	0.13504604	0.42694125	0.40819429	Myo5b	1
GO:0120255	olefinic compound biosynthetic process	1/95	22/14464	0.04545455	6.92057416	2.25963982	0.13504604	0.42694125	0.40819429	Clcn2	1
GO:1904030	negative regulation of cyclin-dependent protein kinase activity	1/95	22/14464	0.04545455	6.92057416	2.25963982	0.13504604	0.42694125	0.40819429	Inca1	1
GO:2000042	negative regulation of double-strand break repair via homologous recombination	1/95	22/14464	0.04545455	6.92057416	2.25963982	0.13504604	0.42694125	0.40819429	Abl1	1
GO:0045637	regulation of myeloid cell differentiation	3/95	194/14464	0.01546392	2.35442214	1.54426079	0.13531174	0.42707769	0.40832473	Trem2/Lrrc17/Lyn	3
GO:0014706	striated muscle tissue development	5/95	414/14464	0.01207729	1.8387999	1.40798524	0.13648925	0.43008682	0.41120173	Nr1d2/Hlf/Gpcpd1/Abl1/Arid2	5
GO:0045582	positive regulation of T cell differentiation	2/95	99/14464	0.02020202	3.07581074	1.68511778	0.13789224	0.43121469	0.41228008	Gimap5/Arid2	2
GO:1903432	regulation of TORC1 signaling	2/95	99/14464	0.02020202	3.07581074	1.68511778	0.13789224	0.43121469	0.41228008	Sar1b/Peli1	2
GO:0051348	negative regulation of transferase activity	3/95	196/14464	0.01530612	2.33039742	1.52477444	0.138311	0.43121469	0.41228008	Inca1/Abl1/Lyn	3
GO:0097529	myeloid leukocyte migration	3/95	196/14464	0.01530612	2.33039742	1.52477444	0.138311	0.43121469	0.41228008	Trem2/Serpine1/Lyn	3
GO:0060538	skeletal muscle organ development	3/95	197/14464	0.01522843	2.31856799	1.51512002	0.13981921	0.43121469	0.41228008	Nr1d2/Hlf/Gpcpd1	3
GO:0055017	cardiac muscle tissue growth	2/95	100/14464	0.02	3.04505263	1.66857032	0.1401332	0.43121469	0.41228008	Abl1/Arid2	2
GO:0002504	antigen processing and presentation of peptide or polysaccharide antigen via MHC class II	1/95	23/14464	0.04347826	6.61967963	2.19308046	0.14073573	0.43121469	0.41228008	Trem2	1
GO:0006312	mitotic recombination	1/95	23/14464	0.04347826	6.61967963	2.19308046	0.14073573	0.43121469	0.41228008	Xrcc3	1
GO:0007064	mitotic sister chromatid cohesion	1/95	23/14464	0.04347826	6.61967963	2.19308046	0.14073573	0.43121469	0.41228008	Naa50	1
GO:0010737	protein kinase A signaling	1/95	23/14464	0.04347826	6.61967963	2.19308046	0.14073573	0.43121469	0.41228008	Tcim	1
GO:0043032	positive regulation of macrophage activation	1/95	23/14464	0.04347826	6.61967963	2.19308046	0.14073573	0.43121469	0.41228008	Trem2	1
GO:2000773	negative regulation of cellular senescence	1/95	23/14464	0.04347826	6.61967963	2.19308046	0.14073573	0.43121469	0.41228008	Abl1	1
GO:0002688	regulation of leukocyte chemotaxis	2/95	101/14464	0.01980198	3.0149036	1.65222847	0.14238214	0.43121469	0.41228008	Serpine1/Lyn	2
GO:0048534	hematopoietic or lymphoid organ development	2/95	102/14464	0.01960784	2.98534572	1.63608737	0.14463887	0.43121469	0.41228008	Lrrc17/Abl1	2
GO:1903707	negative regulation of hemopoiesis	2/95	102/14464	0.01960784	2.98534572	1.63608737	0.14463887	0.43121469	0.41228008	Lrrc17/Lyn	2
GO:0016358	dendrite development	4/95	309/14464	0.01294498	1.97090785	1.40274923	0.14570637	0.43121469	0.41228008	Fzd4/Myo5b/Rere/Abl1	4
GO:0000302	response to reactive oxygen species	3/95	201/14464	0.01492537	2.27242734	1.47707757	0.14590791	0.43121469	0.41228008	Net1/ND5/Abl1	3
GO:0009636	response to toxic substance	3/95	201/14464	0.01492537	2.27242734	1.47707757	0.14590791	0.43121469	0.41228008	Inmt/Mt1/Lyn	3
GO:0001783	B cell apoptotic process	1/95	24/14464	0.04166667	6.34385965	2.13036879	0.1463884	0.43121469	0.41228008	Lyn	1
GO:0006882	intracellular zinc ion homeostasis	1/95	24/14464	0.04166667	6.34385965	2.13036879	0.1463884	0.43121469	0.41228008	Mt1	1
GO:0007413	axonal fasciculation	1/95	24/14464	0.04166667	6.34385965	2.13036879	0.1463884	0.43121469	0.41228008	Celsr3	1
GO:0008299	isoprenoid biosynthetic process	1/95	24/14464	0.04166667	6.34385965	2.13036879	0.1463884	0.43121469	0.41228008	Lss	1
GO:0015812	gamma-aminobutyric acid transport	1/95	24/14464	0.04166667	6.34385965	2.13036879	0.1463884	0.43121469	0.41228008	Slc6a6	1
GO:0030808	regulation of nucleotide biosynthetic process	1/95	24/14464	0.04166667	6.34385965	2.13036879	0.1463884	0.43121469	0.41228008	Trem2	1
GO:0031365	N-terminal protein amino acid modification	1/95	24/14464	0.04166667	6.34385965	2.13036879	0.1463884	0.43121469	0.41228008	Naa50	1
GO:0034138	toll-like receptor 3 signaling pathway	1/95	24/14464	0.04166667	6.34385965	2.13036879	0.1463884	0.43121469	0.41228008	Peli1	1
GO:0045540	regulation of cholesterol biosynthetic process	1/95	24/14464	0.04166667	6.34385965	2.13036879	0.1463884	0.43121469	0.41228008	Por	1
GO:0046184	aldehyde biosynthetic process	1/95	24/14464	0.04166667	6.34385965	2.13036879	0.1463884	0.43121469	0.41228008	Clcn2	1
GO:0046688	response to copper ion	1/95	24/14464	0.04166667	6.34385965	2.13036879	0.1463884	0.43121469	0.41228008	Mt1	1
GO:0050820	positive regulation of coagulation	1/95	24/14464	0.04166667	6.34385965	2.13036879	0.1463884	0.43121469	0.41228008	Serpine1	1
GO:0060544	regulation of necroptotic process	1/95	24/14464	0.04166667	6.34385965	2.13036879	0.1463884	0.43121469	0.41228008	Peli1	1
GO:0061098	positive regulation of protein tyrosine kinase activity	1/95	24/14464	0.04166667	6.34385965	2.13036879	0.1463884	0.43121469	0.41228008	Agrn	1
GO:0090330	regulation of platelet aggregation	1/95	24/14464	0.04166667	6.34385965	2.13036879	0.1463884	0.43121469	0.41228008	Lyn	1
GO:0097062	dendritic spine maintenance	1/95	24/14464	0.04166667	6.34385965	2.13036879	0.1463884	0.43121469	0.41228008	Trem2	1
GO:0106030	neuron projection fasciculation	1/95	24/14464	0.04166667	6.34385965	2.13036879	0.1463884	0.43121469	0.41228008	Celsr3	1
GO:0106118	regulation of sterol biosynthetic process	1/95	24/14464	0.04166667	6.34385965	2.13036879	0.1463884	0.43121469	0.41228008	Por	1
GO:0140112	extracellular vesicle biogenesis	1/95	24/14464	0.04166667	6.34385965	2.13036879	0.1463884	0.43121469	0.41228008	Myo5b	1
GO:1900371	regulation of purine nucleotide biosynthetic process	1/95	24/14464	0.04166667	6.34385965	2.13036879	0.1463884	0.43121469	0.41228008	Trem2	1
GO:1902993	positive regulation of amyloid precursor protein catabolic process	1/95	24/14464	0.04166667	6.34385965	2.13036879	0.1463884	0.43121469	0.41228008	Lyn	1
GO:2001014	regulation of skeletal muscle cell differentiation	1/95	24/14464	0.04166667	6.34385965	2.13036879	0.1463884	0.43121469	0.41228008	Nr1d2	1
GO:0031346	positive regulation of cell projection organization	5/95	426/14464	0.01173709	1.78700272	1.34062136	0.14855129	0.43121469	0.41228008	Fzd4/Myo5b/Abl1/Agrn/Lyn	5
GO:0031396	regulation of protein ubiquitination	3/95	204/14464	0.01470588	2.23900929	1.44913086	0.15053144	0.43121469	0.41228008	Abl1/Peli1/Paxip1	3
GO:0038061	non-canonical NF-kappaB signal transduction	2/95	105/14464	0.01904762	2.90005013	1.58882232	0.15145378	0.43121469	0.41228008	Trem2/Tcim	2
GO:0046632	alpha-beta T cell differentiation	2/95	105/14464	0.01904762	2.90005013	1.58882232	0.15145378	0.43121469	0.41228008	Gimap5/Abl1	2
GO:0003416	endochondral bone growth	1/95	25/14464	0.04	6.09010526	2.0711232	0.15200426	0.43121469	0.41228008	Por	1
GO:0009595	detection of biotic stimulus	1/95	25/14464	0.04	6.09010526	2.0711232	0.15200426	0.43121469	0.41228008	Trem2	1
GO:0010875	positive regulation of cholesterol efflux	1/95	25/14464	0.04	6.09010526	2.0711232	0.15200426	0.43121469	0.41228008	Trem2	1
GO:0010955	negative regulation of protein processing	1/95	25/14464	0.04	6.09010526	2.0711232	0.15200426	0.43121469	0.41228008	Serpine1	1
GO:0030431	sleep	1/95	25/14464	0.04	6.09010526	2.0711232	0.15200426	0.43121469	0.41228008	Per3	1
GO:0032689	negative regulation of type II interferon production	1/95	25/14464	0.04	6.09010526	2.0711232	0.15200426	0.43121469	0.41228008	Gimap5	1
GO:0035335	peptidyl-tyrosine dephosphorylation	1/95	25/14464	0.04	6.09010526	2.0711232	0.15200426	0.43121469	0.41228008	Tns2	1
GO:0045830	positive regulation of isotype switching	1/95	25/14464	0.04	6.09010526	2.0711232	0.15200426	0.43121469	0.41228008	Paxip1	1
GO:0048311	mitochondrion distribution	1/95	25/14464	0.04	6.09010526	2.0711232	0.15200426	0.43121469	0.41228008	Tspan4	1
GO:0098877	neurotransmitter receptor transport to plasma membrane	1/95	25/14464	0.04	6.09010526	2.0711232	0.15200426	0.43121469	0.41228008	Myo5b	1

GO:0099159 regulation of modification of postsynaptic structure	1/95	25/14464	0.04	6.09010526	2.0711232	0.15200426	0.43121469	0.41228008	Myo5b	1
GO:1903318 negative regulation of protein maturation	1/95	25/14464	0.04	6.09010526	2.0711232	0.15200426	0.43121469	0.41228008	Serpine1	1
GO:1903975 regulation of glial cell migration	1/95	25/14464	0.04	6.09010526	2.0711232	0.15200426	0.43121469	0.41228008	Trem2	1
GO:0000819 sister chromatid segregation	3/95	205/14464	0.01463415	2.22808729	1.43992329	0.15208317	0.43121469	0.41228008	Xrcc3/Ncapd3/Arid2	3
GO:0002253 activation of immune response	5/95	431/14464	0.01160093	1.76627183	1.31317908	0.15370915	0.43121469	0.41228008	Trem2/Alpk1/Abl1/Peli1/Lyn	5
GO:0009206 purine ribonucleoside triphosphate biosynthetic process	2/95	106/14464	0.01886792	2.87269116	1.57343875	0.15373969	0.43121469	0.41228008	Trem2/ND5	2
GO:0097061 dendritic spine organization	2/95	106/14464	0.01886792	2.87269116	1.57343875	0.15373969	0.43121469	0.41228008	Trem2/Myo5b	2
GO:1902749 regulation of cell cycle G2/M phase transition	2/95	106/14464	0.01886792	2.87269116	1.57343875	0.15373969	0.43121469	0.41228008	Wee1/Paxip1	2
GO:0043087 regulation of GTPase activity	3/95	207/14464	0.01449275	2.20655988	1.4216664	0.15520209	0.43121469	0.41228008	Net1/Adap2/Agrn	3
GO:1903039 positive regulation of leukocyte cell-cell adhesion	3/95	207/14464	0.01449275	2.20655988	1.4216664	0.15520209	0.43121469	0.41228008	Gimap5/Abl1/Arid2	3
GO:0002683 negative regulation of immune system process	5/95	433/14464	0.01154734	1.75811353	1.3023017	0.15579342	0.43121469	0.41228008	Trem2/Gimap5/Lrrc17/Peli1/Lyn	5
GO:0009145 purine nucleoside triphosphate biosynthetic process	2/95	107/14464	0.01869159	2.84584358	1.55823393	0.15603242	0.43121469	0.41228008	Trem2/ND5	2
GO:0030518 nuclear receptor-mediated steroid hormone signaling pathway	2/95	107/14464	0.01869159	2.84584358	1.55823393	0.15603242	0.43121469	0.41228008	Per1/Klf9	2
GO:0032963 collagen metabolic process	2/95	107/14464	0.01869159	2.84584358	1.55823393	0.15603242	0.43121469	0.41228008	Tns2/Serpine1	2
GO:0060419 heart growth	2/95	107/14464	0.01869159	2.84584358	1.55823393	0.15603242	0.43121469	0.41228008	Abl1/Arid2	2
GO:0006282 regulation of DNA repair	3/95	208/14464	0.01442308	2.19595142	1.41261588	0.15676918	0.43121469	0.41228008	Abl1/Peli1/Arid2	3
GO:0002861 regulation of inflammatory response to antigenic stimulus	1/95	26/14464	0.03846154	5.85587045	2.01501355	0.15758357	0.43121469	0.41228008	Trem2	1
GO:0010893 positive regulation of steroid biosynthetic process	1/95	26/14464	0.03846154	5.85587045	2.01501355	0.15758357	0.43121469	0.41228008	Por	1
GO:0030149 sphingolipid catabolic process	1/95	26/14464	0.03846154	5.85587045	2.01501355	0.15758357	0.43121469	0.41228008	Acer2	1
GO:0032878 regulation of establishment or maintenance of cell polarity	1/95	26/14464	0.03846154	5.85587045	2.01501355	0.15758357	0.43121469	0.41228008	Abl1	1
GO:0033688 regulation of osteoblast proliferation	1/95	26/14464	0.03846154	5.85587045	2.01501355	0.15758357	0.43121469	0.41228008	Abl1	1
GO:0043372 positive regulation of CD4-positive, alpha-beta T cell differentiation	1/95	26/14464	0.03846154	5.85587045	2.01501355	0.15758357	0.43121469	0.41228008	Gimap5	1
GO:0048714 positive regulation of oligodendrocyte differentiation	1/95	26/14464	0.03846154	5.85587045	2.01501355	0.15758357	0.43121469	0.41228008	Clcn2	1
GO:0050995 negative regulation of lipid catabolic process	1/95	26/14464	0.03846154	5.85587045	2.01501355	0.15758357	0.43121469	0.41228008	Gimap5	1
GO:0060055 angiogenesis involved in wound healing	1/95	26/14464	0.03846154	5.85587045	2.01501355	0.15758357	0.43121469	0.41228008	Serpine1	1
GO:0060142 regulation of syncytium formation by plasma membrane fusion	1/95	26/14464	0.03846154	5.85587045	2.01501355	0.15758357	0.43121469	0.41228008	Trem2	1
GO:0070269 pyroptotic inflammatory response	1/95	26/14464	0.03846154	5.85587045	2.01501355	0.15758357	0.43121469	0.41228008	Trem2	1
GO:0070498 interleukin-1-mediated signaling pathway	1/95	26/14464	0.03846154	5.85587045	2.01501355	0.15758357	0.43121469	0.41228008	Rps6ka5	1
GO:0071276 cellular response to cadmium ion	1/95	26/14464	0.03846154	5.85587045	2.01501355	0.15758357	0.43121469	0.41228008	Mt1	1
GO:0097049 motor neuron apoptotic process	1/95	26/14464	0.03846154	5.85587045	2.01501355	0.15758357	0.43121469	0.41228008	Agrn	1
GO:0150077 regulation of neuroinflammatory response	1/95	26/14464	0.03846154	5.85587045	2.01501355	0.15758357	0.43121469	0.41228008	Trem2	1
GO:1902042 negative regulation of extrinsic apoptotic signaling pathway via death domain receptors	1/95	26/14464	0.03846154	5.85587045	2.01501355	0.15758357	0.43121469	0.41228008	Serpine1	1
GO:1905523 positive regulation of macrophage migration	1/95	26/14464	0.03846154	5.85587045	2.01501355	0.15758357	0.43121469	0.41228008	Trem2	1
GO:0060537 muscle tissue development	5/95	435/14464	0.01149425	1.75003025	1.29148026	0.15788958	0.43121469	0.41228008	Nr1d2/Hlf/Gpcpd1/Abl1/Arid2	5
GO:0008585 female gonad development	2/95	108/14464	0.01851852	2.81949318	1.54320388	0.1583318	0.43121469	0.41228008	Fzd4/Serpine1	2
GO:0030316 osteoclast differentiation	2/95	108/14464	0.01851852	2.81949318	1.54320388	0.1583318	0.43121469	0.41228008	Trem2/Lrrc17	2
GO:0002697 regulation of immune effector process	4/95	320/14464	0.0125	1.90315789	1.3284043	0.15919557	0.43121469	0.41228008	Trem2/Gimap5/Lyn/Paxip1	4
GO:0007160 cell-matrix adhesion	3/95	210/14464	0.01428571	2.17503759	1.39466774	0.1599183	0.43121469	0.41228008	Serpine1/Acer2/Abl1	3
GO:0042391 regulation of membrane potential	5/95	437/14464	0.01144165	1.74202096	1.28071417	0.15999753	0.43121469	0.41228008	Trem2/Gimap5/Tspan4/Clcn2/Agrn	5
GO:0008037 cell recognition	2/95	109/14464	0.01834862	2.79362627	1.52834475	0.16063762	0.43121469	0.41228008	Trem2/Celsr3	2
GO:0039531 regulation of cytoplasmic pattern recognition receptor signaling pathway	2/95	109/14464	0.01834862	2.79362627	1.52834475	0.16063762	0.43121469	0.41228008	Trem2/Peli1	2
GO:0002702 positive regulation of production of molecular mediator of immune response	2/95	110/14464	0.01818182	2.76822967	1.5136528	0.16294972	0.43121469	0.41228008	Gimap5/Paxip1	2
GO:0046545 development of primary female sexual characteristics	2/95	110/14464	0.01818182	2.76822967	1.5136528	0.16294972	0.43121469	0.41228008	Fzd4/Serpine1	2
GO:0050868 negative regulation of T cell activation	2/95	110/14464	0.01818182	2.76822967	1.5136528	0.16294972	0.43121469	0.41228008	Gimap5/Peli1	2
GO:0061387 regulation of extent of cell growth	2/95	110/14464	0.01818182	2.76822967	1.5136528	0.16294972	0.43121469	0.41228008	Myo5b/Abl1	2
GO:0009100 glycoprotein metabolic process	4/95	323/14464	0.0123839	1.88548151	1.30863466	0.16295355	0.43121469	0.41228008	Acer2/Galnt15/Tet3/Alg10b	4
GO:0002699 positive regulation of immune effector process	3/95	212/14464	0.01415094	2.15451837	1.37691964	0.16308697	0.43121469	0.41228008	Trem2/Gimap5/Paxip1	3
GO:0006813 potassium ion transport	3/95	212/14464	0.01415094	2.15451837	1.37691964	0.16308697	0.43121469	0.41228008	Trem2/Agrn/Alg10b	3
GO:1905475 regulation of protein localization to membrane	3/95	212/14464	0.01415094	2.15451837	1.37691964	0.16308697	0.43121469	0.41228008	Trem2/Myo5b/Agrn	3
GO:0001773 myeloid dendritic cell activation	1/95	27/14464	0.03703704	5.63898635	1.96175253	0.16312655	0.43121469	0.41228008	Gimap5	1
GO:0001779 natural killer cell differentiation	1/95	27/14464	0.03703704	5.63898635	1.96175253	0.16312655	0.43121469	0.41228008	Il15ra	1
GO:0006376 mRNA splice site recognition	1/95	27/14464	0.03703704	5.63898635	1.96175253	0.16312655	0.43121469	0.41228008	Rbmx	1
GO:0007608 sensory perception of smell	1/95	27/14464	0.03703704	5.63898635	1.96175253	0.16312655	0.43121469	0.41228008	Or8b53	1
GO:0034311 diol metabolic process	1/95	27/14464	0.03703704	5.63898635	1.96175253	0.16312655	0.43121469	0.41228008	Acer2	1
GO:0035929 steroid hormone secretion	1/95	27/14464	0.03703704	5.63898635	1.96175253	0.16312655	0.43121469	0.41228008	Fzd4	1
GO:0045730 respiratory burst	1/95	27/14464	0.03703704	5.63898635	1.96175253	0.16312655	0.43121469	0.41228008	Trem2	1
GO:0051984 positive regulation of chromosome segregation	1/95	27/14464	0.03703704	5.63898635	1.96175253	0.16312655	0.43121469	0.41228008	Ncapd3	1
GO:0060252 positive regulation of glial cell proliferation	1/95	27/14464	0.03703704	5.63898635	1.96175253	0.16312655	0.43121469	0.41228008	Lyn	1
GO:0060977 coronary vasculature morphogenesis	1/95	27/14464	0.03703704	5.63898635	1.96175253	0.16312655	0.43121469	0.41228008	Arid2	1
GO:2000050 regulation of non-canonical Wnt signaling pathway	1/95	27/14464	0.03703704	5.63898635	1.96175253	0.16312655	0.43121469	0.41228008	Abl1	1
GO:0002274 myeloid leukocyte activation	3/95	213/14464	0.01408451	2.14440326	1.36811921	0.16467849	0.43121469	0.41228008	Trem2/Gimap5/Lyn	3
GO:0045930 negative regulation of mitotic cell cycle	3/95	213/14464	0.01408451	2.14440326	1.36811921	0.16467849	0.43121469	0.41228008	Wee1/Abl1/Xrcc3	3
GO:0009201 ribonucleoside triphosphate biosynthetic process	2/95	111/14464	0.01801802	2.74329066	1.4991244	0.16526792	0.43121469	0.41228008	Trem2/ND5	2
GO:0031398 positive regulation of protein ubiquitination	2/95	111/14464	0.01801802	2.74329066	1.4991244	0.16526792	0.43121469	0.41228008	Peli1/Paxip1	2
GO:0034764 positive regulation of transmembrane transport	3/95	214/14464	0.01401869	2.13438269	1.35936714	0.16627473	0.43121469	0.41228008	Trem2/Gimap5/Abl1	3
GO:0051052 regulation of DNA metabolic process	5/95	443/14464	0.01128668	1.71842699	1.24874204	0.16639085	0.43121469	0.41228008	Abl1/Peli1/Paxip1/Ylpm1/Arid2	5
GO:0001841 neural tube formation	2/95	112/14464	0.01785714	2.71879699	1.48475606	0.16759203	0.43121469	0.41228008	Abl1/Brpf1	2
GO:0051209 release of sequestered calcium ion into cytosol	2/95	112/14464	0.01785714	2.71879699	1.48475606	0.16759203	0.43121469	0.41228008	Abl1/Lyn	2
GO:0002443 leukocyte mediated immunity	4/95	327/14464	0.01223242	1.86241751	1.28259794	0.16801472	0.43121469	0.41228008	Trem2/Gimap5/Lyn/Paxip1	4
GO:0009069 serine family amino acid metabolic process	1/95	28/14464	0.03571429	5.43759398	1.9110888	0.16863344	0.43121469	0.41228008	Tha1	1
GO:0014850 response to muscle activity	1/95	28/14464	0.03571429	5.43759398	1.9110888	0.16863344	0.43121469	0.41228008	Tns2	1
GO:0032692 negative regulation of interleukin-1 production	1/95	28/14464	0.03571429	5.43759398	1.9110888	0.16863344	0.43121469	0.41228008	Trem2	1
GO:0034381 plasma lipoprotein particle clearance	1/95	28/14464	0.03571429	5.43759398	1.9110888	0.16863344	0.43121469	0.41228008	Trem2	1
GO:0035459 vesicle cargo loading	1/95	28/14464	0.03571429	5.43759398	1.9110888	0.16863344	0.43121469	0.41228008	Sar1b	1
GO:0043304 regulation of mast cell degranulation	1/95	28/14464	0.03571429	5.43759398	1.9110888	0.16863344	0.43121469	0.41228008	Lyn	1
GO:0045686 negative regulation of glial cell differentiation	1/95	28/14464	0.03571429	5.43759398	1.9110888	0.16863344	0.43121469	0.41228008	Trem2	1
GO:0048199 vesicle targeting, to, from or within Golgi	1/95	28/14464	0.03571429	5.43759398	1.9110888	0.16863344	0.43121469	0.41228008	Sar1b	1
GO:0050901 leukocyte tethering or rolling	1/95	28/14464	0.03571429	5.43759398	1.9110888	0.16863344	0.43121469	0.41228008	Jam2	1
GO:0060117 auditory receptor cell development	1/95	28/14464	0.03571429	5.43759398	1.9110888	0.16863344	0.43121469	0.41228008	Alg10b	1
GO:0060402 calcium ion transport into cytosol	1/95	28/14464	0.03571429	5.43759398	1.9110888	0.16863344	0.43121469	0.41228008	Gimap5	1
GO:0061036 positive regulation of cartilage development	1/95	28/14464	0.03571429	5.43759398	1.9110888	0.16863344	0.43121469	0.41228008	Por	1
GO:0099150 regulation of postsynaptic specialization assembly	1/95	28/14464	0.03571429	5.43759398	1.9110888	0.16863344	0.43121469	0.41228008	Abl1	1
GO:1902932 positive regulation of alcohol biosynthetic process	1/95	28/14464	0.03571429	5.43759398	1.9110888	0.16863344	0.43121469	0.41228008	Por	1
GO:1903055 positive regulation of extracellular matrix organization	1/95	28/14464	0.03571429	5.43759398	1.9110888	0.16863344	0.43121469	0.41228008	Abl1	1
GO:1903909 regulation of receptor clustering	1/95	28/14464	0.03571429	5.43759398	1.9110888	0.16863344	0.43121469	0.41228008	Agrn	1
GO:1905820 positive regulation of chromosome separation	1/95	28/14464	0.03571429	5.43759398	1.9110888	0.16863344	0.43121469	0.41228008	Ncapd3	1
GO:2000819 regulation of nucleotide-excision repair	1/95	28/14464	0.03571429	5.43759398	1.9110888	0.16863344	0.43121469	0.41228008	Arid2	1
GO:0022900 electron transport chain	2/95	113/14464	0.01769912	2.69473684	1.47054437	0.16992189	0.43121469	0.41228008	Por/ND5	2
GO:0051283 negative regulation of sequestering of calcium ion	2/95	113/14464	0.01769912	2.69473684	1.47054437	0.16992189	0.43121469	0.41228008	Abl1/Lyn	2
GO:0034762 regulation of transmembrane transport	5/95	447/14464	0.01118568	1.70304957	1.22769333	0.17070978	0.43121469	0.41228008	Trem2/Gimap5/Abl1/Agrn/Lyn	5
GO:1903076 regulation of protein localization to plasma membrane	2/95	114/14464	0.01754386	2.6710988	1.45648604	0.17225731	0.43121469	0.41228008	Trem2/Myo5b	2
GO:0052548 regulation of endopeptidase activity	3/95	218/14464	0.01376147	2.0952197	1.32483181	0.17270565	0.43121469	0.41228008	Serpine1/Acer2/Lyn	3
GO:0048638 regulation of developmental growth	4/95	331/14464	0.01208459	1.83991096	1.25692043	0.17313205	0.43121469	0.41228008	Serpine1/Myo5b/Abl1/Agrn	4
GO:0007346 regulation of mitotic cell cycle	5/95	450/14464	0.01111111	1.69169591	1.21204328	0.17397806	0.43121469	0.41228008	Wee1/Ccdc57/Abl1/Xrcc3/Arid2	5
GO:0006120 mitochondrial electron transport, NADH to ubiquinone	1/95	29/14464	0.03448276	5.25009074	1.86280129	0.17410447	0.43121469	0.41228008	ND5	1
GO:0006779 porphyrin-containing compound biosynthetic process	1/95	29/14464	0.03448276	5.25009074	1.86280129	0.17410447	0.43121469	0.41228008	Rsad1	1
GO:0032733 positive regulation of interleukin-10 production	1/95	29/14464	0.03448276	5.25009074	1.86280129	0.17410447	0.43121469	0.41228008	Trem2	1
GO:0033014 tetrapyrrole biosynthetic process	1/95	29/14464	0.03448276	5.25009074	1.86280129	0.17410447	0.43121469	0.41228008	Rsad1	1
GO:0042269 regulation of natural killer cell mediated cytotoxicity	1/95	29/14464	0.03448276	5.25009074	1.86280129	0.17410447	0.43121469	0.41228008	Gimap5	1
GO:0045671 negative regulation of osteoclast differentiation	1/95	29/14464	0.03448276	5.25009074	1.86280129	0.17410447	0.43121469	0.41228008	Lrrc17	1
GO:0045940 positive regulation of steroid metabolic process	1/95	29/14464	0.03448276	5.25009074	1.86280129	0.17410447	0.43121469	0.41228008	Por	1
GO:0046466 membrane lipid catabolic process	1/95	29/14464	0.03448276	5.25009074	1.86280129	0.17410447	0.43121469	0.41228008	Acer2	1
GO:0051450 myoblast proliferation	1/95	29/14464	0.03448276	5.25009074	1.86280129	0.17410447	0.43121469	0.41228008	Abl1	1
GO:0051894 positive regulation of focal adhesion assembly	1/95	29/14464	0.03448276	5.25009074	1.86280129	0.17410447	0.43121469	0.41228008	Abl1	1
GO:0062098 regulation of programmed necrotic cell death	1/95	29/14464	0.03448276	5.25009074	1.86280129	0.17410447	0.43121469	0.41228008	Peli1	1
GO:0086010 membrane depolarization during action potential	1/95	29/14464	0.03448276	5.25009074	1.86280129	0.17410447	0.43121469	0.41228008	Clcn2	1
GO:0090382 phagosome maturation	1/95	29/14464	0.03448276	5.25009074	1.86280129	0.17410447	0.43121469	0.41228008	Rab34	1
GO:0098868 bone growth	1/95	29/14464	0.03448276	5.25009074	1.86280129	0.17410447	0.43121469	0.41228008	Por	1
GO:0120178 steroid hormone biosynthetic process	1/95	29/14464	0.03448276	5.25009074	1.86280129	0.17410447	0.43121469	0.41228008	Clcn2	1
GO:2000406 positive regulation of T cell migration	1/95	29/14464	0.03448276	5.25009074	1.86280129	0.17410447	0.43121469	0.41228008	Abl1	1
GO:0031589 cell-substrate adhesion	4/95	332/14464	0.01204819	1.83436906	1.25055599	0.17441996	0.43121469	0.41228008	Fzd4/Serpine1/Acer2/Abl1	4
GO:0043200 response to amino acid	2/95	115/14464	0.0173913	2.64787185	1.44257786	0.17459813	0.43121469	0.41228008	Gdf10/Lyn	2
GO:0050729 positive regulation of inflammatory response	2/95	115/14464	0.0173913	2.64787185	1.44257786	0.17459813	0.43121469	0.41228008	Trem2/Serpine1	2
GO:0051282 regulation of sequestering of calcium ion	2/95	115/14464	0.0173913	2.64787185	1.44257786	0.17459813	0.43121469	0.41228008	Abl1/Lyn	2
GO:0043401 steroid hormone receptor signaling pathway	2/95	116/14464	0.01724138	2.62504537	1.42881672	0.17694418	0.43477066	0.41567991	Per1/Klf9	2
GO:0045931 positive regulation of mitotic cell cycle	2/95	116/14464	0.01724138	2.62504537	1.42881672	0.17694418	0.43477066	0.41567991	Ccdc57/Abl1	2
GO:0048709 oligodendrocyte differentiation	2/95	116/14464	0.01724138	2.62504537	1.42881672	0.17694418	0.43477066	0.41567991	Clcn2/Lyn	2
GO:0051208 sequestering of calcium ion	2/95	116/14464	0.01724138	2.62504537	1.42881672	0.17694418	0.43477066	0.41567991	Abl1/Lyn	2
GO:0106027 neuron projection organization	2/95	117/14464	0.01709402	2.60260909	1.41519963	0.17929529	0.43612282	0.41697269	Trem2/Myo5b	2
GO:0030261 chromosome condensation	1/95	30/14464	0.03333333	5.07508772	1.81669475	0.17953988	0.43612282	0.41697269	Ncapd3	1
GO:0032814 regulation of natural killer cell activation	1/95	30/14464	0.03333333	5.07508772	1.81669475	0.17953988	0.43612282	0.41697269	Il15ra	1
GO:0042745 circadian sleep/wake cycle	1/95	30/14464	0.03333333	5.07508772	1.81669475	0.17953988	0.43612282	0.41697269	Per3	1
GO:0045672 positive regulation of osteoclast differentiation	1/95	30/14464	0.03333333	5.07508772	1.81669475	0.17953988	0.43612282	0.41697269	Trem2	1
GO:0048026 positive regulation of mRNA splicing, via spliceosome	1/95	30/14464	0.03333333	5.07508772	1.81669475	0.17953988	0.43612282	0.41697269	Rbmx	1
GO:0071404 cellular response to low-density lipoprotein particle stimulus	1/95	30/14464	0.03333333	5.07508772	1.81669475	0.17953988	0.43612282	0.41697269	Trem2	1
GO:0150146 cell junction disassembly	1/95	30/14464	0.03333333	5.07508772	1.81669475	0.17953988	0.43612282	0.41697269	Trem2	1
GO:2000108 positive regulation of leukocyte apoptotic process	1/95	30/14464	0.03333333	5.07508772	1.81669475	0.17953988	0.43612282	0.41697269	Lyn	1
GO:0002250 adaptive immune response	4/95	337/14464	0.01186944	1.8071529	1.21905559	0.18090957	0.4388944	0.41962257	Trem2/Gimap5/Lyn/Paxip1	4
GO:0008203 cholesterol metabolic process	2/95	118/14464	0.01694915	2.58055308	1.40172364	0.1816513	0.43958241	0.42028038	Por/Lss	2
GO:0009142 nucleoside triphosphate biosynthetic process	2/95	118/14464	0.01694915	2.58055308	1.40172364	0.1816513	0.43958241	0.42028038	Trem2/ND5	2
GO:0051402 neuron apoptotic process	4/95	339/14464	0.01179941	1.79649123	1.20660284	0.18352828	0.44196717	0.42256042	Mt1/Trem2/Abl1/Agrn	4
GO:0060271 cilium assembly	4/95	339/14464	0.01179941	1.79649123	1.20660284	0.18352828	0.44196717	0.42256042	Ccdc57/Rab34/Alpk1/Celsr3	4
GO:0030195 negative regulation of blood coagulation	1/95	31/14464	0.03225806	4.91137521	1.77259591	0.18493989	0.44196717	0.42256042	Serpine1	1
GO:0032967 positive regulation of collagen biosynthetic process	1/95	31/14464	0.03225806	4.91137521	1.77259591	0.18493989	0.44196717	0.42256042	Serpine1	1
GO:0033687 osteoblast proliferation	1/95	31/14464	0.03225806	4.91137521	1.77259591	0.18493989	0.44196717	0.42256042	Abl1	1
GO:0043267 negative regulation of potassium ion transport	1/95	31/14464	0.03225806	4.91137521	1.77259591	0.18493989	0.44196717	0.42256042	Agrn	1
GO:0045191 regulation of isotype switching	1/95	31/14464	0.03225806	4.91137521	1.77259591	0.18493989	0.44196717	0.42256042	Paxip1	1
GO:0055094 response to lipoprotein particle	1/95	31/14464	0.03225806	4.91137521	1.77259591	0.18493989	0.44196717	0.42256042	Trem2	1
GO:1990000 amyloid fibril formation	1/95	31/14464	0.03225806	4.91137521	1.77259591	0.18493989	0.44196717	0.42256042	Trem2	1
GO:2000780 negative regulation of double-strand break repair	1/95	31/14464	0.03225806	4.91137521	1.77259591	0.18493989	0.44196717	0.42256042	Abl1	1
GO:0048675 axon extension	2/95	120/14464	0.01666667	2.53754386	1.37518372	0.18637736	0.44481701	0.42528512	Myo5b/Abl1	2
GO:0051056 regulation of small GTPase mediated signal transduction	3/95	227/14464	0.01321586	2.01214932	1.2497582	0.18742861	0.44481701	0.42528512	Net1/Abl1/Agrn	3
GO:0006869 lipid transport	4/95	342/14464	0.01169591	1.78073253	1.18807799	0.18748015	0.44481701	0.42528512	Fzd4/Trem2/Sar1b/Lyn	4
GO:1901890 positive regulation of cell junction assembly	2/95	121/14464	0.01652893	2.51657242	1.36211434	0.18874708	0.44481701	0.42528512	Abl1/Agrn	2
GO:0042110 T cell activation	5/95	464/14464	0.01077586	1.64065336	1.14050135	0.18954664	0.44481701	0.42528512	Trem2/Gimap5/Abl1/Peli1/Arid2	5
GO:0002715 regulation of natural killer cell mediated immunity	1/95	32/14464	0.03125	4.75789474	1.73035042	0.19030472	0.44481701	0.42528512	Gimap5	1
GO:0006901 vesicle coating	1/95	32/14464	0.03125	4.75789474	1.73035042	0.19030472	0.44481701	0.42528512	Sar1b	1
GO:0031116 positive regulation of microtubule polymerization	1/95	32/14464	0.03125	4.75789474	1.73035042	0.19030472	0.44481701	0.42528512	Nav3	1
GO:0032373 positive regulation of sterol transport	1/95	32/14464	0.03125	4.75789474	1.73035042	0.19030472	0.44481701	0.42528512	Trem2	1
GO:0032376 positive regulation of cholesterol transport	1/95	32/14464	0.03125	4.75789474	1.73035042	0.19030472	0.44481701	0.42528512	Trem2	1
GO:0032743 positive regulation of interleukin-2 production	1/95	32/14464	0.03125	4.75789474	1.73035042	0.19030472	0.44481701	0.42528512	Abl1	1
GO:0043045 epigenetic programming of gene expression	1/95	32/14464	0.03125	4.75789474	1.73035042	0.19030472	0.44481701	0.42528512	Tet3	1
GO:0045589 regulation of regulatory T cell differentiation	1/95	32/14464	0.03125	4.75789474	1.73035042	0.19030472	0.44481701	0.42528512	Gimap5	1
GO:0045738 negative regulation of DNA repair	1/95	32/14464	0.03125	4.75789474	1.73035042	0.19030472	0.44481701	0.42528512	Abl1	1
GO:0097242 amyloid-beta clearance	1/95	32/14464	0.03125	4.75789474	1.73035042	0.19030472	0.44481701	0.42528512	Trem2	1
GO:0140058 neuron projection arborization	1/95	32/14464	0.03125	4.75789474	1.73035042	0.19030472	0.44481701	0.42528512	Fzd4	1
GO:1900047 negative regulation of hemostasis	1/95	32/14464	0.03125	4.75789474	1.73035042	0.19030472	0.44481701	0.42528512	Serpine1	1
GO:1901381 positive regulation of potassium ion transmembrane transport	1/95	32/14464	0.03125	4.75789474	1.73035042	0.19030472	0.44481701	0.42528512	Trem2	1
GO:0046660 female sex differentiation	2/95	122/14464	0.01639344	2.49594478	1.34917518	0.19112106	0.44618166	0.42658985	Fzd4/Serpine1	2
GO:0016125 sterol metabolic process	2/95	123/14464	0.01626016	2.47565255	1.33636371	0.19349914	0.44746464	0.4278165	Por/Lss	2
GO:0032368 regulation of lipid transport	2/95	123/14464	0.01626016	2.47565255	1.33636371	0.19349914	0.44746464	0.4278165	Trem2/Sar1b	2
GO:0051384 response to glucocorticoid	2/95	123/14464	0.01626016	2.47565255	1.33636371	0.19349914	0.44746464	0.4278165	Por/Klf9	2
GO:0070665 positive regulation of leukocyte proliferation	2/95	123/14464	0.01626016	2.47565255	1.33636371	0.19349914	0.44746464	0.4278165	Peli1/Lyn	2
GO:1903038 negative regulation of leukocyte cell-cell adhesion	2/95	123/14464	0.01626016	2.47565255	1.33636371	0.19349914	0.44746464	0.4278165	Gimap5/Peli1	2
GO:0000462 maturation of SSU-rRNA from tricistronic rRNA transcript (SSU-rRNA, 5.8S rRNA, LSU-rRNA)	1/95	33/14464	0.03030303	4.61371611	1.68982026	0.19563462	0.44746464	0.4278165	Wdr46	1
GO:0000731 DNA synthesis involved in DNA repair	1/95	33/14464	0.03030303	4.61371611	1.68982026	0.19563462	0.44746464	0.4278165	Pole	1

GO:0030947	regulation of vascular endothelial growth factor receptor signaling pathway	1/95	33/14464	0.03030303	4.61371611	1.68982026	0.19563462	0.44746464	0.4278165	Fzd4	1
GO:0035020	regulation of Rac protein signal transduction	1/95	33/14464	0.03030303	4.61371611	1.68982026	0.19563462	0.44746464	0.4278165	Agrn	1
GO:0046329	negative regulation of JNK cascade	1/95	33/14464	0.03030303	4.61371611	1.68982026	0.19563462	0.44746464	0.4278165	Per1	1
GO:0048710	regulation of astrocyte differentiation	1/95	33/14464	0.03030303	4.61371611	1.68982026	0.19563462	0.44746464	0.4278165	Trem2	1
GO:0050685	positive regulation of mRNA processing	1/95	33/14464	0.03030303	4.61371611	1.68982026	0.19563462	0.44746464	0.4278165	Rbmx	1
GO:0050819	negative regulation of coagulation	1/95	33/14464	0.03030303	4.61371611	1.68982026	0.19563462	0.44746464	0.4278165	Serpine1	1
GO:0050869	negative regulation of B cell activation	1/95	33/14464	0.03030303	4.61371611	1.68982026	0.19563462	0.44746464	0.4278165	Lyn	1
GO:0051491	positive regulation of filopodium assembly	1/95	33/14464	0.03030303	4.61371611	1.68982026	0.19563462	0.44746464	0.4278165	Agrn	1
GO:0060351	cartilage development involved in endochondral bone morphogenesis	1/95	33/14464	0.03030303	4.61371611	1.68982026	0.19563462	0.44746464	0.4278165	Por	1
GO:2000516	positive regulation of CD4-positive, alpha-beta T cell activation	1/95	33/14464	0.03030303	4.61371611	1.68982026	0.19563462	0.44746464	0.4278165	Gimap5	1
GO:0044283	small molecule biosynthetic process	5/95	470/14464	0.0106383	1.61970885	1.11056355	0.1963712	0.44861469	0.42891605	Acer2/Por/Lss/Clcn2/Tha1	5
GO:0044843	cell cycle G1/S phase transition	3/95	233/14464	0.01287554	1.96033431	1.20160139	0.19742265	0.44992331	0.43016721	Tcim/Pole/Arid2	3
GO:0000086	G2/M transition of mitotic cell cycle	2/95	125/14464	0.016	2.43604211	1.31111405	0.19826698	0.44992331	0.43016721	Wee1/Ccdc57	2
GO:0010950	positive regulation of endopeptidase activity	2/95	125/14464	0.016	2.43604211	1.31111405	0.19826698	0.44992331	0.43016721	Acer2/Lyn	2
GO:0009743	response to carbohydrate	3/95	234/14464	0.01282051	1.95195682	1.19371445	0.19910118	0.44992331	0.43016721	Trem2/Rab34/Lyn	3
GO:0001838	embryonic epithelial tube formation	2/95	126/14464	0.01587302	2.41670844	1.29867114	0.20065644	0.44992331	0.43016721	Abl1/Brpf1	2
GO:0006959	humoral immune response	2/95	126/14464	0.01587302	2.41670844	1.29867114	0.20065644	0.44992331	0.43016721	Trem2/Gimap5	2
GO:0014812	muscle cell migration	2/95	126/14464	0.01587302	2.41670844	1.29867114	0.20065644	0.44992331	0.43016721	Serpine1/Net1	2
GO:0050728	negative regulation of inflammatory response	2/95	126/14464	0.01587302	2.41670844	1.29867114	0.20065644	0.44992331	0.43016721	Nr1d2/Trem2	2
GO:0007431	salivary gland development	1/95	34/14464	0.02941176	4.47801858	1.65088155	0.2009298	0.44992331	0.43016721	Clcn2	1
GO:0010043	response to zinc ion	1/95	34/14464	0.02941176	4.47801858	1.65088155	0.2009298	0.44992331	0.43016721	Mt1	1
GO:0010714	positive regulation of collagen metabolic process	1/95	34/14464	0.02941176	4.47801858	1.65088155	0.2009298	0.44992331	0.43016721	Serpine1	1
GO:0034110	regulation of homotypic cell-cell adhesion	1/95	34/14464	0.02941176	4.47801858	1.65088155	0.2009298	0.44992331	0.43016721	Lyn	1
GO:0045745	positive regulation of G protein-coupled receptor signaling pathway	1/95	34/14464	0.02941176	4.47801858	1.65088155	0.2009298	0.44992331	0.43016721	Trem2	1
GO:0051654	establishment of mitochondrion localization	1/95	34/14464	0.02941176	4.47801858	1.65088155	0.2009298	0.44992331	0.43016721	Tspan4	1
GO:0150117	positive regulation of cell-substrate junction organization	1/95	34/14464	0.02941176	4.47801858	1.65088155	0.2009298	0.44992331	0.43016721	Abl1	1
GO:1902175	regulation of oxidative stress-induced intrinsic apoptotic signaling pathway	1/95	34/14464	0.02941176	4.47801858	1.65088155	0.2009298	0.44992331	0.43016721	Trem2	1
GO:1905332	positive regulation of morphogenesis of an epithelium	1/95	34/14464	0.02941176	4.47801858	1.65088155	0.2009298	0.44992331	0.43016721	Abl1	1
GO:0002449	lymphocyte mediated immunity	3/95	236/14464	0.01271186	1.93541481	1.17805628	0.20246875	0.45284095	0.43295674	Trem2/Gimap5/Paxip1	3
GO:0050921	positive regulation of chemotaxis	2/95	127/14464	0.01574803	2.39767924	1.28634646	0.20304939	0.45308346	0.4331886	Trem2/Serpine1	2
GO:1902652	secondary alcohol metabolic process	2/95	127/14464	0.01574803	2.39767924	1.28634646	0.20304939	0.45308346	0.4331886	Por/Lss	2
GO:0014902	myotube differentiation	2/95	128/14464	0.015625	2.37894737	1.27413781	0.20544568	0.45343617	0.43352582	Bhlhe41/Jam2	2
GO:0006956	complement activation	1/95	35/14464	0.02857143	4.35007519	1.61342278	0.20619048	0.45343617	0.43352582	Trem2	1
GO:0010874	regulation of cholesterol efflux	1/95	35/14464	0.02857143	4.35007519	1.61342278	0.20619048	0.45343617	0.43352582	Trem2	1
GO:0014912	negative regulation of smooth muscle cell migration	1/95	35/14464	0.02857143	4.35007519	1.61342278	0.20619048	0.45343617	0.43352582	Serpine1	1
GO:0018279	protein N-linked glycosylation via asparagine	1/95	35/14464	0.02857143	4.35007519	1.61342278	0.20619048	0.45343617	0.43352582	Alg10b	1
GO:0021680	cerebellar Purkinje cell layer development	1/95	35/14464	0.02857143	4.35007519	1.61342278	0.20619048	0.45343617	0.43352582	Rere	1
GO:0022616	DNA strand elongation	1/95	35/14464	0.02857143	4.35007519	1.61342278	0.20619048	0.45343617	0.43352582	Pole	1
GO:0033144	negative regulation of intracellular steroid hormone receptor signaling pathway	1/95	35/14464	0.02857143	4.35007519	1.61342278	0.20619048	0.45343617	0.43352582	Per1	1
GO:0045066	regulatory T cell differentiation	1/95	35/14464	0.02857143	4.35007519	1.61342278	0.20619048	0.45343617	0.43352582	Gimap5	1
GO:0045746	negative regulation of Notch signaling pathway	1/95	35/14464	0.02857143	4.35007519	1.61342278	0.20619048	0.45343617	0.43352582	Tcim	1
GO:0051281	positive regulation of release of sequestered calcium ion into cytosol	1/95	35/14464	0.02857143	4.35007519	1.61342278	0.20619048	0.45343617	0.43352582	Abl1	1
GO:0070316	regulation of G0 to G1 transition	1/95	35/14464	0.02857143	4.35007519	1.61342278	0.20619048	0.45343617	0.43352582	Arid2	1
GO:0006935	chemotaxis	4/95	356/14464	0.01123596	1.71070373	1.10397335	0.2062792	0.45343617	0.43352582	Trem2/Serpine1/Agrn/Lyn	4
GO:0001818	negative regulation of cytokine production	3/95	239/14464	0.0125523	1.9111209	1.15485257	0.20754558	0.45487144	0.43489806	Trem2/Gimap5/Nav3	3
GO:1903320	regulation of protein modification by small protein conjugation or removal	3/95	239/14464	0.0125523	1.9111209	1.15485257	0.20754558	0.45487144	0.43489806	Abl1/Peli1/Paxip1	3
GO:0042330	taxis	4/95	357/14464	0.01120448	1.70591184	1.09810777	0.20764324	0.45487144	0.43489806	Trem2/Serpine1/Agrn/Lyn	4
GO:1901988	negative regulation of cell cycle phase transition	3/95	240/14464	0.0125	1.90315789	1.14719212	0.20924443	0.45785641	0.43775196	Wee1/Xrcc3/Lyn	3
GO:0007266	Rho protein signal transduction	2/95	130/14464	0.01538462	2.34234818	1.25006005	0.21024774	0.45791086	0.43780402	Net1/Abl1	2
GO:0170039	proteinogenic amino acid metabolic process	2/95	130/14464	0.01538462	2.34234818	1.25006005	0.21024774	0.45791086	0.43780402	Pipox/Tha1	2
GO:0030098	lymphocyte differentiation	4/95	359/14464	0.01114206	1.69640815	1.08643125	0.21037936	0.45791086	0.43780402	Gimap5/Il15ra/Abl1/Arid2	4
GO:0007026	negative regulation of microtubule depolymerization	1/95	36/14464	0.02777778	4.22923977	1.5773432	0.21141689	0.45791086	0.43780402	Nav3	1
GO:0014002	astrocyte development	1/95	36/14464	0.02777778	4.22923977	1.5773432	0.21141689	0.45791086	0.43780402	Trem2	1
GO:0050798	activated T cell proliferation	1/95	36/14464	0.02777778	4.22923977	1.5773432	0.21141689	0.45791086	0.43780402	Abl1	1
GO:0070266	necroptotic process	1/95	36/14464	0.02777778	4.22923977	1.5773432	0.21141689	0.45791086	0.43780402	Peli1	1
GO:0071402	cellular response to lipoprotein particle stimulus	1/95	36/14464	0.02777778	4.22923977	1.5773432	0.21141689	0.45791086	0.43780402	Trem2	1
GO:1900225	regulation of NLRP3 inflammasome complex assembly	1/95	36/14464	0.02777778	4.22923977	1.5773432	0.21141689	0.45791086	0.43780402	Trem2	1
GO:0007098	centrosome cycle	2/95	131/14464	0.01526718	2.32446766	1.23818682	0.21265321	0.46006934	0.43986772	Ccdc57/Xrcc3	2
GO:0001101	response to acid chemical	2/95	132/14464	0.01515152	2.30685805	1.22642137	0.21506145	0.46288771	0.44256234	Gdf10/Lyn	2
GO:0010952	positive regulation of peptidase activity	2/95	132/14464	0.01515152	2.30685805	1.22642137	0.21506145	0.46288771	0.44256234	Acer2/Lyn	2
GO:1901873	regulation of post-translational protein modification	3/95	244/14464	0.01229508	1.87195858	1.11691133	0.21607125	0.46288771	0.44256234	Abl1/Peli1/Paxip1	3
GO:0006084	acetyl-CoA metabolic process	1/95	37/14464	0.02702703	4.11493599	1.54255158	0.21660925	0.46288771	0.44256234	Pipox	1
GO:0010837	regulation of keratinocyte proliferation	1/95	37/14464	0.02702703	4.11493599	1.54255158	0.21660925	0.46288771	0.44256234	Klf9	1
GO:0043277	apoptotic cell clearance	1/95	37/14464	0.02702703	4.11493599	1.54255158	0.21660925	0.46288771	0.44256234	Trem2	1
GO:0045023	G0 to G1 transition	1/95	37/14464	0.02702703	4.11493599	1.54255158	0.21660925	0.46288771	0.44256234	Arid2	1
GO:0051154	negative regulation of striated muscle cell differentiation	1/95	37/14464	0.02702703	4.11493599	1.54255158	0.21660925	0.46288771	0.44256234	Bhlhe41	1
GO:0090114	COPII-coated vesicle budding	1/95	37/14464	0.02702703	4.11493599	1.54255158	0.21660925	0.46288771	0.44256234	Sar1b	1
GO:0099637	neurotransmitter receptor transport	1/95	37/14464	0.02702703	4.11493599	1.54255158	0.21660925	0.46288771	0.44256234	Myo5b	1
GO:1904646	cellular response to amyloid-beta	1/95	37/14464	0.02702703	4.11493599	1.54255158	0.21660925	0.46288771	0.44256234	Trem2	1
GO:0045739	positive regulation of DNA repair	2/95	134/14464	0.01492537	2.27242734	1.20320608	0.21988572	0.46714861	0.44663614	Peli1/Arid2	2
GO:0042063	gliogenesis	4/95	366/14464	0.01092896	1.66396319	1.04612484	0.22003718	0.46714861	0.44663614	Trem2/Clcn2/Abl1/Lyn	4
GO:0001569	branching involved in blood vessel morphogenesis	1/95	38/14464	0.02631579	4.0066482	1.50896502	0.22176779	0.46714861	0.44663614	Abl1	1
GO:0006778	porphyrin-containing compound metabolic process	1/95	38/14464	0.02631579	4.0066482	1.50896502	0.22176779	0.46714861	0.44663614	Rsad1	1
GO:0007020	microtubule nucleation	1/95	38/14464	0.02631579	4.0066482	1.50896502	0.22176779	0.46714861	0.44663614	Ccdc57	1
GO:0009593	detection of chemical stimulus	1/95	38/14464	0.02631579	4.0066482	1.50896502	0.22176779	0.46714861	0.44663614	Trem2	1
GO:0031112	positive regulation of microtubule polymerization or depolymerization	1/95	38/14464	0.02631579	4.0066482	1.50896502	0.22176779	0.46714861	0.44663614	Nav3	1
GO:0044546	NLRP3 inflammasome complex assembly	1/95	38/14464	0.02631579	4.0066482	1.50896502	0.22176779	0.46714861	0.44663614	Trem2	1
GO:0090049	regulation of cell migration involved in sprouting angiogenesis	1/95	38/14464	0.02631579	4.0066482	1.50896502	0.22176779	0.46714861	0.44663614	Abl1	1
GO:0140632	canonical inflammasome complex assembly	1/95	38/14464	0.02631579	4.0066482	1.50896502	0.22176779	0.46714861	0.44663614	Trem2	1
GO:1900026	positive regulation of substrate adhesion-dependent cell spreading	1/95	38/14464	0.02631579	4.0066482	1.50896502	0.22176779	0.46714861	0.44663614	Abl1	1
GO:2000404	regulation of T cell migration	1/95	38/14464	0.02631579	4.0066482	1.50896502	0.22176779	0.46714861	0.44663614	Abl1	1
GO:2000649	regulation of sodium ion transmembrane transporter activity	1/95	38/14464	0.02631579	4.0066482	1.50896502	0.22176779	0.46714861	0.44663614	Agrn	1
GO:0071902	positive regulation of protein serine/threonine kinase activity	2/95	135/14464	0.01481481	2.25559454	1.19175254	0.22230147	0.46775934	0.44722006	Fzd4/Tcim	2
GO:0044782	cilium organization	4/95	369/14464	0.01084011	1.65043503	1.02911116	0.22421335	0.47078234	0.45011032	Ccdc57/Rab34/Alpk1/Celsr3	4
GO:0070372	regulation of ERK1 and ERK2 cascade	3/95	249/14464	0.01204819	1.83436906	1.07984682	0.22467141	0.47078234	0.45011032	Trem2/Abl1/Lyn	3
GO:0071559	response to transforming growth factor beta	3/95	249/14464	0.01204819	1.83436906	1.07984682	0.22467141	0.47078234	0.45011032	Serpine1/Gdf10/Abl1	3
GO:0072175	epithelial tube formation	2/95	136/14464	0.01470588	2.23900929	1.18039933	0.22471945	0.47078234	0.45011032	Abl1/Brpf1	2
GO:0010559	regulation of glycoprotein biosynthetic process	1/95	39/14464	0.02564103	3.90391363	1.47650802	0.2268927	0.47173033	0.45101669	Acer2	1
GO:0010830	regulation of myotube differentiation	1/95	39/14464	0.02564103	3.90391363	1.47650802	0.2268927	0.47173033	0.45101669	Bhlhe41	1
GO:0030890	positive regulation of B cell proliferation	1/95	39/14464	0.02564103	3.90391363	1.47650802	0.2268927	0.47173033	0.45101669	Peli1	1
GO:0043001	Golgi to plasma membrane protein transport	1/95	39/14464	0.02564103	3.90391363	1.47650802	0.2268927	0.47173033	0.45101669	Rab34	1
GO:0090279	regulation of calcium ion import	1/95	39/14464	0.02564103	3.90391363	1.47650802	0.2268927	0.47173033	0.45101669	Serpine1	1
GO:1903580	positive regulation of ATP metabolic process	1/95	39/14464	0.02564103	3.90391363	1.47650802	0.2268927	0.47173033	0.45101669	Trem2	1
GO:1905521	regulation of macrophage migration	1/95	39/14464	0.02564103	3.90391363	1.47650802	0.2268927	0.47173033	0.45101669	Trem2	1
GO:0035725	sodium ion transmembrane transport	2/95	137/14464	0.01459854	2.22266615	1.1691447	0.22713953	0.47173242	0.45101868	Slc6a6/Agrn	2
GO:0031960	response to corticosteroid	2/95	138/14464	0.01449275	2.20655988	1.15798697	0.22956158	0.47431181	0.45348482	Por/Klf9	2
GO:0050851	antigen receptor-mediated signaling pathway	2/95	138/14464	0.01449275	2.20655988	1.15798697	0.22956158	0.47431181	0.45348482	Abl1/Lyn	2
GO:0051099	positive regulation of binding	2/95	138/14464	0.01449275	2.20655988	1.15798697	0.22956158	0.47431181	0.45348482	Abl1/Agrn	2
GO:1903322	positive regulation of protein modification by small protein conjugation or removal	2/95	138/14464	0.01449275	2.20655988	1.15798697	0.22956158	0.47431181	0.45348482	Peli1/Paxip1	2
GO:0010721	negative regulation of cell development	3/95	252/14464	0.01190476	1.81253133	1.05801179	0.22986451	0.47431181	0.45348482	Trem2/Lrrc17/Lyn	3
GO:0022409	positive regulation of cell-cell adhesion	3/95	252/14464	0.01190476	1.81253133	1.05801179	0.22986451	0.47431181	0.45348482	Gimap5/Abl1/Arid2	3
GO:0046651	lymphocyte proliferation	3/95	253/14464	0.01185771	1.80536717	1.05079885	0.23160072	0.47612847	0.4552217	Abl1/Peli1/Lyn	3
GO:0007099	centriole replication	1/95	40/14464	0.025	3.80631579	1.44511165	0.23198422	0.47612847	0.4552217	Ccdc57	1
GO:0090181	regulation of cholesterol metabolic process	1/95	40/14464	0.025	3.80631579	1.44511165	0.23198422	0.47612847	0.4552217	Por	1
GO:0140894	endolysosomal toll-like receptor signaling pathway	1/95	40/14464	0.025	3.80631579	1.44511165	0.23198422	0.47612847	0.4552217	Peli1	1
GO:1901875	positive regulation of post-translational protein modification	2/95	139/14464	0.01438849	2.19068535	1.14692447	0.23198547	0.47612847	0.4552217	Peli1/Paxip1	2
GO:0007204	positive regulation of cytosolic calcium ion concentration	2/95	140/14464	0.01428571	2.17503759	1.13595557	0.23441108	0.47681832	0.45588126	Gimap5/Abl1	2
GO:0021782	glial cell development	2/95	140/14464	0.01428571	2.17503759	1.13595557	0.23441108	0.47681832	0.45588126	Trem2/Lyn	2
GO:0043405	regulation of MAP kinase activity	2/95	140/14464	0.01428571	2.17503759	1.13595557	0.23441108	0.47681832	0.45588126	Fzd4/Lyn	2
GO:0006816	calcium ion transport	4/95	377/14464	0.01061008	1.61541254	0.98447679	0.23545131	0.47681832	0.45588126	Serpine1/Gimap5/Abl1/Lyn	4
GO:0006367	transcription initiation at RNA polymerase II promoter	2/95	141/14464	0.0141844	2.1596118	1.12507871	0.23683827	0.47681832	0.45588126	Tet3/Paxip1	2
GO:0007605	sensory perception of sound	2/95	141/14464	0.0141844	2.1596118	1.12507871	0.23683827	0.47681832	0.45588126	Fzd4/Alg10b	2
GO:0031023	microtubule organizing center organization	2/95	141/14464	0.0141844	2.1596118	1.12507871	0.23683827	0.47681832	0.45588126	Ccdc57/Xrcc3	2
GO:0006284	base-excision repair	1/95	41/14464	0.02439024	3.71347882	1.41471281	0.23704256	0.47681832	0.45588126	Pole	1
GO:0007094	mitotic spindle assembly checkpoint signaling	1/95	41/14464	0.02439024	3.71347882	1.41471281	0.23704256	0.47681832	0.45588126	Xrcc3	1
GO:0007520	myoblast fusion	1/95	41/14464	0.02439024	3.71347882	1.41471281	0.23704256	0.47681832	0.45588126	Jam2	1
GO:0010543	regulation of platelet activation	1/95	41/14464	0.02439024	3.71347882	1.41471281	0.23704256	0.47681832	0.45588126	Lyn	1
GO:0043268	positive regulation of potassium ion transport	1/95	41/14464	0.02439024	3.71347882	1.41471281	0.23704256	0.47681832	0.45588126	Trem2	1
GO:0043331	response to dsRNA	1/95	41/14464	0.02439024	3.71347882	1.41471281	0.23704256	0.47681832	0.45588126	Peli1	1
GO:0043507	positive regulation of JUN kinase activity	1/95	41/14464	0.02439024	3.71347882	1.41471281	0.23704256	0.47681832	0.45588126	Fzd4	1
GO:0045907	positive regulation of vasoconstriction	1/95	41/14464	0.02439024	3.71347882	1.41471281	0.23704256	0.47681832	0.45588126	Abl1	1
GO:0045910	negative regulation of DNA recombination	1/95	41/14464	0.02439024	3.71347882	1.41471281	0.23704256	0.47681832	0.45588126	Abl1	1
GO:0048536	spleen development	1/95	41/14464	0.02439024	3.71347882	1.41471281	0.23704256	0.47681832	0.45588126	Abl1	1
GO:0071173	spindle assembly checkpoint signaling	1/95	41/14464	0.02439024	3.71347882	1.41471281	0.23704256	0.47681832	0.45588126	Xrcc3	1
GO:1902991	regulation of amyloid precursor protein catabolic process	1/95	41/14464	0.02439024	3.71347882	1.41471281	0.23704256	0.47681832	0.45588126	Lyn	1
GO:0010212	response to ionizing radiation	2/95	142/14464	0.01408451	2.14440326	1.11429235	0.23926693	0.47840374	0.45739706	Net1/Paxip1	2
GO:0045927	positive regulation of growth	3/95	258/14464	0.01162791	1.77037944	1.01521109	0.24031833	0.47840374	0.45739706	Serpine1/Myo5b/Agrn	3
GO:0000723	telomere maintenance	2/95	143/14464	0.01398601	2.12940743	1.10359497	0.24169693	0.47840374	0.45739706	Xrcc3/Ylpm1	2
GO:0007009	plasma membrane organization	2/95	143/14464	0.01398601	2.12940743	1.10359497	0.24169693	0.47840374	0.45739706	Spaca6/Agrn	2
GO:0000079	regulation of cyclin-dependent protein serine/threonine kinase activity	1/95	42/14464	0.02380952	3.62506266	1.38525363	0.24206794	0.47840374	0.45739706	Inca1	1
GO:0030851	granulocyte differentiation	1/95	42/14464	0.02380952	3.62506266	1.38525363	0.24206794	0.47840374	0.45739706	Lyn	1
GO:0031114	regulation of microtubule depolymerization	1/95	42/14464	0.02380952	3.62506266	1.38525363	0.24206794	0.47840374	0.45739706	Nav3	1
GO:0045981	positive regulation of nucleotide metabolic process	1/95	42/14464	0.02380952	3.62506266	1.38525363	0.24206794	0.47840374	0.45739706	Trem2	1
GO:0046627	negative regulation of insulin receptor signaling pathway	1/95	42/14464	0.02380952	3.62506266	1.38525363	0.24206794	0.47840374	0.45739706	Tns2	1
GO:0046638	positive regulation of alpha-beta T cell differentiation	1/95	42/14464	0.02380952	3.62506266	1.38525363	0.24206794	0.47840374	0.45739706	Gimap5	1
GO:0048147	negative regulation of fibroblast proliferation	1/95	42/14464	0.02380952	3.62506266	1.38525363	0.24206794	0.47840374	0.45739706	Inca1	1
GO:0070192	chromosome organization involved in meiotic cell cycle	1/95	42/14464	0.02380952	3.62506266	1.38525363	0.24206794	0.47840374	0.45739706	Ncapd3	1
GO:0071174	mitotic spindle checkpoint signaling	1/95	42/14464	0.02380952	3.62506266	1.38525363	0.24206794	0.47840374	0.45739706	Xrcc3	1
GO:0098534	centriole assembly	1/95	42/14464	0.02380952	3.62506266	1.38525363	0.24206794	0.47840374	0.45739706	Ccdc57	1
GO:1900544	positive regulation of purine nucleotide metabolic process	1/95	42/14464	0.02380952	3.62506266	1.38525363	0.24206794	0.47840374	0.45739706	Trem2	1
GO:1904645	response to amyloid-beta	1/95	42/14464	0.02380952	3.62506266	1.38525363	0.24206794	0.47840374	0.45739706	Trem2	1
GO:0032943	mononuclear cell proliferation	3/95	259/14464	0.01158301	1.763544	1.00818691	0.2420688	0.47840374	0.45739706	Abl1/Peli1/Lyn	3
GO:0007596	blood coagulation	2/95	145/14464	0.0137931	2.1000363	1.08246134	0.24656048	0.48297854	0.46177099	Serpine1/Lyn	2
GO:0032653	regulation of interleukin-10 production	1/95	43/14464	0.02325581	3.54075887	1.35668091	0.24706056	0.48297854	0.46177099	Trem2	1
GO:0033046	negative regulation of sister chromatid segregation	1/95	43/14464	0.02325581	3.54075887	1.35668091	0.24706056	0.48297854	0.46177099	Xrcc3	1
GO:0033048	negative regulation of mitotic sister chromatid segregation	1/95	43/14464	0.02325581	3.54075887	1.35668091	0.24706056	0.48297854	0.46177099	Xrcc3	1
GO:0042181	ketone biosynthetic process	1/95	43/14464	0.02325581	3.54075887	1.35668091	0.24706056	0.48297854	0.46177099	Clcn2	1
GO:0045841	negative regulation of mitotic metaphase/anaphase transition	1/95	43/14464	0.02325581	3.54075887	1.35668091	0.24706056	0.48297854	0.46177099	Xrcc3	1
GO:0061756	leukocyte adhesion to vascular endothelial cell	1/95	43/14464	0.02325581	3.54075887	1.35668091	0.24706056	0.48297854	0.46177099	Jam2	1
GO:1902041	regulation of extrinsic apoptotic signaling pathway via death domain receptors	1/95	43/14464	0.02325581	3.54075887	1.35668091	0.24706056	0.48297854	0.46177099	Serpine1	1
GO:1903018	regulation of glycoprotein metabolic process	1/95	43/14464	0.02325581	3.54075887	1.35668091	0.24706056	0.48297854	0.46177099	Acer2	1
GO:2000816	negative regulation of mitotic sister chromatid separation	1/95	43/14464	0.02325581	3.54075887	1.35668091	0.24706056	0.48297854	0.46177099	Xrcc3	1
GO:0052547	regulation of peptidase activity	3/95	262/14464	0.01145038	1.74335074	0.98729604	0.24733319	0.48297854	0.46177099	Serpine1/Acer2/Lyn	3
GO:0002460	adaptive immune response based on somatic recombination of immune receptors built from immunoglobu	3/95	263/14464	0.01140684	1.73672203	0.98039208	0.24909215	0.48297854	0.46177099	Trem2/Gimap5/Paxip1	3
GO:0030217	T cell differentiation	3/95	263/14464	0.01140684	1.73672203	0.98039208	0.24909215	0.48297854	0.46177099	Gimap5/Abl1/Arid2	3
GO:0098813	nuclear chromosome segregation	3/95	264/14464	0.01136364	1.73014354	0.9735175	0.2508531	0.48297854	0.46177099	Xrcc3/Ncapd3/Arid2	3
GO:0009152	purine ribonucleotide biosynthetic process	2/95	147/14464	0.01360544	2.07146438	1.0616665	0.25142796	0.48297854	0.46177099	Trem2/ND5	2
GO:0170033	L-amino acid metabolic process	2/95	147/14464	0.01360544	2.07146438	1.0616665	0.25142796	0.48297854	0.46177099	Pipox/Tha1	2
GO:0001774	microglial cell activation	1/95	44/14464	0.02272727	3.46028708	1.32894564	0.25202063	0.48297854	0.46177099	Trem2	1
GO:0030858	positive regulation of epithelial cell differentiation	1/95	44/14464	0.02272727	3.46028708	1.32894564	0.25202063	0.48297854	0.46177099	Serpine1	1
GO:0043300	regulation of leukocyte degranulation	1/95	44/14464	0.02272727	3.46028708	1.32894564	0.25202063	0.48297854	0.46177099	Lyn	1
GO:0043370	regulation of CD4-positive, alpha-beta T cell differentiation	1/95	44/14464	0.02272727	3.46028708	1.32894564	0.25202063	0.48297854	0.46177099	Gimap5	1
GO:0051438	regulation of ubiquitin-protein transferase activity	1/95	44/14464	0.02272727	3.46028708	1.32894564	0.25202063	0.48297854	0.46177099	Abl1	1
GO:0071300	cellular response to retinoic acid	1/95	44/14464	0.02272727	3.46028708	1.32894564	0.25202063	0.48297854	0.46177099	Lyn	1
GO:0141085	regulation of inflammasome-mediated signaling pathway	1/95	44/14464	0.02272727	3.46028708	1.32894564	0.25202063	0.48297854	0.46177099	Trem2	1
GO:1901976	regulation of cell cycle checkpoint	1/95	44/14464	0.02272727	3.46028708	1.32894564	0.25202063	0.48297854	0.46177099	Xrcc3	1

GO:1902100	negative regulation of metaphase/anaphase transition of cell cycle	1/95	44/14464	0.02272727	3.46028708	1.32894564	0.25202063	0.48297854	0.46177099	Xrcc3	1
GO:1905819	negative regulation of chromosome separation	1/95	44/14464	0.02272727	3.46028708	1.32894564	0.25202063	0.48297854	0.46177099	Xrcc3	1
GO:0002244	hematopoietic progenitor cell differentiation	2/95	148/14464	0.01351351	2.05746799	1.05139273	0.25386289	0.48297854	0.46177099	Arl11/Lyn	2
GO:0007599	hemostasis	2/95	148/14464	0.01351351	2.05746799	1.05139273	0.25386289	0.48297854	0.46177099	Serpine1/Lyn	2
GO:0034614	cellular response to reactive oxygen species	2/95	148/14464	0.01351351	2.05746799	1.05139273	0.25386289	0.48297854	0.46177099	Net1/Abl1	2
GO:0035148	tube formation	2/95	148/14464	0.01351351	2.05746799	1.05139273	0.25386289	0.48297854	0.46177099	Abl1/Brpf1	2
GO:0045766	positive regulation of angiogenesis	2/95	148/14464	0.01351351	2.05746799	1.05139273	0.25386289	0.48297854	0.46177099	Serpine1/Abl1	2
GO:0016331	morphogenesis of embryonic epithelium	2/95	149/14464	0.01342282	2.04365948	1.04119963	0.25629846	0.48297854	0.46177099	Abl1/Brpf1	2
GO:0032200	telomere organization	2/95	149/14464	0.01342282	2.04365948	1.04119963	0.25629846	0.48297854	0.46177099	Xrcc3/Ylpm1	2
GO:0046631	alpha-beta T cell activation	2/95	149/14464	0.01342282	2.04365948	1.04119963	0.25629846	0.48297854	0.46177099	Gimap5/Abl1	2
GO:0002204	somatic recombination of immunoglobulin genes involved in immune response	1/95	45/14464	0.02222222	3.38339181	1.3020026	0.25694837	0.48297854	0.46177099	Paxip1	1
GO:0002208	somatic diversification of immunoglobulins involved in immune response	1/95	45/14464	0.02222222	3.38339181	1.3020026	0.25694837	0.48297854	0.46177099	Paxip1	1
GO:0002639	positive regulation of immunoglobulin production	1/95	45/14464	0.02222222	3.38339181	1.3020026	0.25694837	0.48297854	0.46177099	Paxip1	1
GO:0006509	membrane protein ectodomain proteolysis	1/95	45/14464	0.02222222	3.38339181	1.3020026	0.25694837	0.48297854	0.46177099	Rbmx	1
GO:0007606	sensory perception of chemical stimulus	1/95	45/14464	0.02222222	3.38339181	1.3020026	0.25694837	0.48297854	0.46177099	Or8b53	1
GO:0008038	neuron recognition	1/95	45/14464	0.02222222	3.38339181	1.3020026	0.25694837	0.48297854	0.46177099	Celsr3	1
GO:0031638	zymogen activation	1/95	45/14464	0.02222222	3.38339181	1.3020026	0.25694837	0.48297854	0.46177099	Serpine1	1
GO:0043029	T cell homeostasis	1/95	45/14464	0.02222222	3.38339181	1.3020026	0.25694837	0.48297854	0.46177099	Gimap5	1
GO:0043407	negative regulation of MAP kinase activity	1/95	45/14464	0.02222222	3.38339181	1.3020026	0.25694837	0.48297854	0.46177099	Lyn	1
GO:0045190	isotype switching	1/95	45/14464	0.02222222	3.38339181	1.3020026	0.25694837	0.48297854	0.46177099	Paxip1	1
GO:0098698	postsynaptic specialization assembly	1/95	45/14464	0.02222222	3.38339181	1.3020026	0.25694837	0.48297854	0.46177099	Abl1	1
GO:0099010	modification of postsynaptic structure	1/95	45/14464	0.02222222	3.38339181	1.3020026	0.25694837	0.48297854	0.46177099	Myo5b	1
GO:1905953	negative regulation of lipid localization	1/95	45/14464	0.02222222	3.38339181	1.3020026	0.25694837	0.48297854	0.46177099	Trem2	1
GO:2001259	positive regulation of cation channel activity	1/95	45/14464	0.02222222	3.38339181	1.3020026	0.25694837	0.48297854	0.46177099	Trem2	1
GO:0010876	lipid localization	4/95	393/14464	0.01017812	1.54964511	0.89824308	0.25832268	0.48297854	0.46177099	Fzd4/Trem2/Sar1b/Lyn	4
GO:0050817	coagulation	2/95	150/14464	0.01333333	2.03003509	1.03108594	0.25873455	0.48297854	0.46177099	Serpine1/Lyn	2
GO:0007224	smoothened signaling pathway	2/95	151/14464	0.01324503	2.01659115	1.02105041	0.26117106	0.48297854	0.46177099	Por/Rab34	2
GO:0141193	nuclear receptor-mediated signaling pathway	2/95	151/14464	0.01324503	2.01659115	1.02105041	0.26117106	0.48297854	0.46177099	Per1/Klf9	2
GO:1904018	positive regulation of vasculature development	2/95	151/14464	0.01324503	2.01659115	1.02105041	0.26117106	0.48297854	0.46177099	Serpine1/Abl1	2
GO:0001912	positive regulation of leukocyte mediated cytotoxicity	1/95	46/14464	0.02173913	3.30983982	1.27580997	0.26184399	0.48297854	0.46177099	Gimap5	1
GO:0010824	regulation of centrosome duplication	1/95	46/14464	0.02173913	3.30983982	1.27580997	0.26184399	0.48297854	0.46177099	Xrcc3	1
GO:0014014	negative regulation of gliogenesis	1/95	46/14464	0.02173913	3.30983982	1.27580997	0.26184399	0.48297854	0.46177099	Trem2	1
GO:0031577	spindle checkpoint signaling	1/95	46/14464	0.02173913	3.30983982	1.27580997	0.26184399	0.48297854	0.46177099	Xrcc3	1
GO:0032613	interleukin-10 production	1/95	46/14464	0.02173913	3.30983982	1.27580997	0.26184399	0.48297854	0.46177099	Trem2	1
GO:0032965	regulation of collagen biosynthetic process	1/95	46/14464	0.02173913	3.30983982	1.27580997	0.26184399	0.48297854	0.46177099	Serpine1	1
GO:0033013	tetrapyrrole metabolic process	1/95	46/14464	0.02173913	3.30983982	1.27580997	0.26184399	0.48297854	0.46177099	Rsad1	1
GO:0035272	exocrine system development	1/95	46/14464	0.02173913	3.30983982	1.27580997	0.26184399	0.48297854	0.46177099	Clcn2	1
GO:0043303	mast cell degranulation	1/95	46/14464	0.02173913	3.30983982	1.27580997	0.26184399	0.48297854	0.46177099	Lyn	1
GO:0043616	keratinocyte proliferation	1/95	46/14464	0.02173913	3.30983982	1.27580997	0.26184399	0.48297854	0.46177099	Klf9	1
GO:0048713	regulation of oligodendrocyte differentiation	1/95	46/14464	0.02173913	3.30983982	1.27580997	0.26184399	0.48297854	0.46177099	Clcn2	1
GO:0051985	negative regulation of chromosome segregation	1/95	46/14464	0.02173913	3.30983982	1.27580997	0.26184399	0.48297854	0.46177099	Xrcc3	1
GO:1904029	regulation of cyclin-dependent protein kinase activity	1/95	46/14464	0.02173913	3.30983982	1.27580997	0.26184399	0.48297854	0.46177099	Inca1	1
GO:2000772	regulation of cellular senescence	1/95	46/14464	0.02173913	3.30983982	1.27580997	0.26184399	0.48297854	0.46177099	Abl1	1
GO:0030336	negative regulation of cell migration	3/95	271/14464	0.01107011	1.68545349	0.92619676	0.2632315	0.48297854	0.46177099	Serpine1/Nav3/Arid2	3
GO:0042445	hormone metabolic process	2/95	152/14464	0.01315789	2.0033241	1.01109182	0.26360788	0.48297854	0.46177099	Por/Clcn2	2
GO:0050714	positive regulation of protein secretion	2/95	152/14464	0.01315789	2.0033241	1.01109182	0.26360788	0.48297854	0.46177099	Trem2/Rab34	2
GO:1905952	regulation of lipid localization	2/95	152/14464	0.01315789	2.0033241	1.01109182	0.26360788	0.48297854	0.46177099	Trem2/Sar1b	2
GO:0043086	negative regulation of catalytic activity	4/95	398/14464	0.01005025	1.5301772	0.87207734	0.26556426	0.48297854	0.46177099	Serpine1/Inca1/Abl1/Lyn	4
GO:0002269	leukocyte activation involved in inflammatory response	1/95	47/14464	0.0212766	3.23941769	1.25032901	0.26670769	0.48297854	0.46177099	Trem2	1
GO:0007062	sister chromatid cohesion	1/95	47/14464	0.0212766	3.23941769	1.25032901	0.26670769	0.48297854	0.46177099	Naa50	1
GO:0007566	embryo implantation	1/95	47/14464	0.0212766	3.23941769	1.25032901	0.26670769	0.48297854	0.46177099	Klf9	1
GO:0010883	regulation of lipid storage	1/95	47/14464	0.0212766	3.23941769	1.25032901	0.26670769	0.48297854	0.46177099	Trem2	1
GO:0030514	negative regulation of BMP signaling pathway	1/95	47/14464	0.0212766	3.23941769	1.25032901	0.26670769	0.48297854	0.46177099	Abl1	1
GO:0032330	regulation of chondrocyte differentiation	1/95	47/14464	0.0212766	3.23941769	1.25032901	0.26670769	0.48297854	0.46177099	Por	1
GO:0033120	positive regulation of RNA splicing	1/95	47/14464	0.0212766	3.23941769	1.25032901	0.26670769	0.48297854	0.46177099	Rbmx	1
GO:0033344	cholesterol efflux	1/95	47/14464	0.0212766	3.23941769	1.25032901	0.26670769	0.48297854	0.46177099	Trem2	1
GO:0045646	regulation of erythrocyte differentiation	1/95	47/14464	0.0212766	3.23941769	1.25032901	0.26670769	0.48297854	0.46177099	Lyn	1
GO:0045663	positive regulation of myoblast differentiation	1/95	47/14464	0.0212766	3.23941769	1.25032901	0.26670769	0.48297854	0.46177099	Arid2	1
GO:0046686	response to cadmium ion	1/95	47/14464	0.0212766	3.23941769	1.25032901	0.26670769	0.48297854	0.46177099	Mt1	1
GO:0071526	semaphorin-plexin signaling pathway	1/95	47/14464	0.0212766	3.23941769	1.25032901	0.26670769	0.48297854	0.46177099	Trem2	1
GO:0141084	inflammasome-mediated signaling pathway	1/95	47/14464	0.0212766	3.23941769	1.25032901	0.26670769	0.48297854	0.46177099	Trem2	1
GO:0170038	proteinogenic amino acid biosynthetic process	1/95	47/14464	0.0212766	3.23941769	1.25032901	0.26670769	0.48297854	0.46177099	Tha1	1
GO:1900077	negative regulation of cellular response to insulin stimulus	1/95	47/14464	0.0212766	3.23941769	1.25032901	0.26670769	0.48297854	0.46177099	Tns2	1
GO:0051258	protein polymerization	3/95	273/14464	0.01098901	1.67310584	0.91292669	0.26678335	0.48297854	0.46177099	Ccdc57/Abl1/Nav3	3
GO:0070371	ERK1 and ERK2 cascade	3/95	273/14464	0.01098901	1.67310584	0.91292669	0.26678335	0.48297854	0.46177099	Trem2/Abl1/Lyn	3
GO:0062013	positive regulation of small molecule metabolic process	2/95	154/14464	0.01298701	1.9773069	0.99140073	0.26848199	0.48468198	0.46339963	Trem2/Por	2
GO:0098876	vesicle-mediated transport to the plasma membrane	2/95	154/14464	0.01298701	1.9773069	0.99140073	0.26848199	0.48468198	0.46339963	Rab34/Myo5b	2
GO:1904375	regulation of protein localization to cell periphery	2/95	154/14464	0.01298701	1.9773069	0.99140073	0.26848199	0.48468198	0.46339963	Trem2/Myo5b	2
GO:0071333	cellular response to glucose stimulus	2/95	155/14464	0.01290323	1.96455008	0.98166592	0.27091909	0.48518123	0.46387696	Trem2/Rab34	2
GO:0072331	signal transduction by p53 class mediator	2/95	155/14464	0.01290323	1.96455008	0.98166592	0.27091909	0.48518123	0.46387696	Acer2/Paxip1	2
GO:0002448	mast cell mediated immunity	1/95	48/14464	0.02083333	3.17192982	1.22552378	0.27153968	0.48518123	0.46387696	Lyn	1
GO:0015804	neutral amino acid transport	1/95	48/14464	0.02083333	3.17192982	1.22552378	0.27153968	0.48518123	0.46387696	Slc6a6	1
GO:0030193	regulation of blood coagulation	1/95	48/14464	0.02083333	3.17192982	1.22552378	0.27153968	0.48518123	0.46387696	Serpine1	1
GO:0042491	inner ear auditory receptor cell differentiation	1/95	48/14464	0.02083333	3.17192982	1.22552378	0.27153968	0.48518123	0.46387696	Alg10b	1
GO:0045687	positive regulation of glial cell differentiation	1/95	48/14464	0.02083333	3.17192982	1.22552378	0.27153968	0.48518123	0.46387696	Clcn2	1
GO:0045815	transcription initiation-coupled chromatin remodeling	1/95	48/14464	0.02083333	3.17192982	1.22552378	0.27153968	0.48518123	0.46387696	Tet3	1
GO:0061045	negative regulation of wound healing	1/95	48/14464	0.02083333	3.17192982	1.22552378	0.27153968	0.48518123	0.46387696	Serpine1	1
GO:0071548	response to dexamethasone	1/95	48/14464	0.02083333	3.17192982	1.22552378	0.27153968	0.48518123	0.46387696	Por	1
GO:0170034	L-amino acid biosynthetic process	1/95	48/14464	0.02083333	3.17192982	1.22552378	0.27153968	0.48518123	0.46387696	Tha1	1
GO:0030010	establishment of cell polarity	2/95	156/14464	0.01282051	1.95195682	0.97200344	0.27335606	0.48740511	0.46600319	Wee1/Abl1	2
GO:0010948	negative regulation of cell cycle process	3/95	277/14464	0.01083032	1.64894547	0.8867078	0.27390479	0.48740511	0.46600319	Wee1/Xrcc3/Lyn	3
GO:0002700	regulation of production of molecular mediator of immune response	2/95	157/14464	0.01273885	1.93952397	0.96241221	0.27579282	0.48740511	0.46600319	Gimap5/Paxip1	2
GO:0006721	terpenoid metabolic process	1/95	49/14464	0.02040816	3.10719656	1.20136089	0.27634016	0.48740511	0.46600319	Lss	1
GO:0022602	ovulation cycle process	1/95	49/14464	0.02040816	3.10719656	1.20136089	0.27634016	0.48740511	0.46600319	Fzd4	1
GO:0031111	negative regulation of microtubule polymerization or depolymerization	1/95	49/14464	0.02040816	3.10719656	1.20136089	0.27634016	0.48740511	0.46600319	Nav3	1
GO:0032729	positive regulation of type II interferon production	1/95	49/14464	0.02040816	3.10719656	1.20136089	0.27634016	0.48740511	0.46600319	Abl1	1
GO:0032757	positive regulation of interleukin-8 production	1/95	49/14464	0.02040816	3.10719656	1.20136089	0.27634016	0.48740511	0.46600319	Serpine1	1
GO:0042267	natural killer cell mediated cytotoxicity	1/95	49/14464	0.02040816	3.10719656	1.20136089	0.27634016	0.48740511	0.46600319	Gimap5	1
GO:0043030	regulation of macrophage activation	1/95	49/14464	0.02040816	3.10719656	1.20136089	0.27634016	0.48740511	0.46600319	Trem2	1
GO:0048010	vascular endothelial growth factor receptor signaling pathway	1/95	49/14464	0.02040816	3.10719656	1.20136089	0.27634016	0.48740511	0.46600319	Fzd4	1
GO:0050850	positive regulation of calcium-mediated signaling	1/95	49/14464	0.02040816	3.10719656	1.20136089	0.27634016	0.48740511	0.46600319	Trem2	1
GO:0061097	regulation of protein tyrosine kinase activity	1/95	49/14464	0.02040816	3.10719656	1.20136089	0.27634016	0.48740511	0.46600319	Agrn	1
GO:1900046	regulation of hemostasis	1/95	49/14464	0.02040816	3.10719656	1.20136089	0.27634016	0.48740511	0.46600319	Serpine1	1
GO:0071331	cellular response to hexose stimulus	2/95	158/14464	0.01265823	1.9272485	0.95289116	0.27822925	0.48774757	0.46633061	Trem2/Rab34	2
GO:0061025	membrane fusion	2/95	159/14464	0.01257862	1.91512744	0.94343923	0.28066526	0.48774757	0.46633061	Rab34/Spaca6	2
GO:0071326	cellular response to monosaccharide stimulus	2/95	159/14464	0.01257862	1.91512744	0.94343923	0.28066526	0.48774757	0.46633061	Trem2/Rab34	2
GO:0001933	negative regulation of protein phosphorylation	3/95	281/14464	0.01067616	1.62547294	0.86090525	0.28104736	0.48774757	0.46633061	Inca1/Abl1/Lyn	3
GO:0000245	spliceosomal complex assembly	1/95	50/14464	0.02	3.04505263	1.17780924	0.28110934	0.48774757	0.46633061	Rbmx	1
GO:0002279	mast cell activation involved in immune response	1/95	50/14464	0.02	3.04505263	1.17780924	0.28110934	0.48774757	0.46633061	Lyn	1
GO:0003254	regulation of membrane depolarization	1/95	50/14464	0.02	3.04505263	1.17780924	0.28110934	0.48774757	0.46633061	Clcn2	1
GO:0010634	positive regulation of epithelial cell migration	1/95	50/14464	0.02	3.04505263	1.17780924	0.28110934	0.48774757	0.46633061	Serpine1	1
GO:0030490	maturation of SSU-rRNA	1/95	50/14464	0.02	3.04505263	1.17780924	0.28110934	0.48774757	0.46633061	Wdr46	1
GO:0032371	regulation of sterol transport	1/95	50/14464	0.02	3.04505263	1.17780924	0.28110934	0.48774757	0.46633061	Trem2	1
GO:0032374	regulation of cholesterol transport	1/95	50/14464	0.02	3.04505263	1.17780924	0.28110934	0.48774757	0.46633061	Trem2	1
GO:0032413	negative regulation of ion transmembrane transporter activity	1/95	50/14464	0.02	3.04505263	1.17780924	0.28110934	0.48774757	0.46633061	Agrn	1
GO:0033047	regulation of mitotic sister chromatid segregation	1/95	50/14464	0.02	3.04505263	1.17780924	0.28110934	0.48774757	0.46633061	Xrcc3	1
GO:0048538	thymus development	1/95	50/14464	0.02	3.04505263	1.17780924	0.28110934	0.48774757	0.46633061	Abl1	1
GO:0060251	regulation of glial cell proliferation	1/95	50/14464	0.02	3.04505263	1.17780924	0.28110934	0.48774757	0.46633061	Lyn	1
GO:0072678	T cell migration	1/95	50/14464	0.02	3.04505263	1.17780924	0.28110934	0.48774757	0.46633061	Abl1	1
GO:0090303	positive regulation of wound healing	1/95	50/14464	0.02	3.04505263	1.17780924	0.28110934	0.48774757	0.46633061	Serpine1	1
GO:0097300	programmed necrotic cell death	1/95	50/14464	0.02	3.04505263	1.17780924	0.28110934	0.48774757	0.46633061	Peli1	1
GO:2000146	negative regulation of cell motility	3/95	282/14464	0.0106383	1.61970885	0.85451803	0.28283595	0.49030008	0.46877104	Serpine1/Nav3/Arid2	3
GO:0009416	response to light stimulus	3/95	283/14464	0.01060071	1.61398549	0.84815577	0.28462564	0.49284922	0.47120824	Per3/Per1/Bhlhe40	3
GO:0009266	response to temperature stimulus	2/95	161/14464	0.01242236	1.89133704	0.92473873	0.28553564	0.49284922	0.47120824	Tcim/Lyn	2
GO:0001961	positive regulation of cytokine-mediated signaling pathway	1/95	51/14464	0.01960784	2.98534572	1.15483985	0.28584741	0.49284922	0.47120824	Trem2	1
GO:0046470	phosphatidylcholine metabolic process	1/95	51/14464	0.01960784	2.98534572	1.15483985	0.28584741	0.49284922	0.47120824	Pnpla7	1
GO:0060986	endocrine hormone secretion	1/95	51/14464	0.01960784	2.98534572	1.15483985	0.28584741	0.49284922	0.47120824	Fzd4	1
GO:0141137	positive regulation of gene expression, epigenetic	1/95	51/14464	0.01960784	2.98534572	1.15483985	0.28584741	0.49284922	0.47120824	Tet3	1
GO:0043254	regulation of protein-containing complex assembly	4/95	413/14464	0.00968523	1.47460176	0.79566174	0.28751013	0.49299226	0.47134501	Trem2/Sar1b/Abl1/Nav3	4
GO:0009260	ribonucleotide biosynthetic process	2/95	162/14464	0.01234568	1.87966212	0.91548816	0.2879698	0.49299226	0.47134501	Trem2/ND5	2
GO:0021915	neural tube development	2/95	162/14464	0.01234568	1.87966212	0.91548816	0.2879698	0.49299226	0.47134501	Abl1/Brpf1	2
GO:0070374	positive regulation of ERK1 and ERK2 cascade	2/95	162/14464	0.01234568	1.87966212	0.91548816	0.2879698	0.49299226	0.47134501	Trem2/Abl1	2
GO:1901991	negative regulation of mitotic cell cycle phase transition	2/95	163/14464	0.01226994	1.86813045	0.90630278	0.29040316	0.49299226	0.47134501	Wee1/Xrcc3	2
GO:0002042	cell migration involved in sprouting angiogenesis	1/95	52/14464	0.01923077	2.92793522	1.13242567	0.29055458	0.49299226	0.47134501	Abl1	1
GO:0002228	natural killer cell mediated immunity	1/95	52/14464	0.01923077	2.92793522	1.13242567	0.29055458	0.49299226	0.47134501	Gimap5	1
GO:0006749	glutathione metabolic process	1/95	52/14464	0.01923077	2.92793522	1.13242567	0.29055458	0.49299226	0.47134501	Gsta4	1
GO:0010965	regulation of mitotic sister chromatid separation	1/95	52/14464	0.01923077	2.92793522	1.13242567	0.29055458	0.49299226	0.47134501	Xrcc3	1
GO:0021587	cerebellum morphogenesis	1/95	52/14464	0.01923077	2.92793522	1.13242567	0.29055458	0.49299226	0.47134501	Abl1	1
GO:0031343	positive regulation of cell killing	1/95	52/14464	0.01923077	2.92793522	1.13242567	0.29055458	0.49299226	0.47134501	Gimap5	1
GO:0035315	hair cell differentiation	1/95	52/14464	0.01923077	2.92793522	1.13242567	0.29055458	0.49299226	0.47134501	Alg10b	1
GO:0045005	DNA-templated DNA replication maintenance of fidelity	1/95	52/14464	0.01923077	2.92793522	1.13242567	0.29055458	0.49299226	0.47134501	Pole	1
GO:0045773	positive regulation of axon extension	1/95	52/14464	0.01923077	2.92793522	1.13242567	0.29055458	0.49299226	0.47134501	Myo5b	1
GO:0051489	regulation of filopodium assembly	1/95	52/14464	0.01923077	2.92793522	1.13242567	0.29055458	0.49299226	0.47134501	Agrn	1
GO:0089718	amino acid import across plasma membrane	1/95	52/14464	0.01923077	2.92793522	1.13242567	0.29055458	0.49299226	0.47134501	Slc6a6	1
GO:0097006	regulation of plasma lipoprotein particle levels	1/95	52/14464	0.01923077	2.92793522	1.13242567	0.29055458	0.49299226	0.47134501	Trem2	1
GO:1902305	regulation of sodium ion transmembrane transport	1/95	52/14464	0.01923077	2.92793522	1.13242567	0.29055458	0.49299226	0.47134501	Agrn	1
GO:0051054	positive regulation of DNA metabolic process	3/95	287/14464	0.01045296	1.59149092	0.82295215	0.29179437	0.49465848	0.47293806	Peli1/Paxip1/Arid2	3
GO:1901615	organic hydroxy compound metabolic process	4/95	417/14464	0.00959233	1.4604569	0.77578779	0.29341038	0.49564806	0.47388419	Acer2/Por/Lss/Clcn2	4
GO:0001941	postsynaptic membrane organization	1/95	53/14464	0.01886792	2.87269116	1.11054142	0.29523106	0.49564806	0.47388419	Agrn	1
GO:0002067	glandular epithelial cell differentiation	1/95	53/14464	0.01886792	2.87269116	1.11054142	0.29523106	0.49564806	0.47388419	Clcn2	1
GO:0008631	intrinsic apoptotic signaling pathway in response to oxidative stress	1/95	53/14464	0.01886792	2.87269116	1.11054142	0.29523106	0.49564806	0.47388419	Trem2	1
GO:0014003	oligodendrocyte development	1/95	53/14464	0.01886792	2.87269116	1.11054142	0.29523106	0.49564806	0.47388419	Lyn	1
GO:0045839	negative regulation of mitotic nuclear division	1/95	53/14464	0.01886792	2.87269116	1.11054142	0.29523106	0.49564806	0.47388419	Xrcc3	1
GO:0048002	antigen processing and presentation of peptide antigen	1/95	53/14464	0.01886792	2.87269116	1.11054142	0.29523106	0.49564806	0.47388419	Trem2	1
GO:0050994	regulation of lipid catabolic process	1/95	53/14464	0.01886792	2.87269116	1.11054142	0.29523106	0.49564806	0.47388419	Gimap5	1
GO:0070527	platelet aggregation	1/95	53/14464	0.01886792	2.87269116	1.11054142	0.29523106	0.49564806	0.47388419	Lyn	1
GO:1901607	alpha-amino acid biosynthetic process	1/95	53/14464	0.01886792	2.87269116	1.11054142	0.29523106	0.49564806	0.47388419	Tha1	1
GO:0001666	response to hypoxia	3/95	289/14464	0.01038062	1.58047714	0.81049481	0.29538412	0.49564806	0.47388419	Fzd4/Trem2/ND5	3
GO:0043410	positive regulation of MAPK cascade	4/95	419/14464	0.00954654	1.45348574	0.76592721	0.296367	0.49564806	0.47388419	Fzd4/Trem2/Abl1/Lyn	4
GO:0048545	response to steroid hormone	3/95	290/14464	0.01034483	1.57502722	0.80430157	0.29718017	0.49564806	0.47388419	Per1/Por/Klf9	3
GO:0070588	calcium ion transmembrane transport	3/95	290/14464	0.01034483	1.57502722	0.80430157	0.29718017	0.49564806	0.47388419	Gimap5/Abl1/Lyn	3
GO:0002381	immunoglobulin production involved in immunoglobulin-mediated immune response	1/95	54/14464	0.01851852	2.81949318	1.08916341	0.29987702	0.49564806	0.47388419	Paxip1	1
GO:0006998	nuclear envelope organization	1/95	54/14464	0.01851852	2.81949318	1.08916341	0.29987702	0.49564806	0.47388419	Cdan1	1
GO:0010712	regulation of collagen metabolic process	1/95	54/14464	0.01851852	2.81949318	1.08916341	0.29987702	0.49564806	0.47388419	Serpine1	1
GO:0016447	somatic recombination of immunoglobulin gene segments	1/95	54/14464	0.01851852	2.81949318	1.08916341	0.29987702	0.49564806	0.47388419	Paxip1	1
GO:0021575	hindbrain morphogenesis	1/95	54/14464	0.01851852	2.81949318	1.08916341	0.29987702	0.49564806	0.47388419	Abl1	1
GO:0031641	regulation of myelination	1/95	54/14464	0.01851852	2.81949318	1.08916341	0.29987702	0.49564806	0.47388419	Jam2	1
GO:0032720	negative regulation of tumor necrosis factor production	1/95	54/14464	0.01851852	2.81949318	1.08916341	0.29987702	0.49564806	0.47388419	Trem2	1
GO:0043506	regulation of JUN kinase activity	1/95	54/14464	0.01851852	2.81949318	1.08916341	0.29987702	0.49564806	0.47388419	Fzd4	1
GO:0046173	polyol biosynthetic process	1/95	54/14464	0.01851852	2.81949318	1.08916341	0.29987702	0.49564806	0.47388419	Acer2	1
GO:0050818	regulation of coagulation	1/95	54/14464	0.01851852	2.81949318	1.08916341	0.29987702	0.49564806	0.47388419	Serpine1	1
GO:0051496	positive regulation of stress fiber assembly	1/95	54/14464	0.01851852	2.81949318	1.08916341	0.29987702	0.49564806	0.47388419	Abl1	1
GO:0060119	inner ear receptor cell development	1/95	54/14464	0.01851852	2.81949318	1.08916341	0.29987702	0.49564806	0.47388419	Alg10b	1
GO:0061900	glial cell activation	1/95	54/14464	0.01851852	2.81949318	1.08916341	0.29987702	0.49564806	0.47388419	Trem2	1
GO:1900024	regulation of substrate adhesion-dependent cell spreading	1/95	54/14464	0.01851852	2.81949318	1.08916341	0.29987702	0.49564806	0.47388419	Abl1	1
GO:0071248	cellular response to metal ion	2/95	167/14464	0.01197605	1.82338481	0.87019453	0.30012665	0.49564806	0.47388419	Mt1/Cpne7	2

GO:1902905 positive regulation of supramolecular fiber organization	2/95	167/14464	0.01197605	1.82338481	0.87019453	0.30012665	0.49564806	0.47388419	Abl1/Nav3	2
GO:0072659 protein localization to plasma membrane	3/95	292/14464	0.01027397	1.56423937	0.79198501	0.30077445	0.49629078	0.47449868	Trem2/Rab34/Myo5b	3
GO:0000070 mitotic sister chromatid segregation	2/95	168/14464	0.01190476	1.81253133	0.86132134	0.30255459	0.49795955	0.47609418	Xrcc3/Ncapd3	2
GO:0046390 ribose phosphate biosynthetic process	2/95	168/14464	0.01190476	1.81253133	0.86132134	0.30255459	0.49795955	0.47609418	Trem2/ND5	2
GO:0046605 regulation of centrosome cycle	1/95	55/14464	0.01818182	2.76822967	1.06826948	0.30449269	0.49795955	0.47609418	Xrcc3	1
GO:0051306 mitotic sister chromatid separation	1/95	55/14464	0.01818182	2.76822967	1.06826948	0.30449269	0.49795955	0.47609418	Xrcc3	1
GO:0060688 regulation of morphogenesis of a branching structure	1/95	55/14464	0.01818182	2.76822967	1.06826948	0.30449269	0.49795955	0.47609418	Abl1	1
GO:1905517 macrophage migration	1/95	55/14464	0.01818182	2.76822967	1.06826948	0.30449269	0.49795955	0.47609418	Trem2	1
GO:0009952 anterior/posterior pattern specification	2/95	169/14464	0.01183432	1.80180629	0.85250798	0.30498118	0.49795955	0.47609418	Bhlhe40/Bhlhe41	2
GO:0022412 cellular process involved in reproduction in multicellular organism	3/95	295/14464	0.01016949	1.54833185	0.77368219	0.30617061	0.49795955	0.47609418	Fzd4/Spaca6/Jam2	3
GO:0046890 regulation of lipid biosynthetic process	2/95	170/14464	0.01176471	1.79120743	0.84375363	0.30740634	0.49795955	0.47609418	Por/Clcn2	2
GO:0045862 positive regulation of proteolysis	3/95	296/14464	0.01013514	1.543101	0.7676264	0.30797043	0.49795955	0.47609418	Trem2/Acer2/Lyn	3
GO:0002437 inflammatory response to antigenic stimulus	1/95	56/14464	0.01785714	2.71879699	1.04783879	0.30907824	0.49795955	0.47609418	Trem2	1
GO:0002886 regulation of myeloid leukocyte mediated immunity	1/95	56/14464	0.01785714	2.71879699	1.04783879	0.30907824	0.49795955	0.47609418	Lyn	1
GO:0007019 microtubule depolymerization	1/95	56/14464	0.01785714	2.71879699	1.04783879	0.30907824	0.49795955	0.47609418	Nav3	1
GO:0032964 collagen biosynthetic process	1/95	56/14464	0.01785714	2.71879699	1.04783879	0.30907824	0.49795955	0.47609418	Serpine1	1
GO:0034198 cellular response to amino acid starvation	1/95	56/14464	0.01785714	2.71879699	1.04783879	0.30907824	0.49795955	0.47609418	Sar1b	1
GO:0039532 negative regulation of cytoplasmic pattern recognition receptor signaling pathway	1/95	56/14464	0.01785714	2.71879699	1.04783879	0.30907824	0.49795955	0.47609418	Trem2	1
GO:0042987 amyloid precursor protein catabolic process	1/95	56/14464	0.01785714	2.71879699	1.04783879	0.30907824	0.49795955	0.47609418	Lyn	1
GO:0043462 regulation of ATP-dependent activity	1/95	56/14464	0.01785714	2.71879699	1.04783879	0.30907824	0.49795955	0.47609418	Agm	1
GO:0043536 positive regulation of blood vessel endothelial cell migration	1/95	56/14464	0.01785714	2.71879699	1.04783879	0.30907824	0.49795955	0.47609418	Abl1	1
GO:0048260 positive regulation of receptor-mediated endocytosis	1/95	56/14464	0.01785714	2.71879699	1.04783879	0.30907824	0.49795955	0.47609418	Serpine1	1
GO:0050854 regulation of antigen receptor-mediated signaling pathway	1/95	56/14464	0.01785714	2.71879699	1.04783879	0.30907824	0.49795955	0.47609418	Lyn	1
GO:0051646 mitochondrion localization	1/95	56/14464	0.01785714	2.71879699	1.04783879	0.30907824	0.49795955	0.47609418	Tspan4	1
GO:0051784 negative regulation of nuclear division	1/95	56/14464	0.01785714	2.71879699	1.04783879	0.30907824	0.49795955	0.47609418	Xrcc3	1
GO:0072348 sulfur compound transport	1/95	56/14464	0.01785714	2.71879699	1.04783879	0.30907824	0.49795955	0.47609418	Slc6a6	1
GO:0072698 protein localization to microtubule cytoskeleton	1/95	56/14464	0.01785714	2.71879699	1.04783879	0.30907824	0.49795955	0.47609418	Abl1	1
GO:1903556 negative regulation of tumor necrosis factor superfamily cytokine production	1/95	56/14464	0.01785714	2.71879699	1.04783879	0.30907824	0.49795955	0.47609418	Trem2	1
GO:0030099 myeloid cell differentiation	4/95	428/14464	0.00934579	1.42292179	0.72216758	0.30971904	0.49795955	0.47609418	Trem2/Gimap5/Lrrc17/Lyn	4
GO:0040013 negative regulation of locomotion	3/95	297/14464	0.01010101	1.53790537	0.76159289	0.30977072	0.49795955	0.47609418	Serpine1/Nav3/Arid2	3
GO:0001678 intracellular glucose homeostasis	2/95	171/14464	0.01169591	1.78073253	0.83505746	0.30982997	0.49795955	0.47609418	Trem2/Rab34	2
GO:0071322 cellular response to carbohydrate stimulus	2/95	171/14464	0.01169591	1.78073253	0.83505746	0.30982997	0.49795955	0.47609418	Trem2/Rab34	2
GO:1901605 alpha-amino acid metabolic process	2/95	171/14464	0.01169591	1.78073253	0.83505746	0.30982997	0.49795955	0.47609418	Pipox/Tha1	2
GO:1902904 negative regulation of supramolecular fiber organization	2/95	172/14464	0.01162791	1.77037944	0.82641868	0.31225199	0.49988654	0.47793656	Trem2/Nav3	2
GO:0001954 positive regulation of cell-matrix adhesion	1/95	57/14464	0.01754386	2.6710988	1.0278518	0.31363387	0.49988654	0.47793656	Abl1	1
GO:0006473 protein acetylation	1/95	57/14464	0.01754386	2.6710988	1.0278518	0.31363387	0.49988654	0.47793656	Naa50	1
GO:0016601 Rac protein signal transduction	1/95	57/14464	0.01754386	2.6710988	1.0278518	0.31363387	0.49988654	0.47793656	Agm	1
GO:0032410 negative regulation of transporter activity	1/95	57/14464	0.01754386	2.6710988	1.0278518	0.31363387	0.49988654	0.47793656	Agm	1
GO:0033619 membrane protein proteolysis	1/95	57/14464	0.01754386	2.6710988	1.0278518	0.31363387	0.49988654	0.47793656	Rbmx	1
GO:0046635 positive regulation of alpha-beta T cell activation	1/95	57/14464	0.01754386	2.6710988	1.0278518	0.31363387	0.49988654	0.47793656	Gimap5	1
GO:0046902 regulation of mitochondrial membrane permeability	1/95	57/14464	0.01754386	2.6710988	1.0278518	0.31363387	0.49988654	0.47793656	Gimap5	1
GO:0071470 cellular response to osmotic stress	1/95	57/14464	0.01754386	2.6710988	1.0278518	0.31363387	0.49988654	0.47793656	Clcn2	1
GO:1900271 regulation of long-term synaptic potentiation	1/95	57/14464	0.01754386	2.6710988	1.0278518	0.31363387	0.49988654	0.47793656	Abl1	1
GO:0043393 regulation of protein binding	2/95	173/14464	0.01156069	1.76014603	0.81783651	0.31467233	0.50112547	0.47912109	Abl1/Agm	2
GO:0042326 negative regulation of phosphorylation	3/95	301/14464	0.00996678	1.51746809	0.73767814	0.31697583	0.50375299	0.48163323	Inca1/Abl1/Lyn	3
GO:0006805 xenobiotic metabolic process	1/95	58/14464	0.01724138	2.62504537	1.00829011	0.31815978	0.50375299	0.48163323	Gsta4	1
GO:0010171 body morphogenesis	1/95	58/14464	0.01724138	2.62504537	1.00829011	0.31815978	0.50375299	0.48163323	Zfp281	1
GO:0046637 regulation of alpha-beta T cell differentiation	1/95	58/14464	0.01724138	2.62504537	1.00829011	0.31815978	0.50375299	0.48163323	Gimap5	1
GO:0055117 regulation of cardiac muscle contraction	1/95	58/14464	0.01724138	2.62504537	1.00829011	0.31815978	0.50375299	0.48163323	Agm	1
GO:0060760 positive regulation of response to cytokine stimulus	1/95	58/14464	0.01724138	2.62504537	1.00829011	0.31815978	0.50375299	0.48163323	Trem2	1
GO:0070613 regulation of protein processing	1/95	58/14464	0.01724138	2.62504537	1.00829011	0.31815978	0.50375299	0.48163323	Serpine1	1
GO:0046777 protein autophosphorylation	2/95	175/14464	0.01142857	1.74003008	0.80083897	0.31950761	0.50505362	0.48287675	Abl1/Lyn	2
GO:1902806 regulation of cell cycle G1/S phase transition	2/95	175/14464	0.01142857	1.74003008	0.80083897	0.31950761	0.50505362	0.48287675	Tcim/Arid2	2
GO:0006164 purine nucleotide biosynthetic process	2/95	176/14464	0.01136364	1.73014354	0.79242211	0.32192239	0.50669163	0.48444283	Trem2/ND5	2
GO:0050954 sensory perception of mechanical stimulus	2/95	176/14464	0.01136364	1.73014354	0.79242211	0.32192239	0.50669163	0.48444283	Fzd4/Alg10b	2
GO:0036293 response to decreased oxygen levels	3/95	304/14464	0.00986842	1.50249307	0.71996763	0.32238264	0.50669163	0.48444283	Fzd4/Trem2/ND5	3
GO:1901990 regulation of mitotic cell cycle phase transition	3/95	304/14464	0.00986842	1.50249307	0.71996763	0.32238264	0.50669163	0.48444283	Wee1/Xrcc3/Arid2	3
GO:0000381 regulation of alternative mRNA splicing, via spliceosome	1/95	59/14464	0.01694915	2.58055308	0.9891364	0.32265616	0.50669163	0.48444283	Rbmx	1
GO:0002763 positive regulation of myeloid leukocyte differentiation	1/95	59/14464	0.01694915	2.58055308	0.9891364	0.32265616	0.50669163	0.48444283	Trem2	1
GO:0006903 vesicle targeting	1/95	59/14464	0.01694915	2.58055308	0.9891364	0.32265616	0.50669163	0.48444283	Sar1b	1
GO:0035176 social behavior	1/95	59/14464	0.01694915	2.58055308	0.9891364	0.32265616	0.50669163	0.48444283	Trem2	1
GO:0062197 cellular response to chemical stress	3/95	305/14464	0.00983607	1.49756687	0.7141062	0.32418526	0.50788787	0.48558655	Net1/Clcn2/Abl1	3
GO:0006352 DNA-templated transcription initiation	2/95	177/14464	0.01129944	1.72036872	0.78405888	0.32433517	0.50788787	0.48558655	Tet3/Paxip1	2
GO:0034765 regulation of monoatomic ion transmembrane transporter activity	2/95	177/14464	0.01129944	1.72036872	0.78405888	0.32433517	0.50788787	0.48558655	Trem2/Agm	2
GO:0051495 positive regulation of cytoskeleton organization	2/95	178/14464	0.01123596	1.71070373	0.77574859	0.32674586	0.50788787	0.48558655	Abl1/Nav3	2
GO:0010257 NADH dehydrogenase complex assembly	1/95	60/14464	0.01666667	2.53754386	0.97037434	0.32712319	0.50788787	0.48558655	ND5	1
GO:0016445 somatic diversification of immunoglobulins	1/95	60/14464	0.01666667	2.53754386	0.97037434	0.32712319	0.50788787	0.48558655	Paxip1	1
GO:0030101 natural killer cell activation	1/95	60/14464	0.01666667	2.53754386	0.97037434	0.32712319	0.50788787	0.48558655	Il15ra	1
GO:0032147 activation of protein kinase activity	1/95	60/14464	0.01666667	2.53754386	0.97037434	0.32712319	0.50788787	0.48558655	Abl1	1
GO:0032981 mitochondrial respiratory chain complex I assembly	1/95	60/14464	0.01666667	2.53754386	0.97037434	0.32712319	0.50788787	0.48558655	ND5	1
GO:0048008 platelet-derived growth factor receptor signaling pathway	1/95	60/14464	0.01666667	2.53754386	0.97037434	0.32712319	0.50788787	0.48558655	Abl1	1
GO:0051703 biological process involved in intraspecies interaction between organisms	1/95	60/14464	0.01666667	2.53754386	0.97037434	0.32712319	0.50788787	0.48558655	Trem2	1
GO:0070228 regulation of lymphocyte apoptotic process	1/95	60/14464	0.01666667	2.53754386	0.97037434	0.32712319	0.50788787	0.48558655	Lyn	1
GO:0071385 cellular response to glucocorticoid stimulus	1/95	60/14464	0.01666667	2.53754386	0.97037434	0.32712319	0.50788787	0.48558655	Klf9	1
GO:1905330 regulation of morphogenesis of an epithelium	1/95	60/14464	0.01666667	2.53754386	0.97037434	0.32712319	0.50788787	0.48558655	Abl1	1
GO:0051235 maintenance of location	3/95	307/14464	0.00977199	1.48781073	0.70244552	0.32779078	0.50851294	0.48618417	Trem2/Abl1/Lyn	3
GO:0030595 leukocyte chemotaxis	2/95	179/14464	0.01117318	1.70114672	0.76749053	0.32915438	0.50926259	0.4869009	Serpine1/Lyn	2
GO:0051091 positive regulation of DNA-binding transcription factor activity	2/95	179/14464	0.01117318	1.70114672	0.76749053	0.32915438	0.50926259	0.4869009	Fzd4/Rps6ka5	2
GO:0032102 negative regulation of response to external stimulus	3/95	308/14464	0.00974026	1.48298018	0.69664594	0.32959361	0.50926259	0.4869009	Nr1d2/Trem2/Serpine1	3
GO:0051656 establishment of organelle localization	4/95	442/14464	0.00904977	1.37785187	0.65600244	0.3306092	0.50926259	0.4869009	Tspan4/Sar1b/Myo5b/Lyn	4
GO:0002931 response to ischemia	1/95	61/14464	0.01639344	2.49594478	0.95198855	0.33156108	0.50926259	0.4869009	Trem2	1
GO:0032677 regulation of interleukin-8 production	1/95	61/14464	0.01639344	2.49594478	0.95198855	0.33156108	0.50926259	0.4869009	Serpine1	1
GO:0034308 primary alcohol metabolic process	1/95	61/14464	0.01639344	2.49594478	0.95198855	0.33156108	0.50926259	0.4869009	Clcn2	1
GO:0060350 endochondral bone morphogenesis	1/95	61/14464	0.01639344	2.49594478	0.95198855	0.33156108	0.50926259	0.4869009	Por	1
GO:0099518 vesicle cytoskeletal trafficking	1/95	61/14464	0.01639344	2.49594478	0.95198855	0.33156108	0.50926259	0.4869009	Myo5b	1
GO:1903053 regulation of extracellular matrix organization	1/95	61/14464	0.01639344	2.49594478	0.95198855	0.33156108	0.50926259	0.4869009	Abl1	1
GO:1990928 response to amino acid starvation	1/95	61/14464	0.01639344	2.49594478	0.95198855	0.33156108	0.50926259	0.4869009	Sar1b	1
GO:0030100 regulation of endocytosis	3/95	310/14464	0.00967742	1.47341256	0.68510755	0.33319919	0.50926259	0.4869009	Trem2/Serpine1/Il15ra	3
GO:0000075 cell cycle checkpoint signaling	2/95	181/14464	0.01104972	1.68234952	0.7511284	0.33396465	0.50926259	0.4869009	Xrcc3/Lyn	2
GO:0043010 camera-type eye development	3/95	311/14464	0.0096463	1.4686749	0.67936843	0.33500187	0.50926259	0.4869009	Per1/Fzd4/Clcn2	3
GO:0002562 somatic diversification of immune receptors via germline recombination within a single locus	1/95	62/14464	0.01612903	2.45568761	0.93396446	0.33597	0.50926259	0.4869009	Paxip1	1
GO:0006487 protein N-linked glycosylation	1/95	62/14464	0.01612903	2.45568761	0.93396446	0.33597	0.50926259	0.4869009	Alg10b	1
GO:0006893 Golgi to plasma membrane transport	1/95	62/14464	0.01612903	2.45568761	0.93396446	0.33597	0.50926259	0.4869009	Rab34	1
GO:0008652 amino acid biosynthetic process	1/95	62/14464	0.01612903	2.45568761	0.93396446	0.33597	0.50926259	0.4869009	Tha1	1
GO:0016444 somatic cell DNA recombination	1/95	62/14464	0.01612903	2.45568761	0.93396446	0.33597	0.50926259	0.4869009	Paxip1	1
GO:0019646 aerobic electron transport chain	1/95	62/14464	0.01612903	2.45568761	0.93396446	0.33597	0.50926259	0.4869009	ND5	1
GO:0021695 cerebellar cortex development	1/95	62/14464	0.01612903	2.45568761	0.93396446	0.33597	0.50926259	0.4869009	Rere	1
GO:0032233 positive regulation of actin filament bundle assembly	1/95	62/14464	0.01612903	2.45568761	0.93396446	0.33597	0.50926259	0.4869009	Abl1	1
GO:0032637 interleukin-8 production	1/95	62/14464	0.01612903	2.45568761	0.93396446	0.33597	0.50926259	0.4869009	Serpine1	1
GO:0042130 negative regulation of T cell proliferation	1/95	62/14464	0.01612903	2.45568761	0.93396446	0.33597	0.50926259	0.4869009	Peli1	1
GO:0051148 negative regulation of muscle cell differentiation	1/95	62/14464	0.01612903	2.45568761	0.93396446	0.33597	0.50926259	0.4869009	Bhlhe41	1
GO:0051893 regulation of focal adhesion assembly	1/95	62/14464	0.01612903	2.45568761	0.93396446	0.33597	0.50926259	0.4869009	Abl1	1
GO:0090109 regulation of cell-substrate junction assembly	1/95	62/14464	0.01612903	2.45568761	0.93396446	0.33597	0.50926259	0.4869009	Abl1	1
GO:1903317 regulation of protein maturation	1/95	62/14464	0.01612903	2.45568761	0.93396446	0.33597	0.50926259	0.4869009	Serpine1	1
GO:2000514 regulation of CD4-positive, alpha-beta T cell activation	1/95	62/14464	0.01612903	2.45568761	0.93396446	0.33597	0.50926259	0.4869009	Gimap5	1
GO:0007409 axonogenesis	4/95	446/14464	0.00896861	1.36549445	0.637504	0.33659684	0.50981005	0.48742432	Myo5b/Abl1/Agm/Celsr3	4
GO:0071383 cellular response to steroid hormone stimulus	2/95	183/14464	0.01092896	1.66396319	0.7349672	0.33876538	0.51228587	0.48979143	Per1/Klf9	2
GO:1990138 neuron projection extension	2/95	183/14464	0.01092896	1.66396319	0.7349672	0.33876538	0.51228587	0.48979143	Myo5b/Abl1	2
GO:0006081 cellular aldehyde metabolic process	1/95	63/14464	0.01587302	2.41670844	0.91628833	0.34035015	0.51266243	0.49015145	Clcn2	1
GO:0006919 activation of cysteine-type endopeptidase activity involved in apoptotic process	1/95	63/14464	0.01587302	2.41670844	0.91628833	0.34035015	0.51266243	0.49015145	Acer2	1
GO:0045669 positive regulation of osteoblast differentiation	1/95	63/14464	0.01587302	2.41670844	0.91628833	0.34035015	0.51266243	0.49015145	Gdf10	1
GO:0051898 negative regulation of phosphatidylinositol 3-kinase/protein kinase B signal transduction	1/95	63/14464	0.01587302	2.41670844	0.91628833	0.34035015	0.51266243	0.49015145	Trem2	1
GO:0061035 regulation of cartilage development	1/95	63/14464	0.01587302	2.41670844	0.91628833	0.34035015	0.51266243	0.49015145	Por	1
GO:1903532 positive regulation of secretion by cell	3/95	315/14464	0.00952381	1.45002506	0.656609	0.34221094	0.51437213	0.49178609	Trem2/Rab34/Myo5b	3
GO:0042098 T cell proliferation	2/95	185/14464	0.01081081	1.6459744	0.71900183	0.34355601	0.51437213	0.49178609	Abl1/Peli1	2
GO:0002637 regulation of immunoglobulin production	1/95	64/14464	0.015625	2.37894737	0.89894714	0.3447017	0.51437213	0.49178609	Paxip1	1
GO:0003407 neural retina development	1/95	64/14464	0.015625	2.37894737	0.89894714	0.3447017	0.51437213	0.49178609	Per1	1
GO:0007528 neuromuscular junction development	1/95	64/14464	0.015625	2.37894737	0.89894714	0.3447017	0.51437213	0.49178609	Agm	1
GO:0010972 negative regulation of G2/M transition of mitotic cell cycle	1/95	64/14464	0.015625	2.37894737	0.89894714	0.3447017	0.51437213	0.49178609	Wee1	1
GO:0042776 proton motive force-driven mitochondrial ATP synthesis	1/95	64/14464	0.015625	2.37894737	0.89894714	0.3447017	0.51437213	0.49178609	ND5	1
GO:0044380 protein localization to cytoskeleton	1/95	64/14464	0.015625	2.37894737	0.89894714	0.3447017	0.51437213	0.49178609	Abl1	1
GO:0045428 regulation of nitric oxide biosynthetic process	1/95	64/14464	0.015625	2.37894737	0.89894714	0.3447017	0.51437213	0.49178609	Gimap5	1
GO:0050885 neuromuscular process controlling balance	1/95	64/14464	0.015625	2.37894737	0.89894714	0.3447017	0.51437213	0.49178609	Abl1	1
GO:0062208 positive regulation of pattern recognition receptor signaling pathway	1/95	64/14464	0.015625	2.37894737	0.89894714	0.3447017	0.51437213	0.49178609	Peli1	1
GO:2000179 positive regulation of neural precursor cell proliferation	1/95	64/14464	0.015625	2.37894737	0.89894714	0.3447017	0.51437213	0.49178609	Lyn	1
GO:0022898 regulation of transmembrane transporter activity	2/95	186/14464	0.01075269	1.63712507	0.71109103	0.34594735	0.51542932	0.49279686	Trem2/Agm	2
GO:0099175 regulation of postsynapse organization	2/95	186/14464	0.01075269	1.63712507	0.71109103	0.34594735	0.51542932	0.49279686	Rps6ka5/Rbmx	2
GO:0001910 regulation of leukocyte mediated cytotoxicity	1/95	65/14464	0.01538462	2.34234818	0.88192857	0.34902485	0.51840456	0.49564145	Gimap5	1
GO:0045576 mast cell activation	1/95	65/14464	0.01538462	2.34234818	0.88192857	0.34902485	0.51840456	0.49564145	Lyn	1
GO:0060261 positive regulation of transcription initiation by RNA polymerase II	1/95	65/14464	0.01538462	2.34234818	0.88192857	0.34902485	0.51840456	0.49564145	Paxip1	1
GO:0140115 export across plasma membrane	1/95	65/14464	0.01538462	2.34234818	0.88192857	0.34902485	0.51840456	0.49564145	Agm	1
GO:0062012 regulation of small molecule metabolic process	3/95	319/14464	0.00940439	1.43184293	0.63415789	0.34941559	0.51858353	0.49581257	Trem2/Por/Clcn2	3
GO:0010817 regulation of hormone levels	4/95	455/14464	0.00879121	1.33848467	0.59651156	0.35008746	0.51917917	0.49638205	Fzd4/Por/Clcn2/Lyn	4
GO:0061351 neural precursor cell proliferation	2/95	189/14464	0.01058201	1.61113896	0.68763906	0.35310472	0.52195585	0.4990368	Rere/Lyn	2
GO:0002720 positive regulation of cytokine production involved in immune response	1/95	66/14464	0.01515152	2.30685805	0.86522095	0.35331978	0.52195585	0.4990368	Gimap5	1
GO:0060976 coronary vasculature development	1/95	66/14464	0.01515152	2.30685805	0.86522095	0.35331978	0.52195585	0.4990368	Arid2	1
GO:0071384 cellular response to corticosteroid stimulus	1/95	66/14464	0.01515152	2.30685805	0.86522095	0.35331978	0.52195585	0.4990368	Klf9	1
GO:1902750 negative regulation of cell cycle G2/M phase transition	1/95	66/14464	0.01515152	2.30685805	0.86522095	0.35331978	0.52195585	0.4990368	Wee1	1
GO:0007517 muscle organ development	3/95	323/14464	0.00928793	1.41411113	0.61200621	0.35661343	0.52422368	0.50120506	Nr1d2/Hlf/Gpcpd1	3
GO:0032088 negative regulation of NF-kappaB transcription factor activity	1/95	67/14464	0.01492537	2.27242734	0.84881318	0.35758666	0.52422368	0.50120506	Peli1	1
GO:0042246 tissue regeneration	1/95	67/14464	0.01492537	2.27242734	0.84881318	0.35758666	0.52422368	0.50120506	Serpine1	1
GO:0045123 cellular extravasation	1/95	67/14464	0.01492537	2.27242734	0.84881318	0.35758666	0.52422368	0.50120506	Jam2	1
GO:0045132 meiotic chromosome segregation	1/95	67/14464	0.01492537	2.27242734	0.84881318	0.35758666	0.52422368	0.50120506	Ncapd3	1
GO:0060612 adipose tissue development	1/95	67/14464	0.01492537	2.27242734	0.84881318	0.35758666	0.52422368	0.50120506	Paxip1	1
GO:0070534 protein K63-linked ubiquitination	1/95	67/14464	0.01492537	2.27242734	0.84881318	0.35758666	0.52422368	0.50120506	Peli1	1
GO:0080164 regulation of nitric oxide metabolic process	1/95	67/14464	0.01492537	2.27242734	0.84881318	0.35758666	0.52422368	0.50120506	Gimap5	1
GO:0150116 regulation of cell-substrate junction organization	1/95	67/14464	0.01492537	2.27242734	0.84881318	0.35758666	0.52422368	0.50120506	Abl1	1
GO:1903036 positive regulation of response to wounding	1/95	67/14464	0.01492537	2.27242734	0.84881318	0.35758666	0.52422368	0.50120506	Serpine1	1
GO:0032271 regulation of protein polymerization	2/95	192/14464	0.01041667	1.58596491	0.66459571	0.36023571	0.52689964	0.50376351	Abl1/Nav3	2
GO:0050670 regulation of lymphocyte proliferation	2/95	192/14464	0.01041667	1.58596491	0.66459571	0.36023571	0.52689964	0.50376351	Peli1/Lyn	2
GO:1901654 response to ketone	2/95	192/14464	0.01041667	1.58596491	0.66459571	0.36023571	0.52689964	0.50376351	Por/Klf9	2
GO:0006720 isoprenoid metabolic process	1/95	68/14464	0.01470588	2.23900929	0.83269474	0.36182569	0.52841971	0.50521683	Lss	1
GO:0071806 protein transmembrane transport	1/95	68/14464	0.01470588	2.23900929	0.83269474	0.36182569	0.52841971	0.50521683	Myo5b	1
GO:0070482 response to oxygen levels	3/95	327/14464	0.00917431	1.39681313	0.59014538	0.36380219	0.53013213	0.50685406	Fzd4/Trem2/ND5	3
GO:0001763 morphogenesis of a branching structure	2/95	194/14464	0.01030928	1.56961476	0.64945316	0.3649742	0.53013213	0.50685406	Rere/Abl1	2
GO:0051650 establishment of vesicle localization	2/95	194/14464	0.01030928	1.56961476	0.64945316	0.3649742	0.53013213	0.50685406	Sar1b/Myo5b	2
GO:0009611 response to wounding	4/95	465/14464	0.00860215	1.30970006	0.55194649	0.3650904	0.53013213	0.50685406	Trem2/Serpine1/Mir22hg/Lyn	4
GO:0002200 somatic diversification of immune receptors	1/95	69/14464	0.01449275	2.20655988	0.81685564	0.36603704	0.53013213	0.50685406	Paxip1	1
GO:0008347 glial cell migration	1/95	69/14464	0.01449275	2.20655988	0.81685564	0.36603704	0.53013213	0.50685406	Trem2	1
GO:0010632 regulation of epithelial cell migration	1/95	69/14464	0.01449275	2.20655988	0.81685564	0.36603704	0.53013213	0.50685406	Serpine1	1
GO:0015986 proton motive force-driven ATP synthesis	1/95	69/14464	0.01449275	2.20655988	0.81685564	0.36603704	0.53013213	0.50685406	ND5	1
GO:0042475 odontogenesis of dentin-containing tooth	1/95	69/14464	0.01449275	2.20655988	0.81685564	0.36603704	0.53013213	0.50685406	Serpine1	1
GO:0048844 artery morphogenesis	1/95	69/14464	0.01449275	2.20655988	0.81685564	0.36603704	0.53013213	0.50685406	Arid2	1
GO:0070509 calcium ion import	1/95	69/14464	0.01449275	2.20655988	0.81685564	0.36603704	0.53013213	0.50685406	Serpine1	1
GO:0008361 regulation of cell size	2/95	195/14464	0.01025641	1.56156545	0.64194621	0.36733861	0.53161598	0.50827276	Myo5b/Abl1	2
GO:0032409 regulation of transporter activity	2/95	196/14464	0.01020408	1.55359828	0.63448147	0.36969972	0.53297365	0.50957081	Trem2/Agm	2

GO:0032944 regulation of mononuclear cell proliferation	2/95	196/14464	0.01020408	1.55359828	0.63448147	0.36969972	0.53297365	0.50957081 Peli1/Lyn	2
GO:0001960 negative regulation of cytokine-mediated signaling pathway	1/95	70/14464	0.01428571	2.17503759	0.80128637	0.37022088	0.53297365	0.50957081 Trem2	1
GO:0032008 positive regulation of TOR signaling	1/95	70/14464	0.01428571	2.17503759	0.80128637	0.37022088	0.53297365	0.50957081 Trem2	1
GO:0033143 regulation of intracellular steroid hormone receptor signaling pathway	1/95	70/14464	0.01428571	2.17503759	0.80128637	0.37022088	0.53297365	0.50957081 Per1	1
GO:0046626 regulation of insulin receptor signaling pathway	1/95	70/14464	0.01428571	2.17503759	0.80128637	0.37022088	0.53297365	0.50957081 Tns2	1
GO:1903035 negative regulation of response to wounding	1/95	70/14464	0.01428571	2.17503759	0.80128637	0.37022088	0.53297365	0.50957081 Serpine1	1
GO:0034728 nucleosome organization	1/95	71/14464	0.01408451	2.14440326	0.78597788	0.37437741	0.53734499	0.5137502 Arid2	1
GO:0046847 filopodium assembly	1/95	71/14464	0.01408451	2.14440326	0.78597788	0.37437741	0.53734499	0.5137502 Agrn	1
GO:0060113 inner ear receptor cell differentiation	1/95	71/14464	0.01408451	2.14440326	0.78597788	0.37437741	0.53734499	0.5137502 Alg10b	1
GO:2000144 positive regulation of DNA-templated transcription initiation	1/95	71/14464	0.01408451	2.14440326	0.78597788	0.37437741	0.53734499	0.5137502 Paxip1	1
GO:0051047 positive regulation of secretion	3/95	334/14464	0.00898204	1.36753861	0.55256432	0.37635399	0.53977825	0.51607663 Trem2/Rab34/Myo5b	3
GO:0015800 acidic amino acid transport	1/95	72/14464	0.01388889	2.11461988	0.77092154	0.37850679	0.54004054	0.51632739 Slc6a6	1
GO:0042775 mitochondrial ATP synthesis coupled electron transport	1/95	72/14464	0.01388889	2.11461988	0.77092154	0.37850679	0.54004054	0.51632739 ND5	1
GO:0042982 amyloid precursor protein metabolic process	1/95	72/14464	0.01388889	2.11461988	0.77092154	0.37850679	0.54004054	0.51632739 Lyn	1
GO:0043299 leukocyte degranulation	1/95	72/14464	0.01388889	2.11461988	0.77092154	0.37850679	0.54004054	0.51632739 Lyn	1
GO:0051591 response to cAMP	1/95	72/14464	0.01388889	2.11461988	0.77092154	0.37850679	0.54004054	0.51632739 Per1	1
GO:0090559 regulation of membrane permeability	1/95	72/14464	0.01388889	2.11461988	0.77092154	0.37850679	0.54004054	0.51632739 Gimap5	1
GO:0099084 postsynaptic specialization organization	1/95	72/14464	0.01388889	2.11461988	0.77092154	0.37850679	0.54004054	0.51632739 Abl1	1
GO:0008406 gonad development	2/95	200/14464	0.01	1.52252632	0.60503462	0.37910998	0.5404993	0.51676601 Fzd4/Serpine1	2
GO:0009749 response to glucose	2/95	201/14464	0.00995025	1.51495156	0.59777354	0.38145372	0.54343704	0.51957476 Trem2/Rab34	2
GO:0042773 ATP synthesis coupled electron transport	1/95	73/14464	0.01369863	2.08565249	0.75610916	0.38260919	0.54387188	0.51999051 ND5	1
GO:0071277 cellular response to calcium ion	1/95	73/14464	0.01369863	2.08565249	0.75610916	0.38260919	0.54387188	0.51999051 Cpne7	1
GO:1902476 chloride transmembrane transport	1/95	73/14464	0.01369863	2.08565249	0.75610916	0.38260919	0.54387188	0.51999051 Clcn2	1
GO:0017157 regulation of exocytosis	2/95	202/14464	0.00990099	1.5074518	0.59055179	0.38379382	0.54515199	0.52121441 Myo5b/Lyn	2
GO:0045137 development of primary sexual characteristics	2/95	203/14464	0.00985222	1.50002593	0.5833689	0.38613023	0.54562363	0.52166534 Fzd4/Serpine1	2
GO:0006942 regulation of striated muscle contraction	1/95	74/14464	0.01351351	2.05746799	0.74153289	0.38668481	0.54562363	0.52166534 Agrn	1
GO:0014823 response to activity	1/95	74/14464	0.01351351	2.05746799	0.74153289	0.38668481	0.54562363	0.52166534 Tns2	1
GO:0019229 regulation of vasoconstriction	1/95	74/14464	0.01351351	2.05746799	0.74153289	0.38668481	0.54562363	0.52166534 Abl1	1
GO:0032651 regulation of interleukin-1 beta production	1/95	74/14464	0.01351351	2.05746799	0.74153289	0.38668481	0.54562363	0.52166534 Trem2	1
GO:0034109 homotypic cell-cell adhesion	1/95	74/14464	0.01351351	2.05746799	0.74153289	0.38668481	0.54562363	0.52166534 Lyn	1
GO:0060260 regulation of transcription initiation by RNA polymerase II	1/95	74/14464	0.01351351	2.05746799	0.74153289	0.38668481	0.54562363	0.52166534 Paxip1	1
GO:0060761 negative regulation of response to cytokine stimulus	1/95	74/14464	0.01351351	2.05746799	0.74153289	0.38668481	0.54562363	0.52166534 Trem2	1
GO:0071479 cellular response to ionizing radiation	1/95	74/14464	0.01351351	2.05746799	0.74153289	0.38668481	0.54562363	0.52166534 Net1	1
GO:0016054 organic acid catabolic process	2/95	204/14464	0.00980392	1.49267286	0.57622445	0.38846289	0.54732767	0.52329455 Pipox/Tha1	2
GO:0046395 carboxylic acid catabolic process	2/95	204/14464	0.00980392	1.49267286	0.57622445	0.38846289	0.54732767	0.52329455 Pipox/Tha1	2
GO:0008625 extrinsic apoptotic signaling pathway via death domain receptors	1/95	75/14464	0.01333333	2.03003509	0.72718526	0.39073379	0.54939881	0.52527474 Serpine1	1
GO:0009746 response to hexose	2/95	205/14464	0.0097561	1.48539153	0.56911799	0.39079175	0.54939881	0.52527474 Trem2/Rab34	2
GO:0051651 maintenance of location in cell	2/95	205/14464	0.0097561	1.48539153	0.56911799	0.39079175	0.54939881	0.52527474 Abl1/Lyn	2
GO:0043113 receptor clustering	1/95	76/14464	0.01315789	2.0033241	0.71305914	0.39475633	0.55345031	0.52914834 Agrn	1
GO:0045661 regulation of myoblast differentiation	1/95	76/14464	0.01315789	2.0033241	0.71305914	0.39475633	0.55345031	0.52914834 Arid2	1
GO:0050886 endocrine process	1/95	76/14464	0.01315789	2.0033241	0.71305914	0.39475633	0.55345031	0.52914834 Fzd4	1
GO:0000082 G1/S transition of mitotic cell cycle	2/95	207/14464	0.00966184	1.47103992	0.55501732	0.39543789	0.55345031	0.52914834 Pole/Arid2	2
GO:0007411 axon guidance	2/95	207/14464	0.00966184	1.47103992	0.55501732	0.39543789	0.55345031	0.52914834 Agrn/Celsr3	2
GO:0060348 bone development	2/95	207/14464	0.00966184	1.47103992	0.55501732	0.39543789	0.55345031	0.52914834 Por/Lrrc17	2
GO:0010563 negative regulation of phosphorus metabolic process	3/95	345/14464	0.00869565	1.32393593	0.49515871	0.39598086	0.55345031	0.52914834 Inca1/Abl1/Lyn	3
GO:0045936 negative regulation of phosphate metabolic process	3/95	345/14464	0.00869565	1.32393593	0.49515871	0.39598086	0.55345031	0.52914834 Inca1/Abl1/Lyn	3
GO:1903829 positive regulation of protein localization	4/95	486/14464	0.00823045	1.25310808	0.46150812	0.39654756	0.55383898	0.52951995 Trem2/Rab34/Myo5b/Agrn	4
GO:0097485 neuron projection guidance	2/95	208/14464	0.00961538	1.46396761	0.54802228	0.39775506	0.55449726	0.53014932 Agrn/Celsr3	2
GO:0000380 alternative mRNA splicing, via spliceosome	1/95	77/14464	0.01298701	1.9773069	0.6991477	0.39875259	0.55449726	0.53014932 Rbmx	1
GO:0032370 positive regulation of lipid transport	1/95	77/14464	0.01298701	1.9773069	0.6991477	0.39875259	0.55449726	0.53014932 Trem2	1
GO:0032414 positive regulation of ion transmembrane transporter activity	1/95	77/14464	0.01298701	1.9773069	0.6991477	0.39875259	0.55449726	0.53014932 Trem2	1
GO:0042490 mechanoreceptor differentiation	1/95	77/14464	0.01298701	1.9773069	0.6991477	0.39875259	0.55449726	0.53014932 Alg10b	1
GO:1901880 negative regulation of protein depolymerization	1/95	77/14464	0.01298701	1.9773069	0.6991477	0.39875259	0.55449726	0.53014932 Nav3	1
GO:0007163 establishment or maintenance of cell polarity	2/95	210/14464	0.00952381	1.45002506	0.53414073	0.40237737	0.55719173	0.53272548 Wee1/Abl1	2
GO:0031348 negative regulation of defense response	2/95	210/14464	0.00952381	1.45002506	0.53414073	0.40237737	0.55719173	0.53272548 Nr1d2/Trem2	2
GO:0043542 endothelial cell migration	2/95	210/14464	0.00952381	1.45002506	0.53414073	0.40237737	0.55719173	0.53272548 Abl1/Paxip1	2
GO:0031341 regulation of cell killing	1/95	78/14464	0.01282051	1.95195682	0.68544442	0.40272274	0.55719173	0.53272548 Gimap5	1
GO:0043367 CD4-positive, alpha-beta T cell differentiation	1/95	78/14464	0.01282051	1.95195682	0.68544442	0.40272274	0.55719173	0.53272548 Gimap5	1
GO:0050795 regulation of behavior	1/95	78/14464	0.01282051	1.95195682	0.68544442	0.40272274	0.55719173	0.53272548 Per3	1
GO:0060997 dendritic spine morphogenesis	1/95	78/14464	0.01282051	1.95195682	0.68544442	0.40272274	0.55719173	0.53272548 Myo5b	1
GO:0034284 response to monosaccharide	2/95	211/14464	0.00947867	1.44315291	0.52725343	0.40468242	0.55942212	0.53485794 Trem2/Rab34	2
GO:0001654 eye development	3/95	351/14464	0.00854701	1.30130454	0.46465144	0.40662347	0.55942212	0.53485794 Per1/Fzd4/Clcn2	3
GO:0031397 negative regulation of protein ubiquitination	1/95	79/14464	0.01265823	1.9272485	0.67194306	0.40666694	0.55942212	0.53485794 Abl1	1
GO:0032611 interleukin-1 beta production	1/95	79/14464	0.01265823	1.9272485	0.67194306	0.40666694	0.55942212	0.53485794 Trem2	1
GO:0051965 positive regulation of synapse assembly	1/95	79/14464	0.01265823	1.9272485	0.67194306	0.40666694	0.55942212	0.53485794 Agrn	1
GO:0097009 energy homeostasis	1/95	79/14464	0.01265823	1.9272485	0.67194306	0.40666694	0.55942212	0.53485794 Nr1d2	1
GO:1903578 regulation of ATP metabolic process	1/95	79/14464	0.01265823	1.9272485	0.67194306	0.40666694	0.55942212	0.53485794 Trem2	1
GO:1904063 negative regulation of cation transmembrane transport	1/95	79/14464	0.01265823	1.9272485	0.67194306	0.40666694	0.55942212	0.53485794 Agrn	1
GO:0070663 regulation of leukocyte proliferation	2/95	213/14464	0.00938967	1.42960217	0.51358381	0.40928005	0.56261348	0.53790916 Peli1/Lyn	2
GO:0006900 vesicle budding from membrane	1/95	80/14464	0.0125	1.90315789	0.65863766	0.41058537	0.56319752	0.53846756 Sar1b	1
GO:0010507 negative regulation of autophagy	1/95	80/14464	0.0125	1.90315789	0.65863766	0.41058537	0.56319752	0.53846756 Trem2	1
GO:0070227 lymphocyte apoptotic process	1/95	80/14464	0.0125	1.90315789	0.65863766	0.41058537	0.56319752	0.53846756 Lyn	1
GO:0009165 nucleotide biosynthetic process	2/95	214/14464	0.00934579	1.42292179	0.50680074	0.41157256	0.56414838	0.53937667 Trem2/ND5	2
GO:0150063 visual system development	3/95	354/14464	0.00847458	1.29027654	0.44960153	0.41192545	0.56422907	0.53945381 Per1/Fzd4/Clcn2	3
GO:0051648 vesicle localization	2/95	215/14464	0.00930233	1.41630355	0.50005166	0.41386079	0.56570673	0.54086659 Sar1b/Myo5b	2
GO:0002690 positive regulation of leukocyte chemotaxis	1/95	81/14464	0.01234568	1.87966212	0.6455225	0.4144782	0.56570673	0.54086659 Serpine1	1
GO:0033077 T cell differentiation in thymus	1/95	81/14464	0.01234568	1.87966212	0.6455225	0.4144782	0.56570673	0.54086659 Abl1	1
GO:0071103 DNA conformation change	1/95	81/14464	0.01234568	1.87966212	0.6455225	0.4144782	0.56570673	0.54086659 Abl1	1
GO:2000142 regulation of DNA-templated transcription initiation	1/95	81/14464	0.01234568	1.87966212	0.6455225	0.4144782	0.56570673	0.54086659 Paxip1	1
GO:0032411 positive regulation of transporter activity	1/95	82/14464	0.01219512	1.85673941	0.63259208	0.41834558	0.57017413	0.54513782 Trem2	1
GO:0032642 regulation of chemokine production	1/95	82/14464	0.01219512	1.85673941	0.63259208	0.41834558	0.57017413	0.54513782 Trem2	1
GO:0048880 sensory system development	3/95	359/14464	0.00835655	1.27230611	0.42481104	0.42073078	0.57301801	0.54785683 Per1/Fzd4/Clcn2	3
GO:0006637 acyl-CoA metabolic process	1/95	83/14464	0.01204819	1.83436906	0.61984116	0.42218769	0.5733745	0.54819767 Pipox	1
GO:0030510 regulation of BMP signaling pathway	1/95	83/14464	0.01204819	1.83436906	0.61984116	0.42218769	0.5733745	0.54819767 Abl1	1
GO:0035383 thioester metabolic process	1/95	83/14464	0.01204819	1.83436906	0.61984116	0.42218769	0.5733745	0.54819767 Pipox	1
GO:1900076 regulation of cellular response to insulin stimulus	1/95	83/14464	0.01204819	1.83436906	0.61984116	0.42218769	0.5733745	0.54819767 Tns2	1
GO:0048872 homeostasis of number of cells	3/95	360/14464	0.00833333	1.26877193	0.419896	0.42248691	0.57337509	0.54819823 Gimap5/Abl1/Lyn	3
GO:0002440 production of molecular mediator of immune response	2/95	219/14464	0.00913242	1.39043499	0.47338805	0.42297019	0.57362529	0.54843745 Gimap5/Paxip1	2
GO:0010976 positive regulation of neuron projection development	2/95	220/14464	0.00909091	1.38411483	0.46680356	0.42523645	0.57570632	0.5504271 Abl1/Lyn	2
GO:0046034 ATP metabolic process	2/95	220/14464	0.00909091	1.38411483	0.46680356	0.42523645	0.57570632	0.5504271 Trem2/ND5	2
GO:0007600 sensory perception	3/95	362/14464	0.00828729	1.26176214	0.41010823	0.42599402	0.57570632	0.5504271 Fzd4/Or8b53/Alg10b	3
GO:0008593 regulation of Notch signaling pathway	1/95	84/14464	0.01190476	1.81253133	0.6072647	0.42600468	0.57570632	0.5504271 Tcim	1
GO:0034766 negative regulation of monoatomic ion transmembrane transport	1/95	84/14464	0.01190476	1.81253133	0.6072647	0.42600468	0.57570632	0.5504271 Agrn	1
GO:0007059 chromosome segregation	3/95	364/14464	0.00824176	1.25482938	0.4003763	0.42949408	0.57879292	0.55337816 Xrcc3/Ncapd3/Arid2	3
GO:0046434 organophosphate catabolic process	2/95	222/14464	0.00900901	1.37164533	0.4537299	0.42975538	0.57879292	0.55337816 Pnpla7/Gpcpd1	2
GO:0090068 positive regulation of cell cycle process	2/95	222/14464	0.00900901	1.37164533	0.4537299	0.42975538	0.57879292	0.55337816 Xrcc3/Ncapd3	2
GO:0019915 lipid storage	1/95	85/14464	0.01176471	1.79120743	0.59485786	0.42979672	0.57879292	0.55337816 Trem2	1
GO:0048041 focal adhesion assembly	1/95	85/14464	0.01176471	1.79120743	0.59485786	0.42979672	0.57879292	0.55337816 Abl1	1
GO:0006672 ceramide metabolic process	1/95	86/14464	0.01162791	1.77037944	0.58261598	0.43356397	0.5806066	0.55511221 Acer2	1
GO:0006821 chloride transport	1/95	86/14464	0.01162791	1.77037944	0.58261598	0.43356397	0.5806066	0.55511221 Clcn2	1
GO:0030168 platelet activation	1/95	86/14464	0.01162791	1.77037944	0.58261598	0.43356397	0.5806066	0.55511221 Lyn	1
GO:0032273 positive regulation of protein polymerization	1/95	86/14464	0.01162791	1.77037944	0.58261598	0.43356397	0.5806066	0.55511221 Nav3	1
GO:0032456 endocytic recycling	1/95	86/14464	0.01162791	1.77037944	0.58261598	0.43356397	0.5806066	0.55511221 Myo5b	1
GO:0043242 negative regulation of protein-containing complex disassembly	1/95	86/14464	0.01162791	1.77037944	0.58261598	0.43356397	0.5806066	0.55511221 Nav3	1
GO:0050848 regulation of calcium-mediated signaling	1/95	86/14464	0.01162791	1.77037944	0.58261598	0.43356397	0.5806066	0.55511221 Trem2	1
GO:2001237 negative regulation of extrinsic apoptotic signaling pathway	1/95	86/14464	0.01162791	1.77037944	0.58261598	0.43356397	0.5806066	0.55511221 Serpine1	1
GO:1903522 regulation of blood circulation	2/95	224/14464	0.00892857	1.3593985	0.44078113	0.43425594	0.58112772	0.55561045 Abl1/Agrn	2
GO:0015908 fatty acid transport	1/95	87/14464	0.01149425	1.75003025	0.57053461	0.43730659	0.58398842	0.55834553 Lyn	1
GO:0051881 regulation of mitochondrial membrane potential	1/95	87/14464	0.01149425	1.75003025	0.57053461	0.43730659	0.58398842	0.55834553 Tspan4	1
GO:0090288 negative regulation of cellular response to growth factor stimulus	1/95	87/14464	0.01149425	1.75003025	0.57053461	0.43730659	0.58398842	0.55834553 Abl1	1
GO:0002286 T cell activation involved in immune response	1/95	88/14464	0.01136364	1.73014354	0.55860945	0.44102475	0.58407625	0.5584295 Trem2	1
GO:0030148 sphingolipid biosynthetic process	1/95	88/14464	0.01136364	1.73014354	0.55860945	0.44102475	0.58407625	0.5584295 Acer2	1
GO:0030301 cholesterol transport	1/95	88/14464	0.01136364	1.73014354	0.55860945	0.44102475	0.58407625	0.5584295 Trem2	1
GO:0032652 regulation of interleukin-1 production	1/95	88/14464	0.01136364	1.73014354	0.55860945	0.44102475	0.58407625	0.5584295 Trem2	1
GO:0043406 positive regulation of MAP kinase activity	1/95	88/14464	0.01136364	1.73014354	0.55860945	0.44102475	0.58407625	0.5584295 Fzd4	1
GO:0043543 protein acylation	1/95	88/14464	0.01136364	1.73014354	0.55860945	0.44102475	0.58407625	0.5584295 Naa50	1
GO:0043954 cellular component maintenance	1/95	88/14464	0.01136364	1.73014354	0.55860945	0.44102475	0.58407625	0.5584295 Trem2	1
GO:0045833 negative regulation of lipid metabolic process	1/95	88/14464	0.01136364	1.73014354	0.55860945	0.44102475	0.58407625	0.5584295 Gimap5	1
GO:0051153 regulation of striated muscle cell differentiation	1/95	88/14464	0.01136364	1.73014354	0.55860945	0.44102475	0.58407625	0.5584295 Bhlhe41	1
GO:0120254 olefinic compound metabolic process	1/95	88/14464	0.01136364	1.73014354	0.55860945	0.44102475	0.58407625	0.5584295 Clcn2	1
GO:1900542 regulation of purine nucleotide metabolic process	1/95	88/14464	0.01136364	1.73014354	0.55860945	0.44102475	0.58407625	0.5584295 Trem2	1
GO:2000106 regulation of leukocyte apoptotic process	1/95	88/14464	0.01136364	1.73014354	0.55860945	0.44102475	0.58407625	0.5584295 Lyn	1
GO:0072522 purine-containing compound biosynthetic process	2/95	228/14464	0.00877193	1.3355494	0.41524807	0.44320079	0.5865395	0.56078459 Trem2/ND5	2
GO:0006140 regulation of nucleotide metabolic process	1/95	89/14464	0.01123596	1.71070373	0.54683637	0.44471859	0.5865395	0.56078459 Trem2	1
GO:0007156 homophilic cell adhesion via plasma membrane adhesion molecules	1/95	89/14464	0.01123596	1.71070373	0.54683637	0.44471859	0.5865395	0.56078459 Celsr3	1
GO:0098661 inorganic anion transmembrane transport	1/95	89/14464	0.01123596	1.71070373	0.54683637	0.44471859	0.5865395	0.56078459 Clcn2	1
GO:1903321 negative regulation of protein modification by small protein conjugation or removal	1/95	89/14464	0.01123596	1.71070373	0.54683637	0.44471859	0.5865395	0.56078459 Abl1	1
GO:2001251 negative regulation of chromosome organization	1/95	89/14464	0.01123596	1.71070373	0.54683637	0.44471859	0.5865395	0.56078459 Xrcc3	1
GO:1902903 regulation of supramolecular fiber organization	3/95	374/14464	0.00802139	1.22127779	0.35252982	0.4468817	0.58898762	0.56312522 Trem2/Abl1/Nav3	3
GO:0010951 negative regulation of endopeptidase activity	1/95	90/14464	0.01111111	1.69169591	0.53521141	0.44838827	0.58975812	0.56386188 Serpine1	1
GO:0043535 regulation of blood vessel endothelial cell migration	1/95	90/14464	0.01111111	1.69169591	0.53521141	0.44838827	0.58975812	0.56386188 Abl1	1
GO:0046634 regulation of alpha-beta T cell activation	1/95	90/14464	0.01111111	1.69169591	0.53521141	0.44838827	0.58975812	0.56386188 Gimap5	1
GO:0043603 amide metabolic process	3/95	376/14464	0.00797872	1.21478163	0.34311849	0.45033532	0.5905059	0.56457683 Acer2/Pipox/Gsta4	3
GO:0006970 response to osmotic stress	1/95	91/14464	0.01098901	1.67310584	0.52373074	0.45203396	0.5905059	0.56457683 Clcn2	1
GO:0010469 regulation of signaling receptor activity	1/95	91/14464	0.01098901	1.67310584	0.52373074	0.45203396	0.5905059	0.56457683 Serpine1	1
GO:0019751 polyol metabolic process	1/95	91/14464	0.01098901	1.67310584	0.52373074	0.45203396	0.5905059	0.56457683 Acer2	1
GO:0032602 chemokine production	1/95	91/14464	0.01098901	1.67310584	0.52373074	0.45203396	0.5905059	0.56457683 Trem2	1
GO:0042310 vasoconstriction	1/95	91/14464	0.01098901	1.67310584	0.52373074	0.45203396	0.5905059	0.56457683 Abl1	1
GO:0045921 positive regulation of exocytosis	1/95	91/14464	0.01098901	1.67310584	0.52373074	0.45203396	0.5905059	0.56457683 Myo5b	1
GO:0090398 cellular senescence	1/95	91/14464	0.01098901	1.67310584	0.52373074	0.45203396	0.5905059	0.56457683 Abl1	1
GO:1904019 epithelial cell apoptotic process	1/95	91/14464	0.01098901	1.67310584	0.52373074	0.45203396	0.5905059	0.56457683 Abl1	1
GO:2001257 regulation of cation channel activity	1/95	91/14464	0.01098901	1.67310584	0.52373074	0.45203396	0.5905059	0.56457683 Trem2	1
GO:0031400 negative regulation of protein modification process	3/95	378/14464	0.00793651	1.20835422	0.3337583	0.45378053	0.59198154	0.56598767 Inca1/Abl1/Lyn	3
GO:1990778 protein localization to cell periphery	3/95	378/14464	0.00793651	1.20835422	0.3337583	0.45378053	0.59198154	0.56598767 Trem2/Rab34/Myo5b	3
GO:2001233 regulation of apoptotic signaling pathway	3/95	379/14464	0.00791557	1.20516595	0.3290972	0.45549993	0.59241429	0.56640142 Trem2/Serpine1/Inca1	3
GO:0007173 epidermal growth factor receptor signaling pathway	1/95	92/14464	0.01086957	1.65491991	0.51239068	0.45565581	0.59241429	0.56640142 Abl1	1
GO:0015918 sterol transport	1/95	92/14464	0.01086957	1.65491991	0.51239068	0.45565581	0.59241429	0.56640142 Trem2	1
GO:0046889 positive regulation of lipid biosynthetic process	1/95	92/14464	0.01086957	1.65491991	0.51239068	0.45565581	0.59241429	0.56640142 Por	1
GO:0051899 membrane depolarization	1/95	92/14464	0.01086957	1.65491991	0.51239068	0.45565581	0.59241429	0.56640142 Clcn2	1
GO:0045333 cellular respiration	2/95	234/14464	0.00854701	1.30130454	0.37782343	0.45647323	0.59307524	0.56703335 ND5/Coq10b	2
GO:0010631 epithelial cell migration	1/95	93/14464	0.01075269	1.63712507	0.50118769	0.45925396	0.59387355	0.56779661 Serpine1	1
GO:0032612 interleukin-1 production	1/95	93/14464	0.01075269	1.63712507	0.50118769	0.45925396	0.59387355	0.56779661 Trem2	1
GO:0042116 macrophage activation	1/95	93/14464	0.01075269	1.63712507	0.50118769	0.45925396	0.59387355	0.56779661 Trem2	1
GO:0043280 positive regulation of cysteine-type endopeptidase activity involved in apoptotic process	1/95	93/14464	0.01075269	1.63712507	0.50118769	0.45925396	0.59387355	0.56779661 Acer2	1
GO:0090132 epithelium migration	1/95	93/14464	0.01075269	1.63712507	0.50118769	0.45925396	0.59387355	0.56779661 Serpine1	1
GO:1901874 negative regulation of post-translational protein modification	1/95	93/14464	0.01075269	1.63712507	0.50118769	0.45925396	0.59387355	0.56779661 Abl1	1
GO:1901879 regulation of protein depolymerization	1/95	93/14464	0.01075269	1.63712507	0.50118769	0.45925396	0.59387355	0.56779661 Nav3	1
GO:0001843 neural tube closure	1/95	94/14464	0.0106383	1.61970885	0.49011835	0.46282858	0.59516302	0.56902946 Abl1	1
GO:0007229 integrin-mediated signaling pathway	1/95	94/14464	0.0106383	1.61970885	0.49011835	0.46282858	0.59516302	0.56902946 Abl1	1
GO:0009308 amine metabolic process	1/95	94/14464	0.0106383	1.61970885	0.49011835	0.46282858	0.59516302	0.56902946 Inmt	1
GO:0010389 regulation of G2/M transition of mitotic cell cycle	1/95	94/14464	0.0106383	1.61970885	0.49011835	0.46282858	0.59516302	0.56902946 Wee1	1
GO:0051492 regulation of stress fiber assembly	1/95	94/14464	0.0106383	1.61970885	0.49011835	0.46282858	0.59516302	0.56902946 Abl1	1
GO:0060349 bone morphogenesis	1/95	94/14464	0.0106383	1.61970885	0.49011835	0.46282858	0.59516302	0.56902946 Por	1
GO:0060606 tube closure	1/95	94/14464	0.0106383	1.61970885	0.49011835	0.46282858	0.59516302	0.56902946 Abl1	1
GO:0007548 sex differentiation	2/95	237/14464	0.00843882	1.28483233	0.3594881	0.46304242	0.59516302	0.56902946 Fzd4/Serpine1	2
GO:0048738 cardiac muscle tissue development	2/95	237/14464	0.00843882	1.28483233	0.3594881	0.46304242	0.59516302	0.56902946 Abl1/Arid2	2

GO:0006520	amino acid metabolic process	2/95	238/14464	0.00840336	1.27943388	0.35343036	0.46522201	0.59745185	0.57121778	Pipox/Tha1	2
GO:0016055	Wnt signaling pathway	3/95	385/14464	0.00779221	1.18638414	0.3013916	0.46577001	0.59745185	0.57121778	Fzd4/Abl1/Celsr3	3
GO:0007044	cell-substrate junction assembly	1/95	95/14464	0.01052632	1.60265928	0.47917936	0.46637982	0.59745185	0.57121778	Abl1	1
GO:0045639	positive regulation of myeloid cell differentiation	1/95	95/14464	0.01052632	1.60265928	0.47917936	0.46637982	0.59745185	0.57121778	Trem2	1
GO:0070936	protein K48-linked ubiquitination	1/95	95/14464	0.01052632	1.60265928	0.47917936	0.46637982	0.59745185	0.57121778	Peli1	1
GO:0007626	locomotory behavior	2/95	240/14464	0.00833333	1.26877193	0.34139439	0.46956584	0.60113199	0.57473632	Ciart/Fzd4	2
GO:0003333	amino acid transmembrane transport	1/95	96/14464	0.01041667	1.58596491	0.46836754	0.46990783	0.60116874	0.57477147	Slc6a6	1
GO:0006979	response to oxidative stress	3/95	388/14464	0.00773196	1.17721107	0.28770327	0.47087432	0.60200387	0.57556992	Net1/ND5/Abl1	3
GO:0050772	positive regulation of axonogenesis	1/95	97/14464	0.01030928	1.56961476	0.45767983	0.47341275	0.60384662	0.57733175	Myo5b	1
GO:0090130	tissue migration	1/95	97/14464	0.01030928	1.56961476	0.45767983	0.47341275	0.60384662	0.57733175	Serpine1	1
GO:0098656	monoatomic anion transmembrane transport	1/95	97/14464	0.01030928	1.56961476	0.45767983	0.47341275	0.60384662	0.57733175	Clcn2	1
GO:0071560	cellular response to transforming growth factor beta stimulus	2/95	242/14464	0.00826446	1.25828621	0.32946273	0.47388901	0.60384662	0.57733175	Serpine1/Abl1	2
GO:0140014	mitotic nuclear division	2/95	242/14464	0.00826446	1.25828621	0.32946273	0.47388901	0.60384662	0.57733175	Xrcc3/Ncapd3	2
GO:0009205	purine ribonucleoside triphosphate metabolic process	2/95	243/14464	0.00823045	1.25310808	0.32353539	0.47604278	0.60446566	0.57792362	Trem2/ND5	2
GO:0034599	cellular response to oxidative stress	2/95	243/14464	0.00823045	1.25310808	0.32353539	0.47604278	0.60446566	0.57792362	Net1/Abl1	2
GO:0002718	regulation of cytokine production involved in immune response	1/95	98/14464	0.01020408	1.55359828	0.44711327	0.47689474	0.60446566	0.57792362	Gimap5	1
GO:0007040	lysosome organization	1/95	98/14464	0.01020408	1.55359828	0.44711327	0.47689474	0.60446566	0.57792362	Rab34	1
GO:0032204	regulation of telomere maintenance	1/95	98/14464	0.01020408	1.55359828	0.44711327	0.47689474	0.60446566	0.57792362	Ylpm1	1
GO:0036465	synaptic vesicle recycling	1/95	98/14464	0.01020408	1.55359828	0.44711327	0.47689474	0.60446566	0.57792362	Myo5b	1
GO:0080171	lytic vacuole organization	1/95	98/14464	0.01020408	1.55359828	0.44711327	0.47689474	0.60446566	0.57792362	Rab34	1
GO:1905954	positive regulation of lipid localization	1/95	98/14464	0.01020408	1.55359828	0.44711327	0.47689474	0.60446566	0.57792362	Trem2	1
GO:0060326	cell chemotaxis	2/95	245/14464	0.00816327	1.24287863	0.31175651	0.48033456	0.60684611	0.58019953	Serpine1/Lyn	2
GO:0002444	myeloid leukocyte mediated immunity	1/95	99/14464	0.01010101	1.53790537	0.436665	0.48035395	0.60684611	0.58019953	Lyn	1
GO:0014020	primary neural tube formation	1/95	99/14464	0.01010101	1.53790537	0.436665	0.48035395	0.60684611	0.58019953	Abl1	1
GO:0033108	mitochondrial respiratory chain complex assembly	1/95	99/14464	0.01010101	1.53790537	0.436665	0.48035395	0.60684611	0.58019953	ND5	1
GO:0061041	regulation of wound healing	1/95	99/14464	0.01010101	1.53790537	0.436665	0.48035395	0.60684611	0.58019953	Serpine1	1
GO:0007088	regulation of mitotic nuclear division	1/95	100/14464	0.01	1.52252632	0.42633226	0.48379053	0.61038397	0.58358205	Xrcc3	1
GO:0014910	regulation of smooth muscle cell migration	1/95	100/14464	0.01	1.52252632	0.42633226	0.48379053	0.61038397	0.58358205	Serpine1	1
GO:0006650	glycerophospholipid metabolic process	2/95	247/14464	0.00809717	1.23281483	0.3000771	0.48460517	0.61060888	0.58379709	Pnpla7/Gpcpd1	2
GO:0032984	protein-containing complex disassembly	2/95	247/14464	0.00809717	1.23281483	0.3000771	0.48460517	0.61060888	0.58379709	Nav3/Arid2	2
GO:0009199	ribonucleoside triphosphate metabolic process	2/95	248/14464	0.00806452	1.2278438	0.29427413	0.48673249	0.61187542	0.58500801	Trem2/ND5	2
GO:0007338	single fertilization	1/95	101/14464	0.00990099	1.5074518	0.41611237	0.48720461	0.61187542	0.58500801	Spaca6	1
GO:0035710	CD4-positive, alpha-beta T cell activation	1/95	101/14464	0.00990099	1.5074518	0.41611237	0.48720461	0.61187542	0.58500801	Gimap5	1
GO:0050852	T cell receptor signaling pathway	1/95	101/14464	0.00990099	1.5074518	0.41611237	0.48720461	0.61187542	0.58500801	Abl1	1
GO:0097306	cellular response to alcohol	1/95	101/14464	0.00990099	1.5074518	0.41611237	0.48720461	0.61187542	0.58500801	Klf9	1
GO:0033002	muscle cell proliferation	2/95	249/14464	0.00803213	1.2229127	0.28849533	0.48885444	0.61354589	0.58660513	Abl1/Arid2	2
GO:0001909	leukocyte mediated cytotoxicity	1/95	102/14464	0.00980392	1.49267286	0.40600276	0.49059635	0.61372516	0.58677653	Gimap5	1
GO:0010595	positive regulation of endothelial cell migration	1/95	102/14464	0.00980392	1.49267286	0.40600276	0.49059635	0.61372516	0.58677653	Abl1	1
GO:0042476	odontogenesis	1/95	102/14464	0.00980392	1.49267286	0.40600276	0.49059635	0.61372516	0.58677653	Serpine1	1
GO:0099068	postsynapse assembly	1/95	102/14464	0.00980392	1.49267286	0.40600276	0.49059635	0.61372516	0.58677653	Abl1	1
GO:0150115	cell-substrate junction organization	1/95	102/14464	0.00980392	1.49267286	0.40600276	0.49059635	0.61372516	0.58677653	Abl1	1
GO:0022411	cellular component disassembly	3/95	400/14464	0.0075	1.14189474	0.234002	0.49107396	0.61392243	0.58696513	Trem2/Nav3/Arid2	3
GO:0009144	purine nucleoside triphosphate metabolic process	2/95	251/14464	0.00796813	1.21316838	0.27700939	0.49308217	0.61553282	0.58850482	Trem2/ND5	2
GO:0006892	post-Golgi vesicle-mediated transport	1/95	103/14464	0.00970874	1.47818089	0.39600093	0.49396589	0.61553282	0.58850482	Rab34	1
GO:0042303	molting cycle	1/95	103/14464	0.00970874	1.47818089	0.39600093	0.49396589	0.61553282	0.58850482	Per1	1
GO:0042633	hair cycle	1/95	103/14464	0.00970874	1.47818089	0.39600093	0.49396589	0.61553282	0.58850482	Per1	1
GO:0140029	exocytic process	1/95	103/14464	0.00970874	1.47818089	0.39600093	0.49396589	0.61553282	0.58850482	Lyn	1
GO:0031330	negative regulation of cellular catabolic process	1/95	104/14464	0.00961538	1.46396761	0.38610448	0.49731338	0.61849927	0.59134101	Trem2	1
GO:0098754	detoxification	1/95	104/14464	0.00961538	1.46396761	0.38610448	0.49731338	0.61849927	0.59134101	Mt1	1
GO:1901655	cellular response to ketone	1/95	104/14464	0.00961538	1.46396761	0.38610448	0.49731338	0.61849927	0.59134101	Klf9	1
GO:0048588	developmental cell growth	2/95	254/14464	0.00787402	1.19883962	0.25995637	0.49938304	0.61902458	0.59184325	Myo5b/Abl1	2
GO:0060560	developmental growth involved in morphogenesis	2/95	254/14464	0.00787402	1.19883962	0.25995637	0.49938304	0.61902458	0.59184325	Myo5b/Abl1	2
GO:0002377	immunoglobulin production	1/95	105/14464	0.00952381	1.45002506	0.37631105	0.50063895	0.61902458	0.59184325	Paxip1	1
GO:0019395	fatty acid oxidation	1/95	105/14464	0.00952381	1.45002506	0.37631105	0.50063895	0.61902458	0.59184325	Por	1
GO:0022904	respiratory electron transport chain	1/95	105/14464	0.00952381	1.45002506	0.37631105	0.50063895	0.61902458	0.59184325	ND5	1
GO:0045445	myoblast differentiation	1/95	105/14464	0.00952381	1.45002506	0.37631105	0.50063895	0.61902458	0.59184325	Arid2	1
GO:0051092	positive regulation of NF-kappaB transcription factor activity	1/95	105/14464	0.00952381	1.45002506	0.37631105	0.50063895	0.61902458	0.59184325	Rps6ka5	1
GO:0110020	regulation of actomyosin structure organization	1/95	105/14464	0.00952381	1.45002506	0.37631105	0.50063895	0.61902458	0.59184325	Abl1	1
GO:1901800	positive regulation of proteasomal protein catabolic process	1/95	105/14464	0.00952381	1.45002506	0.37631105	0.50063895	0.61902458	0.59184325	Trem2	1
GO:0050878	regulation of body fluid levels	2/95	256/14464	0.0078125	1.18947368	0.24870249	0.50355623	0.62110863	0.59383579	Serpine1/Lyn	2
GO:0090150	establishment of protein localization to membrane	2/95	256/14464	0.0078125	1.18947368	0.24870249	0.50355623	0.62110863	0.59383579	Rab34/Myo5b	2
GO:0016525	negative regulation of angiogenesis	1/95	106/14464	0.00943396	1.43634558	0.3666184	0.50394275	0.62110863	0.59383579	Serpine1	1
GO:0048024	regulation of mRNA splicing, via spliceosome	1/95	106/14464	0.00943396	1.43634558	0.3666184	0.50394275	0.62110863	0.59383579	Rbmx	1
GO:0060840	artery development	1/95	106/14464	0.00943396	1.43634558	0.3666184	0.50394275	0.62110863	0.59383579	Arid2	1
GO:0002062	chondrocyte differentiation	1/95	107/14464	0.00934579	1.42292179	0.35702434	0.50722492	0.62355197	0.59617185	Por	1
GO:0008306	associative learning	1/95	107/14464	0.00934579	1.42292179	0.35702434	0.50722492	0.62355197	0.59617185	Abl1	1
GO:0042274	ribosomal small subunit biogenesis	1/95	107/14464	0.00934579	1.42292179	0.35702434	0.50722492	0.62355197	0.59617185	Wdr46	1
GO:0097250	mitochondrial respirasome assembly	1/95	107/14464	0.00934579	1.42292179	0.35702434	0.50722492	0.62355197	0.59617185	ND5	1
GO:0032231	regulation of actin filament bundle assembly	1/95	108/14464	0.00925926	1.40974659	0.34752674	0.51048561	0.62555676	0.5980886	Abl1	1
GO:0050671	positive regulation of lymphocyte proliferation	1/95	108/14464	0.00925926	1.40974659	0.34752674	0.51048561	0.62555676	0.5980886	Peli1	1
GO:0120034	positive regulation of plasma membrane bounded cell projection assembly	1/95	108/14464	0.00925926	1.40974659	0.34752674	0.51048561	0.62555676	0.5980886	Agrn	1
GO:2000181	negative regulation of blood vessel morphogenesis	1/95	108/14464	0.00925926	1.40974659	0.34752674	0.51048561	0.62555676	0.5980886	Serpine1	1
GO:2001056	positive regulation of cysteine-type endopeptidase activity	1/95	108/14464	0.00925926	1.40974659	0.34752674	0.51048561	0.62555676	0.5980886	Acer2	1
GO:0034440	lipid oxidation	1/95	109/14464	0.00917431	1.39681313	0.33812354	0.51372494	0.6279224	0.60035037	Por	1
GO:0038127	ERBB signaling pathway	1/95	109/14464	0.00917431	1.39681313	0.33812354	0.51372494	0.6279224	0.60035037	Abl1	1
GO:0046683	response to organophosphorus	1/95	109/14464	0.00917431	1.39681313	0.33812354	0.51372494	0.6279224	0.60035037	Per1	1
GO:1901343	negative regulation of vasculature development	1/95	109/14464	0.00917431	1.39681313	0.33812354	0.51372494	0.6279224	0.60035037	Serpine1	1
GO:0051222	positive regulation of protein transport	2/95	262/14464	0.00763359	1.16223383	0.21547408	0.51594273	0.63023177	0.60255833	Trem2/Rab34	2
GO:0030048	actin filament-based movement	1/95	110/14464	0.00909091	1.38411483	0.32881277	0.51694306	0.63065082	0.60295898	Myo5b	1
GO:0045446	endothelial cell differentiation	1/95	110/14464	0.00909091	1.38411483	0.32881277	0.51694306	0.63065082	0.60295898	Fzd4	1
GO:0050708	regulation of protein secretion	2/95	263/14464	0.00760456	1.15781469	0.21001173	0.51798757	0.63134021	0.6036181	Trem2/Rab34	2
GO:0007006	mitochondrial membrane organization	1/95	111/14464	0.00900901	1.37164533	0.31959248	0.52014011	0.63134021	0.6036181	Gimap5	1
GO:0014909	smooth muscle cell migration	1/95	111/14464	0.00900901	1.37164533	0.31959248	0.52014011	0.63134021	0.6036181	Serpine1	1
GO:0030038	contractile actin filament bundle assembly	1/95	111/14464	0.00900901	1.37164533	0.31959248	0.52014011	0.63134021	0.6036181	Abl1	1
GO:0032946	positive regulation of mononuclear cell proliferation	1/95	111/14464	0.00900901	1.37164533	0.31959248	0.52014011	0.63134021	0.6036181	Peli1	1
GO:0043149	stress fiber assembly	1/95	111/14464	0.00900901	1.37164533	0.31959248	0.52014011	0.63134021	0.6036181	Abl1	1
GO:0060291	long-term synaptic potentiation	1/95	111/14464	0.00900901	1.37164533	0.31959248	0.52014011	0.63134021	0.6036181	Abl1	1
GO:2000177	regulation of neural precursor cell proliferation	1/95	111/14464	0.00900901	1.37164533	0.31959248	0.52014011	0.63134021	0.6036181	Lyn	1
GO:0009141	nucleoside triphosphate metabolic process	2/95	265/14464	0.00754717	1.14907646	0.19915025	0.5220603	0.63247078	0.60469903	Trem2/ND5	2
GO:0032868	response to insulin	2/95	265/14464	0.00754717	1.14907646	0.19915025	0.5220603	0.63247078	0.60469903	Tns2/Lyn	2
GO:0042593	glucose homeostasis	2/95	265/14464	0.00754717	1.14907646	0.19915025	0.5220603	0.63247078	0.60469903	Trem2/Rab34	2
GO:0009791	post-embryonic development	1/95	112/14464	0.00892857	1.3593985	0.3104608	0.52331622	0.63319283	0.60538937	Abl1	1
GO:0071887	leukocyte apoptotic process	1/95	112/14464	0.00892857	1.3593985	0.3104608	0.52331622	0.63319283	0.60538937	Lyn	1
GO:0007264	small GTPase-mediated signal transduction	3/95	420/14464	0.00714286	1.0875188	0.14799884	0.5238988	0.63332822	0.60551882	Net1/Abl1/Agrn	3
GO:0033500	carbohydrate homeostasis	2/95	266/14464	0.0075188	1.14475663	0.19375076	0.52408818	0.63332822	0.60551882	Trem2/Rab34	2
GO:2001252	positive regulation of chromosome organization	1/95	113/14464	0.00884956	1.34736842	0.30141593	0.52647152	0.63580796	0.60788968	Ncapd3	1
GO:0045785	positive regulation of cell adhesion	3/95	422/14464	0.007109	1.08236468	0.13962413	0.5271194	0.63619001	0.60825495	Gimap5/Abl1/Arid2	3
GO:0022604	regulation of cell morphogenesis	2/95	268/14464	0.00746269	1.13621367	0.18301337	0.52812687	0.63660519	0.6086519	Fzd4/Myo5b	2
GO:0048608	reproductive structure development	2/95	268/14464	0.00746269	1.13621367	0.18301337	0.52812687	0.63660519	0.6086519	Fzd4/Serpine1	2
GO:0000077	DNA damage checkpoint signaling	1/95	114/14464	0.00877193	1.3355494	0.29245609	0.52960616	0.63678836	0.60882703	Lyn	1
GO:0007259	cell surface receptor signaling pathway via JAK-STAT	1/95	114/14464	0.00877193	1.3355494	0.29245609	0.52960616	0.63678836	0.60882703	Il15ra	1
GO:0060048	cardiac muscle contraction	1/95	114/14464	0.00877193	1.3355494	0.29245609	0.52960616	0.63678836	0.60882703	Agrn	1
GO:1990830	cellular response to leukemia inhibitory factor	1/95	114/14464	0.00877193	1.3355494	0.29245609	0.52960616	0.63678836	0.60882703	Fzd4	1
GO:0045765	regulation of angiogenesis	2/95	270/14464	0.00740741	1.12779727	0.17235694	0.53214274	0.63943764	0.61135998	Serpine1/Abl1	2
GO:0051053	negative regulation of DNA metabolic process	1/95	115/14464	0.00869565	1.32393593	0.28357957	0.53272027	0.63973104	0.61164049	Abl1	1
GO:0034763	negative regulation of transmembrane transport	1/95	116/14464	0.00862069	1.31252269	0.27478472	0.53581397	0.64222494	0.61402489	Agrn	1
GO:1990823	response to leukemia inhibitory factor	1/95	116/14464	0.00862069	1.31252269	0.27478472	0.53581397	0.64222494	0.61402489	Fzd4	1
GO:0061448	connective tissue development	2/95	272/14464	0.00735294	1.11950464	0.16178012	0.53613567	0.64222494	0.61402489	Por/Paxip1	2
GO:0061458	reproductive system development	2/95	272/14464	0.00735294	1.11950464	0.16178012	0.53613567	0.64222494	0.61402489	Fzd4/Serpine1	2
GO:0000375	RNA splicing, via transesterification reactions	2/95	273/14464	0.00732601	1.11540389	0.15652114	0.53812351	0.64271283	0.61449135	Srrm2/Rbmx	2
GO:0000377	RNA splicing, via transesterification reactions with bulged adenosine as nucleophile	2/95	273/14464	0.00732601	1.11540389	0.15652114	0.53812351	0.64271283	0.61449135	Srrm2/Rbmx	2
GO:0000398	mRNA splicing, via spliceosome	2/95	273/14464	0.00732601	1.11540389	0.15652114	0.53812351	0.64271283	0.61449135	Srrm2/Rbmx	2
GO:0006820	monoatomic anion transport	1/95	117/14464	0.00854701	1.30130454	0.26606992	0.53888741	0.64271283	0.61449135	Clcn2	1
GO:0015695	organic cation transport	1/95	117/14464	0.00854701	1.30130454	0.26606992	0.53888741	0.64271283	0.61449135	Slc6a6	1
GO:0015698	inorganic anion transport	1/95	117/14464	0.00854701	1.30130454	0.26606992	0.53888741	0.64271283	0.61449135	Clcn2	1
GO:0043534	blood vessel endothelial cell migration	1/95	117/14464	0.00854701	1.30130454	0.26606992	0.53888741	0.64271283	0.61449135	Abl1	1
GO:0002367	cytokine production involved in immune response	1/95	118/14464	0.00847458	1.29027654	0.25743361	0.54194071	0.64555197	0.61720583	Gimap5	1
GO:0043271	negative regulation of monoatomic ion transport	1/95	118/14464	0.00847458	1.29027654	0.25743361	0.54194071	0.64555197	0.61720583	Agrn	1
GO:0006888	endoplasmic reticulum to Golgi vesicle-mediated transport	1/95	119/14464	0.00840336	1.27943388	0.24887426	0.544974	0.64795856	0.61950674	Sar1b	1
GO:0030278	regulation of ossification	1/95	119/14464	0.00840336	1.27943388	0.24887426	0.544974	0.64795856	0.61950674	Gdf10	1
GO:1903313	positive regulation of mRNA metabolic process	1/95	119/14464	0.00840336	1.27943388	0.24887426	0.544974	0.64795856	0.61950674	Rbmx	1
GO:1901342	regulation of vasculature development	2/95	277/14464	0.00722022	1.09929698	0.13567766	0.5460171	0.64879679	0.62030817	Serpine1/Abl1	2
GO:0006874	intracellular calcium ion homeostasis	2/95	278/14464	0.00719424	1.09534267	0.13051411	0.547976	0.64993069	0.62139228	Abl1/Lyn	2
GO:0090287	regulation of cellular response to growth factor stimulus	2/95	278/14464	0.00719424	1.09534267	0.13051411	0.547976	0.64993069	0.62139228	Fzd4/Abl1	2
GO:0051783	regulation of nuclear division	1/95	120/14464	0.00833333	1.26877193	0.2403904	0.54798742	0.64993069	0.62139228	Xrcc3	1
GO:0030509	BMP signaling pathway	1/95	121/14464	0.00826446	1.25828621	0.23198058	0.55098109	0.65186974	0.62324618	Abl1	1
GO:0031570	DNA integrity checkpoint signaling	1/95	121/14464	0.00826446	1.25828621	0.23198058	0.55098109	0.65186974	0.62324618	Lyn	1
GO:0046467	membrane lipid biosynthetic process	1/95	121/14464	0.00826446	1.25828621	0.23198058	0.55098109	0.65186974	0.62324618	Acer2	1
GO:0051261	protein depolymerization	1/95	121/14464	0.00826446	1.25828621	0.23198058	0.55098109	0.65186974	0.62324618	Nav3	1
GO:0046942	carboxylic acid transport	2/95	281/14464	0.00711744	1.08364862	0.11513454	0.55381777	0.65458123	0.62583861	Slc6a6/Lyn	2
GO:0045834	positive regulation of lipid metabolic process	1/95	122/14464	0.00819672	1.24797239	0.22364341	0.55395514	0.65458123	0.62583861	Por	1
GO:0007416	synapse assembly	2/95	283/14464	0.00706714	1.07599033	0.10497257	0.55768309	0.6581758	0.62927535	Abl1/Agrn	2
GO:0015849	organic acid transport	2/95	283/14464	0.00706714	1.07599033	0.10497257	0.55768309	0.6581758	0.62927535	Slc6a6/Lyn	2
GO:0048193	Golgi vesicle transport	2/95	284/14464	0.00704225	1.07220163	0.09991847	0.55960697	0.658298	0.62939218	Sar1b/Rab34	2
GO:0002040	sprouting angiogenesis	1/95	124/14464	0.00806452	1.2278438	0.20718161	0.55984489	0.658298	0.62939218	Abl1	1
GO:0008286	insulin receptor signaling pathway	1/95	124/14464	0.00806452	1.2278438	0.20718161	0.55984489	0.658298	0.62939218	Tns2	1
GO:0045667	regulation of osteoblast differentiation	1/95	124/14464	0.00806452	1.2278438	0.20718161	0.55984489	0.658298	0.62939218	Gdf10	1
GO:0097696	cell surface receptor signaling pathway via STAT	1/95	124/14464	0.00806452	1.2278438	0.20718161	0.55984489	0.658298	0.62939218	Il15ra	1
GO:1903052	positive regulation of proteolysis involved in protein catabolic process	1/95	124/14464	0.00806452	1.2278438	0.20718161	0.55984489	0.658298	0.62939218	Trem2	1
GO:0006790	sulfur compound metabolic process	2/95	285/14464	0.00701754	1.06843952	0.0948821	0.56152498	0.65986922	0.63089441	Pipox/Gsta4	2
GO:1901293	nucleoside phosphate biosynthetic process	2/95	286/14464	0.00699301	1.06470372	0.08986333	0.56343713	0.66171104	0.63265536	Trem2/ND5	2
GO:0010466	negative regulation of peptidase activity	1/95	126/14464	0.00793651	1.20835422	0.19099456	0.56565768	0.66269663	0.63359767	Serpine1	1
GO:0010811	positive regulation of cell-substrate adhesion	1/95	126/14464	0.00793651	1.20835422	0.19099456	0.56565768	0.66269663	0.63359767	Abl1	1
GO:0015718	monocarboxylic acid transport	1/95	126/14464	0.00793651	1.20835422	0.19099456	0.56565768	0.66269663	0.63359767	Lyn	1
GO:0090101	negative regulation of transmembrane receptor protein serine/threonine kinase signaling pathway	1/95	126/14464	0.00793651	1.20835422	0.19099456	0.56565768	0.66269663	0.63359767	Abl1	1
GO:0030003	intracellular monoatomic cation homeostasis	3/95	447/14464	0.00671141	1.02182974	0.03811997	0.56635578	0.66310966	0.63399256	Mt1/Abl1/Lyn	3
GO:1901888	regulation of cell junction assembly	2/95	288/14464	0.00694444	1.05730994	0.07987798	0.56724377	0.66374439	0.63459942	Abl1/Agrn	2
GO:0007041	lysosomal transport	1/95	127/14464	0.00787402	1.19883962	0.18300096	0.56853552	0.66404118	0.63488318	Rab34	1
GO:0010565	regulation of cellular ketone metabolic process	1/95	127/14464	0.00787402	1.19883962	0.18300096	0.56853552	0.66404118	0.63488318	Clcn2	1
GO:0030177	positive regulation of Wnt signaling pathway	1/95	127/14464	0.00787402	1.19883962	0.18300096	0.56853552	0.66404118	0.63488318	Abl1	1
GO:0006091	generation of precursor metabolites and energy	3/95	449/14464	0.00668151	1.01727816	0.0302414	0.56941003	0.66465806	0.63547297	Por/ND5/Coq10b	3
GO:1014074	response to purine-containing compound	1/95	128/14464	0.0078125	1.18947368	0.17507237	0.57139449	0.66575958	0.63652613	Per1	1
GO:0030856	regulation of epithelial cell differentiation	1/95	128/14464	0.0078125	1.18947368	0.17507237	0.57139449	0.66575958	0.63652613	Serpine1	1
GO:0046328	regulation of JNK cascade	1/95	128/14464	0.0078125	1.18947368	0.17507237	0.57139449	0.66575958	0.63652613	Per1	1
GO:0006665	sphingolipid metabolic process	1/95	129/14464	0.00775194	1.18025296	0.16720764	0.57423472	0.66744787	0.63814028	Acer2	1
GO:0030183	B cell differentiation	1/95	129/14464	0.00775194	1.18025296	0.16720764	0.57423472	0.66744787	0.63814028	Abl1	1
GO:0048259	regulation of receptor-mediated endocytosis	1/95	129/14464	0.00775194	1.18025296	0.16720764	0.57423472	0.66744787	0.63814028	Serpine1	1
GO:0050684	regulation of mRNA processing	1/95	129/14464	0.00775194	1.18025296	0.16720764	0.57423472	0.66744787	0.63814028	Rbmx	1
GO:0006873	intracellular monoatomic ion homeostasis	3/95	454/14464	0.00660793	1.00607466	0.01069322	0.57698892	0.6691064	0.63972598	Mt1/Abl1/Lyn	3
GO:0098771	inorganic ion homeostasis	3/95	454/14464	0.00660793	1.00607466	0.01069322	0.57698892	0.6691064	0.63972598	Mt1/Abl1/Lyn	3
GO:0006906	vesicle fusion	1/95	130/14464	0.00769231	1.17117409	0.15940566	0.57705633	0.6691064	0.63972598	Rab34	1
GO:0051592	response to calcium ion	1/95	130/14464	0.00769231	1.17117409	0.15940566	0.57705633	0.6691064	0.63972598	Cpne7	1
GO:0009566	fertilization	1/95	131/14464	0.00763359	1.16223383	0.1516653	0.57985943	0.6705308	0.64108783	Spaca6	1
GO:0043244	regulation of protein-containing complex disassembly	1/95	131/14464	0.00763359	1.16223383	0.1516653	0.57985943	0.6705308	0.64108783	Nav3	1
GO:0071772	response to BMP	1/95	131/14464	0.00763359	1.16223383	0.1516653	0.57985943	0.6705308	0.64108783	Abl1	1
GO:0071773	cellular response to BMP stimulus	1/95	131/14464	0.00763359	1.16223383	0.1516653	0.57985943	0.6705308	0.64108783	Abl1	1

GO:0010506	regulation of autophagy	2/95	295/14464	0.00677966	1.03222123	0.04546333	0.58038127	0.6705308	0.64108783	Trem2/Acer2	2
GO:0055074	calcium ion homeostasis	2/95	295/14464	0.00677966	1.03222123	0.04546333	0.58038127	0.6705308	0.64108783	Abl1/Lyn	2
GO:0044282	small molecule catabolic process	2/95	296/14464	0.00675676	1.028734	0.04061303	0.5822344	0.67226704	0.64274784	Pipox/Tha1	2
GO:0090174	organelle membrane fusion	1/95	132/14464	0.00757576	1.15342903	0.14398552	0.58264415	0.67233561	0.6428134	Rab34	1
GO:0032675	regulation of interleukin-6 production	1/95	133/14464	0.0075188	1.14475663	0.13636526	0.5854106	0.67512196	0.6454774	Trem2	1
GO:0001959	regulation of cytokine-mediated signaling pathway	1/95	134/14464	0.00746269	1.13621367	0.12880352	0.58815891	0.67707075	0.64734062	Trem2	1
GO:0003158	endothelium development	1/95	134/14464	0.00746269	1.13621367	0.12880352	0.58815891	0.67707075	0.64734062	Fzd4	1
GO:0060996	dendritic spine development	1/95	134/14464	0.00746269	1.13621367	0.12880352	0.58815891	0.67707075	0.64734062	Myo5b	1
GO:0008277	regulation of G protein-coupled receptor signaling pathway	1/95	135/14464	0.00740741	1.12779727	0.12129929	0.59088919	0.67959164	0.64975081	Trem2	1
GO:0071214	cellular response to abiotic stimulus	2/95	301/14464	0.00664452	1.01164539	0.01660112	0.59141117	0.67959164	0.64975081	Net1/Clcn2	2
GO:0104004	cellular response to environmental stimulus	2/95	301/14464	0.00664452	1.01164539	0.01660112	0.59141117	0.67959164	0.64975081	Net1/Clcn2	2
GO:0007093	mitotic cell cycle checkpoint signaling	1/95	136/14464	0.00735294	1.11950464	0.11385161	0.59360155	0.68088547	0.65098783	Xrcc3	1
GO:0007565	female pregnancy	1/95	136/14464	0.00735294	1.11950464	0.11385161	0.59360155	0.68088547	0.65098783	Klf9	1
GO:1903825	organic acid transmembrane transport	1/95	136/14464	0.00735294	1.11950464	0.11385161	0.59360155	0.68088547	0.65098783	Slc6a6	1
GO:0007005	mitochondrion organization	3/95	466/14464	0.00643777	0.98016716	-0.0353853	0.59484222	0.68190097	0.65195875	Gimap5/Tspan4/ND5	3
GO:0006470	protein dephosphorylation	1/95	137/14464	0.00729927	1.11133308	0.10645954	0.59629613	0.68275195	0.65277236	Tns2	1
GO:1902600	proton transmembrane transport	1/95	137/14464	0.00729927	1.11133308	0.10645954	0.59629613	0.68275195	0.65277236	ND5	1
GO:0006119	oxidative phosphorylation	1/95	138/14464	0.00724638	1.10327994	0.09912216	0.59897302	0.68459156	0.65453119	ND5	1
GO:0050768	negative regulation of neurogenesis	1/95	138/14464	0.00724638	1.10327994	0.09912216	0.59897302	0.68459156	0.65453119	Trem2	1
GO:0051606	detection of stimulus	1/95	138/14464	0.00724638	1.10327994	0.09912216	0.59897302	0.68459156	0.65453119	Trem2	1
GO:0006997	nucleus organization	1/95	139/14464	0.00719424	1.09534267	0.09183857	0.60163235	0.6868129	0.65665499	Cdan1	1
GO:0032635	interleukin-6 production	1/95	139/14464	0.00719424	1.09534267	0.09183857	0.60163235	0.6868129	0.65665499	Trem2	1
GO:0007611	learning or memory	2/95	307/14464	0.00651466	0.99187382	-0.0117017	0.60222743	0.68708349	0.6569137	Trem2/Abl1	2
GO:0051098	regulation of binding	2/95	308/14464	0.00649351	0.98865345	-0.0163662	0.60400935	0.68859991	0.65836354	Abl1/Agrn	2
GO:0071456	cellular response to hypoxia	1/95	140/14464	0.00714286	1.0875188	0.0846079	0.60427423	0.68859991	0.65836354	Trem2	1
GO:0006261	DNA-templated DNA replication	1/95	141/14464	0.0070922	1.0798059	0.07742929	0.60689878	0.69046174	0.66014362	Pole	1
GO:0031644	regulation of nervous system process	1/95	141/14464	0.0070922	1.0798059	0.07742929	0.60689878	0.69046174	0.66014362	Jam2	1
GO:0009150	purine ribonucleotide metabolic process	2/95	311/14464	0.00643087	0.9791166	-0.0302716	0.60931947	0.69046174	0.66014362	Trem2/ND5	2
GO:0010038	response to metal ion	2/95	311/14464	0.00643087	0.9791166	-0.0302716	0.60931947	0.69046174	0.66014362	Mt1/Cpne7	2
GO:0051146	striated muscle cell differentiation	2/95	311/14464	0.00643087	0.9791166	-0.0302716	0.60931947	0.69046174	0.66014362	Bhlhe41/Jam2	2
GO:0006937	regulation of muscle contraction	1/95	142/14464	0.00704225	1.07220163	0.0703019	0.60950609	0.69046174	0.66014362	Agrn	1
GO:0009913	epidermal cell differentiation	1/95	142/14464	0.00704225	1.07220163	0.0703019	0.60950609	0.69046174	0.66014362	Alg10b	1
GO:0050680	negative regulation of epithelial cell proliferation	1/95	142/14464	0.00704225	1.07220163	0.0703019	0.60950609	0.69046174	0.66014362	Klf9	1
GO:0140013	meiotic nuclear division	1/95	142/14464	0.00704225	1.07220163	0.0703019	0.60950609	0.69046174	0.66014362	Ncapd3	1
GO:1903034	regulation of response to wounding	1/95	142/14464	0.00704225	1.07220163	0.0703019	0.60950609	0.69046174	0.66014362	Serpine1	1
GO:0001890	placenta development	1/95	143/14464	0.00699301	1.06470372	0.06322492	0.6120963	0.69217018	0.66177704	Serpine1	1
GO:0042129	regulation of T cell proliferation	1/95	143/14464	0.00699301	1.06470372	0.06322492	0.6120963	0.69217018	0.66177704	Peli1	1
GO:1903305	regulation of regulated secretory pathway	1/95	143/14464	0.00699301	1.06470372	0.06322492	0.6120963	0.69217018	0.66177704	Lyn	1
GO:0046879	hormone secretion	2/95	314/14464	0.00636943	0.96976198	-0.0440471	0.61457614	0.6938534	0.66338634	Fzd4/Lyn	2
GO:0043281	regulation of cysteine-type endopeptidase activity involved in apoptotic process	1/95	144/14464	0.00694444	1.05730994	0.05619756	0.6146695	0.6938534	0.66338634	Acer2	1
GO:0051147	regulation of muscle cell differentiation	1/95	144/14464	0.00694444	1.05730994	0.05619756	0.6146695	0.6938534	0.66338634	Bhlhe41	1
GO:0060759	regulation of response to cytokine stimulus	1/95	145/14464	0.00689655	1.05001815	0.04921902	0.61722582	0.69632942	0.66575364	Trem2	1
GO:1904951	positive regulation of establishment of protein localization	2/95	316/14464	0.00632911	0.96362425	-0.0531601	0.61805091	0.69685059	0.66625193	Trem2/Rab34	2
GO:0007286	spermatid development	1/95	146/14464	0.00684932	1.04282624	0.04228857	0.61976535	0.69796344	0.66731591	Jam2	1
GO:2001235	positive regulation of apoptotic signaling pathway	1/95	146/14464	0.00684932	1.04282624	0.04228857	0.61976535	0.69796344	0.66731591	Inca1	1
GO:0051961	negative regulation of nervous system development	1/95	147/14464	0.00680272	1.03573219	0.03540544	0.62228821	0.69998304	0.66924683	Trem2	1
GO:2001236	regulation of extrinsic apoptotic signaling pathway	1/95	147/14464	0.00680272	1.03573219	0.03540544	0.62228821	0.69998304	0.66924683	Serpine1	1
GO:0006644	phospholipid metabolic process	2/95	319/14464	0.00626959	0.95456195	-0.0667254	0.62321859	0.70061891	0.66985478	Pnpla7/Gpcpd1	2
GO:0003002	regionalization	2/95	320/14464	0.00625	0.95157895	-0.0712198	0.6249293	0.70213076	0.67130024	Bhlhe40/Bhlhe41	2
GO:0009914	hormone transport	2/95	321/14464	0.00623053	0.94861453	-0.0757007	0.62663409	0.70313005	0.67225566	Fzd4/Lyn	2
GO:0042060	wound healing	2/95	321/14464	0.00623053	0.94861453	-0.0757007	0.62663409	0.70313005	0.67225566	Serpine1/Lyn	2
GO:0030218	erythrocyte differentiation	1/95	149/14464	0.00671141	1.02182974	0.02177829	0.62728434	0.70313005	0.67225566	Lyn	1
GO:0036294	cellular response to decreased oxygen levels	1/95	149/14464	0.00671141	1.02182974	0.02177829	0.62728434	0.70313005	0.67225566	Trem2	1
GO:0006941	striated muscle contraction	1/95	150/14464	0.00666667	1.01501754	0.01503286	0.62975783	0.70455696	0.67361991	Agrn	1
GO:0010977	negative regulation of neuron projection development	1/95	150/14464	0.00666667	1.01501754	0.01503286	0.62975783	0.70455696	0.67361991	Il15ra	1
GO:0048754	branching morphogenesis of an epithelial tube	1/95	150/14464	0.00666667	1.01501754	0.01503286	0.62975783	0.70455696	0.67361991	Abl1	1
GO:0060562	epithelial tube morphogenesis	2/95	323/14464	0.00619195	0.94274075	-0.0846222	0.63002592	0.70455696	0.67361991	Abl1/Brpf1	2
GO:0031647	regulation of protein stability	2/95	324/14464	0.00617284	0.93983106	-0.0890631	0.63171296	0.70603214	0.67503031	Per3/Lss	2
GO:2000045	regulation of G1/S transition of mitotic cell cycle	1/95	151/14464	0.00662252	1.00829557	0.00833196	0.63221507	0.70618203	0.67517362	Arid2	1
GO:0046486	glycerolipid metabolic process	2/95	325/14464	0.00615385	0.93693927	-0.0934908	0.6333941	0.70708742	0.67603926	Pnpla7/Gpcpd1	2
GO:0044703	multi-organism reproductive process	1/95	152/14464	0.00657895	1.00166205	0.00167492	0.63465618	0.70808442	0.67699248	Klf9	1
GO:0031333	negative regulation of protein-containing complex assembly	1/95	153/14464	0.00653595	0.99511524	-0.0049389	0.63708125	0.70996453	0.67879004	Trem2	1
GO:0044706	multi-multicellular organism process	1/95	153/14464	0.00653595	0.99511524	-0.0049389	0.63708125	0.70996453	0.67879004	Klf9	1
GO:0006575	cellular modified amino acid metabolic process	1/95	154/14464	0.00649351	0.98865345	-0.0115102	0.6394904	0.71058603	0.67938425	Gsta4	1
GO:0035150	regulation of tube size	1/95	154/14464	0.00649351	0.98865345	-0.0115102	0.6394904	0.71058603	0.67938425	Abl1	1
GO:0035296	regulation of tube diameter	1/95	154/14464	0.00649351	0.98865345	-0.0115102	0.6394904	0.71058603	0.67938425	Abl1	1
GO:0048515	spermatid differentiation	1/95	154/14464	0.00649351	0.98865345	-0.0115102	0.6394904	0.71058603	0.67938425	Jam2	1
GO:0097746	blood vessel diameter maintenance	1/95	154/14464	0.00649351	0.98865345	-0.0115102	0.6394904	0.71058603	0.67938425	Abl1	1
GO:0009895	negative regulation of catabolic process	2/95	330/14464	0.00606061	0.92274322	-0.1154353	0.64171133	0.71159726	0.68035107	Trem2/Gimap5	2
GO:0015980	energy derivation by oxidation of organic compounds	2/95	330/14464	0.00606061	0.92274322	-0.1154353	0.64171133	0.71159726	0.68035107	ND5/Coq10b	2
GO:0001508	action potential	1/95	155/14464	0.00645161	0.98227504	-0.0180395	0.64188372	0.71159726	0.68035107	Clcn2	1
GO:0050777	negative regulation of immune response	1/95	155/14464	0.00645161	0.98227504	-0.0180395	0.64188372	0.71159726	0.68035107	Trem2	1
GO:0001906	cell killing	1/95	156/14464	0.00641026	0.97597841	-0.0245274	0.64426132	0.71299739	0.68168972	Gimap5	1
GO:0007254	JNK cascade	1/95	156/14464	0.00641026	0.97597841	-0.0245274	0.64426132	0.71299739	0.68168972	Per1	1
GO:0007613	memory	1/95	156/14464	0.00641026	0.97597841	-0.0245274	0.64426132	0.71299739	0.68168972	Trem2	1
GO:0009259	ribonucleotide metabolic process	2/95	332/14464	0.0060241	0.91718453	-0.1241242	0.64499703	0.71340017	0.68207482	Trem2/ND5	2
GO:0071478	cellular response to radiation	1/95	157/14464	0.00636943	0.96976198	-0.0309747	0.64662329	0.71478692	0.68340068	Net1	1
GO:0006865	amino acid transport	1/95	158/14464	0.00632911	0.96362425	-0.0373817	0.64896975	0.71696774	0.68548573	Slc6a6	1
GO:0010594	regulation of endothelial cell migration	1/95	159/14464	0.00628931	0.95756372	-0.0437492	0.65130079	0.71830243	0.68676182	Abl1	1
GO:0030041	actin filament polymerization	1/95	159/14464	0.00628931	0.95756372	-0.0437492	0.65130079	0.71830243	0.68676182	Abl1	1
GO:0071897	DNA biosynthetic process	1/95	159/14464	0.00628931	0.95756372	-0.0437492	0.65130079	0.71830243	0.68676182	Pole	1
GO:0032680	regulation of tumor necrosis factor production	1/95	161/14464	0.00621118	0.94566852	-0.0563675	0.65591702	0.72256301	0.69083532	Trem2	1
GO:0051897	positive regulation of phosphatidylinositol 3-kinase/protein kinase B signal transduction	1/95	161/14464	0.00621118	0.94566852	-0.0563675	0.65591702	0.72256301	0.69083532	Trem2	1
GO:0019693	ribose phosphate metabolic process	2/95	339/14464	0.00589971	0.89824561	-0.1541475	0.65631235	0.72258371	0.69085511	Trem2/ND5	2
GO:0007034	vacuolar transport	1/95	162/14464	0.00617284	0.93983106	-0.0626194	0.65820241	0.72341949	0.69165419	Rab34	1
GO:0007219	Notch signaling pathway	1/95	162/14464	0.00617284	0.93983106	-0.0626194	0.65820241	0.72341949	0.69165419	Tcim	1
GO:0034101	erythrocyte homeostasis	1/95	162/14464	0.00617284	0.93983106	-0.0626194	0.65820241	0.72341949	0.69165419	Lyn	1
GO:0015711	organic anion transport	2/95	341/14464	0.0058651	0.89297731	-0.1626175	0.65949276	0.72425557	0.69245356	Slc6a6/Lyn	2
GO:0051017	actin filament bundle assembly	1/95	163/14464	0.00613497	0.93406522	-0.068834	0.66047278	0.72425557	0.69245356	Abl1	1
GO:1903555	regulation of tumor necrosis factor superfamily cytokine production	1/95	163/14464	0.00613497	0.93406522	-0.068834	0.66047278	0.72425557	0.69245356	Trem2	1
GO:2000116	regulation of cysteine-type endopeptidase activity	1/95	163/14464	0.00613497	0.93406522	-0.068834	0.66047278	0.72425557	0.69245356	Acer2	1
GO:0050890	cognition	2/95	344/14464	0.00581395	0.88518972	-0.1752348	0.66421977	0.72793793	0.69597423	Trem2/Abl1	2
GO:0008016	regulation of heart contraction	1/95	165/14464	0.00606061	0.92274322	-0.0811528	0.66496883	0.72793793	0.69597423	Agrn	1
GO:0071453	cellular response to oxygen levels	1/95	165/14464	0.00606061	0.92274322	-0.0811528	0.66496883	0.72793793	0.69597423	Trem2	1
GO:0032640	tumor necrosis factor production	1/95	166/14464	0.0060241	0.91718453	-0.0872581	0.66719472	0.72912681	0.6971109	Trem2	1
GO:0061572	actin filament bundle organization	1/95	166/14464	0.0060241	0.91718453	-0.0872581	0.66719472	0.72912681	0.6971109	Abl1	1
GO:1903046	meiotic cell cycle process	1/95	166/14464	0.0060241	0.91718453	-0.0872581	0.66719472	0.72912681	0.6971109	Ncapd3	1
GO:0098739	import across plasma membrane	1/95	167/14464	0.00598802	0.9116924	-0.0933279	0.66940598	0.73112696	0.69902322	Slc6a6	1
GO:0071706	tumor necrosis factor superfamily cytokine production	1/95	168/14464	0.00595238	0.90626566	-0.0993628	0.67160269	0.73310897	0.7009182	Trem2	1
GO:0051170	import into nucleus	1/95	169/14464	0.00591716	0.90090315	-0.1053632	0.67378496	0.73507296	0.70279596	Cdan1	1
GO:0016050	vesicle organization	2/95	351/14464	0.00569801	0.86753636	-0.2042764	0.67504707	0.73603144	0.70371235	Sar1b/Rab34	2
GO:0006887	exocytosis	2/95	352/14464	0.00568182	0.86507177	-0.2083807	0.67657081	0.7368555	0.70450023	Myo5b/Lyn	2
GO:0009306	protein secretion	2/95	352/14464	0.00568182	0.86507177	-0.2083807	0.67657081	0.7368555	0.70450023	Trem2/Rab34	2
GO:0042552	myelination	1/95	171/14464	0.00584795	0.89036627	-0.1172623	0.67810655	0.73727278	0.70489919	Jam2	1
GO:0050770	regulation of axonogenesis	1/95	171/14464	0.00584795	0.89036627	-0.1172623	0.67810655	0.73727278	0.70489919	Myo5b	1
GO:0051494	negative regulation of cytoskeleton organization	1/95	171/14464	0.00584795	0.89036627	-0.1172623	0.67810655	0.73727278	0.70489919	Nav3	1
GO:0035592	establishment of protein localization to extracellular region	2/95	354/14464	0.00564972	0.86018436	-0.2165567	0.67960112	0.73834398	0.70592335	Trem2/Rab34	2
GO:0006643	membrane lipid metabolic process	1/95	172/14464	0.00581395	0.88518972	-0.1231619	0.68024605	0.73834398	0.70592335	Acer2	1
GO:2001242	regulation of intrinsic apoptotic signaling pathway	1/95	172/14464	0.00581395	0.88518972	-0.1231619	0.68024605	0.73834398	0.70592335	Trem2	1
GO:0010639	negative regulation of organelle organization	2/95	355/14464	0.0056338	0.8577613	-0.2206285	0.6811077	0.73886132	0.70641797	Nav3/Xrcc3	2
GO:0030307	positive regulation of cell growth	1/95	173/14464	0.00578035	0.88007301	-0.1290287	0.68237147	0.73917993	0.70672259	Myo5b	1
GO:0007389	pattern specification process	2/95	356/14464	0.00561798	0.85535186	-0.2246895	0.68260858	0.73917993	0.70672259	Bhlhe40/Bhlhe41	2
GO:0071692	protein localization to extracellular region	2/95	357/14464	0.00560224	0.85295592	-0.22874	0.68410376	0.73917993	0.70672259	Trem2/Rab34	2
GO:0001659	temperature homeostasis	1/95	174/14464	0.00574713	0.87501512	-0.1348633	0.68448292	0.73917993	0.70672259	Gimap5	1
GO:0007272	ensheathment of neurons	1/95	174/14464	0.00574713	0.87501512	-0.1348633	0.68448292	0.73917993	0.70672259	Jam2	1
GO:0007612	learning	1/95	174/14464	0.00574713	0.87501512	-0.1348633	0.68448292	0.73917993	0.70672259	Abl1	1
GO:0008366	axon ensheathment	1/95	174/14464	0.00574713	0.87501512	-0.1348633	0.68448292	0.73917993	0.70672259	Jam2	1
GO:0043484	regulation of RNA splicing	1/95	174/14464	0.00574713	0.87501512	-0.1348633	0.68448292	0.73917993	0.70672259	Rbmx	1
GO:0006839	mitochondrial transport	1/95	175/14464	0.00571429	0.87001504	-0.1406659	0.68658048	0.74102809	0.7084896	Gimap5	1
GO:0000280	nuclear division	2/95	360/14464	0.00555556	0.84584795	-0.2408281	0.68855524	0.74244196	0.70984138	Xrcc3/Ncapd3	2
GO:0048284	organelle fusion	1/95	176/14464	0.00568182	0.86507177	-0.1464371	0.68866424	0.74244196	0.70984138	Rab34	1
GO:0022618	protein-RNA complex assembly	1/95	177/14464	0.00564972	0.86018436	-0.1521772	0.69073429	0.74383789	0.71117602	Rbmx	1
GO:0061138	morphogenesis of a branching epithelium	1/95	177/14464	0.00564972	0.86018436	-0.1521772	0.69073429	0.74383789	0.71117602	Abl1	1
GO:0001764	neuron migration	1/95	178/14464	0.00561798	0.85535186	-0.1578866	0.69279073	0.74521603	0.71249365	Celsr3	1
GO:0030258	lipid modification	1/95	178/14464	0.00561798	0.85535186	-0.1578866	0.69279073	0.74521603	0.71249365	Por	1
GO:0051346	negative regulation of hydrolase activity	1/95	179/14464	0.00558659	0.85057336	-0.1635657	0.69483363	0.74699481	0.71419432	Serpine1	1
GO:0032535	regulation of cellular component size	2/95	365/14464	0.00547945	0.83426099	-0.2607683	0.69586137	0.74768084	0.71485022	Myo5b/Abl1	2
GO:0009060	aerobic respiration	1/95	180/14464	0.00555556	0.84584795	-0.1692148	0.69686309	0.74791961	0.71507851	ND5	1
GO:0071824	protein-DNA complex organization	1/95	180/14464	0.00555556	0.84584795	-0.1692148	0.69686309	0.74791961	0.71507851	Arid2	1
GO:0019722	calcium-mediated signaling	1/95	181/14464	0.00552486	0.84117476	-0.1748344	0.69887919	0.74924534	0.71634603	Trem2	1
GO:0048839	inner ear development	1/95	181/14464	0.00552486	0.84117476	-0.1748344	0.69887919	0.74924534	0.71634603	Alg10b	1
GO:0098742	cell-cell adhesion via plasma-membrane adhesion molecules	1/95	182/14464	0.00549451	0.83655292	-0.1804247	0.70088202	0.75097298	0.7179978	Celsr3	1
GO:0008154	actin polymerization or depolymerization	1/95	183/14464	0.00546448	0.83198159	-0.1859862	0.70287167	0.75184545	0.71883197	Abl1	1
GO:0009267	cellular response to starvation	1/95	183/14464	0.00546448	0.83198159	-0.1859862	0.70287167	0.75184545	0.71883197	Sar1b	1
GO:0042180	cellular ketone metabolic process	1/95	183/14464	0.00546448	0.83198159	-0.1859862	0.70287167	0.75184545	0.71883197	Clcn2	1
GO:0061136	regulation of proteasomal protein catabolic process	1/95	184/14464	0.00543478	0.82745995	-0.1915193	0.70484823	0.75353969	0.72045182	Trem2	1
GO:0001649	osteoblast differentiation	1/95	186/14464	0.00537634	0.81856254	-0.2025011	0.70876239	0.75645997	0.72324387	Gdf10	1
GO:0051216	cartilage development	1/95	186/14464	0.00537634	0.81856254	-0.2025011	0.70876239	0.75645997	0.72324387	Por	1
GO:0071826	protein-RNA complex organization	1/95	186/14464	0.00537634	0.81856254	-0.2025011	0.70876239	0.75645997	0.72324387	Rbmx	1
GO:0043524	negative regulation of neuron apoptotic process	1/95	188/14464	0.00531915	0.80985442	-0.2133731	0.71262519	0.75973763	0.7263776	Mt1	1
GO:0050905	neuromuscular process	1/95	188/14464	0.00531915	0.80985442	-0.2133731	0.71262519	0.75973763	0.7263776	Abl1	1
GO:0050806	positive regulation of synaptic transmission	1/95	189/14464	0.00529101	0.80556948	-0.2187686	0.71453753	0.76135343	0.72792245	Abl1	1
GO:0032259	methylation	1/95	190/14464	0.00526316	0.80132964	-0.2241376	0.71643728	0.76253086	0.72904818	Inmt	1
GO:0120032	regulation of plasma membrane bounded cell projection assembly	1/95	190/14464	0.00526316	0.80132964	-0.2241376	0.71643728	0.76253086	0.72904818	Agrn	1
GO:0031099	regeneration	1/95	191/14464	0.0052356	0.7971342	-0.2294804	0.71832452	0.76411572	0.73056345	Serpine1	1
GO:0031334	positive regulation of protein-containing complex assembly	1/95	192/14464	0.00520833	0.79298246	-0.2347973	0.72019933	0.76483814	0.73125415	Nav3	1
GO:0045732	positive regulation of protein catabolic process	1/95	192/14464	0.00520833	0.79298246	-0.2347973	0.72019933	0.76483814	0.73125415	Trem2	1
GO:0060491	regulation of cell projection assembly	1/95	192/14464	0.00520833	0.79298246	-0.2347973	0.72019933	0.76483814	0.73125415	Agrn	1
GO:0030324	lung development	1/95	194/14464	0.00515464	0.78480738	-0.2453545	0.72391199	0.7683557	0.73461725	Clcn2	1
GO:0031329	regulation of cellular catabolic process	2/95	388/14464	0.00515464	0.78480738	-0.3493666	0.72768489	0.77193328	0.73803775	Trem2/Acer2	2
GO:0001558	regulation of cell growth	2/95	389/14464	0.00514139	0.78278988	-0.3531087	0.72900331	0.77203472	0.73813473	Myo5b/Abl1	2
GO:0030323	respiratory tube development	1/95	197/14464	0.00507614	0.772856	-0.2610032	0.72938976	0.77203472	0.73813473	Clcn2	1
GO:0050821	protein stabilization	1/95	197/14464	0.00507614	0.772856	-0.2610032	0.72938976	0.77203472	0.73813473	Per3	1
GO:0051963	regulation of synapse assembly	1/95	197/14464	0.00507614	0.772856	-0.2610032	0.72938976	0.77203472	0.73813473	Agrn	1
GO:0030705	cytoskeleton-dependent intracellular transport	1/95	198/14464	0.00505051	0.76895268	-0.2661706	0.73119168	0.77351535	0.73955034	Myo5b	1
GO:0031345	negative regulation of cell projection organization	1/95	201/14464	0.00497512	0.75747578	-0.2815305	0.73652651	0.77859219	0.74440426	Il15ra	1
GO:0006163	purine nucleotide metabolic process	2/95	395/14464	0.00506329	0.7708994	-0.3753798	0.7368022	0.77859219	0.74440426	Trem2/ND5	2
GO:0002262	myeloid cell homeostasis	1/95	202/14464	0.0049505	0.7537259	-0.2866038	0.7382814	0.77929703	0.74507815	Lyn	1
GO:0035264	multicellular organism growth	1/95	202/14464	0.0049505	0.7537259	-0.2866038	0.7382814	0.77929703	0.74507815	Tns2	1
GO:0060047	heart contraction	1/95	203/14464	0.00492611	0.75001296	-0.2916542	0.74002472	0.78070777	0.74642695	Agrn	1
GO:0003018	vascular process in circulatory system	1/95	207/14464	0.00483092	0.73551996	-0.3116318	0.74688385	0.7866466	0.752105	Abl1	1
GO:0043583	ear development	1/95	207/14464	0.00483092	0.73551996	-0.3116318	0.74688385	0.7866466	0.752105	Alg10b	1
GO:0097191	extrinsic apoptotic signaling pathway	1/95	207/14464	0.00483092	0.73551996	-0.3116318	0.74688385	0.7866466	0.752105	Serpine1	1
GO:0016311	dephosphorylation	1/95	209/14464	0.00478469	0.72848149	-0.3214894	0.75024595	0.78975424	0.75507619	Tns2	1
GO:0048732	gland development	2/95	408/14464	0.00490196	0.74633643	-0.4226054	0.75305381	0.79227536	0.75748661	Clcn2/Abl1	2
GO:0051321	meiotic cell cycle	1/95	211/14464	0.00473934	0.72157645	-0.3312621	0.75356386	0.79237756	0.75758432	Ncapd3	1

GO:0031032 actomyosin structure organization	1/95	212/14464	0.00471698	0.71817279	-0.3361171	0.75520642	0.7930855	0.75826117	Abl1	1
GO:0032869 cellular response to insulin stimulus	1/95	212/14464	0.00471698	0.71817279	-0.3361171	0.75520642	0.7930855	0.75826117	Tns2	1
GO:0043434 response to peptide hormone	2/95	410/14464	0.00487805	0.74269576	-0.4297507	0.75547696	0.7930855	0.75826117	Tns2/Lyn	2
GO:0003015 heart process	1/95	213/14464	0.00469484	0.71480109	-0.3409516	0.75683815	0.79408005	0.75921205	Agrn	1
GO:0006364 rRNA processing	1/95	214/14464	0.0046729	0.7114609	-0.3457657	0.75845912	0.79491155	0.76000704	Wdr46	1
GO:0048705 skeletal system morphogenesis	1/95	214/14464	0.0046729	0.7114609	-0.3457657	0.75845912	0.79491155	0.76000704	Por	1
GO:0042594 response to starvation	1/95	216/14464	0.00462963	0.70487329	-0.3553335	0.76166904	0.79740474	0.76239075	Sar1b	1
GO:0045926 negative regulation of growth	1/95	216/14464	0.00462963	0.70487329	-0.3553335	0.76166904	0.79740474	0.76239075	Mt1	1
GO:0048285 organelle fission	2/95	416/14464	0.00480769	0.73198381	-0.4510017	0.76262533	0.79797056	0.76293172	Xrcc3/Ncapd3	2
GO:0008380 RNA splicing	2/95	417/14464	0.00479616	0.73022845	-0.454517	0.7637992	0.79876331	0.76368966	Srrm2/Rbmx	2
GO:0048863 stem cell differentiation	1/95	218/14464	0.00458716	0.69840657	-0.3648223	0.76483675	0.79897753	0.76389448	Zfp281	1
GO:1903050 regulation of proteolysis involved in protein catabolic process	1/95	218/14464	0.00458716	0.69840657	-0.3648223	0.76483675	0.79897753	0.76389448	Trem2	1
GO:0006836 neurotransmitter transport	1/95	219/14464	0.00456621	0.6952175	-0.3695376	0.76640494	0.80018013	0.76504428	Slc6a6	1
GO:0016485 protein processing	1/95	220/14464	0.00454545	0.69205742	-0.3742336	0.76796279	0.8009351	0.7657661	Serpine1	1
GO:0060541 respiratory system development	1/95	220/14464	0.00454545	0.69205742	-0.3742336	0.76796279	0.8009351	0.7657661	Clcn2	1
GO:0048568 embryonic organ development	2/95	421/14464	0.00475059	0.72329041	-0.4685037	0.76844515	0.80100285	0.76583087	Pole/Arid2	2
GO:0042692 muscle cell differentiation	2/95	423/14464	0.00472813	0.7198706	-0.475453	0.7707386	0.80295731	0.76769951	Bhlhe41/Jam2	2
GO:0007033 vacuole organization	1/95	223/14464	0.0044843	0.68274723	-0.3882088	0.77257491	0.80399743	0.76869396	Rab34	1
GO:0090092 regulation of transmembrane receptor protein serine/threonine kinase signaling pathway	1/95	223/14464	0.0044843	0.68274723	-0.3882088	0.77257491	0.80399743	0.76869396	Abl1	1
GO:0045861 negative regulation of proteolysis	1/95	224/14464	0.00446429	0.67969925	-0.39283	0.77409204	0.80513963	0.76978601	Serpine1	1
GO:2001234 negative regulation of apoptotic signaling pathway	1/95	225/14464	0.00444444	0.67667836	-0.3974331	0.77559915	0.80627019	0.77086692	Serpine1	1
GO:0007015 actin filament organization	2/95	429/14464	0.004662	0.70980248	-0.4961281	0.77750212	0.80781081	0.77233989	Myo5b/Abl1	2
GO:0040029 epigenetic regulation of gene expression	1/95	230/14464	0.00434783	0.66196796	-0.4201806	0.78298676	0.81262931	0.77694681	Tet3	1
GO:1902115 regulation of organelle assembly	1/95	230/14464	0.00434783	0.66196796	-0.4201806	0.78298676	0.81262931	0.77694681	Abl1	1
GO:0001667 ameboidal-type cell migration	1/95	231/14464	0.004329	0.6591023	-0.4246778	0.78443514	0.81325285	0.77754298	Serpine1	1
GO:0045055 regulated exocytosis	1/95	231/14464	0.004329	0.6591023	-0.4246778	0.78443514	0.81325285	0.77754298	Lyn	1
GO:0022613 ribonucleoprotein complex biogenesis	2/95	436/14464	0.00458716	0.69840657	-0.5199301	0.78517466	0.81358	0.77785576	Rbmx/Wdr46	2
GO:0046394 carboxylic acid biosynthetic process	1/95	232/14464	0.00431034	0.65626134	-0.4291579	0.78587395	0.81386515	0.77812838	Tha1	1
GO:0015850 organic hydroxy compound transport	1/95	235/14464	0.00425532	0.64788354	-0.4424975	0.79013363	0.8169539	0.78108151	Trem2	1
GO:0016053 organic acid biosynthetic process	1/95	235/14464	0.00425532	0.64788354	-0.4424975	0.79013363	0.8169539	0.78108151	Tha1	1
GO:0090257 regulation of muscle system process	1/95	235/14464	0.00425532	0.64788354	-0.4424975	0.79013363	0.8169539	0.78108151	Agrn	1
GO:0008015 blood circulation	2/95	443/14464	0.00451467	0.6873708	-0.5434	0.79261681	0.81908006	0.78311431	Abl1/Agrn	2
GO:0048167 regulation of synaptic plasticity	1/95	239/14464	0.0041841	0.6370403	-0.4600541	0.79568302	0.82180609	0.78572065	Abl1	1
GO:0051896 regulation of phosphatidylinositol 3-kinase/protein kinase B signal transduction	1/95	240/14464	0.00416667	0.63438596	-0.4644033	0.79704753	0.82277257	0.78664469	Trem2	1
GO:0031669 cellular response to nutrient levels	1/95	241/14464	0.00414938	0.63175366	-0.4687368	0.79840302	0.82328608	0.78713565	Sar1b	1
GO:0045444 fat cell differentiation	1/95	241/14464	0.00414938	0.63175366	-0.4687368	0.79840302	0.82328608	0.78713565	Gdf10	1
GO:0071695 anatomical structure maturation	1/95	242/14464	0.00413223	0.62914311	-0.4730548	0.79974955	0.82387574	0.78769941	Rere	1
GO:0009117 nucleotide metabolic process	2/95	450/14464	0.00444444	0.67667836	-0.5665493	0.7998335	0.82387574	0.78769941	Trem2/ND5	2
GO:0000209 protein polyubiquitination	1/95	243/14464	0.00411523	0.62655404	-0.4773573	0.80108718	0.8242822	0.78808803	Peli1	1
GO:0003007 heart morphogenesis	1/95	243/14464	0.00411523	0.62655404	-0.4773573	0.80108718	0.8242822	0.78808803	Arid2	1
GO:0051223 regulation of protein transport	2/95	452/14464	0.00442478	0.67368421	-0.573106	0.80185469	0.82462977	0.78842034	Trem2/Rab34	2
GO:0006260 DNA replication	1/95	245/14464	0.00408163	0.62143931	-0.4859166	0.80373598	0.82612177	0.78984682	Pole	1
GO:0006397 mRNA processing	2/95	455/14464	0.0043956	0.66924234	-0.5828942	0.80485298	0.82638463	0.79009813	Srrm2/Rbmx	2
GO:0031667 response to nutrient levels	2/95	455/14464	0.0043956	0.66924234	-0.5828942	0.80485298	0.82638463	0.79009813	Sar1b/Il15ra	2
GO:0016072 rRNA metabolic process	1/95	247/14464	0.00404858	0.61640742	-0.4944159	0.80634987	0.82703656	0.79072144	Wdr46	1
GO:0097305 response to alcohol	1/95	247/14464	0.00404858	0.61640742	-0.4944159	0.80634987	0.82703656	0.79072144	Klf9	1
GO:0099504 synaptic vesicle cycle	1/95	249/14464	0.00401606	0.61145635	-0.5028562	0.80892931	0.82923896	0.79282714	Myo5b	1
GO:0031503 protein-containing complex localization	1/95	251/14464	0.00398406	0.60658419	-0.5112385	0.81147474	0.83118573	0.79468842	Myo5b	1
GO:0071407 cellular response to organic cyclic compound	2/95	462/14464	0.004329	0.6591023	-0.605519	0.81169466	0.83118573	0.79468842	Per1/Klf9	2
GO:0006898 receptor-mediated endocytosis	1/95	254/14464	0.00393701	0.59941981	-0.5237054	0.81523012	0.83420105	0.79757134	Serpine1	1
GO:0001501 skeletal system development	2/95	466/14464	0.00429185	0.65344477	-0.6183155	0.81550869	0.83420105	0.79757134	Por/Lrrc17	2
GO:0034976 response to endoplasmic reticulum stress	1/95	256/14464	0.00390625	0.59473684	-0.531947	0.81769248	0.83598927	0.79928104	Abl1	1
GO:0002009 morphogenesis of an epithelium	2/95	470/14464	0.00425532	0.64788354	-0.6310184	0.81925441	0.83669463	0.79995543	Abl1/Brpf1	2
GO:0003013 circulatory system process	2/95	470/14464	0.00425532	0.64788354	-0.6310184	0.81925441	0.83669463	0.79995543	Abl1/Agrn	2
GO:0110053 regulation of actin filament organization	1/95	258/14464	0.00387597	0.59012648	-0.540134	0.82012237	0.83713554	0.80037698	Abl1	1
GO:0008544 epidermis development	1/95	259/14464	0.003861	0.587848	-0.5442073	0.82132526	0.83791769	0.80112478	Alg10b	1
GO:0016049 cell growth	2/95	476/14464	0.00420168	0.63971694	-0.649901	0.82474694	0.84096142	0.80403486	Myo5b/Abl1	2
GO:0034329 cell junction assembly	2/95	485/14464	0.00412371	0.6278459	-0.6778496	0.83270832	0.84794851	0.81071515	Abl1/Agrn	2
GO:0060070 canonical Wnt signaling pathway	1/95	269/14464	0.00371747	0.56599491	-0.5842261	0.83292493	0.84794851	0.81071515	Fzd4	1
GO:1903311 regulation of mRNA metabolic process	1/95	269/14464	0.00371747	0.56599491	-0.5842261	0.83292493	0.84794851	0.81071515	Rbmx	1
GO:0007281 germ cell development	1/95	276/14464	0.00362319	0.55163997	-0.611503	0.84059816	0.85349623	0.81601927	Jam2	1
GO:0016197 endosomal transport	1/95	276/14464	0.00362319	0.55163997	-0.611503	0.84059816	0.85349623	0.81601927	Myo5b	1
GO:0030198 extracellular matrix organization	1/95	276/14464	0.00362319	0.55163997	-0.611503	0.84059816	0.85349623	0.81601927	Abl1	1
GO:0043062 extracellular structure organization	1/95	276/14464	0.00362319	0.55163997	-0.611503	0.84059816	0.85349623	0.81601927	Abl1	1
GO:0045229 external encapsulating structure organization	1/95	276/14464	0.00362319	0.55163997	-0.611503	0.84059816	0.85349623	0.81601927	Abl1	1
GO:0043491 phosphatidylinositol 3-kinase/protein kinase B signal transduction	1/95	278/14464	0.00359712	0.54767134	-0.6191905	0.84272574	0.85520396	0.81765201	Trem2	1
GO:0051604 protein maturation	2/95	498/14464	0.00401606	0.61145635	-0.7174575	0.84363948	0.85567873	0.81810594	Serpine1/Naa50	2
GO:0006936 muscle contraction	1/95	281/14464	0.00355872	0.54182431	-0.6306362	0.84586452	0.85748231	0.81983032	Agrn	1
GO:0030111 regulation of Wnt signaling pathway	1/95	282/14464	0.0035461	0.53990295	-0.6344289	0.84689694	0.85807563	0.82039759	Abl1	1
GO:0071375 cellular response to peptide hormone stimulus	1/95	292/14464	0.00342466	0.52141312	-0.6717603	0.85685213	0.86724643	0.8291657	Tns2	1
GO:0141091 transforming growth factor beta receptor superfamily signaling pathway	1/95	292/14464	0.00342466	0.52141312	-0.6717603	0.85685213	0.86724643	0.8291657	Abl1	1
GO:0097193 intrinsic apoptotic signaling pathway	1/95	302/14464	0.00331126	0.50414779	-0.7080618	0.86616637	0.87619594	0.83772223	Trem2	1
GO:0001822 kidney development	1/95	303/14464	0.00330033	0.50248393	-0.7116381	0.86706414	0.87619594	0.83772223	Tns2	1
GO:0099003 vesicle-mediated transport in synapse	1/95	303/14464	0.00330033	0.50248393	-0.7116381	0.86706414	0.87619594	0.83772223	Myo5b	1
GO:0042254 ribosome biogenesis	1/95	309/14464	0.00323625	0.49272696	-0.7328985	0.87232696	0.88105023	0.84236338	Wdr46	1
GO:0007178 cell surface receptor protein serine/threonine kinase signaling pathway	1/95	313/14464	0.00319489	0.48643013	-0.7468887	0.87572043	0.88401237	0.84519545	Abl1	1
GO:0072001 renal system development	1/95	315/14464	0.0031746	0.48334169	-0.7538302	0.87738354	0.88522556	0.84635537	Tns2	1
GO:0021700 developmental maturation	1/95	323/14464	0.00309598	0.47137038	-0.7812507	0.88381861	0.89110011	0.85197197	Rere	1
GO:0006913 nucleocytoplasmic transport	1/95	324/14464	0.00308642	0.46991553	-0.7846403	0.88459912	0.89110011	0.85197197	Cdan1	1
GO:0051169 nuclear transport	1/95	324/14464	0.00308642	0.46991553	-0.7846403	0.88459912	0.89110011	0.85197197	Cdan1	1
GO:0032956 regulation of actin cytoskeleton organization	1/95	328/14464	0.00304878	0.46418485	-0.7981166	0.88766963	0.89372404	0.85448068	Abl1	1
GO:0006631 fatty acid metabolic process	1/95	335/14464	0.00298507	0.45448547	-0.8213925	0.89284971	0.89846806	0.85901639	Por	1
GO:0042176 regulation of protein catabolic process	1/95	344/14464	0.00290698	0.44259486	-0.8507669	0.89916384	0.9043477	0.86463786	Trem2	1
GO:0050678 regulation of epithelial cell proliferation	1/95	346/14464	0.00289017	0.44003651	-0.8572133	0.90051619	0.9052334	0.86548467	Klf9	1
GO:0001503 ossification	1/95	359/14464	0.00278552	0.42410204	-0.8984294	0.90887931	0.91316199	0.87306511	Gdf10	1
GO:0032970 regulation of actin filament-based process	1/95	368/14464	0.00271739	0.41372998	-0.9262994	0.91425775	0.9180851	0.87777205	Abl1	1
GO:0003012 muscle system process	1/95	395/14464	0.00253165	0.3854497	-1.0069365	0.92857885	0.93197846	0.89105535	Agrn	1
GO:0140694 non-membrane-bounded organelle assembly	1/95	400/14464	0.0025	0.38063158	-1.0214153	0.93095817	0.93387806	0.89287154	Ccdc57	1
GO:0043161 proteasome-mediated ubiquitin-dependent protein catabolic process	1/95	412/14464	0.00242718	0.36954522	-1.0556287	0.93635329	0.93879936	0.89757674	Peli1	1
GO:0051051 negative regulation of transport	1/95	415/14464	0.00240964	0.36687381	-1.0640679	0.93763555	0.93959405	0.89833654	Agrn	1
GO:0050673 epithelial cell proliferation	1/95	417/14464	0.00239808	0.36511422	-1.0696694	0.93847615	0.93994558	0.89867263	Klf9	1
GO:0007283 spermatogenesis	1/95	423/14464	0.00236407	0.3599353	-1.0863575	0.94093126	0.94191293	0.9005536	Jam2	1
GO:0048232 male gamete generation	1/95	437/14464	0.00228833	0.34840419	-1.1246421	0.94628925	0.94678263	0.90520947	Jam2	1
GO:0010608 post-transcriptional regulation of gene expression	1/95	468/14464	0.00213675	0.32532614	-1.2064034	0.95650177	0.95650177	0.91450184	Per1	1

ID	Description	GeneRatio	BgRatio	RichFactor	FoldEnrichm	zScore	pvalue	p.adjust	qvalue	geneID	Count
GO:1902475	L-alpha-amino acid transmembrane transport	4/47	68/14464	0.05882353	18.1026283	8.07118063	6.8433E-05	0.07876637	0.07318728	Per2/Slc1a5/Slc7a2/Slc25a15	4
GO:0015807	L-amino acid transport	4/47	87/14464	0.04597701	14.1491807	7.02368227	0.00017879	0.10021232	0.0931142	Per2/Slc1a5/Slc7a2/Slc25a15	4
GO:0003333	amino acid transmembrane transport	4/47	96/14464	0.04166667	12.822695	6.6358203	0.0002612	0.10021232	0.0931142	Per2/Slc1a5/Slc7a2/Slc25a15	4
GO:0089718	amino acid import across plasma membrane	3/47	52/14464	0.05769231	17.7545008	6.91053564	0.0006356	0.11052783	0.10269905	Per2/Slc1a5/Slc7a2	3
GO:2000107	negative regulation of leukocyte apoptotic process	3/47	53/14464	0.05660377	17.4195102	6.83741196	0.00067221	0.11052783	0.10269905	Tsc22d3/Mertk/Bcl2l1	3
GO:0009404	toxin metabolic process	2/47	13/14464	0.15384615	47.3453355	9.54485706	0.00078794	0.11052783	0.10269905	Fmo2/Fmo1	2
GO:0006805	xenobiotic metabolic process	3/47	58/14464	0.05172414	15.9178283	6.49962902	0.0008754	0.11052783	0.10269905	Fmo2/Fmo1/Cyp2f2	3
GO:1905039	carboxylic acid transmembrane transport	4/47	133/14464	0.03007519	9.25547912	5.46097928	0.00089727	0.11052783	0.10269905	Per2/Slc1a5/Slc7a2/Slc25a15	4
GO:1903826	L-arginine transmembrane transport	2/47	14/14464	0.14285714	43.9635258	9.18270605	0.00091736	0.11052783	0.10269905	Slc7a2/Slc25a15	2
GO:1903825	organic acid transmembrane transport	4/47	136/14464	0.02941176	9.05131414	5.3862203	0.00097505	0.11052783	0.10269905	Per2/Slc1a5/Slc7a2/Slc25a15	4
GO:1990822	basic amino acid transmembrane transport	2/47	15/14464	0.13333333	41.0326241	8.85689438	0.0010563	0.11052783	0.10269905	Slc7a2/Slc25a15	2
GO:0046322	negative regulation of fatty acid oxidation	2/47	16/14464	0.125	38.4680851	8.56166628	0.00120471	0.11555151	0.1073669	Fmo2/Fmo1	2
GO:0015802	basic amino acid transport	2/47	18/14464	0.11111111	34.1938534	8.04564363	0.00152966	0.12575951	0.11685186	Slc7a2/Slc25a15	2
GO:1902236	negative regulation of endoplasmic reticulum stress-induced intrinsic apoptotic signaling pathway	2/47	18/14464	0.11111111	34.1938534	8.04564363	0.00152966	0.12575951	0.11685186	Bcl2l1/Herpud1	2
GO:0006865	amino acid transport	4/47	158/14464	0.02531646	7.79100458	4.90054215	0.00169671	0.13019388	0.12097213	Per2/Slc1a5/Slc7a2/Slc25a15	4
GO:2000106	regulation of leukocyte apoptotic process	3/47	88/14464	0.03409091	10.4912959	5.09904086	0.00290845	0.20922667	0.19440697	Tsc22d3/Mertk/Bcl2l1	3
GO:1902235	regulation of endoplasmic reticulum stress-induced intrinsic apoptotic signaling pathway	2/47	29/14464	0.06896552	21.2237711	6.22434159	0.00396782	0.24805124	0.23048156	Bcl2l1/Herpud1	2
GO:1903077	negative regulation of protein localization to plasma membrane	2/47	30/14464	0.06666667	20.5163121	6.10950048	0.00424246	0.24805124	0.23048156	Bcl2l1/Ppfia1	2
GO:0006631	fatty acid metabolic process	5/47	335/14464	0.01492537	4.59320419	3.79917467	0.00447121	0.24805124	0.23048156	Fmo2/Per2/Fmo1/Cyp2f2/Acsf3	5
GO:0045922	negative regulation of fatty acid metabolic process	2/47	31/14464	0.06451613	19.8544955	6.00009506	0.0045257	0.24805124	0.23048156	Fmo2/Fmo1	2
GO:1904376	negative regulation of protein localization to cell periphery	2/47	31/14464	0.06451613	19.8544955	6.00009506	0.0045257	0.24805124	0.23048156	Bcl2l1/Ppfia1	2
GO:0071887	leukocyte apoptotic process	3/47	112/14464	0.02678571	8.24316109	4.39361113	0.00572162	0.29934452	0.27814169	Tsc22d3/Mertk/Bcl2l1	3
GO:0015695	organic cation transport	3/47	117/14464	0.02564103	7.89088925	4.27295506	0.00645688	0.29939616	0.27818967	Slc1a5/Slc7a2/Slc25a15	3
GO:1905476	negative regulation of protein localization to membrane	2/47	38/14464	0.05263158	16.1970885	5.3557451	0.006744	0.29939616	0.27818967	Bcl2l1/Ppfia1	2
GO:0016053	organic acid biosynthetic process	4/47	235/14464	0.01702128	5.23820733	3.73998287	0.00701172	0.29939616	0.27818967	Per2/Fmo1/Acsf3/Rdh10	4
GO:0046320	regulation of fatty acid oxidation	2/47	39/14464	0.05128205	15.7817785	5.27766422	0.00709391	0.29939616	0.27818967	Fmo2/Fmo1	2
GO:0051896	regulation of phosphatidylinositol 3-kinase/protein kinase B signal transduction	4/47	240/14464	0.01666667	5.12907801	3.68288799	0.00754259	0.29939616	0.27818967	Xdh/Mertk/Phlda3/Sesn1	4
GO:0071466	cellular response to xenobiotic stimulus	3/47	124/14464	0.02419355	7.44543583	4.11556276	0.00757717	0.29939616	0.27818967	Fmo2/Fmo1/Cyp2f2	3
GO:0019748	secondary metabolic process	2/47	41/14464	0.04878049	15.0119357	5.12982953	0.00781793	0.29939616	0.27818967	Fmo2/Fmo1	2
GO:0010565	regulation of cellular ketone metabolic process	3/47	127/14464	0.02362205	7.26955939	4.0518225	0.00809032	0.29939616	0.27818967	Fmo2/Fmo1/Rdh10	3
GO:1903573	negative regulation of response to endoplasmic reticulum stress	2/47	42/14464	0.04761905	14.6545086	5.05974521	0.00819194	0.29939616	0.27818967	Bcl2l1/Herpud1	2
GO:0001558	regulation of cell growth	5/47	389/14464	0.01285347	3.95558716	3.37392203	0.00832379	0.29939616	0.27818967	Fam107a/Trip10/Crtf3/Sesn1/Ip6k2	5
GO:0006739	NADP metabolic process	2/47	49/14464	0.04081633	12.5610074	4.62835932	0.01102898	0.36409233	0.33830335	Fmo2/Fmo1	2
GO:0043491	phosphatidylinositol 3-kinase/protein kinase B signal transduction	4/47	278/14464	0.01438849	4.42798102	3.29512202	0.01246126	0.36409233	0.33830335	Xdh/Mertk/Phlda3/Sesn1	4
GO:0046942	carboxylic acid transport	4/47	281/14464	0.01423488	4.3807072	3.26751312	0.01291964	0.36409233	0.33830335	Per2/Slc1a5/Slc7a2/Slc25a15	4
GO:0015849	organic acid transport	4/47	283/14464	0.01413428	4.34974814	3.24932099	0.01323117	0.36409233	0.33830335	Per2/Slc1a5/Slc7a2/Slc25a15	4
GO:0072330	monocarboxylic acid biosynthetic process	3/47	156/14464	0.01923077	5.91816694	3.52627982	0.01410922	0.36409233	0.33830335	Per2/Acsf3/Rdh10	3
GO:2000134	negative regulation of G1/S transition of mitotic cell cycle	2/47	59/14464	0.03389831	10.4320231	4.14490806	0.01572021	0.36409233	0.33830335	Fam107a/Crtf3	2
GO:0019216	regulation of lipid metabolic process	4/47	298/14464	0.01342282	4.13080109	3.11803267	0.01572185	0.36409233	0.33830335	Fmo2/Fmo1/Rdh10/Mtmr2	4
GO:0097193	intrinsic apoptotic signaling pathway	4/47	302/14464	0.01324503	4.07608849	3.08447087	0.01643277	0.36409233	0.33830335	Prodh/Phlda3/Bcl2l1/Herpud1	4
GO:0001701	in utero embryonic development	5/47	461/14464	0.01084599	3.3377948	2.91264114	0.01643344	0.36409233	0.33830335	Otud7b/Bcl2l1/Rdh10/Mxi1/Ints1	5
GO:0002931	response to ischemia	2/47	61/14464	0.03278689	10.0899895	4.06202408	0.01674472	0.36409233	0.33830335	Per2/Bcl2l1	2
GO:0098739	import across plasma membrane	3/47	167/14464	0.01796407	5.52834756	3.3605952	0.01690845	0.36409233	0.33830335	Per2/Slc1a5/Slc7a2	3
GO:0070059	intrinsic apoptotic signaling pathway in response to endoplasmic reticulum stress	2/47	62/14464	0.03225806	9.92724777	4.02200589	0.01726743	0.36409233	0.33830335	Bcl2l1/Herpud1	2
GO:0044283	small molecule biosynthetic process	5/47	470/14464	0.0106383	3.27387958	2.86144979	0.01772229	0.36409233	0.33830335	Per2/Fmo1/Acsf3/Ip6k2/Rdh10	5
GO:0051898	negative regulation of phosphatidylinositol 3-kinase/protein kinase B signal transduction	2/47	63/14464	0.03174603	9.76967241	3.98288707	0.01779706	0.36409233	0.33830335	Xdh/Phlda3	2
GO:0030308	negative regulation of cell growth	3/47	171/14464	0.01754386	5.39902949	3.30395333	0.01799817	0.36409233	0.33830335	Crtf3/Sesn1/Ip6k2	3
GO:2001242	regulation of intrinsic apoptotic signaling pathway	3/47	172/14464	0.01744186	5.36763978	3.29007051	0.01827661	0.36409233	0.33830335	Prodh/Bcl2l1/Herpud1	3
GO:0016049	cell growth	5/47	476/14464	0.0105042	3.23261219	2.82800146	0.01861806	0.36409233	0.33830335	Fam107a/Trip10/Crtf3/Sesn1/Ip6k2	5
GO:0062012	regulation of small molecule metabolic process	4/47	319/14464	0.01253918	3.85886747	2.94800735	0.01967947	0.36409233	0.33830335	Fmo2/Fmo1/Bcl2l1/Rdh10	4
GO:1902807	negative regulation of cell cycle G1/S phase transition	2/47	67/14464	0.02985075	9.18640838	3.83473606	0.01998368	0.36409233	0.33830335	Fam107a/Crtf3	2
GO:0030258	lipid modification	3/47	178/14464	0.01685393	5.1867081	3.20898757	0.01999791	0.36409233	0.33830335	Fmo2/Fmo1/Mtmr2	3
GO:0042180	cellular ketone metabolic process	3/47	183/14464	0.01639344	5.04499477	3.14416169	0.02149874	0.36409233	0.33830335	Fmo2/Fmo1/Rdh10	3
GO:0015800	acidic amino acid transport	2/47	72/14464	0.02777778	8.54846336	3.66610498	0.02286667	0.36409233	0.33830335	Per2/Slc1a5	2
GO:0015711	organic anion transport	4/47	341/14464	0.01173021	3.60990828	2.78470778	0.02443691	0.36409233	0.33830335	Per2/Slc1a5/Slc7a2/Slc25a15	4
GO:0097009	energy homeostasis	2/47	79/14464	0.02531646	7.79100458	3.45567832	0.02717188	0.36409233	0.33830335	Fmo2/Fmo1	2
GO:0071230	cellular response to amino acid stimulus	2/47	80/14464	0.025	7.69361702	3.42773066	0.02781173	0.36409233	0.33830335	Sesn1/Bcl2l1	2
GO:1905897	regulation of response to endoplasmic reticulum stress	2/47	81/14464	0.02469136	7.5986341	3.40026284	0.02845766	0.36409233	0.33830335	Bcl2l1/Herpud1	2
GO:0031396	regulation of protein ubiquitination	3/47	204/14464	0.01470588	4.52565707	2.89558329	0.02846311	0.36409233	0.33830335	Per2/Fam107a/Herpud1	3
GO:0043648	dicarboxylic acid metabolic process	2/47	83/14464	0.02409639	7.41553448	3.34670952	0.02976756	0.36409233	0.33830335	Prodh/Acsf3	2
GO:0048041	focal adhesion assembly	2/47	85/14464	0.02352941	7.24105131	3.29490993	0.03110122	0.36409233	0.33830335	Fam107a/Tesk2	2
GO:0035871	protein K11-linked deubiquitination	1/47	10/14464	0.1	30.7744681	5.3776268	0.03203323	0.36409233	0.33830335	Otud7b	1
GO:0036466	synaptic vesicle recycling via endosome	1/47	10/14464	0.1	30.7744681	5.3776268	0.03203323	0.36409233	0.33830335	Bcl2l1	1
GO:0070344	regulation of fat cell proliferation	1/47	10/14464	0.1	30.7744681	5.3776268	0.03203323	0.36409233	0.33830335	Per2	1
GO:1901031	regulation of response to reactive oxygen species	1/47	10/14464	0.1	30.7744681	5.3776268	0.03203323	0.36409233	0.33830335	Sesn1	1
GO:2000807	regulation of synaptic vesicle clustering	1/47	10/14464	0.1	30.7744681	5.3776268	0.03203323	0.36409233	0.33830335	Bcl2l1	1
GO:0090288	negative regulation of cellular response to growth factor stimulus	2/47	87/14464	0.02298851	7.07459036	3.24476433	0.03245829	0.36409233	0.33830335	Xdh/Fstl3	2
GO:0045926	negative regulation of growth	3/47	216/14464	0.01388889	4.27423168	2.76821617	0.032921	0.36409233	0.33830335	Crtf3/Sesn1/Ip6k2	3
GO:0006835	dicarboxylic acid transport	2/47	88/14464	0.02272727	6.99419729	3.22028278	0.0331455	0.36409233	0.33830335	Per2/Slc1a5	2
GO:0045833	negative regulation of lipid metabolic process	2/47	88/14464	0.02272727	6.99419729	3.22028278	0.0331455	0.36409233	0.33830335	Fmo2/Fmo1	2
GO:0120254	olefinic compound metabolic process	2/47	88/14464	0.02272727	6.99419729	3.22028278	0.0331455	0.36409233	0.33830335	Cyp2f2/Rdh10	2
GO:0019217	regulation of fatty acid metabolic process	2/47	89/14464	0.02247191	6.91561081	3.19618083	0.03383843	0.36409233	0.33830335	Fmo2/Fmo1	2
GO:2001233	regulation of apoptotic signaling pathway	4/47	379/14464	0.01055409	3.24796497	2.53204483	0.03418455	0.36409233	0.33830335	G0s2/Prodh/Bcl2l1/Herpud1	4
GO:1903828	negative regulation of protein localization	3/47	221/14464	0.01357466	4.1775296	2.71785121	0.03488047	0.36409233	0.33830335	Otud7b/Bcl2l1/Ppfia1	3
GO:0009120	deoxyribonucleoside metabolic process	1/47	11/14464	0.09090909	27.9767892	5.11032207	0.03518077	0.36409233	0.33830335	Xdh	1
GO:0009169	purine ribonucleoside monophosphate catabolic process	1/47	11/14464	0.09090909	27.9767892	5.11032207	0.03518077	0.36409233	0.33830335	Xdh	1
GO:0016114	terpenoid biosynthetic process	1/47	11/14464	0.09090909	27.9767892	5.11032207	0.03518077	0.36409233	0.33830335	Rdh10	1
GO:0042573	retinoic acid metabolic process	1/47	11/14464	0.09090909	27.9767892	5.11032207	0.03518077	0.36409233	0.33830335	Rdh10	1
GO:0045602	negative regulation of endothelial cell differentiation	1/47	11/14464	0.09090909	27.9767892	5.11032207	0.03518077	0.36409233	0.33830335	Xdh	1
GO:0075522	IRES-dependent viral translational initiation	1/47	11/14464	0.09090909	27.9767892	5.11032207	0.03518077	0.36409233	0.33830335	Pcbp2	1
GO:1902548	negative regulation of cellular response to vascular endothelial growth factor stimulus	1/47	11/14464	0.09090909	27.9767892	5.11032207	0.03518077	0.36409233	0.33830335	Xdh	1
GO:0071229	cellular response to acid chemical	2/47	91/14464	0.02197802	6.76361936	3.14907459	0.03524129	0.36409233	0.33830335	Sesn1/Bcl2l1	2
GO:0120162	positive regulation of cold-induced thermogenesis	2/47	91/14464	0.02197802	6.76361936	3.14907459	0.03524129	0.36409233	0.33830335	Per2/G0s2	2
GO:0007044	cell-substrate junction assembly	2/47	95/14464	0.02105263	6.47883539	3.05898576	0.03811381	0.36409233	0.33830335	Fam107a/Tesk2	2
GO:0009128	purine nucleoside monophosphate catabolic process	1/47	12/14464	0.08333333	25.6453901	4.87644147	0.03831828	0.36409233	0.33830335	Xdh	1
GO:0009155	purine deoxyribonucleotide catabolic process	1/47	12/14464	0.08333333	25.6453901	4.87644147	0.03831828	0.36409233	0.33830335	Xdh	1
GO:0009170	purine deoxyribonucleoside monophosphate metabolic process	1/47	12/14464	0.08333333	25.6453901	4.87644147	0.03831828	0.36409233	0.33830335	Xdh	1
GO:0032717	negative regulation of interleukin-8 production	1/47	12/14464	0.08333333	25.6453901	4.87644147	0.03831828	0.36409233	0.33830335	Otud7b	1
GO:0043584	nose development	1/47	12/14464	0.08333333	25.6453901	4.87644147	0.03831828	0.36409233	0.33830335	Rdh10	1
GO:0046085	adenosine metabolic process	1/47	12/14464	0.08333333	25.6453901	4.87644147	0.03831828	0.36409233	0.33830335	Xdh	1
GO:0071233	cellular response to L-leucine	1/47	12/14464	0.08333333	25.6453901	4.87644147	0.03831828	0.36409233	0.33830335	Sesn1	1
GO:0090394	negative regulation of excitatory postsynaptic potential	1/47	12/14464	0.08333333	25.6453901	4.87644147	0.03831828	0.36409233	0.33830335	Mtmr2	1
GO:1900244	positive regulation of synaptic vesicle endocytosis	1/47	12/14464	0.08333333	25.6453901	4.87644147	0.03831828	0.36409233	0.33830335	Bcl2l1	1
GO:1901029	negative regulation of mitochondrial outer membrane permeabilization involved in apoptotic signaling pat	1/47	12/14464	0.08333333	25.6453901	4.87644147	0.03831828	0.36409233	0.33830335	Bcl2l1	1
GO:1990253	cellular response to leucine starvation	1/47	12/14464	0.08333333	25.6453901	4.87644147	0.03831828	0.36409233	0.33830335	Sesn1	1
GO:2000644	regulation of receptor catabolic process	1/47	12/14464	0.08333333	25.6453901	4.87644147	0.03831828	0.36409233	0.33830335	Mtmr2	1
GO:2000756	regulation of peptidyl-lysine acetylation	1/47	12/14464	0.08333333	25.6453901	4.87644147	0.03831828	0.36409233	0.33830335	Klf15	1
GO:0006163	purine nucleotide metabolic process	4/47	395/14464	0.01012658	3.11640183	2.43503795	0.0388823	0.36409233	0.33830335	Fmo2/Fmo1/Xdh/Bcl2l1	4
GO:0046394	carboxylic acid biosynthetic process	3/47	232/14464	0.01293103	3.97945708	2.61209418	0.03940082	0.36409233	0.33830335	Per2/Acsf3/Rdh10	3
GO:0062014	negative regulation of small molecule metabolic process	2/47	98/14464	0.02040816	6.28050369	2.99475384	0.04032535	0.36409233	0.33830335	Fmo2/Fmo1	2
GO:2001243	negative regulation of intrinsic apoptotic signaling pathway	2/47	99/14464	0.02020202	6.21706426	2.97393616	0.04107319	0.36409233	0.33830335	Bcl2l1/Herpud1	2
GO:0006924	activation-induced cell death of T cells	1/47	13/14464	0.07692308	23.6726678	4.66945315	0.04144581	0.36409233	0.33830335	Tsc22d3	1
GO:0030540	female genitalia development	1/47	13/14464	0.07692308	23.6726678	4.66945315	0.04144581	0.36409233	0.33830335	Mertk	1
GO:0042454	ribonucleoside catabolic process	1/47	13/14464	0.07692308	23.6726678	4.66945315	0.04144581	0.36409233	0.33830335	Xdh	1
GO:0042754	negative regulation of circadian rhythm	1/47	13/14464	0.07692308	23.6726678	4.66945315	0.04144581	0.36409233	0.33830335	Per2	1
GO:0043605	amide catabolic process	1/47	13/14464	0.07692308	23.6726678	4.66945315	0.04144581	0.36409233	0.33830335	Xdh	1
GO:0055057	neuroblast division	1/47	13/14464	0.07692308	23.6726678	4.66945315	0.04144581	0.36409233	0.33830335	Tead3	1
GO:0070341	fat cell proliferation	1/47	13/14464	0.07692308	23.6726678	4.66945315	0.04144581	0.36409233	0.33830335	Per2	1
GO:0007548	sex differentiation	3/47	237/14464	0.01265823	3.89550229	2.56615032	0.04155005	0.36409233	0.33830335	Mertk/Bcl2l1/Rdh10	3
GO:1903320	regulation of protein modification by small protein conjugation or removal	3/47	239/14464	0.0125523	3.86290394	2.54812226	0.04242617	0.36409233	0.33830335	Per2/Fam107a/Herpud1	3
GO:0003300	cardiac muscle hypertrophy	2/47	101/14464	0.01980198	6.09395408	2.93314684	0.04258463	0.36409233	0.33830335	Klf15/Trip10	2
GO:0150115	cell-substrate junction organization	2/47	102/14464	0.01960784	6.03420943	2.9131616	0.04334815	0.36409233	0.33830335	Fam107a/Tesk2	2
GO:0002385	mucosal immune response	1/47	14/14464	0.07142857	21.9817629	4.48448672	0.04456338	0.36409233	0.33830335	Otud7b	1
GO:0035234	ectopic germ cell programmed cell death	1/47	14/14464	0.07142857	21.9817629	4.48448672	0.04456338	0.36409233	0.33830335	Bcl2l1	1
GO:0043201	response to L-leucine	1/47	14/14464	0.07142857	21.9817629	4.48448672	0.04456338	0.36409233	0.33830335	Sesn1	1
GO:0043649	dicarboxylic acid catabolic process	1/47	14/14464	0.07142857	21.9817629	4.48448672	0.04456338	0.36409233	0.33830335	Acsf3	1
GO:0060572	morphogenesis of an epithelial bud	1/47	14/14464	0.07142857	21.9817629	4.48448672	0.04456338	0.36409233	0.33830335	Rdh10	1
GO:0061051	positive regulation of cell growth involved in cardiac muscle cell development	1/47	14/14464	0.07142857	21.9817629	4.48448672	0.04456338	0.36409233	0.33830335	Trip10	1
GO:0070207	protein homotrimerization	1/47	14/14464	0.07142857	21.9817629	4.48448672	0.04456338	0.36409233	0.33830335	Slc1a5	1
GO:1900272	negative regulation of long-term synaptic potentiation	1/47	14/14464	0.07142857	21.9817629	4.48448672	0.04456338	0.36409233	0.33830335	Fam107a	1
GO:2001171	positive regulation of ATP biosynthetic process	1/47	14/14464	0.07142857	21.9817629	4.48448672	0.04456338	0.36409233	0.33830335	Bcl2l1	1
GO:1901873	regulation of post-translational protein modification	3/47	244/14464	0.01229508	3.78374608	2.50389092	0.04465729	0.36409233	0.33830335	Per2/Fam107a/Herpud1	3
GO:0014897	striated muscle hypertrophy	2/47	104/14464	0.01923077	5.91816694	2.87397766	0.04489057	0.36409233	0.33830335	Klf15/Trip10	2
GO:0008630	intrinsic apoptotic signaling pathway in response to DNA damage	2/47	105/14464	0.01904762	5.86180344	2.8547667	0.0456694	0.36409233	0.33830335	Phlda3/Bcl2l1	2
GO:0019395	fatty acid oxidation	2/47	105/14464	0.01904762	5.86180344	2.8547667	0.0456694	0.36409233	0.33830335	Fmo2/Fmo1	2
GO:0014896	muscle hypertrophy	2/47	106/14464	0.01886792	5.80650341	2.83580188	0.04645325	0.36409233	0.33830335	Klf15/Trip10	2
GO:0001833	inner cell mass cell proliferation	1/47	15/14464	0.06666667	20.5163121	4.31782623	0.04767103	0.36409233	0.33830335	Ints1	1
GO:0032926	negative regulation of activin receptor signaling pathway	1/47	15/14464	0.06666667	20.5163121	4.31782623	0.04767103	0.36409233	0.33830335	Fstl3	1
GO:0099532	synaptic vesicle endosomal processing	1/47	15/14464	0.06666667	20.5163121	4.31782623	0.04767103	0.36409233	0.33830335	Bcl2l1	1
GO:0160049	negative regulation of cGAS/STING signaling pathway	1/47	15/14464	0.06666667	20.5163121	4.31782623	0.04767103	0.36409233	0.33830335	Pcbp2	1
GO:1901653	cellular response to peptide	1/47	15/14464	0.06666667	20.5163121	4.31782623	0.04767103	0.36409233	0.33830335	Klf15	1
GO:2000811	negative regulation of anoikis	1/47	15/14464	0.06666667	20.5163121	4.31782623	0.04767103	0.36409233	0.33830335	Bcl2l1	1
GO:2001212	regulation of vasculogenesis	1/47	15/14464	0.06666667	20.5163121	4.31782623	0.04767103	0.36409233	0.33830335	Xdh	1
GO:0007283	spermatogenesis	4/47	423/14464	0.00945626	2.91011519	2.2765196	0.04796006	0.36409233	0.33830335	Cfap157/Mertk/Tesk2/Bcl2l1	4
GO:0034440	lipid oxidation	2/47	109/14464	0.01834862	5.64669139	2.78032877	0.04883456	0.36409233	0.33830335	Fmo2/Fmo1	2
GO:0032946	positive regulation of mononuclear cell proliferation	2/47	111/14464	0.01801802	5.5449492	2.74447863	0.05044651	0.36409233	0.33830335	Bcl2l1/Icosl	2
GO:0002251	organ or tissue specific immune response	1/47	16/14464	0.0625	19.2340426	4.16658035	0.05076879	0.36409233	0.33830335	Otud7b	1
GO:0009159	deoxyribonucleoside monophosphate catabolic process	1/47	16/14464	0.0625	19.2340426	4.16658035	0.05076879	0.36409233	0.33830335	Xdh	1
GO:0030810	positive regulation of nucleotide biosynthetic process	1/47	16/14464	0.0625	19.2340426	4.16658035	0.05076879	0.36409233	0.33830335	Bcl2l1	1
GO:0032753	positive regulation of interleukin-4 production	1/47	16/14464	0.0625	19.2340426	4.16658035	0.05076879	0.36409233	0.33830335	Icosl	1
GO:0051895	negative regulation of focal adhesion assembly	1/47	16/14464	0.0625	19.2340426	4.16658035	0.05076879	0.36409233	0.33830335	Fam107a	1
GO:0098712	L-glutamate import across plasma membrane	1/47	16/14464	0.0625	19.2340426	4.16658035	0.05076879	0.36409233	0.33830335	Per2	1
GO:0150118	negative regulation of cell-substrate junction organization	1/47	16/14464	0.0625	19.2340426	4.16658035	0.05076879	0.36409233	0.33830335	Fam107a	1
GO:1900373	positive regulation of purine nucleotide biosynthetic process	1/47	16/14464	0.0625	19.2340426	4.16658035	0.05076879	0.36409233	0.33830335	Bcl2l1	1
GO:1903423	positive regulation of synaptic vesicle recycling	1/47	16/14464	0.0625	19.2340426	4.16658035	0.05076879	0.36409233	0.33830335	Bcl2l1	1
GO:0002683	negative regulation of immune system process	4/47	433/14464	0.00923788	2.84290698	2.22302181	0.05146669	0.36409233	0.33830335	Tsc22d3/Mertk/Fstl3/Pcbp2	4
GO:1903076	regulation of protein localization to plasma membrane	2/47	114/14464	0.01754386	5.39902949	2.69230353	0.05290027	0.36409233	0.33830335	Bcl2l1/Ppfia1	2
GO:0048232	male gamete generation	4/47	437/14464	0.00915332	2.81688495	2.20204617	0.05290824	0.36409233	0.33830335	Cfap157/Mertk/Tesk2/Bcl2l1	4
GO:0043200	response to amino acid	2/47	115/14464	0.0173913	5.35208141	2.6753203	0.0537276	0.36409233	0.33830335	Sesn1/Bcl2l1	2
GO:0002091	negative regulation of receptor internalization	1/47	17/14464	0.05882353	18.1026283	4.02846091	0.05385668	0.36409233	0.33830335	Mtmr2	1
GO:0008356	asymmetric cell division	1/47	17/14464	0.05882353	18.1026283	4.02846091	0.05385668	0.36409233	0.33830335	Tead3	1
GO:0032352	positive regulation of hormone metabolic process	1/47	17/14464	0.05882353	18.1026283	4.02846091	0.05385668	0.36409233	0.33830335	Rdh10	1
GO:0050687	negative regulation of defense response to virus	1/47	17/14464	0.05882353	18.1026283	4.02846091	0.05385668	0.36409233	0.33830335	Pcbp2	1
GO:0060602	branch elongation of an epithelium	1/47	17/14464	0.05882353	18.1026283	4.02846091	0.05385668	0.36409233	0.33830335	Rdh10	1
GO:1903376	regulation of oxidative stress-induced neuron intrinsic apoptotic signaling pathway	1/47	17/14464	0.05882353	18.1026283	4.02846091	0.05385668	0.36409233	0.33830335	Prodh	1
GO:0001776	leukocyte homeostasis	2/47	117/14464	0.01709402	5.26059284	2.64194083	0.05539613	0.36409233	0.33830335	Tsc22d3/Mertk	2
GO:0048608	reproductive structure development	3/47	268/14464	0.01119403	3.44490314	2.30667673	0.0561655	0.36409233	0.33830335	Mertk/Bcl2l1/Rdh10	3
GO:0051588	regulation of neurotransmitter transport	2/47	118/14464	0.01694915	5.21601154	2.62553646	0.05623726	0.36409233	0.33830335	Per2/Bcl2l1	2
GO:0009158	ribonucleoside monophosphate catabolic process	1/47	18/14464	0.05555556	17.0969267	3.90163004	0.05693474	0.36409233	0.33830335	Xdh	1
GO:0009164	nucleoside catabolic process	1/47	18/14464	0.05555556	17.0969267	3.90163004	0.05693474	0.36409233	0.33830335	Xdh	1
GO:0010447	response to acidic pH	1/47	18/14464	0.05555556	17.0969267	3.90163004	0.05693474	0.36409233	0.33830335	Kcnk4	1
GO:0015810	aspartate transmembrane transport	1/47	18/14464	0.05555556	17.0969267	3.90163004	0.05693474	0.36409233	0.33830335	Slc1a5	1
GO:0031645	negative regulation of nervous system process	1/47	18/14464	0.05555556	17.0969267	3.90163004	0.05693474	0.36409233	0.33830335	Mtmr2	1
GO:0042104	positive regulation of activated T cell proliferation	1/47	18/14464	0.05555556	17.0969267	3.90163004	0.05693474	0.36409233	0.33830335	Icosl	1
GO:0051580	regulation of neurotransmitter uptake	1/47	18/14464	0.05555556	17.0969267	3.90163004	0.05693474	0.36409233	0.33830335	Per2	1

GO:0070206	protein trimerization	1/47	18/14464	0.05555556	17.0969267	3.90163004	0.05693474	0.36409233	0.33830335	Slc1a5	1
GO:0071467	cellular response to pH	1/47	18/14464	0.05555556	17.0969267	3.90163004	0.05693474	0.36409233	0.33830335	Kcnk4	1
GO:0009117	nucleotide metabolic process	4/47	450/14464	0.00888889	2.73550827	2.13546551	0.05774633	0.36409233	0.33830335	Fmo2/Fmo1/Xdh/Bcl2l1	4
GO:0061458	reproductive system development	3/47	272/14464	0.01102941	3.3942428	2.2759961	0.05820958	0.36409233	0.33830335	Mertk/Bcl2l1/Rdh10	3
GO:0046660	female sex differentiation	2/47	122/14464	0.01639344	5.04499477	2.561732	0.05964671	0.36409233	0.33830335	Mertk/Bcl2l1	2
GO:0006144	purine nucleobase metabolic process	1/47	19/14464	0.05263158	16.1970885	3.78459222	0.060003	0.36409233	0.33830335	Xdh	1
GO:0006817	phosphate ion transport	1/47	19/14464	0.05263158	16.1970885	3.78459222	0.060003	0.36409233	0.33830335	Ip6k2	1
GO:0009151	purine deoxyribonucleotide metabolic process	1/47	19/14464	0.05263158	16.1970885	3.78459222	0.060003	0.36409233	0.33830335	Xdh	1
GO:0036480	neuron intrinsic apoptotic signaling pathway in response to oxidative stress	1/47	19/14464	0.05263158	16.1970885	3.78459222	0.060003	0.36409233	0.33830335	Prodh	1
GO:0150104	transport across blood-brain barrier	1/47	19/14464	0.05263158	16.1970885	3.78459222	0.060003	0.36409233	0.33830335	Slc1a5	1
GO:1900452	regulation of long-term synaptic depression	1/47	19/14464	0.05263158	16.1970885	3.78459222	0.060003	0.36409233	0.33830335	Bcl2l1	1
GO:0070665	positive regulation of leukocyte proliferation	2/47	123/14464	0.01626016	5.00397855	2.54621606	0.06051012	0.36409233	0.33830335	Bcl2l1/Icosl	2
GO:0006089	lactate metabolic process	1/47	20/14464	0.05	15.387234	3.67611627	0.06306149	0.36409233	0.33830335	Per2	1
GO:0009065	glutamine family amino acid catabolic process	1/47	20/14464	0.05	15.387234	3.67611627	0.06306149	0.36409233	0.33830335	Prodh	1
GO:0009125	nucleoside monophosphate catabolic process	1/47	20/14464	0.05	15.387234	3.67611627	0.06306149	0.36409233	0.33830335	Xdh	1
GO:0010232	vascular transport	1/47	20/14464	0.05	15.387234	3.67611627	0.06306149	0.36409233	0.33830335	Slc1a5	1
GO:0010958	regulation of amino acid import across plasma membrane	1/47	20/14464	0.05	15.387234	3.67611627	0.06306149	0.36409233	0.33830335	Per2	1
GO:0017121	plasma membrane phospholipid scrambling	1/47	20/14464	0.05	15.387234	3.67611627	0.06306149	0.36409233	0.33830335	Plscr4	1
GO:0034472	snRNA 3'-end processing	1/47	20/14464	0.05	15.387234	3.67611627	0.06306149	0.36409233	0.33830335	Ints1	1
GO:0035795	negative regulation of mitochondrial membrane permeability	1/47	20/14464	0.05	15.387234	3.67611627	0.06306149	0.36409233	0.33830335	Bcl2l1	1
GO:0097091	synaptic vesicle clustering	1/47	20/14464	0.05	15.387234	3.67611627	0.06306149	0.36409233	0.33830335	Bcl2l1	1
GO:0140896	cGAS/STING signaling pathway	1/47	20/14464	0.05	15.387234	3.67611627	0.06306149	0.36409233	0.33830335	Pcbp2	1
GO:1900746	regulation of vascular endothelial growth factor signaling pathway	1/47	20/14464	0.05	15.387234	3.67611627	0.06306149	0.36409233	0.33830335	Xdh	1
GO:1901028	regulation of mitochondrial outer membrane permeabilization involved in apoptotic signaling pathway	1/47	20/14464	0.05	15.387234	3.67611627	0.06306149	0.36409233	0.33830335	Bcl2l1	1
GO:1903789	regulation of amino acid transmembrane transport	1/47	20/14464	0.05	15.387234	3.67611627	0.06306149	0.36409233	0.33830335	Per2	1
GO:1904294	positive regulation of ERAD pathway	1/47	20/14464	0.05	15.387234	3.67611627	0.06306149	0.36409233	0.33830335	Herpud1	1
GO:2000641	regulation of early endosome to late endosome transport	1/47	20/14464	0.05	15.387234	3.67611627	0.06306149	0.36409233	0.33830335	Mtmr2	1
GO:0043500	muscle adaptation	2/47	126/14464	0.01587302	4.8848362	2.5006584	0.06312627	0.36409233	0.33830335	Klf15/Trip10	2
GO:0001824	blastocyst development	2/47	127/14464	0.01574803	4.84637293	2.48579196	0.06400685	0.36409233	0.33830335	Mxi1/Ints1	2
GO:0019081	viral translation	1/47	21/14464	0.04761905	14.6545086	3.57517817	0.06611023	0.36409233	0.33830335	Pcbp2	1
GO:0034656	nucleobase-containing small molecule catabolic process	1/47	21/14464	0.04761905	14.6545086	3.57517817	0.06611023	0.36409233	0.33830335	Xdh	1
GO:0046040	IMP metabolic process	1/47	21/14464	0.04761905	14.6545086	3.57517817	0.06611023	0.36409233	0.33830335	Xdh	1
GO:0050872	white fat cell differentiation	1/47	21/14464	0.04761905	14.6545086	3.57517817	0.06611023	0.36409233	0.33830335	Per2	1
GO:0060571	morphogenesis of an epithelial fold	1/47	21/14464	0.04761905	14.6545086	3.57517817	0.06611023	0.36409233	0.33830335	Rdh10	1
GO:0090201	negative regulation of release of cytochrome c from mitochondria	1/47	21/14464	0.04761905	14.6545086	3.57517817	0.06611023	0.36409233	0.33830335	Bcl2l1	1
GO:2000209	regulation of anoikis	1/47	21/14464	0.04761905	14.6545086	3.57517817	0.06611023	0.36409233	0.33830335	Bcl2l1	1
GO:2001169	regulation of ATP biosynthetic process	1/47	21/14464	0.04761905	14.6545086	3.57517817	0.06611023	0.36409233	0.33830335	Bcl2l1	1
GO:0001101	response to acid chemical	2/47	132/14464	0.01515152	4.66279819	2.4137219	0.06847207	0.36409233	0.33830335	Sesn1/Bcl2l1	2
GO:0007288	sperm axoneme assembly	1/47	22/14464	0.04545455	13.9883946	3.48091819	0.06914927	0.36409233	0.33830335	Cfap157	1
GO:0010623	programmed cell death involved in cell development	1/47	22/14464	0.04545455	13.9883946	3.48091819	0.06914927	0.36409233	0.33830335	Bcl2l1	1
GO:0032673	regulation of interleukin-4 production	1/47	22/14464	0.04545455	13.9883946	3.48091819	0.06914927	0.36409233	0.33830335	Icosl	1
GO:0032925	regulation of activin receptor signaling pathway	1/47	22/14464	0.04545455	13.9883946	3.48091819	0.06914927	0.36409233	0.33830335	Fstl3	1
GO:0043153	entrainment of circadian clock by photoperiod	1/47	22/14464	0.04545455	13.9883946	3.48091819	0.06914927	0.36409233	0.33830335	Per2	1
GO:0061318	renal filtration cell differentiation	1/47	22/14464	0.04545455	13.9883946	3.48091819	0.06914927	0.36409233	0.33830335	Klf15	1
GO:0072112	podocyte differentiation	1/47	22/14464	0.04545455	13.9883946	3.48091819	0.06914927	0.36409233	0.33830335	Klf15	1
GO:1902547	regulation of cellular response to vascular endothelial growth factor stimulus	1/47	22/14464	0.04545455	13.9883946	3.48091819	0.06914927	0.36409233	0.33830335	Xdh	1
GO:1905709	negative regulation of membrane permeability	1/47	22/14464	0.04545455	13.9883946	3.48091819	0.06914927	0.36409233	0.33830335	Bcl2l1	1
GO:0044282	small molecule catabolic process	3/47	296/14464	0.01013514	3.11903393	2.10314959	0.07120337	0.36409233	0.33830335	Xdh/Prodh/Acsf3	3
GO:0009264	deoxyribonucleotide catabolic process	1/47	23/14464	0.04347826	13.3802035	3.39260846	0.07217863	0.36409233	0.33830335	Xdh	1
GO:0009648	photoperiodism	1/47	23/14464	0.04347826	13.3802035	3.39260846	0.07217863	0.36409233	0.33830335	Per2	1
GO:0009649	entrainment of circadian clock	1/47	23/14464	0.04347826	13.3802035	3.39260846	0.07217863	0.36409233	0.33830335	Per2	1
GO:0015740	C4-dicarboxylate transport	1/47	23/14464	0.04347826	13.3802035	3.39260846	0.07217863	0.36409233	0.33830335	Slc1a5	1
GO:0046037	GMP metabolic process	1/47	23/14464	0.04347826	13.3802035	3.39260846	0.07217863	0.36409233	0.33830335	Xdh	1
GO:0046386	deoxyribose phosphate catabolic process	1/47	23/14464	0.04347826	13.3802035	3.39260846	0.07217863	0.36409233	0.33830335	Xdh	1
GO:0072311	glomerular epithelial cell differentiation	1/47	23/14464	0.04347826	13.3802035	3.39260846	0.07217863	0.36409233	0.33830335	Klf15	1
GO:0046488	phosphatidylinositol metabolic process	2/47	137/14464	0.01459854	4.49262308	2.34517386	0.073038	0.36409233	0.33830335	Ip6k2/Mtmr2	2
GO:0120161	regulation of cold-induced thermogenesis	2/47	139/14464	0.01438849	4.42798102	2.31867119	0.07489162	0.36409233	0.33830335	Per2/G0s2	2
GO:0001956	positive regulation of neurotransmitter secretion	1/47	24/14464	0.04166667	12.822695	3.30962802	0.07519834	0.36409233	0.33830335	Bcl2l1	1
GO:0008299	isoprenoid biosynthetic process	1/47	24/14464	0.04166667	12.822695	3.30962802	0.07519834	0.36409233	0.33830335	Rdh10	1
GO:0030808	regulation of nucleotide biosynthetic process	1/47	24/14464	0.04166667	12.822695	3.30962802	0.07519834	0.36409233	0.33830335	Bcl2l1	1
GO:0032958	inositol phosphate biosynthetic process	1/47	24/14464	0.04166667	12.822695	3.30962802	0.07519834	0.36409233	0.33830335	Ip6k2	1
GO:0070233	negative regulation of T cell apoptotic process	1/47	24/14464	0.04166667	12.822695	3.30962802	0.07519834	0.36409233	0.33830335	Tsc22d3	1
GO:0097062	dendritic spine maintenance	1/47	24/14464	0.04166667	12.822695	3.30962802	0.07519834	0.36409233	0.33830335	Mtmr2	1
GO:1900371	regulation of purine nucleotide biosynthetic process	1/47	24/14464	0.04166667	12.822695	3.30962802	0.07519834	0.36409233	0.33830335	Bcl2l1	1
GO:1901658	glycosyl compound catabolic process	1/47	24/14464	0.04166667	12.822695	3.30962802	0.07519834	0.36409233	0.33830335	Xdh	1
GO:1901983	regulation of protein acetylation	1/47	24/14464	0.04166667	12.822695	3.30962802	0.07519834	0.36409233	0.33830335	Klf15	1
GO:0001832	blastocyst growth	1/47	25/14464	0.04	12.3097872	3.2314434	0.07820843	0.36409233	0.33830335	Ints1	1
GO:0006353	DNA-templated transcription termination	1/47	25/14464	0.04	12.3097872	3.2314434	0.07820843	0.36409233	0.33830335	Per2	1
GO:0039694	viral RNA genome replication	1/47	25/14464	0.04	12.3097872	3.2314434	0.07820843	0.36409233	0.33830335	Pcbp2	1
GO:0046033	AMP metabolic process	1/47	25/14464	0.04	12.3097872	3.2314434	0.07820843	0.36409233	0.33830335	Xdh	1
GO:0046128	purine ribonucleoside metabolic process	1/47	25/14464	0.04	12.3097872	3.2314434	0.07820843	0.36409233	0.33830335	Xdh	1
GO:0097284	hepatocyte apoptotic process	1/47	25/14464	0.04	12.3097872	3.2314434	0.07820843	0.36409233	0.33830335	Bcl2l1	1
GO:0098810	neurotransmitter reuptake	1/47	25/14464	0.04	12.3097872	3.2314434	0.07820843	0.36409233	0.33830335	Per2	1
GO:1900117	regulation of execution phase of apoptosis	1/47	25/14464	0.04	12.3097872	3.2314434	0.07820843	0.36409233	0.33830335	Bcl2l1	1
GO:1902230	negative regulation of intrinsic apoptotic signaling pathway in response to DNA damage	1/47	25/14464	0.04	12.3097872	3.2314434	0.07820843	0.36409233	0.33830335	Bcl2l1	1
GO:0106106	cold-induced thermogenesis	2/47	143/14464	0.01398601	4.30412141	2.26713831	0.07864408	0.36409233	0.33830335	Per2/G0s2	2
GO:0030100	regulation of endocytosis	3/47	310/14464	0.00967742	2.97817433	2.01023341	0.07934097	0.36409233	0.33830335	Mertk/Bcl2l1/Mtmr2	3
GO:0043281	regulation of cysteine-type endopeptidase activity involved in apoptotic process	2/47	144/14464	0.01388889	4.27423168	2.25454971	0.07959143	0.36409233	0.33830335	Xdh/Ints1	2
GO:0003299	muscle hypertrophy in response to stress	1/47	26/14464	0.03846154	11.8363339	3.1575934	0.08120892	0.36409233	0.33830335	Klf15	1
GO:0014898	cardiac muscle hypertrophy in response to stress	1/47	26/14464	0.03846154	11.8363339	3.1575934	0.08120892	0.36409233	0.33830335	Klf15	1
GO:0035115	embryonic forelimb morphogenesis	1/47	26/14464	0.03846154	11.8363339	3.1575934	0.08120892	0.36409233	0.33830335	Rdh10	1
GO:0070536	protein K63-linked deubiquitination	1/47	26/14464	0.03846154	11.8363339	3.1575934	0.08120892	0.36409233	0.33830335	Otud7b	1
GO:0071108	protein K48-linked deubiquitination	1/47	26/14464	0.03846154	11.8363339	3.1575934	0.08120892	0.36409233	0.33830335	Otud7b	1
GO:0072010	glomerular epithelium development	1/47	26/14464	0.03846154	11.8363339	3.1575934	0.08120892	0.36409233	0.33830335	Klf15	1
GO:1900745	positive regulation of p38MAPK cascade	1/47	26/14464	0.03846154	11.8363339	3.1575934	0.08120892	0.36409233	0.33830335	Xdh	1
GO:1902042	negative regulation of extrinsic apoptotic signaling pathway via death domain receptors	1/47	26/14464	0.03846154	11.8363339	3.1575934	0.08120892	0.36409233	0.33830335	Bcl2l1	1
GO:2001236	regulation of extrinsic apoptotic signaling pathway	2/47	147/14464	0.01360544	4.18700246	2.21745965	0.08245511	0.36409233	0.33830335	G0s2/Bcl2l1	2
GO:0001779	natural killer cell differentiation	1/47	27/14464	0.03703704	11.3979511	3.0876769	0.08419986	0.36409233	0.33830335	Mertk	1
GO:0006536	glutamate metabolic process	1/47	27/14464	0.03703704	11.3979511	3.0876769	0.08419986	0.36409233	0.33830335	Prodh	1
GO:0006953	acute-phase response	1/47	27/14464	0.03703704	11.3979511	3.0876769	0.08419986	0.36409233	0.33830335	Cd163	1
GO:0014887	cardiac muscle adaptation	1/47	27/14464	0.03703704	11.3979511	3.0876769	0.08419986	0.36409233	0.33830335	Klf15	1
GO:0018394	peptidyl-lysine acetylation	1/47	27/14464	0.03703704	11.3979511	3.0876769	0.08419986	0.36409233	0.33830335	Klf15	1
GO:0030970	retrograde protein transport, ER to cytosol	1/47	27/14464	0.03703704	11.3979511	3.0876769	0.08419986	0.36409233	0.33830335	Herpud1	1
GO:0032288	myelin assembly	1/47	27/14464	0.03703704	11.3979511	3.0876769	0.08419986	0.36409233	0.33830335	Mtmr2	1
GO:0032633	interleukin-4 production	1/47	27/14464	0.03703704	11.3979511	3.0876769	0.08419986	0.36409233	0.33830335	Icosl	1
GO:0032801	receptor catabolic process	1/47	27/14464	0.03703704	11.3979511	3.0876769	0.08419986	0.36409233	0.33830335	Mtmr2	1
GO:0042572	retinol metabolic process	1/47	27/14464	0.03703704	11.3979511	3.0876769	0.08419986	0.36409233	0.33830335	Rdh10	1
GO:1903513	endoplasmic reticulum to cytosol transport	1/47	27/14464	0.03703704	11.3979511	3.0876769	0.08419986	0.36409233	0.33830335	Herpud1	1
GO:1904292	regulation of ERAD pathway	1/47	27/14464	0.03703704	11.3979511	3.0876769	0.08419986	0.36409233	0.33830335	Herpud1	1
GO:2000045	regulation of G1/S transition of mitotic cell cycle	2/47	151/14464	0.01324503	4.07608849	2.16951489	0.08632262	0.36409233	0.33830335	Fam107a/Crlf3	2
GO:0001780	neutrophil homeostasis	1/47	28/14464	0.03571429	10.9908815	3.02134318	0.08718127	0.36409233	0.33830335	Mertk	1
GO:0003401	axis elongation	1/47	28/14464	0.03571429	10.9908815	3.02134318	0.08718127	0.36409233	0.33830335	Rdh10	1
GO:0009162	deoxyribonucleoside monophosphate metabolic process	1/47	28/14464	0.03571429	10.9908815	3.02134318	0.08718127	0.36409233	0.33830335	Xdh	1
GO:0016226	iron-sulfur cluster assembly	1/47	28/14464	0.03571429	10.9908815	3.02134318	0.08718127	0.36409233	0.33830335	Xdh	1
GO:0031163	metallo-sulfur cluster assembly	1/47	28/14464	0.03571429	10.9908815	3.02134318	0.08718127	0.36409233	0.33830335	Xdh	1
GO:0032469	endoplasmic reticulum calcium ion homeostasis	1/47	28/14464	0.03571429	10.9908815	3.02134318	0.08718127	0.36409233	0.33830335	Herpud1	1
GO:0035303	regulation of dephosphorylation	1/47	28/14464	0.03571429	10.9908815	3.02134318	0.08718127	0.36409233	0.33830335	Mtmr2	1
GO:0046856	phosphatidylinositol dephosphorylation	1/47	28/14464	0.03571429	10.9908815	3.02134318	0.08718127	0.36409233	0.33830335	Mtmr2	1
GO:0048261	negative regulation of receptor-mediated endocytosis	1/47	28/14464	0.03571429	10.9908815	3.02134318	0.08718127	0.36409233	0.33830335	Mtmr2	1
GO:0097345	mitochondrial outer membrane permeabilization	1/47	28/14464	0.03571429	10.9908815	3.02134318	0.08718127	0.36409233	0.33830335	Bcl2l1	1
GO:0062013	positive regulation of small molecule metabolic process	2/47	154/14464	0.01298701	3.99668417	2.13462794	0.0892591	0.36409233	0.33830335	Bcl2l1/Rdh10	2
GO:1904375	regulation of protein localization to cell periphery	2/47	154/14464	0.01298701	3.99668417	2.13462794	0.0892591	0.36409233	0.33830335	Bcl2l1/Ppfia1	2
GO:0015813	L-glutamate transmembrane transport	1/47	29/14464	0.03448276	10.6118855	2.95828403	0.09015318	0.36409233	0.33830335	Per2	1
GO:0019369	arachidonate metabolic process	1/47	29/14464	0.03448276	10.6118855	2.95828403	0.09015318	0.36409233	0.33830335	Cyp2f2	1
GO:0043276	anoikis	1/47	29/14464	0.03448276	10.6118855	2.95828403	0.09015318	0.36409233	0.33830335	Bcl2l1	1
GO:0045671	negative regulation of osteoclast differentiation	1/47	29/14464	0.03448276	10.6118855	2.95828403	0.09015318	0.36409233	0.33830335	Fstl3	1
GO:0046006	regulation of activated T cell proliferation	1/47	29/14464	0.03448276	10.6118855	2.95828403	0.09015318	0.36409233	0.33830335	Icosl	1
GO:0060259	regulation of feeding behavior	1/47	29/14464	0.03448276	10.6118855	2.95828403	0.09015318	0.36409233	0.33830335	Mchr1	1
GO:0060441	epithelial tube branching involved in lung morphogenesis	1/47	29/14464	0.03448276	10.6118855	2.95828403	0.09015318	0.36409233	0.33830335	Rdh10	1
GO:0071480	cellular response to gamma radiation	1/47	29/14464	0.03448276	10.6118855	2.95828403	0.09015318	0.36409233	0.33830335	Bcl2l1	1
GO:1990845	adaptive thermogenesis	2/47	157/14464	0.01273885	3.92031441	2.1006119	0.09222539	0.36957269	0.34339554	Per2/G0s2	2
GO:0031589	cell-substrate adhesion	3/47	332/14464	0.00903614	2.78082543	1.87425746	0.09291418	0.36957269	0.34339554	Fam107a/Mertk/Tesk2	3
GO:0046427	positive regulation of receptor signaling pathway via JAK-STAT	1/47	30/14464	0.03333333	10.258156	2.89822732	0.09311562	0.36957269	0.34339554	Crlf3	1
GO:0051590	positive regulation of neurotransmitter transport	1/47	30/14464	0.03333333	10.258156	2.89822732	0.09311562	0.36957269	0.34339554	Bcl2l1	1
GO:0061050	regulation of cell growth involved in cardiac muscle cell development	1/47	30/14464	0.03333333	10.258156	2.89822732	0.09311562	0.36957269	0.34339554	Trip10	1
GO:0009119	ribonucleoside metabolic process	1/47	31/14464	0.03225806	9.92724777	2.84093176	0.09606862	0.37868144	0.35185911	Xdh	1
GO:0042278	purine nucleoside metabolic process	1/47	31/14464	0.03225806	9.92724777	2.84093176	0.09606862	0.37868144	0.35185911	Xdh	1
GO:0051017	actin filament bundle assembly	2/47	163/14464	0.01226994	3.77600835	2.03503574	0.09824405	0.37896252	0.35212028	Fam107a/Ppfia1	2
GO:1901991	negative regulation of mitotic cell cycle phase transition	2/47	163/14464	0.01226994	3.77600835	2.03503574	0.09824405	0.37896252	0.35212028	Fam107a/Crlf3	2
GO:2000116	regulation of cysteine-type endopeptidase activity	2/47	163/14464	0.01226994	3.77600835	2.03503574	0.09824405	0.37896252	0.35212028	Xdh/Ints1	2
GO:0009112	nucleobase metabolic process	1/47	32/14464	0.03125	9.61702128	2.78618255	0.0990122	0.37896252	0.35212028	Xdh	1
GO:0016180	snRNA processing	1/47	32/14464	0.03125	9.61702128	2.78618255	0.0990122	0.37896252	0.35212028	Ints1	1
GO:1900242	regulation of synaptic vesicle endocytosis	1/47	32/14464	0.03125	9.61702128	2.78618255	0.0990122	0.37896252	0.35212028	Bcl2l1	1
GO:1902110	positive regulation of mitochondrial membrane permeability involved in apoptotic process	1/47	32/14464	0.03125	9.61702128	2.78618255	0.0990122	0.37896252	0.35212028	Bcl2l1	1
GO:2000677	regulation of transcription regulatory region DNA binding	1/47	32/14464	0.03125	9.61702128	2.78618255	0.0990122	0.37896252	0.35212028	Per2	1
GO:0061572	actin filament bundle organization	2/47	166/14464	0.01204819	3.70776724	2.00340337	0.10129482	0.37896252	0.35212028	Fam107a/Ppfia1	2
GO:0051497	negative regulation of stress fiber assembly	1/47	33/14464	0.03030303	9.32559639	2.73378768	0.10194641	0.37896252	0.35212028	Ppfia1	1
GO:1903725	regulation of phospholipid metabolic process	1/47	33/14464	0.03030303	9.32559639	2.73378768	0.10194641	0.37896252	0.35212028	Mtmr2	1
GO:0045807	positive regulation of endocytosis	2/47	167/14464	0.01197605	3.68556504	1.99302215	0.10231768	0.37896252	0.35212028	Mertk/Bcl2l1	2
GO:0009410	response to xenobiotic stimulus	3/47	348/14464	0.00862069	2.65297139	1.78213174	0.10335638	0.37896252	0.35212028	Fmo2/Fmo1/Cyp2f2	3
GO:0019362	pyridine nucleotide metabolic process	2/47	169/14464	0.01183432	3.64194889	1.97249702	0.10437215	0.37896252	0.35212028	Fmo2/Fmo1	2
GO:0046496	nicotinamide nucleotide metabolic process	2/47	169/14464	0.01183432	3.64194889	1.97249702	0.10437215	0.37896252	0.35212028	Fmo2/Fmo1	2
GO:0001953	negative regulation of cell-matrix adhesion	1/47	34/14464	0.02941176	9.05131414	2.683575	0.10487126	0.37896252	0.35212028	Fam107a	1
GO:0010667	negative regulation of cardiac muscle cell apoptotic process	1/47	34/14464	0.02941176	9.05131414	2.683575	0.10487126	0.37896252	0.35212028	Trip10	1
GO:0035136	forelimb morphogenesis	1/47	34/14464	0.02941176	9.05131414	2.683575	0.10487126	0.37896252	0.35212028	Rdh10	1
GO:0048806	genitalia development	1/47	34/14464	0.02941176	9.05131414	2.683575	0.10487126	0.37896252	0.35212028	Mertk	1
GO:0071398	cellular response to fatty acid	1/47	34/14464	0.02941176	9.05131414	2.683575	0.10487126	0.37896252	0.35212028	Kcnk4	1
GO:1902175	regulation of oxidative stress-induced intrinsic apoptotic signaling pathway	1/47	34/14464	0.02941176	9.05131414	2.683575	0.10487126	0.37896252	0.35212028	Prodh	1
GO:1902686	mitochondrial outer membrane permeabilization involved in programmed cell death	1/47	34/14464	0.02941176	9.05131414	2.683575	0.10487126	0.37896252	0.35212028	Bcl2l1	1
GO:0007595	lactation	1/47	35/14464	0.02857143	8.79270517	2.63538957	0.10778679	0.37896252	0.35212028	Xdh	1
GO:0009268	response to pH	1/47	35/14464	0.02857143	8.79270517	2.63538957	0.10778679	0.37896252	0.35212028	Kcnk4	1
GO:0031076	embryonic camera-type eye development	1/47	35/14464	0.02857143	8.79270517	2.63538957	0.10778679	0.37896252	0.35212028	Rdh10	1
GO:0043392	negative regulation of DNA binding	1/47	35/14464	0.02857143	8.79270517	2.63538957	0.10778679	0.37896252	0.35212028	Per2	1
GO:0070229	negative regulation of lymphocyte apoptotic process	1/47	35/14464	0.02857143	8.79270517	2.63538957	0.10778679	0.37896252	0.35212028	Tsc22d3	1
GO:1905898	positive regulation of response to endoplasmic reticulum stress	1/47	35/14464	0.02857143	8.79270517	2.63538957	0.10778679	0.37896252	0.35212028	Herpud1	1
GO:0001659	temperature homeostasis	2/47	174/14464	0.01149425	3.53729518	1.92251695	0.10955798	0.37896252	0.35212028	Per2/G0s2	2
GO:0006839	mitochondrial transport	2/47	175/14464	0.01142857	3.51708207	1.91274094	0.11060343	0.37896252	0.35212028	Bcl2l1/Slc25a15	2
GO:1902806	regulation of cell cycle G1/S phase transition	2/47	175/14464	0.01142857	3.51708207	1.91274094	0.11060343	0.37896252	0.35212028	Fam107a/Crlf3	2
GO:0001523	retinoid metabolic process	1/47	36/14464	0.02777778	8.54846336	2.58909155	0.11069302	0.37896252	0.35212028	Rdh10	1
GO:0009167	purine ribonucleoside monophosphate metabolic process	1/47	36/14464	0.02777778	8.54846336	2.58909155	0.11069302	0.37896252	0.35212028	Xdh	1
GO:0010664	negative regulation of striated muscle cell apoptotic process	1/47	36/14464	0.02777778	8.54846336	2.58909155	0.11069302	0.37896252	0.35212028	Trip10	1
GO:0032350	regulation of hormone metabolic process	1/47	36/14464	0.02777778	8.54846336	2.58909155	0.11069302	0.37896252	0.35212028	Rdh10	1
GO:0043647	inositol phosphate metabolic process	1/47	36/14464	0.02777778	8.54846336	2.58909155	0.11069302	0.37896252	0.35212028	Ip6k2	1
GO:0050798	activated T cell proliferation	1/47	36/14464	0.02777778	8.54846336	2.58909155	0.11069302	0.37896252	0.35212028	Icosl	1
GO:0050951	sensory perception of temperature stimulus	1/47	36/14464	0.02777778	8.54846336	2.58909155	0.11069302	0.37896252	0.35212028	Kcnk4	1
GO:0060292	long-term synaptic depression	1/47	36/14464	0.02777778	8.54846336	2.58909155	0.11069302	0.37896252	0.35212028	Bcl2l1	1

GO:1901889	negative regulation of cell junction assembly	1/47	36/14464	0.02777778	8.54846336	2.58909155	0.11069302	0.37896252	0.35212028 Fam107a	1
GO:1902229	regulation of intrinsic apoptotic signaling pathway in response to DNA damage	1/47	36/14464	0.02777778	8.54846336	2.58909155	0.11069302	0.37896252	0.35212028 Bcl2l1	1
GO:0048872	homeostasis of number of cells	3/47	360/14464	0.00833333	2.56453901	1.7163552	0.11148549	0.37896252	0.35212028 Tsc22d3/Slc1a5/Mertk	3
GO:0072524	pyridine-containing compound metabolic process	2/47	176/14464	0.01136364	3.49709865	1.90303592	0.11165158	0.37896252	0.35212028 Fmo2/Fmo1	2
GO:0001504	neurotransmitter uptake	1/47	37/14464	0.02702703	8.31742381	2.54455433	0.11358998	0.37896252	0.35212028 Per2	1
GO:0010613	positive regulation of cardiac muscle hypertrophy	1/47	37/14464	0.02702703	8.31742381	2.54455433	0.11358998	0.37896252	0.35212028 Trip10	1
GO:0014742	positive regulation of muscle hypertrophy	1/47	37/14464	0.02702703	8.31742381	2.54455433	0.11358998	0.37896252	0.35212028 Trip10	1
GO:0035794	positive regulation of mitochondrial membrane permeability	1/47	37/14464	0.02702703	8.31742381	2.54455433	0.11358998	0.37896252	0.35212028 Bcl2l1	1
GO:0038084	vascular endothelial growth factor signaling pathway	1/47	37/14464	0.02702703	8.31742381	2.54455433	0.11358998	0.37896252	0.35212028 Xdh	1
GO:0043277	apoptotic cell clearance	1/47	37/14464	0.02702703	8.31742381	2.54455433	0.11358998	0.37896252	0.35212028 Mertk	1
GO:0045601	regulation of endothelial cell differentiation	1/47	37/14464	0.02702703	8.31742381	2.54455433	0.11358998	0.37896252	0.35212028 Xdh	1
GO:0046326	positive regulation of D-glucose import	1/47	37/14464	0.02702703	8.31742381	2.54455433	0.11358998	0.37896252	0.35212028 Klf15	1
GO:0055023	positive regulation of cardiac muscle tissue growth	1/47	37/14464	0.02702703	8.31742381	2.54455433	0.11358998	0.37896252	0.35212028 Trip10	1
GO:1904894	positive regulation of receptor signaling pathway via STAT	1/47	37/14464	0.02702703	8.31742381	2.54455433	0.11358998	0.37896252	0.35212028 Crlf3	1
GO:0032924	activin receptor signaling pathway	1/47	38/14464	0.02631579	8.09854423	2.50166297	0.11647771	0.38414283	0.35693366 Fstl3	1
GO:0051938	L-glutamate import	1/47	38/14464	0.02631579	8.09854423	2.50166297	0.11647771	0.38414283	0.35693366 Per2	1
GO:0071312	cellular response to alkaloid	1/47	38/14464	0.02631579	8.09854423	2.50166297	0.11647771	0.38414283	0.35693366 Bcl2l1	1
GO:0120316	sperm flagellum assembly	1/47	38/14464	0.02631579	8.09854423	2.50166297	0.11647771	0.38414283	0.35693366 Cfap157	1
GO:0009126	purine nucleoside monophosphate metabolic process	1/47	39/14464	0.02564103	7.89088925	2.46031285	0.11935623	0.38589614	0.35856278 Xdh	1
GO:0032232	negative regulation of actin filament bundle assembly	1/47	39/14464	0.02564103	7.89088925	2.46031285	0.11935623	0.38589614	0.35856278 Ppfia1	1
GO:0060421	positive regulation of heart growth	1/47	39/14464	0.02564103	7.89088925	2.46031285	0.11935623	0.38589614	0.35856278 Trip10	1
GO:1900744	regulation of p38MAPK cascade	1/47	39/14464	0.02564103	7.89088925	2.46031285	0.11935623	0.38589614	0.35856278 Xdh	1
GO:1902108	regulation of mitochondrial membrane permeability involved in apoptotic process	1/47	39/14464	0.02564103	7.89088925	2.46031285	0.11935623	0.38589614	0.35856278 Bcl2l1	1
GO:1902882	regulation of response to oxidative stress	1/47	39/14464	0.02564103	7.89088925	2.46031285	0.11935623	0.38589614	0.35856278 Sesn1	1
GO:1903580	positive regulation of ATP metabolic process	1/47	39/14464	0.02564103	7.89088925	2.46031285	0.11935623	0.38589614	0.35856278 Bcl2l1	1
GO:0016101	diterpenoid metabolic process	1/47	40/14464	0.025	7.69361702	2.42040851	0.12222558	0.39017268	0.36253641 Rdh10	1
GO:1900181	negative regulation of protein localization to nucleus	1/47	40/14464	0.025	7.69361702	2.42040851	0.12222558	0.39017268	0.36253641 Otud7b	1
GO:1901617	organic hydroxy compound biosynthetic process	2/47	187/14464	0.01069519	3.29138696	1.80070029	0.12335143	0.39017268	0.36253641 Per2/Ip6k2	2
GO:0031400	negative regulation of protein modification process	3/47	378/14464	0.00793651	2.4424181	1.62249697	0.12412974	0.39017268	0.36253641 Per2/Klf15/Xdh	3
GO:0003298	physiological muscle hypertrophy	1/47	41/14464	0.02439024	7.50596783	2.38186263	0.12508577	0.39017268	0.36253641 Trip10	1
GO:0003301	physiological cardiac muscle hypertrophy	1/47	41/14464	0.02439024	7.50596783	2.38186263	0.12508577	0.39017268	0.36253641 Trip10	1
GO:0009394	2'-deoxyribonucleotide metabolic process	1/47	41/14464	0.02439024	7.50596783	2.38186263	0.12508577	0.39017268	0.36253641 Xdh	1
GO:0032527	protein exit from endoplasmic reticulum	1/47	41/14464	0.02439024	7.50596783	2.38186263	0.12508577	0.39017268	0.36253641 Herpud1	1
GO:0045047	protein targeting to ER	1/47	41/14464	0.02439024	7.50596783	2.38186263	0.12508577	0.39017268	0.36253641 Herpud1	1
GO:0046839	phospholipid dephosphorylation	1/47	41/14464	0.02439024	7.50596783	2.38186263	0.12508577	0.39017268	0.36253641 Mtmr2	1
GO:0048701	embryonic cranial skeleton morphogenesis	1/47	41/14464	0.02439024	7.50596783	2.38186263	0.12508577	0.39017268	0.36253641 Rdh10	1
GO:0061049	cell growth involved in cardiac muscle cell development	1/47	41/14464	0.02439024	7.50596783	2.38186263	0.12508577	0.39017268	0.36253641 Trip10	1
GO:1903421	regulation of synaptic vesicle recycling	1/47	41/14464	0.02439024	7.50596783	2.38186263	0.12508577	0.39017268	0.36253641 Bcl2l1	1
GO:0050806	positive regulation of synaptic transmission	2/47	189/14464	0.01058201	3.25655747	1.78291203	0.12551059	0.39043969	0.36278451 Fam107a/Bcl2l1	2
GO:0001937	negative regulation of endothelial cell proliferation	1/47	42/14464	0.02380952	7.32725431	2.34459515	0.12793684	0.39199586	0.36423046 Xdh	1
GO:0019692	deoxyribose phosphate metabolic process	1/47	42/14464	0.02380952	7.32725431	2.34459515	0.12793684	0.39199586	0.36423046 Xdh	1
GO:0045981	positive regulation of nucleotide metabolic process	1/47	42/14464	0.02380952	7.32725431	2.34459515	0.12793684	0.39199586	0.36423046 Bcl2l1	1
GO:0050974	detection of mechanical stimulus involved in sensory perception	1/47	42/14464	0.02380952	7.32725431	2.34459515	0.12793684	0.39199586	0.36423046 Kcnk4	1
GO:1900544	positive regulation of purine nucleotide metabolic process	1/47	42/14464	0.02380952	7.32725431	2.34459515	0.12793684	0.39199586	0.36423046 Bcl2l1	1
GO:0005978	glycogen biosynthetic process	1/47	43/14464	0.02325581	7.15685304	2.30853254	0.13077881	0.39199586	0.36423046 Per2	1
GO:0009250	glucan biosynthetic process	1/47	43/14464	0.02325581	7.15685304	2.30853254	0.13077881	0.39199586	0.36423046 Per2	1
GO:0009262	deoxyribonucleotide metabolic process	1/47	43/14464	0.02325581	7.15685304	2.30853254	0.13077881	0.39199586	0.36423046 Xdh	1
GO:0035850	epithelial cell differentiation involved in kidney development	1/47	43/14464	0.02325581	7.15685304	2.30853254	0.13077881	0.39199586	0.36423046 Klf15	1
GO:0042771	intrinsic apoptotic signaling pathway in response to DNA damage by p53 class mediator	1/47	43/14464	0.02325581	7.15685304	2.30853254	0.13077881	0.39199586	0.36423046 Phlda3	1
GO:0045022	early endosome to late endosome transport	1/47	43/14464	0.02325581	7.15685304	2.30853254	0.13077881	0.39199586	0.36423046 Mtmr2	1
GO:0070232	regulation of T cell apoptotic process	1/47	43/14464	0.02325581	7.15685304	2.30853254	0.13077881	0.39199586	0.36423046 Tsc22d3	1
GO:1902041	regulation of extrinsic apoptotic signaling pathway via death domain receptors	1/47	43/14464	0.02325581	7.15685304	2.30853254	0.13077881	0.39199586	0.36423046 Bcl2l1	1
GO:1905710	positive regulation of membrane permeability	1/47	43/14464	0.02325581	7.15685304	2.30853254	0.13077881	0.39199586	0.36423046 Bcl2l1	1
GO:0032944	regulation of mononuclear cell proliferation	2/47	196/14464	0.01020408	3.14025185	1.72247147	0.13313948	0.39738266	0.36923571 Bcl2l1/Icosl	2
GO:0010828	positive regulation of D-glucose transmembrane transport	1/47	44/14464	0.02272727	6.99419729	2.27360709	0.13361172	0.39738266	0.36923571 Klf15	1
GO:0030857	negative regulation of epithelial cell differentiation	1/47	44/14464	0.02272727	6.99419729	2.27360709	0.13361172	0.39738266	0.36923571 Xdh	1
GO:0002204	somatic recombination of immunoglobulin genes involved in immune response	1/47	45/14464	0.02222222	6.83877069	2.23975636	0.13643559	0.39756296	0.36940323 Icosl	1
GO:0002208	somatic diversification of immunoglobulins involved in immune response	1/47	45/14464	0.02222222	6.83877069	2.23975636	0.13643559	0.39756296	0.36940323 Icosl	1
GO:0043029	T cell homeostasis	1/47	45/14464	0.02222222	6.83877069	2.23975636	0.13643559	0.39756296	0.36940323 Tsc22d3	1
GO:0045190	isotype switching	1/47	45/14464	0.02222222	6.83877069	2.23975636	0.13643559	0.39756296	0.36940323 Icosl	1
GO:0045332	phospholipid translocation	1/47	45/14464	0.02222222	6.83877069	2.23975636	0.13643559	0.39756296	0.36940323 Plscr4	1
GO:0046854	phosphatidylinositol phosphate biosynthetic process	1/47	45/14464	0.02222222	6.83877069	2.23975636	0.13643559	0.39756296	0.36940323 Ip6k2	1
GO:0072599	establishment of protein localization to endoplasmic reticulum	1/47	45/14464	0.02222222	6.83877069	2.23975636	0.13643559	0.39756296	0.36940323 Herpud1	1
GO:1904262	negative regulation of TORC1 signaling	1/47	45/14464	0.02222222	6.83877069	2.23975636	0.13643559	0.39756296	0.36940323 Sesn1	1
GO:0008406	gonad development	2/47	200/14464	0.01	3.07744681	1.68913717	0.13754664	0.39978833	0.37147098 Bcl2l1/Rdh10	2
GO:0009636	response to toxic substance	2/47	201/14464	0.00995025	3.06213613	1.68093373	0.13865361	0.40075208	0.37236647 Cyp2f2/Sesn1	2
GO:0009116	nucleoside metabolic process	1/47	46/14464	0.02173913	6.69010176	2.20692265	0.13925046	0.40075208	0.37236647 Xdh	1
GO:0051155	positive regulation of striated muscle cell differentiation	1/47	46/14464	0.02173913	6.69010176	2.20692265	0.13925046	0.40075208	0.37236647 Trip10	1
GO:0002262	myeloid cell homeostasis	2/47	202/14464	0.00990099	3.04697704	1.6727811	0.13976261	0.40075208	0.37236647 Slc1a5/Mertk	2
GO:0045137	development of primary sexual characteristics	2/47	203/14464	0.00985222	3.0319673	1.66467868	0.1408736	0.40075208	0.37236647 Bcl2l1/Rdh10	2
GO:0016054	organic acid catabolic process	2/47	204/14464	0.00980392	3.01710471	1.65662587	0.14198658	0.40075208	0.37236647 Prodh/Acsf3	2
GO:0046395	carboxylic acid catabolic process	2/47	204/14464	0.00980392	3.01710471	1.65662587	0.14198658	0.40075208	0.37236647 Prodh/Acsf3	2
GO:0007566	embryo implantation	1/47	47/14464	0.0212766	6.54775917	2.17505254	0.14205634	0.40075208	0.37236647 Ints1	1
GO:0030514	negative regulation of BMP signaling pathway	1/47	47/14464	0.0212766	6.54775917	2.17505254	0.14205634	0.40075208	0.37236647 Fstl3	1
GO:0051955	regulation of amino acid transport	1/47	47/14464	0.0212766	6.54775917	2.17505254	0.14205634	0.40075208	0.37236647 Per2	1
GO:0090199	regulation of release of cytochrome c from mitochondria	1/47	47/14464	0.0212766	6.54775917	2.17505254	0.14205634	0.40075208	0.37236647 Bcl2l1	1
GO:1902459	positive regulation of stem cell population maintenance	1/47	47/14464	0.0212766	6.54775917	2.17505254	0.14205634	0.40075208	0.37236647 Tead3	1
GO:0015804	neutral amino acid transport	1/47	48/14464	0.02083333	6.41134752	2.14409651	0.14485327	0.40460741	0.37594872 Slc1a5	1
GO:0000082	G1/S transition of mitotic cell cycle	2/47	207/14464	0.00966184	2.97337856	1.63275934	0.14533713	0.40460741	0.37594872 Fam107a/Crlf3	2
GO:0003018	vascular process in circulatory system	2/47	207/14464	0.00966184	2.97337856	1.63275934	0.14533713	0.40460741	0.37594872 Per2/Slc1a5	2
GO:0097191	extrinsic apoptotic signaling pathway	2/47	207/14464	0.00966184	2.97337856	1.63275934	0.14533713	0.40460741	0.37594872 G0s2/Bcl2l1	2
GO:0001825	blastocyst formation	1/47	49/14464	0.02040816	6.28050369	2.11400855	0.14764128	0.40460741	0.37594872 Mxi1	1
GO:0006721	terpenoid metabolic process	1/47	49/14464	0.02040816	6.28050369	2.11400855	0.14764128	0.40460741	0.37594872 Rdh10	1
GO:0014888	striated muscle adaptation	1/47	49/14464	0.02040816	6.28050369	2.11400855	0.14764128	0.40460741	0.37594872 Klf15	1
GO:0034204	lipid translocation	1/47	49/14464	0.02040816	6.28050369	2.11400855	0.14764128	0.40460741	0.37594872 Plscr4	1
GO:0043030	regulation of macrophage activation	1/47	49/14464	0.02040816	6.28050369	2.11400855	0.14764128	0.40460741	0.37594872 Slc7a2	1
GO:0046622	positive regulation of organ growth	1/47	49/14464	0.02040816	6.28050369	2.11400855	0.14764128	0.40460741	0.37594872 Trip10	1
GO:0098927	vesicle-mediated transport between endosomal compartments	1/47	49/14464	0.02040816	6.28050369	2.11400855	0.14764128	0.40460741	0.37594872 Mtmr2	1
GO:0170040	proteinogenic amino acid catabolic process	1/47	49/14464	0.02040816	6.28050369	2.11400855	0.14764128	0.40460741	0.37594872 Prodh	1
GO:0007160	cell-matrix adhesion	2/47	210/14464	0.00952381	2.93090172	1.6093189	0.14870462	0.40655349	0.37775696 Fam107a/Tesk2	2
GO:0002762	negative regulation of myeloid leukocyte differentiation	1/47	50/14464	0.02	6.15489362	2.08474588	0.15042039	0.4073738	0.37851917 Fstl3	1
GO:0016073	snRNA metabolic process	1/47	50/14464	0.02	6.15489362	2.08474588	0.15042039	0.4073738	0.37851917 Ints1	1
GO:0043154	negative regulation of cysteine-type endopeptidase activity involved in apoptotic process	1/47	50/14464	0.02	6.15489362	2.08474588	0.15042039	0.4073738	0.37851917 Ints1	1
GO:0061005	cell differentiation involved in kidney development	1/47	50/14464	0.02	6.15489362	2.08474588	0.15042039	0.4073738	0.37851917 Klf15	1
GO:1905475	regulation of protein localization to membrane	2/47	212/14464	0.00943396	2.90325171	1.59392148	0.15095872	0.40787204	0.37898211 Bcl2l1/Ppfia1	2
GO:0045930	negative regulation of mitotic cell cycle	2/47	213/14464	0.00938967	2.88962142	1.58629008	0.15208843	0.40805079	0.3791482 Fam107a/Crlf3	2
GO:0070663	regulation of leukocyte proliferation	2/47	213/14464	0.00938967	2.88962142	1.58629008	0.15208843	0.40805079	0.3791482 Bcl2l1/Icosl	2
GO:0072593	reactive oxygen species metabolic process	2/47	213/14464	0.00938967	2.88962142	1.58629008	0.15208843	0.40805079	0.3791482 Xdh/Sesn1	2
GO:0048645	animal organ formation	1/47	51/14464	0.01960784	6.03420943	2.05626861	0.15319063	0.40914186	0.38016199 Rdh10	1
GO:1901615	organic hydroxy compound metabolic process	3/47	417/14464	0.00719424	2.21399051	1.43625714	0.15320603	0.40914186	0.38016199 Per2/Ip6k2/Rdh10	3
GO:2000242	negative regulation of reproductive process	1/47	52/14464	0.01923077	5.91816694	2.02853957	0.15595203	0.41551107	0.38608007 Bcl2l1	1
GO:0052548	regulation of endopeptidase activity	2/47	218/14464	0.00917431	2.8233457	1.54878618	0.15776272	0.41567466	0.38623207 Xdh/Ints1	2
GO:0008631	intrinsic apoptotic signaling pathway in response to oxidative stress	1/47	53/14464	0.01886792	5.80650341	2.00152401	0.15870461	0.41567466	0.38623207 Prodh	1
GO:0009161	ribonucleoside monophosphate metabolic process	1/47	53/14464	0.01886792	5.80650341	2.00152401	0.15870461	0.41567466	0.38623207 Xdh	1
GO:0042149	cellular response to glucose starvation	1/47	53/14464	0.01886792	5.80650341	2.00152401	0.15870461	0.41567466	0.38623207 Sesn1	1
GO:0050982	detection of mechanical stimulus	1/47	53/14464	0.01886792	5.80650341	2.00152401	0.15870461	0.41567466	0.38623207 Kcnk4	1
GO:0170035	L-amino acid catabolic process	1/47	53/14464	0.01886792	5.80650341	2.00152401	0.15870461	0.41567466	0.38623207 Prodh	1
GO:0006836	neurotransmitter transport	2/47	219/14464	0.00913242	2.81045371	1.54141268	0.15890256	0.41567466	0.38623207 Per2/Bcl2l1	2
GO:0048762	mesenchymal cell differentiation	2/47	219/14464	0.00913242	2.81045371	1.54141268	0.15890256	0.41567466	0.38623207 Trip10/Rdh10	2
GO:0002381	immunoglobulin production involved in immunoglobulin-mediated immune response	1/47	54/14464	0.01851852	5.69897557	1.97518942	0.16144841	0.41665274	0.38714087 Icosl	1
GO:0016447	somatic recombination of immunoglobulin gene segments	1/47	54/14464	0.01851852	5.69897557	1.97518942	0.16144841	0.41665274	0.38714087 Icosl	1
GO:0031641	regulation of myelination	1/47	54/14464	0.01851852	5.69897557	1.97518942	0.16144841	0.41665274	0.38714087 Mtmr2	1
GO:0035329	hippo signaling	1/47	54/14464	0.01851852	5.69897557	1.97518942	0.16144841	0.41665274	0.38714087 Tead3	1
GO:0046173	polyol biosynthetic process	1/47	54/14464	0.01851852	5.69897557	1.97518942	0.16144841	0.41665274	0.38714087 Ip6k2	1
GO:0097035	regulation of membrane lipid distribution	1/47	54/14464	0.01851852	5.69897557	1.97518942	0.16144841	0.41665274	0.38714087 Plscr4	1
GO:0010332	response to gamma radiation	1/47	55/14464	0.01818182	5.59535783	1.94950535	0.16418345	0.41673131	0.38721387 Bcl2l1	1
GO:0010812	negative regulation of cell-substrate adhesion	1/47	55/14464	0.01818182	5.59535783	1.94950535	0.16418345	0.41673131	0.38721387 Fam107a	1
GO:0019228	neuronal action potential	1/47	55/14464	0.01818182	5.59535783	1.94950535	0.16418345	0.41673131	0.38721387 Kcnk4	1
GO:0060425	lung morphogenesis	1/47	55/14464	0.01818182	5.59535783	1.94950535	0.16418345	0.41673131	0.38721387 Rdh10	1
GO:0070542	response to fatty acid	1/47	55/14464	0.01818182	5.59535783	1.94950535	0.16418345	0.41673131	0.38721387 Kcnk4	1
GO:0150052	regulation of postsynapse assembly	1/47	55/14464	0.01818182	5.59535783	1.94950535	0.16418345	0.41673131	0.38721387 Fam107a	1
GO:2001234	negative regulation of apoptotic signaling pathway	2/47	225/14464	0.00888889	2.73550827	1.49802436	0.16577471	0.41673131	0.38721387 Bcl2l1/Herpud1	2
GO:0034198	cellular response to amino acid starvation	1/47	56/14464	0.01785714	5.49544073	1.92444327	0.16690976	0.41673131	0.38721387 Sesn1	1
GO:0035924	cellular response to vascular endothelial growth factor stimulus	1/47	56/14464	0.01785714	5.49544073	1.92444327	0.16690976	0.41673131	0.38721387 Xdh	1
GO:0038066	p38MAPK cascade	1/47	56/14464	0.01785714	5.49544073	1.92444327	0.16690976	0.41673131	0.38721387 Xdh	1
GO:0039532	negative regulation of cytoplasmic pattern recognition receptor signaling pathway	1/47	56/14464	0.01785714	5.49544073	1.92444327	0.16690976	0.41673131	0.38721387 Pcbp2	1
GO:0043124	negative regulation of canonical NF-kappaB signal transduction	1/47	56/14464	0.01785714	5.49544073	1.92444327	0.16690976	0.41673131	0.38721387 Otud7b	1
GO:0050688	regulation of defense response to virus	1/47	56/14464	0.01785714	5.49544073	1.92444327	0.16690976	0.41673131	0.38721387 Pcbp2	1
GO:1903307	positive regulation of regulated secretory pathway	1/47	56/14464	0.01785714	5.49544073	1.92444327	0.16690976	0.41673131	0.38721387 Bcl2l1	1
GO:2001238	positive regulation of extrinsic apoptotic signaling pathway	1/47	56/14464	0.01785714	5.49544073	1.92444327	0.16690976	0.41673131	0.38721387 G0s2	1
GO:0042391	regulation of membrane potential	3/47	437/14464	0.00686499	2.11266371	1.34853716	0.16890421	0.41718183	0.38763248 Kcnk4/Bcl2l1/Mtmr2	3
GO:0006473	protein acetylation	1/47	57/14464	0.01754386	5.39902949	1.89997635	0.16962736	0.41718183	0.38763248 Klf15	1
GO:0009064	glutamine family amino acid metabolic process	1/47	57/14464	0.01754386	5.39902949	1.89997635	0.16962736	0.41718183	0.38763248 Prodh	1
GO:0010665	regulation of cardiac muscle cell apoptotic process	1/47	57/14464	0.01754386	5.39902949	1.89997635	0.16962736	0.41718183	0.38763248 Trip10	1
GO:0046902	regulation of mitochondrial membrane permeability	1/47	57/14464	0.01754386	5.39902949	1.89997635	0.16962736	0.41718183	0.38763248 Bcl2l1	1
GO:0098815	modulation of excitatory postsynaptic potential	1/47	57/14464	0.01754386	5.39902949	1.89997635	0.16962736	0.41718183	0.38763248 Mtmr2	1
GO:1900271	regulation of long-term synaptic potentiation	1/47	57/14464	0.01754386	5.39902949	1.89997635	0.16962736	0.41718183	0.38763248 Fam107a	1
GO:0046324	regulation of D-glucose import	1/47	58/14464	0.01724138	5.30594277	1.87607941	0.17233629	0.42204058	0.39214708 Klf15	1
GO:2000117	negative regulation of cysteine-type endopeptidase activity	1/47	58/14464	0.01724138	5.30594277	1.87607941	0.17233629	0.42204058	0.39214708 Ints1	1
GO:0044843	cell cycle G1/S phase transition	2/47	233/14464	0.00858369	2.64158524	1.44232909	0.17501983	0.42503605	0.39493039 Fam107a/Crlf3	2
GO:0010662	regulation of striated muscle cell apoptotic process	1/47	59/14464	0.01694915	5.21601154	1.85272874	0.17503657	0.42503605	0.39493039 Trip10	1
GO:0044458	motile cilium assembly	1/47	59/14464	0.01694915	5.21601154	1.85272874	0.17503657	0.42503605	0.39493039 Cfap157	1
GO:0070231	T cell apoptotic process	1/47	59/14464	0.01694915	5.21601154	1.85272874	0.17503657	0.42503605	0.39493039 Tsc22d3	1
GO:0007218	neuropeptide signaling pathway	1/47	60/14464	0.01666667	5.12907801	1.82990199	0.17772822	0.42617747	0.39599095 Mchr1	1
GO:0016445	somatic diversification of immunoglobulins	1/47	60/14464	0.01666667	5.12907801	1.82990199	0.17772822	0.42617747	0.39599095 Icosl	1
GO:0030101	natural killer cell activation	1/47	60/14464	0.01666667	5.12907801	1.82990199	0.17772822	0.42617747	0.39599095 Mertk	1
GO:0070228	regulation of lymphocyte apoptotic process	1/47	60/14464	0.01666667	5.12907801	1.82990199	0.17772822	0.42617747	0.39599095 Tsc22d3	1
GO:0071385	cellular response to glucocorticoid stimulus	1/47	60/14464	0.01666667	5.12907801	1.82990199	0.17772822	0.42617747	0.39599095 Fam107a	1
GO:2000300	regulation of synaptic vesicle exocytosis	1/47	60/14464	0.01666667	5.12907801	1.82990199	0.17772822	0.42617747	0.39599095 Bcl2l1	1
GO:0032677	regulation of interleukin-8 production	1/47	61/14464	0.01639344	5.04499477	1.80757811	0.18041128	0.42760045	0.39731315 Otud7b	1
GO:0034308	primary alcohol metabolic process	1/47	61/14464	0.01639344	5.04499477	1.80757811	0.18041128	0.42760045	0.39731315 Rdh10	1
GO:1990928	response to amino acid starvation	1/47	61/14464	0.01639344	5.04499477	1.80757811	0.18041128	0.42760045	0.39731315 Sesn1	1
GO:0001818	negative regulation of cytokine production	2/47	239/14464	0.0083682	2.5752693	1.40206547	0.18200955	0.42760045	0.39731315 Mertk/Otud7b	2
GO:0048167	regulation of synaptic plasticity	2/47	239/14464	0.0083682	2.5752693	1.40206547	0.18200955	0.42760045	0.39731315 Fam107a/Bcl2l1	2
GO:0000271	polysaccharide biosynthetic process	1/47	62/14464	0.01612903	4.96362388	1.78573722	0.18308577	0.42760045	0.39731315 Per2	1
GO:0002562	somatic diversification of immune receptors via germline recombination within a single locus	1/47	62/14464	0.01612903	4.96362388	1.78573722	0.18308577	0.42760045	0.39731315 Icosl	1
GO:0016444	somatic cell DNA recombination	1/47	62/14464	0.01612903	4.96362388	1.78573722	0.18308577	0.42760045	0.39731315 Icosl	1
GO:0032637	interleukin-8 production	1/47	62/14464	0.01612903	4.96362388	1.78573722	0.18308577	0.42760045	0.39731315 Otud7b	1
GO:0050805	negative regulation of synaptic transmission	1/47	62/14464	0.01612903	4.96362388	1.78573722	0.18308577	0.42760045	0.39731315 Bcl2l1	1
GO:0051893	regulation of focal adhesion assembly	1/47	62/14464	0.01612903	4.96362388	1.78573722	0.18308577	0.42760045	0.39731315 Fam107a	1
GO:0090109	regulation of cell-substrate junction assembly	1/47	62/14464	0.01612903	4.96362388	1.78573722	0.18308577	0.42760045	0.39731315 Fam107a	1
GO:1901988	negative regulation of cell cycle phase transition	2/47	240/14464	0.00833333	2.56453901	1.39547422	0.18317881	0.42760045	0.39731315 Fam107a/Crlf3	2
GO:0031669	cellular response to nutrient levels	2/47	241/14464	0.00829876	2.55389777	1.38891616	0.18434924	0.42760045	0.39731315 Fam107a/Sesn1	2
GO:0001836	release of cytochrome c from mitochondria	1/47	63/14464	0.01587302	4.8848362	1.76436053	0.18575172	0.42760045	0.39731315 Bcl2l1	1
GO:0006081	cellular aldehyde metabolic process	1/47	63/14464	0.01587302	4.8848362	1.76436053	0.18575172	0.42760045	0.39731315 Rdh10	1
GO:0006919	activation of cysteine-type endopeptidase activity involved in apoptotic process	1/47	63/14464	0.01587302	4.8848362	1.76436053	0.18575172	0.42760045	0.39731315 Xdh	1

GO:0010656	negative regulation of muscle cell apoptotic process	1/47	63/14464	0.01587302	4.8848362	1.76436053	0.18575172	0.42760045	0.39731315	Trip10	1
GO:0097192	extrinsic apoptotic signaling pathway in absence of ligand	1/47	63/14464	0.01587302	4.8848362	1.76436053	0.18575172	0.42760045	0.39731315	Bcl2l1	1
GO:1901657	glycosyl compound metabolic process	1/47	63/14464	0.01587302	4.8848362	1.76436053	0.18575172	0.42760045	0.39731315	Xdh	1
GO:0001541	ovarian follicle development	1/47	64/14464	0.015625	4.80851064	1.74343029	0.18840915	0.43280204	0.4021463	Bcl2l1	1
GO:0010659	cardiac muscle cell apoptotic process	1/47	65/14464	0.01538462	4.73453355	1.72292967	0.19105809	0.43280204	0.4021463	Trip10	1
GO:0032007	negative regulation of TOR signaling	1/47	65/14464	0.01538462	4.73453355	1.72292967	0.19105809	0.43280204	0.4021463	Sesn1	1
GO:0032922	circadian regulation of gene expression	1/47	65/14464	0.01538462	4.73453355	1.72292967	0.19105809	0.43280204	0.4021463	Per2	1
GO:0038034	signal transduction in absence of ligand	1/47	65/14464	0.01538462	4.73453355	1.72292967	0.19105809	0.43280204	0.4021463	Bcl2l1	1
GO:0046425	regulation of receptor signaling pathway via JAK-STAT	1/47	65/14464	0.01538462	4.73453355	1.72292967	0.19105809	0.43280204	0.4021463	Crlf3	1
GO:0050766	positive regulation of phagocytosis	1/47	65/14464	0.01538462	4.73453355	1.72292967	0.19105809	0.43280204	0.4021463	Mertk	1
GO:1904888	cranial skeletal system development	1/47	65/14464	0.01538462	4.73453355	1.72292967	0.19105809	0.43280204	0.4021463	Rdh10	1
GO:0006650	glycerophospholipid metabolic process	2/47	247/14464	0.00809717	2.49185976	1.35024684	0.19139552	0.43280204	0.4021463	Ip6k2/Mtmr2	2
GO:0051607	defense response to virus	2/47	248/14464	0.00806452	2.48181194	1.3439122	0.19257366	0.43459465	0.40381194	Bcl2l1/Pcbp2	2
GO:0002694	regulation of leukocyte activation	3/47	467/14464	0.00642398	1.97694656	1.22532823	0.19329829	0.43459465	0.40381194	Mertk/Slc7a2/Icosl	3
GO:0071384	cellular response to corticosteroid stimulus	1/47	66/14464	0.01515152	4.66279819	1.70284276	0.19369857	0.43459465	0.40381194	Fam107a	1
GO:0098869	cellular oxidant detoxification	1/47	66/14464	0.01515152	4.66279819	1.70284276	0.19369857	0.43459465	0.40381194	Sesn1	1
GO:0006766	vitamin metabolic process	1/47	67/14464	0.01492537	4.59320419	1.68315445	0.19633061	0.43624814	0.40534831	Rdh10	1
GO:0010658	striated muscle cell apoptotic process	1/47	67/14464	0.01492537	4.59320419	1.68315445	0.19633061	0.43624814	0.40534831	Trip10	1
GO:0097479	synaptic vesicle localization	1/47	67/14464	0.01492537	4.59320419	1.68315445	0.19633061	0.43624814	0.40534831	Bcl2l1	1
GO:0150116	regulation of cell-substrate junction organization	1/47	67/14464	0.01492537	4.59320419	1.68315445	0.19633061	0.43624814	0.40534831	Fam107a	1
GO:2000036	regulation of stem cell population maintenance	1/47	67/14464	0.01492537	4.59320419	1.68315445	0.19633061	0.43624814	0.40534831	Tead3	1
GO:0022409	positive regulation of cell-cell adhesion	2/47	252/14464	0.00793651	2.4424181	1.31887765	0.1972963	0.43701592	0.4060617	Fstl3/Icosl	2
GO:0006720	isoprenoid metabolic process	1/47	68/14464	0.01470588	4.52565707	1.66385041	0.19895425	0.43701592	0.4060617	Rdh10	1
GO:0033138	positive regulation of peptidyl-serine phosphorylation	1/47	68/14464	0.01470588	4.52565707	1.66385041	0.19895425	0.43701592	0.4060617	Ip6k2	1
GO:0045806	negative regulation of endocytosis	1/47	68/14464	0.01470588	4.52565707	1.66385041	0.19895425	0.43701592	0.4060617	Mtmr2	1
GO:0055021	regulation of cardiac muscle tissue growth	1/47	68/14464	0.01470588	4.52565707	1.66385041	0.19895425	0.43701592	0.4060617	Trip10	1
GO:1901606	alpha-amino acid catabolic process	1/47	68/14464	0.01470588	4.52565707	1.66385041	0.19895425	0.43701592	0.4060617	Prodh	1
GO:0002200	somatic diversification of immune receptors	1/47	69/14464	0.01449275	4.46006784	1.64491703	0.2015695	0.44041918	0.40922391	Icosl	1
GO:0071260	cellular response to mechanical stimulus	1/47	69/14464	0.01449275	4.46006784	1.64491703	0.2015695	0.44041918	0.40922391	Kcnk4	1
GO:0034976	response to endoplasmic reticulum stress	2/47	256/14464	0.0078125	2.40425532	1.29431582	0.20203417	0.44041918	0.40922391	Bcl2l1/Herpud1	2
GO:0050878	regulation of body fluid levels	2/47	256/14464	0.0078125	2.40425532	1.29431582	0.20203417	0.44041918	0.40922391	Xdh/Mertk	2
GO:0001676	long-chain fatty acid metabolic process	1/47	70/14464	0.01428571	4.39635258	1.62634138	0.20417639	0.44257444	0.41122651	Cyp2f2	1
GO:0032835	glomerulus development	1/47	70/14464	0.01428571	4.39635258	1.62634138	0.20417639	0.44257444	0.41122651	Klf15	1
GO:0045670	regulation of osteoclast differentiation	1/47	70/14464	0.01428571	4.39635258	1.62634138	0.20417639	0.44257444	0.41122651	Fstl3	1
GO:0032943	mononuclear cell proliferation	2/47	259/14464	0.00772201	2.3764068	1.27619414	0.20559687	0.44461137	0.41311916	Bcl2l1/Icosl	2
GO:0009123	nucleoside monophosphate metabolic process	1/47	71/14464	0.01408451	4.33443212	1.60811116	0.20677496	0.44461137	0.41311916	Xdh	1
GO:0071496	cellular response to external stimulus	1/47	71/14464	0.01408451	4.33443212	1.60811116	0.20677496	0.44461137	0.41311916	Kcnk4	1
GO:2000379	positive regulation of reactive oxygen species metabolic process	1/47	71/14464	0.01408451	4.33443212	1.60811116	0.20677496	0.44461137	0.41311916	Xdh	1
GO:0052547	regulation of peptidase activity	2/47	262/14464	0.00763359	2.34919604	1.25832151	0.20916702	0.44461137	0.41311916	Xdh/Ints1	2
GO:0014032	neural crest cell development	1/47	72/14464	0.01388889	4.27423168	1.59021466	0.20936521	0.44461137	0.41311916	Rdh10	1
GO:0019080	viral gene expression	1/47	72/14464	0.01388889	4.27423168	1.59021466	0.20936521	0.44461137	0.41311916	Pcbp2	1
GO:0030968	endoplasmic reticulum unfolded protein response	1/47	72/14464	0.01388889	4.27423168	1.59021466	0.20936521	0.44461137	0.41311916	Herpud1	1
GO:0051149	positive regulation of muscle cell differentiation	1/47	72/14464	0.01388889	4.27423168	1.59021466	0.20936521	0.44461137	0.41311916	Trip10	1
GO:0090559	regulation of membrane permeability	1/47	72/14464	0.01388889	4.27423168	1.59021466	0.20936521	0.44461137	0.41311916	Bcl2l1	1
GO:0097194	execution phase of apoptosis	1/47	72/14464	0.01388889	4.27423168	1.59021466	0.20936521	0.44461137	0.41311916	Bcl2l1	1
GO:0010611	regulation of cardiac muscle hypertrophy	1/47	73/14464	0.01369863	4.21568056	1.57264072	0.21194719	0.44619011	0.41458609	Trip10	1
GO:0032092	positive regulation of protein binding	1/47	73/14464	0.01369863	4.21568056	1.57264072	0.21194719	0.44619011	0.41458609	Ip6k2	1
GO:0060420	regulation of heart growth	1/47	73/14464	0.01369863	4.21568056	1.57264072	0.21194719	0.44619011	0.41458609	Trip10	1
GO:1904892	regulation of receptor signaling pathway via STAT	1/47	73/14464	0.01369863	4.21568056	1.57264072	0.21194719	0.44619011	0.41458609	Crlf3	1
GO:0002312	B cell activation involved in immune response	1/47	74/14464	0.01351351	4.1587119	1.55537872	0.21452092	0.44619011	0.41458609	Icosl	1
GO:0006690	icosanoid metabolic process	1/47	74/14464	0.01351351	4.1587119	1.55537872	0.21452092	0.44619011	0.41458609	Cyp2f2	1
GO:0019229	regulation of vasoconstriction	1/47	74/14464	0.01351351	4.1587119	1.55537872	0.21452092	0.44619011	0.41458609	Per2	1
GO:0042102	positive regulation of T cell proliferation	1/47	74/14464	0.01351351	4.1587119	1.55537872	0.21452092	0.44619011	0.41458609	Icosl	1
GO:0071479	cellular response to ionizing radiation	1/47	74/14464	0.01351351	4.1587119	1.55537872	0.21452092	0.44619011	0.41458609	Bcl2l1	1
GO:0060485	mesenchyme development	2/47	267/14464	0.00749064	2.3052036	1.22906903	0.21513253	0.44619011	0.41458609	Trip10/Rdh10	2
GO:0001656	metanephros development	1/47	75/14464	0.01333333	4.10326241	1.53841852	0.21708642	0.44619011	0.41458609	Rdh10	1
GO:0002526	acute inflammatory response	1/47	75/14464	0.01333333	4.10326241	1.53841852	0.21708642	0.44619011	0.41458609	Cd163	1
GO:0008625	extrinsic apoptotic signaling pathway via death domain receptors	1/47	75/14464	0.01333333	4.10326241	1.53841852	0.21708642	0.44619011	0.41458609	Bcl2l1	1
GO:0010827	regulation of D-glucose transmembrane transport	1/47	75/14464	0.01333333	4.10326241	1.53841852	0.21708642	0.44619011	0.41458609	Klf15	1
GO:0014743	regulation of muscle hypertrophy	1/47	75/14464	0.01333333	4.10326241	1.53841852	0.21708642	0.44619011	0.41458609	Trip10	1
GO:0032465	regulation of cytokinesis	1/47	75/14464	0.01333333	4.10326241	1.53841852	0.21708642	0.44619011	0.41458609	Bcl2l1	1
GO:0035082	axoneme assembly	1/47	75/14464	0.01333333	4.10326241	1.53841852	0.21708642	0.44619011	0.41458609	Cfap157	1
GO:0046323	D-glucose import	1/47	75/14464	0.01333333	4.10326241	1.53841852	0.21708642	0.44619011	0.41458609	Klf15	1
GO:0006809	nitric oxide biosynthetic process	1/47	76/14464	0.01315789	4.04927212	1.52175044	0.21964371	0.45064155	0.41872223	Slc7a2	1
GO:0071396	cellular response to lipid	3/47	499/14464	0.00601202	1.85016842	1.1035079	0.2202543	0.45104753	0.41909945	Fam107a/Kcnk4/Plscr4	3
GO:0007589	body fluid secretion	1/47	77/14464	0.01298701	3.99668417	1.50536525	0.22219283	0.45104753	0.41909945	Xdh	1
GO:0032890	regulation of organic acid transport	1/47	77/14464	0.01298701	3.99668417	1.50536525	0.22219283	0.45104753	0.41909945	Per2	1
GO:0033559	unsaturated fatty acid metabolic process	1/47	77/14464	0.01298701	3.99668417	1.50536525	0.22219283	0.45104753	0.41909945	Cyp2f2	1
GO:0043112	receptor metabolic process	1/47	77/14464	0.01298701	3.99668417	1.50536525	0.22219283	0.45104753	0.41909945	Mtmr2	1
GO:0070972	protein localization to endoplasmic reticulum	1/47	77/14464	0.01298701	3.99668417	1.50536525	0.22219283	0.45104753	0.41909945	Herpud1	1
GO:0006066	alcohol metabolic process	2/47	275/14464	0.00727273	2.23814313	1.18359189	0.2247124	0.45178568	0.41978532	Ip6k2/Rdh10	2
GO:0048864	stem cell development	1/47	78/14464	0.01282051	3.94544463	1.48925411	0.2247338	0.45178568	0.41978532	Rdh10	1
GO:0050795	regulation of behavior	1/47	78/14464	0.01282051	3.94544463	1.48925411	0.2247338	0.45178568	0.41978532	Mchr1	1
GO:0050906	detection of stimulus involved in sensory perception	1/47	78/14464	0.01282051	3.94544463	1.48925411	0.2247338	0.45178568	0.41978532	Kcnk4	1
GO:0007281	germ cell development	2/47	276/14464	0.00724638	2.23003392	1.17801742	0.22591257	0.45178568	0.41978532	Cfap157/Bcl2l1	2
GO:0016197	endosomal transport	2/47	276/14464	0.00724638	2.23003392	1.17801742	0.22591257	0.45178568	0.41978532	Bcl2l1/Mtmr2	2
GO:0010948	negative regulation of cell cycle process	2/47	277/14464	0.00722022	2.22198326	1.17246673	0.22711329	0.45178568	0.41978532	Fam107a/Crlf3	2
GO:0007405	neuroblast proliferation	1/47	79/14464	0.01265823	3.89550229	1.47340859	0.22726665	0.45178568	0.41978532	Tead3	1
GO:0009063	amino acid catabolic process	1/47	79/14464	0.01265823	3.89550229	1.47340859	0.22726665	0.45178568	0.41978532	Prodh	1
GO:0015914	phospholipid transport	1/47	79/14464	0.01265823	3.89550229	1.47340859	0.22726665	0.45178568	0.41978532	Plscr4	1
GO:0031397	negative regulation of protein ubiquitination	1/47	79/14464	0.01265823	3.89550229	1.47340859	0.22726665	0.45178568	0.41978532	Per2	1
GO:1903578	regulation of ATP metabolic process	1/47	79/14464	0.01265823	3.89550229	1.47340859	0.22726665	0.45178568	0.41978532	Bcl2l1	1
GO:0090287	regulation of cellular response to growth factor stimulus	2/47	278/14464	0.00719424	2.21399051	1.16693962	0.22831453	0.45308625	0.42099376	Xdh/Fstl3	2
GO:0002260	lymphocyte homeostasis	1/47	80/14464	0.0125	3.84680851	1.45782061	0.22979139	0.45367049	0.42153662	Tsc22d3	1
GO:0002532	production of molecular mediator involved in inflammatory response	1/47	80/14464	0.0125	3.84680851	1.45782061	0.22979139	0.45367049	0.42153662	Slc7a2	1
GO:0070227	lymphocyte apoptotic process	1/47	80/14464	0.0125	3.84680851	1.45782061	0.22979139	0.45367049	0.42153662	Tsc22d3	1
GO:0007601	visual perception	1/47	81/14464	0.01234568	3.79931705	1.44248243	0.23230806	0.4562911	0.42397161	Rdh10	1
GO:0045638	negative regulation of myeloid cell differentiation	1/47	81/14464	0.01234568	3.79931705	1.44248243	0.23230806	0.4562911	0.42397161	Fstl3	1
GO:0046209	nitric oxide metabolic process	1/47	81/14464	0.01234568	3.79931705	1.44248243	0.23230806	0.4562911	0.42397161	Slc7a2	1
GO:0032496	response to lipopolysaccharide	2/47	283/14464	0.00706714	2.17487407	1.13965075	0.23432812	0.45869331	0.42620367	Fmo1/Plscr4	2
GO:0043523	regulation of neuron apoptotic process	2/47	283/14464	0.00706714	2.17487407	1.13965075	0.23432812	0.45869331	0.42620367	Prodh/Bcl2l1	2
GO:0014033	neural crest cell differentiation	1/47	83/14464	0.01204819	3.70776724	1.41252618	0.23731729	0.46140574	0.42872397	Rdh10	1
GO:0030510	regulation of BMP signaling pathway	1/47	83/14464	0.01204819	3.70776724	1.41252618	0.23731729	0.46140574	0.42872397	Fstl3	1
GO:0050953	sensory perception of light stimulus	1/47	83/14464	0.01204819	3.70776724	1.41252618	0.23731729	0.46140574	0.42872397	Rdh10	1
GO:2001057	reactive nitrogen species metabolic process	1/47	83/14464	0.01204819	3.70776724	1.41252618	0.23731729	0.46140574	0.42872397	Slc7a2	1
GO:0070661	leukocyte proliferation	2/47	287/14464	0.00696864	2.14456224	1.11822414	0.23914692	0.46157388	0.42888021	Bcl2l1/Icosl	2
GO:0005977	glycogen metabolic process	1/47	84/14464	0.01190476	3.66362715	1.39789421	0.23980989	0.46157388	0.42888021	Per2	1
GO:0006094	gluconeogenesis	1/47	84/14464	0.01190476	3.66362715	1.39789421	0.23980989	0.46157388	0.42888021	Per2	1
GO:0016579	protein deubiquitination	1/47	84/14464	0.01190476	3.66362715	1.39789421	0.23980989	0.46157388	0.42888021	Otud7b	1
GO:0034620	cellular response to unfolded protein	1/47	84/14464	0.01190476	3.66362715	1.39789421	0.23980989	0.46157388	0.42888021	Herpud1	1
GO:0048488	synaptic vesicle endocytosis	1/47	84/14464	0.01190476	3.66362715	1.39789421	0.23980989	0.46157388	0.42888021	Bcl2l1	1
GO:0044042	glucan metabolic process	1/47	85/14464	0.01176471	3.62052566	1.38348422	0.24229451	0.46557761	0.43260034	Per2	1
GO:0001570	vasculogenesis	1/47	86/14464	0.01162791	3.57842652	1.36928994	0.24477119	0.46563304	0.43265185	Xdh	1
GO:0002090	regulation of receptor internalization	1/47	86/14464	0.01162791	3.57842652	1.36928994	0.24477119	0.46563304	0.43265185	Mtmr2	1
GO:0030168	platelet activation	1/47	86/14464	0.01162791	3.57842652	1.36928994	0.24477119	0.46563304	0.43265185	Mertk	1
GO:0043242	negative regulation of protein-containing complex disassembly	1/47	86/14464	0.01162791	3.57842652	1.36928994	0.24477119	0.46563304	0.43265185	Per2	1
GO:0072332	intrinsic apoptotic signaling pathway by p53 class mediator	1/47	86/14464	0.01162791	3.57842652	1.36928994	0.24477119	0.46563304	0.43265185	Phlda3	1
GO:2001237	negative regulation of extrinsic apoptotic signaling pathway	1/47	86/14464	0.01162791	3.57842652	1.36928994	0.24477119	0.46563304	0.43265185	Bcl2l1	1
GO:0072659	protein localization to plasma membrane	2/47	292/14464	0.00684932	2.10784028	1.09192718	0.24517875	0.46563304	0.43265185	Bcl2l1/Ppfia1	2
GO:0006661	phosphatidylinositol biosynthetic process	1/47	87/14464	0.01149425	3.53729518	1.35530537	0.24723995	0.46563304	0.43265185	Ip6k2	1
GO:0051881	regulation of mitochondrial membrane potential	1/47	87/14464	0.01149425	3.53729518	1.35530537	0.24723995	0.46563304	0.43265185	Bcl2l1	1
GO:0022412	cellular process involved in reproduction in multicellular organism	2/47	295/14464	0.00677966	2.08640462	1.07639998	0.24880159	0.46563304	0.43265185	Cfap157/Bcl2l1	2
GO:0019319	hexose biosynthetic process	1/47	88/14464	0.01136364	3.49709865	1.34152471	0.2497008	0.46563304	0.43265185	Per2	1
GO:0031123	RNA 3'-end processing	1/47	88/14464	0.01136364	3.49709865	1.34152471	0.2497008	0.46563304	0.43265185	Ints1	1
GO:0043543	protein acylation	1/47	88/14464	0.01136364	3.49709865	1.34152471	0.2497008	0.46563304	0.43265185	Klf15	1
GO:0043954	cellular component maintenance	1/47	88/14464	0.01136364	3.49709865	1.34152471	0.2497008	0.46563304	0.43265185	Mtmr2	1
GO:0048704	embryonic skeletal system morphogenesis	1/47	88/14464	0.01136364	3.49709865	1.34152471	0.2497008	0.46563304	0.43265185	Rdh10	1
GO:0051153	regulation of striated muscle cell differentiation	1/47	88/14464	0.01136364	3.49709865	1.34152471	0.2497008	0.46563304	0.43265185	Trip10	1
GO:1900542	regulation of purine nucleotide metabolic process	1/47	88/14464	0.01136364	3.49709865	1.34152471	0.2497008	0.46563304	0.43265185	Bcl2l1	1
GO:1990748	cellular detoxification	1/47	88/14464	0.01136364	3.49709865	1.34152471	0.2497008	0.46563304	0.43265185	Sesn1	1
GO:0045862	positive regulation of proteolysis	2/47	296/14464	0.00675676	2.07935595	1.07126507	0.25000975	0.46563304	0.43265185	Xdh/Herpud1	2
GO:0002237	response to molecule of bacterial origin	2/47	297/14464	0.00673401	2.07235475	1.0661503	0.25121814	0.46702295	0.43394331	Fmo1/Plscr4	2
GO:0006140	regulation of nucleotide metabolic process	1/47	89/14464	0.01123596	3.4578054	1.32794242	0.25215378	0.46702295	0.43394331	Bcl2l1	1
GO:1903321	negative regulation of protein modification by small protein conjugation or removal	1/47	89/14464	0.01123596	3.4578054	1.32794242	0.25215378	0.46702295	0.43394331	Per2	1
GO:0010951	negative regulation of endopeptidase activity	1/47	90/14464	0.01111111	3.41938534	1.31455316	0.25459892	0.46702295	0.43394331	Ints1	1
GO:0019233	sensory perception of pain	1/47	90/14464	0.01111111	3.41938534	1.31455316	0.25459892	0.46702295	0.43394331	Kcnk4	1
GO:0051101	regulation of DNA binding	1/47	90/14464	0.01111111	3.41938534	1.31455316	0.25459892	0.46702295	0.43394331	Per2	1
GO:0140238	presynaptic endocytosis	1/47	90/14464	0.01111111	3.41938534	1.31455316	0.25459892	0.46702295	0.43394331	Bcl2l1	1
GO:0071214	cellular response to abiotic stimulus	2/47	301/14464	0.00664452	2.04481516	1.04588939	0.2560539	0.46702295	0.43394331	Kcnk4/Bcl2l1	2
GO:0104004	cellular response to environmental stimulus	2/47	301/14464	0.00664452	2.04481516	1.04588939	0.2560539	0.46702295	0.43394331	Kcnk4/Bcl2l1	2
GO:0006970	response to osmotic stress	1/47	91/14464	0.01098901	3.38180968	1.30135179	0.25703622	0.46702295	0.43394331	Tsc22d3	1
GO:0019751	polyol metabolic process	1/47	91/14464	0.01098901	3.38180968	1.30135179	0.25703622	0.46702295	0.43394331	Ip6k2	1
GO:0042310	vasoconstriction	1/47	91/14464	0.01098901	3.38180968	1.30135179	0.25703622	0.46702295	0.43394331	Per2	1
GO:0045921	positive regulation of exocytosis	1/47	91/14464	0.01098901	3.38180968	1.30135179	0.25703622	0.46702295	0.43394331	Bcl2l1	1
GO:0051952	regulation of amine transport	1/47	91/14464	0.01098901	3.38180968	1.30135179	0.25703622	0.46702295	0.43394331	Per2	1
GO:1904019	epithelial cell apoptotic process	1/47	91/14464	0.01098901	3.38180968	1.30135179	0.25703622	0.46702295	0.43394331	Bcl2l1	1
GO:0001822	kidney development	2/47	303/14464	0.00660066	2.03131803	1.03587567	0.25847289	0.46702295	0.43394331	Klf15/Rdh10	2
GO:0099173	postsynapse organization	2/47	303/14464	0.00660066	2.03131803	1.03587567	0.25847289	0.46702295	0.43394331	Fam107a/Mtmr2	2
GO:0007631	feeding behavior	1/47	92/14464	0.01086957	3.34505088	1.28833336	0.25946573	0.46702295	0.43394331	Mchr1	1
GO:0009581	detection of external stimulus	1/47	92/14464	0.01086957	3.34505088	1.28833336	0.25946573	0.46702295	0.43394331	Kcnk4	1
GO:0009582	detection of abiotic stimulus	1/47	92/14464	0.01086957	3.34505088	1.28833336	0.25946573	0.46702295	0.43394331	Kcnk4	1
GO:0046889	positive regulation of lipid biosynthetic process	1/47	92/14464	0.01086957	3.34505088	1.28833336	0.25946573	0.46702295	0.43394331	Rdh10	1
GO:1901990	regulation of mitotic cell cycle phase transition	2/47	304/14464	0.00657895	2.02463606	1.03089747	0.25968261	0.46702295	0.43394331	Fam107a/Crlf3	2
GO:0006112	energy reserve metabolic process	1/47	93/14464	0.01075269	3.30908259	1.27549312	0.26188746	0.46733716	0.43423527	Per2	1
GO:0042116	macrophage activation	1/47	93/14464	0.01075269	3.30908259	1.27549312	0.26188746	0.46733716	0.43423527	Slc7a2	1
GO:0043280	positive regulation of cysteine-type endopeptidase activity involved in apoptotic process	1/47	93/14464	0.01075269	3.30908259	1.27549312	0.26188746	0.46733716	0.43423527	Xdh	1
GO:0046364	monosaccharide biosynthetic process	1/47	93/14464	0.01075269	3.30908259	1.27549312	0.26188746	0.46733716	0.43423527	Per2	1
GO:1901874	negative regulation of post-translational protein modification	1/47	93/14464	0.01075269	3.30908259	1.27549312	0.26188746	0.46733716	0.43423527	Per2	1
GO:0034446	substrate adhesion-dependent cell spreading	1/47	94/14464	0.0106383	3.27387958	1.26282647	0.26430144	0.46985442	0.43657423	Mertk	1
GO:0051492	regulation of stress fiber assembly	1/47	94/14464	0.0106383	3.27387958	1.26282647	0.26430144	0.46985442	0.43657423	Ppfia1	1
GO:0051098	regulation of binding	2/47	308/14464	0.00649351	1.99834208	1.01117223	0.26452273	0.46985442	0.43657423	Per2/Ip6k2	2
GO:0006754	ATP biosynthetic process	1/47	95/14464	0.01052632	3.23941769	1.25032901	0.26670769	0.47082907	0.43747984	Bcl2l1	1
GO:0019226	transmission of nerve impulse	1/47	95/14464	0.01052632	3.23941769	1.25032901	0.26670769	0.47082907	0.43747984	Kcnk4	1
GO:1990542	mitochondrial transmembrane transport	1/47	95/14464	0.01052632	3.23941769	1.25032901	0.26670769	0.47082907	0.43747984	Slc25a15	1
GO:2000060	positive regulation of ubiquitin-dependent protein catabolic process	1/47	95/14464	0.01052632	3.23941769	1.25032901	0.26670769	0.47082907	0.43747984	Herpud1	1
GO:0009150	purine ribonucleotide metabolic process	2/47	311/14464	0.00643087	1.97906547	0.99657124	0.26815373	0.47189979	0.43847472	Xdh/Bcl2l1	2
GO:0043010	camera-type eye development	2/47	311/14464	0.00643087	1.97906547	0.99657124	0.26815373	0.47189979	0.43847472	Mertk/Rdh10	2
GO:0050764	regulation of phagocytosis	1/47	96/14464	0.01041667	3.20567376	1.23799647	0.26910624	0.47189979	0.43847472	Mertk	1
GO:1902106	negative regulation of leukocyte differentiation	1/47	96/14464	0.01041667	3.20567376	1.23799647	0.26910624	0.47189979	0.43847472	Fstl3	1
GO:0009615	response to virus	2/47	312/14464	0.00641026	1.97272231	0.99174025	0.26936417	0.47189979	0.43847472	Bcl2l1/Pcbp2	2
GO:0016239	positive regulation of macroautophagy	1/47	97/14464	0.01030928	3.17262558	1.22582474	0.27149711	0.47491363	0.44127509	Sesn1	1
GO:0072001	renal system development	2/47	315/14464	0.00634921	1.95393448	0.97735339	0.27299562	0.47681025	0.44303737	Klf15/Rdh10	2
GO:0036465	synaptic vesicle recycling	1/47	98/14464	0.01020408	3.14025185	1.21380986	0.27388032	0.47690809	0.44312829	Bcl2l1	1
GO:0055013	cardiac muscle cell development	1/47	98/14464	0.01020408	3.14025185	1.21380986	0.27388032	0.47690809	0.44312829	Trip10	1
GO:0033135	regulation of peptidyl-serine phosphorylation	1/47	99/14464	0.01010101	3.10853213	1.201948	0.2762559	0.47793754	0.44408481	Ip6k2	1
GO:0046620	regulation of organ growth	1/47	99/14464	0.01010101	3.10853213	1.201948	0.2762559	0.47793754	0.44408481	Trip10	1

GO:1903432 regulation of TORC1 signaling	1/47	99/14464	0.01010101	3.10853213	1.201948	0.2762559	0.47793754	0.44408481	Sesn1	1
GO:0006644 phospholipid metabolic process	2/47	319/14464	0.00626959	1.92943374	0.95841361	0.27783739	0.47793754	0.44408481	Ip6k2/Mtmr2	2
GO:0008645 hexose transmembrane transport	1/47	100/14464	0.01	3.07744681	1.19023547	0.27862388	0.47793754	0.44408481	Klf15	1
GO:0035967 cellular response to topologically incorrect protein	1/47	100/14464	0.01	3.07744681	1.19023547	0.27862388	0.47793754	0.44408481	Herpud1	1
GO:0046928 regulation of neurotransmitter secretion	1/47	100/14464	0.01	3.07744681	1.19023547	0.27862388	0.47793754	0.44408481	Bcl2l1	1
GO:0055017 cardiac muscle tissue growth	1/47	100/14464	0.01	3.07744681	1.19023547	0.27862388	0.47793754	0.44408481	Trip10	1
GO:0060079 excitatory postsynaptic potential	1/47	100/14464	0.01	3.07744681	1.19023547	0.27862388	0.47793754	0.44408481	Mtmr2	1
GO:1904659 D-glucose transmembrane transport	1/47	100/14464	0.01	3.07744681	1.19023547	0.27862388	0.47793754	0.44408481	Klf15	1
GO:0010660 regulation of muscle cell apoptotic process	1/47	101/14464	0.00990099	3.04697704	1.1786687	0.28098427	0.47913022	0.44519302	Trip10	1
GO:0015837 amine transport	1/47	101/14464	0.00990099	3.04697704	1.1786687	0.28098427	0.47913022	0.44519302	Per2	1
GO:0050852 T cell receptor signaling pathway	1/47	101/14464	0.00990099	3.04697704	1.1786687	0.28098427	0.47913022	0.44519302	Icosl	1
GO:0097237 cellular response to toxic substance	1/47	101/14464	0.00990099	3.04697704	1.1786687	0.28098427	0.47913022	0.44519302	Sesn1	1
GO:0015749 monosaccharide transmembrane transport	1/47	102/14464	0.00980392	3.01710471	1.16724425	0.28333711	0.48100444	0.44693448	Klf15	1
GO:0099068 postsynapse assembly	1/47	102/14464	0.00980392	3.01710471	1.16724425	0.28333711	0.48100444	0.44693448	Fam107a	1
GO:1903707 negative regulation of hemopoiesis	1/47	102/14464	0.00980392	3.01710471	1.16724425	0.28333711	0.48100444	0.44693448	Fstl3	1
GO:0031647 regulation of protein stability	2/47	324/14464	0.00617284	1.89965852	0.93511747	0.28388825	0.4812303	0.44714435	Fam107a/Ip6k2	2
GO:0046486 glycerolipid metabolic process	2/47	325/14464	0.00615385	1.89381342	0.9305075	0.28509812	0.48157762	0.44746706	Ip6k2/Mtmr2	2
GO:0042752 regulation of circadian rhythm	1/47	103/14464	0.00970874	2.98781244	1.15595878	0.2856824	0.48157762	0.44746706	Per2	1
GO:0043502 regulation of muscle adaptation	1/47	103/14464	0.00970874	2.98781244	1.15595878	0.2856824	0.48157762	0.44746706	Trip10	1
GO:0031330 negative regulation of cellular catabolic process	1/47	104/14464	0.00961538	2.95908347	1.14480907	0.28802019	0.48157762	0.44746706	Mtmr2	1
GO:0043279 response to alkaloid	1/47	104/14464	0.00961538	2.95908347	1.14480907	0.28802019	0.48157762	0.44746706	Bcl2l1	1
GO:0070646 protein modification by small protein removal	1/47	104/14464	0.00961538	2.95908347	1.14480907	0.28802019	0.48157762	0.44746706	Otud7b	1
GO:0072009 nephron epithelium development	1/47	104/14464	0.00961538	2.95908347	1.14480907	0.28802019	0.48157762	0.44746706	Klf15	1
GO:0098754 detoxification	1/47	104/14464	0.00961538	2.95908347	1.14480907	0.28802019	0.48157762	0.44746706	Sesn1	1
GO:0032956 regulation of actin cytoskeleton organization	2/47	328/14464	0.00609756	1.87649196	0.91677394	0.28872691	0.48157762	0.44746706	Fam107a/Ppfia1	2
GO:0002377 immunoglobulin production	1/47	105/14464	0.00952381	2.93090172	1.13379202	0.29035048	0.48157762	0.44746706	Icosl	1
GO:0002832 negative regulation of response to biotic stimulus	1/47	105/14464	0.00952381	2.93090172	1.13379202	0.29035048	0.48157762	0.44746706	Pcbp2	1
GO:0036503 ERAD pathway	1/47	105/14464	0.00952381	2.93090172	1.13379202	0.29035048	0.48157762	0.44746706	Herpud1	1
GO:0110020 regulation of actomyosin structure organization	1/47	105/14464	0.00952381	2.93090172	1.13379202	0.29035048	0.48157762	0.44746706	Ppfia1	1
GO:1901800 positive regulation of proteasomal protein catabolic process	1/47	105/14464	0.00952381	2.93090172	1.13379202	0.29035048	0.48157762	0.44746706	Herpud1	1
GO:0009206 purine ribonucleoside triphosphate biosynthetic process	1/47	106/14464	0.00943396	2.90325171	1.12290459	0.29267331	0.48157762	0.44746706	Bcl2l1	1
GO:0097061 dendritic spine organization	1/47	106/14464	0.00943396	2.90325171	1.12290459	0.29267331	0.48157762	0.44746706	Mtmr2	1
GO:0009259 ribonucleotide metabolic process	2/47	332/14464	0.0060241	1.85388362	0.89868312	0.29356296	0.48157762	0.44746706	Xdh/Bcl2l1	2
GO:0005976 polysaccharide metabolic process	1/47	107/14464	0.00934579	2.87611851	1.11214389	0.2949887	0.48157762	0.44746706	Per2	1
GO:0009145 purine nucleoside triphosphate biosynthetic process	1/47	107/14464	0.00934579	2.87611851	1.11214389	0.2949887	0.48157762	0.44746706	Bcl2l1	1
GO:0016064 immunoglobulin mediated immune response	1/47	107/14464	0.00934579	2.87611851	1.11214389	0.2949887	0.48157762	0.44746706	Icosl	1
GO:0055006 cardiac cell development	1/47	107/14464	0.00934579	2.87611851	1.11214389	0.2949887	0.48157762	0.44746706	Trip10	1
GO:0060419 heart growth	1/47	107/14464	0.00934579	2.87611851	1.11214389	0.2949887	0.48157762	0.44746706	Trip10	1
GO:0008585 female gonad development	1/47	108/14464	0.00925926	2.84948779	1.10150708	0.29729667	0.48157762	0.44746706	Bcl2l1	1
GO:0019079 viral genome replication	1/47	108/14464	0.00925926	2.84948779	1.10150708	0.29729667	0.48157762	0.44746706	Pcbp2	1
GO:0030316 osteoclast differentiation	1/47	108/14464	0.00925926	2.84948779	1.10150708	0.29729667	0.48157762	0.44746706	Fstl3	1
GO:0032231 regulation of actin filament bundle assembly	1/47	108/14464	0.00925926	2.84948779	1.10150708	0.29729667	0.48157762	0.44746706	Ppfia1	1
GO:0050671 positive regulation of lymphocyte proliferation	1/47	108/14464	0.00925926	2.84948779	1.10150708	0.29729667	0.48157762	0.44746706	Icosl	1
GO:2001056 positive regulation of cysteine-type endopeptidase activity	1/47	108/14464	0.00925926	2.84948779	1.10150708	0.29729667	0.48157762	0.44746706	Xdh	1
GO:0001578 microtubule bundle formation	1/47	109/14464	0.00917431	2.8233457	1.09099143	0.29959725	0.48157762	0.44746706	Cfap157	1
GO:0030326 embryonic limb morphogenesis	1/47	109/14464	0.00917431	2.8233457	1.09099143	0.29959725	0.48157762	0.44746706	Rdh10	1
GO:0035113 embryonic appendage morphogenesis	1/47	109/14464	0.00917431	2.8233457	1.09099143	0.29959725	0.48157762	0.44746706	Rdh10	1
GO:0039531 regulation of cytoplasmic pattern recognition receptor signaling pathway	1/47	109/14464	0.00917431	2.8233457	1.09099143	0.29959725	0.48157762	0.44746706	Pcbp2	1
GO:0002250 adaptive immune response	2/47	337/14464	0.00593472	1.82637793	0.87641411	0.29960324	0.48157762	0.44746706	Otud7b/Icosl	2
GO:0006986 response to unfolded protein	1/47	110/14464	0.00909091	2.79767892	1.08059429	0.30189045	0.48157762	0.44746706	Herpud1	1
GO:0019724 B cell mediated immunity	1/47	110/14464	0.00909091	2.79767892	1.08059429	0.30189045	0.48157762	0.44746706	Icosl	1
GO:0034219 carbohydrate transmembrane transport	1/47	110/14464	0.00909091	2.79767892	1.08059429	0.30189045	0.48157762	0.44746706	Klf15	1
GO:0045446 endothelial cell differentiation	1/47	110/14464	0.00909091	2.79767892	1.08059429	0.30189045	0.48157762	0.44746706	Xdh	1
GO:0046545 development of primary female sexual characteristics	1/47	110/14464	0.00909091	2.79767892	1.08059429	0.30189045	0.48157762	0.44746706	Bcl2l1	1
GO:0019693 ribose phosphate metabolic process	2/47	339/14464	0.00589971	1.81560284	0.86761094	0.30201758	0.48157762	0.44746706	Xdh/Bcl2l1	2
GO:0051402 neuron apoptotic process	2/47	339/14464	0.00589971	1.81560284	0.86761094	0.30201758	0.48157762	0.44746706	Prodh/Bcl2l1	2
GO:0007006 mitochondrial membrane organization	1/47	111/14464	0.00900901	2.7724746	1.0703131	0.30417631	0.48157762	0.44746706	Bcl2l1	1
GO:0009201 ribonucleoside triphosphate biosynthetic process	1/47	111/14464	0.00900901	2.7724746	1.0703131	0.30417631	0.48157762	0.44746706	Bcl2l1	1
GO:0030038 contractile actin filament bundle assembly	1/47	111/14464	0.00900901	2.7724746	1.0703131	0.30417631	0.48157762	0.44746706	Ppfia1	1
GO:0031398 positive regulation of protein ubiquitination	1/47	111/14464	0.00900901	2.7724746	1.0703131	0.30417631	0.48157762	0.44746706	Fam107a	1
GO:0043149 stress fiber assembly	1/47	111/14464	0.00900901	2.7724746	1.0703131	0.30417631	0.48157762	0.44746706	Ppfia1	1
GO:0051928 positive regulation of calcium ion transport	1/47	111/14464	0.00900901	2.7724746	1.0703131	0.30417631	0.48157762	0.44746706	Mchr1	1
GO:0060291 long-term synaptic potentiation	1/47	111/14464	0.00900901	2.7724746	1.0703131	0.30417631	0.48157762	0.44746706	Fam107a	1
GO:0099565 chemical synaptic transmission, postsynaptic	1/47	111/14464	0.00900901	2.7724746	1.0703131	0.30417631	0.48157762	0.44746706	Mtmr2	1
GO:0010657 muscle cell apoptotic process	1/47	112/14464	0.00892857	2.74772036	1.06014536	0.30645483	0.48446641	0.45015124	Trip10	1
GO:0045786 negative regulation of cell cycle	2/47	343/14464	0.0058309	1.79442963	0.85017913	0.30684275	0.48446641	0.45015124	Fam107a/Crlf3	2
GO:0038202 TORC1 signaling	1/47	113/14464	0.00884956	2.72340426	1.05008867	0.30872606	0.48677218	0.45229369	Sesn1	1
GO:0001952 regulation of cell-matrix adhesion	1/47	114/14464	0.00877193	2.69951474	1.04014069	0.31099	0.48700612	0.45251106	Fam107a	1
GO:0007259 cell surface receptor signaling pathway via JAK-STAT	1/47	114/14464	0.00877193	2.69951474	1.04014069	0.31099	0.48700612	0.45251106	Crlf3	1
GO:0008637 apoptotic mitochondrial changes	1/47	114/14464	0.00877193	2.69951474	1.04014069	0.31099	0.48700612	0.45251106	Bcl2l1	1
GO:0008643 carbohydrate transport	1/47	114/14464	0.00877193	2.69951474	1.04014069	0.31099	0.48700612	0.45251106	Klf15	1
GO:1990830 cellular response to leukemia inhibitory factor	1/47	114/14464	0.00877193	2.69951474	1.04014069	0.31099	0.48700612	0.45251106	Mynn	1
GO:1990823 response to leukemia inhibitory factor	1/47	116/14464	0.00862069	2.65297139	1.02056183	0.31549615	0.49339139	0.45844406	Mynn	1
GO:0001654 eye development	2/47	351/14464	0.00569801	1.75353095	0.81599153	0.31647669	0.494212	0.45920654	Mertk/Rdh10	2
GO:0008584 male gonad development	1/47	117/14464	0.00854701	2.63029642	1.01092661	0.31773839	0.494212	0.45920654	Bcl2l1	1
GO:0015698 inorganic anion transport	1/47	117/14464	0.00854701	2.63029642	1.01092661	0.31773839	0.494212	0.45920654	Ip6k2	1
GO:0106027 neuron projection organization	1/47	117/14464	0.00854701	2.63029642	1.01092661	0.31773839	0.494212	0.45920654	Mtmr2	1
GO:0009142 nucleoside triphosphate biosynthetic process	1/47	118/14464	0.00847458	2.60800577	1.00139142	0.31997344	0.49518207	0.4601079	Bcl2l1	1
GO:0010821 regulation of mitochondrion organization	1/47	118/14464	0.00847458	2.60800577	1.00139142	0.31997344	0.49518207	0.4601079	Bcl2l1	1
GO:0046546 development of primary male sexual characteristics	1/47	118/14464	0.00847458	2.60800577	1.00139142	0.31997344	0.49518207	0.4601079	Bcl2l1	1
GO:0150063 visual system development	2/47	354/14464	0.00564972	1.73867051	0.80339581	0.32008293	0.49518207	0.4601079	Mertk/Rdh10	2
GO:0010639 negative regulation of organelle organization	2/47	355/14464	0.0056338	1.73377285	0.79922373	0.32128415	0.49637323	0.46121469	Bcl2l1/Ppfia1	2
GO:0016079 synaptic vesicle exocytosis	1/47	119/14464	0.00840336	2.58608976	0.99195424	0.32220132	0.49645746	0.46129296	Bcl2l1	1
GO:0030317 flagellated sperm motility	1/47	119/14464	0.00840336	2.58608976	0.99195424	0.32220132	0.49645746	0.46129296	Cfap157	1
GO:0048706 embryonic skeletal system development	1/47	120/14464	0.00833333	2.56453901	0.98261311	0.32442207	0.49921096	0.46385142	Rdh10	1
GO:0048880 sensory system development	2/47	359/14464	0.00557103	1.71445505	0.78266563	0.32608443	0.49927978	0.46391537	Mertk/Rdh10	2
GO:0002761 regulation of myeloid leukocyte differentiation	1/47	121/14464	0.00826446	2.54334447	0.97336613	0.32663569	0.49927978	0.46391537	Fstl3	1
GO:0030509 BMP signaling pathway	1/47	121/14464	0.00826446	2.54334447	0.97336613	0.32663569	0.49927978	0.46391537	Fstl3	1
GO:0046165 alcohol biosynthetic process	1/47	121/14464	0.00826446	2.54334447	0.97336613	0.32663569	0.49927978	0.46391537	Ip6k2	1
GO:0097722 sperm motility	1/47	121/14464	0.00826446	2.54334447	0.97336613	0.32663569	0.49927978	0.46391537	Cfap157	1
GO:0030879 mammary gland development	1/47	122/14464	0.00819672	2.52249738	0.96421145	0.32884221	0.50132103	0.46581203	Xdh	1
GO:0045834 positive regulation of lipid metabolic process	1/47	122/14464	0.00819672	2.52249738	0.96421145	0.32884221	0.50132103	0.46581203	Rdh10	1
GO:0007600 sensory perception	2/47	362/14464	0.00552486	1.70024686	0.77038137	0.32967956	0.50193277	0.46638045	Kcnk4/Rdh10	2
GO:0006633 fatty acid biosynthetic process	1/47	123/14464	0.00813008	2.50198928	0.95514728	0.33104165	0.5026767	0.46707168	Acsf3	1
GO:0051384 response to glucocorticoid	1/47	123/14464	0.00813008	2.50198928	0.95514728	0.33104165	0.5026767	0.46707168	Fam107a	1
GO:0051100 negative regulation of binding	1/47	124/14464	0.00806452	2.48181194	0.94617187	0.33323404	0.50401101	0.46831148	Per2	1
GO:0097696 cell surface receptor signaling pathway via STAT	1/47	124/14464	0.00806452	2.48181194	0.94617187	0.33323404	0.50401101	0.46831148	Crlf3	1
GO:1903052 positive regulation of proteolysis involved in protein catabolic process	1/47	124/14464	0.00806452	2.48181194	0.94617187	0.33323404	0.50401101	0.46831148	Herpud1	1
GO:0010950 positive regulation of endopeptidase activity	1/47	125/14464	0.008	2.46195745	0.93728352	0.33541939	0.50665055	0.47076406	Xdh	1
GO:0032970 regulation of actin filament-based process	2/47	368/14464	0.00543478	1.67252544	0.74614864	0.33685568	0.50794117	0.47196326	Fam107a/Ppfia1	2
GO:0010466 negative regulation of peptidase activity	1/47	126/14464	0.00793651	2.4424181	0.92848057	0.33759774	0.50794117	0.47196326	Ints1	1
GO:0090101 negative regulation of transmembrane receptor protein serine/threonine kinase signaling pathway	1/47	126/14464	0.00793651	2.4424181	0.92848057	0.33759774	0.50794117	0.47196326	Fstl3	1
GO:0001936 regulation of endothelial cell proliferation	1/47	127/14464	0.00787402	2.42318646	0.91976143	0.3397691	0.50987514	0.47376024	Xdh	1
GO:0035966 response to topologically incorrect protein	1/47	127/14464	0.00787402	2.42318646	0.91976143	0.3397691	0.50987514	0.47376024	Herpud1	1
GO:0010927 cellular component assembly involved in morphogenesis	1/47	128/14464	0.0078125	2.40425532	0.91112453	0.34193348	0.51046101	0.47430462	Mtmr2	1
GO:0030856 regulation of epithelial cell differentiation	1/47	128/14464	0.0078125	2.40425532	0.91112453	0.34193348	0.51046101	0.47430462	Xdh	1
GO:0032989 cellular anatomical entity morphogenesis	1/47	128/14464	0.0078125	2.40425532	0.91112453	0.34193348	0.51046101	0.47430462	Mtmr2	1
GO:0060294 cilium movement involved in cell motility	1/47	128/14464	0.0078125	2.40425532	0.91112453	0.34193348	0.51046101	0.47430462	Cfap157	1
GO:0015748 organophosphate ester transport	1/47	129/14464	0.00775194	2.38561768	0.90256834	0.34409093	0.51235273	0.47606235	Plscr4	1
GO:0048259 regulation of receptor-mediated endocytosis	1/47	129/14464	0.00775194	2.38561768	0.90256834	0.34409093	0.51235273	0.47606235	Mtmr2	1
GO:0018205 peptidyl-lysine modification	1/47	130/14464	0.00769231	2.36726678	0.89409138	0.34624145	0.51399953	0.4775925	Klf15	1
GO:0170039 proteinogenic amino acid metabolic process	1/47	130/14464	0.00769231	2.36726678	0.89409138	0.34624145	0.51399953	0.4775925	Prodh	1
GO:0009154 purine ribonucleotide catabolic process	1/47	131/14464	0.00763359	2.34919604	0.88569221	0.34838507	0.51399953	0.4775925	Xdh	1
GO:0009566 fertilization	1/47	131/14464	0.00763359	2.34919604	0.88569221	0.34838507	0.51399953	0.4775925	Bcl2l1	1
GO:0043244 regulation of protein-containing complex disassembly	1/47	131/14464	0.00763359	2.34919604	0.88569221	0.34838507	0.51399953	0.4775925	Per2	1
GO:0071772 response to BMP	1/47	131/14464	0.00763359	2.34919604	0.88569221	0.34838507	0.51399953	0.4775925	Fstl3	1
GO:0071773 cellular response to BMP stimulus	1/47	131/14464	0.00763359	2.34919604	0.88569221	0.34838507	0.51399953	0.4775925	Fstl3	1
GO:1990778 protein localization to cell periphery	2/47	378/14464	0.00529101	1.62827873	0.70671627	0.34876945	0.51399953	0.4775925	Bcl2l1/Ppfia1	2
GO:0001539 cilium or flagellum-dependent cell motility	1/47	132/14464	0.00757576	2.3313991	0.87736943	0.35052181	0.51460536	0.47815543	Cfap157	1
GO:0010952 positive regulation of peptidase activity	1/47	132/14464	0.00757576	2.3313991	0.87736943	0.35052181	0.51460536	0.47815543	Xdh	1
GO:0060285 cilium-dependent cell motility	1/47	132/14464	0.00757576	2.3313991	0.87736943	0.35052181	0.51460536	0.47815543	Cfap157	1
GO:0002757 immune response-activating signaling pathway	2/47	380/14464	0.00526316	1.61970885	0.69896778	0.35114461	0.51486299	0.47839481	Icosl/Pcbp2	2
GO:0055007 cardiac muscle cell differentiation	1/47	133/14464	0.0075188	2.31386978	0.86912165	0.35265169	0.51641489	0.47983678	Trip10	1
GO:0003158 endothelium development	1/47	134/14464	0.00746269	2.2966021	0.86094755	0.35477474	0.51820524	0.48150031	Xdh	1
GO:0046661 male sex differentiation	1/47	134/14464	0.00746269	2.2966021	0.86094755	0.35477474	0.51820524	0.48150031	Bcl2l1	1
GO:0071902 positive regulation of protein serine/threonine kinase activity	1/47	135/14464	0.00740741	2.27959023	0.85284583	0.35689097	0.52063562	0.48375856	Ip6k2	1
GO:0007565 female pregnancy	1/47	136/14464	0.00735294	2.26282854	0.84481522	0.35900041	0.52238871	0.48538747	Ints1	1
GO:2000241 regulation of reproductive process	1/47	136/14464	0.00735294	2.26282854	0.84481522	0.35900041	0.52238871	0.48538747	Bcl2l1	1
GO:0031329 regulation of cellular catabolic process	2/47	388/14464	0.00515464	1.58631279	0.66841513	0.36061781	0.52320655	0.48614738	Sesn1/Mtmr2	2
GO:0072073 kidney epithelium development	1/47	137/14464	0.00729927	2.24631154	0.83685447	0.36110307	0.52320655	0.48614738	Klf15	1
GO:0031960 response to corticosteroid	1/47	138/14464	0.00724638	2.23003392	0.8289624	0.36319899	0.52320655	0.48614738	Fam107a	1
GO:0050851 antigen receptor-mediated signaling pathway	1/47	138/14464	0.00724638	2.23003392	0.8289624	0.36319899	0.52320655	0.48614738	Icosl	1
GO:0051099 positive regulation of binding	1/47	138/14464	0.00724638	2.23003392	0.8289624	0.36319899	0.52320655	0.48614738	Ip6k2	1
GO:0051606 detection of stimulus	1/47	138/14464	0.00724638	2.23003392	0.8289624	0.36319899	0.52320655	0.48614738	Kcnk4	1
GO:0060078 regulation of postsynaptic membrane potential	1/47	138/14464	0.00724638	2.23003392	0.8289624	0.36319899	0.52320655	0.48614738	Mtmr2	1
GO:1903322 positive regulation of protein modification by small protein conjugation or removal	1/47	138/14464	0.00724638	2.23003392	0.8289624	0.36319899	0.52320655	0.48614738	Fam107a	1
GO:0035107 appendage morphogenesis	1/47	139/14464	0.00719424	2.21399051	0.82113781	0.36528817	0.52359487	0.4865082	Rdh10	1
GO:0035108 limb morphogenesis	1/47	139/14464	0.00719424	2.21399051	0.82113781	0.36528817	0.52359487	0.4865082	Rdh10	1
GO:0044272 sulfur compound biosynthetic process	1/47	139/14464	0.00719424	2.21399051	0.82113781	0.36528817	0.52359487	0.4865082	Fmo1	1
GO:1901875 positive regulation of post-translational protein modification	1/47	139/14464	0.00719424	2.21399051	0.82113781	0.36528817	0.52359487	0.4865082	Fam107a	1
GO:0007204 positive regulation of cytosolic calcium ion concentration	1/47	140/14464	0.00714286	2.19817629	0.81337956	0.36737065	0.52368577	0.48659266	Mchr1	1
GO:0009261 ribonucleotide catabolic process	1/47	140/14464	0.00714286	2.19817629	0.81337956	0.36737065	0.52368577	0.48659266	Xdh	1
GO:0051250 negative regulation of lymphocyte activation	1/47	140/14464	0.00714286	2.19817629	0.81337956	0.36737065	0.52368577	0.48659266	Mertk	1
GO:0071456 cellular response to hypoxia	1/47	140/14464	0.00714286	2.19817629	0.81337956	0.36737065	0.52368577	0.48659266	Hif3a	1
GO:0009314 response to radiation	2/47	394/14464	0.00507614	1.56215574	0.64594923	0.36769211	0.52368577	0.48659266	Per2/Bcl2l1	2
GO:0003012 muscle system process	2/47	395/14464	0.00506329	1.55820092	0.64224118	0.36886847	0.52368577	0.48659266	Klf15/Trip10	2
GO:0006195 purine nucleotide catabolic process	1/47	141/14464	0.0070922	2.18258639	0.80568654	0.36944643	0.52368577	0.48659266	Xdh	1
GO:0031644 regulation of nervous system process	1/47	141/14464	0.0070922	2.18258639	0.80568654	0.36944643	0.52368577	0.48659266	Mtmr2	1
GO:2000377 regulation of reactive oxygen species metabolic process	1/47	141/14464	0.0070922	2.18258639	0.80568654	0.36944643	0.52368577	0.48659266	Xdh	1
GO:0002764 immune response-regulating signaling pathway	2/47	397/14464	0.00503778	1.55035104	0.63485562	0.37121883	0.52373729	0.48664052	Icosl/Pcbp2	2
GO:0010212 response to ionizing radiation	1/47	142/14464	0.00704225	2.16721606	0.79805765	0.37151555	0.52373729	0.48664052	Bcl2l1	1
GO:0050680 negative regulation of epithelial cell proliferation	1/47	142/14464	0.00704225	2.16721606	0.79805765	0.37151555	0.52373729	0.48664052	Xdh	1
GO:0022407 regulation of cell-cell adhesion	2/47	398/14464	0.00502513	1.54645568	0.63117799	0.37239282	0.52373729	0.48664052	Fstl3/Icosl	2
GO:0044772 mitotic cell cycle phase transition	2/47	399/14464	0.00501253	1.54257985	0.62751038	0.37356599	0.52373729	0.48664052	Fam107a/Crlf3	2
GO:0007009 plasma membrane organization	1/47	143/14464	0.00699301	2.15206071	0.79049183	0.37357803	0.52373729	0.48664052	Plscr4	1
GO:0032006 regulation of TOR signaling	1/47	143/14464	0.00699301	2.15206071	0.79049183	0.37357803	0.52373729	0.48664052	Sesn1	1
GO:0042129 regulation of T cell proliferation	1/47	143/14464	0.00699301	2.15206071	0.79049183	0.37357803	0.52373729	0.48664052	Icosl	1
GO:1903305 regulation of regulated secretory pathway	1/47	143/14464	0.00699301	2.15206071	0.79049183	0.37357803	0.52373729	0.48664052	Bcl2l1	1
GO:0016241 regulation of macroautophagy	1/47	144/14464	0.00694444	2.13711584	0.78298802	0.37563388	0.52470218	0.48753707	Sesn1	1
GO:0051147 regulation of muscle cell differentiation	1/47	144/14464	0.00694444	2.13711584	0.78298802	0.37563388	0.52470218	0.48753707	Trip10	1
GO:0072006 nephron development	1/47	144/14464	0.00694444	2.13711584	0.78298802	0.37563388	0.52470218	0.48753707	Klf15	1
GO:1901987 regulation of cell cycle phase transition	2/47	402/14464	0.00497512	1.53106806	0.61656709	0.3770806	0.52565088	0.48841858	Fam107a/Crlf3	2
GO:0007596 blood coagulation	1/47	145/14464	0.00689655	2.12237711	0.77554523	0.37768313	0.52565088	0.48841858	Mertk	1
GO:0032388 positive regulation of intracellular transport	1/47	145/14464	0.00689655	2.12237711	0.77554523	0.37768313	0.52565088	0.48841858	Mtmr2	1
GO:0007286 spermatid development	1/47	146/14464	0.00684932	2.10784028	0.76816245	0.37972579	0.52658359	0.48928523	Cfap157	1
GO:1900180 regulation of protein localization to nucleus	1/47	146/14464	0.00684932	2.10784028	0.76816245	0.37972579	0.52658359	0.48928523	Otud7b	1

GO:2001235 positive regulation of apoptotic signaling pathway	1/47	146/14464	0.00684932	2.10784028	0.76816245	0.37972579	0.52658359	0.48928523	G0s2	1
GO:0009152 purine ribonucleotide biosynthetic process	1/47	147/14464	0.00680272	2.09350123	0.76083872	0.38176189	0.52750052	0.49013721	Bcl2l1	1
GO:0060041 retina development in camera-type eye	1/47	147/14464	0.00680272	2.09350123	0.76083872	0.38176189	0.52750052	0.49013721	Mertk	1
GO:0170033 L-amino acid metabolic process	1/47	147/14464	0.00680272	2.09350123	0.76083872	0.38176189	0.52750052	0.49013721	Prodh	1
GO:0001935 endothelial cell proliferation	1/47	148/14464	0.00675676	2.07935595	0.75357308	0.38379145	0.5280291	0.49062835	Xdh	1
GO:0002244 hematopoietic progenitor cell differentiation	1/47	148/14464	0.00675676	2.07935595	0.75357308	0.38379145	0.5280291	0.49062835	Fstl3	1
GO:0007599 hemostasis	1/47	148/14464	0.00675676	2.07935595	0.75357308	0.38379145	0.5280291	0.49062835	Mertk	1
GO:0051249 regulation of lymphocyte activation	2/47	409/14464	0.00488998	1.50486397	0.59137271	0.38525177	0.5280291	0.49062835	Mertk/Icosl	2
GO:0001837 epithelial to mesenchymal transition	1/47	149/14464	0.00671141	2.06540054	0.74636462	0.38581449	0.5280291	0.49062835	Trip10	1
GO:0016331 morphogenesis of embryonic epithelium	1/47	149/14464	0.00671141	2.06540054	0.74636462	0.38581449	0.5280291	0.49062835	Rdh10	1
GO:0030218 erythrocyte differentiation	1/47	149/14464	0.00671141	2.06540054	0.74636462	0.38581449	0.5280291	0.49062835	Slc1a5	1
GO:0036294 cellular response to decreased oxygen levels	1/47	149/14464	0.00671141	2.06540054	0.74636462	0.38581449	0.5280291	0.49062835	Hif3a	1
GO:0031623 receptor internalization	1/47	150/14464	0.00666667	2.05163121	0.73921243	0.38783102	0.52890226	0.49143966	Mtmr2	1
GO:0048754 branching morphogenesis of an epithelial tube	1/47	150/14464	0.00666667	2.05163121	0.73921243	0.38783102	0.52890226	0.49143966	Rdh10	1
GO:0050817 coagulation	1/47	150/14464	0.00666667	2.05163121	0.73921243	0.38783102	0.52890226	0.49143966	Mertk	1
GO:0042445 hormone metabolic process	1/47	152/14464	0.00657895	2.02463606	0.72507334	0.39184467	0.53248314	0.4947669	Rdh10	1
GO:0044703 multi-organism reproductive process	1/47	152/14464	0.00657895	2.02463606	0.72507334	0.39184467	0.53248314	0.4947669	Ints1	1
GO:0051302 regulation of cell division	1/47	152/14464	0.00657895	2.02463606	0.72507334	0.39184467	0.53248314	0.4947669	Bcl2l1	1
GO:0044706 multi-multicellular organism process	1/47	153/14464	0.00653595	2.01140314	0.71808474	0.39384183	0.53349331	0.49570552	Ints1	1
GO:0046474 glycerophospholipid biosynthetic process	1/47	153/14464	0.00653595	2.01140314	0.71808474	0.39384183	0.53349331	0.49570552	Ip6k2	1
GO:0050673 epithelial cell proliferation	2/47	417/14464	0.00479616	1.47599367	0.56314222	0.39453711	0.53349331	0.49570552	Xdh/Bcl2l1	2
GO:0035150 regulation of tube size	1/47	154/14464	0.00649351	1.99834208	0.71114899	0.39583257	0.53349331	0.49570552	Per2	1
GO:0035296 regulation of tube diameter	1/47	154/14464	0.00649351	1.99834208	0.71114899	0.39583257	0.53349331	0.49570552	Per2	1
GO:0048515 spermatid differentiation	1/47	154/14464	0.00649351	1.99834208	0.71114899	0.39583257	0.53349331	0.49570552	Cfap157	1
GO:0097746 blood vessel diameter maintenance	1/47	154/14464	0.00649351	1.99834208	0.71114899	0.39583257	0.53349331	0.49570552	Per2	1
GO:0001508 action potential	1/47	155/14464	0.00645161	1.98544955	0.70426528	0.3978169	0.53491502	0.49702653	Kcnk4	1
GO:0072331 signal transduction by p53 class mediator	1/47	155/14464	0.00645161	1.98544955	0.70426528	0.3978169	0.53491502	0.49702653	Phlda3	1
GO:0048568 embryonic organ development	2/47	421/14464	0.00475059	1.46196998	0.54924426	0.39915756	0.5358438	0.49788952	Tead3/Rdh10	2
GO:0045785 positive regulation of cell adhesion	2/47	422/14464	0.00473934	1.4585056	0.54579186	0.40031029	0.5358438	0.49788952	Fstl3/Icosl	2
GO:0002753 cytoplasmic pattern recognition receptor signaling pathway	1/47	157/14464	0.00636943	1.9601572	0.69065086	0.40176646	0.5358438	0.49788952	Pcbp2	1
GO:0062207 regulation of pattern recognition receptor signaling pathway	1/47	157/14464	0.00636943	1.9601572	0.69065086	0.40176646	0.5358438	0.49788952	Pcbp2	1
GO:0071478 cellular response to radiation	1/47	157/14464	0.00636943	1.9601572	0.69065086	0.40176646	0.5358438	0.49788952	Bcl2l1	1
GO:0072523 purine-containing compound catabolic process	1/47	157/14464	0.00636943	1.9601572	0.69065086	0.40176646	0.5358438	0.49788952	Xdh	1
GO:2000058 regulation of ubiquitin-dependent protein catabolic process	1/47	157/14464	0.00636943	1.9601572	0.69065086	0.40176646	0.5358438	0.49788952	Herpud1	1
GO:0009166 nucleotide catabolic process	1/47	158/14464	0.00632911	1.94775114	0.68391861	0.40373172	0.53784168	0.49974589	Xdh	1
GO:0003341 cilium movement	1/47	159/14464	0.00628931	1.93550114	0.67723536	0.40569066	0.53858126	0.50043309	Cfap157	1
GO:0030041 actin filament polymerization	1/47	159/14464	0.00628931	1.93550114	0.67723536	0.40569066	0.53858126	0.50043309	Fam107a	1
GO:0099072 regulation of postsynaptic membrane neurotransmitter receptor levels	1/47	159/14464	0.00628931	1.93550114	0.67723536	0.40569066	0.53858126	0.50043309	Ppfia1	1
GO:0030099 myeloid cell differentiation	2/47	428/14464	0.0046729	1.43805926	0.52525929	0.4072062	0.53992801	0.50168445	Slc1a5/Fstl3	2
GO:0070507 regulation of microtubule cytoskeleton organization	1/47	160/14464	0.00625	1.92340426	0.67060036	0.4076433	0.53992801	0.50168445	Fam107a	1
GO:0007015 actin filament organization	2/47	429/14464	0.004662	1.43470714	0.52186705	0.40835205	0.54002028	0.50177018	Fam107a/Ppfia1	2
GO:0002695 negative regulation of leukocyte activation	1/47	161/14464	0.00621118	1.91145765	0.66401293	0.40958967	0.54002028	0.50177018	Mertk	1
GO:0009266 response to temperature stimulus	1/47	161/14464	0.00621118	1.91145765	0.66401293	0.40958967	0.54002028	0.50177018	Kcnk4	1
GO:0051897 positive regulation of phosphatidylinositol 3-kinase/protein kinase B signal transduction	1/47	161/14464	0.00621118	1.91145765	0.66401293	0.40958967	0.54002028	0.50177018	Mertk	1
GO:0002253 activation of immune response	2/47	431/14464	0.00464037	1.42804956	0.51510777	0.41064074	0.54072005	0.50242039	Icosl/Pcbp2	2
GO:0009260 ribonucleotide biosynthetic process	1/47	162/14464	0.00617284	1.89965852	0.65747235	0.41152977	0.54072005	0.50242039	Bcl2l1	1
GO:0034101 erythrocyte homeostasis	1/47	162/14464	0.00617284	1.89965852	0.65747235	0.41152977	0.54072005	0.50242039	Slc1a5	1
GO:0002285 lymphocyte activation involved in immune response	1/47	163/14464	0.00613497	1.88800418	0.65097795	0.41346363	0.54202351	0.50363152	Icosl	1
GO:0010508 positive regulation of autophagy	1/47	163/14464	0.00613497	1.88800418	0.65097795	0.41346363	0.54202351	0.50363152	Sesn1	1
GO:0035051 cardiocyte differentiation	1/47	165/14464	0.00606061	1.86511928	0.63812506	0.41731271	0.54582606	0.50716473	Trip10	1
GO:0071453 cellular response to oxygen levels	1/47	165/14464	0.00606061	1.86511928	0.63812506	0.41731271	0.54582606	0.50716473	Hif3a	1
GO:0000910 cytokinesis	1/47	168/14464	0.00595238	1.83181358	0.61917591	0.42304003	0.55206244	0.51295939	Bcl2l1	1
GO:0046390 ribose phosphate biosynthetic process	1/47	168/14464	0.00595238	1.83181358	0.61917591	0.42304003	0.55206244	0.51295939	Bcl2l1	1
GO:0007269 neurotransmitter secretion	1/47	170/14464	0.00588235	1.81026283	0.60675611	0.42682759	0.55430435	0.5150425	Bcl2l1	1
GO:0046890 regulation of lipid biosynthetic process	1/47	170/14464	0.00588235	1.81026283	0.60675611	0.42682759	0.55430435	0.5150425	Rdh10	1
GO:0099643 signal release from synapse	1/47	170/14464	0.00588235	1.81026283	0.60675611	0.42682759	0.55430435	0.5150425	Bcl2l1	1
GO:0001678 intracellular glucose homeostasis	1/47	171/14464	0.00584795	1.7996765	0.60060835	0.42871223	0.55430435	0.5150425	Klf15	1
GO:0042552 myelination	1/47	171/14464	0.00584795	1.7996765	0.60060835	0.42871223	0.55430435	0.5150425	Mtmr2	1
GO:0051494 negative regulation of cytoskeleton organization	1/47	171/14464	0.00584795	1.7996765	0.60060835	0.42871223	0.55430435	0.5150425	Ppfia1	1
GO:1901605 alpha-amino acid metabolic process	1/47	171/14464	0.00584795	1.7996765	0.60060835	0.42871223	0.55430435	0.5150425	Prodh	1
GO:0034762 regulation of transmembrane transport	2/47	447/14464	0.00447427	1.3769337	0.46220204	0.42880026	0.55430435	0.5150425	Per2/Klf15	2
GO:0031929 TOR signaling	1/47	172/14464	0.00581395	1.78921326	0.59450124	0.43059081	0.55430435	0.5150425	Sesn1	1
GO:0048736 appendage development	1/47	172/14464	0.00581395	1.78921326	0.59450124	0.43059081	0.55430435	0.5150425	Rdh10	1
GO:0060173 limb development	1/47	172/14464	0.00581395	1.78921326	0.59450124	0.43059081	0.55430435	0.5150425	Rdh10	1
GO:1902904 negative regulation of supramolecular fiber organization	1/47	172/14464	0.00581395	1.78921326	0.59450124	0.43059081	0.55430435	0.5150425	Ppfia1	1
GO:0007346 regulation of mitotic cell cycle	2/47	450/14464	0.00444444	1.36775414	0.45250506	0.43217446	0.55430435	0.5150425	Fam107a/Crlf3	2
GO:0030307 positive regulation of cell growth	1/47	173/14464	0.00578035	1.77887099	0.58843425	0.43246334	0.55430435	0.5150425	Trip10	1
GO:0043393 regulation of protein binding	1/47	173/14464	0.00578035	1.77887099	0.58843425	0.43246334	0.55430435	0.5150425	Ip6k2	1
GO:0071222 cellular response to lipopolysaccharide	1/47	173/14464	0.00578035	1.77887099	0.58843425	0.43246334	0.55430435	0.5150425	Plscr4	1
GO:0007272 ensheathment of neurons	1/47	174/14464	0.00574713	1.76864759	0.58240682	0.43432985	0.55545962	0.51611594	Mtmr2	1
GO:0008366 axon ensheathment	1/47	174/14464	0.00574713	1.76864759	0.58240682	0.43432985	0.55545962	0.51611594	Mtmr2	1
GO:0010817 regulation of hormone levels	2/47	455/14464	0.0043956	1.35272387	0.43649338	0.43777579	0.55711561	0.51765463	Per2/Rdh10	2
GO:0031667 response to nutrient levels	2/47	455/14464	0.0043956	1.35272387	0.43649338	0.43777579	0.55711561	0.51765463	Fam107a/Sesn1	2
GO:0006164 purine nucleotide biosynthetic process	1/47	176/14464	0.00568182	1.74854932	0.57046855	0.43804485	0.55711561	0.51765463	Bcl2l1	1
GO:0050954 sensory perception of mechanical stimulus	1/47	176/14464	0.00568182	1.74854932	0.57046855	0.43804485	0.55711561	0.51765463	Kcnk4	1
GO:1901292 nucleoside phosphate catabolic process	1/47	176/14464	0.00568182	1.74854932	0.57046855	0.43804485	0.55711561	0.51765463	Xdh	1
GO:0061138 morphogenesis of a branching epithelium	1/47	177/14464	0.00564972	1.73867051	0.56455667	0.43989339	0.55884911	0.51926535	Rdh10	1
GO:0050866 negative regulation of cell activation	1/47	178/14464	0.00561798	1.7289027	0.5586823	0.44173598	0.56057123	0.52086549	Mertk	1
GO:0019827 stem cell population maintenance	1/47	179/14464	0.00558659	1.71924403	0.55284493	0.44357263	0.56104626	0.52130687	Tead3	1
GO:0050796 regulation of insulin secretion	1/47	179/14464	0.00558659	1.71924403	0.55284493	0.44357263	0.56104626	0.52130687	Per2	1
GO:0051346 negative regulation of hydrolase activity	1/47	179/14464	0.00558659	1.71924403	0.55284493	0.44357263	0.56104626	0.52130687	Ints1	1
GO:0035265 organ growth	1/47	180/14464	0.00555556	1.70969267	0.54704408	0.44540336	0.5621264	0.5223105	Trip10	1
GO:0071219 cellular response to molecule of bacterial origin	1/47	180/14464	0.00555556	1.70969267	0.54704408	0.44540336	0.5621264	0.5223105	Plscr4	1
GO:0051260 protein homooligomerization	1/47	181/14464	0.00552486	1.70024686	0.54127929	0.44722821	0.56381124	0.52387601	Slc1a5	1
GO:0071805 potassium ion transmembrane transport	1/47	182/14464	0.00549451	1.69090484	0.53555008	0.44904717	0.56406541	0.52411218	Kcnk4	1
GO:0002520 immune system development	1/47	183/14464	0.00546448	1.68166492	0.529856	0.45086028	0.56406541	0.52411218	Icosl	1
GO:0008154 actin polymerization or depolymerization	1/47	183/14464	0.00546448	1.68166492	0.529856	0.45086028	0.56406541	0.52411218	Fam107a	1
GO:0009267 cellular response to starvation	1/47	183/14464	0.00546448	1.68166492	0.529856	0.45086028	0.56406541	0.52411218	Sesn1	1
GO:0016051 carbohydrate biosynthetic process	1/47	183/14464	0.00546448	1.68166492	0.529856	0.45086028	0.56406541	0.52411218	Per2	1
GO:0048639 positive regulation of developmental growth	1/47	183/14464	0.00546448	1.68166492	0.529856	0.45086028	0.56406541	0.52411218	Trip10	1
GO:0071383 cellular response to steroid hormone stimulus	1/47	183/14464	0.00546448	1.68166492	0.529856	0.45086028	0.56406541	0.52411218	Fam107a	1
GO:0061136 regulation of proteasomal protein catabolic process	1/47	184/14464	0.00543478	1.67252544	0.5241966	0.45266754	0.56571156	0.52564172	Herpud1	1
GO:0003013 circulatory system process	2/47	470/14464	0.00425532	1.30955183	0.38953999	0.45440713	0.56611884	0.52602016	Per2/Slc1a5	2
GO:0042098 T cell proliferation	1/47	185/14464	0.00540541	1.66348476	0.51857144	0.45446899	0.56611884	0.52602016	Icosl	1
GO:0098727 maintenance of cell number	1/47	185/14464	0.00540541	1.66348476	0.51857144	0.45446899	0.56611884	0.52602016	Tead3	1
GO:0099175 regulation of postsynapse organization	1/47	186/14464	0.00537634	1.65454129	0.51298009	0.45626463	0.56774118	0.52752758	Fam107a	1
GO:0018105 peptidyl-serine phosphorylation	1/47	188/14464	0.00531915	1.63693979	0.50189713	0.45983857	0.56972465	0.52937056	Ip6k2	1
GO:0043524 negative regulation of neuron apoptotic process	1/47	188/14464	0.00531915	1.63693979	0.50189713	0.45983857	0.56972465	0.52937056	Bcl2l1	1
GO:0050870 positive regulation of T cell activation	1/47	188/14464	0.00531915	1.63693979	0.50189713	0.45983857	0.56972465	0.52937056	Icosl	1
GO:0055002 striated muscle cell development	1/47	188/14464	0.00531915	1.63693979	0.50189713	0.45983857	0.56972465	0.52937056	Trip10	1
GO:0061351 neural precursor cell proliferation	1/47	189/14464	0.00529101	1.62827873	0.49640469	0.46161691	0.57131298	0.53084639	Tead3	1
GO:0045732 positive regulation of protein catabolic process	1/47	192/14464	0.00520833	1.60283688	0.48011877	0.46691762	0.5760152	0.53521555	Herpud1	1
GO:0050670 regulation of lymphocyte proliferation	1/47	192/14464	0.00520833	1.60283688	0.48011877	0.46691762	0.5760152	0.53521555	Icosl	1
GO:1901654 response to ketone	1/47	192/14464	0.00520833	1.60283688	0.48011877	0.46691762	0.5760152	0.53521555	Bcl2l1	1
GO:0001763 morphogenesis of a branching structure	1/47	194/14464	0.00515464	1.58631279	0.46941711	0.47042302	0.57767027	0.53675339	Rdh10	1
GO:0006909 phagocytosis	1/47	194/14464	0.00515464	1.58631279	0.46941711	0.47042302	0.57767027	0.53675339	Mertk	1
GO:0030324 lung development	1/47	194/14464	0.00515464	1.58631279	0.46941711	0.47042302	0.57767027	0.53675339	Rdh10	1
GO:0045637 regulation of myeloid cell differentiation	1/47	194/14464	0.00515464	1.58631279	0.46941711	0.47042302	0.57767027	0.53675339	Fstl3	1
GO:0034329 cell junction assembly	2/47	485/14464	0.00412371	1.26905023	0.34411758	0.47076864	0.57767027	0.53675339	Fam107a/Tesk2	2
GO:0045165 cell fate commitment	1/47	195/14464	0.00512821	1.57817785	0.46411185	0.47216725	0.57876944	0.5377747	Tead3	1
GO:0045017 glycerolipid biosynthetic process	1/47	196/14464	0.00510204	1.57012592	0.45883649	0.47390585	0.57993677	0.53885936	Ip6k2	1
GO:0006006 glucose metabolic process	1/47	197/14464	0.00507614	1.56215574	0.45359066	0.47563885	0.57993677	0.53885936	Per2	1
GO:0030323 respiratory tube development	1/47	197/14464	0.00507614	1.56215574	0.45359066	0.47563885	0.57993677	0.53885936	Rdh10	1
GO:0050821 protein stabilization	1/47	197/14464	0.00507614	1.56215574	0.45359066	0.47563885	0.57993677	0.53885936	Ip6k2	1
GO:0051963 regulation of synapse assembly	1/47	197/14464	0.00507614	1.56215574	0.45359066	0.47563885	0.57993677	0.53885936	Fam107a	1
GO:0018209 peptidyl-serine modification	1/47	199/14464	0.00502513	1.54645568	0.44318624	0.4790881	0.58352424	0.54219271	Ip6k2	1
GO:0000302 response to reactive oxygen species	1/47	201/14464	0.00497512	1.53106806	0.43289587	0.48251514	0.58645716	0.5449179	Sesn1	1
GO:0008654 phospholipid biosynthetic process	1/47	201/14464	0.00497512	1.53106806	0.43289587	0.48251514	0.58645716	0.5449179	Ip6k2	1
GO:0009612 response to mechanical stimulus	1/47	202/14464	0.0049505	1.52348852	0.42779263	0.48422038	0.58727746	0.54568009	Kcnk4	1
GO:0017157 regulation of exocytosis	1/47	202/14464	0.0049505	1.52348852	0.42779263	0.48422038	0.58727746	0.54568009	Bcl2l1	1
GO:0044770 cell cycle phase transition	2/47	498/14464	0.00401606	1.23592241	0.30590637	0.48472075	0.58727746	0.54568009	Fam107a/Crlf3	2
GO:0007623 circadian rhythm	1/47	204/14464	0.00490196	1.50855236	0.41766844	0.48761437	0.59016208	0.5483604	Per2	1
GO:0071216 cellular response to biotic stimulus	1/47	205/14464	0.00487805	1.50119357	0.41264687	0.48930316	0.59158397	0.54968157	Plscr4	1
GO:0010810 regulation of cell-substrate adhesion	1/47	206/14464	0.00485437	1.49390622	0.4076519	0.4909865	0.5923747	0.55041629	Fam107a	1
GO:0043270 positive regulation of monoatomic ion transport	1/47	206/14464	0.00485437	1.49390622	0.4076519	0.4909865	0.5923747	0.55041629	Mchr1	1
GO:0043583 ear development	1/47	207/14464	0.00483092	1.48668928	0.40268324	0.49266441	0.59315558	0.55114186	Rdh10	1
GO:1903039 positive regulation of leukocyte cell-cell adhesion	1/47	207/14464	0.00483092	1.48668928	0.40268324	0.49266441	0.59315558	0.55114186	Icosl	1
GO:0016311 dephosphorylation	1/47	209/14464	0.00478469	1.47246259	0.39282365	0.496004	0.59655236	0.55429804	Mtmr2	1
GO:0031348 negative regulation of defense response	1/47	210/14464	0.0047619	1.46545086	0.38793216	0.49766572	0.59730266	0.5549952	Pcbp2	1
GO:0090276 regulation of peptide hormone secretion	1/47	210/14464	0.0047619	1.46545086	0.38793216	0.49766572	0.59730266	0.5549952	Per2	1
GO:0006813 potassium ion transport	1/47	212/14464	0.00471698	1.45162585	0.37822436	0.50097309	0.60002084	0.55752085	Kcnk4	1
GO:0031032 actomyosin structure organization	1/47	212/14464	0.00471698	1.45162585	0.37822436	0.50097309	0.60002084	0.55752085	Ppfia1	1
GO:0002274 myeloid leukocyte activation	1/47	213/14464	0.00469484	1.44481071	0.3734075	0.50261877	0.60011847	0.55761157	Slc7a2	1
GO:0030073 insulin secretion	1/47	213/14464	0.00469484	1.44481071	0.3734075	0.50261877	0.60011847	0.55761157	Per2	1
GO:0043122 regulation of canonical NF-kappaB signal transduction	1/47	213/14464	0.00469484	1.44481071	0.3734075	0.50261877	0.60011847	0.55761157	Otud7b	1
GO:0009165 nucleotide biosynthetic process	1/47	214/14464	0.0046729	1.43805926	0.36861499	0.50425914	0.60020918	0.55769585	Bcl2l1	1
GO:0034764 positive regulation of transmembrane transport	1/47	214/14464	0.0046729	1.43805926	0.36861499	0.50425914	0.60020918	0.55769585	Klf15	1
GO:0048705 skeletal system morphogenesis	1/47	214/14464	0.0046729	1.43805926	0.36861499	0.50425914	0.60020918	0.55769585	Rdh10	1
GO:0002791 regulation of peptide secretion	1/47	215/14464	0.00465116	1.43137061	0.36384655	0.50589422	0.60029304	0.55777377	Per2	1
GO:0051648 vesicle localization	1/47	215/14464	0.00465116	1.43137061	0.36384655	0.50589422	0.60029304	0.55777377	Bcl2l1	1
GO:0090087 regulation of peptide transport	1/47	215/14464	0.00465116	1.43137061	0.36384655	0.50589422	0.60029304	0.55777377	Per2	1
GO:0002429 immune response-activating cell surface receptor signaling pathway	1/47	216/14464	0.00462963	1.42474389	0.35910193	0.50752402	0.6009878	0.55841932	Icosl	1
GO:0042594 response to starvation	1/47	216/14464	0.00462963	1.42474389	0.35910193	0.50752402	0.6009878	0.55841932	Sesn1	1
GO:0048863 stem cell differentiation	1/47	218/14464	0.00458716	1.41167285	0.34968312	0.51076784	0.60296798	0.56025924	Rdh10	1
GO:0055001 muscle cell development	1/47	218/14464	0.00458716	1.41167285	0.34968312	0.51076784	0.60296798	0.56025924	Trip10	1
GO:1903050 regulation of proteolysis involved in protein catabolic process	1/47	218/14464	0.00458716	1.41167285	0.34968312	0.51076784	0.60296798	0.56025924	Herpud1	1
GO:0002440 production of molecular mediator of immune response	1/47	219/14464	0.00456621	1.40522685	0.34500844	0.5123819	0.60425366	0.56145385	Icosl	1
GO:0046034 ATP metabolic process	1/47	220/14464	0.00454545	1.39883946	0.34035658	0.51399075	0.6049114	0.56206501	Bcl2l1	1
GO:0060541 respiratory system development	1/47	220/14464	0.00454545	1.39883946	0.34035658	0.51399075	0.6049114	0.56206501	Rdh10	1
GO:0046434 organophosphate catabolic process	1/47	222/14464	0.0045045	1.3862373	0.33112036	0.51719288	0.60805822	0.56498894	Xdh	1
GO:0090092 regulation of transmembrane receptor protein serine/threonine kinase signaling pathway	1/47	223/14464	0.0044843	1.38002099	0.32653553	0.51878619	0.60930908	0.5661512	Fstl3	1
GO:0045861 negative regulation of proteolysis	1/47	224/14464	0.00446429	1.37386018	0.32197259	0.52037435	0.60992961	0.56672777	Ints1	1
GO:1903522 regulation of blood circulation	1/47	224/14464	0.00446429	1.37386018	0.32197259	0.52037435	0.60992961	0.56672777	Per2	1
GO:0002768 immune response-regulating cell surface receptor signaling pathway	1/47	227/14464	0.00440529	1.35570344	0.30841282	0.52510813	0.61422709	0.57072086	Icosl	1
GO:0019058 viral life cycle	1/47	227/14464	0.00440529	1.35570344	0.30841282	0.52510813	0.61422709	0.57072086	Pcbp2	1
GO:0072522 purine-containing compound biosynthetic process	1/47	228/14464	0.00438596	1.34975737	0.30393519	0.52667587	0.61543546	0.57184364	Bcl2l1	1
GO:1901136 carbohydrate derivative catabolic process	1/47	229/14464	0.00436681	1.34386324	0.29947834	0.52823854	0.61663546	0.57295864	Xdh	1
GO:0019318 hexose metabolic process	1/47	230/14464	0.00434783	1.33802035	0.29504208	0.52979617	0.61720181	0.57348488	Per2	1
GO:0031331 positive regulation of cellular catabolic process	1/47	230/14464	0.00434783	1.33802035	0.29504208	0.52979617	0.61720181	0.57348488	Sesn1	1
GO:0045055 regulated exocytosis	1/47	231/14464	0.004329	1.33222806	0.29062619	0.53134876	0.61838465	0.57458394	Bcl2l1	1
GO:0002221 pattern recognition receptor signaling pathway	1/47	232/14464	0.00431034	1.32648569	0.28623046	0.53289633	0.61893408	0.57509445	Pcbp2	1
GO:0002573 myeloid leukocyte differentiation	1/47	232/14464	0.00431034	1.32648569	0.28623046	0.53289633	0.61893408	0.57509445	Fstl3	1
GO:0002758 innate immune response-activating signaling pathway	1/47	235/14464	0.00425532	1.30955183	0.27316232	0.5375091	0.62240742	0.57832177	Pcbp2	1
GO:0071900 regulation of protein serine/threonine kinase activity	1/47	235/14464	0.00425532	1.30955183	0.27316232	0.5375091	0.62240742	0.57832177	Ip6k2	1
GO:0090257 regulation of muscle system process	1/47	235/14464	0.00425532	1.30955183	0.27316232	0.5375091	0.62240742	0.57832177	Trip10	1
GO:0002449 lymphocyte mediated immunity	1/47	236/14464	0.00423729	1.30400288	0.2688453	0.53903676	0.62292301	0.57880084	Icosl	1

GO:0007249 canonical NF-kappaB signal transduction	1/47	236/14464	0.00423729	1.30400288	0.2688453	0.53903676	0.62292301	0.57880084	Otud7b	1
GO:0048738 cardiac muscle tissue development	1/47	237/14464	0.00421941	1.29850076	0.26454748	0.54055948	0.62405613	0.5798537	Trip10	1
GO:0006520 amino acid metabolic process	1/47	238/14464	0.00420168	1.29304488	0.26026866	0.54207728	0.62518131	0.58089918	Prodh	1
GO:0042113 B cell activation	1/47	240/14464	0.00416667	1.2822695	0.25176733	0.54509817	0.62740799	0.58296815	Icosl	1
GO:0051924 regulation of calcium ion transport	1/47	240/14464	0.00416667	1.2822695	0.25176733	0.54509817	0.62740799	0.58296815	Mchr1	1
GO:0002366 leukocyte activation involved in immune response	1/47	241/14464	0.00414938	1.27694888	0.24754446	0.54660129	0.62788232	0.58340888	Icosl	1
GO:0045444 fat cell differentiation	1/47	241/14464	0.00414938	1.27694888	0.24754446	0.54660129	0.62788232	0.58340888	Per2	1
GO:0009205 purine ribonucleoside triphosphate metabolic process	1/47	243/14464	0.00411523	1.26643902	0.2391534	0.54959296	0.63068943	0.58601716	Bcl2l1	1
GO:0051259 protein complex oligomerization	1/47	244/14464	0.00409836	1.26124869	0.23498488	0.55108154	0.63176778	0.58701913	Slc1a5	1
GO:0002263 cell activation involved in immune response	1/47	245/14464	0.00408163	1.25610074	0.23083413	0.55256531	0.63220941	0.58742948	Icosl	1
GO:0032886 regulation of microtubule-based process	1/47	245/14464	0.00408163	1.25610074	0.23083413	0.55256531	0.63220941	0.58742948	Fam107a	1
GO:0045860 positive regulation of protein kinase activity	1/47	246/14464	0.00406504	1.25099464	0.22670099	0.55404428	0.63327206	0.58841686	Ip6k2	1
GO:0032984 protein-containing complex disassembly	1/47	247/14464	0.00404858	1.24592988	0.22258529	0.55551846	0.63369846	0.58881305	Per2	1
GO:0097305 response to alcohol	1/47	247/14464	0.00404858	1.24592988	0.22258529	0.55551846	0.63369846	0.58881305	Bcl2l1	1
GO:0009199 ribonucleoside triphosphate metabolic process	1/47	248/14464	0.00403226	1.24090597	0.21848688	0.55698787	0.63474558	0.58978601	Bcl2l1	1
GO:0099504 synaptic vesicle cycle	1/47	249/14464	0.00401606	1.23592241	0.2144056	0.55845253	0.63578522	0.59075201	Bcl2l1	1
GO:0005996 monosaccharide metabolic process	1/47	250/14464	0.004	1.23097872	0.21034127	0.55991245	0.63681742	0.59171109	Per2	1
GO:0009144 purine nucleoside triphosphate metabolic process	1/47	251/14464	0.00398406	1.22607443	0.20629376	0.56136764	0.6378422	0.59266329	Bcl2l1	1
GO:0010721 negative regulation of cell development	1/47	252/14464	0.00396825	1.22120905	0.2022629	0.56281812	0.63885962	0.59360865	Fstl3	1
GO:0046651 lymphocyte proliferation	1/47	253/14464	0.00395257	1.21638214	0.19824855	0.56426391	0.63898575	0.59372584	Icosl	1
GO:0006898 receptor-mediated endocytosis	1/47	254/14464	0.00393701	1.21159323	0.19425055	0.56570502	0.63898575	0.59372584	Mtmr2	1
GO:0048562 embryonic organ morphogenesis	1/47	254/14464	0.00393701	1.21159323	0.19425055	0.56570502	0.63898575	0.59372584	Rdh10	1
GO:0048588 developmental cell growth	1/47	254/14464	0.00393701	1.21159323	0.19425055	0.56570502	0.63898575	0.59372584	Trip10	1
GO:0060560 developmental growth involved in morphogenesis	1/47	254/14464	0.00393701	1.21159323	0.19425055	0.56570502	0.63898575	0.59372584	Rdh10	1
GO:0030072 peptide hormone secretion	1/47	256/14464	0.00390625	1.20212766	0.18630303	0.56857326	0.6415959	0.59615111	Per2	1
GO:0002218 activation of innate immune response	1/47	258/14464	0.00387597	1.19280884	0.17841919	0.57142295	0.64229279	0.59679864	Pcbp2	1
GO:0033674 positive regulation of kinase activity	1/47	258/14464	0.00387597	1.19280884	0.17841919	0.57142295	0.64229279	0.59679864	Ip6k2	1
GO:0045927 positive regulation of growth	1/47	258/14464	0.00387597	1.19280884	0.17841919	0.57142295	0.64229279	0.59679864	Trip10	1
GO:0110053 regulation of actin filament organization	1/47	258/14464	0.00387597	1.19280884	0.17841919	0.57142295	0.64229279	0.59679864	Ppfia1	1
GO:0051251 positive regulation of lymphocyte activation	1/47	260/14464	0.00384615	1.18363339	0.17059789	0.57425422	0.64484547	0.59917051	Icosl	1
GO:0046883 regulation of hormone secretion	1/47	261/14464	0.00383142	1.17909839	0.16671035	0.57566298	0.64579736	0.60005498	Per2	1
GO:0002460 adaptive immune response based on somatic recombination of immune receptors built from immunoglobu	1/47	263/14464	0.00380228	1.17013187	0.15898083	0.57846683	0.64642264	0.60063598	Icosl	1
GO:0002790 peptide secretion	1/47	263/14464	0.00380228	1.17013187	0.15898083	0.57846683	0.64642264	0.60063598	Per2	1
GO:0007162 negative regulation of cell adhesion	1/47	263/14464	0.00380228	1.17013187	0.15898083	0.57846683	0.64642264	0.60063598	Fam107a	1
GO:0050708 regulation of protein secretion	1/47	263/14464	0.00380228	1.17013187	0.15898083	0.57846683	0.64642264	0.60063598	Per2	1
GO:0009141 nucleoside triphosphate metabolic process	1/47	265/14464	0.00377358	1.16130068	0.15131115	0.58125255	0.64764926	0.6017757	Bcl2l1	1
GO:0032868 response to insulin	1/47	265/14464	0.00377358	1.16130068	0.15131115	0.58125255	0.64764926	0.6017757	Klf15	1
GO:0042593 glucose homeostasis	1/47	265/14464	0.00377358	1.16130068	0.15131115	0.58125255	0.64764926	0.6017757	Klf15	1
GO:0033500 carbohydrate homeostasis	1/47	266/14464	0.0037594	1.15693489	0.14749844	0.58263864	0.64856584	0.60262736	Klf15	1
GO:0010001 glial cell differentiation	1/47	272/14464	0.00367647	1.13141427	0.12492399	0.59086139	0.65644928	0.60995242	Klf15	1
GO:0015833 peptide transport	1/47	272/14464	0.00367647	1.13141427	0.12492399	0.59086139	0.65644928	0.60995242	Per2	1
GO:0051258 protein polymerization	1/47	273/14464	0.003663	1.12726989	0.12121076	0.59221634	0.65732016	0.61076161	Fam107a	1
GO:0032386 regulation of intracellular transport	1/47	277/14464	0.00361011	1.11099163	0.10649409	0.59759238	0.66264819	0.61571226	Mtmr2	1
GO:0006605 protein targeting	1/47	278/14464	0.0035971	1.10699525	0.10284842	0.59892551	0.66284929	0.61589911	Herpud1	1
GO:0006874 intracellular calcium ion homeostasis	1/47	278/14464	0.0035971	1.10699525	0.10284842	0.59892551	0.66284929	0.61589911	Herpud1	1
GO:0006310 DNA recombination	1/47	279/14464	0.00358423	1.10302753	0.09921593	0.60025432	0.66368177	0.61667262	Icosl	1
GO:0001933 negative regulation of protein phosphorylation	1/47	281/14464	0.00355872	1.0951768	0.09199006	0.60289903	0.6659662	0.61879524	Xdh	1
GO:0030111 regulation of Wnt signaling pathway	1/47	282/14464	0.0035461	1.09129319	0.08839646	0.60421495	0.66631083	0.61911546	Klf15	1
GO:0002696 positive regulation of leukocyte activation	1/47	283/14464	0.00353357	1.08743703	0.08481563	0.60552661	0.66631083	0.61911546	Icosl	1
GO:0007416 synapse assembly	1/47	283/14464	0.00353357	1.08743703	0.08481563	0.60552661	0.66631083	0.61911546	Fam107a	1
GO:0009416 response to light stimulus	1/47	283/14464	0.00353357	1.08743703	0.08481563	0.60552661	0.66631083	0.61911546	Per2	1
GO:0006790 sulfur compound metabolic process	1/47	285/14464	0.00350877	1.0798059	0.07769182	0.60813717	0.6685443	0.62119073	Fmo1	1
GO:1901293 nucleoside phosphate biosynthetic process	1/47	286/14464	0.0034965	1.07603035	0.07414864	0.6094361	0.6686949	0.62133067	Bcl2l1	1
GO:1902105 regulation of leukocyte differentiation	1/47	286/14464	0.0034965	1.07603035	0.07414864	0.6094361	0.6686949	0.62133067	Fstl3	1
GO:1901888 regulation of cell junction assembly	1/47	288/14464	0.00347222	1.06855792	0.06709925	0.61202133	0.67089196	0.6233721	Fam107a	1
GO:0001666 response to hypoxia	1/47	289/14464	0.00346021	1.06486049	0.06359285	0.61330766	0.67166234	0.62408792	Hif3a	1
GO:0048545 response to steroid hormone	1/47	290/14464	0.00344828	1.06118855	0.0600985	0.61458982	0.67242669	0.62479813	Fam107a	1
GO:0048511 rhythmic process	1/47	291/14464	0.00343643	1.05754186	0.05661613	0.61586781	0.67318504	0.62550277	Per2	1
GO:0141091 transforming growth factor beta receptor superfamily signaling pathway	1/47	292/14464	0.00342466	1.05392014	0.05314563	0.61714165	0.67329862	0.6256083	Fstl3	1
GO:1903037 regulation of leukocyte cell-cell adhesion	1/47	292/14464	0.00342466	1.05392014	0.05314563	0.61714165	0.67329862	0.6256083	Icosl	1
GO:0050727 regulation of inflammatory response	1/47	294/14464	0.00340136	1.04675062	0.04623988	0.61967695	0.67542441	0.62758352	Slc7a2	1
GO:0010506 regulation of autophagy	1/47	295/14464	0.00338983	1.04320231	0.04280446	0.62093844	0.67551998	0.62767232	Sesn1	1
GO:0055074 calcium ion homeostasis	1/47	295/14464	0.00338983	1.04320231	0.04280446	0.62093844	0.67551998	0.62767232	Herpud1	1
GO:0050863 regulation of T cell activation	1/47	297/14464	0.003367	1.03617738	0.03596806	0.62344912	0.6776109	0.62961514	Icosl	1
GO:0016032 viral process	1/47	301/14464	0.00332226	1.02240758	0.02243062	0.62842177	0.68172805	0.63344066	Pcbp2	1
GO:0042326 negative regulation of phosphorylation	1/47	301/14464	0.00332226	1.02240758	0.02243062	0.62842177	0.68172805	0.63344066	Xdh	1
GO:0050867 positive regulation of cell activation	1/47	303/14464	0.00330033	1.01565901	0.01572821	0.63088393	0.68311139	0.63472603	Icosl	1
GO:0099003 vesicle-mediated transport in synapse	1/47	303/14464	0.00330033	1.01565901	0.01572821	0.63088393	0.68311139	0.63472603	Bcl2l1	1
GO:0036293 response to decreased oxygen levels	1/47	304/14464	0.00328947	1.01231803	0.0123933	0.63210902	0.68379463	0.63536087	Hif3a	1
GO:0034504 protein localization to nucleus	1/47	306/14464	0.00326797	1.00570157	0.00575566	0.63454727	0.68514438	0.63661501	Otud7b	1
GO:0051347 positive regulation of transferase activity	1/47	306/14464	0.00326797	1.00570157	0.00575566	0.63454727	0.68514438	0.63661501	Ip6k2	1
GO:0032102 negative regulation of response to external stimulus	1/47	308/14464	0.00324675	0.99917104	-0.0008396	0.6369697	0.68711539	0.63844642	Pcbp2	1
GO:0016236 macroautophagy	1/47	309/14464	0.00323625	0.99593748	-0.0041216	0.63817501	0.68777101	0.6390556	Sesn1	1
GO:0045089 positive regulation of innate immune response	1/47	310/14464	0.00322581	0.99272478	-0.0073931	0.63937641	0.68842118	0.63965972	Pcbp2	1
GO:0010038 response to metal ion	1/47	311/14464	0.00321543	0.98953274	-0.0106544	0.6405739	0.68842256	0.639661	Xdh	1
GO:0051146 striated muscle cell differentiation	1/47	311/14464	0.00321543	0.98953274	-0.0106544	0.6405739	0.68842256	0.639661	Trip10	1
GO:0007178 cell surface receptor protein serine/threonine kinase signaling pathway	1/47	313/14464	0.00319489	0.98320984	-0.0171464	0.64295722	0.69032465	0.64142837	Fstl3	1
GO:0042886 amide transport	1/47	314/14464	0.00318471	0.9800786	-0.0203773	0.64414307	0.69032465	0.64142837	Per2	1
GO:0046879 hormone secretion	1/47	314/14464	0.00318471	0.9800786	-0.0203773	0.64414307	0.69032465	0.64142837	Per2	1
GO:1903532 positive regulation of secretion by cell	1/47	315/14464	0.0031746	0.97696724	-0.0235982	0.64532507	0.69094805	0.64200761	Bcl2l1	1
GO:0051345 positive regulation of hydrolase activity	1/47	317/14464	0.00315457	0.97080341	-0.0300103	0.64767755	0.69282236	0.64374916	Xdh	1
GO:0030522 intracellular receptor signaling pathway	1/47	318/14464	0.00314465	0.96775057	-0.0332016	0.64884806	0.69343001	0.64431376	Pcbp2	1
GO:0009914 hormone transport	1/47	321/14464	0.00311526	0.95870617	-0.0427177	0.65233679	0.69586621	0.64657741	Per2	1
GO:0042060 wound healing	1/47	321/14464	0.00311526	0.95870617	-0.0427177	0.65233679	0.69586621	0.64657741	Mertk	1
GO:0060562 epithelial tube morphogenesis	1/47	323/14464	0.00309598	0.95276991	-0.0490141	0.65464374	0.6976805	0.6482632	Rdh10	1
GO:0007159 leukocyte cell-cell adhesion	1/47	326/14464	0.00306748	0.94400209	-0.0583885	0.6580761	0.7006038	0.65097943	Icosl	1
GO:0002443 leukocyte mediated immunity	1/47	327/14464	0.0030581	0.94111523	-0.0614949	0.65921278	0.7006038	0.65097943	Icosl	1
GO:0070482 response to oxygen levels	1/47	327/14464	0.0030581	0.94111523	-0.0614949	0.65921278	0.7006038	0.65097943	Hif3a	1
GO:0009895 negative regulation of catabolic process	1/47	330/14464	0.0030303	0.93255964	-0.0707595	0.66260069	0.70280107	0.65302107	Mtmr2	1
GO:0015980 energy derivation by oxidation of organic compounds	1/47	330/14464	0.0030303	0.93255964	-0.0707595	0.66260069	0.70280107	0.65302107	Per2	1
GO:0002833 positive regulation of response to biotic stimulus	1/47	331/14464	0.00302115	0.92974224	-0.0738298	0.66372264	0.70280107	0.65302107	Pcbp2	1
GO:0048638 regulation of developmental growth	1/47	331/14464	0.00302115	0.92974224	-0.0738298	0.66372264	0.70280107	0.65302107	Trip10	1
GO:0051047 positive regulation of secretion	1/47	334/14464	0.00299401	0.92139126	-0.0829877	0.66706666	0.70569276	0.65570794	Bcl2l1	1
GO:0060271 cilium assembly	1/47	339/14464	0.00294985	0.90780142	-0.0980778	0.67256783	0.71085911	0.66050835	Cfap157	1
GO:0006869 lipid transport	1/47	342/14464	0.00292398	0.89983825	-0.1070305	0.67582572	0.71364716	0.66309892	Plscr4	1
GO:0042176 regulation of protein catabolic process	1/47	344/14464	0.00290698	0.89460663	-0.1129577	0.67798	0.71443209	0.66382826	Herpud1	1
GO:0050890 cognition	1/47	344/14464	0.00290698	0.89460663	-0.1129577	0.67798	0.71443209	0.66382826	Fam107a	1
GO:0010563 negative regulation of phosphorus metabolic process	1/47	345/14464	0.00289855	0.89201357	-0.1159091	0.67905188	0.71443209	0.66382826	Xdh	1
GO:0045936 negative regulation of phosphate metabolic process	1/47	345/14464	0.00289855	0.89201357	-0.1159091	0.67905188	0.71443209	0.66382826	Xdh	1
GO:0050678 regulation of epithelial cell proliferation	1/47	346/14464	0.00289017	0.88943549	-0.1188524	0.68012027	0.71490267	0.6642655	Xdh	1
GO:0006887 exocytosis	1/47	352/14464	0.00284091	0.87427466	-0.1363454	0.68645787	0.72024887	0.66923303	Bcl2l1	1
GO:0009306 protein secretion	1/47	352/14464	0.00284091	0.87427466	-0.1363454	0.68645787	0.72024887	0.66923303	Per2	1
GO:0035592 establishment of protein localization to extracellular region	1/47	354/14464	0.00282486	0.86933526	-0.1421141	0.68854297	0.72177865	0.67065445	Per2	1
GO:0006935 chemotaxis	1/47	356/14464	0.00280899	0.86445135	-0.1478523	0.6906145	0.72305506	0.67184045	Maco1	1
GO:0042330 taxis	1/47	357/14464	0.00280112	0.86202992	-0.1507101	0.6916452	0.72305506	0.67184045	Maco1	1
GO:0071692 protein localization to extracellular region	1/47	357/14464	0.00280112	0.86202992	-0.1507101	0.6916452	0.72305506	0.67184045	Per2	1
GO:0001503 ossification	1/47	359/14464	0.00278552	0.85722752	-0.1564033	0.69369653	0.7232289	0.67200198	Fstl3	1
GO:0007018 microtubule-based movement	1/47	359/14464	0.00278552	0.85722752	-0.1564033	0.69369653	0.7232289	0.67200198	Cfap157	1
GO:0030098 lymphocyte differentiation	1/47	359/14464	0.00278552	0.85722752	-0.1564033	0.69369653	0.7232289	0.67200198	Mertk	1
GO:1903706 regulation of hemopoiesis	1/47	362/14464	0.00276243	0.85012343	-0.1648877	0.6967485	0.72575342	0.67434768	Fstl3	1
GO:0042063 gliogenesis	1/47	366/14464	0.00273224	0.84083246	-0.176099	0.70077154	0.72928395	0.67762814	Klf15	1
GO:0044782 cilium organization	1/47	369/14464	0.00271003	0.83399643	-0.1844331	0.7037545	0.73172667	0.67989784	Cfap157	1
GO:0050807 regulation of synapse organization	1/47	370/14464	0.0027027	0.83174238	-0.1871971	0.70474233	0.73209244	0.6802377	Fam107a	1
GO:0045088 regulation of innate immune response	1/47	374/14464	0.0026738	0.82284674	-0.1981847	0.70866153	0.73483732	0.68278816	Pcbp2	1
GO:1902903 regulation of supramolecular fiber organization	1/47	374/14464	0.0026738	0.82284674	-0.1981847	0.70866153	0.73483732	0.68278816	Ppfia1	1
GO:0010959 regulation of metal ion transport	1/47	375/14464	0.00266667	0.82065248	-0.2009146	0.70963335	0.73486334	0.68281234	Mchr1	1
GO:0043603 amide metabolic process	1/47	376/14464	0.00265957	0.8184699	-0.2036379	0.710602	0.73486334	0.68281234	Xdh	1
GO:0050803 regulation of synapse structure or activity	1/47	376/14464	0.00265957	0.8184699	-0.2036379	0.710602	0.73486334	0.68281234	Fam107a	1
GO:0006816 calcium ion transport	1/47	377/14464	0.00265252	0.81629889	-0.2063545	0.71156748	0.73520123	0.68312629	Mchr1	1
GO:0016055 Wnt signaling pathway	1/47	385/14464	0.0025974	0.79933683	-0.227852	0.71917868	0.74239879	0.68981405	Klf15	1
GO:0001819 positive regulation of cytokine production	1/47	386/14464	0.00259067	0.79726601	-0.2305103	0.72011614	0.74270043	0.69009432	Icosl	1
GO:0006979 response to oxidative stress	1/47	388/14464	0.00257732	0.79315639	-0.2358083	0.7219819	0.74395807	0.69126288	Sesn1	1
GO:0010876 lipid localization	1/47	393/14464	0.00254453	0.78306535	-0.248945	0.72659319	0.74804004	0.69505572	Plscr4	1
GO:0043086 negative regulation of catalytic activity	1/47	398/14464	0.00251256	0.77322784	-0.2619308	0.7311296	0.75203769	0.69877021	Ints1	1
GO:0022411 cellular component disassembly	1/47	400/14464	0.0025	0.7693617	-0.2670839	0.73292346	0.75320974	0.69985925	Per2	1
GO:0048732 gland development	1/47	408/14464	0.00245098	0.75427618	-0.2874664	0.73998251	0.75978579	0.70596951	Xdh	1
GO:0031349 positive regulation of defense response	1/47	410/14464	0.00243902	0.75059678	-0.2925059	0.74171854	0.76021197	0.70636551	Pcbp2	1
GO:0043434 response to peptide hormone	1/47	410/14464	0.00243902	0.75059678	-0.2925059	0.74171854	0.76021197	0.70636551	Klf15	1
GO:0043161 proteasome-mediated ubiquitin-dependent protein catabolic process	1/47	412/14464	0.00242718	0.74695311	-0.2975235	0.74344323	0.76130174	0.70737809	Pcbp2	1
GO:0014706 striated muscle tissue development	1/47	414/14464	0.00241546	0.74334464	-0.3025193	0.74515664	0.76189576	0.70793003	Trip10	1
GO:0051051 negative regulation of transport	1/47	415/14464	0.00240964	0.74155345	-0.3050091	0.74600914	0.76189576	0.70793003	Mtmr2	1
GO:0072594 establishment of protein localization to organelle	1/47	415/14464	0.00240964	0.74155345	-0.3050091	0.74600914	0.76189576	0.70793003	Herpud1	1
GO:0050767 regulation of neurogenesis	1/47	418/14464	0.00239234	0.73623129	-0.3124466	0.74854993	0.76381292	0.70971139	Per2	1
GO:0043410 positive regulation of MAPK cascade	1/47	419/14464	0.00238663	0.73447418	-0.3149152	0.74939132	0.76399416	0.7098798	Xdh	1
GO:0042692 muscle cell differentiation	1/47	423/14464	0.00236407	0.7275288	-0.3247375	0.75272941	0.76671818	0.71241088	Trip10	1
GO:0045859 regulation of protein kinase activity	1/47	426/14464	0.00234742	0.72240535	-0.3320503	0.75520438	0.76855901	0.71412132	Ip6k2	1
GO:0060537 muscle tissue development	1/47	435/14464	0.00229885	0.70745904	-0.3537192	0.76248471	0.7752826	0.72036867	Trip10	1
GO:0043269 regulation of monoatomic ion transport	1/47	440/14464	0.00227273	0.69941973	-0.3655877	0.76643732	0.77861373	0.72346386	Mchr1	1
GO:0008015 blood circulation	1/47	443/14464	0.00225734	0.69468325	-0.3726522	0.76877791	0.7803028	0.72503329	Per2	1
GO:0002831 regulation of response to biotic stimulus	1/47	446/14464	0.00224215	0.6900105	-0.3796751	0.77109554	0.78136705	0.72602215	Pcbp2	1
GO:0030003 intracellular monoatomic cation homeostasis	1/47	447/14464	0.00223714	0.68846685	-0.3820069	0.77186302	0.78136705	0.72602215	Herpud1	1
GO:1903131 mononuclear cell differentiation	1/47	447/14464	0.00223714	0.68846685	-0.3820069	0.77186302	0.78136705	0.72602215	Mertk	1
GO:0006091 generation of precursor metabolites and energy	1/47	449/14464	0.00222717	0.68540018	-0.3866569	0.77339043	0.78222529	0.7268196	Per2	1
GO:0051223 regulation of protein transport	1/47	452/14464	0.00221239	0.68085106	-0.3935983	0.77566279	0.78383483	0.72831514	Per2	1
GO:0006873 intracellular monoatomic ion homeostasis	1/47	454/14464	0.00220264	0.67785172	-0.3982038	0.7771653	0.78397656	0.72844683	Herpud1	1
GO:0098771 inorganic ion homeostasis	1/47	454/14464	0.00220264	0.67785172	-0.3982038	0.7771653	0.78397656	0.72844683	Herpud1	1
GO:0043549 regulation of kinase activity	1/47	456/14464	0.00219298	0.67487869	-0.4027917	0.77865795	0.78479448	0.72920682	Ip6k2	1
GO:0071407 cellular response to organic cyclic compound	1/47	462/14464	0.0021645	0.66611403	-0.4164519	0.78307744	0.78855829	0.73270403	Fam107a	1
GO:0042110 T cell activation	1/47	464/14464	0.00215517	0.66324285	-0.4209713	0.78453132	0.78871664	0.73285116	Icosl	1
GO:0009611 response to wounding	1/47	465/14464	0.00215054	0.66181652	-0.4232247	0.78525467	0.78871664	0.73285116	Mertk	1
GO:0001501 skeletal system development	1/47	466/14464	0.00214592	0.66039631	-0.425474	0.78597566	0.78871664	0.73285116	Rdh10	1
GO:0007005 mitochondrion organization	1/47	466/14464	0.00214592	0.66039631	-0.425474	0.78597566	0.78871664	0.73285116	Bcl2l1	1
GO:0002009 morphogenesis of an epithelium	1/47	470/14464	0.00212766	0.65477592	-0.4344299	0.78883597	0.79089739	0.73487745	Rdh10	1
GO:0034655 nucleobase-containing compound catabolic process	1/47	471/14464	0.00212314	0.65338573	-0.4366587	0.78954518	0.7909195	0.73489799	Xdh	1
GO:0005975 carbohydrate metabolic process	1/47	491/14464	0.00203666	0.62677124	-0.4804096	0.80324965	0.80394813	0.74700379	Per2	1
GO:0051604 protein maturation	1/47	498/14464	0.00200803	0.61796121	-0.4953667	0.80783671	0.80783671	0.75061694	Pcbp2	1

ID	Description	GeneRatio	BgRatio	RichFactor	FoldEnrichm	zScore	pvalue	p.adjust	qvalue	geneID	Count
GO:0036499	PERK-mediated unfolded protein response	2/40	16/14464	0.125	45.2	9.31525453	0.00087319	0.34729843	0.32084266	Bok/Ptpn1	2
GO:0097193	intrinsic apoptotic signaling pathway	5/40	302/14464	0.01655629	5.98675497	4.61185386	0.00138771	0.34729843	0.32084266	Bok/Rrp8/Pnp/Ptpn1/Fbh1	5
GO:0035561	regulation of chromatin binding	2/40	21/14464	0.0952381	34.4380952	8.0749307	0.00151479	0.34729843	0.32084266	Ctbp2/Fbh1	2
GO:0140467	integrated stress response signaling	2/40	25/14464	0.08	28.928	7.35965767	0.00214891	0.34729843	0.32084266	Bok/Ptpn1	2
GO:1900101	regulation of endoplasmic reticulum unfolded protein response	2/40	26/14464	0.07692308	27.8153846	7.20665145	0.00232393	0.34729843	0.32084266	Bok/Ptpn1	2
GO:0097345	mitochondrial outer membrane permeabilization	2/40	28/14464	0.07142857	25.8285714	6.92506163	0.00269348	0.34729843	0.32084266	Bok/Rhot1	2
GO:1902325	regulation of endoplasmic reticulum stress-induced intrinsic apoptotic signaling pathway	2/40	29/14464	0.06896552	24.937931	6.79506395	0.00288795	0.34729843	0.32084266	Bok/Ptpn1	2
GO:0008630	intrinsic apoptotic signaling pathway in response to DNA damage	3/40	105/14464	0.02857143	10.3314286	5.05355527	0.0030219	0.34729843	0.32084266	Bok/Pnp/Fbh1	3
GO:1902110	positive regulation of mitochondrial membrane permeability involved in apoptotic process	2/40	32/14464	0.0625	22.6	6.44142289	0.00350972	0.34729843	0.32084266	Bok/Rhot1	2
GO:0008637	apoptotic mitochondrial changes	3/40	114/14464	0.02631579	9.51578947	4.80692918	0.00381044	0.34729843	0.32084266	Bok/Pnp/Rhot1	3
GO:1902686	mitochondrial outer membrane permeabilization involved in programmed cell death	2/40	34/14464	0.05882353	21.2705882	6.23144802	0.00395584	0.34729843	0.32084266	Bok/Rhot1	2
GO:0035794	positive regulation of mitochondrial membrane permeability	2/40	37/14464	0.05405405	19.5459459	5.94809872	0.00467174	0.34729843	0.32084266	Bok/Rhot1	2
GO:1902108	regulation of mitochondrial membrane permeability involved in apoptotic process	2/40	39/14464	0.05128205	18.5435897	5.77709072	0.00517975	0.34729843	0.32084266	Bok/Rhot1	2
GO:0006984	ER-nucleus signaling pathway	2/40	43/14464	0.04651163	16.8186047	5.47042469	0.00626834	0.34729843	0.32084266	Bok/Ptpn1	2
GO:1905710	positive regulation of membrane permeability	2/40	43/14464	0.04651163	16.8186047	5.47042469	0.00626834	0.34729843	0.32084266	Bok/Rhot1	2
GO:0046902	regulation of mitochondrial membrane permeability	2/40	57/14464	0.03508772	12.6877193	4.65582614	0.01081245	0.34729843	0.32084266	Bok/Rhot1	2
GO:1904035	regulation of epithelial cell apoptotic process	2/40	59/14464	0.03389831	12.2576271	4.56281191	0.01155136	0.34729843	0.32084266	Bok/Capn10	2
GO:2001242	regulation of intrinsic apoptotic signaling pathway	3/40	172/14464	0.01744186	6.30697674	3.68706875	0.01182761	0.34729843	0.32084266	Bok/Ptpn1/Fbh1	3
GO:1902806	regulation of cell cycle G1/S phase transition	3/40	175/14464	0.01714286	6.19885714	3.64369756	0.01239049	0.34729843	0.32084266	Plk5/Fam83d/Ctdspl	3
GO:0070059	intrinsic apoptotic signaling pathway in response to endoplasmic reticulum stress	2/40	62/14464	0.03225806	11.6645161	4.43140995	0.01270027	0.34729843	0.32084266	Bok/Ptpn1	2
GO:2001244	positive regulation of intrinsic apoptotic signaling pathway	2/40	62/14464	0.03225806	11.6645161	4.43140995	0.01270027	0.34729843	0.32084266	Bok/Fbh1	2
GO:0031102	neuron projection regeneration	2/40	71/14464	0.02816901	10.1859155	4.08594286	0.01643123	0.34729843	0.32084266	Matn2/Cntf	2
GO:0030968	endoplasmic reticulum unfolded protein response	2/40	72/14464	0.02777778	10.0444444	4.05138859	0.01687144	0.34729843	0.32084266	Bok/Ptpn1	2
GO:0090559	regulation of membrane permeability	2/40	72/14464	0.02777778	10.0444444	4.05138859	0.01687144	0.34729843	0.32084266	Bok/Rhot1	2
GO:0042063	gliogenesis	4/40	366/14464	0.01092896	3.95191257	3.01218479	0.01799287	0.34729843	0.32084266	Matn2/Bok/Cntf/Arhgef10	4
GO:0031400	negative regulation of protein modification process	4/40	378/14464	0.01058201	3.82645503	2.93231397	0.02000407	0.34729843	0.32084266	Cry1/Pkib/Ctdspl/Ptpn1	4
GO:1905897	regulation of response to endoplasmic reticulum stress	2/40	81/14464	0.02469136	8.92839506	3.76807366	0.02105576	0.34729843	0.32084266	Bok/Ptpn1	2
GO:0034620	cellular response to unfolded protein	2/40	84/14464	0.02380952	8.60952381	3.68327387	0.02253723	0.34729843	0.32084266	Bok/Ptpn1	2
GO:0071901	negative regulation of protein serine/threonine kinase activity	2/40	84/14464	0.02380952	8.60952381	3.68327387	0.02253723	0.34729843	0.32084266	Pkib/Ptpn1	2
GO:0072332	intrinsic apoptotic signaling pathway by p53 class mediator	2/40	86/14464	0.02325581	8.40930233	3.6290557	0.02354829	0.34729843	0.32084266	Bok/Rrp8	2
GO:1901987	regulation of cell cycle phase transition	4/40	402/14464	0.00995025	3.59800995	2.78193271	0.02443334	0.34729843	0.32084266	Plk5/Cry1/Fam83d/Ctdspl	4
GO:1904019	epithelial cell apoptotic process	2/40	91/14464	0.02197802	7.94725275	3.50087347	0.02615625	0.34729843	0.32084266	Bok/Capn10	2
GO:0044843	cell cycle G1/S phase transition	3/40	233/14464	0.01287554	4.65579399	2.96249958	0.02632204	0.34729843	0.32084266	Plk5/Fam83d/Ctdspl	3
GO:0048678	response to axon injury	2/40	92/14464	0.02173913	7.86086957	3.47640851	0.02669138	0.34729843	0.32084266	Matn2/Cntf	2
GO:0002357	defense response to tumor cell	1/40	10/14464	0.1	36.16	5.85694537	0.02732163	0.34729843	0.32084266	Plk5	1
GO:0032263	GMP salvage	1/40	10/14464	0.1	36.16	5.85694537	0.02732163	0.34729843	0.32084266	Pnp	1
GO:0042451	purine nucleoside biosynthetic process	1/40	10/14464	0.1	36.16	5.85694537	0.02732163	0.34729843	0.32084266	Pnp	1
GO:0042455	ribonucleoside biosynthetic process	1/40	10/14464	0.1	36.16	5.85694537	0.02732163	0.34729843	0.32084266	Pnp	1
GO:0046129	purine ribonucleoside biosynthetic process	1/40	10/14464	0.1	36.16	5.85694537	0.02732163	0.34729843	0.32084266	Pnp	1
GO:1902231	positive regulation of intrinsic apoptotic signaling pathway in response to DNA damage	1/40	10/14464	0.1	36.16	5.85694537	0.02732163	0.34729843	0.32084266	Fbh1	1
GO:1990700	nucleolar chromatin organization	1/40	10/14464	0.1	36.16	5.85694537	0.02732163	0.34729843	0.32084266	Rrp8	1
GO:0006183	GTP biosynthetic process	1/40	11/14464	0.09090909	32.8727273	5.56868817	0.03001342	0.34729843	0.32084266	Pnp	1
GO:0009120	deoxyribonucleoside metabolic process	1/40	11/14464	0.09090909	32.8727273	5.56868817	0.03001342	0.34729843	0.32084266	Pnp	1
GO:0009169	purine ribonucleoside monophosphate catabolic process	1/40	11/14464	0.09090909	32.8727273	5.56868817	0.03001342	0.34729843	0.32084266	Pnp	1
GO:0009215	purine deoxyribonucleoside triphosphate metabolic process	1/40	11/14464	0.09090909	32.8727273	5.56868817	0.03001342	0.34729843	0.32084266	Pnp	1
GO:0046015	regulation of transcription by glucose	1/40	11/14464	0.09090909	32.8727273	5.56868817	0.03001342	0.34729843	0.32084266	Rrp8	1
GO:0048012	hepatocyte growth factor receptor signaling pathway	1/40	11/14464	0.09090909	32.8727273	5.56868817	0.03001342	0.34729843	0.32084266	Ptpn1	1
GO:0070493	thrombin-activated receptor signaling pathway	1/40	11/14464	0.09090909	32.8727273	5.56868817	0.03001342	0.34729843	0.32084266	Iqgap2	1
GO:1902513	regulation of organelle transport along microtubule	1/40	11/14464	0.09090909	32.8727273	5.56868817	0.03001342	0.34729843	0.32084266	Rhot1	1
GO:1990709	presynaptic active zone organization	1/40	11/14464	0.09090909	32.8727273	5.56868817	0.03001342	0.34729843	0.32084266	Ctbp2	1
GO:0035967	cellular response to topologically incorrect protein	2/40	100/14464	0.02	7.232	3.29310879	0.03113038	0.34729843	0.32084266	Bok/Ptpn1	2
GO:0009128	purine nucleoside monophosphate catabolic process	1/40	12/14464	0.08333333	30.1333333	5.31658983	0.03269795	0.34729843	0.32084266	Pnp	1
GO:0009155	purine deoxyribonucleotide catabolic process	1/40	12/14464	0.08333333	30.1333333	5.31658983	0.03269795	0.34729843	0.32084266	Pnp	1
GO:0009170	purine deoxyribonucleoside monophosphate metabolic process	1/40	12/14464	0.08333333	30.1333333	5.31658983	0.03269795	0.34729843	0.32084266	Pnp	1
GO:0030948	negative regulation of vascular endothelial growth factor receptor signaling pathway	1/40	12/14464	0.08333333	30.1333333	5.31658983	0.03269795	0.34729843	0.32084266	Ptpn1	1
GO:0035563	positive regulation of chromatin binding	1/40	12/14464	0.08333333	30.1333333	5.31658983	0.03269795	0.34729843	0.32084266	Ctbp2	1
GO:0035791	platelet-derived growth factor receptor-beta signaling pathway	1/40	12/14464	0.08333333	30.1333333	5.31658983	0.03269795	0.34729843	0.32084266	Ptpn1	1
GO:0046085	adenosine metabolic process	1/40	12/14464	0.08333333	30.1333333	5.31658983	0.03269795	0.34729843	0.32084266	Pnp	1
GO:1901029	negative regulation of mitochondrial outer membrane permeabilization involved in apoptotic signaling pat	1/40	12/14464	0.08333333	30.1333333	5.31658983	0.03269795	0.34729843	0.32084266	Bok	1
GO:2000035	regulation of stem cell division	1/40	12/14464	0.08333333	30.1333333	5.31658983	0.03269795	0.34729843	0.32084266	Prdm15	1
GO:2000322	regulation of glucocorticoid receptor signaling pathway	1/40	12/14464	0.08333333	30.1333333	5.31658983	0.03269795	0.34729843	0.32084266	Cry1	1
GO:2000644	regulation of receptor catabolic process	1/40	12/14464	0.08333333	30.1333333	5.31658983	0.03269795	0.34729843	0.32084266	Ptpn1	1
GO:2000674	regulation of type B pancreatic cell apoptotic process	1/40	12/14464	0.08333333	30.1333333	5.31658983	0.03269795	0.34729843	0.32084266	Capn10	1
GO:2000849	regulation of glucocorticoid secretion	1/40	12/14464	0.08333333	30.1333333	5.31658983	0.03269795	0.34729843	0.32084266	Cry1	1
GO:0046883	regulation of hormone secretion	3/40	261/14464	0.01149425	4.15632184	2.70974125	0.03514751	0.34729843	0.32084266	Cry1/Cntf/Capn10	3
GO:0009200	deoxyribonucleoside triphosphate metabolic process	1/40	13/14464	0.07692308	27.8153846	5.09357949	0.03537523	0.34729843	0.32084266	Pnp	1
GO:0035933	glucocorticoid secretion	1/40	13/14464	0.07692308	27.8153846	5.09357949	0.03537523	0.34729843	0.32084266	Cry1	1
GO:0042454	ribonucleoside catabolic process	1/40	13/14464	0.07692308	27.8153846	5.09357949	0.03537523	0.34729843	0.32084266	Pnp	1
GO:0042754	negative regulation of circadian rhythm	1/40	13/14464	0.07692308	27.8153846	5.09357949	0.03537523	0.34729843	0.32084266	Cry1	1
GO:0043605	amide catabolic process	1/40	13/14464	0.07692308	27.8153846	5.09357949	0.03537523	0.34729843	0.32084266	Pnp	1
GO:0048385	regulation of retinoic acid receptor signaling pathway	1/40	13/14464	0.07692308	27.8153846	5.09357949	0.03537523	0.34729843	0.32084266	Ctbp2	1
GO:1904753	negative regulation of vascular associated smooth muscle cell migration	1/40	13/14464	0.07692308	27.8153846	5.09357949	0.03537523	0.34729843	0.32084266	Ptpn1	1
GO:0032868	response to insulin	3/40	265/14464	0.01132075	4.09358491	2.67653193	0.03651932	0.34729843	0.32084266	Cry1/Capn10/Ptpn1	3
GO:0006986	response to unfolded protein	2/40	110/14464	0.01818182	6.57454545	3.09054627	0.03705868	0.34729843	0.32084266	Bok/Ptpn1	2
GO:0007006	mitochondrial membrane organization	2/40	111/14464	0.01801802	6.51531532	3.07168605	0.03767379	0.34729843	0.32084266	Bok/Rhot1	2
GO:0015721	bile acid and bile salt transport	1/40	14/14464	0.07142857	25.8285714	4.89438533	0.03804529	0.34729843	0.32084266	Slc10a6	1
GO:0036498	IRE1-mediated unfolded protein response	1/40	14/14464	0.07142857	25.8285714	4.89438533	0.03804529	0.34729843	0.32084266	Ptpn1	1
GO:0048240	sperm capacitation	1/40	14/14464	0.07142857	25.8285714	4.89438533	0.03804529	0.34729843	0.32084266	Catsperz	1
GO:0048680	positive regulation of axon regeneration	1/40	14/14464	0.07142857	25.8285714	4.89438533	0.03804529	0.34729843	0.32084266	Cntf	1
GO:0010001	glial cell differentiation	3/40	272/14464	0.01102941	3.98823529	2.61995465	0.03898615	0.34729843	0.32084266	Bok/Cntf/Arhgef10	3
GO:0007005	mitochondrion organization	4/40	466/14464	0.00858369	3.10386266	2.43104883	0.03899502	0.34729843	0.32084266	Bok/Capn10/Pnp/Rhot1	4
GO:0001732	formation of cytoplasmic translation initiation complex	1/40	15/14464	0.06666667	24.1066667	4.71498554	0.04070814	0.34729843	0.32084266	Eif3j1	1
GO:0006167	AMP biosynthetic process	1/40	15/14464	0.06666667	24.1066667	4.71498554	0.04070814	0.34729843	0.32084266	Pnp	1
GO:0006188	IMP biosynthetic process	1/40	15/14464	0.06666667	24.1066667	4.71498554	0.04070814	0.34729843	0.32084266	Pnp	1
GO:0009163	nucleoside biosynthetic process	1/40	15/14464	0.06666667	24.1066667	4.71498554	0.04070814	0.34729843	0.32084266	Pnp	1
GO:0034404	nucleobase-containing small molecule biosynthetic process	1/40	15/14464	0.06666667	24.1066667	4.71498554	0.04070814	0.34729843	0.32084266	Pnp	1
GO:0070572	positive regulation of neuron projection regeneration	1/40	15/14464	0.06666667	24.1066667	4.71498554	0.04070814	0.34729843	0.32084266	Cntf	1
GO:0090308	regulation of DNA methylation-dependent heterochromatin formation	1/40	15/14464	0.06666667	24.1066667	4.71498554	0.04070814	0.34729843	0.32084266	Samd1	1
GO:0097050	type B pancreatic cell apoptotic process	1/40	15/14464	0.06666667	24.1066667	4.71498554	0.04070814	0.34729843	0.32084266	Capn10	1
GO:1900102	negative regulation of endoplasmic reticulum unfolded protein response	1/40	15/14464	0.06666667	24.1066667	4.71498554	0.04070814	0.34729843	0.32084266	Ptpn1	1
GO:0032386	regulation of intracellular transport	3/40	277/14464	0.01083032	3.91624549	2.58068509	0.04079948	0.34729843	0.32084266	Capn10/Rhot1/Ptpn1	3
GO:0010821	regulation of mitochondrion organization	2/40	118/14464	0.01694915	6.12881356	2.94583783	0.04208873	0.34729843	0.32084266	Bok/Rhot1	2
GO:0001933	negative regulation of protein phosphorylation	3/40	281/14464	0.01067616	3.86049822	2.54992333	0.04228077	0.34729843	0.32084266	Pkib/Ctdspl/Ptpn1	3
GO:0006177	GMP biosynthetic process	1/40	16/14464	0.0625	22.6	4.55225108	0.0433638	0.34729843	0.32084266	Pnp	1
GO:0009159	deoxyribonucleoside monophosphate catabolic process	1/40	16/14464	0.0625	22.6	4.55225108	0.0433638	0.34729843	0.32084266	Pnp	1
GO:0016081	synaptic vesicle docking	1/40	16/14464	0.0625	22.6	4.55225108	0.0433638	0.34729843	0.32084266	Ctbp2	1
GO:0042921	nuclear receptor-mediated glucocorticoid signaling pathway	1/40	16/14464	0.0625	22.6	4.55225108	0.0433638	0.34729843	0.32084266	Cry1	1
GO:0045721	negative regulation of gluconeogenesis	1/40	16/14464	0.0625	22.6	4.55225108	0.0433638	0.34729843	0.32084266	Cry1	1
GO:0070314	G1 to G0 transition	1/40	16/14464	0.0625	22.6	4.55225108	0.0433638	0.34729843	0.32084266	Rrp8	1
GO:0106380	purine ribonucleotide salvage	1/40	16/14464	0.0625	22.6	4.55225108	0.0433638	0.34729843	0.32084266	Pnp	1
GO:0140718	facultative heterochromatin formation	1/40	16/14464	0.0625	22.6	4.55225108	0.0433638	0.34729843	0.32084266	Rrp8	1
GO:1904355	positive regulation of telomere capping	1/40	16/14464	0.0625	22.6	4.55225108	0.0433638	0.34729843	0.32084266	Pkib	1
GO:0031507	heterochromatin formation	2/40	120/14464	0.01666667	6.02666667	2.91173538	0.04338447	0.34729843	0.32084266	Rrp8/Samd1	2
GO:0007052	mitotic spindle organization	2/40	121/14464	0.01652893	5.9768595	2.89497221	0.04403794	0.34729843	0.32084266	Plk5/Arhgef10	2
GO:0045579	positive regulation of B cell differentiation	1/40	17/14464	0.05882353	21.2705882	4.40370591	0.0460123	0.34729843	0.32084266	Pnp	1
GO:0060546	negative regulation of necroptotic process	1/40	17/14464	0.05882353	21.2705882	4.40370591	0.0460123	0.34729843	0.32084266	Bok	1
GO:0071732	cellular response to nitric oxide	1/40	17/14464	0.05882353	21.2705882	4.40370591	0.0460123	0.34729843	0.32084266	Ptpn1	1
GO:2000846	regulation of corticosteroid hormone secretion	1/40	17/14464	0.05882353	21.2705882	4.40370591	0.0460123	0.34729843	0.32084266	Cry1	1
GO:0015718	monocarboxylic acid transport	2/40	126/14464	0.01587302	5.73968254	2.81388646	0.0473603	0.34729843	0.32084266	Slc10a6/Slc43a3	2
GO:0044770	cell cycle phase transition	4/40	498/14464	0.00803213	2.90441767	2.27749371	0.04781458	0.34729843	0.32084266	Plk5/Cry1/Fam83d/Ctdspl	4
GO:0035966	response to topologically incorrect protein	2/40	127/14464	0.01574803	5.69448819	2.79819065	0.04803561	0.34729843	0.32084266	Bok/Ptpn1	2
GO:0006614	SRP-dependent cotranslational protein targeting to membrane	1/40	18/14464	0.05555556	20.0888889	4.26736145	0.04865364	0.34729843	0.32084266	Zfand2b	1
GO:0009158	ribonucleoside monophosphate catabolic process	1/40	18/14464	0.05555556	20.0888889	4.26736145	0.04865364	0.34729843	0.32084266	Pnp	1
GO:0009164	nucleoside catabolic process	1/40	18/14464	0.05555556	20.0888889	4.26736145	0.04865364	0.34729843	0.32084266	Pnp	1
GO:0031958	nuclear receptor-mediated corticosteroid signaling pathway	1/40	18/14464	0.05555556	20.0888889	4.26736145	0.04865364	0.34729843	0.32084266	Cry1	1
GO:0043567	regulation of insulin-like growth factor receptor signaling pathway	1/40	18/14464	0.05555556	20.0888889	4.26736145	0.04865364	0.34729843	0.32084266	Zfand2b	1
GO:0060081	membrane hyperpolarization	1/40	18/14464	0.05555556	20.0888889	4.26736145	0.04865364	0.34729843	0.32084266	Cntf	1
GO:0060221	retinal rod cell differentiation	1/40	18/14464	0.05555556	20.0888889	4.26736145	0.04865364	0.34729843	0.32084266	Cntf	1
GO:0060795	cell fate commitment involved in formation of primary germ layer	1/40	18/14464	0.05555556	20.0888889	4.26736145	0.04865364	0.34729843	0.32084266	Smad1	1
GO:1900119	positive regulation of execution phase of apoptosis	1/40	18/14464	0.05555556	20.0888889	4.26736145	0.04865364	0.34729843	0.32084266	Bok	1
GO:1902236	negative regulation of endoplasmic reticulum stress-induced intrinsic apoptotic signaling pathway	1/40	18/14464	0.05555556	20.0888889	4.26736145	0.04865364	0.34729843	0.32084266	Ptpn1	1
GO:1903232	melanosome assembly	1/40	18/14464	0.05555556	20.0888889	4.26736145	0.04865364	0.34729843	0.32084266	Hps5	1
GO:0042326	negative regulation of phosphorylation	3/40	301/14464	0.00996678	3.60398671	2.40414532	0.05009094	0.34729843	0.32084266	Pkib/Ctdspl/Ptpn1	3
GO:0003084	positive regulation of systemic arterial blood pressure	1/40	19/14464	0.05263158	19.0315789	4.14159967	0.05128785	0.34729843	0.32084266	Ptpn1	1
GO:0006144	purine nucleobase metabolic process	1/40	19/14464	0.05263158	19.0315789	4.14159967	0.05128785	0.34729843	0.32084266	Pnp	1
GO:0006613	cotranslational protein targeting to membrane	1/40	19/14464	0.05263158	19.0315789	4.14159967	0.05128785	0.34729843	0.32084266	Zfand2b	1
GO:0007000	nucleolus organization	1/40	19/14464	0.05263158	19.0315789	4.14159967	0.05128785	0.34729843	0.32084266	Rrp8	1
GO:0009151	purine deoxyribonucleotide metabolic process	1/40	19/14464	0.05263158	19.0315789	4.14159967	0.05128785	0.34729843	0.32084266	Pnp	1
GO:0032261	purine nucleotide salvage	1/40	19/14464	0.05263158	19.0315789	4.14159967	0.05128785	0.34729843	0.32084266	Pnp	1
GO:0035930	corticosteroid hormone secretion	1/40	19/14464	0.05263158	19.0315789	4.14159967	0.05128785	0.34729843	0.32084266	Cry1	1
GO:0048333	mesodermal cell differentiation	1/40	19/14464	0.05263158	19.0315789	4.14159967	0.05128785	0.34729843	0.32084266	Smad1	1
GO:0062099	negative regulation of programmed necrotic cell death	1/40	19/14464	0.05263158	19.0315789	4.14159967	0.05128785	0.34729843	0.32084266	Bok	1
GO:1902018	negative regulation of cilium assembly	1/40	19/14464	0.05263158	19.0315789	4.14159967	0.05128785	0.34729843	0.32084266	Tchp	1
GO:1902170	cellular response to reactive nitrogen species	1/40	19/14464	0.05263158	19.0315789	4.14159967	0.05128785	0.34729843	0.32084266	Ptpn1	1
GO:2000353	positive regulation of endothelial cell apoptotic process	1/40	19/14464	0.05263158	19.0315789	4.14159967	0.05128785	0.34729843	0.32084266	Ptpn1	1
GO:0050731	positive regulation of peptidyl-tyrosine phosphorylation	2/40	133/14464	0.01503759	5.43759398	2.70739404	0.05216129	0.34729843	0.32084266	Cntf/Ptpn1	2
GO:0009125	nucleoside monophosphate catabolic process	1/40	20/14464	0.05	18.08	4.02508871	0.05391496	0.34729843	0.32084266	Pnp	1
GO:0033762	response to glucagon	1/40	20/14464	0.05	18.08	4.02508871	0.05391496	0.34729843	0.32084266	Cry1	1
GO:0035795	negative regulation of mitochondrial membrane permeability	1/40	20/14464	0.05	18.08	4.02508871	0.05391496	0.34729843	0.32084266	Bok	1
GO:0048143	astrocyte activation	1/40	20/14464	0.05	18.08	4.02508871	0.05391496	0.34729843	0.32084266	Cntf	1
GO:0060155	platelet dense granule organization	1/40	20/14464	0.05	18.08	4.02508871	0.05391496	0.34729843	0.32084266	Hps5	1
GO:1901028	regulation of mitochondrial outer membrane permeabilization involved in apoptotic signaling pathway	1/40	20/14464	0.05	18.08	4.02508871	0.05391496	0.34729843	0.32084266	Bok	1
GO:1901659	glycosyl compound biosynthetic process	1/40	20/14464	0.05	18.08	4.02508871	0.05391496	0.34729843	0.32084266	Pnp	1
GO:0046879	hormone secretion	3/40	314/14464	0.00955414	3.45477707	2.31587436	0.05552242	0.34729843	0.32084266	Cry1/Cntf/Capn10	3
GO:0031445	regulation of heterochromatin formation	1/40	21/14464	0.04761905	17.2190476	3.91672087	0.05653497	0.34729843	0.32084266	Samd1	1
GO:0034656	nucleobase-containing small molecule catabolic process	1/40	21/14464	0.04761905	17.2190476	3.91672087	0.05653497	0.34729843	0.32084266	Pnp	1
GO:0043101	purine-containing compound salvage	1/40	21/14464	0.04761905	17.2190476	3.91672087	0.05653497	0.34729843	0.32084266	Pnp	1
GO:0046040	IMP metabolic process	1/40	21/14464	0.04761905	17.2190476	3.91672087	0.05653497	0.34729843	0.32084266	Pnp	1
GO:0050872	white fat cell differentiation	1/40	21/14464	0.04761905	17.2190476	3.91672087	0.05653497	0.34729843	0.32084266	Ctbp2	1
GO:0071731	response to nitric oxide	1/40	21/14464	0.04761905	17.2190476	3.91672087	0.05653497	0.34729843	0.32084266	Ptpn1	1
GO:2000001	regulation of DNA damage checkpoint	1/40	21/14464	0.04761905	17.2190476	3.91672087	0.05653497	0.34729843	0.32084266	Cry1	1
GO:0021782	glial cell development	2/40	140/14464	0.01428571	5.16571429	2.60818068	0.05712962	0.34729843	0.32084266	Cntf/Arhgef10	2
GO:0009914	hormone transport	3/40	321/14464	0.00934579	3.37943925	2.27024485	0.05856053	0.34729843	0.32084266	Cry1/Cntf/Capn10	3
GO:0010623	programmed cell death involved in cell development	1/40	22/14464	0.04545455	16.4363636	3.81556629	0.0591479	0.34729843	0.32084266	Cntf	1
GO:0043153	entrainment of circadian clock by photoperiod	1/40	22/14464	0.04545455	16.4363636	3.81556629	0.0591479	0.34729843	0.32084266	Cry1	1
GO:0051900	regulation of mitochondrial depolarization	1/40	22/14464	0.04545455	16.4363636	3.81556629	0.0591479	0.34729843	0.32084266	Bok	1
GO:1903672	positive regulation of sprouting angiogenesis	1/40	22/14464	0.04545455	16.4363636	3.81556629	0.0591479	0.34729843	0.32084266	Smad1	1
GO:1905709	negative regulation of membrane permeability	1/40	22/14464	0.04545455	16.4363636	3.81556629	0.0591479	0.34729843	0.32084266	Bok	1
GO:2000042	negative regulation of double-strand break repair via homologous recombination	1/40	22/14464	0.04545455	16.4363636	3.81556629	0.0591479	0.34729843	0.32084266	Fbh1	1
GO:2001235	positive regulation of apoptotic signaling pathway	2/40	146/14464	0.01369863	4.95342466	2.52827929	0.06151542	0.34729843	0.32084266	Bok/Fbh1	2
GO:0032956	regulation of actin cytoskeleton organization	3/40	328/14464	0.00914634	3.30731707	2.22585703	0.06167683	0.34729843	0.32084266	Iqgap2/Arhgef10/Capn10	3
GO:0009168	purine ribonucleoside monophosphate biosynthetic process	1/40	23/14464	0.04347826	15.7217391	3.72083776	0.06175378	0.34729843	0.32084266	Pnp	1
GO:0009264	deoxyribonucleotide catabolic process	1/40	23/14464	0.04347826	15.7217391	3.72083776	0.06175378	0.34729843	0.32084266	Pnp	1
GO:0009648	photoperiodism	1/40	23/14464	0.04347826	15.7217391	3.72083776	0.06175378	0.34729843	0.32084266	Cry1	1
GO:0009649	entrainment of circadian clock	1/40	23/14464	0.04347826	15.7217391	3.72083776	0.06175378	0.34729843	0.32084266	Cry1	1

GO:0010666	positive regulation of cardiac muscle cell apoptotic process	1/40	23/14464	0.04347826	15.7217391	3.72083776	0.06175378	0.34729843	0.32084266	Ptpn1	1
GO:0046037	GMP metabolic process	1/40	23/14464	0.04347826	15.7217391	3.72083776	0.06175378	0.34729843	0.32084266	Pnp	1
GO:0046386	deoxyribose phosphate catabolic process	1/40	23/14464	0.04347826	15.7217391	3.72083776	0.06175378	0.34729843	0.32084266	Pnp	1
GO:2000831	regulation of steroid hormone secretion	1/40	23/14464	0.04347826	15.7217391	3.72083776	0.06175378	0.34729843	0.32084266	Cry1	1
GO:0010663	positive regulation of striated muscle cell apoptotic process	1/40	24/14464	0.04166667	15.0666667	3.63186379	0.06435262	0.34729843	0.32084266	Ptpn1	1
GO:0060544	regulation of necroptotic process	1/40	24/14464	0.04166667	15.0666667	3.63186379	0.06435262	0.34729843	0.32084266	Bok	1
GO:0061098	positive regulation of protein tyrosine kinase activity	1/40	24/14464	0.04166667	15.0666667	3.63186379	0.06435262	0.34729843	0.32084266	Ptpn1	1
GO:0070233	negative regulation of T cell apoptotic process	1/40	24/14464	0.04166667	15.0666667	3.63186379	0.06435262	0.34729843	0.32084266	Pnp	1
GO:0072530	purine-containing compound transmembrane transport	1/40	24/14464	0.04166667	15.0666667	3.63186379	0.06435262	0.34729843	0.32084266	Slc43a3	1
GO:1901658	glycosyl compound catabolic process	1/40	24/14464	0.04166667	15.0666667	3.63186379	0.06435262	0.34729843	0.32084266	Pnp	1
GO:1904037	positive regulation of epithelial cell apoptotic process	1/40	24/14464	0.04166667	15.0666667	3.63186379	0.06435262	0.34729843	0.32084266	Capn10	1
GO:0141193	nuclear receptor-mediated signaling pathway	2/40	151/14464	0.01324503	4.78940397	2.46496284	0.0652563	0.34729843	0.32084266	Cry1/Ctbp2	2
GO:1902850	microtubule cytoskeleton organization involved in mitosis	2/40	151/14464	0.01324503	4.78940397	2.46496284	0.0652563	0.34729843	0.32084266	Plk5/Arhgef10	2
GO:2000045	regulation of G1/S transition of mitotic cell cycle	2/40	151/14464	0.01324503	4.78940397	2.46496284	0.0652563	0.34729843	0.32084266	Plk5/Ctdspl	2
GO:0045814	negative regulation of gene expression, epigenetic	2/40	153/14464	0.0130719	4.72679739	2.44041024	0.06677389	0.34729843	0.32084266	Rrp8/Samd1	2
GO:0046887	positive regulation of hormone secretion	2/40	153/14464	0.0130719	4.72679739	2.44041024	0.06677389	0.34729843	0.32084266	Cntf/Capn10	2
GO:0009127	purine nucleoside monophosphate biosynthetic process	1/40	25/14464	0.04	14.464	3.54806756	0.06694444	0.34729843	0.32084266	Pnp	1
GO:0035335	peptidyl-tyrosine dephosphorylation	1/40	25/14464	0.04	14.464	3.54806756	0.06694444	0.34729843	0.32084266	Ptpn1	1
GO:0046033	AMP metabolic process	1/40	25/14464	0.04	14.464	3.54806756	0.06694444	0.34729843	0.32084266	Pnp	1
GO:0046128	purine ribonucleoside metabolic process	1/40	25/14464	0.04	14.464	3.54806756	0.06694444	0.34729843	0.32084266	Pnp	1
GO:1900117	regulation of execution phase of apoptosis	1/40	25/14464	0.04	14.464	3.54806756	0.06694444	0.34729843	0.32084266	Bok	1
GO:1904353	regulation of telomere capping	1/40	25/14464	0.04	14.464	3.54806756	0.06694444	0.34729843	0.32084266	Pkib	1
GO:0006869	lipid transport	3/40	342/14464	0.00877193	3.17192982	2.14055835	0.06813997	0.34729843	0.32084266	Cry1/Slc10a6/Slc43a3	3
GO:0072331	signal transduction by p53 class mediator	2/40	155/14464	0.01290323	4.66580645	2.41627895	0.06830336	0.34729843	0.32084266	Bok/Rrp8	2
GO:0045786	negative regulation of cell cycle	3/40	343/14464	0.00874636	3.16268222	2.13463376	0.06861322	0.34729843	0.32084266	Cry1/Rrp8/Ctdspl	3
GO:0003299	muscle hypertrophy in response to stress	1/40	26/14464	0.03846154	13.9076923	3.46895041	0.06952926	0.34729843	0.32084266	Smad1	1
GO:0010460	positive regulation of heart rate	1/40	26/14464	0.03846154	13.9076923	3.46895041	0.06952926	0.34729843	0.32084266	Ptpn1	1
GO:0014898	cardiac muscle hypertrophy in response to stress	1/40	26/14464	0.03846154	13.9076923	3.46895041	0.06952926	0.34729843	0.32084266	Smad1	1
GO:0034643	establishment of mitochondrion localization, microtubule-mediated	1/40	26/14464	0.03846154	13.9076923	3.46895041	0.06952926	0.34729843	0.32084266	Rhot1	1
GO:0046039	GTP metabolic process	1/40	26/14464	0.03846154	13.9076923	3.46895041	0.06952926	0.34729843	0.32084266	Pnp	1
GO:0046628	positive regulation of insulin receptor signaling pathway	1/40	26/14464	0.03846154	13.9076923	3.46895041	0.06952926	0.34729843	0.32084266	Ptpn1	1
GO:0047497	mitochondrion transport along microtubule	1/40	26/14464	0.03846154	13.9076923	3.46895041	0.06952926	0.34729843	0.32084266	Rhot1	1
GO:0010563	negative regulation of phosphorus metabolic process	3/40	345/14464	0.00869565	3.14434783	2.12284919	0.06956429	0.34729843	0.32084266	Pkib/Ctdspl/Ptpn1	3
GO:0045936	negative regulation of phosphate metabolic process	3/40	345/14464	0.00869565	3.14434783	2.12284919	0.06956429	0.34729843	0.32084266	Pkib/Ctdspl/Ptpn1	3
GO:0043409	negative regulation of MAPK cascade	2/40	157/14464	0.01273885	4.60636943	2.39255584	0.06984454	0.34729843	0.32084266	Prdm15/Ptpn1	2
GO:0006308	DNA catabolic process	1/40	27/14464	0.03703704	13.3925926	3.3940787	0.0721071	0.34729843	0.32084266	Fbh1	1
GO:0006921	cellular component disassembly involved in execution phase of apoptosis	1/40	27/14464	0.03703704	13.3925926	3.3940787	0.0721071	0.34729843	0.32084266	Bok	1
GO:0014887	cardiac muscle adaptation	1/40	27/14464	0.03703704	13.3925926	3.3940787	0.0721071	0.34729843	0.32084266	Smad1	1
GO:0032801	receptor catabolic process	1/40	27/14464	0.03703704	13.3925926	3.3940787	0.0721071	0.34729843	0.32084266	Ptpn1	1
GO:0035929	steroid hormone secretion	1/40	27/14464	0.03703704	13.3925926	3.3940787	0.0721071	0.34729843	0.32084266	Cry1	1
GO:0036120	cellular response to platelet-derived growth factor stimulus	1/40	27/14464	0.03703704	13.3925926	3.3940787	0.0721071	0.34729843	0.32084266	Ptpn1	1
GO:0045722	positive regulation of gluconeogenesis	1/40	27/14464	0.03703704	13.3925926	3.3940787	0.0721071	0.34729843	0.32084266	Cry1	1
GO:0006469	negative regulation of protein kinase activity	2/40	161/14464	0.01242236	4.49192547	2.34628447	0.07296135	0.349549	0.32292179	Pkib/Ptpn1	2
GO:0001916	positive regulation of T cell mediated cytotoxicity	1/40	28/14464	0.03571429	12.9142857	3.3230733	0.07467797	0.349549	0.32292179	Pnp	1
GO:0009162	deoxyribonucleoside monophosphate metabolic process	1/40	28/14464	0.03571429	12.9142857	3.3230733	0.07467797	0.349549	0.32292179	Pnp	1
GO:0060219	camera-type eye photoreceptor cell differentiation	1/40	28/14464	0.03571429	12.9142857	3.3230733	0.07467797	0.349549	0.32292179	Cntf	1
GO:0061036	positive regulation of cartilage development	1/40	28/14464	0.03571429	12.9142857	3.3230733	0.07467797	0.349549	0.32292179	Smad1	1
GO:0090077	foam cell differentiation	1/40	28/14464	0.03571429	12.9142857	3.3230733	0.07467797	0.349549	0.32292179	Samd1	1
GO:0016339	calcium-dependent cell-cell adhesion via plasma membrane cell adhesion molecules	1/40	29/14464	0.03448276	12.4689655	3.25560108	0.0772419	0.35119634	0.32444364	Cdh19	1
GO:0030901	midbrain development	1/40	29/14464	0.03448276	12.4689655	3.25560108	0.0772419	0.35119634	0.32444364	Smad1	1
GO:0032369	negative regulation of lipid transport	1/40	29/14464	0.03448276	12.4689655	3.25560108	0.0772419	0.35119634	0.32444364	Cry1	1
GO:0043173	nucleotide salvage	1/40	29/14464	0.03448276	12.4689655	3.25560108	0.0772419	0.35119634	0.32444364	Pnp	1
GO:0051882	mitochondrial depolarization	1/40	29/14464	0.03448276	12.4689655	3.25560108	0.0772419	0.35119634	0.32444364	Bok	1
GO:0062098	regulation of programmed necrotic cell death	1/40	29/14464	0.03448276	12.4689655	3.25560108	0.0772419	0.35119634	0.32444364	Bok	1
GO:0009755	hormone-mediated signaling pathway	2/40	169/14464	0.01183432	4.27928994	2.2581221	0.07932819	0.35119634	0.32444364	Cry1/Ctbp2	2
GO:0036119	response to platelet-derived growth factor	1/40	30/14464	0.03333333	12.0533333	3.19136793	0.0797989	0.35119634	0.32444364	Ptpn1	1
GO:0046427	positive regulation of receptor signaling pathway via JAK-STAT	1/40	30/14464	0.03333333	12.0533333	3.19136793	0.0797989	0.35119634	0.32444364	Cntf	1
GO:0048384	retinoic acid receptor signaling pathway	1/40	30/14464	0.03333333	12.0533333	3.19136793	0.0797989	0.35119634	0.32444364	Ctbp2	1
GO:0007269	neurotransmitter secretion	2/40	170/14464	0.01176471	4.25411765	2.24748682	0.08013613	0.35119634	0.32444364	Ctbp2/Rhot1	2
GO:0099643	signal release from synapse	2/40	170/14464	0.01176471	4.25411765	2.24748682	0.08013613	0.35119634	0.32444364	Ctbp2/Rhot1	2
GO:0032970	regulation of actin filament-based process	3/40	368/14464	0.00815217	2.94782609	1.99316143	0.08093229	0.35119634	0.32444364	Iqgap2/Arhgef10/Capn10	3
GO:0009119	ribonucleoside metabolic process	1/40	31/14464	0.03225806	11.6645161	3.13011309	0.082349	0.35119634	0.32444364	Pnp	1
GO:0042278	purine nucleoside metabolic process	1/40	31/14464	0.03225806	11.6645161	3.13011309	0.082349	0.35119634	0.32444364	Pnp	1
GO:0065002	intracellular protein transmembrane transport	1/40	31/14464	0.03225806	11.6645161	3.13011309	0.082349	0.35119634	0.32444364	Zfand2b	1
GO:1900078	positive regulation of cellular response to insulin stimulus	1/40	31/14464	0.03225806	11.6645161	3.13011309	0.082349	0.35119634	0.32444364	Ptpn1	1
GO:2000780	negative regulation of double-strand break repair	1/40	31/14464	0.03225806	11.6645161	3.13011309	0.082349	0.35119634	0.32444364	Fbh1	1
GO:0033673	negative regulation of kinase activity	2/40	173/14464	0.01156069	4.18034682	2.21606532	0.08257561	0.35119634	0.32444364	Pkib/Ptpn1	2
GO:0006839	mitochondrial transport	2/40	175/14464	0.01142857	4.13257143	2.19551001	0.08421477	0.35119634	0.32444364	Bok/Rhot1	2
GO:0002347	response to tumor cell	1/40	32/14464	0.03125	11.3	3.07160443	0.0848922	0.35119634	0.32444364	Plk5	1
GO:0009112	nucleobase metabolic process	1/40	32/14464	0.03125	11.3	3.07160443	0.0848922	0.35119634	0.32444364	Pnp	1
GO:0032743	positive regulation of interleukin-2 production	1/40	32/14464	0.03125	11.3	3.07160443	0.0848922	0.35119634	0.32444364	Pnp	1
GO:0045577	regulation of B cell differentiation	1/40	32/14464	0.03125	11.3	3.07160443	0.0848922	0.35119634	0.32444364	Pnp	1
GO:0045738	negative regulation of DNA repair	1/40	32/14464	0.03125	11.3	3.07160443	0.0848922	0.35119634	0.32444364	Fbh1	1
GO:2001233	regulation of apoptotic signaling pathway	3/40	379/14464	0.00791557	2.86226913	1.93463908	0.08664146	0.35119634	0.32444364	Bok/Ptpn1/Fbh1	3
GO:0006346	DNA methylation-dependent heterochromatin formation	1/40	33/14464	0.03030303	10.9575758	3.01563453	0.08742853	0.35119634	0.32444364	Samd1	1
GO:0022011	myelination in peripheral nervous system	1/40	33/14464	0.03030303	10.9575758	3.01563453	0.08742853	0.35119634	0.32444364	Arhgef10	1
GO:0030947	regulation of vascular endothelial growth factor receptor signaling pathway	1/40	33/14464	0.03030303	10.9575758	3.01563453	0.08742853	0.35119634	0.32444364	Ptpn1	1
GO:0032292	peripheral nervous system axon ensheathment	1/40	33/14464	0.03030303	10.9575758	3.01563453	0.08742853	0.35119634	0.32444364	Arhgef10	1
GO:1902410	mitotic cytokinetic process	1/40	33/14464	0.03030303	10.9575758	3.01563453	0.08742853	0.35119634	0.32444364	Iqgap2	1
GO:1904385	cellular response to angiotensin	1/40	33/14464	0.03030303	10.9575758	3.01563453	0.08742853	0.35119634	0.32444364	Ptpn1	1
GO:0001914	regulation of T cell mediated cytotoxicity	1/40	34/14464	0.02941176	10.6352941	2.96201737	0.089958	0.35119634	0.32444364	Pnp	1
GO:0042531	positive regulation of tyrosine phosphorylation of STAT protein	1/40	34/14464	0.02941176	10.6352941	2.96201737	0.089958	0.35119634	0.32444364	Cntf	1
GO:0051654	establishment of mitochondrion localization	1/40	34/14464	0.02941176	10.6352941	2.96201737	0.089958	0.35119634	0.32444364	Rhot1	1
GO:1902275	regulation of chromatin organization	1/40	34/14464	0.02941176	10.6352941	2.96201737	0.089958	0.35119634	0.32444364	Samd1	1
GO:1904752	regulation of vascular associated smooth muscle cell migration	1/40	34/14464	0.02941176	10.6352941	2.96201737	0.089958	0.35119634	0.32444364	Ptpn1	1
GO:0007140	male meiotic nuclear division	1/40	35/14464	0.02857143	10.3314286	2.91058558	0.09248064	0.35119634	0.32444364	Catsperz	1
GO:0014912	negative regulation of smooth muscle cell migration	1/40	35/14464	0.02857143	10.3314286	2.91058558	0.09248064	0.35119634	0.32444364	Ptpn1	1
GO:0032212	positive regulation of telomere maintenance via telomerase	1/40	35/14464	0.02857143	10.3314286	2.91058558	0.09248064	0.35119634	0.32444364	Pkib	1
GO:0032438	melanosome organization	1/40	35/14464	0.02857143	10.3314286	2.91058558	0.09248064	0.35119634	0.32444364	Hps5	1
GO:0033144	negative regulation of intracellular steroid hormone receptor signaling pathway	1/40	35/14464	0.02857143	10.3314286	2.91058558	0.09248064	0.35119634	0.32444364	Cry1	1
GO:0048679	regulation of axon regeneration	1/40	35/14464	0.02857143	10.3314286	2.91058558	0.09248064	0.35119634	0.32444364	Cntf	1
GO:0070229	negative regulation of lymphocyte apoptotic process	1/40	35/14464	0.02857143	10.3314286	2.91058558	0.09248064	0.35119634	0.32444364	Pnp	1
GO:1905898	positive regulation of response to endoplasmic reticulum stress	1/40	35/14464	0.02857143	10.3314286	2.91058558	0.09248064	0.35119634	0.32444364	Bok	1
GO:0010876	lipid localization	3/40	393/14464	0.00763359	2.76030534	1.86310844	0.09415229	0.35119634	0.32444364	Cry1/Slc10a6/Slc43a3	3
GO:0007051	spindle organization	2/40	187/14464	0.01069519	3.86737968	2.07827904	0.09425619	0.35119634	0.32444364	Plk5/Arhgef10	2
GO:0009156	ribonucleoside monophosphate biosynthetic process	1/40	36/14464	0.02777778	10.0444444	2.86118811	0.09499646	0.35119634	0.32444364	Pnp	1
GO:0009167	purine ribonucleoside monophosphate metabolic process	1/40	36/14464	0.02777778	10.0444444	2.86118811	0.09499646	0.35119634	0.32444364	Pnp	1
GO:0014002	astrocyte development	1/40	36/14464	0.02777778	10.0444444	2.86118811	0.09499646	0.35119634	0.32444364	Cntf	1
GO:0042908	xenobiotic transport	1/40	36/14464	0.02777778	10.0444444	2.86118811	0.09499646	0.35119634	0.32444364	Slc43a3	1
GO:0045823	positive regulation of heart contraction	1/40	36/14464	0.02777778	10.0444444	2.86118811	0.09499646	0.35119634	0.32444364	Ptpn1	1
GO:0048753	pigment granule organization	1/40	36/14464	0.02777778	10.0444444	2.86118811	0.09499646	0.35119634	0.32444364	Hps5	1
GO:0070266	necroptotic process	1/40	36/14464	0.02777778	10.0444444	2.86118811	0.09499646	0.35119634	0.32444364	Bok	1
GO:0120033	negative regulation of plasma membrane bounded cell projection assembly	1/40	36/14464	0.02777778	10.0444444	2.86118811	0.09499646	0.35119634	0.32444364	Tchp	1
GO:1902229	regulation of intrinsic apoptotic signaling pathway in response to DNA damage	1/40	36/14464	0.02777778	10.0444444	2.86118811	0.09499646	0.35119634	0.32444364	Fbh1	1
GO:0043524	negative regulation of neuron apoptotic process	2/40	188/14464	0.0106383	3.84680851	2.06895118	0.09510834	0.35119634	0.32444364	Bok/Cntf	2
GO:0032259	methylation	2/40	190/14464	0.01052632	3.80631579	2.05048608	0.09681944	0.3528454	0.32596709	Rrp8/Prdm15	2
GO:0016233	telomere capping	1/40	37/14464	0.02702703	9.77297297	2.8136882	0.09750548	0.3528454	0.32596709	Pkib	1
GO:0046326	positive regulation of D-glucose import	1/40	37/14464	0.02702703	9.77297297	2.8136882	0.09750548	0.3528454	0.32596709	Capn10	1
GO:1904894	positive regulation of receptor signaling pathway via STAT	1/40	37/14464	0.02702703	9.77297297	2.8136882	0.09750548	0.3528454	0.32596709	Cntf	1
GO:0031099	regeneration	2/40	191/14464	0.0104712	3.78638743	2.04134724	0.09767837	0.3528454	0.32596709	Matn2/Cntf	2
GO:0050730	regulation of peptidyl-tyrosine phosphorylation	2/40	191/14464	0.0104712	3.78638743	2.04134724	0.09767837	0.3528454	0.32596709	Cntf/Ptpn1	2
GO:0044331	cell-cell adhesion mediated by cadherin	1/40	38/14464	0.02631579	9.51578947	2.76796174	0.10000772	0.35598503	0.32886755	Cdh19	1
GO:0045165	cell fate commitment	2/40	195/14464	0.01025641	3.70871795	2.00539835	0.10113609	0.35598503	0.32886755	Prdm15/Smad1	2
GO:0051348	negative regulation of transferase activity	2/40	196/14464	0.01020408	3.68979592	1.996559	0.10200594	0.35598503	0.32886755	Pkib/Ptpn1	2
GO:0006904	vesicle docking involved in exocytosis	1/40	39/14464	0.02564103	9.27179487	2.72389574	0.10250319	0.35598503	0.32886755	Ctbp2	1
GO:0009126	purine nucleoside monophosphate metabolic process	1/40	39/14464	0.02564103	9.27179487	2.72389574	0.10250319	0.35598503	0.32886755	Pnp	1
GO:0010661	positive regulation of muscle cell apoptotic process	1/40	39/14464	0.02564103	9.27179487	2.72389574	0.10250319	0.35598503	0.32886755	Ptpn1	1
GO:0017145	stem cell division	1/40	39/14464	0.02564103	9.27179487	2.72389574	0.10250319	0.35598503	0.32886755	Prdm15	1
GO:0043094	cellular metabolic compound salvage	1/40	39/14464	0.02564103	9.27179487	2.72389574	0.10250319	0.35598503	0.32886755	Pnp	1
GO:0060632	regulation of microtubule-based movement	1/40	39/14464	0.02564103	9.27179487	2.72389574	0.10250319	0.35598503	0.32886755	Rhot1	1
GO:1904358	positive regulation of telomere maintenance via telomere lengthening	1/40	39/14464	0.02564103	9.27179487	2.72389574	0.10250319	0.35598503	0.32886755	Pkib	1
GO:1904738	vascular associated smooth muscle cell migration	1/40	39/14464	0.02564103	9.27179487	2.72389574	0.10250319	0.35598503	0.32886755	Ptpn1	1
GO:0051963	regulation of synapse assembly	2/40	197/14464	0.01015228	3.67106599	1.98777732	0.10287793	0.35598503	0.32886755	Lrrn3/Ptpn1	2
GO:0043434	response to peptide hormone	3/40	410/14464	0.00731707	2.64585366	1.78032587	0.10362676	0.35598503	0.32886755	Cry1/Capn10/Ptpn1	3
GO:0099558	maintenance of synapse structure	1/40	40/14464	0.025	9.04	2.68138712	0.10499192	0.35598503	0.32886755	Ctbp2	1
GO:1903524	positive regulation of blood circulation	1/40	40/14464	0.025	9.04	2.68138712	0.10499192	0.35598503	0.32886755	Ptpn1	1
GO:0031345	negative regulation of cell projection organization	2/40	201/14464	0.00995025	3.59800995	1.95321356	0.10638683	0.35598503	0.32886755	Tchp/Ptpn1	2
GO:0001754	eye photoreceptor cell differentiation	1/40	41/14464	0.02439024	8.8195122	2.64034161	0.10747392	0.35598503	0.32886755	Cntf	1
GO:0009394	2'-deoxyribonucleotide metabolic process	1/40	41/14464	0.02439024	8.8195122	2.64034161	0.10747392	0.35598503	0.32886755	Pnp	1
GO:0030866	cortical actin cytoskeleton organization	1/40	41/14464	0.02439024	8.8195122	2.64034161	0.10747392	0.35598503	0.32886755	Iqgap2	1
GO:0034314	Arp2/3 complex-mediated actin nucleation	1/40	41/14464	0.02439024	8.8195122	2.64034161	0.10747392	0.35598503	0.32886755	Iqgap2	1
GO:0043507	positive regulation of JUN kinase activity	1/40	41/14464	0.02439024	8.8195122	2.64034161	0.10747392	0.35598503	0.32886755	Ptpn1	1
GO:0044060	regulation of endocrine process	1/40	41/14464	0.02439024	8.8195122	2.64034161	0.10747392	0.35598503	0.32886755	Cry1	1
GO:0045047	protein targeting to ER	1/40	41/14464	0.02439024	8.8195122	2.64034161	0.10747392	0.35598503	0.32886755	Zfand2b	1
GO:0045910	negative regulation of DNA recombination	1/40	41/14464	0.02439024	8.8195122	2.64034161	0.10747392	0.35598503	0.32886755	Fbh1	1
GO:1990776	response to angiotensin	1/40	41/14464	0.02439024	8.8195122	2.64034161	0.10747392	0.35598503	0.32886755	Ptpn1	1
GO:0019692	deoxyribose phosphate metabolic process	1/40	42/14464	0.02380952	8.60952381	2.60067278	0.1099492	0.3570663	0.32986645	Pnp	1
GO:0045777	positive regulation of blood pressure	1/40	42/14464	0.02380952	8.60952381	2.60067278	0.1099492	0.3570663	0.32986645	Ptpn1	1
GO:0046638	positive regulation of alpha-beta T cell differentiation	1/40	42/14464	0.02380952	8.60952381	2.60067278	0.1099492	0.3570663	0.32986645	Pnp	1
GO:0070570	regulation of neuron projection regeneration	1/40	42/14464	0.02380952	8.60952381	2.60067278	0.1099492	0.3570663	0.32986645	Cntf	1
GO:0090630	activation of GTPase activity	1/40	42/14464	0.02380952	8.60952381	2.60067278	0.1099492	0.3570663	0.32986645	Arhgef10	1
GO:1903573	negative regulation of response to endoplasmic reticulum stress	1/40	42/14464	0.02380952	8.60952381	2.60067278	0.1099492	0.3570663	0.32986645	Ptpn1	1
GO:0000082	G1/S transition of mitotic cell cycle	2/40	207/14464	0.00966184	3.49371981	1.90298377	0.11171118	0.35774606	0.33049443	Plk5/Ctdspl	2
GO:0009262	deoxyribonucleotide metabolic process	1/40	43/14464	0.02325581	8.40930233	2.56230124	0.11241779	0.35774606	0.33049443	Pnp	1
GO:0050732	negative regulation of peptidyl-tyrosine phosphorylation	1/40	43/14464	0.02325581	8.40930233	2.56230124	0.11241779	0.35774606	0.33049443	Ptpn1	1
GO:0070232	regulation of T cell apoptotic process	1/40	43/14464	0.02325581	8.40930233	2.56230124	0.11241779	0.35774606	0.33049443	Pnp	1
GO:0140719	constitutive heterochromatin formation	1/40	43/14464	0.02325581	8.40930233	2.56230124	0.11241779	0.35774606	0.33049443	Samd1	1
GO:0006282	regulation of DNA repair	2/40	208/14464	0.00961538	3.47692308	1.89479258	0.11260547	0.35774606	0.33049443	Pnp/Fbh1	2
GO:0001913	T cell mediated cytotoxicity	1/40	44/14464	0.02272727	8.21818182	2.52515393	0.11487971	0.35774606	0.33049443	Pnp	1
GO:0002183	cytoplasmic translational initiation	1/40	44/14464	0.02272727	8.21818182	2.52515393	0.11487971	0.35774606	0.33049443	Eif3j1	1
GO:0010828	positive regulation of D-glucose transmembrane transport	1/40	44/14464	0.02272727	8.21818182	2.52515393	0.11487971	0.35774606	0.33049443	Capn10	1
GO:0042509	regulation of tyrosine phosphorylation of STAT protein	1/40	44/14464	0.02272727	8.21818182	2.52515393	0.11487971	0.35774606	0.33049443	Cntf	1
GO:1901976	regulation of cell cycle checkpoint	1/40	44/14464	0.02272727	8.21818182	2.52515393	0.11487971	0.35774606	0.33049443	Cry1	1
GO:1902116	negative regulation of organelle assembly	1/40	44/14464	0.02272727	8.21818182	2.52515393	0.11487971	0.35774606	0.33049443	Tchp	1
GO:1903670	regulation of sprouting angiogenesis	1/40	44/14464	0.02272727	8.21818182	2.52515393	0.11487971	0.35774606	0.33049443	Smad1	1
GO:0031032	actomyosin structure organization	2/40	212/14464	0.00943396	3.41132075	1.86252169	0.1162017	0.35774606	0.33049443	Iqgap2/Arhgef10	2
GO:0032869	cellular response to insulin stimulus	2/40	212/14464	0.00943396	3.41132075	1.86252169	0.1162017	0.35774606	0.33049443	Capn10/Ptpn1	2
GO:0009124	nucleoside monophosphate biosynthetic process	1/40	45/14464	0.02222222	8.03555556	2.48916345	0.11733497	0.35774606	0.33049443	Pnp	1
GO:0043407	negative regulation of MAP kinase activity	1/40	45/14464	0.02222222	8.03555556	2.48916345	0.11733497	0.35774606	0.33049443	Ptpn1	1
GO:0048009	insulin-like growth factor receptor signaling pathway	1/40	45/14464	0.02222222	8.03555556	2.48916345	0.11733497	0.35774606	0.33049443	Zfand2b	1
GO:0072599	establishment of protein localization to endoplasmic reticulum	1/40	45/14464	0.02222222	8.03555556	2.48916345	0.11733497	0.35774606	0.33049443	Zfand2b	1
GO:1905953	negative regulation of lipid localization	1/40	45/14464	0.02222222	8.03555556	2.48916345	0.11733497	0.35774606	0.33049443	Cry1	1
GO:0001912	positive regulation of leukocyte mediated cytotoxicity	1/40	46/14464	0.02173913	7.86086957	2.45426752	0.11978358	0.36189161	0.33432419	Pnp	1
GO:0009116	nucleoside metabolic process	1/40	46/14464	0.02173913	7.86086957	2.45426752	0.11978358	0.36189161	0.33432419	Pnp	1
GO:2000351	regulation of endothelial cell apoptotic process	1/40	46/14464	0.02173913	7.86086957	2.45426752	0.11978358	0.36189161	0.33432419	Ptpn1	1
GO:0007260	tyrosine phosphorylation of STAT protein	1/40	47/14464	0.0212766	7.69361702	2.42040851	0.12222558	0.36477112	0.33698435	Cntf	1

GO:0014044	Schwann cell development	1/40	47/14464	0.0212766	7.69361702	2.42040851	0.12222558	0.36477112	0.33698435	Arhgef10	1
GO:0031297	replication fork processing	1/40	47/14464	0.0212766	7.69361702	2.42040851	0.12222558	0.36477112	0.33698435	Fbh1	1
GO:0048066	developmental pigmentation	1/40	47/14464	0.0212766	7.69361702	2.42040851	0.12222558	0.36477112	0.33698435	Hps5	1
GO:0006836	neurotransmitter transport	2/40	219/14464	0.00913242	3.30228311	1.80786455	0.12256602	0.36477112	0.33698435	Ctbp2/Rhot1	2
GO:0051052	regulation of DNA metabolic process	3/40	443/14464	0.00677201	2.44875847	1.63089006	0.12305726	0.36514311	0.337328	Pkib/Pnp/Fbh1	3
GO:0010976	positive regulation of neuron projection development	2/40	220/14464	0.00909091	3.28727273	1.80023683	0.12348232	0.36531712	0.33748876	Plk5/Cntf	2
GO:0032506	cytokinetic process	1/40	48/14464	0.02083333	7.53333333	2.38753299	0.12466096	0.36662826	0.33870002	Iqgap2	1
GO:0140353	lipid export from cell	1/40	48/14464	0.02083333	7.53333333	2.38753299	0.12466096	0.36662826	0.33870002	Cry1	1
GO:0014888	striated muscle adaptation	1/40	49/14464	0.02040816	7.37959184	2.3555913	0.12708976	0.36923085	0.34110435	Smad1	1
GO:0048010	vascular endothelial growth factor receptor signaling pathway	1/40	49/14464	0.02040816	7.37959184	2.3555913	0.12708976	0.36923085	0.34110435	Ptpn1	1
GO:0061097	regulation of protein tyrosine kinase activity	1/40	49/14464	0.02040816	7.37959184	2.3555913	0.12708976	0.36923085	0.34110435	Ptpn1	1
GO:1902895	positive regulation of miRNA transcription	1/40	49/14464	0.02040816	7.37959184	2.3555913	0.12708976	0.36923085	0.34110435	Smad1	1
GO:2001234	negative regulation of apoptotic signaling pathway	2/40	225/14464	0.00888889	3.21422222	1.76274321	0.1280894	0.36923085	0.34110435	Bok/Ptpn1	2
GO:0003254	regulation of membrane depolarization	1/40	50/14464	0.02	7.232	2.3245373	0.12951199	0.36923085	0.34110435	Bok	1
GO:0032663	regulation of interleukin-2 production	1/40	50/14464	0.02	7.232	2.3245373	0.12951199	0.36923085	0.34110435	Pnp	1
GO:0072577	endothelial cell apoptotic process	1/40	50/14464	0.02	7.232	2.3245373	0.12951199	0.36923085	0.34110435	Ptpn1	1
GO:0097300	programmed necrotic cell death	1/40	50/14464	0.02	7.232	2.3245373	0.12951199	0.36923085	0.34110435	Bok	1
GO:0010817	regulation of hormone levels	3/40	455/14464	0.00659341	2.38417582	1.57982769	0.13043999	0.36923085	0.34110435	Cry1/Cntf/Capn10	3
GO:0031667	response to nutrient levels	3/40	455/14464	0.00659341	2.38417582	1.57982769	0.13043999	0.36923085	0.34110435	Rrp8/Capn10/Ptpn1	3
GO:0002711	positive regulation of T cell mediated immunity	1/40	51/14464	0.01960784	7.09019608	2.29432798	0.13192766	0.36923085	0.34110435	Pnp	1
GO:0060986	endocrine hormone secretion	1/40	51/14464	0.01960784	7.09019608	2.29432798	0.13192766	0.36923085	0.34110435	Cry1	1
GO:0040029	epigenetic regulation of gene expression	2/40	230/14464	0.00869565	3.14434783	1.72628303	0.13273772	0.36923085	0.34110435	Rrp8/Samd1	2
GO:0001707	mesoderm formation	1/40	52/14464	0.01923077	6.95384615	2.26492323	0.1343368	0.36923085	0.34110435	Smad1	1
GO:0006111	regulation of gluconeogenesis	1/40	52/14464	0.01923077	6.95384615	2.26492323	0.1343368	0.36923085	0.34110435	Cry1	1
GO:0031343	positive regulation of cell killing	1/40	52/14464	0.01923077	6.95384615	2.26492323	0.1343368	0.36923085	0.34110435	Pnp	1
GO:0032210	regulation of telomere maintenance via telomerase	1/40	52/14464	0.01923077	6.95384615	2.26492323	0.1343368	0.36923085	0.34110435	Pkib	1
GO:0045005	DNA-templated DNA replication maintenance of fidelity	1/40	52/14464	0.01923077	6.95384615	2.26492323	0.1343368	0.36923085	0.34110435	Fbh1	1
GO:0018108	peptidyl-tyrosine phosphorylation	2/40	233/14464	0.00858369	3.10386266	1.7048806	0.13554571	0.36923085	0.34110435	Cntf/Ptpn1	2
GO:0009611	response to wounding	3/40	465/14464	0.00645161	2.33290323	1.53848378	0.13671302	0.36923085	0.34110435	Matn2/Cntf/Hps5	3
GO:0001881	receptor recycling	1/40	53/14464	0.01886792	6.82264151	2.23628556	0.13673942	0.36923085	0.34110435	Ptpn1	1
GO:0009161	ribonucleoside monophosphate metabolic process	1/40	53/14464	0.01886792	6.82264151	2.23628556	0.13673942	0.36923085	0.34110435	Pnp	1
GO:0033059	cellular pigmentation	1/40	53/14464	0.01886792	6.82264151	2.23628556	0.13673942	0.36923085	0.34110435	Hps5	1
GO:0042149	cellular response to glucose starvation	1/40	53/14464	0.01886792	6.82264151	2.23628556	0.13673942	0.36923085	0.34110435	Rrp8	1
GO:2000107	negative regulation of leukocyte apoptotic process	1/40	53/14464	0.01886792	6.82264151	2.23628556	0.13673942	0.36923085	0.34110435	Pnp	1
GO:0071900	regulation of protein serine/threonine kinase activity	2/40	235/14464	0.00851064	3.07744681	1.69080291	0.13742536	0.36923085	0.34110435	Pkib/Ptpn1	2
GO:0014037	Schwann cell differentiation	1/40	54/14464	0.01851852	6.6962963	2.20837994	0.13913553	0.36923085	0.34110435	Arhgef10	1
GO:0034332	adherens junction organization	1/40	54/14464	0.01851852	6.6962963	2.20837994	0.13913553	0.36923085	0.34110435	Cdh19	1
GO:0043506	regulation of JUN kinase activity	1/40	54/14464	0.01851852	6.6962963	2.20837994	0.13913553	0.36923085	0.34110435	Ptpn1	1
GO:0051496	positive regulation of stress fiber assembly	1/40	54/14464	0.01851852	6.6962963	2.20837994	0.13913553	0.36923085	0.34110435	Arhgef10	1
GO:0061900	glial cell activation	1/40	54/14464	0.01851852	6.6962963	2.20837994	0.13913553	0.36923085	0.34110435	Cntf	1
GO:1990090	cellular response to nerve growth factor stimulus	1/40	54/14464	0.01851852	6.6962963	2.20837994	0.13913553	0.36923085	0.34110435	Ptpn1	1
GO:0018212	peptidyl-tyrosine modification	2/40	237/14464	0.00843882	3.05147679	1.67687406	0.139311	0.36923085	0.34110435	Cntf/Ptpn1	2
GO:0010332	response to gamma radiation	1/40	55/14464	0.01818182	6.57454545	2.18117352	0.14152516	0.36923085	0.34110435	Pnp	1
GO:0010812	negative regulation of cell-substrate adhesion	1/40	55/14464	0.01818182	6.57454545	2.18117352	0.14152516	0.36923085	0.34110435	Ptpn1	1
GO:0010907	positive regulation of glucose metabolic process	1/40	55/14464	0.01818182	6.57454545	2.18117352	0.14152516	0.36923085	0.34110435	Cry1	1
GO:0030865	cortical cytoskeleton organization	1/40	55/14464	0.01818182	6.57454545	2.18117352	0.14152516	0.36923085	0.34110435	Iqgap2	1
GO:0150052	regulation of postsynapse assembly	1/40	55/14464	0.01818182	6.57454545	2.18117352	0.14152516	0.36923085	0.34110435	Ptpn1	1
GO:0051896	regulation of phosphatidylinositol 3-kinase/protein kinase B signal transduction	2/40	240/14464	0.00833333	3.01333333	1.65625333	0.14215042	0.36923085	0.34110435	Cntf/Ptpn1	2
GO:1901988	negative regulation of cell cycle phase transition	2/40	240/14464	0.00833333	3.01333333	1.65625333	0.14215042	0.36923085	0.34110435	Cry1/Ctdspl	2
GO:0048332	mesoderm morphogenesis	1/40	56/14464	0.01785714	6.45714286	2.15463554	0.14390833	0.36923085	0.34110435	Smad1	1
GO:0051646	mitochondrion localization	1/40	56/14464	0.01785714	6.45714286	2.15463554	0.14390833	0.36923085	0.34110435	Rhot1	1
GO:0060042	retina morphogenesis in camera-type eye	1/40	56/14464	0.01785714	6.45714286	2.15463554	0.14390833	0.36923085	0.34110435	Cntf	1
GO:0072698	protein localization to microtubule cytoskeleton	1/40	56/14464	0.01785714	6.45714286	2.15463554	0.14390833	0.36923085	0.34110435	Fam83d	1
GO:1990089	response to nerve growth factor	1/40	56/14464	0.01785714	6.45714286	2.15463554	0.14390833	0.36923085	0.34110435	Ptpn1	1
GO:2000630	positive regulation of miRNA metabolic process	1/40	56/14464	0.01785714	6.45714286	2.15463554	0.14390833	0.36923085	0.34110435	Smad1	1
GO:0051259	protein complex oligomerization	2/40	244/14464	0.00819672	2.96393443	1.62925263	0.14595618	0.36923085	0.34110435	Bok/Samd1	2
GO:0010665	regulation of cardiac muscle cell apoptotic process	1/40	57/14464	0.01754386	6.34385965	2.12873708	0.14628504	0.36923085	0.34110435	Ptpn1	1
GO:0032623	interleukin-2 production	1/40	57/14464	0.01754386	6.34385965	2.12873708	0.14628504	0.36923085	0.34110435	Pnp	1
GO:0033363	secretory granule organization	1/40	57/14464	0.01754386	6.34385965	2.12873708	0.14628504	0.36923085	0.34110435	Hps5	1
GO:0045010	actin nucleation	1/40	57/14464	0.01754386	6.34385965	2.12873708	0.14628504	0.36923085	0.34110435	Iqgap2	1
GO:0046635	positive regulation of alpha-beta T cell activation	1/40	57/14464	0.01754386	6.34385965	2.12873708	0.14628504	0.36923085	0.34110435	Pnp	1
GO:0048278	vesicle docking	1/40	57/14464	0.01754386	6.34385965	2.12873708	0.14628504	0.36923085	0.34110435	Ctbp2	1
GO:0060038	cardiac muscle cell proliferation	1/40	57/14464	0.01754386	6.34385965	2.12873708	0.14628504	0.36923085	0.34110435	Smad1	1
GO:1902808	positive regulation of cell cycle G1/S phase transition	1/40	57/14464	0.01754386	6.34385965	2.12873708	0.14628504	0.36923085	0.34110435	Fam83d	1
GO:0045912	negative regulation of carbohydrate metabolic process	1/40	58/14464	0.01724138	6.23448276	2.10345099	0.14865532	0.36994133	0.34176072	Cry1	1
GO:0046324	regulation of D-glucose import	1/40	58/14464	0.01724138	6.23448276	2.10345099	0.14865532	0.36994133	0.34176072	Capn10	1
GO:0046530	photoreceptor cell differentiation	1/40	58/14464	0.01724138	6.23448276	2.10345099	0.14865532	0.36994133	0.34176072	Cntf	1
GO:0046637	regulation of alpha-beta T cell differentiation	1/40	58/14464	0.01724138	6.23448276	2.10345099	0.14865532	0.36994133	0.34176072	Pnp	1
GO:0034329	cell junction assembly	3/40	485/14464	0.00618557	2.23670103	1.45886086	0.14957059	0.36994133	0.34176072	Cdh19/Lrrn3/Ptpn1	3
GO:0070372	regulation of ERK1 and ERK2 cascade	2/40	249/14464	0.00803213	2.90441767	1.59626421	0.15074399	0.36994133	0.34176072	Fam83d/Ptpn1	2
GO:0006278	RNA-templated DNA biosynthetic process	1/40	59/14464	0.01694915	6.12881356	2.07875171	0.15101918	0.36994133	0.34176072	Pkib	1
GO:0007004	telomere maintenance via telomerase	1/40	59/14464	0.01694915	6.12881356	2.07875171	0.15101918	0.36994133	0.34176072	Pkib	1
GO:0010662	regulation of striated muscle cell apoptotic process	1/40	59/14464	0.01694915	6.12881356	2.07875171	0.15101918	0.36994133	0.34176072	Ptpn1	1
GO:0031103	axon regeneration	1/40	59/14464	0.01694915	6.12881356	2.07875171	0.15101918	0.36994133	0.34176072	Cntf	1
GO:0070231	T cell apoptotic process	1/40	59/14464	0.01694915	6.12881356	2.07875171	0.15101918	0.36994133	0.34176072	Pnp	1
GO:2000134	negative regulation of G1/S transition of mitotic cell cycle	1/40	59/14464	0.01694915	6.12881356	2.07875171	0.15101918	0.36994133	0.34176072	Ctdspl	1
GO:0009880	embryonic pattern specification	1/40	60/14464	0.01666667	6.02666667	2.05461516	0.15337664	0.37205964	0.34371766	Smad1	1
GO:0034331	cell junction maintenance	1/40	60/14464	0.01666667	6.02666667	2.05461516	0.15337664	0.37205964	0.34371766	Ctbp2	1
GO:0048008	platelet-derived growth factor receptor signaling pathway	1/40	60/14464	0.01666667	6.02666667	2.05461516	0.15337664	0.37205964	0.34371766	Ptpn1	1
GO:0070228	regulation of lymphocyte apoptotic process	1/40	60/14464	0.01666667	6.02666667	2.05461516	0.15337664	0.37205964	0.34371766	Pnp	1
GO:1904356	regulation of telomere maintenance via telomere lengthening	1/40	61/14464	0.01639344	5.92786885	2.03101865	0.15572772	0.37684596	0.34813938	Pkib	1
GO:0034976	response to endoplasmic reticulum stress	2/40	256/14464	0.0078125	2.825	1.55143187	0.15750092	0.37808407	0.34928318	Bok/Ptpn1	2
GO:0032233	positive regulation of actin filament bundle assembly	1/40	62/14464	0.01612903	5.83225806	2.00794075	0.15807243	0.37808407	0.34928318	Arhgef10	1
GO:0045744	negative regulation of G protein-coupled receptor signaling pathway	1/40	62/14464	0.01612903	5.83225806	2.00794075	0.15807243	0.37808407	0.34928318	Cry1	1
GO:0051148	negative regulation of muscle cell differentiation	1/40	62/14464	0.01612903	5.83225806	2.00794075	0.15807243	0.37808407	0.34928318	Smad1	1
GO:0001836	release of cytochrome c from mitochondria	1/40	63/14464	0.01587302	5.73968254	1.98536122	0.16041079	0.37808407	0.34928318	Bok	1
GO:0006919	activation of cysteine-type endopeptidase activity involved in apoptotic process	1/40	63/14464	0.01587302	5.73968254	1.98536122	0.16041079	0.37808407	0.34928318	Bok	1
GO:0045669	positive regulation of osteoblast differentiation	1/40	63/14464	0.01587302	5.73968254	1.98536122	0.16041079	0.37808407	0.34928318	Smad1	1
GO:0051898	negative regulation of phosphatidylinositol 3-kinase/protein kinase B signal transduction	1/40	63/14464	0.01587302	5.73968254	1.98536122	0.16041079	0.37808407	0.34928318	Ptpn1	1
GO:0061035	regulation of cartilage development	1/40	63/14464	0.01587302	5.73968254	1.98536122	0.16041079	0.37808407	0.34928318	Smad1	1
GO:0097192	extrinsic apoptotic signaling pathway in absence of ligand	1/40	63/14464	0.01587302	5.73968254	1.98536122	0.16041079	0.37808407	0.34928318	Bok	1
GO:1901657	glycosyl compound metabolic process	1/40	63/14464	0.01587302	5.73968254	1.98536122	0.16041079	0.37808407	0.34928318	Pnp	1
GO:0003407	neural retina development	1/40	64/14464	0.015625	5.65	1.96326094	0.16274282	0.38177552	0.35269343	Cntf	1
GO:0044380	protein localization to cytoskeleton	1/40	64/14464	0.015625	5.65	1.96326094	0.16274282	0.38177552	0.35269343	Fam83d	1
GO:0001910	regulation of leukocyte mediated cytotoxicity	1/40	65/14464	0.01538462	5.56307692	1.94162178	0.16506854	0.38236397	0.35323705	Pnp	1
GO:0010659	cardiac muscle cell apoptotic process	1/40	65/14464	0.01538462	5.56307692	1.94162178	0.16506854	0.38236397	0.35323705	Ptpn1	1
GO:0032922	circadian regulation of gene expression	1/40	65/14464	0.01538462	5.56307692	1.94162178	0.16506854	0.38236397	0.35323705	Cry1	1
GO:0038034	signal transduction in absence of ligand	1/40	65/14464	0.01538462	5.56307692	1.94162178	0.16506854	0.38236397	0.35323705	Bok	1
GO:0046425	regulation of receptor signaling pathway via JAK-STAT	1/40	65/14464	0.01538462	5.56307692	1.94162178	0.16506854	0.38236397	0.35323705	Cntf	1
GO:0098813	nuclear chromosome segregation	2/40	264/14464	0.00757576	2.73939394	1.50200741	0.16529476	0.38236397	0.35323705	Arhgef10/Fam83d	2
GO:0032508	DNA duplex unwinding	1/40	66/14464	0.01515152	5.47878788	1.92042658	0.16738795	0.38541753	0.356058	Fbh1	1
GO:2000573	positive regulation of DNA biosynthetic process	1/40	66/14464	0.01515152	5.47878788	1.92042658	0.16738795	0.38541753	0.356058	Pkib	1
GO:0010658	striated muscle cell apoptotic process	1/40	67/14464	0.01492537	5.39701493	1.89965909	0.16970109	0.38805501	0.35849457	Ptpn1	1
GO:1902807	negative regulation of cell cycle G1/S phase transition	1/40	67/14464	0.01492537	5.39701493	1.89965909	0.16970109	0.38805501	0.35849457	Ctdspl	1
GO:1903036	positive regulation of response to wounding	1/40	67/14464	0.01492537	5.39701493	1.89965909	0.16970109	0.38805501	0.35849457	Cntf	1
GO:0060395	SMAD protein signal transduction	1/40	68/14464	0.01470588	5.31764706	1.87930387	0.17200795	0.39064221	0.36088469	Smad1	1
GO:0071806	protein transmembrane transport	1/40	68/14464	0.01470588	5.31764706	1.87930387	0.17200795	0.39064221	0.36088469	Zfand2b	1
GO:1902893	regulation of miRNA transcription	1/40	68/14464	0.01470588	5.31764706	1.87930387	0.17200795	0.39064221	0.36088469	Smad1	1
GO:0070371	ERK1 and ERK2 cascade	2/40	273/14464	0.00732601	2.64908425	1.4485517	0.17414603	0.39407175	0.36405299	Fam83d/Ptpn1	2
GO:0008347	glial cell migration	1/40	69/14464	0.01449275	5.24057971	1.85934626	0.17430857	0.39407175	0.36405299	Matn2	1
GO:0033143	regulation of intracellular steroid hormone receptor signaling pathway	1/40	70/14464	0.01428571	5.16571429	1.83977233	0.17660295	0.39478284	0.3647099	Cry1	1
GO:0046626	regulation of insulin receptor signaling pathway	1/40	70/14464	0.01428571	5.16571429	1.83977233	0.17660295	0.39478284	0.3647099	Ptpn1	1
GO:0051480	regulation of cytosolic calcium ion concentration	1/40	70/14464	0.01428571	5.16571429	1.83977233	0.17660295	0.39478284	0.3647099	Bok	1
GO:0061614	miRNA transcription	1/40	70/14464	0.01428571	5.16571429	1.83977233	0.17660295	0.39478284	0.3647099	Smad1	1
GO:1902017	regulation of cilium assembly	1/40	70/14464	0.01428571	5.16571429	1.83977233	0.17660295	0.39478284	0.3647099	Tchp	1
GO:0010948	negative regulation of cell cycle process	2/40	277/14464	0.00722022	2.61083032	1.42547869	0.17810588	0.39723157	0.3669721	Cry1/Ctdspl	2
GO:0009123	nucleoside monophosphate metabolic process	1/40	71/14464	0.01408451	5.09295775	1.82056884	0.17889112	0.39723157	0.3669721	Pnp	1
GO:0043491	phosphatidylinositol 3-kinase/protein kinase B signal transduction	2/40	278/14464	0.00719424	2.60143885	1.41977365	0.17909818	0.39723157	0.3669721	Cntf/Ptpn1	2
GO:0010833	telomere maintenance via telomere lengthening	1/40	72/14464	0.01388889	5.02222222	1.80172318	0.18117309	0.39723157	0.3669721	Pkib	1
GO:0032392	DNA geometric change	1/40	72/14464	0.01388889	5.02222222	1.80172318	0.18117309	0.39723157	0.3669721	Fbh1	1
GO:0070373	negative regulation of ERK1 and ERK2 cascade	1/40	72/14464	0.01388889	5.02222222	1.80172318	0.18117309	0.39723157	0.3669721	Ptpn1	1
GO:0090307	mitotic spindle assembly	1/40	72/14464	0.01388889	5.02222222	1.80172318	0.18117309	0.39723157	0.3669721	Arhgef10	1
GO:0097194	execution phase of apoptosis	1/40	72/14464	0.01388889	5.02222222	1.80172318	0.18117309	0.39723157	0.3669721	Bok	1
GO:0046942	carboxylic acid transport	2/40	281/14464	0.00711744	2.57366548	1.40280679	0.18208055	0.39723157	0.3669721	Slc10a6/Slc43a3	2
GO:0032206	positive regulation of telomere maintenance	1/40	73/14464	0.01369863	4.95342466	1.78322335	0.18344887	0.39723157	0.3669721	Pkib	1
GO:0045665	negative regulation of neuron differentiation	1/40	73/14464	0.01369863	4.95342466	1.78322335	0.18344887	0.39723157	0.3669721	Cntf	1
GO:0048644	muscle organ morphogenesis	1/40	73/14464	0.01369863	4.95342466	1.78322335	0.18344887	0.39723157	0.3669721	Cntf	1
GO:1904892	regulation of receptor signaling pathway via STAT	1/40	73/14464	0.01369863	4.95342466	1.78322335	0.18344887	0.39723157	0.3669721	Cntf	1
GO:0007416	synapse assembly	2/40	283/14464	0.00706714	2.55547703	1.39161699	0.18407321	0.39723157	0.3669721	Lrrn3/Ptpn1	2
GO:0015849	organic acid transport	2/40	283/14464	0.00706714	2.55547703	1.39161699	0.18407321	0.39723157	0.3669721	Slc10a6/Slc43a3	2
GO:0043523	regulation of neuron apoptotic process	2/40	283/14464	0.00706714	2.55547703	1.39161699	0.18407321	0.39723157	0.3669721	Bok/Cntf	2
GO:0014823	response to activity	1/40	74/14464	0.01351351	4.88648649	1.7650579	0.18571849	0.39809865	0.36777313	Cry1	1
GO:0042102	positive regulation of T cell proliferation	1/40	74/14464	0.01351351	4.88648649	1.7650579	0.18571849	0.39809865	0.36777313	Pnp	1
GO:0051298	centrosome duplication	1/40	74/14464	0.01351351	4.88648649	1.7650579	0.18571849	0.39809865	0.36777313	Arhgef10	1
GO:0002709	regulation of T cell mediated immunity	1/40	75/14464	0.01333333	4.82133333	1.74721593	0.18798195	0.39809865	0.36777313	Pnp	1
GO:0010569	regulation of double-strand break repair via homologous recombination	1/40	75/14464	0.01333333	4.82133333	1.74721593	0.18798195	0.39809865	0.36777313	Fbh1	1
GO:0010827	regulation of D-glucose transmembrane transport	1/40	75/14464	0.01333333	4.82133333	1.74721593	0.18798195	0.39809865	0.36777313	Capn10	1
GO:0046323	D-glucose import	1/40	75/14464	0.01333333	4.82133333	1.74721593	0.18798195	0.39809865	0.36777313	Capn10	1
GO:0150076	neuroinflammatory response	1/40	75/14464	0.01333333	4.82133333	1.74721593	0.18798195	0.39809865	0.36777313	Cntf	1
GO:0051054	positive regulation of DNA metabolic process	2/40	287/14464	0.00696864	2.51986063	1.36952156	0.18806867	0.39809865	0.36777313	Pkib/Pnp	2
GO:1901888	regulation of cell junction assembly	2/40	288/14464	0.00694444	2.51111111	1.36405576	0.18906958	0.39936944	0.36894711	Lrrn3/Ptpn1	2
GO:0050886	endocrine process	1/40	76/14464	0.01315789	4.75789474	1.72968701	0.19023928	0.40014465	0.36966327	Cry1	1
GO:0140056	organelle localization by membrane tethering	1/40	76/14464	0.01315789	4.75789474	1.72968701	0.19023928	0.40014465	0.36966327	Ctbp2	1
GO:0043112	receptor metabolic process	1/40	77/14464	0.01298701	4.6961039	1.71246121	0.19249049	0.40233338	0.37168527	Ptpn1	1
GO:0046888	negative regulation of hormone secretion	1/40	77/14464	0.01298701	4.6961039	1.71246121	0.19249049	0.40233338	0.37168527	Cry1	1
GO:0070972	protein localization to endoplasmic reticulum	1/40	77/14464	0.01298701	4.6961039	1.71246121	0.19249049	0.40233338	0.37168527	Zfand2b	1
GO:0071375	cellular response to peptide hormone stimulus	2/40	292/14464	0.00684932	2.47671233	1.34241899	0.19308107	0.40272349	0.37204566	Capn10/Ptpn1	2
GO:0007422	peripheral nervous system development	1/40	78/14464	0.01282051	4.63589744	1.69552903	0.1947356	0.40364116	0.37289343	Arhgef10	1
GO:0031341	regulation of cell killing	1/40	78/14464	0.01282051	4.63589744	1.69552903	0.1947356	0.40364116	0.37289343	Pnp	1
GO:0050871	positive regulation of B cell activation	1/40	78/14464	0.01282051	4.63589744	1.69552903	0.1947356	0.40364116	0.37289343	Pnp	1
GO:0031397	negative regulation of protein ubiquitination	1/40	79/14464	0.01265823	4.57721519	1.67888137	0.19697463	0.40575145	0.37484297	Cry1	1
GO:0051965	positive regulation of synapse assembly	1/40	79/14464	0.01265823	4.57721519	1.67888137	0.19697463	0.40575145	0.37484297	Lrrn3	1
GO:0097009	energy homeostasis	1/40	79/14464	0.01265823	4.57721519	1.67888137	0.19697463	0.40575145	0.37484297	Rrp8	1
GO:0070227	lymphocyte apoptotic process	1/40	80/14464	0.0125	4.52	1.66250956	0.19920758	0.40950506	0.37831065	Pnp	1
GO:0071103	DNA conformation change	1/40	81/14464	0.01234568	4.46419753	1.64640528	0.20143448	0.41323081	0.38175258	Fbh1	1
GO:0014855	striated muscle cell proliferation	1/40	82/14464	0.01219512	4.4097561	1.63056055	0.20365533	0.41607453	0.38437968	Smad1	1
GO:2000628	regulation of miRNA metabolic process	1/40	82/14464	0.01219512	4.4097561	1.63056055	0.20365533	0.41607453	0.38437968	Smad1	1
GO:0001822	kidney development	2/40	303/14464	0.00660066	2.38679868	1.28470704	0.20417227	0.41627761	0.38456729	Cys1/Smad1	2
GO:1901990	regulation of mitotic cell cycle phase transition	2/40	304/14464	0.00657895	2.37894737	1.27958514	0.20518454	0.41748772	0.38568522	Plk5/Ctdspl	2
GO:1900076	regulation of cellular response to insulin stimulus	1/40	83/14464	0.01204819	4.35662651	1.61496775	0.20587017	0.41802966	0.38618587	Ptpn1	1
GO:0006094	gluconeogenesis	1/40	84/14464	0.01190476	4.3047619	1.59961957	0.208079	0.42014228	0.38813756	Cry1	1
GO:0042157	lipoprotein metabolic process	1/40	84/14464	0.01190476	4.3047619	1.59961957	0.208079	0.42014228	0.38813756	Samd1	1
GO:0051098	regulation of binding	2/40	308/14464	0.00649351	2.34805195	1.25929663	0.20923965	0.42014228	0.38813756	Ctbp2/Fbh1	2
GO:0003073	regulation of systemic arterial blood pressure	1/40	85/14464	0.01176471	4.25411765	1.58450897	0.21028184	0.42014228	0.38813756	Ptpn1	1
GO:0019915	lipid storage	1/40	85/14464	0.01176471	4.25411765	1.58450897	0.21028184	0.42014228	0.38813756	Cry1	1
GO:0022406	membrane docking	1/40	85/14464	0.01176471	4.25411765	1.58450897	0.21028184	0.42014228	0.38813756	Ctbp2	1

GO:0048708 astrocyte differentiation	1/40	85/14464	0.01176471	4.25411765	1.58450897	0.21028184	0.42014228	0.38813756	Cntf	1
GO:0051310 metaphase chromosome alignment	1/40	85/14464	0.01176471	4.25411765	1.58450897	0.21028184	0.42014228	0.38813756	Fam83d	1
GO:0000281 mitotic cytokinesis	1/40	87/14464	0.01149425	4.15632184	1.55497389	0.21466961	0.42392944	0.39163623	Iqgap2	1
GO:0015908 fatty acid transport	1/40	87/14464	0.01149425	4.15632184	1.55497389	0.21466961	0.42392944	0.39163623	Slc43a3	1
GO:0051881 regulation of mitochondrial membrane potential	1/40	87/14464	0.01149425	4.15632184	1.55497389	0.21466961	0.42392944	0.39163623	Bok	1
GO:0072384 organelle transport along microtubule	1/40	87/14464	0.01149425	4.15632184	1.55497389	0.21466961	0.42392944	0.39163623	Rhot1	1
GO:0090288 negative regulation of cellular response to growth factor stimulus	1/40	87/14464	0.01149425	4.15632184	1.55497389	0.21466961	0.42392944	0.39163623	Ptpn1	1
GO:0072001 renal system development	2/40	315/14464	0.00634921	2.29587302	1.22453494	0.21635775	0.42392944	0.39163623	Cys1/Smad1	2
GO:1903532 positive regulation of secretion by cell	2/40	315/14464	0.00634921	2.29587302	1.22453494	0.21635775	0.42392944	0.39163623	Cntf/Capn10	2
GO:0019319 hexose biosynthetic process	1/40	88/14464	0.01136364	4.10909091	1.54053673	0.21685458	0.42392944	0.39163623	Cry1	1
GO:0043406 positive regulation of MAP kinase activity	1/40	88/14464	0.01136364	4.10909091	1.54053673	0.21685458	0.42392944	0.39163623	Ptpn1	1
GO:0043954 cellular component maintenance	1/40	88/14464	0.01136364	4.10909091	1.54053673	0.21685458	0.42392944	0.39163623	Ctbp2	1
GO:2000106 regulation of leukocyte apoptotic process	1/40	88/14464	0.01136364	4.10909091	1.54053673	0.21685458	0.42392944	0.39163623	Pnp	1
GO:0051345 positive regulation of hydrolase activity	2/40	317/14464	0.00630915	2.28138801	1.21477108	0.21839614	0.42559885	0.39317848	Bok/Arhgef10	2
GO:0045913 positive regulation of carbohydrate metabolic process	1/40	89/14464	0.01123596	4.06292135	1.52631177	0.21903361	0.42559885	0.39317848	Cry1	1
GO:1903321 negative regulation of protein modification by small protein conjugation or removal	1/40	89/14464	0.01123596	4.06292135	1.52631177	0.21903361	0.42559885	0.39317848	Cry1	1
GO:0030522 intracellular receptor signaling pathway	2/40	318/14464	0.00628931	2.27421384	1.20991645	0.21941606	0.42559885	0.39317848	Cry1/Ctbp2	2
GO:0001657 ureteric bud development	1/40	90/14464	0.01111111	4.01777778	1.51229328	0.22120674	0.42575891	0.39332635	Smad1	1
GO:0002027 regulation of heart rate	1/40	90/14464	0.01111111	4.01777778	1.51229328	0.22120674	0.42575891	0.39332635	Ptpn1	1
GO:0007498 mesoderm development	1/40	90/14464	0.01111111	4.01777778	1.51229328	0.22120674	0.42575891	0.39332635	Smad1	1
GO:0046634 regulation of alpha-beta T cell activation	1/40	90/14464	0.01111111	4.01777778	1.51229328	0.22120674	0.42575891	0.39332635	Pnp	1
GO:0072163 mesonephric epithelium development	1/40	91/14464	0.01098901	3.97362637	1.49847574	0.22337396	0.42827662	0.39565226	Smad1	1
GO:0072164 mesonephric tubule development	1/40	91/14464	0.01098901	3.97362637	1.49847574	0.22337396	0.42827662	0.39565226	Smad1	1
GO:0001823 mesonephros development	1/40	92/14464	0.01086957	3.93043478	1.48485382	0.22553531	0.42994016	0.39718909	Smad1	1
GO:0044344 cellular response to fibroblast growth factor stimulus	1/40	92/14464	0.01086957	3.93043478	1.48485382	0.22553531	0.42994016	0.39718909	Ptpn1	1
GO:0051899 membrane depolarization	1/40	92/14464	0.01086957	3.93043478	1.48485382	0.22553531	0.42994016	0.39718909	Bok	1
GO:0043280 positive regulation of cysteine-type endopeptidase activity involved in apoptotic process	1/40	93/14464	0.01075269	3.88817204	1.47142243	0.22769079	0.43075469	0.39794156	Bok	1
GO:0046364 monosaccharide biosynthetic process	1/40	93/14464	0.01075269	3.88817204	1.47142243	0.22769079	0.43075469	0.39794156	Cry1	1
GO:0051303 establishment of chromosome localization	1/40	93/14464	0.01075269	3.88817204	1.47142243	0.22769079	0.43075469	0.39794156	Fam83d	1
GO:1901874 negative regulation of post-translational protein modification	1/40	93/14464	0.01075269	3.88817204	1.47142243	0.22769079	0.43075469	0.39794156	Cry1	1
GO:0051492 regulation of stress fiber assembly	1/40	94/14464	0.0106383	3.84680851	1.45817662	0.22984043	0.43399793	0.40093774	Arhgef10	1
GO:0002708 positive regulation of lymphocyte mediated immunity	1/40	95/14464	0.01052632	3.80631579	1.44511165	0.23198422	0.43639297	0.40315034	Pnp	1
GO:0010586 miRNA metabolic process	1/40	95/14464	0.01052632	3.80631579	1.44511165	0.23198422	0.43639297	0.40315034	Smad1	1
GO:0001704 formation of primary germ layer	1/40	96/14464	0.01041667	3.76666667	1.43222294	0.2341222	0.43958538	0.40609957	Smad1	1
GO:0051047 positive regulation of secretion	2/40	334/14464	0.00598802	2.16526946	1.13460541	0.23579182	0.44175767	0.40810638	Cntf/Capn10	2
GO:0032024 positive regulation of insulin secretion	1/40	97/14464	0.01030928	3.72783505	1.41950609	0.23625438	0.44175767	0.40810638	Capn10	1
GO:0032204 regulation of telomere maintenance	1/40	98/14464	0.01020408	3.68979592	1.40695682	0.23838077	0.44175767	0.40810638	Pkib	1
GO:0043473 pigmentation	1/40	98/14464	0.01020408	3.68979592	1.40695682	0.23838077	0.44175767	0.40810638	Hps5	1
GO:0062014 negative regulation of small molecule metabolic process	1/40	98/14464	0.01020408	3.68979592	1.40695682	0.23838077	0.44175767	0.40810638	Cry1	1
GO:0071774 response to fibroblast growth factor	1/40	98/14464	0.01020408	3.68979592	1.40695682	0.23838077	0.44175767	0.40810638	Ptpn1	1
GO:0090263 positive regulation of canonical Wnt signaling pathway	1/40	98/14464	0.01020408	3.68979592	1.40695682	0.23838077	0.44175767	0.40810638	Prdm15	1
GO:0045582 positive regulation of T cell differentiation	1/40	99/14464	0.01010101	3.65252525	1.39457104	0.24050138	0.44207085	0.4083957	Pnp	1
GO:2001243 negative regulation of intrinsic apoptotic signaling pathway	1/40	99/14464	0.01010101	3.65252525	1.39457104	0.24050138	0.44207085	0.4083957	Ptpn1	1
GO:0051402 neuron apoptotic process	2/40	339/14464	0.00589971	2.13333333	1.11193555	0.24092755	0.44207085	0.4083957	Bok/Cntf	2
GO:0008645 hexose transmembrane transport	1/40	100/14464	0.01	3.616	1.38234477	0.24261624	0.44207085	0.4083957	Capn10	1
GO:0014910 regulation of smooth muscle cell migration	1/40	100/14464	0.01	3.616	1.38234477	0.24261624	0.44207085	0.4083957	Ptpn1	1
GO:0046928 regulation of neurotransmitter secretion	1/40	100/14464	0.01	3.616	1.38234477	0.24261624	0.44207085	0.4083957	Rhot1	1
GO:0050000 chromosome localization	1/40	100/14464	0.01	3.616	1.38234477	0.24261624	0.44207085	0.4083957	Fam83d	1
GO:0055017 cardiac muscle tissue growth	1/40	100/14464	0.01	3.616	1.38234477	0.24261624	0.44207085	0.4083957	Smad1	1
GO:1904659 D-glucose transmembrane transport	1/40	100/14464	0.01	3.616	1.38234477	0.24261624	0.44207085	0.4083957	Capn10	1
GO:0015711 organic anion transport	2/40	341/14464	0.0058651	2.12082111	1.10297672	0.24298377	0.44207085	0.4083957	Slc10a6/Slc43a3	2
GO:0003300 cardiac muscle hypertrophy	1/40	101/14464	0.00990099	3.58019802	1.37027419	0.24472536	0.44260479	0.40888897	Smad1	1
GO:0010660 regulation of muscle cell apoptotic process	1/40	101/14464	0.00990099	3.58019802	1.37027419	0.24472536	0.44260479	0.40888897	Ptpn1	1
GO:0043255 regulation of carbohydrate biosynthetic process	1/40	101/14464	0.00990099	3.58019802	1.37027419	0.24472536	0.44260479	0.40888897	Cry1	1
GO:0001909 leukocyte mediated cytotoxicity	1/40	102/14464	0.00980392	3.54509804	1.35835559	0.24682875	0.44260479	0.40888897	Pnp	1
GO:0002824 positive regulation of adaptive immune response based on somatic recombination of immune receptors b	1/40	102/14464	0.00980392	3.54509804	1.35835559	0.24682875	0.44260479	0.40888897	Pnp	1
GO:0015749 monosaccharide transmembrane transport	1/40	102/14464	0.00980392	3.54509804	1.35835559	0.24682875	0.44260479	0.40888897	Capn10	1
GO:0099068 postsynapse assembly	1/40	102/14464	0.00980392	3.54509804	1.35835559	0.24682875	0.44260479	0.40888897	Ptpn1	1
GO:1901989 positive regulation of cell cycle phase transition	1/40	102/14464	0.00980392	3.54509804	1.35835559	0.24682875	0.44260479	0.40888897	Fam83d	1
GO:0042752 regulation of circadian rhythm	1/40	103/14464	0.00970874	3.51067961	1.34658539	0.24892643	0.44397075	0.41015088	Cry1	1
GO:0140029 exocytic process	1/40	103/14464	0.00970874	3.51067961	1.34658539	0.24892643	0.44397075	0.41015088	Ctbp2	1
GO:2000278 regulation of DNA biosynthetic process	1/40	103/14464	0.00970874	3.51067961	1.34658539	0.24892643	0.44397075	0.41015088	Pkib	1
GO:0009410 response to xenobiotic stimulus	2/40	348/14464	0.00574713	2.07816092	1.0720961	0.25018796	0.44542393	0.41149336	Pnp/Smad1	2
GO:0014897 striated muscle hypertrophy	1/40	104/14464	0.00961538	3.47692308	1.33496015	0.25101841	0.4461058	0.41212329	Smad1	1
GO:0010906 regulation of glucose metabolic process	1/40	105/14464	0.00952381	3.44380952	1.32347651	0.25310471	0.44742091	0.41333822	Cry1	1
GO:0046632 alpha-beta T cell differentiation	1/40	105/14464	0.00952381	3.44380952	1.32347651	0.25310471	0.44742091	0.41333822	Pnp	1
GO:0110020 regulation of actomyosin structure organization	1/40	105/14464	0.00952381	3.44380952	1.32347651	0.25310471	0.44742091	0.41333822	Arhgef10	1
GO:0009206 purine ribonucleoside triphosphate biosynthetic process	1/40	106/14464	0.00943396	3.41132075	1.31213124	0.25518534	0.44943809	0.41520174	Pnp	1
GO:0014896 muscle hypertrophy	1/40	106/14464	0.00943396	3.41132075	1.31213124	0.25518534	0.44943809	0.41520174	Smad1	1
GO:0002456 T cell mediated immunity	1/40	107/14464	0.00934579	3.37943925	1.3009212	0.25726032	0.44943809	0.41520174	Pnp	1
GO:0009145 purine nucleoside triphosphate biosynthetic process	1/40	107/14464	0.00934579	3.37943925	1.3009212	0.25726032	0.44943809	0.41520174	Pnp	1
GO:0030518 nuclear receptor-mediated steroid hormone signaling pathway	1/40	107/14464	0.00934579	3.37943925	1.3009212	0.25726032	0.44943809	0.41520174	Cry1	1
GO:0060419 heart growth	1/40	107/14464	0.00934579	3.37943925	1.3009212	0.25726032	0.44943809	0.41520174	Smad1	1
GO:0010639 negative regulation of organelle organization	2/40	355/14464	0.0056338	2.0371831	1.04192827	0.25740135	0.44943809	0.41520174	Bok/Tchp	2
GO:0032231 regulation of actin filament bundle assembly	1/40	108/14464	0.00925926	3.34814815	1.28984338	0.25932967	0.44965509	0.41540222	Arhgef10	1
GO:0050671 positive regulation of lymphocyte proliferation	1/40	108/14464	0.00925926	3.34814815	1.28984338	0.25932967	0.44965509	0.41540222	Pnp	1
GO:0072526 pyridine-containing compound catabolic process	1/40	108/14464	0.00925926	3.34814815	1.28984338	0.25932967	0.44965509	0.41540222	Pnp	1
GO:2001056 positive regulation of cysteine-type endopeptidase activity	1/40	108/14464	0.00925926	3.34814815	1.28984338	0.25932967	0.44965509	0.41540222	Bok	1
GO:0034440 lipid oxidation	1/40	109/14464	0.00917431	3.31743119	1.27889483	0.26139339	0.45058839	0.41626442	Samd1	1
GO:0007018 microtubule-based movement	2/40	359/14464	0.00557103	2.01448468	1.02499679	0.26152629	0.45058839	0.41626442	Catsperz/Rhot1	2
GO:0000280 nuclear division	2/40	360/14464	0.00555556	2.00888889	1.02079791	0.26255778	0.45058839	0.41626442	Catsperz/Arhgef10	2
GO:0002821 positive regulation of adaptive immune response	1/40	110/14464	0.00909091	3.28727273	1.26807271	0.26345151	0.45058839	0.41626442	Pnp	1
GO:0034219 carbohydrate transmembrane transport	1/40	110/14464	0.00909091	3.28727273	1.26807271	0.26345151	0.45058839	0.41626442	Capn10	1
GO:0009201 ribonucleoside triphosphate biosynthetic process	1/40	111/14464	0.00900901	3.25765766	1.25737426	0.26550403	0.45058839	0.41626442	Pnp	1
GO:0014909 smooth muscle cell migration	1/40	111/14464	0.00900901	3.25765766	1.25737426	0.26550403	0.45058839	0.41626442	Ptpn1	1
GO:0030038 contractile actin filament bundle assembly	1/40	111/14464	0.00900901	3.25765766	1.25737426	0.26550403	0.45058839	0.41626442	Arhgef10	1
GO:0031398 positive regulation of protein ubiquitination	1/40	111/14464	0.00900901	3.25765766	1.25737426	0.26550403	0.45058839	0.41626442	Cry1	1
GO:0032946 positive regulation of mononuclear cell proliferation	1/40	111/14464	0.00900901	3.25765766	1.25737426	0.26550403	0.45058839	0.41626442	Pnp	1
GO:0043149 stress fiber assembly	1/40	111/14464	0.00900901	3.25765766	1.25737426	0.26550403	0.45058839	0.41626442	Arhgef10	1
GO:0007059 chromosome segregation	2/40	364/14464	0.00549451	1.98681319	1.00413571	0.26668452	0.45058839	0.41626442	Arhgef10/Fam83d	2
GO:0006413 translational initiation	1/40	112/14464	0.00892857	3.22857143	1.24679683	0.26755098	0.45058839	0.41626442	Eif3j1	1
GO:0006612 protein targeting to membrane	1/40	112/14464	0.00892857	3.22857143	1.24679683	0.26755098	0.45058839	0.41626442	Zfand2b	1
GO:0010657 muscle cell apoptotic process	1/40	112/14464	0.00892857	3.22857143	1.24679683	0.26755098	0.45058839	0.41626442	Ptpn1	1
GO:0061640 cytoskeleton-dependent cytokinesis	1/40	112/14464	0.00892857	3.22857143	1.24679683	0.26755098	0.45058839	0.41626442	Iqgap2	1
GO:0071887 leukocyte apoptotic process	1/40	112/14464	0.00892857	3.22857143	1.24679683	0.26755098	0.45058839	0.41626442	Pnp	1
GO:2001252 positive regulation of chromosome organization	1/40	113/14464	0.00884956	3.2	1.23633781	0.26959237	0.45286508	0.41836768	Pkib	1
GO:0000077 DNA damage checkpoint signaling	1/40	114/14464	0.00877193	3.17192982	1.22599471	0.2716282	0.45286508	0.41836768	Cry1	1
GO:0007259 cell surface receptor signaling pathway via JAK-STAT	1/40	114/14464	0.00877193	3.17192982	1.22599471	0.2716282	0.45286508	0.41836768	Cntf	1
GO:0008643 carbohydrate transport	1/40	114/14464	0.00877193	3.17192982	1.22599471	0.2716282	0.45286508	0.41836768	Capn10	1
GO:0045621 positive regulation of lymphocyte differentiation	1/40	114/14464	0.00877193	3.17192982	1.22599471	0.2716282	0.45286508	0.41836768	Pnp	1
GO:1990830 cellular response to leukemia inhibitory factor	1/40	114/14464	0.00877193	3.17192982	1.22599471	0.2716282	0.45286508	0.41836768	Ctbp2	1
GO:0050807 regulation of synapse organization	2/40	370/14464	0.00540541	1.95459459	0.97953291	0.2728761	0.4539726	0.41939083	Lrrn3/Ptpn1	2
GO:0002705 positive regulation of leukocyte mediated immunity	1/40	115/14464	0.00869565	3.14434783	1.21576508	0.27365851	0.4539726	0.41939083	Pnp	1
GO:0051053 negative regulation of DNA metabolic process	1/40	115/14464	0.00869565	3.14434783	1.21576508	0.27365851	0.4539726	0.41939083	Fbh1	1
GO:0043401 steroid hormone receptor signaling pathway	1/40	116/14464	0.00862069	3.11724138	1.20564657	0.27568329	0.45506001	0.4203954	Cry1	1
GO:0048709 oligodendrocyte differentiation	1/40	116/14464	0.00862069	3.11724138	1.20564657	0.27568329	0.45506001	0.4203954	Bok	1
GO:1990823 response to leukemia inhibitory factor	1/40	116/14464	0.00862069	3.11724138	1.20564657	0.27568329	0.45506001	0.4203954	Ctbp2	1
GO:0000018 regulation of DNA recombination	1/40	117/14464	0.00854701	3.09059829	1.19563689	0.27770258	0.45612763	0.4213817	Fbh1	1
GO:0008584 male gonad development	1/40	117/14464	0.00854701	3.09059829	1.19563689	0.27770258	0.45612763	0.4213817	Bok	1
GO:0015698 inorganic anion transport	1/40	117/14464	0.00854701	3.09059829	1.19563689	0.27770258	0.45612763	0.4213817	Slc25a30	1
GO:0050803 regulation of synapse structure or activity	2/40	376/14464	0.00531915	1.92340426	0.95538288	0.27906793	0.45642753	0.42165876	Lrrn3/Ptpn1	2
GO:0009142 nucleoside triphosphate biosynthetic process	1/40	118/14464	0.00847458	3.06440678	1.1857338	0.27971637	0.45642753	0.42165876	Pnp	1
GO:0046546 development of primary male sexual characteristics	1/40	118/14464	0.00847458	3.06440678	1.1857338	0.27971637	0.45642753	0.42165876	Bok	1
GO:0051588 regulation of neurotransmitter transport	1/40	118/14464	0.00847458	3.06440678	1.1857338	0.27971637	0.45642753	0.42165876	Rhot1	1
GO:0016079 synaptic vesicle exocytosis	1/40	119/14464	0.00840336	3.03865546	1.17593515	0.28172469	0.4574585	0.42261118	Ctbp2	1
GO:0030317 flagellated sperm motility	1/40	119/14464	0.00840336	3.03865546	1.17593515	0.28172469	0.4574585	0.42261118	Catsperz	1
GO:0048593 camera-type eye morphogenesis	1/40	119/14464	0.00840336	3.03865546	1.17593515	0.28172469	0.4574585	0.42261118	Cntf	1
GO:0007043 cell-cell junction assembly	1/40	121/14464	0.00826446	2.98842975	1.15664283	0.28572496	0.45946417	0.42446408	Cdh19	1
GO:0030509 BMP signaling pathway	1/40	121/14464	0.00826446	2.98842975	1.15664283	0.28572496	0.45946417	0.42446408	Smad1	1
GO:0031570 DNA integrity checkpoint signaling	1/40	121/14464	0.00826446	2.98842975	1.15664283	0.28572496	0.45946417	0.42446408	Cry1	1
GO:0090277 positive regulation of peptide hormone secretion	1/40	121/14464	0.00826446	2.98842975	1.15664283	0.28572496	0.45946417	0.42446408	Capn10	1
GO:0097722 sperm motility	1/40	121/14464	0.00826446	2.98842975	1.15664283	0.28572496	0.45946417	0.42446408	Catsperz	1
GO:1901890 positive regulation of cell junction assembly	1/40	121/14464	0.00826446	2.98842975	1.15664283	0.28572496	0.45946417	0.42446408	Lrrn3	1
GO:0032368 regulation of lipid transport	1/40	123/14464	0.00813008	2.9398374	1.13774385	0.28970351	0.46233644	0.42711755	Cry1	1
GO:0050864 regulation of B cell activation	1/40	123/14464	0.00813008	2.9398374	1.13774385	0.28970351	0.46233644	0.42711755	Pnp	1
GO:0070665 positive regulation of leukocyte proliferation	1/40	123/14464	0.00813008	2.9398374	1.13774385	0.28970351	0.46233644	0.42711755	Pnp	1
GO:0031329 regulation of cellular catabolic process	2/40	388/14464	0.00515464	1.86391753	0.90837432	0.29144578	0.46233644	0.42711755	Bok/Ptpn1	2
GO:0002040 sprouting angiogenesis	1/40	124/14464	0.00806452	2.91612903	1.12843708	0.29168467	0.46233644	0.42711755	Smad1	1
GO:0008286 insulin receptor signaling pathway	1/40	124/14464	0.00806452	2.91612903	1.12843708	0.29168467	0.46233644	0.42711755	Ptpn1	1
GO:0045667 regulation of osteoblast differentiation	1/40	124/14464	0.00806452	2.91612903	1.12843708	0.29168467	0.46233644	0.42711755	Smad1	1
GO:0051100 negative regulation of binding	1/40	124/14464	0.00806452	2.91612903	1.12843708	0.29168467	0.46233644	0.42711755	Fbh1	1
GO:0097696 cell surface receptor signaling pathway via STAT	1/40	124/14464	0.00806452	2.91612903	1.12843708	0.29168467	0.46233644	0.42711755	Cntf	1
GO:0001558 regulation of cell growth	2/40	389/14464	0.00514139	1.85912596	0.90453167	0.29247662	0.46285585	0.42759739	Cntf/Tchp	2
GO:0002793 positive regulation of peptide secretion	1/40	125/14464	0.008	2.8928	1.11922302	0.29366045	0.46325865	0.42796951	Capn10	1
GO:0010950 positive regulation of endopeptidase activity	1/40	125/14464	0.008	2.8928	1.11922302	0.29366045	0.46325865	0.42796951	Bok	1
GO:0014812 muscle cell migration	1/40	126/14464	0.00793651	2.86984127	1.11009988	0.29563085	0.46416371	0.42880563	Ptpn1	1
GO:0043500 muscle adaptation	1/40	126/14464	0.00793651	2.86984127	1.11009988	0.29563085	0.46416371	0.42880563	Smad1	1
GO:0051225 spindle assembly	1/40	126/14464	0.00793651	2.86984127	1.11009988	0.29563085	0.46416371	0.42880563	Arhgef10	1
GO:0030177 positive regulation of Wnt signaling pathway	1/40	127/14464	0.00787402	2.84724409	1.10106596	0.29759589	0.46583324	0.43034797	Prdm15	1
GO:0009314 response to radiation	2/40	394/14464	0.00507614	1.83553299	0.88548457	0.29762866	0.46583324	0.43034797	Cry1/Pnp	2
GO:0060294 cilium movement involved in cell motility	1/40	128/14464	0.0078125	2.825	1.09211957	0.29955559	0.4681143	0.43245527	Catsperz	1
GO:0030183 B cell differentiation	1/40	129/14464	0.00775194	2.80310078	1.08325908	0.30150995	0.47006573	0.43425805	Pnp	1
GO:0043086 negative regulation of catalytic activity	2/40	398/14464	0.00502513	1.81708543	0.87044238	0.30174731	0.47006573	0.43425805	Pkib/Ptpn1	2
GO:0044772 mitotic cell cycle phase transition	2/40	399/14464	0.00501253	1.81253133	0.86670844	0.3027765	0.47093318	0.43505943	Plk5/Ctdspl	2
GO:0043433 negative regulation of DNA-binding transcription factor activity	1/40	130/14464	0.00769231	2.78153846	1.07448291	0.303459	0.47125954	0.43536092	Cys1	1
GO:0007098 centrosome cycle	1/40	131/14464	0.00763359	2.76030534	1.0657895	0.30540275	0.47134139	0.43543654	Arhgef10	1
GO:0009154 purine ribonucleotide catabolic process	1/40	131/14464	0.00763359	2.76030534	1.0657895	0.30540275	0.47134139	0.43543654	Pnp	1
GO:0071772 response to BMP	1/40	131/14464	0.00763359	2.76030534	1.0657895	0.30540275	0.47134139	0.43543654	Smad1	1
GO:0071773 cellular response to BMP stimulus	1/40	131/14464	0.00763359	2.76030534	1.0657895	0.30540275	0.47134139	0.43543654	Smad1	1
GO:0001539 cilium or flagellum-dependent cell motility	1/40	132/14464	0.00757576	2.73939394	1.05717735	0.3073412	0.47214049	0.43617476	Catsperz	1
GO:0010952 positive regulation of peptidase activity	1/40	132/14464	0.00757576	2.73939394	1.05717735	0.3073412	0.47214049	0.43617476	Bok	1
GO:0060285 cilium-dependent cell motility	1/40	132/14464	0.00757576	2.73939394	1.05717735	0.3073412	0.47214049	0.43617476	Catsperz	1
GO:2000779 regulation of double-strand break repair	1/40	133/14464	0.0075188	2.71879699	1.048645	0.30927438	0.47437932	0.43824305	Fbh1	1
GO:0045739 positive regulation of DNA repair	1/40	134/14464	0.00746269	2.69850746	1.04019101	0.3112023	0.47587223	0.43962224	Pnp	1
GO:0046661 male sex differentiation	1/40	134/14464	0.00746269	2.69850746	1.04019101	0.3112023	0.47587223	0.43962224	Bok	1
GO:0008277 regulation of G protein-coupled receptor signaling pathway	1/40	135/14464	0.00740741	2.67851852	1.03181399	0.31312498	0.47734801	0.4409856	Cry1	1
GO:0071902 positive regulation of protein serine/threonine kinase activity	1/40	135/14464	0.00740741	2.67851852	1.03181399	0.31312498	0.47734801	0.4409856	Ptpn1	1
GO:0043161 proteasome-mediated ubiquitin-dependent protein catabolic process	2/40	412/14464	0.00485437	1.75533981	0.81910248	0.31613527	0.48097987	0.4443408	Lrrc75a/Zfand2b	2
GO:0006470 protein dephosphorylation	1/40	137/14464	0.00729927	2.63941606	1.01528547	0.31695464	0.48097987	0.4443408	Ptpn1	1
GO:0072073 kidney epithelium development	1/40	137/14464	0.00729927	2.63941606	1.01528547	0.31695464	0.48097987	0.4443408	Smad1	1
GO:0051099 positive regulation of binding	1/40	138/14464	0.00724638	2.62028986	1.00713135	0.31886165	0.48144767	0.44477296	Ctbp2	1
GO:1903322 positive regulation of protein modification by small protein conjugation or removal	1/40	138/14464	0.00724638	2.62028986	1.00713135	0.31886165	0.48144767	0.44477296	Cry1	1
GO:0048285 organelle fission	2/40	416/14464	0.00480769	1.73846154	0.80479196	0.32023661	0.48144767	0.44477296	Catsperz/Arhgef10	2
GO:0006997 nucleus organization	1/40	139/14464	0.00719424	2.60143885	0.99904897	0.32076348	0.48144767	0.44477296	Rrp8	1
GO:1901875 positive regulation of post-translational protein modification	1/40	139/14464	0.00719424	2.60143885	0.99904897	0.32076348	0.48144767	0.44477296	Cry1	1
GO:0002706 regulation of lymphocyte mediated immunity	1/40	140/14464	0.00714286	2.58285714	0.9910371	0.32266012	0.48144767	0.44477296	Pnp	1

GO:0009261	ribonucleotide catabolic process	1/40	140/14464	0.00714286	2.58285714	0.9910371	0.32266012	0.48144767	0.44477296	Pnp	1
GO:0043405	regulation of MAP kinase activity	1/40	140/14464	0.00714286	2.58285714	0.9910371	0.32266012	0.48144767	0.44477296	Ptpn1	1
GO:0071456	cellular response to hypoxia	1/40	140/14464	0.00714286	2.58285714	0.9910371	0.32266012	0.48144767	0.44477296	Ptpn1	1
GO:0043410	positive regulation of MAPK cascade	2/40	419/14464	0.00477327	1.72601432	0.79415936	0.32330939	0.48144767	0.44477296	Cntf/Ptpn1	2
GO:0007264	small GTPase-mediated signal transduction	2/40	420/14464	0.0047619	1.72190476	0.79063398	0.32433301	0.48144767	0.44477296	Sipa1l2/Rhot1	2
GO:0006195	purine nucleotide catabolic process	1/40	141/14464	0.0070922	2.56453901	0.98309455	0.32455161	0.48144767	0.44477296	Pnp	1
GO:0006261	DNA-templated DNA replication	1/40	141/14464	0.0070922	2.56453901	0.98309455	0.32455161	0.48144767	0.44477296	Fbh1	1
GO:0031023	microtubule organizing center organization	1/40	141/14464	0.0070922	2.56453901	0.98309455	0.32455161	0.48144767	0.44477296	Arhgef10	1
GO:0055088	lipid homeostasis	1/40	141/14464	0.0070922	2.56453901	0.98309455	0.32455161	0.48144767	0.44477296	Tlcd3a	1
GO:0010212	response to ionizing radiation	1/40	142/14464	0.00704225	2.54647887	0.97522014	0.32643794	0.48144767	0.44477296	Pnp	1
GO:0048592	eye morphogenesis	1/40	142/14464	0.00704225	2.54647887	0.97522014	0.32643794	0.48144767	0.44477296	Cntf	1
GO:0140013	meiotic nuclear division	1/40	142/14464	0.00704225	2.54647887	0.97522014	0.32643794	0.48144767	0.44477296	Catsperz	1
GO:1903034	regulation of response to wounding	1/40	142/14464	0.00704225	2.54647887	0.97522014	0.32643794	0.48144767	0.44477296	Cntf	1
GO:0000723	telomere maintenance	1/40	143/14464	0.00699301	2.52867133	0.96741273	0.32831913	0.48208273	0.44535965	Pkib	1
GO:0032006	regulation of TOR signaling	1/40	143/14464	0.00699301	2.52867133	0.96741273	0.32831913	0.48208273	0.44535965	Fam83d	1
GO:0042129	regulation of T cell proliferation	1/40	143/14464	0.00699301	2.52867133	0.96741273	0.32831913	0.48208273	0.44535965	Pnp	1
GO:0043281	regulation of cysteine-type endopeptidase activity involved in apoptotic process	1/40	144/14464	0.00694444	2.51111111	0.95967121	0.33019521	0.48239543	0.44564853	Bok	1
GO:0051147	regulation of muscle cell differentiation	1/40	144/14464	0.00694444	2.51111111	0.95967121	0.33019521	0.48239543	0.44564853	Smad1	1
GO:0031346	positive regulation of cell projection organization	2/40	426/14464	0.00469484	1.69765258	0.7696756	0.33046748	0.48239543	0.44564853	Plk5/Cntf	2
GO:0045859	regulation of protein kinase activity	2/40	426/14464	0.00469484	1.69765258	0.7696756	0.33046748	0.48239543	0.44564853	Pkib/Ptpn1	2
GO:0007596	blood coagulation	1/40	145/14464	0.00689655	2.4937931	0.9519945	0.33206617	0.48331383	0.44649696	Hps5	1
GO:0032388	positive regulation of intracellular transport	1/40	145/14464	0.00689655	2.4937931	0.9519945	0.33206617	0.48331383	0.44649696	Capn10	1
GO:0007015	actin filament organization	2/40	429/14464	0.004662	1.68578089	0.75931879	0.33352983	0.48461461	0.44769866	Iqgap2/Arhgef10	2
GO:0007286	spermatid development	1/40	146/14464	0.00684932	2.47671233	0.94438152	0.33393204	0.48461461	0.44769866	Catsperz	1
GO:0009152	purine ribonucleotide biosynthetic process	1/40	147/14464	0.00680272	2.45986395	0.93683125	0.33579282	0.48519629	0.44823603	Pnp	1
GO:0043547	positive regulation of GTPase activity	1/40	147/14464	0.00680272	2.45986395	0.93683125	0.33579282	0.48519629	0.44823603	Arhgef10	1
GO:0060041	retina development in camera-type eye	1/40	147/14464	0.00680272	2.45986395	0.93683125	0.33579282	0.48519629	0.44823603	Cntf	1
GO:0007599	hemostasis	1/40	148/14464	0.00675676	2.44324324	0.92934267	0.33764854	0.48632285	0.44927677	Hps5	1
GO:0045766	positive regulation of angiogenesis	1/40	148/14464	0.00675676	2.44324324	0.92934267	0.33764854	0.48632285	0.44927677	Smad1	1
GO:0001837	epithelial to mesenchymal transition	1/40	149/14464	0.00671141	2.42684564	0.92191479	0.3394992	0.48632285	0.44927677	Fam83d	1
GO:0032200	telomere organization	1/40	149/14464	0.00671141	2.42684564	0.92191479	0.3394992	0.48632285	0.44927677	Pkib	1
GO:0036294	cellular response to decreased oxygen levels	1/40	149/14464	0.00671141	2.42684564	0.92191479	0.3394992	0.48632285	0.44927677	Ptpn1	1
GO:0046631	alpha-beta T cell activation	1/40	149/14464	0.00671141	2.42684564	0.92191479	0.3394992	0.48632285	0.44927677	Pnp	1
GO:0022613	ribonucleoprotein complex biogenesis	2/40	436/14464	0.00458716	1.6587156	0.73546099	0.34066157	0.48664814	0.44957728	Rrp8/Eif3j1	2
GO:0010977	negative regulation of neuron projection development	1/40	150/14464	0.00666667	2.41066667	0.91454664	0.34134482	0.48664814	0.44957728	Ptpn1	1
GO:0050817	coagulation	1/40	150/14464	0.00666667	2.41066667	0.91454664	0.34134482	0.48664814	0.44957728	Hps5	1
GO:0042391	regulation of membrane potential	2/40	437/14464	0.00457666	1.65491991	0.73208725	0.34167874	0.48664814	0.44957728	Bok/Cntf	2
GO:0007224	smoothened signaling pathway	1/40	151/14464	0.00662252	2.39470199	0.90723728	0.34318541	0.48670818	0.44963275	Evc2	1
GO:0045580	regulation of T cell differentiation	1/40	151/14464	0.00662252	2.39470199	0.90723728	0.34318541	0.48670818	0.44963275	Pnp	1
GO:1904018	positive regulation of vasculature development	1/40	151/14464	0.00662252	2.39470199	0.90723728	0.34318541	0.48670818	0.44963275	Smad1	1
GO:0050714	positive regulation of protein secretion	1/40	152/14464	0.00657895	2.37894737	0.89998579	0.34502098	0.48723218	0.45011683	Capn10	1
GO:0051302	regulation of cell division	1/40	152/14464	0.00657895	2.37894737	0.89998579	0.34502098	0.48723218	0.45011683	Prdm15	1
GO:1905952	regulation of lipid localization	1/40	152/14464	0.00657895	2.37894737	0.89998579	0.34502098	0.48723218	0.45011683	Cry1	1
GO:0051656	establishment of organelle localization	2/40	442/14464	0.00452489	1.6361991	0.71534498	0.34675804	0.4889926	0.45174315	Fam83d/Rhot1	2
GO:0048515	spermatid differentiation	1/40	154/14464	0.00649351	2.34805195	0.88565281	0.34867714	0.48962128	0.45232394	Catsperz	1
GO:0062013	positive regulation of small molecule metabolic process	1/40	154/14464	0.00649351	2.34805195	0.88565281	0.34867714	0.48962128	0.45232394	Cry1	1
GO:1903531	negative regulation of secretion by cell	1/40	154/14464	0.00649351	2.34805195	0.88565281	0.34867714	0.48962128	0.45232394	Cry1	1
GO:0001906	cell killing	1/40	156/14464	0.00641026	2.31794872	0.87154071	0.3523134	0.49333772	0.45575727	Pnp	1
GO:0002822	regulation of adaptive immune response based on somatic recombination of immune receptors built from	1/40	156/14464	0.00641026	2.31794872	0.87154071	0.3523134	0.49333772	0.45575727	Pnp	1
GO:0072523	purine-containing compound catabolic process	1/40	157/14464	0.00636943	2.30318471	0.86456539	0.35412409	0.49517773	0.45745712	Pnp	1
GO:0007346	regulation of mitotic cell cycle	2/40	450/14464	0.00444444	1.60711111	0.68898492	0.35486099	0.49551317	0.45776701	Plk5/Ctdspl	2
GO:0009166	nucleotide catabolic process	1/40	158/14464	0.00632911	2.28860759	0.85764281	0.35592985	0.49631058	0.45850368	Pnp	1
GO:0051223	regulation of protein transport	2/40	452/14464	0.00442478	1.6	0.68247498	0.35688187	0.49673747	0.45889805	Capn10/Ptpn1	2
GO:0003341	cilium movement	1/40	159/14464	0.00628931	2.27421384	0.85077218	0.35773069	0.49673747	0.45889805	Catsperz	1
GO:0071897	DNA biosynthetic process	1/40	159/14464	0.00628931	2.27421384	0.85077218	0.35773069	0.49673747	0.45889805	Pkib	1
GO:0002181	cytoplasmic translation	1/40	160/14464	0.00625	2.26	0.84395272	0.35952662	0.49824855	0.46029402	Eif3j1	1
GO:0043549	regulation of kinase activity	2/40	456/14464	0.00438596	1.58596491	0.6695489	0.36091752	0.49824855	0.46029402	Pkib/Ptpn1	2
GO:0008217	regulation of blood pressure	1/40	161/14464	0.00621118	2.24596273	0.83718368	0.36131766	0.49824855	0.46029402	Ptpn1	1
GO:0033157	regulation of intracellular protein transport	1/40	161/14464	0.00621118	2.24596273	0.83718368	0.36131766	0.49824855	0.46029402	Ptpn1	1
GO:0051897	positive regulation of phosphatidylinositol 3-kinase/protein kinase B signal transduction	1/40	161/14464	0.00621118	2.24596273	0.83718368	0.36131766	0.49824855	0.46029402	Cntf	1
GO:0007369	gastrulation	1/40	162/14464	0.00617284	2.23209877	0.83046431	0.36310381	0.49933034	0.4612934	Smad1	1
GO:0009260	ribonucleotide biosynthetic process	1/40	162/14464	0.00617284	2.23209877	0.83046431	0.36310381	0.49933034	0.4612934	Pnp	1
GO:0051017	actin filament bundle assembly	1/40	163/14464	0.00613497	2.21840491	0.8237939	0.36488508	0.49971213	0.46164611	Arhgef10	1
GO:1901991	negative regulation of mitotic cell cycle phase transition	1/40	163/14464	0.00613497	2.21840491	0.8237939	0.36488508	0.49971213	0.46164611	Ctdspl	1
GO:2000116	regulation of cysteine-type endopeptidase activity	1/40	163/14464	0.00613497	2.21840491	0.8237939	0.36488508	0.49971213	0.46164611	Bok	1
GO:0000724	double-strand break repair via homologous recombination	1/40	164/14464	0.00609756	2.20487805	0.81717172	0.36666151	0.50117024	0.46299315	Fbh1	1
GO:0071407	cellular response to organic cyclic compound	2/40	462/14464	0.004329	1.56536797	0.65038944	0.36695514	0.50117024	0.46299315	Cry1/Smad1	2
GO:0008016	regulation of heart contraction	1/40	165/14464	0.00606061	2.19151515	0.81059709	0.36843308	0.50181391	0.46358779	Ptpn1	1
GO:0071453	cellular response to oxygen levels	1/40	165/14464	0.00606061	2.19151515	0.81059709	0.36843308	0.50181391	0.46358779	Ptpn1	1
GO:0061572	actin filament bundle organization	1/40	166/14464	0.0060241	2.17831325	0.80406932	0.37019983	0.50284636	0.46454159	Arhgef10	1
GO:1903046	meiotic cell cycle process	1/40	166/14464	0.0060241	2.17831325	0.80406932	0.37019983	0.50284636	0.46454159	Catsperz	1
GO:1902905	positive regulation of supramolecular fiber organization	1/40	167/14464	0.00598802	2.16526946	0.79758775	0.37196175	0.5045522	0.46611748	Arhgef10	1
GO:0000070	mitotic sister chromatid segregation	1/40	168/14464	0.00595238	2.15238095	0.79115172	0.37371887	0.50487495	0.46641565	Arhgef10	1
GO:0000910	cytokinesis	1/40	168/14464	0.00595238	2.15238095	0.79115172	0.37371887	0.50487495	0.46641565	Iqgap2	1
GO:0046390	ribose phosphate biosynthetic process	1/40	168/14464	0.00595238	2.15238095	0.79115172	0.37371887	0.50487495	0.46641565	Pnp	1
GO:0044283	small molecule biosynthetic process	2/40	470/14464	0.00425532	1.5387234	0.62525931	0.37497416	0.50511315	0.4666357	Cry1/Pnp	2
GO:0000725	recombinational repair	1/40	169/14464	0.00591716	2.13964497	0.78476059	0.3754712	0.50511315	0.4666357	Fbh1	1
GO:0034655	nucleobase-containing compound catabolic process	2/40	471/14464	0.00424628	1.53545648	0.62215063	0.37597393	0.50511315	0.4666357	Pnp/Fbh1	2
GO:0002819	regulation of adaptive immune response	1/40	170/14464	0.00588235	2.12705882	0.77841373	0.37721874	0.50511315	0.4666357	Pnp	1
GO:0030902	hindbrain development	1/40	170/14464	0.00588235	2.12705882	0.77841373	0.37721874	0.50511315	0.4666357	Smad1	1
GO:0051048	negative regulation of secretion	1/40	170/14464	0.00588235	2.12705882	0.77841373	0.37721874	0.50511315	0.4666357	Cry1	1
GO:0030308	negative regulation of cell growth	1/40	171/14464	0.00584795	2.11461988	0.77211053	0.37896152	0.50511315	0.4666357	Tchp	1
GO:0042552	myelination	1/40	171/14464	0.00584795	2.11461988	0.77211053	0.37896152	0.50511315	0.4666357	Arhgef10	1
GO:1902107	positive regulation of leukocyte differentiation	1/40	171/14464	0.00584795	2.11461988	0.77211053	0.37896152	0.50511315	0.4666357	Pnp	1
GO:1903708	positive regulation of hemopoiesis	1/40	171/14464	0.00584795	2.11461988	0.77211053	0.37896152	0.50511315	0.4666357	Pnp	1
GO:0031929	TOR signaling	1/40	172/14464	0.00581395	2.10232558	0.76585039	0.38069954	0.50642787	0.46785028	Fam83d	1
GO:0016049	cell growth	2/40	476/14464	0.00420168	1.51932773	0.60671329	0.3809638	0.50642787	0.46785028	Cntf/Tchp	2
GO:0030307	positive regulation of cell growth	1/40	173/14464	0.00578035	2.09017341	0.75963271	0.38243282	0.50770375	0.46902897	Cntf	1
GO:0007272	ensheathment of neurons	1/40	174/14464	0.00574713	2.07816092	0.75345692	0.38416137	0.50864393	0.46989753	Arhgef10	1
GO:0008366	axon ensheathment	1/40	174/14464	0.00574713	2.07816092	0.75345692	0.38416137	0.50864393	0.46989753	Arhgef10	1
GO:0042770	signal transduction in response to DNA damage	1/40	175/14464	0.00571429	2.06628571	0.74732245	0.3858852	0.5091456	0.47036098	Cry1	1
GO:0048469	cell maturation	1/40	175/14464	0.00571429	2.06628571	0.74732245	0.3858852	0.5091456	0.47036098	Catsperz	1
GO:0006164	purine nucleotide biosynthetic process	1/40	176/14464	0.00568182	2.05454545	0.74122874	0.38760432	0.5091456	0.47036098	Pnp	1
GO:0045216	cell-cell junction organization	1/40	176/14464	0.00568182	2.05454545	0.74122874	0.38760432	0.5091456	0.47036098	Cdh19	1
GO:0072524	pyridine-containing compound metabolic process	1/40	176/14464	0.00568182	2.05454545	0.74122874	0.38760432	0.5091456	0.47036098	Pnp	1
GO:1901292	nucleoside phosphate catabolic process	1/40	176/14464	0.00568182	2.05454545	0.74122874	0.38760432	0.5091456	0.47036098	Pnp	1
GO:0010970	transport along microtubule	1/40	177/14464	0.00564972	2.04293785	0.73517524	0.38931876	0.50961493	0.47079456	Rhot1	1
GO:0022618	protein-RNA complex assembly	1/40	177/14464	0.00564972	2.04293785	0.73517524	0.38931876	0.50961493	0.47079456	Eif3j1	1
GO:0001764	neuron migration	1/40	178/14464	0.00561798	2.03146067	0.72916142	0.39102851	0.50961493	0.47079456	Matn2	1
GO:0030258	lipid modification	1/40	178/14464	0.00561798	2.03146067	0.72916142	0.39102851	0.50961493	0.47079456	Samd1	1
GO:0045619	regulation of lymphocyte differentiation	1/40	178/14464	0.00561798	2.03146067	0.72916142	0.39102851	0.50961493	0.47079456	Pnp	1
GO:0051495	positive regulation of cytoskeleton organization	1/40	178/14464	0.00561798	2.03146067	0.72916142	0.39102851	0.50961493	0.47079456	Arhgef10	1
GO:0050796	regulation of insulin secretion	1/40	179/14464	0.00558659	2.02011173	0.72318676	0.3927336	0.51116892	0.47223017	Capn10	1
GO:0035265	organ growth	1/40	180/14464	0.00555556	2.00888889	0.71725074	0.39443403	0.51271281	0.47365645	Smad1	1
GO:0000075	cell cycle checkpoint signaling	1/40	181/14464	0.00552486	1.99779006	0.71135284	0.39612982	0.51291094	0.47383949	Cry1	1
GO:0048839	inner ear development	1/40	181/14464	0.00552486	1.99779006	0.71135284	0.39612982	0.51291094	0.47383949	Cys1	1
GO:0051260	protein homooligomerization	1/40	181/14464	0.00552486	1.99779006	0.71135284	0.39612982	0.51291094	0.47383949	Samd1	1
GO:0098742	cell-cell adhesion via plasma-membrane adhesion molecules	1/40	182/14464	0.00549451	1.98681319	0.70549259	0.39782097	0.51394708	0.4747967	Cdh19	1
GO:0006109	regulation of carbohydrate metabolic process	1/40	183/14464	0.00546448	1.97595628	0.69966948	0.39950751	0.51394708	0.4747967	Cry1	1
GO:0009267	cellular response to starvation	1/40	183/14464	0.00546448	1.97595628	0.69966948	0.39950751	0.51394708	0.4747967	Rrp8	1
GO:0016051	carbohydrate biosynthetic process	1/40	183/14464	0.00546448	1.97595628	0.69966948	0.39950751	0.51394708	0.4747967	Cry1	1
GO:0071383	cellular response to steroid hormone stimulus	1/40	183/14464	0.00546448	1.97595628	0.69966948	0.39950751	0.51394708	0.4747967	Cry1	1
GO:0006814	sodium ion transport	1/40	185/14464	0.00540541	1.95459459	0.68813282	0.40286679	0.51576206	0.47647342	Slc10a6	1
GO:0042098	T cell proliferation	1/40	185/14464	0.00540541	1.95459459	0.68813282	0.40286679	0.51576206	0.47647342	Pnp	1
GO:0045664	regulation of neuron differentiation	1/40	185/14464	0.00540541	1.95459459	0.68813282	0.40286679	0.51576206	0.47647342	Cntf	1
GO:0001649	osteoblast differentiation	1/40	186/14464	0.00537634	1.94408602	0.68241833	0.40453955	0.51576206	0.47647342	Smad1	1
GO:0051216	cartilage development	1/40	186/14464	0.00537634	1.94408602	0.68241833	0.40453955	0.51576206	0.47647342	Smad1	1
GO:0071826	protein-RNA complex organization	1/40	186/14464	0.00537634	1.94408602	0.68241833	0.40453955	0.51576206	0.47647342	Eif3j1	1
GO:0099175	regulation of postsynapse organization	1/40	186/14464	0.00537634	1.94408602	0.68241833	0.40453955	0.51576206	0.47647342	Ptpn1	1
GO:0050870	positive regulation of T cell activation	1/40	188/14464	0.00531915	1.92340426	0.67109478	0.40787137	0.51934579	0.47978416	Pnp	1
GO:0007179	transforming growth factor beta receptor signaling pathway	1/40	190/14464	0.00526316	1.90315789	0.65990887	0.41118501	0.52223116	0.48244973	Smad1	1
GO:0120032	regulation of plasma membrane bounded cell projection assembly	1/40	190/14464	0.00526316	1.90315789	0.65990887	0.41118501	0.52223116	0.48244973	Tchp	1
GO:0050670	regulation of lymphocyte proliferation	1/40	192/14464	0.00520833	1.88333333	0.64885723	0.41448058	0.52507895	0.48508059	Pnp	1
GO:0060491	regulation of cell projection assembly	1/40	192/14464	0.00520833	1.88333333	0.64885723	0.41448058	0.52507895	0.48508059	Tchp	1
GO:0032944	regulation of mononuclear cell proliferation	1/40	196/14464	0.00510204	1.84489796	0.62714372	0.42101783	0.53268373	0.49210607	Pnp	1
GO:0002703	regulation of leukocyte mediated immunity	1/40	197/14464	0.00507614	1.83553299	0.62179428	0.422641	0.53338364	0.49275266	Pnp	1
GO:0006006	glucose metabolic process	1/40	197/14464	0.00507614	1.83553299	0.62179428	0.422641	0.53338364	0.49275266	Cry1	1
GO:0030705	cytoskeleton-dependent intracellular transport	1/40	198/14464	0.00505051	1.82626263	0.61647565	0.42425972	0.53474961	0.49401458	Rhot1	1
GO:0008406	gonad development	1/40	200/14464	0.005	1.808	0.6059294	0.42748391	0.53813315	0.49714038	Bok	1
GO:0045137	development of primary sexual characteristics	1/40	203/14464	0.00492611	1.78128079	0.59033225	0.43228721	0.542809	0.50146004	Bok	1
GO:0060047	heart contraction	1/40	203/14464	0.00492611	1.78128079	0.59033225	0.43228721	0.542809	0.50146004	Ptpn1	1
GO:0007623	circadian rhythm	1/40	204/14464	0.00490196	1.77254902	0.58519108	0.43387956	0.54324799	0.50186559	Cry1	1
GO:0031396	regulation of protein ubiquitination	1/40	204/14464	0.00490196	1.77254902	0.58519108	0.43387956	0.54324799	0.50186559	Cry1	1
GO:0000819	sister chromatid segregation	1/40	205/14464	0.00487805	1.76390244	0.58007831	0.43546755	0.54324799	0.50186559	Arhgef10	1
GO:0071216	cellular response to biotic stimulus	1/40	205/14464	0.00487805	1.76390244	0.58007831	0.43546755	0.54324799	0.50186559	Fbh1	1
GO:0010810	regulation of cell-substrate adhesion	1/40	206/14464	0.00485437	1.75533981	0.5749936	0.43705121	0.54324799	0.50186559	Ptpn1	1
GO:0007411	axon guidance	1/40	207/14464	0.00483092	1.7468599	0.56993664	0.43863053	0.54324799	0.50186559	Matn2	1
GO:0043087	regulation of GTPase activity	1/40	207/14464	0.00483092	1.7468599	0.56993664	0.43863053	0.54324799	0.50186559	Arhgef10	1
GO:0043583	ear development	1/40	207/14464	0.00483092	1.7468599	0.56993664	0.43863053	0.54324799	0.50186559	Cys1	1
GO:0060348	bone development	1/40	207/14464	0.00483092	1.7468599	0.56993664	0.43863053	0.54324799	0.50186559	Smad1	1
GO:0097191	extrinsic apoptotic signaling pathway	1/40	207/14464	0.00483092	1.7468599	0.56993664	0.43863053	0.54324799	0.50186559	Bok	1
GO:1903039	positive regulation of leukocyte cell-cell adhesion	1/40	207/14464	0.00483092	1.7468599	0.56993664	0.43863053	0.54324799	0.50186559	Pnp	1
GO:0097485	neuron projection guidance	1/40	208/14464	0.00480769	1.73846154	0.5649071	0.44020553	0.54452222	0.50304275	Matn2	1
GO:0016311	dephosphorylation	1/40	209/14464	0.00478469	1.73014354	0.5599047	0.44177622	0.54578797	0.50421208	Ptpn1	1
GO:0090276	regulation of peptide hormone secretion	1/40	210/14464	0.0047619	1.72190476	0.55492911	0.44334261	0.54704528	0.50537362	Capn10	1
GO:0051321	meiotic cell cycle	1/40	211/14464	0.00473934	1.71374408	0.54998005	0.44490472	0.54794224	0.50620225	Catsperz	1
GO:0002699	positive regulation of immune effector process	1/40	212/14464	0.00471698	1.70566038	0.54505721	0.44646256	0.54794224	0.50620225	Pnp	1
GO:0099111	microtubule-based transport	1/40	212/14464	0.00471698	1.70566038	0.54505721	0.44646256	0.54794224	0.50620225	Rhot1	1
GO:0003015	heart process	1/40	213/14464	0.00469484	1.69765258	0.54016031	0.44801613	0.54794224	0.50620225	Ptpn1	1
GO:0030073	insulin secretion	1/40	213/14464	0.00469484	1.69765258	0.54016031	0.44801613	0.54794224	0.50620225	Capn10	1
GO:0045930	negative regulation of mitotic cell cycle	1/40	213/14464	0.00469484	1.69765258	0.54016031	0.44801613	0.54794224	0.50620225	Ctdspl	1
GO:0070663	regulation of leukocyte proliferation	1/40	213/14464	0.00469484	1.69765258	0.54016031	0.44801613	0.54794224	0.50620225	Pnp	1
GO:0006364	rRNA processing	1/40	214/14464	0.0046729	1.68971963	0.53528906	0.44956545	0.54794224	0.50620225	Rrp8	1
GO:0009165	nucleotide biosynthetic process	1/40	214/14464	0.0046729	1.68971963	0.53528906	0.44956545	0.54794224	0.50620225	Pnp	1
GO:0034764	positive regulation of transmembrane transport	1/40	214/14464	0.0046729	1.68971963	0.53528906	0.44956545	0.54794224	0.50620225	Capn10	1
GO:0002791	regulation of peptide secretion	1/40	215/14464	0.00465116	1.68186047	0.53044319	0.45111053	0.54848438	0.50670309	Capn10	1
GO:0090087	regulation of peptide transport	1/40	215/14464	0.00465116	1.68186047	0.53044319	0.45111053	0.54848438	0.50670309	Capn10	1
GO:0042594	response to starvation	1/40	216/14464	0.00462963	1.67407407	0.52562242	0.45265138	0.54901876	0.50719677	Rrp8	1
GO:0045926	negative regulation of growth	1/40	216/14464	0.00462963	1.67407407	0.52562242	0.45265138	0.54901876	0.50719677	Tchp	1
GO:0052548	regulation of endopeptidase activity	1/40	218/14464	0.00458716	1.6587156	0.51605508	0.45572044	0.55206959	0.5100152	Bok	1
GO:0048762	mesenchymal cell differentiation	1/40	219/14464	0.00456621	1.65114155	0.51130798	0.45724867	0.55324869	0.51110448	Fam83d	1
GO:0046434	organophosphate catabolic process	1/40	222/14464	0.0045045	1.62882883	0.49720991	0.4618083	0.55741268	0.51495127	Pnp	1
GO:0090068	positive regulation of cell cycle process	1/40	222/14464	0.0045045	1.62882883	0.49720991	0.4618083	0.55741268	0.51495127	Fam83d	1
GO:1903522	regulation of blood circulation	1/40	224/14464	0.00446429	1.61428571	0.48792802	0.46482729	0.56037824	0.51769093	Ptpn1	1
GO:0060828	regulation of canonical Wnt signaling pathway	1/40	226/14464	0.00442478	1.6	0.47873735	0.46782976	0.56331675	0.52040559	Prdm15	1
GO:0051056	regulation of small GTPase mediated signal transduction	1/40	227/14464	0.00440529	1.59295154	0.47417562	0.46932483	0.56443529	0.52143892	Sipa1l2	1

GO:0072522 purine-containing compound biosynthetic process	1/40	228/14464	0.00438596	1.58596491	0.469636	0.4708158	0.56554621	0.52246522	Pnp	1
GO:1901136 carbohydrate derivative catabolic process	1/40	229/14464	0.00436681	1.5790393	0.46511825	0.47230269	0.56638388	0.52323908	Pnp	1
GO:0019318 hexose metabolic process	1/40	230/14464	0.00434783	1.57217391	0.46062215	0.47378551	0.56638388	0.52323908	Cry1	1
GO:0031331 positive regulation of cellular catabolic process	1/40	230/14464	0.00434783	1.57217391	0.46062215	0.47378551	0.56638388	0.52323908	Ptpn1	1
GO:1902115 regulation of organelle assembly	1/40	230/14464	0.00434783	1.57217391	0.46062215	0.47378551	0.56638388	0.52323908	Tchp	1
GO:0045055 regulated exocytosis	1/40	231/14464	0.004329	1.56536797	0.4561475	0.47526426	0.56747122	0.52424359	Ctbp2	1
GO:0015850 organic hydroxy compound transport	1/40	235/14464	0.00425532	1.5387234	0.43845893	0.48113887	0.57379838	0.53008878	Slc10a6	1
GO:0002449 lymphocyte mediated immunity	1/40	236/14464	0.00423729	1.53220339	0.43408826	0.48259747	0.57416429	0.53042681	Pnp	1
GO:0033044 regulation of chromosome organization	1/40	236/14464	0.00423729	1.53220339	0.43408826	0.48259747	0.57416429	0.53042681	Pkib	1
GO:0007548 sex differentiation	1/40	237/14464	0.00421941	1.5257384	0.42973778	0.48405207	0.57452371	0.53075885	Bok	1
GO:0048738 cardiac muscle tissue development	1/40	237/14464	0.00421941	1.5257384	0.42973778	0.48405207	0.57452371	0.53075885	Smad1	1
GO:1903320 regulation of protein modification by small protein conjugation or removal	1/40	239/14464	0.0041841	1.51297071	0.42109658	0.48694933	0.57727524	0.53330078	Cry1	1
GO:0042113 B cell activation	1/40	240/14464	0.00416667	1.50666667	0.41680547	0.488392	0.57758455	0.53358653	Pnp	1
GO:0031669 cellular response to nutrient levels	1/40	241/14464	0.00414938	1.50041494	0.41253378	0.48983072	0.57758455	0.53358653	Rrp8	1
GO:0045444 fat cell differentiation	1/40	241/14464	0.00414938	1.50041494	0.41253378	0.48983072	0.57758455	0.53358653	Ctbp2	1
GO:0071560 cellular response to transforming growth factor beta stimulus	1/40	242/14464	0.00413223	1.49421488	0.4082813	0.49126549	0.57758455	0.53358653	Smad1	1
GO:0071695 anatomical structure maturation	1/40	242/14464	0.00413223	1.49421488	0.4082813	0.49126549	0.57758455	0.53358653	Catsperz	1
GO:0090596 sensory organ morphogenesis	1/40	242/14464	0.00413223	1.49421488	0.4082813	0.49126549	0.57758455	0.53358653	Cntf	1
GO:0140014 mitotic nuclear division	1/40	242/14464	0.00413223	1.49421488	0.4082813	0.49126549	0.57758455	0.53358653	Arhgef10	1
GO:0009205 purine ribonucleoside triphosphate metabolic process	1/40	243/14464	0.00411523	1.48806584	0.40404786	0.49269633	0.5785845	0.53451031	Pnp	1
GO:1901873 regulation of post-translational protein modification	1/40	244/14464	0.00409836	1.48196721	0.39983328	0.49412324	0.5795775	0.53542766	Cry1	1
GO:0006260 DNA replication	1/40	245/14464	0.00408163	1.47591837	0.39563738	0.49554624	0.57988217	0.53570912	Fbh1	1
GO:0032886 regulation of microtubule-based process	1/40	245/14464	0.00408163	1.47591837	0.39563738	0.49554624	0.57988217	0.53570912	Rhot1	1
GO:0045860 positive regulation of protein kinase activity	1/40	246/14464	0.00406504	1.4699187	0.39145997	0.49696534	0.58086101	0.53661341	Ptpn1	1
GO:0016072 rRNA metabolic process	1/40	247/14464	0.00404858	1.46396761	0.38730089	0.49838055	0.58183303	0.53751137	Rrp8	1
GO:0009199 ribonucleoside triphosphate metabolic process	1/40	248/14464	0.00403226	1.45806452	0.38315996	0.49979187	0.58239595	0.53803141	Pnp	1
GO:0033002 muscle cell proliferation	1/40	249/14464	0.00401606	1.45220884	0.37903701	0.50119932	0.58239595	0.53803141	Smad1	1
GO:0071559 response to transforming growth factor beta	1/40	249/14464	0.00401606	1.45220884	0.37903701	0.50119932	0.58239595	0.53803141	Smad1	1
GO:0099504 synaptic vesicle cycle	1/40	249/14464	0.00401606	1.45220884	0.37903701	0.50119932	0.58239595	0.53803141	Ctbp2	1
GO:0005996 monosaccharide metabolic process	1/40	250/14464	0.004	1.4464	0.37493188	0.50260291	0.58334703	0.53891005	Cry1	1
GO:0009144 purine nucleoside triphosphate metabolic process	1/40	251/14464	0.00398406	1.44063745	0.3708444	0.50400265	0.58429144	0.53978252	Pnp	1
GO:0022409 positive regulation of cell-cell adhesion	1/40	252/14464	0.00396825	1.43492063	0.3667744	0.50539855	0.58522921	0.54064885	Pnp	1
GO:0046651 lymphocyte proliferation	1/40	253/14464	0.00395257	1.42924901	0.36272173	0.50679061	0.58616037	0.54150908	Pnp	1
GO:0030072 peptide hormone secretion	1/40	256/14464	0.00390625	1.4125	0.35066611	0.51094393	0.58891457	0.54405347	Capn10	1
GO:0050878 regulation of body fluid levels	1/40	256/14464	0.00390625	1.4125	0.35066611	0.51094393	0.58891457	0.54405347	Hps5	1
GO:0090150 establishment of protein localization to membrane	1/40	256/14464	0.00390625	1.4125	0.35066611	0.51094393	0.58891457	0.54405347	Zfand2b	1
GO:0033674 positive regulation of kinase activity	1/40	258/14464	0.00387597	1.40155039	0.3427128	0.51369385	0.59003775	0.5450911	Ptpn1	1
GO:0045927 positive regulation of growth	1/40	258/14464	0.00387597	1.40155039	0.3427128	0.51369385	0.59003775	0.5450911	Cntf	1
GO:0110053 regulation of actin filament organization	1/40	258/14464	0.00387597	1.40155039	0.3427128	0.51369385	0.59003775	0.5450911	Arhgef10	1
GO:0032943 mononuclear cell proliferation	1/40	259/14464	0.003861	1.396139	0.33876083	0.51506315	0.59092976	0.54591515	Pnp	1
GO:0051251 positive regulation of lymphocyte activation	1/40	260/14464	0.00384615	1.39076923	0.33482512	0.51642869	0.5917233	0.54664825	Pnp	1
GO:0051222 positive regulation of protein transport	1/40	262/14464	0.00381679	1.38015267	0.3270019	0.51914852	0.5917233	0.54664825	Capn10	1
GO:0052547 regulation of peptidase activity	1/40	262/14464	0.00381679	1.38015267	0.3270019	0.51914852	0.5917233	0.54664825	Bok	1
GO:0002460 adaptive immune response based on somatic recombination of immune receptors built from immunoglobu	1/40	263/14464	0.00380228	1.37490494	0.3231141	0.52050284	0.5917233	0.54664825	Pnp	1
GO:0002790 peptide secretion	1/40	263/14464	0.00380228	1.37490494	0.3231141	0.52050284	0.5917233	0.54664825	Capn10	1
GO:0007162 negative regulation of cell adhesion	1/40	263/14464	0.00380228	1.37490494	0.3231141	0.52050284	0.5917233	0.54664825	Ptpn1	1
GO:0030217 T cell differentiation	1/40	263/14464	0.00380228	1.37490494	0.3231141	0.52050284	0.5917233	0.54664825	Pnp	1
GO:0050708 regulation of protein secretion	1/40	263/14464	0.00380228	1.37490494	0.3231141	0.52050284	0.5917233	0.54664825	Capn10	1
GO:0009141 nucleoside triphosphate metabolic process	1/40	265/14464	0.00377358	1.3645283	0.31538544	0.52320034	0.59343656	0.548231	Pnp	1
GO:0042593 glucose homeostasis	1/40	265/14464	0.00377358	1.3645283	0.31538544	0.52320034	0.59343656	0.548231	Cry1	1
GO:0033500 carbohydrate homeostasis	1/40	266/14464	0.0037594	1.3593985	0.31154431	0.52454353	0.59428398	0.54901386	Cry1	1
GO:0060485 mesenchyme development	1/40	267/14464	0.00374532	1.35430712	0.30771846	0.52588303	0.59512529	0.54979109	Fam83d	1
GO:0048608 reproductive structure development	1/40	268/14464	0.00373134	1.34925373	0.30390776	0.52721886	0.59596054	0.55056271	Bok	1
GO:0060070 canonical Wnt signaling pathway	1/40	269/14464	0.00371747	1.34423792	0.30011208	0.52855101	0.59678976	0.55132876	Prdm15	1
GO:0045765 regulation of angiogenesis	1/40	270/14464	0.0037037	1.33925926	0.2963313	0.5298795	0.59761297	0.55208926	Smad1	1
GO:0030336 negative regulation of cell migration	1/40	271/14464	0.00369004	1.33431734	0.29256528	0.53120434	0.59789186	0.55234691	Ptpn1	1
GO:0015833 peptide transport	1/40	272/14464	0.00367647	1.32941176	0.2888139	0.53252555	0.59789186	0.55234691	Capn10	1
GO:0061448 connective tissue development	1/40	272/14464	0.00367647	1.32941176	0.2888139	0.53252555	0.59789186	0.55234691	Smad1	1
GO:0061458 reproductive system development	1/40	272/14464	0.00367647	1.32941176	0.2888139	0.53252555	0.59789186	0.55234691	Bok	1
GO:0007281 germ cell development	1/40	276/14464	0.00362319	1.31014493	0.27395231	0.53777415	0.60107716	0.55528957	Catsperz	1
GO:0030198 extracellular matrix organization	1/40	276/14464	0.00362319	1.31014493	0.27395231	0.53777415	0.60107716	0.55528957	Matn2	1
GO:0043062 extracellular structure organization	1/40	276/14464	0.00362319	1.31014493	0.27395231	0.53777415	0.60107716	0.55528957	Matn2	1
GO:0045229 external encapsulating structure organization	1/40	276/14464	0.00362319	1.31014493	0.27395231	0.53777415	0.60107716	0.55528957	Matn2	1
GO:1901342 regulation of vasculature development	1/40	277/14464	0.00361011	1.30541516	0.27027229	0.53907729	0.60128987	0.55548608	Smad1	1
GO:0006605 protein targeting	1/40	278/14464	0.00359712	1.30071942	0.26660619	0.54037686	0.60128987	0.55548608	Zfand2b	1
GO:0006874 intracellular calcium ion homeostasis	1/40	278/14464	0.00359712	1.30071942	0.26660619	0.54037686	0.60128987	0.55548608	Bok	1
GO:0090287 regulation of cellular response to growth factor stimulus	1/40	278/14464	0.00359712	1.30071942	0.26660619	0.54037686	0.60128987	0.55548608	Ptpn1	1
GO:0006310 DNA recombination	1/40	279/14464	0.00358423	1.29605735	0.26295389	0.54167285	0.60206001	0.55619755	Fbh1	1
GO:0006302 double-strand break repair	1/40	281/14464	0.00355872	1.28683274	0.25569025	0.54425415	0.60425544	0.55822574	Fbh1	1
GO:0030111 regulation of Wnt signaling pathway	1/40	282/14464	0.0035461	1.2822695	0.25207867	0.54553948	0.60433651	0.55830064	Prdm15	1
GO:2000146 negative regulation of cell motility	1/40	282/14464	0.0035461	1.2822695	0.25207867	0.54553948	0.60433651	0.55830064	Ptpn1	1
GO:0002696 positive regulation of leukocyte activation	1/40	283/14464	0.00353357	1.27773852	0.24848045	0.54682127	0.60441331	0.55837159	Pnp	1
GO:0009416 response to light stimulus	1/40	283/14464	0.00353357	1.27773852	0.24848045	0.54682127	0.60441331	0.55837159	Cry1	1
GO:1901293 nucleoside phosphate biosynthetic process	1/40	286/14464	0.0034965	1.26433566	0.23776484	0.55064555	0.60729382	0.56103267	Pnp	1
GO:1902105 regulation of leukocyte differentiation	1/40	286/14464	0.0034965	1.26433566	0.23776484	0.55064555	0.60729382	0.56103267	Pnp	1
GO:0070661 leukocyte proliferation	1/40	287/14464	0.00348432	1.25993031	0.23421896	0.5519133	0.6080194	0.56170298	Pnp	1
GO:0045787 positive regulation of cell cycle	1/40	288/14464	0.00347222	1.25555556	0.2306859	0.55317756	0.60873955	0.56236827	Fam83d	1
GO:0001666 response to hypoxia	1/40	289/14464	0.00346021	1.25121107	0.22716556	0.55443835	0.60945428	0.56302855	Ptpn1	1
GO:0048545 response to steroid hormone	1/40	290/14464	0.00344828	1.24689655	0.22365785	0.55569566	0.61016363	0.56368387	Cry1	1
GO:0048511 rhythmic process	1/40	291/14464	0.00343643	1.24261168	0.22016265	0.55694952	0.61019634	0.56371409	Cry1	1
GO:0141188 nucleic acid catabolic process	1/40	291/14464	0.00343643	1.24261168	0.22016265	0.55694952	0.61019634	0.56371409	Fbh1	1
GO:0141091 transforming growth factor beta receptor superfamily signaling pathway	1/40	292/14464	0.00342466	1.23835616	0.21667987	0.55819993	0.61022514	0.56374069	Smad1	1
GO:1903037 regulation of leukocyte cell-cell adhesion	1/40	292/14464	0.00342466	1.23835616	0.21667987	0.55819993	0.61022514	0.56374069	Pnp	1
GO:0010506 regulation of autophagy	1/40	295/14464	0.00338983	1.22576271	0.20630509	0.56193054	0.61228934	0.56564765	Bok	1
GO:0022412 cellular process involved in reproduction in multicellular organism	1/40	295/14464	0.00338983	1.22576271	0.20630509	0.56193054	0.61228934	0.56564765	Catsperz	1
GO:0055074 calcium ion homeostasis	1/40	295/14464	0.00338983	1.22576271	0.20630509	0.56193054	0.61228934	0.56564765	Bok	1
GO:0044282 small molecule catabolic process	1/40	296/14464	0.00337838	1.22162162	0.20287103	0.56316724	0.61229851	0.56565613	Pnp	1
GO:0045862 positive regulation of proteolysis	1/40	296/14464	0.00337838	1.22162162	0.20287103	0.56316724	0.61229851	0.56565613	Bok	1
GO:0040013 negative regulation of locomotion	1/40	297/14464	0.003367	1.21750842	0.19944892	0.56440053	0.61230395	0.56566115	Ptpn1	1
GO:0050863 regulation of T cell activation	1/40	297/14464	0.003367	1.21750842	0.19944892	0.56440053	0.61230395	0.56566115	Pnp	1
GO:0050867 positive regulation of cell activation	1/40	303/14464	0.00330033	1.19339934	0.17916191	0.57172932	0.61823658	0.57114186	Pnp	1
GO:0099003 vesicle-mediated transport in synapse	1/40	303/14464	0.00330033	1.19339934	0.17916191	0.57172932	0.61823658	0.57114186	Ctbp2	1
GO:0099173 postsynapse organization	1/40	303/14464	0.00330033	1.19339934	0.17916191	0.57172932	0.61823658	0.57114186	Ptpn1	1
GO:0036293 response to decreased oxygen levels	1/40	304/14464	0.00328947	1.18947368	0.17582086	0.57293904	0.61887348	0.57173024	Ptpn1	1
GO:0051347 positive regulation of transferase activity	1/40	306/14464	0.00326797	1.18169935	0.16917238	0.57534849	0.62080351	0.57351325	Ptpn1	1
GO:0051235 maintenance of location	1/40	307/14464	0.00325733	1.17785016	0.16586478	0.57654824	0.62142551	0.57408786	Cry1	1
GO:0051090 regulation of DNA-binding transcription factor activity	1/40	308/14464	0.00324675	1.17402597	0.16256816	0.57774468	0.62198386	0.57460368	Cys1	1
GO:0016358 dendrite development	1/40	309/14464	0.00323625	1.17022654	0.15928244	0.57893783	0.62198386	0.57460368	Matn2	1
GO:0042254 ribosome biogenesis	1/40	309/14464	0.00323625	1.17022654	0.15928244	0.57893783	0.62198386	0.57460368	Rrp8	1
GO:0030100 regulation of endocytosis	1/40	310/14464	0.00322581	1.16645161	0.15600754	0.58012769	0.62252452	0.57510316	Ptpn1	1
GO:0009150 purine ribonucleotide metabolic process	1/40	311/14464	0.00321543	1.16270096	0.15274338	0.58131428	0.62252452	0.57510316	Pnp	1
GO:0043010 camera-type eye development	1/40	311/14464	0.00321543	1.16270096	0.15274338	0.58131428	0.62252452	0.57510316	Cntf	1
GO:0007178 cell surface receptor protein serine/threonine kinase signaling pathway	1/40	313/14464	0.00319489	1.15527157	0.14624697	0.58367764	0.62438477	0.5768217	Smad1	1
GO:0042886 amide transport	1/40	314/14464	0.00318471	1.15159236	0.14301456	0.58485444	0.62497307	0.57736519	Capn10	1
GO:1904951 positive regulation of establishment of protein localization	1/40	316/14464	0.00316456	1.1443038	0.13658094	0.58719832	0.62680591	0.57905841	Capn10	1
GO:0062012 regulation of small molecule metabolic process	1/40	319/14464	0.0031348	1.13354232	0.12700743	0.59068996	0.62985871	0.58187866	Cry1	1
GO:0002697 regulation of immune effector process	1/40	320/14464	0.003125	1.13	0.12383647	0.59184743	0.63041868	0.58239598	Pnp	1
GO:0042060 wound healing	1/40	321/14464	0.00311526	1.12647975	0.12067549	0.59300171	0.63097407	0.58290905	Hps5	1
GO:0007517 muscle organ development	1/40	323/14464	0.00309598	1.11950464	0.11438322	0.59530072	0.63207116	0.58392257	Cntf	1
GO:0021700 developmental maturation	1/40	323/14464	0.00309598	1.11950464	0.11438322	0.59530072	0.63207116	0.58392257	Catsperz	1
GO:0007159 leukocyte cell-cell adhesion	1/40	326/14464	0.00306748	1.10920245	0.10501795	0.59872552	0.63488455	0.58652165	Pnp	1
GO:0002443 leukocyte mediated immunity	1/40	327/14464	0.0030581	1.1058104	0.10191543	0.59986083	0.63488455	0.58652165	Pnp	1
GO:0070482 response to oxygen levels	1/40	327/14464	0.0030581	1.1058104	0.10191543	0.59986083	0.63488455	0.58652165	Ptpn1	1
GO:0009259 ribonucleotide metabolic process	1/40	332/14464	0.00301205	1.08915663	0.08654404	0.60549056	0.63948526	0.5907719	Pnp	1
GO:0031589 cell-substrate adhesion	1/40	332/14464	0.00301205	1.08915663	0.08654404	0.60549056	0.63948526	0.5907719	Ptpn1	1
GO:0002250 adaptive immune response	1/40	337/14464	0.00296736	1.07299703	0.07140219	0.61104303	0.64466656	0.59555851	Pnp	1
GO:0019693 ribose phosphate metabolic process	1/40	339/14464	0.00294985	1.06666667	0.06540797	0.61324262	0.64562079	0.59644005	Pnp	1
GO:0060271 cilium assembly	1/40	339/14464	0.00294985	1.06666667	0.06540797	0.61324262	0.64562079	0.59644005	Tchp	1
GO:0042176 regulation of protein catabolic process	1/40	344/14464	0.00290698	1.05116279	0.0505746	0.61868867	0.6506673	0.60110214	Fam83d	1
GO:0001654 eye development	1/40	351/14464	0.002849	1.03019943	0.03016192	0.6261877	0.65620718	0.60622001	Cntf	1
GO:0016050 vesicle organization	1/40	351/14464	0.002849	1.03019943	0.03016192	0.6261877	0.65620718	0.60622001	Hps5	1
GO:0051962 positive regulation of nervous system development	1/40	351/14464	0.002849	1.03019943	0.03016192	0.6261877	0.65620718	0.60622001	Lrrn3	1
GO:0006887 exocytosis	1/40	352/14464	0.00284091	1.02727273	0.02727859	0.62724719	0.65620718	0.60622001	Ctbp2	1
GO:0009306 protein secretion	1/40	352/14464	0.00284091	1.02727273	0.02727859	0.62724719	0.65620718	0.60622001	Capn10	1
GO:0019221 cytokine-mediated signaling pathway	1/40	353/14464	0.00283286	1.02436261	0.0244033	0.62830374	0.65634865	0.6063507	Cntf	1
GO:0035592 establishment of protein localization to extracellular region	1/40	354/14464	0.00282486	1.02146893	0.02153599	0.62935738	0.65634865	0.6063507	Capn10	1
GO:0150063 visual system development	1/40	354/14464	0.00282486	1.02146893	0.02153599	0.62935738	0.65634865	0.6063507	Cntf	1
GO:0007389 pattern specification process	1/40	356/14464	0.00280899	1.01573034	0.01582511	0.63145592	0.65784906	0.60773682	Smad1	1
GO:0071692 protein localization to extracellular region	1/40	357/14464	0.00280112	1.01288515	0.01298144	0.63250084	0.65824983	0.60810706	Capn10	1
GO:0001503 ossification	1/40	359/14464	0.00278552	1.00724234	0.00731739	0.63458203	0.65835409	0.60820338	Smad1	1
GO:0030098 lymphocyte differentiation	1/40	359/14464	0.00278552	1.00724234	0.00731739	0.63458203	0.65835409	0.60820338	Pnp	1
GO:0048880 sensory system development	1/40	359/14464	0.00278552	1.00724234	0.00731739	0.63458203	0.65835409	0.60820338	Cntf	1
GO:1903706 regulation of hemopoiesis	1/40	362/14464	0.00276243	0.99889503	-0.0011212	0.63768228	0.66088278	0.61053944	Pnp	1
GO:0044782 cilium organization	1/40	369/14464	0.00271003	0.9799458	-0.0205496	0.6448168	0.66758291	0.61672919	Tchp	1
GO:1902903 regulation of supramolecular fiber organization	1/40	374/14464	0.0026738	0.96684492	-0.0342095	0.64982883	0.672074	0.62087816	Arhgef10	1
GO:0043603 amide metabolic process	1/40	376/14464	0.00265957	0.96170213	-0.0396242	0.65181427	0.67342884	0.62212979	Pnp	1
GO:0016055 Wnt signaling pathway	1/40	385/14464	0.0025974	0.93922078	-0.0636526	0.66061378	0.6818136	0.62987584	Prdm15	1
GO:0001819 positive regulation of cytokine production	1/40	386/14464	0.00259067	0.93678756	-0.0662891	0.66157801	0.68210267	0.63014288	Pnp	1
GO:0003012 muscle system process	1/40	395/14464	0.00253165	0.91544304	-0.089729	0.67013678	0.68950089	0.63697754	Smad1	1
GO:0006163 purine nucleotide metabolic process	1/40	395/14464	0.00253165	0.91544304	-0.089729	0.67013678	0.68950089	0.63697754	Pnp	1
GO:0022407 regulation of cell-cell adhesion	1/40	398/14464	0.00251256	0.90854271	-0.0974296	0.67294251	0.6916739	0.63898502	Pnp	1
GO:0022411 cellular component disassembly	1/40	400/14464	0.0025	0.904	-0.1025329	0.67480007	0.69215604	0.63943043	Bok	1
GO:0140694 non-membrane-bounded organelle assembly	1/40	400/14464	0.0025	0.904	-0.1025329	0.67480007	0.69215604	0.63943043	Arhgef10	1
GO:0051249 regulation of lymphocyte activation	1/40	409/14464	0.00244499	0.88410758	-0.1252038	0.68303254	0.6998802	0.6465662	Pnp	1
GO:0014706 striated muscle tissue development	1/40	414/14464	0.00241546	0.87342995	-0.1375971	0.68751796	0.70321964	0.64965125	Smad1	1
GO:0051051 negative regulation of transport	1/40	415/14464	0.00240964	0.8713253	-0.1400589	0.68840759	0.70321964	0.64965125	Cry1	1
GO:0072594 establishment of protein localization to organelle	1/40	415/14464	0.00240964	0.8713253	-0.1400589	0.68840759	0.70321964	0.64965125	Zfand2b	1
GO:0045785 positive regulation of cell adhesion	1/40	422/14464	0.00236967	0.85687204	-0.1571385	0.69456621	0.70822264	0.65427314	Pnp	1
GO:0007283 spermatogenesis	1/40	423/14464	0.00236407	0.85484634	-0.1595569	0.69543627	0.70822264	0.65427314	Catsperz	1
GO:0042692 muscle cell differentiation	1/40	423/14464	0.00236407	0.85484634	-0.1595569	0.69543627	0.70822264	0.65427314	Smad1	1
GO:0060537 muscle tissue development	1/40	435/14464	0.00229885	0.83126437	-0.1881717	0.70569032	0.71793189	0.66324278	Smad1	1
GO:0048232 male gamete generation	1/40	437/14464	0.00228833	0.82745995	-0.1928699	0.70736628	0.71890335	0.66414024	Catsperz	1
GO:0008015 blood circulation	1/40	443/14464	0.00225734	0.81625282	-0.2068471	0.71233852	0.72321946	0.66812757	Ptpn1	1
GO:0007409 axonogenesis	1/40	446/14464	0.00224215	0.81076233	-0.2137708	0.71479366	0.72359094	0.66847075	Matn2	1
GO:0030003 intracellular monoatomic cation homeostasis	1/40	447/14464	0.00223714	0.80894855	-0.2160692	0.71560749	0.72359094	0.66847075	Bok	1
GO:0034762 regulation of transmembrane transport	1/40	447/14464	0.00223714	0.80894855	-0.2160692	0.71560749	0.72359094	0.66847075	Capn10	1
GO:1903131 mononuclear cell differentiation	1/40	447/14464	0.00223714	0.80894855	-0.2160692	0.71560749	0.72359094	0.66847075	Pnp	1
GO:0009117 nucleotide metabolic process	1/40	450/14464	0.00222222	0.80355556	-0.2229366	0.71803541	0.72531034	0.67005918	Pnp	1
GO:0006873 intracellular monoatomic ion homeostasis	1/40	454/14464	0.00220264	0.79647577	-0.2320285	0.72124123	0.72707534	0.67168972	Bok	1
GO:0098771 inorganic ion homeostasis	1/40	454/14464	0.00220264	0.79647577	-0.2320285	0.72124123	0.72707534	0.67168972	Bok	1
GO:0042110 T cell activation	1/40	464/14464	0.00215517	0.77931034	-0.2544446	0.72910109	0.73425635	0.67832372	Pnp	1
GO:0001501 skeletal system development	1/40	466/14464	0.00214592	0.77596567	-0.2588755	0.73064693	0.73507063	0.67907596	Smad1	1
GO:0002694 regulation of leukocyte activation	1/40	467/14464	0.00214133	0.77430407	-0.2610845	0.73141662	0.7351032	0.67910605	Pnp	1
GO:0003013 circulatory system process	1/40	470/14464	0.00212766	0.7693617	-0.2676861	0.73371285	0.73666839	0.68055201	Ptpn1	1
GO:0010720 positive regulation of cell development	1/40	473/14464	0.00211416	0.76448203	-0.27425	0.73598993	0.73821123	0.68197733	Pnp	1
GO:1903829 positive regulation of protein localization	1/40	486/14464	0.00205761	0.74403292	-0.3022707	0.74563983	0.7471386	0.69022465	Capn10	1

GO:0005975	carbohydrate metabolic process	1/40	491/14464	0.00203666	0.73645621	-0.3128708	0.74925901	0.75001127	0.69287849	Cry1	1
GO:0071396	cellular response to lipid	1/40	499/14464	0.00200401	0.7246493	-0.3296342	0.75494551	0.75494551	0.69743686	Cry1	1

ID	Description	GeneRatio	BgRatio	RichFactor	FoldEnrichm	zScore	pvalue	p.adjust	qvalue	geneID	Count
GO:0033152	immunoglobulin V(D)J recombination	2/97	10/14464	0.2	29.8226804	7.49156161	0.00193413	0.50874395	0.5048838	Lig4/Nhej1	2
GO:0033197	response to vitamin E	2/97	10/14464	0.2	29.8226804	7.49156161	0.00193413	0.50874395	0.5048838	Fkbp1b/Becn1	2
GO:0050821	protein stabilization	6/97	197/14464	0.03045685	4.54152494	4.11234082	0.00209363	0.50874395	0.5048838	Hspa1b/Ptges3/Atp1b3/Ctnnd1/Caml/Cct7	6
GO:0071838	cell proliferation in bone marrow	2/97	11/14464	0.18181818	27.1115276	7.11838767	0.00235363	0.50874395	0.5048838	Shc1/Lipa	2
GO:1903265	positive regulation of tumor necrosis factor-mediated signaling pathway	2/97	11/14464	0.18181818	27.1115276	7.11838767	0.00235363	0.50874395	0.5048838	Hspa1b/Traf2	2
GO:0051084	'de novo' post-translational protein folding	3/97	41/14464	0.07317073	10.9107367	5.22158174	0.00259125	0.50874395	0.5048838	Hspa1b/Ptges3/Cct7	3
GO:0006458	'de novo' protein folding	3/97	43/14464	0.06976744	10.4032606	5.07395985	0.00297101	0.50874395	0.5048838	Hspa1b/Ptges3/Cct7	3
GO:0006266	DNA ligation	2/97	13/14464	0.15384615	22.9405234	6.50281986	0.00330884	0.50874395	0.5048838	Lig4/Nhej1	2
GO:0007040	lysosome organization	4/97	98/14464	0.04081633	6.08626131	4.15122038	0.0042438	0.50874395	0.5048838	Rab39/Lipa/Cln5/Becn1	4
GO:0080171	lytic vacuole organization	4/97	98/14464	0.04081633	6.08626131	4.15122038	0.0042438	0.50874395	0.5048838	Rab39/Lipa/Cln5/Becn1	4
GO:0006644	phospholipid metabolic process	7/97	319/14464	0.02194357	3.27208092	3.37170582	0.00561474	0.50874395	0.5048838	Pcsk9/Pmvk/Smpd4/Sphk2/Becn1/Pip4k2c/Pigc	7
GO:0051604	protein maturation	9/97	498/14464	0.01807229	2.69482052	3.16252801	0.00618475	0.50874395	0.5048838	Hspa1b/Tspan14/Pcsk9/Ptges3/Ctnnd1/Cln5/Cct7/Aph1b/Pigc	9
GO:0033151	V(D)J recombination	2/97	18/14464	0.11111111	16.5681558	5.43039762	0.00635046	0.50874395	0.5048838	Lig4/Nhej1	2
GO:0006352	DNA-templated transcription initiation	5/97	177/14464	0.02824859	4.212243	3.53310477	0.00675494	0.50874395	0.5048838	Sphk2/Ubtf/Taf4b/Maz/Eloa	5
GO:0032412	regulation of monoatomic ion transmembrane transporter activity	5/97	177/14464	0.02824859	4.212243	3.53310477	0.00675494	0.50874395	0.5048838	Gpr35/Fkbp1b/Pcsk9/Atp1b3/Sphk2	5
GO:0034612	response to tumor necrosis factor	5/97	180/14464	0.02777778	4.14203895	3.48541832	0.00724004	0.50874395	0.5048838	Hspa1b/Commd7/Smpd4/Traf2/Tuba1a	5
GO:2000786	positive regulation of autophagosome assembly	2/97	20/14464	0.1	14.9113402	5.11531337	0.00781784	0.50874395	0.5048838	Becn1/Pip4k2c	2
GO:0022898	regulation of transmembrane transporter activity	5/97	186/14464	0.02688172	4.00842479	3.39307888	0.00828243	0.50874395	0.5048838	Gpr35/Fkbp1b/Pcsk9/Atp1b3/Sphk2	5
GO:0061077	chaperone-mediated protein folding	3/97	62/14464	0.0483871	7.21516462	4.02966638	0.00830821	0.50874395	0.5048838	Hspa1b/Ptges3/Cct7	3
GO:0007339	binding of sperm to zona pellucida	2/97	22/14464	0.09090909	13.5557638	4.8425356	0.00942255	0.50874395	0.5048838	Hspa1b/Cct7	2
GO:0032409	regulation of transporter activity	5/97	196/14464	0.0255102	3.80391332	3.24745456	0.01024426	0.50874395	0.5048838	Gpr35/Fkbp1b/Pcsk9/Atp1b3/Sphk2	5
GO:0033273	response to vitamin	3/97	67/14464	0.04477612	6.6767195	3.82675667	0.01027399	0.50874395	0.5048838	Fkbp1b/Lipa/Becn1	3
GO:0008654	phospholipid biosynthetic process	5/97	201/14464	0.02487562	3.70928861	3.17819012	0.01133576	0.50874395	0.5048838	Pmvk/Sphk2/Becn1/Pip4k2c/Pigc	5
GO:0035036	sperm-egg recognition	2/97	26/14464	0.07692308	11.4702617	4.3905882	0.01302862	0.50874395	0.5048838	Hspa1b/Cct7	2
GO:0070536	protein K63-linked deubiquitination	2/97	26/14464	0.07692308	11.4702617	4.3905882	0.01302862	0.50874395	0.5048838	Stambpl1/Otub2	2
GO:0032801	receptor catabolic process	2/97	27/14464	0.07407407	11.0454372	4.29283564	0.01401004	0.50874395	0.5048838	Pcsk9/Becn1	2
GO:0044090	positive regulation of vacuole organization	2/97	27/14464	0.07407407	11.0454372	4.29283564	0.01401004	0.50874395	0.5048838	Becn1/Pip4k2c	2
GO:0006367	transcription initiation at RNA polymerase II promoter	4/97	141/14464	0.02836879	4.23016743	3.16701127	0.01491489	0.50874395	0.5048838	Sphk2/Taf4b/Maz/Eloa	4
GO:0043112	receptor metabolic process	3/97	77/14464	0.03896104	5.80961307	3.47698161	0.01495428	0.50874395	0.5048838	Pcsk9/Becn1/Caml	3
GO:0034381	plasma lipoprotein particle clearance	2/97	28/14464	0.07142857	10.6509573	4.2000841	0.0150225	0.50874395	0.5048838	Pcsk9/Lipa	2
GO:0045648	positive regulation of erythrocyte differentiation	2/97	28/14464	0.07142857	10.6509573	4.2000841	0.0150225	0.50874395	0.5048838	Hspa1b/Ankrd54	2
GO:1904063	negative regulation of cation transmembrane transport	3/97	79/14464	0.03797468	5.66253426	3.41438636	0.01601248	0.50874395	0.5048838	Gpr35/Fkbp1b/Pcsk9	3
GO:0009314	response to radiation	7/97	394/14464	0.0177665	2.64922288	2.72717136	0.01666341	0.50874395	0.5048838	Cdkn1a/Mettl3/Tuba1a/Lig4/Nhej1/Pde6d/Smc1a	7
GO:0007033	vacuole organization	5/97	223/14464	0.02242152	3.34334982	2.89768604	0.01708352	0.50874395	0.5048838	Rab39/Lipa/Cln5/Becn1/Pip4k2c	5
GO:0051085	chaperone cofactor-dependent protein refolding	2/97	30/14464	0.06666667	9.94089347	4.02791421	0.01713873	0.50874395	0.5048838	Hspa1b/Ptges3	2
GO:0002244	hematopoietic progenitor cell differentiation	4/97	148/14464	0.02702703	4.03009195	3.04444291	0.01751425	0.50874395	0.5048838	Lipa/Mettl3/Lig4/Dnai4	4
GO:0033209	tumor necrosis factor-mediated signaling pathway	3/97	82/14464	0.03658537	5.45536837	3.32439726	0.01767703	0.50874395	0.5048838	Hspa1b/Commd7/Traf2	3
GO:0034766	negative regulation of monoatomic ion transmembrane transport	3/97	84/14464	0.03571429	5.32547865	3.26682888	0.01883842	0.50874395	0.5048838	Gpr35/Fkbp1b/Pcsk9	3
GO:0001964	startle response	2/97	32/14464	0.0625	9.31958763	3.87119942	0.01937377	0.50874395	0.5048838	Tuba1a/Aph1b	2
GO:0006661	phosphatidylinositol biosynthetic process	3/97	87/14464	0.03448276	5.14184145	3.18383809	0.02065827	0.50874395	0.5048838	Becn1/Pip4k2c/Pigc	3
GO:0030301	cholesterol transport	3/97	88/14464	0.03409091	5.08341143	3.15702091	0.02128566	0.50874395	0.5048838	Pcsk9/Lipa/Stard3	3
GO:0031647	regulation of protein stability	6/97	324/14464	0.01851852	2.7613593	2.63467937	0.02176333	0.50874395	0.5048838	Hspa1b/Ptges3/Atp1b3/Ctnnd1/Caml/Cct7	6
GO:0046486	glycerolipid metabolic process	6/97	325/14464	0.01846154	2.75286281	2.62610613	0.02205603	0.50874395	0.5048838	Pcsk9/Lipa/Smpd4/Becn1/Pip4k2c/Pigc	6
GO:0071897	DNA biosynthetic process	4/97	159/14464	0.02515723	3.75128056	2.86629921	0.02214075	0.50874395	0.5048838	Cdkn1a/Ptges3/Lig4/Cct7	4
GO:0071356	cellular response to tumor necrosis factor	4/97	160/14464	0.025	3.72783505	2.85089591	0.02259491	0.50874395	0.5048838	Hspa1b/Commd7/Smpd4/Traf2	4
GO:0032212	positive regulation of telomere maintenance via telomerase	2/97	35/14464	0.05714286	8.52076583	3.66024357	0.02294147	0.50874395	0.5048838	Ptges3/Cct7	2
GO:0007173	epidermal growth factor receptor signaling pathway	3/97	92/14464	0.0326087	4.86239355	3.05368226	0.02389915	0.50874395	0.5048838	Shc1/Nrg2/Caml	3
GO:0015918	sterol transport	3/97	92/14464	0.0326087	4.86239355	3.05368226	0.02389915	0.50874395	0.5048838	Pcsk9/Lipa/Stard3	3
GO:0016239	positive regulation of macroautophagy	3/97	97/14464	0.03092784	4.6117547	2.9326017	0.02739978	0.50874395	0.5048838	Becn1/Pafah1b2/Pip4k2c	3
GO:1904358	positive regulation of telomere maintenance via telomere lengthening	2/97	39/14464	0.05128205	7.64684113	3.41524375	0.02808226	0.50874395	0.5048838	Ptges3/Cct7	2
GO:0034976	response to endoplasmic reticulum stress	5/97	256/14464	0.01953125	2.91237113	2.53663338	0.02888869	0.50874395	0.5048838	Pdia6/Edem1/Uggt1/Nhlrc1/Rnf103	5
GO:0051452	intracellular pH reduction	2/97	40/14464	0.05	7.4556701	3.35939044	0.02943326	0.50874395	0.5048838	Rab39/Cln5	2
GO:0007338	single fertilization	3/97	101/14464	0.02970297	4.42911095	2.84152616	0.0303868	0.50874395	0.5048838	Hspa1b/Antxr2/Cct7	3
GO:0009988	cell-cell recognition	2/97	41/14464	0.04878049	7.27382449	3.30543412	0.03080977	0.50874395	0.5048838	Hspa1b/Cct7	2
GO:0019221	cytokine-mediated signaling pathway	6/97	353/14464	0.01699717	2.53450542	2.39832976	0.0313654	0.50874395	0.5048838	Hspa1b/Gpr35/Mettl3/Commd7/Gpr75/Traf2	6
GO:2000278	regulation of DNA biosynthetic process	3/97	103/14464	0.02912621	4.3431088	2.7977493	0.03194222	0.50874395	0.5048838	Cdkn1a/Ptges3/Cct7	3
GO:0000079	regulation of cyclin-dependent protein serine/threonine kinase activity	2/97	42/14464	0.04761905	7.10063819	3.25326307	0.03221141	0.50874395	0.5048838	Cdkn1a/Sfn	2
GO:0019827	stem cell population maintenance	4/97	179/14464	0.02234637	3.33214306	2.57972609	0.0323163	0.50874395	0.5048838	Mettl3/Lig4/Sap30l/Smc1a	4
GO:0002377	immunoglobulin production	3/97	105/14464	0.02857143	4.26038292	2.75507341	0.03353874	0.50874395	0.5048838	Traf2/Lig4/Nhej1	3
GO:0036503	ERAD pathway	3/97	105/14464	0.02857143	4.26038292	2.75507341	0.03353874	0.50874395	0.5048838	Edem1/Uggt1/Rnf103	3
GO:0005978	glycogen biosynthetic process	2/97	43/14464	0.04651163	6.93550707	3.20277464	0.03363777	0.50874395	0.5048838	Ptges3/Nhlrc1	2
GO:0009250	glucan biosynthetic process	2/97	43/14464	0.04651163	6.93550707	3.20277464	0.03363777	0.50874395	0.5048838	Ptges3/Nhlrc1	2
GO:0010803	regulation of tumor necrosis factor-mediated signaling pathway	2/97	43/14464	0.04651163	6.93550707	3.20277464	0.03363777	0.50874395	0.5048838	Hspa1b/Traf2	2
GO:0009206	purine ribonucleoside triphosphate biosynthetic process	3/97	106/14464	0.02830189	4.22019062	2.73413248	0.03435237	0.50874395	0.5048838	Lipa/Sphk2/Nme1	3
GO:0048873	homeostasis of number of cells within a tissue	2/97	44/14464	0.04545455	6.77788191	3.15387433	0.03508847	0.50874395	0.5048838	Lipa/Tuba1a	2
GO:0009145	purine nucleoside triphosphate biosynthetic process	3/97	107/14464	0.02803738	4.18074959	2.71344819	0.03517622	0.50874395	0.5048838	Lipa/Sphk2/Nme1	3
GO:0098727	maintenance of cell number	4/97	185/14464	0.02162162	3.22407356	2.5016015	0.03582419	0.50874395	0.5048838	Mettl3/Lig4/Sap30l/Smc1a	4
GO:0046854	phosphatidylinositol phosphate biosynthetic process	2/97	45/14464	0.04444444	6.62726231	3.10647498	0.03656313	0.50874395	0.5048838	Becn1/Pip4k2c	2
GO:0038127	ERBB signaling pathway	3/97	109/14464	0.02752294	4.10403859	2.67282655	0.03685448	0.50874395	0.5048838	Shc1/Nrg2/Caml	3
GO:0006066	alcohol metabolic process	5/97	275/14464	0.01818182	2.71115276	2.35402597	0.03757541	0.50874395	0.5048838	Pcsk9/Lipa/Pmvk/Sphk2/Nudt3	5
GO:0043616	keratinocyte proliferation	2/97	46/14464	0.04347826	6.48319139	3.06049606	0.03806137	0.50874395	0.5048838	Cdkn1a/Sfn	2
GO:1904029	regulation of cyclin-dependent protein kinase activity	2/97	46/14464	0.04347826	6.48319139	3.06049606	0.03806137	0.50874395	0.5048838	Cdkn1a/Sfn	2
GO:2000785	regulation of autophagosome assembly	2/97	46/14464	0.04347826	6.48319139	3.06049606	0.03806137	0.50874395	0.5048838	Becn1/Pip4k2c	2
GO:0009201	ribonucleoside triphosphate biosynthetic process	3/97	111/14464	0.02702703	4.03009195	2.63316436	0.03857333	0.50874395	0.5048838	Lipa/Sphk2/Nme1	3
GO:0034655	nucleobase-containing compound catabolic process	7/97	471/14464	0.014862	2.21612275	2.20477595	0.03887305	0.50874395	0.5048838	Hspa1b/Dnase1l3/Lipa/Mettl3/Traf2/Nudt3/Hbs1l	7
GO:0045646	regulation of erythrocyte differentiation	2/97	47/14464	0.04255319	6.34525115	3.015863	0.03958281	0.50874395	0.5048838	Hspa1b/Ankrd54	2
GO:2001252	positive regulation of chromosome organization	3/97	113/14464	0.02654867	3.95876289	2.5944202	0.04033256	0.50874395	0.5048838	Ptges3/Lig4/Cct7	3
GO:0072089	stem cell proliferation	3/97	114/14464	0.02631579	3.9240369	2.57538011	0.04122726	0.50874395	0.5048838	Sfn/Lig4/Taf4b	3
GO:1903170	negative regulation of calcium ion transmembrane transport	2/97	49/14464	0.04081633	6.08626131	2.93036285	0.04269382	0.50874395	0.5048838	Gpr35/Fkbp1b	2
GO:0034763	negative regulation of transmembrane transport	3/97	116/14464	0.02586207	3.85638109	2.53794072	0.04304665	0.50874395	0.5048838	Gpr35/Fkbp1b/Pcsk9	3
GO:0032413	negative regulation of ion transmembrane transporter activity	2/97	50/14464	0.04	5.96453608	2.8893718	0.04428265	0.50874395	0.5048838	Gpr35/Pcsk9	2
GO:0008203	cholesterol metabolic process	3/97	118/14464	0.02542373	3.7910187	2.50132631	0.04490583	0.50874395	0.5048838	Pcsk9/Lipa/Pmvk	3
GO:0009142	nucleoside triphosphate biosynthetic process	3/97	118/14464	0.02542373	3.7910187	2.50132631	0.04490583	0.50874395	0.5048838	Lipa/Sphk2/Nme1	3
GO:0043271	negative regulation of monoatomic ion transport	3/97	118/14464	0.02542373	3.7910187	2.50132631	0.04490583	0.50874395	0.5048838	Gpr35/Fkbp1b/Pcsk9	3
GO:0001961	positive regulation of cytokine-mediated signaling pathway	2/97	51/14464	0.03921569	5.84758439	2.84947787	0.04589323	0.50874395	0.5048838	Hspa1b/Traf2	2
GO:0006695	cholesterol biosynthetic process	2/97	51/14464	0.03921569	5.84758439	2.84947787	0.04589323	0.50874395	0.5048838	Lipa/Pmvk	2
GO:0070098	chemokine-mediated signaling pathway	2/97	51/14464	0.03921569	5.84758439	2.84947787	0.04589323	0.50874395	0.5048838	Gpr35/Gpr75	2
GO:1902653	secondary alcohol biosynthetic process	2/97	51/14464	0.03921569	5.84758439	2.84947787	0.04589323	0.50874395	0.5048838	Lipa/Pmvk	2
GO:0141188	nucleic acid catabolic process	5/97	291/14464	0.01718213	2.56208595	2.21183405	0.04601688	0.50874395	0.5048838	Hspa1b/Dnase1l3/Mettl3/Traf2/Hbs1l	5
GO:0032210	regulation of telomere maintenance via telomerase	2/97	52/14464	0.03846154	5.73513085	2.81062914	0.04752519	0.50874395	0.5048838	Ptges3/Cct7	2
GO:0097006	regulation of plasma lipoprotein particle levels	2/97	52/14464	0.03846154	5.73513085	2.81062914	0.04752519	0.50874395	0.5048838	Pcsk9/Lipa	2
GO:1902305	regulation of sodium ion transmembrane transport	2/97	52/14464	0.03846154	5.73513085	2.81062914	0.04752519	0.50874395	0.5048838	Pcsk9/Atp1b3	2
GO:1904062	regulation of monoatomic cation transmembrane transport	5/97	294/14464	0.0170068	2.53594221	2.1862289	0.04771621	0.50874395	0.5048838	Gpr35/Fkbp1b/Pcsk9/Atp1b3/Sphk2	5
GO:0007052	mitotic spindle organization	3/97	121/14464	0.02479339	3.6970265	2.44787881	0.04776866	0.50874395	0.5048838	Hspa1b/Efhc1/Smc1a	3
GO:0046165	alcohol biosynthetic process	3/97	121/14464	0.02479339	3.6970265	2.44787881	0.04776866	0.50874395	0.5048838	Lipa/Pmvk/Sphk2	3
GO:0046467	membrane lipid biosynthetic process	3/97	121/14464	0.02479339	3.6970265	2.44787881	0.04776866	0.50874395	0.5048838	Smpd4/Sphk2/Pigc	3
GO:0001738	morphogenesis of a polarized epithelium	2/97	53/14464	0.03773585	5.62692083	2.7727771	0.04917818	0.50874395	0.5048838	Sfrp5/Ctnnd1	2
GO:0001881	receptor recycling	2/97	53/14464	0.03773585	5.62692083	2.7727771	0.04917818	0.50874395	0.5048838	Pcsk9/Caml	2
GO:0050435	amyloid-beta metabolic process	2/97	53/14464	0.03773585	5.62692083	2.7727771	0.04917818	0.50874395	0.5048838	Becn1/Aph1b	2
GO:0016125	sterol metabolic process	3/97	123/14464	0.02439024	3.63691225	2.41318451	0.04972615	0.50874395	0.5048838	Pcsk9/Lipa/Pmvk	3
GO:0016447	somatic recombination of immunoglobulin gene segments	2/97	54/14464	0.03703704	5.52271859	2.73587638	0.05085185	0.50874395	0.5048838	Lig4/Nhej1	2
GO:0043506	regulation of JUN kinase activity	2/97	54/14464	0.03703704	5.52271859	2.73587638	0.05085185	0.50874395	0.5048838	Sfrp5/Traf2	2
GO:0010332	response to gamma radiation	2/97	55/14464	0.03636364	5.42230553	2.69988448	0.05254586	0.50874395	0.5048838	Cdkn1a/Lig4	2
GO:0070085	glycosylation	4/97	210/14464	0.01904762	2.84025528	2.20724914	0.05273437	0.50874395	0.5048838	Mlec/Uggt1/Cln5/Fut11	4
GO:0006694	steroid biosynthetic process	3/97	126/14464	0.02380952	3.5503191	2.36247663	0.05273509	0.50874395	0.5048838	Lipa/Pmvk/Stard3	3
GO:0007041	lysosomal transport	3/97	127/14464	0.02362205	3.52236383	2.34591603	0.05375729	0.50874395	0.5048838	Pcsk9/Lipa/Becn1	3
GO:1902652	secondary alcohol metabolic process	3/97	127/14464	0.02362205	3.52236383	2.34591603	0.05375729	0.50874395	0.5048838	Pcsk9/Lipa/Pmvk	3
GO:0002886	regulation of myeloid leukocyte mediated immunity	2/97	56/14464	0.03571429	5.32547865	2.66476153	0.05425986	0.50874395	0.5048838	Dnase1l3/Sphk2	2
GO:0016126	sterol biosynthetic process	2/97	56/14464	0.03571429	5.32547865	2.66476153	0.05425986	0.50874395	0.5048838	Lipa/Pmvk	2
GO:0034198	cellular response to amino acid starvation	2/97	56/14464	0.03571429	5.32547865	2.66476153	0.05425986	0.50874395	0.5048838	Cdkn1a/Becn1	2
GO:1903409	reactive oxygen species biosynthetic process	2/97	56/14464	0.03571429	5.32547865	2.66476153	0.05425986	0.50874395	0.5048838	Lipa/Sphk2	2
GO:0072593	reactive oxygen species metabolic process	4/97	213/14464	0.01877934	2.80025168	2.17486534	0.05501184	0.50874395	0.5048838	Shc1/Cdkn1a/Lipa/Sphk2	4
GO:0032410	negative regulation of transporter activity	2/97	57/14464	0.03508772	5.2320492	2.63047011	0.05599352	0.50874395	0.5048838	Gpr35/Pcsk9	2
GO:0044088	regulation of vacuole organization	2/97	58/14464	0.03448276	5.14184145	2.59697504	0.0577465	0.50874395	0.5048838	Becn1/Pip4k2c	2
GO:0060760	positive regulation of response to cytokine stimulus	2/97	58/14464	0.03448276	5.14184145	2.59697504	0.0577465	0.50874395	0.5048838	Hspa1b/Traf2	2
GO:0009566	fertilization	3/97	131/14464	0.02290076	3.41481073	2.281299	0.05794115	0.50874395	0.5048838	Hspa1b/Antxr2/Cct7	3
GO:0009150	purine ribonucleotide metabolic process	5/97	311/14464	0.01607717	2.39732158	2.046842	0.05804645	0.50874395	0.5048838	Hspa1b/Lipa/Sphk2/Nudt3/Nme1	5
GO:0006278	RNA-templated DNA biosynthetic process	2/97	59/14464	0.03389831	5.0546916	2.56424322	0.05951848	0.50874395	0.5048838	Ptges3/Cct7	2
GO:0007004	telomere maintenance via telomerase	2/97	59/14464	0.03389831	5.0546916	2.56424322	0.05951848	0.50874395	0.5048838	Ptges3/Cct7	2
GO:0002440	production of molecular mediator of immune response	4/97	219/14464	0.01826484	2.72353246	2.11174907	0.05972495	0.50874395	0.5048838	Traf2/Sphk2/Lig4/Nhej1	4
GO:0016485	protein processing	4/97	220/14464	0.01818182	2.71115276	2.10143594	0.06053087	0.50874395	0.5048838	Pcsk9/Ctnnd1/Cln5/Aph1b	4
GO:0001959	regulation of cytokine-mediated signaling pathway	3/97	134/14464	0.02238806	3.33835975	2.23446031	0.06117764	0.50874395	0.5048838	Hspa1b/Mettl3/Traf2	3
GO:0016445	somatic diversification of immunoglobulins	2/97	60/14464	0.03333333	4.97044674	2.53224346	0.06130911	0.50874395	0.5048838	Lig4/Nhej1	2
GO:0046513	ceramide biosynthetic process	2/97	60/14464	0.03333333	4.97044674	2.53224346	0.06130911	0.50874395	0.5048838	Smpd4/Sphk2	2
GO:1904356	regulation of telomere maintenance via telomere lengthening	2/97	61/14464	0.03278689	4.888964	2.50094635	0.06311809	0.50874395	0.5048838	Ptges3/Cct7	2
GO:1990868	response to chemokine	2/97	61/14464	0.03278689	4.888964	2.50094635	0.06311809	0.50874395	0.5048838	Gpr35/Gpr75	2
GO:1990869	cellular response to chemokine	2/97	61/14464	0.03278689	4.888964	2.50094635	0.06311809	0.50874395	0.5048838	Gpr35/Gpr75	2
GO:1990928	response to amino acid starvation	2/97	61/14464	0.03278689	4.888964	2.50094635	0.06311809	0.50874395	0.5048838	Cdkn1a/Becn1	2
GO:0046488	phosphatidylinositol metabolic process	3/97	137/14464	0.02189781	3.26525698	2.18893137	0.06449729	0.50874395	0.5048838	Becn1/Pip4k2c/Pigc	3
GO:0008202	steroid metabolic process	4/97	225/14464	0.01777778	2.65090493	2.05071634	0.06464741	0.50874395	0.5048838	Pcsk9/Lipa/Pmvk/Stard3	4
GO:0000271	polysaccharide biosynthetic process	2/97	62/14464	0.03225806	4.81010974	2.47032413	0.06494509	0.50874395	0.5048838	Ptges3/Nhlrc1	2
GO:0002562	somatic diversification of immune receptors via germline recombination within a single locus	2/97	62/14464	0.03225806	4.81010974	2.47032413	0.06494509	0.50874395	0.5048838	Lig4/Nhej1	2
GO:0006487	protein N-linked glycosylation	2/97	62/14464	0.03225806	4.81010974	2.47032413	0.06494509	0.50874395	0.5048838	Mlec/Uggt1	2
GO:0016444	somatic cell DNA recombination	2/97	62/14464	0.03225806	4.81010974	2.47032413	0.06494509	0.50874395	0.5048838	Lig4/Nhej1	2
GO:0030199	collagen fibril organization	2/97	62/14464	0.03225806	4.81010974	2.47032413	0.06494509	0.50874395	0.5048838	P4ha1/Antxr2	2
GO:0006517	protein deglycosylation	1/97	10/14464	0.1	14.9113402	3.61582132	0.06509462	0.50874395	0.5048838	Edem1	1
GO:0007220	Notch receptor processing	1/97	10/14464	0.1	14.9113402	3.61582132	0.06509462	0.50874395	0.5048838	Aph1b	1
GO:0007602	phototransduction	1/97	10/14464	0.1	14.9113402	3.61582132	0.06509462	0.50874395	0.5048838	Pde6d	1
GO:0009221	pyrimidine deoxyribonucleotide biosynthetic process	1/97	10/14464	0.1	14.9113402	3.61582132	0.06509462	0.50874395	0.5048838	Nme1	1
GO:0010881	regulation of cardiac muscle contraction by regulation of the release of sequestered calcium ion	1/97	10/14464	0.1	14.9113402	3.61582132	0.06509462	0.50874395	0.5048838	Fkbp1b	1
GO:0019511	peptidyl-proline hydroxylation	1/97	10/14464	0.1	14.9113402	3.61582132	0.06509462	0.50874395	0.5048838	P4ha1	1
GO:0031424	keratinization	1/97	10/14464	0.1	14.9113402	3.61582132	0.06509462	0.50874395	0.5048838	Sfn	1
GO:0033189	response to vitamin A	1/97	10/14464	0.1	14.9113402	3.61582132	0.06509462	0.50874395	0.5048838	Lipa	1
GO:0035871	protein K11-linked deubiquitination	1/97	10/14464	0.1	14.9113402	3.61582132	0.06509462	0.50874395	0.5048838	Otub2	1
GO:0046051	UTP metabolic process	1/97	10/14464	0.1	14.9113402	3.61582132	0.06509462	0.50874395	0.5048838	Nme1	1
GO:0048853	forebrain morphogenesis	1/97	10/14464	0.1	14.9113402	3.61582132	0.06509462	0.50874395	0.5048838	Tuba1a	1
GO:0051775	response to redox state	1/97	10/14464	0.1	14.9113402	3.61582132	0.06509462	0.50874395	0.5048838	Fkbp1b	1
GO:0070424	regulation of nucleotide-binding domain, leucine rich repeat containing receptor signaling pathway	1/97	10/14464	0.1	14.9113402	3.61582132	0.06509462	0.50874395	0.5048838	Hspa1b	1
GO:0070863	positive regulation of protein exit from endoplasmic reticulum	1/97	10/14464	0.1	14.9113402	3.61582132	0.06509462	0.50874395	0.5048838	Edem1	1
GO:0070942	neutrophil mediated cytotoxicity	1/97	10/14464	0.1	14.9113402	3.61582132	0.06509462	0.50874395	0.5048838	Dnase1l3	1
GO:0072102	glomerulus morphogenesis	1/97	10/14464	0.1	14.9113402	3.61582132	0.06509462	0.50874395	0.5048838	Ctnnd1	1
GO:0090649	response to oxygen-glucose deprivation	1/97	10/14464	0.1	14.9113402	3.61582132	0.06509462	0.50874395	0.5048838	Becn1	1
GO:0097400	interleukin-17-mediated signaling pathway	1/97	10/14464	0.1	14.9113402	3.61582132	0.06509462	0.50874395	0.5048838	Traf2	1
GO:0098908	regulation of neuronal action potential	1/97	10/14464	0.1	14.9113402	3.61582132	0.06509462	0.50874395	0.5048838	Gpr35	1
GO:1902423	regulation of attachment of mitotic spindle microtubules to kinetochore	1/97	10/14464	0.1	14.9113402	3.61582132	0.06509462	0.50874395	0.5048838	Becn1	1
GO:1903278	positive regulation of sodium ion export across plasma membrane	1/97	10/14464	0.1	14.9113402	3.61582132	0.06509462	0.50874395	0.5048838	Atp1b3	1
GO:1904627	response to phorbol 13-acetate 12-myristate	1/97	10/14464	0.1	14.9113402	3.61582132	0.06509462	0.50874395	0.5048838	Sphk2	1
GO:1904628	cellular response to phorbol 13-acetate 12-myristate	1/97	10/14464	0.1	14.9113402	3.61582132	0.06509462	0.50874395	0.5048838	Sphk2	1
GO:1904851	positive regulation of establishment of protein localization to telomere	1/97	10/14464	0.1	14.9113402	3.61582132	0.06509462	0.50874395	0.5048838	Cct7	1
GO:2000650	negative regulation of sodium ion transmembrane transporter activity	1/97	10/14464	0.1	14.9113402	3.61582132	0.06509462	0.50874395	0.5048838	Pcsk9	1
GO:0006402	mRNA catabolic process	4/97	226/14464	0.01769912	2.63917526	2.04073743	0.06548802	0.50874395	0.5048838	Hspa1b/Mettl3/Traf2/Hbs1l	4
GO:0048144	fibroblast proliferation	3/97	138/14464	0.02173913	3.2415957	2.17403418	0.06562211	0.50874395	0.5048838	Cdkn1a/Ptges3/Lig4	3
GO:0034765	regulation of monoatomic ion transmembrane transport	5/97	323/14464	0.01547988	2.308257	1.95382778	0.06605639	0.50874395	0.5048838	Gpr35/Fkbp1b/Pcsk9/Atp1b3/Sphk2	5
GO:0045859	regulation of protein kinase activity	6/97	426/14464	0.01408451	2.10018876	1.89387538	0.06660823	0.50874395	0.5048838	Cdkn1a/Sfrp5/Sfn/Ankrd54/Traf2/Nhlrc1	6
GO:0007584	response to nutrient	3/97	139/14464	0.02158273	3.21827486	2.15927252	0.06675597	0.50874395	0.5048838	Fkbp1b/Lipa/Becn1	3

GO:0051453 regulation of intracellular pH	2/97	63/14464	0.03174603	4.7337588	2.44035059	0.06678979	0.50874395	0.5048838	Rab39/Cln5	2
GO:1901019 regulation of calcium ion transmembrane transporter activity	2/97	63/14464	0.03174603	4.7337588	2.44035059	0.06678979	0.50874395	0.5048838	Gpr35/Fkbp1b	2
GO:0031331 positive regulation of cellular catabolic process	4/97	230/14464	0.0173913	2.59327656	2.00135264	0.06890781	0.50874395	0.5048838	Pcsk9/Becn1/Pafah1b2/Pip4k2c	4
GO:2000377 regulation of reactive oxygen species metabolic process	3/97	141/14464	0.0212766	3.17262558	2.13014645	0.06905067	0.50874395	0.5048838	Shc1/Cdkn1a/Sphk2	3
GO:0010212 response to ionizing radiation	3/97	142/14464	0.02112676	3.15028314	2.11577754	0.0702114	0.50874395	0.5048838	Cdkn1a/Lig4/Nhej1	3
GO:0006303 double-strand break repair via nonhomologous end joining	2/97	65/14464	0.03076923	4.58810468	2.38225162	0.07053103	0.50874395	0.5048838	Lig4/Nhej1	2
GO:0051926 negative regulation of calcium ion transport	2/97	65/14464	0.03076923	4.58810468	2.38225162	0.07053103	0.50874395	0.5048838	Gpr35/Fkbp1b	2
GO:0140115 export across plasma membrane	2/97	65/14464	0.03076923	4.58810468	2.38225162	0.07053103	0.50874395	0.5048838	Abcc5/Atp1b3	2
GO:0003334 keratinocyte development	1/97	11/14464	0.09090909	13.5557638	3.42288646	0.07136872	0.50874395	0.5048838	Sfn	1
GO:0006183 GTP biosynthetic process	1/97	11/14464	0.09090909	13.5557638	3.42288646	0.07136872	0.50874395	0.5048838	Nme1	1
GO:0006241 CTP biosynthetic process	1/97	11/14464	0.09090909	13.5557638	3.42288646	0.07136872	0.50874395	0.5048838	Nme1	1
GO:0006361 transcription initiation at RNA polymerase I promoter	1/97	11/14464	0.09090909	13.5557638	3.42288646	0.07136872	0.50874395	0.5048838	Ubtf	1
GO:0006977 DNA damage response, signal transduction by p53 class mediator resulting in cell cycle arrest	1/97	11/14464	0.09090909	13.5557638	3.42288646	0.07136872	0.50874395	0.5048838	Cdkn1a	1
GO:0009157 deoxyribonucleoside monophosphate biosynthetic process	1/97	11/14464	0.09090909	13.5557638	3.42288646	0.07136872	0.50874395	0.5048838	Nme1	1
GO:0010968 regulation of microtubule nucleation	1/97	11/14464	0.09090909	13.5557638	3.42288646	0.07136872	0.50874395	0.5048838	Hspa1b	1
GO:0010988 regulation of low-density lipoprotein particle clearance	1/97	11/14464	0.09090909	13.5557638	3.42288646	0.07136872	0.50874395	0.5048838	Pcsk9	1
GO:0030007 intracellular potassium ion homeostasis	1/97	11/14464	0.09090909	13.5557638	3.42288646	0.07136872	0.50874395	0.5048838	Atp1b3	1
GO:0031293 membrane protein intracellular domain proteolysis	1/97	11/14464	0.09090909	13.5557638	3.42288646	0.07136872	0.50874395	0.5048838	Aph1b	1
GO:0034351 negative regulation of glial cell apoptotic process	1/97	11/14464	0.09090909	13.5557638	3.42288646	0.07136872	0.50874395	0.5048838	Traf2	1
GO:0034635 glutathione transport	1/97	11/14464	0.09090909	13.5557638	3.42288646	0.07136872	0.50874395	0.5048838	Abcc5	1
GO:0036065 fucosylation	1/97	11/14464	0.09090909	13.5557638	3.42288646	0.07136872	0.50874395	0.5048838	Fut11	1
GO:0043508 negative regulation of JUN kinase activity	1/97	11/14464	0.09090909	13.5557638	3.42288646	0.07136872	0.50874395	0.5048838	Sfrp5	1
GO:0043652 engulfment of apoptotic cell	1/97	11/14464	0.09090909	13.5557638	3.42288646	0.07136872	0.50874395	0.5048838	Becn1	1
GO:0051987 positive regulation of attachment of spindle microtubules to kinetochore	1/97	11/14464	0.09090909	13.5557638	3.42288646	0.07136872	0.50874395	0.5048838	Becn1	1
GO:0060837 blood vessel endothelial cell differentiation	1/97	11/14464	0.09090909	13.5557638	3.42288646	0.07136872	0.50874395	0.5048838	Lipa	1
GO:0062028 regulation of stress granule assembly	1/97	11/14464	0.09090909	13.5557638	3.42288646	0.07136872	0.50874395	0.5048838	Becn1	1
GO:0071493 cellular response to UV-B	1/97	11/14464	0.09090909	13.5557638	3.42288646	0.07136872	0.50874395	0.5048838	Cdkn1a	1
GO:1900121 negative regulation of receptor binding	1/97	11/14464	0.09090909	13.5557638	3.42288646	0.07136872	0.50874395	0.5048838	Pcsk9	1
GO:1901678 iron coordination entity transport	1/97	11/14464	0.09090909	13.5557638	3.42288646	0.07136872	0.50874395	0.5048838	Abcc5	1
GO:1902306 negative regulation of sodium ion transmembrane transport	1/97	11/14464	0.09090909	13.5557638	3.42288646	0.07136872	0.50874395	0.5048838	Pcsk9	1
GO:1903276 regulation of sodium ion export across plasma membrane	1/97	11/14464	0.09090909	13.5557638	3.42288646	0.07136872	0.50874395	0.5048838	Atp1b3	1
GO:0002720 positive regulation of cytokine production involved in immune response	2/97	66/14464	0.03030303	4.51858794	2.35408049	0.07242697	0.50874395	0.5048838	Traf2/Sphk2	2
GO:2000573 positive regulation of DNA biosynthetic process	2/97	66/14464	0.03030303	4.51858794	2.35408049	0.07242697	0.50874395	0.5048838	Ptges3/Cct7	2
GO:0009259 ribonucleotide metabolic process	5/97	332/14464	0.01506024	2.24568377	1.88671823	0.07245211	0.50874395	0.5048838	Hspa1b/Lipa/Sphk2/Nudt3/Nme1	5
GO:0016241 regulation of macroautophagy	3/97	144/14464	0.02083333	3.10652921	2.0874171	0.07255937	0.50874395	0.5048838	Becn1/Pafah1b2/Pip4k2c	3
GO:0071900 regulation of protein serine/threonine kinase activity	4/97	235/14464	0.01702128	2.53810046	1.9532753	0.07331059	0.50874395	0.5048838	Cdkn1a/Sfrp5/Sfn/Traf2	4
GO:0060759 regulation of response to cytokine stimulus	3/97	145/14464	0.02068966	3.08510487	2.07342139	0.07374651	0.50874395	0.5048838	Hspa1b/Mettl3/Traf2	3
GO:0033044 regulation of chromosome organization	4/97	236/14464	0.01694915	2.5273458	1.94380843	0.07420809	0.50874395	0.5048838	Ptges3/Lig4/Becn1/Cct7	4
GO:0006766 vitamin metabolic process	2/97	67/14464	0.02985075	4.45114633	2.32646641	0.07433937	0.50874395	0.5048838	Lipa/Mmaa	2
GO:0043269 regulation of monoatomic ion transport	6/97	440/14464	0.01363636	2.03336457	1.80873928	0.07527238	0.50874395	0.5048838	Gpr35/Trpv2/Fkbp1b/Pcsk9/Atp1b3/Sphk2	6
GO:0009152 purine ribonucleotide biosynthetic process	3/97	147/14464	0.02040816	3.04313065	2.04578886	0.07614685	0.50874395	0.5048838	Lipa/Sphk2/Nme1	3
GO:0006720 isoprenoid metabolic process	2/97	68/14464	0.02941176	4.3856883	2.29938939	0.07626793	0.50874395	0.5048838	Lipa/Pmvk	2
GO:0035019 somatic stem cell population maintenance	2/97	68/14464	0.02941176	4.3856883	2.29938939	0.07626793	0.50874395	0.5048838	Lig4/Smc1a	2
GO:0048146 positive regulation of fibroblast proliferation	2/97	68/14464	0.02941176	4.3856883	2.29938939	0.07626793	0.50874395	0.5048838	Cdkn1a/Lig4	2
GO:0000012 single strand break repair	1/97	12/14464	0.08333333	12.4261168	3.25354877	0.07760114	0.50874395	0.5048838	Lig4	1
GO:0002679 respiratory burst involved in defense response	1/97	12/14464	0.08333333	12.4261168	3.25354877	0.07760114	0.50874395	0.5048838	Lipa	1
GO:0009209 pyrimidine ribonucleoside triphosphate biosynthetic process	1/97	12/14464	0.08333333	12.4261168	3.25354877	0.07760114	0.50874395	0.5048838	Nme1	1
GO:0019227 neuronal action potential propagation	1/97	12/14464	0.08333333	12.4261168	3.25354877	0.07760114	0.50874395	0.5048838	Fkbp1b	1
GO:0032736 positive regulation of interleukin-13 production	1/97	12/14464	0.08333333	12.4261168	3.25354877	0.07760114	0.50874395	0.5048838	Sphk2	1
GO:0042939 tripeptide transport	1/97	12/14464	0.08333333	12.4261168	3.25354877	0.07760114	0.50874395	0.5048838	Abcc5	1
GO:0046036 CTP metabolic process	1/97	12/14464	0.08333333	12.4261168	3.25354877	0.07760114	0.50874395	0.5048838	Nme1	1
GO:0046073 dTMP metabolic process	1/97	12/14464	0.08333333	12.4261168	3.25354877	0.07760114	0.50874395	0.5048838	Nme1	1
GO:0046512 sphingosine biosynthetic process	1/97	12/14464	0.08333333	12.4261168	3.25354877	0.07760114	0.50874395	0.5048838	Sphk2	1
GO:0046520 sphingoid biosynthetic process	1/97	12/14464	0.08333333	12.4261168	3.25354877	0.07760114	0.50874395	0.5048838	Sphk2	1
GO:0070203 regulation of establishment of protein localization to telomere	1/97	12/14464	0.08333333	12.4261168	3.25354877	0.07760114	0.50874395	0.5048838	Cct7	1
GO:0071233 cellular response to L-leucine	1/97	12/14464	0.08333333	12.4261168	3.25354877	0.07760114	0.50874395	0.5048838	Stambpl1	1
GO:0090385 phagosome-lysosome fusion	1/97	12/14464	0.08333333	12.4261168	3.25354877	0.07760114	0.50874395	0.5048838	Rab39	1
GO:1904152 regulation of retrograde protein transport, ER to cytosol	1/97	12/14464	0.08333333	12.4261168	3.25354877	0.07760114	0.50874395	0.5048838	Edem1	1
GO:1904424 regulation of GTP binding	1/97	12/14464	0.08333333	12.4261168	3.25354877	0.07760114	0.50874395	0.5048838	Cln5	1
GO:2000303 regulation of ceramide biosynthetic process	1/97	12/14464	0.08333333	12.4261168	3.25354877	0.07760114	0.50874395	0.5048838	Sphk2	1
GO:2000644 regulation of receptor catabolic process	1/97	12/14464	0.08333333	12.4261168	3.25354877	0.07760114	0.50874395	0.5048838	Pcsk9	1
GO:0019693 ribose phosphate metabolic process	5/97	339/14464	0.01474926	2.19931271	1.83598899	0.07765489	0.50874395	0.5048838	Hspa1b/Lipa/Sphk2/Nudt3/Nme1	5
GO:0002200 somatic diversification of immune receptors	2/97	69/14464	0.02898551	4.3221276	2.27283042	0.07821236	0.50874395	0.5048838	Lig4/Nhej1	2
GO:0030641 regulation of cellular pH	2/97	69/14464	0.02898551	4.3221276	2.27283042	0.07821236	0.50874395	0.5048838	Rab39/Cln5	2
GO:0009205 purine ribonucleoside triphosphate metabolic process	4/97	243/14464	0.01646091	2.4545416	1.87886782	0.08064691	0.50874395	0.5048838	Hspa1b/Lipa/Sphk2/Nme1	4
GO:1902850 microtubule cytoskeleton organization involved in mitosis	3/97	151/14464	0.01986755	2.96251792	1.99190574	0.08105025	0.50874395	0.5048838	Hspa1b/Efhc1/Smc1a	3
GO:0051302 regulation of cell division	3/97	152/14464	0.01973684	2.94302767	1.97871219	0.08229719	0.50874395	0.5048838	Sfn/Efhc1/Becn1	3
GO:0046474 glycerophospholipid biosynthetic process	3/97	153/14464	0.01960784	2.9237922	1.96562604	0.08355245	0.50874395	0.5048838	Becn1/Pip4k2c/Pigc	3
GO:0002328 pro-B cell differentiation	1/97	13/14464	0.07692308	11.4702617	3.10321794	0.08379217	0.50874395	0.5048838	Lig4	1
GO:0003376 sphingosine-1-phosphate receptor signaling pathway	1/97	13/14464	0.07692308	11.4702617	3.10321794	0.08379217	0.50874395	0.5048838	Sphk2	1
GO:0010459 negative regulation of heart rate	1/97	13/14464	0.07692308	11.4702617	3.10321794	0.08379217	0.50874395	0.5048838	Fkbp1b	1
GO:0023035 CD40 signaling pathway	1/97	13/14464	0.07692308	11.4702617	3.10321794	0.08379217	0.50874395	0.5048838	Traf2	1
GO:0038133 ERBB2-ERBB3 signaling pathway	1/97	13/14464	0.07692308	11.4702617	3.10321794	0.08379217	0.50874395	0.5048838	Nrg2	1
GO:0051280 negative regulation of release of sequestered calcium ion into cytosol	1/97	13/14464	0.07692308	11.4702617	3.10321794	0.08379217	0.50874395	0.5048838	Fkbp1b	1
GO:0070202 regulation of establishment of protein localization to chromosome	1/97	13/14464	0.07692308	11.4702617	3.10321794	0.08379217	0.50874395	0.5048838	Cct7	1
GO:0070341 fat cell proliferation	1/97	13/14464	0.07692308	11.4702617	3.10321794	0.08379217	0.50874395	0.5048838	Lipa	1
GO:0097384 cellular lipid biosynthetic process	1/97	13/14464	0.07692308	11.4702617	3.10321794	0.08379217	0.50874395	0.5048838	Lipa	1
GO:0098870 action potential propagation	1/97	13/14464	0.07692308	11.4702617	3.10321794	0.08379217	0.50874395	0.5048838	Fkbp1b	1
GO:1901386 negative regulation of voltage-gated calcium channel activity	1/97	13/14464	0.07692308	11.4702617	3.10321794	0.08379217	0.50874395	0.5048838	Gpr35	1
GO:1902902 negative regulation of autophagosome assembly	1/97	13/14464	0.07692308	11.4702617	3.10321794	0.08379217	0.50874395	0.5048838	Becn1	1
GO:1904816 positive regulation of protein localization to chromosome, telomeric region	1/97	13/14464	0.07692308	11.4702617	3.10321794	0.08379217	0.50874395	0.5048838	Cct7	1
GO:1905671 regulation of lysosome organization	1/97	13/14464	0.07692308	11.4702617	3.10321794	0.08379217	0.50874395	0.5048838	Becn1	1
GO:0010833 telomere maintenance via telomere lengthening	2/97	72/14464	0.02777778	4.14203895	2.19608553	0.08413794	0.50874395	0.5048838	Ptges3/Cct7	2
GO:0030968 endoplasmic reticulum unfolded protein response	2/97	72/14464	0.02777778	4.14203895	2.19608553	0.08413794	0.50874395	0.5048838	Pdia6/Edem1	2
GO:0032091 negative regulation of protein binding	2/97	72/14464	0.02777778	4.14203895	2.19608553	0.08413794	0.50874395	0.5048838	Cdkn1a/Pcsk9	2
GO:0090307 mitotic spindle assembly	2/97	72/14464	0.02777778	4.14203895	2.19608553	0.08413794	0.50874395	0.5048838	Hspa1b/Smc1a	2
GO:0006650 glycerophospholipid metabolic process	4/97	247/14464	0.01619433	2.41479194	1.84276131	0.08444769	0.50874395	0.5048838	Smpd4/Becn1/Pip4k2c/Pigc	4
GO:0009199 ribonucleoside triphosphate metabolic process	4/97	248/14464	0.01612903	2.40505487	1.83384419	0.08541152	0.50874395	0.5048838	Hspa1b/Lipa/Sphk2/Nme1	4
GO:0043549 regulation of kinase activity	6/97	456/14464	0.01315789	1.96201845	1.71518026	0.08593023	0.50874395	0.5048838	Cdkn1a/Sfrp5/Sfn/Ankrd54/Traf2/Nhlrc1	6
GO:0032206 positive regulation of telomere maintenance	2/97	73/14464	0.02739726	4.08529869	2.17142669	0.08614293	0.50874395	0.5048838	Ptges3/Cct7	2
GO:0007254 JNK cascade	3/97	156/14464	0.01923077	2.86756542	1.92699538	0.08736766	0.50874395	0.5048838	Sfrp5/Traf2/Becn1	3
GO:0006690 icosanoid metabolic process	2/97	74/14464	0.02702703	4.03009195	2.14720389	0.08816235	0.50874395	0.5048838	Cyp2j13/Ptges3	2
GO:0071479 cellular response to ionizing radiation	2/97	74/14464	0.02702703	4.03009195	2.14720389	0.08816235	0.50874395	0.5048838	Cdkn1a/Lig4	2
GO:0009144 purine nucleoside triphosphate metabolic process	4/97	251/14464	0.01593625	2.3763092	1.80734894	0.08833546	0.50874395	0.5048838	Hspa1b/Lipa/Sphk2/Nme1	4
GO:0071478 cellular response to radiation	3/97	157/14464	0.01910828	2.84930068	1.9143224	0.08865568	0.50874395	0.5048838	Cdkn1a/Mettl3/Lig4	3
GO:0006700 C21-steroid hormone biosynthetic process	1/97	14/14464	0.07142857	10.6509573	2.96846889	0.08994206	0.50874395	0.5048838	Stard3	1
GO:0009208 pyrimidine ribonucleoside triphosphate metabolic process	1/97	14/14464	0.07142857	10.6509573	2.96846889	0.08994206	0.50874395	0.5048838	Nme1	1
GO:0023019 signal transduction involved in regulation of gene expression	1/97	14/14464	0.07142857	10.6509573	2.96846889	0.08994206	0.50874395	0.5048838	Traf2	1
GO:0030213 hyaluronan biosynthetic process	1/97	14/14464	0.07142857	10.6509573	2.96846889	0.08994206	0.50874395	0.5048838	Abcc5	1
GO:0036498 IRE1-mediated unfolded protein response	1/97	14/14464	0.07142857	10.6509573	2.96846889	0.08994206	0.50874395	0.5048838	Pdia6	1
GO:0038129 ERBB3 signaling pathway	1/97	14/14464	0.07142857	10.6509573	2.96846889	0.08994206	0.50874395	0.5048838	Nrg2	1
GO:0043201 response to L-leucine	1/97	14/14464	0.07142857	10.6509573	2.96846889	0.08994206	0.50874395	0.5048838	Stambpl1	1
GO:0048680 positive regulation of axon regeneration	1/97	14/14464	0.07142857	10.6509573	2.96846889	0.08994206	0.50874395	0.5048838	Fkbp1b	1
GO:0071280 cellular response to copper ion	1/97	14/14464	0.07142857	10.6509573	2.96846889	0.08994206	0.50874395	0.5048838	Becn1	1
GO:0071285 cellular response to lithium ion	1/97	14/14464	0.07142857	10.6509573	2.96846889	0.08994206	0.50874395	0.5048838	Lig4	1
GO:0072393 microtubule anchoring at microtubule organizing center	1/97	14/14464	0.07142857	10.6509573	2.96846889	0.08994206	0.50874395	0.5048838	Bicd2	1
GO:0090153 regulation of sphingolipid biosynthetic process	1/97	14/14464	0.07142857	10.6509573	2.96846889	0.08994206	0.50874395	0.5048838	Sphk2	1
GO:1901018 positive regulation of potassium ion transmembrane transporter activity	1/97	14/14464	0.07142857	10.6509573	2.96846889	0.08994206	0.50874395	0.5048838	Atp1b3	1
GO:0002526 acute inflammatory response	2/97	75/14464	0.02666667	3.97635739	2.12340296	0.09019592	0.50874395	0.5048838	Dnase1l3/Lipa	2
GO:0003341 cilium movement	3/97	159/14464	0.01886792	2.81346042	1.88927303	0.0912558	0.50874395	0.5048838	Efhc1/Tuba1a/Dnai4	3
GO:0042110 T cell activation	6/97	464/14464	0.01293103	1.92819054	1.66979873	0.0915589	0.50874395	0.5048838	Fkbp1b/Lipa/Mettl3/Traf2/Lig4/Nhej1	6
GO:0009266 response to temperature stimulus	3/97	161/14464	0.01863354	2.7785106	1.86460928	0.09388766	0.50874395	0.5048838	Hspa1b/Trpv2/Lipa	3
GO:0033559 unsaturated fatty acid metabolic process	2/97	77/14464	0.02597403	3.87307538	2.07701329	0.09430441	0.50874395	0.5048838	Cyp2j13/Ptges3	2
GO:0061178 regulation of insulin secretion involved in cellular response to glucose stimulus	2/97	77/14464	0.02597403	3.87307538	2.07701329	0.09430441	0.50874395	0.5048838	Myt1/Fkbp1b	2
GO:0048872 homeostasis of number of cells	5/97	360/14464	0.01388889	2.07101947	1.69086713	0.09444245	0.50874395	0.5048838	Hspa1b/Lipa/Ankrd54/Tuba1a/Caml	5
GO:0007034 vacuolar transport	3/97	162/14464	0.01851852	2.7613593	1.85241842	0.09521535	0.50874395	0.5048838	Pcsk9/Lipa/Becn1	3
GO:0007219 Notch signaling pathway	3/97	162/14464	0.01851852	2.7613593	1.85241842	0.09521535	0.50874395	0.5048838	Tspan14/Mettl3/Aph1b	3
GO:0009260 ribonucleotide biosynthetic process	3/97	162/14464	0.01851852	2.7613593	1.85241842	0.09521535	0.50874395	0.5048838	Lipa/Sphk2/Nme1	3
GO:0006465 signal peptide processing	1/97	15/14464	0.06666667	9.94089347	2.84668668	0.0960511	0.50874395	0.5048838	Cln5	1
GO:0007042 lysosomal lumen acidification	1/97	15/14464	0.06666667	9.94089347	2.84668668	0.0960511	0.50874395	0.5048838	Cln5	1
GO:0009176 pyrimidine deoxyribonucleoside monophosphate metabolic process	1/97	15/14464	0.06666667	9.94089347	2.84668668	0.0960511	0.50874395	0.5048838	Nme1	1
GO:0010635 regulation of mitochondrial fusion	1/97	15/14464	0.06666667	9.94089347	2.84668668	0.0960511	0.50874395	0.5048838	Mief1	1
GO:0010882 regulation of cardiac muscle contraction by calcium ion signaling	1/97	15/14464	0.06666667	9.94089347	2.84668668	0.0960511	0.50874395	0.5048838	Fkbp1b	1
GO:0010984 regulation of lipoprotein particle clearance	1/97	15/14464	0.06666667	9.94089347	2.84668668	0.0960511	0.50874395	0.5048838	Pcsk9	1
GO:0042159 lipoprotein catabolic process	1/97	15/14464	0.06666667	9.94089347	2.84668668	0.0960511	0.50874395	0.5048838	Lipa	1
GO:0051284 positive regulation of sequestering of calcium ion	1/97	15/14464	0.06666667	9.94089347	2.84668668	0.0960511	0.50874395	0.5048838	Fkbp1b	1
GO:0060026 convergent extension	1/97	15/14464	0.06666667	9.94089347	2.84668668	0.0960511	0.50874395	0.5048838	Sfrp5	1
GO:0070572 positive regulation of neuron projection regeneration	1/97	15/14464	0.06666667	9.94089347	2.84668668	0.0960511	0.50874395	0.5048838	Fkbp1b	1
GO:0090136 epithelial cell-cell adhesion	1/97	15/14464	0.06666667	9.94089347	2.84668668	0.0960511	0.50874395	0.5048838	Serpinb8	1
GO:0090235 regulation of metaphase plate congression	1/97	15/14464	0.06666667	9.94089347	2.84668668	0.0960511	0.50874395	0.5048838	Becn1	1
GO:0090520 sphingolipid mediated signaling pathway	1/97	15/14464	0.06666667	9.94089347	2.84668668	0.0960511	0.50874395	0.5048838	Sphk2	1
GO:1900102 negative regulation of endoplasmic reticulum unfolded protein response	1/97	15/14464	0.06666667	9.94089347	2.84668668	0.0960511	0.50874395	0.5048838	Pdia6	1
GO:1902036 regulation of hematopoietic stem cell differentiation	1/97	15/14464	0.06666667	9.94089347	2.84668668	0.0960511	0.50874395	0.5048838	Mettl3	1
GO:0010508 positive regulation of autophagy	3/97	163/14464	0.01840491	2.74441844	1.84031973	0.0965508	0.50874395	0.5048838	Becn1/Pafah1b2/Pip4k2c	3
GO:0002028 regulation of sodium ion transport	2/97	79/14464	0.02531646	3.77502284	2.03215668	0.09846622	0.50874395	0.5048838	Pcsk9/Atp1b3	2
GO:0030216 keratinocyte differentiation	2/97	79/14464	0.02531646	3.77502284	2.03215668	0.09846622	0.50874395	0.5048838	Cdkn1a/Sfn	2
GO:0031397 negative regulation of protein ubiquitination	2/97	79/14464	0.02531646	3.77502284	2.03215668	0.09846622	0.50874395	0.5048838	Hspa1b/Caml	2
GO:0016049 cell growth	6/97	476/14464	0.01260504	1.8795807	1.60336858	0.10037177	0.50874395	0.5048838	Hspa1b/Cdkn1a/Sfn/Trpv2/Sphk2/Raph1	6
GO:0030217 T cell differentiation	4/97	263/14464	0.01520913	2.26788444	1.70502243	0.10050887	0.50874395	0.5048838	Lipa/Mettl3/Lig4/Nhej1	4
GO:0006885 regulation of pH	2/97	80/14464	0.025	3.72783505	2.01027409	0.10056645	0.50874395	0.5048838	Rab39/Cln5	2
GO:0006457 protein folding	3/97	166/14464	0.01807229	2.69482052	1.80456332	0.10060312	0.50874395	0.5048838	Hspa1b/Ptges3/Cct7	3
GO:0071248 cellular response to metal ion	3/97	167/14464	0.01796407	2.67868387	1.79282008	0.10196902	0.50874395	0.5048838	Tuba1a/Lig4/Becn1	3
GO:0006309 apoptotic DNA fragmentation	1/97	16/14464	0.0625	9.31958763	2.73583524	0.10211955	0.50874395	0.5048838	Dnase1l3	1
GO:0006883 intracellular sodium ion homeostasis	1/97	16/14464	0.0625	9.31958763	2.73583524	0.10211955	0.50874395	0.5048838	Atp1b3	1
GO:0006978 DNA damage response, signal transduction by p53 class mediator resulting in transcription of p21 class r	1/97	16/14464	0.0625	9.31958763	2.73583524	0.10211955	0.50874395	0.5048838	Cdkn1a	1
GO:0009148 pyrimidine nucleoside triphosphate biosynthetic process	1/97	16/14464	0.0625	9.31958763	2.73583524	0.10211955	0.50874395	0.5048838	Nme1	1
GO:0010766 negative regulation of sodium ion transport	1/97	16/14464	0.0625	9.31958763	2.73583524	0.10211955	0.50874395	0.5048838	Pcsk9	1
GO:0031053 primary miRNA processing	1/97	16/14464	0.0625	9.31958763	2.73583524	0.10211955	0.50874395	0.5048838	Mettl3	1
GO:0034350 regulation of glial cell apoptotic process	1/97	16/14464	0.0625	9.31958763	2.73583524	0.10211955	0.50874395	0.5048838	Traf2	1
GO:0035461 vitamin transmembrane transport	1/97	16/14464	0.0625	9.31958763	2.73583524	0.10211955	0.50874395	0.5048838	Abcc5	1
GO:0035672 oligopeptide transmembrane transport	1/97	16/14464	0.0625	9.31958763	2.73583524	0.10211955	0.50874395	0.5048838	Abcc5	1
GO:0042921 nuclear receptor-mediated glucocorticoid signaling pathway	1/97	16/14464	0.0625	9.31958763	2.73583524	0.10211955	0.50874395	0.5048838	Ptges3	1
GO:0050862 positive regulation of T cell receptor signaling pathway	1/97	16/14464	0.0625	9.31958763	2.73583524	0.10211955	0.50874395	0.5048838	Lipa	1
GO:0060134 prepulse inhibition	1/97	16/14464	0.0625	9.31958763	2.73583524	0.10211955	0.50874395	0.5048838	Aph1b	1
GO:1902065 response to L-glutamate	1/97	16/14464	0.0625	9.31958763	2.73583524	0.10211955	0.50874395	0.5048838	Tuba1a	1
GO:1904814 regulation of protein localization to chromosome, telomeric region	1/97	16/14464	0.0625	9.31958763	2.73583524	0.10211955	0.50874395	0.5048838	Cct7	1
GO:0009141 nucleoside triphosphate metabolic process	4/97	265/14464	0.01509434	2.25076833	1.68850732	0.1026105	0.50874395	0.5048838	Hspa1b/Lipa/Sphk2/Nme1	4
GO:0007601 visual perception	2/97	81/14464	0.02469136	3.6818124	1.98874077	0.10267923	0.50874395	0.5048838	Cln5/Pde6d	2
GO:0045824 negative regulation of innate immune response	2/97	81/14464	0.02469136	3.6818124	1.98874077	0.10267923	0.50874395	0.5048838	Sfn/Mettl3	2
GO:0072091 regulation of stem cell proliferation	2/97	81/14464	0.02469136	3.6818124	1.98874077	0.10267923	0.50874395	0.5048838	Sfn/Taf4b	2
GO:0000070 mitotic sister chromatid segregation	3/97	168/14464	0.01785714	2.66273932	1.78116254	0.1033424	0.50874395	0.5048838	Hspa1b/Becn1/Smc1a	3
GO:0046390 ribose phosphate biosynthetic process	3/97	168/14464	0.01785714	2.66273932	1.78116254	0.1033424	0.50874395	0.5048838	Lipa/Sphk2/Nme1	3
GO:0006401 RNA catabolic process	4/97	268/14464	0.01492537	2.22557317	1.66400896	0.105801	0.50874395	0.5048838	Hspa1b/Mettl3/Traf2/Hbs1l	4
GO:0050953 sensory perception of light stimulus	2/97	83/14464	0.02409639	3.59309403	1.94668096	0.10694136	0.50874395	0.5048838	Cln5/Pde6d	2
GO:0010959 regulation of metal ion transport	5/97	375/14464	0.01333333	1.98817869	1.59310004	0.107488	0.50874395	0.5048838	Gpr35/Trpv2/Fkbp1b/Pcsk9/Atp1b3	5
GO:0030308 negative regulation of cell growth	3/97	171/14464	0.01754386	2.6160246	1.74669203	0.10750672	0.50874395	0.5048838	Hspa1b/Cdkn1a/Sphk2	3

GO:0001845 phagolysosome assembly	1/97	17/14464	0.05882353	8.77137659	2.63430219	0.10814768	0.50874395	0.5048838	Rab39	1
GO:0002091 negative regulation of receptor internalization	1/97	17/14464	0.05882353	8.77137659	2.63430219	0.10814768	0.50874395	0.5048838	Pcsk9	1
GO:0002726 positive regulation of T cell cytokine production	1/97	17/14464	0.05882353	8.77137659	2.63430219	0.10814768	0.50874395	0.5048838	Traf2	1
GO:0007614 short-term memory	1/97	17/14464	0.05882353	8.77137659	2.63430219	0.10814768	0.50874395	0.5048838	Aph1b	1
GO:0009130 pyrimidine nucleoside monophosphate biosynthetic process	1/97	17/14464	0.05882353	8.77137659	2.63430219	0.10814768	0.50874395	0.5048838	Nme1	1
GO:0009263 deoxyribonucleotide biosynthetic process	1/97	17/14464	0.05882353	8.77137659	2.63430219	0.10814768	0.50874395	0.5048838	Nme1	1
GO:0009265 2'-deoxyribonucleotide biosynthetic process	1/97	17/14464	0.05882353	8.77137659	2.63430219	0.10814768	0.50874395	0.5048838	Nme1	1
GO:0010224 response to UV-B	1/97	17/14464	0.05882353	8.77137659	2.63430219	0.10814768	0.50874395	0.5048838	Cdkn1a	1
GO:0010226 response to lithium ion	1/97	17/14464	0.05882353	8.77137659	2.63430219	0.10814768	0.50874395	0.5048838	Lig4	1
GO:0032656 regulation of interleukin-13 production	1/97	17/14464	0.05882353	8.77137659	2.63430219	0.10814768	0.50874395	0.5048838	Sphk2	1
GO:0036092 phosphatidylinositol-3-phosphate biosynthetic process	1/97	17/14464	0.05882353	8.77137659	2.63430219	0.10814768	0.50874395	0.5048838	Becn1	1
GO:0036376 sodium ion export across plasma membrane	1/97	17/14464	0.05882353	8.77137659	2.63430219	0.10814768	0.50874395	0.5048838	Atp1b3	1
GO:0042772 DNA damage response, signal transduction resulting in transcription	1/97	17/14464	0.05882353	8.77137659	2.63430219	0.10814768	0.50874395	0.5048838	Cdkn1a	1
GO:0045472 response to ether	1/97	17/14464	0.05882353	8.77137659	2.63430219	0.10814768	0.50874395	0.5048838	Lipa	1
GO:0045606 positive regulation of epidermal cell differentiation	1/97	17/14464	0.05882353	8.77137659	2.63430219	0.10814768	0.50874395	0.5048838	Sfn	1
GO:0046385 deoxyribose phosphate biosynthetic process	1/97	17/14464	0.05882353	8.77137659	2.63430219	0.10814768	0.50874395	0.5048838	Nme1	1
GO:0051969 regulation of transmission of nerve impulse	1/97	17/14464	0.05882353	8.77137659	2.63430219	0.10814768	0.50874395	0.5048838	Gpr35	1
GO:0060019 radial glial cell differentiation	1/97	17/14464	0.05882353	8.77137659	2.63430219	0.10814768	0.50874395	0.5048838	Mettl3	1
GO:0060314 regulation of ryanodine-sensitive calcium-release channel activity	1/97	17/14464	0.05882353	8.77137659	2.63430219	0.10814768	0.50874395	0.5048838	Fkbp1b	1
GO:0061179 negative regulation of insulin secretion involved in cellular response to glucose stimulus	1/97	17/14464	0.05882353	8.77137659	2.63430219	0.10814768	0.50874395	0.5048838	Fkbp1b	1
GO:0070131 positive regulation of mitochondrial translation	1/97	17/14464	0.05882353	8.77137659	2.63430219	0.10814768	0.50874395	0.5048838	Mrps27	1
GO:0071732 cellular response to nitric oxide	1/97	17/14464	0.05882353	8.77137659	2.63430219	0.10814768	0.50874395	0.5048838	Traf2	1
GO:0071816 tail-anchored membrane protein insertion into ER membrane	1/97	17/14464	0.05882353	8.77137659	2.63430219	0.10814768	0.50874395	0.5048838	Caml	1
GO:0043603 amide metabolic process	5/97	376/14464	0.01329787	1.98289098	1.58674309	0.10838807	0.50874395	0.5048838	Pmvk/Smpd4/Sphk2/Becn1/Aph1b	5
GO:0006643 membrane lipid metabolic process	3/97	172/14464	0.01744186	2.60081515	1.73536535	0.10890937	0.50874395	0.5048838	Smpd4/Sphk2/Pigc	3
GO:0031929 TOR signaling	3/97	172/14464	0.01744186	2.60081515	1.73536535	0.10890937	0.50874395	0.5048838	Stambpl1/Lipa/Tbck	3
GO:0005977 glycogen metabolic process	2/97	84/14464	0.02380952	3.5503191	1.92613506	0.10909021	0.50874395	0.5048838	Ptges3/Nhlrc1	2
GO:0008593 regulation of Notch signaling pathway	2/97	84/14464	0.02380952	3.5503191	1.92613506	0.10909021	0.50874395	0.5048838	Tspan14/Mettl3	2
GO:0016579 protein deubiquitination	2/97	84/14464	0.02380952	3.5503191	1.92613506	0.10909021	0.50874395	0.5048838	Stambpl1/Otub2	2
GO:0034620 cellular response to unfolded protein	2/97	84/14464	0.02380952	3.5503191	1.92613506	0.10909021	0.50874395	0.5048838	Pdia6/Edem1	2
GO:0042157 lipoprotein metabolic process	2/97	84/14464	0.02380952	3.5503191	1.92613506	0.10909021	0.50874395	0.5048838	Pcsk9/Lipa	2
GO:0071901 negative regulation of protein serine/threonine kinase activity	2/97	84/14464	0.02380952	3.5503191	1.92613506	0.10909021	0.50874395	0.5048838	Cdkn1a/Sfrp5	2
GO:0044042 glucan metabolic process	2/97	85/14464	0.02352941	3.50855064	1.90589955	0.11125059	0.50874395	0.5048838	Ptges3/Nhlrc1	2
GO:0006672 ceramide metabolic process	2/97	86/14464	0.02325581	3.46775354	1.88596567	0.11342224	0.50874395	0.5048838	Smpd4/Sphk2	2
GO:0006670 sphingosine metabolic process	1/97	18/14464	0.05555556	8.28407789	2.54079159	0.11413575	0.50874395	0.5048838	Sphk2	1
GO:0021859 pyramidal neuron differentiation	1/97	18/14464	0.05555556	8.28407789	2.54079159	0.11413575	0.50874395	0.5048838	Tuba1a	1
GO:0031645 negative regulation of nervous system process	1/97	18/14464	0.05555556	8.28407789	2.54079159	0.11413575	0.50874395	0.5048838	Gpr35	1
GO:0031958 nuclear receptor-mediated corticosteroid signaling pathway	1/97	18/14464	0.05555556	8.28407789	2.54079159	0.11413575	0.50874395	0.5048838	Ptges3	1
GO:0032616 interleukin-13 production	1/97	18/14464	0.05555556	8.28407789	2.54079159	0.11413575	0.50874395	0.5048838	Sphk2	1
GO:0033003 regulation of mast cell activation	1/97	18/14464	0.05555556	8.28407789	2.54079159	0.11413575	0.50874395	0.5048838	Sphk2	1
GO:0034383 low-density lipoprotein particle clearance	1/97	18/14464	0.05555556	8.28407789	2.54079159	0.11413575	0.50874395	0.5048838	Pcsk9	1
GO:0038083 peptidyl-tyrosine autophosphorylation	1/97	18/14464	0.05555556	8.28407789	2.54079159	0.11413575	0.50874395	0.5048838	Ctnnd1	1
GO:0043306 positive regulation of mast cell degranulation	1/97	18/14464	0.05555556	8.28407789	2.54079159	0.11413575	0.50874395	0.5048838	Sphk2	1
GO:0070200 establishment of protein localization to telomere	1/97	18/14464	0.05555556	8.28407789	2.54079159	0.11413575	0.50874395	0.5048838	Cct7	1
GO:0090083 regulation of inclusion body assembly	1/97	18/14464	0.05555556	8.28407789	2.54079159	0.11413575	0.50874395	0.5048838	Hspa1b	1
GO:0097398 cellular response to interleukin-17	1/97	18/14464	0.05555556	8.28407789	2.54079159	0.11413575	0.50874395	0.5048838	Traf2	1
GO:0110154 RNA decapping	1/97	18/14464	0.05555556	8.28407789	2.54079159	0.11413575	0.50874395	0.5048838	Nudt3	1
GO:0141156 cAMP/PKA signal transduction	1/97	18/14464	0.05555556	8.28407789	2.54079159	0.11413575	0.50874395	0.5048838	Sfn	1
GO:1905038 regulation of membrane lipid metabolic process	1/97	18/14464	0.05555556	8.28407789	2.54079159	0.11413575	0.50874395	0.5048838	Sphk2	1
GO:0030198 extracellular matrix organization	4/97	276/14464	0.01449275	2.1610638	1.60022868	0.11452812	0.50874395	0.5048838	P4ha1/Vwa1/Adamts8/Antxr2	4
GO:0043062 extracellular structure organization	4/97	276/14464	0.01449275	2.1610638	1.60022868	0.11452812	0.50874395	0.5048838	P4ha1/Vwa1/Adamts8/Antxr2	4
GO:0045229 external encapsulating structure organization	4/97	276/14464	0.01449275	2.1610638	1.60022868	0.11452812	0.50874395	0.5048838	P4ha1/Vwa1/Adamts8/Antxr2	4
GO:0006164 purine nucleotide biosynthetic process	3/97	176/14464	0.01704545	2.54170572	1.69084622	0.11459102	0.50874395	0.5048838	Lipa/Sphk2/Nme1	3
GO:0035773 insulin secretion involved in cellular response to glucose stimulus	2/97	87/14464	0.02298851	3.4278943	1.86632503	0.11560492	0.50874395	0.5048838	Myt1/Fkbp1b	2
GO:0048477 oogenesis	2/97	87/14464	0.02298851	3.4278943	1.86632503	0.11560492	0.50874395	0.5048838	Mettl3/Taf4b	2
GO:0072384 organelle transport along microtubule	2/97	87/14464	0.02298851	3.4278943	1.86632503	0.11560492	0.50874395	0.5048838	Bicd2/Tuba1a	2
GO:1902117 positive regulation of organelle assembly	2/97	87/14464	0.02298851	3.4278943	1.86632503	0.11560492	0.50874395	0.5048838	Becn1/Pip4k2c	2
GO:0006641 triglyceride metabolic process	2/97	88/14464	0.02272727	3.38894096	1.84696955	0.11779839	0.50874395	0.5048838	Pcsk9/Lipa	2
GO:0030148 sphingolipid biosynthetic process	2/97	88/14464	0.02272727	3.38894096	1.84696955	0.11779839	0.50874395	0.5048838	Smpd4/Sphk2	2
GO:0034644 cellular response to UV	2/97	88/14464	0.02272727	3.38894096	1.84696955	0.11779839	0.50874395	0.5048838	Cdkn1a/Mettl3	2
GO:0120254 olefinic compound metabolic process	2/97	88/14464	0.02272727	3.38894096	1.84696955	0.11779839	0.50874395	0.5048838	Cyp2j13/Stard3	2
GO:0031329 regulation of cellular catabolic process	5/97	388/14464	0.0128866	1.92156446	1.51193794	0.11947716	0.50874395	0.5048838	Mtcl3/Pcsk9/Becn1/Pafah1b2/Pip4k2c	5
GO:0002275 myeloid cell activation involved in immune response	2/97	89/14464	0.02247191	3.35086297	1.82789147	0.12000241	0.50874395	0.5048838	Dnase1l3/Sphk2	2
GO:1903321 negative regulation of protein modification by small protein conjugation or removal	2/97	89/14464	0.02247191	3.35086297	1.82789147	0.12000241	0.50874395	0.5048838	Hspa1b/Caml	2
GO:0002283 neutrophil activation involved in immune response	1/97	19/14464	0.05263158	7.84807379	2.45424816	0.12008403	0.50874395	0.5048838	Dnase1l3	1
GO:0006857 oligopeptide transport	1/97	19/14464	0.05263158	7.84807379	2.45424816	0.12008403	0.50874395	0.5048838	Abcc5	1
GO:0010288 response to lead ion	1/97	19/14464	0.05263158	7.84807379	2.45424816	0.12008403	0.50874395	0.5048838	Becn1	1
GO:0051988 regulation of attachment of spindle microtubules to kinetochore	1/97	19/14464	0.05263158	7.84807379	2.45424816	0.12008403	0.50874395	0.5048838	Becn1	1
GO:0070431 nucleotide-binding oligomerization domain containing 2 signaling pathway	1/97	19/14464	0.05263158	7.84807379	2.45424816	0.12008403	0.50874395	0.5048838	Hspa1b	1
GO:0090280 positive regulation of calcium ion import	1/97	19/14464	0.05263158	7.84807379	2.45424816	0.12008403	0.50874395	0.5048838	Trpv2	1
GO:1900120 regulation of receptor binding	1/97	19/14464	0.05263158	7.84807379	2.45424816	0.12008403	0.50874395	0.5048838	Pcsk9	1
GO:1902170 cellular response to reactive nitrogen species	1/97	19/14464	0.05263158	7.84807379	2.45424816	0.12008403	0.50874395	0.5048838	Traf2	1
GO:0001558 regulation of cell growth	5/97	389/14464	0.01285347	1.91662471	1.50582385	0.12042494	0.50874395	0.5048838	Hspa1b/Cdkn1a/Sfn/Trpv2/Sphk2	5
GO:0010951 negative regulation of endopeptidase activity	2/97	90/14464	0.02222222	3.31363116	1.80908333	0.12221675	0.50874395	0.5048838	Hspa1b/Serpinb8	2
GO:0009416 response to light stimulus	4/97	283/14464	0.01413428	2.10760992	1.54617498	0.12241848	0.50874395	0.5048838	Cdkn1a/Mettl3/Tuba1a/Pde6d	4
GO:0019751 polyol metabolic process	2/97	91/14464	0.02197802	3.27721763	1.79053797	0.12444117	0.50874395	0.5048838	Sphk2/Nudt3	2
GO:0046785 microtubule polymerization	2/97	91/14464	0.02197802	3.27721763	1.79053797	0.12444117	0.50874395	0.5048838	Hspa1b/Tuba1a	2
GO:2001257 regulation of cation channel activity	2/97	91/14464	0.02197802	3.27721763	1.79053797	0.12444117	0.50874395	0.5048838	Gpr35/Fkbp1b	2
GO:0002520 immune system development	3/97	183/14464	0.01639344	2.444482	1.61581035	0.12479817	0.50874395	0.5048838	Lipa/Lig4/Nhej1	3
GO:0009267 cellular response to starvation	3/97	183/14464	0.01639344	2.444482	1.61581035	0.12479817	0.50874395	0.5048838	Cdkn1a/Pcsk9/Becn1	3
GO:0006684 sphingomyelin metabolic process	1/97	20/14464	0.05	7.4556701	2.3738026	0.12599278	0.50874395	0.5048838	Smpd4	1
GO:0006775 fat-soluble vitamin metabolic process	1/97	20/14464	0.05	7.4556701	2.3738026	0.12599278	0.50874395	0.5048838	Lipa	1
GO:0007289 spermatid nucleus differentiation	1/97	20/14464	0.05	7.4556701	2.3738026	0.12599278	0.50874395	0.5048838	Agfg1	1
GO:0009147 pyrimidine nucleoside triphosphate metabolic process	1/97	20/14464	0.05	7.4556701	2.3738026	0.12599278	0.50874395	0.5048838	Nme1	1
GO:0009219 pyrimidine deoxyribonucleotide metabolic process	1/97	20/14464	0.05	7.4556701	2.3738026	0.12599278	0.50874395	0.5048838	Nme1	1
GO:0010155 regulation of proton transport	1/97	20/14464	0.05	7.4556701	2.3738026	0.12599278	0.50874395	0.5048838	Sphk2	1
GO:0010878 cholesterol storage	1/97	20/14464	0.05	7.4556701	2.3738026	0.12599278	0.50874395	0.5048838	Lipa	1
GO:0031954 positive regulation of protein autophosphorylation	1/97	20/14464	0.05	7.4556701	2.3738026	0.12599278	0.50874395	0.5048838	Ctnnd1	1
GO:0034312 diol biosynthetic process	1/97	20/14464	0.05	7.4556701	2.3738026	0.12599278	0.50874395	0.5048838	Sphk2	1
GO:0035751 regulation of lysosomal lumen pH	1/97	20/14464	0.05	7.4556701	2.3738026	0.12599278	0.50874395	0.5048838	Cln5	1
GO:0036342 post-anal tail morphogenesis	1/97	20/14464	0.05	7.4556701	2.3738026	0.12599278	0.50874395	0.5048838	Sfrp5	1
GO:0045736 negative regulation of cyclin-dependent protein serine/threonine kinase activity	1/97	20/14464	0.05	7.4556701	2.3738026	0.12599278	0.50874395	0.5048838	Cdkn1a	1
GO:0046519 sphingoid metabolic process	1/97	20/14464	0.05	7.4556701	2.3738026	0.12599278	0.50874395	0.5048838	Sphk2	1
GO:0051131 chaperone-mediated protein complex assembly	1/97	20/14464	0.05	7.4556701	2.3738026	0.12599278	0.50874395	0.5048838	Ptges3	1
GO:0006163 purine nucleotide metabolic process	5/97	395/14464	0.01265823	1.88751142	1.46951119	0.12618647	0.50874395	0.5048838	Hspa1b/Lipa/Sphk2/Nudt3/Nme1	5
GO:0006112 energy reserve metabolic process	2/97	93/14464	0.02150538	3.20673983	1.75420816	0.12891932	0.50874395	0.5048838	Ptges3/Nhlrc1	2
GO:0098659 inorganic cation import across plasma membrane	2/97	93/14464	0.02150538	3.20673983	1.75420816	0.12891932	0.50874395	0.5048838	Trpv2/Atp1b3	2
GO:0099587 inorganic ion import across plasma membrane	2/97	93/14464	0.02150538	3.20673983	1.75420816	0.12891932	0.50874395	0.5048838	Trpv2/Atp1b3	2
GO:1901874 negative regulation of post-translational protein modification	2/97	93/14464	0.02150538	3.20673983	1.75420816	0.12891932	0.50874395	0.5048838	Hspa1b/Caml	2
GO:0007051 spindle organization	3/97	187/14464	0.01604278	2.39219362	1.5744685	0.1307751	0.50874395	0.5048838	Hspa1b/Efhc1/Smc1a	3
GO:1901617 organic hydroxy compound biosynthetic process	3/97	187/14464	0.01604278	2.39219362	1.5744685	0.1307751	0.50874395	0.5048838	Lipa/Pmvk/Sphk2	3
GO:0009220 pyrimidine ribonucleotide biosynthetic process	1/97	21/14464	0.04761905	7.10063819	2.29873139	0.13186225	0.50874395	0.5048838	Nme1	1
GO:0009583 detection of light stimulus	1/97	21/14464	0.04761905	7.10063819	2.29873139	0.13186225	0.50874395	0.5048838	Pde6d	1
GO:0010954 positive regulation of protein processing	1/97	21/14464	0.04761905	7.10063819	2.29873139	0.13186225	0.50874395	0.5048838	Ctnnd1	1
GO:0021542 dentate gyrus development	1/97	21/14464	0.04761905	7.10063819	2.29873139	0.13186225	0.50874395	0.5048838	Tuba1a	1
GO:0035641 locomotory exploration behavior	1/97	21/14464	0.04761905	7.10063819	2.29873139	0.13186225	0.50874395	0.5048838	Tuba1a	1
GO:0038128 ERBB2 signaling pathway	1/97	21/14464	0.04761905	7.10063819	2.29873139	0.13186225	0.50874395	0.5048838	Nrg2	1
GO:0045684 positive regulation of epidermis development	1/97	21/14464	0.04761905	7.10063819	2.29873139	0.13186225	0.50874395	0.5048838	Sfn	1
GO:0045948 positive regulation of translational initiation	1/97	21/14464	0.04761905	7.10063819	2.29873139	0.13186225	0.50874395	0.5048838	Mettl3	1
GO:0046827 positive regulation of protein export from nucleus	1/97	21/14464	0.04761905	7.10063819	2.29873139	0.13186225	0.50874395	0.5048838	Sfn	1
GO:0060065 uterus development	1/97	21/14464	0.04761905	7.10063819	2.29873139	0.13186225	0.50874395	0.5048838	Antxr2	1
GO:0070861 regulation of protein exit from endoplasmic reticulum	1/97	21/14464	0.04761905	7.10063819	2.29873139	0.13186225	0.50874395	0.5048838	Edem1	1
GO:0071731 response to nitric oxide	1/97	21/14464	0.04761905	7.10063819	2.29873139	0.13186225	0.50874395	0.5048838	Traf2	1
GO:0097396 response to interleukin-17	1/97	21/14464	0.04761905	7.10063819	2.29873139	0.13186225	0.50874395	0.5048838	Traf2	1
GO:1902455 negative regulation of stem cell population maintenance	1/97	21/14464	0.04761905	7.10063819	2.29873139	0.13186225	0.50874395	0.5048838	Sap30l	1
GO:2000647 negative regulation of stem cell proliferation	1/97	21/14464	0.04761905	7.10063819	2.29873139	0.13186225	0.50874395	0.5048838	Sfn	1
GO:2001169 regulation of ATP biosynthetic process	1/97	21/14464	0.04761905	7.10063819	2.29873139	0.13186225	0.50874395	0.5048838	Sphk2	1
GO:0072659 protein localization to plasma membrane	4/97	292/14464	0.01369863	2.04264934	1.47892497	0.13289776	0.50874395	0.5048838	Tspan14/Sfn/Nhlrc1/Atp1b3	4
GO:0006754 ATP biosynthetic process	2/97	95/14464	0.02105263	3.13922952	1.7188498	0.13343506	0.50874395	0.5048838	Lipa/Sphk2	2
GO:0019226 transmission of nerve impulse	2/97	95/14464	0.02105263	3.13922952	1.7188498	0.13343506	0.50874395	0.5048838	Gpr35/Fkbp1b	2
GO:0045639 positive regulation of myeloid cell differentiation	2/97	95/14464	0.02105263	3.13922952	1.7188498	0.13343506	0.50874395	0.5048838	Hspa1b/Ankrd54	2
GO:0071482 cellular response to light stimulus	2/97	95/14464	0.02105263	3.13922952	1.7188498	0.13343506	0.50874395	0.5048838	Cdkn1a/Mettl3	2
GO:0010506 regulation of autophagy	4/97	295/14464	0.01355932	2.02187664	1.4570414	0.1364716	0.50874395	0.5048838	Mtcl3/Becn1/Pafah1b2/Pip4k2c	4
GO:0001675 acrosome assembly	1/97	22/14464	0.04545455	6.77788191	2.22842666	0.13769272	0.50874395	0.5048838	Agfg1	1
GO:0008207 C21-steroid hormone metabolic process	1/97	22/14464	0.04545455	6.77788191	2.22842666	0.13769272	0.50874395	0.5048838	Stard3	1
GO:0010623 programmed cell death involved in cell development	1/97	22/14464	0.04545455	6.77788191	2.22842666	0.13769272	0.50874395	0.5048838	Dnase1l3	1
GO:0010839 negative regulation of keratinocyte proliferation	1/97	22/14464	0.04545455	6.77788191	2.22842666	0.13769272	0.50874395	0.5048838	Sfn	1
GO:0016556 mRNA modification	1/97	22/14464	0.04545455	6.77788191	2.22842666	0.13769272	0.50874395	0.5048838	Mettl3	1
GO:0031571 mitotic G1 DNA damage checkpoint signaling	1/97	22/14464	0.04545455	6.77788191	2.22842666	0.13769272	0.50874395	0.5048838	Cdkn1a	1
GO:0034349 glial cell apoptotic process	1/97	22/14464	0.04545455	6.77788191	2.22842666	0.13769272	0.50874395	0.5048838	Traf2	1
GO:0035872 nucleotide-binding domain, leucine rich repeat containing receptor signaling pathway	1/97	22/14464	0.04545455	6.77788191	2.22842666	0.13769272	0.50874395	0.5048838	Hspa1b	1
GO:0045822 negative regulation of heart contraction	1/97	22/14464	0.04545455	6.77788191	2.22842666	0.13769272	0.50874395	0.5048838	Fkbp1b	1
GO:0046579 positive regulation of Ras protein signal transduction	1/97	22/14464	0.04545455	6.77788191	2.22842666	0.13769272	0.50874395	0.5048838	Rasgef1a	1
GO:0050849 negative regulation of calcium-mediated signaling	1/97	22/14464	0.04545455	6.77788191	2.22842666	0.13769272	0.50874395	0.5048838	Fkbp1b	1
GO:0120255 olefinic compound biosynthetic process	1/97	22/14464	0.04545455	6.77788191	2.22842666	0.13769272	0.50874395	0.5048838	Stard3	1
GO:0140354 lipid import into cell	1/97	22/14464	0.04545455	6.77788191	2.22842666	0.13769272	0.50874395	0.5048838	Lipa	1
GO:1904030 negative regulation of cyclin-dependent protein kinase activity	1/97	22/14464	0.04545455	6.77788191	2.22842666	0.13769272	0.50874395	0.5048838	Cdkn1a	1
GO:0007029 endoplasmic reticulum organization	2/97	97/14464	0.02061856	3.07450314	1.68441434	0.13798661	0.50874395	0.5048838	Smpd4/Caml	2
GO:0002718 regulation of cytokine production involved in immune response	2/97	98/14464	0.02040816	3.04313065	1.66752848	0.14027527	0.50874395	0.5048838	Traf2/Sphk2	2
GO:0032204 regulation of telomere maintenance	2/97	98/14464	0.02040816	3.04313065	1.66752848	0.14027527	0.50874395	0.5048838	Ptges3/Cct7	2
GO:0006486 protein glycosylation	3/97	194/14464	0.01546392	2.30587735	1.50460771	0.14147292	0.50874395	0.5048838	Mlec/Uggt1/Fut11	3
GO:0043413 macromolecule glycosylation	3/97	194/14464	0.01546392	2.30587735	1.50460771	0.14147292	0.50874395	0.5048838	Mlec/Uggt1/Fut11	3
GO:0045637 regulation of myeloid cell differentiation	3/97	194/14464	0.01546392	2.30587735	1.50460771	0.14147292	0.50874395	0.5048838	Hspa1b/Ankrd54/Nme1	3
GO:0002444 myeloid leukocyte mediated immunity	2/97	99/14464	0.02020202	3.01239196	1.65085668	0.14257223	0.50874395	0.5048838	Dnase1l3/Sphk2	2
GO:0001963 synaptic transmission, dopaminergic	1/97	23/14464	0.04347826	6.48319139	2.16237347	0.14348442	0.50874395	0.5048838	Aph1b	1
GO:0002320 lymphoid progenitor cell differentiation	1/97	23/14464	0.04347826	6.48319139	2.16237347	0.14348442	0.50874395	0.5048838	Lig4	1
GO:0009048 dosage compensation by inactivation of X chromosome	1/97	23/14464	0.04347826	6.48319139	2.16237347	0.14348442	0.50874395	0.5048838	Mettl3	1
GO:0009129 pyrimidine nucleoside monophosphate metabolic process	1/97	23/14464	0.04347826	6.48319139	2.16237347	0.14348442	0.50874395	0.5048838	Nme1	1
GO:0010165 response to X-ray	1/97	23/14464	0.04347826	6.48319139	2.16237347	0.14348442	0.50874395	0.5048838	Lig4	1
GO:0016540 protein autoprocessing	1/97	23/14464	0.04347826	6.48319139	2.16237347	0.14348442	0.50874395	0.5048838	Pcsk9	1
GO:0032482 Rab protein signal transduction	1/97	23/14464	0.04347826	6.48319139	2.16237347	0.14348442	0.50874395	0.5048838	Rab39	1
GO:0033617 mitochondrial cytochrome c oxidase assembly	1/97	23/14464	0.04347826	6.48319139	2.16237347	0.14348442	0.50874395	0.5048838	Coa4	1
GO:0042026 protein refolding	1/97	23/14464	0.04347826	6.48319139	2.16237347	0.14348442	0.50874395	0.5048838	Hspa1b	1
GO:0044819 mitotic G1/S transition checkpoint signaling	1/97	23/14464	0.04347826	6.48319139	2.16237347	0.14348442	0.50874395	0.5048838	Cdkn1a	1
GO:0045109 intermediate filament organization	1/97	23/14464	0.04347826	6.48319139	2.16237347	0.14348442	0.50874395	0.5048838	Agfg1	1
GO:0046164 alcohol catabolic process	1/97	23/14464	0.04347826	6.48319139	2.16237347	0.14348442	0.50874395	0.5048838	Nudt3	1
GO:0050857 positive regulation of antigen receptor-mediated signaling pathway	1/97	23/14464	0.04347826	6.48319139	2.16237347	0.14348442	0.50874395	0.5048838	Lipa	1
GO:0051315 attachment of mitotic spindle microtubules to kinetochore	1/97	23/14464	0.04347826	6.48319139	2.16237347	0.14348442	0.50874395	0.5048838	Becn1	1
GO:0060339 negative regulation of type I interferon-mediated signaling pathway	1/97	23/14464	0.04347826	6.48319139	2.16237347	0.14348442	0.50874395	0.5048838	Mettl3	1
GO:1901673 regulation of mitotic spindle assembly	1/97	23/14464	0.04347826	6.48319139	2.16237347	0.14348442	0.50874395	0.5048838	Hspa1b	1
GO:1902307 positive regulation of sodium ion transmembrane transport	1/97	23/14464	0.04347826	6.48319139	2.16237347	0.14348442	0.50874395	0.5048838	Atp1b3	1
GO:1903319 positive regulation of protein maturation	1/97	23/14464	0.04347826	6.48319139	2.16237347	0.14348442	0.50874395	0.5048838	Ctnnd1	1
GO:0045017 glycerolipid biosynthetic process	3/97	196/14464	0.01530612	2.28234799	1.48519814	0.1445827	0.50944436	0.50557889	Becn1/Pip4k2c/Pigc	3
GO:0035967 cellular response to topologically incorrect protein	2/97	100/14464	0.02	2.98226804	1.63439376	0.14487729	0.50944436	0.50557889	Pdia6/Edem1	2
GO:0002703 regulation of leukocyte mediated immunity	3/97	197/14464	0.01522843	2.27076247	1.47558141	0.14614616	0.50944436	0.50557889	Dnase1l3/Traf2/Sphk2	3
GO:1901615 organic hydroxy compound metabolic process	5/97	417/14464	0.01199041	1.78793048	1.34151691	0.14837165	0.50944436	0.50557889	Pcsk9/Lipa/Pmvk/Sphk2/Nudt3	5

GO:0001516 prostaglandin biosynthetic process	1/97	24/14464	0.04166667	6.21305842	2.10013218	0.14923763	0.50944436	0.50557889	Ptges3	1
GO:0007035 vacuolar acidification	1/97	24/14464	0.04166667	6.21305842	2.10013218	0.14923763	0.50944436	0.50557889	Cln5	1
GO:0008299 isoprenoid biosynthetic process	1/97	24/14464	0.04166667	6.21305842	2.10013218	0.14923763	0.50944436	0.50557889	Pmvk	1
GO:0010880 regulation of release of sequestered calcium ion into cytosol by sarcoplasmic reticulum	1/97	24/14464	0.04166667	6.21305842	2.10013218	0.14923763	0.50944436	0.50557889	Fkbp1b	1
GO:0018126 protein hydroxylation	1/97	24/14464	0.04166667	6.21305842	2.10013218	0.14923763	0.50944436	0.50557889	P4ha1	1
GO:0030808 regulation of nucleotide biosynthetic process	1/97	24/14464	0.04166667	6.21305842	2.10013218	0.14923763	0.50944436	0.50557889	Sphk2	1
GO:0034453 microtubule anchoring	1/97	24/14464	0.04166667	6.21305842	2.10013218	0.14923763	0.50944436	0.50557889	Bicd2	1
GO:0046457 prostanoid biosynthetic process	1/97	24/14464	0.04166667	6.21305842	2.10013218	0.14923763	0.50944436	0.50557889	Ptges3	1
GO:0046475 glycerophospholipid catabolic process	1/97	24/14464	0.04166667	6.21305842	2.10013218	0.14923763	0.50944436	0.50557889	Smpd4	1
GO:0046688 response to copper ion	1/97	24/14464	0.04166667	6.21305842	2.10013218	0.14923763	0.50944436	0.50557889	Becn1	1
GO:0055075 potassium ion homeostasis	1/97	24/14464	0.04166667	6.21305842	2.10013218	0.14923763	0.50944436	0.50557889	Atp1b3	1
GO:0070723 response to cholesterol	1/97	24/14464	0.04166667	6.21305842	2.10013218	0.14923763	0.50944436	0.50557889	Pmvk	1
GO:0090141 positive regulation of mitochondrial fission	1/97	24/14464	0.04166667	6.21305842	2.10013218	0.14923763	0.50944436	0.50557889	Mief1	1
GO:1900371 regulation of purine nucleotide biosynthetic process	1/97	24/14464	0.04166667	6.21305842	2.10013218	0.14923763	0.50944436	0.50557889	Sphk2	1
GO:1903523 negative regulation of blood circulation	1/97	24/14464	0.04166667	6.21305842	2.10013218	0.14923763	0.50944436	0.50557889	Fkbp1b	1
GO:0006939 smooth muscle contraction	2/97	102/14464	0.01960784	2.9237922	1.60207483	0.14951084	0.50944436	0.50557889	Shc1/Fkbp1b	2
GO:0051098 regulation of binding	4/97	308/14464	0.01298701	1.93653769	1.36509336	0.15240194	0.51519346	0.51128436	Cdkn1a/Pcsk9/Cln5/Nme1	4
GO:0002262 myeloid cell homeostasis	3/97	202/14464	0.01485149	2.21455548	1.42835066	0.15404666	0.51519346	0.51128436	Hspa1b/Lipa/Ankrd54	3
GO:0070646 protein modification by small protein removal	2/97	104/14464	0.01923077	2.86756542	1.57053381	0.1541743	0.51519346	0.51128436	Stambpl1/Otub2	2
GO:0090316 positive regulation of intracellular protein transport	2/97	104/14464	0.01923077	2.86756542	1.57053381	0.1541743	0.51519346	0.51128436	Sfn/Edem1	2
GO:0002724 regulation of T cell cytokine production	1/97	25/14464	0.04	5.96453608	2.04132489	0.15495258	0.51519346	0.51128436	Traf2	1
GO:0006353 DNA-templated transcription termination	1/97	25/14464	0.04	5.96453608	2.04132489	0.15495258	0.51519346	0.51128436	Maz	1
GO:0007549 sex-chromosome dosage compensation	1/97	25/14464	0.04	5.96453608	2.04132489	0.15495258	0.51519346	0.51128436	Mettl3	1
GO:0010039 response to iron ion	1/97	25/14464	0.04	5.96453608	2.04132489	0.15495258	0.51519346	0.51128436	Becn1	1
GO:0034755 iron ion transmembrane transport	1/97	25/14464	0.04	5.96453608	2.04132489	0.15495258	0.51519346	0.51128436	Abcc5	1
GO:0070199 establishment of protein localization to chromosome	1/97	25/14464	0.04	5.96453608	2.04132489	0.15495258	0.51519346	0.51128436	Cct7	1
GO:1901099 negative regulation of signal transduction in absence of ligand	1/97	25/14464	0.04	5.96453608	2.04132489	0.15495258	0.51519346	0.51128436	Hspa1b	1
GO:2001240 negative regulation of extrinsic apoptotic signaling pathway in absence of ligand	1/97	25/14464	0.04	5.96453608	2.04132489	0.15495258	0.51519346	0.51128436	Hspa1b	1
GO:0002832 negative regulation of response to biotic stimulus	2/97	105/14464	0.01904762	2.84025528	1.55504384	0.15651674	0.51519346	0.51128436	Sfn/Mettl3	2
GO:0008630 intrinsic apoptotic signaling pathway in response to DNA damage	2/97	105/14464	0.01904762	2.84025528	1.55504384	0.15651674	0.51519346	0.51128436	Cdkn1a/Sfn	2
GO:0051092 positive regulation of NF-kappaB transcription factor activity	2/97	105/14464	0.01904762	2.84025528	1.55504384	0.15651674	0.51519346	0.51128436	Hspa1b/Traf2	2
GO:0031396 regulation of protein ubiquitination	3/97	204/14464	0.01470588	2.19284415	1.40984393	0.15724452	0.51519346	0.51128436	Hspa1b/Nhlrc1/Caml	3
GO:0000819 sister chromatid segregation	3/97	205/14464	0.01463415	2.18214735	1.40067063	0.15885127	0.51519346	0.51128436	Hspa1b/Becn1/Smc1a	3
GO:0008535 respiratory chain complex IV assembly	1/97	26/14464	0.03846154	5.73513085	1.98562464	0.16062954	0.51519346	0.51128436	Coa4	1
GO:0015868 purine ribonucleotide transport	1/97	26/14464	0.03846154	5.73513085	1.98562464	0.16062954	0.51519346	0.51128436	Abcc5	1
GO:0030149 sphingolipid catabolic process	1/97	26/14464	0.03846154	5.73513085	1.98562464	0.16062954	0.51519346	0.51128436	Smpd4	1
GO:0030262 apoptotic nuclear changes	1/97	26/14464	0.03846154	5.73513085	1.98562464	0.16062954	0.51519346	0.51128436	Dnase1l3	1
GO:0042790 nucleolar large rRNA transcription by RNA polymerase I	1/97	26/14464	0.03846154	5.73513085	1.98562464	0.16062954	0.51519346	0.51128436	Ubtf	1
GO:0043302 positive regulation of leukocyte degranulation	1/97	26/14464	0.03846154	5.73513085	1.98562464	0.16062954	0.51519346	0.51128436	Sphk2	1
GO:0045324 late endosome to vacuole transport	1/97	26/14464	0.03846154	5.73513085	1.98562464	0.16062954	0.51519346	0.51128436	Becn1	1
GO:0046039 GTP metabolic process	1/97	26/14464	0.03846154	5.73513085	1.98562464	0.16062954	0.51519346	0.51128436	Nme1	1
GO:0061436 establishment of skin barrier	1/97	26/14464	0.03846154	5.73513085	1.98562464	0.16062954	0.51519346	0.51128436	Sfn	1
GO:0070129 regulation of mitochondrial translation	1/97	26/14464	0.03846154	5.73513085	1.98562464	0.16062954	0.51519346	0.51128436	Mrps27	1
GO:0071108 protein K48-linked deubiquitination	1/97	26/14464	0.03846154	5.73513085	1.98562464	0.16062954	0.51519346	0.51128436	Otub2	1
GO:0098885 modification of postsynaptic actin cytoskeleton	1/97	26/14464	0.03846154	5.73513085	1.98562464	0.16062954	0.51519346	0.51128436	Cap1	1
GO:1900101 regulation of endoplasmic reticulum unfolded protein response	1/97	26/14464	0.03846154	5.73513085	1.98562464	0.16062954	0.51519346	0.51128436	Pdia6	1
GO:1901020 negative regulation of calcium ion transmembrane transporter activity	1/97	26/14464	0.03846154	5.73513085	1.98562464	0.16062954	0.51519346	0.51128436	Gpr35	1
GO:1901385 regulation of voltage-gated calcium channel activity	1/97	26/14464	0.03846154	5.73513085	1.98562464	0.16062954	0.51519346	0.51128436	Gpr35	1
GO:0005976 polysaccharide metabolic process	2/97	107/14464	0.01869159	2.78716639	1.52460396	0.16122205	0.51520319	0.51129402	Ptges3/Nhlrc1	2
GO:0042542 response to hydrogen peroxide	2/97	107/14464	0.01869159	2.78716639	1.52460396	0.16122205	0.51520319	0.51129402	Fkbp1b/Becn1	2
GO:1904951 positive regulation of establishment of protein localization	4/97	316/14464	0.01265823	1.88751142	1.31069602	0.16254527	0.51779006	0.51386127	Sfn/Edem1/Cct7/Mief1	4
GO:0008037 cell recognition	2/97	109/14464	0.01834862	2.73602573	1.49485759	0.16595332	0.51779006	0.51386127	Hspa1b/Cct7	2
GO:0006308 DNA catabolic process	1/97	27/14464	0.03703704	5.52271859	1.9327469	0.16626875	0.51779006	0.51386127	Dnase1l3	1
GO:0006516 glycoprotein catabolic process	1/97	27/14464	0.03703704	5.52271859	1.9327469	0.16626875	0.51779006	0.51386127	Edem1	1
GO:0006921 cellular component disassembly involved in execution phase of apoptosis	1/97	27/14464	0.03703704	5.52271859	1.9327469	0.16626875	0.51779006	0.51386127	Dnase1l3	1
GO:0014808 release of sequestered calcium ion into cytosol by sarcoplasmic reticulum	1/97	27/14464	0.03703704	5.52271859	1.9327469	0.16626875	0.51779006	0.51386127	Fkbp1b	1
GO:0030970 retrograde protein transport, ER to cytosol	1/97	27/14464	0.03703704	5.52271859	1.9327469	0.16626875	0.51779006	0.51386127	Edem1	1
GO:0034311 diol metabolic process	1/97	27/14464	0.03703704	5.52271859	1.9327469	0.16626875	0.51779006	0.51386127	Sphk2	1
GO:0045730 respiratory burst	1/97	27/14464	0.03703704	5.52271859	1.9327469	0.16626875	0.51779006	0.51386127	Lipa	1
GO:0051503 adenine nucleotide transport	1/97	27/14464	0.03703704	5.52271859	1.9327469	0.16626875	0.51779006	0.51386127	Abcc5	1
GO:0051984 positive regulation of chromosome segregation	1/97	27/14464	0.03703704	5.52271859	1.9327469	0.16626875	0.51779006	0.51386127	Becn1	1
GO:0070841 inclusion body assembly	1/97	27/14464	0.03703704	5.52271859	1.9327469	0.16626875	0.51779006	0.51386127	Hspa1b	1
GO:1903513 endoplasmic reticulum to cytosol transport	1/97	27/14464	0.03703704	5.52271859	1.9327469	0.16626875	0.51779006	0.51386127	Edem1	1
GO:2000050 regulation of non-canonical Wnt signaling pathway	1/97	27/14464	0.03703704	5.52271859	1.9327469	0.16626875	0.51779006	0.51386127	Sfrp5	1
GO:0002702 positive regulation of production of molecular mediator of immune response	2/97	110/14464	0.01818182	2.71115276	1.480235	0.16832822	0.51779006	0.51386127	Traf2/Sphk2	2
GO:0006986 response to unfolded protein	2/97	110/14464	0.01818182	2.71115276	1.480235	0.16832822	0.51779006	0.51386127	Pdia6/Edem1	2
GO:0032869 cellular response to insulin stimulus	3/97	212/14464	0.01415094	2.11009531	1.33789585	0.17023885	0.51779006	0.51386127	Shc1/Pcsk9/Pip4k2c	3
GO:1905475 regulation of protein localization to membrane	3/97	212/14464	0.01415094	2.11009531	1.33789585	0.17023885	0.51779006	0.51386127	Sfn/Nhlrc1/Mief1	3
GO:0009100 glycoprotein metabolic process	4/97	323/14464	0.0123839	1.8466056	1.26437049	0.17161951	0.51779006	0.51386127	Mlec/Edem1/Uggt1/Fut11	4
GO:0003401 axis elongation	1/97	28/14464	0.03571429	5.32547865	1.8824427	0.17187046	0.51779006	0.51386127	Sfrp5	1
GO:0006221 pyrimidine nucleotide biosynthetic process	1/97	28/14464	0.03571429	5.32547865	1.8824427	0.17187046	0.51779006	0.51386127	Nme1	1
GO:0006622 protein targeting to lysosome	1/97	28/14464	0.03571429	5.32547865	1.8824427	0.17187046	0.51779006	0.51386127	Becn1	1
GO:0009162 deoxyribonucleoside monophosphate metabolic process	1/97	28/14464	0.03571429	5.32547865	1.8824427	0.17187046	0.51779006	0.51386127	Nme1	1
GO:0015865 purine nucleotide transport	1/97	28/14464	0.03571429	5.32547865	1.8824427	0.17187046	0.51779006	0.51386127	Abcc5	1
GO:0036314 response to sterol	1/97	28/14464	0.03571429	5.32547865	1.8824427	0.17187046	0.51779006	0.51386127	Pmvk	1
GO:0043304 regulation of mast cell degranulation	1/97	28/14464	0.03571429	5.32547865	1.8824427	0.17187046	0.51779006	0.51386127	Sphk2	1
GO:0048261 negative regulation of receptor-mediated endocytosis	1/97	28/14464	0.03571429	5.32547865	1.8824427	0.17187046	0.51779006	0.51386127	Pcsk9	1
GO:0055078 sodium ion homeostasis	1/97	28/14464	0.03571429	5.32547865	1.8824427	0.17187046	0.51779006	0.51386127	Atp1b3	1
GO:0070286 axonemal dynein complex assembly	1/97	28/14464	0.03571429	5.32547865	1.8824427	0.17187046	0.51779006	0.51386127	Dnai4	1
GO:1903514 release of sequestered calcium ion into cytosol by endoplasmic reticulum	1/97	28/14464	0.03571429	5.32547865	1.8824427	0.17187046	0.51779006	0.51386127	Fkbp1b	1
GO:0007265 Ras protein signal transduction	2/97	112/14464	0.01785714	2.66273932	1.45147306	0.17309561	0.51779006	0.51386127	Cdkn1a/Rasgef1a	2
GO:0048145 regulation of fibroblast proliferation	2/97	112/14464	0.01785714	2.66273932	1.45147306	0.17309561	0.51779006	0.51386127	Cdkn1a/Lig4	2
GO:0009165 nucleotide biosynthetic process	3/97	214/14464	0.01401869	2.0903748	1.32040534	0.17353569	0.51779006	0.51386127	Lipa/Sphk2/Nme1	3
GO:0051656 establishment of organelle localization	5/97	442/14464	0.01131222	1.68680319	1.20495401	0.1754542	0.51779006	0.51386127	Bicd2/Sphk2/Tuba1a/Stard3/Becn1	5
GO:0006417 regulation of translation	4/97	326/14464	0.01226994	1.8296123	1.24486428	0.17556271	0.51779006	0.51386127	Mettl3/Nhlrc1/Mrps27/Hbs1l	4
GO:0042594 response to starvation	3/97	216/14464	0.01388889	2.07101947	1.30310468	0.17685086	0.51779006	0.51386127	Cdkn1a/Pcsk9/Becn1	3
GO:0045926 negative regulation of growth	3/97	216/14464	0.01388889	2.07101947	1.30310468	0.17685086	0.51779006	0.51386127	Hspa1b/Cdkn1a/Sphk2	3
GO:0002443 leukocyte mediated immunity	4/97	327/14464	0.01223242	1.82401715	1.23840733	0.17688413	0.51779006	0.51386127	Dnase1l3/Traf2/Sphk2/Lig4	4
GO:0001919 regulation of receptor recycling	1/97	29/14464	0.03448276	5.14184145	1.83449313	0.17743492	0.51779006	0.51386127	Pcsk9	1
GO:0002092 positive regulation of receptor internalization	1/97	29/14464	0.03448276	5.14184145	1.83449313	0.17743492	0.51779006	0.51386127	Pcsk9	1
GO:0006506 GPI anchor biosynthetic process	1/97	29/14464	0.03448276	5.14184145	1.83449313	0.17743492	0.51779006	0.51386127	Pigc	1
GO:0009218 pyrimidine ribonucleotide metabolic process	1/97	29/14464	0.03448276	5.14184145	1.83449313	0.17743492	0.51779006	0.51386127	Nme1	1
GO:0019369 arachidonate metabolic process	1/97	29/14464	0.03448276	5.14184145	1.83449313	0.17743492	0.51779006	0.51386127	Cyp2j13	1
GO:0032369 negative regulation of lipid transport	1/97	29/14464	0.03448276	5.14184145	1.83449313	0.17743492	0.51779006	0.51386127	Pcsk9	1
GO:0045048 protein insertion into ER membrane	1/97	29/14464	0.03448276	5.14184145	1.83449313	0.17743492	0.51779006	0.51386127	Caml	1
GO:0046466 membrane lipid catabolic process	1/97	29/14464	0.03448276	5.14184145	1.83449313	0.17743492	0.51779006	0.51386127	Smpd4	1
GO:0048266 behavioral response to pain	1/97	29/14464	0.03448276	5.14184145	1.83449313	0.17743492	0.51779006	0.51386127	Vwa1	1
GO:0071480 cellular response to gamma radiation	1/97	29/14464	0.03448276	5.14184145	1.83449313	0.17743492	0.51779006	0.51386127	Cdkn1a	1
GO:0072576 liver morphogenesis	1/97	29/14464	0.03448276	5.14184145	1.83449313	0.17743492	0.51779006	0.51386127	Lipa	1
GO:0090382 phagosome maturation	1/97	29/14464	0.03448276	5.14184145	1.83449313	0.17743492	0.51779006	0.51386127	Rab39	1
GO:0120178 steroid hormone biosynthetic process	1/97	29/14464	0.03448276	5.14184145	1.83449313	0.17743492	0.51779006	0.51386127	Stard3	1
GO:2001258 negative regulation of cation channel activity	1/97	29/14464	0.03448276	5.14184145	1.83449313	0.17743492	0.51779006	0.51386127	Gpr35	1
GO:1903076 regulation of protein localization to plasma membrane	2/97	114/14464	0.01754386	2.6160246	1.4233328	0.17788525	0.51823902	0.51430682	Sfn/Nhlrc1	2
GO:0052548 regulation of endopeptidase activity	3/97	218/14464	0.01376147	2.05201929	1.28598972	0.18018386	0.51827196	0.51433951	Hspa1b/Serpinb8/Aph1b	3
GO:0006639 acylglycerol metabolic process	2/97	115/14464	0.0173913	2.59327656	1.40948768	0.18028796	0.51827196	0.51433951	Pcsk9/Lipa	2
GO:0043200 response to amino acid	2/97	115/14464	0.0173913	2.59327656	1.40948768	0.18028796	0.51827196	0.51433951	Stambpl1/Tuba1a	2
GO:0030003 intracellular monoatomic cation homeostasis	5/97	447/14464	0.01118568	1.66793515	1.17867083	0.18108954	0.51827196	0.51433951	Rab39/Efhc1/Fkbp1b/Atp1b3/Cln5	5
GO:0034762 regulation of transmembrane transport	5/97	447/14464	0.01118568	1.66793515	1.17867083	0.18108954	0.51827196	0.51433951	Gpr35/Fkbp1b/Pcsk9/Atp1b3/Sphk2	5
GO:0006638 neutral lipid metabolic process	2/97	116/14464	0.01724138	2.57092073	1.39578843	0.1826957	0.51827196	0.51433951	Pcsk9/Lipa	2
GO:0006505 GPI anchor metabolic process	1/97	30/14464	0.03333333	4.97044674	1.78870477	0.18296238	0.51827196	0.51433951	Pigc	1
GO:0007588 excretion	1/97	30/14464	0.03333333	4.97044674	1.78870477	0.18296238	0.51827196	0.51433951	Lipa	1
GO:0034250 positive regulation of amide metabolic process	1/97	30/14464	0.03333333	4.97044674	1.78870477	0.18296238	0.51827196	0.51433951	Sphk2	1
GO:0090128 regulation of synapse maturation	1/97	30/14464	0.03333333	4.97044674	1.78870477	0.18296238	0.51827196	0.51433951	Nrg2	1
GO:0090314 positive regulation of protein targeting to membrane	1/97	30/14464	0.03333333	4.97044674	1.78870477	0.18296238	0.51827196	0.51433951	Mief1	1
GO:0099022 vesicle tethering	1/97	30/14464	0.03333333	4.97044674	1.78870477	0.18296238	0.51827196	0.51433951	Stard3	1
GO:1903077 negative regulation of protein localization to plasma membrane	1/97	30/14464	0.03333333	4.97044674	1.78870477	0.18296238	0.51827196	0.51433951	Sfn	1
GO:1904706 negative regulation of vascular associated smooth muscle cell proliferation	1/97	30/14464	0.03333333	4.97044674	1.78870477	0.18296238	0.51827196	0.51433951	Cdkn1a	1
GO:0046034 ATP metabolic process	3/97	220/14464	0.01363636	2.03336457	1.26905645	0.18353422	0.51827196	0.51433951	Hspa1b/Lipa/Sphk2	3
GO:0060541 respiratory system development	3/97	220/14464	0.01363636	2.03336457	1.26905645	0.18353422	0.51827196	0.51433951	Myt1/Lipa/Ptges3	3
GO:0009117 nucleotide metabolic process	5/97	450/14464	0.01111111	1.65681558	1.16305609	0.18450358	0.51827196	0.51433951	Hspa1b/Lipa/Sphk2/Nudt3/Nme1	5
GO:0001776 leukocyte homeostasis	2/97	117/14464	0.01709402	2.54894704	1.38223207	0.1851083	0.51827196	0.51433951	Lipa/Caml	2
GO:0006354 DNA-templated transcription elongation	2/97	117/14464	0.01709402	2.54894704	1.38223207	0.1851083	0.51827196	0.51433951	Ubtf/Eloa	2
GO:0043588 skin development	3/97	222/14464	0.01351351	2.01504597	1.25230099	0.18690145	0.51827196	0.51433951	Cdkn1a/Sfn/Ptges3	3
GO:0046434 organophosphate catabolic process	3/97	222/14464	0.01351351	2.01504597	1.25230099	0.18690145	0.51827196	0.51433951	Lipa/Smpd4/Nudt3	3
GO:0000045 autophagosome assembly	2/97	118/14464	0.01694915	2.5273458	1.3688157	0.18752558	0.51827196	0.51433951	Becn1/Pip4k2c	2
GO:0002367 cytokine production involved in immune response	2/97	118/14464	0.01694915	2.5273458	1.3688157	0.18752558	0.51827196	0.51433951	Traf2/Sphk2	2
GO:0001736 establishment of planar polarity	1/97	31/14464	0.03225806	4.81010974	1.74490602	0.18845307	0.51827196	0.51433951	Sfrp5	1
GO:0007164 establishment of tissue polarity	1/97	31/14464	0.03225806	4.81010974	1.74490602	0.18845307	0.51827196	0.51433951	Sfrp5	1
GO:0030212 hyaluronan metabolic process	1/97	31/14464	0.03225806	4.81010974	1.74490602	0.18845307	0.51827196	0.51433951	Abcc5	1
GO:0032367 intracellular cholesterol transport	1/97	31/14464	0.03225806	4.81010974	1.74490602	0.18845307	0.51827196	0.51433951	Pcsk9	1
GO:0034067 protein localization to Golgi apparatus	1/97	31/14464	0.03225806	4.81010974	1.74490602	0.18845307	0.51827196	0.51433951	Bicd2	1
GO:0060218 hematopoietic stem cell differentiation	1/97	31/14464	0.03225806	4.81010974	1.74490602	0.18845307	0.51827196	0.51433951	Mettl3	1
GO:0072337 modified amino acid transport	1/97	31/14464	0.03225806	4.81010974	1.74490602	0.18845307	0.51827196	0.51433951	Abcc5	1
GO:0098703 calcium ion import across plasma membrane	1/97	31/14464	0.03225806	4.81010974	1.74490602	0.18845307	0.51827196	0.51433951	Trpv2	1
GO:1902656 calcium ion import into cytosol	1/97	31/14464	0.03225806	4.81010974	1.74490602	0.18845307	0.51827196	0.51433951	Trpv2	1
GO:1904376 negative regulation of protein localization to cell periphery	1/97	31/14464	0.03225806	4.81010974	1.74490602	0.18845307	0.51827196	0.51433951	Sfn	1
GO:2000279 negative regulation of DNA biosynthetic process	1/97	31/14464	0.03225806	4.81010974	1.74490602	0.18845307	0.51827196	0.51433951	Cdkn1a	1
GO:0006873 intracellular monoatomic ion homeostasis	5/97	454/14464	0.01101322	1.64221808	1.1424137	0.18909301	0.51827196	0.51433951	Rab39/Efhc1/Fkbp1b/Atp1b3/Cln5	5
GO:0030317 flagellated sperm motility	2/97	119/14464	0.01680672	2.5061076	1.35553651	0.18994738	0.51827196	0.51433951	Efhc1/Tuba1a	2
GO:0090090 negative regulation of canonical Wnt signaling pathway	2/97	119/14464	0.01680672	2.5061076	1.35553651	0.18994738	0.51827196	0.51433951	Sfrp5/Ctnnd1	2
GO:0031667 response to nutrient levels	5/97	455/14464	0.01098901	1.63860881	1.13728432	0.19024692	0.51827196	0.51433951	Cdkn1a/Fkbp1b/Pcsk9/Lipa/Becn1	5
GO:0045861 negative regulation of proteolysis	3/97	224/14464	0.01339286	1.99705449	1.23571957	0.19028507	0.51827196	0.51433951	Hspa1b/Serpinb8/Caml	3
GO:0048675 axon extension	2/97	120/14464	0.01666667	2.48522337	1.34239174	0.19237353	0.51827196	0.51433951	Trpv2/Raph1	2
GO:0051402 neuron apoptotic process	4/97	339/14464	0.01179941	1.75945017	1.16261767	0.19300287	0.51827196	0.51433951	Pcsk9/Ptges3/Tuba1a/Lig4	4
GO:0002021 response to dietary excess	1/97	32/14464	0.03125	4.65979381	1.70294398	0.19390724	0.51827196	0.51433951	Lipa	1
GO:0002446 neutrophil mediated immunity	1/97	32/14464	0.03125	4.65979381	1.70294398	0.19390724	0.51827196	0.51433951	Dnase1l3	1
GO:0007435 salivary gland morphogenesis	1/97	32/14464	0.03125	4.65979381	1.70294398	0.19390724	0.51827196	0.51433951	Ctnnd1	1
GO:0031116 positive regulation of microtubule polymerization	1/97	32/14464	0.03125	4.65979381	1.70294398	0.19390724	0.51827196	0.51433951	Hspa1b	1
GO:0032743 positive regulation of interleukin-2 production	1/97	32/14464	0.03125	4.65979381	1.70294398	0.19390724	0.51827196	0.51433951	Traf2	1
GO:0051180 vitamin transport	1/97	32/14464	0.03125	4.65979381	1.70294398	0.19390724	0.51827196	0.51433951	Abcc5	1
GO:0070198 protein localization to chromosome, telomeric region	1/97	32/14464	0.03125	4.65979381	1.70294398	0.19390724	0.51827196	0.51433951	Cct7	1
GO:0090169 regulation of spindle assembly	1/97	32/14464	0.03125	4.65979381	1.70294398	0.19390724	0.51827196	0.51433951	Hspa1b	1
GO:0090322 regulation of superoxide metabolic process	1/97	32/14464	0.03125	4.65979381	1.70294398	0.19390724	0.51827196	0.51433951	Shc1	1
GO:0140058 neuron projection arborization	1/97	32/14464	0.03125	4.65979381	1.70294398	0.19390724	0.51827196	0.51433951	Tuba1a	1
GO:0180046 GPI anchored protein biosynthesis	1/97	32/14464	0.03125	4.65979381	1.70294398	0.19390724	0.51827196	0.51433951	Pigc	1
GO:1901381 positive regulation of potassium ion transmembrane transport	1/97	32/14464	0.03125	4.65979381	1.70294398	0.19390724	0.51827196	0.51433951	Atp1b3	1
GO:2000765 regulation of cytoplasmic translation	1/97	32/14464	0.03125	4.65979381	1.70294398	0.19390724	0.51827196	0.51433951	Mettl3	1
GO:0032434 regulation of proteasomal ubiquitin-dependent protein catabolic process	2/97	121/14464	0.01652893	2.46468433	1.32937876	0.19480387	0.51908104	0.51514245	Hspa1b/Caml	2
GO:0097722 sperm motility	2/97	121/14464	0.01652893	2.46468433	1.32937876	0.19480387	0.51908104	0.51514245	Efhc1/Tuba1a	2
GO:0072522 purine-containing compound biosynthetic process	3/97	228/14464	0.01315789	1.96201845	1.20306432	0.19709957	0.52158732	0.51762972	Lipa/Sphk2/Nme1	3
GO:1901136 carbohydrate derivative catabolic process	3/97	229/14464	0.01310044	1.95345068	1.19500371	0.19881269	0.52158732	0.51762972	Lipa/Edem1/Nudt3	3
GO:0006862 nucleotide transport	1/97	33/14464	0.03030303	4.51858794	1.66268195	0.19932513	0.52158732	0.51762972	Abcc5	1
GO:0018208 peptidyl-proline modification	1/97	33/14464	0.03030303	4.51858794	1.66268195	0.19932513	0.52158732	0.51762972	P4ha1	1
GO:0031018 endocrine pancreas development	1/97	33/14464	0.03030303	4.51858794	1.66268195	0.19932513	0.52158732	0.51762972	Myt1	1
GO:0032781 positive regulation of ATP-dependent activity	1/97	33/14464	0.03030303	4.51858794	1.66268195	0.19932513	0.52158732	0.51762972	Atp1b3	1
GO:0045943 positive regulation of transcription by RNA polymerase I	1/97	33/14464	0.03030303	4.51858794	1.66268195	0.19932513	0.52158732	0.51762972	Ubtf	1

GO:0046329	negative regulation of JNK cascade	1/97	33/14464	0.03030303	4.51858794	1.66268195	0.19932513	0.52158732	0.51762972	Sfrp5	1
GO:0046456	icosanoid biosynthetic process	1/97	33/14464	0.03030303	4.51858794	1.66268195	0.19932513	0.52158732	0.51762972	Ptges3	1
GO:0048854	brain morphogenesis	1/97	33/14464	0.03030303	4.51858794	1.66268195	0.19932513	0.52158732	0.51762972	Tuba1a	1
GO:0051642	centrosome localization	1/97	33/14464	0.03030303	4.51858794	1.66268195	0.19932513	0.52158732	0.51762972	Bicd2	1
GO:0070296	sarcoplasmic reticulum calcium ion transport	1/97	33/14464	0.03030303	4.51858794	1.66268195	0.19932513	0.52158732	0.51762972	Fkbp1b	1
GO:1902115	regulation of organelle assembly	3/97	230/14464	0.01304348	1.94495742	1.18698352	0.2005295	0.5239545	0.51997893	Hspa1b/Becn1/Pip4k2c	3
GO:0008286	insulin receptor signaling pathway	2/97	124/14464	0.01612903	2.40505487	1.29110501	0.20211836	0.52453241	0.52055246	Shc1/Pip4k2c	2
GO:0021987	cerebral cortex development	2/97	124/14464	0.01612903	2.40505487	1.29110501	0.20211836	0.52453241	0.52055246	Efhc1/Tuba1a	2
GO:0051100	negative regulation of binding	2/97	124/14464	0.01612903	2.40505487	1.29110501	0.20211836	0.52453241	0.52055246	Cdkn1a/Pcsk9	2
GO:0045727	positive regulation of translation	2/97	125/14464	0.016	2.38581443	1.27859402	0.20456382	0.52453241	0.52055246	Mettl3/Mrps27	2
GO:0002369	T cell cytokine production	1/97	34/14464	0.02941176	4.3856883	1.62399721	0.20470698	0.52453241	0.52055246	Traf2	1
GO:0007431	salivary gland development	1/97	34/14464	0.02941176	4.3856883	1.62399721	0.20470698	0.52453241	0.52055246	Ctnnd1	1
GO:0032366	intracellular sterol transport	1/97	34/14464	0.02941176	4.3856883	1.62399721	0.20470698	0.52453241	0.52055246	Pcsk9	1
GO:0034063	stress granule assembly	1/97	34/14464	0.02941176	4.3856883	1.62399721	0.20470698	0.52453241	0.52055246	Becn1	1
GO:0042119	neutrophil activation	1/97	34/14464	0.02941176	4.3856883	1.62399721	0.20470698	0.52453241	0.52055246	Dnase1l3	1
GO:0061842	microtubule organizing center localization	1/97	34/14464	0.02941176	4.3856883	1.62399721	0.20470698	0.52453241	0.52055246	Bicd2	1
GO:0010608	post-transcriptional regulation of gene expression	5/97	468/14464	0.01068376	1.5930919	1.07170429	0.2054771	0.52453241	0.52055246	Mettl3/Traf2/Nhlrc1/Mrps27/Hbs1l	5
GO:0010466	negative regulation of peptidase activity	2/97	126/14464	0.01587302	2.3668794	1.26620259	0.20701267	0.52453241	0.52055246	Hspa1b/Serpinb8	2
GO:0051225	spindle assembly	2/97	126/14464	0.01587302	2.3668794	1.26620259	0.20701267	0.52453241	0.52055246	Hspa1b/Smc1a	2
GO:1905037	autophagosome organization	2/97	126/14464	0.01587302	2.3668794	1.26620259	0.20701267	0.52453241	0.52055246	Becn1/Pip4k2c	2
GO:0044283	small molecule biosynthetic process	5/97	470/14464	0.0106383	1.58631279	1.06179182	0.20785659	0.52453241	0.52055246	Lipa/Ptges3/Pmvk/Sphk2/Stard3	5
GO:0015850	organic hydroxy compound transport	3/97	235/14464	0.01276596	1.90357535	1.14747472	0.20916669	0.52453241	0.52055246	Pcsk9/Lipa/Stard3	3
GO:0090257	regulation of muscle system process	3/97	235/14464	0.01276596	1.90357535	1.14747472	0.20916669	0.52453241	0.52055246	Shc1/Fkbp1b/Becn1	3
GO:0035966	response to topologically incorrect protein	2/97	127/14464	0.01574803	2.34824255	1.25392847	0.20946475	0.52453241	0.52055246	Pdia6/Edem1	2
GO:0002673	regulation of acute inflammatory response	1/97	35/14464	0.02857143	4.26038292	1.58677931	0.21005303	0.52453241	0.52055246	Dnase1l3	1
GO:0007595	lactation	1/97	35/14464	0.02857143	4.26038292	1.58677931	0.21005303	0.52453241	0.52055246	Nme1	1
GO:0008053	mitochondrial fusion	1/97	35/14464	0.02857143	4.26038292	1.58677931	0.21005303	0.52453241	0.52055246	Mief1	1
GO:0016242	negative regulation of macroautophagy	1/97	35/14464	0.02857143	4.26038292	1.58677931	0.21005303	0.52453241	0.52055246	Becn1	1
GO:0018279	protein N-linked glycosylation via asparagine	1/97	35/14464	0.02857143	4.26038292	1.58677931	0.21005303	0.52453241	0.52055246	Uggt1	1
GO:0045746	negative regulation of Notch signaling pathway	1/97	35/14464	0.02857143	4.26038292	1.58677931	0.21005303	0.52453241	0.52055246	Mettl3	1
GO:0048546	digestive tract morphogenesis	1/97	35/14464	0.02857143	4.26038292	1.58677931	0.21005303	0.52453241	0.52055246	Sfrp5	1
GO:0048679	regulation of axon regeneration	1/97	35/14464	0.02857143	4.26038292	1.58677931	0.21005303	0.52453241	0.52055246	Fkbp1b	1
GO:0062125	regulation of mitochondrial gene expression	1/97	35/14464	0.02857143	4.26038292	1.58677931	0.21005303	0.52453241	0.52055246	Mrps27	1
GO:0072344	rescue of stalled ribosome	1/97	35/14464	0.02857143	4.26038292	1.58677931	0.21005303	0.52453241	0.52055246	Hbs1l	1
GO:0072528	pyrimidine-containing compound biosynthetic process	1/97	35/14464	0.02857143	4.26038292	1.58677931	0.21005303	0.52453241	0.52055246	Nme1	1
GO:0090313	regulation of protein targeting to membrane	1/97	35/14464	0.02857143	4.26038292	1.58677931	0.21005303	0.52453241	0.52055246	Mief1	1
GO:1901616	organic hydroxy compound catabolic process	1/97	35/14464	0.02857143	4.26038292	1.58677931	0.21005303	0.52453241	0.52055246	Nudt3	1
GO:0022612	gland morphogenesis	2/97	128/14464	0.015625	2.32989691	1.24176947	0.21191992	0.52693603	0.52293784	Lipa/Ctnnd1	2
GO:0046328	regulation of JNK cascade	2/97	128/14464	0.015625	2.32989691	1.24176947	0.21191992	0.52693603	0.52293784	Sfrp5/Traf2	2
GO:0060294	cilium movement involved in cell motility	2/97	128/14464	0.015625	2.32989691	1.24176947	0.21191992	0.52693603	0.52293784	Efhc1/Tuba1a	2
GO:0006665	sphingolipid metabolic process	2/97	129/14464	0.01550388	2.31183569	1.22972347	0.21437803	0.52741674	0.5234149	Smpd4/Sphk2	2
GO:0001523	retinoid metabolic process	1/97	36/14464	0.02777778	4.14203895	1.55092845	0.2153635	0.52741674	0.5234149	Lipa	1
GO:0031952	regulation of protein autophosphorylation	1/97	36/14464	0.02777778	4.14203895	1.55092845	0.2153635	0.52741674	0.5234149	Ctnnd1	1
GO:0032350	regulation of hormone metabolic process	1/97	36/14464	0.02777778	4.14203895	1.55092845	0.2153635	0.52741674	0.5234149	Myt1	1
GO:0042908	xenobiotic transport	1/97	36/14464	0.02777778	4.14203895	1.55092845	0.2153635	0.52741674	0.5234149	Abcc5	1
GO:0043647	inositol phosphate metabolic process	1/97	36/14464	0.02777778	4.14203895	1.55092845	0.2153635	0.52741674	0.5234149	Nudt3	1
GO:0045747	positive regulation of Notch signaling pathway	1/97	36/14464	0.02777778	4.14203895	1.55092845	0.2153635	0.52741674	0.5234149	Tspan14	1
GO:0061744	motor behavior	1/97	36/14464	0.02777778	4.14203895	1.55092845	0.2153635	0.52741674	0.5234149	Tuba1a	1
GO:0090140	regulation of mitochondrial fission	1/97	36/14464	0.02777778	4.14203895	1.55092845	0.2153635	0.52741674	0.5234149	Mief1	1
GO:1903320	regulation of protein modification by small protein conjugation or removal	3/97	239/14464	0.0125523	1.87171634	1.11655473	0.21613676	0.52741674	0.5234149	Hspa1b/Nhlrc1/Caml	3
GO:0042113	B cell activation	3/97	240/14464	0.0125	1.86391753	1.10891702	0.21788717	0.52741674	0.5234149	Cdkn1a/Lig4/Nhej1	3
GO:0051924	regulation of calcium ion transport	3/97	240/14464	0.0125	1.86391753	1.10891702	0.21788717	0.52741674	0.5234149	Gpr35/Trpv2/Fkbp1b	3
GO:0009154	purine ribonucleotide catabolic process	2/97	131/14464	0.01526718	2.27654049	1.20596223	0.21930245	0.52741674	0.5234149	Lipa/Nudt3	2
GO:0002366	leukocyte activation involved in immune response	3/97	241/14464	0.01244813	1.85618343	1.10131549	0.21964063	0.52741674	0.5234149	Dnase1l3/Sphk2/Lig4	3
GO:0031669	cellular response to nutrient levels	3/97	241/14464	0.01244813	1.85618343	1.10131549	0.21964063	0.52741674	0.5234149	Cdkn1a/Pcsk9/Becn1	3
GO:0006084	acetyl-CoA metabolic process	1/97	37/14464	0.02702703	4.03009195	1.51635423	0.22063864	0.52741674	0.5234149	Pmvk	1
GO:0007095	mitotic G2 DNA damage checkpoint signaling	1/97	37/14464	0.02702703	4.03009195	1.51635423	0.22063864	0.52741674	0.5234149	Cdkn1a	1
GO:0010613	positive regulation of cardiac muscle hypertrophy	1/97	37/14464	0.02702703	4.03009195	1.51635423	0.22063864	0.52741674	0.5234149	Becn1	1
GO:0010837	regulation of keratinocyte proliferation	1/97	37/14464	0.02702703	4.03009195	1.51635423	0.22063864	0.52741674	0.5234149	Sfn	1
GO:0014742	positive regulation of muscle hypertrophy	1/97	37/14464	0.02702703	4.03009195	1.51635423	0.22063864	0.52741674	0.5234149	Becn1	1
GO:0017004	cytochrome complex assembly	1/97	37/14464	0.02702703	4.03009195	1.51635423	0.22063864	0.52741674	0.5234149	Coa4	1
GO:0043277	apoptotic cell clearance	1/97	37/14464	0.02702703	4.03009195	1.51635423	0.22063864	0.52741674	0.5234149	Becn1	1
GO:0046825	regulation of protein export from nucleus	1/97	37/14464	0.02702703	4.03009195	1.51635423	0.22063864	0.52741674	0.5234149	Sfn	1
GO:0048246	macrophage chemotaxis	1/97	37/14464	0.02702703	4.03009195	1.51635423	0.22063864	0.52741674	0.5234149	Gpr35	1
GO:1990573	potassium ion import across plasma membrane	1/97	37/14464	0.02702703	4.03009195	1.51635423	0.22063864	0.52741674	0.5234149	Atp1b3	1
GO:0007018	microtubule-based movement	4/97	359/14464	0.01114206	1.66143066	1.04274236	0.2208377	0.52741674	0.5234149	Bicd2/Efhc1/Tuba1a/Dnai4	4
GO:0030098	lymphocyte differentiation	4/97	359/14464	0.01114206	1.66143066	1.04274236	0.2208377	0.52741674	0.5234149	Lipa/Mettl3/Lig4/Nhej1	4
GO:0140014	mitotic nuclear division	3/97	242/14464	0.01239669	1.84851325	1.0937498	0.22139708	0.52741674	0.5234149	Hspa1b/Becn1/Smc1a	3
GO:0001101	response to acid chemical	2/97	132/14464	0.01515152	2.25929397	1.194243	0.22176848	0.52741674	0.5234149	Stambpl1/Tuba1a	2
GO:0001539	cilium or flagellum-dependent cell motility	2/97	132/14464	0.01515152	2.25929397	1.194243	0.22176848	0.52741674	0.5234149	Efhc1/Tuba1a	2
GO:0031109	microtubule polymerization or depolymerization	2/97	132/14464	0.01515152	2.25929397	1.194243	0.22176848	0.52741674	0.5234149	Hspa1b/Tuba1a	2
GO:0060285	cilium-dependent cell motility	2/97	132/14464	0.01515152	2.25929397	1.194243	0.22176848	0.52741674	0.5234149	Efhc1/Tuba1a	2
GO:0007292	female gamete generation	2/97	133/14464	0.01503759	2.2423068	1.18262879	0.22423686	0.52998113	0.52595983	Mettl3/Taf4b	2
GO:1901873	regulation of post-translational protein modification	3/97	244/14464	0.01229508	1.8333615	1.07872455	0.2249187	0.52998113	0.52595983	Hspa1b/Nhlrc1/Caml	3
GO:1903706	regulation of hemopoiesis	4/97	362/14464	0.01104972	1.6476619	1.02540232	0.22510566	0.52998113	0.52595983	Hspa1b/Mettl3/Ankrd54/Nme1	4
GO:0007020	microtubule nucleation	1/97	38/14464	0.02631579	3.9240369	1.48297455	0.22587868	0.52998113	0.52595983	Hspa1b	1
GO:0009303	rRNA transcription	1/97	38/14464	0.02631579	3.9240369	1.48297455	0.22587868	0.52998113	0.52595983	Ubtf	1
GO:0031112	positive regulation of microtubule polymerization or depolymerization	1/97	38/14464	0.02631579	3.9240369	1.48297455	0.22587868	0.52998113	0.52595983	Hspa1b	1
GO:0044331	cell-cell adhesion mediated by cadherin	1/97	38/14464	0.02631579	3.9240369	1.48297455	0.22587868	0.52998113	0.52595983	Ctnnd1	1
GO:1905476	negative regulation of protein localization to membrane	1/97	38/14464	0.02631579	3.9240369	1.48297455	0.22587868	0.52998113	0.52595983	Sfn	1
GO:2000649	regulation of sodium ion transmembrane transporter activity	1/97	38/14464	0.02631579	3.9240369	1.48297455	0.22587868	0.52998113	0.52595983	Pcsk9	1
GO:2001239	regulation of extrinsic apoptotic signaling pathway in absence of ligand	1/97	38/14464	0.02631579	3.9240369	1.48297455	0.22587868	0.52998113	0.52595983	Hspa1b	1
GO:0002263	cell activation involved in immune response	3/97	245/14464	0.0122449	1.82587839	1.0712643	0.22668378	0.53115716	0.52712694	Dnase1l3/Sphk2/Lig4	3
GO:0043604	amide biosynthetic process	2/97	135/14464	0.01481481	2.20908744	1.15970804	0.22918012	0.53628762	0.53221848	Smpd4/Sphk2	2
GO:0097305	response to alcohol	3/97	247/14464	0.01214575	1.81109395	1.0564469	0.23022215	0.5364337	0.53236344	Lipa/Pmvk/Sphk2	3
GO:0006693	prostaglandin metabolic process	1/97	39/14464	0.02564103	3.82342057	1.4507146	0.23108385	0.5364337	0.53236344	Ptges3	1
GO:0030890	positive regulation of B cell proliferation	1/97	39/14464	0.02564103	3.82342057	1.4507146	0.23108385	0.5364337	0.53236344	Cdkn1a	1
GO:0032435	negative regulation of proteasomal ubiquitin-dependent protein catabolic process	1/97	39/14464	0.02564103	3.82342057	1.4507146	0.23108385	0.5364337	0.53236344	Caml	1
GO:0033028	myeloid cell apoptotic process	1/97	39/14464	0.02564103	3.82342057	1.4507146	0.23108385	0.5364337	0.53236344	Lipa	1
GO:0090279	regulation of calcium ion import	1/97	39/14464	0.02564103	3.82342057	1.4507146	0.23108385	0.5364337	0.53236344	Trpv2	1
GO:0035725	sodium ion transmembrane transport	2/97	137/14464	0.01459854	2.17683799	1.13718558	0.23413113	0.53791601	0.5338345	Pcsk9/Atp1b3	2
GO:0001782	B cell homeostasis	1/97	40/14464	0.025	3.72783505	1.41950609	0.23625438	0.53791601	0.5338345	Caml	1
GO:0006220	pyrimidine nucleotide metabolic process	1/97	40/14464	0.025	3.72783505	1.41950609	0.23625438	0.53791601	0.5338345	Nme1	1
GO:0006636	unsaturated fatty acid biosynthetic process	1/97	40/14464	0.025	3.72783505	1.41950609	0.23625438	0.53791601	0.5338345	Ptges3	1
GO:0006692	prostanoid metabolic process	1/97	40/14464	0.025	3.72783505	1.41950609	0.23625438	0.53791601	0.5338345	Ptges3	1
GO:0016101	diterpenoid metabolic process	1/97	40/14464	0.025	3.72783505	1.41950609	0.23625438	0.53791601	0.5338345	Lipa	1
GO:0035196	miRNA processing	1/97	40/14464	0.025	3.72783505	1.41950609	0.23625438	0.53791601	0.5338345	Mettl3	1
GO:0036230	granulocyte activation	1/97	40/14464	0.025	3.72783505	1.41950609	0.23625438	0.53791601	0.5338345	Dnase1l3	1
GO:0043388	positive regulation of DNA binding	1/97	40/14464	0.025	3.72783505	1.41950609	0.23625438	0.53791601	0.5338345	Nme1	1
GO:0051445	regulation of meiotic cell cycle	1/97	40/14464	0.025	3.72783505	1.41950609	0.23625438	0.53791601	0.5338345	Mettl3	1
GO:0060338	regulation of type I interferon-mediated signaling pathway	1/97	40/14464	0.025	3.72783505	1.41950609	0.23625438	0.53791601	0.5338345	Mettl3	1
GO:1901016	regulation of potassium ion transmembrane transporter activity	1/97	40/14464	0.025	3.72783505	1.41950609	0.23625438	0.53791601	0.5338345	Atp1b3	1
GO:1901532	regulation of hematopoietic progenitor cell differentiation	1/97	40/14464	0.025	3.72783505	1.41950609	0.23625438	0.53791601	0.5338345	Mettl3	1
GO:0051099	positive regulation of binding	2/97	138/14464	0.01449275	2.1610638	1.12606943	0.23660921	0.53791601	0.5338345	Cln5/Nme1	2
GO:0006997	nucleus organization	2/97	139/14464	0.01438849	2.14551658	1.11504778	0.23908882	0.53791601	0.5338345	Agfg1/Golm1	2
GO:0046651	lymphocyte proliferation	3/97	253/14464	0.01185771	1.76814311	1.01279627	0.24089914	0.53791601	0.5338345	Cdkn1a/Fkbp1b/Lipa	3
GO:0008608	attachment of spindle microtubules to kinetochore	1/97	41/14464	0.02439024	3.63691225	1.38928651	0.2413905	0.53791601	0.5338345	Becn1	1
GO:0009394	2'-deoxyribonucleotide metabolic process	1/97	41/14464	0.02439024	3.63691225	1.38928651	0.2413905	0.53791601	0.5338345	Nme1	1
GO:0009395	phospholipid catabolic process	1/97	41/14464	0.02439024	3.63691225	1.38928651	0.2413905	0.53791601	0.5338345	Smpd4	1
GO:0010765	positive regulation of sodium ion transport	1/97	41/14464	0.02439024	3.63691225	1.38928651	0.2413905	0.53791601	0.5338345	Atp1b3	1
GO:0032527	protein exit from endoplasmic reticulum	1/97	41/14464	0.02439024	3.63691225	1.38928651	0.2413905	0.53791601	0.5338345	Edem1	1
GO:0043268	positive regulation of potassium ion transport	1/97	41/14464	0.02439024	3.63691225	1.38928651	0.2413905	0.53791601	0.5338345	Atp1b3	1
GO:0043507	positive regulation of JUN kinase activity	1/97	41/14464	0.02439024	3.63691225	1.38928651	0.2413905	0.53791601	0.5338345	Traf2	1
GO:0045047	protein targeting to ER	1/97	41/14464	0.02439024	3.63691225	1.38928651	0.2413905	0.53791601	0.5338345	Edem1	1
GO:0045104	intermediate filament cytoskeleton organization	1/97	41/14464	0.02439024	3.63691225	1.38928651	0.2413905	0.53791601	0.5338345	Agfg1	1
GO:0045907	positive regulation of vasoconstriction	1/97	41/14464	0.02439024	3.63691225	1.38928651	0.2413905	0.53791601	0.5338345	Shc1	1
GO:0048536	spleen development	1/97	41/14464	0.02439024	3.63691225	1.38928651	0.2413905	0.53791601	0.5338345	Lipa	1
GO:0051205	protein insertion into membrane	1/97	41/14464	0.02439024	3.63691225	1.38928651	0.2413905	0.53791601	0.5338345	Caml	1
GO:0060236	regulation of mitotic spindle organization	1/97	41/14464	0.02439024	3.63691225	1.38928651	0.2413905	0.53791601	0.5338345	Hspa1b	1
GO:0007204	positive regulation of cytosolic calcium ion concentration	2/97	140/14464	0.01428571	2.13019146	1.10411903	0.24156983	0.53791601	0.5338345	Gpr35/Fkbp1b	2
GO:0009261	ribonucleotide catabolic process	2/97	140/14464	0.01428571	2.13019146	1.10411903	0.24156983	0.53791601	0.5338345	Lipa/Nudt3	2
GO:0043405	regulation of MAP kinase activity	2/97	140/14464	0.01428571	2.13019146	1.10411903	0.24156983	0.53791601	0.5338345	Sfrp5/Traf2	2
GO:0045088	regulation of innate immune response	4/97	374/14464	0.01069519	1.59479574	0.95759125	0.24238845	0.53800769	0.53392549	Hspa1b/Sfn/Mettl3/Becn1	4
GO:0060560	developmental growth involved in morphogenesis	3/97	254/14464	0.01181102	1.76118191	1.00563348	0.24268708	0.53800769	0.53392549	Sfrp5/Trpv2/Raph1	3
GO:0006195	purine nucleotide catabolic process	2/97	141/14464	0.0141844	2.11508372	1.09328162	0.24405212	0.53800769	0.53392549	Lipa/Nudt3	2
GO:0055088	lipid homeostasis	2/97	141/14464	0.0141844	2.11508372	1.09328162	0.24405212	0.53800769	0.53392549	Pcsk9/Lipa	2
GO:0009225	nucleotide-sugar metabolic process	1/97	42/14464	0.02380952	3.5503191	1.35999852	0.24649242	0.53800769	0.53392549	Uggt1	1
GO:0019692	deoxyribose phosphate metabolic process	1/97	42/14464	0.02380952	3.5503191	1.35999852	0.24649242	0.53800769	0.53392549	Nme1	1
GO:0032790	ribosome disassembly	1/97	42/14464	0.02380952	3.5503191	1.35999852	0.24649242	0.53800769	0.53392549	Hbs1l	1
GO:0042446	hormone biosynthetic process	1/97	42/14464	0.02380952	3.5503191	1.35999852	0.24649242	0.53800769	0.53392549	Stard3	1
GO:0045103	intermediate filament-based process	1/97	42/14464	0.02380952	3.5503191	1.35999852	0.24649242	0.53800769	0.53392549	Agfg1	1
GO:0045604	regulation of epidermal cell differentiation	1/97	42/14464	0.02380952	3.5503191	1.35999852	0.24649242	0.53800769	0.53392549	Sfn	1
GO:0046627	negative regulation of insulin receptor signaling pathway	1/97	42/14464	0.02380952	3.5503191	1.35999852	0.24649242	0.53800769	0.53392549	Pip4k2c	1
GO:0070570	regulation of neuron projection regeneration	1/97	42/14464	0.02380952	3.5503191	1.35999852	0.24649242	0.53800769	0.53392549	Fkbp1b	1
GO:0086009	membrane repolarization	1/97	42/14464	0.02380952	3.5503191	1.35999852	0.24649242	0.53800769	0.53392549	Atp1b3	1
GO:1903573	negative regulation of response to endoplasmic reticulum stress	1/97	42/14464	0.02380952	3.5503191	1.35999852	0.24649242	0.53800769	0.53392549	Pdia6	1
GO:0006937	regulation of muscle contraction	2/97	142/14464	0.01408451	2.10018876	1.08253403	0.24653556	0.53800769	0.53392549	Shc1/Fkbp1b	2
GO:0009913	epidermal cell differentiation	2/97	142/14464	0.01408451	2.10018876	1.08253403	0.24653556	0.53800769	0.53392549	Cdkn1a/Sfn	2
GO:0031400	negative regulation of protein modification process	4/97	378/14464	0.01058201	1.5779196	0.93551659	0.24821846	0.53843486	0.53434942	Hspa1b/Cdkn1a/Sfrp5/Caml	4
GO:1990778	protein localization to cell periphery	4/97	378/14464	0.01058201	1.5779196	0.93551659	0.24821846	0.53843486	0.53434942	Tspan14/Sfn/Nhlrc1/Atp1b3	4
GO:0000723	telomere maintenance	2/97	143/14464	0.01398601	2.08550213	1.07187475	0.24902002	0.53843486	0.53434942	Ptges3/Cct7	2
GO:0032006	regulation of TOR signaling	2/97	143/14464	0.01398601	2.08550213	1.07187475	0.24902002	0.53843486	0.53434942	Stambpl1/Tbck	2
GO:2001233	regulation of apoptotic signaling pathway	4/97	379/14464	0.01055409	1.57375622	0.9300378	0.24968094	0.53843486	0.53434942	Hspa1b/Traf2/Becn1/Maz	4
GO:0030178	negative regulation of Wnt signaling pathway	2/97	144/14464	0.01388889	2.07101947	1.06130233	0.25150538	0.53843486	0.53434942	Sfrp5/Ctnnd1	2
GO:0009262	deoxyribonucleotide metabolic process	1/97	43/14464	0.02325581	3.46775354	1.33158942	0.25156039	0.53843486	0.53434942	Nme1	1
GO:0021696	cerebellar cortex morphogenesis	1/97	43/14464	0.02325581	3.46775354	1.33158942	0.25156039	0.53843486	0.53434942	Tuba1a	1
GO:0021872	forebrain generation of neurons	1/97	43/14464	0.02325581	3.46775354	1.33158942	0.25156039	0.53843486	0.53434942	Mettl3	1
GO:0032365	intracellular lipid transport	1/97	43/14464	0.02325581	3.46775354	1.33158942	0.25156039	0.53843486	0.53434942	Pcsk9	1
GO:0042181	ketone biosynthetic process	1/97	43/14464	0.02325581	3.46775354	1.33158942	0.25156039	0.53843486	0.53434942	Stard3	1
GO:0042771	intrinsic apoptotic signaling pathway in response to DNA damage by p53 class mediator	1/97	43/14464	0.02325581	3.46775354	1.33158942	0.25156039	0.53843486	0.53434942	Cdkn1a	1
GO:0045022	early endosome to late endosome transport	1/97	43/14464	0.02325581	3.46775354	1.33158942	0.25156039	0.53843486	0.53434942	Becn1	1
GO:0050856	regulation of T cell receptor signaling pathway	1/97	43/14464	0.02325581	3.46775354	1.33158942	0.25156039	0.53843486	0.53434942	Lipa	1
GO:0016042	lipid catabolic process	3/97	259/14464	0.01158301	1.72718226	0.9702967	0.25165977	0.53843486	0.53434942	Lipa/Smpd4/Pafah1b2	3
GO:0032943	mononuclear cell proliferation	3/97	259/14464	0.01158301	1.72718226	0.9702967	0.25165977	0.53843486	0.53434942	Cdkn1a/Fkbp1b/Lipa	3
GO:0032388	positive regulation of intracellular transport	2/97	145/14464	0.0137931	2.05673658	1.05081536	0.25399152	0.54114633	0.53704032	Sfn/Edem1	2
GO:2001235	positive regulation of apoptotic signaling pathway	2/97	146/14464	0.01369863	2.04264934	1.04041244	0.25647832	0.54114633	0.53704032	Traf2/Becn1	2
GO:0002183	cytoplasmic translational initiation	1/97	44/14464	0.02272727	3.38894096	1.30401065	0.25659462	0.54114633	0.53704032	Mettl3	1
GO:0006356	regulation of transcription by RNA polymerase I	1/97	44/14464	0.02272727	3.38894096	1.30401065	0.25659462	0.54114633	0.53704032	Ubtf	1
GO:0006767	water-soluble vitamin metabolic process	1/97	44/14464	0.02272727	3.38894096	1.30401065	0.25659462	0.54114633	0.53704032	Mmaa	1
GO:0030858	positive regulation of epithelial cell differentiation	1/97	44/14464	0.02272727	3.38894096	1.30401065	0.25659462	0.54114633	0.53704032	Sfn	1
GO:0034205	amyloid-beta formation	1/97	44/14464	0.02272727	3.38894096	1.30401065	0.25659462	0.54114633	0.53704032	Aph1b	1
GO:0043300	regulation of leukocyte degranulation	1/97	44/14464	0.02272727	3.38894096	1.30401065	0.25659462	0.54114633	0.53704032	Sphk2	1
GO:0048265	response to pain	1/97	44/14464	0.02272727	3.38894096	1.30401065	0.25659462	0.54114633	0.53704032	Vwa1	1
GO:1902116	negative regulation of organelle assembly	1/97	44/14464	0.02272727	3.38894096	1.30401065	0.25659462	0.54114633	0.53704032	Becn1	1
GO:0052547	regulation of peptidase activity	3/97	262/14464	0.01145038	1.70740537	0.94945909	0.25706784	0.54114633	0.53704032	Hspa1b/Serpinb8/Aph1b	3
GO:0043488	regulation of mRNA stability	2/97	147/14464	0.01360544	2.02875377	1.03009222	0.25896565	0.54114633	0.53704032	Mettl3/Traf2	2

GO:2001236	regulation of extrinsic apoptotic signaling pathway	2/97	147/14464	0.01360544	2.02875377	1.03009222	0.25896565	0.54114633	0.53704032	Hspa1b/Traf2	2
GO:0098813	nuclear chromosome segregation	3/97	264/14464	0.01136364	1.69447048	0.93571501	0.26068253	0.54114633	0.53704032	Hspa1b/Becn1/Smc1a	3
GO:0009411	response to UV	2/97	148/14464	0.01351351	2.01504597	1.01985338	0.2614534	0.54114633	0.53704032	Cdkn1a/Mettl3	2
GO:0002204	somatic recombination of immunoglobulin genes involved in immune response	1/97	45/14464	0.02222222	3.31363116	1.27721739	0.26159534	0.54114633	0.53704032	Lig4	1
GO:0002208	somatic diversification of immunoglobulins involved in immune response	1/97	45/14464	0.02222222	3.31363116	1.27721739	0.26159534	0.54114633	0.53704032	Lig4	1
GO:0006509	membrane protein ectodomain proteolysis	1/97	45/14464	0.02222222	3.31363116	1.27721739	0.26159534	0.54114633	0.53704032	Aph1b	1
GO:0006623	protein targeting to vacuole	1/97	45/14464	0.02222222	3.31363116	1.27721739	0.26159534	0.54114633	0.53704032	Becn1	1
GO:0009124	nucleoside monophosphate biosynthetic process	1/97	45/14464	0.02222222	3.31363116	1.27721739	0.26159534	0.54114633	0.53704032	Nme1	1
GO:0043407	negative regulation of MAP kinase activity	1/97	45/14464	0.02222222	3.31363116	1.27721739	0.26159534	0.54114633	0.53704032	Sfrp5	1
GO:0045190	isotype switching	1/97	45/14464	0.02222222	3.31363116	1.27721739	0.26159534	0.54114633	0.53704032	Lig4	1
GO:0048009	insulin-like growth factor receptor signaling pathway	1/97	45/14464	0.02222222	3.31363116	1.27721739	0.26159534	0.54114633	0.53704032	Shc1	1
GO:0072599	establishment of protein localization to endoplasmic reticulum	1/97	45/14464	0.02222222	3.31363116	1.27721739	0.26159534	0.54114633	0.53704032	Edem1	1
GO:0090224	regulation of spindle organization	1/97	45/14464	0.02222222	3.31363116	1.27721739	0.26159534	0.54114633	0.53704032	Hspa1b	1
GO:0099010	modification of postsynaptic structure	1/97	45/14464	0.02222222	3.31363116	1.27721739	0.26159534	0.54114633	0.53704032	Cap1	1
GO:1905953	negative regulation of lipid localization	1/97	45/14464	0.02222222	3.31363116	1.27721739	0.26159534	0.54114633	0.53704032	Pcsk9	1
GO:2000648	positive regulation of stem cell proliferation	1/97	45/14464	0.02222222	3.31363116	1.27721739	0.26159534	0.54114633	0.53704032	Taf4b	1
GO:0032868	response to insulin	3/97	265/14464	0.01132075	1.68807625	0.92888647	0.26249252	0.54236043	0.53824521	Shc1/Pcsk9/Pip4k2c	3
GO:0030218	erythrocyte differentiation	2/97	149/14464	0.01342282	2.00152218	1.00969462	0.26394145	0.54306726	0.53894667	Hspa1b/Ankrd54	2
GO:0032200	telomere organization	2/97	149/14464	0.01342282	2.00152218	1.00969462	0.26394145	0.54306726	0.53894667	Ptges3/Cct7	2
GO:0036294	cellular response to decreased oxygen levels	2/97	149/14464	0.01342282	2.00152218	1.00969462	0.26394145	0.54306726	0.53894667	Becn1/Mief1	2
GO:0001889	liver development	2/97	150/14464	0.01333333	1.98817869	0.99961468	0.26642969	0.54306726	0.53894667	Pcsk9/Lipa	2
GO:0006890	retrograde vesicle-mediated transport, Golgi to endoplasmic reticulum	1/97	46/14464	0.02173913	3.2415957	1.25116822	0.26656276	0.54306726	0.53894667	Bicd2	1
GO:0008340	determination of adult lifespan	1/97	46/14464	0.02173913	3.2415957	1.25116822	0.26656276	0.54306726	0.53894667	Lipa	1
GO:0033013	tetrapyrrole metabolic process	1/97	46/14464	0.02173913	3.2415957	1.25116822	0.26656276	0.54306726	0.53894667	Mmaa	1
GO:0035272	exocrine system development	1/97	46/14464	0.02173913	3.2415957	1.25116822	0.26656276	0.54306726	0.53894667	Ctnnd1	1
GO:0043303	mast cell degranulation	1/97	46/14464	0.02173913	3.2415957	1.25116822	0.26656276	0.54306726	0.53894667	Sphk2	1
GO:0046503	glycerolipid catabolic process	1/97	46/14464	0.02173913	3.2415957	1.25116822	0.26656276	0.54306726	0.53894667	Smpd4	1
GO:0071364	cellular response to epidermal growth factor stimulus	1/97	46/14464	0.02173913	3.2415957	1.25116822	0.26656276	0.54306726	0.53894667	Becn1	1
GO:1903426	regulation of reactive oxygen species biosynthetic process	1/97	46/14464	0.02173913	3.2415957	1.25116822	0.26656276	0.54306726	0.53894667	Sphk2	1
GO:0042445	hormone metabolic process	2/97	152/14464	0.01315789	1.96201845	0.97968632	0.27140628	0.54801033	0.54385223	Myt1/Stard3	2
GO:0007062	sister chromatid cohesion	1/97	47/14464	0.0212766	3.17262558	1.22582474	0.27149711	0.54801033	0.54385223	Smc1a	1
GO:0021795	cerebral cortex cell migration	1/97	47/14464	0.0212766	3.17262558	1.22582474	0.27149711	0.54801033	0.54385223	Efhc1	1
GO:0033344	cholesterol efflux	1/97	47/14464	0.0212766	3.17262558	1.22582474	0.27149711	0.54801033	0.54385223	Lipa	1
GO:0035640	exploration behavior	1/97	47/14464	0.0212766	3.17262558	1.22582474	0.27149711	0.54801033	0.54385223	Tuba1a	1
GO:0042789	mRNA transcription by RNA polymerase II	1/97	47/14464	0.0212766	3.17262558	1.22582474	0.27149711	0.54801033	0.54385223	Taf4b	1
GO:1900077	negative regulation of cellular response to insulin stimulus	1/97	47/14464	0.0212766	3.17262558	1.22582474	0.27149711	0.54801033	0.54385223	Pip4k2c	1
GO:1902459	positive regulation of stem cell population maintenance	1/97	47/14464	0.0212766	3.17262558	1.22582474	0.27149711	0.54801033	0.54385223	Sap30l	1
GO:0061008	hepaticobiliary system development	2/97	153/14464	0.0130719	1.9491948	0.96983553	0.27389441	0.55216543	0.54797581	Pcsk9/Lipa	2
GO:0009101	glycoprotein biosynthetic process	3/97	272/14464	0.01102941	1.64463311	0.88187549	0.27520691	0.55216543	0.54797581	Mlec/Uggt1/Fut11	3
GO:1904375	regulation of protein localization to cell periphery	2/97	154/14464	0.01298701	1.93653769	0.96005877	0.27638228	0.55216543	0.54797581	Sfn/Nhlrc1	2
GO:0002448	mast cell mediated immunity	1/97	48/14464	0.02083333	3.10652921	1.20115134	0.2763986	0.55216543	0.54797581	Sphk2	1
GO:0006911	phagocytosis, engulfment	1/97	48/14464	0.02083333	3.10652921	1.20115134	0.2763986	0.55216543	0.54797581	Becn1	1
GO:0009409	response to cold	1/97	48/14464	0.02083333	3.10652921	1.20115134	0.2763986	0.55216543	0.54797581	Lipa	1
GO:0045682	regulation of epidermis development	1/97	48/14464	0.02083333	3.10652921	1.20115134	0.2763986	0.55216543	0.54797581	Sfn	1
GO:0045815	transcription initiation-coupled chromatin remodeling	1/97	48/14464	0.02083333	3.10652921	1.20115134	0.2763986	0.55216543	0.54797581	Sphk2	1
GO:0051341	regulation of oxidoreductase activity	1/97	48/14464	0.02083333	3.10652921	1.20115134	0.2763986	0.55216543	0.54797581	Sphk2	1
GO:0043086	negative regulation of catalytic activity	4/97	398/14464	0.01005025	1.49862716	0.82882754	0.27780308	0.55243184	0.5482402	Hspa1b/Cdkn1a/Sfrp5/Serpinb8	4
GO:0001508	action potential	2/97	155/14464	0.01290323	1.9240439	0.95035491	0.27886979	0.55243184	0.5482402	Gpr35/Fkbp1b	2
GO:0050777	negative regulation of immune response	2/97	155/14464	0.01290323	1.9240439	0.95035491	0.27886979	0.55243184	0.5482402	Sfn/Mettl3	2
GO:0071333	cellular response to glucose stimulus	2/97	155/14464	0.01290323	1.9240439	0.95035491	0.27886979	0.55243184	0.5482402	Myt1/Fkbp1b	2
GO:0000266	mitochondrial fission	1/97	49/14464	0.02040816	3.04313065	1.17711494	0.28126745	0.55243184	0.5482402	Mief1	1
GO:0006721	terpenoid metabolic process	1/97	49/14464	0.02040816	3.04313065	1.17711494	0.28126745	0.55243184	0.5482402	Lipa	1
GO:0032757	positive regulation of interleukin-8 production	1/97	49/14464	0.02040816	3.04313065	1.17711494	0.28126745	0.55243184	0.5482402	Hspa1b	1
GO:0060074	synapse maturation	1/97	49/14464	0.02040816	3.04313065	1.17711494	0.28126745	0.55243184	0.5482402	Nrg2	1
GO:0098927	vesicle-mediated transport between endosomal compartments	1/97	49/14464	0.02040816	3.04313065	1.17711494	0.28126745	0.55243184	0.5482402	Becn1	1
GO:0007613	memory	2/97	156/14464	0.01282051	1.91171028	0.94072287	0.28135683	0.55243184	0.5482402	Tuba1a/Aph1b	2
GO:0007281	germ cell development	3/97	276/14464	0.01086957	1.62079785	0.85561062	0.28250281	0.55243184	0.5482402	Agfg1/Mettl3/Taf4b	3
GO:0002700	regulation of production of molecular mediator of immune response	2/97	157/14464	0.01273885	1.89953378	0.93116154	0.2838433	0.55243184	0.5482402	Traf2/Sphk2	2
GO:0002753	cytoplasmic pattern recognition receptor signaling pathway	2/97	157/14464	0.01273885	1.89953378	0.93116154	0.2838433	0.55243184	0.5482402	Hspa1b/Becn1	2
GO:0072523	purine-containing compound catabolic process	2/97	157/14464	0.01273885	1.89953378	0.93116154	0.2838433	0.55243184	0.5482402	Lipa/Nudt3	2
GO:1990845	adaptive thermogenesis	2/97	157/14464	0.01273885	1.89953378	0.93116154	0.2838433	0.55243184	0.5482402	Trpv2/Lipa	2
GO:2000058	regulation of ubiquitin-dependent protein catabolic process	2/97	157/14464	0.01273885	1.89953378	0.93116154	0.2838433	0.55243184	0.5482402	Hspa1b/Caml	2
GO:0032386	regulation of intracellular transport	3/97	277/14464	0.01083032	1.61494659	0.84911011	0.28432977	0.55243184	0.5482402	Sfn/Pcsk9/Edem1	3
GO:0002279	mast cell activation involved in immune response	1/97	50/14464	0.02	2.98226804	1.15368471	0.28610387	0.55243184	0.5482402	Sphk2	1
GO:0002762	negative regulation of myeloid leukocyte differentiation	1/97	50/14464	0.02	2.98226804	1.15368471	0.28610387	0.55243184	0.5482402	Nme1	1
GO:0006826	iron ion transport	1/97	50/14464	0.02	2.98226804	1.15368471	0.28610387	0.55243184	0.5482402	Abcc5	1
GO:0032371	regulation of sterol transport	1/97	50/14464	0.02	2.98226804	1.15368471	0.28610387	0.55243184	0.5482402	Pcsk9	1
GO:0032374	regulation of cholesterol transport	1/97	50/14464	0.02	2.98226804	1.15368471	0.28610387	0.55243184	0.5482402	Pcsk9	1
GO:0032663	regulation of interleukin-2 production	1/97	50/14464	0.02	2.98226804	1.15368471	0.28610387	0.55243184	0.5482402	Traf2	1
GO:0033047	regulation of mitotic sister chromatid segregation	1/97	50/14464	0.02	2.98226804	1.15368471	0.28610387	0.55243184	0.5482402	Becn1	1
GO:0043154	negative regulation of cysteine-type endopeptidase activity involved in apoptotic process	1/97	50/14464	0.02	2.98226804	1.15368471	0.28610387	0.55243184	0.5482402	Hspa1b	1
GO:0070849	response to epidermal growth factor	1/97	50/14464	0.02	2.98226804	1.15368471	0.28610387	0.55243184	0.5482402	Becn1	1
GO:0070918	regulatory ncRNA processing	1/97	50/14464	0.02	2.98226804	1.15368471	0.28610387	0.55243184	0.5482402	Mettl3	1
GO:0097300	programmed necrotic cell death	1/97	50/14464	0.02	2.98226804	1.15368471	0.28610387	0.55243184	0.5482402	Traf2	1
GO:0006605	protein targeting	3/97	278/14464	0.01079137	1.60913743	0.84263544	0.28615782	0.55243184	0.5482402	Edem1/Becn1/Mief1	3
GO:0009166	nucleotide catabolic process	2/97	158/14464	0.01265823	1.88751142	0.92166988	0.28632909	0.55243184	0.5482402	Lipa/Nudt3	2
GO:0071331	cellular response to hexose stimulus	2/97	158/14464	0.01265823	1.88751142	0.92166988	0.28632909	0.55243184	0.5482402	Myt1/Fkbp1b	2
GO:0071326	cellular response to monosaccharide stimulus	2/97	159/14464	0.01257862	1.87564028	0.91224686	0.2888141	0.55599895	0.55178024	Myt1/Fkbp1b	2
GO:0097553	calcium ion transmembrane import into cytosol	2/97	159/14464	0.01257862	1.87564028	0.91224686	0.2888141	0.55599895	0.55178024	Trpv2/Fkbp1b	2
GO:0002711	positive regulation of T cell mediated immunity	1/97	51/14464	0.01960784	2.9237922	1.13083194	0.29090808	0.55696312	0.5527371	Traf2	1
GO:0009299	mRNA transcription	1/97	51/14464	0.01960784	2.9237922	1.13083194	0.29090808	0.55696312	0.5527371	Taf4b	1
GO:0034605	cellular response to heat	1/97	51/14464	0.01960784	2.9237922	1.13083194	0.29090808	0.55696312	0.5527371	Hspa1b	1
GO:0046676	negative regulation of insulin secretion	1/97	51/14464	0.01960784	2.9237922	1.13083194	0.29090808	0.55696312	0.5527371	Fkbp1b	1
GO:0141137	positive regulation of gene expression, epigenetic	1/97	51/14464	0.01960784	2.9237922	1.13083194	0.29090808	0.55696312	0.5527371	Sphk2	1
GO:0070507	regulation of microtubule cytoskeleton organization	2/97	160/14464	0.0125	1.86391753	0.90289145	0.29129823	0.5570999	0.55287284	Hspa1b/Bicd2	2
GO:0048732	gland development	4/97	408/14464	0.00980392	1.4618961	0.77763449	0.29281873	0.55939578	0.5551513	Pcsk9/Lipa/Ctnnd1/Nme1	4
GO:0006469	negative regulation of protein kinase activity	2/97	161/14464	0.01242236	1.8523404	0.89360267	0.29378138	0.55940072	0.5551562	Cdkn1a/Sfrp5	2
GO:0033157	regulation of intracellular protein transport	2/97	161/14464	0.01242236	1.8523404	0.89360267	0.29378138	0.55940072	0.5551562	Sfn/Edem1	2
GO:0061013	regulation of mRNA catabolic process	2/97	161/14464	0.01242236	1.8523404	0.89360267	0.29378138	0.55940072	0.5551562	Mettl3/Traf2	2
GO:0021587	cerebellum morphogenesis	1/97	52/14464	0.01923077	2.86756542	1.10852983	0.2956803	0.56046377	0.55621119	Tuba1a	1
GO:0032387	negative regulation of intracellular transport	1/97	52/14464	0.01923077	2.86756542	1.10852983	0.2956803	0.56046377	0.55621119	Pcsk9	1
GO:0044818	mitotic G2/M transition checkpoint	1/97	52/14464	0.01923077	2.86756542	1.10852983	0.2956803	0.56046377	0.55621119	Cdkn1a	1
GO:0045773	positive regulation of axon extension	1/97	52/14464	0.01923077	2.86756542	1.10852983	0.2956803	0.56046377	0.55621119	Trpv2	1
GO:0034101	erythrocyte homeostasis	2/97	162/14464	0.01234568	1.8409062	0.88437954	0.29626346	0.56046377	0.55621119	Hspa1b/Ankrd54	2
GO:0043487	regulation of RNA stability	2/97	162/14464	0.01234568	1.8409062	0.88437954	0.29626346	0.56046377	0.55621119	Mettl3/Traf2	2
GO:0006024	glycosaminoglycan biosynthetic process	1/97	53/14464	0.01886792	2.81346042	1.08675331	0.30042072	0.56526956	0.56098051	Abcc5	1
GO:0042149	cellular response to glucose starvation	1/97	53/14464	0.01886792	2.81346042	1.08675331	0.30042072	0.56526956	0.56098051	Becn1	1
GO:0046578	regulation of Ras protein signal transduction	1/97	53/14464	0.01886792	2.81346042	1.08675331	0.30042072	0.56526956	0.56098051	Rasgef1a	1
GO:0070527	platelet aggregation	1/97	53/14464	0.01886792	2.81346042	1.08675331	0.30042072	0.56526956	0.56098051	Pdia6	1
GO:0099563	modification of synaptic structure	1/97	53/14464	0.01886792	2.81346042	1.08675331	0.30042072	0.56526956	0.56098051	Cap1	1
GO:1901293	nucleoside phosphate biosynthetic process	3/97	286/14464	0.01048951	1.5641266	0.79174279	0.30081587	0.56540445	0.56111438	Lipa/Sphk2/Nme1	3
GO:0070661	leukocyte proliferation	3/97	287/14464	0.01045296	1.55867668	0.78549122	0.30265163	0.56801543	0.56370555	Cdkn1a/Fkbp1b/Lipa	3
GO:0072594	establishment of protein localization to organelle	4/97	415/14464	0.00963855	1.43723761	0.74259303	0.30339787	0.56801543	0.56370555	Cdkn1a/Edem1/Becn1/Cct7	4
GO:0071453	cellular response to oxygen levels	2/97	165/14464	0.01212121	1.80743518	0.85709472	0.30370227	0.56801543	0.56370555	Becn1/Mief1	2
GO:0048285	organelle fission	4/97	416/14464	0.00961538	1.43378271	0.73763866	0.3049131	0.56801543	0.56370555	Hspa1b/Becn1/Mief1/Smc1a	4
GO:0002381	immunoglobulin production involved in immunoglobulin-mediated immune response	1/97	54/14464	0.01851852	2.7613593	1.06547892	0.30512957	0.56801543	0.56370555	Lig4	1
GO:0021575	hindbrain morphogenesis	1/97	54/14464	0.01851852	2.7613593	1.06547892	0.30512957	0.56801543	0.56370555	Tuba1a	1
GO:0035567	non-canonical Wnt signaling pathway	1/97	54/14464	0.01851852	2.7613593	1.06547892	0.30512957	0.56801543	0.56370555	Sfrp5	1
GO:0046173	polyol biosynthetic process	1/97	54/14464	0.01851852	2.7613593	1.06547892	0.30512957	0.56801543	0.56370555	Sphk2	1
GO:0061462	protein localization to lysosome	1/97	54/14464	0.01851852	2.7613593	1.06547892	0.30512957	0.56801543	0.56370555	Becn1	1
GO:0050673	epithelial cell proliferation	4/97	417/14464	0.00959233	1.43034438	0.73269696	0.30642924	0.569828	0.56550436	Cdkn1a/Sfn/Lipa/Nme1	4
GO:0070588	calcium ion transmembrane transport	3/97	290/14464	0.01034483	1.54255244	0.76687842	0.30816249	0.57004497	0.56571968	Gpr35/Trpv2/Fkbp1b	3
GO:0098739	import across plasma membrane	2/97	167/14464	0.01197605	1.78578925	0.83921623	0.30865436	0.57004497	0.56571968	Trpv2/Atp1b3	2
GO:1903169	regulation of calcium ion transmembrane transport	2/97	167/14464	0.01197605	1.78578925	0.83921623	0.30865436	0.57004497	0.56571968	Gpr35/Fkbp1b	2
GO:0007080	mitotic metaphase chromosome alignment	1/97	55/14464	0.01818182	2.71115276	1.04468467	0.30980705	0.57004497	0.56571968	Becn1	1
GO:0019228	neuronal action potential	1/97	55/14464	0.01818182	2.71115276	1.04468467	0.30980705	0.57004497	0.56571968	Gpr35	1
GO:0072527	pyrimidine-containing compound metabolic process	1/97	55/14464	0.01818182	2.71115276	1.04468467	0.30980705	0.57004497	0.56571968	Nme1	1
GO:1901799	negative regulation of proteasomal protein catabolic process	1/97	55/14464	0.01818182	2.71115276	1.04468467	0.30980705	0.57004497	0.56571968	Caml	1
GO:1904263	positive regulation of TORC1 signaling	1/97	55/14464	0.01818182	2.71115276	1.04468467	0.30980705	0.57004497	0.56571968	Stambpl1	1
GO:1905517	macrophage migration	1/97	55/14464	0.01818182	2.71115276	1.04468467	0.30980705	0.57004497	0.56571968	Gpr35	1
GO:2000059	negative regulation of ubiquitin-dependent protein catabolic process	1/97	55/14464	0.01818182	2.71115276	1.04468467	0.30980705	0.57004497	0.56571968	Caml	1
GO:0007264	small GTPase-mediated signal transduction	4/97	420/14464	0.00952381	1.42012764	0.71794719	0.31098281	0.57127284	0.56693824	Cdkn1a/Rab39/Lipa/Rasgef1a	4
GO:0000910	cytokinesis	2/97	168/14464	0.01190476	1.77515955	0.83036777	0.311128	0.57127284	0.56693824	Efhc1/Becn1	2
GO:0071375	cellular response to peptide hormone stimulus	3/97	292/14464	0.01027397	1.53198701	0.75458626	0.31183891	0.57137679	0.5670414	Shc1/Pcsk9/Pip4k2c	3
GO:0038066	p38MAPK cascade	1/97	56/14464	0.01785714	2.66273932	1.02434994	0.31445336	0.57137679	0.5670414	Becn1	1
GO:0042987	amyloid precursor protein catabolic process	1/97	56/14464	0.01785714	2.66273932	1.02434994	0.31445336	0.57137679	0.5670414	Aph1b	1
GO:0043462	regulation of ATP-dependent activity	1/97	56/14464	0.01785714	2.66273932	1.02434994	0.31445336	0.57137679	0.5670414	Atp1b3	1
GO:0048260	positive regulation of receptor-mediated endocytosis	1/97	56/14464	0.01785714	2.66273932	1.02434994	0.31445336	0.57137679	0.5670414	Pcsk9	1
GO:0050854	regulation of antigen receptor-mediated signaling pathway	1/97	56/14464	0.01785714	2.66273932	1.02434994	0.31445336	0.57137679	0.5670414	Lipa	1
GO:0072348	sulfur compound transport	1/97	56/14464	0.01785714	2.66273932	1.02434994	0.31445336	0.57137679	0.5670414	Abcc5	1
GO:1903307	positive regulation of regulated secretory pathway	1/97	56/14464	0.01785714	2.66273932	1.02434994	0.31445336	0.57137679	0.5670414	Sphk2	1
GO:1903533	regulation of protein targeting	1/97	56/14464	0.01785714	2.66273932	1.02434994	0.31445336	0.57137679	0.5670414	Mief1	1
GO:2001238	positive regulation of extrinsic apoptotic signaling pathway	1/97	56/14464	0.01785714	2.66273932	1.02434994	0.31445336	0.57137679	0.5670414	Traf2	1
GO:0007283	spermatogenesis	4/97	423/14464	0.00945626	1.41005581	0.70330896	0.31554355	0.57203841	0.567698	Agfg1/Mettl3/Pafah1b2/Taf4b	4
GO:0022412	cellular process involved in reproduction in multicellular organism	3/97	295/14464	0.01016949	1.51640748	0.7363189	0.31735645	0.57203841	0.567698	Agfg1/Mettl3/Taf4b	3
GO:0001678	intracellular glucose homeostasis	2/97	171/14464	0.01169591	1.7440164	0.80417558	0.31853822	0.57203841	0.567698	Myt1/Fkbp1b	2
GO:0071322	cellular response to carbohydrate stimulus	2/97	171/14464	0.01169591	1.7440164	0.80417558	0.31853822	0.57203841	0.567698	Myt1/Fkbp1b	2
GO:0031113	regulation of microtubule polymerization	1/97	57/14464	0.01754386	2.6160246	1.00445534	0.31906871	0.57203841	0.567698	Hspa1b	1
GO:0032623	interleukin-2 production	1/97	57/14464	0.01754386	2.6160246	1.00445534	0.31906871	0.57203841	0.567698	Traf2	1
GO:0033363	secretory granule organization	1/97	57/14464	0.01754386	2.6160246	1.00445534	0.31906871	0.57203841	0.567698	Agfg1	1
GO:0033619	membrane protein proteolysis	1/97	57/14464	0.01754386	2.6160246	1.00445534	0.31906871	0.57203841	0.567698	Aph1b	1
GO:0048286	lung alveolus development	1/97	57/14464	0.01754386	2.6160246	1.00445534	0.31906871	0.57203841	0.567698	Ptges3	1
GO:0051123	RNA polymerase II preinitiation complex assembly	1/97	57/14464	0.01754386	2.6160246	1.00445534	0.31906871	0.57203841	0.567698	Taf4b	1
GO:0099024	plasma membrane invagination	1/97	57/14464	0.01754386	2.6160246	1.00445534	0.31906871	0.57203841	0.567698	Becn1	1
GO:0045862	positive regulation of proteolysis	3/97	296/14464	0.01013514	1.51128448	0.73027462	0.31919623	0.57203841	0.567698	Hspa1b/Ctnnd1/Aph1b	3
GO:0048660	regulation of smooth muscle cell proliferation	2/97	172/14464	0.01162791	1.73387677	0.79555988	0.32100444	0.57203841	0.567698	Shc1/Cdkn1a	2
GO:0030099	myeloid cell differentiation	4/97	428/14464	0.00934579	1.3935832	0.67915479	0.3231588	0.57203841	0.567698	Hspa1b/Lipa/Ankrd54/Nme1	4
GO:0030307	positive regulation of cell growth	2/97	173/14464	0.01156069	1.72385436	0.7870004	0.3234686	0.57203841	0.567698	Sfn/Trpv2	2
GO:0033673	negative regulation of kinase activity	2/97	173/14464	0.01156069	1.72385436	0.7870004	0.3234686	0.57203841	0.567698	Cdkn1a/Sfrp5	2
GO:0043393	regulation of protein binding	2/97	173/14464	0.01156069	1.72385436	0.7870004	0.3234686	0.57203841	0.567698	Cdkn1a/Pcsk9	2
GO:0006023	aminoglycan biosynthetic process	1/97	58/14464	0.01724138	2.57092073	0.98498264	0.32365331	0.57203841	0.567698	Abcc5	1
GO:0006805	xenobiotic metabolic process	1/97	58/14464	0.01724138	2.57092073	0.98498264	0.32365331	0.57203841	0.567698	Cyp2j13	1
GO:0006940	regulation of smooth muscle contraction	1/97	58/14464	0.01724138	2.57092073	0.98498264	0.32365331	0.57203841	0.567698	Shc1	1
GO:0055117	regulation of cardiac muscle contraction	1/97	58/14464	0.01724138	2.57092073	0.98498264	0.32365331	0.57203841	0.567698	Fkbp1b	1
GO:0060337	type I interferon-mediated signaling pathway	1/97	58/14464	0.01724138	2.57092073	0.98498264	0.32365331	0.57203841	0.567698	Mettl3	1
GO:0070613	regulation of protein processing	1/97	58/14464	0.01724138	2.57092073	0.98498264	0.32365331	0.57203841	0.567698	Ctnnd1	1
GO:0071357	cellular response to type I interferon	1/97	58/14464	0.01724138	2.57092073	0.98498264	0.32365331	0.57203841	0.567698	Mettl3	1
GO:0072666	establishment of protein localization to vacuole	1/97	58/14464	0.01724138	2.57092073	0.98498264	0.32365331	0.57203841	0.567698	Becn1	1
GO:0090278	negative regulation of peptide hormone secretion	1/97	58/14464	0.01724138	2.57092073	0.98498264	0.32365331	0.57203841	0.567698	Fkbp1b	1
GO:2000117	negative regulation of cysteine-type endopeptidase activity	1/97	58/14464	0.01724138	2.57092073	0.98498264	0.32365331	0.57203841	0.567698	Hspa1b	1
GO:0006903	vesicle targeting	1/97	59/14464	0.01694915	2.5273458	0.96591467	0.32820736	0.5746137	0.57025375	Stard3	1
GO:0022029	telencephalon cell migration	1/97	59/14464	0.01694915	2.5273458	0.96591467	0.32820736	0.5746137	0.57025375	Efhc1	1
GO:0031103	axon regeneration	1/97	59/14464	0.01694915	2.5273458	0.96591467	0.32820736	0.5746137	0.57025375	Fkbp1b	1
GO:0048255	mRNA stabilization	1/97	59/14464	0.01694915	2.5273458	0.96591467	0.32820736	0.5746137	0.57025375	Traf2	1
GO:0070231	T cell apoptotic process	1/97	59/14464	0.01694915	2.5273458	0.96591467	0.32820736	0.5746137	0.57025375	Lipa	1
GO:0098900	regulation of action potential	1/97	59/14464	0.01694915	2.5273458	0.96591467	0.32820736	0.5746137	0.57025375	Gpr35	1

GO:2000134	negative regulation of G1/S transition of mitotic cell cycle	1/97	59/14464	0.01694915	2.5273458	0.96591467	0.32820736	0.5746137	0.57025375	Cdkn1a	1
GO:0021543	pallium development	2/97	175/14464	0.01142857	1.70415317	0.77004704	0.32839038	0.5746137	0.57025375	Efhc1/Tuba1a	2
GO:0071214	cellular response to abiotic stimulus	3/97	301/14464	0.00996678	1.48618009	0.70038172	0.32839765	0.5746137	0.57025375	Cdkn1a/Mettl3/Lig4	3
GO:0104004	cellular response to environmental stimulus	3/97	301/14464	0.00996678	1.48618009	0.70038172	0.32839765	0.5746137	0.57025375	Cdkn1a/Mettl3/Lig4	3
GO:0097193	intrinsic apoptotic signaling pathway	3/97	302/14464	0.00993377	1.48125896	0.69446765	0.33023814	0.57716769	0.57278836	Cdkn1a/Sfn/Becn1	3
GO:0048284	organelle fusion	2/97	176/14464	0.01136364	1.69447048	0.7616517	0.33084784	0.57716769	0.57278836	Rab39/Mief1	2
GO:1901292	nucleoside phosphate catabolic process	2/97	176/14464	0.01136364	1.69447048	0.7616517	0.33084784	0.57716769	0.57278836	Lipa/Nudt3	2
GO:0002792	negative regulation of peptide secretion	1/97	60/14464	0.01666667	2.48522337	0.94723526	0.33273106	0.57814503	0.57375828	Fkbp1b	1
GO:0019933	cAMP-mediated signaling	1/97	60/14464	0.01666667	2.48522337	0.94723526	0.33273106	0.57814503	0.57375828	Cap1	1
GO:0032147	activation of protein kinase activity	1/97	60/14464	0.01666667	2.48522337	0.94723526	0.33273106	0.57814503	0.57375828	Traf2	1
GO:0048662	negative regulation of smooth muscle cell proliferation	1/97	60/14464	0.01666667	2.48522337	0.94723526	0.33273106	0.57814503	0.57375828	Cdkn1a	1
GO:0010970	transport along microtubule	2/97	177/14464	0.01129944	1.6848972	0.75330962	0.33330292	0.57856355	0.57417363	Bicd2/Tuba1a	2
GO:0042391	regulation of membrane potential	4/97	437/14464	0.00915332	1.3648824	0.63641916	0.33690122	0.58187338	0.57745835	Gpr35/Fkbp1b/Lipa/Atp1b3	4
GO:0048232	male gamete generation	4/97	437/14464	0.00915332	1.3648824	0.63641916	0.33690122	0.58187338	0.57745835	Agfg1/Mettl3/Pafah1b2/Taf4b	4
GO:0009247	glycolipid biosynthetic process	1/97	61/14464	0.01639344	2.444482	0.92892914	0.33722461	0.58187338	0.57745835	Pigc	1
GO:0021885	forebrain cell migration	1/97	61/14464	0.01639344	2.444482	0.92892914	0.33722461	0.58187338	0.57745835	Efhc1	1
GO:0030888	regulation of B cell proliferation	1/97	61/14464	0.01639344	2.444482	0.92892914	0.33722461	0.58187338	0.57745835	Cdkn1a	1
GO:0032677	regulation of interleukin-8 production	1/97	61/14464	0.01639344	2.444482	0.92892914	0.33722461	0.58187338	0.57745835	Hspa1b	1
GO:0050796	regulation of insulin secretion	2/97	179/14464	0.01117318	1.66607153	0.73678246	0.33820558	0.58187338	0.57745835	Myt1/Fkbp1b	2
GO:0051091	positive regulation of DNA-binding transcription factor activity	2/97	179/14464	0.01117318	1.66607153	0.73678246	0.33820558	0.58187338	0.57745835	Hspa1b/Traf2	2
GO:0051346	negative regulation of hydrolase activity	2/97	179/14464	0.01117318	1.66607153	0.73678246	0.33820558	0.58187338	0.57745835	Hspa1b/Serpinb8	2
GO:0048659	smooth muscle cell proliferation	2/97	180/14464	0.01111111	1.65681558	0.72859601	0.34065302	0.58213547	0.57771845	Shc1/Cdkn1a	2
GO:0032102	negative regulation of response to external stimulus	3/97	308/14464	0.00974026	1.45240327	0.65942108	0.34127908	0.58213547	0.57771845	Sfn/Mettl3/Aph1b	3
GO:0051090	regulation of DNA-binding transcription factor activity	3/97	308/14464	0.00974026	1.45240327	0.65942108	0.34127908	0.58213547	0.57771845	Hspa1b/Commd7/Traf2	3
GO:0006611	protein export from nucleus	1/97	62/14464	0.01612903	2.40505487	0.91098188	0.34168821	0.58213547	0.57771845	Sfn	1
GO:0006801	superoxide metabolic process	1/97	62/14464	0.01612903	2.40505487	0.91098188	0.34168821	0.58213547	0.57771845	Shc1	1
GO:0021695	cerebellar cortex development	1/97	62/14464	0.01612903	2.40505487	0.91098188	0.34168821	0.58213547	0.57771845	Tuba1a	1
GO:0031016	pancreas development	1/97	62/14464	0.01612903	2.40505487	0.91098188	0.34168821	0.58213547	0.57771845	Myt1	1
GO:0032637	interleukin-8 production	1/97	62/14464	0.01612903	2.40505487	0.91098188	0.34168821	0.58213547	0.57771845	Hspa1b	1
GO:1903317	regulation of protein maturation	1/97	62/14464	0.01612903	2.40505487	0.91098188	0.34168821	0.58213547	0.57771845	Ctnnd1	1
GO:2001244	positive regulation of intrinsic apoptotic signaling pathway	1/97	62/14464	0.01612903	2.40505487	0.91098188	0.34168821	0.58213547	0.57771845	Becn1	1
GO:0016236	macroautophagy	3/97	309/14464	0.00970874	1.44770293	0.65365149	0.34311852	0.5840031	0.5795719	Becn1/Pafah1b2/Pip4k2c	3
GO:0071805	potassium ion transmembrane transport	2/97	182/14464	0.01098901	1.63860881	0.71237407	0.34553974	0.58569347	0.58124945	Kcnk10/Atp1b3	2
GO:0001836	release of cytochrome c from mitochondria	1/97	63/14464	0.01587302	2.3668794	0.89337987	0.34612206	0.58569347	0.58124945	Sfn	1
GO:0051898	negative regulation of phosphatidylinositol 3-kinase/protein kinase B signal transduction	1/97	63/14464	0.01587302	2.3668794	0.89337987	0.34612206	0.58569347	0.58124945	Sfrp5	1
GO:0097192	extrinsic apoptotic signaling pathway in absence of ligand	1/97	63/14464	0.01587302	2.3668794	0.89337987	0.34612206	0.58569347	0.58124945	Hspa1b	1
GO:0097352	autophagosome maturation	1/97	63/14464	0.01587302	2.3668794	0.89337987	0.34612206	0.58569347	0.58124945	Becn1	1
GO:0170041	non-proteinogenic amino acid metabolic process	1/97	63/14464	0.01587302	2.3668794	0.89337987	0.34612206	0.58569347	0.58124945	P4ha1	1
GO:0010038	response to metal ion	3/97	311/14464	0.0096463	1.43839295	0.64217222	0.34679643	0.58626708	0.58181871	Tuba1a/Lig4/Becn1	3
GO:0016051	carbohydrate biosynthetic process	2/97	183/14464	0.01092896	1.62965467	0.70433728	0.34797887	0.58713038	0.58267546	Ptges3/Nhlrc1	2
GO:1990138	neuron projection extension	2/97	183/14464	0.01092896	1.62965467	0.70433728	0.34797887	0.58713038	0.58267546	Trpv2/Raph1	2
GO:0061136	regulation of proteasomal protein catabolic process	2/97	184/14464	0.01086957	1.62079785	0.69634911	0.35041509	0.58883415	0.5843663	Hspa1b/Caml	2
GO:0002637	regulation of immunoglobulin production	1/97	64/14464	0.015625	2.32989691	0.87611019	0.35052634	0.58883415	0.5843663	Traf2	1
GO:0010972	negative regulation of G2/M transition of mitotic cell cycle	1/97	64/14464	0.015625	2.32989691	0.87611019	0.35052634	0.58883415	0.5843663	Cdkn1a	1
GO:0062208	positive regulation of pattern recognition receptor signaling pathway	1/97	64/14464	0.015625	2.32989691	0.87611019	0.35052634	0.58883415	0.5843663	Hspa1b	1
GO:0002831	regulation of response to biotic stimulus	4/97	446/14464	0.00896861	1.33733993	0.59459899	0.35067297	0.58883415	0.5843663	Hspa1b/Sfn/Mettl3/Becn1	4
GO:1903131	mononuclear cell differentiation	4/97	447/14464	0.00894855	1.33434812	0.59000694	0.3522042	0.59021903	0.58574067	Lipa/Mettl3/Lig4/Nhej1	4
GO:0042886	amide transport	3/97	314/14464	0.00955414	1.42465034	0.62510085	0.35231027	0.59021903	0.58574067	Myt1/Abcc5/Fkbp1b	3
GO:0006814	sodium ion transport	2/97	185/14464	0.01081081	1.61203678	0.68840894	0.35284833	0.59021903	0.58574067	Pcsk9/Atp1b3	2
GO:0042098	T cell proliferation	2/97	185/14464	0.01081081	1.61203678	0.68840894	0.35284833	0.59021903	0.58574067	Fkbp1b/Lipa	2
GO:0001910	regulation of leukocyte mediated cytotoxicity	1/97	65/14464	0.01538462	2.29405234	0.85916063	0.35490127	0.59026396	0.58578526	Dnase1l3	1
GO:0010324	membrane invagination	1/97	65/14464	0.01538462	2.29405234	0.85916063	0.35490127	0.59026396	0.58578526	Becn1	1
GO:0034340	response to type I interferon	1/97	65/14464	0.01538462	2.29405234	0.85916063	0.35490127	0.59026396	0.58578526	Mettl3	1
GO:0038034	signal transduction in absence of ligand	1/97	65/14464	0.01538462	2.29405234	0.85916063	0.35490127	0.59026396	0.58578526	Hspa1b	1
GO:0045576	mast cell activation	1/97	65/14464	0.01538462	2.29405234	0.85916063	0.35490127	0.59026396	0.58578526	Sphk2	1
GO:0060261	positive regulation of transcription initiation by RNA polymerase II	1/97	65/14464	0.01538462	2.29405234	0.85916063	0.35490127	0.59026396	0.58578526	Taf4b	1
GO:0006091	generation of precursor metabolites and energy	4/97	449/14464	0.00890869	1.32840447	0.58085495	0.35526698	0.59031054	0.58583149	Lipa/Ptges3/Nhlrc1/Sphk2	4
GO:0034248	regulation of amide metabolic process	1/97	66/14464	0.01515152	2.25929397	0.84251962	0.35924703	0.59282607	0.58832793	Sphk2	1
GO:0060976	coronary vasculature development	1/97	66/14464	0.01515152	2.25929397	0.84251962	0.35924703	0.59282607	0.58832793	Dctn5	1
GO:0070897	transcription preinitiation complex assembly	1/97	66/14464	0.01515152	2.25929397	0.84251962	0.35924703	0.59282607	0.58832793	Taf4b	1
GO:0140962	multicellular organismal-level chemical homeostasis	1/97	66/14464	0.01515152	2.25929397	0.84251962	0.35924703	0.59282607	0.58832793	Lipa	1
GO:1902373	negative regulation of mRNA catabolic process	1/97	66/14464	0.01515152	2.25929397	0.84251962	0.35924703	0.59282607	0.58832793	Traf2	1
GO:1902750	negative regulation of cell cycle G2/M phase transition	1/97	66/14464	0.01515152	2.25929397	0.84251962	0.35924703	0.59282607	0.58832793	Cdkn1a	1
GO:0030522	intracellular receptor signaling pathway	3/97	318/14464	0.00943396	1.40673021	0.60260839	0.35965479	0.59282607	0.58832793	Hspa1b/Ptges3/Becn1	3
GO:0051223	regulation of protein transport	4/97	452/14464	0.00884956	1.31958763	0.56720641	0.35986163	0.59282607	0.58832793	Sfn/Myt1/Fkbp1b/Edem1	4
GO:0050905	neuromuscular process	2/97	188/14464	0.0106383	1.58631279	0.66487046	0.36012946	0.59282607	0.58832793	Tuba1a/Aph1b	2
GO:0002697	regulation of immune effector process	3/97	320/14464	0.009375	1.39793814	0.59147508	0.36332326	0.59282607	0.58832793	Dnase1l3/Traf2/Sphk2	3
GO:0001510	RNA methylation	1/97	67/14464	0.01492537	2.22557317	0.82617616	0.36356381	0.59282607	0.58832793	Mettl3	1
GO:0030330	DNA damage response, signal transduction by p53 class mediator	1/97	67/14464	0.01492537	2.22557317	0.82617616	0.36356381	0.59282607	0.58832793	Cdkn1a	1
GO:0032088	negative regulation of NF-kappaB transcription factor activity	1/97	67/14464	0.01492537	2.22557317	0.82617616	0.36356381	0.59282607	0.58832793	Commd7	1
GO:0035904	aorta development	1/97	67/14464	0.01492537	2.22557317	0.82617616	0.36356381	0.59282607	0.58832793	Dctn5	1
GO:0042246	tissue regeneration	1/97	67/14464	0.01492537	2.22557317	0.82617616	0.36356381	0.59282607	0.58832793	Cdkn1a	1
GO:0060612	adipose tissue development	1/97	67/14464	0.01492537	2.22557317	0.82617616	0.36356381	0.59282607	0.58832793	Lipa	1
GO:0070534	protein K63-linked ubiquitination	1/97	67/14464	0.01492537	2.22557317	0.82617616	0.36356381	0.59282607	0.58832793	Traf2	1
GO:1902807	negative regulation of cell cycle G1/S phase transition	1/97	67/14464	0.01492537	2.22557317	0.82617616	0.36356381	0.59282607	0.58832793	Cdkn1a	1
GO:1903036	positive regulation of response to wounding	1/97	67/14464	0.01492537	2.22557317	0.82617616	0.36356381	0.59282607	0.58832793	Fkbp1b	1
GO:2000036	regulation of stem cell population maintenance	1/97	67/14464	0.01492537	2.22557317	0.82617616	0.36356381	0.59282607	0.58832793	Sap30l	1
GO:0031099	regeneration	2/97	191/14464	0.0104712	1.56139688	0.64174288	0.3673813	0.59592675	0.59140509	Cdkn1a/Fkbp1b	2
GO:0006414	translational elongation	1/97	68/14464	0.01470588	2.19284415	0.81011982	0.36785181	0.59592675	0.59140509	Hbs1l	1
GO:0006446	regulation of translational initiation	1/97	68/14464	0.01470588	2.19284415	0.81011982	0.36785181	0.59592675	0.59140509	Mettl3	1
GO:0008542	visual learning	1/97	68/14464	0.01470588	2.19284415	0.81011982	0.36785181	0.59592675	0.59140509	Tuba1a	1
GO:0045806	negative regulation of endocytosis	1/97	68/14464	0.01470588	2.19284415	0.81011982	0.36785181	0.59592675	0.59140509	Pcsk9	1
GO:0051865	protein autoubiquitination	1/97	68/14464	0.01470588	2.19284415	0.81011982	0.36785181	0.59592675	0.59140509	Traf2	1
GO:0060395	SMAD protein signal transduction	1/97	68/14464	0.01470588	2.19284415	0.81011982	0.36785181	0.59592675	0.59140509	Becn1	1
GO:0015931	nucleobase-containing compound transport	2/97	192/14464	0.01041667	1.5532646	0.6341224	0.36979177	0.59740852	0.59287561	Bicd2/Abcc5	2
GO:0045732	positive regulation of protein catabolic process	2/97	192/14464	0.01041667	1.5532646	0.6341224	0.36979177	0.59740852	0.59287561	Hspa1b/Pcsk9	2
GO:1901654	response to ketone	2/97	192/14464	0.01041667	1.5532646	0.6341224	0.36979177	0.59740852	0.59287561	Lipa/Sphk2	2
GO:0006913	nucleocytoplasmic transport	3/97	324/14464	0.00925926	1.38067965	0.56942886	0.37065115	0.59769207	0.59315701	Cdkn1a/Sfn/Ankrd54	3
GO:0051169	nuclear transport	3/97	324/14464	0.00925926	1.38067965	0.56942886	0.37065115	0.59769207	0.59315701	Cdkn1a/Sfn/Ankrd54	3
GO:0006360	transcription by RNA polymerase I	1/97	69/14464	0.01449275	2.1610638	0.79434069	0.37211121	0.59784044	0.59330426	Ubtf	1
GO:0008333	endosome to lysosome transport	1/97	69/14464	0.01449275	2.1610638	0.79434069	0.37211121	0.59784044	0.59330426	Lipa	1
GO:0070509	calcium ion import	1/97	69/14464	0.01449275	2.1610638	0.79434069	0.37211121	0.59784044	0.59330426	Trpv2	1
GO:1901264	carbohydrate derivative transport	1/97	69/14464	0.01449275	2.1610638	0.79434069	0.37211121	0.59784044	0.59330426	Abcc5	1
GO:0001701	in utero embryonic development	4/97	461/14464	0.00867679	1.29382561	0.52681919	0.37364308	0.59967747	0.59512735	Cdkn1a/Smpd4/Lig4/Rundc1	4
GO:0006909	phagocytosis	2/97	194/14464	0.01030928	1.53725157	0.61901131	0.37460213	0.59967747	0.59512735	Rab39/Becn1	2
GO:0030324	lung development	2/97	194/14464	0.01030928	1.53725157	0.61901131	0.37460213	0.59967747	0.59512735	Lipa/Ptges3	2
GO:0001676	long-chain fatty acid metabolic process	1/97	70/14464	0.01428571	2.13019146	0.77882934	0.37634221	0.59967747	0.59512735	Cyp2j13	1
GO:0001960	negative regulation of cytokine-mediated signaling pathway	1/97	70/14464	0.01428571	2.13019146	0.77882934	0.37634221	0.59967747	0.59512735	Mettl3	1
GO:0032008	positive regulation of TOR signaling	1/97	70/14464	0.01428571	2.13019146	0.77882934	0.37634221	0.59967747	0.59512735	Stambpl1	1
GO:0032835	glomerulus development	1/97	70/14464	0.01428571	2.13019146	0.77882934	0.37634221	0.59967747	0.59512735	Ctnnd1	1
GO:0043489	RNA stabilization	1/97	70/14464	0.01428571	2.13019146	0.77882934	0.37634221	0.59967747	0.59512735	Traf2	1
GO:0046626	regulation of insulin receptor signaling pathway	1/97	70/14464	0.01428571	2.13019146	0.77882934	0.37634221	0.59967747	0.59512735	Pip4k2c	1
GO:0051348	negative regulation of transferase activity	2/97	196/14464	0.01020408	1.52156533	0.60406992	0.37939797	0.60144	0.59687651	Cdkn1a/Sfrp5	2
GO:0003281	ventricular septum development	1/97	71/14464	0.01408451	2.10018876	0.76357681	0.38054499	0.60144	0.59687651	Dctn5	1
GO:0007632	visual behavior	1/97	71/14464	0.01408451	2.10018876	0.76357681	0.38054499	0.60144	0.59687651	Tuba1a	1
GO:0009123	nucleoside monophosphate metabolic process	1/97	71/14464	0.01408451	2.10018876	0.76357681	0.38054499	0.60144	0.59687651	Nme1	1
GO:0031102	neuron projection regeneration	1/97	71/14464	0.01408451	2.10018876	0.76357681	0.38054499	0.60144	0.59687651	Fkbp1b	1
GO:1903051	negative regulation of proteolysis involved in protein catabolic process	1/97	71/14464	0.01408451	2.10018876	0.76357681	0.38054499	0.60144	0.59687651	Caml	1
GO:1904705	regulation of vascular associated smooth muscle cell proliferation	1/97	71/14464	0.01408451	2.10018876	0.76357681	0.38054499	0.60144	0.59687651	Cdkn1a	1
GO:2000144	positive regulation of DNA-templated transcription initiation	1/97	71/14464	0.01408451	2.10018876	0.76357681	0.38054499	0.60144	0.59687651	Taf4b	1
GO:2000379	positive regulation of reactive oxygen species metabolic process	1/97	71/14464	0.01408451	2.10018876	0.76357681	0.38054499	0.60144	0.59687651	Cdkn1a	1
GO:0007005	mitochondrion organization	4/97	466/14464	0.00858369	1.27994337	0.50473353	0.38129329	0.60177587	0.59720982	Sfn/Coa4/Lipa/Mief1	4
GO:0009895	negative regulation of catabolic process	3/97	330/14464	0.00909091	1.35557638	0.53689499	0.38161603	0.60177587	0.59720982	Traf2/Becn1/Caml	3
GO:0030323	respiratory tube development	2/97	197/14464	0.01015228	1.51384165	0.59666161	0.38179029	0.60177587	0.59720982	Lipa/Ptges3	2
GO:0030705	cytoskeleton-dependent intracellular transport	2/97	198/14464	0.01010101	1.50619598	0.58929423	0.38417882	0.60205023	0.5974821	Bicd2/Tuba1a	2
GO:0022600	digestive system process	1/97	72/14464	0.01388889	2.07101947	0.74857454	0.38471974	0.60205023	0.5974821	Lipa	1
GO:0042982	amyloid precursor protein metabolic process	1/97	72/14464	0.01388889	2.07101947	0.74857454	0.38471974	0.60205023	0.5974821	Aph1b	1
GO:0043299	leukocyte degranulation	1/97	72/14464	0.01388889	2.07101947	0.74857454	0.38471974	0.60205023	0.5974821	Sphk2	1
GO:0051057	positive regulation of small GTPase mediated signal transduction	1/97	72/14464	0.01388889	2.07101947	0.74857454	0.38471974	0.60205023	0.5974821	Rasgef1a	1
GO:0097194	execution phase of apoptosis	1/97	72/14464	0.01388889	2.07101947	0.74857454	0.38471974	0.60205023	0.5974821	Dnase1l3	1
GO:1903008	organelle disassembly	1/97	72/14464	0.01388889	2.07101947	0.74857454	0.38471974	0.60205023	0.5974821	Hbs1l	1
GO:2000027	regulation of animal organ morphogenesis	1/97	72/14464	0.01388889	2.07101947	0.74857454	0.38471974	0.60205023	0.5974821	Sfrp5	1
GO:0006289	nucleotide-excision repair	1/97	73/14464	0.01369863	2.04264934	0.7338144	0.38886664	0.60523287	0.60064059	Lig4	1
GO:0010611	regulation of cardiac muscle hypertrophy	1/97	73/14464	0.01369863	2.04264934	0.7338144	0.38886664	0.60523287	0.60064059	Becn1	1
GO:0046824	positive regulation of nucleocytoplasmic transport	1/97	73/14464	0.01369863	2.04264934	0.7338144	0.38886664	0.60523287	0.60064059	Sfn	1
GO:0071277	cellular response to calcium ion	1/97	73/14464	0.01369863	2.04264934	0.7338144	0.38886664	0.60523287	0.60064059	Tuba1a	1
GO:0140888	interferon-mediated signaling pathway	1/97	73/14464	0.01369863	2.04264934	0.7338144	0.38886664	0.60523287	0.60064059	Mettl3	1
GO:1903510	mucopolysaccharide metabolic process	1/97	73/14464	0.01369863	2.04264934	0.7338144	0.38886664	0.60523287	0.60064059	Abcc5	1
GO:0006631	fatty acid metabolic process	3/97	335/14464	0.00895522	1.3353439	0.51025717	0.39072397	0.60523287	0.60064059	Cyp2j13/Lipa/Ptges3	3
GO:0000302	response to reactive oxygen species	2/97	201/14464	0.00995025	1.48371544	0.56743296	0.39132098	0.60523287	0.60064059	Fkbp1b/Becn1	2
GO:0009749	response to glucose	2/97	201/14464	0.00995025	1.48371544	0.56743296	0.39132098	0.60523287	0.60064059	Myt1/Fkbp1b	2
GO:0002312	B cell activation involved in immune response	1/97	74/14464	0.01351351	2.01504597	0.71928862	0.39298587	0.60523287	0.60064059	Lig4	1
GO:0006942	regulation of striated muscle contraction	1/97	74/14464	0.01351351	2.01504597	0.71928862	0.39298587	0.60523287	0.60064059	Fkbp1b	1
GO:0019229	regulation of vasoconstriction	1/97	74/14464	0.01351351	2.01504597	0.71928862	0.39298587	0.60523287	0.60064059	Shc1	1
GO:0032436	positive regulation of proteasomal ubiquitin-dependent protein catabolic process	1/97	74/14464	0.01351351	2.01504597	0.71928862	0.39298587	0.60523287	0.60064059	Hspa1b	1
GO:0034109	homotypic cell-cell adhesion	1/97	74/14464	0.01351351	2.01504597	0.71928862	0.39298587	0.60523287	0.60064059	Pdia6	1
GO:0051279	regulation of release of sequestered calcium ion into cytosol	1/97	74/14464	0.01351351	2.01504597	0.71928862	0.39298587	0.60523287	0.60064059	Fkbp1b	1
GO:0060260	regulation of transcription initiation by RNA polymerase II	1/97	74/14464	0.01351351	2.01504597	0.71928862	0.39298587	0.60523287	0.60064059	Taf4b	1
GO:0060761	negative regulation of response to cytokine stimulus	1/97	74/14464	0.01351351	2.01504597	0.71928862	0.39298587	0.60523287	0.60064059	Mettl3	1
GO:0070301	cellular response to hydrogen peroxide	1/97	74/14464	0.01351351	2.01504597	0.71928862	0.39298587	0.60523287	0.60064059	Becn1	1
GO:0002709	regulation of T cell mediated immunity	1/97	75/14464	0.01333333	1.98817869	0.70498979	0.39707763	0.60778608	0.60317443	Traf2	1
GO:0014743	regulation of muscle hypertrophy	1/97	75/14464	0.01333333	1.98817869	0.70498979	0.39707763	0.60778608	0.60317443	Becn1	1
GO:0032465	regulation of cytokinesis	1/97	75/14464	0.01333333	1.98817869	0.70498979	0.39707763	0.60778608	0.60317443	Becn1	1
GO:0035082	axoneme assembly	1/97	75/14464	0.01333333	1.98817869	0.70498979	0.39707763	0.60778608	0.60317443	Dnai4	1
GO:0042632	cholesterol homeostasis	1/97	75/14464	0.01333333	1.98817869	0.70498979	0.39707763	0.60778608	0.60317443	Pcsk9	1
GO:0044773	mitotic DNA damage checkpoint signaling	1/97	75/14464	0.01333333	1.98817869	0.70498979	0.39707763	0.60778608	0.60317443	Cdkn1a	1
GO:1990874	vascular associated smooth muscle cell proliferation	1/97	75/14464	0.01333333	1.98817869	0.70498979	0.39707763	0.60778608	0.60317443	Cdkn1a	1
GO:0009746	response to hexose	2/97	205/14464	0.0097561	1.4547649	0.53882948	0.40078728	0.61239858	0.60775193	Myt1/Fkbp1b	2
GO:0050709	negative regulation of protein secretion	1/97	76/14464	0.01315789	1.96201845	0.69091082	0.40114209	0.61239858	0.60775193	Fkbp1b	1
GO:0055092	sterol homeostasis	1/97	76/14464	0.01315789	1.96201845	0.69091082	0.40114209	0.61239858	0.60775193	Pcsk9	1
GO:0043270	positive regulation of monoatomic ion transport	2/97	206/14464	0.00970874	1.44770293	0.53177297	0.4031434	0.61368456	0.60902816	Trpv2/Atp1b3	2
GO:0006869	lipid transport	3/97	342/14464	0.00877193	1.3080123	0.47365816	0.40342172	0.61368456	0.60902816	Pcsk9/Lipa/Stard3	3
GO:0007586	digestion	1/97	77/14464	0.01298701	1.93653769	0.67704497	0.40517943	0.61368456	0.60902816	Lipa	1
GO:0007589	body fluid secretion	1/97	77/14464	0.01298701	1.93653769	0.67704497	0.40517943	0.61368456	0.60902816	Nme1	1
GO:0032414	positive regulation of ion transmembrane transporter activity	1/97	77/14464	0.01298701	1.93653769	0.67704497	0.40517943	0.61368456	0.60902816	Atp1b3	1
GO:0046888	negative regulation of hormone secretion	1/97	77/14464	0.01298701	1.93653769	0.67704497	0.40517943	0.61368456	0.60902816	Fkbp1b	1
GO:0070972	protein localization to endoplasmic reticulum	1/97	77/14464	0.01298701	1.93653769	0.67704497	0.40517943	0.61368456	0.60902816	Edem1	1
GO:1901379	regulation of potassium ion transmembrane transport	1/97	77/14464	0.01298701	1.93653769	0.67704497	0.40517943	0.61368456	0.60902816	Atp1b3	1
GO:2000736	regulation of stem cell differentiation	1/97	77/14464	0.01298701	1.93653769	0.67704497	0.40517943	0.61368456	0.60902816	Mettl3	1
GO:0097191	extrinsic apoptotic signaling pathway	2/97	207/14464	0.00966184	1.4407092	0.52475336	0.40549523	0.61368456	0.60902816	Hspa1b/Traf2	2
GO:0042176	regulation of protein catabolic process	3/97	344/14464	0.00872093	1.30040758	0.46334531	0.40703688	0.61548483	0.61081477	Hspa1b/Pcsk9/Caml	3
GO:0031341	regulation of cell killing	1/97	78/14464	0.01282051	1.91171028	0.66338575	0.40918982	0.61607563	0.61140109	Dnase1l3	1
GO:0042100	B cell proliferation	1/97	78/14464	0.01282051	1.91171028	0.66338575	0.40918982	0.61607563	0.61140109	Cdkn1a	1
GO:0044774	mitotic DNA integrity checkpoint signaling	1/97	78/14464	0.01282051	1.91171028	0.66338575	0.40918982	0.61607563	0.61140109	Cdkn1a	1
GO:0050871	positive regulation of B cell activation	1/97	78/14464	0.01282051	1.91171028	0.66338575	0.40918982	0.61607563	0.61140109	Cdkn1a	1
GO:0072665	protein localization to vacuole	1/97	78/14464	0.01282051	1.91171028	0.66338575	0.40918982	0.61607563	0.61140109	Becn1	1

GO:1903829 positive regulation of protein localization	4/97	486/14464	0.00823045	1.2272708	0.41876586	0.41178813	0.61748139	0.61279618	Sfn/Edem1/Cct7/Mief1	4
GO:0031348 negative regulation of defense response	2/97	210/14464	0.00952381	1.42012764	0.50391181	0.41252457	0.61748139	0.61279618	Sfn/Mettl3	2
GO:0090276 regulation of peptide hormone secretion	2/97	210/14464	0.00952381	1.42012764	0.50391181	0.41252457	0.61748139	0.61279618	Myt1/Fkbp1b	2
GO:0032418 lysosome localization	1/97	79/14464	0.01265823	1.88751142	0.649927	0.41317346	0.61748139	0.61279618	Sphk2	1
GO:0097009 energy homeostasis	1/97	79/14464	0.01265823	1.88751142	0.649927	0.41317346	0.61748139	0.61279618	Lipa	1
GO:1903578 regulation of ATP metabolic process	1/97	79/14464	0.01265823	1.88751142	0.649927	0.41317346	0.61748139	0.61279618	Sphk2	1
GO:1990849 vacuolar localization	1/97	79/14464	0.01265823	1.88751142	0.649927	0.41317346	0.61748139	0.61279618	Sphk2	1
GO:0009410 response to xenobiotic stimulus	3/97	348/14464	0.00862069	1.28546036	0.44290606	0.41424856	0.61748139	0.61279618	Cyp2j13/Lipa/Becn1	3
GO:0034284 response to monosaccharide	2/97	211/14464	0.00947867	1.41339718	0.49703572	0.4148588	0.61748139	0.61279618	Myt1/Fkbp1b	2
GO:0051321 meiotic cell cycle	2/97	211/14464	0.00947867	1.41339718	0.49703572	0.4148588	0.61748139	0.61279618	Mettl3/Smc1a	2
GO:0002260 lymphocyte homeostasis	1/97	80/14464	0.0125	1.86391753	0.63666277	0.41713051	0.61748139	0.61279618	Caml	1
GO:0002532 production of molecular mediator involved in inflammatory response	1/97	80/14464	0.0125	1.86391753	0.63666277	0.41713051	0.61748139	0.61279618	Lipa	1
GO:0010507 negative regulation of autophagy	1/97	80/14464	0.0125	1.86391753	0.63666277	0.41713051	0.61748139	0.61279618	Becn1	1
GO:0021766 hippocampus development	1/97	80/14464	0.0125	1.86391753	0.63666277	0.41713051	0.61748139	0.61279618	Tuba1a	1
GO:0070227 lymphocyte apoptotic process	1/97	80/14464	0.0125	1.86391753	0.63666277	0.41713051	0.61748139	0.61279618	Lipa	1
GO:0071230 cellular response to amino acid stimulus	1/97	80/14464	0.0125	1.86391753	0.63666277	0.41713051	0.61748139	0.61279618	Stambpl1	1
GO:1902369 negative regulation of RNA catabolic process	1/97	80/14464	0.0125	1.86391753	0.63666277	0.41713051	0.61748139	0.61279618	Traf2	1
GO:0002699 positive regulation of immune effector process	2/97	212/14464	0.00943396	1.40673021	0.49019454	0.41718851	0.61748139	0.61279618	Traf2/Sphk2	2
GO:0006813 potassium ion transport	2/97	212/14464	0.00943396	1.40673021	0.49019454	0.41718851	0.61748139	0.61279618	Kcnk10/Atp1b3	2
GO:0099111 microtubule-based transport	2/97	212/14464	0.00943396	1.40673021	0.49019454	0.41718851	0.61748139	0.61279618	Bicd2/Tuba1a	2
GO:0005975 carbohydrate metabolic process	4/97	491/14464	0.00814664	1.21477313	0.39783948	0.41937231	0.61882691	0.61413149	Lipa/Ptges3/Edem1/Nhlrc1	4
GO:0002274 myeloid leukocyte activation	2/97	213/14464	0.00938967	1.40012584	0.48338787	0.41951367	0.61882691	0.61413149	Dnase1l3/Sphk2	2
GO:0030073 insulin secretion	2/97	213/14464	0.00938967	1.40012584	0.48338787	0.41951367	0.61882691	0.61413149	Myt1/Fkbp1b	2
GO:0043122 regulation of canonical NF-kappaB signal transduction	2/97	213/14464	0.00938967	1.40012584	0.48338787	0.41951367	0.61882691	0.61413149	Traf2/Sphk2	2
GO:0033077 T cell differentiation in thymus	1/97	81/14464	0.01234568	1.8409062	0.62358741	0.42106115	0.61902009	0.61432321	Lig4	1
GO:0045638 negative regulation of myeloid cell differentiation	1/97	81/14464	0.01234568	1.8409062	0.62358741	0.42106115	0.61902009	0.61432321	Nme1	1
GO:1905897 regulation of response to endoplasmic reticulum stress	1/97	81/14464	0.01234568	1.8409062	0.62358741	0.42106115	0.61902009	0.61432321	Pdia6	1
GO:2000142 regulation of DNA-templated transcription initiation	1/97	81/14464	0.01234568	1.8409062	0.62358741	0.42106115	0.61902009	0.61432321	Taf4b	1
GO:0002791 regulation of peptide secretion	2/97	215/14464	0.00930233	1.38710141	0.46987659	0.42415011	0.62251419	0.61779079	Myt1/Fkbp1b	2
GO:0090087 regulation of peptide transport	2/97	215/14464	0.00930233	1.38710141	0.46987659	0.42415011	0.62251419	0.61779079	Myt1/Fkbp1b	2
GO:0032411 positive regulation of transporter activity	1/97	82/14464	0.01219512	1.81845612	0.61069547	0.42496556	0.62266538	0.61794083	Atp1b3	1
GO:0061157 mRNA destabilization	1/97	82/14464	0.01219512	1.81845612	0.61069547	0.42496556	0.62266538	0.61794083	Mettl3	1
GO:0006637 acyl-CoA metabolic process	1/97	83/14464	0.01204819	1.79654701	0.59798175	0.4288439	0.62677185	0.62201615	Pmvk	1
GO:0035383 thioester metabolic process	1/97	83/14464	0.01204819	1.79654701	0.59798175	0.4288439	0.62677185	0.62201615	Pmvk	1
GO:1900076 regulation of cellular response to insulin stimulus	1/97	83/14464	0.01204819	1.79654701	0.59798175	0.4288439	0.62677185	0.62201615	Pip4k2c	1
GO:1903050 regulation of proteolysis involved in protein catabolic process	2/97	218/14464	0.00917431	1.36801286	0.44985939	0.4310695	0.62949832	0.62472192	Hspa1b/Caml	2
GO:0010976 positive regulation of neuron projection development	2/97	220/14464	0.00909091	1.35557638	0.43667696	0.43565843	0.63428293	0.62947023	Fkbp1b/Nme1	2
GO:0000280 nuclear division	3/97	360/14464	0.00833333	1.24261168	0.38302209	0.43571779	0.63428293	0.62947023	Hspa1b/Becn1/Smc1a	3
GO:0006664 glycolipid metabolic process	1/97	85/14464	0.01176471	1.75427532	0.57306915	0.43652309	0.63428293	0.62947023	Pigc	1
GO:0019915 lipid storage	1/97	85/14464	0.01176471	1.75427532	0.57306915	0.43652309	0.63428293	0.62947023	Lipa	1
GO:0030203 glycosaminoglycan metabolic process	1/97	85/14464	0.01176471	1.75427532	0.57306915	0.43652309	0.63428293	0.62947023	Abcc5	1
GO:0051310 metaphase chromosome alignment	1/97	85/14464	0.01176471	1.75427532	0.57306915	0.43652309	0.63428293	0.62947023	Becn1	1
GO:1903828 negative regulation of protein localization	2/97	221/14464	0.00904977	1.34944255	0.43013344	0.43794557	0.6345316	0.62971702	Sfn/Fkbp1b	2
GO:0000041 transition metal ion transport	1/97	86/14464	0.01162791	1.73387677	0.56086087	0.44032428	0.6345316	0.62971702	Abcc5	1
GO:0002090 regulation of receptor internalization	1/97	86/14464	0.01162791	1.73387677	0.56086087	0.44032428	0.6345316	0.62971702	Pcsk9	1
GO:0030168 platelet activation	1/97	86/14464	0.01162791	1.73387677	0.56086087	0.44032428	0.6345316	0.62971702	Pdia6	1
GO:0030512 negative regulation of transforming growth factor beta receptor signaling pathway	1/97	86/14464	0.01162791	1.73387677	0.56086087	0.44032428	0.6345316	0.62971702	Sap30l	1
GO:0032273 positive regulation of protein polymerization	1/97	86/14464	0.01162791	1.73387677	0.56086087	0.44032428	0.6345316	0.62971702	Hspa1b	1
GO:0050848 regulation of calcium-mediated signaling	1/97	86/14464	0.01162791	1.73387677	0.56086087	0.44032428	0.6345316	0.62971702	Fkbp1b	1
GO:0072332 intrinsic apoptotic signaling pathway by p53 class mediator	1/97	86/14464	0.01162791	1.73387677	0.56086087	0.44032428	0.6345316	0.62971702	Cdkn1a	1
GO:1903509 liposaccharide metabolic process	1/97	86/14464	0.01162791	1.73387677	0.56086087	0.44032428	0.6345316	0.62971702	Pigc	1
GO:2001237 negative regulation of extrinsic apoptotic signaling pathway	1/97	86/14464	0.01162791	1.73387677	0.56086087	0.44032428	0.6345316	0.62971702	Hspa1b	1
GO:0007059 chromosome segregation	3/97	364/14464	0.00824176	1.22895661	0.36351835	0.44281302	0.63577964	0.63095559	Hspa1b/Becn1/Smc1a	3
GO:0000281 mitotic cytokinesis	1/97	87/14464	0.01149425	1.71394715	0.54881196	0.44410008	0.63577964	0.63095559	Efhc1	1
GO:0000423 mitophagy	1/97	87/14464	0.01149425	1.71394715	0.54881196	0.44410008	0.63577964	0.63095559	Becn1	1
GO:0009408 response to heat	1/97	87/14464	0.01149425	1.71394715	0.54881196	0.44410008	0.63577964	0.63095559	Hspa1b	1
GO:0032755 positive regulation of interleukin-6 production	1/97	87/14464	0.01149425	1.71394715	0.54881196	0.44410008	0.63577964	0.63095559	Sphk2	1
GO:0050779 RNA destabilization	1/97	87/14464	0.01149425	1.71394715	0.54881196	0.44410008	0.63577964	0.63095559	Mettl3	1
GO:0051881 regulation of mitochondrial membrane potential	1/97	87/14464	0.01149425	1.71394715	0.54881196	0.44410008	0.63577964	0.63095559	Lipa	1
GO:0061014 positive regulation of mRNA catabolic process	1/97	87/14464	0.01149425	1.71394715	0.54881196	0.44410008	0.63577964	0.63095559	Mettl3	1
GO:1903522 regulation of blood circulation	2/97	224/14464	0.00892857	1.33136966	0.41068966	0.4447773	0.63622809	0.63140063	Shc1/Fkbp1b	2
GO:2001234 negative regulation of apoptotic signaling pathway	2/97	225/14464	0.00888889	1.32545246	0.40426961	0.44704451	0.63853424	0.63368929	Hspa1b/Maz	2
GO:0006835 dicarboxylic acid transport	1/97	88/14464	0.01136364	1.69447048	0.53691818	0.44785068	0.63853424	0.63368929	Abcc5	1
GO:0043406 positive regulation of MAP kinase activity	1/97	88/14464	0.01136364	1.69447048	0.53691818	0.44785068	0.63853424	0.63368929	Traf2	1
GO:1900542 regulation of purine nucleotide metabolic process	1/97	88/14464	0.01136364	1.69447048	0.53691818	0.44785068	0.63853424	0.63368929	Sphk2	1
GO:0060828 regulation of canonical Wnt signaling pathway	2/97	226/14464	0.00884956	1.31958763	0.39787962	0.44930666	0.64008805	0.6352313	Sfrp5/Ctnnd1	2
GO:0006140 regulation of nucleotide metabolic process	1/97	89/14464	0.01123596	1.67543148	0.52517542	0.45157623	0.6422744	0.63740107	Sphk2	1
GO:1903312 negative regulation of mRNA metabolic process	1/97	89/14464	0.01123596	1.67543148	0.52517542	0.45157623	0.6422744	0.63740107	Traf2	1
GO:0002027 regulation of heart rate	1/97	90/14464	0.01111111	1.65681558	0.51357975	0.45527689	0.64593364	0.64103254	Fkbp1b	1
GO:0051101 regulation of DNA binding	1/97	90/14464	0.01111111	1.65681558	0.51357975	0.45527689	0.64593364	0.64103254	Nme1	1
GO:0001894 tissue homeostasis	2/97	230/14464	0.00869565	1.29663828	0.37261441	0.45830382	0.64593364	0.64103254	Lipa/Tuba1a	2
GO:0040029 epigenetic regulation of gene expression	2/97	230/14464	0.00869565	1.29663828	0.37261441	0.45830382	0.64593364	0.64103254	Mettl3/Sphk2	2
GO:0060249 anatomical structure homeostasis	2/97	230/14464	0.00869565	1.29663828	0.37261441	0.45830382	0.64593364	0.64103254	Lipa/Tuba1a	2
GO:0030900 forebrain development	3/97	373/14464	0.0080429	1.1993035	0.32042799	0.4586528	0.64593364	0.64103254	Efhc1/Mettl3/Tuba1a	3
GO:0010469 regulation of signaling receptor activity	1/97	91/14464	0.01098901	1.63860881	0.50212738	0.45895285	0.64593364	0.64103254	Pcsk9	1
GO:0042310 vasoconstriction	1/97	91/14464	0.01098901	1.63860881	0.50212738	0.45895285	0.64593364	0.64103254	Shc1	1
GO:0043525 positive regulation of neuron apoptotic process	1/97	91/14464	0.01098901	1.63860881	0.50212738	0.45895285	0.64593364	0.64103254	Pcsk9	1
GO:0045921 positive regulation of exocytosis	1/97	91/14464	0.01098901	1.63860881	0.50212738	0.45895285	0.64593364	0.64103254	Sphk2	1
GO:0071229 cellular response to acid chemical	1/97	91/14464	0.01098901	1.63860881	0.50212738	0.45895285	0.64593364	0.64103254	Stambpl1	1
GO:0090398 cellular senescence	1/97	91/14464	0.01098901	1.63860881	0.50212738	0.45895285	0.64593364	0.64103254	Cdkn1a	1
GO:0120162 positive regulation of cold-induced thermogenesis	1/97	91/14464	0.01098901	1.63860881	0.50212738	0.45895285	0.64593364	0.64103254	Trpv2	1
GO:0009581 detection of external stimulus	1/97	92/14464	0.01086957	1.62079785	0.49081466	0.46260425	0.64765725	0.64274307	Pde6d	1
GO:0009582 detection of abiotic stimulus	1/97	92/14464	0.01086957	1.62079785	0.49081466	0.46260425	0.64765725	0.64274307	Pde6d	1
GO:0042147 retrograde transport, endosome to Golgi	1/97	92/14464	0.01086957	1.62079785	0.49081466	0.46260425	0.64765725	0.64274307	Cln5	1
GO:0046889 positive regulation of lipid biosynthetic process	1/97	92/14464	0.01086957	1.62079785	0.49081466	0.46260425	0.64765725	0.64274307	Sphk2	1
GO:0048565 digestive tract development	1/97	92/14464	0.01086957	1.62079785	0.49081466	0.46260425	0.64765725	0.64274307	Sfrp5	1
GO:0048678 response to axon injury	1/97	92/14464	0.01086957	1.62079785	0.49081466	0.46260425	0.64765725	0.64274307	Fkbp1b	1
GO:0002221 pattern recognition receptor signaling pathway	2/97	232/14464	0.00862069	1.28546036	0.36015451	0.46277111	0.64765725	0.64274307	Hspa1b/Becn1	2
GO:0006816 calcium ion transport	3/97	377/14464	0.00795756	1.1865788	0.30161693	0.46563382	0.65114233	0.64620171	Gpr35/Trpv2/Fkbp1b	3
GO:0051303 establishment of chromosome localization	1/97	93/14464	0.01075269	1.60336991	0.47963807	0.46623126	0.65145662	0.64651362	Becn1	1
GO:0009743 response to carbohydrate	2/97	234/14464	0.00854701	1.27447352	0.3478067	0.46721721	0.65231284	0.64736334	Myt1/Fkbp1b	2
GO:0002758 innate immune response-activating signaling pathway	2/97	235/14464	0.00851064	1.26905023	0.34167415	0.46943223	0.65335712	0.64839969	Hspa1b/Becn1	2
GO:0010389 regulation of G2/M transition of mitotic cell cycle	1/97	94/14464	0.0106383	1.58631279	0.46859422	0.46983404	0.65335712	0.64839969	Cdkn1a	1
GO:0030516 regulation of axon extension	1/97	94/14464	0.0106383	1.58631279	0.46859422	0.46983404	0.65335712	0.64839969	Trpv2	1
GO:0031110 regulation of microtubule polymerization or depolymerization	1/97	94/14464	0.0106383	1.58631279	0.46859422	0.46983404	0.65335712	0.64839969	Hspa1b	1
GO:0033045 regulation of sister chromatid segregation	1/97	94/14464	0.0106383	1.58631279	0.46859422	0.46983404	0.65335712	0.64839969	Becn1	1
GO:0002757 immune response-activating signaling pathway	3/97	380/14464	0.00789474	1.17721107	0.28764157	0.47084452	0.65424182	0.64927769	Hspa1b/Lipa/Becn1	3
GO:0002449 lymphocyte mediated immunity	2/97	236/14464	0.00847458	1.2636729	0.33556881	0.47164187	0.65430952	0.64934487	Traf2/Lig4	2
GO:0007249 canonical NF-kappaB signal transduction	2/97	236/14464	0.00847458	1.2636729	0.33556881	0.47164187	0.65430952	0.64934487	Traf2/Sphk2	2
GO:0002708 positive regulation of lymphocyte mediated immunity	1/97	95/14464	0.01052632	1.56961476	0.45767983	0.47341275	0.65572543	0.65075003	Traf2	1
GO:2000060 positive regulation of ubiquitin-dependent protein catabolic process	1/97	95/14464	0.01052632	1.56961476	0.45767983	0.47341275	0.65572543	0.65075003	Hspa1b	1
GO:0006022 aminoglycan metabolic process	1/97	96/14464	0.01041667	1.5532646	0.44689177	0.47696755	0.65960386	0.65459904	Abcc5	1
GO:1902106 negative regulation of leukocyte differentiation	1/97	96/14464	0.01041667	1.5532646	0.44689177	0.47696755	0.65960386	0.65459904	Nme1	1
GO:0007626 locomotory behavior	2/97	240/14464	0.00833333	1.24261168	0.31141425	0.48042603	0.66186884	0.65684683	Tuba1a/Aph1b	2
GO:0051896 regulation of phosphatidylinositol 3-kinase/protein kinase B signal transduction	2/97	240/14464	0.00833333	1.24261168	0.31141425	0.48042603	0.66186884	0.65684683	Sfrp5/Maz	2
GO:0008344 adult locomotory behavior	1/97	97/14464	0.01030928	1.53725157	0.43622698	0.4804986	0.66186884	0.65684683	Tuba1a	1
GO:0043266 regulation of potassium ion transport	1/97	97/14464	0.01030928	1.53725157	0.43622698	0.4804986	0.66186884	0.65684683	Atp1b3	1
GO:0050772 positive regulation of axonogenesis	1/97	97/14464	0.01030928	1.53725157	0.43622698	0.4804986	0.66186884	0.65684683	Trpv2	1
GO:0001819 positive regulation of cytokine production	3/97	386/14464	0.00777202	1.15891245	0.26002381	0.48119891	0.66231157	0.6572862	Hspa1b/Traf2/Sphk2	3
GO:0000209 protein polyubiquitination	2/97	243/14464	0.00823045	1.2272708	0.29357138	0.48695628	0.66781556	0.66274843	Traf2/Nhlrc1	2
GO:0006096 glycolytic process	1/97	99/14464	0.01010101	1.50619598	0.41525559	0.48749008	0.66781556	0.66274843	Lipa	1
GO:0032760 positive regulation of tumor necrosis factor production	1/97	99/14464	0.01010101	1.50619598	0.41525559	0.48749008	0.66781556	0.66274843	Sphk2	1
GO:0033108 mitochondrial respiratory chain complex assembly	1/97	99/14464	0.01010101	1.50619598	0.41525559	0.48749008	0.66781556	0.66274843	Coa4	1
GO:0048640 negative regulation of developmental growth	1/97	99/14464	0.01010101	1.50619598	0.41525559	0.48749008	0.66781556	0.66274843	Cdkn1a	1
GO:1903432 regulation of TORC1 signaling	1/97	99/14464	0.01010101	1.50619598	0.41525559	0.48749008	0.66781556	0.66274843	Stambpl1	1
GO:0035270 endocrine system development	1/97	100/14464	0.01	1.49113402	0.40494341	0.49095082	0.6709068	0.66581622	Myt1	1
GO:0050000 chromosome localization	1/97	100/14464	0.01	1.49113402	0.40494341	0.49095082	0.6709068	0.66581622	Becn1	1
GO:1903557 positive regulation of tumor necrosis factor superfamily cytokine production	1/97	100/14464	0.01	1.49113402	0.40494341	0.49095082	0.6709068	0.66581622	Sphk2	1
GO:0032886 regulation of microtubule-based process	2/97	245/14464	0.00816327	1.21725226	0.2818025	0.49128187	0.6709068	0.66581622	Hspa1b/Bicd2	2
GO:0010876 lipid localization	3/97	393/14464	0.00763359	1.13827024	0.22834659	0.49316114	0.67147705	0.66638214	Pcsk9/Lipa/Stard3	3
GO:0045860 positive regulation of protein kinase activity	2/97	246/14464	0.00813008	1.21230408	0.27595525	0.49343621	0.67147705	0.66638214	Cdkn1a/Traf2	2
GO:0003300 cardiac muscle hypertrophy	1/97	101/14464	0.00990099	1.47637032	0.39474334	0.49438842	0.67147705	0.66638214	Becn1	1
GO:0006368 transcription elongation by RNA polymerase II	1/97	101/14464	0.00990099	1.47637032	0.39474334	0.49438842	0.67147705	0.66638214	Eloa	1
GO:0021761 limbic system development	1/97	101/14464	0.00990099	1.47637032	0.39474334	0.49438842	0.67147705	0.66638214	Tuba1a	1
GO:0050852 T cell receptor signaling pathway	1/97	101/14464	0.00990099	1.47637032	0.39474334	0.49438842	0.67147705	0.66638214	Lipa	1
GO:0097306 cellular response to alcohol	1/97	101/14464	0.00990099	1.47637032	0.39474334	0.49438842	0.67147705	0.66638214	Sphk2	1
GO:0003012 muscle system process	3/97	395/14464	0.00759494	1.13250685	0.21940033	0.49655472	0.67297736	0.66787107	Shc1/Fkbp1b/Becn1	3
GO:0000422 autophagy of mitochondrion	1/97	102/14464	0.00980392	1.4618961	0.38465283	0.49780305	0.67297736	0.66787107	Becn1	1
GO:0001909 leukocyte mediated cytotoxicity	1/97	102/14464	0.00980392	1.4618961	0.38465283	0.49780305	0.67297736	0.66787107	Dnase1l3	1
GO:0002824 positive regulation of adaptive immune response based on somatic recombination of immune receptors b	1/97	102/14464	0.00980392	1.4618961	0.38465283	0.49780305	0.67297736	0.66787107	Traf2	1
GO:0048534 hematopoietic or lymphoid organ development	1/97	102/14464	0.00980392	1.4618961	0.38465283	0.49780305	0.67297736	0.66787107	Lipa	1
GO:1903707 negative regulation of hemopoiesis	1/97	102/14464	0.00980392	1.4618961	0.38465283	0.49780305	0.67297736	0.66787107	Nme1	1
GO:0033002 muscle cell proliferation	2/97	249/14464	0.00803213	1.19769801	0.25855953	0.49986521	0.67481881	0.66969854	Shc1/Cdkn1a	2
GO:0002764 immune response-regulating signaling pathway	3/97	397/14464	0.00755668	1.12680153	0.21049947	0.49993727	0.67481881	0.66969854	Hspa1b/Lipa/Becn1	3
GO:0033555 multicellular organismal response to stress	1/97	103/14464	0.00970874	1.44770293	0.3746694	0.50119486	0.6749527	0.66983142	Vwa1	1
GO:0043502 regulation of muscle adaptation	1/97	103/14464	0.00970874	1.44770293	0.3746694	0.50119486	0.6749527	0.66983142	Becn1	1
GO:0046032 ADP catabolic process	1/97	103/14464	0.00970874	1.44770293	0.3746694	0.50119486	0.6749527	0.66983142	Lipa	1
GO:0014897 striated muscle hypertrophy	1/97	104/14464	0.00961538	1.43378271	0.36479065	0.50456399	0.6769346	0.67179827	Becn1	1
GO:0031330 negative regulation of cellular catabolic process	1/97	104/14464	0.00961538	1.43378271	0.36479065	0.50456399	0.6769346	0.67179827	Becn1	1
GO:0055123 digestive system development	1/97	104/14464	0.00961538	1.43378271	0.36479065	0.50456399	0.6769346	0.67179827	Sfrp5	1
GO:1901655 cellular response to ketone	1/97	104/14464	0.00961538	1.43378271	0.36479065	0.50456399	0.6769346	0.67179827	Sphk2	1
GO:0022411 cellular component disassembly	3/97	400/14464	0.0075	1.11835052	0.19723216	0.50499011	0.6769346	0.67179827	Dnase1l3/Becn1/Hbs1l	3
GO:0140694 non-membrane-bounded organelle assembly	3/97	400/14464	0.0075	1.11835052	0.19723216	0.50499011	0.6769346	0.67179827	Hspa1b/Becn1/Smc1a	3
GO:0003279 cardiac septum development	1/97	105/14464	0.00952381	1.42012764	0.35501428	0.5079106	0.67721413	0.67207568	Dctn5	1
GO:0009137 purine nucleoside diphosphate catabolic process	1/97	105/14464	0.00952381	1.42012764	0.35501428	0.5079106	0.67721413	0.67207568	Lipa	1
GO:0009181 purine ribonucleoside diphosphate catabolic process	1/97	105/14464	0.00952381	1.42012764	0.35501428	0.5079106	0.67721413	0.67207568	Lipa	1
GO:0019364 pyridine nucleotide catabolic process	1/97	105/14464	0.00952381	1.42012764	0.35501428	0.5079106	0.67721413	0.67207568	Lipa	1
GO:0031047 regulatory ncRNA-mediated gene silencing	1/97	105/14464	0.00952381	1.42012764	0.35501428	0.5079106	0.67721413	0.67207568	Mettl3	1
GO:0038061 non-canonical NF-kappaB signal transduction	1/97	105/14464	0.00952381	1.42012764	0.35501428	0.5079106	0.67721413	0.67207568	Traf2	1
GO:1901800 positive regulation of proteasomal protein catabolic process	1/97	105/14464	0.00952381	1.42012764	0.35501428	0.5079106	0.67721413	0.67207568	Hspa1b	1
GO:0006898 receptor-mediated endocytosis	2/97	254/14464	0.00787402	1.17412128	0.23004017	0.51046548	0.67845731	0.67330944	Pcsk9/Cap1	2
GO:0048588 developmental cell growth	2/97	254/14464	0.00787402	1.17412128	0.23004017	0.51046548	0.67845731	0.67330944	Trpv2/Raph1	2
GO:0014896 muscle hypertrophy	1/97	106/14464	0.00943396	1.40673021	0.34533803	0.51123483	0.67845731	0.67330944	Becn1	1
GO:0016525 negative regulation of angiogenesis	1/97	106/14464	0.00943396	1.40673021	0.34533803	0.51123483	0.67845731	0.67330944	Shc1	1
GO:0060840 artery development	1/97	106/14464	0.00943396	1.40673021	0.34533803	0.51123483	0.67845731	0.67330944	Dctn5	1
GO:1902749 regulation of cell cycle G2/M phase transition	1/97	106/14464	0.00943396	1.40673021	0.34533803	0.51123483	0.67845731	0.67330944	Cdkn1a	1
GO:0002456 T cell mediated immunity	1/97	107/14464	0.00934579	1.3935832	0.33575973	0.51453684	0.67845731	0.67330944	Traf2	1
GO:0008306 associative learning	1/97	107/14464	0.00934579	1.3935832	0.33575973	0.51453684	0.67845731	0.67330944	Tuba1a	1
GO:0016064 immunoglobulin mediated immune response	1/97	107/14464	0.00934579	1.3935832	0.33575973	0.51453684	0.67845731	0.67330944	Lig4	1
GO:0030518 nuclear receptor-mediated steroid hormone signaling pathway	1/97	107/14464	0.00934579	1.3935832	0.33575973	0.51453684	0.67845731	0.67330944	Ptges3	1
GO:0042274 ribosomal small subunit biogenesis	1/97	107/14464	0.00934579	1.3935832	0.33575973	0.51453684	0.67845731	0.67330944	Rps19bp1	1
GO:0097250 mitochondrial respirasome assembly	1/97	107/14464	0.00934579	1.3935832	0.33575973	0.51453684	0.67845731	0.67330944	Coa4	1
GO:0030072 peptide hormone secretion	2/97	256/14464	0.0078125	1.16494845	0.21879318	0.51466499	0.67845731	0.67330944	Myt1/Fkbp1b	2
GO:0050878 regulation of body fluid levels	2/97	256/14464	0.0078125	1.16494845	0.21879318	0.51466499	0.67845731	0.67330944	Pdia6/Nme1	2
GO:0090150 establishment of protein localization to membrane	2/97	256/14464	0.0078125	1.16494845	0.21879318	0.51466499	0.67845731	0.67330944	Caml/Mief1	2
GO:0021537 telencephalon development	2/97	257/14464	0.0077821	1.16041558	0.2132033	0.51675597	0.67913331	0.6739803	Efhc1/Tuba1a	2

GO:0042177	negative regulation of protein catabolic process	1/97	108/14464	0.00925926	1.38067965	0.32627728	0.51781676	0.67913331	0.6739803	Caml	1
GO:0046031	ADP metabolic process	1/97	108/14464	0.00925926	1.38067965	0.32627728	0.51781676	0.67913331	0.6739803	Lipa	1
GO:0050671	positive regulation of lymphocyte proliferation	1/97	108/14464	0.00925926	1.38067965	0.32627728	0.51781676	0.67913331	0.6739803	Cdkn1a	1
GO:0072526	pyridine-containing compound catabolic process	1/97	108/14464	0.00925926	1.38067965	0.32627728	0.51781676	0.67913331	0.6739803	Lipa	1
GO:2000181	negative regulation of blood vessel morphogenesis	1/97	108/14464	0.00925926	1.38067965	0.32627728	0.51781676	0.67913331	0.6739803	Shc1	1
GO:0002218	activation of innate immune response	2/97	258/14464	0.00775194	1.15591785	0.20763559	0.51884109	0.67913331	0.6739803	Hspa1b/Becn1	2
GO:0033674	positive regulation of kinase activity	2/97	258/14464	0.00775194	1.15591785	0.20763559	0.51884109	0.67913331	0.6739803	Cdkn1a/Traf2	2
GO:0045927	positive regulation of growth	2/97	258/14464	0.00775194	1.15591785	0.20763559	0.51884109	0.67913331	0.6739803	Sfn/Trpv2	2
GO:0008544	epidermis development	2/97	259/14464	0.00772201	1.15145484	0.20208982	0.52092032	0.67913331	0.6739803	Cdkn1a/Sfn	2
GO:0001578	microtubule bundle formation	1/97	109/14464	0.00917431	1.36801286	0.31688863	0.52107476	0.67913331	0.6739803	Dnai4	1
GO:0009191	ribonucleoside diphosphate catabolic process	1/97	109/14464	0.00917431	1.36801286	0.31688863	0.52107476	0.67913331	0.6739803	Lipa	1
GO:0039531	regulation of cytoplasmic pattern recognition receptor signaling pathway	1/97	109/14464	0.00917431	1.36801286	0.31688863	0.52107476	0.67913331	0.6739803	Hspa1b	1
GO:1901343	negative regulation of vasculature development	1/97	109/14464	0.00917431	1.36801286	0.31688863	0.52107476	0.67913331	0.6739803	Shc1	1
GO:0043434	response to peptide hormone	3/97	410/14464	0.00731707	1.09107367	0.1537152	0.52164544	0.67913331	0.6739803	Shc1/Pcsk9/Pip4k2c	3
GO:0002821	positive regulation of adaptive immune response	1/97	110/14464	0.00909091	1.35557638	0.30759183	0.52431097	0.67913331	0.6739803	Traf2	1
GO:0019724	B cell mediated immunity	1/97	110/14464	0.00909091	1.35557638	0.30759183	0.52431097	0.67913331	0.6739803	Lig4	1
GO:0045446	endothelial cell differentiation	1/97	110/14464	0.00909091	1.35557638	0.30759183	0.52431097	0.67913331	0.6739803	Lipa	1
GO:0061387	regulation of extent of cell growth	1/97	110/14464	0.00909091	1.35557638	0.30759183	0.52431097	0.67913331	0.6739803	Trpv2	1
GO:0043161	proteasome-mediated ubiquitin-dependent protein catabolic process	3/97	412/14464	0.00728155	1.0857772	0.14513876	0.52494094	0.67913331	0.6739803	Hspa1b/Nhlrc1/Caml	3
GO:0046883	regulation of hormone secretion	2/97	261/14464	0.00766284	1.14263143	0.19106339	0.52506107	0.67913331	0.6739803	Myt1/Fkbp1b	2
GO:0051222	positive regulation of protein transport	2/97	262/14464	0.00763359	1.13827024	0.18558234	0.52712256	0.67913331	0.6739803	Sfn/Edem1	2
GO:0009134	nucleoside diphosphate catabolic process	1/97	111/14464	0.00900901	1.34336398	0.29838494	0.52752553	0.67913331	0.6739803	Lipa	1
GO:0031398	positive regulation of protein ubiquitination	1/97	111/14464	0.00900901	1.34336398	0.29838494	0.52752553	0.67913331	0.6739803	Nhlrc1	1
GO:0032946	positive regulation of mononuclear cell proliferation	1/97	111/14464	0.00900901	1.34336398	0.29838494	0.52752553	0.67913331	0.6739803	Cdkn1a	1
GO:0034502	protein localization to chromosome	1/97	111/14464	0.00900901	1.34336398	0.29838494	0.52752553	0.67913331	0.6739803	Cct7	1
GO:0048661	positive regulation of smooth muscle cell proliferation	1/97	111/14464	0.00900901	1.34336398	0.29838494	0.52752553	0.67913331	0.6739803	Shc1	1
GO:0051028	mRNA transport	1/97	111/14464	0.00900901	1.34336398	0.29838494	0.52752553	0.67913331	0.6739803	Bicd2	1
GO:0051928	positive regulation of calcium ion transport	1/97	111/14464	0.00900901	1.34336398	0.29838494	0.52752553	0.67913331	0.6739803	Trpv2	1
GO:0002460	adaptive immune response based on somatic recombination of immune receptors built from immunoglobu	2/97	263/14464	0.00760456	1.13394222	0.18012248	0.5291781	0.67913331	0.6739803	Traf2/Lig4	2
GO:0002790	peptide secretion	2/97	263/14464	0.00760456	1.13394222	0.18012248	0.5291781	0.67913331	0.6739803	Myt1/Fkbp1b	2
GO:0050708	regulation of protein secretion	2/97	263/14464	0.00760456	1.13394222	0.18012248	0.5291781	0.67913331	0.6739803	Myt1/Fkbp1b	2
GO:0051051	negative regulation of transport	3/97	415/14464	0.00722892	1.07792821	0.13235125	0.52986137	0.67913331	0.6739803	Gpr35/Fkbp1b/Pcsk9	3
GO:0006413	translational initiation	1/97	112/14464	0.00892857	1.33136966	0.28926611	0.53071859	0.67913331	0.6739803	Mettl3	1
GO:0006612	protein targeting to membrane	1/97	112/14464	0.00892857	1.33136966	0.28926611	0.53071859	0.67913331	0.6739803	Mief1	1
GO:0009791	post-embryonic development	1/97	112/14464	0.00892857	1.33136966	0.28926611	0.53071859	0.67913331	0.6739803	Myt1	1
GO:0035249	synaptic transmission, glutamatergic	1/97	112/14464	0.00892857	1.33136966	0.28926611	0.53071859	0.67913331	0.6739803	Aph1b	1
GO:0051209	release of sequestered calcium ion into cytosol	1/97	112/14464	0.00892857	1.33136966	0.28926611	0.53071859	0.67913331	0.6739803	Fkbp1b	1
GO:0061640	cytoskeleton-dependent cytokinesis	1/97	112/14464	0.00892857	1.33136966	0.28926611	0.53071859	0.67913331	0.6739803	Efhc1	1
GO:0071887	leukocyte apoptotic process	1/97	112/14464	0.00892857	1.33136966	0.28926611	0.53071859	0.67913331	0.6739803	Lipa	1
GO:0008380	RNA splicing	3/97	417/14464	0.00719424	1.07275829	0.12387701	0.53312629	0.67970884	0.67455147	Mettl3/Fam50a/Sugp2	3
GO:0042593	glucose homeostasis	2/97	265/14464	0.00754717	1.12538417	0.16926562	0.5332713	0.67970884	0.67455147	Myt1/Fkbp1b	2
GO:0009135	purine nucleoside diphosphate metabolic process	1/97	113/14464	0.00884956	1.31958763	0.28023354	0.5338903	0.67970884	0.67455147	Lipa	1
GO:0009179	purine ribonucleoside diphosphate metabolic process	1/97	113/14464	0.00884956	1.31958763	0.28023354	0.5338903	0.67970884	0.67455147	Lipa	1
GO:0038202	TORC1 signaling	1/97	113/14464	0.00884956	1.31958763	0.28023354	0.5338903	0.67970884	0.67455147	Stambpl1	1
GO:0051283	negative regulation of sequestering of calcium ion	1/97	113/14464	0.00884956	1.31958763	0.28023354	0.5338903	0.67970884	0.67455147	Fkbp1b	1
GO:1905477	positive regulation of protein localization to membrane	1/97	113/14464	0.00884956	1.31958763	0.28023354	0.5338903	0.67970884	0.67455147	Mief1	1
GO:0033500	carbohydrate homeostasis	2/97	266/14464	0.0075188	1.1211534	0.16386826	0.53530893	0.68074495	0.67557972	Myt1/Fkbp1b	2
GO:0000077	DNA damage checkpoint signaling	1/97	114/14464	0.00877193	1.3080123	0.27128548	0.53704078	0.68074495	0.67557972	Cdkn1a	1
GO:0008637	apoptotic mitochondrial changes	1/97	114/14464	0.00877193	1.3080123	0.27128548	0.53704078	0.68074495	0.67557972	Sfn	1
GO:0051983	regulation of chromosome segregation	1/97	114/14464	0.00877193	1.3080123	0.27128548	0.53704078	0.68074495	0.67557972	Becn1	1
GO:0060048	cardiac muscle contraction	1/97	114/14464	0.00877193	1.3080123	0.27128548	0.53704078	0.68074495	0.67557972	Fkbp1b	1
GO:1990830	cellular response to leukemia inhibitory factor	1/97	114/14464	0.00877193	1.3080123	0.27128548	0.53704078	0.68074495	0.67557972	Ubtf	1
GO:0000956	nuclear-transcribed mRNA catabolic process	1/97	115/14464	0.00869565	1.29663828	0.26242023	0.54017019	0.68273138	0.67755107	Hbs1l	1
GO:0002705	positive regulation of leukocyte mediated immunity	1/97	115/14464	0.00869565	1.29663828	0.26242023	0.54017019	0.68273138	0.67755107	Traf2	1
GO:0051053	negative regulation of DNA metabolic process	1/97	115/14464	0.00869565	1.29663828	0.26242023	0.54017019	0.68273138	0.67755107	Cdkn1a	1
GO:0051282	regulation of sequestering of calcium ion	1/97	115/14464	0.00869565	1.29663828	0.26242023	0.54017019	0.68273138	0.67755107	Fkbp1b	1
GO:0060070	canonical Wnt signaling pathway	2/97	269/14464	0.00743494	1.10864983	0.14779832	0.54138579	0.68327968	0.67809522	Sfrp5/Ctnnd1	2
GO:1903311	regulation of mRNA metabolic process	2/97	269/14464	0.00743494	1.10864983	0.14779832	0.54138579	0.68327968	0.67809522	Mettl3/Traf2	2
GO:0043401	steroid hormone receptor signaling pathway	1/97	116/14464	0.00862069	1.28546036	0.25363614	0.54327867	0.68418668	0.67899533	Ptges3	1
GO:0051208	sequestering of calcium ion	1/97	116/14464	0.00862069	1.28546036	0.25363614	0.54327867	0.68418668	0.67899533	Fkbp1b	1
GO:1990823	response to leukemia inhibitory factor	1/97	116/14464	0.00862069	1.28546036	0.25363614	0.54327867	0.68418668	0.67899533	Ubtf	1
GO:0017148	negative regulation of translation	1/97	117/14464	0.00854701	1.27447352	0.24493162	0.54636634	0.68708516	0.68187182	Nhlrc1	1
GO:0021549	cerebellum development	1/97	117/14464	0.00854701	1.27447352	0.24493162	0.54636634	0.68708516	0.68187182	Tuba1a	1
GO:0010001	glial cell differentiation	2/97	272/14464	0.00735294	1.09642207	0.131908	0.54740838	0.6872327	0.68201825	Mettl3/Tuba1a	2
GO:0015833	peptide transport	2/97	272/14464	0.00735294	1.09642207	0.131908	0.54740838	0.6872327	0.68201825	Myt1/Fkbp1b	2
GO:0031346	positive regulation of cell projection organization	3/97	426/14464	0.00704225	1.05009438	0.08623294	0.54766313	0.6872327	0.68201825	Trpv2/Fkbp1b/Nme1	3
GO:0051258	protein polymerization	2/97	273/14464	0.00732601	1.09240588	0.12665036	0.5494038	0.68797243	0.68275236	Hspa1b/Tuba1a	2
GO:0010821	regulation of mitochondrion organization	1/97	118/14464	0.00847458	1.2636729	0.2363051	0.54943336	0.68797243	0.68275236	Mief1	1
GO:0098773	skin epidermis development	1/97	118/14464	0.00847458	1.2636729	0.2363051	0.54943336	0.68797243	0.68275236	Sfn	1
GO:1903313	positive regulation of mRNA metabolic process	1/97	119/14464	0.00840336	1.2530538	0.22775508	0.55247985	0.69129189	0.68604664	Mettl3	1
GO:0016197	endosomal transport	2/97	276/14464	0.00724638	1.0805319	0.11099257	0.5553536	0.69324479	0.68798471	Cln5/Becn1	2
GO:0031507	heterochromatin formation	1/97	120/14464	0.00833333	1.24261168	0.2192801	0.55550595	0.69324479	0.68798471	Mettl3	1
GO:0051224	negative regulation of protein transport	1/97	120/14464	0.00833333	1.24261168	0.2192801	0.55550595	0.69324479	0.68798471	Fkbp1b	1
GO:0002253	activation of immune response	3/97	431/14464	0.00696056	1.03791231	0.06565619	0.55562697	0.69324479	0.68798471	Hspa1b/Lipa/Becn1	3
GO:0002761	regulation of myeloid leukocyte differentiation	1/97	121/14464	0.00826446	1.23234217	0.21087871	0.5585118	0.69483889	0.68956672	Nme1	1
GO:0031570	DNA integrity checkpoint signaling	1/97	121/14464	0.00826446	1.23234217	0.21087871	0.5585118	0.69483889	0.68956672	Cdkn1a	1
GO:0043414	macromolecule methylation	1/97	121/14464	0.00826446	1.23234217	0.21087871	0.5585118	0.69483889	0.68956672	Mettl3	1
GO:0002683	negative regulation of immune system process	3/97	433/14464	0.00692841	1.03311826	0.05749091	0.55878963	0.69483889	0.68956672	Sfn/Mettl3/Nme1	3
GO:0006874	intracellular calcium ion homeostasis	2/97	278/14464	0.00719424	1.07275829	0.10064841	0.55928966	0.69483889	0.68956672	Efhc1/Fkbp1b	2
GO:0043491	phosphatidylinositol 3-kinase/protein kinase B signal transduction	2/97	278/14464	0.00719424	1.07275829	0.10064841	0.55928966	0.69483889	0.68956672	Sfrp5/Maz	2
GO:0006310	DNA recombination	2/97	279/14464	0.00716846	1.06891328	0.09550417	0.56124852	0.69560432	0.69032634	Lig4/Nhej1	2
GO:0003231	cardiac ventricle development	1/97	122/14464	0.00819672	1.222241	0.20254954	0.56149754	0.69560432	0.69032634	Dctn5	1
GO:0030879	mammary gland development	1/97	122/14464	0.00819672	1.222241	0.20254954	0.56149754	0.69560432	0.69032634	Nme1	1
GO:0045834	positive regulation of lipid metabolic process	1/97	122/14464	0.00819672	1.222241	0.20254954	0.56149754	0.69560432	0.69032634	Sphk2	1
GO:0006633	fatty acid biosynthetic process	1/97	123/14464	0.00813008	1.21230408	0.19429123	0.56446328	0.69667031	0.69138424	Ptges3	1
GO:0032368	regulation of lipid transport	1/97	123/14464	0.00813008	1.21230408	0.19429123	0.56446328	0.69667031	0.69138424	Pcsk9	1
GO:0050864	regulation of B cell activation	1/97	123/14464	0.00813008	1.21230408	0.19429123	0.56446328	0.69667031	0.69138424	Cdkn1a	1
GO:0070665	positive regulation of leukocyte proliferation	1/97	123/14464	0.00813008	1.21230408	0.19429123	0.56446328	0.69667031	0.69138424	Cdkn1a	1
GO:0001933	negative regulation of protein phosphorylation	2/97	281/14464	0.00711744	1.06130535	0.08527064	0.56514788	0.69667031	0.69138424	Cdkn1a/Sfrp5	2
GO:0006302	double-strand break repair	2/97	281/14464	0.00711744	1.06130535	0.08527064	0.56514788	0.69667031	0.69138424	Lig4/Nhej1	2
GO:0006936	muscle contraction	2/97	281/14464	0.00711744	1.06130535	0.08527064	0.56514788	0.69667031	0.69138424	Shc1/Fkbp1b	2
GO:0030111	regulation of Wnt signaling pathway	2/97	282/14464	0.0070922	1.05754186	0.08018105	0.56708837	0.69749032	0.69219803	Sfrp5/Ctnnd1	2
GO:0009185	ribonucleoside diphosphate metabolic process	1/97	124/14464	0.00806452	1.20252744	0.18610247	0.56740918	0.69749032	0.69219803	Lipa	1
GO:0071466	cellular response to xenobiotic stimulus	1/97	124/14464	0.00806452	1.20252744	0.18610247	0.56740918	0.69749032	0.69219803	Cyp2j13	1
GO:1903052	positive regulation of proteolysis involved in protein catabolic process	1/97	124/14464	0.00806452	1.20252744	0.18610247	0.56740918	0.69749032	0.69219803	Hspa1b	1
GO:0002696	positive regulation of leukocyte activation	2/97	283/14464	0.00706714	1.05380496	0.07510937	0.56902271	0.69849137	0.69319148	Cdkn1a/Sphk2	2
GO:0043523	regulation of neuron apoptotic process	2/97	283/14464	0.00706714	1.05380496	0.07510937	0.56902271	0.69849137	0.69319148	Pcsk9/Lig4	2
GO:0000086	G2/M transition of mitotic cell cycle	1/97	125/14464	0.008	1.19290722	0.17798199	0.57033535	0.69863083	0.69332988	Cdkn1a	1
GO:0010950	positive regulation of endopeptidase activity	1/97	125/14464	0.008	1.19290722	0.17798199	0.57033535	0.69863083	0.69332988	Aph1b	1
GO:0022037	metencephalon development	1/97	125/14464	0.008	1.19290722	0.17798199	0.57033535	0.69863083	0.69332988	Tuba1a	1
GO:0043500	muscle adaptation	1/97	126/14464	0.00793651	1.1834397	0.16992854	0.57324193	0.70120846	0.69588796	Becn1	1
GO:0090101	negative regulation of transmembrane receptor protein serine/threonine kinase signaling pathway	1/97	126/14464	0.00793651	1.1834397	0.16992854	0.57324193	0.70120846	0.69588796	Sap30l	1
GO:0051052	regulation of DNA metabolic process	3/97	443/14464	0.00677201	1.00979731	0.01720882	0.57440315	0.70211805	0.69679064	Cdkn1a/Ptges3/Cct7	3
GO:1902105	regulation of leukocyte differentiation	2/97	286/14464	0.00699301	1.04275106	0.06000044	0.57478886	0.70211805	0.69679064	Mettl3/Nme1	2
GO:0046822	regulation of nucleocytoplasmic transport	1/97	127/14464	0.00787402	1.17412128	0.16194091	0.57612905	0.70326367	0.69792758	Sfn	1
GO:0051054	positive regulation of DNA metabolic process	2/97	287/14464	0.00696864	1.03911778	0.05499903	0.5766986	0.70346765	0.69813001	Ptges3/Cct7	2
GO:0010927	cellular component assembly involved in morphogenesis	1/97	128/14464	0.0078125	1.16494845	0.15401792	0.57899684	0.70385176	0.69851121	Agfg1	1
GO:0030856	regulation of epithelial cell differentiation	1/97	128/14464	0.0078125	1.16494845	0.15401792	0.57899684	0.70385176	0.69851121	Sfn	1
GO:0032989	cellular anatomical entity morphogenesis	1/97	128/14464	0.0078125	1.16494845	0.15401792	0.57899684	0.70385176	0.69851121	Agfg1	1
GO:0001666	response to hypoxia	2/97	289/14464	0.00692042	1.03192666	0.04504772	0.58049957	0.70385176	0.69851121	Becn1/Mief1	2
GO:0006090	pyruvate metabolic process	1/97	129/14464	0.00775194	1.15591785	0.14615842	0.58184542	0.70385176	0.69851121	Lipa	1
GO:0009132	nucleoside diphosphate metabolic process	1/97	129/14464	0.00775194	1.15591785	0.14615842	0.58184542	0.70385176	0.69851121	Lipa	1
GO:0015748	organophosphate ester transport	1/97	129/14464	0.00775194	1.15591785	0.14615842	0.58184542	0.70385176	0.69851121	Abcc5	1
GO:0016482	cytosolic transport	1/97	129/14464	0.00775194	1.15591785	0.14615842	0.58184542	0.70385176	0.69851121	Cln5	1
GO:0017015	regulation of transforming growth factor beta receptor signaling pathway	1/97	129/14464	0.00775194	1.15591785	0.14615842	0.58184542	0.70385176	0.69851121	Sap30l	1
GO:0030183	B cell differentiation	1/97	129/14464	0.00775194	1.15591785	0.14615842	0.58184542	0.70385176	0.69851121	Nhej1	1
GO:0032543	mitochondrial translation	1/97	129/14464	0.00775194	1.15591785	0.14615842	0.58184542	0.70385176	0.69851121	Mrps27	1
GO:0048259	regulation of receptor-mediated endocytosis	1/97	129/14464	0.00775194	1.15591785	0.14615842	0.58184542	0.70385176	0.69851121	Pcsk9	1
GO:0050769	positive regulation of neurogenesis	2/97	291/14464	0.00687285	1.02483438	0.03516421	0.58427585	0.70532214	0.69997042	Trpv2/Lig4	2
GO:0006906	vesicle fusion	1/97	130/14464	0.00769231	1.14702617	0.1383613	0.58467493	0.70532214	0.69997042	Rab39	1
GO:0043433	negative regulation of DNA-binding transcription factor activity	1/97	130/14464	0.00769231	1.14702617	0.1383613	0.58467493	0.70532214	0.69997042	Commd7	1
GO:0051592	response to calcium ion	1/97	130/14464	0.00769231	1.14702617	0.1383613	0.58467493	0.70532214	0.69997042	Tuba1a	1
GO:0007098	centrosome cycle	1/97	131/14464	0.00763359	1.13827024	0.13062546	0.58748549	0.70773579	0.70236576	Tuba1a	1
GO:1903844	regulation of cellular response to transforming growth factor beta stimulus	1/97	131/14464	0.00763359	1.13827024	0.13062546	0.58748549	0.70773579	0.70236576	Sap30l	1
GO:0010952	positive regulation of peptidase activity	1/97	132/14464	0.00757576	1.12964699	0.12294985	0.59027722	0.71003551	0.70464803	Aph1b	1
GO:0090174	organelle membrane fusion	1/97	132/14464	0.00757576	1.12964699	0.12294985	0.59027722	0.71003551	0.70464803	Rab39	1
GO:0098771	inorganic ion homeostasis	3/97	454/14464	0.00660793	0.98533085	-0.0260943	0.59118505	0.71003551	0.70464803	Efhc1/Fkbp1b/Atp1b3	3
GO:0055074	calcium ion homeostasis	2/97	295/14464	0.00677966	1.01093832	0.01559639	0.59175426	0.71003551	0.70464803	Efhc1/Fkbp1b	2
GO:0006397	mRNA processing	3/97	455/14464	0.00659341	0.98316529	-0.0299806	0.59268989	0.71003551	0.70464803	Mettl3/Fam50a/Sugp2	3
GO:0010817	regulation of hormone levels	3/97	455/14464	0.00659341	0.98316529	-0.0299806	0.59268989	0.71003551	0.70464803	Myt1/Fkbp1b/Stard3	3
GO:0032675	regulation of interleukin-6 production	1/97	133/14464	0.0075188	1.1211534	0.11533343	0.59305025	0.71003551	0.70464803	Sphk2	1
GO:0050731	positive regulation of peptidyl-tyrosine phosphorylation	1/97	133/14464	0.0075188	1.1211534	0.11533343	0.59305025	0.71003551	0.70464803	Ctnnd1	1
GO:1905039	carboxylic acid transmembrane transport	1/97	133/14464	0.0075188	1.1211534	0.11533343	0.59305025	0.71003551	0.70464803	Abcc5	1
GO:0003158	endothelium development	1/97	134/14464	0.00746269	1.11278658	0.10777518	0.59580471	0.71284506	0.70743627	Lipa	1
GO:0019216	regulation of lipid metabolic process	2/97	298/14464	0.00671141	1.00076109	0.00109082	0.59729809	0.714143	0.70872436	Sphk2/Golm1	2
GO:0071902	positive regulation of protein serine/threonine kinase activity	1/97	135/14464	0.00740741	1.10454372	0.10027413	0.59854072	0.71465107	0.70922857	Traf2	1
GO:1904950	negative regulation of establishment of protein localization	1/97	135/14464	0.00740741	1.10454372	0.10027413	0.59854072	0.71465107	0.70922857	Fkbp1b	1
GO:0007093	mitotic cell cycle checkpoint signaling	1/97	136/14464	0.00735294	1.09642207	0.09282932	0.60125839	0.71496576	0.70954087	Cdkn1a	1
GO:0050657	nucleic acid transport	1/97	136/14464	0.00735294	1.09642207	0.09282932	0.60125839	0.71496576	0.70954087	Bicd2	1
GO:0050658	RNA transport	1/97	136/14464	0.00735294	1.09642207	0.09282932	0.60125839	0.71496576	0.70954087	Bicd2	1
GO:1903825	organic acid transmembrane transport	1/97	136/14464	0.00735294	1.09642207	0.09282932	0.60125839	0.71496576	0.70954087	Abcc5	1
GO:1904064	positive regulation of cation transmembrane transport	1/97	136/14464	0.00735294	1.09642207	0.09282932	0.60125839	0.71496576	0.70954087	Atp1b3	1
GO:2000241	regulation of reproductive process	1/97	136/14464	0.00735294	1.09642207	0.09282932	0.60125839	0.71496576	0.70954087	Mettl3	1
GO:0042326	negative regulation of phosphorylation	2/97	301/14464	0.00664452	0.99078672	-0.0132725	0.60278621	0.71629524	0.71086026	Cdkn1a/Sfrp5	2
GO:1902600	proton transmembrane transport	1/97	137/14464	0.00729927	1.08841899	0.0854398	0.60395785	0.71719995	0.71175811	Sphk2	1
GO:0001822	kidney development	2/97	303/14464	0.00660066	0.98424688	-0.0227705	0.60641399	0.71794541	0.71249792	Pcsk9/Ctnnd1	2
GO:0050867	positive regulation of cell activation	2/97	303/14464	0.00660066	0.98424688	-0.0227705	0.60641399	0.71794541	0.71249792	Cdkn1a/Sphk2	2
GO:0050851	antigen receptor-mediated signaling pathway	1/97	138/14464	0.00724638	1.0805319	0.07810467	0.60663923	0.71794541	0.71249792	Lipa	1
GO:0051606	detection of stimulus	1/97	138/14464	0.00724638	1.0805319	0.07810467	0.60663923	0.71794541	0.71249792	Pde6d	1
GO:1903322	positive regulation of protein modification by small protein conjugation or removal	1/97	138/14464	0.00724638	1.0805319	0.07810467	0.60663923	0.71794541	0.71249792	Nhlrc1	1
GO:0009611	response to wounding	3/97	465/14464	0.00645161	0.96202195	-0.0683979	0.60754474	0.71818005	0.71273077	Pdia6/Cdkn1a/Fkbp1b	3
GO:0036293	response to decreased oxygen levels	2/97	304/14464	0.00657895	0.98100922	-0.0274967	0.6082186	0.71818005	0.71273077	Becn1/Mief1	2
GO:0032635	interleukin-6 production	1/97	139/14464	0.00719424	1.07275829	0.07082304	0.60930264	0.71818005	0.71273077	Sphk2	1
GO:0051236	establishment of RNA localization	1/97	139/14464	0.00719424	1.07275829	0.07082304	0.60930264	0.71818005	0.71273077	Bicd2	1
GO:0120161	regulation of cold-induced thermogenesis	1/97	139/14464	0.00719424	1.07275829	0.07082304	0.60930264	0.71818005	0.71273077	Trpv2	1
GO:1901875	positive regulation of post-translational protein modification	1/97	139/14464	0.00719424	1.07275829	0.07082304	0.60930264	0.71818005	0.71273077	Nhlrc1	1
GO:0002694	regulation of leukocyte activation	3/97	467/14464	0.00642398	0.95790194	-0.0759863	0.61047316	0.71907485	0.71361879	Cdkn1a/Mettl3/Sphk2	3
GO:0051347	positive regulation of transferase activity	2/97	306/14464	0.00653595	0.9745974	-0.0369037	0.61180923	0.71935807	0.71389986	Cdkn1a/Traf2	2
GO:0002706	regulation of lymphocyte mediated immunity	1/97	140/14464	0.00714286	1.06509573	0.06359403	0.6119482	0.71935807	0.71389986	Traf2	1
GO:0071456	cellular response to hypoxia	1/97	140/14464	0.00714286	1.06509573	0.06359403	0.6119482	0.71935807	0.71389986	Mief1	1
GO:0007611	learning or memory	2/97	307/14464	0.00651466	0.97142281	-0.0415848	0.61359526	0.72002607	0.71456279	Tuba1a/Aph1b	2
GO:0051235	maintenance of location	2/97	307/14464	0.00651466	0.97142281	-0.0415848	0.61359526	0.72002607	0.71456279	Fkbp1b/Lipa	2
GO:0031023	microtubule organizing center organization	1/97	141/14464	0.0070922	1.05754186	0.0564168	0.61457603	0.72002607	0.71456279	Tuba1a	1
GO:0031644	regulation of nervous system process	1/97	141/14464	0.0070922	1.05754186	0.0564168	0.61457603	0.72002607	0.71456279	Gpr35	1
GO:0044839	cell cycle G2/M phase transition	1/97	141/14464	0.0070922	1.05754186	0.0564168	0.61457603	0.72002607	0.71456279	Cdkn1a	1
GO:0050680	negative regulation of epithelial cell proliferation	1/97	142/14464	0.00704225	1.05009438	0.04929052	0.61718625	0.72211617	0.71663703	Sfn	1

GO:1903034 regulation of response to wounding	1/97	142/14464	0.00704225	1.05009438	0.04929052	0.61718625	0.72211617	0.71663703	Fkbp1b	1
GO:0045089 positive regulation of innate immune response	2/97	310/14464	0.00645161	0.96202195	-0.05554	0.61891624	0.72273091	0.7172471	Hspa1b/Becn1	2
GO:0009451 RNA modification	1/97	143/14464	0.00699301	1.04275106	0.04221437	0.61977897	0.72273091	0.7172471	Mettl3	1
GO:0043123 positive regulation of canonical NF-kappaB signal transduction	1/97	143/14464	0.00699301	1.04275106	0.04221437	0.61977897	0.72273091	0.7172471	Traf2	1
GO:0106106 cold-induced thermogenesis	1/97	143/14464	0.00699301	1.04275106	0.04221437	0.61977897	0.72273091	0.7172471	Trpv2	1
GO:1903305 regulation of regulated secretory pathway	1/97	143/14464	0.00699301	1.04275106	0.04221437	0.61977897	0.72273091	0.7172471	Sphk2	1
GO:0043281 regulation of cysteine-type endopeptidase activity involved in apoptotic process	1/97	144/14464	0.00694444	1.03550974	0.03518756	0.62235431	0.7242845	0.71878891	Hspa1b	1
GO:0043467 regulation of generation of precursor metabolites and energy	1/97	144/14464	0.00694444	1.03550974	0.03518756	0.62235431	0.7242845	0.71878891	Sphk2	1
GO:0072006 nephron development	1/97	144/14464	0.00694444	1.03550974	0.03518756	0.62235431	0.7242845	0.71878891	Ctnnd1	1
GO:0007178 cell surface receptor protein serine/threonine kinase signaling pathway	2/97	313/14464	0.00638978	0.95280129	-0.0693651	0.62418158	0.72592775	0.72041969	Becn1/Sap30l	2
GO:0007596 blood coagulation	1/97	145/14464	0.00689655	1.02836829	0.02820933	0.62491238	0.72629444	0.7207836	Pdia6	1
GO:0046879 hormone secretion	2/97	314/14464	0.00636943	0.94976689	-0.073945	0.62592435	0.72698722	0.72147111	Myt1/Fkbp1b	2
GO:0007286 spermatid development	1/97	146/14464	0.00684932	1.02132467	0.02127892	0.6274533	0.72803671	0.72251265	Agfg1	1
GO:0072001 renal system development	2/97	315/14464	0.00634921	0.94675176	-0.0785109	0.62766094	0.72803671	0.72251265	Pcsk9/Ctnnd1	2
GO:0170033 L-amino acid metabolic process	1/97	147/14464	0.00680272	1.01437688	0.01439559	0.62997719	0.73023882	0.72469804	P4ha1	1
GO:0001935 endothelial cell proliferation	1/97	148/14464	0.00675676	1.00752299	0.00755862	0.63248416	0.73168915	0.72613737	Lipa	1
GO:0007599 hemostasis	1/97	148/14464	0.00675676	1.00752299	0.00755862	0.63248416	0.73168915	0.72613737	Pdia6	1
GO:0034614 cellular response to reactive oxygen species	1/97	148/14464	0.00675676	1.00752299	0.00755862	0.63248416	0.73168915	0.72613737	Becn1	1
GO:0006941 striated muscle contraction	1/97	150/14464	0.00666667	0.99408935	-0.0059791	0.63744776	0.73557956	0.72999826	Fkbp1b	1
GO:0031623 receptor internalization	1/97	150/14464	0.00666667	0.99408935	-0.0059791	0.63744776	0.73557956	0.72999826	Pcsk9	1
GO:0050817 coagulation	1/97	150/14464	0.00666667	0.99408935	-0.0059791	0.63744776	0.73557956	0.72999826	Pdia6	1
GO:0009914 hormone transport	2/97	321/14464	0.00623053	0.92905546	-0.1056167	0.63795115	0.73557956	0.72999826	Myt1/Fkbp1b	2
GO:0042060 wound healing	2/97	321/14464	0.00623053	0.92905546	-0.1056167	0.63795115	0.73557956	0.72999826	Pdia6/Cdkn1a	2
GO:0007224 smoothened signaling pathway	1/97	151/14464	0.00662252	0.98750597	-0.0126811	0.63990463	0.73589032	0.73030667	Tuba1a	1
GO:0045580 regulation of T cell differentiation	1/97	151/14464	0.00662252	0.98750597	-0.0126811	0.63990463	0.73589032	0.73030667	Mettl3	1
GO:0141193 nuclear receptor-mediated signaling pathway	1/97	151/14464	0.00662252	0.98750597	-0.0126811	0.63990463	0.73589032	0.73030667	Ptges3	1
GO:2000045 regulation of G1/S transition of mitotic cell cycle	1/97	151/14464	0.00662252	0.98750597	-0.0126811	0.63990463	0.73589032	0.73030667	Cdkn1a	1
GO:1905952 regulation of lipid localization	1/97	152/14464	0.00657895	0.98100922	-0.0193395	0.64234502	0.7382111	0.73260984	Pcsk9	1
GO:0034767 positive regulation of monoatomic ion transmembrane transport	1/97	153/14464	0.00653595	0.9745974	-0.025955	0.64476903	0.73987248	0.73425861	Atp1b3	1
GO:0045814 negative regulation of gene expression, epigenetic	1/97	153/14464	0.00653595	0.9745974	-0.025955	0.64476903	0.73987248	0.73425861	Mettl3	1
GO:0006575 cellular modified amino acid metabolic process	1/97	154/14464	0.00649351	0.96826884	-0.0325281	0.64717679	0.73987248	0.73425861	P4ha1	1
GO:0035150 regulation of tube size	1/97	154/14464	0.00649351	0.96826884	-0.0325281	0.64717679	0.73987248	0.73425861	Shc1	1
GO:0035296 regulation of tube diameter	1/97	154/14464	0.00649351	0.96826884	-0.0325281	0.64717679	0.73987248	0.73425861	Shc1	1
GO:0048515 spermatid differentiation	1/97	154/14464	0.00649351	0.96826884	-0.0325281	0.64717679	0.73987248	0.73425861	Agfg1	1
GO:0097746 blood vessel diameter maintenance	1/97	154/14464	0.00649351	0.96826884	-0.0325281	0.64717679	0.73987248	0.73425861	Shc1	1
GO:1903531 negative regulation of secretion by cell	1/97	154/14464	0.00649351	0.96826884	-0.0325281	0.64717679	0.73987248	0.73425861	Fkbp1b	1
GO:0070482 response to oxygen levels	2/97	327/14464	0.00611621	0.91200858	-0.1322415	0.64802031	0.74035261	0.7347351	Becn1/Mief1	2
GO:0072331 signal transduction by p53 class mediator	1/97	155/14464	0.00645161	0.96202195	-0.0390594	0.64956839	0.74163655	0.73600929	Cdkn1a	1
GO:0001906 cell killing	1/97	156/14464	0.00641026	0.95585514	-0.0455496	0.65194395	0.74289311	0.73725632	Dnase1l3	1
GO:0002822 regulation of adaptive immune response based on somatic recombination of immune receptors built from	1/97	156/14464	0.00641026	0.95585514	-0.0455496	0.65194395	0.74289311	0.73725632	Traf2	1
GO:0072330 monocarboxylic acid biosynthetic process	1/97	156/14464	0.00641026	0.95585514	-0.0455496	0.65194395	0.74289311	0.73725632	Ptges3	1
GO:0015980 energy derivation by oxidation of organic compounds	2/97	330/14464	0.00606061	0.90371759	-0.1453796	0.65297235	0.74302582	0.73738802	Ptges3/Nhlrc1	2
GO:0043409 negative regulation of MAPK cascade	1/97	157/14464	0.00636943	0.94976689	-0.0519993	0.65430357	0.74302582	0.73738802	Sfrp5	1
GO:0051168 nuclear export	1/97	157/14464	0.00636943	0.94976689	-0.0519993	0.65430357	0.74302582	0.73738802	Sfn	1
GO:0062207 regulation of pattern recognition receptor signaling pathway	1/97	157/14464	0.00636943	0.94976689	-0.0519993	0.65430357	0.74302582	0.73738802	Hspa1b	1
GO:0002833 positive regulation of response to biotic stimulus	2/97	331/14464	0.0060423	0.90098732	-0.1497337	0.65461085	0.74302582	0.73738802	Hspa1b/Becn1	2
GO:0048638 regulation of developmental growth	2/97	331/14464	0.0060423	0.90098732	-0.1497337	0.65461085	0.74302582	0.73738802	Cdkn1a/Trpv2	2
GO:0061025 membrane fusion	1/97	159/14464	0.00628931	0.93782014	-0.0647793	0.65897542	0.74700975	0.74134172	Rab39	1
GO:0099072 regulation of postsynaptic membrane neurotransmitter receptor levels	1/97	159/14464	0.00628931	0.93782014	-0.0647793	0.65897542	0.74700975	0.74134172	Ctnnd1	1
GO:0002181 cytoplasmic translation	1/97	160/14464	0.00625	0.93195876	-0.0711108	0.66128785	0.74866008	0.74297953	Mettl3	1
GO:0065004 protein-DNA complex assembly	1/97	160/14464	0.00625	0.93195876	-0.0711108	0.66128785	0.74866008	0.74297953	Taf4b	1
GO:0032680 regulation of tumor necrosis factor production	1/97	161/14464	0.00621118	0.9261702	-0.0774039	0.66358477	0.7502886	0.74459569	Sphk2	1
GO:0051897 positive regulation of phosphatidylinositol 3-kinase/protein kinase B signal transduction	1/97	161/14464	0.00621118	0.9261702	-0.0774039	0.66358477	0.7502886	0.74459569	Maz	1
GO:0002250 adaptive immune response	2/97	337/14464	0.00593472	0.88494601	-0.1755997	0.66431434	0.75057553	0.74488045	Traf2/Lig4	2
GO:0003205 cardiac chamber development	1/97	162/14464	0.00617284	0.9204531	-0.0836593	0.66586627	0.75057553	0.74488045	Dctn5	1
GO:0070374 positive regulation of ERK1 and ERK2 cascade	1/97	162/14464	0.00617284	0.9204531	-0.0836593	0.66586627	0.75057553	0.74488045	Shc1	1
GO:0002285 lymphocyte activation involved in immune response	1/97	163/14464	0.00613497	0.91480615	-0.0898775	0.66813245	0.75057553	0.74488045	Lig4	1
GO:0006403 RNA localization	1/97	163/14464	0.00613497	0.91480615	-0.0898775	0.66813245	0.75057553	0.74488045	Bicd2	1
GO:0006606 protein import into nucleus	1/97	163/14464	0.00613497	0.91480615	-0.0898775	0.66813245	0.75057553	0.74488045	Cdkn1a	1
GO:0071674 mononuclear cell migration	1/97	163/14464	0.00613497	0.91480615	-0.0898775	0.66813245	0.75057553	0.74488045	Gpr35	1
GO:1901991 negative regulation of mitotic cell cycle phase transition	1/97	163/14464	0.00613497	0.91480615	-0.0898775	0.66813245	0.75057553	0.74488045	Cdkn1a	1
GO:1903555 regulation of tumor necrosis factor superfamily cytokine production	1/97	163/14464	0.00613497	0.91480615	-0.0898775	0.66813245	0.75057553	0.74488045	Sphk2	1
GO:2000116 regulation of cysteine-type endopeptidase activity	1/97	163/14464	0.00613497	0.91480615	-0.0898775	0.66813245	0.75057553	0.74488045	Hspa1b	1
GO:0030534 adult behavior	1/97	164/14464	0.00609756	0.90922806	-0.0960589	0.67038342	0.75262057	0.74690997	Tuba1a	1
GO:0008016 regulation of heart contraction	1/97	165/14464	0.00606061	0.90371759	-0.1022041	0.67261928	0.75464603	0.74892006	Fkbp1b	1
GO:0032640 tumor necrosis factor production	1/97	166/14464	0.0060241	0.89827351	-0.1083135	0.67484013	0.75665205	0.75091087	Sphk2	1
GO:0050890 cognition	2/97	344/14464	0.00581395	0.86693838	-0.2052328	0.67536053	0.75669855	0.75095701	Tuba1a/Aph1b	2
GO:0010563 negative regulation of phosphorus metabolic process	2/97	345/14464	0.0057971	0.86442552	-0.2094197	0.67691457	0.75669855	0.75095701	Cdkn1a/Sfrp5	2
GO:0045936 negative regulation of phosphate metabolic process	2/97	345/14464	0.0057971	0.86442552	-0.2094197	0.67691457	0.75669855	0.75095701	Cdkn1a/Sfrp5	2
GO:0045807 positive regulation of endocytosis	1/97	167/14464	0.00598802	0.89289462	-0.1143876	0.67704607	0.75669855	0.75095701	Pcsk9	1
GO:1902905 positive regulation of supramolecular fiber organization	1/97	167/14464	0.00598802	0.89289462	-0.1143876	0.67704607	0.75669855	0.75095701	Hspa1b	1
GO:0050678 regulation of epithelial cell proliferation	2/97	346/14464	0.00578035	0.86192718	-0.2135953	0.67846264	0.75721086	0.75146543	Sfn/Nme1	2
GO:0016052 carbohydrate catabolic process	1/97	168/14464	0.00595238	0.88757977	-0.120427	0.6792372	0.75721086	0.75146543	Lipa	1
GO:0071706 tumor necrosis factor superfamily cytokine production	1/97	168/14464	0.00595238	0.88757977	-0.120427	0.6792372	0.75721086	0.75146543	Sphk2	1
GO:0140053 mitochondrial gene expression	1/97	168/14464	0.00595238	0.88757977	-0.120427	0.6792372	0.75721086	0.75146543	Mrps27	1
GO:0009755 hormone-mediated signaling pathway	1/97	169/14464	0.00591716	0.88232782	-0.126432	0.68141361	0.75770419	0.75195502	Ptges3	1
GO:0019362 pyridine nucleotide metabolic process	1/97	169/14464	0.00591716	0.88232782	-0.126432	0.68141361	0.75770419	0.75195502	Lipa	1
GO:0046496 nicotinamide nucleotide metabolic process	1/97	169/14464	0.00591716	0.88232782	-0.126432	0.68141361	0.75770419	0.75195502	Lipa	1
GO:0051170 import into nucleus	1/97	169/14464	0.00591716	0.88232782	-0.126432	0.68141361	0.75770419	0.75195502	Cdkn1a	1
GO:0002819 regulation of adaptive immune response	1/97	170/14464	0.00588235	0.87713766	-0.1324032	0.68357541	0.75810804	0.75235581	Traf2	1
GO:0030902 hindbrain development	1/97	170/14464	0.00588235	0.87713766	-0.1324032	0.68357541	0.75810804	0.75235581	Tuba1a	1
GO:0046890 regulation of lipid biosynthetic process	1/97	170/14464	0.00588235	0.87713766	-0.1324032	0.68357541	0.75810804	0.75235581	Sphk2	1
GO:0051048 negative regulation of secretion	1/97	170/14464	0.00588235	0.87713766	-0.1324032	0.68357541	0.75810804	0.75235581	Fkbp1b	1
GO:0022408 negative regulation of cell-cell adhesion	1/97	171/14464	0.00584795	0.8720082	-0.1383409	0.68572268	0.75810804	0.75235581	Mettl3	1
GO:0050770 regulation of axonogenesis	1/97	171/14464	0.00584795	0.8720082	-0.1383409	0.68572268	0.75810804	0.75235581	Trpv2	1
GO:0051494 negative regulation of cytoskeleton organization	1/97	171/14464	0.00584795	0.8720082	-0.1383409	0.68572268	0.75810804	0.75235581	Togaram2	1
GO:1901605 alpha-amino acid metabolic process	1/97	171/14464	0.00584795	0.8720082	-0.1383409	0.68572268	0.75810804	0.75235581	P4ha1	1
GO:0016050 vesicle organization	2/97	351/14464	0.00569801	0.84964901	-0.2343053	0.6861138	0.75810804	0.75235581	Rab39/Agfg1	2
GO:0051962 positive regulation of nervous system development	2/97	351/14464	0.00569801	0.84964901	-0.2343053	0.6861138	0.75810804	0.75235581	Trpv2/Lig4	2
GO:0009306 protein secretion	2/97	352/14464	0.00568182	0.84723524	-0.2384143	0.68762626	0.758594	0.75283807	Myt1/Fkbp1b	2
GO:1902904 negative regulation of supramolecular fiber organization	1/97	172/14464	0.00581395	0.86693838	-0.1442456	0.68785554	0.758594	0.75283807	Togaram2	1
GO:2001242 regulation of intrinsic apoptotic signaling pathway	1/97	172/14464	0.00581395	0.86693838	-0.1442456	0.68785554	0.758594	0.75283807	Becn1	1
GO:0035592 establishment of protein localization to extracellular region	2/97	354/14464	0.00564972	0.8424486	-0.2465996	0.69063348	0.76042753	0.7546577	Myt1/Fkbp1b	2
GO:0001659 temperature homeostasis	1/97	174/14464	0.00574713	0.85697358	-0.1559577	0.69207837	0.76042753	0.7546577	Trpv2	1
GO:0007612 learning	1/97	174/14464	0.00574713	0.85697358	-0.1559577	0.69207837	0.76042753	0.7546577	Tuba1a	1
GO:0043484 regulation of RNA splicing	1/97	174/14464	0.00574713	0.85697358	-0.1559577	0.69207837	0.76042753	0.7546577	Fam50a	1
GO:0048771 tissue remodeling	1/97	174/14464	0.00574713	0.85697358	-0.1559577	0.69207837	0.76042753	0.7546577	Lipa	1
GO:0010639 negative regulation of organelle organization	2/97	355/14464	0.0056338	0.8400755	-0.2506762	0.69212826	0.76042753	0.7546577	Becn1/Togaram2	2
GO:0042770 signal transduction in response to DNA damage	1/97	175/14464	0.00571429	0.85207658	-0.1617659	0.69416853	0.76123374	0.75545779	Cdkn1a	1
GO:0046777 protein autophosphorylation	1/97	175/14464	0.00571429	0.85207658	-0.1617659	0.69416853	0.76123374	0.75545779	Ctnnd1	1
GO:1902806 regulation of cell cycle G1/S phase transition	1/97	175/14464	0.00571429	0.85207658	-0.1617659	0.69416853	0.76123374	0.75545779	Cdkn1a	1
GO:0071692 protein localization to extracellular region	2/97	357/14464	0.00560224	0.8353692	-0.2587974	0.69510022	0.76177755	0.75599747	Myt1/Fkbp1b	2
GO:0072524 pyridine-containing compound metabolic process	1/97	176/14464	0.00568182	0.84723524	-0.1675428	0.69624464	0.76255366	0.75676769	Lipa	1
GO:0022618 protein-RNA complex assembly	1/97	177/14464	0.00564972	0.8424486	-0.1732888	0.69830681	0.76433332	0.75853385	Ptges3	1
GO:0001764 neuron migration	1/97	178/14464	0.00561798	0.83771574	-0.1790041	0.70035513	0.76513798	0.7593324	Tuba1a	1
GO:0045619 regulation of lymphocyte differentiation	1/97	178/14464	0.00561798	0.83771574	-0.1790041	0.70035513	0.76513798	0.7593324	Mettl3	1
GO:0051495 positive regulation of cytoskeleton organization	1/97	178/14464	0.00561798	0.83771574	-0.1790041	0.70035513	0.76513798	0.7593324	Hspa1b	1
GO:0030595 leukocyte chemotaxis	1/97	179/14464	0.00558659	0.83303577	-0.1846894	0.70238967	0.76644449	0.760629	Gpr35	1
GO:0007600 sensory perception	2/97	362/14464	0.00552486	0.82383095	-0.2789174	0.70242796	0.76644449	0.760629	Cln5/Pde6d	2
GO:0071824 protein-DNA complex organization	1/97	180/14464	0.00555556	0.82840779	-0.1903448	0.70441055	0.76812828	0.76230002	Taf4b	1
GO:0000075 cell cycle checkpoint signaling	1/97	181/14464	0.00552486	0.82383095	-0.1959707	0.70641784	0.7688782	0.76304424	Cdkn1a	1
GO:0019722 calcium-mediated signaling	1/97	181/14464	0.00552486	0.82383095	-0.1959707	0.70641784	0.7688782	0.76304424	Fkbp1b	1
GO:0051260 protein homooligomerization	1/97	181/14464	0.00552486	0.82383095	-0.1959707	0.70641784	0.7688782	0.76304424	Vwa1	1
GO:0042063 gliogenesis	2/97	366/14464	0.00546448	0.81482733	-0.2948293	0.70818579	0.77008927	0.76424613	Mettl3/Tuba1a	2
GO:0042742 defense response to bacterium	1/97	182/14464	0.00549451	0.81930441	-0.2015676	0.70841164	0.77008927	0.76424613	Shc1	1
GO:0042180 cellular ketone metabolic process	1/97	183/14464	0.00546448	0.81482733	-0.2071358	0.71039204	0.77080403	0.76495546	Stard3	1
GO:0048639 positive regulation of developmental growth	1/97	183/14464	0.00546448	0.81482733	-0.2071358	0.71039204	0.77080403	0.76495546	Trpv2	1
GO:0071383 cellular response to steroid hormone stimulus	1/97	183/14464	0.00546448	0.81482733	-0.2071358	0.71039204	0.77080403	0.76495546	Ptges3	1
GO:0050807 regulation of synapse organization	2/97	370/14464	0.00540541	0.80601839	-0.310582	0.71385166	0.77407735	0.76820395	Nrg2/Tuba1a	2
GO:0044242 cellular lipid catabolic process	1/97	185/14464	0.00540541	0.80601839	-0.2181873	0.71431299	0.7740974	0.76822385	Smpd4	1
GO:0071826 protein-RNA complex organization	1/97	186/14464	0.00537634	0.80168496	-0.2236714	0.71625372	0.77571964	0.76983378	Ptges3	1
GO:0050679 positive regulation of epithelial cell proliferation	1/97	187/14464	0.00534759	0.79739787	-0.2291281	0.7181814	0.77732575	0.7714277	Nme1	1
GO:1902903 regulation of supramolecular fiber organization	2/97	374/14464	0.00534759	0.79739787	-0.3261792	0.71942632	0.77819134	0.77228672	Hspa1b/Togaram2	2
GO:0043524 negative regulation of neuron apoptotic process	1/97	188/14464	0.00531915	0.79315639	-0.2345577	0.72009612	0.77843414	0.77252768	Lig4	1
GO:0050803 regulation of synapse structure or activity	2/97	376/14464	0.00531915	0.79315639	-0.3339207	0.7221797	0.78020403	0.77428414	Nrg2/Tuba1a	2
GO:0007179 transforming growth factor beta receptor signaling pathway	1/97	190/14464	0.00526316	0.78480738	-0.2453372	0.72388701	0.78108302	0.77515646	Sap30l	1
GO:0032259 methylation	1/97	190/14464	0.00526316	0.78480738	-0.2453372	0.72388701	0.78108302	0.77515646	Mettl3	1
GO:0050730 regulation of peptidyl-tyrosine phosphorylation	1/97	191/14464	0.0052356	0.78069844	-0.2506876	0.72576335	0.78262452	0.77668626	Ctnnd1	1
GO:0031334 positive regulation of protein-containing complex assembly	1/97	192/14464	0.00520833	0.7766323	-0.2560122	0.72762708	0.78318481	0.7772423	Hspa1b	1
GO:0032271 regulation of protein polymerization	1/97	192/14464	0.00520833	0.7766323	-0.2560122	0.72762708	0.78318481	0.7772423	Hspa1b	1
GO:0050670 regulation of lymphocyte proliferation	1/97	192/14464	0.00520833	0.7766323	-0.2560122	0.72762708	0.78318481	0.7772423	Cdkn1a	1
GO:0051650 establishment of vesicle localization	1/97	194/14464	0.00515464	0.76862578	-0.2665851	0.731317	0.78667208	0.78070311	Stard3	1
GO:0008361 regulation of cell size	1/97	195/14464	0.00512821	0.76468411	-0.271834	0.73314337	0.78815167	0.78217147	Trpv2	1
GO:0016055 Wnt signaling pathway	2/97	385/14464	0.00519481	0.77461508	-0.3682997	0.73429293	0.78864679	0.78266284	Sfrp5/Ctnnd1	2
GO:0032944 regulation of mononuclear cell proliferation	1/97	196/14464	0.00510204	0.76078266	-0.2770583	0.73495745	0.78864679	0.78266284	Cdkn1a	1
GO:0097529 myeloid leukocyte migration	1/97	196/14464	0.00510204	0.76078266	-0.2770583	0.73495745	0.78864679	0.78266284	Gpr35	1
GO:0006006 glucose metabolic process	1/97	197/14464	0.00507614	0.75692082	-0.2822582	0.73675932	0.78912702	0.78313942	Lipa	1
GO:0051963 regulation of synapse assembly	1/97	197/14464	0.00507614	0.75692082	-0.2822582	0.73675932	0.78912702	0.78313942	Nrg2	1
GO:0060538 skeletal muscle organ development	1/97	197/14464	0.00507614	0.75692082	-0.2822582	0.73675932	0.78912702	0.78313942	Myt1	1
GO:0006979 response to oxidative stress	2/97	388/14464	0.00515464	0.76862578	-0.3795974	0.73823105	0.79007575	0.78408096	Fkbp1b/Becn1	2
GO:0002064 epithelial cell development	1/97	198/14464	0.00505051	0.75309799	-0.287434	0.73854907	0.79007575	0.78408096	Sfn	1
GO:0009612 response to mechanical stimulus	1/97	202/14464	0.0049505	0.73818516	-0.3079015	0.74558843	0.79663116	0.79058662	Tuba1a	1
GO:0017157 regulation of exocytosis	1/97	202/14464	0.0049505	0.73818516	-0.3079015	0.74558843	0.79663116	0.79058662	Sphk2	1
GO:0060047 heart contraction	1/97	203/14464	0.00492611	0.73454878	-0.3129607	0.74731876	0.79799217	0.79193731	Fkbp1b	1
GO:0007623 circadian rhythm	1/97	204/14464	0.00490196	0.73094805	-0.3179973	0.74903743	0.79933909	0.79327401	Mettl3	1
GO:0051651 maintenance of location in cell	1/97	205/14464	0.00487805	0.72738245	-0.3230117	0.75074454	0.79997346	0.79390357	Fkbp1b	1
GO:0071216 cellular response to biotic stimulus	1/97	205/14464	0.00487805	0.72738245	-0.3230117	0.75074454	0.79997346	0.79390357	Smc1a	1
GO:0022407 regulation of cell-cell adhesion	2/97	398/14464	0.00502513	0.74931358	-0.4166957	0.75100483	0.79997346	0.79390357	Sfn/Mettl3	2
GO:0000082 G1/S transition of mitotic cell cycle	1/97	207/14464	0.00483092	0.7203546	-0.3329744	0.75412436	0.80183052	0.79574654	Cdkn1a	1
GO:0003018 vascular process in circulatory system	1/97	207/14464	0.00483092	0.7203546	-0.3329744	0.75412436	0.80183052	0.79574654	Shc1	1
GO:0060348 bone development	1/97	207/14464	0.00483092	0.7203546	-0.3329744	0.75412436	0.80183052	0.79574654	Lipa	1
GO:0003015 heart process	1/97	213/14464	0.00469484	0.70006292	-0.3623509	0.76399402	0.81082715	0.8046749	Fkbp1b	1
GO:0045930 negative regulation of mitotic cell cycle	1/97	213/14464	0.00469484	0.70006292	-0.3623509	0.76399402	0.81082715	0.8046749	Cdkn1a	1
GO:0070663 regulation of leukocyte proliferation	1/97	213/14464	0.00469484	0.70006292	-0.3623509	0.76399402	0.81082715	0.8046749	Cdkn1a	1
GO:0051249 regulation of lymphocyte activation	2/97	409/14464	0.00488998	0.72916089	-0.4565513	0.76444116	0.81082715	0.8046749	Cdkn1a/Mettl3	2
GO:0034764 positive regulation of transmembrane transport	1/97	214/14464	0.0046729	0.6967916	-0.3671747	0.76560041	0.81110519	0.80495084	Atp1b3	1
GO:0031349 positive regulation of defense response	2/97	410/14464	0.00487805	0.72738245	-0.4601271	0.76563133	0.81110519	0.80495084	Hspa1b/Becn1	2
GO:0051648 vesicle localization	1/97	215/14464	0.00465116	0.69355071	-0.3719784	0.76719597	0.81227048	0.80610728	Stard3	1
GO:0002429 immune response-activating cell surface receptor signaling pathway	1/97	216/14464	0.00462963	0.69033982	-0.3767622	0.76878078	0.81337622	0.80720463	Lipa	1
GO:0043254 regulation of protein-containing complex assembly	2/97	413/14464	0.00484262	0.7220988	-0.470808	0.76917099	0.81337622	0.80720463	Hspa1b/Traf2	2
GO:0048863 stem cell differentiation	1/97	218/14464	0.00458716	0.68400643	-0.3862709	0.77191844	0.81578805	0.80959816	Mettl3	1
GO:0050767 regulation of neurogenesis	2/97	418/14464	0.00478469	0.71346125	-0.4884579	0.7749684	0.81851647	0.81230588	Trpv2/Lig4	2
GO:0043410 positive regulation of MAPK cascade	2/97	419/14464	0.00477327	0.71175848	-0.4919655	0.77611271	0.81923009	0.81301408	Shc1/Traf2	2
GO:0090068 positive regulation of cell cycle process	1/97	222/14464	0.0045045	0.67168199	-0.4050585	0.77806789	0.82079823	0.81457033	Becn1	1
GO:0090092 regulation of transmembrane receptor protein serine/threonine kinase signaling pathway	1/97	223/14464	0.0044843	0.66866996	-0.4097085	0.77957944	0.82189678	0.81566054	Sap30l	1
GO:0002768 immune response-regulating cell surface receptor signaling pathway	1/97	227/14464	0.00440529	0.65688723	-0.428127	0.7855244	0.82716665	0.82089043	Lipa	1

GO:0051056	regulation of small GTPase mediated signal transduction	1/97	227/14464	0.00440529	0.65688723	-0.428127	0.7855244	0.82716665	0.82089043	Rasgef1a	1
GO:0019318	hexose metabolic process	1/97	230/14464	0.00434783	0.64831914	-0.4417547	0.78987872	0.83125105	0.82494384	Lipa	1
GO:0001667	ameboidal-type cell migration	1/97	231/14464	0.004329	0.64551256	-0.4462627	0.79131062	0.83175645	0.8254454	Cap1	1
GO:0045055	regulated exocytosis	1/97	231/14464	0.004329	0.64551256	-0.4462627	0.79131062	0.83175645	0.8254454	Sphk2	1
GO:0002573	myeloid leukocyte differentiation	1/97	232/14464	0.00431034	0.64273018	-0.4507538	0.79273287	0.83225049	0.82593569	Nme1	1
GO:0046394	carboxylic acid biosynthetic process	1/97	232/14464	0.00431034	0.64273018	-0.4507538	0.79273287	0.83225049	0.82593569	Ptges3	1
GO:0018108	peptidyl-tyrosine phosphorylation	1/97	233/14464	0.00429185	0.63997168	-0.455228	0.79414553	0.83273328	0.82641482	Ctnnd1	1
GO:0044843	cell cycle G1/S phase transition	1/97	233/14464	0.00429185	0.63997168	-0.455228	0.79414553	0.83273328	0.82641482	Cdkn1a	1
GO:0022613	ribonucleoprotein complex biogenesis	2/97	436/14464	0.00458716	0.68400643	-0.5504978	0.79481017	0.83293057	0.82661061	Rps19bp1/Ptges3	2
GO:0016053	organic acid biosynthetic process	1/97	235/14464	0.00425532	0.63452512	-0.4641264	0.79694231	0.83466457	0.82833145	Ptges3	1
GO:0018212	peptidyl-tyrosine modification	1/97	237/14464	0.00421941	0.62917047	-0.4729594	0.79970149	0.83705282	0.83070158	Ctnnd1	1
GO:0006520	amino acid metabolic process	1/97	238/14464	0.00420168	0.6265269	-0.4773516	0.80106713	0.83798045	0.83162218	P4ha1	1
GO:0008015	blood circulation	2/97	443/14464	0.00451467	0.6731982	-0.5740224	0.80210478	0.83856409	0.83220139	Shc1/Fkbp1b	2
GO:1901988	negative regulation of cell cycle phase transition	1/97	240/14464	0.00416667	0.62130584	-0.4860885	0.80377082	0.83980358	0.83343147	Cdkn1a	1
GO:0007409	axonogenesis	2/97	446/14464	0.0044843	0.66866996	-0.5840059	0.80516091	0.84075345	0.83437413	Trpv2/Raph1	2
GO:0071560	cellular response to transforming growth factor beta stimulus	1/97	242/14464	0.00413223	0.61617108	-0.4947628	0.80643814	0.8415844	0.83519878	Sap30l	1
GO:0034599	cellular response to oxidative stress	1/97	243/14464	0.00411523	0.6136354	-0.4990768	0.80775832	0.84245915	0.83606689	Becn1	1
GO:0051259	protein complex oligomerization	1/97	244/14464	0.00409836	0.6111205	-0.5033756	0.80906958	0.84332357	0.83692475	Vwa1	1
GO:0060326	cell chemotaxis	1/97	245/14464	0.00408163	0.60862613	-0.5076593	0.81037199	0.84417773	0.83777243	Gpr35	1
GO:0016072	rRNA metabolic process	1/97	247/14464	0.00404858	0.60369798	-0.5161819	0.81295048	0.84585562	0.83943759	Ubtf	1
GO:0032984	protein-containing complex disassembly	1/97	247/14464	0.00404858	0.60369798	-0.5161819	0.81295048	0.84585562	0.83943759	Becn1	1
GO:0051607	defense response to virus	1/97	248/14464	0.00403226	0.60126372	-0.5204211	0.81422669	0.84667951	0.84025522	Becn1	1
GO:0070372	regulation of ERK1 and ERK2 cascade	1/97	249/14464	0.00401606	0.598849	-0.5246457	0.81549427	0.84698989	0.84056326	Shc1	1
GO:0071559	response to transforming growth factor beta	1/97	249/14464	0.00401606	0.598849	-0.5246457	0.81549427	0.84698989	0.84056326	Sap30l	1
GO:0005996	monosaccharide metabolic process	1/97	250/14464	0.004	0.59645361	-0.5288559	0.8167533	0.84779381	0.84136107	Lipa	1
GO:0010721	negative regulation of cell development	1/97	252/14464	0.00396825	0.59171985	-0.5372333	0.8192459	0.84987646	0.84342792	Nme1	1
GO:0002009	morphogenesis of an epithelium	2/97	470/14464	0.00425532	0.63452512	-0.6618622	0.82815153	0.85809654	0.85158563	Sfrp5/Ctnnd1	2
GO:0003013	circulatory system process	2/97	470/14464	0.00425532	0.63452512	-0.6618622	0.82815153	0.85809654	0.85158563	Shc1/Fkbp1b	2
GO:0051251	positive regulation of lymphocyte activation	1/97	260/14464	0.00384615	0.57351308	-0.5701893	0.82888513	0.85834787	0.85183506	Cdkn1a	1
GO:0010720	positive regulation of cell development	2/97	473/14464	0.00422833	0.63050064	-0.6713545	0.83084929	0.85987244	0.85334806	Trpv2/Lig4	2
GO:0007162	negative regulation of cell adhesion	1/97	263/14464	0.00380228	0.56697111	-0.5823275	0.83236715	0.8609336	0.85440116	Mettl3	1
GO:0048608	reproductive structure development	1/97	268/14464	0.00373134	0.55639329	-0.6023023	0.83801534	0.86626305	0.85969018	Antxr2	1
GO:0045765	regulation of angiogenesis	1/97	270/14464	0.0037037	0.55227186	-0.6102052	0.84022151	0.86780136	0.86121682	Shc1	1
GO:0030336	negative regulation of cell migration	1/97	271/14464	0.00369004	0.55023396	-0.6141384	0.84131342	0.86780136	0.86121682	Sap30l	1
GO:0061448	connective tissue development	1/97	272/14464	0.00367647	0.54821104	-0.6180595	0.84239794	0.86780136	0.86121682	Lipa	1
GO:0061458	reproductive system development	1/97	272/14464	0.00367647	0.54821104	-0.6180595	0.84239794	0.86780136	0.86121682	Antxr2	1
GO:0000375	RNA splicing, via transesterification reactions	1/97	273/14464	0.003663	0.54620294	-0.6219687	0.84347512	0.86780136	0.86121682	Mettl3	1
GO:0000377	RNA splicing, via transesterification reactions with bulged adenosine as nucleophile	1/97	273/14464	0.003663	0.54620294	-0.6219687	0.84347512	0.86780136	0.86121682	Mettl3	1
GO:0000398	mRNA splicing, via spliceosome	1/97	273/14464	0.003663	0.54620294	-0.6219687	0.84347512	0.86780136	0.86121682	Mettl3	1
GO:0070371	ERK1 and ERK2 cascade	1/97	273/14464	0.003663	0.54620294	-0.6219687	0.84347512	0.86780136	0.86121682	Shc1	1
GO:0010948	negative regulation of cell cycle process	1/97	277/14464	0.00361011	0.53831553	-0.6374878	0.84771148	0.87113443	0.86452459	Cdkn1a	1
GO:1901342	regulation of vasculature development	1/97	277/14464	0.00361011	0.53831553	-0.6374878	0.84771148	0.87113443	0.86452459	Shc1	1
GO:0090287	regulation of cellular response to growth factor stimulus	1/97	278/14464	0.00359712	0.53637914	-0.6413386	0.84875271	0.87169197	0.86507791	Sap30l	1
GO:0046942	carboxylic acid transport	1/97	281/14464	0.00355872	0.53065268	-0.652823	0.85183432	0.87434316	0.86770897	Abcc5	1
GO:0071396	cellular response to lipid	2/97	499/14464	0.00400802	0.59764891	-0.7515658	0.85269257	0.87435642	0.86772214	Ptges3/Sphk2	2
GO:2000146	negative regulation of cell motility	1/97	282/14464	0.0035461	0.52877093	-0.6566288	0.85284765	0.87435642	0.86772214	Sap30l	1
GO:0007416	synapse assembly	1/97	283/14464	0.00353357	0.52690248	-0.6604234	0.85385412	0.87436263	0.8677283	Nrg2	1
GO:0015849	organic acid transport	1/97	283/14464	0.00353357	0.52690248	-0.6604234	0.85385412	0.87436263	0.8677283	Abcc5	1
GO:0048193	Golgi vesicle transport	1/97	284/14464	0.00352113	0.52504719	-0.6642071	0.85485378	0.87487378	0.86823557	Bicd2	1
GO:0006790	sulfur compound metabolic process	1/97	285/14464	0.00350877	0.52320492	-0.6679799	0.85584667	0.8753774	0.86873537	Pmvk	1
GO:0045787	positive regulation of cell cycle	1/97	288/14464	0.00347222	0.51775487	-0.6792338	0.85878519	0.87735623	0.87069919	Becn1	1
GO:1901888	regulation of cell junction assembly	1/97	288/14464	0.00347222	0.51775487	-0.6792338	0.85878519	0.87735623	0.87069919	Nrg2	1
GO:0048545	response to steroid hormone	1/97	290/14464	0.00344828	0.51418415	-0.6866833	0.86071118	0.87881025	0.87214217	Ptges3	1
GO:0048511	rhythmic process	1/97	291/14464	0.00343643	0.51241719	-0.6903924	0.86166441	0.87926993	0.87259836	Mettl3	1
GO:0141091	transforming growth factor beta receptor superfamily signaling pathway	1/97	292/14464	0.00342466	0.51066234	-0.6940912	0.86261118	0.87972248	0.87304749	Sap30l	1
GO:0050727	regulation of inflammatory response	1/97	294/14464	0.00340136	0.50718844	-0.7014579	0.86448552	0.88111993	0.87443433	Dnase1l3	1
GO:0044282	small molecule catabolic process	1/97	296/14464	0.00337838	0.50376149	-0.7087842	0.86633455	0.88239373	0.87569847	Nudt3	1
GO:0040013	negative regulation of locomotion	1/97	297/14464	0.003367	0.50206533	-0.7124324	0.86724968	0.88239373	0.87569847	Sap30l	1
GO:0050863	regulation of T cell activation	1/97	297/14464	0.003367	0.50206533	-0.7124324	0.86724968	0.88239373	0.87569847	Mettl3	1
GO:0050900	leukocyte migration	1/97	304/14464	0.00328947	0.49050461	-0.737696	0.87348426	0.88770377	0.88096821	Gpr35	1
GO:1901990	regulation of mitotic cell cycle phase transition	1/97	304/14464	0.00328947	0.49050461	-0.737696	0.87348426	0.88770377	0.88096821	Cdkn1a	1
GO:0062197	cellular response to chemical stress	1/97	305/14464	0.00327869	0.4888964	-0.7412669	0.87435092	0.88806823	0.8813299	Becn1	1
GO:0034504	protein localization to nucleus	1/97	306/14464	0.00326797	0.4872987	-0.7448283	0.87521172	0.8884263	0.88168526	Cdkn1a	1
GO:0042254	ribosome biogenesis	1/97	309/14464	0.00323625	0.48256764	-0.7554573	0.87775923	0.89049515	0.88373841	Rps19bp1	1
GO:0030100	regulation of endocytosis	1/97	310/14464	0.00322581	0.48101097	-0.7589819	0.87859691	0.89082796	0.88406869	Pcsk9	1
GO:0009615	response to virus	1/97	312/14464	0.00320513	0.47792757	-0.7660042	0.88025526	0.891992	0.8852239	Becn1	1
GO:1903532	positive regulation of secretion by cell	1/97	315/14464	0.0031746	0.47337588	-0.7764709	0.88270084	0.89395196	0.88716899	Sphk2	1
GO:0051345	positive regulation of hydrolase activity	1/97	317/14464	0.00315457	0.47038928	-0.7834048	0.8843037	0.89505667	0.88826532	Aph1b	1
GO:0062012	regulation of small molecule metabolic process	1/97	319/14464	0.0031348	0.46744013	-0.7903042	0.88588488	0.89613818	0.88933862	Sphk2	1
GO:0007517	muscle organ development	1/97	323/14464	0.00309598	0.4616514	-0.8040014	0.88898335	0.89823289	0.89141744	Myt1	1
GO:0021700	developmental maturation	1/97	323/14464	0.00309598	0.4616514	-0.8040014	0.88898335	0.89823289	0.89141744	Nrg2	1
GO:0051047	positive regulation of secretion	1/97	334/14464	0.00299401	0.44644731	-0.8409945	0.89708134	0.9058915	0.89901794	Sphk2	1
GO:0060271	cilium assembly	1/97	339/14464	0.00294985	0.43986254	-0.8574963	0.90056625	0.90888557	0.90198929	Dnai4	1
GO:0015711	organic anion transport	1/97	341/14464	0.00293255	0.4372827	-0.8640443	0.90192728	0.90973393	0.90283121	Abcc5	1
GO:0045786	negative regulation of cell cycle	1/97	343/14464	0.00291545	0.43473295	-0.8705626	0.90326988	0.91056271	0.90365371	Cdkn1a	1
GO:0006887	exocytosis	1/97	352/14464	0.00284091	0.42361762	-0.8995385	0.9090899	0.91590153	0.90895201	Sphk2	1
GO:0006935	chemotaxis	1/97	356/14464	0.00280899	0.41885787	-0.9122348	0.91156402	0.91786515	0.91090074	Gpr35	1
GO:0042330	taxis	1/97	357/14464	0.00280112	0.4176846	-0.9153919	0.91217207	0.91794863	0.91098359	Gpr35	1
GO:0032535	regulation of cellular component size	1/97	365/14464	0.00273973	0.40852987	-0.9404097	0.91688978	0.92216532	0.91516828	Trpv2	1
GO:0044782	cilium organization	1/97	369/14464	0.00271003	0.40410136	-0.9527634	0.91915369	0.92391066	0.91690038	Dnai4	1
GO:0044772	mitotic cell cycle phase transition	1/97	399/14464	0.00250627	0.3737178	-1.0423622	0.9342956	0.93859121	0.93146954	Cdkn1a	1
GO:1901987	regulation of cell cycle phase transition	1/97	402/14464	0.00248756	0.37092886	-1.0510449	0.93564574	0.93940767	0.9322798	Cdkn1a	1
GO:0045785	positive regulation of cell adhesion	1/97	422/14464	0.00236967	0.35334929	-1.1077545	0.94397093	0.94722226	0.9400351	Sfn	1
GO:0007015	actin filament organization	1/97	429/14464	0.002331	0.34758369	-1.1271436	0.94662526	0.94934077	0.94213753	Cap1	1
GO:0007346	regulation of mitotic cell cycle	1/97	450/14464	0.00222222	0.33136312	-1.1839887	0.95386406	0.95605182	0.94879766	Cdkn1a	1
GO:0071407	cellular response to organic cyclic compound	1/97	462/14464	0.0021645	0.32275628	-1.2156343	0.95755492	0.95920115	0.9519231	Ptges3	1
GO:0001501	skeletal system development	1/97	466/14464	0.00214592	0.31998584	-1.2260549	0.95871905	0.95981724	0.95253451	Lipa	1
GO:0034329	cell junction assembly	1/97	485/14464	0.00206186	0.30745031	-1.2747228	0.96383124	0.96438294	0.95706557	Nrg2	1
GO:0044770	cell cycle phase transition	1/97	498/14464	0.00200803	0.2994245	-1.307271	0.96696275	0.96696275	0.95962581	Cdkn1a	1

ID	Description	GeneRatio	BgRatio	RichFactor	FoldEnrichm	zScore	pvalue	p.adjust	qvalue	geneID	Count
GO:0006457	protein folding	14/138	166/14464	0.08433735	8.83953204	9.970405	6.0978E-10	1.2293E-06	1.2074E-06	Sdf2l1/Hspa5/Calr/Chordc1/Hsp90b1/Pdia3/Hspd1/St13/Fkbp2/D	14
GO:0051604	protein maturation	17/138	498/14464	0.03413655	3.57790583	5.7458193	5.2371E-06	0.00527896	0.00518469	Sdf2l1/Hspa5/Calr/Mmp14/Chordc1/Hsp90b1/Pdia3/Hspd1/St13/I	17
GO:0098761	cellular response to interleukin-7	4/138	19/14464	0.21052632	22.0655988	9.01774808	2.751E-05	0.01720348	0.01689628	Stip1/Pdia3/Hspd1/P4hb	4
GO:0098760	response to interleukin-7	4/138	20/14464	0.2	20.9623188	8.76775708	3.4134E-05	0.01720348	0.01689628	Stip1/Pdia3/Hspd1/P4hb	4
GO:0016556	mRNA modification	4/138	22/14464	0.18181818	19.0566535	8.31842676	5.0777E-05	0.02047346	0.02010786	Dnajb11/Hnrnpc/Mettl14/Virma	4
GO:0042026	protein refolding	4/138	23/14464	0.17391304	18.2281033	8.11538289	6.1014E-05	0.02050065	0.02013457	Hspa5/Hspd1/St13/Bag5	4
GO:1903332	regulation of protein folding	3/138	11/14464	0.27272727	28.5849802	8.98248046	0.00013258	0.03818247	0.03750064	Pdia3/St13/Bag5	3
GO:0061077	chaperone-mediated protein folding	5/138	62/14464	0.08064516	8.45254792	5.77159012	0.00030749	0.07748673	0.07610303	Sdf2l1/Hspa5/Chordc1/St13/Fkbp2	5
GO:0034976	response to endoplasmic reticulum stress	9/138	256/14464	0.03515625	3.68478261	4.25371998	0.00078065	0.1748665	0.17174388	Sdf2l1/Hspa5/Manf/Calr/Hsp90b1/Pdia3/Dnajc3/Ppp2cb/P4hb	9
GO:1901570	fatty acid derivative biosynthetic process	3/138	21/14464	0.14285714	14.9730849	6.2889697	0.00099652	0.2008982	0.19731073	Far2/Hmgcll1/Elovl7	3
GO:1900024	regulation of substrate adhesion-dependent cell spreading	4/138	54/14464	0.07407407	7.76382179	4.88723598	0.00173378	0.31009531	0.30455789	Calr/Has2/Pkp2/P4hb	4
GO:0045185	maintenance of protein location	5/138	93/14464	0.05376344	5.63503195	4.40105681	0.00195139	0.31009531	0.30455789	Hspa5/Dand5/Scin/Pkp2/Sri	5
GO:0032410	negative regulation of transporter activity	4/138	57/14464	0.07017544	7.35519959	4.71830605	0.0021184	0.31009531	0.30455789	Rrad/Sri/Ndfip2/Oxsr1	4
GO:0044272	sulfur compound biosynthetic process	6/138	139/14464	0.04316547	4.52424148	4.09762307	0.00215344	0.31009531	0.30455789	Mettl16/Gclm/Ppcs/Elovl7/Pdk3/Enoph1	6
GO:0009451	RNA modification	6/138	143/14464	0.04195804	4.39768927	4.00747911	0.00248306	0.33372349	0.32776415	Mettl16/Dnajb11/Hnrnpc/Tfb1m/Mettl14/Virma	6
GO:0051085	chaperone cofactor-dependent protein refolding	3/138	30/14464	0.1	10.4811594	5.10193579	0.00285729	0.36001825	0.35358935	Sdf2l1/Hspa5/St13	3
GO:1902373	negative regulation of mRNA catabolic process	4/138	66/14464	0.06060606	6.35221783	4.27721348	0.00361852	0.41132523	0.40398013	Mettl16/Hnrnpc/Pkp3/Mettl14	4
GO:0002396	MHC protein complex assembly	2/138	11/14464	0.18181818	19.0566535	5.87977718	0.00469793	0.41132523	0.40398013	Calr/Pdia3	2
GO:0002501	peptide antigen assembly with MHC protein complex	2/138	11/14464	0.18181818	19.0566535	5.87977718	0.00469793	0.41132523	0.40398013	Calr/Pdia3	2
GO:0035384	thioester biosynthetic process	3/138	36/14464	0.08333333	8.73429952	4.5601112	0.00482002	0.41132523	0.40398013	Ppcs/Elovl7/Pdk3	3
GO:0071616	acyl-CoA biosynthetic process	3/138	36/14464	0.08333333	8.73429952	4.5601112	0.00482002	0.41132523	0.40398013	Ppcs/Elovl7/Pdk3	3
GO:0010837	regulation of keratinocyte proliferation	3/138	37/14464	0.08108108	8.49823737	4.48206648	0.00520911	0.41132523	0.40398013	Has2/Ift172/Ift80	3
GO:0038092	nodal signaling pathway	2/138	12/14464	0.16666667	17.468599	5.60130944	0.00560241	0.41132523	0.40398013	Dand5/Smad2	2
GO:0048368	lateral mesoderm development	2/138	12/14464	0.16666667	17.468599	5.60130944	0.00560241	0.41132523	0.40398013	Dand5/Smad2	2
GO:1901841	regulation of high voltage-gated calcium channel activity	2/138	12/14464	0.16666667	17.468599	5.60130944	0.00560241	0.41132523	0.40398013	Rrad/Sri	2
GO:1900026	positive regulation of substrate adhesion-dependent cell spreading	3/138	38/14464	0.07894737	8.27459954	4.40691007	0.00561659	0.41132523	0.40398013	Calr/Has2/P4hb	3
GO:0006790	sulfur compound metabolic process	8/138	285/14464	0.02807018	2.94207984	3.24992225	0.00602309	0.41132523	0.40398013	Mettl16/Hexa/Gclm/Far2/Ppcs/Elovl7/Pdk3/Enoph1	8
GO:0048385	regulation of retinoic acid receptor signaling pathway	2/138	13/14464	0.15384615	16.1248606	5.35451785	0.00657983	0.41132523	0.40398013	Dhrs3/Calr	2
GO:0071677	positive regulation of mononuclear cell migration	4/138	79/14464	0.05063291	5.30691616	3.76730944	0.00686046	0.41132523	0.40398013	Calr/Mmp14/Cd99l2/Oxsr1	4
GO:0045047	protein targeting to ER	3/138	41/14464	0.07317073	7.66914104	4.19701278	0.00695154	0.41132523	0.40398013	Hspa5/Ubl4a/Srprb	3
GO:0051084	'de novo' post-translational protein folding	3/138	41/14464	0.07317073	7.66914104	4.19701278	0.00695154	0.41132523	0.40398013	Sdf2l1/Hspa5/St13	3
GO:1902369	negative regulation of RNA catabolic process	4/138	80/14464	0.05	5.24057971	3.73281658	0.0071691	0.41132523	0.40398013	Mettl16/Hnrnpc/Pkp3/Mettl14	4
GO:0035966	response to topologically incorrect protein	5/138	127/14464	0.03937008	4.12644072	3.47319509	0.00738544	0.41132523	0.40398013	Sdf2l1/Hspa5/Manf/Hspd1/Dnajc3	5
GO:0023019	signal transduction involved in regulation of gene expression	2/138	14/14464	0.14285714	14.9730849	5.13367835	0.00762874	0.41132523	0.40398013	Dand5/Smad2	2
GO:0071895	odontoblast differentiation	2/138	14/14464	0.14285714	14.9730849	5.13367835	0.00762874	0.41132523	0.40398013	Smad2/Ift80	2
GO:0017015	regulation of transforming growth factor beta receptor signaling pathway	5/138	129/14464	0.03875969	4.06246489	3.42904661	0.00787625	0.41132523	0.40398013	Hspa5/Dand5/Smad2/Brms1/Spred3	5
GO:0006458	'de novo' protein folding	3/138	43/14464	0.06976744	7.3124368	4.06855176	0.0079373	0.41132523	0.40398013	Sdf2l1/Hspa5/St13	3
GO:0006637	acyl-CoA metabolic process	4/138	83/14464	0.04819277	5.05116117	3.6327063	0.0081497	0.41132523	0.40398013	Far2/Ppcs/Elovl7/Pdk3	4
GO:0035383	thioester metabolic process	4/138	83/14464	0.04819277	5.05116117	3.6327063	0.0081497	0.41132523	0.40398013	Far2/Ppcs/Elovl7/Pdk3	4
GO:1903844	regulation of cellular response to transforming growth factor beta stimulus	5/138	131/14464	0.03816794	4.00044253	3.38577951	0.00838932	0.41132523	0.40398013	Hspa5/Dand5/Smad2/Brms1/Spred3	5
GO:1903670	regulation of sprouting angiogenesis	3/138	44/14464	0.06818182	7.14624506	4.0073737	0.00845947	0.41132523	0.40398013	Rhoj/Stard13/S2bpcox16	3
GO:0072599	establishment of protein localization to endoplasmic reticulum	3/138	45/14464	0.06666667	6.98743961	3.94808137	0.00900138	0.41132523	0.40398013	Hspa5/Ubl4a/Srprb	3
GO:0030512	negative regulation of transforming growth factor beta receptor signaling pathway	4/138	86/14464	0.04651163	4.87495787	3.53731091	0.00921447	0.41132523	0.40398013	Hspa5/Dand5/Brms1/Spred3	4
GO:0043604	amide biosynthetic process	5/138	135/14464	0.03703704	3.8819109	3.30176192	0.00948418	0.41132523	0.40398013	Gclm/Ppcs/Degs1/Elovl7/Pdk3	5
GO:0043616	keratinocyte proliferation	3/138	46/14464	0.06521739	6.83553875	3.89057351	0.00956319	0.41132523	0.40398013	Has2/Ift172/Ift80	3
GO:1901568	fatty acid derivative metabolic process	3/138	46/14464	0.06521739	6.83553875	3.89057351	0.00956319	0.41132523	0.40398013	Far2/Hmgcll1/Elovl7	3
GO:0035269	protein O-linked mannosylation	2/138	16/14464	0.125	13.1014493	4.75334977	0.0099353	0.41132523	0.40398013	Fktn/Crppa	2
GO:1903671	negative regulation of sprouting angiogenesis	2/138	16/14464	0.125	13.1014493	4.75334977	0.0099353	0.41132523	0.40398013	Stard13/S2bpcox16	2
GO:0043603	amide metabolic process	9/138	376/14464	0.02393617	2.50878816	2.90940232	0.00999749	0.41132523	0.40398013	Hsp90b1/Hexa/Gclm/Far2/Ppcs/Degs1/Elovl7/Pdk3/P4hb	9
GO:1903312	negative regulation of mRNA metabolic process	4/138	89/14464	0.04494382	4.71063345	3.44623901	0.01036611	0.4177655	0.4103054	Mettl16/Hnrnpc/Pkp3/Mettl14	4
GO:0006620	post-translational protein targeting to endoplasmic reticulum membrane	2/138	17/14464	0.11764706	12.3307758	4.5877689	0.01119015	0.4177655	0.4103054	Hspa5/Ubl4a	2
GO:0043171	peptide catabolic process	2/138	17/14464	0.11764706	12.3307758	4.5877689	0.01119015	0.4177655	0.4103054	Lta4h/Trhde	2
GO:0046500	S-adenosylmethionine metabolic process	2/138	17/14464	0.11764706	12.3307758	4.5877689	0.01119015	0.4177655	0.4103054	Mettl16/Enoph1	2
GO:1901142	insulin metabolic process	2/138	17/14464	0.11764706	12.3307758	4.5877689	0.01119015	0.4177655	0.4103054	Hsp90b1/P4hb	2
GO:0032413	negative regulation of ion transmembrane transporter activity	3/138	50/14464	0.06	6.28869565	3.67661015	0.01201206	0.42036537	0.41285884	Rrad/Sri/Oxsr1	3
GO:0034446	substrate adhesion-dependent cell spreading	4/138	94/14464	0.04255319	4.46006784	3.30313573	0.01248538	0.42036537	0.41285884	Calr/Has2/Pkp2/P4hb	4
GO:0060349	bone morphogenesis	4/138	94/14464	0.04255319	4.46006784	3.30313573	0.01248538	0.42036537	0.41285884	Dhrs3/Mmp14/Has2/Ift80	4
GO:0035268	protein mannosylation	2/138	18/14464	0.11111111	11.6457327	4.43551719	0.01251087	0.42036537	0.41285884	Fktn/Crppa	2
GO:0035336	long-chain fatty-acyl-CoA metabolic process	2/138	18/14464	0.11111111	11.6457327	4.43551719	0.01251087	0.42036537	0.41285884	Far2/Elovl7	2
GO:2000561	regulation of CD4-positive, alpha-beta T cell proliferation	2/138	18/14464	0.11111111	11.6457327	4.43551719	0.01251087	0.42036537	0.41285884	Card11/Itch	2
GO:0006518	peptide metabolic process	3/138	52/14464	0.05769231	6.04682274	3.5781938	0.0133591	0.43772795	0.42991138	Gclm/Lta4h/Trhde	3
GO:0035720	intraciliary anterograde transport	2/138	19/14464	0.10526316	11.0327994	4.29483436	0.01389613	0.43772795	0.42991138	Ift172/Ift80	2
GO:0035739	CD4-positive, alpha-beta T cell proliferation	2/138	19/14464	0.10526316	11.0327994	4.29483436	0.01389613	0.43772795	0.42991138	Card11/Itch	2
GO:0097502	mannosylation	2/138	19/14464	0.10526316	11.0327994	4.29483436	0.01389613	0.43772795	0.42991138	Fktn/Crppa	2
GO:0010810	regulation of cell-substrate adhesion	6/138	206/14464	0.02912621	3.05276488	2.91239165	0.01413155	0.43829537	0.43046866	Calr/Mmp14/Has2/Pkp2/Sema3e/P4hb	6
GO:0006958	complement activation, classical pathway	2/138	20/14464	0.1	10.4811594	4.16427086	0.01534457	0.45495979	0.44683551	Ighg2c/Cr1l	2
GO:0035967	cellular response to topologically incorrect protein	4/138	100/14464	0.04	4.19246377	3.1440869	0.01537132	0.45495979	0.44683551	Sdf2l1/Hspa5/Hspd1/Dnajc3	4
GO:0000375	RNA splicing, via transesterification reactions	7/138	273/14464	0.02564103	2.68747677	2.76260562	0.01579721	0.45495979	0.44683551	Mettl16/Obi1/Srpk1/Prpf31/Hnrnpc/Mettl14/Prpf18	7
GO:0000377	RNA splicing, via transesterification reactions with bulged adenosine as nucleophile	7/138	273/14464	0.02564103	2.68747677	2.76260562	0.01579721	0.45495979	0.44683551	Mettl16/Obi1/Srpk1/Prpf31/Hnrnpc/Mettl14/Prpf18	7
GO:0000398	mRNA splicing, via spliceosome	7/138	273/14464	0.02564103	2.68747677	2.76260562	0.01579721	0.45495979	0.44683551	Mettl16/Obi1/Srpk1/Prpf31/Hnrnpc/Mettl14/Prpf18	7
GO:0006085	acetyl-CoA biosynthetic process	2/138	21/14464	0.0952381	9.98205659	4.04262066	0.01685489	0.46547201	0.45716001	Ppcs/Pdk3	2
GO:0042634	regulation of hair cycle	2/138	21/14464	0.0952381	9.98205659	4.04262066	0.01685489	0.46547201	0.45716001	Bmal1/Pkp3	2
GO:0070935	3'-UTR-mediated mRNA stabilization	2/138	21/14464	0.0952381	9.98205659	4.04262066	0.01685489	0.46547201	0.45716001	Mettl16/Hnrnpc	2
GO:0036503	ERAD pathway	4/138	105/14464	0.03809524	3.99282264	3.02078485	0.01807297	0.4762359	0.46773169	Sdf2l1/Hspa5/Calr/Hsp90b1	4
GO:0010594	regulation of endothelial cell migration	5/138	159/14464	0.03144654	3.29596208	2.85710108	0.01816589	0.4762359	0.46773169	Calr/Rhoj/Sash1/Stard13/S2bpcox16	5
GO:0010839	negative regulation of keratinocyte proliferation	2/138	22/14464	0.09090909	9.52832675	3.92887095	0.01842579	0.4762359	0.46773169	Ift172/Ift80	2
GO:0016486	peptide hormone processing	2/138	22/14464	0.09090909	9.52832675	3.92887095	0.01842579	0.4762359	0.46773169	Hsp90b1/P4hb	2
GO:0031167	rRNA methylation	2/138	22/14464	0.09090909	9.52832675	3.92887095	0.01842579	0.4762359	0.46773169	Mettl16/Tfb1m	2
GO:0048255	mRNA stabilization	3/138	59/14464	0.05084746	5.3294031	3.27042098	0.01872904	0.47794615	0.46941139	Mettl16/Hnrnpc/Mettl14	3
GO:0006986	response to unfolded protein	4/138	110/14464	0.03636364	3.8113307	2.90487915	0.02105297	0.53053488	0.52106104	Hspa5/Manf/Hspd1/Dnajc3	4
GO:0071675	regulation of mononuclear cell migration	4/138	114/14464	0.03508772	3.6775998	2.81694524	0.02364195	0.58842181	0.57791427	Calr/Mmp14/Cd99l2/Oxsr1	4
GO:0000096	sulfur amino acid metabolic process	2/138	26/14464	0.07692308	8.06243032	3.53747675	0.02529	0.59981933	0.58910827	Gclm/Enoph1	2
GO:0035337	fatty-acyl-CoA metabolic process	2/138	26/14464	0.07692308	8.06243032	3.53747675	0.02529	0.59981933	0.58910827	Far2/Elovl7	2
GO:1901020	negative regulation of calcium ion transmembrane transporter activity	2/138	26/14464	0.07692308	8.06243032	3.53747675	0.02529	0.59981933	0.58910827	Rrad/Sri	2
GO:1901385	regulation of voltage-gated calcium channel activity	2/138	26/14464	0.07692308	8.06243032	3.53747675	0.02529	0.59981933	0.58910827	Rrad/Sri	2
GO:0001510	RNA methylation	3/138	67/14464	0.04477612	4.69305646	2.97367573	0.026129	0.60547195	0.59465995	Mettl16/Tfb1m/Mettl14	3
GO:0032088	negative regulation of NF-kappaB transcription factor activity	3/138	67/14464	0.04477612	4.69305646	2.97367573	0.026129	0.60547195	0.59465995	Ppp2cb/Brms1/Itch	3
GO:0070293	renal absorption	2/138	27/14464	0.07407407	7.76382179	3.45256468	0.02714505	0.61497013	0.60398852	Has2/Oxsr1	2
GO:0051865	protein autoubiquitination	3/138	68/14464	0.04411765	4.62404092	2.93990227	0.02714898	0.61497013	0.60398852	Sash1/Obi1/Itch	3
GO:0032507	maintenance of protein location in cell	3/138	69/14464	0.04347826	4.55702583	2.90677886	0.02819001	0.63095922	0.6196921	Hspa5/Scin/Pkp2	3
GO:0071560	cellular response to transforming growth factor beta stimulus	6/138	242/14464	0.02479339	2.59863457	2.46140347	0.02854658	0.63095922	0.6196921	Hspa5/Dand5/Smad2/Stk16/Brms1/Spred3	6
GO:0043489	RNA stabilization	3/138	70/14464	0.04285714	4.49192547	2.87428282	0.02925207	0.63095922	0.6196921	Mettl16/Hnrnpc/Mettl14	3
GO:1904377	positive regulation of protein localization to cell periphery	3/138	71/14464	0.04225352	4.42865891	2.84239256	0.03033511	0.63095922	0.6196921	Arhgef16/Rab38/Cnpy4	3
GO:0002040	sprouting angiogenesis	4/138	124/14464	0.03225806	3.38101917	2.6133972	0.03093035	0.63095922	0.6196921	Rhoj/Stard13/S2bpcox16/Sema3e	4
GO:0140448	signaling receptor ligand precursor processing	2/138	29/14464	0.06896552	7.22838581	3.29512864	0.03101358	0.63095922	0.6196921	Hsp90b1/P4hb	2
GO:2000406	positive regulation of T cell migration	2/138	29/14464	0.06896552	7.22838581	3.29512864	0.03101358	0.63095922	0.6196921	Cd99l2/Oxsr1	2
GO:2001258	negative regulation of cation channel activity	2/138	29/14464	0.06896552	7.22838581	3.29512864	0.03101358	0.63095922	0.6196921	Rrad/Sri	2
GO:0071559	response to transforming growth factor beta	6/138	249/14464	0.02409639	2.52558058	2.38323933	0.03217806	0.63095922	0.6196921	Hspa5/Dand5/Smad2/Stk16/Brms1/Spred3	6
GO:0090101	negative regulation of transmembrane receptor protein serine/threonine kinase signaling pathway	4/138	126/14464	0.03174603	3.3273522	2.57519047	0.03252997	0.63095922	0.6196921	Hspa5/Dand5/Brms1/Spred3	4
GO:0002455	humoral immune response mediated by circulating immunoglobulin	2/138	30/14464	0.06666667	6.98743961	3.22191951	0.03302475	0.63095922	0.6196921	Ighg2c/Cr1l	2
GO:0048384	retinoic acid receptor signaling pathway	2/138	30/14464	0.06666667	6.98743961	3.22191951	0.03302475	0.63095922	0.6196921	Dhrs3/Calr	2
GO:0086004	regulation of cardiac muscle cell contraction	2/138	30/14464	0.06666667	6.98743961	3.22191951	0.03302475	0.63095922	0.6196921	Pkp2/Sri	2
GO:0086065	cell communication involved in cardiac conduction	2/138	30/14464	0.06666667	6.98743961	3.22191951	0.03302475	0.63095922	0.6196921	Pkp2/Sri	2
GO:0000154	rRNA modification	2/138	31/14464	0.06451613	6.76203834	3.15199084	0.03508566	0.63095922	0.6196921	Mettl16/Tfb1m	2
GO:0030212	hyaluronan metabolic process	2/138	31/14464	0.06451613	6.76203834	3.15199084	0.03508566	0.63095922	0.6196921	Has2/Hexa	2
GO:0007179	transforming growth factor beta receptor signaling pathway	5/138	190/14464	0.02631579	2.75819985	2.39429939	0.03551923	0.63095922	0.6196921	Hspa5/Dand5/Smad2/Brms1/Spred3	5
GO:0043433	negative regulation of DNA-binding transcription factor activity	4/138	130/14464	0.03076923	3.22497213	2.5010295	0.0358726	0.63095922	0.6196921	Sri/Ppp2cb/Brms1/Itch	4
GO:0016180	snRNA processing	2/138	32/14464	0.0625	6.55072464	3.08508884	0.0371952	0.63095922	0.6196921	Mettl16/Ints10	2
GO:0045577	regulation of B cell differentiation	2/138	32/14464	0.0625	6.55072464	3.08508884	0.0371952	0.63095922	0.6196921	Card11/Mmp14	2
GO:0070972	protein localization to endoplasmic reticulum	3/138	77/14464	0.03896104	4.08356861	2.66268701	0.03727119	0.63095922	0.6196921	Hspa5/Ubl4a/Srprb	3
GO:0002687	positive regulation of leukocyte migration	4/138	133/14464	0.03007519	3.1522284	2.44727173	0.03850547	0.63095922	0.6196921	Calr/Mmp14/Cd99l2/Oxsr1	4
GO:0002474	antigen processing and presentation of peptide antigen via MHC class I	2/138	33/14464	0.06060606	6.35221783	3.02098661	0.03935227	0.63095922	0.6196921	Calr/Pdia3	2
GO:0018208	peptidyl-proline modification	2/138	33/14464	0.06060606	6.35221783	3.02098661	0.03935227	0.63095922	0.6196921	Fkbp2/P4hb	2
GO:0046329	negative regulation of JNK cascade	2/138	33/14464	0.06060606	6.35221783	3.02098661	0.03935227	0.63095922	0.6196921	Fktn/Itch	2
GO:1904063	negative regulation of cation transmembrane transport	3/138	79/14464	0.03797468	3.98018712	2.60680418	0.03974852	0.63095922	0.6196921	Rrad/Sri/Oxsr1	3
GO:0032409	regulation of transporter activity	5/138	196/14464	0.0255102	2.67376516	2.31551325	0.03975579	0.63095922	0.6196921	Slc5a3/Rrad/Sri/Ndfip2/Oxsr1	5
GO:0001953	negative regulation of cell-matrix adhesion	2/138	34/14464	0.05882353	6.16538789	2.95948059	0.04155578	0.63095922	0.6196921	Mmp14/Sema3e	2
GO:0001958	endochondral ossification	2/138	34/14464	0.05882353	6.16538789	2.95948059	0.04155578	0.63095922	0.6196921	Mmp14/Ift80	2
GO:0036075	replacement ossification	2/138	34/14464	0.05882353	6.16538789	2.95948059	0.04155578	0.63095922	0.6196921	Mmp14/Ift80	2
GO:1902175	regulation of oxidative stress-induced intrinsic apoptotic signaling pathway	2/138	34/14464	0.05882353	6.16538789	2.95948059	0.04155578	0.63095922	0.6196921	Bag5/P4hb	2
GO:2000403	positive regulation of lymphocyte migration	2/138	34/14464	0.05882353	6.16538789	2.95948059	0.04155578	0.63095922	0.6196921	Cd99l2/Oxsr1	2
GO:0006956	complement activation	2/138	35/14464	0.05714286	5.98923395	2.90038759	0.04380468	0.63095922	0.6196921	Ighg2c/Cr1l	2
GO:0033144	negative regulation of intracellular steroid hormone receptor signaling pathway	2/138	35/14464	0.05714286	5.98923395	2.90038759	0.04380468	0.63095922	0.6196921	Bmal1/Calr	2
GO:0046640	regulation of alpha-beta T cell proliferation	2/138	35/14464	0.05714286	5.98923395	2.90038759	0.04380468	0.63095922	0.6196921	Card11/Itch	2
GO:1903311	regulation of mRNA metabolic process	6/138	269/14464	0.02230483	2.33780508	2.17373973	0.04416945	0.63095922	0.6196921	Mettl16/Obi1/Srpk1/Hnrnpc/Pkp3/Mettl14	6
GO:0045765	regulation of angiogenesis	6/138	270/14464	0.02222222	2.32914654	2.16375761	0.04483347	0.63095922	0.6196921	Rhoj/Sash1/Stard13/S2bpcox16/Sema3e/Aggf1	6
GO:0034620	cellular response to unfolded protein	3/138	84/14464	0.03571429	3.74327122	2.4747721	0.04629753	0.63095922	0.6196921	Hspa5/Hspd1/Dnajc3	3
GO:0034766	negative regulation of monoatomic ion transmembrane transport	3/138	84/14464	0.03571429	3.74327122	2.4747721	0.04629753	0.63095922	0.6196921	Rrad/Sri/Oxsr1	3
GO:0008380	RNA splicing	8/138	417/14464	0.01918465	2.01077399	2.05559029	0.04646287	0.63095922	0.6196921	Mettl16/Obi1/Srpk1/Prpf31/Hnrnpc/Mettl14/Virma/Prpf18	8
GO:0051651	maintenance of location in cell	5/138	205/14464	0.02439024	2.55638035	2.20269516	0.04666638	0.63095922	0.6196921	Hspa5/Scin/Hexa/Pkp2/Sri	5
GO:0060348	bone development	5/138	207/14464	0.02415459	2.53168102	2.17844044	0.04829336	0.63095922	0.6196921	Dhrs3/Mmp14/Has2/Ift172/Ift80	5
GO:0006084	acetyl-CoA metabolic process	2/138	37/14464	0.05405405	5.66549158	2.78879454	0.04843439	0.63095922	0.6196921	Ppcs/Pdk3	2
GO:0060563	neuroepithelial cell differentiation	2/138	37/14464	0.05405405	5.66549158	2.78879454	0.04843439	0.63095922	0.6196921	Smad2/Ift80	2
GO:0060021	roof of mouth development	3/138	86/14464	0.03488372	3.6562184	2.4247671	0.04905743	0.63095922	0.6196921	Dhrs3/Smad2/Ift172	3
GO:1901342	regulation of vasculature development	6/138	277/14464	0.02166065	2.27028724	2.09509055	0.04965684	0.63095922	0.6196921	Rhoj/Sash1/Stard13/S2bpcox16/Sema3e/Aggf1	6
GO:0043542	endothelial cell migration	5/138	210/14464	0.02380952	2.49551415	2.14258499	0.05079633	0.63095922	0.6196921	Calr/Rhoj/Sash1/Stard13/S2bpcox16	5
GO:0032924	activin receptor signaling pathway	2/138	38/14464	0.05263158	5.51639969	2.73600865	0.05081316	0.63095922	0.6196921	Dand5/Smad2	2
GO:0090049	regulation of cell migration involved in sprouting angiogenesis	2/138	38/14464	0.05263158	5.51639969	2.73600865	0.05081316	0.63095922	0.6196921	Rhoj/Stard13	2
GO:2000404	regulation of T cell migration	2/138	38/14464	0.05263158	5.51639969	2.73600865	0.05081316	0.63095922	0.6196921	Cd99l2/Oxsr1	2
GO:0170033	L-amino acid metabolic process	4/138	147/14464	0.02721088	2.85201617	2.2150508	0.0522221	0.63095922	0.6196921	Gclm/Hmgcll1/Enoph1/P4hb	4
GO:1903115	regulation of actin filament-based movement	2/138	39/14464	0.05128205	5.37495355	2.68506066	0.05323319	0.63095922	0.6196921	Pkp2/Sri	2
GO:0048705	skeletal system morphogenesis	5/138	214/14464	0.02336449	2.44886902	2.09572766	0.0542505	0.63095922	0.6196921	Dhrs3/Mmp14/Has2/Smad2/Ift80	5
GO:0046633	alpha-beta T cell proliferation	2/138	40/14464	0.05	5.24057971	2.6358375	0.05569348	0.63095922	0.6196921	Card11/Itch	2
GO:0141193	nuclear receptor-mediated signaling pathway	4/138	151/14464	0.02649007	2.77646607	2.15370551	0.05657199	0.63095922	0.6196921	Bmal1/Dhrs3/Calr/Pdk3	4
GO:0044703	multi-organism reproductive process	4/138	152/14464	0.02631579	2.75819985	2.13868159	0.05768914	0.63095922	0.6196921	Bmal1/Calr/Cr1l/Tppp3	4
GO:0001706	endoderm formation	2/138	41/14464	0.04878049	5.11276069	2.58823574	0.05819307	0.63095922	0.6196921	Mmp14/Smad2	2
GO:0019748	secondary metabolic process	2/138	41/14464	0.04878049	5.11276069	2.58823574	0.05819307	0.63095922	0.6196921	Rab38/Akr1a1	2
GO:0043507	positive regulation of JUN kinase activity	2/138	41/14464	0.04878049	5.11276069	2.58823574	0.05819307	0.63095922	0.6196921	Fzd8/Sash1	2
GO:0048701	embryonic cranial skeleton morphogenesis	2/138	41/14464	0.04878049	5.11276069	2.58823574	0.05819307	0.63095922	0.6196921	Mmp14/Smad2	2
GO:0044706	multi-multicellular organism process	4/138	153/14464	0.02614379	2.7401724	2.12377871	0.05881811	0.63095922	0.6196921	Bmal1/Calr/Cr1l/Tppp3	4
GO:0046474	glycerophospholipid biosynthetic process	4/138	153/14464	0.02614379	2.7401724	2.12377871	0.05881811	0.63095922	0.6196921	Rab38/Dgkk/Pigg/Selenoi	4
GO:1904375	regulation of protein localization to cell periphery	4/138	154/14464	0.02597403	2.72237907	2.10899498	0.05995887	0.63095922	0.6196921	Mmp14/Arhgef16/Rab38/Cnpy4	4
GO:0071333	cellular response to glucose stimulus	4/138	155/14464	0.02580645	2.70481533	2.09432855	0.06111141	0.63095922	0.6196921	Smad2/Gclm/Sri/Pdk3	4
GO:0070936	protein K48-linked ubiquitination	3/138	95/14464	0.03157895	3.30983982	2.21685178	0.06244008	0.63095922	0.6196921	Dtx4/Itch/Ube3c	3
GO:0007033	vacuole organization	5/138	223/14464	0.02242152	2.35003574	1.99403932	0.06251093	0.63095922	0.6196921	Hexa/Snx18/Aktip/Wdr45b/Sec22b	5
GO:0090092	regulation of transmembrane receptor protein serine/threonine kinase signaling pathway	5/138	223/14464	0.02242152	2.35003574	1.99403932	0.06251093	0.63095922	0.6196921	Hspa5/Dand5/Smad2/Brms1/Spred3	5
GO:0021872	forebrain generation of neurons	2/138	43/14464	0.04651163	4.87495787	2.49752467	0.06330629	0.63095922	0.6196921	Sema3e/Mettl14	2
GO:0043409	negative regulation of MAPK cascade	4/138	157/14464	0.02547771	2.67035909	2.06534042	0.06345167	0.63095922	0.6196921	Fktn/S2bpcox16/Itch/Spred3	4
GO:0071331	cellular response to hexose stimulus	4/138	158/14464	0.02531646	2.65345808	2.05101523	0.06463934	0.63095922	0.6196921	Smad2/Gclm/Sri/Pdk3	4
GO:0071326	cellular response to monosaccharide stimulus	4/138	159/14464	0.02515723	2.63676967	2.03680035	0.06583865	0.63095922	0.6196921	Smad2/Gclm/Sri/Pdk3	4
GO:0061013	regulation of mRNA catabolic process	4/138	161/14464	0.02484472	2.60401476	2.00869497	0.06827209	0.63095922	0.6196921	Mettl16/Hnrnpc/Pkp3/Mettl14	4
GO:0007219	Notch signaling pathway	4/138	162/14464	0.02469136	2.5879406	1.99480127	0.06950614	0.63095922	0.6196921	Mmp14/Dtx4/Ift172/S2bpcox16	4
GO:0006397	mRNA processing	8/138	455/14464	0.01758242	1.84284122	1.79289212	0.06994329	0.63095922	0.6196921	Mettl16/Obi1/Srpk1/Prpf31/Hnrnpc/Mettl14/Virma/Prpf18	8
GO:1990778	protein localization to cell periphery	7/138	378/14464	0.01851852	1.94095545	1.81939661	0.07023276	0.63095922	0.6196921	Mmp14/Arhgef16/Rab38/Cnpy4/Pkp3/Pkp2/Ift80	7
GO:0002065	columnar/cuboidal epithelial cell differentiation	3/138	100/14464	0.03	3.14434783	2.11185354	0.0705323	0.63095922	0.6196921	Bmal1/Smad2/Ift80	3

GO:0071674	mononuclear cell migration	4/138	163/14464	0.02453988	2.57206366	1.98101147	0.07075169	0.63095922	0.6196921	Calr/Mmp14/Cd99l2/Oxsr1	4
GO:0006890	retrograde vesicle-mediated transport, Golgi to endoplasmic reticulum	2/138	46/14464	0.04347826	4.55702583	2.37148121	0.07124724	0.63095922	0.6196921	Lman2/Sec22b	2
GO:1903426	regulation of reactive oxygen species biosynthetic process	2/138	46/14464	0.04347826	4.55702583	2.37148121	0.07124724	0.63095922	0.6196921	Slc5a3/Hspd1	2
GO:2000785	regulation of autophagosome assembly	2/138	46/14464	0.04347826	4.55702583	2.37148121	0.07124724	0.63095922	0.6196921	Snx18/Sec22b	2
GO:0046394	carboxylic acid biosynthetic process	5/138	232/14464	0.02155172	2.25887056	1.89713557	0.07144553	0.63095922	0.6196921	Degs1/Elovl7/Lta4h/Enoph1/Akr1a1	5
GO:0007368	determination of left/right symmetry	3/138	101/14464	0.02970297	3.11321567	2.09164604	0.07220504	0.63095922	0.6196921	Dand5/Smad2/Ift172	3
GO:0051235	maintenance of location	6/138	307/14464	0.01954397	2.04843507	1.82284824	0.07384839	0.63095922	0.6196921	Hspa5/Dand5/Scin/Hexa/Pkp2/Sri	6
GO:0010595	positive regulation of endothelial cell migration	3/138	102/14464	0.02941176	3.08269395	2.07168796	0.07389552	0.63095922	0.6196921	Calr/Rhoj/Sash1	3
GO:0007566	embryo implantation	2/138	47/14464	0.04255319	4.46006784	2.33185937	0.0739629	0.63095922	0.6196921	Calr/Tppp3	2
GO:0042073	intraciliary transport	2/138	47/14464	0.04255319	4.46006784	2.33185937	0.0739629	0.63095922	0.6196921	Ift172/Ift80	2
GO:0016053	organic acid biosynthetic process	5/138	235/14464	0.0212766	2.23003392	1.86582149	0.07457248	0.63095922	0.6196921	Degs1/Elovl7/Lta4h/Enoph1/Akr1a1	5
GO:0009409	response to cold	2/138	48/14464	0.04166667	4.36714976	2.29333198	0.07671141	0.63095922	0.6196921	Hspd1/Dnajc3	2
GO:0090102	cochlea development	2/138	48/14464	0.04166667	4.36714976	2.29333198	0.07671141	0.63095922	0.6196921	Grhl3/Slc17a8	2
GO:0006520	amino acid metabolic process	5/138	238/14464	0.0210084	2.20192425	1.8349762	0.07777324	0.63095922	0.6196921	Gclm/Hmgcll1/Yars2/Enoph1/P4hb	5
GO:0001501	skeletal system development	8/138	466/14464	0.01716738	1.79934067	1.72146477	0.07789467	0.63095922	0.6196921	Dhrs3/Mmp14/Has2/Smad2/Scin/Hexa/Ift172/Ift80	8
GO:0006979	response to oxidative stress	7/138	388/14464	0.01804124	1.89093082	1.74592861	0.07824586	0.63095922	0.6196921	Bmal1/Mmp14/Dgkk/Gclm/Ppp2cb/Msrb2/Oxsr1	7
GO:1903320	regulation of protein modification by small protein conjugation or removal	5/138	239/14464	0.0209205	2.19271118	1.82479619	0.0788565	0.63095922	0.6196921	Hspa5/Sash1/Bag5/Itch/Ndfip2	5
GO:0010596	negative regulation of endothelial cell migration	2/138	49/14464	0.04081633	4.27802425	2.25584441	0.07949192	0.63095922	0.6196921	Stard13/S2bpcox16	2
GO:0061371	determination of heart left/right asymmetry	2/138	49/14464	0.04081633	4.27802425	2.25584441	0.07949192	0.63095922	0.6196921	Dand5/Ift172	2
GO:1903170	negative regulation of calcium ion transmembrane transport	2/138	49/14464	0.04081633	4.27802425	2.25584441	0.07949192	0.63095922	0.6196921	Rrad/Sri	2
GO:0016525	negative regulation of angiogenesis	3/138	106/14464	0.02830189	2.96636587	1.99423599	0.08083193	0.63095922	0.6196921	Stard13/S2bpcox16/Sema3e	3
GO:0001678	intracellular glucose homeostasis	4/138	171/14464	0.02339181	2.4517332	1.87425824	0.08112528	0.63095922	0.6196921	Smad2/Gclm/Sri/Pdk3	4
GO:0071322	cellular response to carbohydrate stimulus	4/138	171/14464	0.02339181	2.4517332	1.87425824	0.08112528	0.63095922	0.6196921	Smad2/Gclm/Sri/Pdk3	4
GO:1901605	alpha-amino acid metabolic process	4/138	171/14464	0.02339181	2.4517332	1.87425824	0.08112528	0.63095922	0.6196921	Gclm/Hmgcll1/Enoph1/P4hb	4
GO:0000959	mitochondrial RNA metabolic process	2/138	50/14464	0.04	4.19246377	2.21934584	0.08230359	0.63095922	0.6196921	Tfb1m/Yars2	2
GO:0016073	snRNA metabolic process	2/138	50/14464	0.04	4.19246377	2.21934584	0.08230359	0.63095922	0.6196921	Mettl16/Ints10	2
GO:0072678	T cell migration	2/138	50/14464	0.04	4.19246377	2.21934584	0.08230359	0.63095922	0.6196921	Cd99l2/Oxsr1	2
GO:0006643	membrane lipid metabolic process	4/138	172/14464	0.02325581	2.43747893	1.86133898	0.08247246	0.63095922	0.6196921	Pigg/Hexa/Degs1/Elovl7	4
GO:0016064	immunoglobulin mediated immune response	3/138	107/14464	0.02803738	2.93864283	1.97544119	0.08260888	0.63095922	0.6196921	Ighg2c/Hspd1/Cr1l	3
GO:0003007	heart morphogenesis	5/138	243/14464	0.02057613	2.15661716	1.78457155	0.0832707	0.63095922	0.6196921	Dhrs3/Has2/Smad2/Ift172/Pkp2	5
GO:1901873	regulation of post-translational protein modification	5/138	244/14464	0.0204918	2.14777857	1.77463683	0.08439446	0.63095922	0.6196921	Hspa5/Sash1/Bag5/Itch/Ndfip2	5
GO:0060972	left/right pattern formation	3/138	108/14464	0.02777778	2.91143317	1.95686351	0.08440264	0.63095922	0.6196921	Dand5/Smad2/Ift172	3
GO:2000181	negative regulation of blood vessel morphogenesis	3/138	108/14464	0.02777778	2.91143317	1.95686351	0.08440264	0.63095922	0.6196921	Stard13/S2bpcox16/Sema3e	3
GO:0009799	specification of symmetry	3/138	109/14464	0.02752294	2.88472278	1.93849816	0.08621307	0.63095922	0.6196921	Dand5/Smad2/Ift172	3
GO:0009855	determination of bilateral symmetry	3/138	109/14464	0.02752294	2.88472278	1.93849816	0.08621307	0.63095922	0.6196921	Dand5/Smad2/Ift172	3
GO:1901343	negative regulation of vasculature development	3/138	109/14464	0.02752294	2.88472278	1.93849816	0.08621307	0.63095922	0.6196921	Stard13/S2bpcox16/Sema3e	3
GO:0002042	cell migration involved in sprouting angiogenesis	2/138	52/14464	0.03846154	4.03121516	2.14912944	0.08801701	0.63095922	0.6196921	Rhoj/Stard13	2
GO:0061337	cardiac conduction	2/138	52/14464	0.03846154	4.03121516	2.14912944	0.08801701	0.63095922	0.6196921	Pkp2/Sri	2
GO:2000401	regulation of lymphocyte migration	2/138	52/14464	0.03846154	4.03121516	2.14912944	0.08801701	0.63095922	0.6196921	Cd99l2/Oxsr1	2
GO:0019724	B cell mediated immunity	3/138	110/14464	0.02727273	2.85849802	1.92034049	0.08803999	0.63095922	0.6196921	Ighg2c/Hspd1/Cr1l	3
GO:0006024	glycosaminoglycan biosynthetic process	2/138	53/14464	0.03773585	3.9551545	2.11532611	0.09091715	0.63095922	0.6196921	Has2/Hexa	2
GO:0006513	protein monoubiquitination	2/138	53/14464	0.03773585	3.9551545	2.11532611	0.09091715	0.63095922	0.6196921	Obi1/Itch	2
